# Coordinated regulation of transcription by CcpA and the *Staphylococcus aureus* two-component system HptRS

**DOI:** 10.1371/journal.pone.0207161

**Published:** 2018-12-12

**Authors:** Joseph M. Reed, Sean Olson, Danielle F. Brees, Caitlin E. Griffin, Ryan A. Grove, Paul J. Davis, Stephen D. Kachman, Jiri Adamec, Greg A. Somerville

**Affiliations:** 1 School of Veterinary Medicine and Biomedical Sciences, University of Nebraska-Lincoln, Lincoln, Nebraska, United States of America; 2 Department of Statistics, University of Nebraska-Lincoln, Lincoln, Nebraska, United States of America; 3 Department of Biochemistry, University of Nebraska-Lincoln, Lincoln, Nebraska, United States of America; 4 Unaffiliated, Honey Creek, Iowa, United States of America; Universitatsklinikum Munster, GERMANY

## Abstract

The success of *Staphylococcus aureus* as a pathogen is due in part to its ability to adapt to changing environmental conditions using signal transduction pathways, such as metabolite- responsive regulators and two-component systems. *S*. *aureus* has a two-component system encoded by the gene pair *sav*0224 (*hptS*) and *sav*0223 (*hptR*) that regulate the hexose phosphate transport (*uhpT)* system in response to extracellular glucose-6-phosphate. Glycolytic intermediates such as glucose-6-phosphate are important carbon sources that also modulate the activity of the global metabolite-responsive transcriptional regulator CcpA. Because *uhpT* has a putative CcpA binding site in its promoter and it is regulated by HptR, it was hypothesized the regulons of CcpA and HptR might intersect. To determine if the regulatory domains of CcpA and HptRS overlap, *ccpA* was deleted in strains SA564 and SA564-Δ*hptRS* and growth, metabolic, proteomic, and transcriptional differences were assessed. As expected, CcpA represses *hptS* and *hptR* in a glucose dependent manner; however, upon CcpA derepression, the HptRS system functions as a transcriptional activator of metabolic genes within the CcpA regulon. Importantly, inactivation of *ccpA* and *hptRS* altered sensitivity to fosfomycin and ampicillin in the absence of exogenous glucose-6-phosphate, indicating that both CcpA and HptRS modulate antibiotic susceptibility.

## Introduction

*Staphylococcus aureus* is a common commensal bacterium and a versatile pathogen that can infect nearly any anatomic site. As a commensal, *S*. *aureus* persistently colonizes the nares of 12–30% of the population, while 16–70% of people are intermittent carriers [1–3]. The prevalence of *S*. *aureus* and its increasing antibiotic resistance contributed to a 50% increase in diagnoses from 9.17 to 13.79 per 1000 hospitalizations from 1999 to 2005 [4]. This prevalence and the success of *S*. *aureus* as a pathogen are strong indicators of its ability to adapt and survive in diverse conditions.

*S*. *aureus* survival in a host requires nutrients, avoidance of the immune system, and proliferation within a changing environment. To survive environmental challenges, *S*. *aureus* must be able to respond to changing environmental cues [5–7]. Rapid responses to environmental challenges are achieved by alterations in enzymatic activity and through transcriptional and translational initiation changes [6]. Feedback and feedforward enzymatic changes are induced by changes in substrate, cofactor, and/or inhibitor concentrations, while transcriptional and translational changes are mediated through metabolite, metal ion, and/or cofactor-responsive regulators (*e*.*g*., CcpA, Fur, Rex), small RNAs (*e*.*g*., RsaE), alternative sigma factors (*e*.*g*., σ^B^), and two- or three-component regulators (*e*.*g*., KdpD-KdpE) [8–17].

The metabolite-responsive carbon catabolite protein A (CcpA) belongs to the LacI protein family and regulates transcription in response to changes in metabolite concentrations such as glucose, fructose, and glycerol [8, 18]. These carbon sources can be converted into the activated glycolytic intermediates glucose-6-phosphate and fructose-1,6-bisphosphate through glycolysis or gluconeogenesis. Glucose-6-phosphate and fructose-1,6-bisphosphate promote phosphorylation and activation of the CcpA co-regulator, the histidine containing protein (HPr) [12, 13, 19]. CcpA activity is also regulated through phosphorylation by serine/threonine protein kinase 1 (Stk1) in response to changes in intracellular organic phosphate [20–22]. Other transcriptional regulators directly bind to metal ions such as Fur (Fe^2+^) or dinucleotides such as Rex (NAD^+^/NADH) [14, 15, 18]. Together these regulators alter transcription of genes involved in diverse cellular processes such as metabolism, virulence factor synthesis, and antibiotic resistance. There are also non-protein regulators that respond to environmental stimuli such as small regulatory RNAs (*e*.*g*., RsaE), which bind to RNA and alter translation initiation or stability of target mRNAs [16, 17]. Interestingly, transcription of several members of the Rsa family of sRNAs (*rsaA*, *rsaD*, *rsaF*, *rsaE*) are regulated by the alternative sigma factor B (σ^B^), which confers promoter specificity for RNA polymerase to target genes, in response to physical and chemical stresses [16, 17, 23, 24]. Environmental stresses also activate other regulators such as two- and three-component systems.

Two- and three-component signal transduction systems, such as the *S*. *aureus* KdpD-KdpE two-component system, serve as a stimulus response coupling mechanism that allows bacteria to alter transcription of genes necessary for survival in response to changing environmental conditions [10, 11]. The two-component systems are generally comprised of a sensor histidine kinase and a response regulator [11]. Signal sensing is through the input domain in the sensor histidine kinase that activates phosphorylation of a histidine in the autokinase domain. The phosphoryl group is subsequently transferred to an aspartate in the receiver domain of the response regulator, increasing output domain activity, and altering the transcription of response-regulated genes [11]. In the example of the KdpD-KdpE two-component system, changes in environmental potassium can alter the concentration of intracellular potassium and phosphate, affecting the ionic strength of the cytoplasm, and/or the ATP concentration [25]. In response, the KdpD-KdpE two-component system alters transcription of potassium ion transport genes (*i*.*e*., *kdpA*, *kdpB*, and *kdpC*) [26, 27]. Activation of KdpD results in autophosphorylation of histidine 673, which then transfers the phosphoryl group to aspartate 52 on the response regulator KdpE [27]. In addition to activating transcription of the *kdpABC* operon, KdpE alters transcription of *S*. *aureus* virulence genes, such as protein A, α-toxin, and aureolysin [27–29]. Interestingly, *kdpDE* transcription is under the control of another two-component system (AgrC-AgrA) that responds to cell density and metabolic signals [29]. In total, *S*. *aureus* are hypothesized to have 16 two-component systems, many of which are named and at least partially described [9, 30]. Of the partially defined two-component systems in *S*. *aureus*, one is encoded within the gene pair *sav*0224 (*hptS*) and *sav*0223 (*hptR*). The HptRS two-component system is in a predicted three-gene operon downstream of *sav*0225 (*hptA*), a putative metal binding protein. Recently, it was suggested that HptS and HptR regulate transcription of the hexose phosphate transport gene (*uhpT)* in response to changes in the availability of exogenous glucose-6-phosphate [31, 32]. The hexose phosphate transport system functions as an inorganic phosphate-coupled hexose phosphate sugar transporter for sugars such as glucose-6-phosphate, fructose-6-phosphate, and mannose-6-phosphate [33, 34]. This transport system is medically relevant because it is one of only two known transporters (*i*.*e*., UhpT and GlpT) that mediate the uptake of the antibiotic fosfomycin [35, 36]. In an *S*. *aureus* USA300 lineage strain, inactivation of either *hptS* or *hptR* increased fosfomycin resistance, reduced transcription of *uhpT*, and reduced growth when cultivated in a chemically defined medium containing glucose-6-phosphate as the primary carbon source [31]. Interestingly, in *S*. *aureus* strain NCTC8325, it was demonstrated that HptA binds to glucose-6-phosphate and may function to activate the HptRS two component system [32]. Although cultivation in a chemically defined medium containing glucose-6-phosphate indicated that HptRS regulated the UhpT transporter, it is likely that cultivation on a single carbohydrate source caused widespread changes in metabolism. Commonly, carbohydrate-dependent metabolic adaptation is regulated in part by CcpA [8, 13], thus linking separate regulatory systems that balance *S*. *aureus* responses to the availability of rapidly catabolizable carbon sources.

Not only is glucose-6-phosphate the proposed effector molecule of HptA, it is also an effector of CcpA [13]. This is noteworthy because the *uhpT* gene promoter contains both a HptR (-67 to -96 bp), and a putative CcpA binding site (*cre* site; -61 to -46 bp), suggesting *uhpT* gene transcription is coordinately regulated by HptR and CcpA [18, 32]. Support for CcpA-mediated transcriptional regulation of *uhpT* was observed when inactivation of CcpA abolished the glucose-dependent increase in *uhpT* mRNA [8]. Interestingly, transcription of the *hptR*, *hptS*, and *hptA* operon is induced in *S*. *aureus* strain COL under anaerobic cultivation, which is consistent with reduced CcpA activity during anaerobic growth due to a decrease in phosphorylation of the HPr co-regulator [8, 37, 38]. These observations indicate the CcpA and HptR regulons overlap. In total, it is likely that CcpA and HptR form an undefined, complex regulatory network that responds to environmental changes to regulate *uhpT* and other genes. To determine if HptRS is within or interacts with the CcpA regulon, Δ*ccpA*, Δ*hptRS*, and Δ*hptRS*/*ccpA* mutants were constructed in *S*. *aureus* strain SA564 and were assessed for growth, metabolic, proteomic, and transcriptional differences.

## Materials and methods

### Bacterial strains and cultivation conditions

Bacterial strains, bacteriophage, and plasmids used in this study are listed in Table 1. *Escherichia coli* strain DH5α was cultivated in lysogeny broth (LB) or on LB containing 1.5% agar. *S*. *aureus* strains were grown in tryptic soy broth (TSB) containing 0.25% dextrose (BD Biosciences), TSB without dextrose (TSB-dex; BD Biosciences), or on TSB containing 1.5% agar (TSA). Bacterial pre-cultures were inoculated 1:100 from overnight cultures into TSB or TSB-dex, incubated at 37 °C, aerated at 225 rpm using a flask-to-medium ratio of 10:1, and grown for 2 h. These exponential growth phase pre-cultures were centrifuged for 5 min at 5,000 rpm (4,272.5 X g) at 22 °C and suspended in 1–2 mL of medium. Primary cultures were inoculated into 100 mL pre-warmed TSB or TSB-dex, at an absorbance at 600 nm (*A*_600_) of 0.01, and incubated at 37 °C, aerated at 225 rpm, with a flask-to-medium ratio of 10:1 (aerobic) or 10:8 (microaerobic/anaerobic). The *A*_600_ and pH were recorded hourly. The data were analyzed using a 2-way ANalysis Of VAriance (ANOVA) using SigmaPlot version 11.1, from Systat Software, Inc. (San Jose California USA).

**Table 1:** Strains and plasmids used in this study.

Strain, Plasmid, Phage	Description	Source
**Strains**		
RN4220	*S*. *aureus* Restriction-deficient mutant of strain 8325–4	[39]
SA564	*S*. *aureus* Clinical isolate wildtype (*blaZ* ^+^)	[7]
SA564 *ccpA*::*ermB*	*S*. *aureus* CcpA knockout	[12]
Newman *ccpA*::*tetM*	*S*. *aureus* CcpA knockout	[40]
SA564 *hptRS*::*ermB*	*S*. *aureus* Two-component system double knockout	This Study
SA564 *hptRS*::*ermB ccpA*::*tetM*	*S*. *aureus* Triple knockout	This Study
DH5(α)	*E*. *coli* cloning host	Invitrogen
**Phages**		
φ11	Transducing Phage	[39]
80α	Transducing Phage	[41]
**Plasmid**		
pBT2	Temperature-sensitive shuttle vector	[42]

For antibiotic disk diffusion assays, bacterial strains were inoculated from a single colony into 2 mL TSB in a 14 mL culture tube and incubated overnight at 37 °C and aerated at 225 rpm. A 2 h pre-culture was prepared as described. After pre-incubation, the *A*_600_ were recorded and bacteria were diluted to 0.5 McFarland units (*i*.*e*., *A*_600_ = 0.08; 1–5 x 10^8^ CFU/mL). This suspension was diluted 1:10 and 100 μL was spread onto TSA plates and antibiotic disks (Becton, Dickinson and Company) were placed on each plate. Plates were incubated at 37 °C overnight. Zone of inhibition was recorded by measurement of the space between the edge of the disk and the first colony. The data were analyzed using a 2-way ANOVA.

For antimicrobial susceptibility tests, the broth microdilution method of the Clinical and Laboratory Standards Institute (CLSI) was utilized, with the exception that no glucose-6-phosphate was added to the medium [43]. Briefly, bacterial strains were inoculated from a single colony on TSA into cation adjusted Mueller Hinton broth (CAMHB) with 50 mg/L Ca^2+^ and 12.5 mg/L Mg^2+^, and incubated at 37 °C under aeration at 225 rpm [43]. 2 h pre-cultures were prepared as described. The pre-cultures were diluted to produce a final inoculum of 1–5 × 10^5^ CFU/mL in 96-well flat bottom cell culture plates. The plates were incubated at 37 °C for 20 h and the absorbance at 595 nm (*A*_595_) was recorded in a plate reader. The minimum inhibitory concentration (MIC), as defined by CLSI, is the lowest concentration of antimicrobial agent that completely inhibits growth of the organism in microdilution wells as detected by the unaided eye [43]. To eliminate human error, an *A*_595_ ≤ 0.05 was considered to reflect no bacterial growth; hence, the MIC was defined as the lowest concentration of antimicrobial agent that inhibits growth at A_595_ ≤ 0.05. The data were analyzed using a 2-way ANOVA.

### Construction of *hptRS* and *ccpA* mutants in *S*. *aureus*

Primers (Table 2) used for PCR amplification of *S*. *aureus* strain SA564 genomic DNA were designed using the SA564 genome sequence (NCBI Reference Sequence: NZ_CP010890.1) as a template. Inactivation of *hptRS* in strain SA564 was accomplished by deleting a 2,380 bp region, which included all of *sav*0223 (*hptR*) and the majority of *sav*0224 (*hptS*), using the gene splicing by overlap extension technique [44]. Primers MUT0223CF2 and MUT0223Y were used to amplify a 1,416 bp region upstream of *sav*0223 (fragment 1) and primers MUT0224AR and MUT0224X were used to amplify a 1,849 bp region that included 147 bp at the beginning of *sav*0224 and the region downstream of *sav*0225 (fragment 2). Primers MUTO224AF and MUT0223CR, were used to sew fragments 1 and 2 together to create the spliced gene fragment with KpnI and BamH1 restriction sites for digestion and ligation into plasmid pBT-2. The size of the spliced gene fragment was confirmed by restriction digestion. The *ermB* cassette from plasmid pEC4 was amplified using primers SAV0224ermB-F and SAV0224ermB-R and inserted between the cloned *sav*0225 and *sav*0222 DNA by digestion with BsiW1 to create plasmid pBT2-Δ*sav*0223/0224::*ermB*. This plasmid was electroporated into *E*. *coli* strain DH5α, harvested, and transformed into *S*. *aureus* strain RN4220. *S*. *aureus* strain RN4220Δ*sav*0224/0223::*ermB* was constructed using the temperature shift protocol of Foster *et al*. [45]. The Δ*sav*0223/0224::*ermB* mutation from strain RN4220-Δ*sav*0224/0223::*ermB* was transduced into *S*. *aureus* strain SA564 using *S*. *aureus* phage φ11 to make strain SA564-Δ*sav*0224/0223::*ermB*. The resulting transductants were selected for erythromycin resistance and confirmed by PCR. To reduce the likelihood of spurious mutations from selection and transduction, the Δ*sav*0224/0223::*ermB* allele was backcrossed into wild-type strain SA564 using transducing phage 80α to create SA564-Δ*hptRS*::*ermB* (Δ*hptRS*). Deletion of genes *sav*0224/0223 were confirmed by DNA sequencing (Eurofins MWG Operon) using primers SAermB375R, SA0222-fseq500, SAV0222-fseq1000, MUT0223CF, SAMUT0223R400, SAMUT0224ermB222F, and SAMUT0224AF. The *ccpA*::*tetM* allele was transduced from *S*. *aureus* strain Newman *ccpA*::*tetM* using phage 80α into strain SA564-Δ*hptRS*::*ermB* to make strain SA564-Δ*hptRS*::*ermB*/*ccpA*::*tetM* (Δ*hptRS*/*ccpA*). Successful transduction was confirmed by PCR of both *ccpA* and *hptRS* mutant regions using primers acuC-R, SAV1652-F and SAV0225-R, SAV0222-F.

**Table 2:** Primers used in this study.

Primers for knockout construction	*S*. *aureus* Mu50 Open Reading Frame	Sequence
MUT0224X	SAV0226	GAAGTTGCTTCTGTTGGTCCTGCAATATCTTGC
MUT0224AR	SAV0224	GATGAATCGTACGATGCTCAATC
MUT0223CF2		ATCGTACGATTCATCGTCGACGGAATTATGATCTGCCCCGAGAC
MUT0223Y	SAV0222	ATTCGAAACAACTTTAAGGCG
MUTO224AF		AGTCAGGGATCCTTGAACCGTTC
MUT0223CR	SAV0222	AGTCAGGGTACCGGTGTTCCAATCAGTTGGTGG
SAV0224-ermB-f		AGTCAGGTCGACGAAGGAGGGATTCGTCATGTTG
SAV0224-ermB-r		AGTCAGGTCGACGCGACTCATAGAATTATTTCCTCCC
PCR and DNA Sequencing confirmation Primers		
SAermB375R		TTTGGTTTAGGATGAAAGCATTC
SA0222-fseq500	SAV0222	CATGACACTTGTAGCATTTGTG
SAV0222-fseq1000	SAV0222	GTGCAACACCACCTGCAATG
MUT0223CF		GGAATTATGATCTGCCCCGAGAC
SAMUT0223R400	SAV0225	CTGATAACACACCACCCATAAAGACGTC
SA0224ermB222F		GCCATACCACAGATGTTC
MUT0224AF		AGTCAGGGATCCTTGAACCGTTCAATATCTTGC
acuc-R	SAV1735	CGGCTATGGACACACTGTAAATG
SAV1652-F		GTGCTGATGGAGTTATGGC
SAV0225-R	SAV0225	CAAGCCGCTCAGTACAACAACG
SAV0222-F	SAV0222	GGAATCGTTGCTTTCTATGAAG
Primers for RT-qPCR		
16S-RTF	SAVrRNA16	GTGGAGGGTCATTGGAAACT
16S-RTR:	SAVrRNA16	CACTGGTGTTCCTCCATATCTC
pflB-F	SAV0226	ACGACTTCAACACGATGTCTAC
pflB-R	SAV0226	TTCTGCTGGACGGCTTAAAT
ADH-F	SAV0605	AATTGGAGACCGTGTGTCTATC
ADH-R	SAV0605	TTCAGCCATTGCACCATCTA
rocA-F	SAV2554	GCTGAAATGGGTGGTAAAGATG
rocA-R	SAV2554	CCAAACGCTGACGTTACAATAG
DHA-1439F	SAV1439	GCTATTGACCAAGGTGGAACTA
DHA-1439R	SAV1439	GGACTGCTCCTGGTTGATTT
ccpA4F	SAV1736	CAGGAACAAATGGTAAGGATGC
ccpA4R	SAV1736	TCTCCACCTACTAAAGCAAATGA
uhpTA2-F	SAV0222	CCCATCGGTGATTGCATTACT
uhpTA2-R	SAV0222	CCGGCTCTTCCCAAATTTCT
SAV0225RTF	SAV0225	CACCCTCATCCTAAGCGTAAA
SAV0225RTR	SAV0225	GCATCGAACTCTGCAACTAATC
SAV0224RTF	SAV0224	GATGCTTCAGCCACTCATAGAA
SAV0224RTR	SAV0224	TGCCAATGTCAGGCGTATAG
SAV0223RTF	SAV0223	ACCAGTAGACCATGCACAATTA
SAV0223RTR	SAV0223	TGACAAGATGCTAAGCTACGG

### Determination of acetate, glucose, and lactate levels in medium supernatant

Bacterial cultures (1 mL) were harvested hourly during cultivation and centrifuged for 5 min at 20,800 x g at 4 °C. The cell-free media were transferred to microfuge tubes and stored at -20 °C until needed. Glucose, acetate, and ammonia concentrations in the culture media were determined (n = 3) with kits purchased from R-biopharm.

### Reverse transcription PCR

For quantification of mRNA by reverse transcription PCR (RT-qPCR), RNA isolation and RT-qPCR were performed as described [46]. Briefly, cDNA (20 ng/reaction) was used for real-time amplification using primers listed in Table 2. 16S rRNA was used as an internal reference and transcript levels relative to 16S RNA were determined by the comparative threshold (ΔΔ*C*_*T*_) method (Bio-Rad) using two technical replicates per plate. All experiments were performed in biological triplicate for each gene. Experimental setup and data analysis were carried out using a Bio-Rad CFX96 real-time PCR detection system and CFX Manager software version 3.1 following the minimum information for the publication of quantitative real-time PCR (MIQE) guidelines [47]. The data for each biological replicate (n = 3) was compiled using the gene study tool within the BioRad CFX manager software package. In order to assess significance, a *p*-value cutoff of 0.05 (*p* ≤ 0.05), and fold change greater than 1.5 (FC ≥ 1.5), were utilized to determine if there was a statistically significant difference between the transcript abundance of the gene when strains were compared (Tables A-D in S5 File).

### Protein collection and processing

Bacteria (10 *A*_600_ units) were harvested after 2 and 6 h cultivation by centrifugation and suspended in 1 mL of lysis buffer containing 50 mM tris-HCL, 8 M urea, and 1.5 mM phenylmethylsulfonyl fluoride. The bacterial suspensions were lysed for 40 s at a setting of 6 m/s in a FastPrep instrument (MP biomedical) and the lysate was clarified by centrifugation for 5 min at 20,800 x g at 4 °C. Cell-free lysates were stored at -80 °C until use. Protein concentrations were determined using a Modified Lowry Protein Assay Kit (Fisher Scientific) and diluted to a final concentration of 235 μg/mL in ultrapure water. Protein samples were mixed with a 3X volume of cold acetone and incubated at -20 °C for 30 min and centrifuged for 2 min at 15,000 x g to pellet the precipitated proteins. Protein pellets were washed twice with cold acetone, dried 15 min in a speed vacuum, and stored at -80 °C. Protein samples were solubilized in 20 μL denaturing buffer (25 mM ammonium bicarbonate, pH 8.0; 10 mM tris (2-carboxyethyl) phosphine hydrochloride (TCEP); 5% sodium deoxycholate) and incubated for 10 min at 60 °C. Thiol alkylation was achieved by adding 5 μl alkylation buffer (100 mM iodoacetamide in water) and incubating the samples for 1 h at room temperature. Following alkylation, samples were diluted with 275 μL dilution buffer (25 mM ammonium bicarbonate, pH 8), 2 μL trypsin solution (1 mg/mL) was added, and incubated overnight at 37 °C. To stop the reaction, 10 μL of 10% trifluoroacetic acid (TFA) was added, incubated for 30 min, and centrifuged at 15,000 x g. Supernatants were transferred to microfuge tubes and used for LC-MS^E^ analysis.

### LC-MS^E^ analysis

All analyses were carried out using a Waters nanoAcquity UPLC System coupled to a Waters Synapt G2 mass spectrometer. The mobile phases were composed of Solvent A (0.1% formic acid in H_2_O) and Solvent B (0.1% formic acid in acetonitrile). Injection volume was 2 μL. Following injection, the peptides were concentrated on a Trap C-18 enrichment column (0.3 x 1 mm, Waters) and washed at 10 μL/min with Solvent A for 3 min. The enrichment column was then switched into the nanoflow path (500 nL/min) and further separated on a C-18 reversed phase nanocolumn (0.075 x 250 mm; Waters) coupled with the nanoelectrospray ionization (nESI) source of Synapt G2 mass spectrometer. Separation of peptides was achieved at the following gradient: T = 0 min: 5% B; T = 95 min: 50% B; T = 96 min: 85% B; T = 97 min: 85% B; T = 98 min: 5% B; T = 99 min: 5% B; T = 100 min: 85%; B; T = 101 min: 85% B T = 102 min: 5% B; and T = 120 min: 5% B (column re-equilibration). MS data were collected in positive, Data Independent Acquisition (MS^E^) mode under a capillary voltage of 2,900 V. The source temperature was set at 70 °C. Cone gas flow was maintained 6 L/min. Acquisition range was 50–2,000 m/z. MS^E^ data was collected with alternating low (4 eV) and elevated (ramp from 17 to 42 eV) energy over a 100–1500 m/z range. Spectra and peptide identification statistical analysis were carried out using Progenesis software (Waters Corporation, MA) and was searched against all *S*. *aureus* Mu50 predicted proteins (NCBI database).

### Power analysis

Experimental variability was assessed using a pilot proteome study of strains SA564 and SA564-Δ*hptRS* cultivated in TSB. A power analysis was conducted to determine the number of biological replicates needed to achieve the probability of detecting a true difference between strains during the exponential growth phase (2 h) and post-exponential growth phase (6 h) when one exists. It was determined that 5 biological replicates of each strain, growth phase (hour), and cultivation condition (media) combination would achieve a minimum of 74% power assuming dispersion (variability) of 0.05 and 99% power assuming dispersion of 0.01 (Table A in S2 File). The power analysis was conducted using SAS software, Version 9.4 of the SAS System for Windows (Copyright 2002–2012, SAS Institute Inc.). SAS and all other SAS Institute Inc. product or service names are registered trademarks or trademarks of SAS Institute Inc., Cary, NC, USA. The supporting information file 1 contains examples of the SAS code corresponding to the power analysis (S1 File).

### Statistical analysis of the total cytosolic proteome

The relative normalized abundance of each peptide was extracted as a .txt file from Progenesis for statistical analysis. Statistical analysis was performed using R 3.3 software, R Core Team (2017) [48]. The supporting information file 1 contains examples of the R code corresponding to the proteomic analysis (S1 File). Final data sets were exported into Microsoft Excel files for final organization and interpretation.

Peptide data were visualized in a histogram and the relative normalized peptide abundance had a unimodal, right skewed, distribution with responses greater than or equal to 0. For this reason, data were modeled using a generalized linear model that assumed the relative normalized peptide abundance followed a gamma distribution with strain (s), growth phase (h), and media (m) as fixed effects and the relative normalized abundance as the response variable [49]. Peptides with a 0 abundance value, due to an abundance not included in the gamma distribution, were assigned a value of 0.0001 to facilitate modeling and statistical analysis. Peptide abundance and variability were combined and protein abundance was modeled and analyzed for interactions between variables of strain (s), media (m), and/or growth phase (h) (Tables B-J in S2 File). A significant interaction would indicate that the effect of one variable depends on the magnitude of change of another variable. For any significant interaction (*p* ≤ 0.2) the simple effects, the effect of a single variable given the level of another variable on protein abundance, were assessed (*p* ≤ 0.05; Figures F-I in S6 File; Tables A-D in S3 File). For all proteins showing no significant interaction between variables, the main effect of each variable was analyzed by protein (*p* ≤ 0.2; Figures J-L in S6 File; Tables E-G in S3 File). A main effect is the effect of a single variable on protein abundance, averaged over all other levels of variables. For any protein with a main effect of strain, the abundance of each protein was analyzed for significant differences between strains SA564, SA564-Δ*hptRS*, SA564-Δ*ccpA* and SA564-Δ*hptRS*/*ccpA* (*p* ≤ 0.05; Figure L in S6 File; Table G in S3 File). In order to quantify any differences between SA564, SA564-Δ*hptRS*, SA564-Δ*ccpA* and SA564-Δ*hptRS*/*ccpA* the relative fold-change between strains was computed and exported for proteins with a significant interaction or main effect that included strain. All proteins that were identified as significantly (*p* ≤ 0.05) different between the strains were contained within the 3-way interaction of variables strain, media, and growth phase (s x m x h), two-way interaction of strain and growth phase averaged over media (s x h | m), and the main effect of strain (Figures F, H, L in S6 File; Tables A-C in S4 File). Only proteins, with at least two peptides identified within the LC-MS^E^ analysis, a significant interaction or a main effect (*p* ≤ 0.2), and with a significant difference (*p* ≤ 0.05) between the strains were taken into account for subsequent interpretation and transcriptional analysis (Table 3; Tables D, E in S4 File).

**Table 3:** Significant protein differences in SA564-Δ*ccpA* relative to SA564-Δ*hptRS*/*ccpA*.

***Three Way Interaction (s x m x h)***								
Protein	CcpA Regulated	E.C.	Protein Function	Peptides/Protein	**FC TSB 2h**	**FC TSB 6h**	**FC TSB-dex 2h**	**FC TSB-dex 6h**
CLPL_STAAM			ATP-dependent Clp protease	60	0.998	1.396	0.938	1.651
DHA1_STAAM	*	EC:1.4.1.1	Alanine dehydrogenase	7	0.944	1.276	1.096	2.550
LDH2_STAAM		EC:1.1.1.27	L-lactate dehydrogenase 2	42	1.114	2.427	1.206	1.478
OTC_STAAM	***	EC:2.1.3.3	Ornithine transcarbamoylase	5	1.024	1.568	1.128	2.291
PFLB_STAAM	***	EC:2.3.1.54	Formate C-acetyltransferase	146	1.049	2.765	1.136	1.908
IDH_STAAM	***	EC:1.1.1.42	Isocitrate dehydrogenase	64	0.693	0.635	0.749	0.869
SYS_STAAM		EC:6.1.1.11	Seryl-tRNA synthetase	69	1.019	1.011	1.119	0.703
***Two Way Interaction (s x h)***								
Protein	CcpA Regulated	E.C.	Protein Function	Peptides/Protein	**FC 2h**	**FC 6h**		
Y840_STAAM			Uncharacterized protein SAV0840	32	1.328	1.712		
ADH_STAAM	*	EC:1.1.1.1	Alcohol dehydrogenase	41	1.009	2.241		
ASP23_STAAM			Alkaline shock protein 23	52	1.416	1.723		
DAPH_STAAM		EC:2.3.1.89	Tetrahydrodipicolinate N-acetyltransferase	2	1.026	1.897		
SBI_STAAM	*		Immunoglobulin-binding protein	9	1.002	1.532		
OHRL_STAAM			Organic hydroperoxide resistance protein-like	3	1.158	2.595		
ACKA_STAAM	*	EC:2.7.2.1	Acetate kinase	73	1.172	1.489		
Y1710_STAAM			Putative universal stress protein SAV1710	30	1.102	1.617		
Y1625_STAAM			Uncharacterized protein SAV1625	20	1.717	2.095		
SYG_STAAM		EC:6.1.1.14	Glycyl-tRNA synthetase	52	0.967	0.769		
PCKA_STAAM	***	EC:4.1.1.49	Phosphoenolpyruvate carboxykinase	73	1.008	0.778		
TKT_STAAM		EC:2.2.1.1	Transketolase	84	0.821	0.697		
FTHS_STAAM	***	EC:6.3.4.3	Formyltetrahydrofolate synthetase	87	0.857	0.672		
FOLD_STAAM	*	EC:1.5.1.5	Methylenetetrahydrofolate dehydrogenase	48	0.936	0.790		
ENO_STAAM	*	EC:4.2.1.11	2-phospho-D-glycerate hydro-lyase	78	0.806	0.740		
TPX_STAAM		EC:1.11.1.15	Probable thiol peroxidase	27	0.794	0.553		
G6PI_STAAM		EC:5.3.1.9	Glucose-6-phosphate isomerase	77	0.896	0.738		
***Main Effect (s)***								
Protein	CcpA Regulated	E.C.	Protein Function	Peptides/Protein	**FC**			
DNAK_STAAM			Chaperone protein	151	1.216			
EFG_STAAM			Elongation factor G	173	1.111			
IMDH_STAAM	*	EC:1.1.1.205	Inosine-5'-monophosphate dehydrogenase	125	1.197			
MQO2_STAAM		EC:1.1.5.4	Probable malate:quinone oxidoreductase 2	125	1.159			
QOX2_STAAM		EC:1.10.3.12	Probable quinol oxidase subunit 2	45	1.238			
RSBW_STAAM	*	EC:2.7.11.1	Serine-protein kinase RsbW	2	1.227			
SARS_STAAM	*		HTH-type transcriptional regulator	6	1.640			
SODM2_STAAM	*	EC:1.15.1.1	Superoxide dismutase	10	1.660			
ALDA_STAAM	***	EC:1.2.1.3	Putative aldehyde dehydrogenase	88	0.784			
CRTN_STAAM		EC:1.3.8.2	Dehydrosqualene desaturase	2	0.684			
CYSK_STAAM		EC:2.5.1.47	Cysteine synthase	66	0.798			
DBH_STAAM			DNA-binding protein HU	46	0.714			
DEOC2_STAAM		EC:4.1.2.4	Deoxyribose-phosphate aldolase 2	41	0.824			
DNLJ_STAAM		EC:6.5.1.2	DNA ligase	7	0.450			
FEMA_STAAM		EC:2.3.2.17	Aminoacyltransferase	2	0.654			
GLYA_STAAM		EC:2.1.2.1	Serine hydroxymethyltransferase	58	0.839			
ISPD2_STAAM		EC:2.7.7.40	2-C-methyl-D-erythritol 4-phosphate cytidylyltransferase 2	18	0.817			
MURA2_STAAM		EC:2.5.1.7	UDP-N-acetylglucosamine 1-carboxyvinyltransferase 2	11	0.784			
RS1_STAAM			30S ribosomal protein S1	58	0.746			
THIO_STAAM			Thioredoxin	46	0.777			
THLA_STAAM			Probable acetyl-CoA acyltransferase	32	0.818			
Y2578_STAAM			HTH-type transcriptional regulator	2	0.753			
Y968_STAAM			Uncharacterized protein SAV0968	44	0.791			

***—Bioinformatically Identified and Experimentally Verified as CcpA regulated [8, 18]

**—Bioinformatically Identified as CcpA regulated through Regprecise database [18]

*—Experimentally Identified as CcpA regulated [8]

Cells with diagonal line backgrounds = Not Significant

Cells with bold borders = Significant (p ≤ 0.05)

FC—Fold Change

## Results and discussion

### Deletion of *hptRS* does not alter growth or sensitivity to stressors

**Fig 1:**
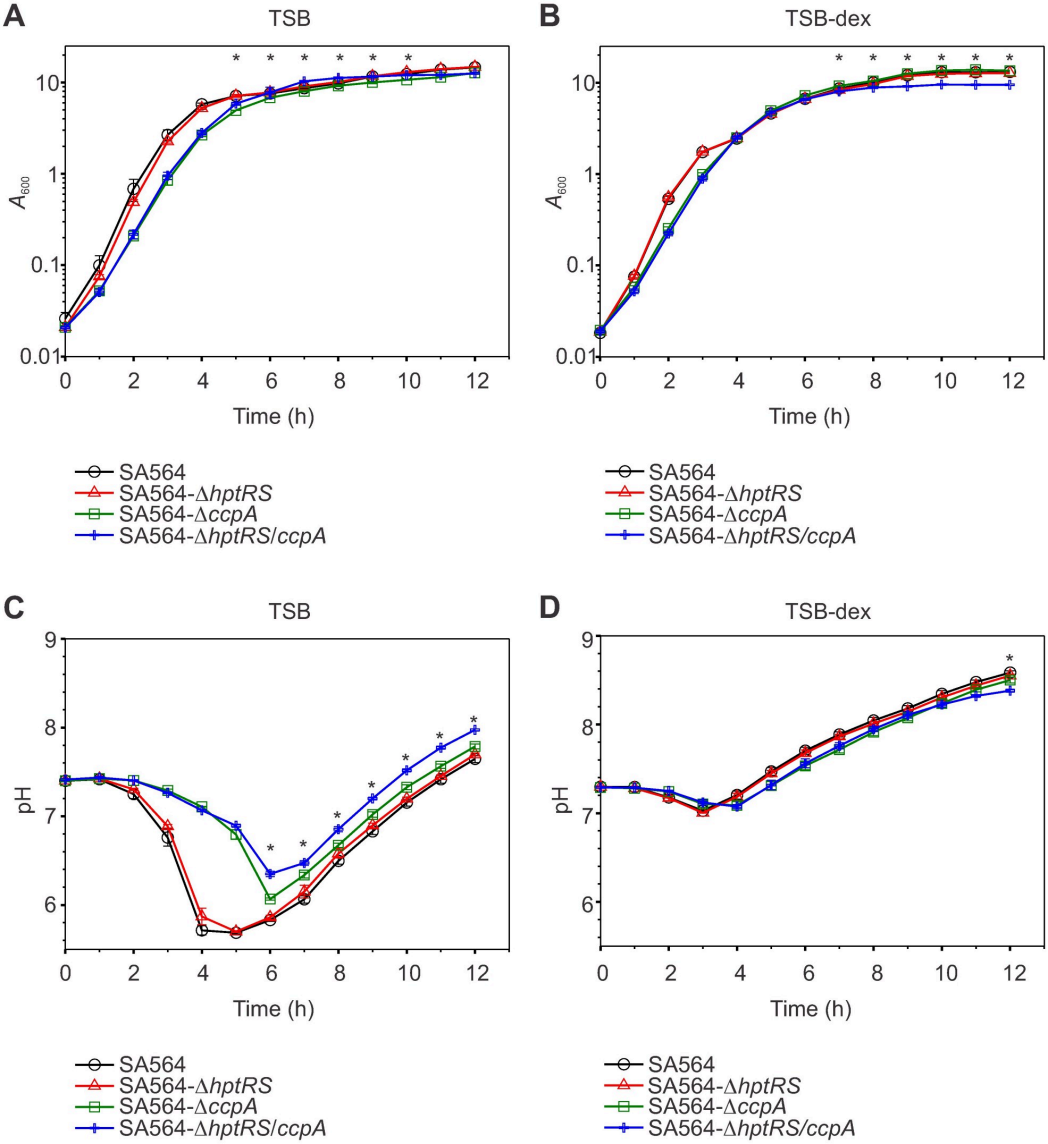
The growth and pH profile of SA564-Δ*hptRS*/*ccpA* is moderately different from SA564, SA564-Δ*hptRS*, and SA564-Δ*ccpA* during the post-exponential phase. The *A*_600_ (A, B) and pH (C, D) were measured every hour for 12 hours. *S*. *aureus* strains SA564 (black symbols), SA564-Δ*hptRS* (red symbols), SA564-Δ*ccpA* (green symbols), and SA564-Δ*hptRS/ccpA* (blue symbols) were cultivated in TSB (A, C) or TSB-dex (B, D). Data are representative of the mean of experiments performed in biological triplicate, with error bars representing the standard error of the mean. A statistically significant difference (*p* ≤ 0.05) between SA564-Δ*ccpA* and SA564-Δ*hptRS*/*ccpA* is represented with an (*).

To assess the growth and viability of strain SA564-Δ*hptRS* relative to the isogenic wild-type strain SA564, the growth and pH profiles were determined (Fig 1). The growth of strains SA564 and SA564-Δ*hptRS* were equivalent when cultivated in TSB and TSB-dex (Fig 1A and 1B). Similarly, strains SA564 and SA564-Δ*hptRS* had equivalent growth when cultivated anaerobically or in iron-limited TSB (Figure A in S6 File). Strains SA564-Δ*hptRS* and SA564 responded comparably when cultivated in TSB with oxidative stress-inducing compounds (*i*.*e*., streptonigrin, hydrogen peroxide, diamide, and paraquat) or membrane de-couplers (*i*.*e*., carbonyl cyanide 3-chlorophenylhydrazon E and nigericin) (Figures B, D in S6 File). In addition, the antibiotic resistance profiles were equivalent when compared using disk diffusion assays (*i*.*e*., ampicillin, penicillin, tetracycline, kanamycin, neomycin, doxycycline, chloramphenicol, vancomycin, minocycline, and oxytetracycline) (Figure C in S6 File). The lack of clear phenotypic differences between strains SA564 and SA564-Δ*hptRS* indicated that this two-component system was inactive under the conditions tested, was non-functional, or that there was overlapping regulation that masks the loss of the *hptRS* two-component system. To determine if and/or when *hptRS* is transcribed, the mRNA abundances of *hptR* and *hptS* were assessed during the exponential (2 h) and post-exponential (6 h) growth phases in strain SA564 during aerobic and anaerobic cultivation (Figure E in S6 File). Transcription of *hptS* was reduced 2.42-fold in the post-exponential growth phase during aerobic cultivation (Figure E in S6 File; *p* ≤ 0.05). In contrast, transcription of *hptR* (1.8-fold) and *hptS* (1.5-fold) was greater in the post-exponential growth phase during anaerobic cultivation (Figure E in S6 File; *p* ≤ 0.05). These data demonstrate that *hptS* and *hptR* are temporally regulated, and indicate that overlapping regulation may mask the effects of *hptRS* deletion.

### Deletion of *ccpA* alters the accumulation and/or depletion of ammonia, acetate, and glucose in the culture media

**Fig 2:**
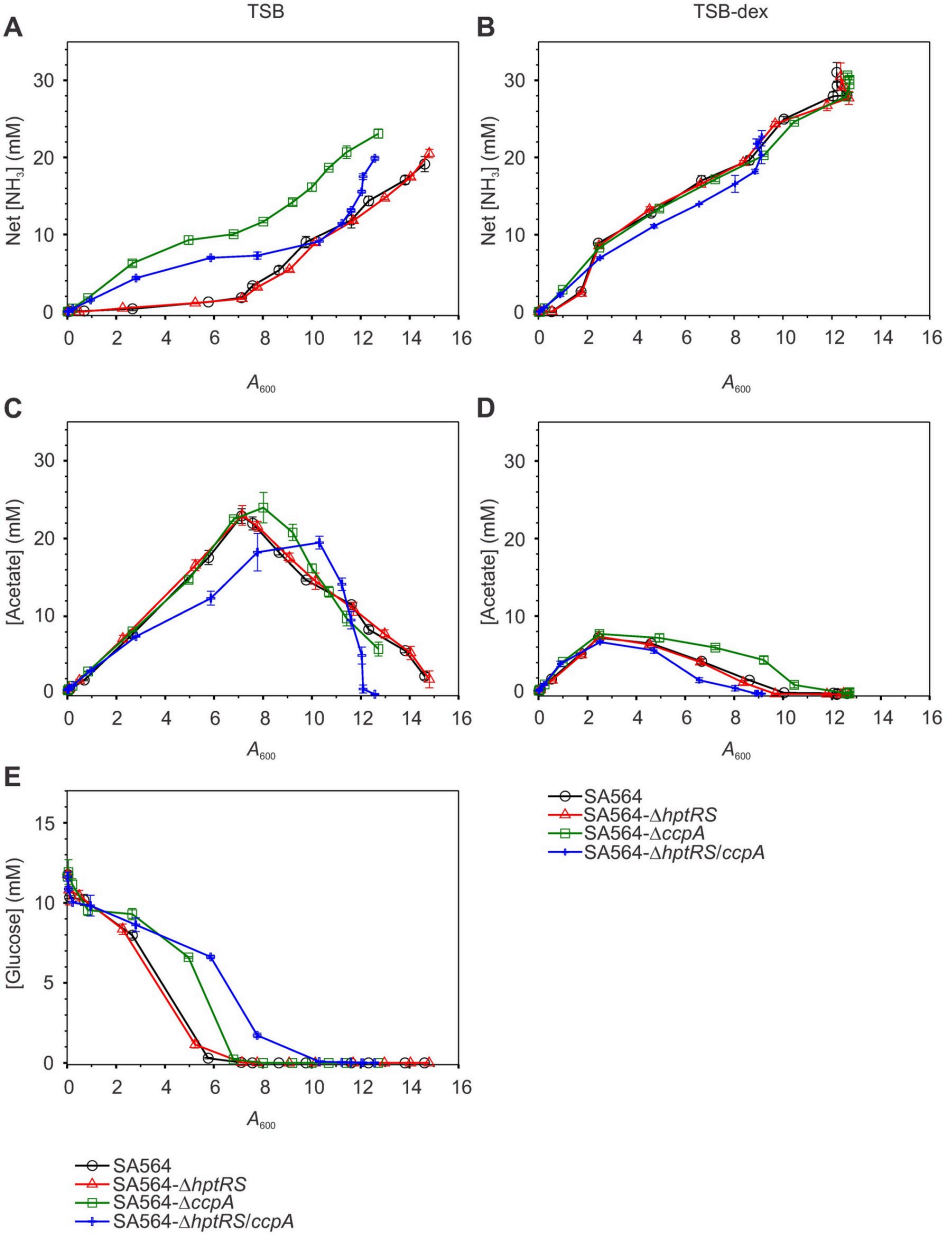
Metabolism of strain SA564-Δ*hptRS*/*ccpA* is different from strains SA564, SA564-Δ*hptRS*, and SA564-Δ*ccpA*. Ammonia accumulation (A, B), acetate accumulation and depletion (C, D), and glucose depletion (E) in the culture media for *S*. *aureus* strains SA564 (black symbols), SA564-Δ*hptRS* (red symbols), SA564-Δ*ccpA* (green symbols), and SA564-Δ*hptRS/ccpA* (blue symbols) cultivated in TSB (A, C, E) and TSB-dex (B, D) are depicted. Data are representative of the mean metabolite concentrations plotted as a function of cell density (*A*_600_) for experiments performed in biological triplicate, with error bars representing the standard error of the mean for absorbance (*A*_600_) and [NH_3_] or [Acetate] or [Glucose].

**Fig 3:**
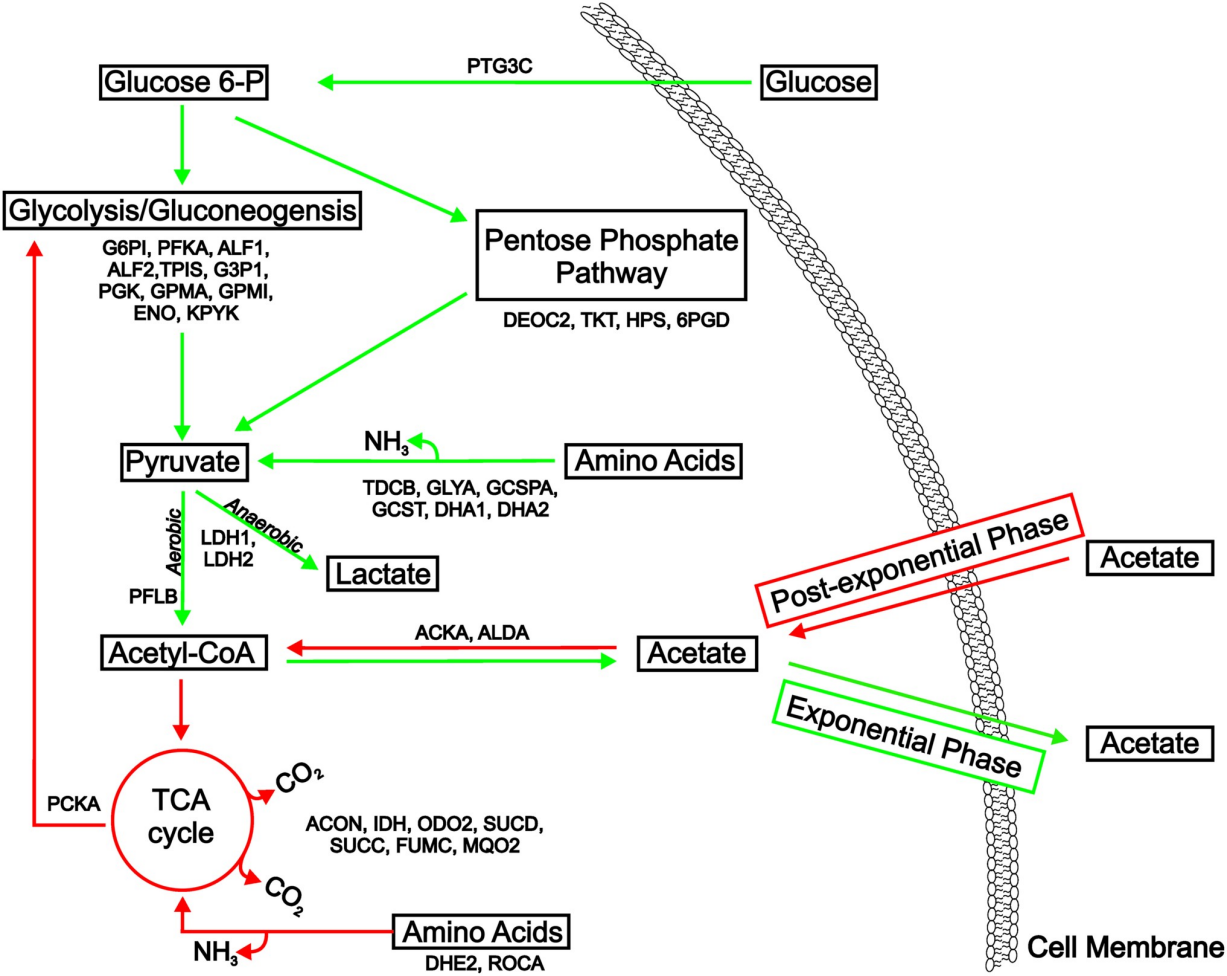
Model of strain SA564 exponential and post-exponential growth phase metabolism during cultivation in TSB, and proteins identified as significantly different during proteomic analysis. Green arrows represent reactions or pathways that occur primarily during the exponential growth phase. Red arrows represent reactions or pathways that occur primarily during the post-exponential growth phase. Proteins within the depicted metabolic pathways, which had significant 3-way (s x m x h) interactions, 2-way (s x h |m) interactions or a main effect of strain are indicated using the protein abbreviations from the proteomic analysis.

When cultivated in TSB, inactivation of *ccpA* slightly reduced the growth rates (μ) of strains SA564-Δ*ccpA* (μ = 1.432 h^-1^) and SA564-Δ*hptRS*/*ccpA* (μ = 1.474 h^-1^) relative to strain SA564 (μ = 1.673 h^-1^; Fig 1A). Concomitant with the reduced growth rates in the *ccpA* inactivated strains, ammonia accumulation was slightly increased and the rate of glucose consumption was slightly decreased during the exponential phase (Fig 2A and 2E), indicating that the reduced growth rate is accompanied by reduced carbon flow through glycolysis and increased amino acid catabolism (Fig 3). Interestingly, while acetate accumulation in strains SA564, SA564-Δ*hptRS* and SA564-Δ*ccpA* cultivated in TSB were similar, strain SA564-Δ*hptRS*/*ccpA* had a significantly altered acetate accumulation and depletion profile (Fig 2C). Similarly, ammonia accumulation was significantly different when strains SA564-Δ*ccpA* and SA564-Δ*hptRS*/*ccpA* were compared (Fig 2A and 2B). The absence of a growth phenotype in strain SA564-Δ*hptRS*, but the presence of a growth phenotype in strain SA564-Δ*hptRS*/*ccpA* that is different from strain SA564-Δ*ccpA*, indicates there is an antagonistic relationship between HptRS and CcpA. As expected, these differences decreased when the strains were cultivated in TSB-dex (Figs 1B, 1D, 2B and 2D). While these observations are consistent with previous reports on CcpA, they establish a previously unobserved linkage between HptRS and CcpA co-regulation [6, 8, 40, 50, 51]. To determine the extent of the regulatory linkage between CcpA and HptRS, total cytosolic proteomes were analyzed by mass spectroscopy during the exponential (2 h) and post-exponential (6 h) growth phases in the presence or absence of glucose.

### Proteomics overview (experimental design and statistical analysis)

**Fig 4:**
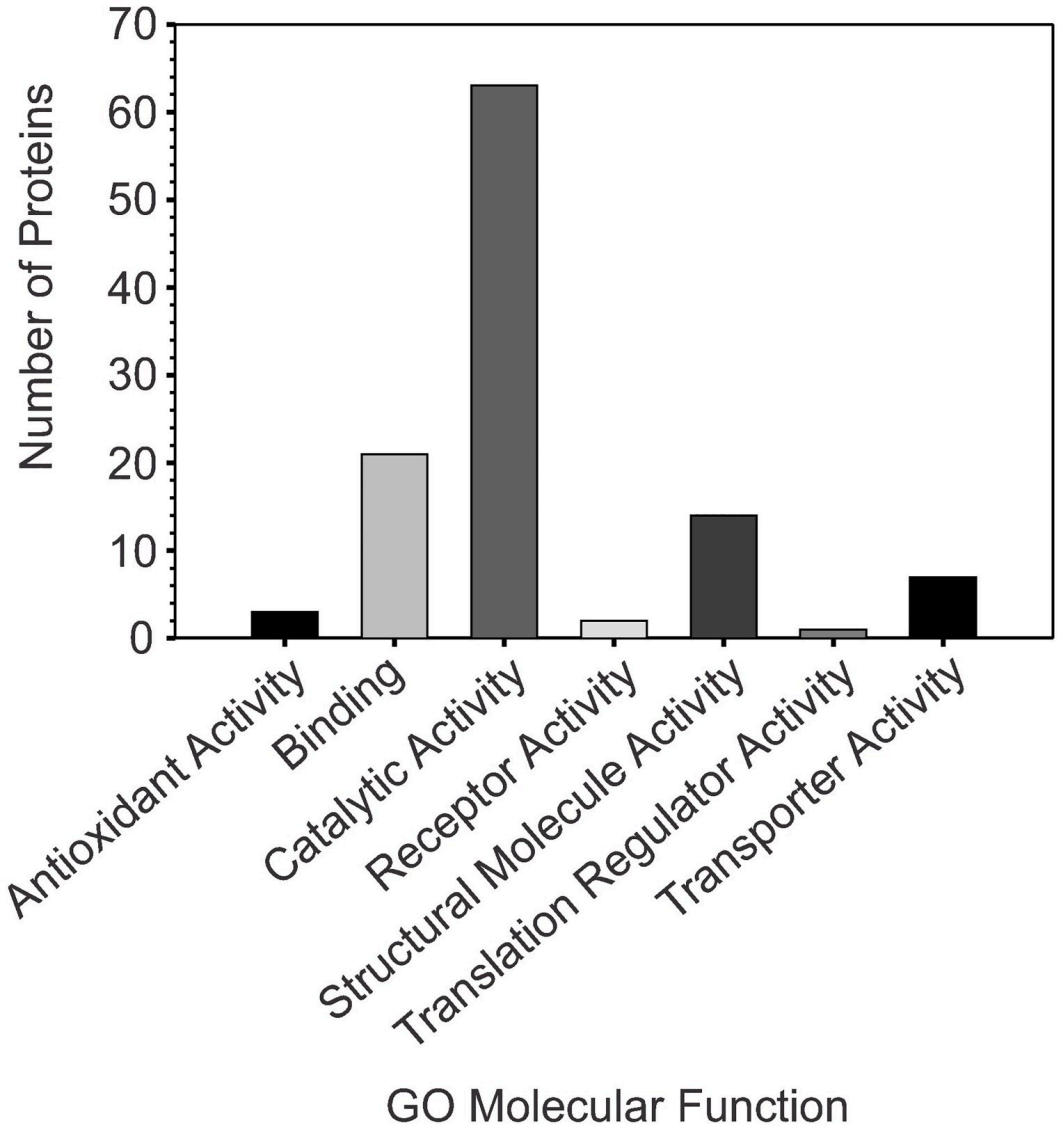
Gene Ontology (GO) analysis of proteins with a significant interaction (s x m x h, s x h | m) or main effect of strain indicates that the majority of differential proteins are involved in catalytic activity. GO analysis was performed by inputting gene symbol abbreviations (*e*.*g*. ADH, PflB, Dha1) into the gene list analysis tab within the PANTHER classification webpage (http://www.pantherdb.org/) and searching against the *S*. *aureus* database within the PANTHER Classification System Database [53].

Cytosolic protein fractions were harvested for five biological replicates for each strain, cultivated in TSB and TSB-dex, during the exponential (2 h) and post-exponential (6 h) growth phases for a total of 80 samples, and analyzed by LC-MS^E^. Using LC-MS^E^, a total of 33,026 spectral peaks were collected and analyzed. The raw data were deposited to the ProteomeXchange Consortium via the PRIDE partner repository with the dataset identifier PXD008708 [52]. The spectra were compared against *S*. *aureus* strain Mu50 predicted proteins (NCBI database) using Progenesis QI, and yielded 11,971 identifiable spectral peaks, with sequences that were assigned to 501 proteins. Comparative quantitative analysis was completed for each protein incorporating the relative normalized abundance for each peptide, yielding a relative abundance for each protein. Proteins identified by two or more peptides (n = 438 proteins) were analyzed for interactions between experimental variables [*i*.*e*., strain (s), media (m), and growth phase (h); Tables B-F in S2 File]. Several groups of proteins emerged within this data set based on the interaction between the variables: (i) Proteins with an abundance that was altered between bacterial strains, cultivation media, and growth phases were defined as having a 3-way interaction (s x m x h) (Table C in S2 File). (ii) Proteins with an abundance that was altered between bacterial strains and cultivation media, irrespective of the growth phase, were defined as having a 2-way interaction averaged over the growth phase (s x m | h) (Table D in S2 File). (iii) Proteins with an abundance that was altered between bacterial strains and within the two growth phases, regardless of the cultivation media were defined as having a 2-way interaction averaged over media (s x h | m) (Table E in S2 File). (iv) Proteins with an abundance that was altered between different cultivation media and within the growth phases, irrespective of the strain, were defined as having a 2-way interaction averaged over strain (m x h | s) (Table F in S2 File). (v) Proteins with an abundance that was altered between the strains but unaffected by growth phase or cultivation media, were defined as having a main effect of strain and no other interactions (Table I in S2 File). A *p-value* cutoff of 0.2, representing that there is 80% confidence in rejecting the null hypothesis that protein abundance was equal between variables, was utilized to determine which proteins would be further analyzed. Using this cutoff, 190 proteins were selected for further analysis. Of these proteins, 105 had designated Kyoto Encyclopedia of Genes and Genomes (KEGG) annotations and enzyme commission (EC) numbers (Table J in S2 File). All proteins that were selected for further analysis were input into the Protein ANnotation THrough Evolutionary Relationship (PANTHER) classification system and sorted by molecular function (Fig 4) [53]. Of the 105 proteins input into PANTHER, the majority were predicted to have catalytic activity (Fig 4) [8, 51, 54].

To determine if proteins with significant interactions or main effects contained statistically significant differences (*p* ≤ 0.05) between the strains, growth phases, growth medium, or a combination of these variables, multiple comparisons were calculated and *p*-values generated for the simple effects (combination of variables) or main effect of each variable (Tables A-G in S3 File). For a given protein, if a significant 3-way interaction was observed, then the effects of each variable were considered (*p* ≤ 0.05; 3-way multiple comparison); if no significant 3-way interaction was observed but a significant 2-way interaction was observed, then the effects of each variable within the 2-way analysis was considered (*p* ≤ 0.05) averaging over the third variable (2-way multiple comparison); and if no significant 3-way or 2-way interactions were observed, then the effect of strain was considered (*p* ≤ 0.05) averaged over media and growth phase (main effect comparison). A *p*-value cutoff of 0.05 was used to determine if there was a statistically significant difference between the effects of strain (s), media (m) and growth phase (h). With this cutoff, it was determined that all proteins identified as being significantly altered were contained within the 3-way interaction (s x m x h), 2-way interaction (s x h | m), and the main effect of strain (s), hence an emphasis was placed on the simple effects or main effect within these categories for comparisons between strains (Table J in S2 File; Tables A, C, G in S3 File). To quantify specific differences in protein abundance between strains SA564, SA564-Δ*hptRS*, SA564-Δ*ccpA* and SA564-Δ*hptRS/ccpA* fold changes (*i*.*e*., SA564/mutant or mutant/mutant) were computed for each protein with a significant interaction or main effect (Table 3; Tables A-E in S4 File). In total, 13 proteins were identified as having significant 3-way interactions, 53 proteins had significant 2-way interactions between strain and growth phase, and 124 proteins were identified for further analysis due to the main effect of strain, providing a high probability of identifying specific proteomic differences within and between strains SA564, SA564-Δ*hptRS*, SA564-Δ*ccpA* and SA564-Δ*hptRS/ccpA* (Table J in S2 File).

### Media and temporal changes in strain SA564

*S*. *aureus* adaptations to glucose-rich and -limited environments have been studied [8, 51, 55]. Cultivation of *S*. *aureus* in glucose-rich media increases the accumulation of proteins involved in glycolysis and protein synthesis. In contrast, cultivation in glucose-limited media increased the abundance of proteins involved in the tricarboxylic acid cycle, amino acid catabolism, and gluconeogenesis. Although less well-studied, temporal protein changes during cultivation in medium containing glucose have also been examined [55]. Temporal changes occur when glucose is depleted and incompletely oxidized carboxylic acids become the primary carbon sources (Fig 3). In response to changing carbon sources, the proteome of *S*. *aureus* must adapt to supply energy and metabolic precursors for growth, which causes a large temporal rearrangement in protein accumulation. Many of these temporal changes are mediated in part by CcpA [8, 12, 51]; hence, several of the significant proteins found in the 3-way and 2-way interactions are translated from genes that are known or predicted to be regulated by CcpA (Tables A-D in S3 File; Table D in S4 File). As examples: (i) Glycolytic/gluconeogenic proteins triosephosphate isomerase (EC:5.3.1.1) and glyceraldehyde 3-phosphate dehydrogenase (EC:1.2.1.12) (s x h | m, m x h | s; *p* ≤ 0.2) are significantly decreased in strain SA564 during the post-exponential growth phase in TSB and increased during the post-exponential phase in TSB-dex (*p* ≤ 0.05); Phosphoglycerate kinase (EC:2.7.2.3) (s x h | m, m x h | s, m x s | h; *p* ≤ 0.2) is increased in strain SA564 cultivated in TSB-dex relative to TSB and increased during the post-exponential phase in TSB-dex (*p* ≤ 0.05); and pyruvate kinase (EC:2.7.1.40) (s x m x h, *p* ≤ 0.2) is significantly decreased in strain SA564 during the post-exponential growth phase in TSB relative to TSB-dex (*p* ≤ 0.05). (ii) TCA cycle proteins aconitate hydratase (EC:4.2.1.3) (s x h | m, m x h | s; *p* ≤ 0.2) and isocitrate dehydrogenase (EC:1.1.1.42) (s x m x h; *p* ≤ 0.2) are significantly increased in both TSB and TSB-dex during the post-exponential growth phase in strain SA564 (*p* ≤ 0.05) relative to the exponential growth phase, and succinyl-coA synthase subunit A (EC:6.2.1.5) (s x h | m, m x h | s, m x s | h; *p* ≤ 0.2) and succinyl-coA synthase subunit B (EC:6.2.1.5) are significantly increased in SA564 during the post-exponential phase in TSB (*p* ≤ 0.05) relative to TSB-dex. (iii) The amino acid catabolic protein alanine dehydrogenase 1 (EC:1.4.1.1) (s x m x h; *p* ≤ 0.2) is increased during the post-exponential growth phase in TSB-dex (*p* ≤ 0.05) relative to TSB in strain SA564, while glutamate dehydrogenase (EC:1.4.1.2) (s x h | m, m x h | s; *p* ≤ 0.2) is increased during the post-exponential growth phase in TSB and TSB-dex (*p* ≤ 0.05) in strain SA564 relative to the exponential growth phase. (iv) The gluconeogenic protein phosphoenolpyruvate carboxykinase (EC: 4.1.1.49) (s x h | m, m x h | s; *p* ≤ 0.2) is significantly increased during the post-exponential growth phase in strain SA564 cultivated in TSB and TSB-dex relative to the exponential phase (*p* ≤ 0.05). The media and temporal differences in strain SA564, and previous observations on cultivation media and temporal differences in other *S*. *aureus* strains [8, 55], establish an important baseline for understanding the proteomic changes associated with CcpA and HptRS transcriptional regulation.

### Protein abundances are similar between strains SA564 and SA564-Δ*hptRS*

Consistent with the growth and pH profiles, deletion of *hptRS* did not significantly (*p* ≥ 0.05) alter cytosolic protein abundance in response to changing growth phases or media composition in strain SA564-Δ*hptRS* relative to strain SA564 for proteins identified as having significant 3-way (s x m x h, *p* ≤ 0.2) or 2-way (s x h | m, *p* ≤ 0.2) interactions, or main effect of strain (s, *p* ≤ 0.2) (Tables A-C in S4 File). These results indicated that HptRS may be inactive under these conditions or the genes regulated by HptRS are also under the control of another glucose-dependent transcriptional regulator, such as CcpA. To determine if CcpA masks the effect of *hptRS* genetic inactivation, the proteomes of strains SA564-Δ*ccpA* and SA564-Δ*hptRS/ccpA* were analyzed.

### Inactivation of *ccpA* decreases the accumulation of glycolytic proteins, and increases TCA cycle and amino acid catabolic proteins

For proteins identified as having significant 3-way (s x m x h, *p* ≤ 0.2), 2-way (s x h | m, *p* ≤ 0.2), or a main effect of strain (s, *p* ≤ 0.2), a total of 56 were significantly different between strains SA564-Δ*ccpA* and SA564 (*p* ≤ 0.05), (Table D in S4 File). Of these proteins, 69.6% have been experimentally identified or predicted by promoter sequence to be regulated by CcpA [8, 12, 18, 19, 51]. These include proteins associated with glycolysis, such as fructose bisphosphate aldolase (EC: 4.1.2.13), glyceraldehyde-3-phosphate dehydrogenase, phosphoglycerate kinase, enolase (EC: 4.2.1.11), and pyruvate kinase, which were decreased in strain SA564-Δ*ccpA* relative to strain SA564, primarily during the exponential growth phase (*p* ≤ 0.05). In contrast, the TCA cycle proteins aconitate hydratase, 2-oxoglutarate dehydrogenase (EC: 2.3.1.61), succinyl-CoA complex proteins, and fumarate hydratase (EC: 4.2.1.2) were increased by *ccpA* inactivation relative to strain SA564 during the exponential growth phase. Amino acid metabolic proteins such as alanine dehydrogenase, threonine deaminase (EC: 4.3.1.19), aminomethyltransferase protein (EC: 2.1.2.10), and glutamate dehydrogenase were also increased during the exponential growth phase in strain SA564-Δ*ccpA* relative to strain SA564. As expected, fewer proteins were significantly different between SA564-Δ*ccpA* and SA564 during the post-exponential growth phase when glucose was depleted; however, CcpA regulated several proteins even in the absence of glucose, such as pyruvate kinase, 1-pyrroline-5-carboxylate dehydrogenase (EC:1.2.1.88), and succinyl-CoA synthetase subunit beta [8, 55]. These data indicate that CcpA-mediated changes in protein abundance were both glucose-dependent and -independent, suggesting factors other than glycolytic intermediates and HPr (*e*.*g*., Stk1), antagonize CcpA transcriptional regulation [20] (Table D in S4 File).

### Many differentially regulated proteins in strain SA564-Δ*hptRS/ccpA* are common to the CcpA regulon

For proteins having significant 3-way (s x m x h, *p* ≤ 0.2) or 2-way (s x h | m, *p* ≤ 0.2) interactions, or a main effect of strain (s, *p* ≤ 0.2), 98 proteins were identified as significantly different between SA564-Δ*hptRS*/*ccpA* and SA564 (*p* ≤ 0.05) (Table E in S4 File). Of these proteins, 46.9% have been experimentally determined or predicted to be regulated by CcpA [8, 18]. The majority (n = 48) of significantly different proteins found in SA564-Δ*ccpA* (n = 56 proteins) were also found in SA564-Δ*hptRS*/*ccpA* relative to SA564 (Tables D, E in S4 File). Interestingly, while CcpA-regulated proteins were common to both SA564-Δ*hptRS*/*ccpA* and SA564-Δ*ccpA*, several proteins (n = 12) had different fold changes in protein abundance relative to SA564. As examples, pyruvate formate lyase (EC: 2.3.1.54) and alcohol dehydrogenase (EC: 1.1.1.1) were present in greater abundance in strain SA564-Δ*ccpA* relative to SA564, yet lower in abundance in strain SA564-Δ*hptRS/ccpA* relative to SA564. These data suggest an antagonistic regulatory relationship between HptRS and CcpA and that certain enzymatic/protein differences caused by inactivation of Δ*hptRS* can only be visualized by removing CcpA-regulated proteins from the proteome of strain SA564-Δ*hptRS*/*ccpA*. To do this, significant differences in protein abundance in strain SA564-Δ*hptRS*/*ccpA* relative to SA564-Δ*ccpA* were analyzed.

### HptRS antagonizes CcpA-dependent regulation

A total of 47 proteins, identified as having significant 3-way (s x m x h, *p* ≤ 0.2), 2-way (s x h | m, *p* ≤ 0.2), or main effect of strain (s, *p* ≤ 0.2), were identified as having significantly altered abundance in strain SA564-Δ*ccpA* relative to SA564-Δ*hptRS*/*ccpA* (*p* ≤ 0.05; Table 3). Of these proteins, 34% are predicted to be regulated by CcpA [8, 18]. Specifically, genes encoding enzymes pyruvate formate lyase B, alanine dehydrogenase, ornithine transcarbamoylase (EC:2.1.3.3), acetate kinase (EC: 2.7.2.1), and alcohol dehydrogenase, have predicted *cre* sites in their promoter regions, and were significantly increased during the post-exponential growth phase in strain SA564-Δ*ccpA* relative to SA564-Δ*hptRS*/*ccpA* [8, 15, 18] (Table 3). These results suggest that the regulons of CcpA and HptRS likely overlap, particularly during the post-exponential growth phase.

Significant differences were also observed within pyruvate, amino acid metabolic, and TCA cycle enzymes during the post-exponential phase (*p* ≤ 0.05; Table 3). As examples, pyruvate formate lyase B, and acetate kinase had significantly increased protein abundances in strain SA564-Δ*ccpA* relative to SA564-Δ*hptRS*/*ccpA*. Amino acid metabolic enzymes such as alanine dehydrogenase, tetrahydrodipicolinate acetyltransferase (EC: 2.3.1.89), and ornithine transcarbamoylase had significantly increased protein abundances in strain SA564-Δ*ccpA* relative to strain SA564-Δ*hptRS*/*ccpA*. These differences in pyruvate metabolic and amino acid catabolic protein abundance are consistent with the physiological differences in acetate and ammonia accumulation between strains SA564-Δ*ccpA* and SA564-Δ*hptRS*/*ccpA* (Figs 1 and 2; Table 3). Additionally, the abundance of the TCA cycle enzyme isocitrate dehydrogenase was significantly decreased in strain SA564-Δ*ccpA* relative to strain SA564-Δ*hptRS*/*ccpA* (*p* ≤ 0.05; Table 3). Taken together, these data suggest that HptR functions to antagonize transcription of a subset of CcpA-regulated genes, which give rise to alterations in protein accumulation. To assess this possibility, the mRNA abundance of select genes was determined to gauge transcriptional regulation of genes encoding proteins that were significantly different in the proteome.

### CcpA and HptRS regulate genes encoding for proteins identified as being significantly different in proteomic analysis

Proteomic analysis demonstrated that genetic inactivation of the genes coding for the transcriptional regulators *ccpA* alone or in combination with *hptRS* resulted in differential protein abundances. The most likely explanation for these results is that transcription of the genes coding for the differentially-regulated proteins was altered. To assess this possibility, mRNA transcript levels were determined for select genes using RT-qPCR. Genes for pyruvate formate lyase B (*pflB*; *sav*0226) and alanine dehydrogenase (*dha1*; *sav*1439) were selected for analysis due to their significant 3-way interaction (s x m x h; *p* ≤ 0.2), and having a protein abundance fold-change greater than 1.5 (FC ≥ 1.5) in strain SA564-Δ*ccpA* relative to SA564-Δ*hptRS*/*ccpA* (Table 3). Alcohol dehydrogenase (*adh*; *sav*0605) was assessed due to its significant 2-way interaction (s x h | m; *p* ≤ 0.2) and having a protein abundance fold-change greater than 1.5 (FC ≥ 1.5) in strain SA564-Δ*ccpA* relative to SA564-Δ*hptRS*/*ccpA* (Table 3). Lastly, the 1-pyrroline-5-carboxylate dehydrogenase gene (*rocA*; *sav*2554) was assessed due to its significant 3-way interaction (s x m x h; *p* ≤ 0.2), and also having a protein abundance fold-change above 1.5 (FC ≥ 1.5) in strains SA564-Δ*ccpA* and SA564-Δ*hptRS*/*ccpA* relative to SA564 (Tables D, E in S4 File). In addition, *rocA* was chosen as a control due to the lack of a difference in protein abundance between strains SA564-Δ*ccpA* and SA564-Δ*hptRS*/*ccpA*, which indicated this effect was caused by CcpA alone. An additional consideration for choosing these genes was that *pflB*, *dha1*, *adh*, and *rocA* have been previously identified or predicted to be transcriptionally regulated by CcpA [18, 51, 56].

**Fig 5:**
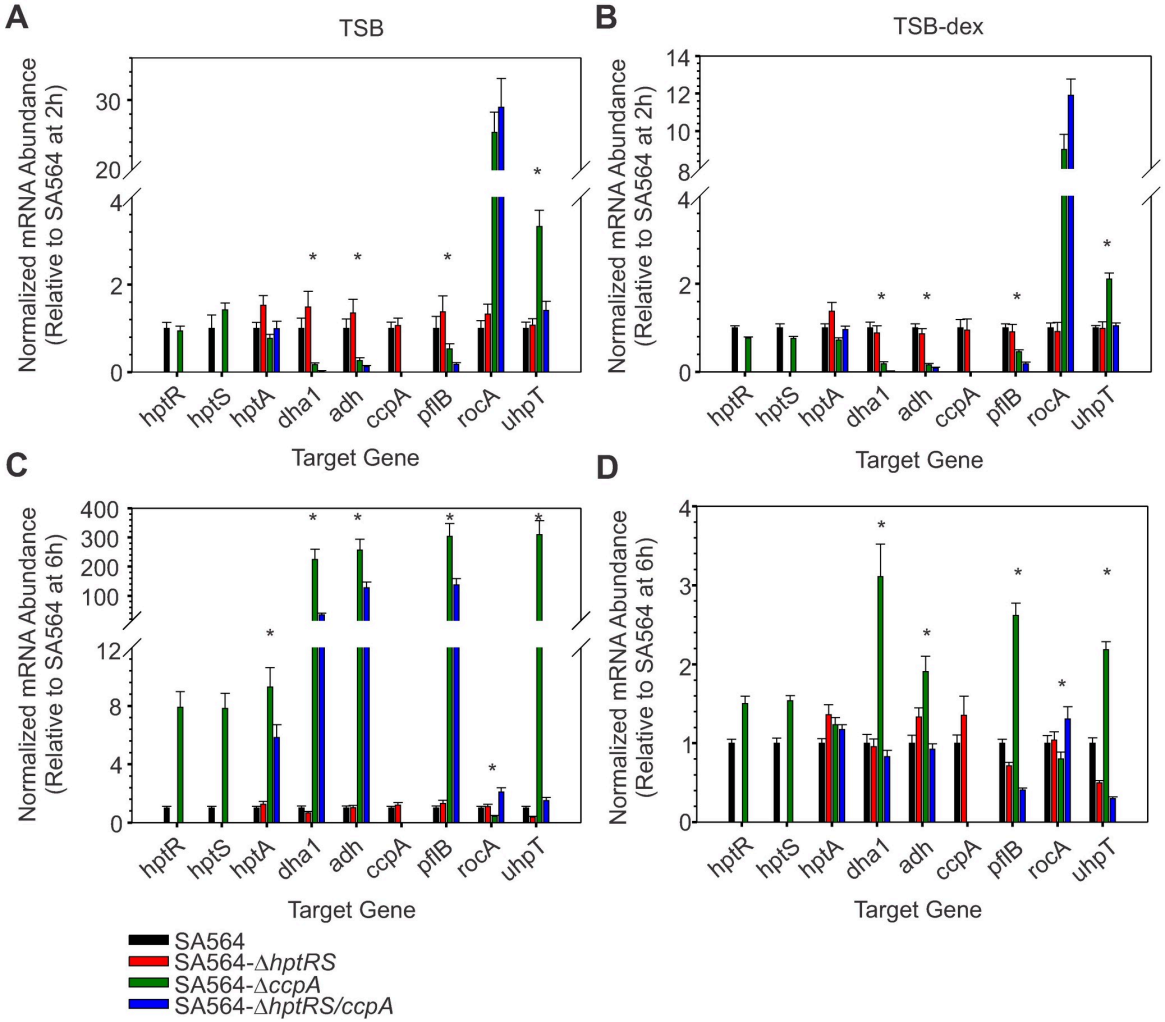
CcpA and HptRS co-regulate transcription of genes during the exponential (2 h) and post-exponential (6 h) growth phases. The relative mRNA abundance during the exponential (A, B) and post-exponential (C, D) growth phases for genes *hptR*, *hptS*, *hptA*, *dha1*, *adh*, *ccpA*, *pflB*, *rocA*, and *uhpT* was determined by RT-qPCR. Data are the mean and standard error of the mean for 3 biological replicates of SA564 (black bars), SA564-Δ*hptRS* (red bars), SA564-Δ*ccpA* (green bars), and SA564-Δ*hptRS/ccpA* (blue bars) cultivated in TSB (A, C) and TSB-dex (B, D). The data were normalized using 16S rRNA and are plotted relative to SA564. A statistically significant difference (*p* ≤ 0.05) between SA564-Δ*ccpA* and SA564-Δ*hptRS*/*ccpA* is represented with an (*).

The mRNA abundances of *pflB*, *dha1*, and *adh* in SA564-Δ*ccpA* were significantly decreased (FC ≥ 1.5; *p* ≤ 0.05) during the exponential growth phase (Fig 5A and 5B) and significantly increased (FC ≥ 1.5; *p* ≤ 0.05) during the post-exponential growth phase (Fig 5C and 5D) relative to SA564 in either TSB or TSB-dex (Tables A, B in S5 File). CcpA is a transcriptional repressor of *pflB*, *adh*, and *dha1*, therefore *ccpA* inactivation should increase mRNA abundance [8]. Interestingly, an increase mRNA abundance was only observed during the post-exponential growth phase for strain SA564-Δ*ccpA* in TSB and TSB-dex (Fig 5C and 5D). As expected, post-exponential growth phase transcription of *pflB*, *adh*, and *dha1* was significantly increased (*p* ≤ 0.05) in strain SA564-Δ*ccpA* cultivated in TSB relative to that cultivated in TSB-dex (Table D in S5 File). Together, these data suggest there are one or more transcriptional activators whose activity is repressed by glucose, or more appropriately glucose depletion, and that activates post-exponential growth phase transcription of *pflB*, *adh*, and *dha1* when CcpA repression is relieved.

Consistent with there being an activator of *pflB*, *dha1*, and *adh* transcription, a significant reduction (FC ≥ 1.5; *p* ≤ 0.05) in mRNA abundance of *pflB*, *dha1*, and *adh* in strain SA564-Δ*hptRS*/*ccpA* relative to strain SA564-Δ*ccpA*, irrespective of media or growth phase, (Fig 5; Table C in S5 File) was observed, indicating that HptRS activated transcription of these genes upon CcpA de-repression or inactivation. During the post-exponential growth phase, when cultivated in TSB, strain SA564-Δ*hptRS*/*ccpA* produced transcripts for *pflB*, *dha1*, and *adh*, indicating that other regulators are also involved in glucose-dependent transcription of these genes (Fig 5C). In contrast to *pflB*, *dha1*, and *adh* mRNA levels, significant exponential growth phase increases (*p* ≤ 0.05) in mRNA abundance of *rocA* were observed in *ccpA* inactivated strains relative to SA564, irrespective of *hptRS* inactivation (Fig 5A and 5B; Tables A, B in S5 File). During the post-exponential growth phase, there were only small differences in *rocA* mRNA levels between strains SA564-Δ*ccpA* and SA564-Δ*hptRS*/*ccpA* (Fig 5C and 5D), confirming that *rocA* is primarily regulated by CcpA. Taken together, these data demonstrate that transcription of *pflB*, *dha1*, and *adh* is co-regulated by CcpA and HptRS, while *rocA* is largely regulated by CcpA. These data also demonstrate the CcpA and HptR regulons intersect and that CcpA functions as a transcriptional repressor, while HptR functions as a transcriptional activator.

### Predicted membrane-associated proteins are also regulated by CcpA and HptRS

Histidine kinase HptS, the protein of unknown function HptA, and UhpT are predicted to be membrane-associated and similar to homologous proteins found in *E*. *coli*, hence they are unlikely to be present in the cytosolic fraction used for proteomic analysis [32, 57]. To determine if CcpA regulates transcription of *hptS*, *hptA*, and *uhpT*, mRNA abundance was assessed in the exponential and post-exponential growth phases from bacteria cultivated in TSB or TSB-dex (Fig 5). Post-exponential growth phase transcription of *hptS* and *hptA* was significantly (*p* ≤ 0.05) increased in strain SA564-Δ*ccpA* relative to strain SA564 cultivated in TSB (Fig 5C; Table A in S5 File). As expected, the magnitude of this effect was lessened when the bacteria were cultivated in TSB-dex (Fig 5D, Table B in S5 File). These results are consistent with a hypothesis put forth previously, whereby CcpA can mediate repression even during cultivation in the absence of glucose [8]. Transcription of *uhpT* was significantly increased (*p* ≤ 0.05) in strain SA564-Δ*ccpA* relative to strain SA564 during the exponential and post-exponential growth phases (Fig 5; Tables A, B in S5 File). Importantly, this transcriptional activation was abolished in strains SA564-Δ*hptRS* and SA564-Δ*hptRS*/*ccpA*, demonstrating the HptRS two-component system activates *uhpT* transcription. In addition, the post-exponential growth phase mRNA level of *uhpT* was increased (FC = 2.92) in strain SA564 cultivated in TSB-dex relative to TSB, suggesting that HptRS transcriptional activation can occur without exogenous glucose (Table D in S5 File). These data reveal a complex regulation of *uhpT* transcription involving CcpA repression and HptRS activation, partially in response to changes in the availability of glucose or glucose-derived metabolites. The significance of these observations relate to the importance of UhpT in susceptibility to the antibiotic fosfomycin [36]. To investigate if genetic inactivation of *ccpA* or *hptRS* altered antibiotic susceptibility through their coordinated regulation of transcription, the antibiotic susceptibility of each strain was analyzed using broth microdilution and disk diffusion assays.

### Inactivation of *ccpA* and/or *hptRS* alters antibiotic susceptibility

**Fig 6:**
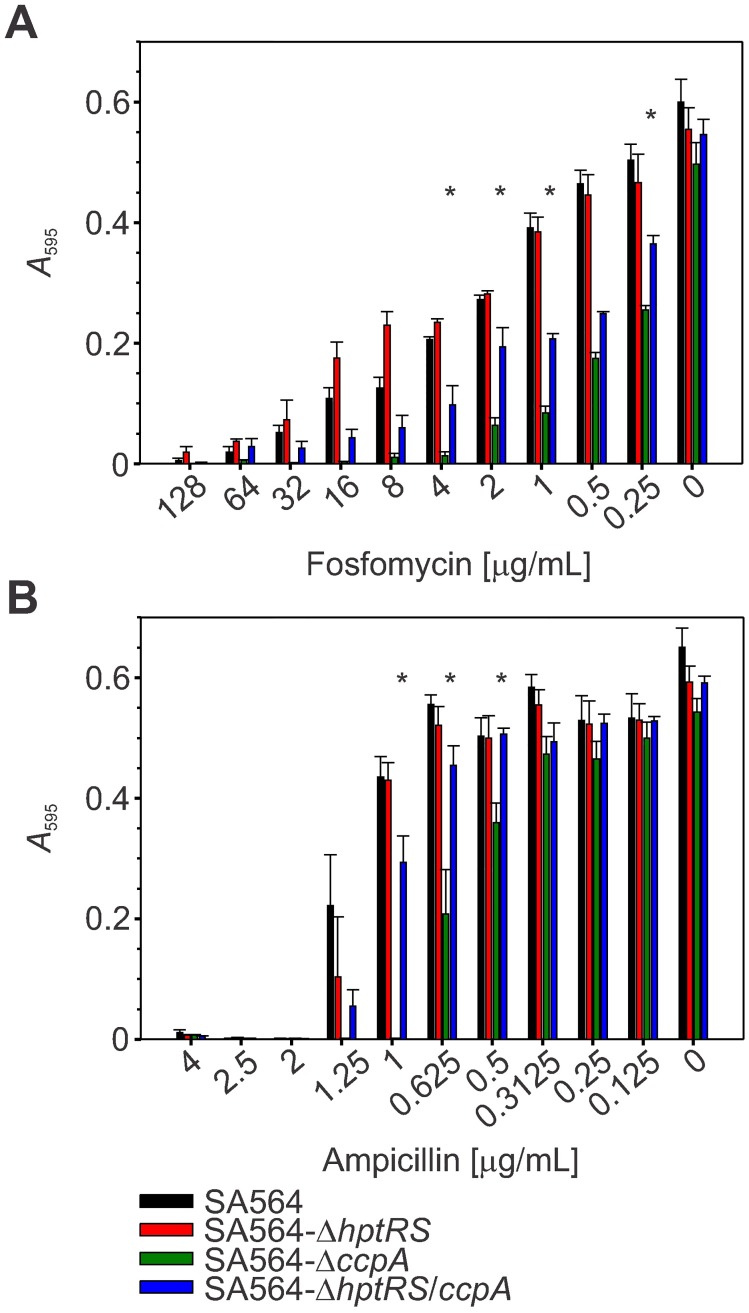
Transcriptional regulation by CcpA and HptRS alter fosfomycin and ampicillin susceptibility. Antibiotic susceptibility to fosfomycin (A) and ampicillin (B) was investigated using broth microdilution. Data are the mean and standard error of the mean for 3 biological replicates, each with 6 technical replicates. Significant differences (*p* ≤ 0.05) between SA564-Δ*ccpA* and SA564-Δ*hptRS*/*ccpA* are represented with an (*).

Previously, susceptibility to fosfomycin was studied in Mueller Hinton medium with added glucose-6-phosphate, which can alter activity of CcpA and subsequent transcription of *hptRS* and *uhpT* [31, 58–60]. To determine if CcpA and/or HptRS mediate susceptibility to fosfomycin and other antibiotics without the addition of glucose-6-phosphate, broth micro-dilution and disc diffusion assays were utilized. As expected, strain SA564-Δ*ccpA* (MIC = 4 μg/mL) was significantly (*p* ≤ 0.05) more susceptible to fosfomycin when compared to strains SA564 (MIC = 64 μg/mL) and SA564-Δ*hptRS* (MIC = 64 μg/mL) (Fig 6A). Interestingly the increase in fosfomycin susceptibility in strain SA564-Δ*ccpA* is similar to other reports where glucose-6-phosphate is added to the culture medium, indicating the addition of exogenous glucose-6-phosphate alters CcpA activity, causing changes in *hptRS* and *uhpT* transcript abundance [58, 60]. Importantly, inactivation of *hptRS* in the *ccpA* mutant strain, SA564-Δ*hptRS*/*ccpA* (MIC = 16 μg/mL), significantly (*p* ≤ 0.05) reduced susceptibility to fosfomycin (Fig 6A). These data demonstrate that fosfomycin susceptibility is at least partially dependent upon transcriptional regulation by CcpA and HptRS.

**Fig 7:**
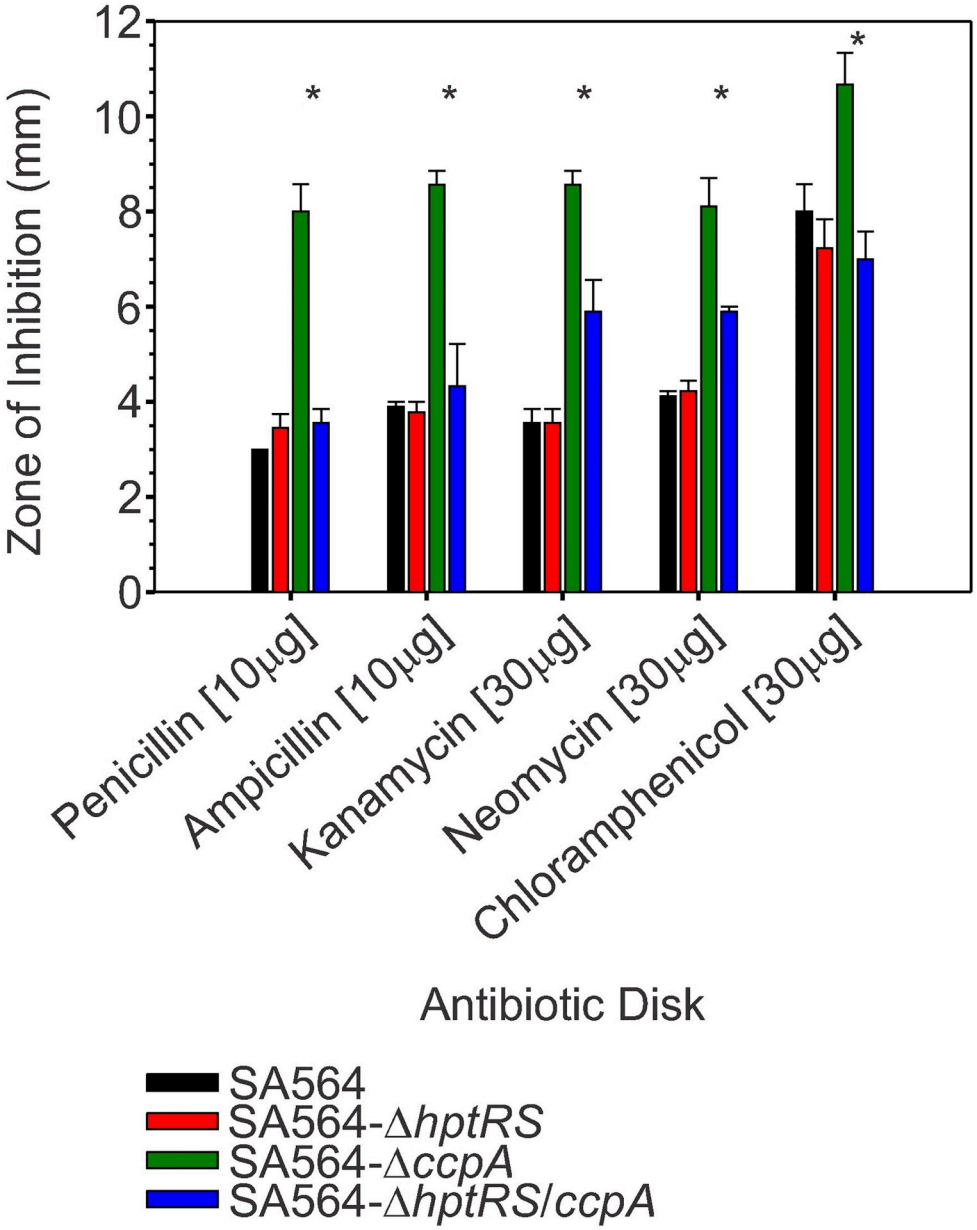
Transcriptional regulation by CcpA and HptRS mediate antibiotic susceptibility. Antibiotic susceptibility was investigated using antibiotic disks on TSA. Data represent the mean and standard error of the mean of the zone of inhibition (edge of the disk to the first colony) for 3 biological replicates. Significant differences (p ≤ 0.05) between SA564-Δ*ccpA* and SA564-Δ*hptRS*/*ccpA* are represented with an (*).

As reported, CcpA mutants also have increased susceptibility to beta-lactam antibiotics [40, 61]. Consistent with this observation, inactivation of *ccpA* increased susceptibility to penicillin and ampicillin, but also to kanamycin, neomycin, and chloramphenicol relative to strains SA564, SA564-Δ*hptRS*, and SA564-Δ*hptRS*/*ccpA* (*p* ≤ 0.05; Figs 6B and 7). Inactivation of *hptRS* in the *ccpA* mutant background restored the resistance profile to a level similar to the wild-type strain, suggesting that HptRS antagonizes CcpA-mediated transcriptional regulation of genes that are involved in antibiotic susceptibility (Figs 6 and 7).

## Conclusions

**Fig 8:**
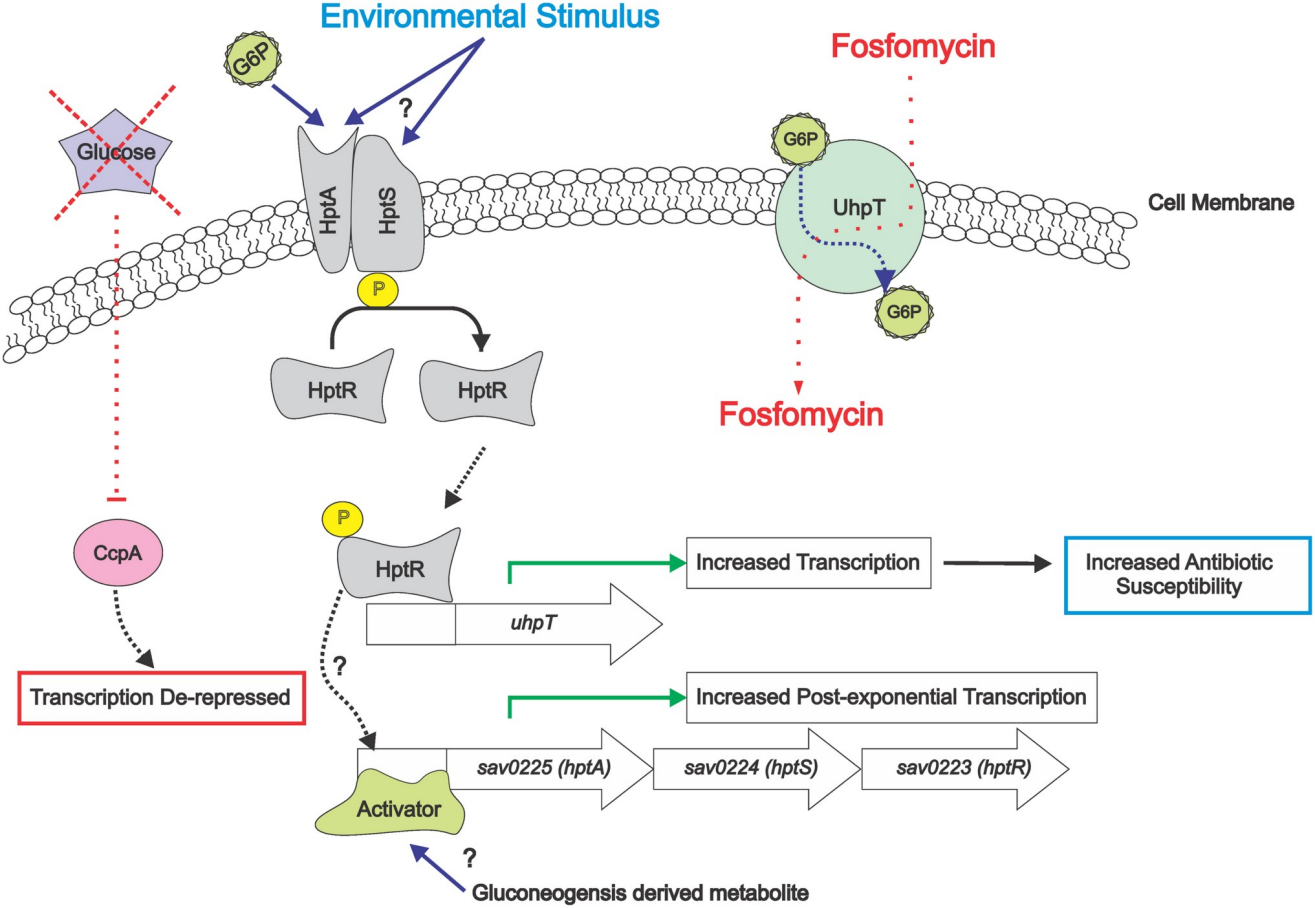
Model for CcpA and HptRS coregulation of *uhpT* transcription and fosfomycin susceptibility. In the absence of glucose, CcpA de-repression increases mRNA abundance of *sav*0225 (*hptA*), *sav*0224 (*hptS*), and *sav*0223 (*hptR*). Transcription is further increased by the presence of metabolites potentially derived from gluconeogensis. Upon activation by glucose-6-phosphate, or another unknown stimulus, the HptRS system functions to activate transcription of genes normally repressed by CcpA, including *uhpT*. Increased transcription of *uhpT* is correlated with increased transport of the antibiotic fosfomycin; hence, coregulation of transcription by CcpA and HptRS directly alter fosfomycin susceptibility, and susceptibility to other antimicrobials.

Gene regulation by multiple transcriptional regulators is common and overlapping regulation impedes the identification of genes controlled by individual regulators. On this latter point, deletion of genes coding for the sensor histidine kinase (*hptS*) and the response regulator (*hptR*) produced no observable phenotypic effects on growth, metabolism, or protein abundance. In contrast, inactivation of both *ccpA* and *hptRS* caused significant changes in pyruvate metabolism, amino acid metabolism, and TCA cycle protein abundance, which affected growth and metabolism relative to the single mutants. By combining rigorous statistical analyses with exhaustive proteomic data, it was possible to determine that HptRS and CcpA function antagonistically to co-regulate transcription of a subset of CcpA-regulated genes (Fig 8). This approach led to the identification of a small group of proteins altered by inactivation of *hptRS*. These data provided focus to determine which protein alterations were the result of transcriptional changes due to *hptRS* inactivation. Lastly, these data provide important information about the complex regulation of antibiotic susceptibility and how “standard” susceptibility assay conditions can influence the outcomes of these assays.

## Supporting information

S1 File Code for power and peptide analysis. (PDF)

S2 File Power analysis and study of interactions. (XLSX)

S3 File Analysis of significantly different proteins. (XLSX)

S4 File Proteomics summary tables. (XLSX)

S5 File Analysis of transcript abundance. (XLSX)

S6 File Phenotypic analysis, anaerobic mRNA abundance, and protein abundance figures. (PDF)
