# Supplementary material for: Coordinated regulation of transcription by CcpA and the Staphylococcus aureus two-component system HptRS
Source: PLoS One. 2018 Dec 12;13(12):e0207161. doi: 10.1371/journal.pone.0207161 (PMC6291074; doi:10.1371/journal.pone.0207161)
Supplement: S6 File — (PDF) [file pone.0207161.s006.pdf]

Table of Contents | Page

Entry	Page
**Figure A:** *S. aureus* strains SA564 and SA564-Δ*hptRS* grow equivalently when cultivated anaerobically or in iron-limited TSB.	**S-2**
**Figure B:** *S. aureus* strains SA564 and SA564-Δ*hptRS* grow equivalently when cultivated in the presence of streptonigrin, carbonyl cyanide 3-chlorophenylhydrazon E, or nigericin.	**S-3**
**Figure C:** Antibiotic resistance profiles are equivalent for SA564 relative to SA564-Δ*hptRS*.	**S-4**
**Figure D:** SA564 and SA564-Δ*hptRS* grow equivalently when cultivated with varying concentrations of diamide, paraquat, or H2O2.	**S-5**
**Figure E:** RT-qPCR analysis of *hptR* and *hptS* mRNA abundance in SA564 cultivated aerobically and anaerobically during the exponential and post-exponential growth phases.	**S-6**
**Figure F:** Mean protein abundance and 95% confidence interval plots for a 3-way interaction (s x m x h)	**S-7**
**Figure G:** Mean protein abundance and 95% confidence interval plots for a 2-way interaction (s x m | h)	**S-509**
**Figure H:** Mean protein abundance and 95% confidence interval plots for a 2-way interaction (s x h | m)	**S-1011**
**Figure I:** Mean protein abundance and 95% confidence interval plots for a 2-way interaction (m x h | s)	**S-1513**
**Figure J:** Mean protein abundance and 95% confidence interval plots for a main effect of hour	**S-2015**
**Figure K:** Mean protein abundance and 95% confidence interval plots for a main effect of media	**S-2517**
**Figure L:** Mean protein abundance and 95% confidence interval plots for a main effect of strain	**S-3019**

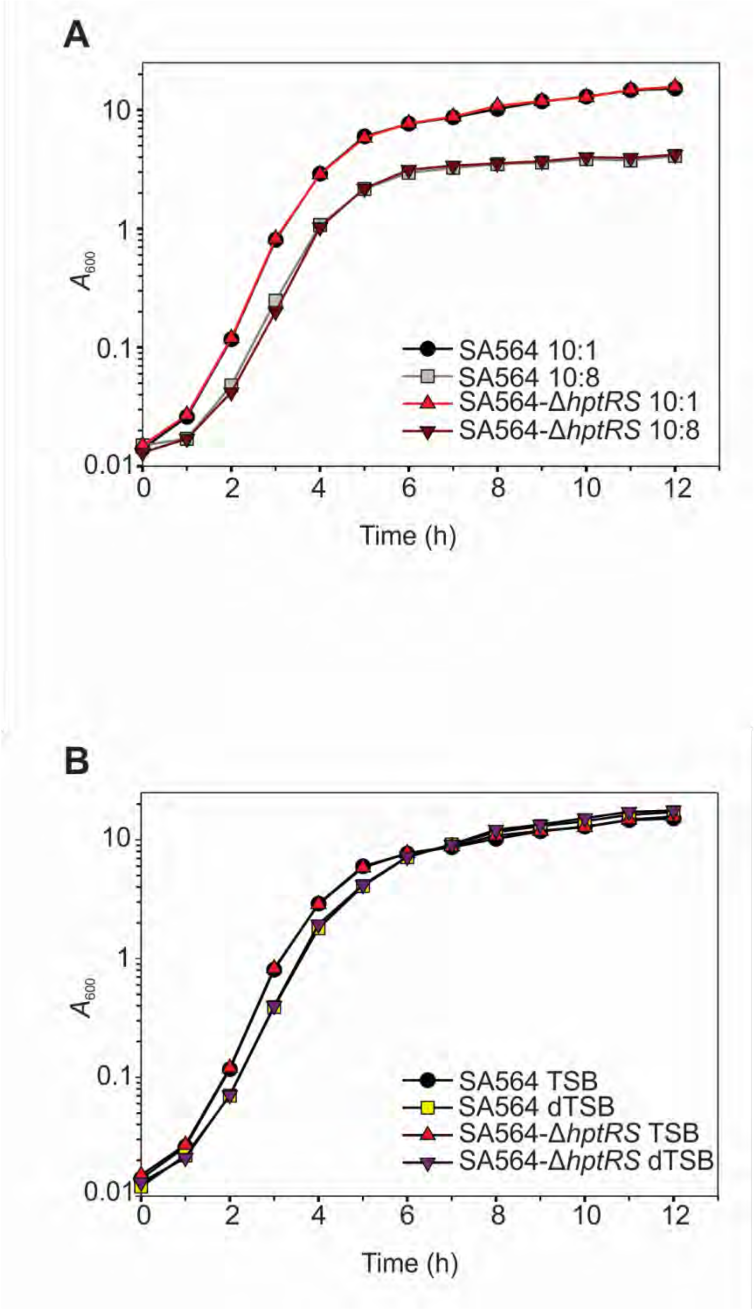

Figure A *S. aureus* strains SA564 and SA564-Δ*hptRS* grow equivalently when cultivated anaerobically (A) or in iron-limited TSB (B). To assess anaerobic growth *S. aureus* strains were cultivated with a flask to medium ratio of 10:1 (aerobic) or 10:8 (anaerobic), at 37 °C under aeration at 225 rpm. To assess growth in iron limited TSB, deferrated TSB (dTSB) was prepared as described [1]. Data is represented as the average of three biological replicates.

(1) Sadykov, M. R.; Zhang, B.; Halouska, S.; Nelson, J. L.; Kreimer, L. W.; Zhu, Y.; Powers, R.; Somerville, G. A. Using NMR metabolomics to investigate tricarboxylic acid cycle-dependent signal transduction in *Staphylococcus epidermidis*. *J. Biol. Chem.* **2010**, *285* (47), 36616–36624.

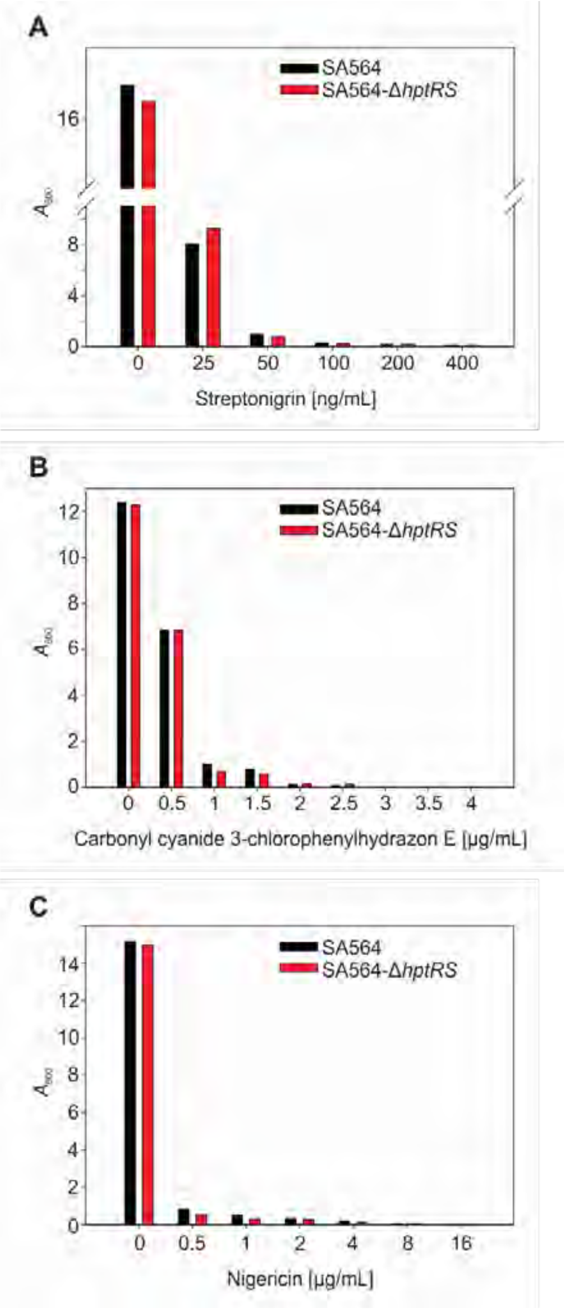

Figure B. *S. aureus* strains SA564 and SA564-Δ*hptRS* grow equivalently when cultivated in the presence of streptonigrin (A),carbonyl cyanide 3-chlorophenylhydrazon E (B), or Nigericin (C). The absorbance was recorded after 18 h of incubation at 37 °C under aeration at 225 rpm with and without streptonigrin, carbonyl cyanide 3-chlorophenylhydrazon E, or nigericin. Data are representative of at least two biological replicates.

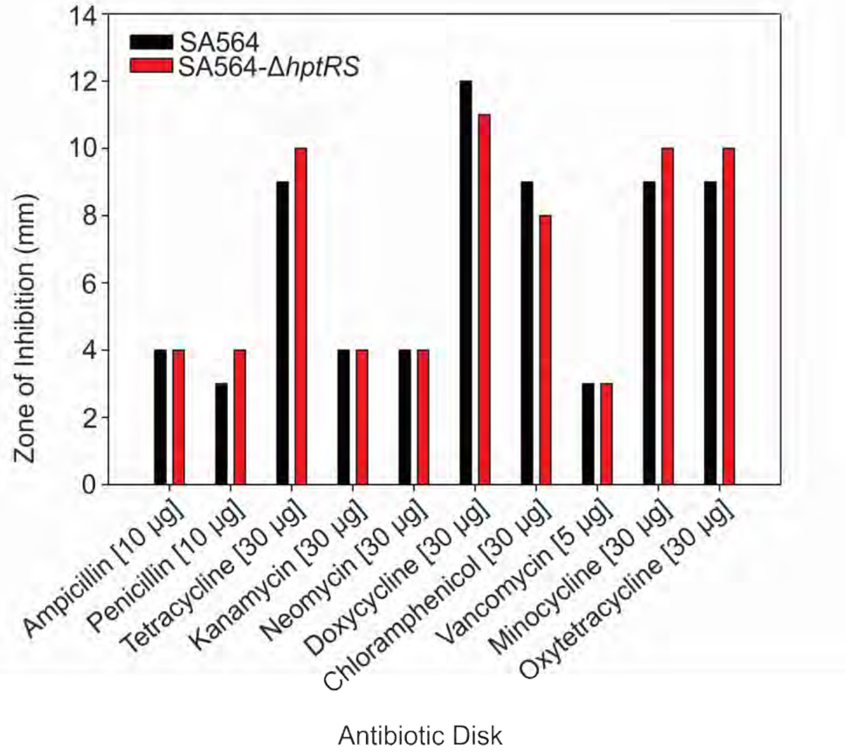

Figure C. Antibiotic resistance profiles are equivalent for SA564 relative to SA564-Δ*hptRS*. Antibiotic susceptibility was investigated using antibiotic disks on TSA. Data represent the mean zone of inhibition for 3 technical replicates.

Figure D. There were no observed growth differences between SA564 and SA564-Δ*hptRS* when diamide, paraquat, or H_2O_2 were added to the culture medium. Bacterial strains were inoculated from a single colony on TSA into TSB and incubated at 37 °C under aeration at 225 rpm. A 2 h pre-culture was prepared as described. After pre-incubation the $A_6$was recorded and bacteria were diluted to .5 McFarland units (i.e., A_6= .8; 1-5 x 18 CFU/mL). This bacterial suspension was used for initial inoculation (2 μL) into 2mL prewarmed TSB, in 17x1 mm test tubes, with or without the listed compounds. Growth was visually inspected at 2 h, 6 h, and 24 h post inoculation and no differences were observed between SA564 and SA564-Δ*hptRS*.

Compound	Concentration
Diamide	10 mM<br>1 mM<br>0.1 mM<br>0.01 mM
Paraquat	50 mM<br>25 mM<br>10 mM<br>5mM<br>1 mM<br>0.1 mM<br>0.01 mM<br>0.001 mM
H_2O_2	2.5 mM<br>5 mM<br>10 mM<br>20 mM<br>30 mM

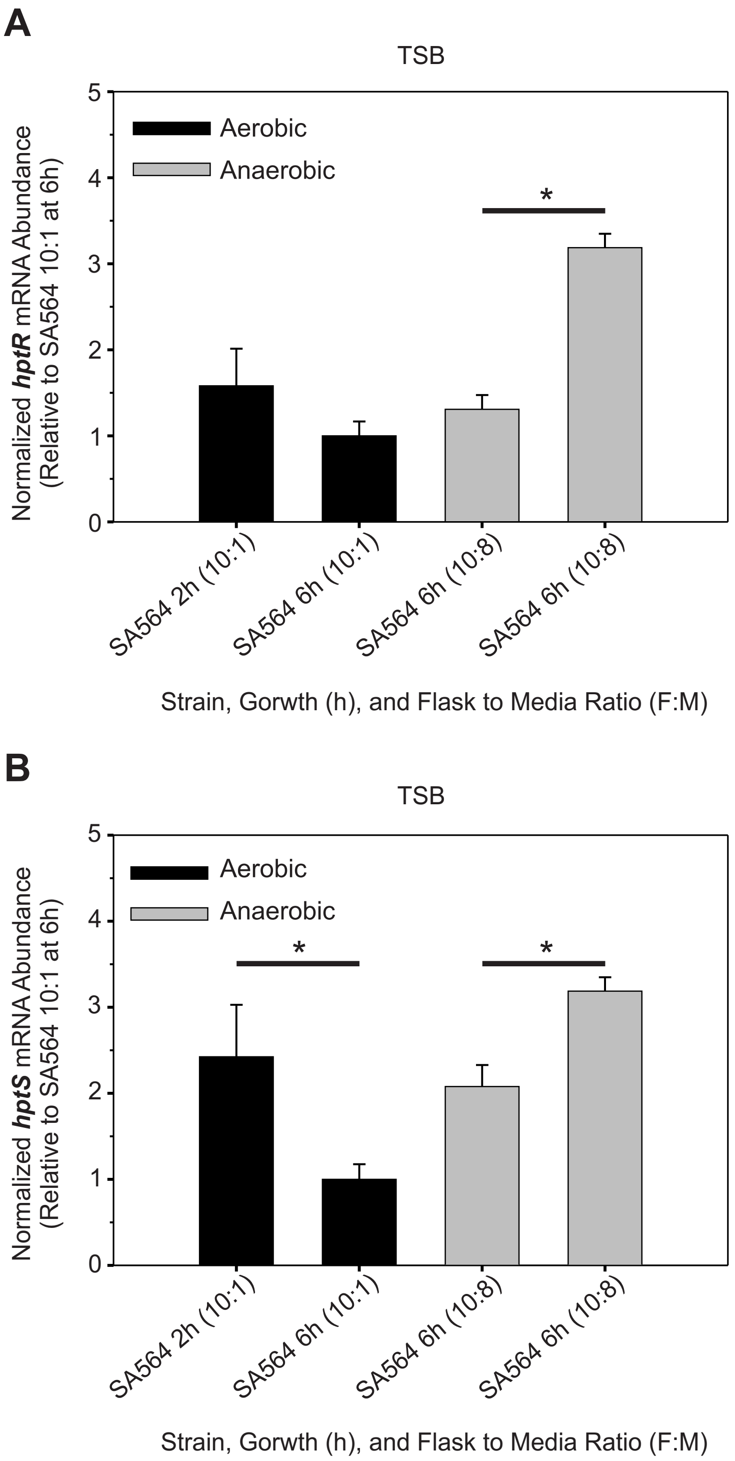

Figure E. Transcript abundance of *hptR* and *hptS* is temporally regulated. Relative mRNA levels during the exponential and post-exponential growth phaseswere determined by RT-qPCR for genes *hptR* (A) and *hptS* (B). The data are represented as the mean and standard error of the mean for 3 biological replicates, each determined in technical duplicate. The data were normalized using 16S rRNA and are plotted relative to SA564 6h (1:1). A statistically significant difference ($p \leq 5$) between exponential (2h) and post-exponential (6h) growth phases is represented with an (*). Bacteria were cultivated in TSB with a flask-to-medium ratio of 1:1, or 1:8.

Figure F.

3-way analysis: s x m x h

Pages: S7-S508

The normalized protein abundance, lower 95% confidence interval, and upper 95% confidence interval were plotted for each protein. These plots were analyzed if a significant three way interaction was identified between strain, media, and hour.

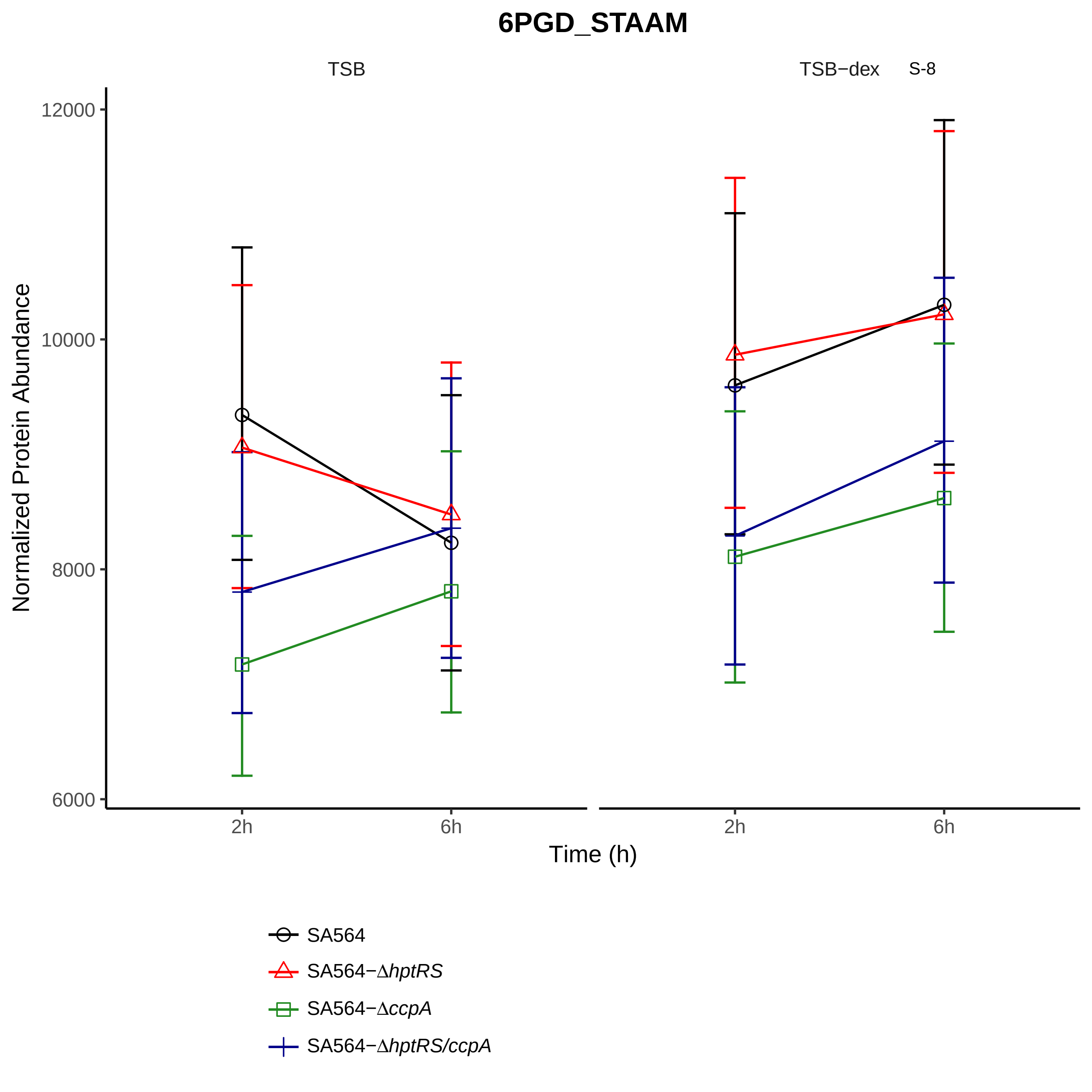

6PGD_STAAM
TSB
TSB−dex
S-8
12000
10000
8000
6000
Normalized Protein Abundance
2h
6h
2h
6h
Time (h)
SA564
SA564−ΔhptRS
SA564−ΔccpA
SA564−ΔhptRS/ccpA

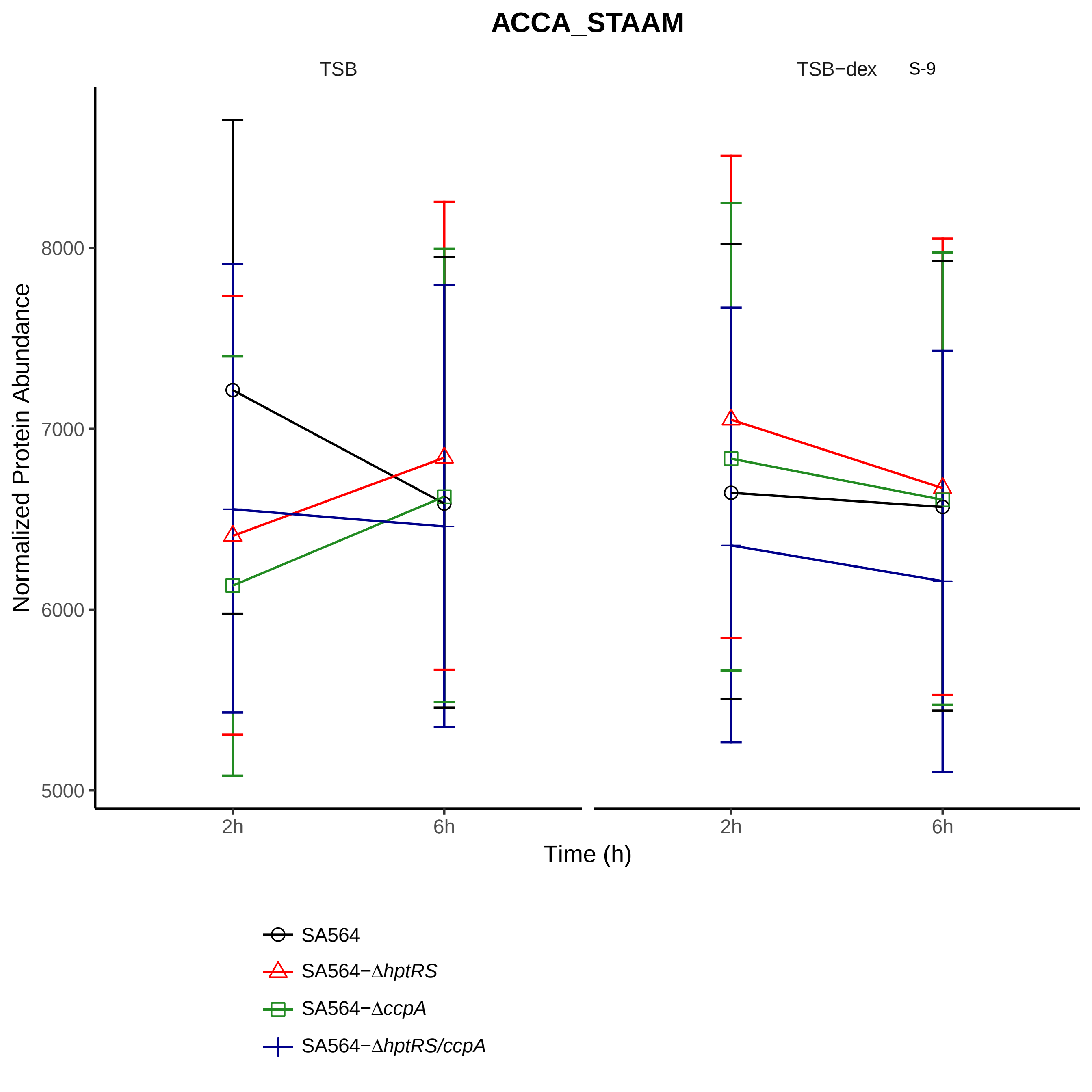

ACCA_STAAM
TSB
TSB−dex
S-9
Normalized Protein Abundance
8000
7000
6000
5000
2h
6h
2h
6h
Time (h)
SA564
SA564−ΔhptRS
SA564−ΔccpA
SA564−ΔhptRS/ccpA

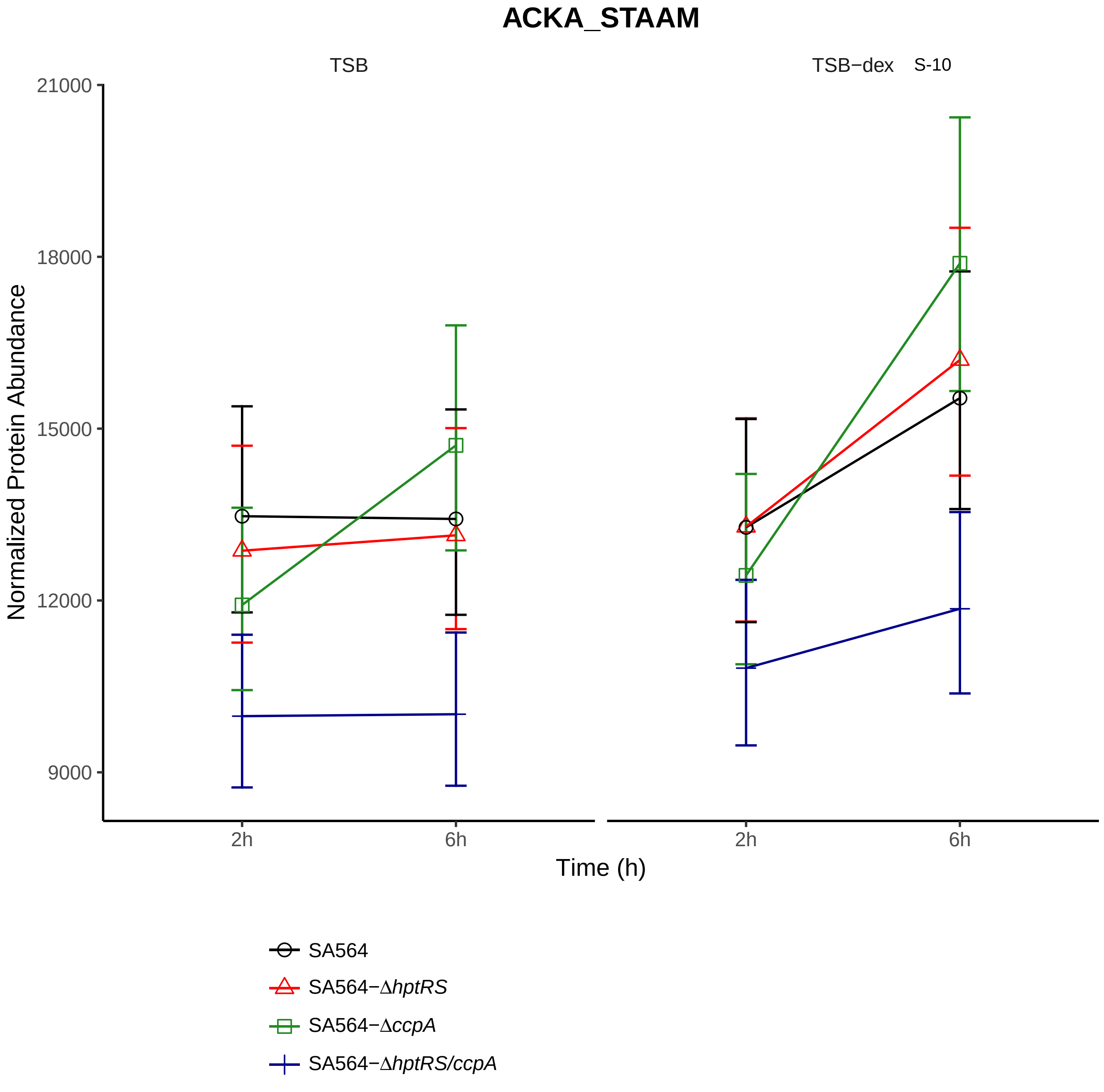

ACKA_STAAM
TSB
TSB−dex
S-10
Normalized Protein Abundance
21000
18000
15000
12000
9000
2h
6h
2h
6h
Time (h)
SA564
SA564−ΔhptRS
SA564−ΔccpA
SA564−ΔhptRS/ccpA

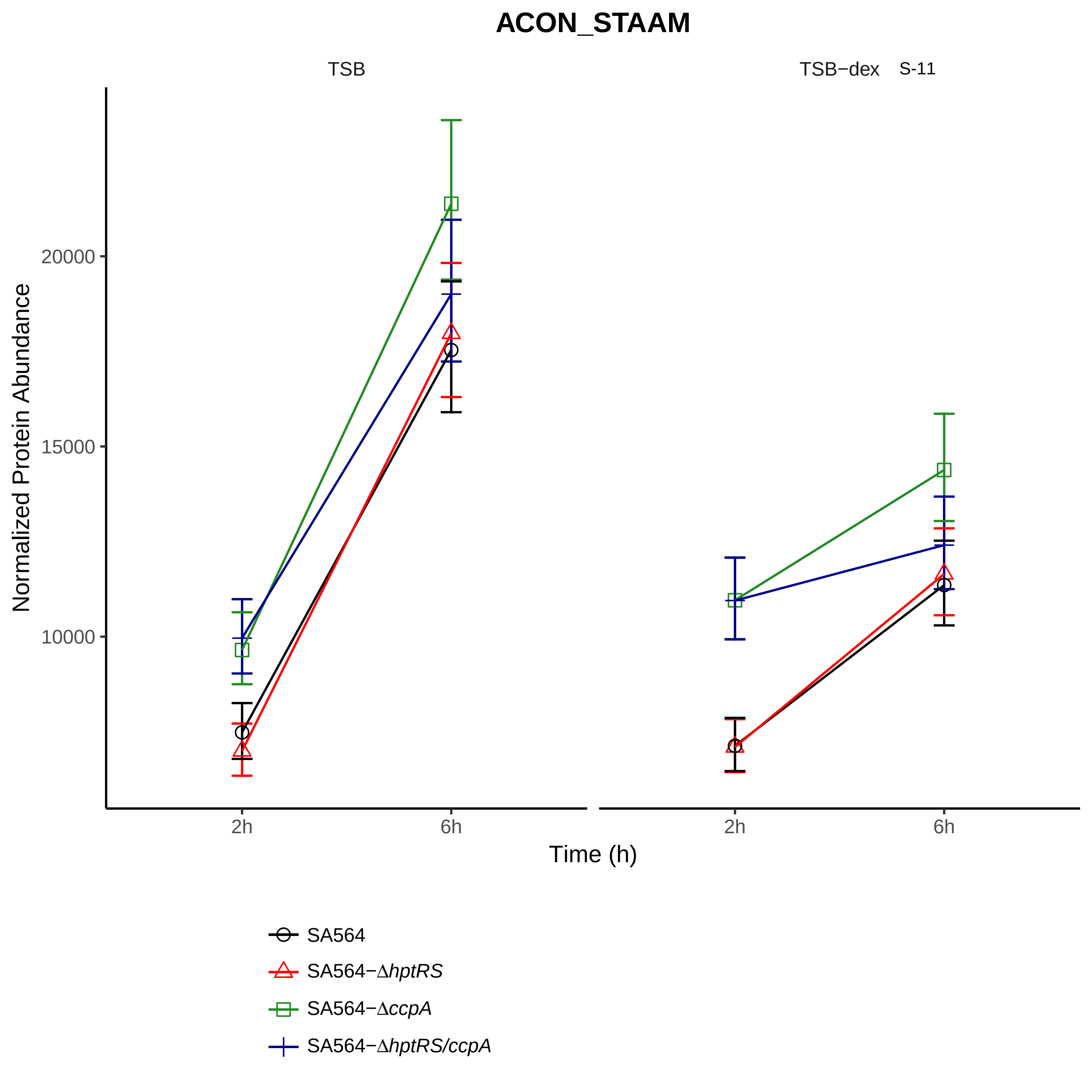

ACON_STAAM
TSB
TSB−dex
S-11
Normalized Protein Abundance
20000
15000
10000
2h
6h
2h
6h
Time (h)
SA564
SA564−Δ*hptRS*
SA564−Δ*ccpA*
SA564−Δ*hptRS/ccpA*

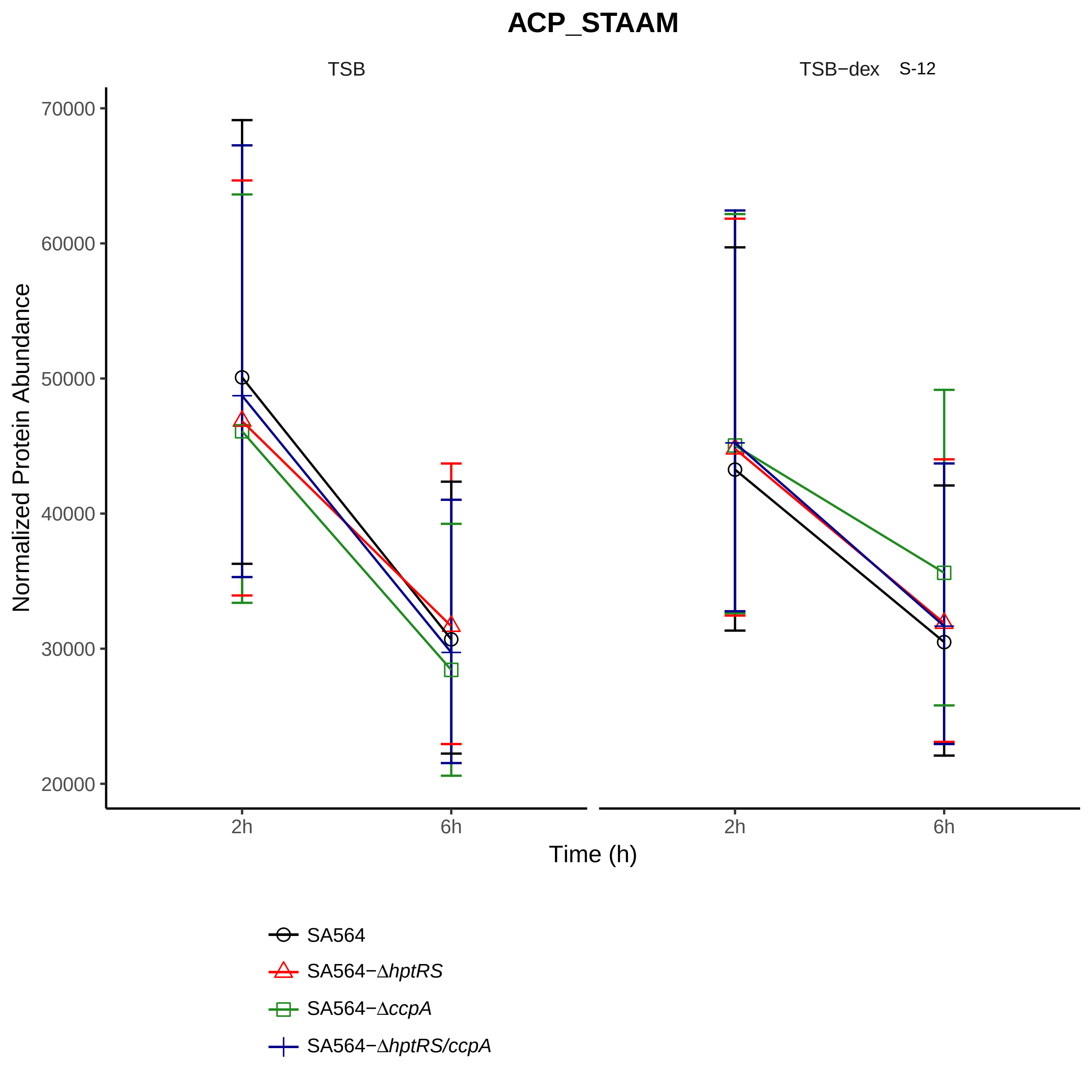
ACP_STAAM
TSB
TSB−dex
S-12
Normalized Protein Abundance
70000
60000
50000
40000
30000
20000
2h
6h
2h
6h
Time (h)
SA564
SA564−ΔhptRS
SA564−ΔccpA
SA564−ΔhptRS/ccpA

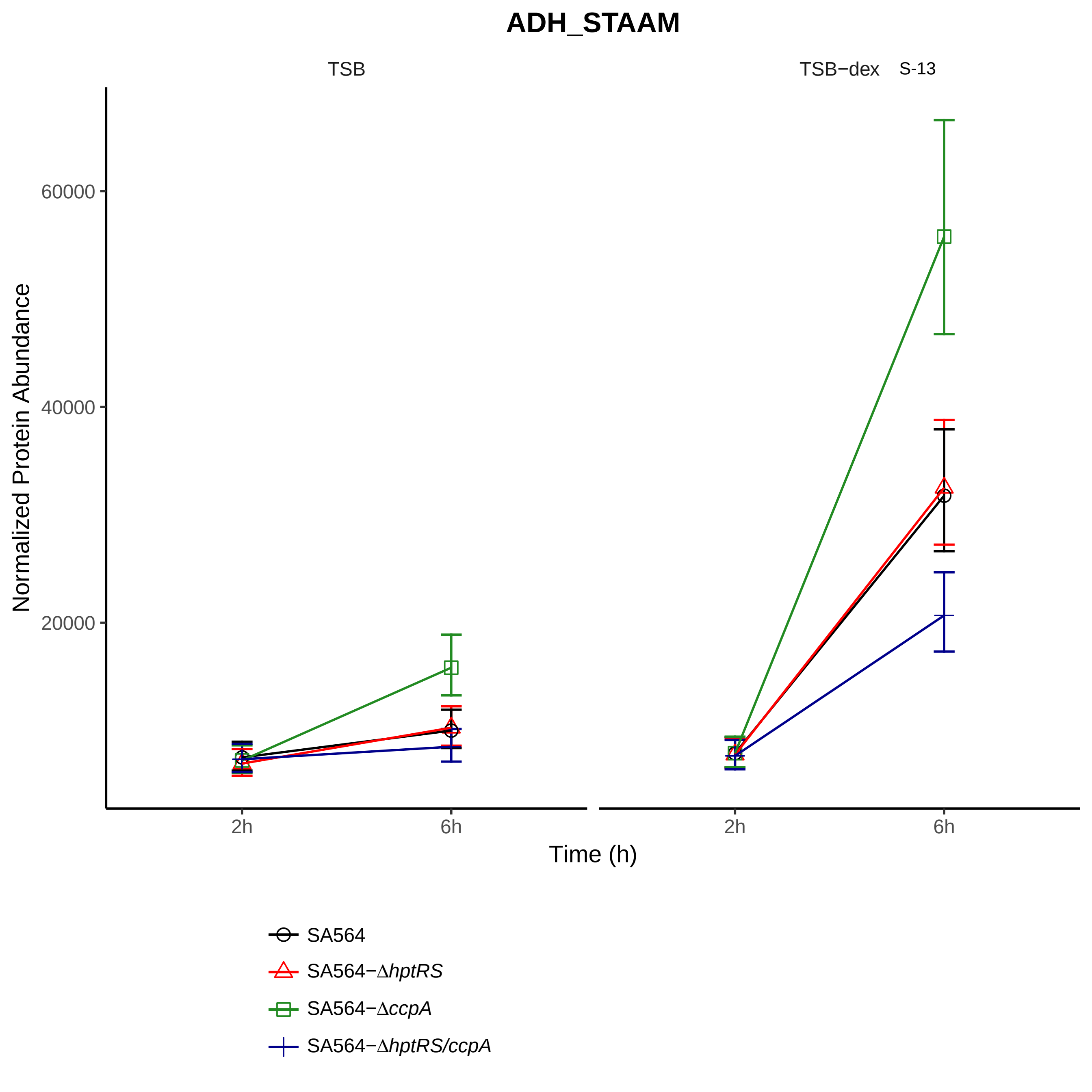
ADH_STAAM
TSB
TSB−dex
S-13
Normalized Protein Abundance
60000
40000
20000
2h
6h
2h
6h
Time (h)
SA564
SA564−Δ*hptRS*
SA564−Δ*ccpA*
SA564−Δ*hptRS/ccpA*

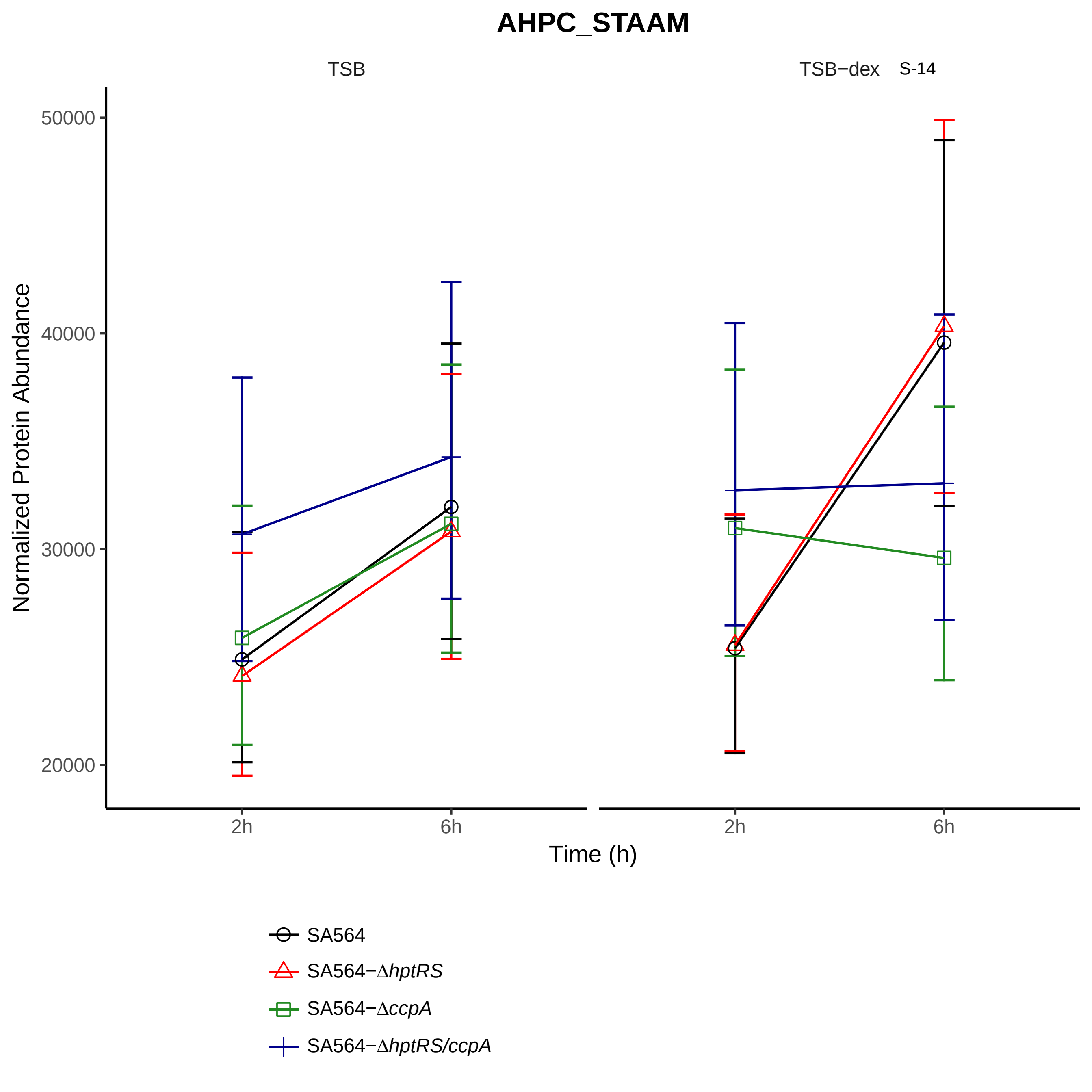
AHPC_STAAM
TSB
TSB−dex
S-14
Normalized Protein Abundance
50000
40000
30000
20000
2h
6h
2h
6h
Time (h)
SA564
SA564−Δ*hptRS*
SA564−Δ*ccpA*
SA564−Δ*hptRS/ccpA*

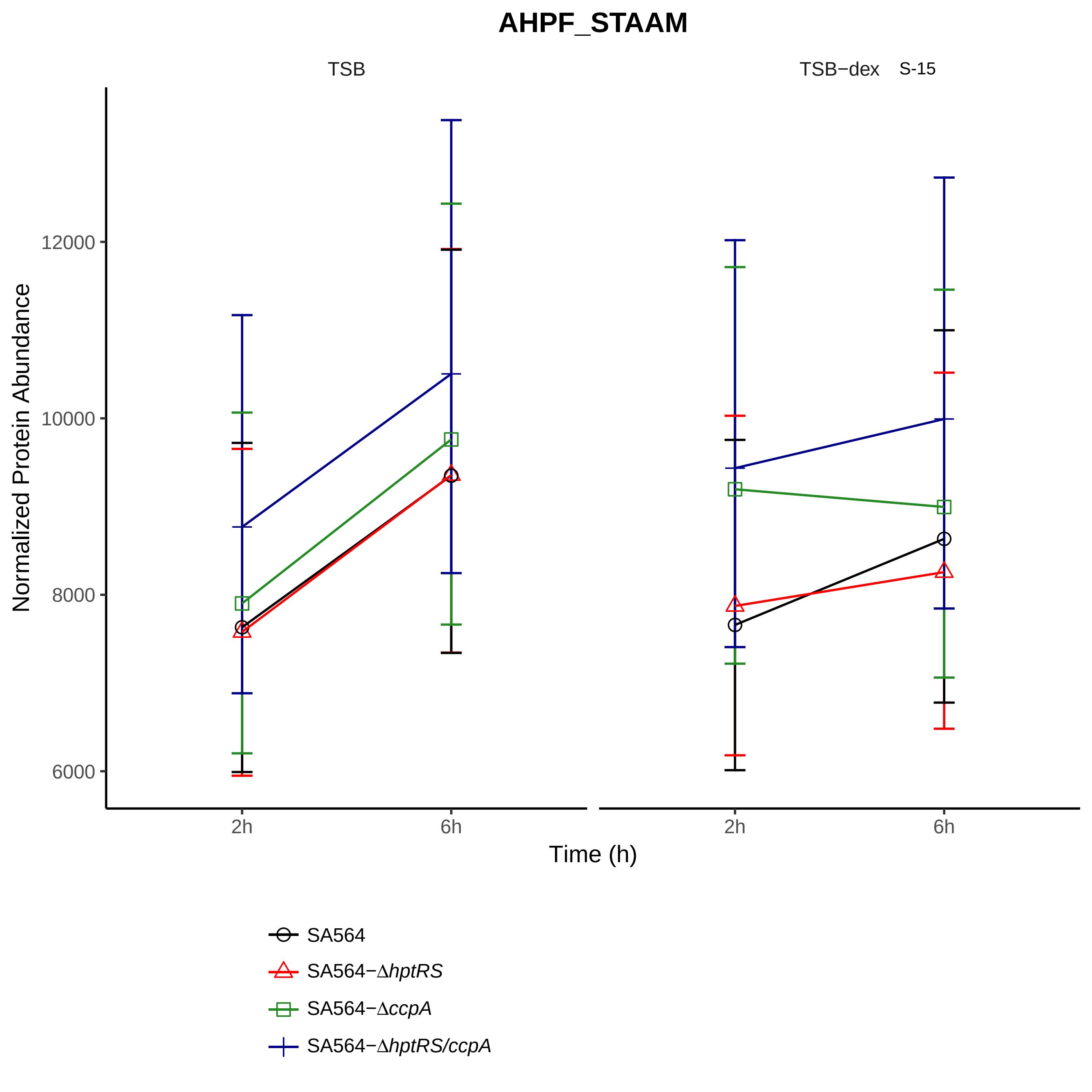

AHPF_STAAM
TSB
TSB−dex
S-15
Normalized Protein Abundance
12000
10000
8000
6000
2h
6h
2h
6h
Time (h)
SA564
SA564−Δ*hptRS*
SA564−Δ*ccpA*
SA564−Δ*hptRS/ccpA*

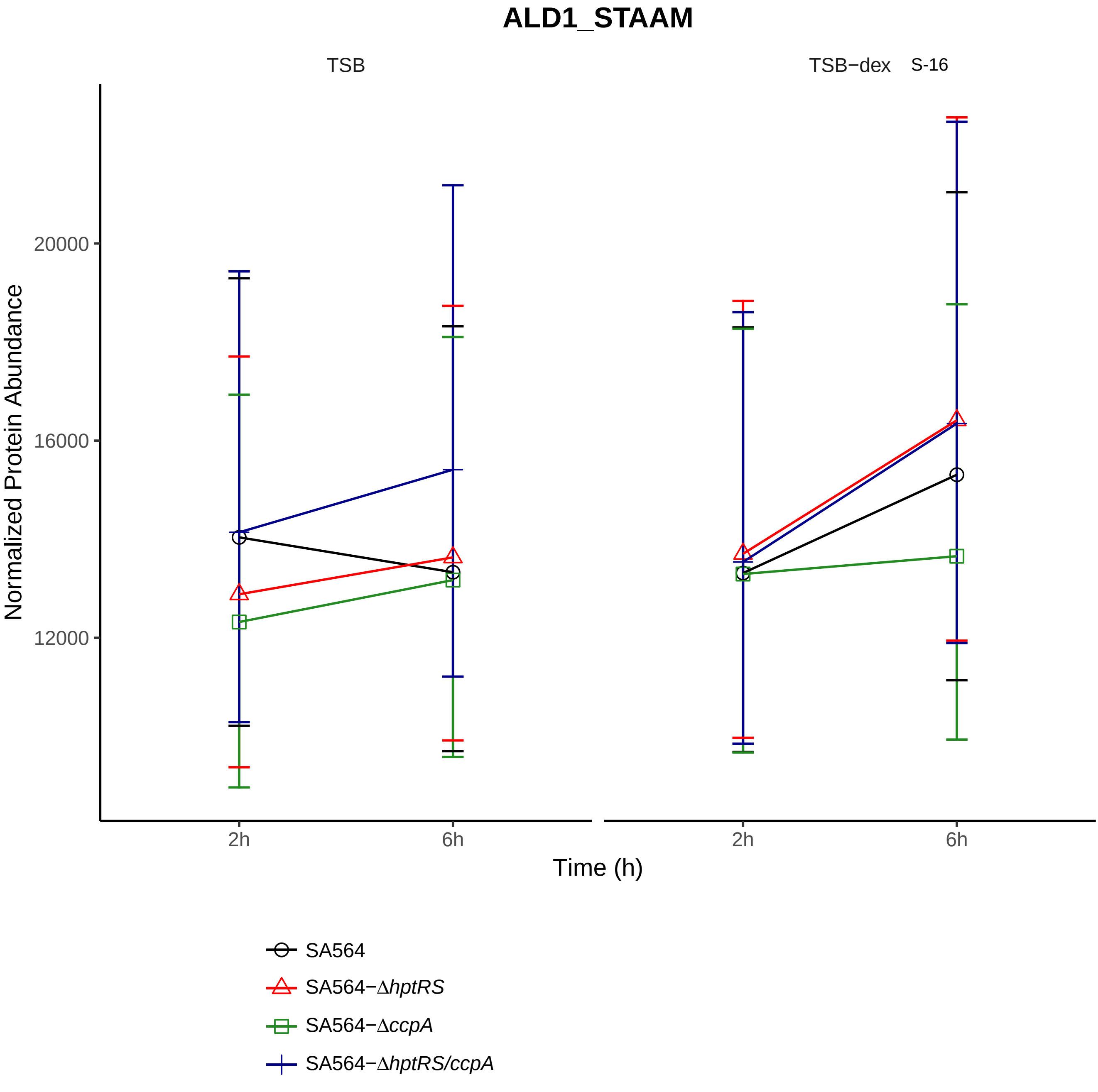

ALD1_STAAM
TSB
TSB−dex
S-16
20000
16000
12000
Normalized Protein Abundance
2h
6h
2h
6h
Time (h)
SA564
SA564−ΔhptRS
SA564−ΔccpA
SA564−ΔhptRS/ccpA

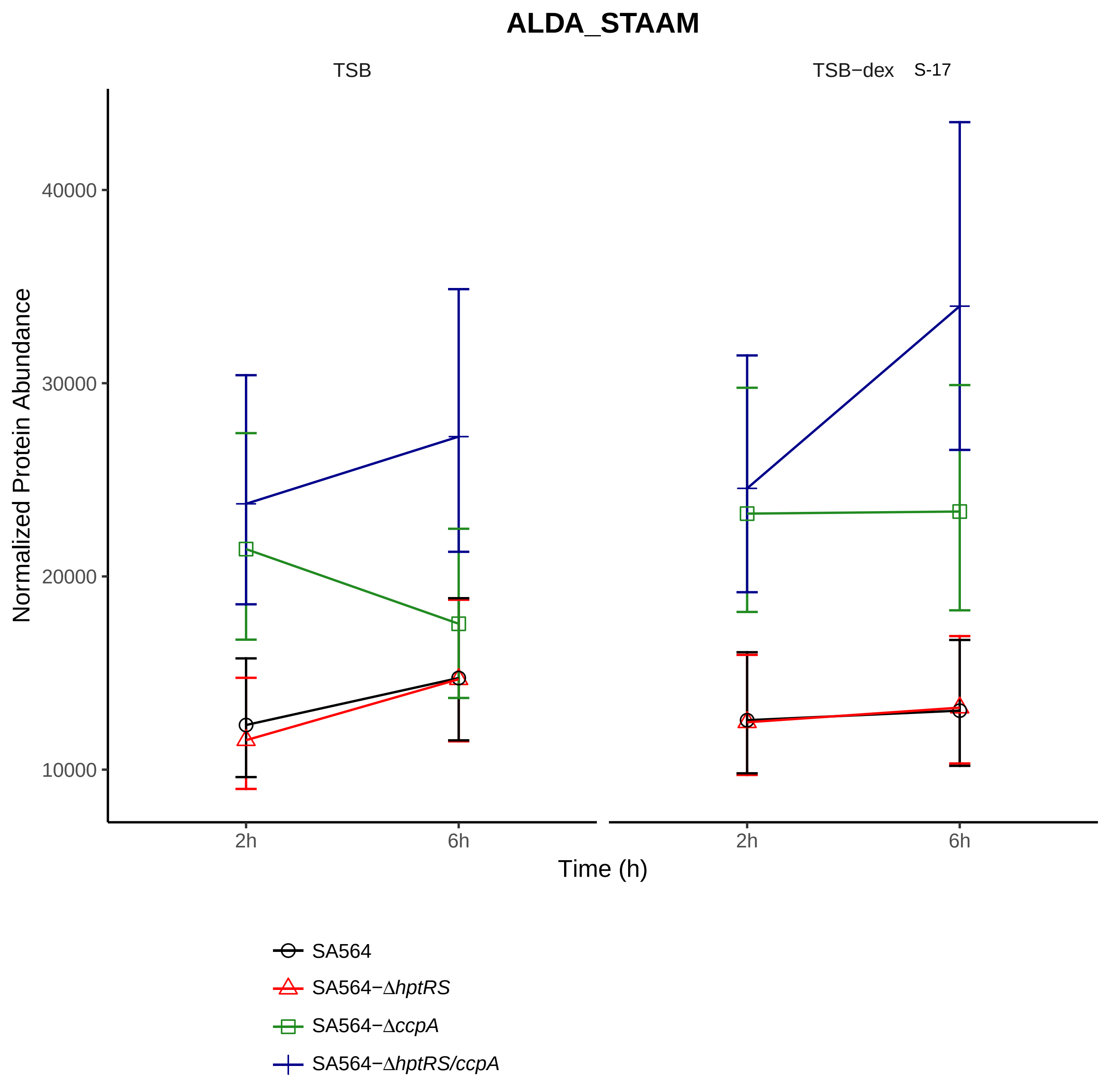

ALDA_STAAM
TSB
TSB−dex
S-17
Normalized Protein Abundance
40000
30000
20000
10000
2h
6h
2h
6h
Time (h)
SA564
SA564−ΔhptRS
SA564−ΔccpA
SA564−ΔhptRS/ccpA

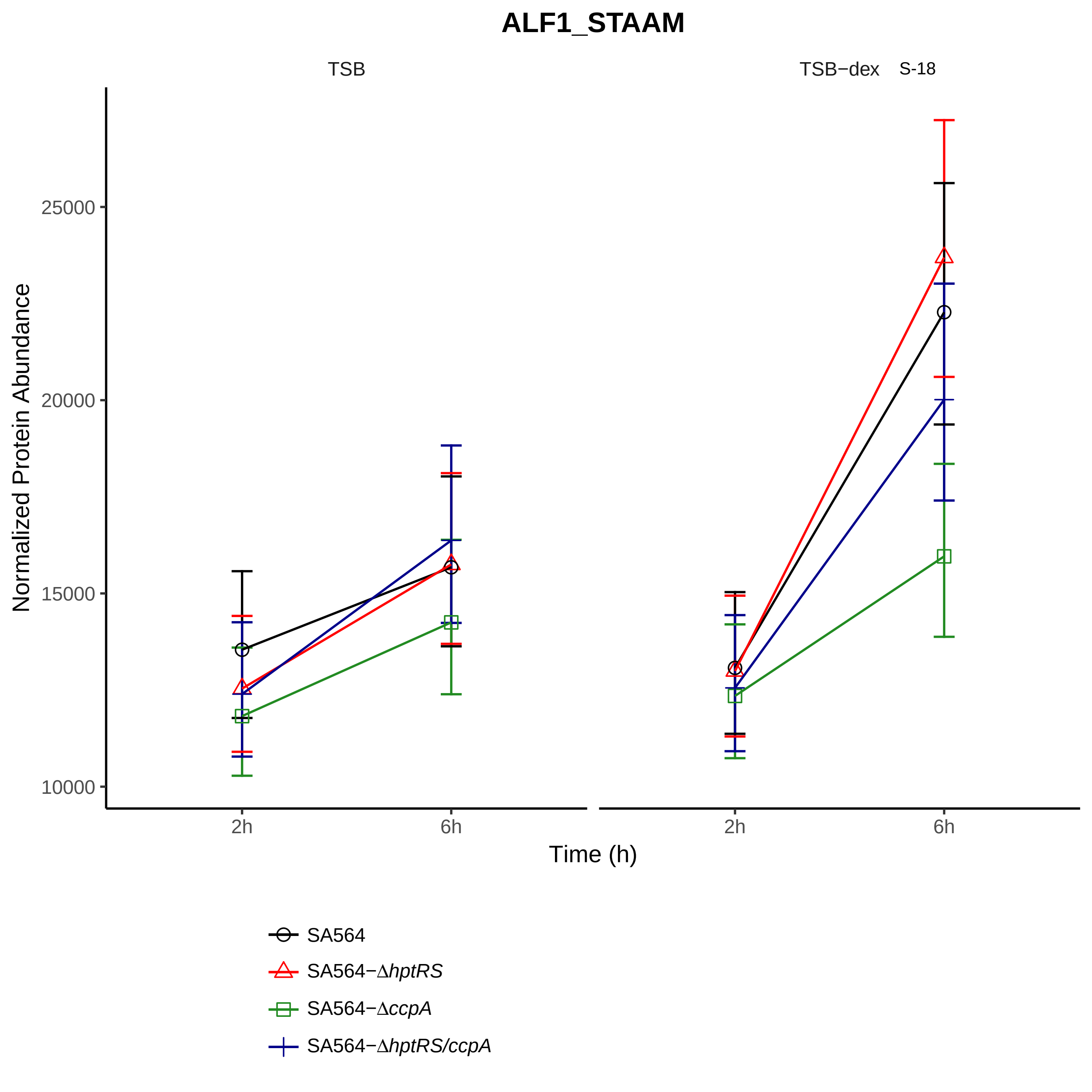

ALF1_STAAM
TSB
TSB−dex
S-18
Normalized Protein Abundance
25000
20000
15000
10000
2h
6h
2h
6h
Time (h)
SA564
SA564−Δ*hptRS*
SA564−Δ*ccpA*
SA564−Δ*hptRS/ccpA*

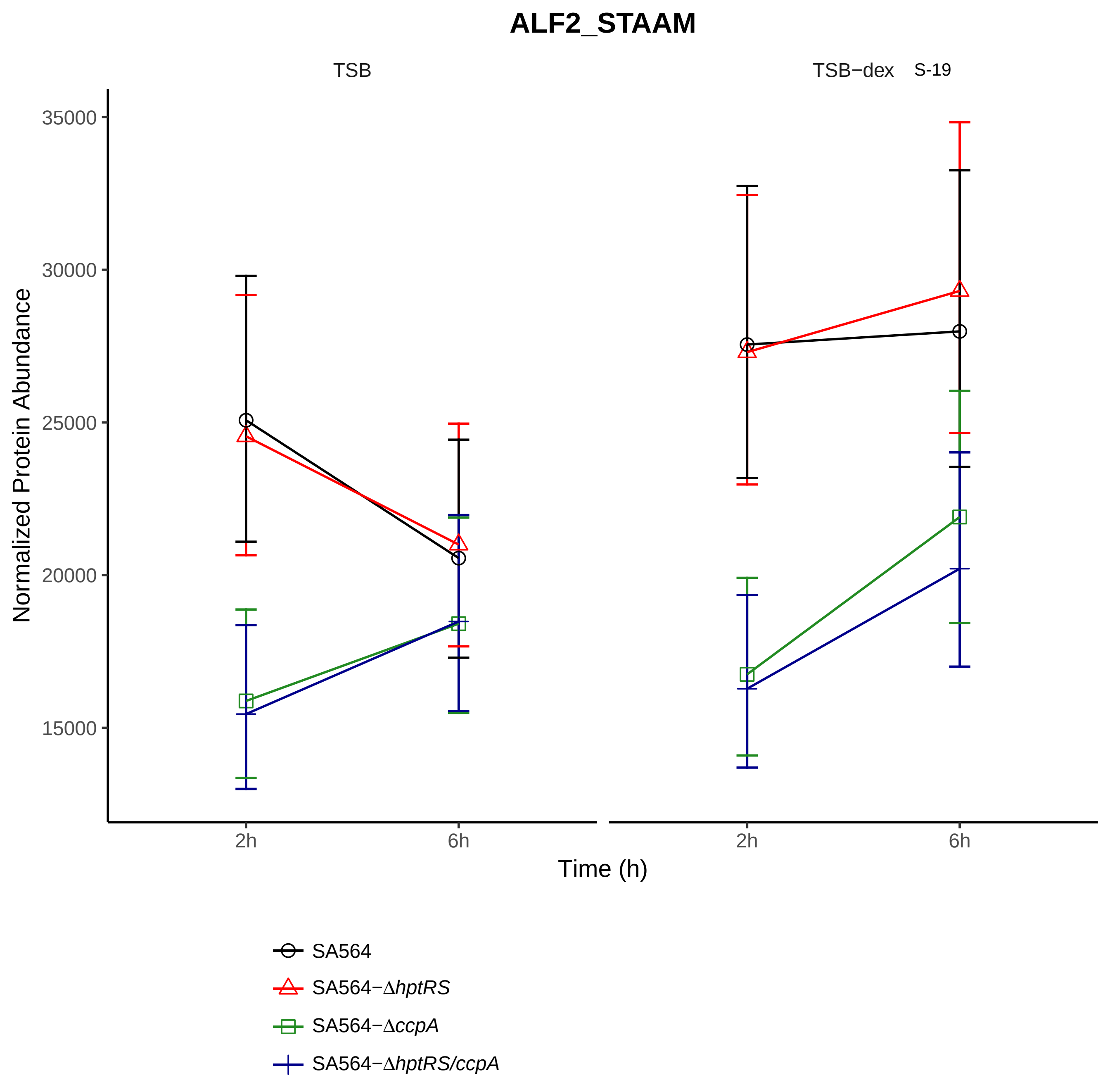
ALF2_STAAM
TSB
TSB−dex
S-19
35000
30000
25000
20000
15000
Normalized Protein Abundance
2h
6h
2h
6h
Time (h)
SA564
SA564−Δ*hptRS*
SA564−Δ*ccpA*
SA564−Δ*hptRS/ccpA*

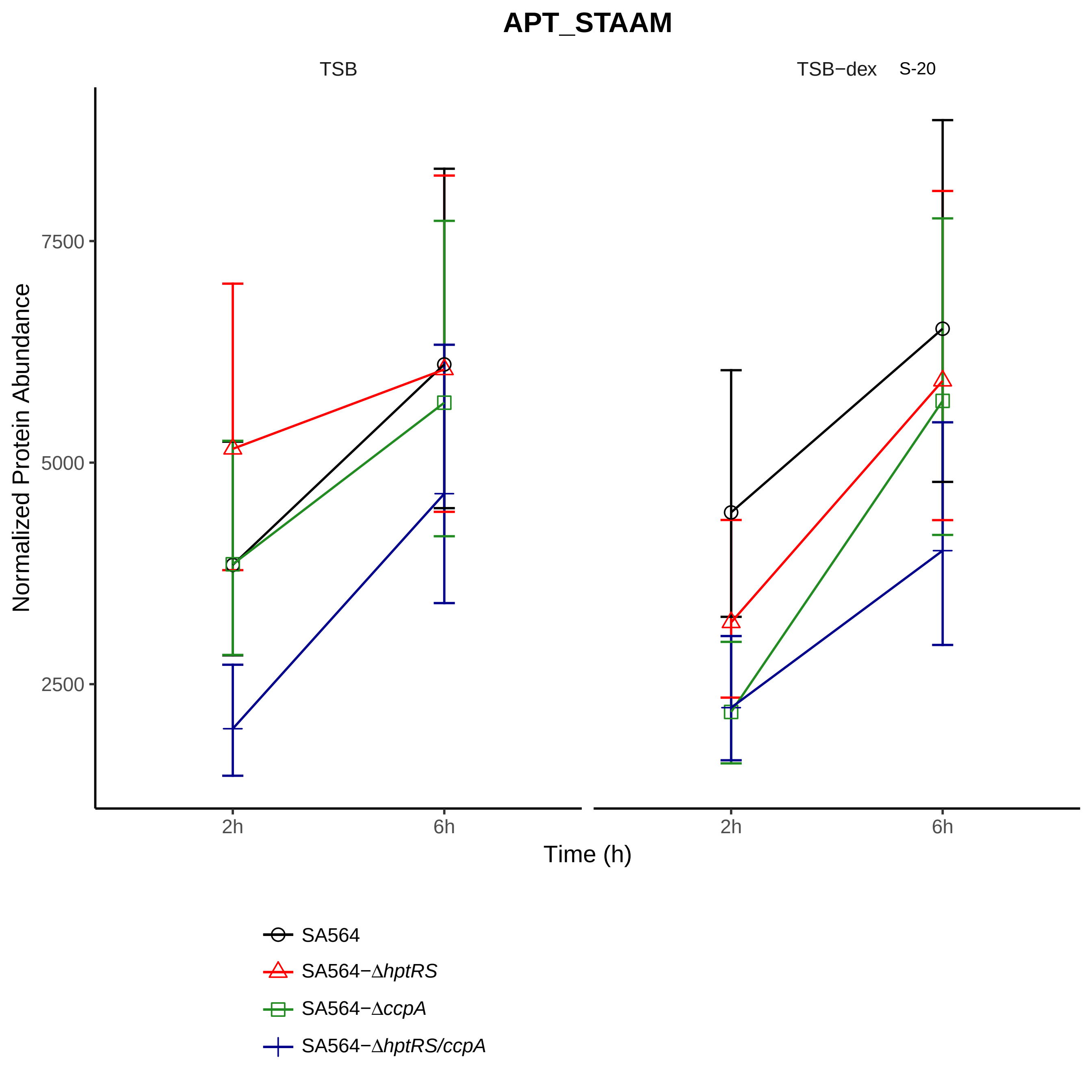

APT_STAAM
TSB
TSB−dex
S-20
Normalized Protein Abundance
7500
5000
2500
2h
6h
2h
6h
Time (h)
SA564
SA564−Δ*hptRS*
SA564−Δ*ccpA*
SA564−Δ*hptRS/ccpA*

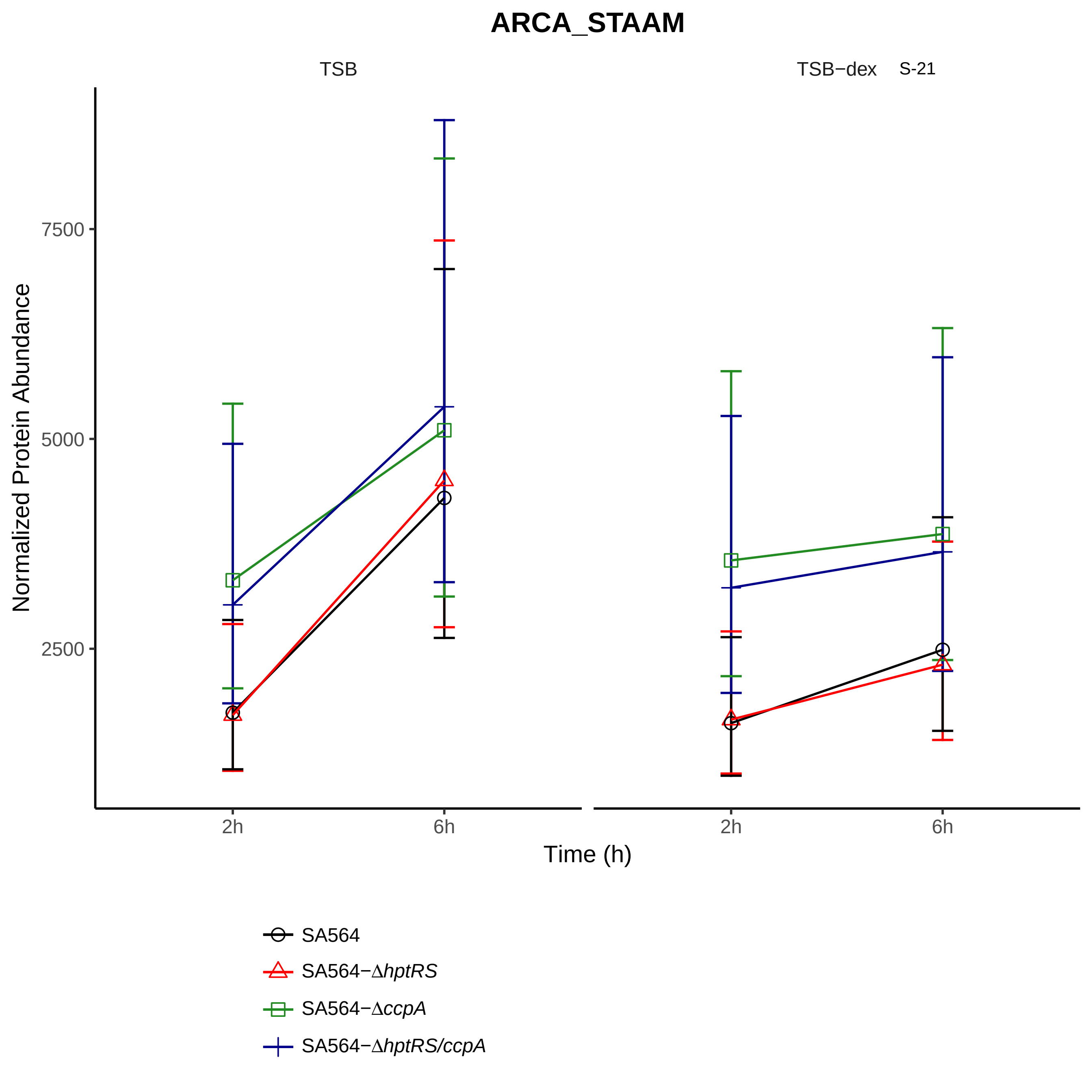
ARCA_STAAM
TSB
TSB−dex
S-21
Normalized Protein Abundance
7500
5000
2500
2h
6h
2h
6h
Time (h)
SA564
SA564−Δ*hptRS*
SA564−Δ*ccpA*
SA564−Δ*hptRS/ccpA*

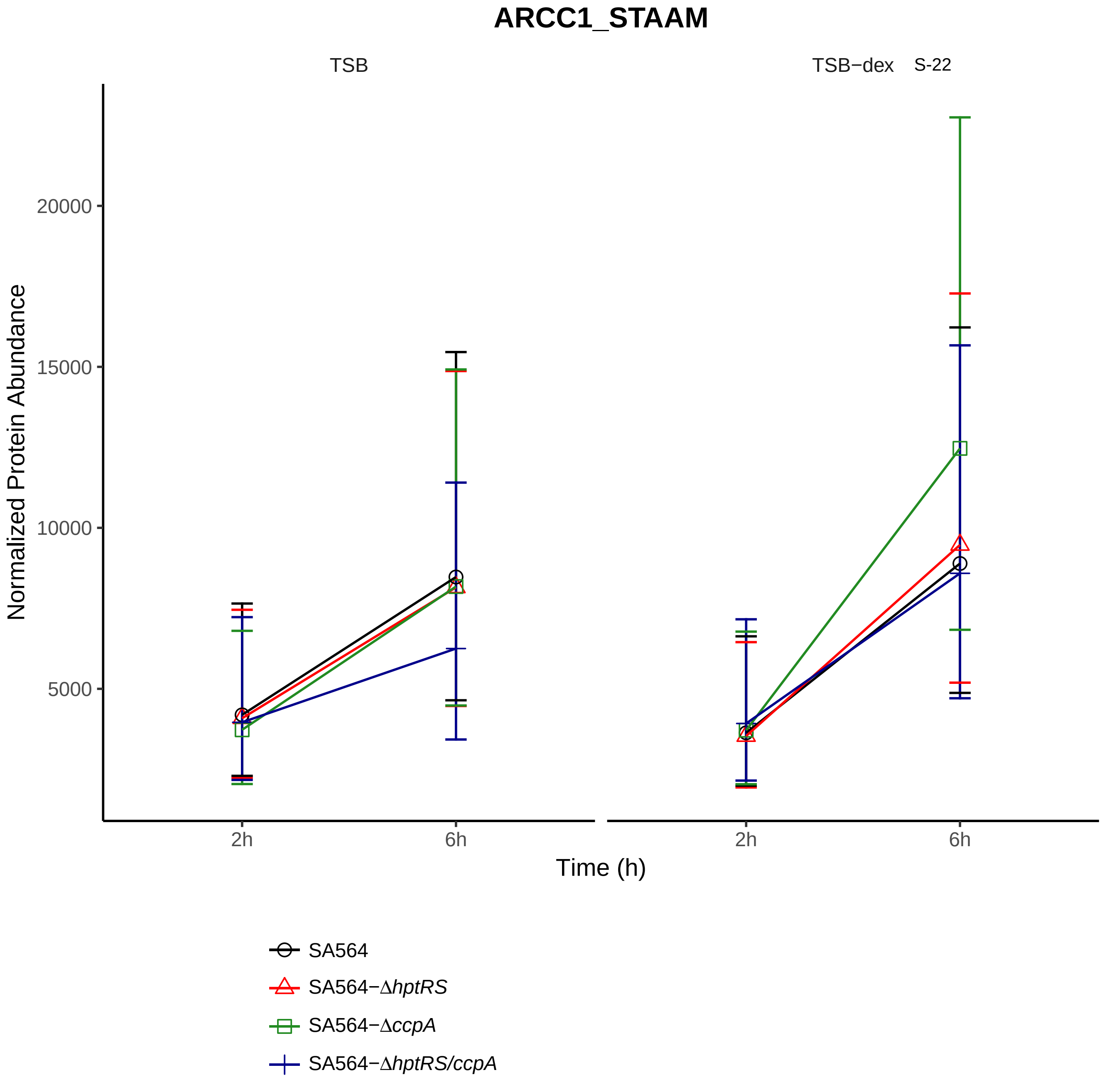

ARCC1_STAAM
TSB
TSB−dex
Normalized Protein Abundance
20000
15000
10000
5000
2h
6h
2h
6h
Time (h)
SA564
SA564−ΔhptRS
SA564−ΔccpA
SA564−ΔhptRS/ccpA

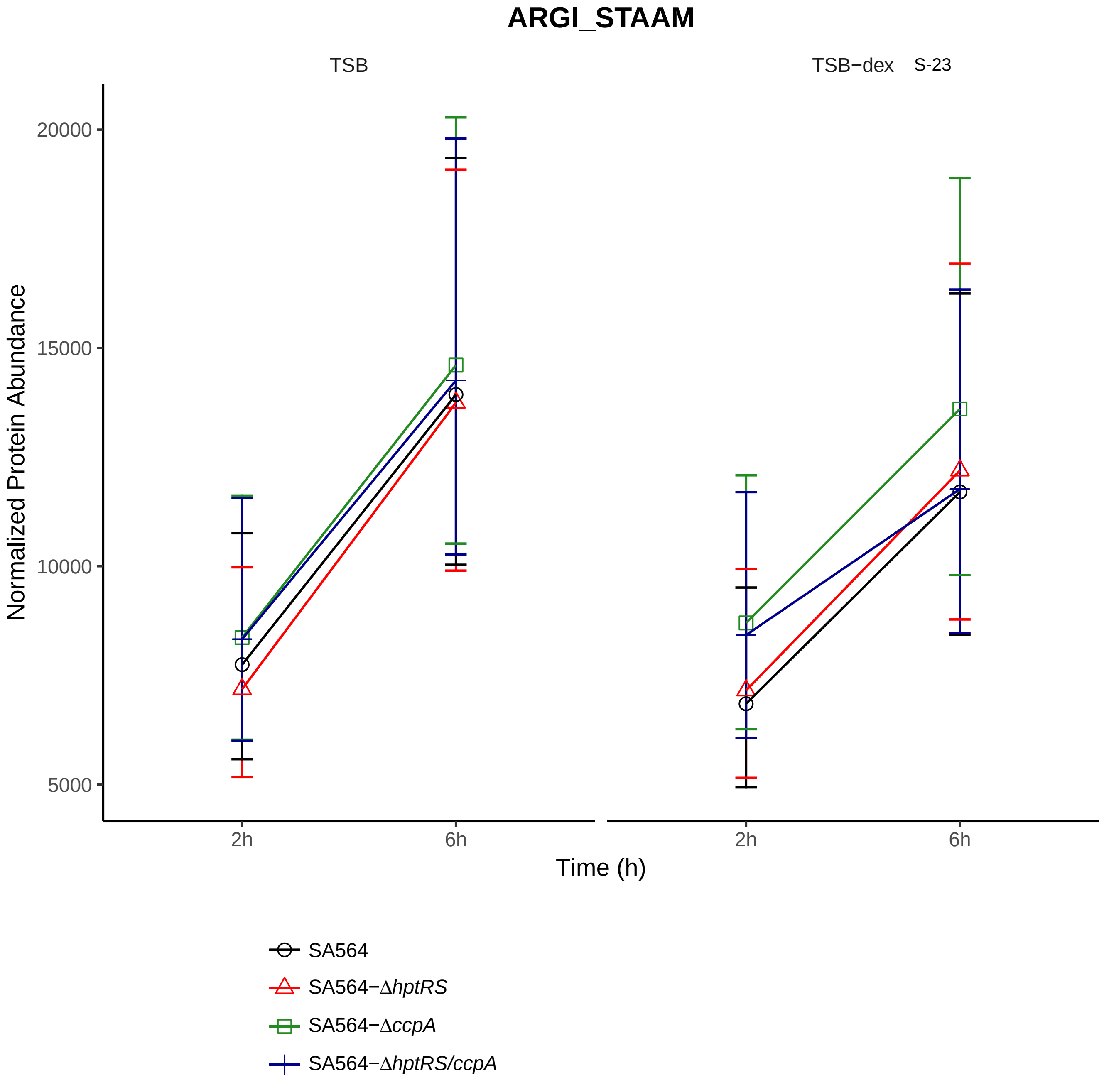
ARGI_STAAM
TSB
TSB−dex
S-23
Normalized Protein Abundance
20000
15000
10000
5000
2h
6h
2h
6h
Time (h)
SA564
SA564−Δ*hptRS*
SA564−Δ*ccpA*
SA564−Δ*hptRS/ccpA*

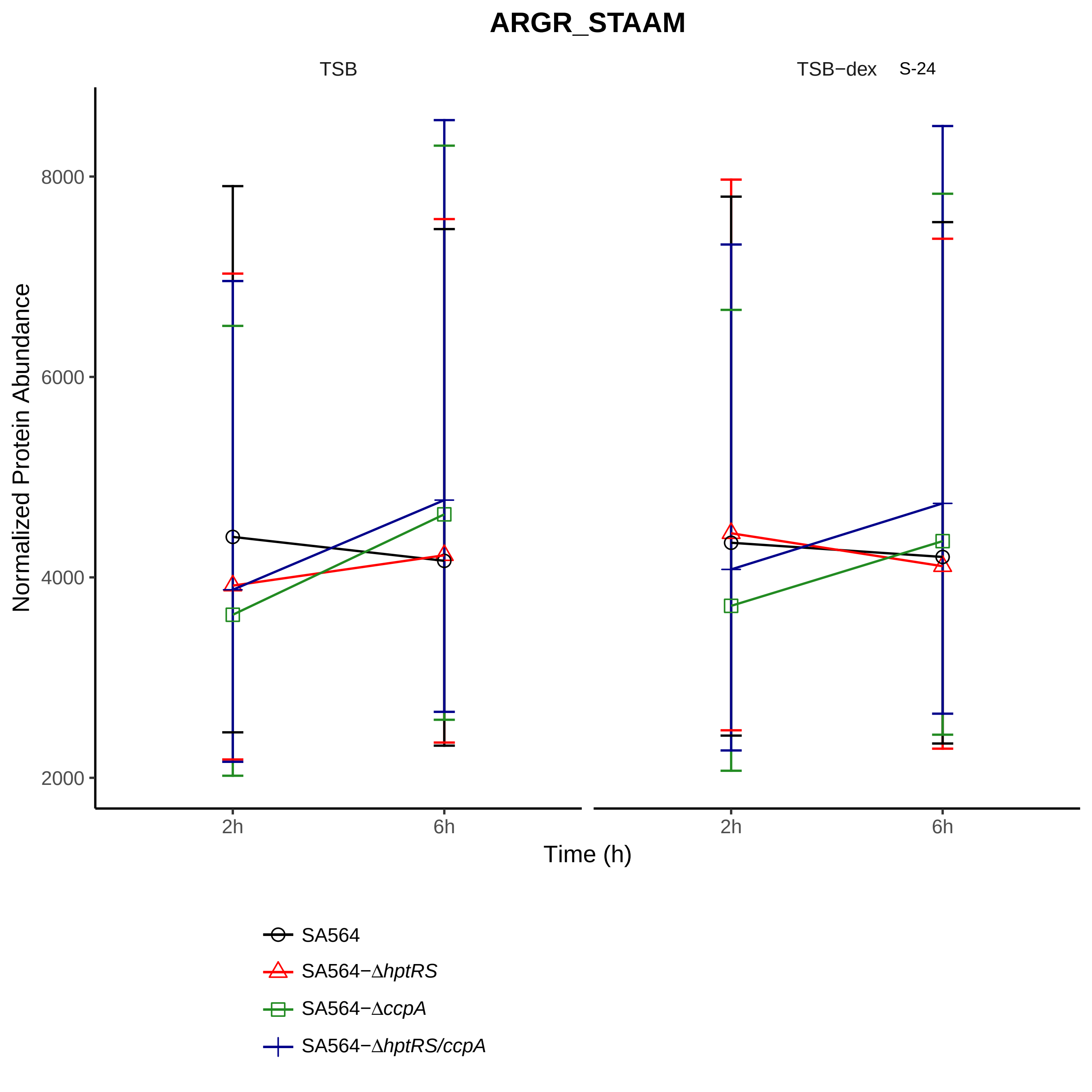

ARGR_STAAM
TSB
TSB−dex
S-24
Normalized Protein Abundance
8000
6000
4000
2000
2h
6h
2h
6h
Time (h)
SA564
SA564−ΔhptRS
SA564−ΔccpA
SA564−ΔhptRS/ccpA

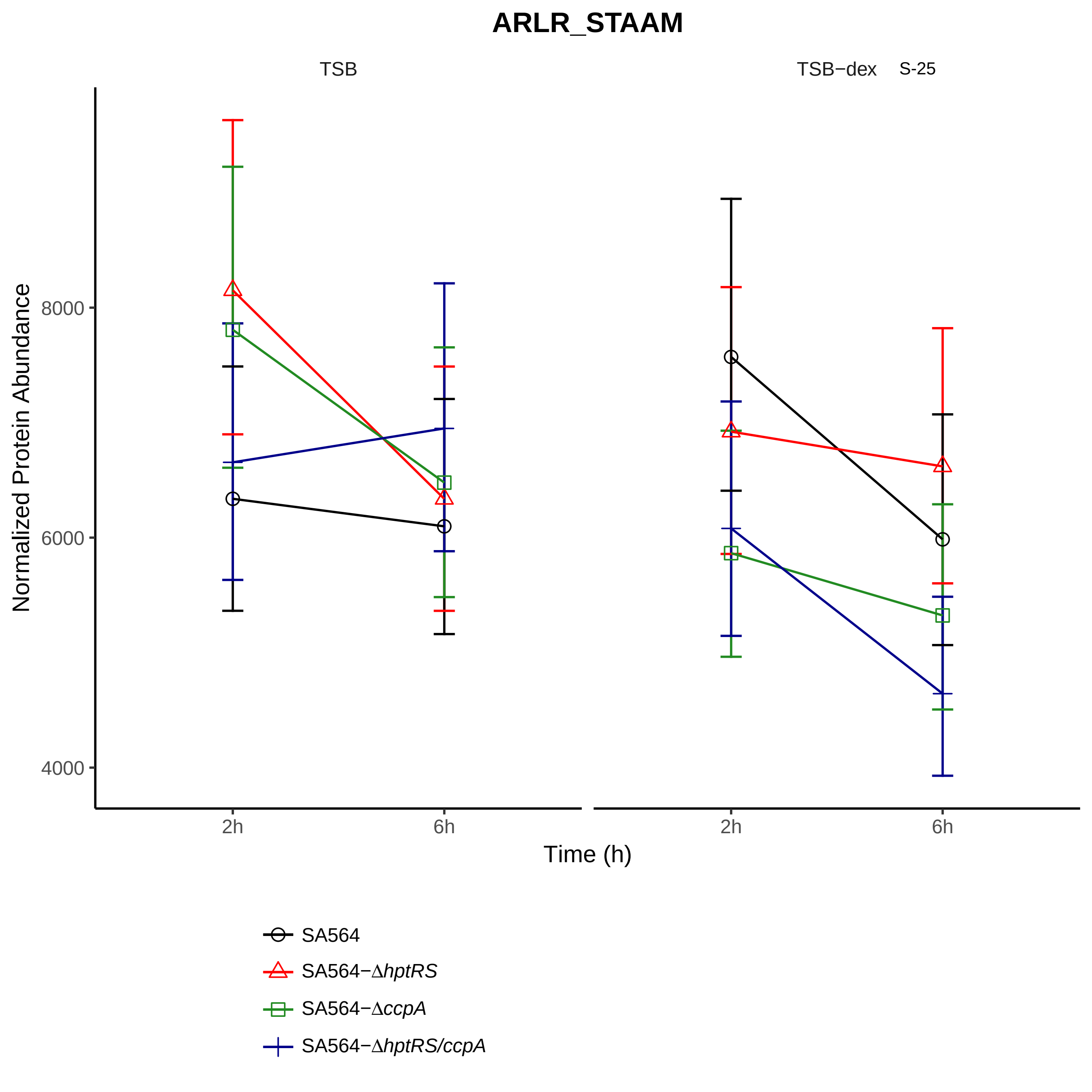

ARLR_STAAM
TSB
TSB−dex
S-25
Normalized Protein Abundance
8000
6000
4000
2h
6h
2h
6h
Time (h)
SA564
SA564−Δ*hptRS*
SA564−Δ*ccpA*
SA564−Δ*hptRS/ccpA*

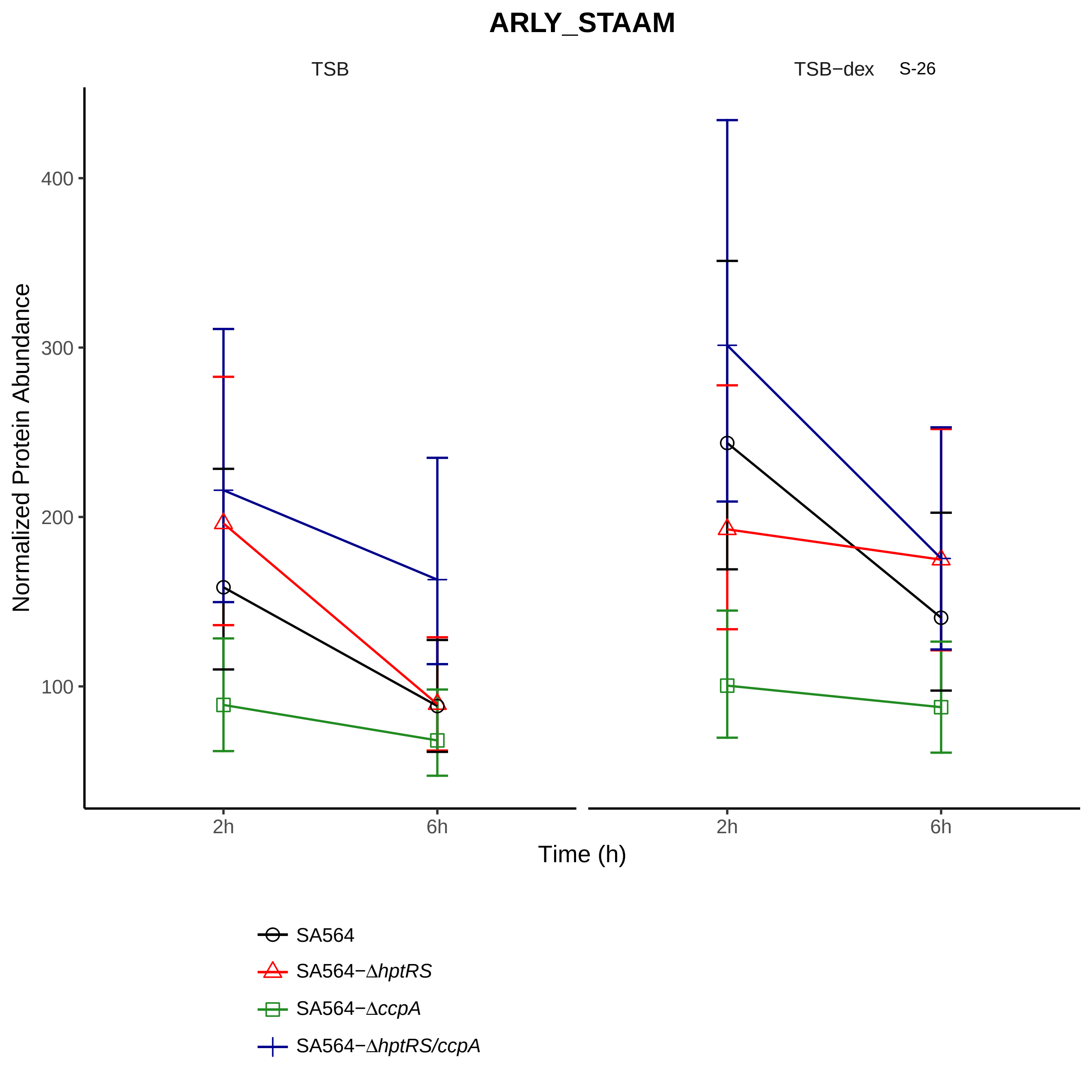

ARLY_STAAM
TSB
TSB−dex
S-26
Normalized Protein Abundance
400
300
200
100
2h
6h
2h
6h
Time (h)
SA564
SA564−ΔhptRS
SA564−ΔccpA
SA564−ΔhptRS/ccpA

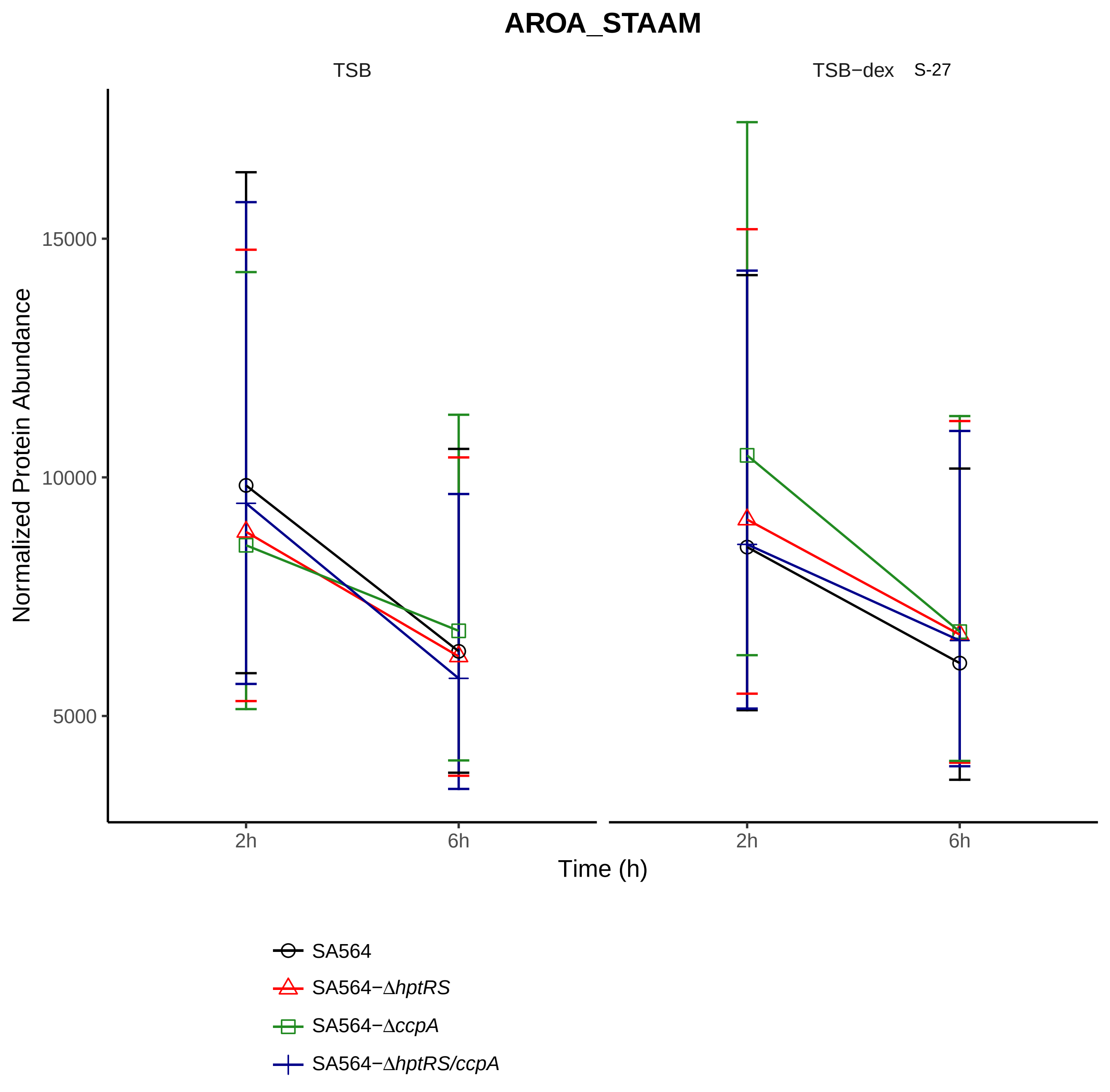

AROA_STAAM
TSB
TSB−dex
S-27
Normalized Protein Abundance
15000
10000
5000
2h
6h
2h
6h
Time (h)
SA564
SA564−Δ*hptRS*
SA564−Δ*ccpA*
SA564−Δ*hptRS/ccpA*

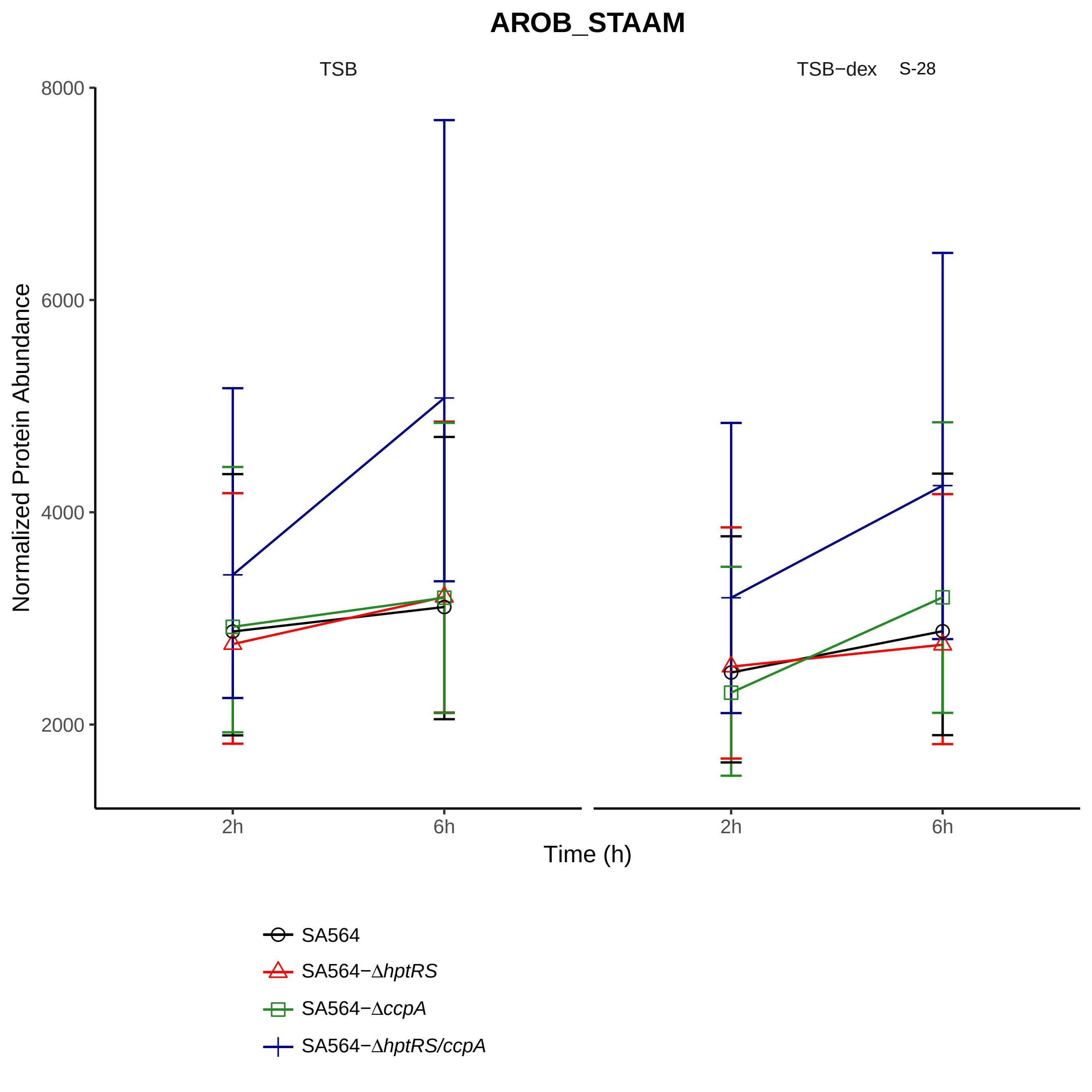
AROB_STAAM
TSB
TSB−dex
S-28
8000
6000
4000
2000
Normalized Protein Abundance
2h
6h
2h
6h
Time (h)
SA564
SA564−ΔhptRS
SA564−ΔccpA
SA564−ΔhptRS/ccpA

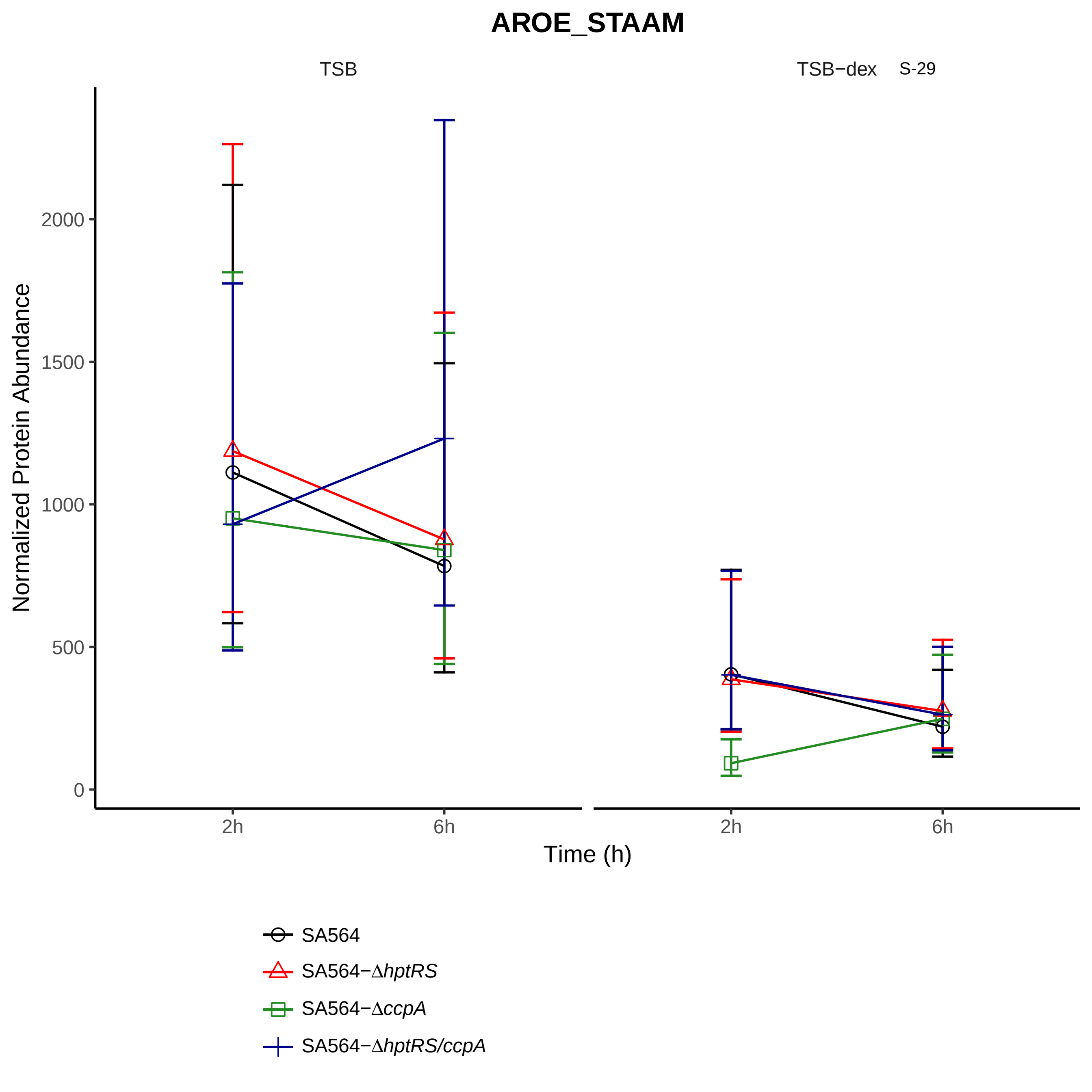
AROE_STAAM
TSB
TSB−dex
S-29
Normalized Protein Abundance
0
500
1000
1500
2000
2h
6h
2h
6h
Time (h)
SA564
SA564−ΔhptRS
SA564−ΔccpA
SA564−ΔhptRS/ccpA

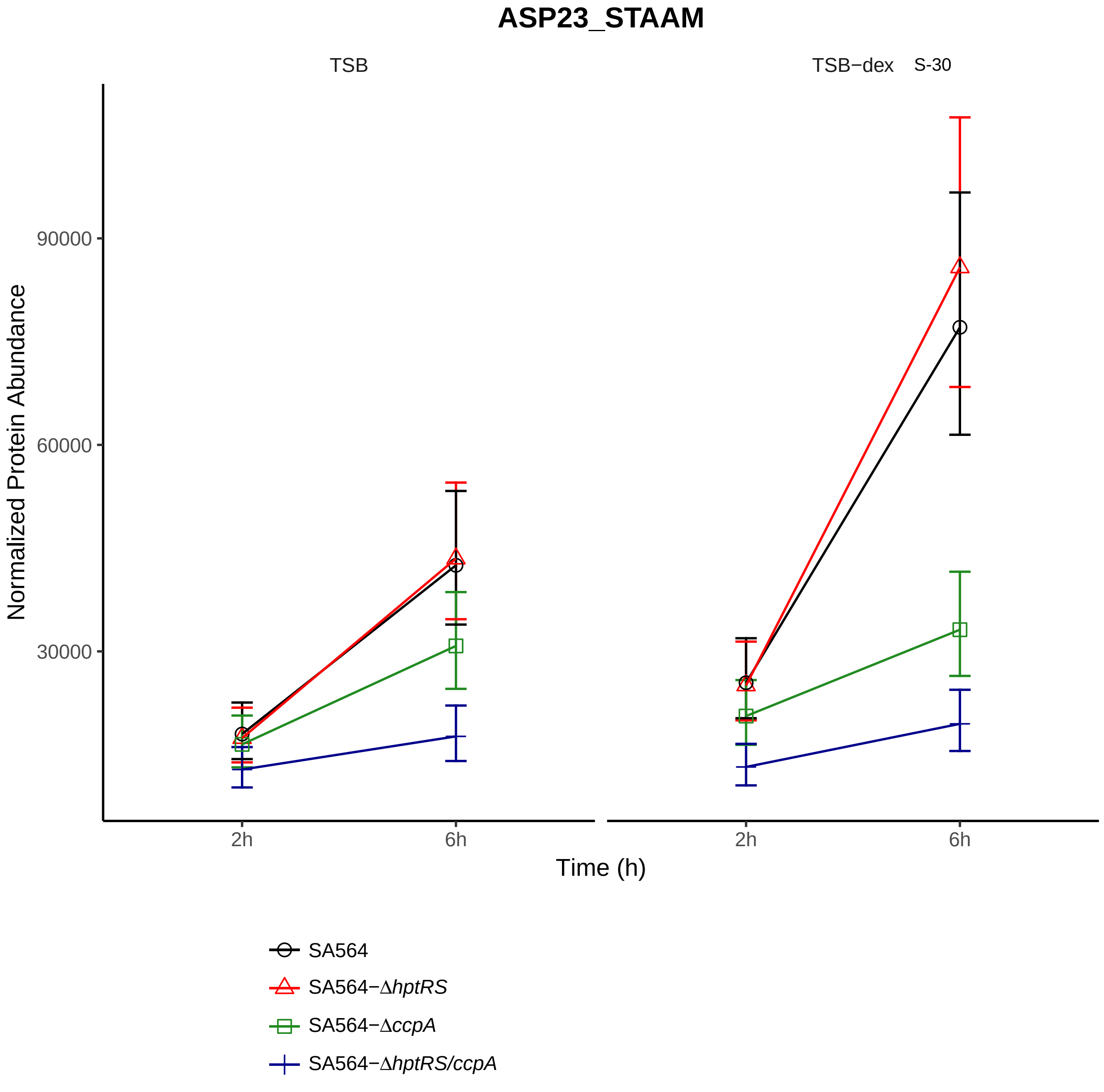

ASP23_STAAM
TSB
TSB−dex
S-30
Normalized Protein Abundance
90000
60000
30000
2h
6h
2h
6h
Time (h)
SA564
SA564−Δ*hptRS*
SA564−Δ*ccpA*
SA564−Δ*hptRS/ccpA*

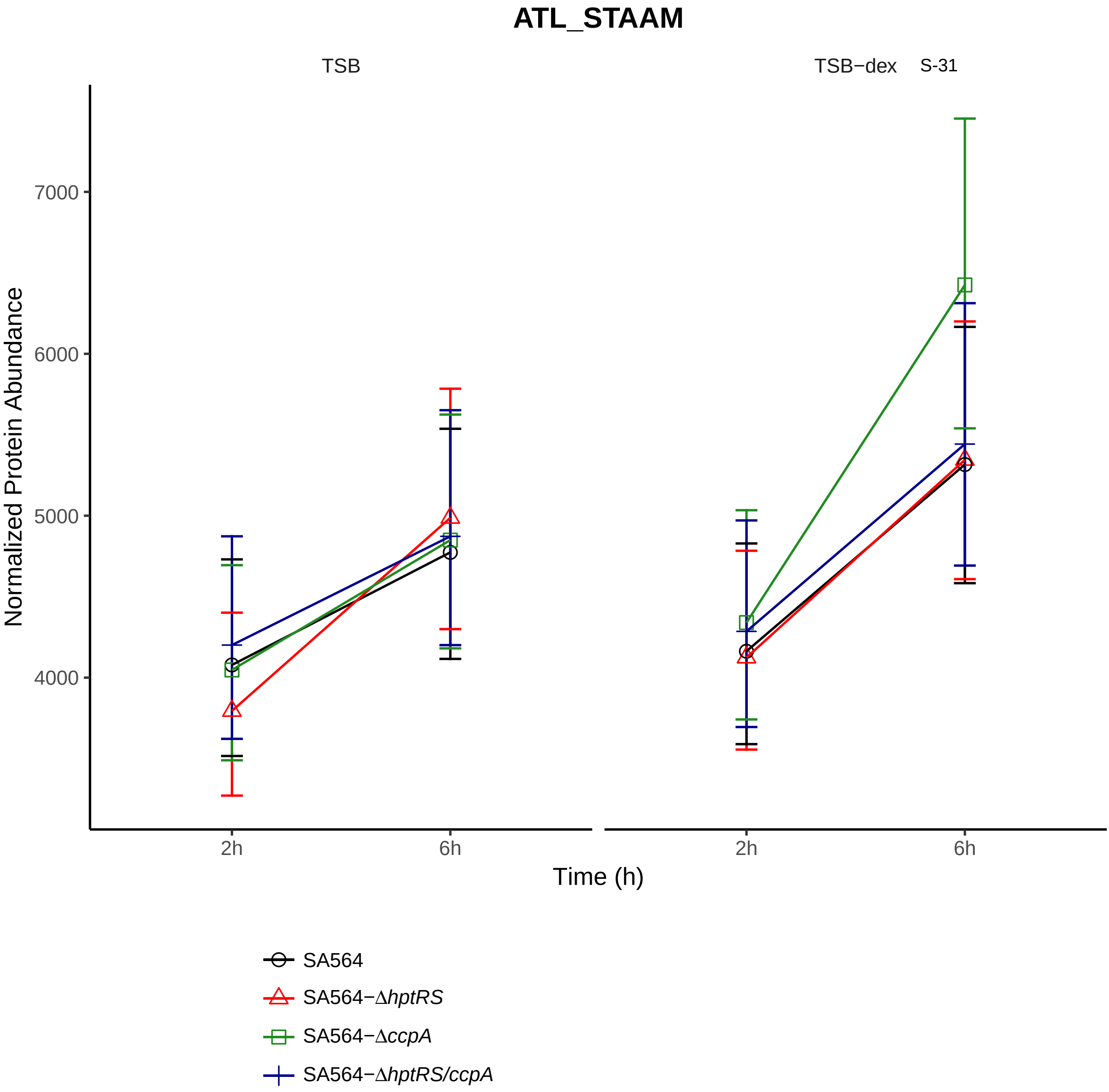

ATL_STAAM
TSB
TSB−dex
7000
6000
5000
4000
Normalized Protein Abundance
2h
6h
2h
6h
Time (h)
SA564
SA564−ΔhptRS
SA564−ΔccpA
SA564−ΔhptRS/ccpA

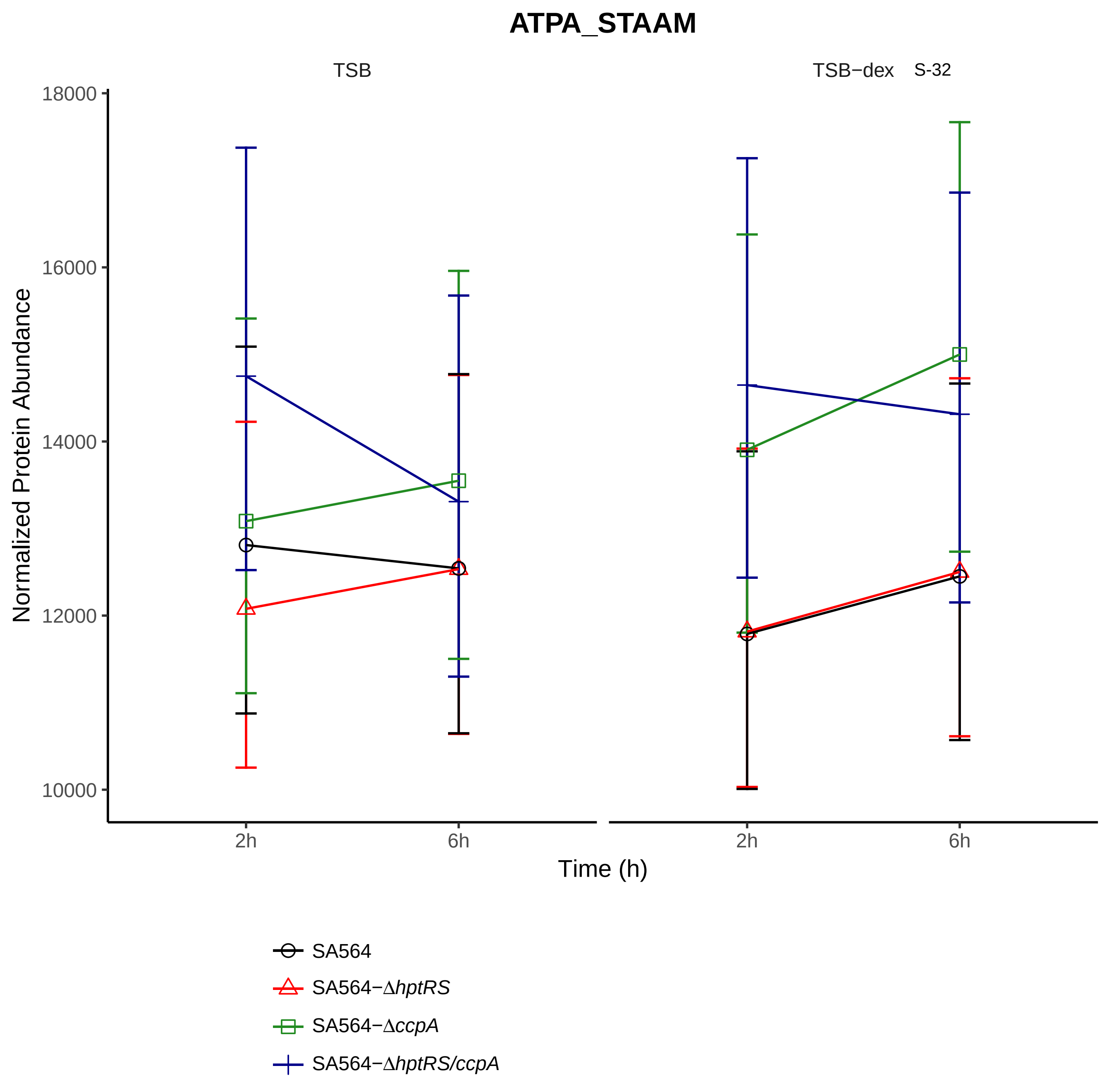
ATPA_STAAM
TSB
TSB−dex
S-32
Normalized Protein Abundance
18000
16000
14000
12000
10000
2h
6h
2h
6h
Time (h)
SA564
SA564−ΔhptRS
SA564−ΔccpA
SA564−ΔhptRS/ccpA

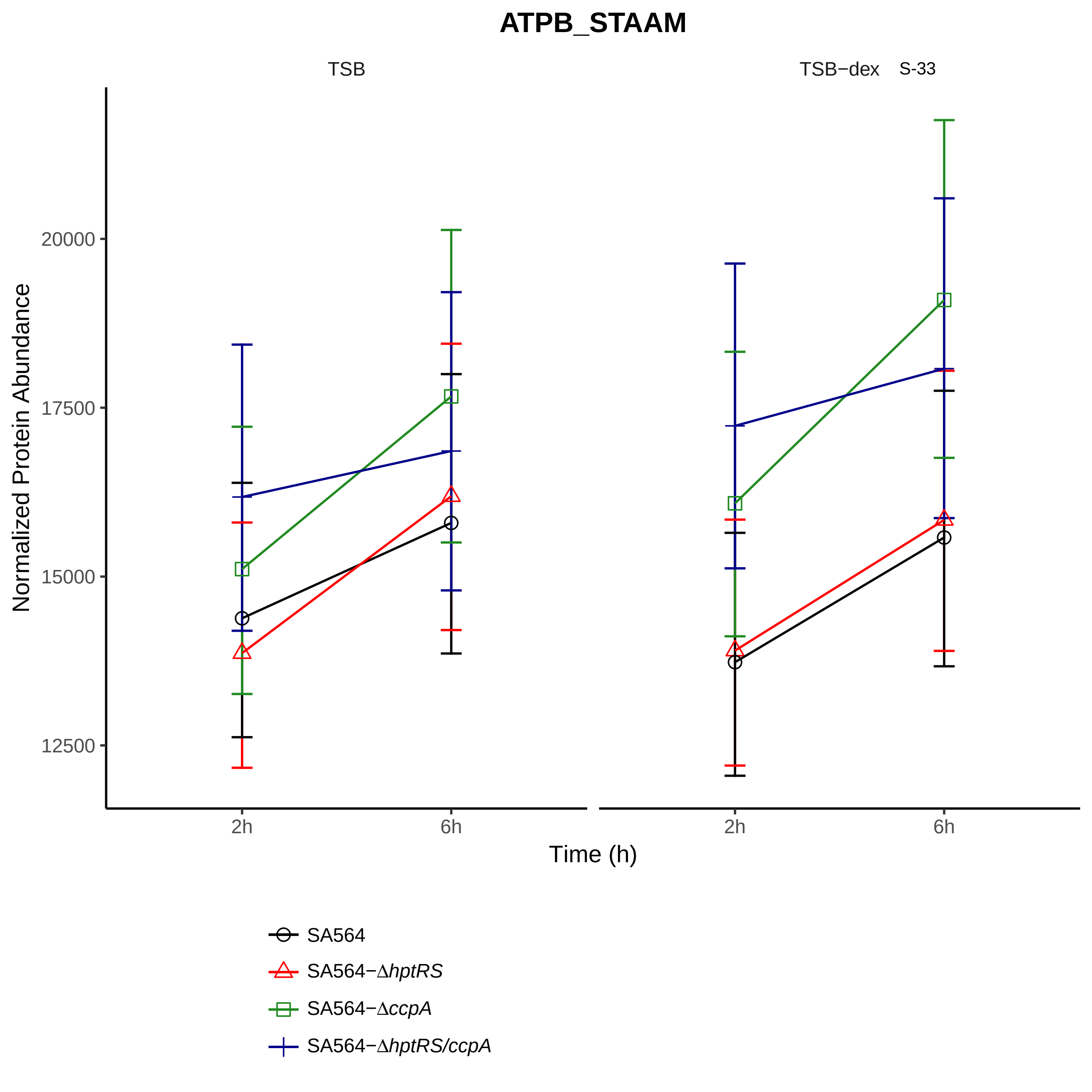
ATPB_STAAM
TSB
TSB−dex
S-33
Normalized Protein Abundance
20000
17500
15000
12500
2h
6h
2h
6h
Time (h)
SA564
SA564−ΔhptRS
SA564−ΔccpA
SA564−ΔhptRS/ccpA

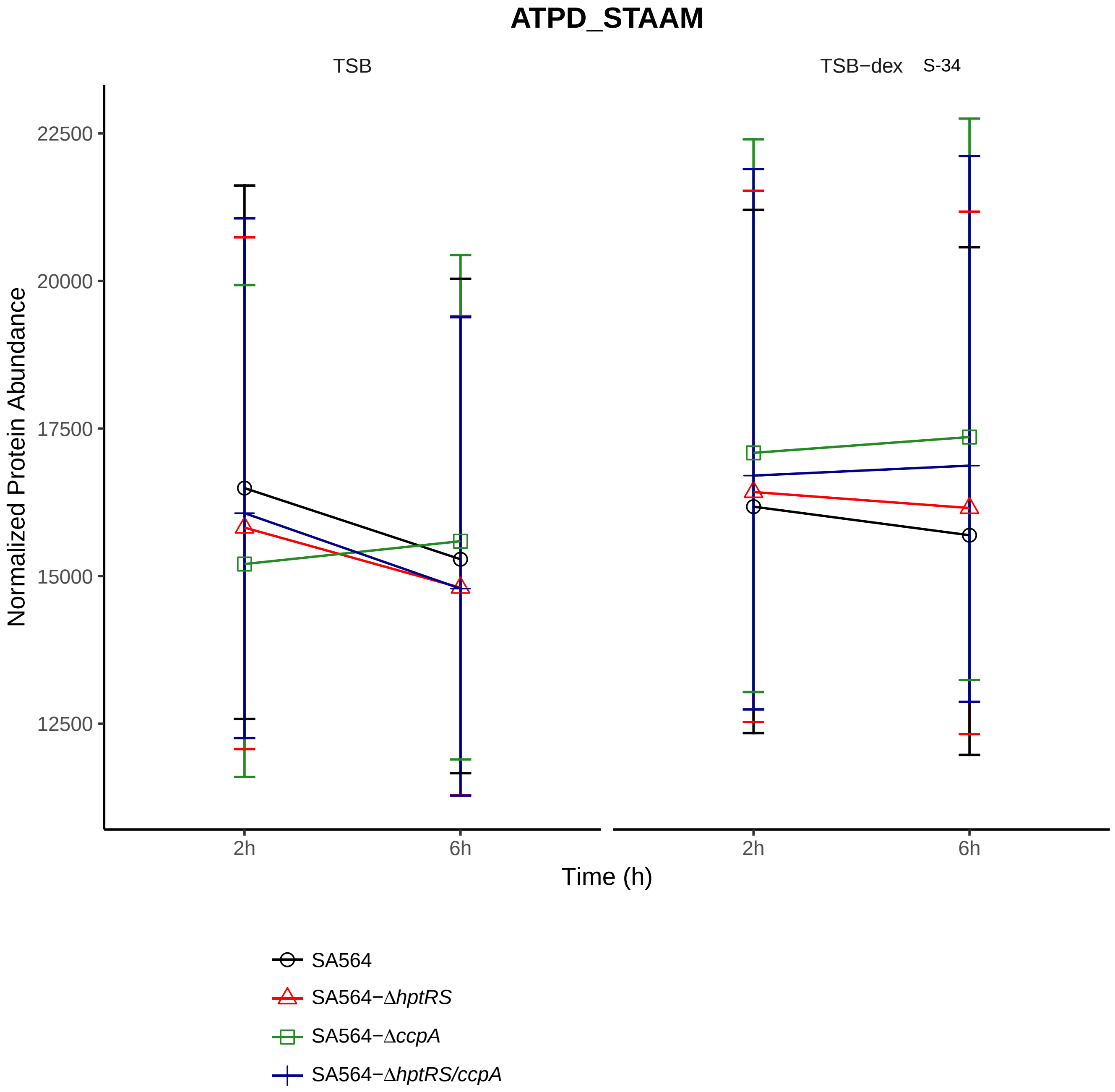
ATPD_STAAM
TSB
TSB−dex
S-34
Normalized Protein Abundance
22500
20000
17500
15000
12500
2h
6h
2h
6h
Time (h)
SA564
SA564−ΔhptRS
SA564−ΔccpA
SA564−ΔhptRS/ccpA

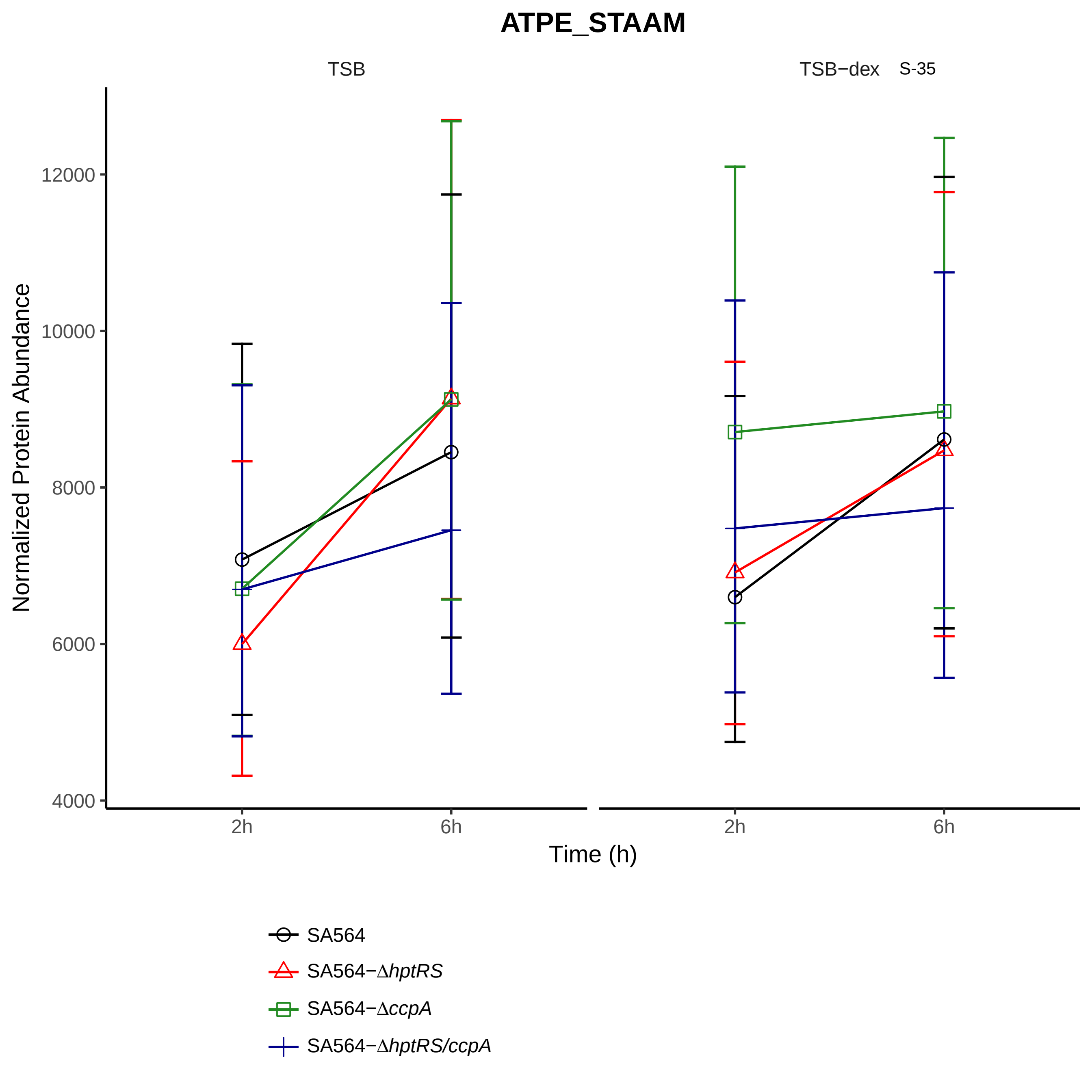

ATPE_STAAM
TSB
TSB−dex
S-35
Normalized Protein Abundance
12000
10000
8000
6000
4000
2h
6h
2h
6h
Time (h)
SA564
SA564−Δ*hptRS*
SA564−Δ*ccpA*
SA564−Δ*hptRS/ccpA*

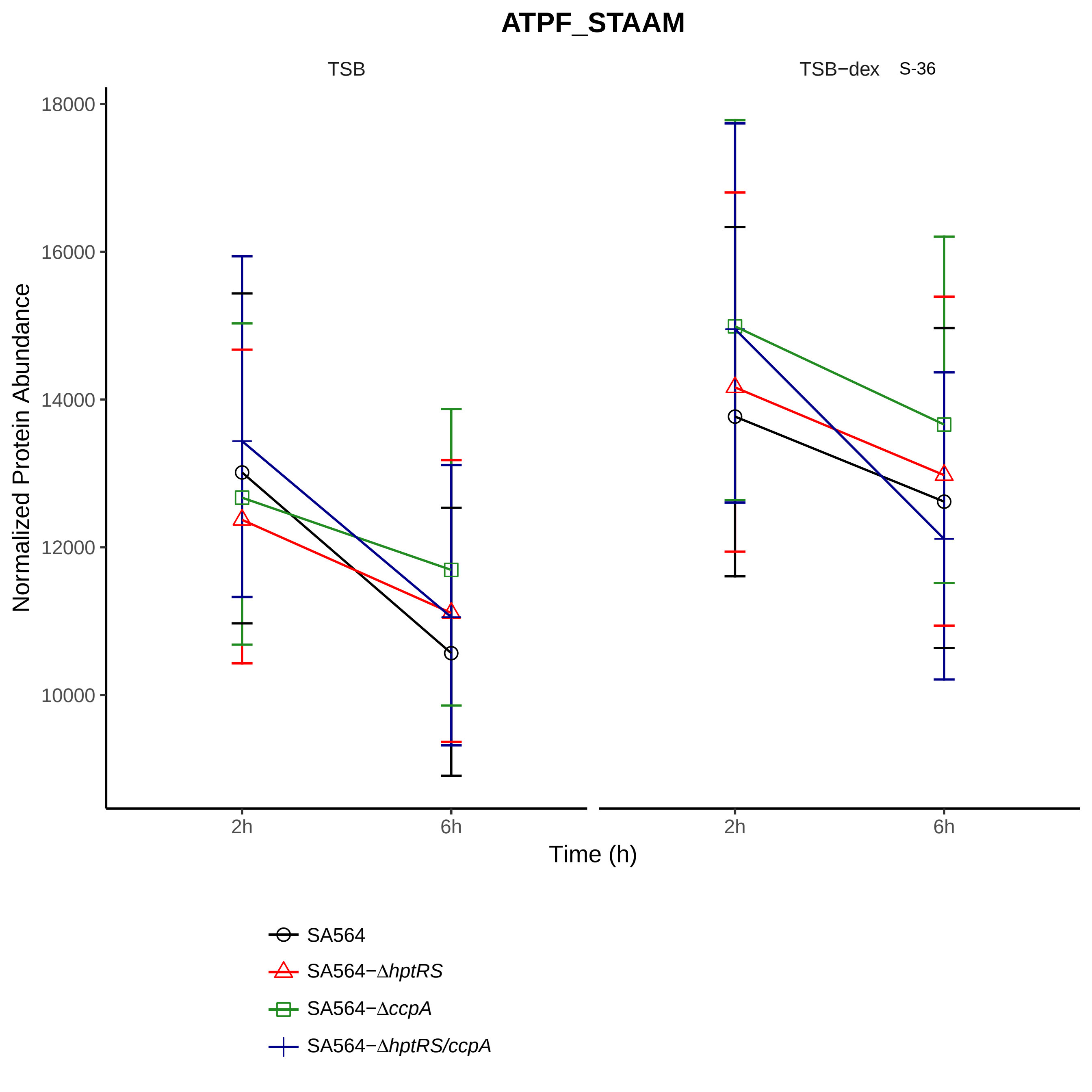

ATPF_STAAM
TSB
TSB−dex
S-36
Normalized Protein Abundance
18000
16000
14000
12000
10000
2h
6h
2h
6h
Time (h)
SA564
SA564−Δ*hptRS*
SA564−Δ*ccpA*
SA564−Δ*hptRS/ccpA*

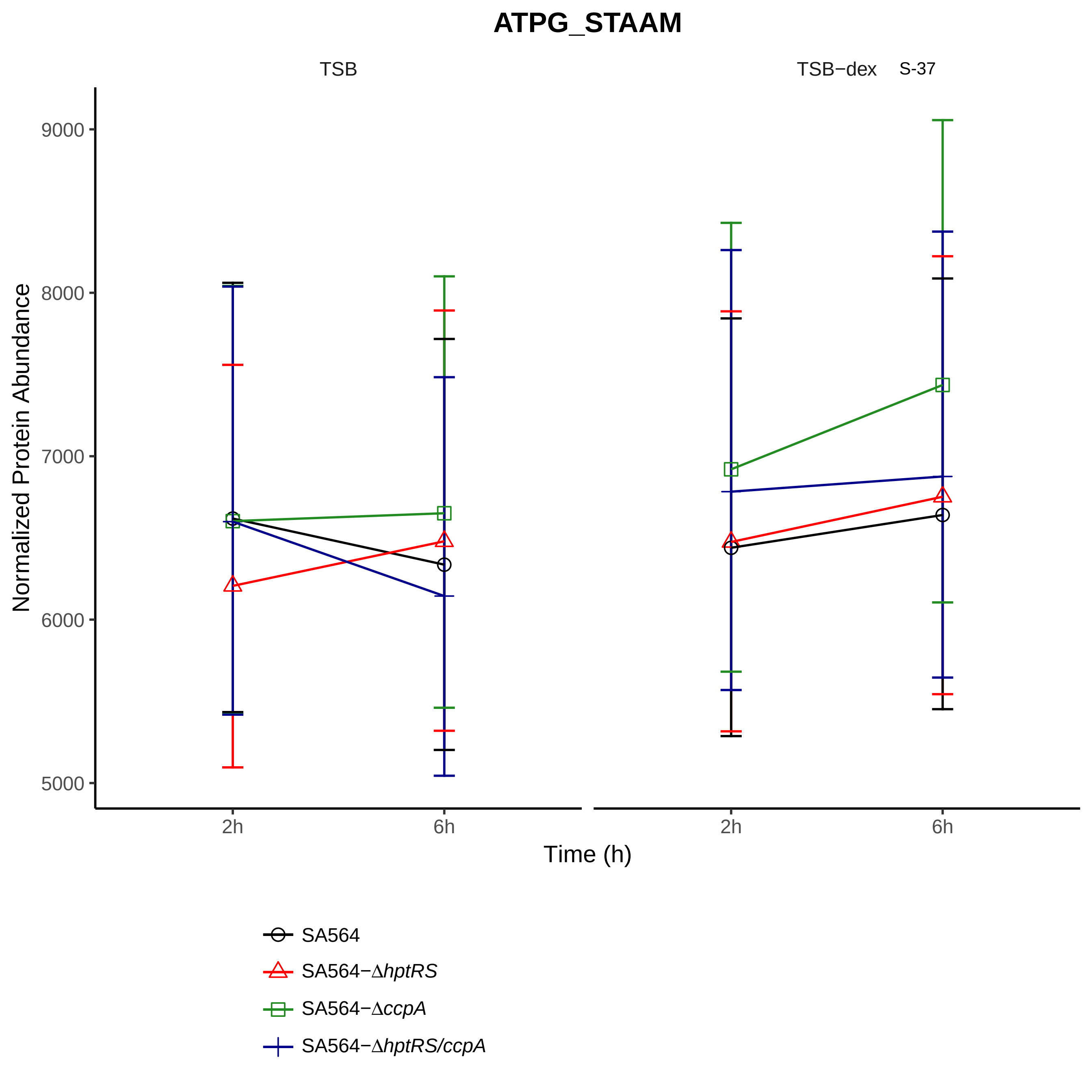

ATPG_STAAM
TSB
TSB−dex
S-37
Normalized Protein Abundance
9000
8000
7000
6000
5000
2h
6h
2h
6h
Time (h)
SA564
SA564−Δ*hptRS*
SA564−Δ*ccpA*
SA564−Δ*hptRS/ccpA*

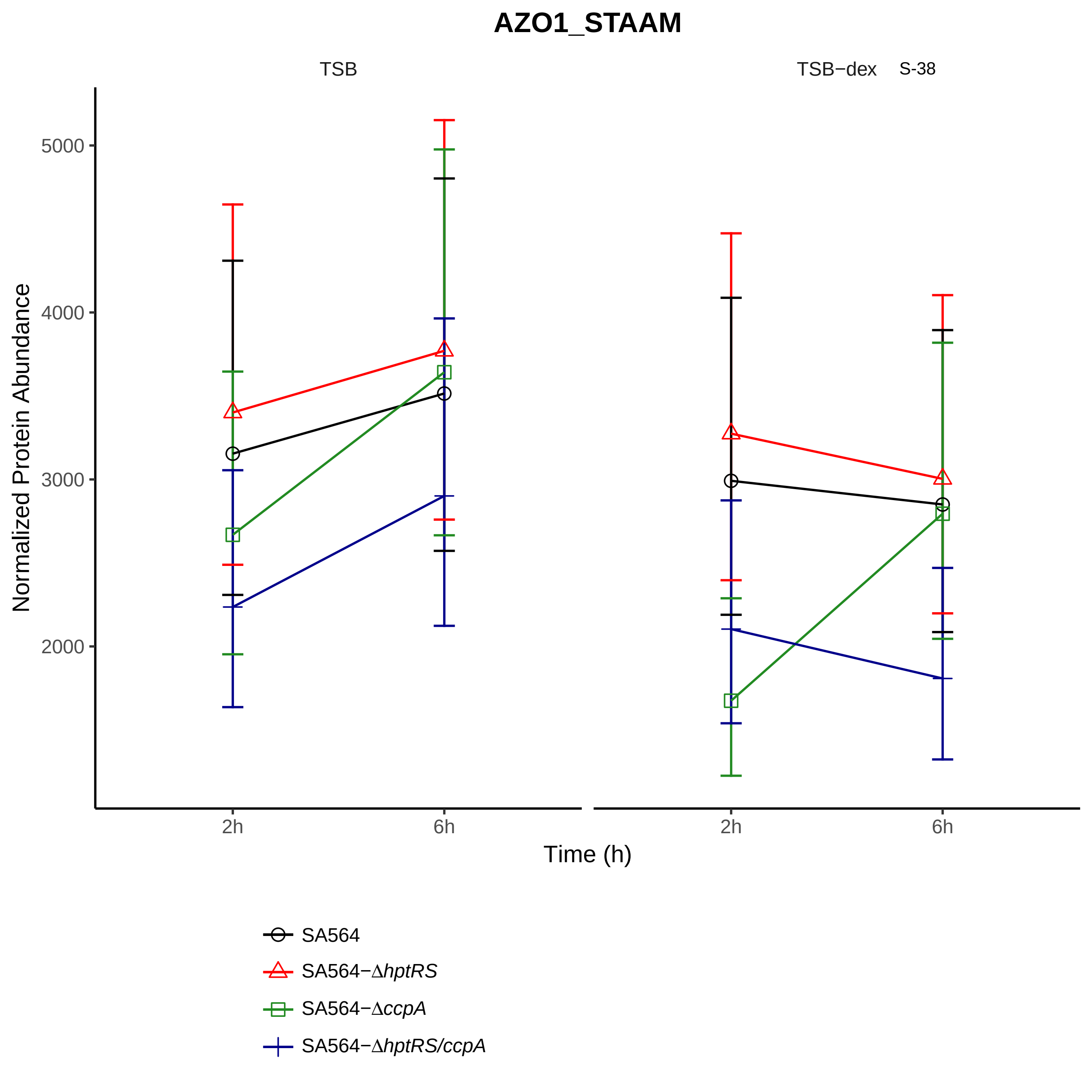

AZO1_STAAM
TSB
TSB−dex
S-38
Normalized Protein Abundance
5000
4000
3000
2000
2h
6h
2h
6h
Time (h)
SA564
SA564−ΔhptRS
SA564−ΔccpA
SA564−ΔhptRS/ccpA

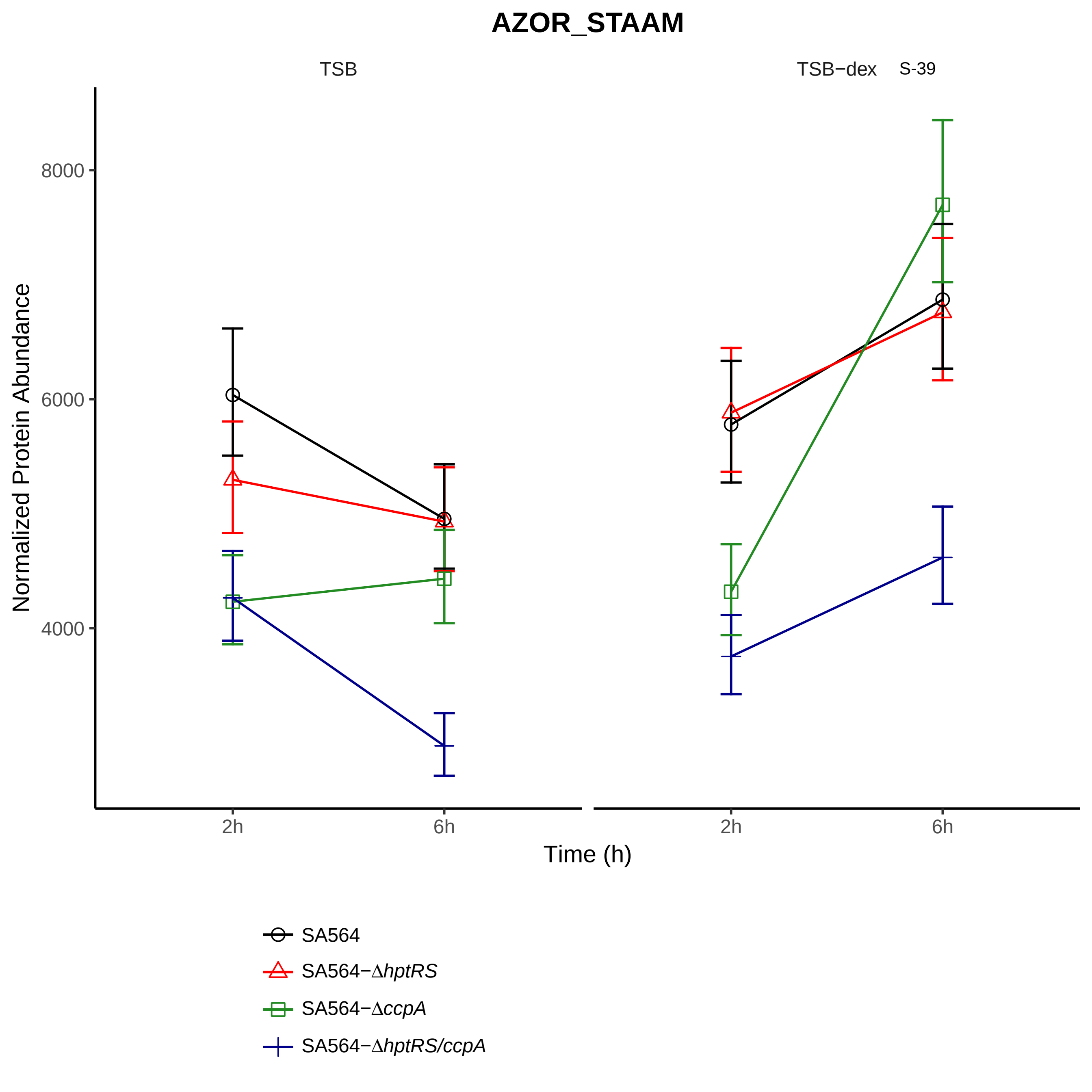
AZOR_STAAM
TSB
TSB−dex
S-39
8000
6000
4000
2h
6h
2h
6h
Time (h)
Normalized Protein Abundance
SA564
SA564−Δ*hptRS*
SA564−Δ*ccpA*
SA564−Δ*hptRS/ccpA*

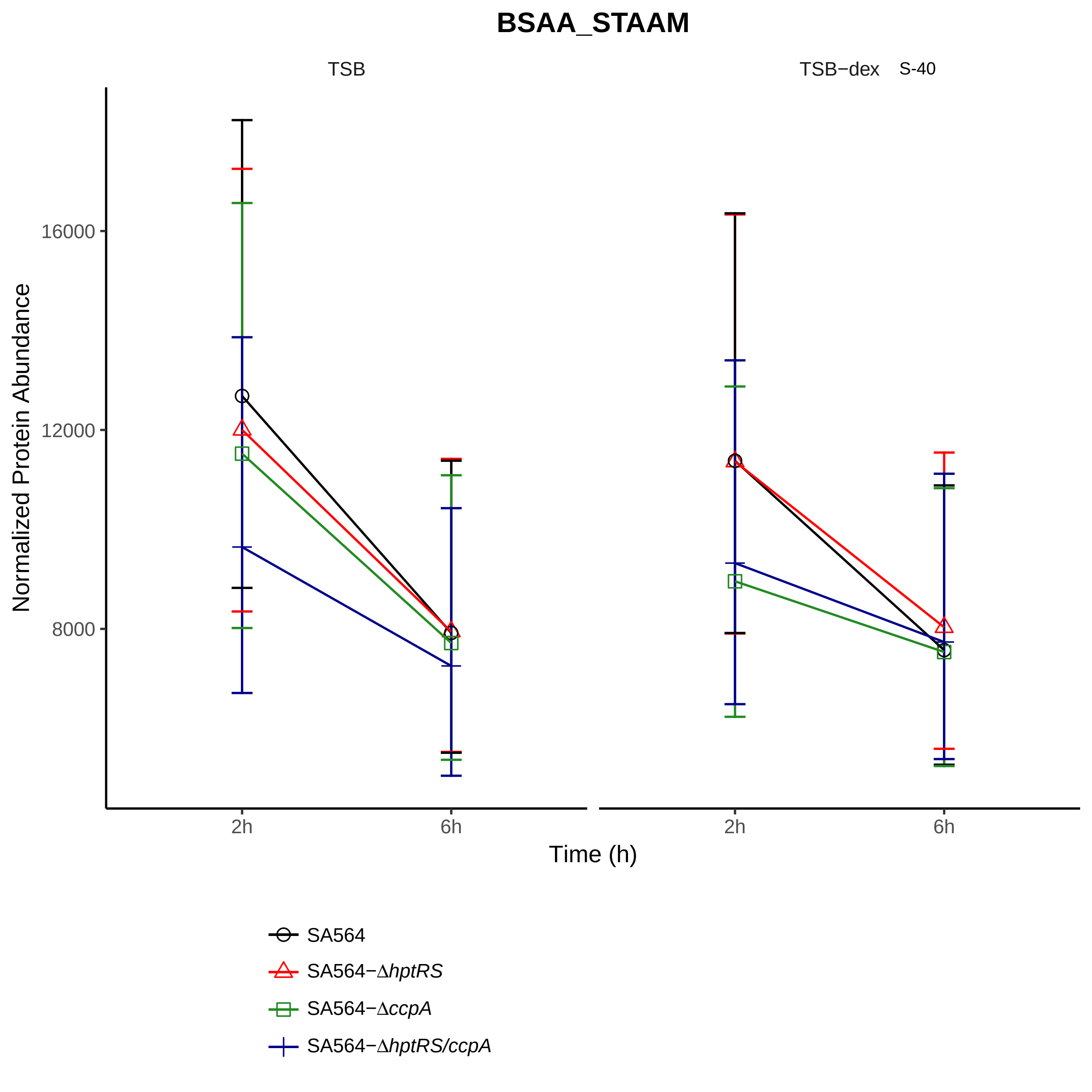

BSAA_STAAM
TSB
TSB−dex
S-40
Normalized Protein Abundance
16000
12000
8000
2h
6h
2h
6h
Time (h)
SA564
SA564−Δ*hptRS*
SA564−Δ*ccpA*
SA564−Δ*hptRS/ccpA*

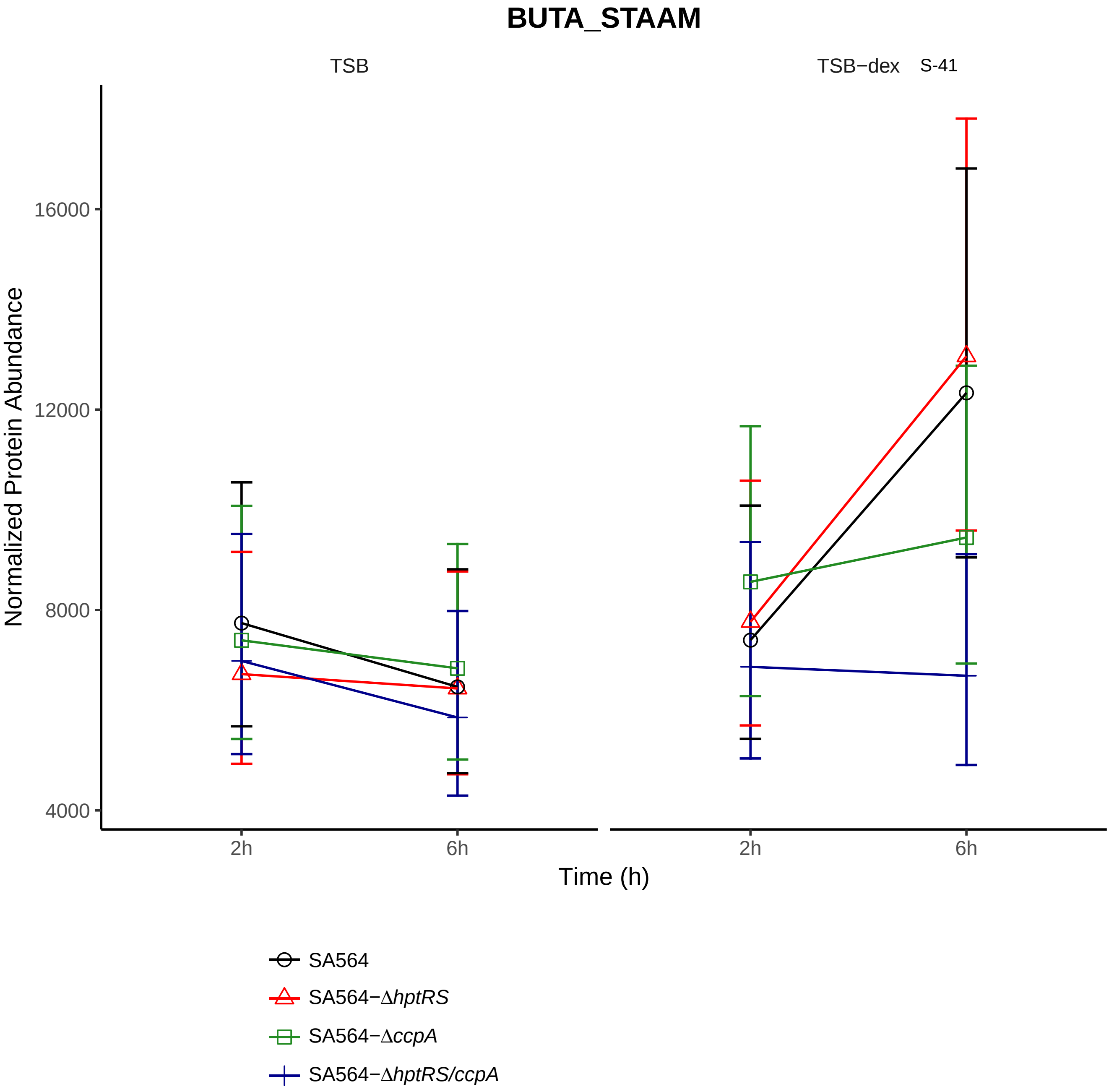

BUTA_STAAM
TSB
TSB−dex
S-41
Normalized Protein Abundance
16000
12000
8000
4000
2h
6h
2h
6h
Time (h)
SA564
SA564−Δ*hptRS*
SA564−Δ*ccpA*
SA564−Δ*hptRS/ccpA*

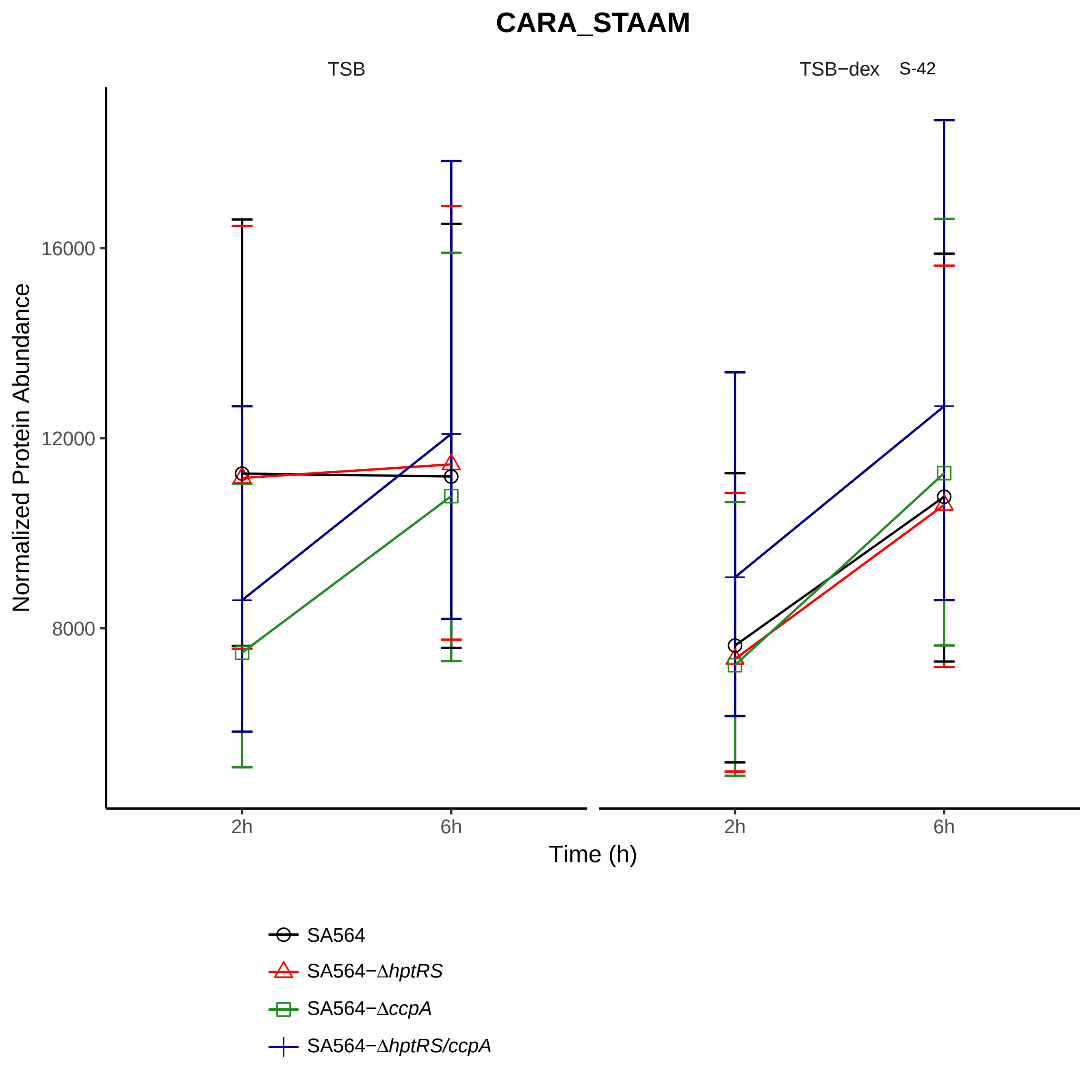

CARA_STAAM
TSB
TSB−dex
S-42
Normalized Protein Abundance
16000
12000
8000
2h
6h
2h
6h
Time (h)
SA564
SA564−ΔhptRS
SA564−ΔccpA
SA564−ΔhptRS/ccpA

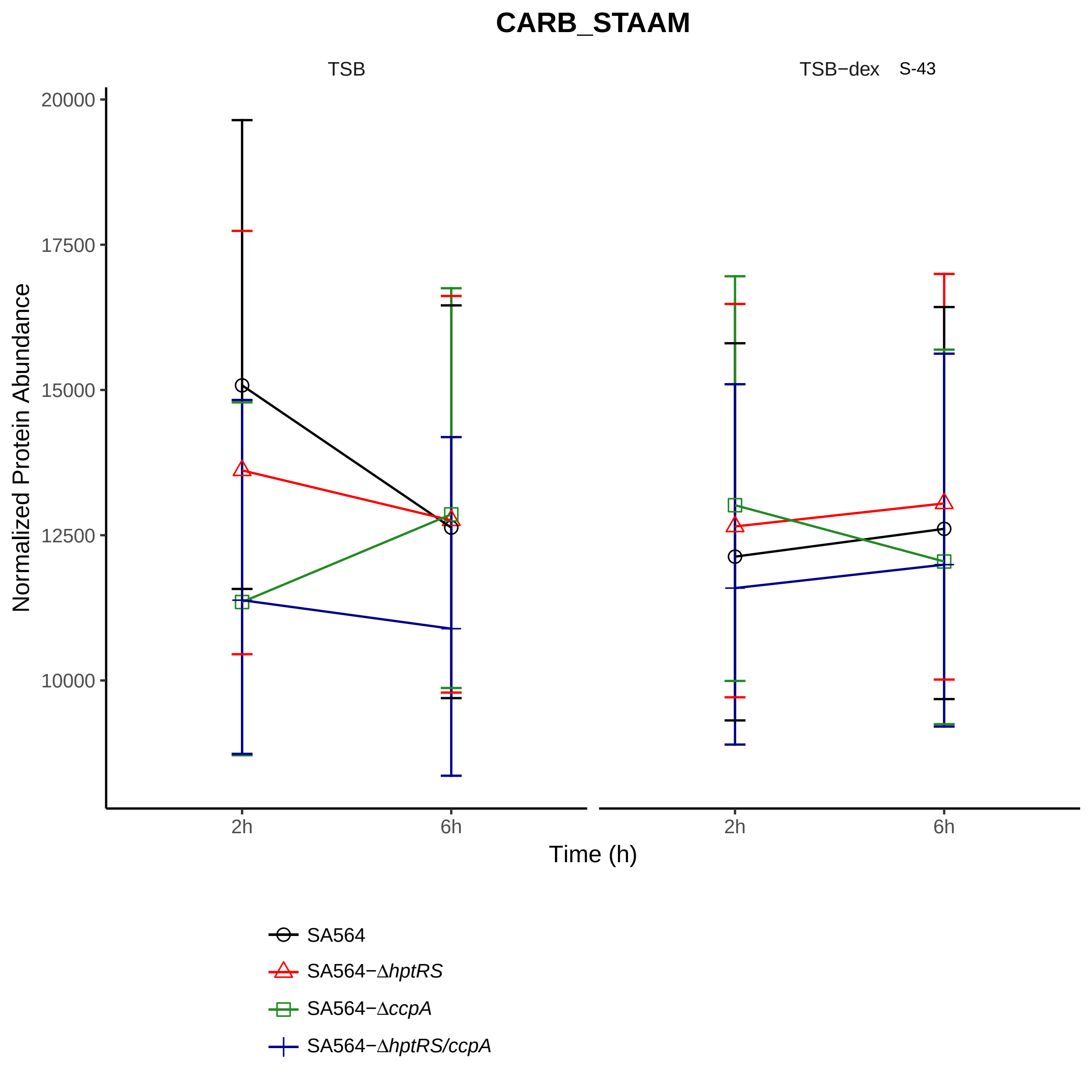

CARB_STAAM
TSB
TSB−dex
S-43
20000
17500
15000
12500
10000
Normalized Protein Abundance
2h
6h
2h
6h
Time (h)
SA564
SA564−Δ*hptRS*
SA564−Δ*ccpA*
SA564−Δ*hptRS/ccpA*

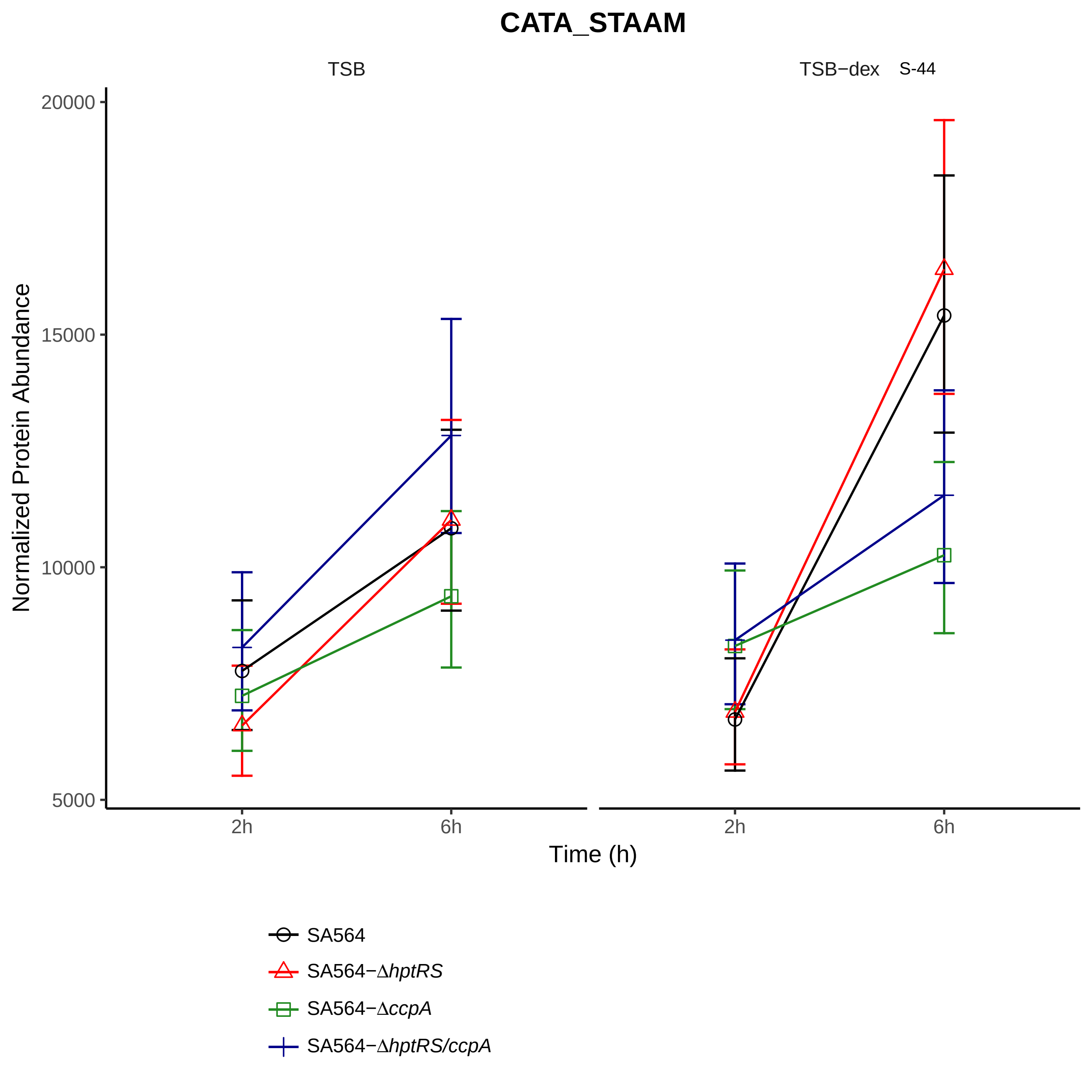

CATA_STAAM
TSB
TSB−dex
S-44
Normalized Protein Abundance
20000
15000
10000
5000
2h
6h
2h
6h
Time (h)
SA564
SA564−ΔhptRS
SA564−ΔccpA
SA564−ΔhptRS/ccpA

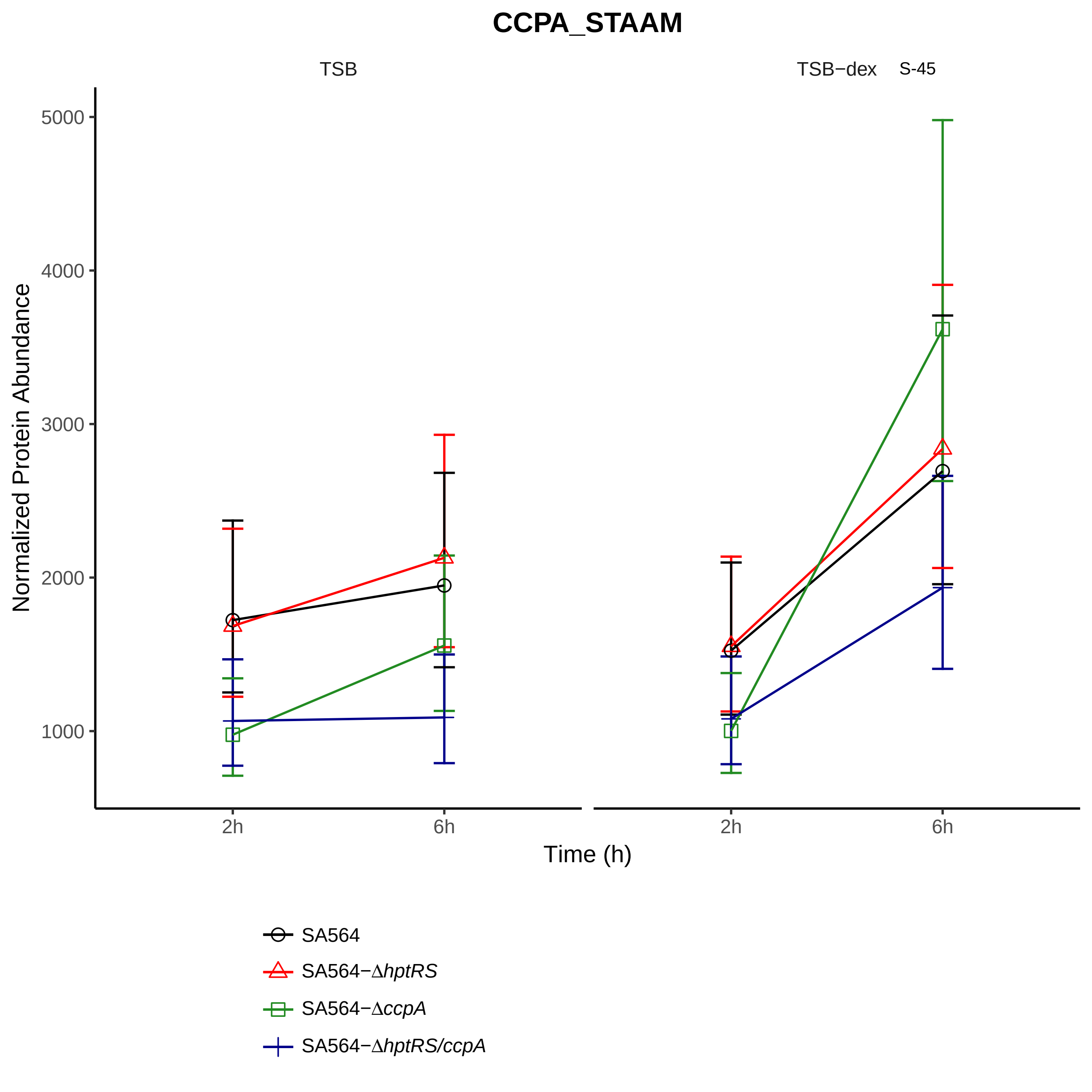

CCPA_STAAM
TSB
TSB−dex
S-45
Normalized Protein Abundance
5000
4000
3000
2000
1000
2h
6h
2h
6h
Time (h)
SA564
SA564−ΔhptRS
SA564−ΔccpA
SA564−ΔhptRS/ccpA

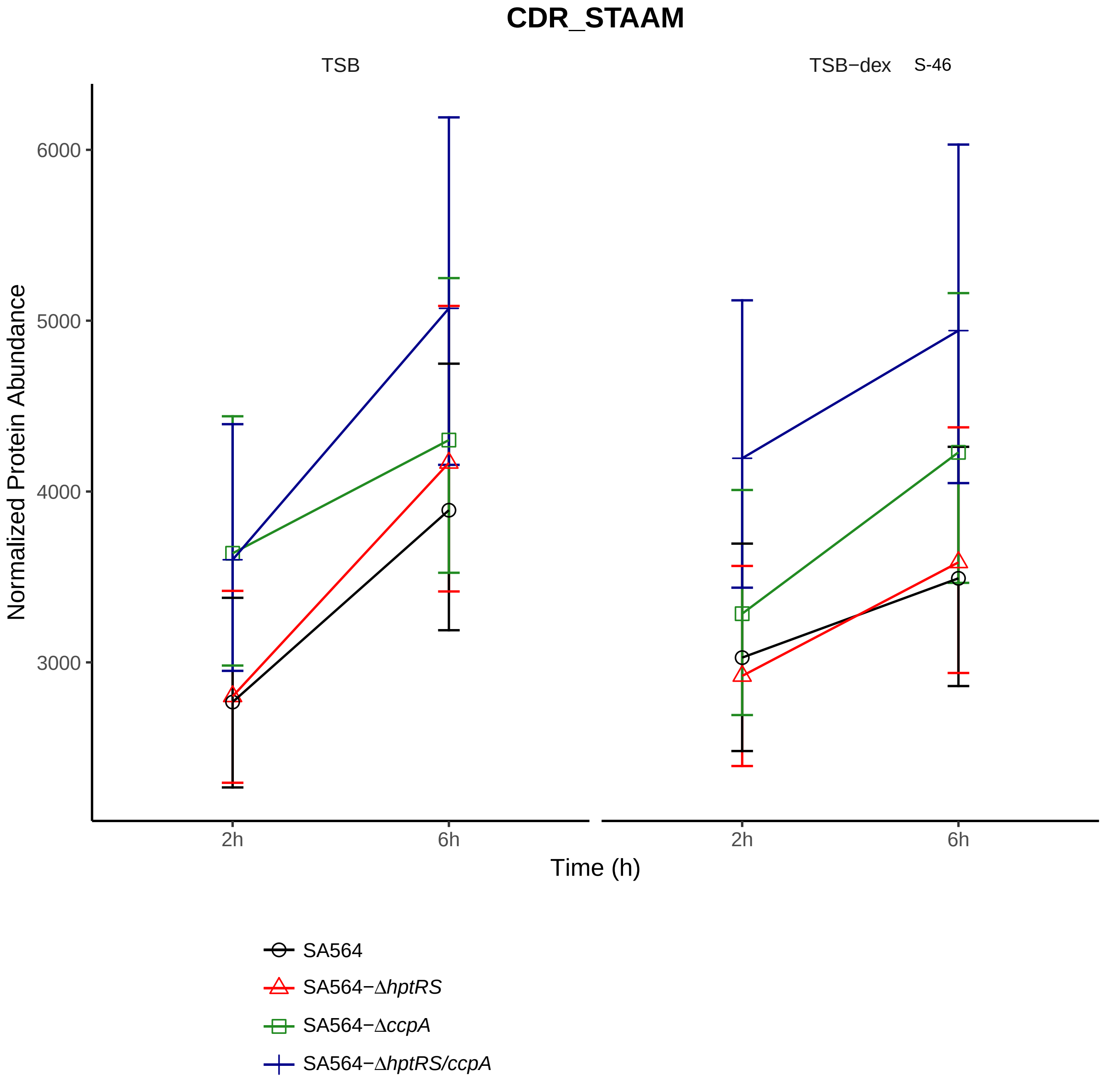

CDR_STAAM
TSB
TSB−dex
S-46
Normalized Protein Abundance
6000
5000
4000
3000
2h
6h
2h
6h
Time (h)
SA564
SA564−Δ*hptRS*
SA564−Δ*ccpA*
SA564−Δ*hptRS/ccpA*

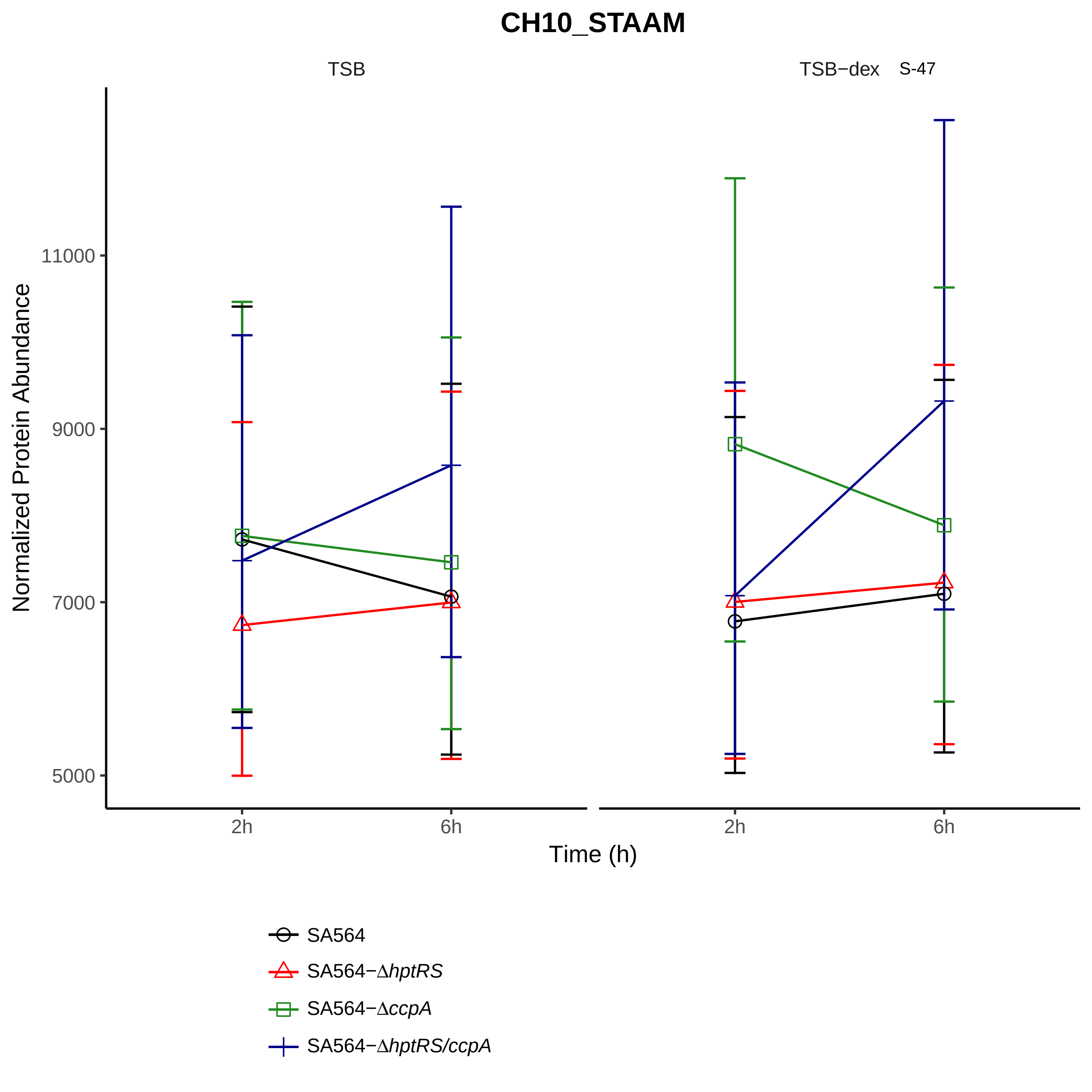
CH10_STAAM
TSB
TSB−dex
S-47
Normalized Protein Abundance
11000
9000
7000
5000
2h
6h
2h
6h
Time (h)
SA564
SA564−ΔhptRS
SA564−ΔccpA
SA564−ΔhptRS/ccpA

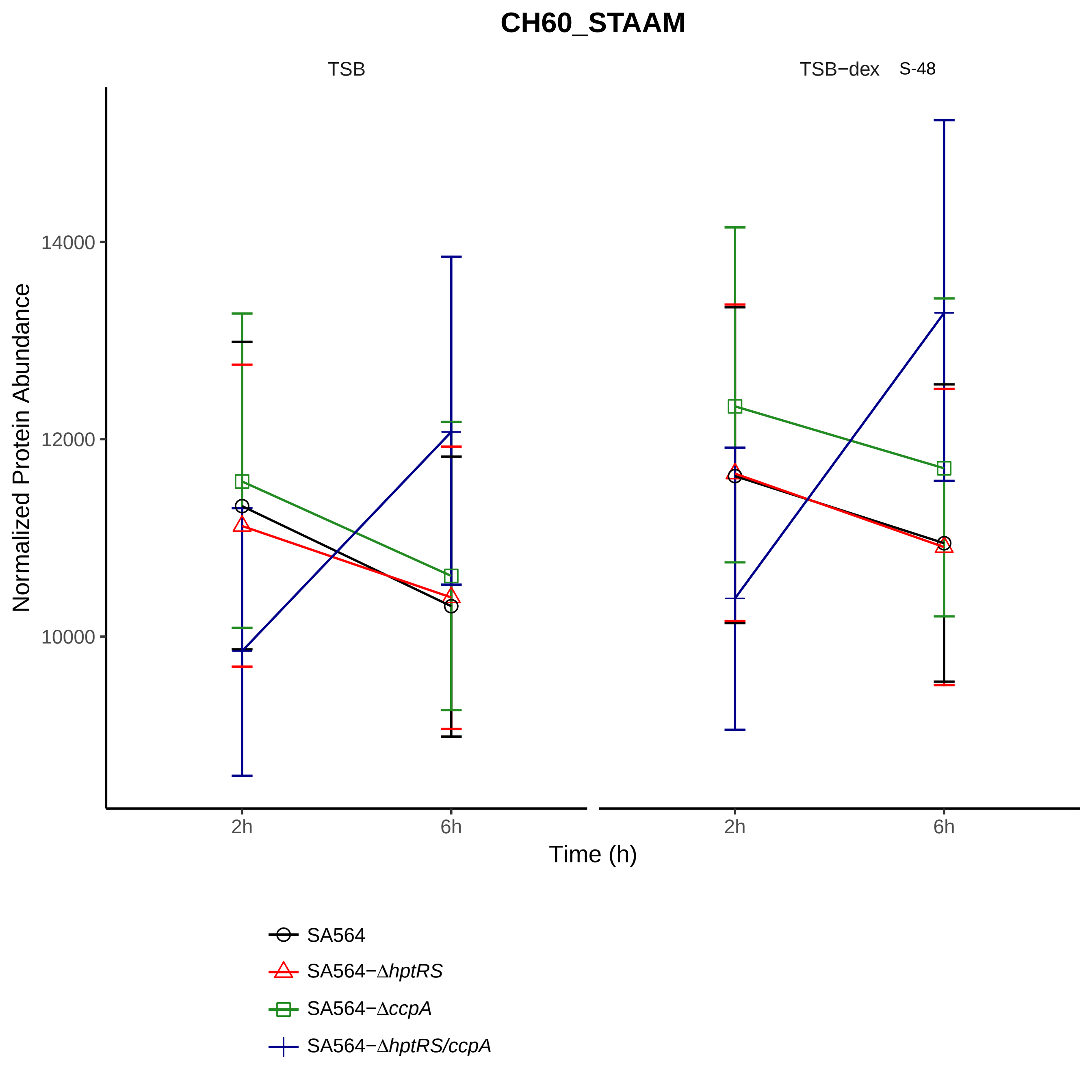

CH60_STAAM
TSB
TSB−dex
S-48
14000
12000
10000
2h
6h
2h
6h
Normalized Protein Abundance
Time (h)
SA564
SA564−Δ*hptRS*
SA564−Δ*ccpA*
SA564−Δ*hptRS/ccpA*

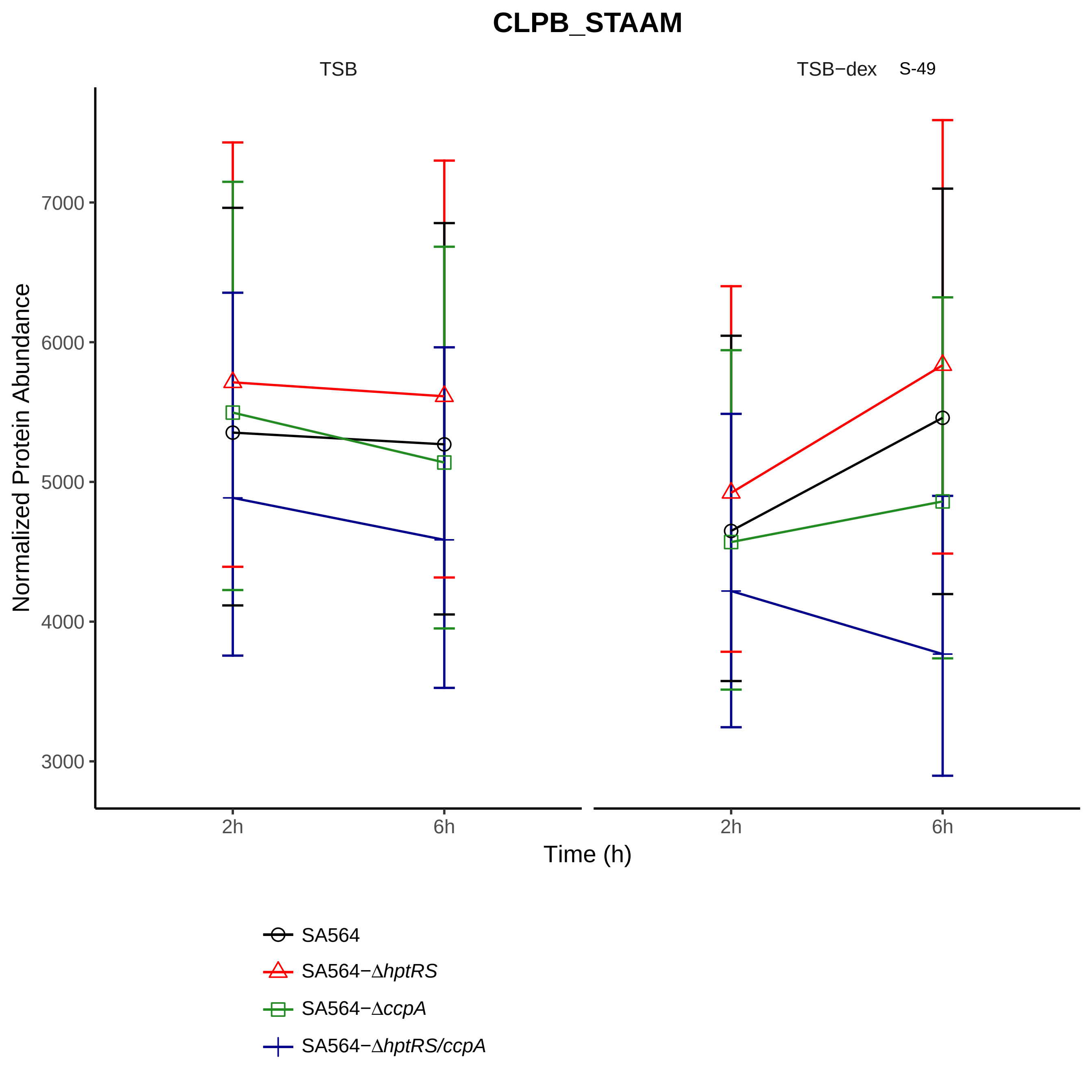
CLPB_STAAM
TSB
TSB−dex
S-49
Normalized Protein Abundance
3000
4000
5000
6000
7000
2h
6h
2h
6h
Time (h)
SA564
SA564−Δ*hptRS*
SA564−Δ*ccpA*
SA564−Δ*hptRS/ccpA*

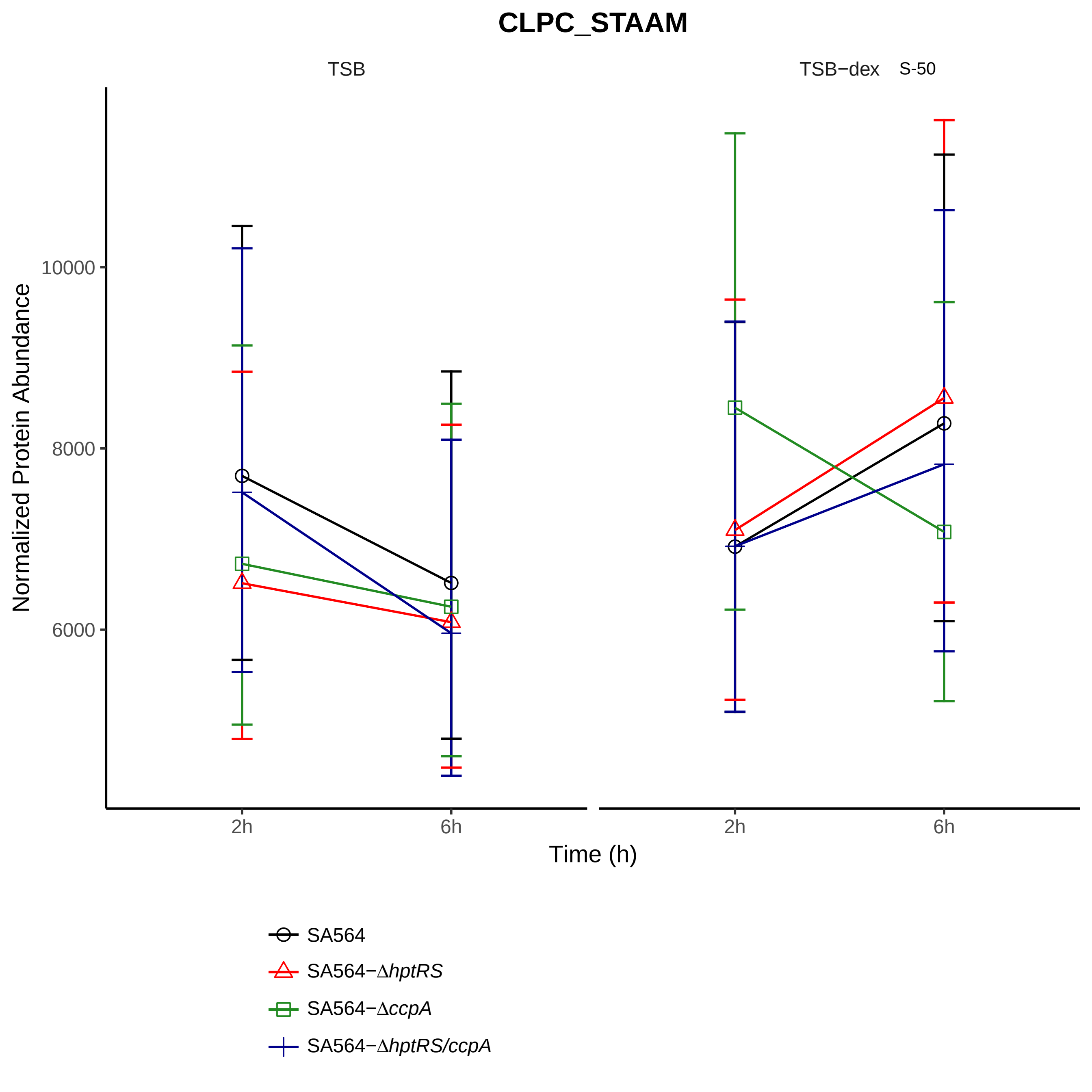

CLPC_STAAM
TSB
TSB−dex
S-50
Normalized Protein Abundance
10000
8000
6000
2h
6h
2h
6h
Time (h)
SA564
SA564−ΔhptRS
SA564−ΔccpA
SA564−ΔhptRS/ccpA

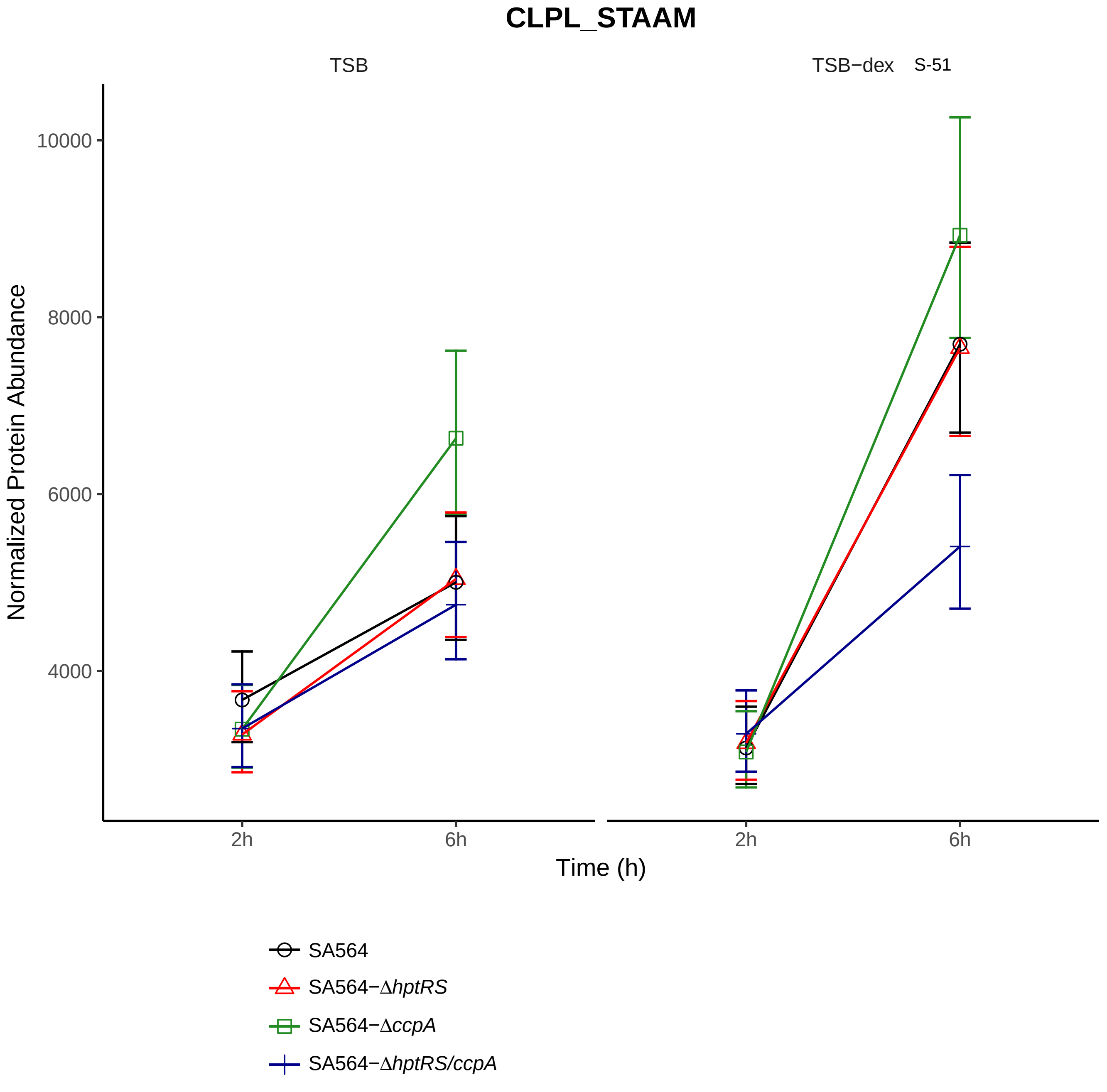

CLPL_STAAM
TSB
TSB−dex
S-51
Normalized Protein Abundance
10000
8000
6000
4000
2h
6h
2h
6h
Time (h)
SA564
SA564−ΔhptRS
SA564−ΔccpA
SA564−ΔhptRS/ccpA

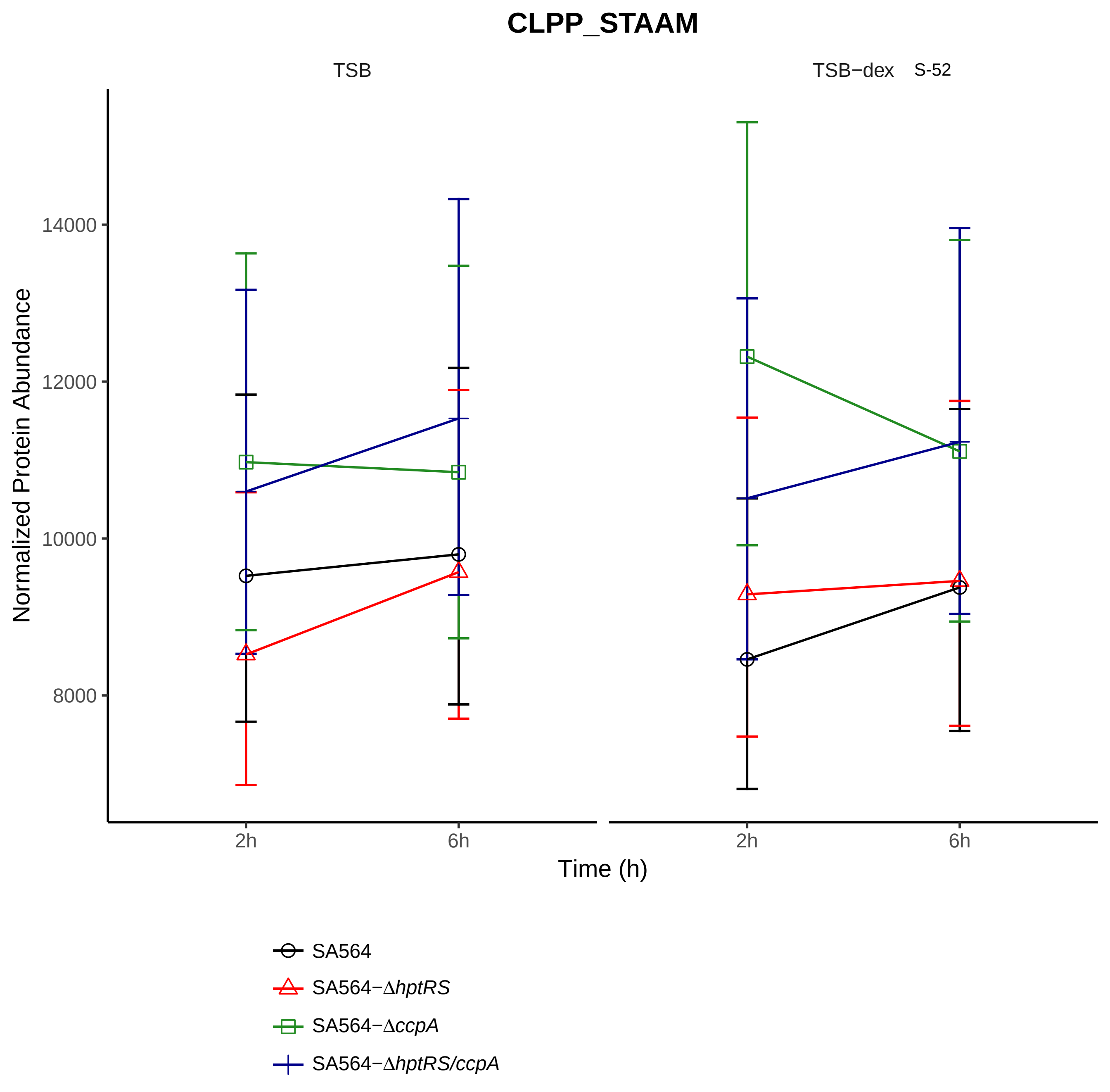

CLPP_STAAM
TSB
TSB−dex
S-52
Normalized Protein Abundance
14000
12000
10000
8000
2h
6h
2h
6h
Time (h)
SA564
SA564−ΔhptRS
SA564−ΔccpA
SA564−ΔhptRS/ccpA

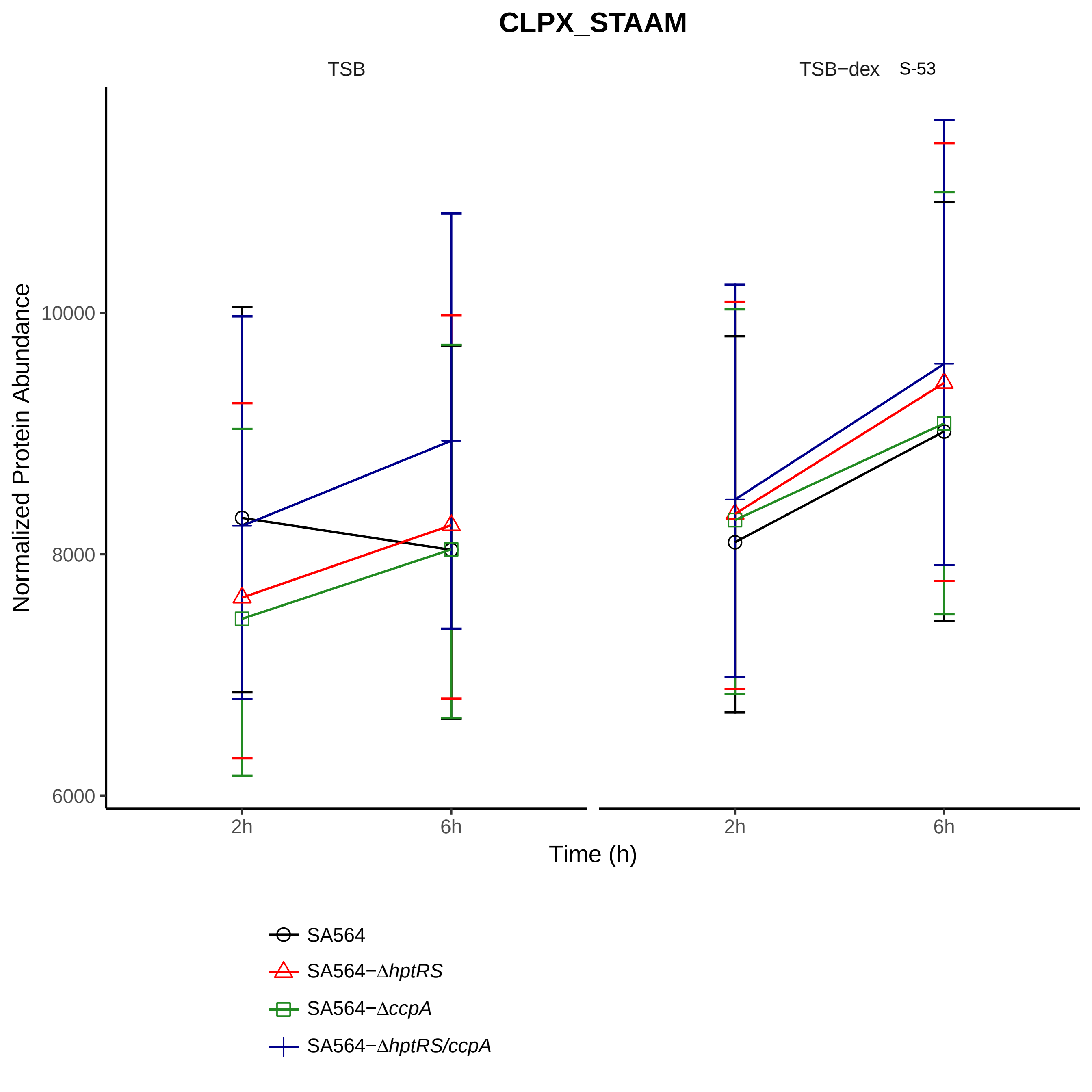
CLPX_STAAM
TSB
TSB−dex
S-53
Normalized Protein Abundance
10000
8000
6000
2h
6h
2h
6h
Time (h)
SA564
SA564−Δ*hptRS*
SA564−Δ*ccpA*
SA564−Δ*hptRS/ccpA*

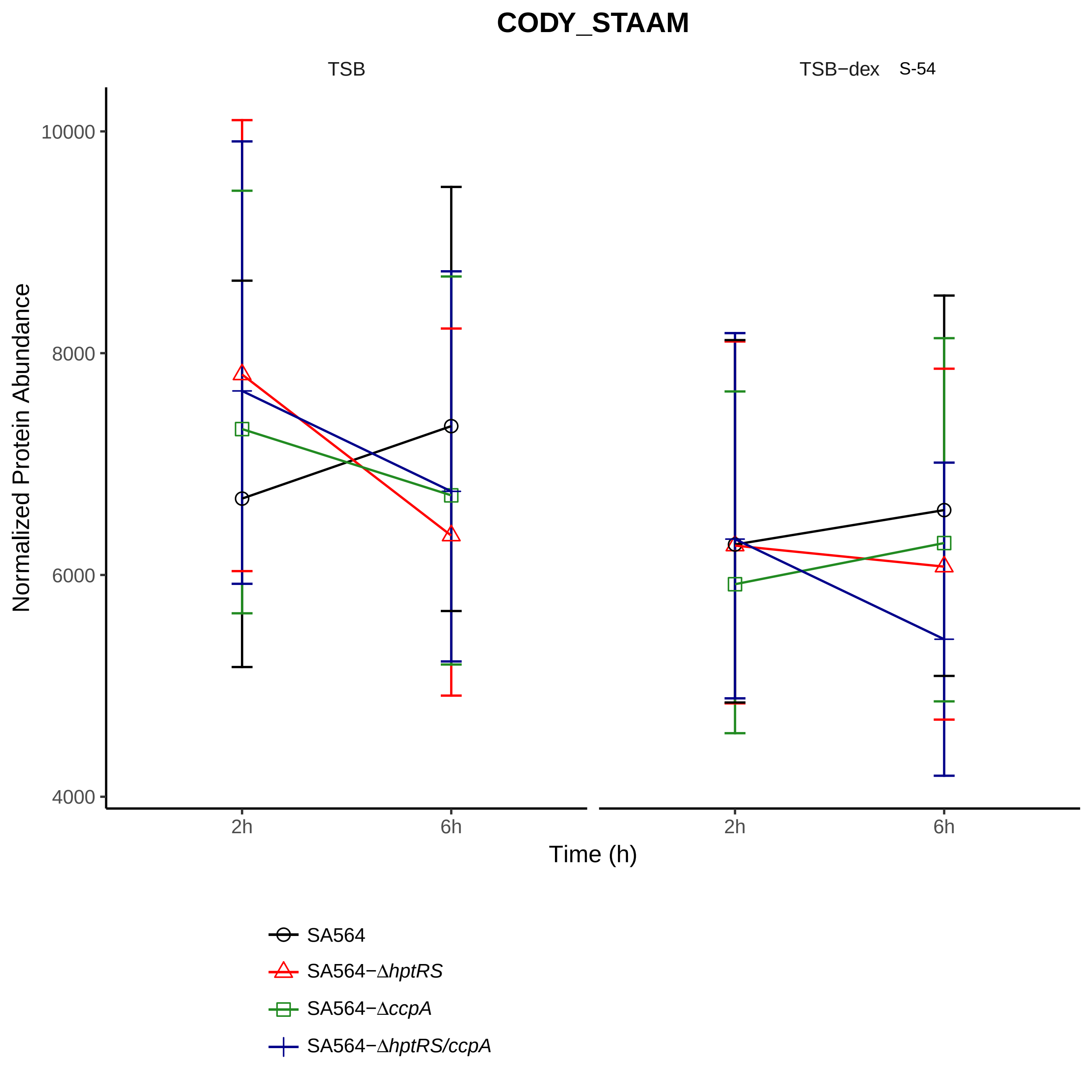

CODY_STAAM
TSB
TSB−dex
S-54
Normalized Protein Abundance
10000
8000
6000
4000
2h
6h
2h
6h
Time (h)
SA564
SA564−ΔhptRS
SA564−ΔccpA
SA564−ΔhptRS/ccpA

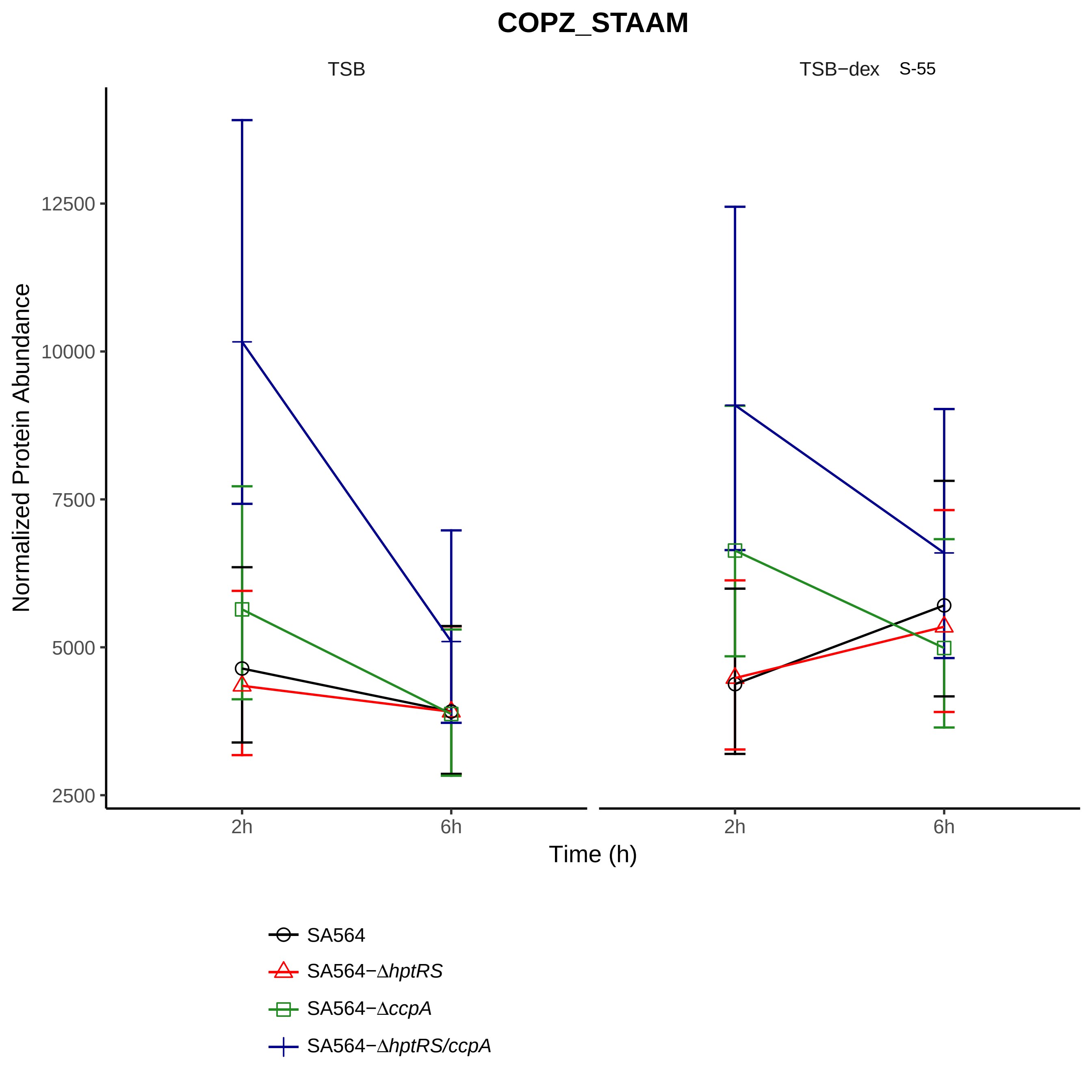

COPZ_STAAM
TSB
TSB−dex
S-55
Normalized Protein Abundance
12500
10000
7500
5000
2500
2h
6h
2h
6h
Time (h)
SA564
SA564−Δ*hptRS*
SA564−Δ*ccpA*
SA564−Δ*hptRS/ccpA*

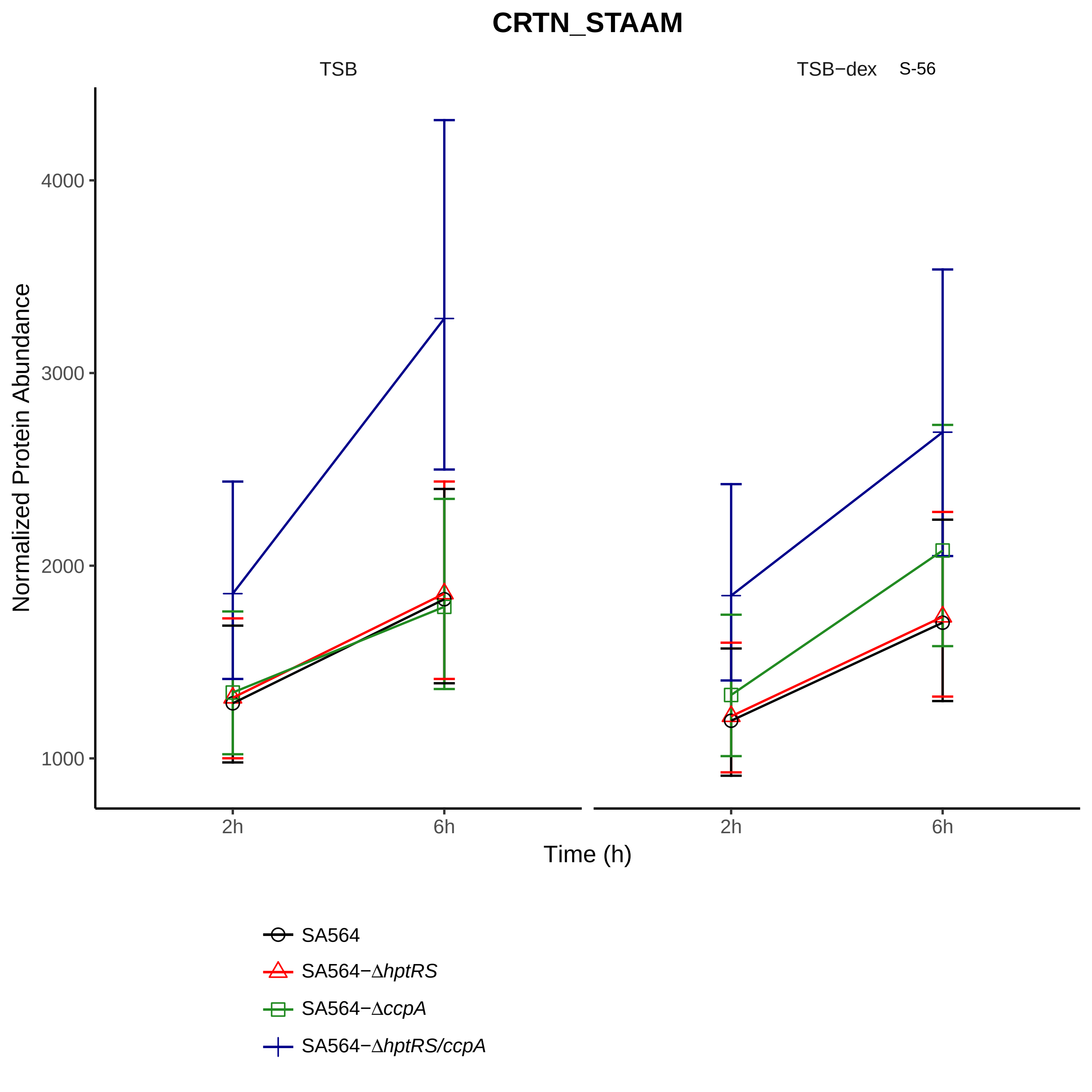
CRTN_STAAM
TSB
TSB−dex
S-56
Normalized Protein Abundance
4000
3000
2000
1000
2h
6h
2h
6h
Time (h)
SA564
SA564−Δ*hptRS*
SA564−Δ*ccpA*
SA564−Δ*hptRS/ccpA*

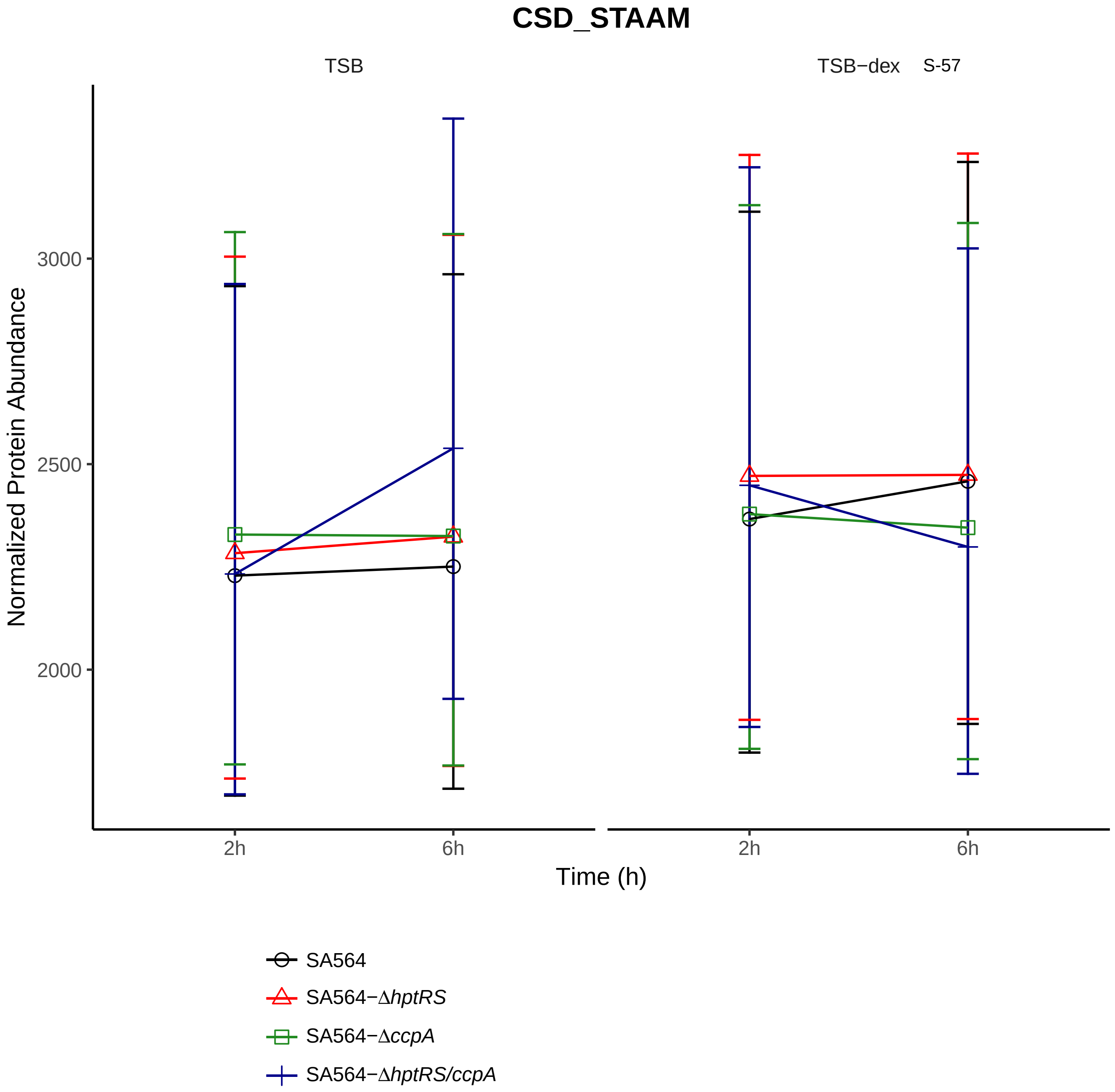

CSD_STAAM
TSB
TSB−dex
S-57
Normalized Protein Abundance
3000
2500
2000
2h
6h
2h
6h
Time (h)
SA564
SA564−ΔhptRS
SA564−ΔccpA
SA564−ΔhptRS/ccpA

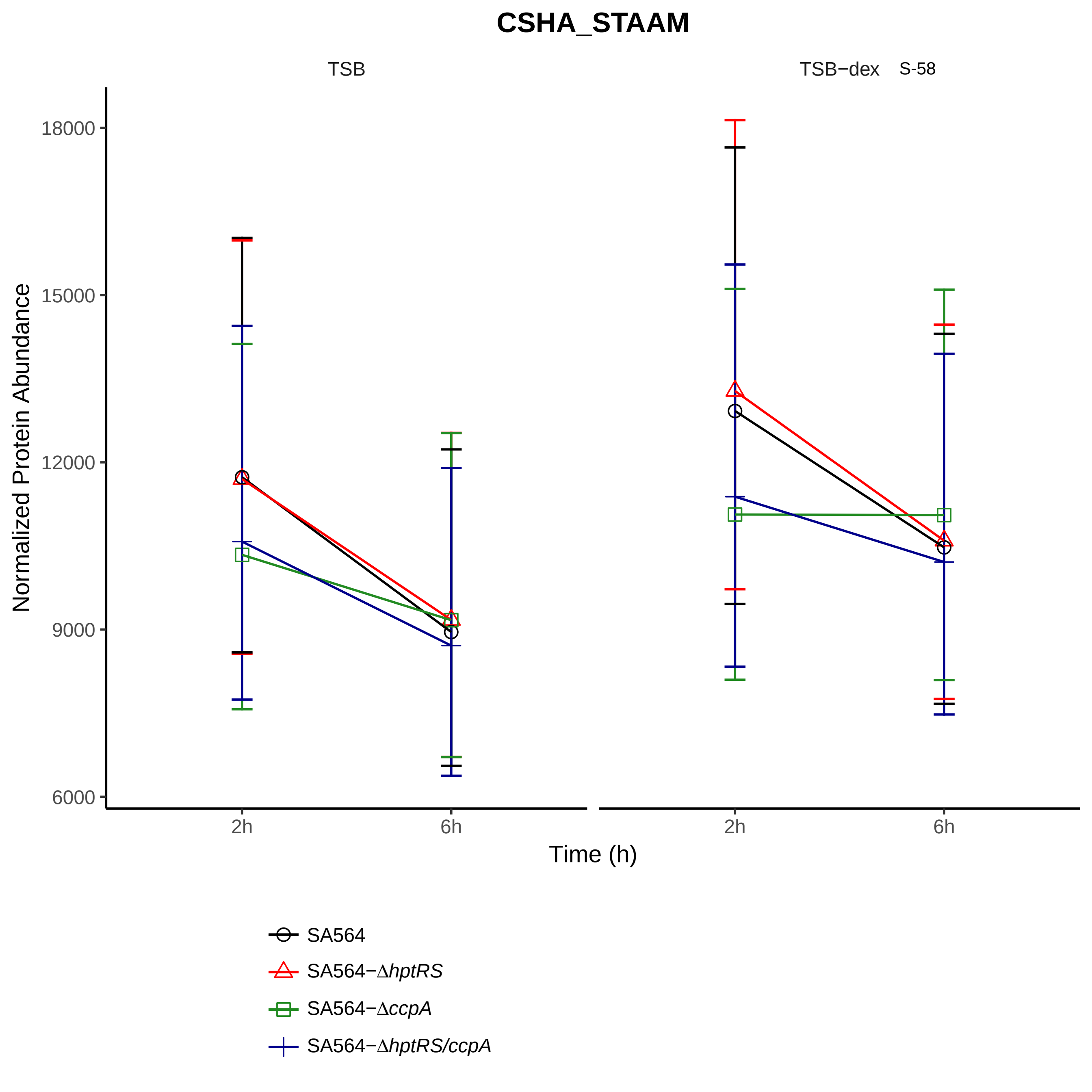

CSHA_STAAM
TSB
TSB−dex
S-58
Normalized Protein Abundance
18000
15000
12000
9000
6000
2h
6h
2h
6h
Time (h)
SA564
SA564−ΔhptRS
SA564−ΔccpA
SA564−ΔhptRS/ccpA

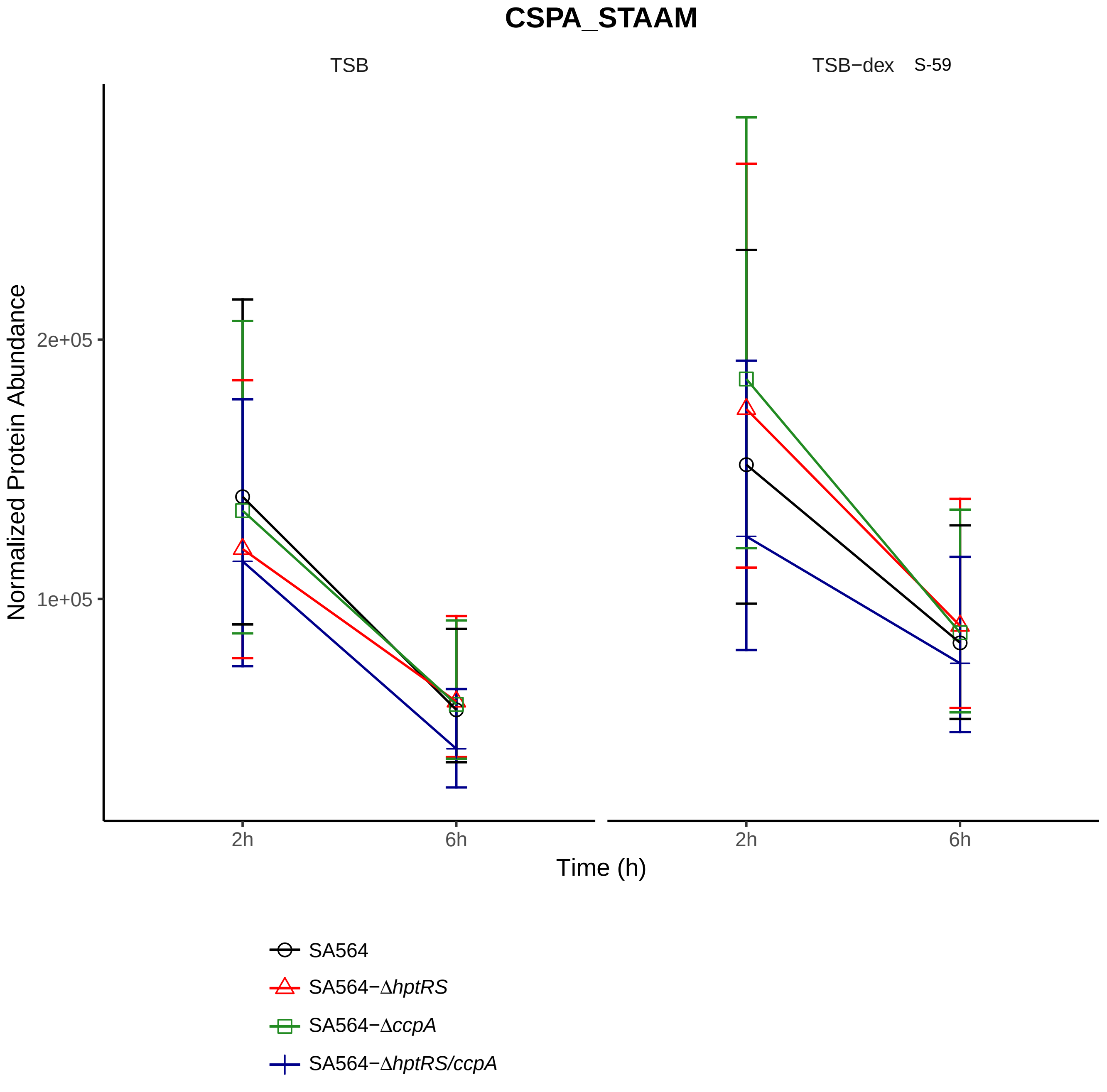
CSPA_STAAM
TSB
TSB−dex
S-59
Normalized Protein Abundance
2e+05
1e+05
2h
6h
2h
6h
Time (h)
SA564
SA564−ΔhptRS
SA564−ΔccpA
SA564−ΔhptRS/ccpA

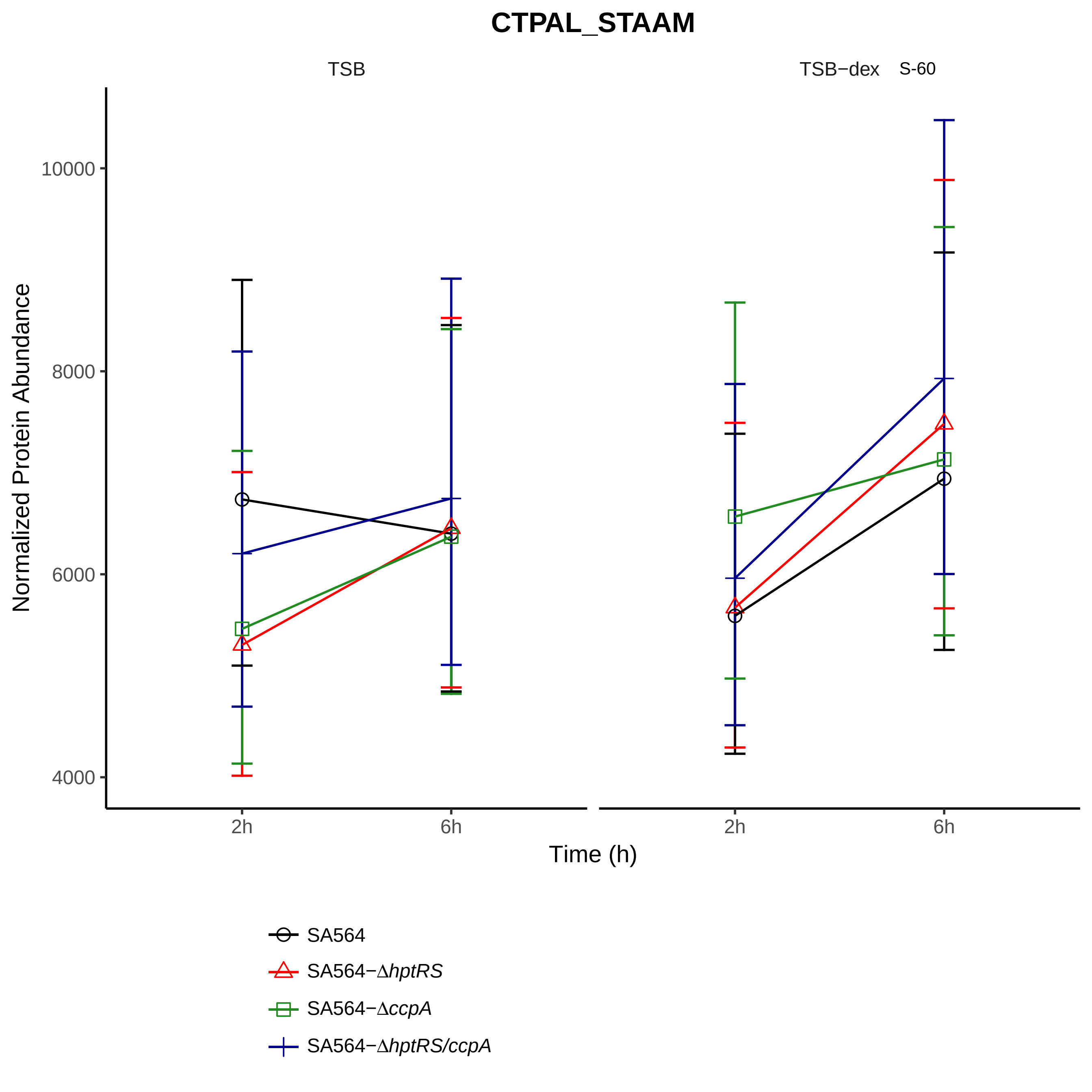

CTPAL_STAAM
TSB
TSB−dex
S-60
Normalized Protein Abundance
10000
8000
6000
4000
2h
6h
2h
6h
Time (h)
SA564
SA564−Δ*hptRS*
SA564−Δ*ccpA*
SA564−Δ*hptRS/ccpA*

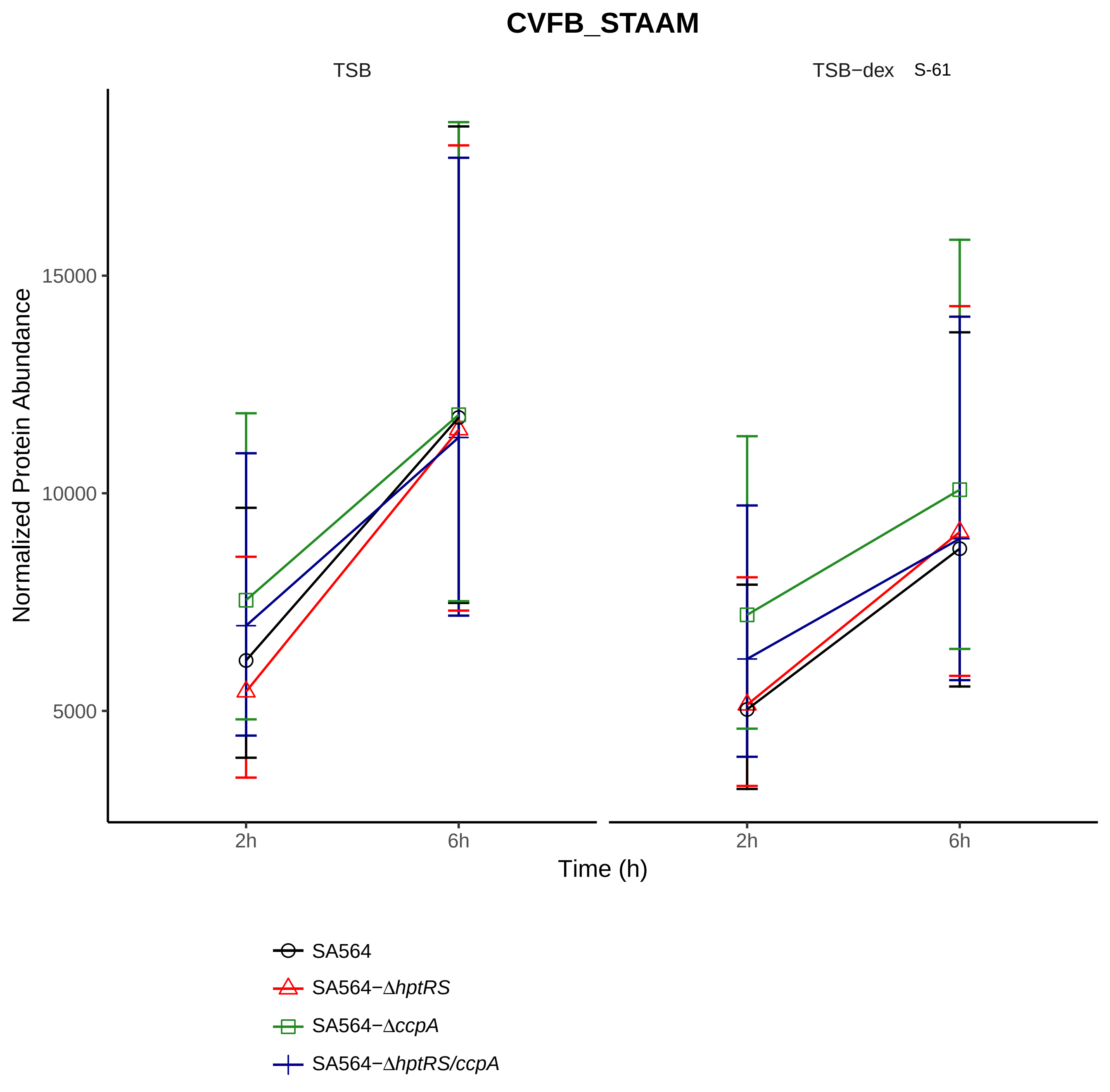

CVFB_STAAM
TSB
TSB−dex
S-61
Normalized Protein Abundance
15000
10000
5000
2h
6h
2h
6h
Time (h)
SA564
SA564−Δ*hptRS*
SA564−Δ*ccpA*
SA564−Δ*hptRS/ccpA*

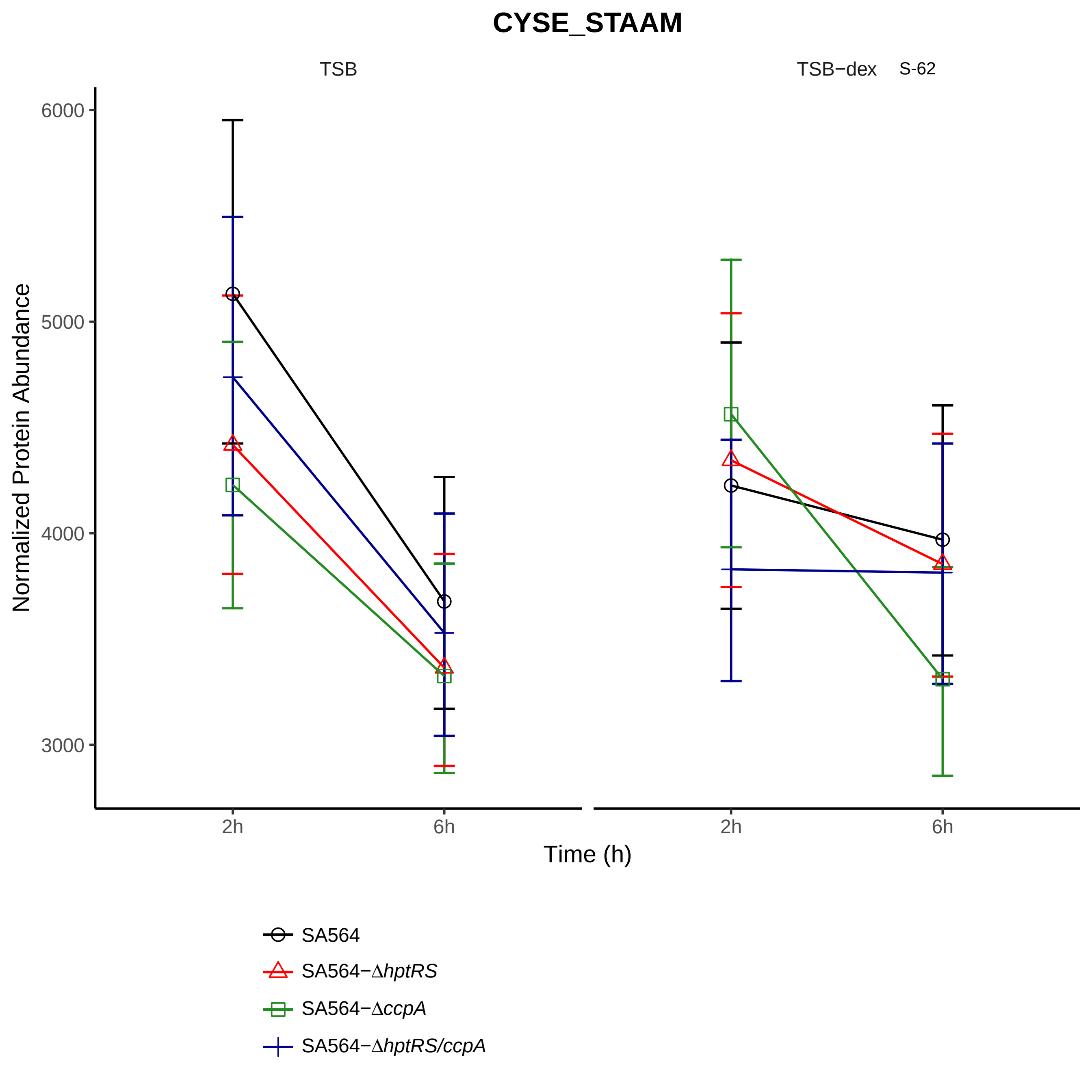

CYSE_STAAM
TSB
TSB−dex
S-62
Normalized Protein Abundance
6000
5000
4000
3000
2h
6h
2h
6h
Time (h)
SA564
SA564−Δ*hptRS*
SA564−Δ*ccpA*
SA564−Δ*hptRS/ccpA*

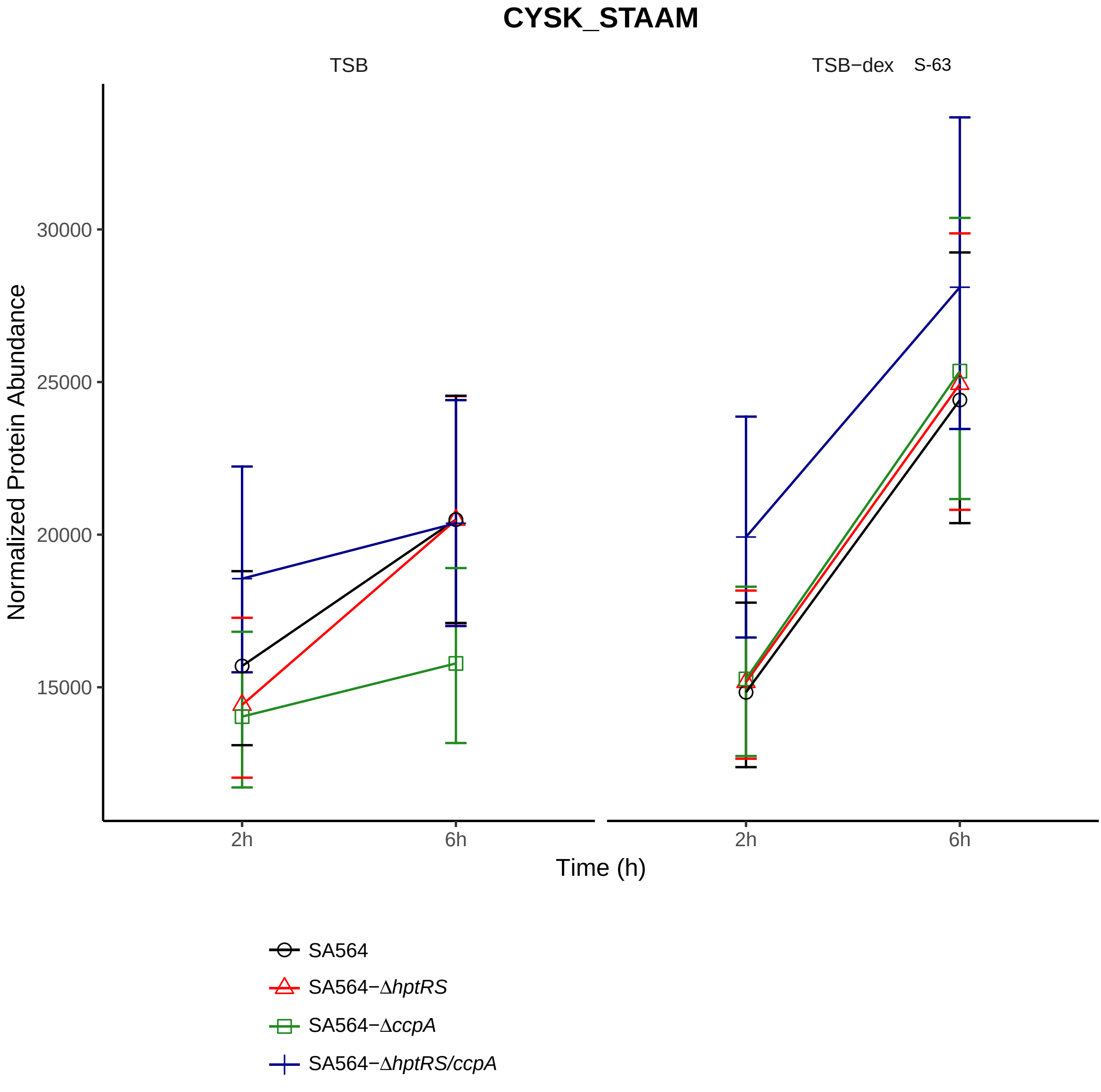

CYSK_STAAM
TSB
TSB−dex
S-63
Normalized Protein Abundance
30000
25000
20000
15000
2h
6h
2h
6h
Time (h)
SA564
SA564−ΔhptRS
SA564−ΔccpA
SA564−ΔhptRS/ccpA

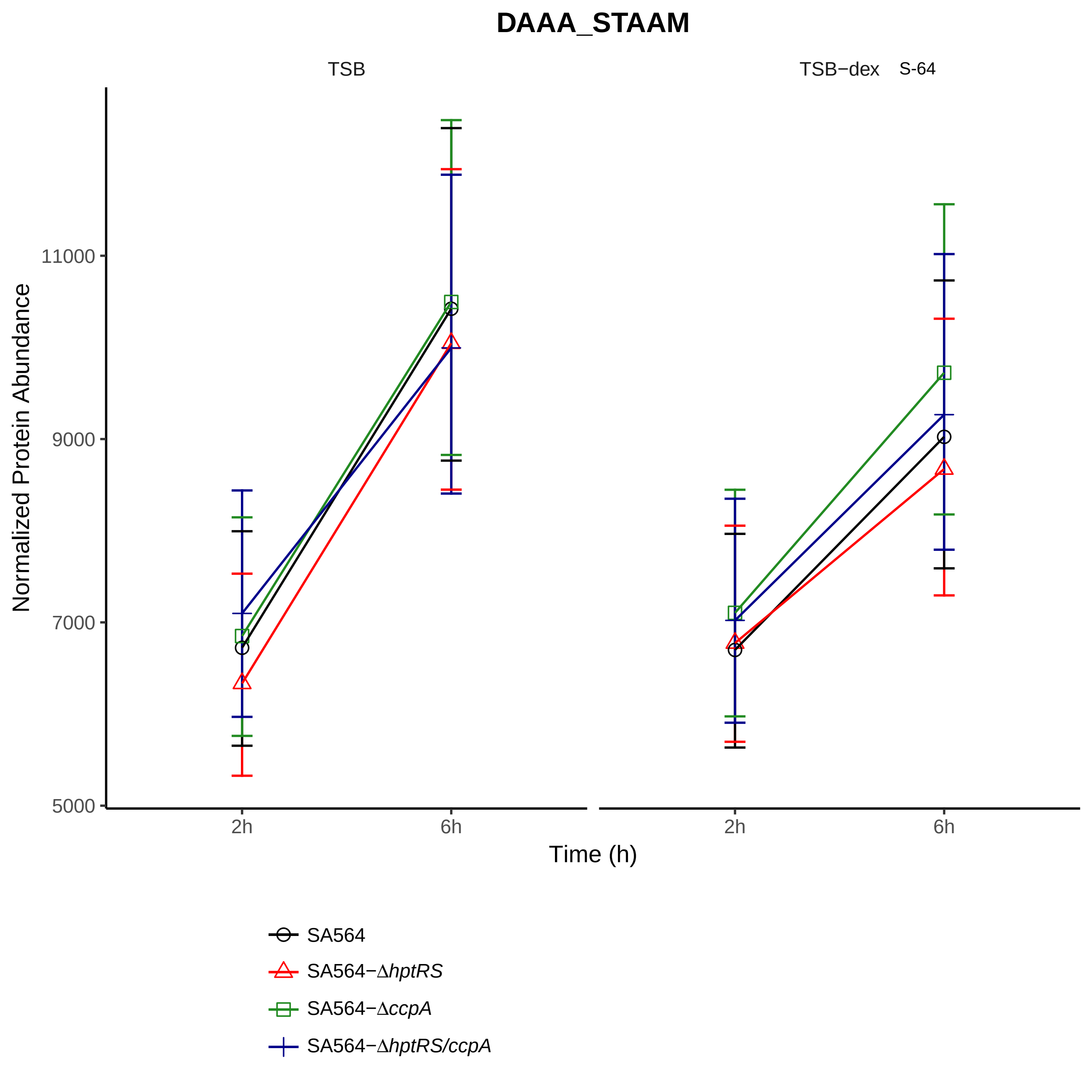

DAAA_STAAM
TSB
TSB−dex
S-64
Normalized Protein Abundance
11000
9000
7000
5000
2h
6h
2h
6h
Time (h)
SA564
SA564−ΔhptRS
SA564−ΔccpA
SA564−ΔhptRS/ccpA

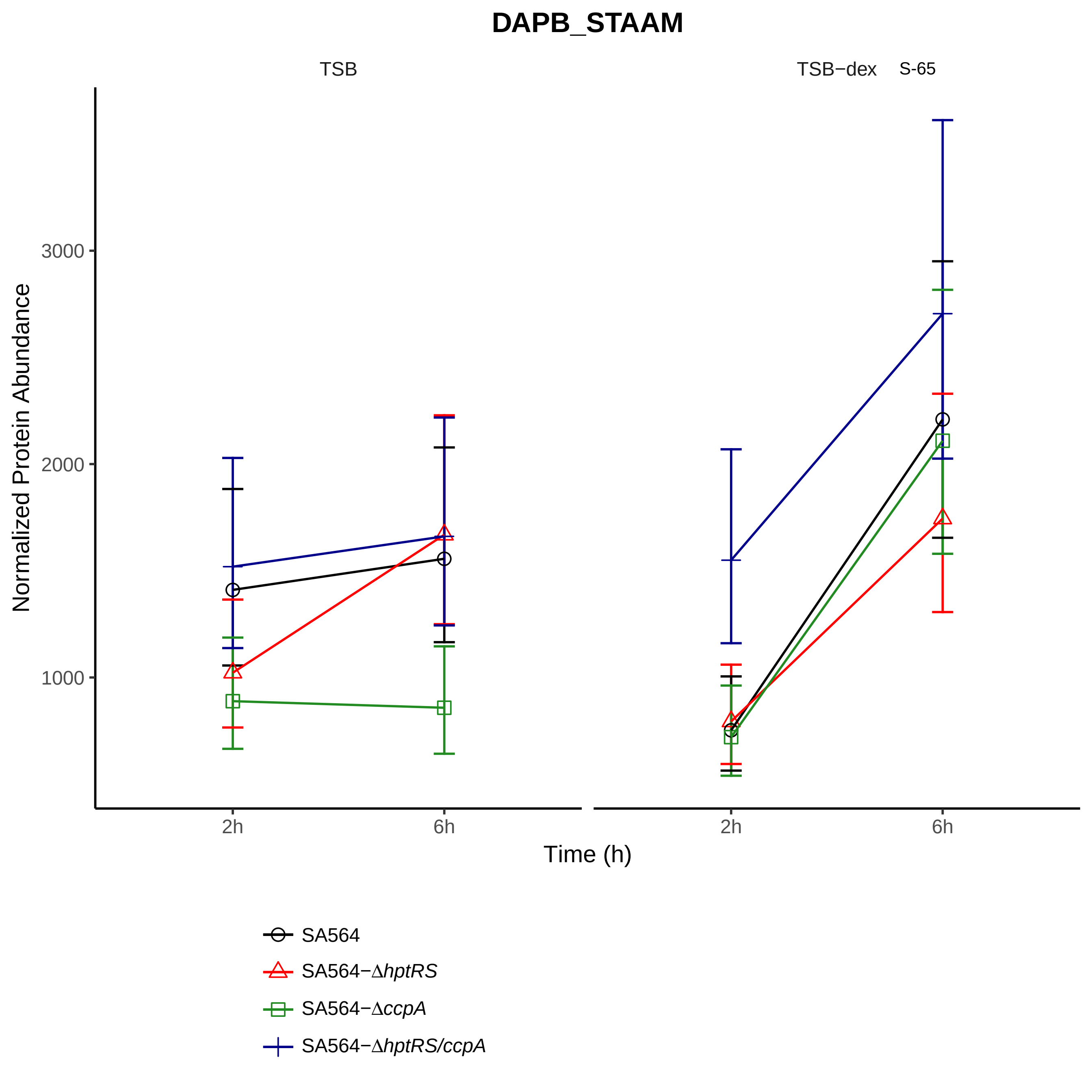

DAPB_STAAM
TSB
TSB−dex
S-65
Normalized Protein Abundance
3000
2000
1000
2h
6h
2h
6h
Time (h)
SA564
SA564−Δ*hptRS*
SA564−Δ*ccpA*
SA564−Δ*hptRS/ccpA*

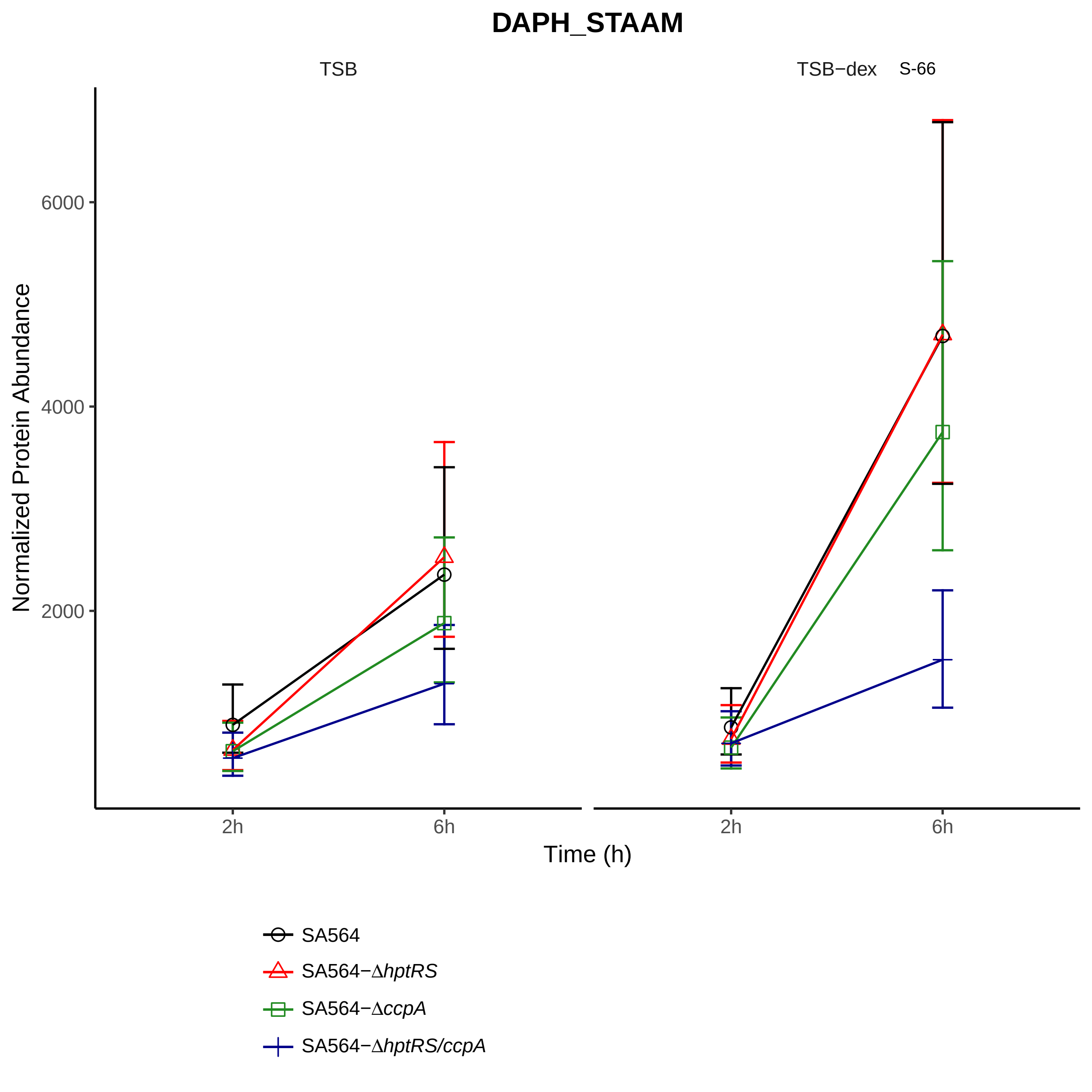

DAPH_STAAM
TSB
TSB−dex
S-66
Normalized Protein Abundance
6000
4000
2000
2h
6h
2h
6h
Time (h)
SA564
SA564−Δ*hptRS*
SA564−Δ*ccpA*
SA564−Δ*hptRS/ccpA*

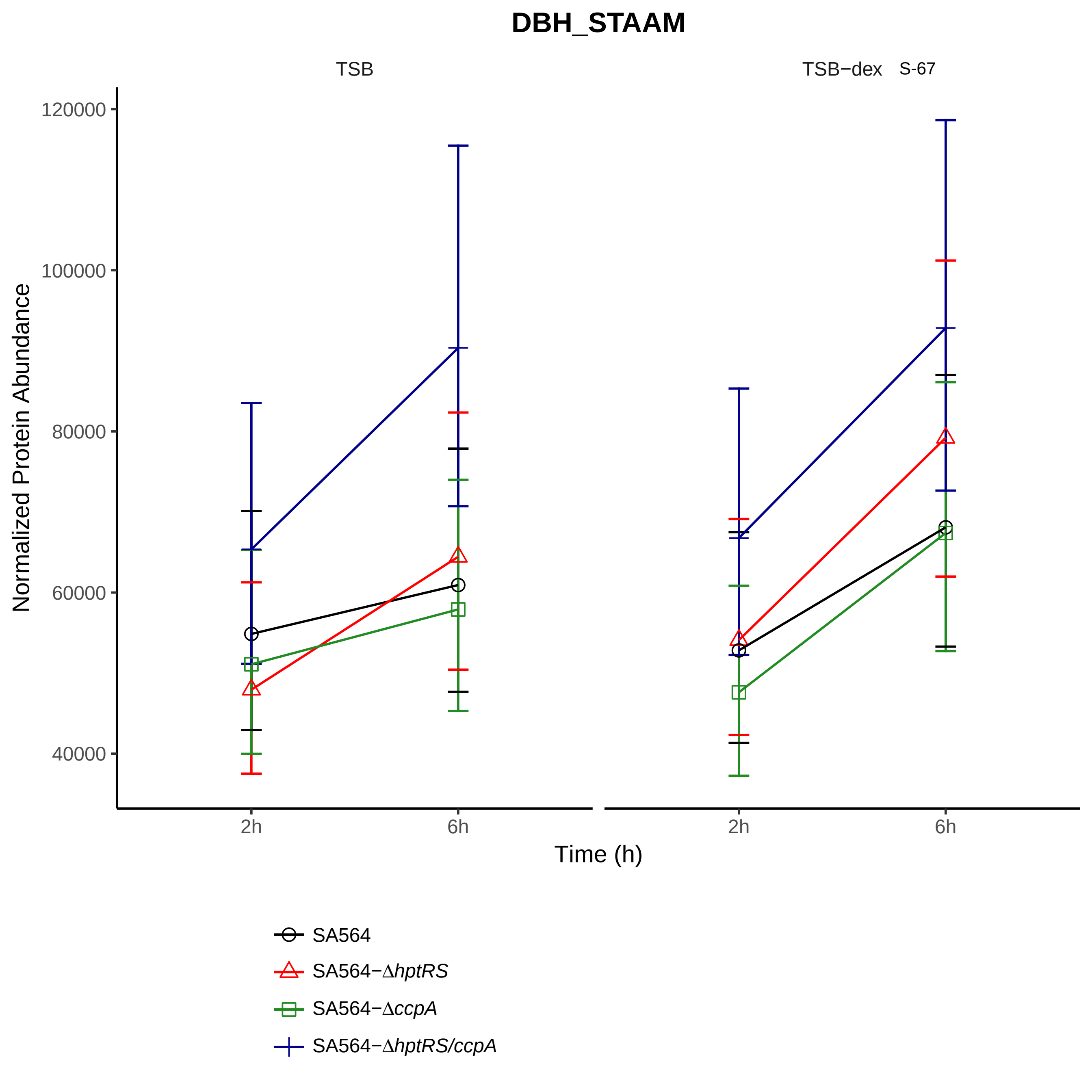

DBH_STAAM
TSB
TSB−dex
S-67
Normalized Protein Abundance
120000
100000
80000
60000
40000
2h
6h
2h
6h
Time (h)
SA564
SA564−Δ*hptRS*
SA564−Δ*ccpA*
SA564−Δ*hptRS/ccpA*

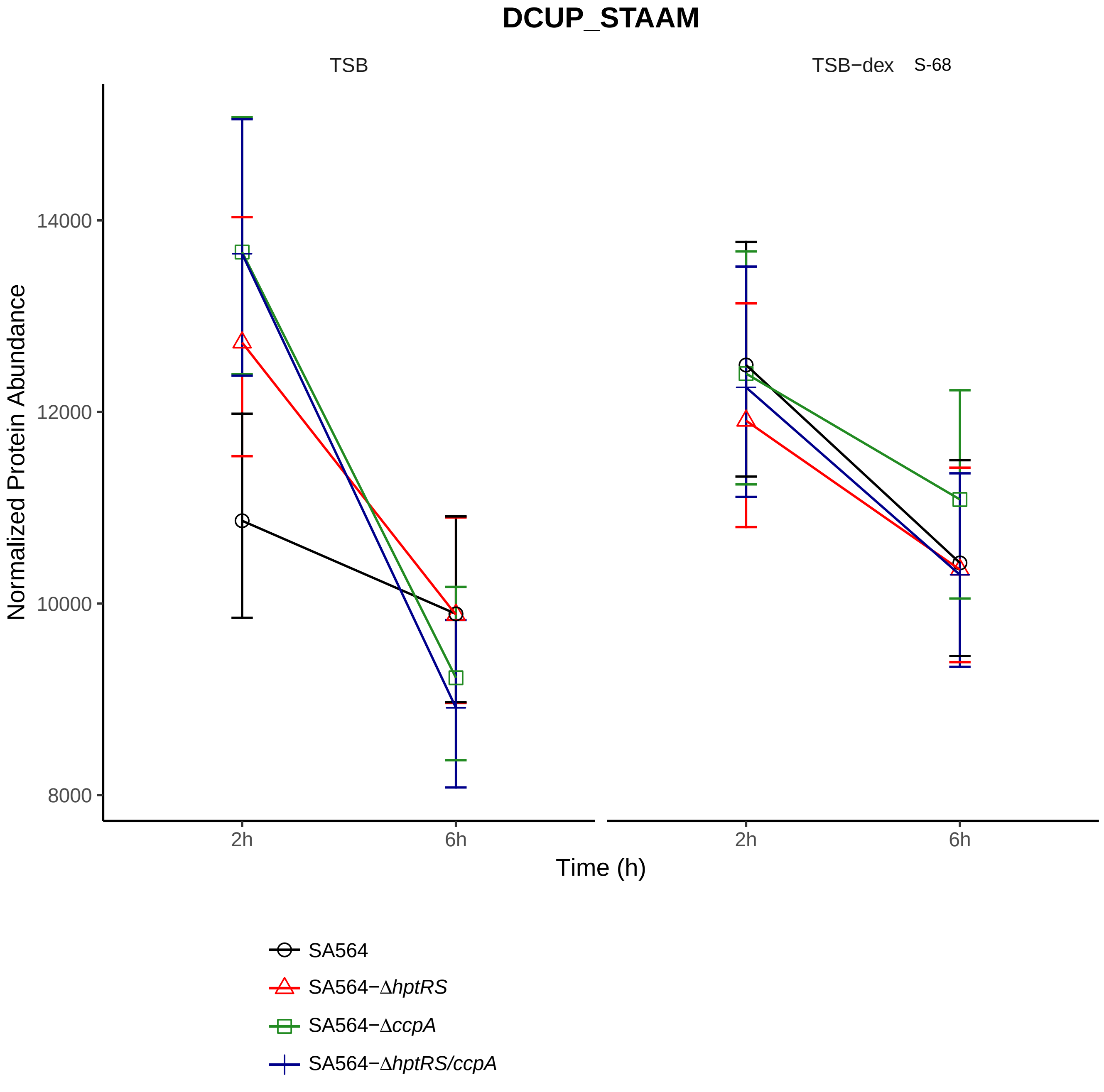
DCUP_STAAM
TSB
TSB−dex
S-68
Normalized Protein Abundance
14000
12000
10000
8000
2h
6h
2h
6h
Time (h)
SA564
SA564−Δ*hptRS*
SA564−Δ*ccpA*
SA564−Δ*hptRS/ccpA*

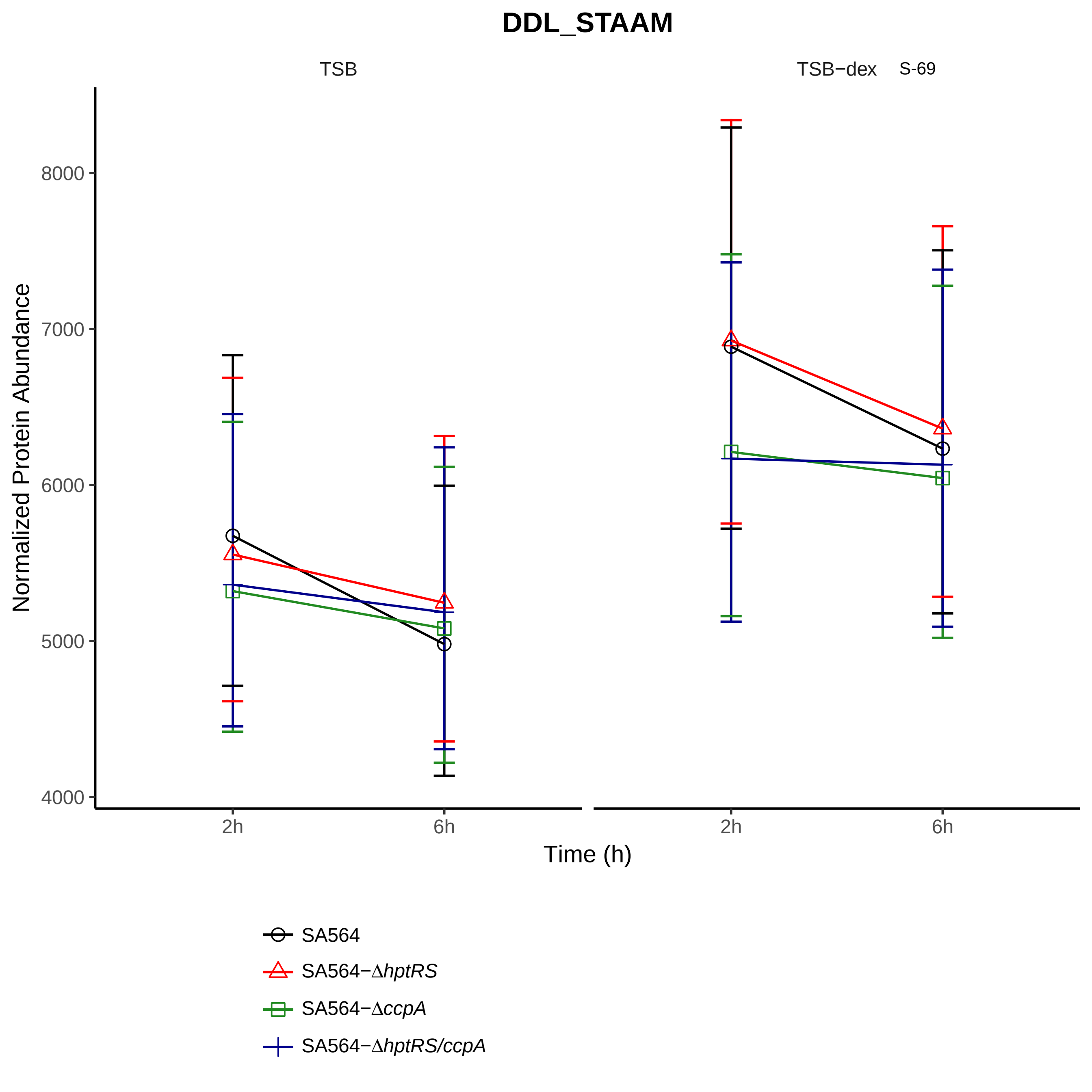

DDL_STAAM
TSB
TSB−dex
S-69
Normalized Protein Abundance
8000
7000
6000
5000
4000
2h
6h
2h
6h
Time (h)
SA564
SA564−ΔhptRS
SA564−ΔccpA
SA564−ΔhptRS/ccpA

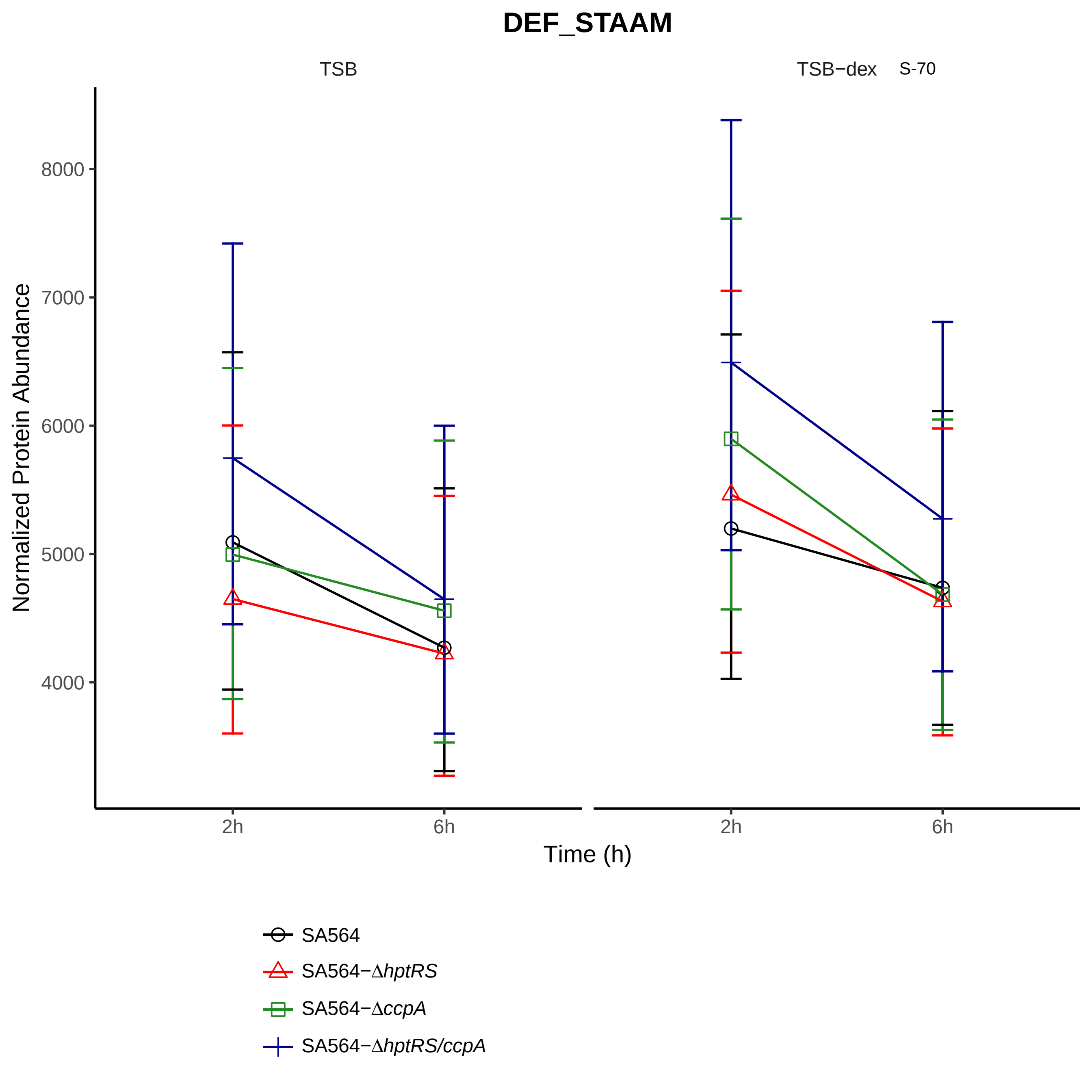

DEF_STAAM
TSB
TSB−dex
S-70
Normalized Protein Abundance
8000
7000
6000
5000
4000
2h
6h
2h
6h
Time (h)
SA564
SA564−ΔhptRS
SA564−ΔccpA
SA564−ΔhptRS/ccpA

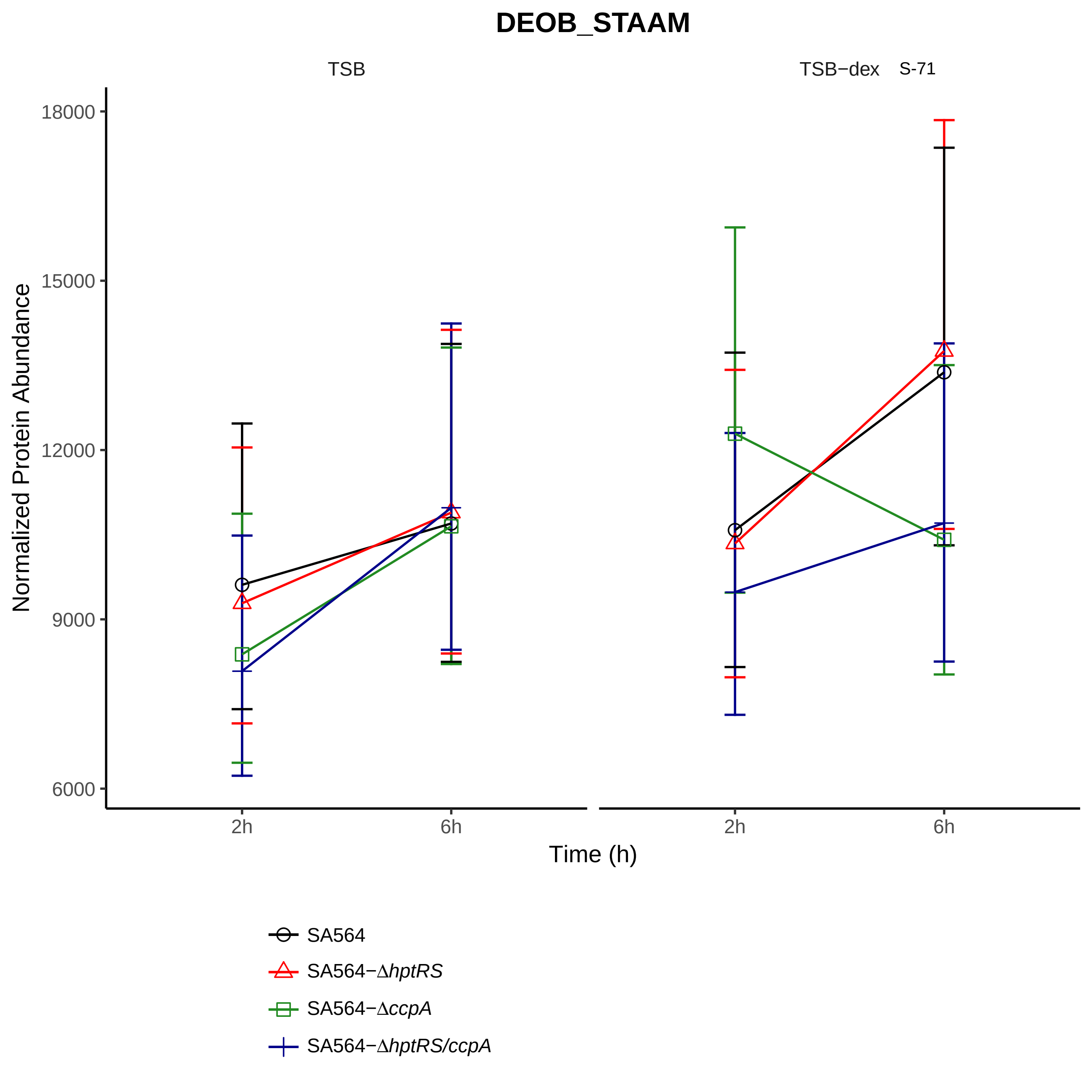

DEOB_STAAM
TSB
TSB−dex
S-71
Normalized Protein Abundance
18000
15000
12000
9000
6000
2h
6h
2h
6h
Time (h)
SA564
SA564−ΔhptRS
SA564−ΔccpA
SA564−ΔhptRS/ccpA

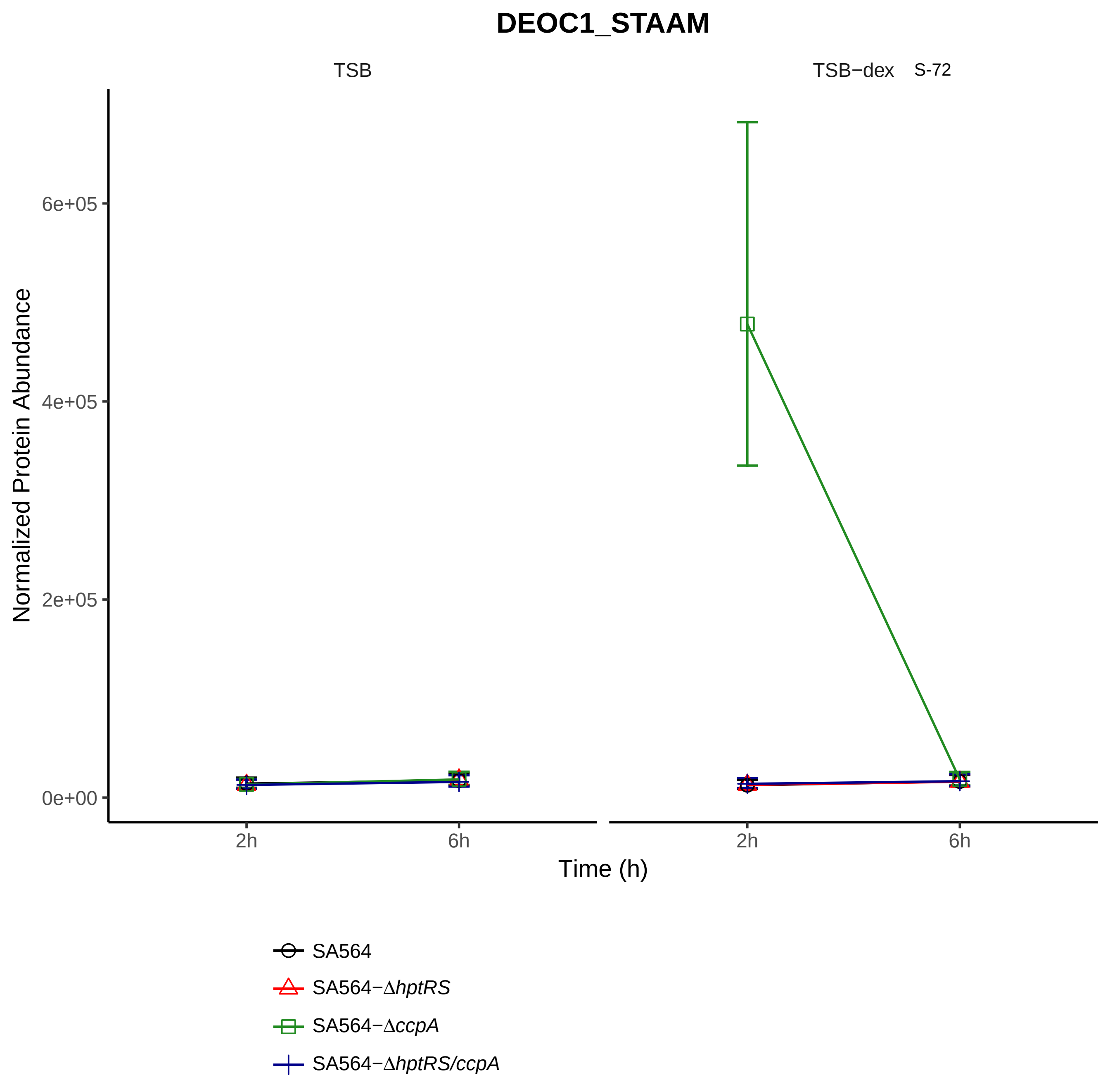

DEOC1_STAAM
TSB
TSB−dex
S-72
Normalized Protein Abundance
6e+05
4e+05
2e+05
0e+00
2h
6h
2h
6h
Time (h)
SA564
SA564−ΔhptRS
SA564−ΔccpA
SA564−ΔhptRS/ccpA

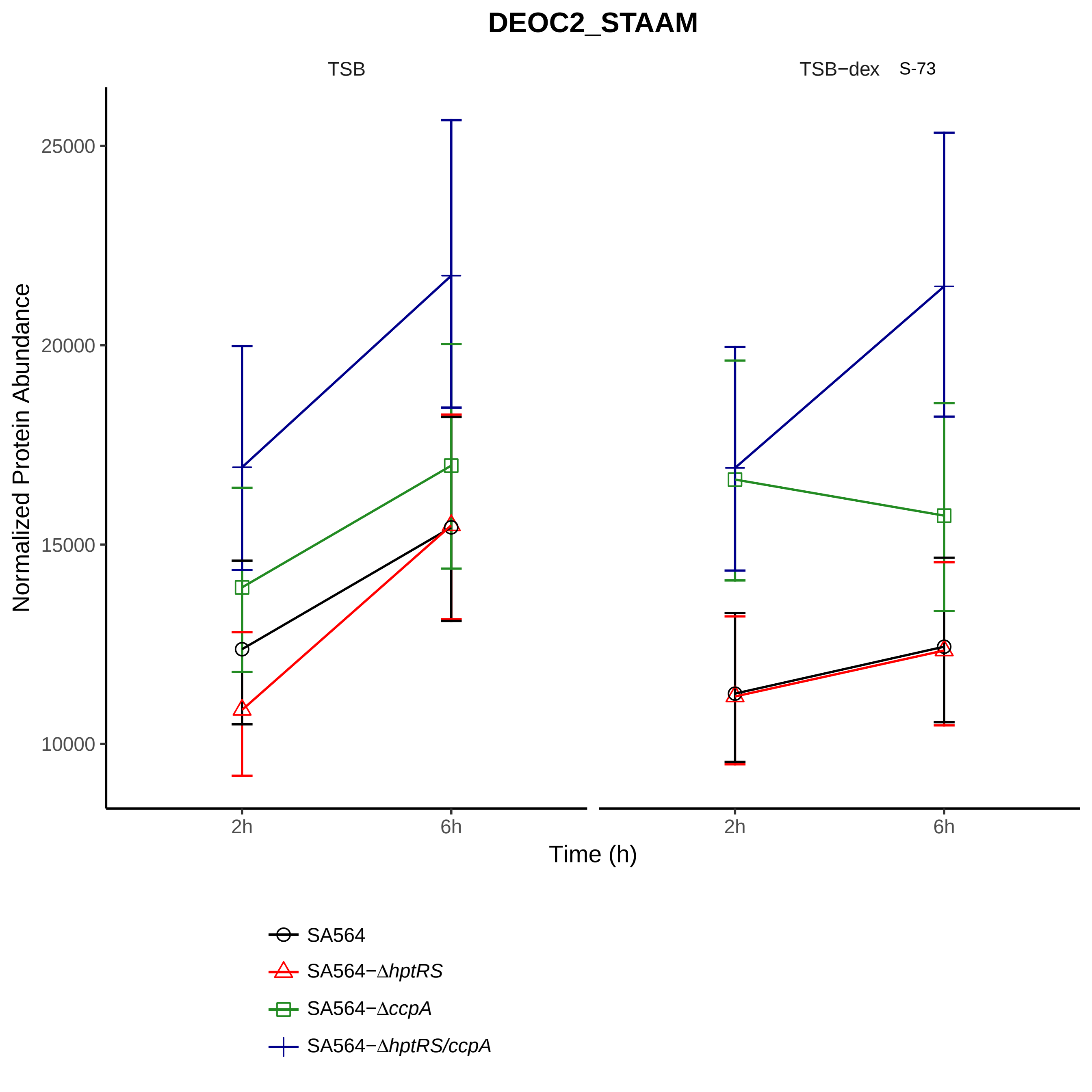

DEOC2_STAAM
TSB
TSB−dex
S-73
Normalized Protein Abundance
25000
20000
15000
10000
2h
6h
2h
6h
Time (h)
SA564
SA564−Δ*hptRS*
SA564−Δ*ccpA*
SA564−Δ*hptRS/ccpA*

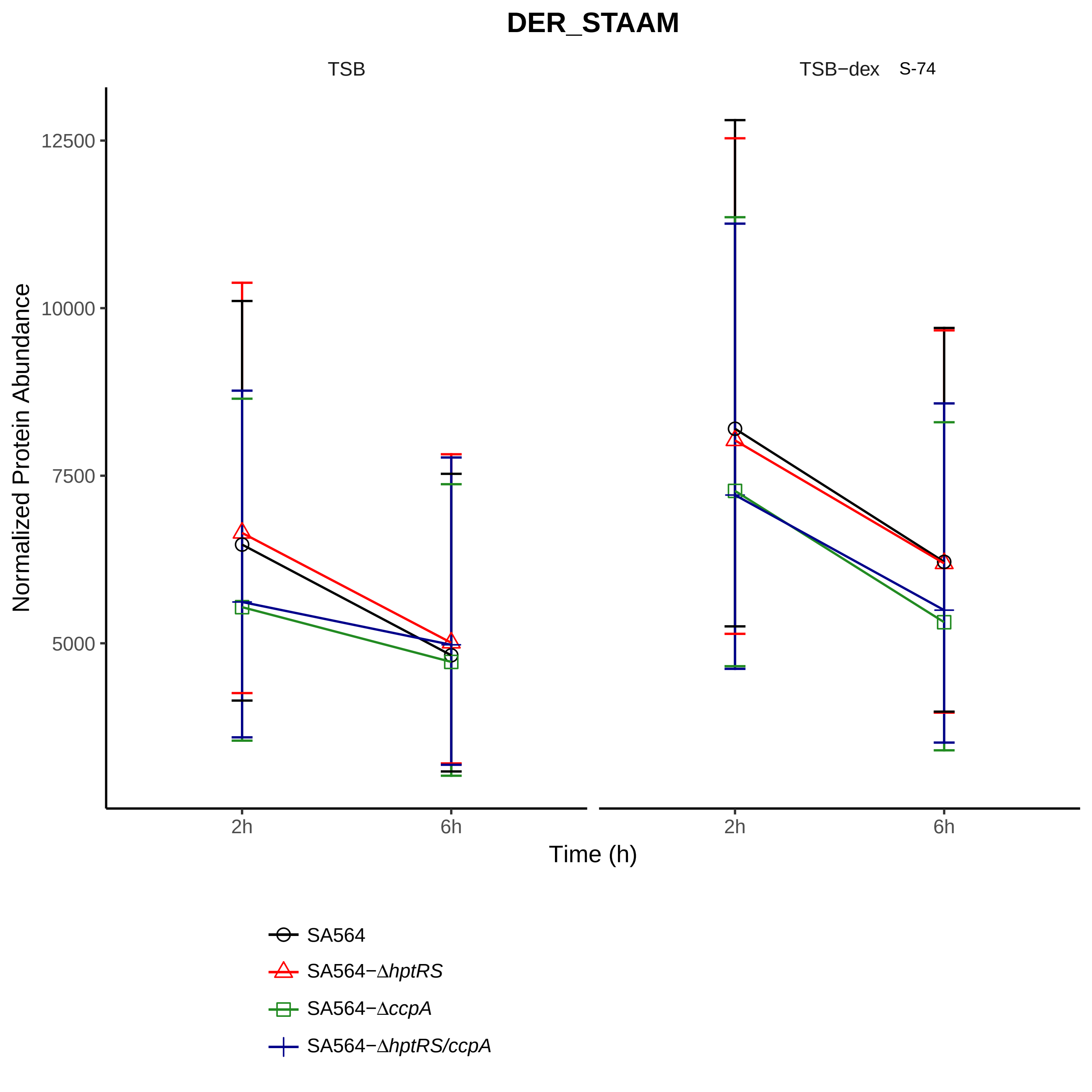
DER_STAAM
TSB
TSB−dex
S-74
12500
10000
7500
5000
Normalized Protein Abundance
2h
6h
2h
6h
Time (h)
SA564
SA564−ΔhptRS
SA564−ΔccpA
SA564−ΔhptRS/ccpA

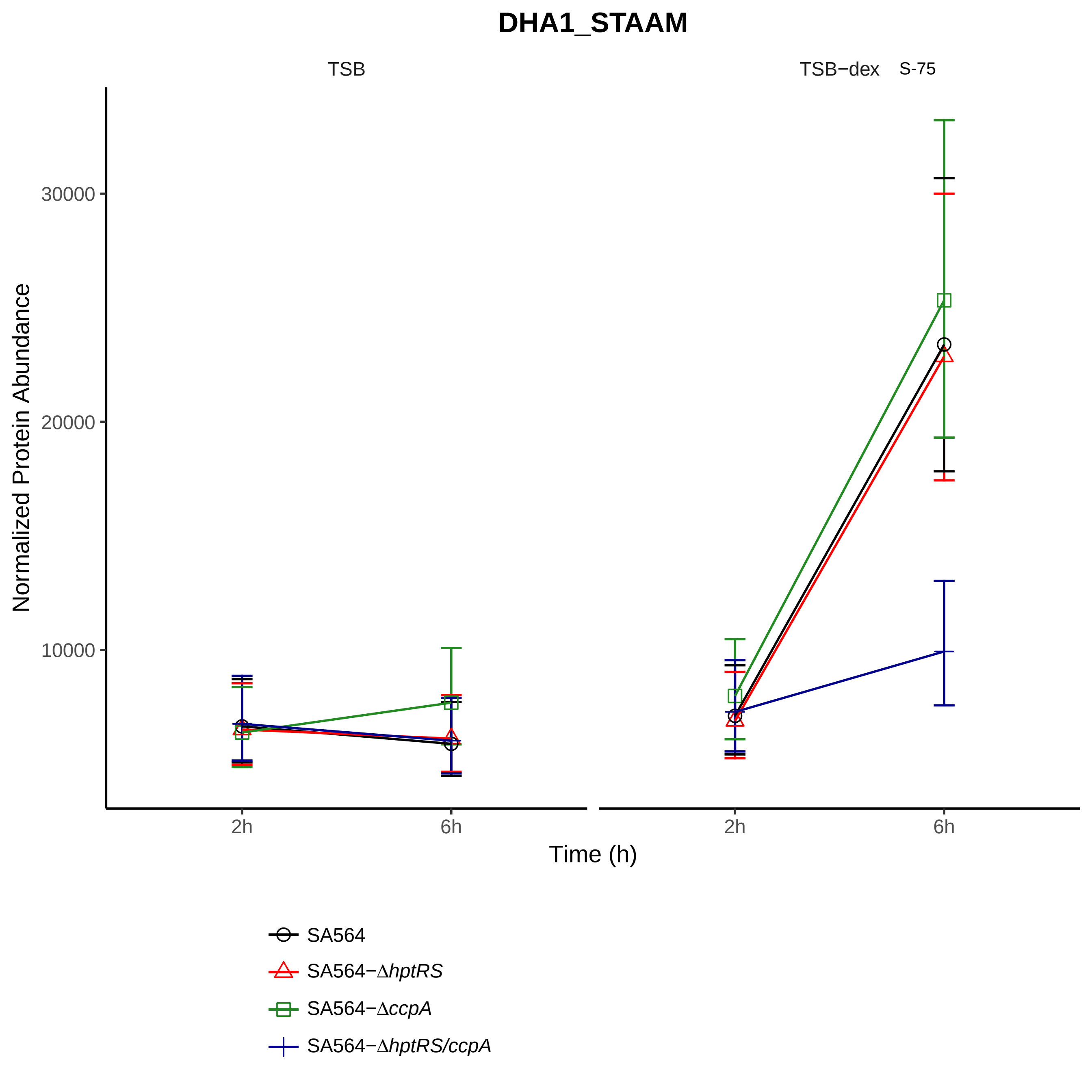

DHA1_STAAM
TSB
TSB−dex
S-75
Normalized Protein Abundance
30000
20000
10000
2h
6h
2h
6h
Time (h)
SA564
SA564−Δ*hptRS*
SA564−Δ*ccpA*
SA564−Δ*hptRS/ccpA*

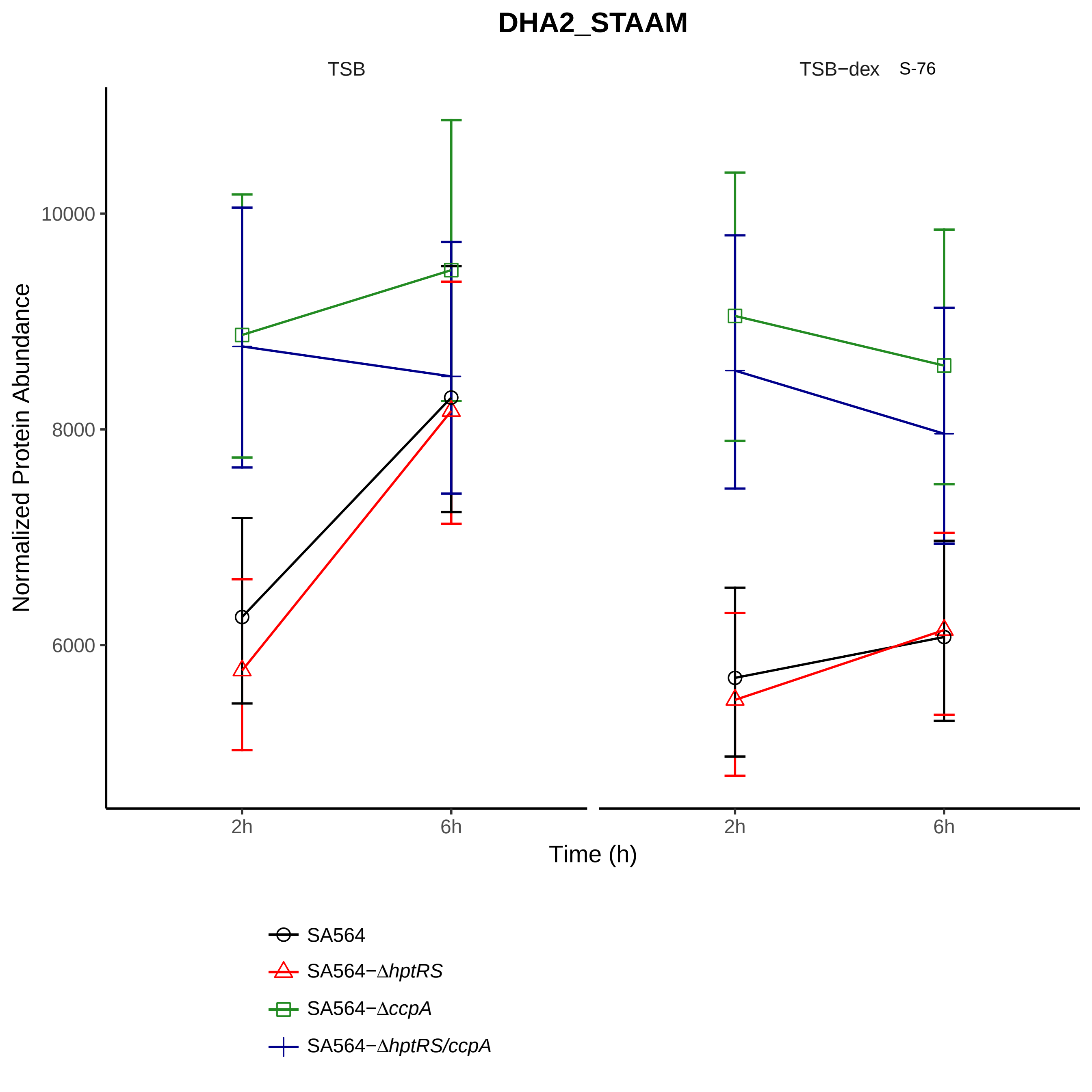
DHA2_STAAM
TSB
TSB−dex
S-76
Normalized Protein Abundance
10000
8000
6000
2h
6h
2h
6h
Time (h)
SA564
SA564−ΔhptRS
SA564−ΔccpA
SA564−ΔhptRS/ccpA

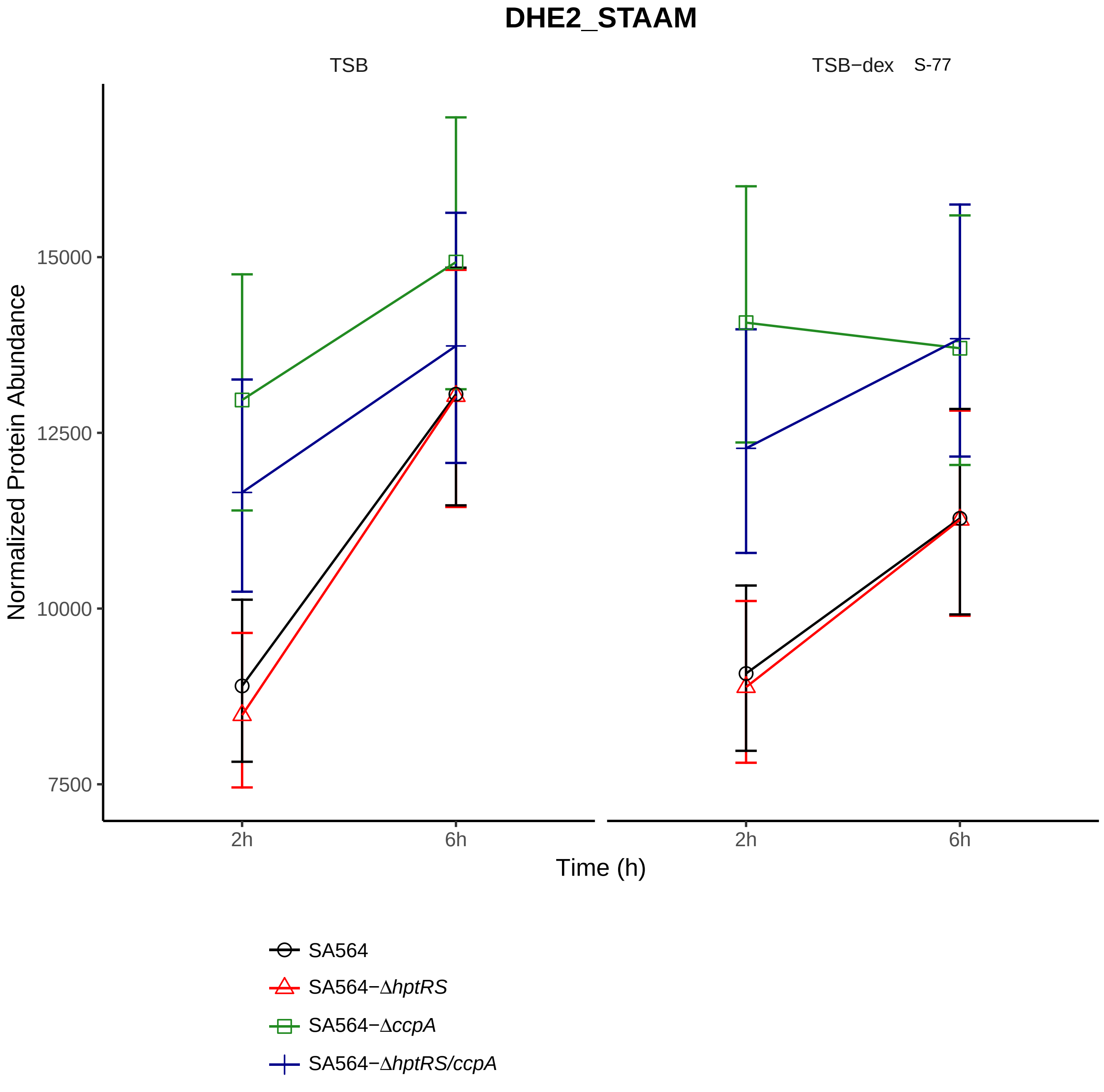

DHE2_STAAM
TSB
TSB−dex
S-77
Normalized Protein Abundance
15000
12500
10000
7500
2h
6h
2h
6h
Time (h)
SA564
SA564−Δ*hptRS*
SA564−Δ*ccpA*
SA564−Δ*hptRS/ccpA*

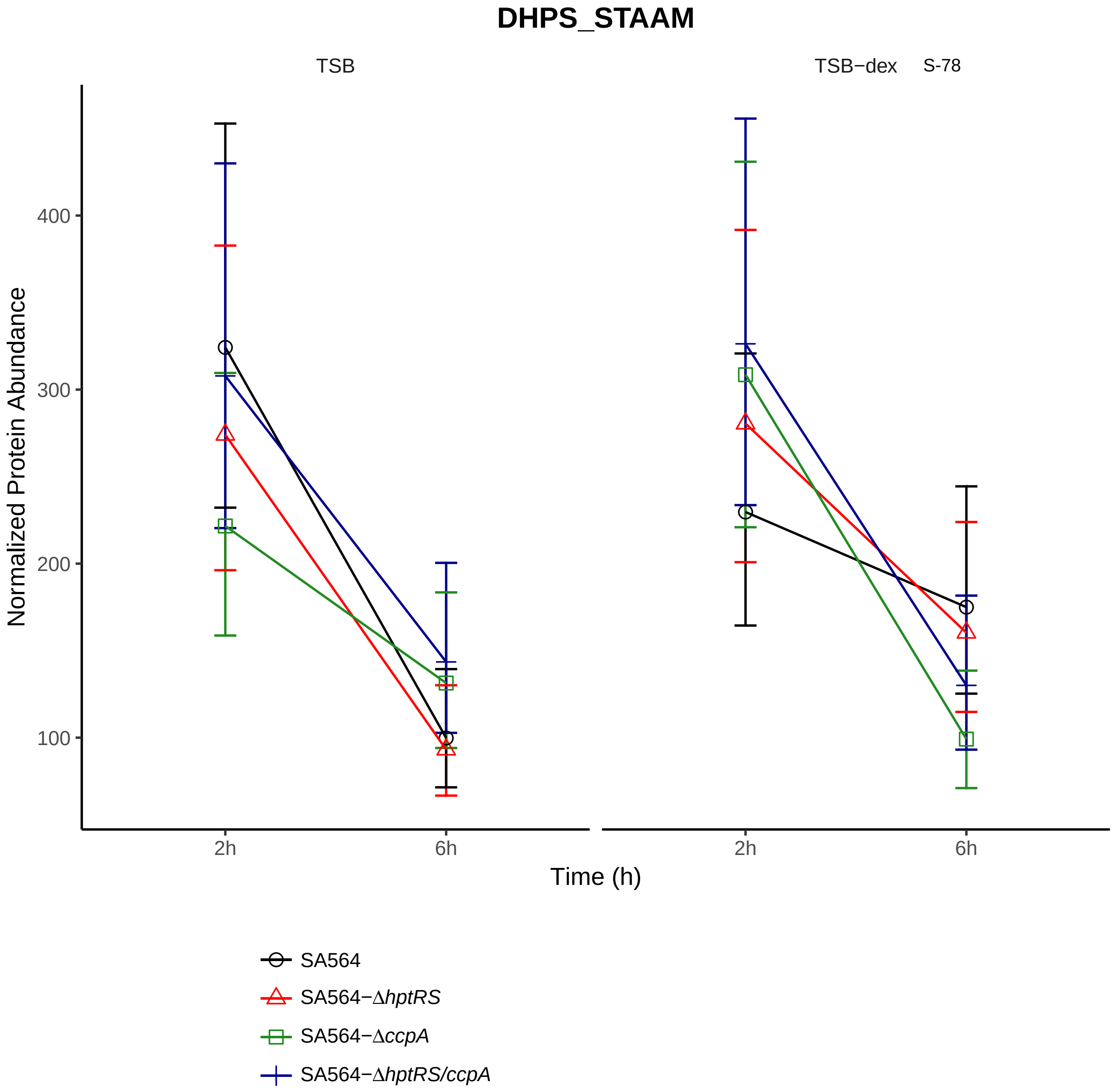

DHPS_STAAM
TSB
TSB−dex
S-78
Normalized Protein Abundance
400
300
200
100
2h
6h
2h
6h
Time (h)
SA564
SA564−ΔhptRS
SA564−ΔccpA
SA564−ΔhptRS/ccpA

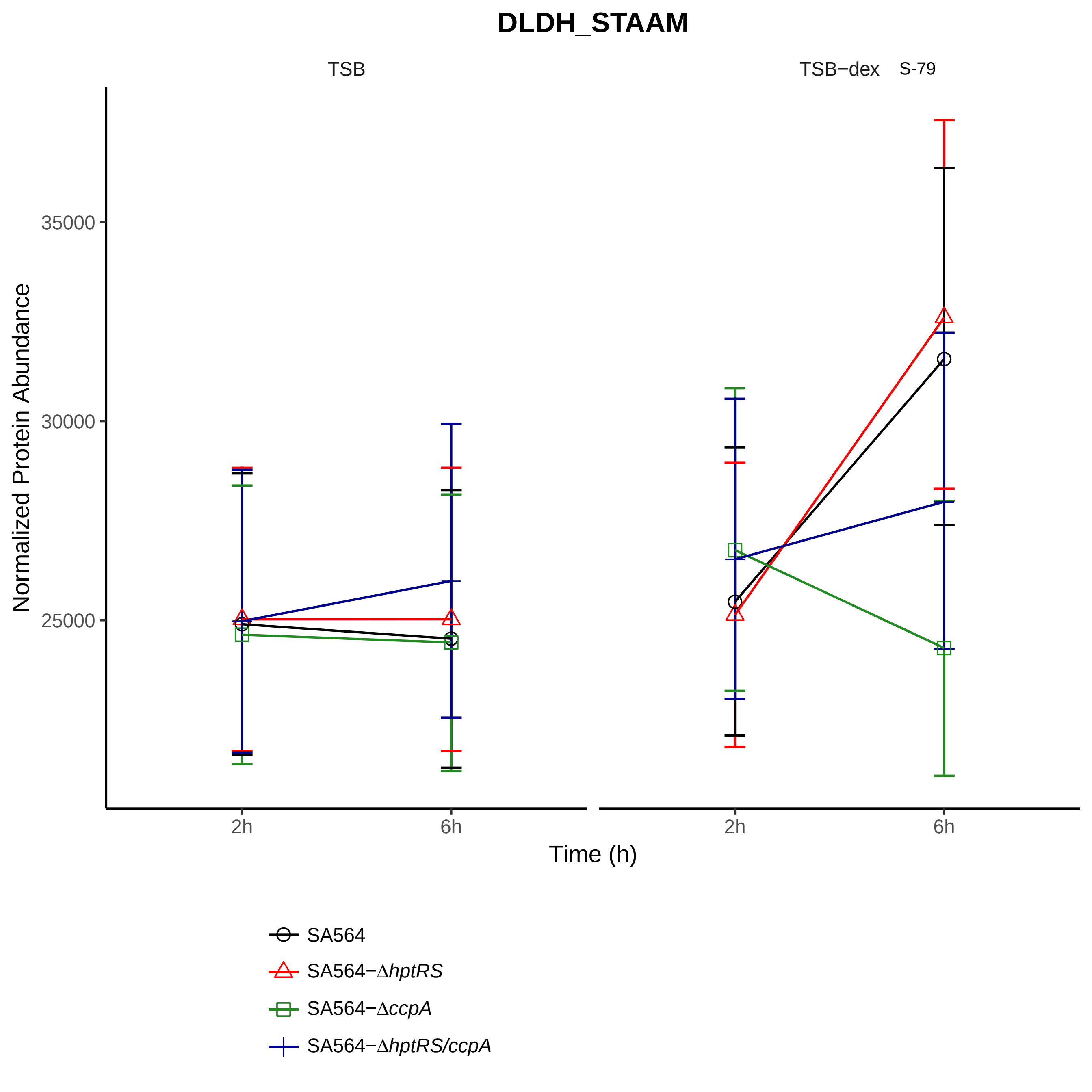
DLDH_STAAM
TSB
TSB−dex
S-79
Normalized Protein Abundance
35000
30000
25000
2h
6h
2h
6h
Time (h)
SA564
SA564−ΔhptRS
SA564−ΔccpA
SA564−ΔhptRS/ccpA

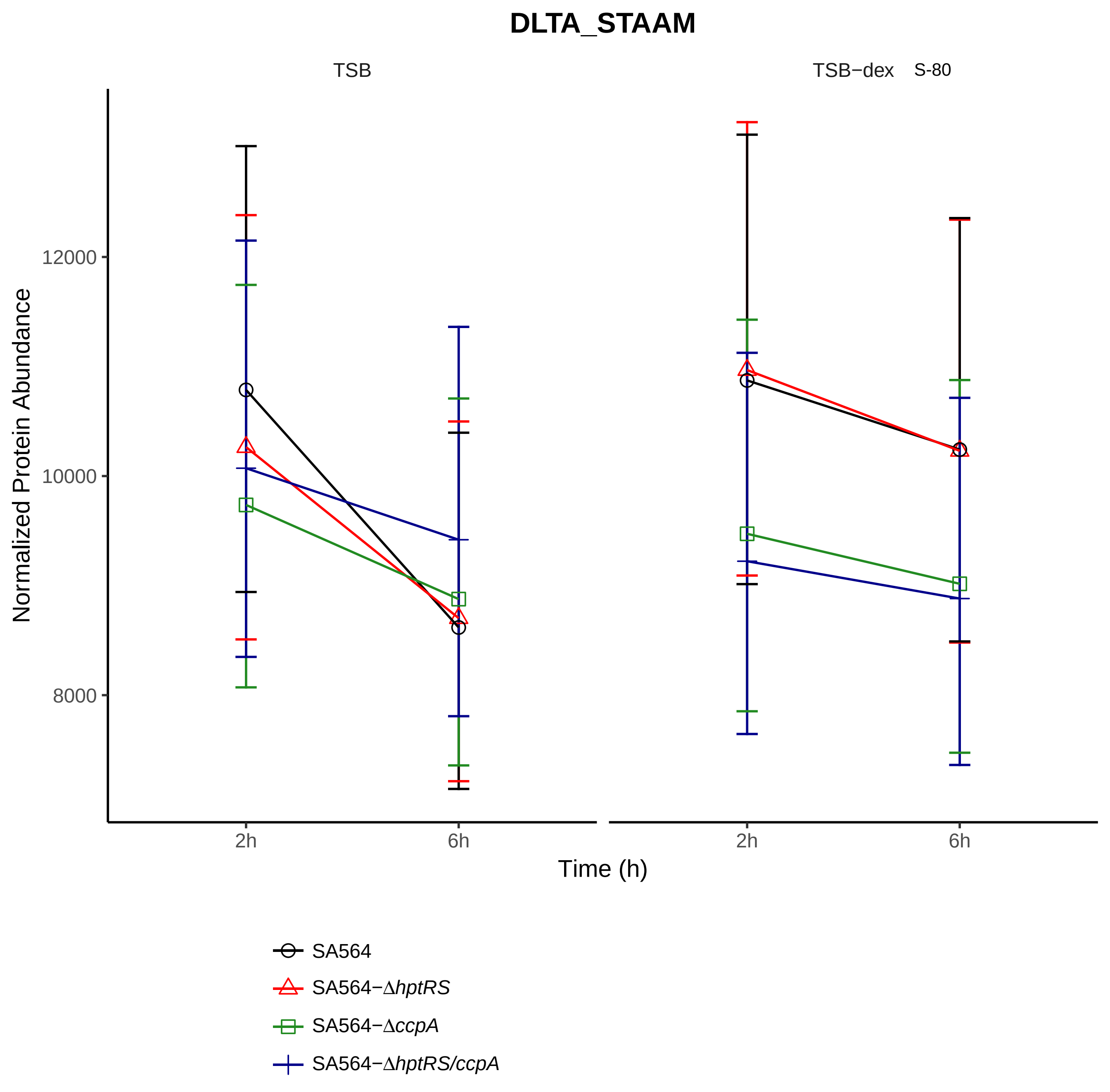

DLTA_STAAM
TSB
TSB−dex
S-80
Normalized Protein Abundance
12000
10000
8000
2h
6h
2h
6h
Time (h)
SA564
SA564−Δ*hptRS*
SA564−Δ*ccpA*
SA564−Δ*hptRS/ccpA*

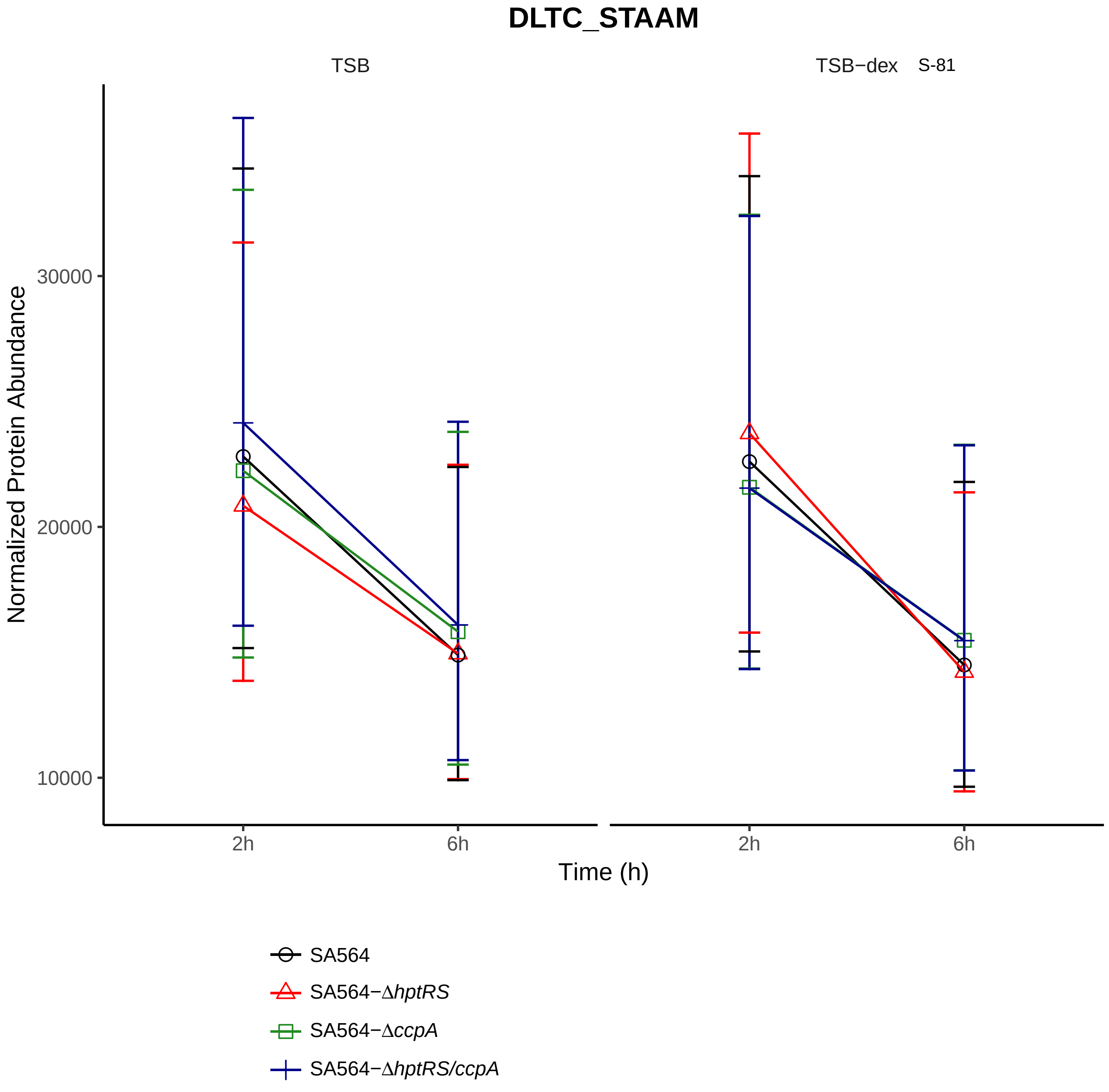
DLTC_STAAM
TSB
TSB−dex
S-81
Normalized Protein Abundance
30000
20000
10000
2h
6h
2h
6h
Time (h)
SA564
SA564−Δ*hptRS*
SA564−Δ*ccpA*
SA564−Δ*hptRS/ccpA*

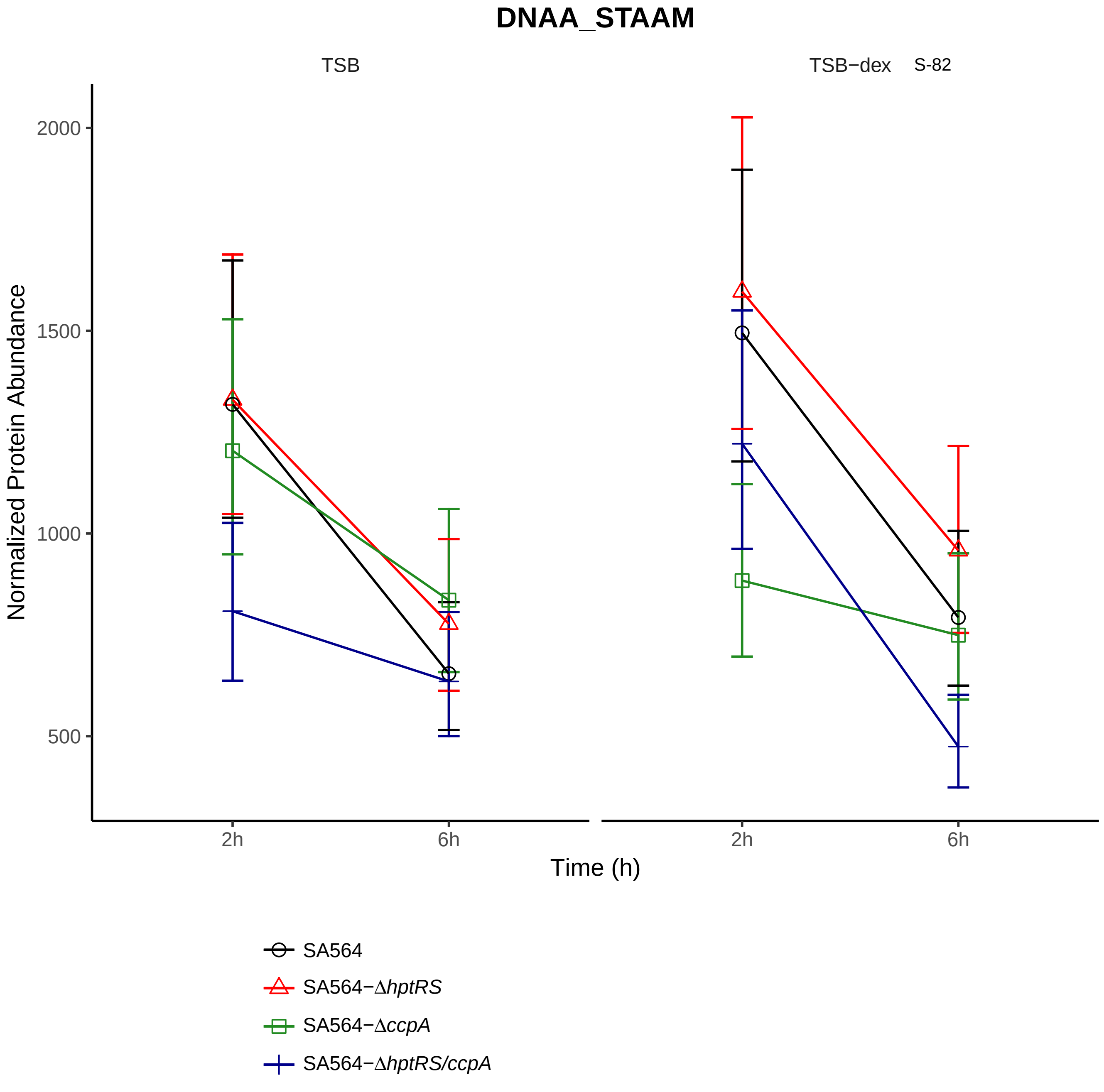
DNAA_STAAM
TSB
TSB−dex
S-82
Normalized Protein Abundance
2000
1500
1000
500
2h
6h
2h
6h
Time (h)
SA564
SA564−ΔhptRS
SA564−ΔccpA
SA564−ΔhptRS/ccpA

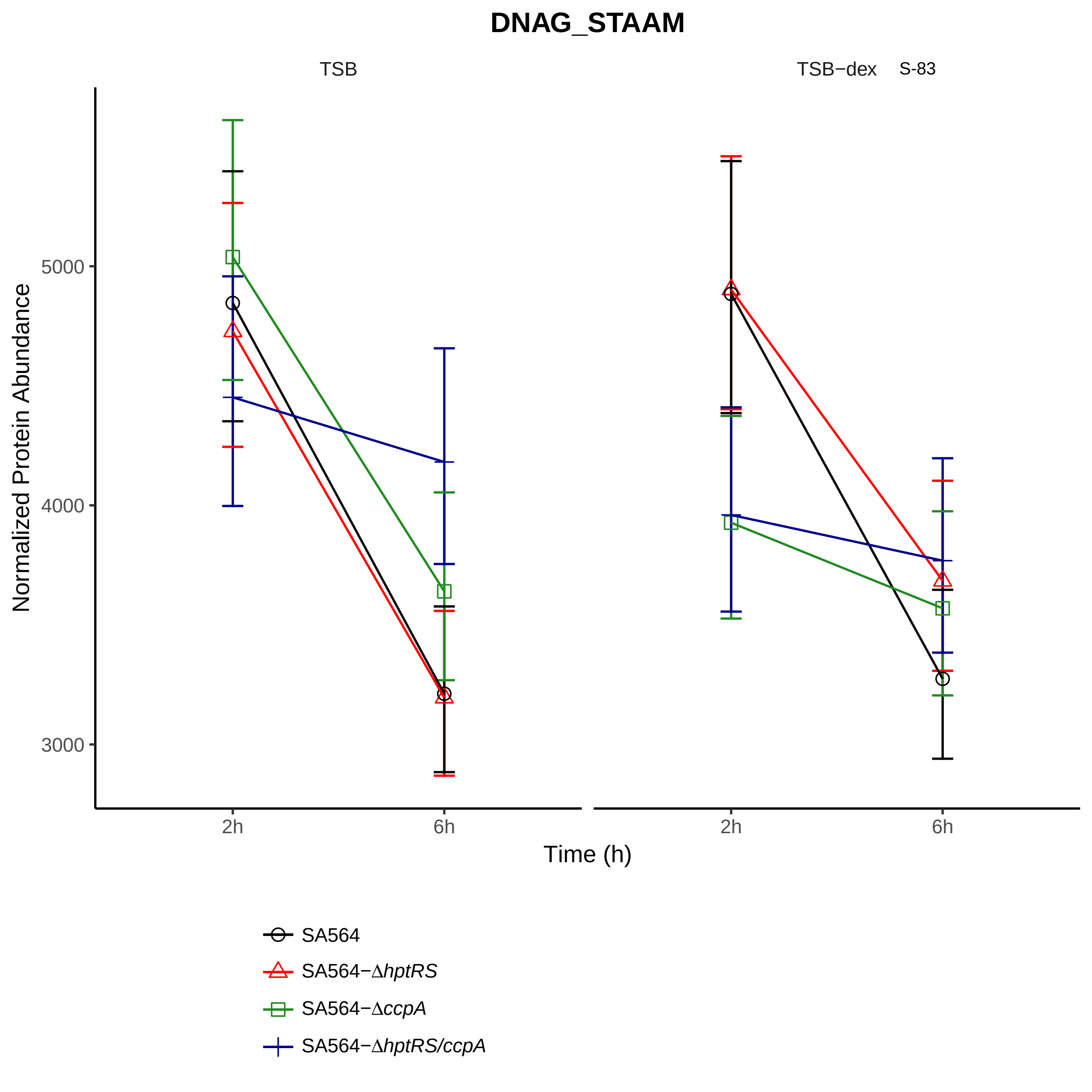

DNAG_STAAM
TSB
TSB−dex
S-83
Normalized Protein Abundance
5000
4000
3000
2h
6h
2h
6h
Time (h)
SA564
SA564−Δ*hptRS*
SA564−Δ*ccpA*
SA564−Δ*hptRS/ccpA*

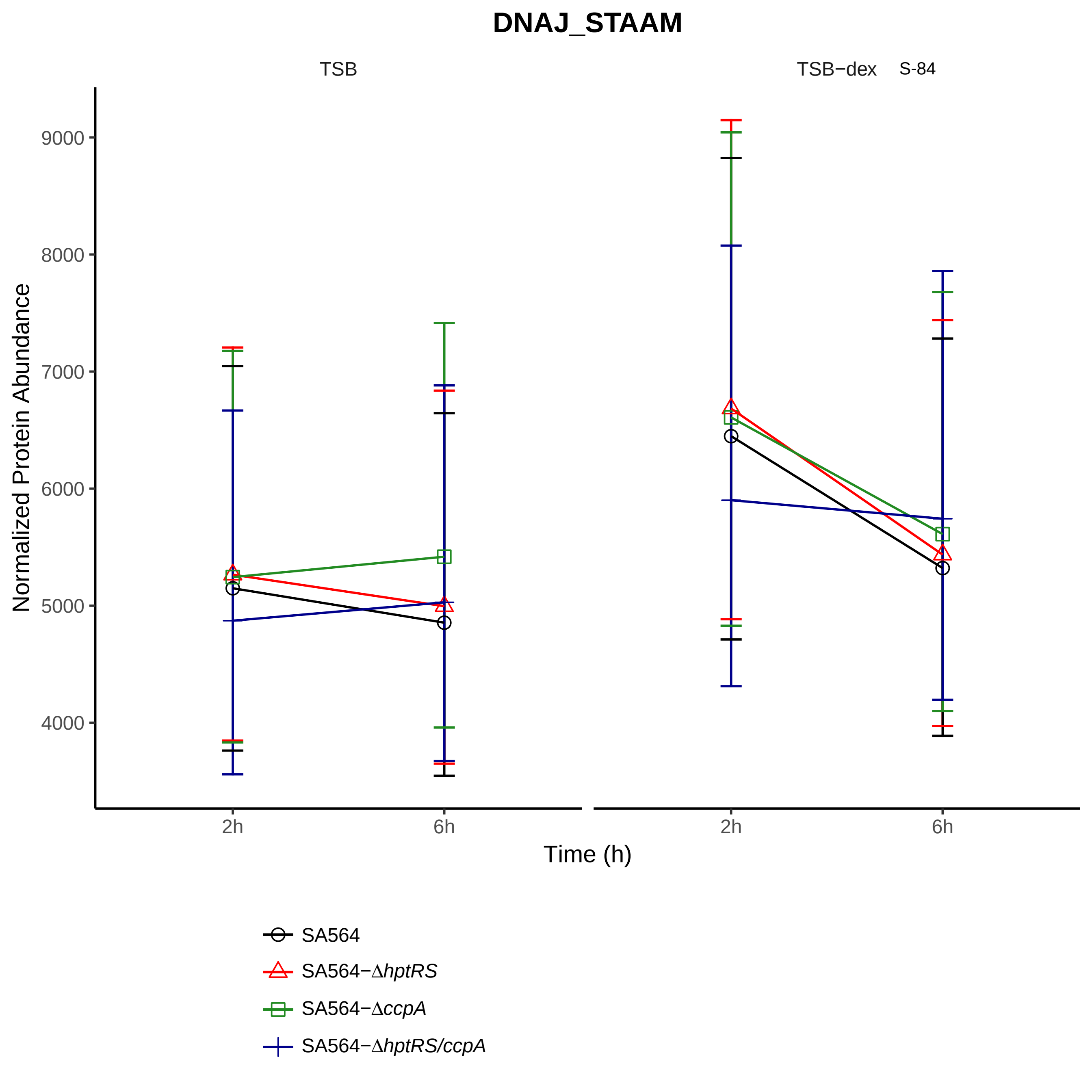
DNAJ_STAAM
TSB
TSB−dex
S-84
Normalized Protein Abundance
9000
8000
7000
6000
5000
4000
2h
6h
2h
6h
Time (h)
SA564
SA564−Δ*hptRS*
SA564−Δ*ccpA*
SA564−Δ*hptRS/ccpA*

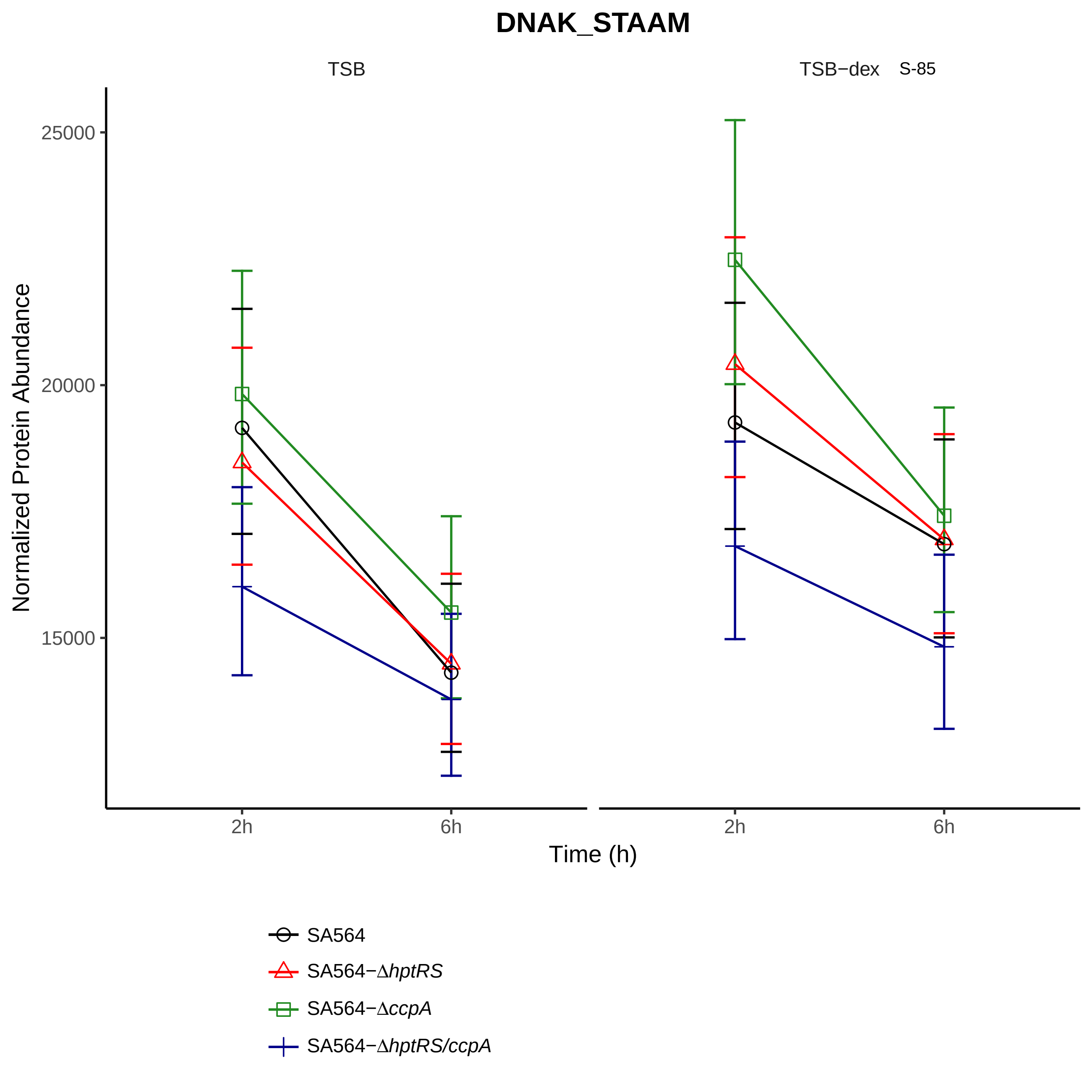

DNAK_STAAM
TSB
TSB−dex
S-85
Normalized Protein Abundance
25000
20000
15000
2h
6h
2h
6h
Time (h)
SA564
SA564−Δ*hptRS*
SA564−Δ*ccpA*
SA564−Δ*hptRS/ccpA*

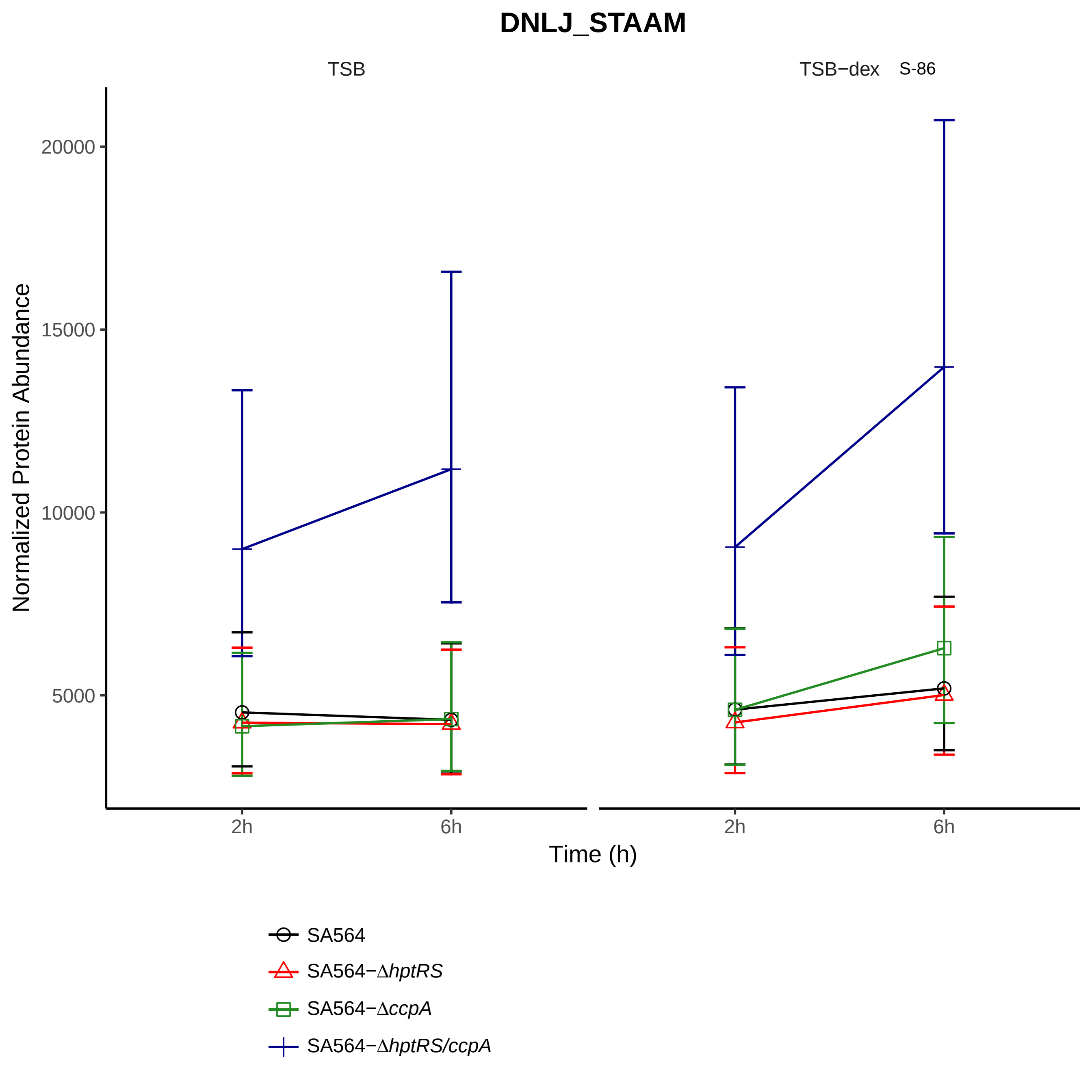
DNLJ_STAAM
TSB
TSB−dex
S-86
Normalized Protein Abundance
20000
15000
10000
5000
2h
6h
2h
6h
Time (h)
SA564
SA564−Δ*hptRS*
SA564−Δ*ccpA*
SA564−Δ*hptRS/ccpA*

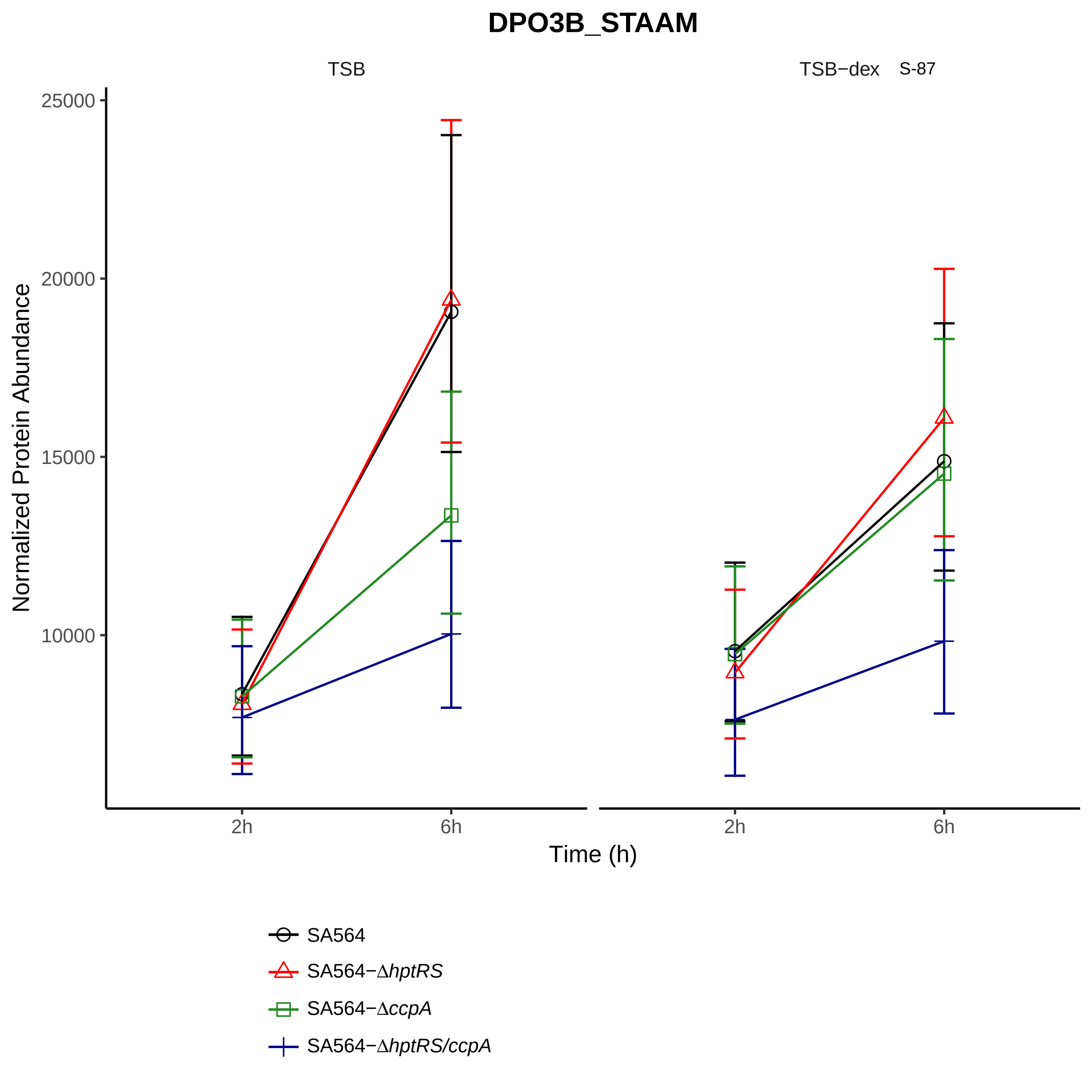
DPO3B_STAAM
TSB
TSB−dex
S-87
Normalized Protein Abundance
25000
20000
15000
10000
2h
6h
2h
6h
Time (h)
SA564
SA564−Δ*hptRS*
SA564−Δ*ccpA*
SA564−Δ*hptRS/ccpA*

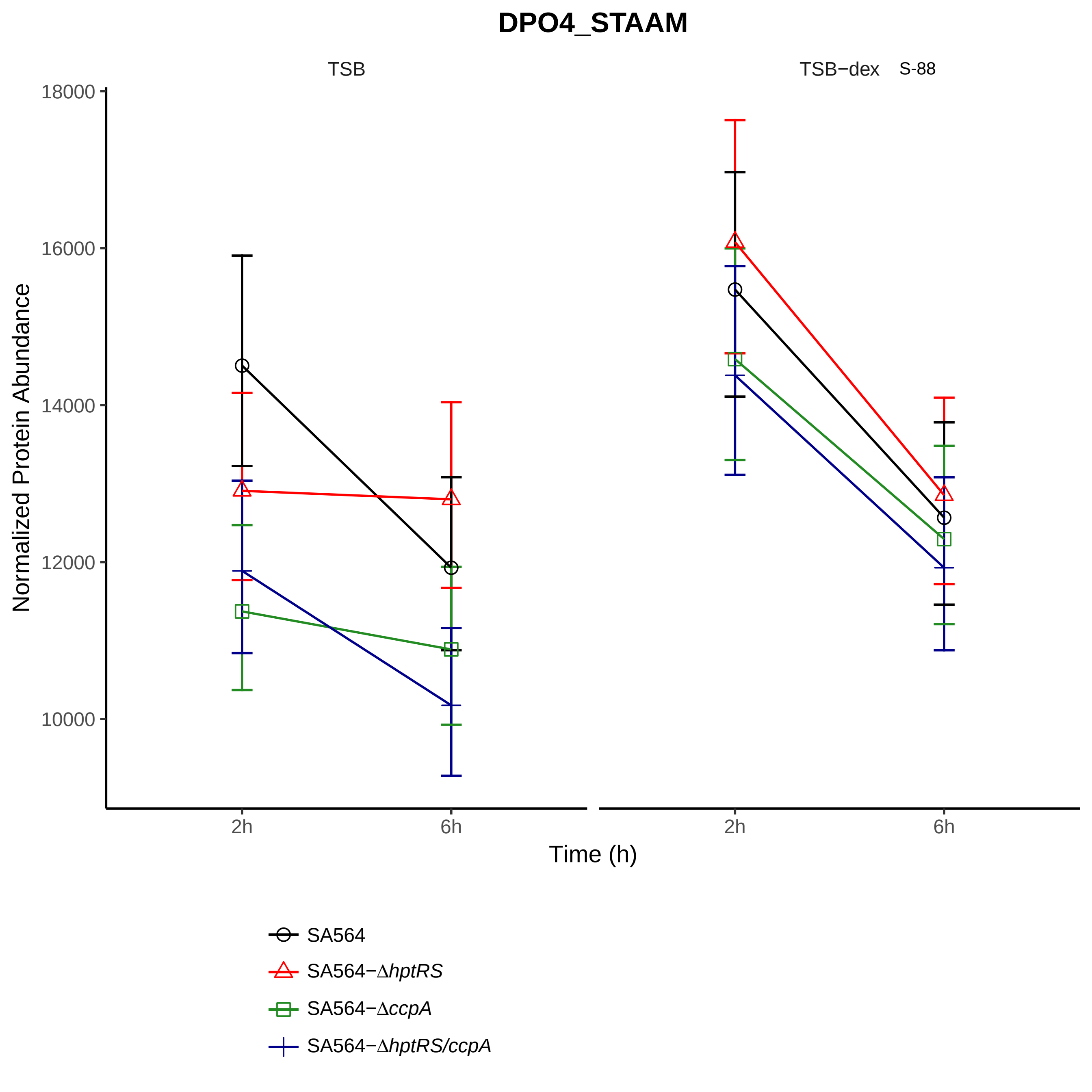

DPO4_STAAM
TSB
TSB−dex
S-88
Normalized Protein Abundance
18000
16000
14000
12000
10000
2h
6h
2h
6h
Time (h)
SA564
SA564−Δ*hptRS*
SA564−Δ*ccpA*
SA564−Δ*hptRS/ccpA*

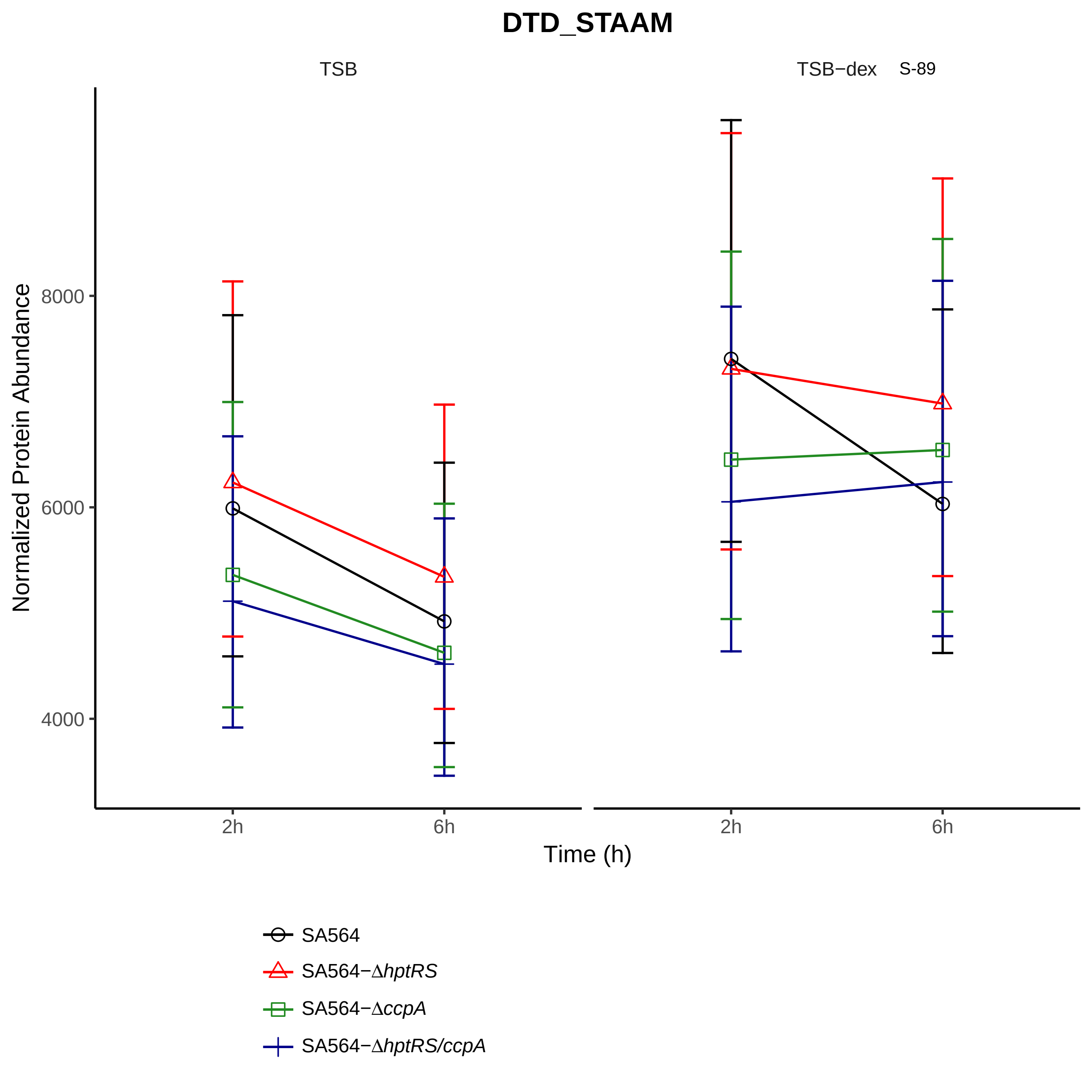

DTD_STAAM
TSB
TSB−dex
S-89
Normalized Protein Abundance
8000
6000
4000
2h
6h
2h
6h
Time (h)
SA564
SA564−Δ*hptRS*
SA564−Δ*ccpA*
SA564−Δ*hptRS/ccpA*

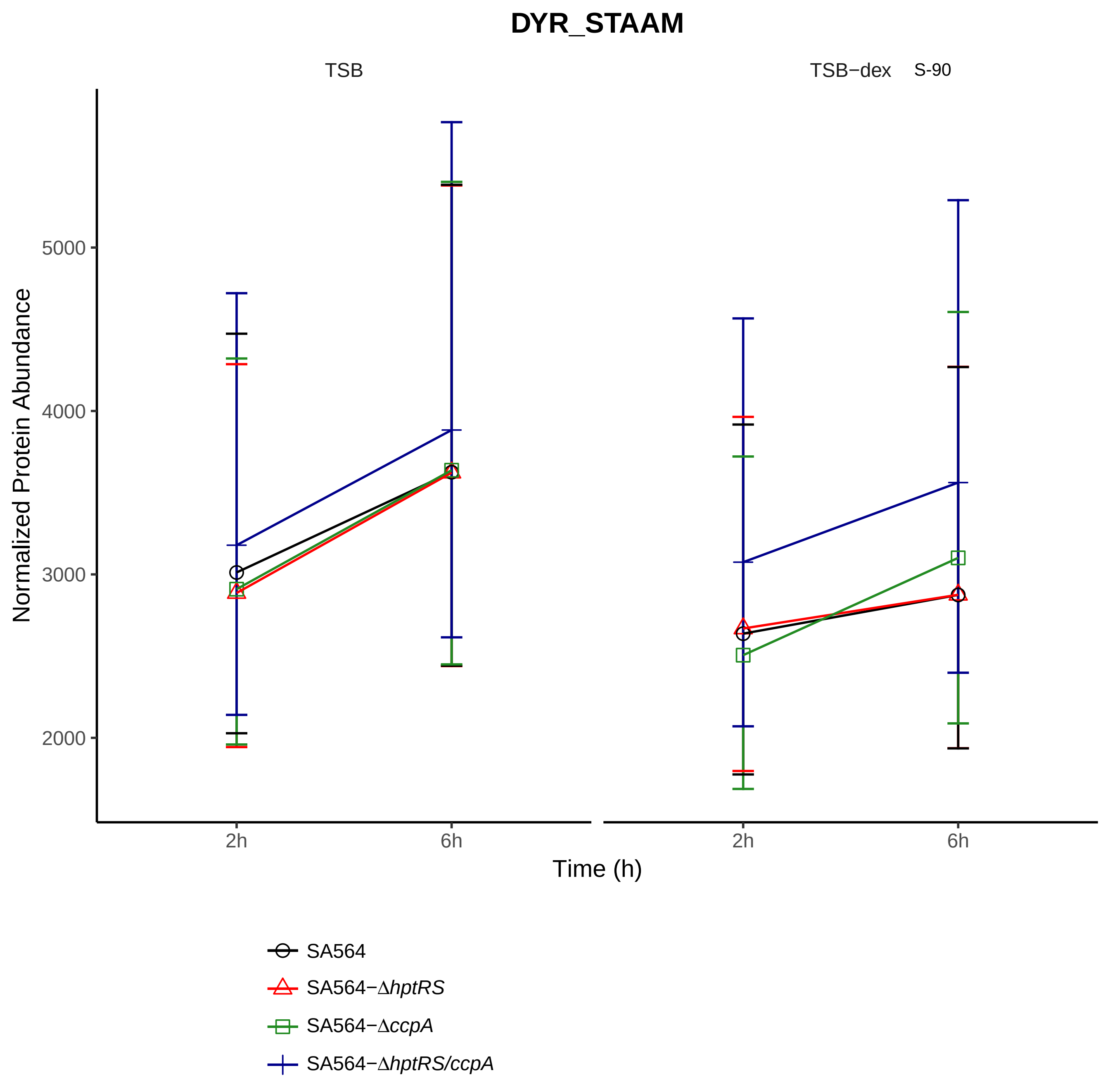

DYR_STAAM
TSB
TSB−dex
S-90
Normalized Protein Abundance
5000
4000
3000
2000
2h
6h
2h
6h
Time (h)
SA564
SA564−ΔhptRS
SA564−ΔccpA
SA564−ΔhptRS/ccpA

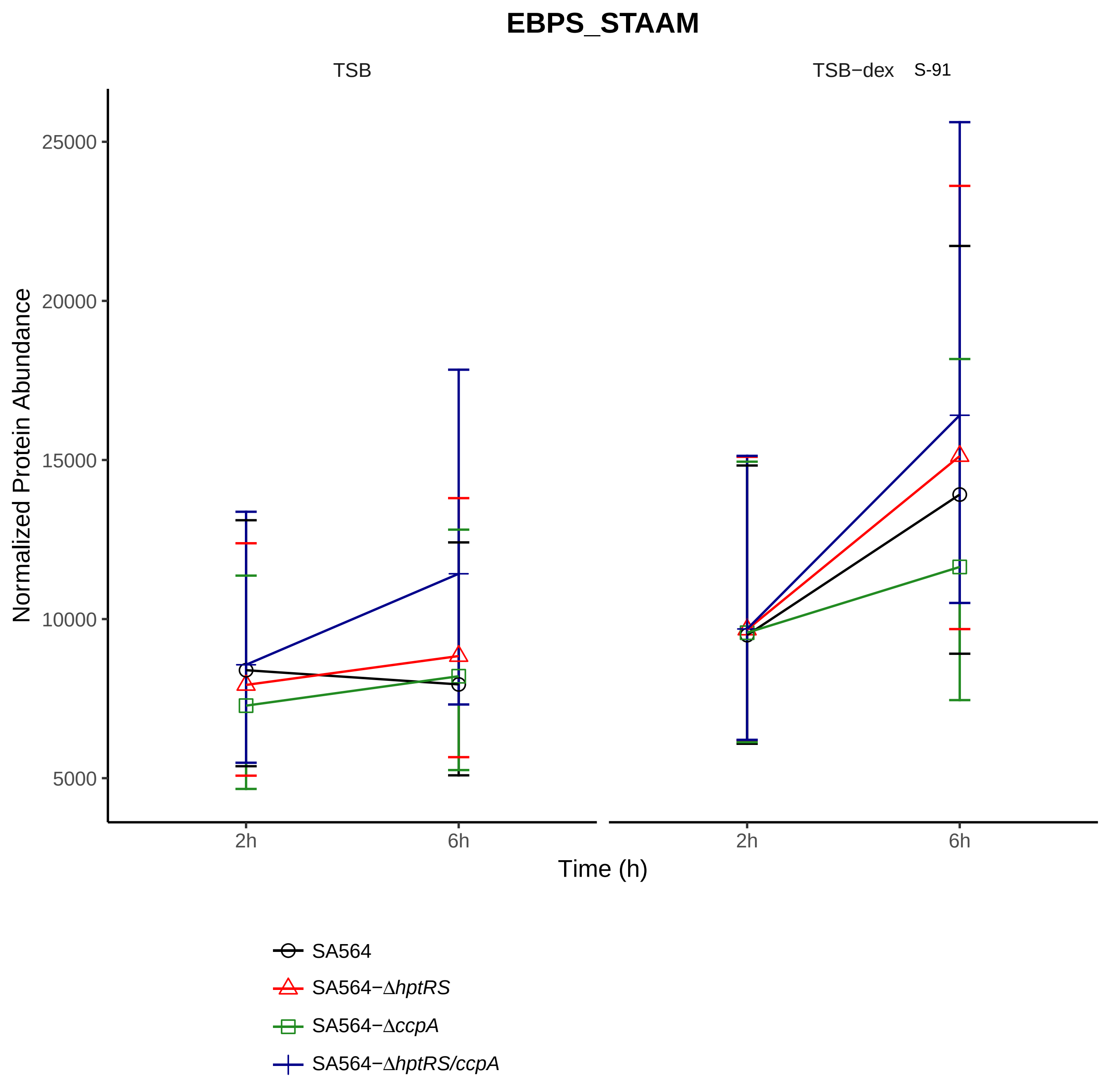
EBPS_STAAM
TSB
TSB−dex
S-91
Normalized Protein Abundance
25000
20000
15000
10000
5000
2h
6h
2h
6h
Time (h)
SA564
SA564−ΔhptRS
SA564−ΔccpA
SA564−ΔhptRS/ccpA

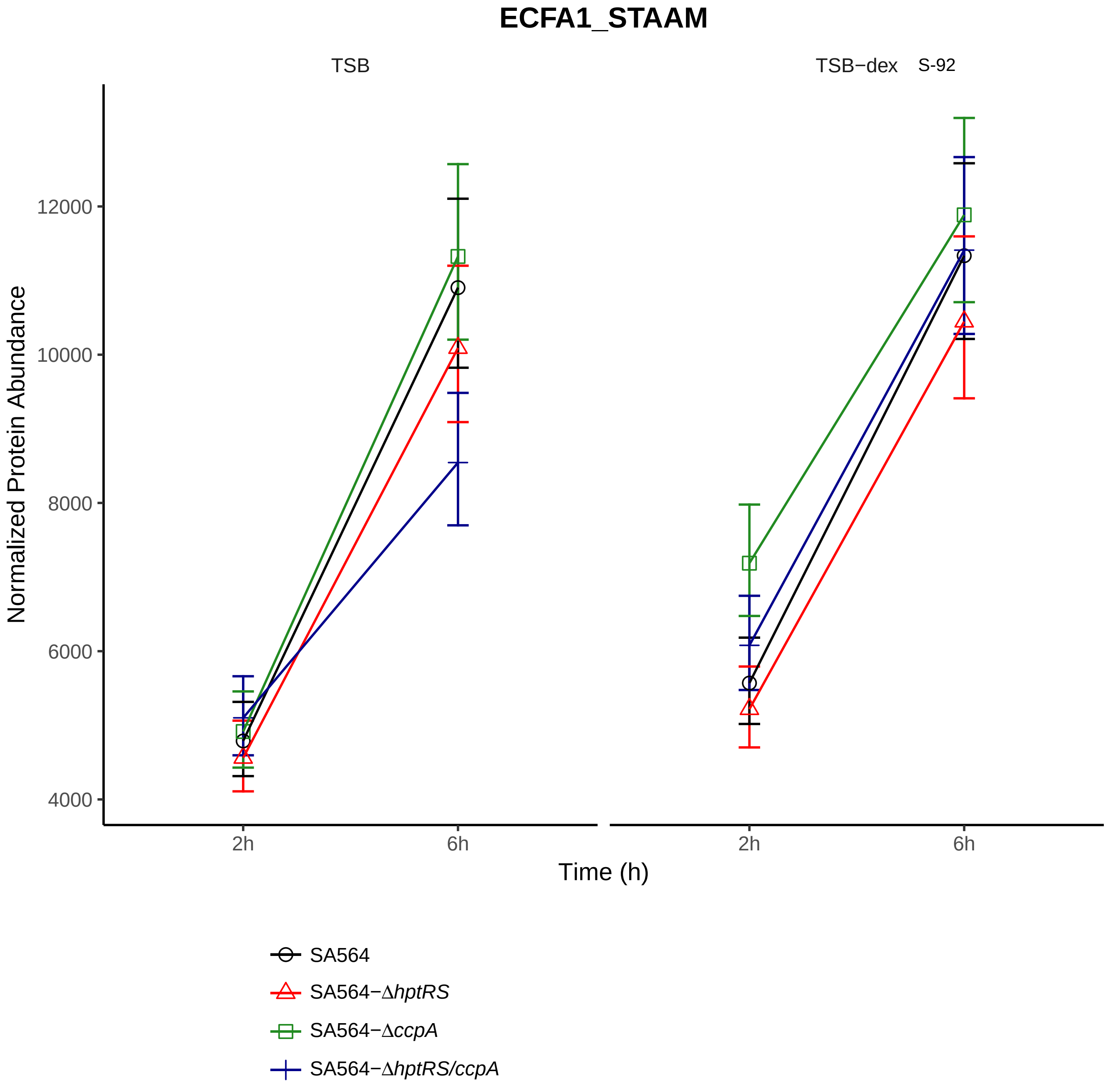

ECFA1_STAAM
TSB
TSB−dex
Normalized Protein Abundance
12000
10000
8000
6000
4000
2h
6h
2h
6h
Time (h)
SA564
SA564−Δ*hptRS*
SA564−Δ*ccpA*
SA564−Δ*hptRS/ccpA*

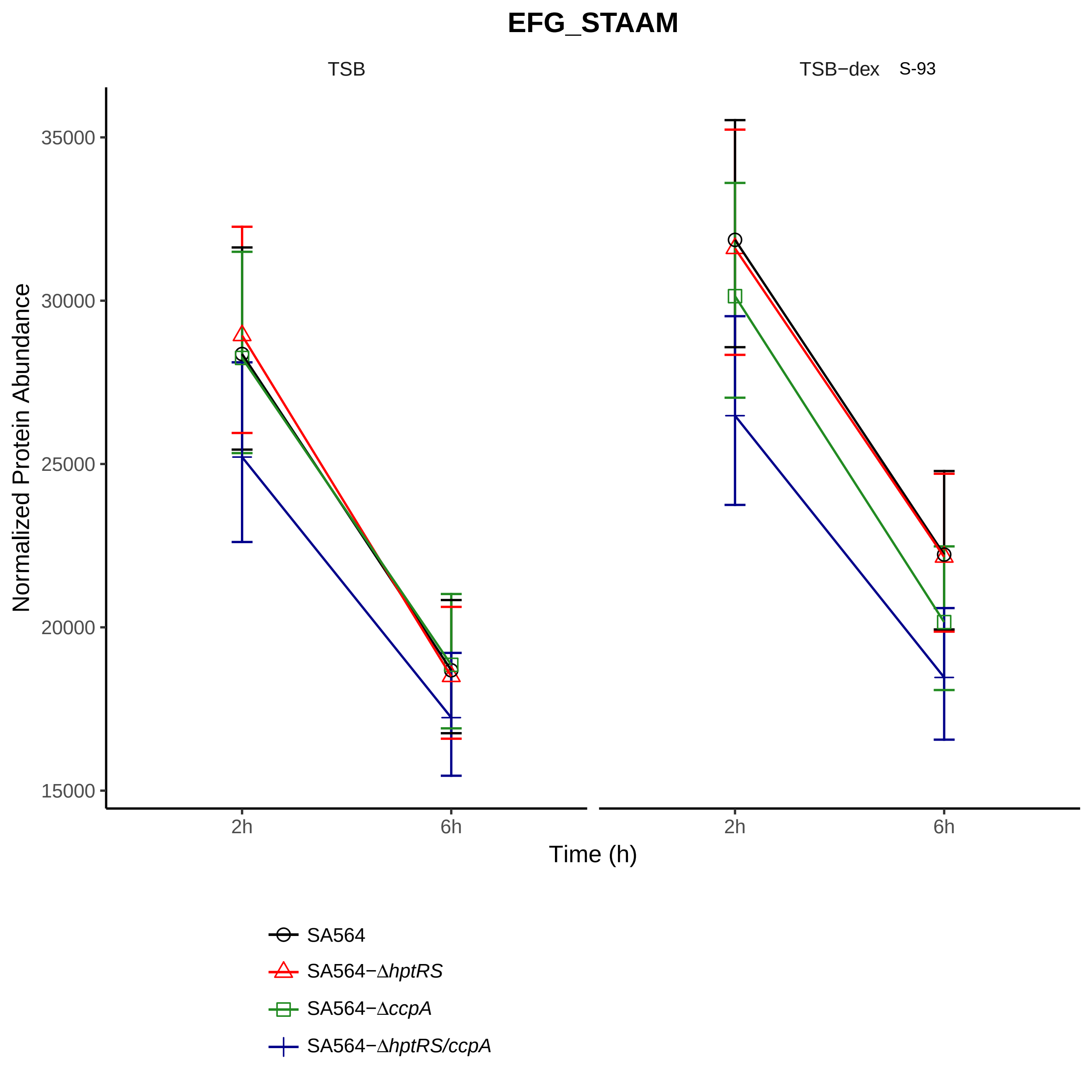
EFG_STAAM
TSB
TSB−dex
S-93
Normalized Protein Abundance
35000
30000
25000
20000
15000
2h
6h
2h
6h
Time (h)
SA564
SA564−ΔhptRS
SA564−ΔccpA
SA564−ΔhptRS/ccpA

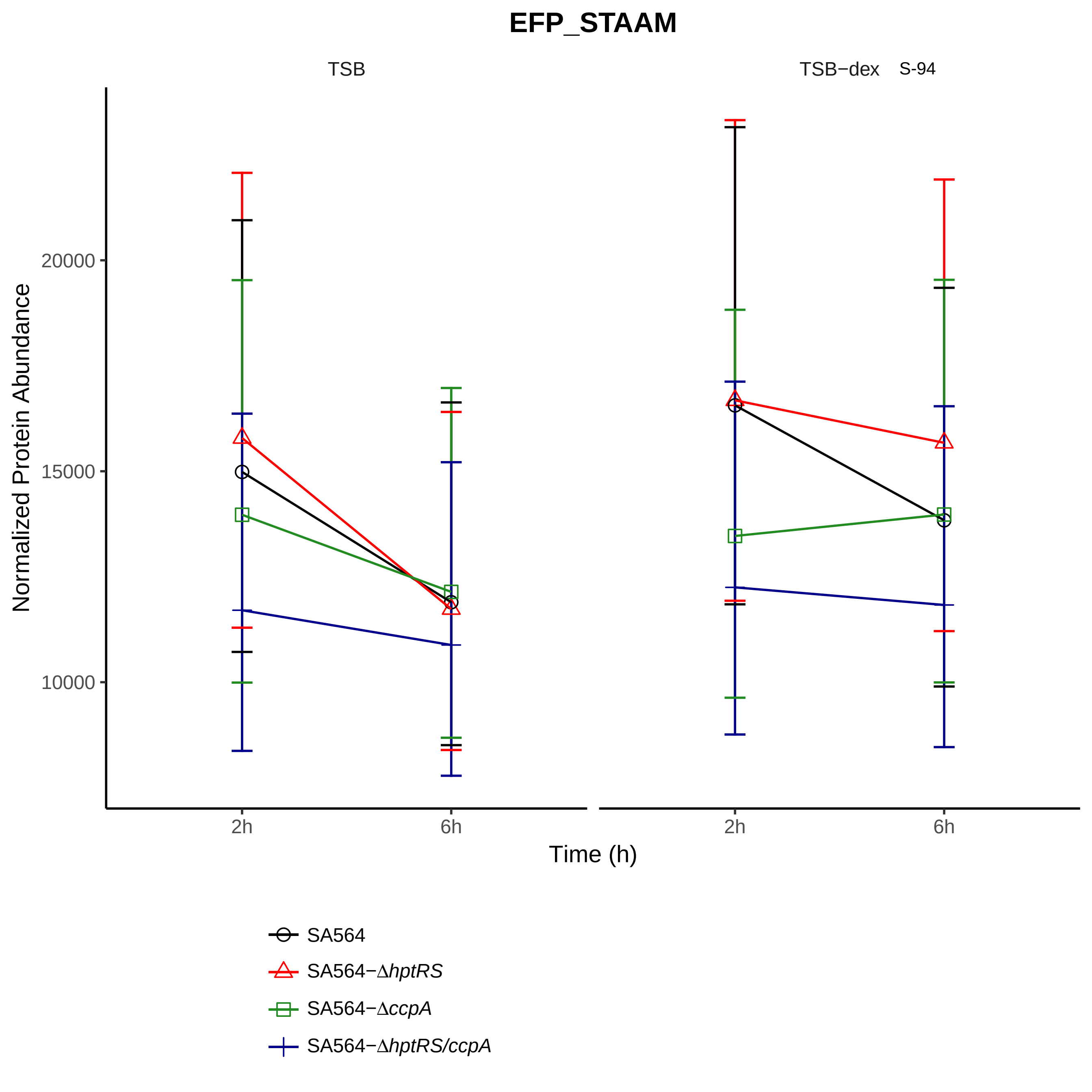

EFP_STAAM
TSB
TSB−dex
S-94
Normalized Protein Abundance
20000
15000
10000
2h
6h
2h
6h
Time (h)
SA564
SA564−Δ*hptRS*
SA564−Δ*ccpA*
SA564−Δ*hptRS/ccpA*

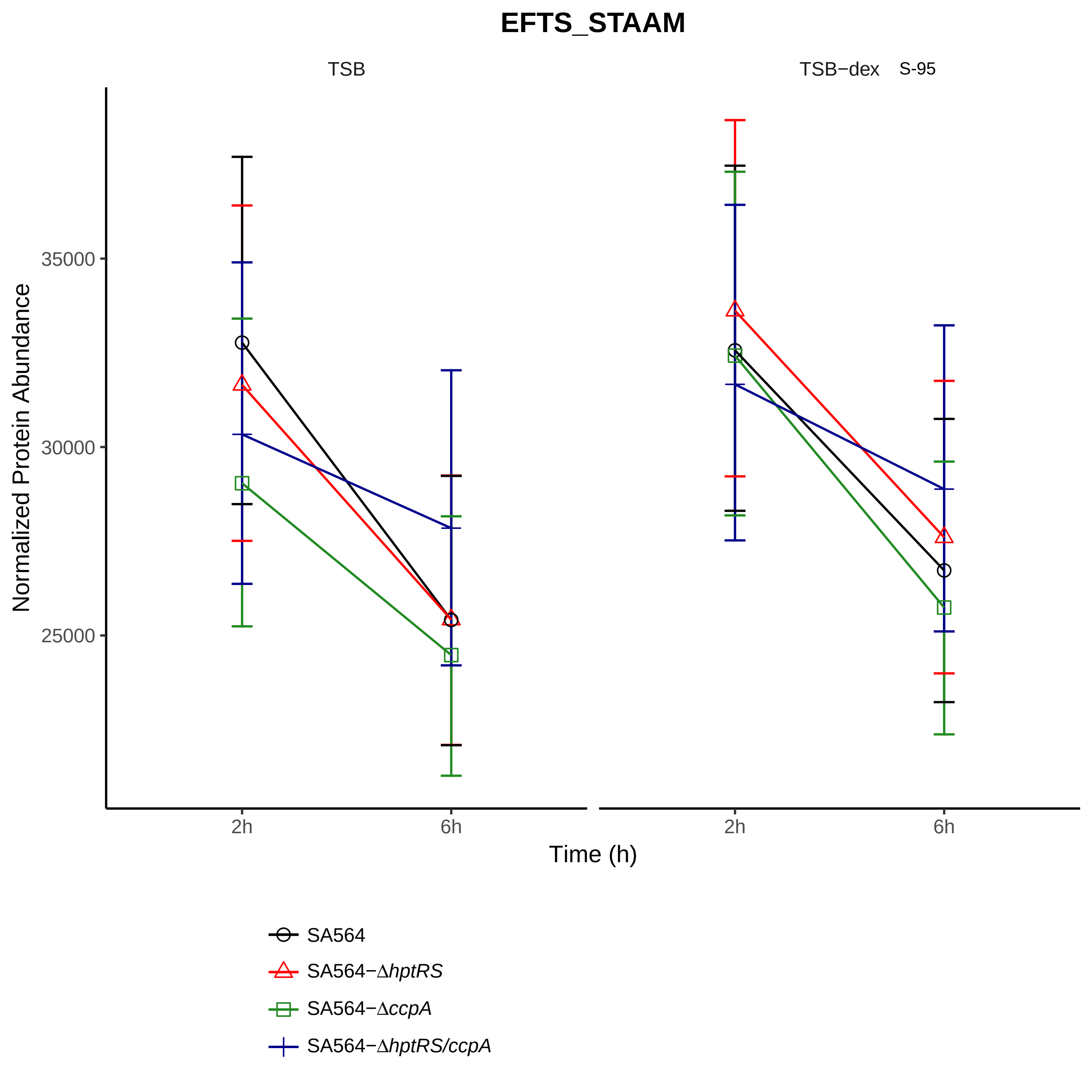

EFTS_STAAM
TSB
TSB−dex
S-95
Normalized Protein Abundance
35000
30000
25000
2h
6h
2h
6h
Time (h)
SA564
SA564−Δ*hptRS*
SA564−Δ*ccpA*
SA564−Δ*hptRS/ccpA*

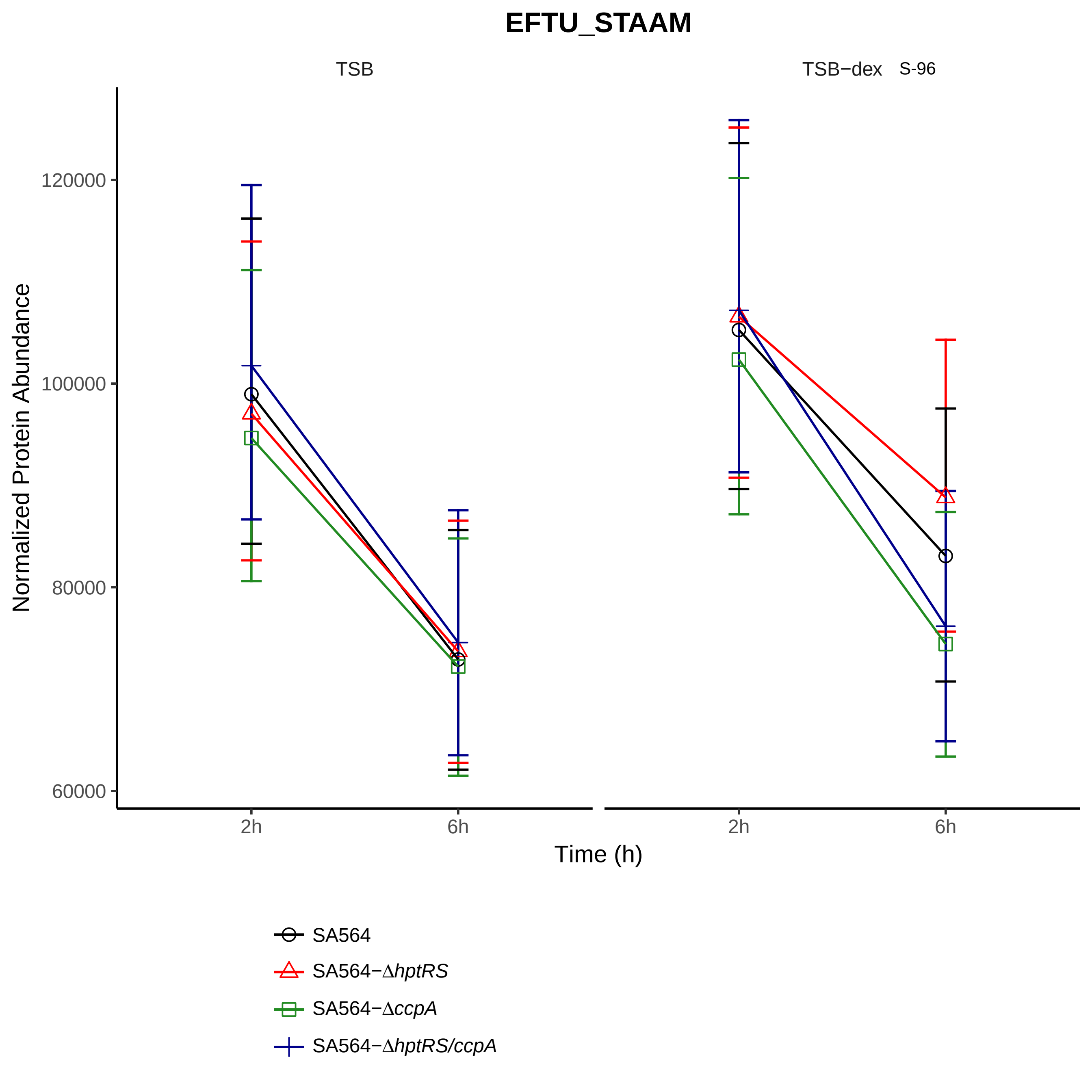

EFTU_STAAM
TSB
TSB−dex
S-96
120000
100000
80000
60000
Normalized Protein Abundance
2h
6h
2h
6h
Time (h)
SA564
SA564−ΔhptRS
SA564−ΔccpA
SA564−ΔhptRS/ccpA

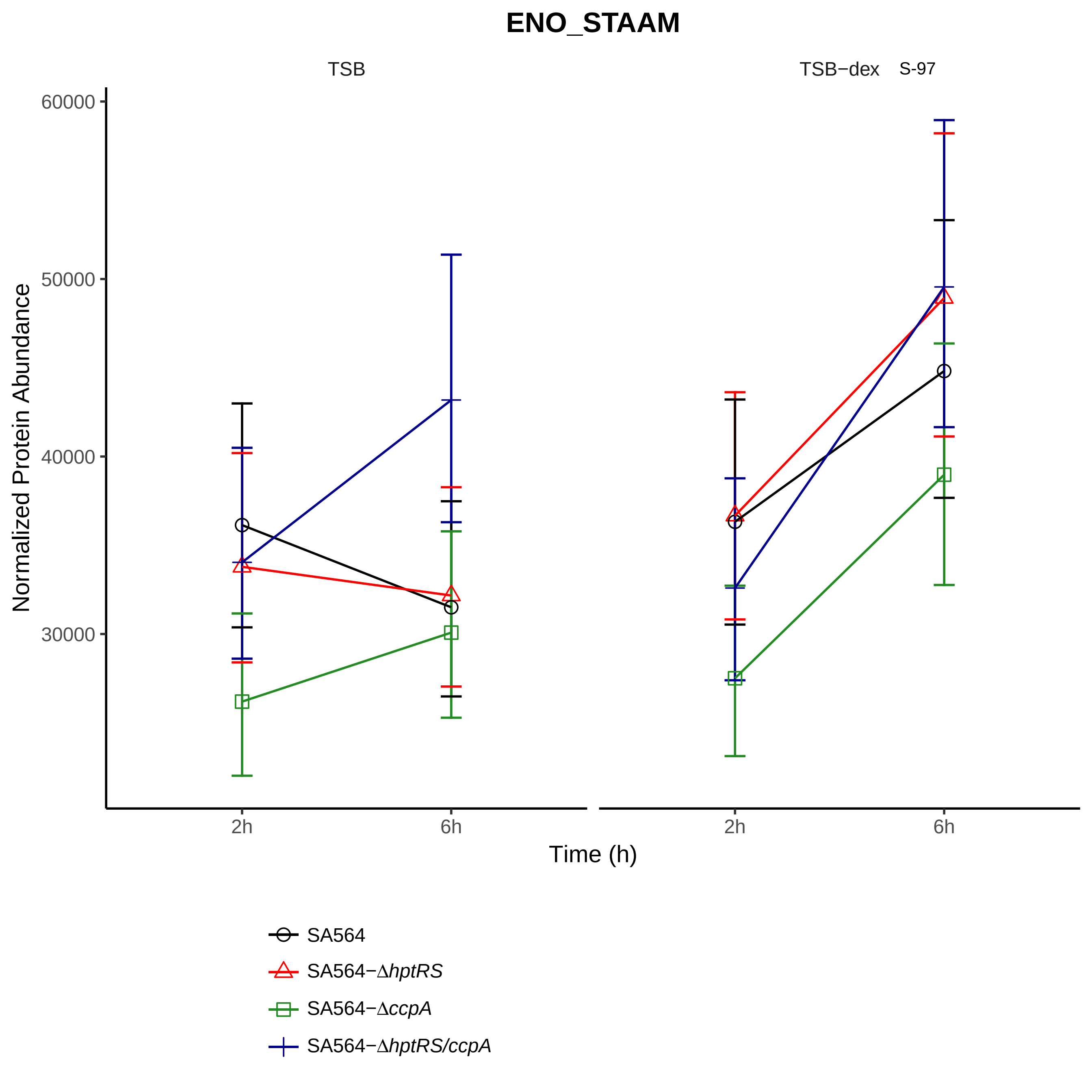

ENO_STAAM
TSB
TSB−dex
S-97
Normalized Protein Abundance
60000
50000
40000
30000
2h
6h
2h
6h
Time (h)
SA564
SA564−Δ*hptRS*
SA564−Δ*ccpA*
SA564−Δ*hptRS/ccpA*

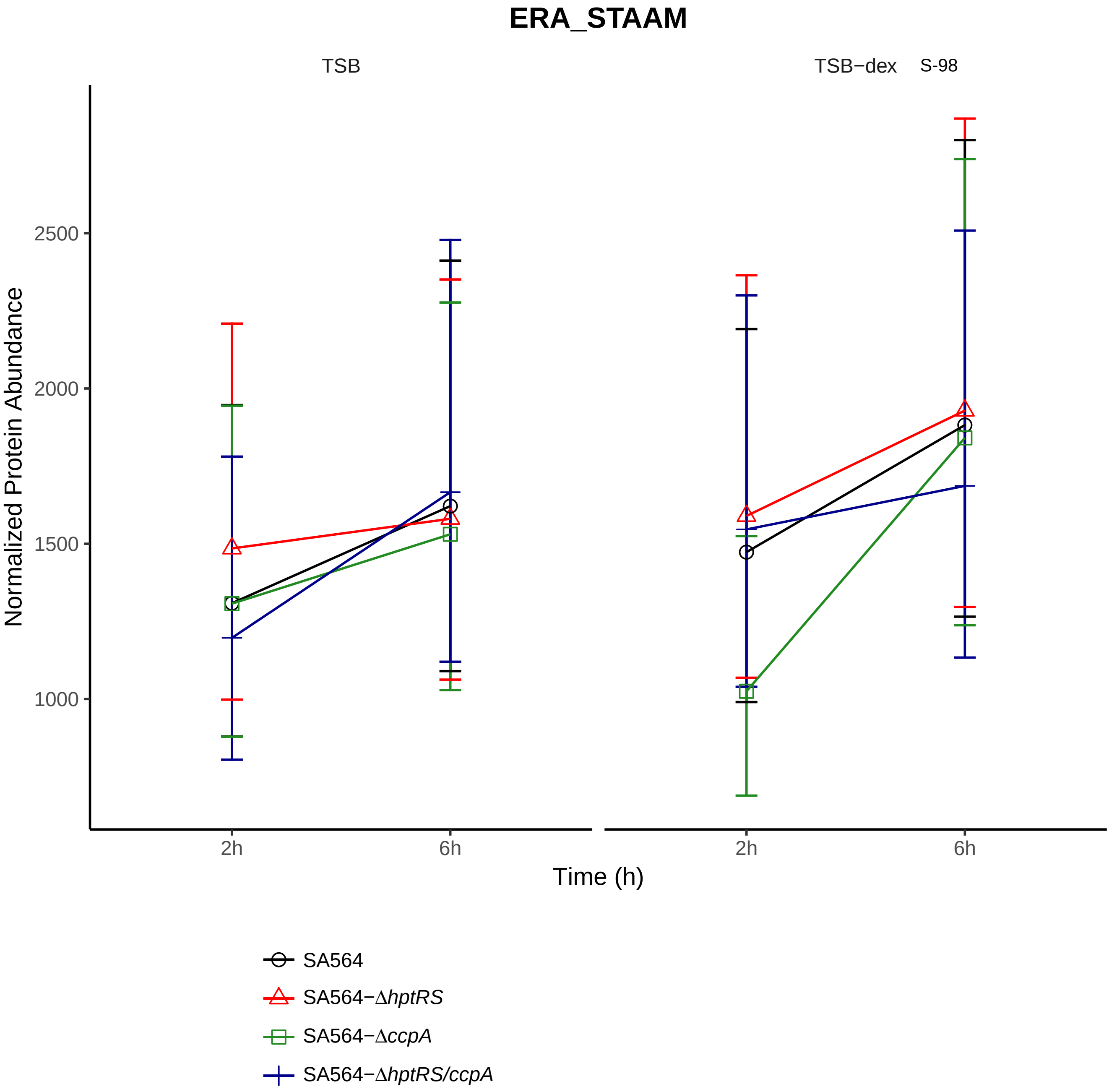

ERA_STAAM
TSB
TSB−dex
S-98
Normalized Protein Abundance
2500
2000
1500
1000
2h
6h
2h
6h
Time (h)
SA564
SA564−Δ*hptRS*
SA564−Δ*ccpA*
SA564−Δ*hptRS/ccpA*

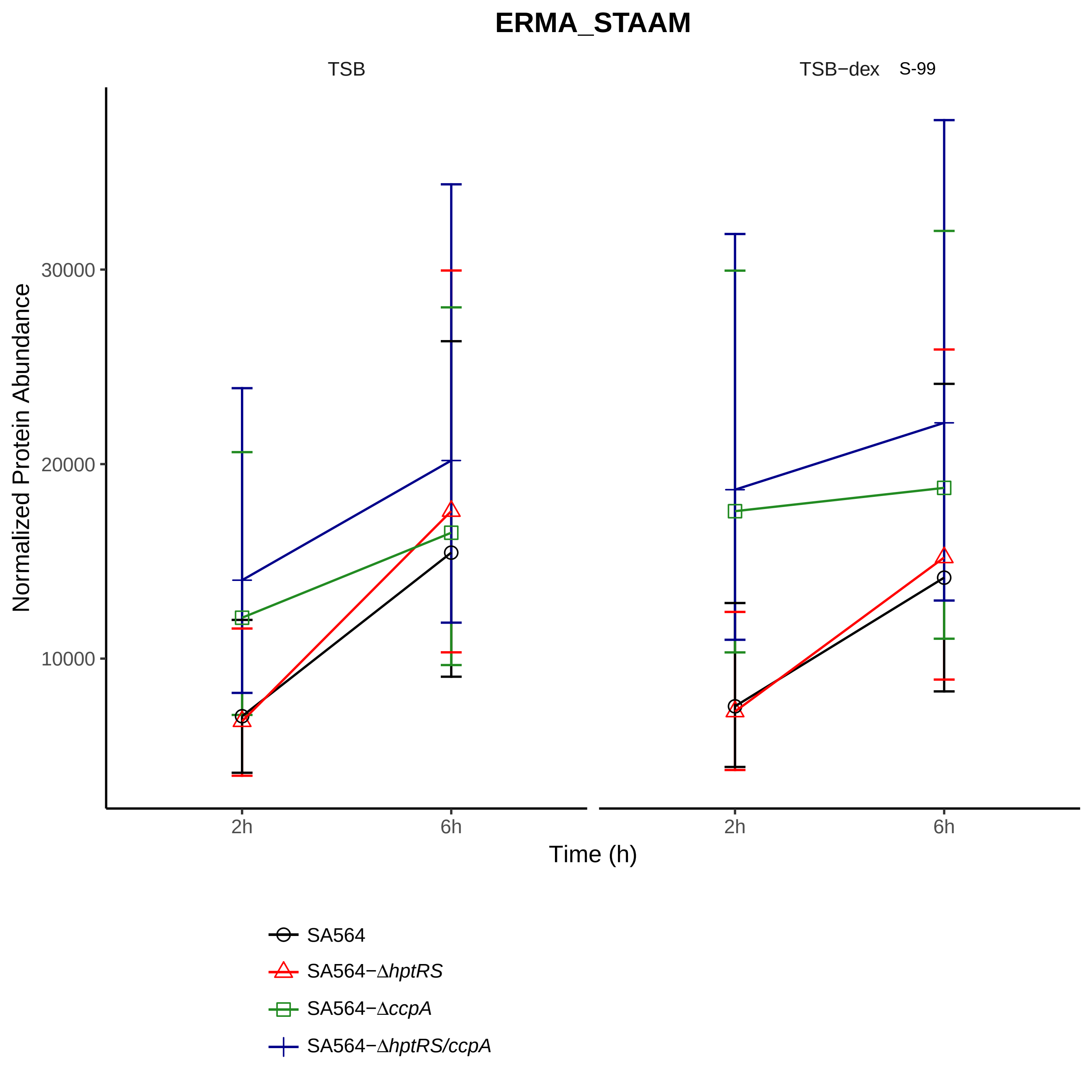
ERMA_STAAM
TSB
TSB−dex
S-99
Normalized Protein Abundance
30000
20000
10000
2h
6h
2h
6h
Time (h)
SA564
SA564−Δ*hptRS*
SA564−Δ*ccpA*
SA564−Δ*hptRS/ccpA*

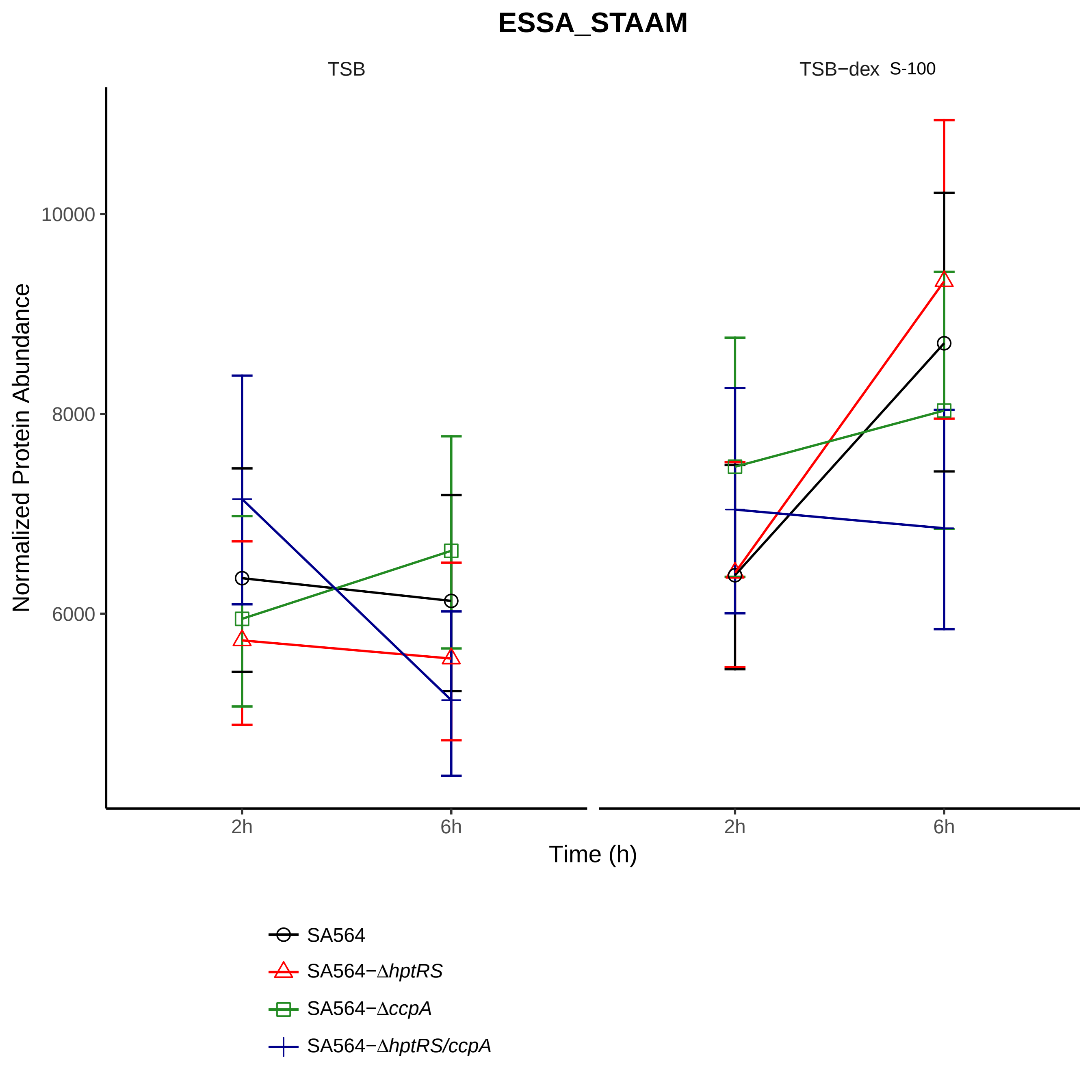

ESSA_STAAM
TSB
TSB−dex
S-100
Normalized Protein Abundance
10000
8000
6000
2h
6h
2h
6h
Time (h)
SA564
SA564−Δ*hptRS*
SA564−Δ*ccpA*
SA564−Δ*hptRS/ccpA*

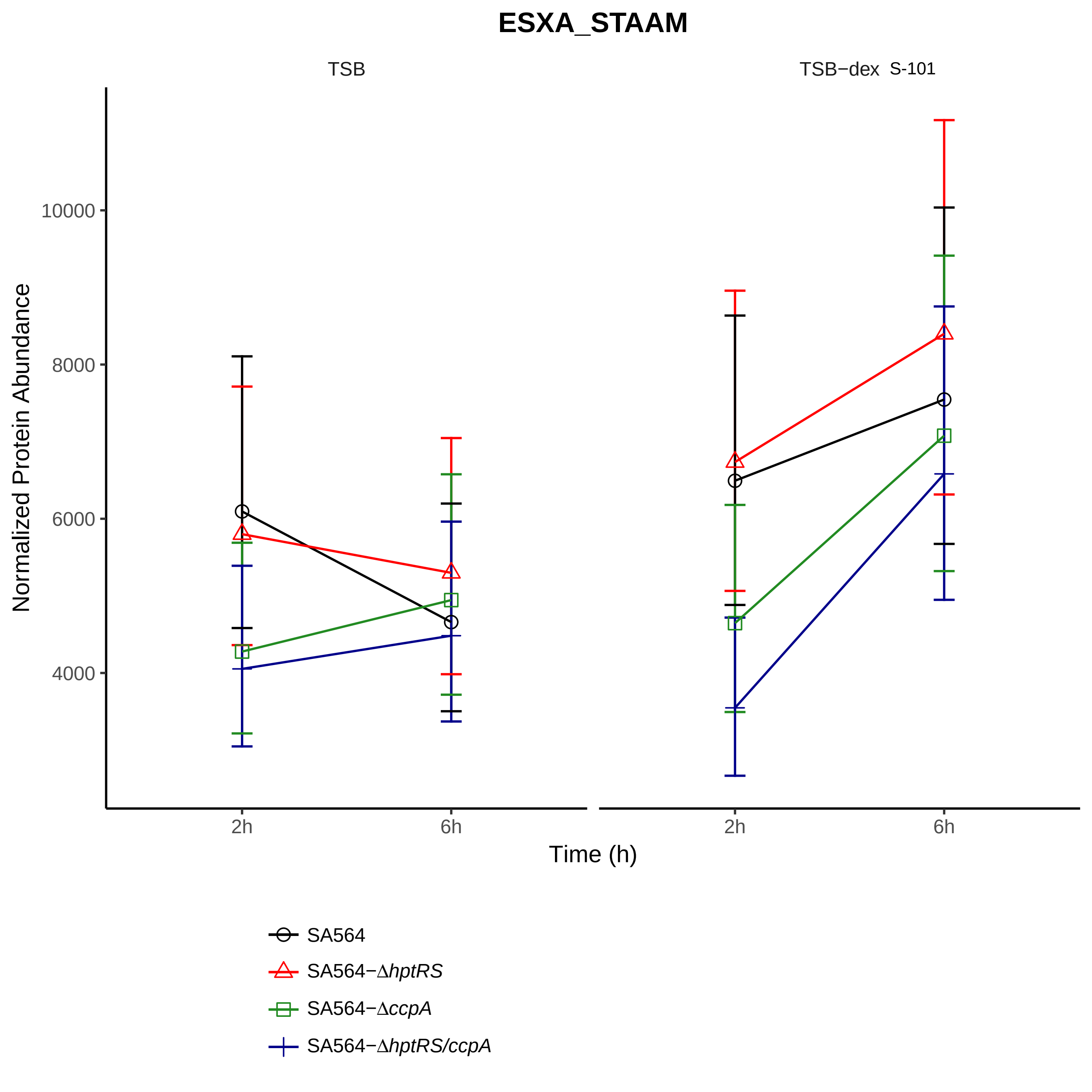

ESXA_STAAM
TSB
TSB−dex
S-101
Normalized Protein Abundance
10000
8000
6000
4000
2h
6h
2h
6h
Time (h)
SA564
SA564−ΔhptRS
SA564−ΔccpA
SA564−ΔhptRS/ccpA

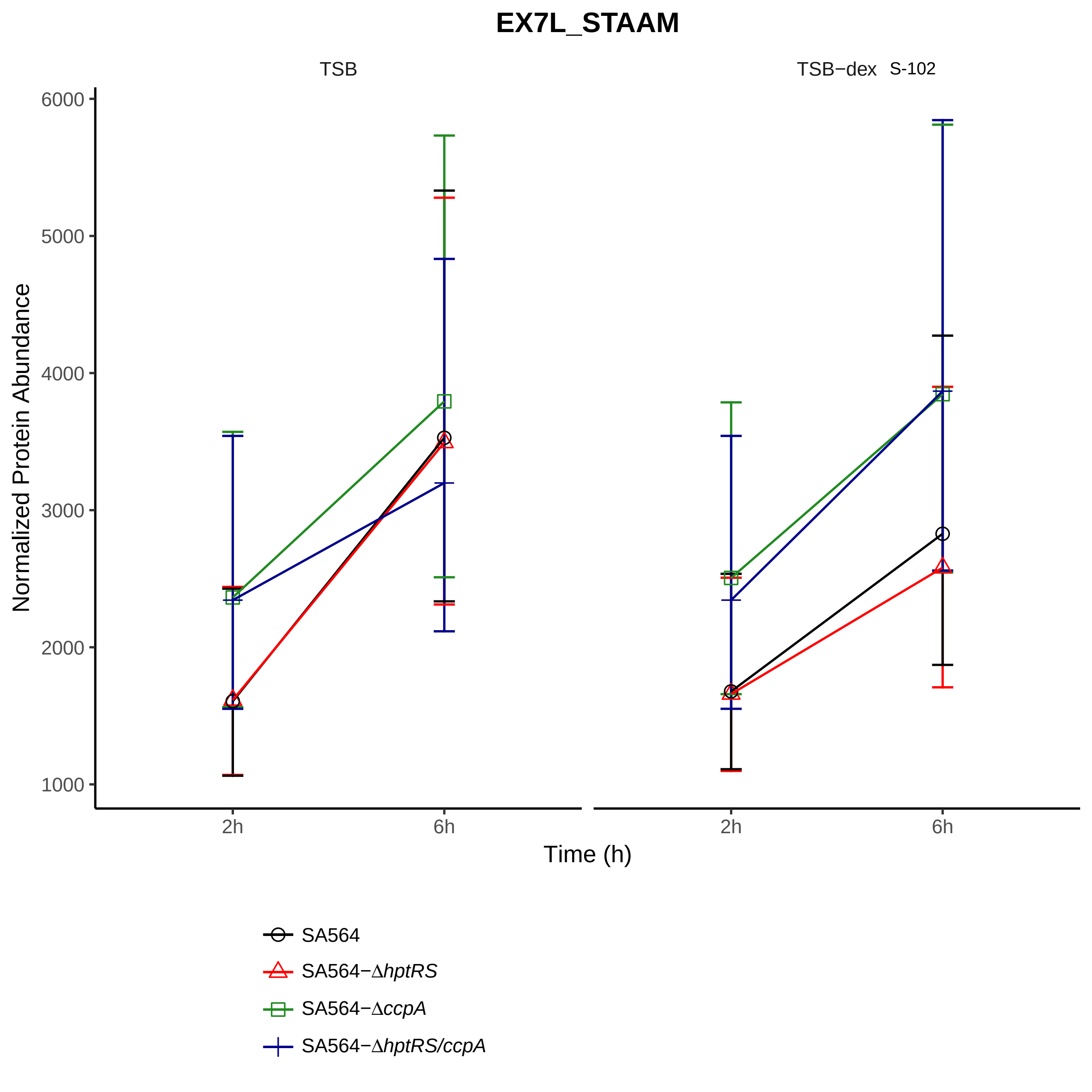

EX7L_STAAM
TSB
TSB−dex
S-102
Normalized Protein Abundance
6000
5000
4000
3000
2000
1000
2h
6h
2h
6h
Time (h)
SA564
SA564−Δ*hptRS*
SA564−Δ*ccpA*
SA564−Δ*hptRS/ccpA*

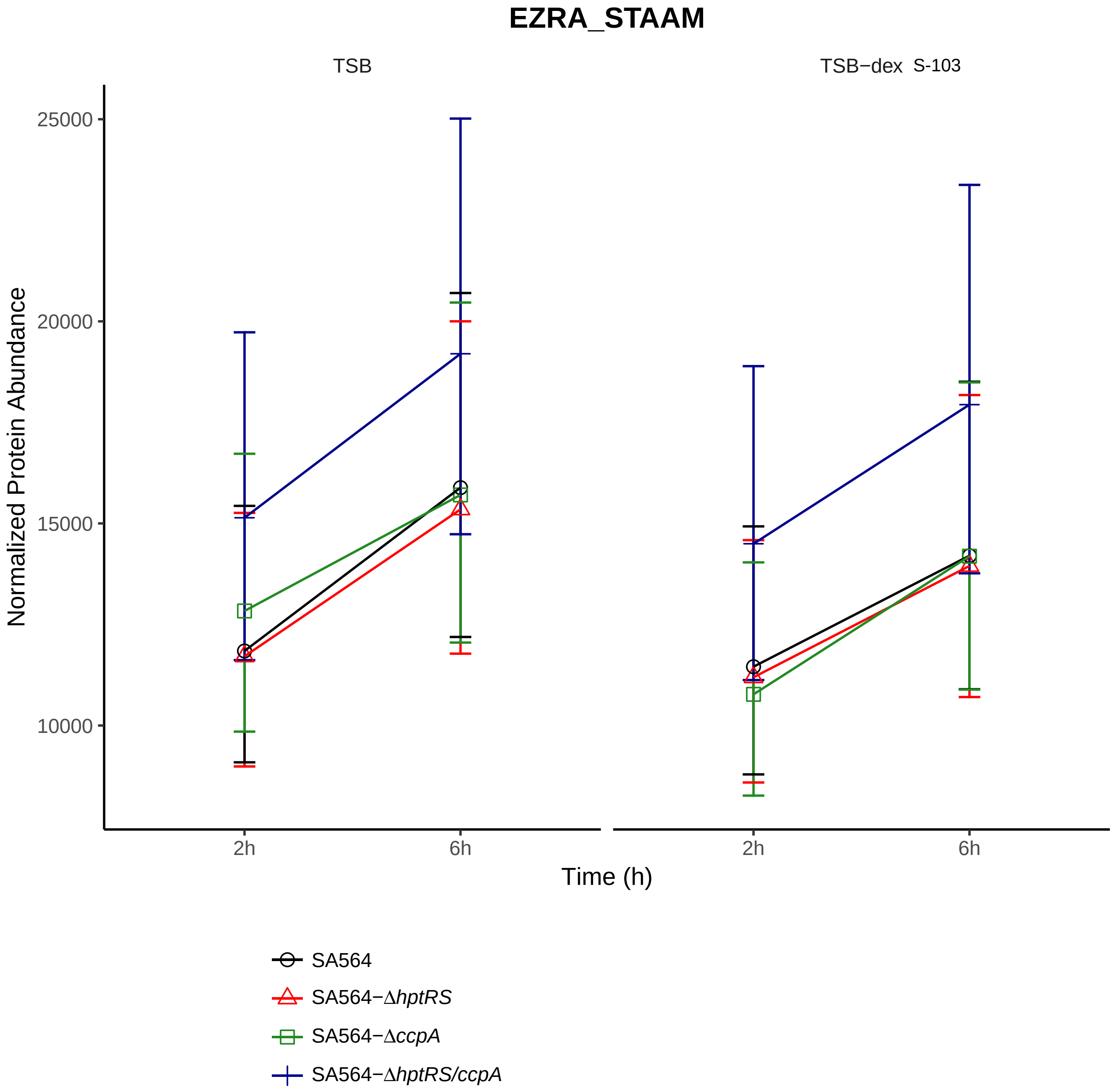

EZRA_STAAM
TSB
TSB−dex
S-103
Normalized Protein Abundance
25000
20000
15000
10000
2h
6h
2h
6h
Time (h)
SA564
SA564−Δ*hptRS*
SA564−Δ*ccpA*
SA564−Δ*hptRS/ccpA*

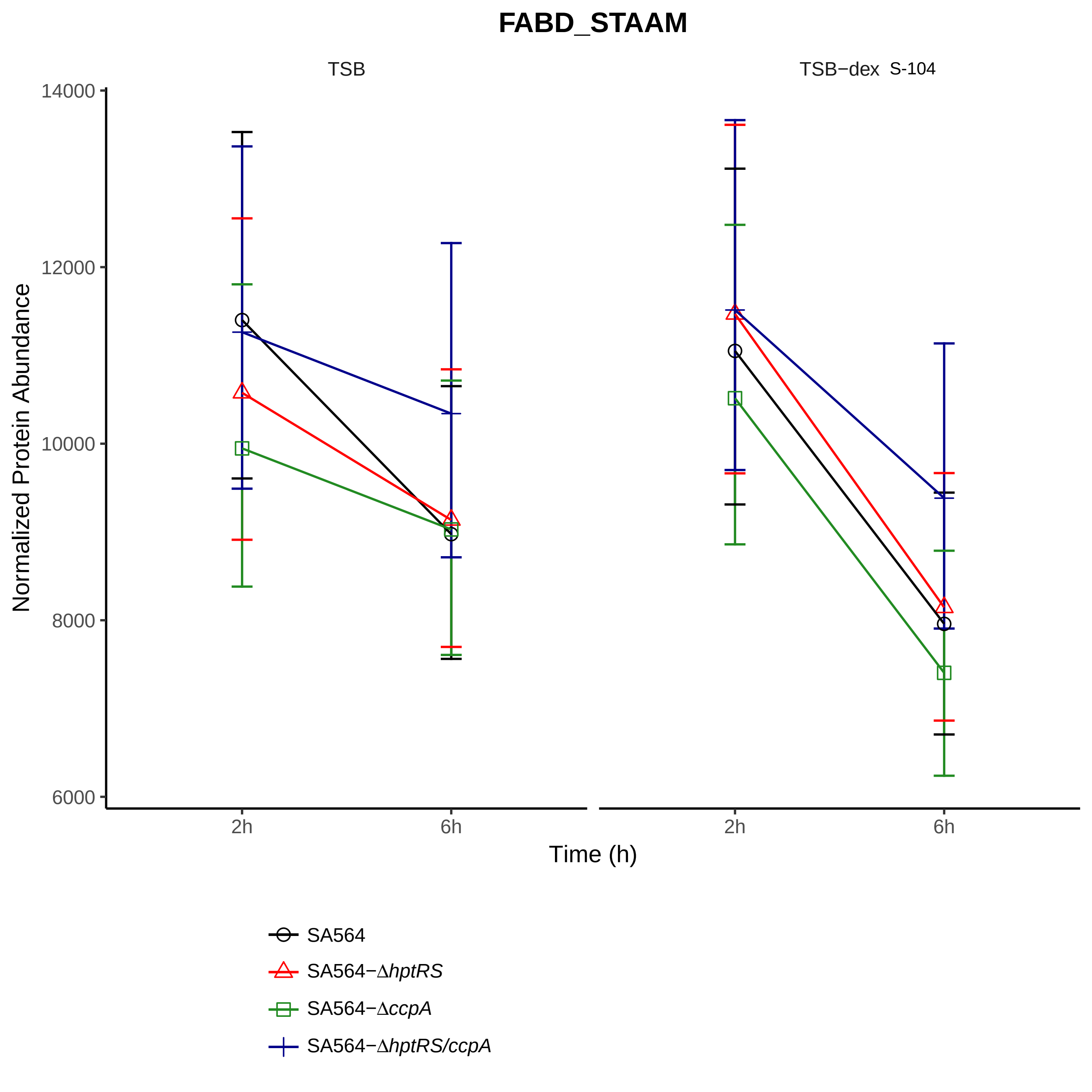

FABD_STAAM
TSB
TSB−dex
S-104
Normalized Protein Abundance
14000
12000
10000
8000
6000
2h
6h
2h
6h
Time (h)
SA564
SA564−Δ*hptRS*
SA564−Δ*ccpA*
SA564−Δ*hptRS/ccpA*

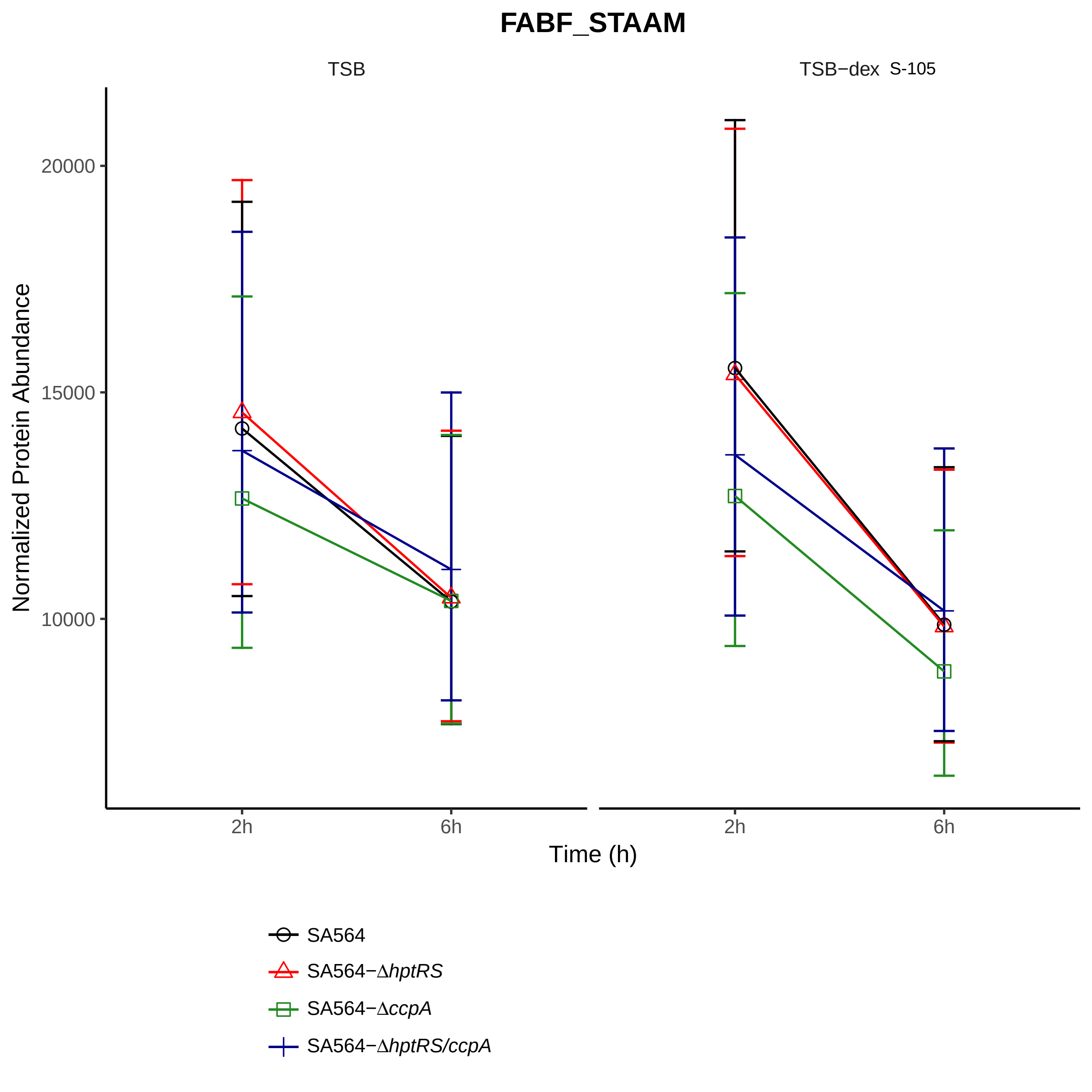

FABF_STAAM
TSB
TSB−dex
S-105
Normalized Protein Abundance
20000
15000
10000
2h
6h
2h
6h
Time (h)
SA564
SA564−ΔhptRS
SA564−ΔccpA
SA564−ΔhptRS/ccpA

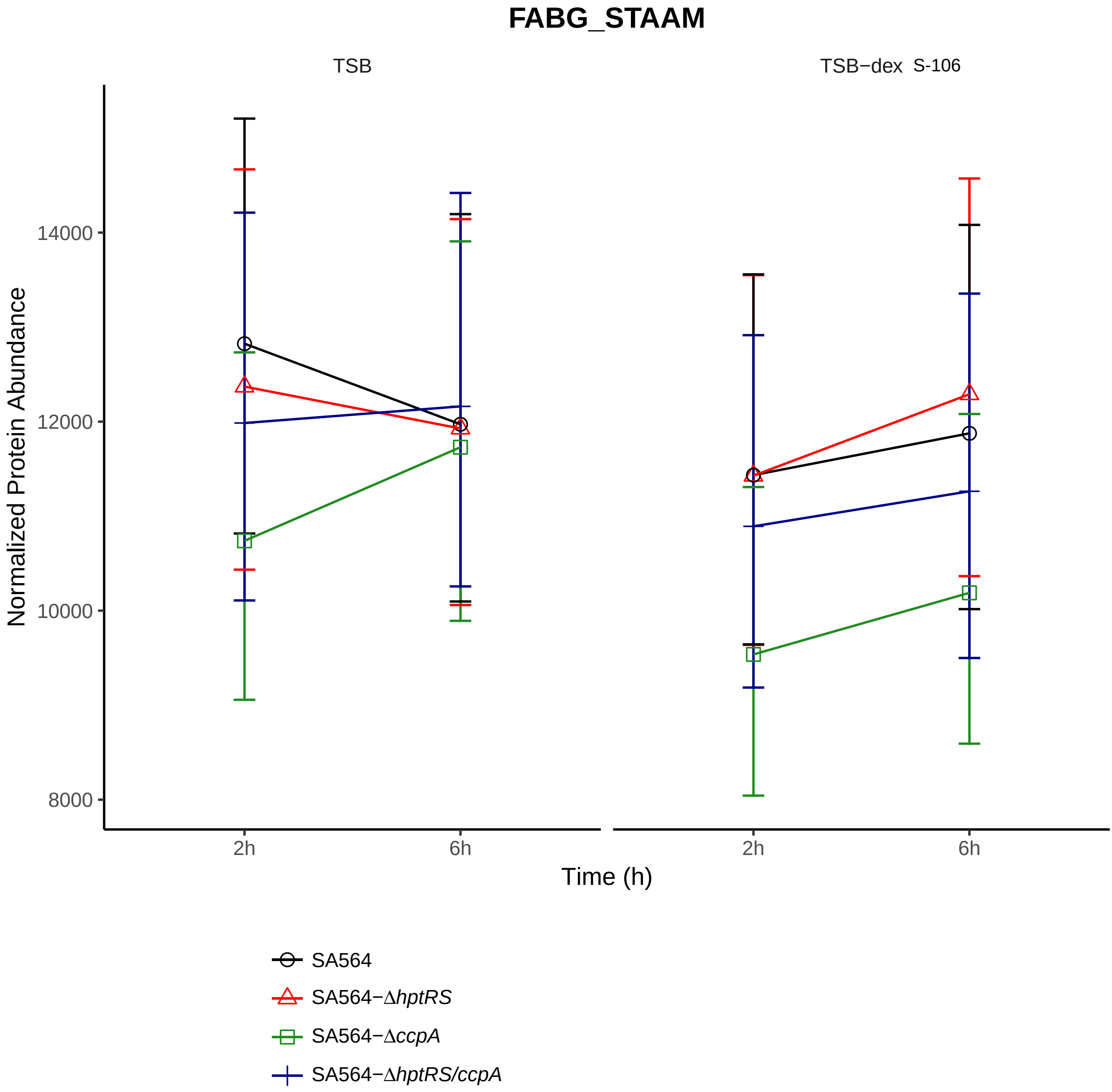
FABG_STAAM
TSB
TSB−dex
S-106
Normalized Protein Abundance
14000
12000
10000
8000
2h
6h
2h
6h
Time (h)
SA564
SA564−ΔhptRS
SA564−ΔccpA
SA564−ΔhptRS/ccpA

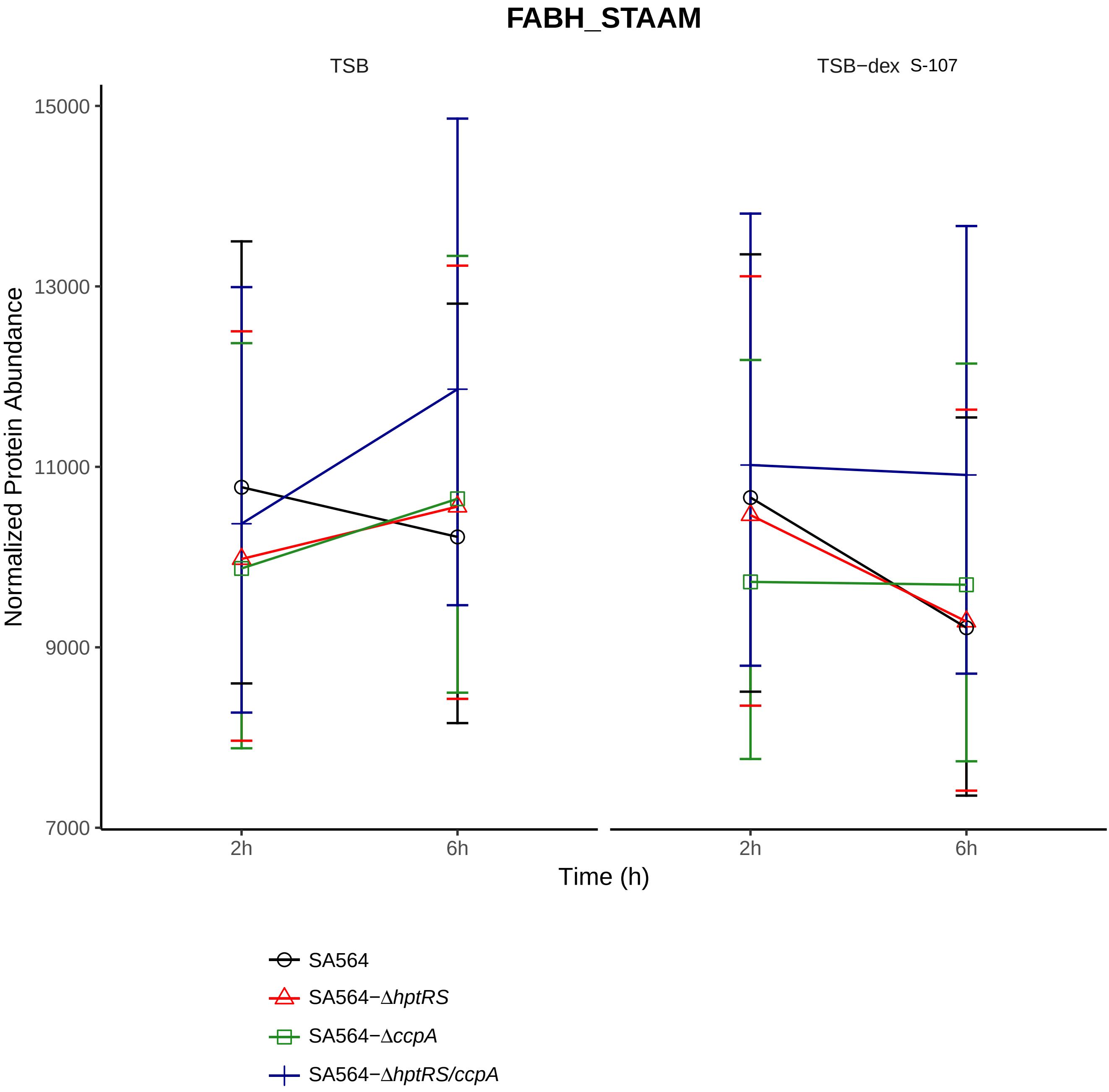

FABH_STAAM
TSB
TSB−dex
S-107
Normalized Protein Abundance
15000
13000
11000
9000
7000
2h
6h
2h
6h
Time (h)
SA564
SA564−Δ*hptRS*
SA564−Δ*ccpA*
SA564−Δ*hptRS/ccpA*

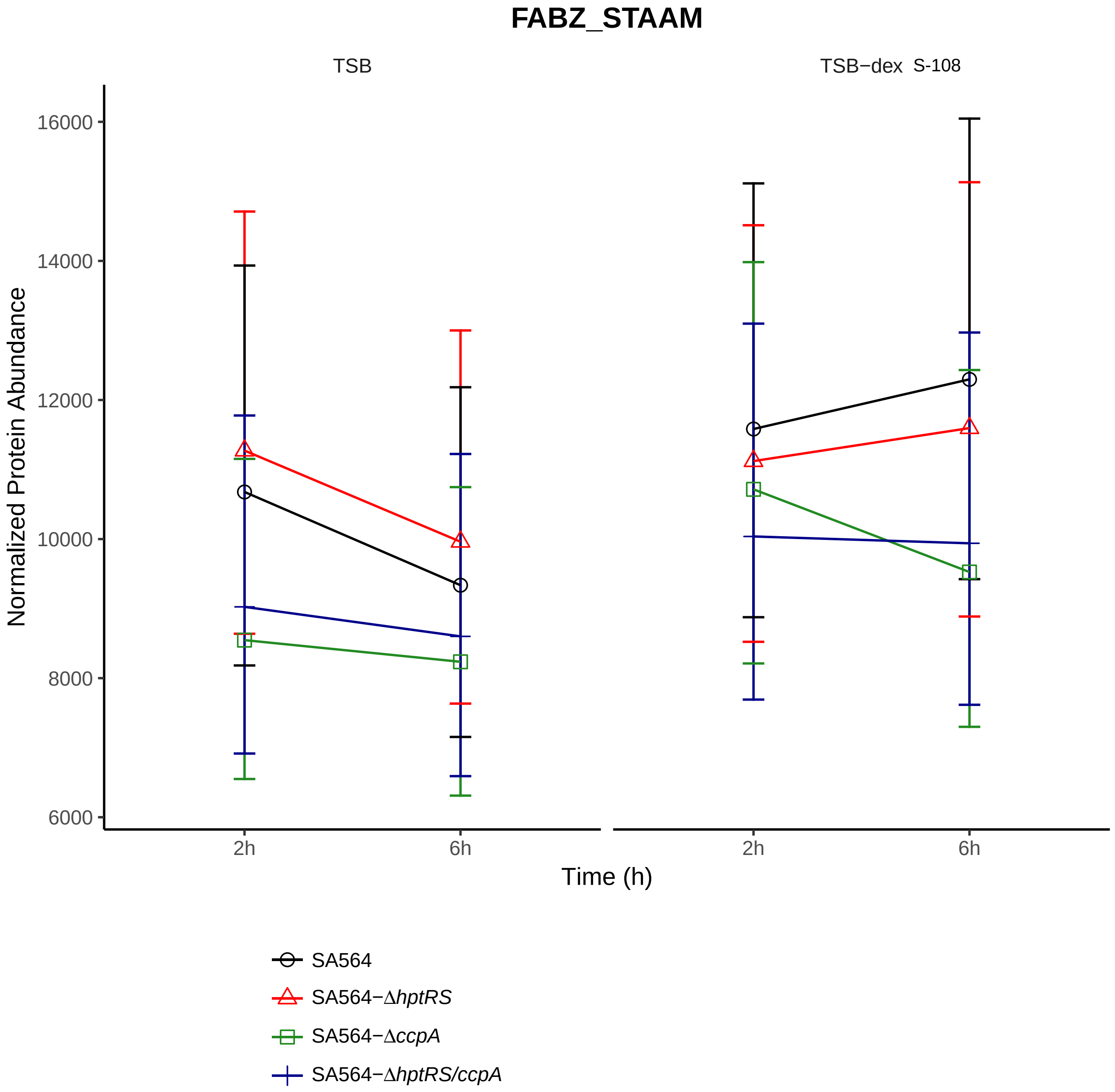

FABZ_STAAM
TSB
TSB−dex
S-108
16000
14000
12000
10000
8000
6000
Normalized Protein Abundance
2h
6h
2h
6h
Time (h)
SA564
SA564−Δ*hptRS*
SA564−Δ*ccpA*
SA564−Δ*hptRS/ccpA*

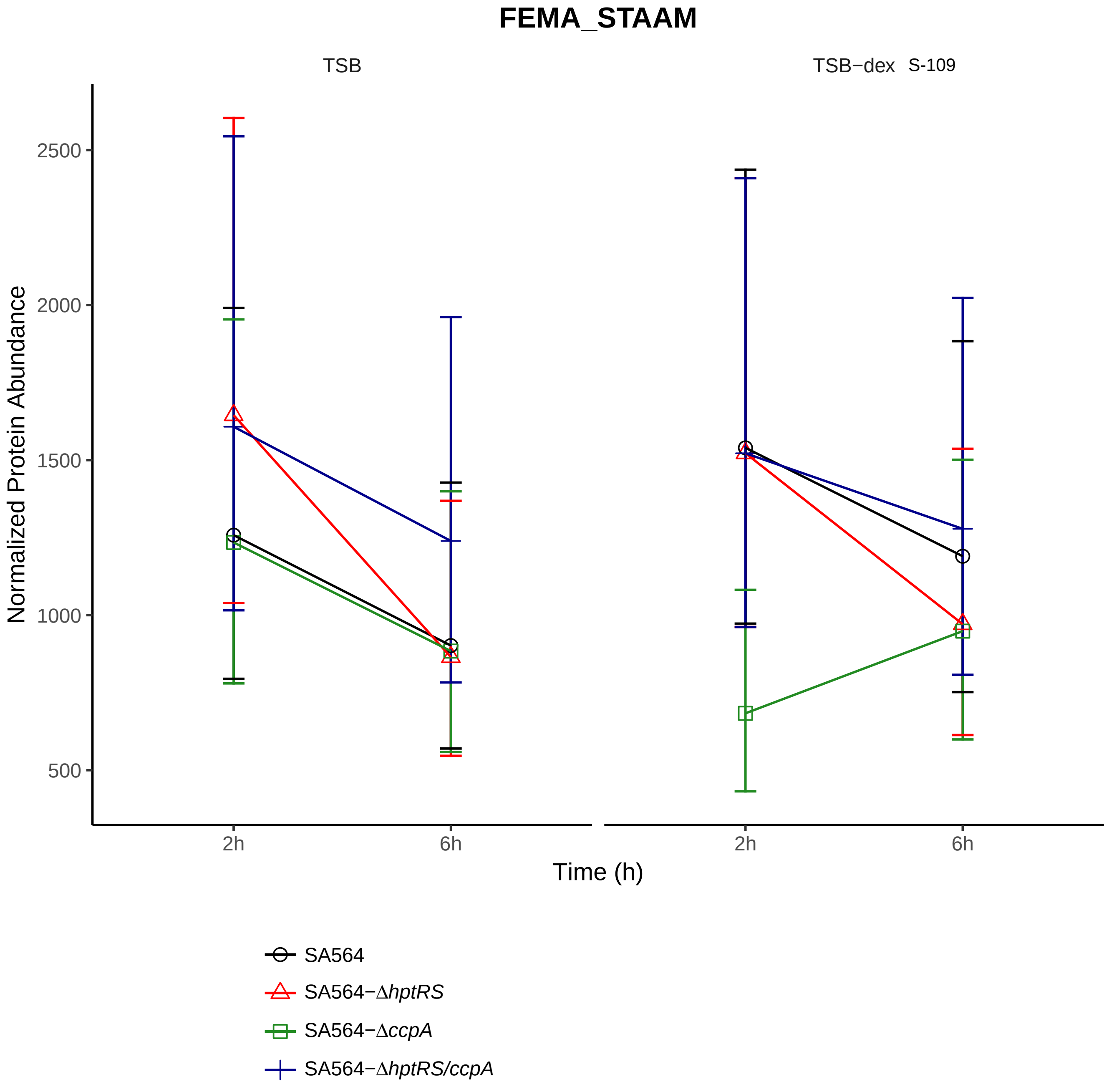
FEMA_STAAM
TSB
TSB−dex
S-109
Normalized Protein Abundance
2500
2000
1500
1000
500
2h
6h
2h
6h
Time (h)
SA564
SA564−ΔhptRS
SA564−ΔccpA
SA564−ΔhptRS/ccpA

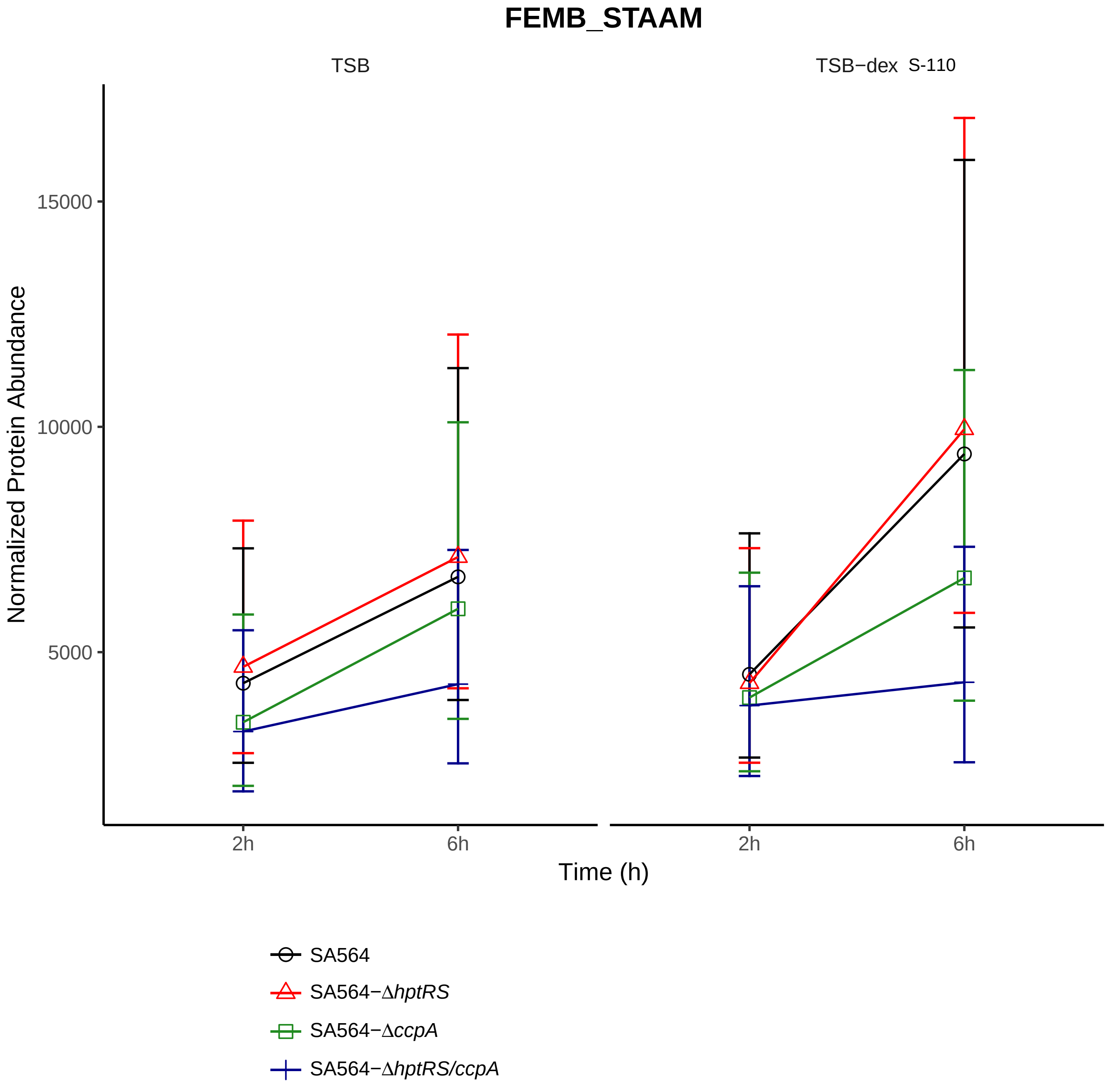
FEMB_STAAM
TSB
TSB−dex
S-110
Normalized Protein Abundance
15000
10000
5000
2h
6h
2h
6h
Time (h)
SA564
SA564−ΔhptRS
SA564−ΔccpA
SA564−ΔhptRS/ccpA

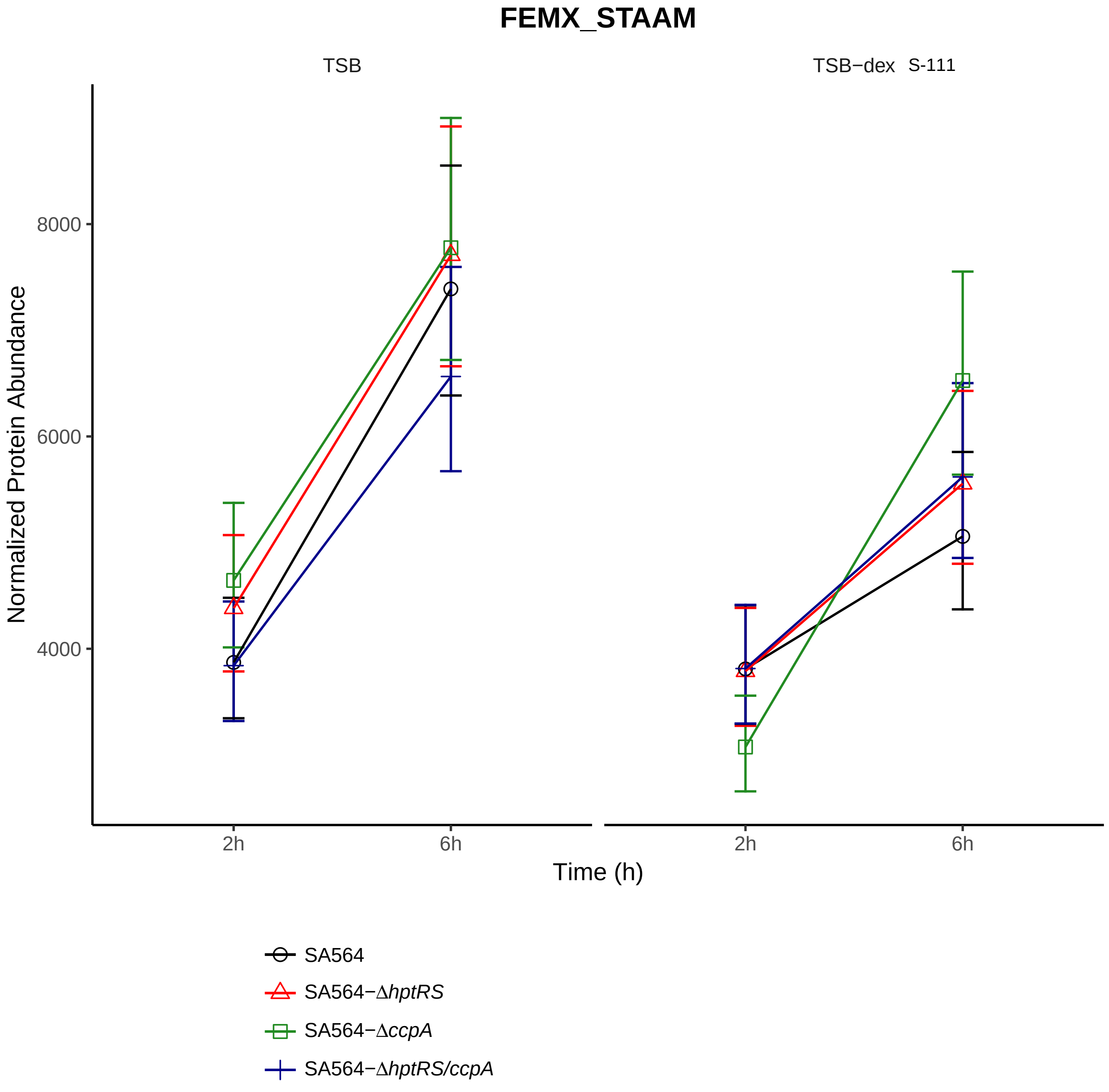
FEMX_STAAM
TSB
TSB−dex
S-111
Normalized Protein Abundance
8000
6000
4000
2h
6h
2h
6h
Time (h)
SA564
SA564−ΔhptRS
SA564−ΔccpA
SA564−ΔhptRS/ccpA

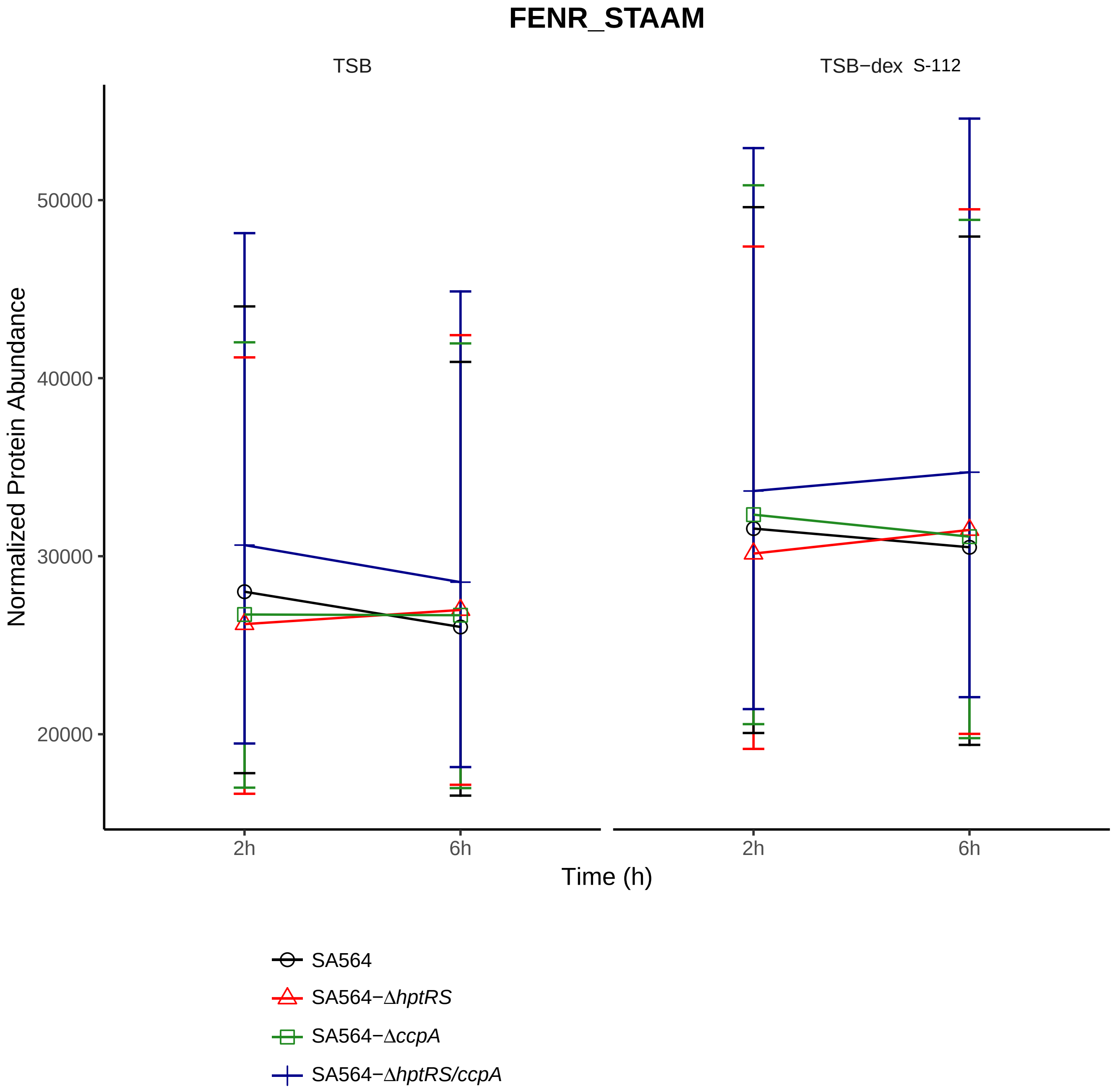

FENR_STAAM
TSB
TSB−dex
S-112
Normalized Protein Abundance
50000
40000
30000
20000
2h
6h
2h
6h
Time (h)
SA564
SA564−ΔhptRS
SA564−ΔccpA
SA564−ΔhptRS/ccpA

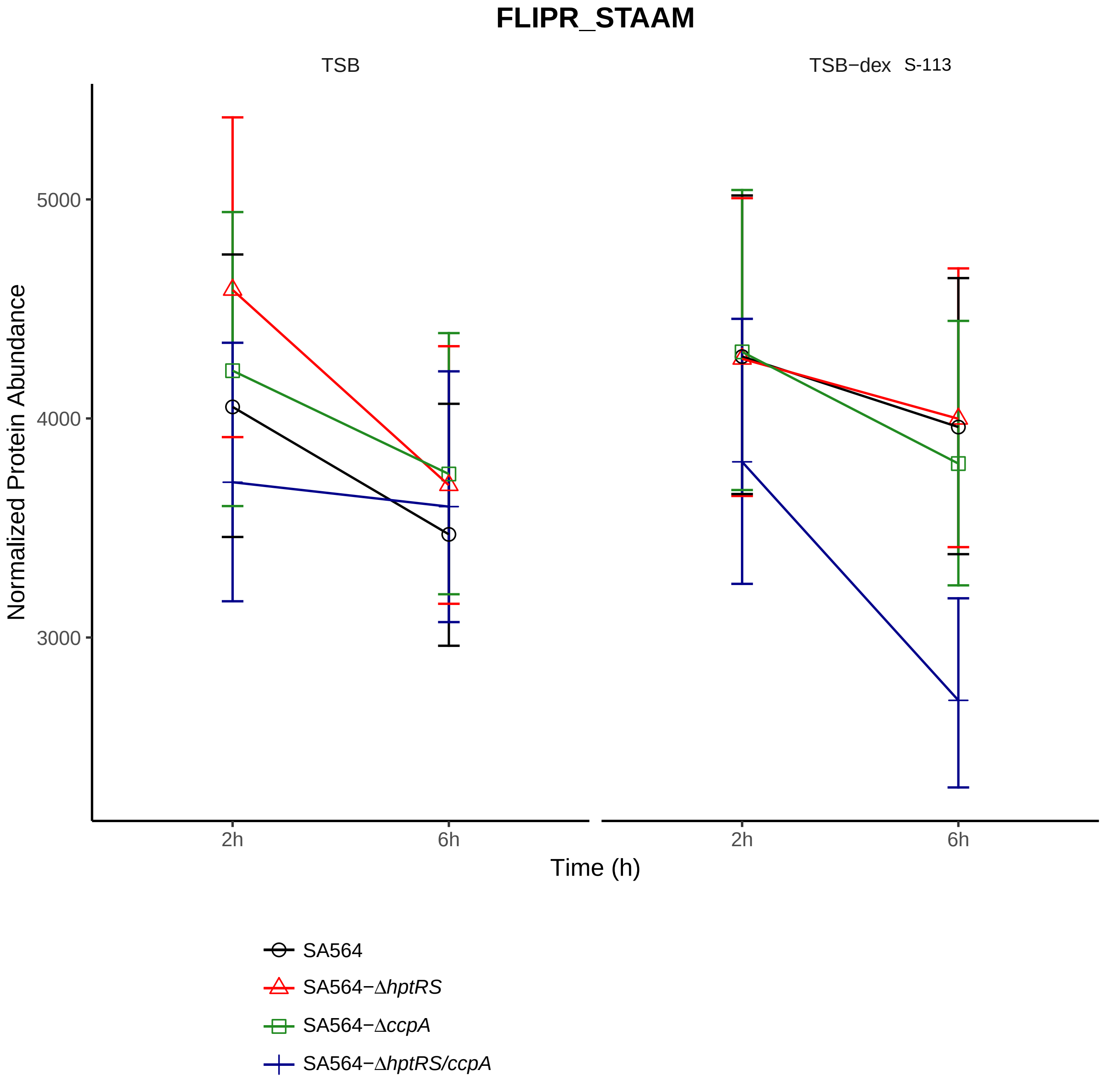

FLIPR_STAAM
TSB
TSB−dex
S-113
Normalized Protein Abundance
5000
4000
3000
2h
6h
2h
6h
Time (h)
SA564
SA564−ΔhptRS
SA564−ΔccpA
SA564−ΔhptRS/ccpA

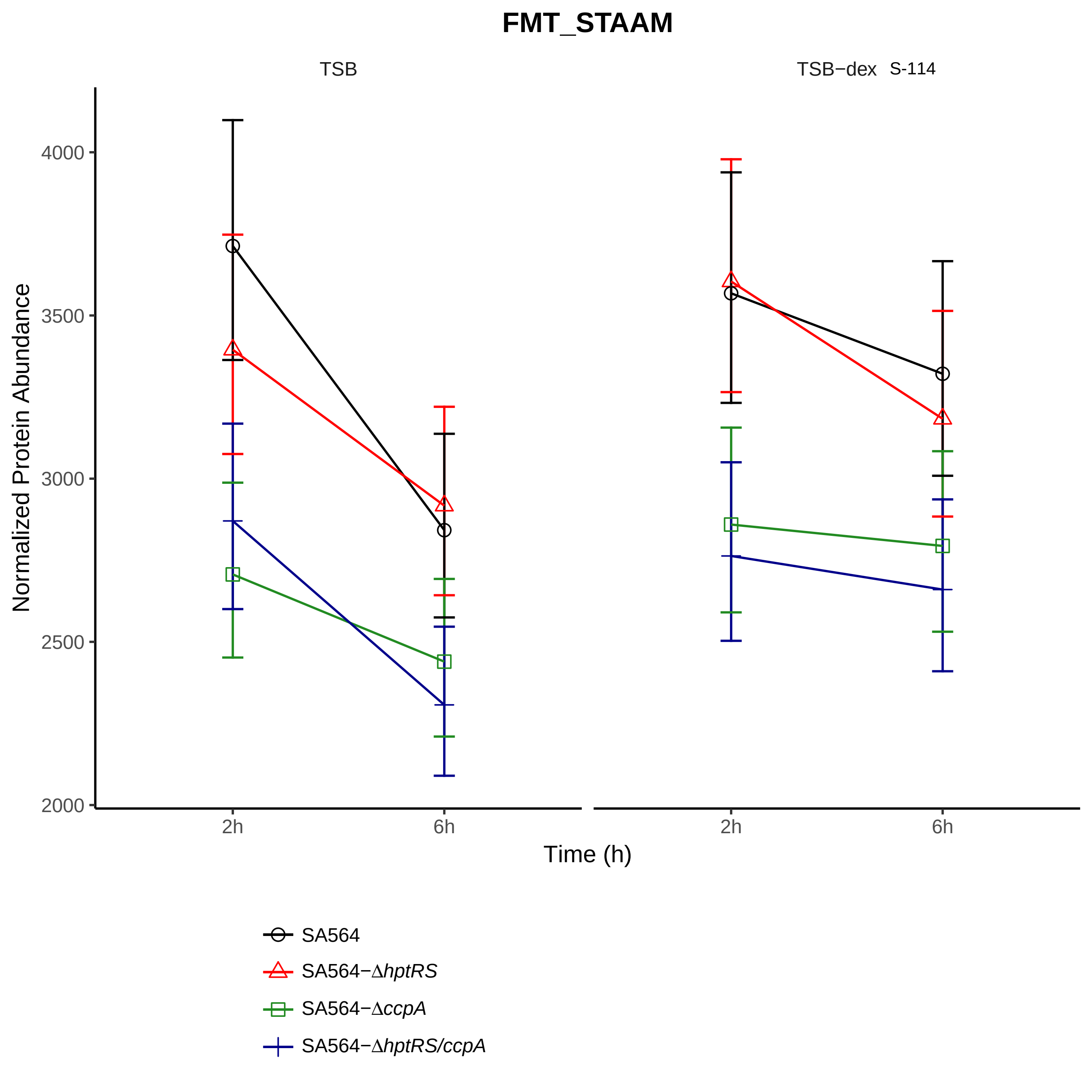
FMT_STAAM
TSB
TSB−dex
S-114
Normalized Protein Abundance
4000
3500
3000
2500
2000
2h
6h
2h
6h
Time (h)
SA564
SA564−Δ*hptRS*
SA564−Δ*ccpA*
SA564−Δ*hptRS/ccpA*

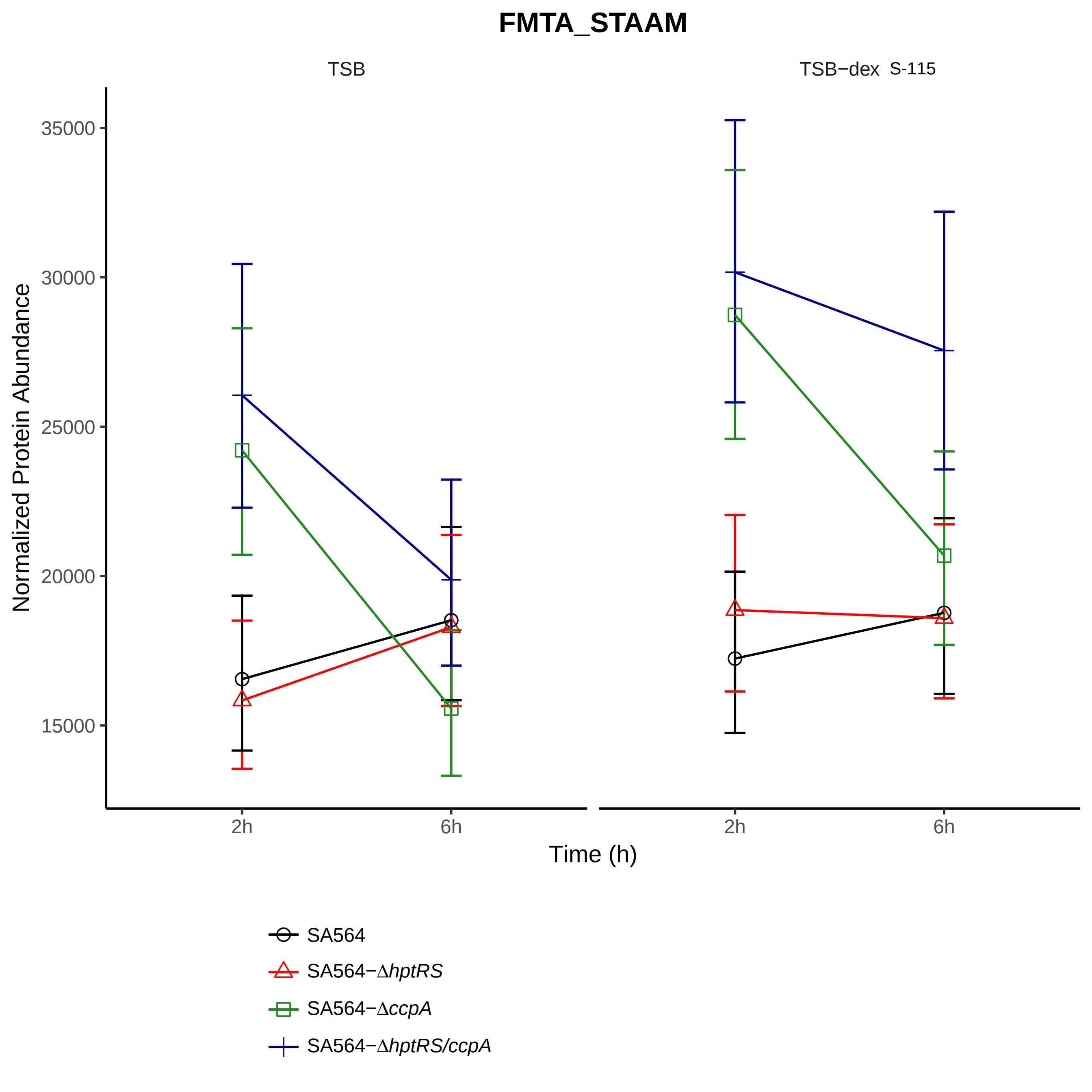
FMTA_STAAM
TSB
TSB−dex
S-115
Normalized Protein Abundance
35000
30000
25000
20000
15000
2h
6h
2h
6h
Time (h)
SA564
SA564−Δ*hptRS*
SA564−Δ*ccpA*
SA564−Δ*hptRS/ccpA*

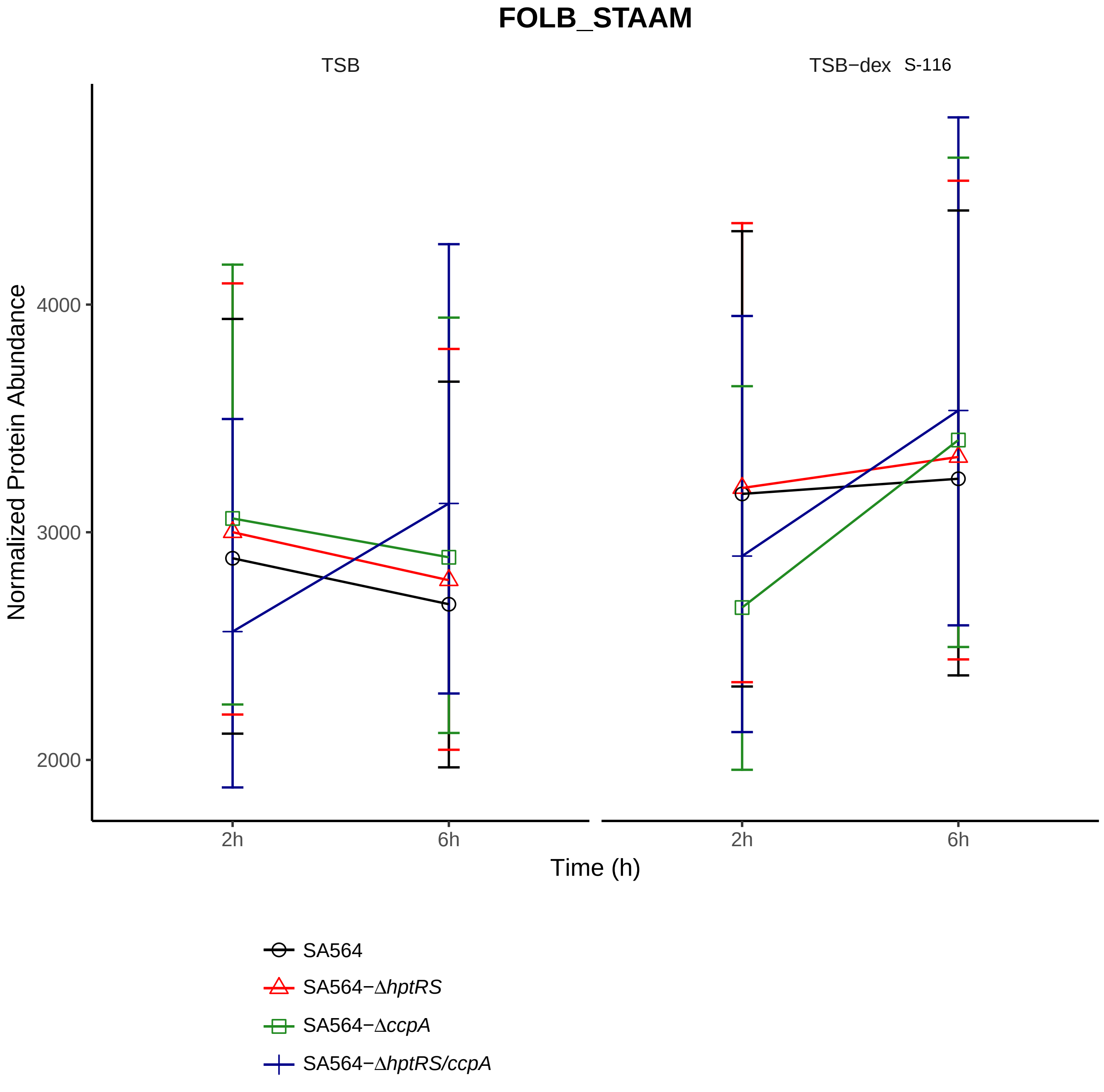
FOLB_STAAM
TSB
TSB−dex
S-116
Normalized Protein Abundance
4000
3000
2000
2h
6h
2h
6h
Time (h)
SA564
SA564−ΔhptRS
SA564−ΔccpA
SA564−ΔhptRS/ccpA

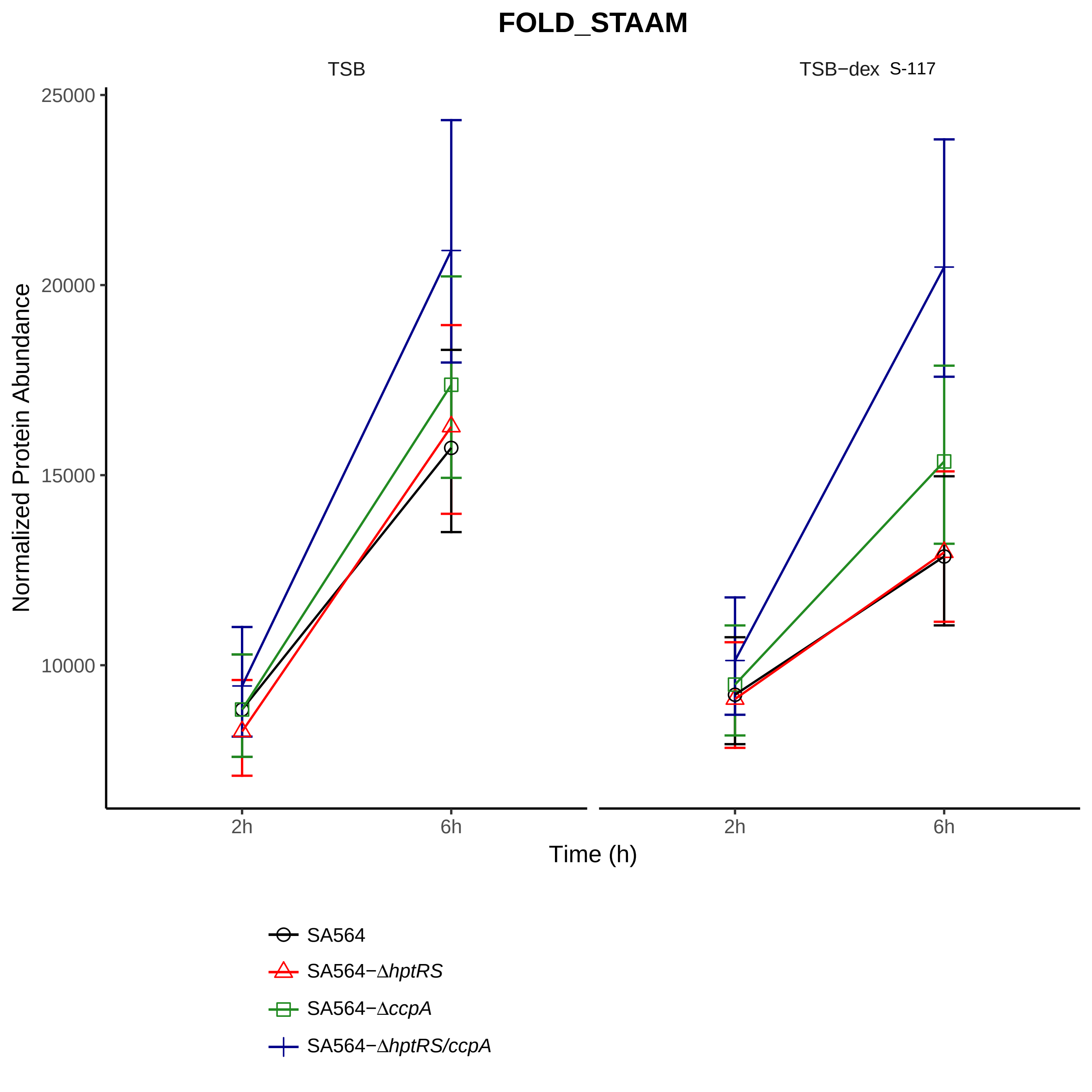
FOLD_STAAM
TSB
TSB−dex
S-117
25000
20000
15000
10000
Normalized Protein Abundance
2h
6h
2h
6h
Time (h)
SA564
SA564−Δ*hptRS*
SA564−Δ*ccpA*
SA564−Δ*hptRS/ccpA*

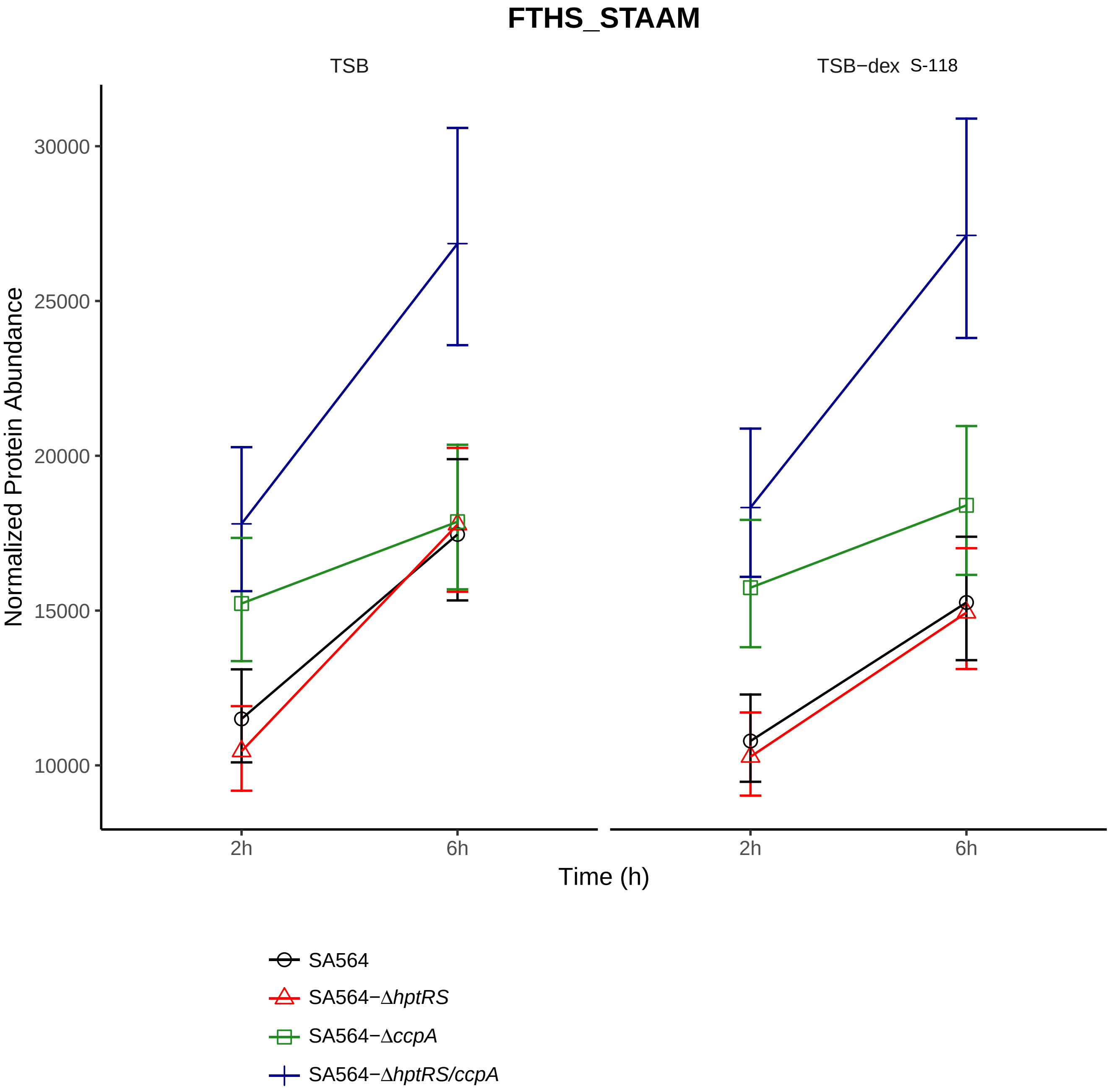
FTHS_STAAM
TSB
TSB−dex
S-118
Normalized Protein Abundance
30000
25000
20000
15000
10000
2h
6h
2h
6h
Time (h)
SA564
SA564−Δ*hptRS*
SA564−Δ*ccpA*
SA564−Δ*hptRS/ccpA*

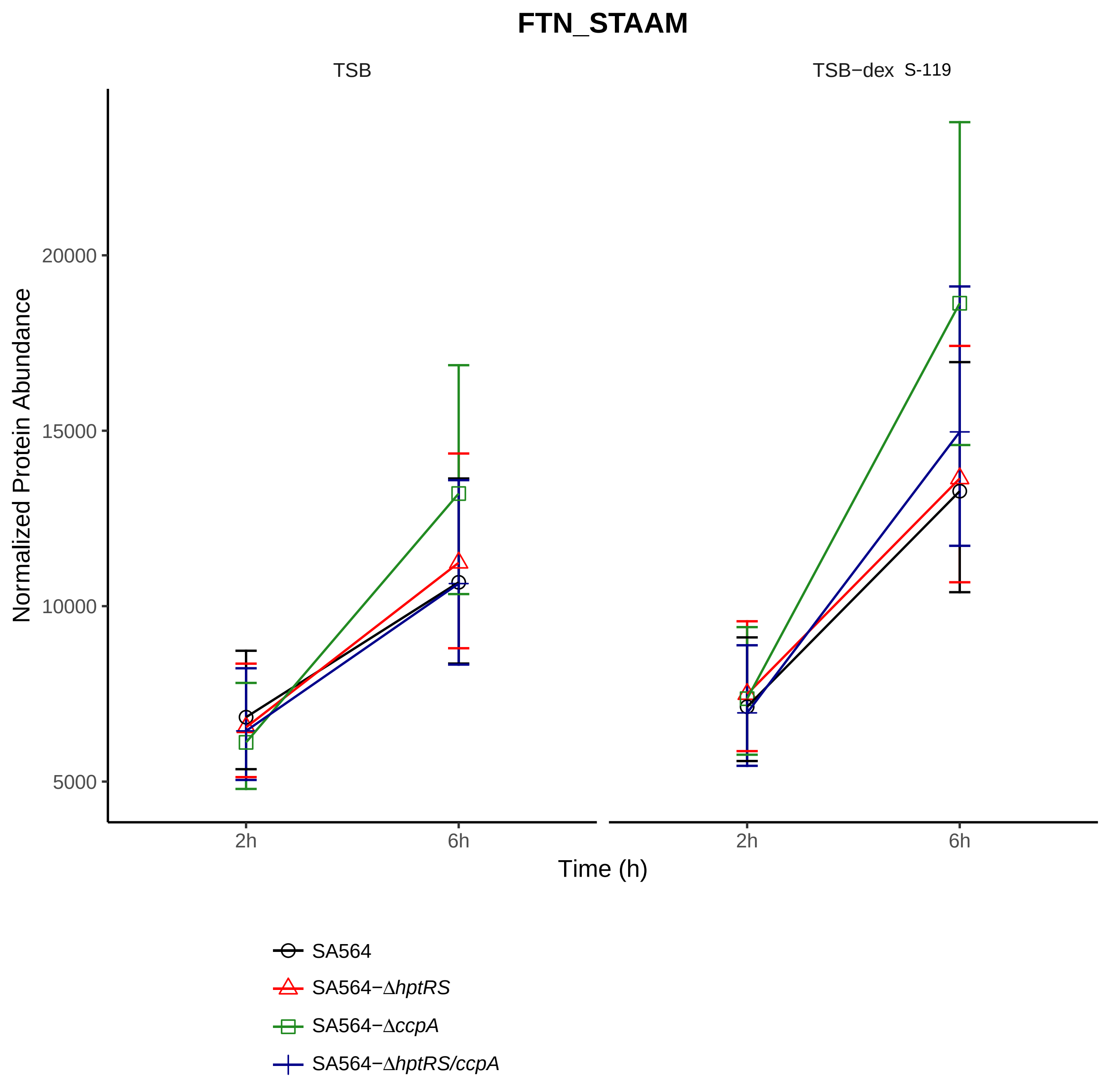

FTN_STAAM
TSB
TSB−dex
S-119
Normalized Protein Abundance
20000
15000
10000
5000
2h
6h
2h
6h
Time (h)
SA564
SA564−ΔhptRS
SA564−ΔccpA
SA564−ΔhptRS/ccpA

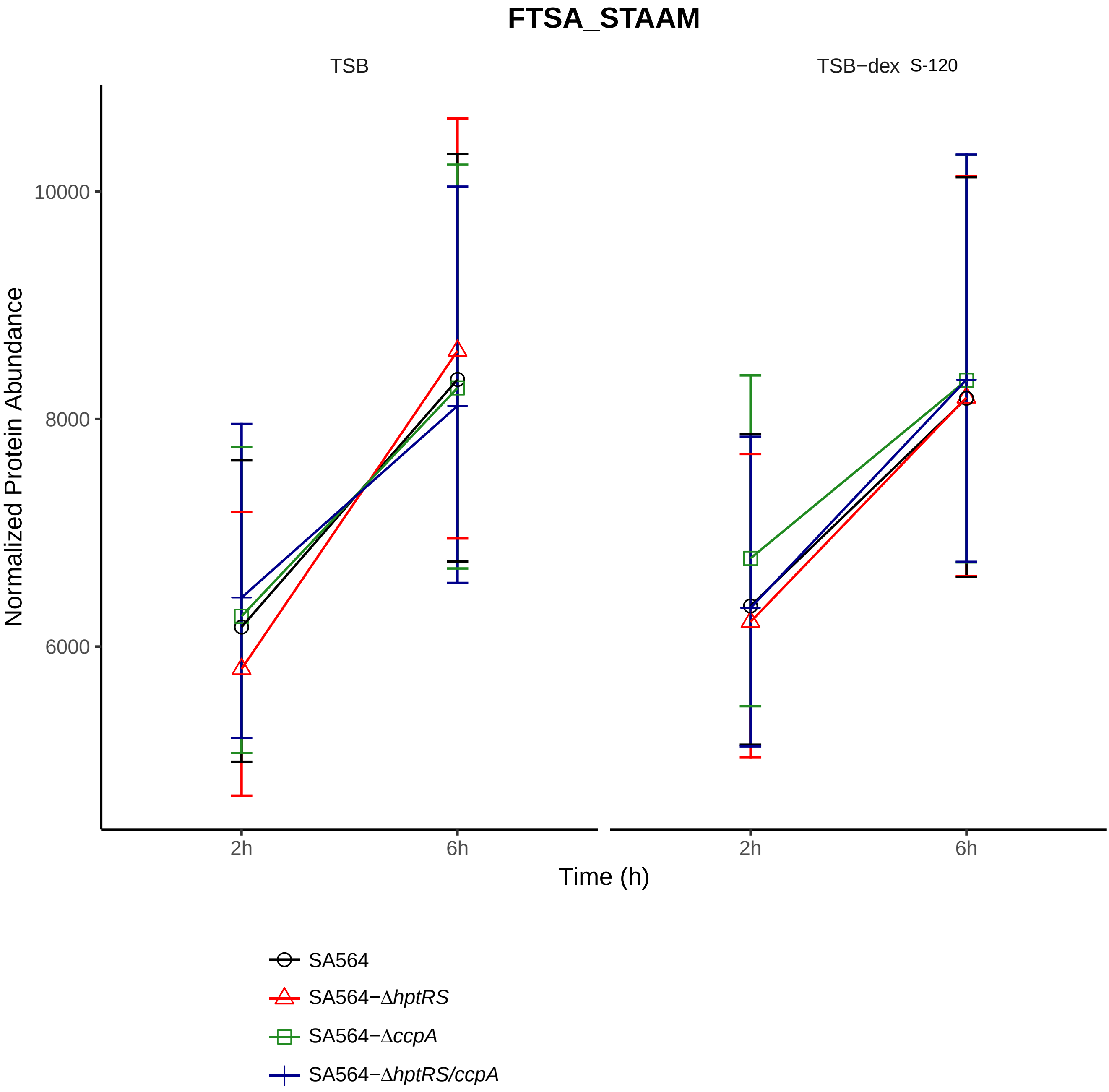

FTSA_STAAM
TSB
TSB−dex
Normalized Protein Abundance
10000
8000
6000
2h
6h
2h
6h
Time (h)
SA564
SA564−Δ*hptRS*
SA564−Δ*ccpA*
SA564−Δ*hptRS/ccpA*

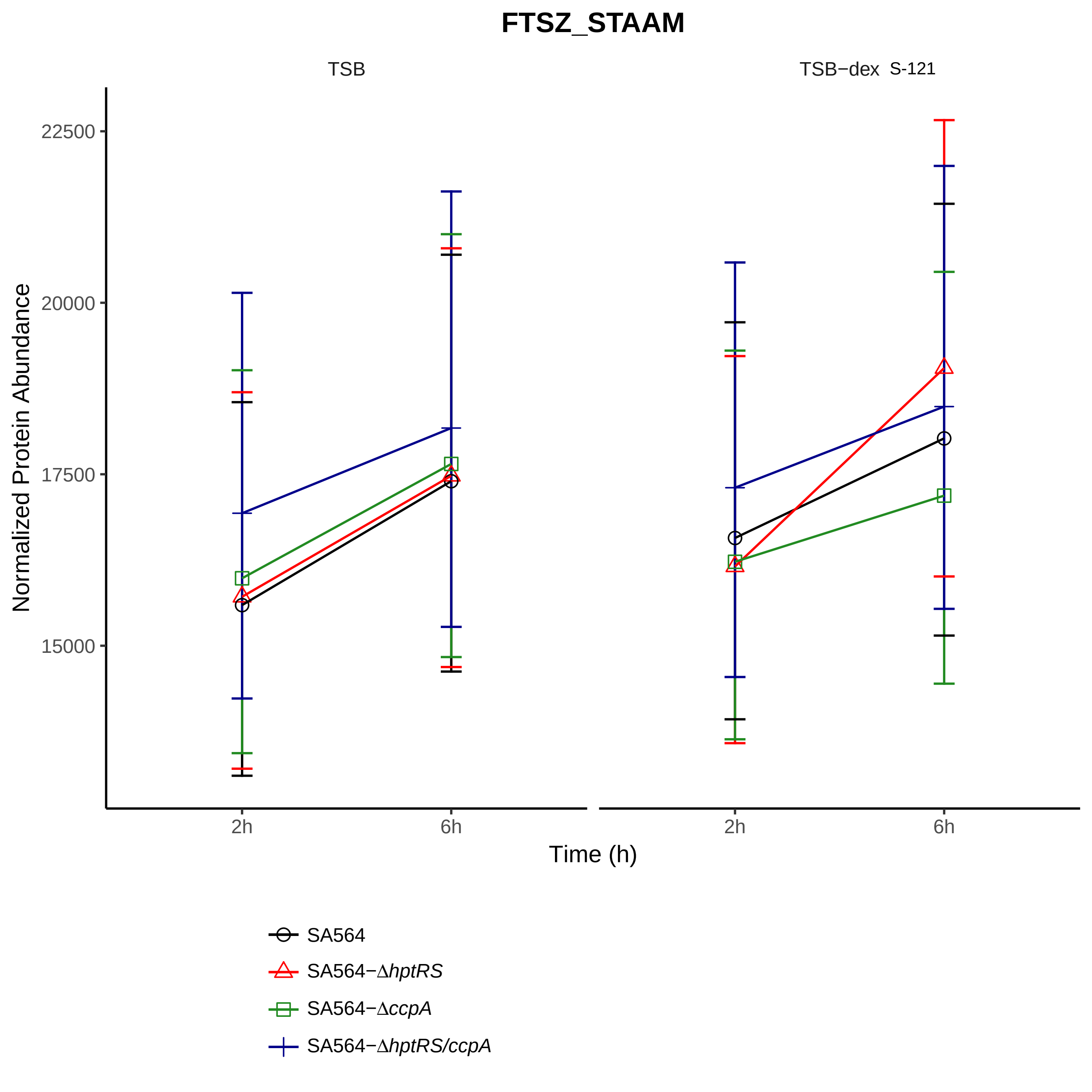
FTSZ_STAAM
TSB
TSB−dex
S-121
22500
20000
17500
15000
Normalized Protein Abundance
2h
6h
2h
6h
Time (h)
SA564
SA564−ΔhptRS
SA564−ΔccpA
SA564−ΔhptRS/ccpA

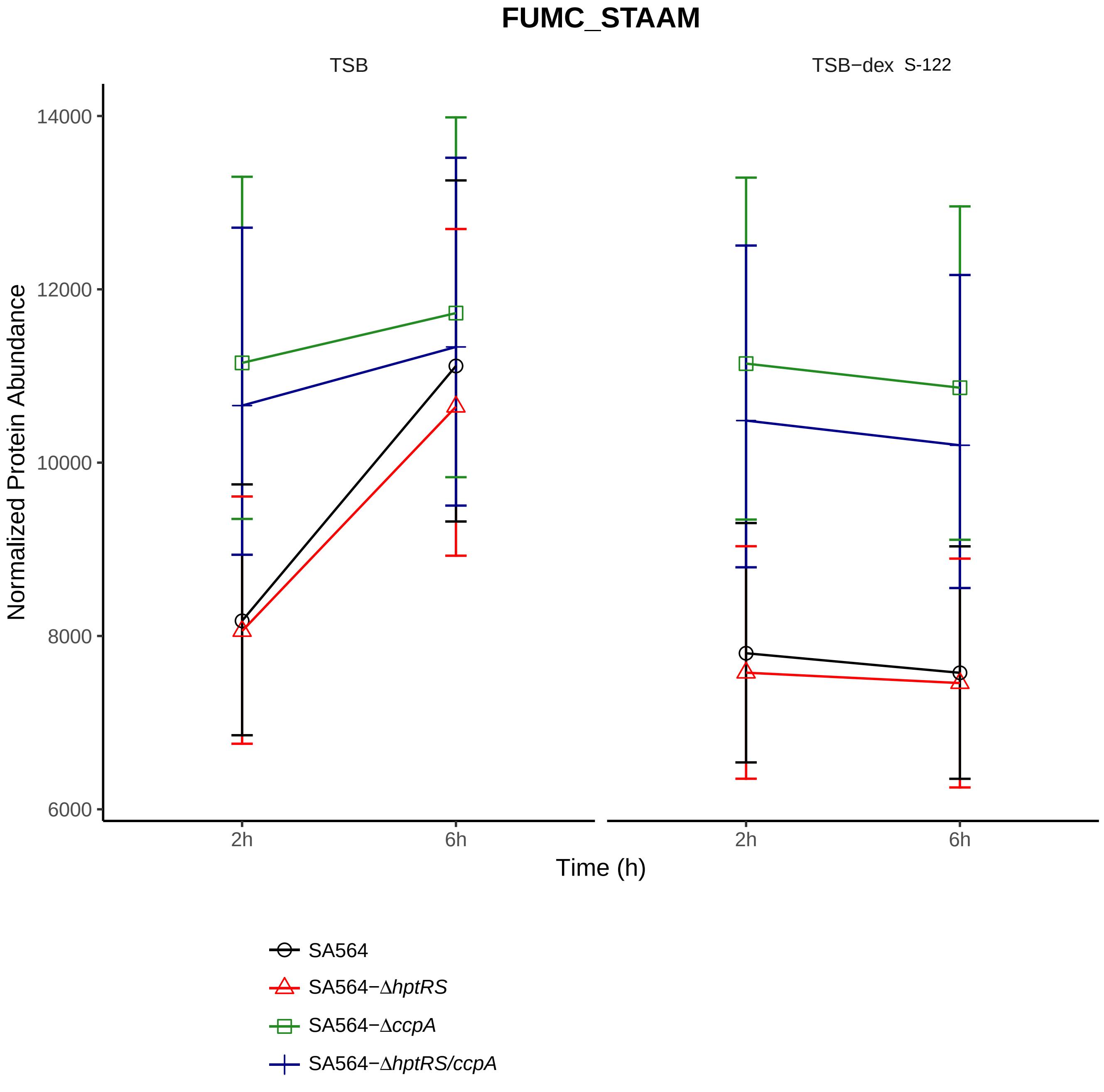
FUMC_STAAM
TSB
TSB−dex
S-122
Normalized Protein Abundance
14000
12000
10000
8000
6000
2h
6h
2h
6h
Time (h)
SA564
SA564−Δ*hptRS*
SA564−Δ*ccpA*
SA564−Δ*hptRS/ccpA*

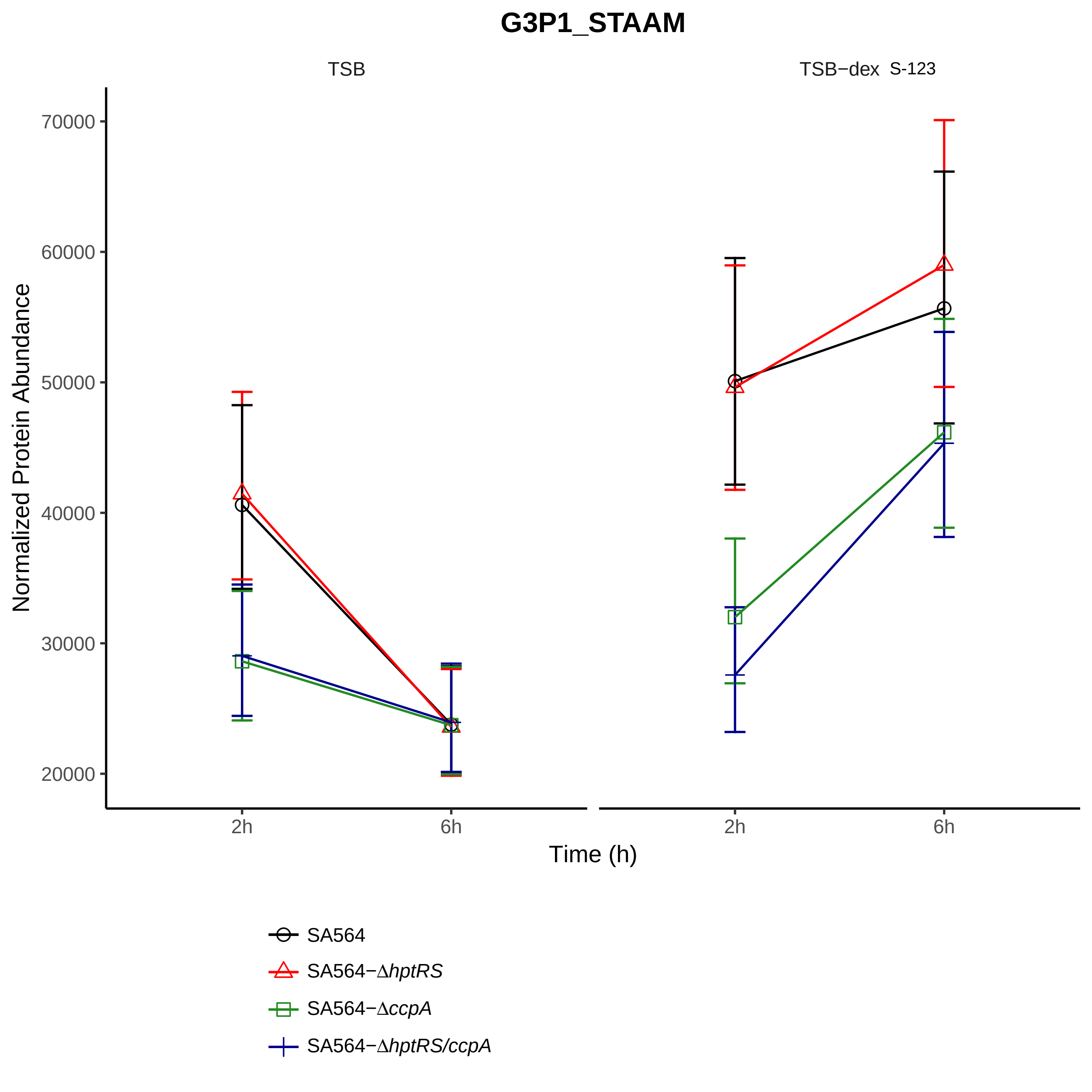

G3P1_STAAM
TSB
TSB−dex
S-123
Normalized Protein Abundance
70000
60000
50000
40000
30000
20000
2h
6h
2h
6h
Time (h)
SA564
SA564−ΔhptRS
SA564−ΔccpA
SA564−ΔhptRS/ccpA

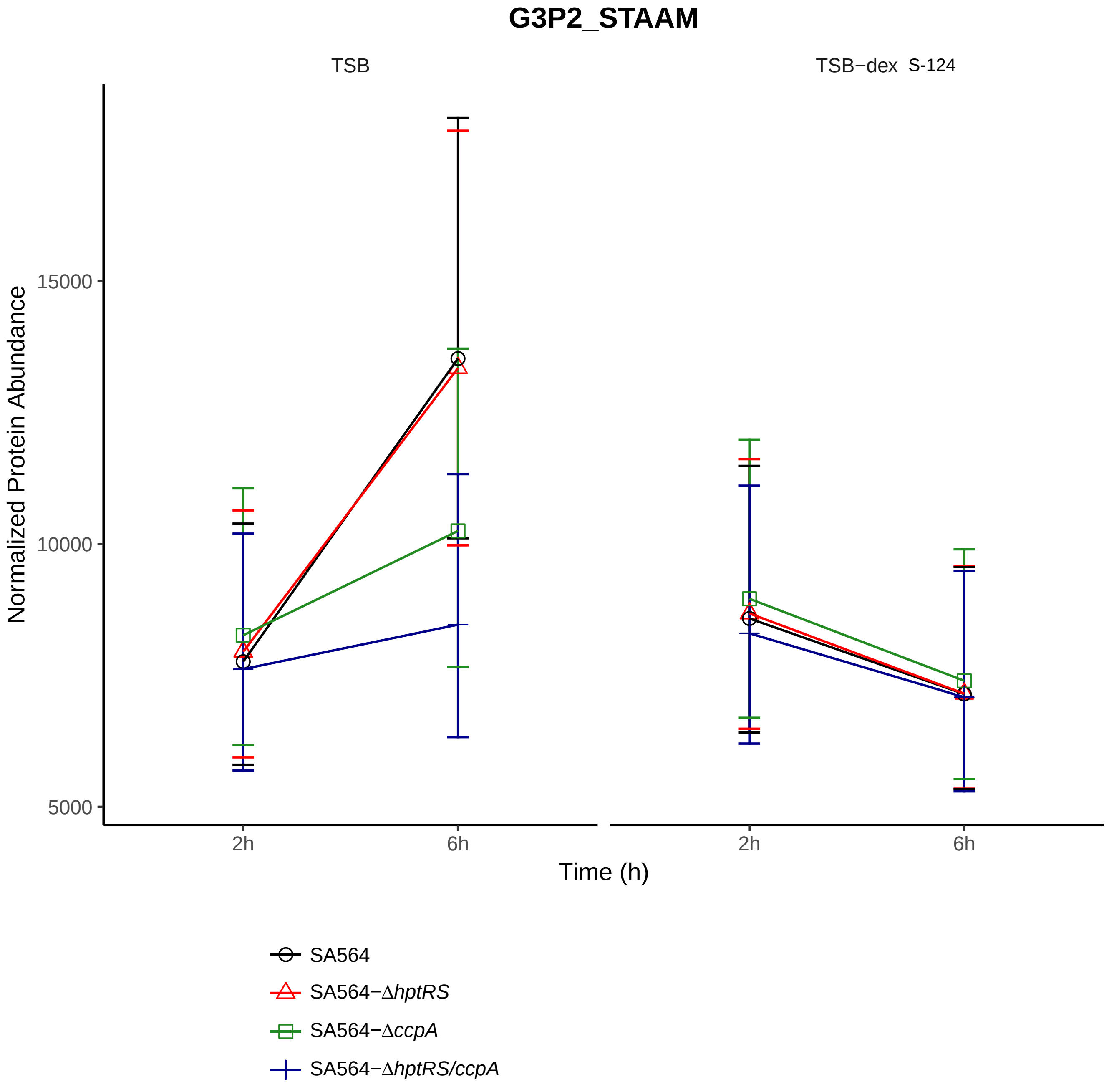

G3P2_STAAM
TSB
TSB−dex
S-124
Normalized Protein Abundance
15000
10000
5000
2h
6h
2h
6h
Time (h)
SA564
SA564−Δ*hptRS*
SA564−Δ*ccpA*
SA564−Δ*hptRS/ccpA*

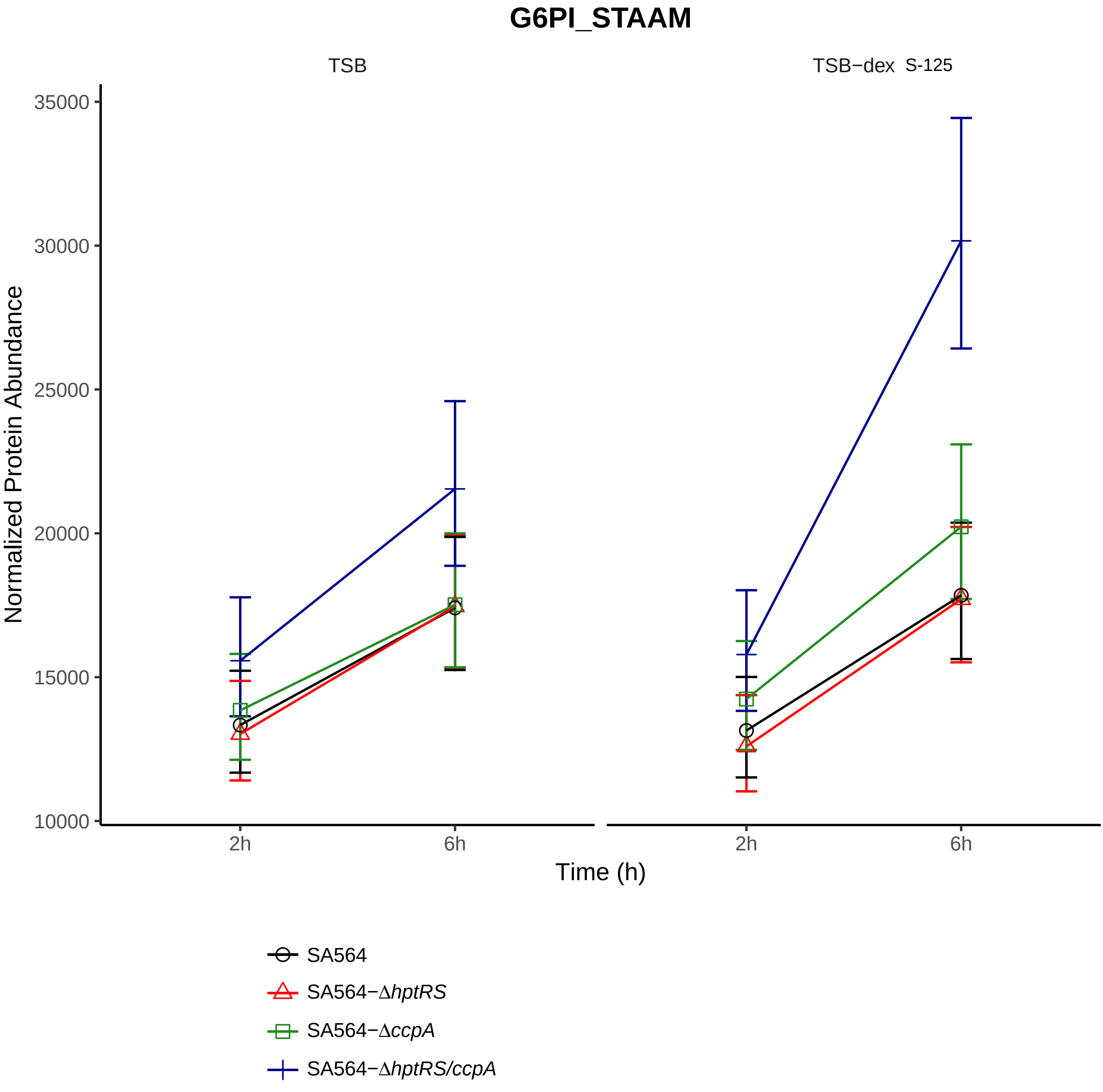

G6PI_STAAM
TSB
TSB−dex
S-125
35000
30000
25000
20000
15000
10000
Normalized Protein Abundance
2h
6h
2h
6h
Time (h)
SA564
SA564−Δ*hptRS*
SA564−Δ*ccpA*
SA564−Δ*hptRS/ccpA*

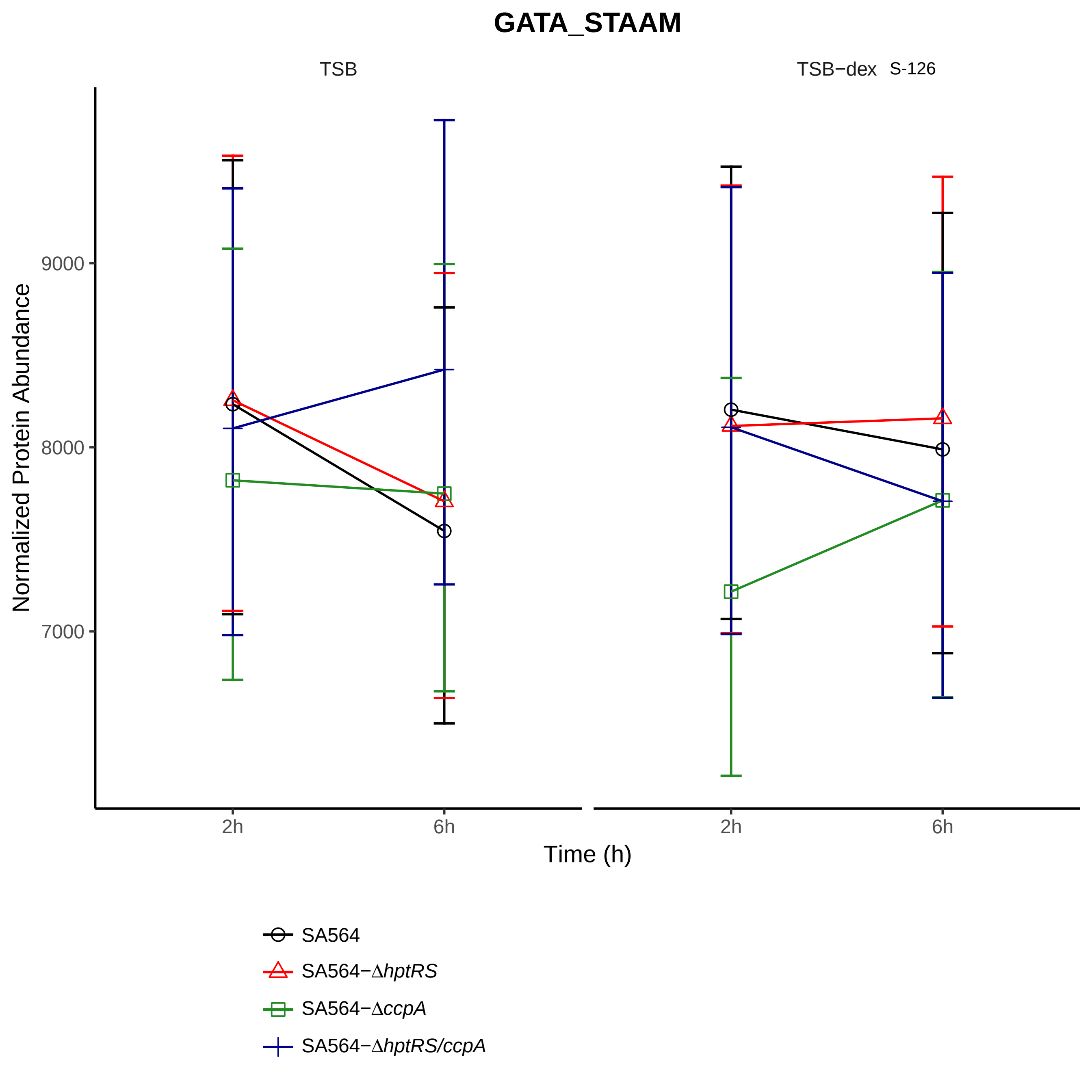

GATA_STAAM
TSB
TSB−dex
S-126
Normalized Protein Abundance
9000
8000
7000
2h
6h
2h
6h
Time (h)
SA564
SA564−ΔhptRS
SA564−ΔccpA
SA564−ΔhptRS/ccpA

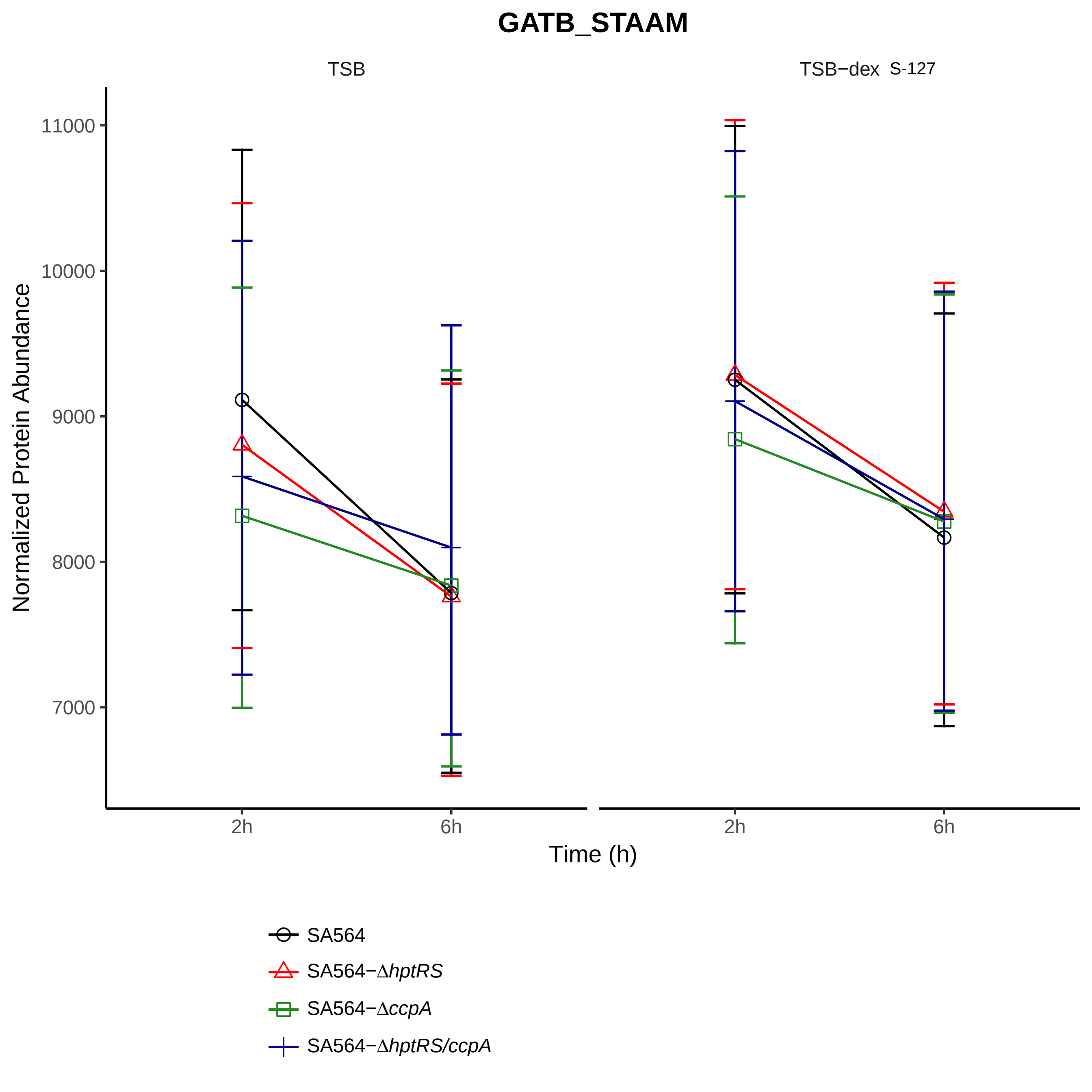
GATB_STAAM
TSB
TSB−dex
S-127
Normalized Protein Abundance
11000
10000
9000
8000
7000
2h
6h
2h
6h
Time (h)
SA564
SA564−Δ*hptRS*
SA564−Δ*ccpA*
SA564−Δ*hptRS/ccpA*

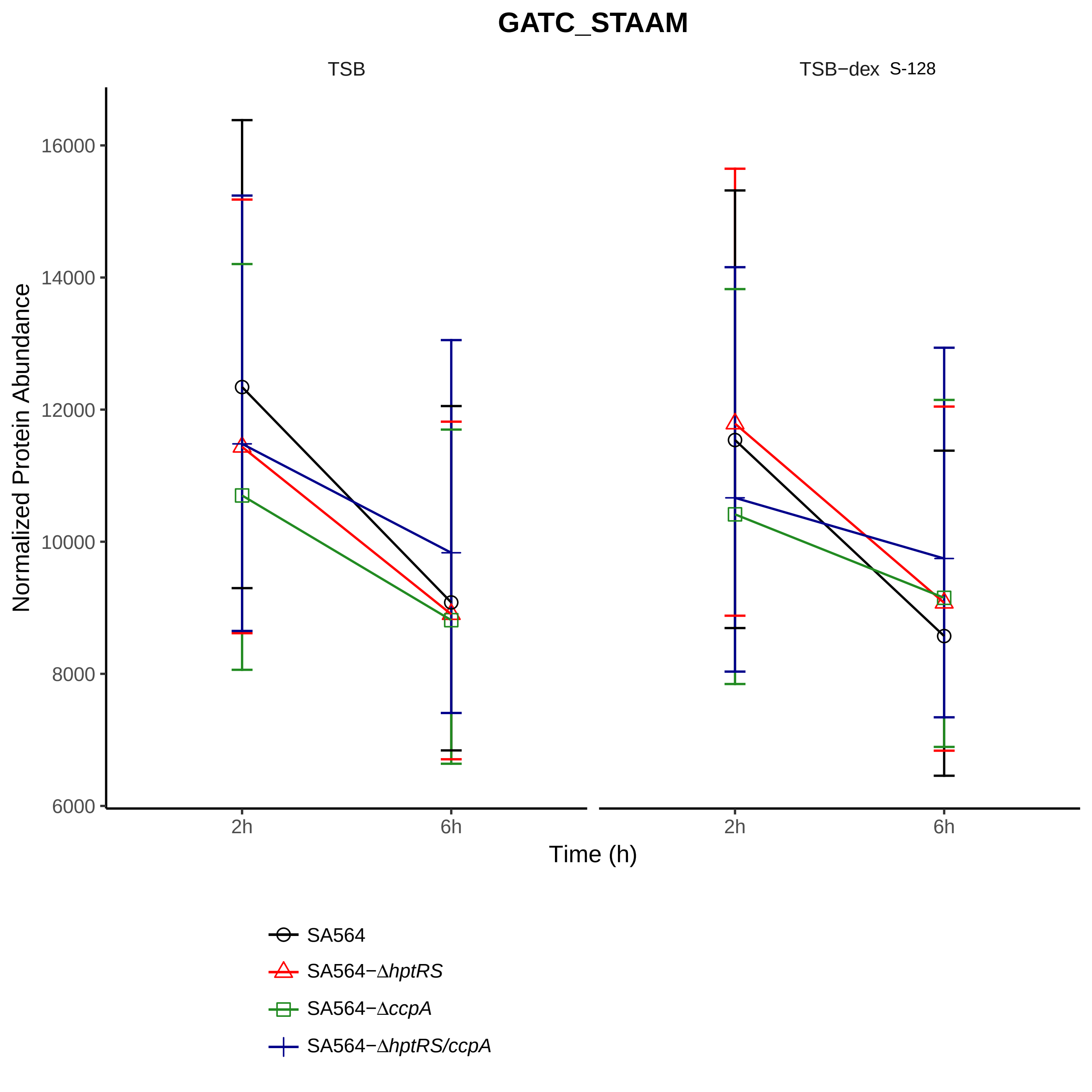

GATC_STAAM
TSB
TSB−dex
S-128
Normalized Protein Abundance
16000
14000
12000
10000
8000
6000
2h
6h
2h
6h
Time (h)
SA564
SA564−Δ*hptRS*
SA564−Δ*ccpA*
SA564−Δ*hptRS/ccpA*

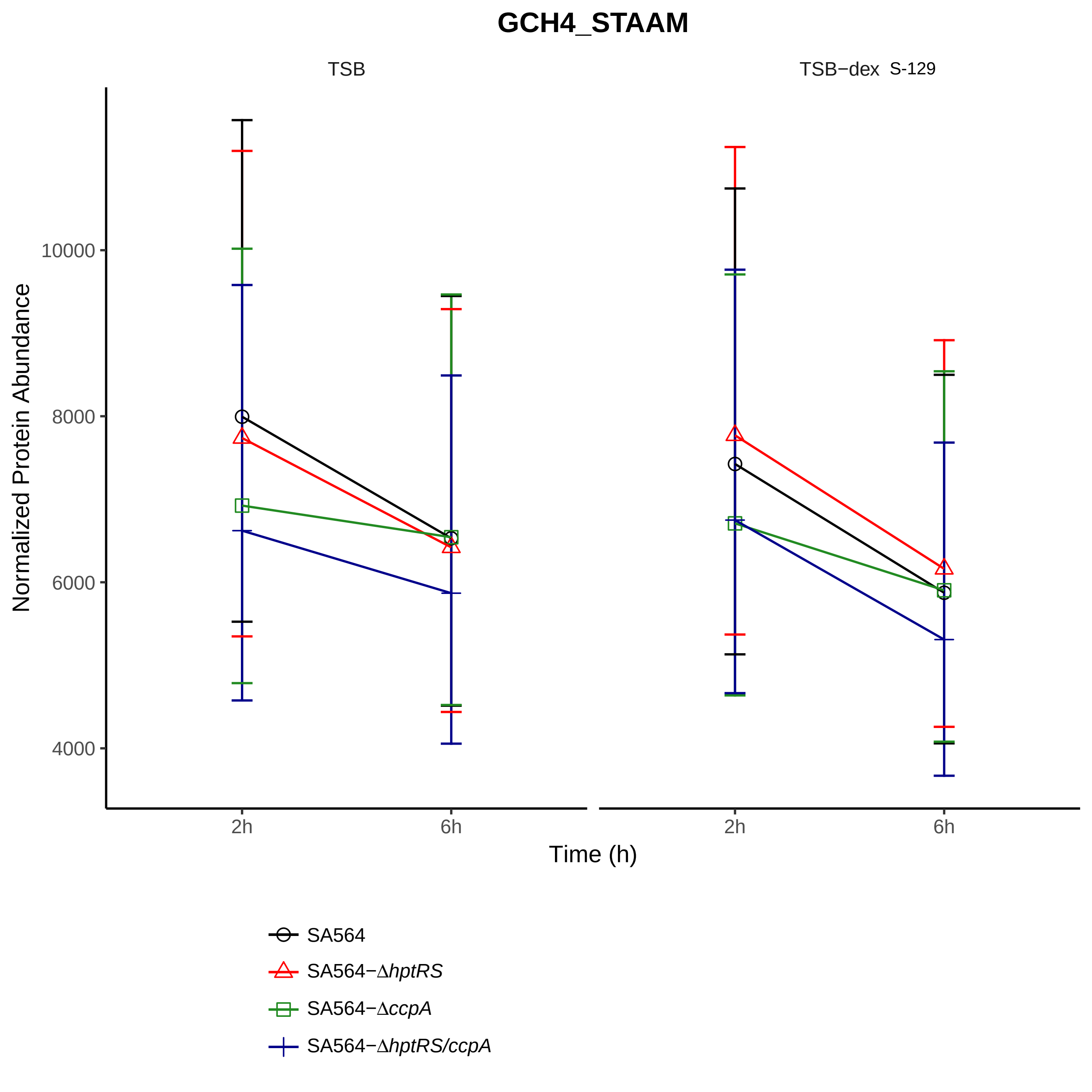
GCH4_STAAM
TSB
TSB−dex
S-129
Normalized Protein Abundance
10000
8000
6000
4000
2h
6h
2h
6h
Time (h)
SA564
SA564−ΔhptRS
SA564−ΔccpA
SA564−ΔhptRS/ccpA

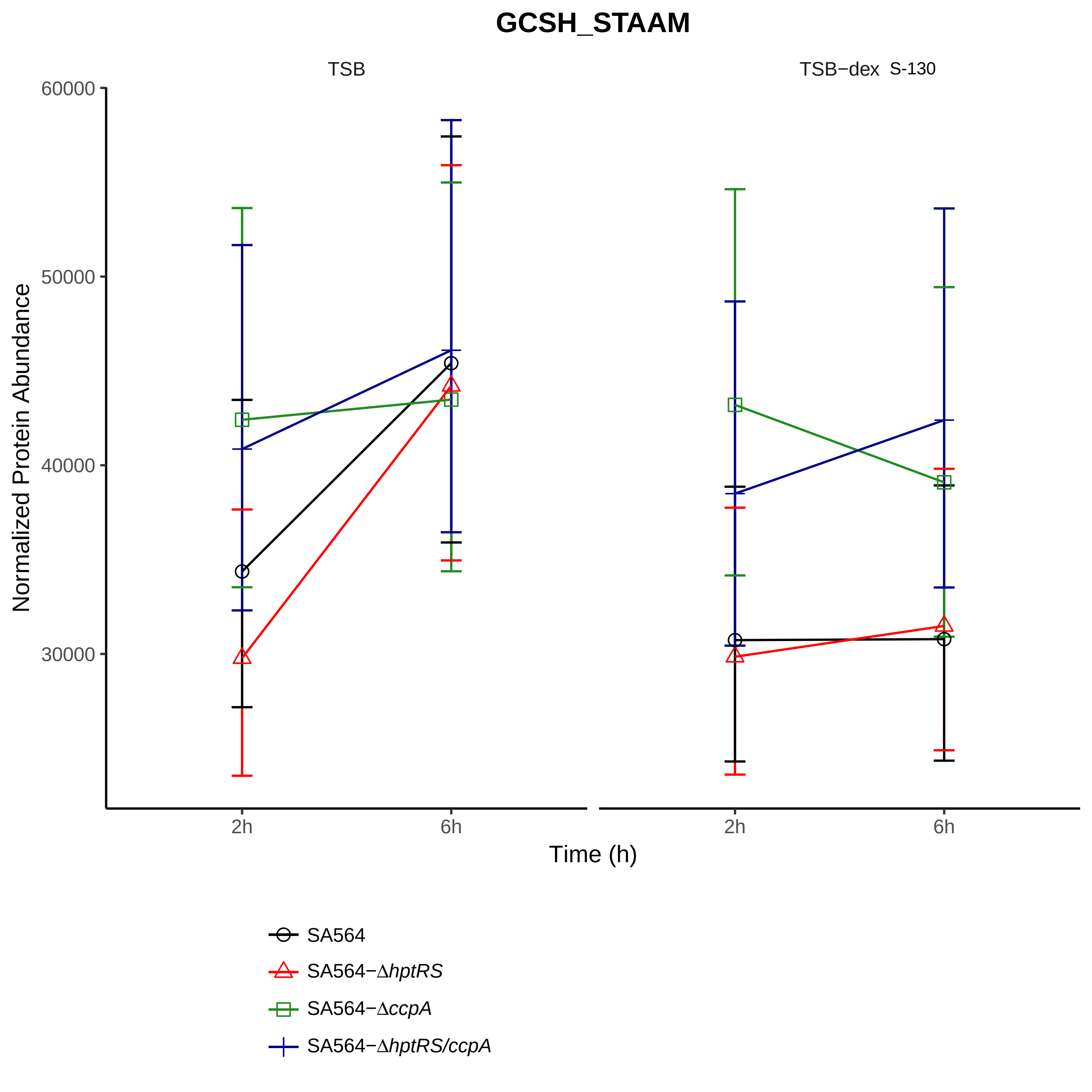

GCSH_STAAM
TSB
TSB−dex
S-130
60000
50000
40000
30000
Normalized Protein Abundance
2h
6h
2h
6h
Time (h)
SA564
SA564−ΔhptRS
SA564−ΔccpA
SA564−ΔhptRS/ccpA

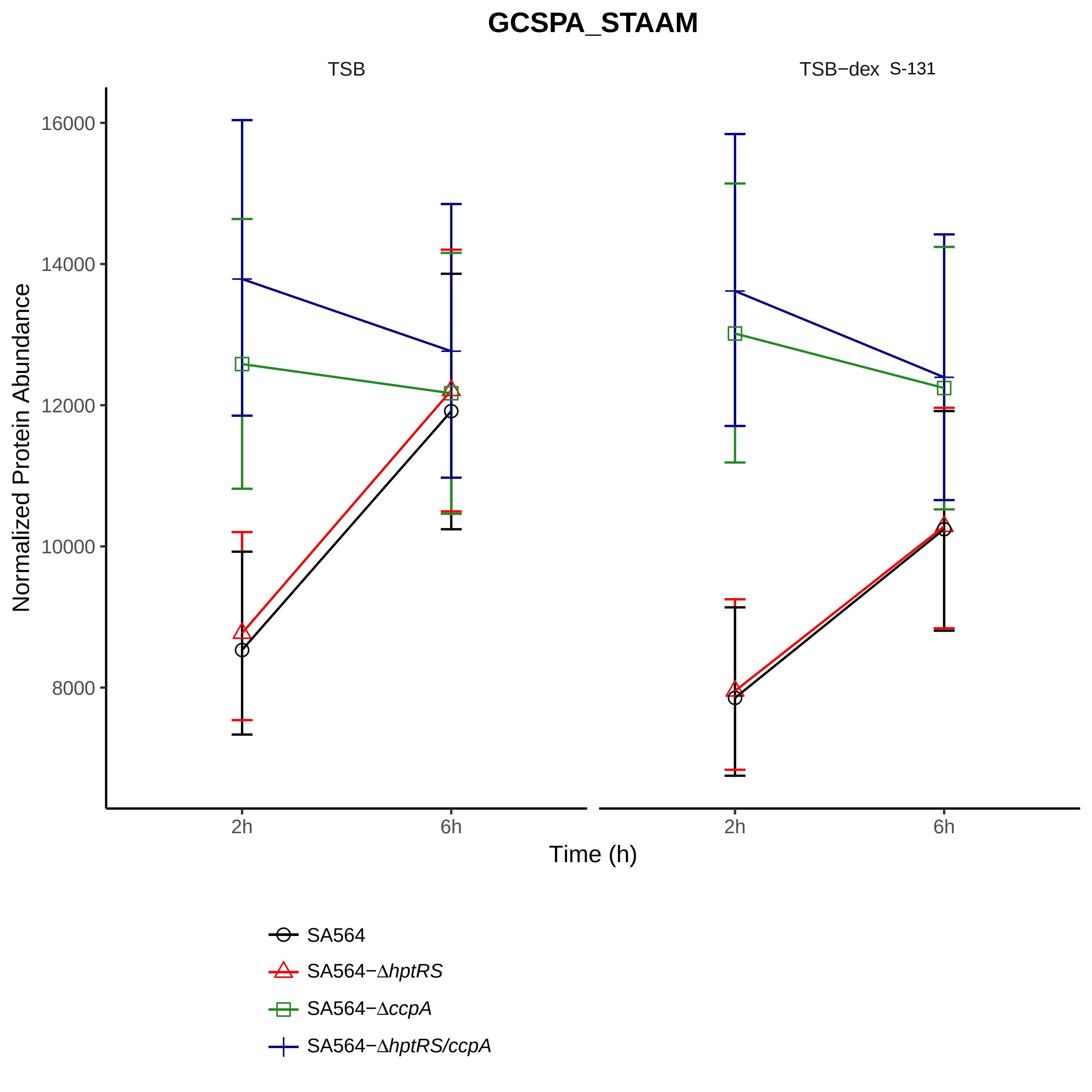

GCSPA_STAAM
TSB
TSB−dex
S-131
16000
14000
12000
10000
8000
Normalized Protein Abundance
2h
6h
2h
6h
Time (h)
SA564
SA564−ΔhptRS
SA564−ΔccpA
SA564−ΔhptRS/ccpA

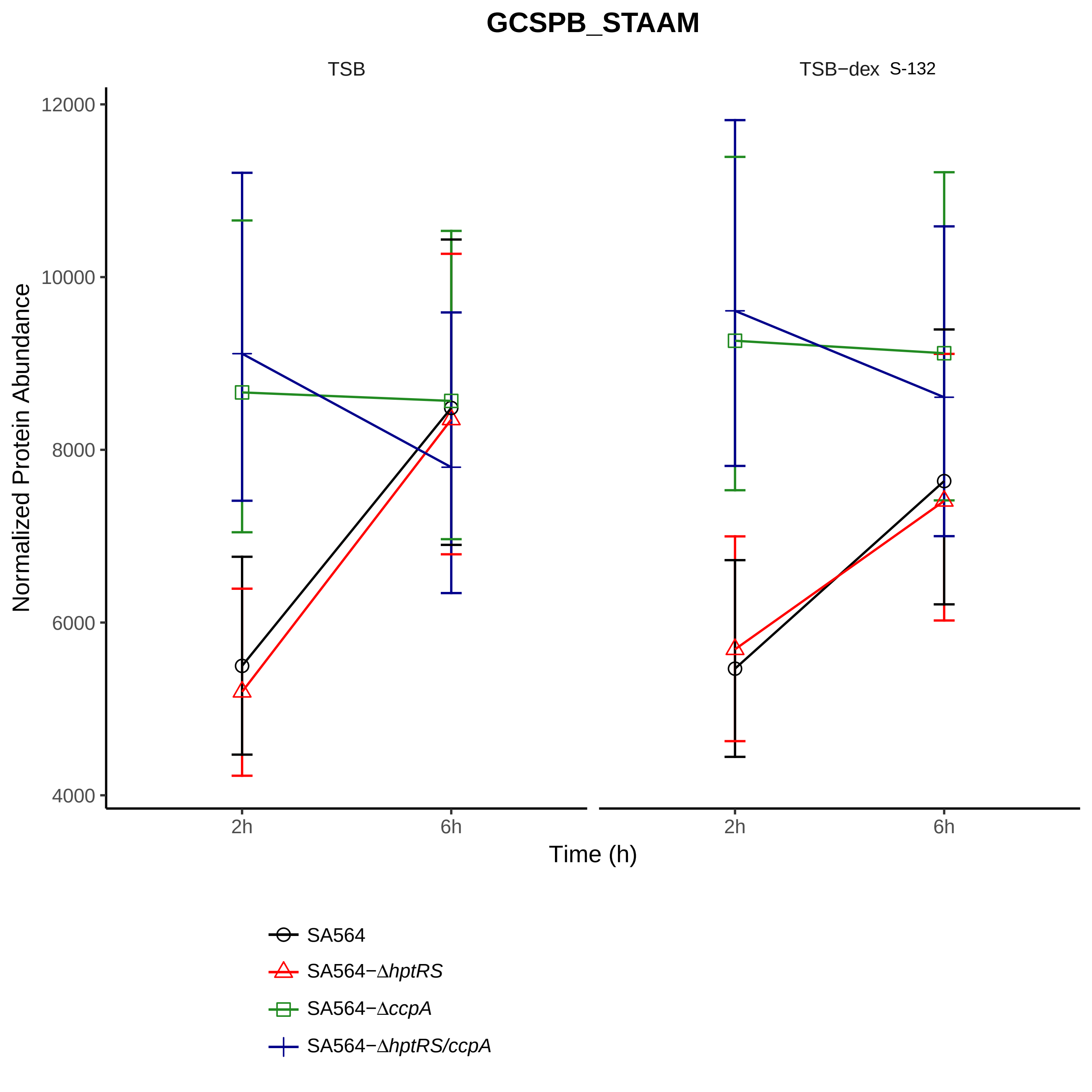

GCSPB_STAAM
TSB
TSB−dex
S-132
Normalized Protein Abundance
12000
10000
8000
6000
4000
2h
6h
2h
6h
Time (h)
SA564
SA564−Δ*hptRS*
SA564−Δ*ccpA*
SA564−Δ*hptRS/ccpA*

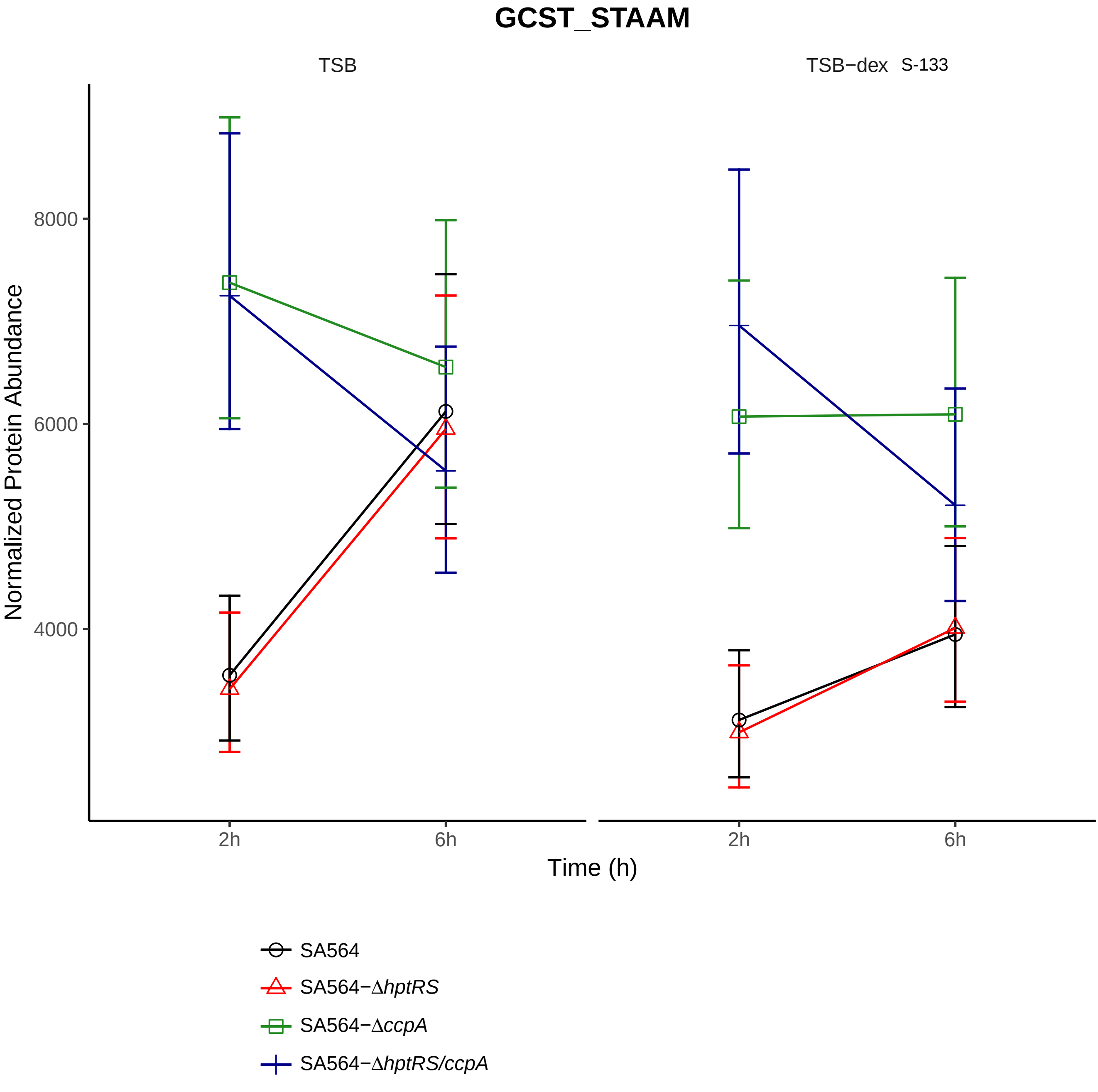
GCST_STAAM
TSB
TSB−dex
S-133
Normalized Protein Abundance
8000
6000
4000
2h
6h
2h
6h
Time (h)
SA564
SA564−Δ*hptRS*
SA564−Δ*ccpA*
SA564−Δ*hptRS/ccpA*

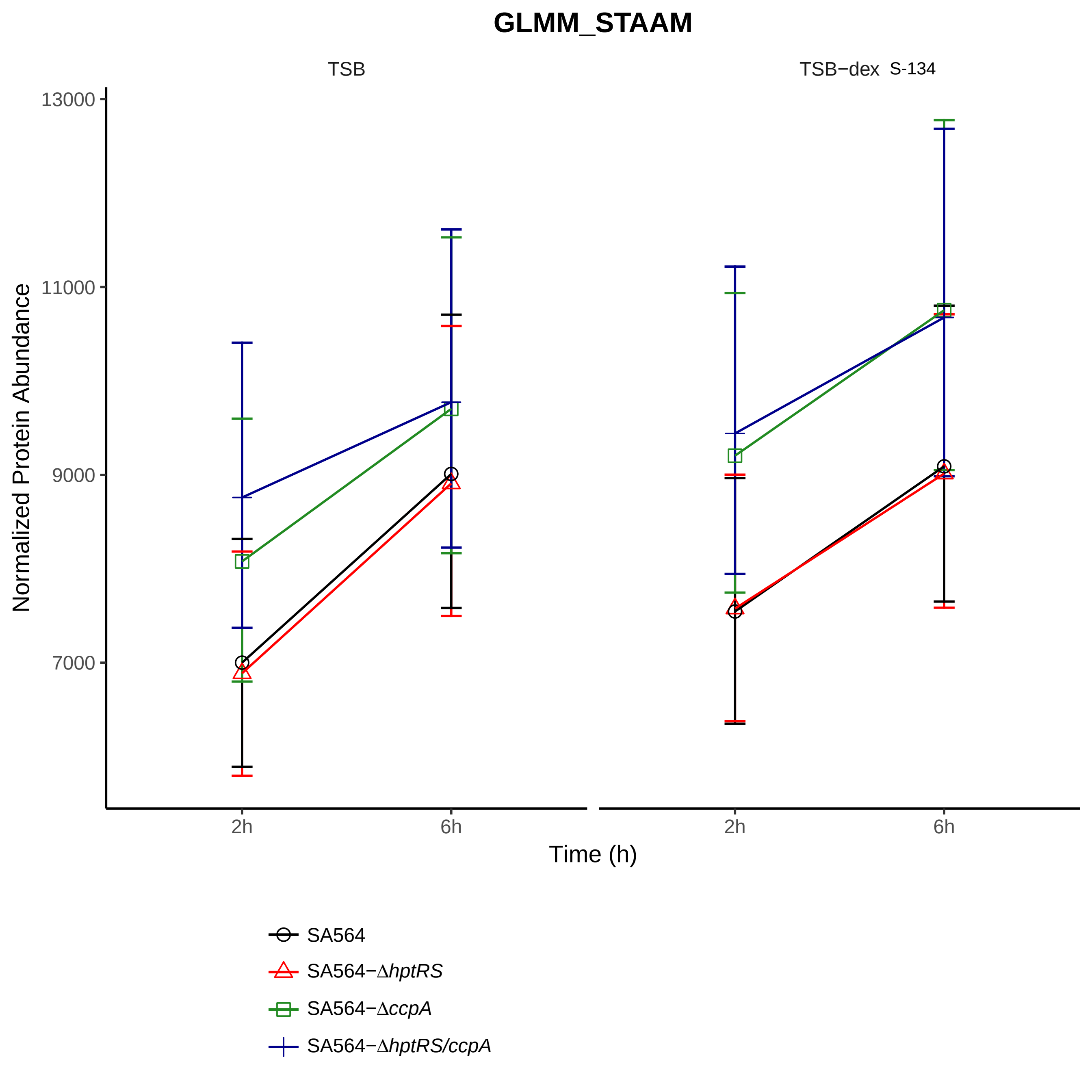
GLMM_STAAM
TSB
TSB−dex
S-134
13000
11000
9000
7000
Normalized Protein Abundance
2h
6h
2h
6h
Time (h)
SA564
SA564−ΔhptRS
SA564−ΔccpA
SA564−ΔhptRS/ccpA

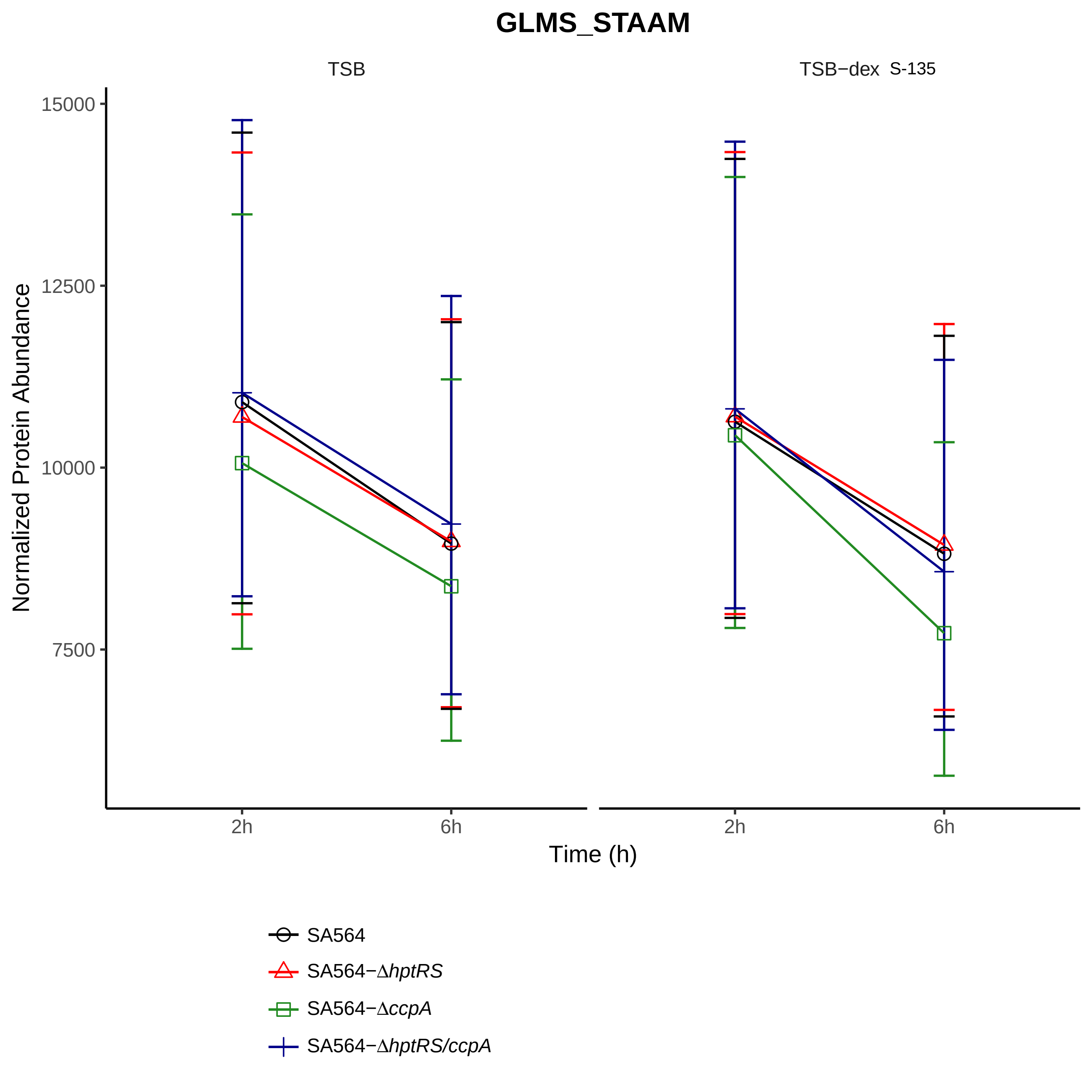

GLMS_STAAM
TSB
TSB−dex
S-135
15000
12500
10000
7500
Normalized Protein Abundance
2h
6h
2h
6h
Time (h)
SA564
SA564−Δ*hptRS*
SA564−Δ*ccpA*
SA564−Δ*hptRS/ccpA*

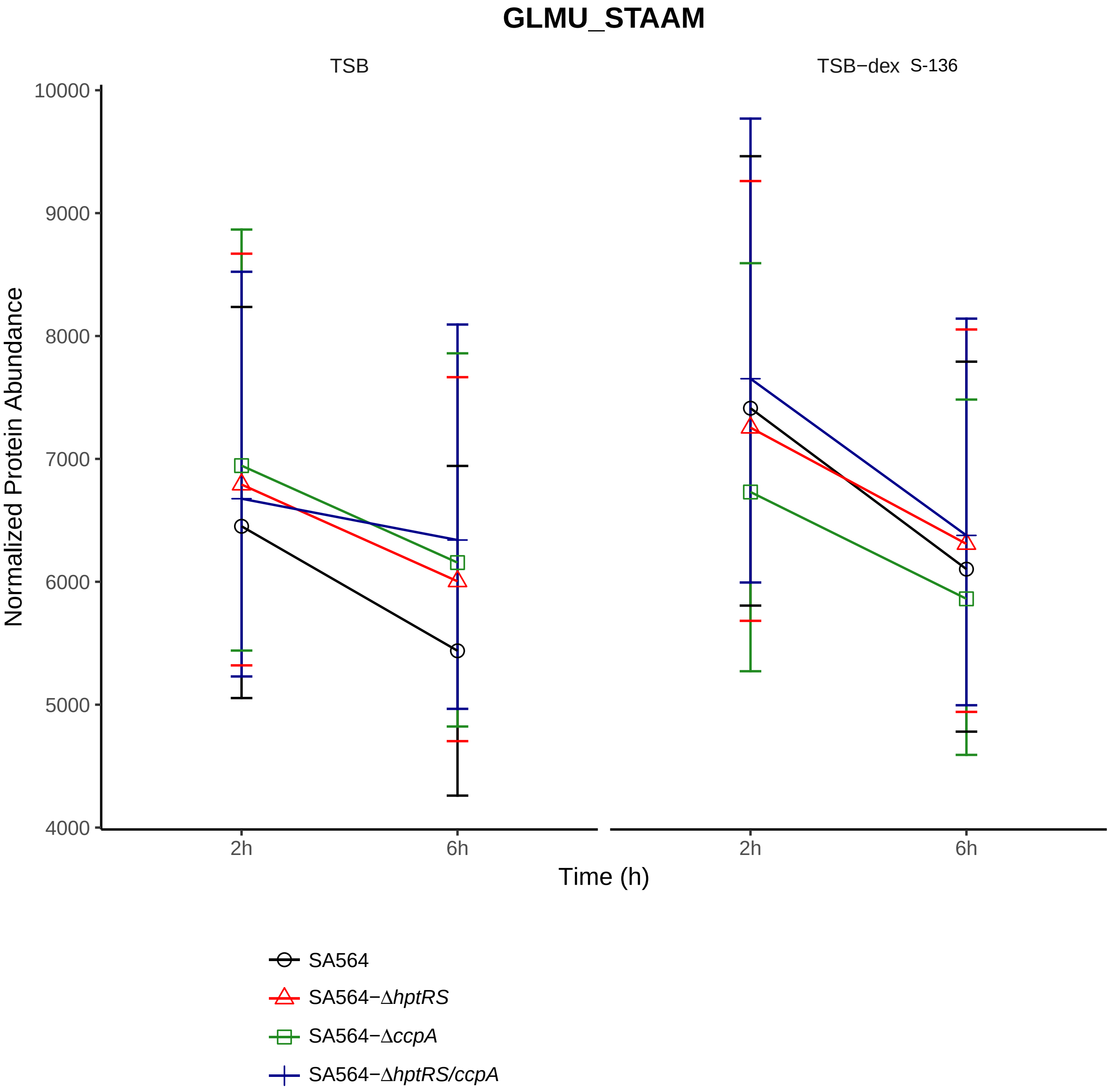

GLMU_STAAM
TSB
TSB−dex
S-136
Normalized Protein Abundance
10000
9000
8000
7000
6000
5000
4000
2h
6h
2h
6h
Time (h)
SA564
SA564−Δ*hptRS*
SA564−Δ*ccpA*
SA564−Δ*hptRS/ccpA*

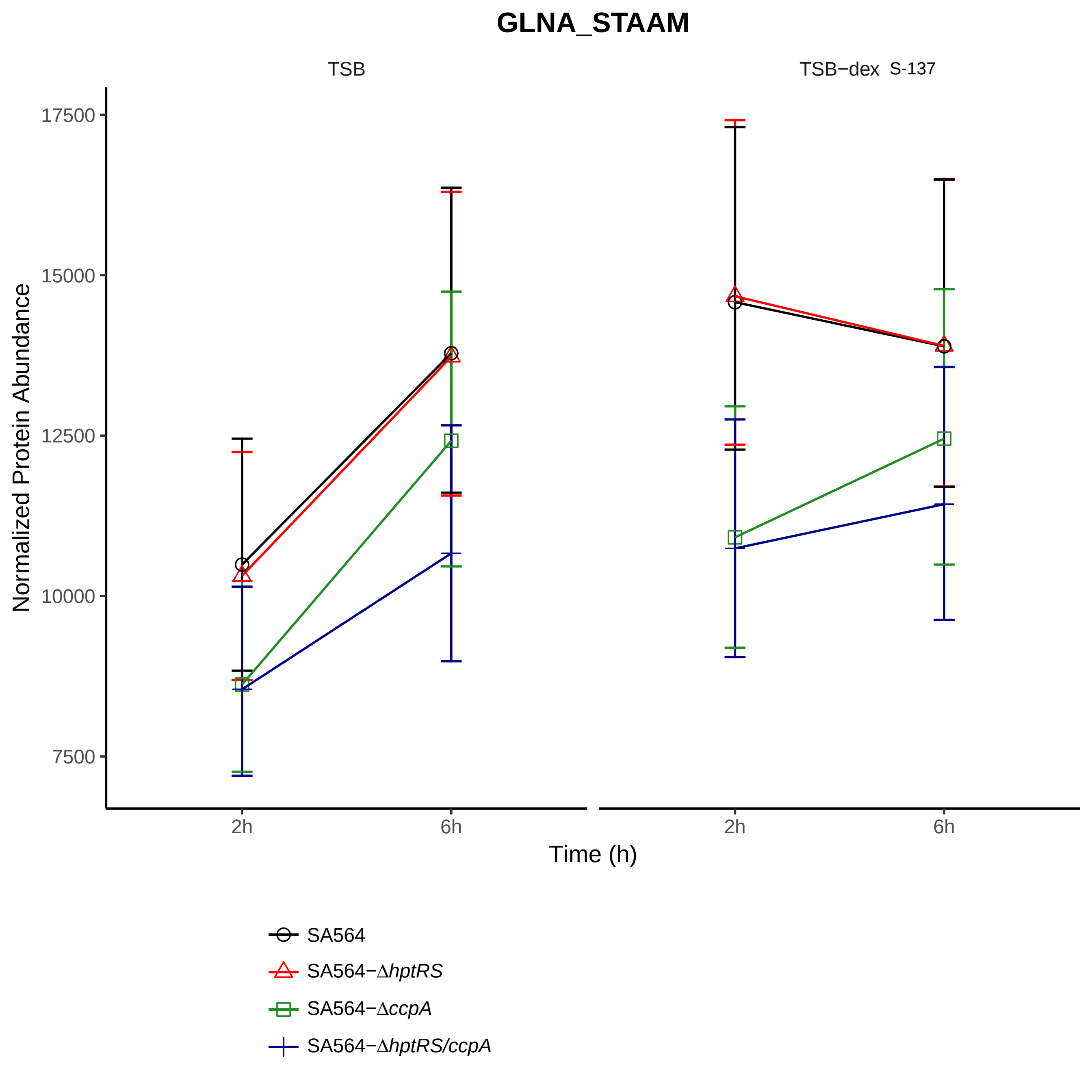

GLNA_STAAM
TSB
TSB−dex
S-137
17500
15000
12500
10000
7500
Normalized Protein Abundance
2h
6h
2h
6h
Time (h)
SA564
SA564−Δ*hptRS*
SA564−Δ*ccpA*
SA564−Δ*hptRS/ccpA*

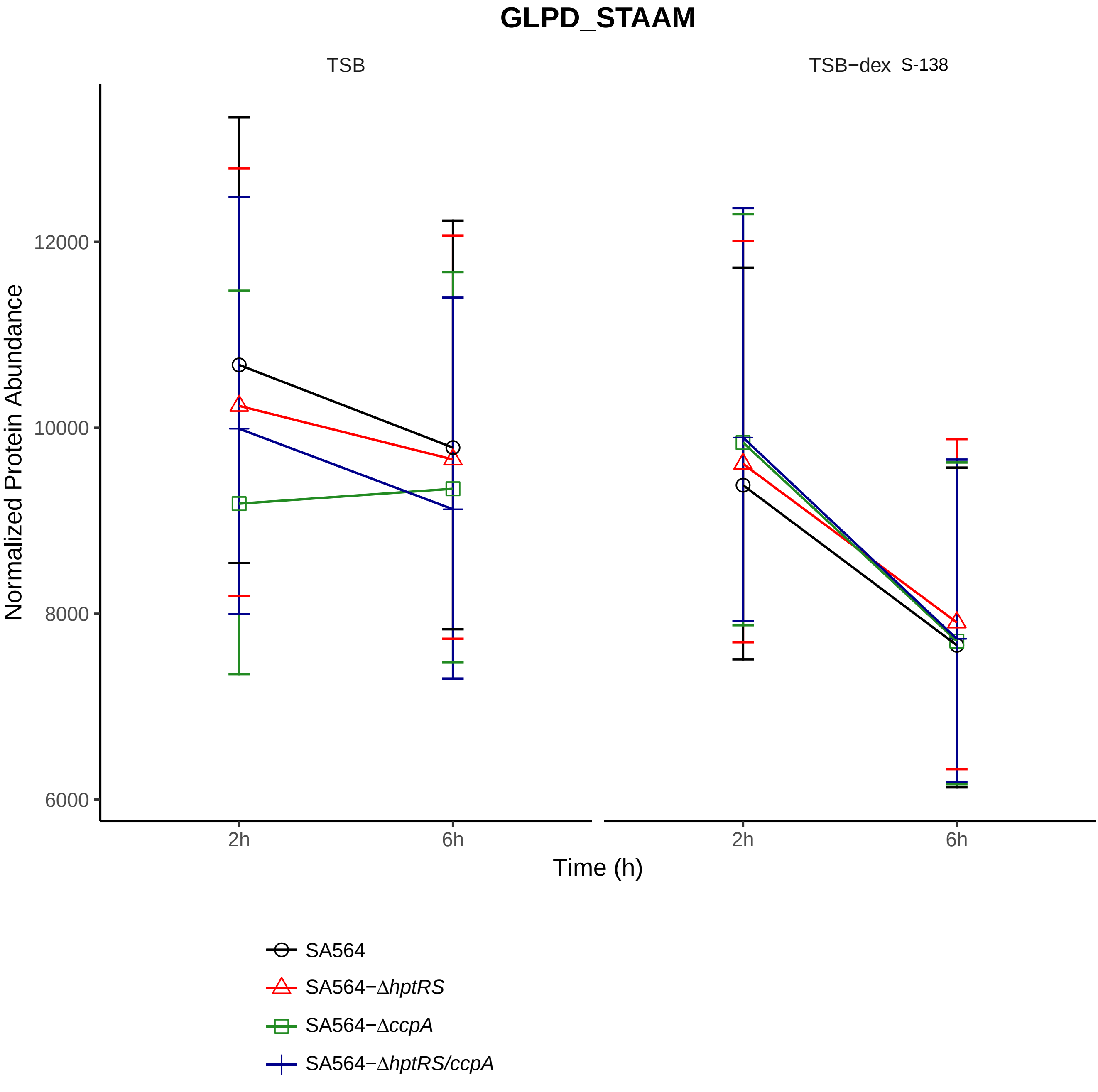
GLPD_STAAM
TSB
TSB−dex
Normalized Protein Abundance
12000
10000
8000
6000
2h
6h
Time (h)
SA564
SA564−ΔhptRS
SA564−ΔccpA
SA564−ΔhptRS/ccpA

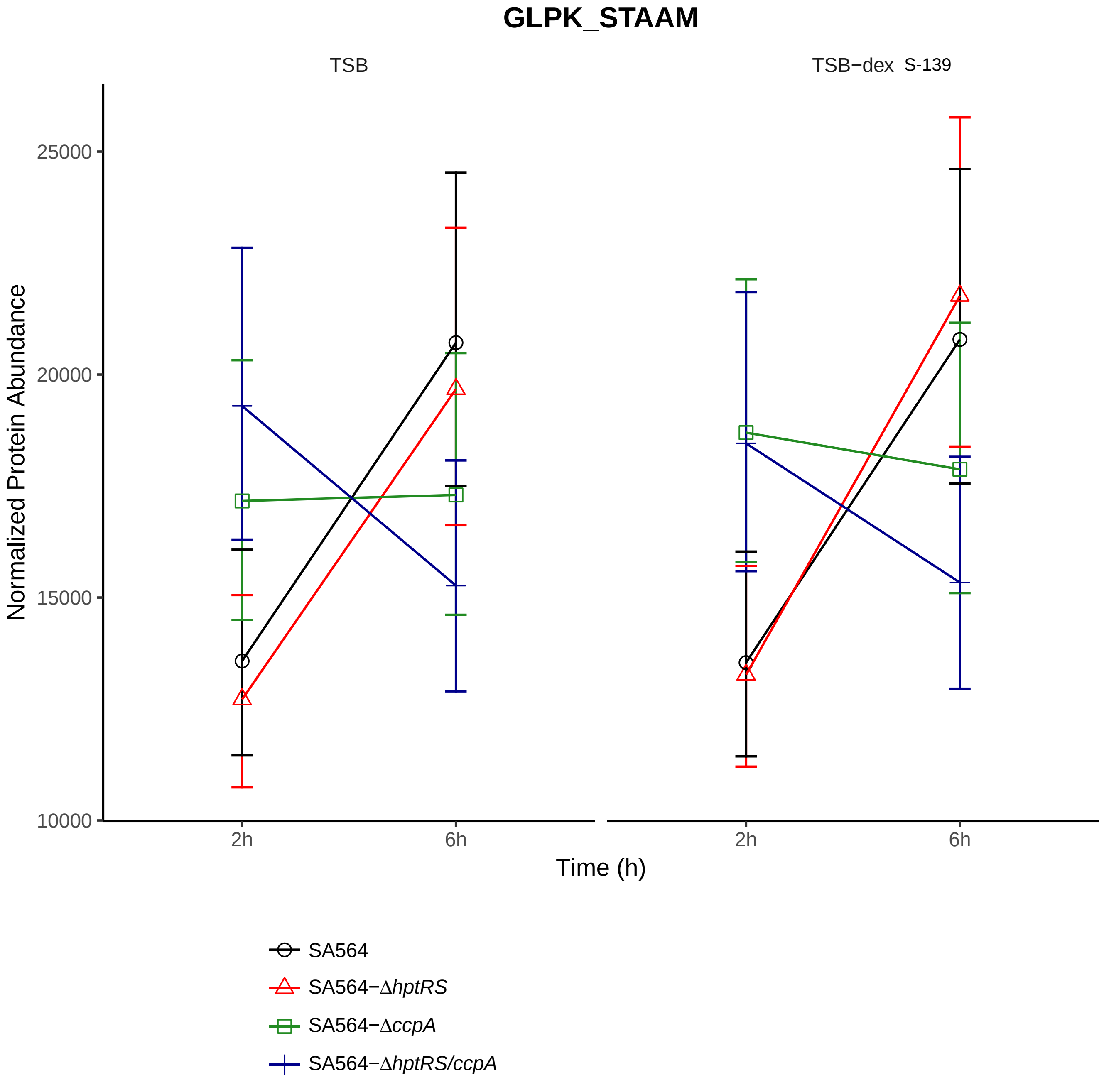

GLPK_STAAM
TSB
TSB−dex
S-139
Normalized Protein Abundance
25000
20000
15000
10000
2h
6h
2h
6h
Time (h)
SA564
SA564−Δ*hptRS*
SA564−Δ*ccpA*
SA564−Δ*hptRS/ccpA*

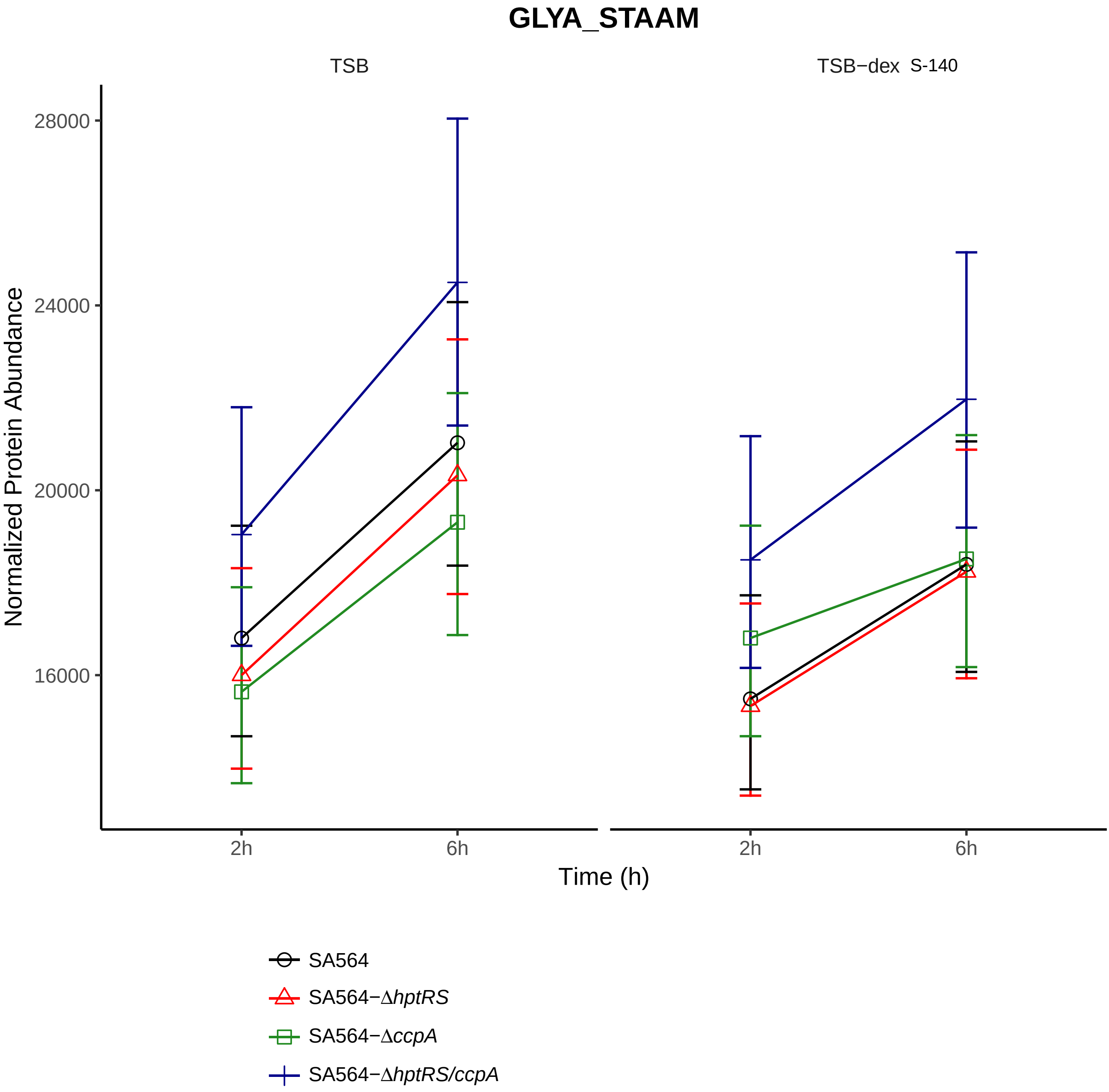

GLYA_STAAM
TSB
TSB−dex
S-140
Normalized Protein Abundance
28000
24000
20000
16000
2h
6h
2h
6h
Time (h)
SA564
SA564−Δ*hptRS*
SA564−Δ*ccpA*
SA564−Δ*hptRS/ccpA*

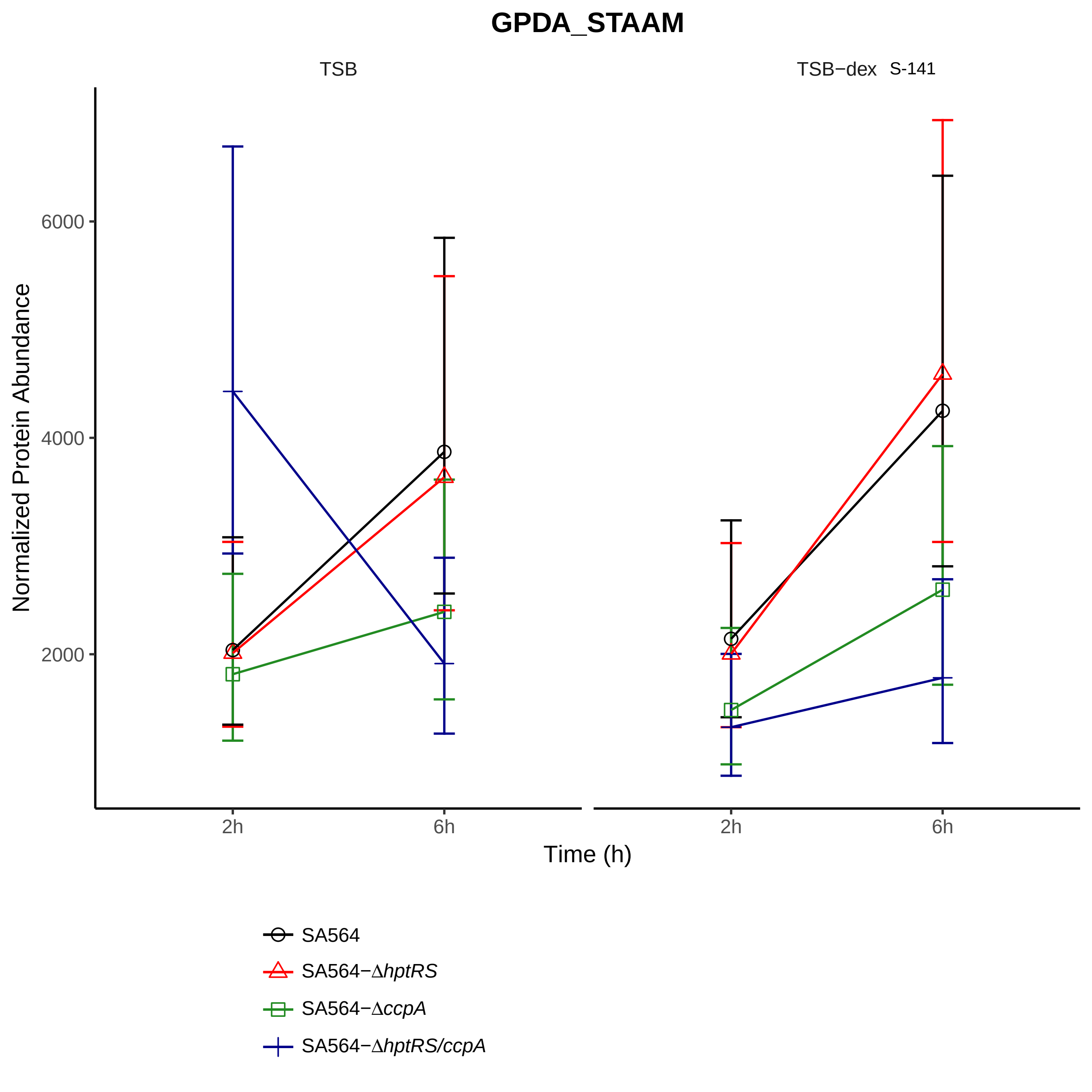

GPDA_STAAM
TSB
TSB−dex
S-141
Normalized Protein Abundance
6000
4000
2000
2h
6h
2h
6h
Time (h)
SA564
SA564−Δ*hptRS*
SA564−Δ*ccpA*
SA564−Δ*hptRS/ccpA*

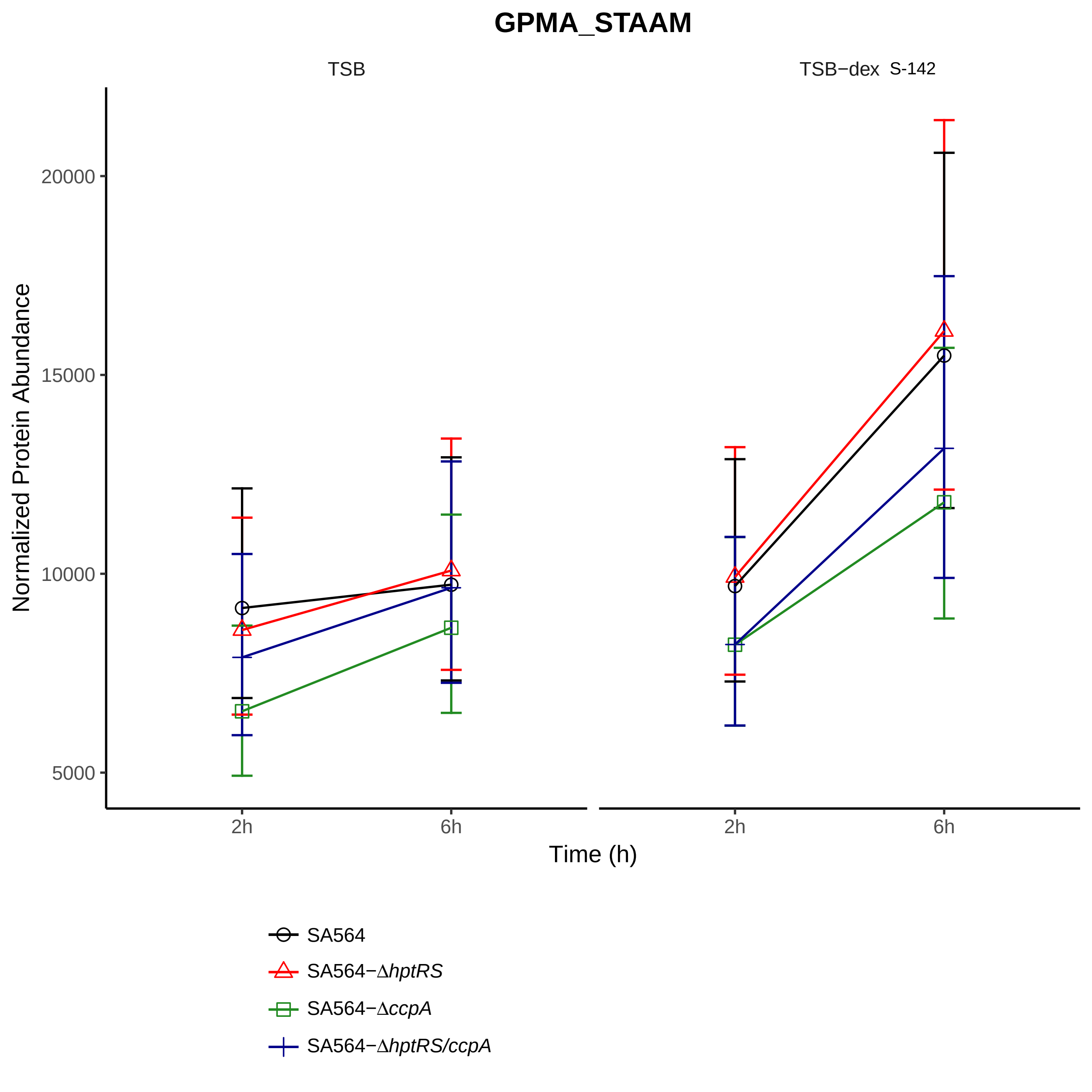

GPMA_STAAM
TSB
TSB−dex
S-142
Normalized Protein Abundance
20000
15000
10000
5000
2h
6h
2h
6h
Time (h)
SA564
SA564−ΔhptRS
SA564−ΔccpA
SA564−ΔhptRS/ccpA

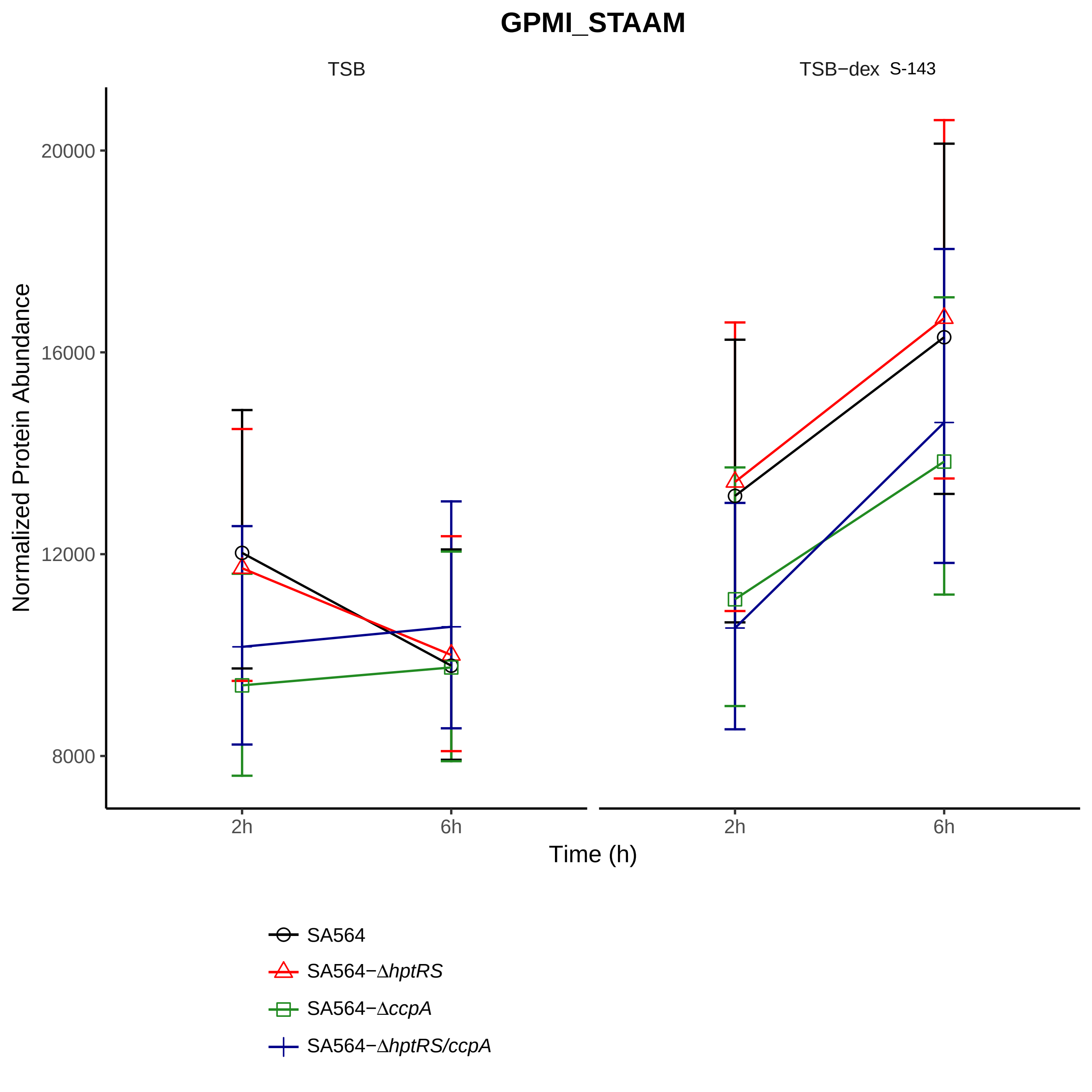

GPMI_STAAM
TSB
TSB−dex
S-143
Normalized Protein Abundance
20000
16000
12000
8000
2h
6h
2h
6h
Time (h)
SA564
SA564−ΔhptRS
SA564−ΔccpA
SA564−ΔhptRS/ccpA

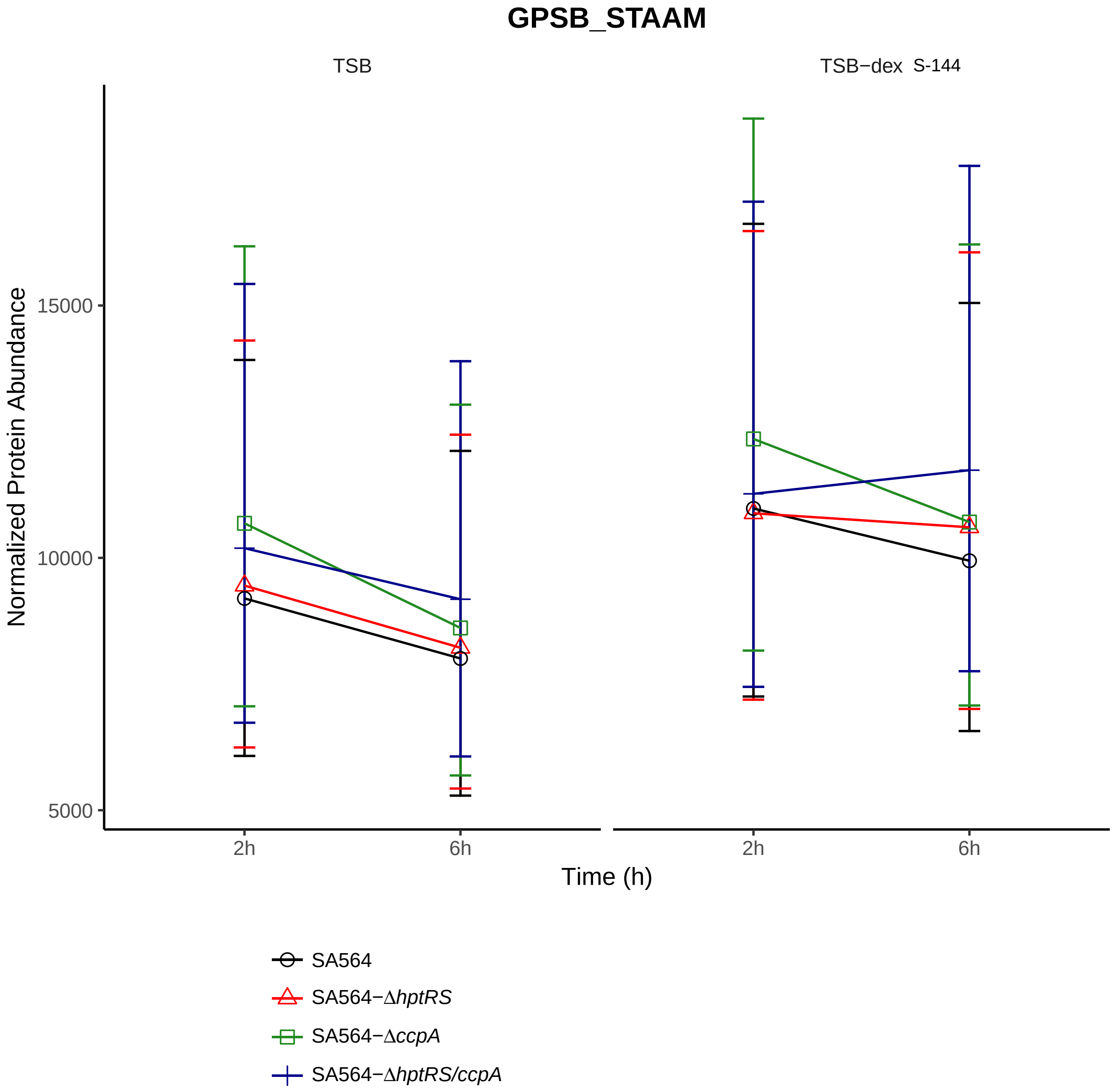

GPSB_STAAM
TSB
TSB−dex
S-144
Normalized Protein Abundance
15000
10000
5000
2h
6h
2h
6h
Time (h)
SA564
SA564−Δ*hptRS*
SA564−Δ*ccpA*
SA564−Δ*hptRS/ccpA*

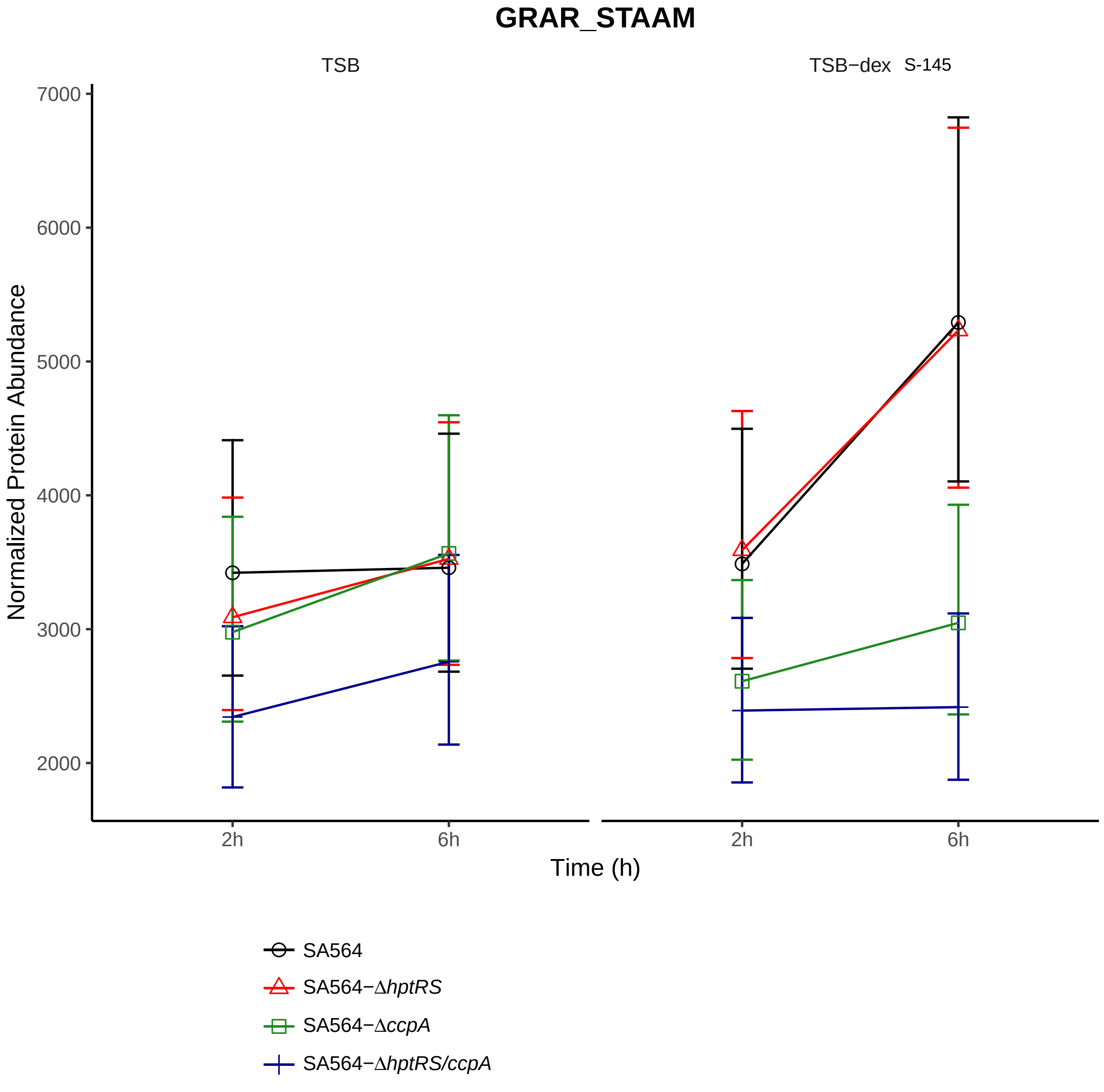

GRAR_STAAM
TSB
TSB−dex
S-145
Normalized Protein Abundance
7000
6000
5000
4000
3000
2000
2h
6h
2h
6h
Time (h)
SA564
SA564−Δ*hptRS*
SA564−Δ*ccpA*
SA564−Δ*hptRS/ccpA*

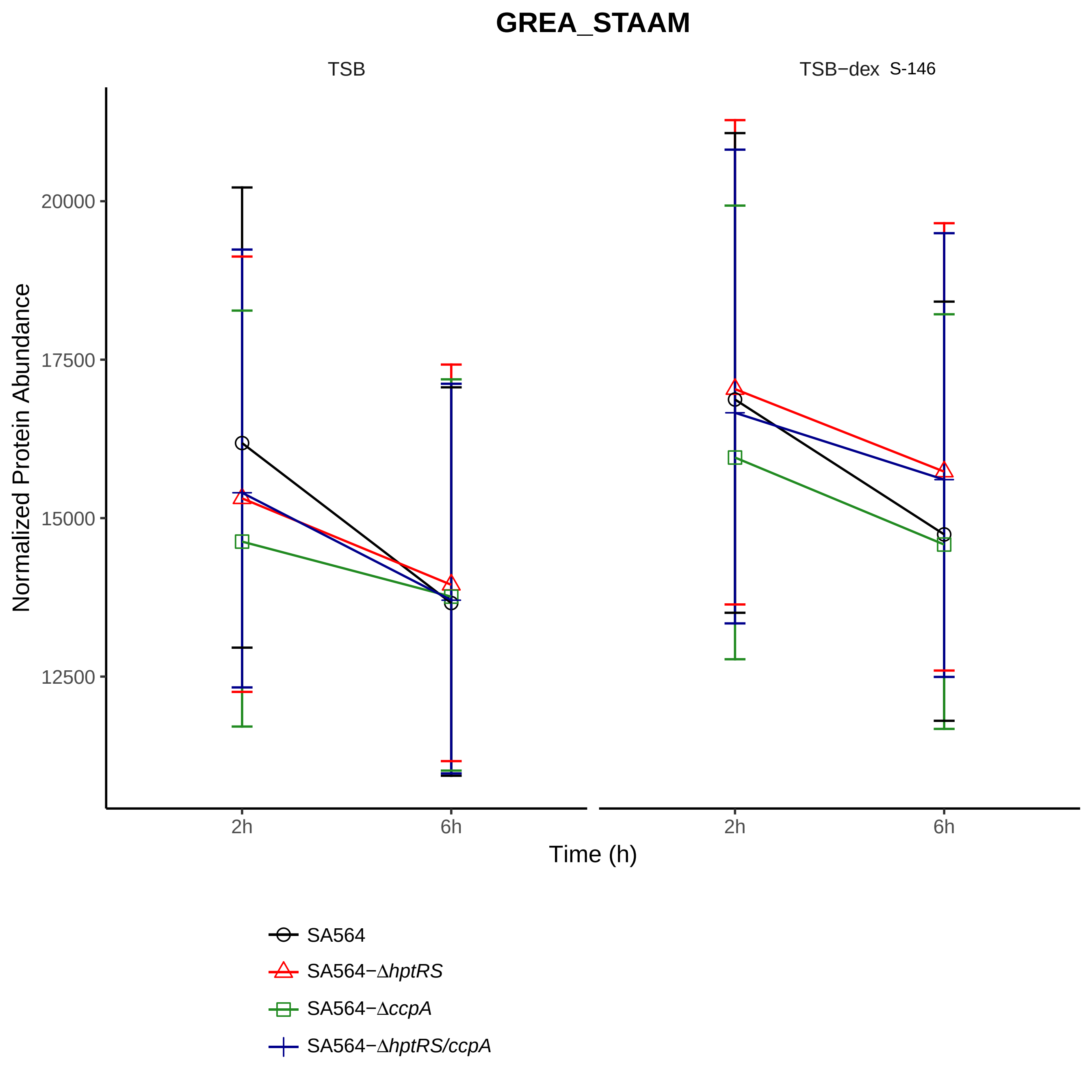

GREA_STAAM
TSB
TSB−dex
S-146
Normalized Protein Abundance
20000
17500
15000
12500
2h
6h
2h
6h
Time (h)
SA564
SA564−ΔhptRS
SA564−ΔccpA
SA564−ΔhptRS/ccpA

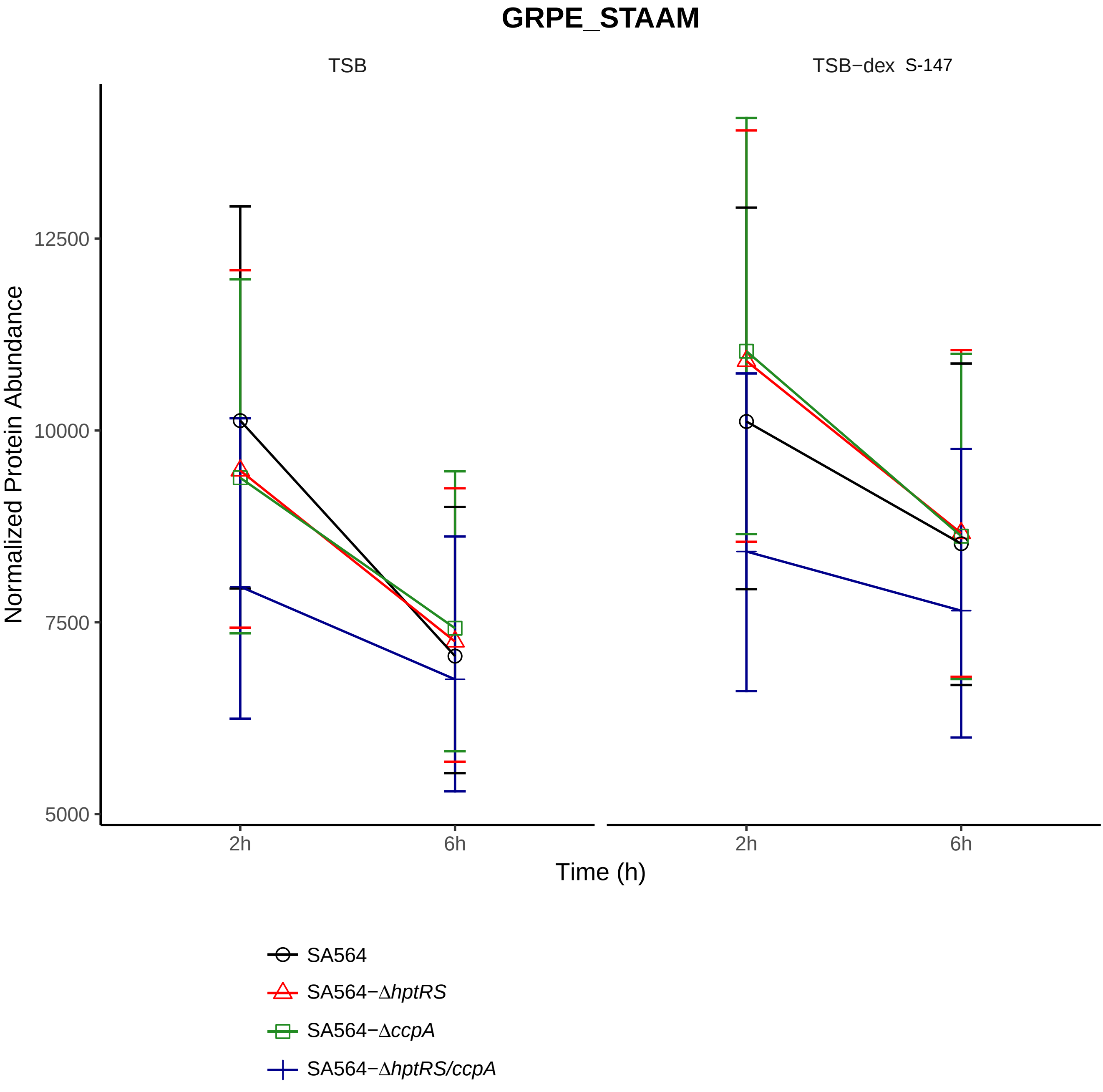
GRPE_STAAM
TSB
TSB−dex
S-147
12500
10000
7500
5000
Normalized Protein Abundance
2h
6h
2h
6h
Time (h)
SA564
SA564−Δ*hptRS*
SA564−Δ*ccpA*
SA564−Δ*hptRS/ccpA*

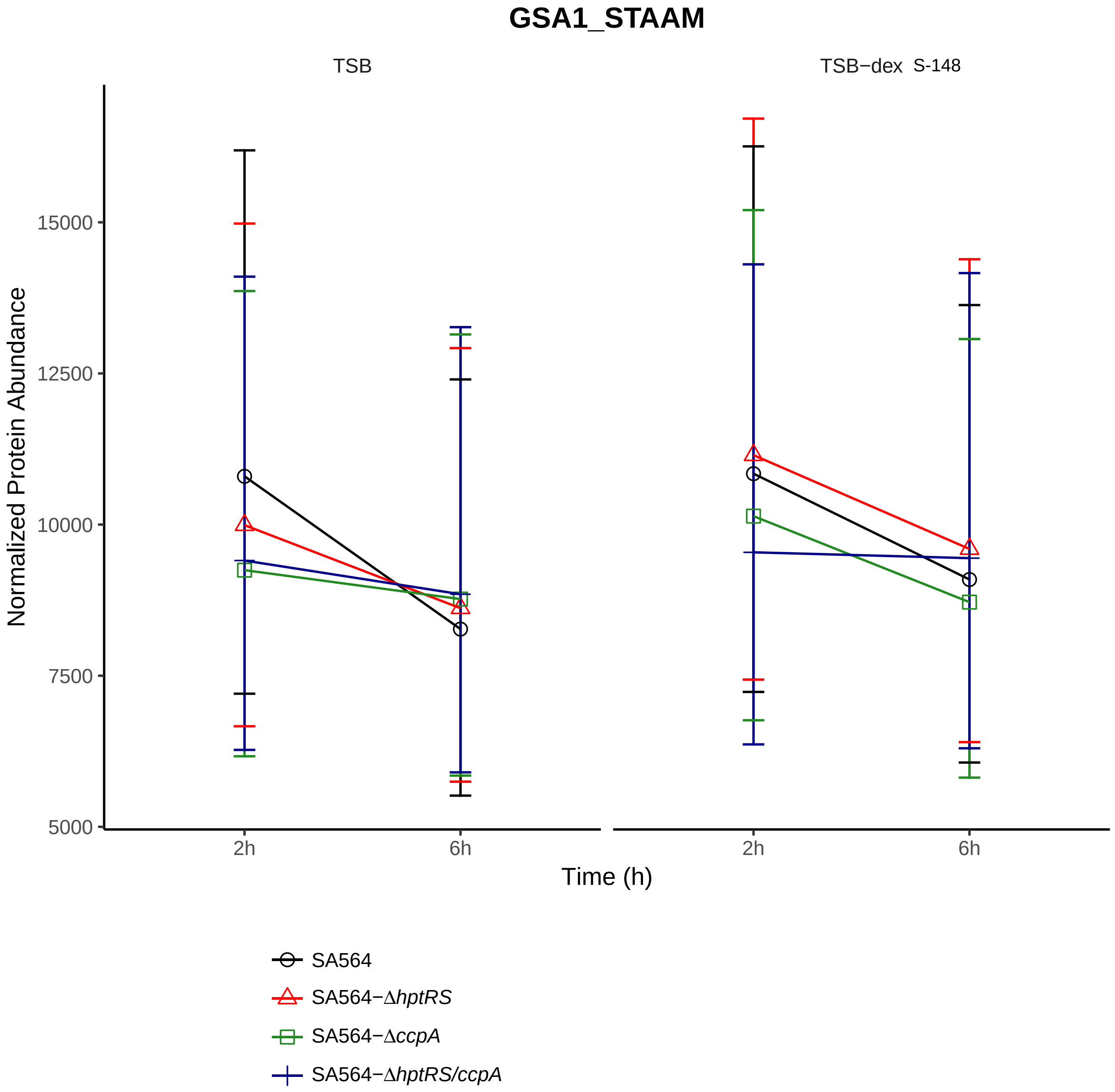

GSA1_STAAM
TSB
TSB−dex
S-148
Normalized Protein Abundance
15000
12500
10000
7500
5000
2h
6h
2h
6h
Time (h)
SA564
SA564−ΔhptRS
SA564−ΔccpA
SA564−ΔhptRS/ccpA

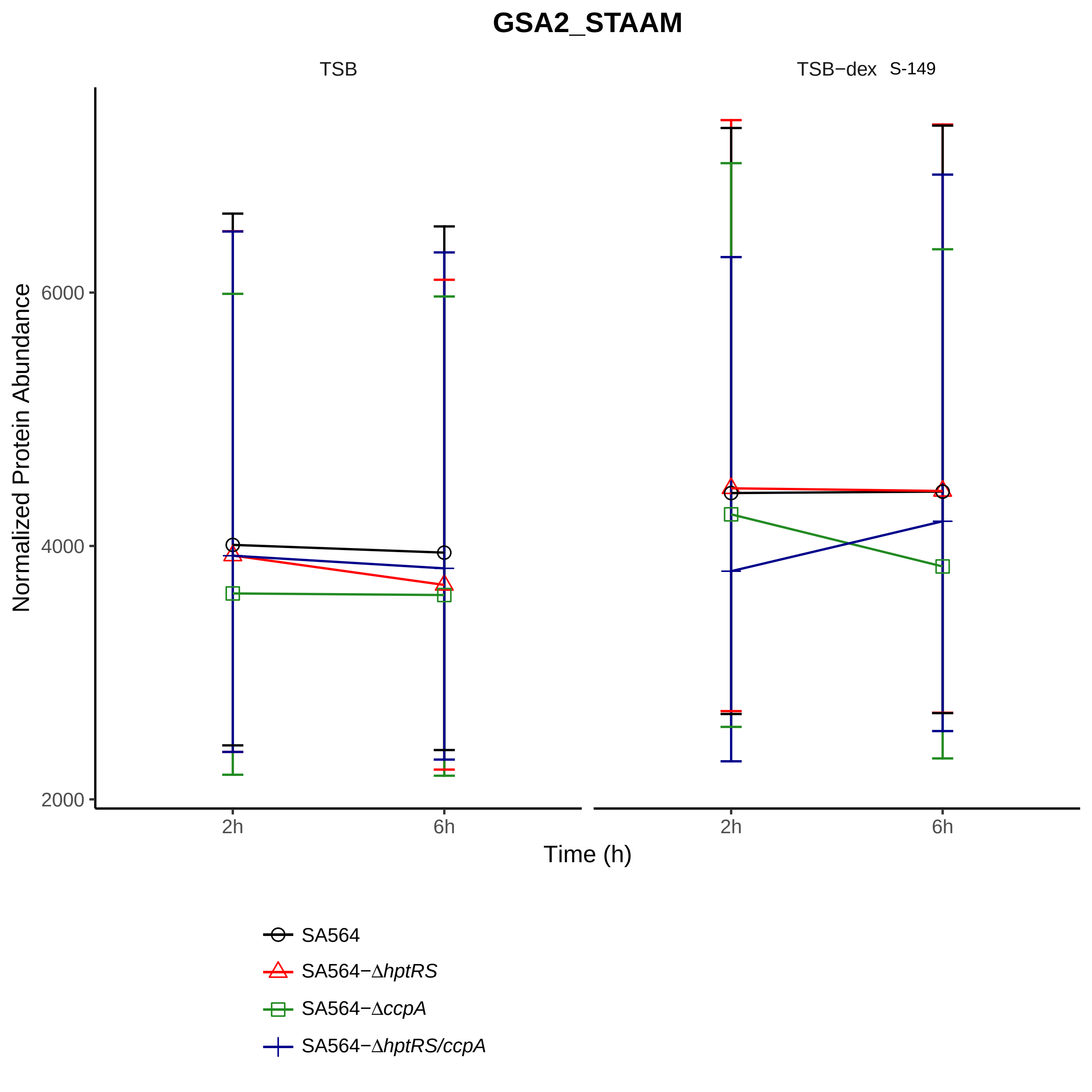

GSA2_STAAM
TSB
TSB−dex
S-149
Normalized Protein Abundance
6000
4000
2000
2h
6h
2h
6h
Time (h)
SA564
SA564−ΔhptRS
SA564−ΔccpA
SA564−ΔhptRS/ccpA

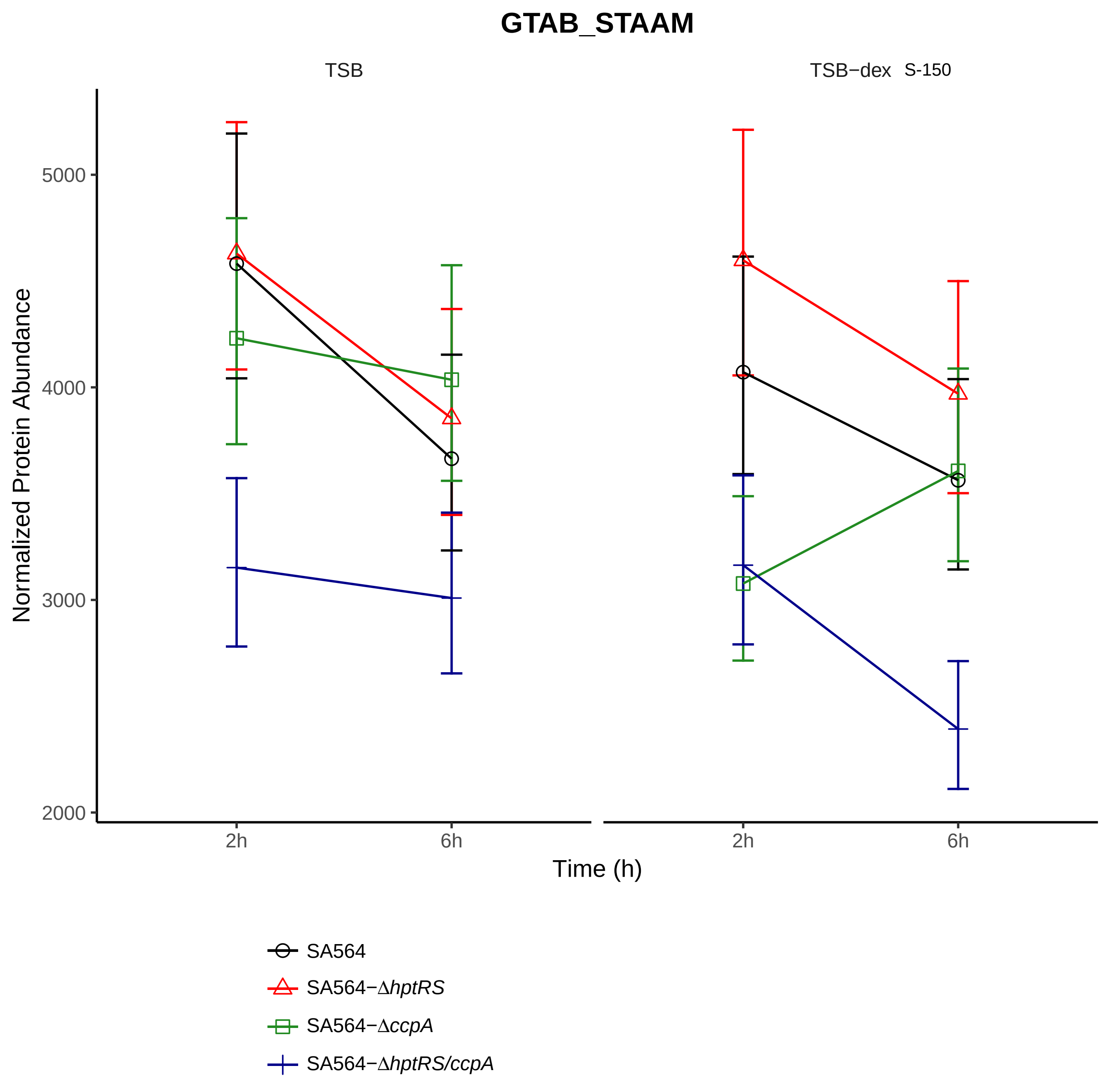
GTAB_STAAM
TSB
TSB−dex
S-150
Normalized Protein Abundance
5000
4000
3000
2000
2h
6h
2h
6h
Time (h)
SA564
SA564−ΔhptRS
SA564−ΔccpA
SA564−ΔhptRS/ccpA

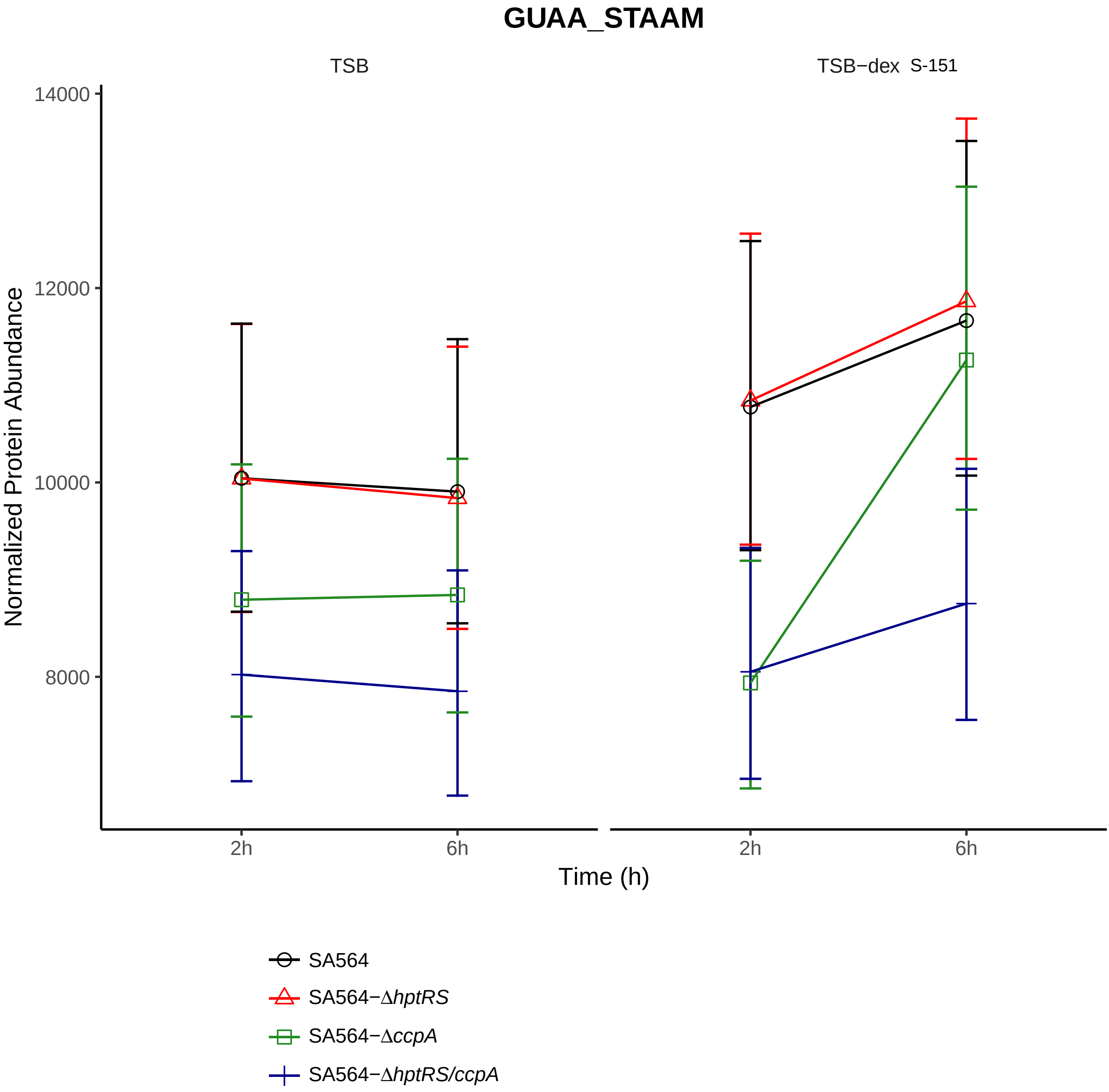

GUAA_STAAM
TSB
TSB−dex
S-151
Normalized Protein Abundance
14000
12000
10000
8000
2h
6h
2h
6h
Time (h)
SA564
SA564−ΔhptRS
SA564−ΔccpA
SA564−ΔhptRS/ccpA

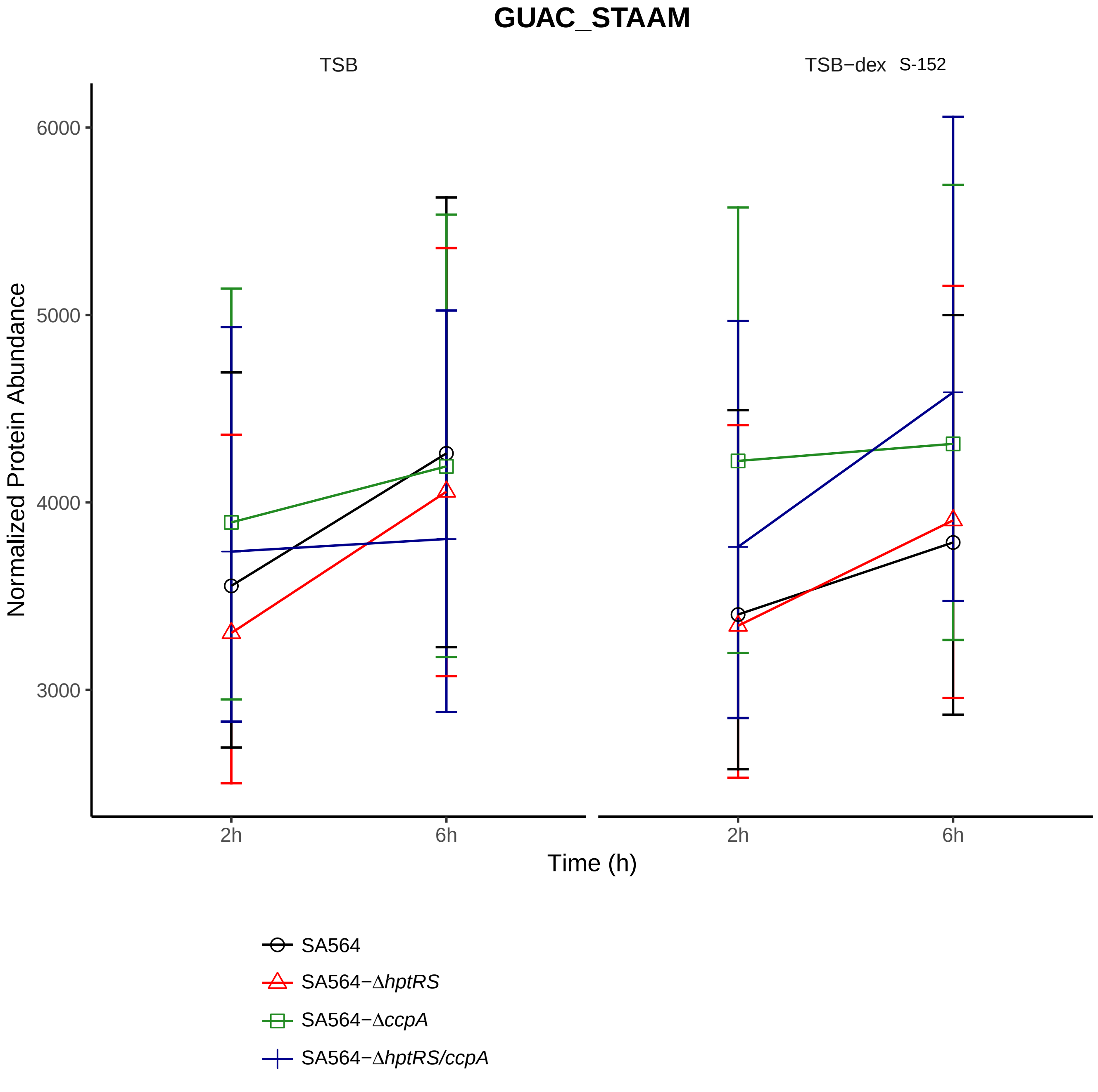

GUAC_STAAM
TSB
TSB−dex
S-152
Normalized Protein Abundance
6000
5000
4000
3000
2h
6h
2h
6h
Time (h)
SA564
SA564−Δ*hptRS*
SA564−Δ*ccpA*
SA564−Δ*hptRS/ccpA*

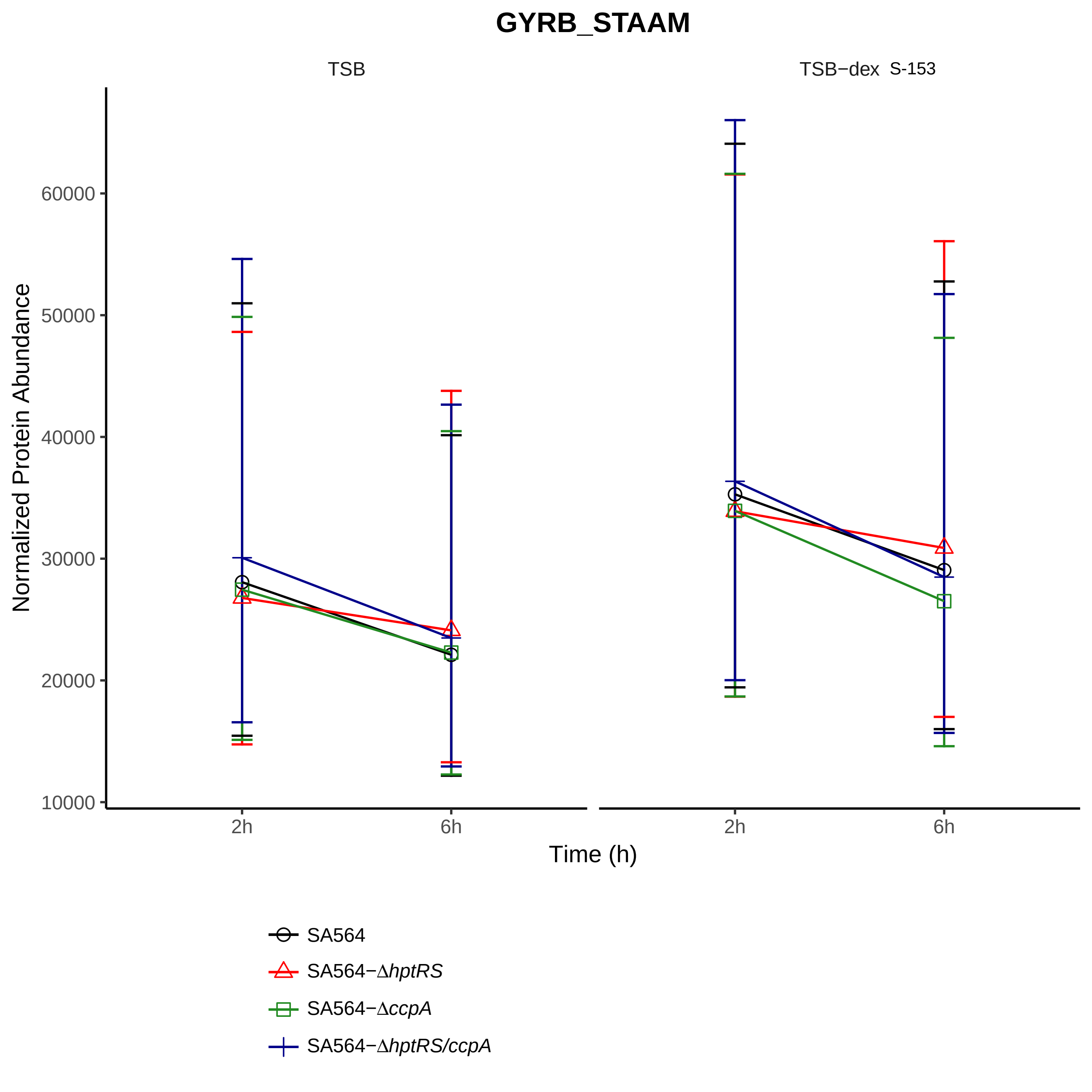
GYRB_STAAM
TSB
TSB−dex
S-153
Normalized Protein Abundance
10000
20000
30000
40000
50000
60000
2h
6h
2h
6h
Time (h)
SA564
SA564−Δ*hptRS*
SA564−Δ*ccpA*
SA564−Δ*hptRS/ccpA*

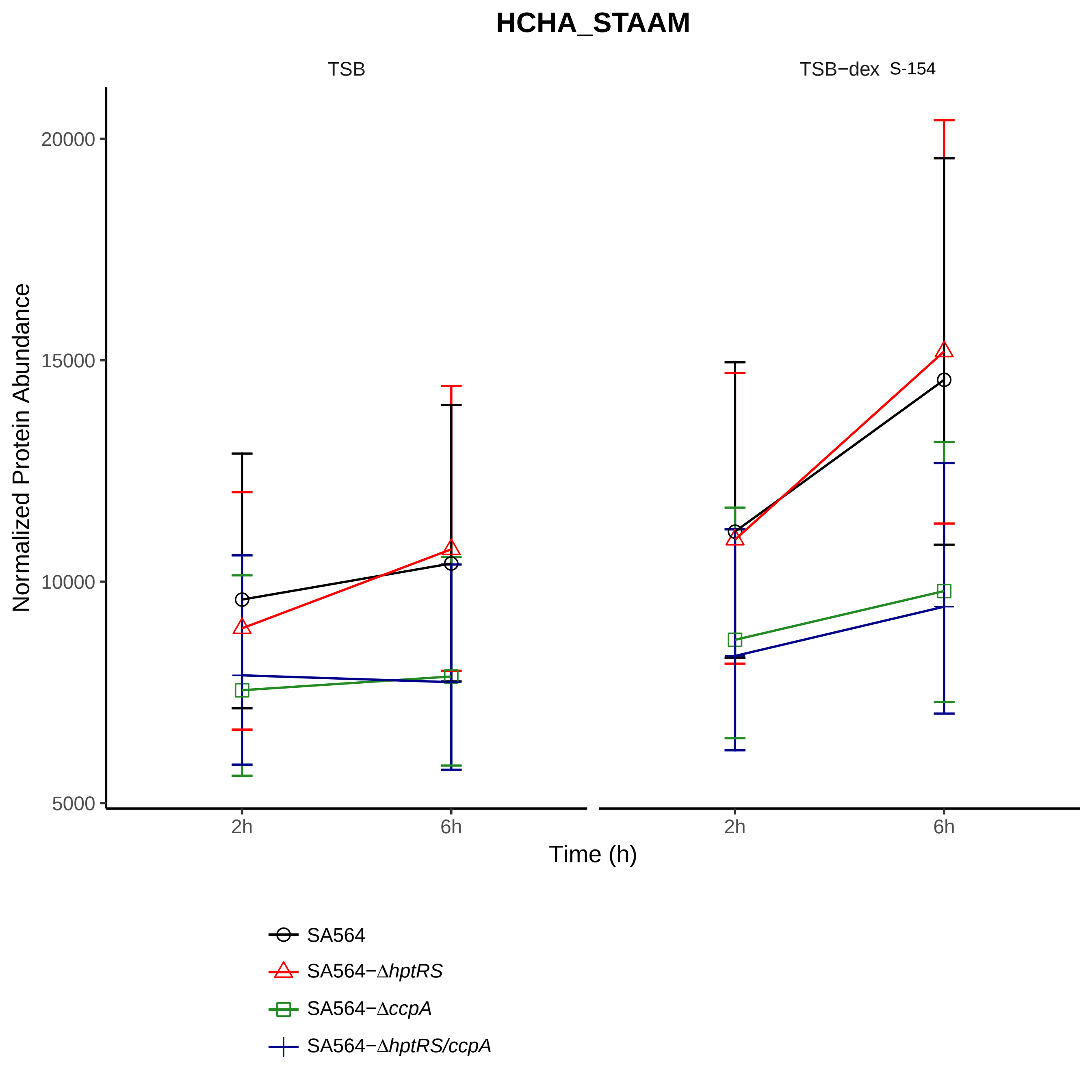

HCHA_STAAM
TSB
TSB−dex
S-154
Normalized Protein Abundance
20000
15000
10000
5000
2h
6h
2h
6h
Time (h)
SA564
SA564−Δ*hptRS*
SA564−Δ*ccpA*
SA564−Δ*hptRS/ccpA*

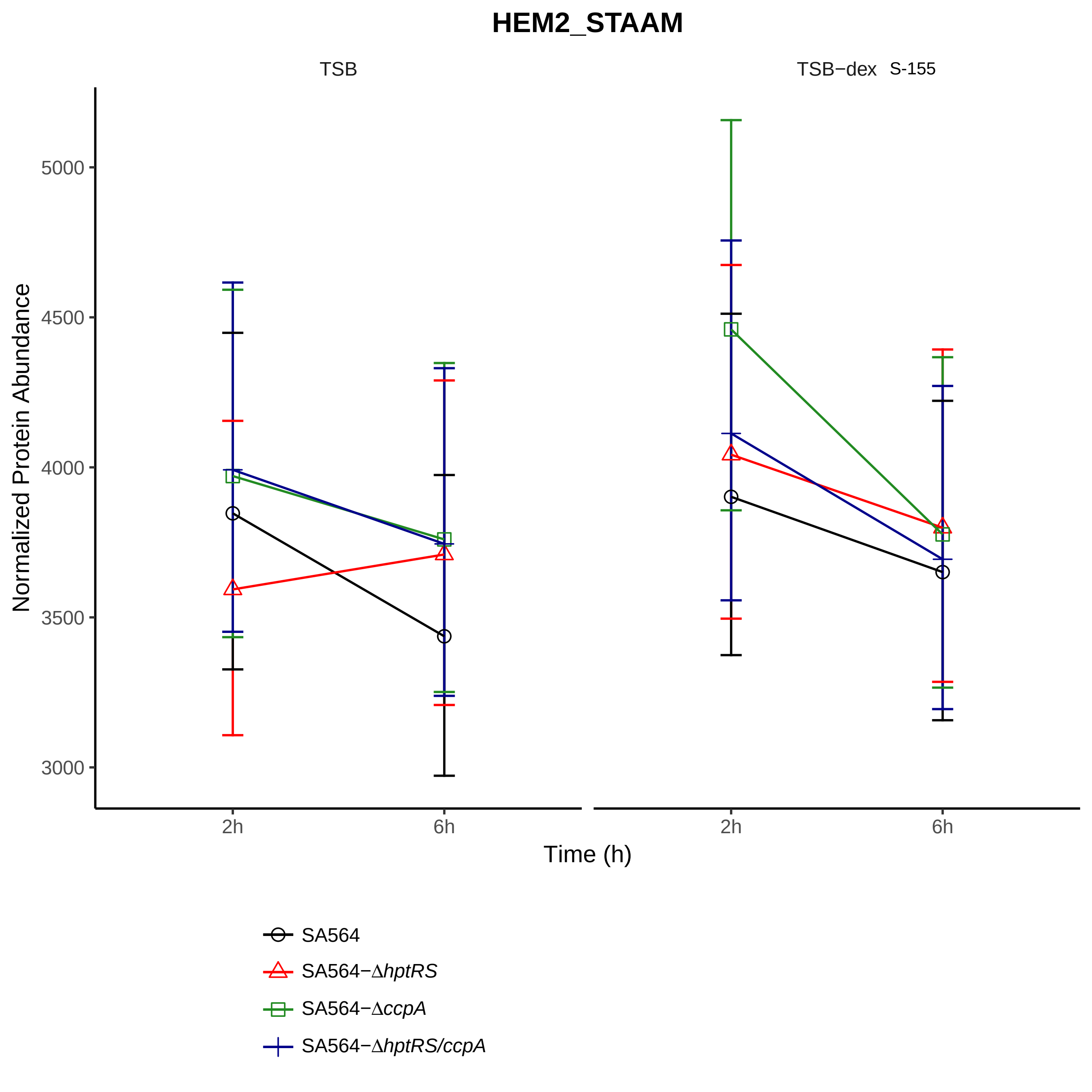

HEM2_STAAM
TSB
TSB−dex
S-155
Normalized Protein Abundance
5000
4500
4000
3500
3000
2h
6h
2h
6h
Time (h)
SA564
SA564−ΔhptRS
SA564−ΔccpA
SA564−ΔhptRS/ccpA

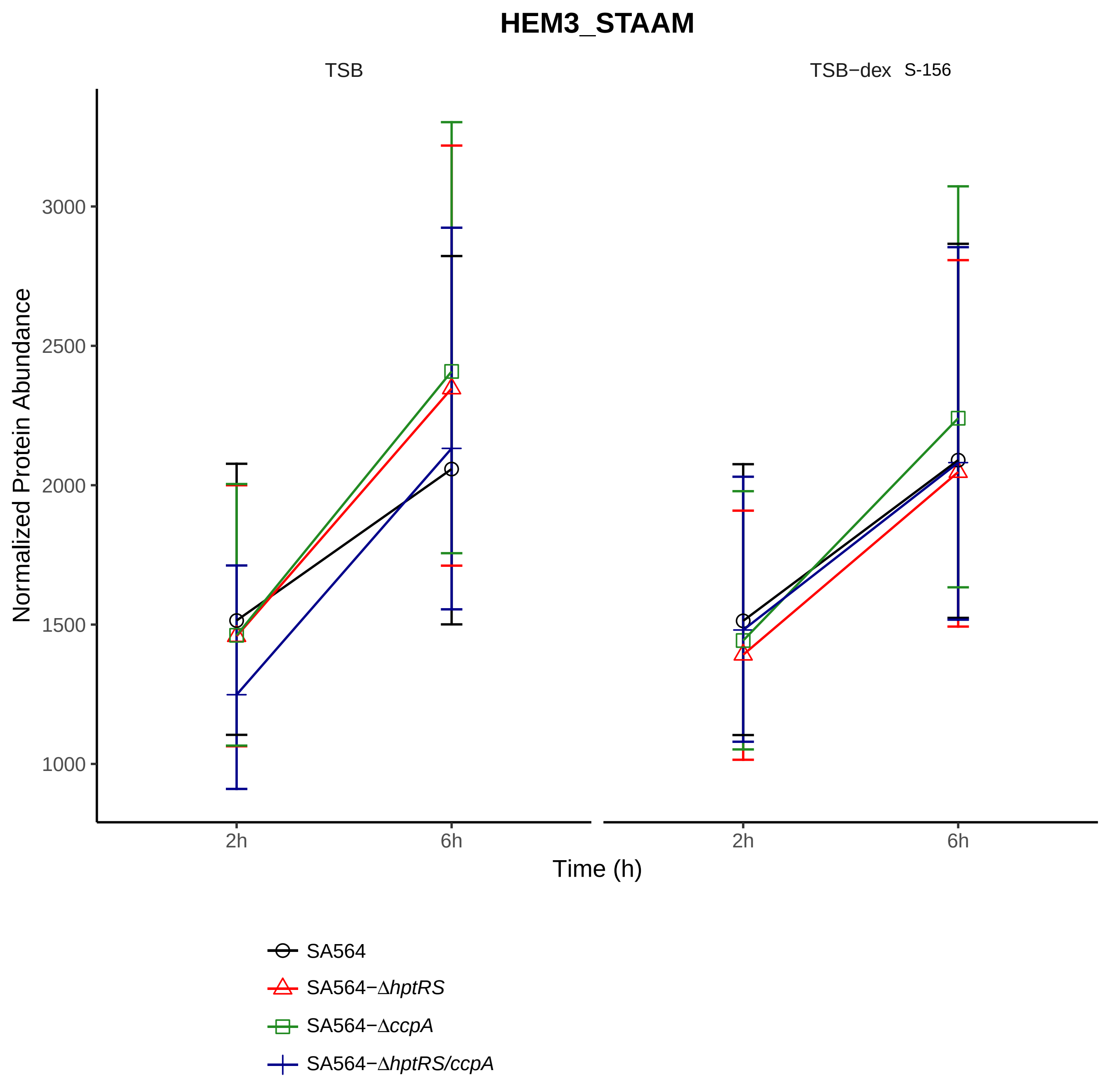

HEM3_STAAM
TSB
TSB−dex
S-156
Normalized Protein Abundance
3000
2500
2000
1500
1000
2h
6h
2h
6h
Time (h)
SA564
SA564−ΔhptRS
SA564−ΔccpA
SA564−ΔhptRS/ccpA

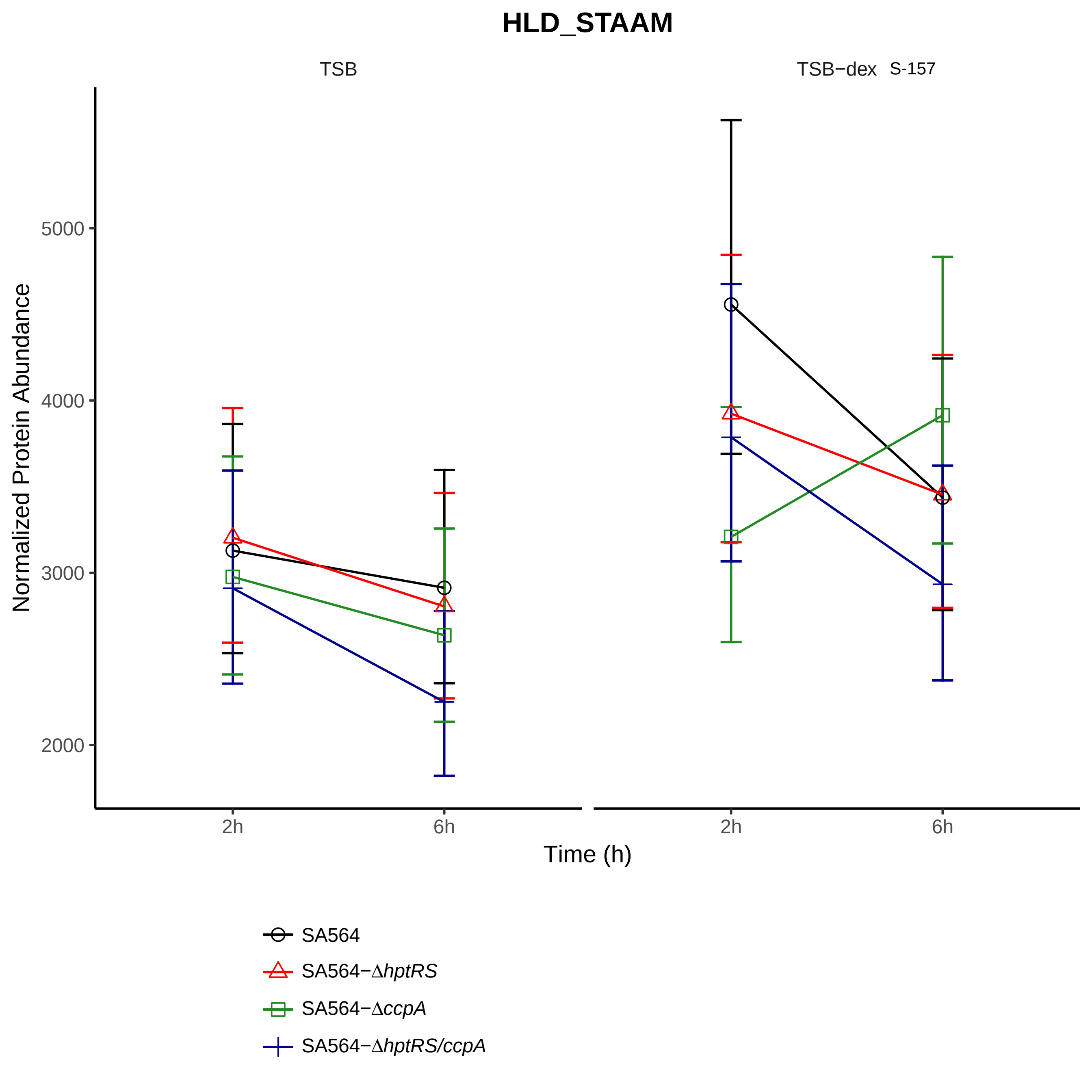

HLD_STAAM
TSB
TSB−dex
S-157
Normalized Protein Abundance
5000
4000
3000
2000
2h
6h
2h
6h
Time (h)
SA564
SA564−Δ*hptRS*
SA564−Δ*ccpA*
SA564−Δ*hptRS/ccpA*

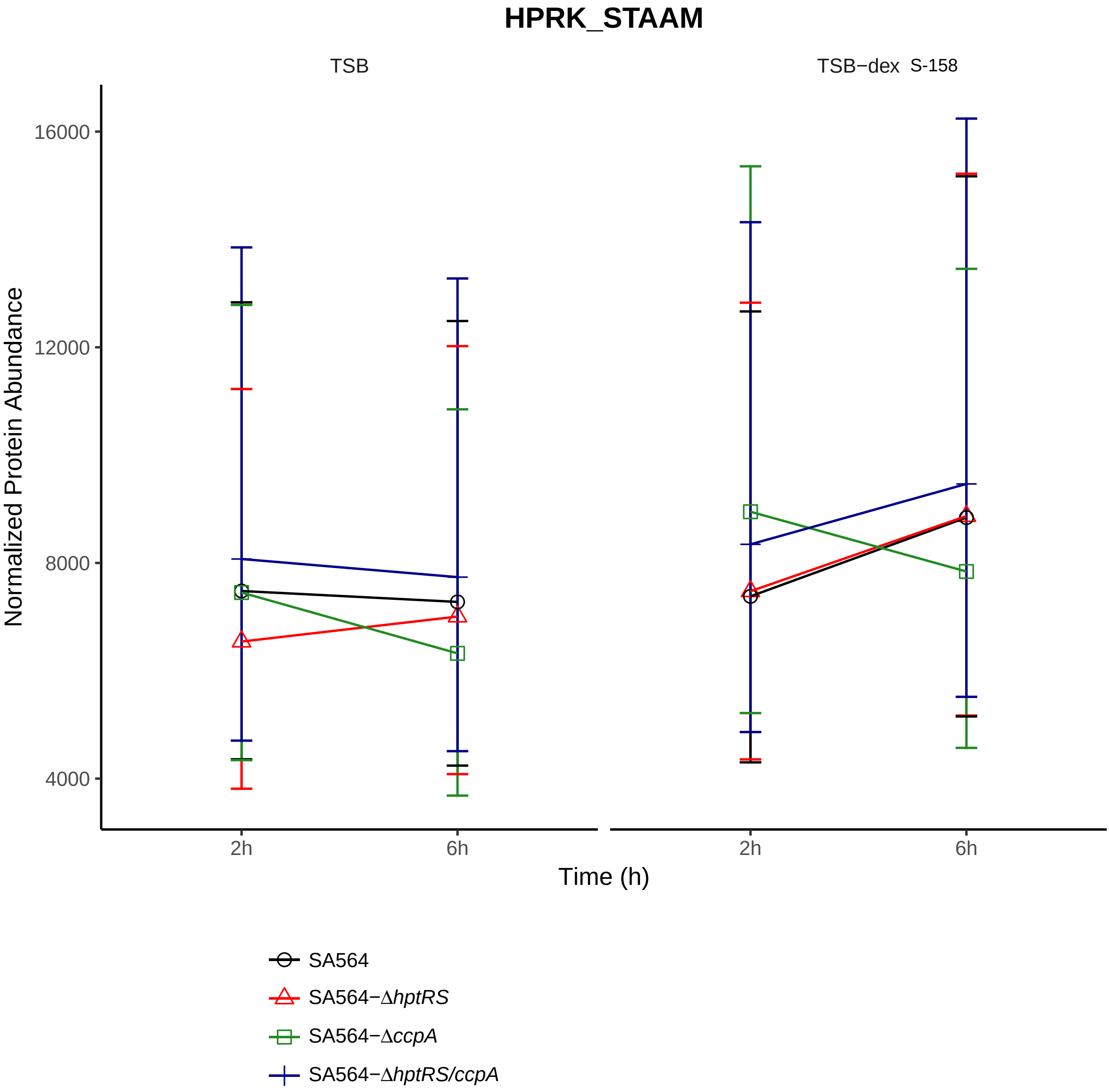

HPRK_STAAM
TSB
TSB−dex
S-158
Normalized Protein Abundance
16000
12000
8000
4000
2h
6h
2h
6h
Time (h)
SA564
SA564−Δ*hptRS*
SA564−Δ*ccpA*
SA564−Δ*hptRS/ccpA*

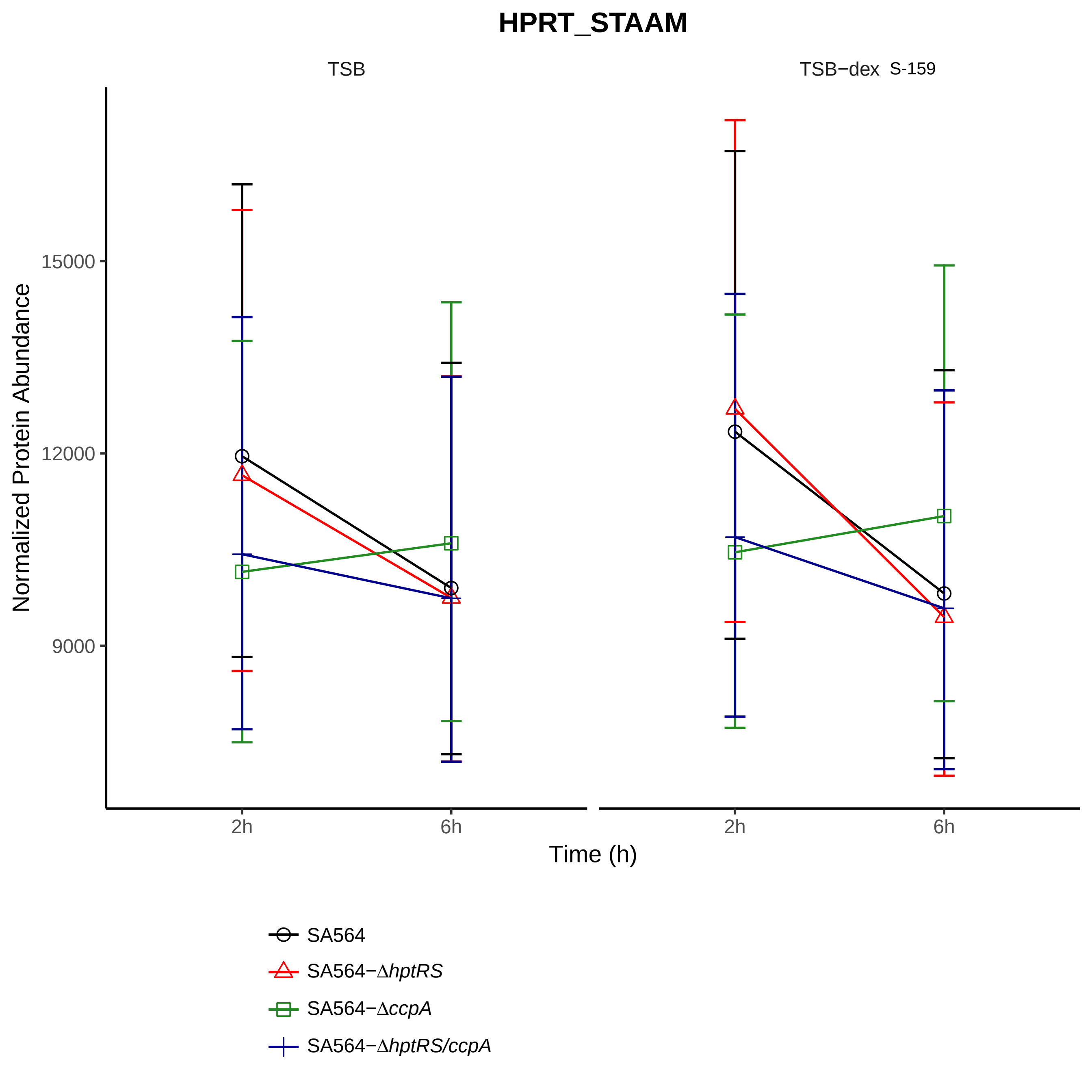

HPRT_STAAM
TSB
TSB−dex
S-159
Normalized Protein Abundance
15000
12000
9000
2h
6h
2h
6h
Time (h)
SA564
SA564−Δ*hptRS*
SA564−Δ*ccpA*
SA564−Δ*hptRS/ccpA*

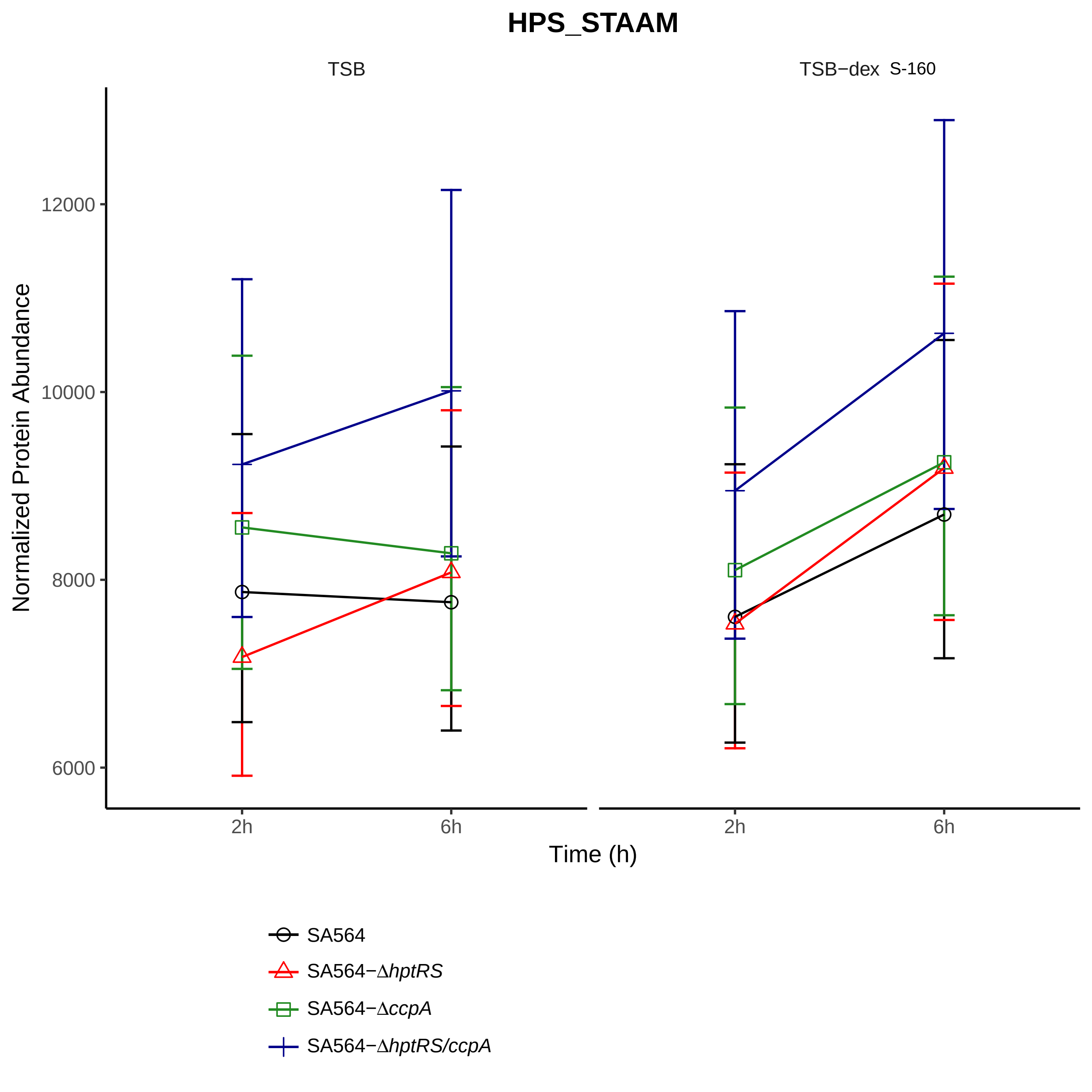

HPS_STAAM
TSB
TSB−dex
S-160
Normalized Protein Abundance
12000
10000
8000
6000
2h
6h
2h
6h
Time (h)
SA564
SA564−Δ*hptRS*
SA564−Δ*ccpA*
SA564−Δ*hptRS/ccpA*

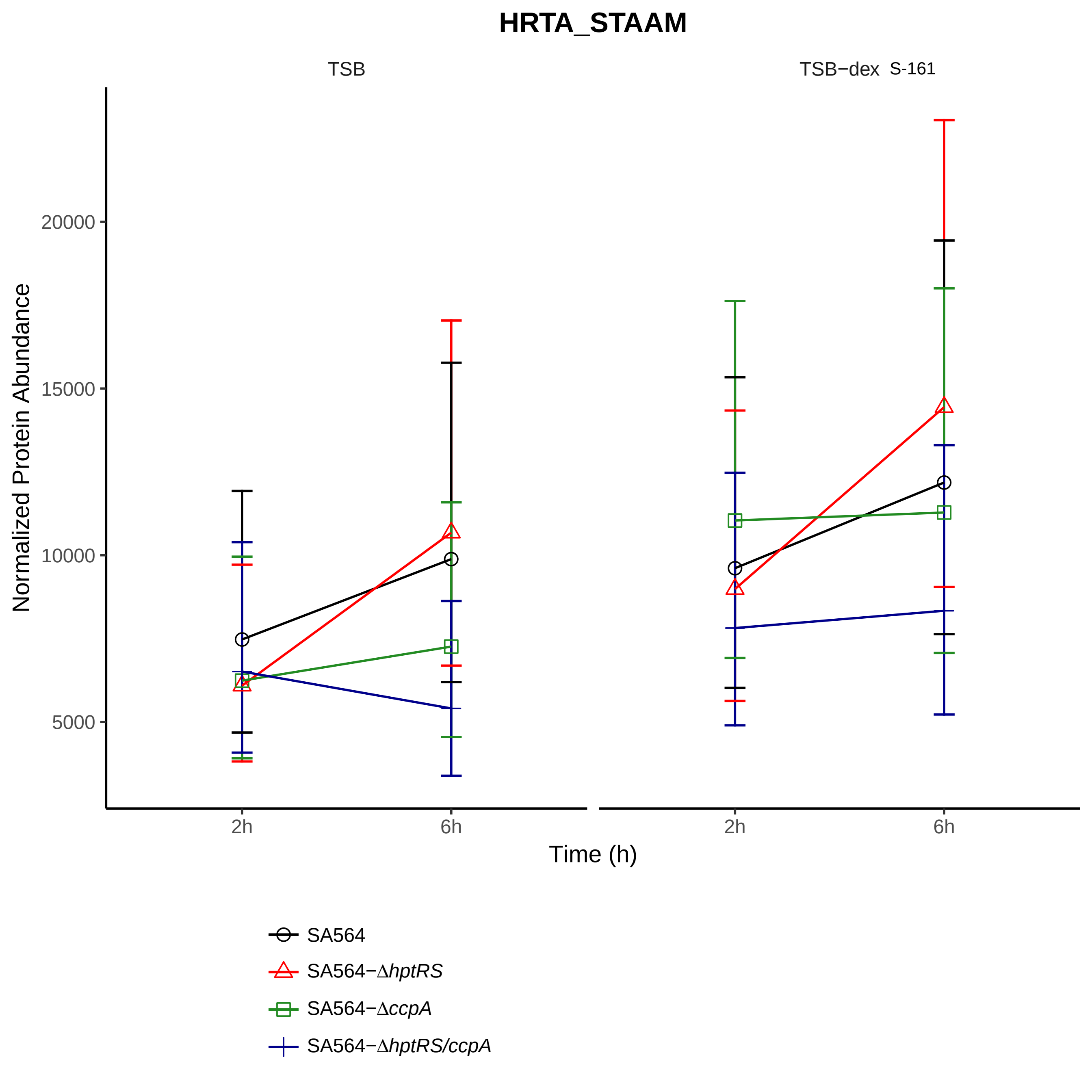
HRTA_STAAM
TSB
TSB−dex
S-161
Normalized Protein Abundance
20000
15000
10000
5000
2h
6h
2h
6h
Time (h)
SA564
SA564−Δ*hptRS*
SA564−Δ*ccpA*
SA564−Δ*hptRS/ccpA*

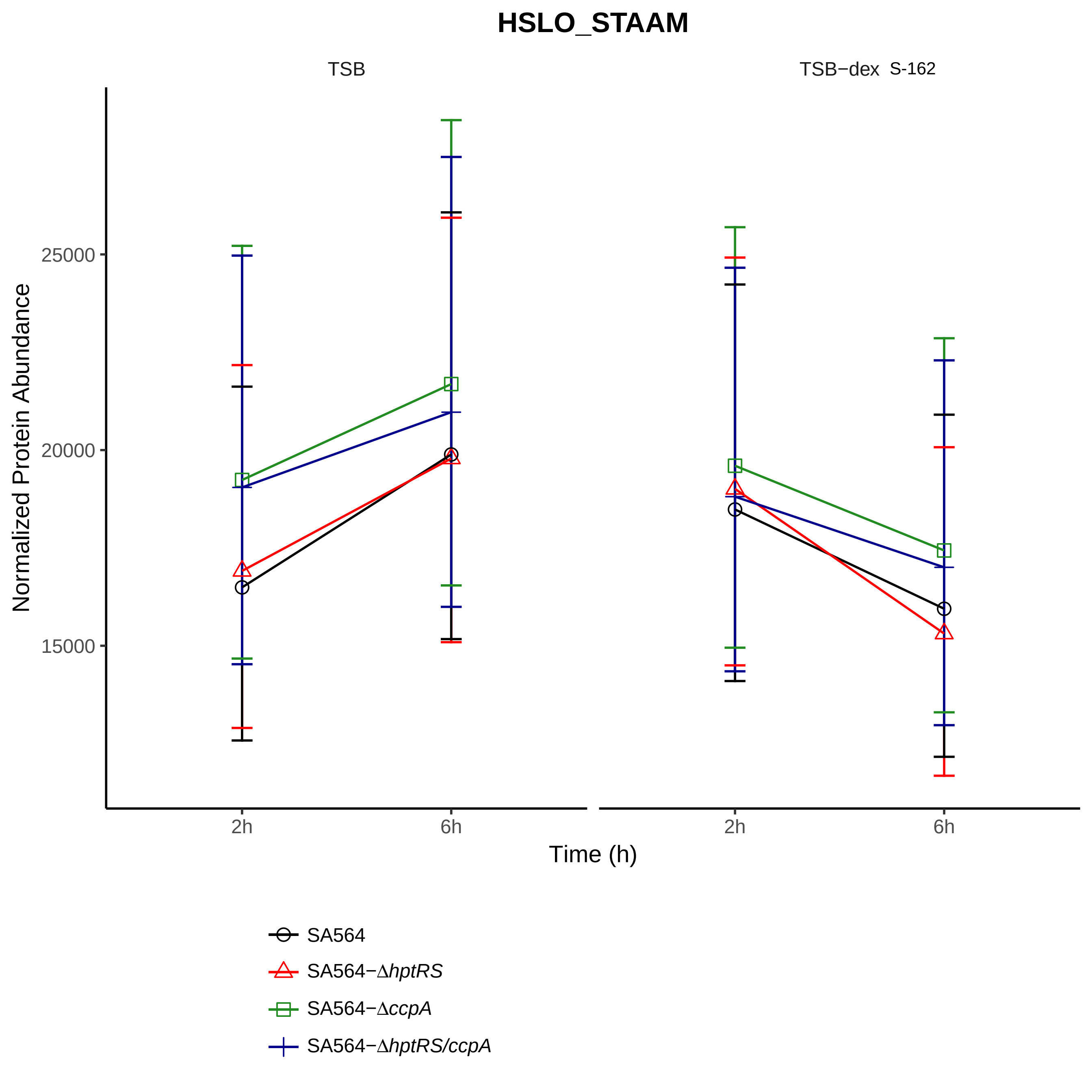
HSLO_STAAM
TSB
TSB−dex
S-162
Normalized Protein Abundance
25000
20000
15000
2h
6h
2h
6h
Time (h)
SA564
SA564−ΔhptRS
SA564−ΔccpA
SA564−ΔhptRS/ccpA

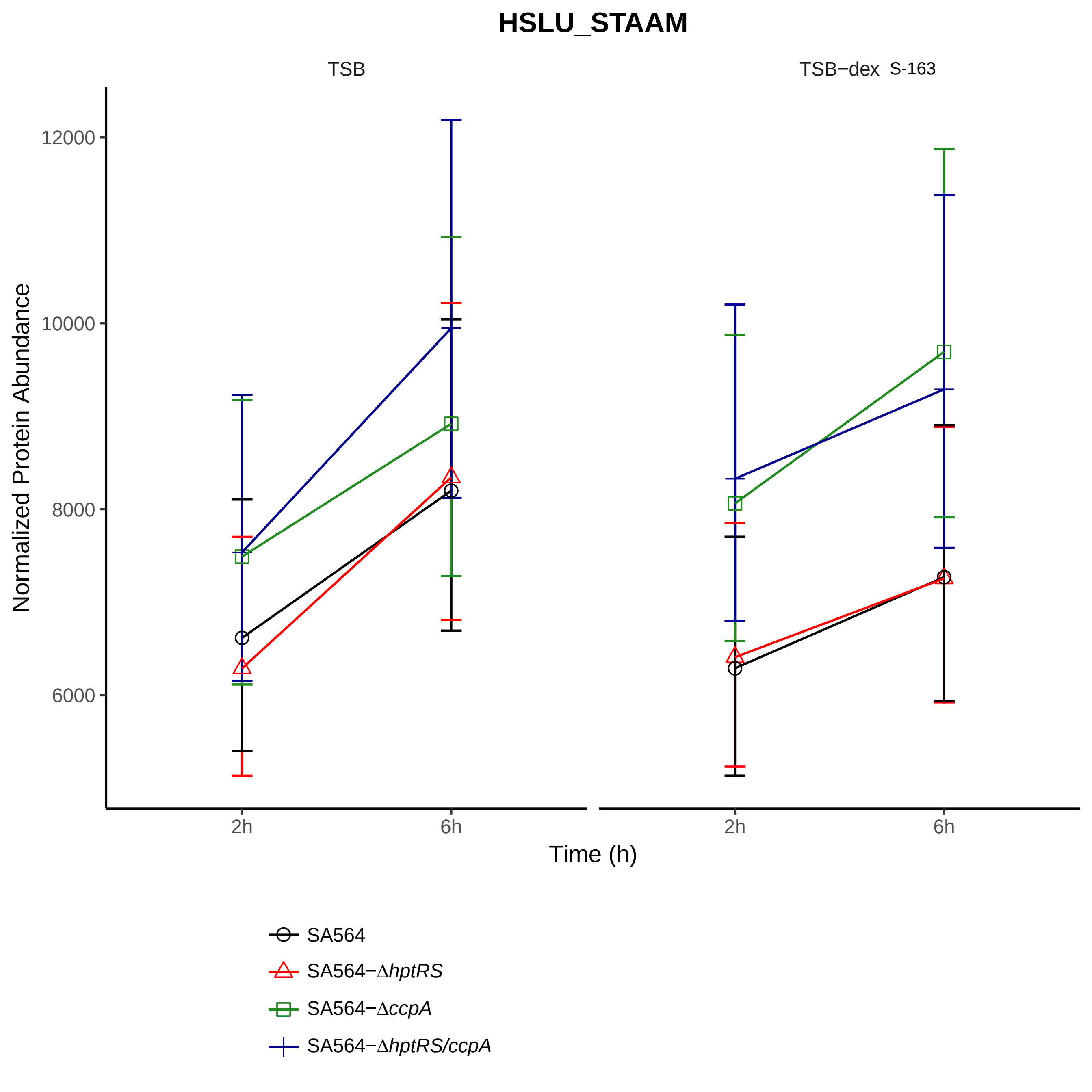
HSLU_STAAM
TSB
TSB−dex
S-163
Normalized Protein Abundance
12000
10000
8000
6000
2h
6h
2h
6h
Time (h)
SA564
SA564−ΔhptRS
SA564−ΔccpA
SA564−ΔhptRS/ccpA

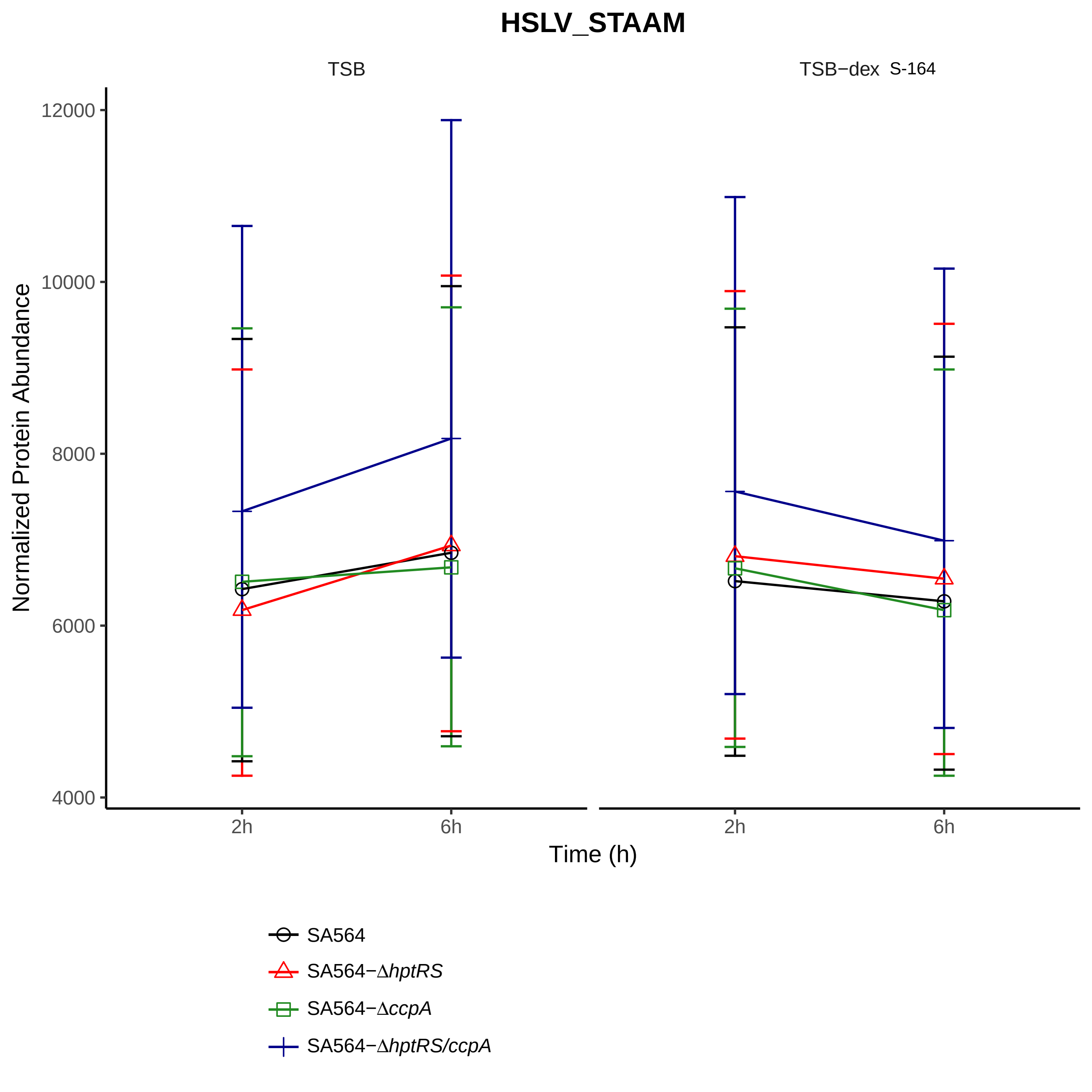

HSLV_STAAM
TSB
TSB−dex
S-164
Normalized Protein Abundance
12000
10000
8000
6000
4000
2h
6h
2h
6h
Time (h)
SA564
SA564−Δ*hptRS*
SA564−Δ*ccpA*
SA564−Δ*hptRS/ccpA*

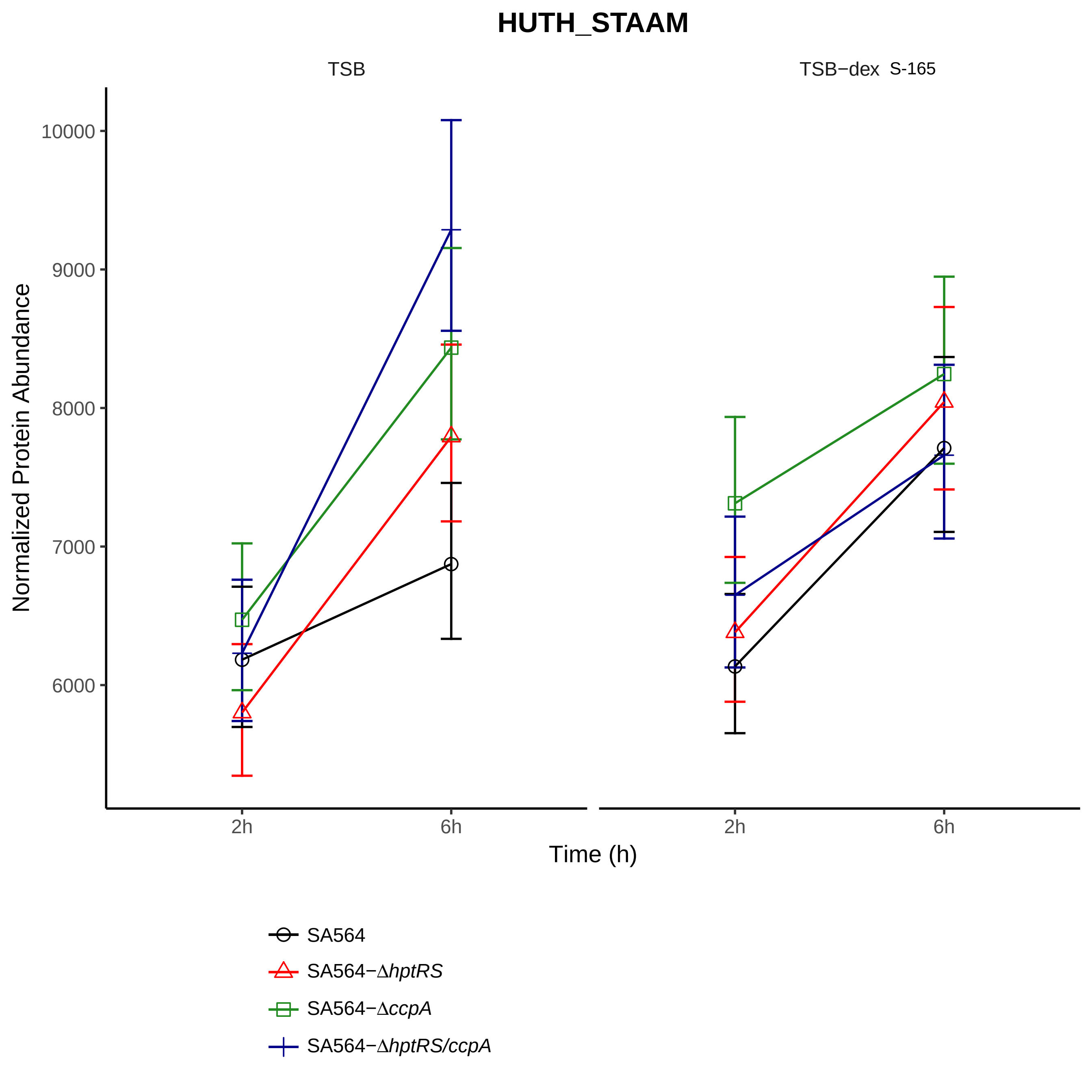

HUTH_STAAM
TSB
TSB−dex
S-165
Normalized Protein Abundance
10000
9000
8000
7000
6000
2h
6h
2h
6h
Time (h)
SA564
SA564−ΔhptRS
SA564−ΔccpA
SA564−ΔhptRS/ccpA

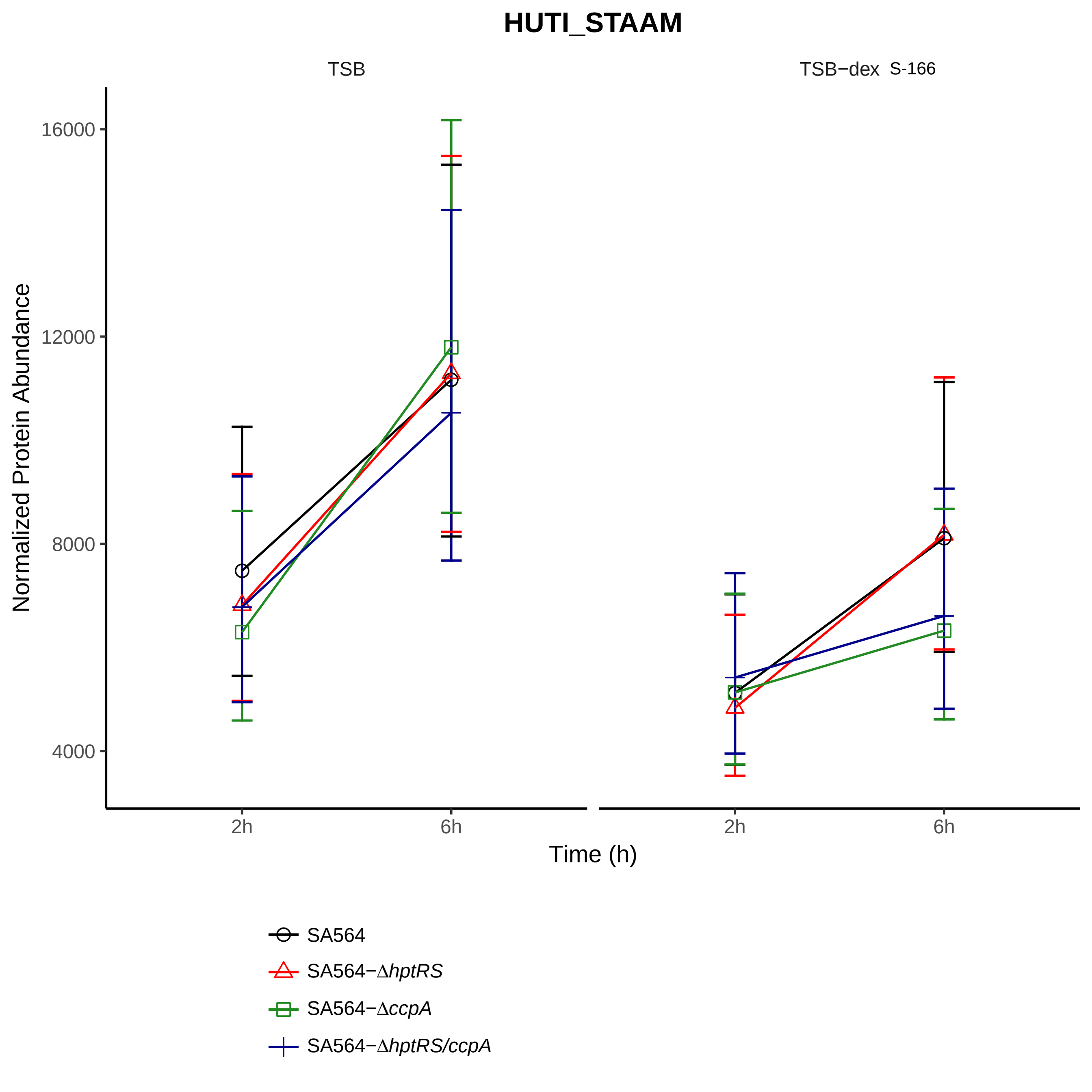

HUTI_STAAM
TSB
TSB−dex
S-166
16000
12000
8000
4000
Normalized Protein Abundance
2h
6h
2h
6h
Time (h)
SA564
SA564−Δ*hptRS*
SA564−Δ*ccpA*
SA564−Δ*hptRS/ccpA*

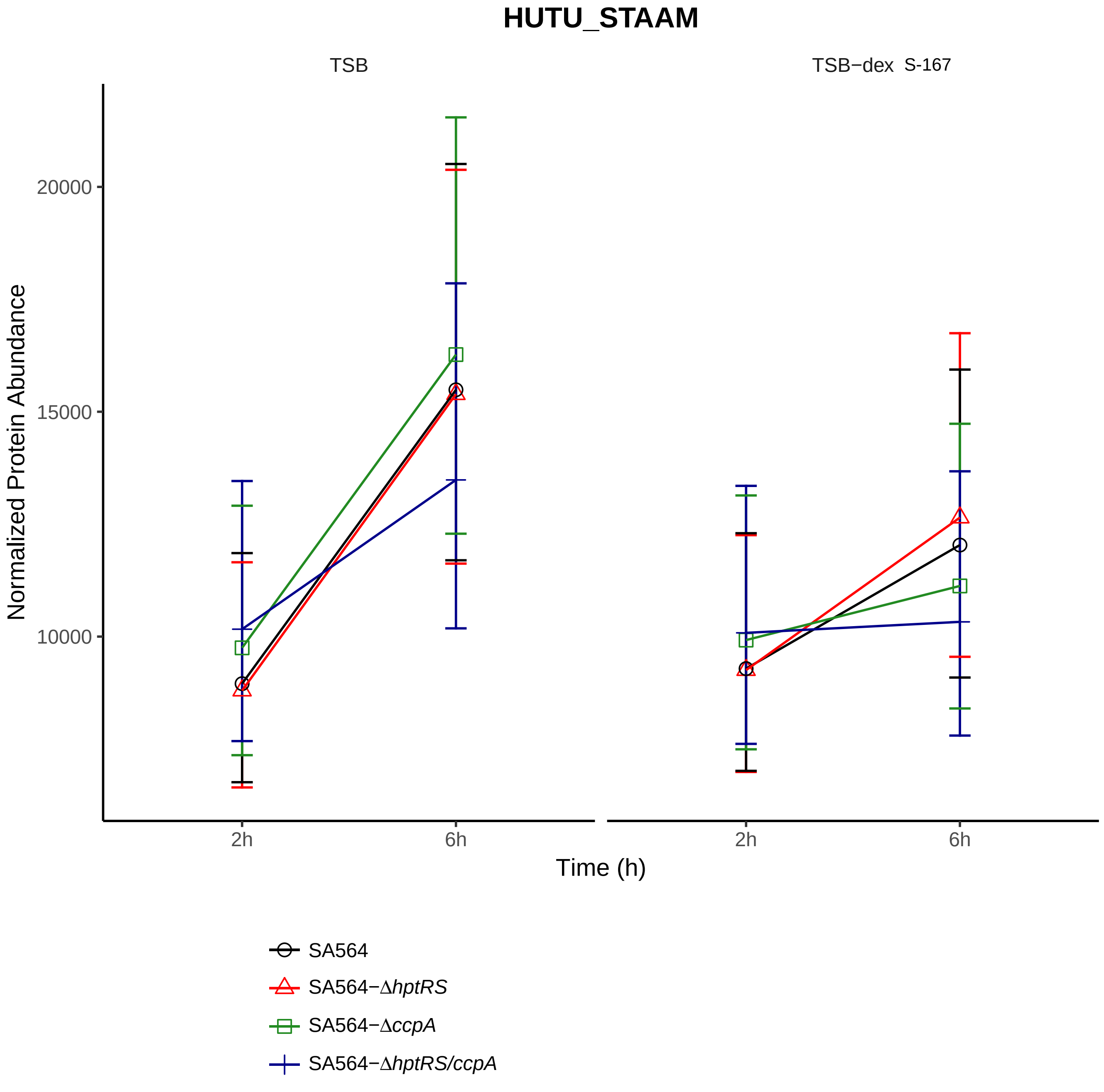

HUTU_STAAM
TSB
TSB−dex
S-167
Normalized Protein Abundance
20000
15000
10000
2h
6h
2h
6h
Time (h)
SA564
SA564−ΔhptRS
SA564−ΔccpA
SA564−ΔhptRS/ccpA

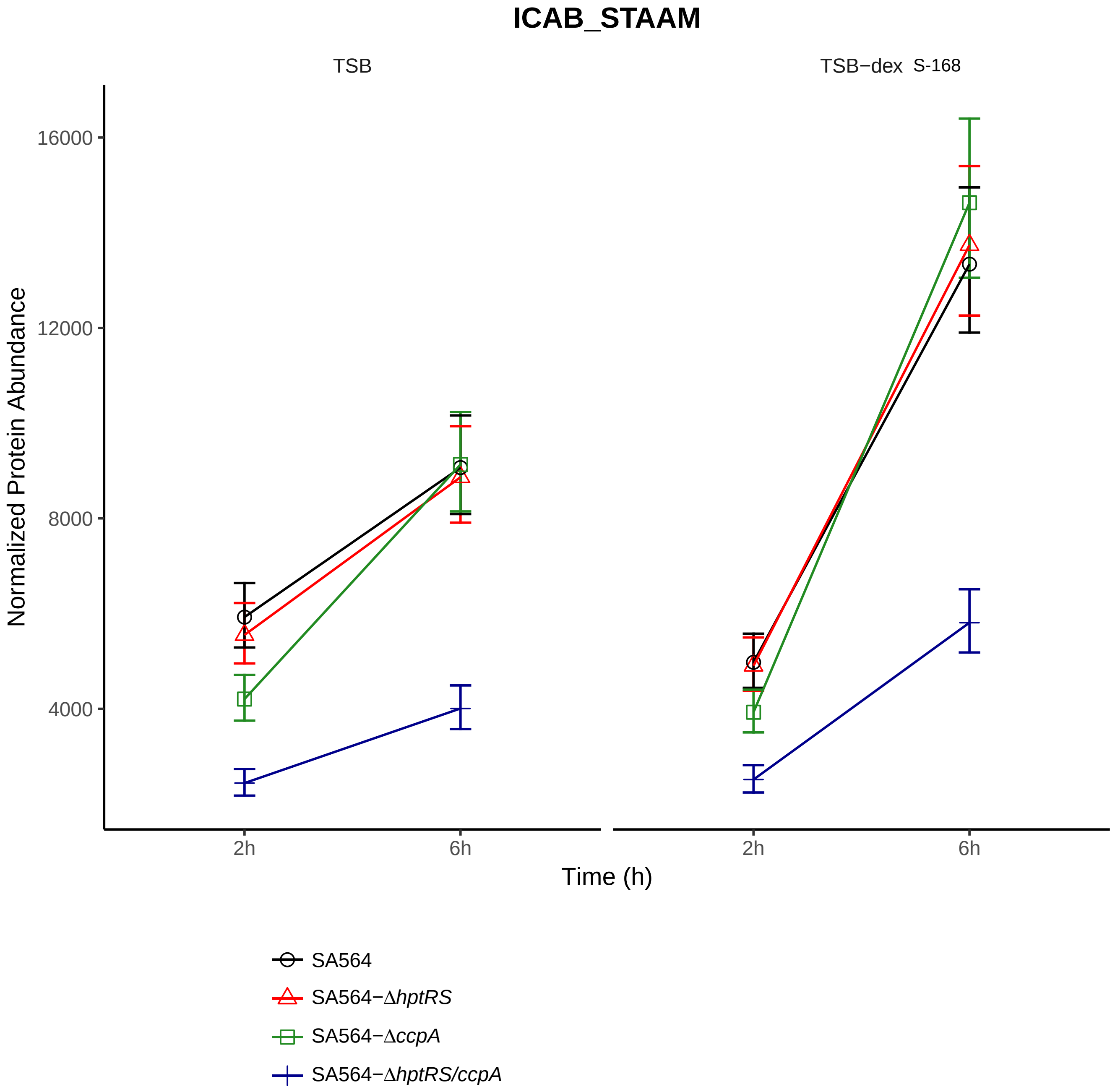

ICAB_STAAM
TSB
TSB−dex
S-168
16000
12000
8000
4000
Normalized Protein Abundance
2h
6h
2h
6h
Time (h)
SA564
SA564−Δ*hptRS*
SA564−Δ*ccpA*
SA564−Δ*hptRS/ccpA*

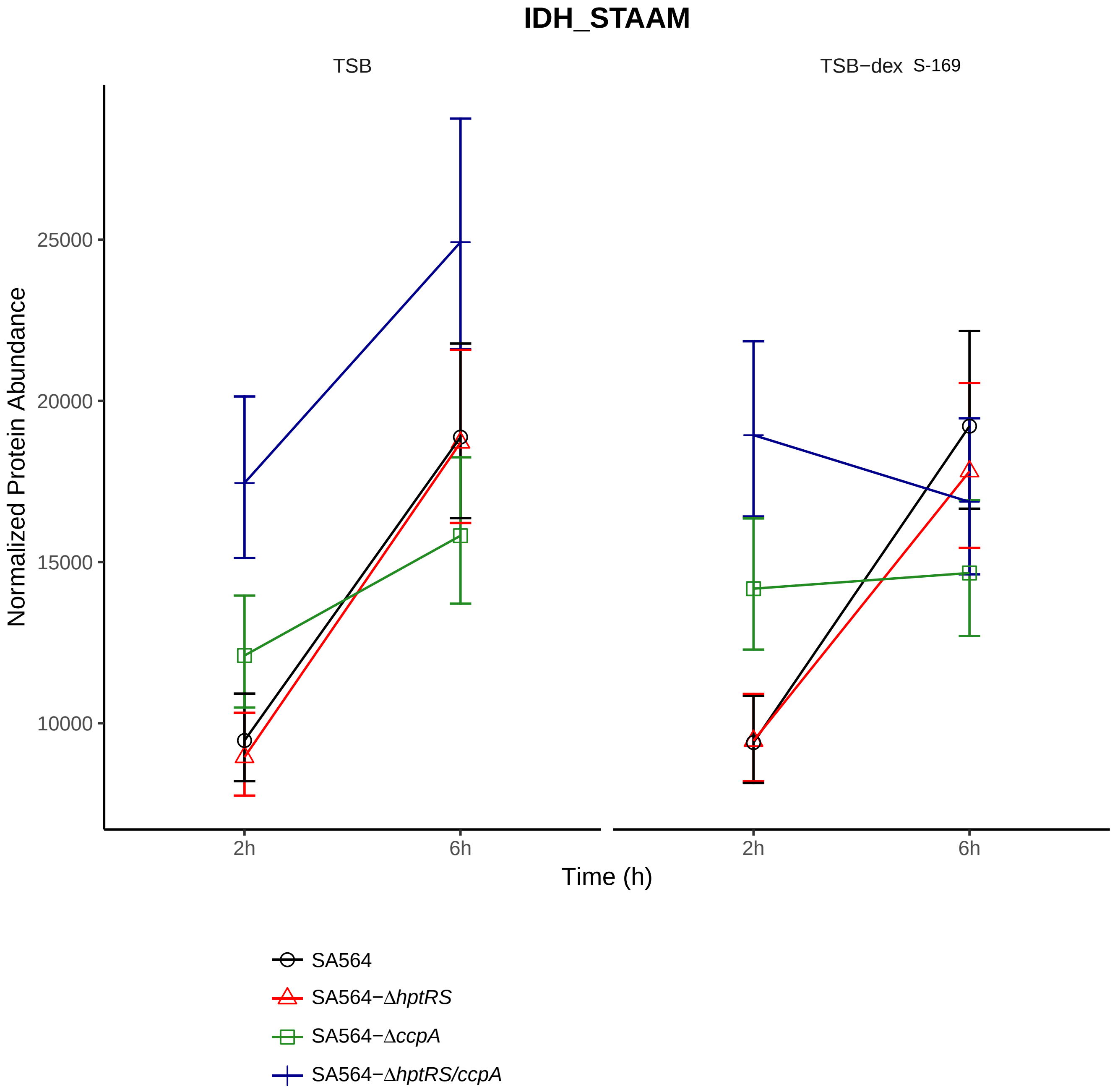

IDH_STAAM
TSB
TSB−dex
S-169
Normalized Protein Abundance
25000
20000
15000
10000
2h
6h
2h
6h
Time (h)
SA564
SA564−ΔhptRS
SA564−ΔccpA
SA564−ΔhptRS/ccpA

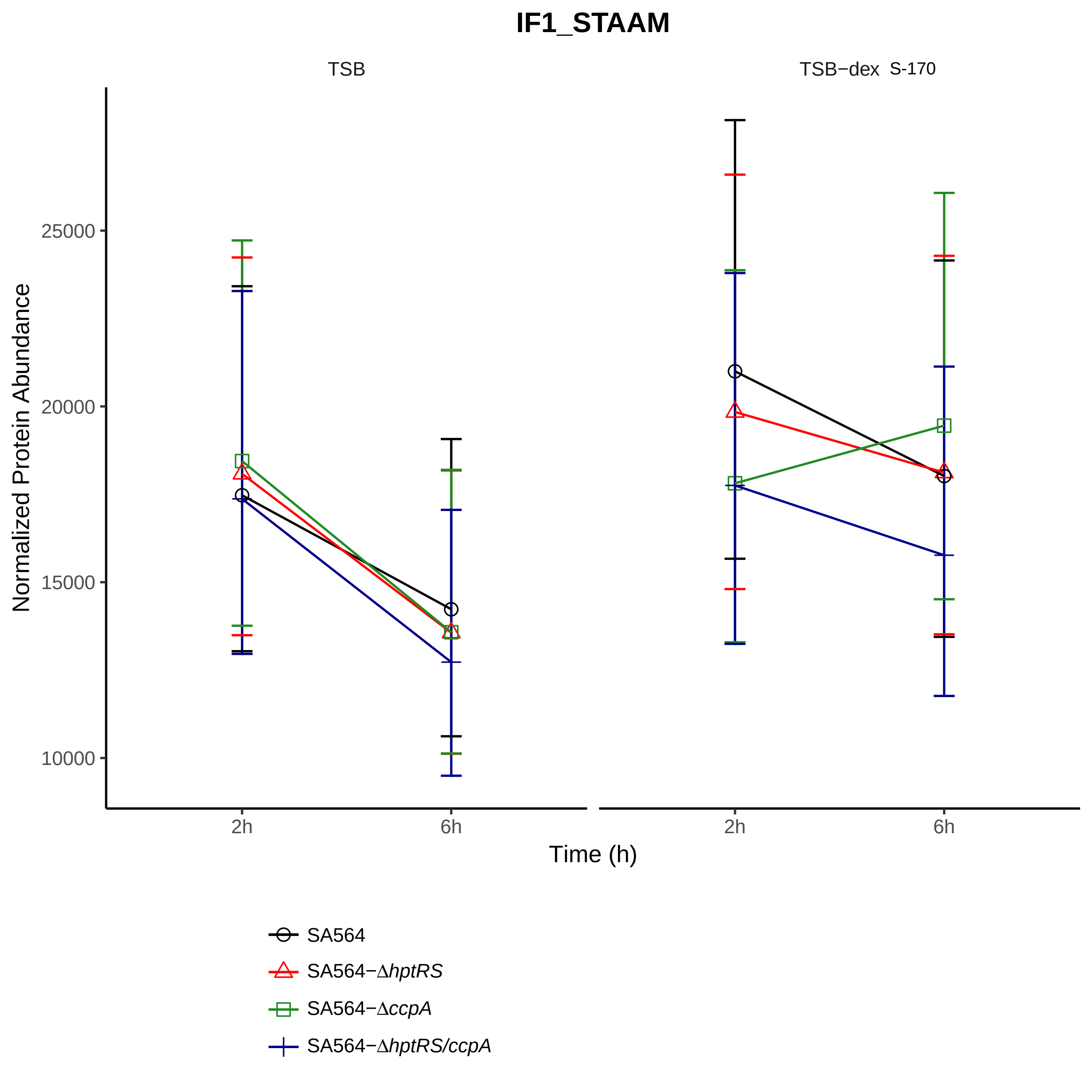
IF1_STAAM
TSB
TSB−dex
S-170
Normalized Protein Abundance
25000
20000
15000
10000
2h
6h
2h
6h
Time (h)
SA564
SA564−ΔhptRS
SA564−ΔccpA
SA564−ΔhptRS/ccpA

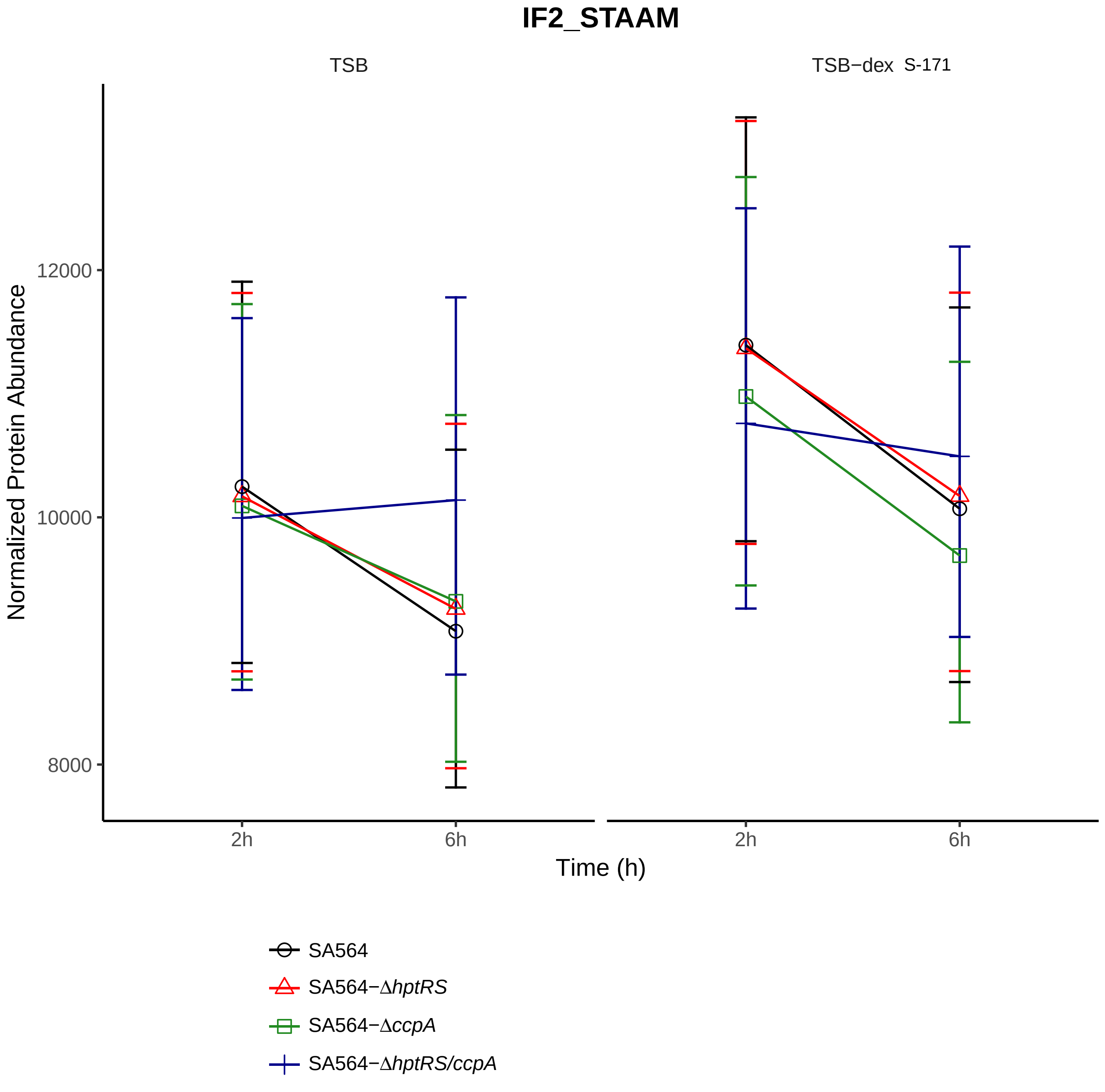

IF2_STAAM
TSB
TSB−dex
S-171
Normalized Protein Abundance
12000
10000
8000
2h
6h
2h
6h
Time (h)
SA564
SA564−ΔhptRS
SA564−ΔccpA
SA564−ΔhptRS/ccpA

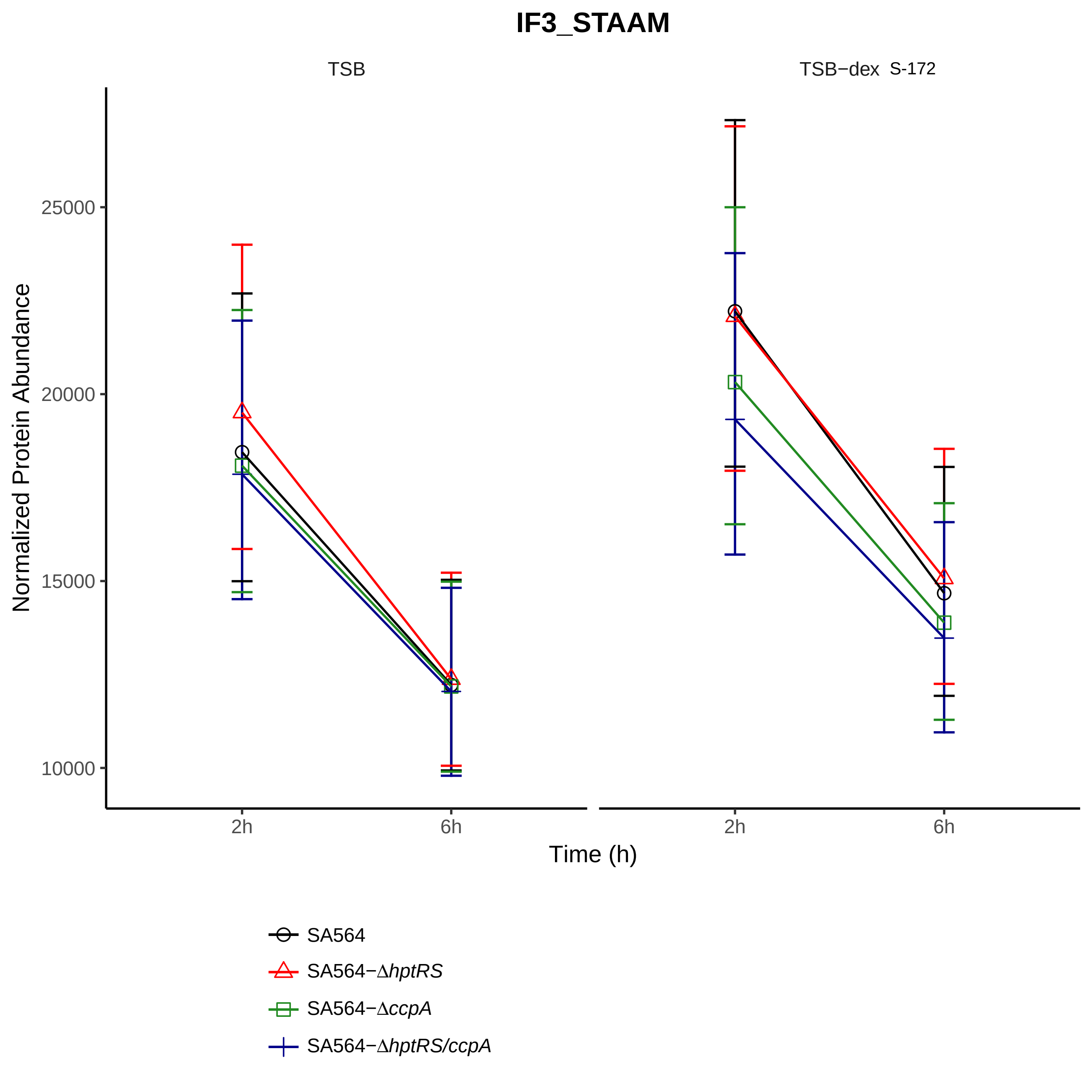

IF3_STAAM
TSB
TSB−dex
S-172
Normalized Protein Abundance
25000
20000
15000
10000
2h
6h
2h
6h
Time (h)
SA564
SA564−ΔhptRS
SA564−ΔccpA
SA564−ΔhptRS/ccpA

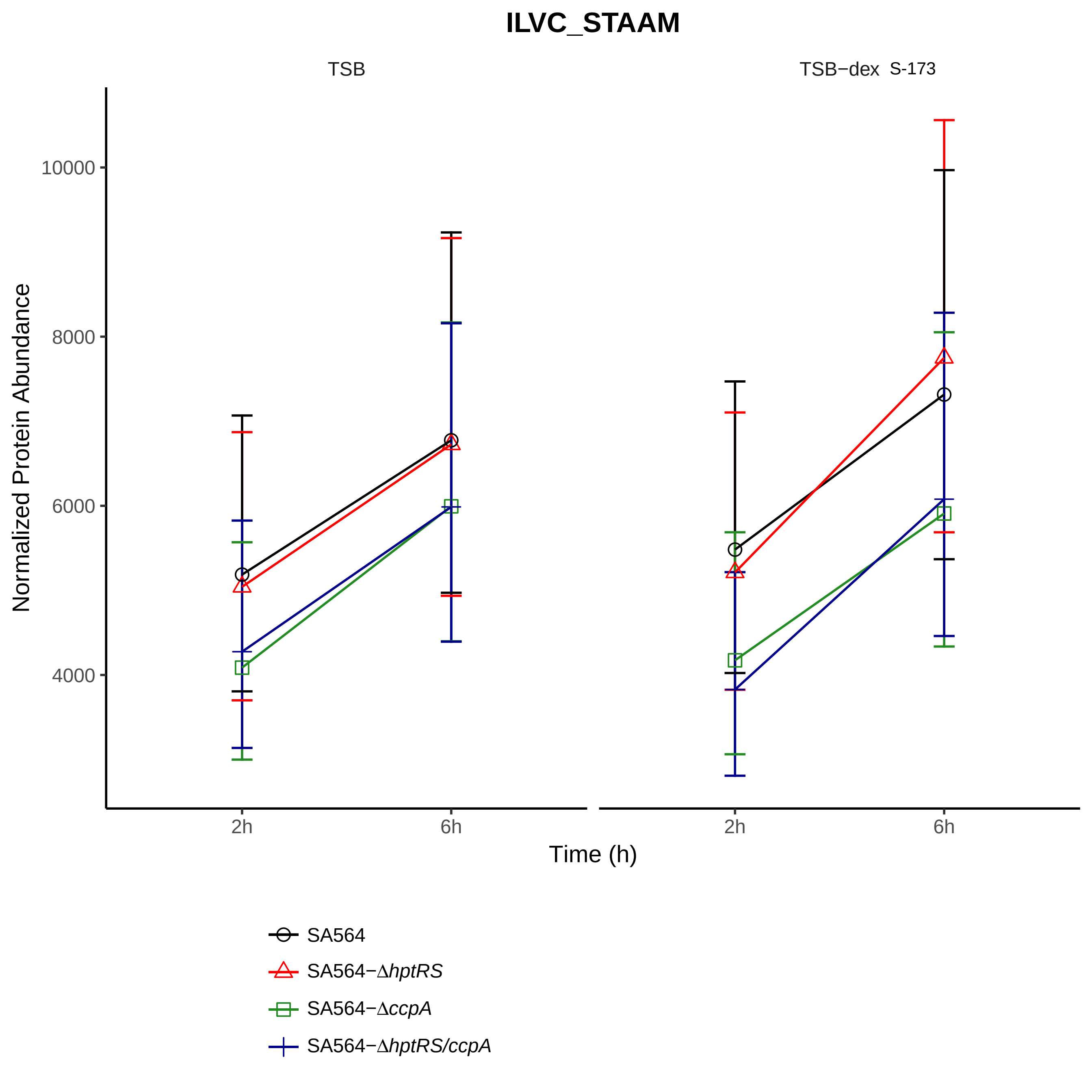

ILVC_STAAM
TSB
TSB−dex
S-173
Normalized Protein Abundance
10000
8000
6000
4000
2h
6h
2h
6h
Time (h)
SA564
SA564−ΔhptRS
SA564−ΔccpA
SA564−ΔhptRS/ccpA

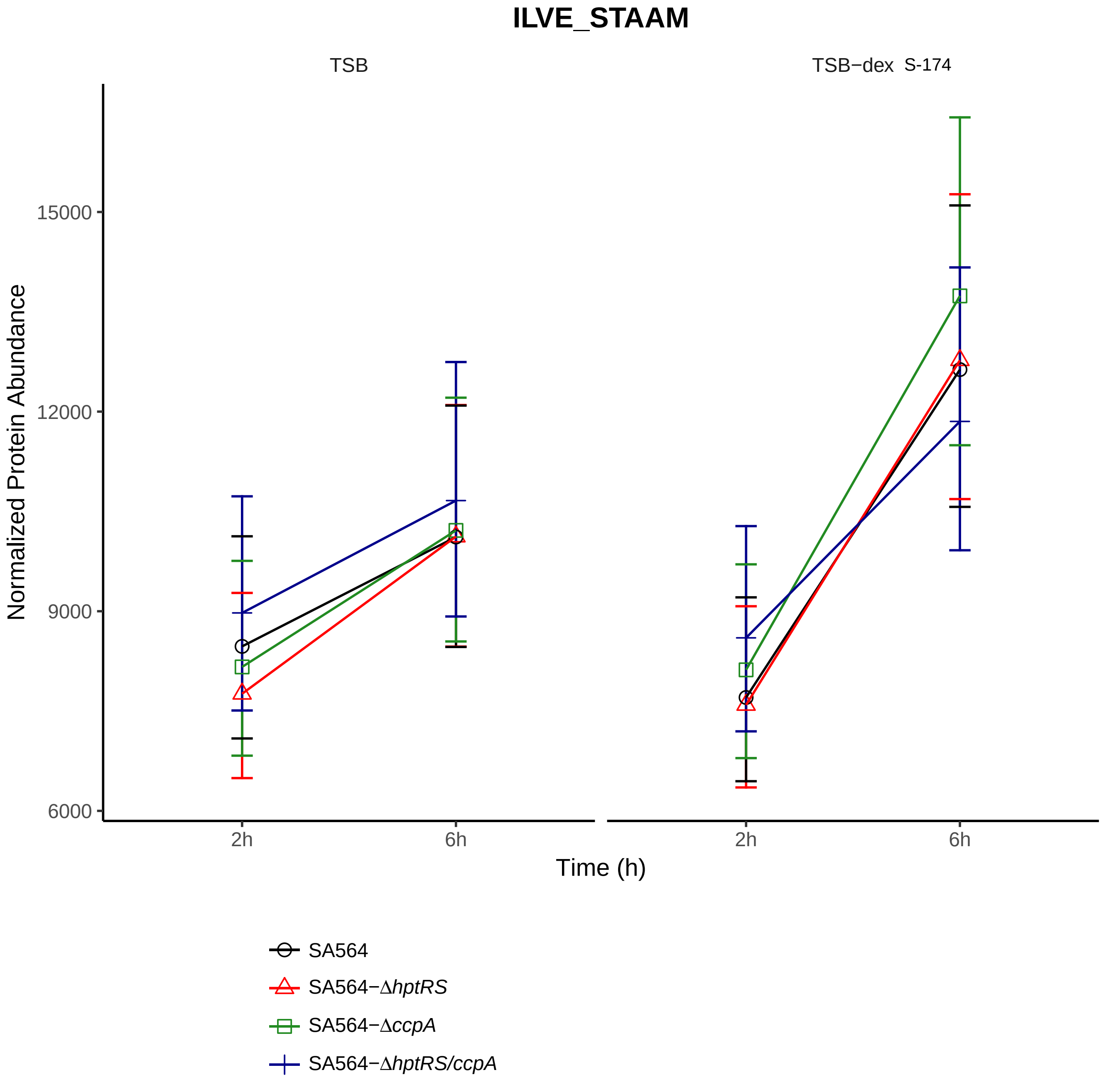
ILVE_STAAM
TSB
TSB−dex
S-174
Normalized Protein Abundance
15000
12000
9000
6000
2h
6h
2h
6h
Time (h)
SA564
SA564−Δ*hptRS*
SA564−Δ*ccpA*
SA564−Δ*hptRS/ccpA*

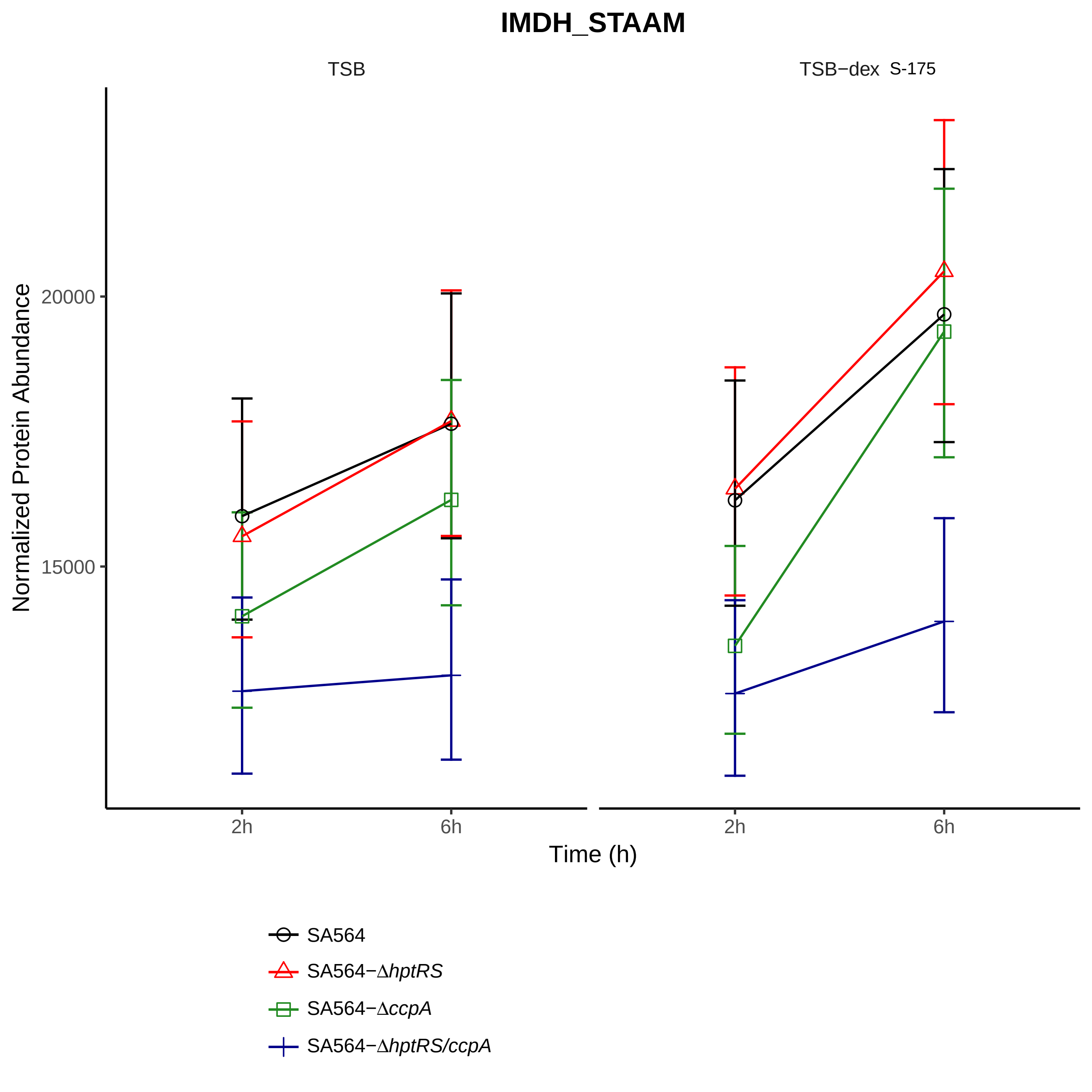
IMDH_STAAM
TSB
TSB−dex
S-175
Normalized Protein Abundance
20000
15000
2h
6h
2h
6h
Time (h)
SA564
SA564−ΔhptRS
SA564−ΔccpA
SA564−ΔhptRS/ccpA

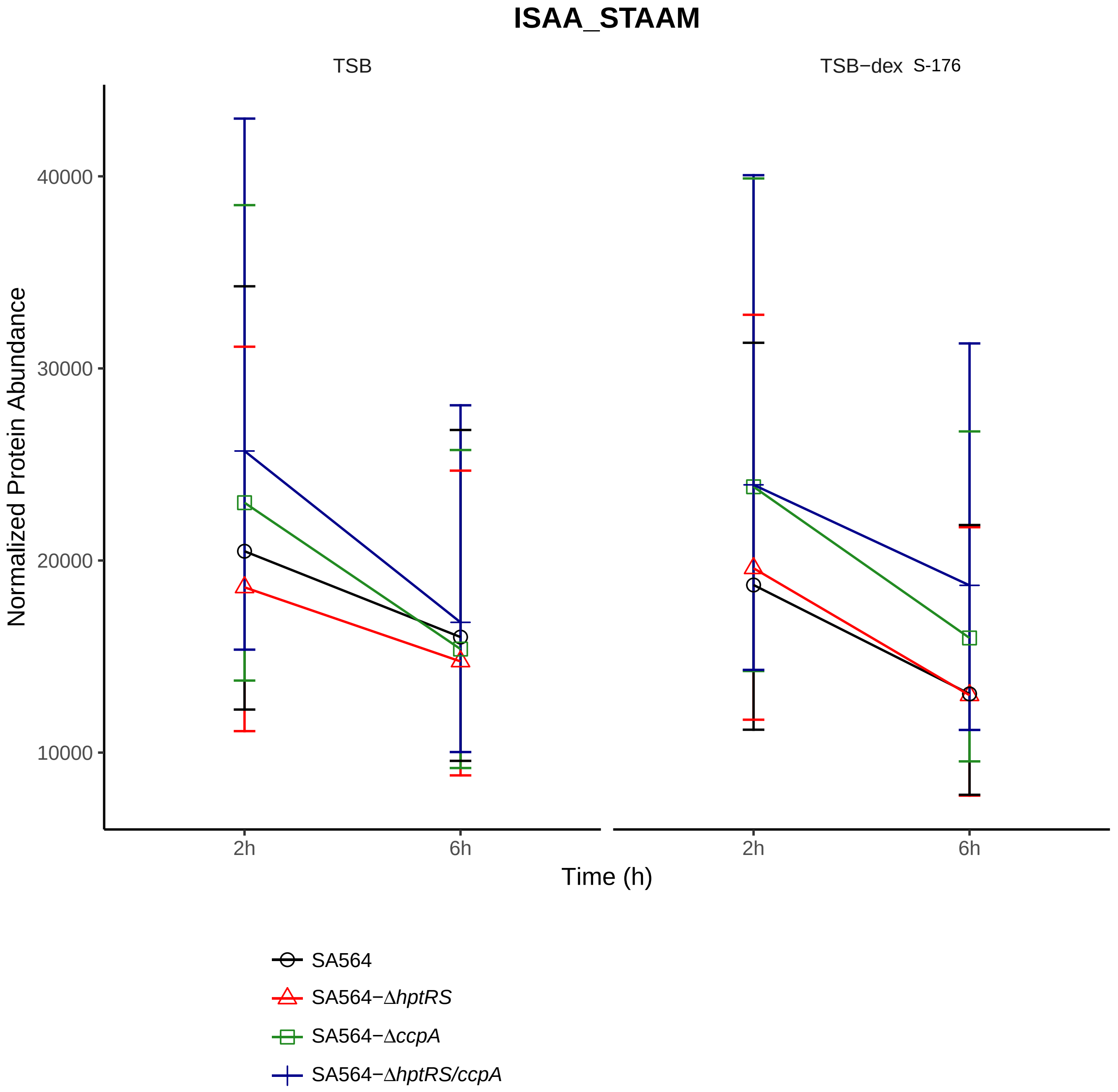

ISAA_STAAM
TSB
TSB−dex
S-176
Normalized Protein Abundance
40000
30000
20000
10000
2h
6h
2h
6h
Time (h)
SA564
SA564−Δ*hptRS*
SA564−Δ*ccpA*
SA564−Δ*hptRS/ccpA*

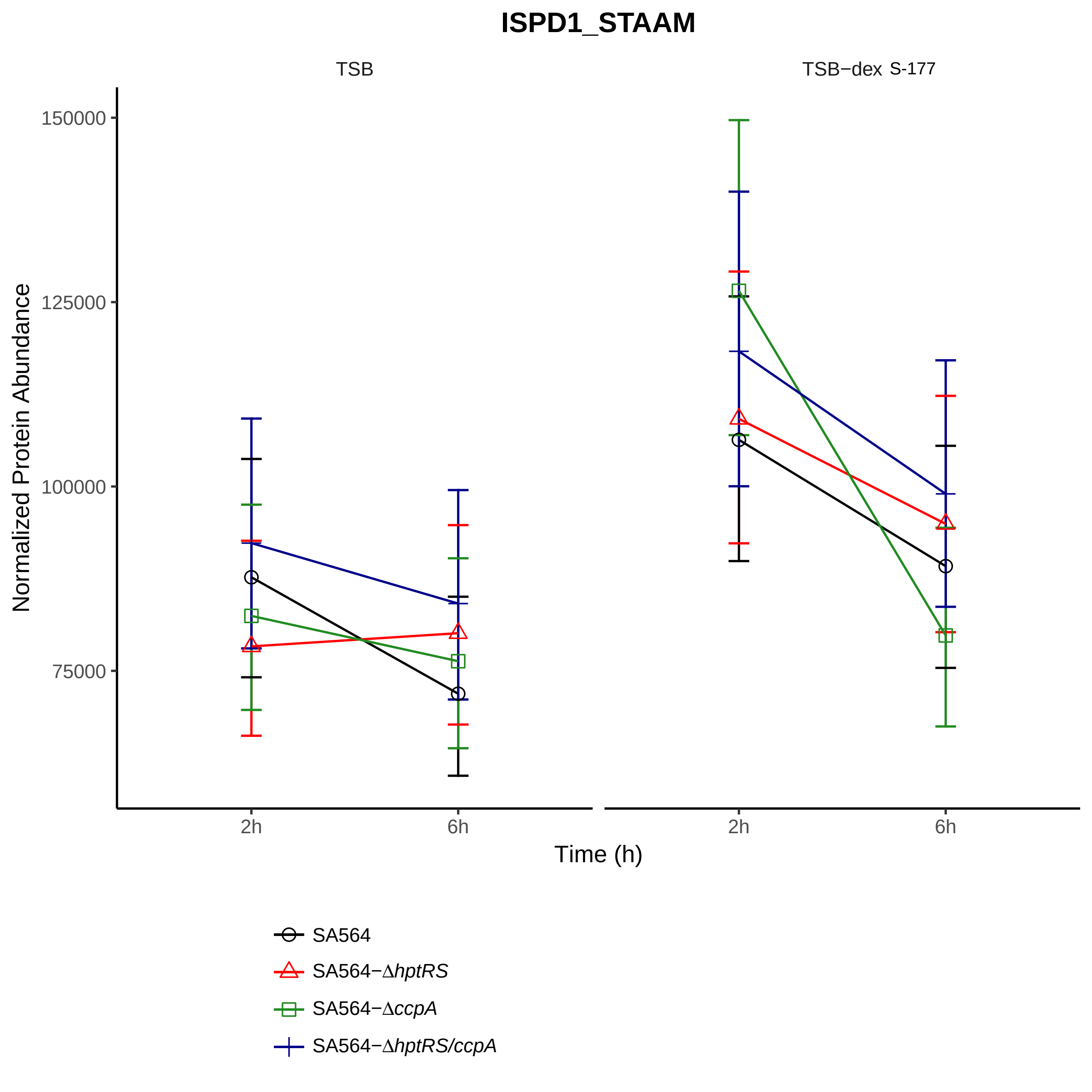

ISPD1_STAAM
TSB
TSB−dex
150000
125000
100000
75000
Normalized Protein Abundance
2h
6h
2h
6h
Time (h)
SA564
SA564−Δ*hptRS*
SA564−Δ*ccpA*
SA564−Δ*hptRS/ccpA*

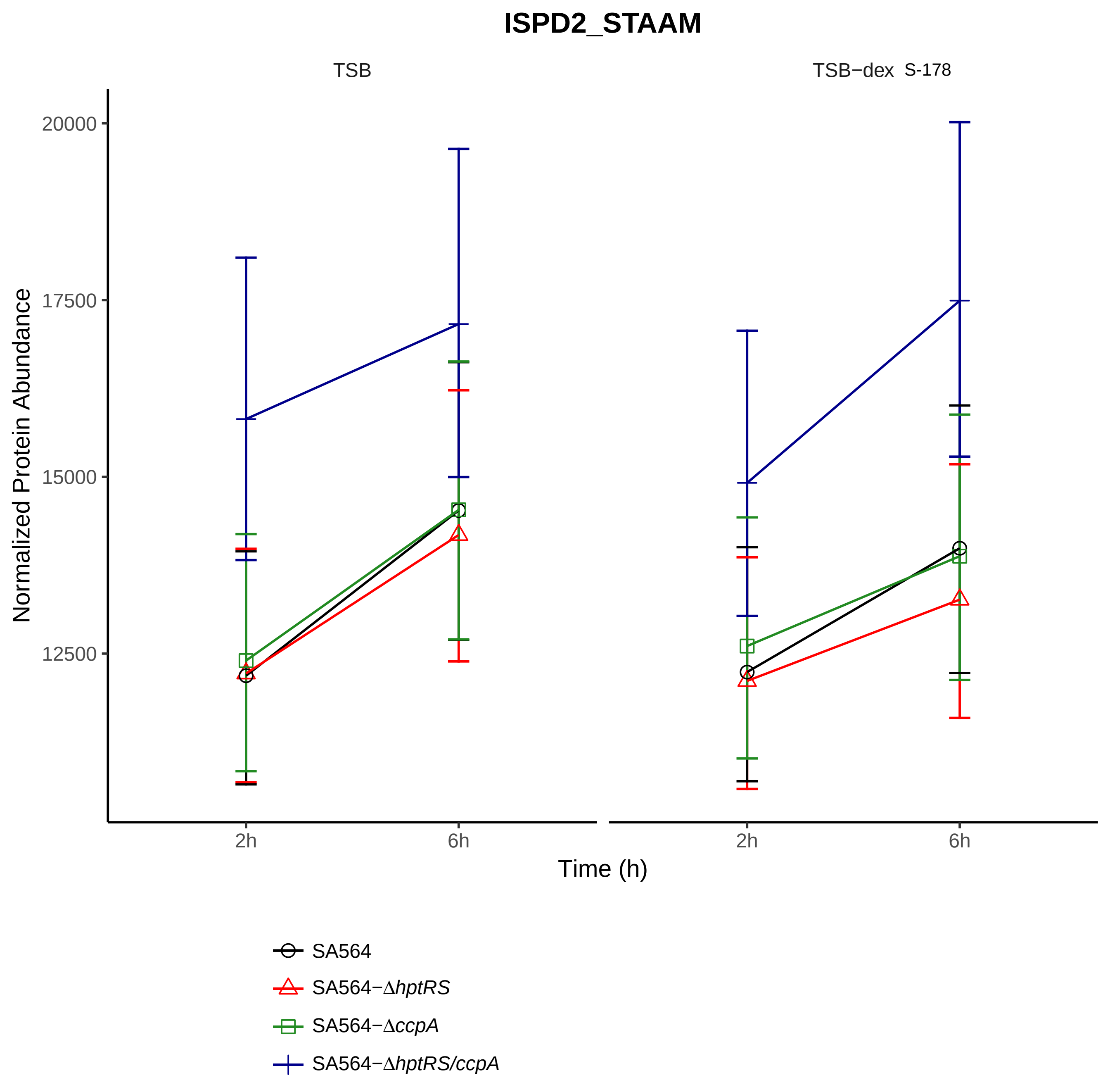
ISPD2_STAAM
TSB
TSB−dex
S-178
Normalized Protein Abundance
20000
17500
15000
12500
2h
6h
2h
6h
Time (h)
SA564
SA564−ΔhptRS
SA564−ΔccpA
SA564−ΔhptRS/ccpA

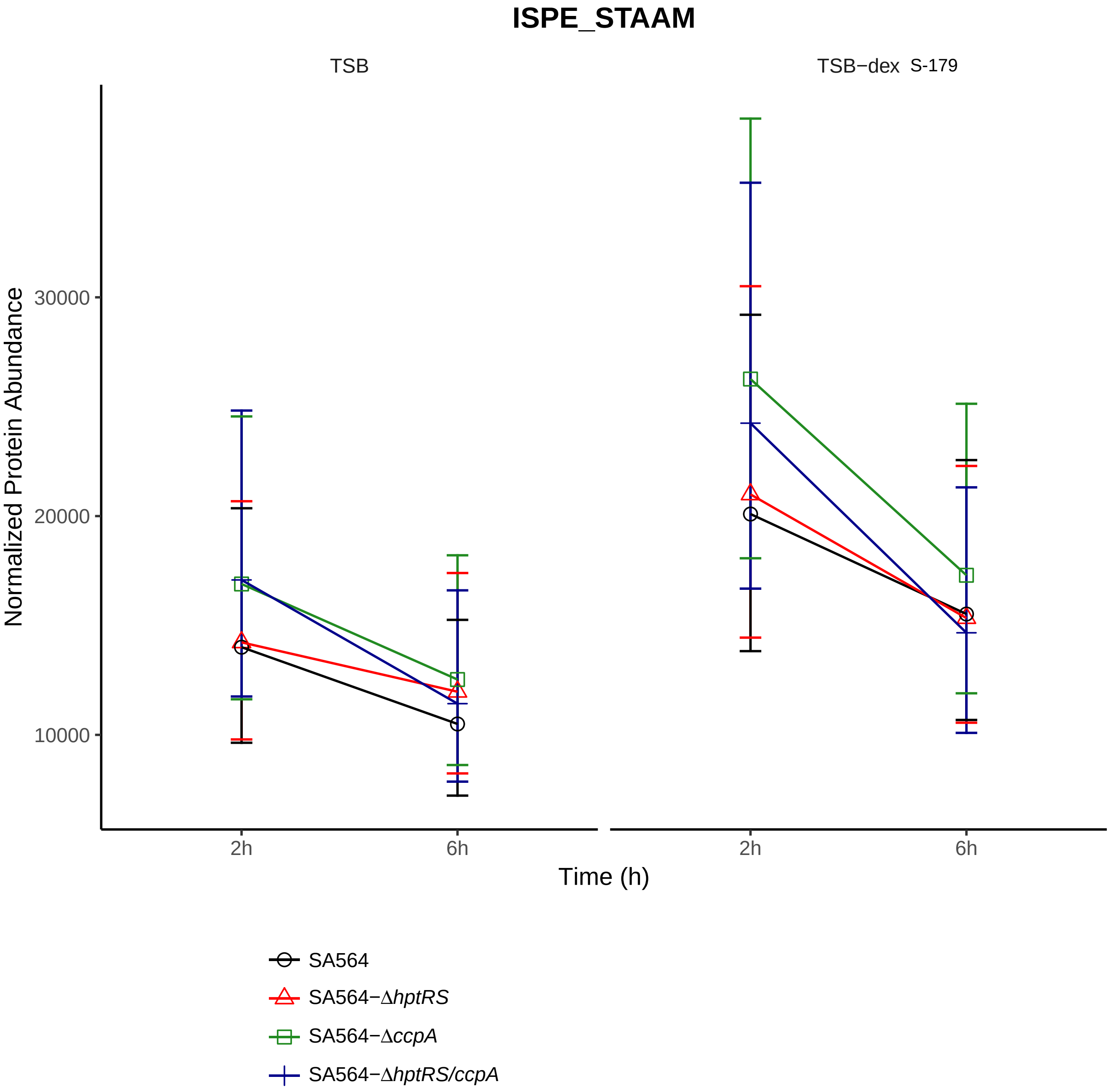

ISPE_STAAM
TSB
TSB−dex
Normalized Protein Abundance
30000
20000
10000
2h
6h
2h
6h
Time (h)
SA564
SA564−Δ*hptRS*
SA564−Δ*ccpA*
SA564−Δ*hptRS/ccpA*

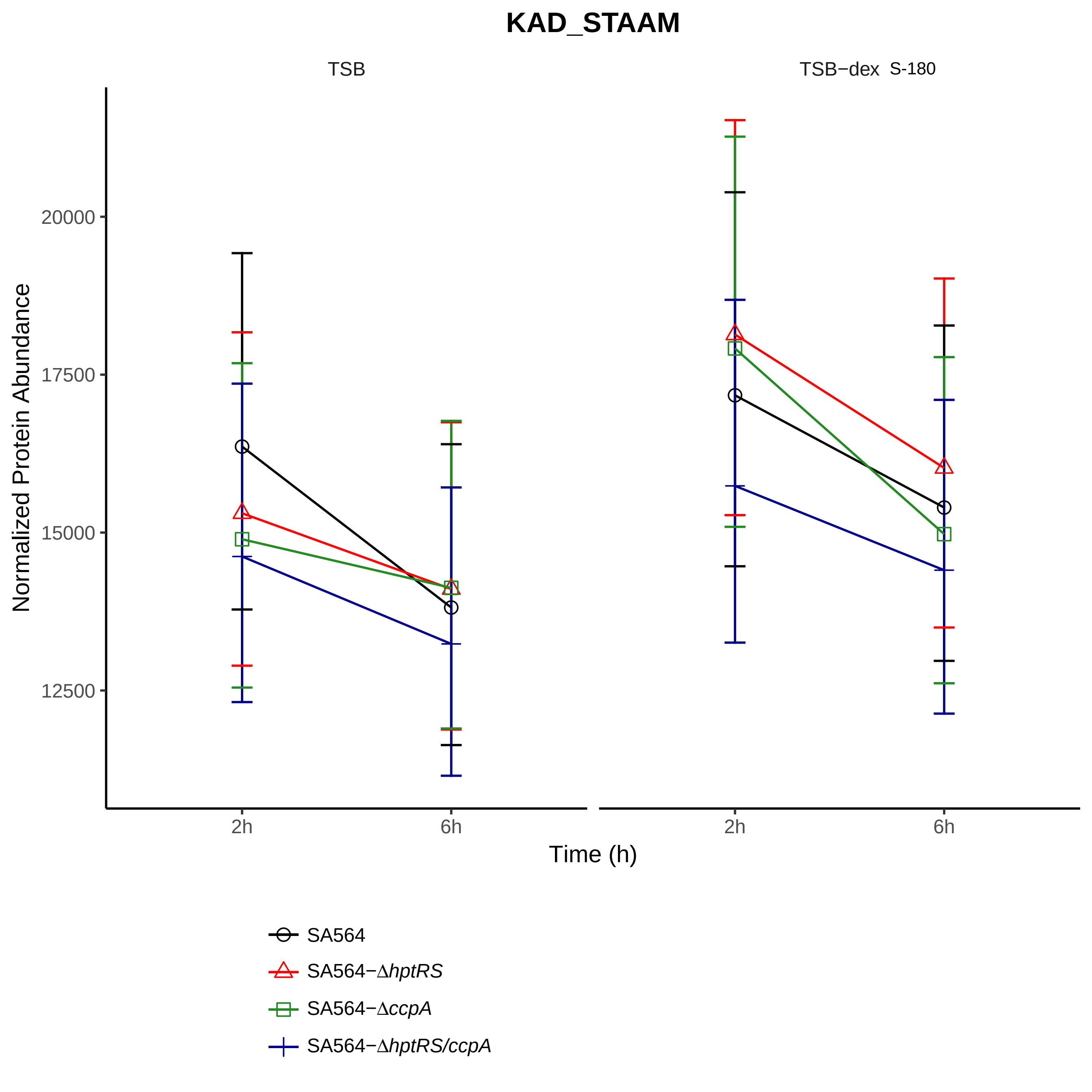

KAD_STAAM
TSB
TSB−dex
S-180
Normalized Protein Abundance
20000
17500
15000
12500
2h
6h
2h
6h
Time (h)
SA564
SA564−ΔhptRS
SA564−ΔccpA
SA564−ΔhptRS/ccpA

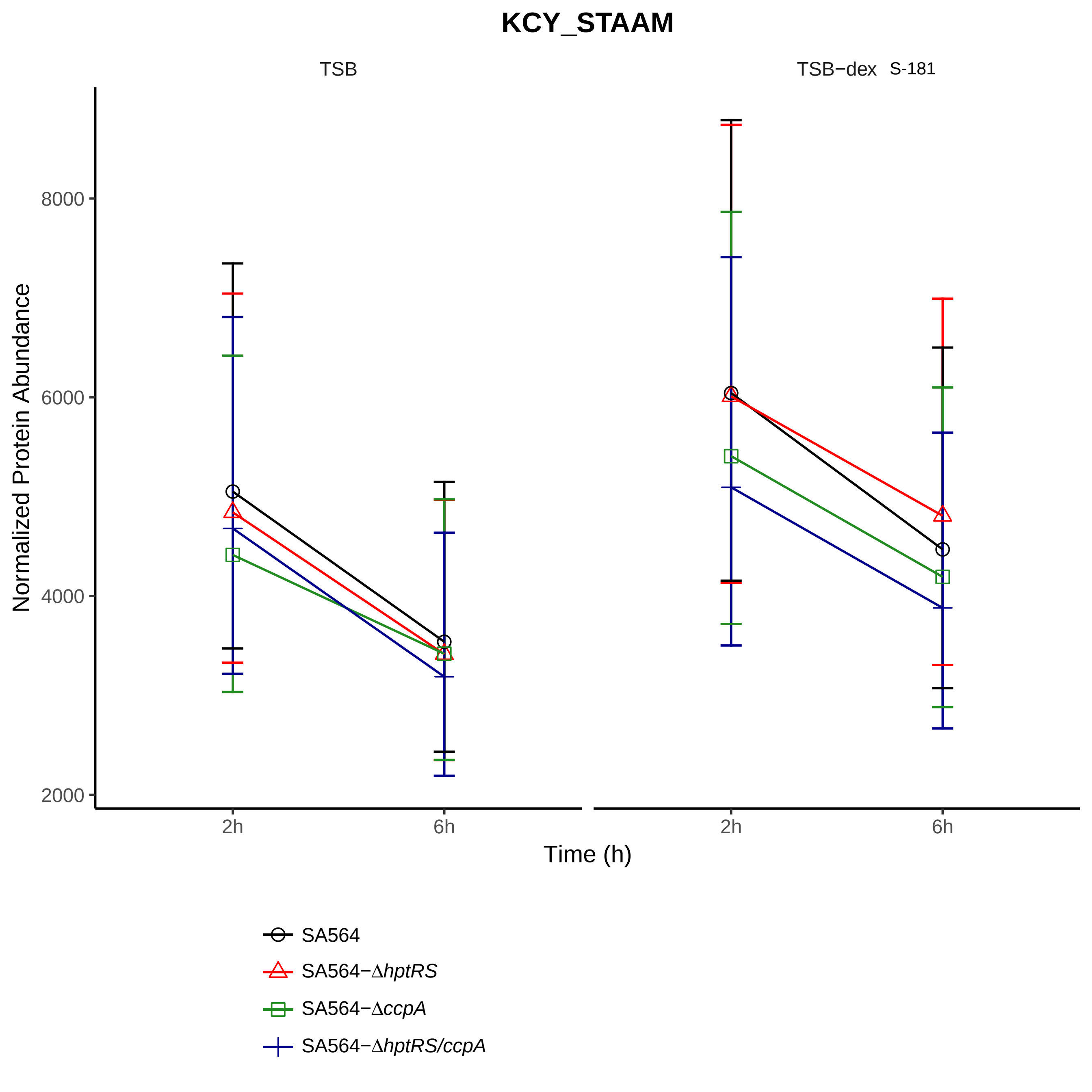
KCY_STAAM
TSB
TSB−dex
S-181
Normalized Protein Abundance
8000
6000
4000
2000
2h
6h
2h
6h
Time (h)
SA564
SA564−Δ*hptRS*
SA564−Δ*ccpA*
SA564−Δ*hptRS/ccpA*

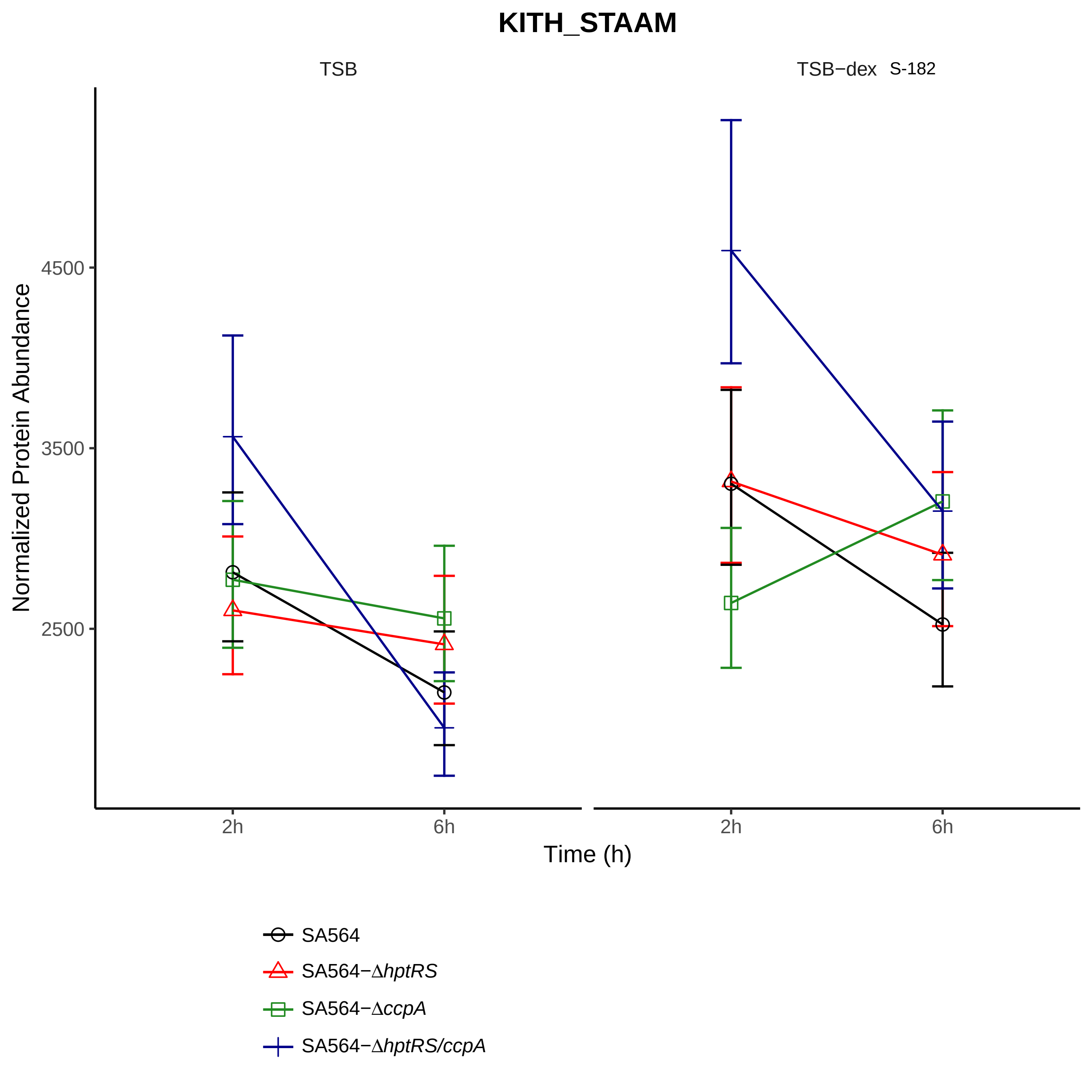
KITH_STAAM
TSB
TSB−dex
S-182
Normalized Protein Abundance
4500
3500
2500
2h
6h
2h
6h
Time (h)
SA564
SA564−Δ*hptRS*
SA564−Δ*ccpA*
SA564−Δ*hptRS/ccpA*

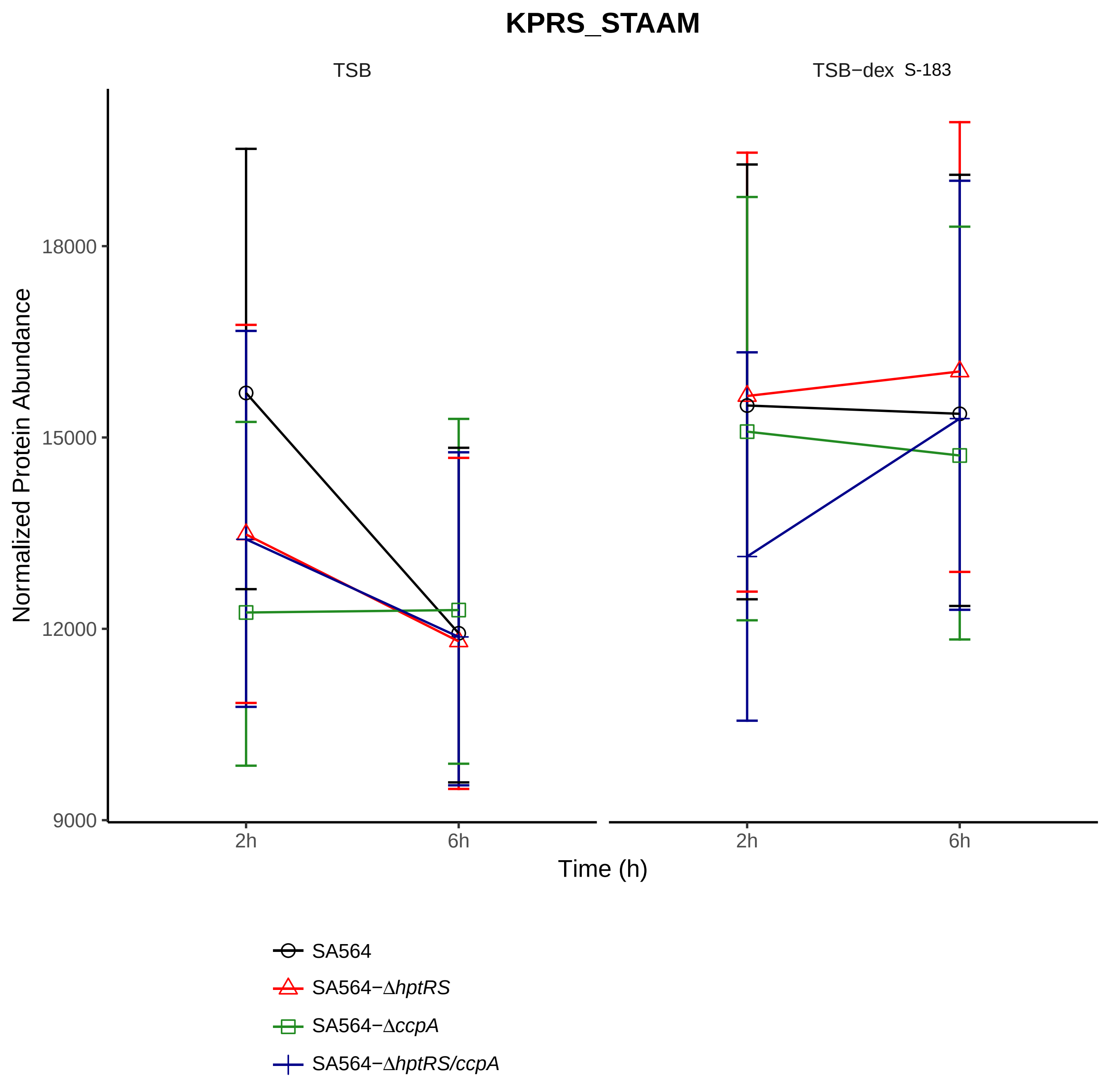

KPRS_STAAM
TSB
TSB−dex
S-183
Normalized Protein Abundance
18000
15000
12000
9000
2h
6h
2h
6h
Time (h)
SA564
SA564−Δ*hptRS*
SA564−Δ*ccpA*
SA564−Δ*hptRS/ccpA*

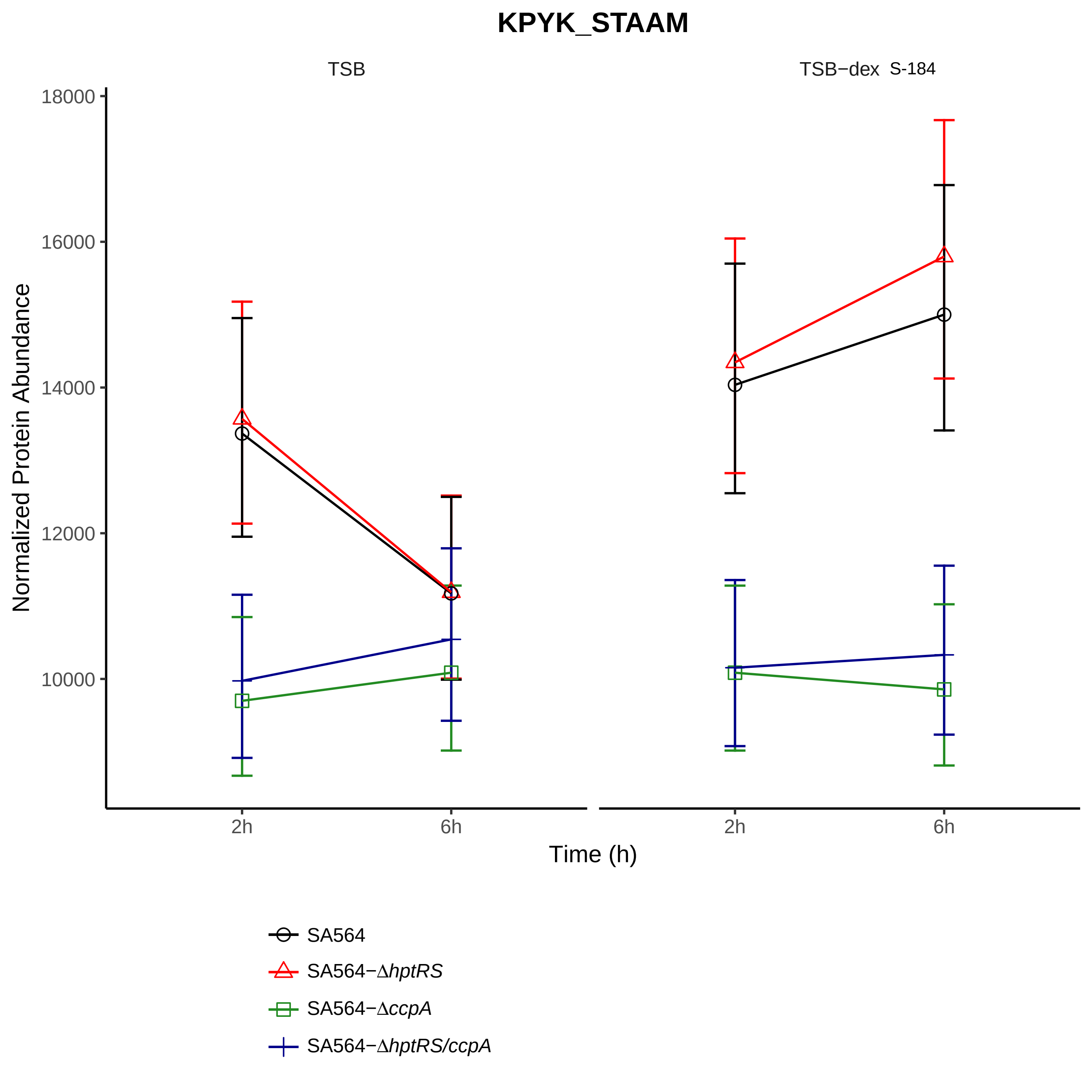

KPYK_STAAM
TSB
TSB−dex
S-184
18000
16000
14000
12000
10000
Normalized Protein Abundance
2h
6h
2h
6h
Time (h)
SA564
SA564−ΔhptRS
SA564−ΔccpA
SA564−ΔhptRS/ccpA

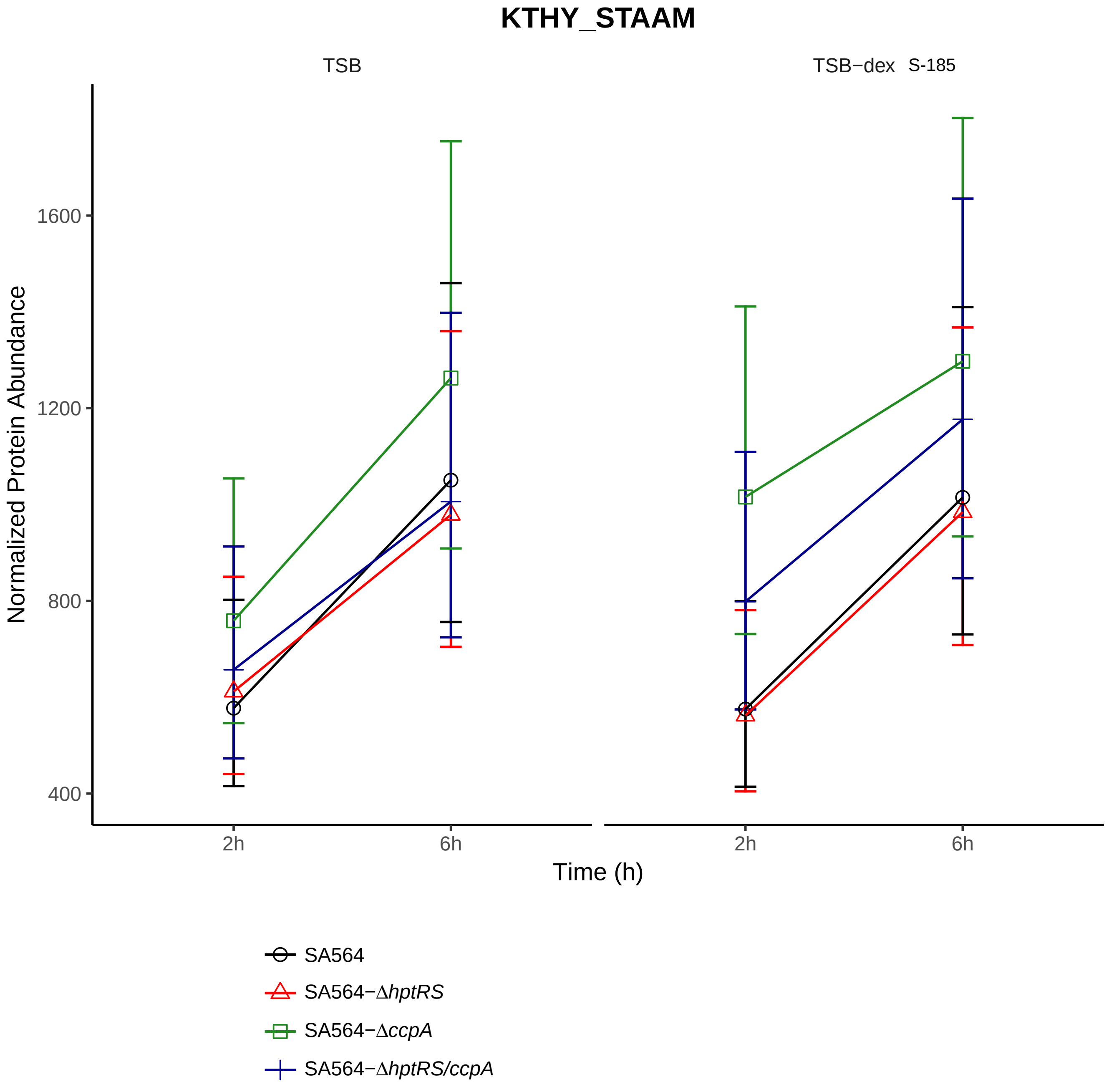
KTHY_STAAM
TSB
TSB−dex
S-185
Normalized Protein Abundance
1600
1200
800
400
2h
6h
2h
6h
Time (h)
SA564
SA564−ΔhptRS
SA564−ΔccpA
SA564−ΔhptRS/ccpA

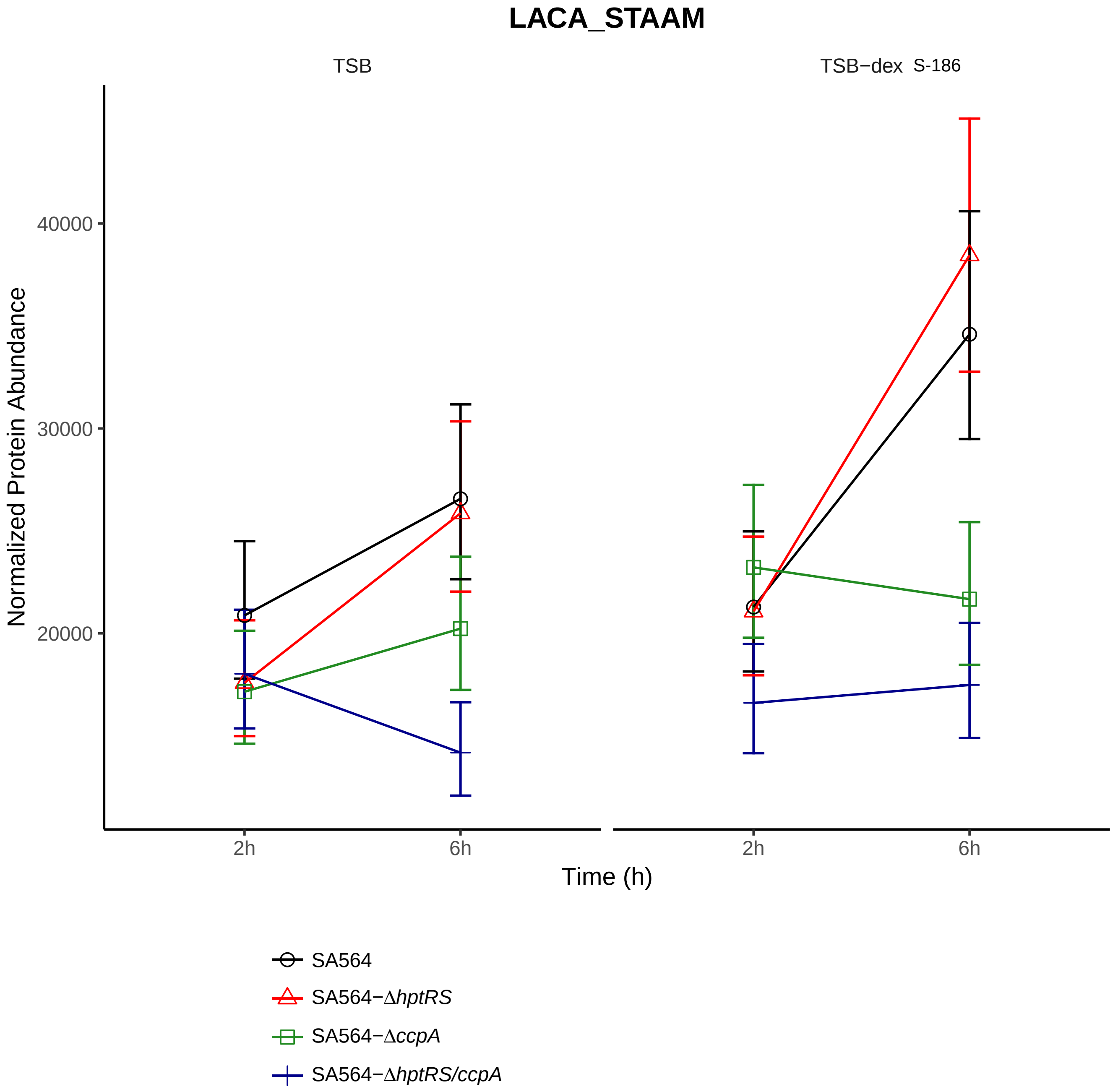

LACA_STAAM
TSB
TSB−dex
S-186
Normalized Protein Abundance
40000
30000
20000
2h
6h
2h
6h
Time (h)
SA564
SA564−Δ*hptRS*
SA564−Δ*ccpA*
SA564−Δ*hptRS/ccpA*

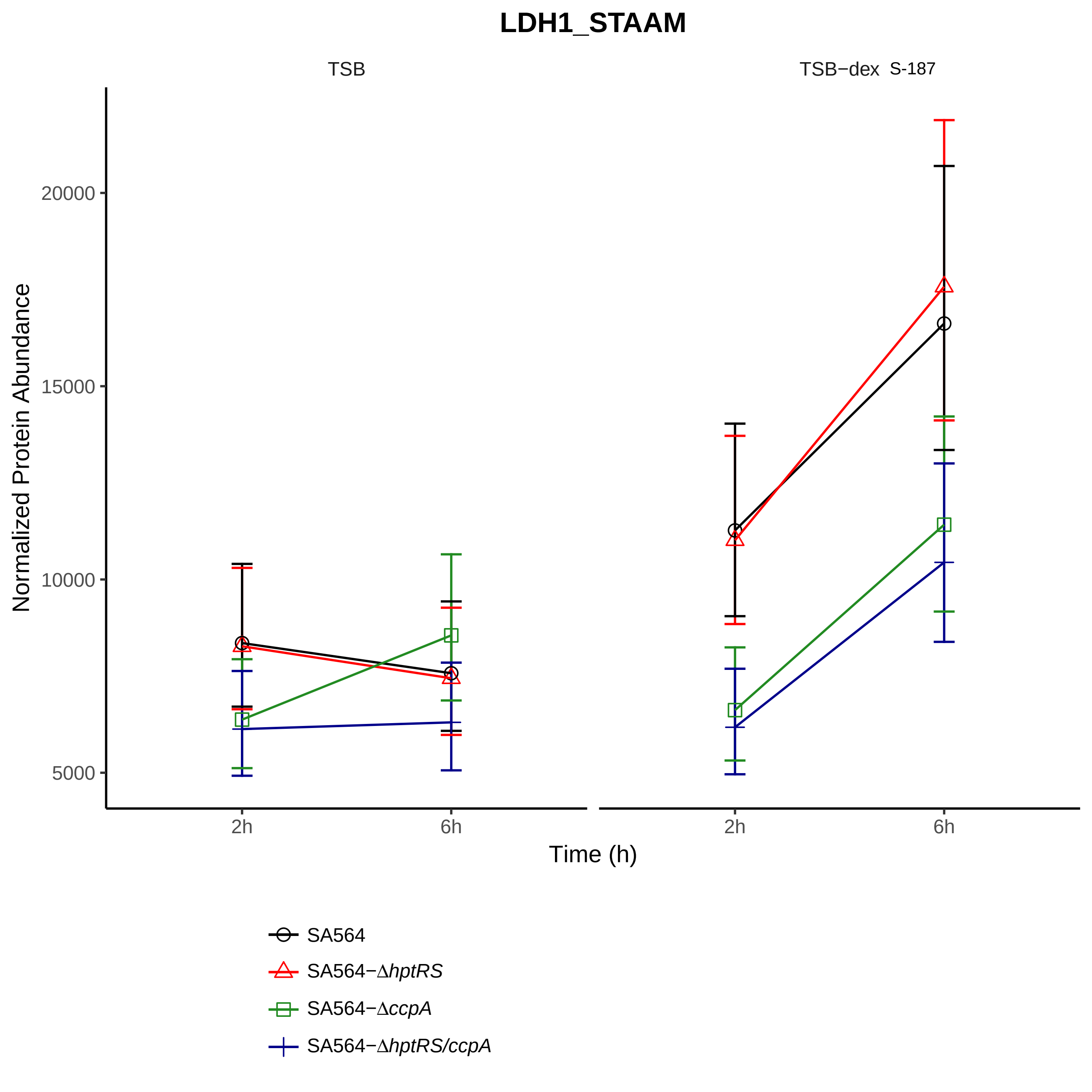

LDH1_STAAM
TSB
TSB−dex
S-187
Normalized Protein Abundance
20000
15000
10000
5000
2h
6h
2h
6h
Time (h)
SA564
SA564−Δ*hptRS*
SA564−Δ*ccpA*
SA564−Δ*hptRS/ccpA*

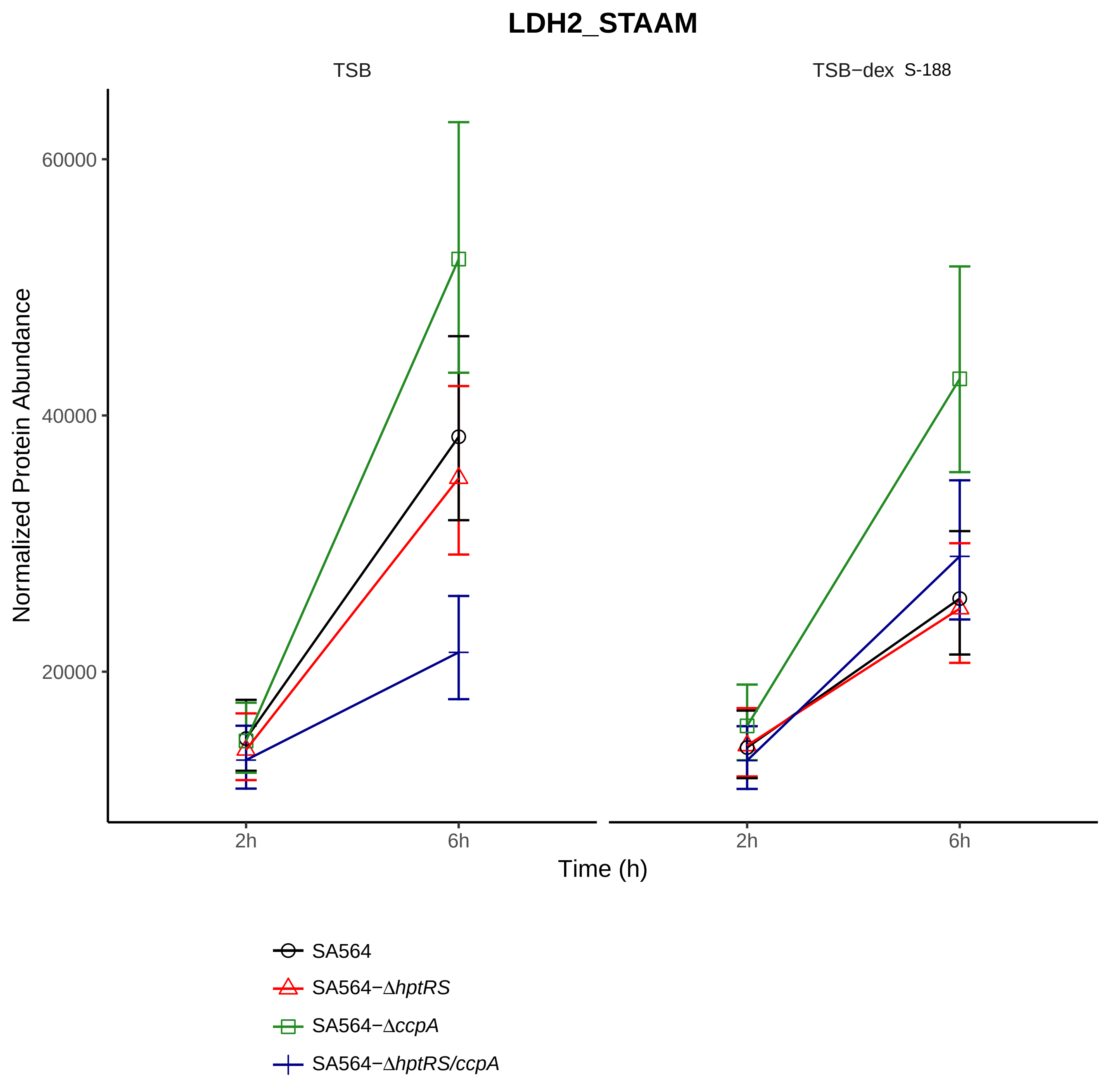

LDH2_STAAM
TSB
TSB−dex
S-188
Normalized Protein Abundance
60000
40000
20000
2h
6h
2h
6h
Time (h)
SA564
SA564−Δ*hptRS*
SA564−Δ*ccpA*
SA564−Δ*hptRS/ccpA*

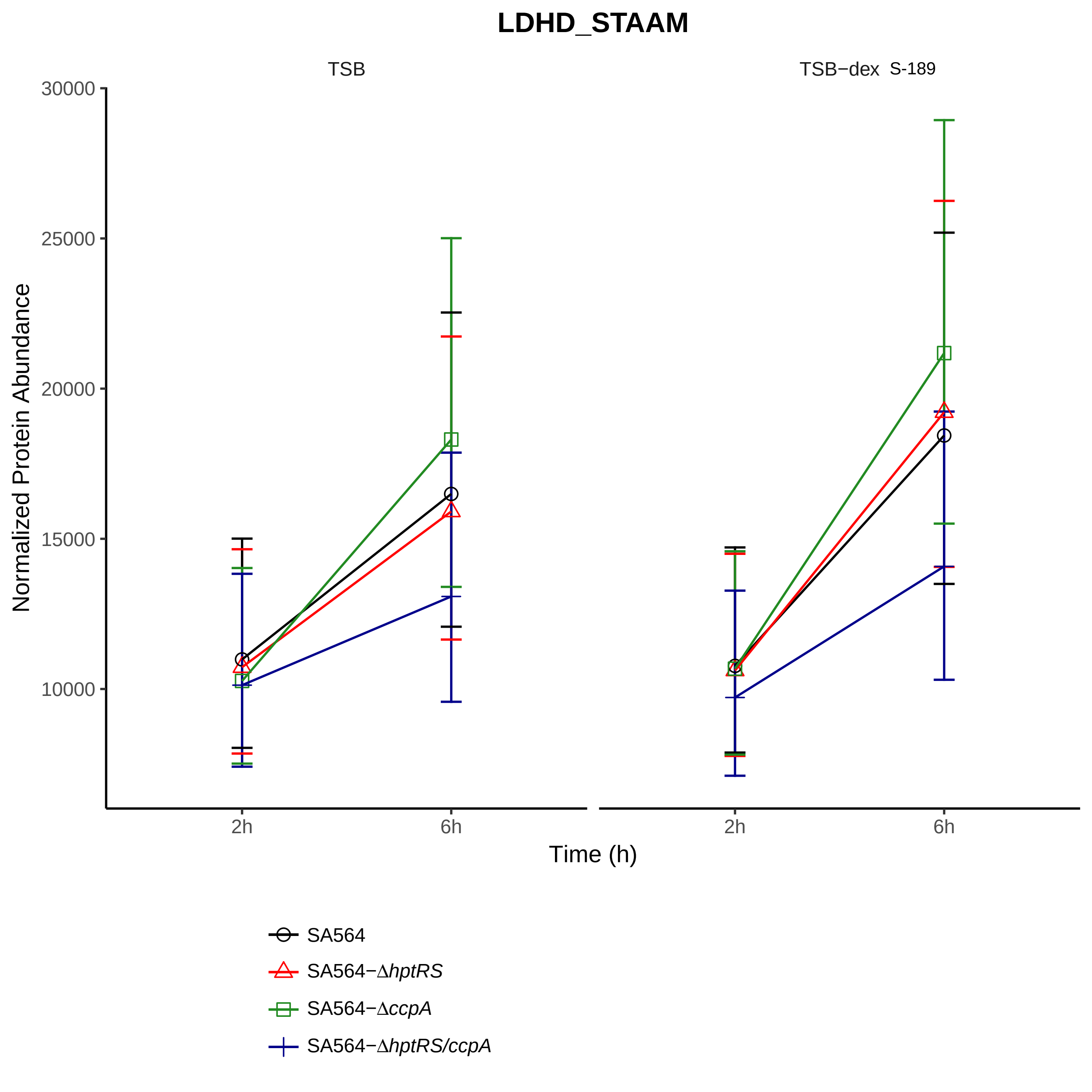

LDHD_STAAM
TSB
TSB−dex
S-189
30000
25000
20000
15000
10000
Normalized Protein Abundance
2h
6h
2h
6h
Time (h)
SA564
SA564−Δ*hptRS*
SA564−Δ*ccpA*
SA564−Δ*hptRS/ccpA*

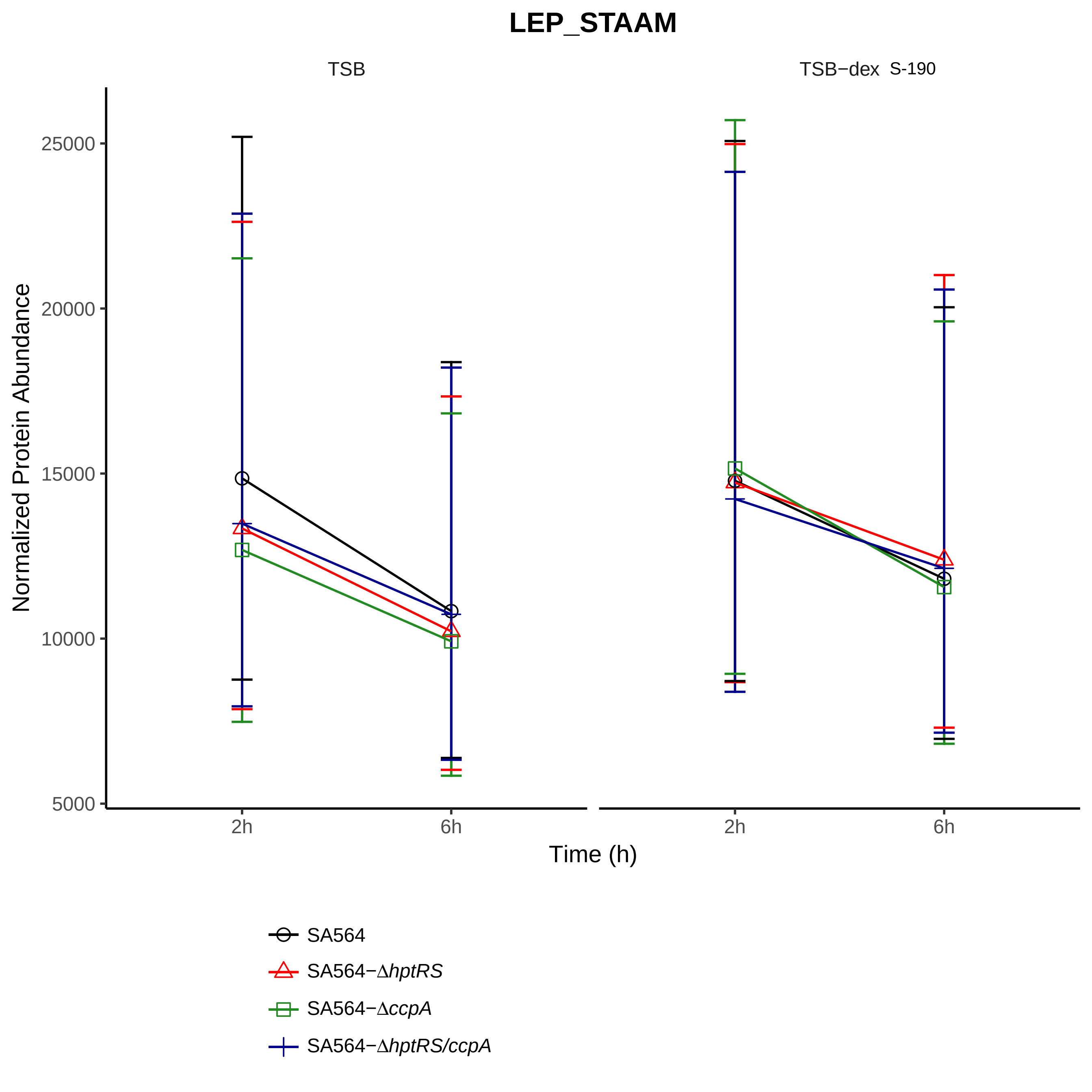

LEP_STAAM
TSB
TSB−dex
S-190
Normalized Protein Abundance
25000
20000
15000
10000
5000
2h
6h
2h
6h
Time (h)
SA564
SA564−ΔhptRS
SA564−ΔccpA
SA564−ΔhptRS/ccpA

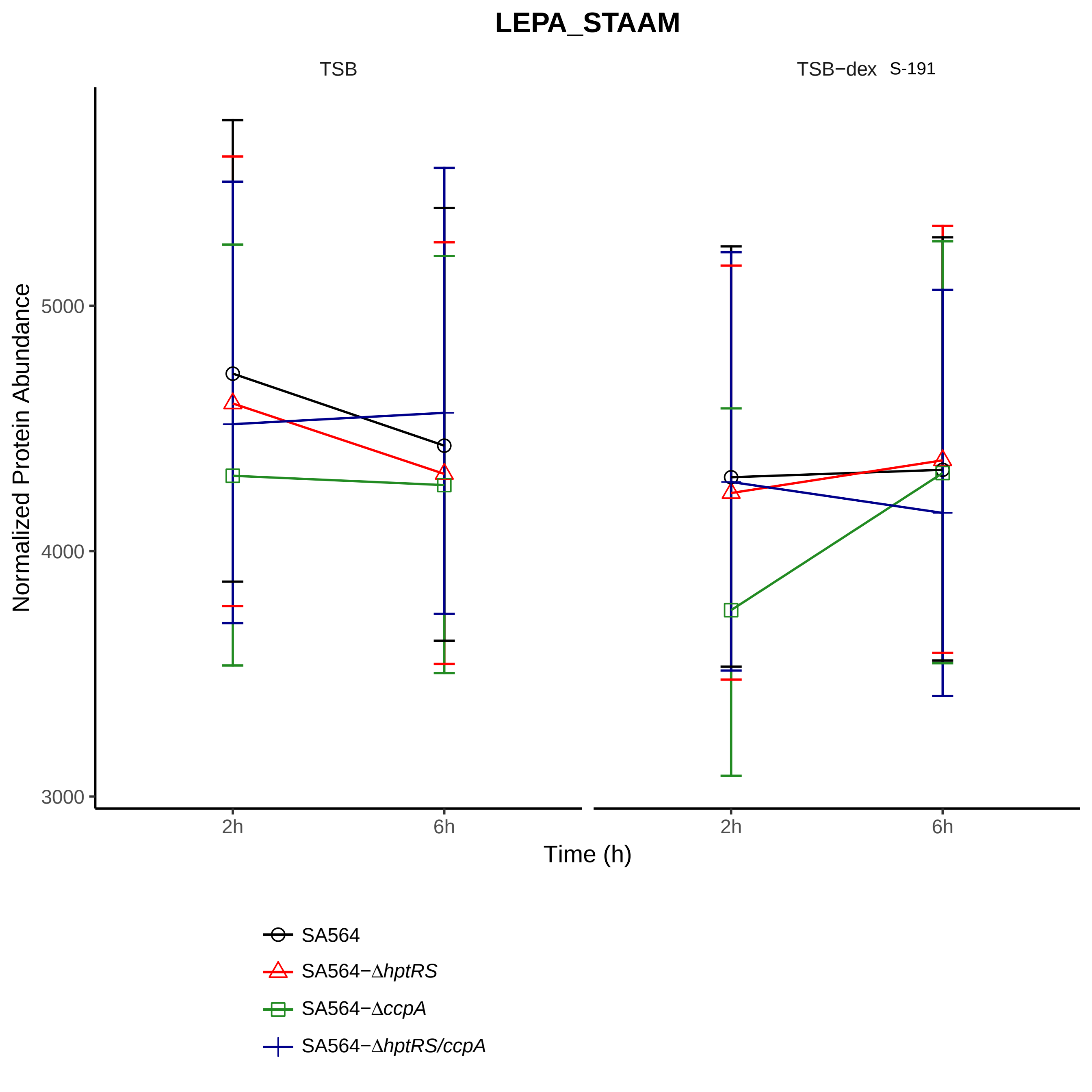

LEPA_STAAM
TSB
TSB−dex
S-191
Normalized Protein Abundance
5000
4000
3000
2h
6h
2h
6h
Time (h)
SA564
SA564−ΔhptRS
SA564−ΔccpA
SA564−ΔhptRS/ccpA

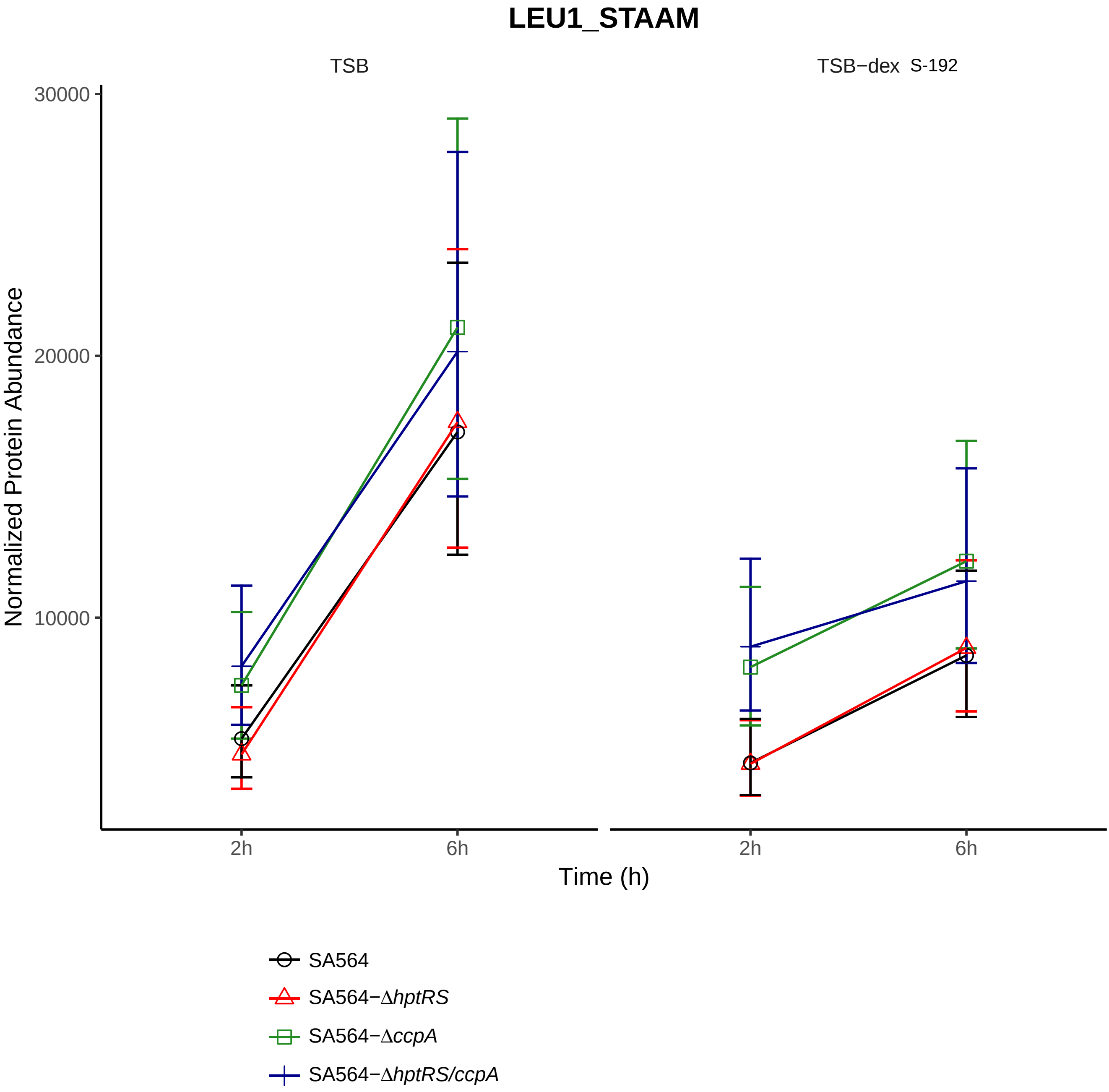

LEU1_STAAM
TSB
TSB−dex
S-192
30000
20000
10000
Normalized Protein Abundance
2h
6h
2h
6h
Time (h)
SA564
SA564−Δ*hptRS*
SA564−Δ*ccpA*
SA564−Δ*hptRS/ccpA*

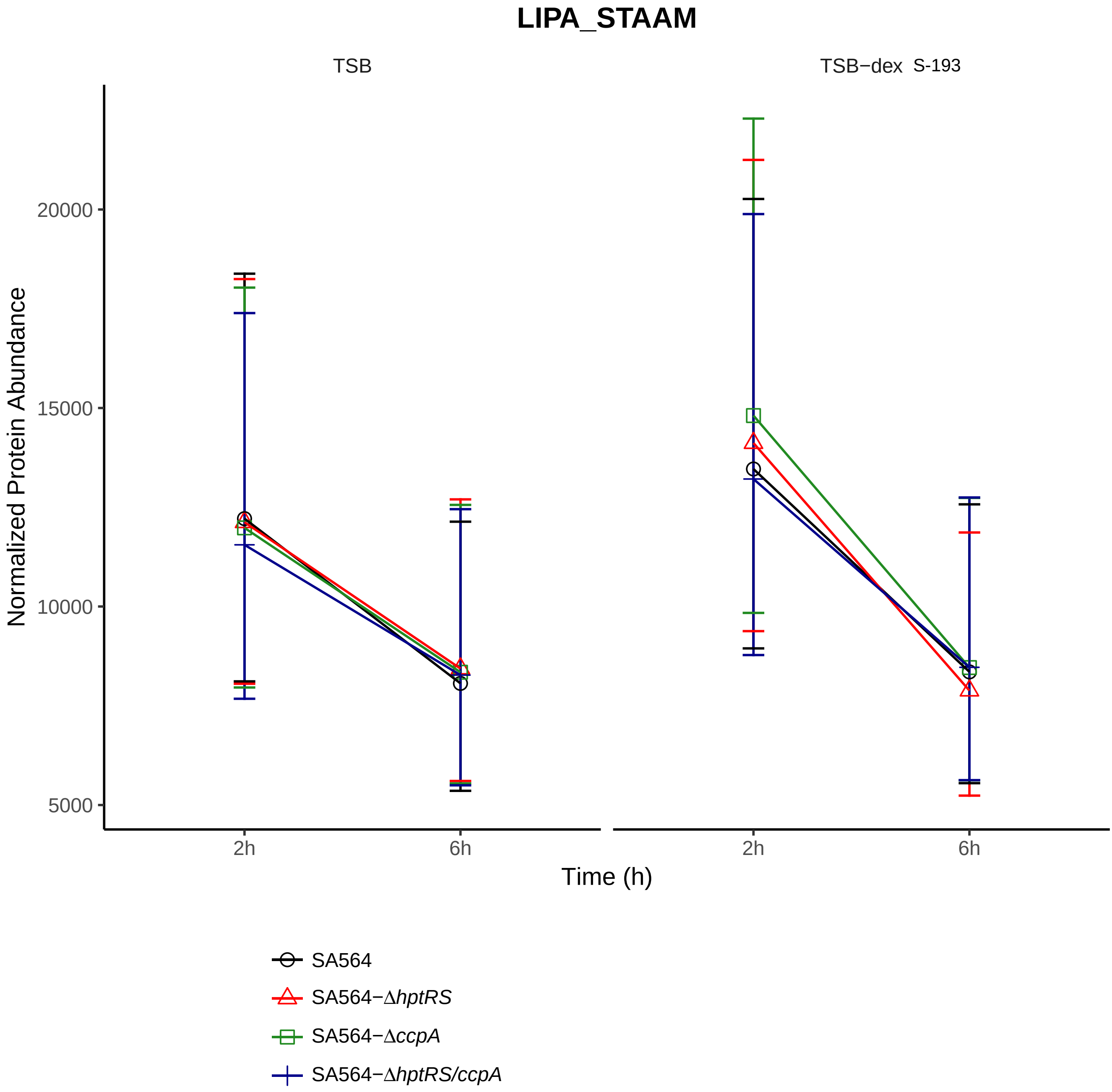

LIPA_STAAM
TSB
TSB−dex
S-193
Normalized Protein Abundance
20000
15000
10000
5000
2h
6h
2h
6h
Time (h)
SA564
SA564−Δ*hptRS*
SA564−Δ*ccpA*
SA564−Δ*hptRS/ccpA*

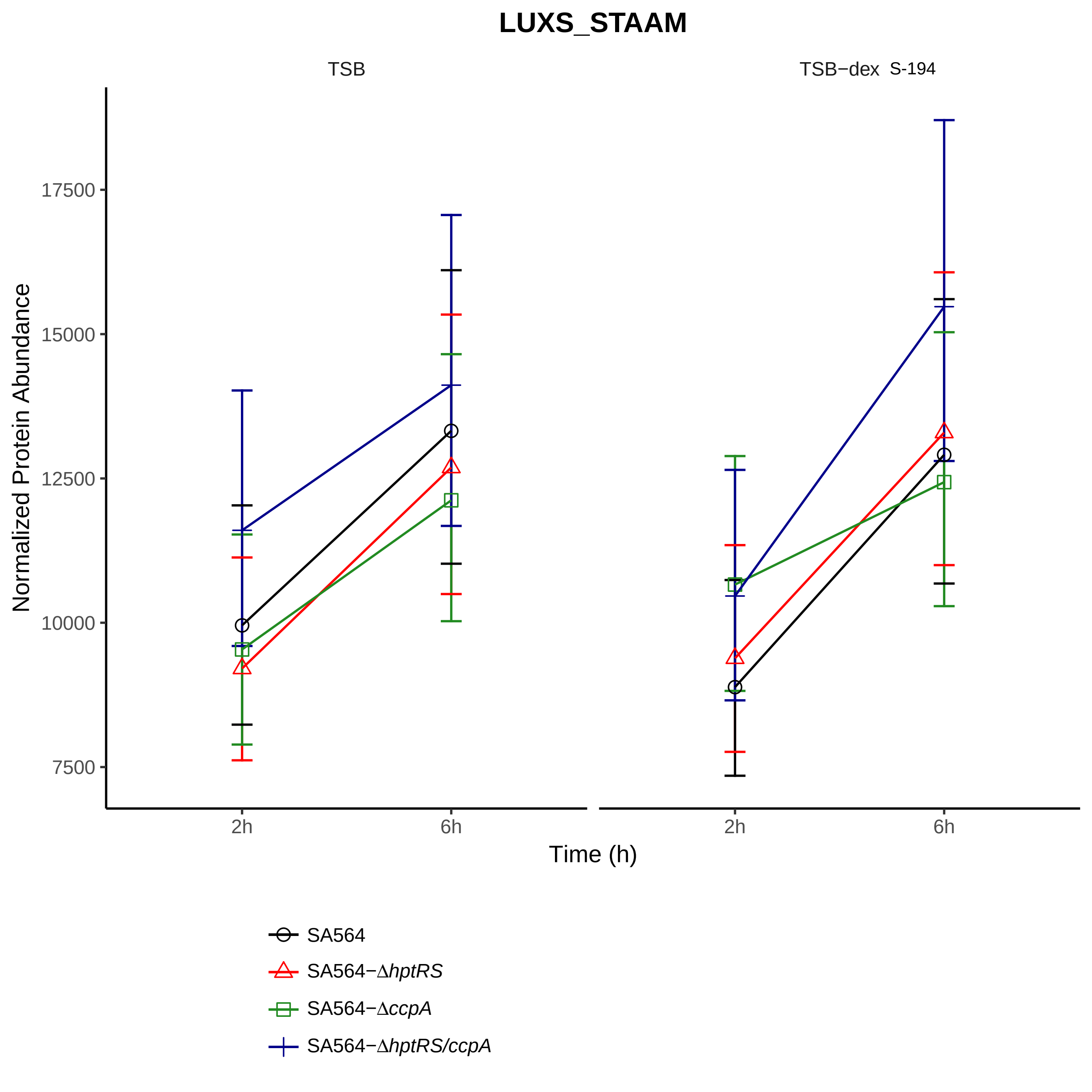

LUXS_STAAM
TSB
TSB−dex
S-194
Normalized Protein Abundance
17500
15000
12500
10000
7500
2h
6h
2h
6h
Time (h)
SA564
SA564−Δ*hptRS*
SA564−Δ*ccpA*
SA564−Δ*hptRS/ccpA*

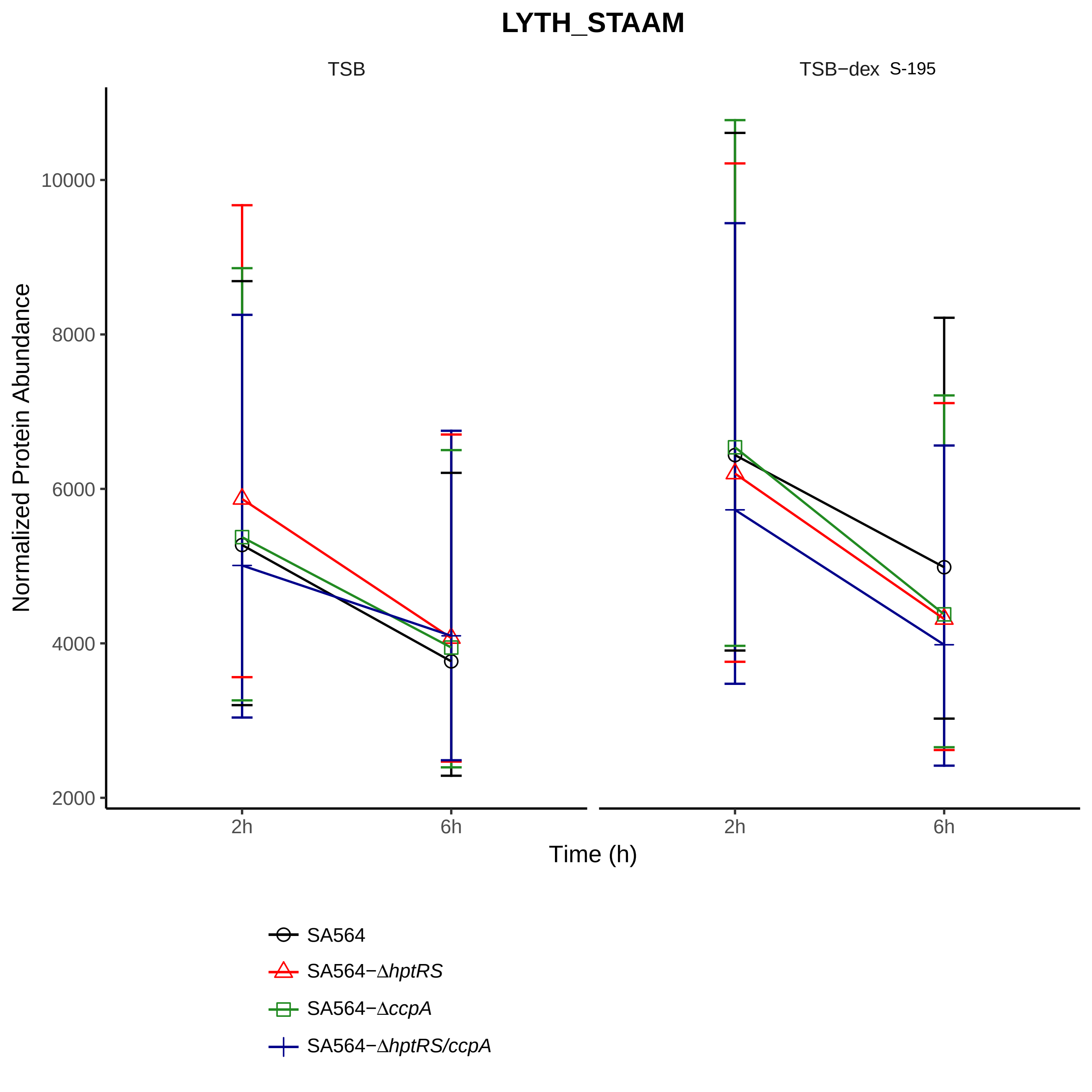

LYTH_STAAM
TSB
TSB−dex
S-195
Normalized Protein Abundance
10000
8000
6000
4000
2000
2h
6h
2h
6h
Time (h)
SA564
SA564−ΔhptRS
SA564−ΔccpA
SA564−ΔhptRS/ccpA

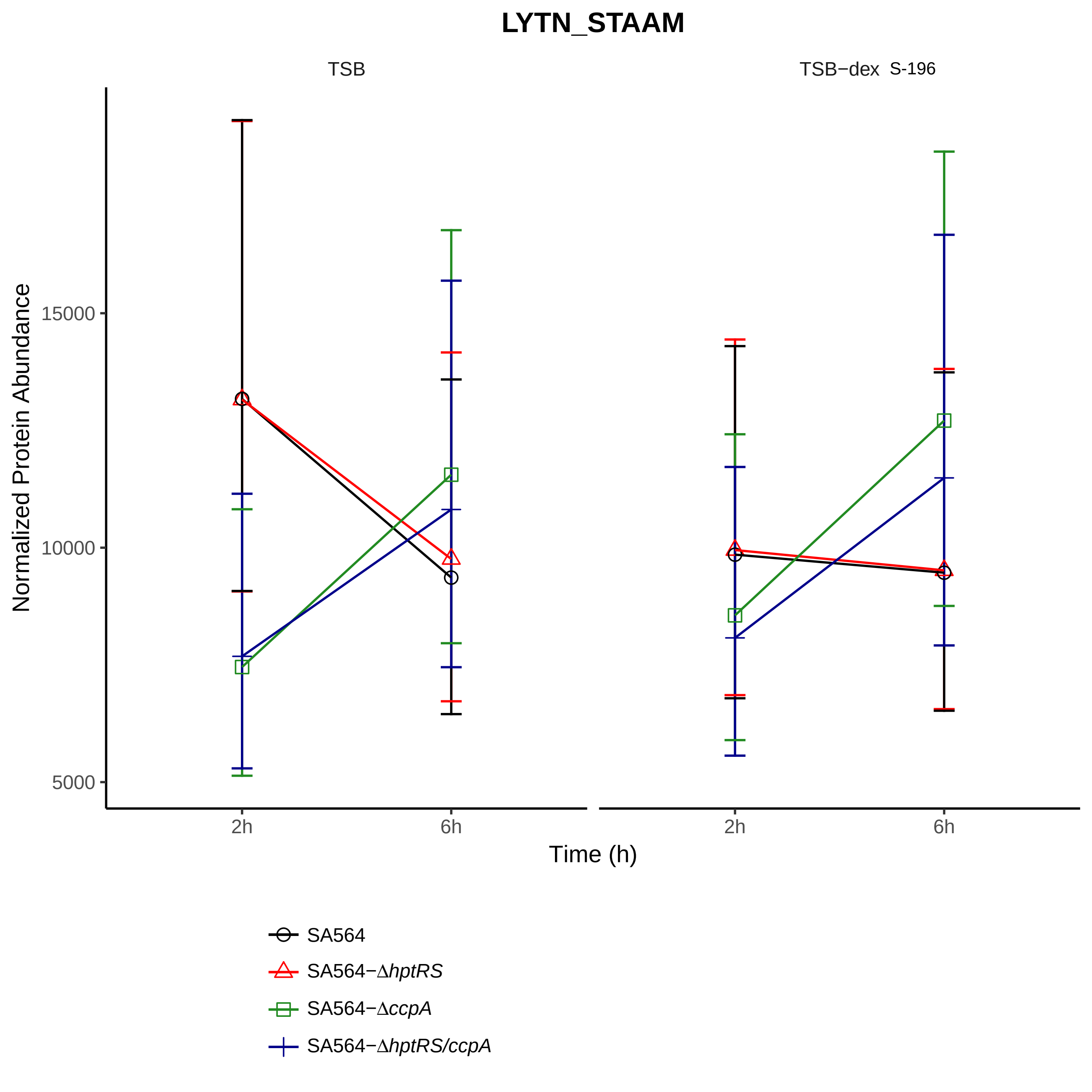

LYTN_STAAM
TSB
TSB−dex
S-196
Normalized Protein Abundance
15000
10000
5000
2h
6h
2h
6h
Time (h)
SA564
SA564−Δ*hptRS*
SA564−Δ*ccpA*
SA564−Δ*hptRS/ccpA*

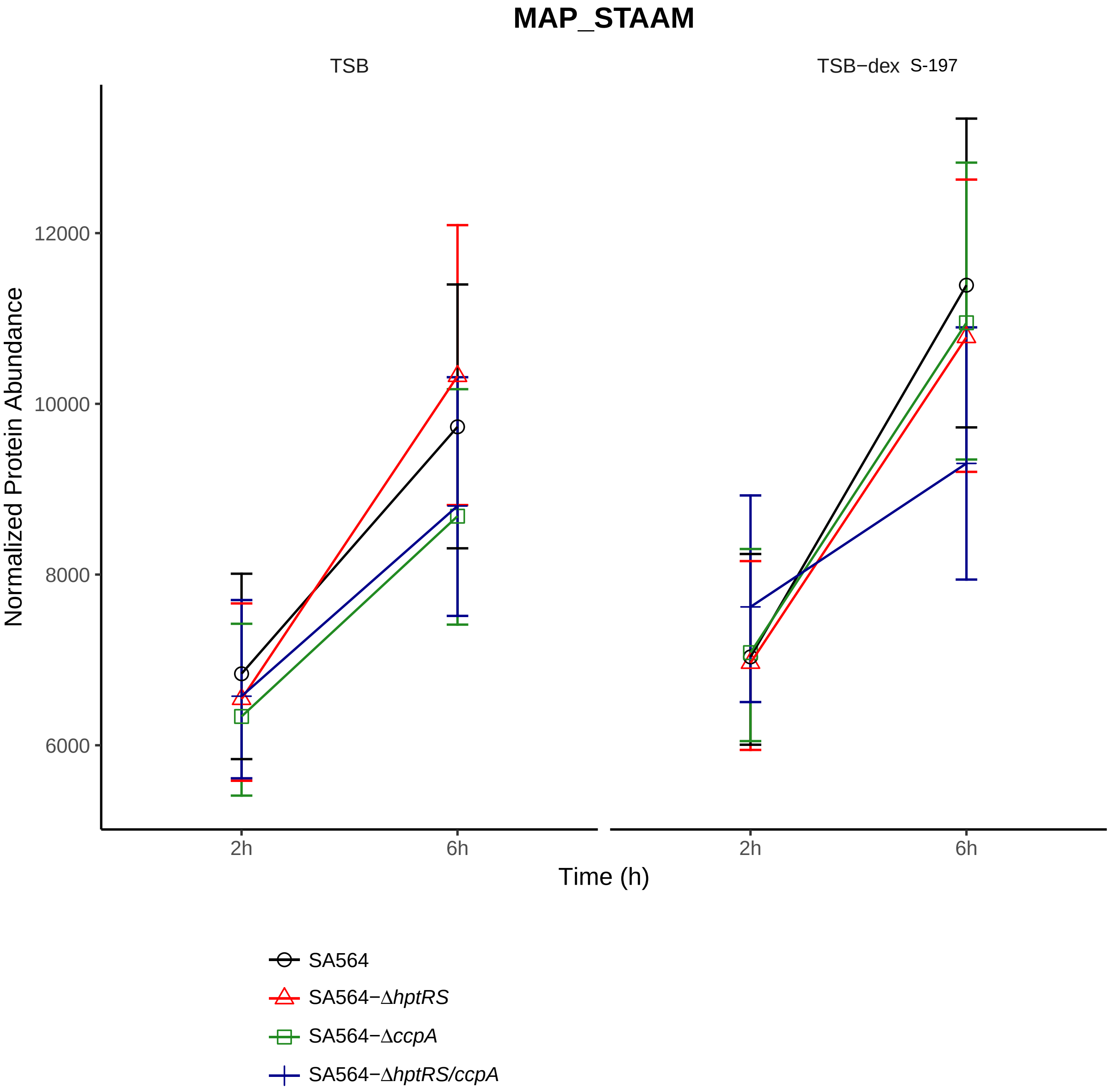

MAP_STAAM
TSB
TSB−dex
Normalized Protein Abundance
12000
10000
8000
6000
2h
6h
2h
6h
Time (h)
SA564
SA564−Δ*hptRS*
SA564−Δ*ccpA*
SA564−Δ*hptRS/ccpA*

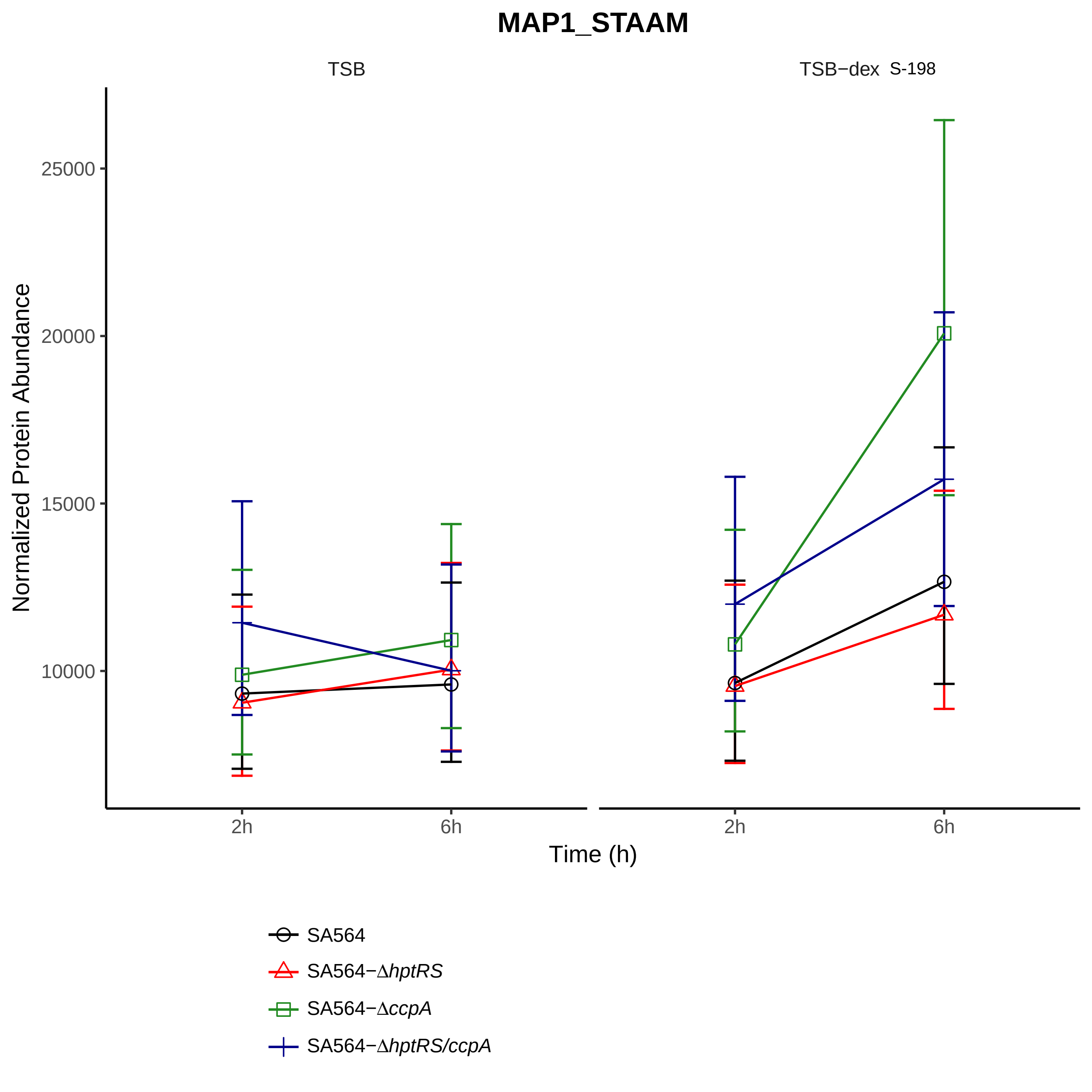

MAP1_STAAM
TSB
TSB−dex
S-198
Normalized Protein Abundance
25000
20000
15000
10000
2h
6h
2h
6h
Time (h)
SA564
SA564−ΔhptRS
SA564−ΔccpA
SA564−ΔhptRS/ccpA

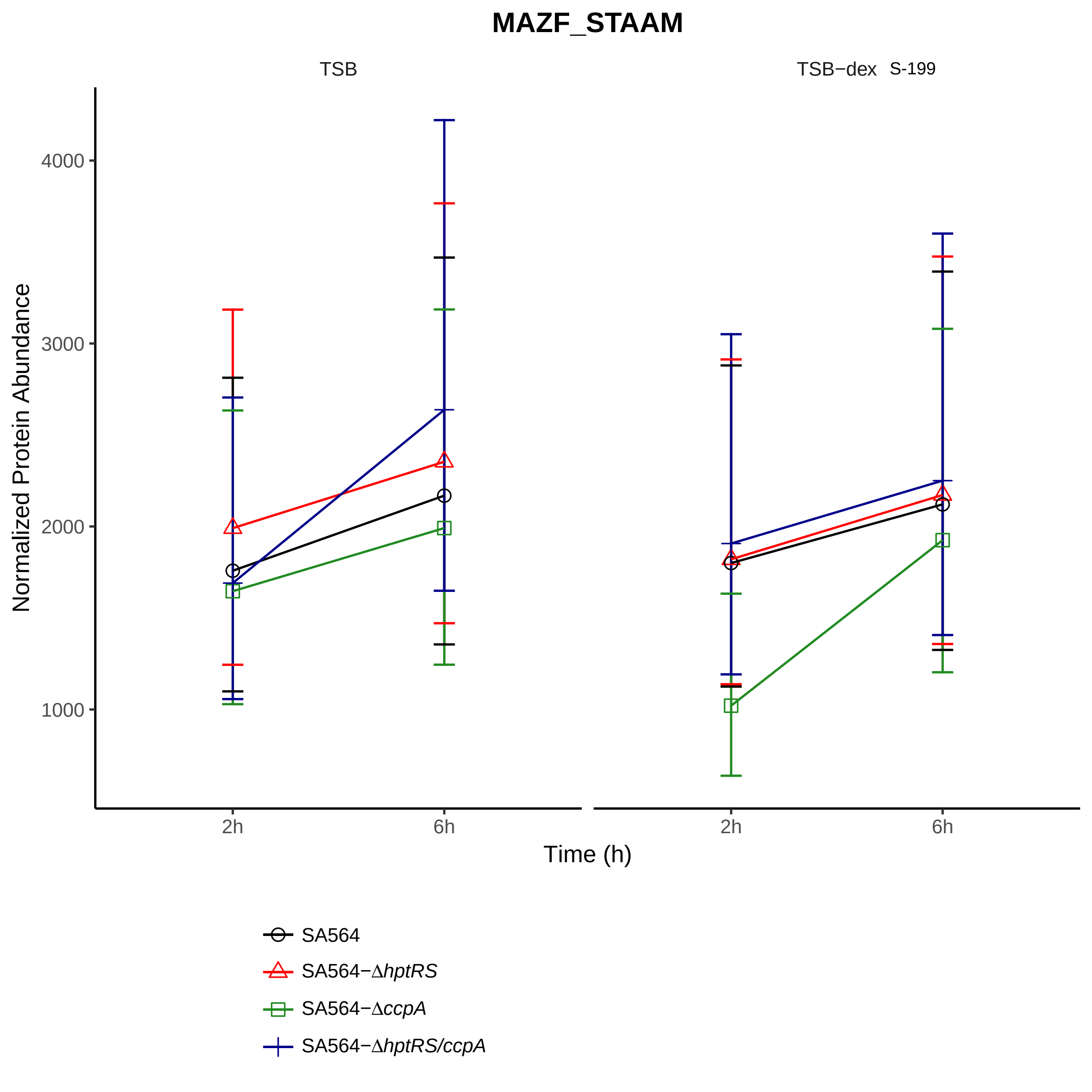
MAZF_STAAM
TSB
TSB−dex
S-199
Normalized Protein Abundance
4000
3000
2000
1000
2h
6h
2h
6h
Time (h)
SA564
SA564−ΔhptRS
SA564−ΔccpA
SA564−ΔhptRS/ccpA

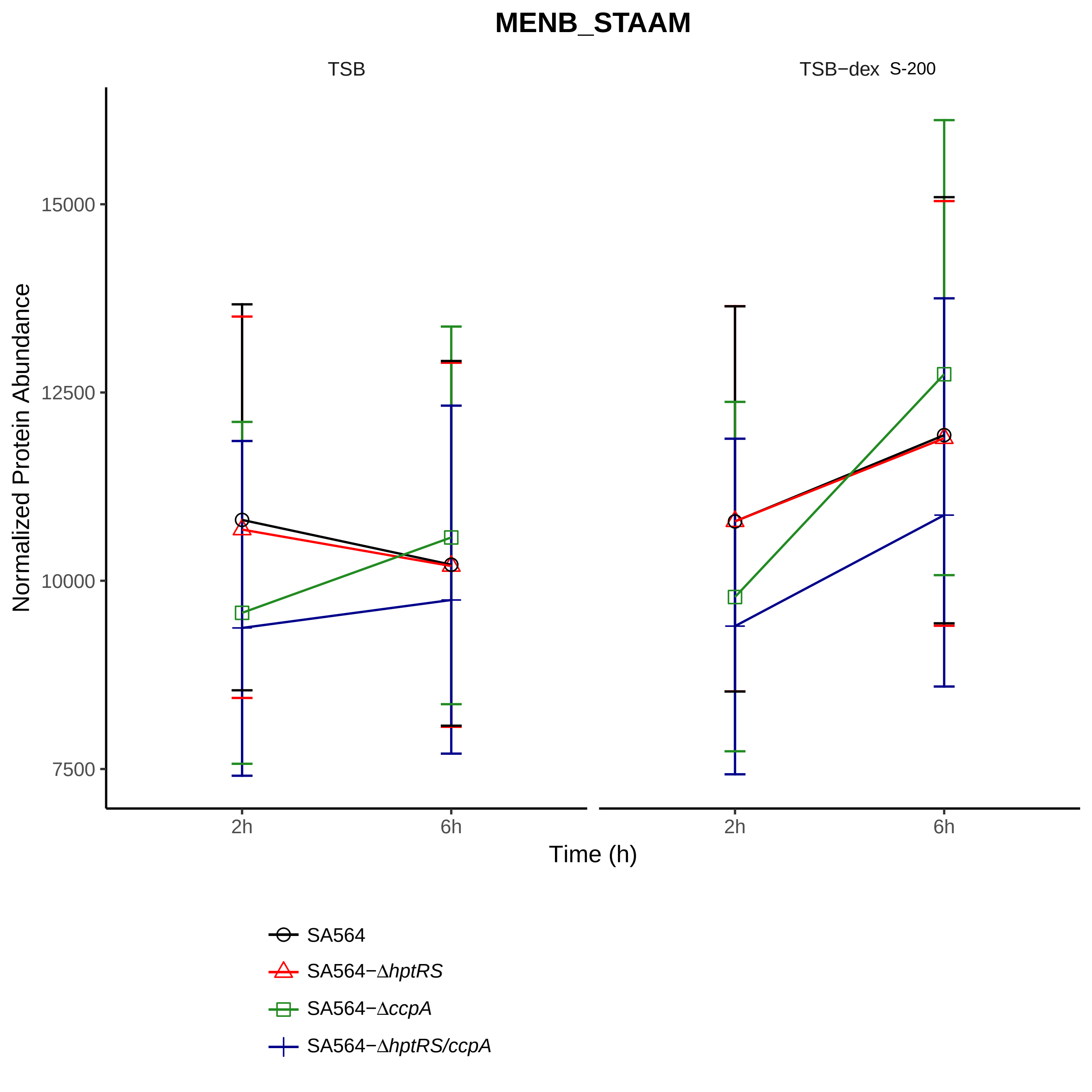

MENB_STAAM
TSB
TSB−dex
S-200
Normalized Protein Abundance
15000
12500
10000
7500
2h
6h
2h
6h
Time (h)
SA564
SA564−Δ*hptRS*
SA564−Δ*ccpA*
SA564−Δ*hptRS/ccpA*

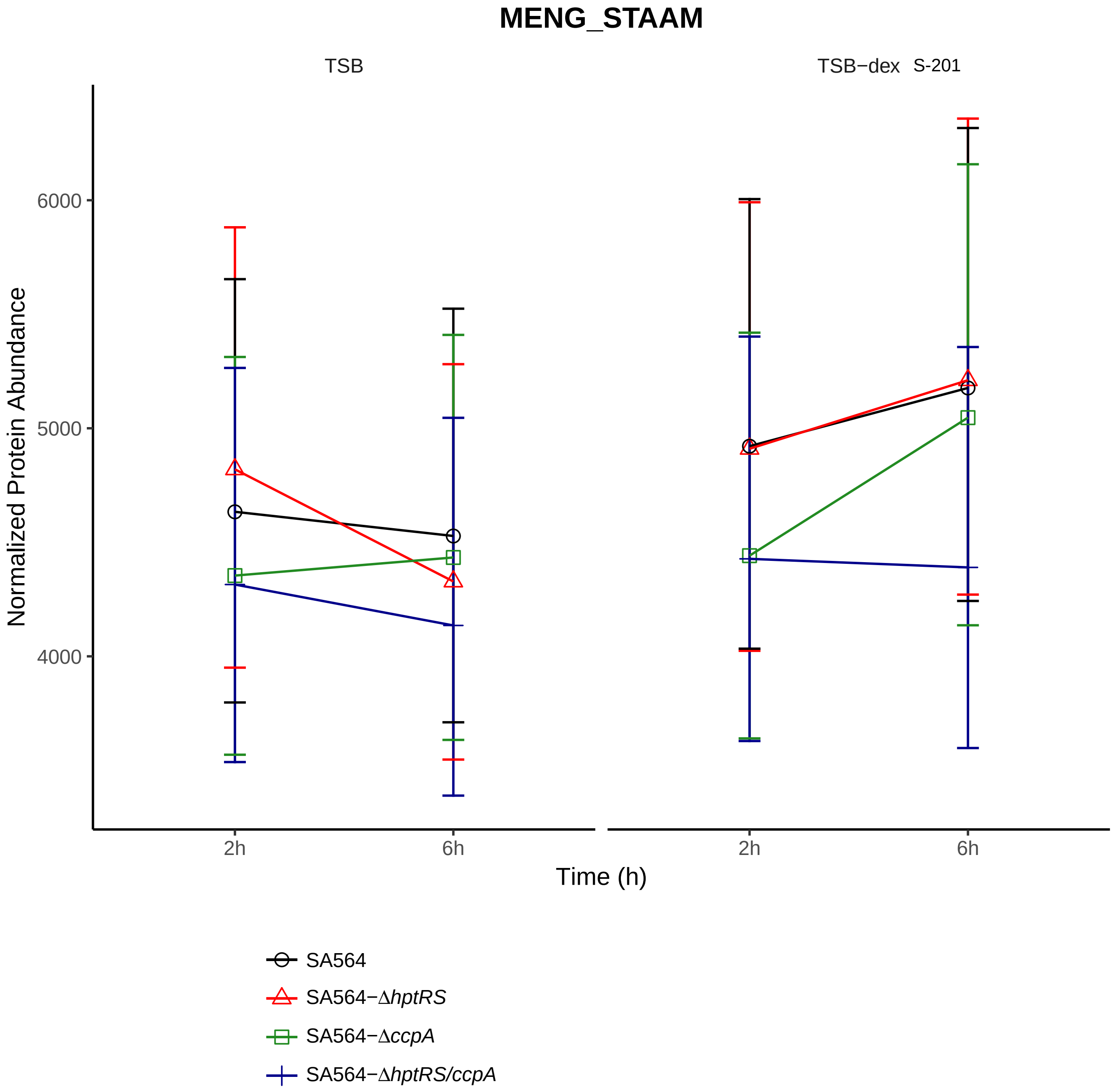
MENG_STAAM
TSB
TSB−dex
S-201
Normalized Protein Abundance
6000
5000
4000
2h
6h
2h
6h
Time (h)
SA564
SA564−Δ*hptRS*
SA564−Δ*ccpA*
SA564−Δ*hptRS/ccpA*

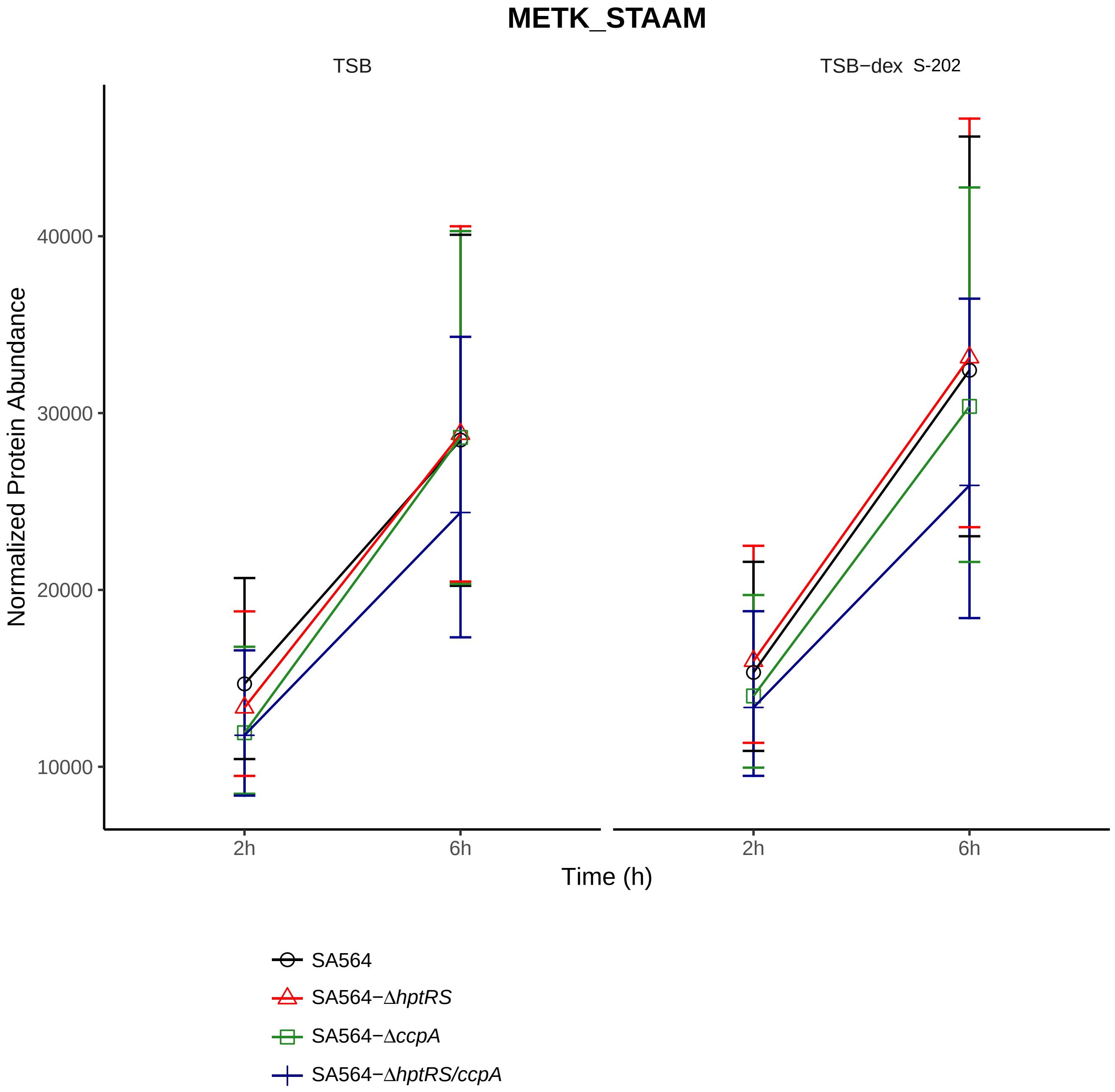

METK_STAAM
TSB
TSB−dex
S-202
Normalized Protein Abundance
40000
30000
20000
10000
2h
6h
2h
6h
Time (h)
SA564
SA564−ΔhptRS
SA564−ΔccpA
SA564−ΔhptRS/ccpA

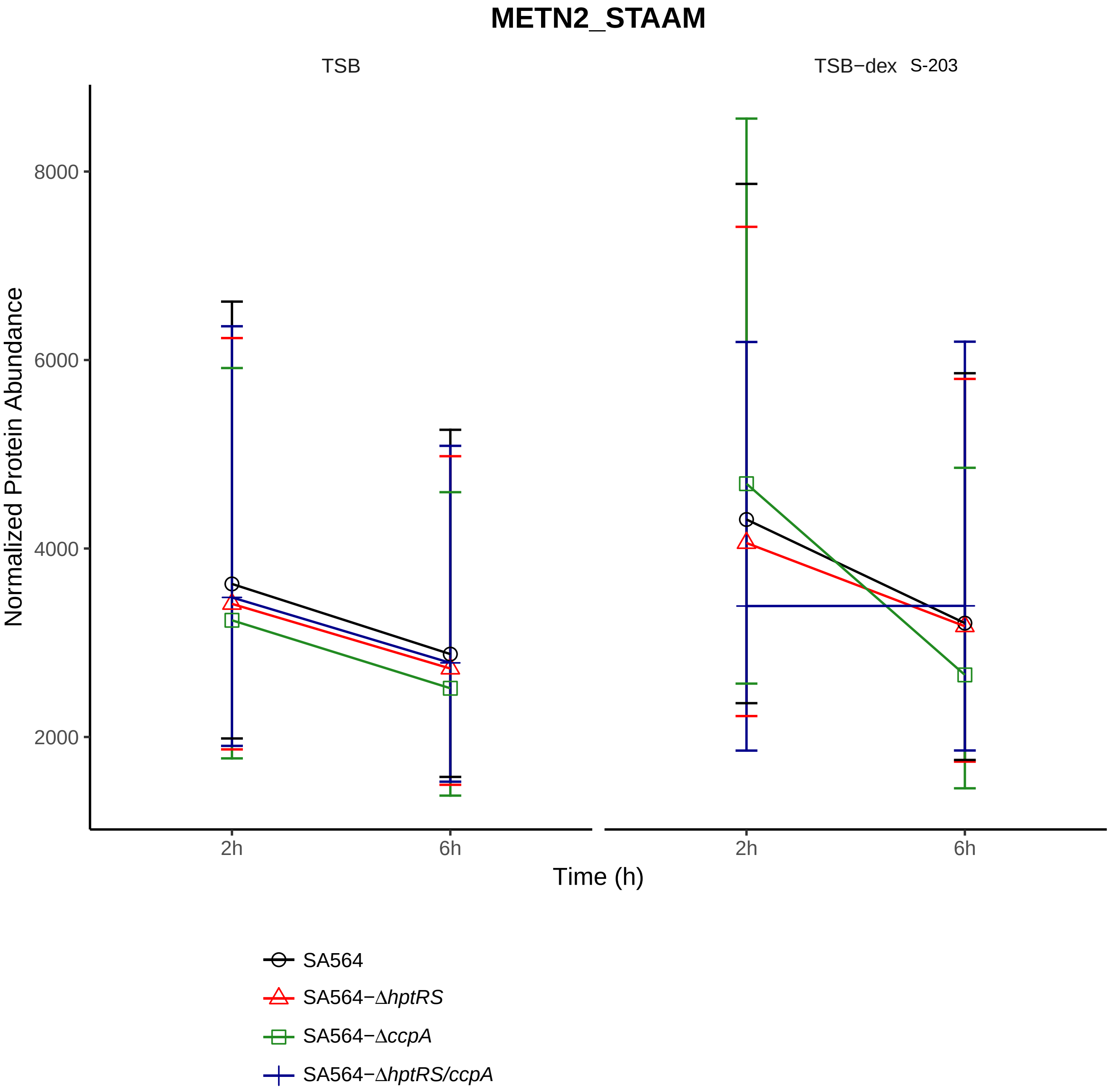

METN2_STAAM
TSB
TSB−dex
S-203
Normalized Protein Abundance
8000
6000
4000
2000
2h
6h
2h
6h
Time (h)
SA564
SA564−Δ*hptRS*
SA564−Δ*ccpA*
SA564−Δ*hptRS/ccpA*

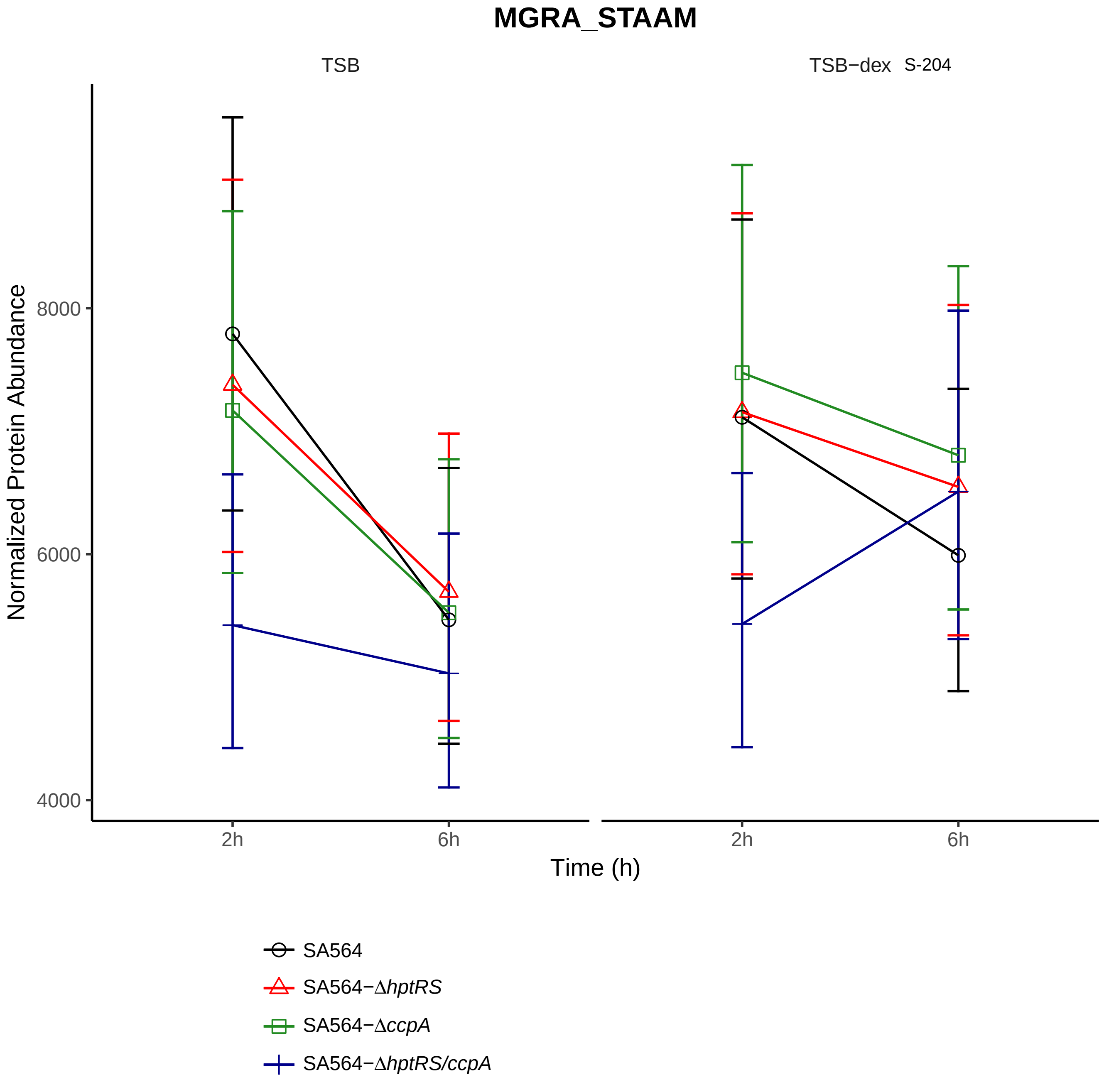

MGRA_STAAM
TSB
TSB−dex
S-204
Normalized Protein Abundance
8000
6000
4000
2h
6h
2h
6h
Time (h)
SA564
SA564−ΔhptRS
SA564−ΔccpA
SA564−ΔhptRS/ccpA

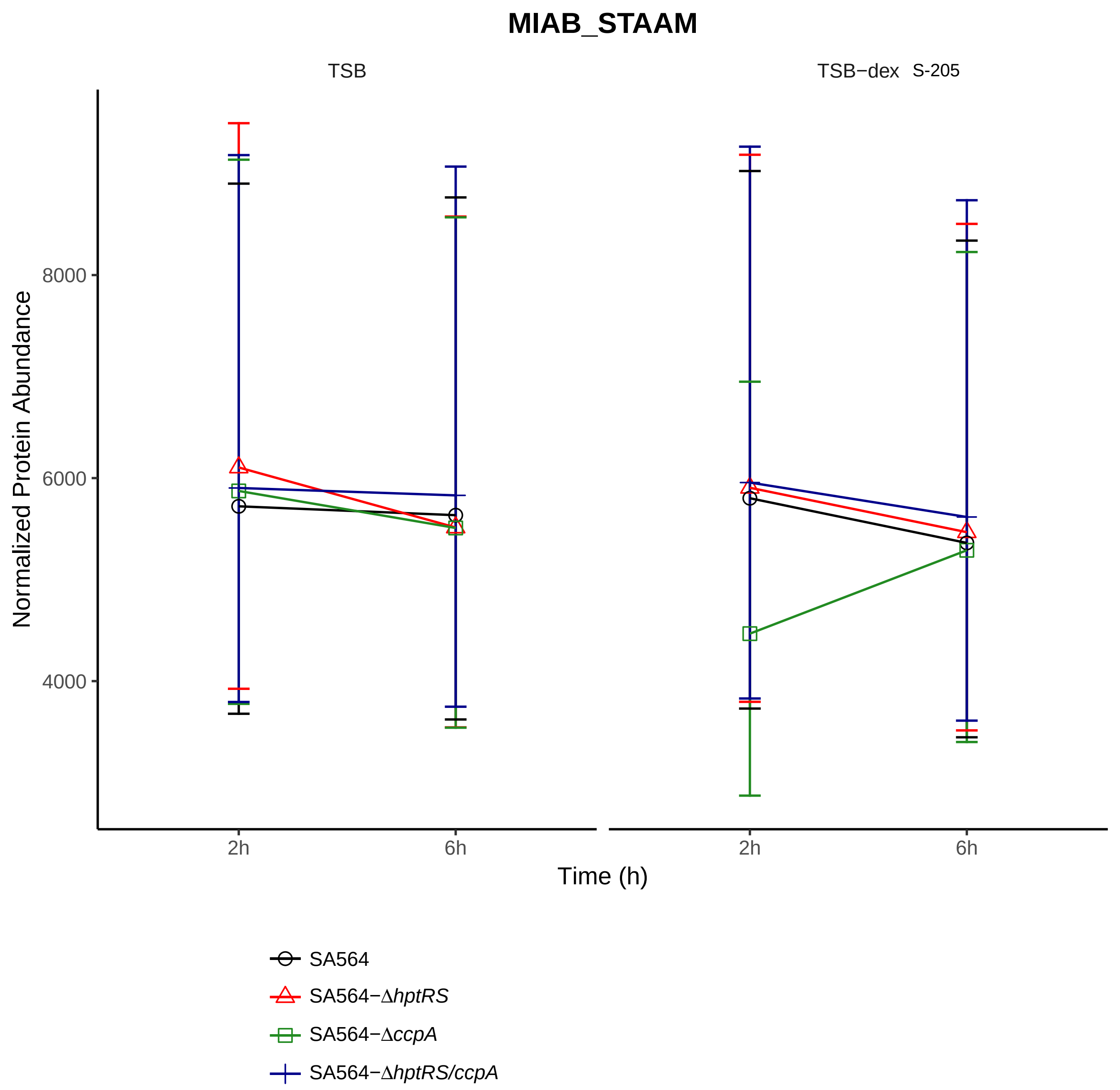
MIAB_STAAM
TSB
TSB−dex
Normalized Protein Abundance
8000
6000
4000
2h
6h
2h
6h
Time (h)
SA564
SA564−ΔhptRS
SA564−ΔccpA
SA564−ΔhptRS/ccpA

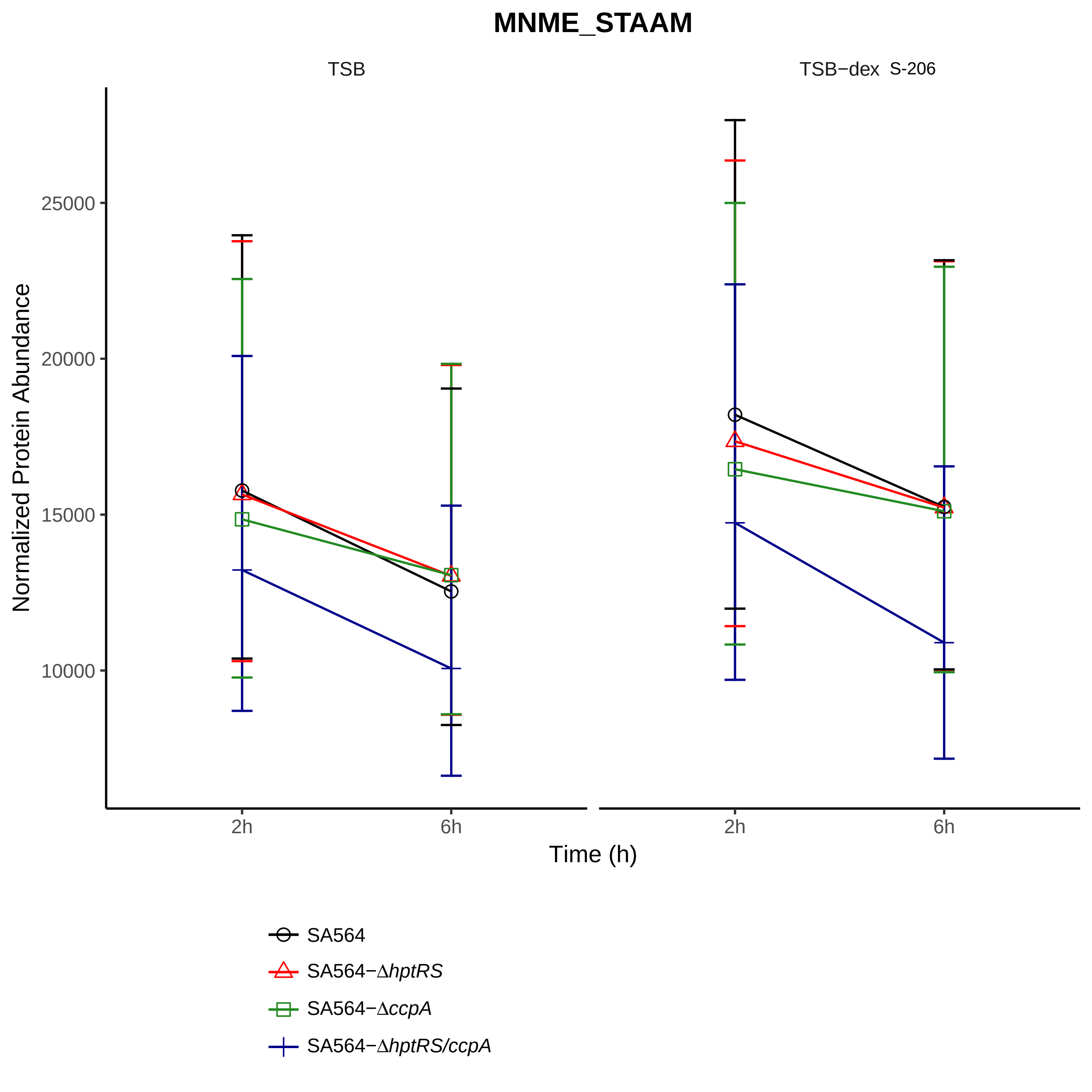

MNME_STAAM
TSB
TSB−dex
S-206
25000
20000
15000
10000
2h
6h
Normalized Protein Abundance
Time (h)
SA564
SA564−ΔhptRS
SA564−ΔccpA
SA564−ΔhptRS/ccpA

MNMG_STAAM

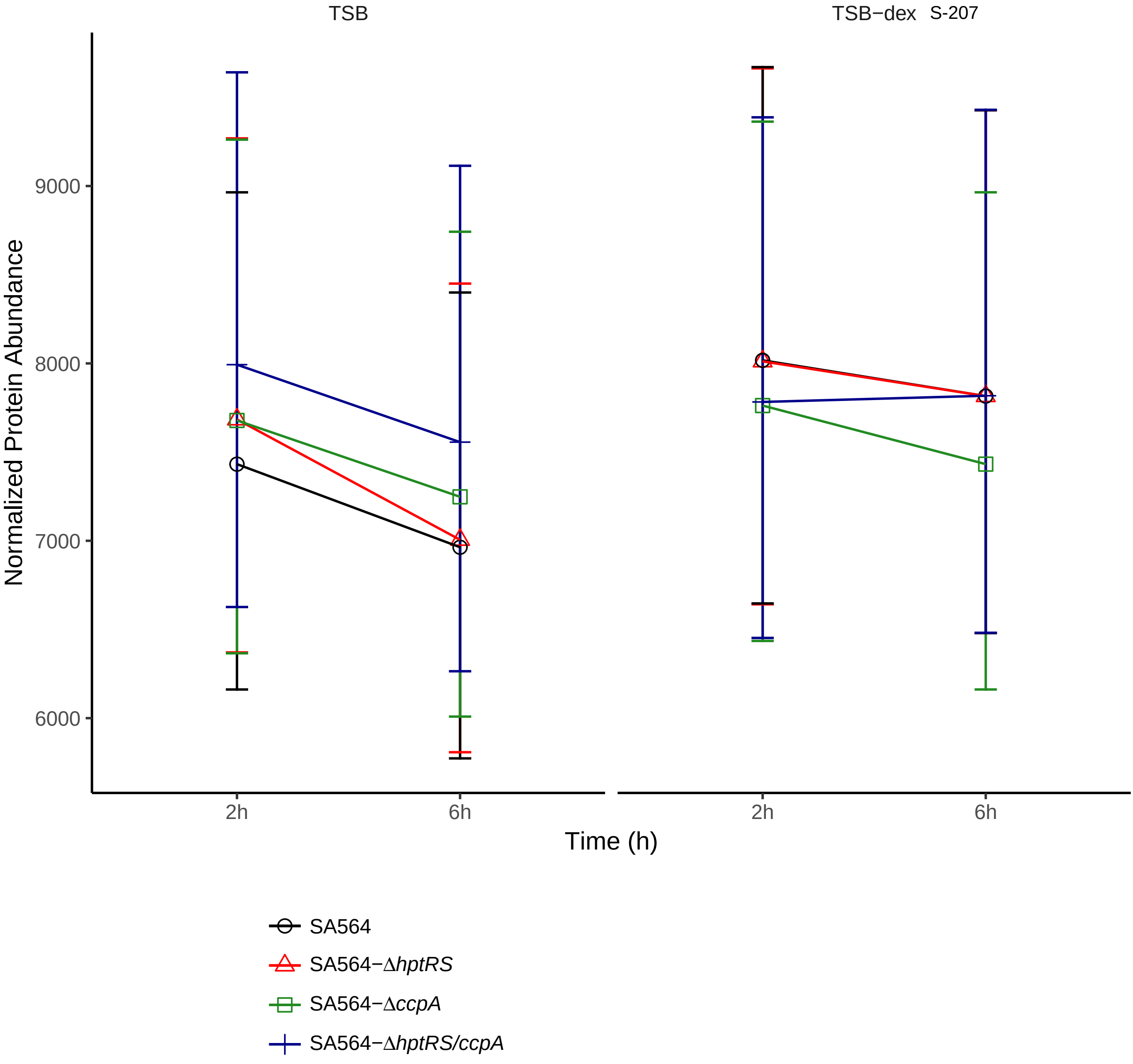

TSB
TSB−dex
S-207
Normalized Protein Abundance
9000
8000
7000
6000
2h
6h
2h
6h
Time (h)
SA564
SA564−Δ*hptRS*
SA564−Δ*ccpA*
SA564−Δ*hptRS/ccpA*

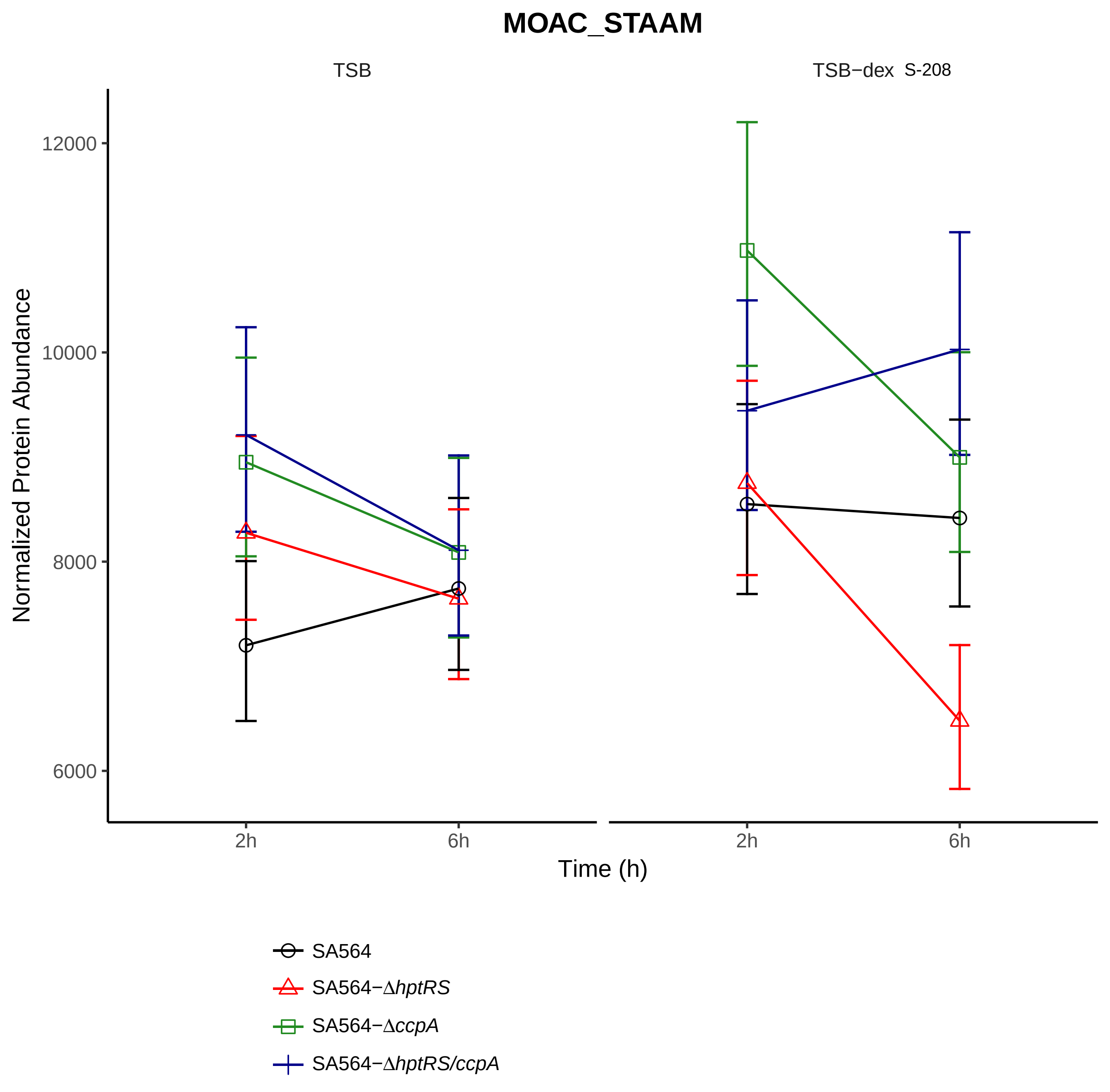
MOAC_STAAM
TSB
TSB−dex
S-208
Normalized Protein Abundance
12000
10000
8000
6000
2h
6h
2h
6h
Time (h)
SA564
SA564−ΔhptRS
SA564−ΔccpA
SA564−ΔhptRS/ccpA

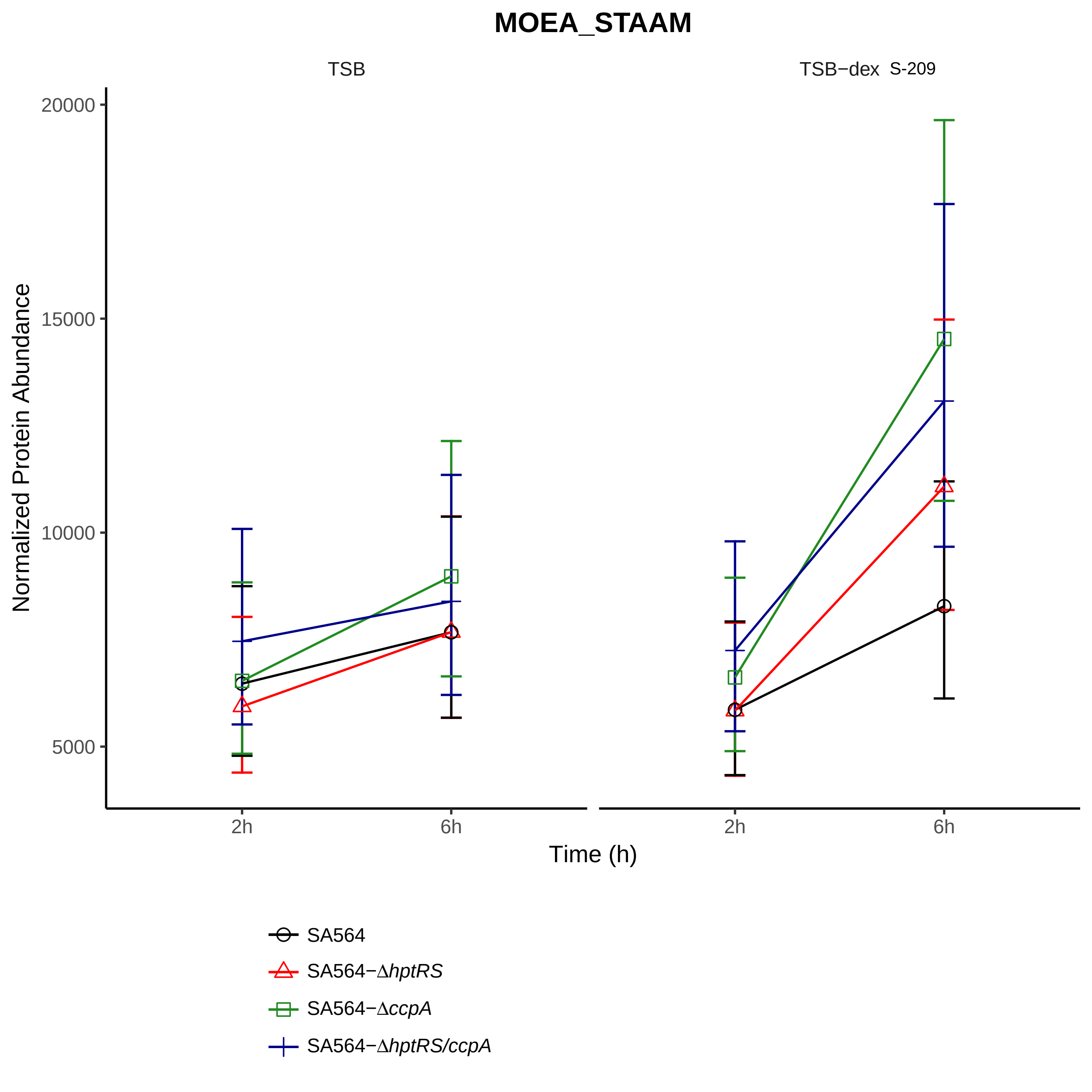

MOEA_STAAM
TSB
TSB−dex
S-209
Normalized Protein Abundance
20000
15000
10000
5000
2h
6h
2h
6h
Time (h)
SA564
SA564−ΔhptRS
SA564−ΔccpA
SA564−ΔhptRS/ccpA

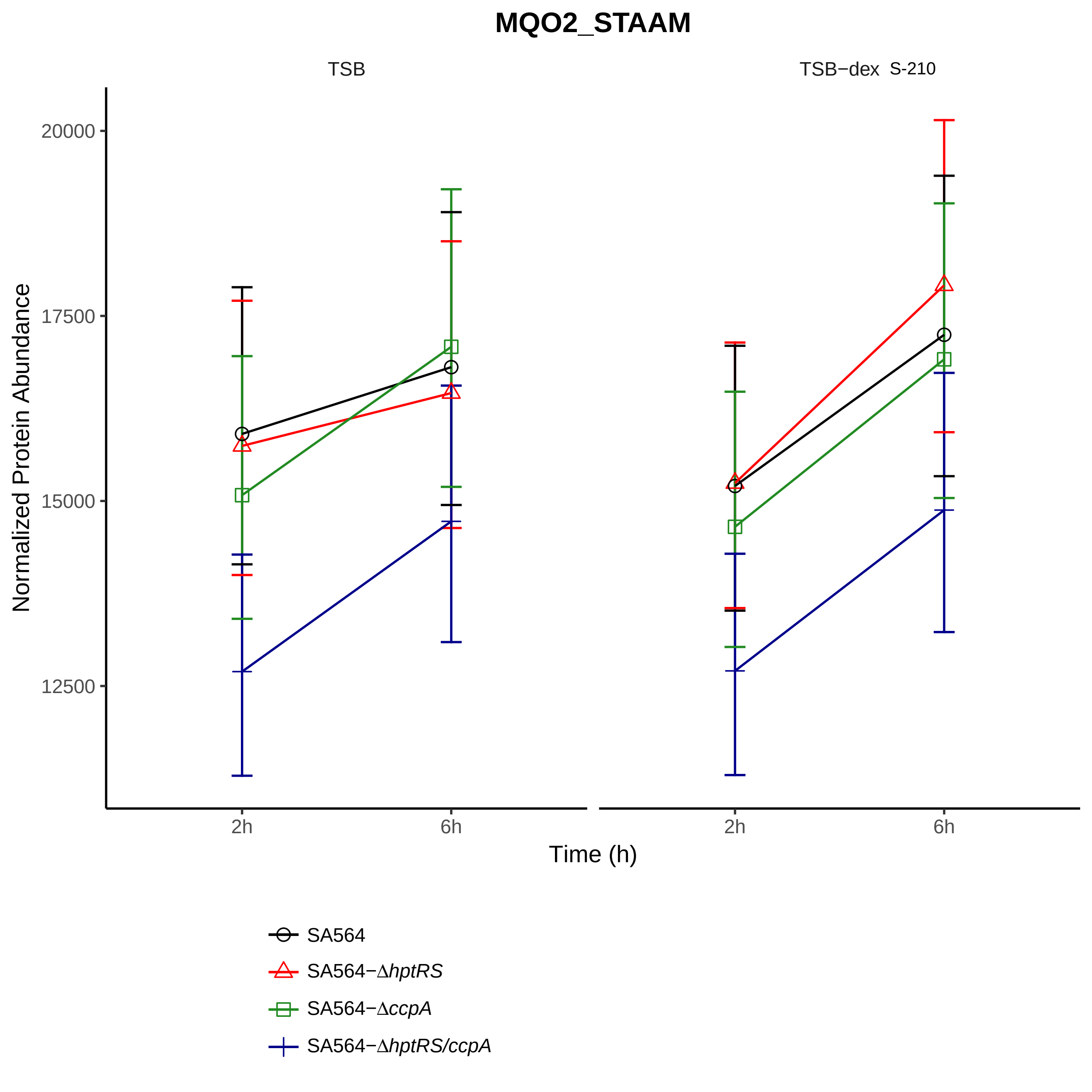

MQO2_STAAM
TSB
TSB−dex
S-210
Normalized Protein Abundance
20000
17500
15000
12500
2h
6h
2h
6h
Time (h)
SA564
SA564−Δ*hptRS*
SA564−Δ*ccpA*
SA564−Δ*hptRS/ccpA*

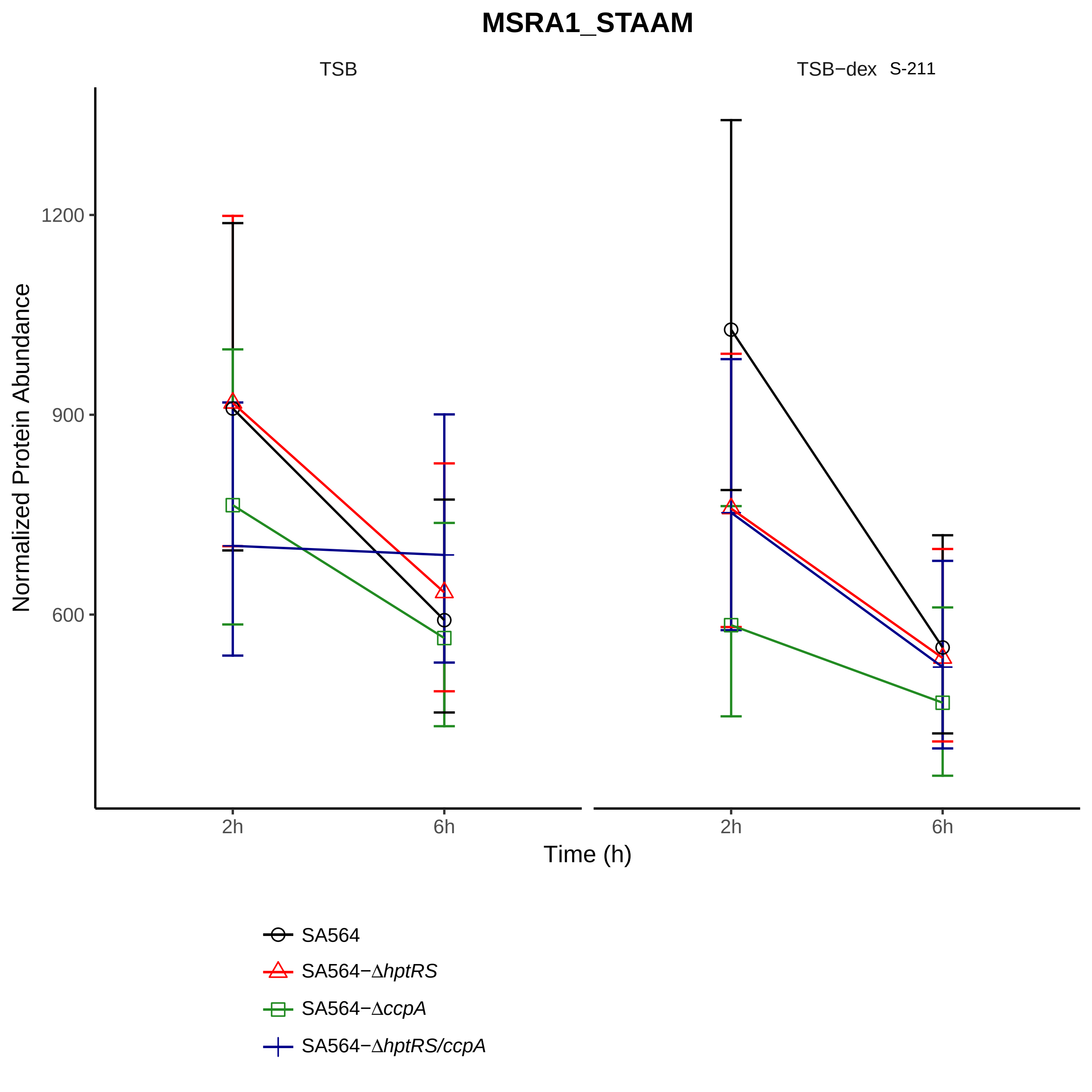

MSRA1_STAAM
TSB
TSB−dex
S-211
Normalized Protein Abundance
1200
900
600
2h
6h
2h
6h
Time (h)
SA564
SA564−ΔhptRS
SA564−ΔccpA
SA564−ΔhptRS/ccpA

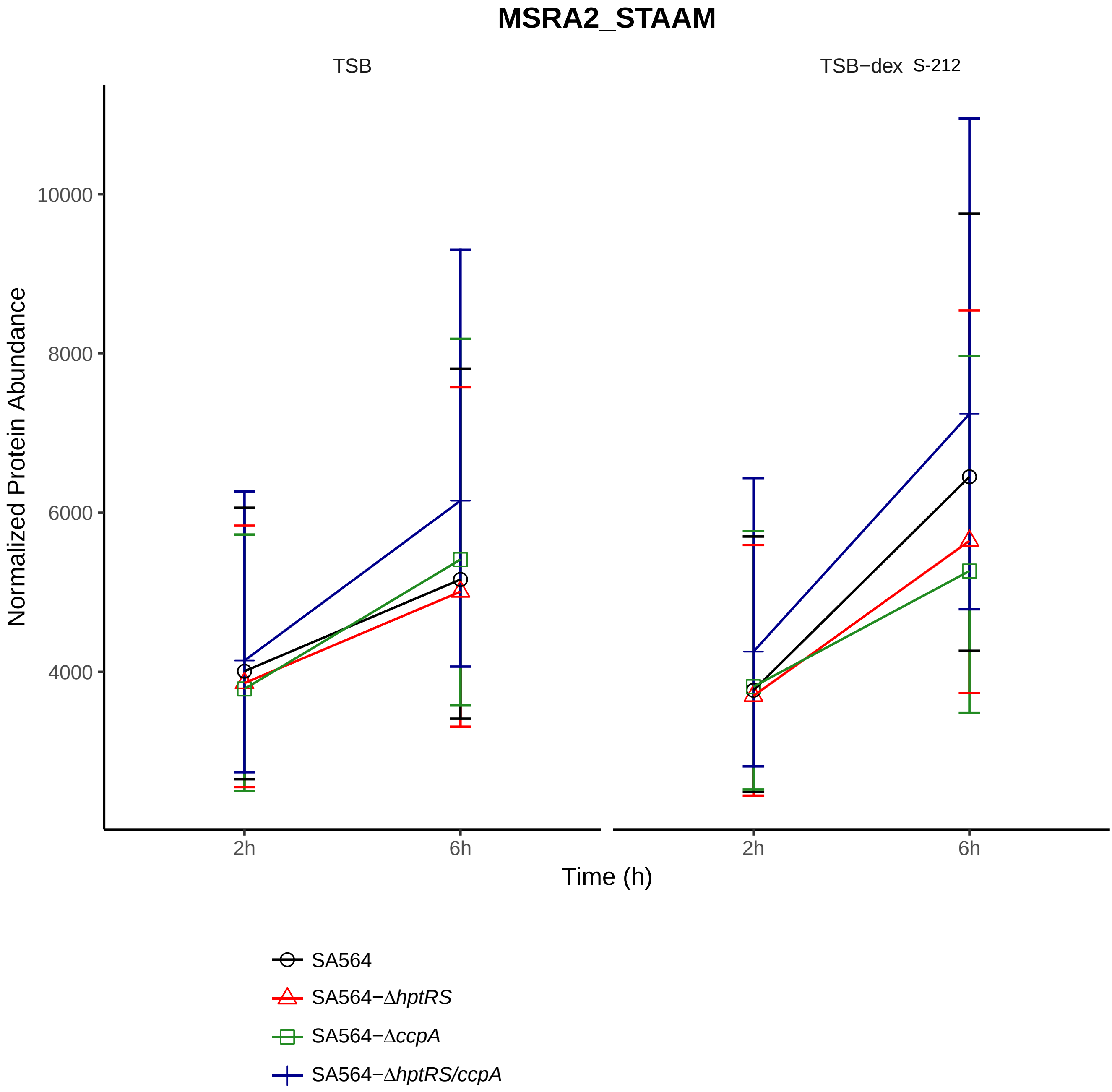

MSRA2_STAAM
TSB
TSB−dex
Normalized Protein Abundance
10000
8000
6000
4000
2h
6h
2h
6h
Time (h)
SA564
SA564−Δ*hptRS*
SA564−Δ*ccpA*
SA564−Δ*hptRS/ccpA*

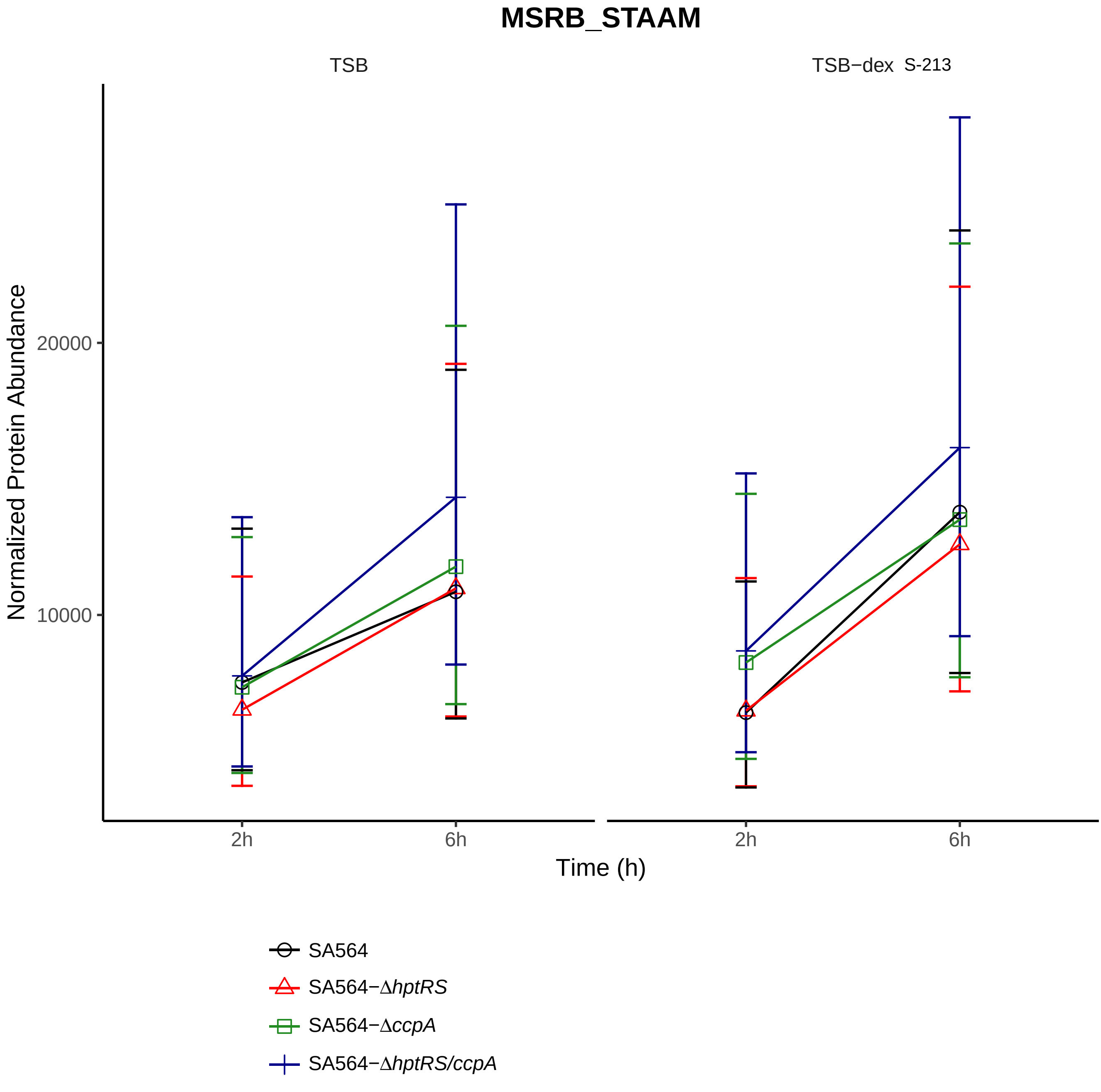

MSRB_STAAM
TSB
TSB−dex
S-213
Normalized Protein Abundance
20000
10000
2h
6h
2h
6h
Time (h)
SA564
SA564−ΔhptRS
SA564−ΔccpA
SA564−ΔhptRS/ccpA

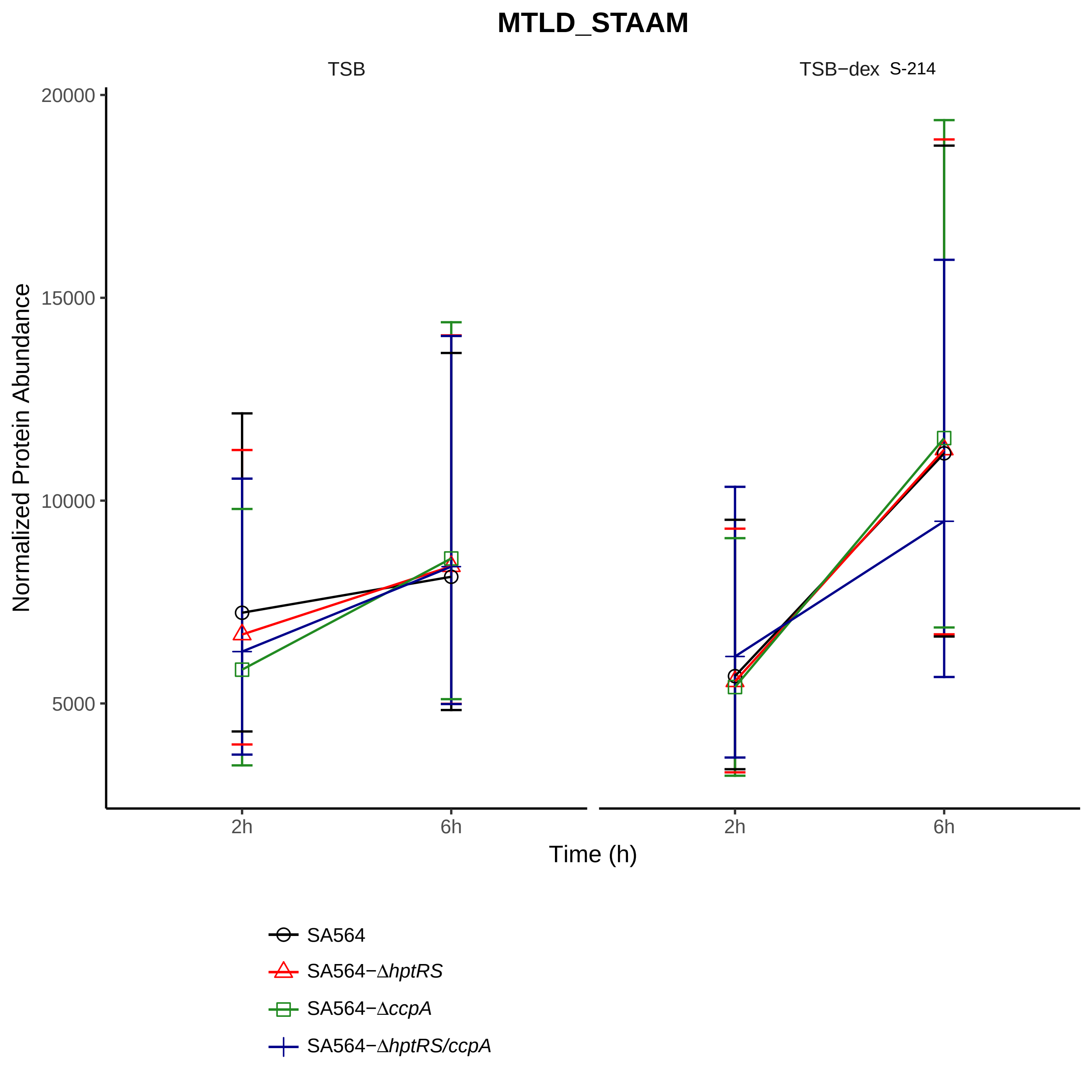

MTLD_STAAM
TSB
TSB−dex
S-214
20000
15000
10000
5000
Normalized Protein Abundance
2h
6h
2h
6h
Time (h)
SA564
SA564−ΔhptRS
SA564−ΔccpA
SA564−ΔhptRS/ccpA

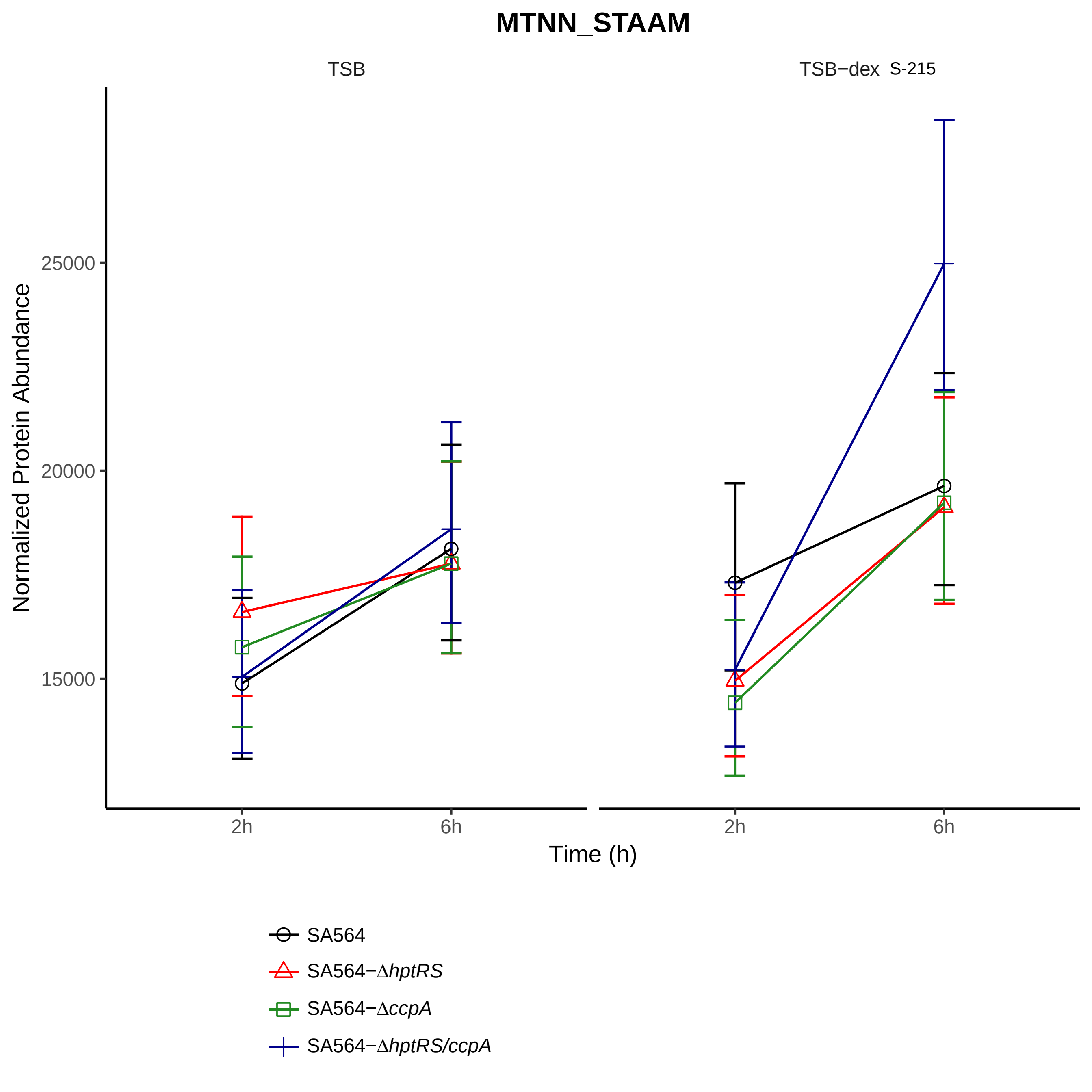

MTNN_STAAM
TSB
TSB−dex
S-215
Normalized Protein Abundance
25000
20000
15000
2h
6h
2h
6h
Time (h)
SA564
SA564−Δ*hptRS*
SA564−Δ*ccpA*
SA564−Δ*hptRS/ccpA*

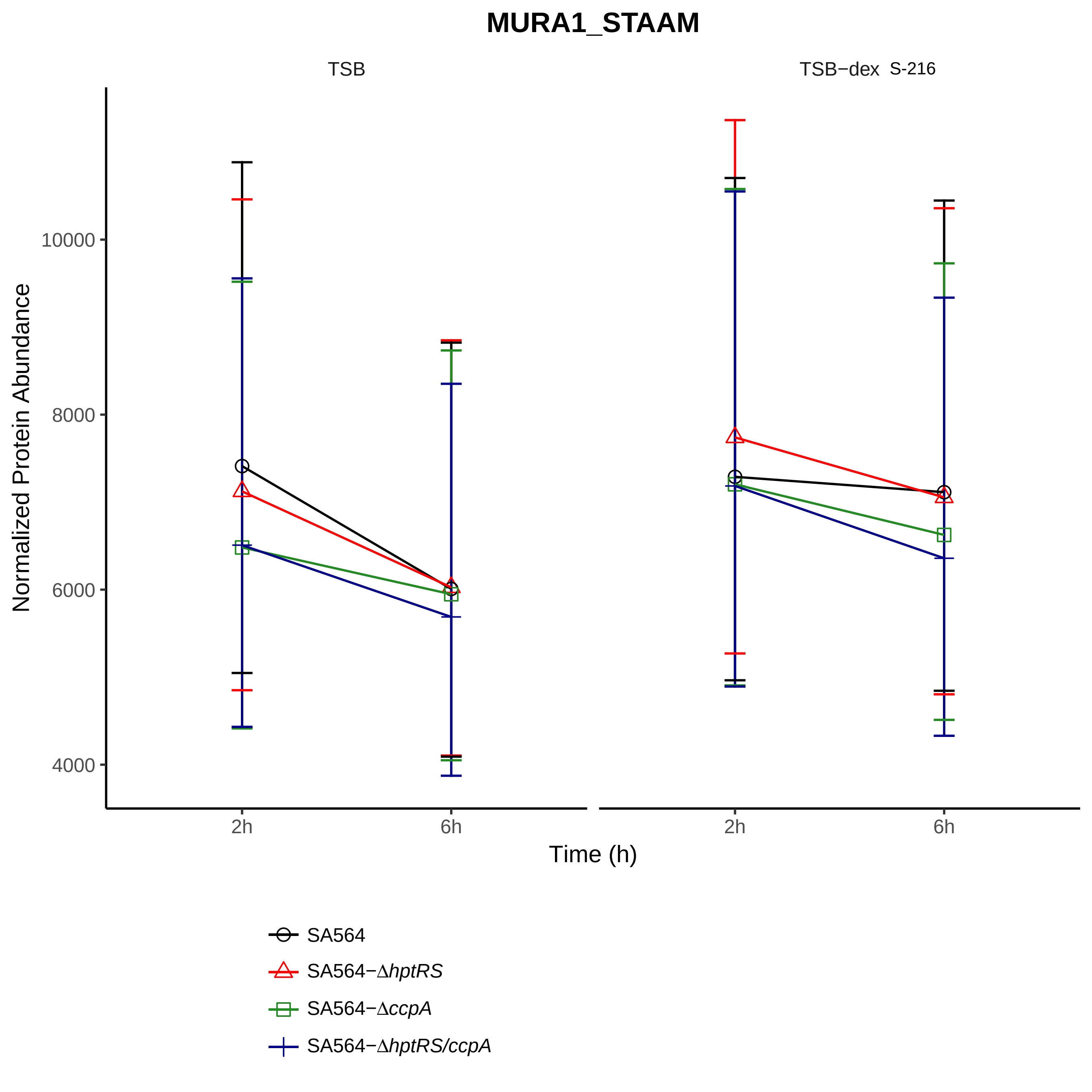

MURA1_STAAM
TSB
TSB−dex
S-216
Normalized Protein Abundance
10000
8000
6000
4000
2h
6h
2h
6h
Time (h)
SA564
SA564−Δ*hptRS*
SA564−Δ*ccpA*
SA564−Δ*hptRS/ccpA*

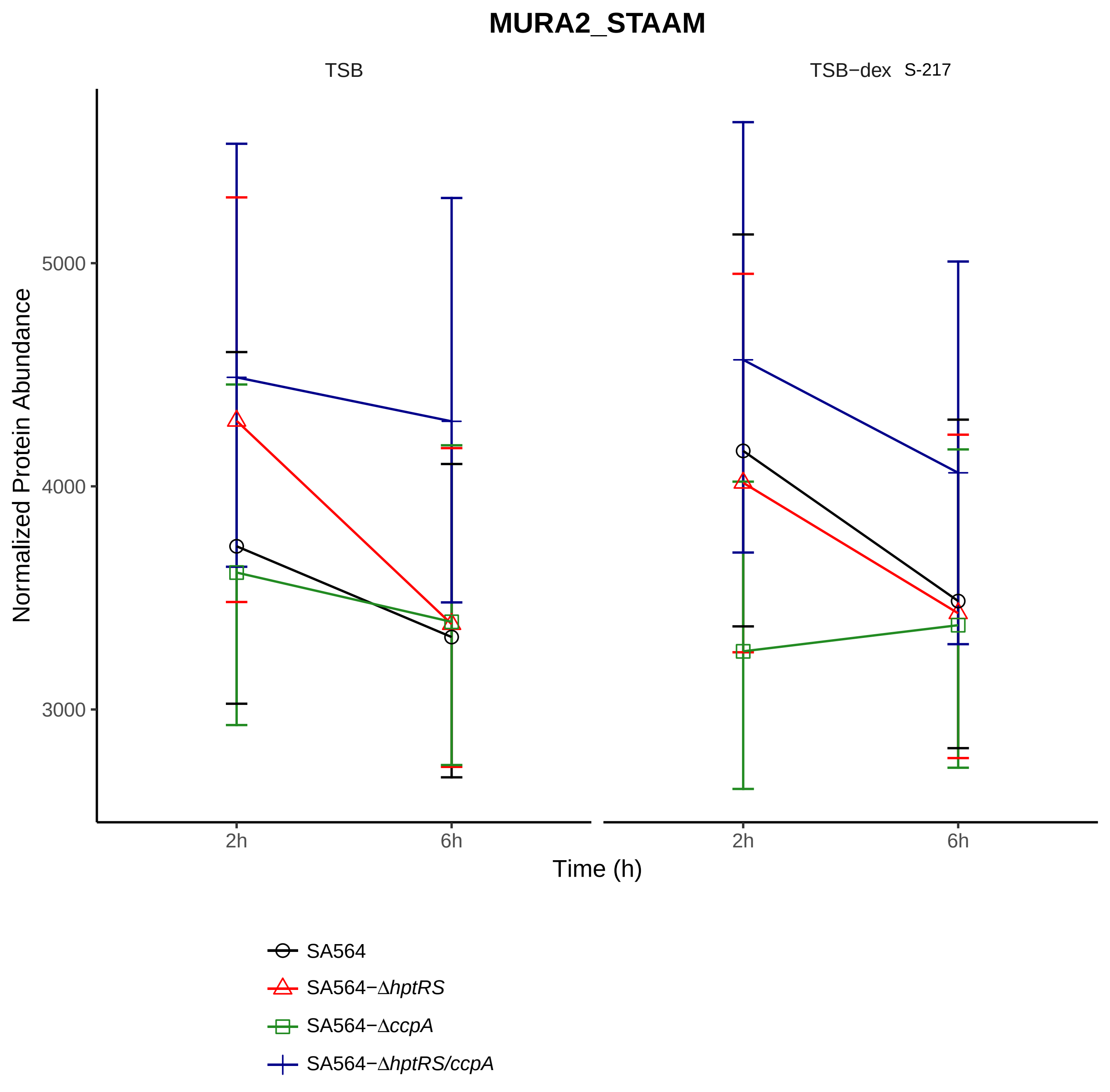

MURA2_STAAM
TSB
TSB−dex
S-217
Normalized Protein Abundance
5000
4000
3000
2h
6h
2h
6h
Time (h)
SA564
SA564−ΔhptRS
SA564−ΔccpA
SA564−ΔhptRS/ccpA

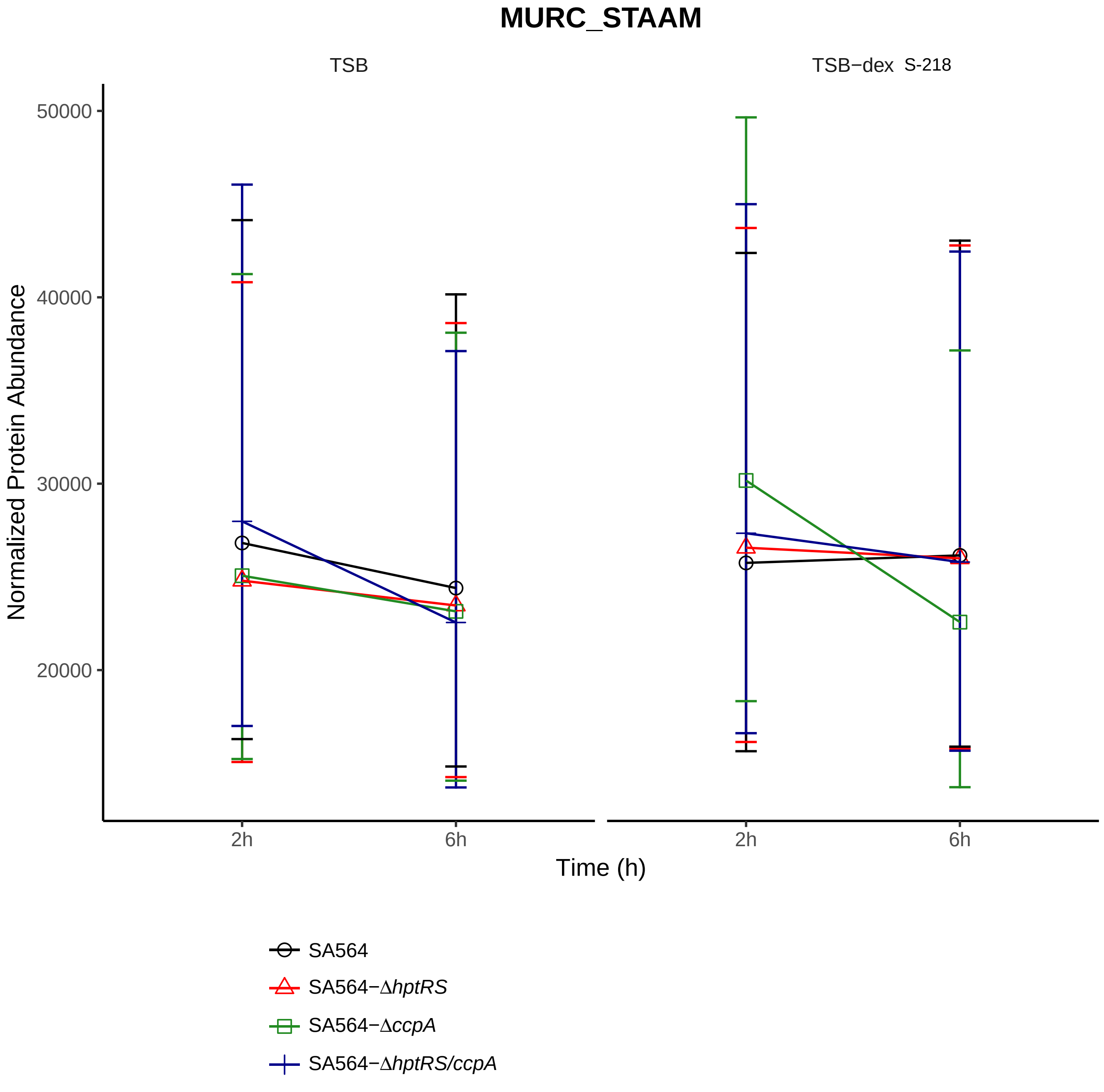
MURC_STAAM
TSB
TSB−dex
S-218
Normalized Protein Abundance
50000
40000
30000
20000
2h
6h
2h
6h
Time (h)
SA564
SA564−Δ*hptRS*
SA564−Δ*ccpA*
SA564−Δ*hptRS/ccpA*

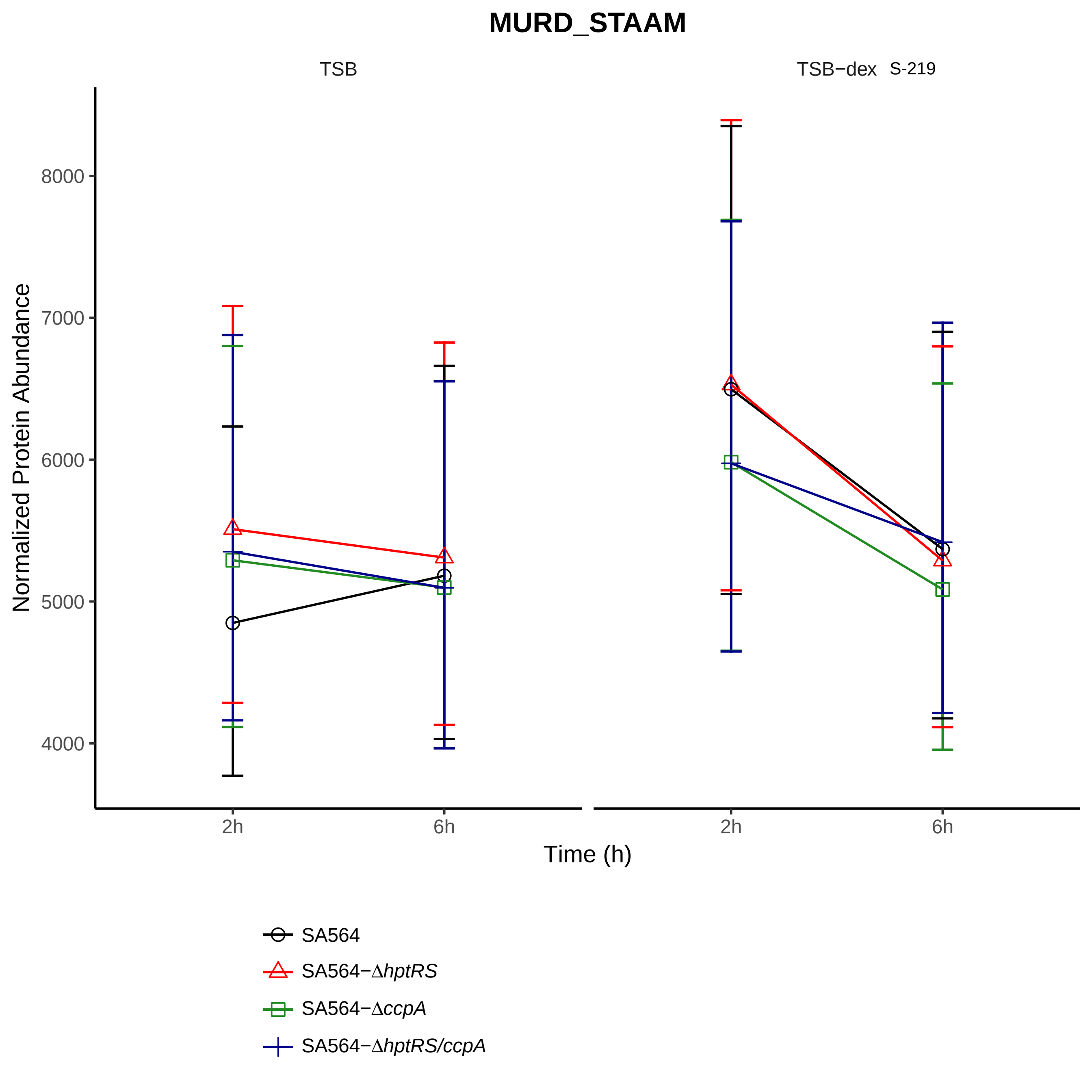
MURD_STAAM
TSB
TSB−dex
S-219
Normalized Protein Abundance
8000
7000
6000
5000
4000
2h
6h
2h
6h
Time (h)
SA564
SA564−ΔhptRS
SA564−ΔccpA
SA564−ΔhptRS/ccpA

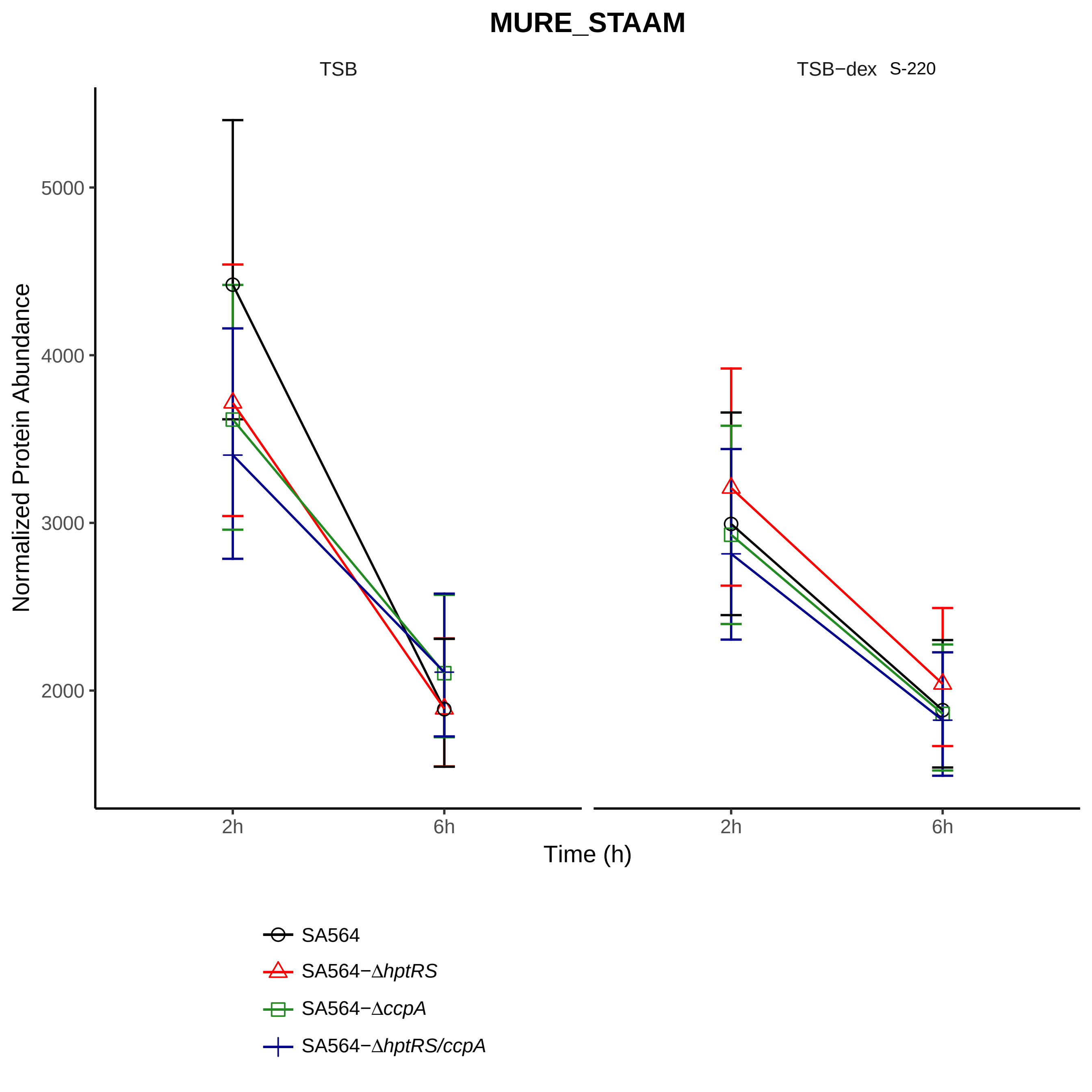
MURE_STAAM
TSB
TSB−dex
S-220
Normalized Protein Abundance
5000
4000
3000
2000
2h
6h
2h
6h
Time (h)
SA564
SA564−Δ*hptRS*
SA564−Δ*ccpA*
SA564−Δ*hptRS/ccpA*

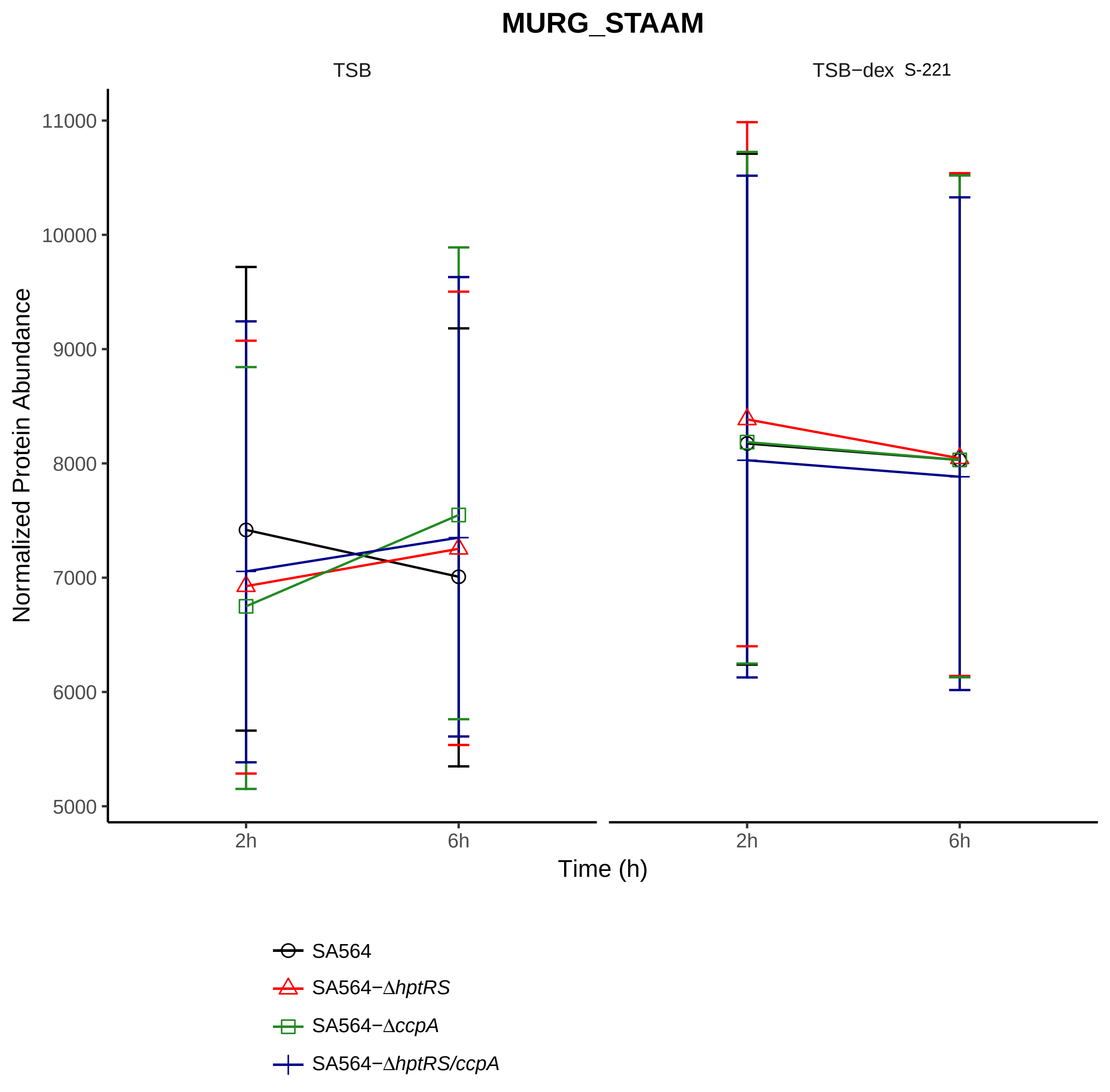

MURG_STAAM
TSB
TSB−dex
S-221
Normalized Protein Abundance
11000
10000
9000
8000
7000
6000
5000
2h
6h
2h
6h
Time (h)
SA564
SA564−Δ*hptRS*
SA564−Δ*ccpA*
SA564−Δ*hptRS/ccpA*

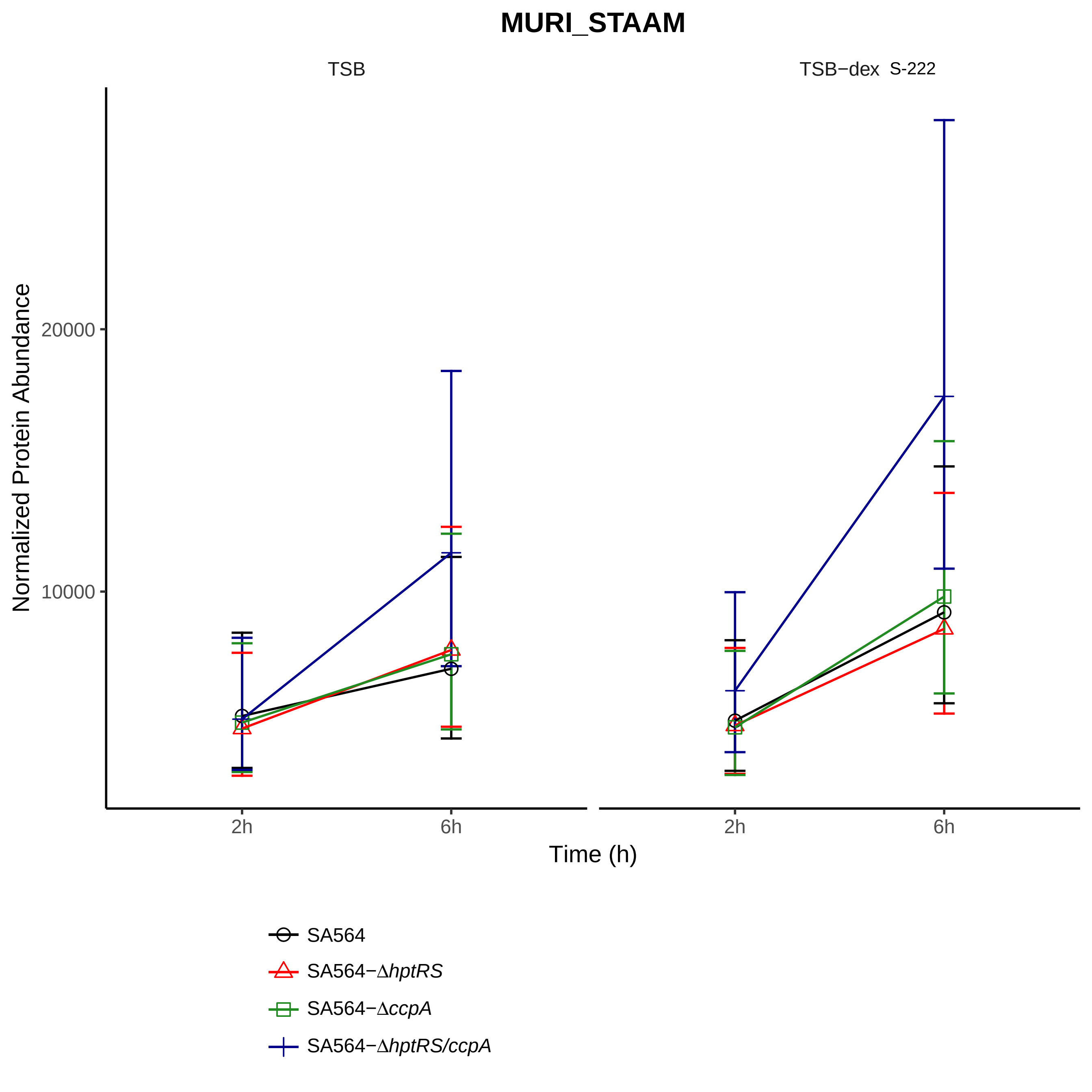

MURI_STAAM
TSB
TSB−dex
S-222
Normalized Protein Abundance
20000
10000
2h
6h
2h
6h
Time (h)
SA564
SA564−Δ*hptRS*
SA564−Δ*ccpA*
SA564−Δ*hptRS/ccpA*

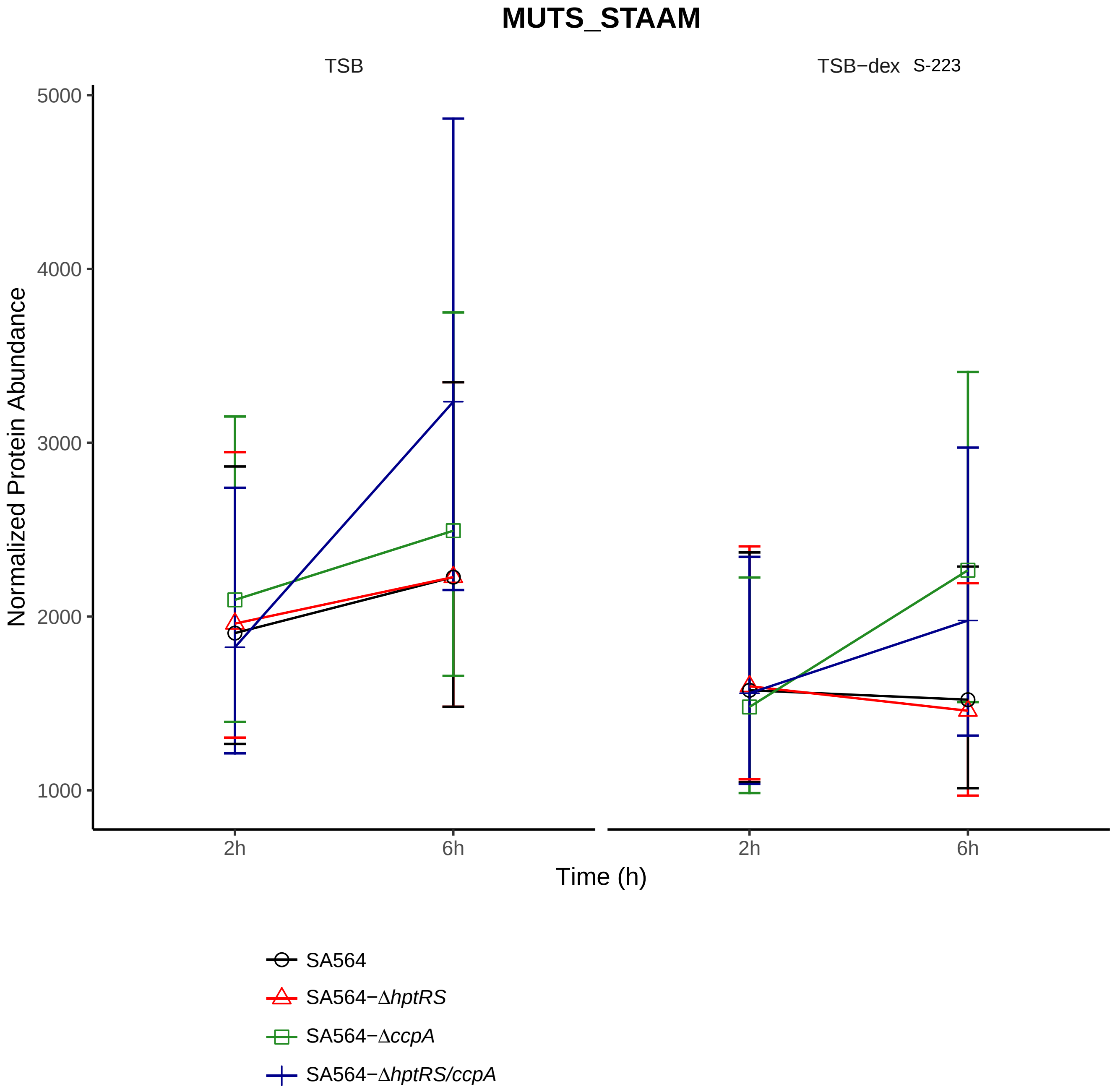

MUTS_STAAM
TSB
TSB−dex
S-223
Normalized Protein Abundance
5000
4000
3000
2000
1000
2h
6h
2h
6h
Time (h)
SA564
SA564−ΔhptRS
SA564−ΔccpA
SA564−ΔhptRS/ccpA

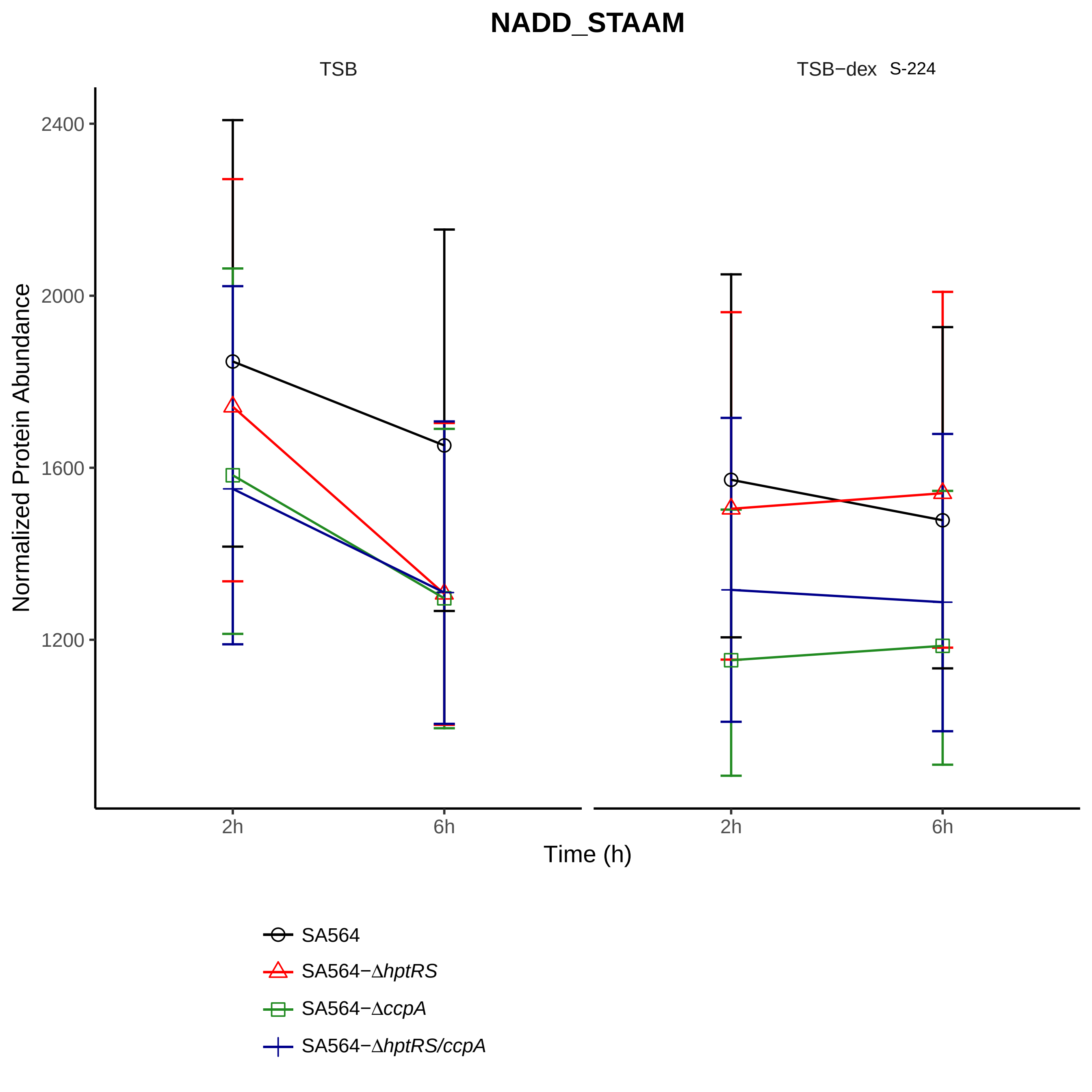

NADD_STAAM
TSB
TSB−dex
S-224
Normalized Protein Abundance
2400
2000
1600
1200
2h
6h
2h
6h
Time (h)
SA564
SA564−ΔhptRS
SA564−ΔccpA
SA564−ΔhptRS/ccpA

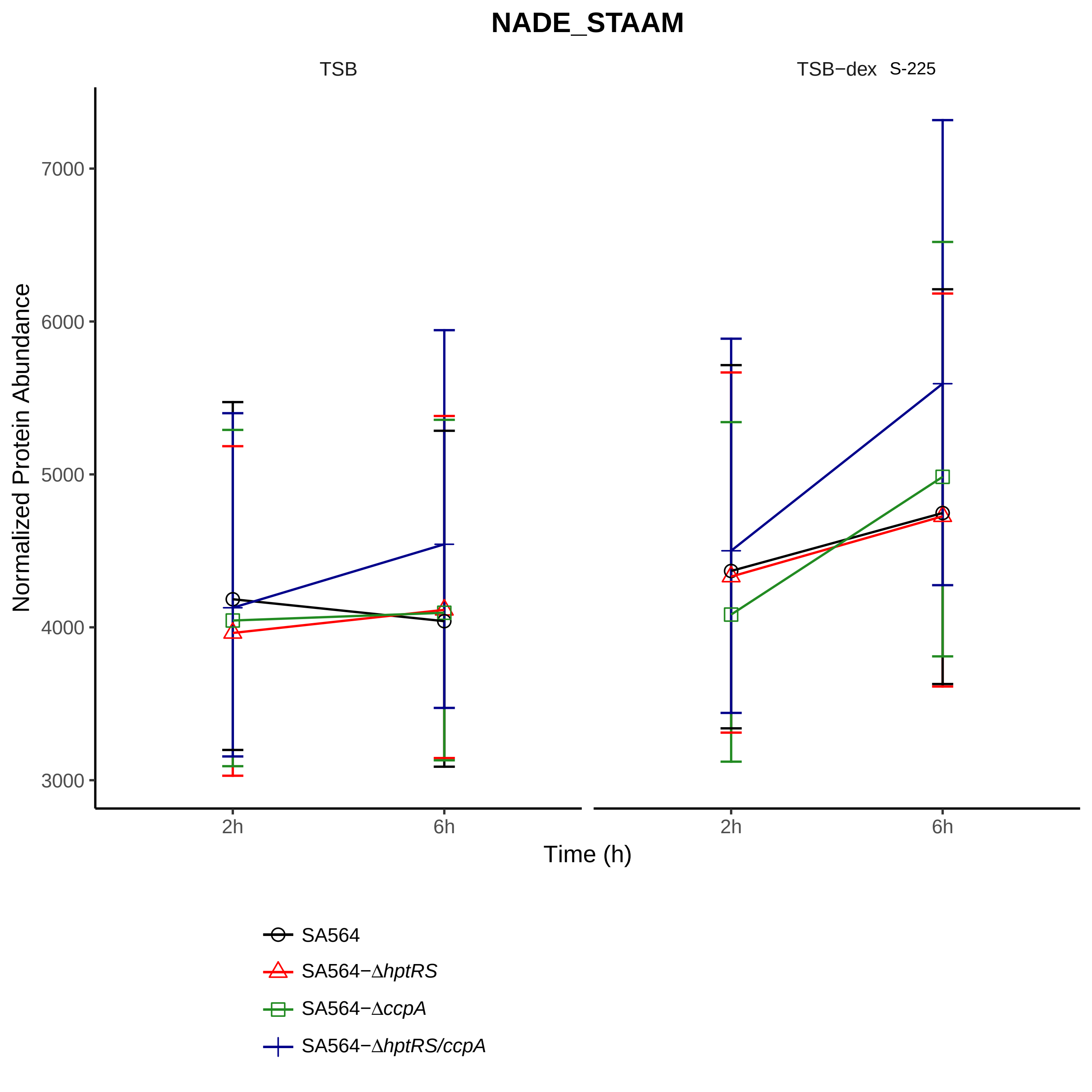

NADE_STAAM
TSB
TSB−dex
S-225
Normalized Protein Abundance
3000
4000
5000
6000
7000
2h
6h
2h
6h
Time (h)
SA564
SA564−Δ*hptRS*
SA564−Δ*ccpA*
SA564−Δ*hptRS/ccpA*

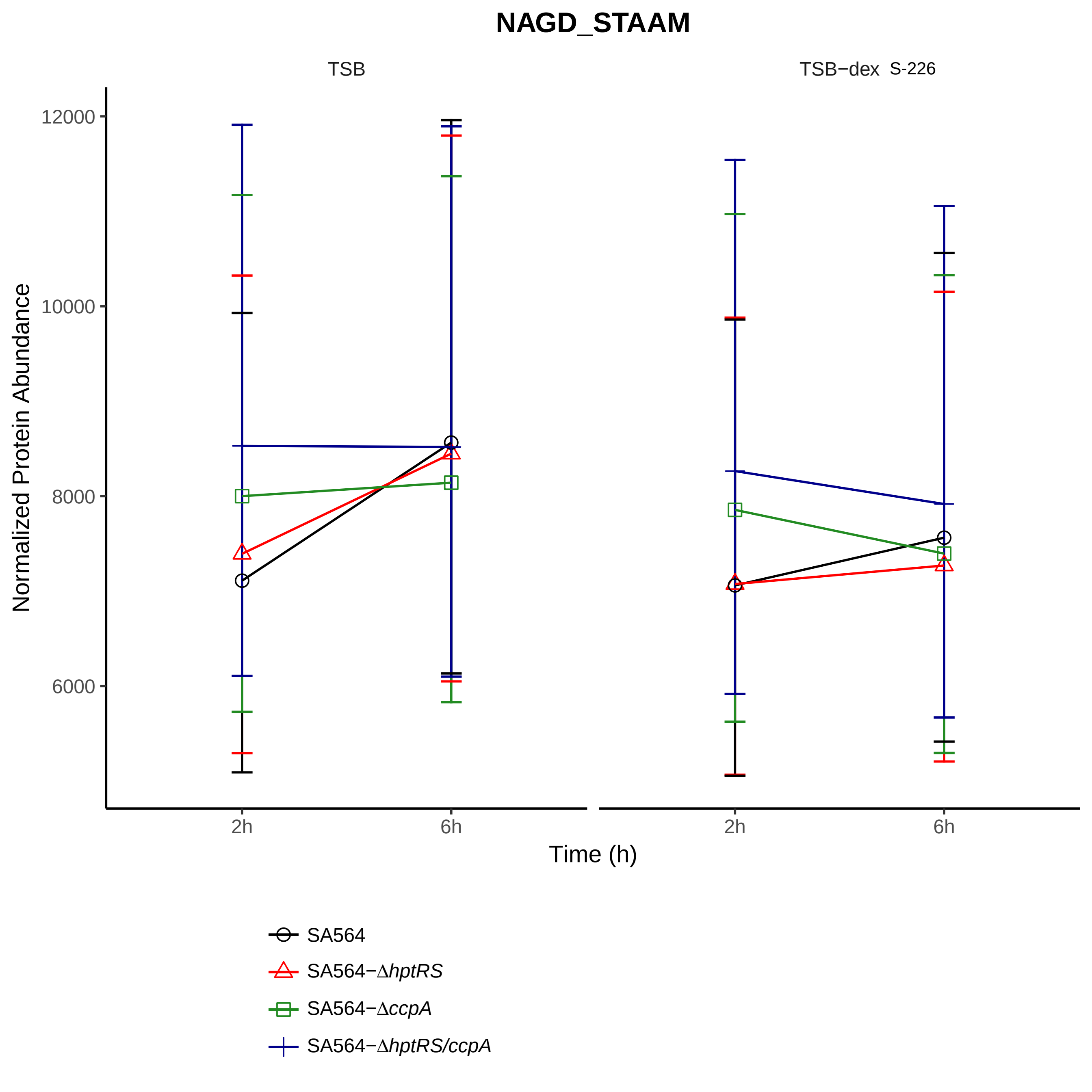

NAGD_STAAM
TSB
TSB−dex
S-226
12000
10000
8000
6000
Normalized Protein Abundance
2h
6h
2h
6h
Time (h)
SA564
SA564−Δ*hptRS*
SA564−Δ*ccpA*
SA564−Δ*hptRS/ccpA*

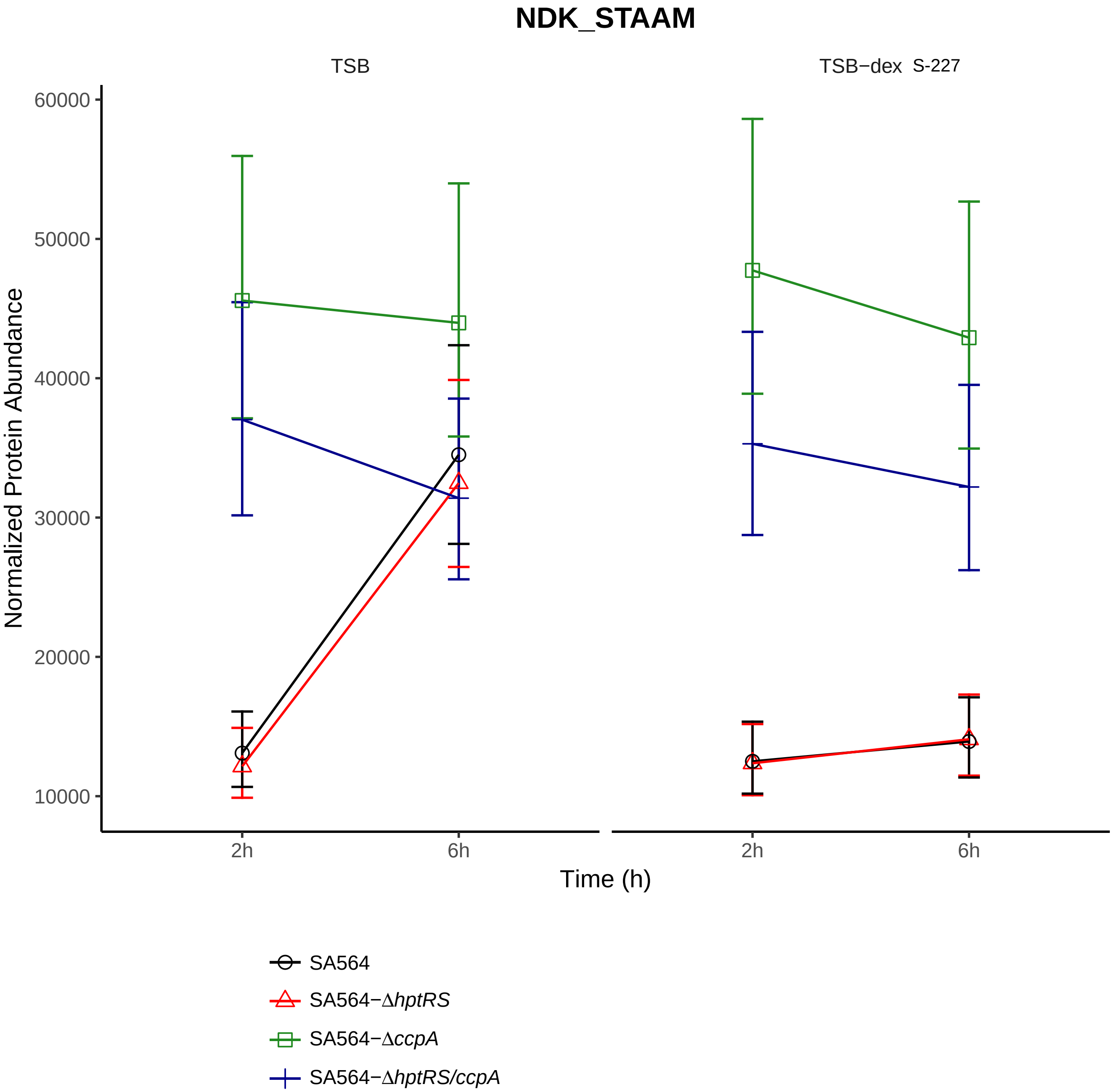

NDK_STAAM
TSB
TSB−dex
S-227
Normalized Protein Abundance
60000
50000
40000
30000
20000
10000
2h
6h
2h
6h
Time (h)
SA564
SA564−Δ*hptRS*
SA564−Δ*ccpA*
SA564−Δ*hptRS/ccpA*

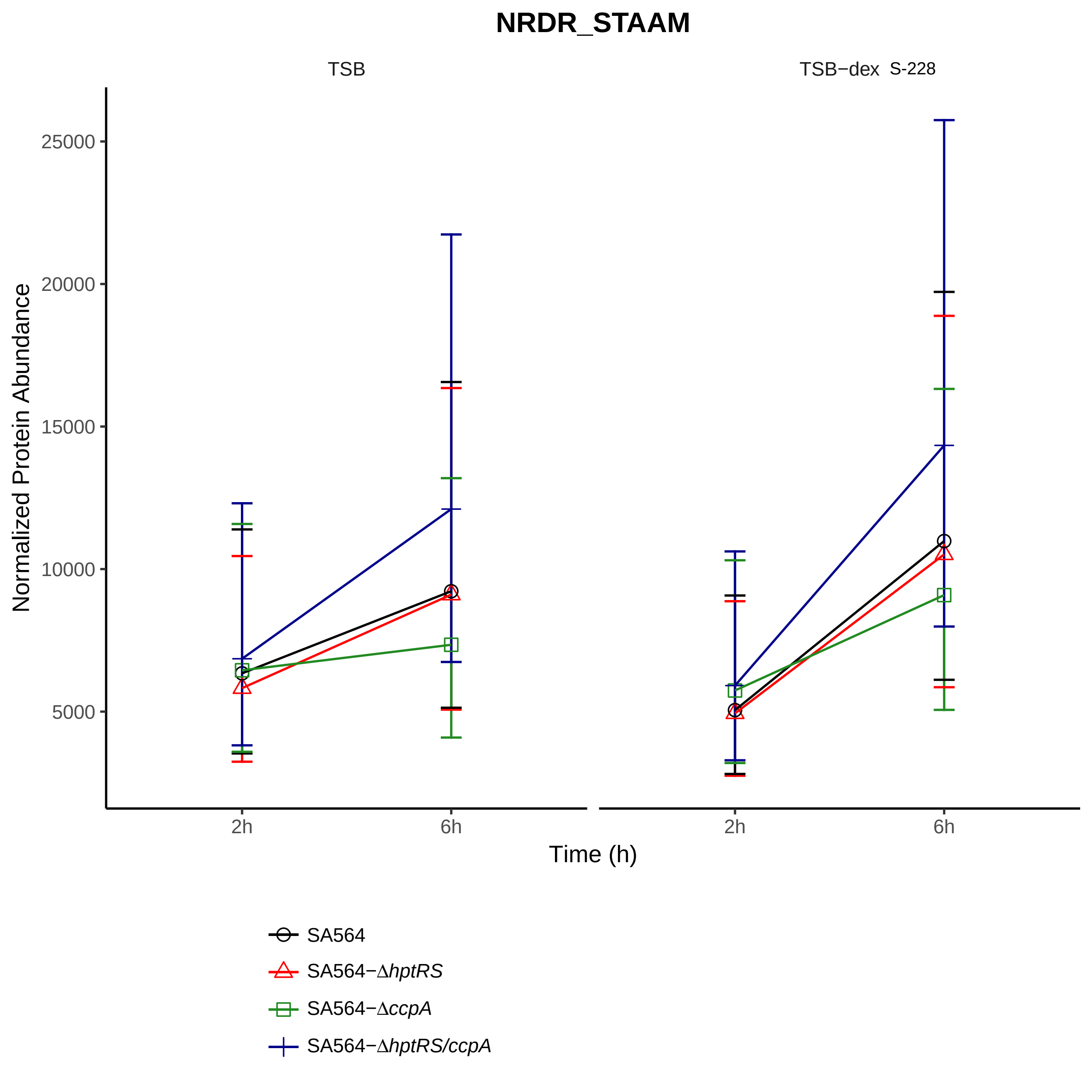

NRDR_STAAM
TSB
TSB−dex
S-228
Normalized Protein Abundance
25000
20000
15000
10000
5000
2h
6h
2h
6h
Time (h)
SA564
SA564−ΔhptRS
SA564−ΔccpA
SA564−ΔhptRS/ccpA

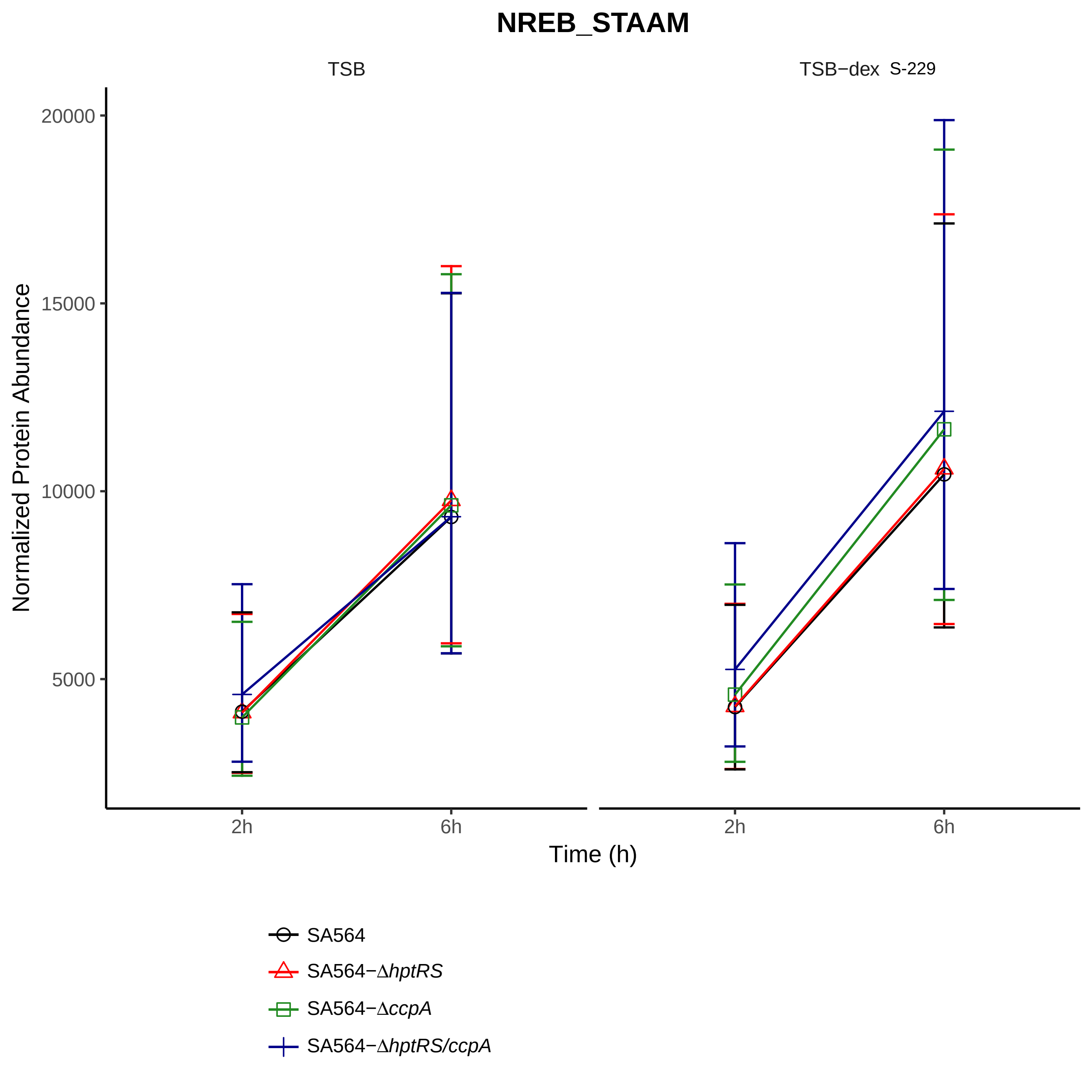

NREB_STAAM
TSB
TSB−dex
S-229
20000
15000
10000
5000
Normalized Protein Abundance
2h
6h
2h
6h
Time (h)
SA564
SA564−ΔhptRS
SA564−ΔccpA
SA564−ΔhptRS/ccpA

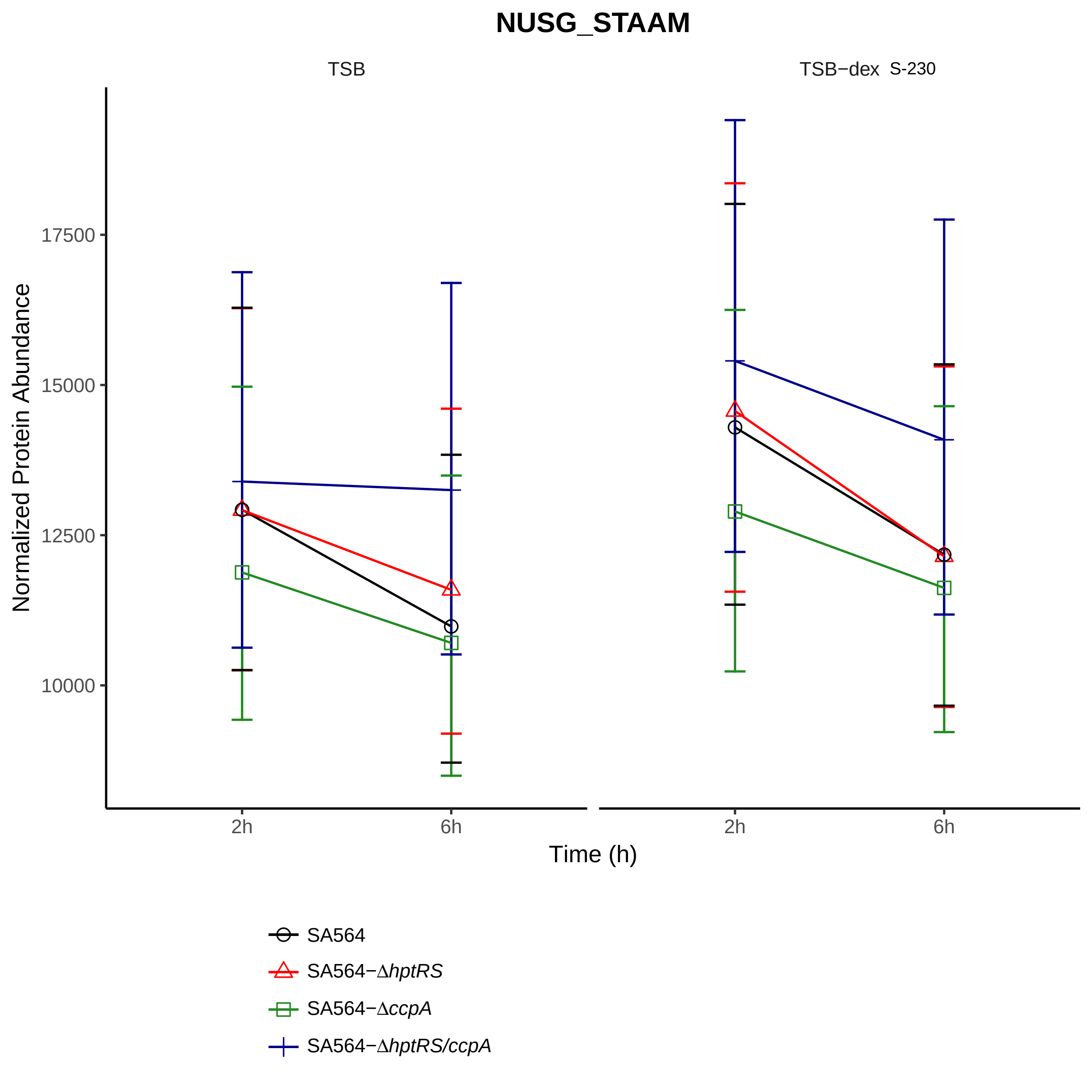

NUSG_STAAM
TSB
TSB−dex
S-230
Normalized Protein Abundance
17500
15000
12500
10000
2h
6h
2h
6h
Time (h)
SA564
SA564−ΔhptRS
SA564−ΔccpA
SA564−ΔhptRS/ccpA

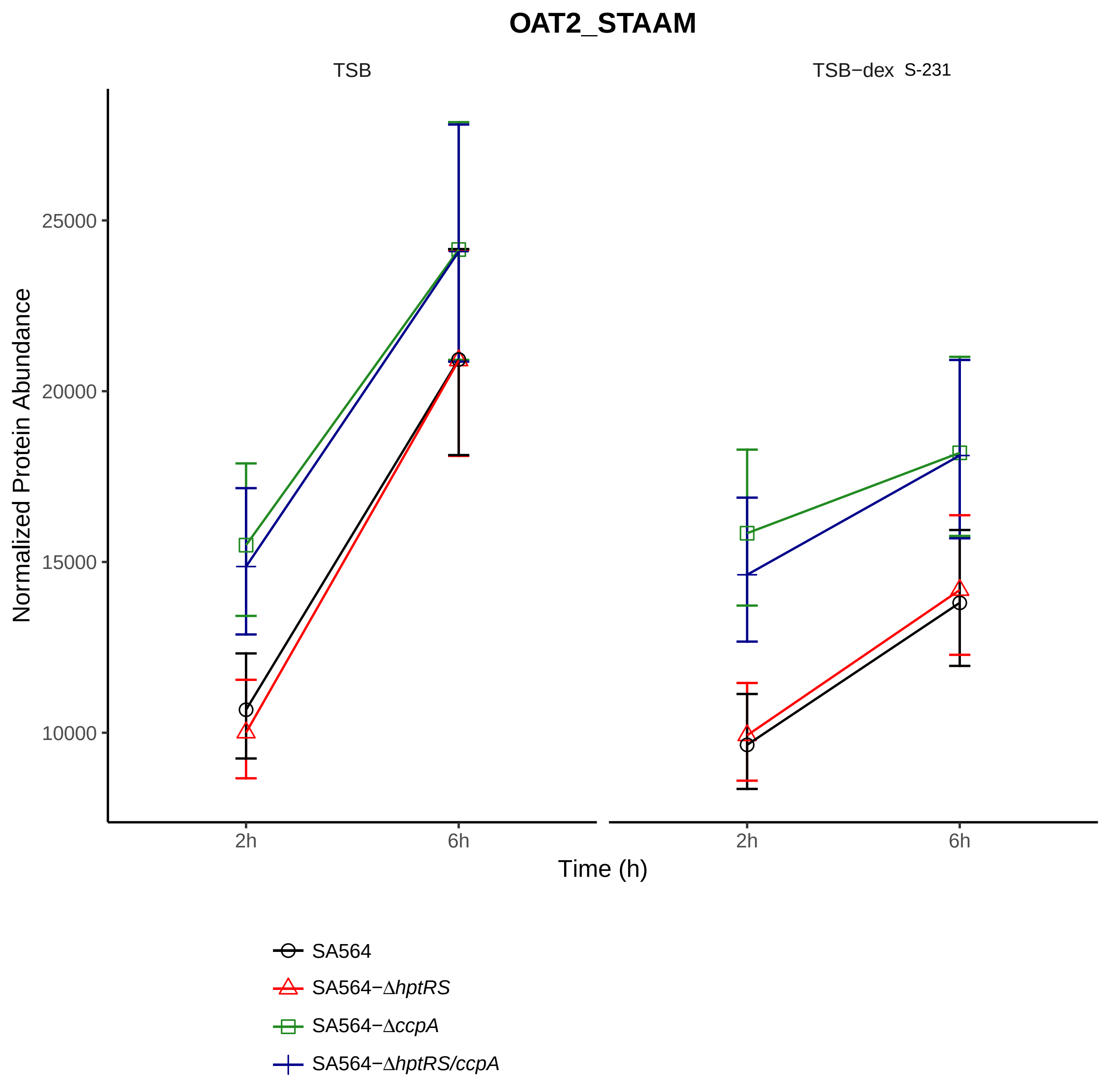
OAT2_STAAM
TSB
TSB−dex
S-231
Normalized Protein Abundance
25000
20000
15000
10000
2h
6h
2h
6h
Time (h)
SA564
SA564−Δ*hptRS*
SA564−Δ*ccpA*
SA564−Δ*hptRS/ccpA*

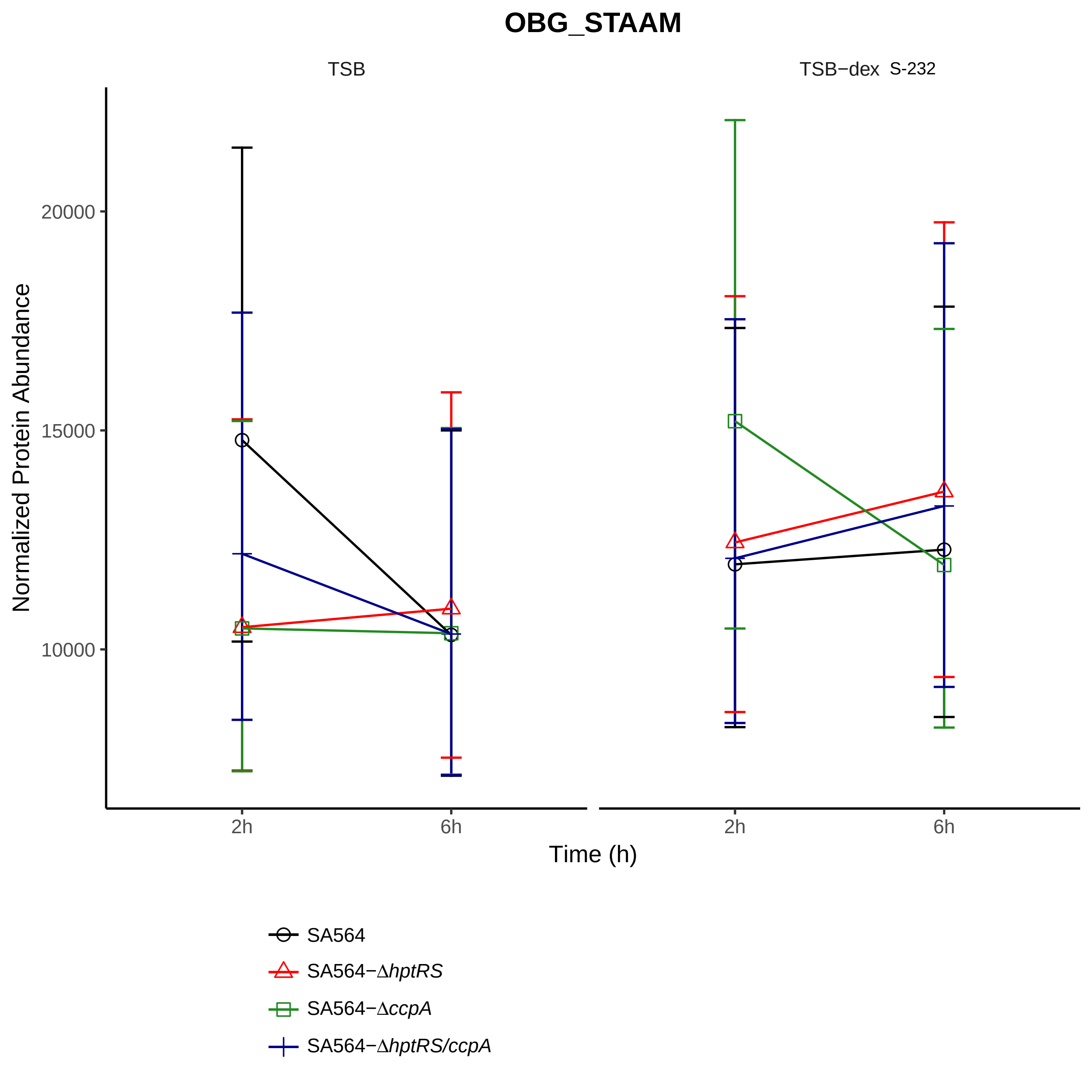

OBG_STAAM
TSB
TSB−dex
S-232
Normalized Protein Abundance
20000
15000
10000
2h
6h
2h
6h
Time (h)
SA564
SA564−Δ*hptRS*
SA564−Δ*ccpA*
SA564−Δ*hptRS/ccpA*

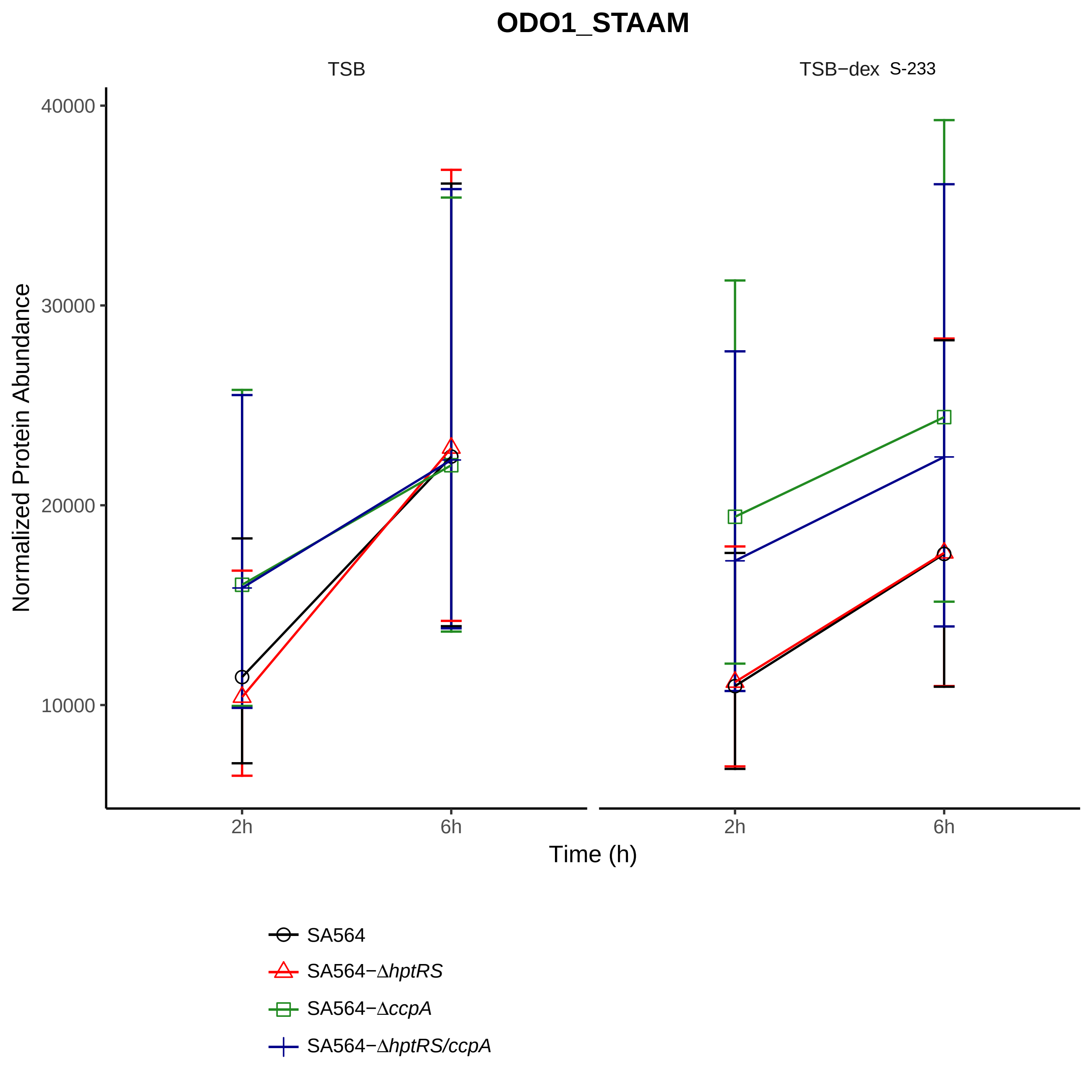

ODO1_STAAM
TSB
TSB−dex
S-233
Normalized Protein Abundance
40000
30000
20000
10000
2h
6h
2h
6h
Time (h)
SA564
SA564−ΔhptRS
SA564−ΔccpA
SA564−ΔhptRS/ccpA

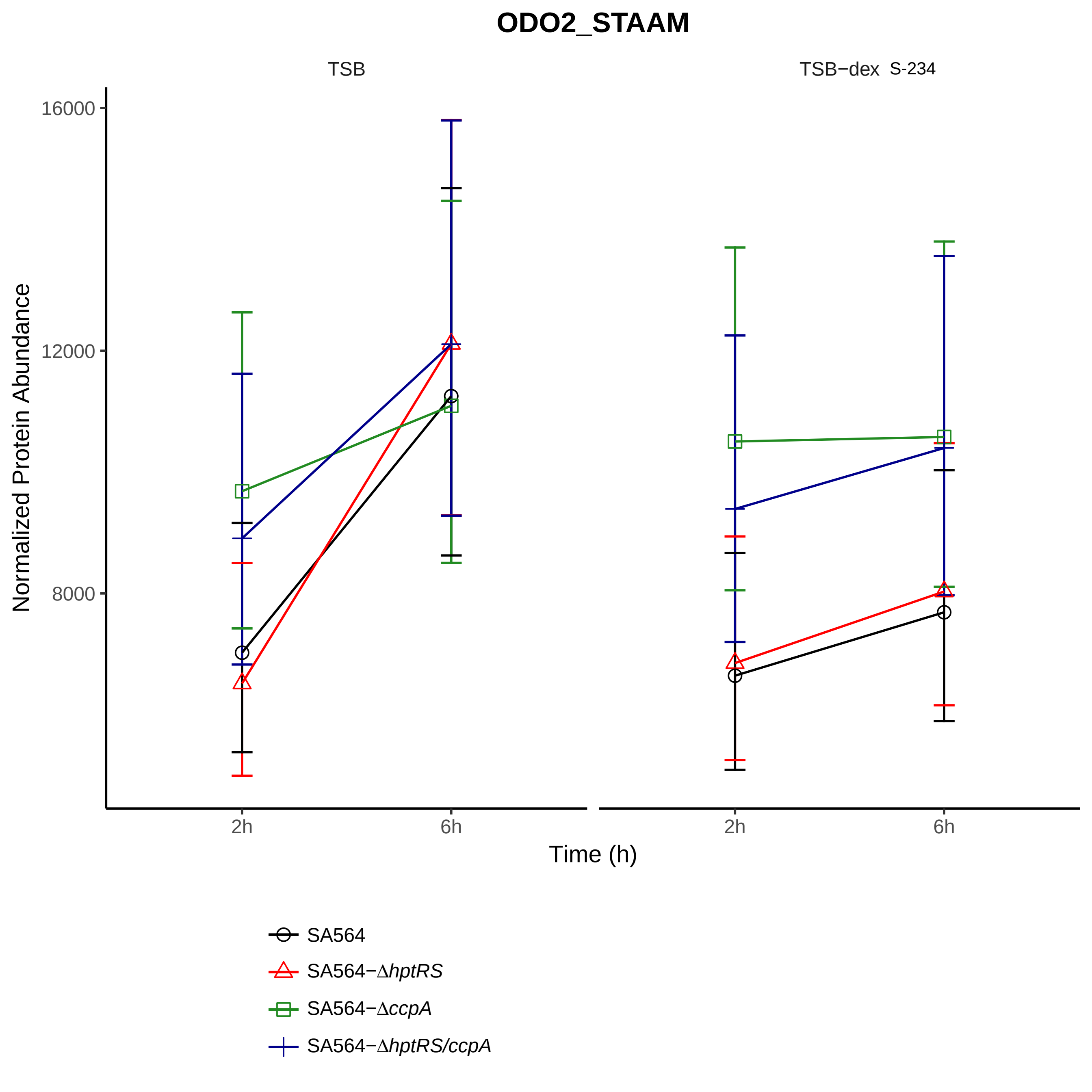
ODO2_STAAM
TSB
TSB−dex
S-234
16000
12000
8000
Normalized Protein Abundance
2h
6h
2h
6h
Time (h)
SA564
SA564−ΔhptRS
SA564−ΔccpA
SA564−ΔhptRS/ccpA

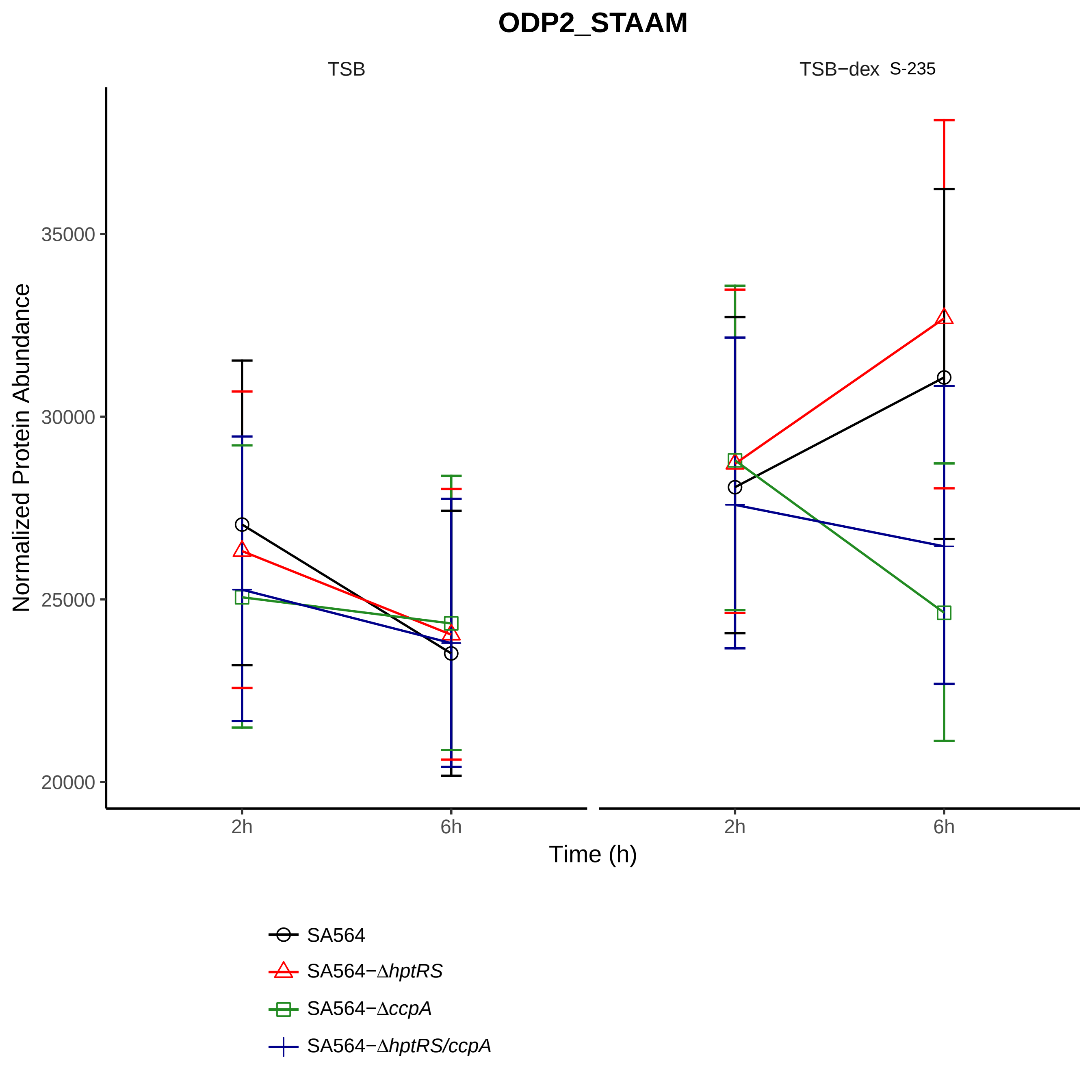

ODP2_STAAM
TSB
TSB−dex
S-235
35000
30000
25000
20000
Normalized Protein Abundance
2h
6h
2h
6h
Time (h)
SA564
SA564−Δ*hptRS*
SA564−Δ*ccpA*
SA564−Δ*hptRS/ccpA*

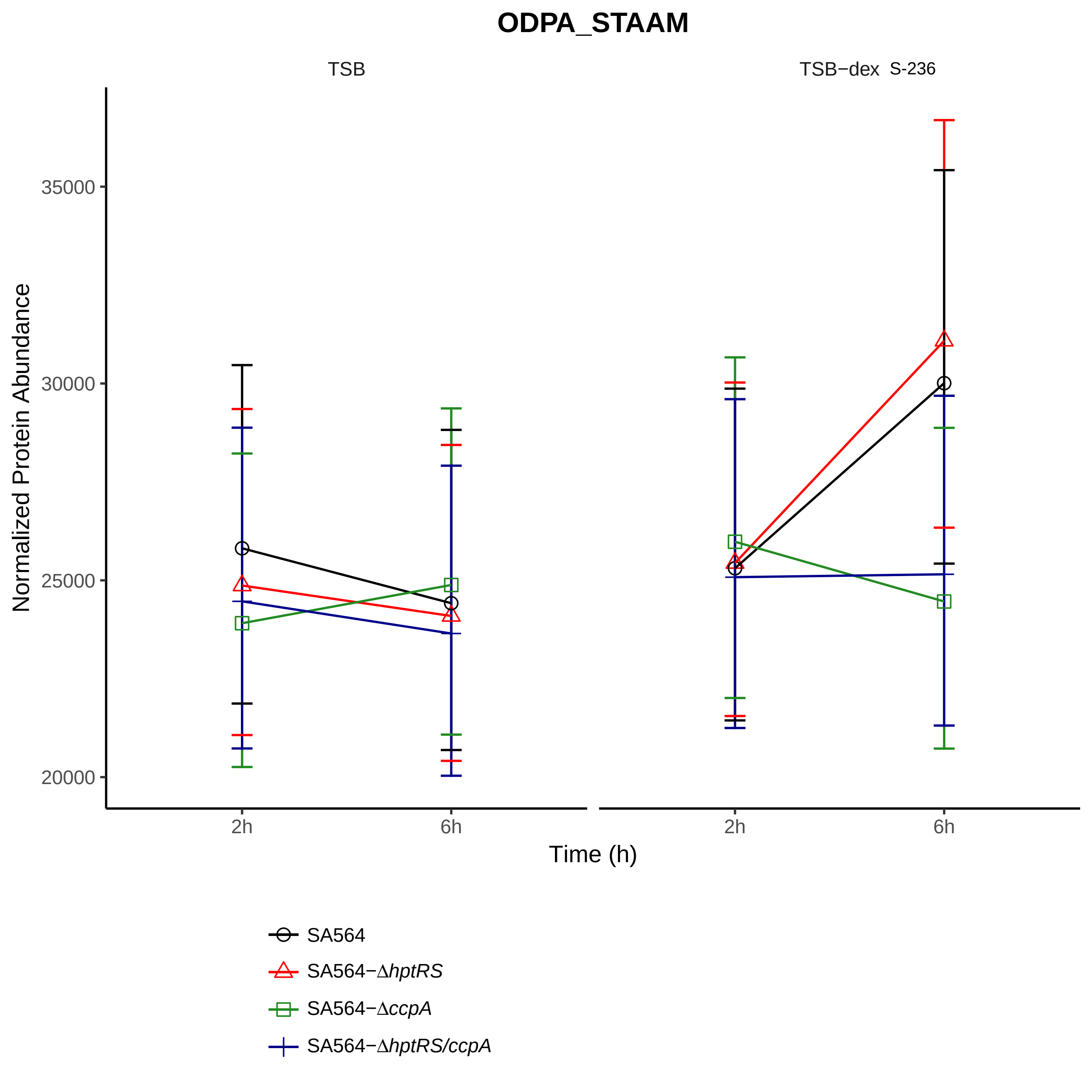

ODPA_STAAM
TSB
TSB−dex
S-236
Normalized Protein Abundance
35000
30000
25000
20000
2h
6h
2h
6h
Time (h)
SA564
SA564−Δ*hptRS*
SA564−Δ*ccpA*
SA564−Δ*hptRS/ccpA*

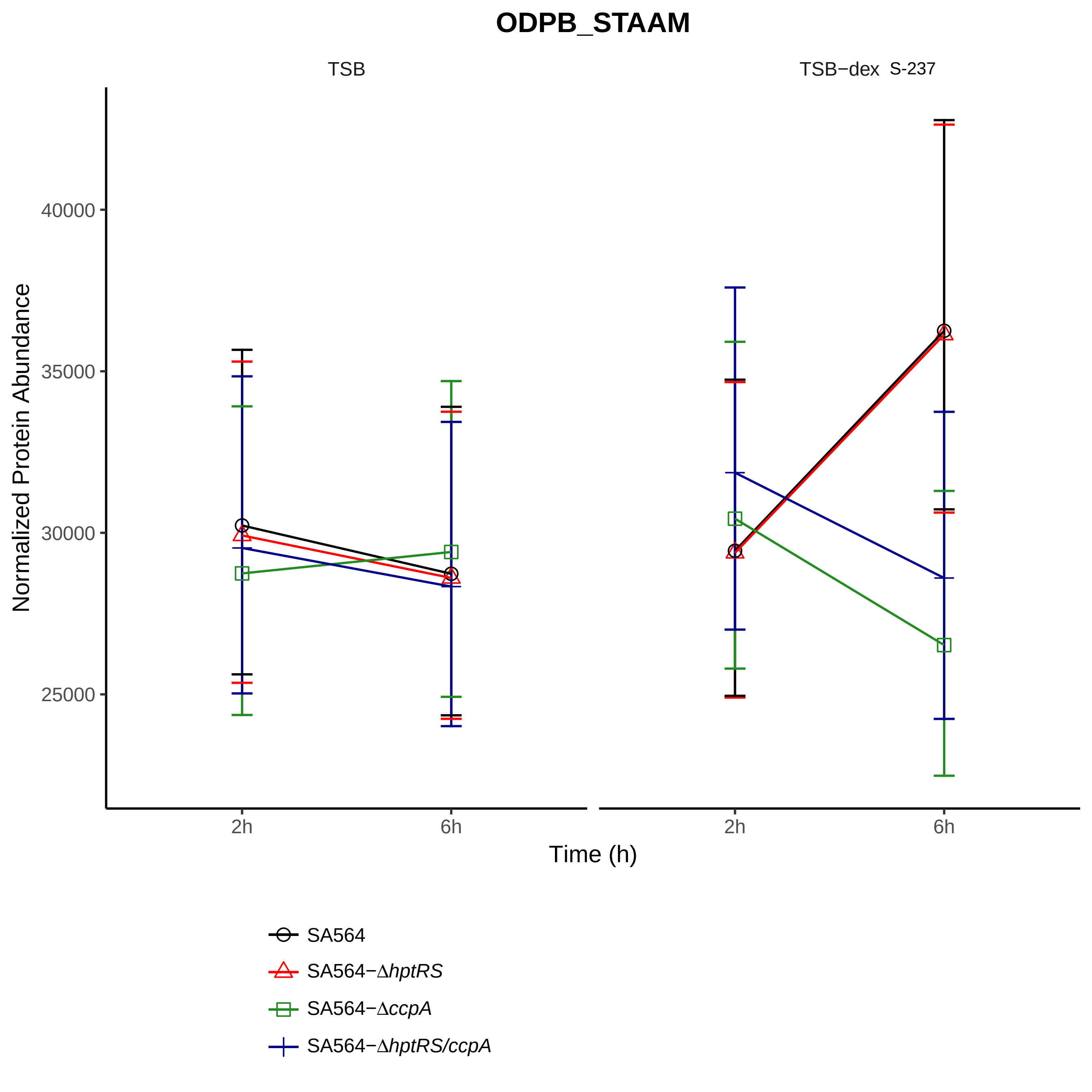

ODPB_STAAM
TSB
TSB−dex
S-237
Normalized Protein Abundance
40000
35000
30000
25000
2h
6h
2h
6h
Time (h)
SA564
SA564−Δ*hptRS*
SA564−Δ*ccpA*
SA564−Δ*hptRS/ccpA*

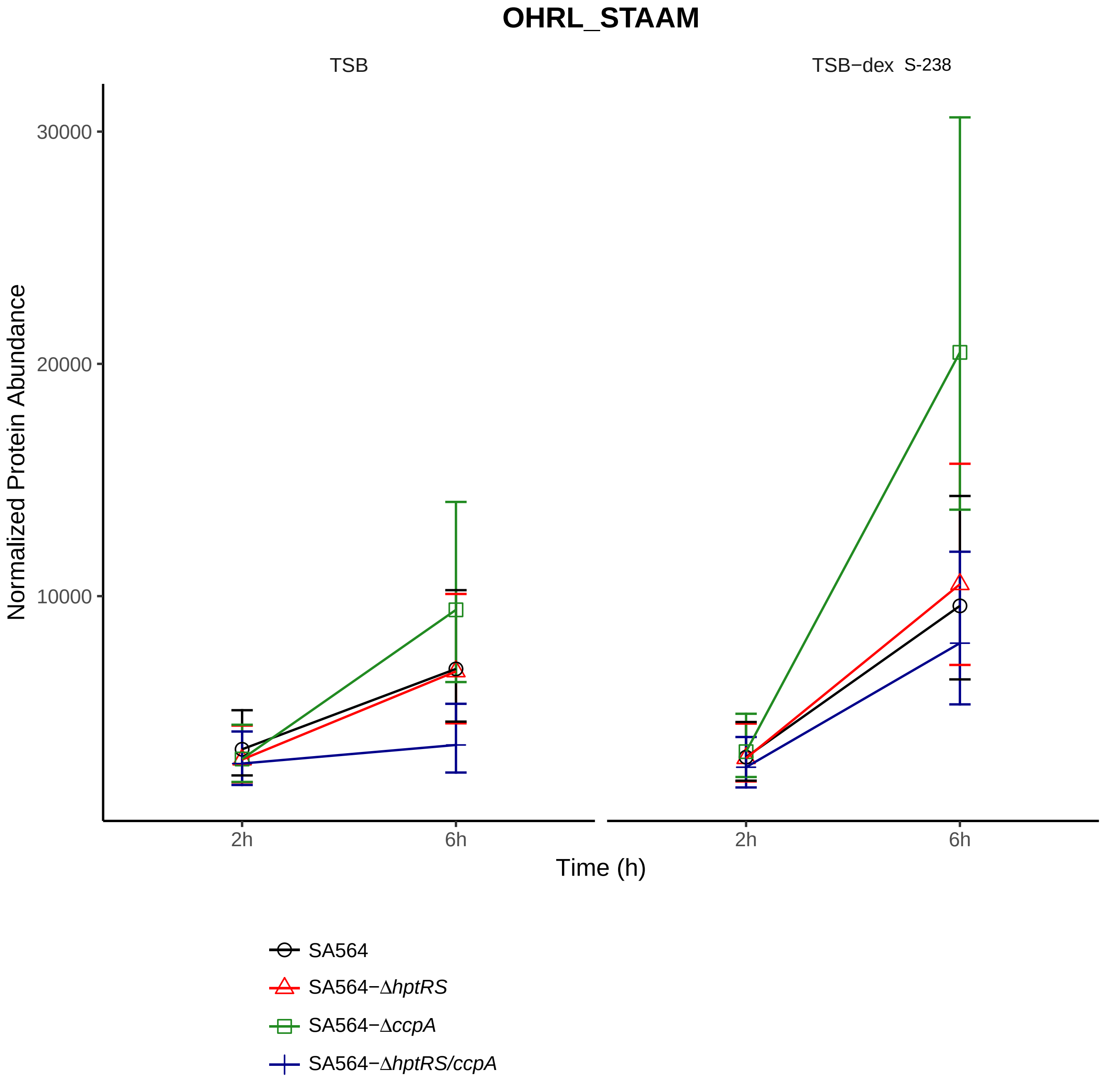
OHRL_STAAM
TSB
TSB−dex
S-238
30000
20000
10000
Normalized Protein Abundance
2h
6h
2h
6h
Time (h)
SA564
SA564−Δ*hptRS*
SA564−Δ*ccpA*
SA564−Δ*hptRS/ccpA*

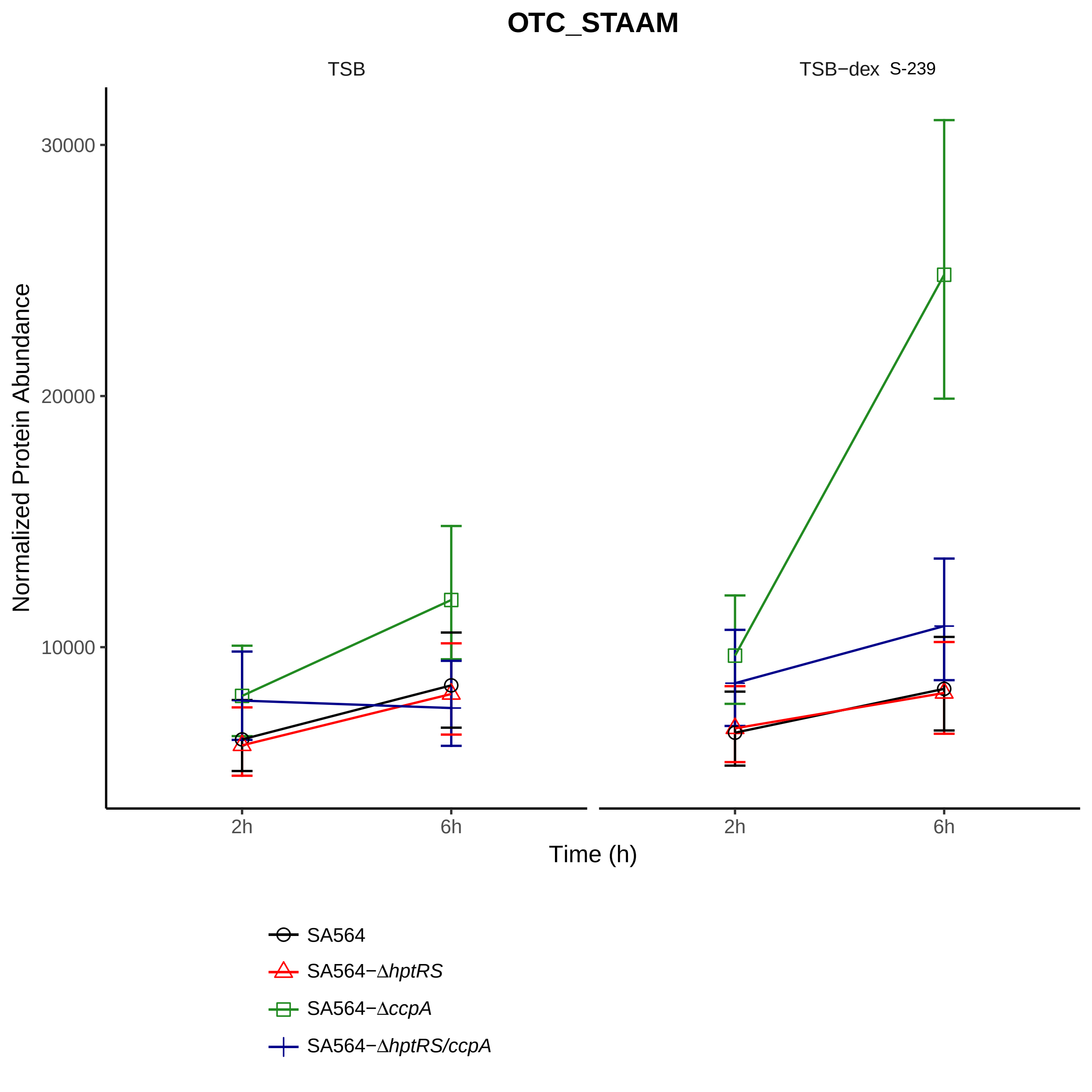
OTC_STAAM
TSB
TSB−dex
S-239
Normalized Protein Abundance
30000
20000
10000
2h
6h
2h
6h
Time (h)
SA564
SA564−Δ*hptRS*
SA564−Δ*ccpA*
SA564−Δ*hptRS/ccpA*

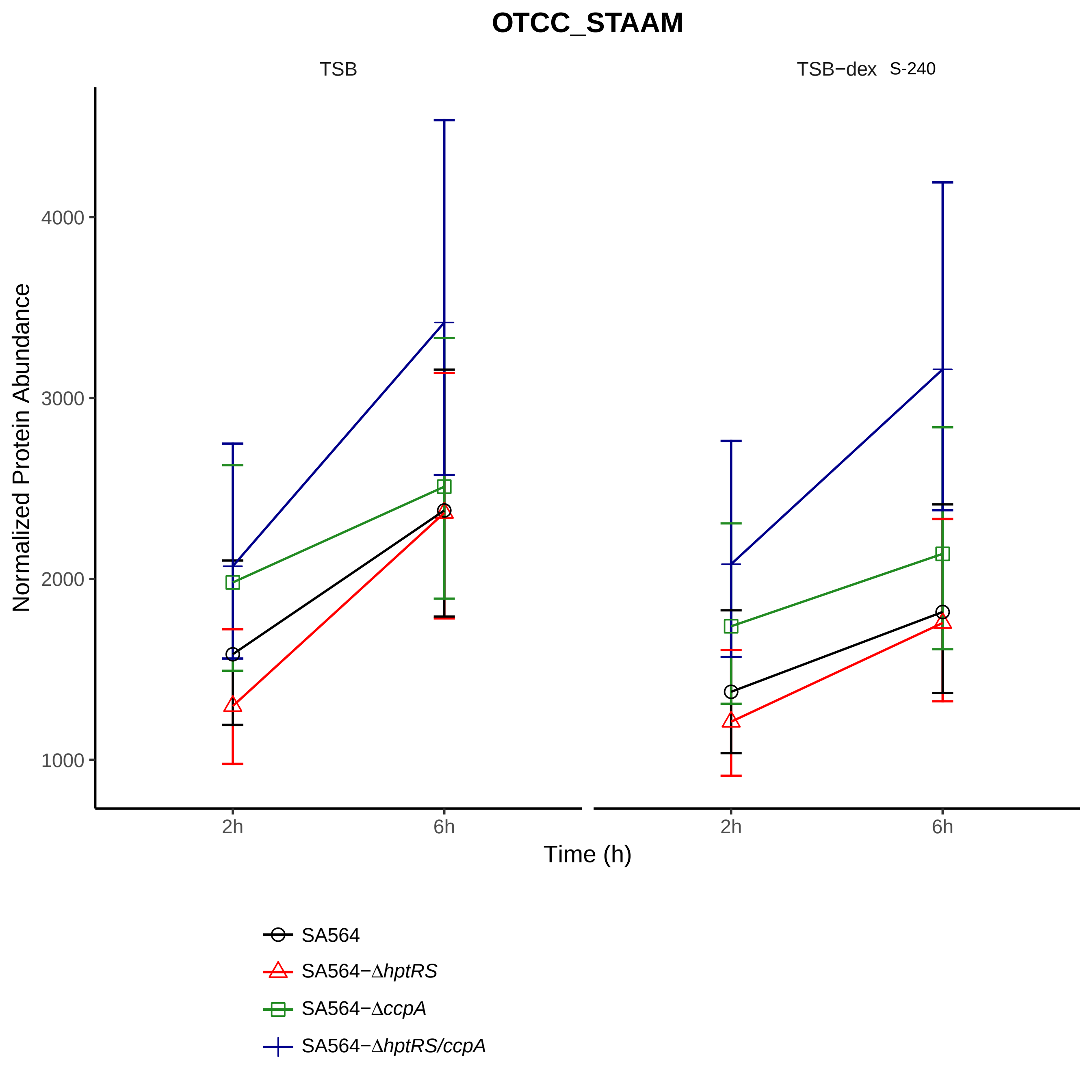

OTCC_STAAM
TSB
TSB−dex
S-240
Normalized Protein Abundance
4000
3000
2000
1000
2h
6h
2h
6h
Time (h)
SA564
SA564−Δ*hptRS*
SA564−Δ*ccpA*
SA564−Δ*hptRS/ccpA*

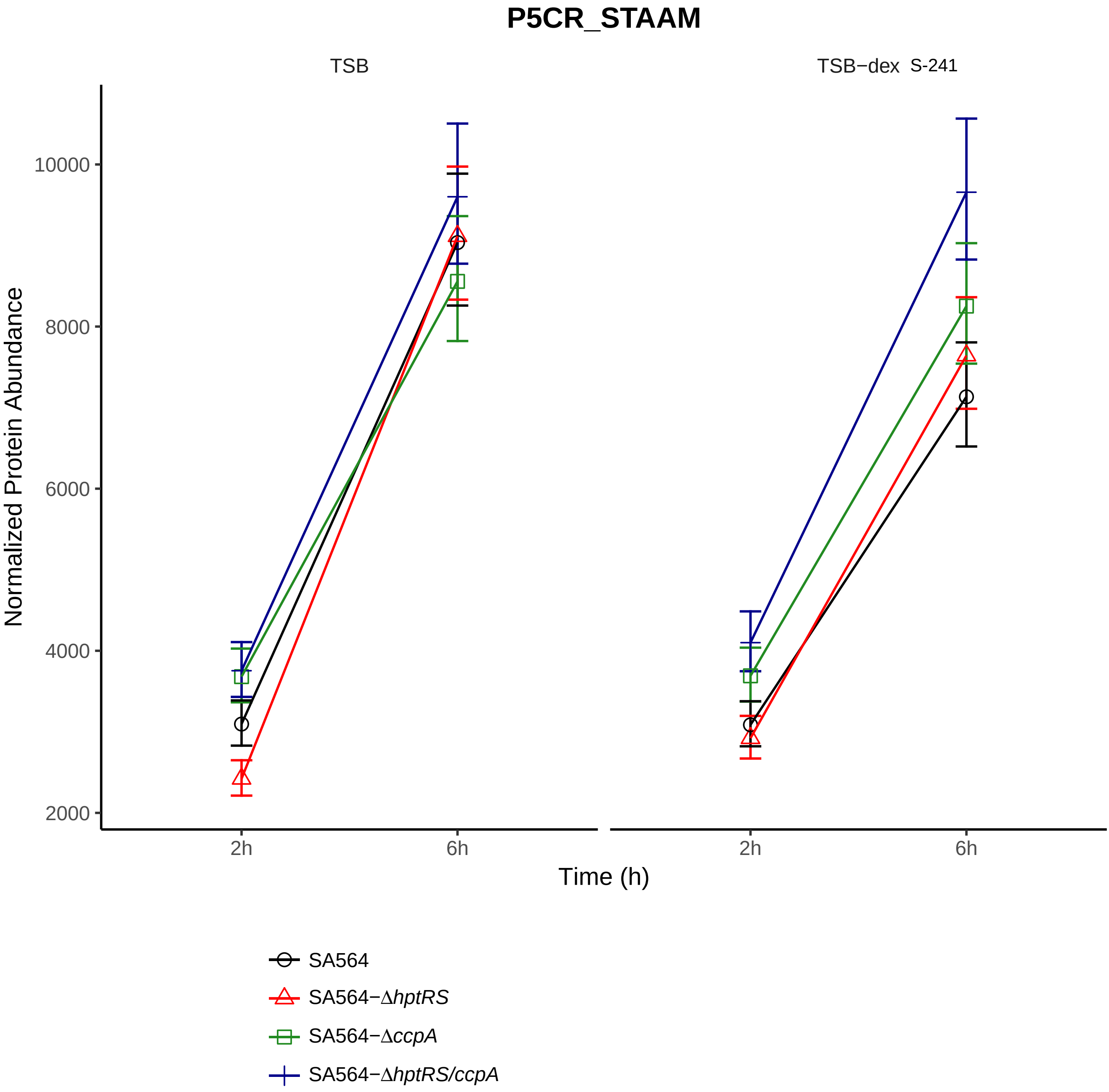

P5CR_STAAM
TSB
TSB−dex
Normalized Protein Abundance
10000
8000
6000
4000
2000
2h
6h
2h
6h
Time (h)
SA564
SA564−Δ*hptRS*
SA564−Δ*ccpA*
SA564−Δ*hptRS/ccpA*

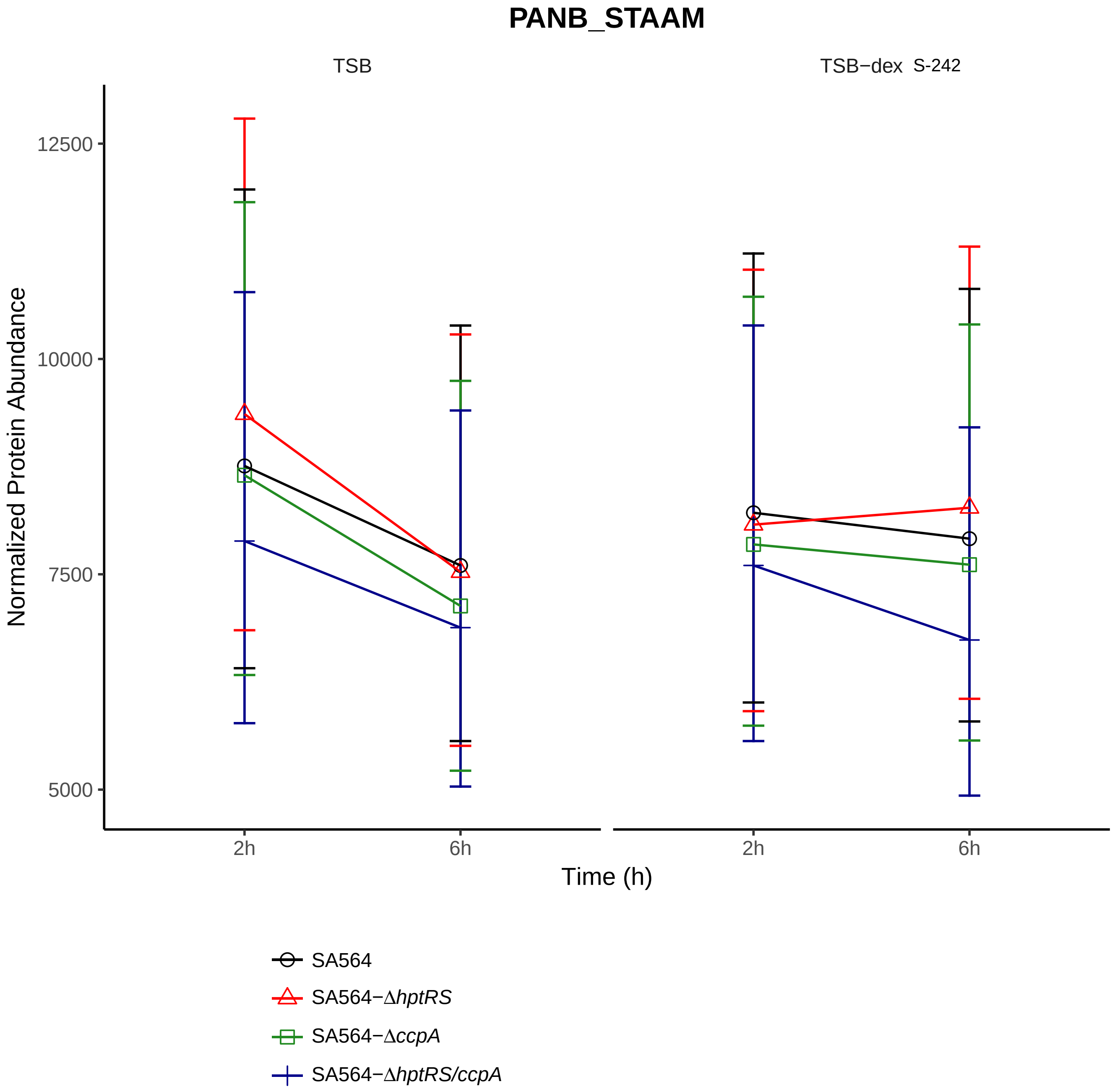

PANB_STAAM
TSB
TSB−dex
S-242
12500
10000
7500
5000
Normalized Protein Abundance
2h
6h
2h
6h
Time (h)
SA564
SA564−Δ*hptRS*
SA564−Δ*ccpA*
SA564−Δ*hptRS/ccpA*

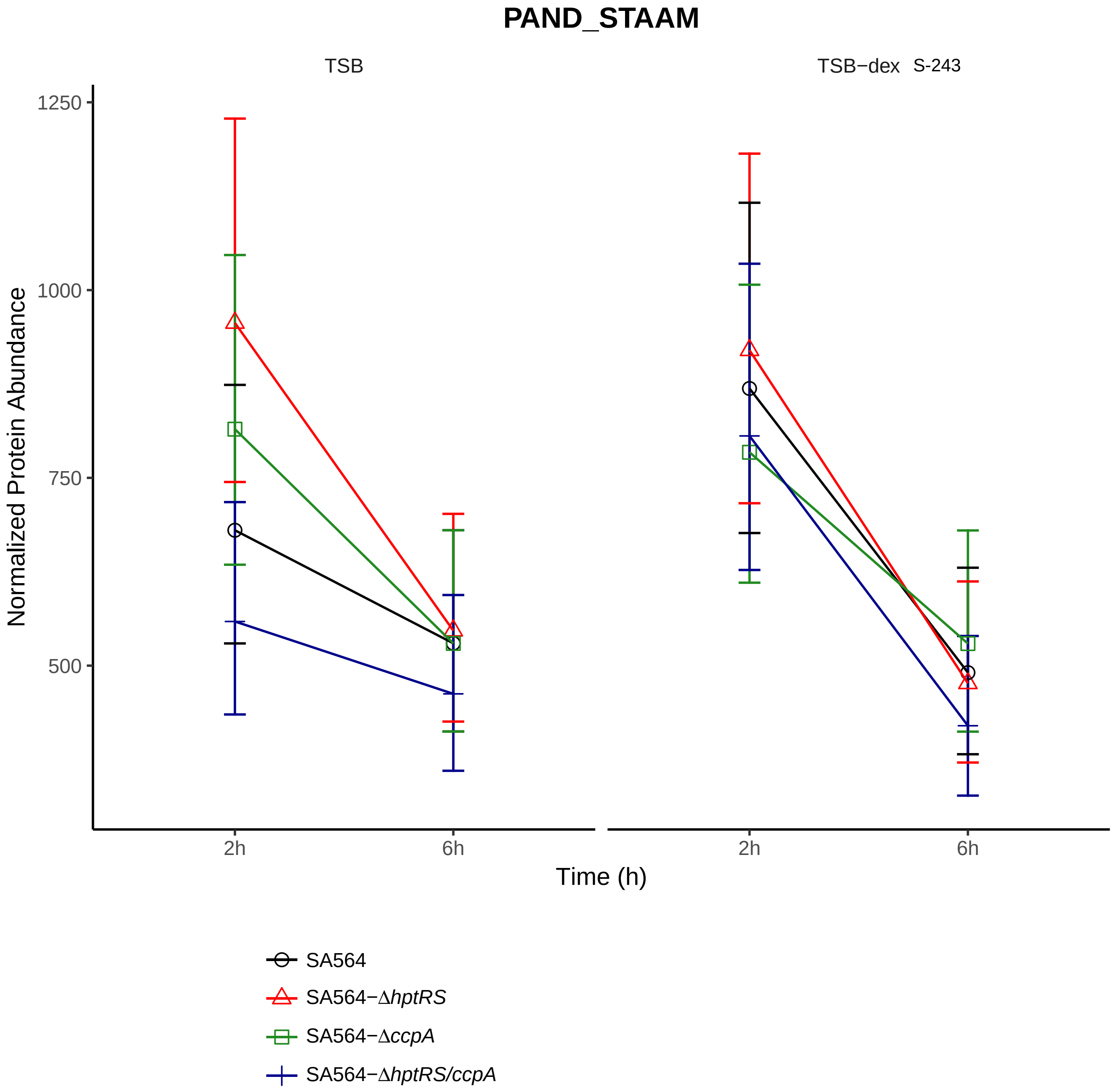

PAND_STAAM
TSB
TSB−dex
S-243
1250
1000
750
500
Normalized Protein Abundance
2h
6h
2h
6h
Time (h)
SA564
SA564−ΔhptRS
SA564−ΔccpA
SA564−ΔhptRS/ccpA

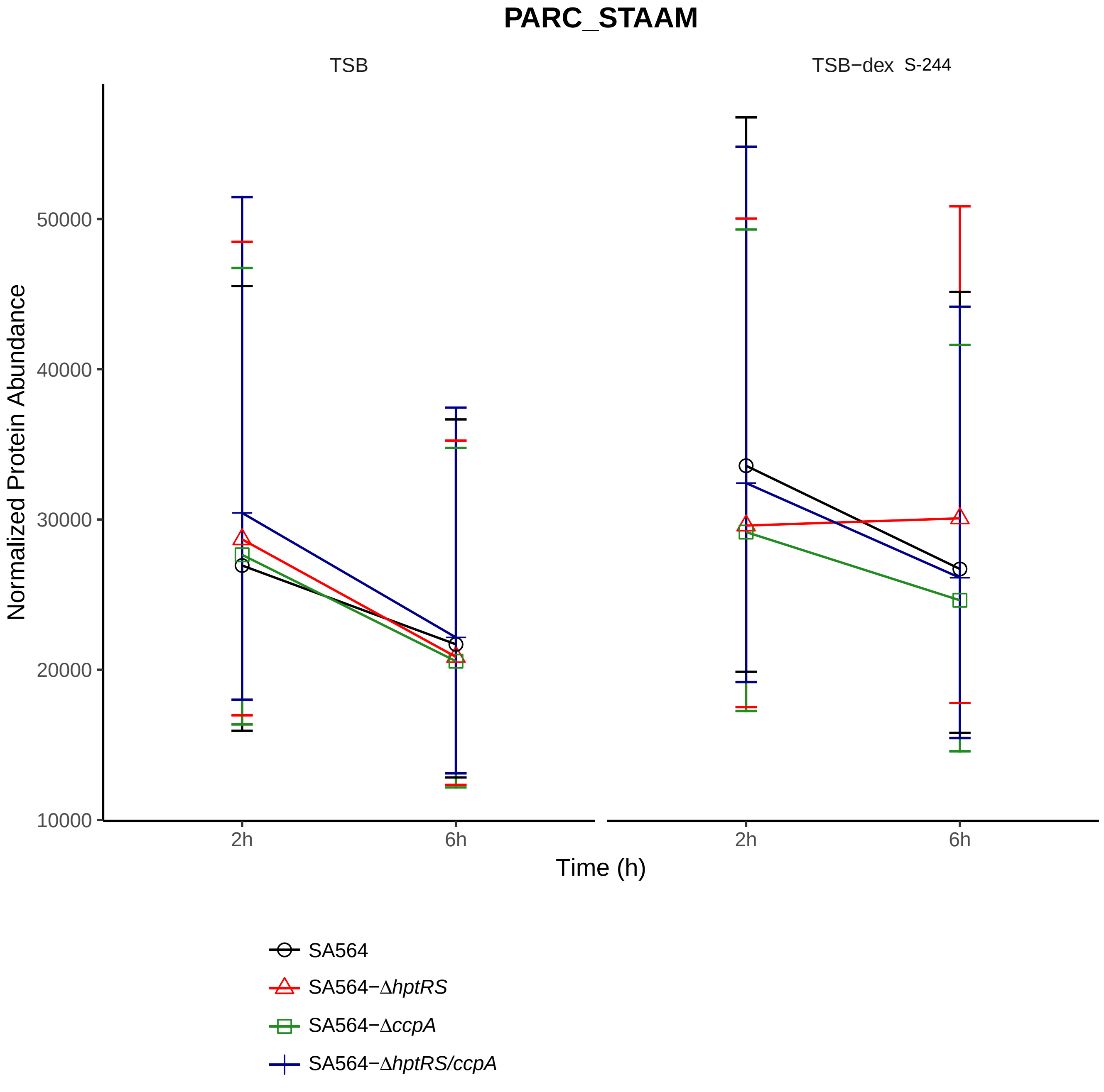

PARC_STAAM
TSB
TSB−dex
S-244
Normalized Protein Abundance
10000
20000
30000
40000
50000
2h
6h
2h
6h
Time (h)
SA564
SA564−ΔhptRS
SA564−ΔccpA
SA564−ΔhptRS/ccpA

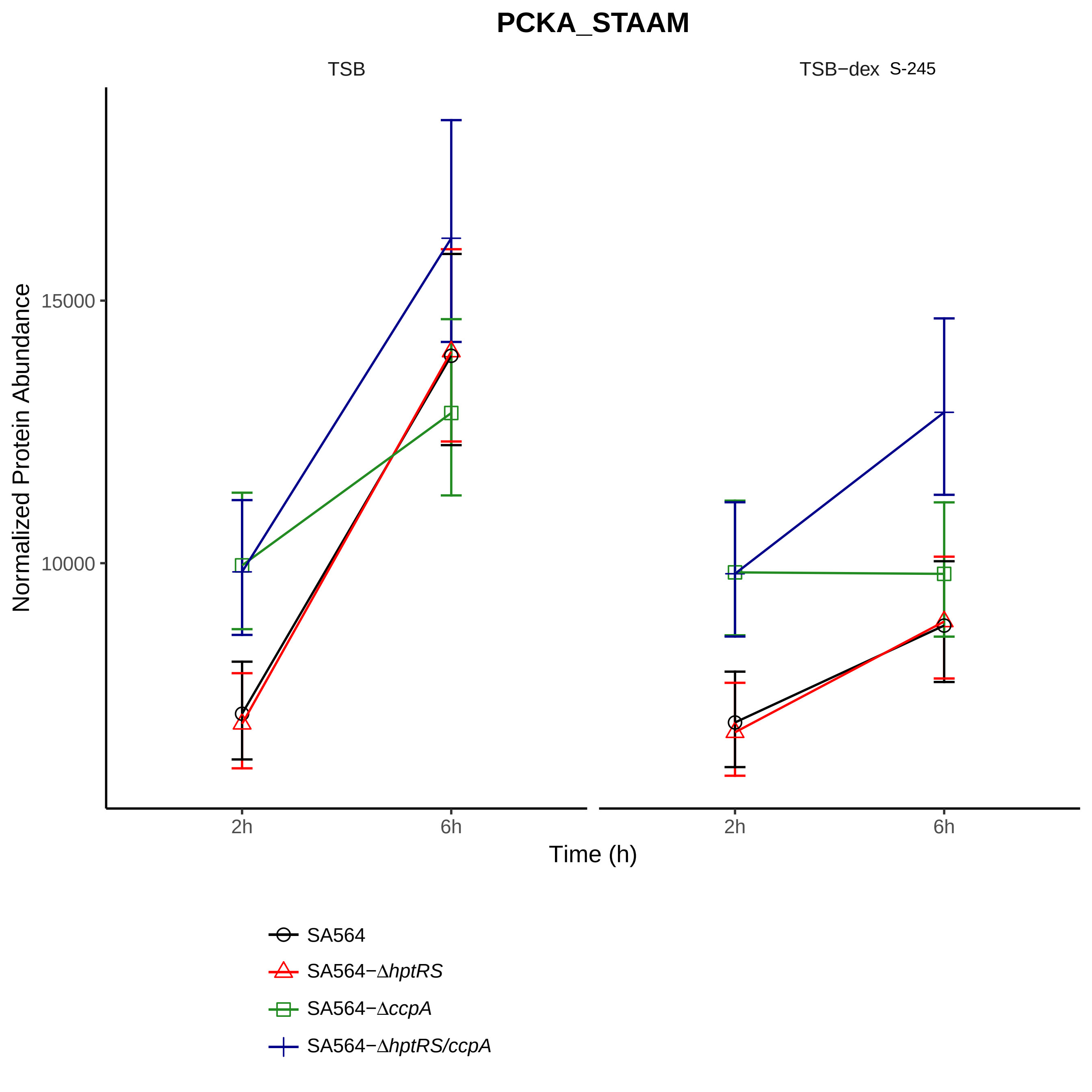

PCKA_STAAM
TSB
TSB−dex
S-245
Normalized Protein Abundance
15000
10000
2h
6h
2h
6h
Time (h)
SA564
SA564−Δ*hptRS*
SA564−Δ*ccpA*
SA564−Δ*hptRS/ccpA*

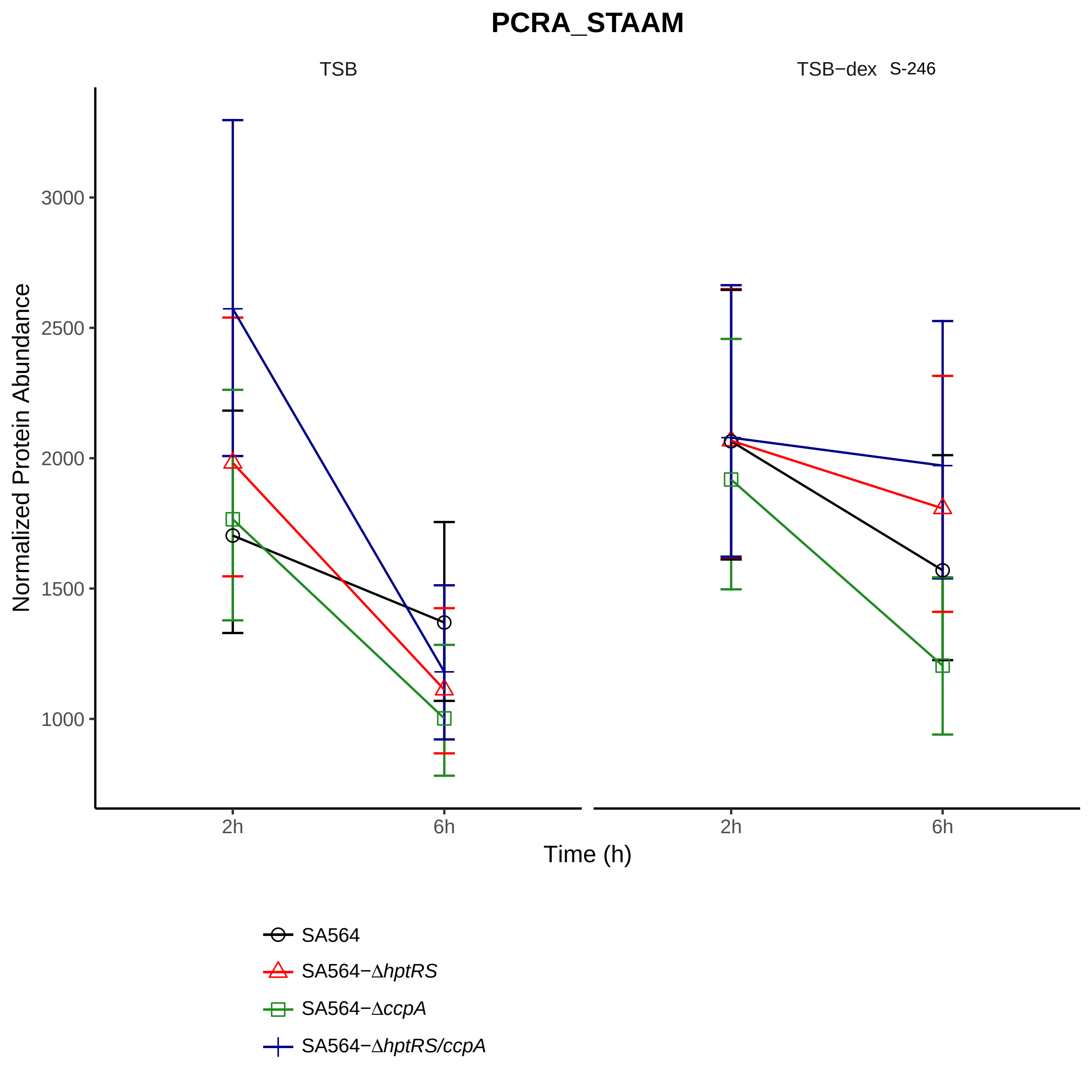
PCRA_STAAM
TSB
TSB−dex
S-246
Normalized Protein Abundance
3000
2500
2000
1500
1000
2h
6h
2h
6h
Time (h)
SA564
SA564−ΔhptRS
SA564−ΔccpA
SA564−ΔhptRS/ccpA

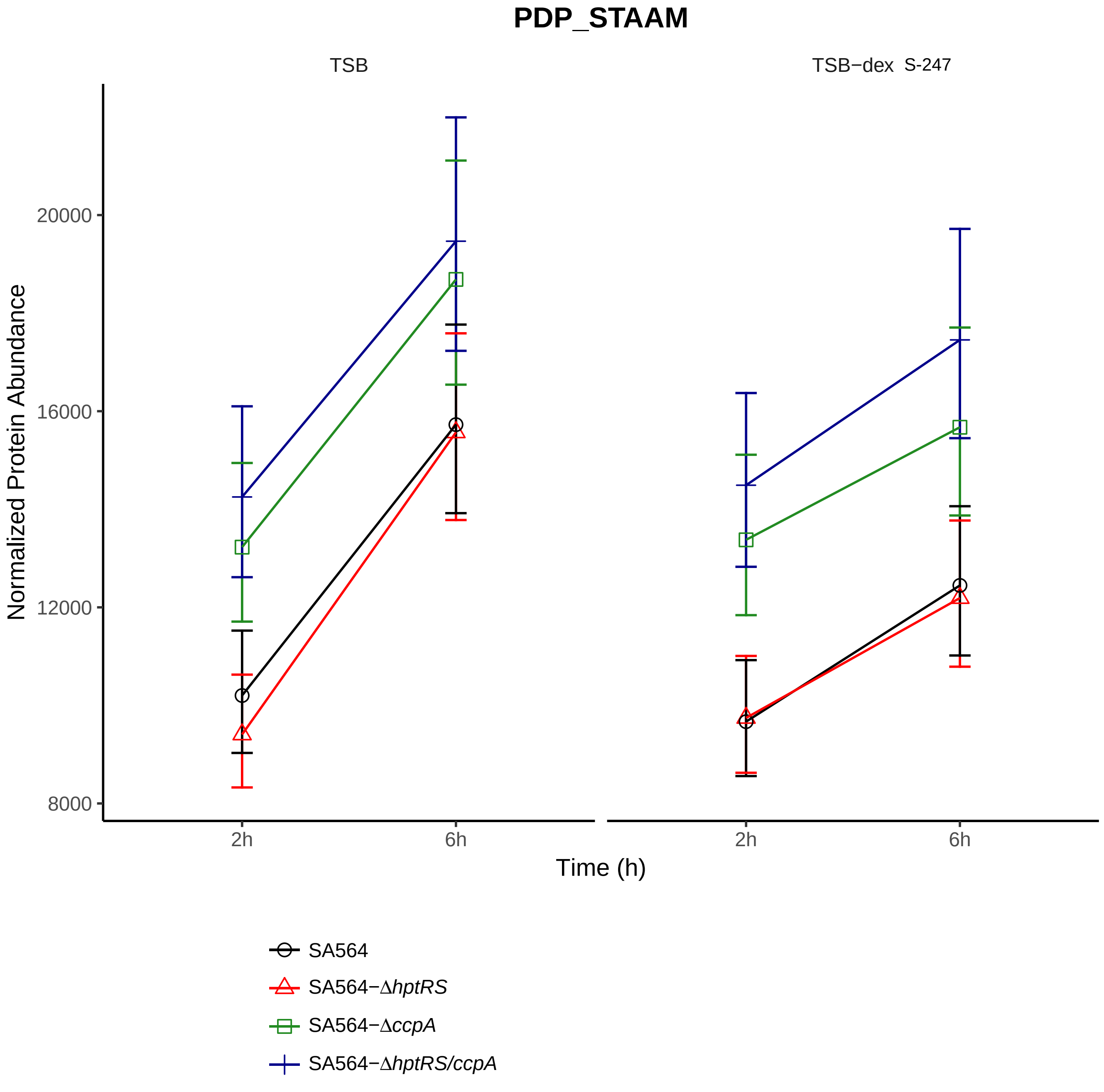

PDP_STAAM
TSB
TSB−dex
S-247
Normalized Protein Abundance
20000
16000
12000
8000
2h
6h
2h
6h
Time (h)
SA564
SA564−ΔhptRS
SA564−ΔccpA
SA564−ΔhptRS/ccpA

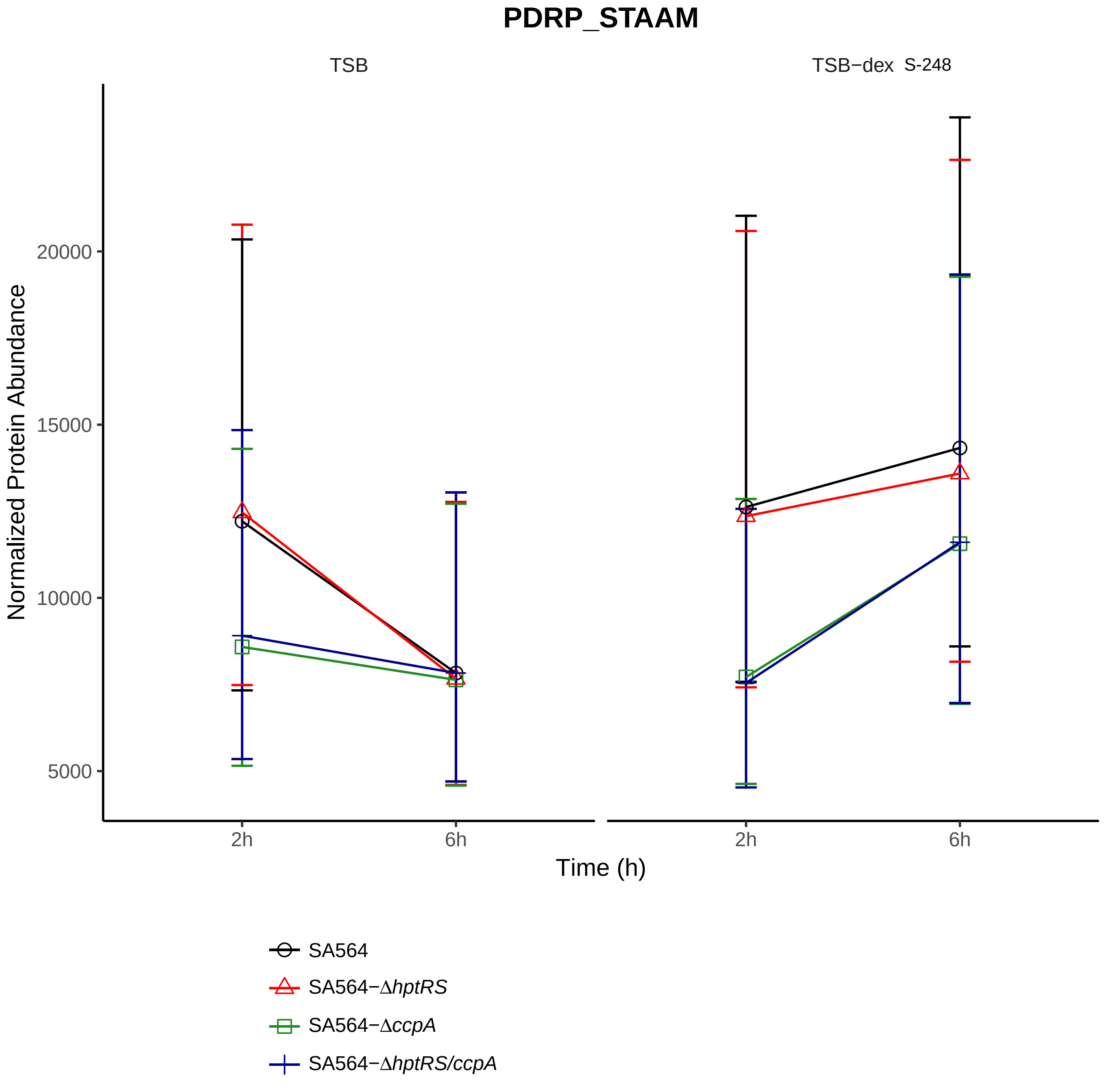

PDRP_STAAM
TSB
TSB−dex
S-248
Normalized Protein Abundance
20000
15000
10000
5000
2h
6h
2h
6h
Time (h)
SA564
SA564−Δ*hptRS*
SA564−Δ*ccpA*
SA564−Δ*hptRS/ccpA*

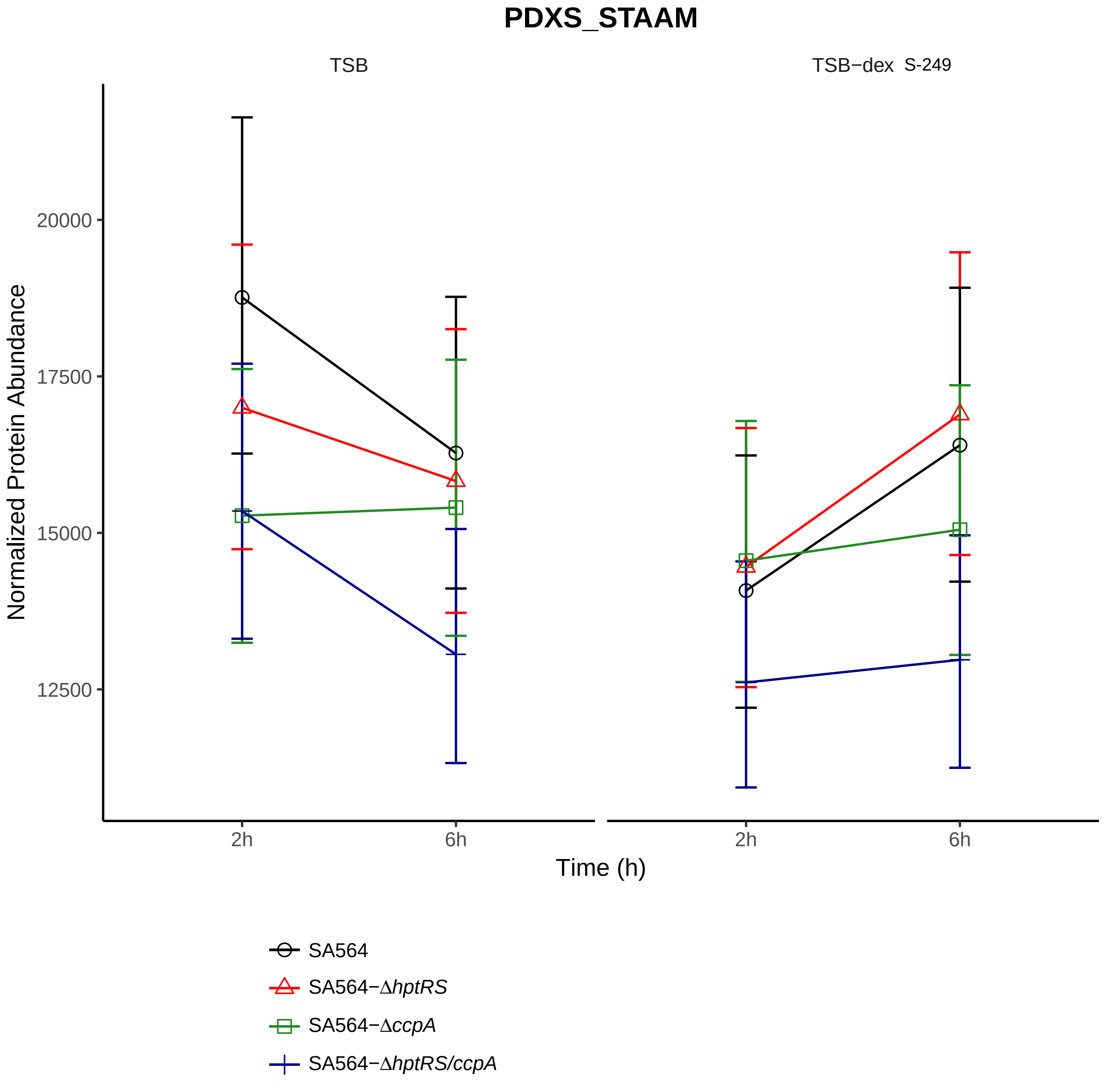
PDXS_STAAM
TSB
TSB−dex
S-249
Normalized Protein Abundance
20000
17500
15000
12500
2h
6h
2h
6h
Time (h)
SA564
SA564−ΔhptRS
SA564−ΔccpA
SA564−ΔhptRS/ccpA

PDXT_STAAM

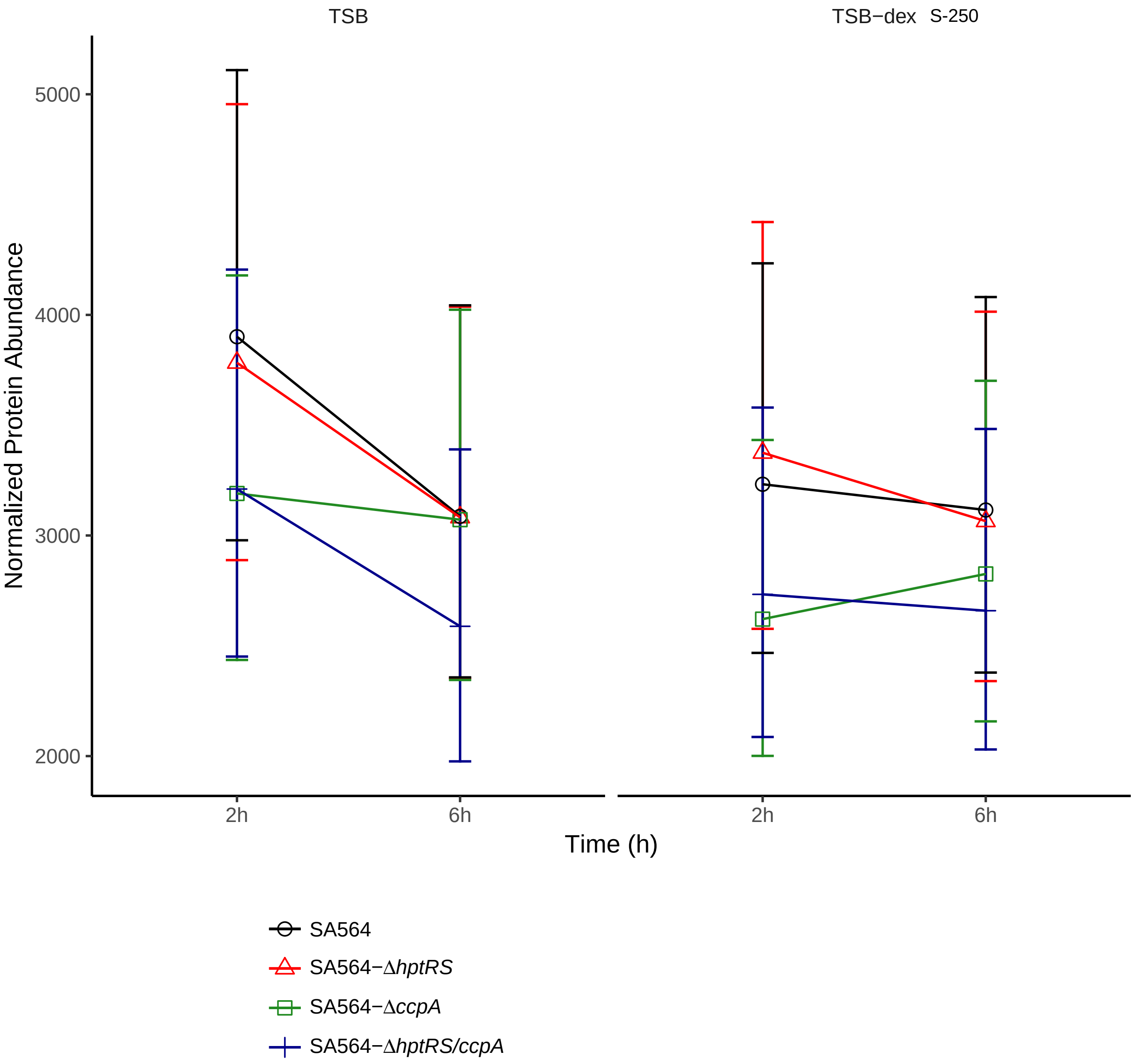

TSB
TSB−dex
S-250
5000
4000
3000
2000
Normalized Protein Abundance
2h
6h
2h
6h
Time (h)
SA564
SA564−Δ*hptRS*
SA564−Δ*ccpA*
SA564−Δ*hptRS/ccpA*

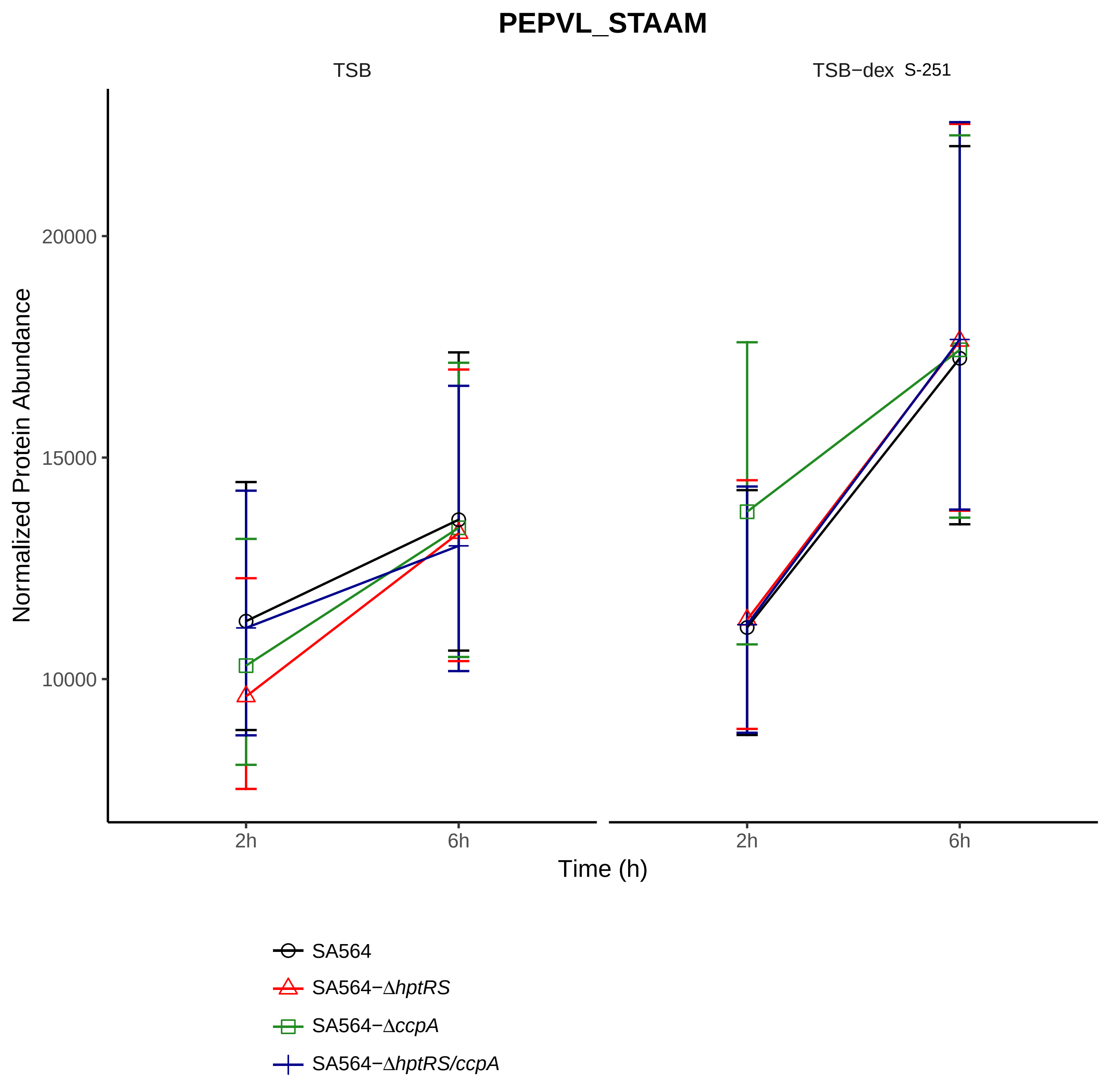

PEPVL_STAAM
TSB
TSB−dex
S-251
Normalized Protein Abundance
20000
15000
10000
2h
6h
2h
6h
Time (h)
SA564
SA564−Δ*hptRS*
SA564−Δ*ccpA*
SA564−Δ*hptRS/ccpA*

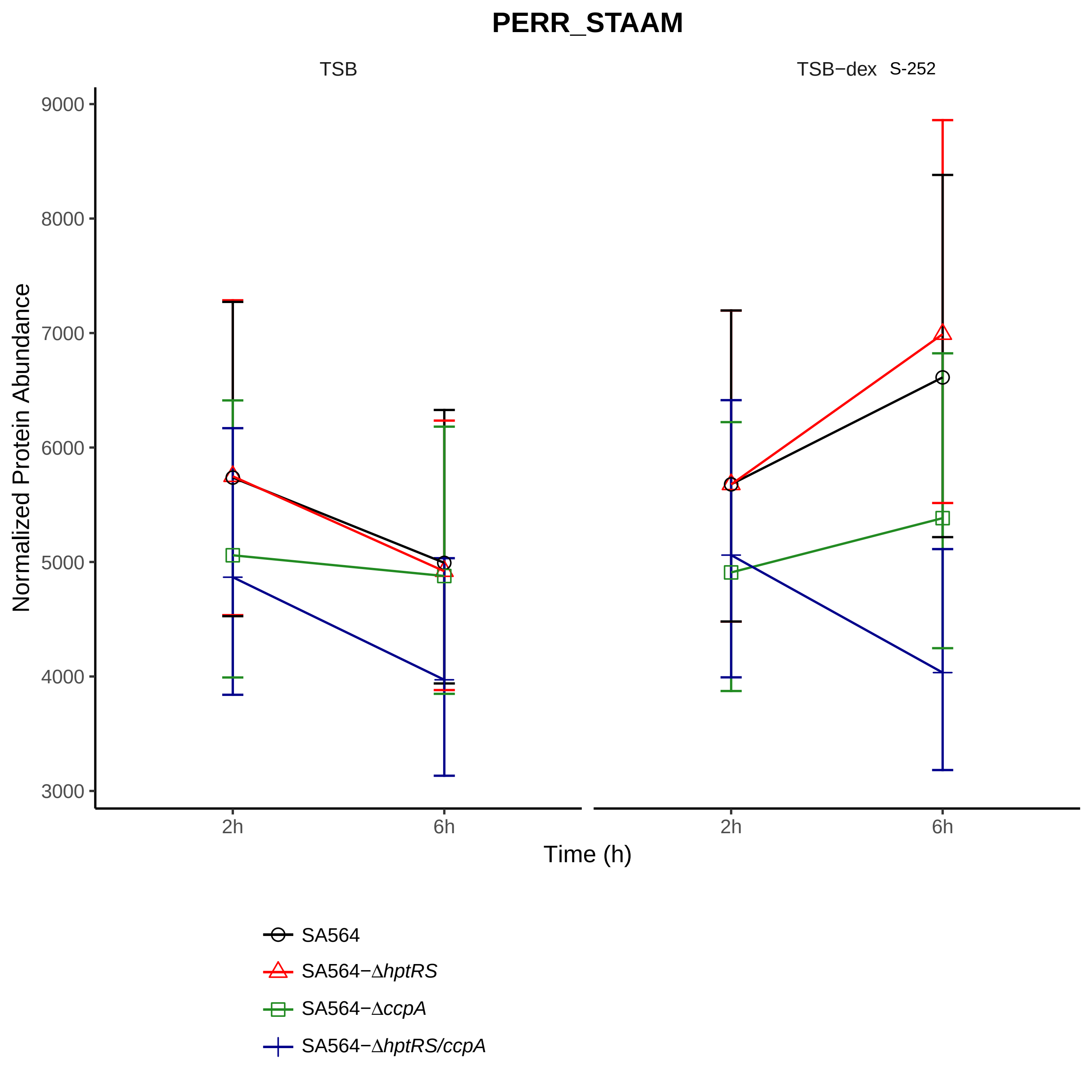

PERR_STAAM
TSB
TSB−dex
S-252
Normalized Protein Abundance
9000
8000
7000
6000
5000
4000
3000
2h
6h
2h
6h
Time (h)
SA564
SA564−Δ*hptRS*
SA564−Δ*ccpA*
SA564−Δ*hptRS/ccpA*

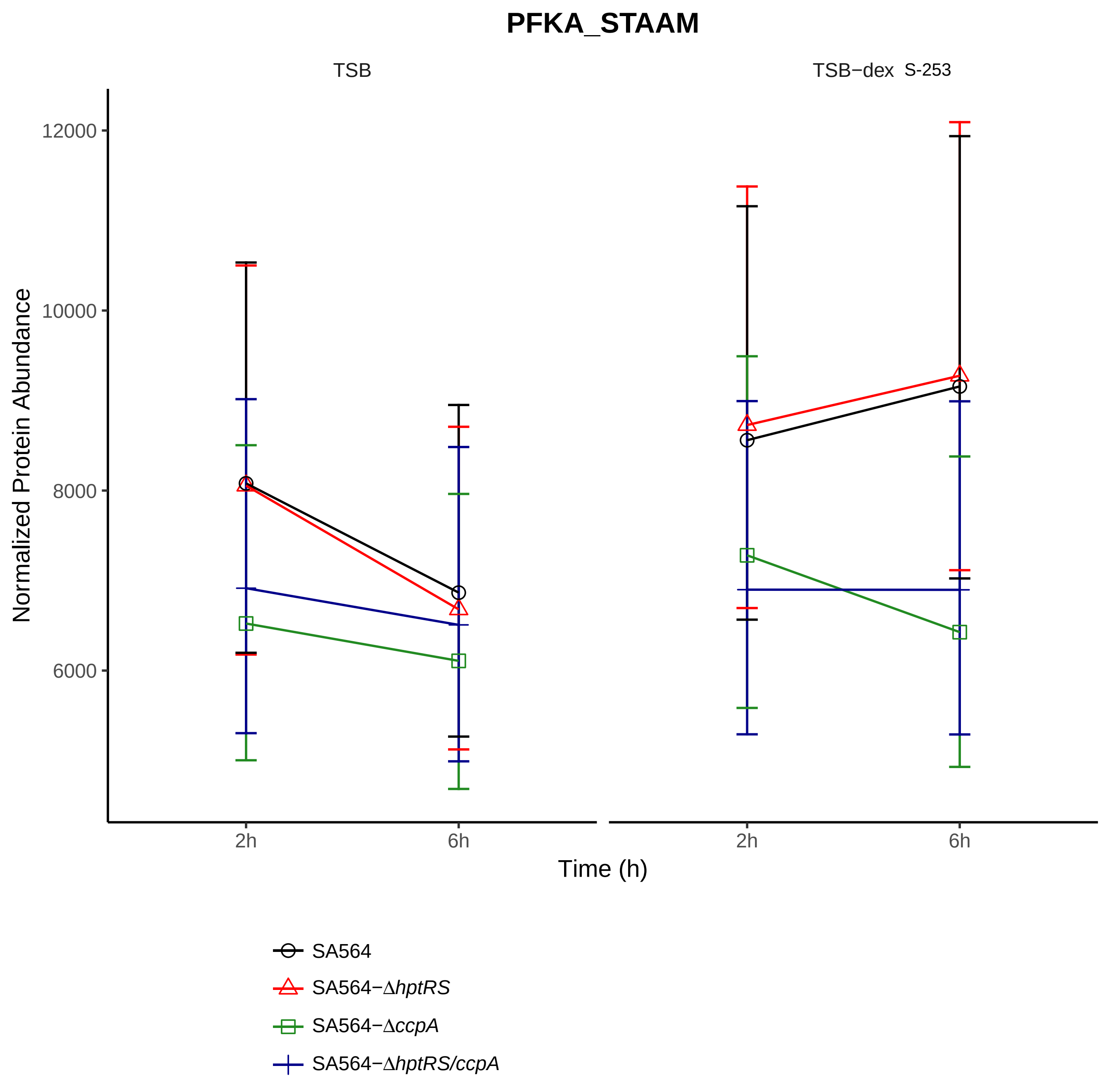

PFKA_STAAM
TSB
TSB−dex
S-253
Normalized Protein Abundance
12000
10000
8000
6000
2h
6h
2h
6h
Time (h)
SA564
SA564−Δ*hptRS*
SA564−Δ*ccpA*
SA564−Δ*hptRS/ccpA*

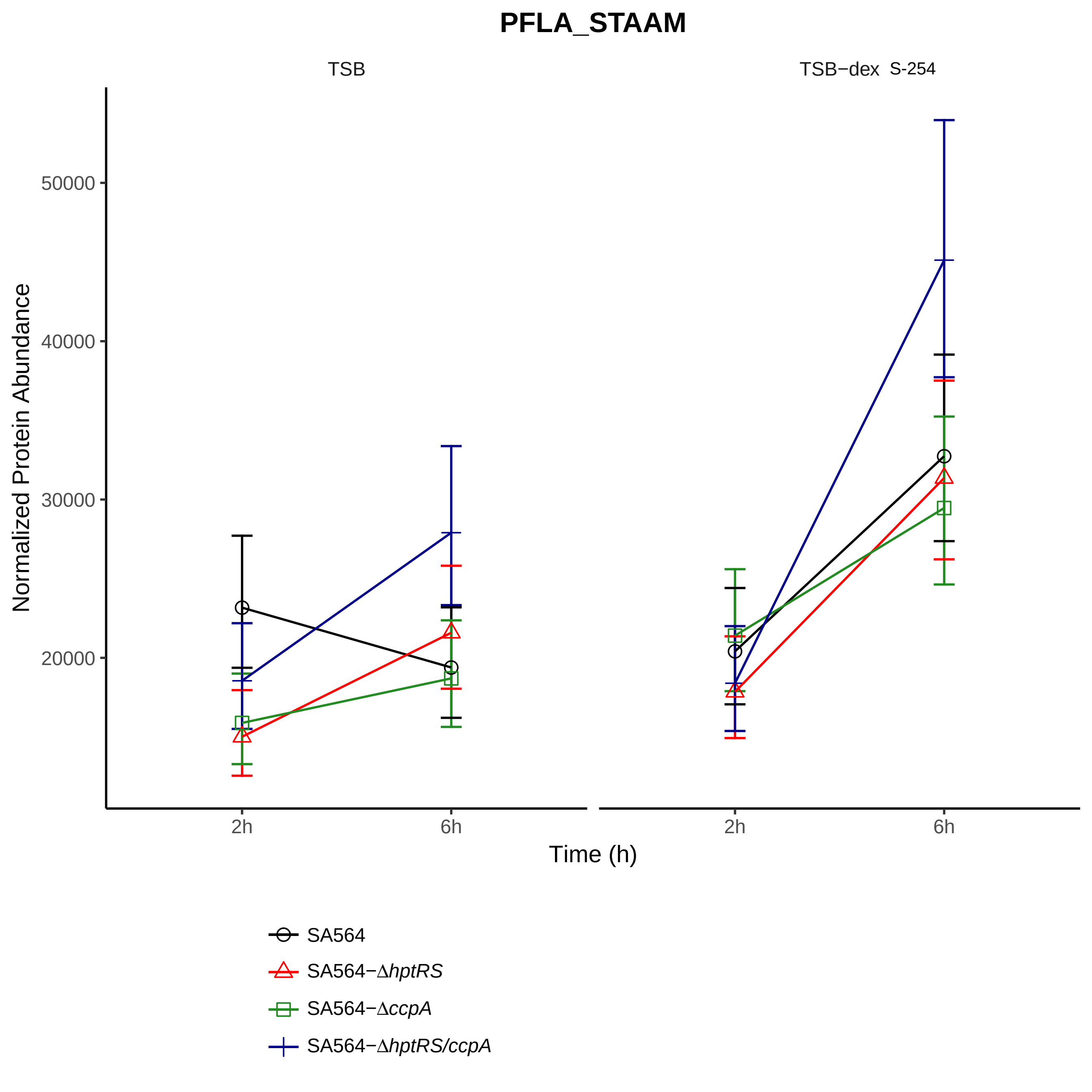

PFLA_STAAM
TSB
TSB−dex
S-254
Normalized Protein Abundance
50000
40000
30000
20000
2h
6h
2h
6h
Time (h)
SA564
SA564−Δ*hptRS*
SA564−Δ*ccpA*
SA564−Δ*hptRS/ccpA*

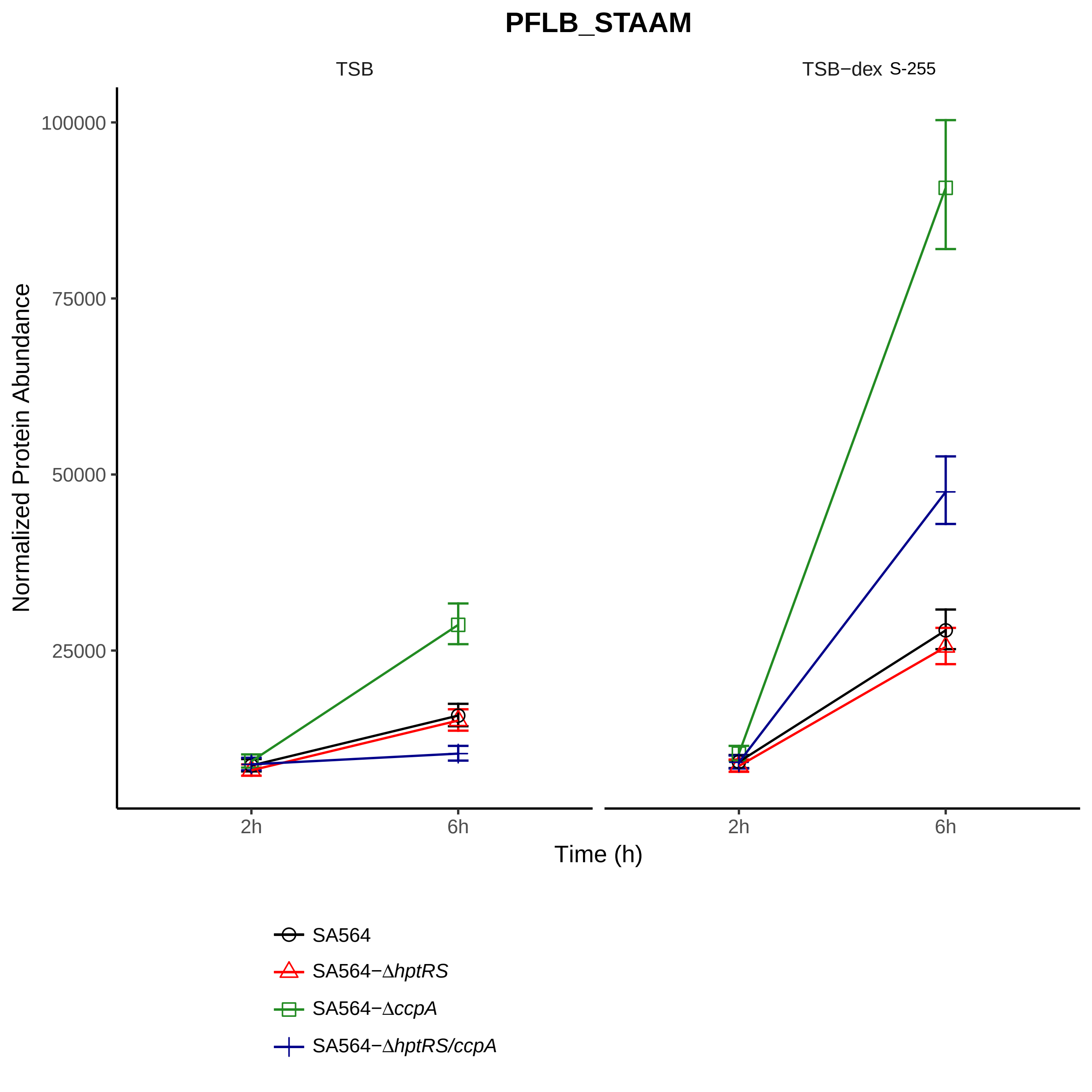

PFLB_STAAM
TSB
TSB−dex
S-255
100000
75000
50000
25000
Normalized Protein Abundance
2h
6h
2h
6h
Time (h)
SA564
SA564−Δ*hptRS*
SA564−Δ*ccpA*
SA564−Δ*hptRS/ccpA*

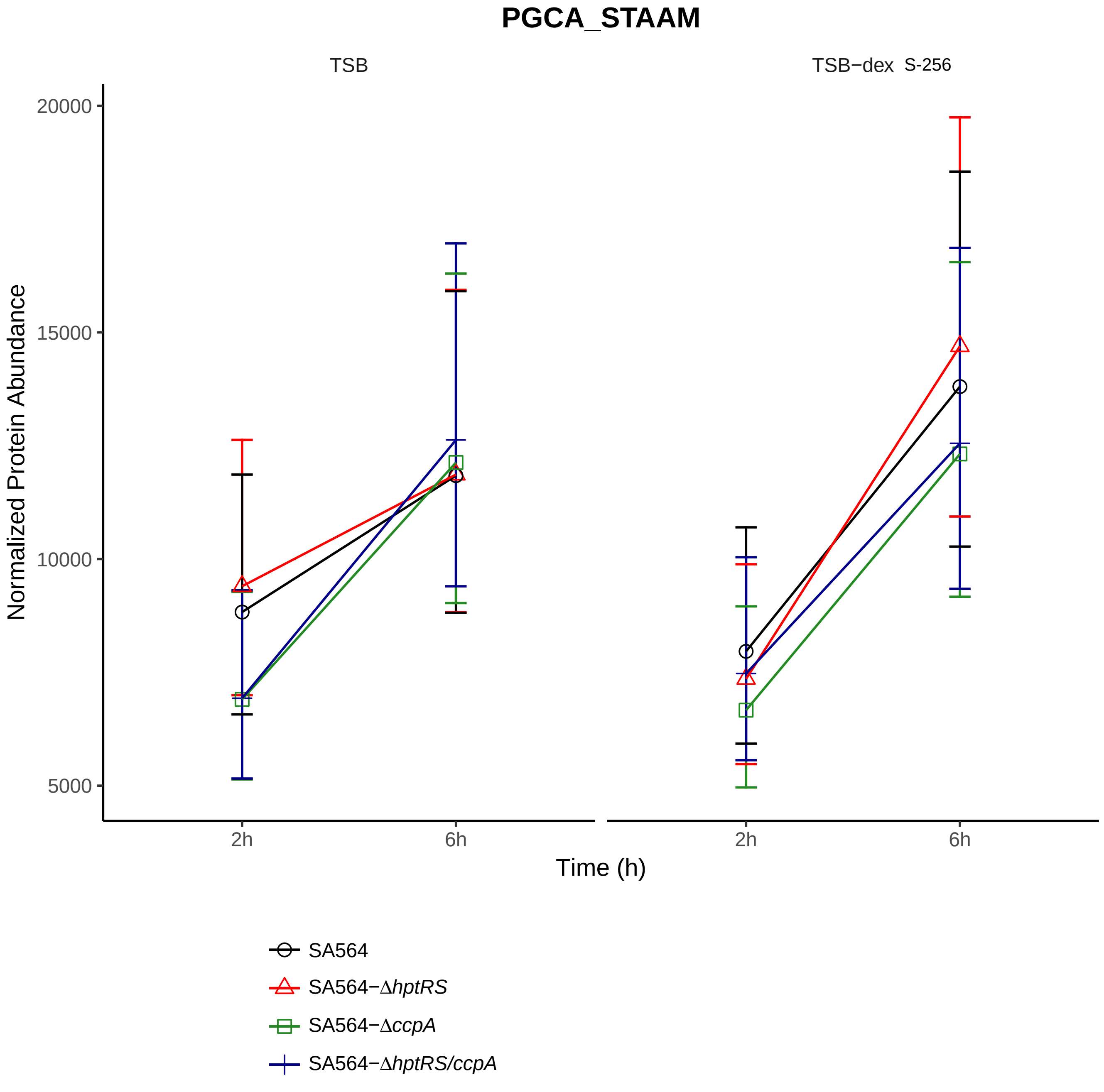

PGCA_STAAM
TSB
TSB−dex
S-256
Normalized Protein Abundance
20000
15000
10000
5000
2h
6h
2h
6h
Time (h)
SA564
SA564−Δ*hptRS*
SA564−Δ*ccpA*
SA564−Δ*hptRS/ccpA*

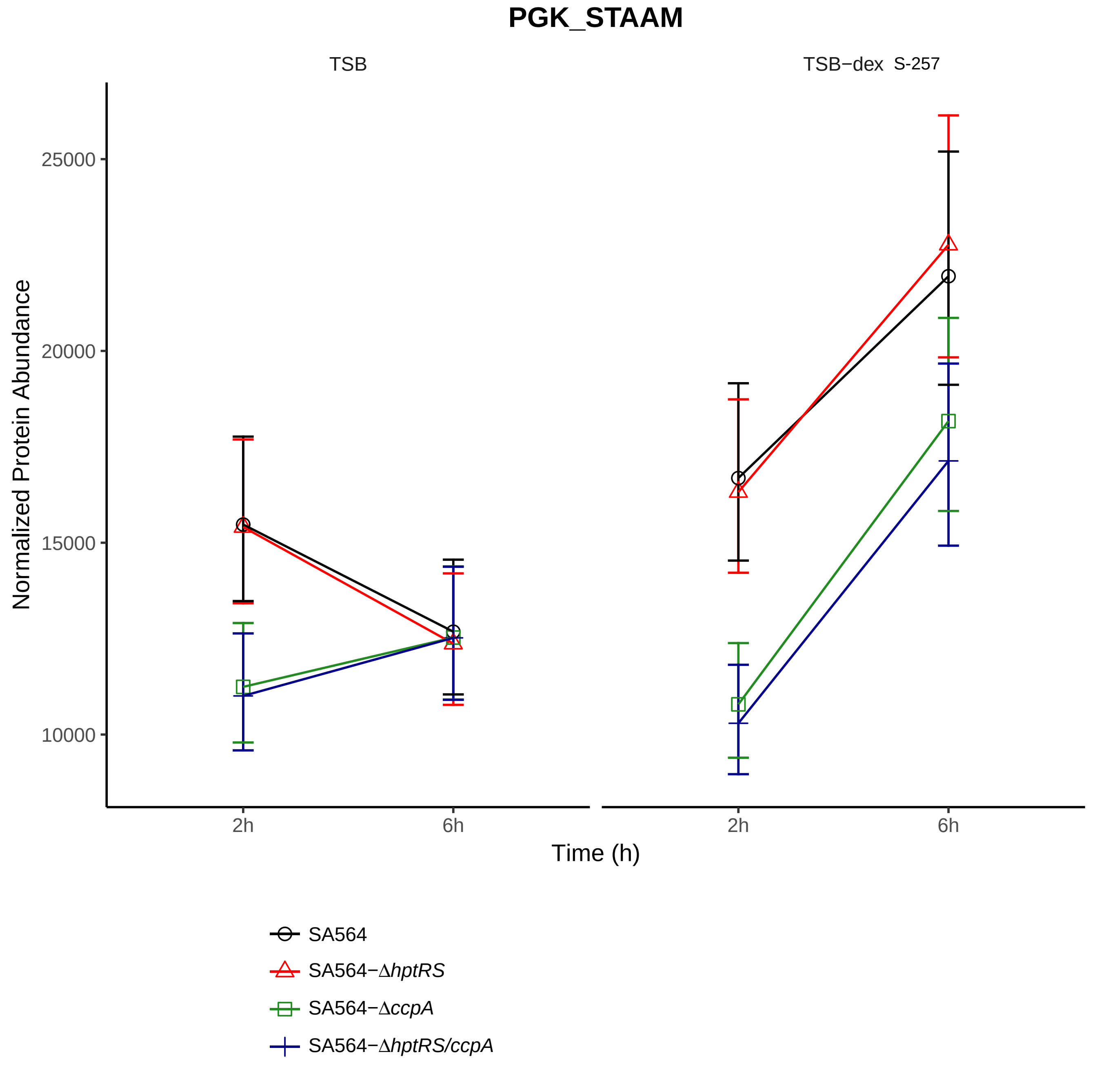

PGK_STAAM
TSB
TSB−dex
S-257
Normalized Protein Abundance
25000
20000
15000
10000
2h
6h
2h
6h
Time (h)
SA564
SA564−Δ*hptRS*
SA564−Δ*ccpA*
SA564−Δ*hptRS/ccpA*

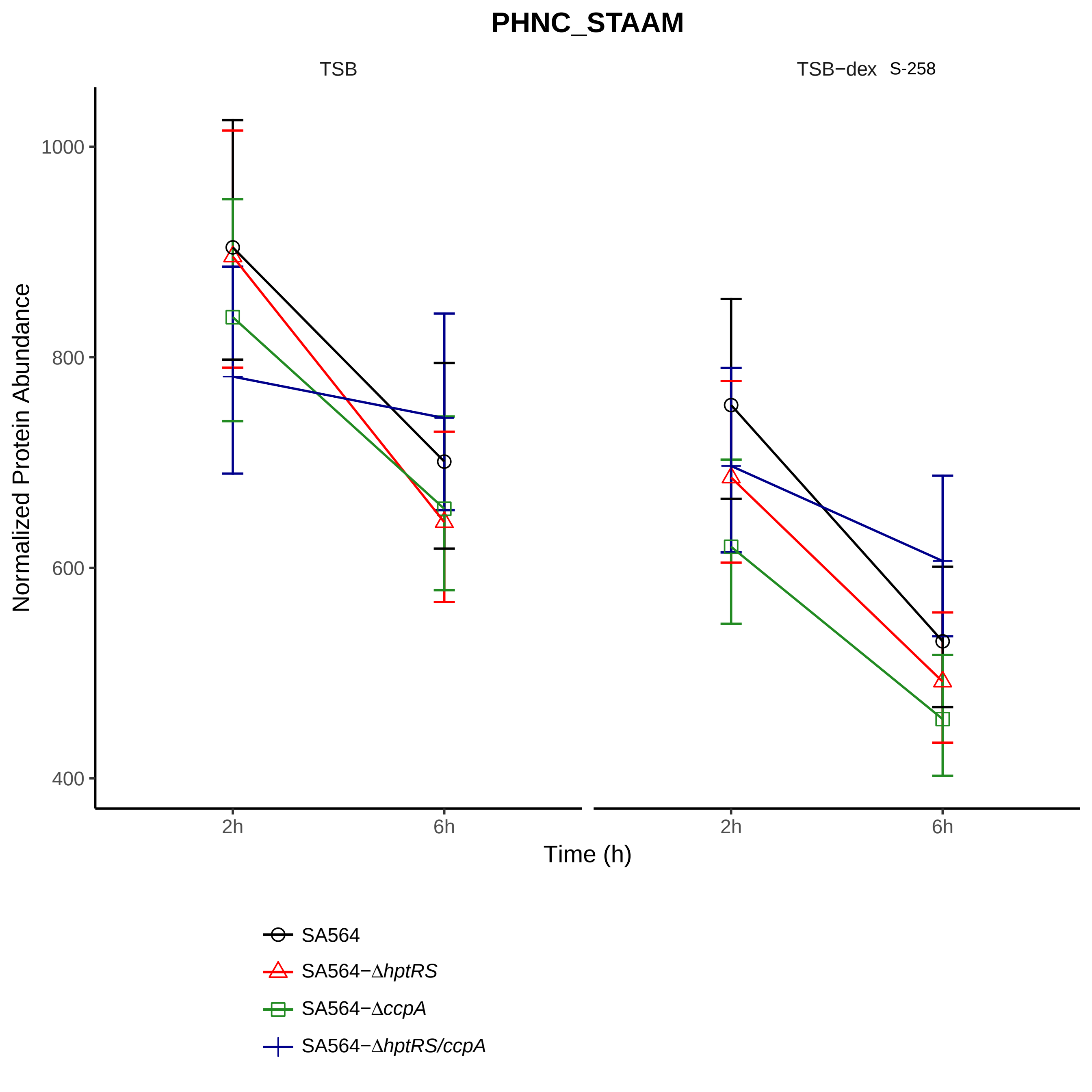

PHNC_STAAM
TSB
TSB−dex
S-258
Normalized Protein Abundance
1000
800
600
400
2h
6h
2h
6h
Time (h)
SA564
SA564−Δ*hptRS*
SA564−Δ*ccpA*
SA564−Δ*hptRS/ccpA*

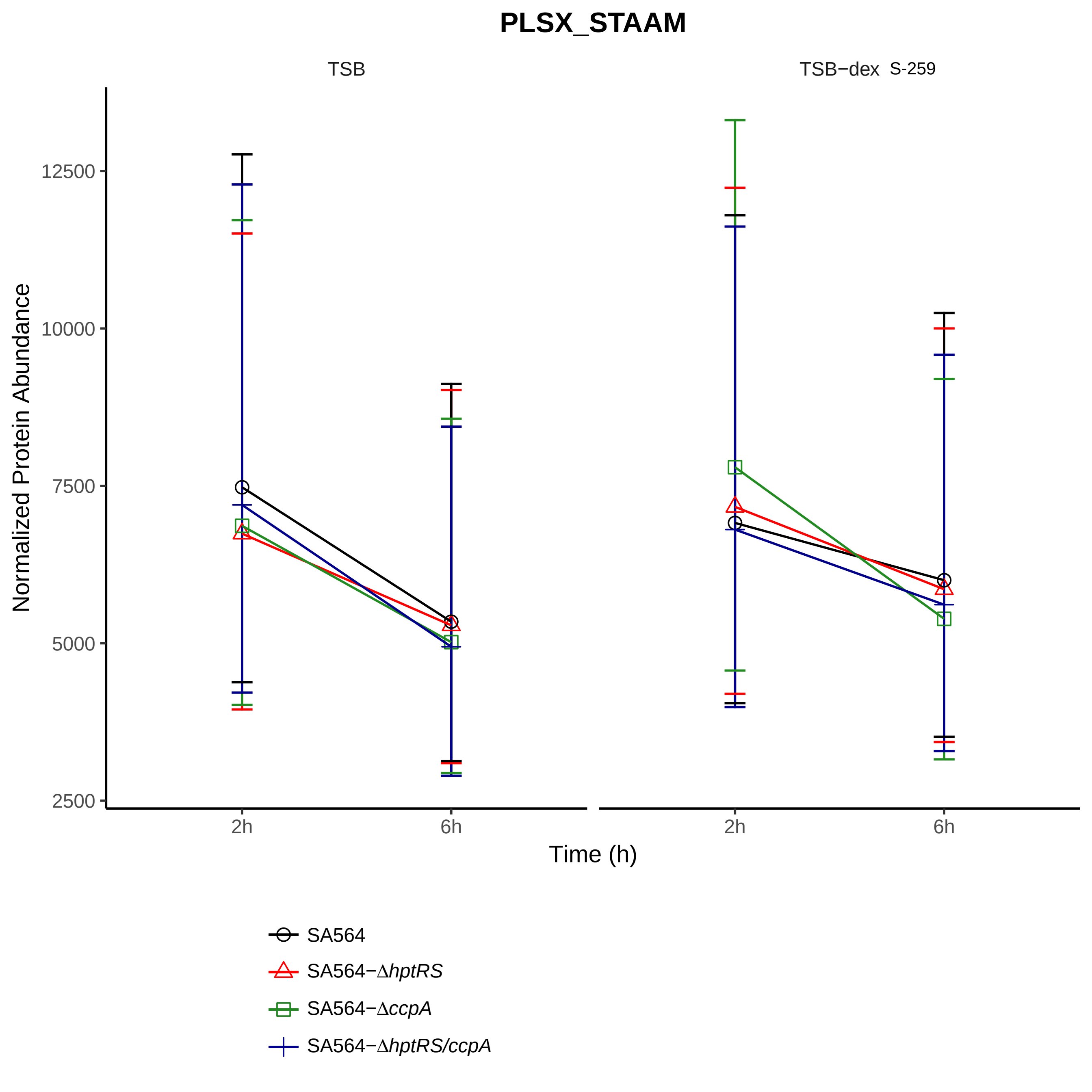

PLSX_STAAM
TSB
TSB−dex
S-259
12500
10000
7500
5000
2500
Normalized Protein Abundance
2h
6h
2h
6h
Time (h)
SA564
SA564−ΔhptRS
SA564−ΔccpA
SA564−ΔhptRS/ccpA

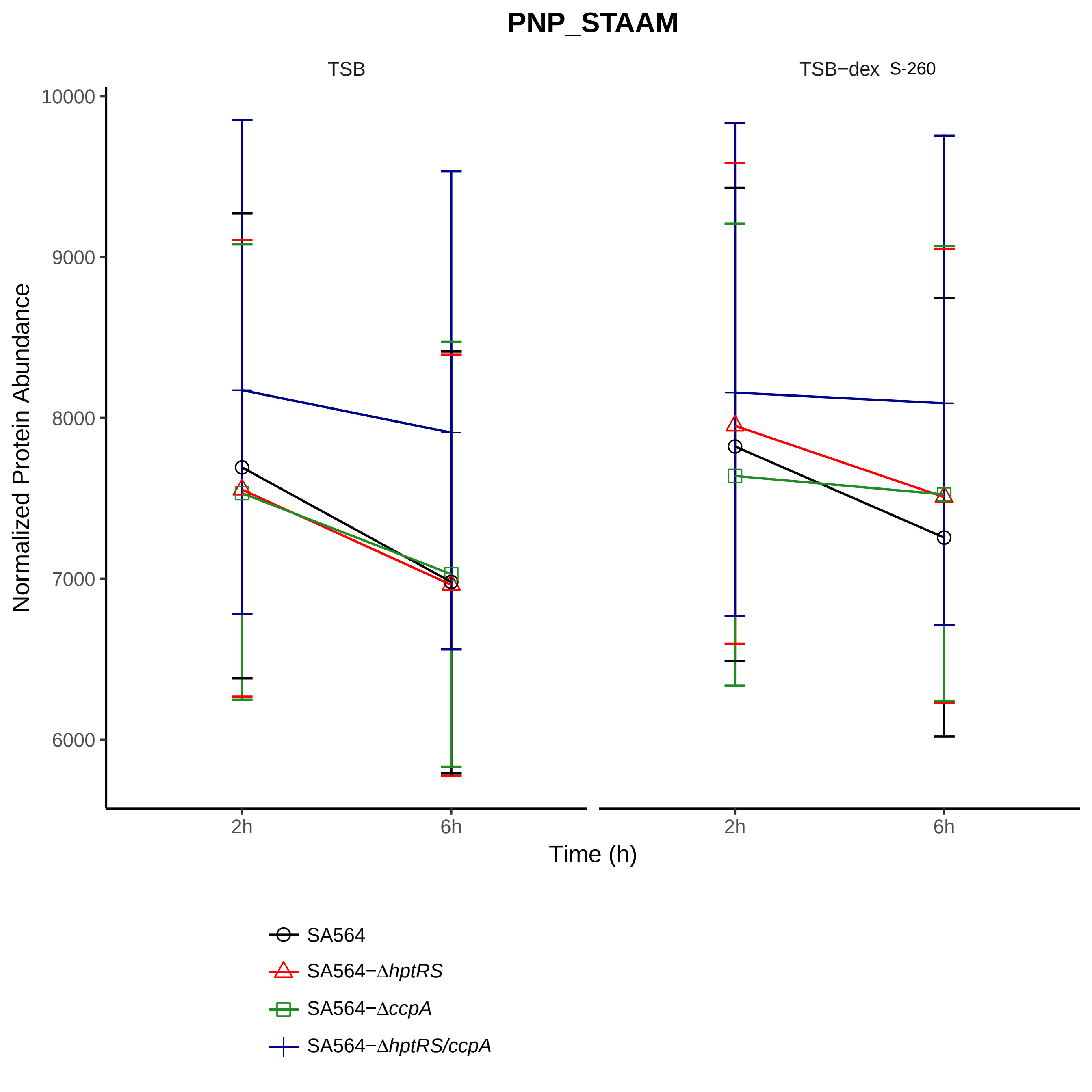

PNP_STAAM
TSB
TSB−dex
S-260
10000
9000
8000
7000
6000
Normalized Protein Abundance
2h
6h
2h
6h
Time (h)
SA564
SA564−Δ*hptRS*
SA564−Δ*ccpA*
SA564−Δ*hptRS/ccpA*

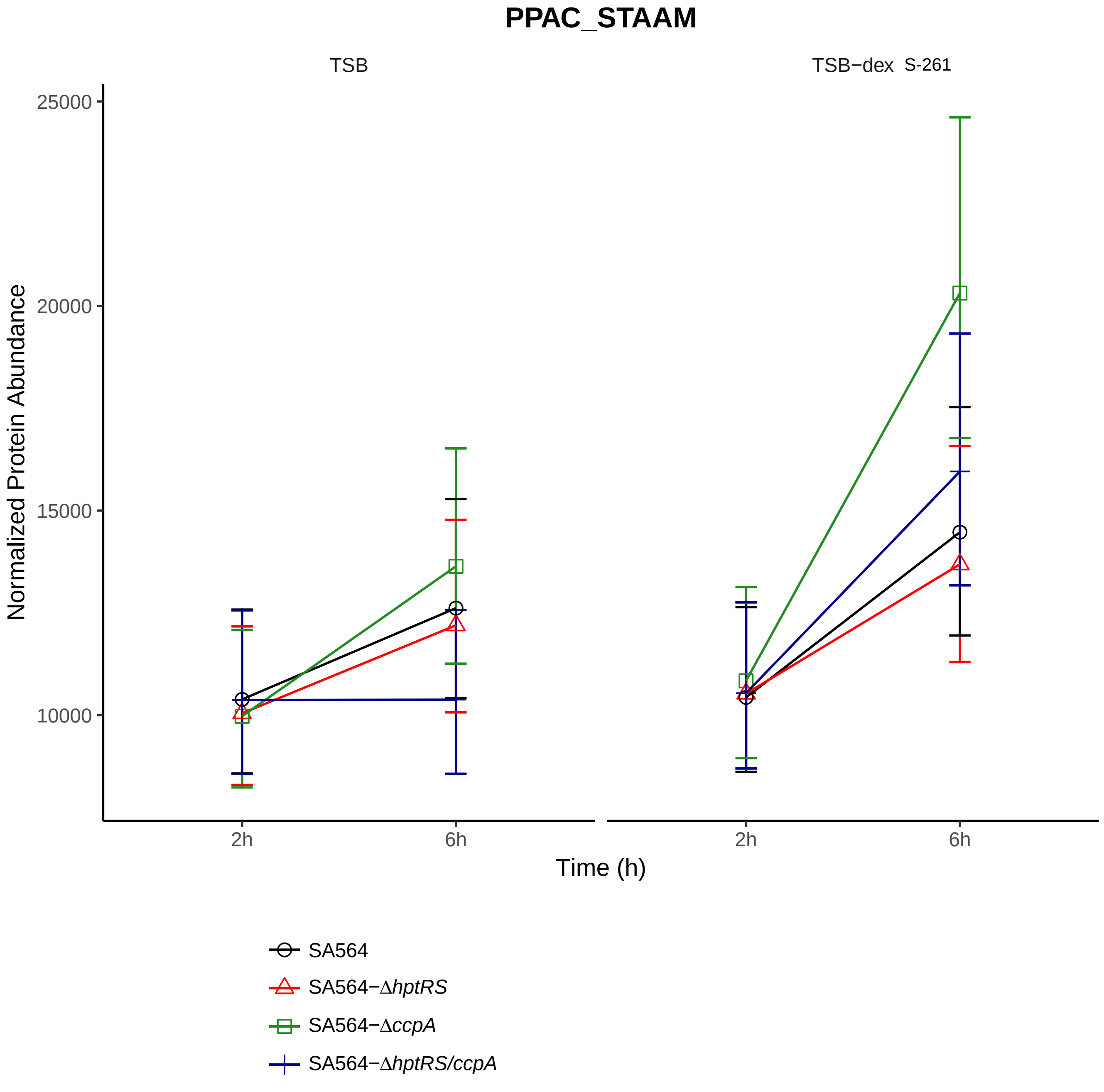

PPAC_STAAM
TSB
TSB−dex
S-261
Normalized Protein Abundance
25000
20000
15000
10000
2h
6h
2h
6h
Time (h)
SA564
SA564−Δ*hptRS*
SA564−Δ*ccpA*
SA564−Δ*hptRS/ccpA*

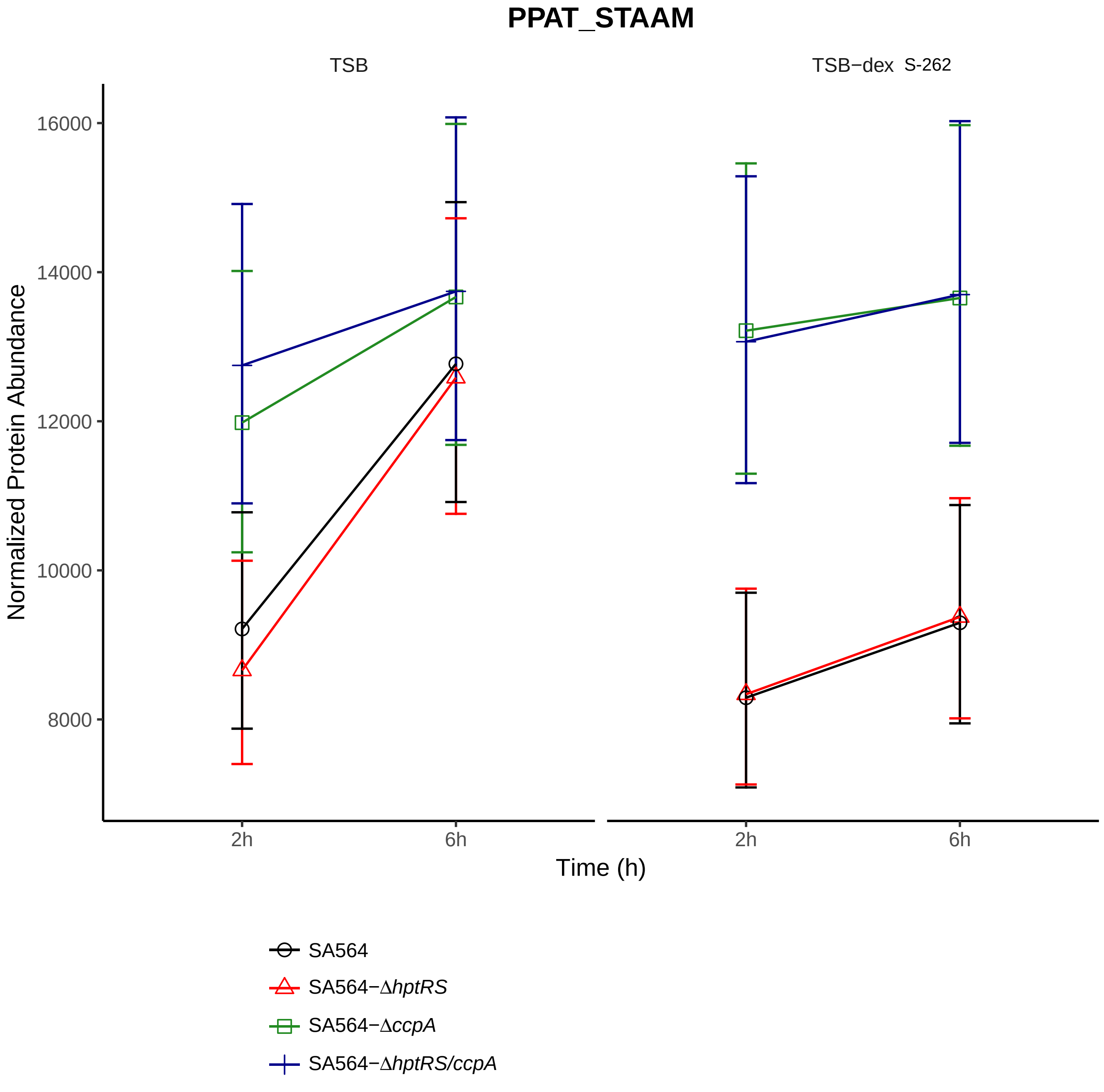

PPAT_STAAM
TSB
TSB−dex
S-262
Normalized Protein Abundance
16000
14000
12000
10000
8000
2h
6h
2h
6h
Time (h)
SA564
SA564−ΔhptRS
SA564−ΔccpA
SA564−ΔhptRS/ccpA

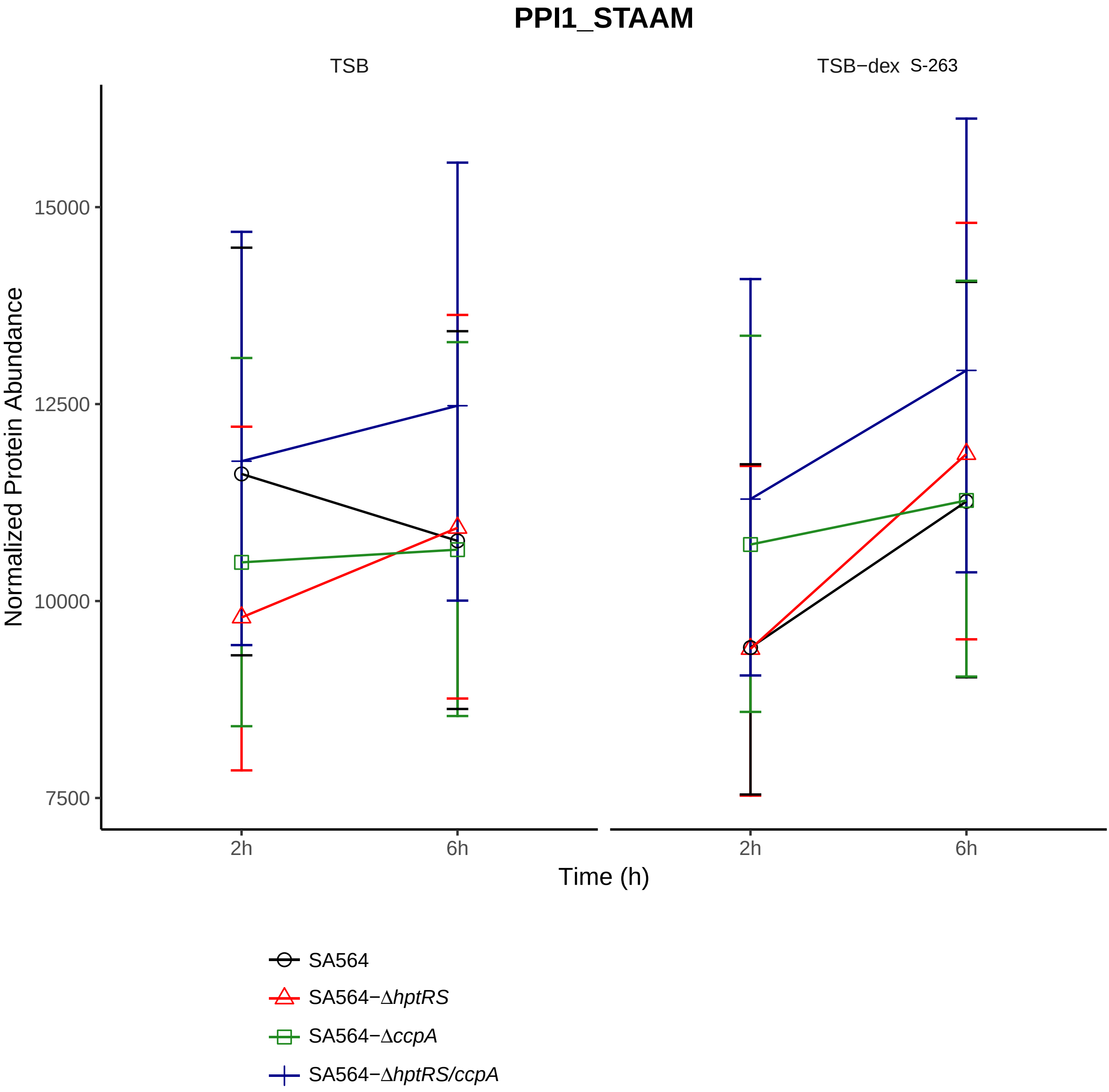

PPI1_STAAM
TSB
TSB−dex
S-263
Normalized Protein Abundance
15000
12500
10000
7500
2h
6h
2h
6h
Time (h)
SA564
SA564−ΔhptRS
SA564−ΔccpA
SA564−ΔhptRS/ccpA

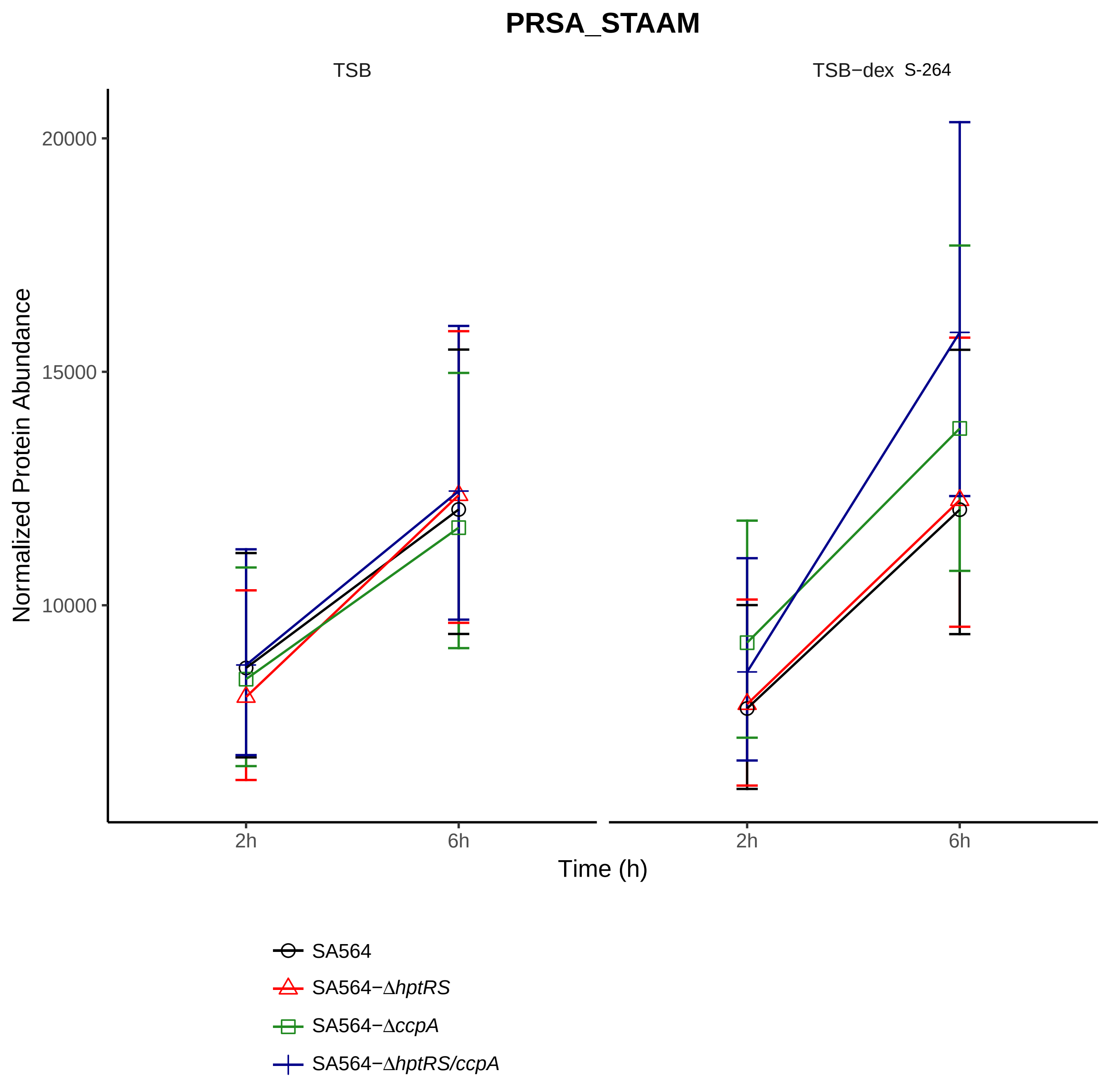

PRSA_STAAM
TSB
TSB−dex
S-264
20000
15000
10000
2h
6h
2h
6h
Normalized Protein Abundance
Time (h)
SA564
SA564−Δ*hptRS*
SA564−Δ*ccpA*
SA564−Δ*hptRS/ccpA*

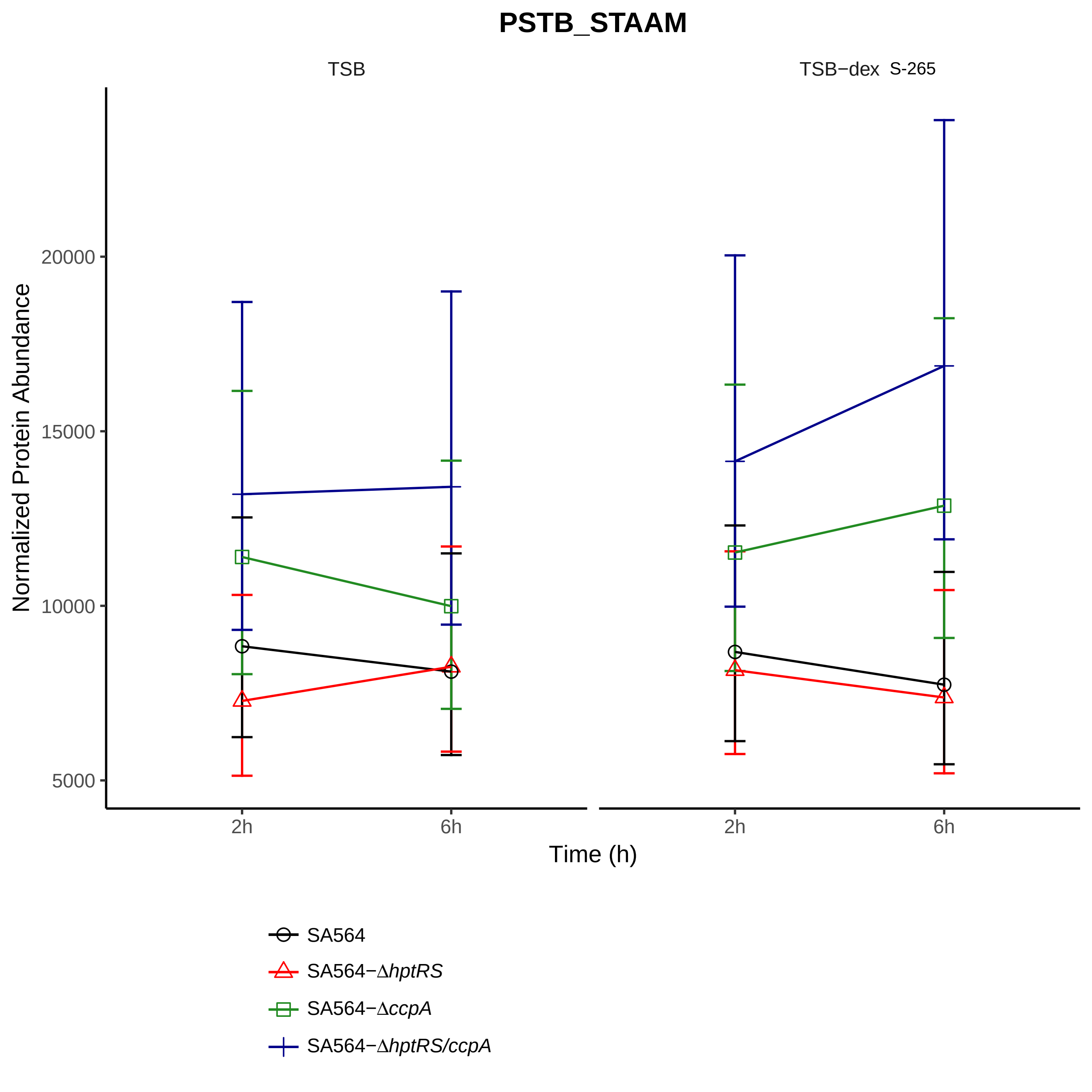
PSTB_STAAM
TSB
TSB−dex
S-265
Normalized Protein Abundance
20000
15000
10000
5000
2h
6h
2h
6h
Time (h)
SA564
SA564−Δ*hptRS*
SA564−Δ*ccpA*
SA564−Δ*hptRS/ccpA*

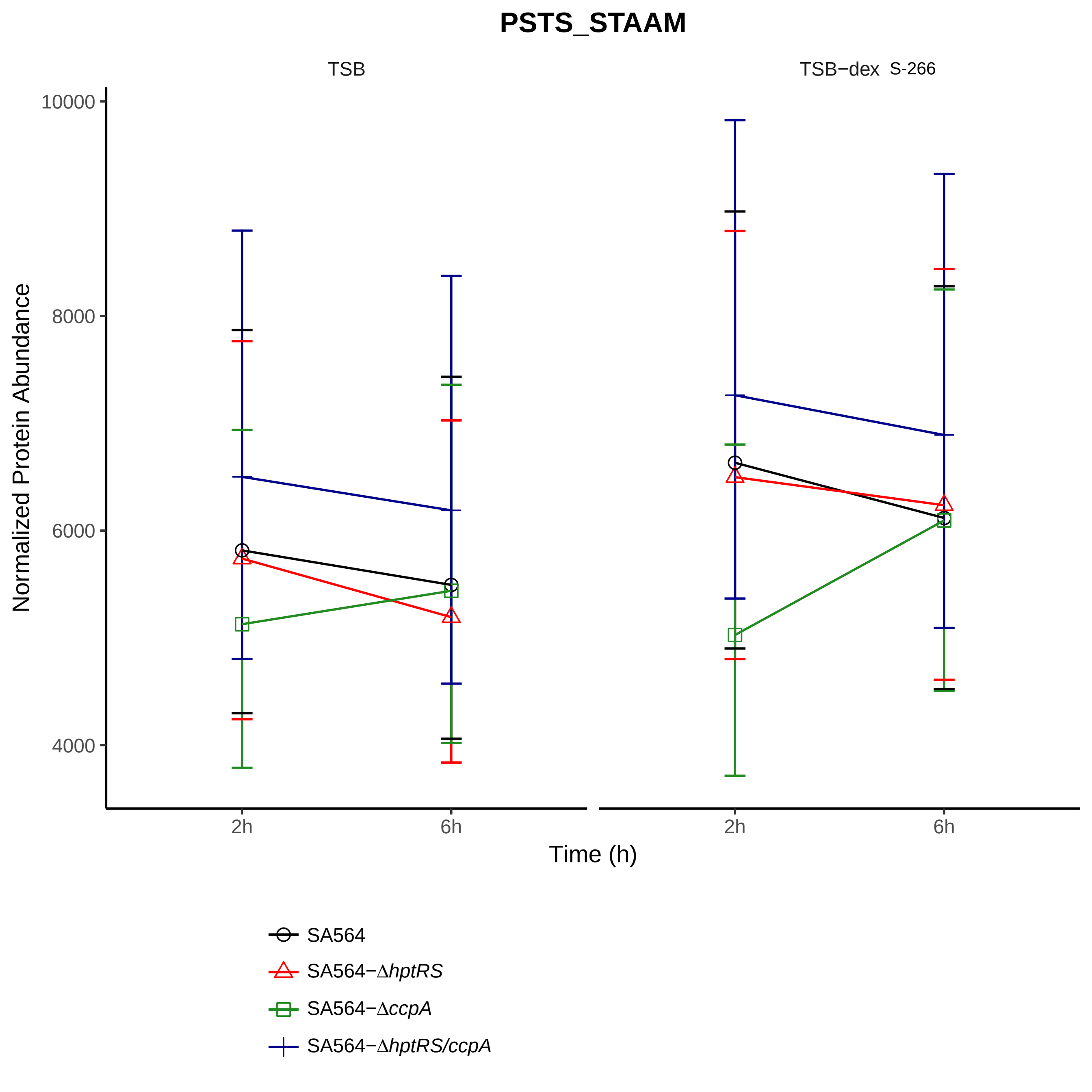

PSTS_STAAM
TSB
TSB−dex
S-266
10000
8000
6000
4000
Normalized Protein Abundance
2h
6h
2h
6h
Time (h)
SA564
SA564−Δ*hptRS*
SA564−Δ*ccpA*
SA564−Δ*hptRS/ccpA*

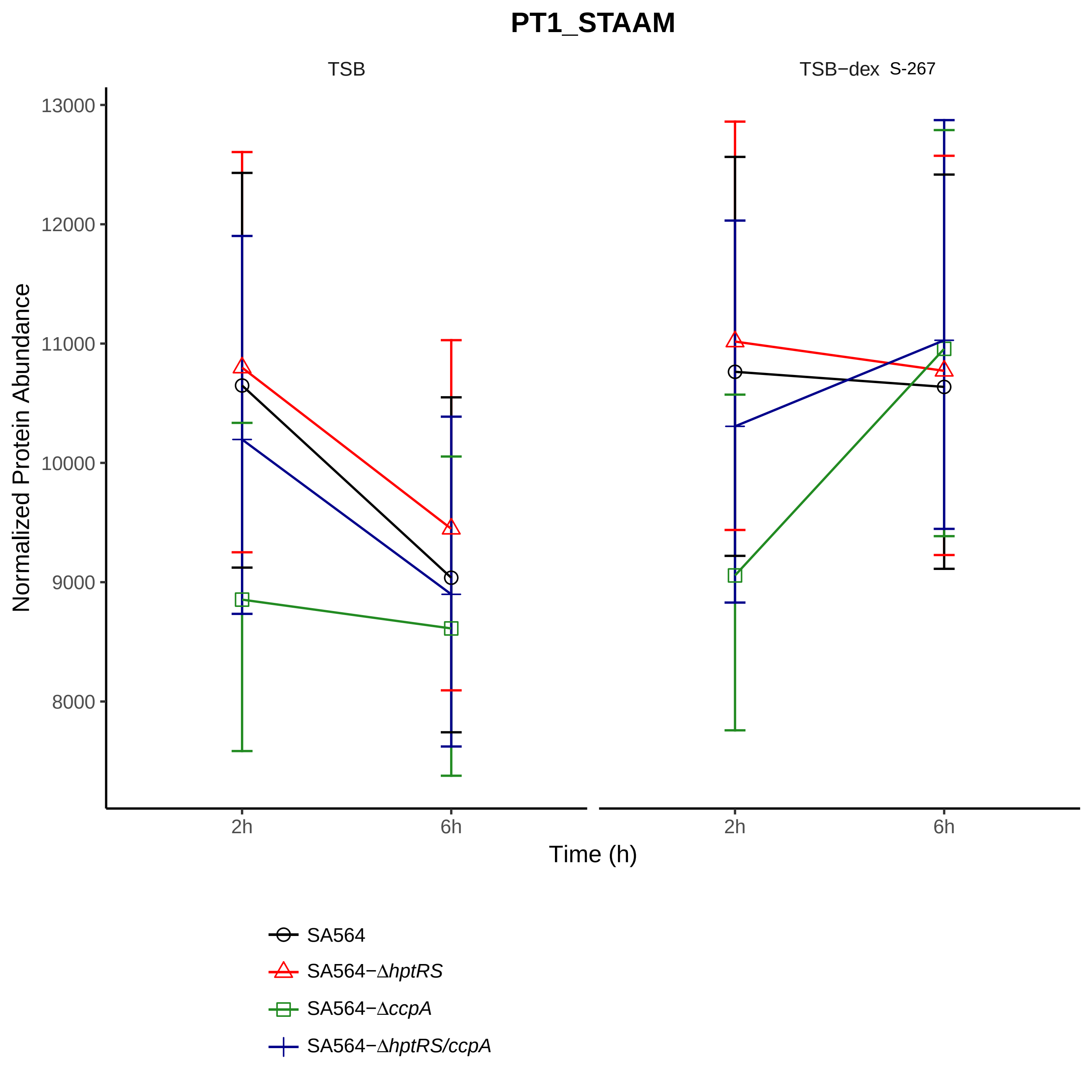

PT1_STAAM
TSB
TSB−dex
S-267
Normalized Protein Abundance
13000
12000
11000
10000
9000
8000
2h
6h
2h
6h
Time (h)
SA564
SA564−Δ*hptRS*
SA564−Δ*ccpA*
SA564−Δ*hptRS/ccpA*

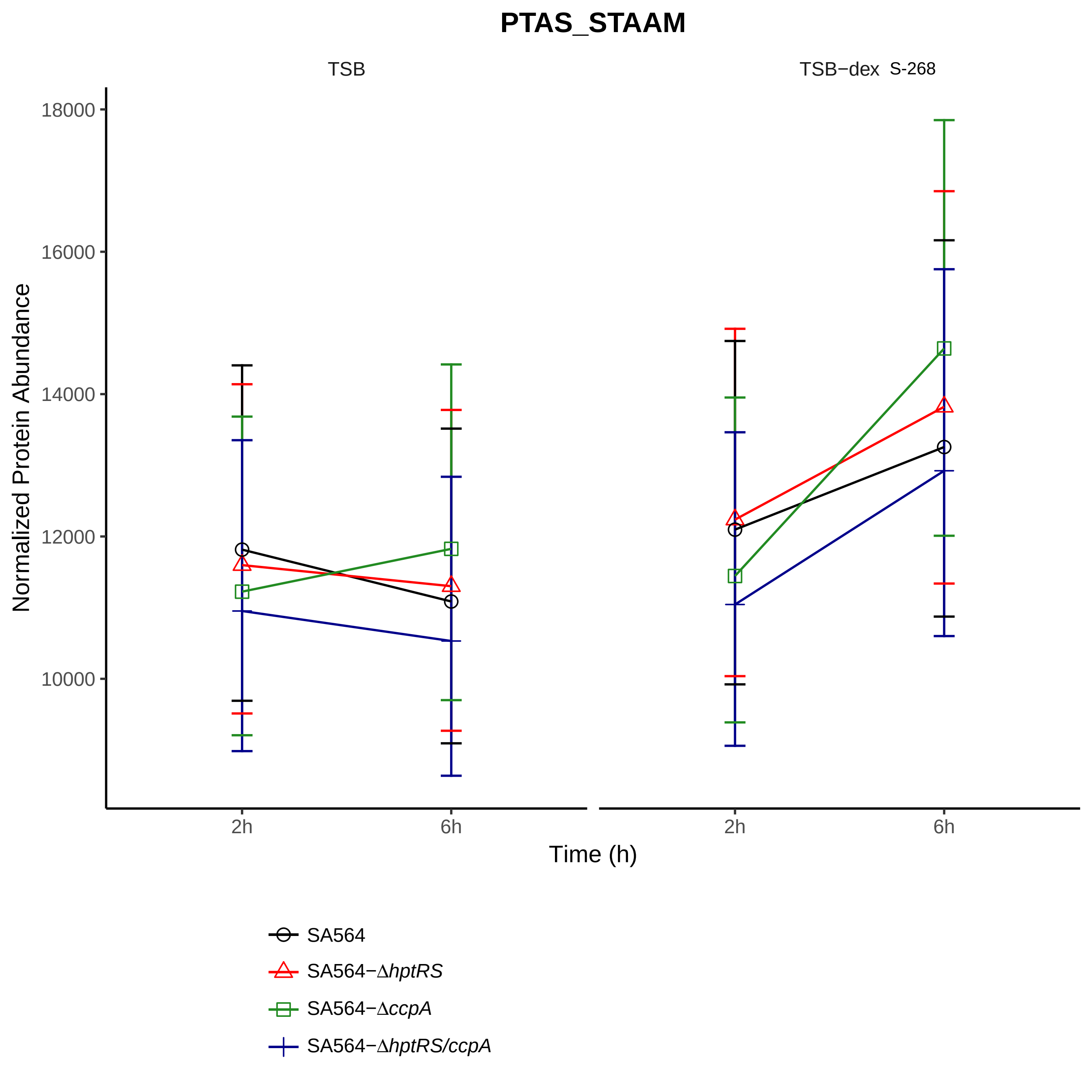
PTAS_STAAM
TSB
TSB−dex
S-268
Normalized Protein Abundance
18000
16000
14000
12000
10000
2h
6h
2h
6h
Time (h)
SA564
SA564−ΔhptRS
SA564−ΔccpA
SA564−ΔhptRS/ccpA

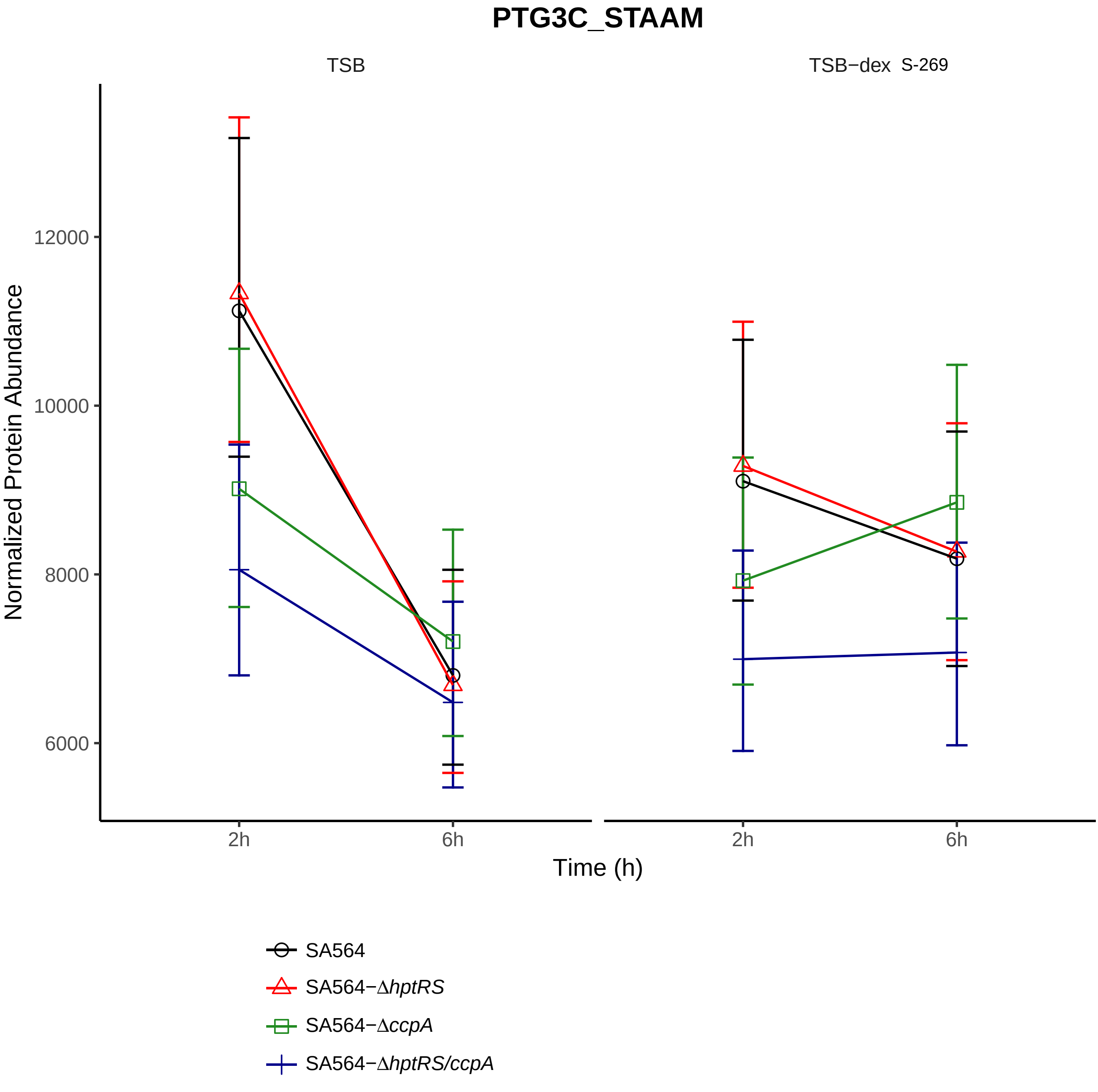

PTG3C_STAAM
TSB
TSB−dex
Normalized Protein Abundance
12000
10000
8000
6000
2h
6h
2h
6h
Time (h)
SA564
SA564−ΔhptRS
SA564−ΔccpA
SA564−ΔhptRS/ccpA

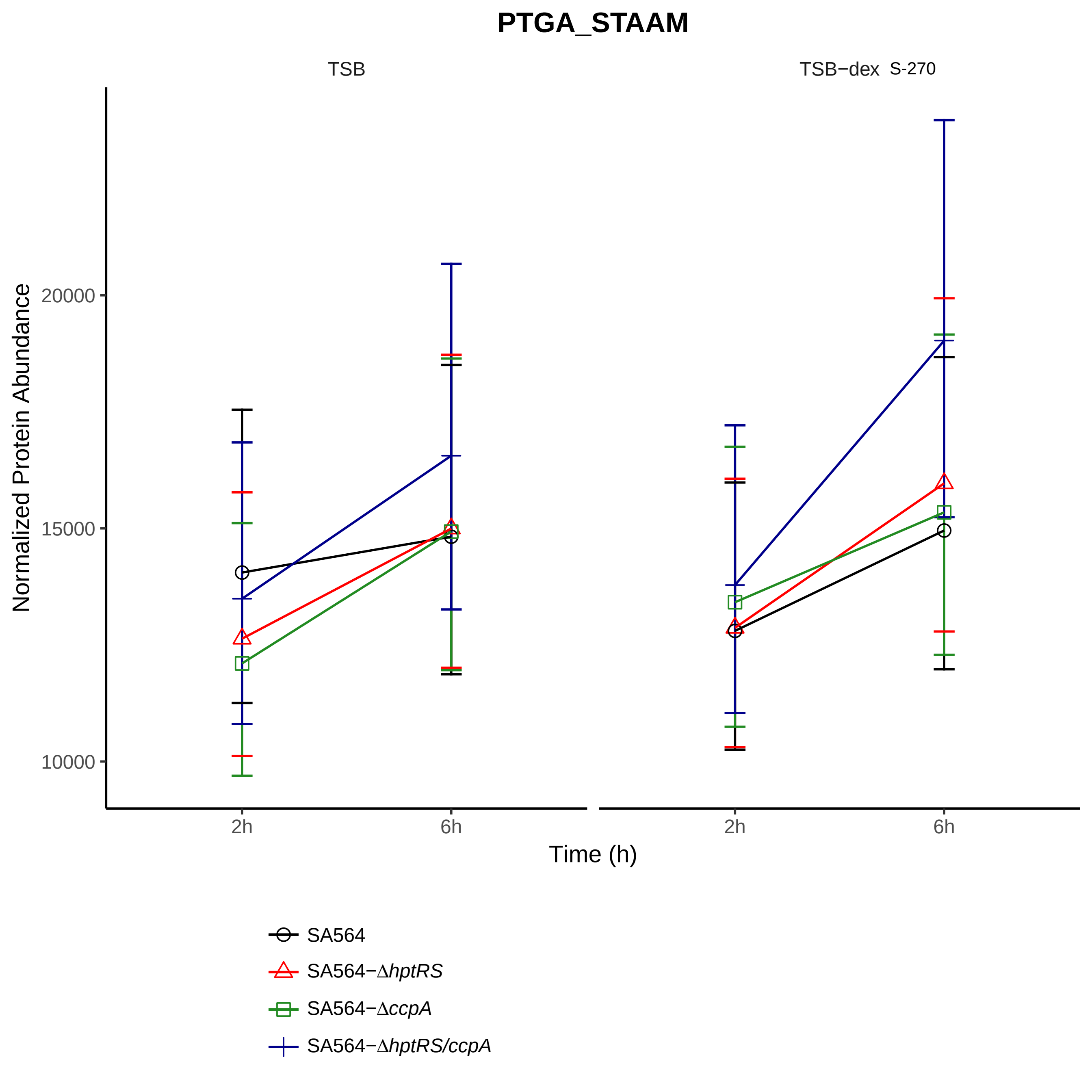
PTGA_STAAM
TSB
TSB−dex
S-270
Normalized Protein Abundance
20000
15000
10000
2h
6h
2h
6h
Time (h)
SA564
SA564−Δ*hptRS*
SA564−Δ*ccpA*
SA564−Δ*hptRS/ccpA*

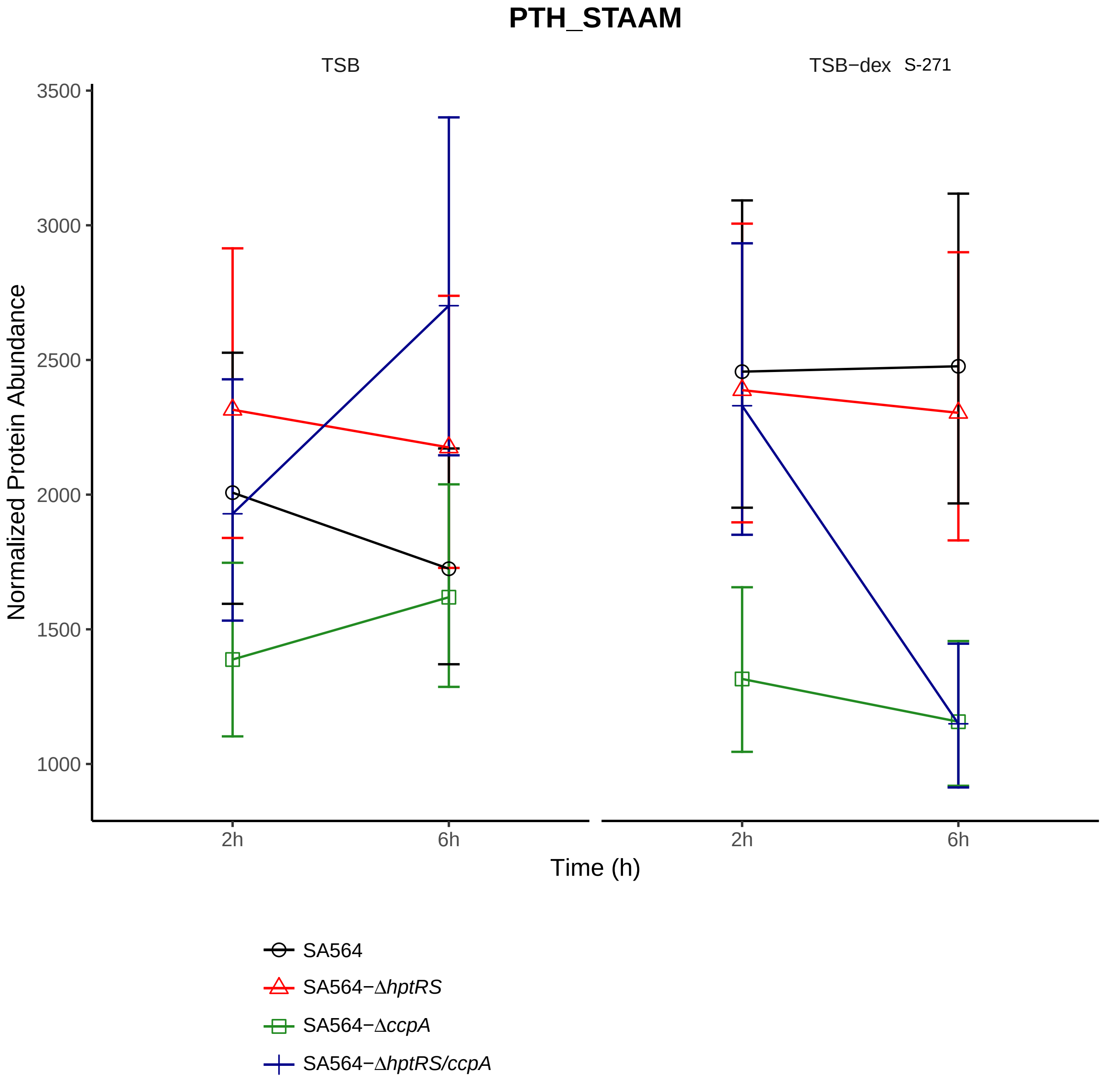

PTH_STAAM
TSB
TSB−dex
S-271
Normalized Protein Abundance
3500
3000
2500
2000
1500
1000
2h
6h
2h
6h
Time (h)
SA564
SA564−Δ*hptRS*
SA564−Δ*ccpA*
SA564−Δ*hptRS/ccpA*

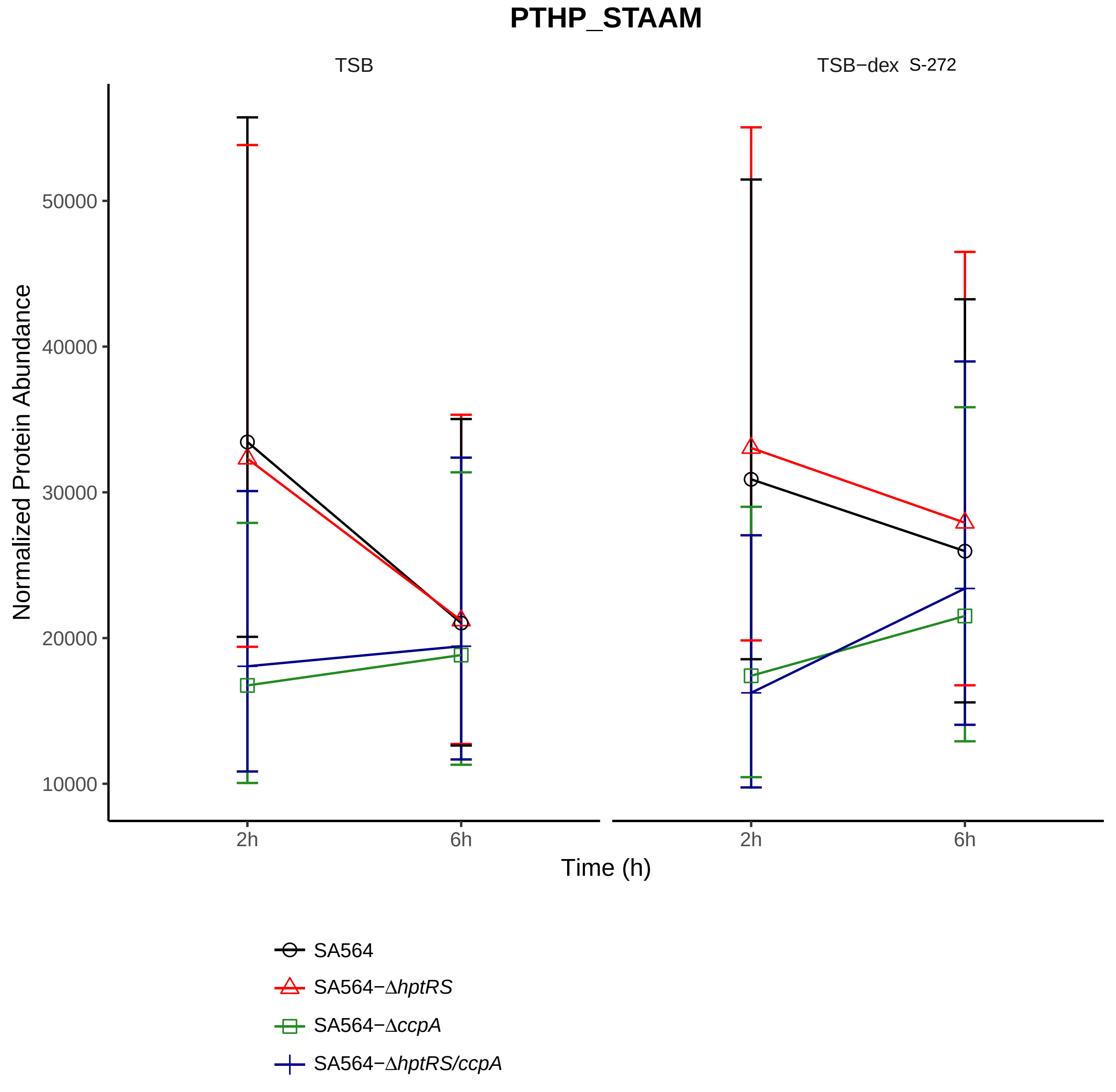

PTHP_STAAM
TSB
TSB−dex
S-272
Normalized Protein Abundance
50000
40000
30000
20000
10000
2h
6h
2h
6h
Time (h)
SA564
SA564−Δ*hptRS*
SA564−Δ*ccpA*
SA564−Δ*hptRS/ccpA*

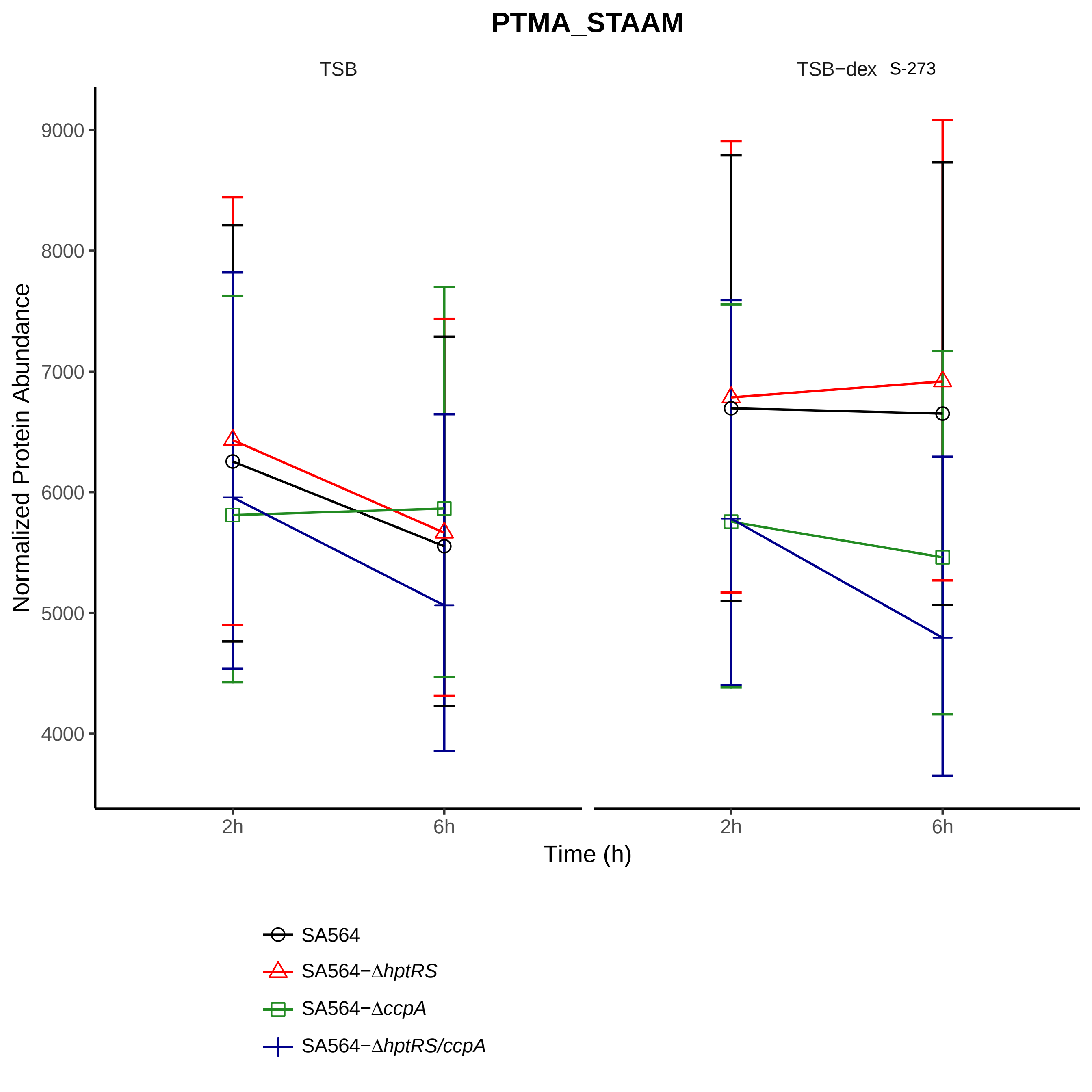
PTMA_STAAM
TSB
TSB−dex
S-273
Normalized Protein Abundance
9000
8000
7000
6000
5000
4000
2h
6h
2h
6h
Time (h)
SA564
SA564−ΔhptRS
SA564−ΔccpA
SA564−ΔhptRS/ccpA

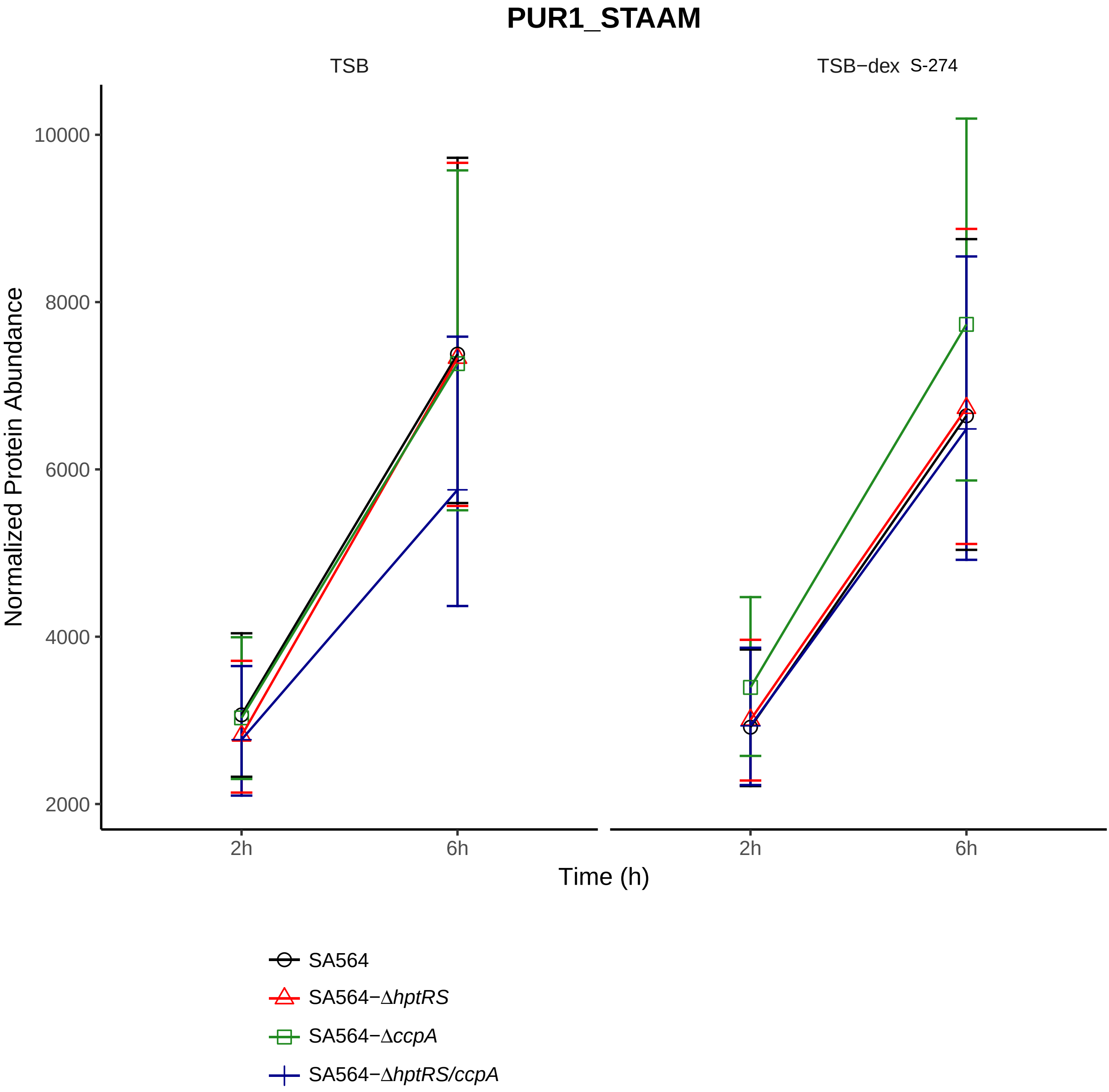

PUR1_STAAM
TSB
TSB−dex
S-274
Normalized Protein Abundance
10000
8000
6000
4000
2000
2h
6h
2h
6h
Time (h)
SA564
SA564−ΔhptRS
SA564−ΔccpA
SA564−ΔhptRS/ccpA

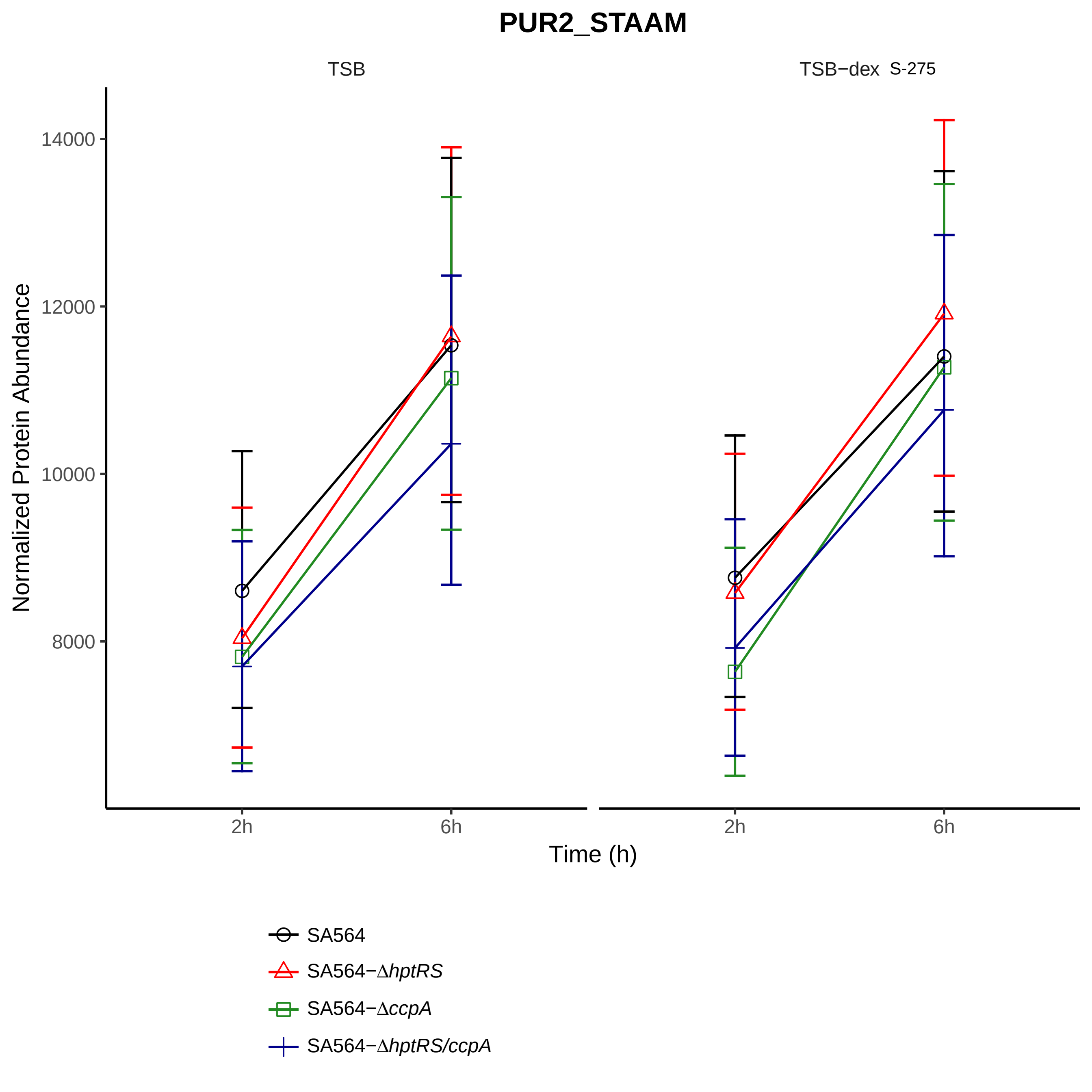

PUR2_STAAM
TSB
TSB−dex
S-275
Normalized Protein Abundance
14000
12000
10000
8000
2h
6h
2h
6h
Time (h)
SA564
SA564−Δ*hptRS*
SA564−Δ*ccpA*
SA564−Δ*hptRS/ccpA*

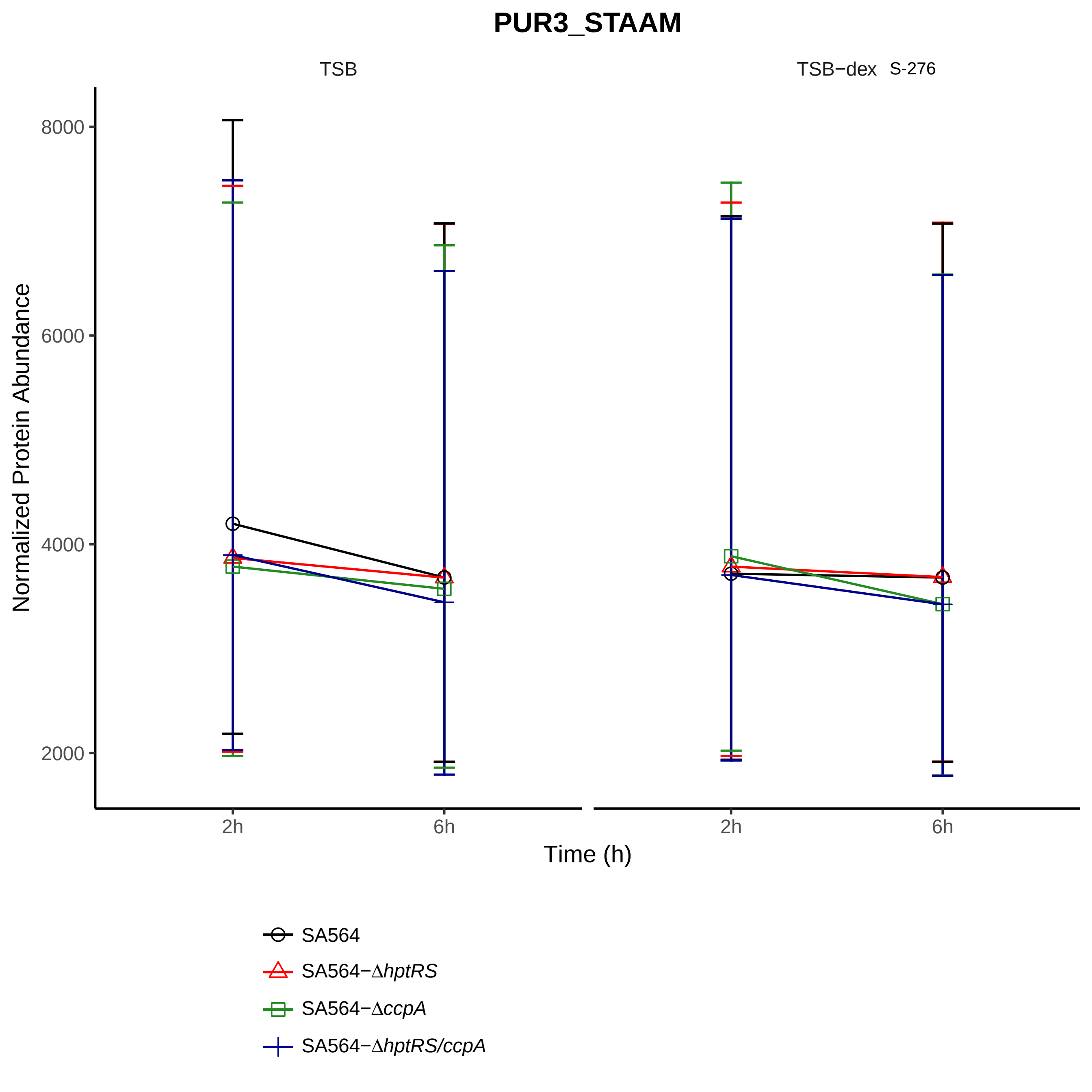
PUR3_STAAM
TSB
TSB−dex
S-276
Normalized Protein Abundance
8000
6000
4000
2000
2h
6h
2h
6h
Time (h)
SA564
SA564−ΔhptRS
SA564−ΔccpA
SA564−ΔhptRS/ccpA

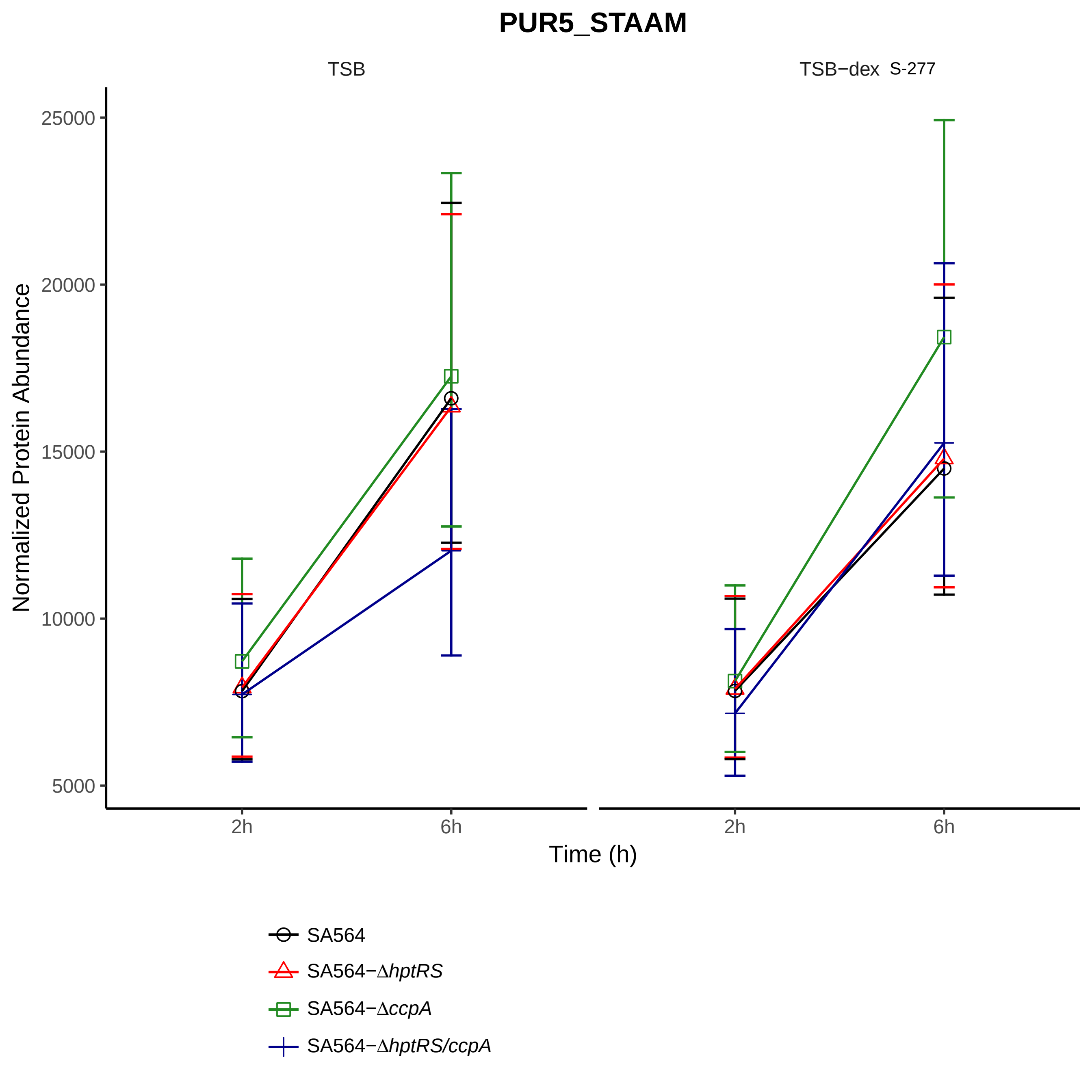

PUR5_STAAM
TSB
TSB−dex
S-277
Normalized Protein Abundance
25000
20000
15000
10000
5000
2h
6h
2h
6h
Time (h)
SA564
SA564−ΔhptRS
SA564−ΔccpA
SA564−ΔhptRS/ccpA

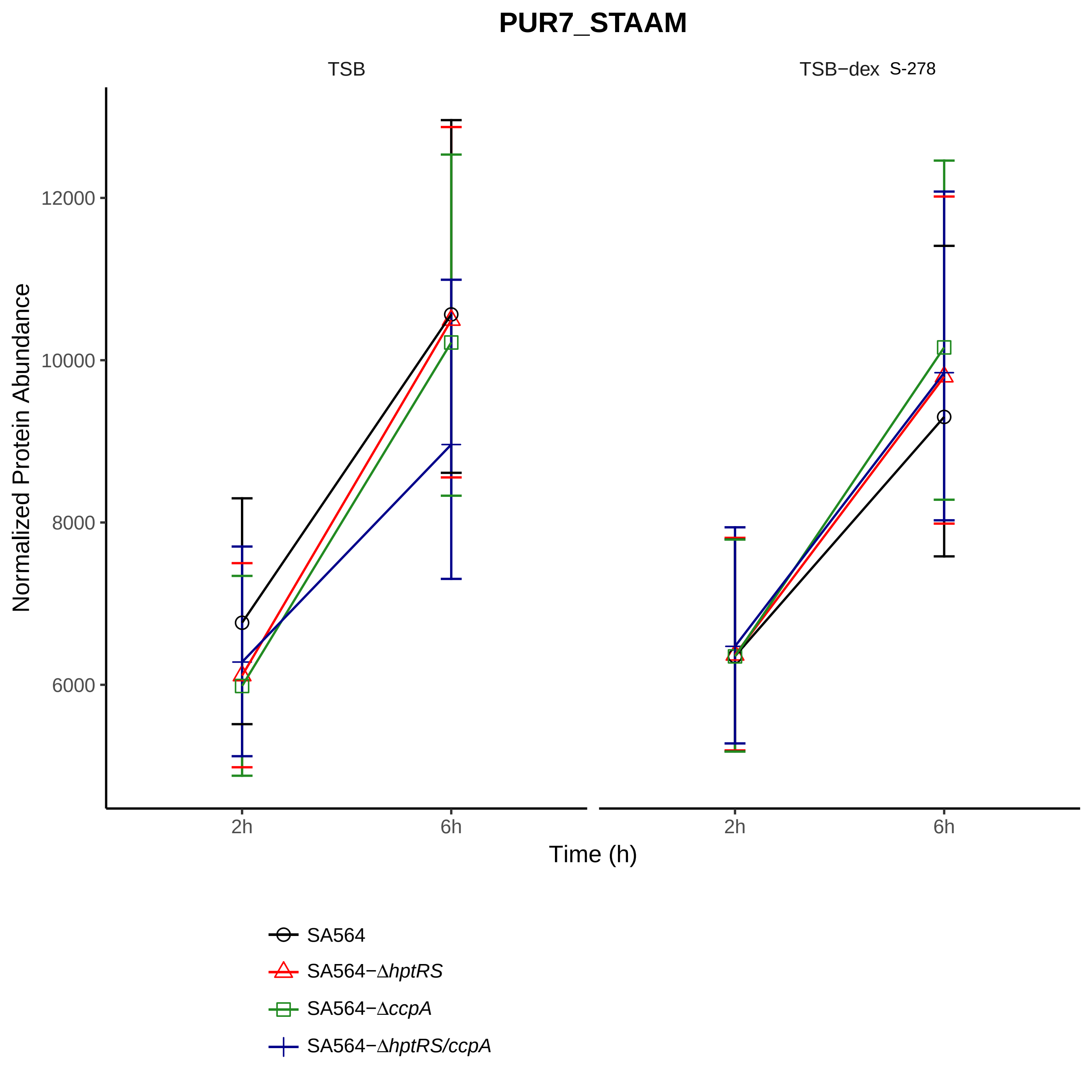
PUR7_STAAM
TSB
TSB−dex
S-278
Normalized Protein Abundance
12000
10000
8000
6000
2h
6h
2h
6h
Time (h)
SA564
SA564−Δ*hptRS*
SA564−Δ*ccpA*
SA564−Δ*hptRS/ccpA*

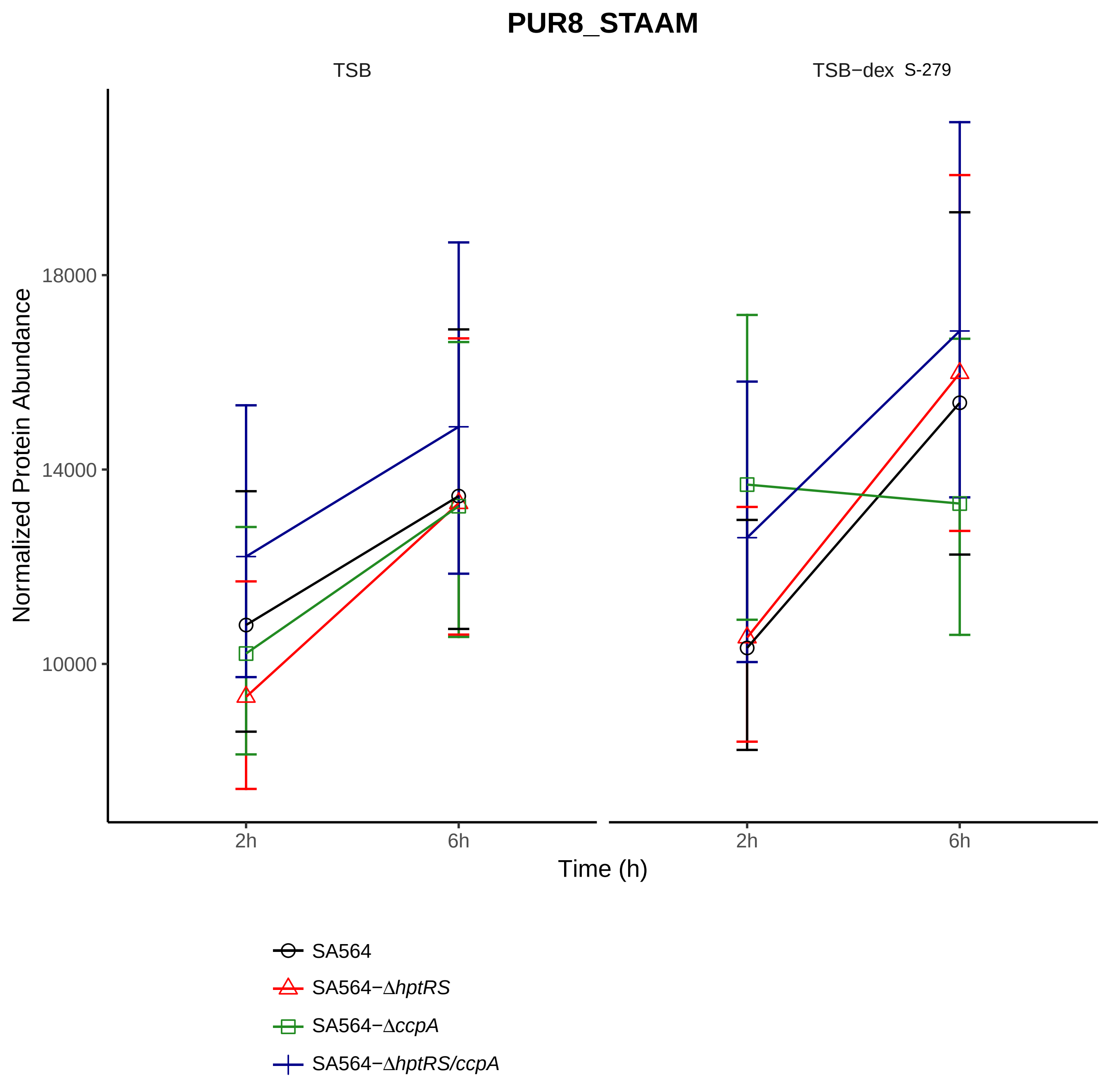

PUR8_STAAM
TSB
TSB−dex
S-279
Normalized Protein Abundance
18000
14000
10000
2h
6h
2h
6h
Time (h)
SA564
SA564−Δ*hptRS*
SA564−Δ*ccpA*
SA564−Δ*hptRS/ccpA*

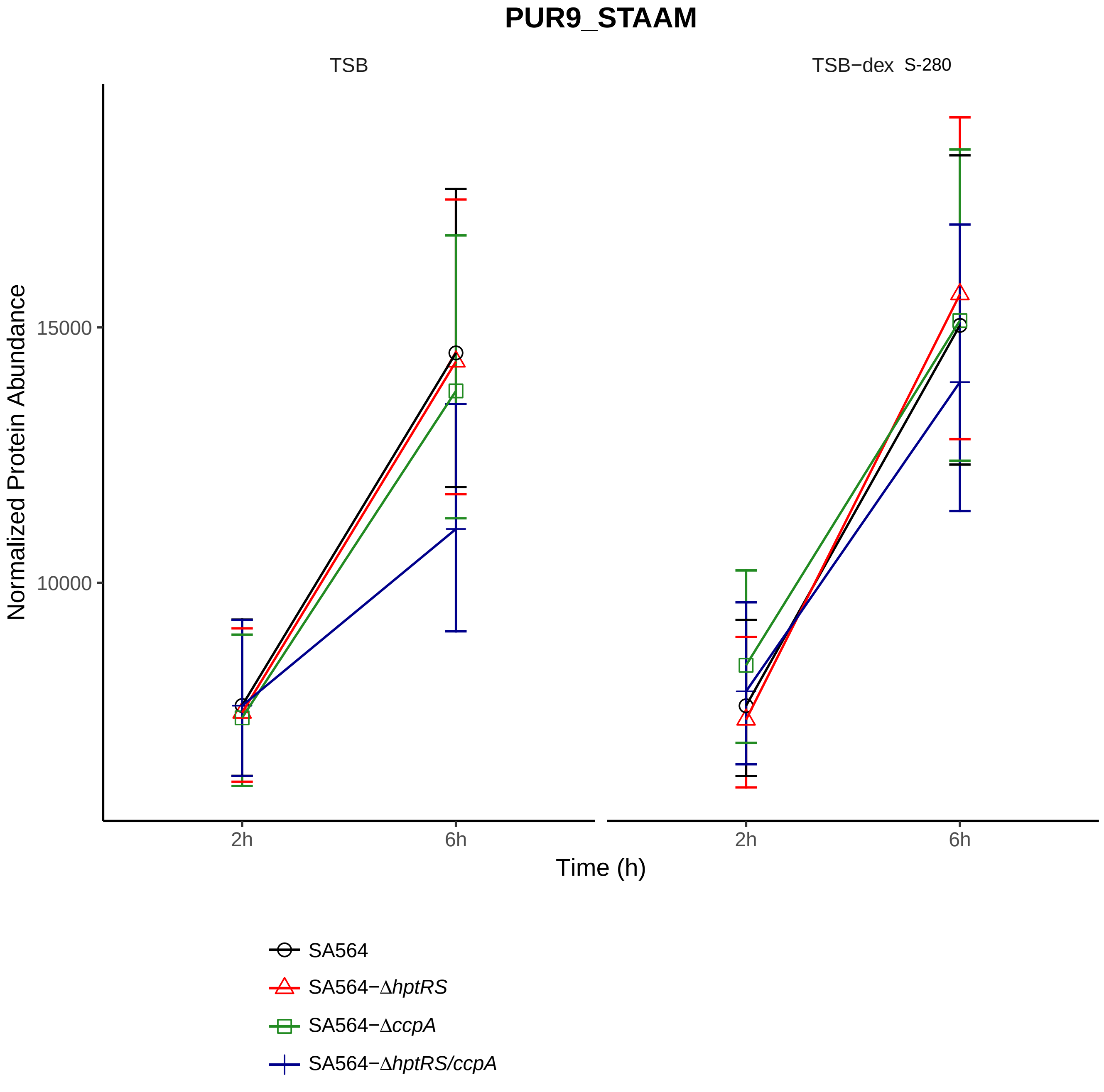
PUR9_STAAM
TSB
TSB−dex
S-280
Normalized Protein Abundance
15000
10000
2h
6h
2h
6h
Time (h)
SA564
SA564−Δ*hptRS*
SA564−Δ*ccpA*
SA564−Δ*hptRS/ccpA*

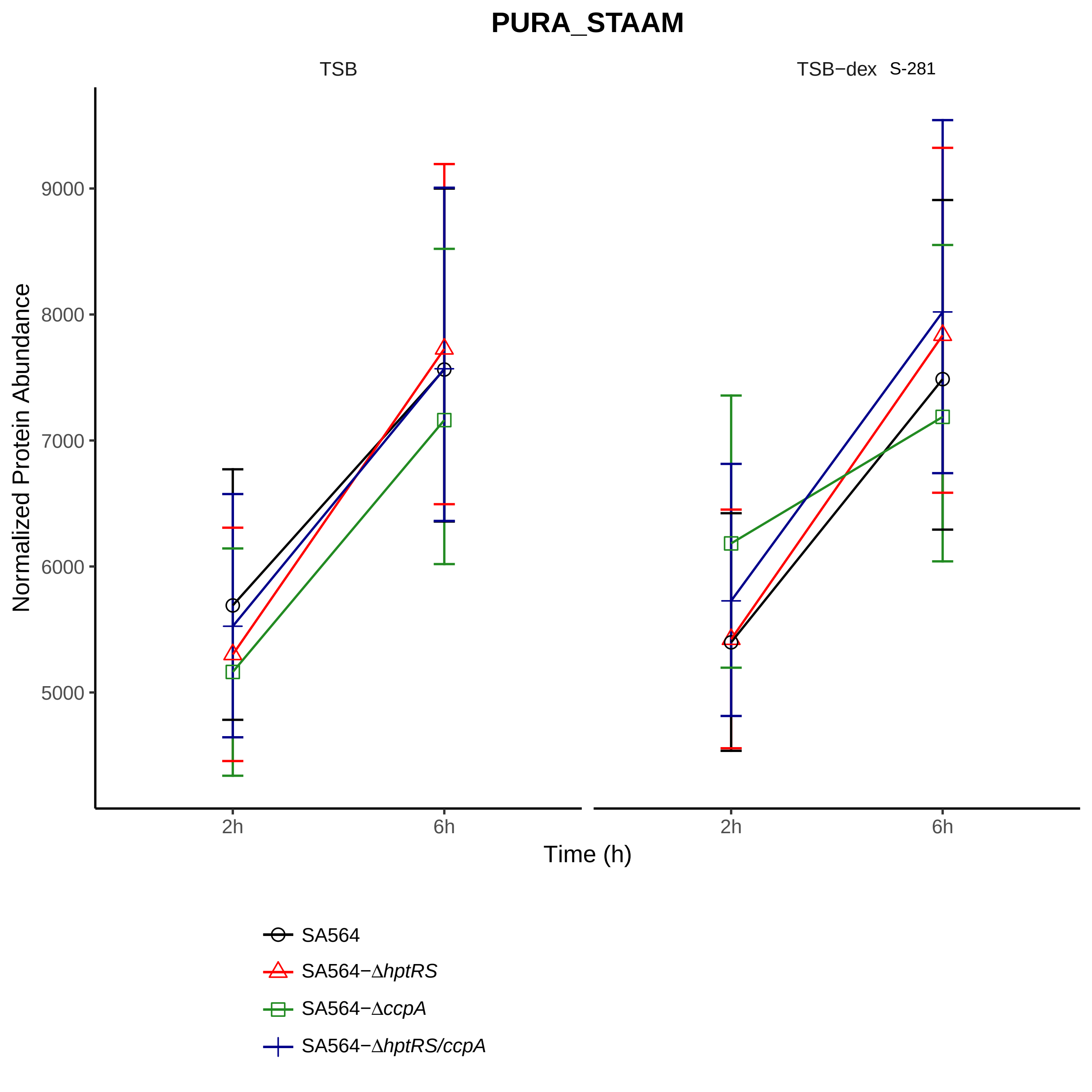
PURA_STAAM
TSB
TSB−dex
S-281
Normalized Protein Abundance
9000
8000
7000
6000
5000
2h
6h
2h
6h
Time (h)
SA564
SA564−Δ*hptRS*
SA564−Δ*ccpA*
SA564−Δ*hptRS/ccpA*

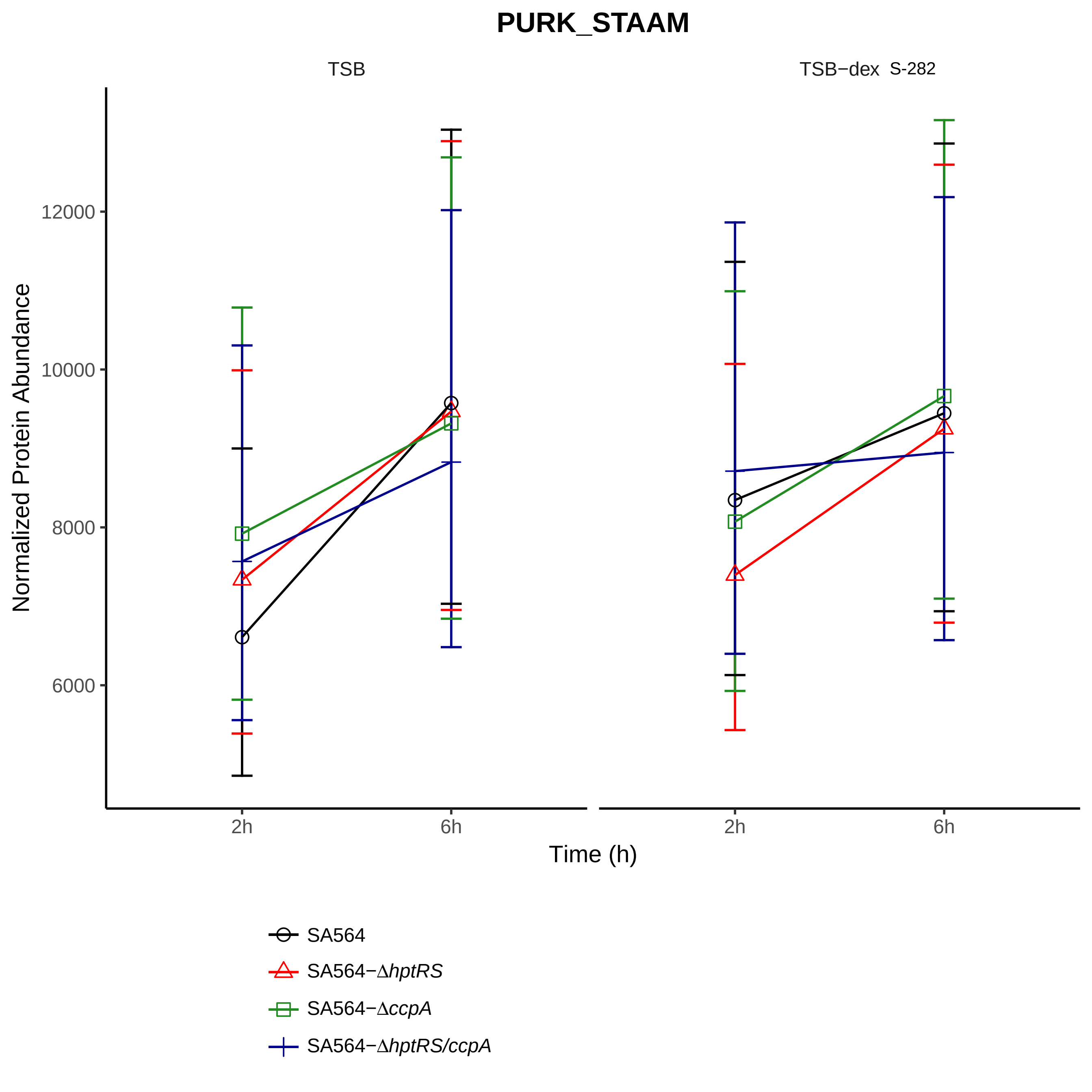
PURK_STAAM
TSB
TSB−dex
S-282
Normalized Protein Abundance
12000
10000
8000
6000
2h
6h
2h
6h
Time (h)
SA564
SA564−ΔhptRS
SA564−ΔccpA
SA564−ΔhptRS/ccpA

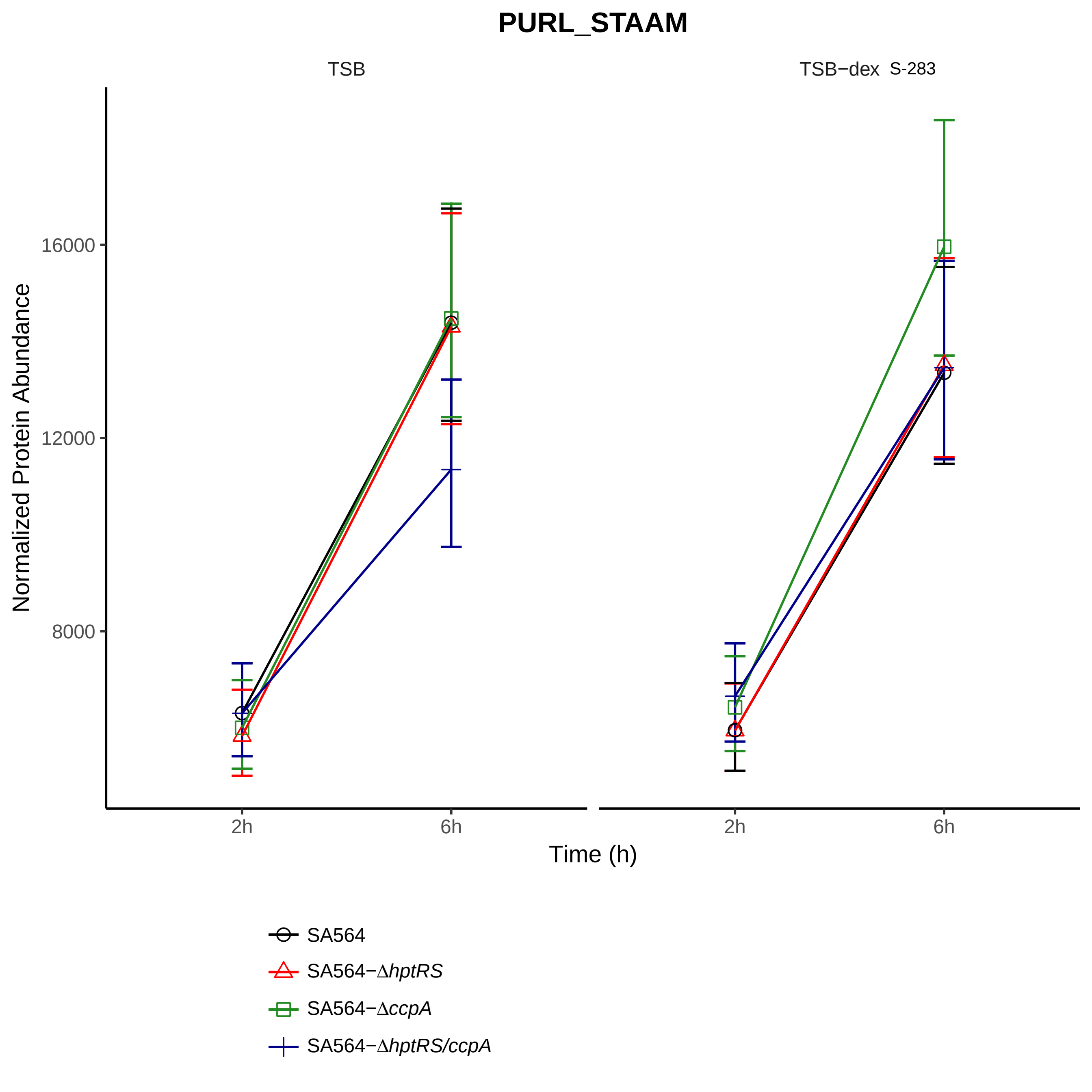
PURL_STAAM
TSB
TSB−dex
S-283
Normalized Protein Abundance
16000
12000
8000
2h
6h
2h
6h
Time (h)
SA564
SA564−Δ*hptRS*
SA564−Δ*ccpA*
SA564−Δ*hptRS/ccpA*

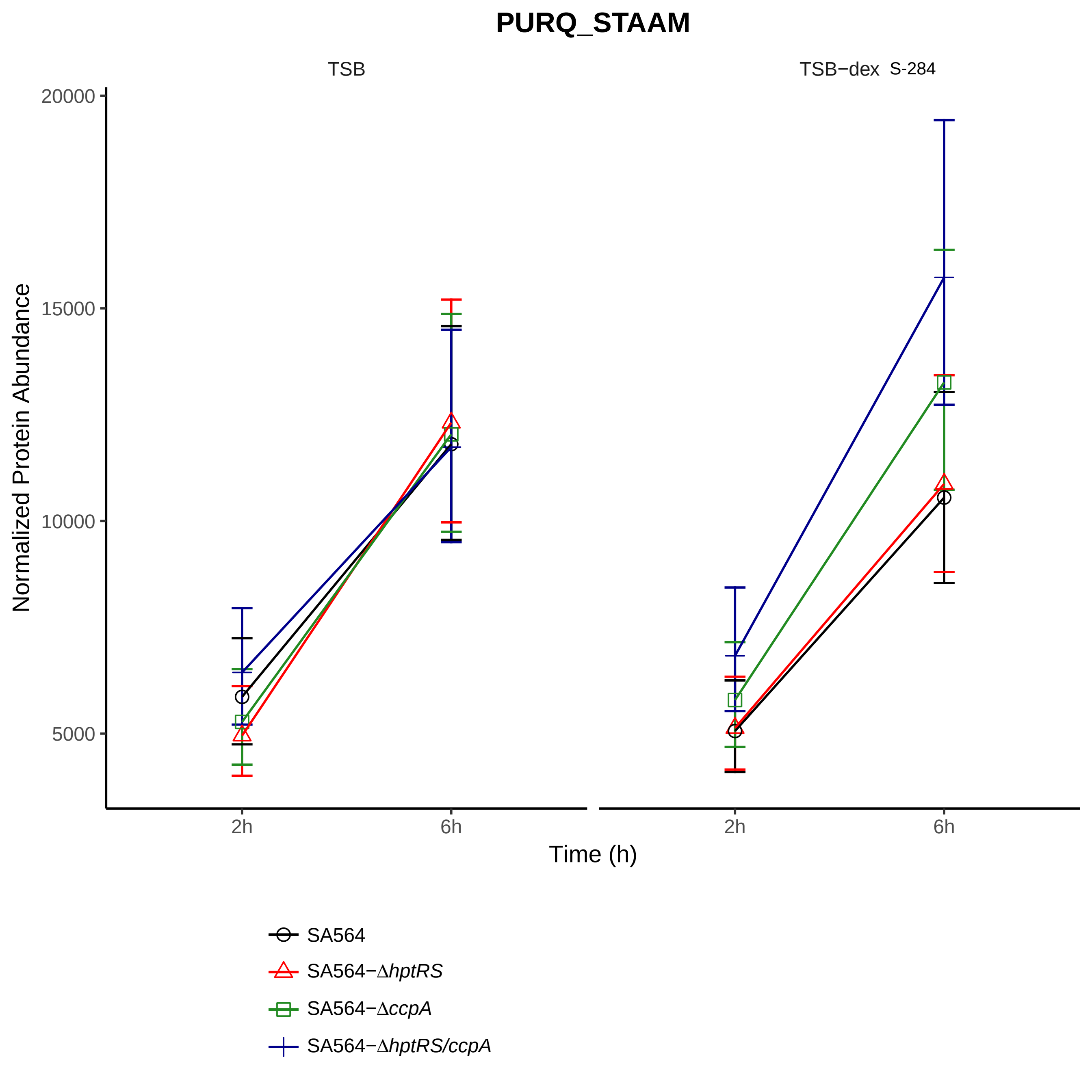
PURQ_STAAM
TSB
TSB−dex
S-284
Normalized Protein Abundance
20000
15000
10000
5000
2h
6h
2h
6h
Time (h)
SA564
SA564−ΔhptRS
SA564−ΔccpA
SA564−ΔhptRS/ccpA

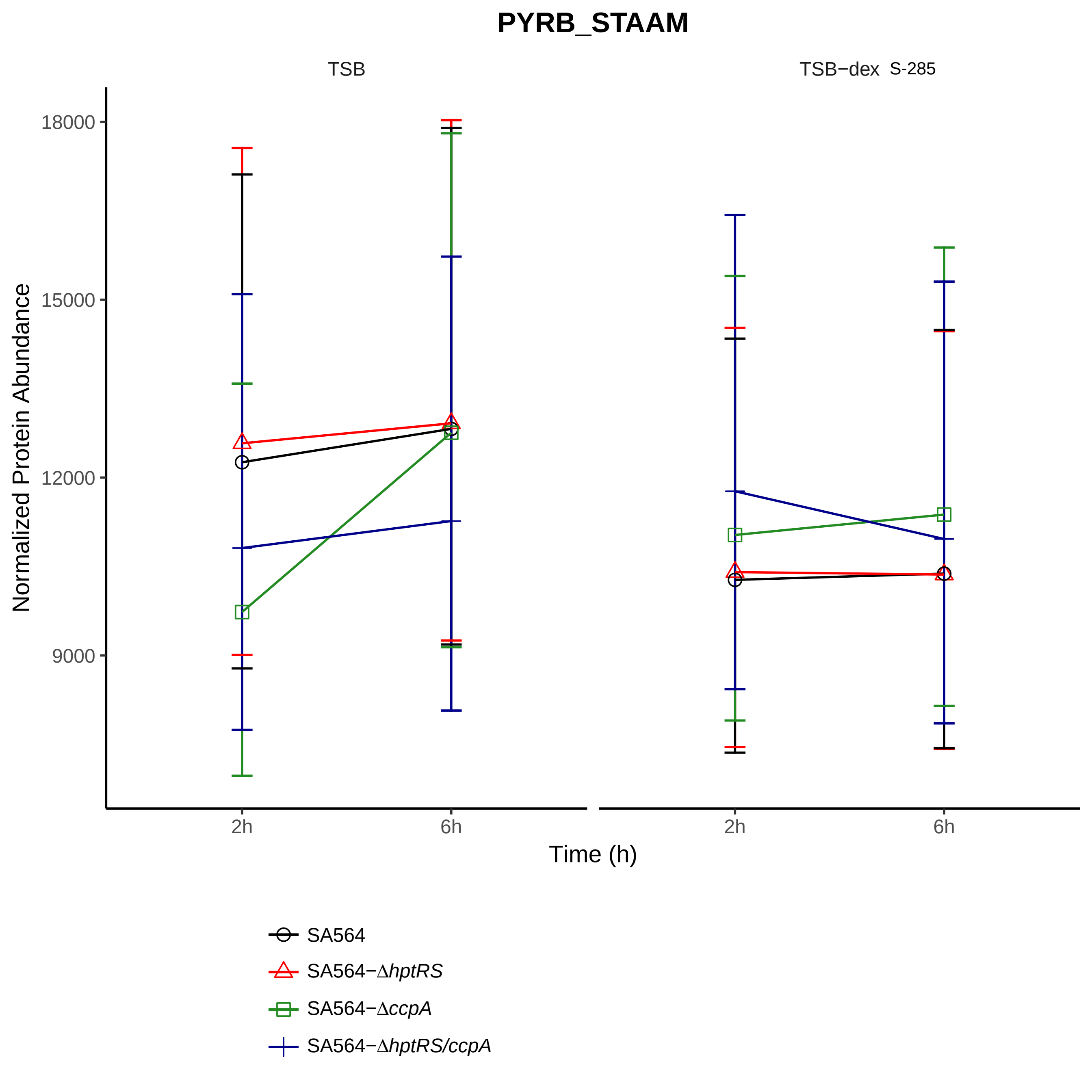

PYRB_STAAM
TSB
TSB−dex
S-285
Normalized Protein Abundance
18000
15000
12000
9000
2h
6h
2h
6h
Time (h)
SA564
SA564−Δ*hptRS*
SA564−Δ*ccpA*
SA564−Δ*hptRS/ccpA*

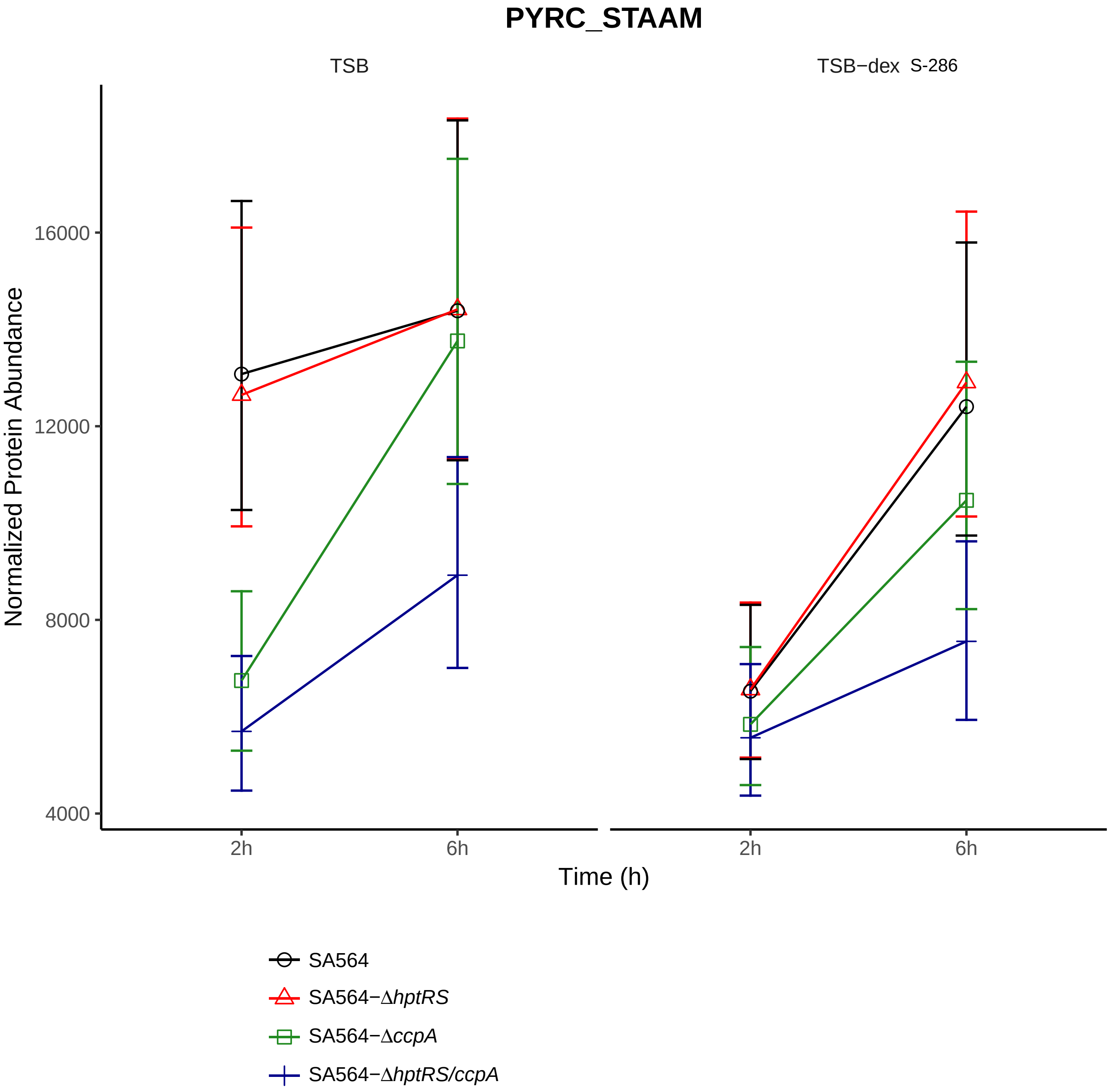
PYRC_STAAM
TSB
TSB−dex
S-286
Normalized Protein Abundance
16000
12000
8000
4000
2h
6h
2h
6h
Time (h)
SA564
SA564−Δ*hptRS*
SA564−Δ*ccpA*
SA564−Δ*hptRS/ccpA*

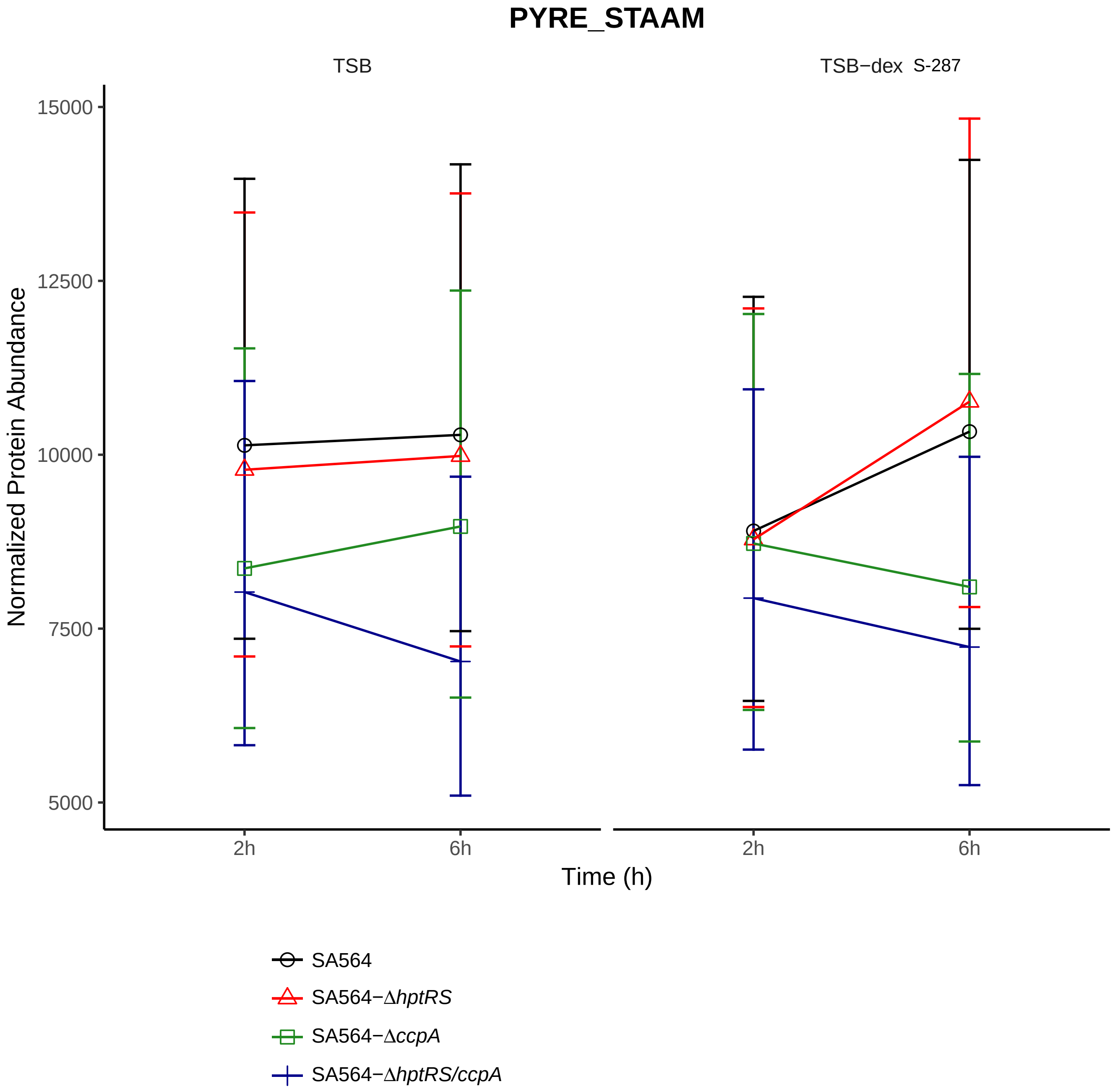

PYRE_STAAM
TSB
TSB−dex
S-287
Normalized Protein Abundance
15000
12500
10000
7500
5000
2h
6h
2h
6h
Time (h)
SA564
SA564−Δ*hptRS*
SA564−Δ*ccpA*
SA564−Δ*hptRS/ccpA*

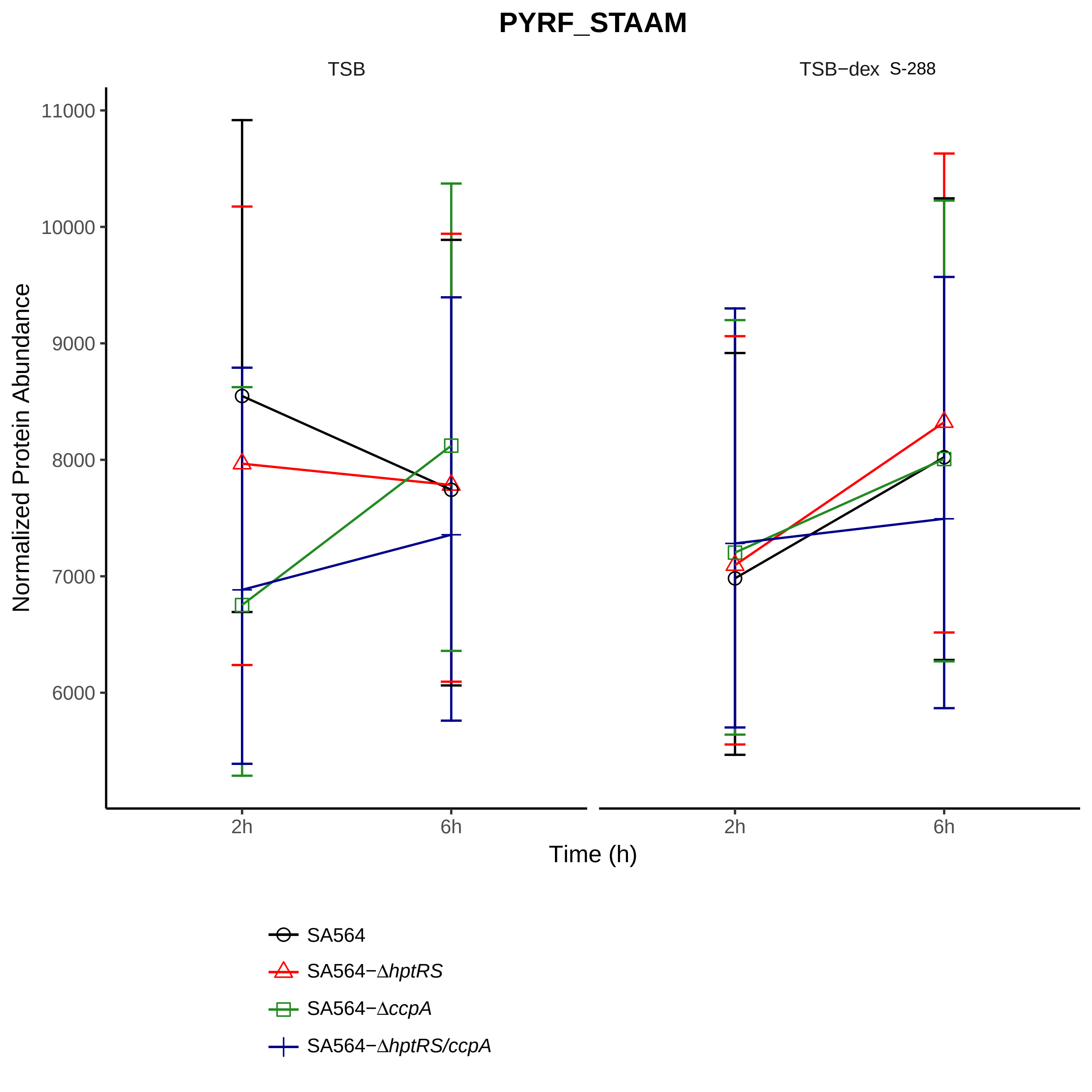

PYRF_STAAM
TSB
TSB−dex
S-288
Normalized Protein Abundance
11000
10000
9000
8000
7000
6000
2h
6h
2h
6h
Time (h)
SA564
SA564−Δ*hptRS*
SA564−Δ*ccpA*
SA564−Δ*hptRS/ccpA*

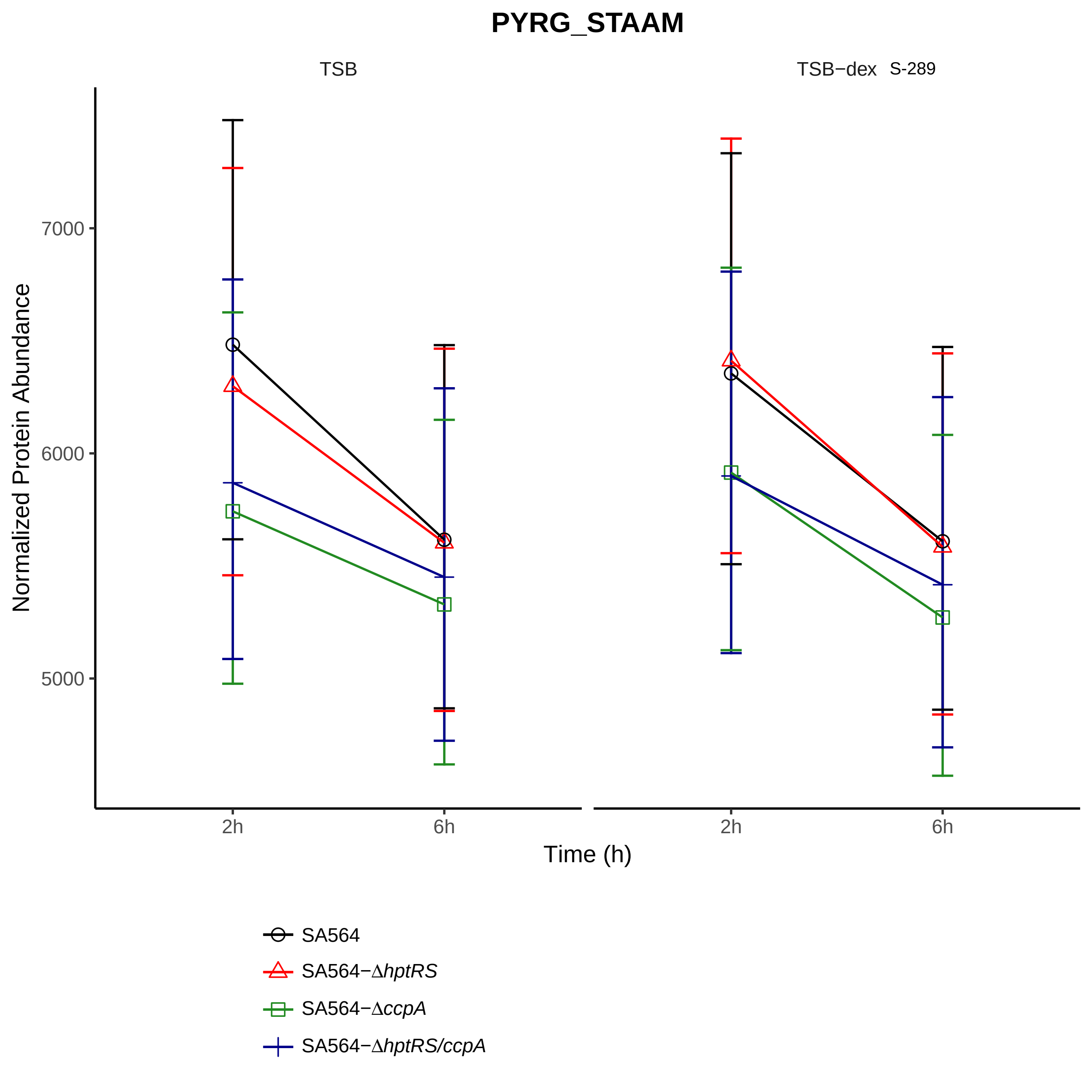
PYRG_STAAM
TSB
TSB−dex
S-289
Normalized Protein Abundance
7000
6000
5000
2h
6h
2h
6h
Time (h)
SA564
SA564−ΔhptRS
SA564−ΔccpA
SA564−ΔhptRS/ccpA

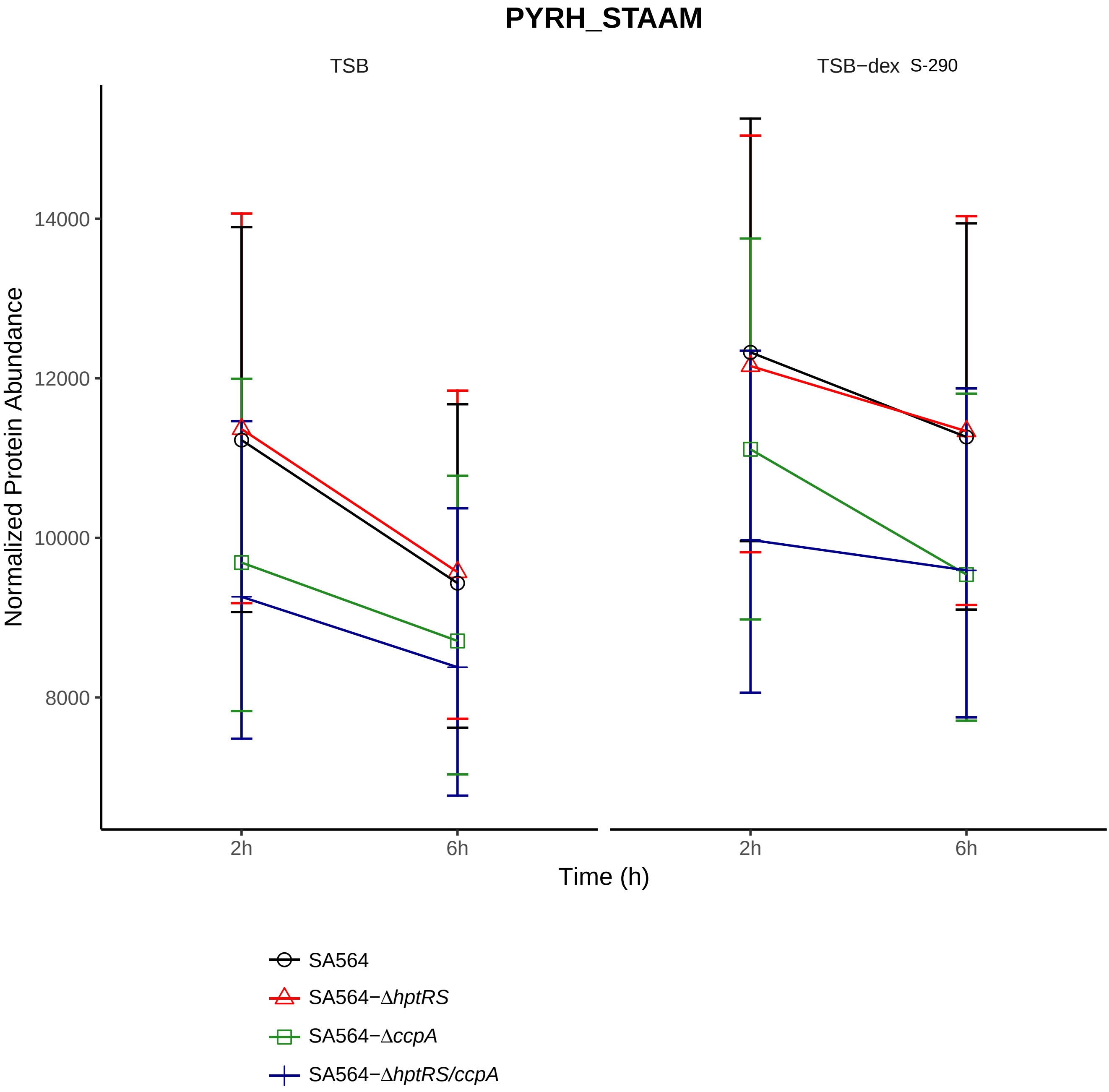

PYRH_STAAM
TSB
TSB−dex
Normalized Protein Abundance
14000
12000
10000
8000
2h
6h
2h
6h
Time (h)
SA564
SA564−Δ*hptRS*
SA564−Δ*ccpA*
SA564−Δ*hptRS/ccpA*

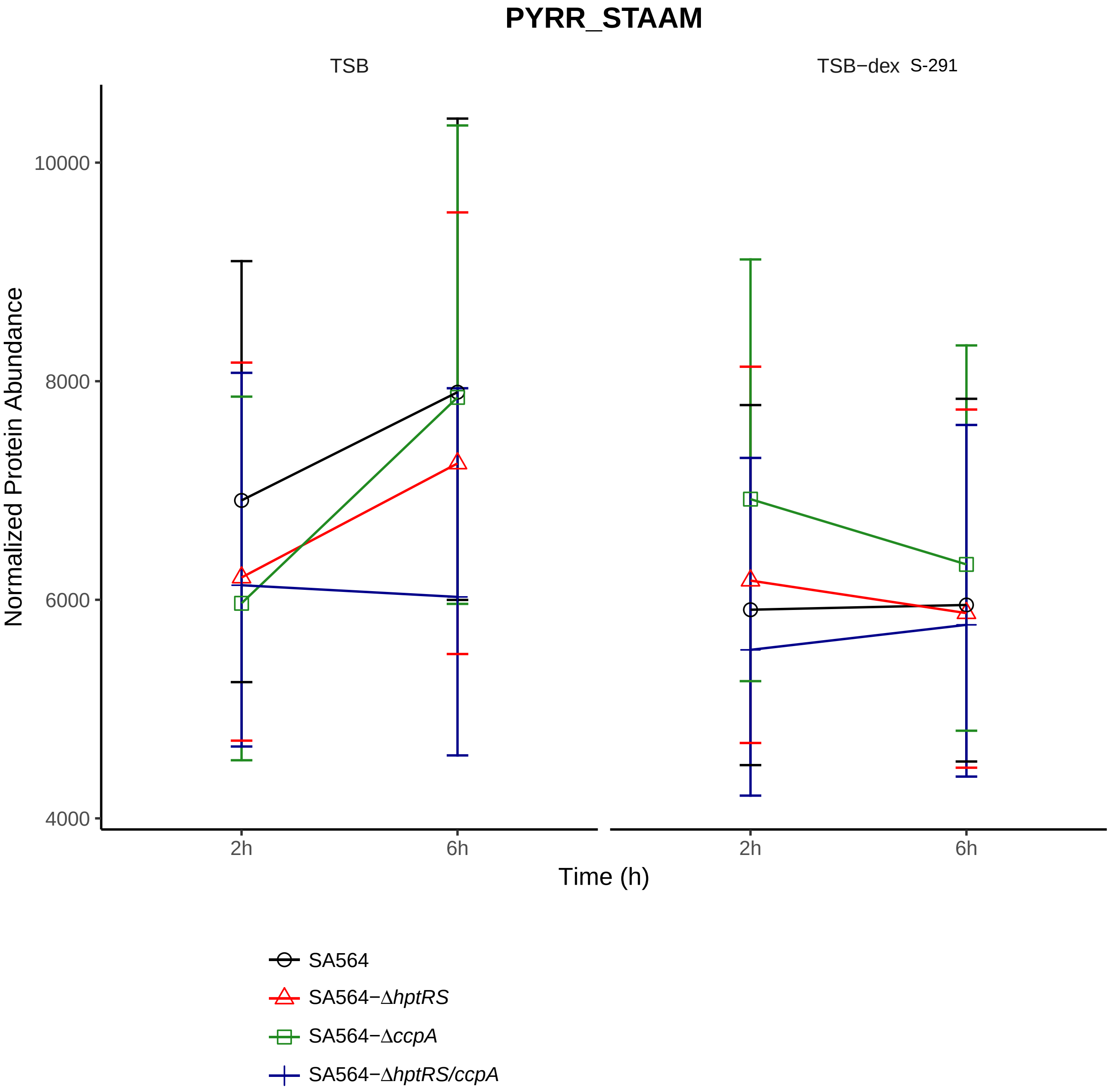
PYRR_STAAM
TSB
TSB−dex
S-291
Normalized Protein Abundance
10000
8000
6000
4000
2h
6h
2h
6h
Time (h)
SA564
SA564−ΔhptRS
SA564−ΔccpA
SA564−ΔhptRS/ccpA

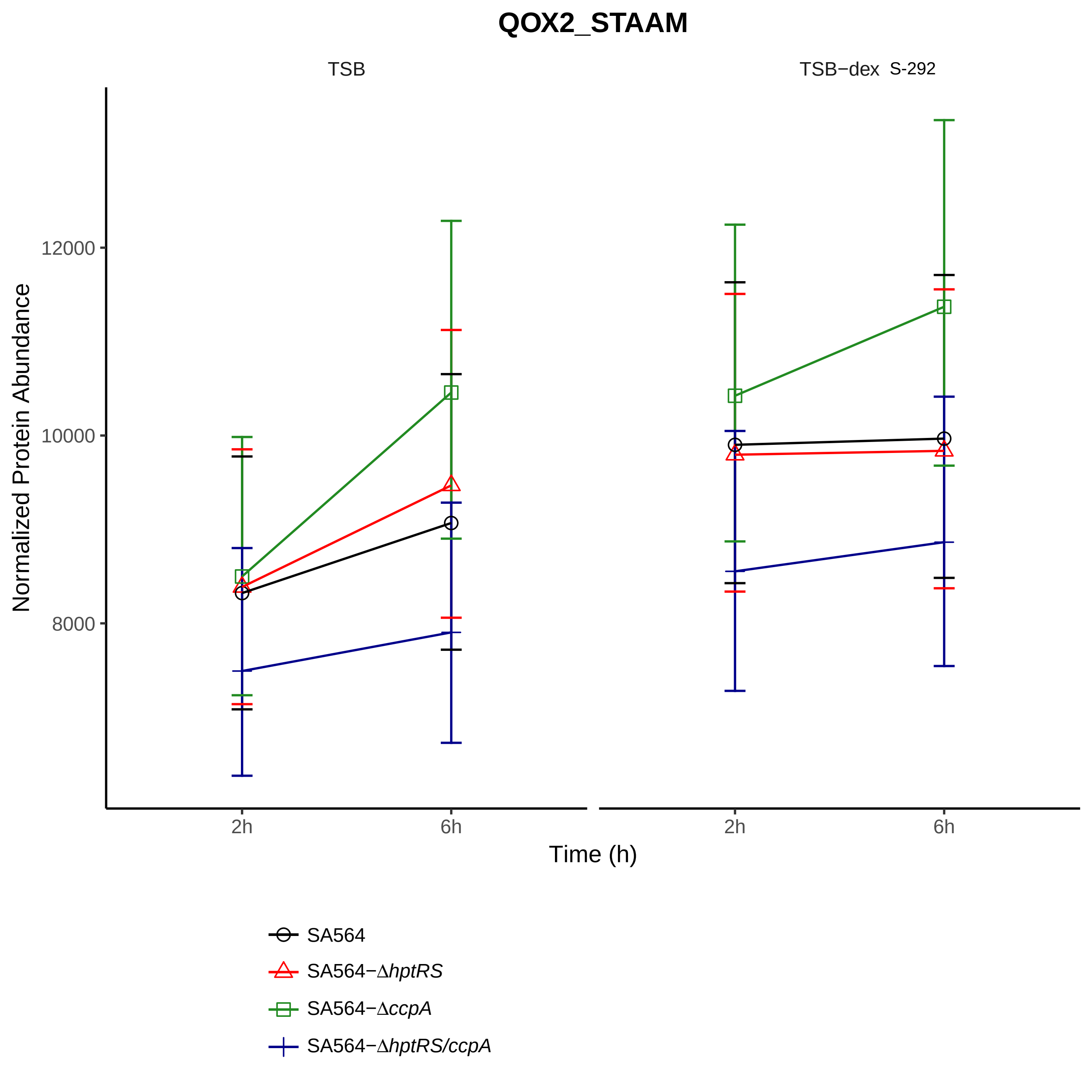

QOX2_STAAM
TSB
TSB−dex
S-292
Normalized Protein Abundance
12000
10000
8000
2h
6h
2h
6h
Time (h)
SA564
SA564−Δ*hptRS*
SA564−Δ*ccpA*
SA564−Δ*hptRS/ccpA*

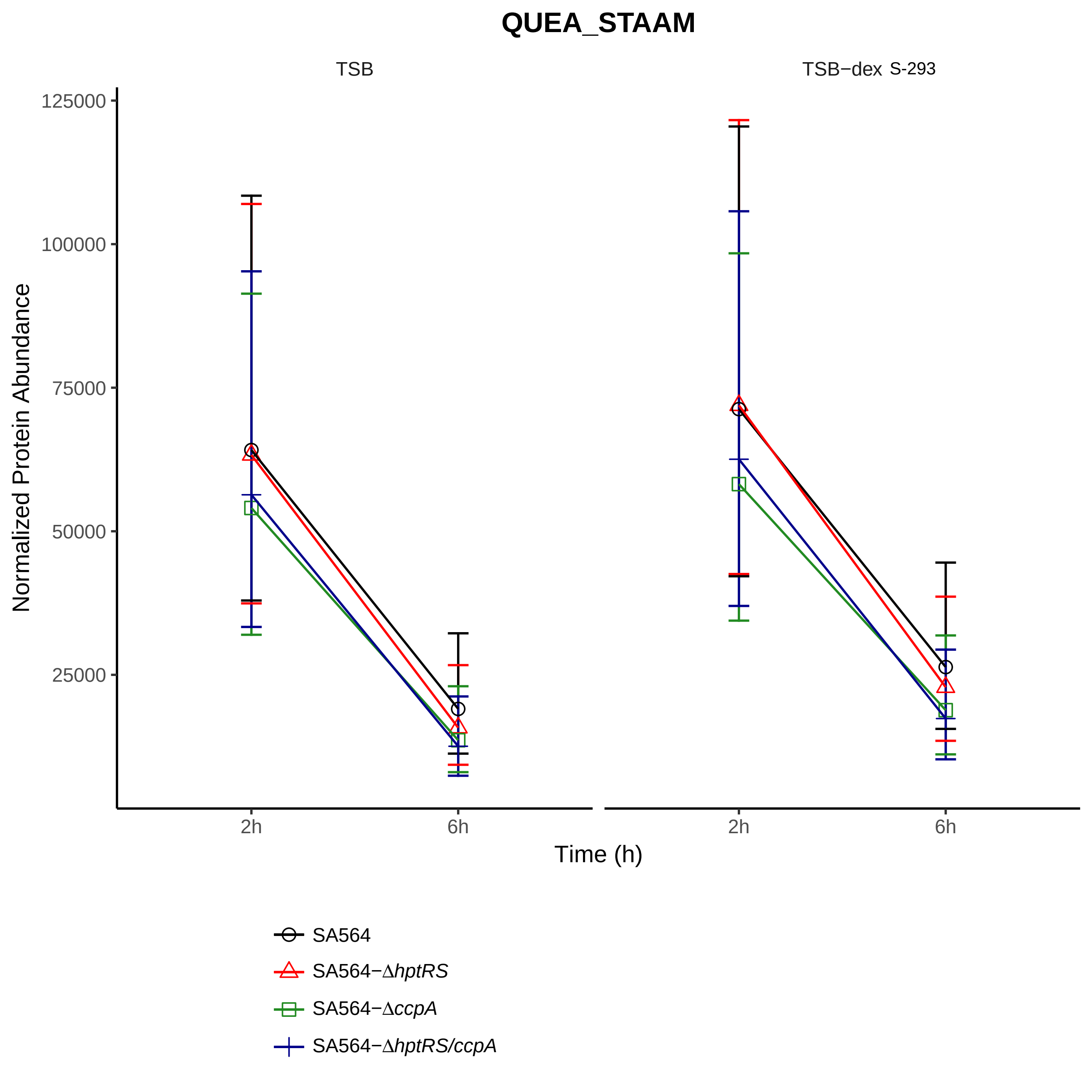

QUEA_STAAM
TSB
TSB−dex
S-293
125000
100000
75000
50000
25000
Normalized Protein Abundance
2h
6h
2h
6h
Time (h)
SA564
SA564−ΔhptRS
SA564−ΔccpA
SA564−ΔhptRS/ccpA

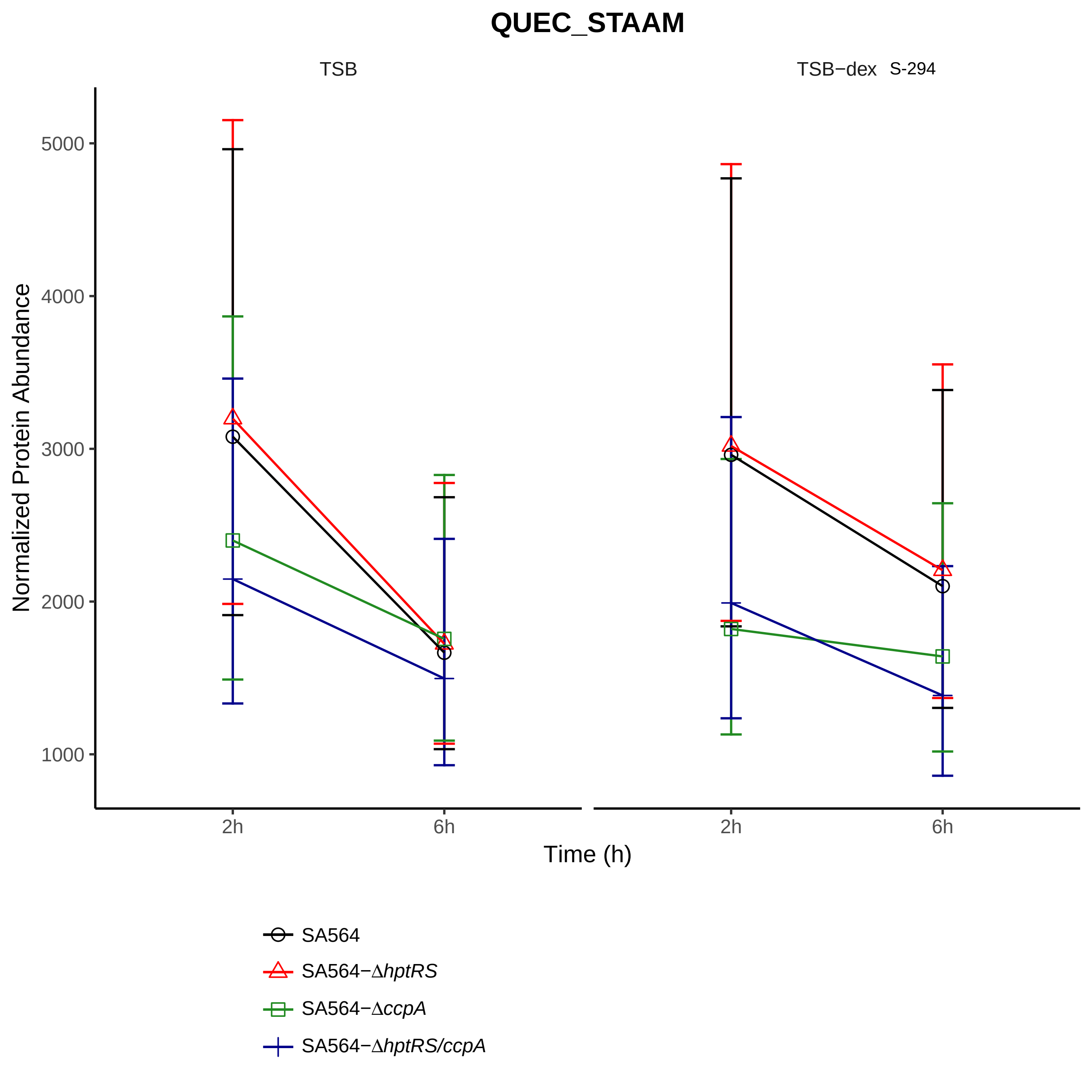

QUEC_STAAM
TSB
TSB−dex
S-294
Normalized Protein Abundance
5000
4000
3000
2000
1000
2h
6h
2h
6h
Time (h)
SA564
SA564−ΔhptRS
SA564−ΔccpA
SA564−ΔhptRS/ccpA

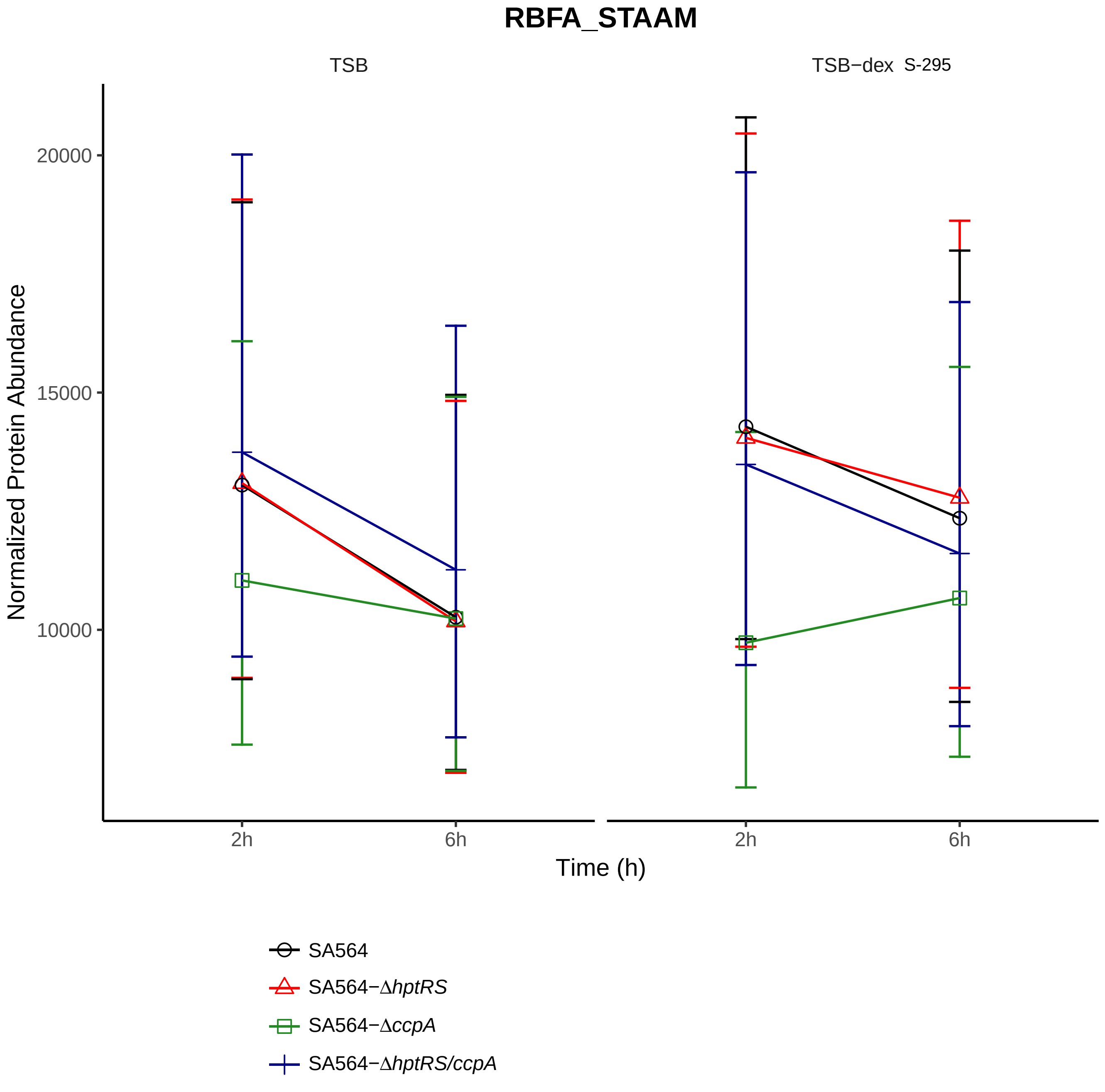

RBFA_STAAM
TSB
TSB−dex
S-295
Normalized Protein Abundance
20000
15000
10000
2h
6h
2h
6h
Time (h)
SA564
SA564−Δ*hptRS*
SA564−Δ*ccpA*
SA564−Δ*hptRS/ccpA*

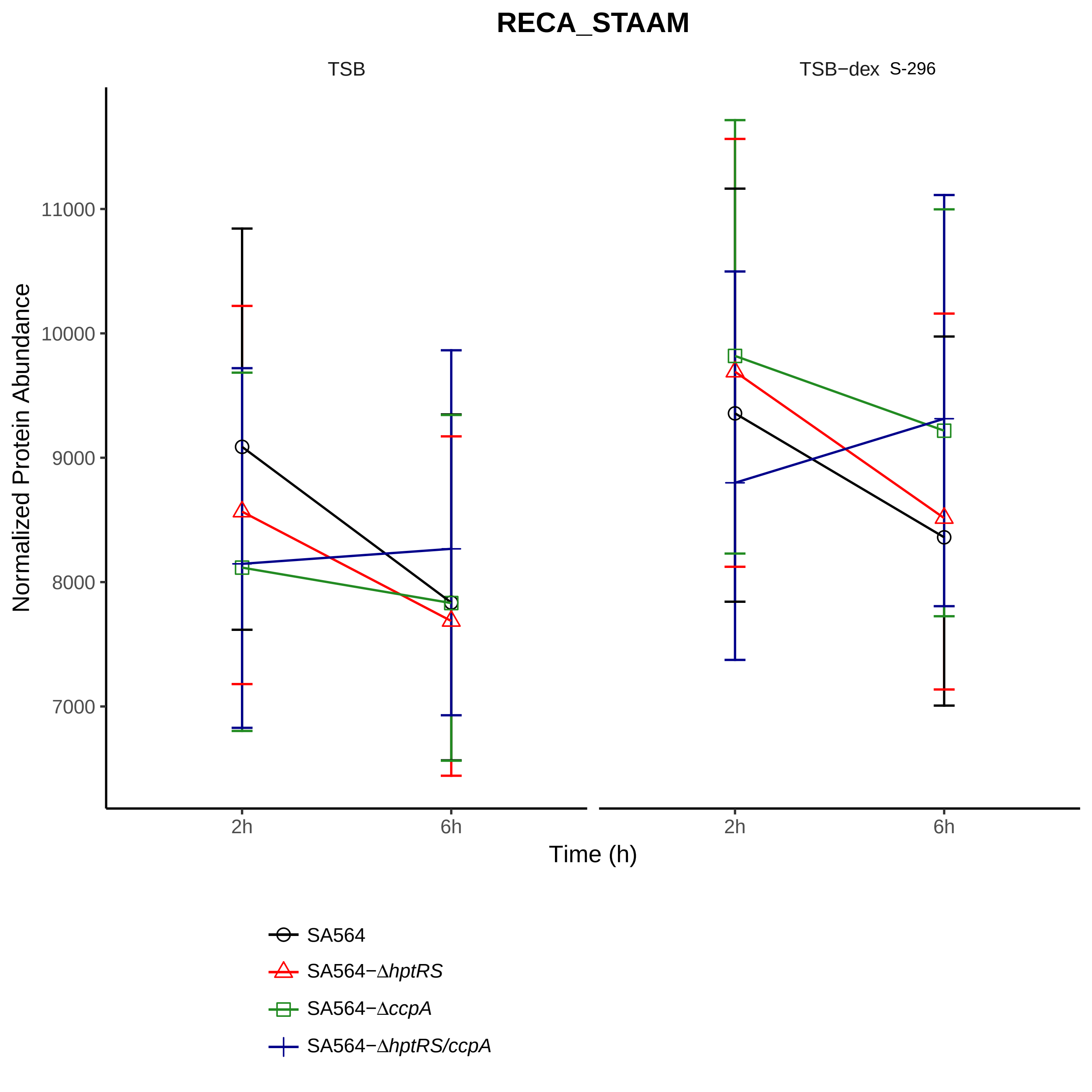

RECA_STAAM
TSB
TSB−dex
S-296
Normalized Protein Abundance
11000
10000
9000
8000
7000
2h
6h
2h
6h
Time (h)
SA564
SA564−ΔhptRS
SA564−ΔccpA
SA564−ΔhptRS/ccpA

RELA_STAAM

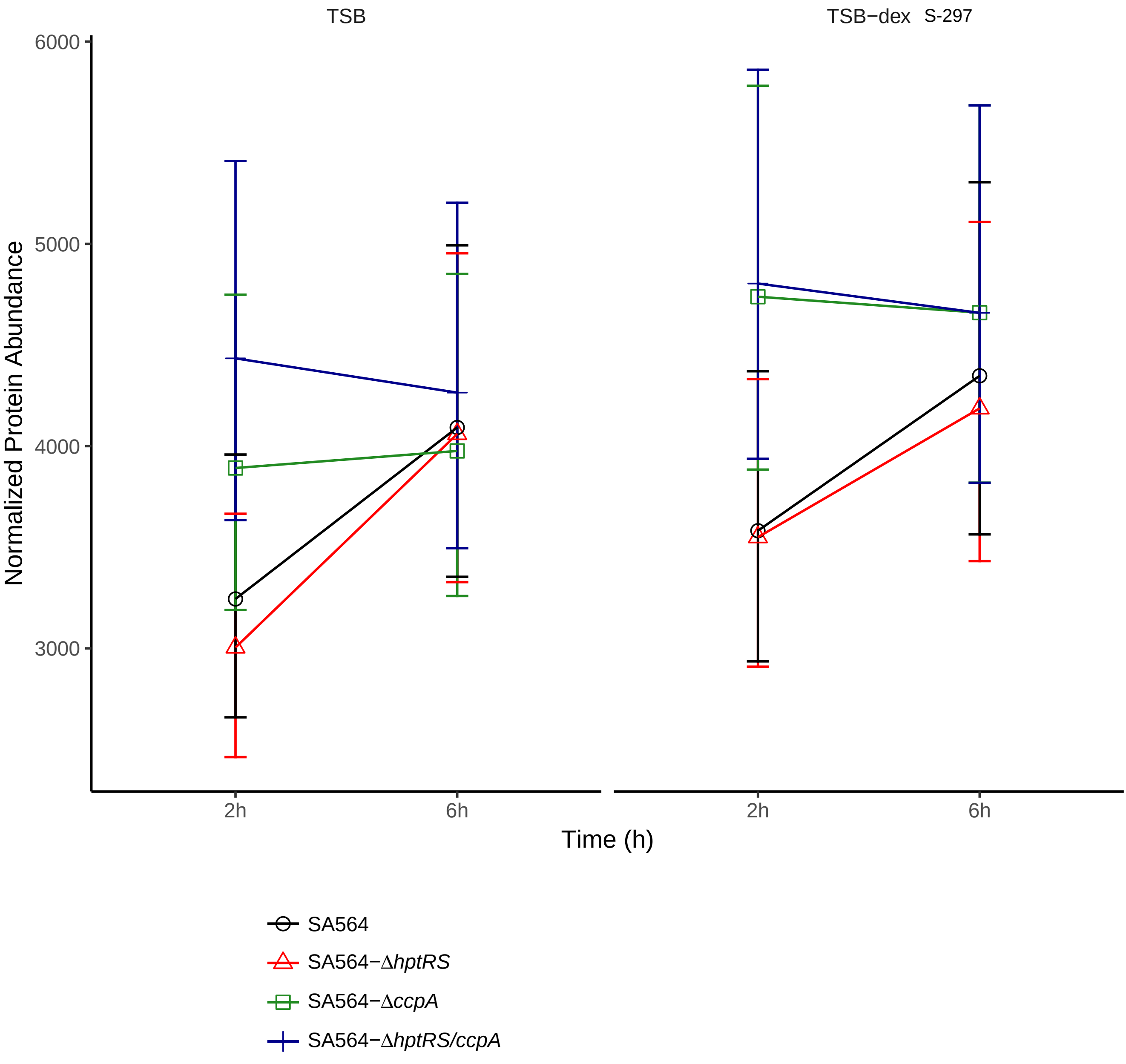

TSB
TSB−dex
S-297
6000
5000
4000
3000
Normalized Protein Abundance
2h
6h
2h
6h
Time (h)
SA564
SA564−Δ*hptRS*
SA564−Δ*ccpA*
SA564−Δ*hptRS/ccpA*

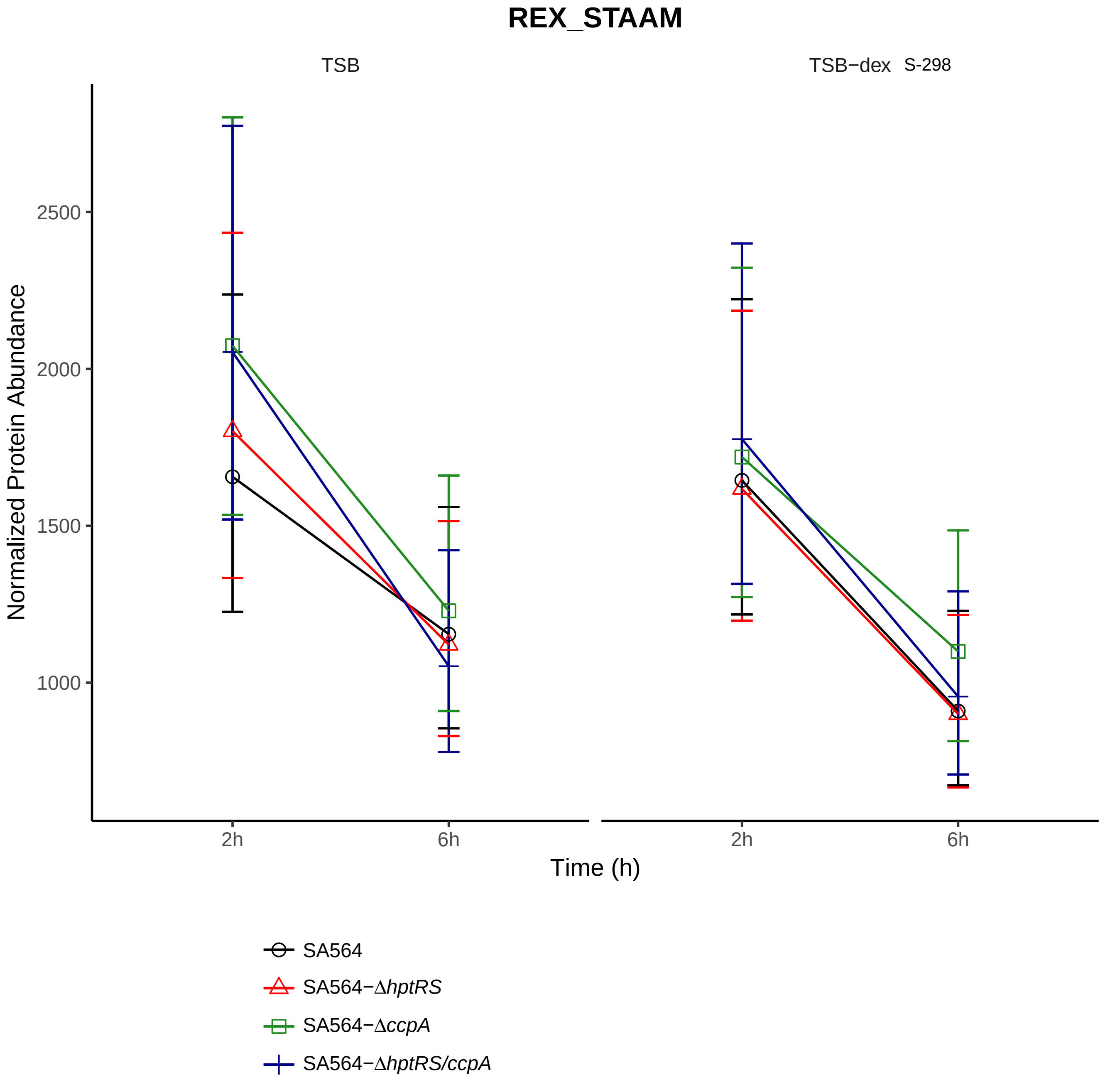
REX_STAAM
TSB
TSB−dex
S-298
Normalized Protein Abundance
2500
2000
1500
1000
2h
6h
2h
6h
Time (h)
SA564
SA564−Δ*hptRS*
SA564−Δ*ccpA*
SA564−Δ*hptRS/ccpA*

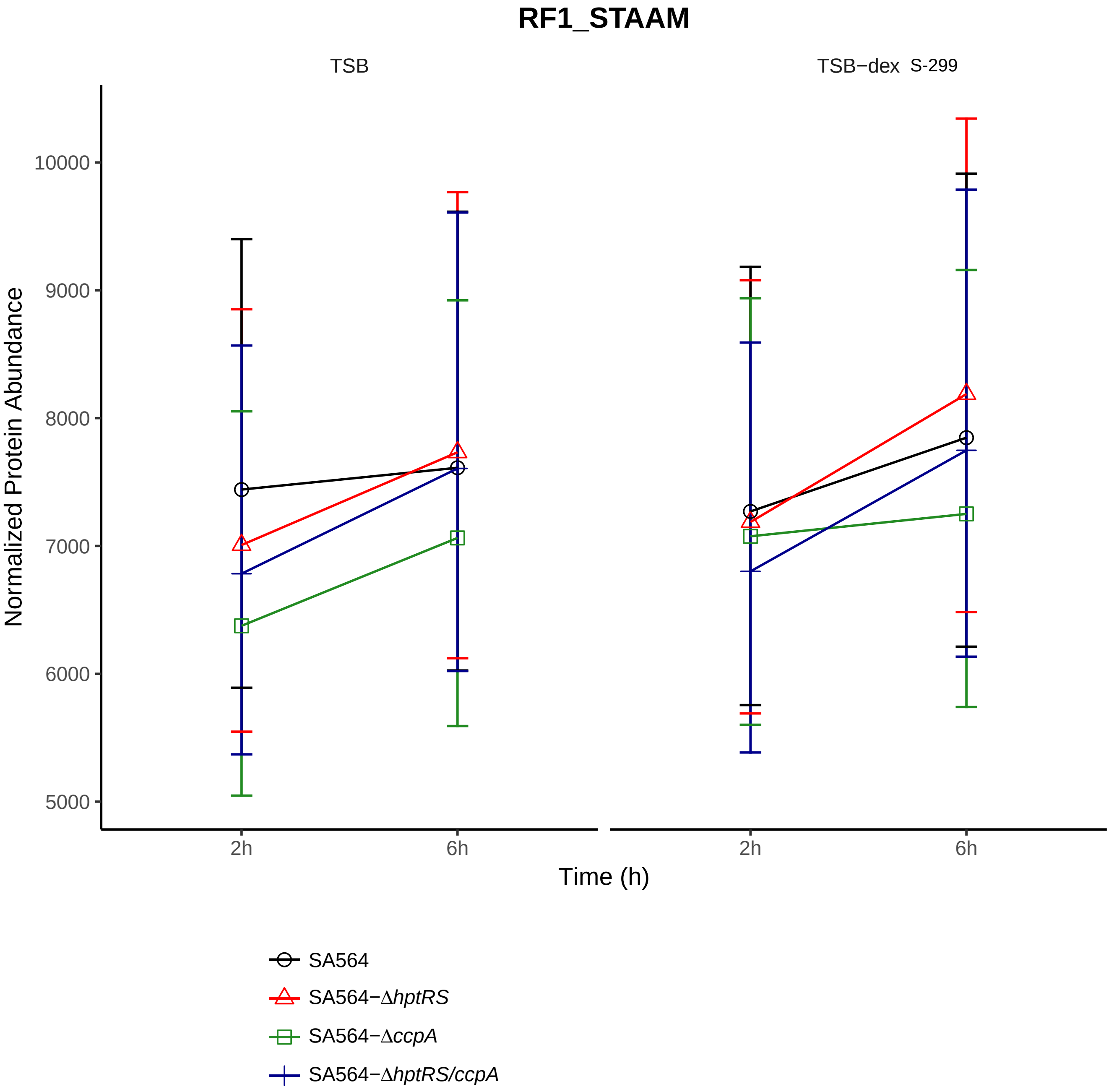

RF1_STAAM
TSB
TSB−dex
Normalized Protein Abundance
10000
9000
8000
7000
6000
5000
2h
6h
2h
6h
Time (h)
SA564
SA564−Δ*hptRS*
SA564−Δ*ccpA*
SA564−Δ*hptRS/ccpA*

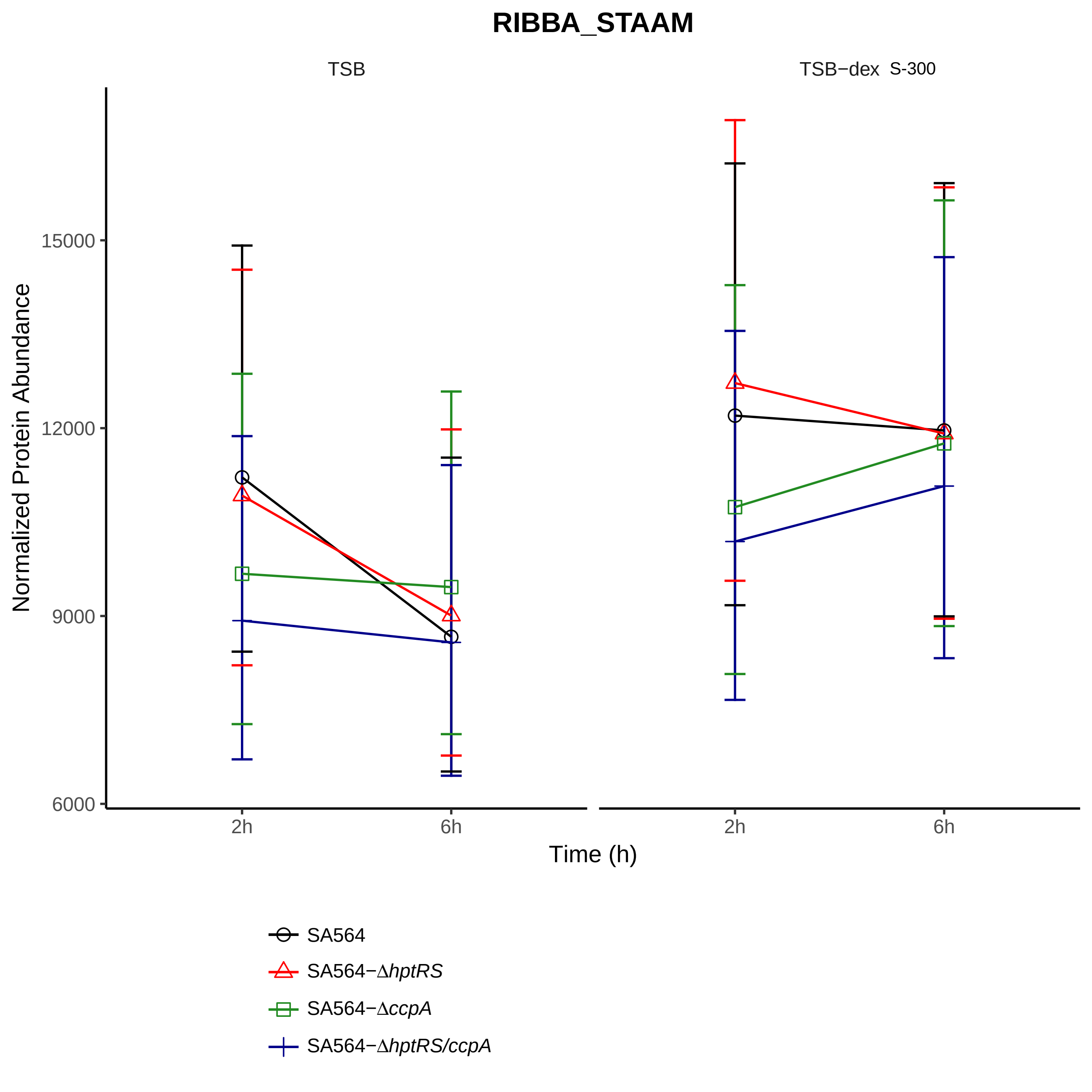

RIBBA_STAAM
TSB
TSB−dex S-300
Normalized Protein Abundance
15000
12000
9000
6000
2h
6h
2h
6h
Time (h)
SA564
SA564−ΔhptRS
SA564−ΔccpA
SA564−ΔhptRS/ccpA

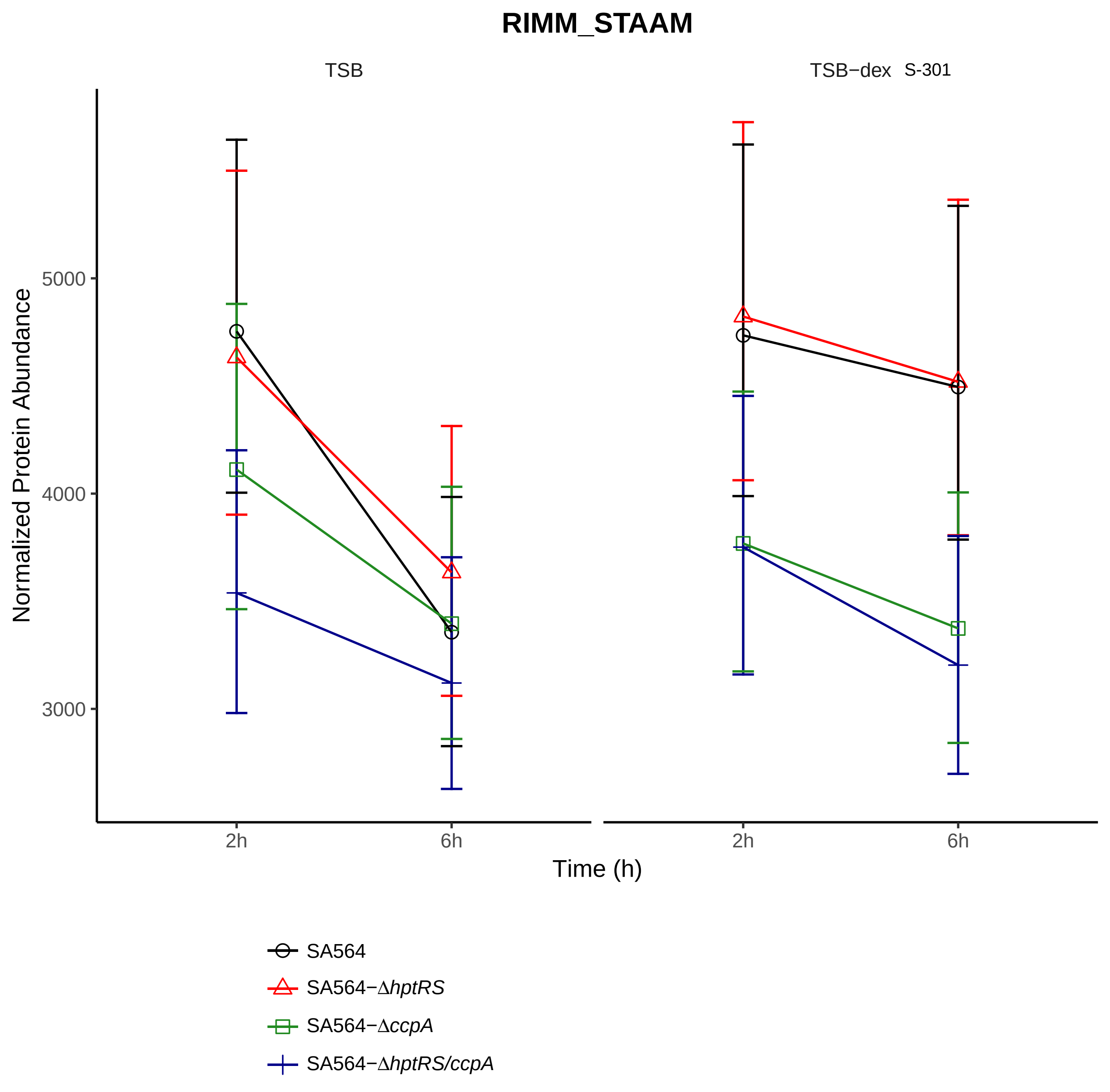

RIMM_STAAM
TSB
TSB−dex
S-301
Normalized Protein Abundance
5000
4000
3000
2h
6h
2h
6h
Time (h)
SA564
SA564−Δ*hptRS*
SA564−Δ*ccpA*
SA564−Δ*hptRS/ccpA*

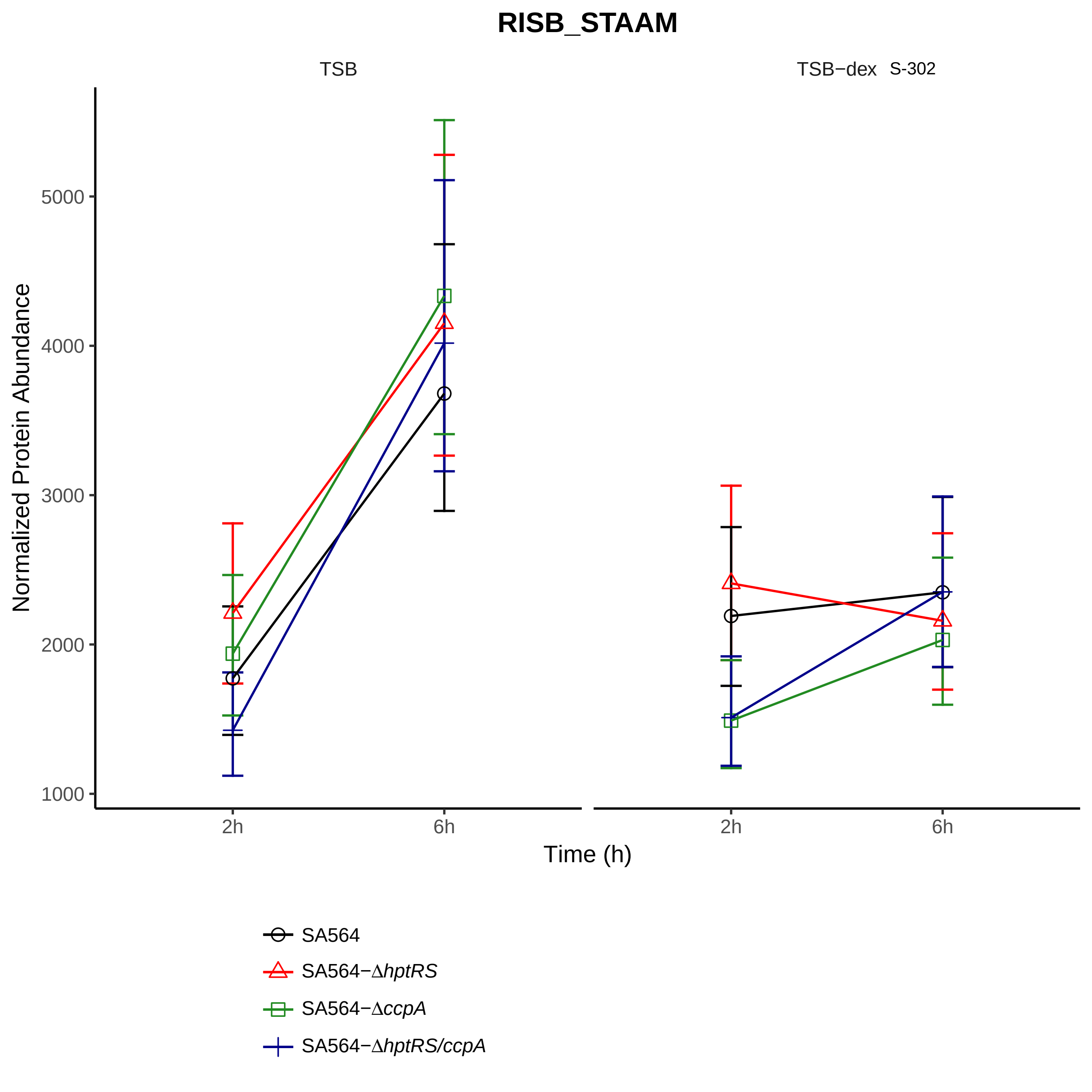
RISB_STAAM
TSB
TSB−dex
S-302
Normalized Protein Abundance
1000
2000
3000
4000
5000
2h
6h
2h
6h
Time (h)
SA564
SA564−Δ*hptRS*
SA564−Δ*ccpA*
SA564−Δ*hptRS/ccpA*

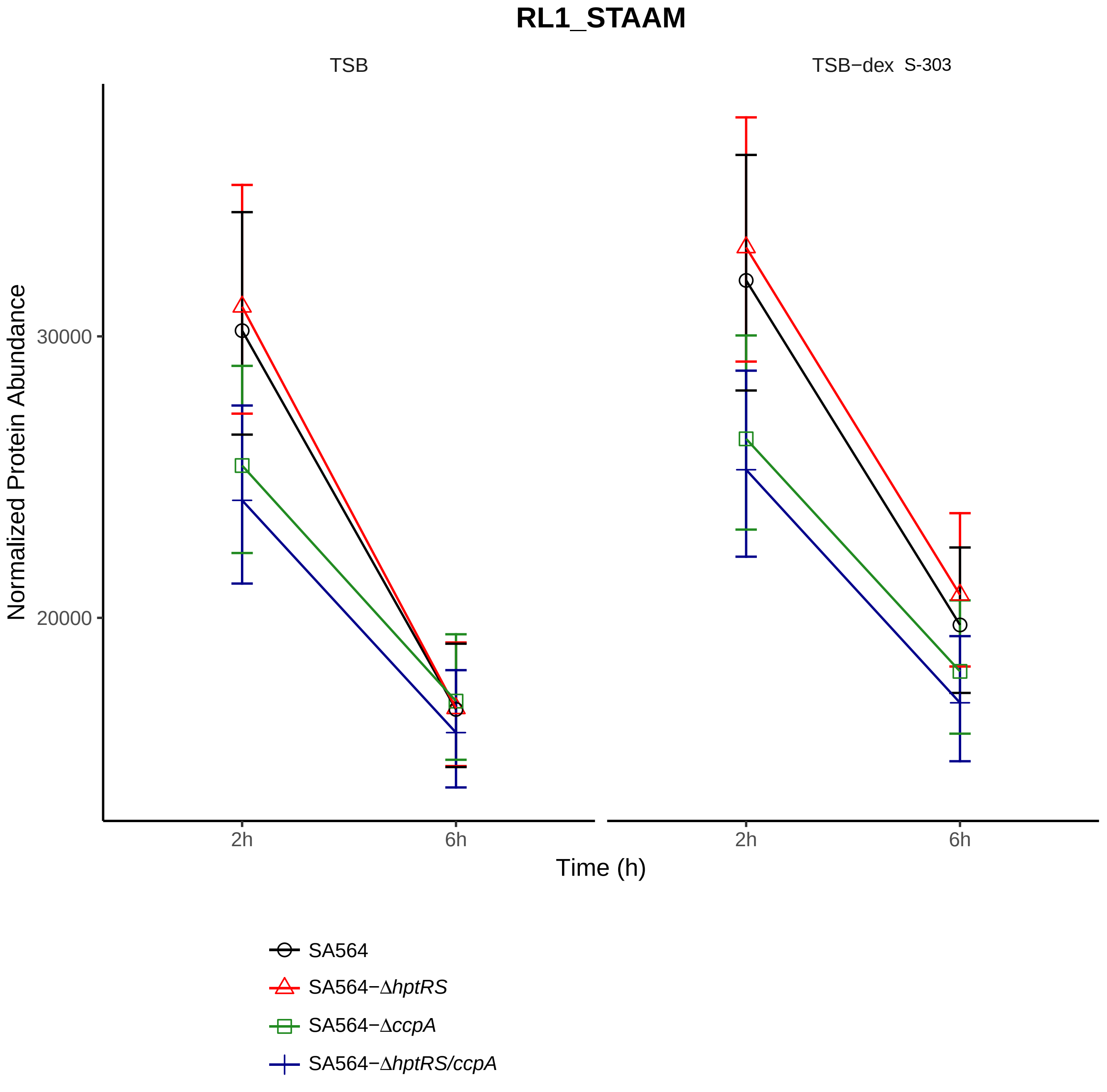

RL1_STAAM
TSB
TSB−dex
S-303
30000
20000
Normalized Protein Abundance
2h
6h
2h
6h
Time (h)
SA564
SA564−Δ*hptRS*
SA564−Δ*ccpA*
SA564−Δ*hptRS/ccpA*

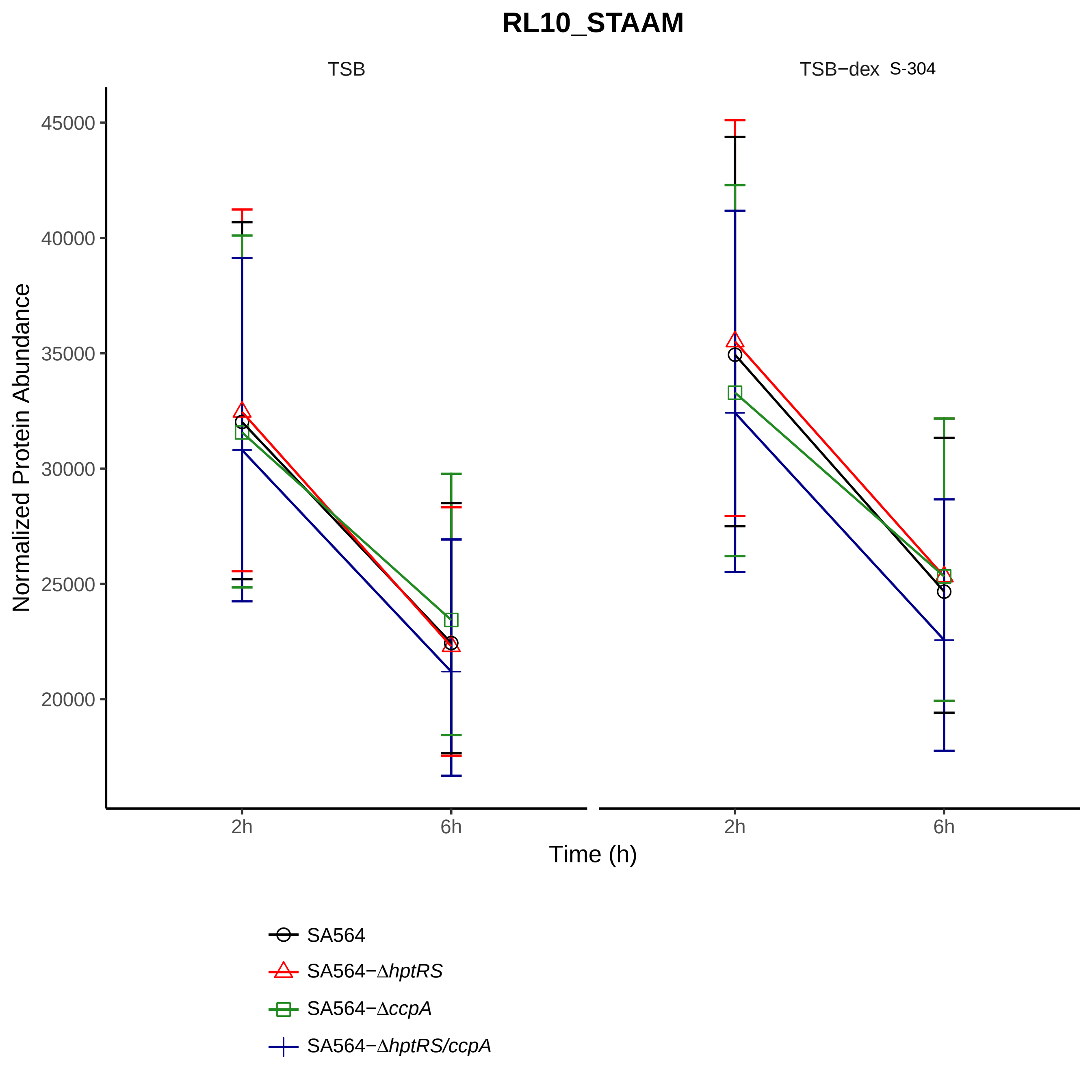

RL10_STAAM
TSB
TSB−dex
S-304
Normalized Protein Abundance
45000
40000
35000
30000
25000
20000
2h
6h
2h
6h
Time (h)
SA564
SA564−ΔhptRS
SA564−ΔccpA
SA564−ΔhptRS/ccpA

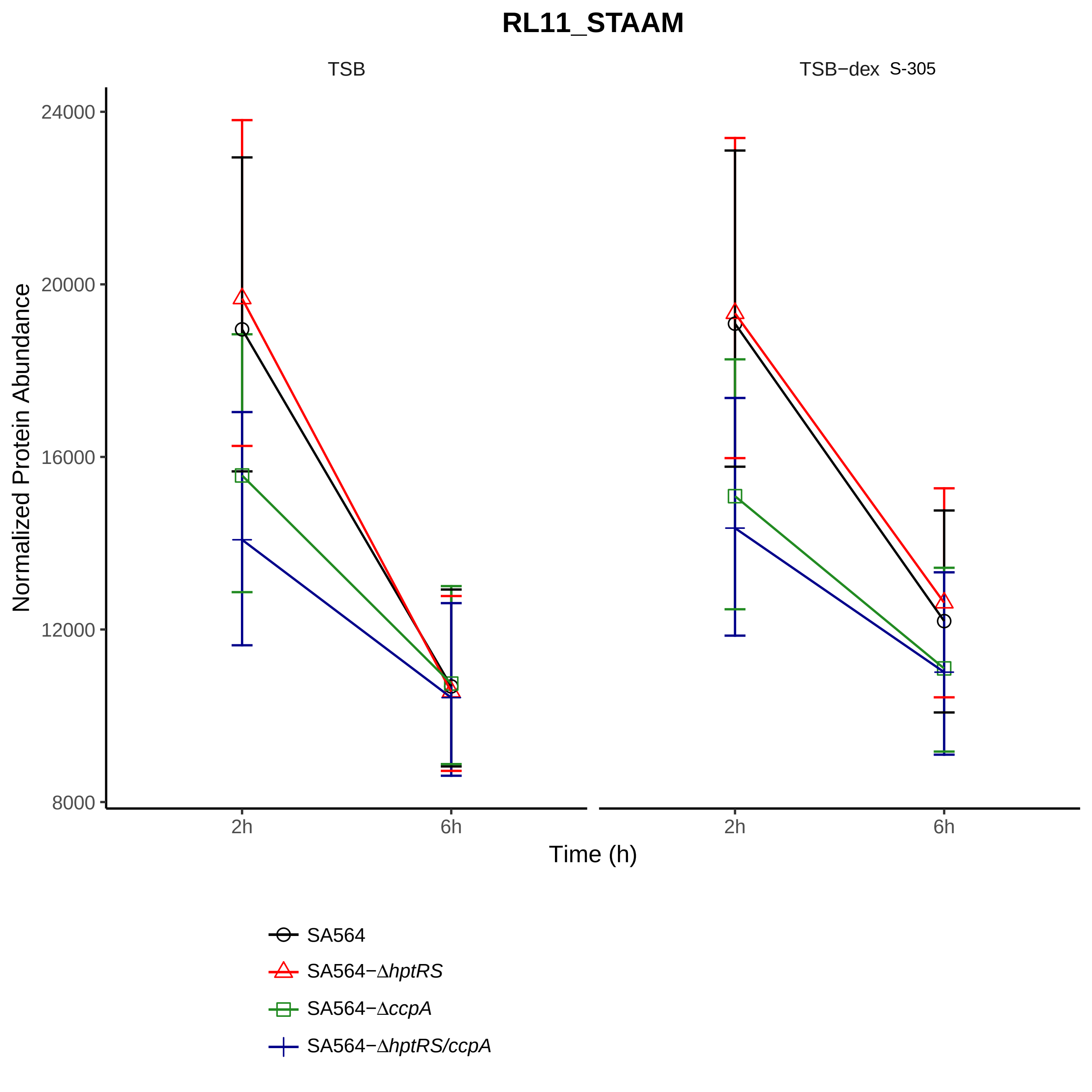

RL11_STAAM
TSB
TSB−dex
S-305
Normalized Protein Abundance
24000
20000
16000
12000
8000
2h
6h
2h
6h
Time (h)
SA564
SA564−ΔhptRS
SA564−ΔccpA
SA564−ΔhptRS/ccpA

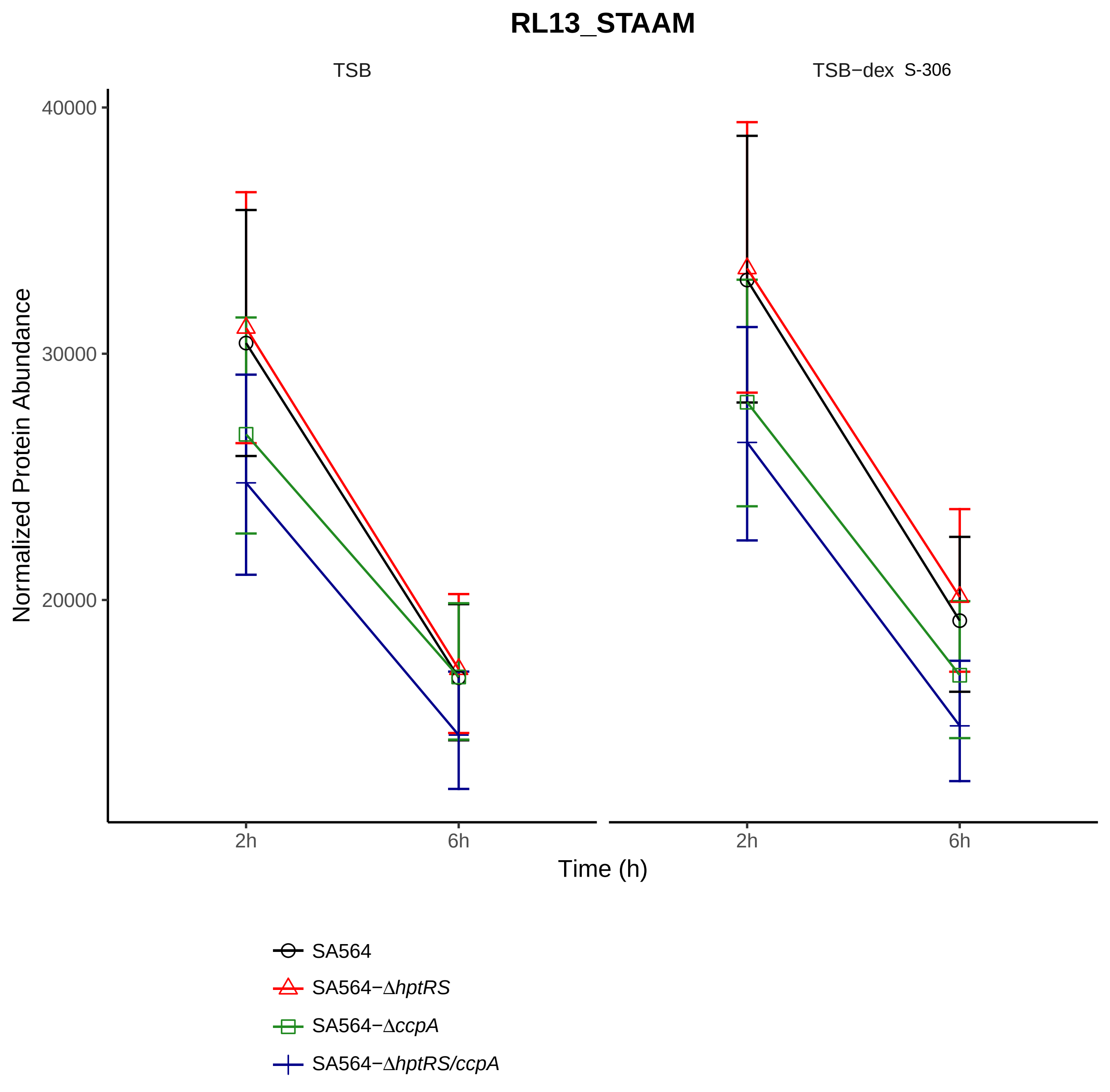
RL13_STAAM
TSB
TSB−dex
S-306
Normalized Protein Abundance
40000
30000
20000
2h
6h
2h
6h
Time (h)
SA564
SA564−ΔhptRS
SA564−ΔccpA
SA564−ΔhptRS/ccpA

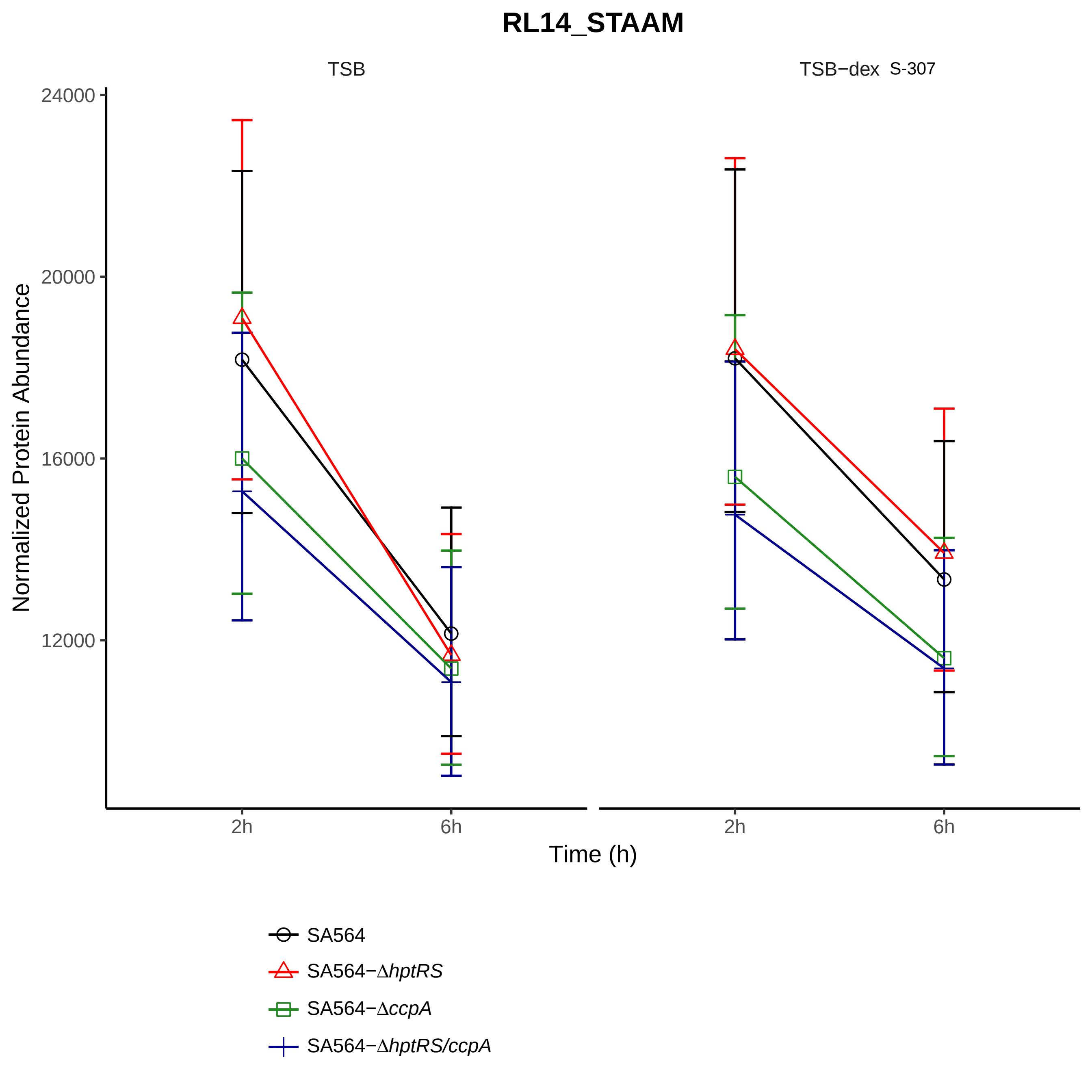
RL14_STAAM
TSB
TSB−dex
S-307
24000
20000
16000
12000
Normalized Protein Abundance
2h
6h
2h
6h
Time (h)
SA564
SA564−ΔhptRS
SA564−ΔccpA
SA564−ΔhptRS/ccpA

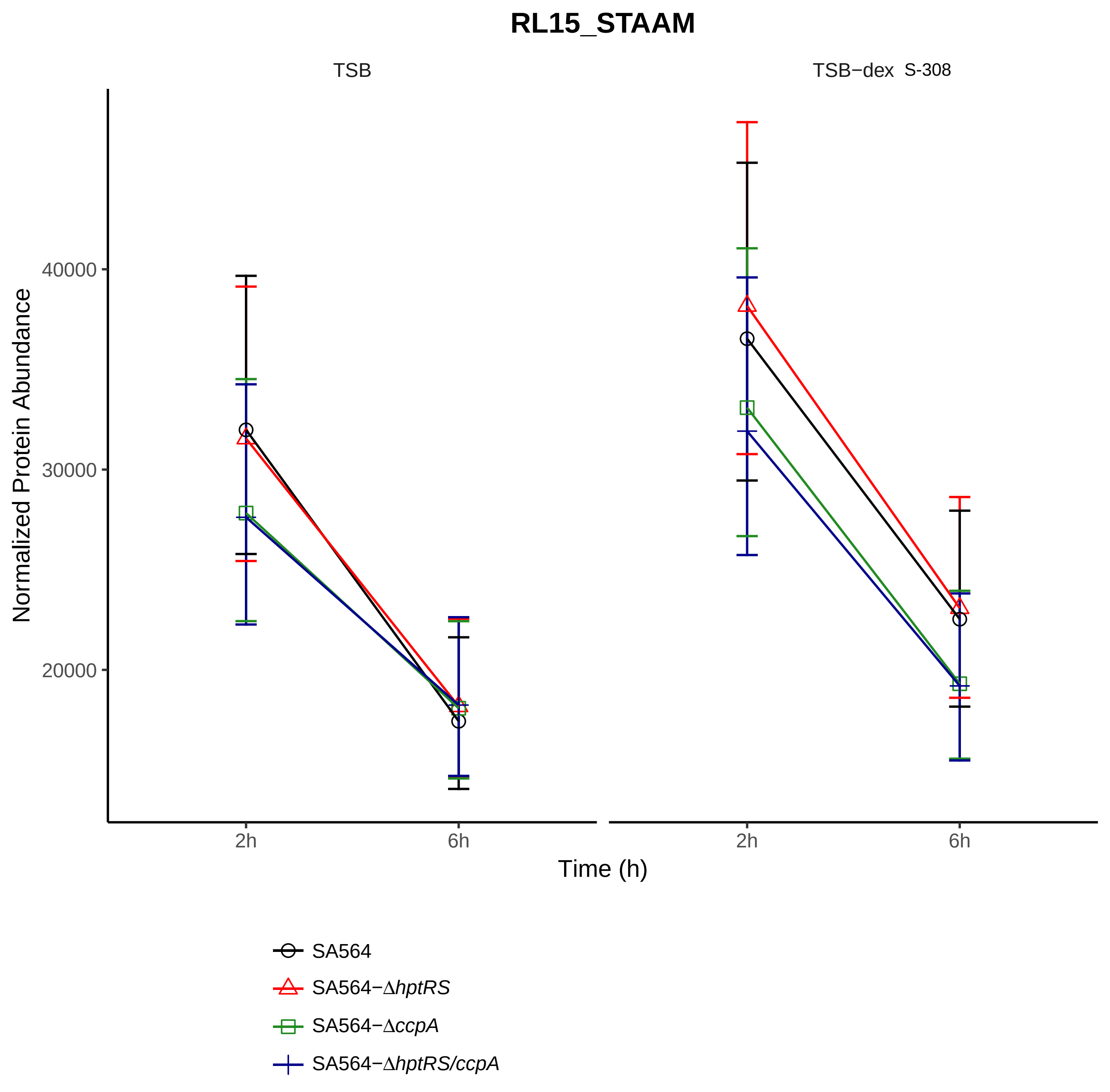

RL15_STAAM
TSB
TSB−dex
Normalized Protein Abundance
40000
30000
20000
2h
6h
2h
6h
Time (h)
SA564
SA564−ΔhptRS
SA564−ΔccpA
SA564−ΔhptRS/ccpA

S-308

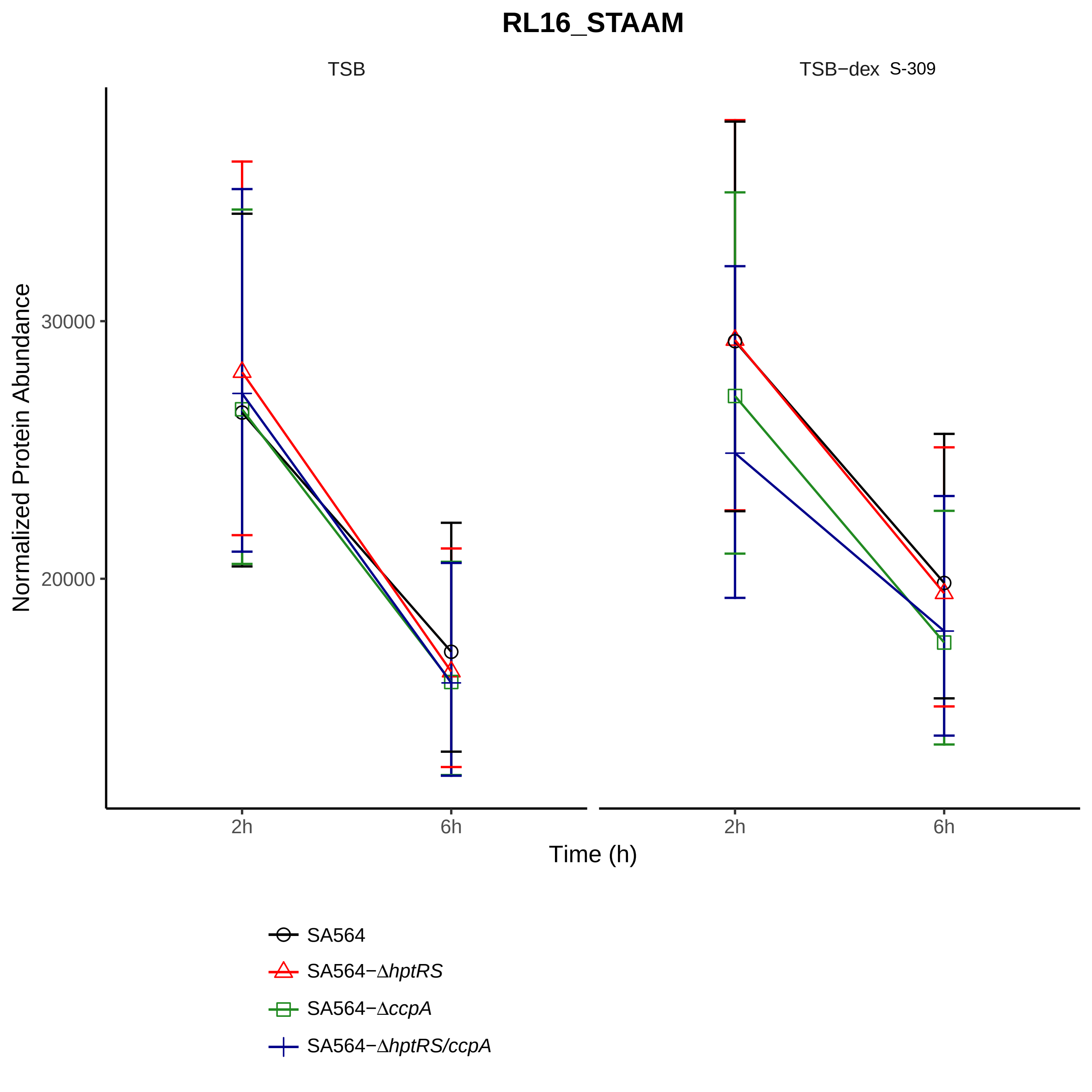

RL16_STAAM
TSB
TSB−dex
S-309
Normalized Protein Abundance
30000
20000
2h
6h
2h
6h
Time (h)
SA564
SA564−Δ*hptRS*
SA564−Δ*ccpA*
SA564−Δ*hptRS/ccpA*

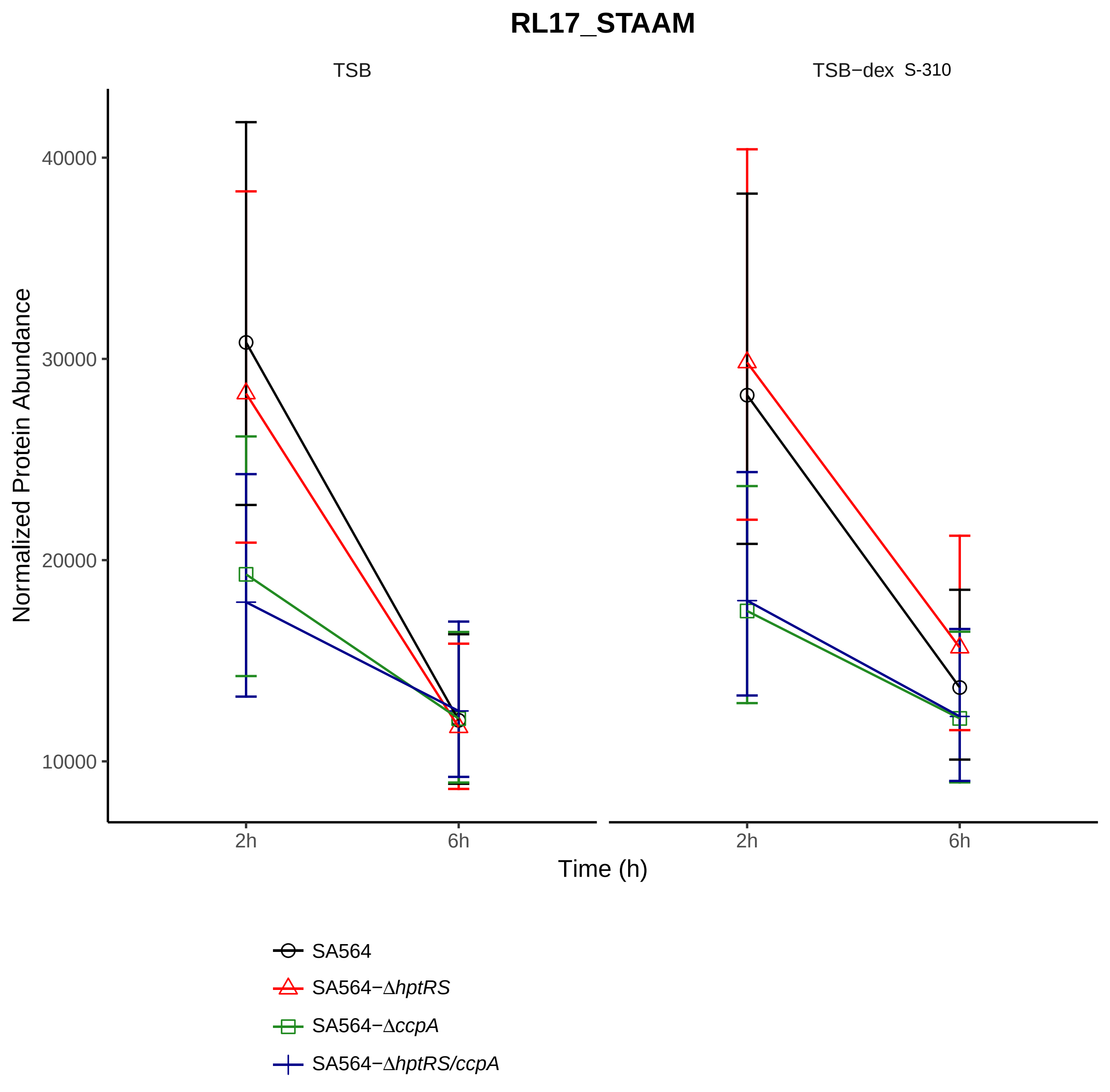

RL17_STAAM
TSB
TSB−dex
S-310
Normalized Protein Abundance
40000
30000
20000
10000
2h
6h
2h
6h
Time (h)
SA564
SA564−Δ*hptRS*
SA564−Δ*ccpA*
SA564−Δ*hptRS/ccpA*

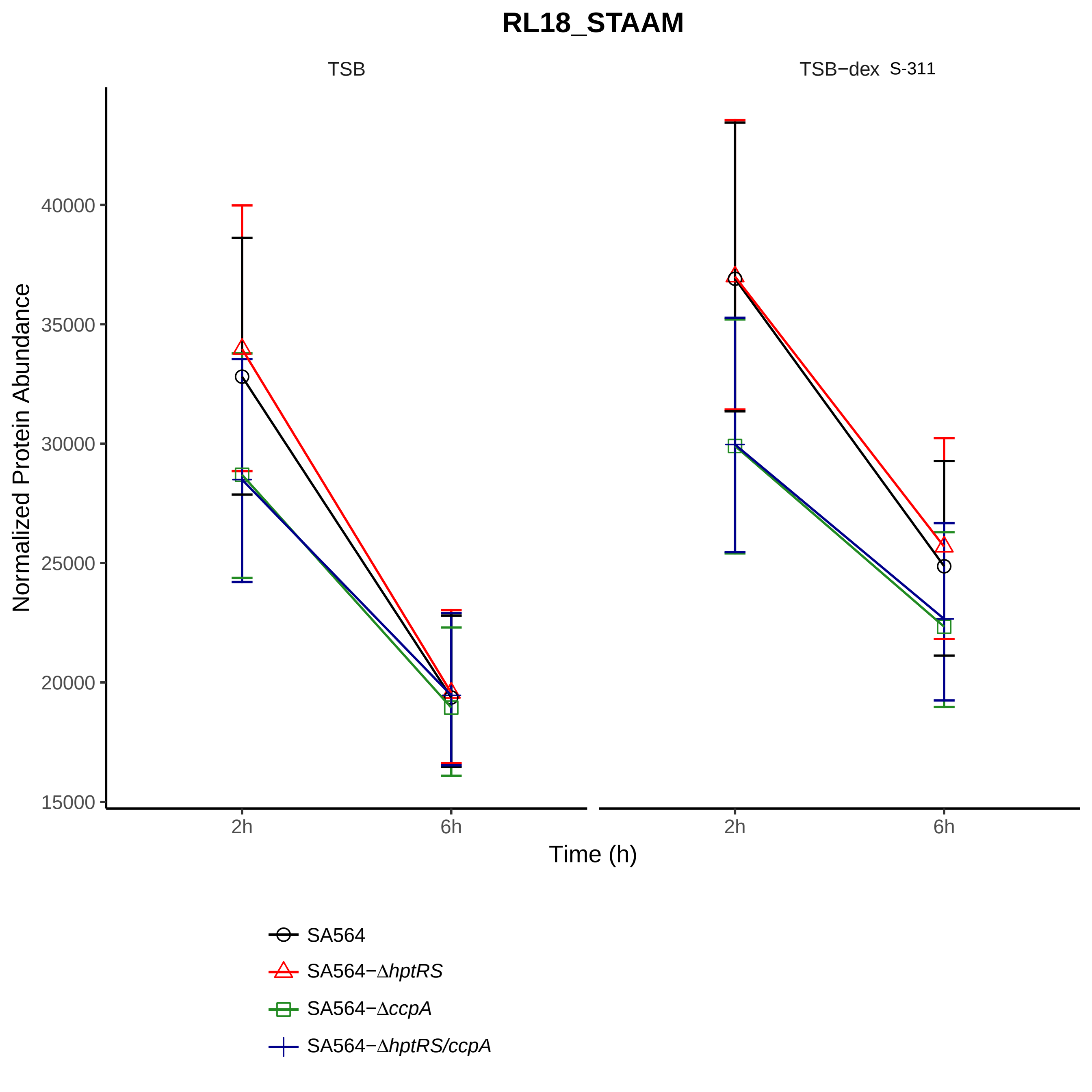
RL18_STAAM
TSB
TSB−dex
S-311
Normalized Protein Abundance
40000
35000
30000
25000
20000
15000
2h
6h
2h
6h
Time (h)
SA564
SA564−ΔhptRS
SA564−ΔccpA
SA564−ΔhptRS/ccpA

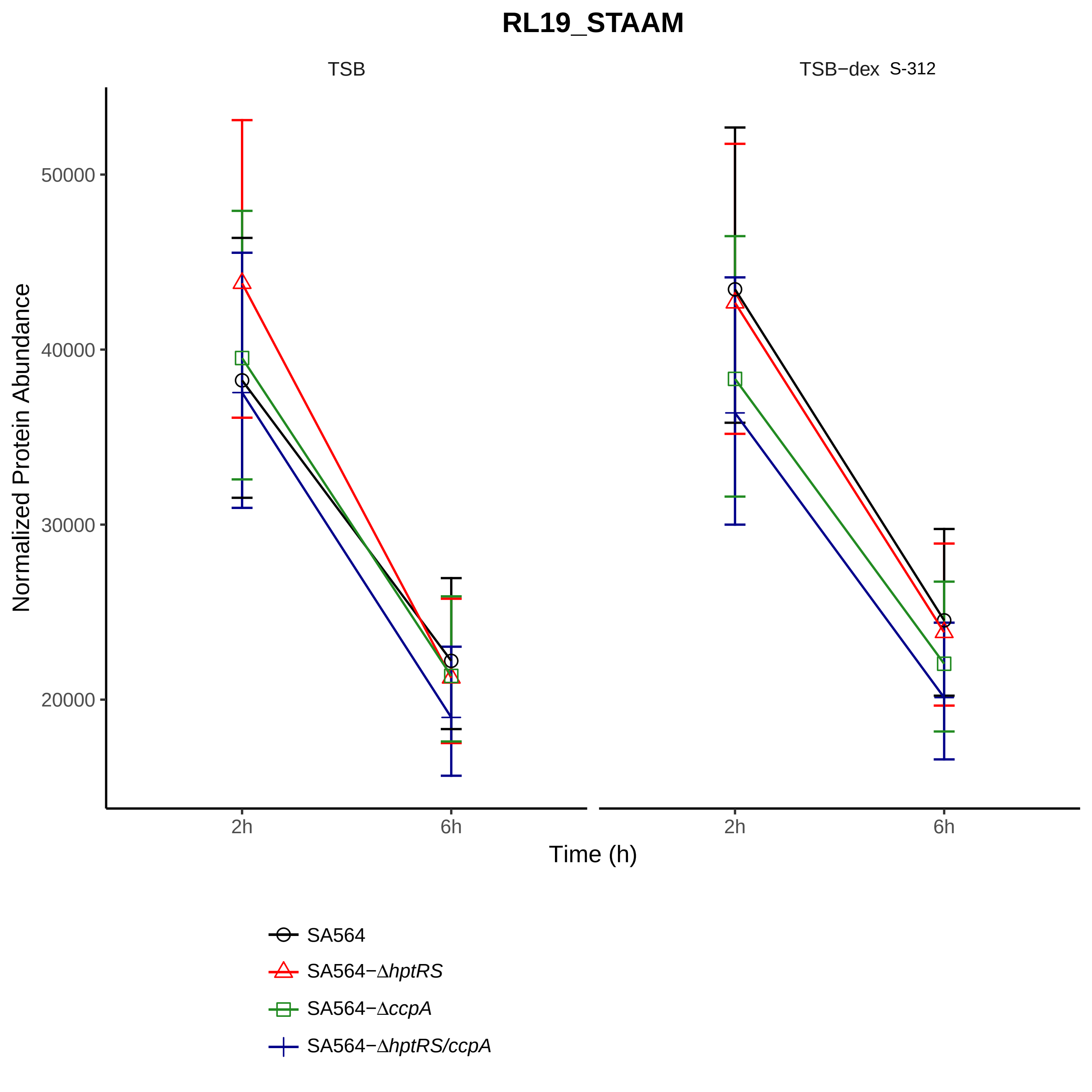

RL19_STAAM
TSB
TSB−dex
S-312
Normalized Protein Abundance
50000
40000
30000
20000
2h
6h
2h
6h
Time (h)
SA564
SA564−Δ*hptRS*
SA564−Δ*ccpA*
SA564−Δ*hptRS/ccpA*

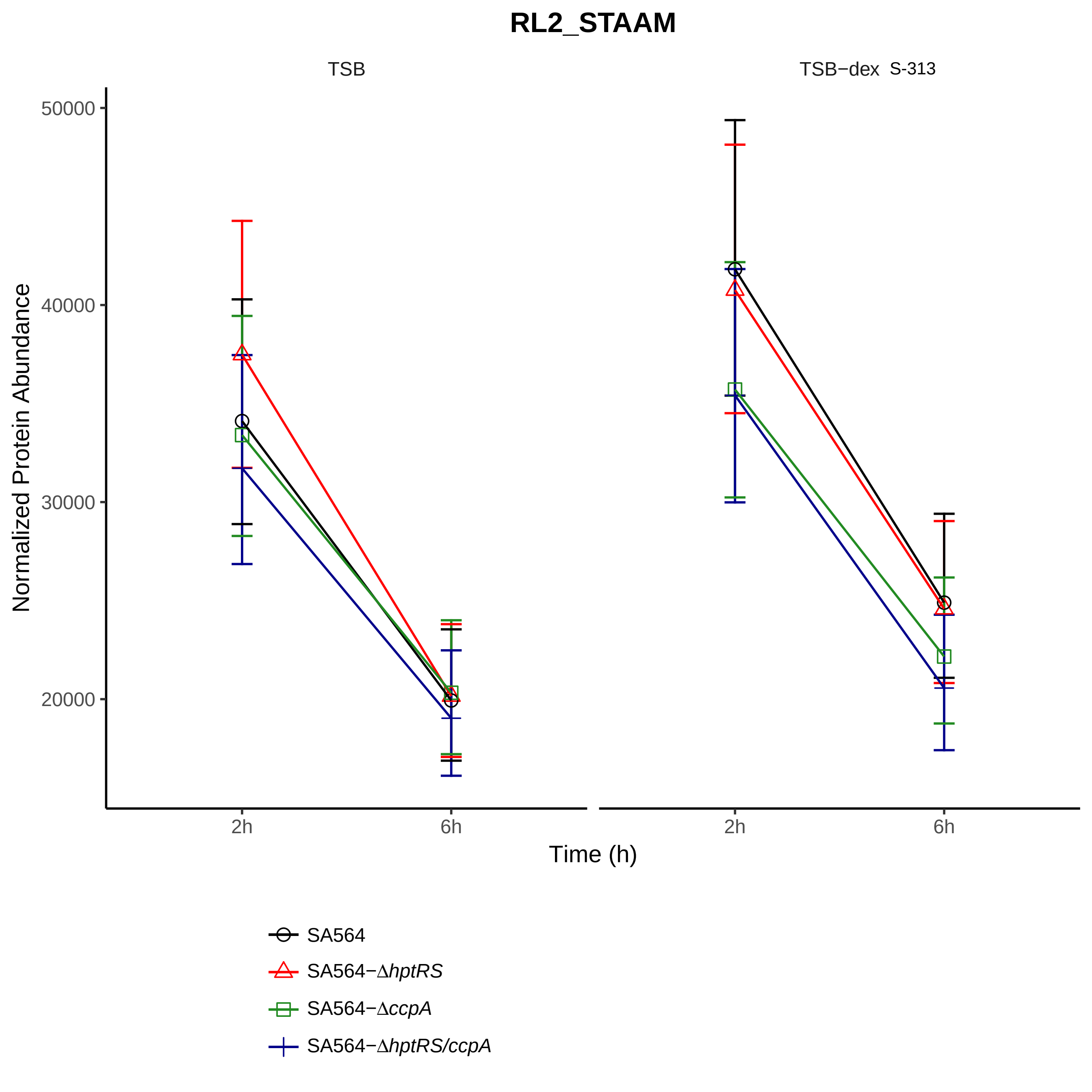
RL2_STAAM
TSB
TSB−dex
S-313
Normalized Protein Abundance
50000
40000
30000
20000
2h
6h
2h
6h
Time (h)
SA564
SA564−ΔhptRS
SA564−ΔccpA
SA564−ΔhptRS/ccpA

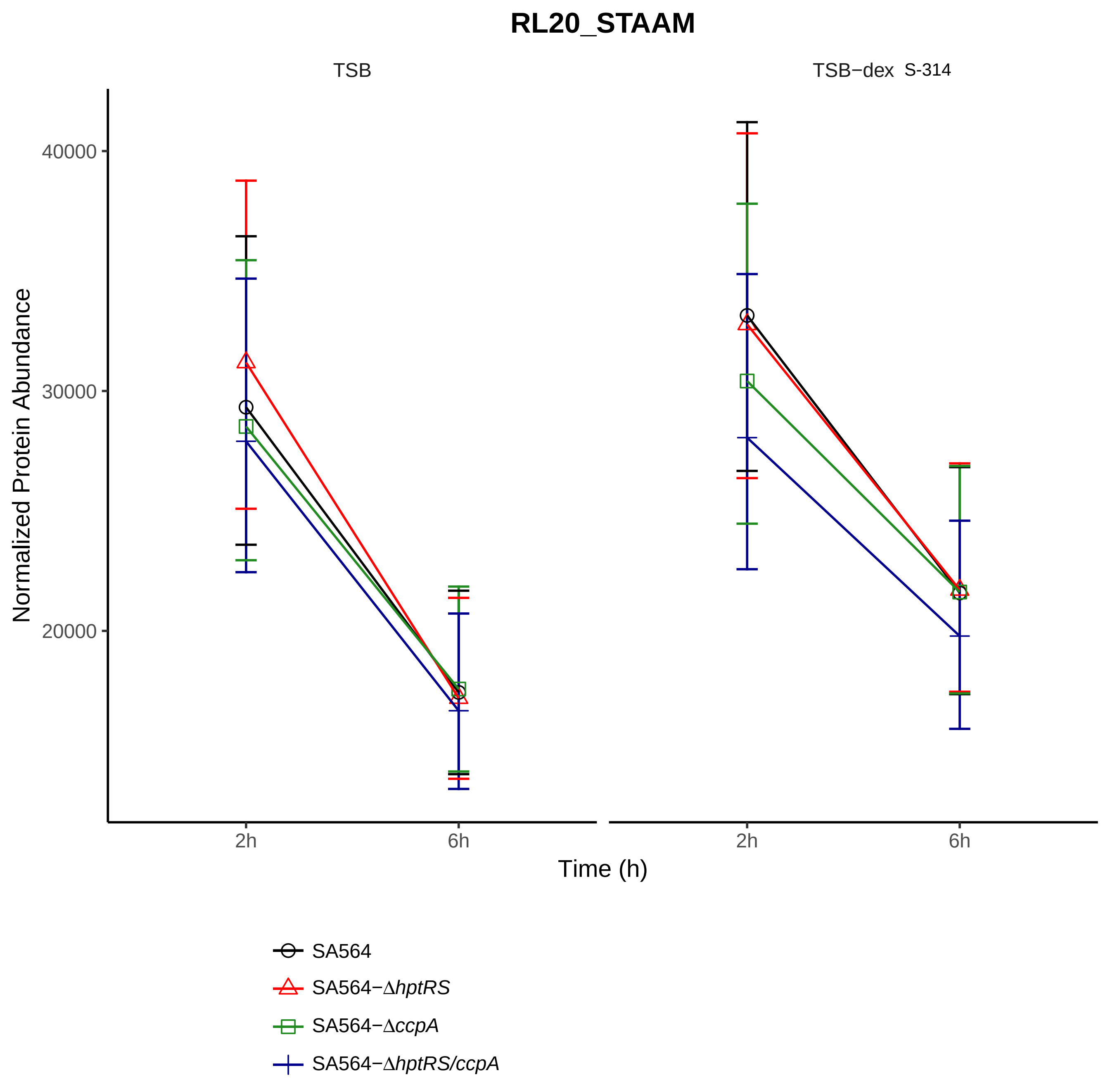

RL20_STAAM
TSB
TSB−dex
Normalized Protein Abundance
40000
30000
20000
2h
6h
2h
6h
Time (h)
SA564
SA564−Δ*hptRS*
SA564−Δ*ccpA*
SA564−Δ*hptRS/ccpA*

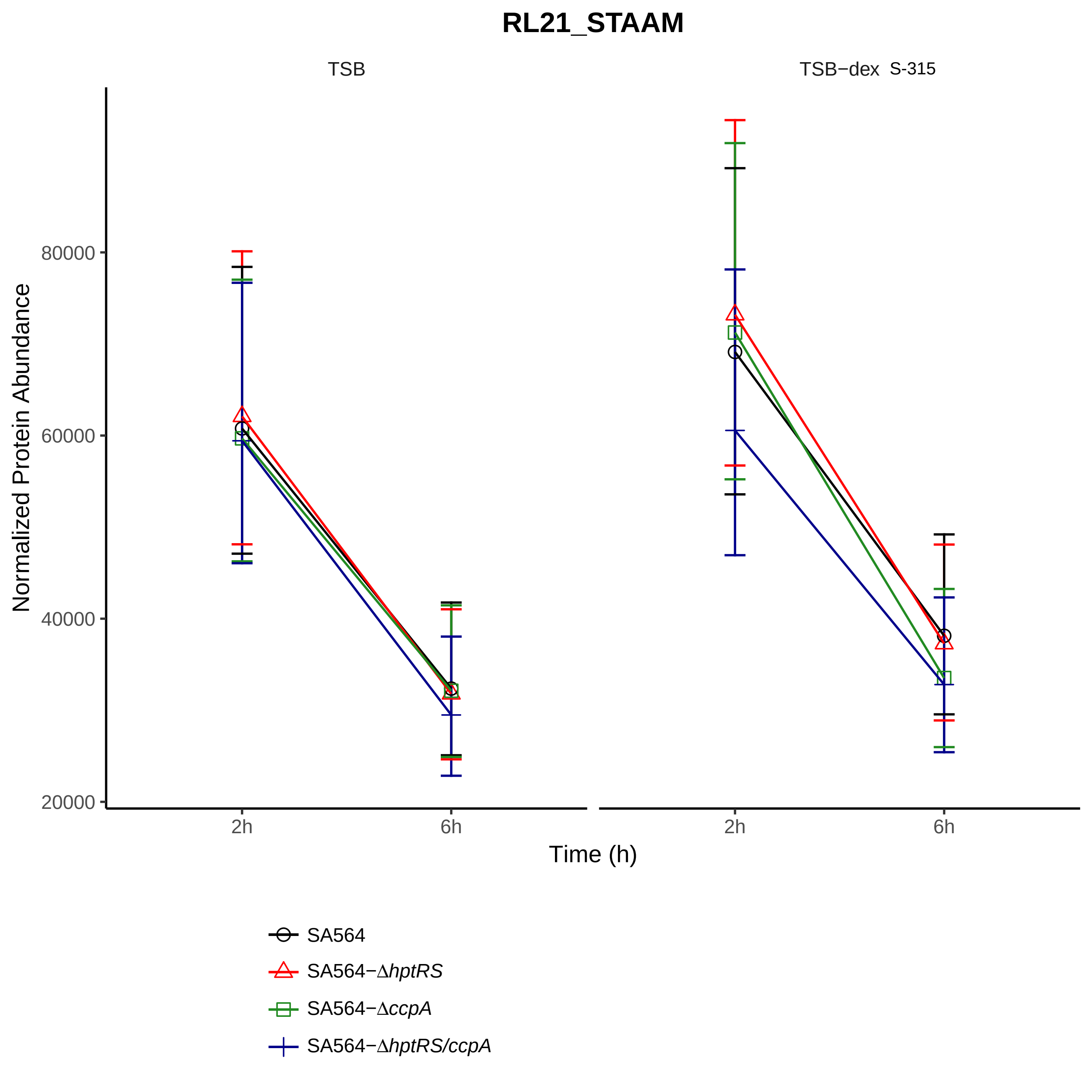

RL21_STAAM
TSB
TSB−dex
S-315
Normalized Protein Abundance
80000
60000
40000
20000
2h
6h
2h
6h
Time (h)
SA564
SA564−ΔhptRS
SA564−ΔccpA
SA564−ΔhptRS/ccpA

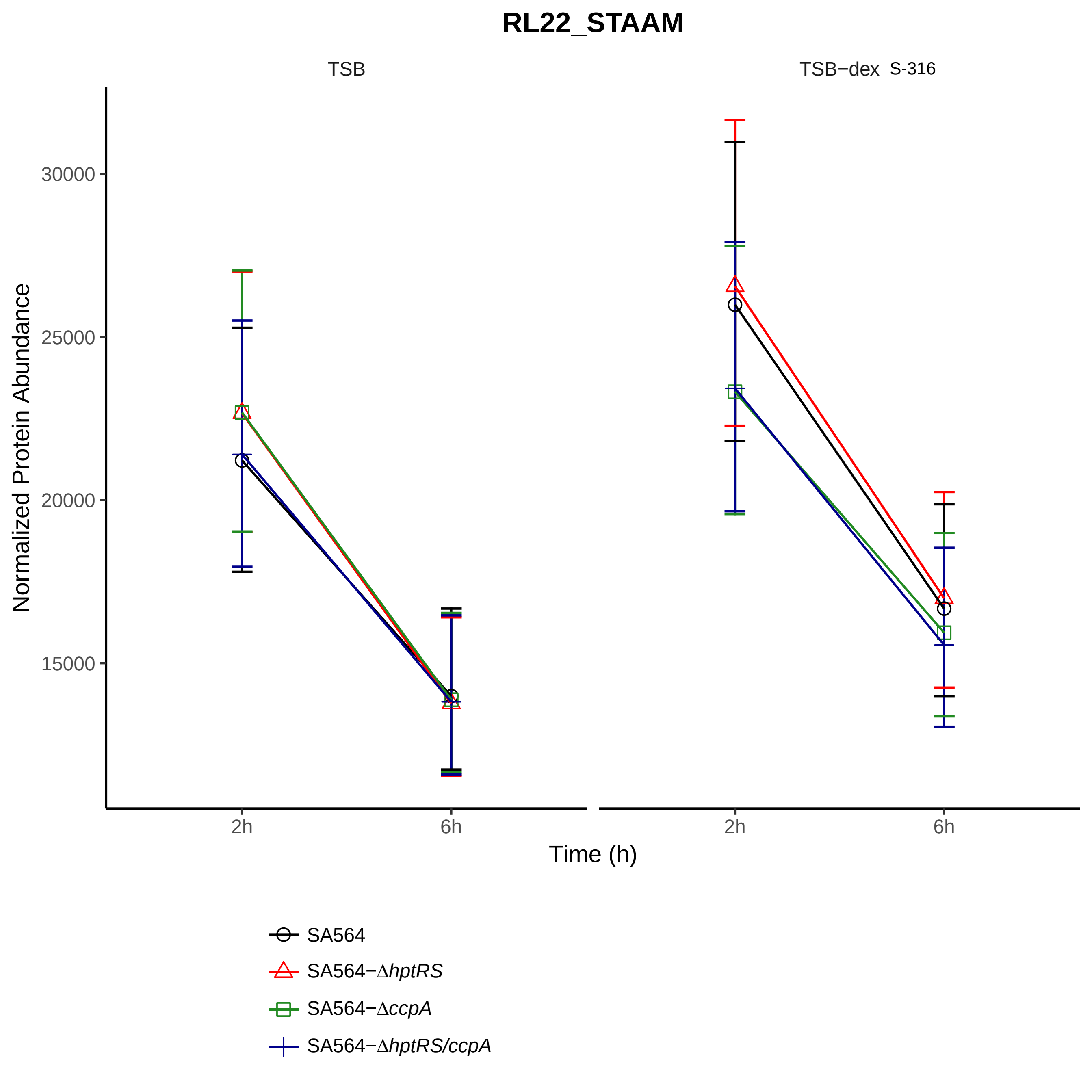
RL22_STAAM
TSB
TSB−dex
S-316
Normalized Protein Abundance
30000
25000
20000
15000
2h
6h
2h
6h
Time (h)
SA564
SA564−ΔhptRS
SA564−ΔccpA
SA564−ΔhptRS/ccpA

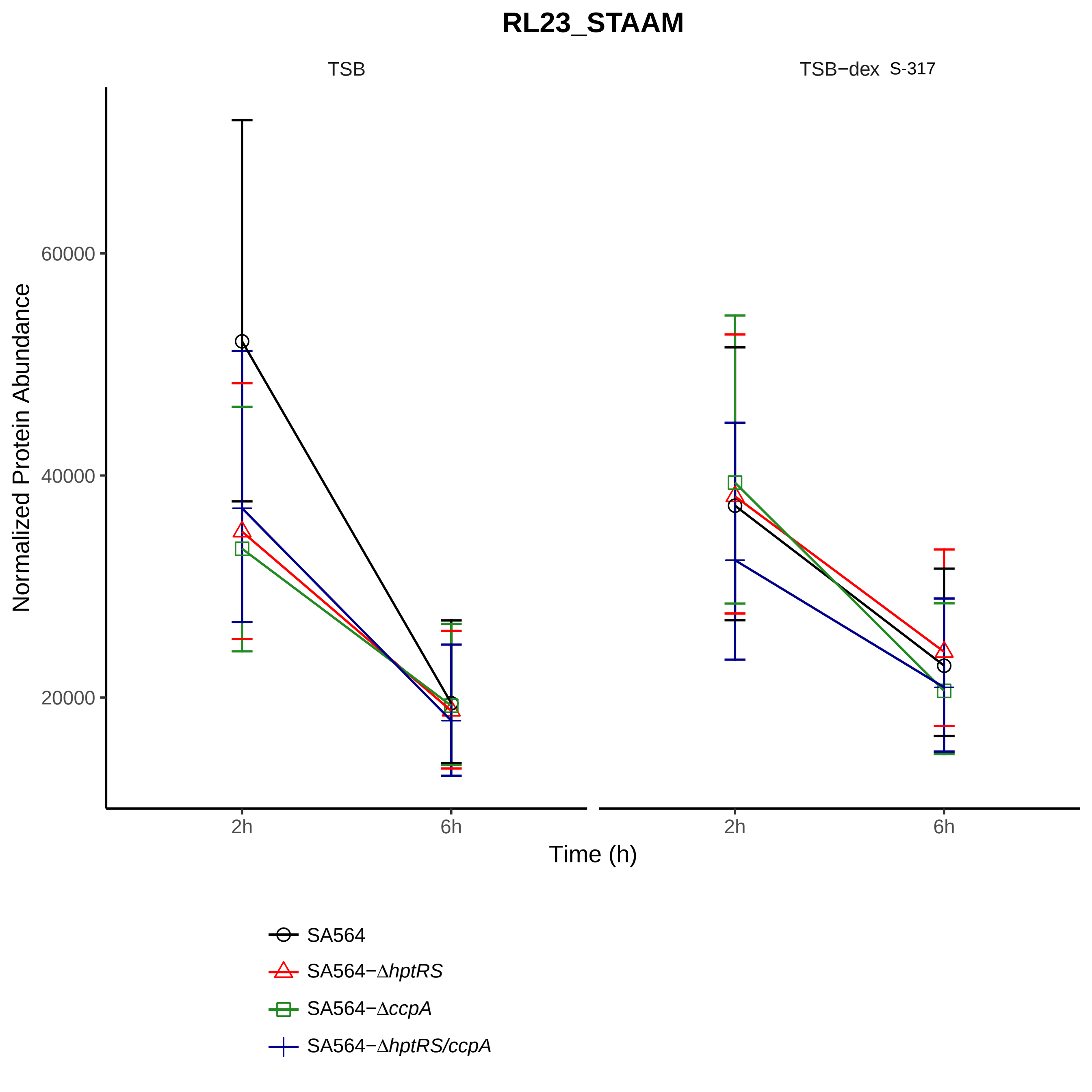

RL23_STAAM
TSB
TSB−dex
S-317
Normalized Protein Abundance
60000
40000
20000
2h
6h
2h
6h
Time (h)
SA564
SA564−ΔhptRS
SA564−ΔccpA
SA564−ΔhptRS/ccpA

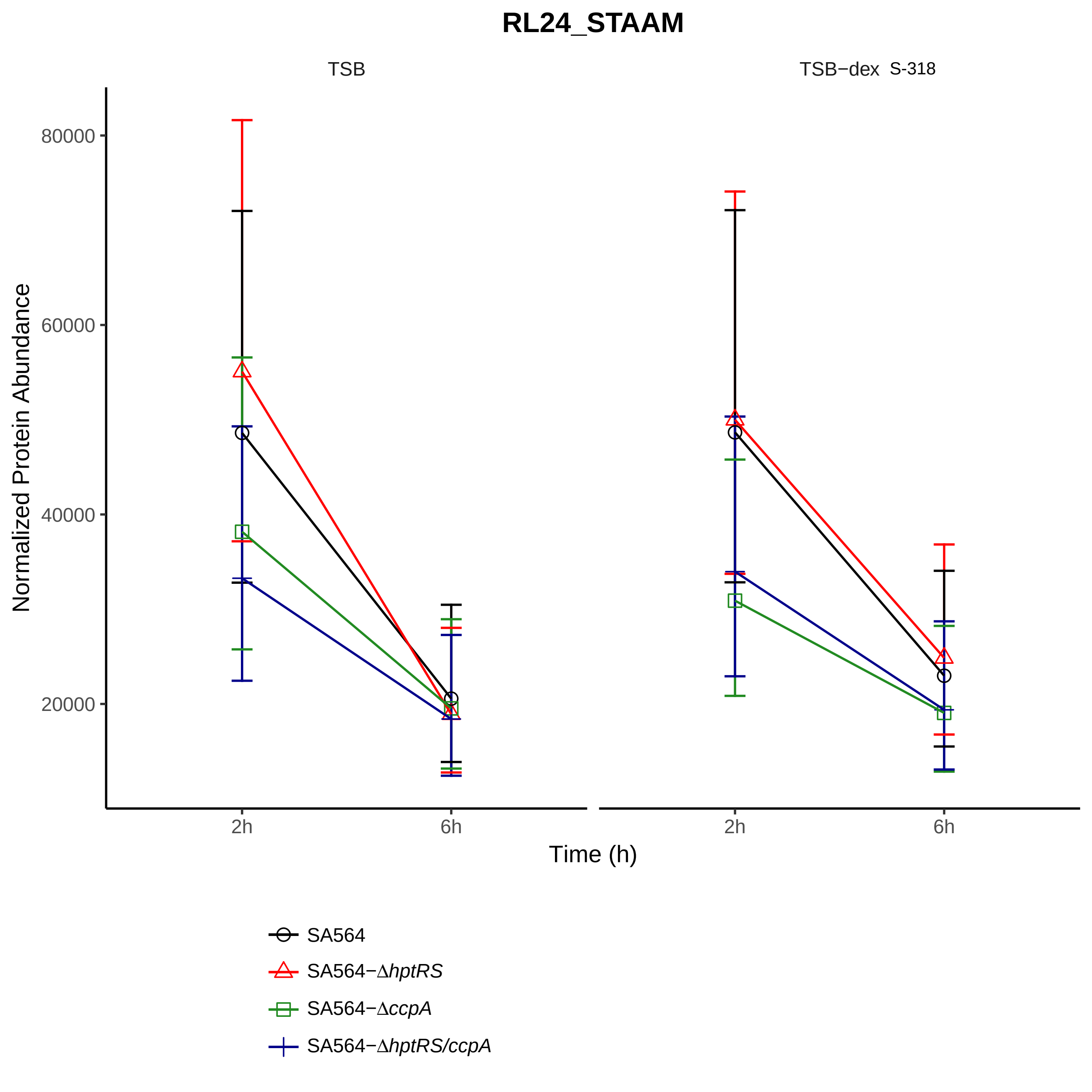

RL24_STAAM
TSB
TSB−dex
S-318
Normalized Protein Abundance
80000
60000
40000
20000
2h
6h
2h
6h
Time (h)
SA564
SA564−Δ*hptRS*
SA564−Δ*ccpA*
SA564−Δ*hptRS/ccpA*

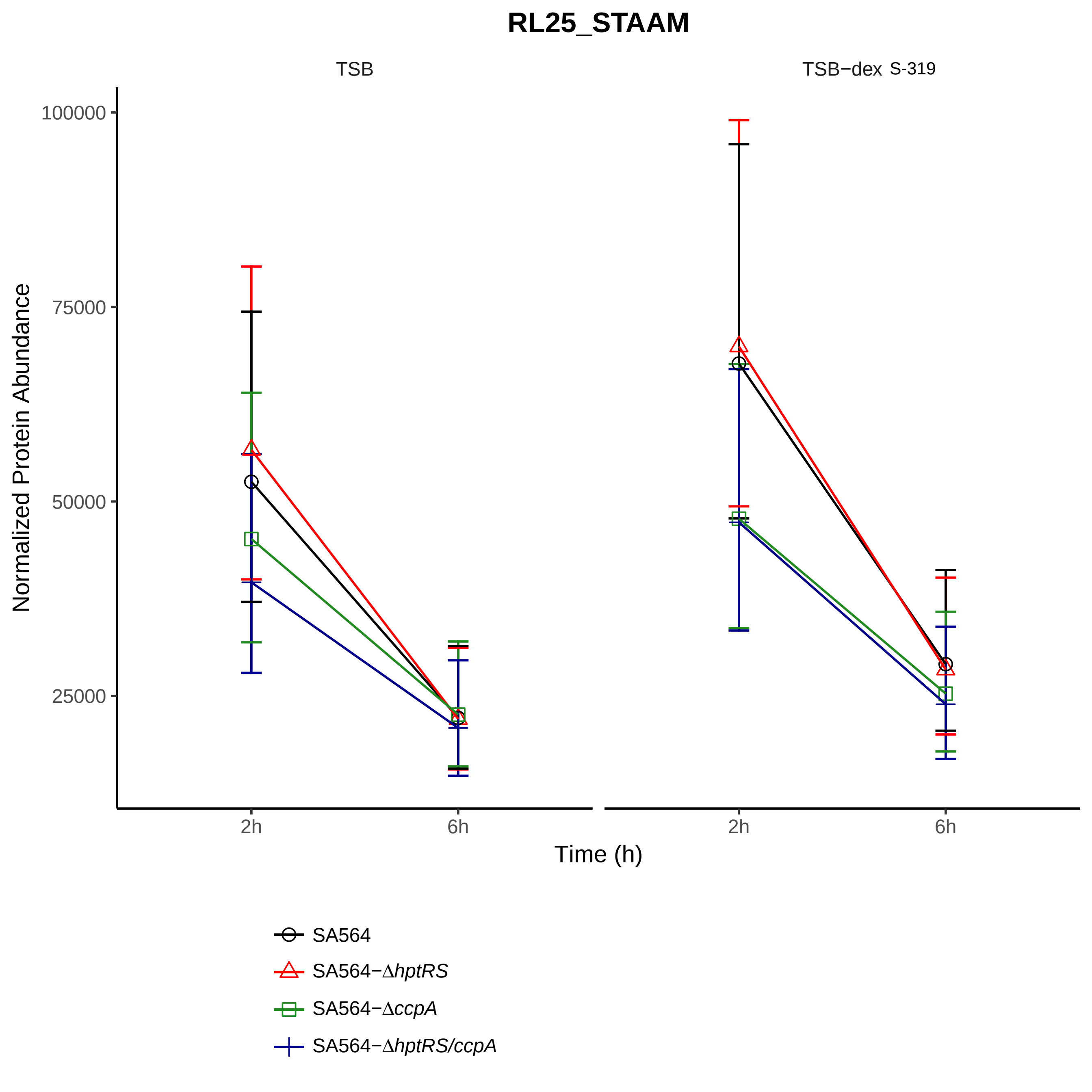
RL25_STAAM
TSB
TSB−dex
S-319
Normalized Protein Abundance
100000
75000
50000
25000
2h
6h
2h
6h
Time (h)
SA564
SA564−ΔhptRS
SA564−ΔccpA
SA564−ΔhptRS/ccpA

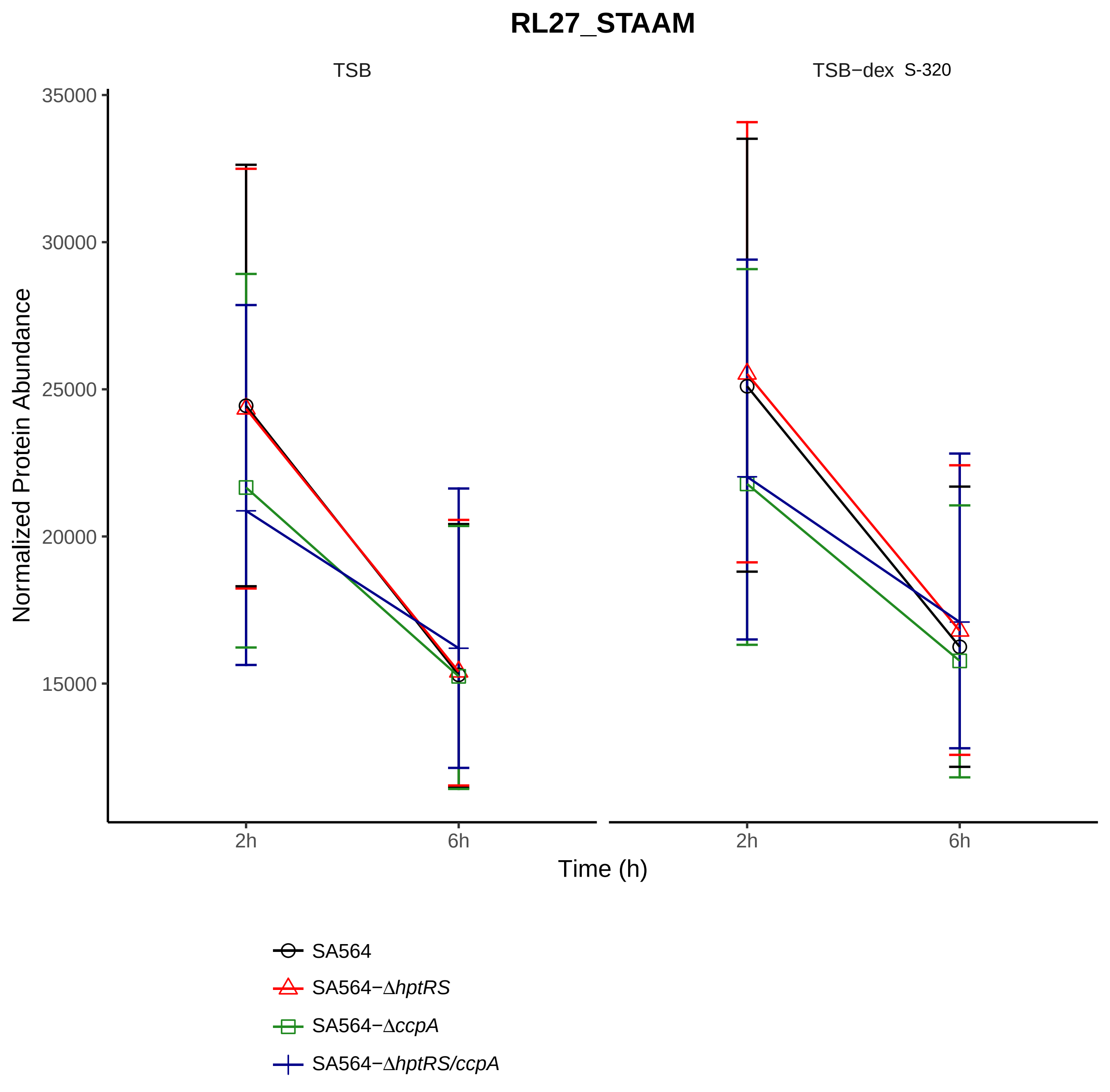

RL27_STAAM
TSB
TSB−dex
S-320
35000
30000
25000
20000
15000
Normalized Protein Abundance
2h
6h
2h
6h
Time (h)
SA564
SA564−Δ*hptRS*
SA564−Δ*ccpA*
SA564−Δ*hptRS/ccpA*

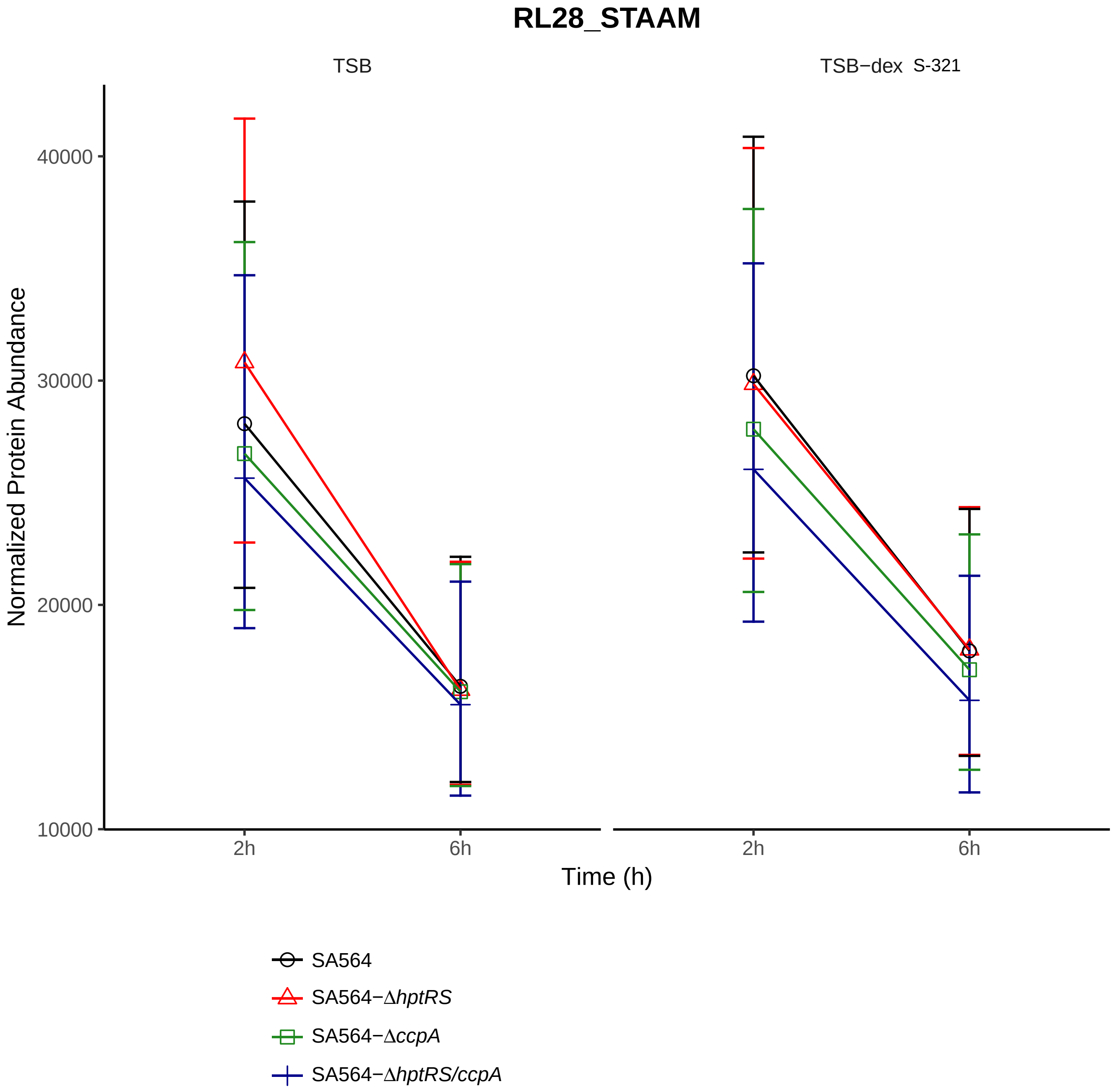
RL28_STAAM
TSB
TSB−dex
S-321
Normalized Protein Abundance
40000
30000
20000
10000
2h
6h
2h
6h
Time (h)
SA564
SA564−ΔhptRS
SA564−ΔccpA
SA564−ΔhptRS/ccpA

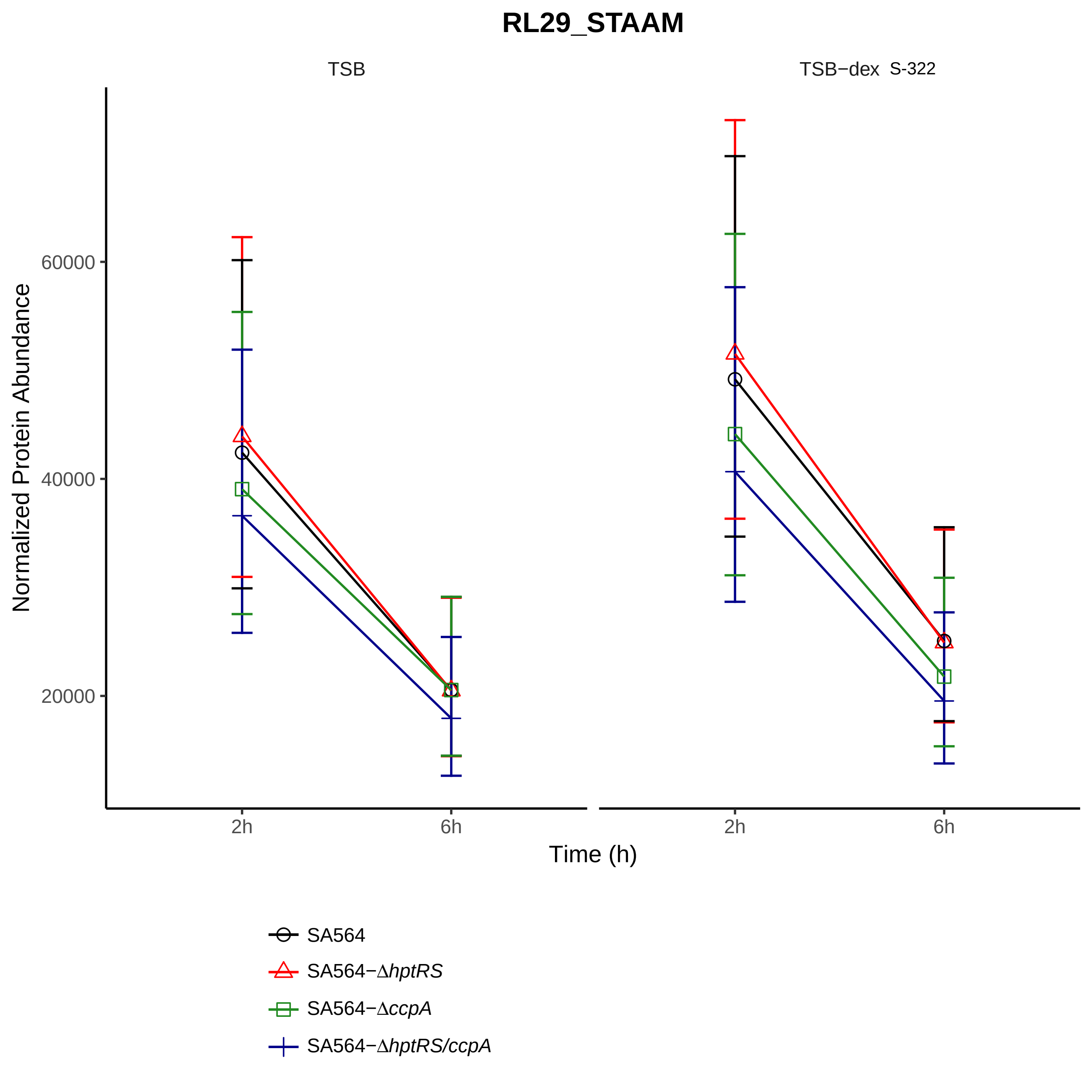

RL29_STAAM
TSB
TSB−dex
S-322
Normalized Protein Abundance
60000
40000
20000
2h
6h
2h
6h
Time (h)
SA564
SA564−Δ*hptRS*
SA564−Δ*ccpA*
SA564−Δ*hptRS/ccpA*

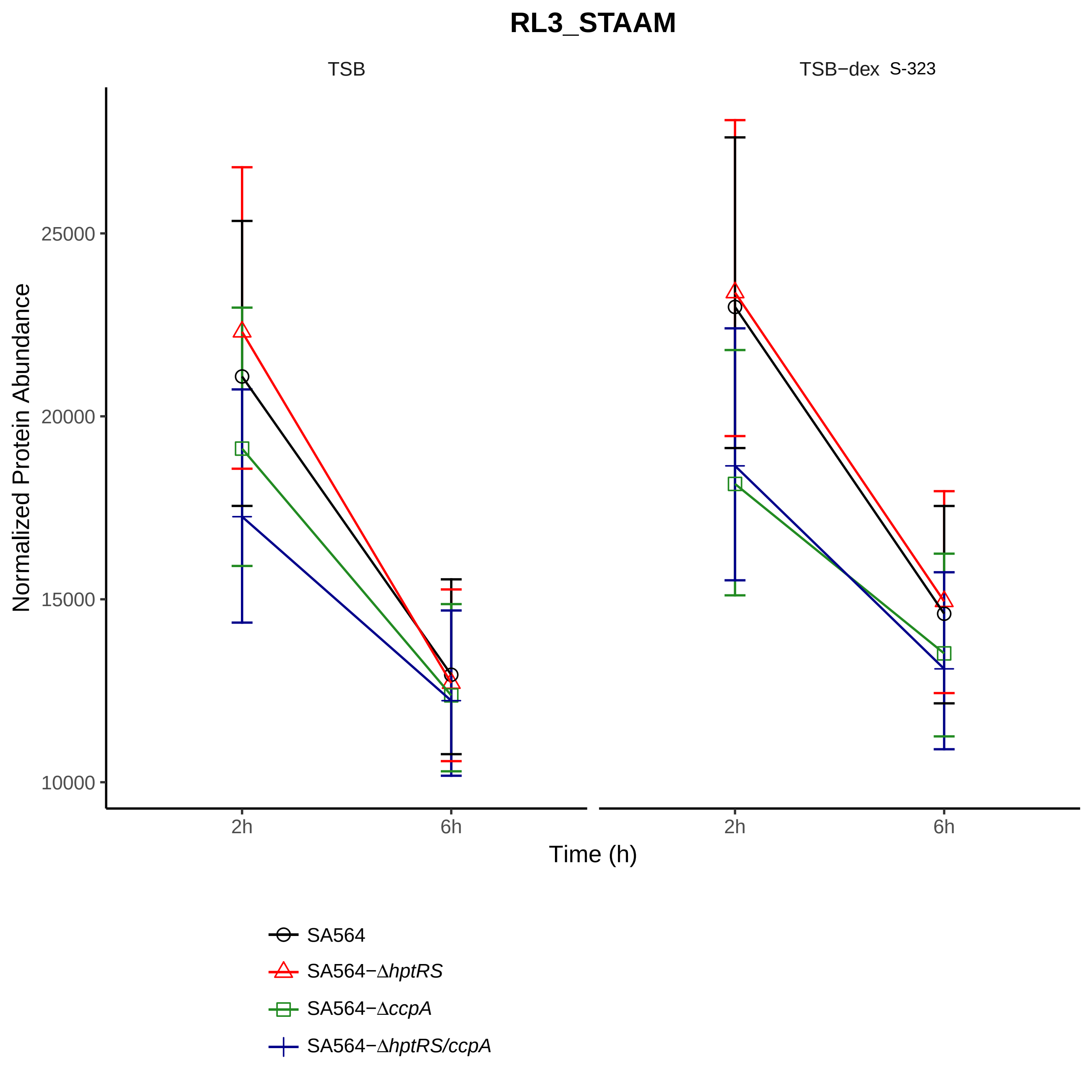

RL3_STAAM
TSB
TSB−dex
S-323
Normalized Protein Abundance
25000
20000
15000
10000
2h
6h
2h
6h
Time (h)
SA564
SA564−Δ*hptRS*
SA564−Δ*ccpA*
SA564−Δ*hptRS/ccpA*

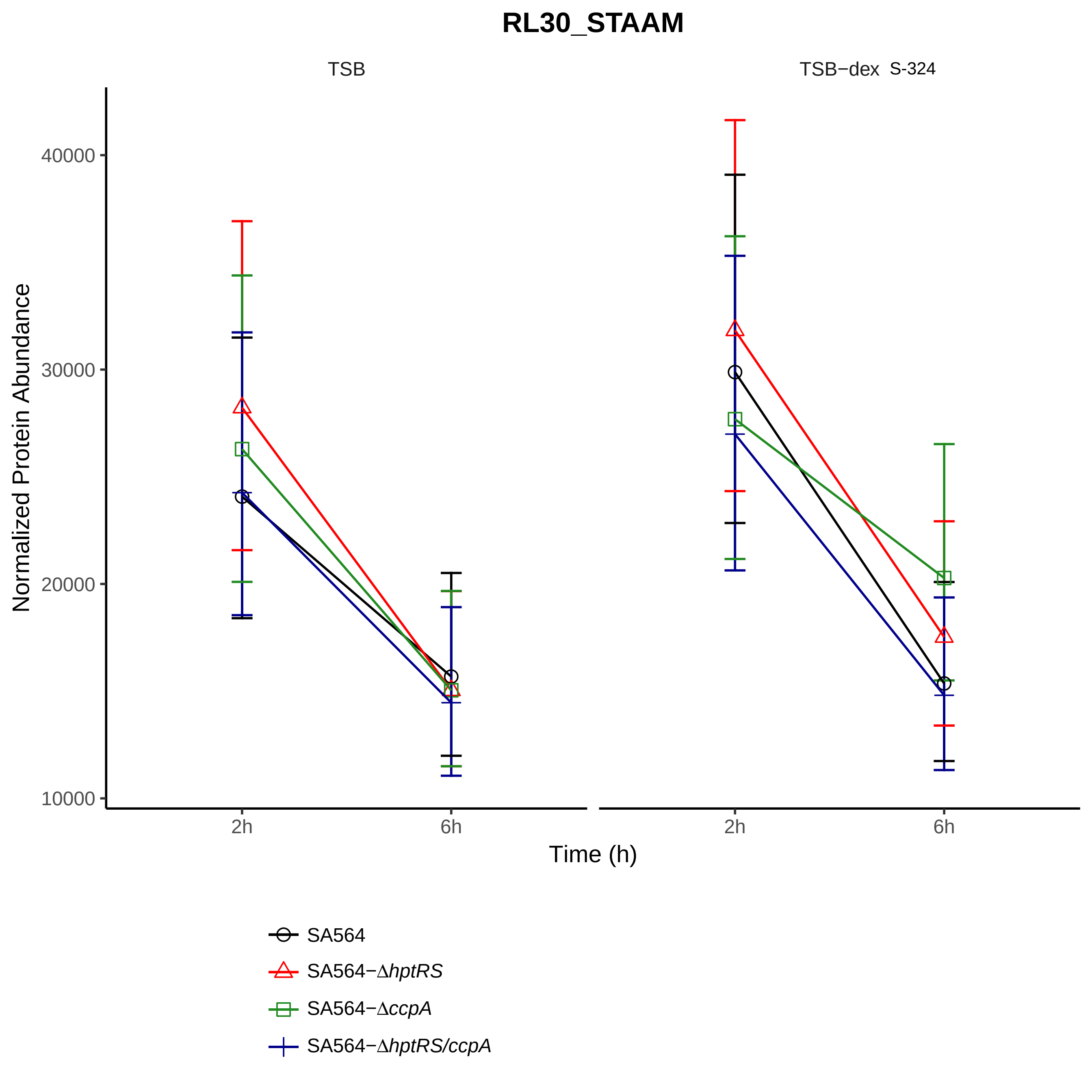

RL30_STAAM
TSB
TSB−dex
S-324
Normalized Protein Abundance
40000
30000
20000
10000
2h
6h
2h
6h
Time (h)
SA564
SA564−Δ*hptRS*
SA564−Δ*ccpA*
SA564−Δ*hptRS/ccpA*

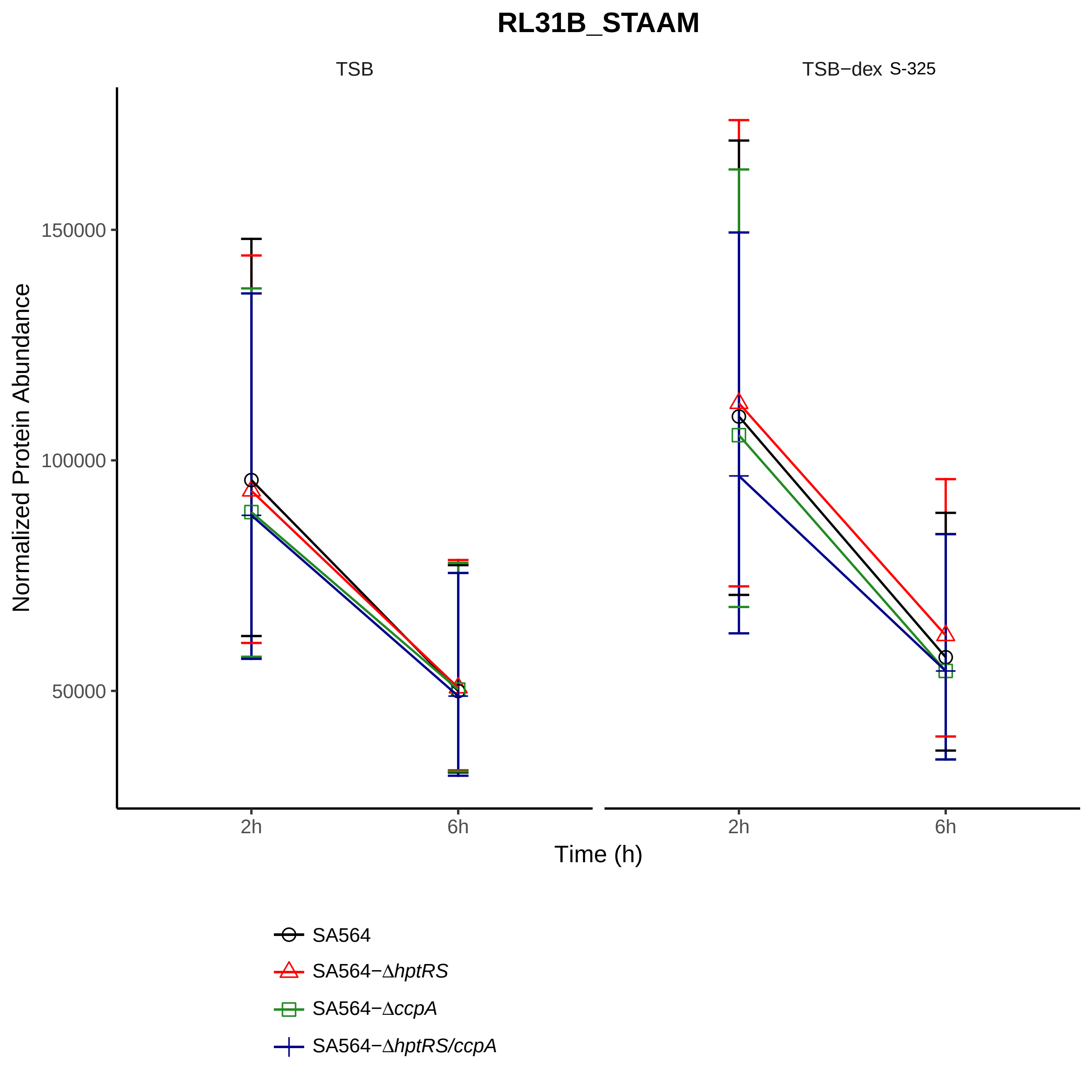

RL31B_STAAM
TSB
TSB−dex
S-325
Normalized Protein Abundance
150000
100000
50000
2h
6h
2h
6h
Time (h)
SA564
SA564−ΔhptRS
SA564−ΔccpA
SA564−ΔhptRS/ccpA

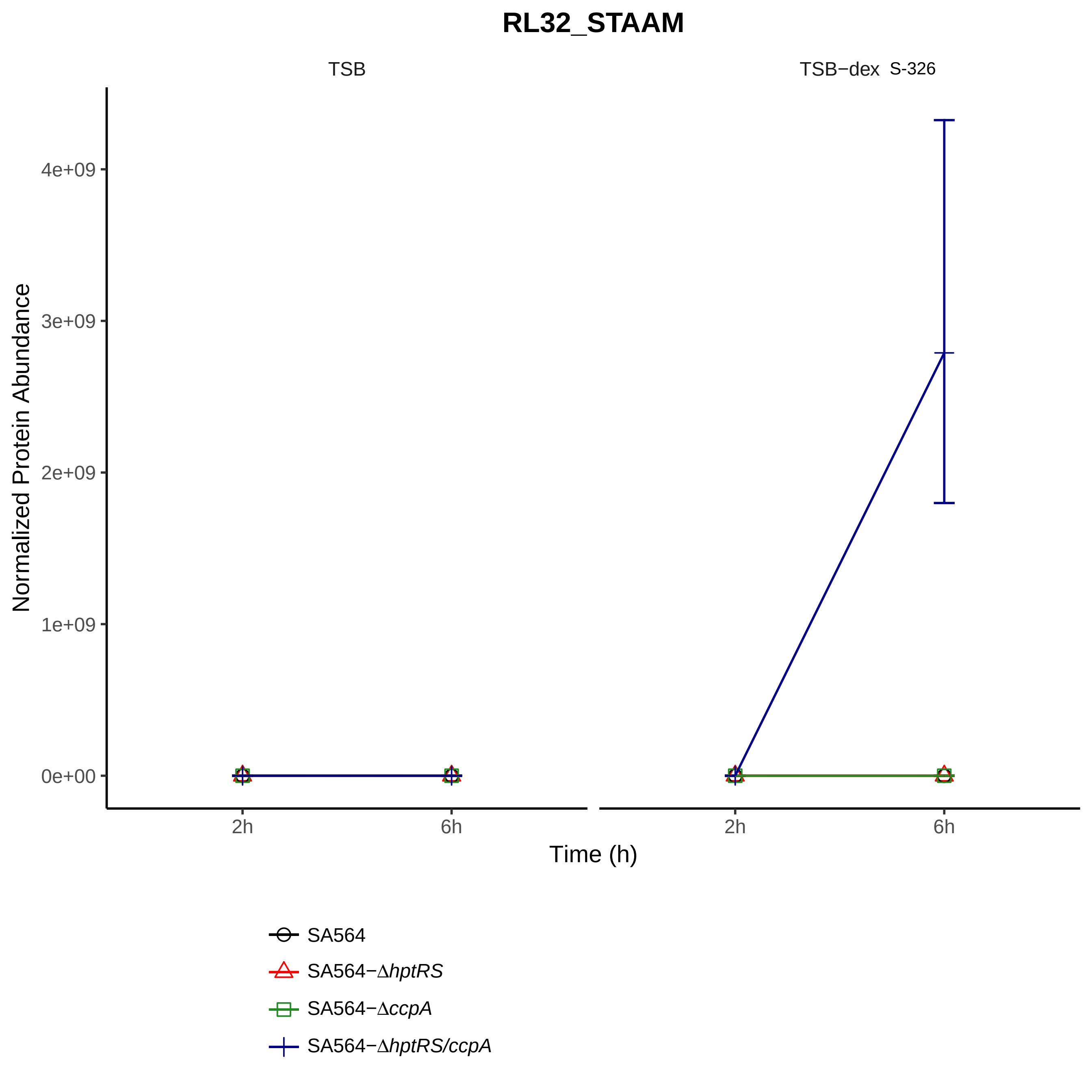

RL32_STAAM
TSB
TSB−dex
S-326
Normalized Protein Abundance
4e+09
3e+09
2e+09
1e+09
0e+00
2h
6h
2h
6h
Time (h)
SA564
SA564−Δ*hptRS*
SA564−Δ*ccpA*
SA564−Δ*hptRS/ccpA*

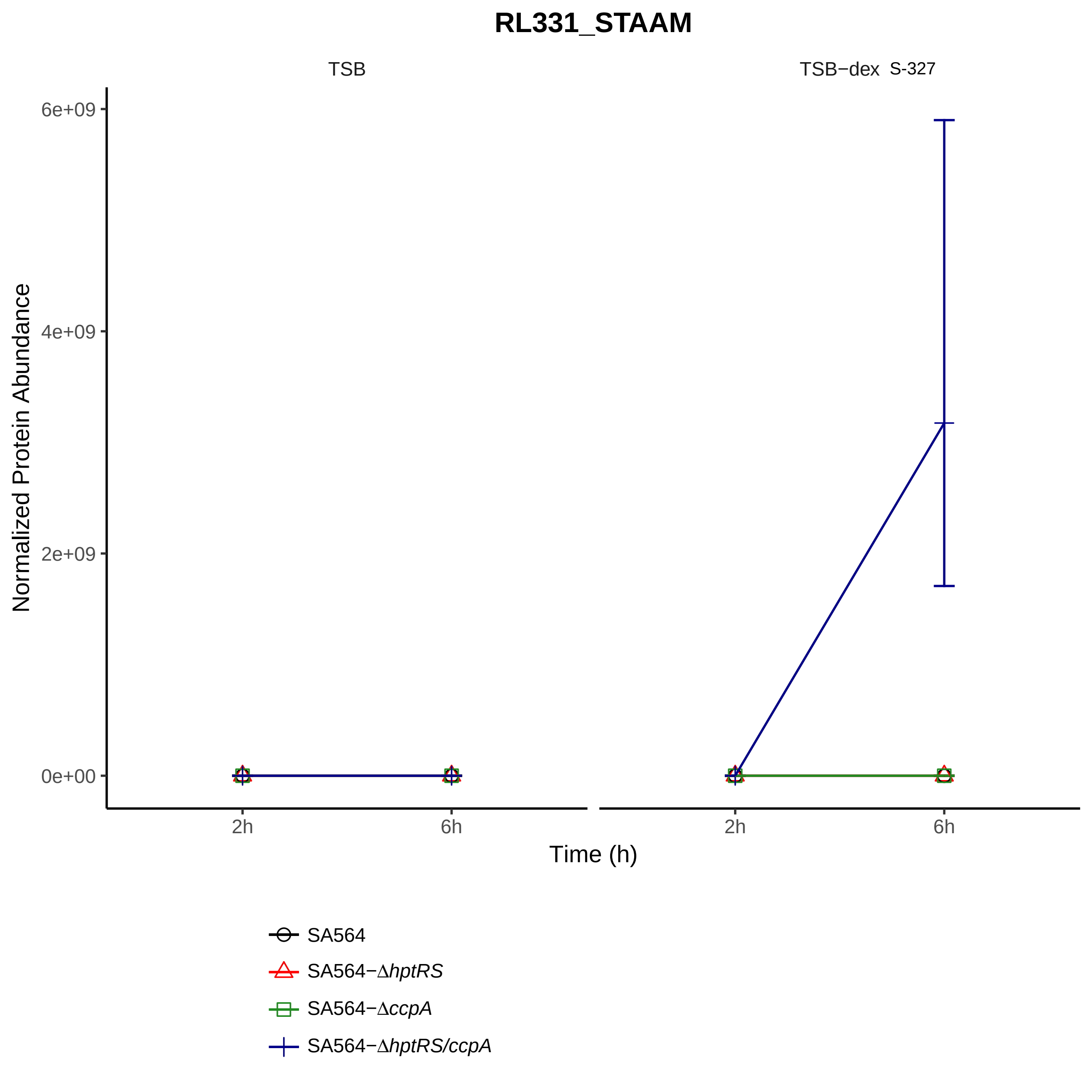
RL331_STAAM
TSB
TSB−dex
S-327
6e+09
4e+09
2e+09
0e+00
Normalized Protein Abundance
2h
6h
2h
6h
Time (h)
SA564
SA564−Δ*hptRS*
SA564−Δ*ccpA*
SA564−Δ*hptRS/ccpA*

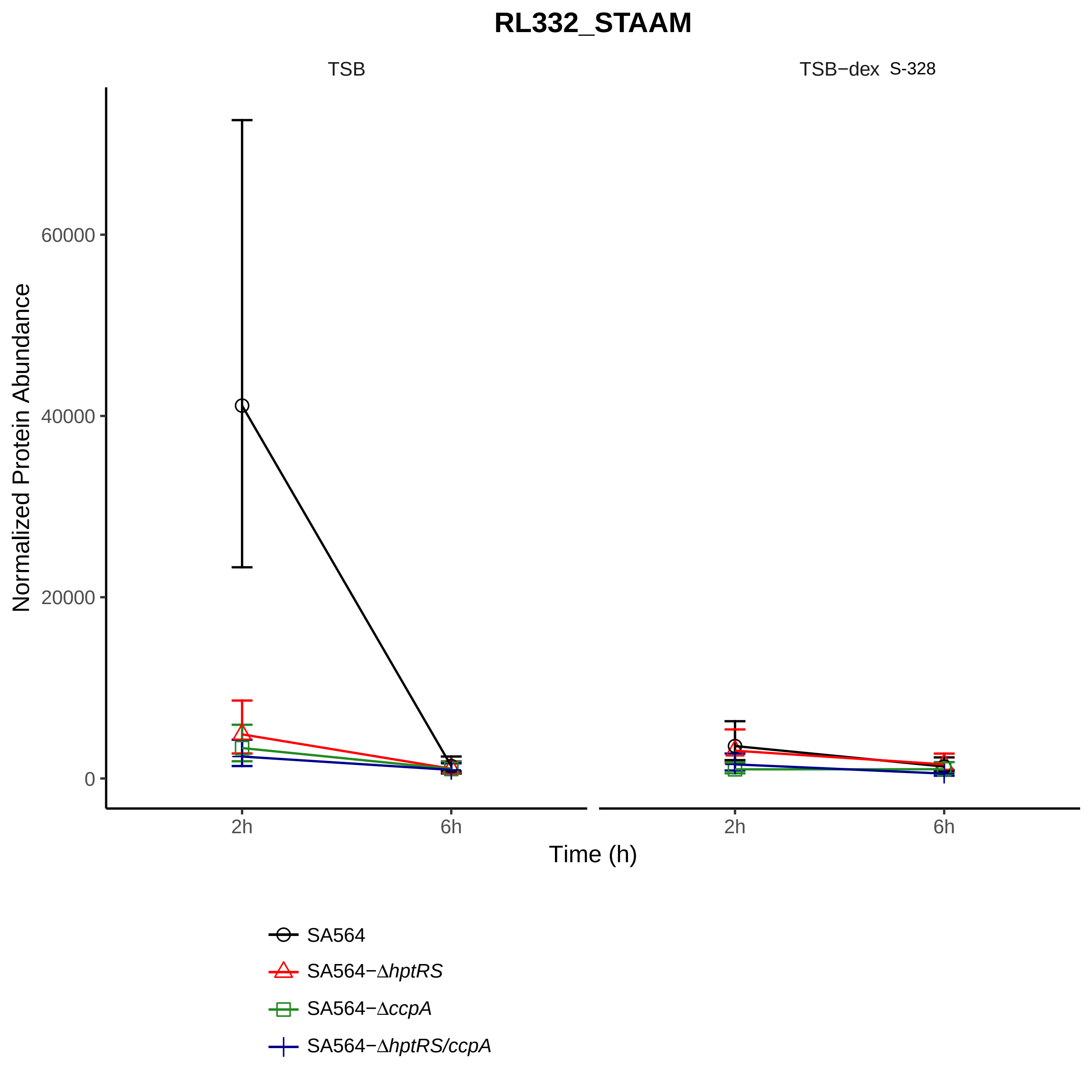
RL332_STAAM
TSB
TSB−dex
S-328
Normalized Protein Abundance
60000
40000
20000
0
2h
6h
2h
6h
Time (h)
SA564
SA564−Δ*hptRS*
SA564−Δ*ccpA*
SA564−Δ*hptRS/ccpA*

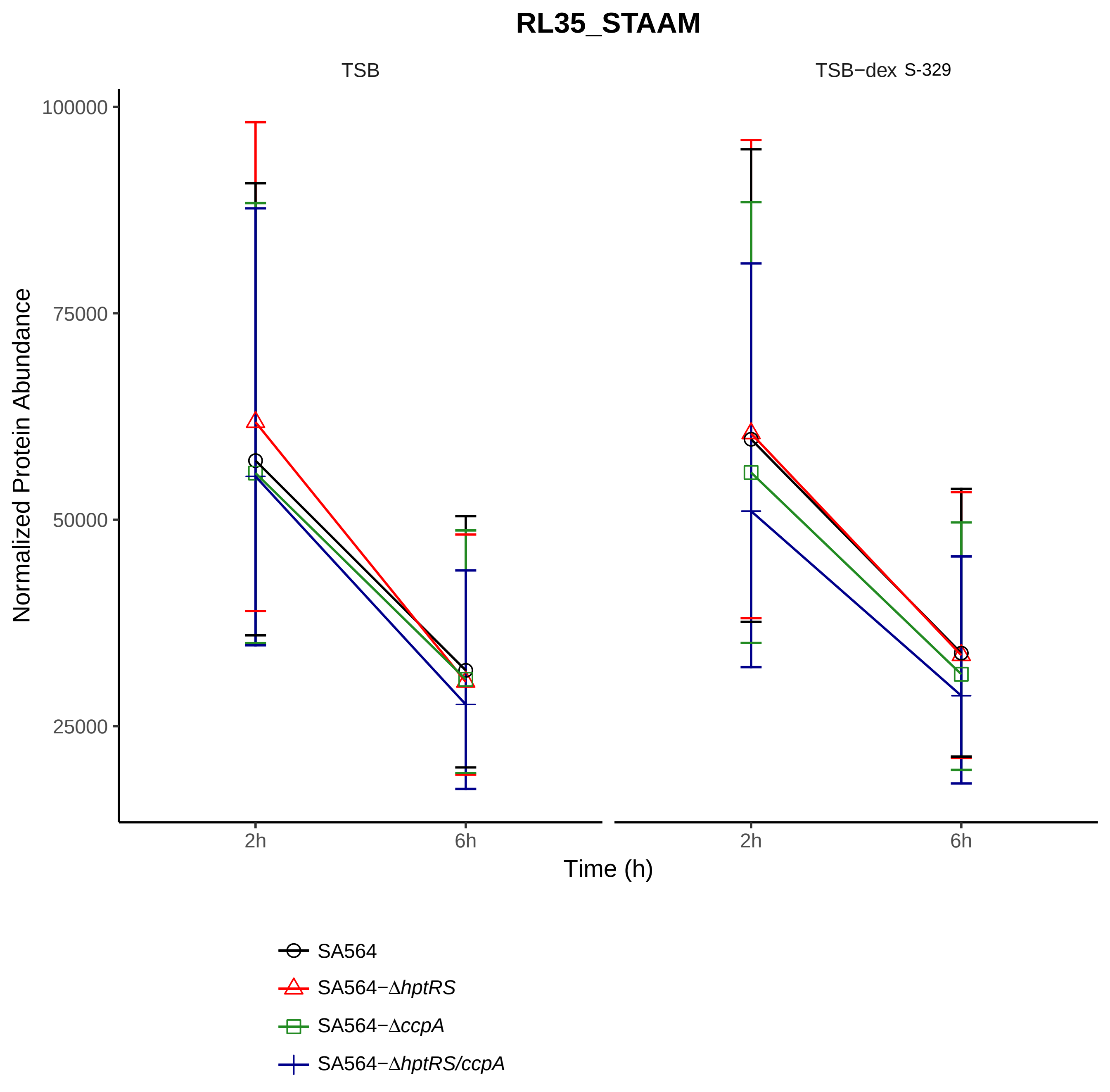

RL35_STAAM
TSB
TSB−dex
Normalized Protein Abundance
100000
75000
50000
25000
2h
6h
2h
6h
Time (h)
SA564
SA564−ΔhptRS
SA564−ΔccpA
SA564−ΔhptRS/ccpA

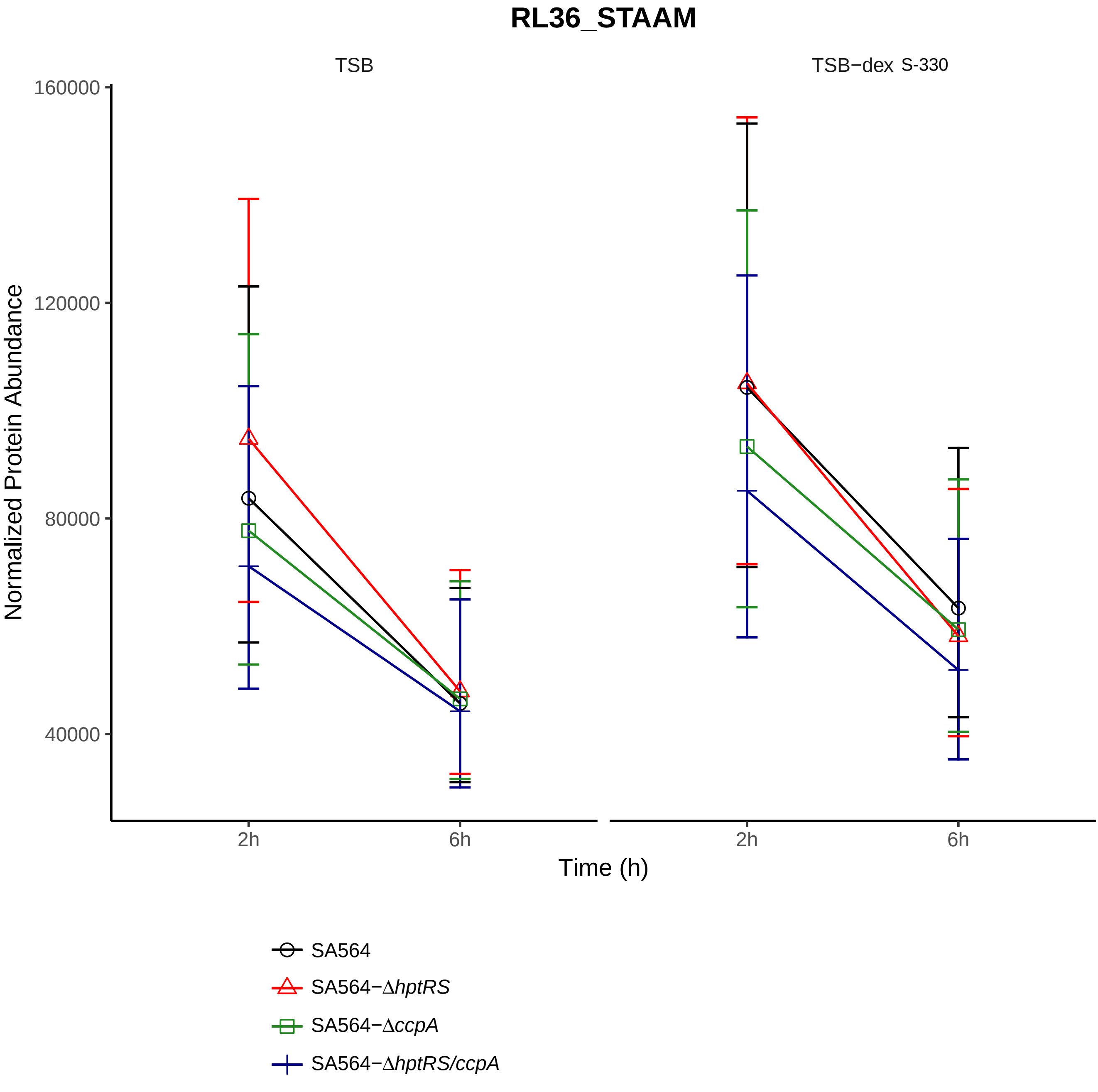

RL36_STAAM
TSB
TSB−dex
Normalized Protein Abundance
160000
120000
80000
40000
2h
6h
2h
6h
Time (h)
SA564
SA564−ΔhptRS
SA564−ΔccpA
SA564−ΔhptRS/ccpA

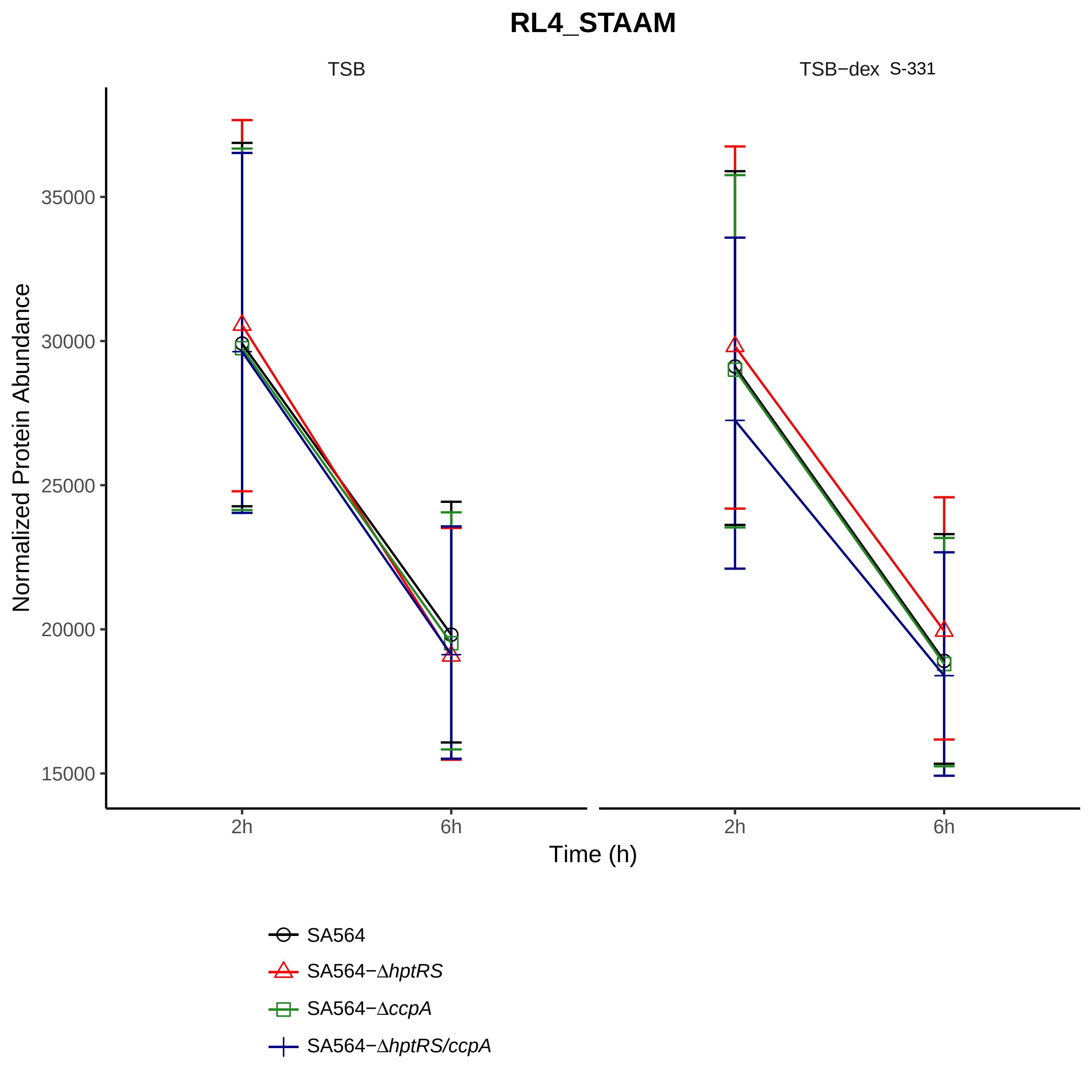

RL4_STAAM
TSB
TSB−dex
S-331
Normalized Protein Abundance
35000
30000
25000
20000
15000
2h
6h
2h
6h
Time (h)
SA564
SA564−Δ*hptRS*
SA564−Δ*ccpA*
SA564−Δ*hptRS/ccpA*

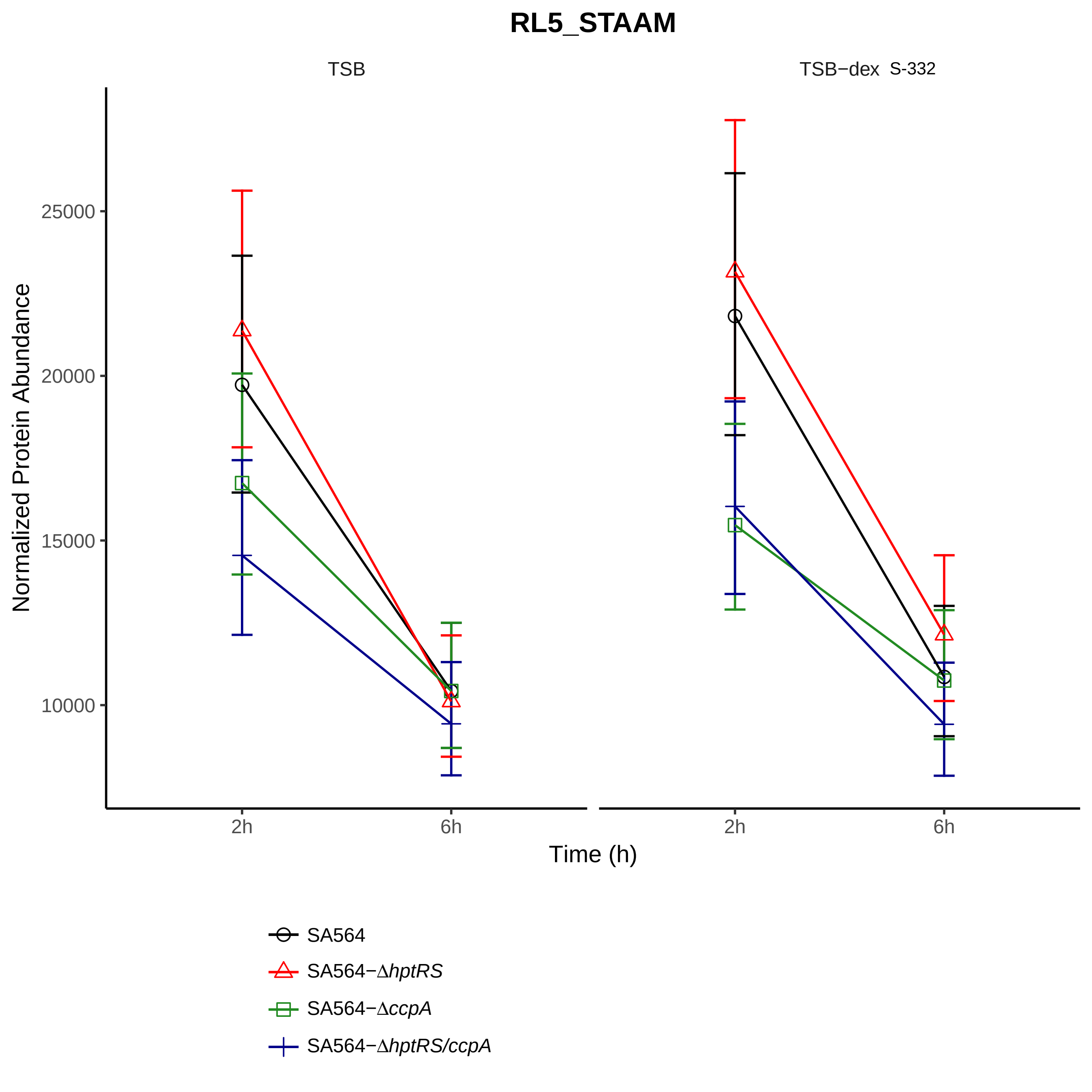

RL5_STAAM
TSB
TSB−dex
S-332
Normalized Protein Abundance
25000
20000
15000
10000
2h
6h
2h
6h
Time (h)
SA564
SA564−ΔhptRS
SA564−ΔccpA
SA564−ΔhptRS/ccpA

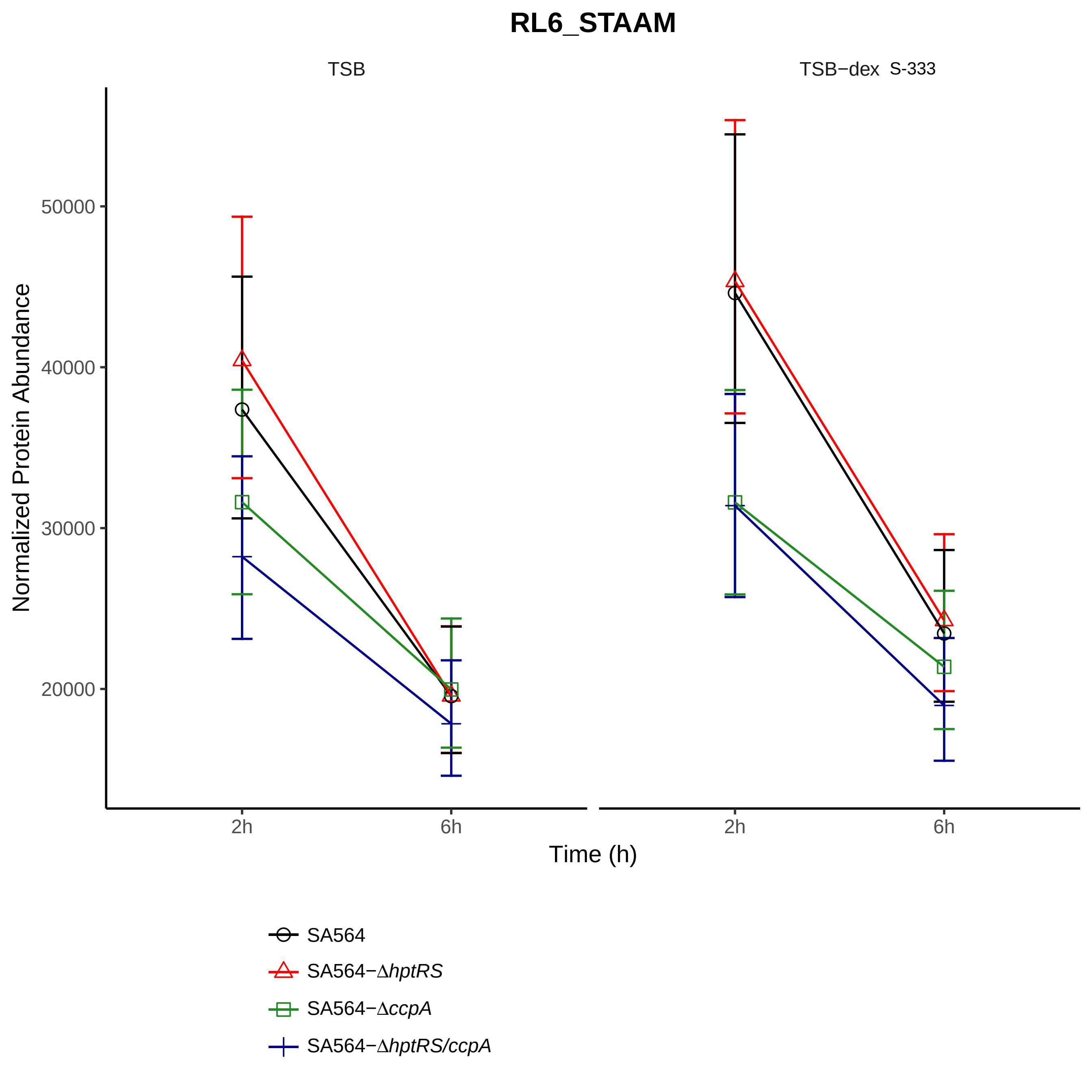

RL6_STAAM
TSB
TSB−dex
S-333
Normalized Protein Abundance
50000
40000
30000
20000
2h
6h
2h
6h
Time (h)
SA564
SA564−Δ*hptRS*
SA564−Δ*ccpA*
SA564−Δ*hptRS/ccpA*

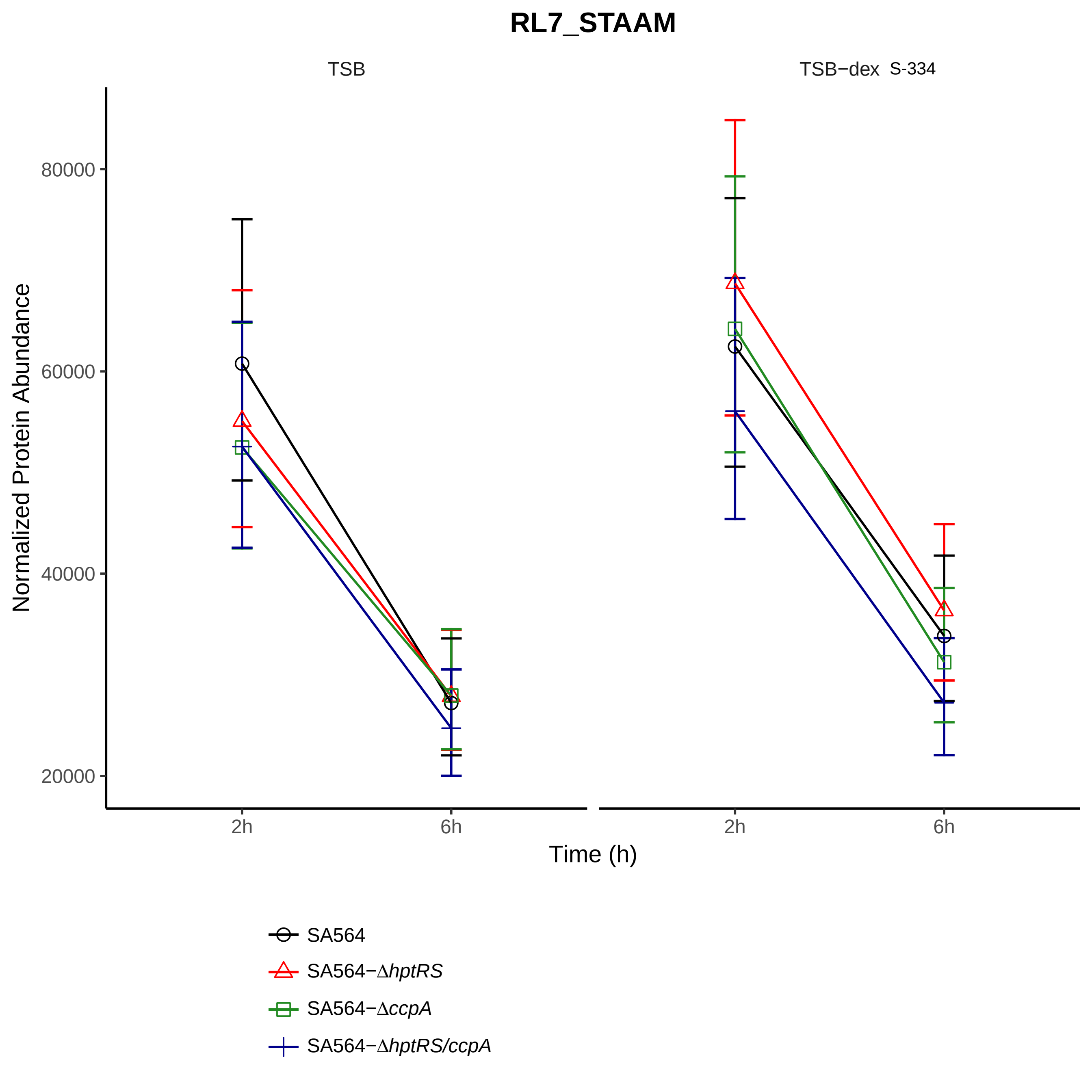

RL7_STAAM
TSB
TSB−dex
S-334
Normalized Protein Abundance
80000
60000
40000
20000
2h
6h
2h
6h
Time (h)
SA564
SA564−ΔhptRS
SA564−ΔccpA
SA564−ΔhptRS/ccpA

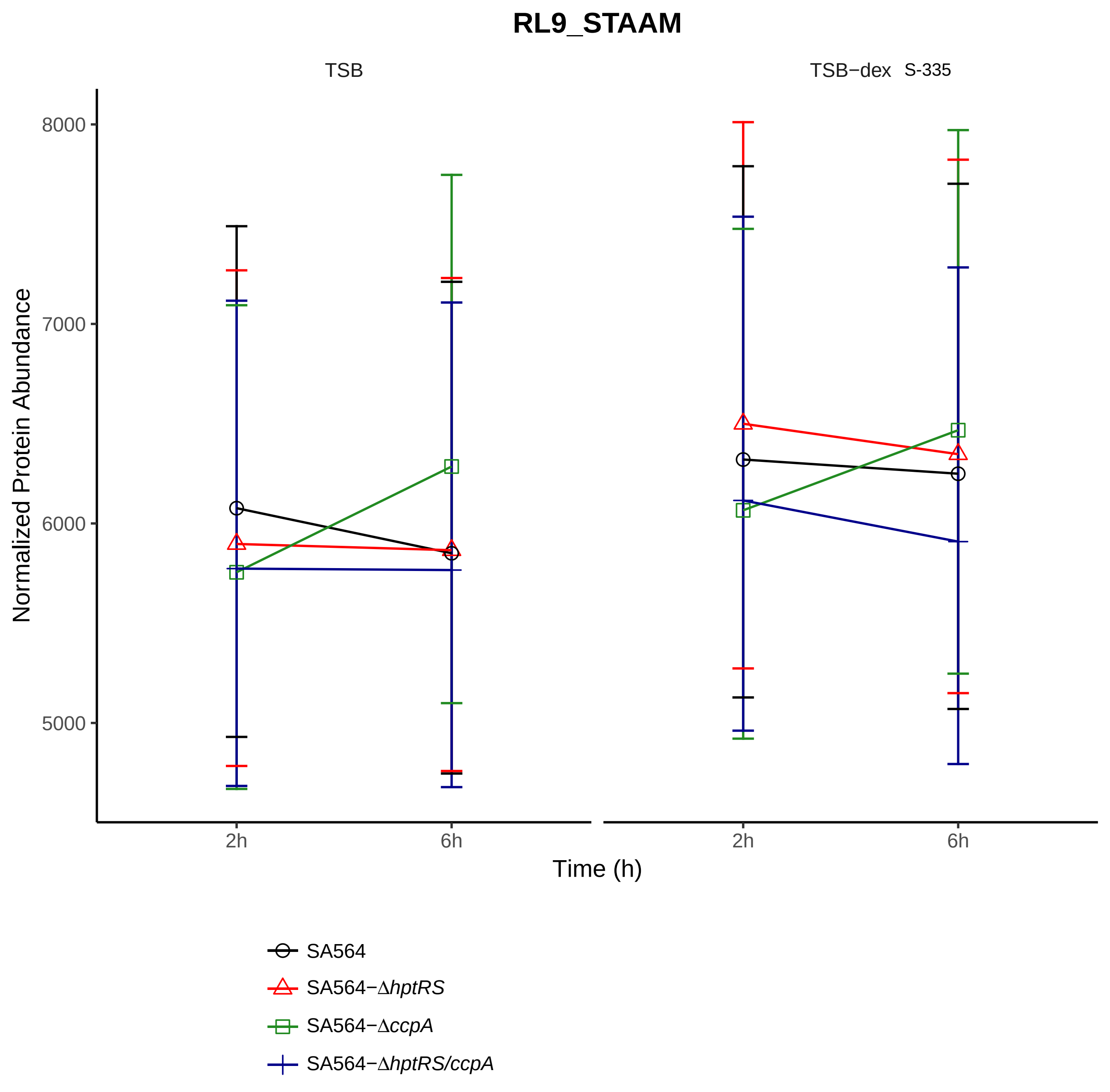

RL9_STAAM
TSB
TSB−dex
S-335
8000
7000
6000
5000
Normalized Protein Abundance
2h
6h
2h
6h
Time (h)
SA564
SA564−ΔhptRS
SA564−ΔccpA
SA564−ΔhptRS/ccpA

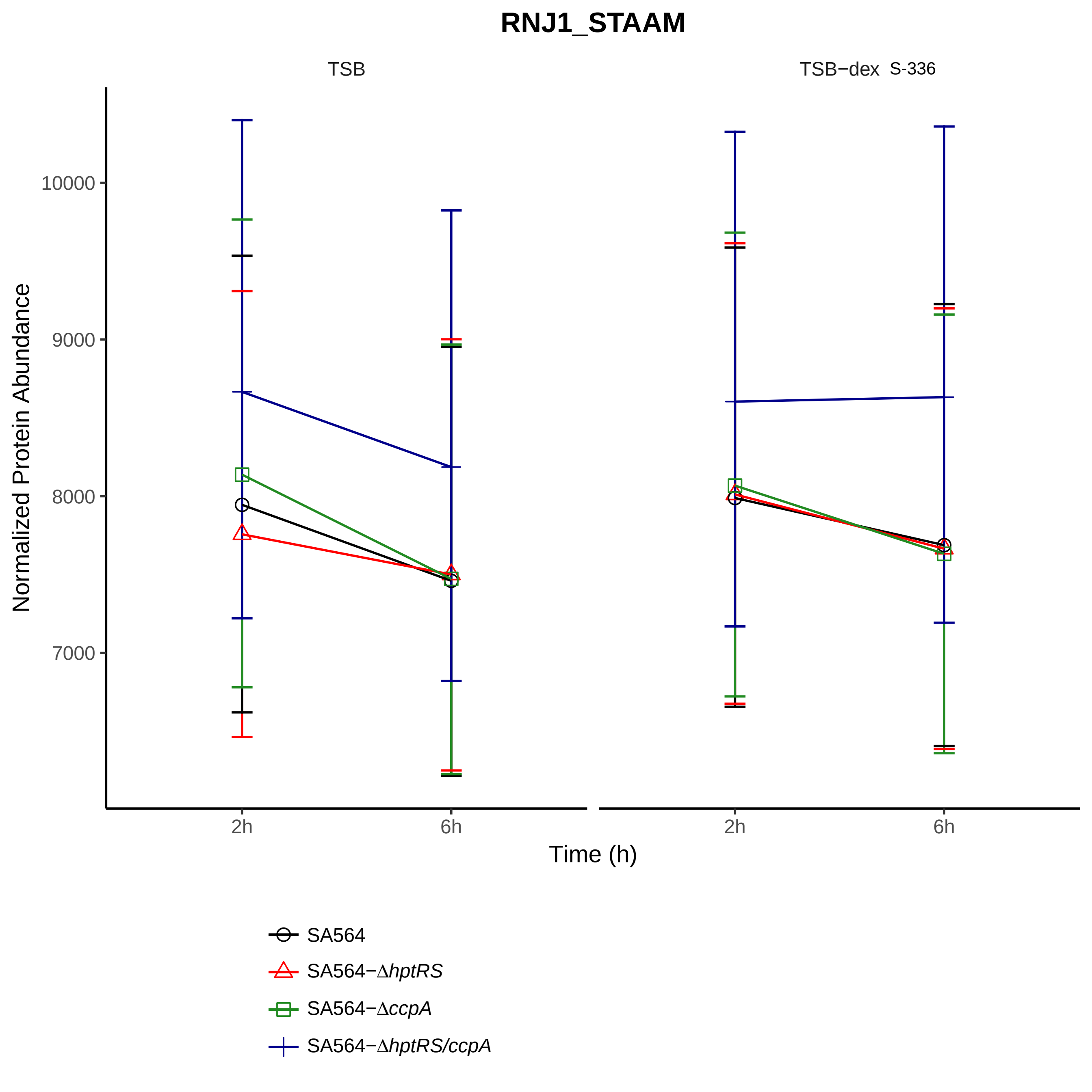
RNJ1_STAAM
TSB
TSB−dex
S-336
Normalized Protein Abundance
10000
9000
8000
7000
2h
6h
2h
6h
Time (h)
SA564
SA564−ΔhptRS
SA564−ΔccpA
SA564−ΔhptRS/ccpA

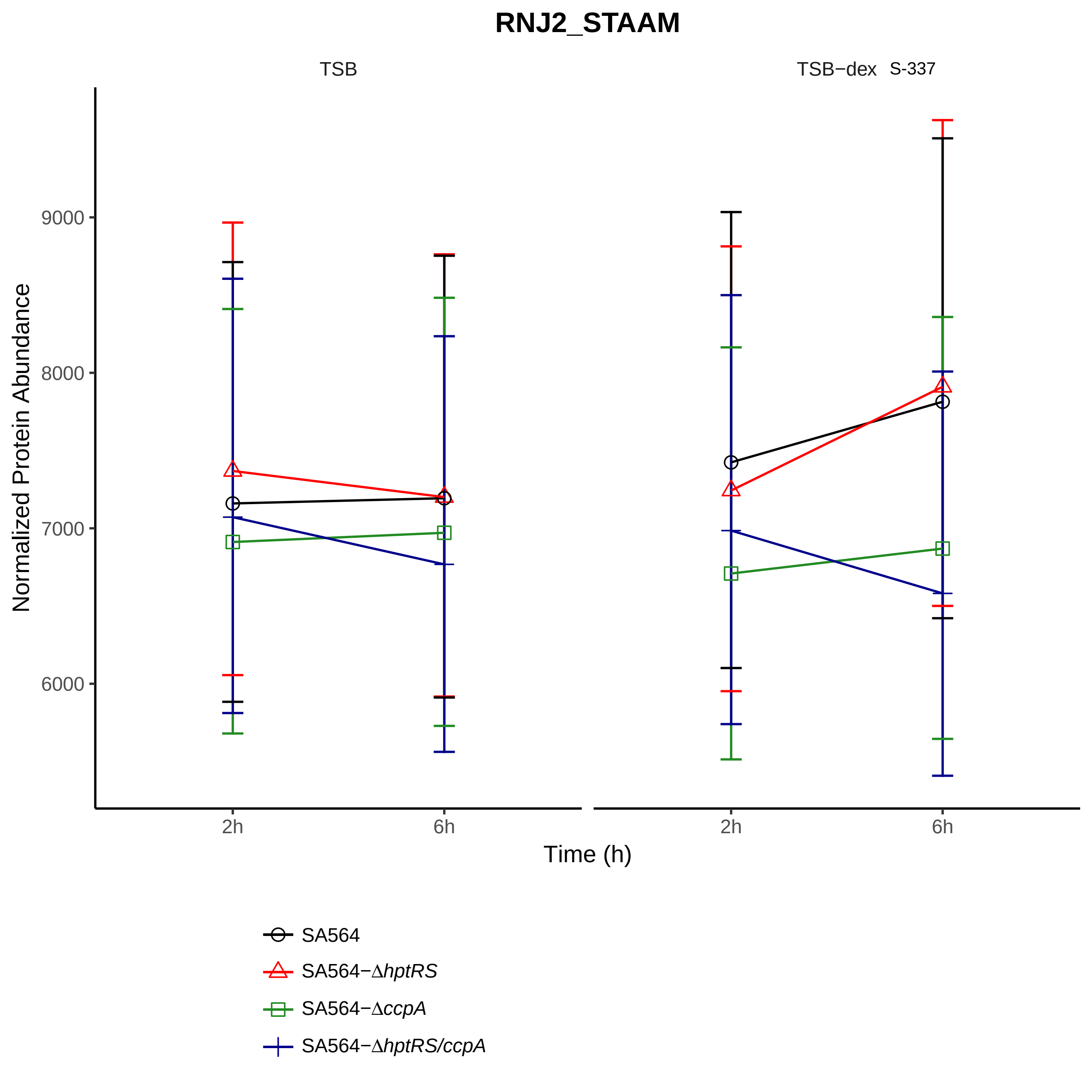

RNJ2_STAAM
TSB
TSB−dex
S-337
Normalized Protein Abundance
9000
8000
7000
6000
2h
6h
2h
6h
Time (h)
SA564
SA564−Δ*hptRS*
SA564−Δ*ccpA*
SA564−Δ*hptRS/ccpA*

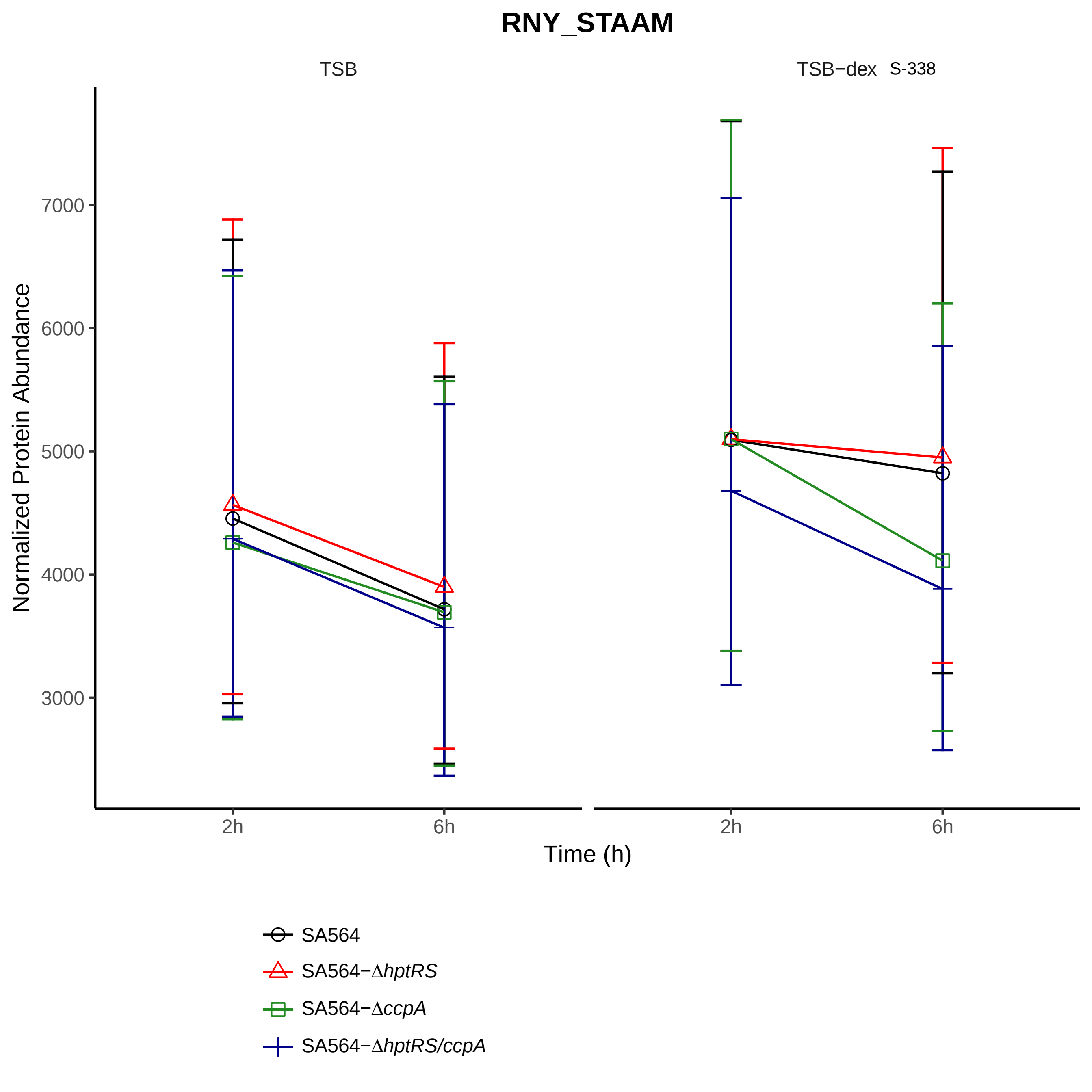
RNY_STAAM
TSB
TSB−dex
S-338
Normalized Protein Abundance
3000
4000
5000
6000
7000
2h
6h
2h
6h
Time (h)
SA564
SA564−ΔhptRS
SA564−ΔccpA
SA564−ΔhptRS/ccpA

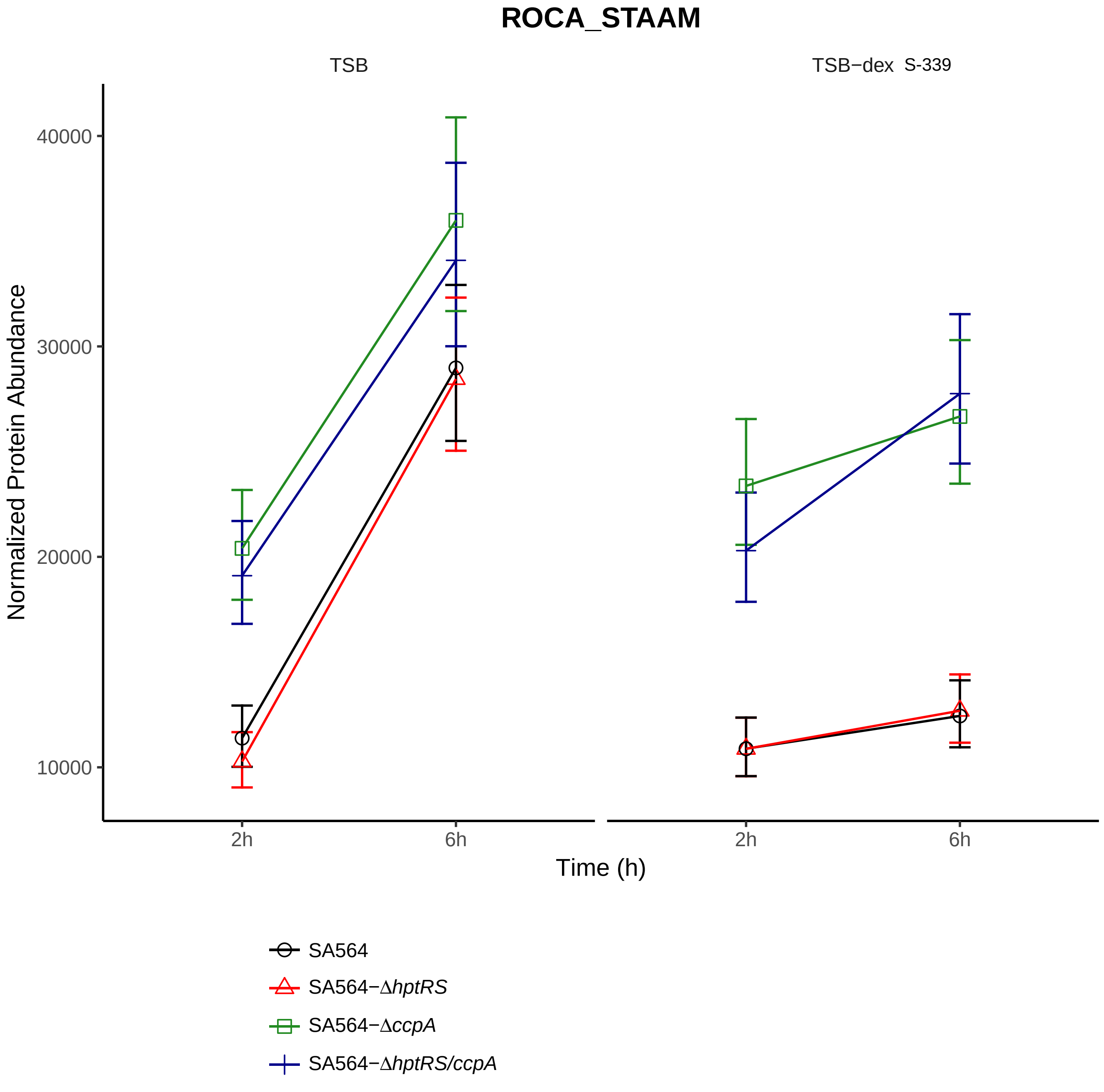
ROCA_STAAM
TSB
TSB−dex
S-339
40000
30000
20000
10000
Normalized Protein Abundance
2h
6h
2h
6h
Time (h)
SA564
SA564−Δ*hptRS*
SA564−Δ*ccpA*
SA564−Δ*hptRS/ccpA*

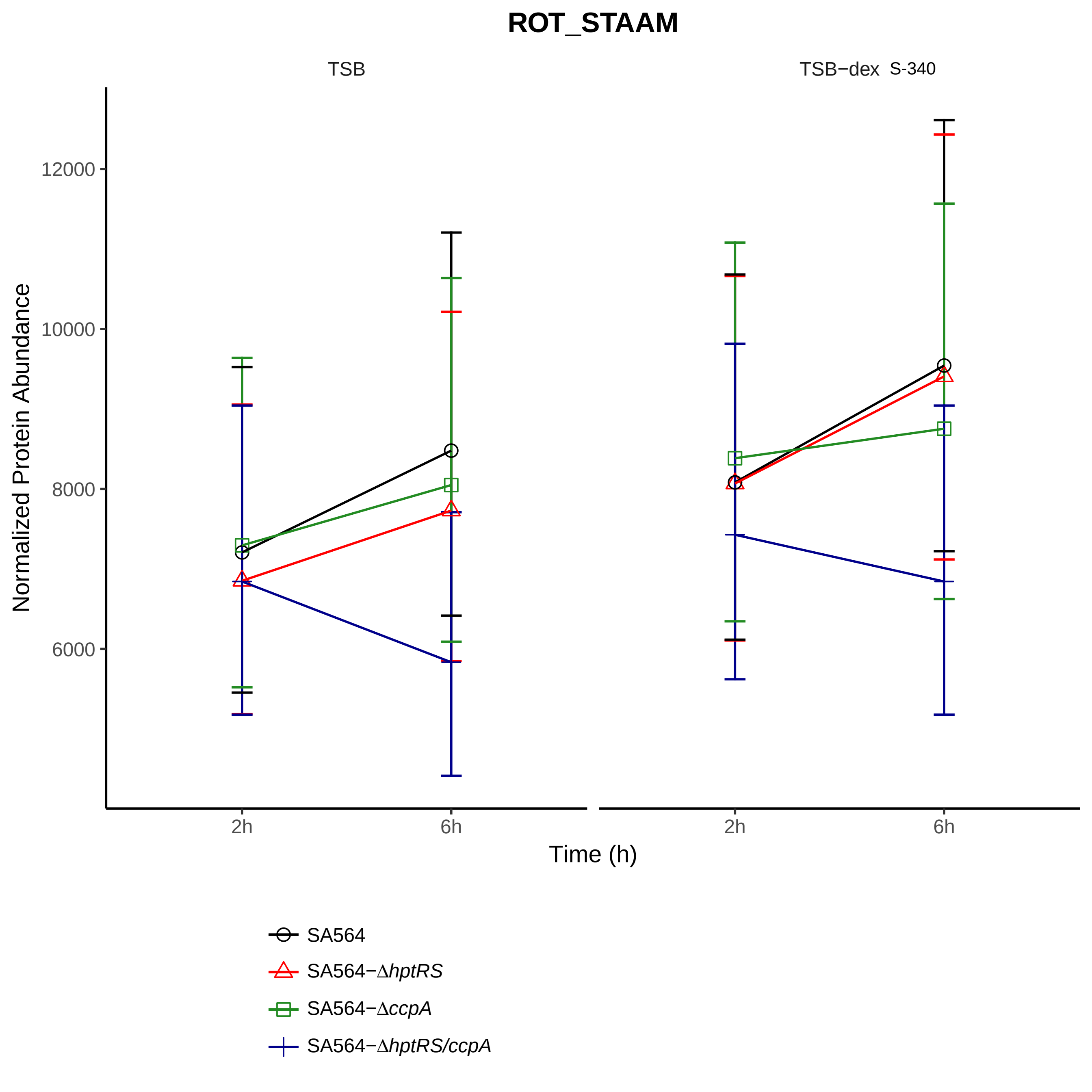
ROT_STAAM
TSB
TSB−dex
S-340
Normalized Protein Abundance
12000
10000
8000
6000
2h
6h
2h
6h
Time (h)
SA564
SA564−ΔhptRS
SA564−ΔccpA
SA564−ΔhptRS/ccpA

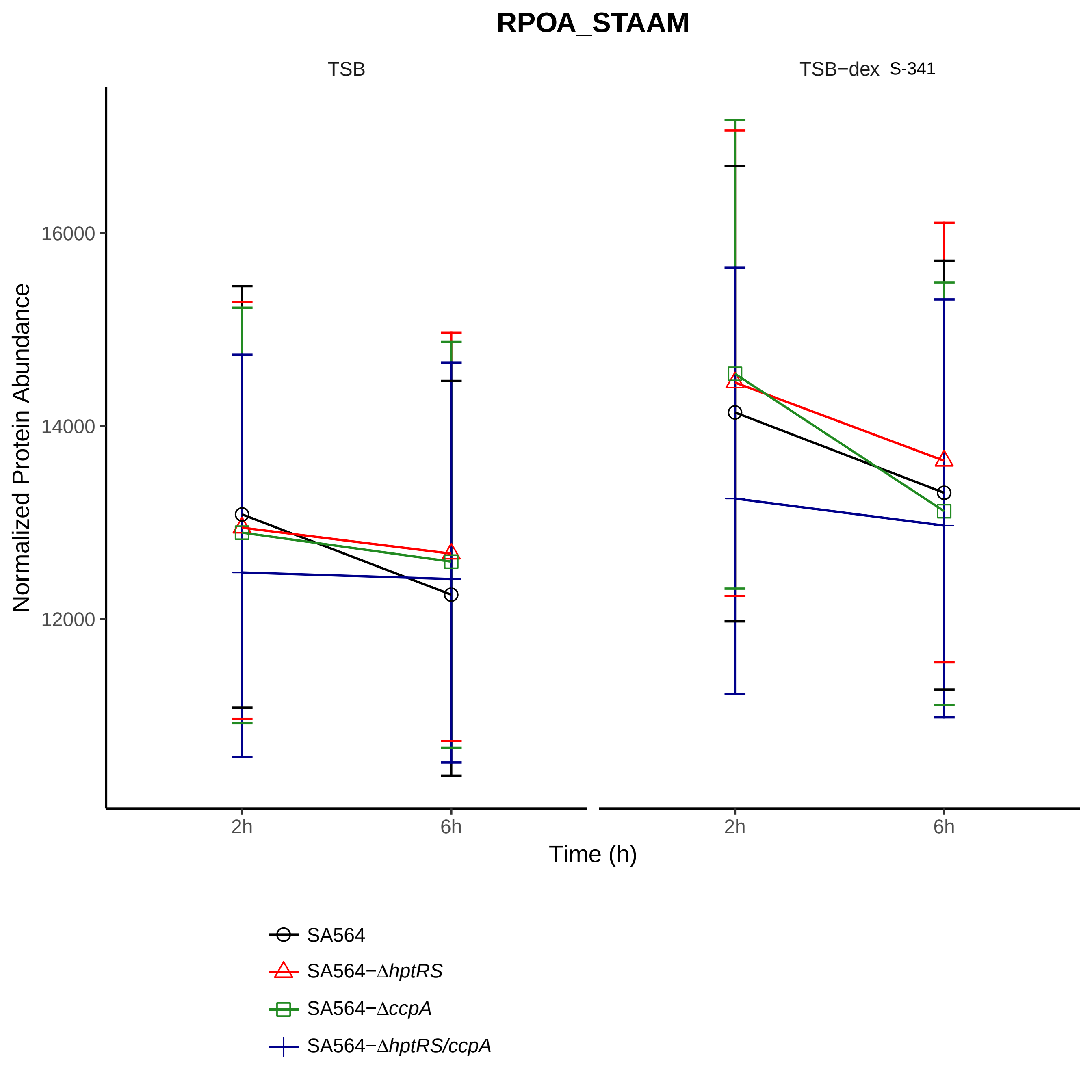

RPOA_STAAM
TSB
TSB−dex
S-341
16000
14000
12000
Normalized Protein Abundance
2h
6h
2h
6h
Time (h)
SA564
SA564−Δ*hptRS*
SA564−Δ*ccpA*
SA564−Δ*hptRS/ccpA*

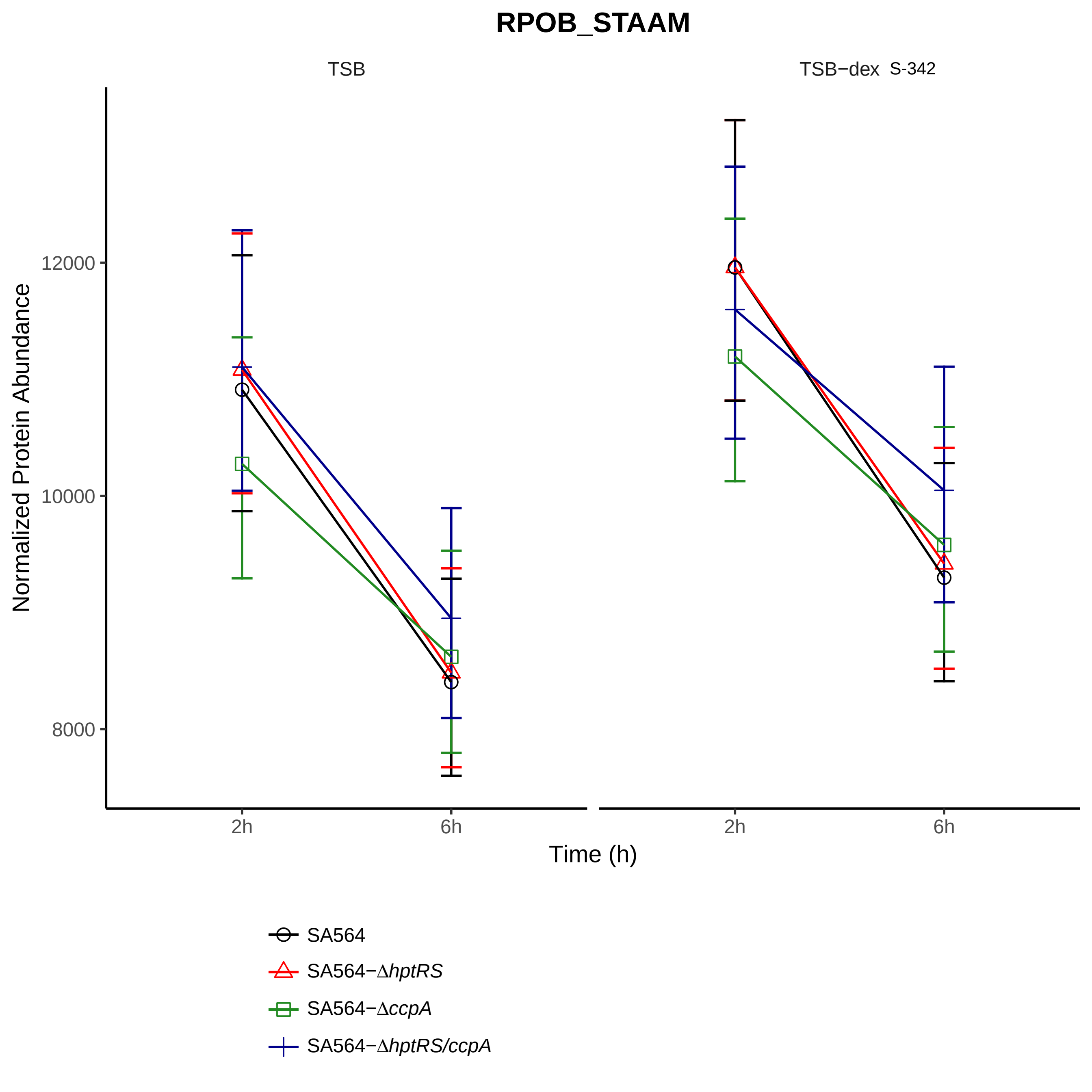

RPOB_STAAM
TSB
TSB−dex
S-342
Normalized Protein Abundance
12000
10000
8000
2h
6h
2h
6h
Time (h)
SA564
SA564−ΔhptRS
SA564−ΔccpA
SA564−ΔhptRS/ccpA

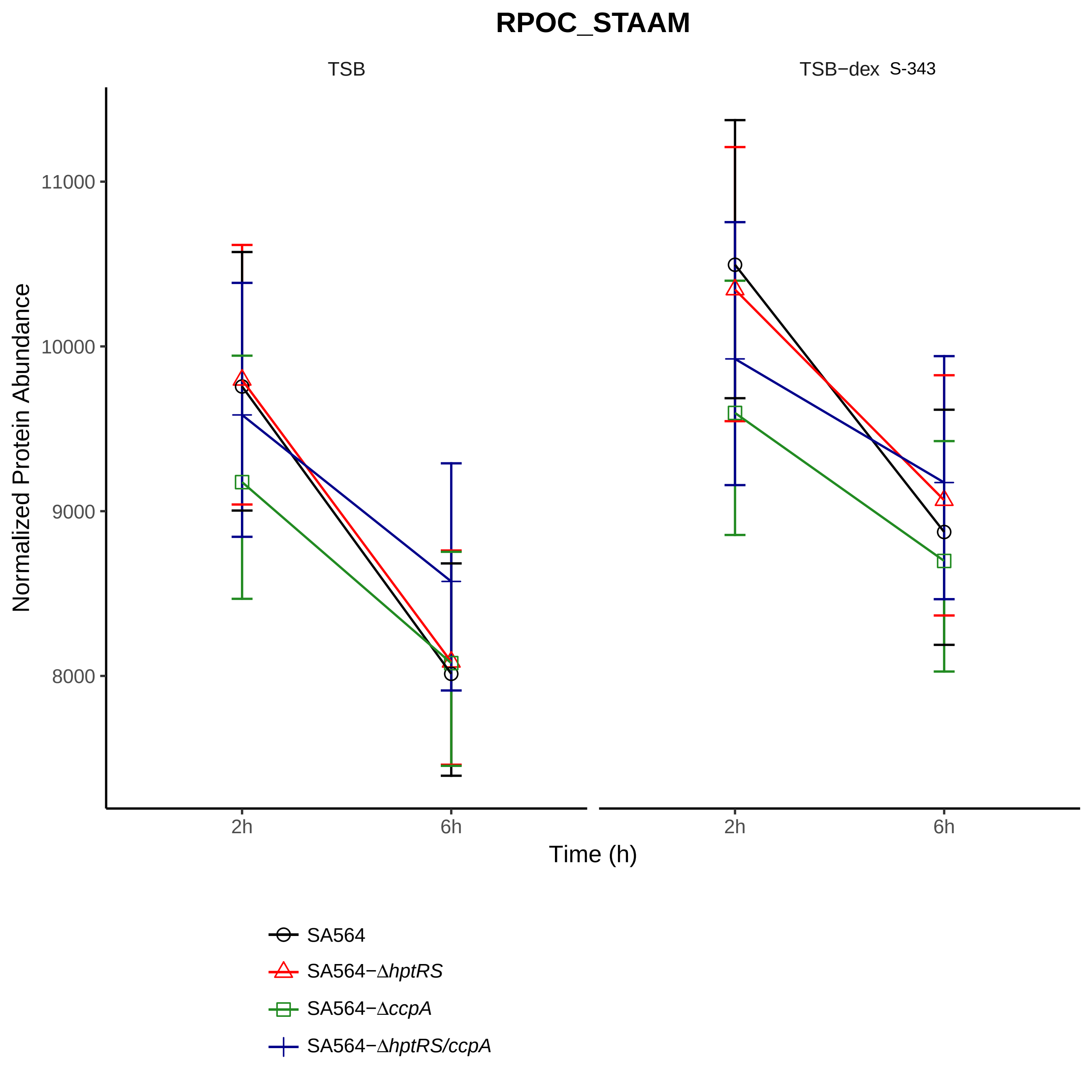

RPOC_STAAM
TSB
TSB−dex
S-343
Normalized Protein Abundance
11000
10000
9000
8000
2h
6h
2h
6h
Time (h)
SA564
SA564−Δ*hptRS*
SA564−Δ*ccpA*
SA564−Δ*hptRS/ccpA*

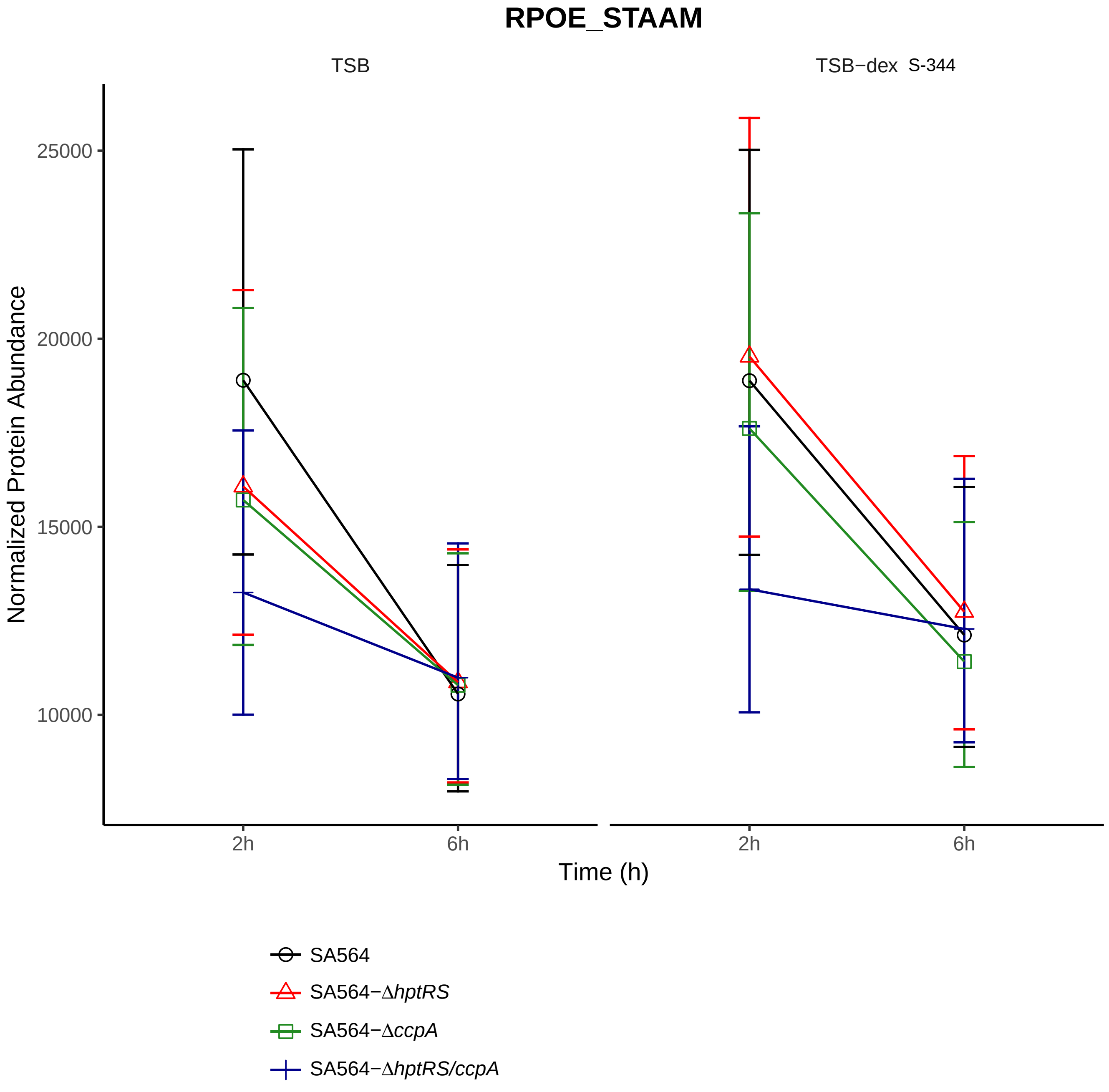

RPOE_STAAM
TSB
TSB−dex
S-344
Normalized Protein Abundance
25000
20000
15000
10000
2h
6h
2h
6h
Time (h)
SA564
SA564−ΔhptRS
SA564−ΔccpA
SA564−ΔhptRS/ccpA

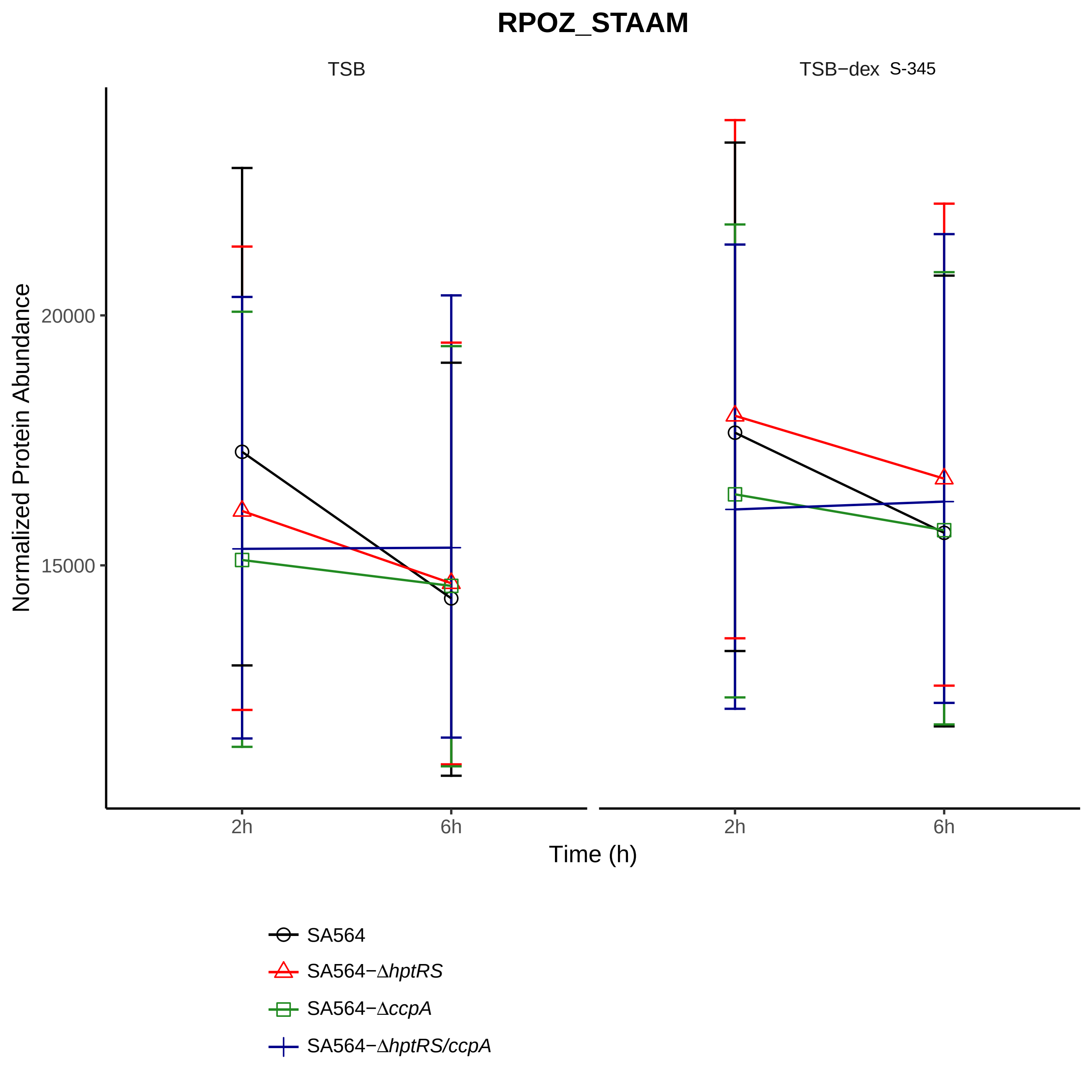
RPOZ_STAAM
TSB
TSB−dex
S-345
Normalized Protein Abundance
20000
15000
2h
6h
2h
6h
Time (h)
SA564
SA564−ΔhptRS
SA564−ΔccpA
SA564−ΔhptRS/ccpA

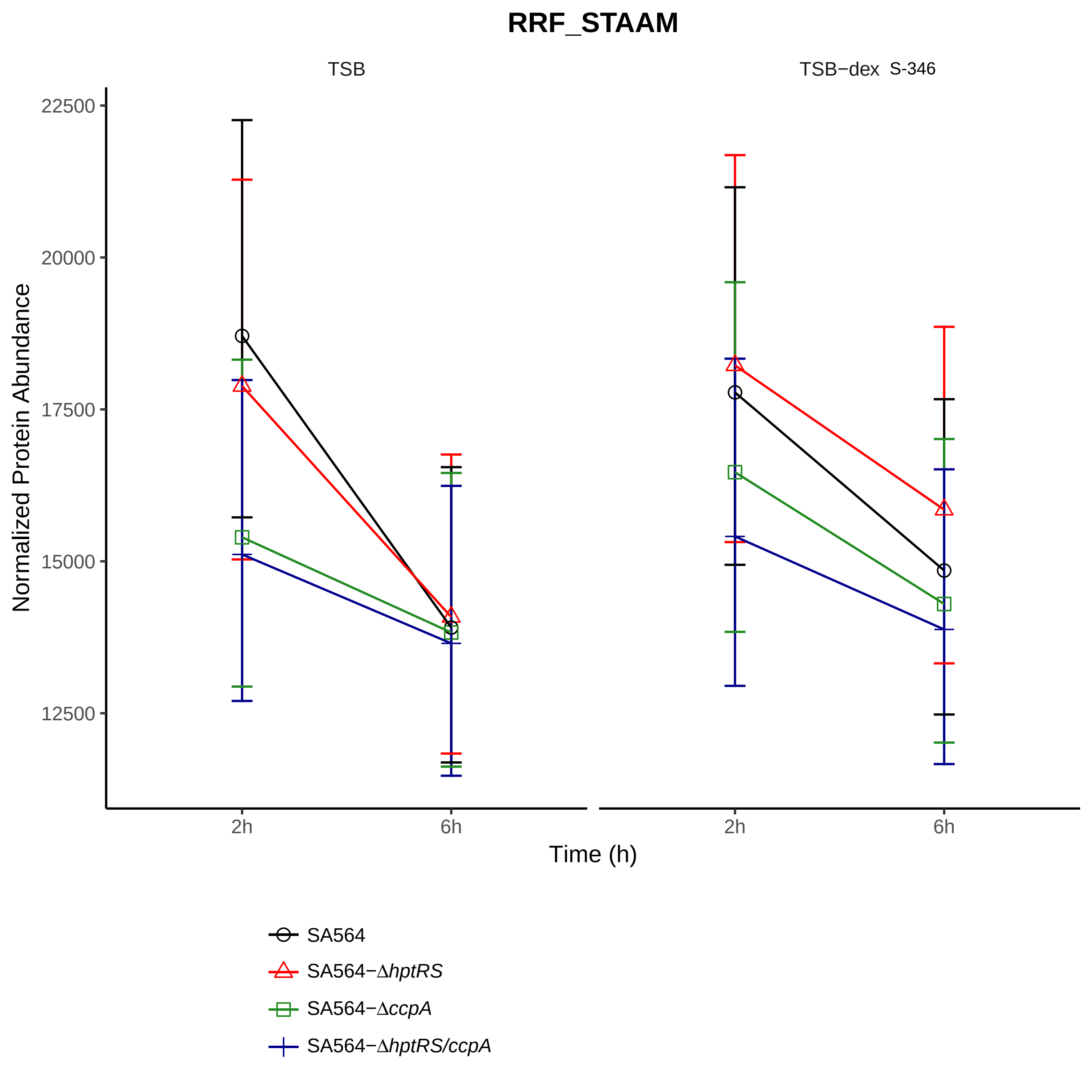

RRF_STAAM
TSB
TSB−dex
S-346
22500
20000
17500
15000
12500
Normalized Protein Abundance
2h
6h
2h
6h
Time (h)
SA564
SA564−ΔhptRS
SA564−ΔccpA
SA564−ΔhptRS/ccpA

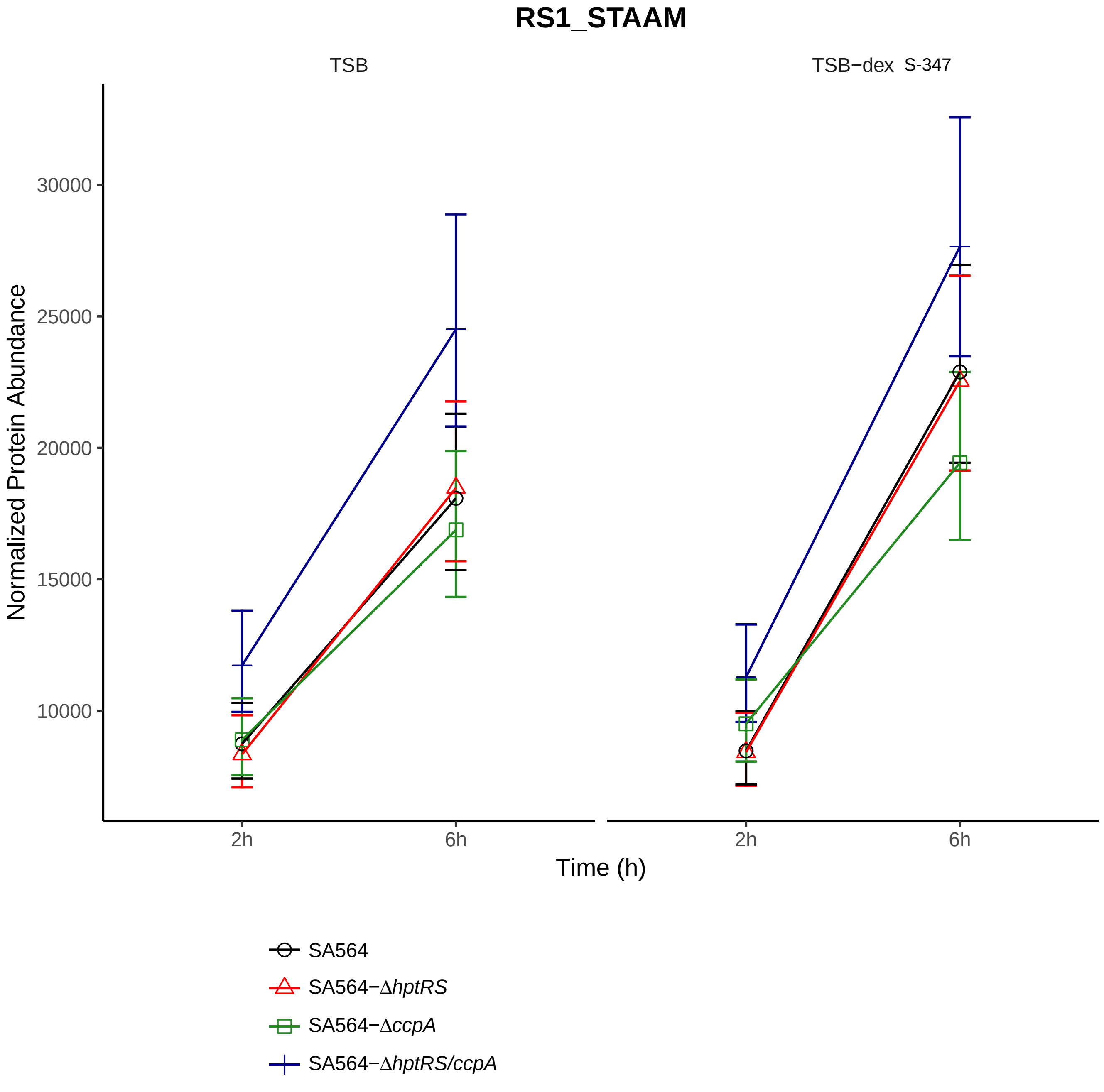

RS1_STAAM
TSB
TSB−dex
S-347
Normalized Protein Abundance
30000
25000
20000
15000
10000
2h
6h
2h
6h
Time (h)
SA564
SA564−ΔhptRS
SA564−ΔccpA
SA564−ΔhptRS/ccpA

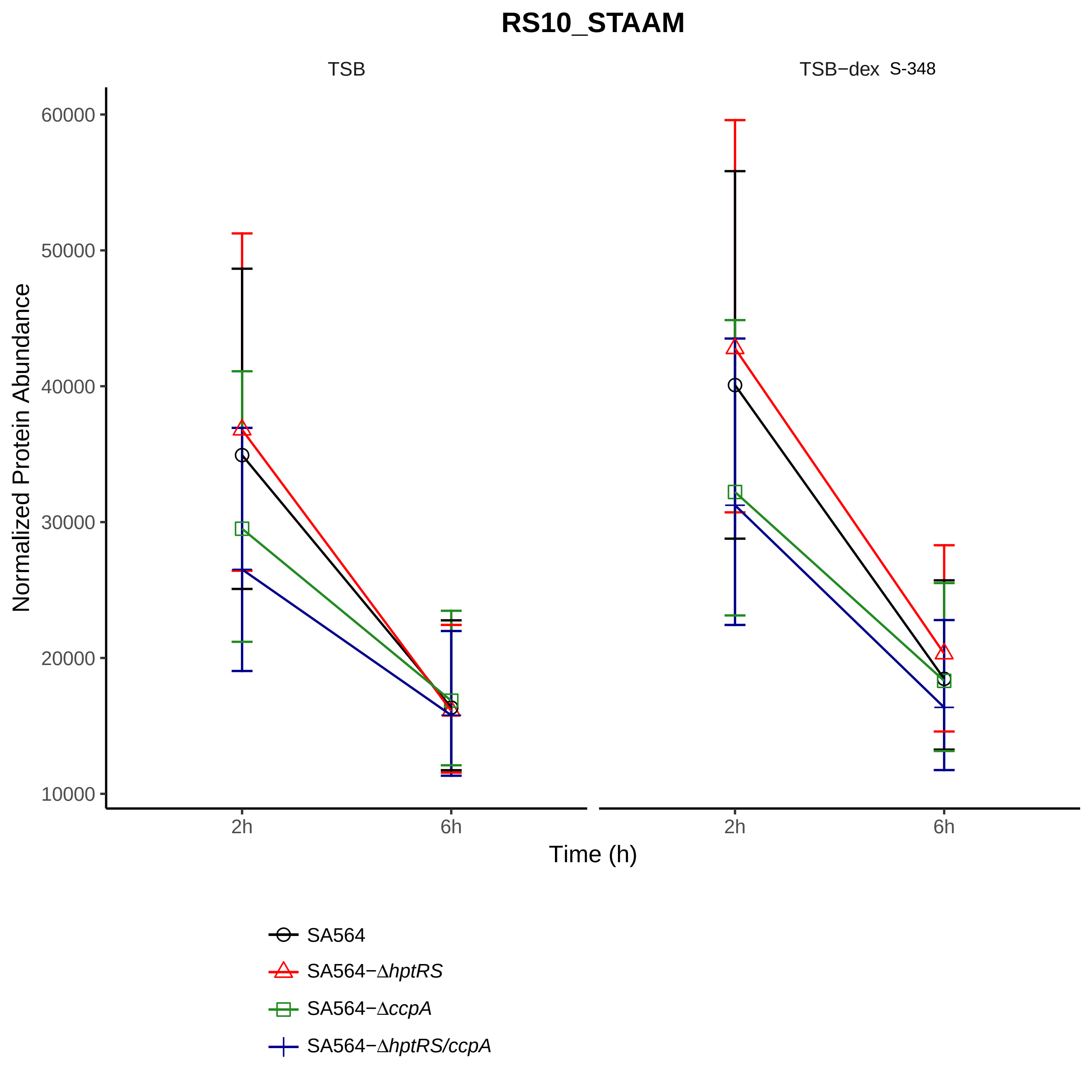

RS10_STAAM
TSB
TSB−dex
S-348
Normalized Protein Abundance
60000
50000
40000
30000
20000
10000
2h
6h
2h
6h
Time (h)
SA564
SA564−Δ*hptRS*
SA564−Δ*ccpA*
SA564−Δ*hptRS/ccpA*

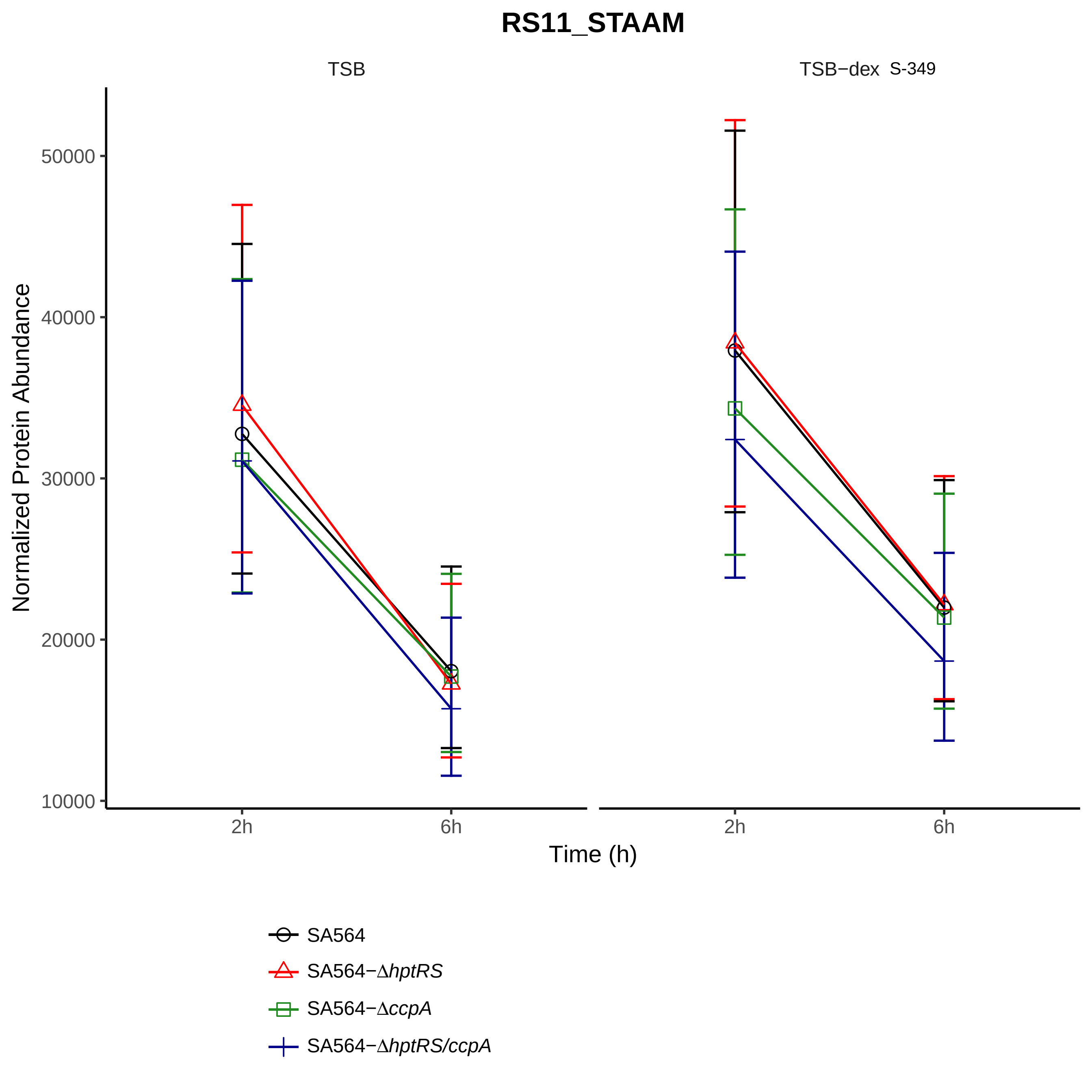
RS11_STAAM
TSB
TSB−dex
S-349
Normalized Protein Abundance
50000
40000
30000
20000
10000
2h
6h
2h
6h
Time (h)
SA564
SA564−ΔhptRS
SA564−ΔccpA
SA564−ΔhptRS/ccpA

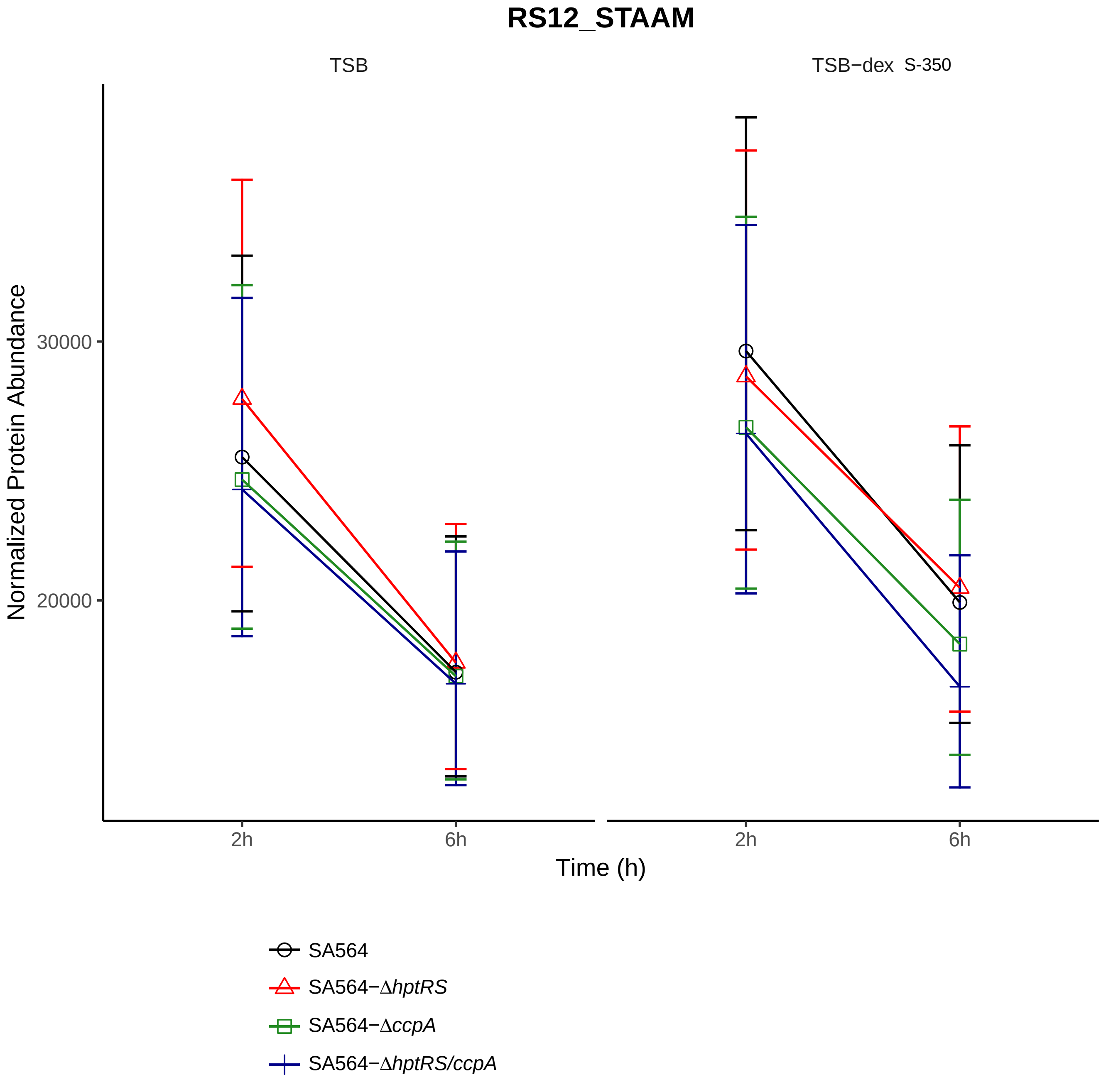

RS12_STAAM
TSB
TSB−dex
S-350
Normalized Protein Abundance
30000
20000
2h
6h
2h
6h
Time (h)
SA564
SA564−Δ*hptRS*
SA564−Δ*ccpA*
SA564−Δ*hptRS/ccpA*

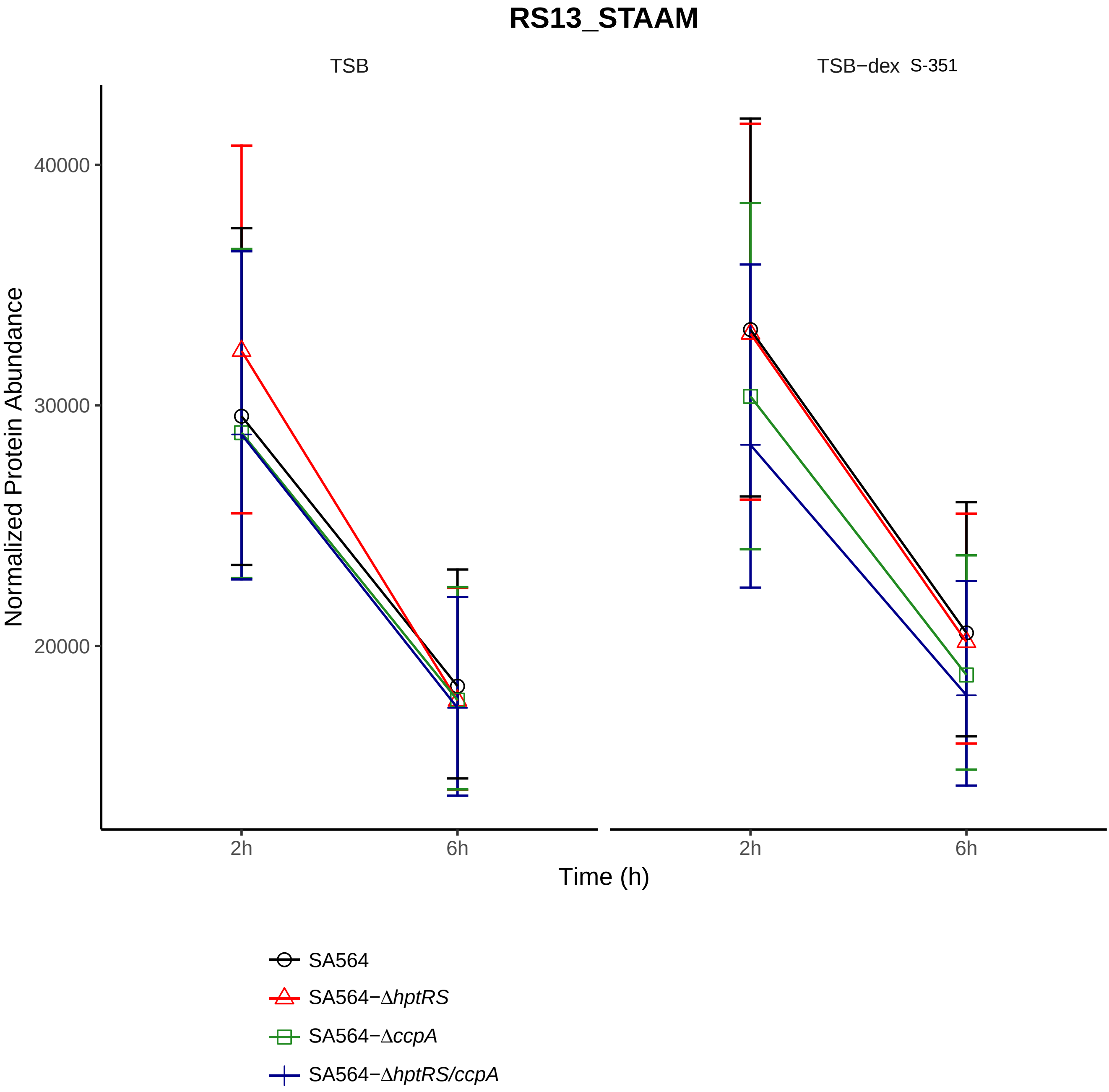

RS13_STAAM
TSB
TSB−dex
S-351
Normalized Protein Abundance
40000
30000
20000
2h
6h
2h
6h
Time (h)
SA564
SA564−ΔhptRS
SA564−ΔccpA
SA564−ΔhptRS/ccpA

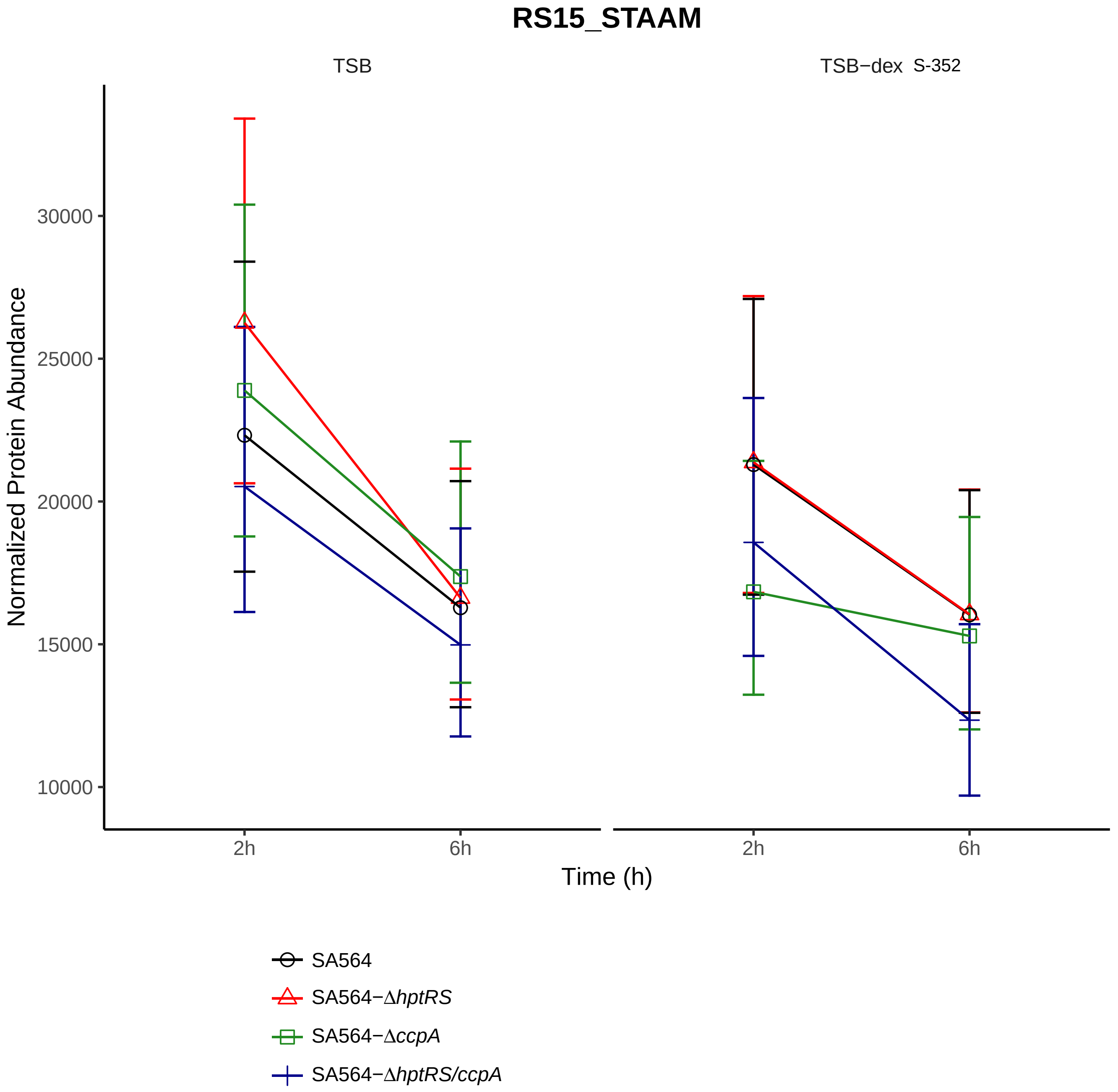
RS15_STAAM
TSB
TSB−dex
S-352
Normalized Protein Abundance
30000
25000
20000
15000
10000
2h
6h
2h
6h
Time (h)
SA564
SA564−ΔhptRS
SA564−ΔccpA
SA564−ΔhptRS/ccpA

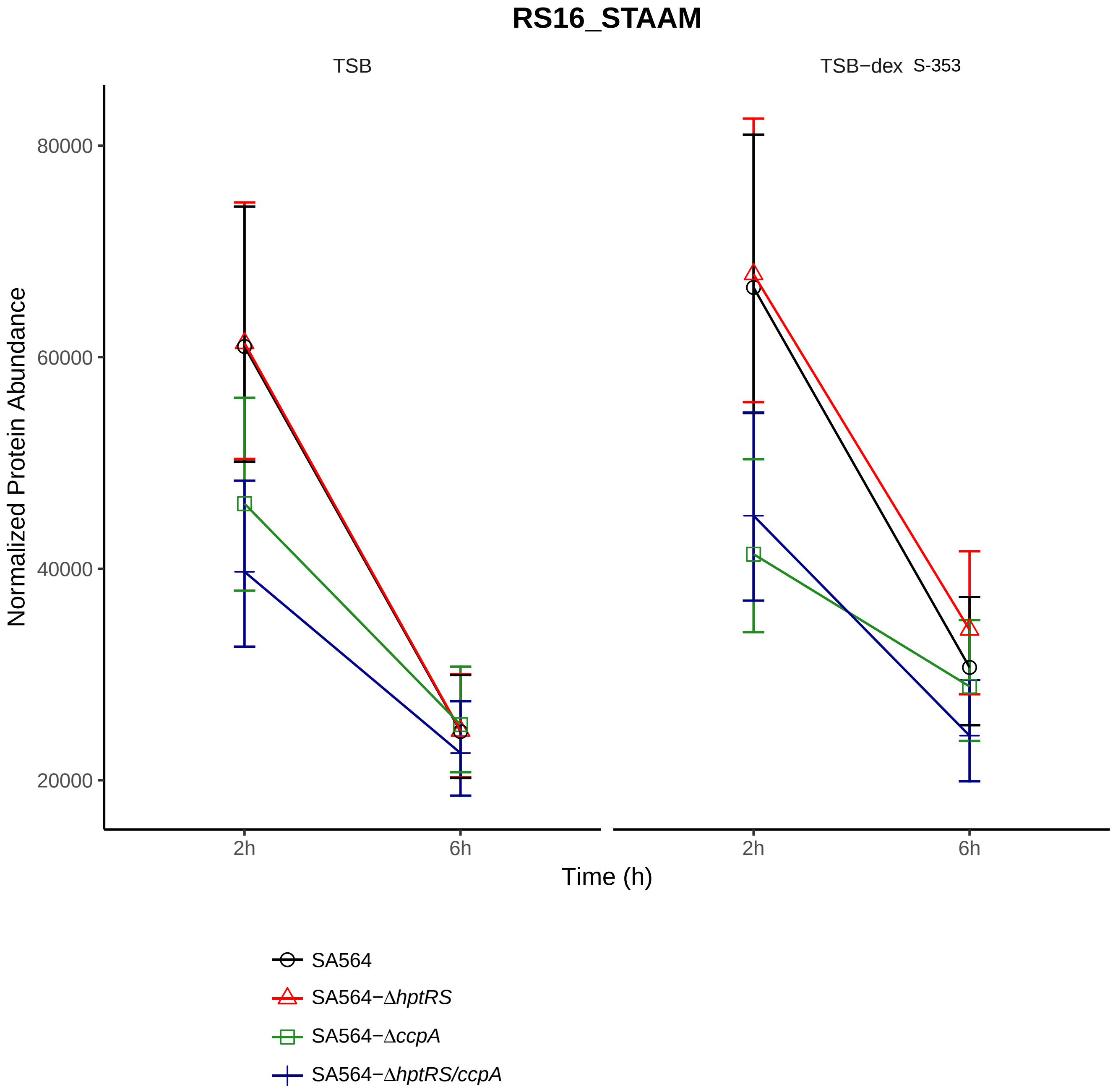
RS16_STAAM
TSB
TSB−dex
S-353
80000
60000
40000
20000
Normalized Protein Abundance
2h
6h
2h
6h
Time (h)
SA564
SA564−ΔhptRS
SA564−ΔccpA
SA564−ΔhptRS/ccpA

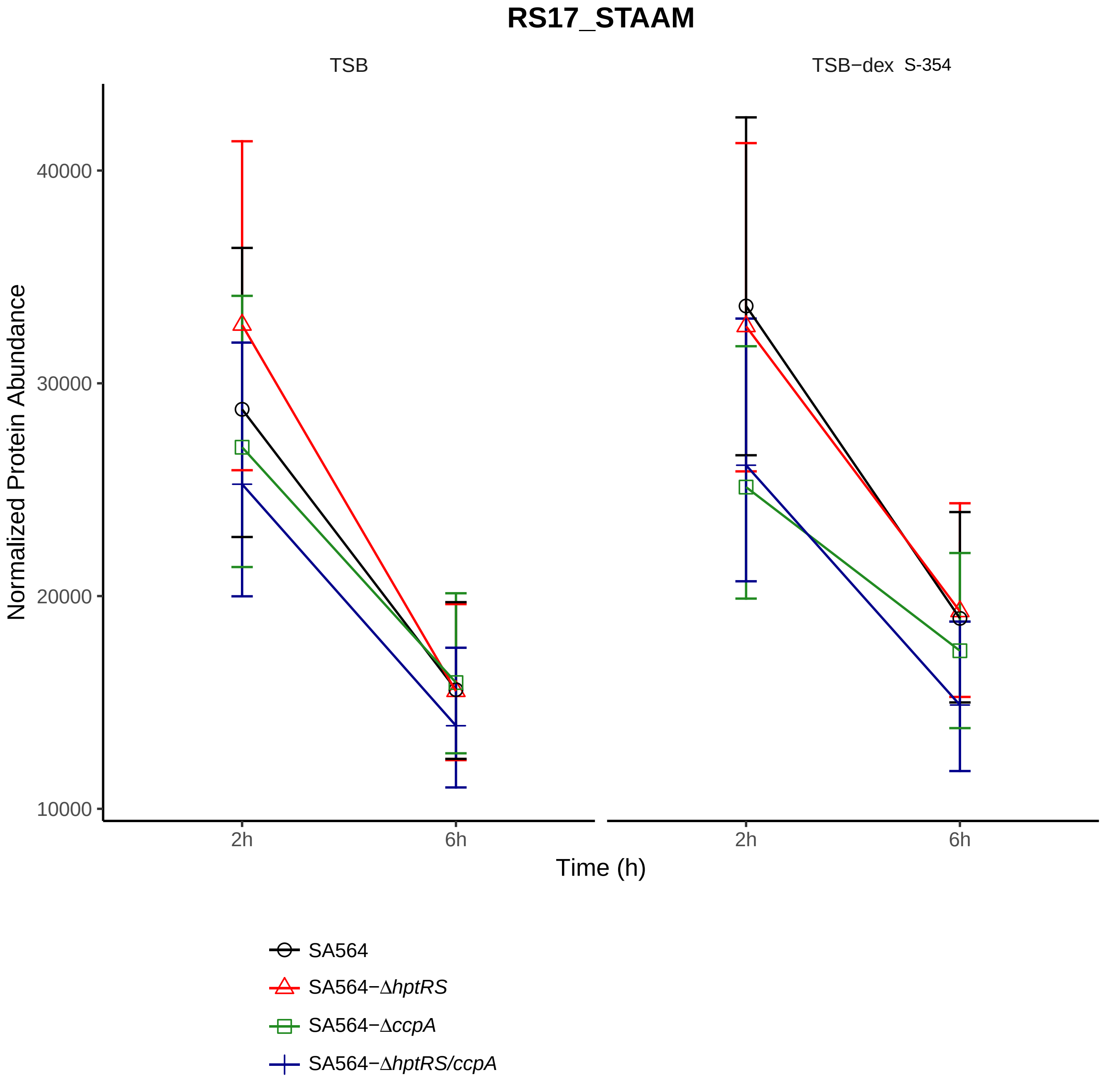
RS17_STAAM
TSB
TSB−dex
S-354
Normalized Protein Abundance
40000
30000
20000
10000
2h
6h
2h
6h
Time (h)
SA564
SA564−ΔhptRS
SA564−ΔccpA
SA564−ΔhptRS/ccpA

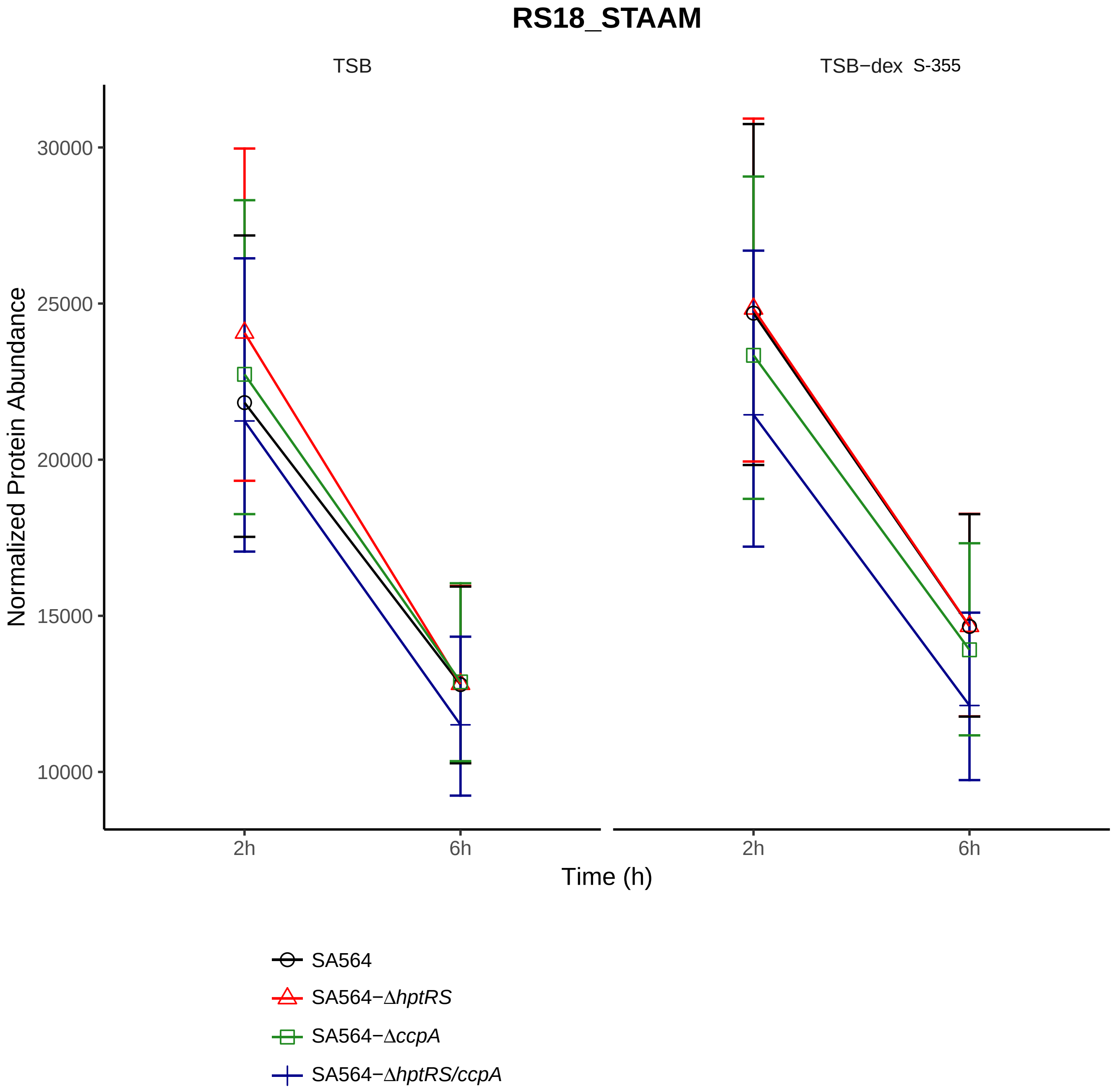

RS18_STAAM
TSB
TSB−dex
S-355
Normalized Protein Abundance
30000
25000
20000
15000
10000
2h
6h
2h
6h
Time (h)
SA564
SA564−Δ*hptRS*
SA564−Δ*ccpA*
SA564−Δ*hptRS/ccpA*

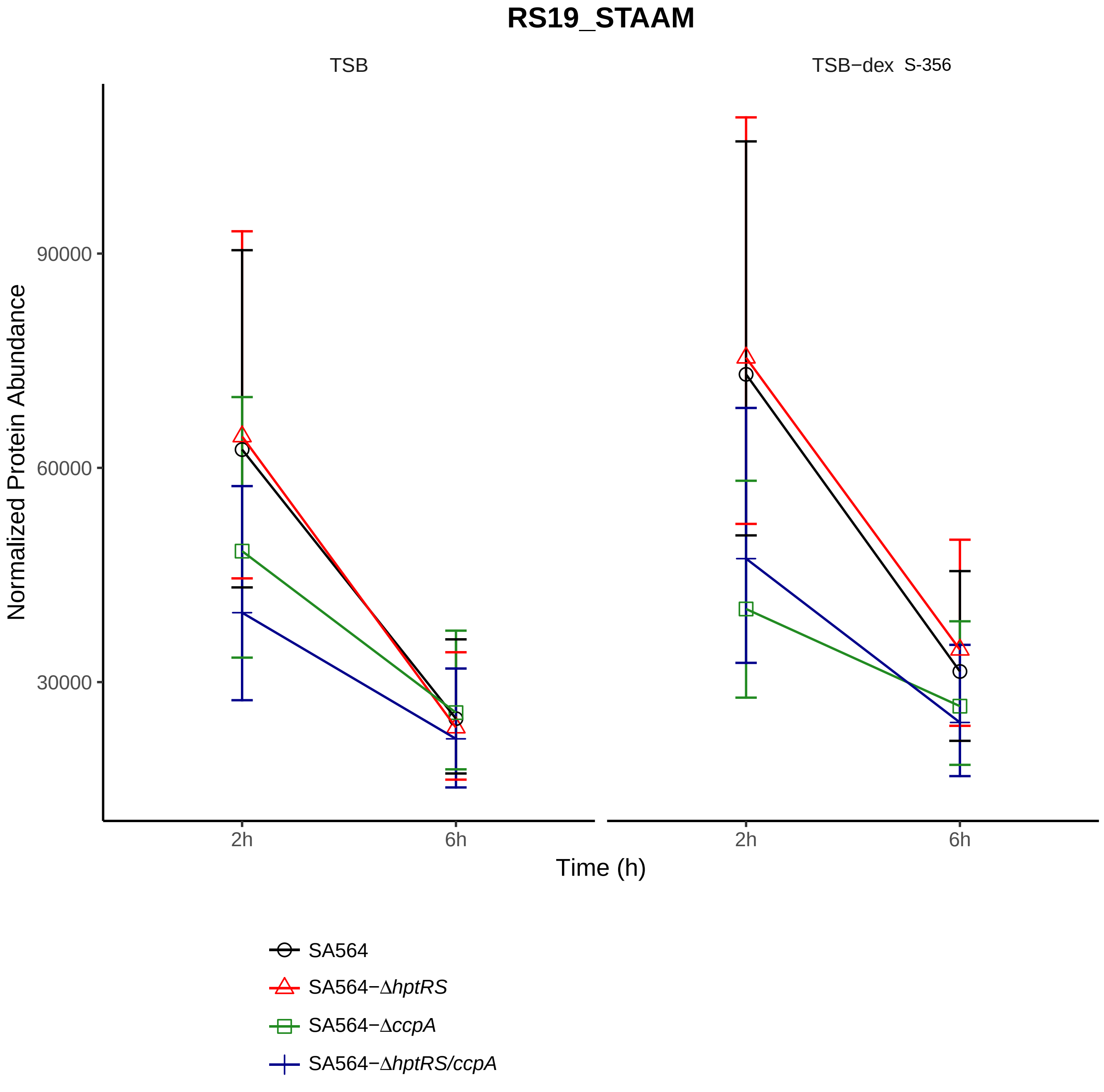

RS19_STAAM
TSB
TSB−dex
S-356
Normalized Protein Abundance
90000
60000
30000
2h
6h
2h
6h
Time (h)
SA564
SA564−ΔhptRS
SA564−ΔccpA
SA564−ΔhptRS/ccpA

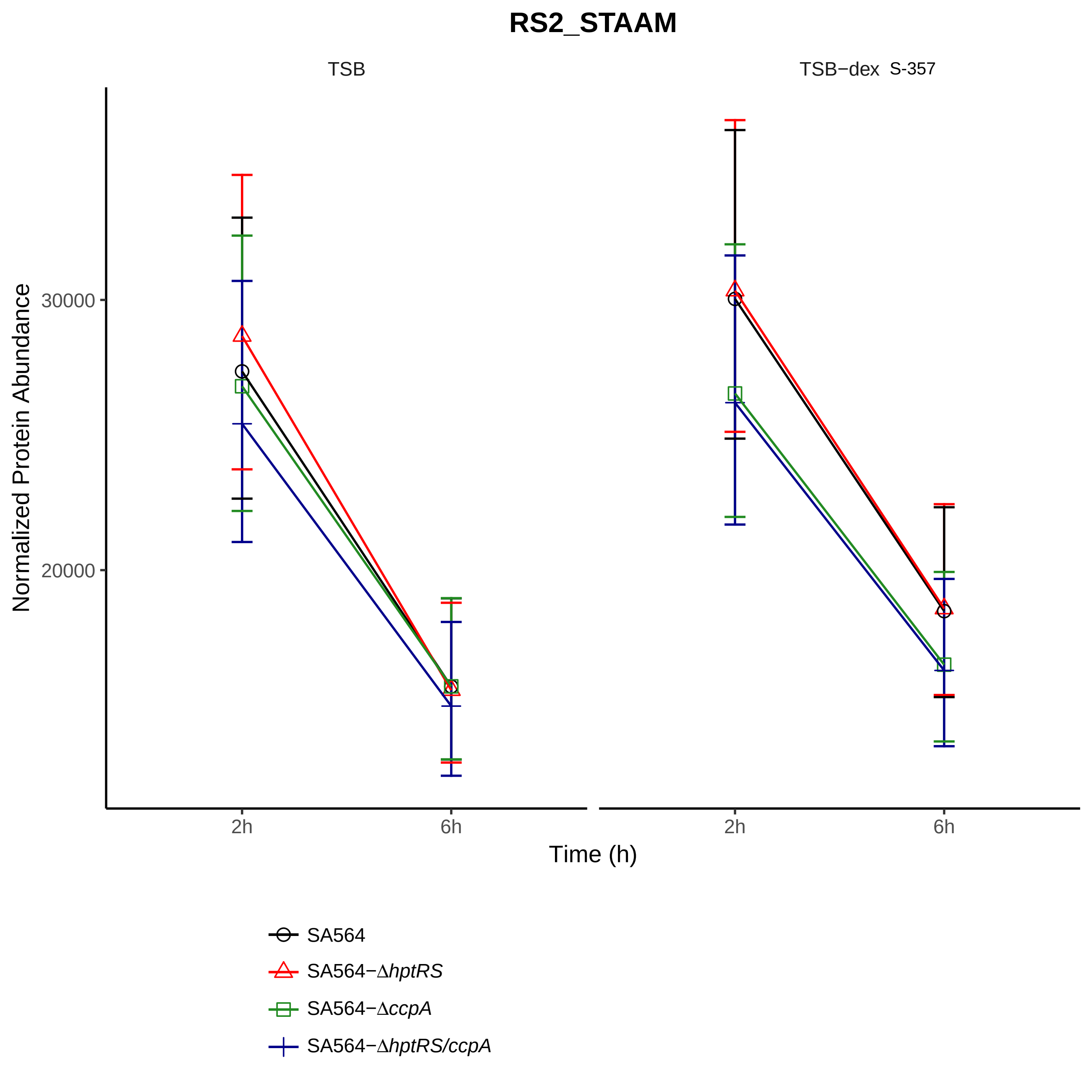

RS2_STAAM
TSB
TSB−dex
S-357
Normalized Protein Abundance
30000
20000
2h
6h
2h
6h
Time (h)
SA564
SA564−ΔhptRS
SA564−ΔccpA
SA564−ΔhptRS/ccpA

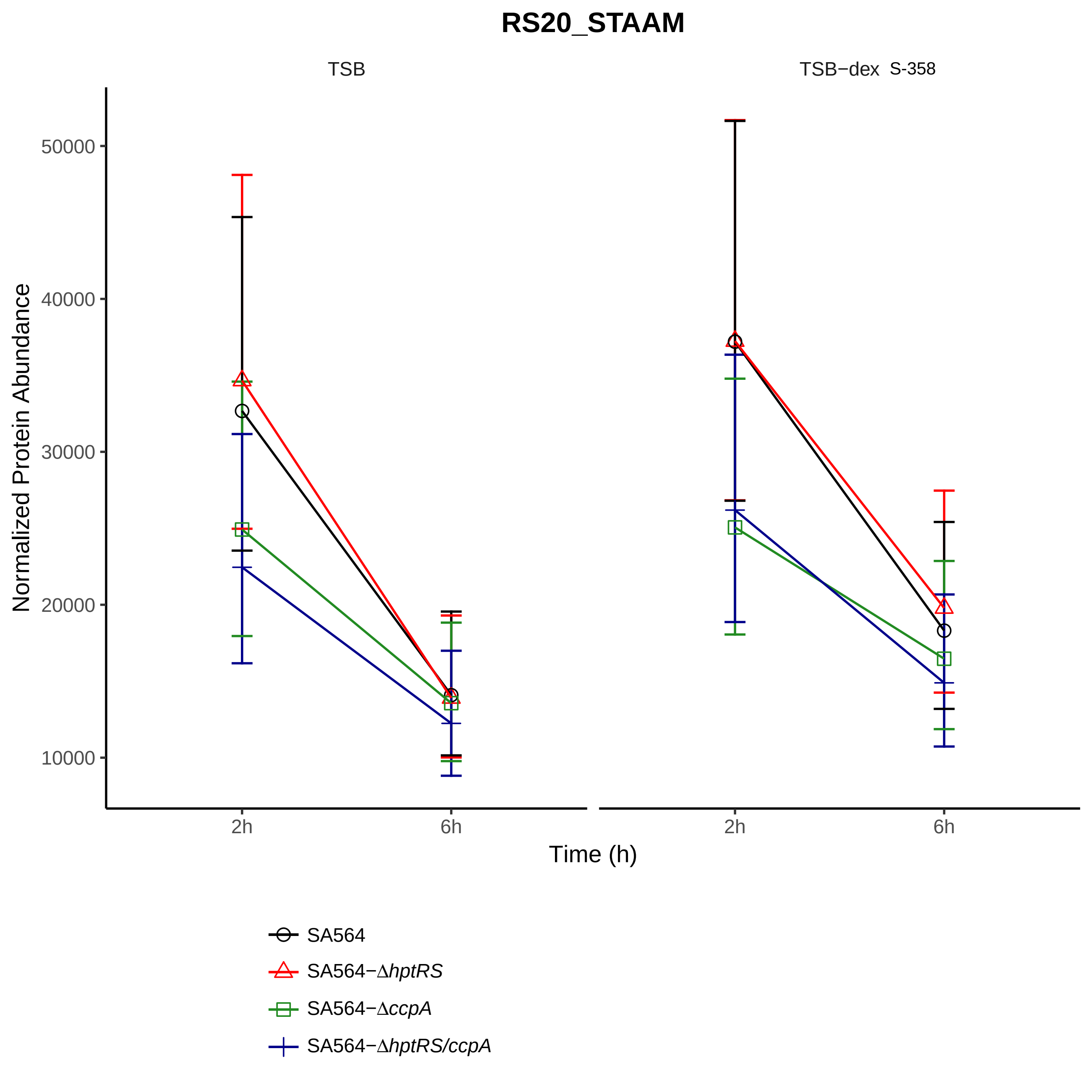

RS20_STAAM
TSB
TSB−dex
S-358
Normalized Protein Abundance
10000
20000
30000
40000
50000
2h
6h
2h
6h
Time (h)
SA564
SA564−Δ*hptRS*
SA564−Δ*ccpA*
SA564−Δ*hptRS/ccpA*

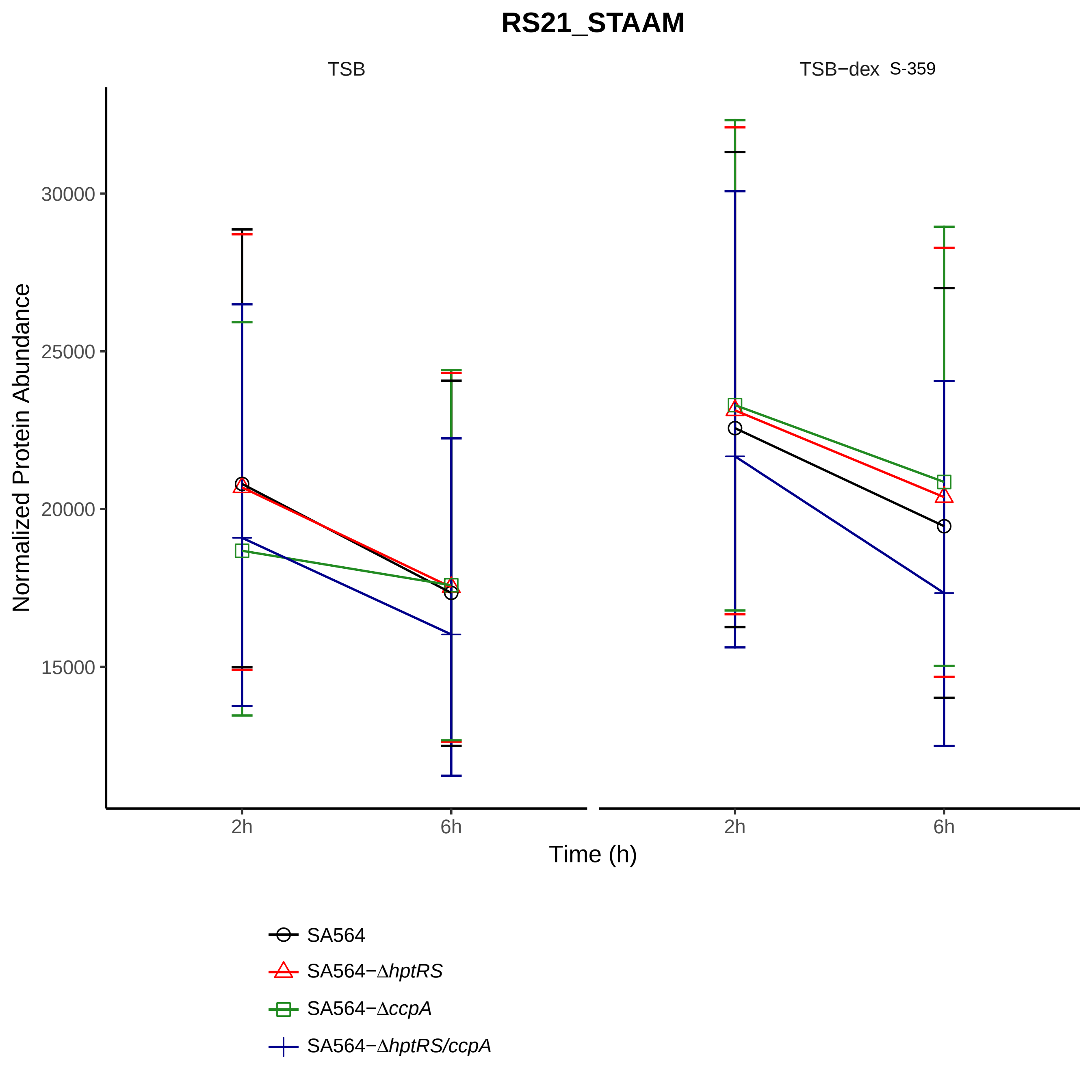

RS21_STAAM
TSB
TSB−dex
S-359
Normalized Protein Abundance
30000
25000
20000
15000
2h
6h
2h
6h
Time (h)
SA564
SA564−Δ*hptRS*
SA564−Δ*ccpA*
SA564−Δ*hptRS/ccpA*

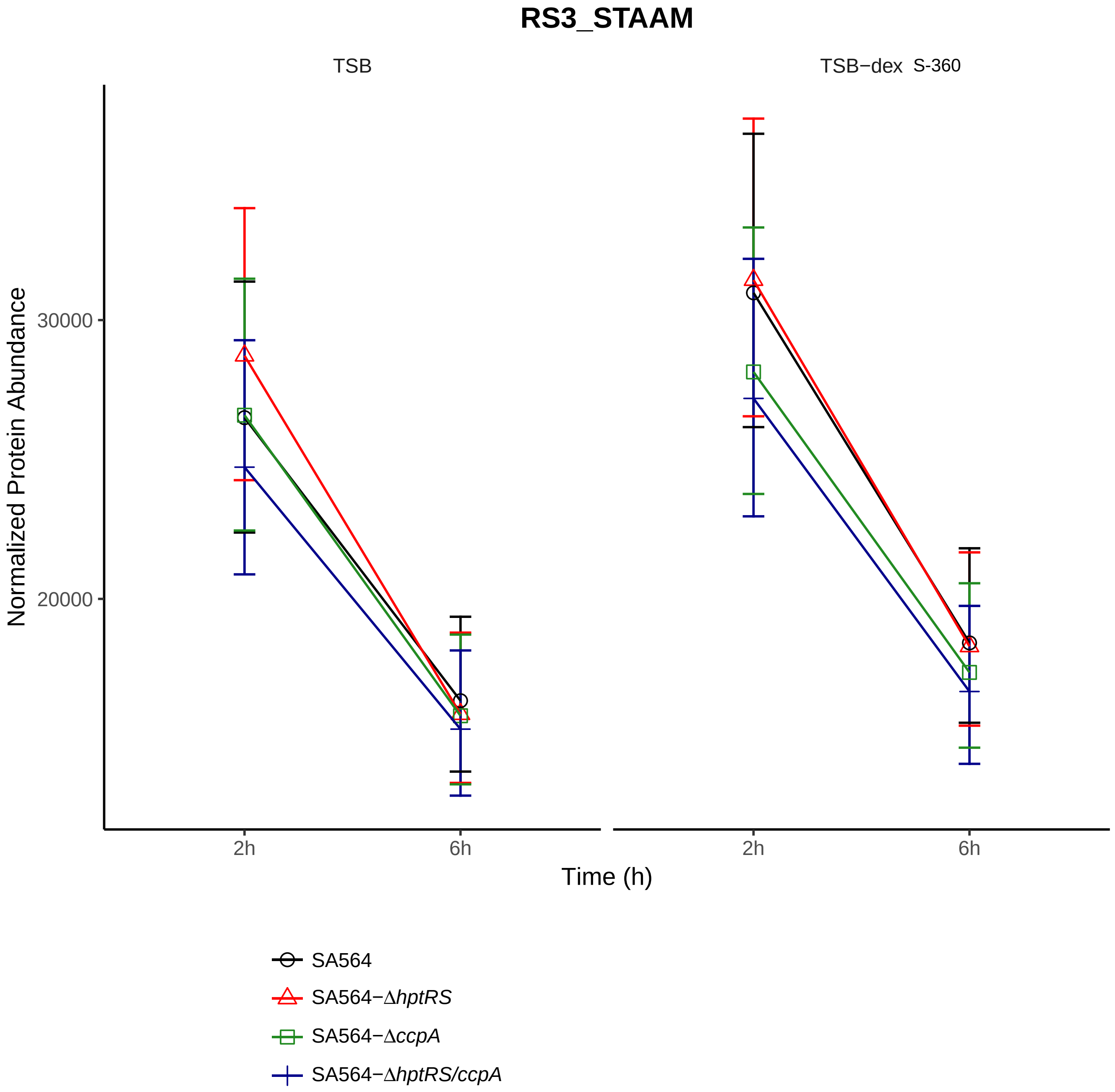
RS3_STAAM
TSB
TSB−dex
S-360
Normalized Protein Abundance
30000
20000
2h
6h
2h
6h
Time (h)
SA564
SA564−Δ*hptRS*
SA564−Δ*ccpA*
SA564−Δ*hptRS/ccpA*

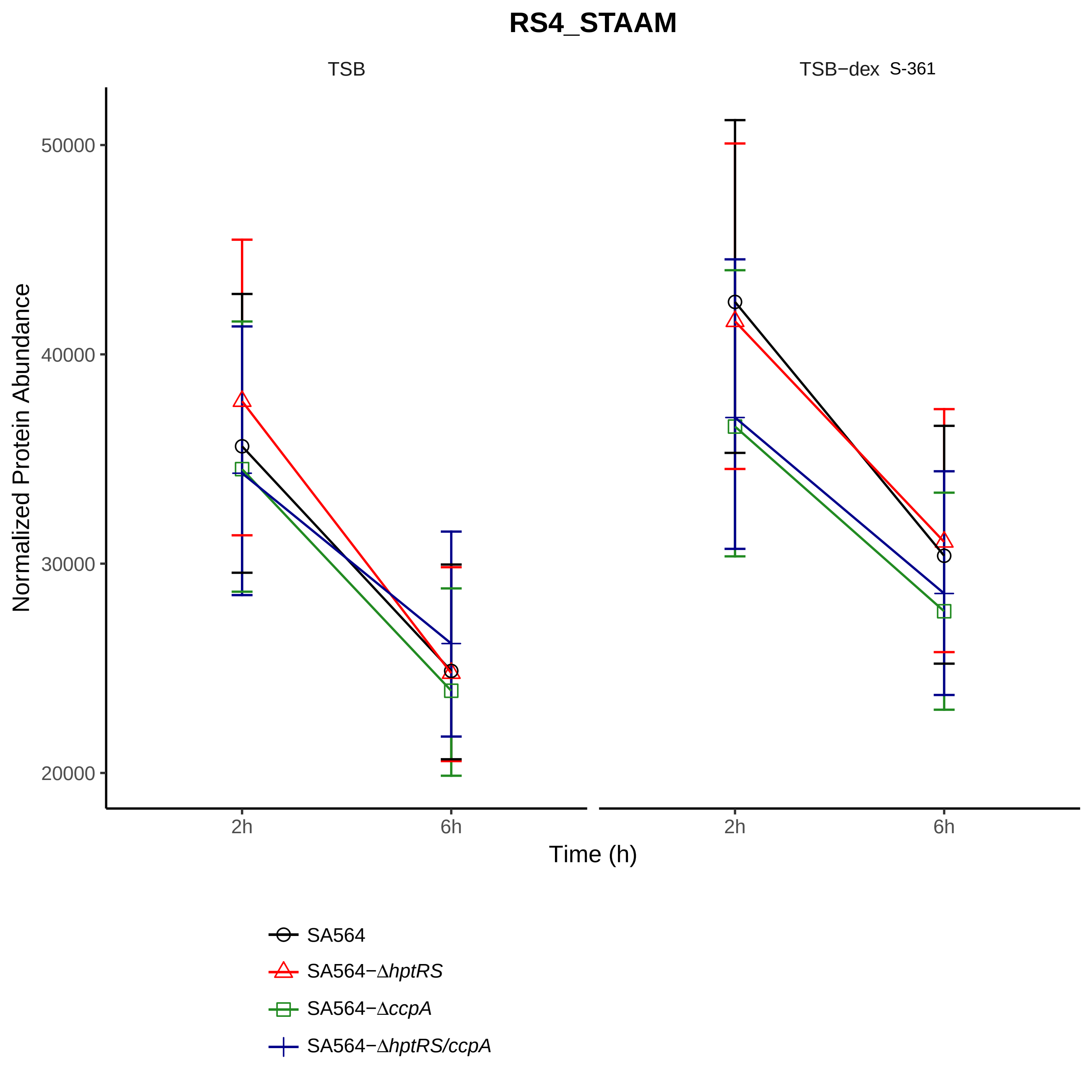

RS4_STAAM
TSB
TSB−dex
S-361
Normalized Protein Abundance
50000
40000
30000
20000
2h
6h
2h
6h
Time (h)
SA564
SA564−Δ*hptRS*
SA564−Δ*ccpA*
SA564−Δ*hptRS/ccpA*

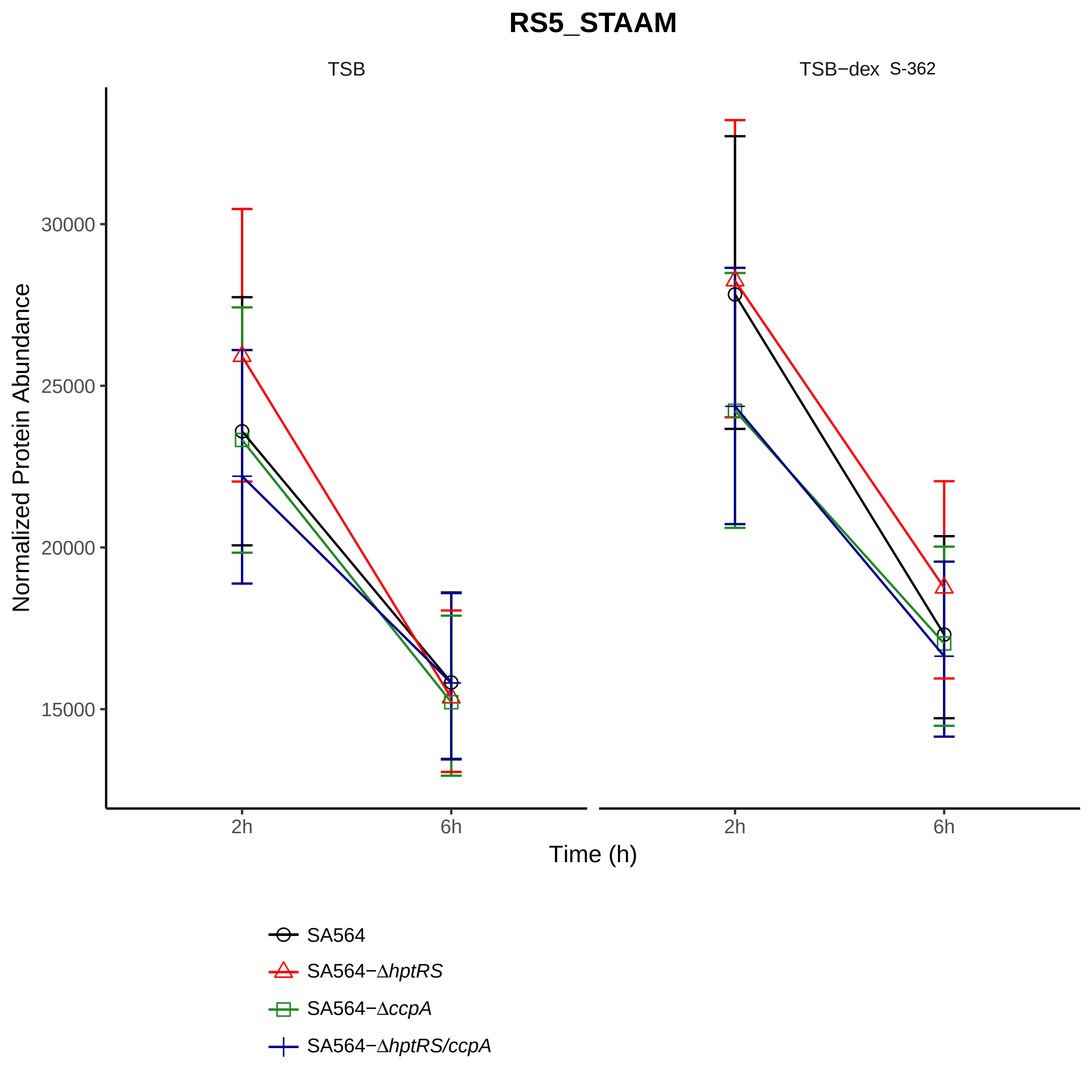

RS5_STAAM
TSB
TSB−dex
S-362
30000
25000
20000
15000
Normalized Protein Abundance
2h
6h
2h
6h
Time (h)
SA564
SA564−ΔhptRS
SA564−ΔccpA
SA564−ΔhptRS/ccpA

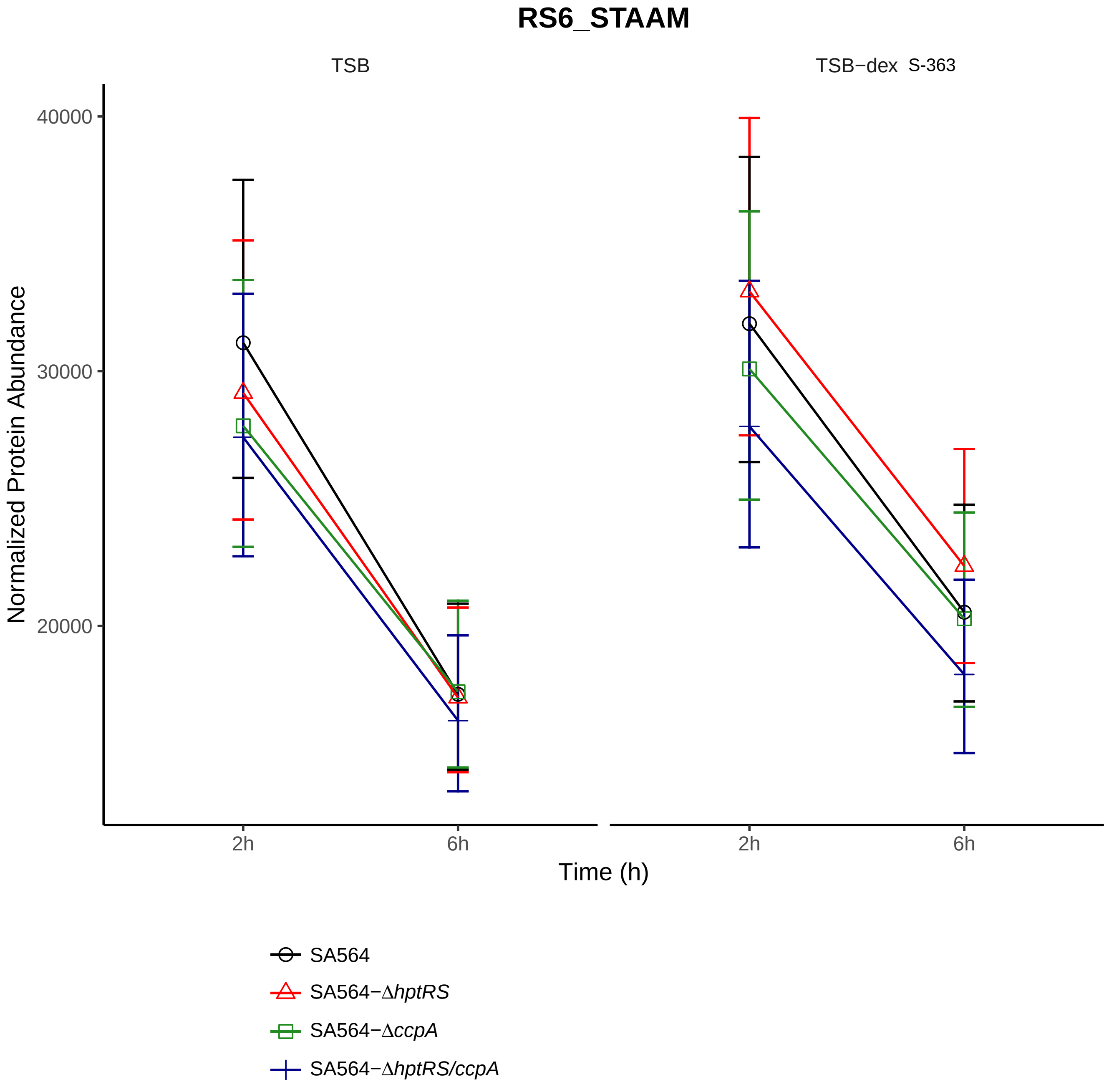
RS6_STAAM
TSB
TSB−dex
S-363
Normalized Protein Abundance
40000
30000
20000
2h
6h
2h
6h
Time (h)
SA564
SA564−ΔhptRS
SA564−ΔccpA
SA564−ΔhptRS/ccpA

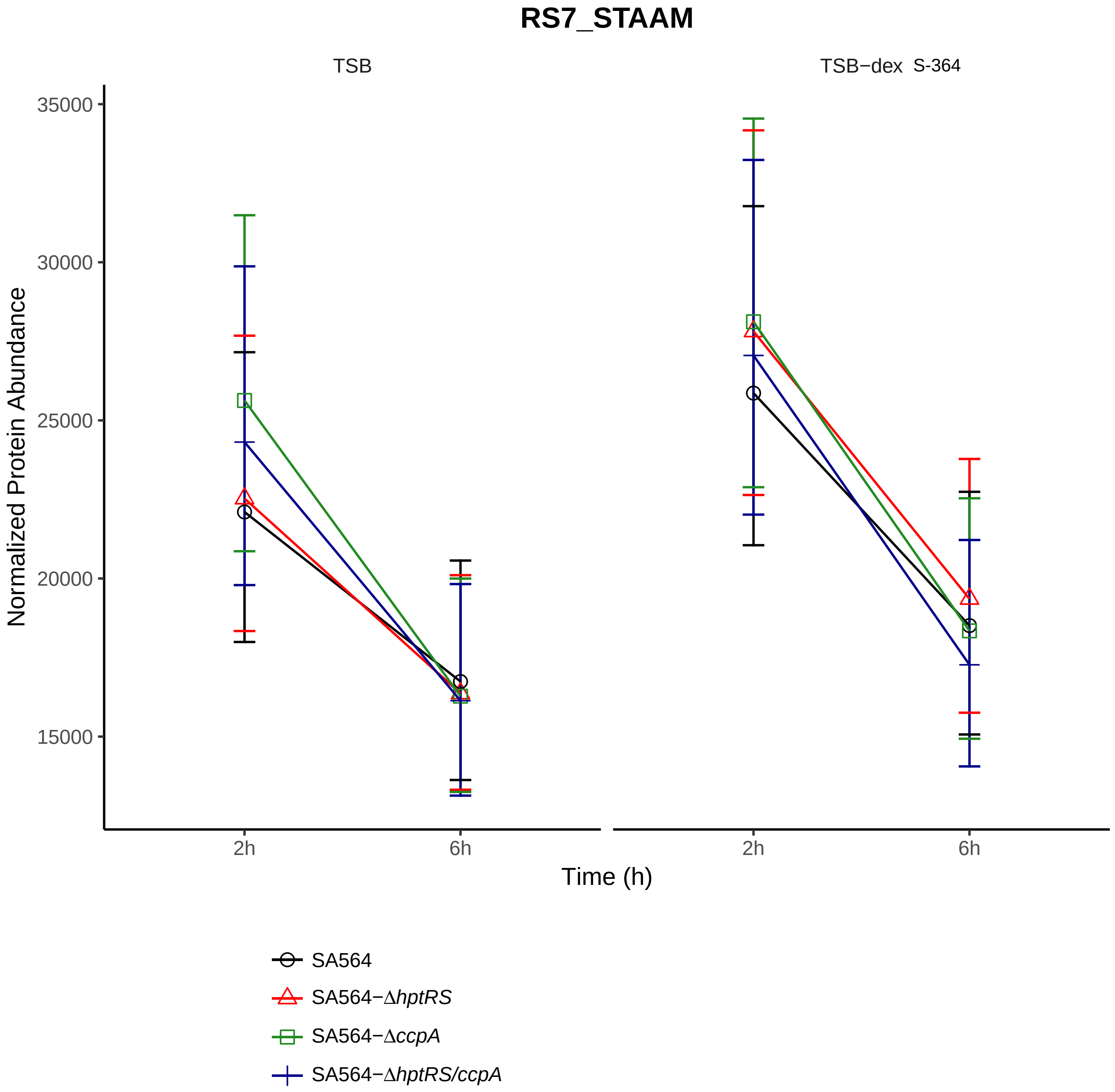

RS7_STAAM
TSB
TSB−dex
S-364
Normalized Protein Abundance
35000
30000
25000
20000
15000
2h
6h
2h
6h
Time (h)
SA564
SA564−Δ*hptRS*
SA564−Δ*ccpA*
SA564−Δ*hptRS/ccpA*

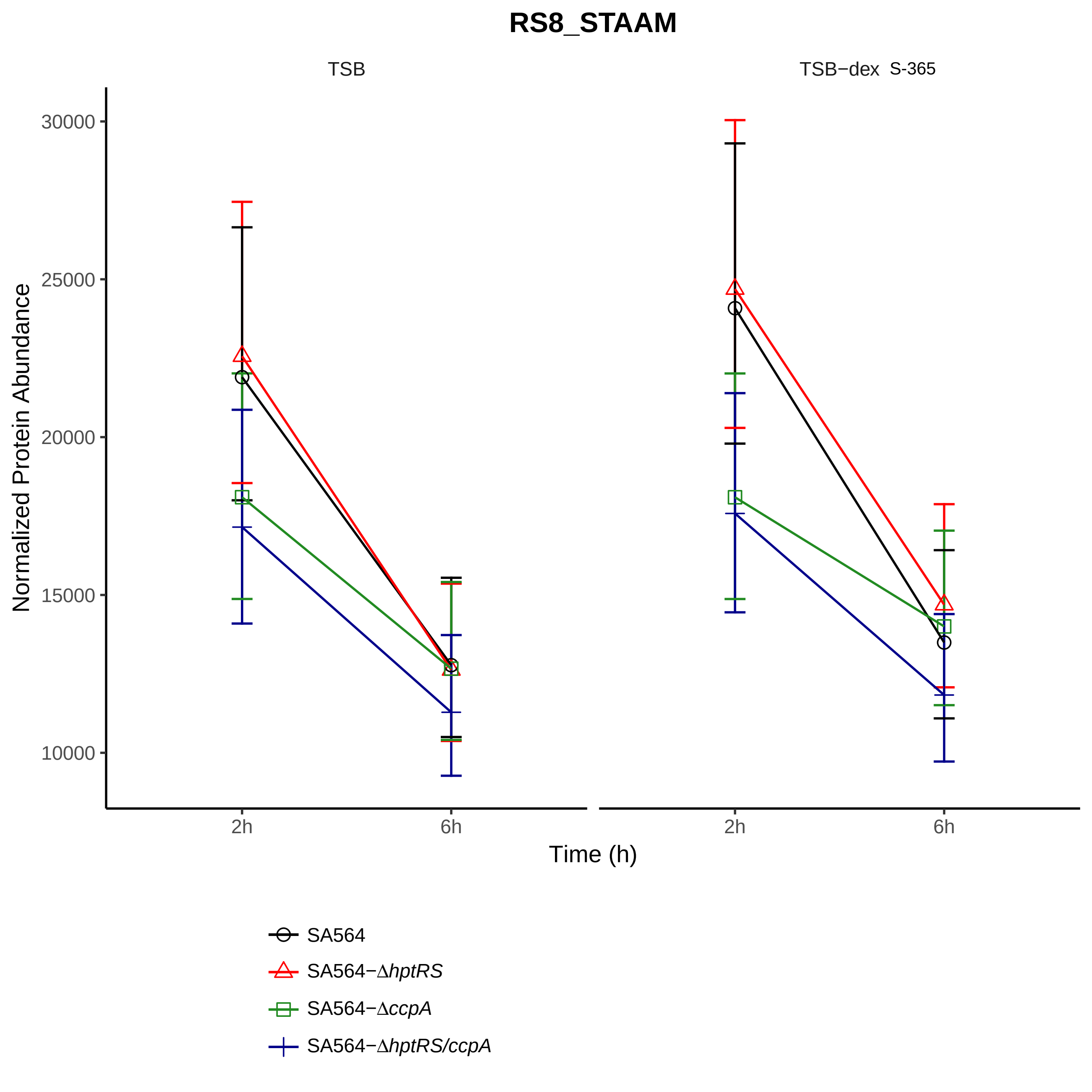

RS8_STAAM
TSB
TSB−dex
S-365
Normalized Protein Abundance
30000
25000
20000
15000
10000
2h
6h
2h
6h
Time (h)
SA564
SA564−Δ*hptRS*
SA564−Δ*ccpA*
SA564−Δ*hptRS/ccpA*

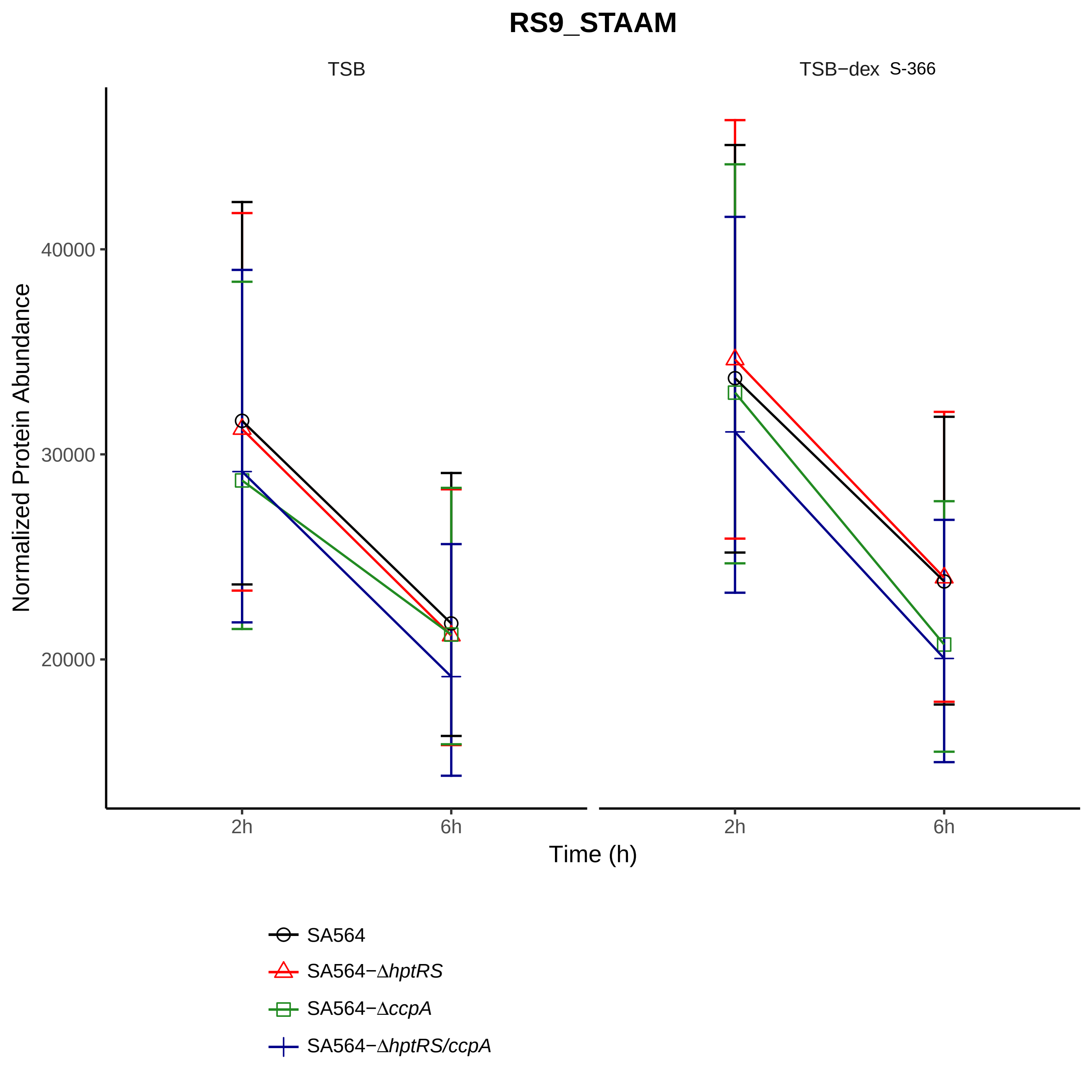

RS9_STAAM
TSB
TSB−dex
S-366
Normalized Protein Abundance
40000
30000
20000
2h
6h
2h
6h
Time (h)
SA564
SA564−Δ*hptRS*
SA564−Δ*ccpA*
SA564−Δ*hptRS/ccpA*

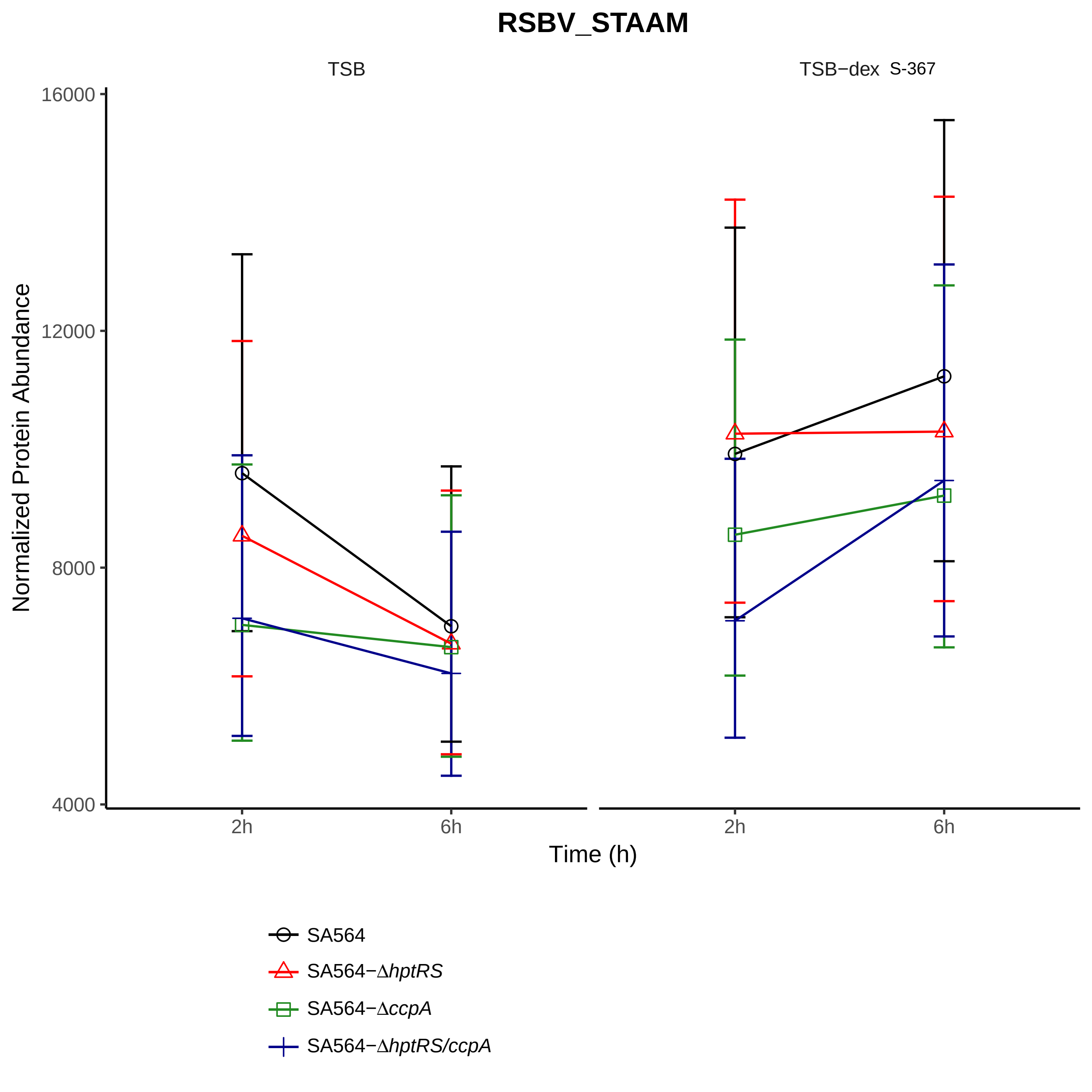

RSBV_STAAM
TSB
TSB−dex
S-367
16000
12000
8000
4000
Normalized Protein Abundance
2h
6h
2h
6h
Time (h)
SA564
SA564−ΔhptRS
SA564−ΔccpA
SA564−ΔhptRS/ccpA

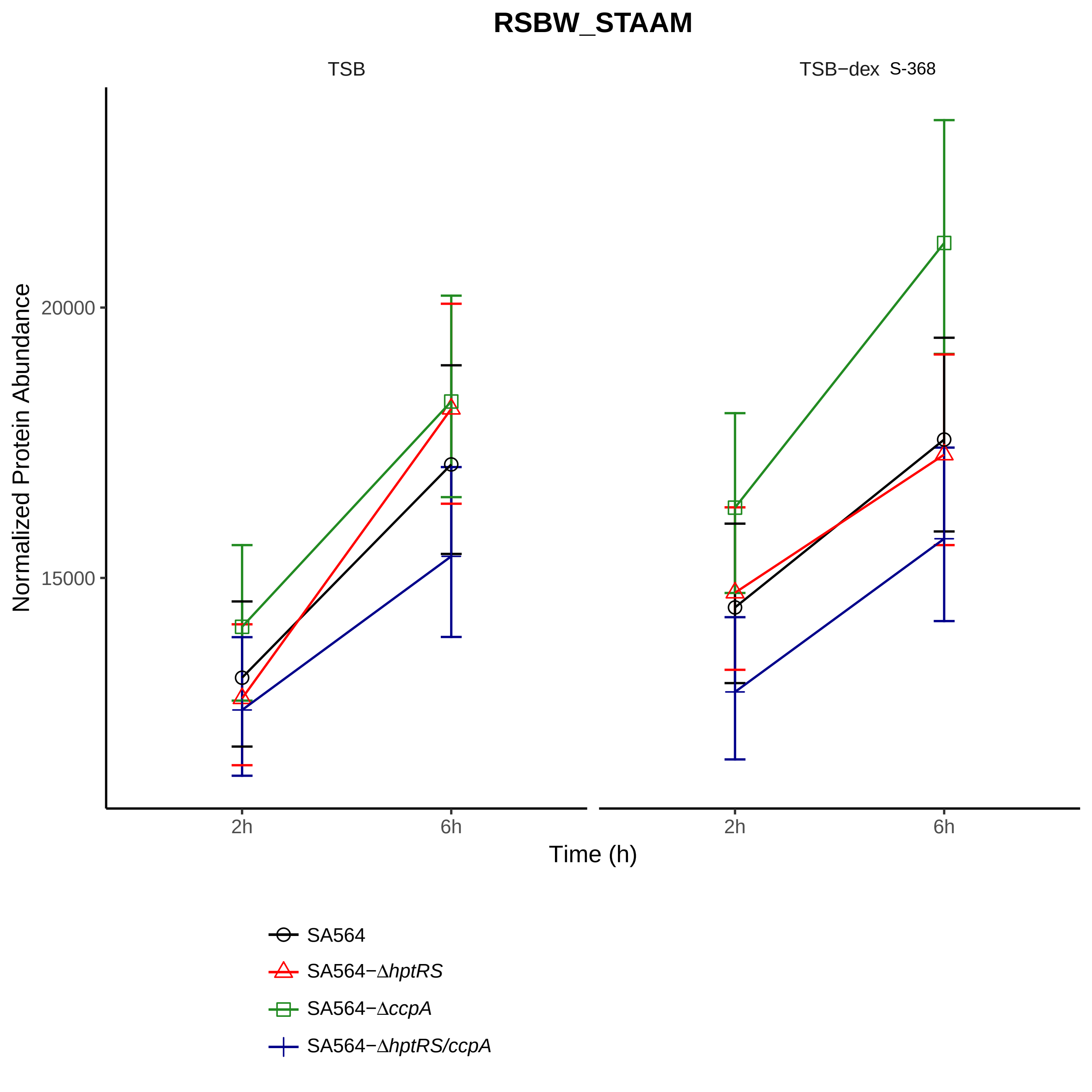

RSBW_STAAM
TSB
TSB−dex
S-368
Normalized Protein Abundance
20000
15000
2h
6h
2h
6h
Time (h)
SA564
SA564−ΔhptRS
SA564−ΔccpA
SA564−ΔhptRS/ccpA

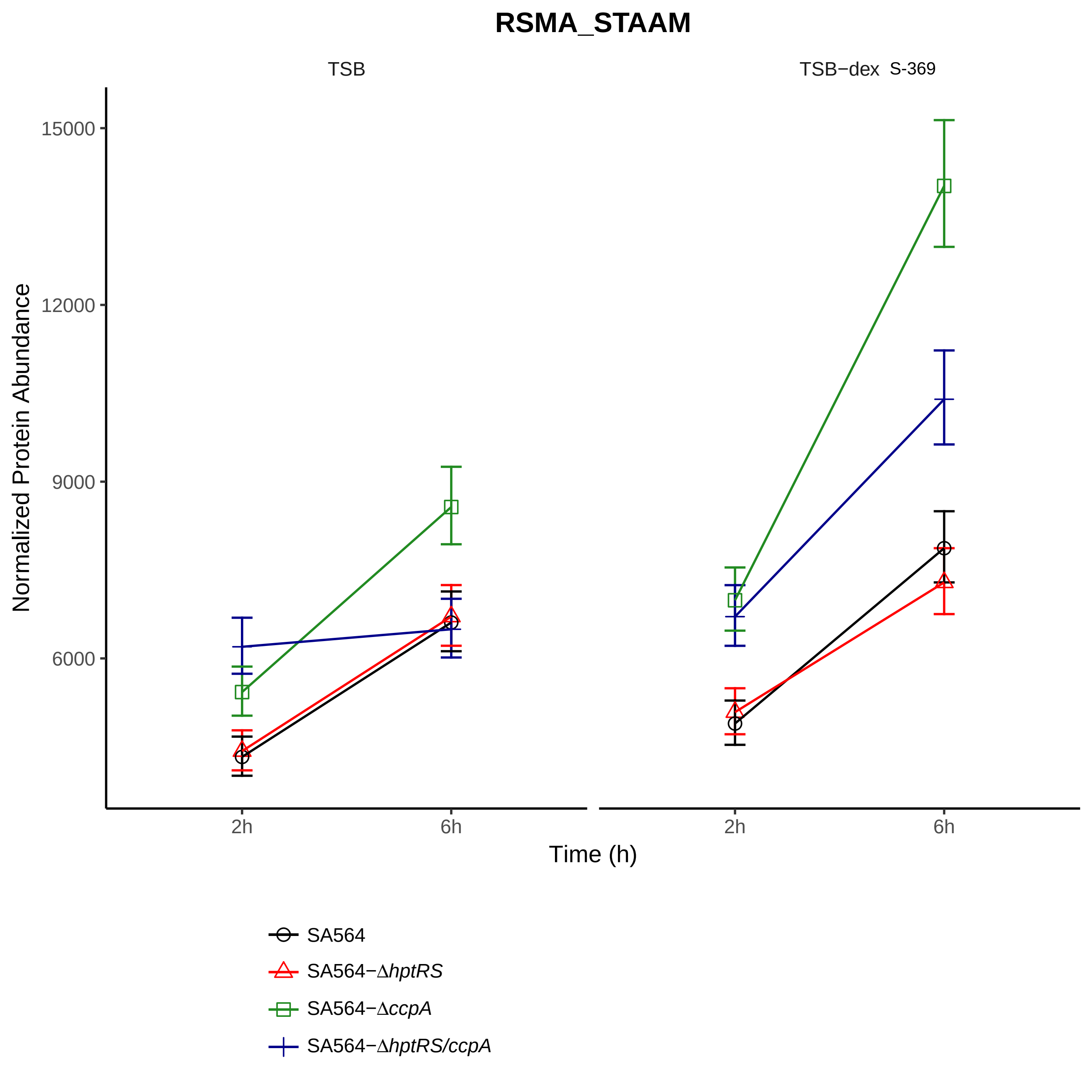

RSMA_STAAM
TSB
TSB−dex
S-369
Normalized Protein Abundance
15000
12000
9000
6000
2h
6h
2h
6h
Time (h)
SA564
SA564−Δ*hptRS*
SA564−Δ*ccpA*
SA564−Δ*hptRS/ccpA*

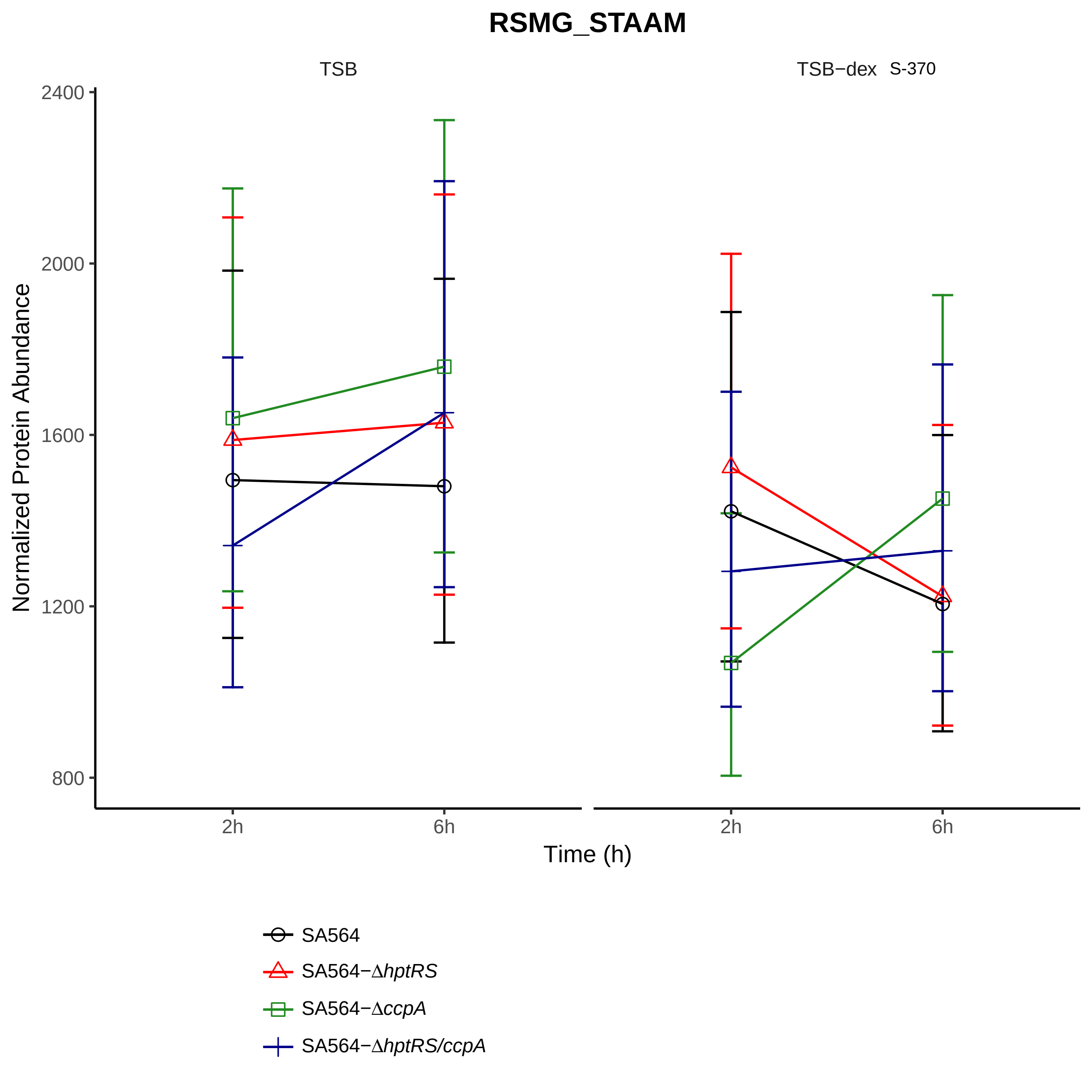

RSMG_STAAM
TSB
TSB−dex
S-370
2400
2000
1600
1200
800
Normalized Protein Abundance
2h
6h
2h
6h
Time (h)
SA564
SA564−ΔhptRS
SA564−ΔccpA
SA564−ΔhptRS/ccpA

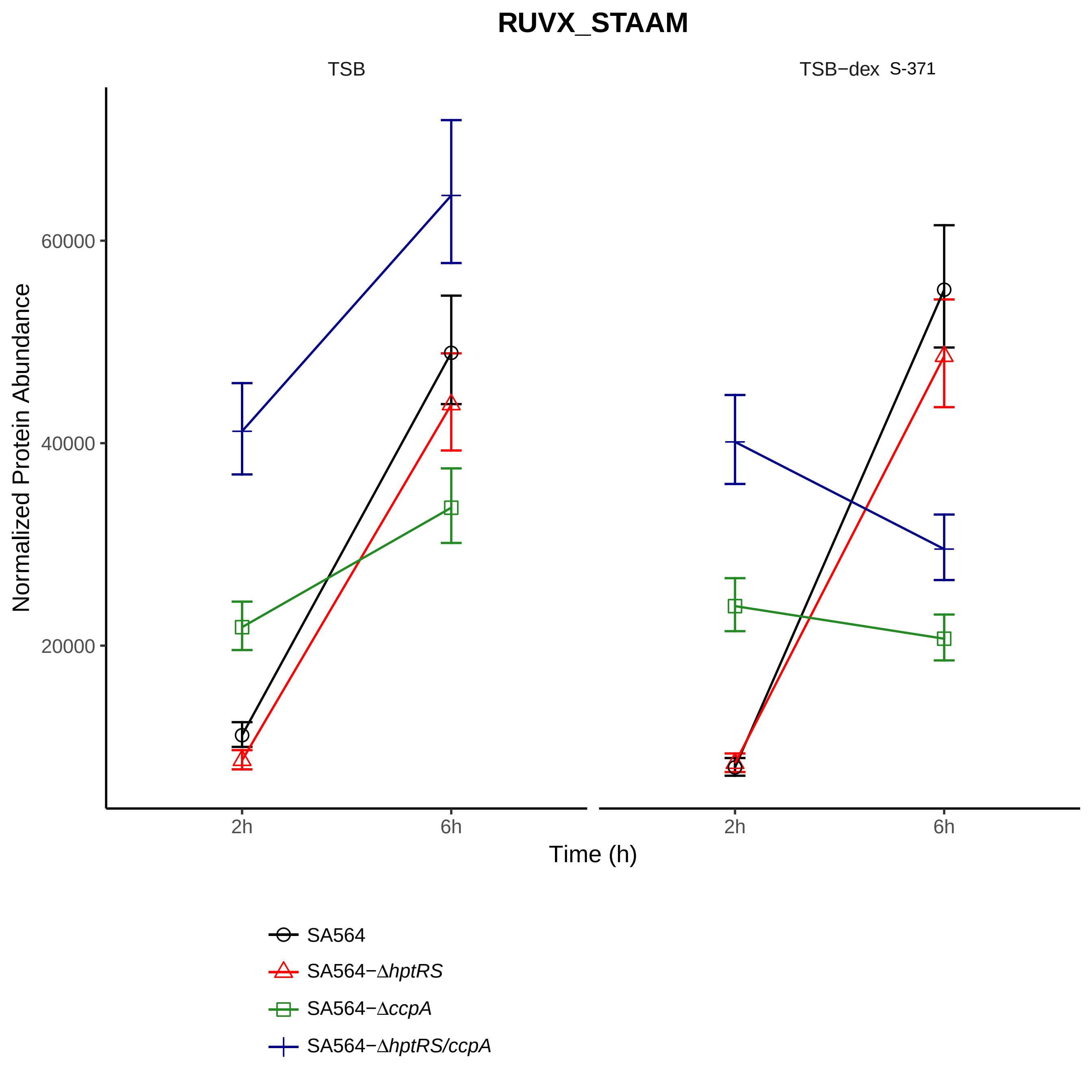
RUVX_STAAM
TSB
TSB−dex
S-371
Normalized Protein Abundance
60000
40000
20000
2h
6h
2h
6h
Time (h)
SA564
SA564−ΔhptRS
SA564−ΔccpA
SA564−ΔhptRS/ccpA

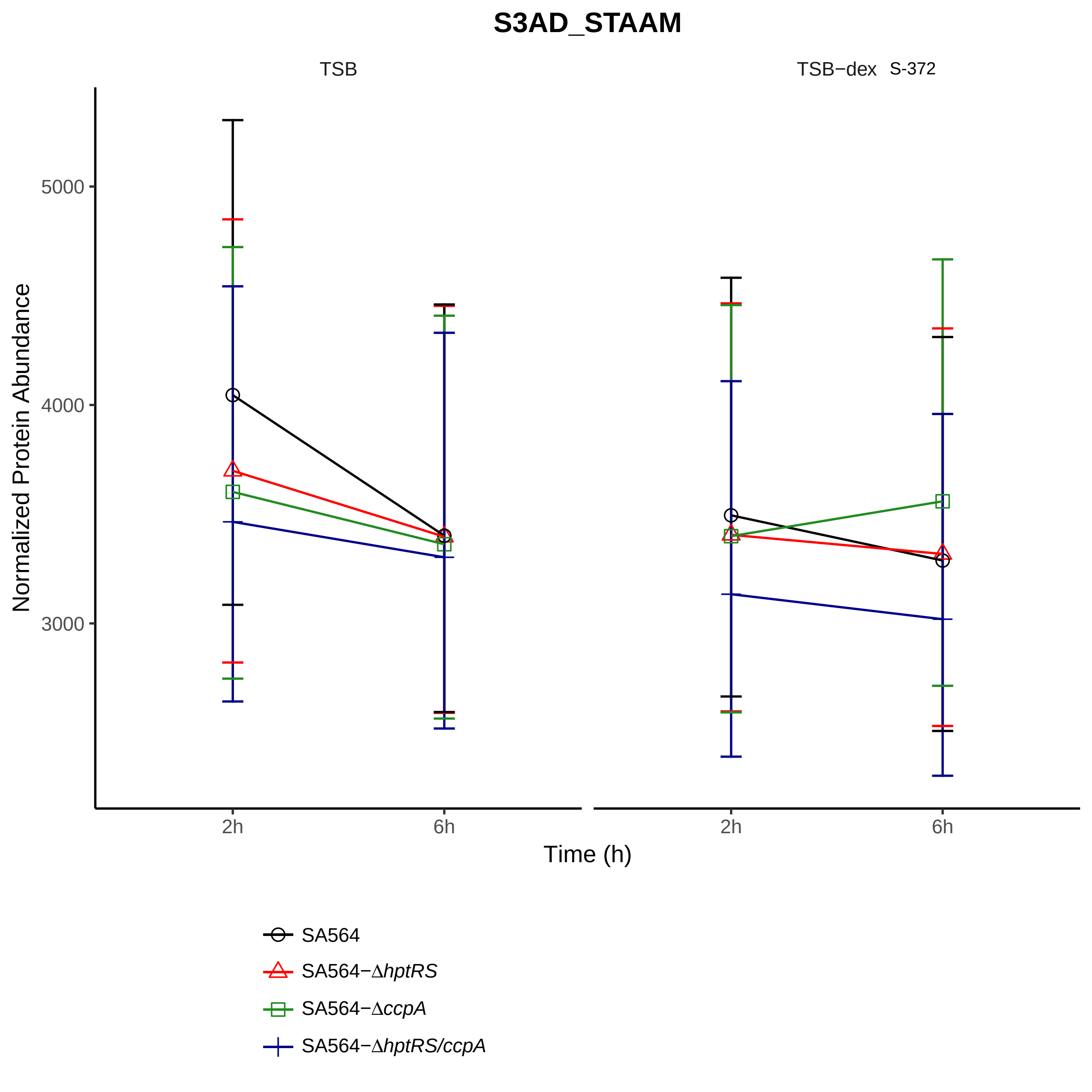

S3AD_STAAM
TSB
TSB−dex
S-372
Normalized Protein Abundance
5000
4000
3000
2h
6h
2h
6h
Time (h)
SA564
SA564−ΔhptRS
SA564−ΔccpA
SA564−ΔhptRS/ccpA

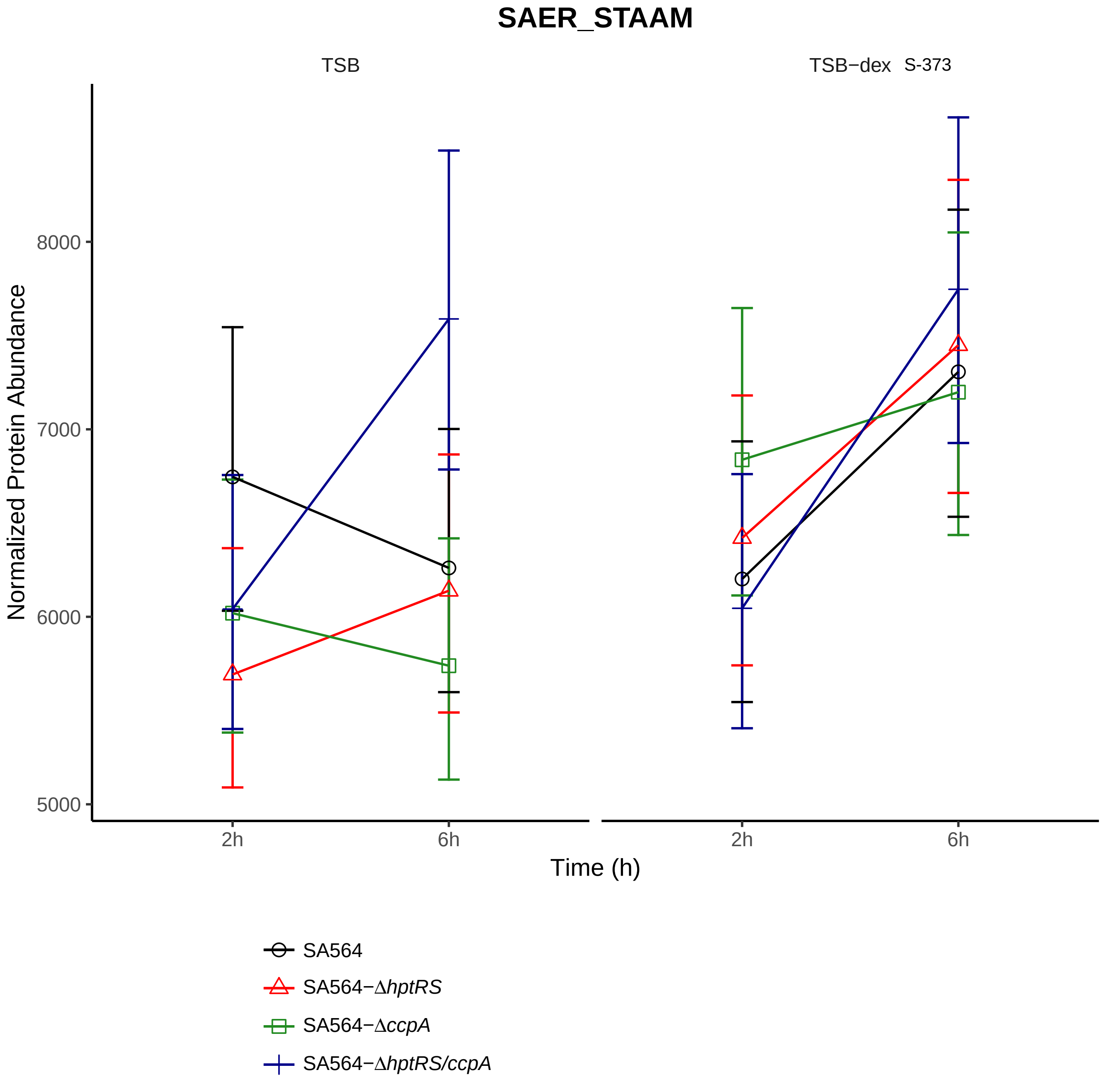

SAER_STAAM
TSB
TSB−dex
S-373
Normalized Protein Abundance
8000
7000
6000
5000
2h
6h
2h
6h
Time (h)
SA564
SA564−ΔhptRS
SA564−ΔccpA
SA564−ΔhptRS/ccpA

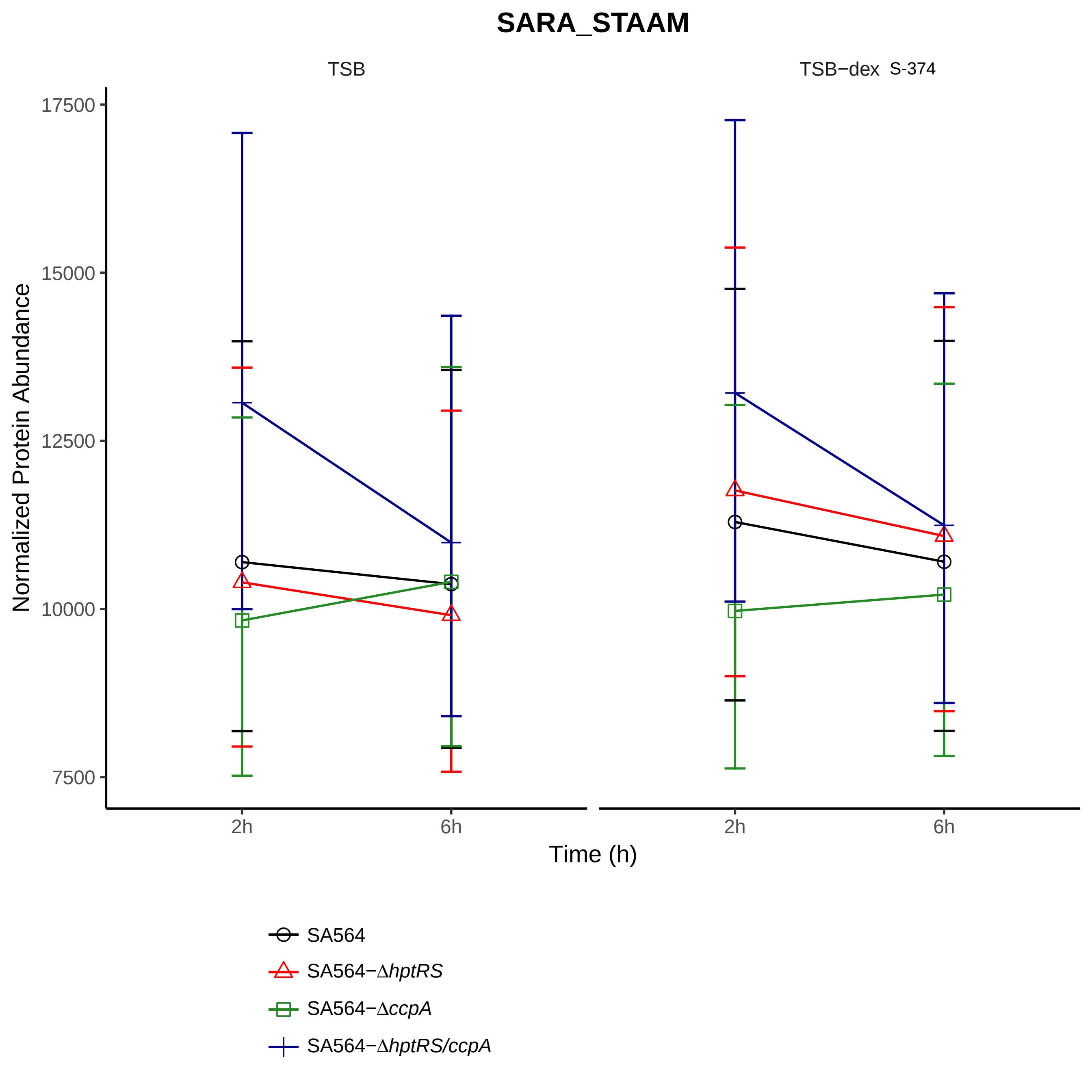

SARA_STAAM
TSB
TSB−dex
S-374
Normalized Protein Abundance
17500
15000
12500
10000
7500
2h
6h
2h
6h
Time (h)
SA564
SA564−ΔhptRS
SA564−ΔccpA
SA564−ΔhptRS/ccpA

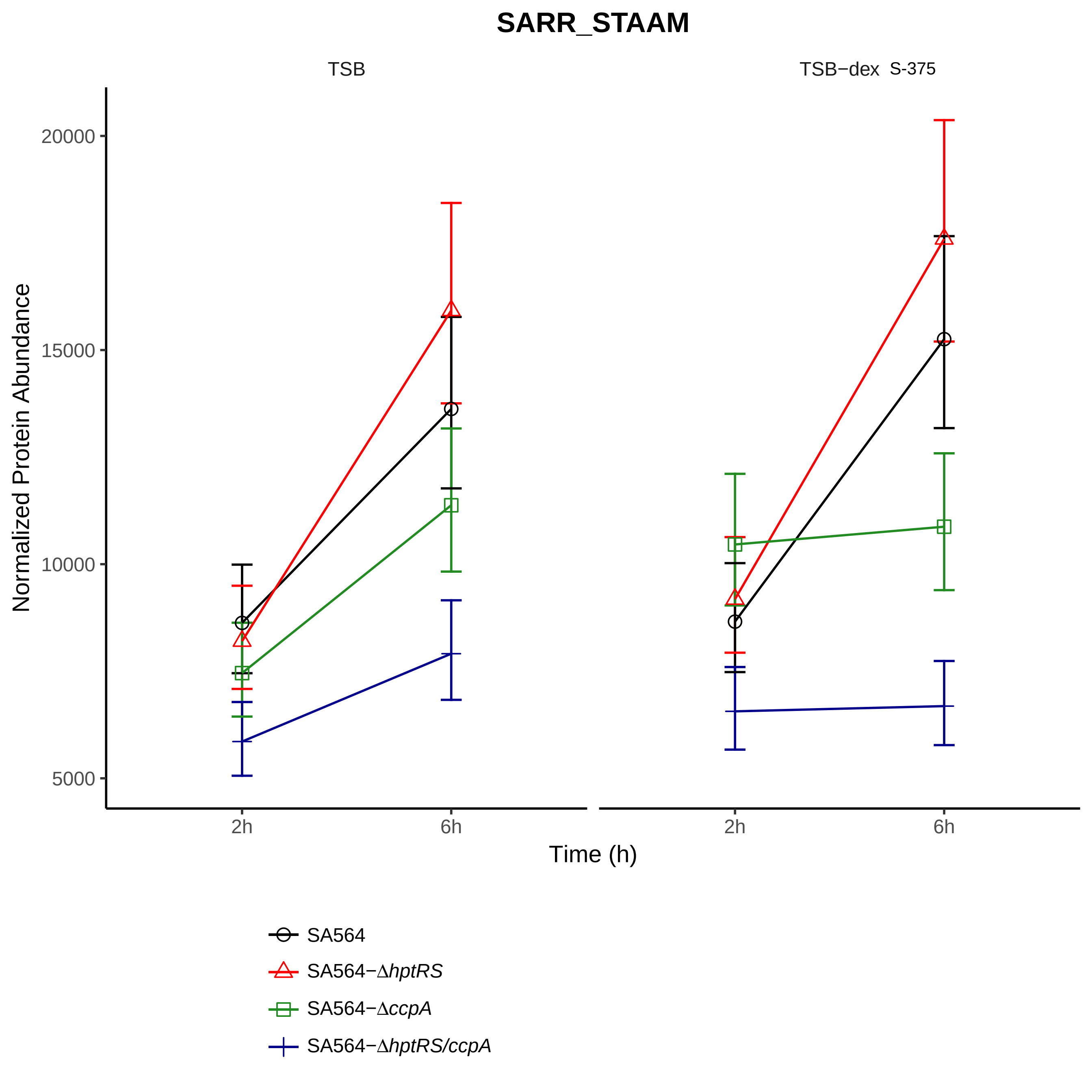

SARR_STAAM
TSB
TSB−dex
S-375
Normalized Protein Abundance
20000
15000
10000
5000
2h
6h
2h
6h
Time (h)
SA564
SA564−ΔhptRS
SA564−ΔccpA
SA564−ΔhptRS/ccpA

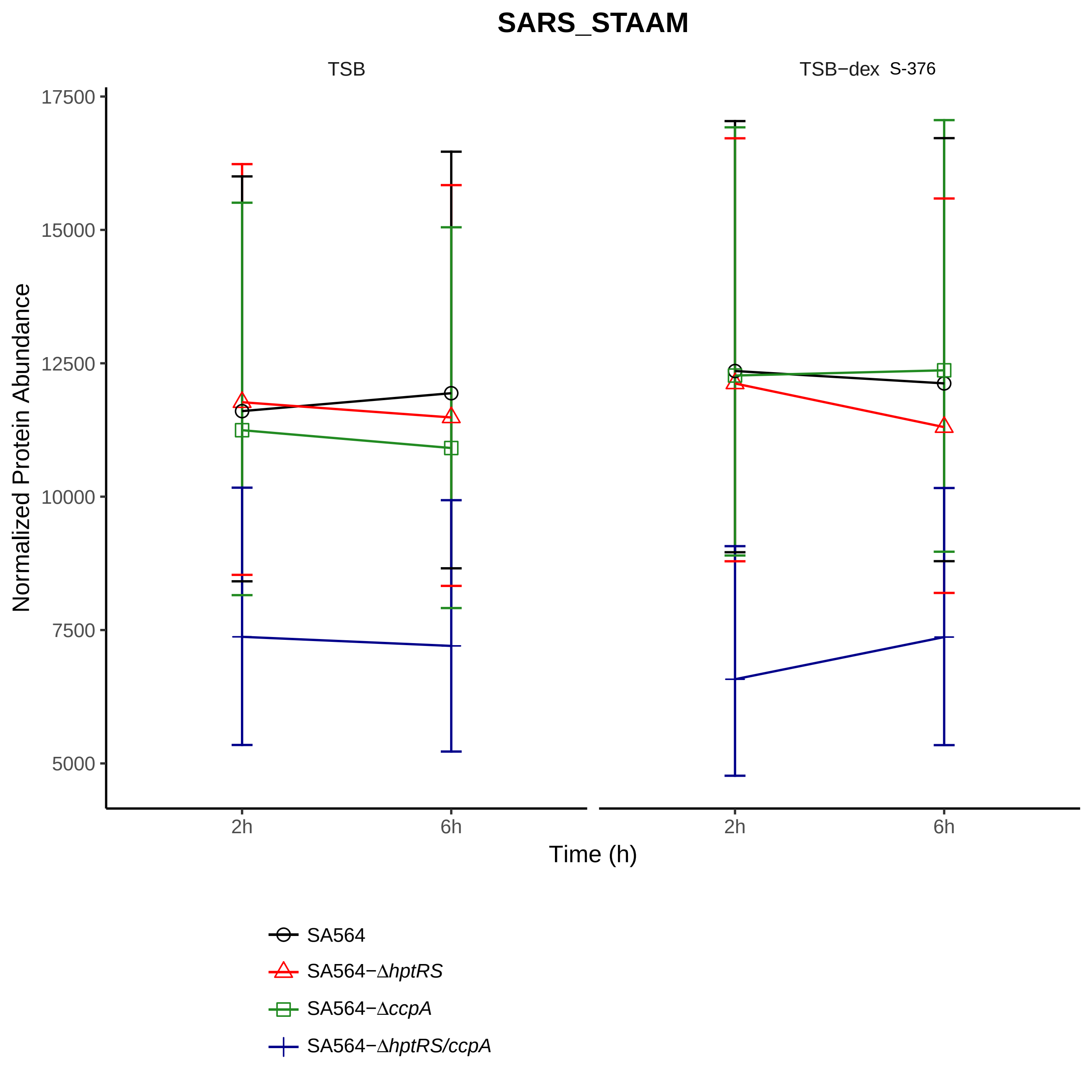

SARS_STAAM
TSB
TSB−dex
S-376
Normalized Protein Abundance
17500
15000
12500
10000
7500
5000
2h
6h
2h
6h
Time (h)
SA564
SA564−ΔhptRS
SA564−ΔccpA
SA564−ΔhptRS/ccpA

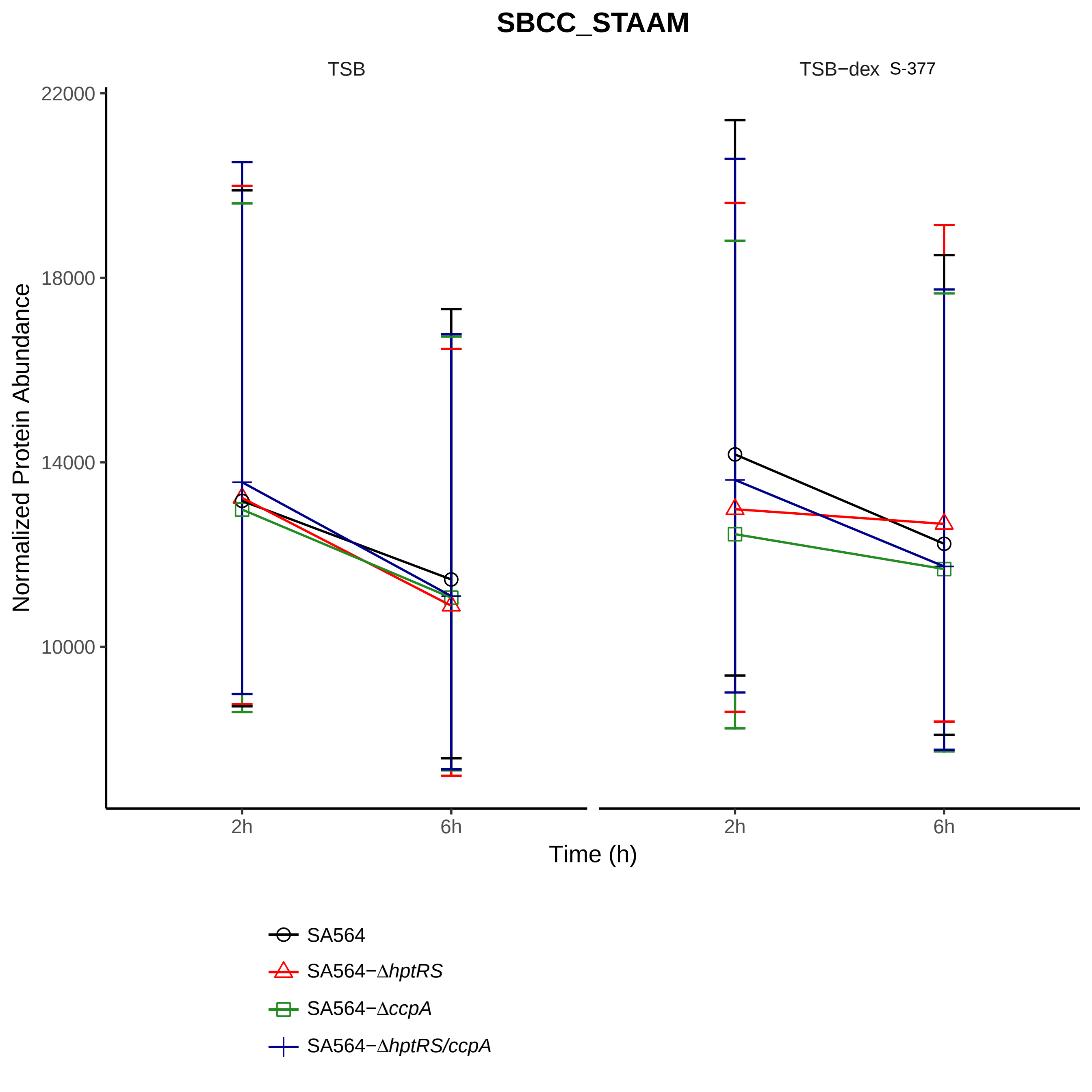
SBCC_STAAM
TSB
TSB−dex
S-377
22000
18000
14000
10000
Normalized Protein Abundance
2h
6h
2h
6h
Time (h)
SA564
SA564−ΔhptRS
SA564−ΔccpA
SA564−ΔhptRS/ccpA

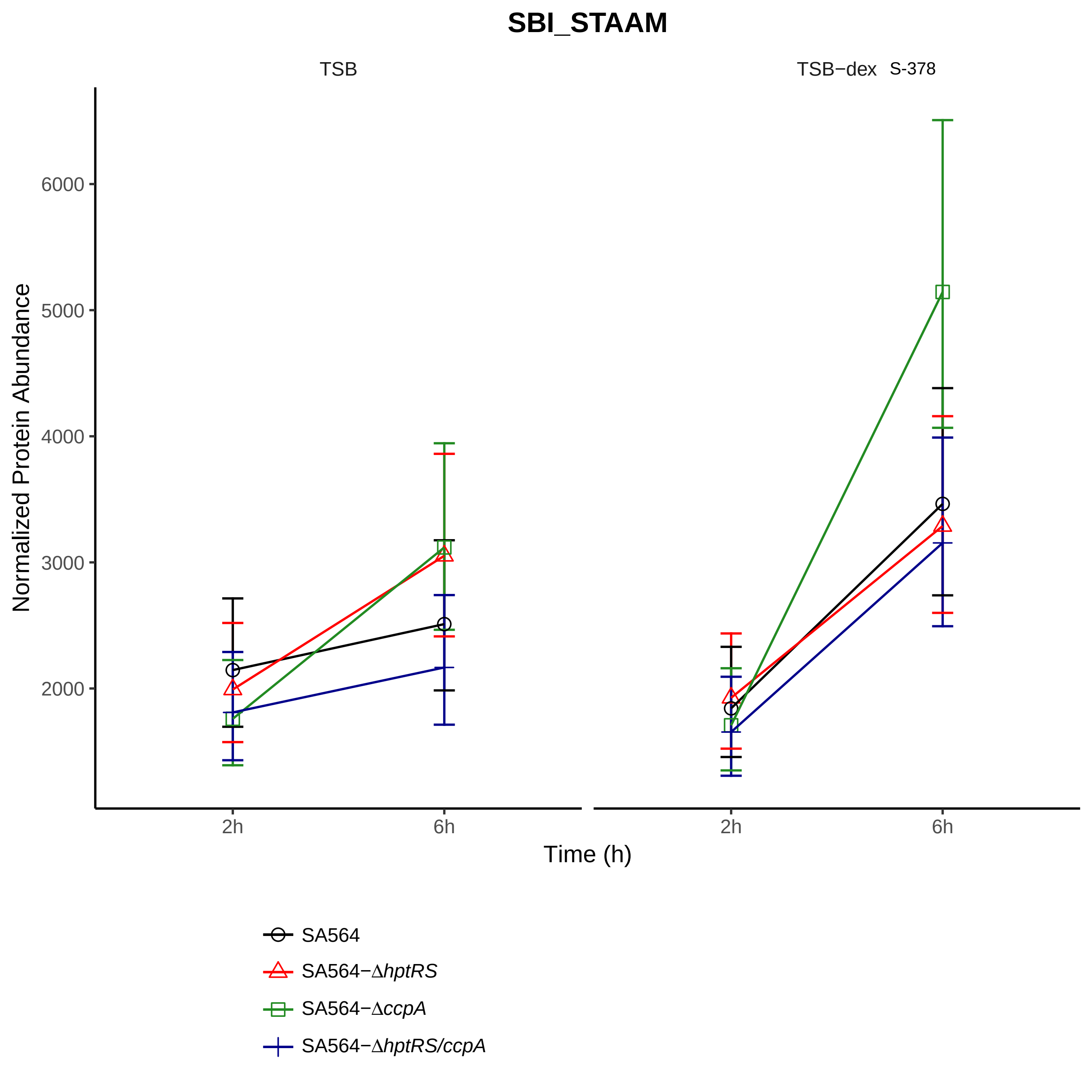

SBI_STAAM
TSB
TSB−dex
S-378
Normalized Protein Abundance
6000
5000
4000
3000
2000
2h
6h
2h
6h
Time (h)
SA564
SA564−ΔhptRS
SA564−ΔccpA
SA564−ΔhptRS/ccpA

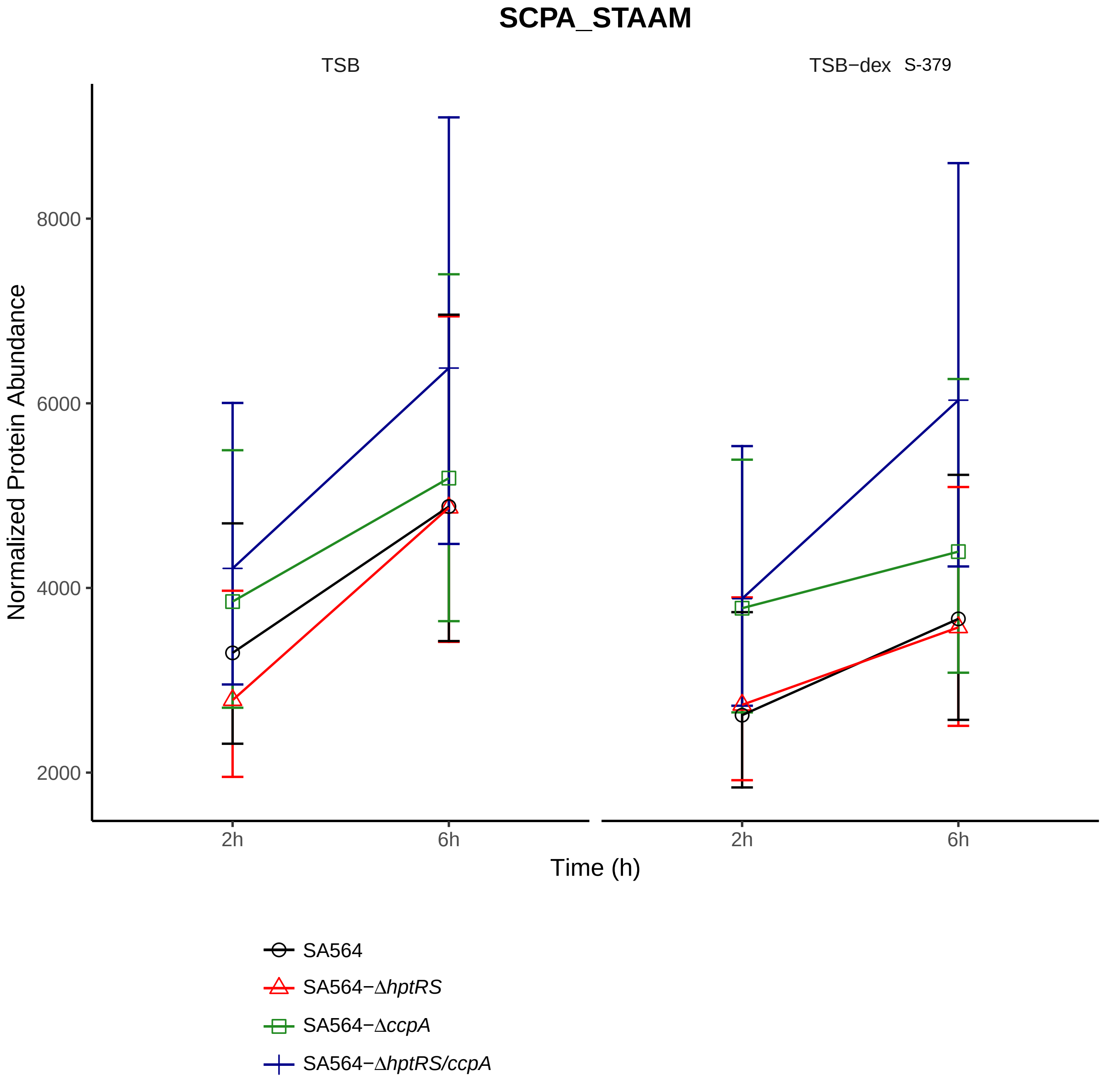

SCPA_STAAM
TSB
TSB−dex
S-379
Normalized Protein Abundance
8000
6000
4000
2000
2h
6h
2h
6h
Time (h)
SA564
SA564−Δ*hptRS*
SA564−Δ*ccpA*
SA564−Δ*hptRS/ccpA*

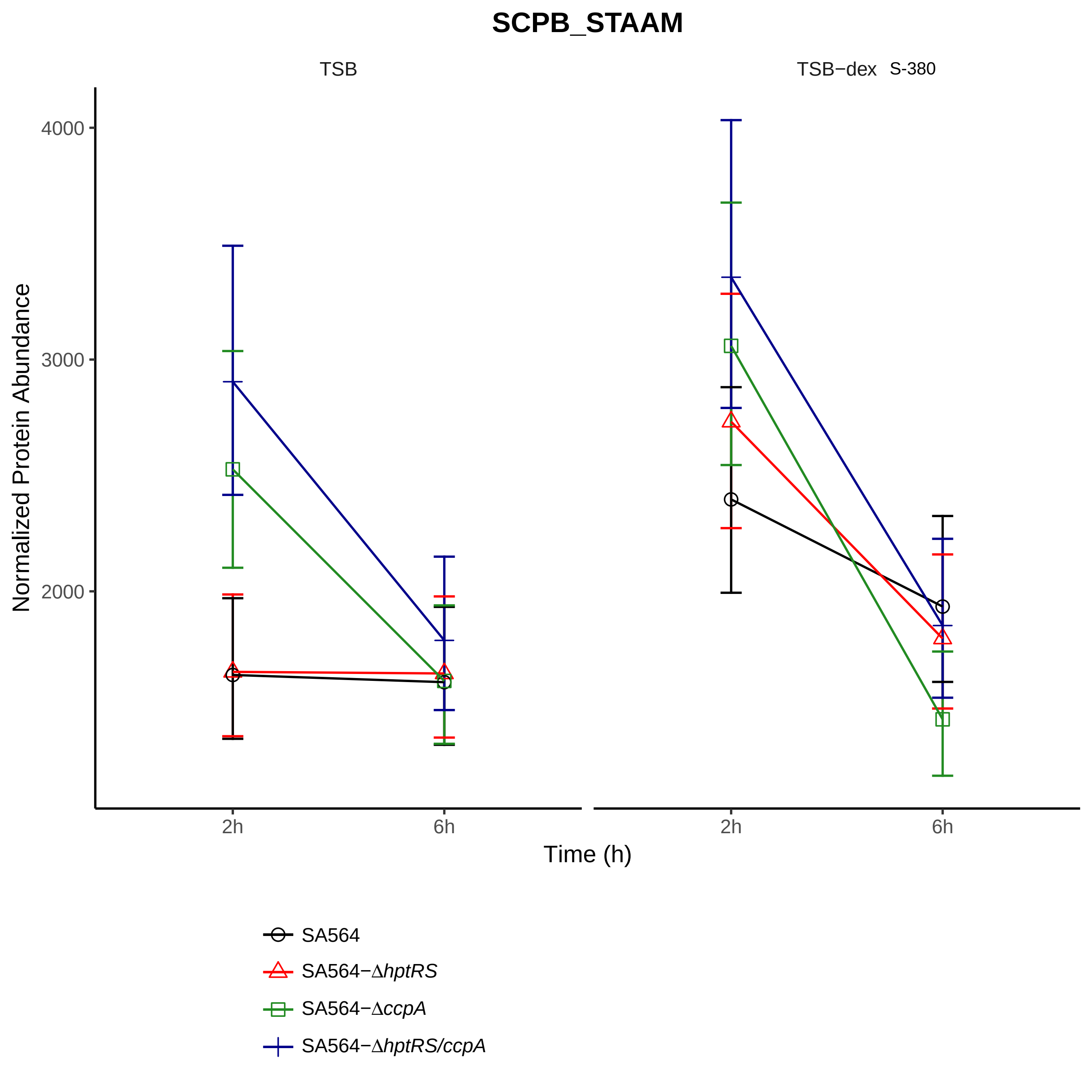
SCPB_STAAM
TSB
TSB−dex
S-380
Normalized Protein Abundance
4000
3000
2000
2h
6h
2h
6h
Time (h)
SA564
SA564−ΔhptRS
SA564−ΔccpA
SA564−ΔhptRS/ccpA

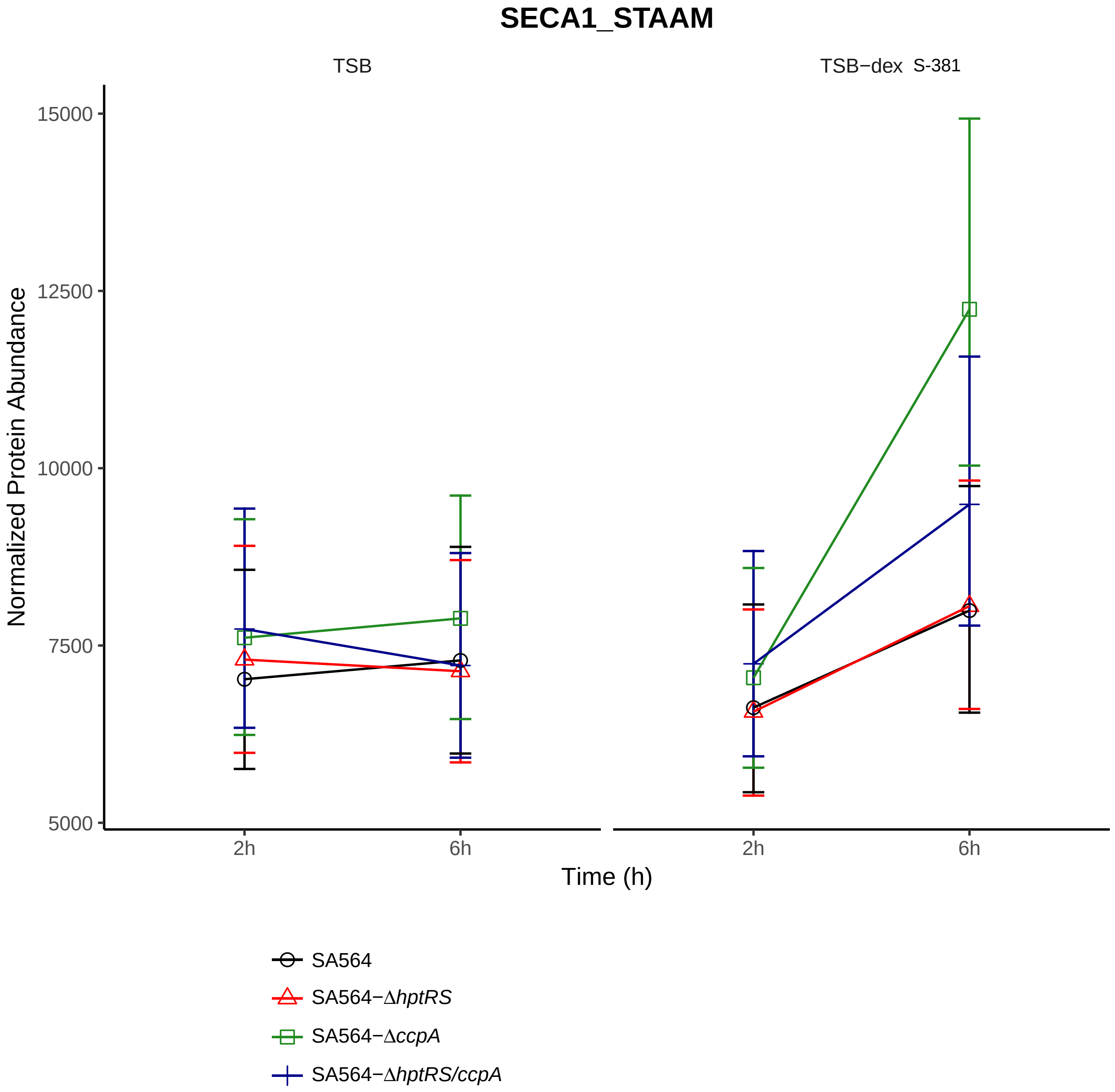

SECA1_STAAM
TSB
TSB−dex
S-381
15000
12500
10000
7500
5000
Normalized Protein Abundance
2h
6h
2h
6h
Time (h)
SA564
SA564−ΔhptRS
SA564−ΔccpA
SA564−ΔhptRS/ccpA

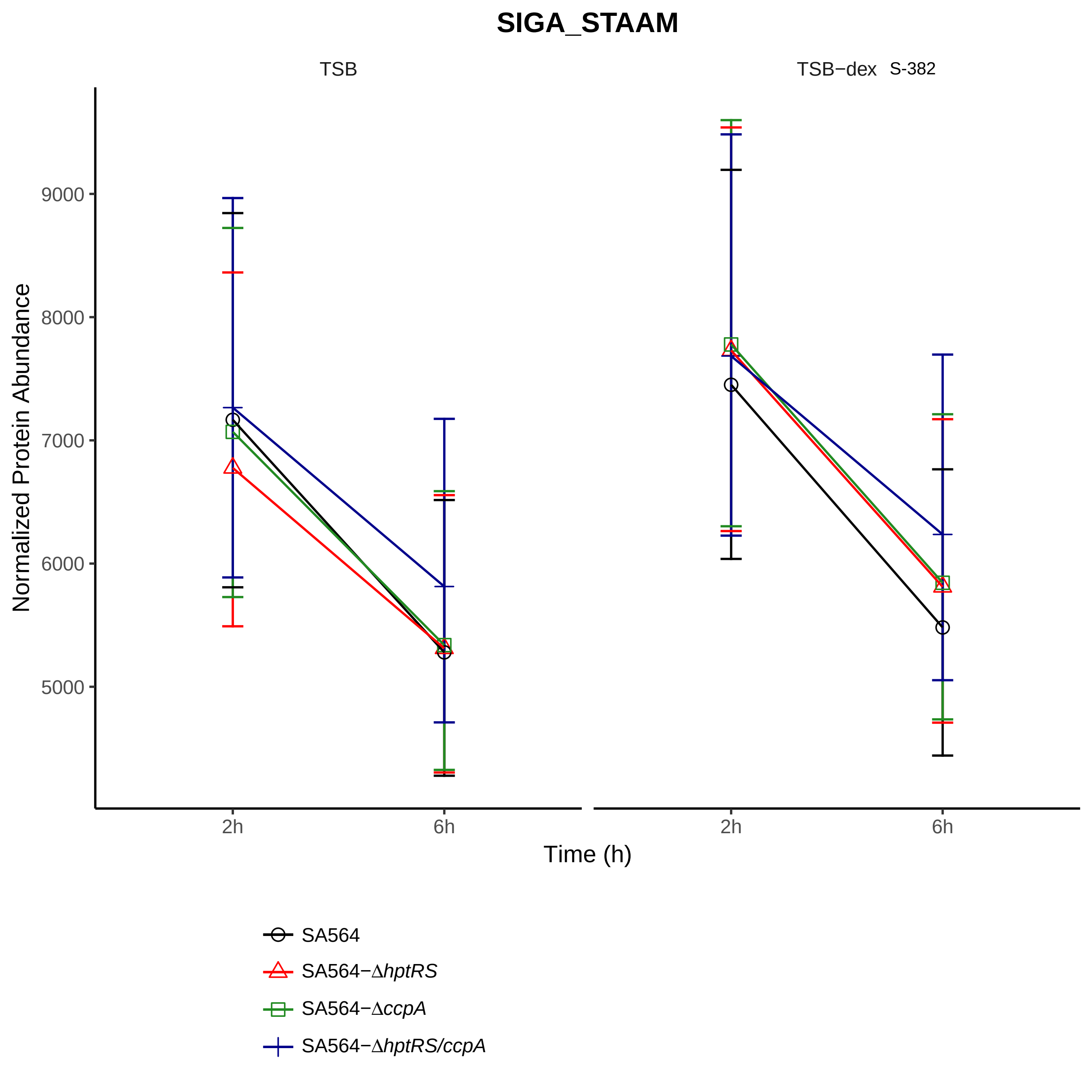
SIGA_STAAM
TSB
TSB−dex
S-382
Normalized Protein Abundance
9000
8000
7000
6000
5000
2h
6h
2h
6h
Time (h)
SA564
SA564−ΔhptRS
SA564−ΔccpA
SA564−ΔhptRS/ccpA

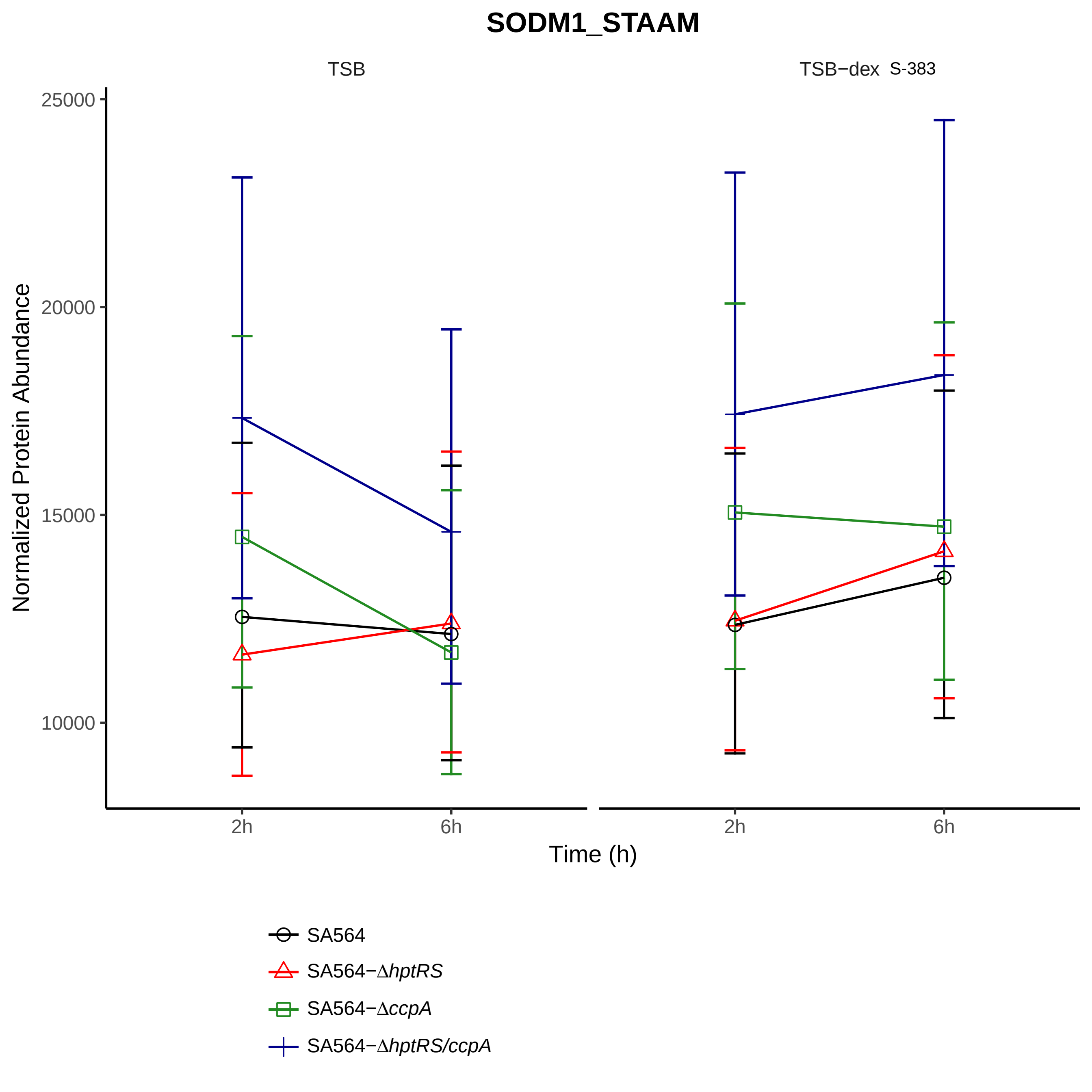

SODM1_STAAM
TSB
TSB−dex
S-383
25000
20000
15000
10000
Normalized Protein Abundance
2h
6h
2h
6h
Time (h)
SA564
SA564−Δ*hptRS*
SA564−Δ*ccpA*
SA564−Δ*hptRS/ccpA*

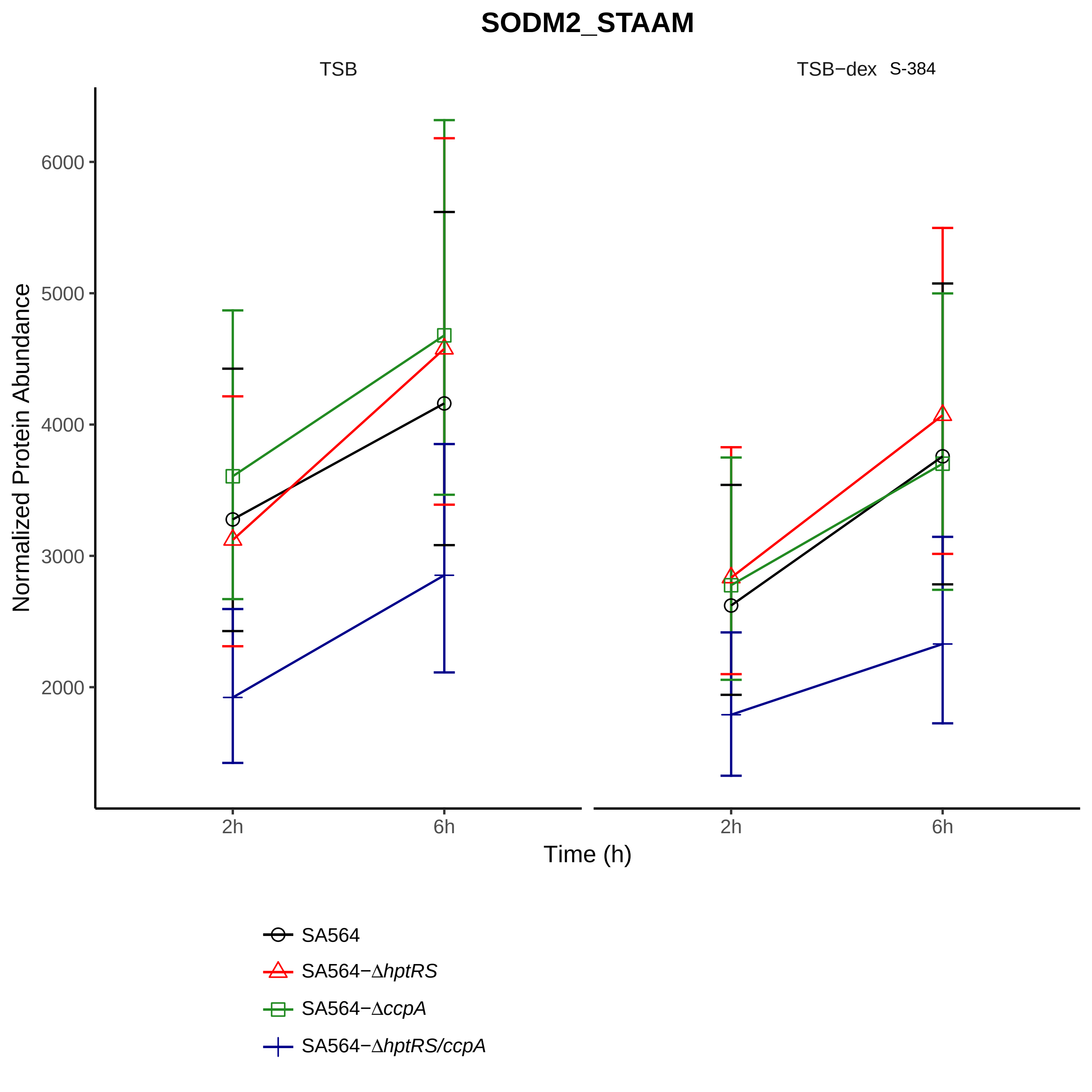

SODM2_STAAM
TSB
TSB−dex
S-384
Normalized Protein Abundance
6000
5000
4000
3000
2000
2h
6h
2h
6h
Time (h)
SA564
SA564−Δ*hptRS*
SA564−Δ*ccpA*
SA564−Δ*hptRS/ccpA*

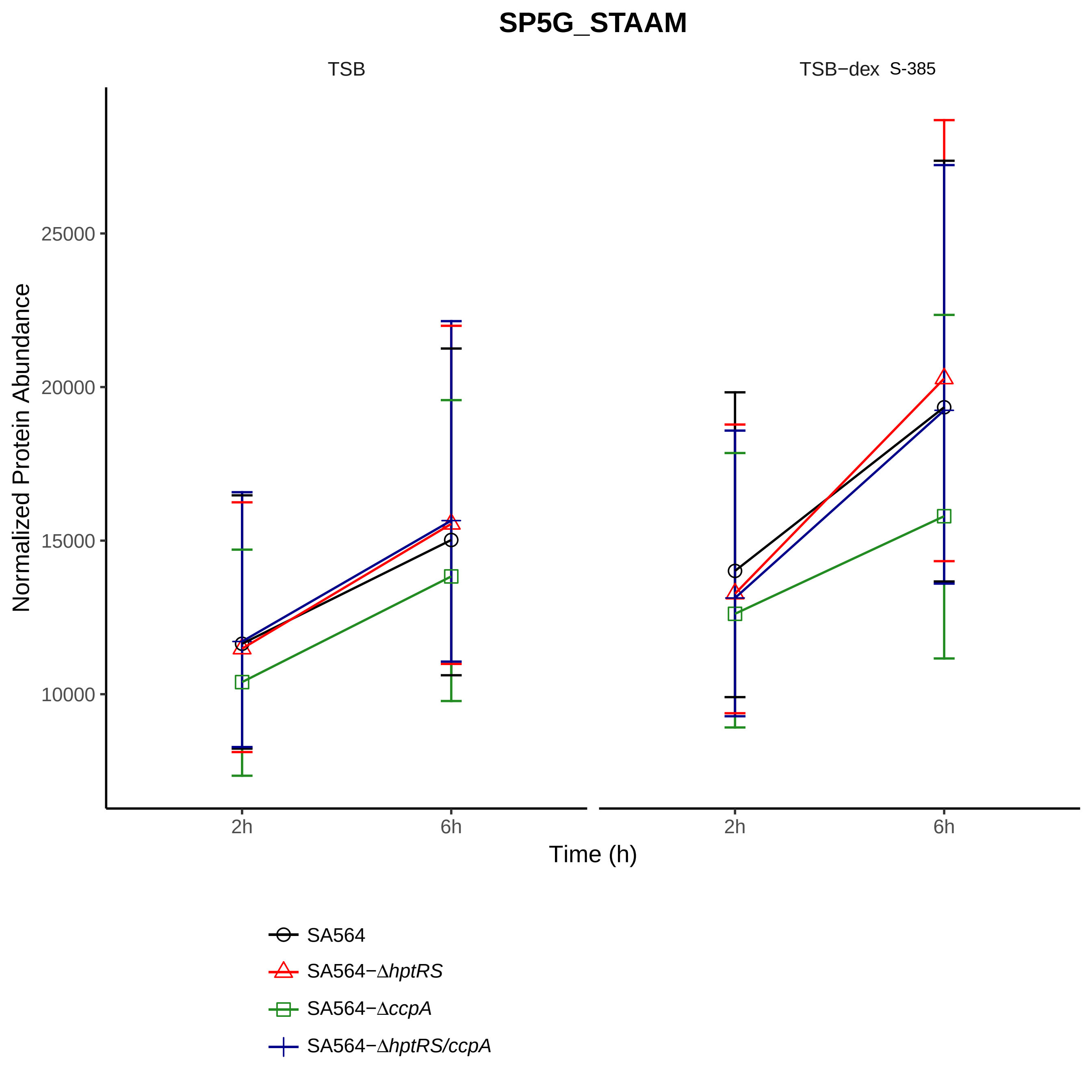

SP5G_STAAM
TSB
TSB−dex
S-385
Normalized Protein Abundance
25000
20000
15000
10000
2h
6h
2h
6h
Time (h)
SA564
SA564−ΔhptRS
SA564−ΔccpA
SA564−ΔhptRS/ccpA

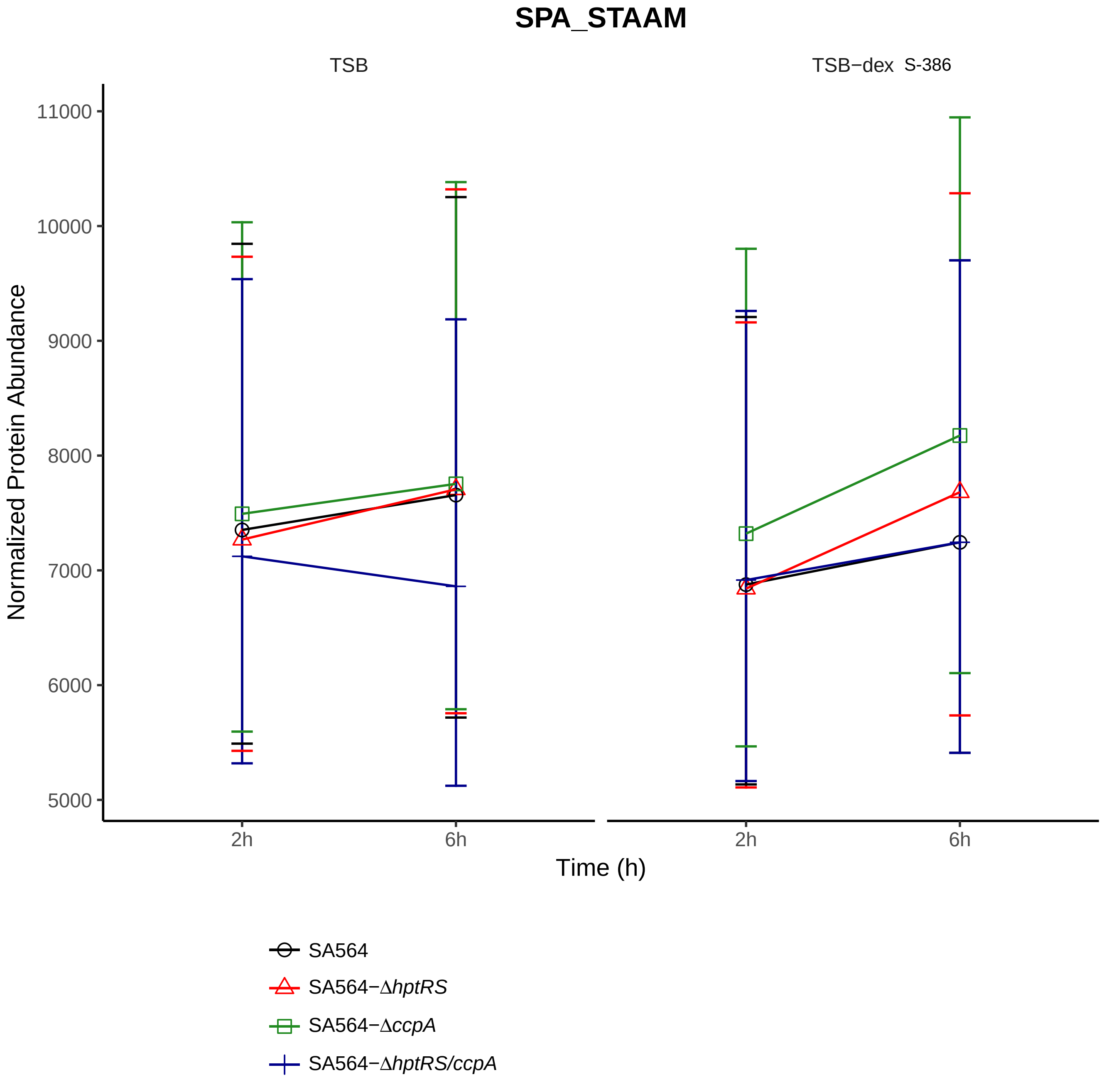

SPA_STAAM
TSB
TSB−dex
S-386
11000
10000
9000
8000
7000
6000
5000
Normalized Protein Abundance
2h
6h
2h
6h
Time (h)
SA564
SA564−ΔhptRS
SA564−ΔccpA
SA564−ΔhptRS/ccpA

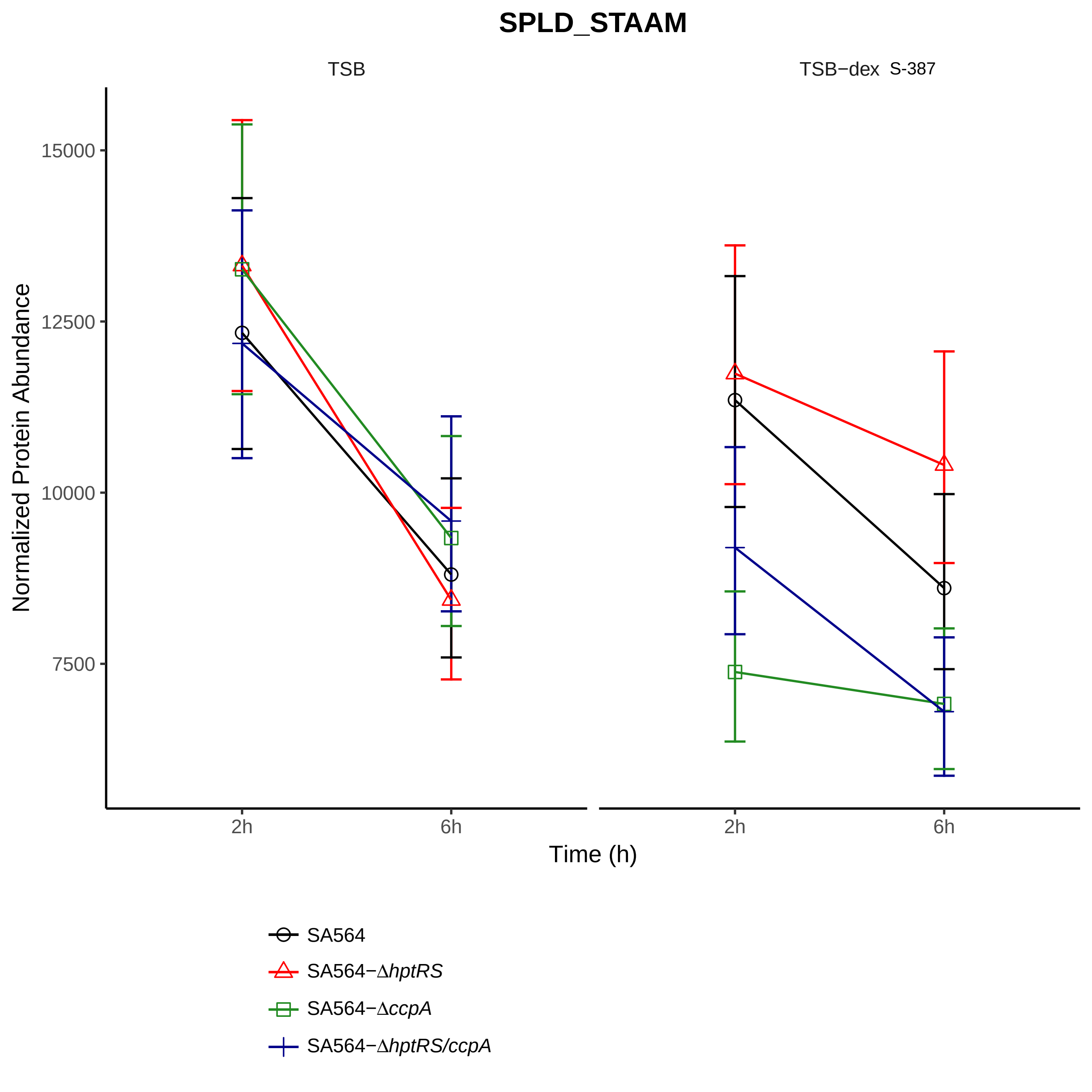

SPLD_STAAM
TSB
TSB−dex
S-387
Normalized Protein Abundance
15000
12500
10000
7500
2h
6h
2h
6h
Time (h)
SA564
SA564−Δ*hptRS*
SA564−Δ*ccpA*
SA564−Δ*hptRS/ccpA*

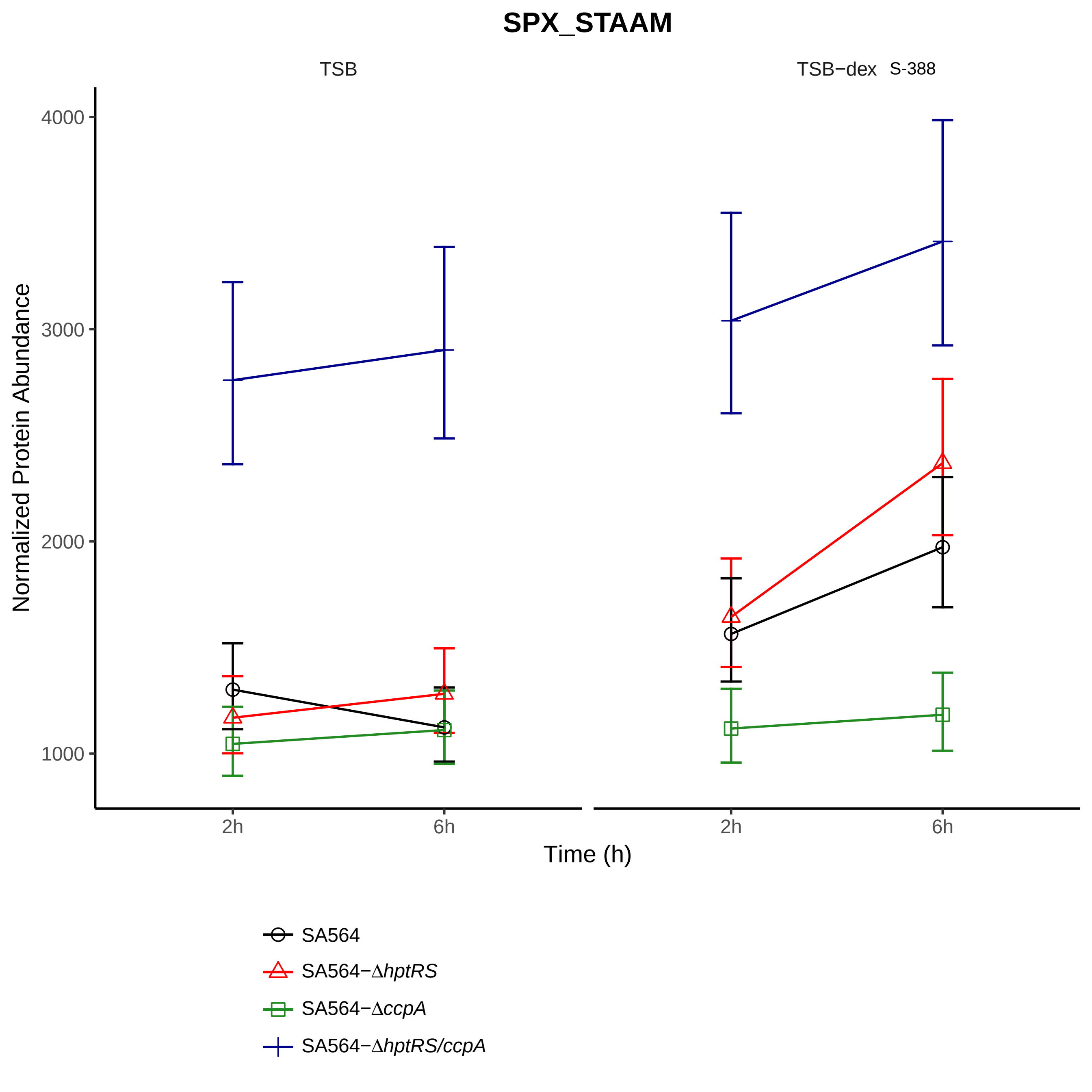

SPX_STAAM
TSB
TSB−dex
S-388
Normalized Protein Abundance
4000
3000
2000
1000
2h
6h
2h
6h
Time (h)
SA564
SA564−ΔhptRS
SA564−ΔccpA
SA564−ΔhptRS/ccpA

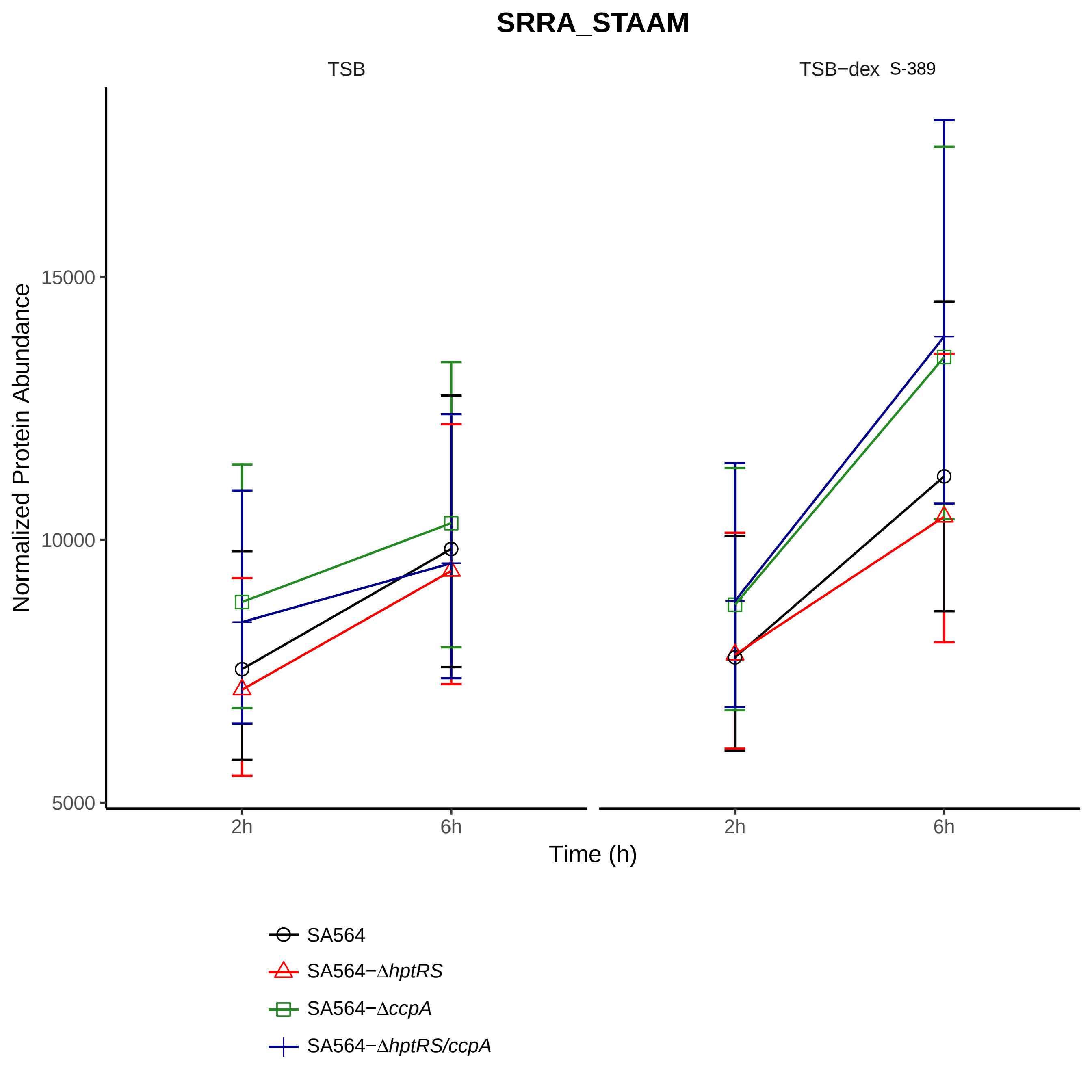
SRRA_STAAM
TSB
TSB−dex
S-389
Normalized Protein Abundance
15000
10000
5000
2h
6h
2h
6h
Time (h)
SA564
SA564−ΔhptRS
SA564−ΔccpA
SA564−ΔhptRS/ccpA

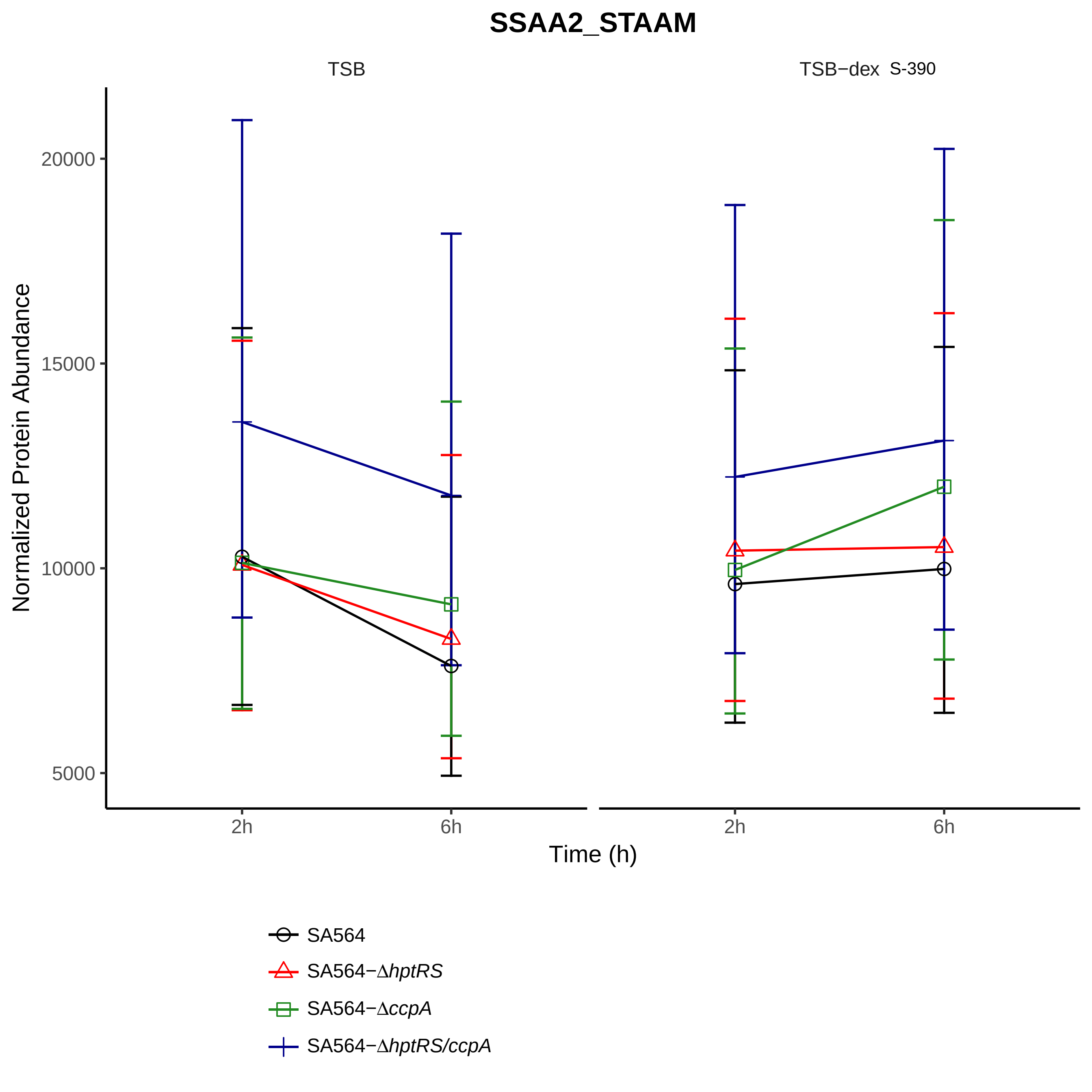

SSAA2_STAAM
TSB
TSB−dex
S-390
Normalized Protein Abundance
20000
15000
10000
5000
2h
6h
2h
6h
Time (h)
SA564
SA564−Δ*hptRS*
SA564−Δ*ccpA*
SA564−Δ*hptRS/ccpA*

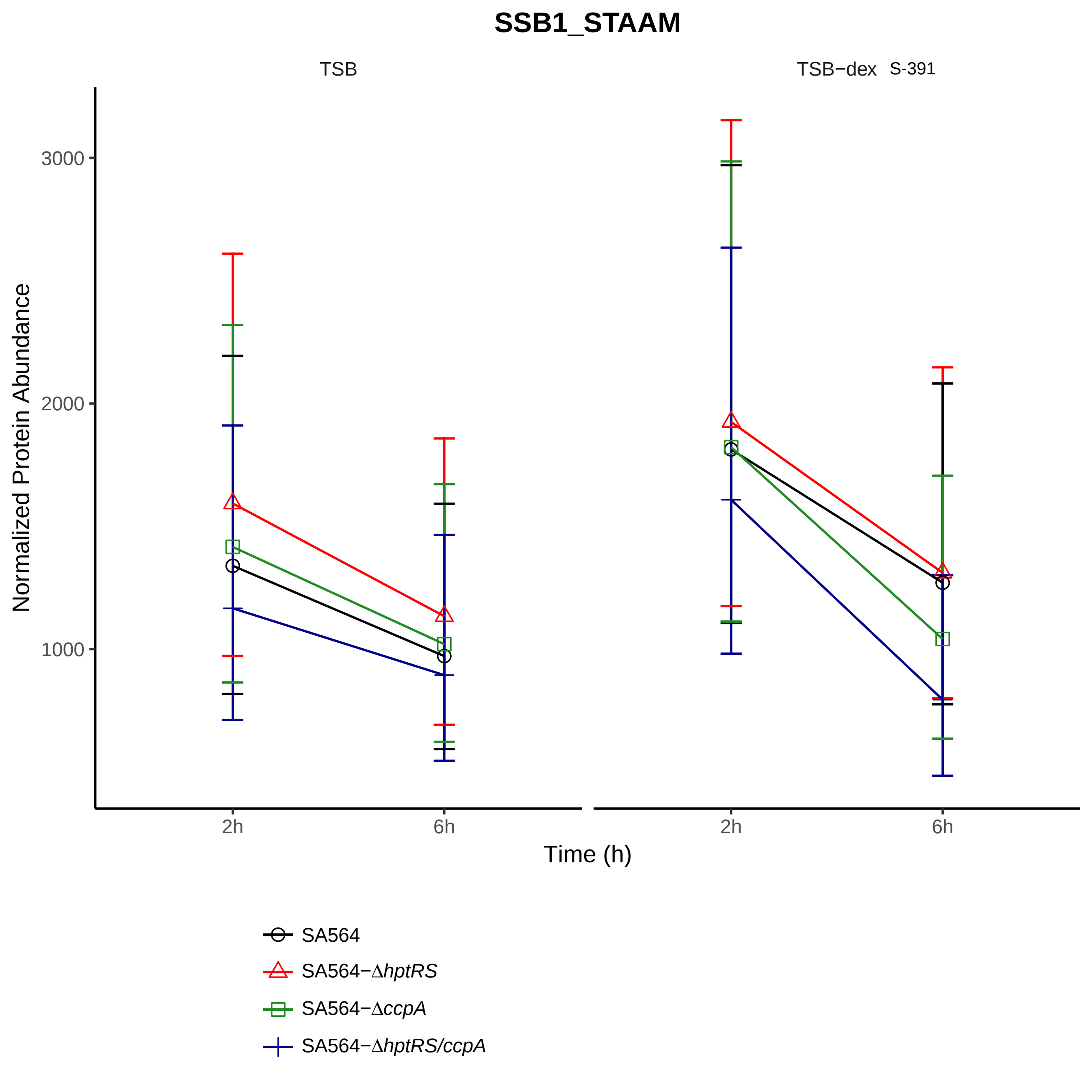

SSB1_STAAM
TSB
TSB−dex
S-391
Normalized Protein Abundance
3000
2000
1000
2h
6h
2h
6h
Time (h)
SA564
SA564−Δ*hptRS*
SA564−Δ*ccpA*
SA564−Δ*hptRS/ccpA*

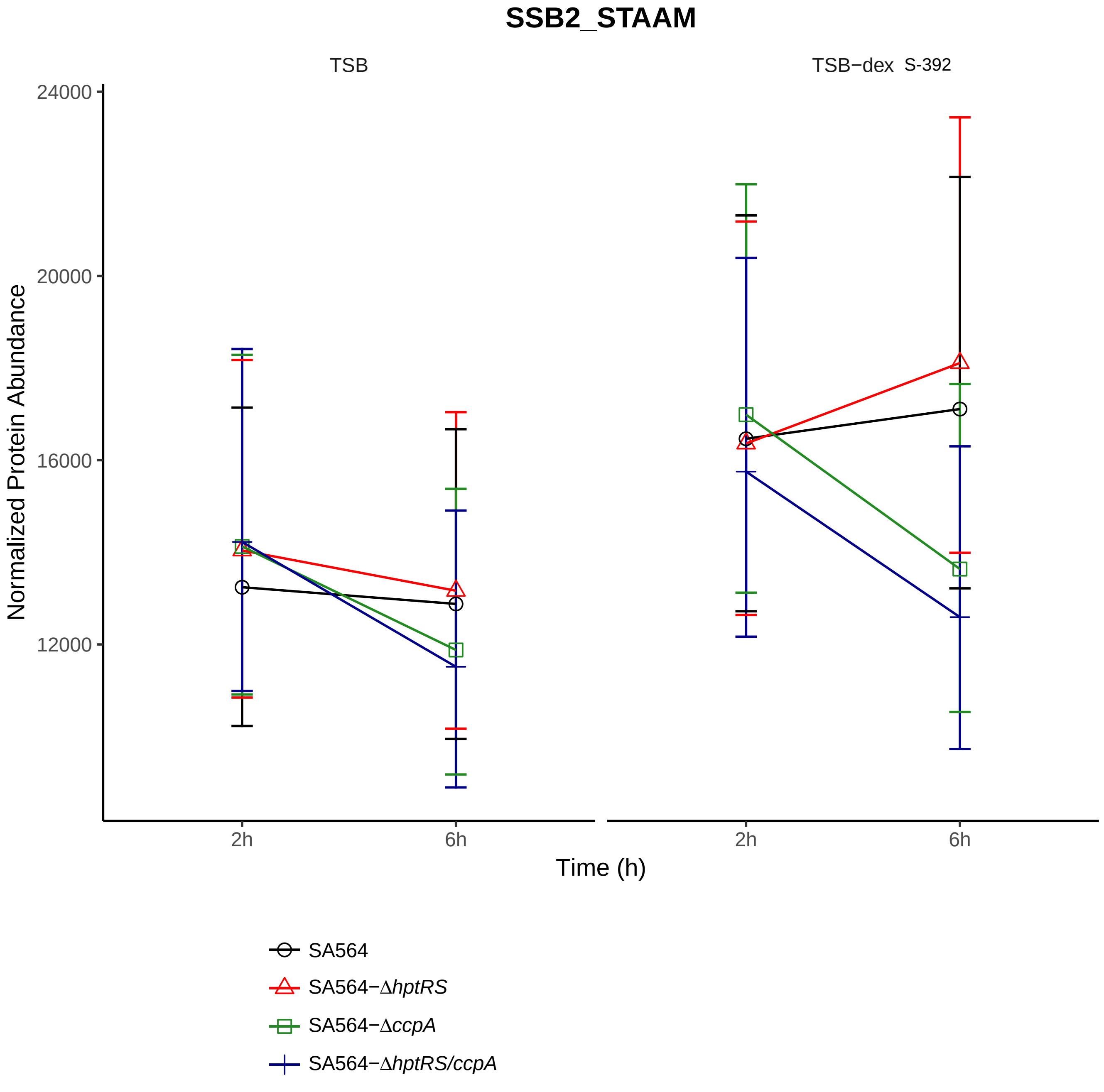
SSB2_STAAM
TSB
TSB−dex
S-392
24000
20000
16000
12000
Normalized Protein Abundance
2h
6h
2h
6h
Time (h)
SA564
SA564−ΔhptRS
SA564−ΔccpA
SA564−ΔhptRS/ccpA

SUCC_STAAM

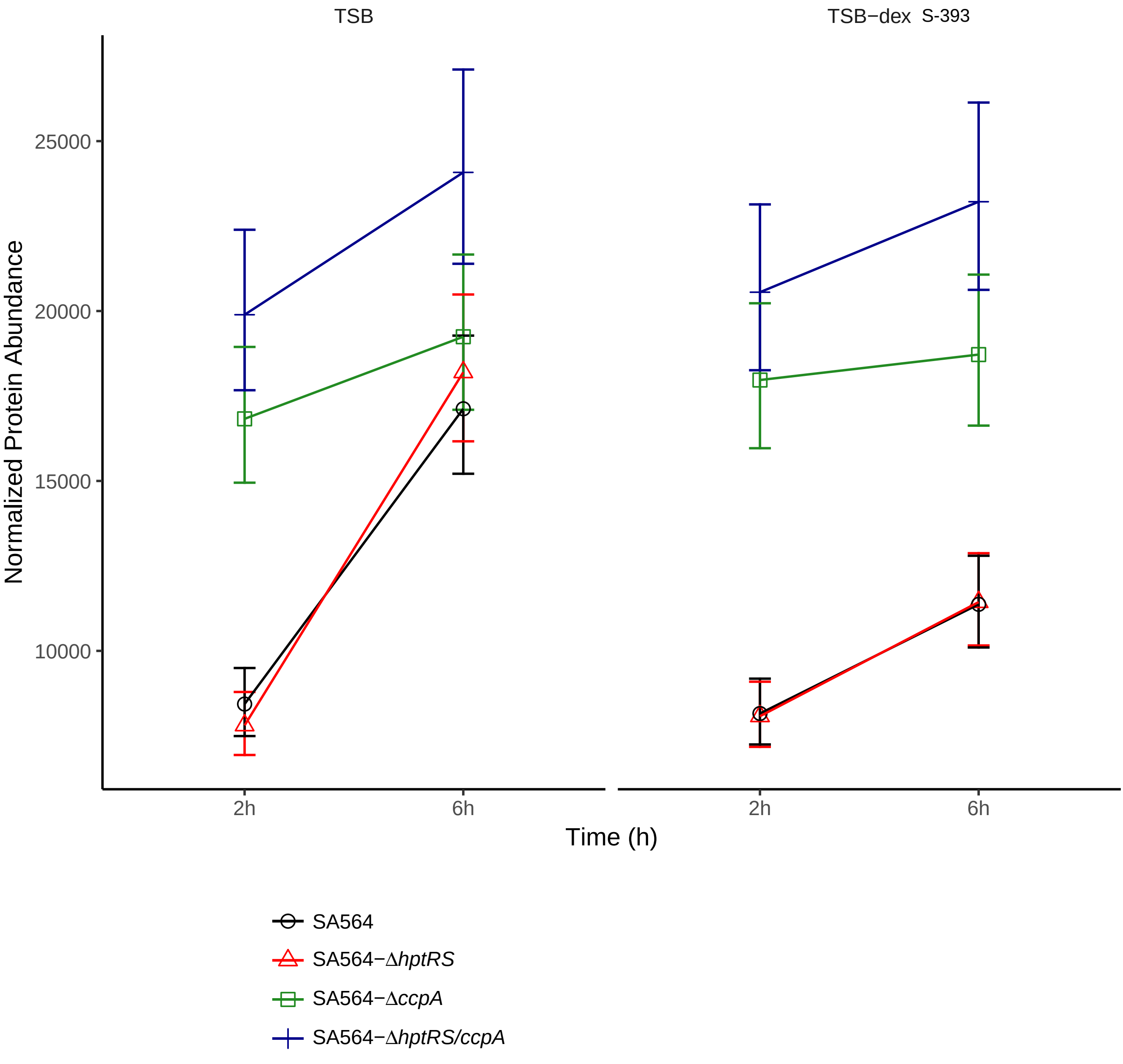

TSB
TSB−dex
S-393
Normalized Protein Abundance
25000
20000
15000
10000
2h
6h
2h
6h
Time (h)
SA564
SA564−Δ*hptRS*
SA564−Δ*ccpA*
SA564−Δ*hptRS/ccpA*

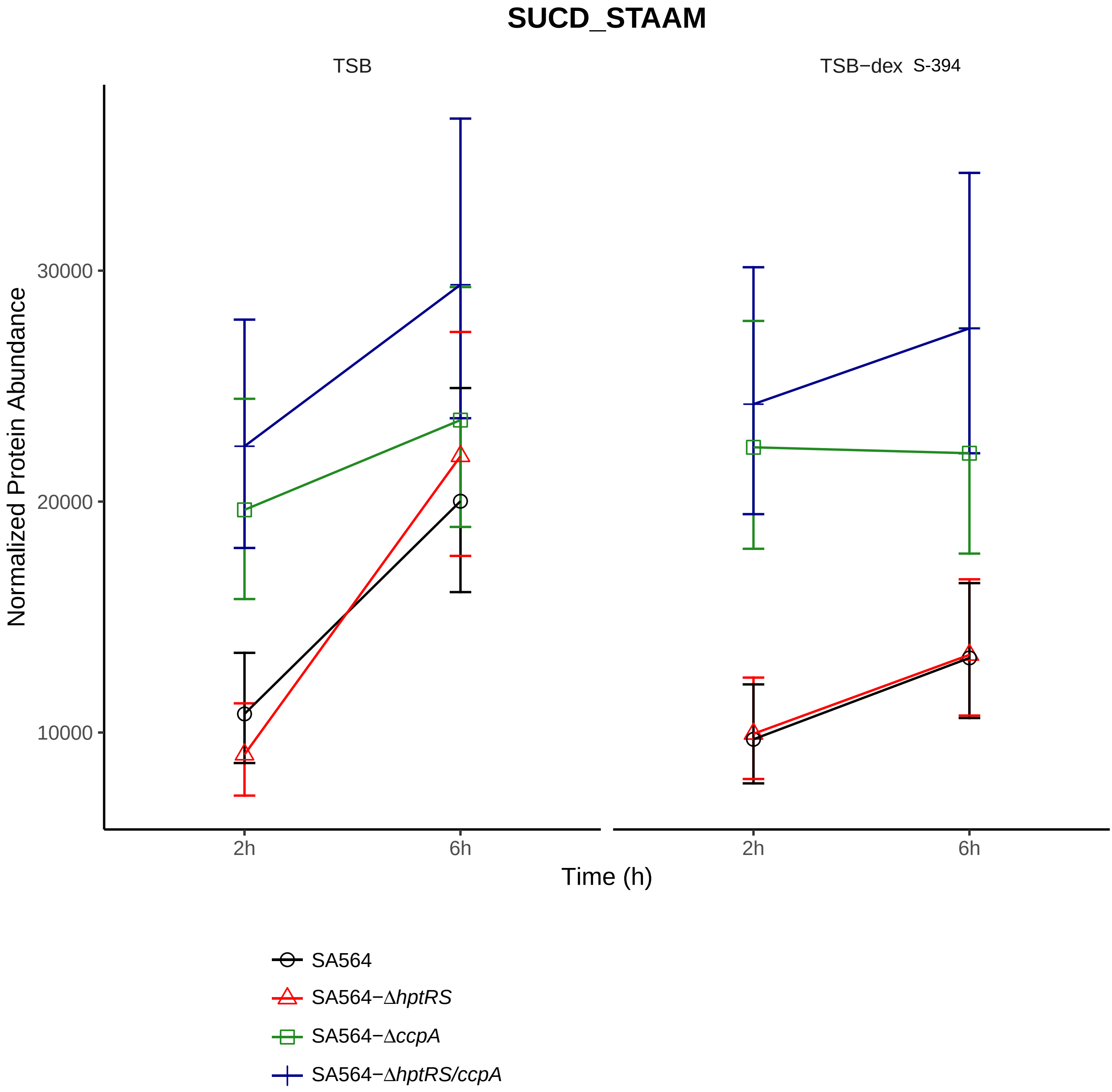

SUCD_STAAM
TSB
TSB−dex
S-394
Normalized Protein Abundance
30000
20000
10000
2h
6h
2h
6h
Time (h)
SA564
SA564−Δ*hptRS*
SA564−Δ*ccpA*
SA564−Δ*hptRS/ccpA*

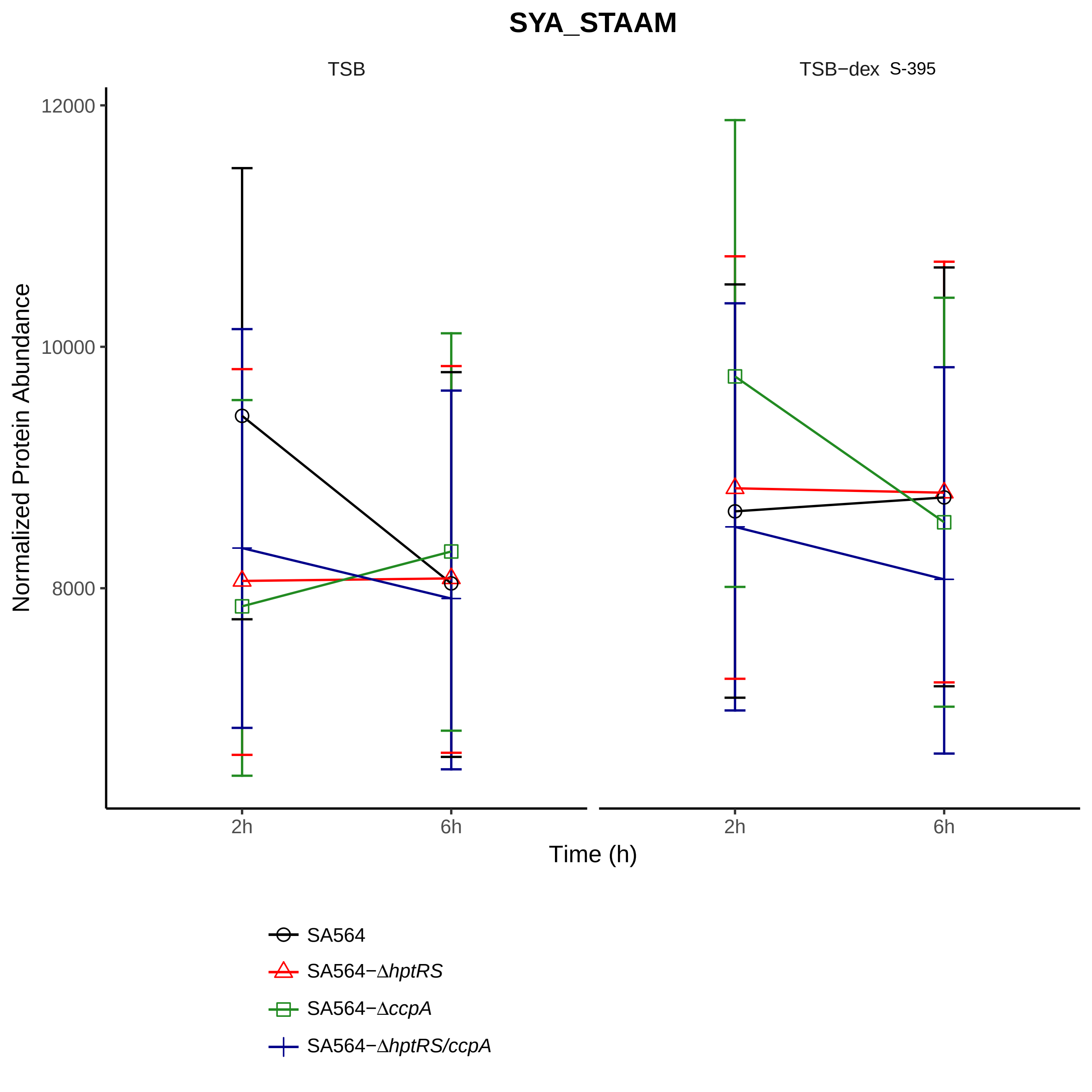

SYA_STAAM
TSB
TSB−dex
S-395
12000
10000
8000
Normalized Protein Abundance
2h
6h
2h
6h
Time (h)
SA564
SA564−Δ*hptRS*
SA564−Δ*ccpA*
SA564−Δ*hptRS/ccpA*

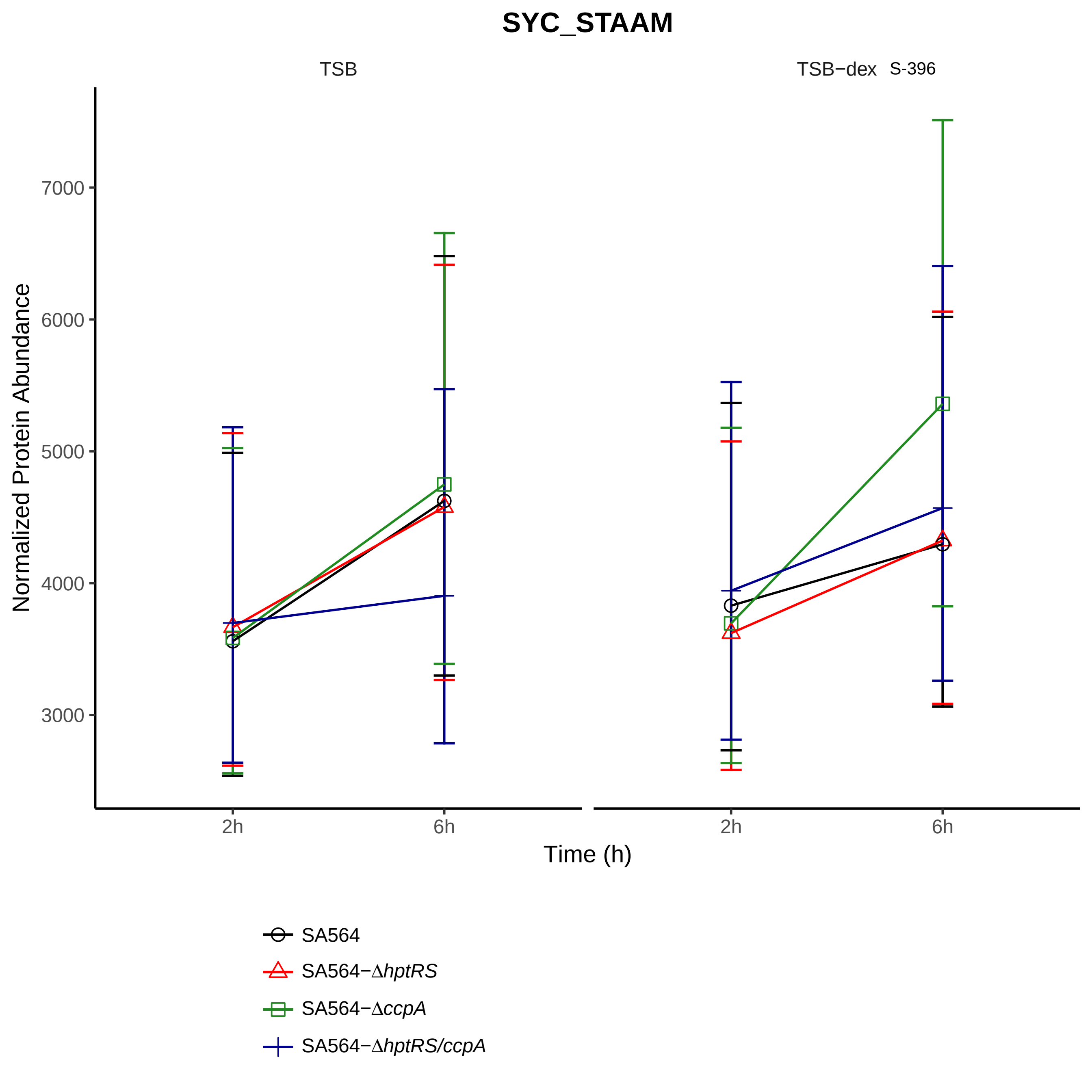
SYC_STAAM
TSB
TSB−dex
S-396
Normalized Protein Abundance
7000
6000
5000
4000
3000
2h
6h
2h
6h
Time (h)
SA564
SA564−ΔhptRS
SA564−ΔccpA
SA564−ΔhptRS/ccpA

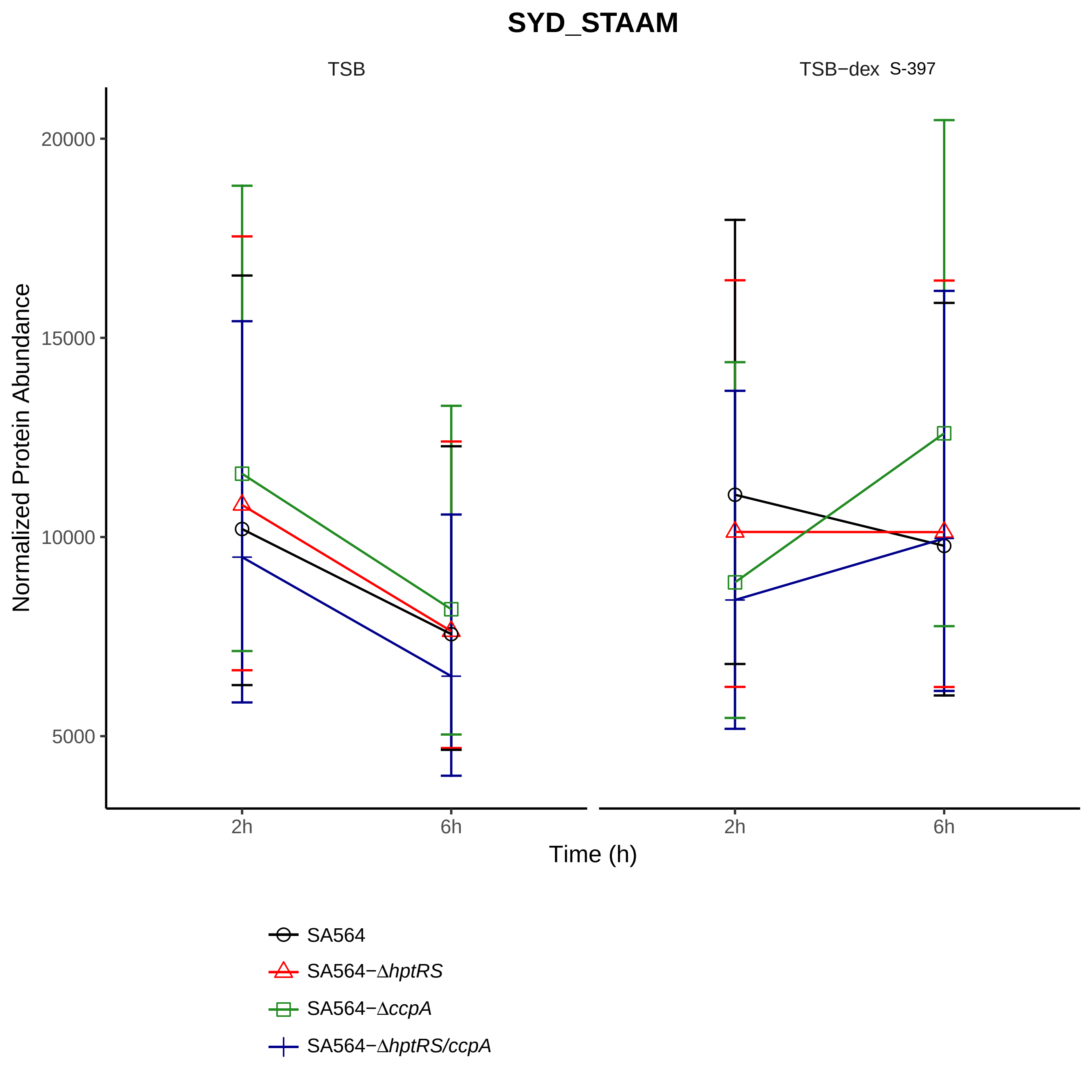
SYD_STAAM
TSB
TSB−dex
S-397
20000
15000
10000
5000
Normalized Protein Abundance
2h
6h
2h
6h
Time (h)
SA564
SA564−ΔhptRS
SA564−ΔccpA
SA564−ΔhptRS/ccpA

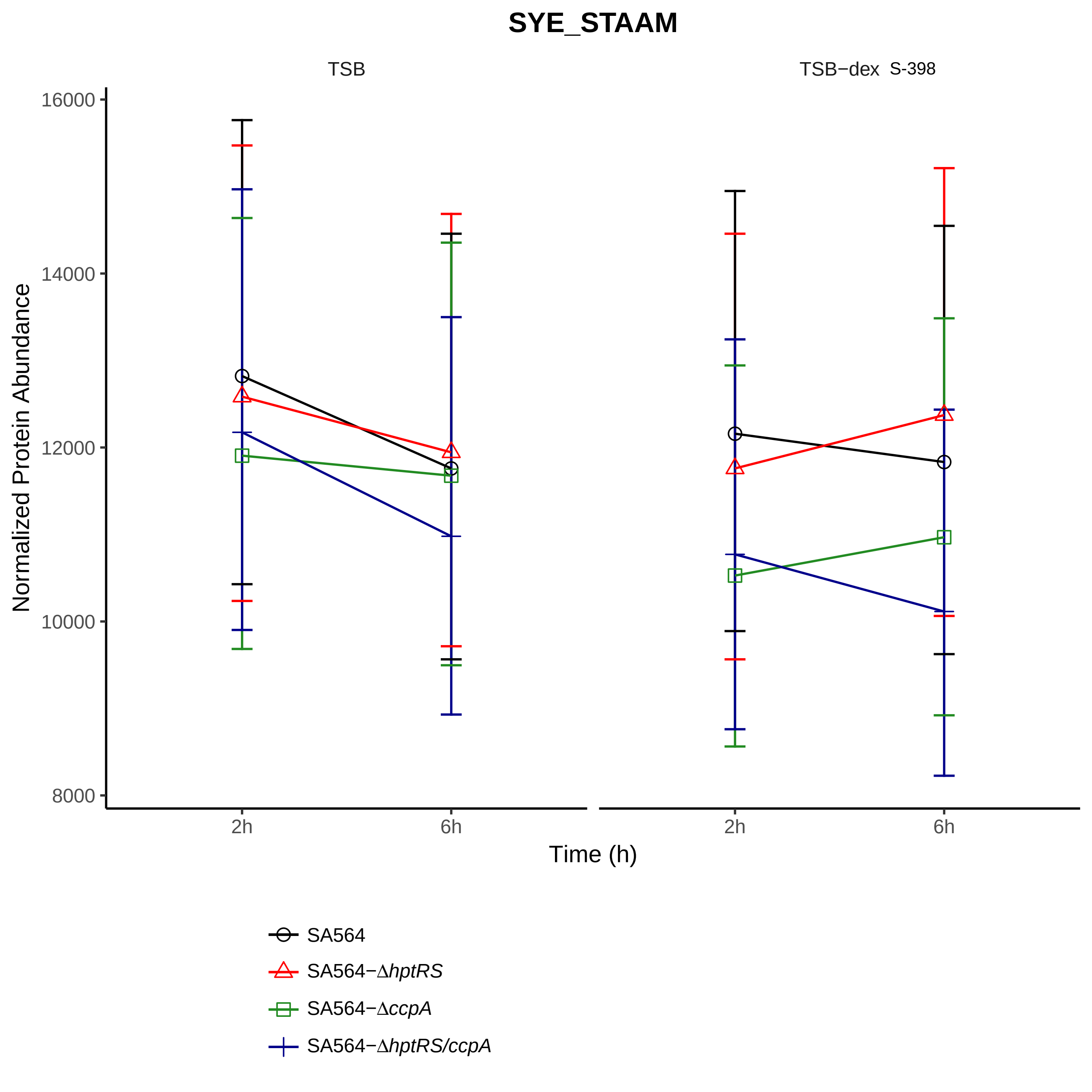

SYE_STAAM
TSB
TSB−dex
S-398
16000
14000
12000
10000
8000
Normalized Protein Abundance
2h
6h
2h
6h
Time (h)
SA564
SA564−ΔhptRS
SA564−ΔccpA
SA564−ΔhptRS/ccpA

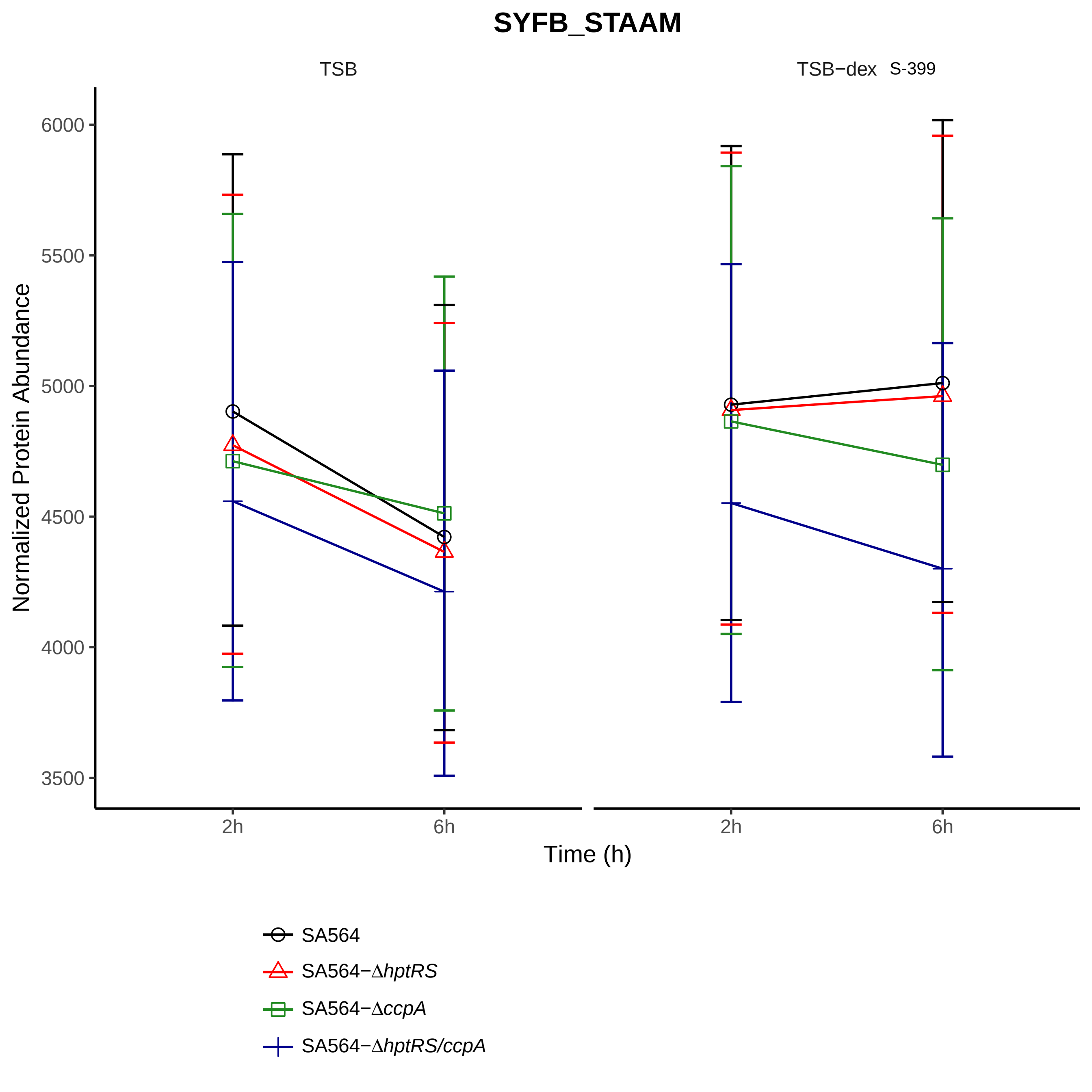

SYFB_STAAM
TSB
TSB−dex
S-399
6000
5500
5000
4500
4000
3500
Normalized Protein Abundance
2h
6h
2h
6h
Time (h)
SA564
SA564−ΔhptRS
SA564−ΔccpA
SA564−ΔhptRS/ccpA

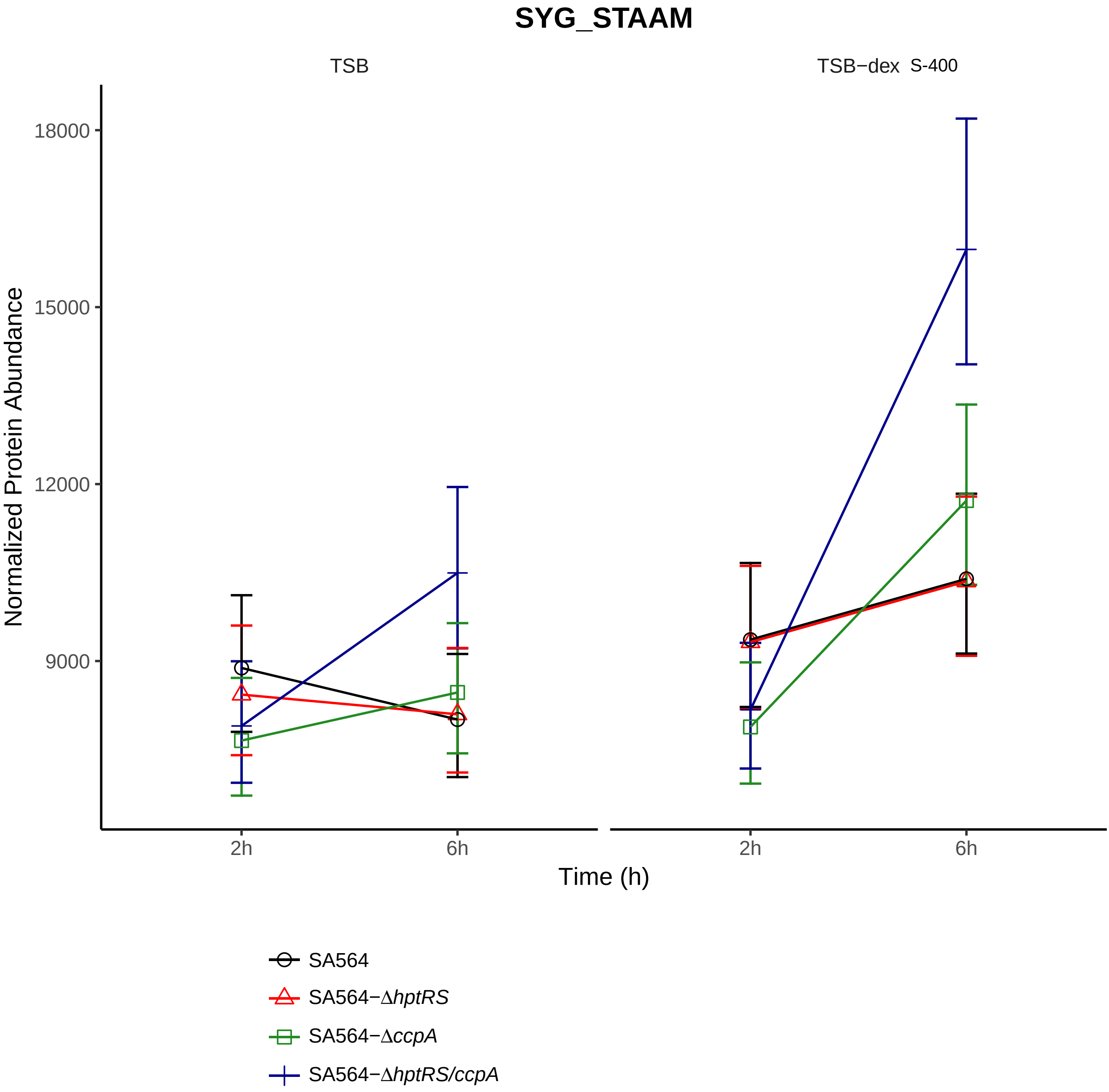

SYG_STAAM
TSB
TSB−dex
S-400
Normalized Protein Abundance
18000
15000
12000
9000
2h
6h
2h
6h
Time (h)
SA564
SA564−ΔhptRS
SA564−ΔccpA
SA564−ΔhptRS/ccpA

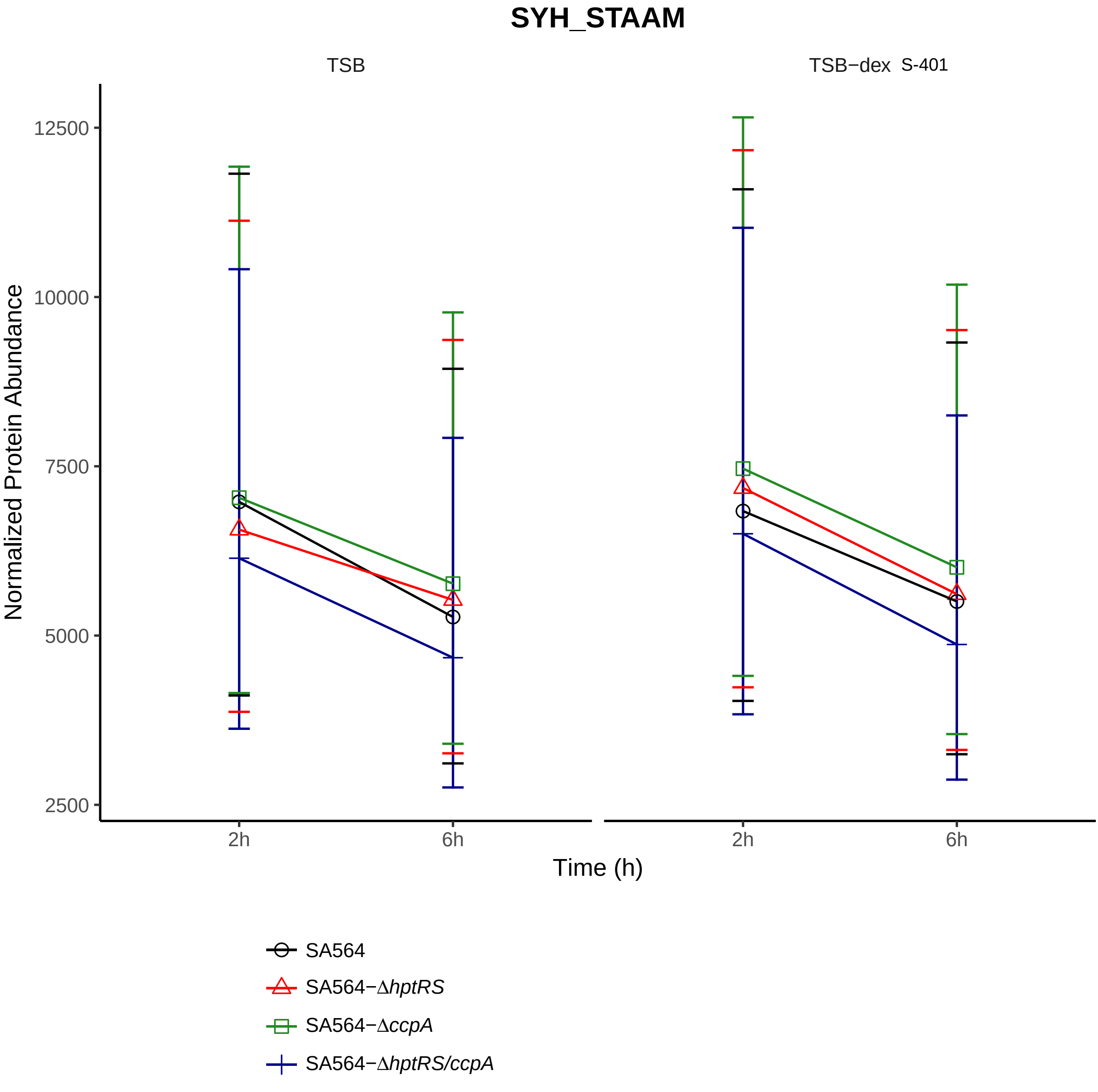
SYH_STAAM
TSB
TSB−dex
S-401
Normalized Protein Abundance
12500
10000
7500
5000
2500
2h
6h
2h
6h
Time (h)
SA564
SA564−ΔhptRS
SA564−ΔccpA
SA564−ΔhptRS/ccpA

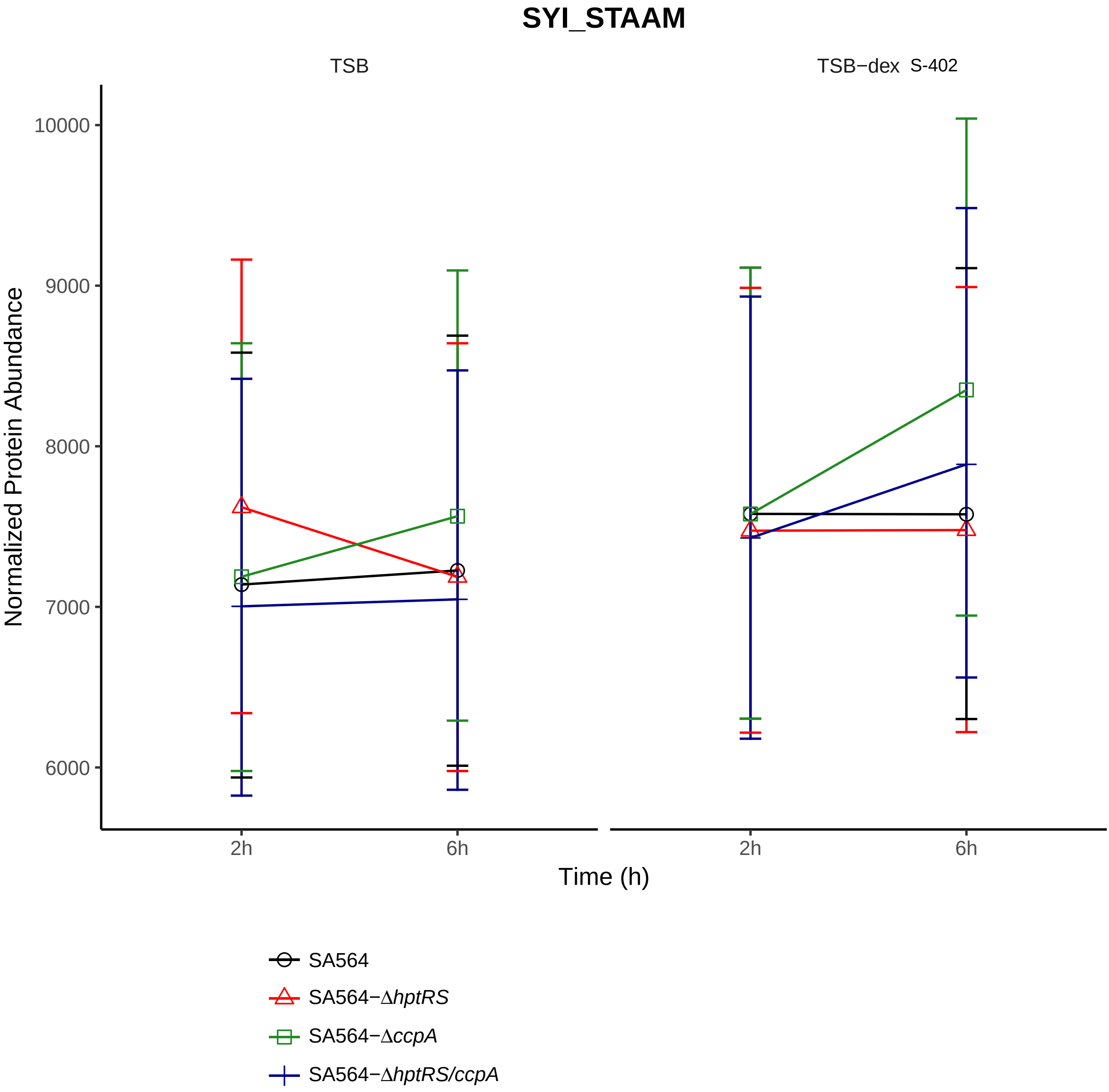
SYI_STAAM
TSB
TSB−dex
S-402
Normalized Protein Abundance
10000
9000
8000
7000
6000
2h
6h
2h
6h
Time (h)
SA564
SA564−Δ*hptRS*
SA564−Δ*ccpA*
SA564−Δ*hptRS/ccpA*

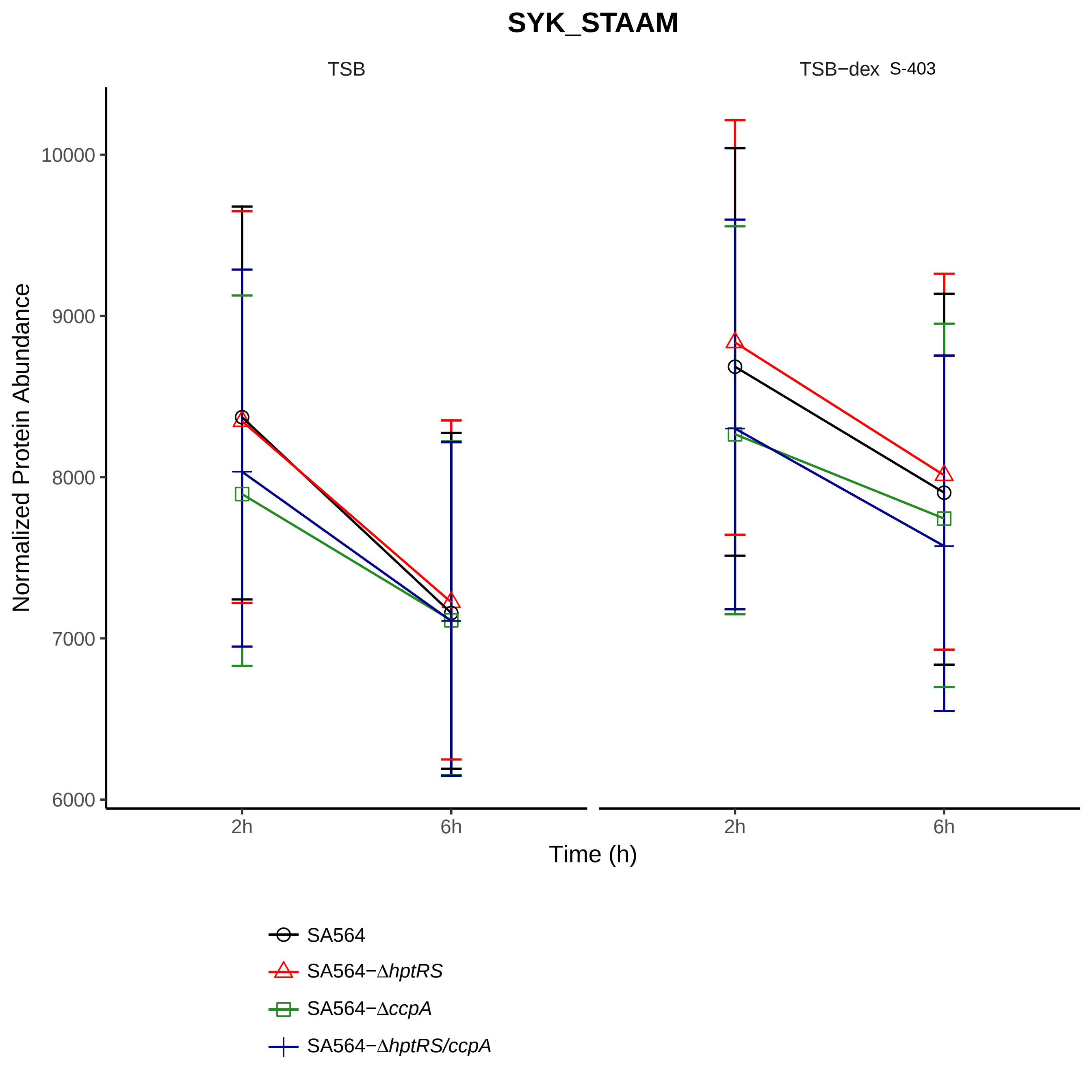

SYK_STAAM
TSB
TSB−dex
S-403
Normalized Protein Abundance
10000
9000
8000
7000
6000
2h
6h
2h
6h
Time (h)
SA564
SA564−Δ*hptRS*
SA564−Δ*ccpA*
SA564−Δ*hptRS/ccpA*

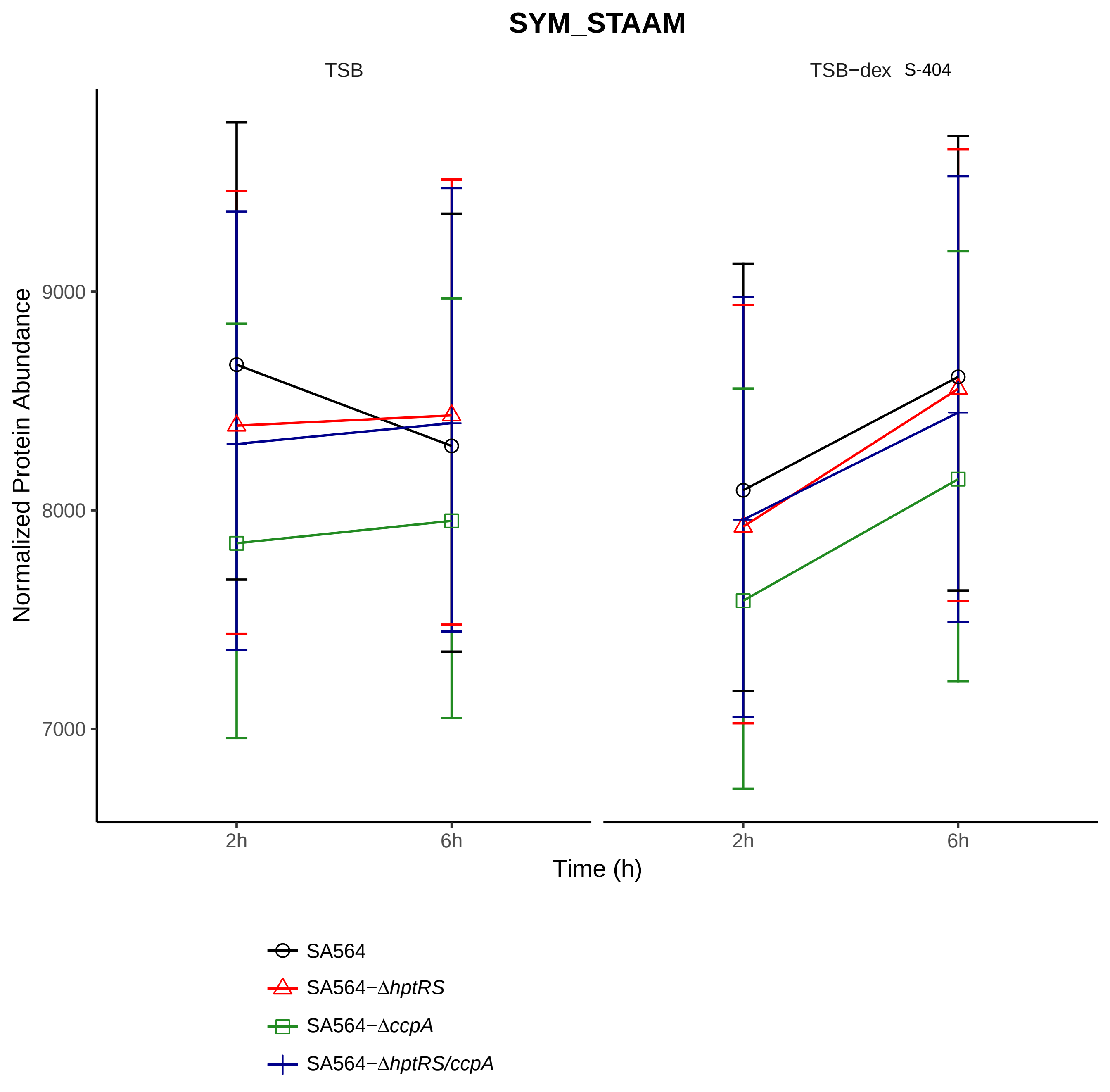
SYM_STAAM
TSB
TSB−dex
S-404
Normalized Protein Abundance
9000
8000
7000
2h
6h
2h
6h
Time (h)
SA564
SA564−Δ*hptRS*
SA564−Δ*ccpA*
SA564−Δ*hptRS/ccpA*

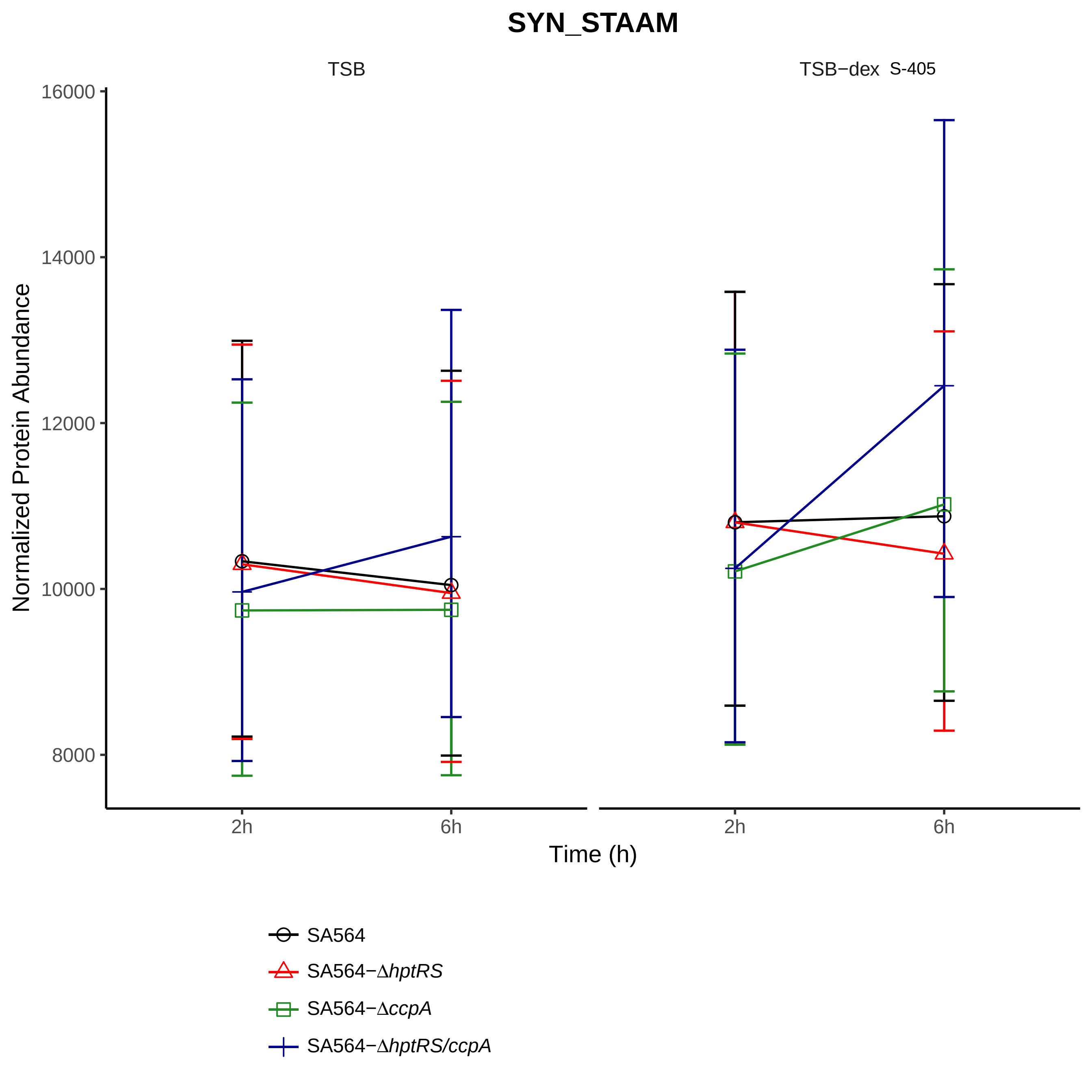

SYN_STAAM
TSB
TSB−dex
S-405
16000
14000
12000
10000
8000
Normalized Protein Abundance
2h
6h
2h
6h
Time (h)
SA564
SA564−Δ*hptRS*
SA564−Δ*ccpA*
SA564−Δ*hptRS/ccpA*

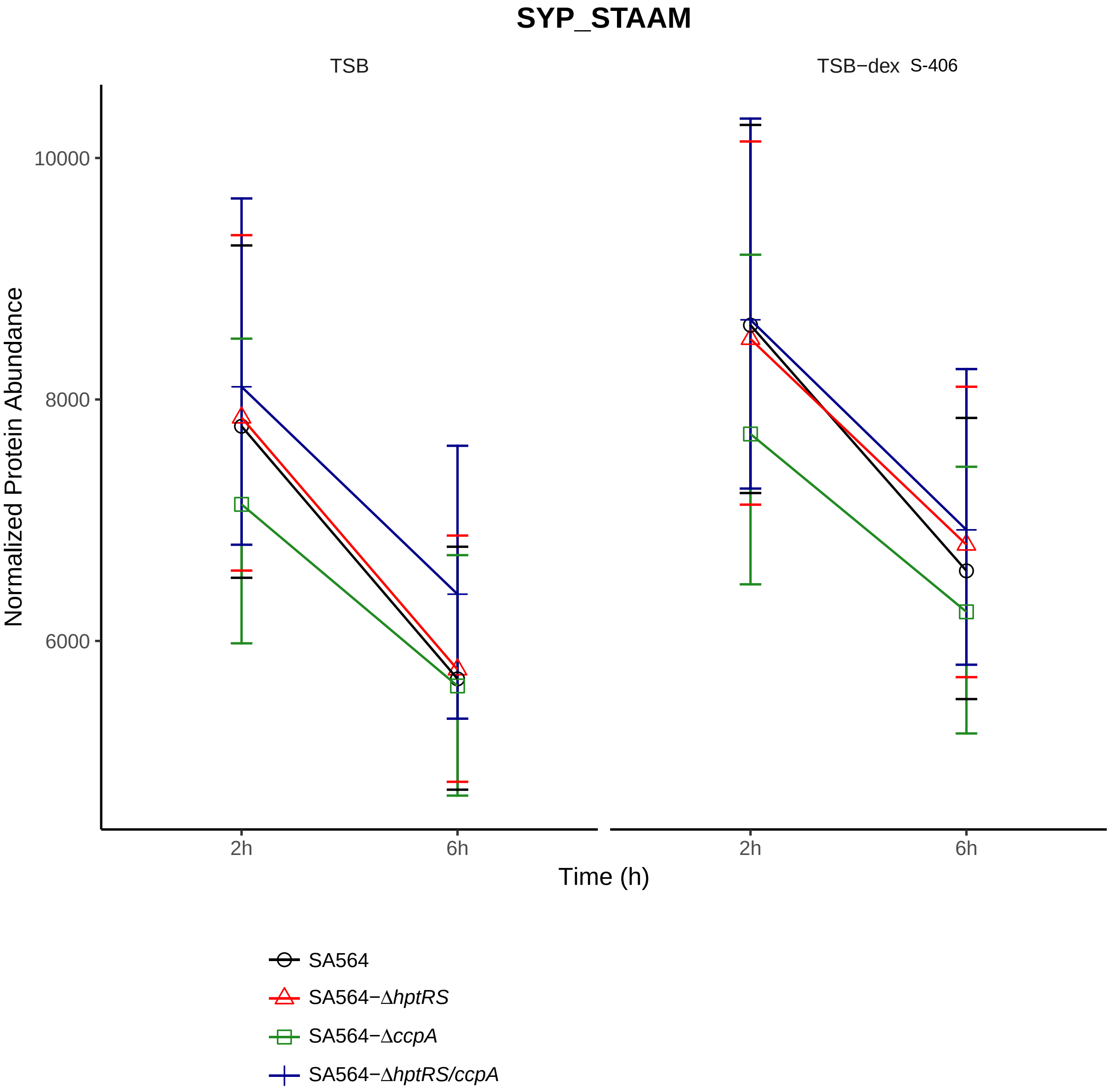

SYP_STAAM
TSB
TSB−dex
S-406
Normalized Protein Abundance
10000
8000
6000
2h
6h
2h
6h
Time (h)
SA564
SA564−Δ*hptRS*
SA564−Δ*ccpA*
SA564−Δ*hptRS/ccpA*

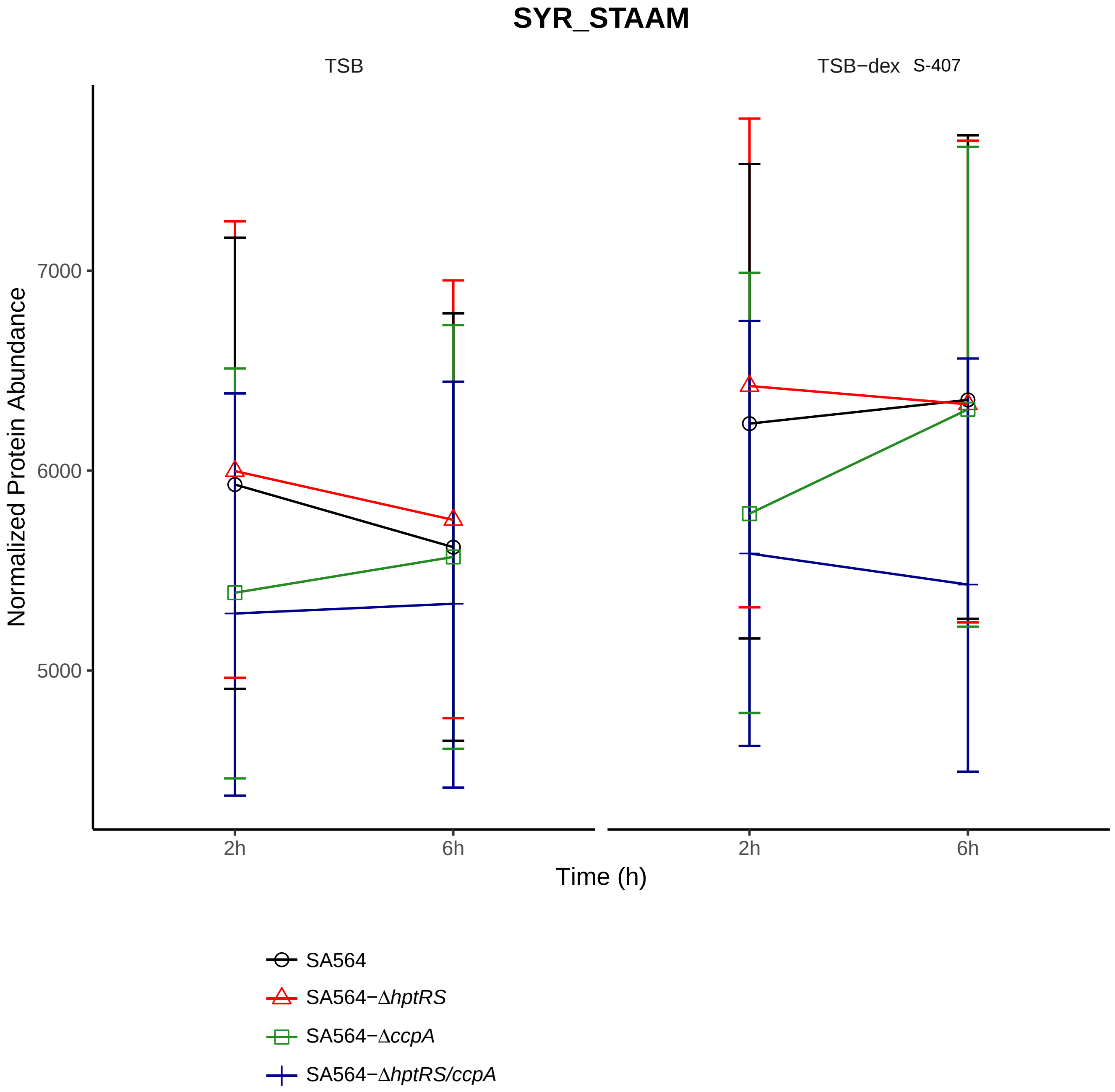
SYR_STAAM
TSB
TSB−dex
S-407
Normalized Protein Abundance
7000
6000
5000
2h
6h
2h
6h
Time (h)
SA564
SA564−Δ*hptRS*
SA564−Δ*ccpA*
SA564−Δ*hptRS/ccpA*

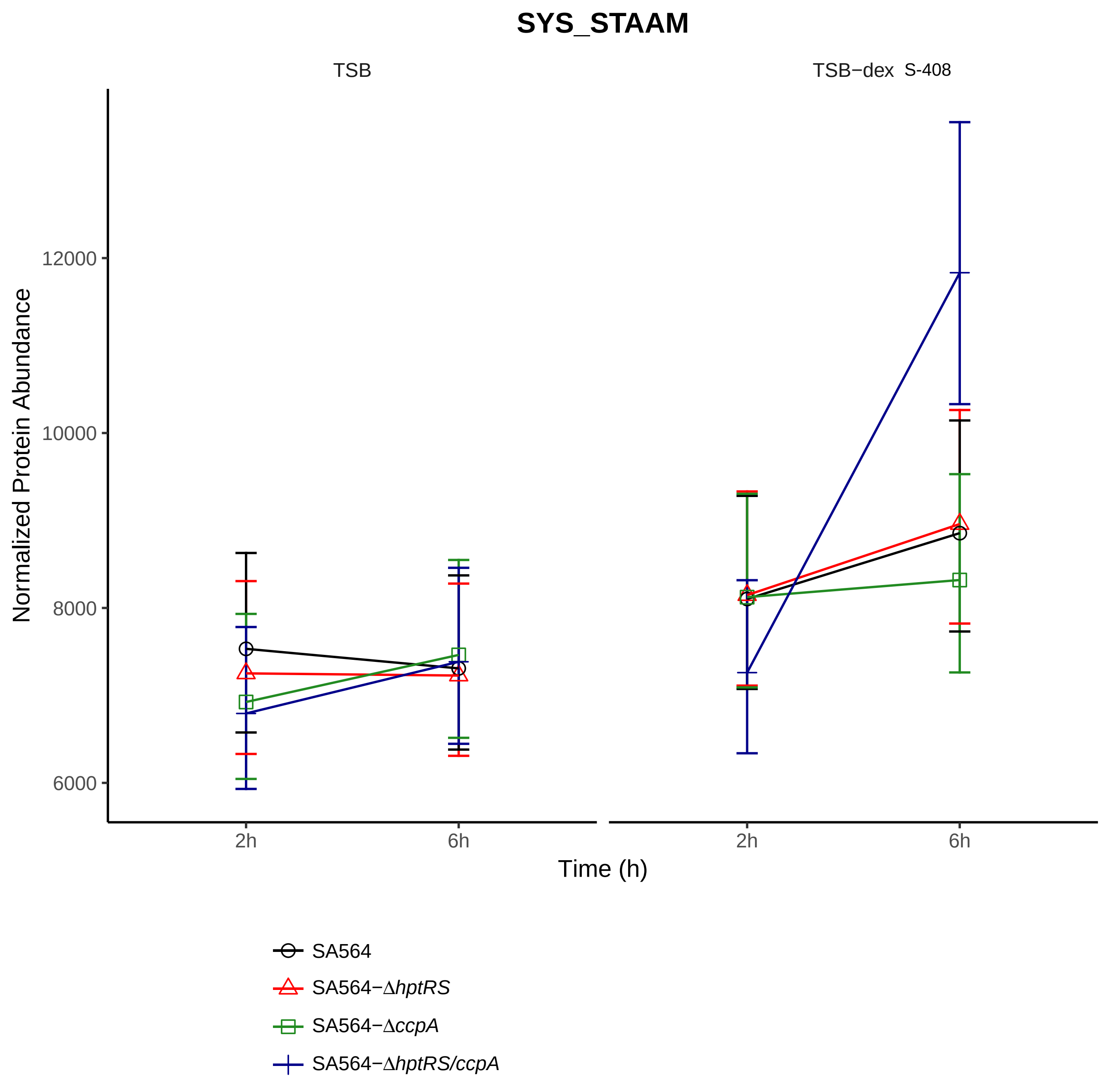

SYS_STAAM
TSB
TSB−dex
S-408
Normalized Protein Abundance
12000
10000
8000
6000
2h
6h
2h
6h
Time (h)
SA564
SA564−ΔhptRS
SA564−ΔccpA
SA564−ΔhptRS/ccpA

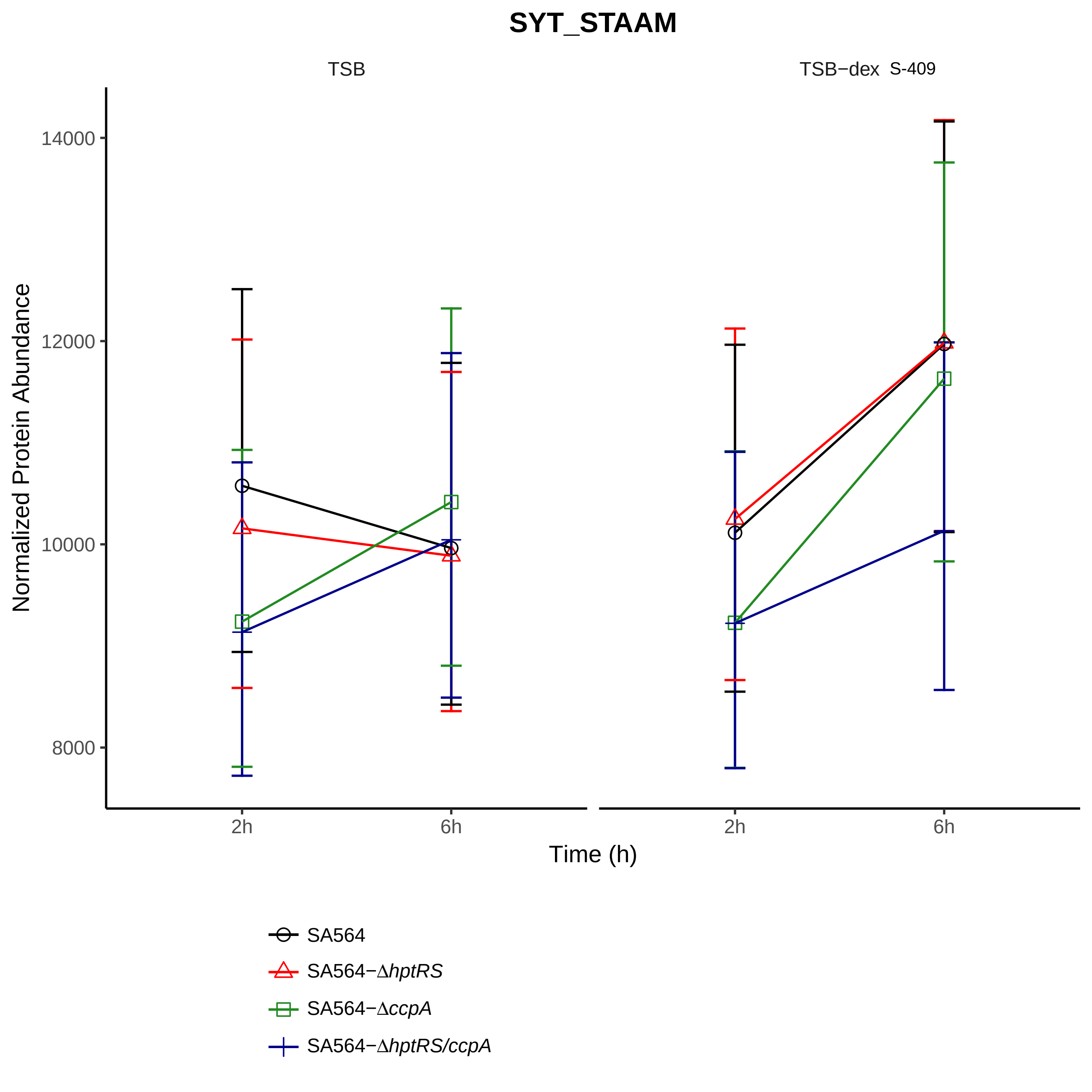

SYT_STAAM
TSB
TSB−dex
S-409
Normalized Protein Abundance
14000
12000
10000
8000
2h
6h
2h
6h
Time (h)
SA564
SA564−ΔhptRS
SA564−ΔccpA
SA564−ΔhptRS/ccpA

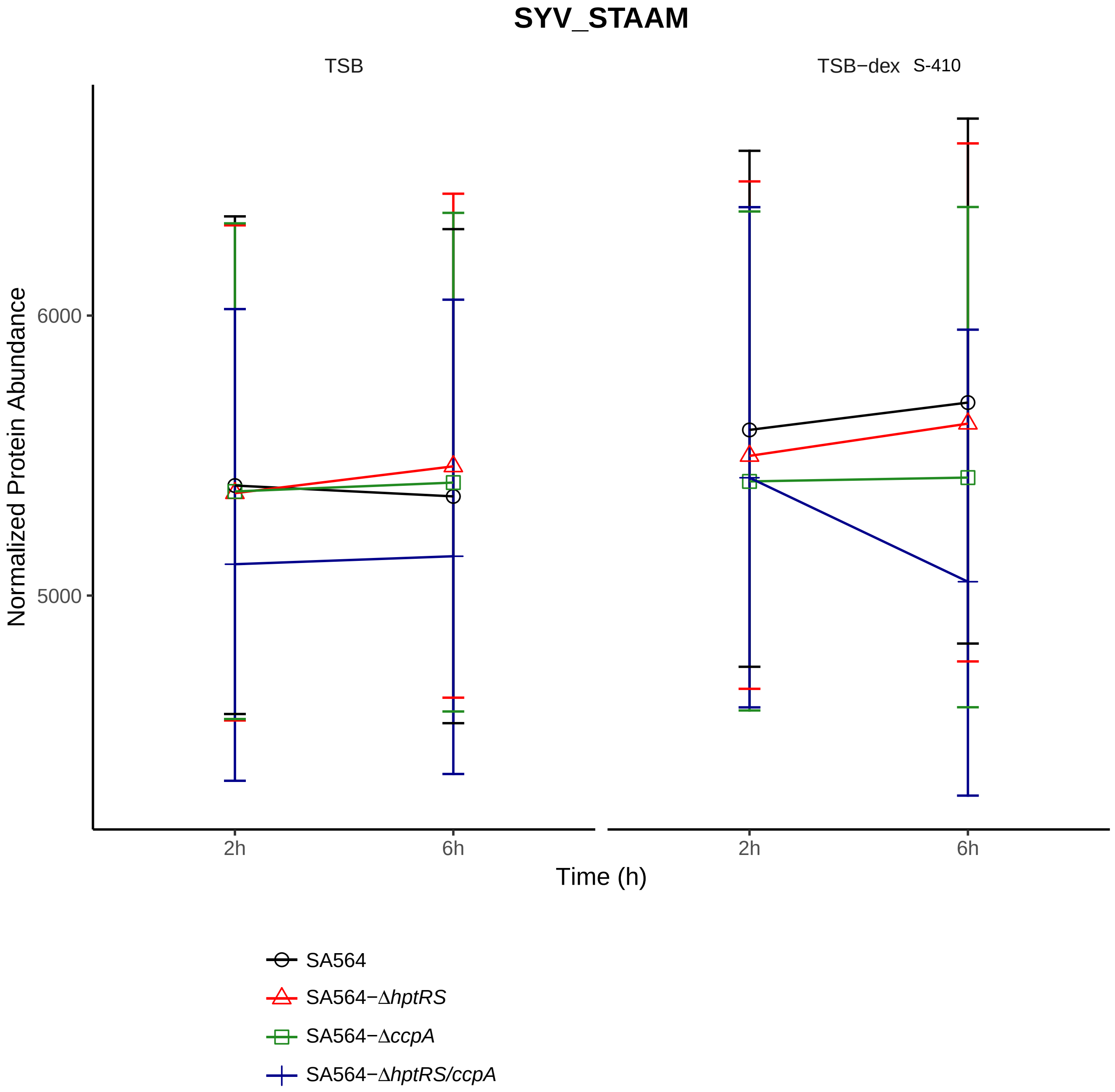

SYV_STAAM
TSB
TSB−dex
S-410
Normalized Protein Abundance
6000
5000
2h
6h
2h
6h
Time (h)
SA564
SA564−ΔhptRS
SA564−ΔccpA
SA564−ΔhptRS/ccpA

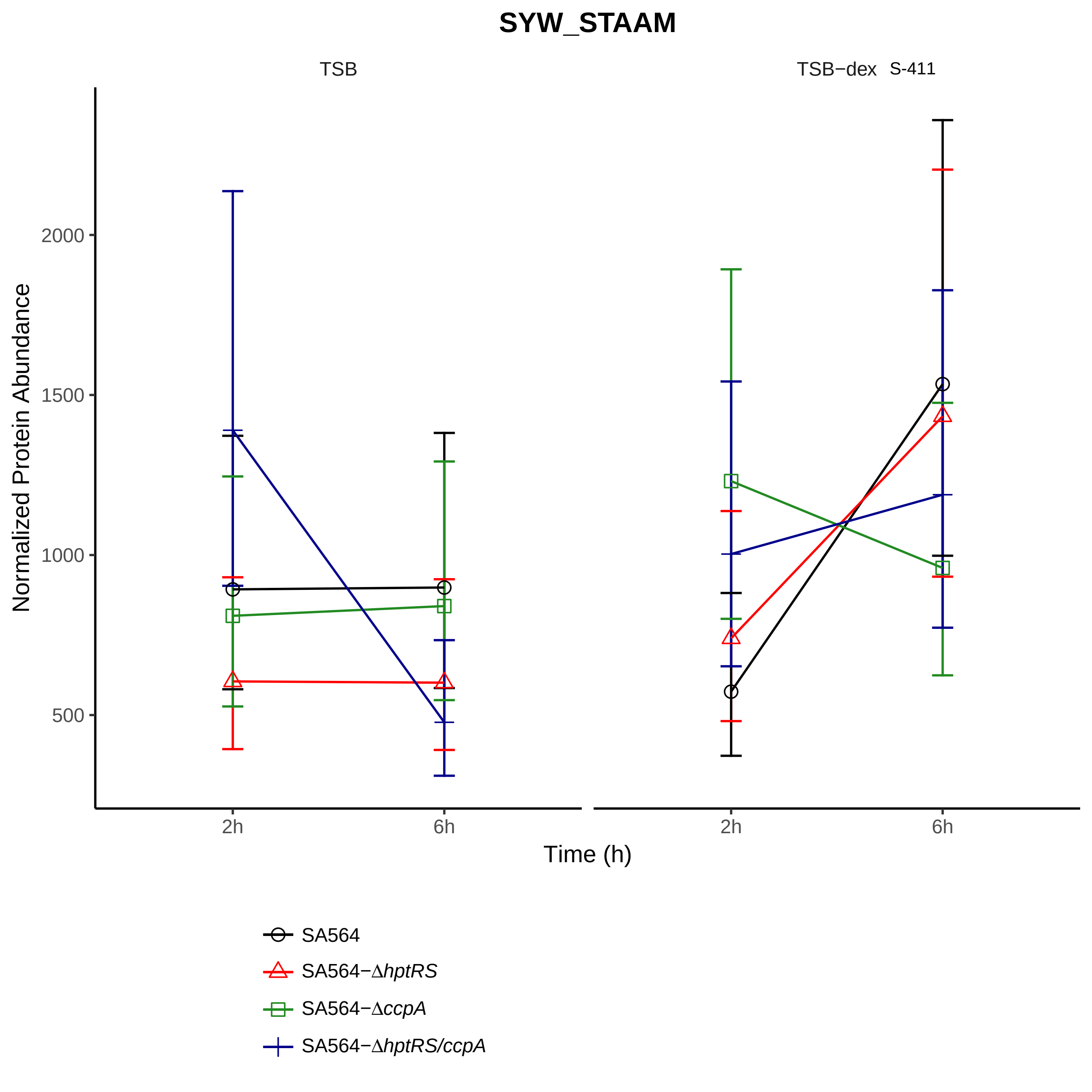

SYW_STAAM
TSB
TSB−dex
S-411
Normalized Protein Abundance
2000
1500
1000
500
2h
6h
2h
6h
Time (h)
SA564
SA564−Δ*hptRS*
SA564−Δ*ccpA*
SA564−Δ*hptRS/ccpA*

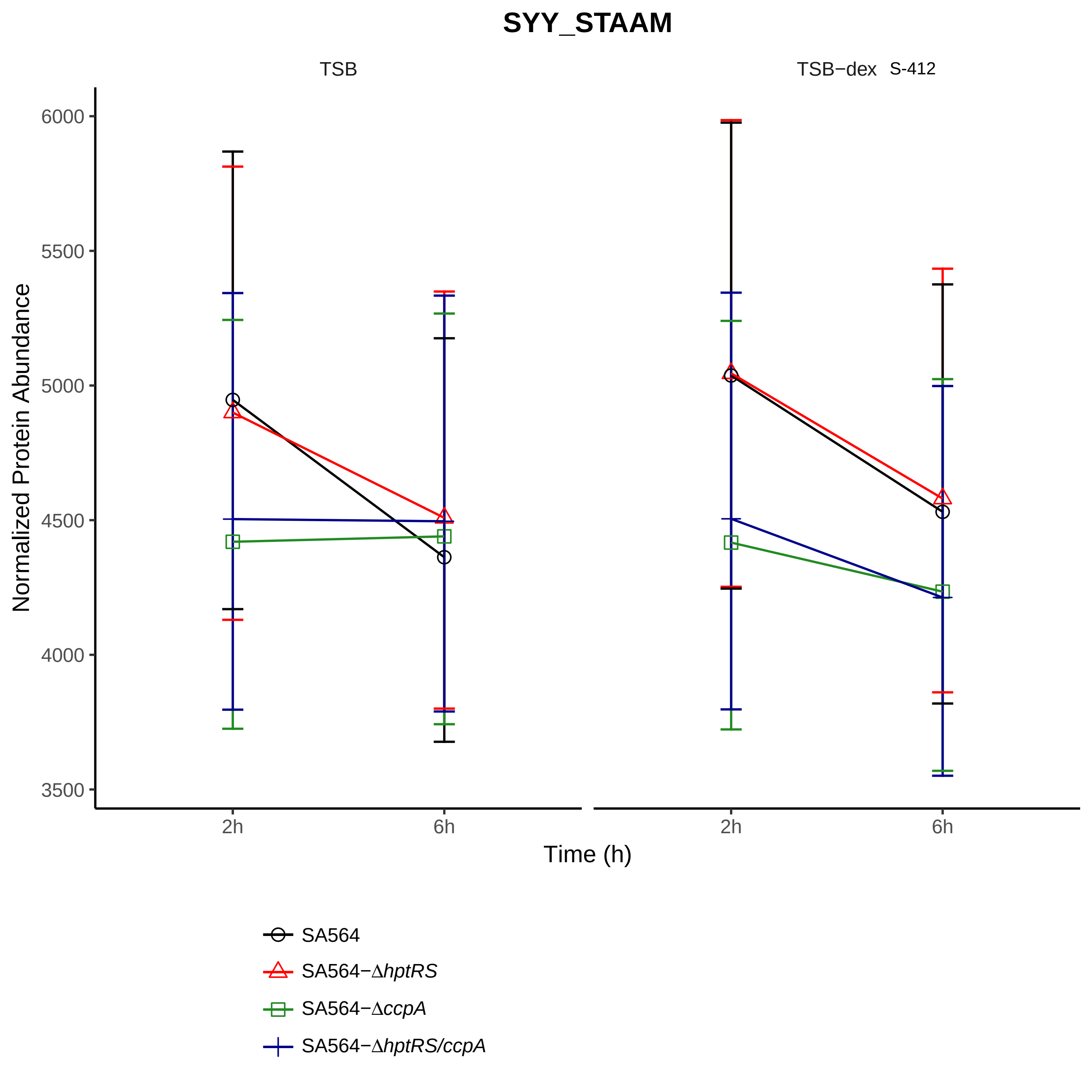

SYY_STAAM
TSB
TSB−dex
S-412
Normalized Protein Abundance
6000
5500
5000
4500
4000
3500
2h
6h
2h
6h
Time (h)
SA564
SA564−Δ*hptRS*
SA564−Δ*ccpA*
SA564−Δ*hptRS/ccpA*

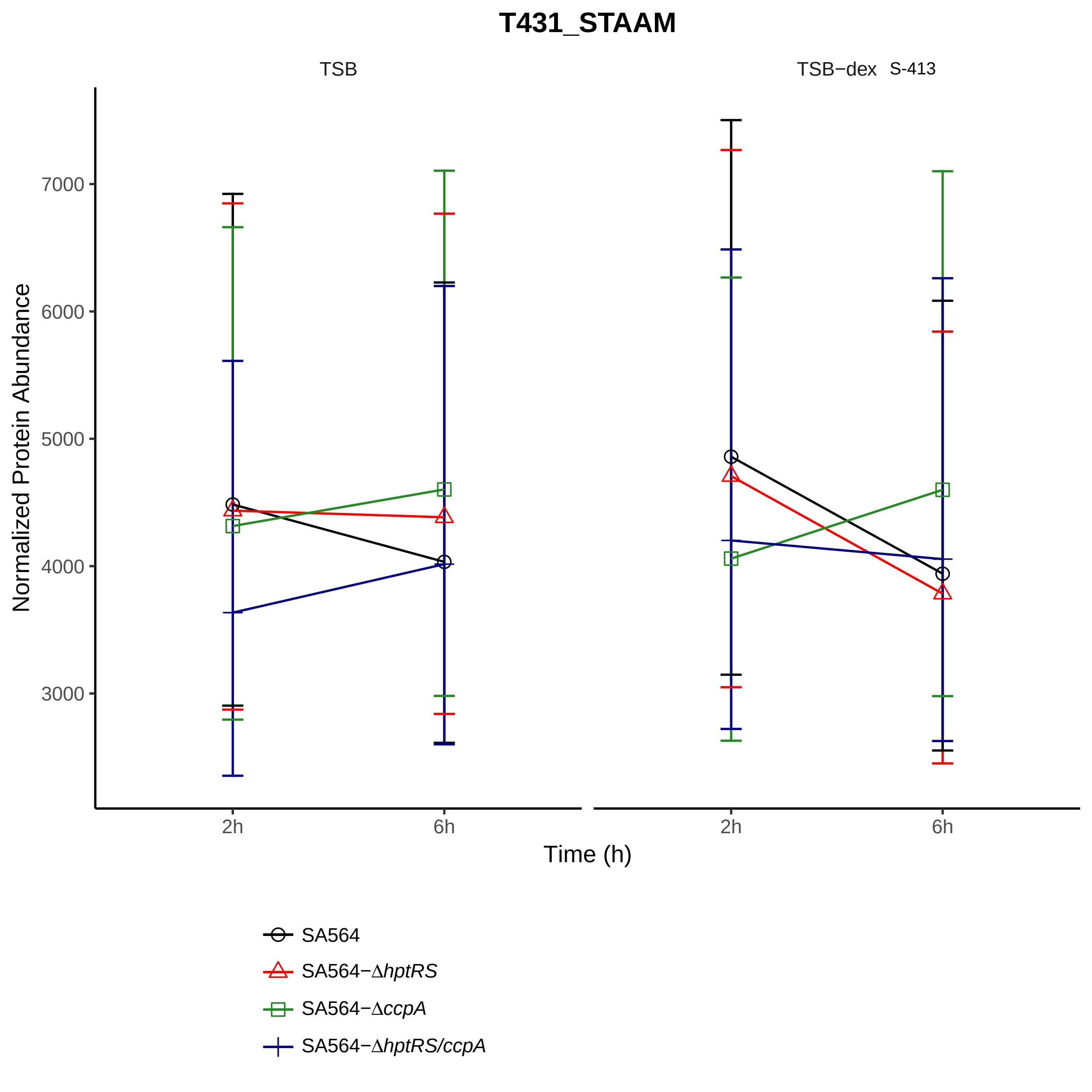

T431_STAAM
TSB
TSB−dex
S-413
Normalized Protein Abundance
7000
6000
5000
4000
3000
2h
6h
2h
6h
Time (h)
SA564
SA564−Δ*hptRS*
SA564−Δ*ccpA*
SA564−Δ*hptRS/ccpA*

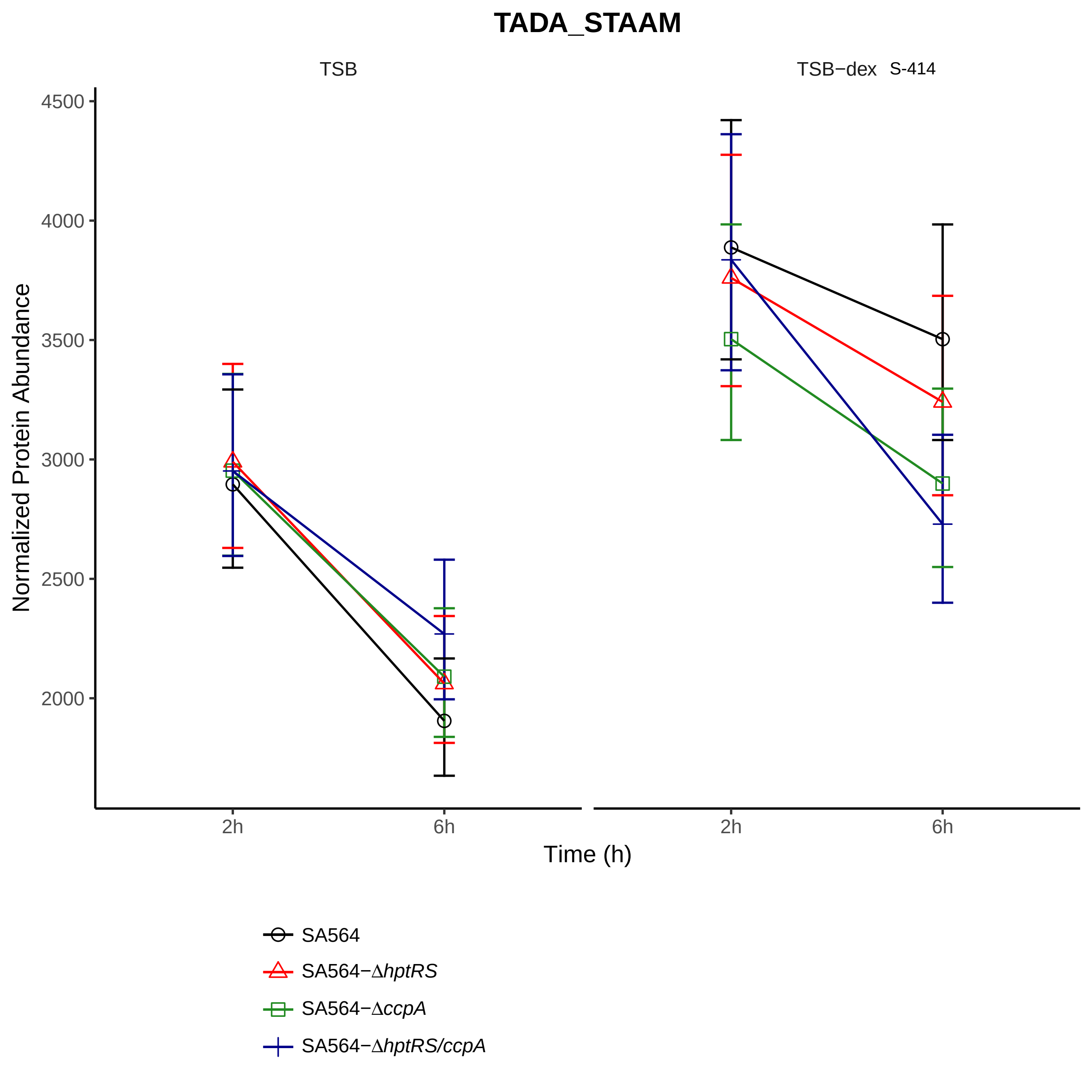

TADA_STAAM
TSB
TSB−dex
S-414
Normalized Protein Abundance
4500
4000
3500
3000
2500
2000
2h
6h
2h
6h
Time (h)
SA564
SA564−ΔhptRS
SA564−ΔccpA
SA564−ΔhptRS/ccpA

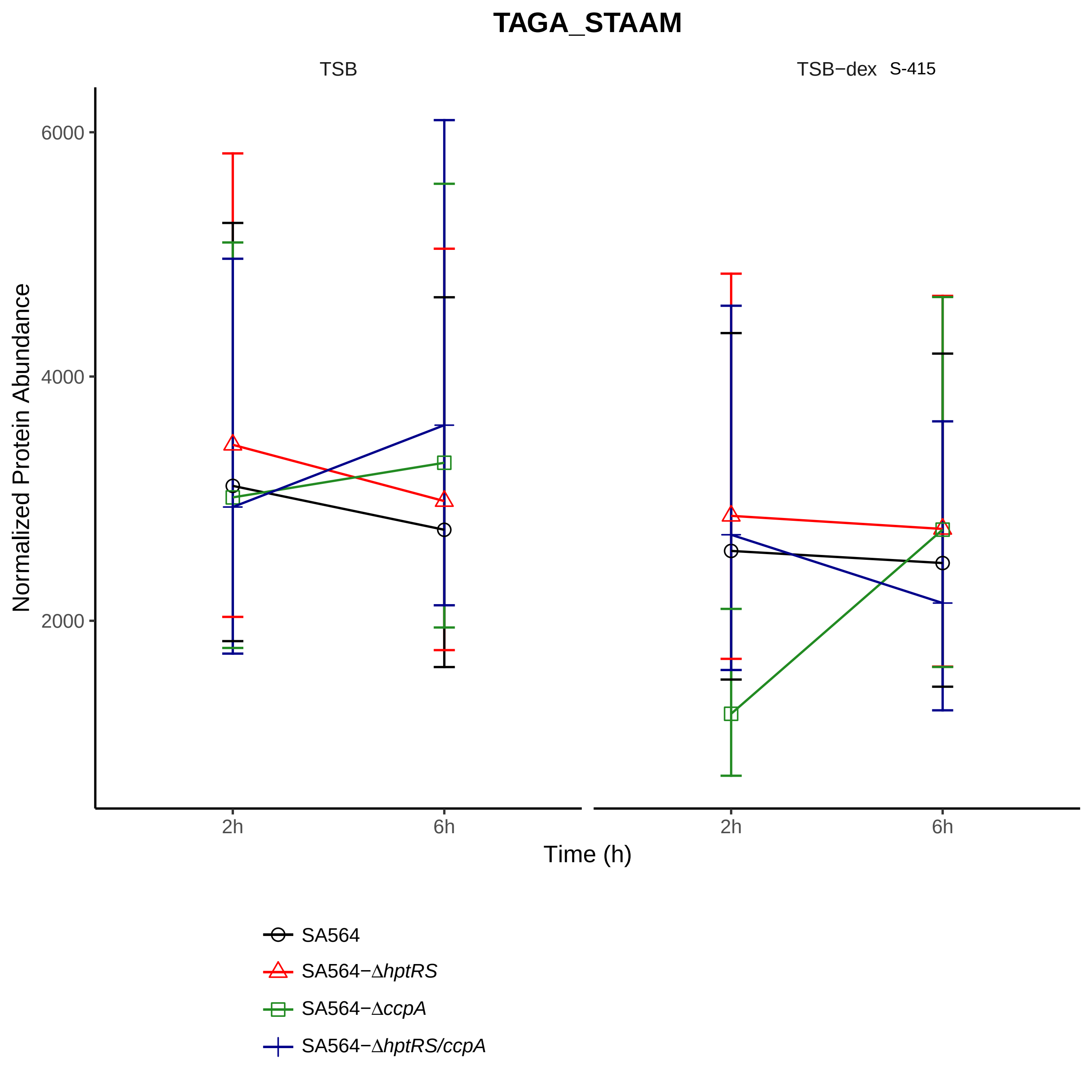
TAGA_STAAM
TSB
TSB−dex
S-415
Normalized Protein Abundance
6000
4000
2000
2h
6h
2h
6h
Time (h)
SA564
SA564−Δ*hptRS*
SA564−Δ*ccpA*
SA564−Δ*hptRS/ccpA*

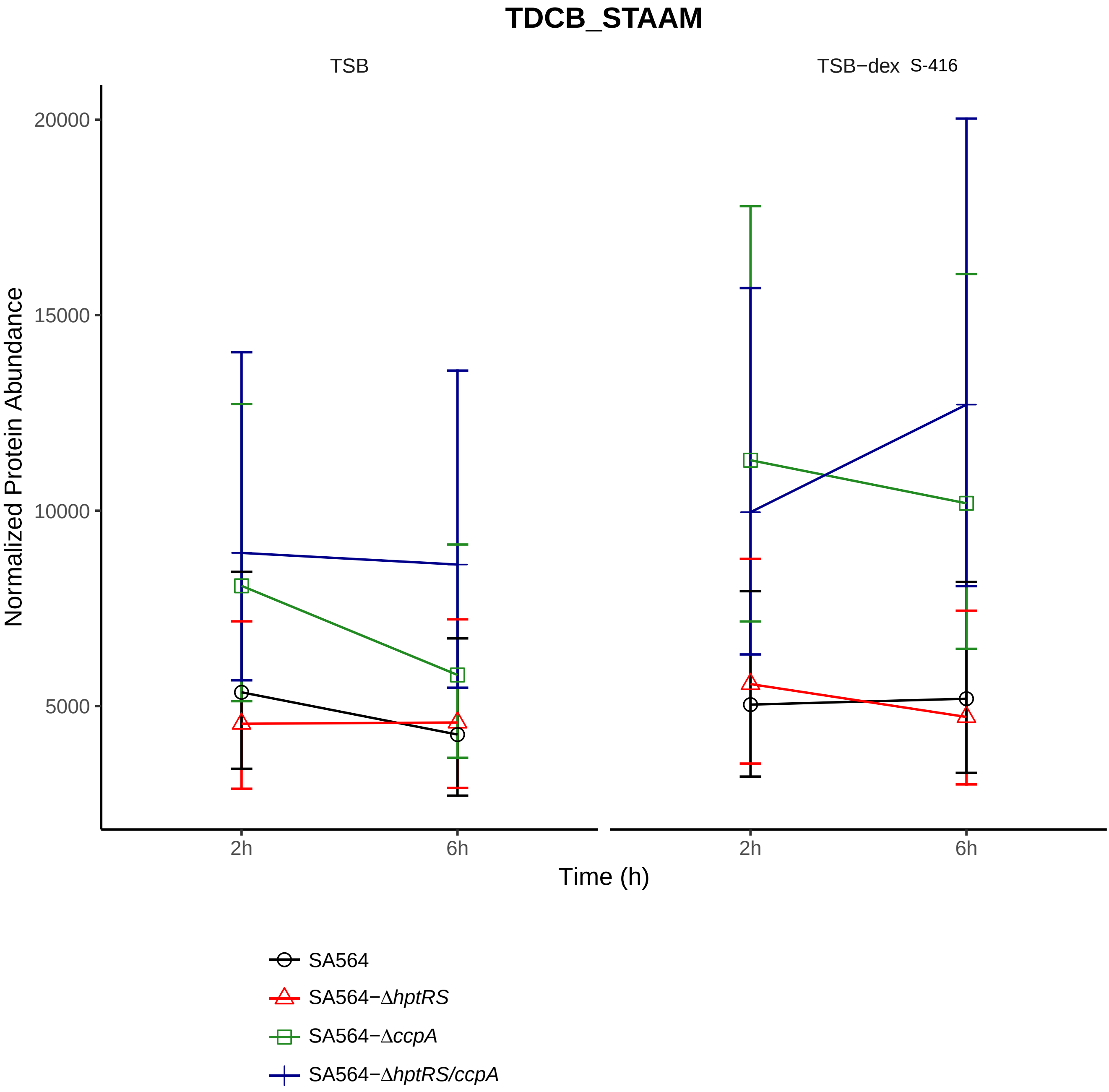
TDCB_STAAM
TSB
TSB−dex
S-416
20000
15000
10000
5000
Normalized Protein Abundance
2h
6h
2h
6h
Time (h)
SA564
SA564−Δ*hptRS*
SA564−Δ*ccpA*
SA564−Δ*hptRS/ccpA*

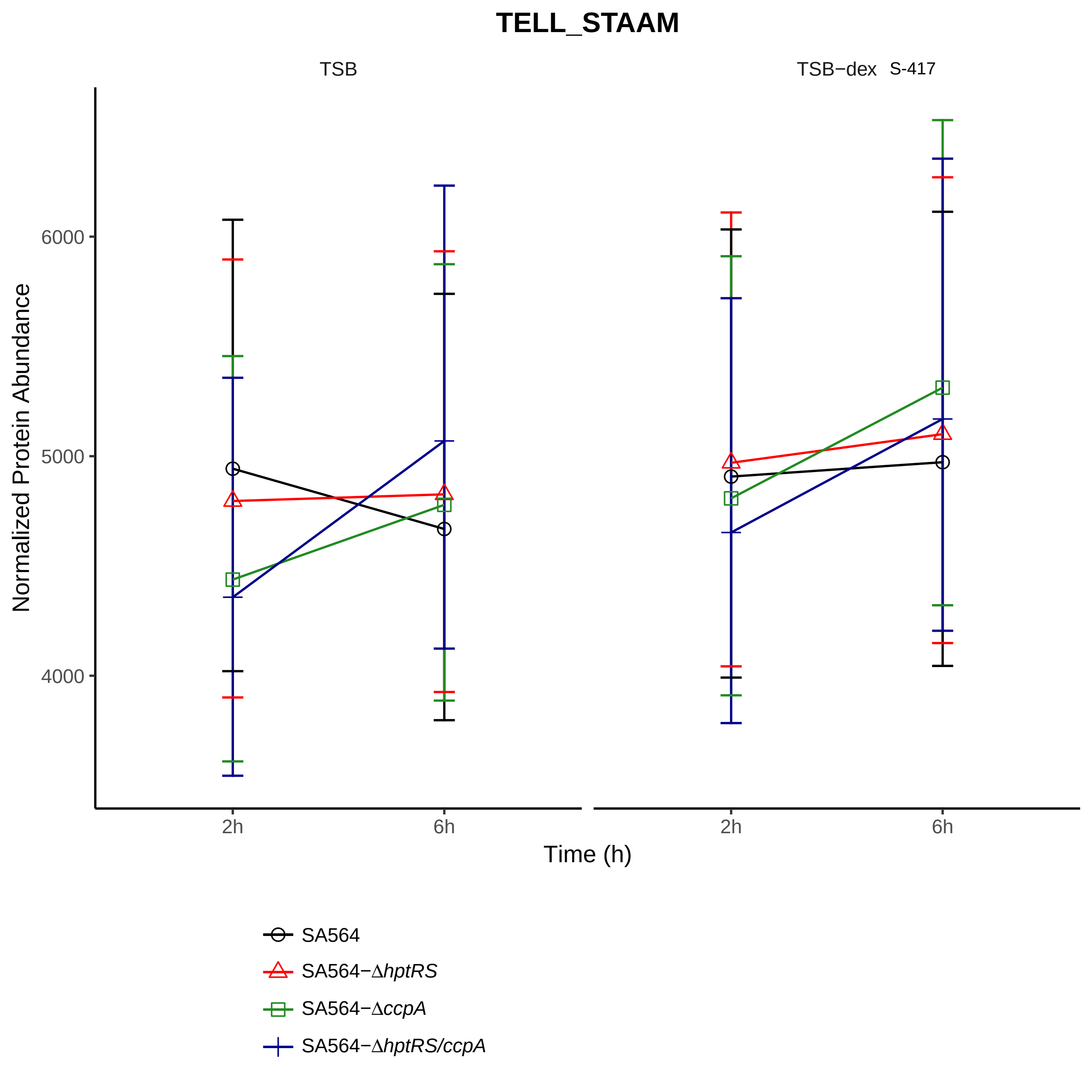

TELL_STAAM
TSB
TSB−dex
S-417
Normalized Protein Abundance
6000
5000
4000
2h
6h
2h
6h
Time (h)
SA564
SA564−Δ*hptRS*
SA564−Δ*ccpA*
SA564−Δ*hptRS/ccpA*

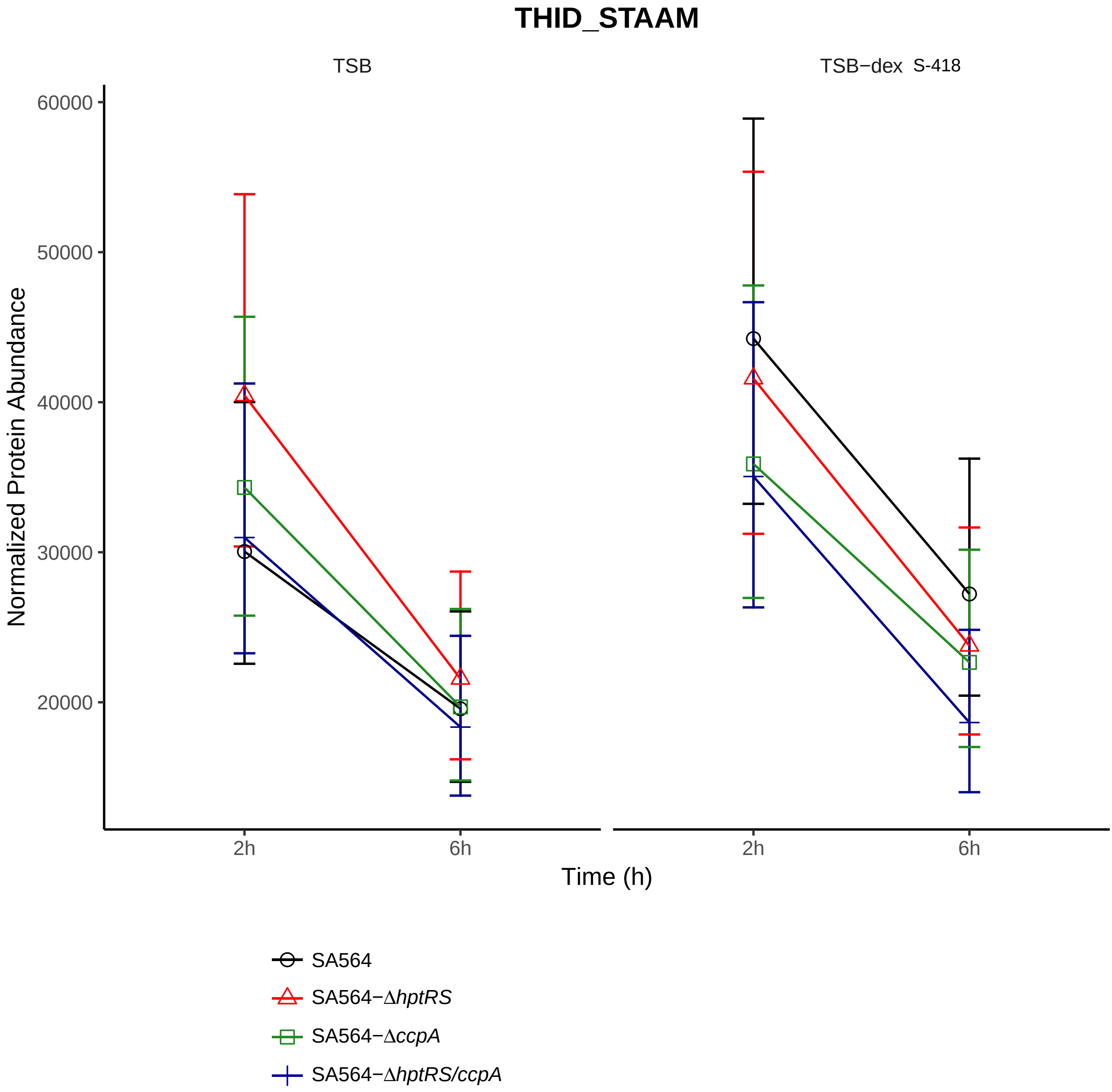

THID_STAAM
TSB
TSB−dex
S-418
Normalized Protein Abundance
60000
50000
40000
30000
20000
2h
6h
2h
6h
Time (h)
SA564
SA564−ΔhptRS
SA564−ΔccpA
SA564−ΔhptRS/ccpA

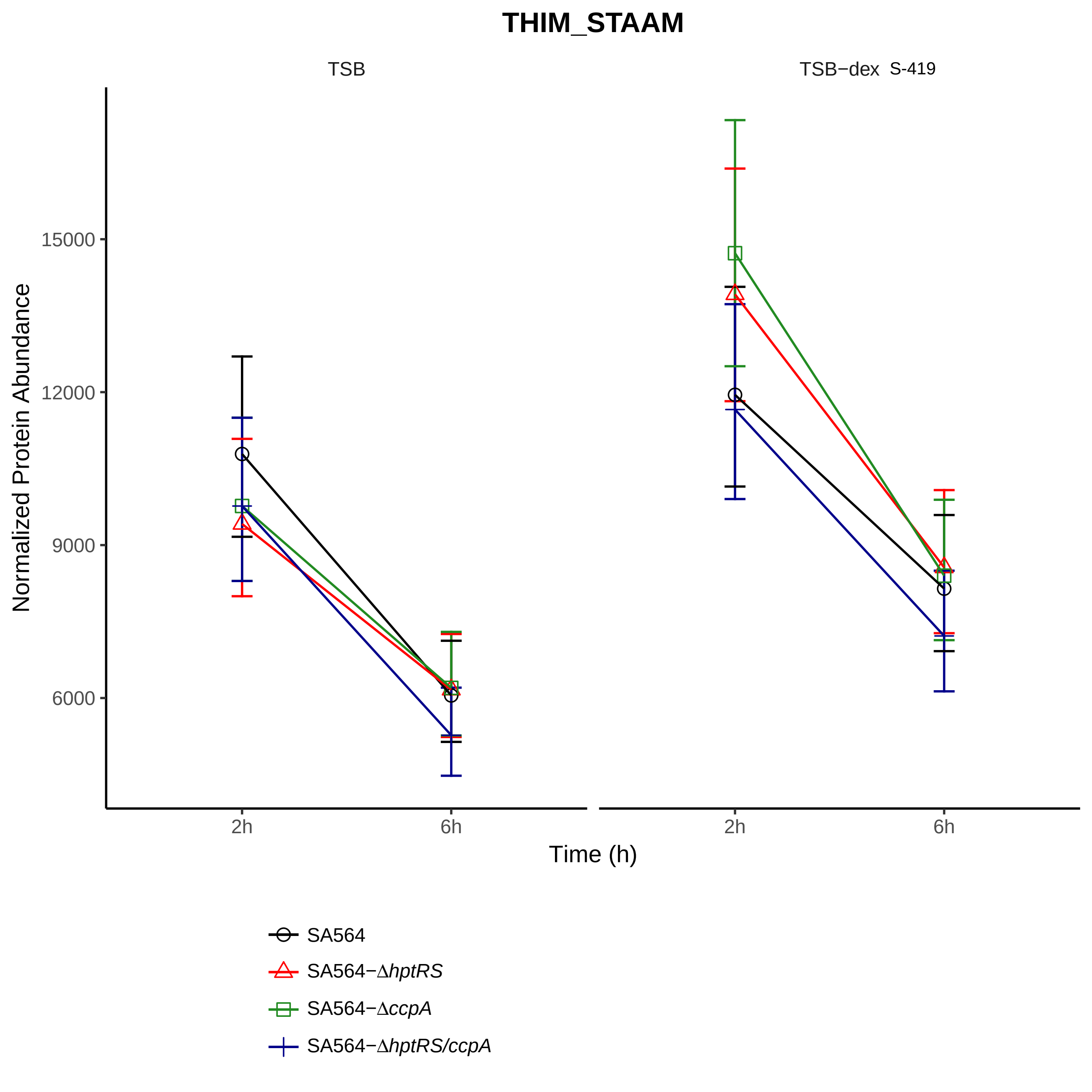

THIM_STAAM
TSB
TSB−dex
S-419
Normalized Protein Abundance
15000
12000
9000
6000
2h
6h
2h
6h
Time (h)
SA564
SA564−Δ*hptRS*
SA564−Δ*ccpA*
SA564−Δ*hptRS/ccpA*

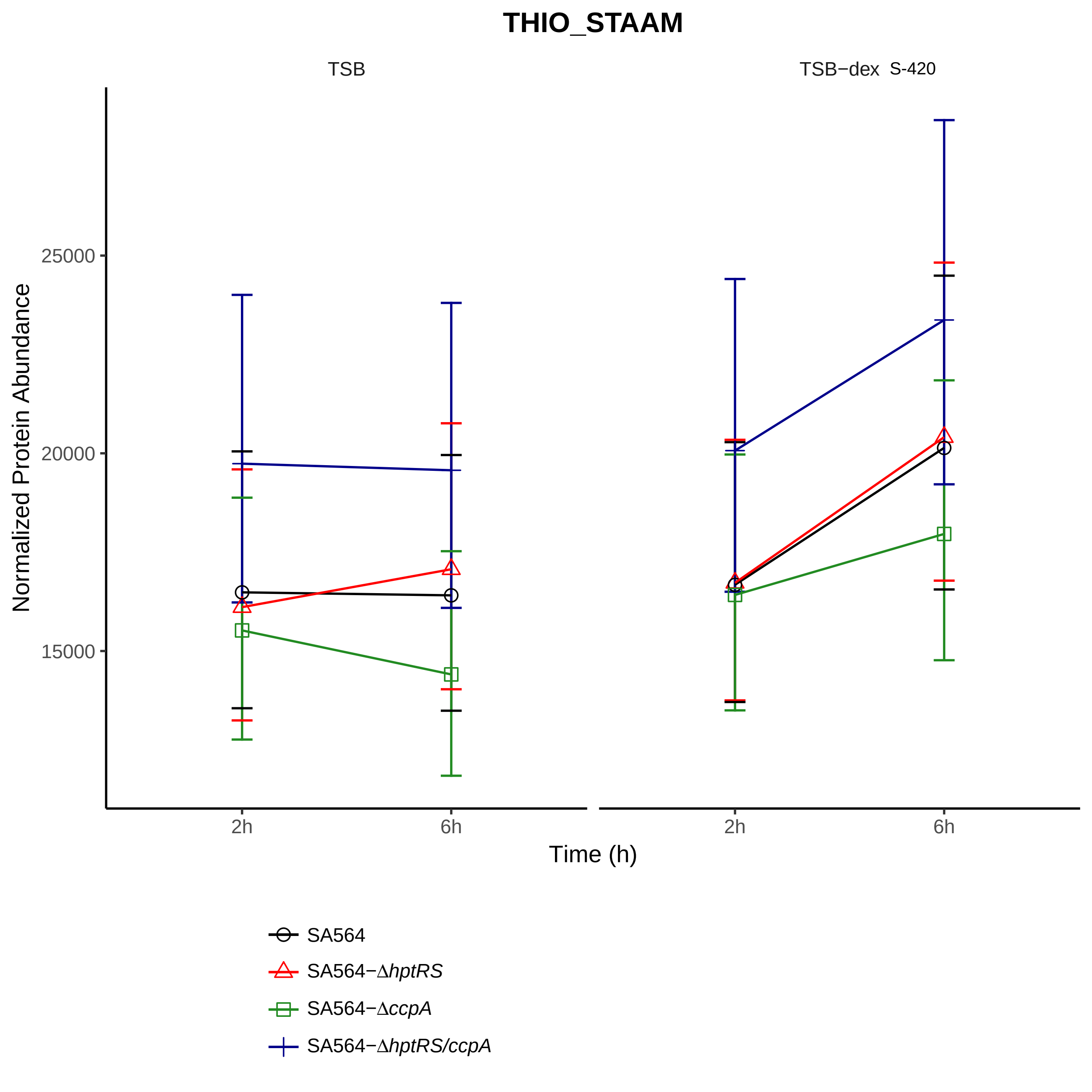
THIO_STAAM
TSB
TSB−dex
S-420
Normalized Protein Abundance
25000
20000
15000
2h
6h
2h
6h
Time (h)
SA564
SA564−ΔhptRS
SA564−ΔccpA
SA564−ΔhptRS/ccpA

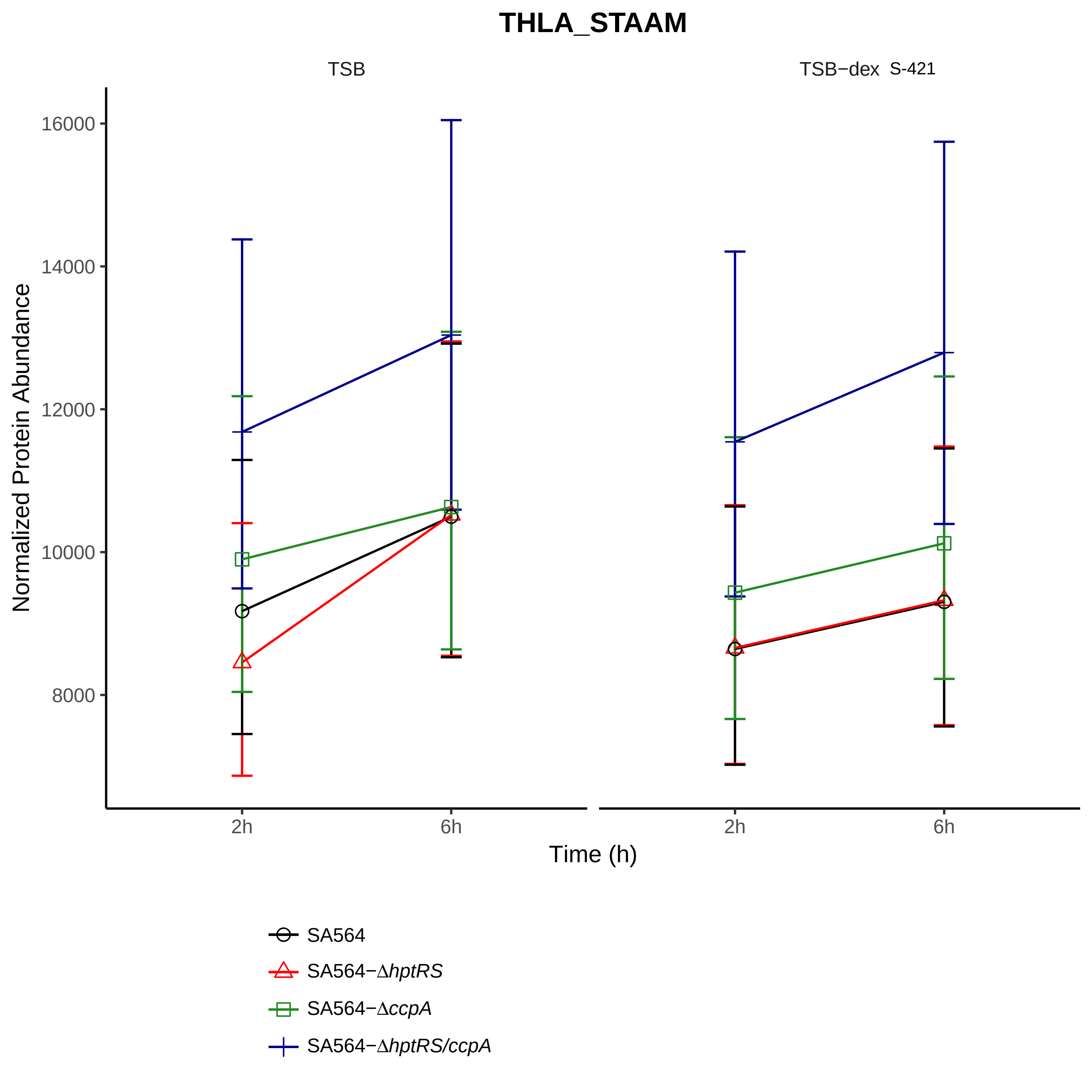

THLA_STAAM
TSB
TSB−dex
S-421
16000
14000
12000
10000
8000
Normalized Protein Abundance
2h
6h
2h
6h
Time (h)
SA564
SA564−Δ*hptRS*
SA564−Δ*ccpA*
SA564−Δ*hptRS/ccpA*

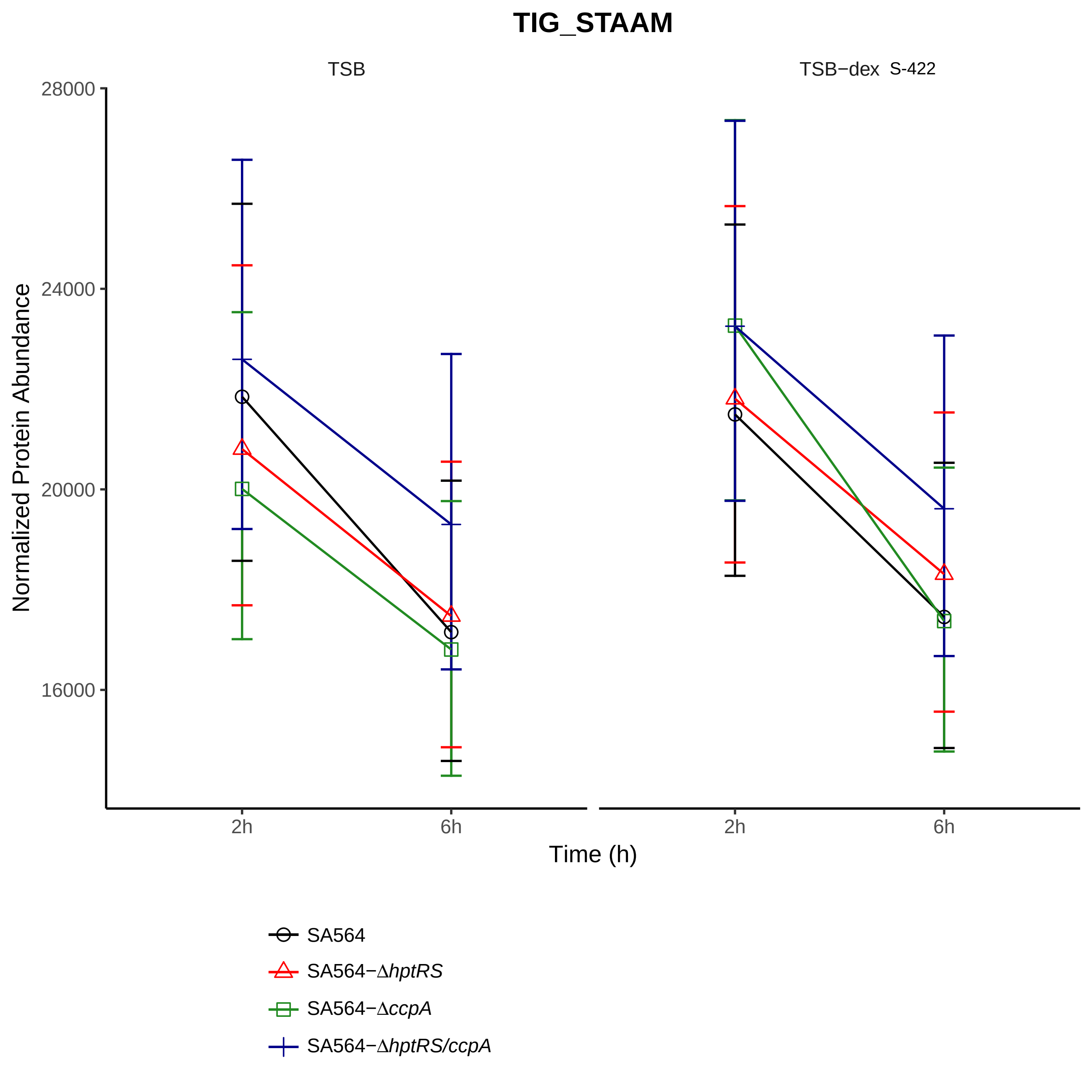
TIG_STAAM
TSB
TSB−dex
S-422
28000
24000
20000
16000
Normalized Protein Abundance
2h
6h
2h
6h
Time (h)
SA564
SA564−Δ*hptRS*
SA564−Δ*ccpA*
SA564−Δ*hptRS/ccpA*

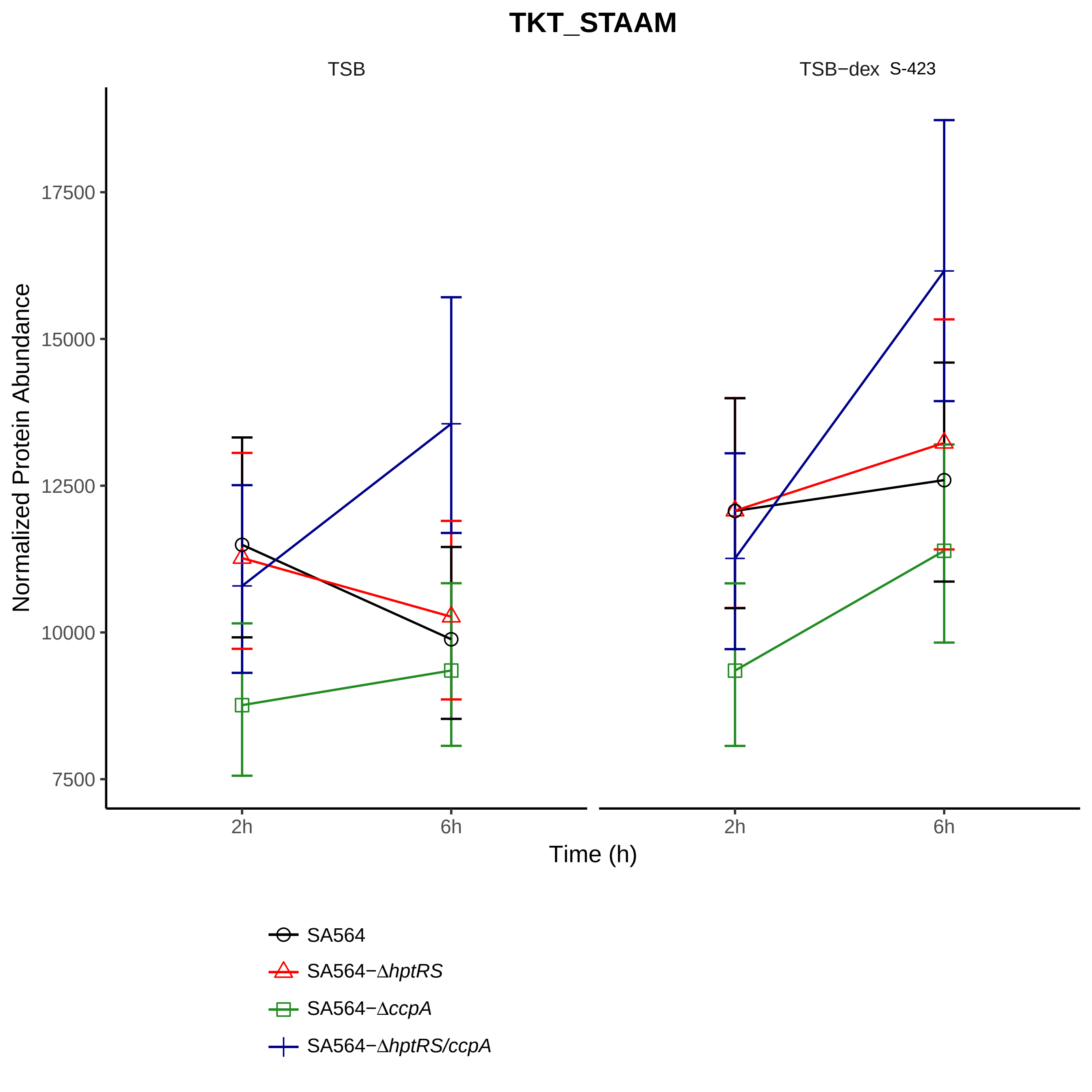
TKT_STAAM
TSB
TSB−dex
S-423
Normalized Protein Abundance
17500
15000
12500
10000
7500
2h
6h
2h
6h
Time (h)
SA564
SA564−ΔhptRS
SA564−ΔccpA
SA564−ΔhptRS/ccpA

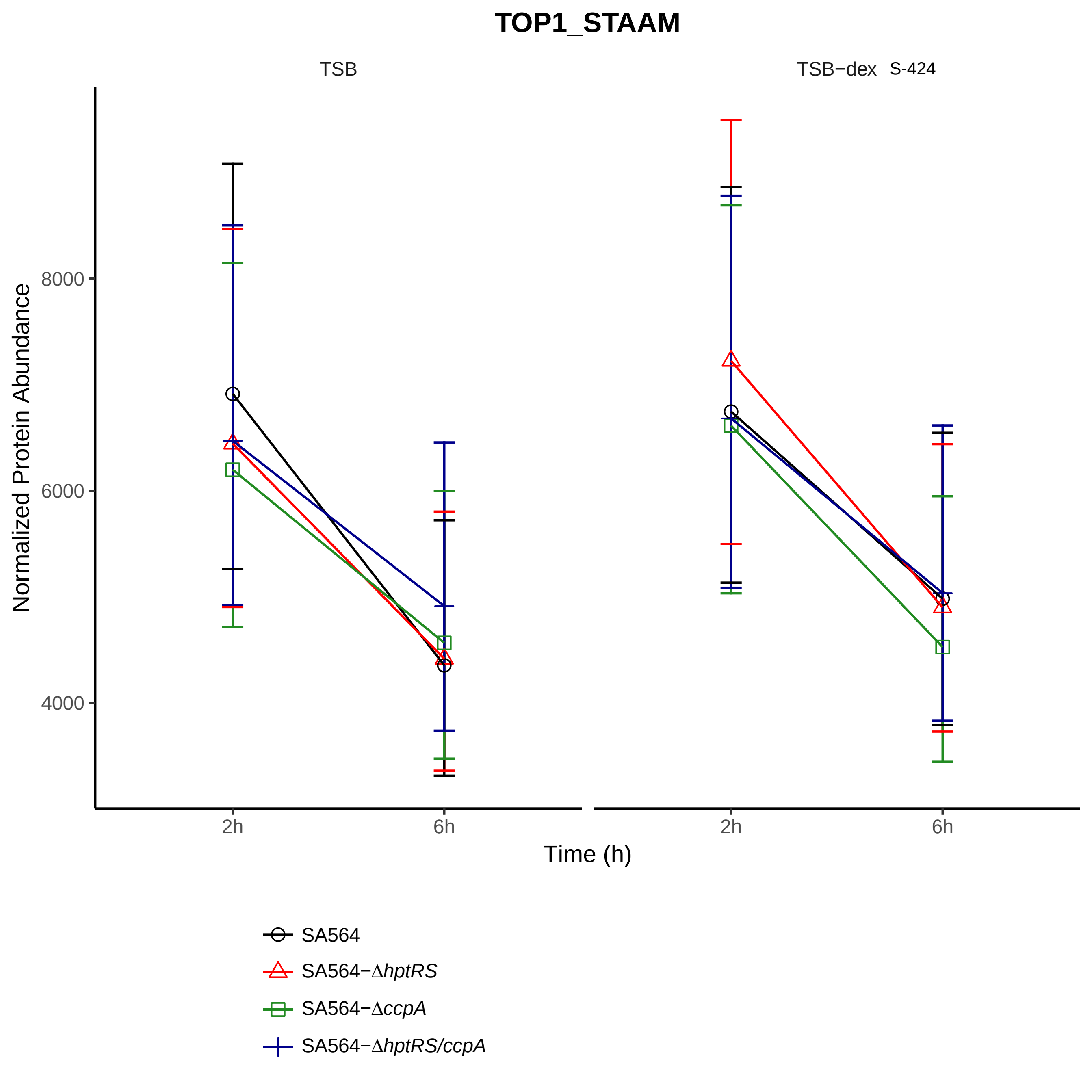

TOP1_STAAM
TSB
TSB−dex
S-424
Normalized Protein Abundance
8000
6000
4000
2h
6h
2h
6h
Time (h)
SA564
SA564−ΔhptRS
SA564−ΔccpA
SA564−ΔhptRS/ccpA

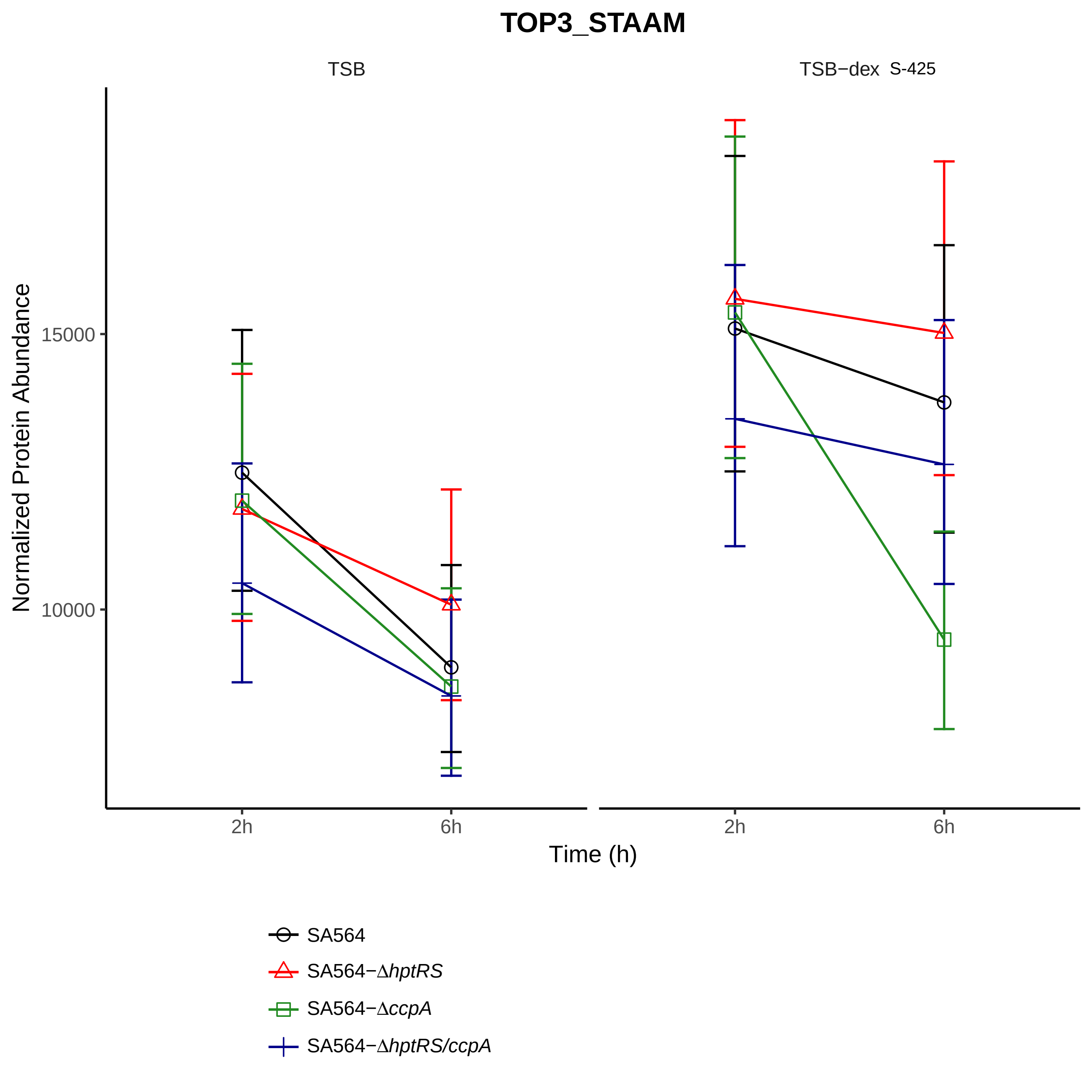

TOP3_STAAM
TSB
TSB−dex
S-425
Normalized Protein Abundance
15000
10000
2h
6h
2h
6h
Time (h)
SA564
SA564−ΔhptRS
SA564−ΔccpA
SA564−ΔhptRS/ccpA

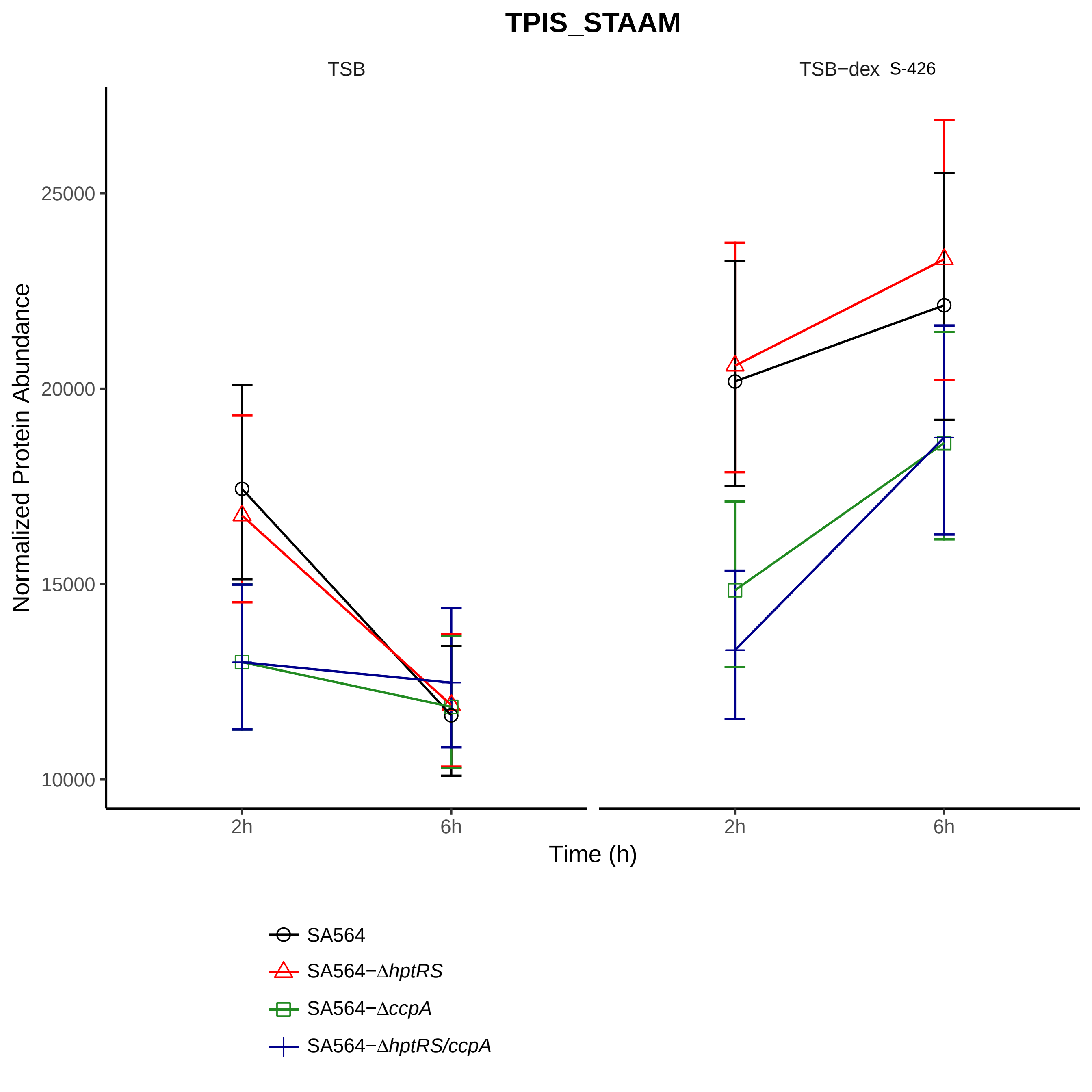
TPIS_STAAM
TSB
TSB−dex
S-426
Normalized Protein Abundance
25000
20000
15000
10000
2h
6h
2h
6h
Time (h)
SA564
SA564−Δ*hptRS*
SA564−Δ*ccpA*
SA564−Δ*hptRS/ccpA*

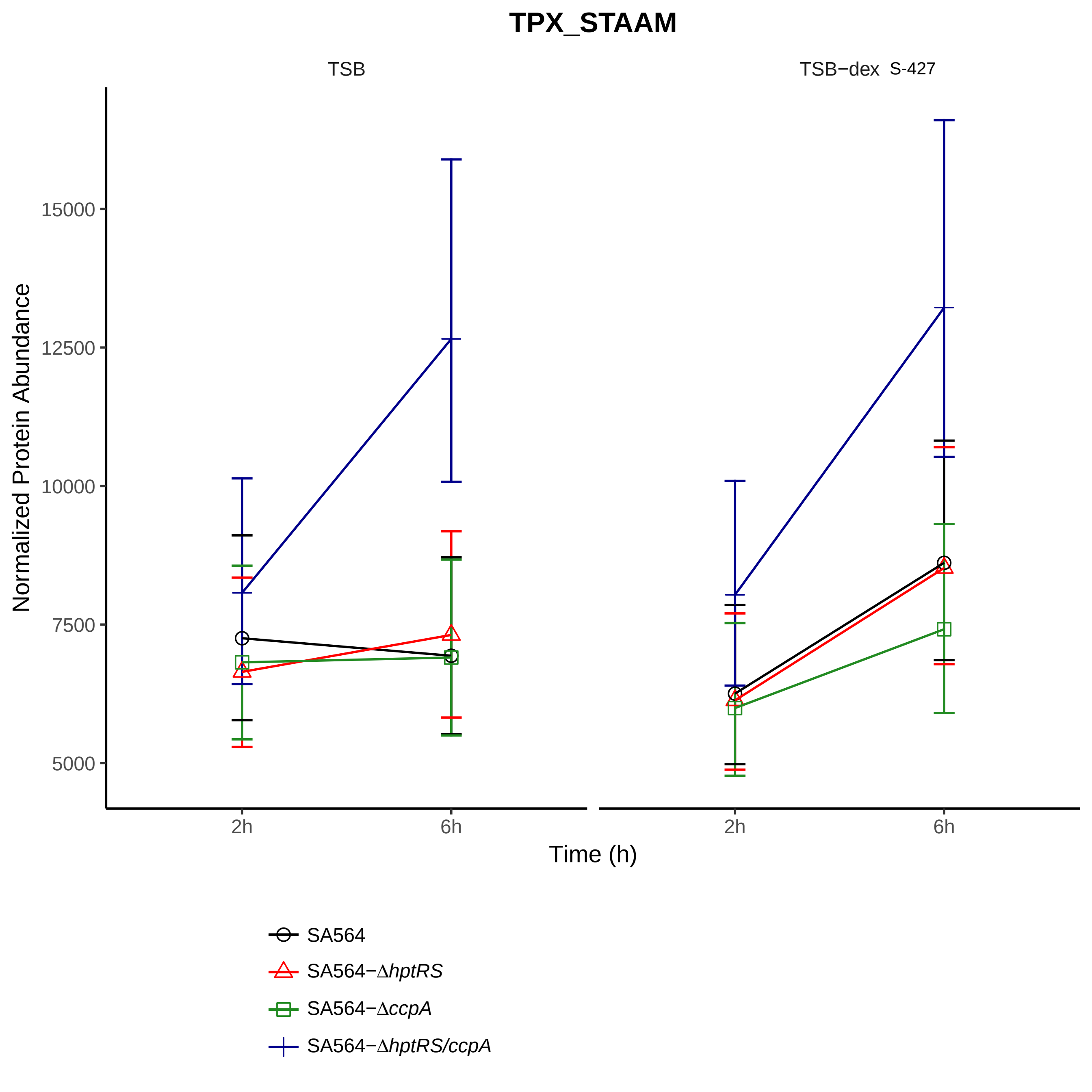
TPX_STAAM
TSB
TSB−dex
S-427
Normalized Protein Abundance
15000
12500
10000
7500
5000
2h
6h
2h
6h
Time (h)
SA564
SA564−ΔhptRS
SA564−ΔccpA
SA564−ΔhptRS/ccpA

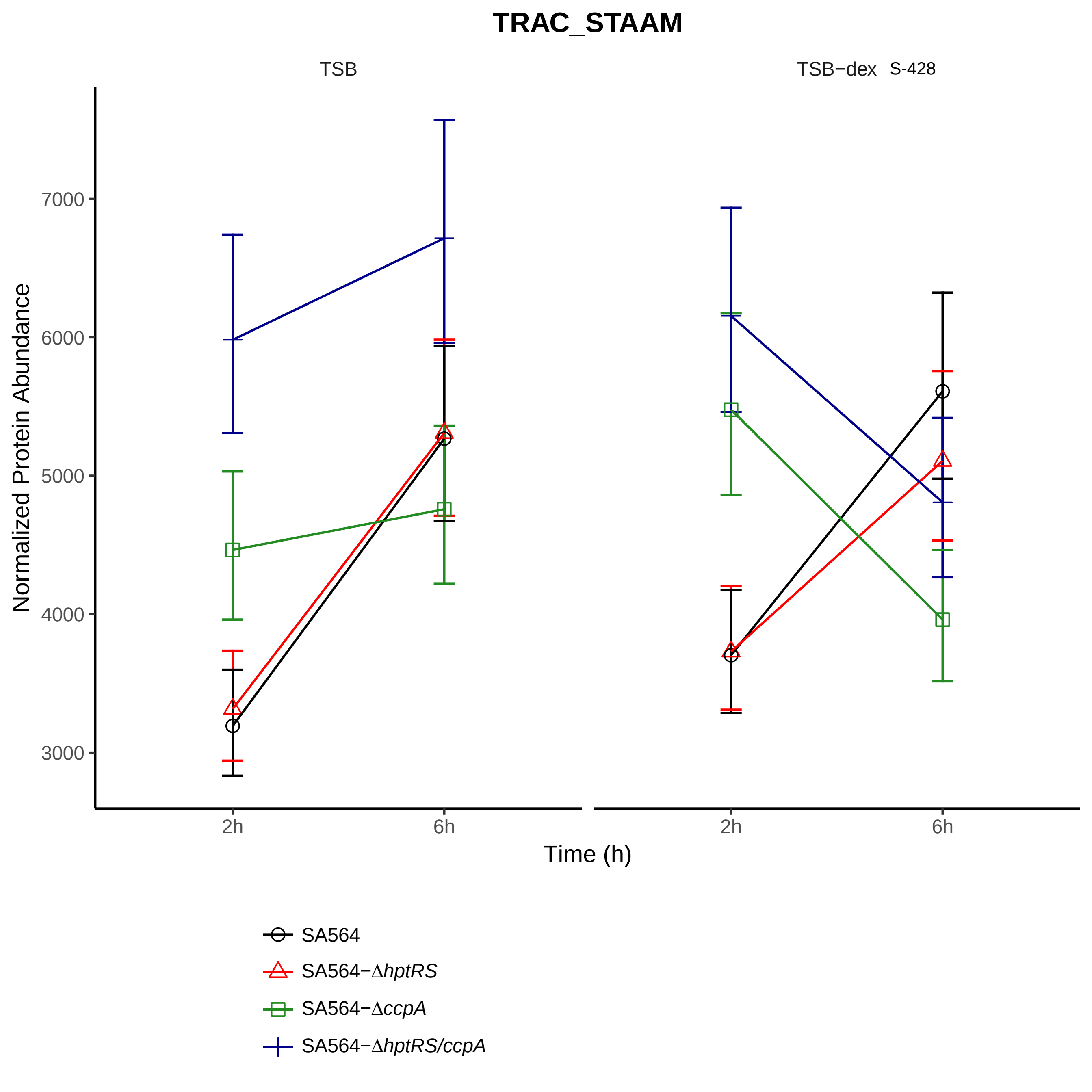
TRAC_STAAM
TSB
TSB−dex
S-428
Normalized Protein Abundance
7000
6000
5000
4000
3000
2h
6h
2h
6h
Time (h)
SA564
SA564−Δ*hptRS*
SA564−Δ*ccpA*
SA564−Δ*hptRS/ccpA*

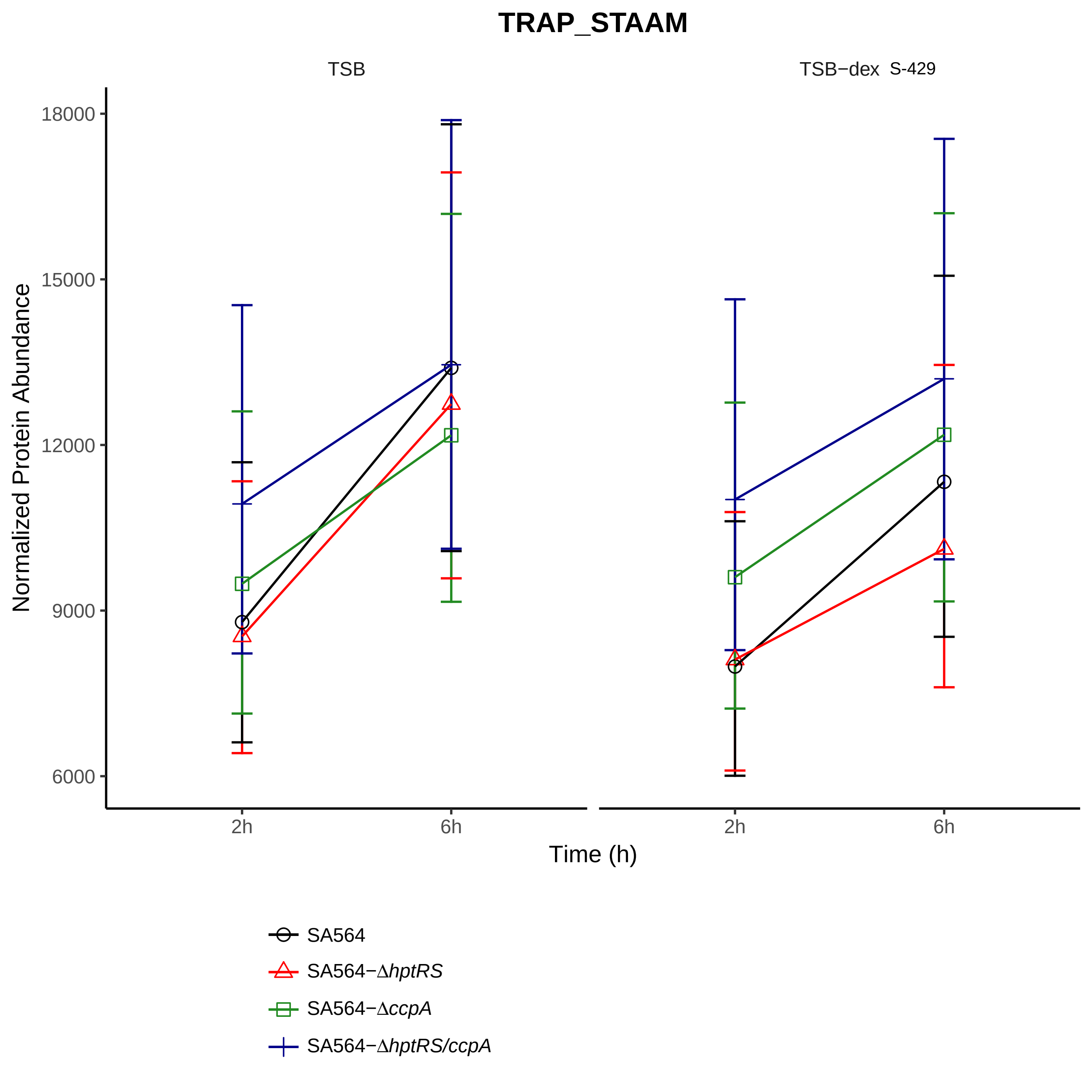

TRAP_STAAM
TSB
TSB−dex
S-429
Normalized Protein Abundance
18000
15000
12000
9000
6000
2h
6h
Time (h)
SA564
SA564−Δ*hptRS*
SA564−Δ*ccpA*
SA564−Δ*hptRS/ccpA*

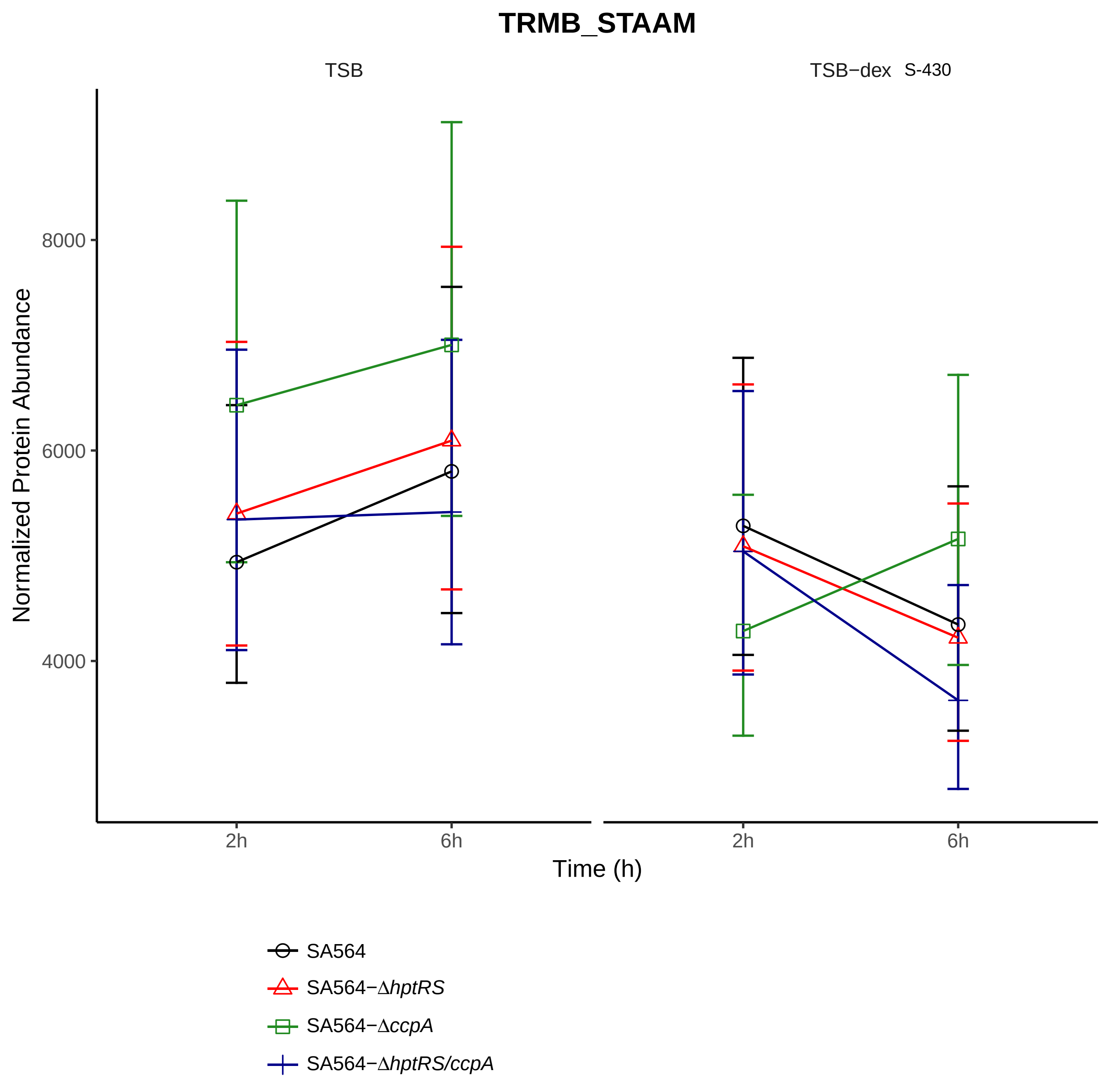

TRMB_STAAM
TSB
TSB−dex
S-430
Normalized Protein Abundance
8000
6000
4000
2h
6h
2h
6h
Time (h)
SA564
SA564−Δ*hptRS*
SA564−Δ*ccpA*
SA564−Δ*hptRS/ccpA*

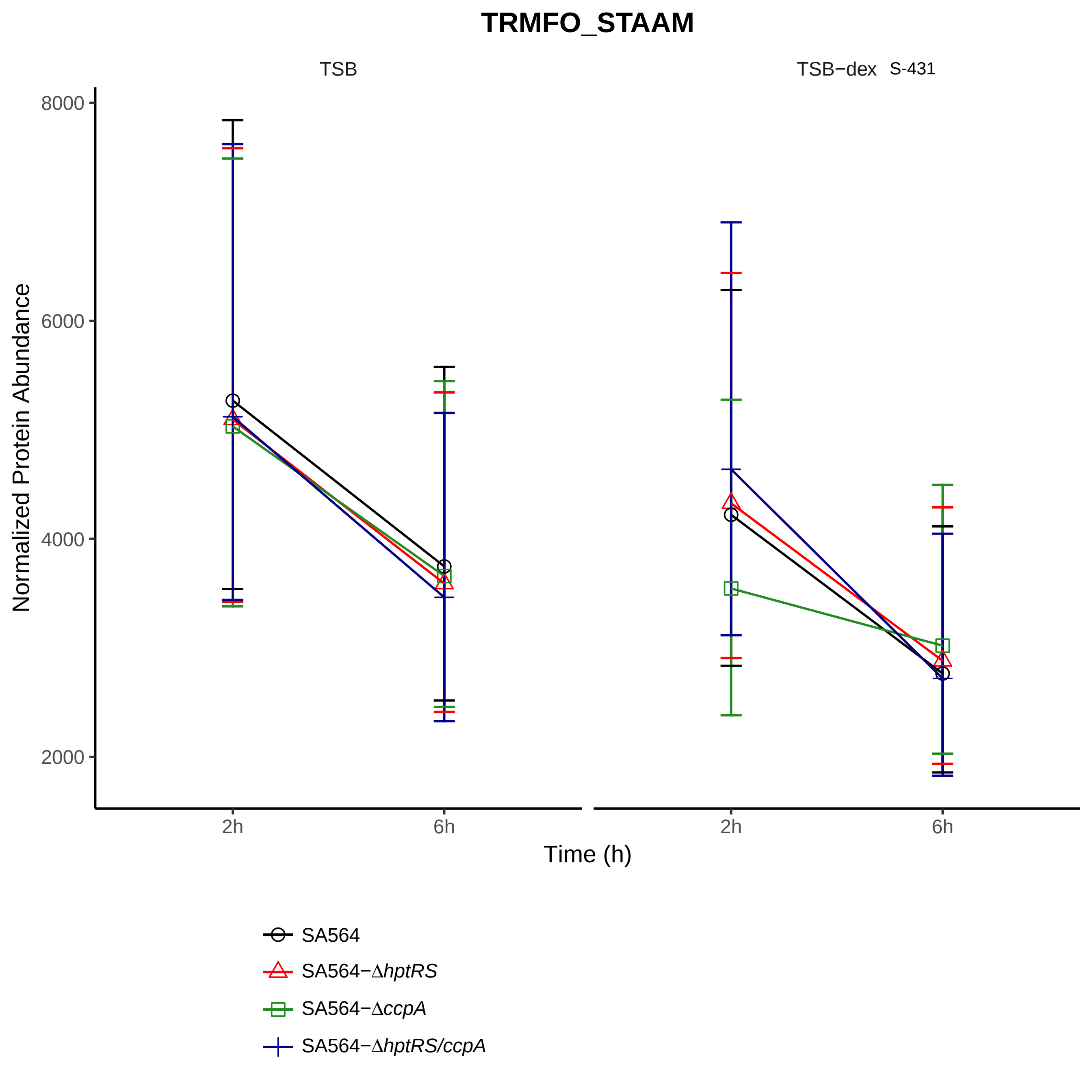
TRMFO_STAAM
TSB
TSB−dex
S-431
Normalized Protein Abundance
8000
6000
4000
2000
2h
6h
2h
6h
Time (h)
SA564
SA564−ΔhptRS
SA564−ΔccpA
SA564−ΔhptRS/ccpA

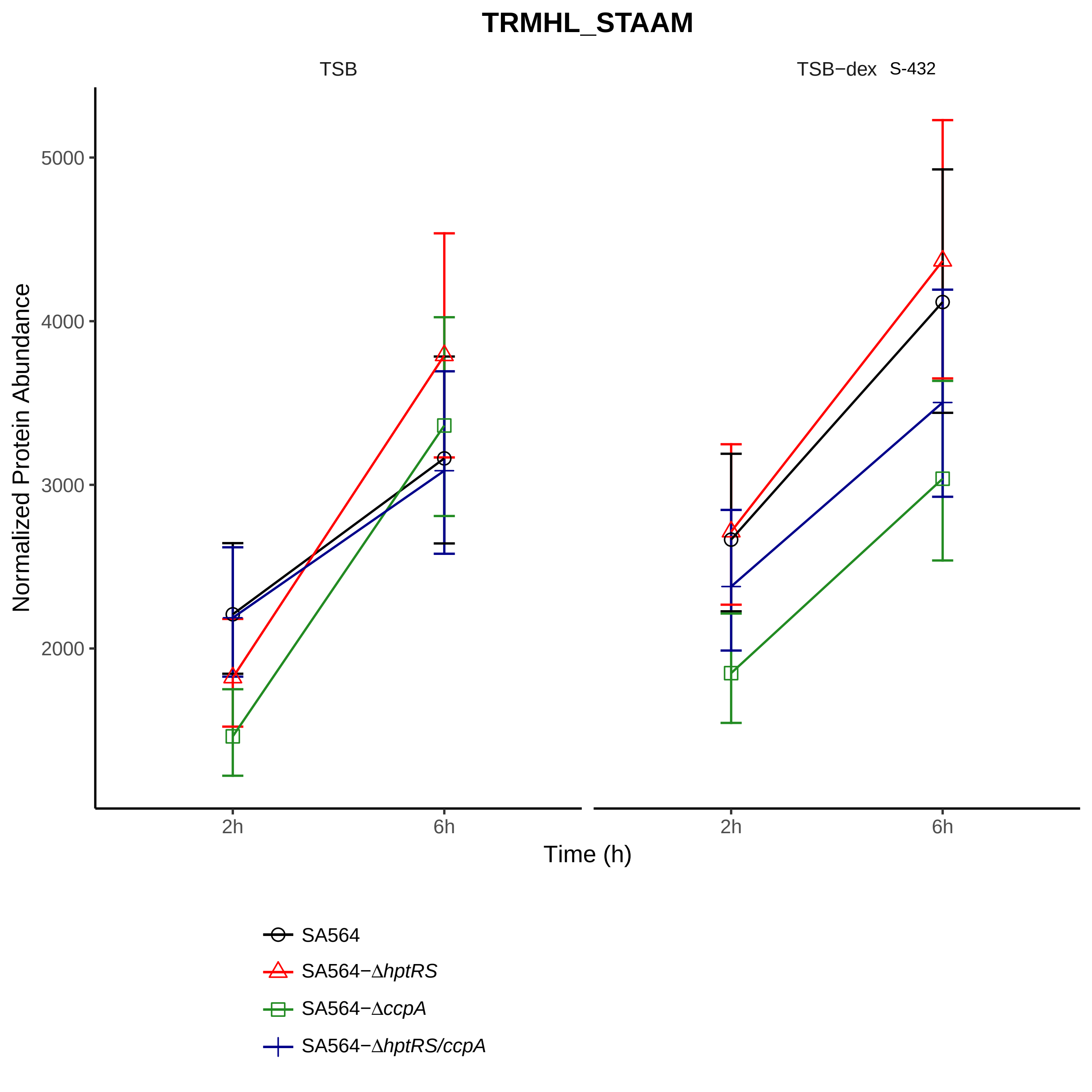
TRMHL_STAAM
TSB
TSB−dex
S-432
Normalized Protein Abundance
5000
4000
3000
2000
2h
6h
2h
6h
Time (h)
SA564
SA564−ΔhptRS
SA564−ΔccpA
SA564−ΔhptRS/ccpA

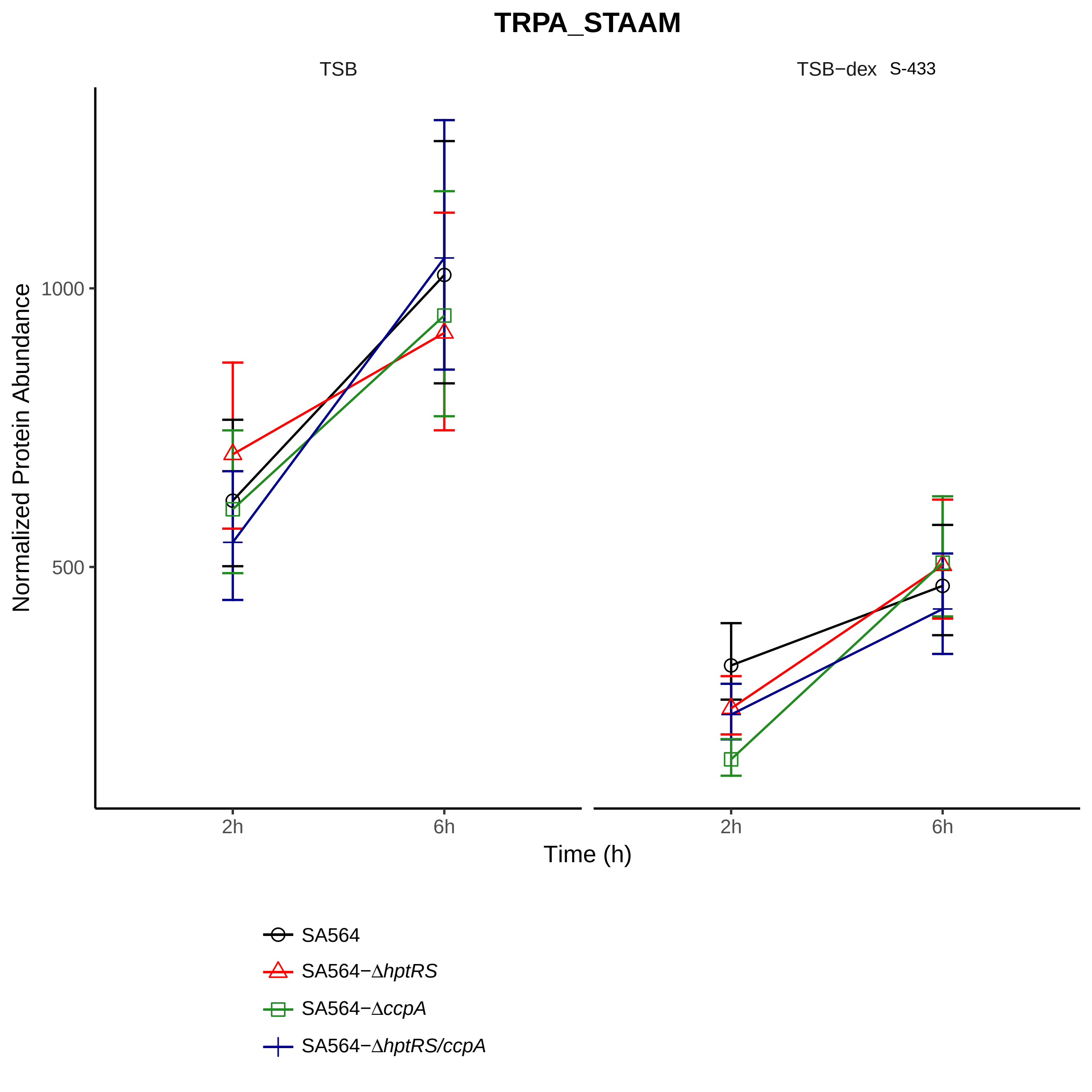
TRPA_STAAM
TSB
TSB−dex
S-433
Normalized Protein Abundance
1000
500
2h
6h
2h
6h
Time (h)
SA564
SA564−Δ*hptRS*
SA564−Δ*ccpA*
SA564−Δ*hptRS/ccpA*

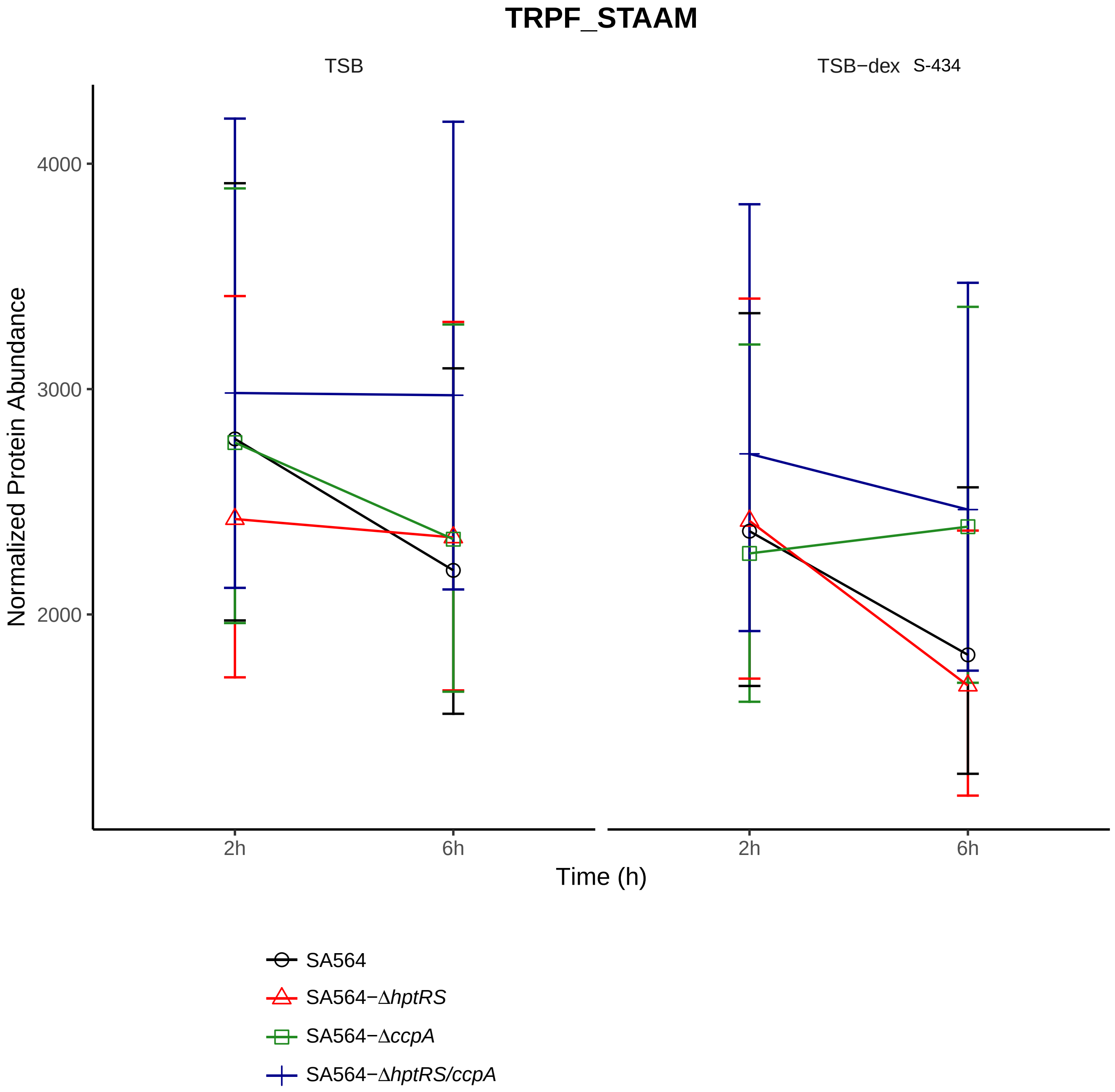

TRPF_STAAM
TSB
TSB−dex
S-434
Normalized Protein Abundance
4000
3000
2000
2h
6h
2h
6h
Time (h)
SA564
SA564−ΔhptRS
SA564−ΔccpA
SA564−ΔhptRS/ccpA

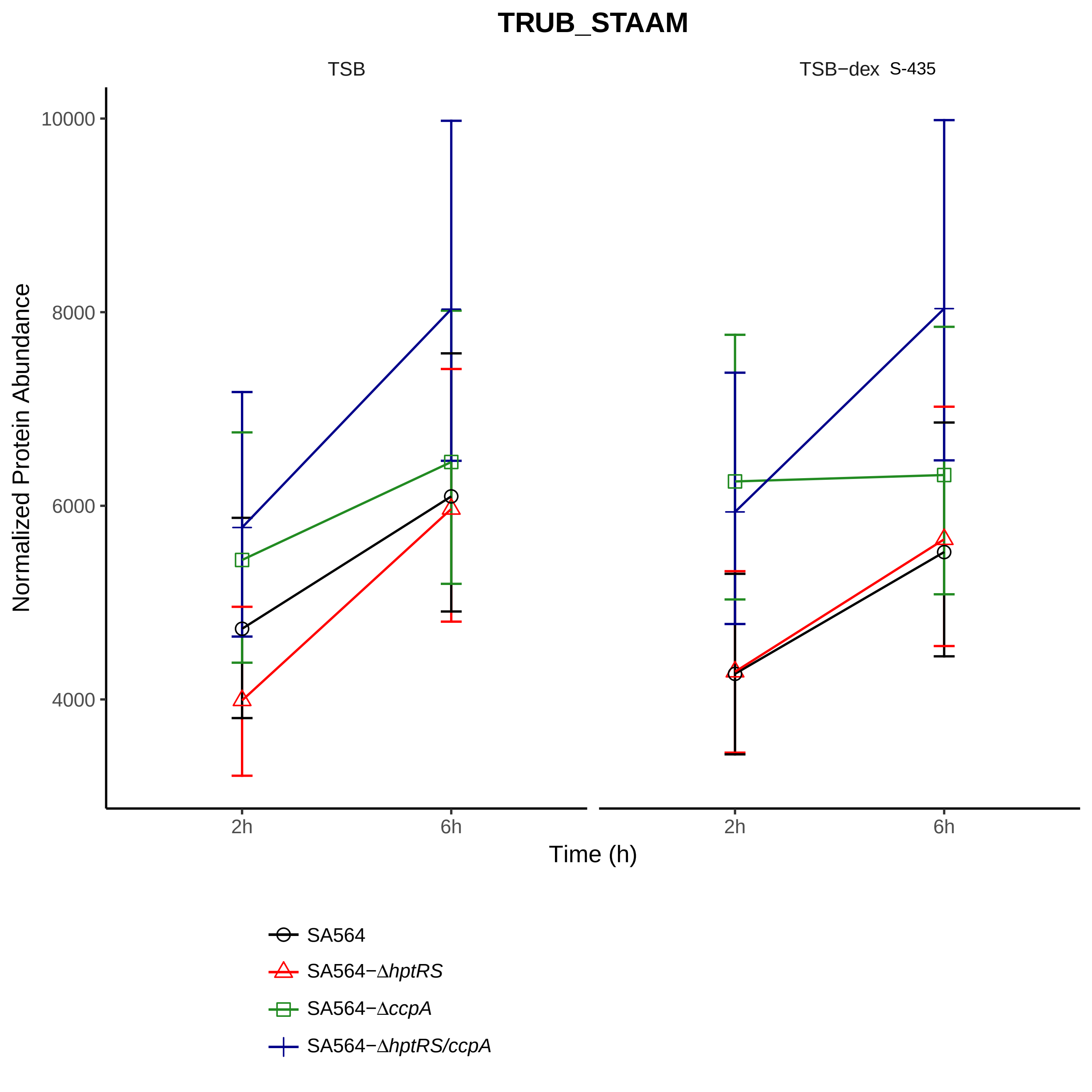

TRUB_STAAM
TSB
TSB−dex
S-435
Normalized Protein Abundance
10000
8000
6000
4000
2h
6h
2h
6h
Time (h)
SA564
SA564−ΔhptRS
SA564−ΔccpA
SA564−ΔhptRS/ccpA

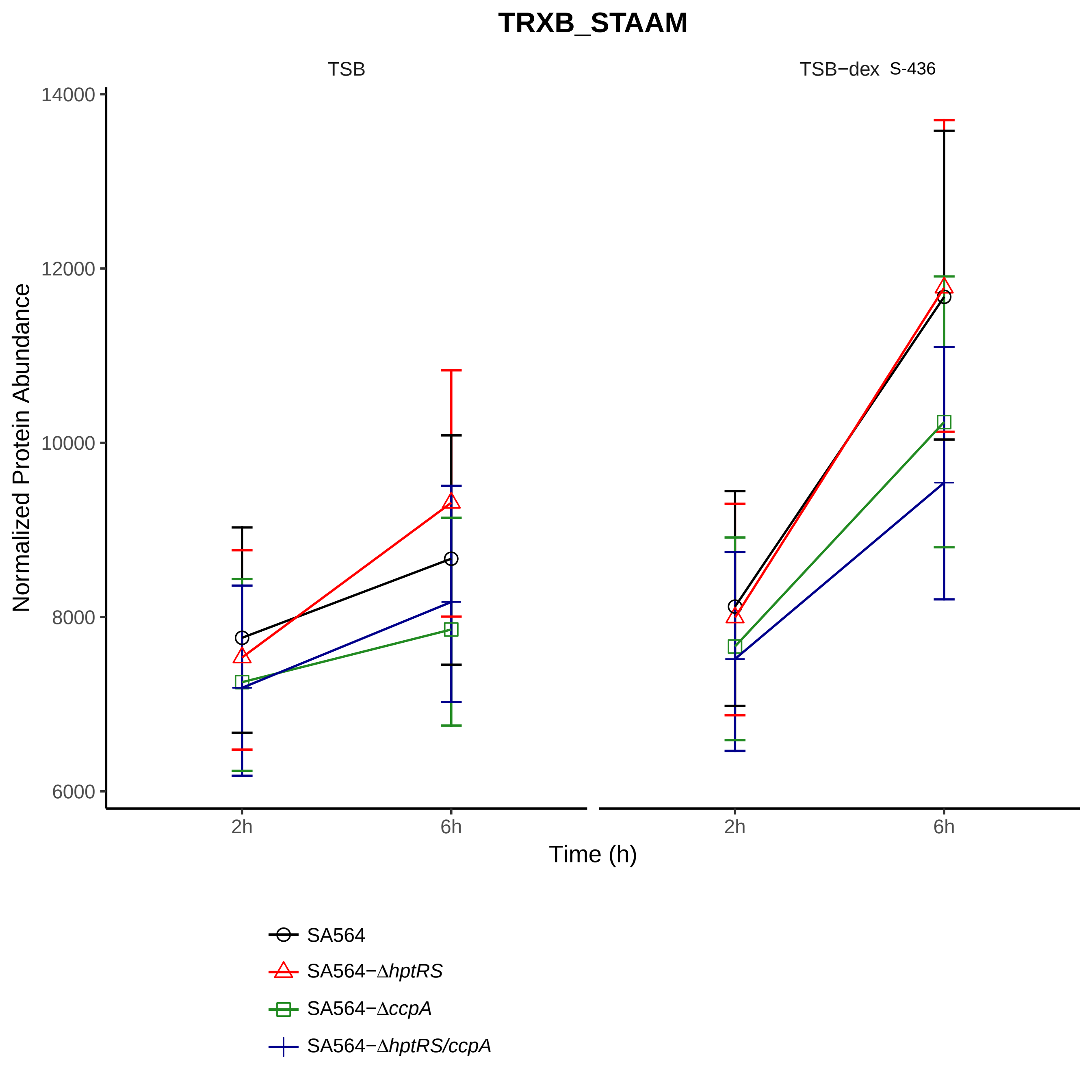

TRXB_STAAM
TSB
TSB−dex
S-436
Normalized Protein Abundance
14000
12000
10000
8000
6000
2h
6h
2h
6h
Time (h)
SA564
SA564−ΔhptRS
SA564−ΔccpA
SA564−ΔhptRS/ccpA

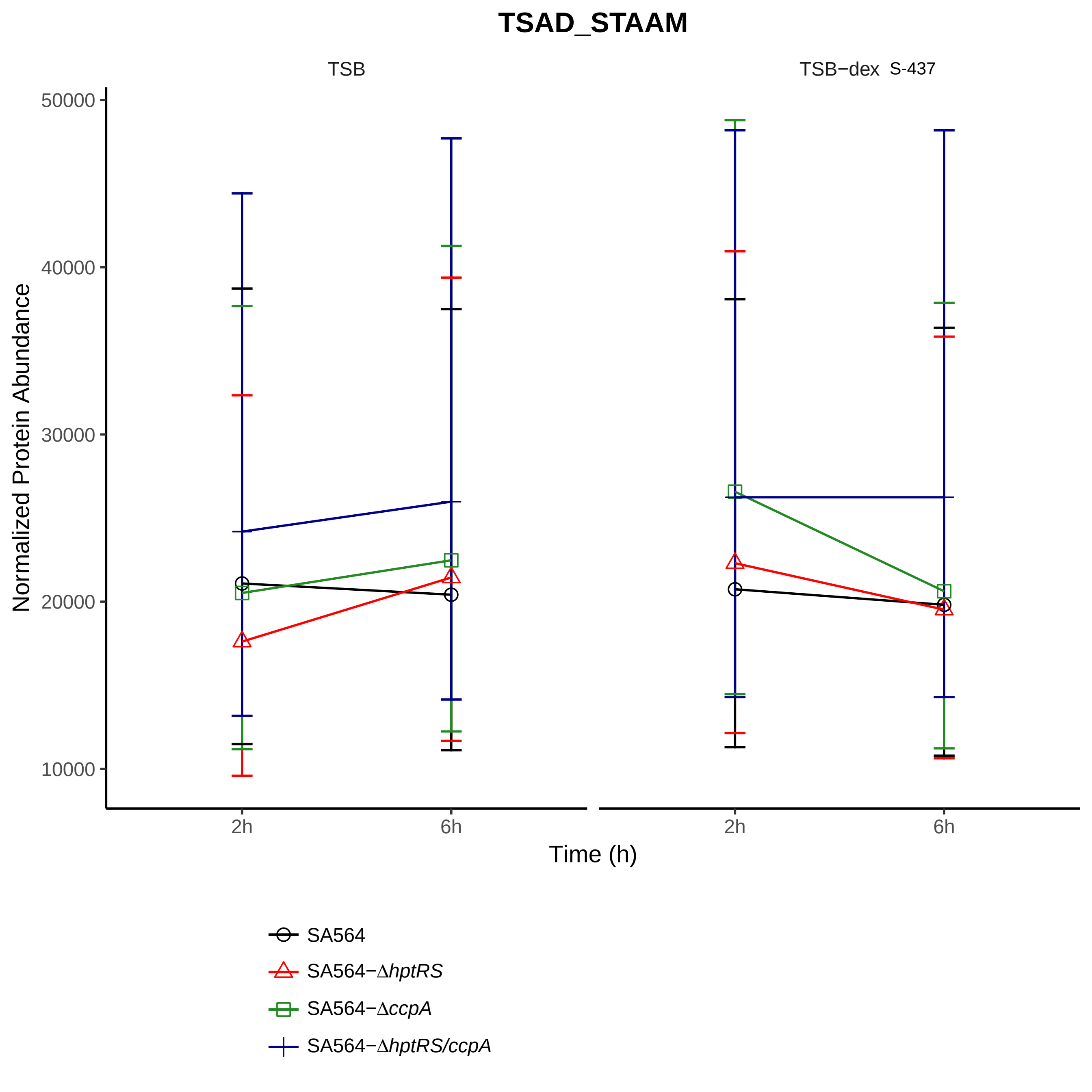

TSAD_STAAM
TSB
TSB−dex
S-437
50000
40000
30000
20000
10000
Normalized Protein Abundance
2h
6h
2h
6h
Time (h)
SA564
SA564−Δ*hptRS*
SA564−Δ*ccpA*
SA564−Δ*hptRS/ccpA*

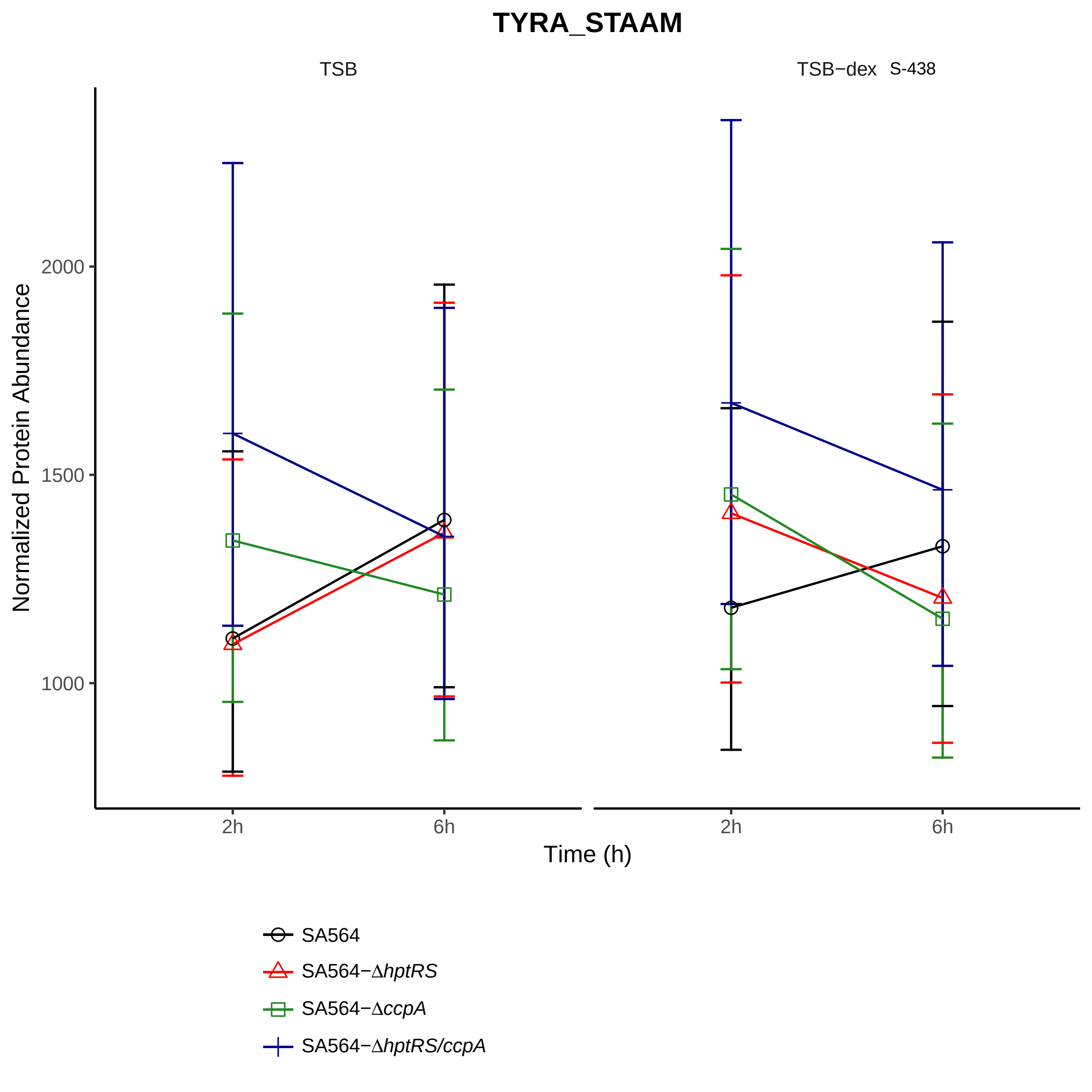
TYRA_STAAM
TSB
TSB−dex
S-438
Normalized Protein Abundance
2000
1500
1000
2h
6h
2h
6h
Time (h)
SA564
SA564−Δ*hptRS*
SA564−Δ*ccpA*
SA564−Δ*hptRS/ccpA*

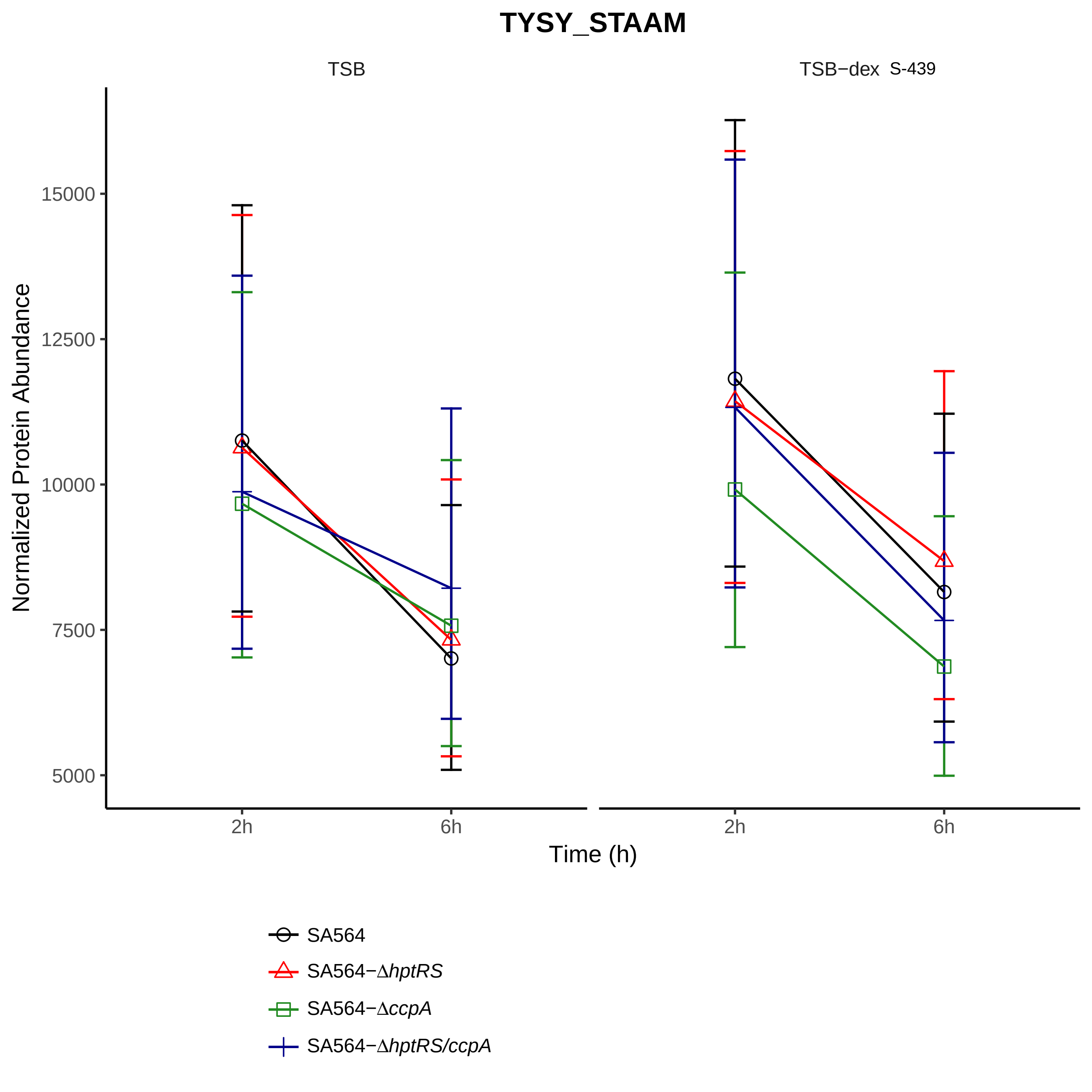

TYSY_STAAM
TSB
TSB−dex
S-439
Normalized Protein Abundance
15000
12500
10000
7500
5000
2h
6h
2h
6h
Time (h)
SA564
SA564−Δ*hptRS*
SA564−Δ*ccpA*
SA564−Δ*hptRS/ccpA*

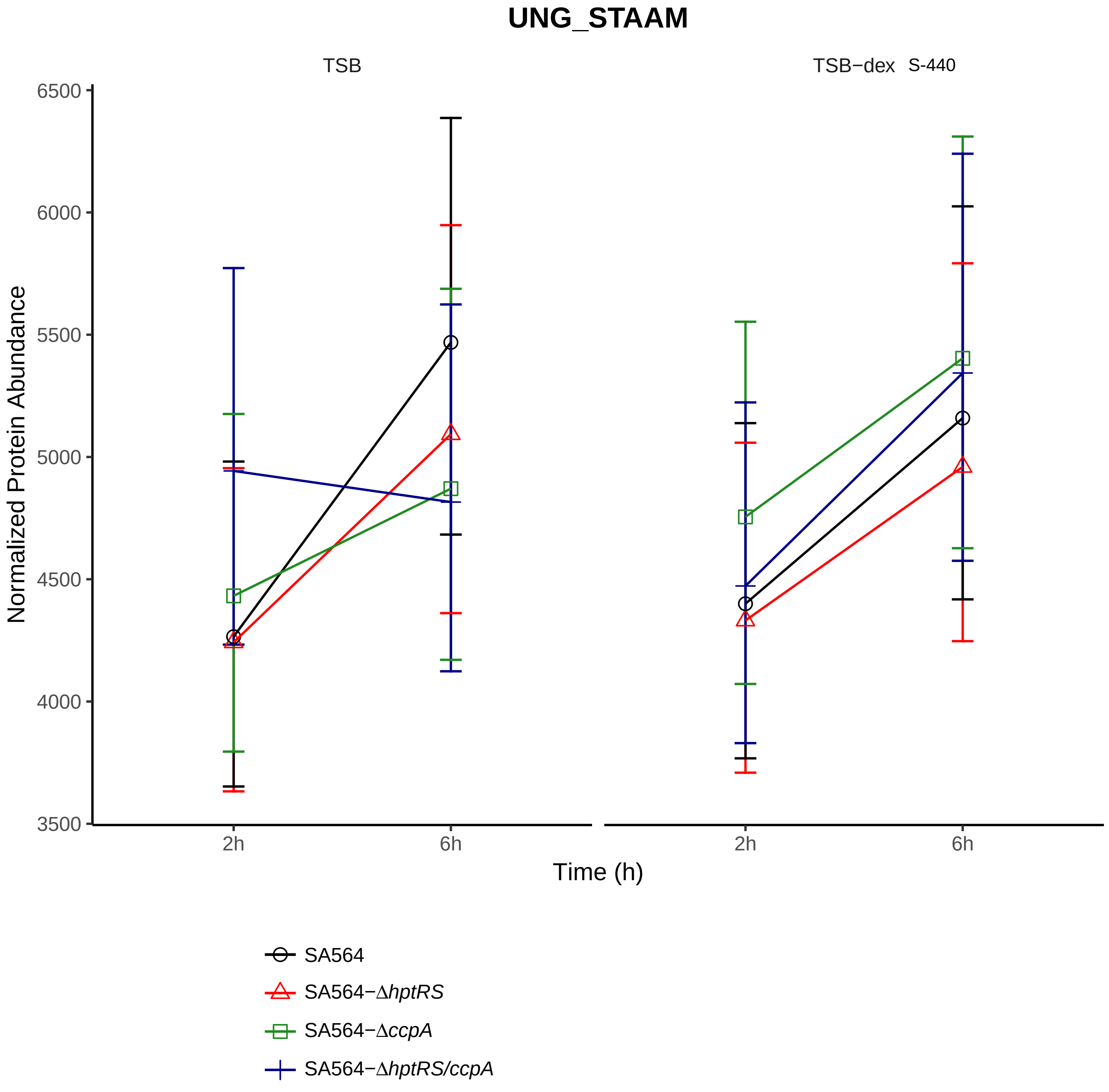
UNG_STAAM
TSB
TSB−dex
S-440
Normalized Protein Abundance
6500
6000
5500
5000
4500
4000
3500
2h
6h
2h
6h
Time (h)
SA564
SA564−ΔhptRS
SA564−ΔccpA
SA564−ΔhptRS/ccpA

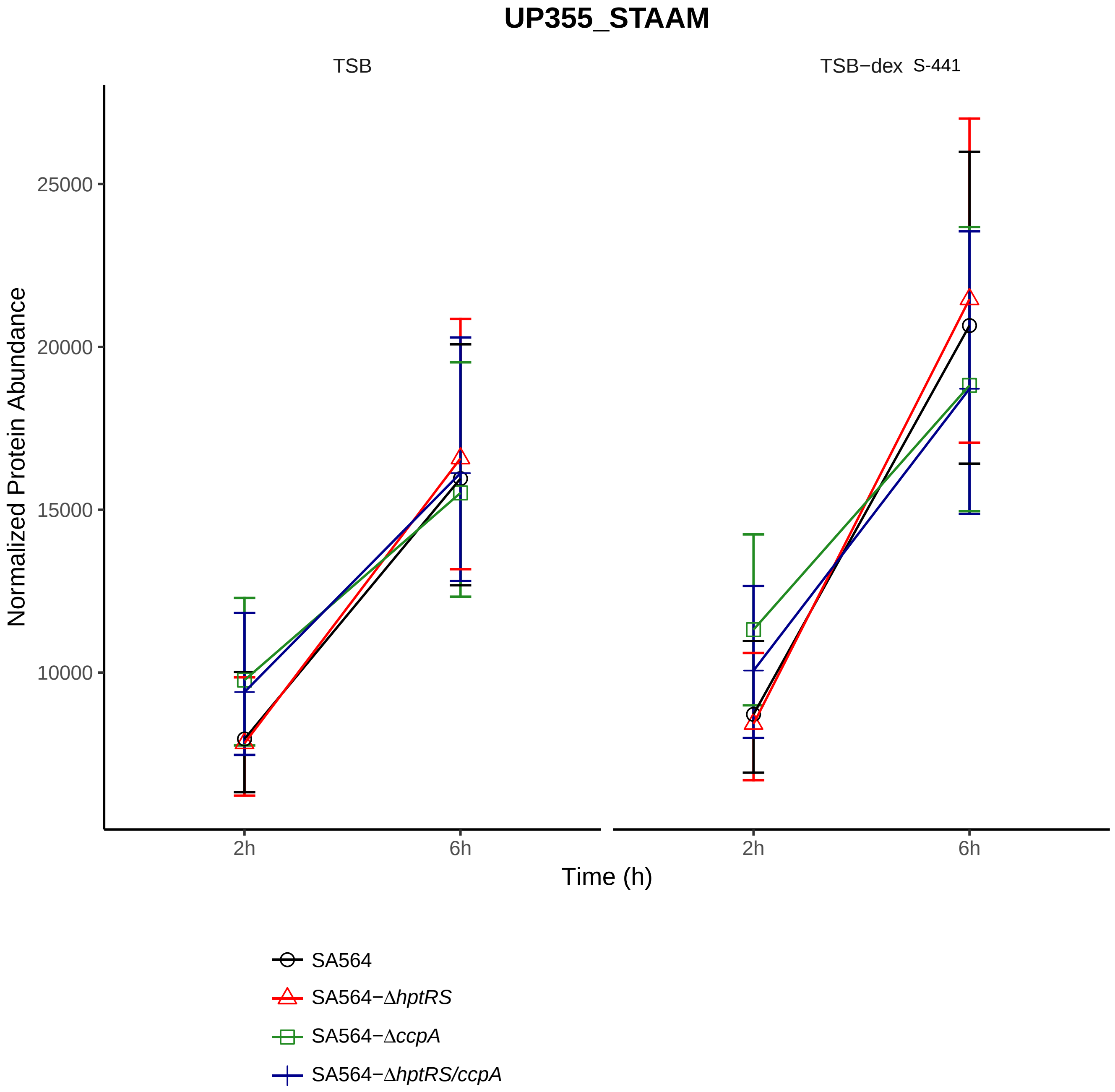

UP355_STAAM
TSB
TSB−dex
S-441
Normalized Protein Abundance
25000
20000
15000
10000
2h
6h
2h
6h
Time (h)
SA564
SA564−Δ*hptRS*
SA564−Δ*ccpA*
SA564−Δ*hptRS/ccpA*

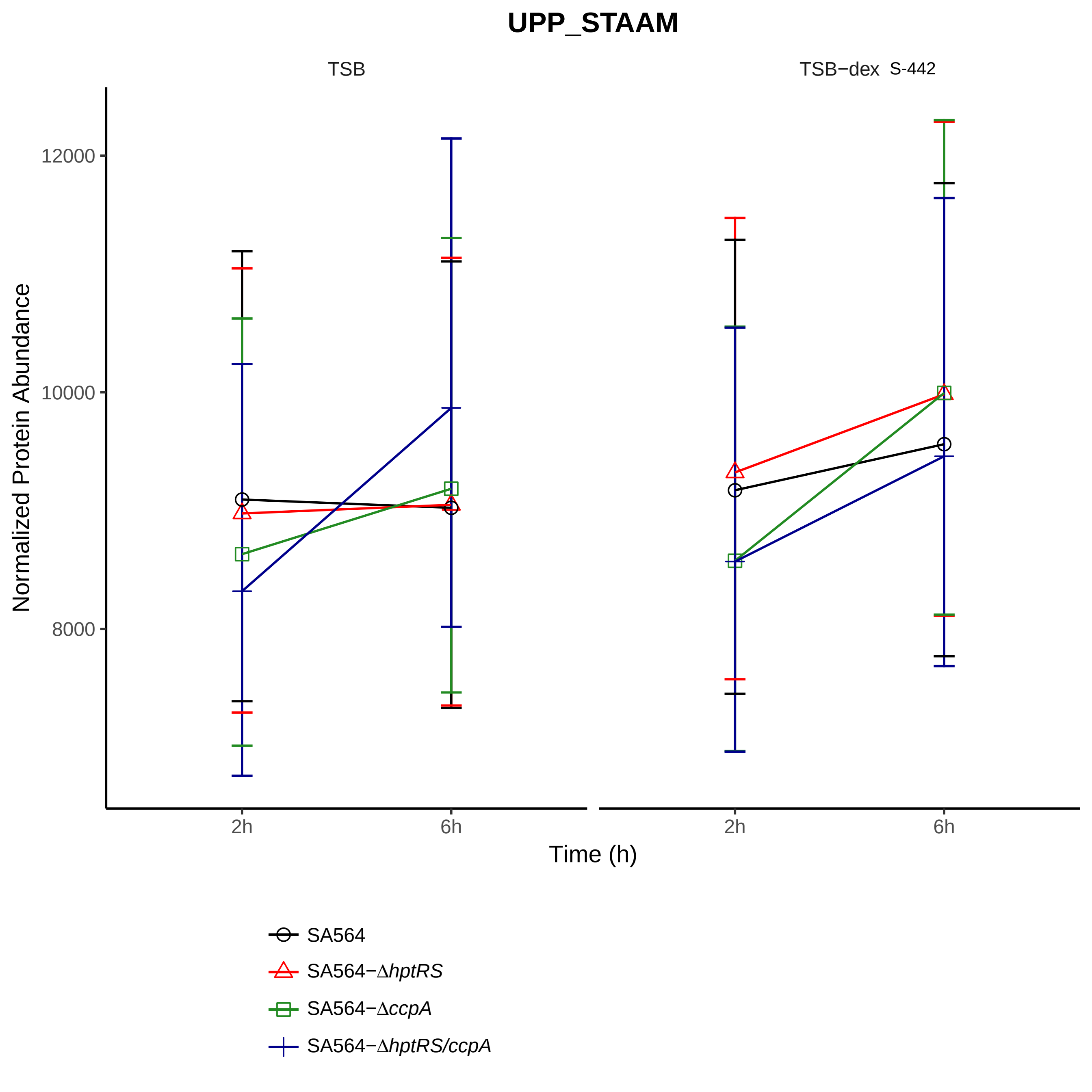
UPP_STAAM
TSB
TSB−dex
S-442
12000
10000
8000
Normalized Protein Abundance
2h
6h
2h
6h
Time (h)
SA564
SA564−ΔhptRS
SA564−ΔccpA
SA564−ΔhptRS/ccpA

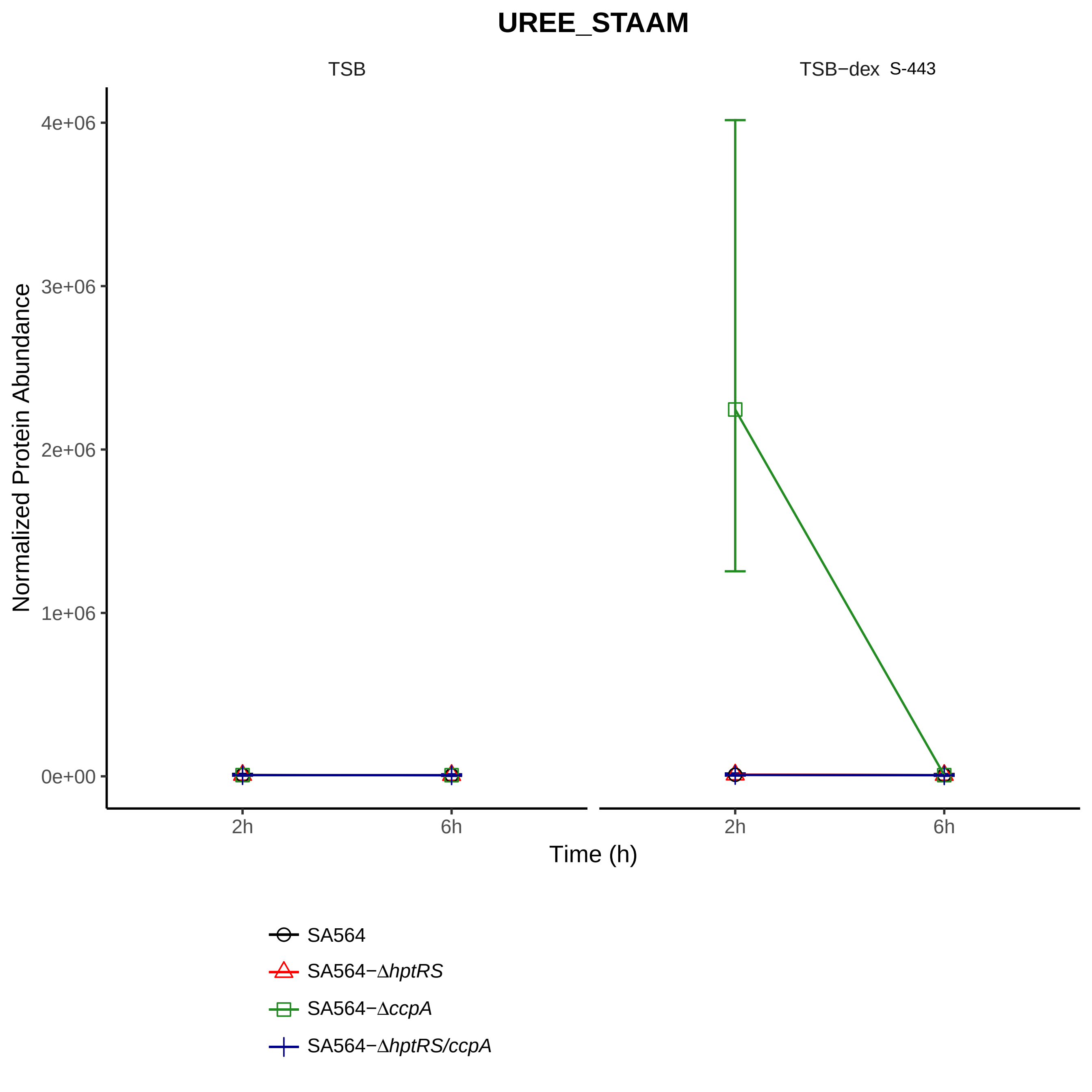

UREE_STAAM
TSB
TSB−dex
S-443
Normalized Protein Abundance
4e+06
3e+06
2e+06
1e+06
0e+00
2h
6h
2h
6h
Time (h)
SA564
SA564−Δ*hptRS*
SA564−Δ*ccpA*
SA564−Δ*hptRS/ccpA*

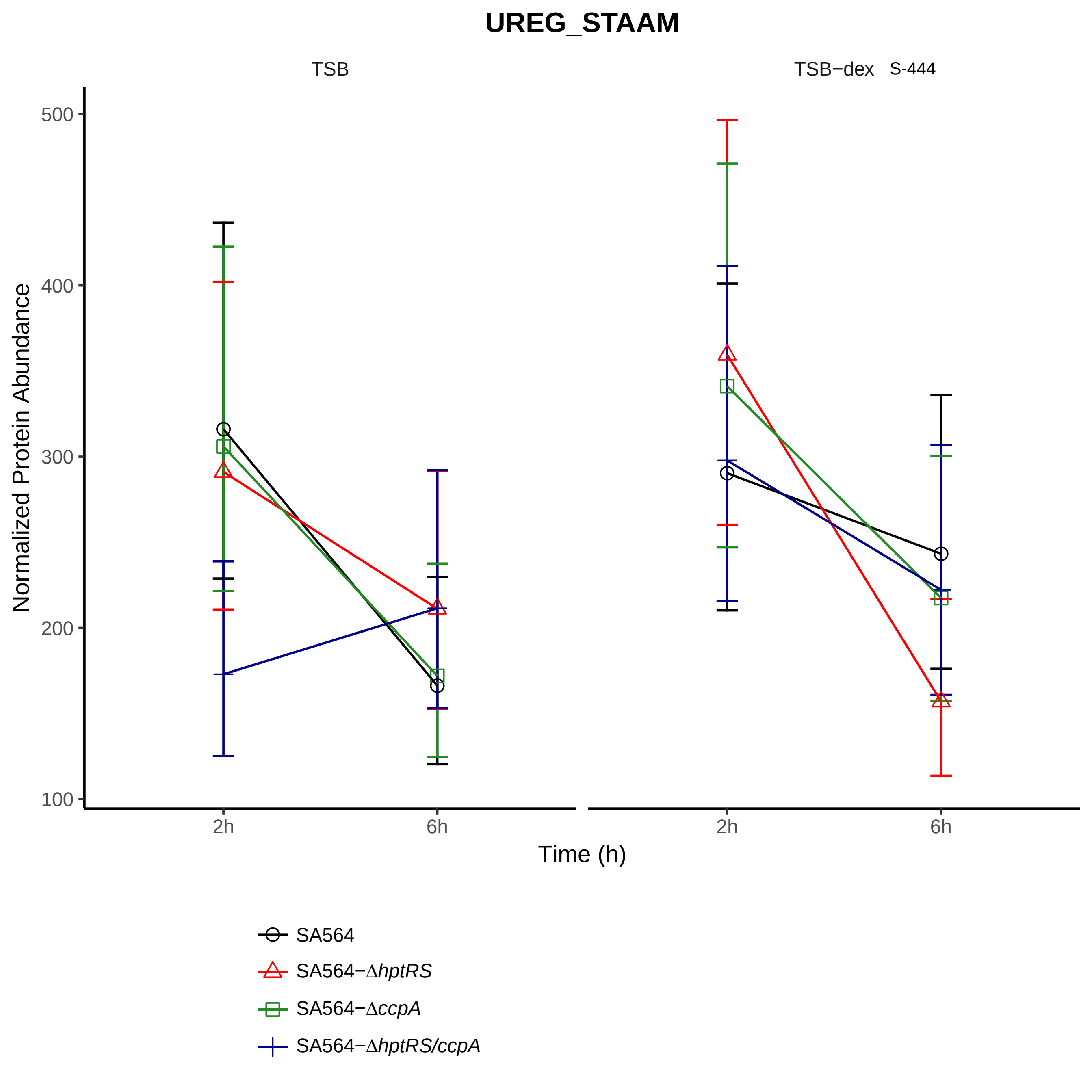

UREG_STAAM
TSB
TSB−dex
S-444
Normalized Protein Abundance
500
400
300
200
100
2h
6h
2h
6h
Time (h)
SA564
SA564−Δ*hptRS*
SA564−Δ*ccpA*
SA564−Δ*hptRS/ccpA*

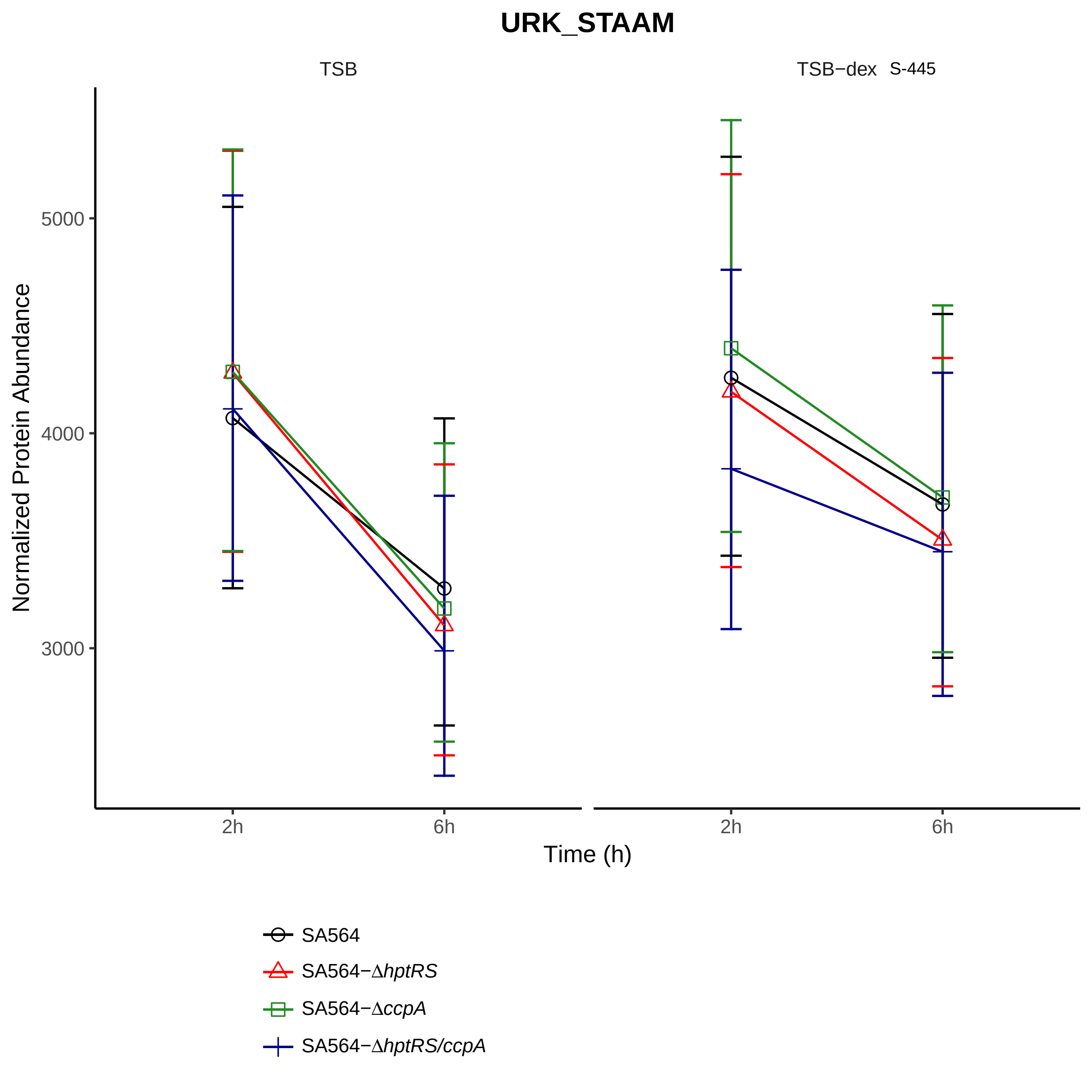

URK_STAAM
TSB
TSB−dex
S-445
Normalized Protein Abundance
5000
4000
3000
2h
6h
2h
6h
Time (h)
SA564
SA564−Δ*hptRS*
SA564−Δ*ccpA*
SA564−Δ*hptRS/ccpA*

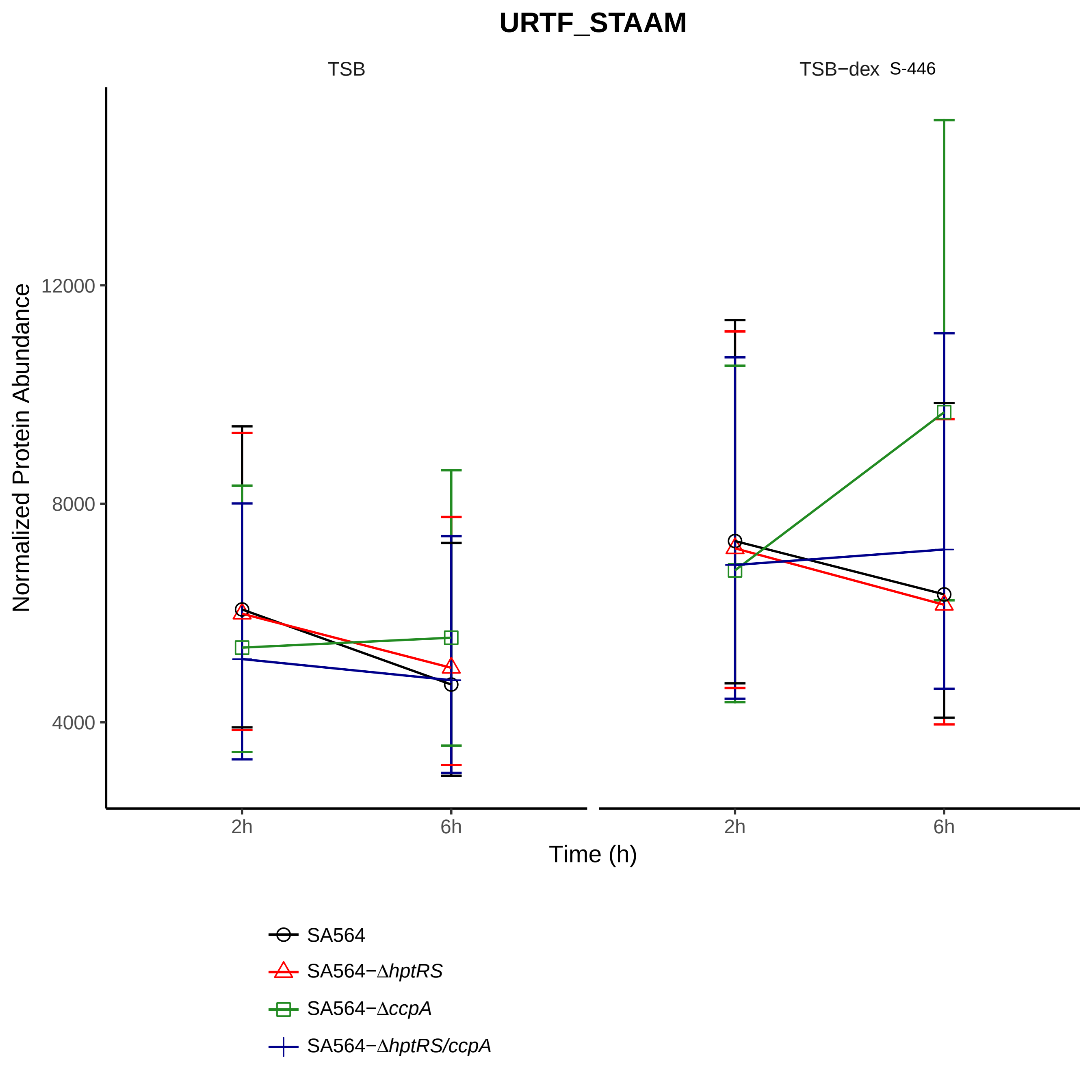

URTF_STAAM
TSB
TSB−dex
S-446
Normalized Protein Abundance
12000
8000
4000
2h
6h
2h
6h
Time (h)
SA564
SA564−Δ*hptRS*
SA564−Δ*ccpA*
SA564−Δ*hptRS/ccpA*

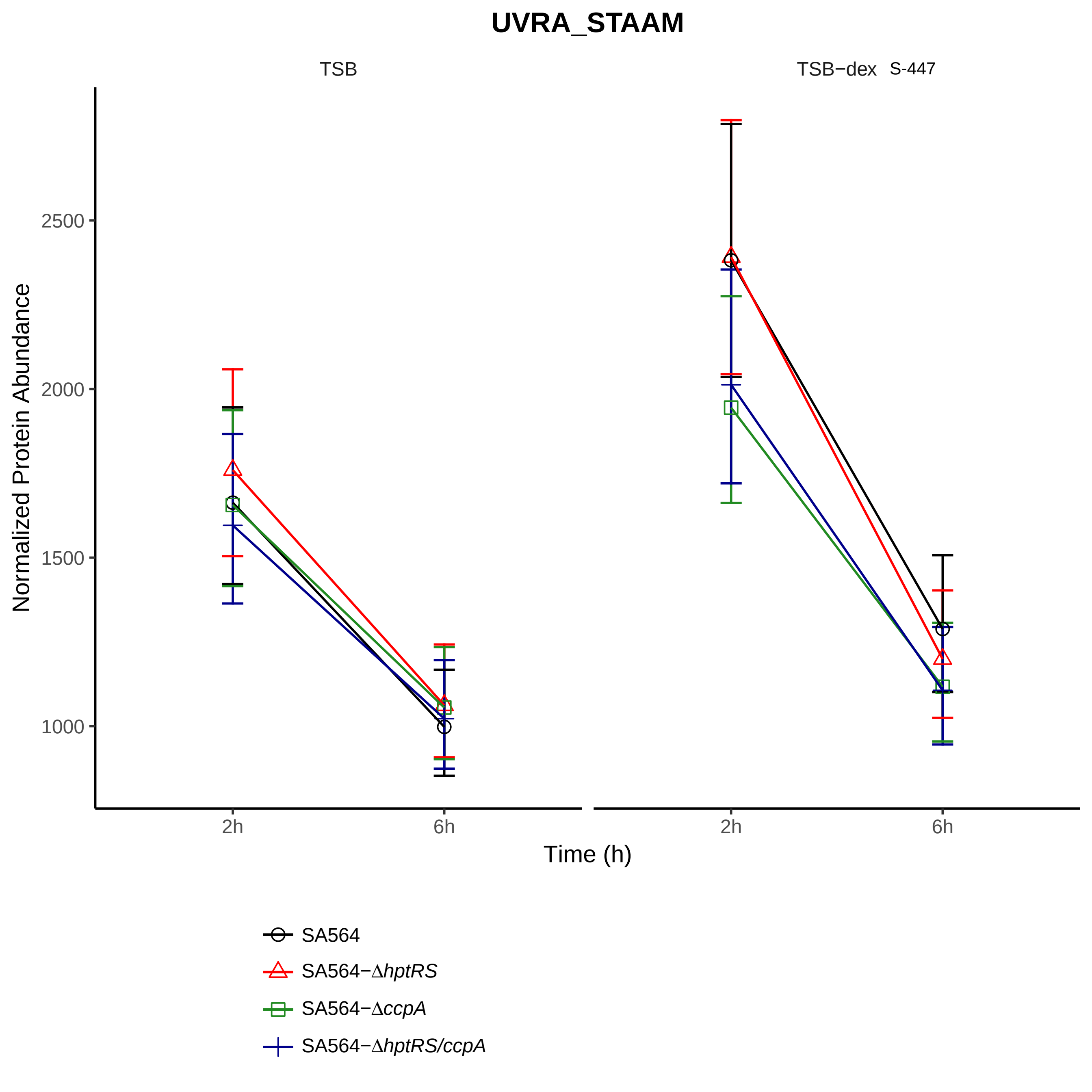

UVRA_STAAM
TSB
TSB−dex
S-447
Normalized Protein Abundance
2500
2000
1500
1000
2h
6h
2h
6h
Time (h)
SA564
SA564−Δ*hptRS*
SA564−Δ*ccpA*
SA564−Δ*hptRS/ccpA*

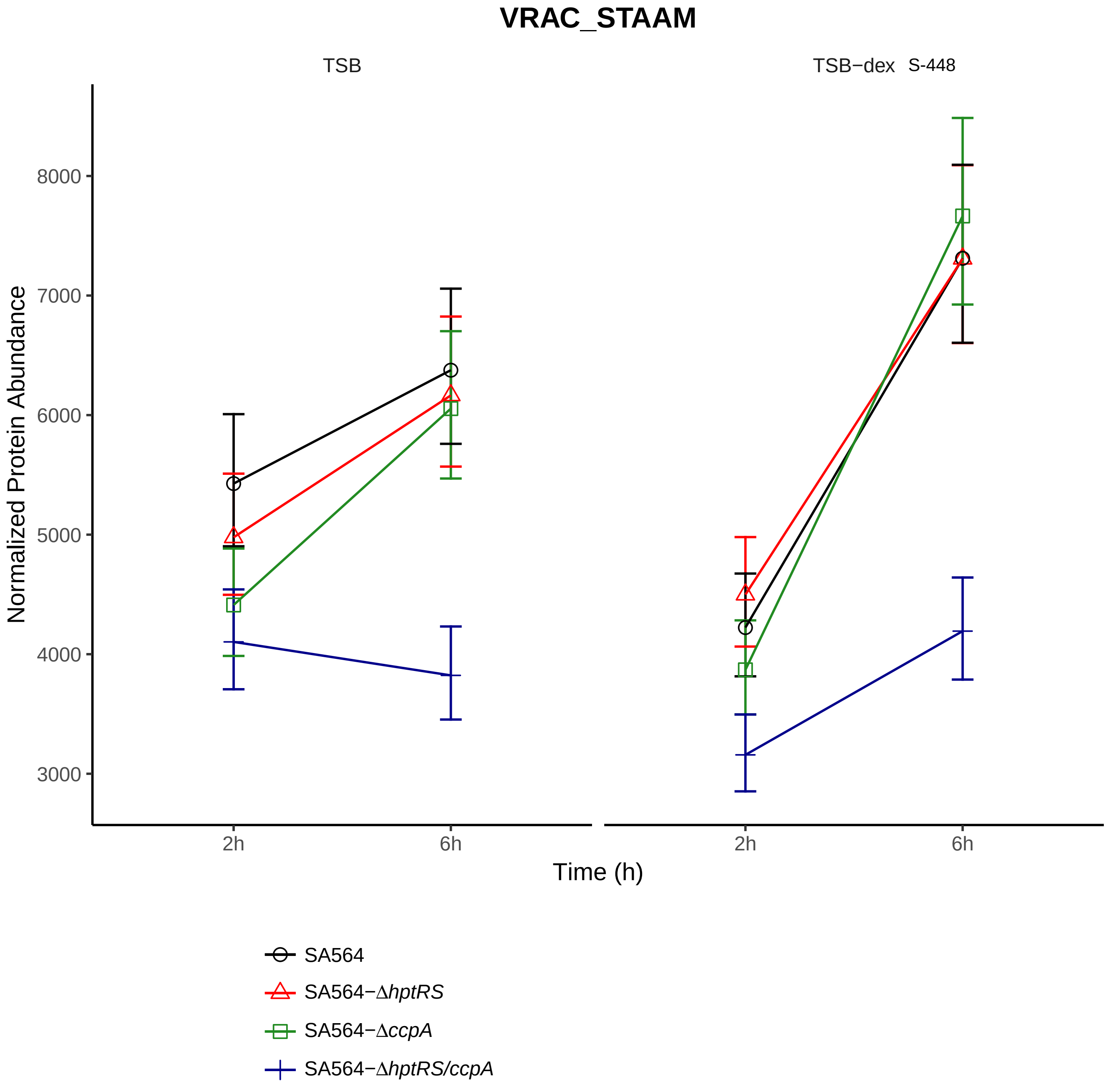

VRAC_STAAM
TSB
TSB−dex
S-448
Normalized Protein Abundance
8000
7000
6000
5000
4000
3000
2h
6h
2h
6h
Time (h)
SA564
SA564−ΔhptRS
SA564−ΔccpA
SA564−ΔhptRS/ccpA

VRAR_STAAM

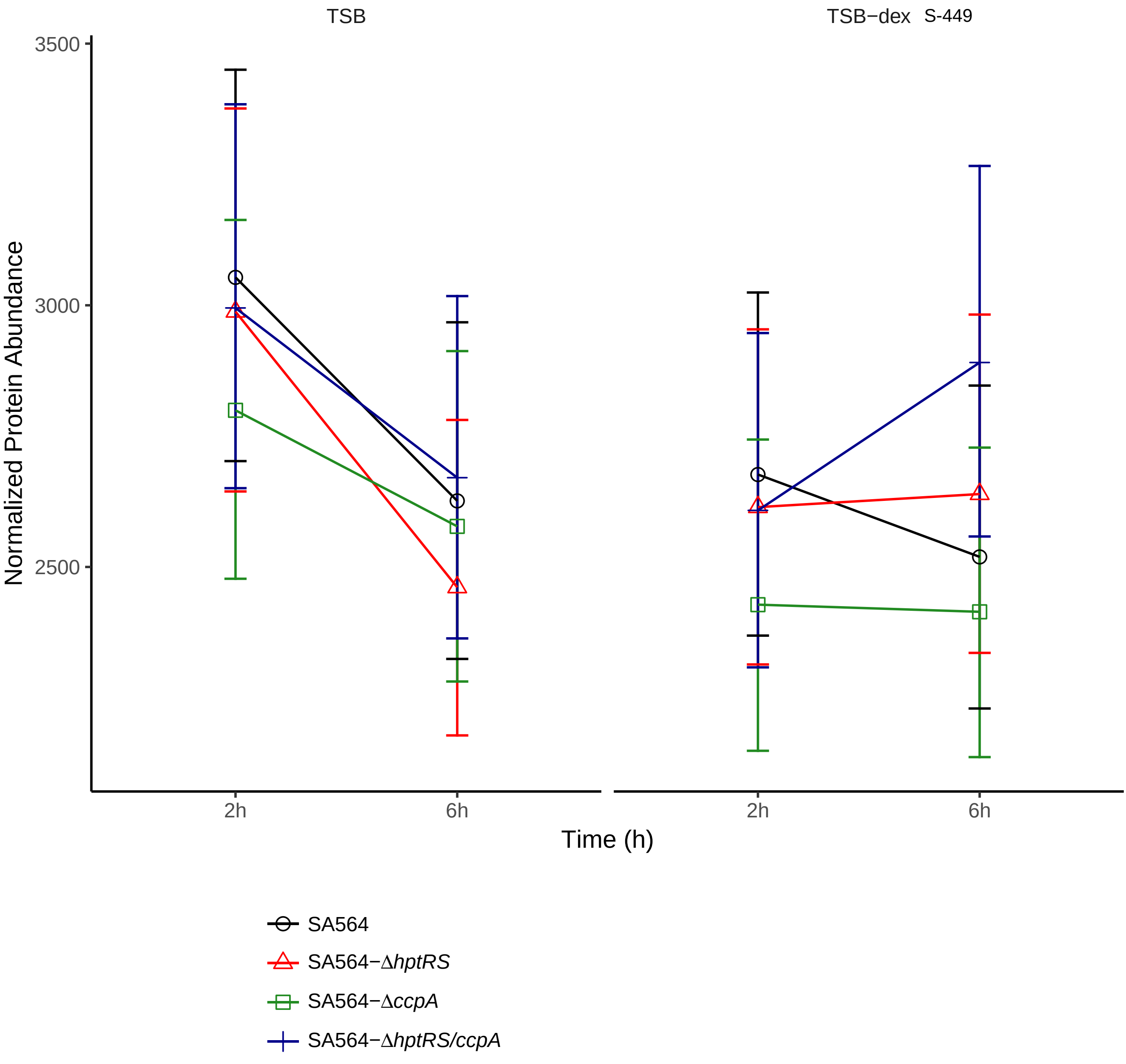

TSB
TSB−dex
S-449
3500
3000
2500
Normalized Protein Abundance
2h
6h
2h
6h
Time (h)
SA564
SA564−Δ*hptRS*
SA564−Δ*ccpA*
SA564−Δ*hptRS/ccpA*

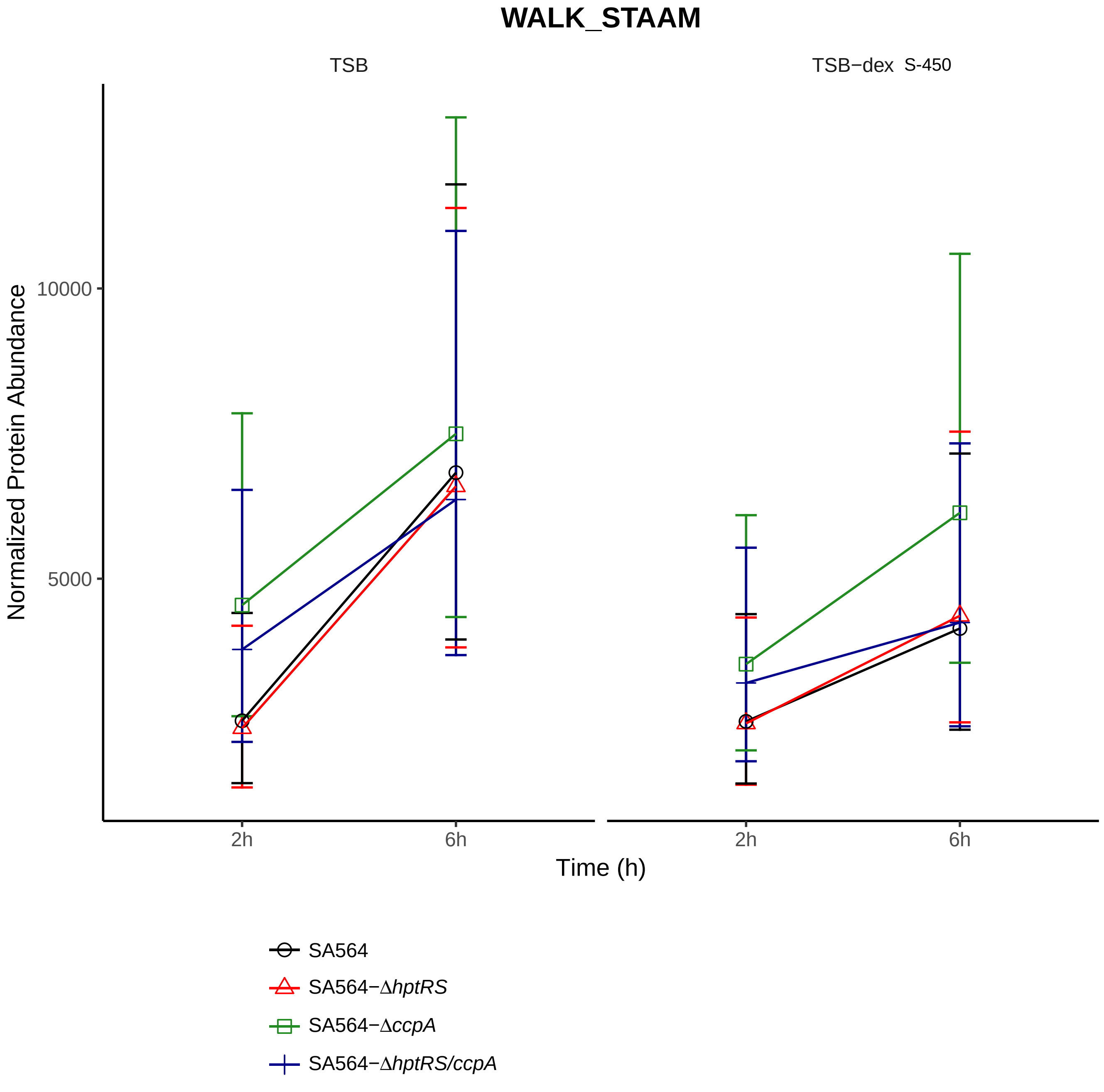
WALK_STAAM
TSB
TSB−dex
S-450
Normalized Protein Abundance
10000
5000
2h
6h
2h
6h
Time (h)
SA564
SA564−Δ*hptRS*
SA564−Δ*ccpA*
SA564−Δ*hptRS/ccpA*

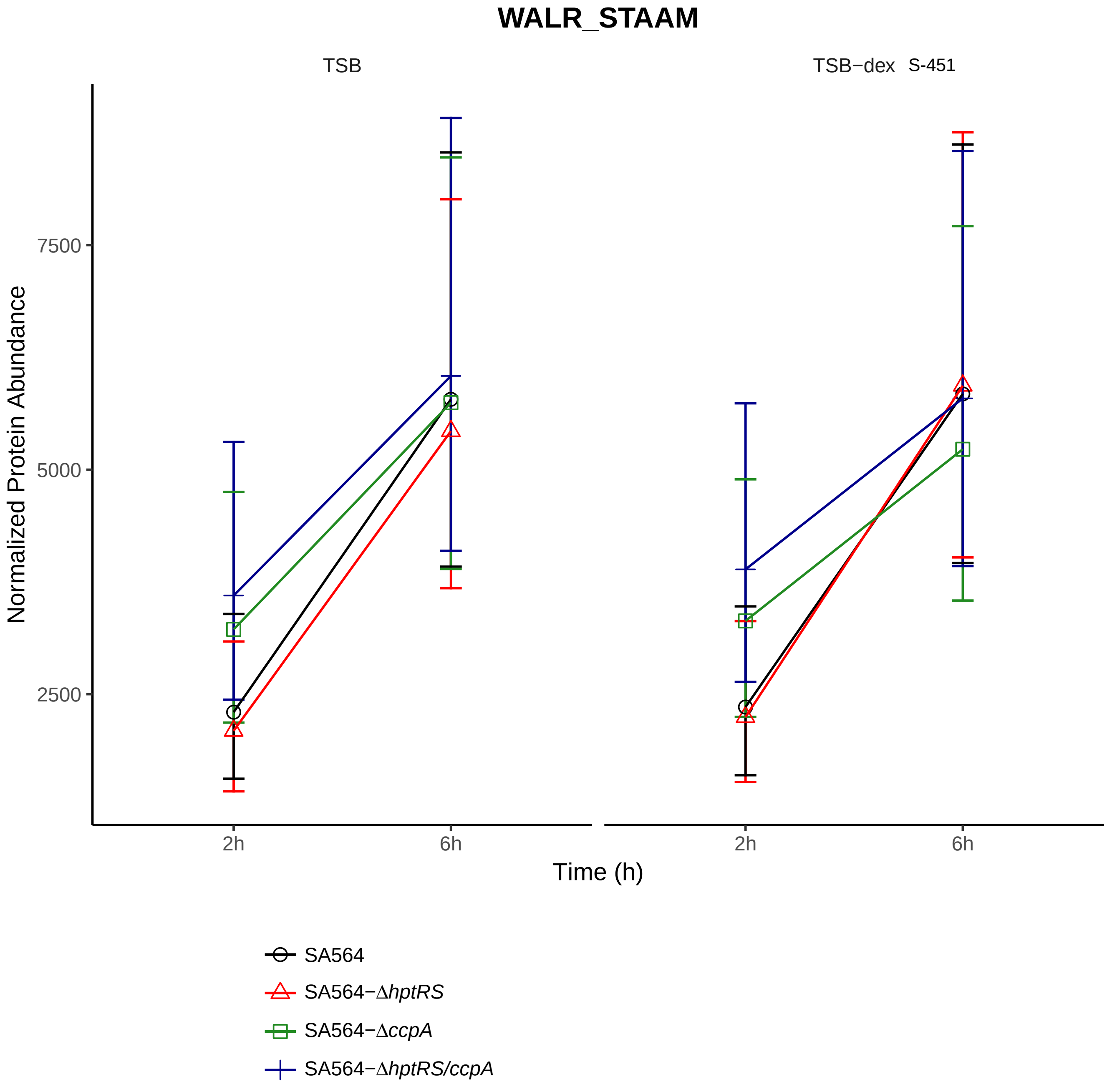

WALR_STAAM
TSB
TSB−dex
S-451
Normalized Protein Abundance
7500
5000
2500
2h
6h
2h
6h
Time (h)
SA564
SA564−Δ*hptRS*
SA564−Δ*ccpA*
SA564−Δ*hptRS/ccpA*

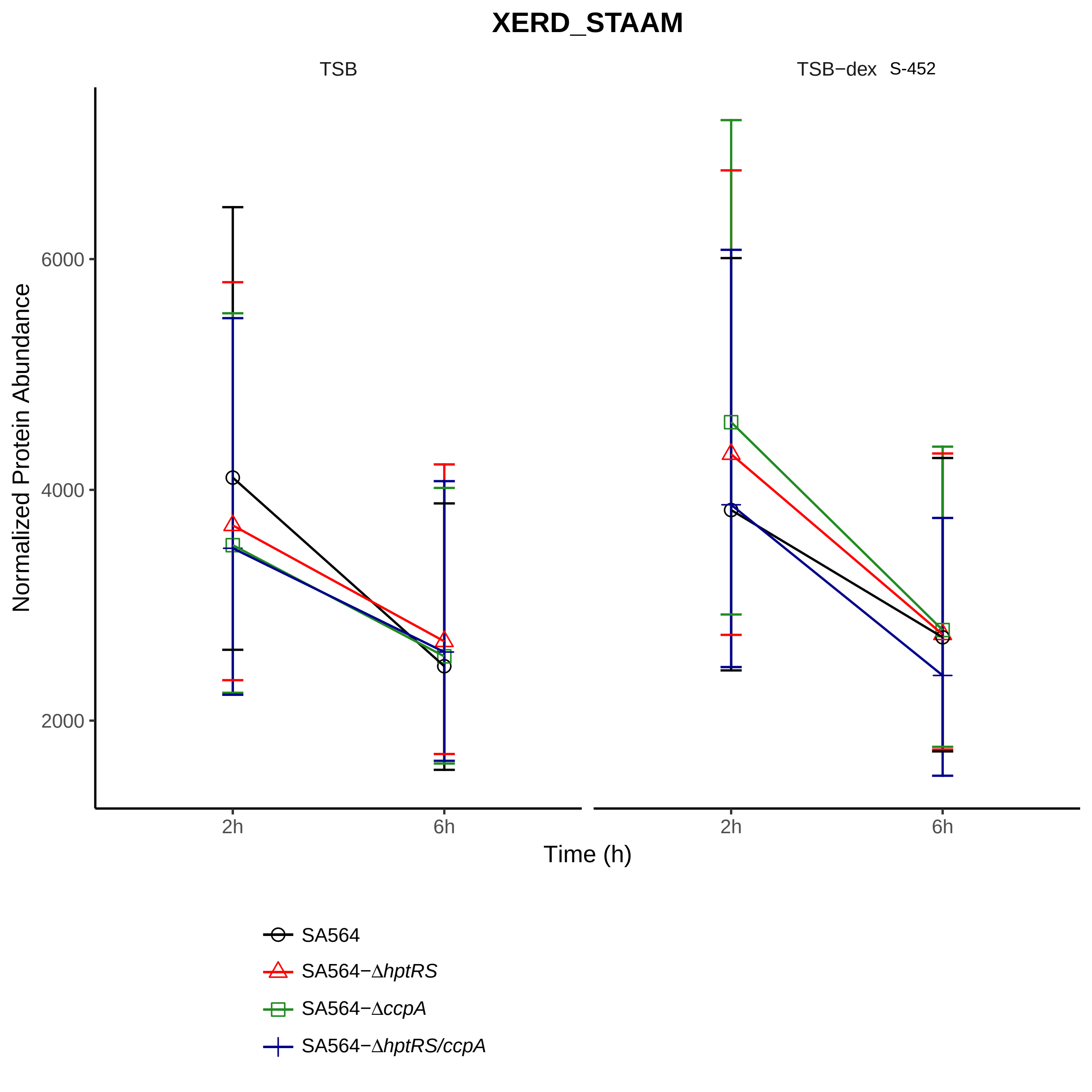
XERD_STAAM
TSB
TSB−dex
S-452
Normalized Protein Abundance
6000
4000
2000
2h
6h
2h
6h
Time (h)
SA564
SA564−ΔhptRS
SA564−ΔccpA
SA564−ΔhptRS/ccpA

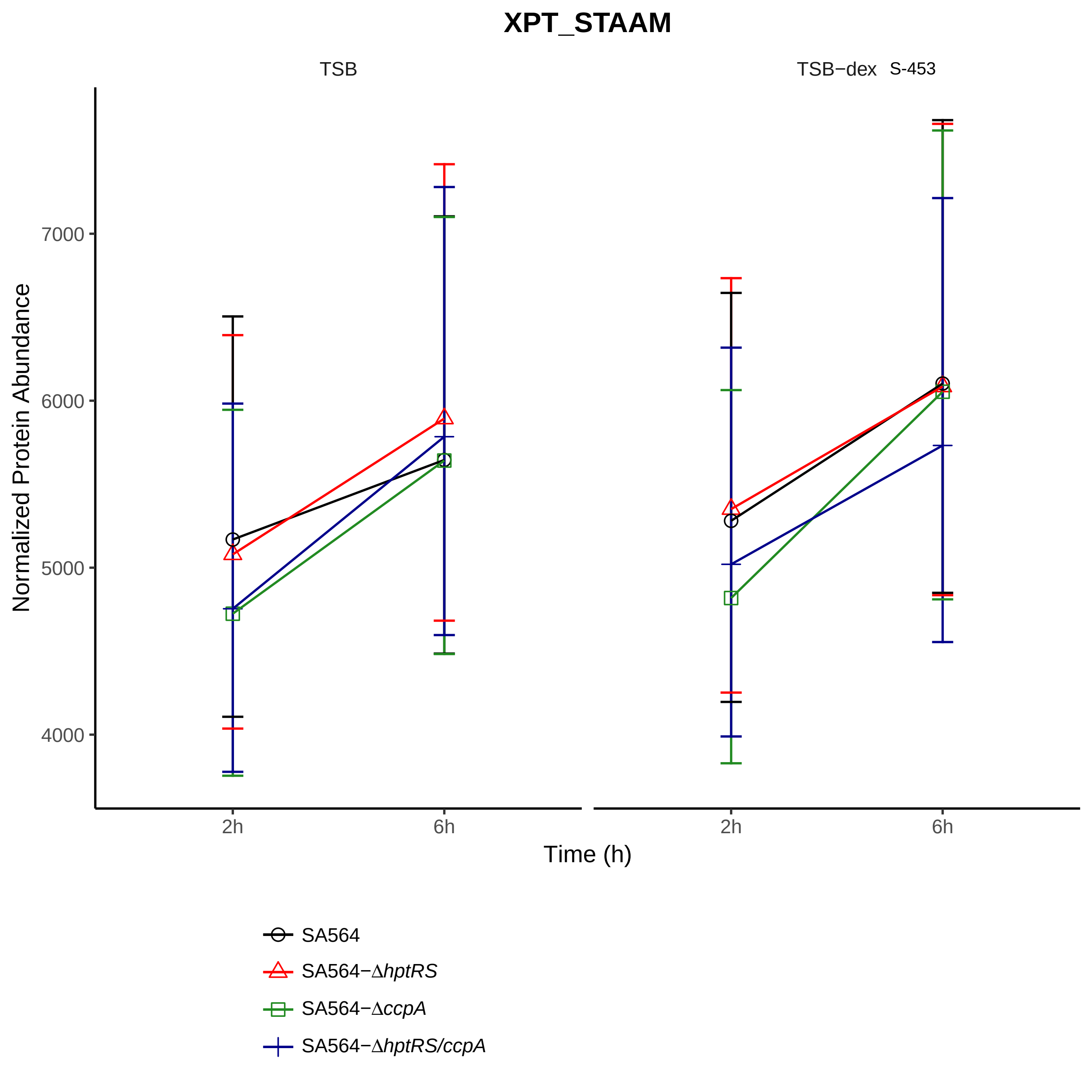

XPT_STAAM
TSB
TSB−dex
S-453
Normalized Protein Abundance
7000
6000
5000
4000
2h
6h
2h
6h
Time (h)
SA564
SA564−ΔhptRS
SA564−ΔccpA
SA564−ΔhptRS/ccpA

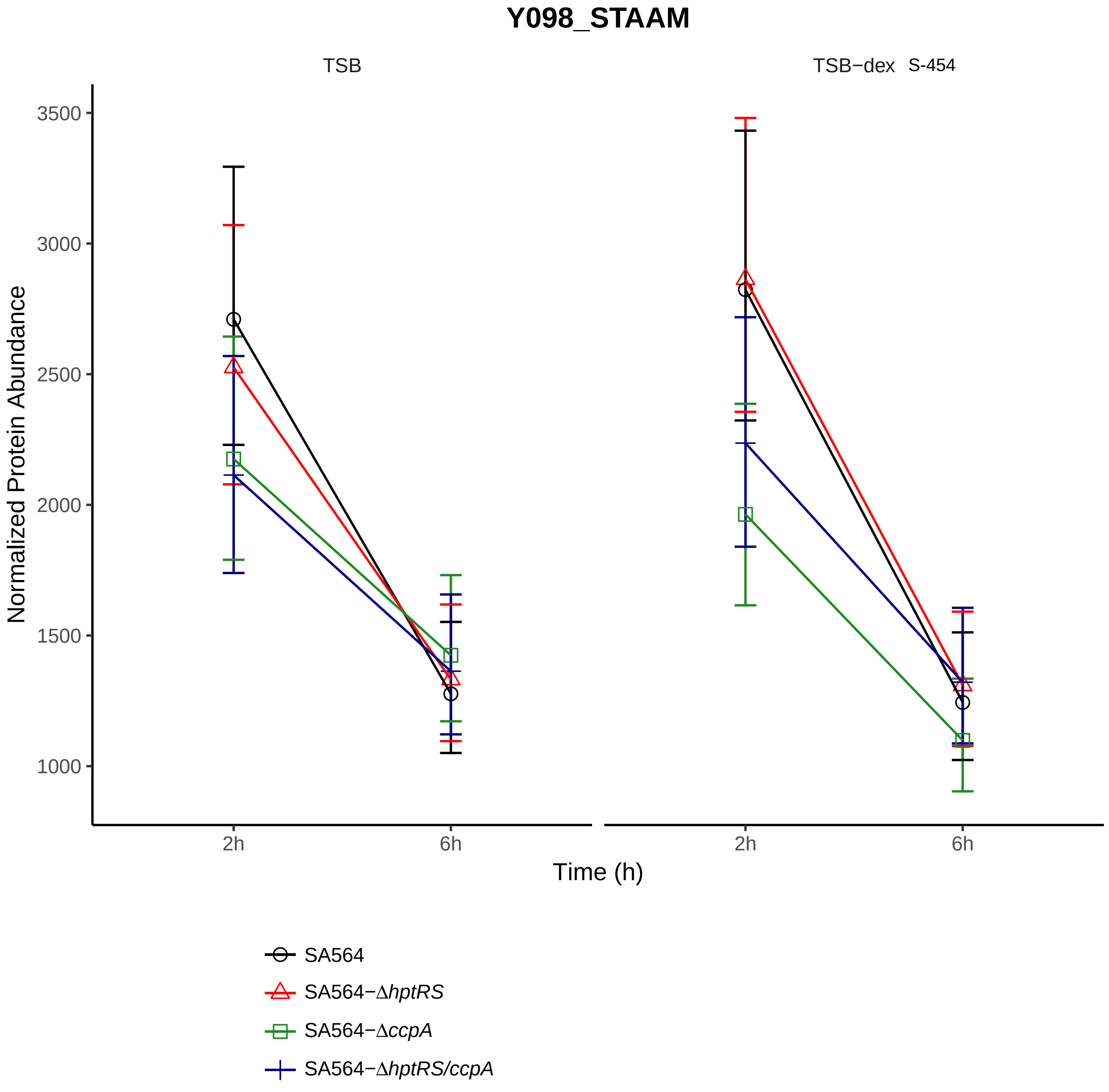

Y098_STAAM
TSB
TSB−dex
S-454
Normalized Protein Abundance
3500
3000
2500
2000
1500
1000
2h
6h
2h
6h
Time (h)
SA564
SA564−Δ*hptRS*
SA564−Δ*ccpA*
SA564−Δ*hptRS/ccpA*

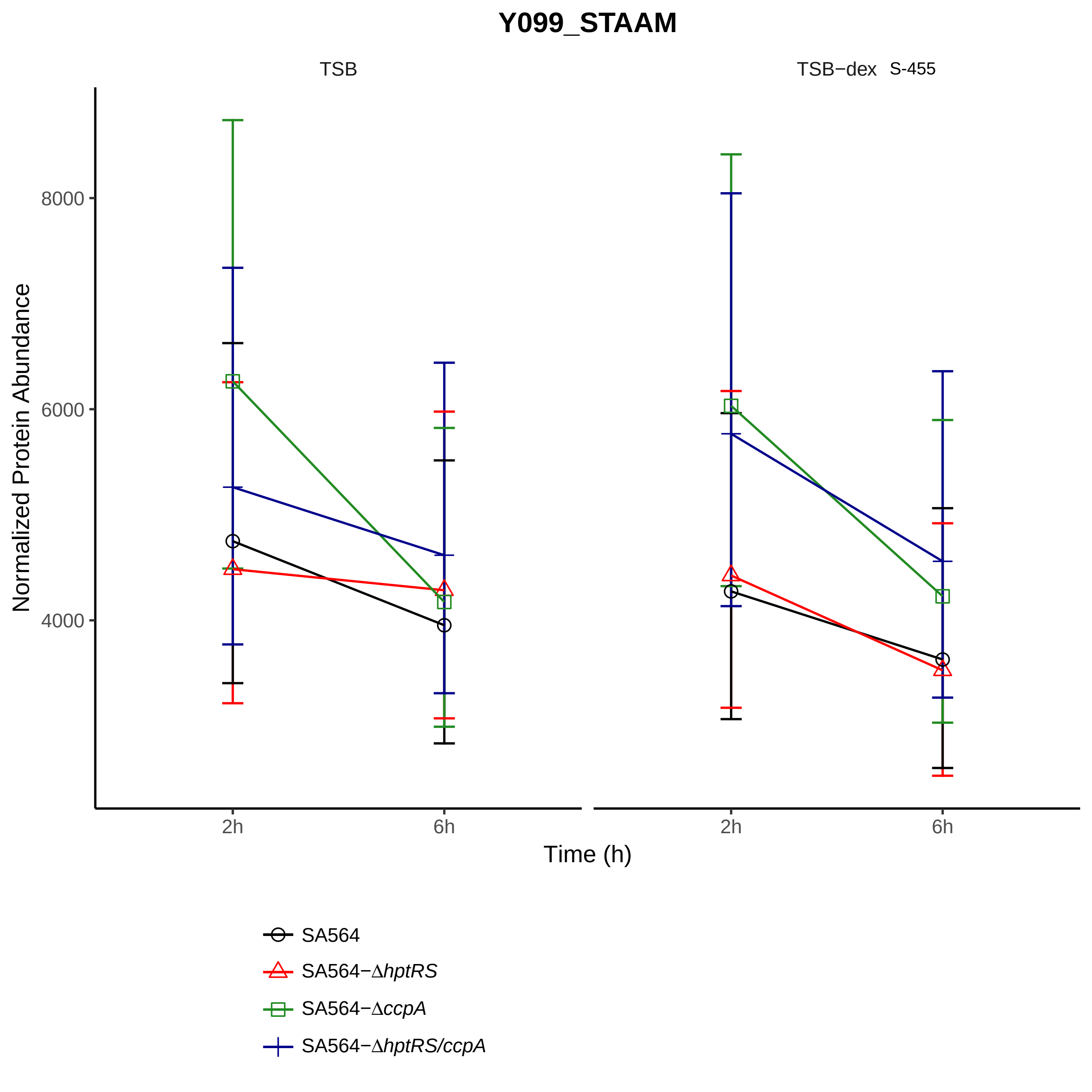

Y099_STAAM
TSB
TSB−dex
S-455
Normalized Protein Abundance
8000
6000
4000
2h
6h
2h
6h
Time (h)
SA564
SA564−ΔhptRS
SA564−ΔccpA
SA564−ΔhptRS/ccpA

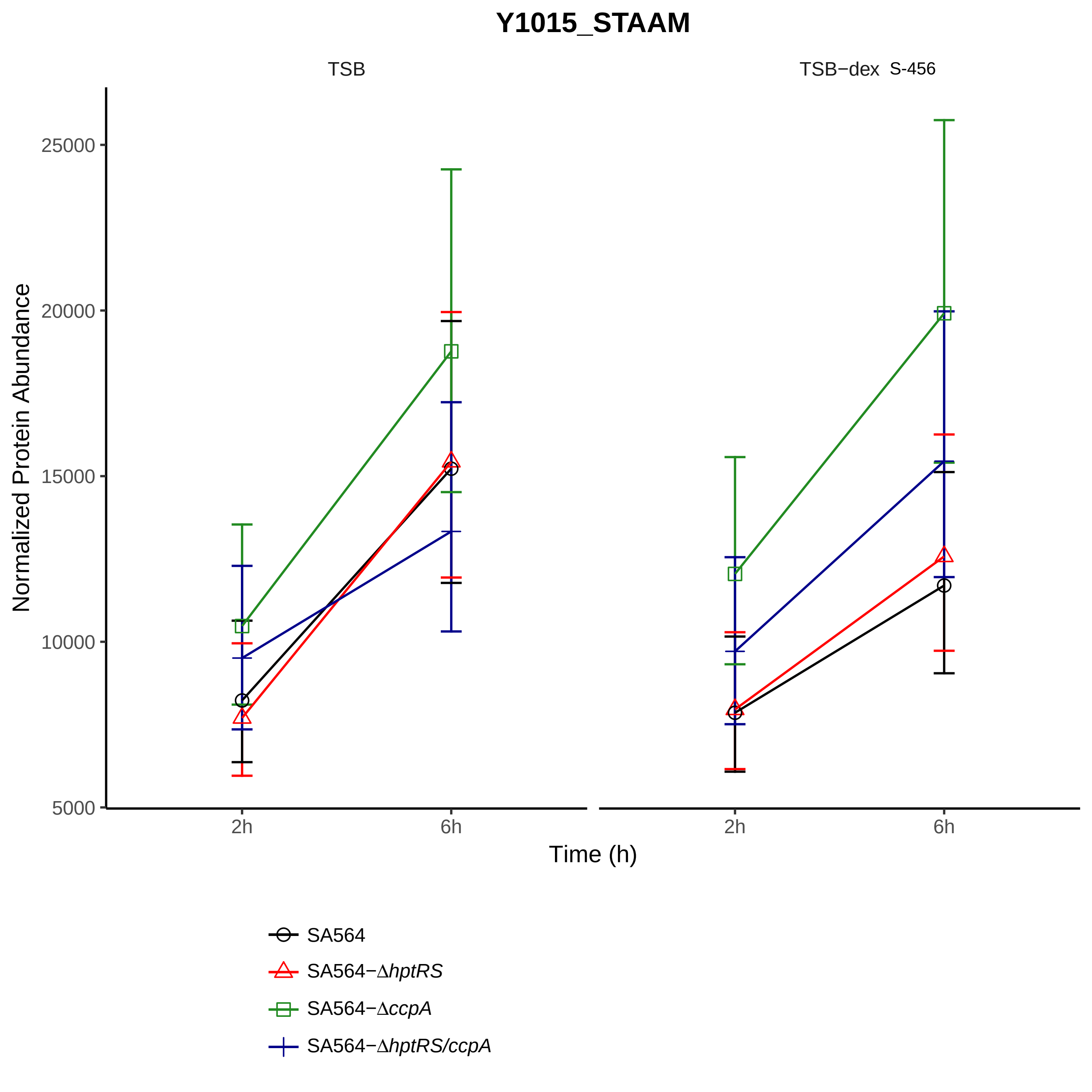

Y1015_STAAM
TSB
TSB−dex
S-456
Normalized Protein Abundance
25000
20000
15000
10000
5000
2h
6h
2h
6h
Time (h)
SA564
SA564−Δ*hptRS*
SA564−Δ*ccpA*
SA564−Δ*hptRS/ccpA*

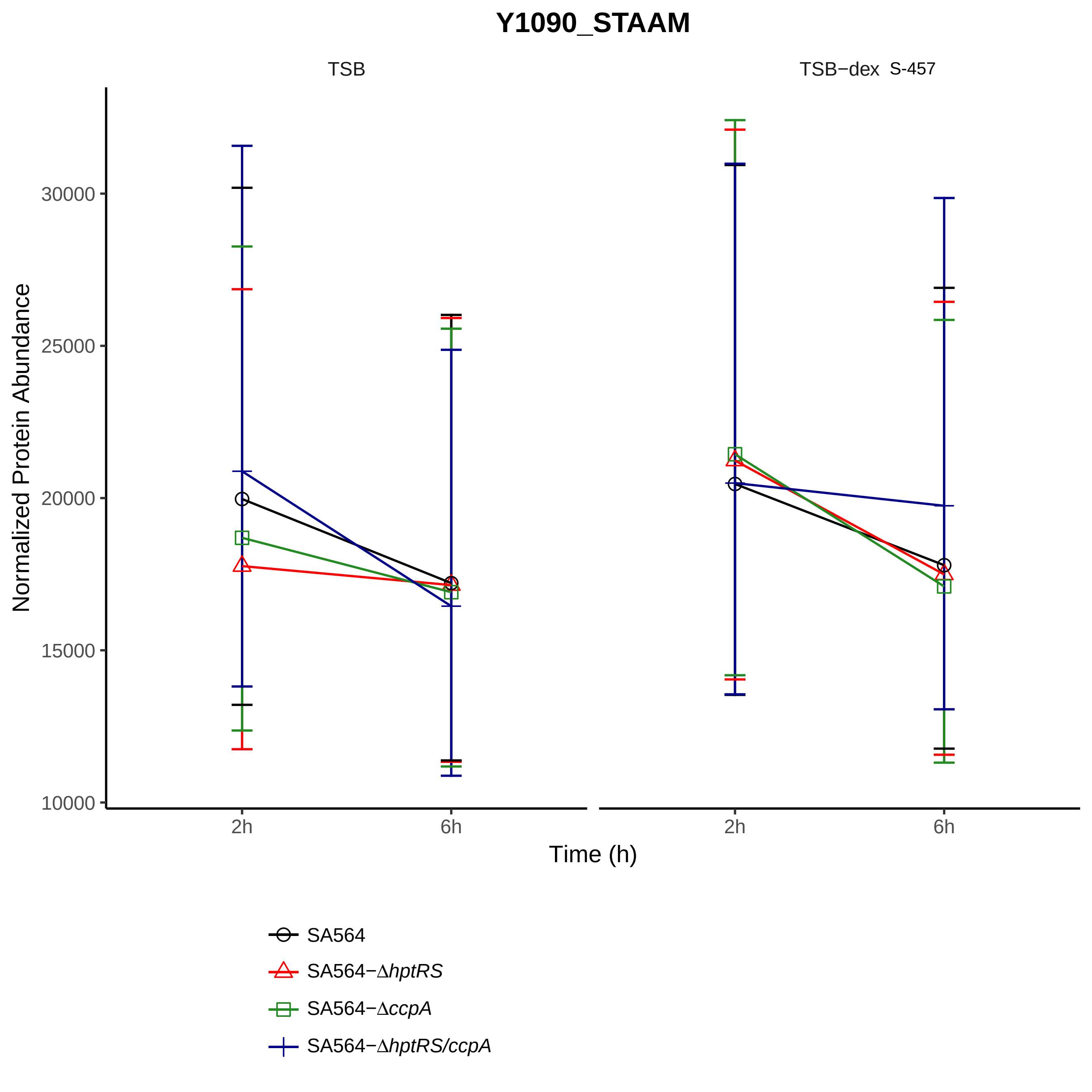

Y1090_STAAM
TSB
TSB−dex
S-457
Normalized Protein Abundance
30000
25000
20000
15000
10000
2h
6h
2h
6h
Time (h)
SA564
SA564−Δ*hptRS*
SA564−Δ*ccpA*
SA564−Δ*hptRS/ccpA*

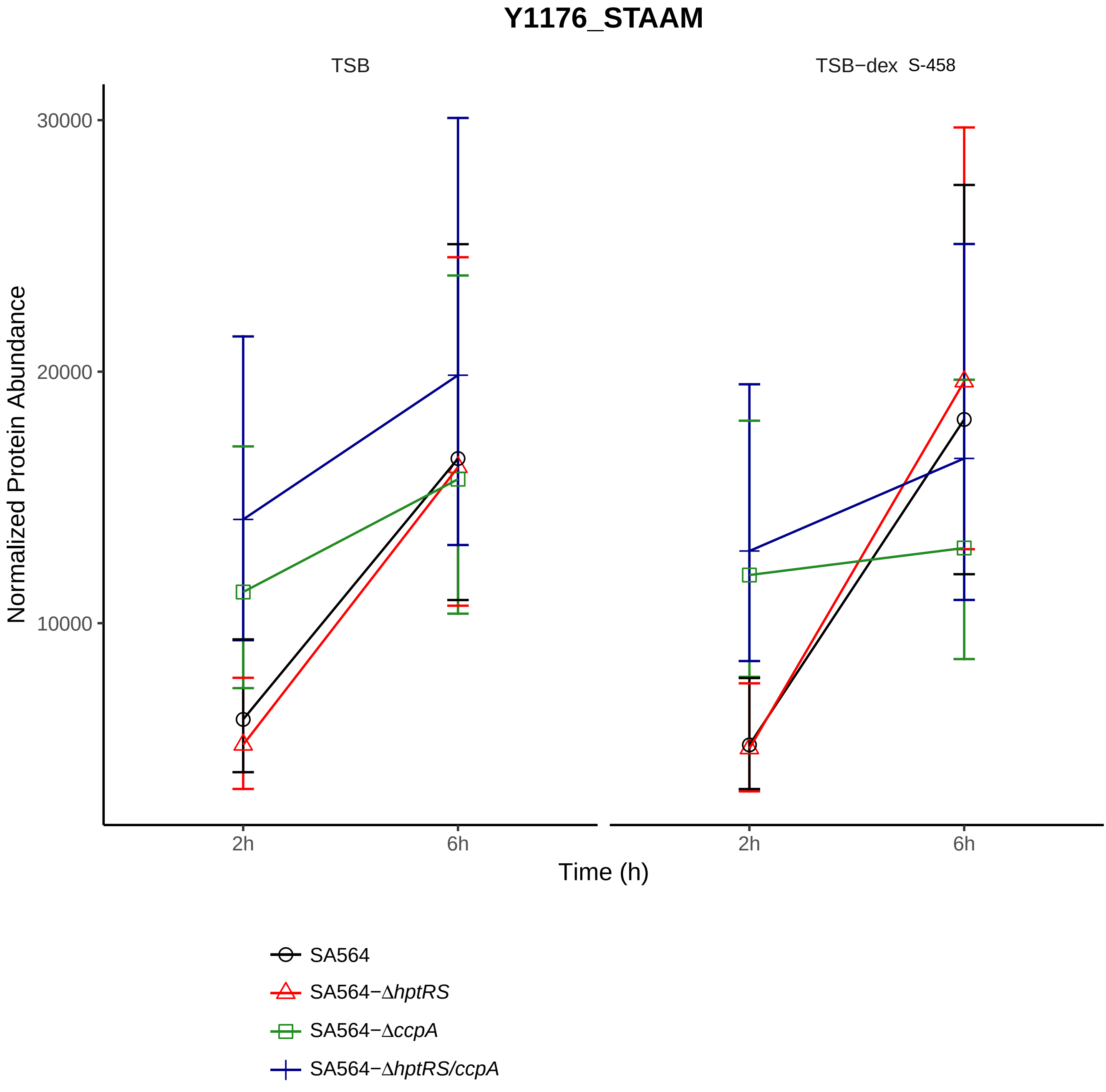

Y1176_STAAM
TSB
TSB−dex
30000
20000
10000
Normalized Protein Abundance
2h
6h
2h
6h
Time (h)
SA564
SA564−Δ*hptRS*
SA564−Δ*ccpA*
SA564−Δ*hptRS/ccpA*

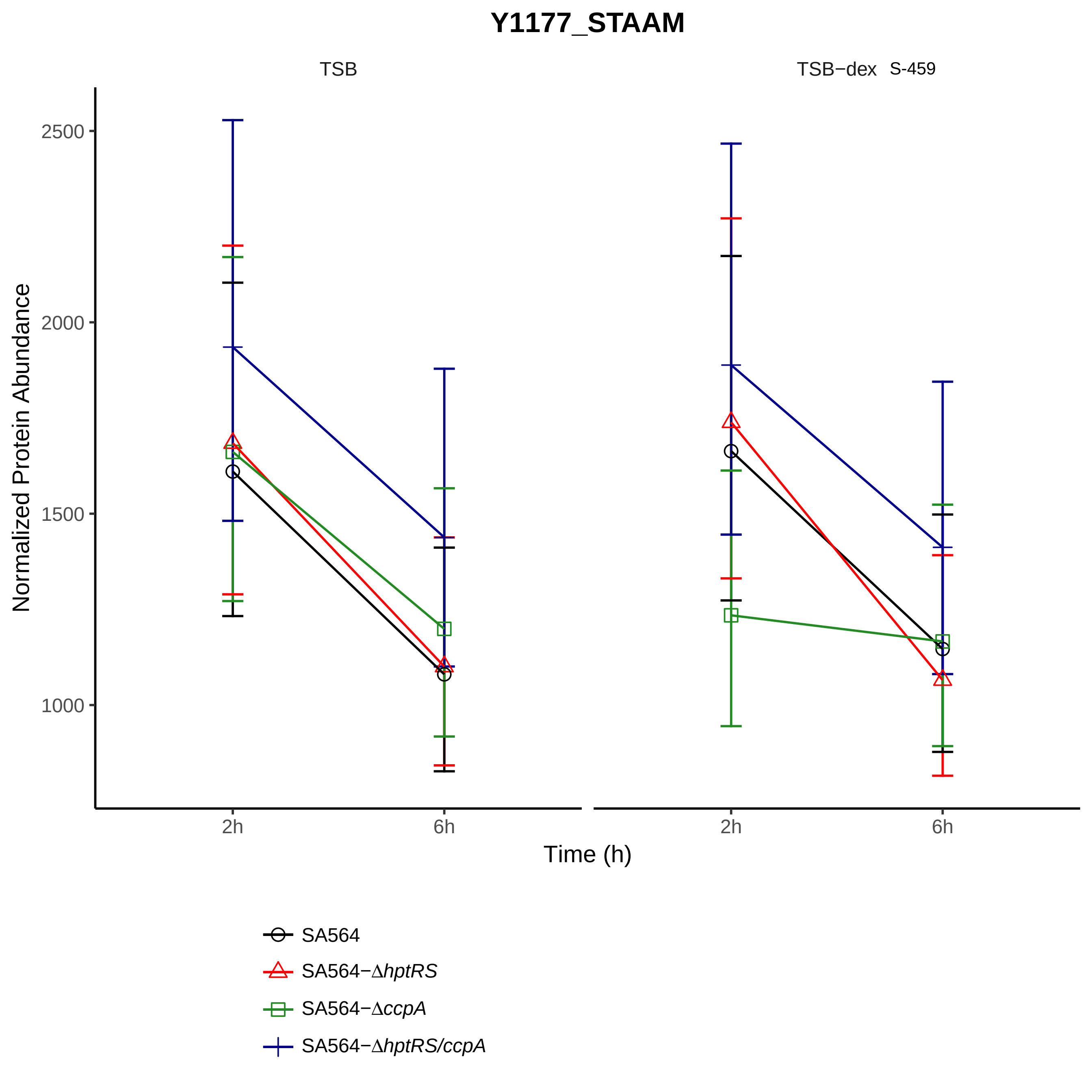

Y1177_STAAM
TSB
TSB−dex
S-459
2500
2000
1500
1000
Normalized Protein Abundance
2h
6h
2h
6h
Time (h)
SA564
SA564−Δ*hptRS*
SA564−Δ*ccpA*
SA564−Δ*hptRS/ccpA*

Y1187_STAAM

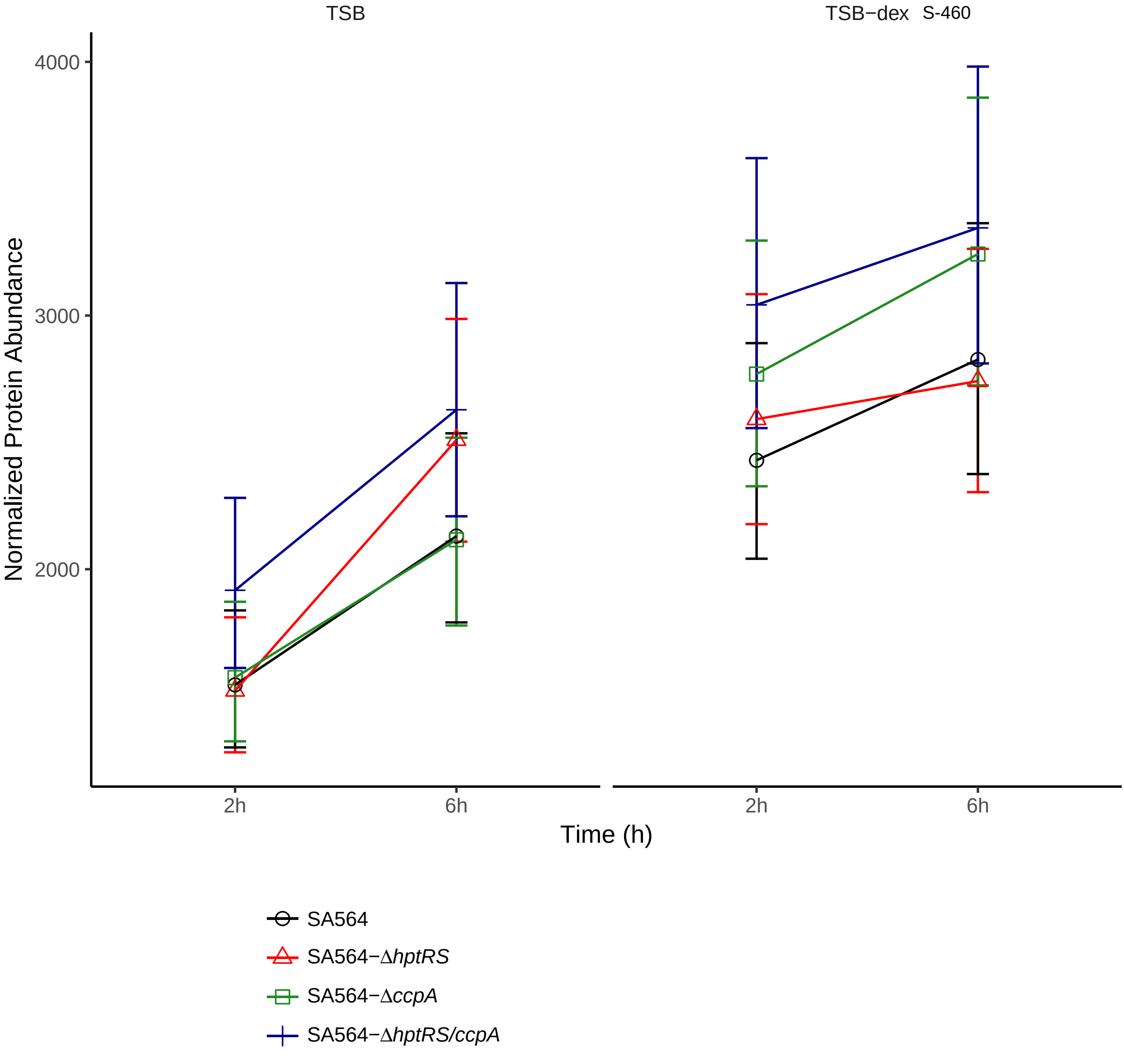

TSB
TSB−dex
S-460
4000
3000
2000
Normalized Protein Abundance
2h
6h
2h
6h
Time (h)
SA564
SA564−ΔhptRS
SA564−ΔccpA
SA564−ΔhptRS/ccpA

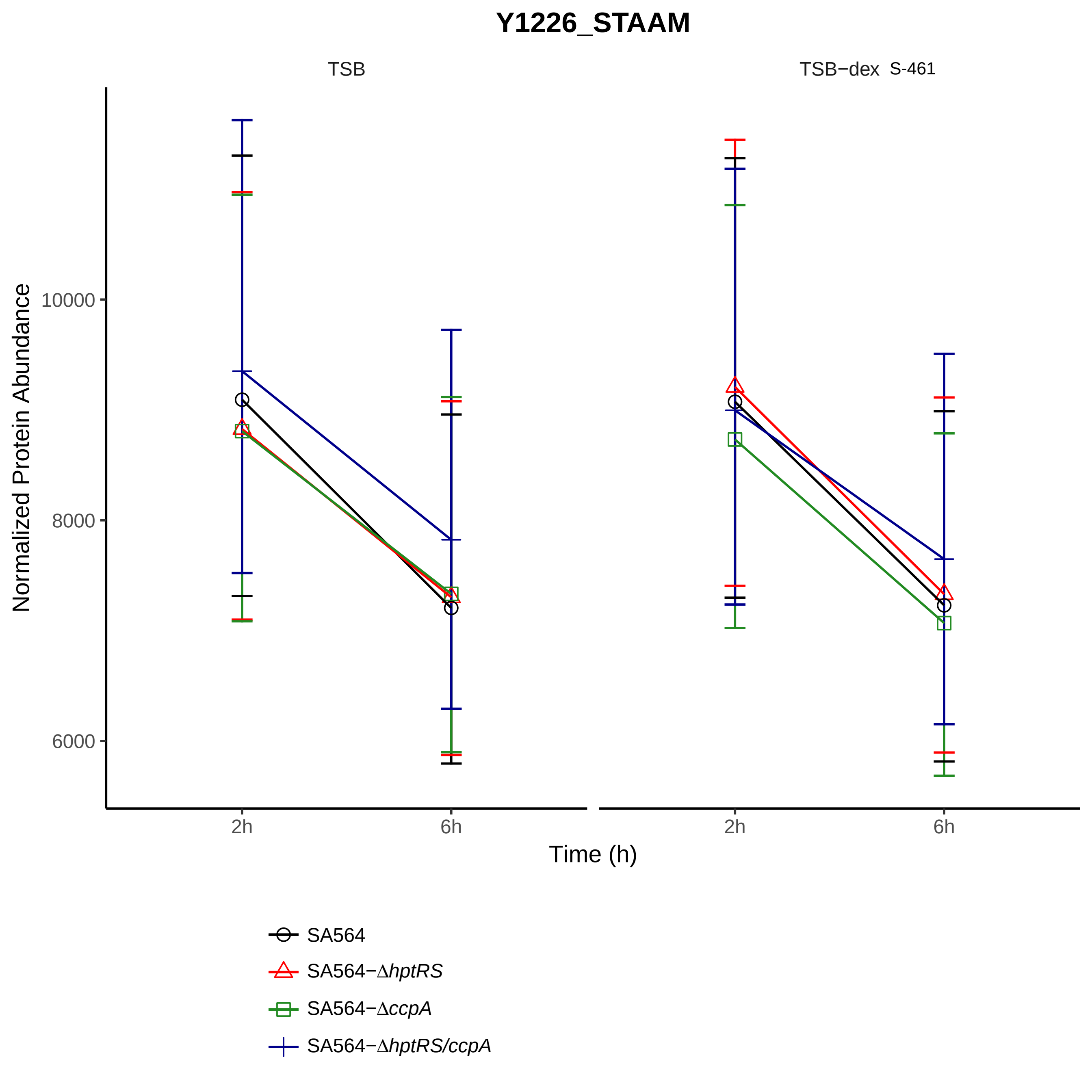

Y1226_STAAM
TSB
TSB−dex
S-461
Normalized Protein Abundance
10000
8000
6000
2h
6h
2h
6h
Time (h)
SA564
SA564−Δ*hptRS*
SA564−Δ*ccpA*
SA564−Δ*hptRS/ccpA*

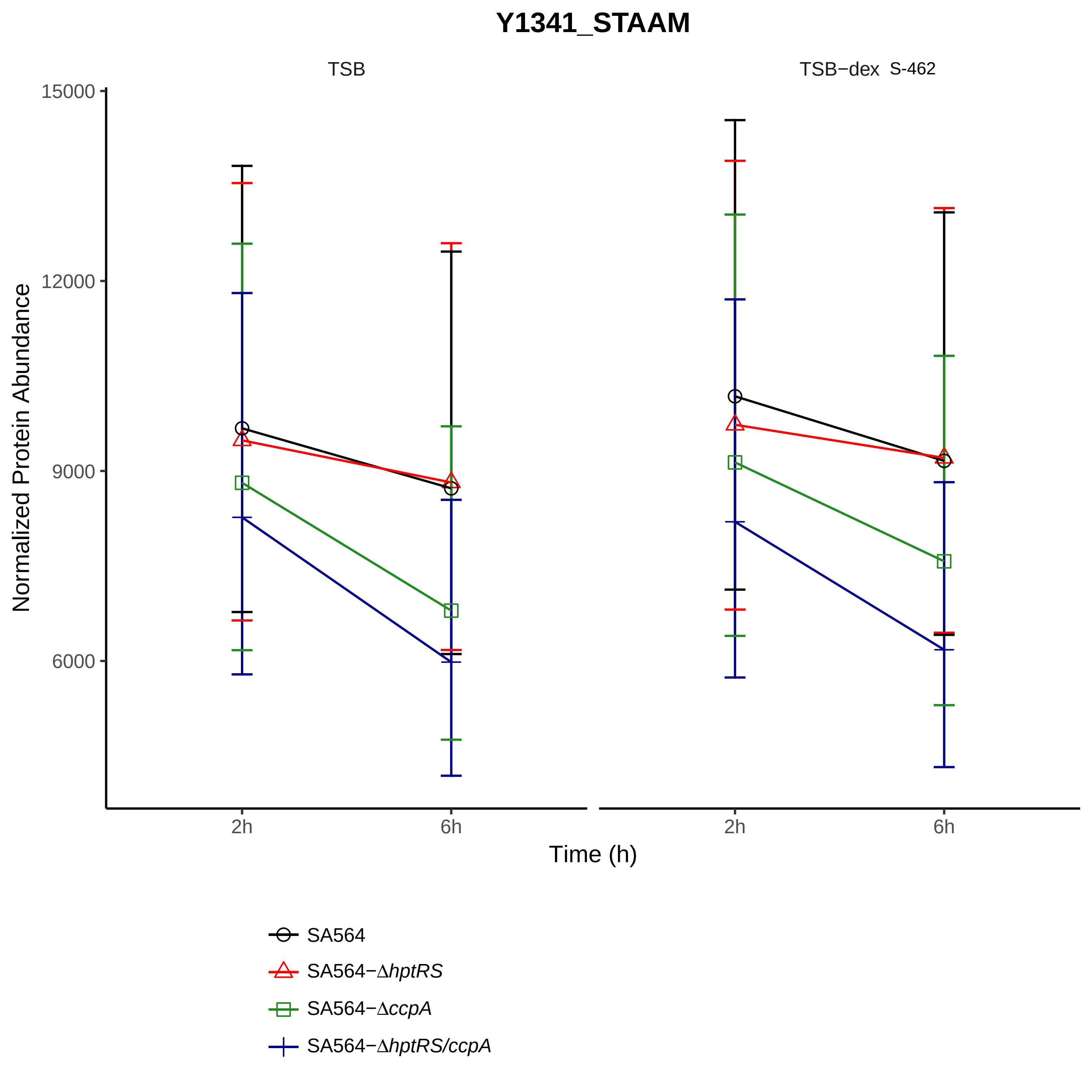

Y1341_STAAM
TSB
TSB−dex
S-462
15000
12000
9000
6000
Normalized Protein Abundance
2h
6h
2h
6h
Time (h)
SA564
SA564−Δ*hptRS*
SA564−Δ*ccpA*
SA564−Δ*hptRS/ccpA*

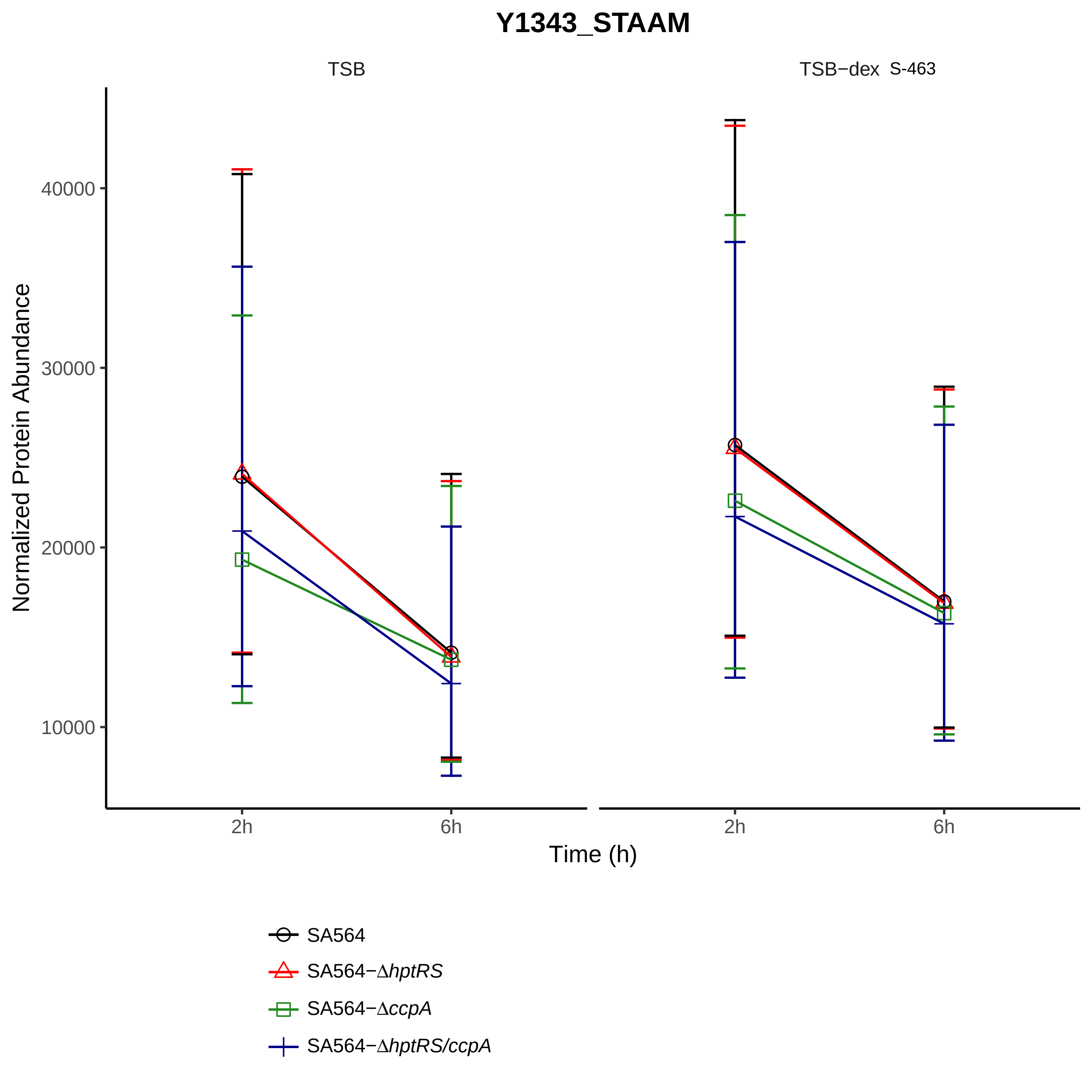

Y1343_STAAM
TSB
TSB−dex
S-463
Normalized Protein Abundance
40000
30000
20000
10000
2h
6h
2h
6h
Time (h)
SA564
SA564−ΔhptRS
SA564−ΔccpA
SA564−ΔhptRS/ccpA

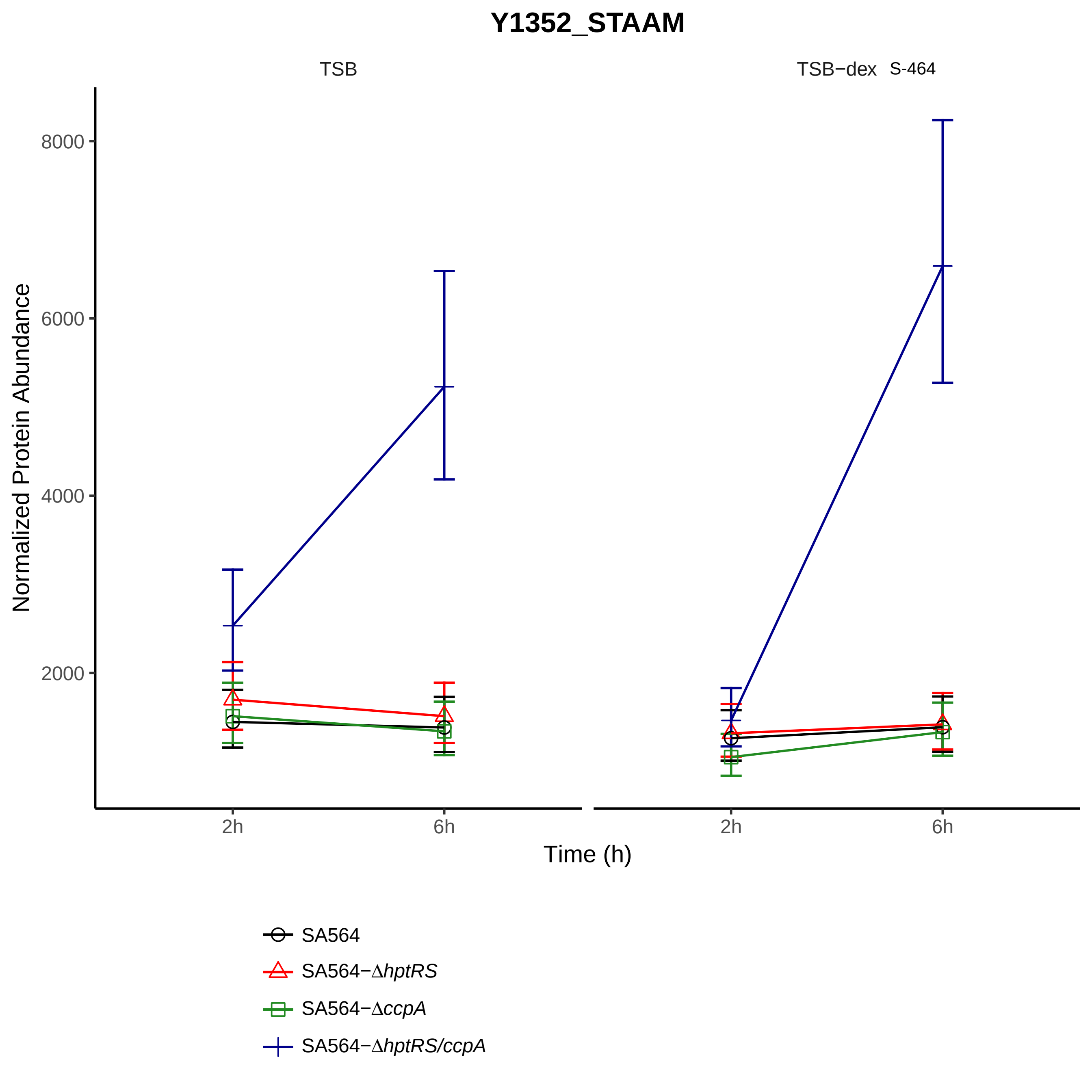

Y1352_STAAM
TSB
TSB−dex
S-464
8000
6000
4000
2000
Normalized Protein Abundance
2h
6h
2h
6h
Time (h)
SA564
SA564−Δ*hptRS*
SA564−Δ*ccpA*
SA564−Δ*hptRS/ccpA*

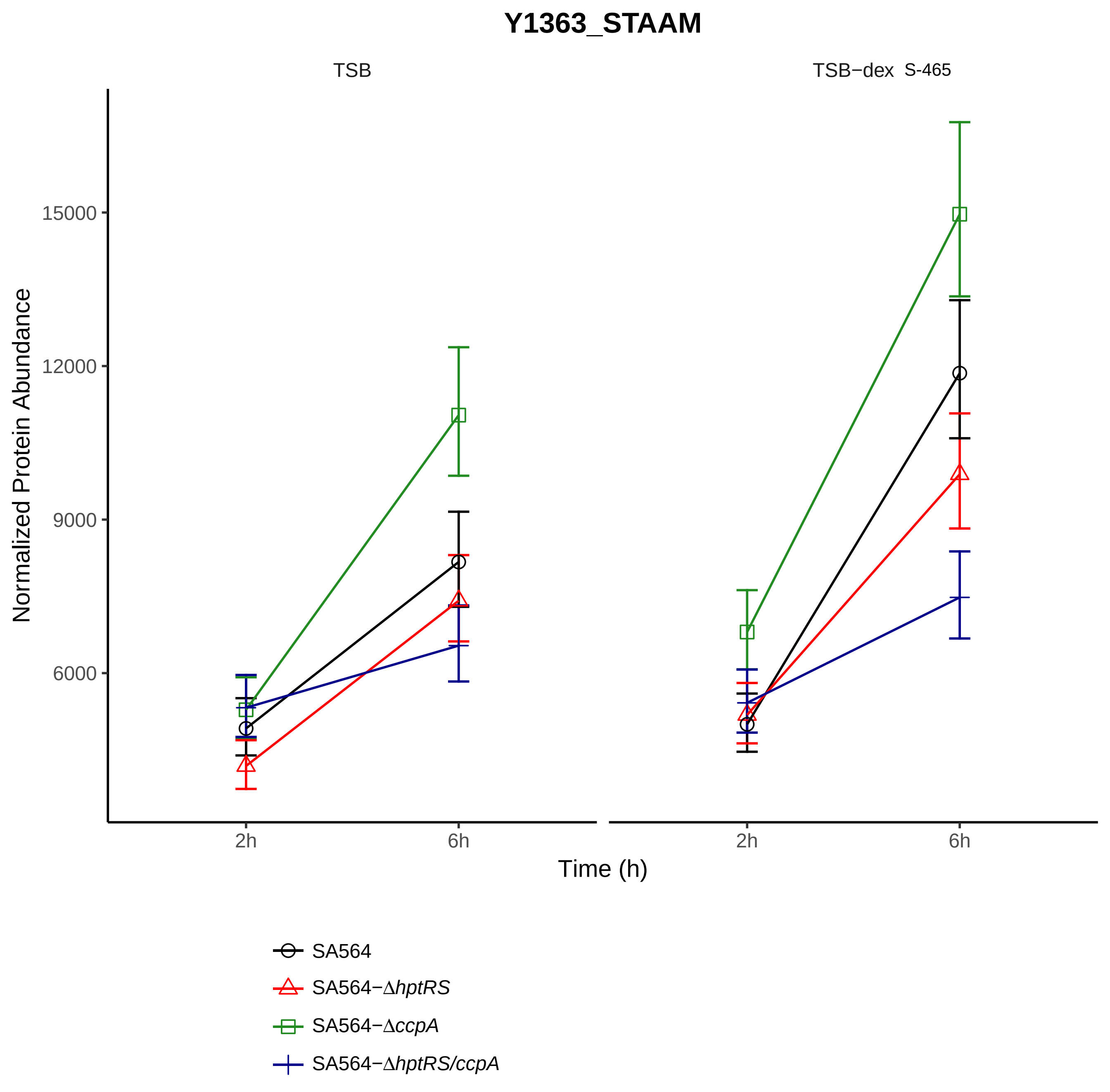
Y1363_STAAM
TSB
TSB−dex
S-465
Normalized Protein Abundance
15000
12000
9000
6000
2h
6h
2h
6h
Time (h)
SA564
SA564−Δ*hptRS*
SA564−Δ*ccpA*
SA564−Δ*hptRS/ccpA*

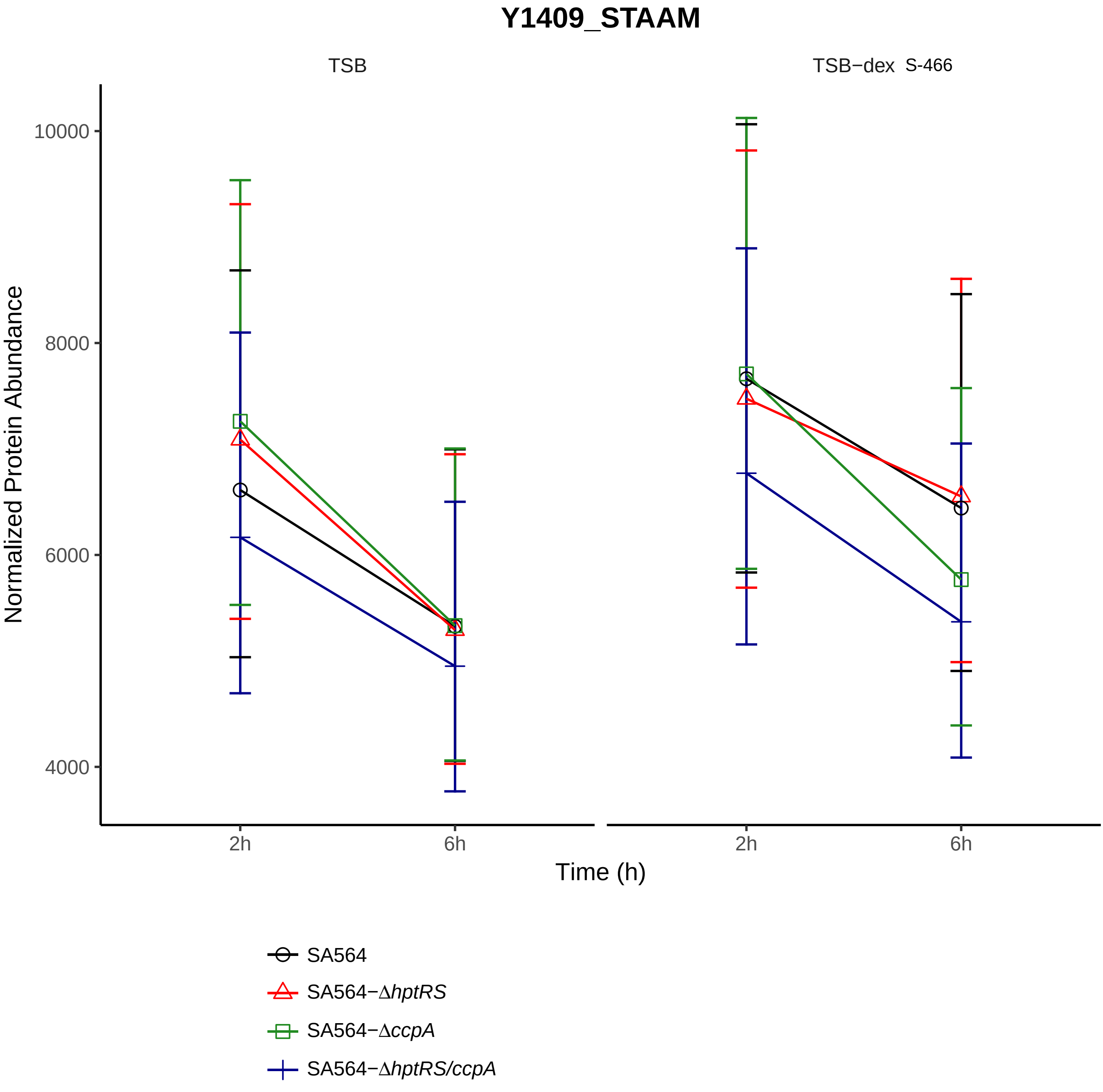
Y1409_STAAM
TSB
TSB−dex
S-466
Normalized Protein Abundance
10000
8000
6000
4000
2h
6h
2h
6h
Time (h)
SA564
SA564−Δ*hptRS*
SA564−Δ*ccpA*
SA564−Δ*hptRS/ccpA*

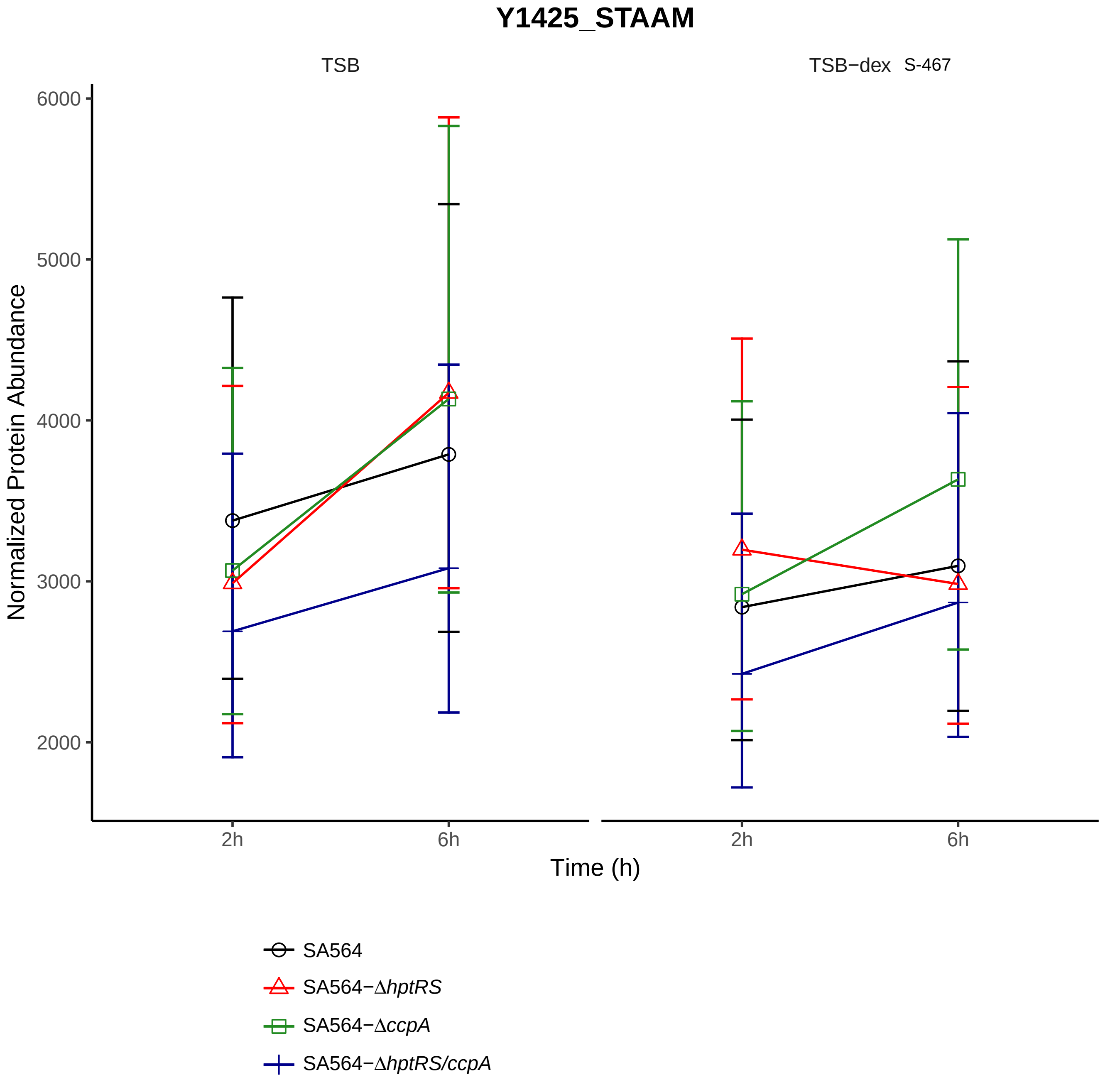

Y1425_STAAM
TSB
TSB−dex
S-467
Normalized Protein Abundance
6000
5000
4000
3000
2000
2h
6h
2h
6h
Time (h)
SA564
SA564−ΔhptRS
SA564−ΔccpA
SA564−ΔhptRS/ccpA

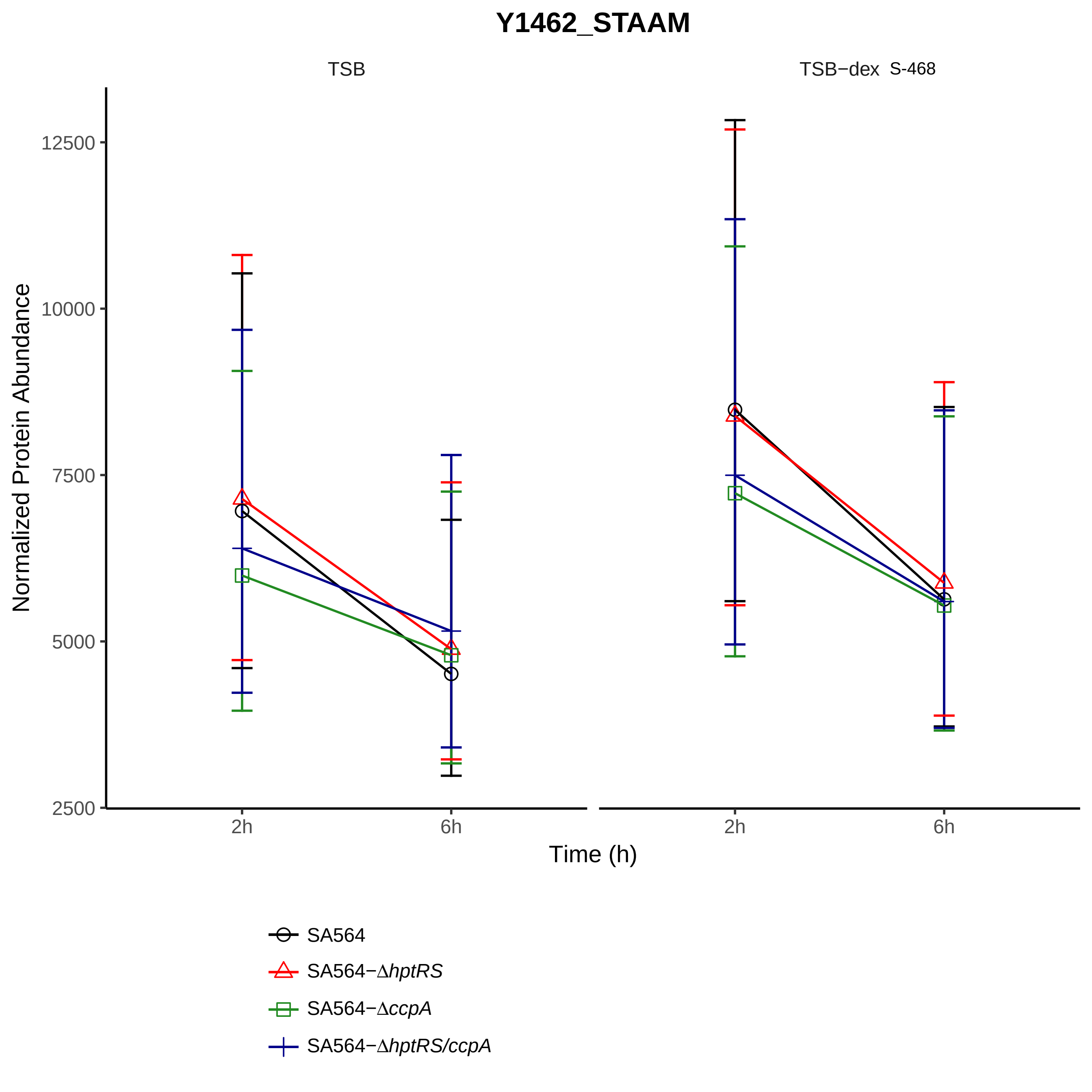

Y1462_STAAM
TSB
TSB−dex
S-468
Normalized Protein Abundance
12500
10000
7500
5000
2500
2h
6h
2h
6h
Time (h)
SA564
SA564−Δ*hptRS*
SA564−Δ*ccpA*
SA564−Δ*hptRS/ccpA*

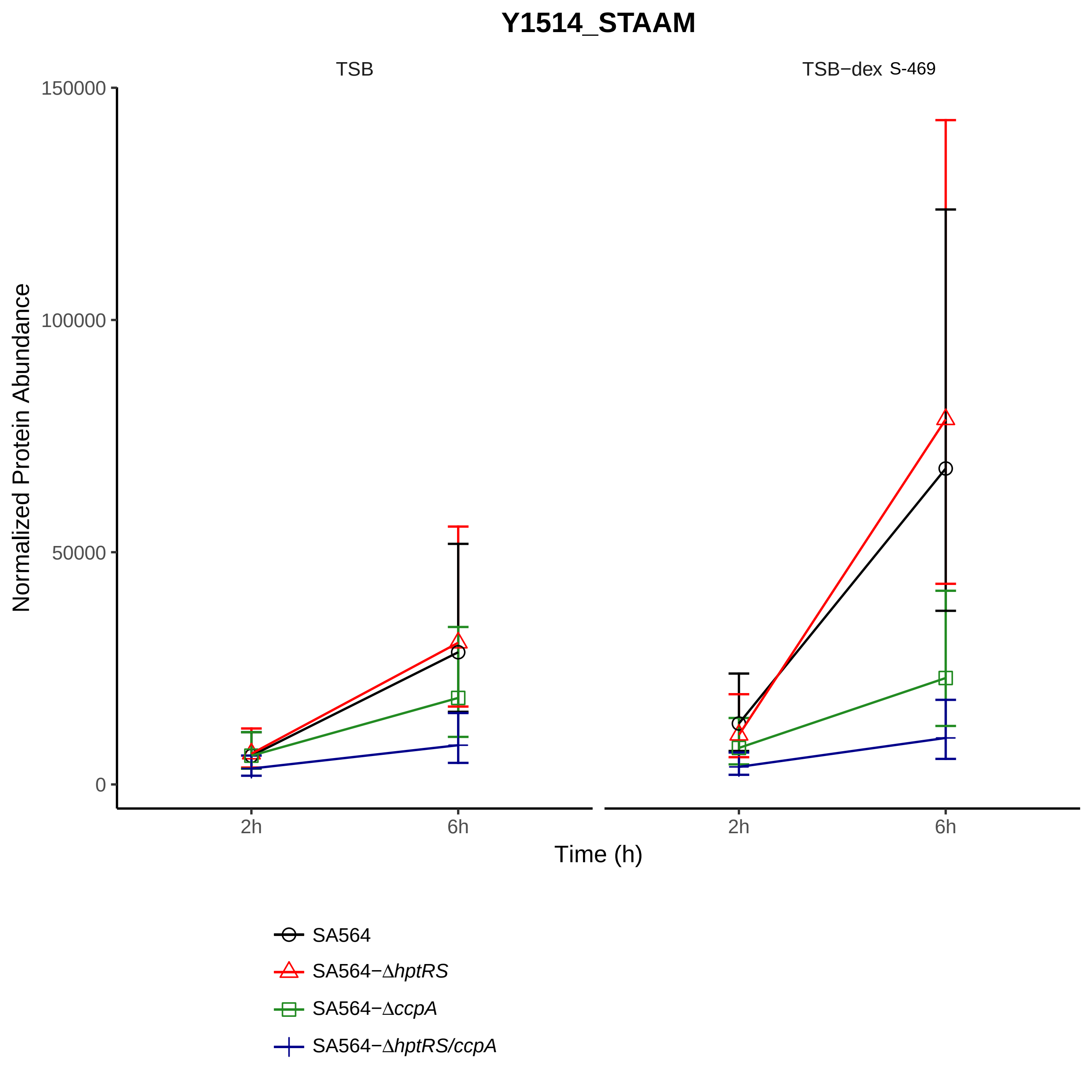

Y1514_STAAM
TSB
TSB−dex
S-469
Normalized Protein Abundance
150000
100000
50000
0
2h
6h
2h
6h
Time (h)
SA564
SA564−Δ*hptRS*
SA564−Δ*ccpA*
SA564−Δ*hptRS/ccpA*

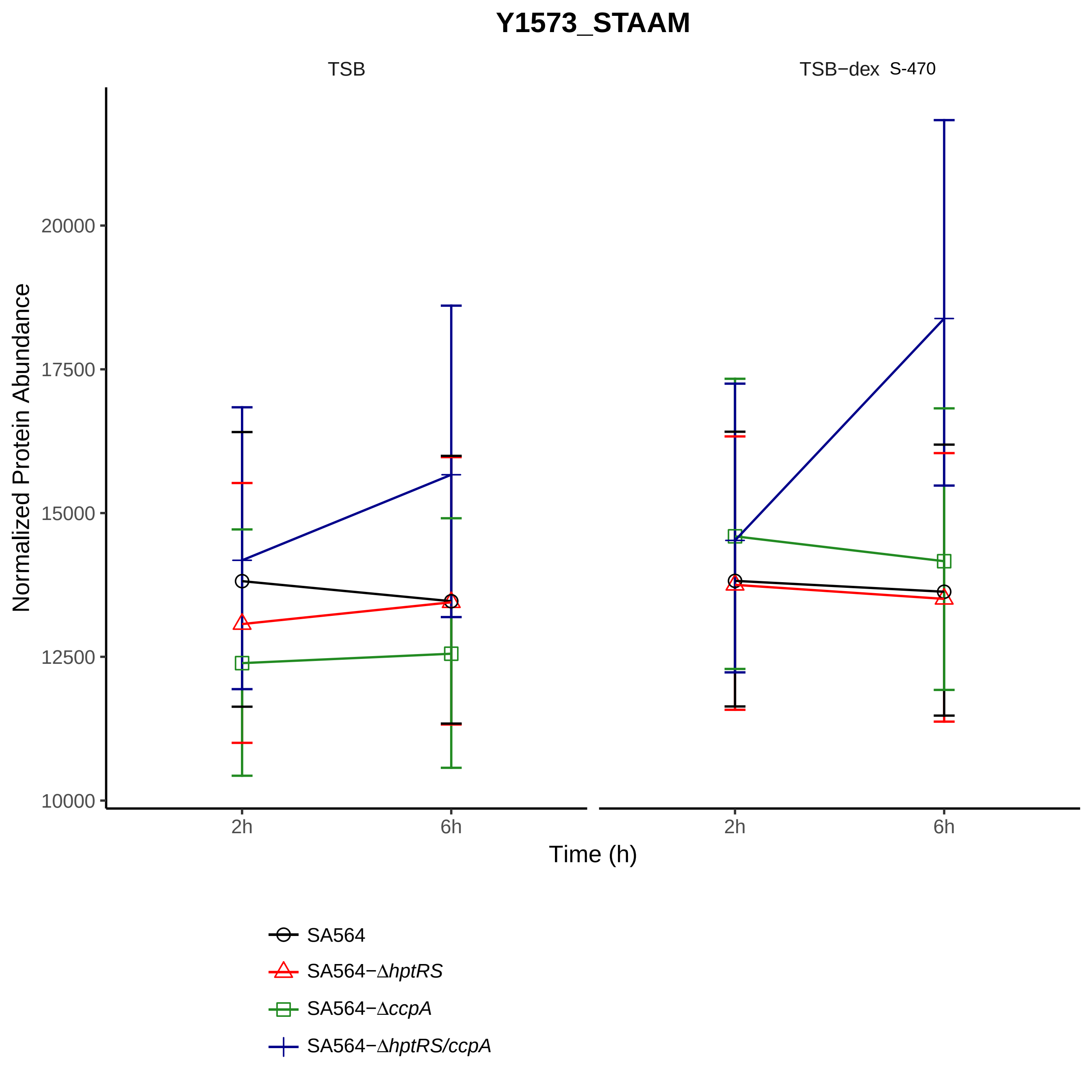

Y1573_STAAM
TSB
TSB−dex
S-470
Normalized Protein Abundance
20000
17500
15000
12500
10000
2h
6h
2h
6h
Time (h)
SA564
SA564−Δ*hptRS*
SA564−Δ*ccpA*
SA564−Δ*hptRS/ccpA*

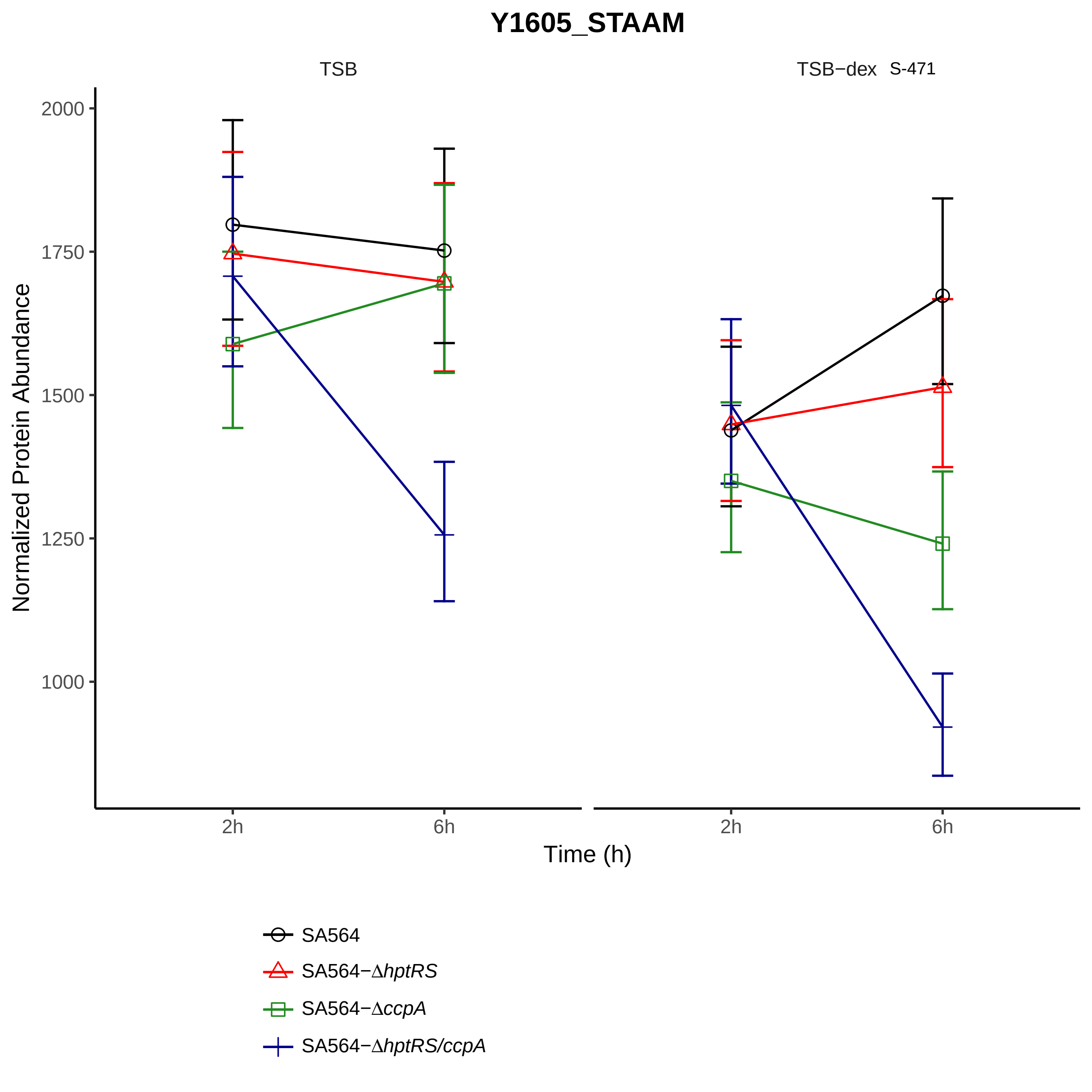

Y1605_STAAM
TSB
TSB−dex
S-471
2000
1750
1500
1250
1000
Normalized Protein Abundance
2h
6h
2h
6h
Time (h)
SA564
SA564−ΔhptRS
SA564−ΔccpA
SA564−ΔhptRS/ccpA

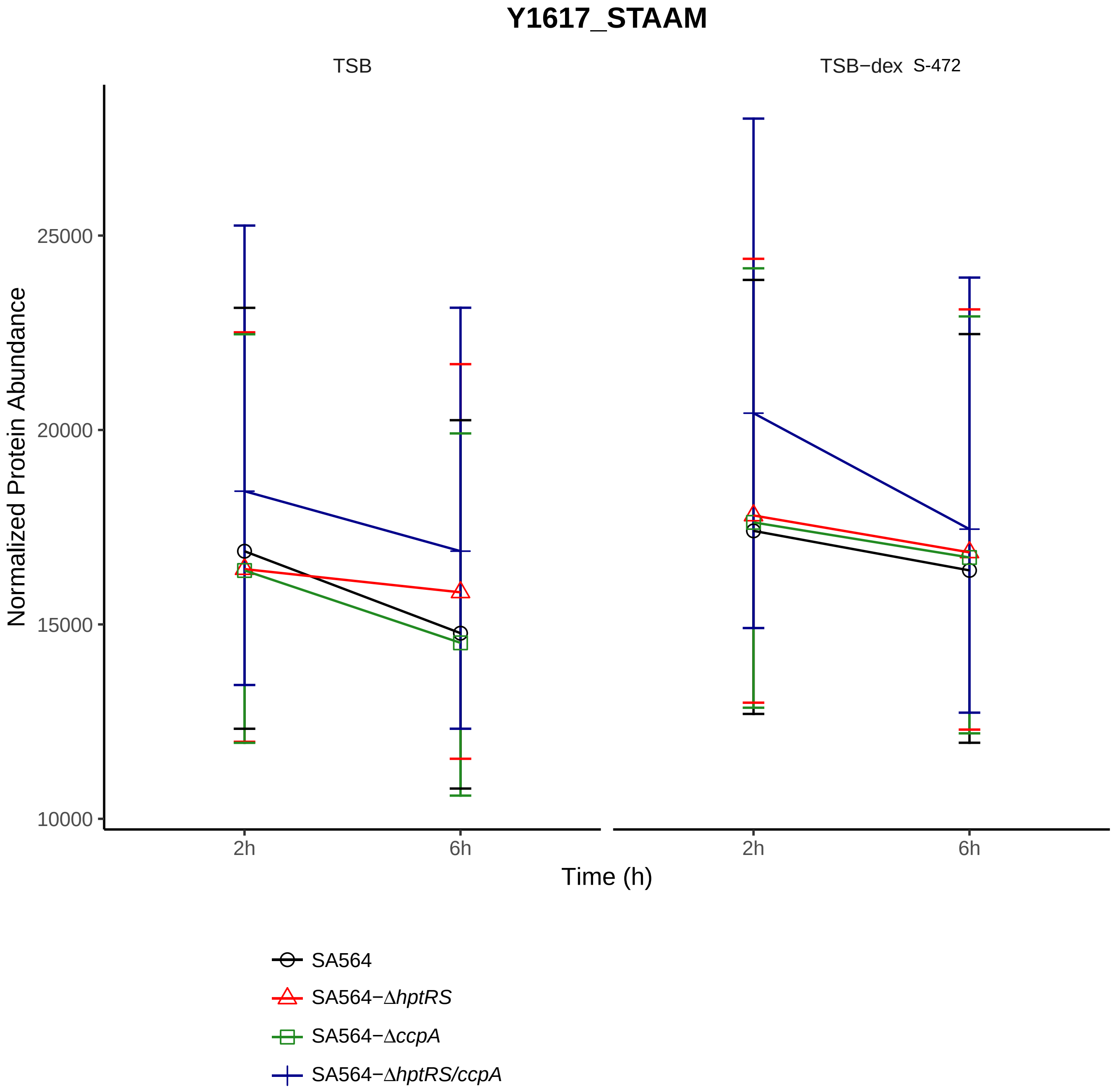

Y1617_STAAM
TSB
TSB−dex
S-472
Normalized Protein Abundance
25000
20000
15000
10000
2h
6h
2h
6h
Time (h)
SA564
SA564−Δ*hptRS*
SA564−Δ*ccpA*
SA564−Δ*hptRS/ccpA*

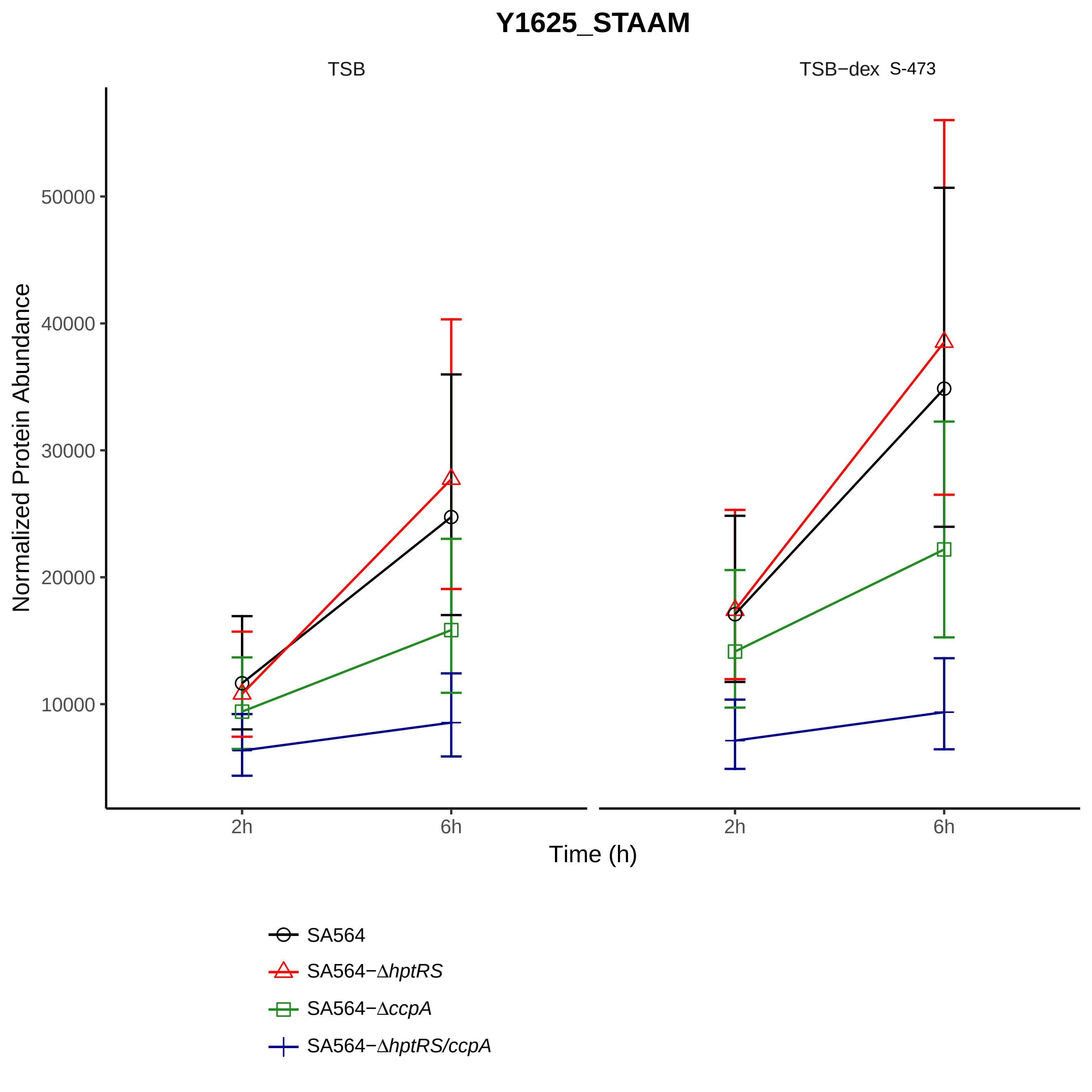

Y1625_STAAM
TSB
TSB−dex
S-473
50000
40000
30000
20000
10000
Normalized Protein Abundance
2h
6h
2h
6h
Time (h)
SA564
SA564−ΔhptRS
SA564−ΔccpA
SA564−ΔhptRS/ccpA

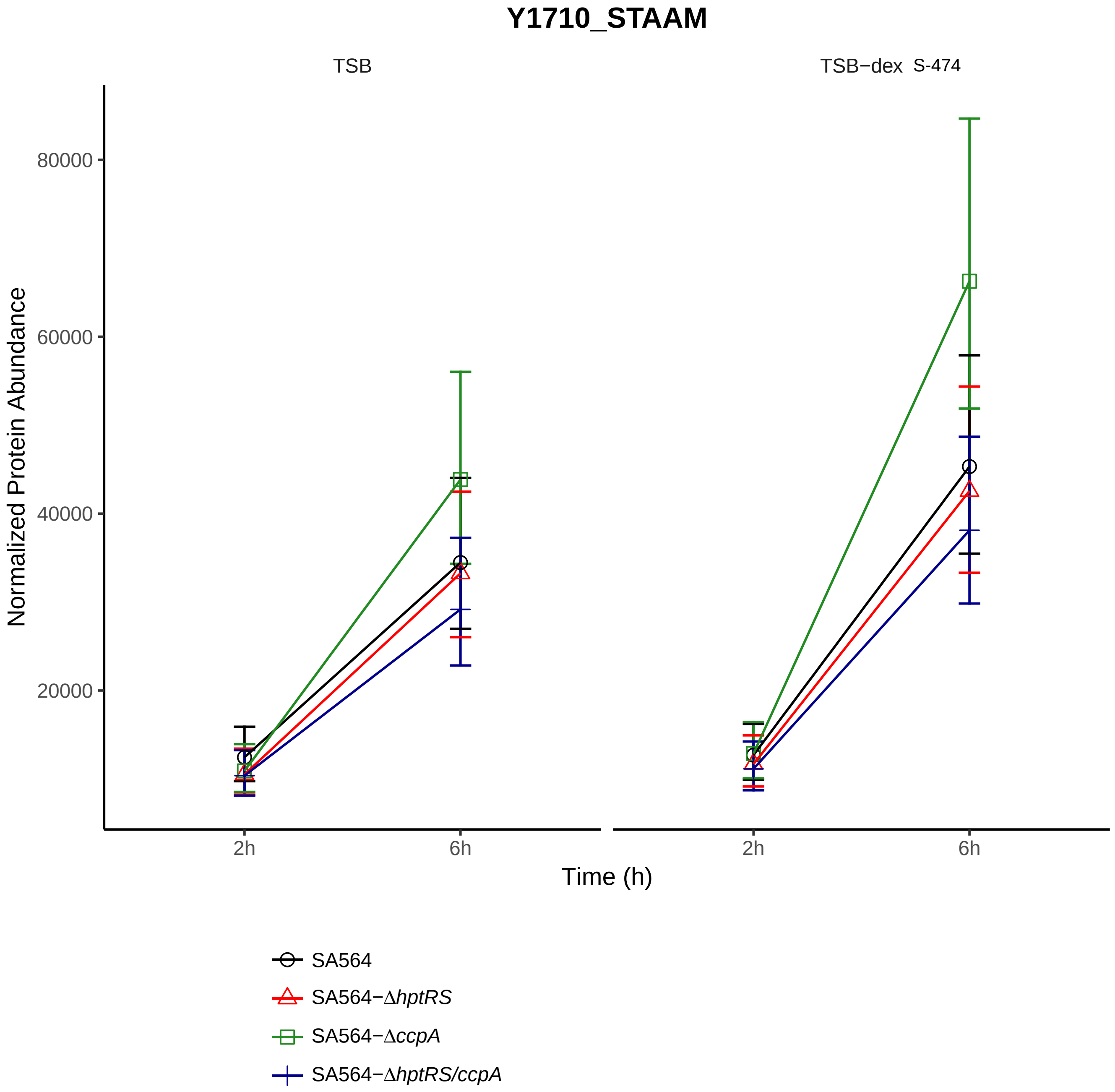

Y1710_STAAM
TSB
TSB−dex
S-474
Normalized Protein Abundance
80000
60000
40000
20000
2h
6h
2h
6h
Time (h)
SA564
SA564−Δ*hptRS*
SA564−Δ*ccpA*
SA564−Δ*hptRS/ccpA*

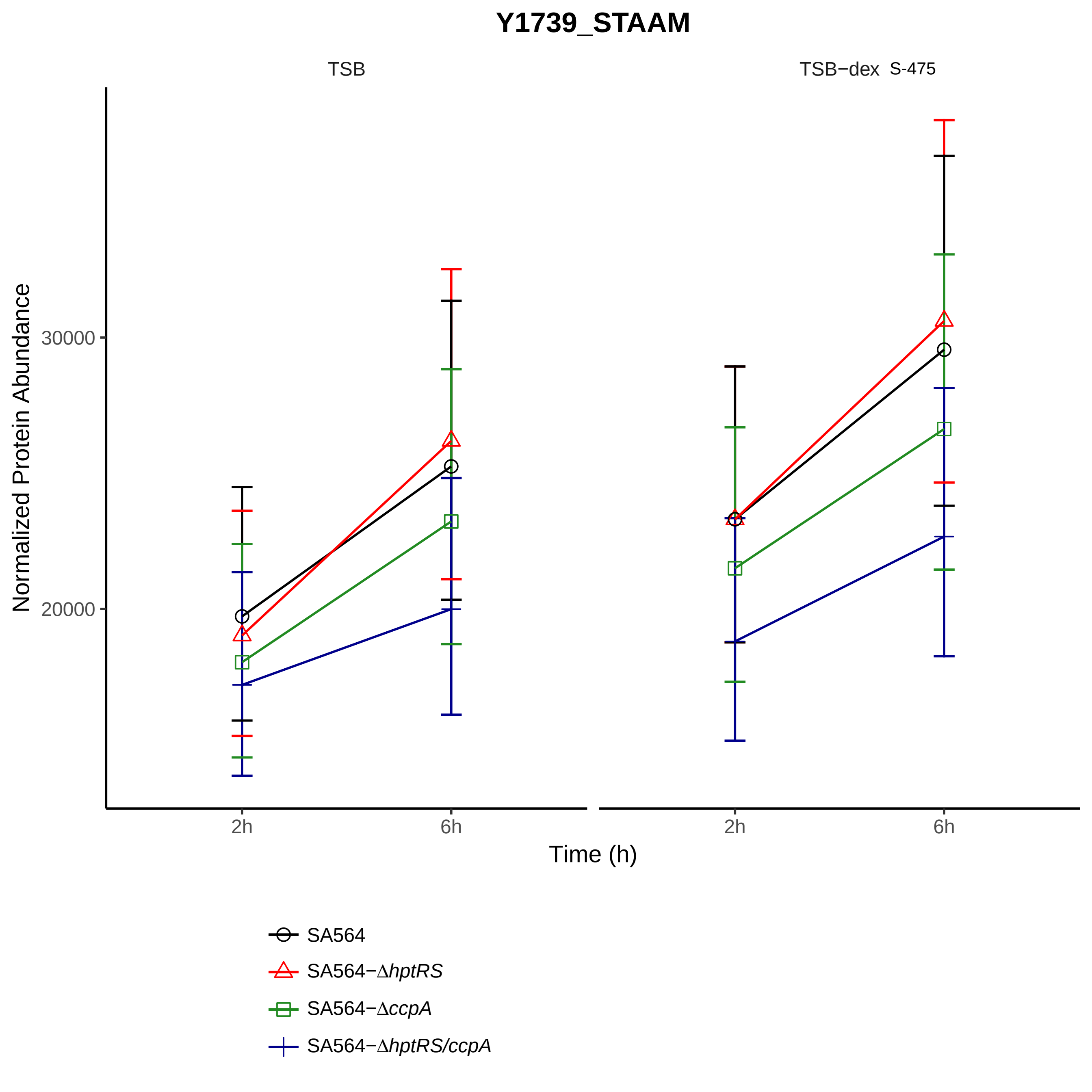
Y1739_STAAM
TSB
TSB−dex
S-475
Normalized Protein Abundance
30000
20000
2h
6h
2h
6h
Time (h)
SA564
SA564−Δ*hptRS*
SA564−Δ*ccpA*
SA564−Δ*hptRS/ccpA*

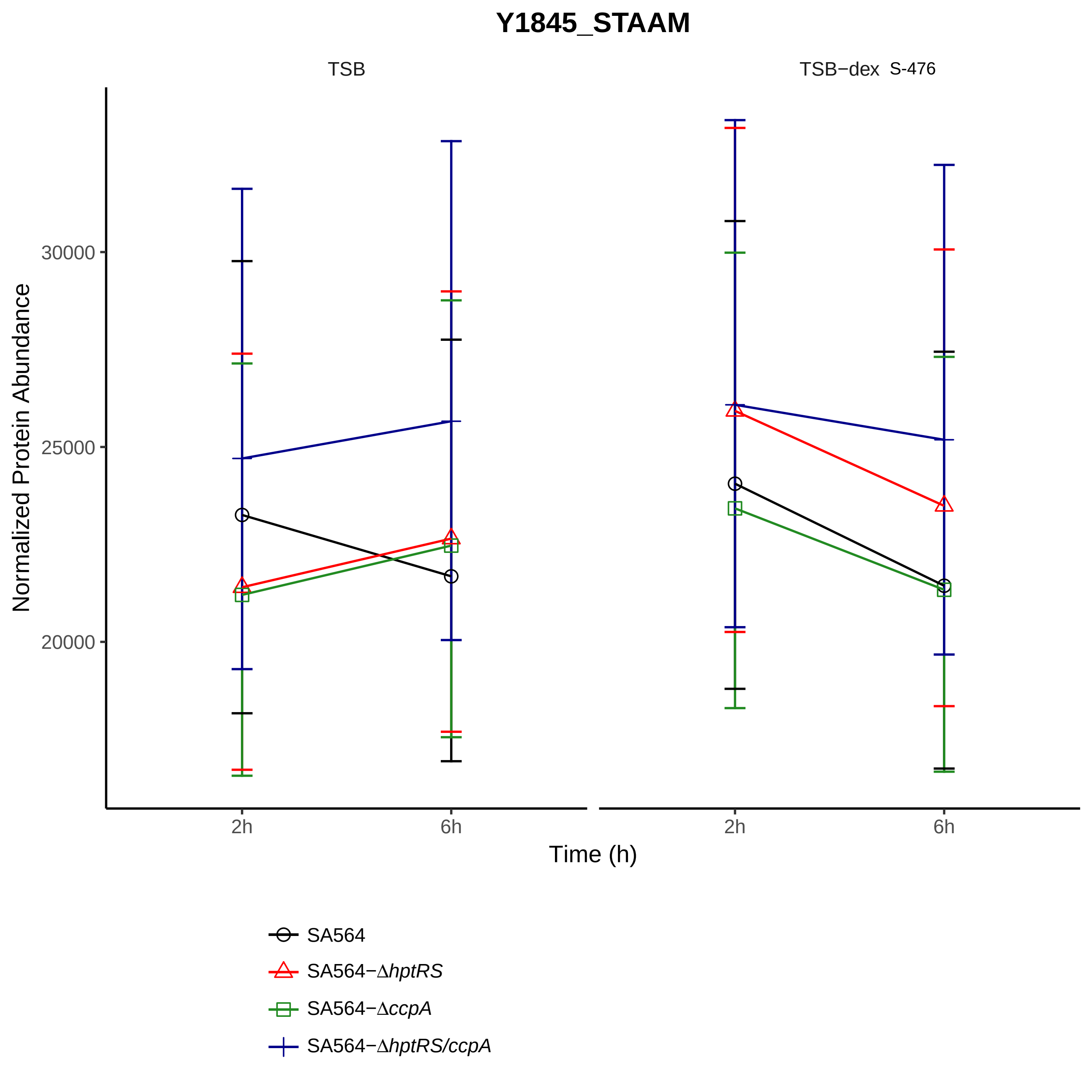

Y1845_STAAM
TSB
TSB−dex
S-476
Normalized Protein Abundance
30000
25000
20000
2h
6h
2h
6h
Time (h)
SA564
SA564−Δ*hptRS*
SA564−Δ*ccpA*
SA564−Δ*hptRS/ccpA*

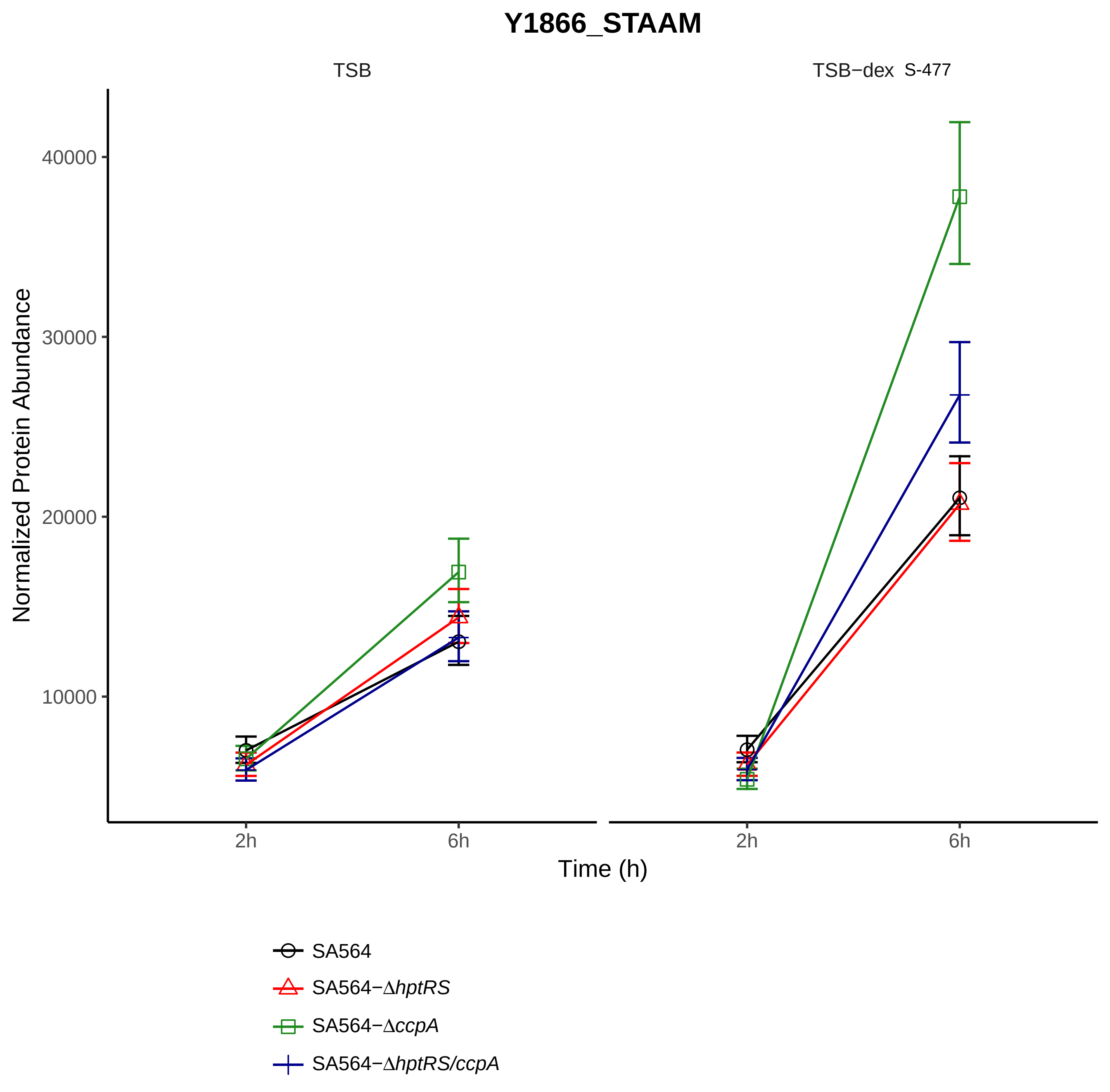

Y1866_STAAM
TSB
TSB−dex
S-477
Normalized Protein Abundance
40000
30000
20000
10000
2h
6h
2h
6h
Time (h)
SA564
SA564−ΔhptRS
SA564−ΔccpA
SA564−ΔhptRS/ccpA

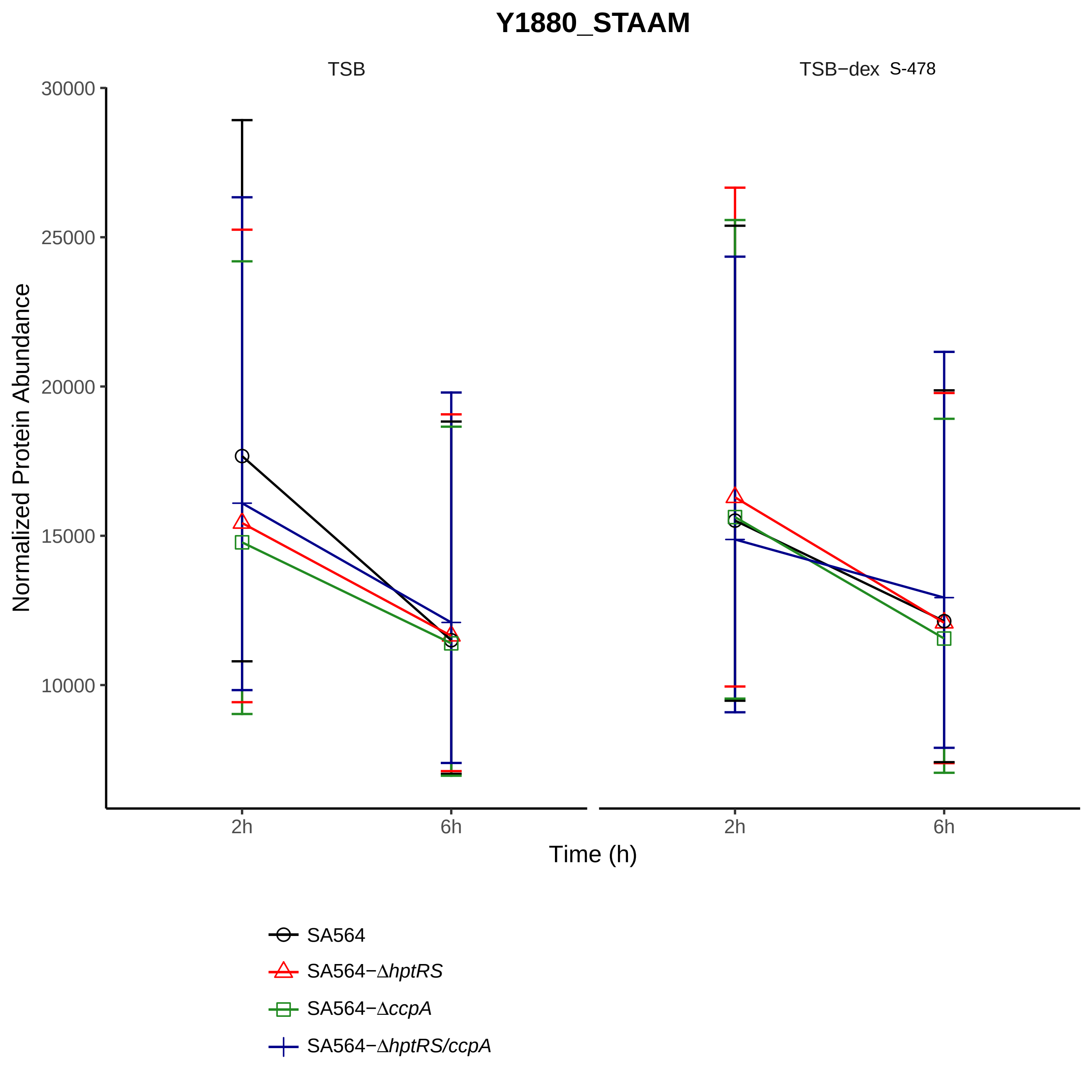

Y1880_STAAM
TSB
TSB−dex
S-478
30000
25000
20000
15000
10000
Normalized Protein Abundance
2h
6h
2h
6h
Time (h)
SA564
SA564−Δ*hptRS*
SA564−Δ*ccpA*
SA564−Δ*hptRS/ccpA*

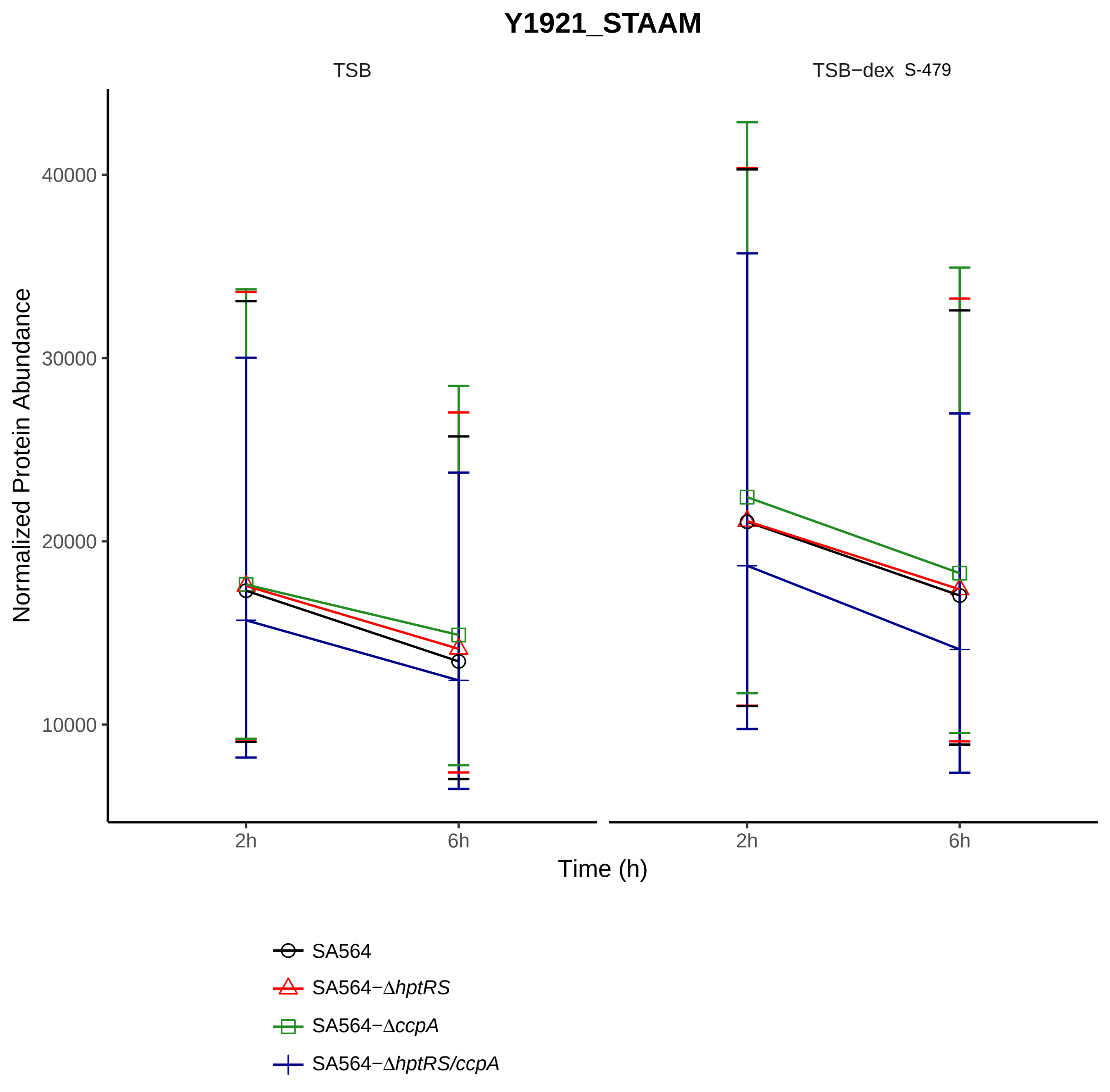
Y1921_STAAM
TSB
TSB−dex
S-479
Normalized Protein Abundance
40000
30000
20000
10000
2h
6h
2h
6h
Time (h)
SA564
SA564−ΔhptRS
SA564−ΔccpA
SA564−ΔhptRS/ccpA

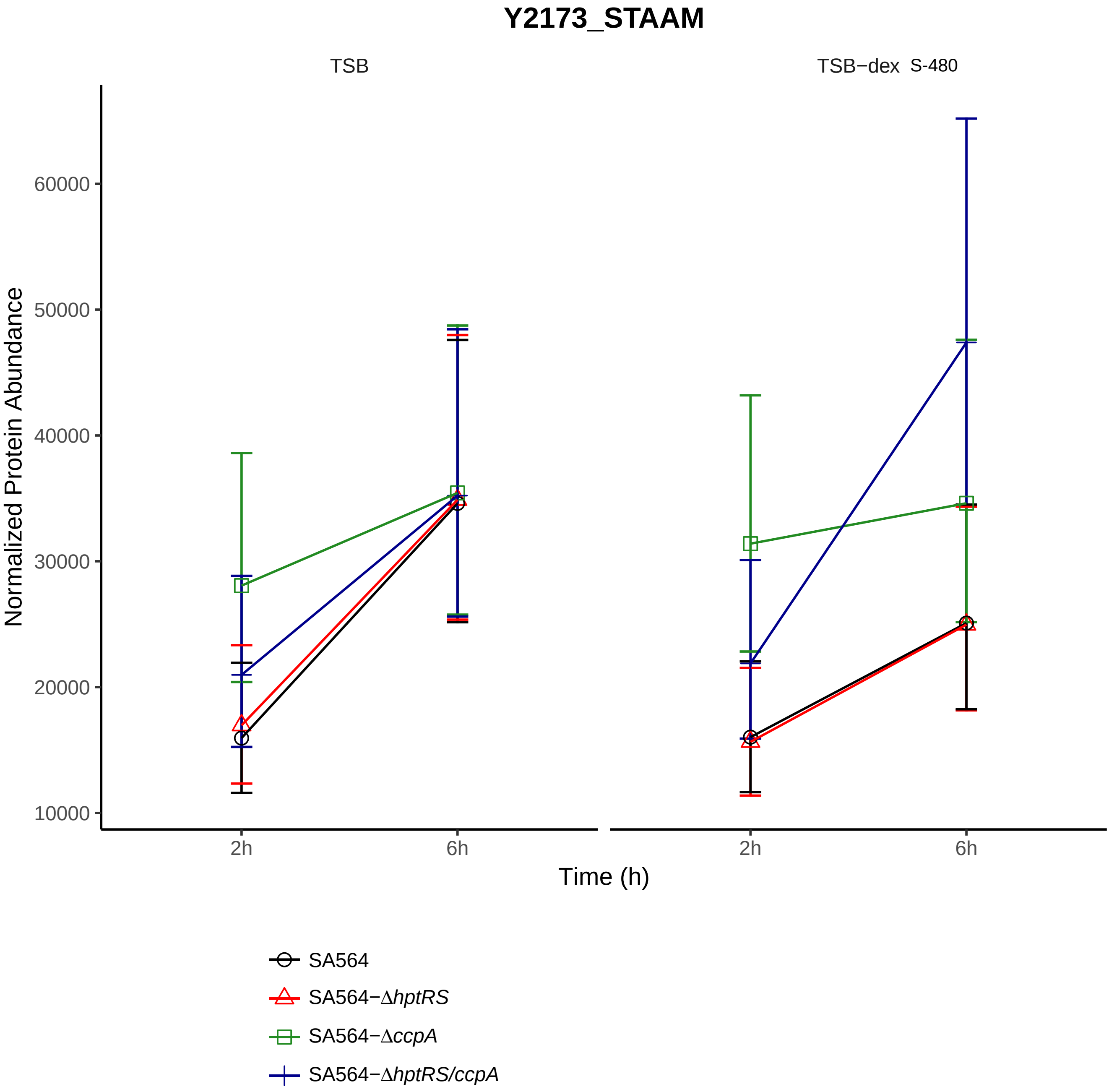

Y2173_STAAM
TSB
TSB−dex
S-480
Normalized Protein Abundance
60000
50000
40000
30000
20000
10000
2h
6h
2h
6h
Time (h)
SA564
SA564−Δ*hptRS*
SA564−Δ*ccpA*
SA564−Δ*hptRS/ccpA*

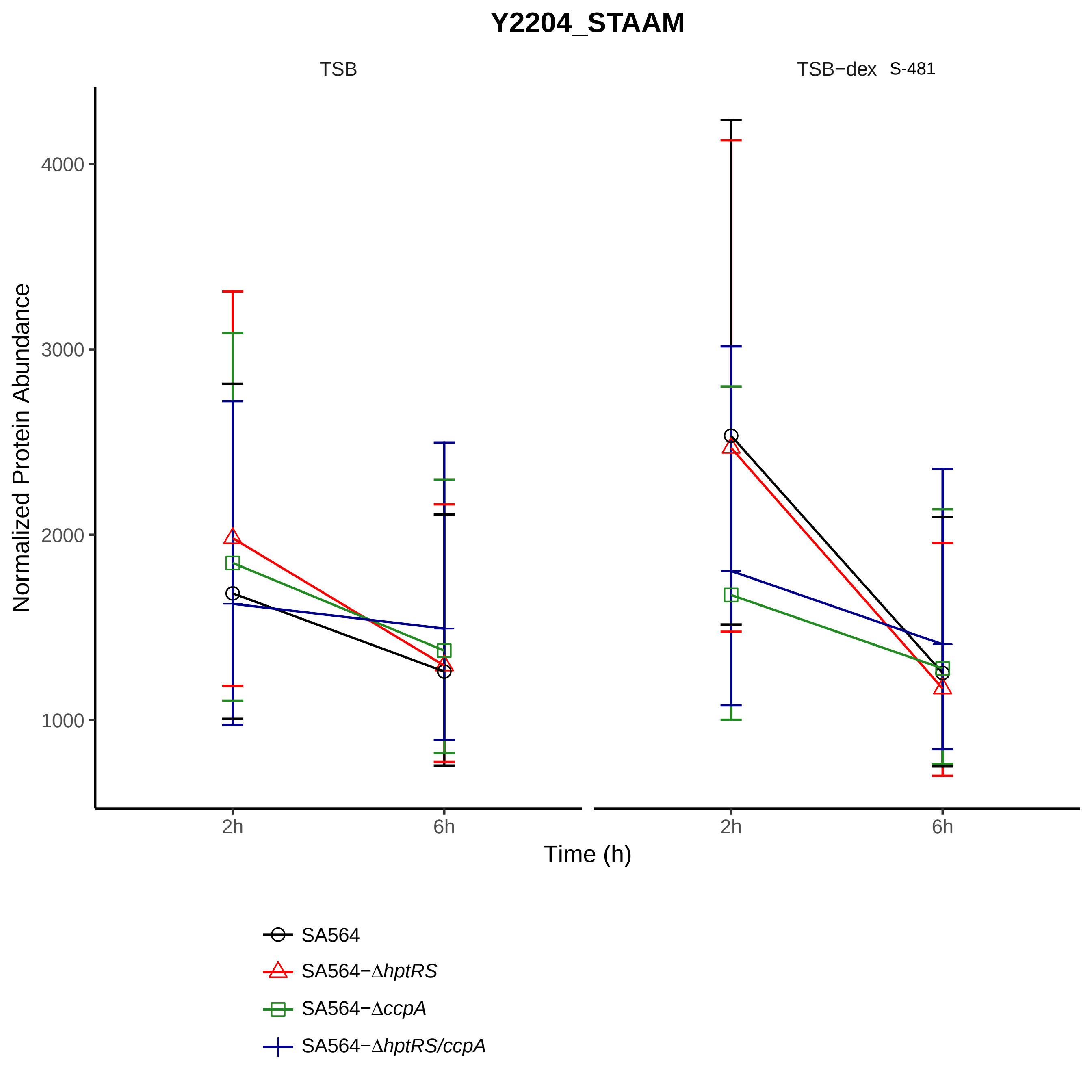

Y2204_STAAM
TSB
TSB−dex
S-481
Normalized Protein Abundance
4000
3000
2000
1000
2h
6h
2h
6h
Time (h)
SA564
SA564−Δ*hptRS*
SA564−Δ*ccpA*
SA564−Δ*hptRS/ccpA*

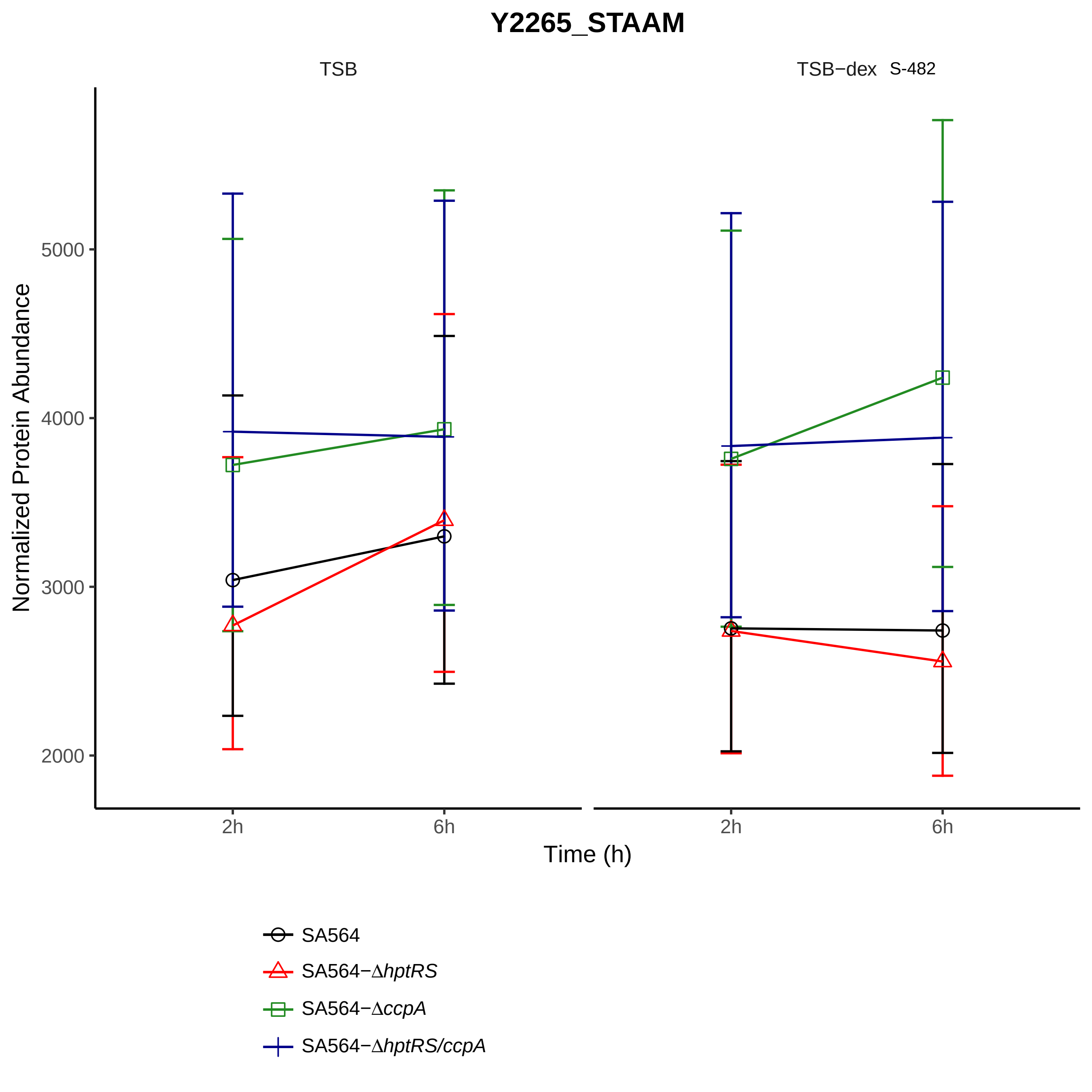
Y2265_STAAM
TSB
TSB−dex
S-482
Normalized Protein Abundance
5000
4000
3000
2000
2h
6h
2h
6h
Time (h)
SA564
SA564−ΔhptRS
SA564−ΔccpA
SA564−ΔhptRS/ccpA

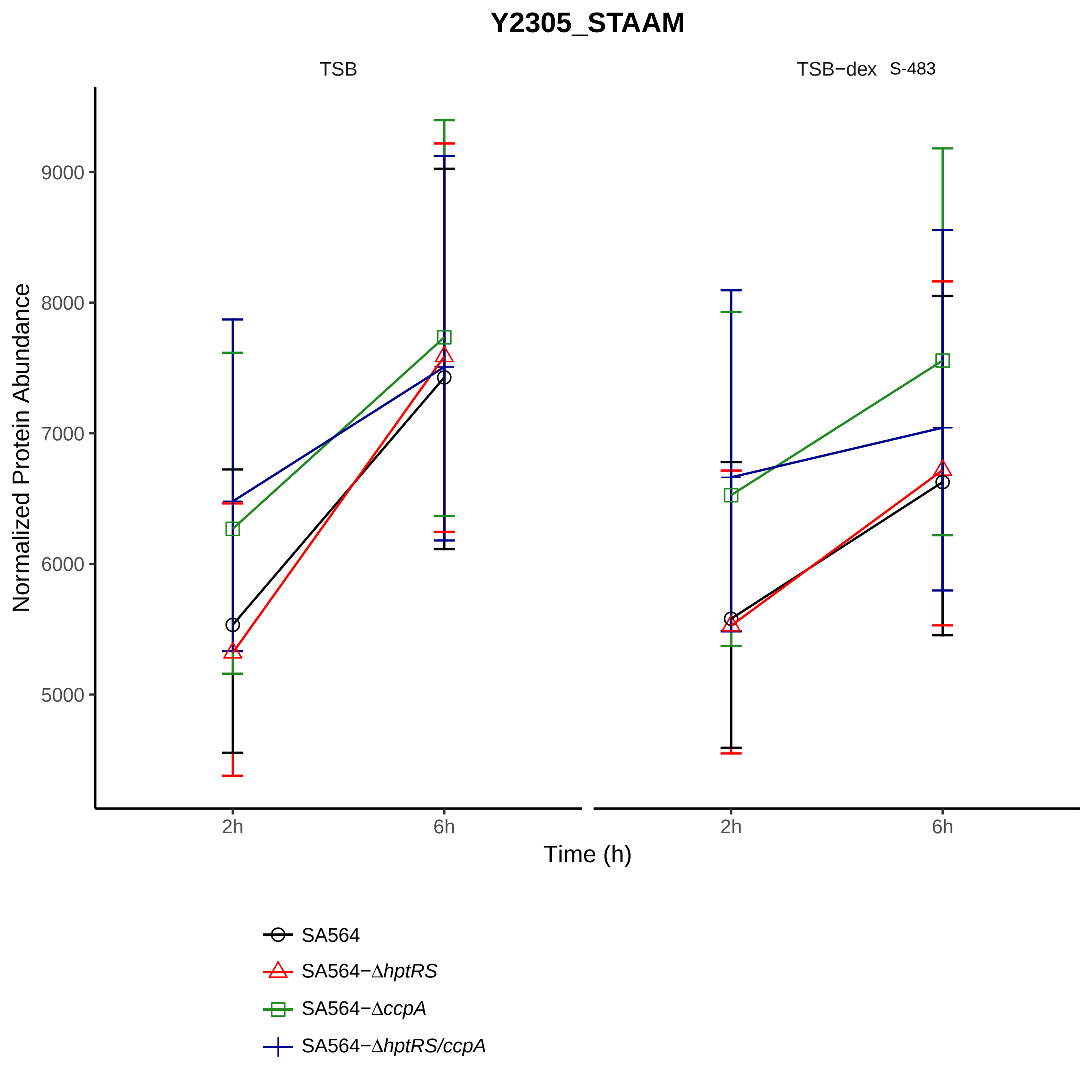

Y2305_STAAM
TSB
TSB−dex
S-483
Normalized Protein Abundance
9000
8000
7000
6000
5000
2h
6h
2h
6h
Time (h)
SA564
SA564−ΔhptRS
SA564−ΔccpA
SA564−ΔhptRS/ccpA

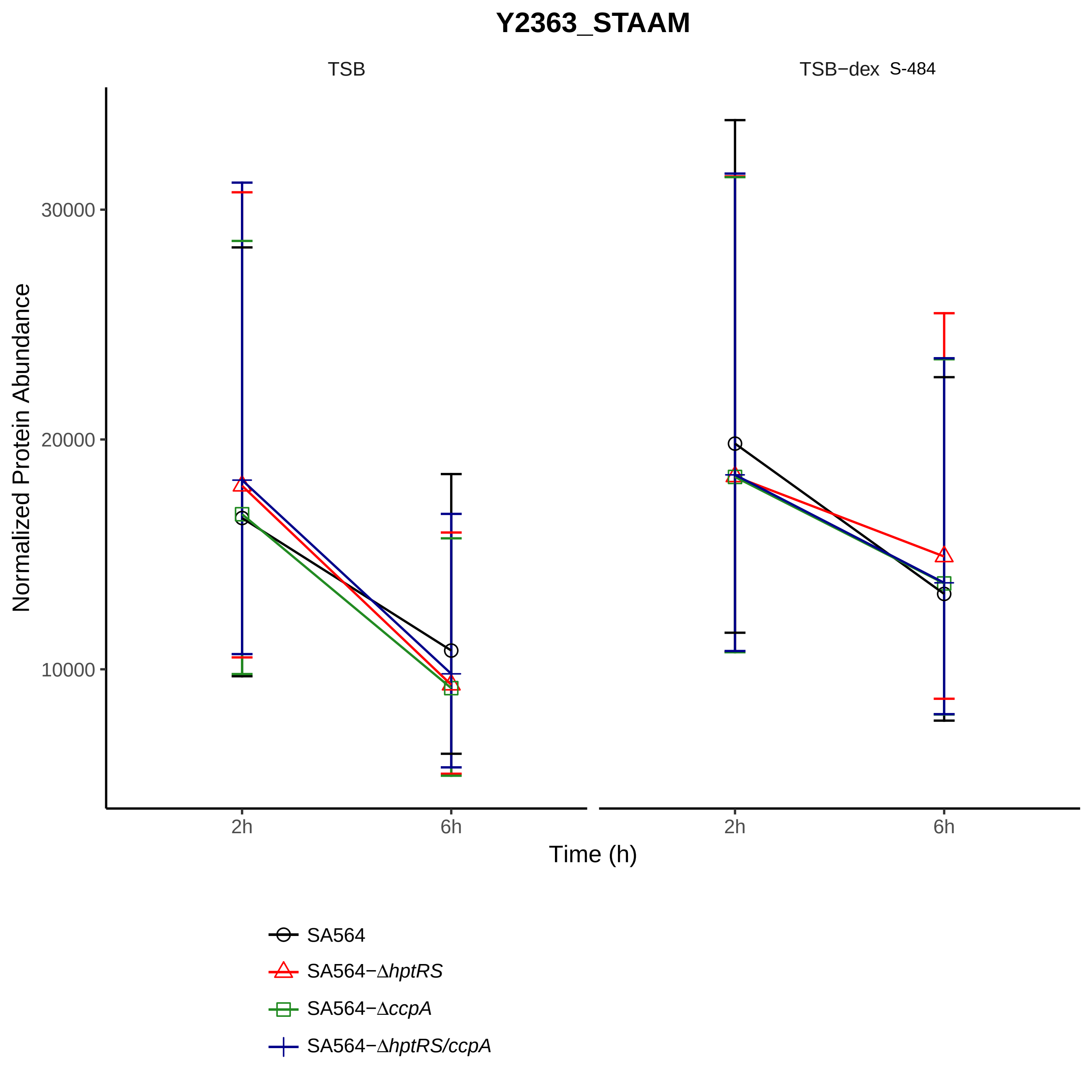
Y2363_STAAM
TSB
TSB−dex
S-484
Normalized Protein Abundance
30000
20000
10000
2h
6h
2h
6h
Time (h)
SA564
SA564−ΔhptRS
SA564−ΔccpA
SA564−ΔhptRS/ccpA

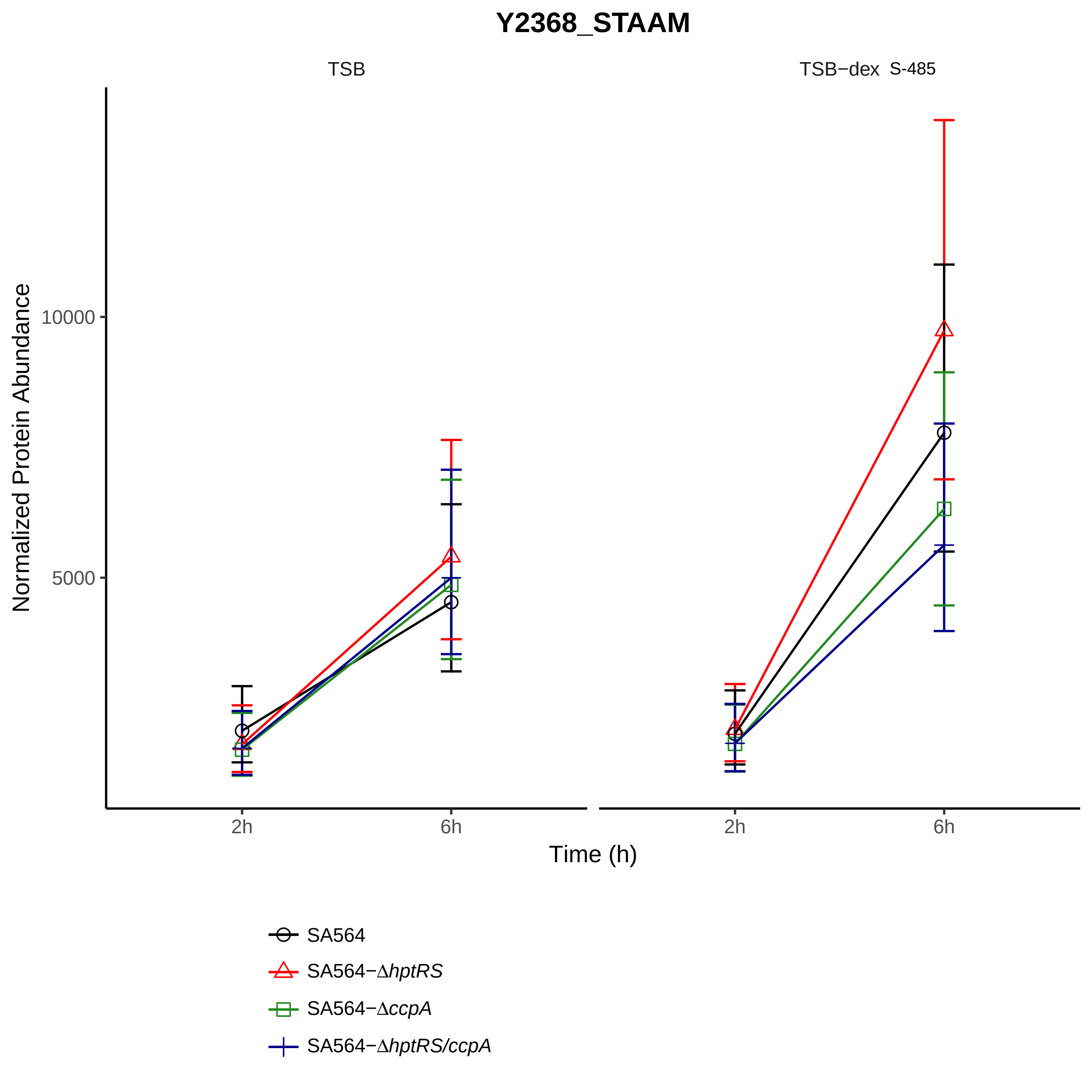

Y2368_STAAM
TSB
TSB−dex
S-485
Normalized Protein Abundance
10000
5000
2h
6h
2h
6h
Time (h)
SA564
SA564−Δ*hptRS*
SA564−Δ*ccpA*
SA564−Δ*hptRS/ccpA*

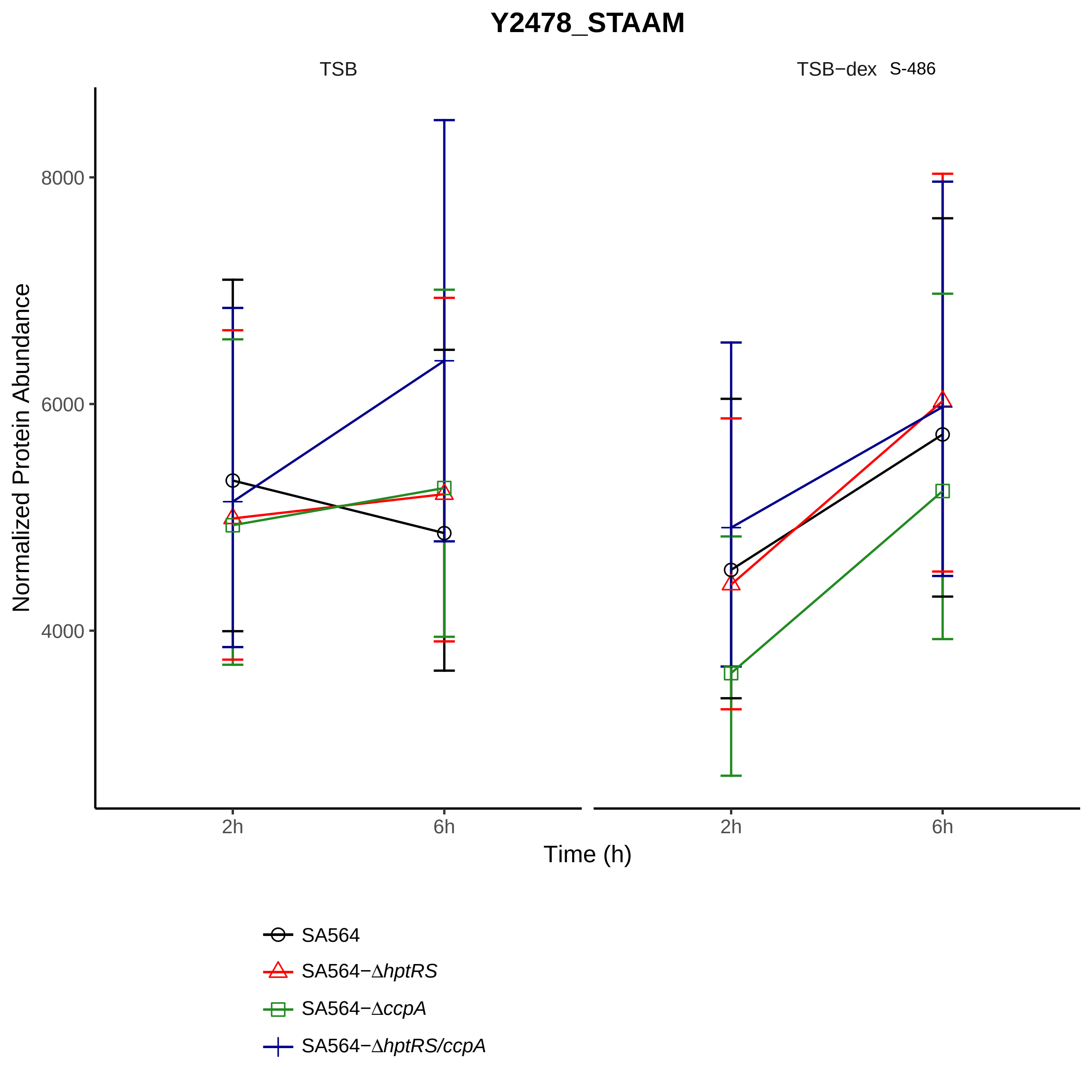

Y2478_STAAM
TSB
TSB−dex
S-486
Normalized Protein Abundance
8000
6000
4000
2h
6h
2h
6h
Time (h)
SA564
SA564−ΔhptRS
SA564−ΔccpA
SA564−ΔhptRS/ccpA

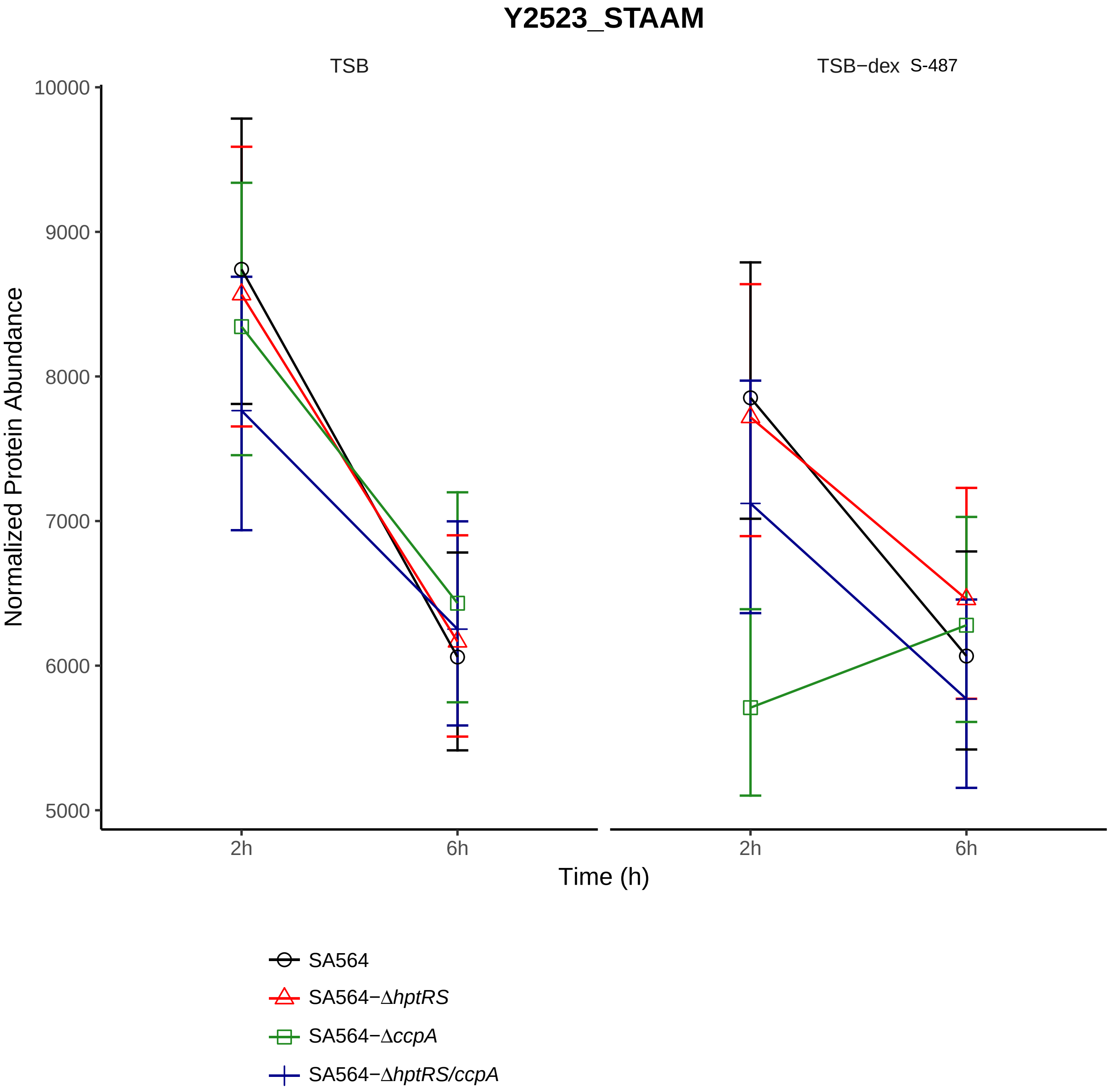

Y2523_STAAM
TSB
TSB−dex
S-487
Normalized Protein Abundance
10000
9000
8000
7000
6000
5000
2h
6h
2h
6h
Time (h)
SA564
SA564−Δ*hptRS*
SA564−Δ*ccpA*
SA564−Δ*hptRS/ccpA*

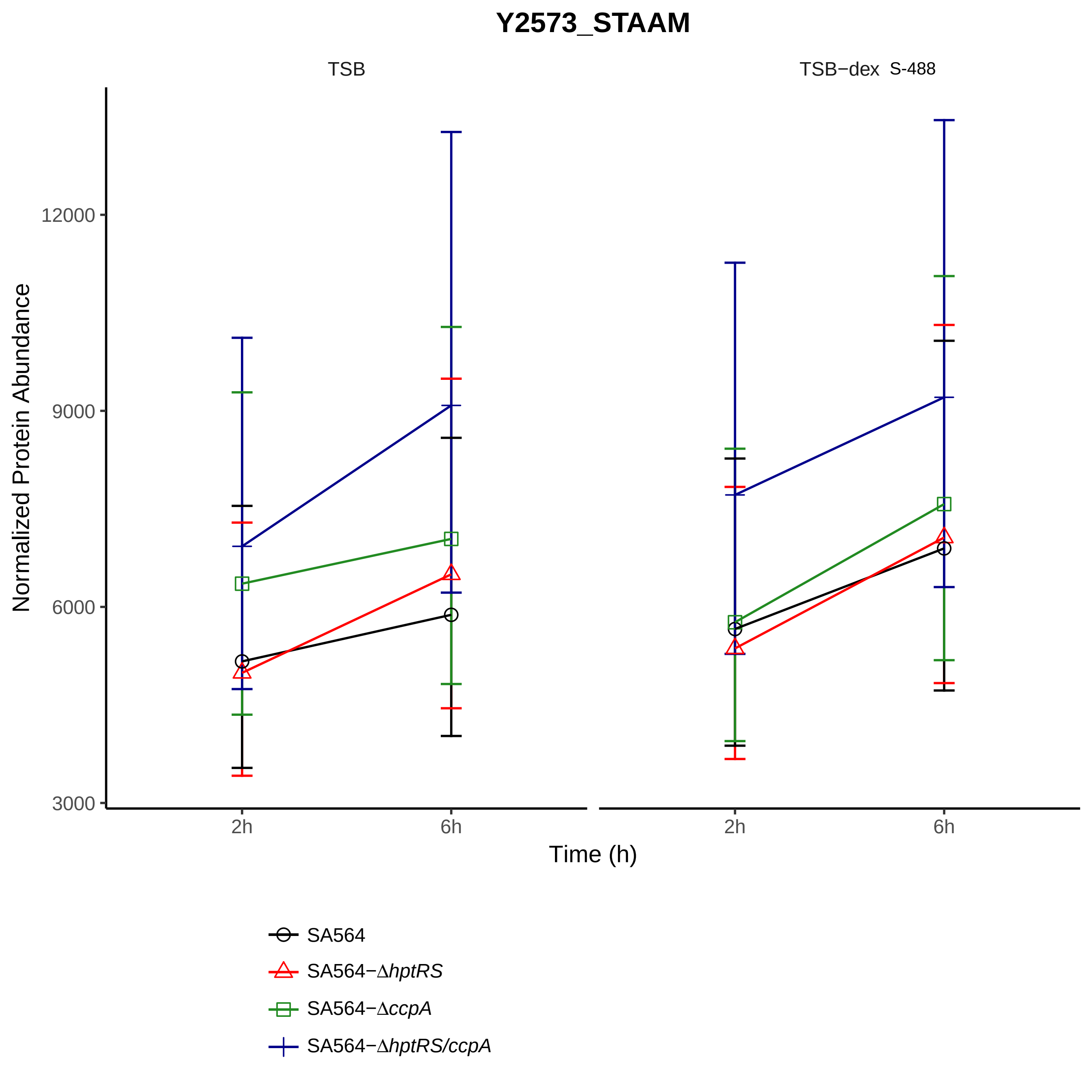
Y2573_STAAM
TSB
TSB−dex
S-488
Normalized Protein Abundance
12000
9000
6000
3000
2h
6h
2h
6h
Time (h)
SA564
SA564−Δ*hptRS*
SA564−Δ*ccpA*
SA564−Δ*hptRS/ccpA*

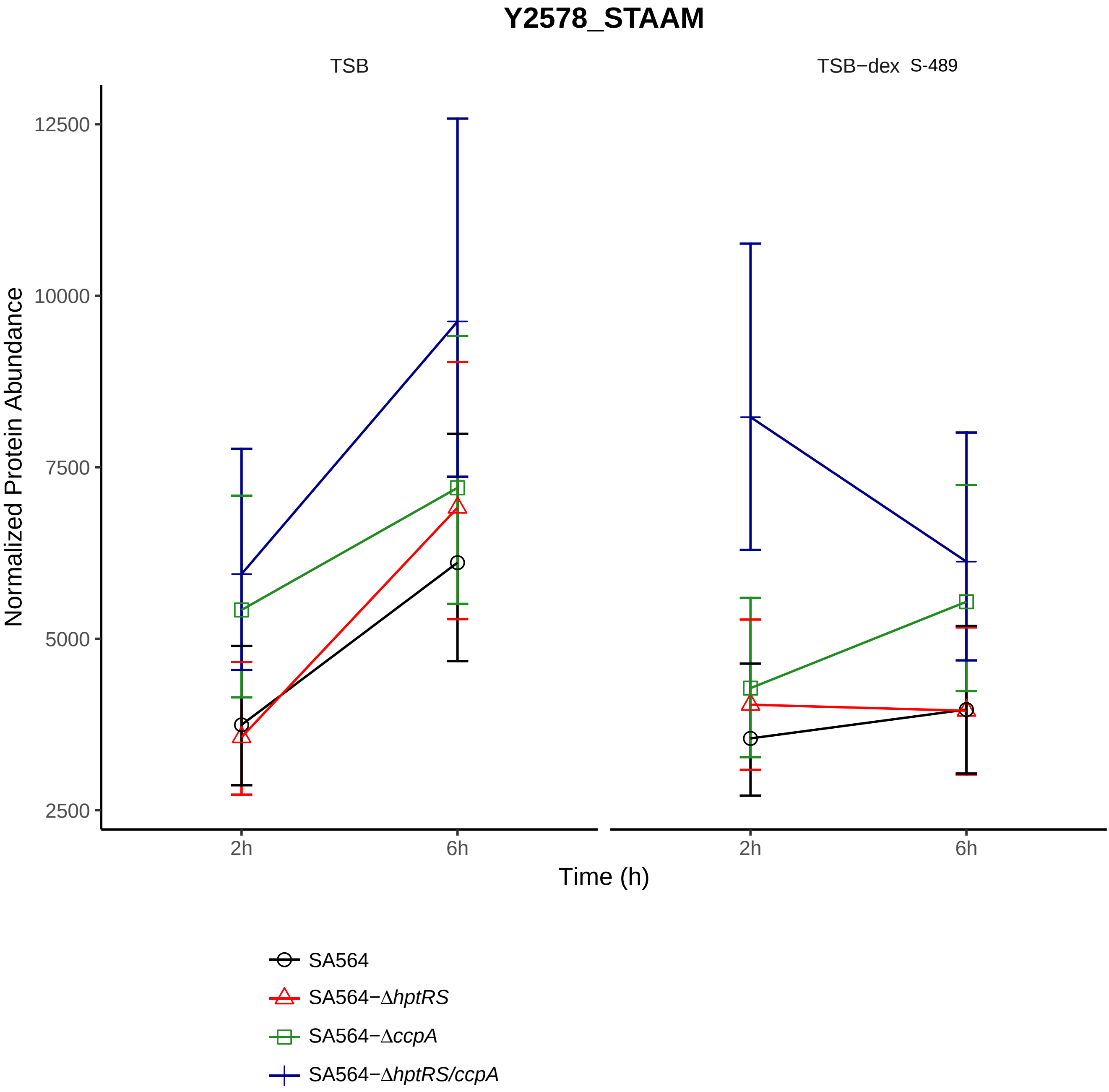

Y2578_STAAM
TSB
TSB−dex
S-489
Normalized Protein Abundance
12500
10000
7500
5000
2500
2h
6h
2h
6h
Time (h)
SA564
SA564−Δ*hptRS*
SA564−Δ*ccpA*
SA564−Δ*hptRS/ccpA*

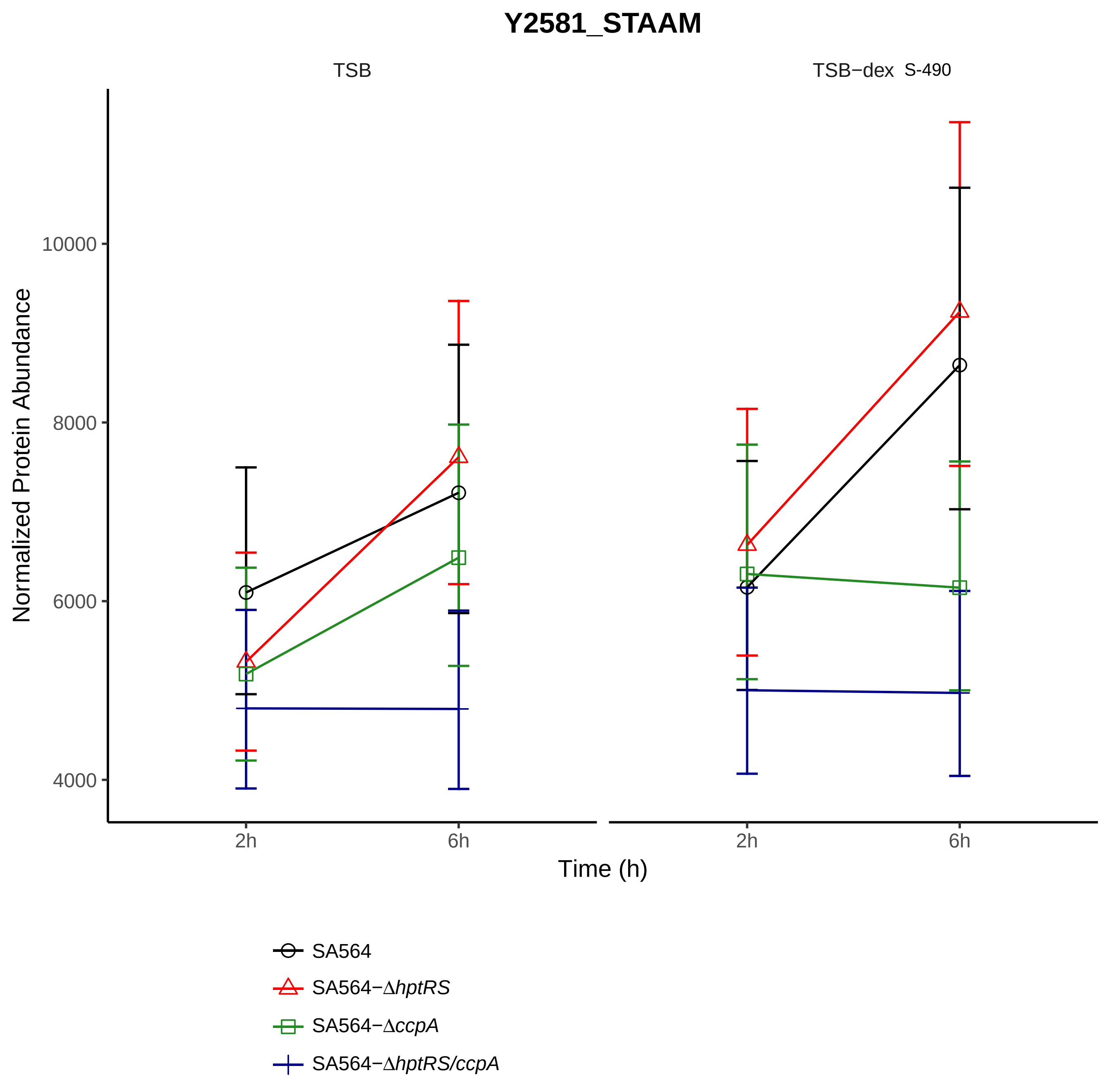

Y2581_STAAM
TSB
TSB−dex
S-490
Normalized Protein Abundance
10000
8000
6000
4000
2h
6h
2h
6h
Time (h)
SA564
SA564−ΔhptRS
SA564−ΔccpA
SA564−ΔhptRS/ccpA

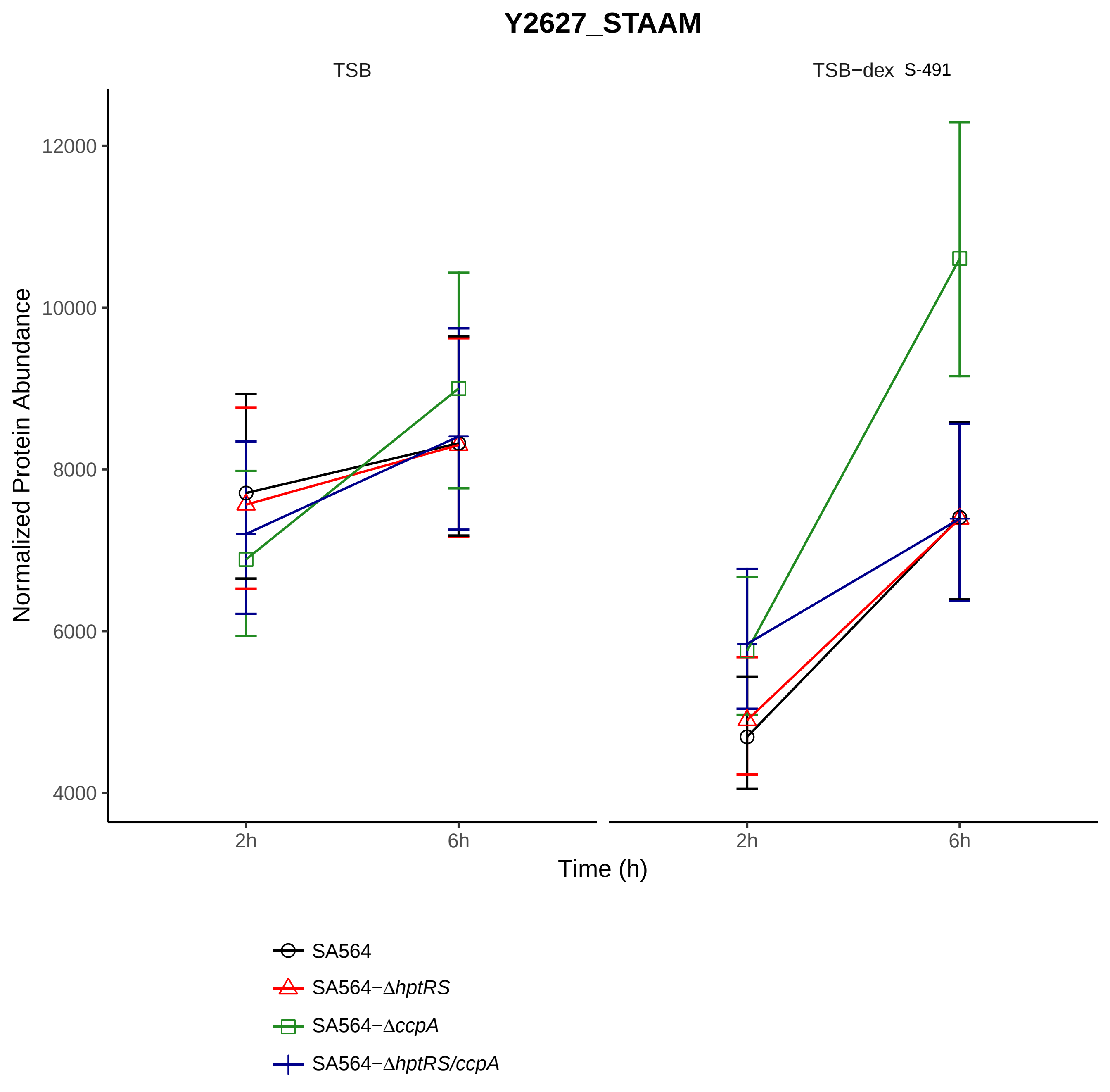

Y2627_STAAM
TSB
TSB−dex
S-491
Normalized Protein Abundance
12000
10000
8000
6000
4000
2h
6h
2h
6h
Time (h)
SA564
SA564−Δ*hptRS*
SA564−Δ*ccpA*
SA564−Δ*hptRS/ccpA*

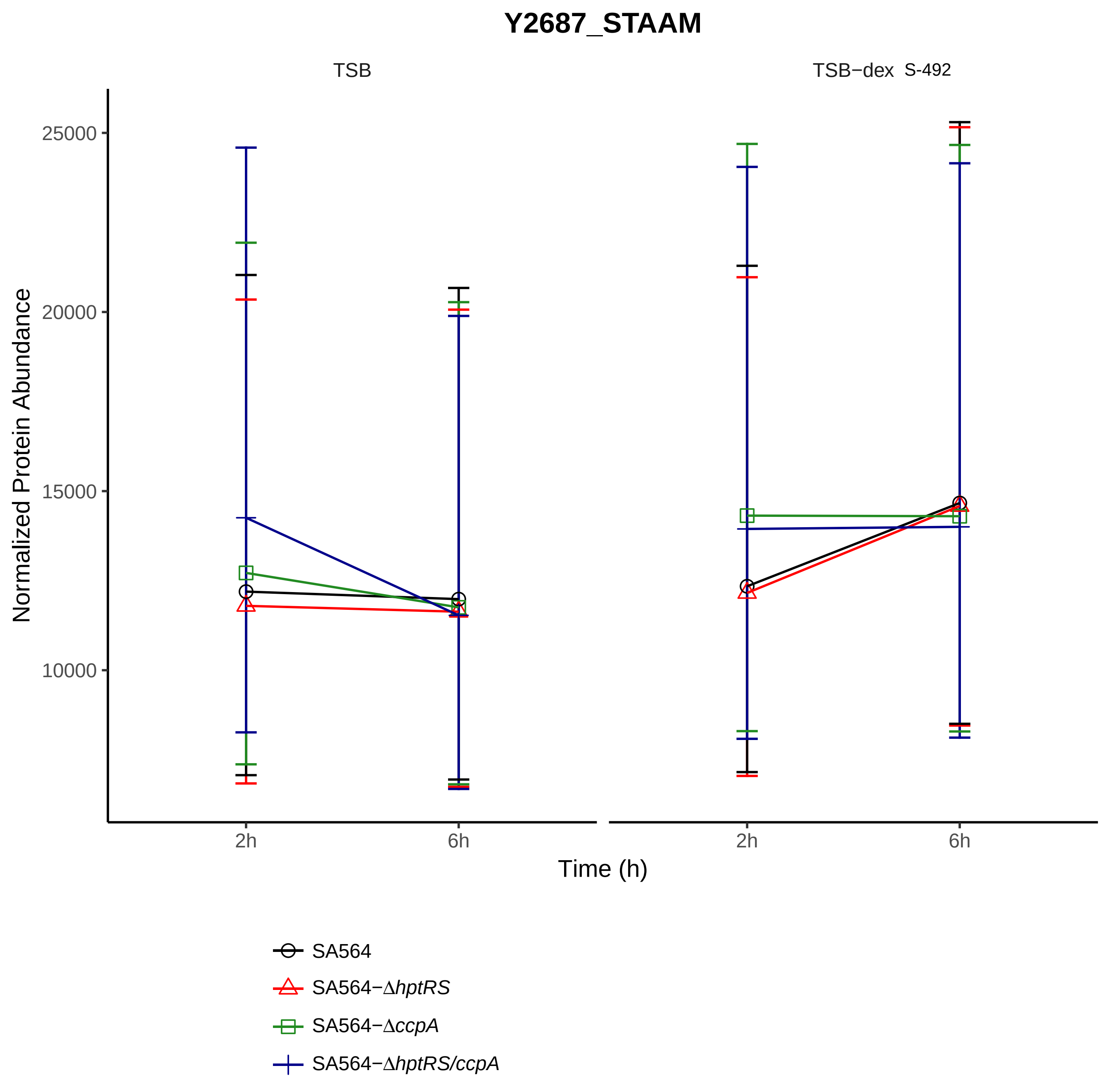

Y2687_STAAM
TSB
TSB−dex
S-492
25000
20000
15000
10000
Normalized Protein Abundance
2h
6h
2h
6h
Time (h)
SA564
SA564−Δ*hptRS*
SA564−Δ*ccpA*
SA564−Δ*hptRS/ccpA*

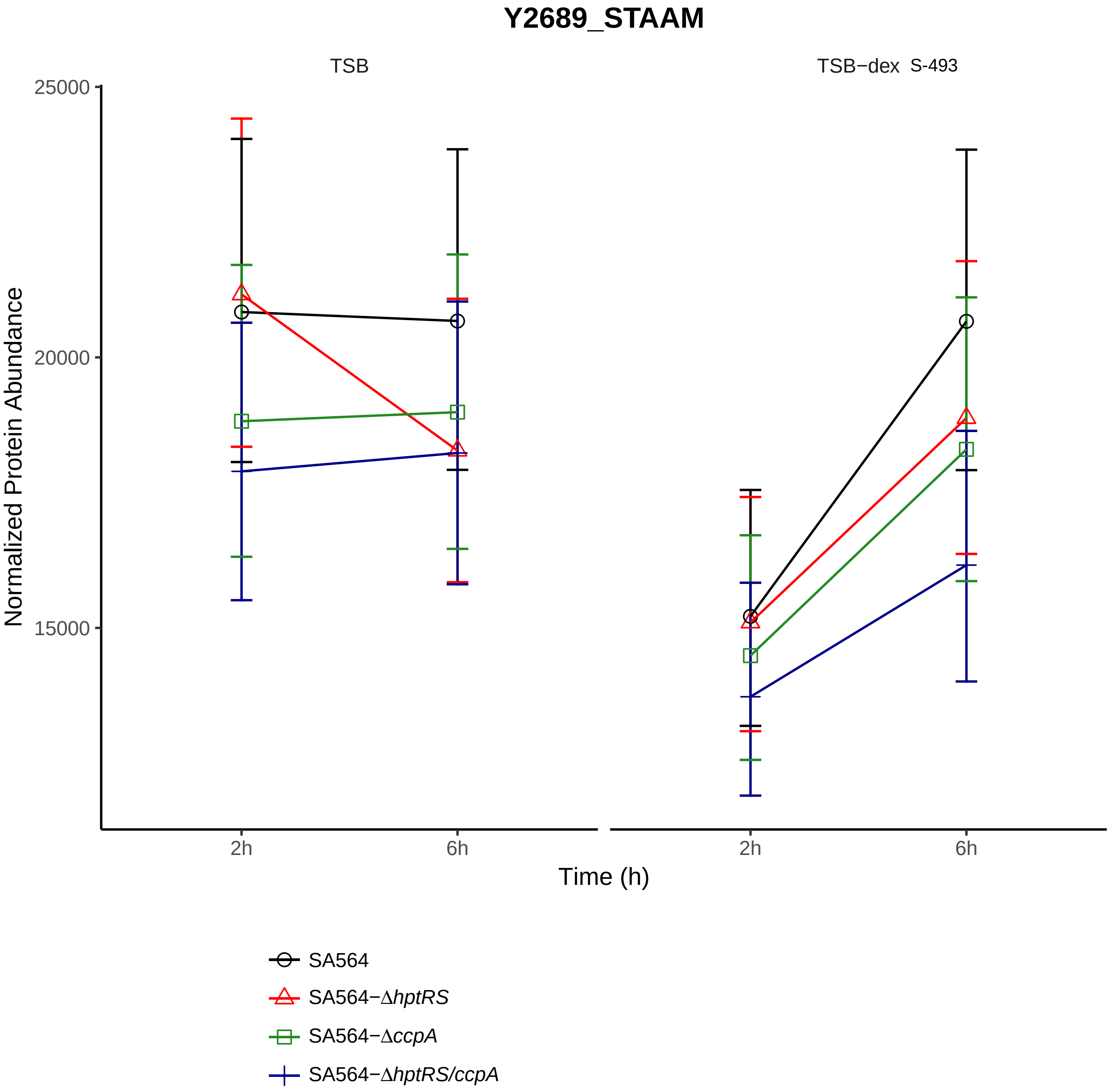
Y2689_STAAM
TSB
TSB−dex
S-493
25000
20000
15000
Normalized Protein Abundance
2h
6h
2h
6h
Time (h)
SA564
SA564−Δ*hptRS*
SA564−Δ*ccpA*
SA564−Δ*hptRS/ccpA*

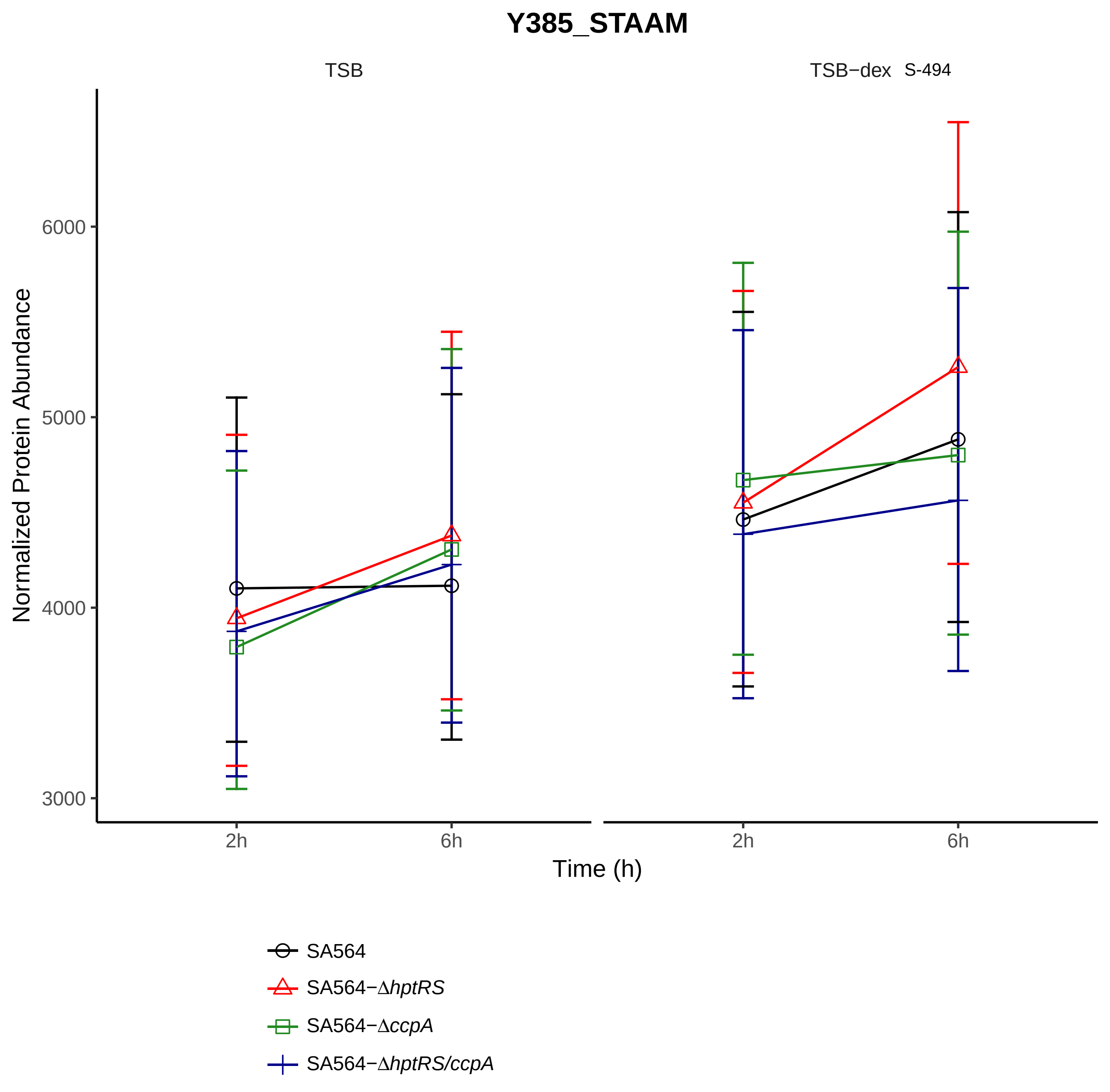
Y385_STAAM
TSB
TSB−dex
S-494
Normalized Protein Abundance
6000
5000
4000
3000
2h
6h
2h
6h
Time (h)
SA564
SA564−ΔhptRS
SA564−ΔccpA
SA564−ΔhptRS/ccpA

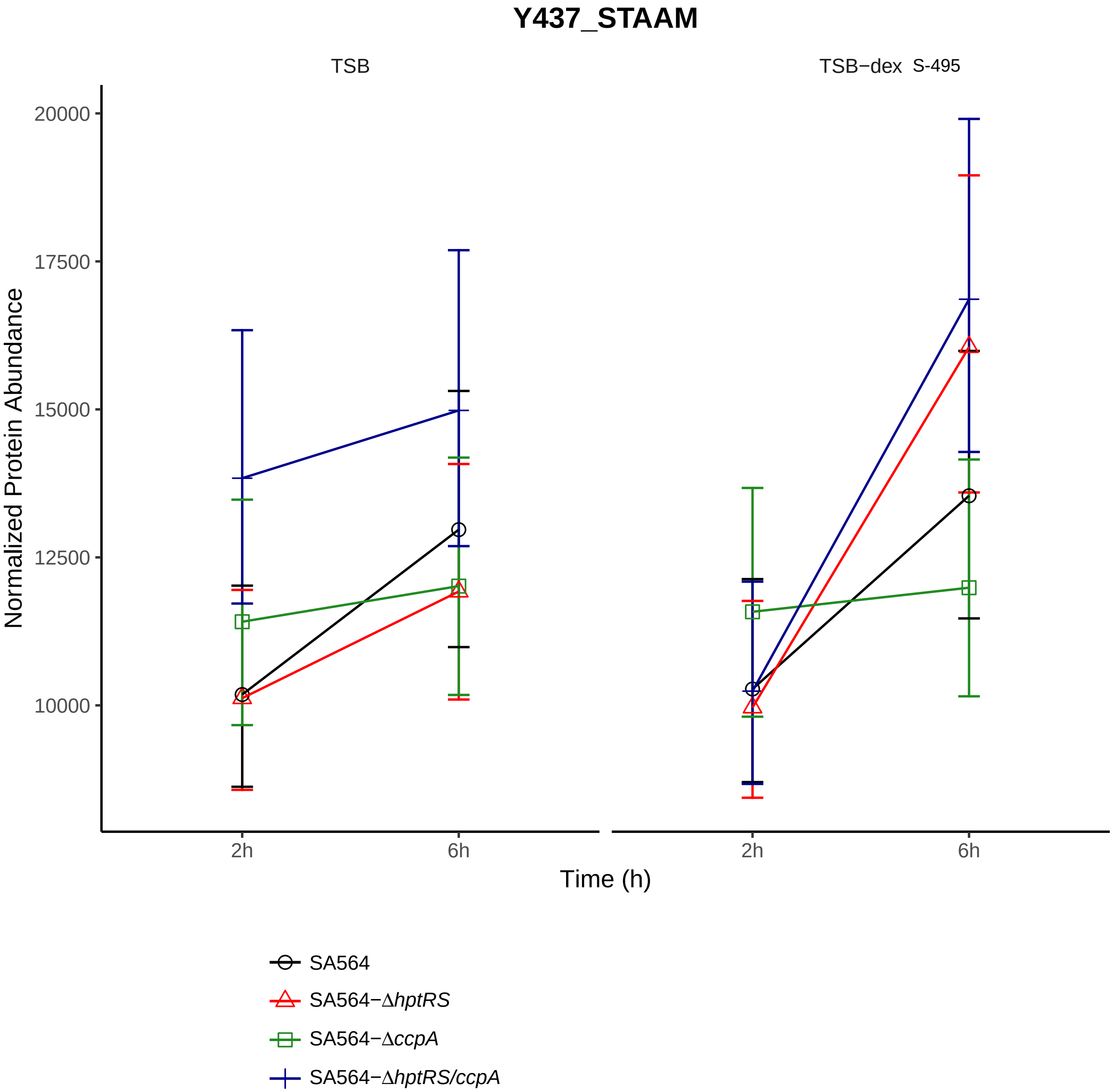

Y437_STAAM
TSB
TSB−dex
S-495
Normalized Protein Abundance
20000
17500
15000
12500
10000
2h
6h
2h
6h
Time (h)
SA564
SA564−Δ*hptRS*
SA564−Δ*ccpA*
SA564−Δ*hptRS/ccpA*

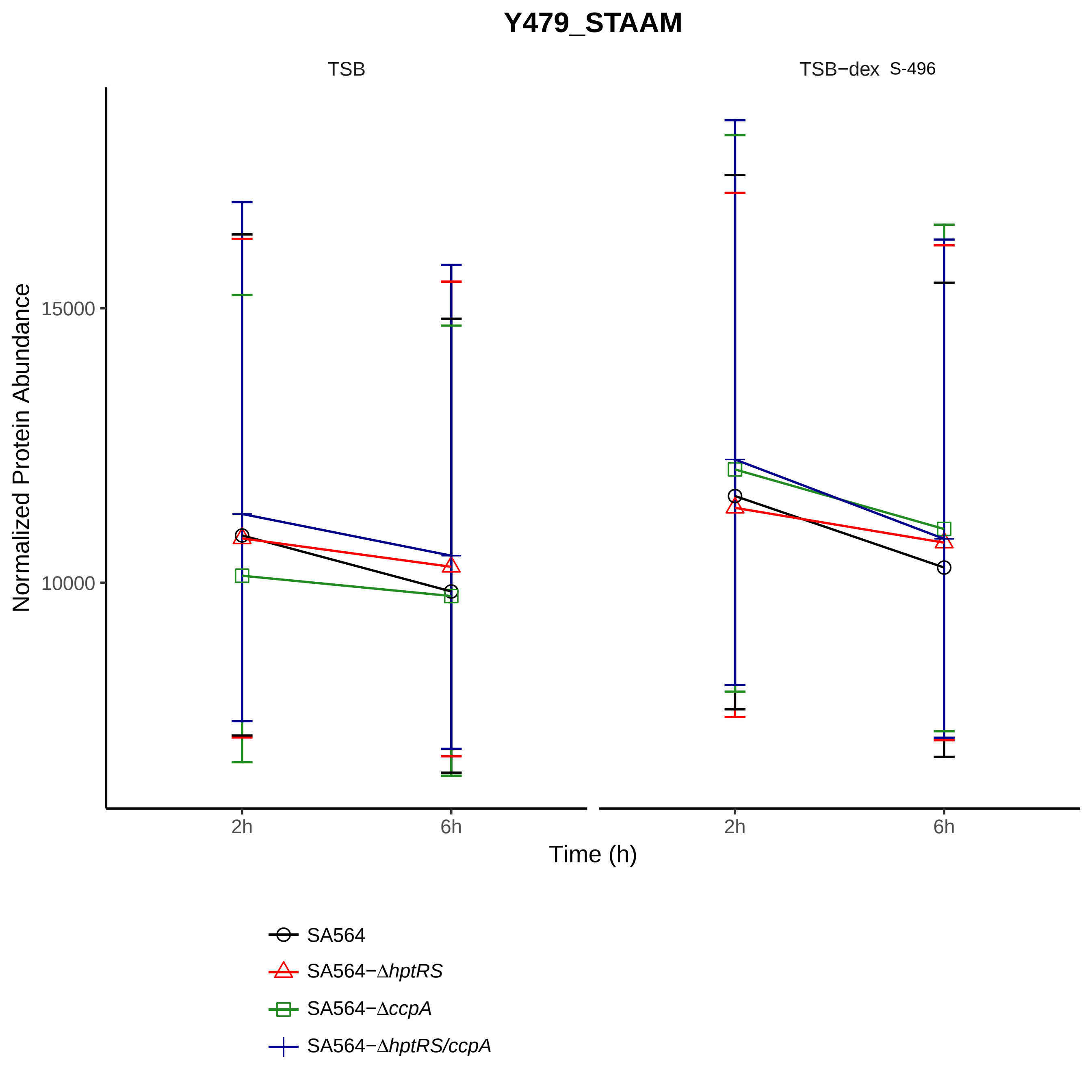

Y479_STAAM
TSB
TSB−dex
S-496
Normalized Protein Abundance
15000
10000
2h
6h
2h
6h
Time (h)
SA564
SA564−ΔhptRS
SA564−ΔccpA
SA564−ΔhptRS/ccpA

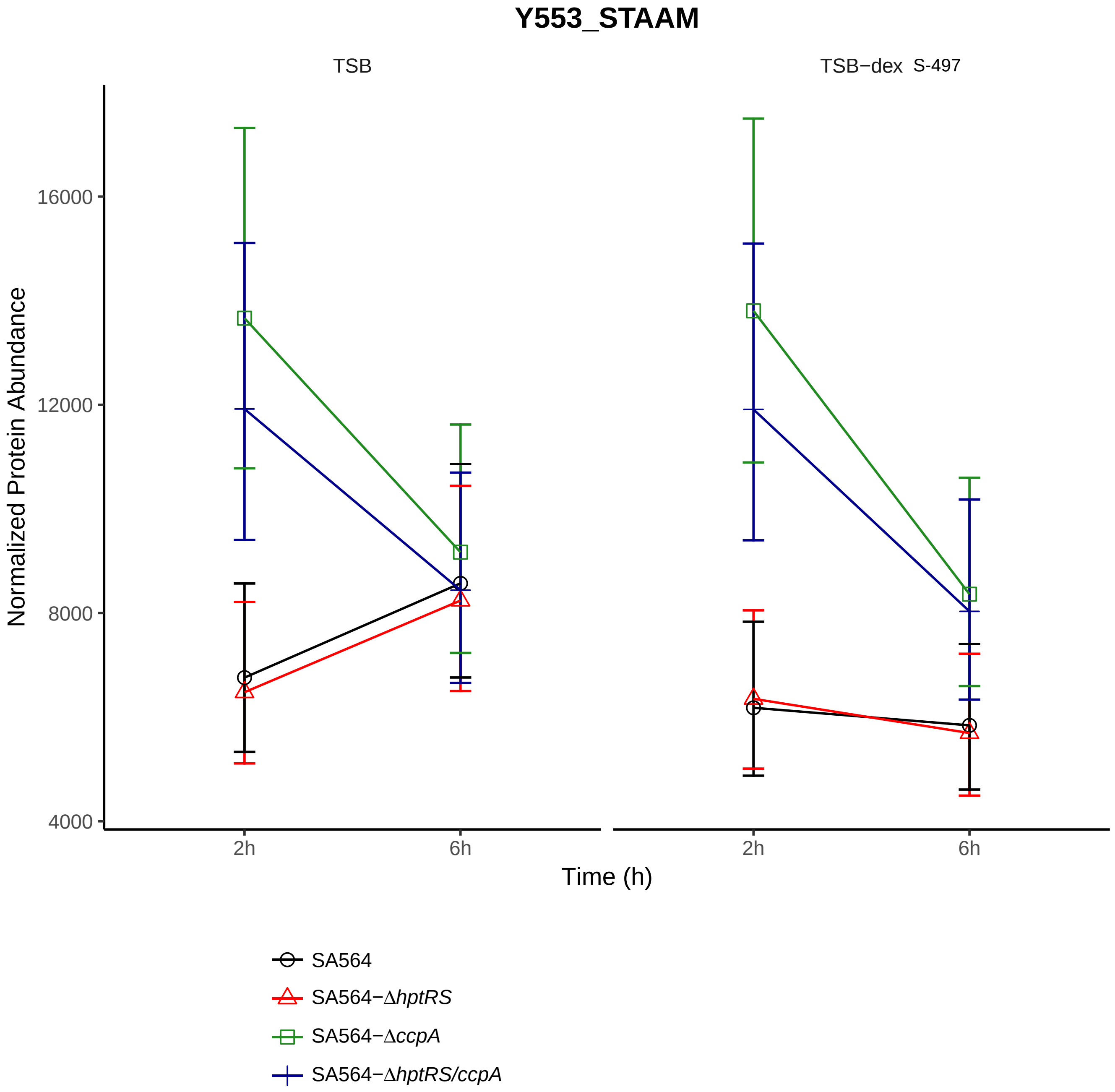

Y553_STAAM
TSB
TSB−dex
S-497
Normalized Protein Abundance
16000
12000
8000
4000
2h
6h
2h
6h
Time (h)
SA564
SA564−Δ*hptRS*
SA564−Δ*ccpA*
SA564−Δ*hptRS/ccpA*

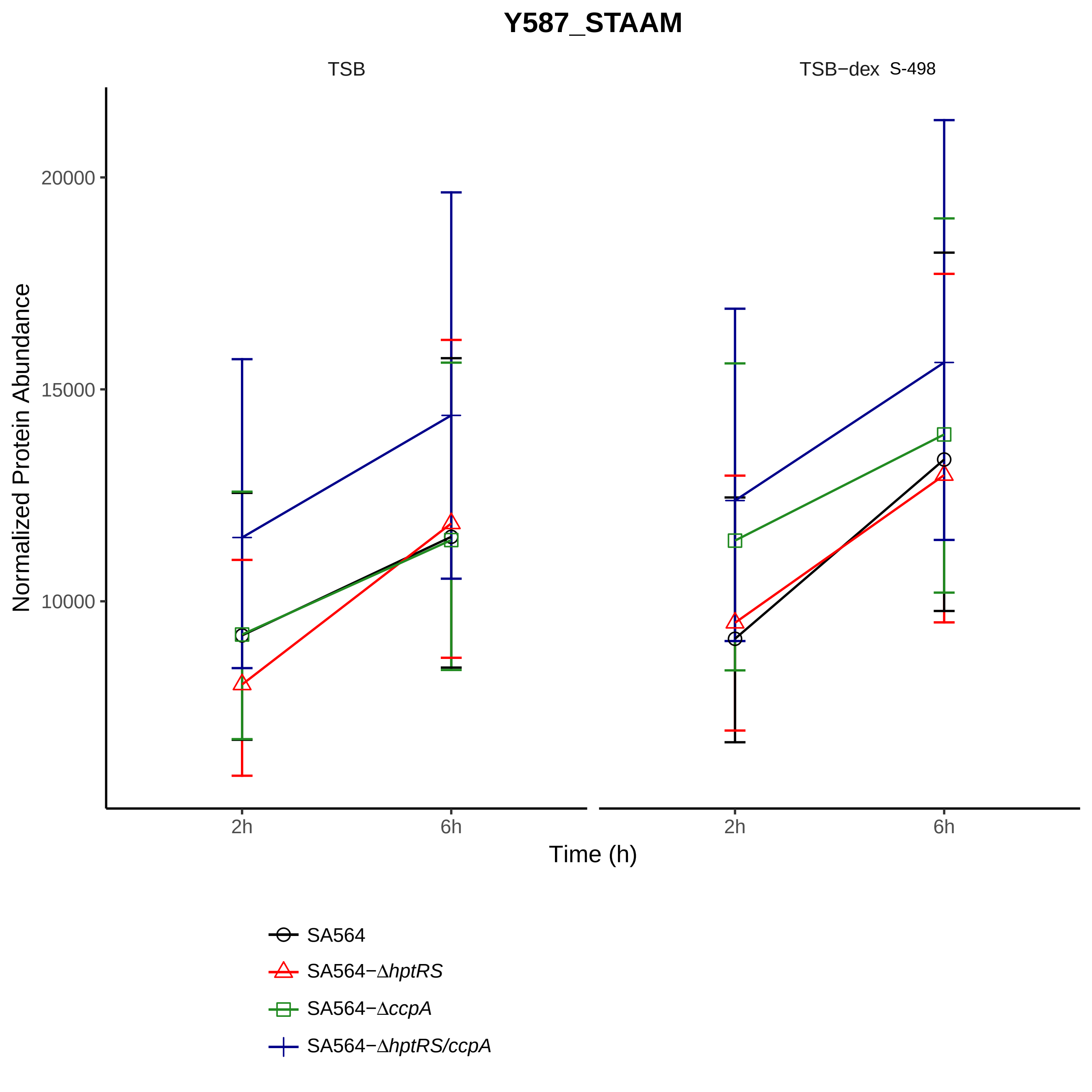

Y587_STAAM
TSB
TSB−dex
S-498
Normalized Protein Abundance
20000
15000
10000
2h
6h
2h
6h
Time (h)
SA564
SA564−ΔhptRS
SA564−ΔccpA
SA564−ΔhptRS/ccpA

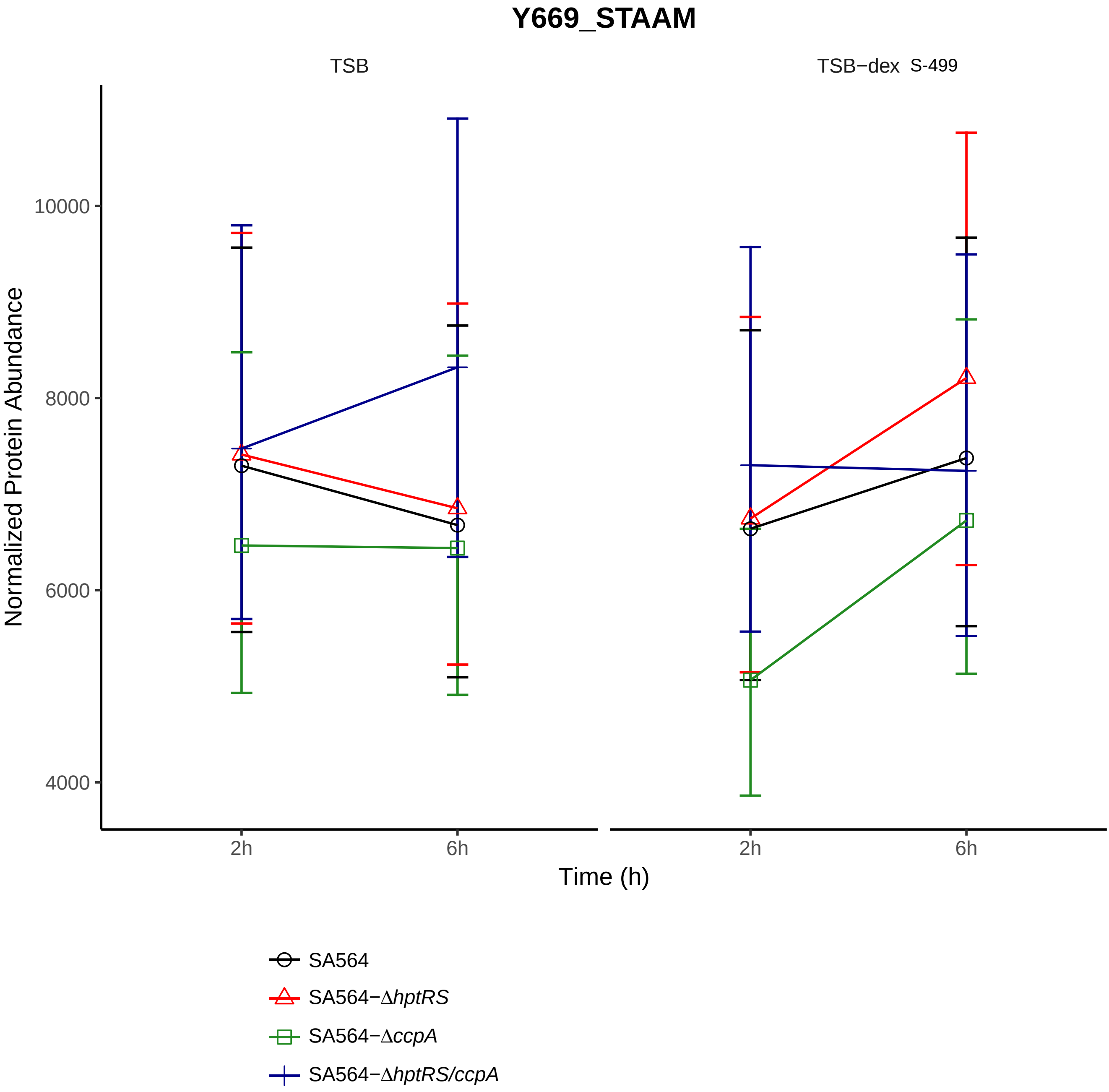
Y669_STAAM
TSB
TSB−dex
S-499
Normalized Protein Abundance
10000
8000
6000
4000
2h
6h
2h
6h
Time (h)
SA564
SA564−Δ*hptRS*
SA564−Δ*ccpA*
SA564−Δ*hptRS/ccpA*

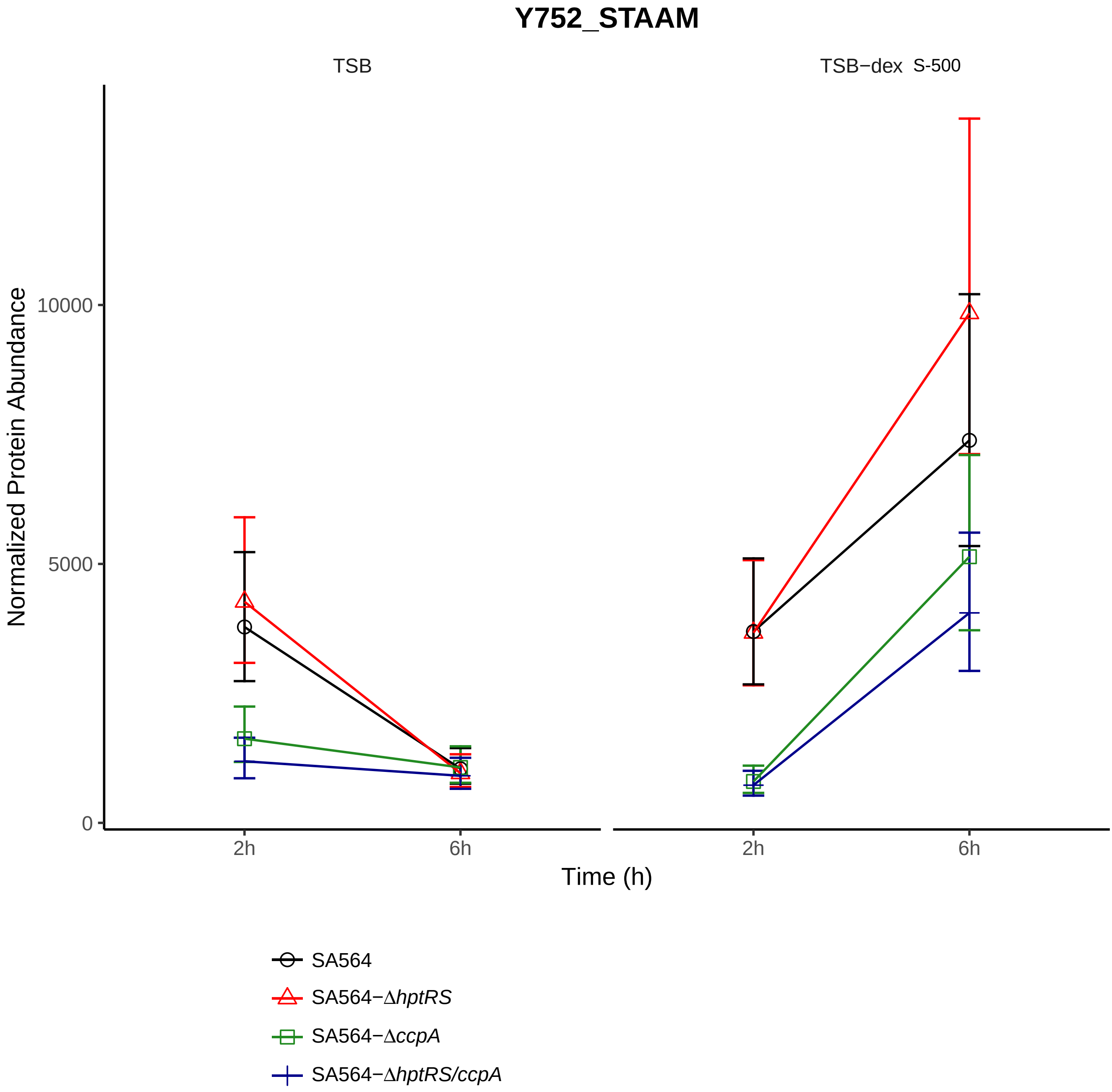
Y752_STAAM
TSB
TSB−dex
S-500
Normalized Protein Abundance
10000
5000
0
2h
6h
2h
6h
Time (h)
SA564
SA564−ΔhptRS
SA564−ΔccpA
SA564−ΔhptRS/ccpA

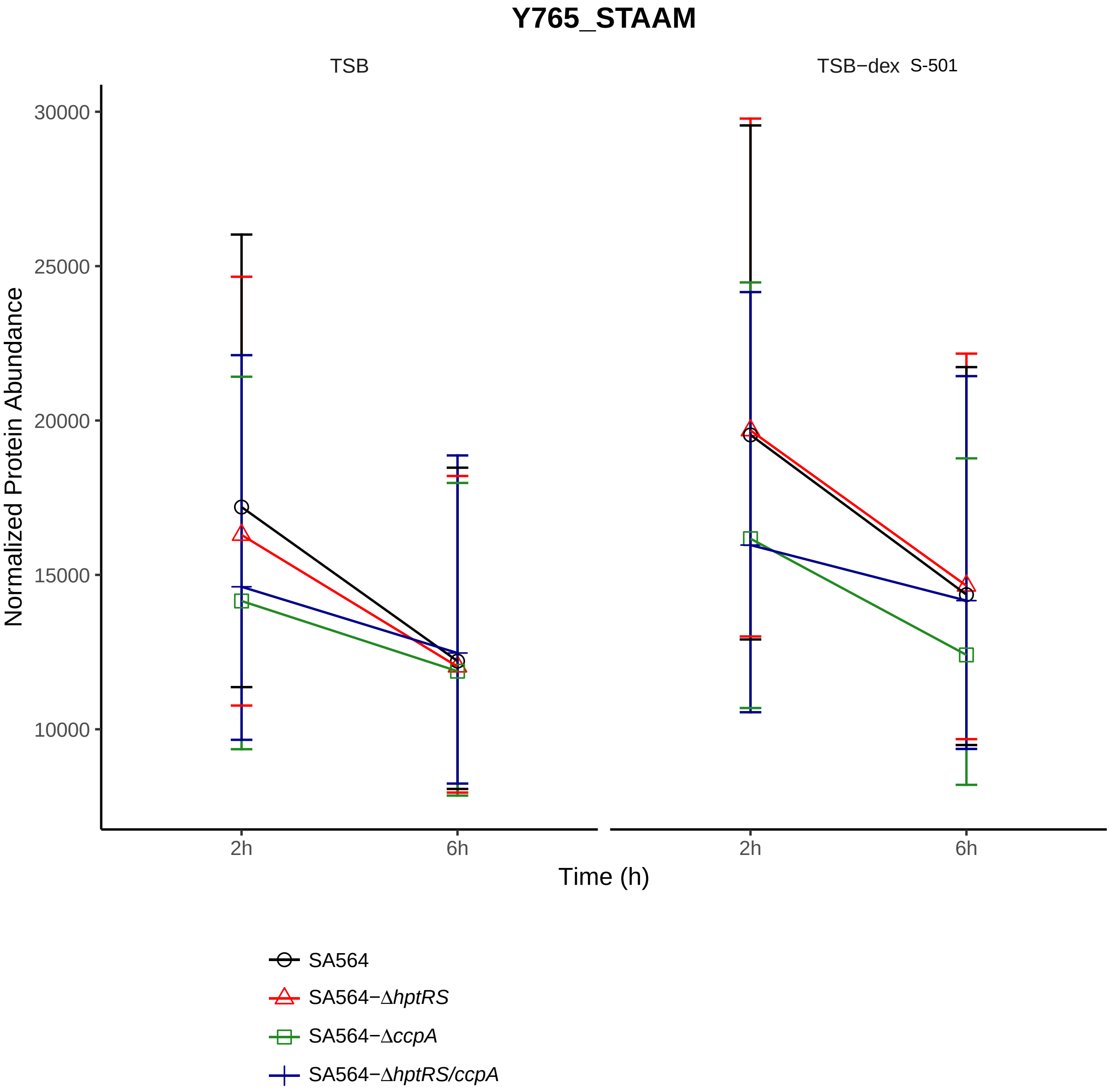

Y765_STAAM
TSB
TSB−dex
Normalized Protein Abundance
30000
25000
20000
15000
10000
2h
6h
2h
6h
Time (h)
SA564
SA564−Δ*hptRS*
SA564−Δ*ccpA*
SA564−Δ*hptRS/ccpA*

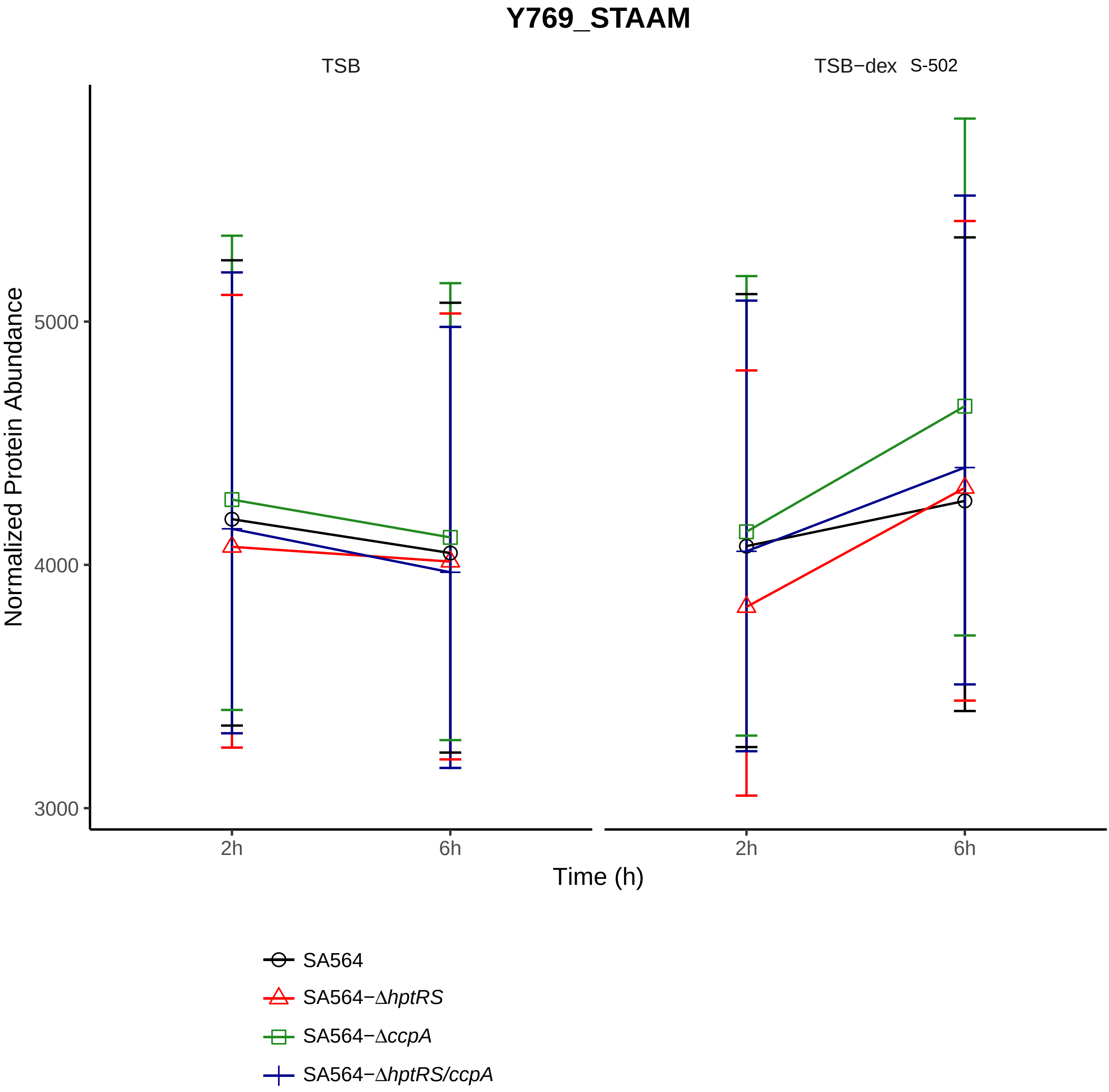

Y769_STAAM
TSB
TSB−dex
Normalized Protein Abundance
5000
4000
3000
2h
6h
2h
6h
Time (h)
SA564
SA564−ΔhptRS
SA564−ΔccpA
SA564−ΔhptRS/ccpA

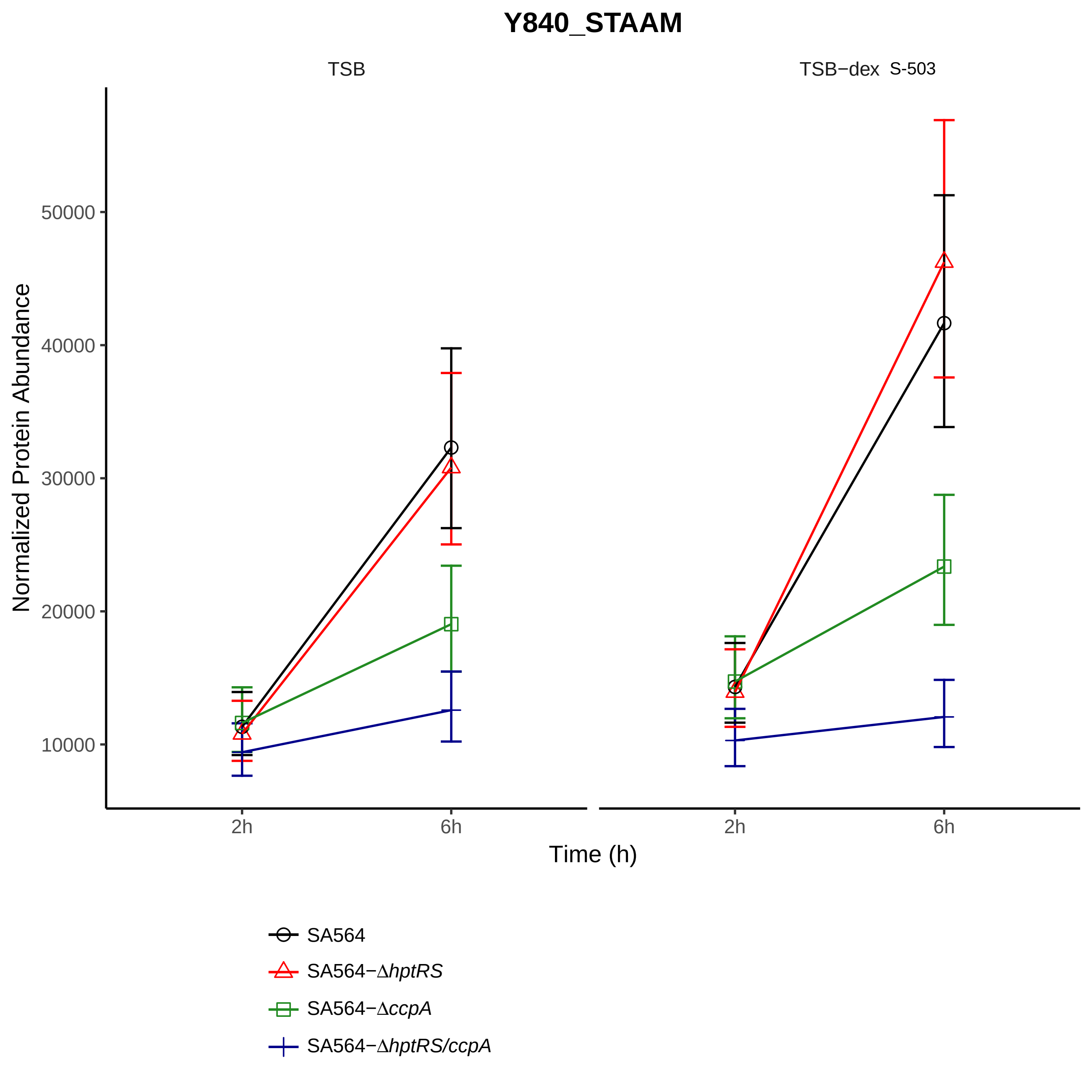
Y840_STAAM
TSB
TSB−dex
S-503
Normalized Protein Abundance
50000
40000
30000
20000
10000
2h
6h
2h
6h
Time (h)
SA564
SA564−ΔhptRS
SA564−ΔccpA
SA564−ΔhptRS/ccpA

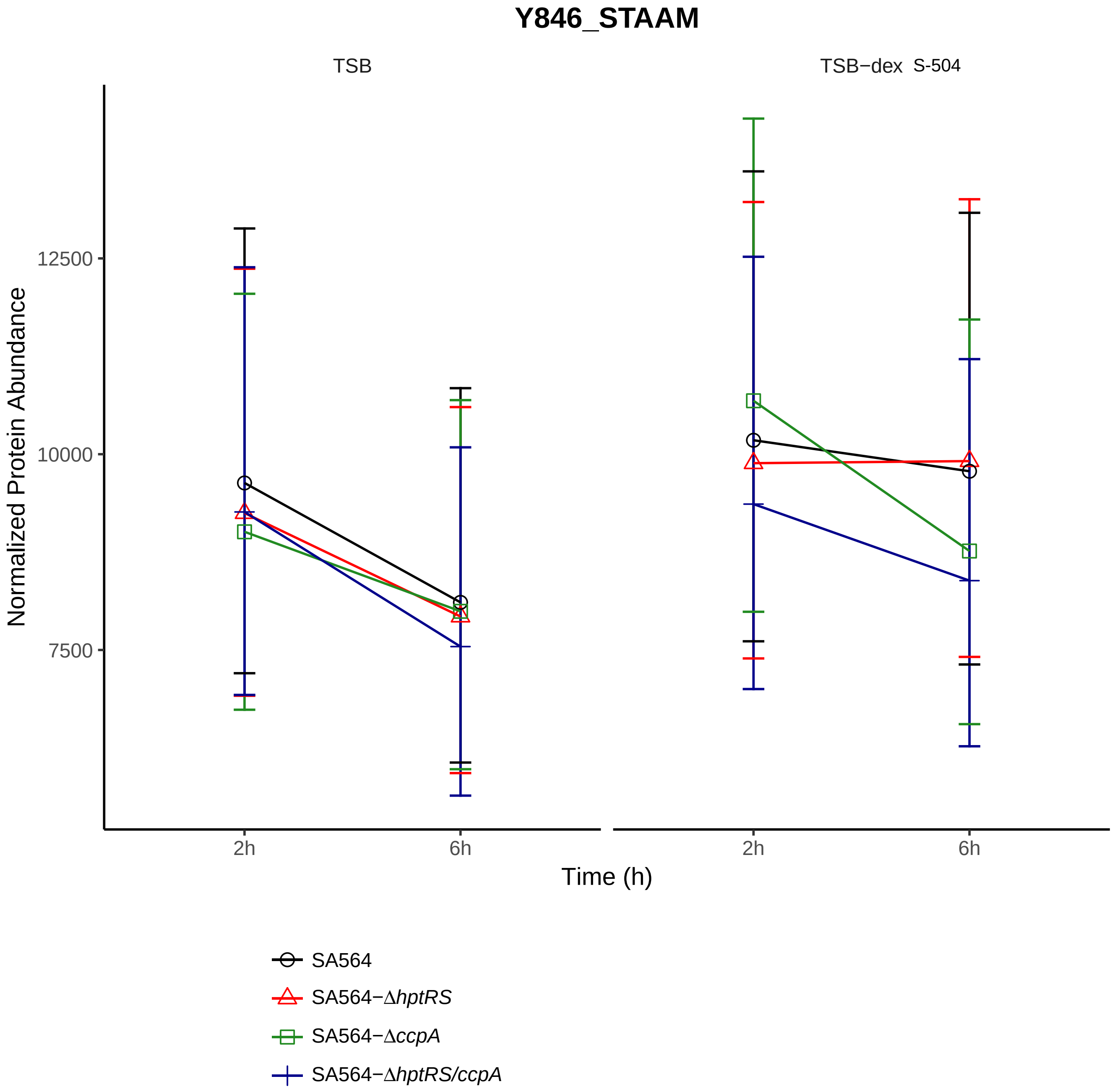

Y846_STAAM
TSB
TSB−dex
S-504
Normalized Protein Abundance
12500
10000
7500
2h
6h
2h
6h
Time (h)
SA564
SA564−ΔhptRS
SA564−ΔccpA
SA564−ΔhptRS/ccpA

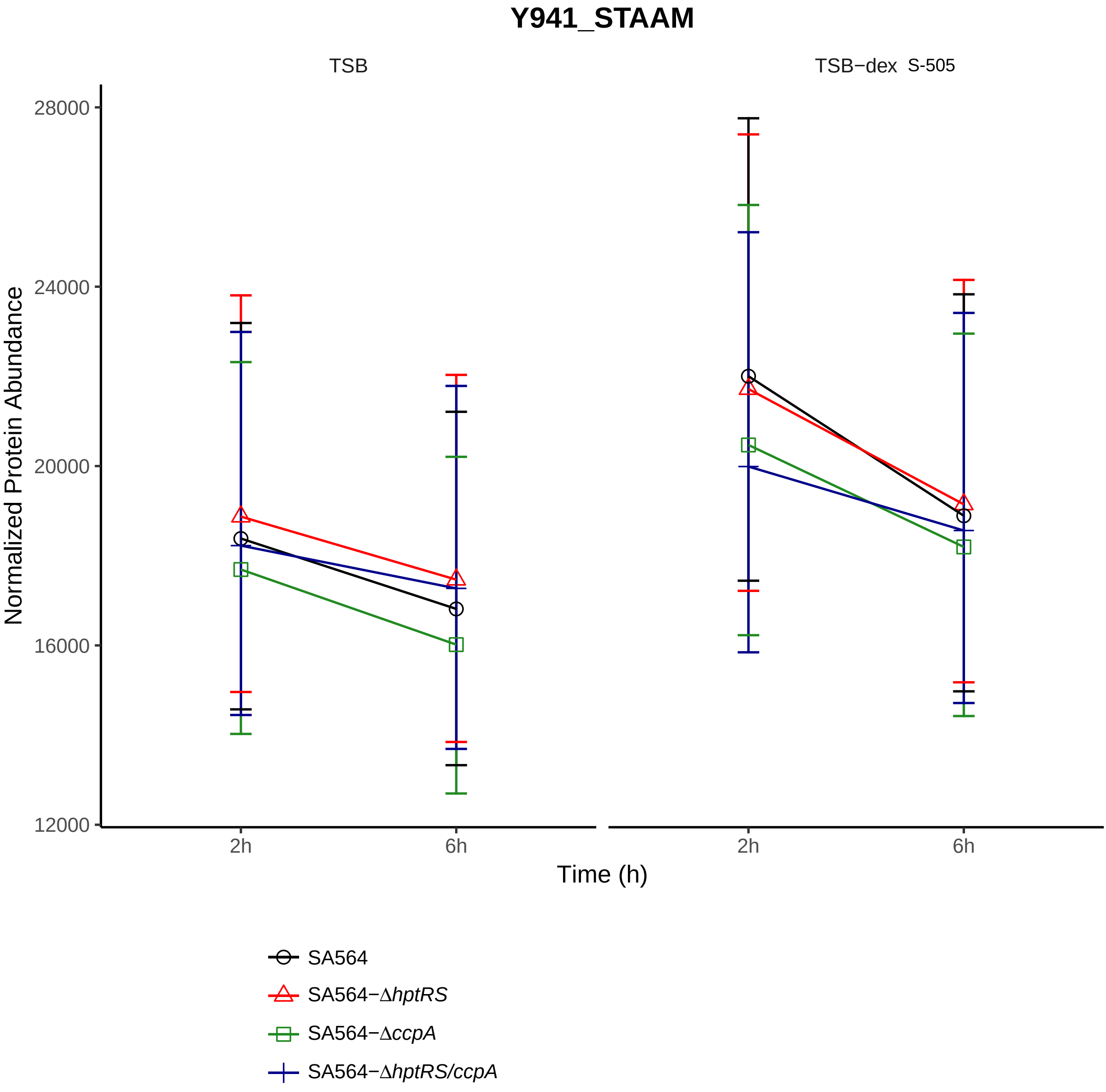

Y941_STAAM
TSB
TSB−dex
Normalized Protein Abundance
28000
24000
20000
16000
12000
2h
6h
2h
6h
Time (h)
SA564
SA564−ΔhptRS
SA564−ΔccpA
SA564−ΔhptRS/ccpA

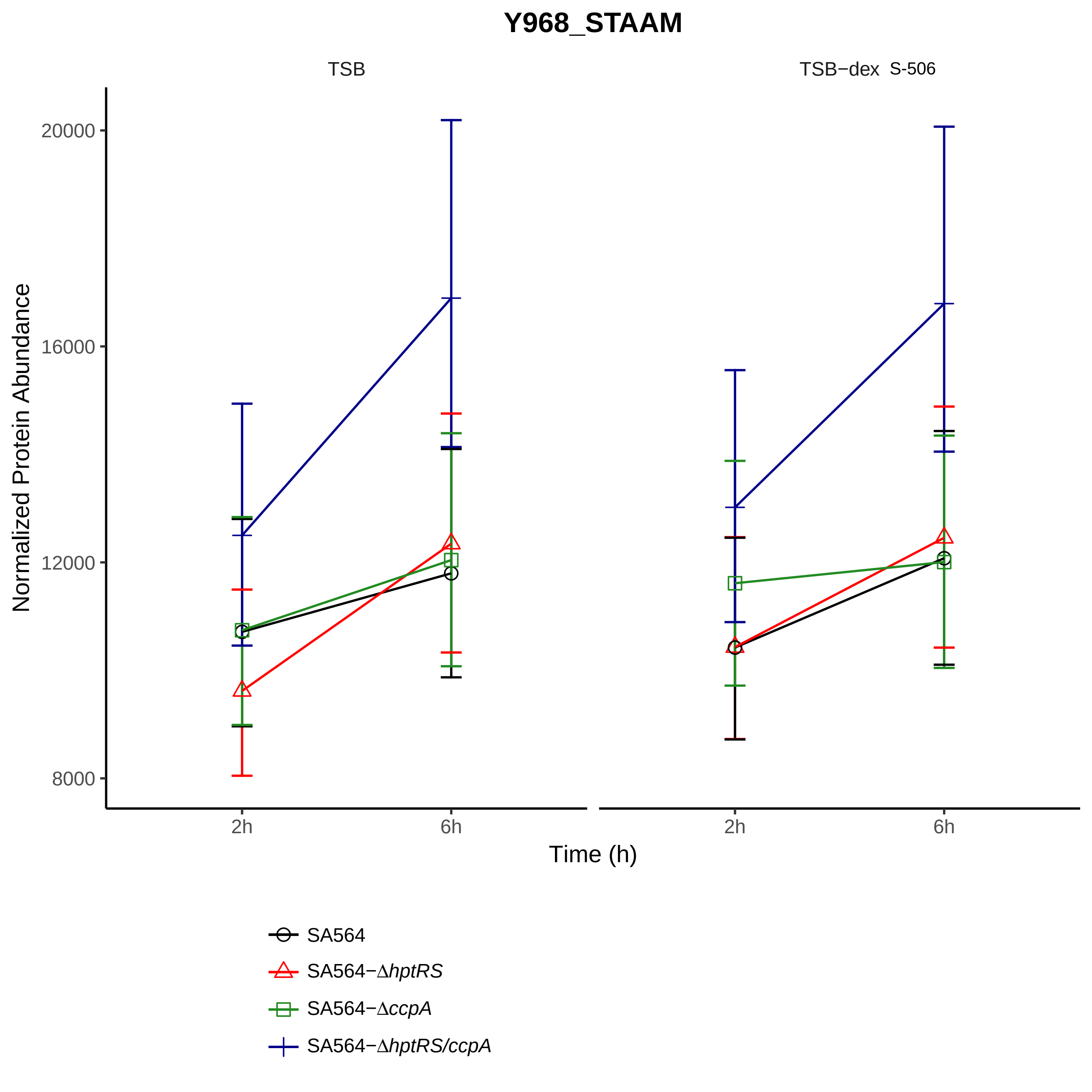

Y968_STAAM
TSB
TSB−dex
S-506
Normalized Protein Abundance
20000
16000
12000
8000
2h
6h
2h
6h
Time (h)
SA564
SA564−Δ*hptRS*
SA564−Δ*ccpA*
SA564−Δ*hptRS/ccpA*

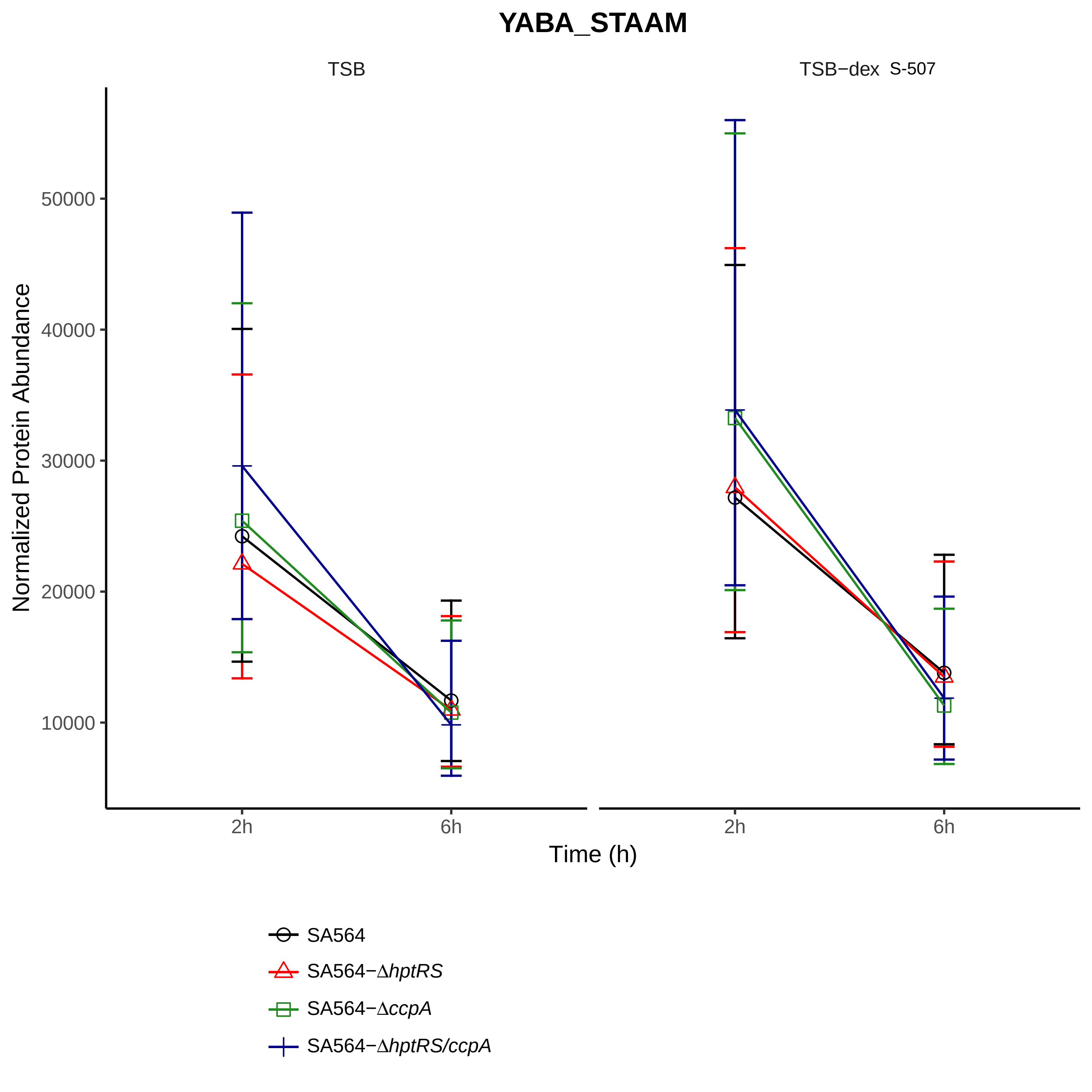

YABA_STAAM
TSB
TSB−dex
S-507
Normalized Protein Abundance
50000
40000
30000
20000
10000
2h
6h
2h
6h
Time (h)
SA564
SA564−Δ*hptRS*
SA564−Δ*ccpA*
SA564−Δ*hptRS/ccpA*

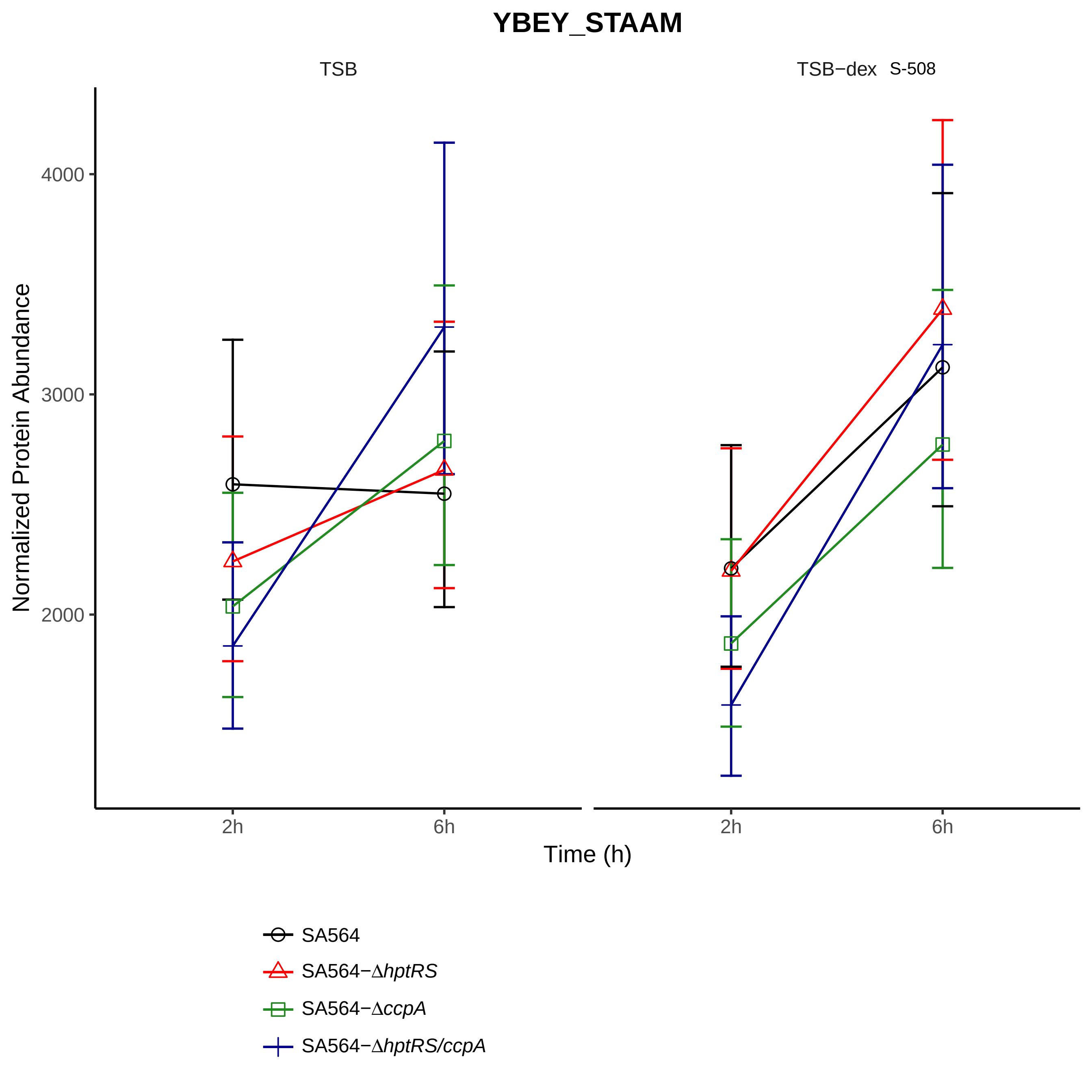

YBEY_STAAM
TSB
TSB−dex
S-508
Normalized Protein Abundance
4000
3000
2000
2h
6h
2h
6h
Time (h)
SA564
SA564−Δ*hptRS*
SA564−Δ*ccpA*
SA564−Δ*hptRS/ccpA*

Figure G.

2-way analysis: s x m | h

Pages: S509-S1010

The normalized protein abundance, lower 95% confidence interval, and upper 95% confidence interval were plotted for each protein. These plots were analyzed if a 3-way interaction was rejected and 2-way interaction was identified between strain and media.

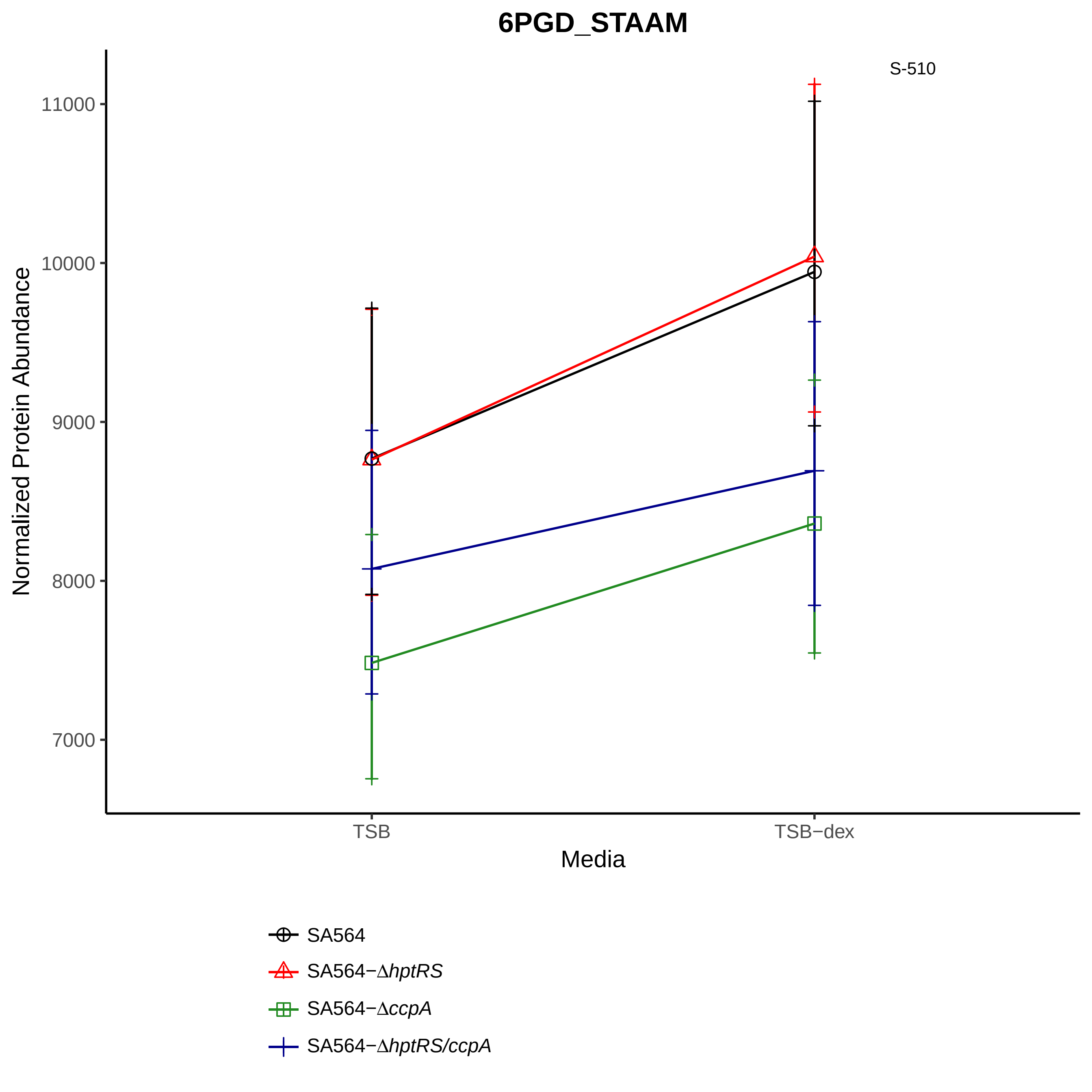
6PGD_STAAM
S-510
Normalized Protein Abundance
11000
10000
9000
8000
7000
TSB
TSB−dex
Media
SA564
SA564−ΔhptRS
SA564−ΔccpA
SA564−ΔhptRS/ccpA

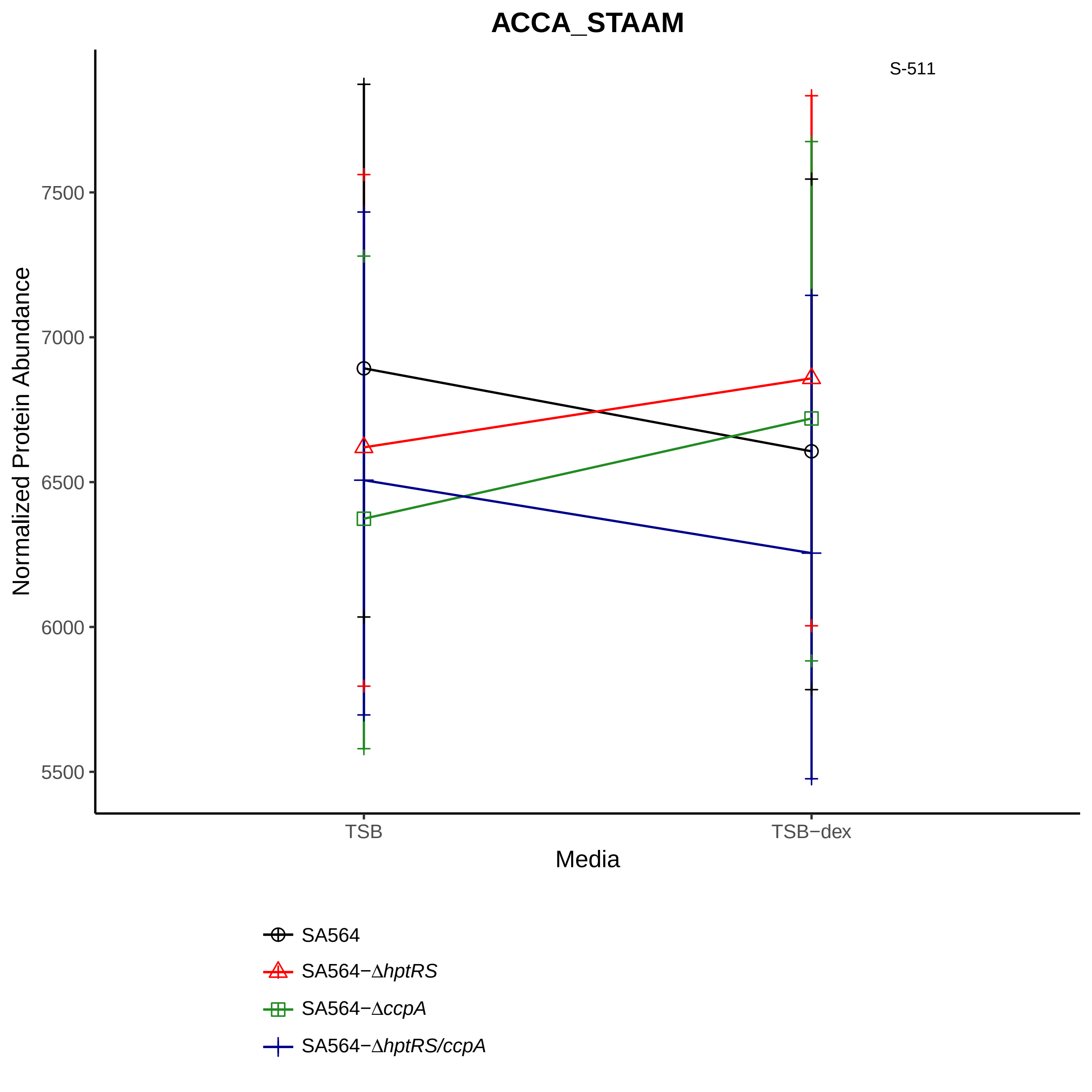
ACCA_STAAM
S-511
Normalized Protein Abundance
7500
7000
6500
6000
5500
TSB
TSB−dex
Media
SA564
SA564−ΔhptRS
SA564−ΔccpA
SA564−ΔhptRS/ccpA

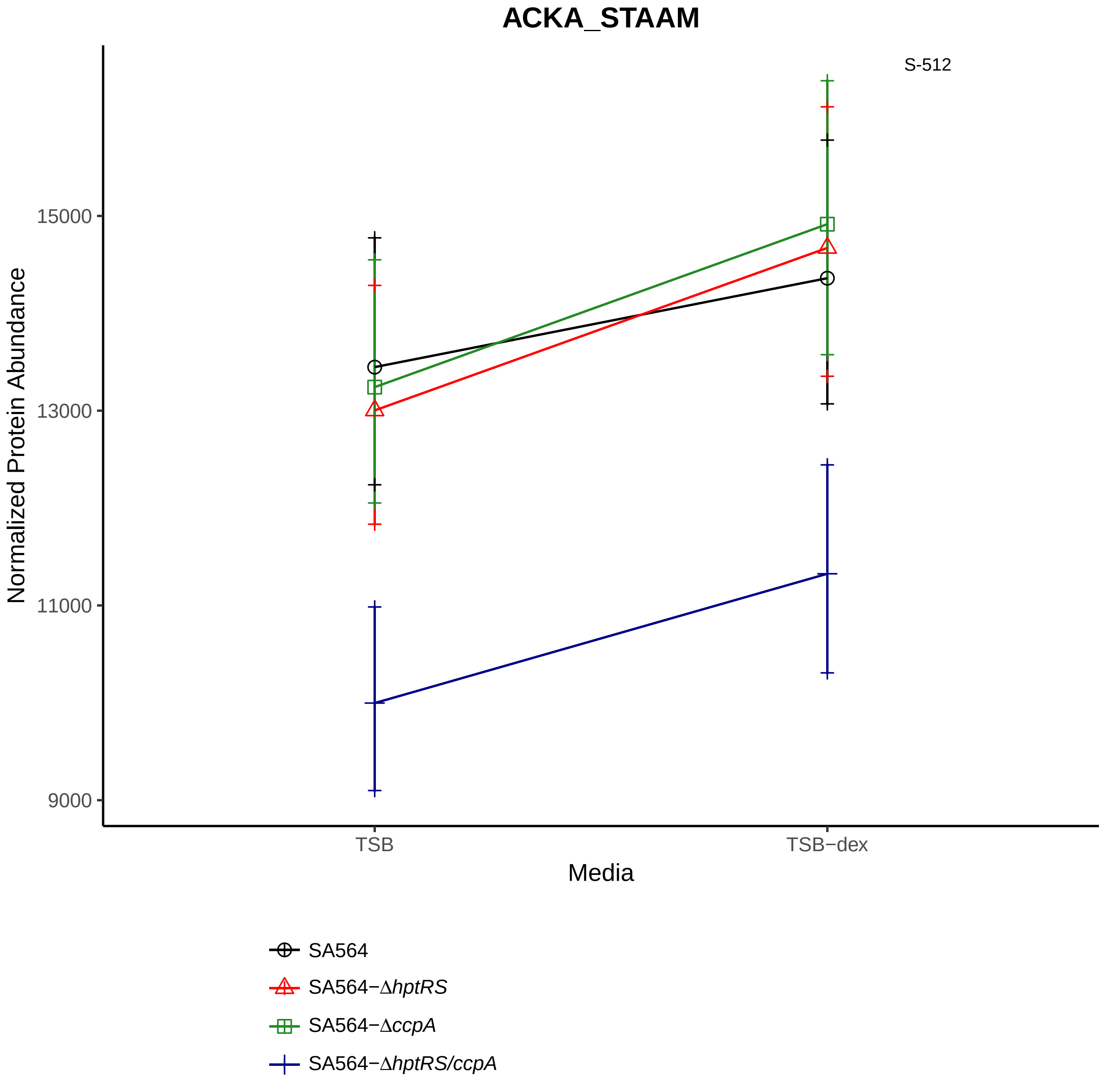

ACKA_STAAM
S-512
Normalized Protein Abundance
15000
13000
11000
9000
TSB
TSB−dex
Media
SA564
SA564−Δ*hptRS*
SA564−Δ*ccpA*
SA564−Δ*hptRS/ccpA*

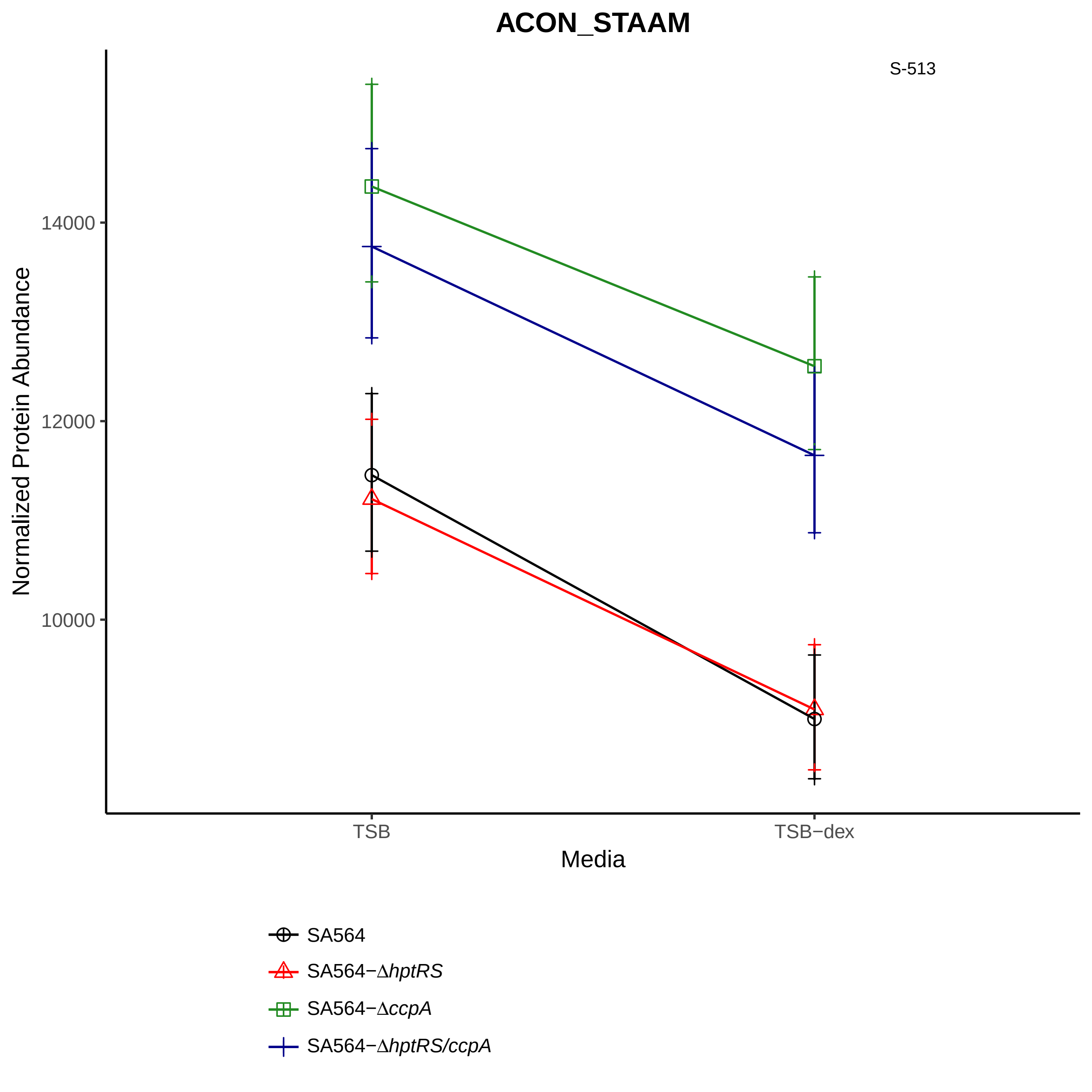

ACON_STAAM
S-513
Normalized Protein Abundance
14000
12000
10000
TSB
TSB−dex
Media
SA564
SA564−Δ*hptRS*
SA564−Δ*ccpA*
SA564−Δ*hptRS/ccpA*

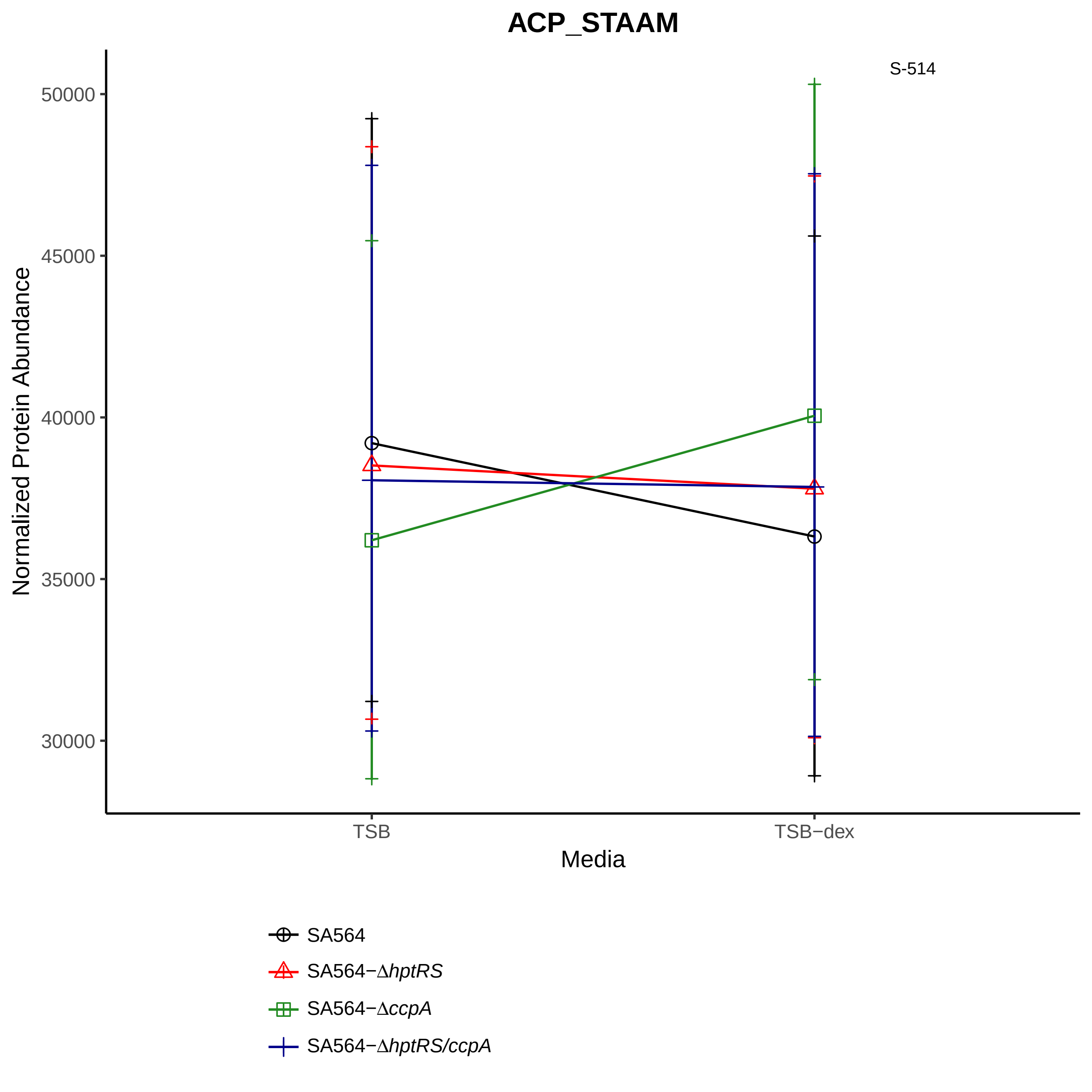
ACP_STAAM
S-514
Normalized Protein Abundance
50000
45000
40000
35000
30000
TSB
TSB−dex
Media
SA564
SA564−Δ*hptRS*
SA564−Δ*ccpA*
SA564−Δ*hptRS/ccpA*

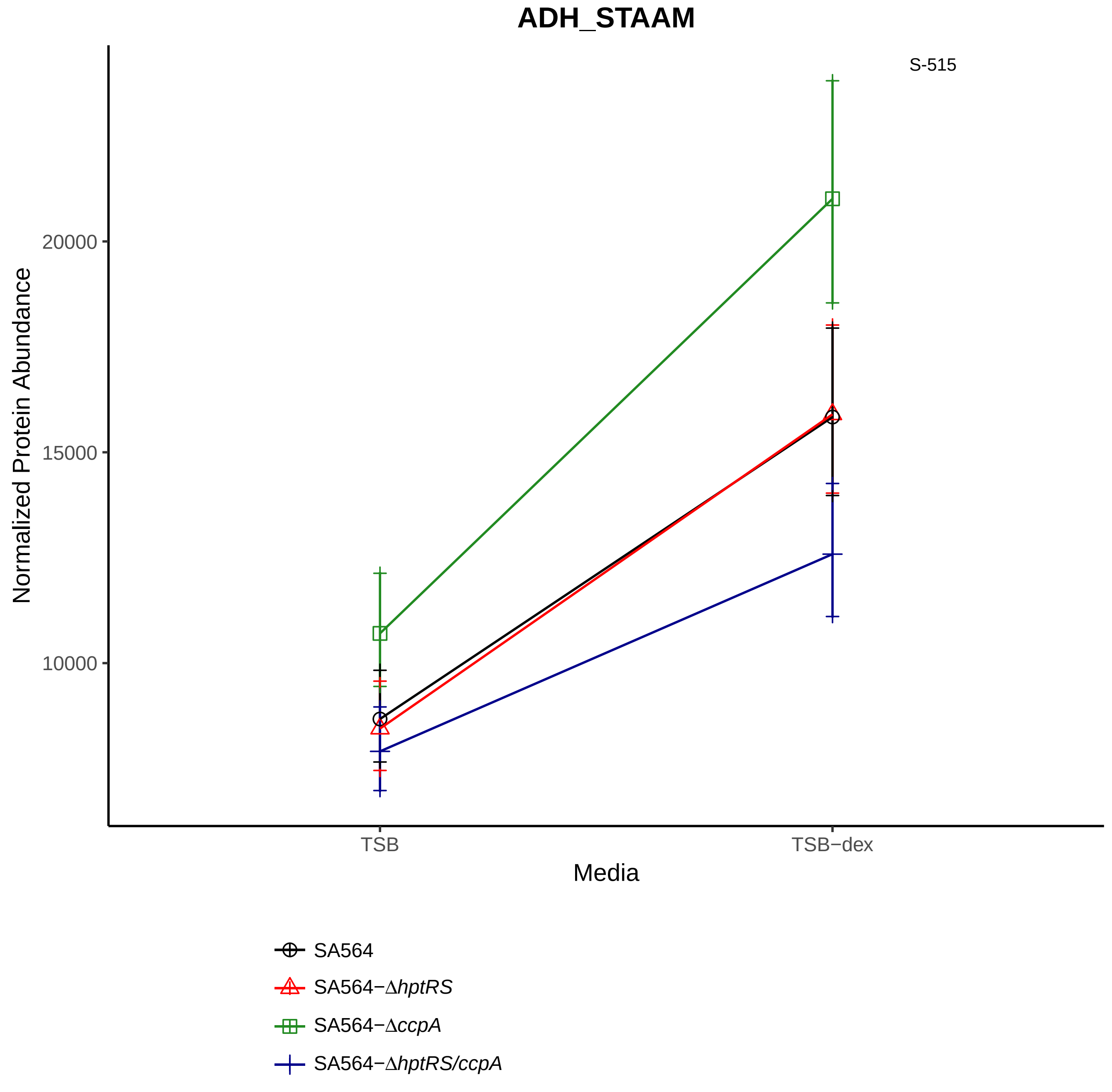

ADH_STAAM
S-515
Normalized Protein Abundance
20000
15000
10000
TSB
TSB−dex
Media
SA564
SA564−Δ*hptRS*
SA564−Δ*ccpA*
SA564−Δ*hptRS/ccpA*

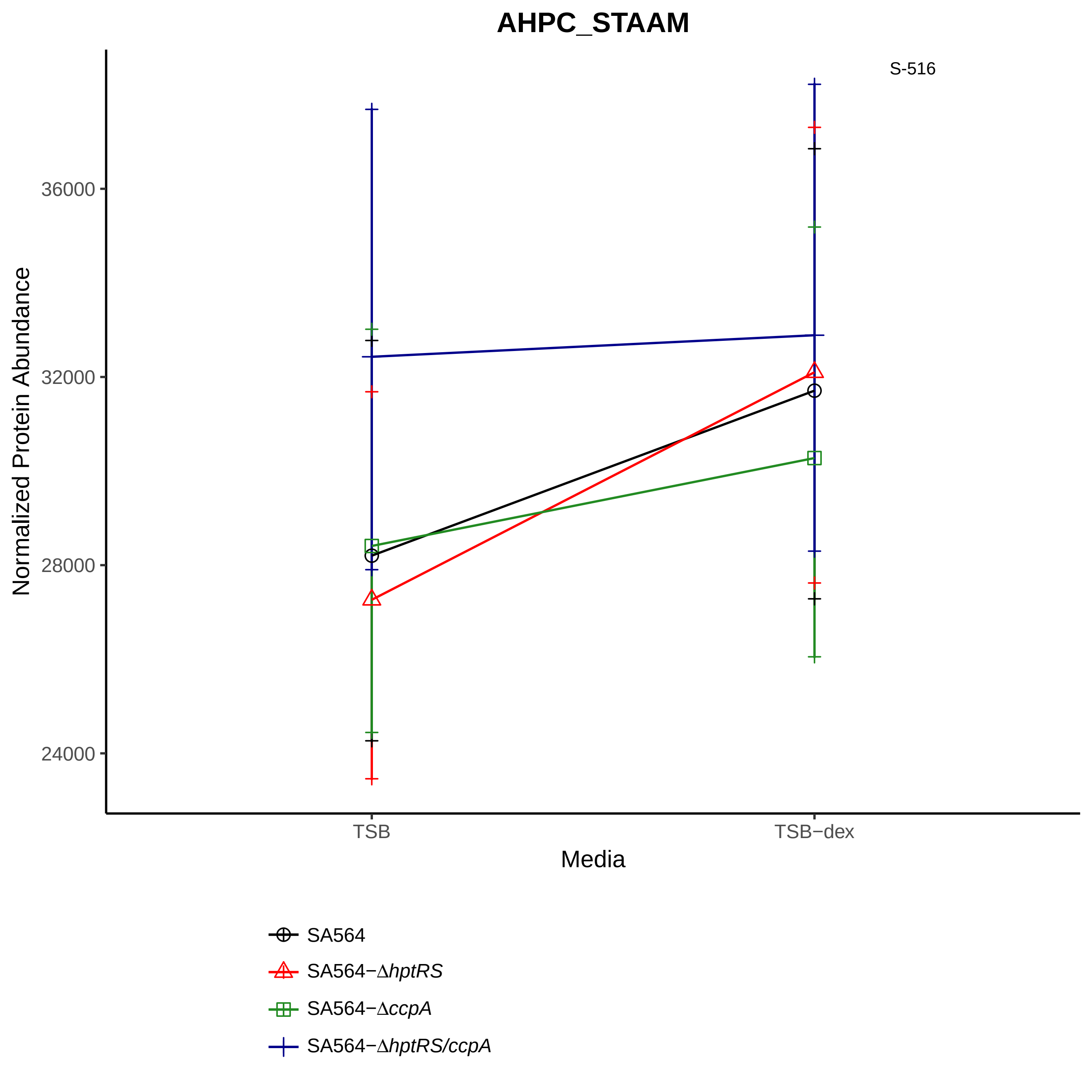

AHPC_STAAM
S-516
Normalized Protein Abundance
36000
32000
28000
24000
TSB
TSB−dex
Media
SA564
SA564−Δ*hptRS*
SA564−Δ*ccpA*
SA564−Δ*hptRS/ccpA*

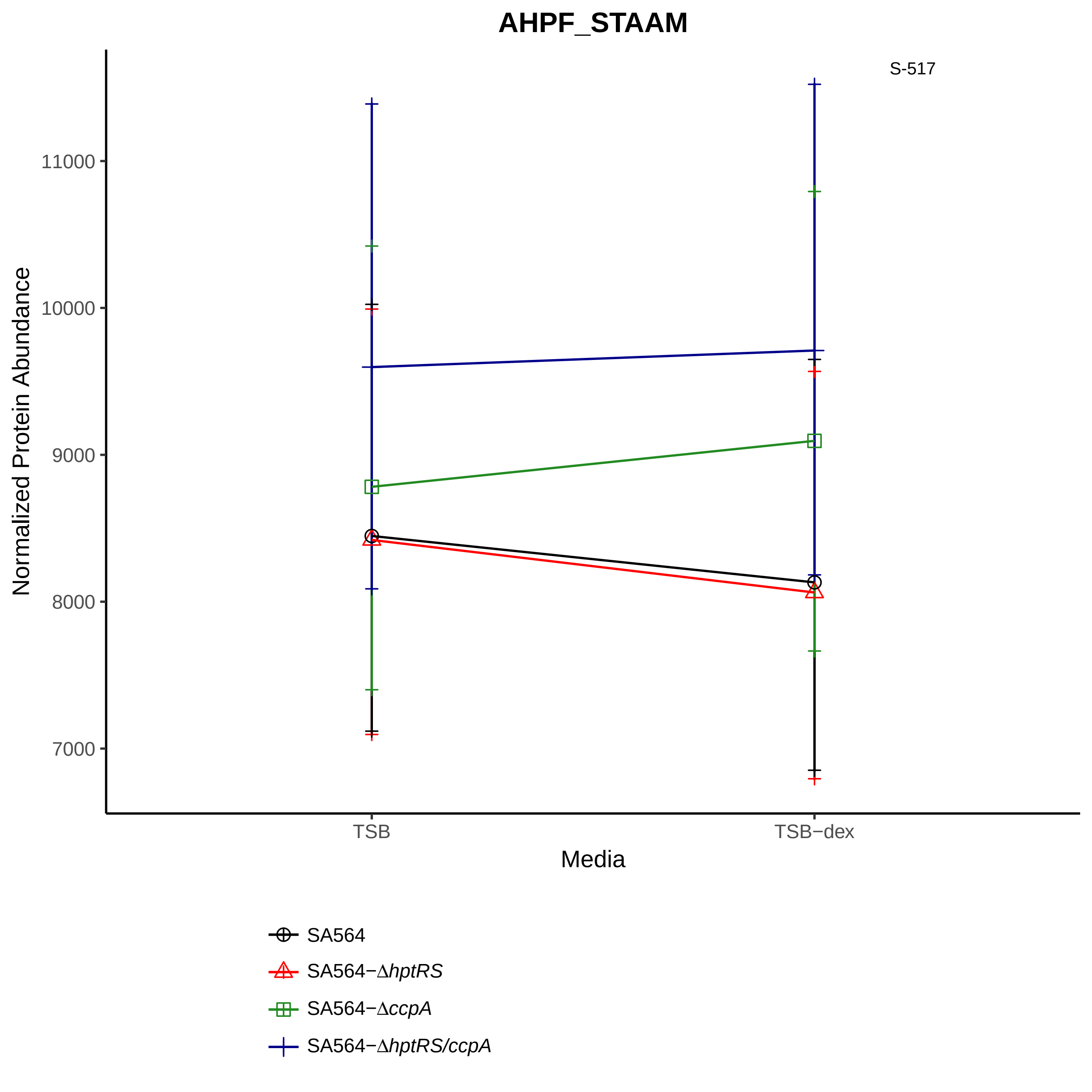
AHPF_STAAM
S-517
Normalized Protein Abundance
11000
10000
9000
8000
7000
TSB
TSB−dex
Media
SA564
SA564−ΔhptRS
SA564−ΔccpA
SA564−ΔhptRS/ccpA

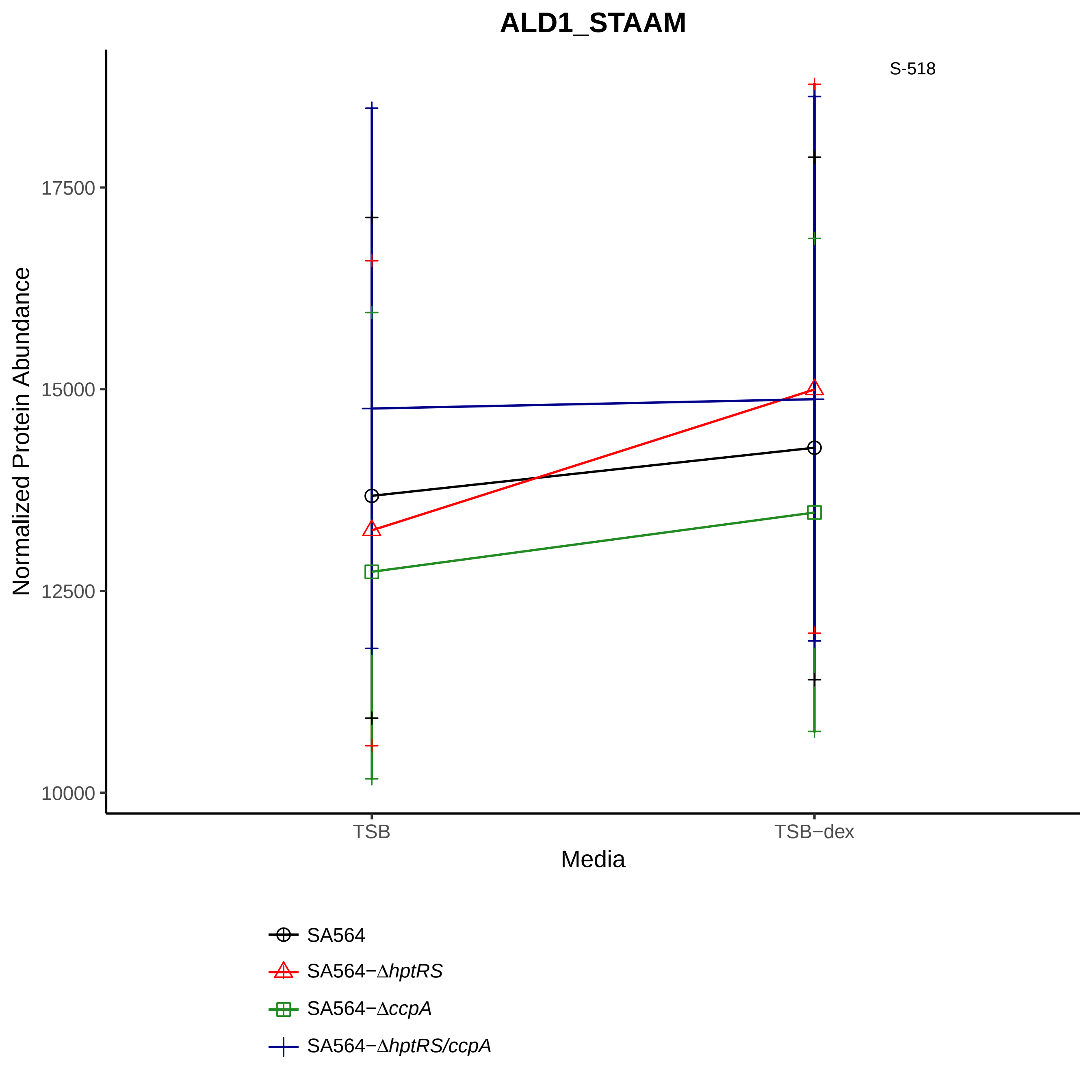
ALD1_STAAM
S-518
17500
15000
12500
10000
Normalized Protein Abundance
TSB
TSB−dex
Media
SA564
SA564−Δ*hptRS*
SA564−Δ*ccpA*
SA564−Δ*hptRS/ccpA*

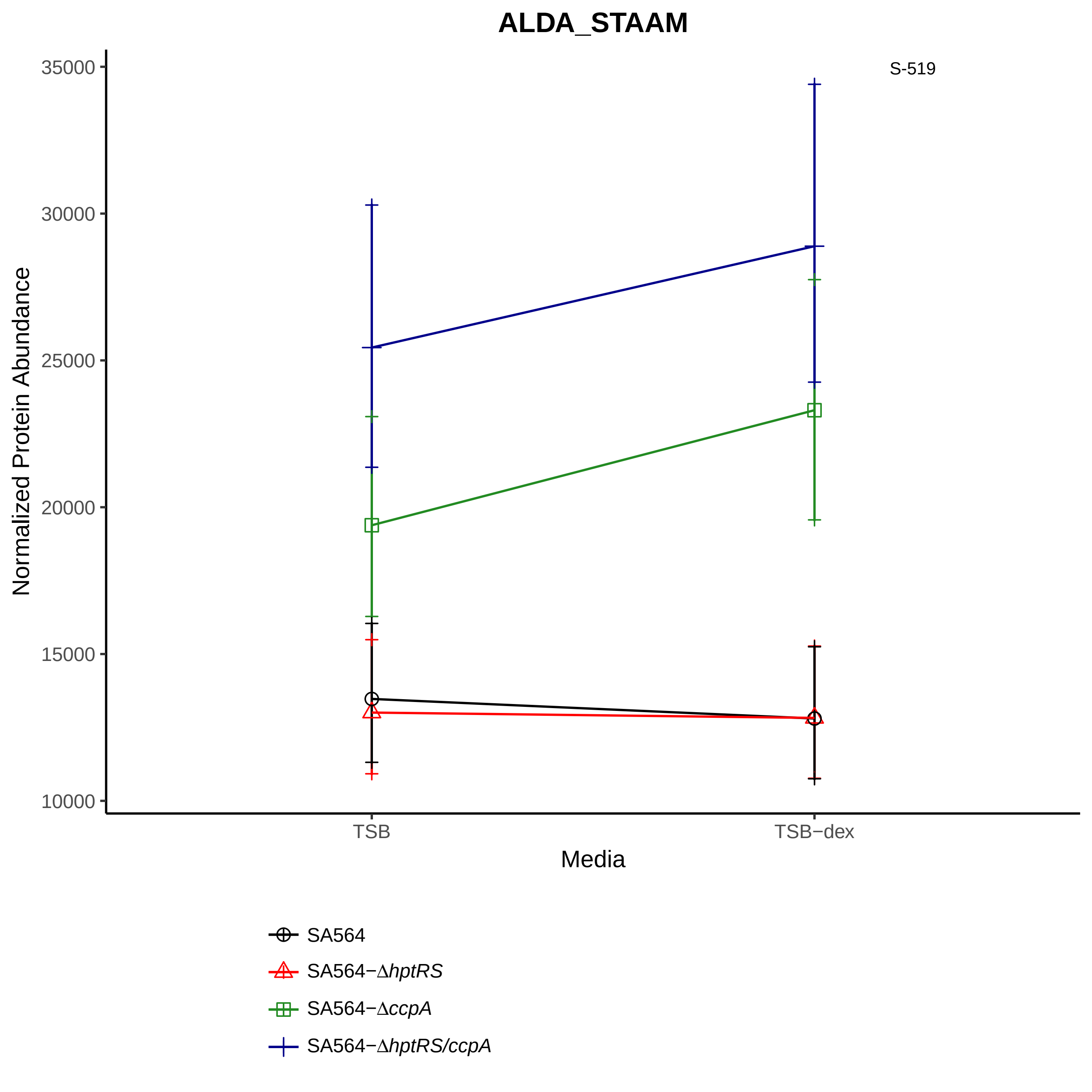

ALDA_STAAM
S-519
Normalized Protein Abundance
35000
30000
25000
20000
15000
10000
TSB
TSB−dex
Media
SA564
SA564−Δ*hptRS*
SA564−Δ*ccpA*
SA564−Δ*hptRS/ccpA*

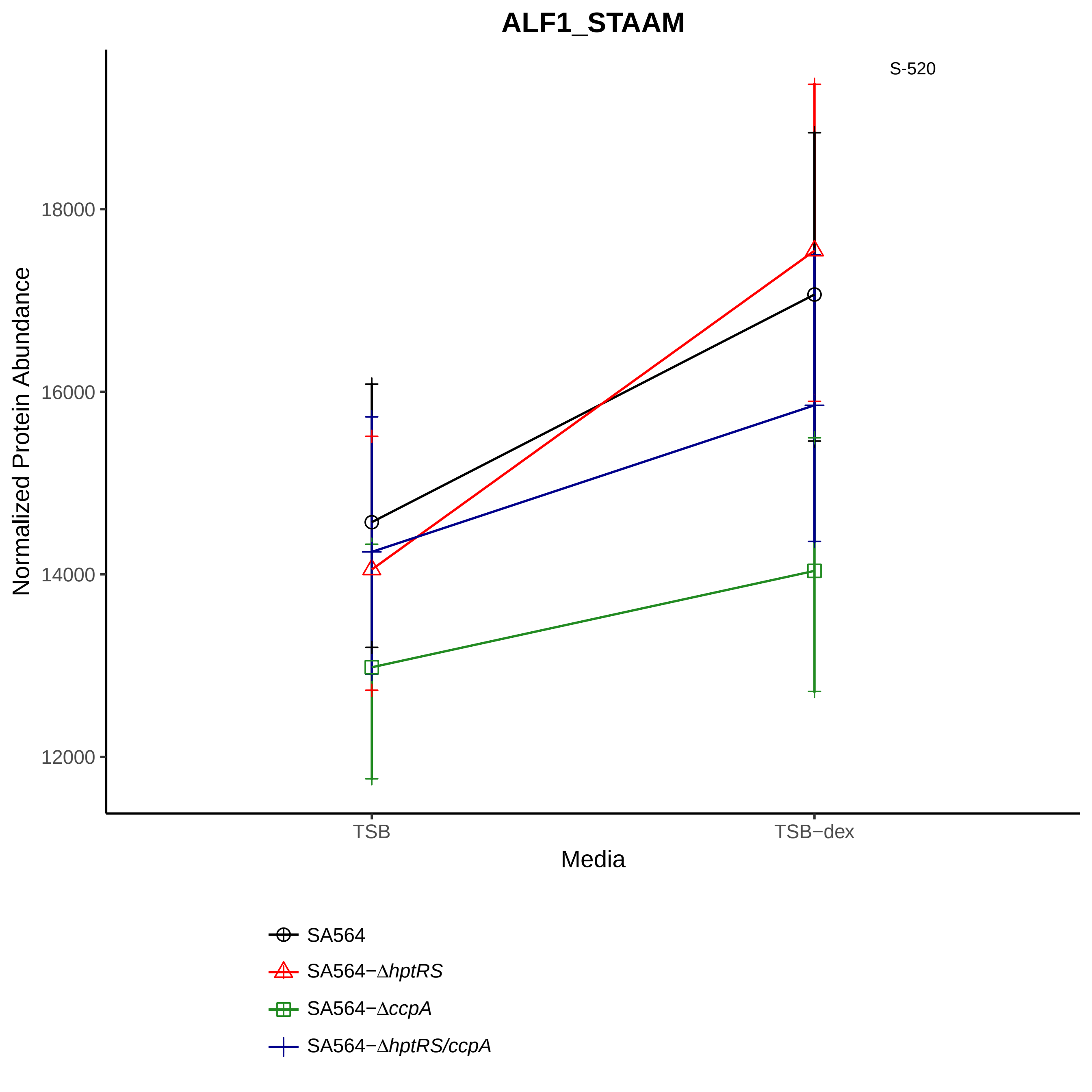

ALF1_STAAM
S-520
Normalized Protein Abundance
18000
16000
14000
12000
TSB
TSB−dex
Media
SA564
SA564−Δ*hptRS*
SA564−Δ*ccpA*
SA564−Δ*hptRS/ccpA*

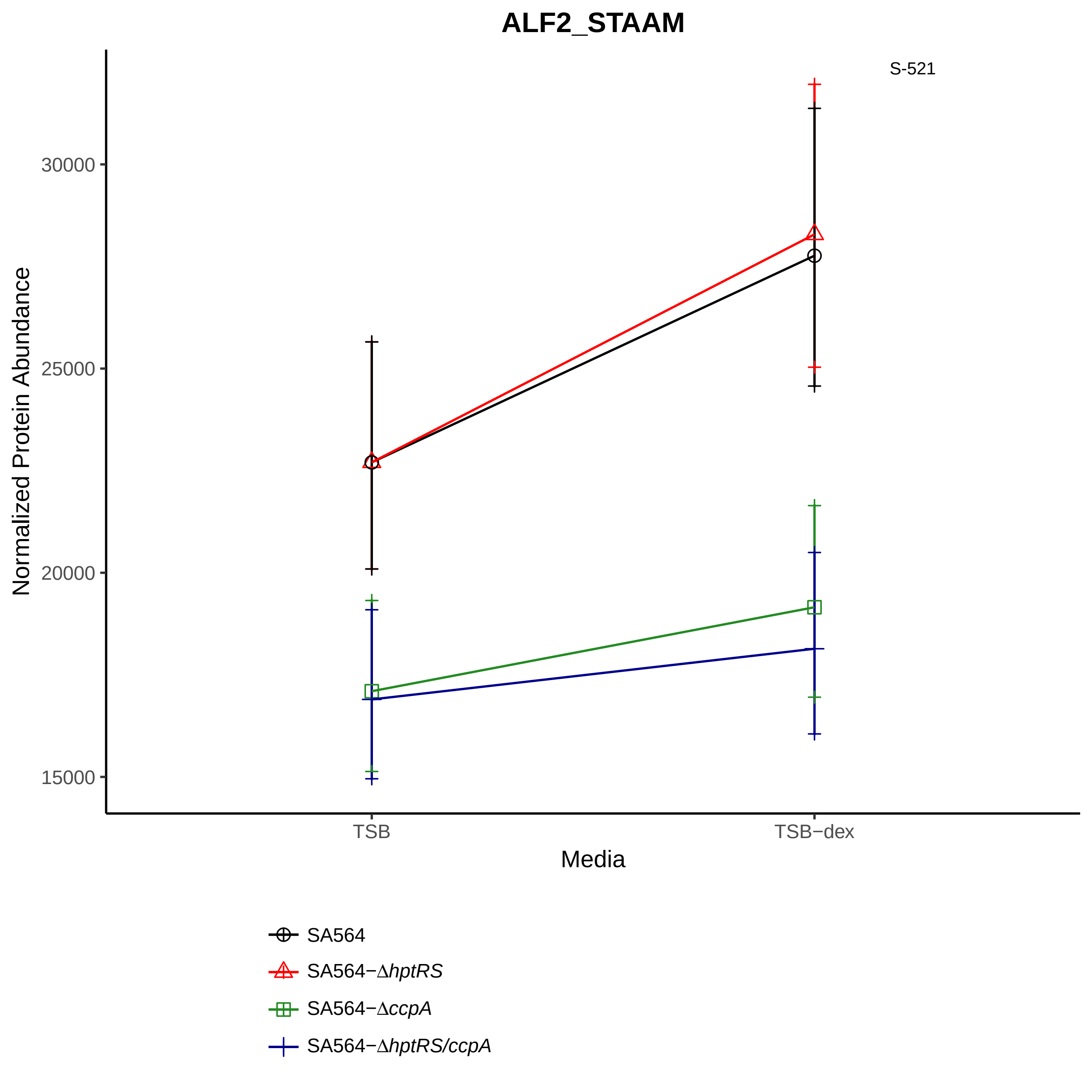

ALF2_STAAM
S-521
Normalized Protein Abundance
30000
25000
20000
15000
TSB
TSB−dex
Media
SA564
SA564−Δ*hptRS*
SA564−Δ*ccpA*
SA564−Δ*hptRS/ccpA*

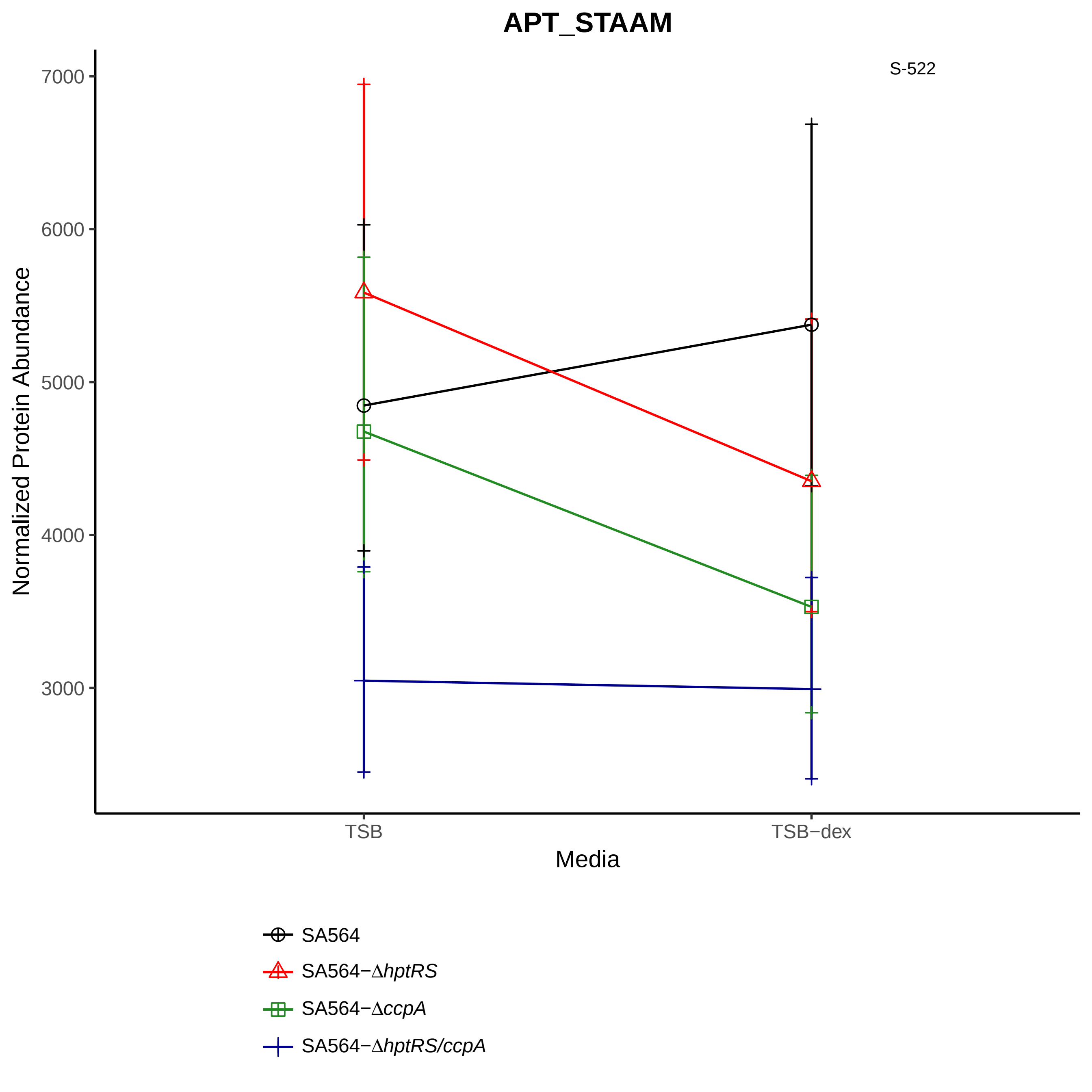

APT_STAAM
S-522
Normalized Protein Abundance
7000
6000
5000
4000
3000
TSB
TSB−dex
Media
SA564
SA564−ΔhptRS
SA564−ΔccpA
SA564−ΔhptRS/ccpA

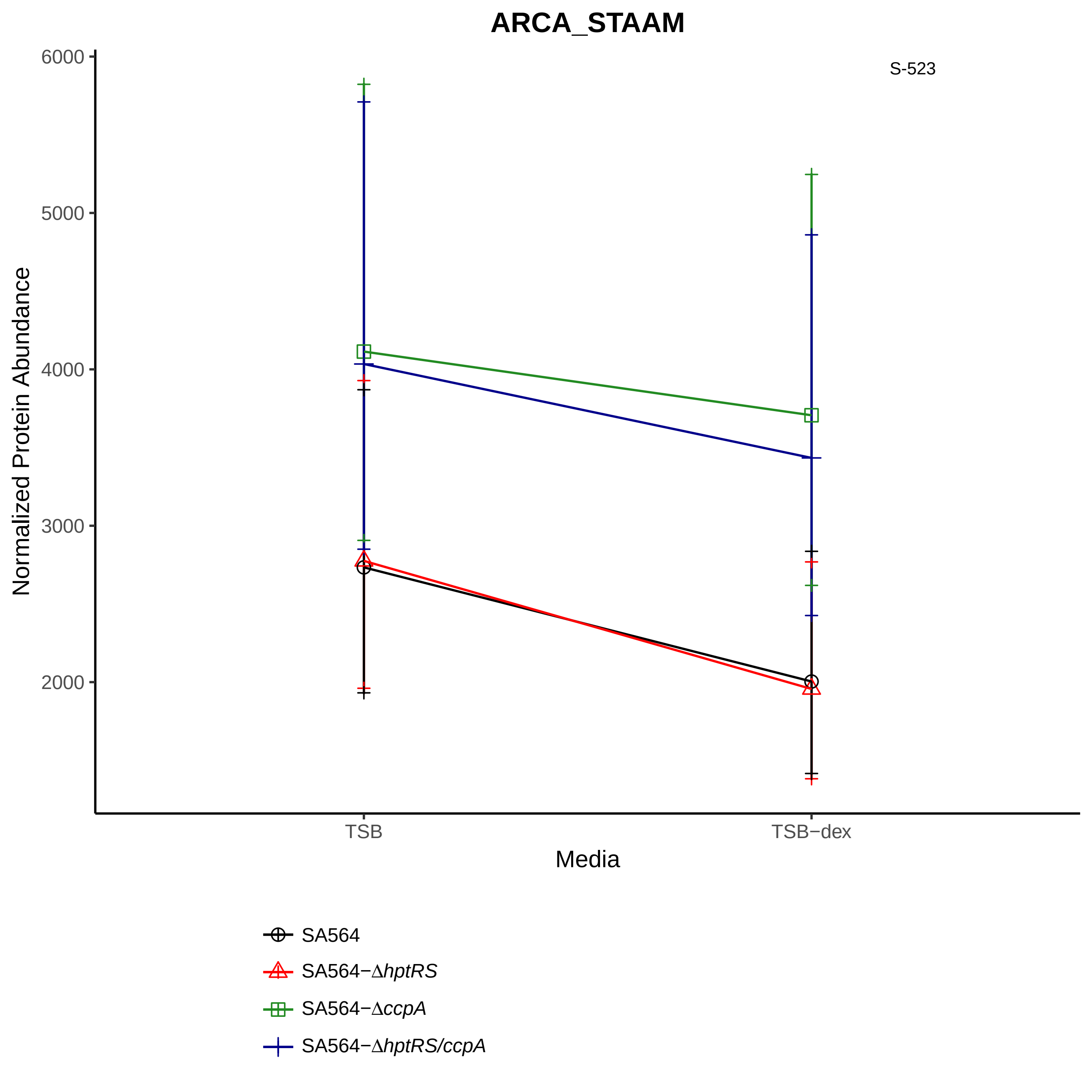
ARCA_STAAM
S-523
Normalized Protein Abundance
6000
5000
4000
3000
2000
TSB
TSB−dex
Media
SA564
SA564−ΔhptRS
SA564−ΔccpA
SA564−ΔhptRS/ccpA

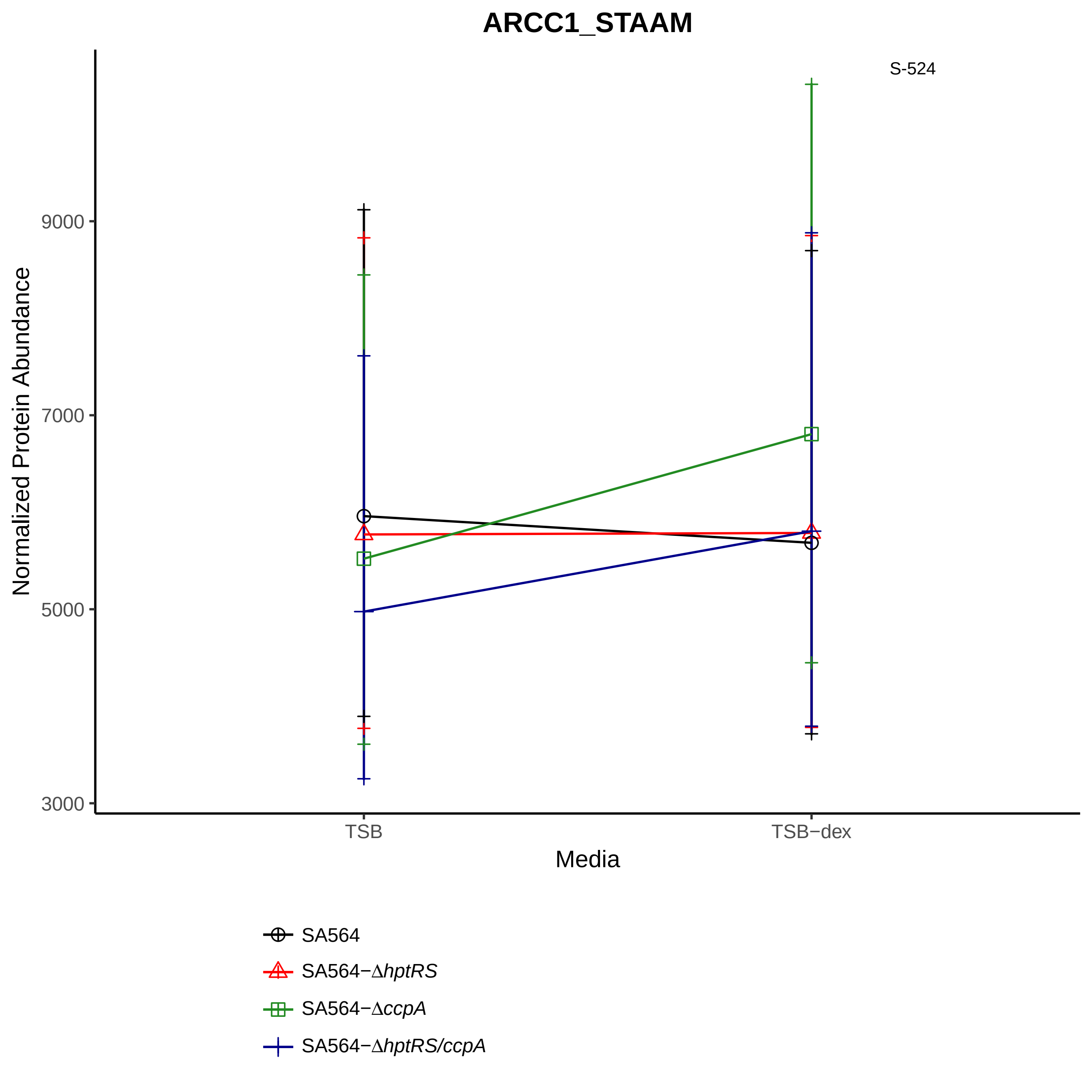

ARCC1_STAAM
S-524
Normalized Protein Abundance
9000
7000
5000
3000
TSB
TSB−dex
Media
SA564
SA564−Δ*hptRS*
SA564−Δ*ccpA*
SA564−Δ*hptRS/ccpA*

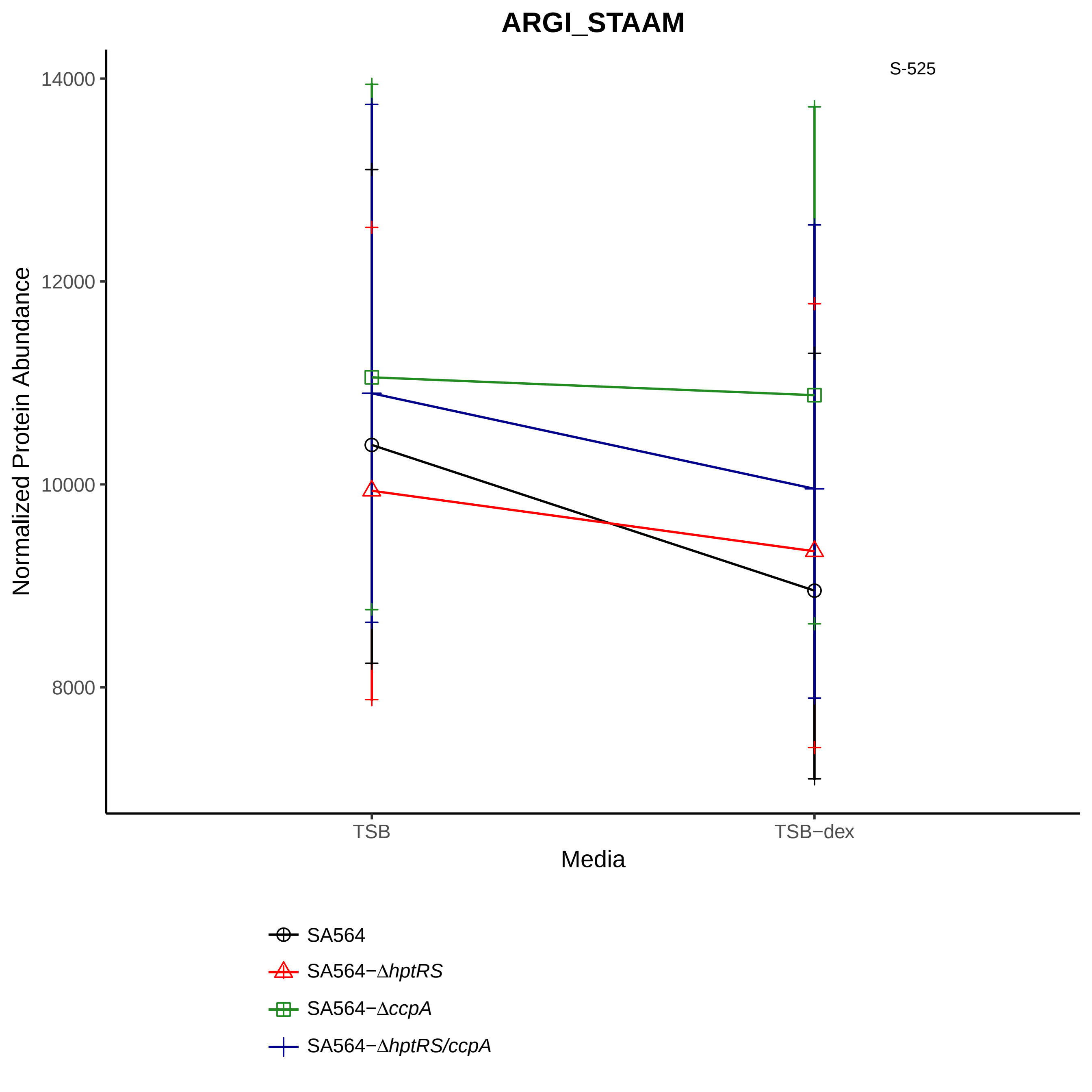

ARGI_STAAM
S-525
Normalized Protein Abundance
14000
12000
10000
8000
TSB
TSB−dex
Media
SA564
SA564−Δ*hptRS*
SA564−Δ*ccpA*
SA564−Δ*hptRS/ccpA*

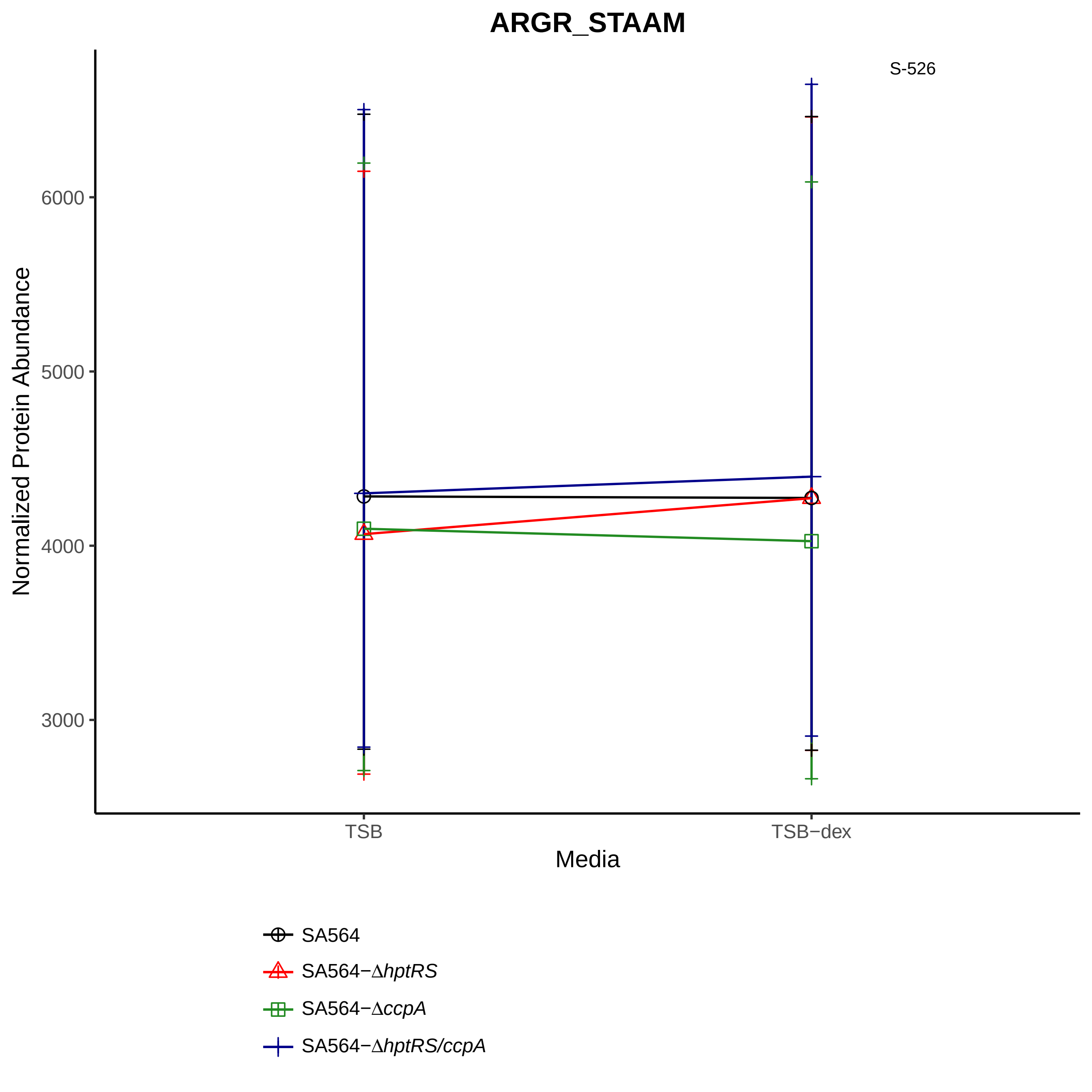
ARGR_STAAM
S-526
Normalized Protein Abundance
6000
5000
4000
3000
TSB
TSB−dex
Media
SA564
SA564−ΔhptRS
SA564−ΔccpA
SA564−ΔhptRS/ccpA

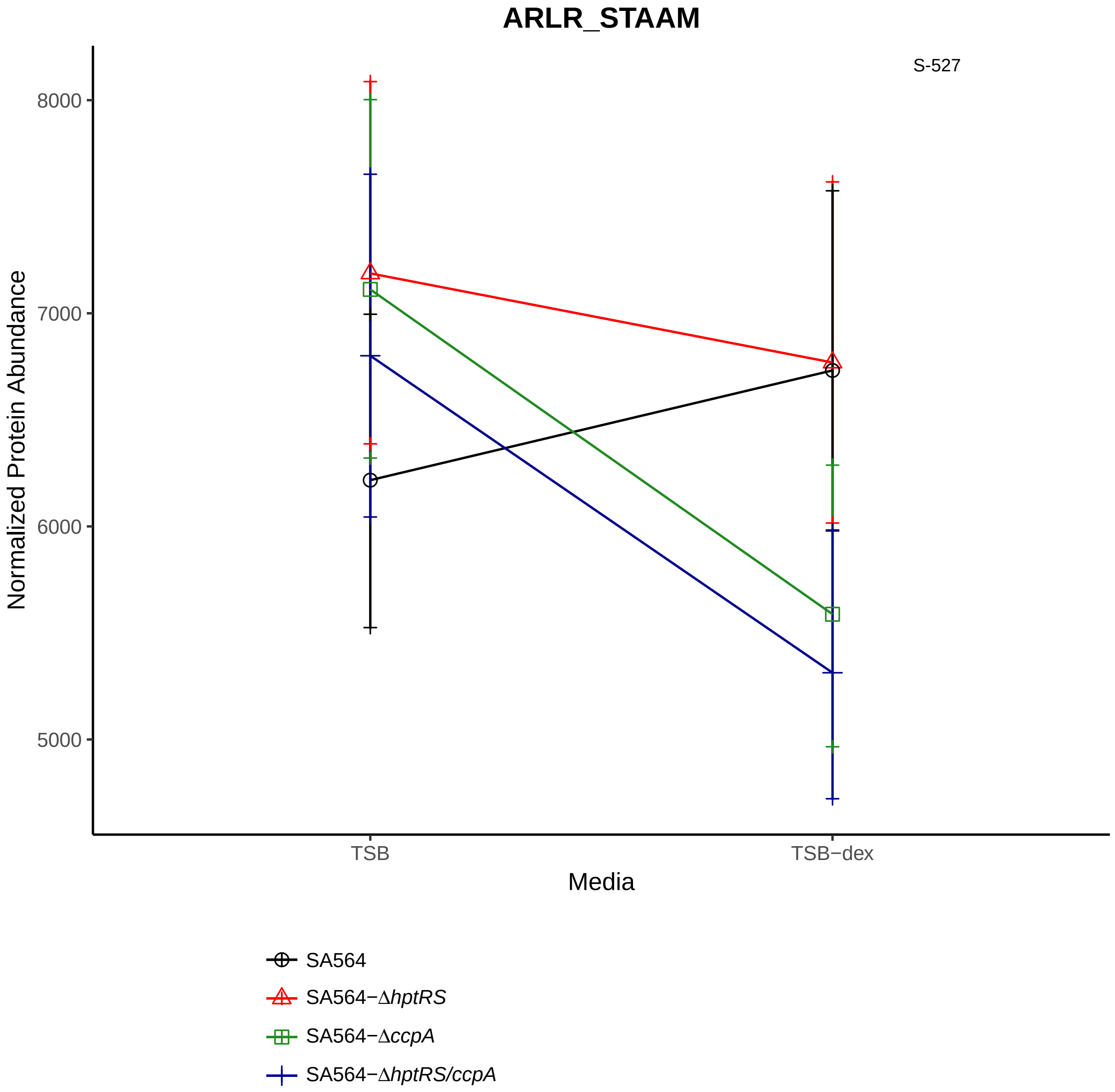

ARLR_STAAM
S-527
Normalized Protein Abundance
8000
7000
6000
5000
TSB
TSB−dex
Media
SA564
SA564−Δ*hptRS*
SA564−Δ*ccpA*
SA564−Δ*hptRS/ccpA*

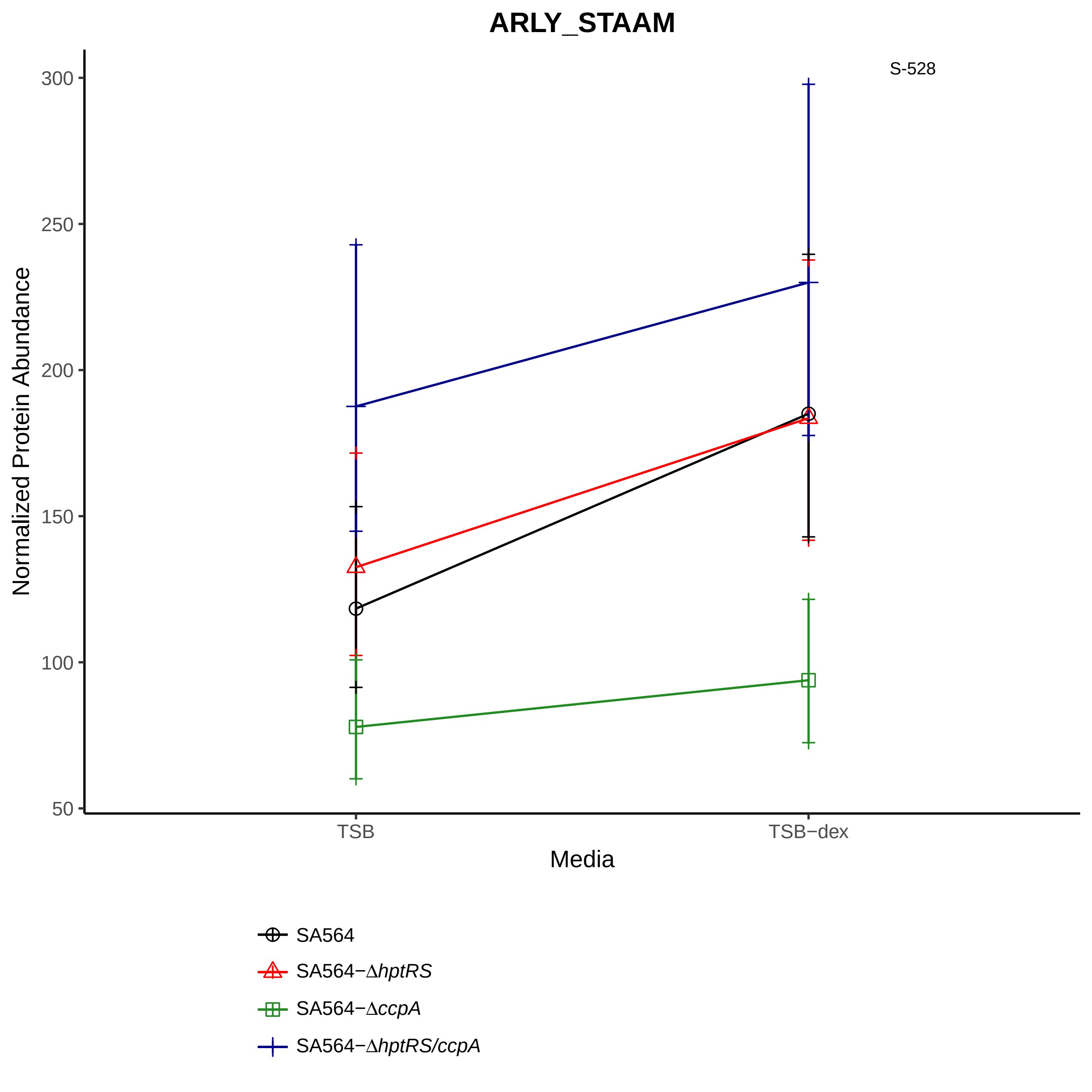
ARLY_STAAM
S-528
Normalized Protein Abundance
300
250
200
150
100
50
TSB
TSB−dex
Media
SA564
SA564−Δ*hptRS*
SA564−Δ*ccpA*
SA564−Δ*hptRS/ccpA*

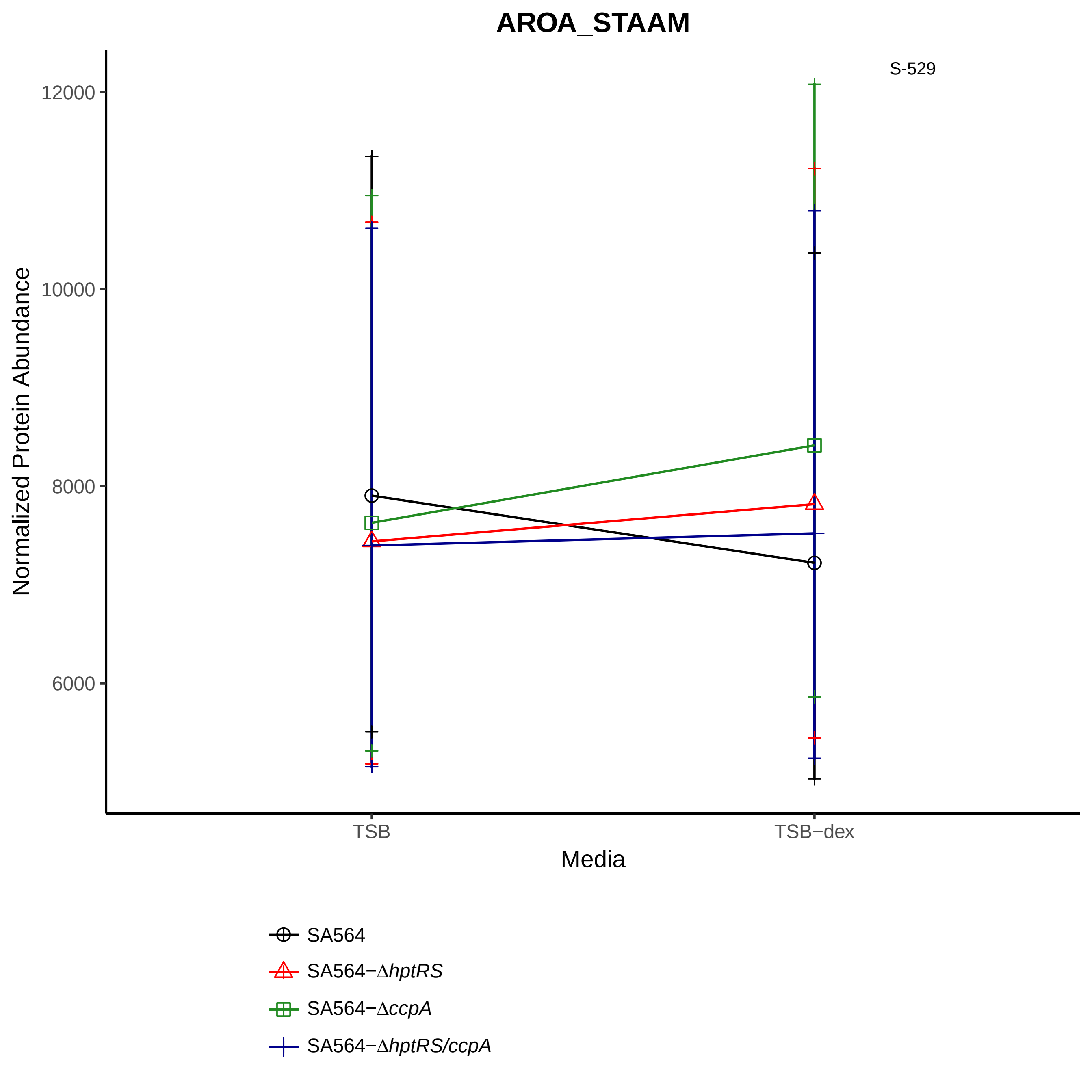

AROA_STAAM
S-529
12000
10000
8000
6000
Normalized Protein Abundance
TSB
TSB−dex
Media
SA564
SA564−Δ*hptRS*
SA564−Δ*ccpA*
SA564−Δ*hptRS/ccpA*

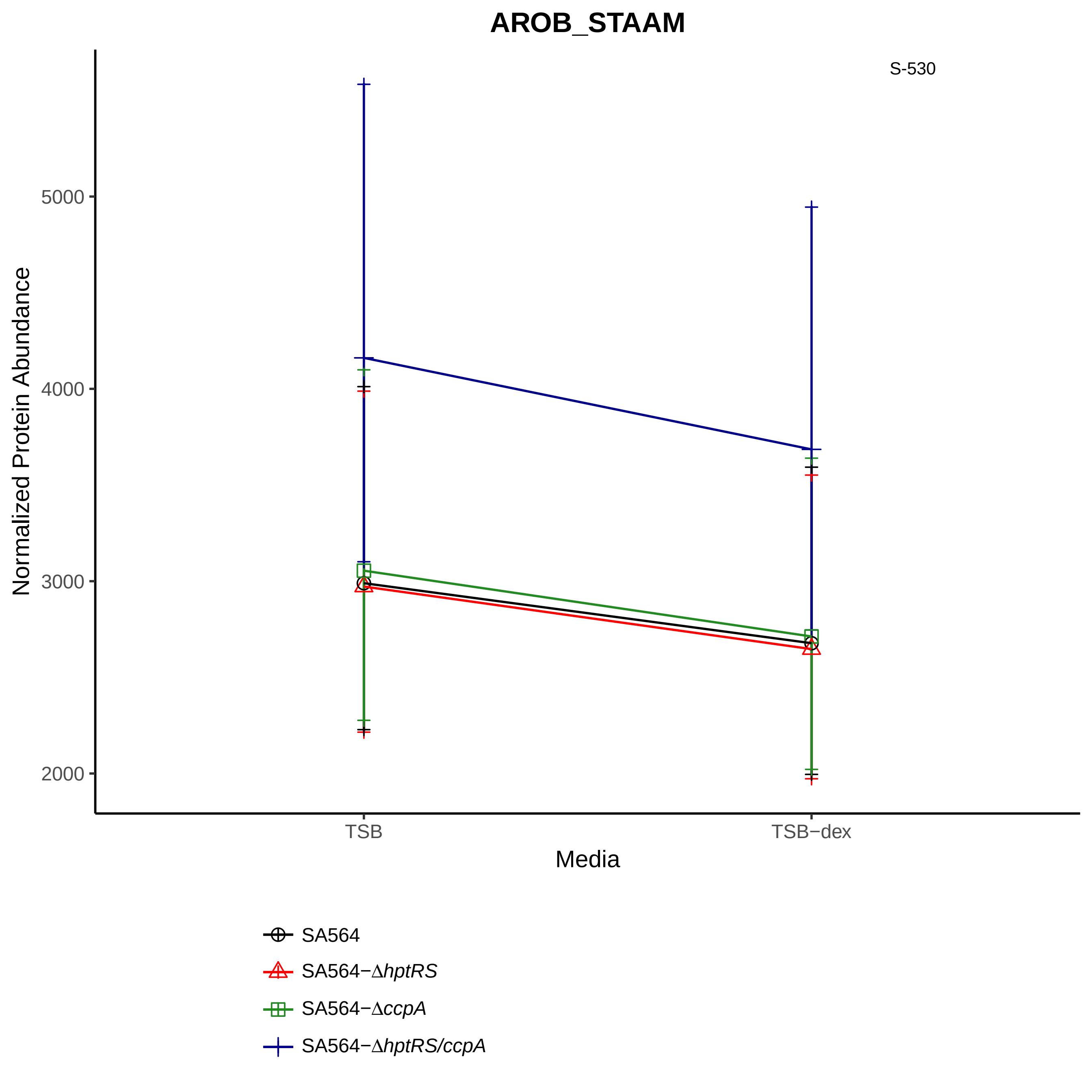

AROB_STAAM
S-530
Normalized Protein Abundance
5000
4000
3000
2000
TSB
TSB−dex
Media
SA564
SA564−Δ*hptRS*
SA564−Δ*ccpA*
SA564−Δ*hptRS/ccpA*

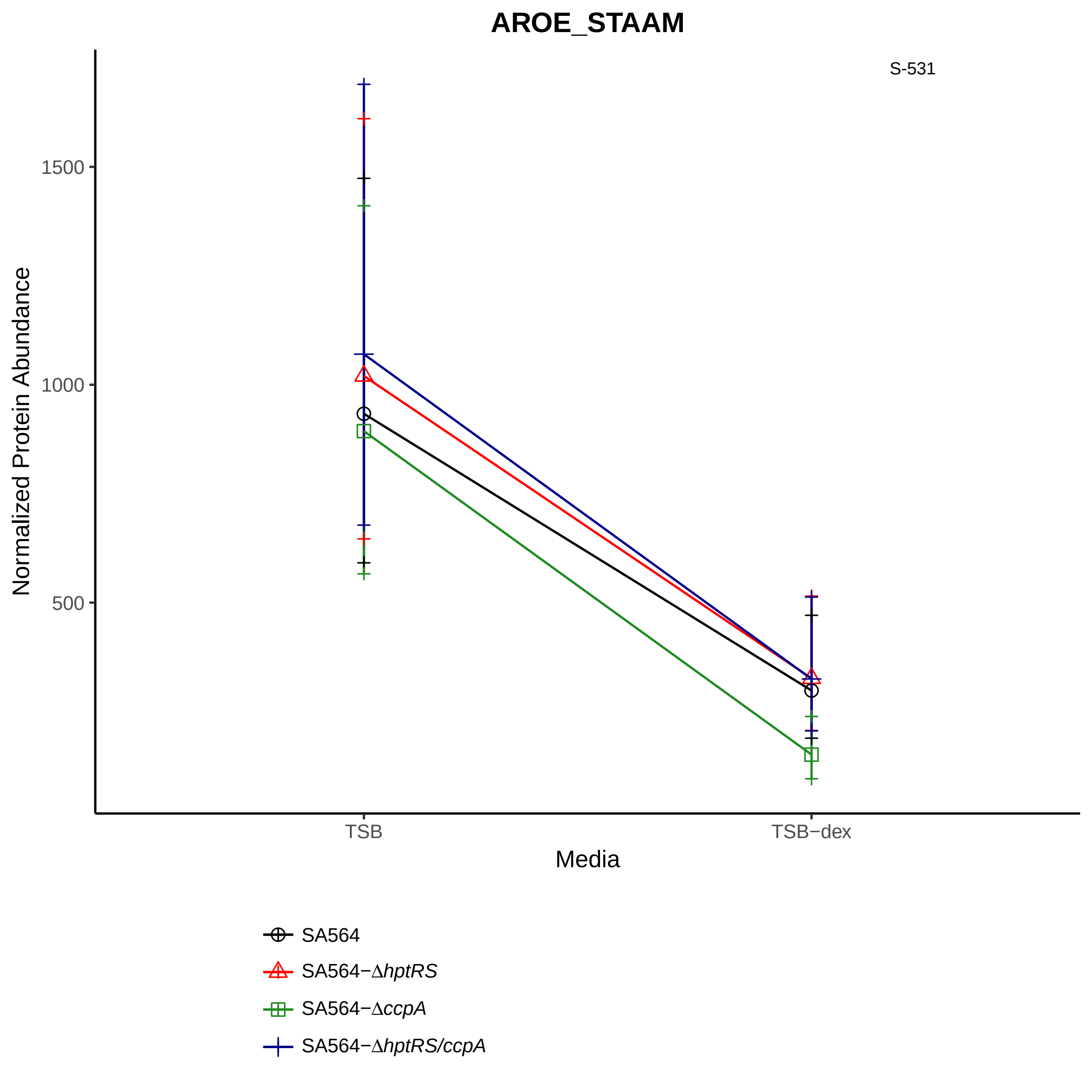

AROE_STAAM
S-531
Normalized Protein Abundance
1500
1000
500
TSB
TSB−dex
Media
SA564
SA564−Δ*hptRS*
SA564−Δ*ccpA*
SA564−Δ*hptRS/ccpA*

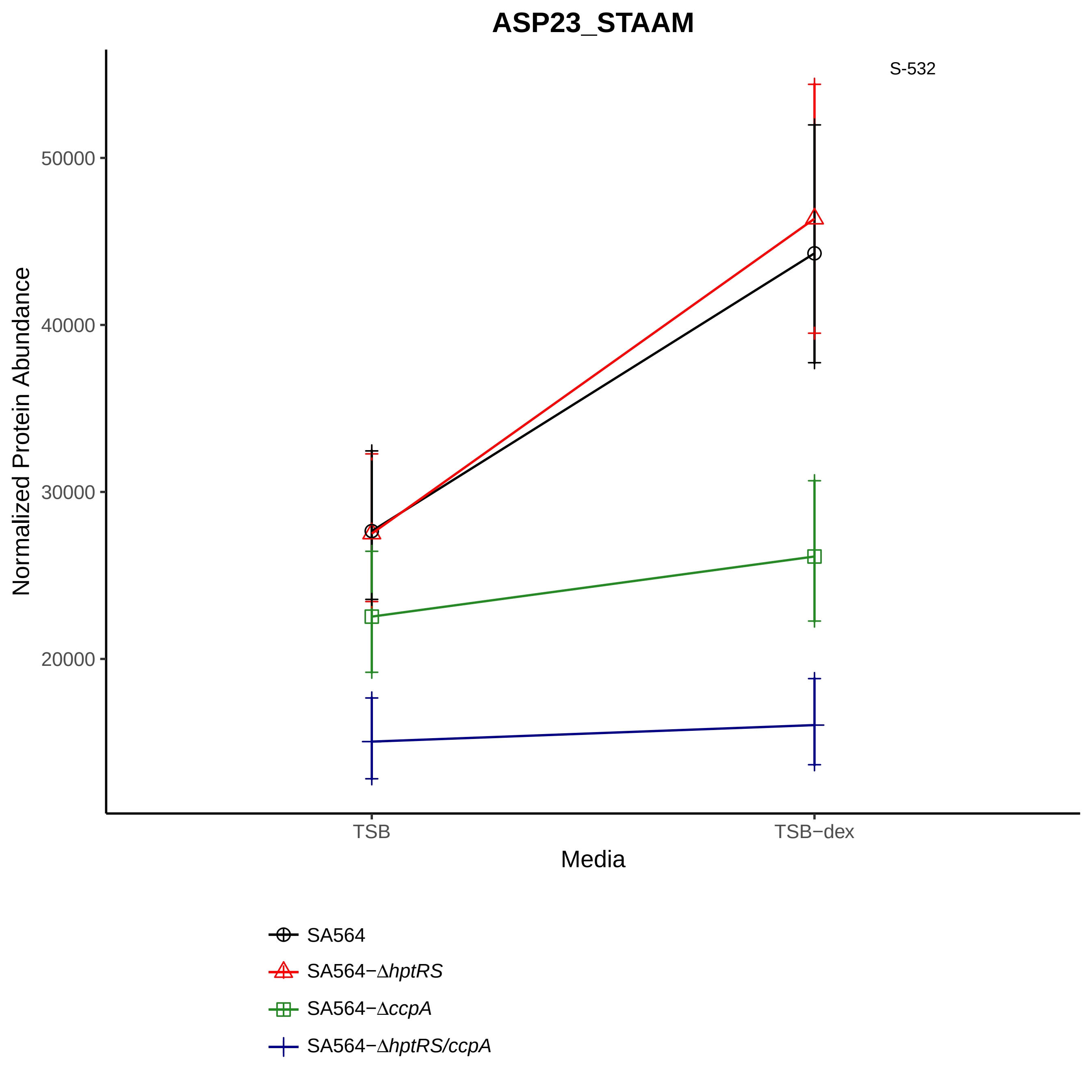

ASP23_STAAM
S-532
Normalized Protein Abundance
50000
40000
30000
20000
TSB
TSB−dex
Media
SA564
SA564−Δ*hptRS*
SA564−Δ*ccpA*
SA564−Δ*hptRS/ccpA*

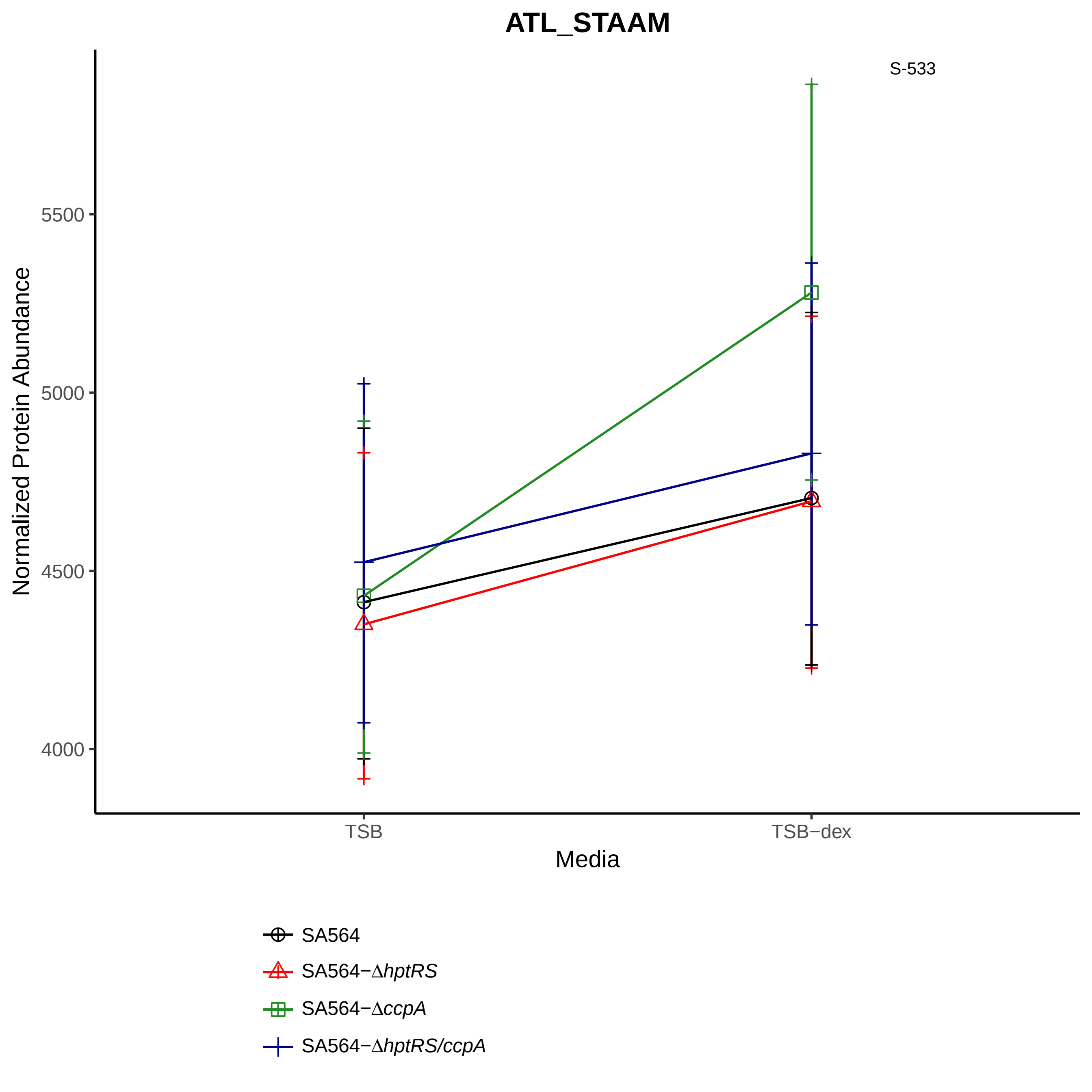
ATL_STAAM
S-533
Normalized Protein Abundance
5500
5000
4500
4000
TSB
TSB−dex
Media
SA564
SA564−ΔhptRS
SA564−ΔccpA
SA564−ΔhptRS/ccpA

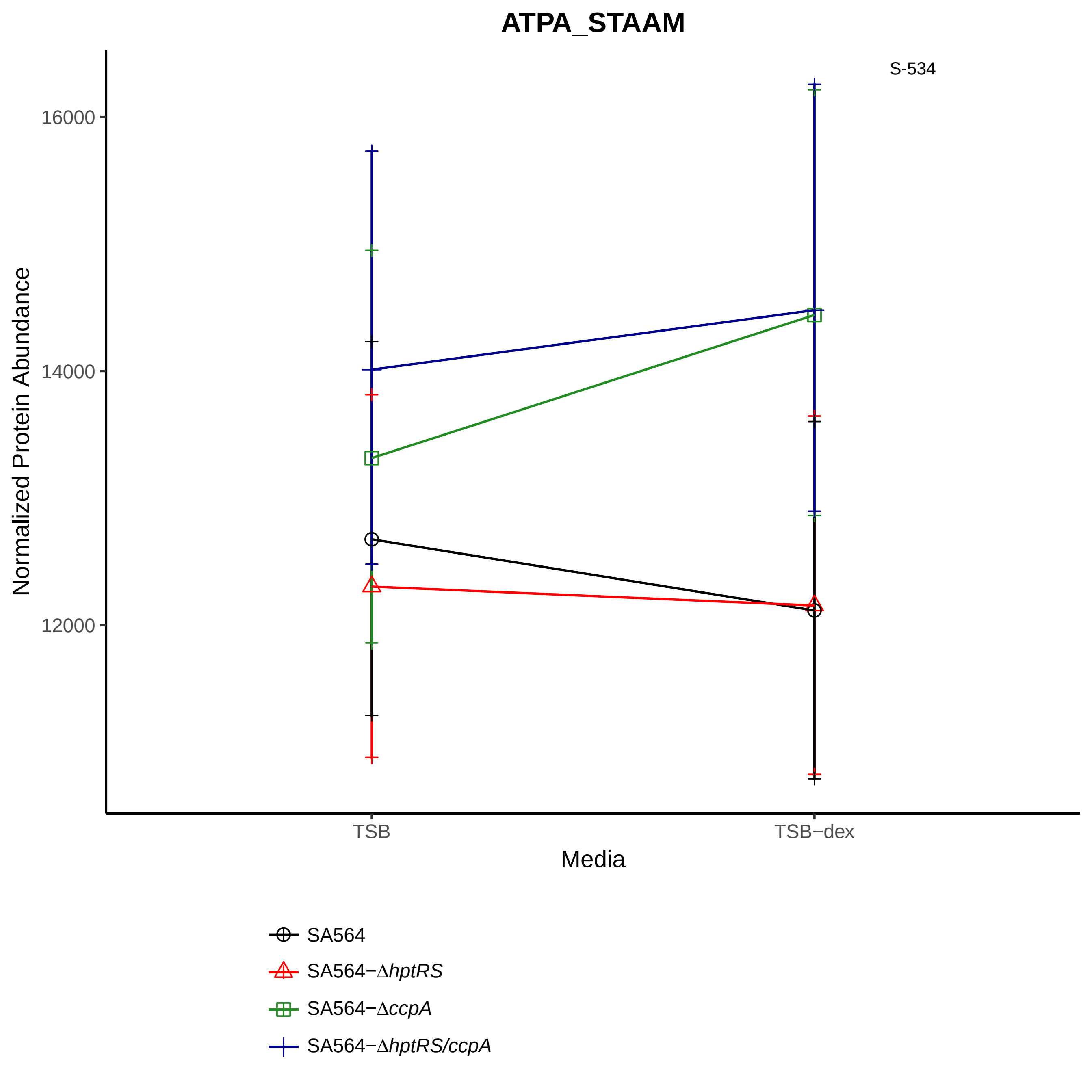
ATPA_STAAM
S-534
16000
14000
12000
Normalized Protein Abundance
TSB
TSB−dex
Media
SA564
SA564−Δ*hptRS*
SA564−Δ*ccpA*
SA564−Δ*hptRS/ccpA*

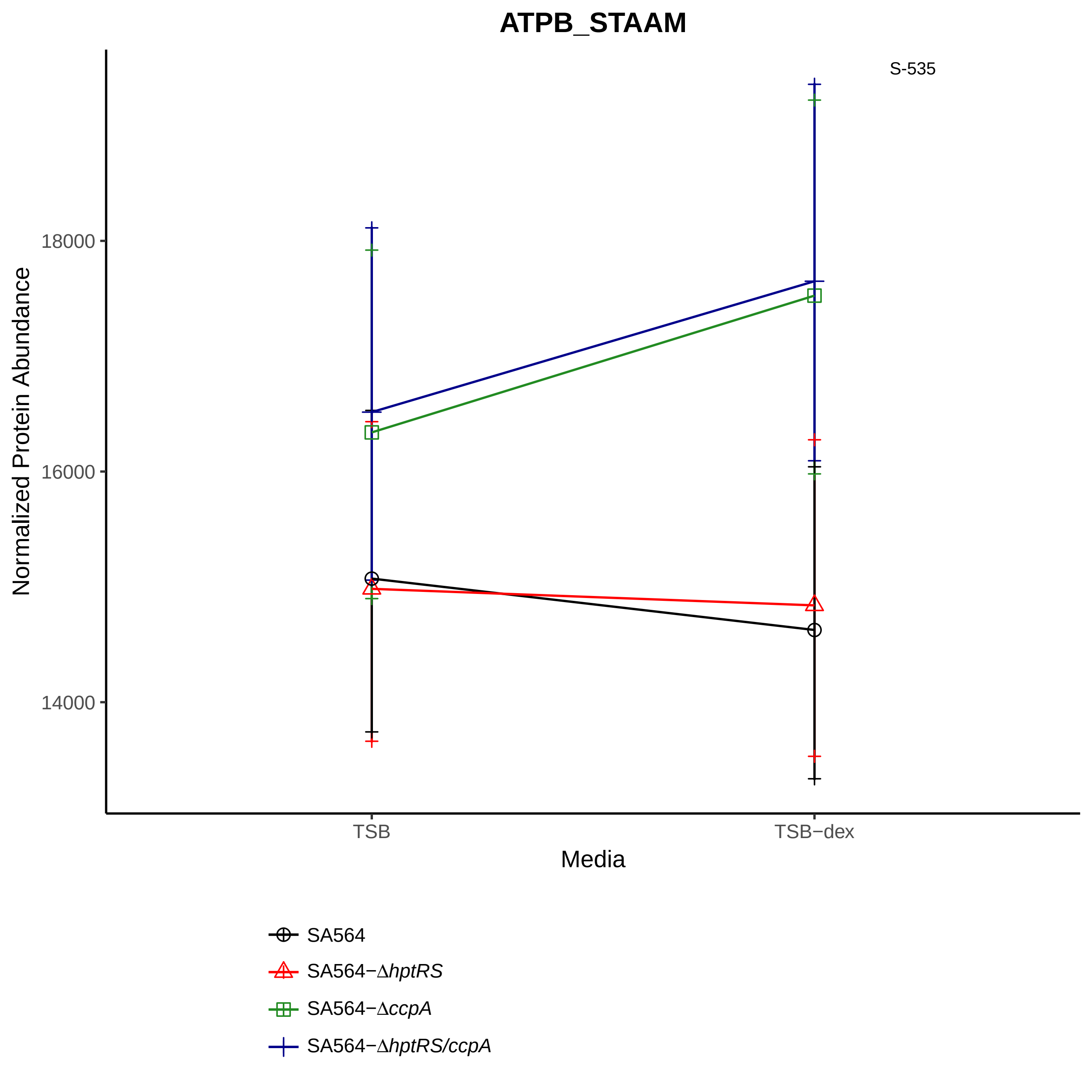

ATPB_STAAM
S-535
Normalized Protein Abundance
18000
16000
14000
TSB
TSB−dex
Media
SA564
SA564−Δ*hptRS*
SA564−Δ*ccpA*
SA564−Δ*hptRS/ccpA*

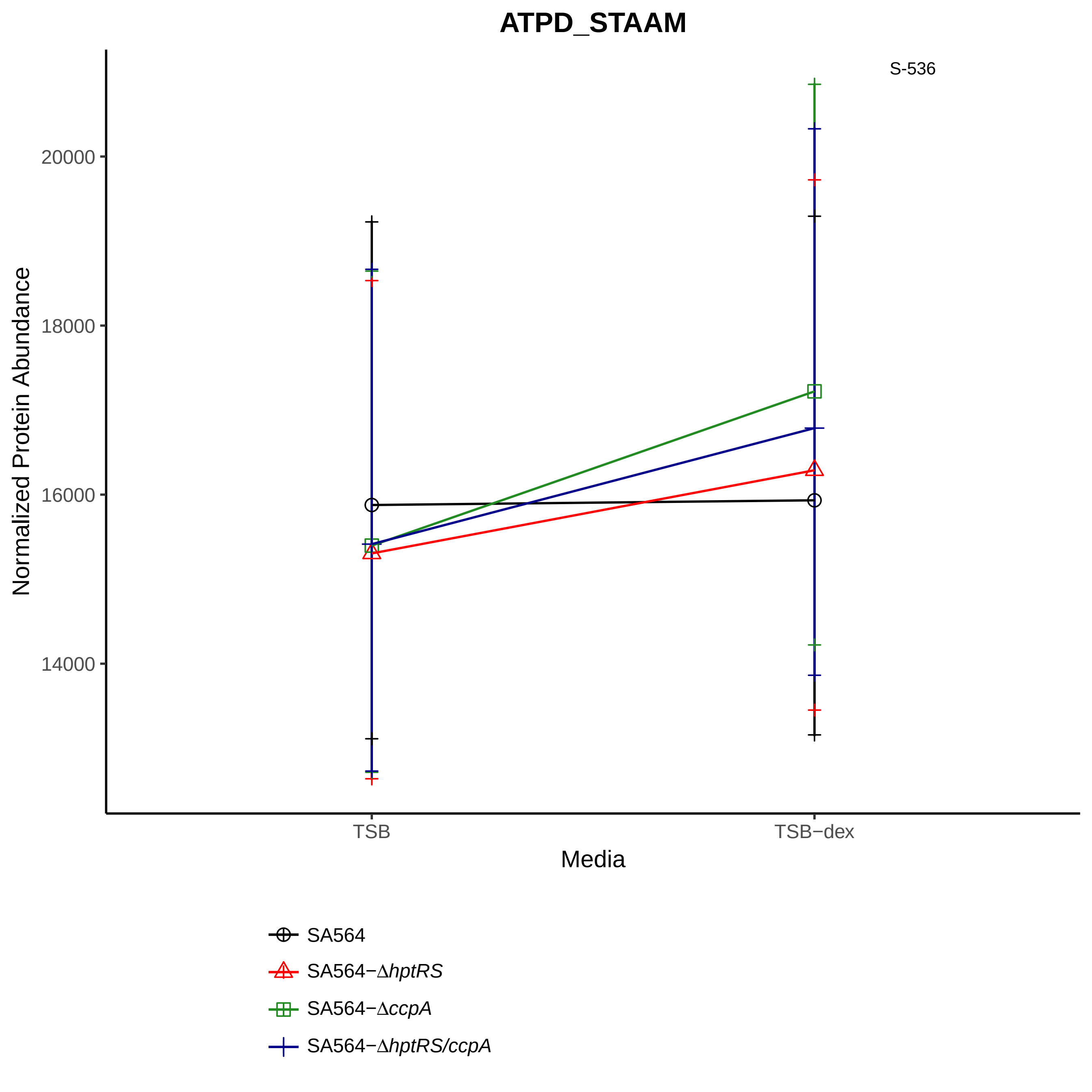

ATPD_STAAM
S-536
Normalized Protein Abundance
20000
18000
16000
14000
TSB
TSB−dex
Media
SA564
SA564−Δ*hptRS*
SA564−Δ*ccpA*
SA564−Δ*hptRS/ccpA*

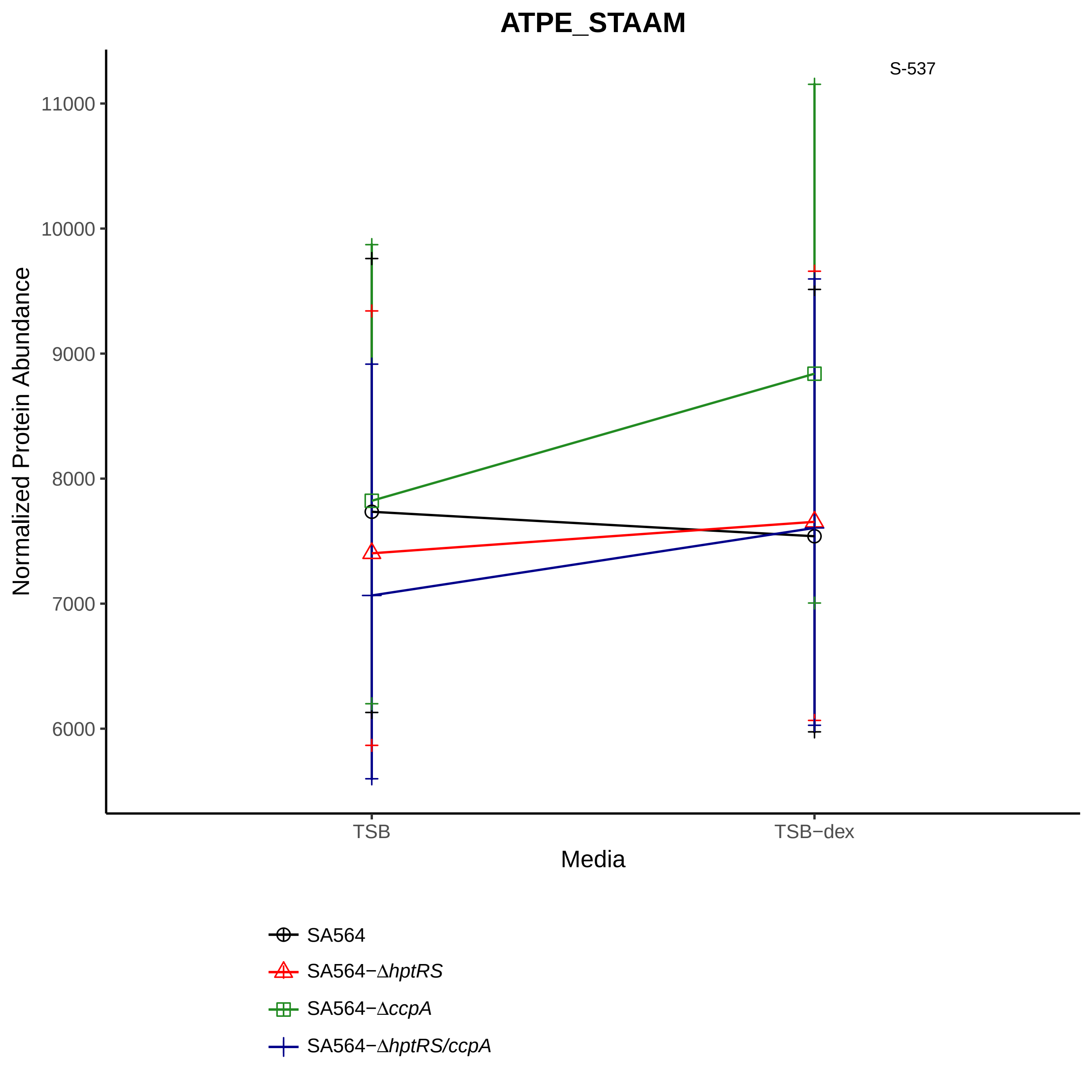
ATPE_STAAM
S-537
Normalized Protein Abundance
11000
10000
9000
8000
7000
6000
TSB
TSB−dex
Media
SA564
SA564−ΔhptRS
SA564−ΔccpA
SA564−ΔhptRS/ccpA

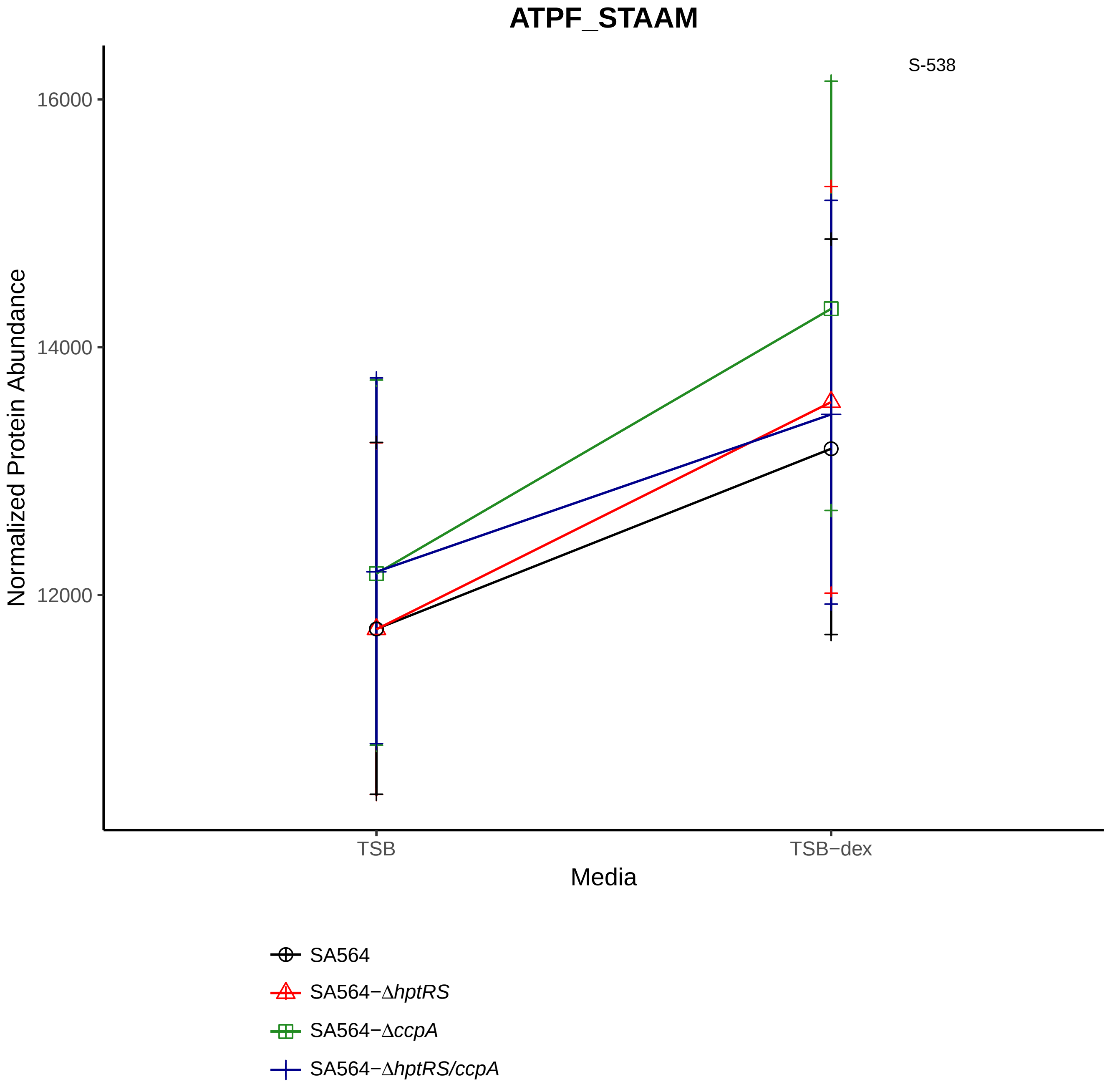

ATPF_STAAM
S-538
16000
14000
12000
Normalized Protein Abundance
TSB
TSB−dex
Media
SA564
SA564−Δ*hptRS*
SA564−Δ*ccpA*
SA564−Δ*hptRS/ccpA*

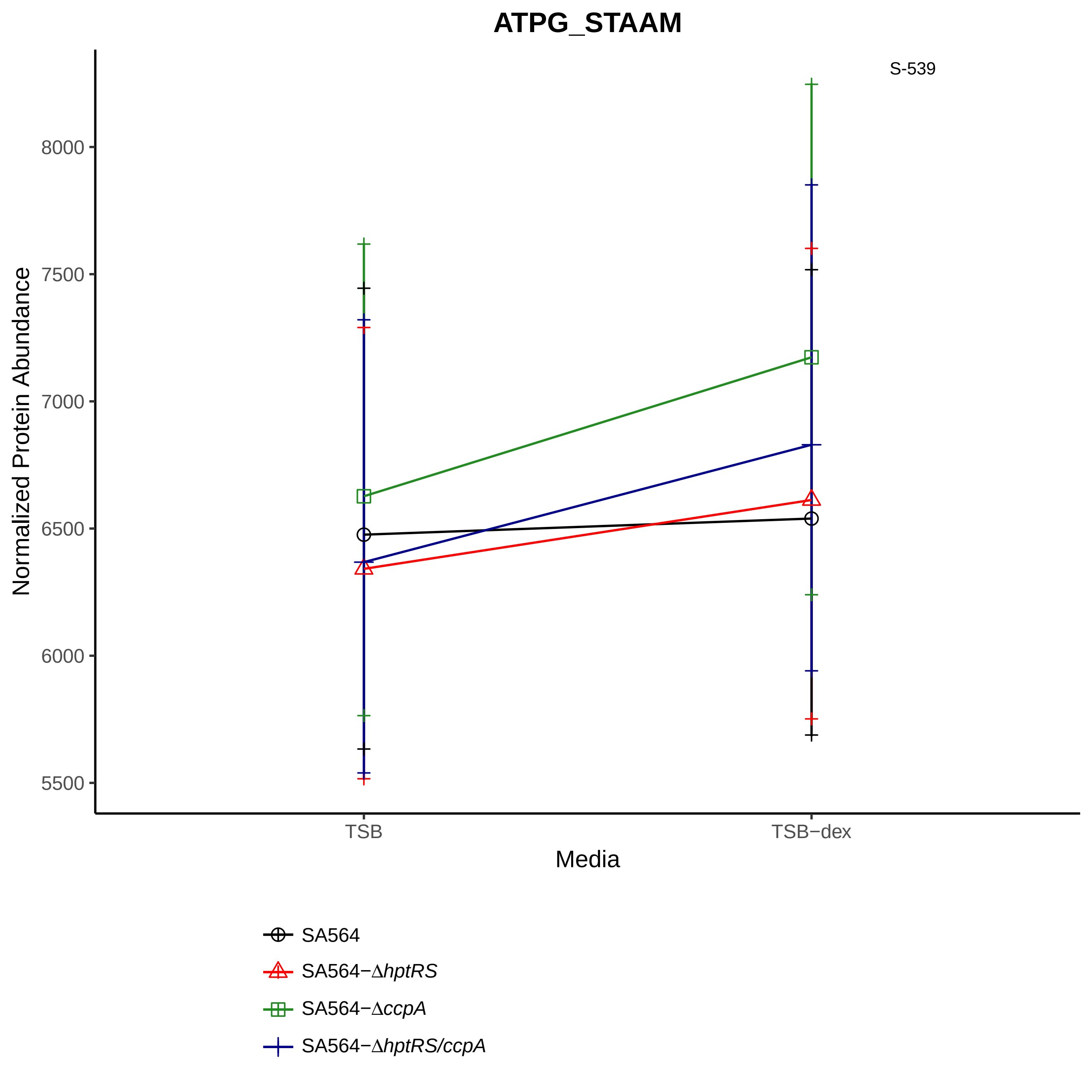

ATPG_STAAM
S-539
Normalized Protein Abundance
8000
7500
7000
6500
6000
5500
TSB
TSB−dex
Media
SA564
SA564−Δ*hptRS*
SA564−Δ*ccpA*
SA564−Δ*hptRS/ccpA*

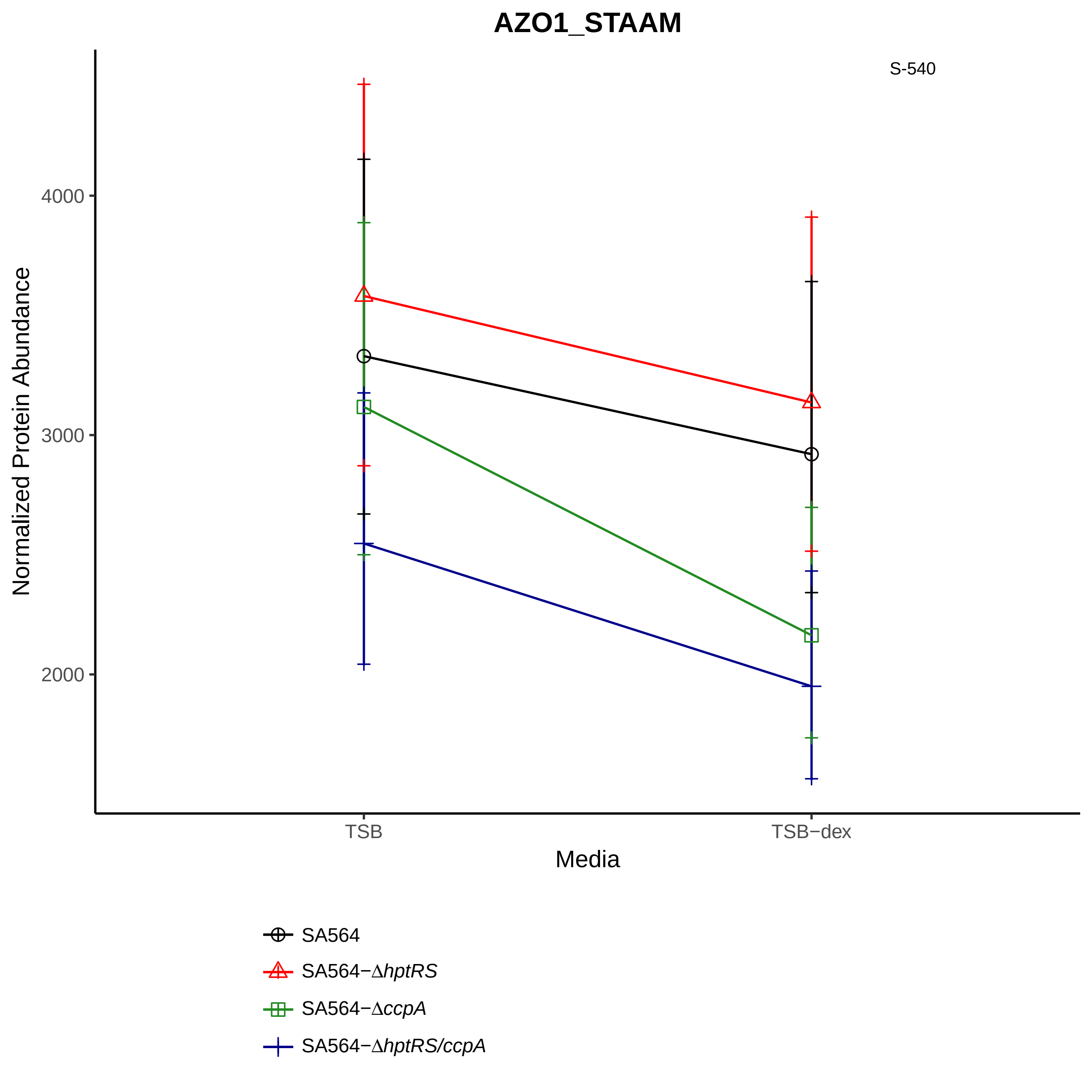

AZO1_STAAM
S-540
Normalized Protein Abundance
4000
3000
2000
TSB
TSB−dex
Media
SA564
SA564−ΔhptRS
SA564−ΔccpA
SA564−ΔhptRS/ccpA

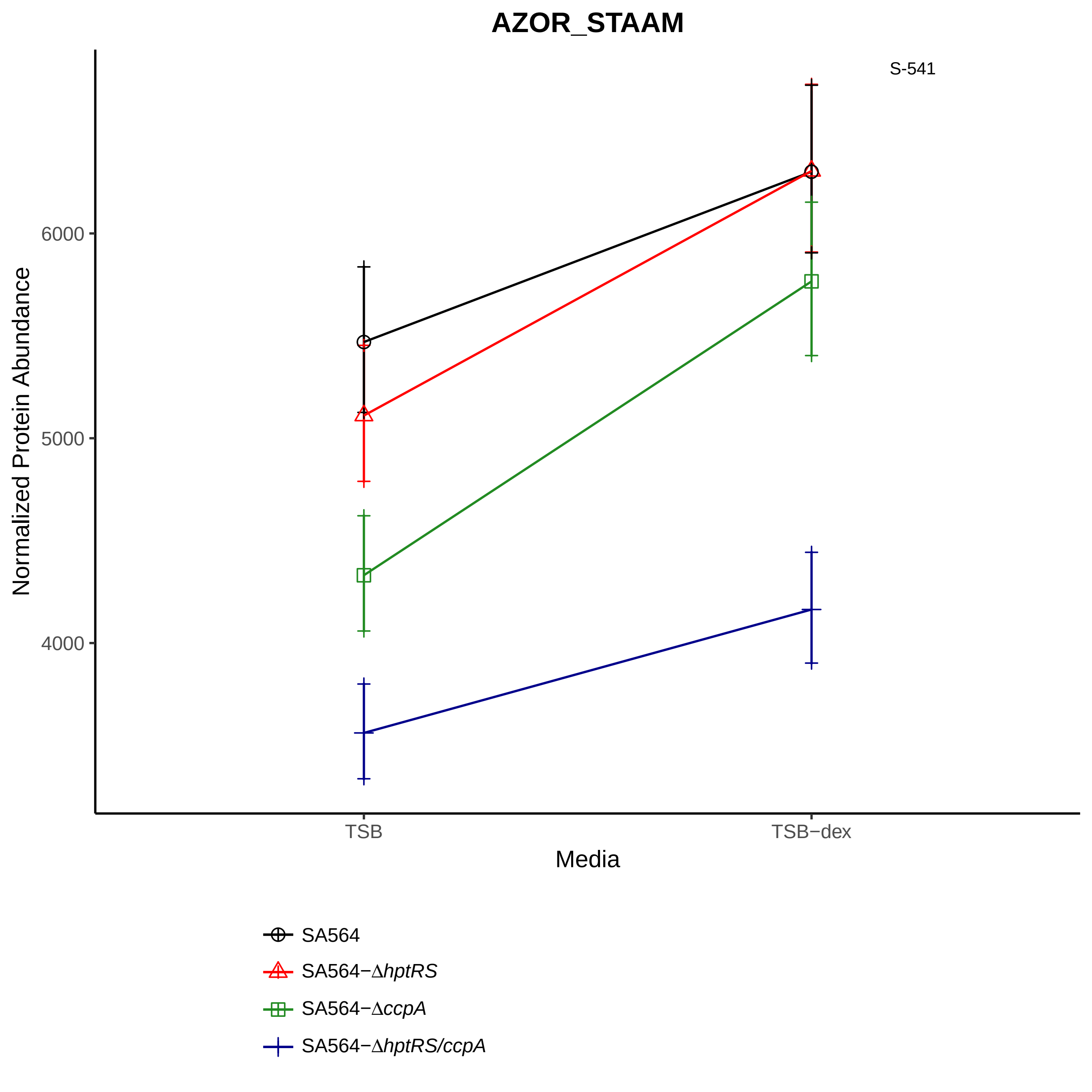

AZOR_STAAM
S-541
Normalized Protein Abundance
6000
5000
4000
TSB
TSB−dex
Media
SA564
SA564−Δ*hptRS*
SA564−Δ*ccpA*
SA564−Δ*hptRS/ccpA*

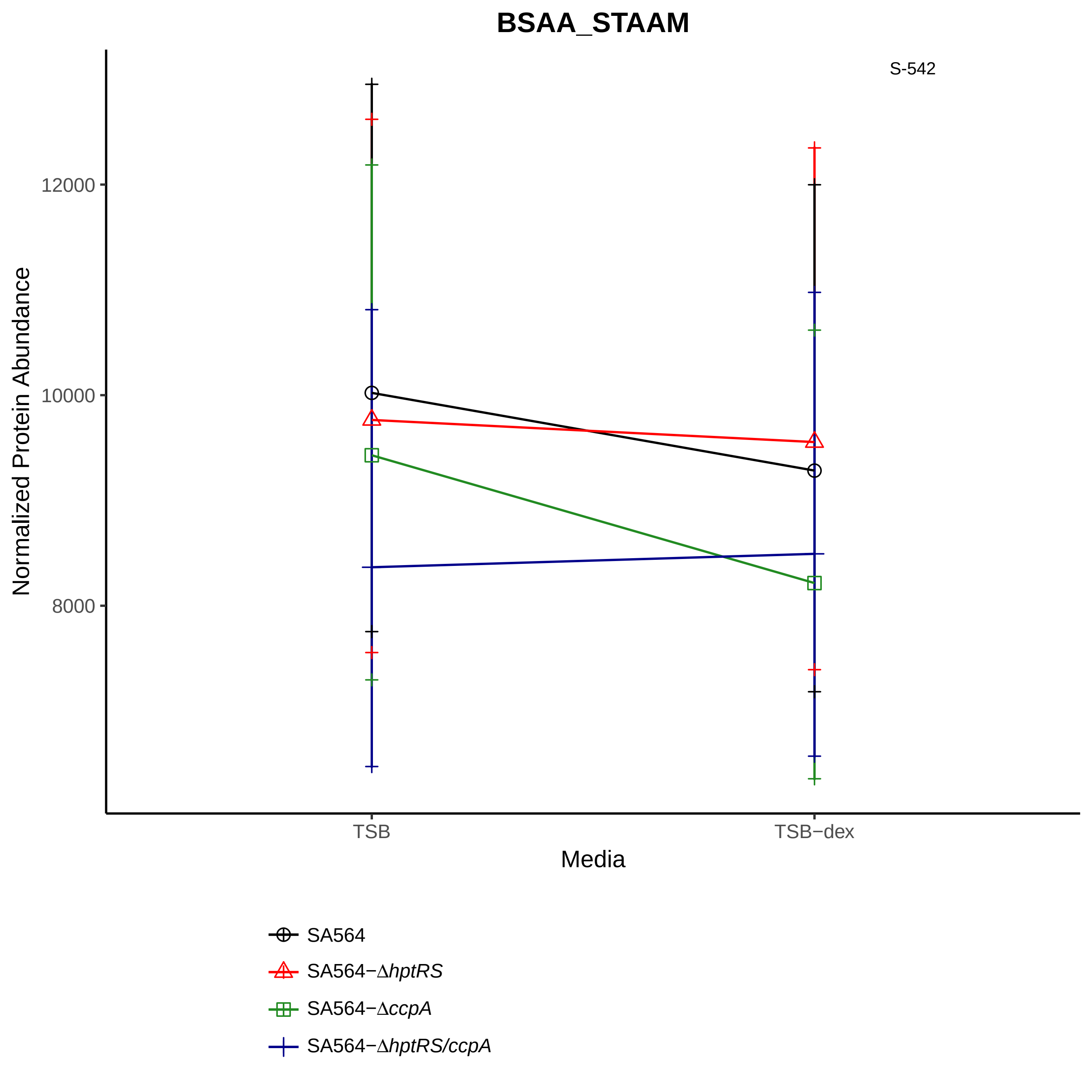

BSAA_STAAM
S-542
12000
10000
8000
Normalized Protein Abundance
TSB
TSB−dex
Media
SA564
SA564−ΔhptRS
SA564−ΔccpA
SA564−ΔhptRS/ccpA

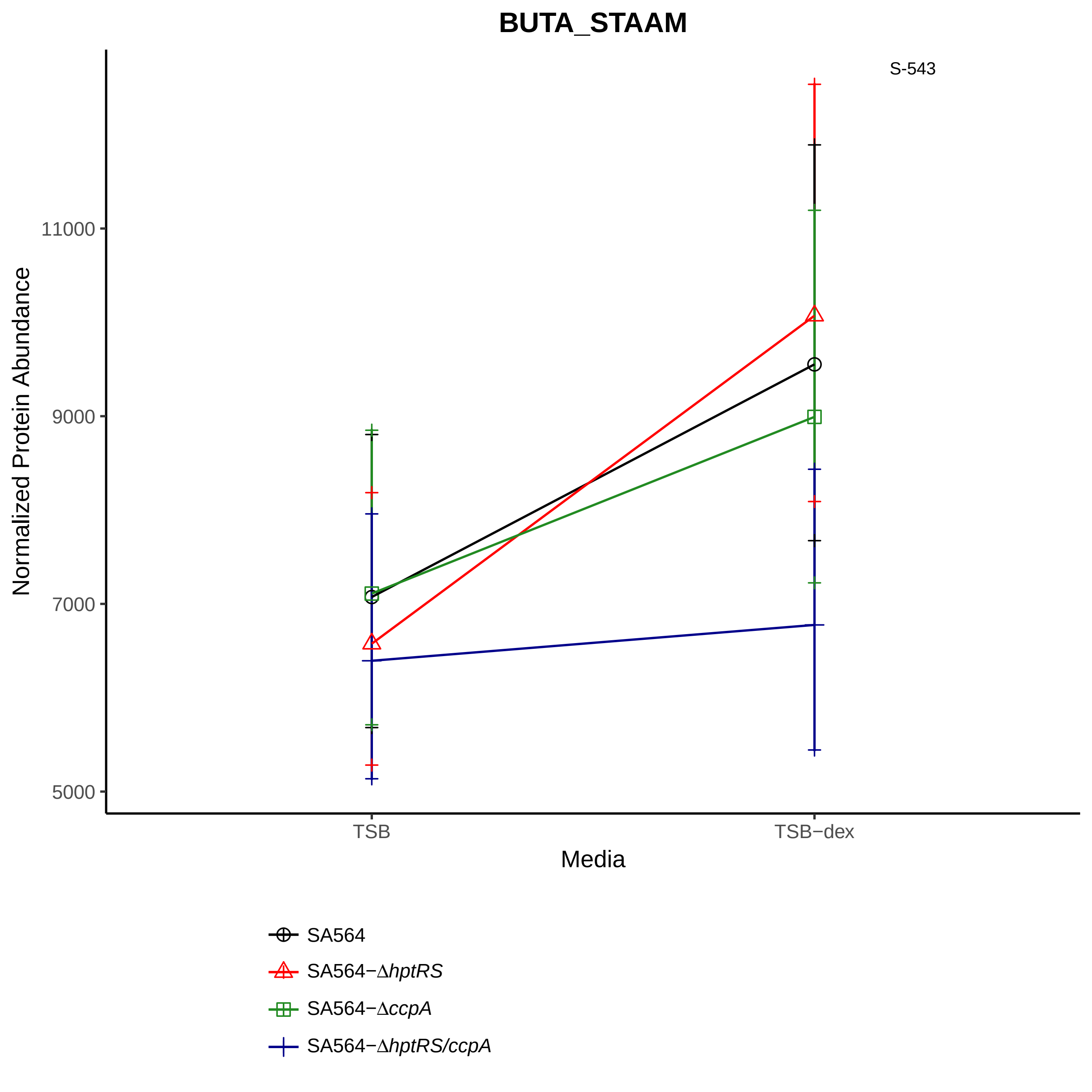
BUTA_STAAM
S-543
Normalized Protein Abundance
11000
9000
7000
5000
TSB
TSB−dex
Media
SA564
SA564−Δ*hptRS*
SA564−Δ*ccpA*
SA564−Δ*hptRS/ccpA*

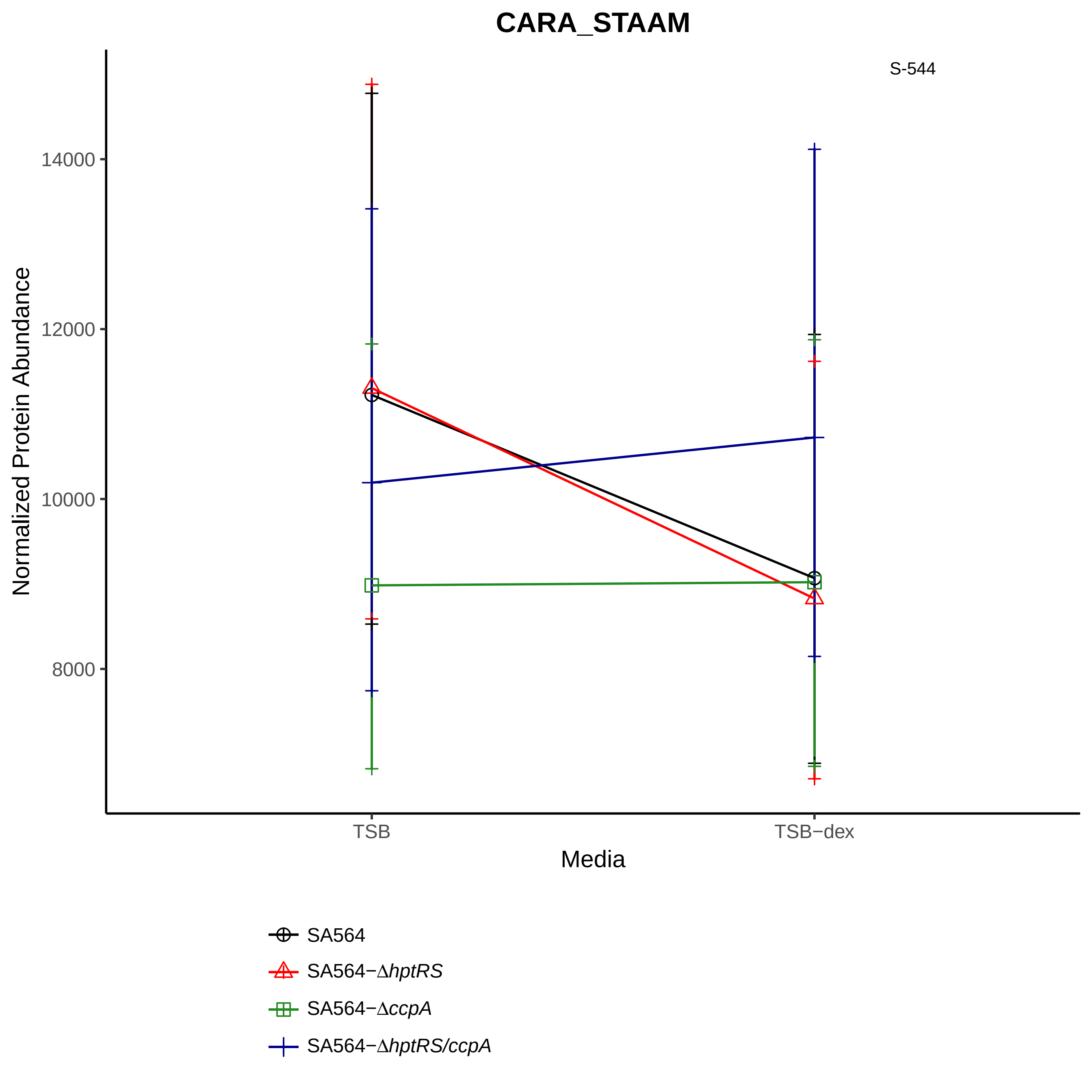

CARA_STAAM
S-544
Normalized Protein Abundance
14000
12000
10000
8000
TSB
TSB−dex
Media
SA564
SA564−Δ*hptRS*
SA564−Δ*ccpA*
SA564−Δ*hptRS/ccpA*

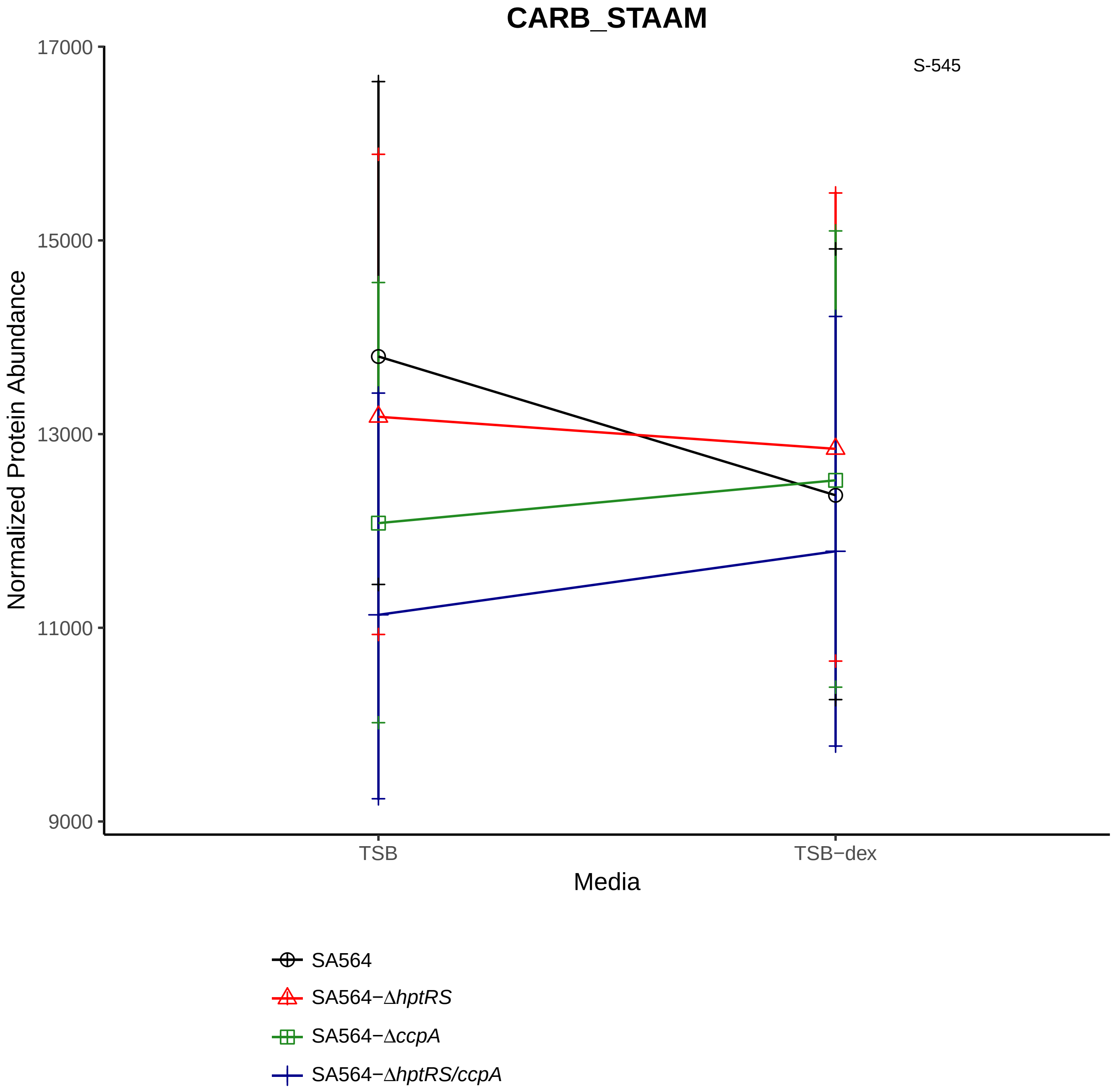
CARB_STAAM
S-545
Normalized Protein Abundance
17000
15000
13000
11000
9000
TSB
TSB−dex
Media
SA564
SA564−Δ*hptRS*
SA564−Δ*ccpA*
SA564−Δ*hptRS/ccpA*

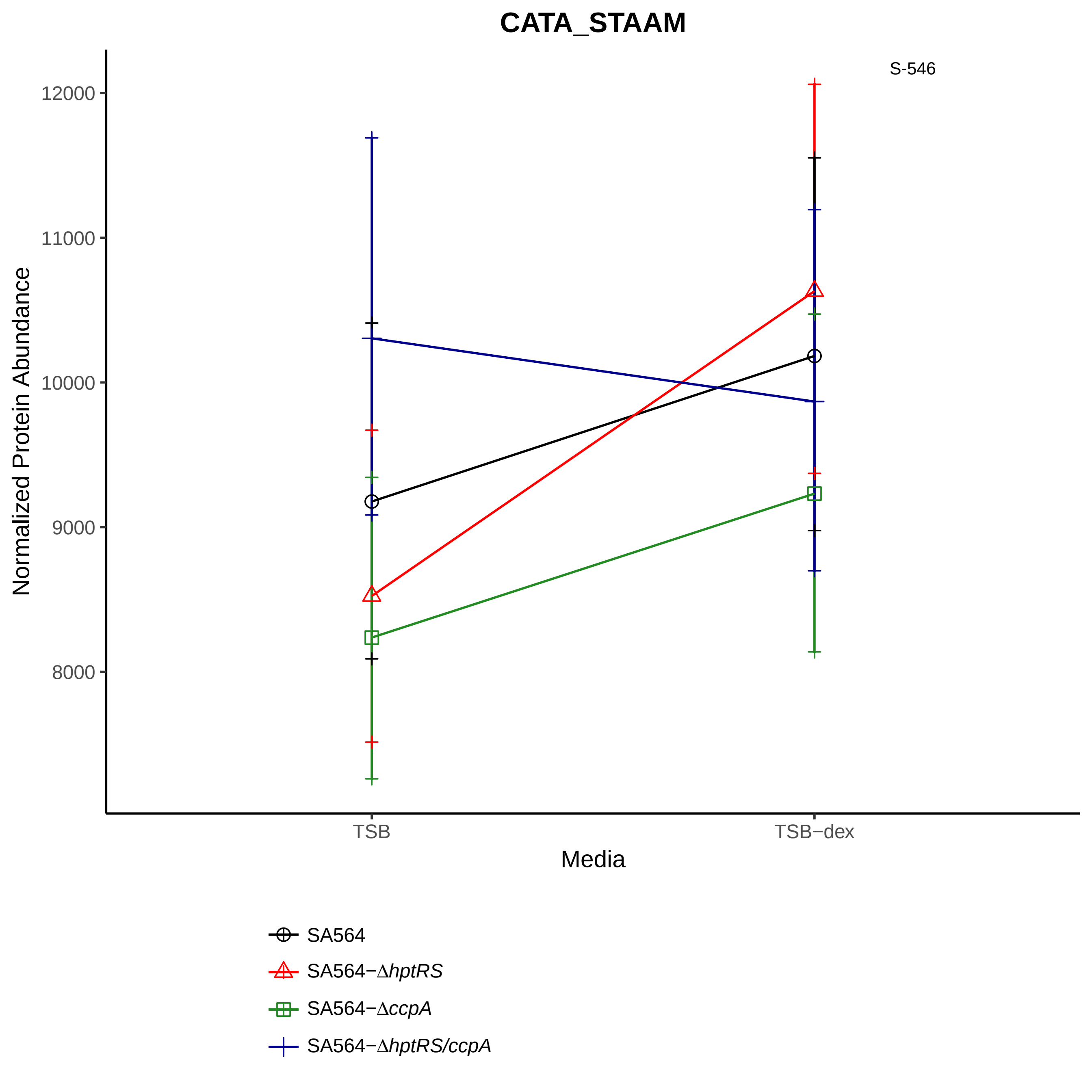

CATA_STAAM
S-546
Normalized Protein Abundance
12000
11000
10000
9000
8000
TSB
TSB−dex
Media
SA564
SA564−ΔhptRS
SA564−ΔccpA
SA564−ΔhptRS/ccpA

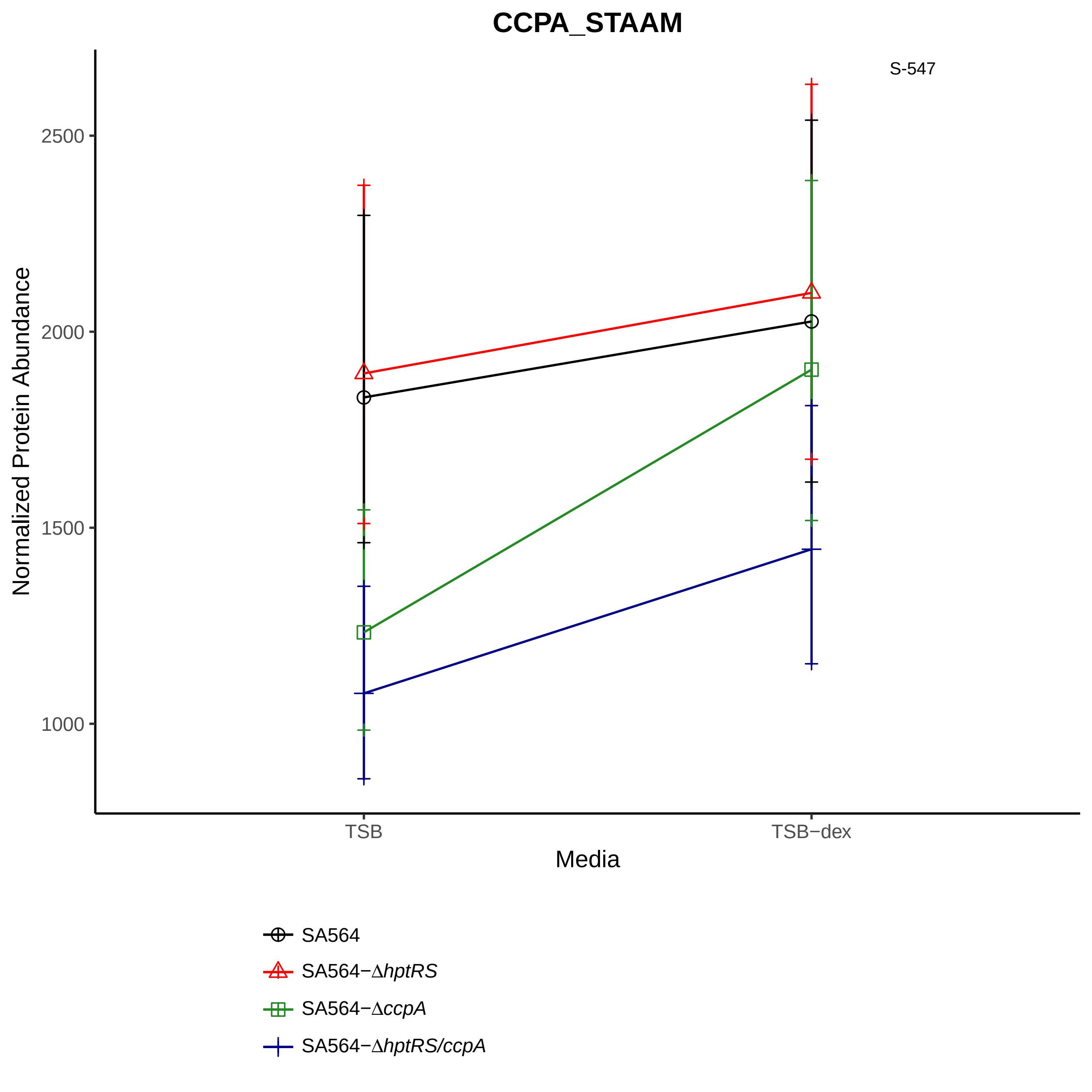

CCPA_STAAM
S-547
2500
2000
1500
1000
Normalized Protein Abundance
TSB
TSB−dex
Media
SA564
SA564−ΔhptRS
SA564−ΔccpA
SA564−ΔhptRS/ccpA

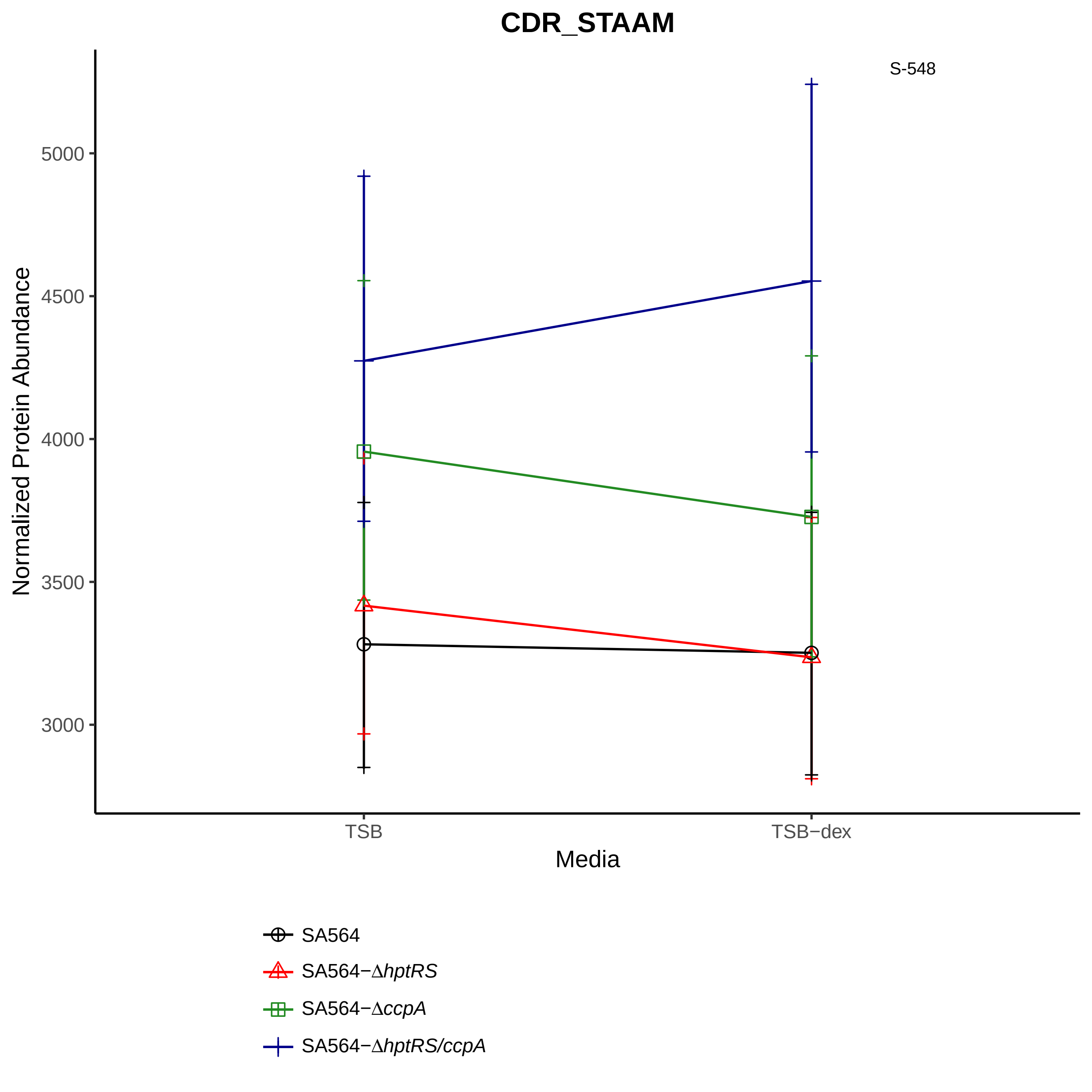
CDR_STAAM
S-548
Normalized Protein Abundance
5000
4500
4000
3500
3000
TSB
TSB−dex
Media
SA564
SA564−ΔhptRS
SA564−ΔccpA
SA564−ΔhptRS/ccpA

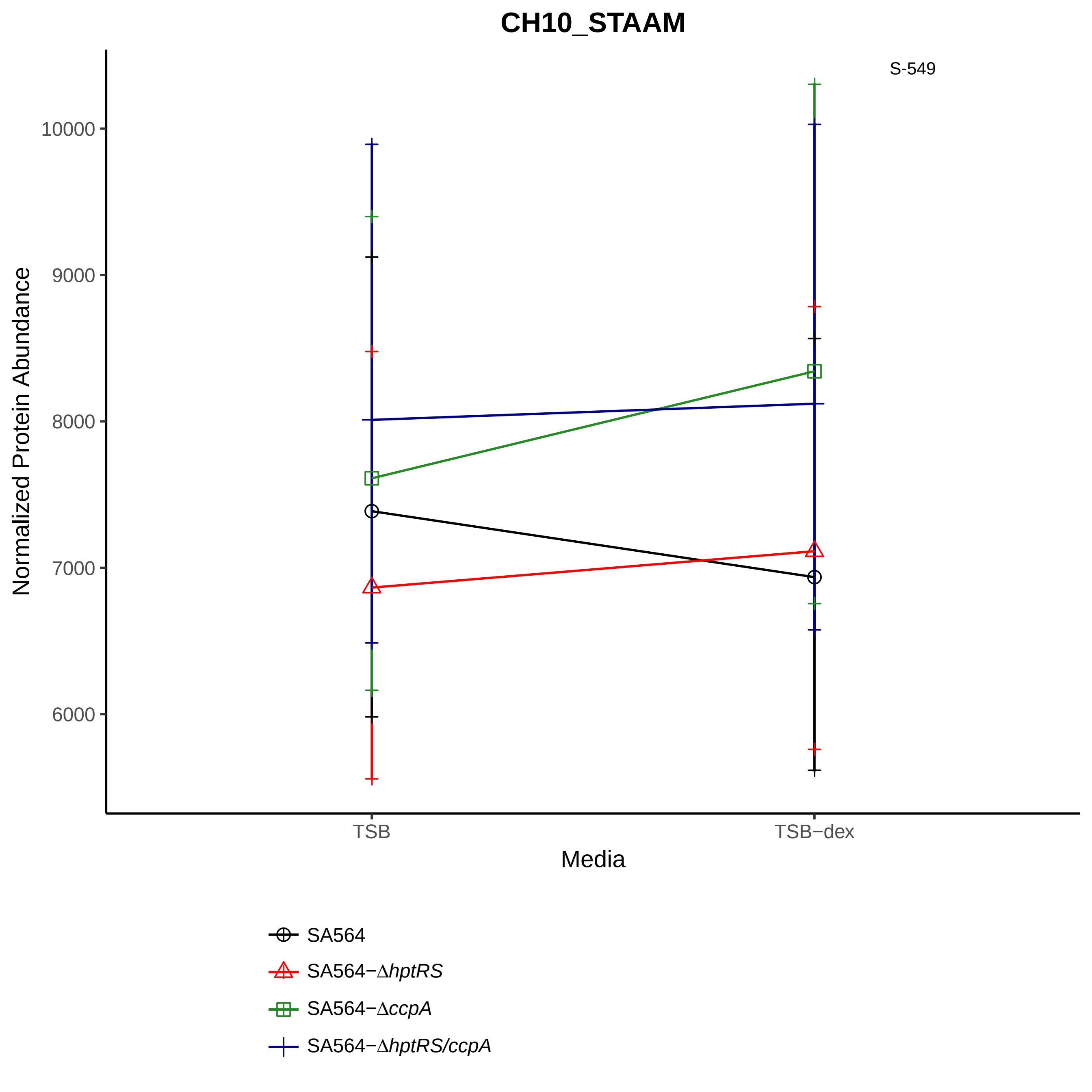

CH10_STAAM
S-549
Normalized Protein Abundance
10000
9000
8000
7000
6000
TSB
TSB−dex
Media
SA564
SA564−Δ*hptRS*
SA564−Δ*ccpA*
SA564−Δ*hptRS/ccpA*

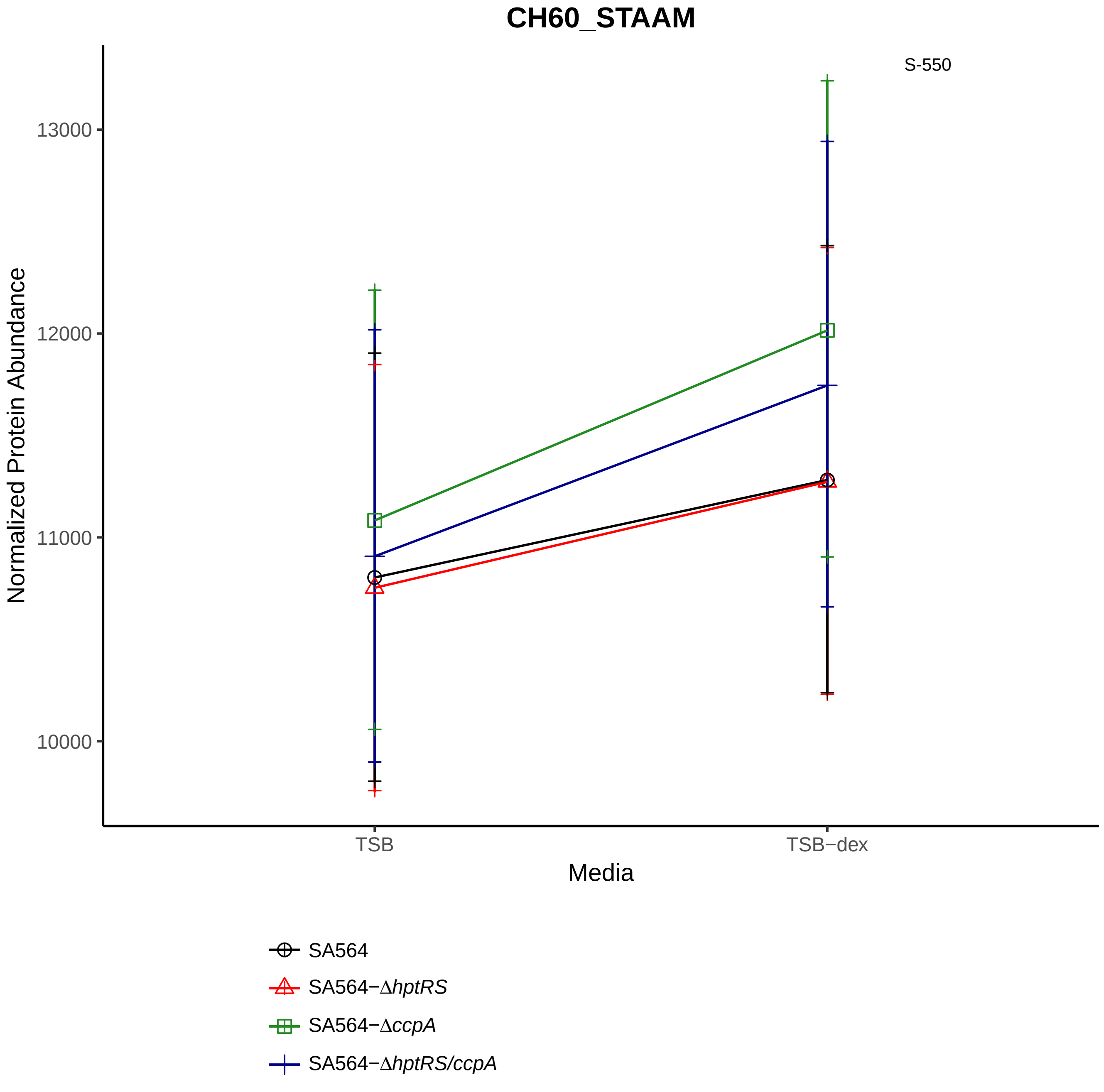

CH60_STAAM
S-550
Normalized Protein Abundance
13000
12000
11000
10000
TSB
TSB−dex
Media
SA564
SA564−Δ*hptRS*
SA564−Δ*ccpA*
SA564−Δ*hptRS/ccpA*

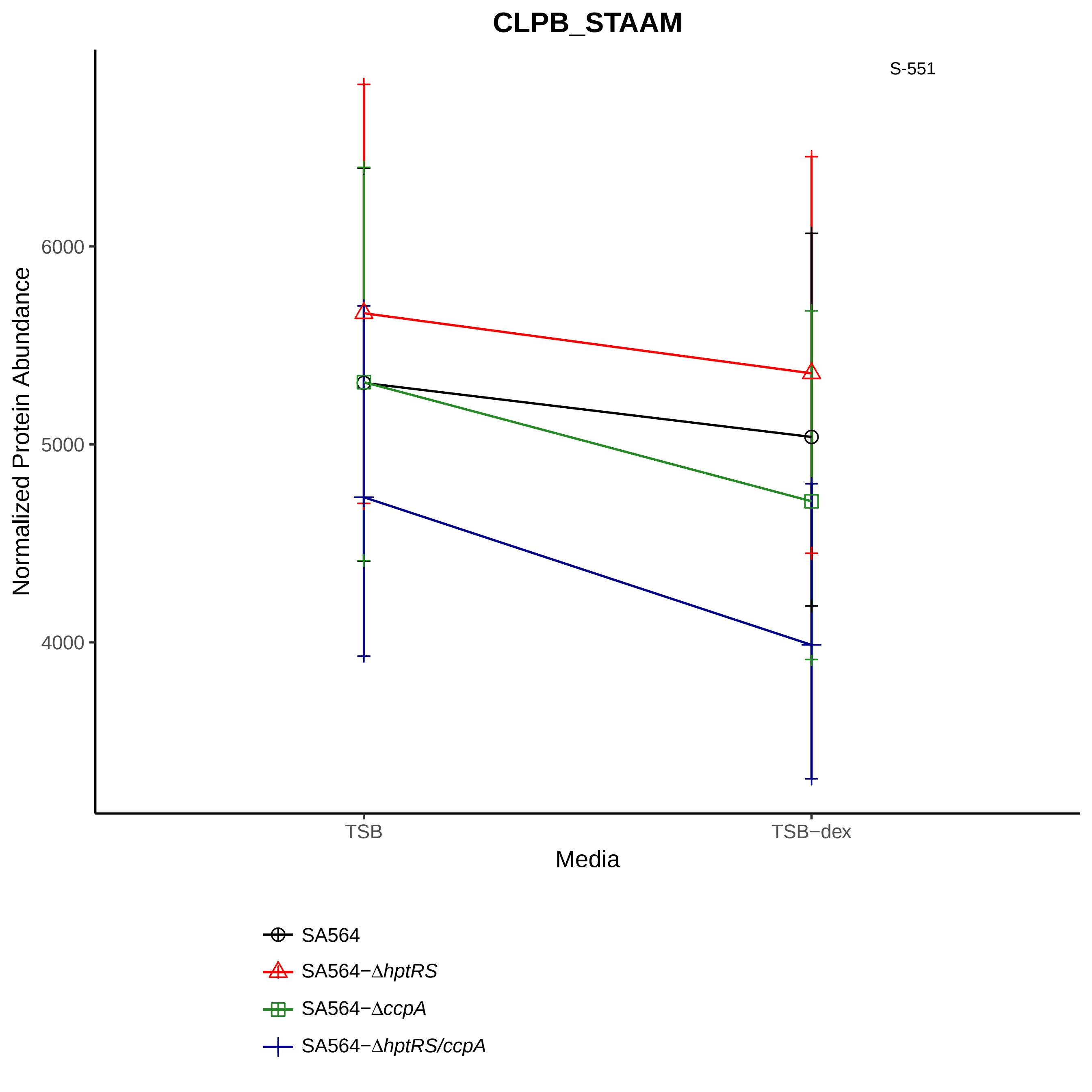

CLPB_STAAM
S-551
Normalized Protein Abundance
6000
5000
4000
TSB
TSB−dex
Media
SA564
SA564−ΔhptRS
SA564−ΔccpA
SA564−ΔhptRS/ccpA

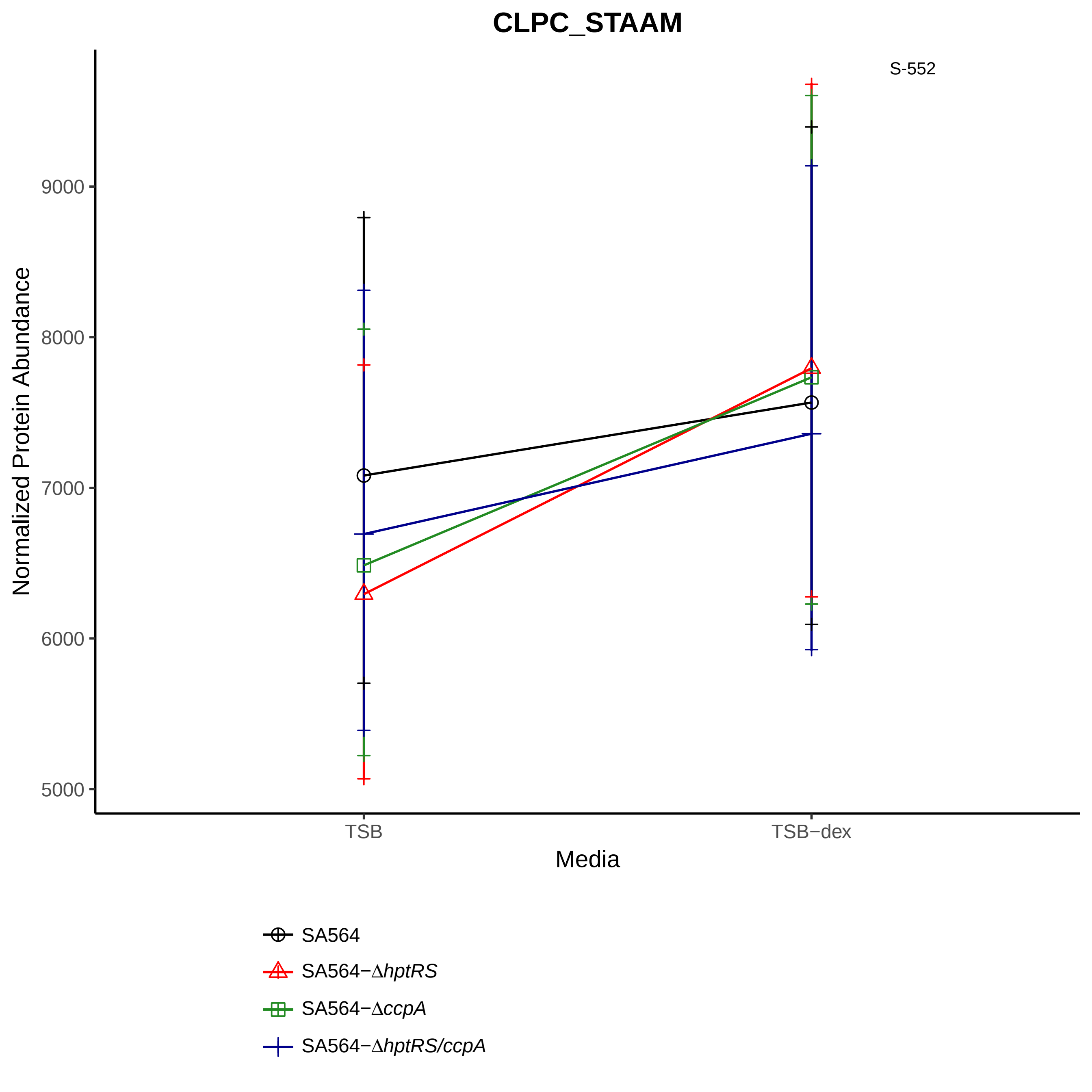

CLPC_STAAM
S-552
Normalized Protein Abundance
9000
8000
7000
6000
5000
TSB
TSB−dex
Media
SA564
SA564−Δ*hptRS*
SA564−Δ*ccpA*
SA564−Δ*hptRS/ccpA*

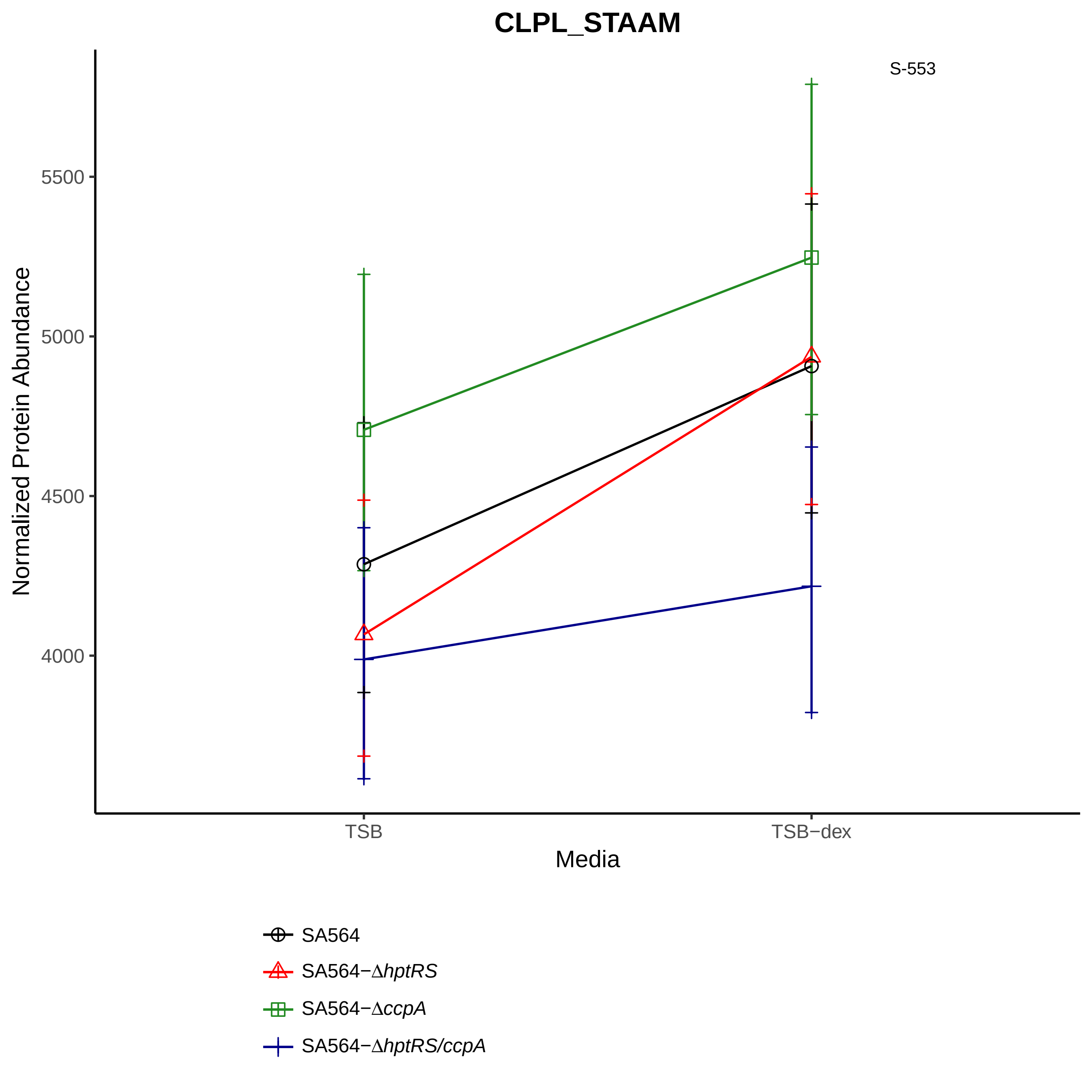
CLPL_STAAM
S-553
Normalized Protein Abundance
5500
5000
4500
4000
TSB
TSB−dex
Media
SA564
SA564−ΔhptRS
SA564−ΔccpA
SA564−ΔhptRS/ccpA

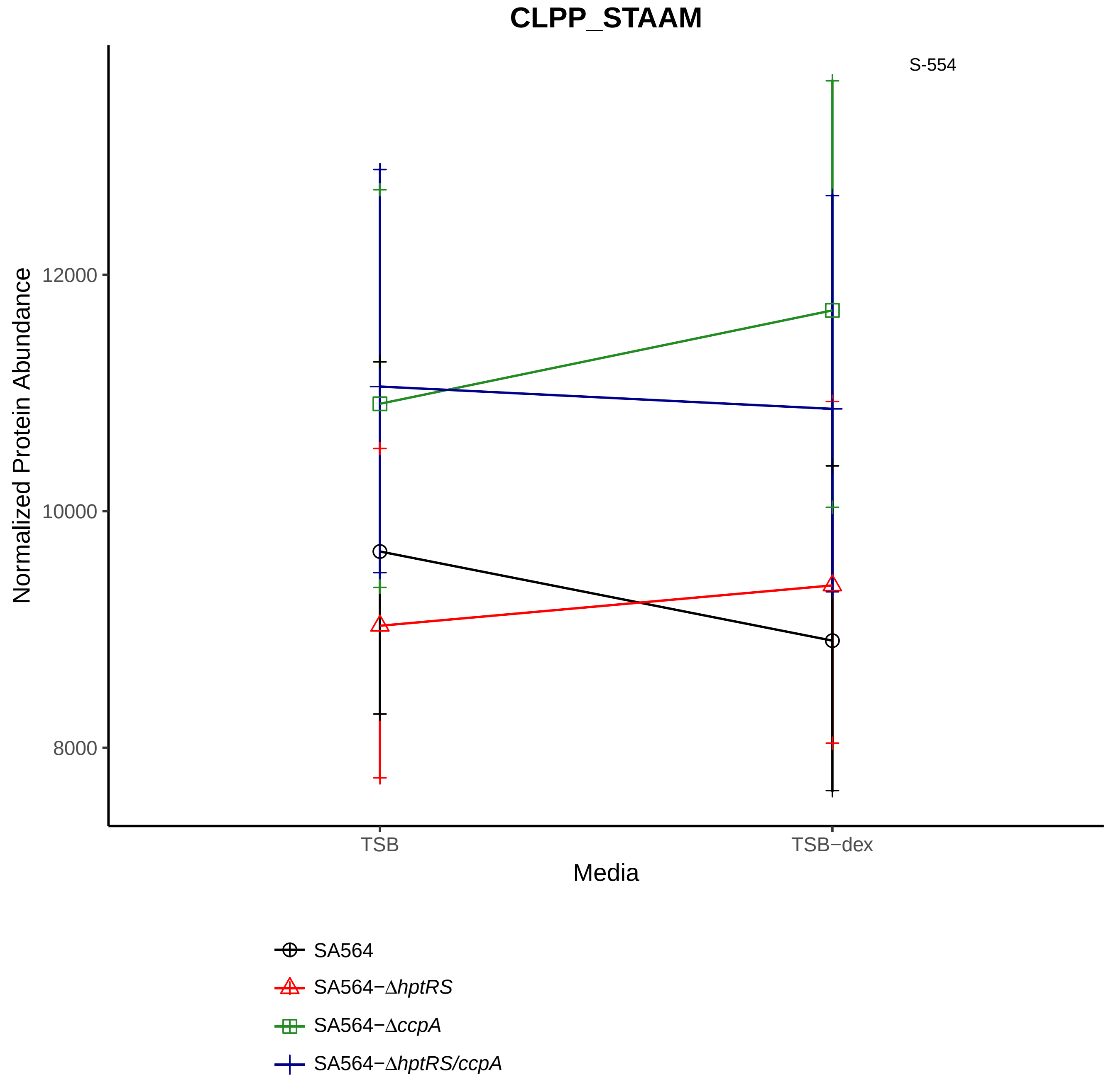

CLPP_STAAM
S-554
Normalized Protein Abundance
12000
10000
8000
TSB
TSB−dex
Media
SA564
SA564−Δ*hptRS*
SA564−Δ*ccpA*
SA564−Δ*hptRS/ccpA*

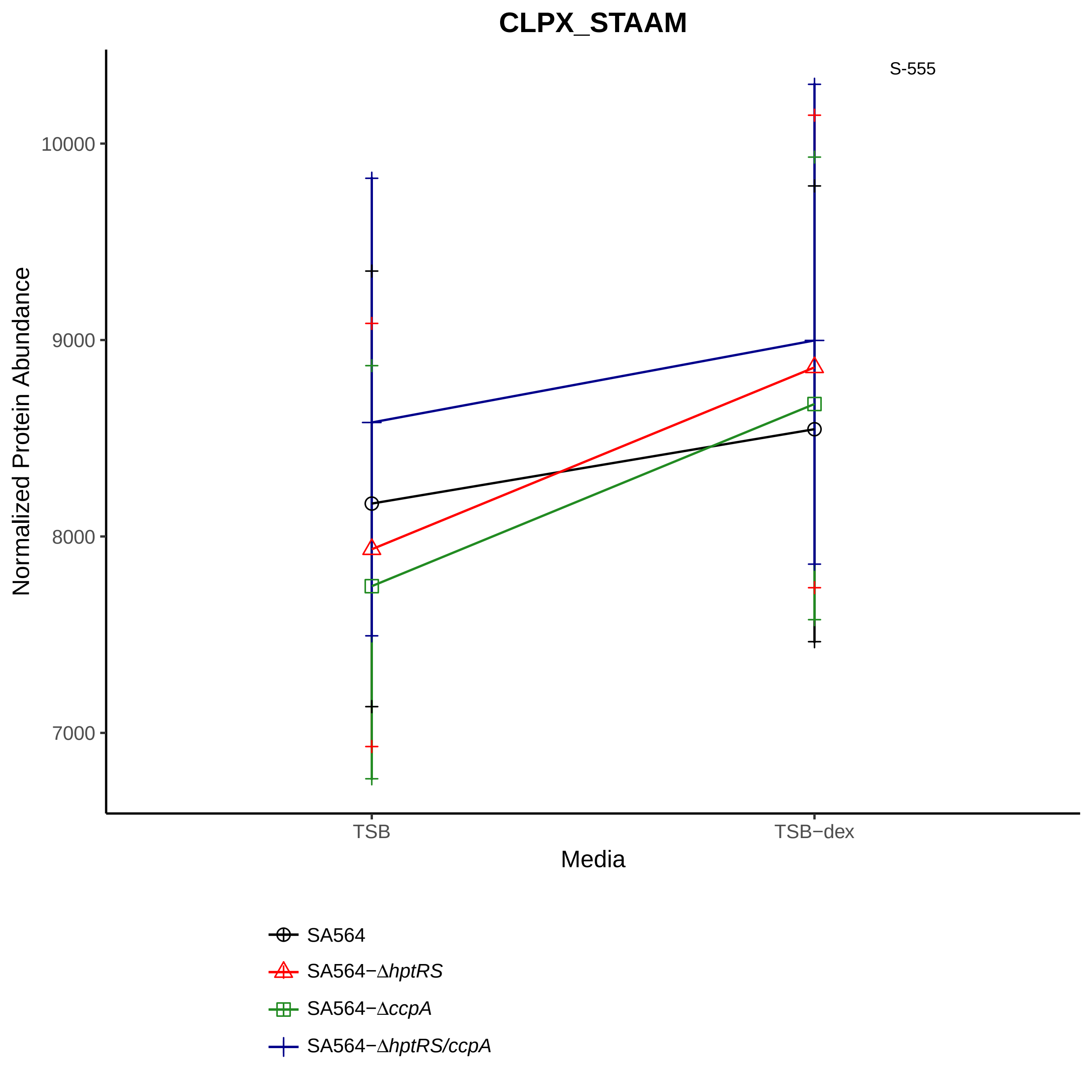

CLPX_STAAM
S-555
Normalized Protein Abundance
10000
9000
8000
7000
TSB
TSB−dex
Media
SA564
SA564−Δ*hptRS*
SA564−Δ*ccpA*
SA564−Δ*hptRS/ccpA*

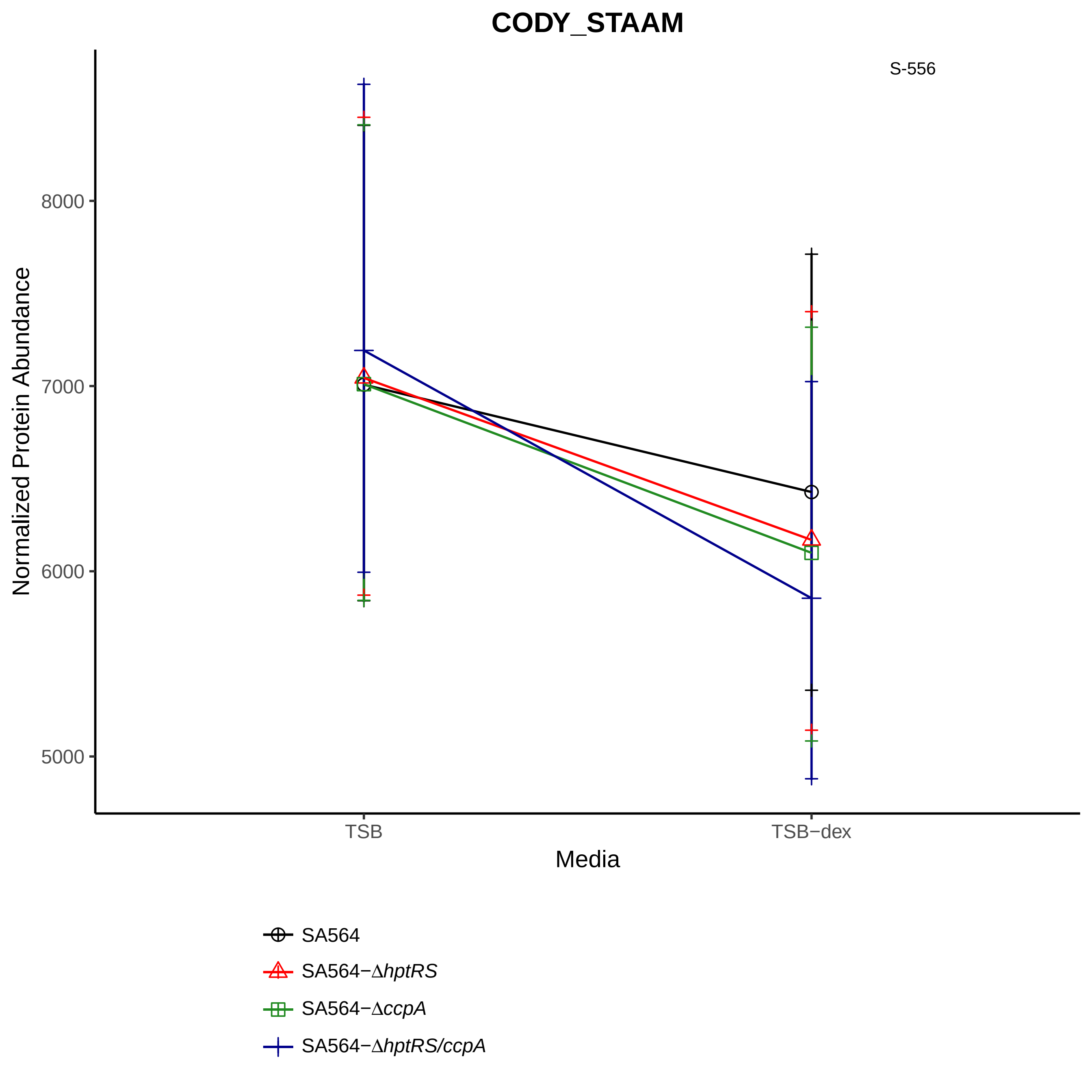
CODY_STAAM
S-556
Normalized Protein Abundance
8000
7000
6000
5000
TSB
TSB−dex
Media
SA564
SA564−ΔhptRS
SA564−ΔccpA
SA564−ΔhptRS/ccpA

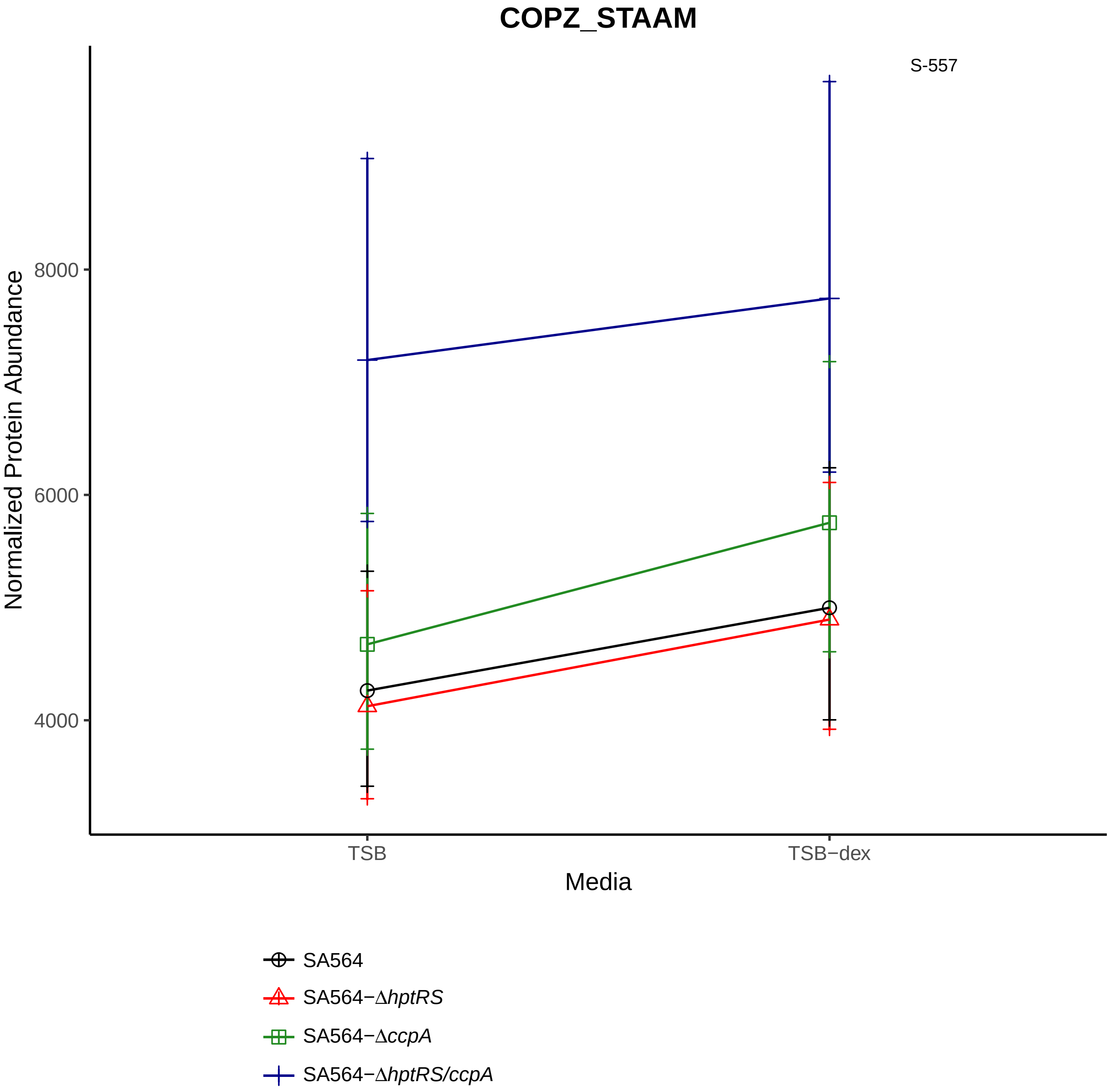
COPZ_STAAM
S-557
Normalized Protein Abundance
8000
6000
4000
TSB
TSB−dex
Media
SA564
SA564−Δ*hptRS*
SA564−Δ*ccpA*
SA564−Δ*hptRS/ccpA*

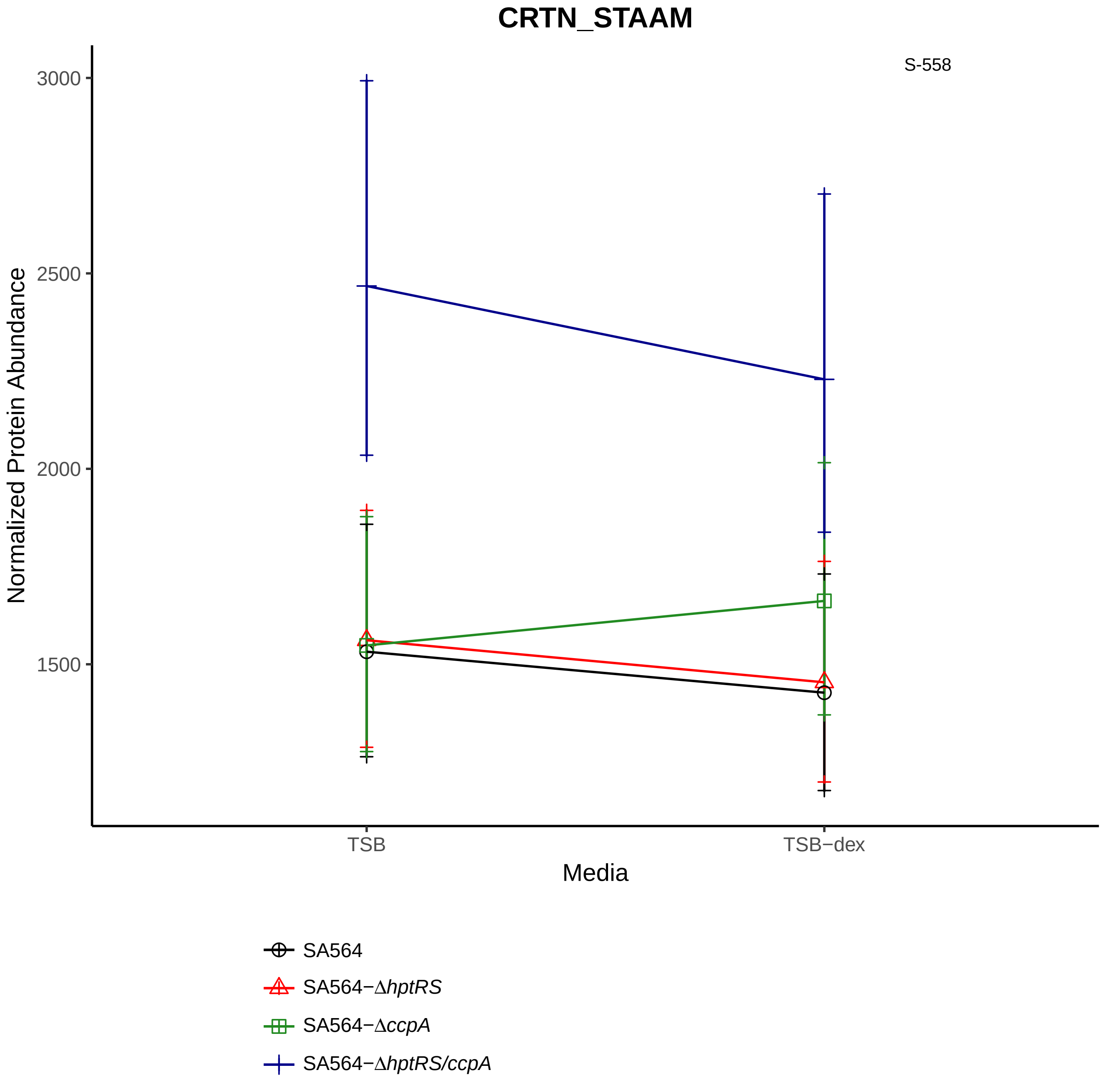

CRTN_STAAM
S-558
Normalized Protein Abundance
3000
2500
2000
1500
TSB
TSB−dex
Media
SA564
SA564−Δ*hptRS*
SA564−Δ*ccpA*
SA564−Δ*hptRS/ccpA*

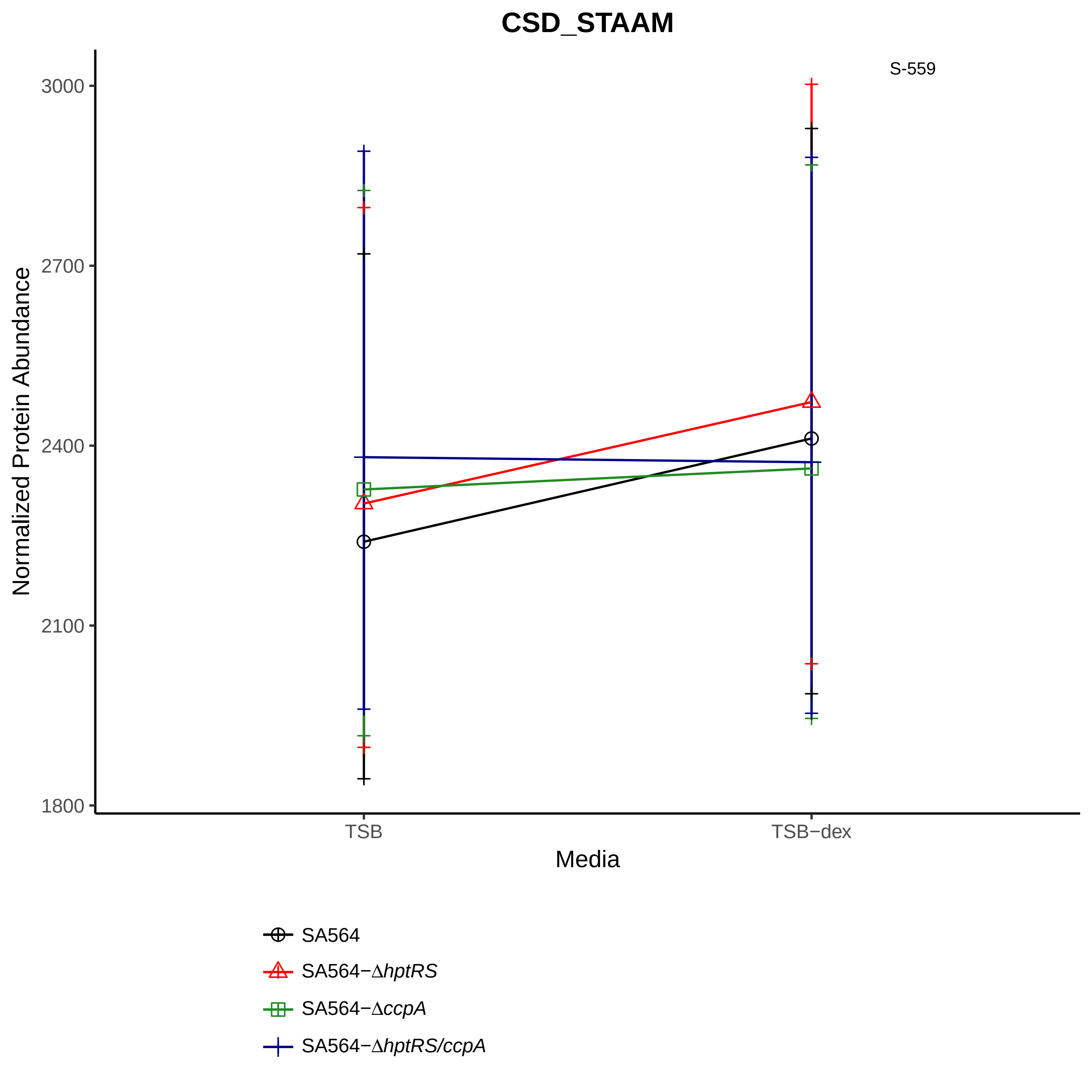

CSD_STAAM
S-559
Normalized Protein Abundance
3000
2700
2400
2100
1800
TSB
TSB−dex
Media
SA564
SA564−Δ*hptRS*
SA564−Δ*ccpA*
SA564−Δ*hptRS/ccpA*

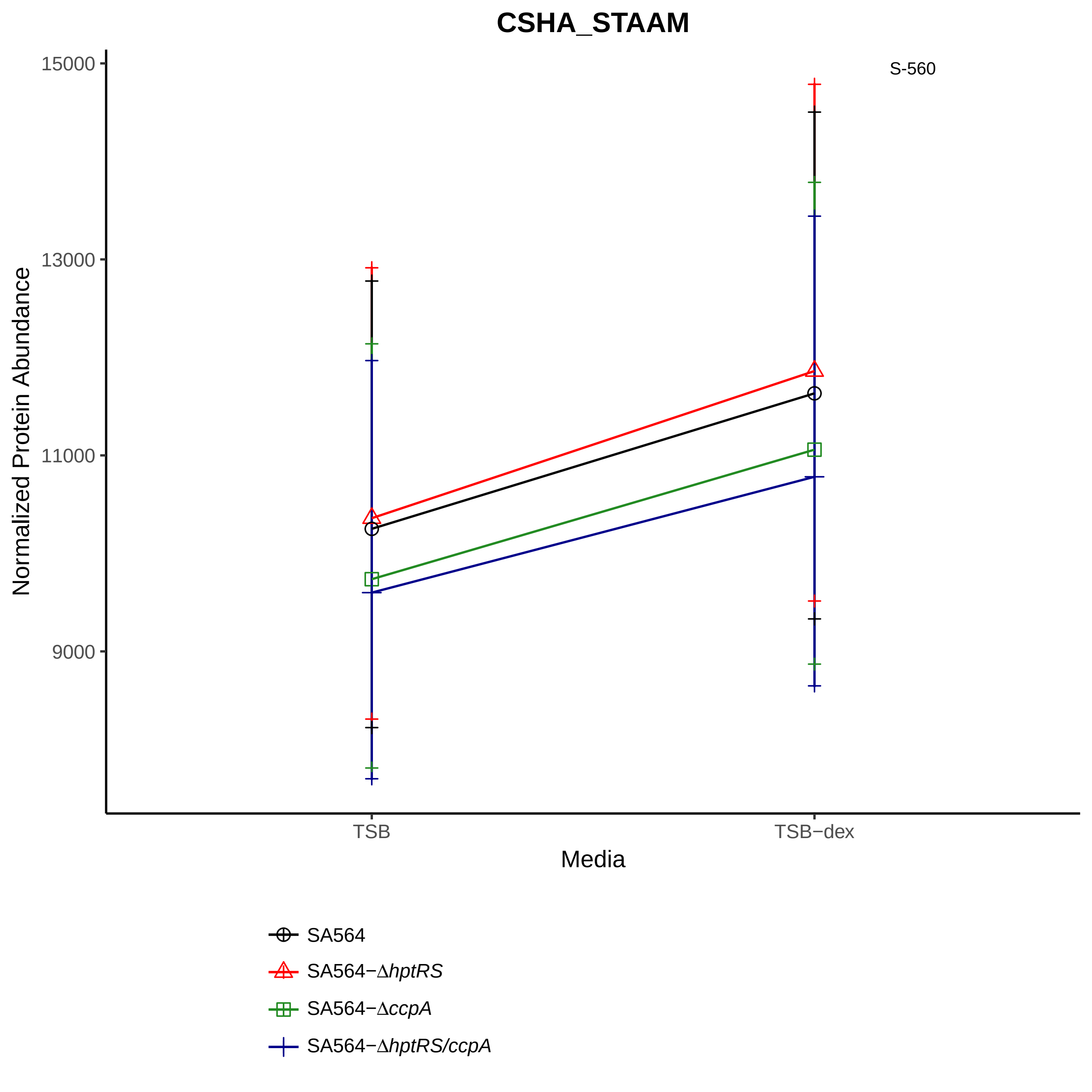
CSHA_STAAM
S-560
Normalized Protein Abundance
15000
13000
11000
9000
TSB
TSB−dex
Media
SA564
SA564−ΔhptRS
SA564−ΔccpA
SA564−ΔhptRS/ccpA

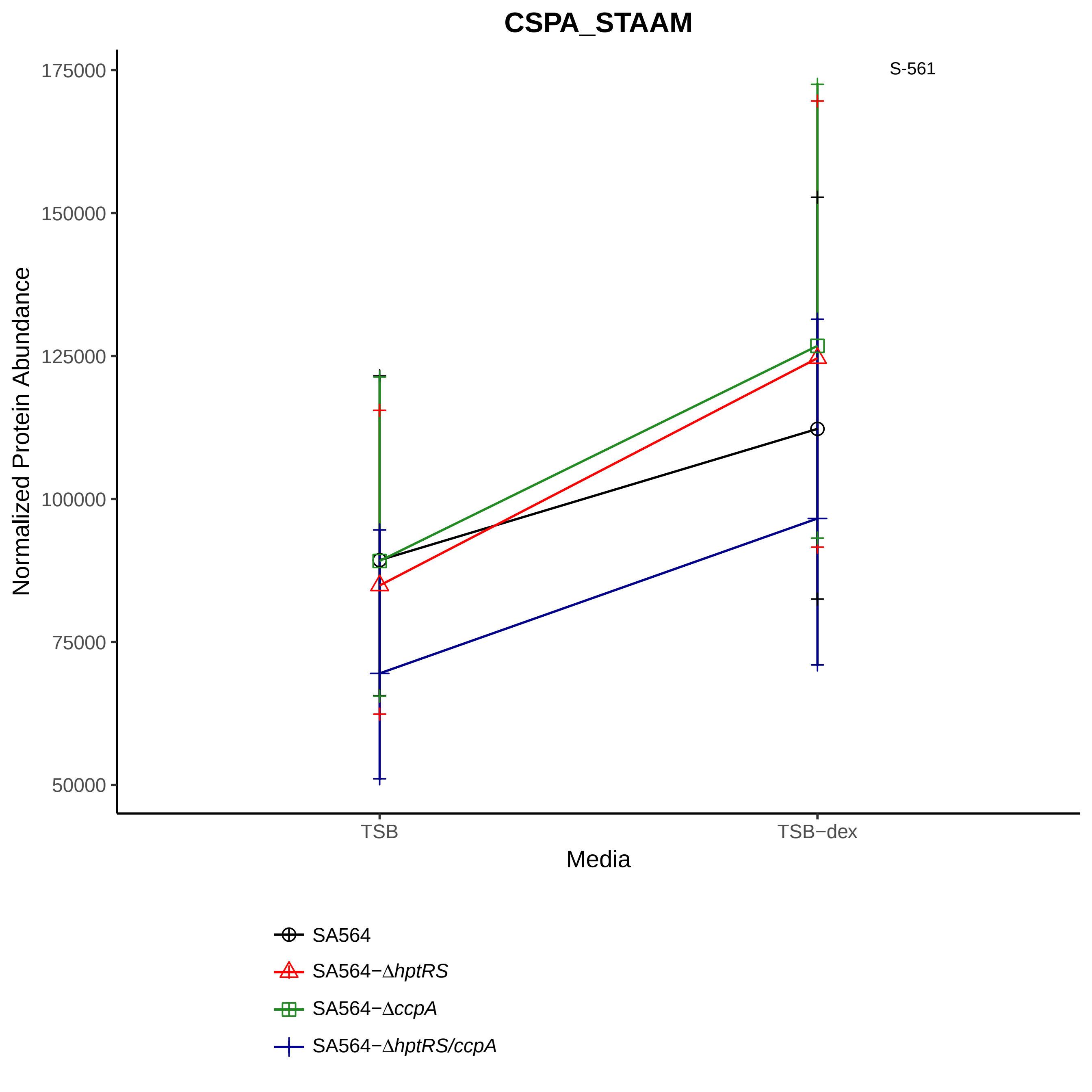

CSPA_STAAM
S-561
Normalized Protein Abundance
175000
150000
125000
100000
75000
50000
TSB
TSB−dex
Media
SA564
SA564−ΔhptRS
SA564−ΔccpA
SA564−ΔhptRS/ccpA

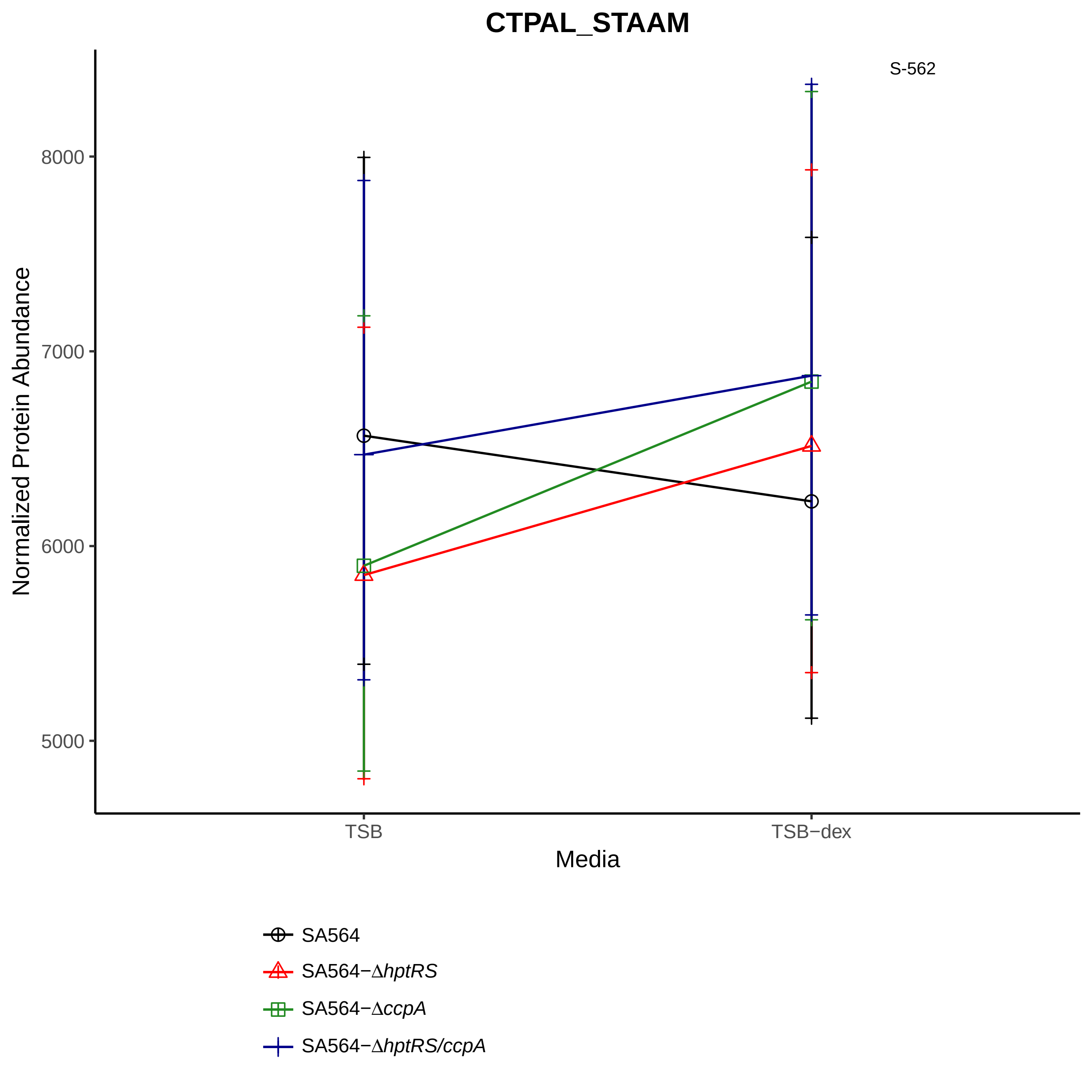

CTPAL_STAAM
S-562
Normalized Protein Abundance
8000
7000
6000
5000
TSB
TSB−dex
Media
SA564
SA564−Δ*hptRS*
SA564−Δ*ccpA*
SA564−Δ*hptRS/ccpA*

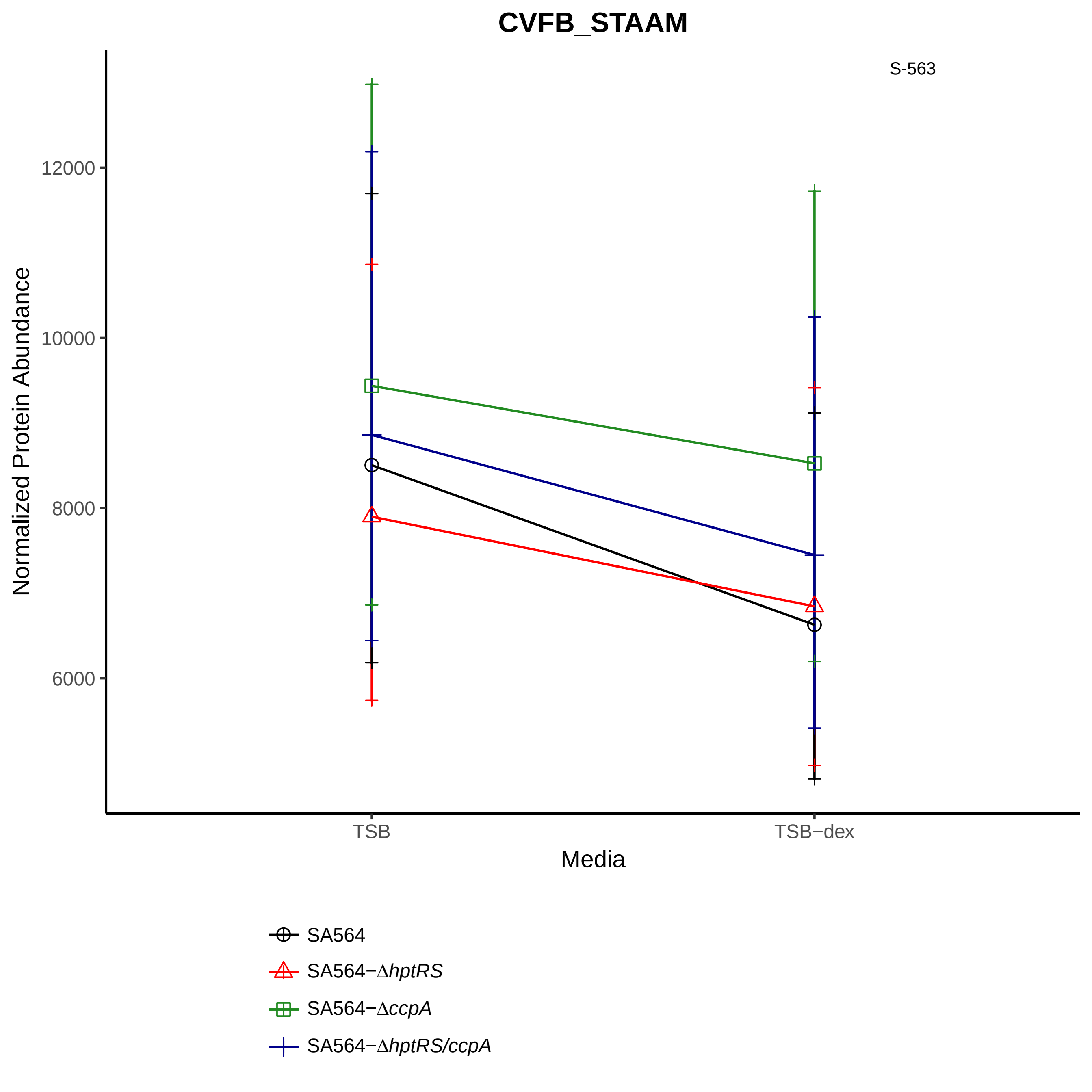

CVFB_STAAM
S-563
Normalized Protein Abundance
12000
10000
8000
6000
TSB
TSB−dex
Media
SA564
SA564−ΔhptRS
SA564−ΔccpA
SA564−ΔhptRS/ccpA

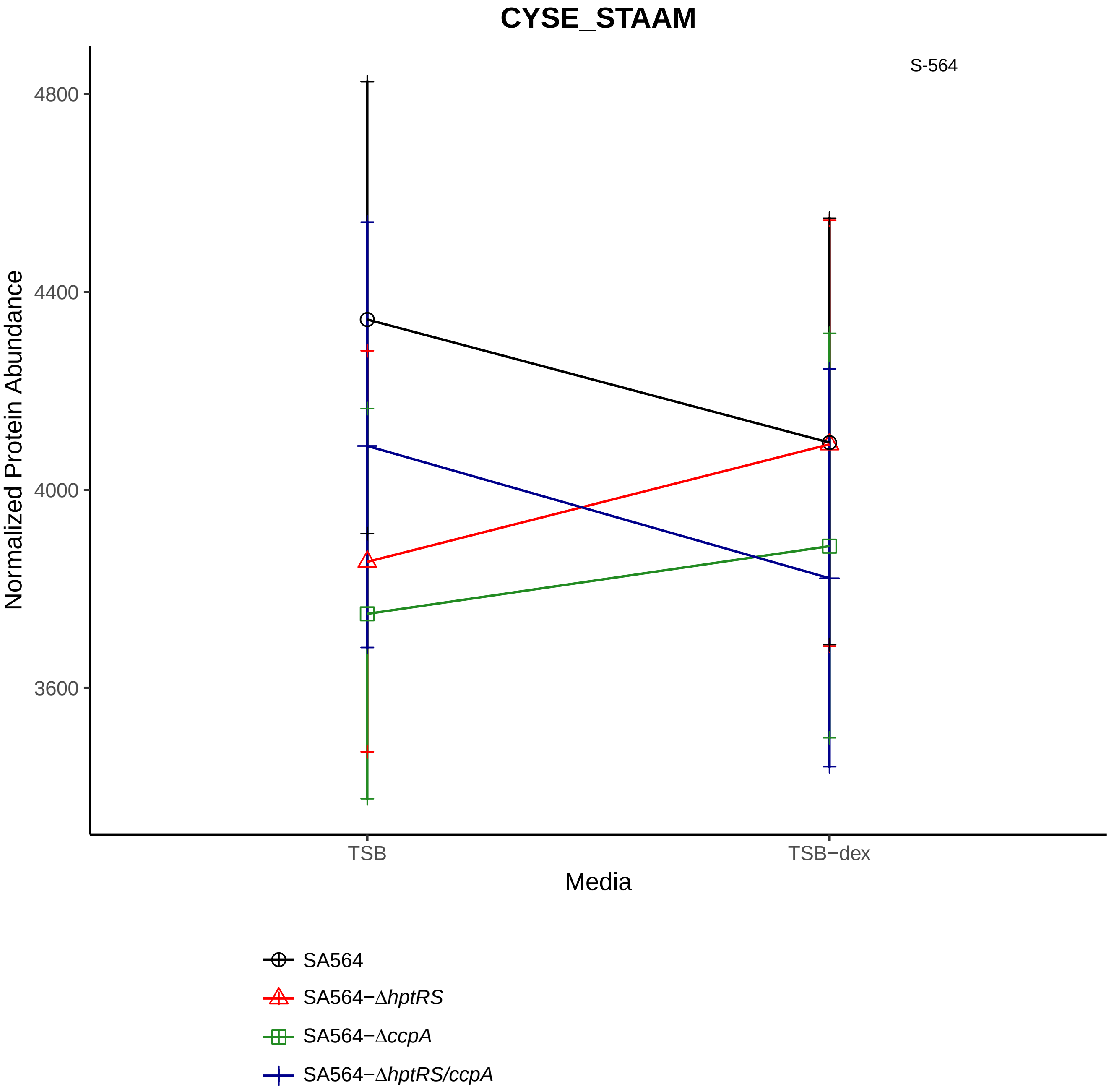

CYSE_STAAM
S-564
Normalized Protein Abundance
4800
4400
4000
3600
TSB
TSB−dex
Media
SA564
SA564−ΔhptRS
SA564−ΔccpA
SA564−ΔhptRS/ccpA

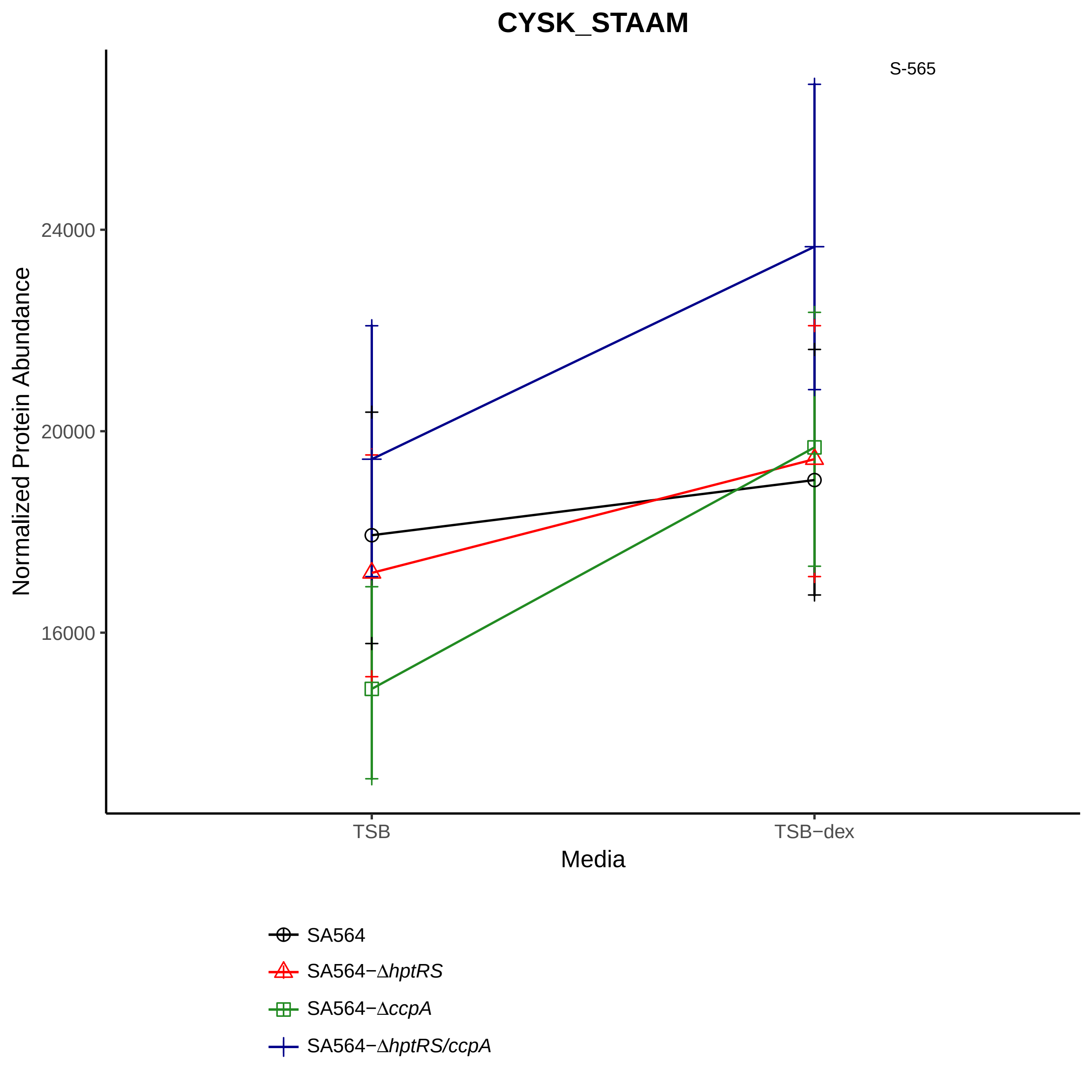
CYSK_STAAM
S-565
Normalized Protein Abundance
24000
20000
16000
TSB
TSB−dex
Media
SA564
SA564−Δ*hptRS*
SA564−Δ*ccpA*
SA564−Δ*hptRS/ccpA*

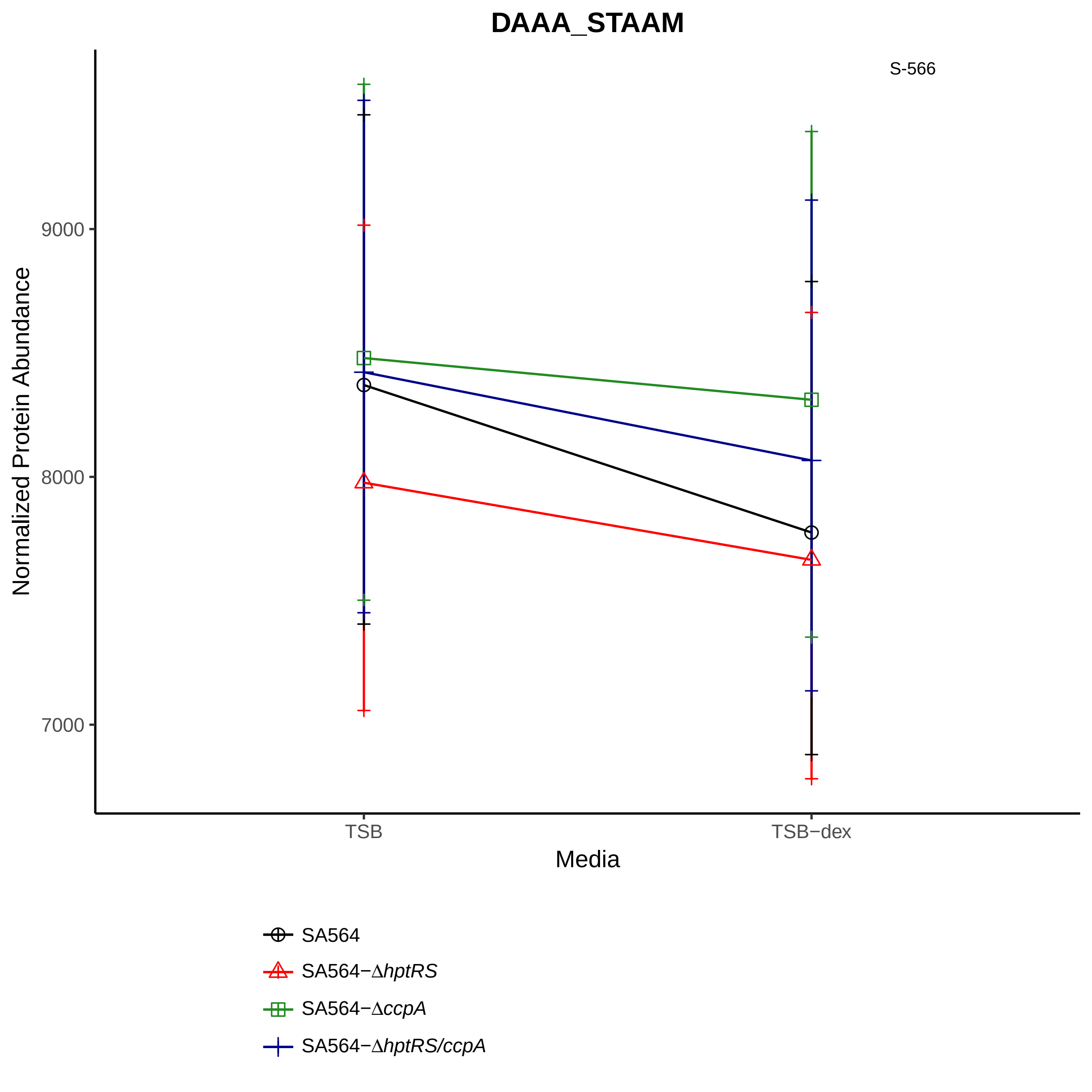

DAAA_STAAM
S-566
Normalized Protein Abundance
9000
8000
7000
TSB
TSB−dex
Media
SA564
SA564−Δ*hptRS*
SA564−Δ*ccpA*
SA564−Δ*hptRS/ccpA*

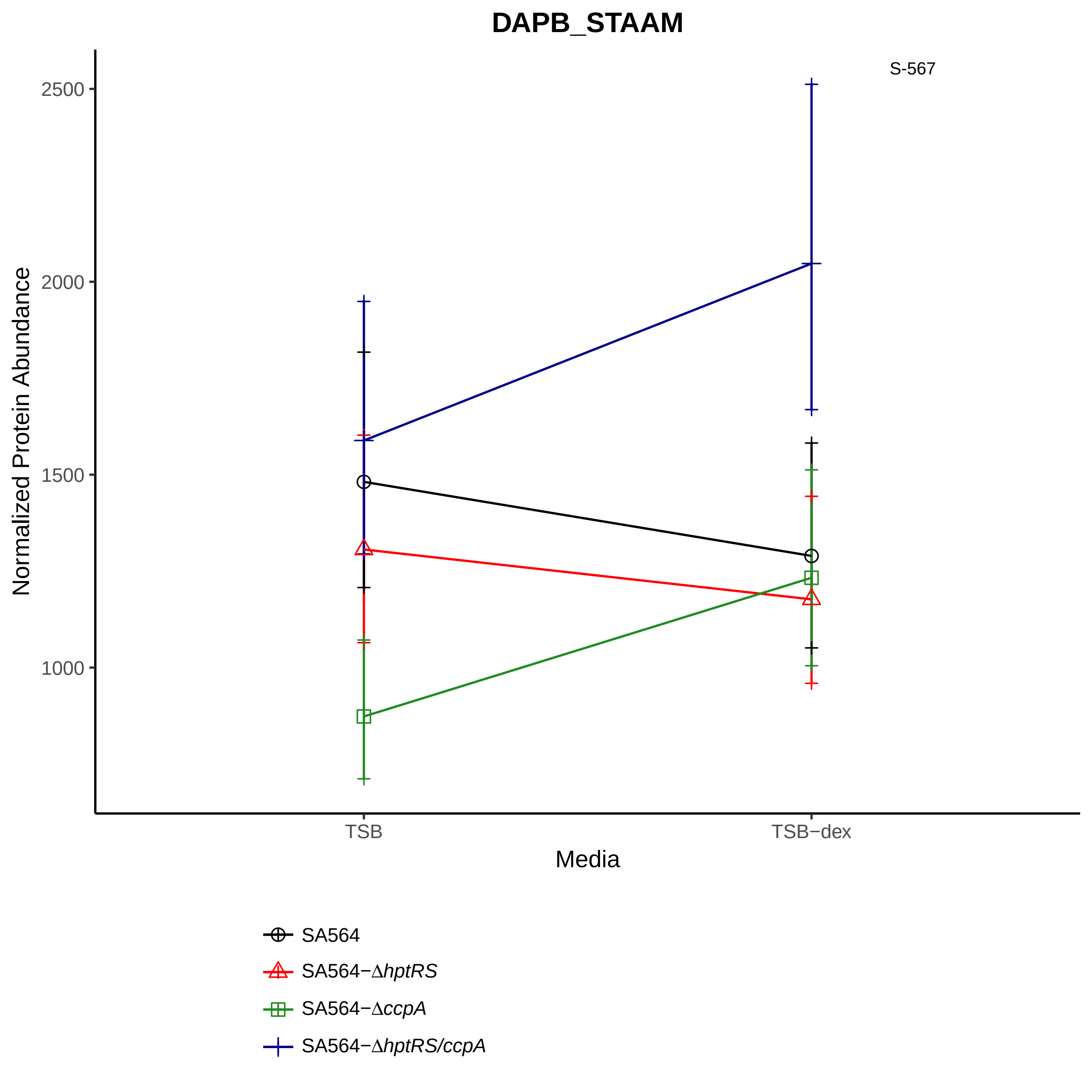

DAPB_STAAM
S-567
Normalized Protein Abundance
2500
2000
1500
1000
TSB
TSB−dex
Media
SA564
SA564−Δ*hptRS*
SA564−Δ*ccpA*
SA564−Δ*hptRS/ccpA*

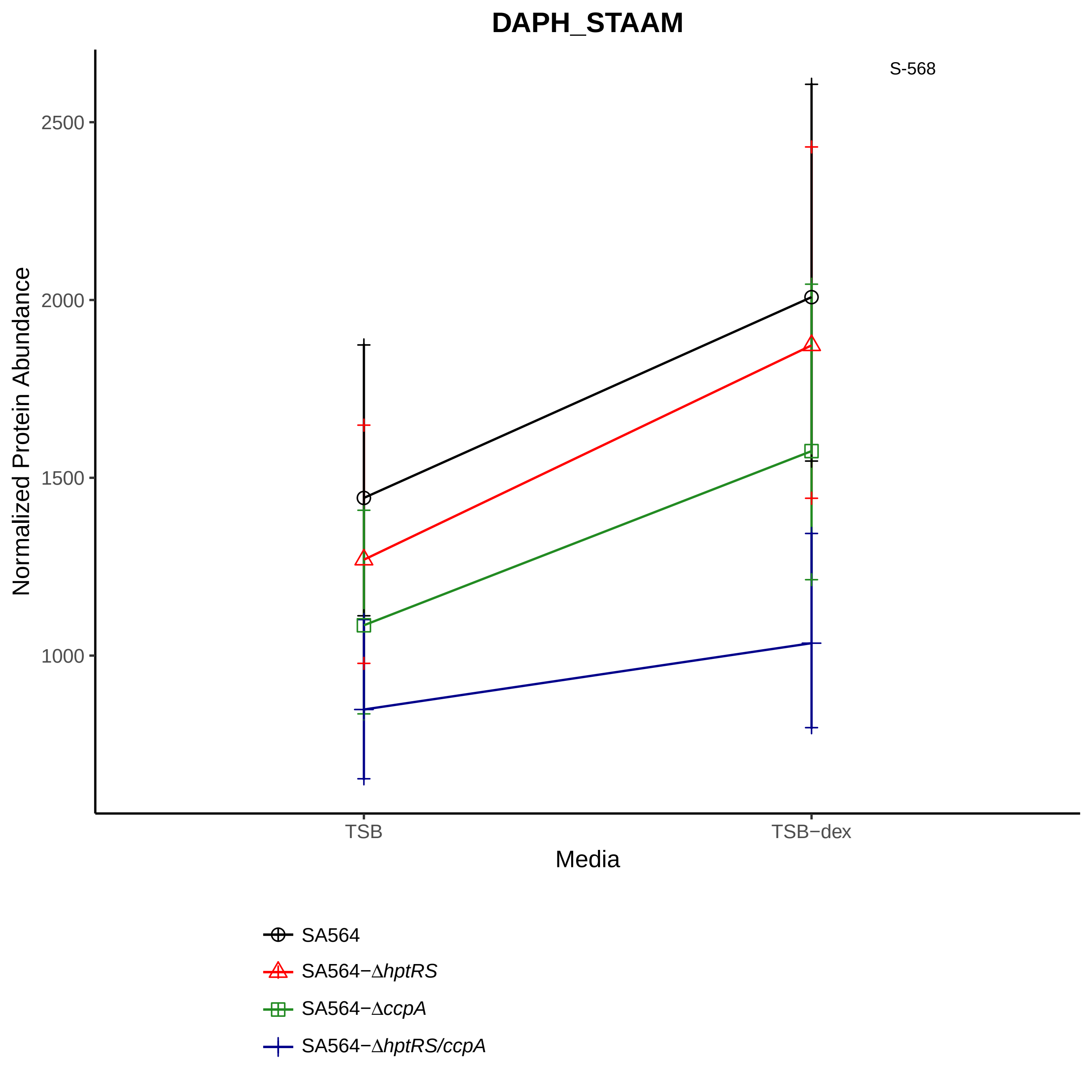
DAPH_STAAM
S-568
Normalized Protein Abundance
2500
2000
1500
1000
TSB
TSB−dex
Media
SA564
SA564−ΔhptRS
SA564−ΔccpA
SA564−ΔhptRS/ccpA

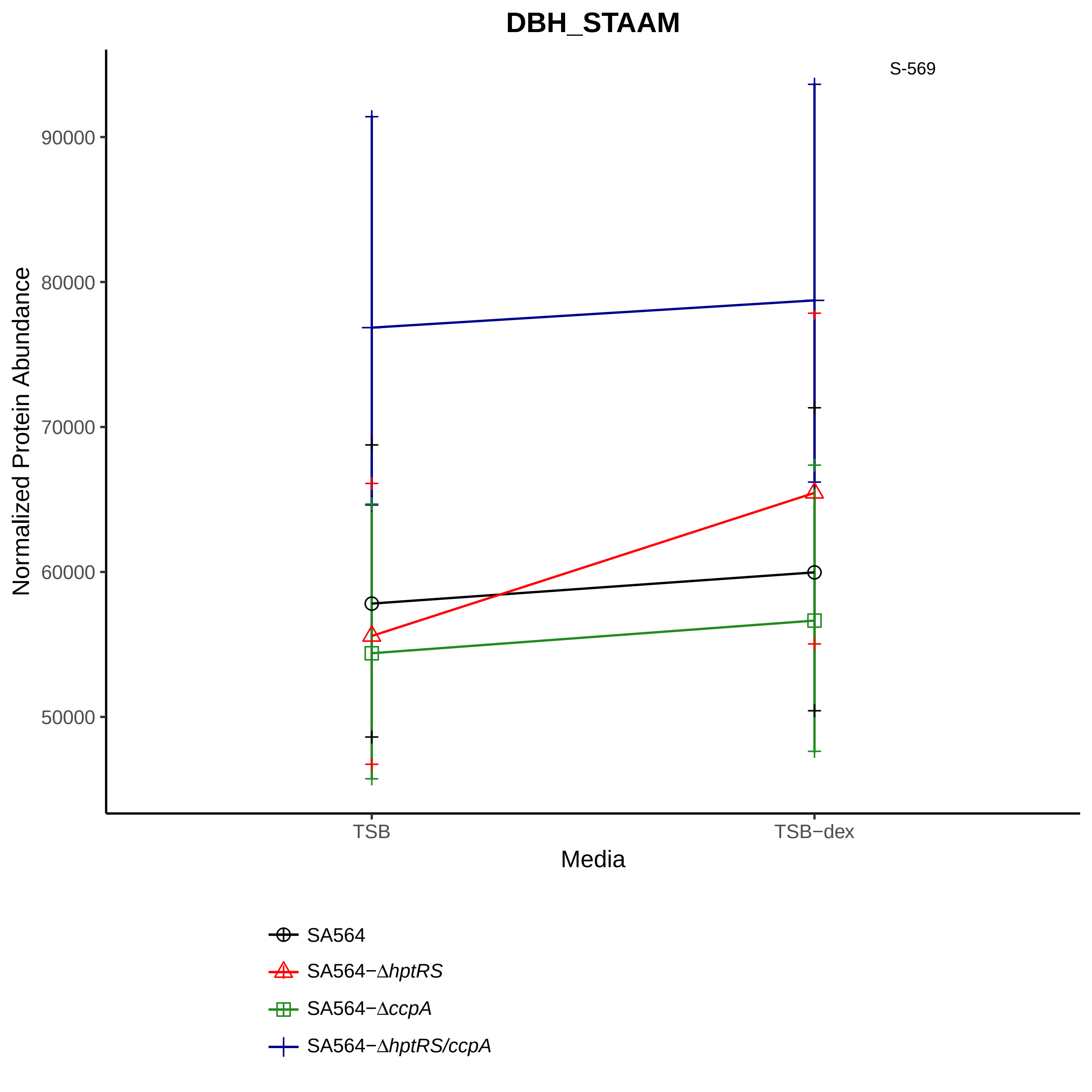
DBH_STAAM
S-569
Normalized Protein Abundance
90000
80000
70000
60000
50000
TSB
TSB−dex
Media
SA564
SA564−ΔhptRS
SA564−ΔccpA
SA564−ΔhptRS/ccpA

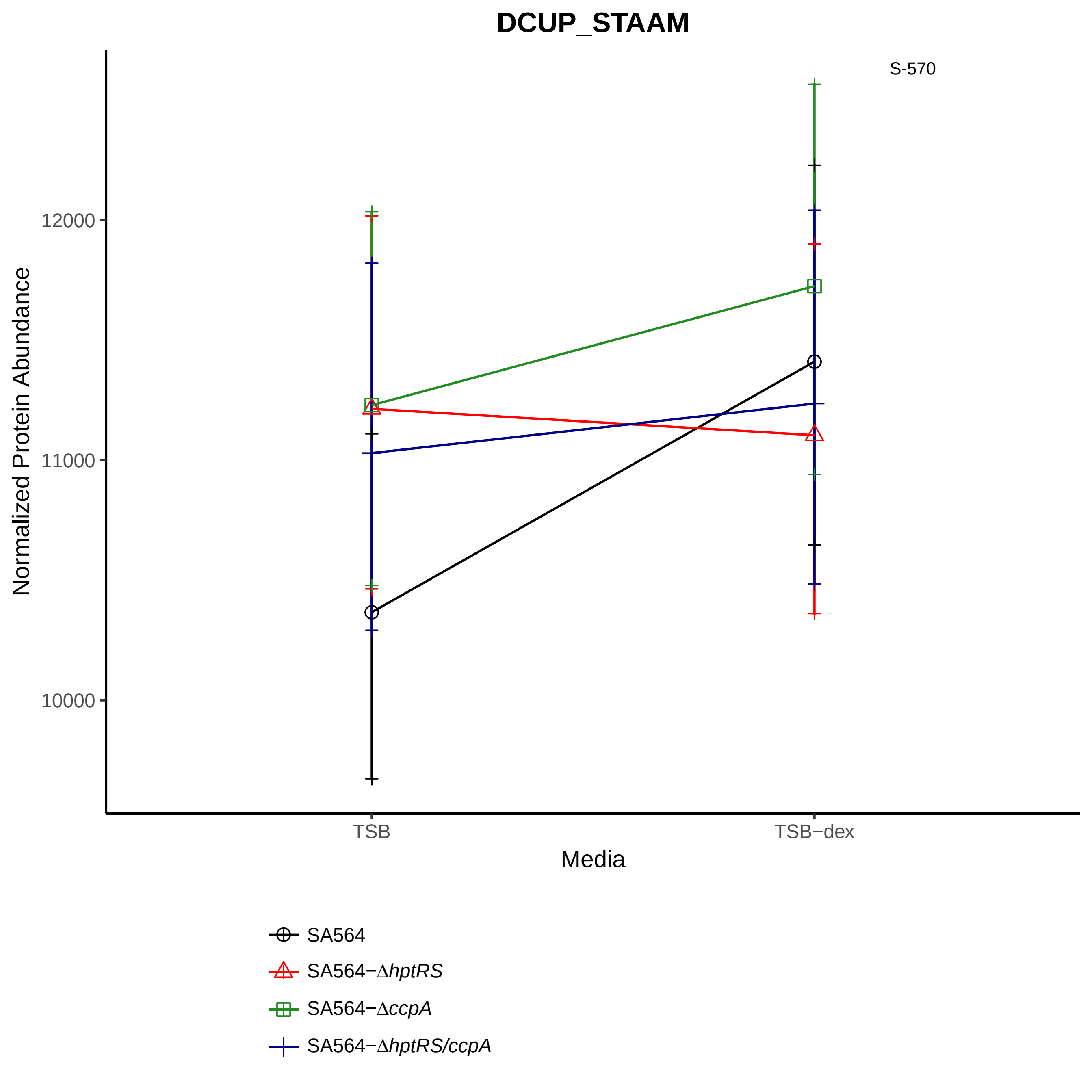
DCUP_STAAM
S-570
Normalized Protein Abundance
12000
11000
10000
TSB
TSB−dex
Media
SA564
SA564−Δ*hptRS*
SA564−Δ*ccpA*
SA564−Δ*hptRS/ccpA*

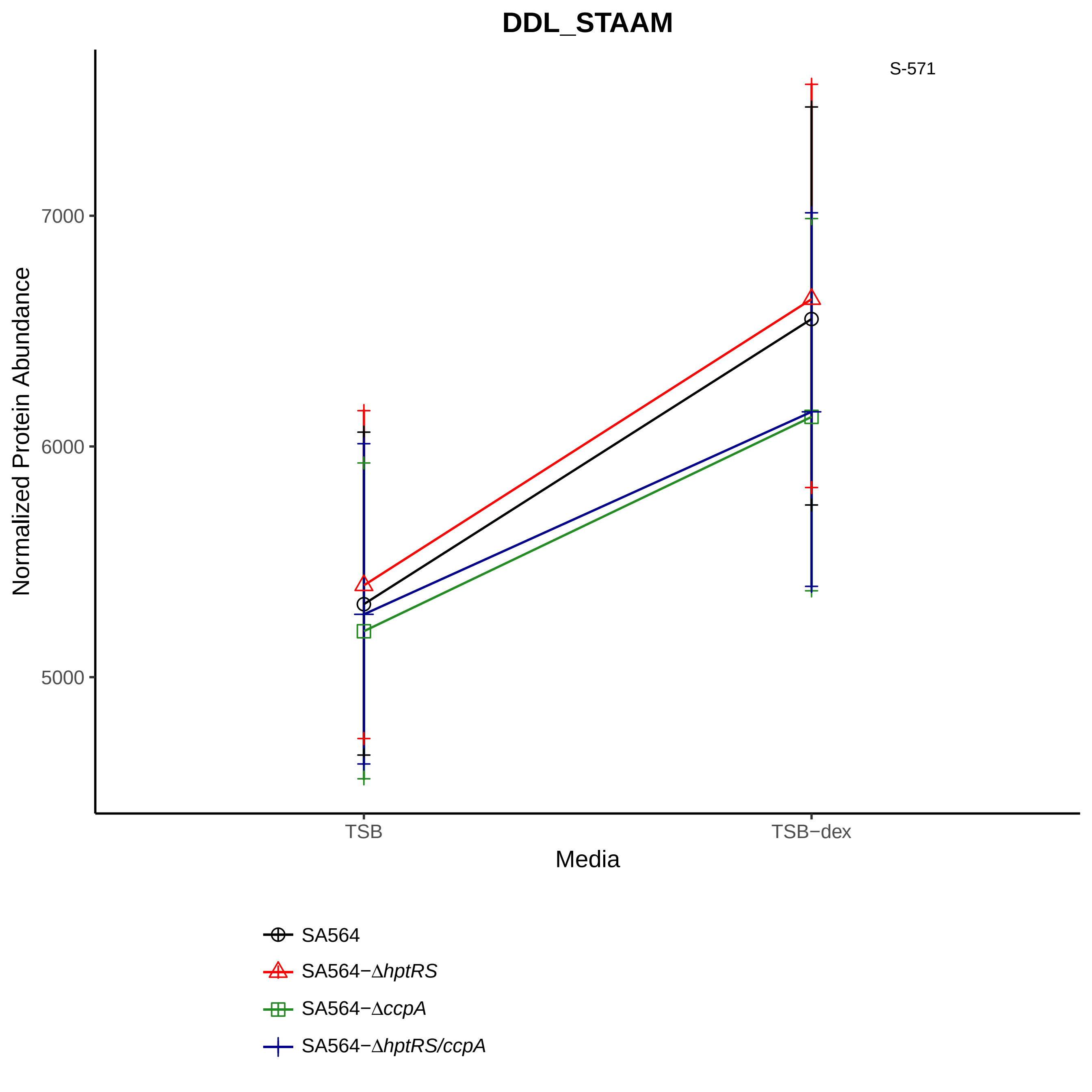

DDL_STAAM
S-571
7000
6000
5000
Normalized Protein Abundance
TSB
TSB−dex
Media
SA564
SA564−Δ*hptRS*
SA564−Δ*ccpA*
SA564−Δ*hptRS/ccpA*

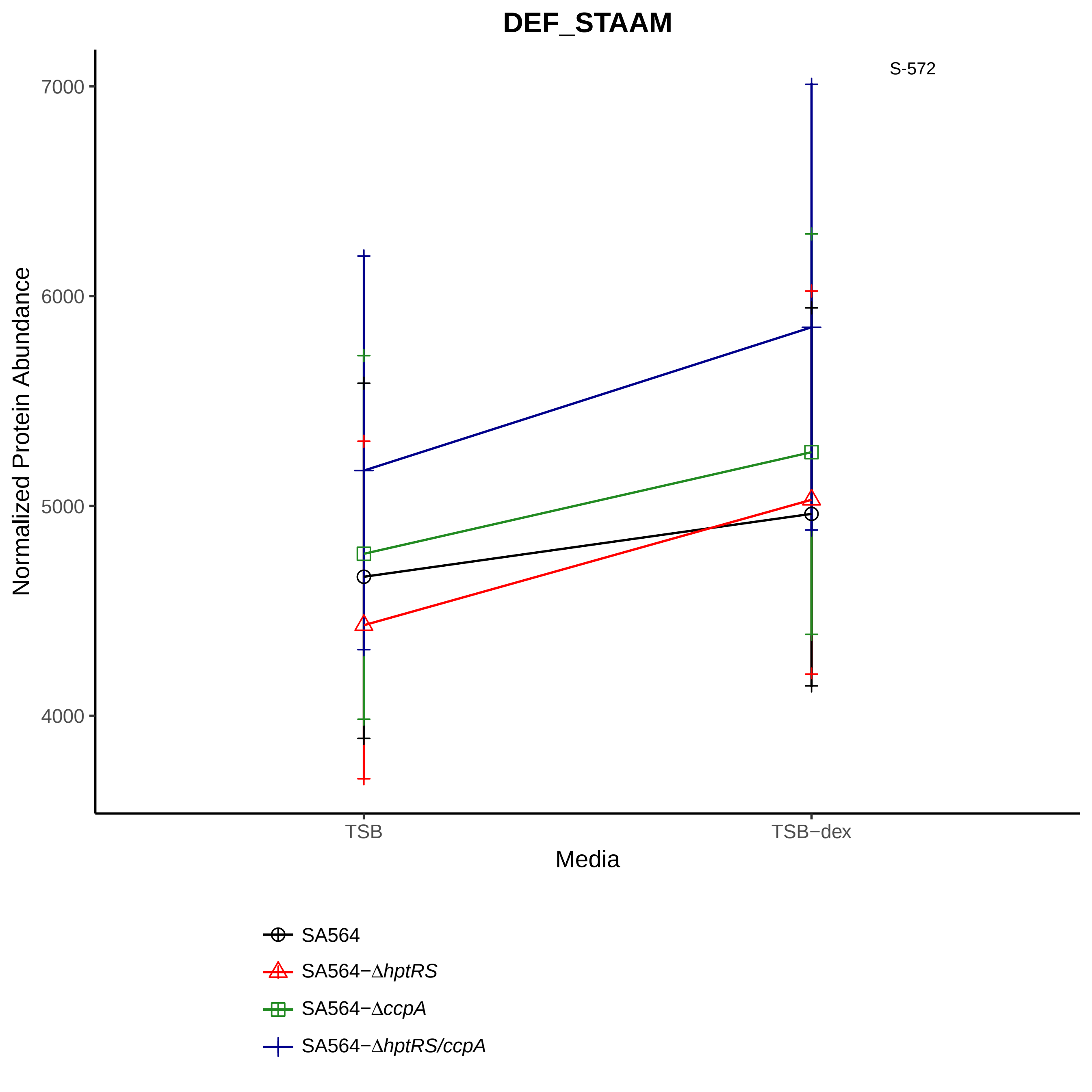

DEF_STAAM
S-572
Normalized Protein Abundance
7000
6000
5000
4000
TSB
TSB−dex
Media
SA564
SA564−Δ*hptRS*
SA564−Δ*ccpA*
SA564−Δ*hptRS/ccpA*

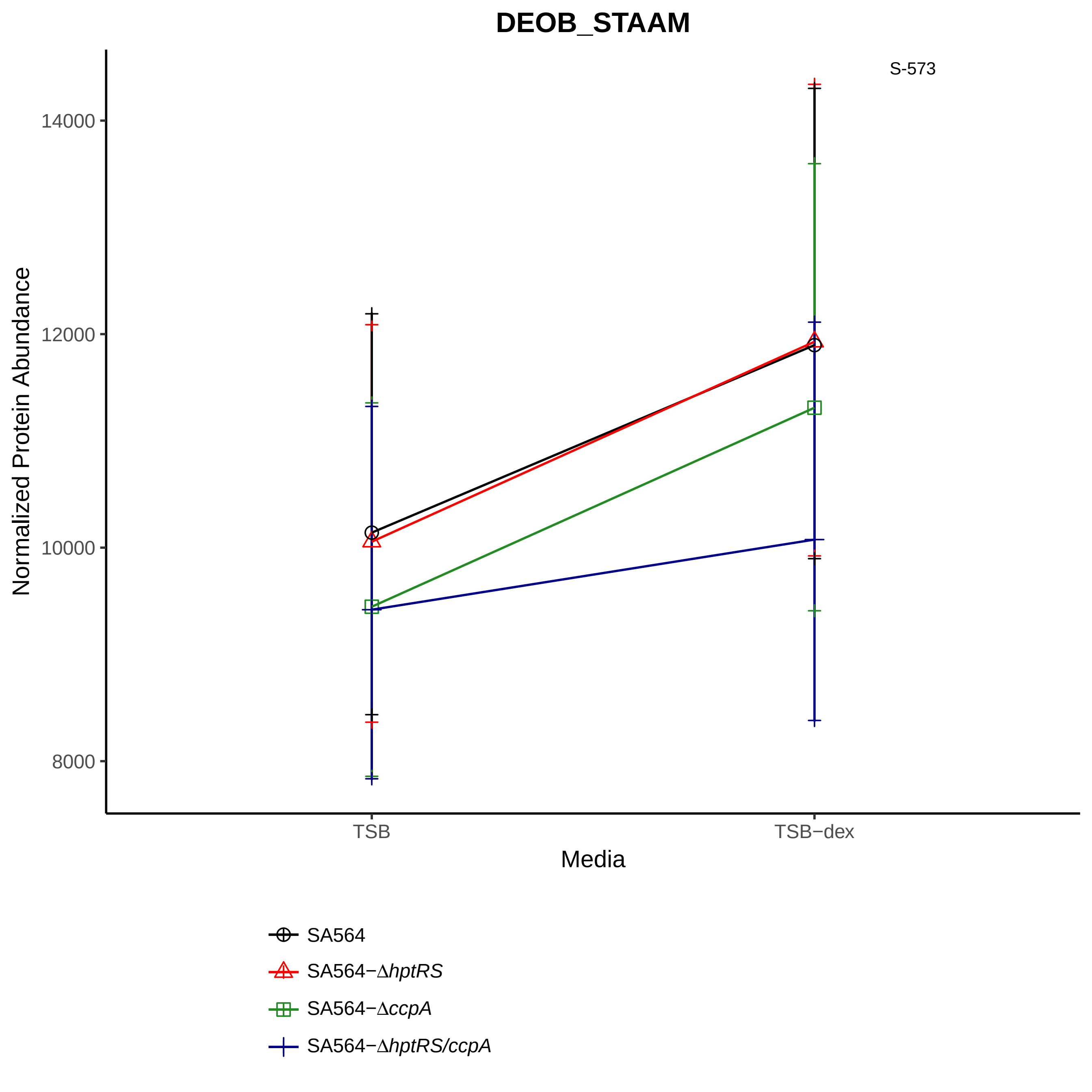

DEOB_STAAM
S-573
Normalized Protein Abundance
14000
12000
10000
8000
TSB
TSB−dex
Media
SA564
SA564−Δ*hptRS*
SA564−Δ*ccpA*
SA564−Δ*hptRS/ccpA*

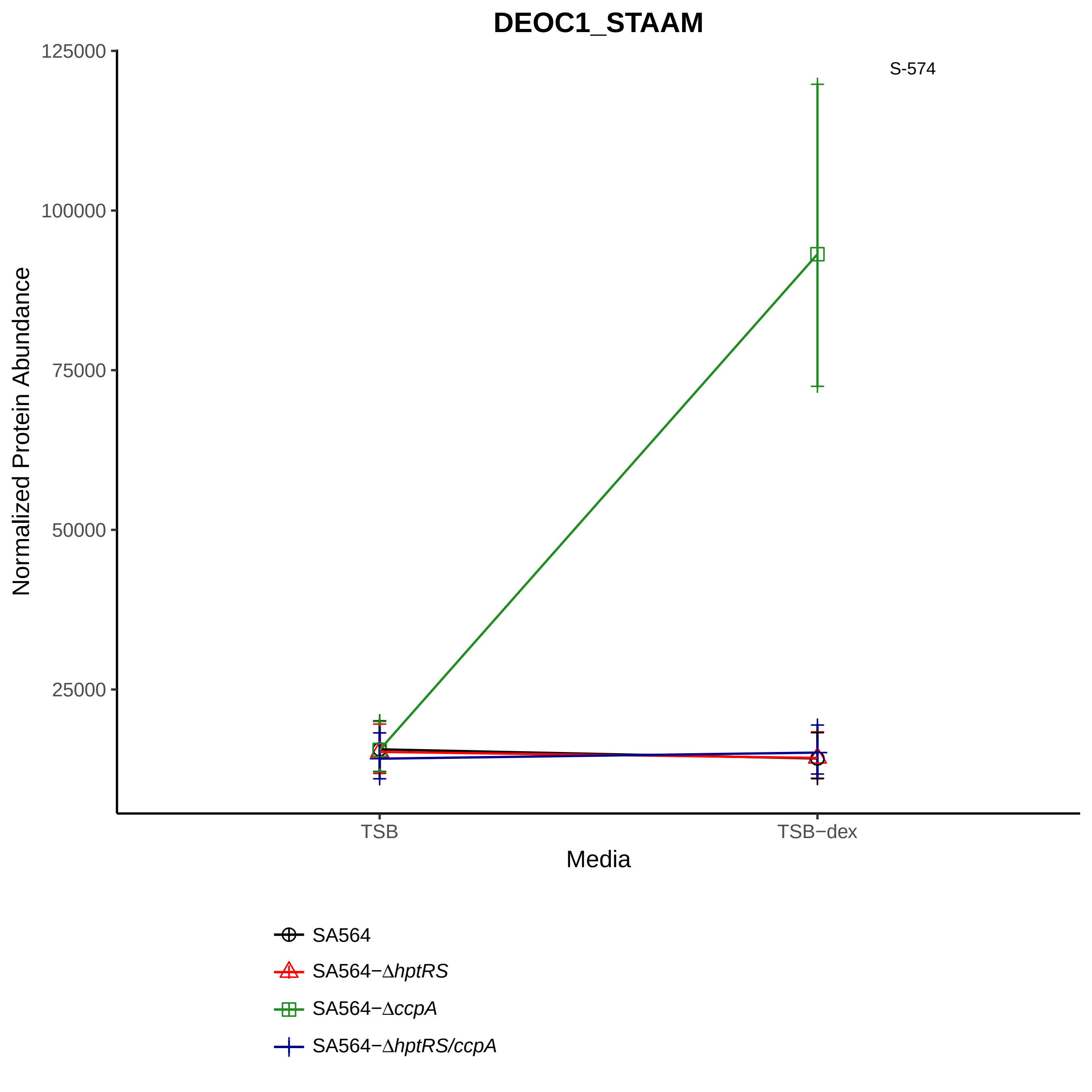

DEOC1_STAAM
S-574
125000
100000
75000
50000
25000
Normalized Protein Abundance
TSB
TSB−dex
Media
SA564
SA564−Δ*hptRS*
SA564−Δ*ccpA*
SA564−Δ*hptRS/ccpA*

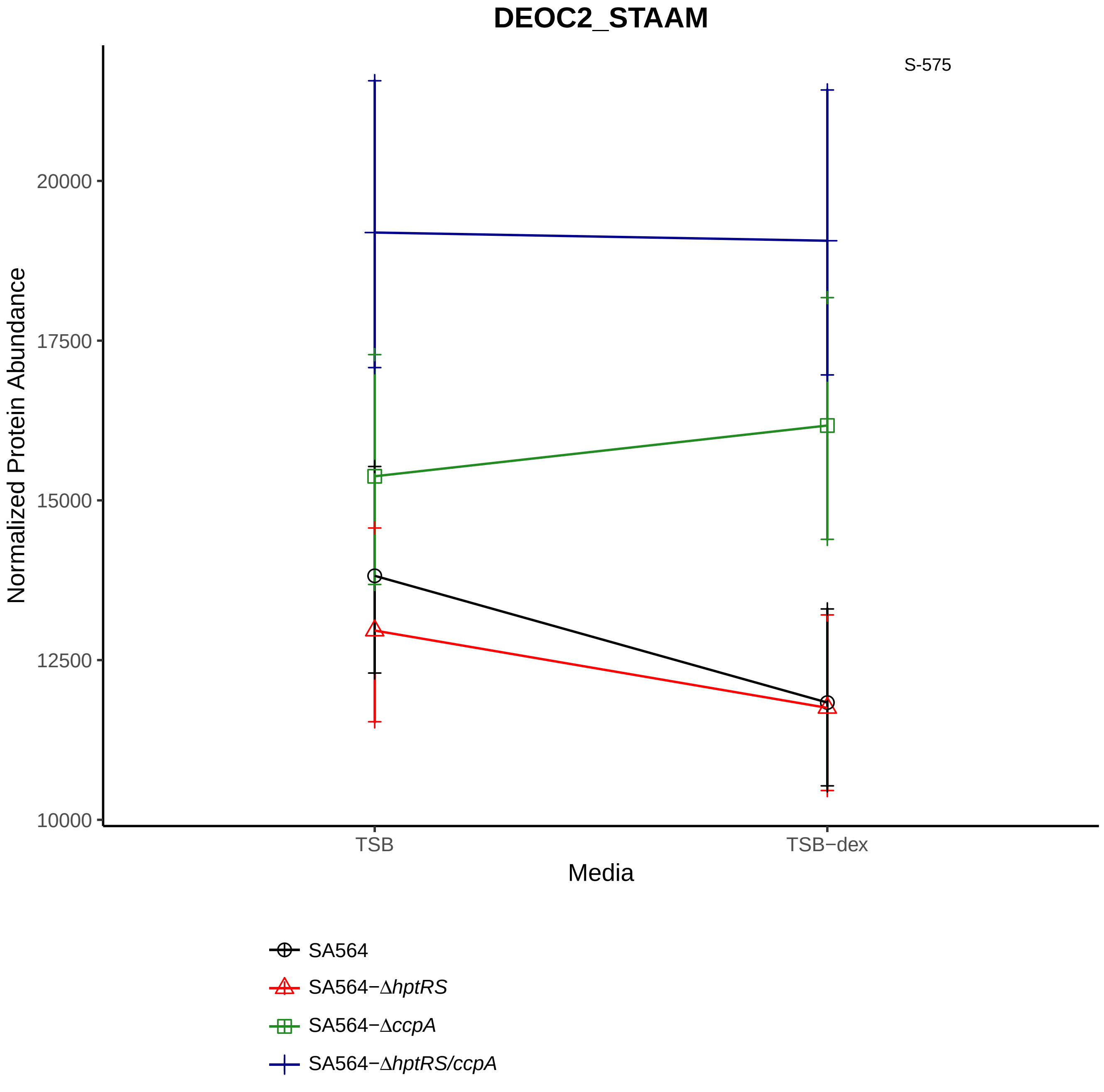

DEOC2_STAAM
S-575
Normalized Protein Abundance
20000
17500
15000
12500
10000
TSB
TSB−dex
Media
SA564
SA564−Δ*hptRS*
SA564−Δ*ccpA*
SA564−Δ*hptRS/ccpA*

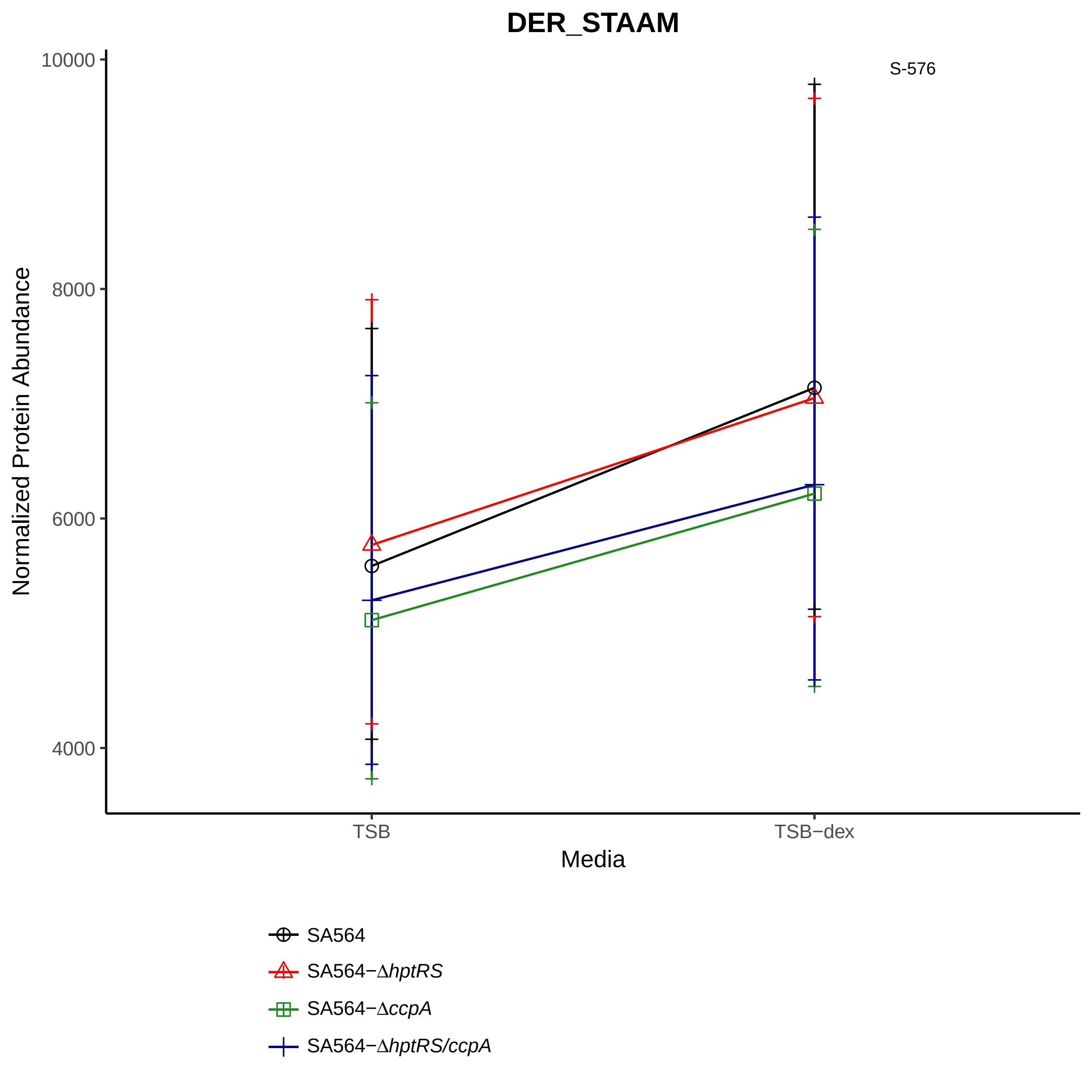

DER_STAAM
S-576
Normalized Protein Abundance
10000
8000
6000
4000
TSB
TSB−dex
Media
SA564
SA564−Δ*hptRS*
SA564−Δ*ccpA*
SA564−Δ*hptRS/ccpA*

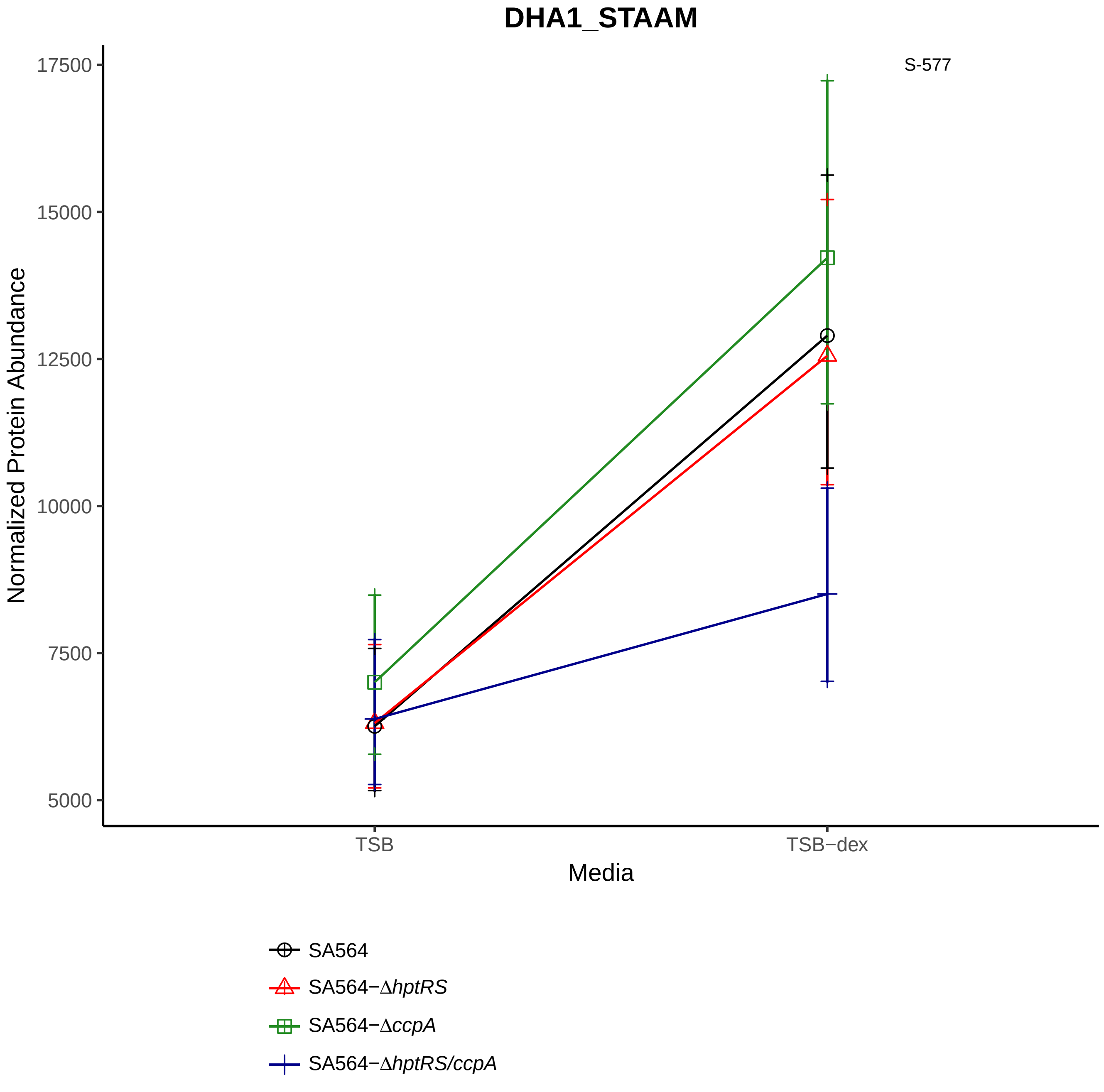
DHA1_STAAM
S-577
Normalized Protein Abundance
17500
15000
12500
10000
7500
5000
TSB
TSB−dex
Media
SA564
SA564−Δ*hptRS*
SA564−Δ*ccpA*
SA564−Δ*hptRS/ccpA*

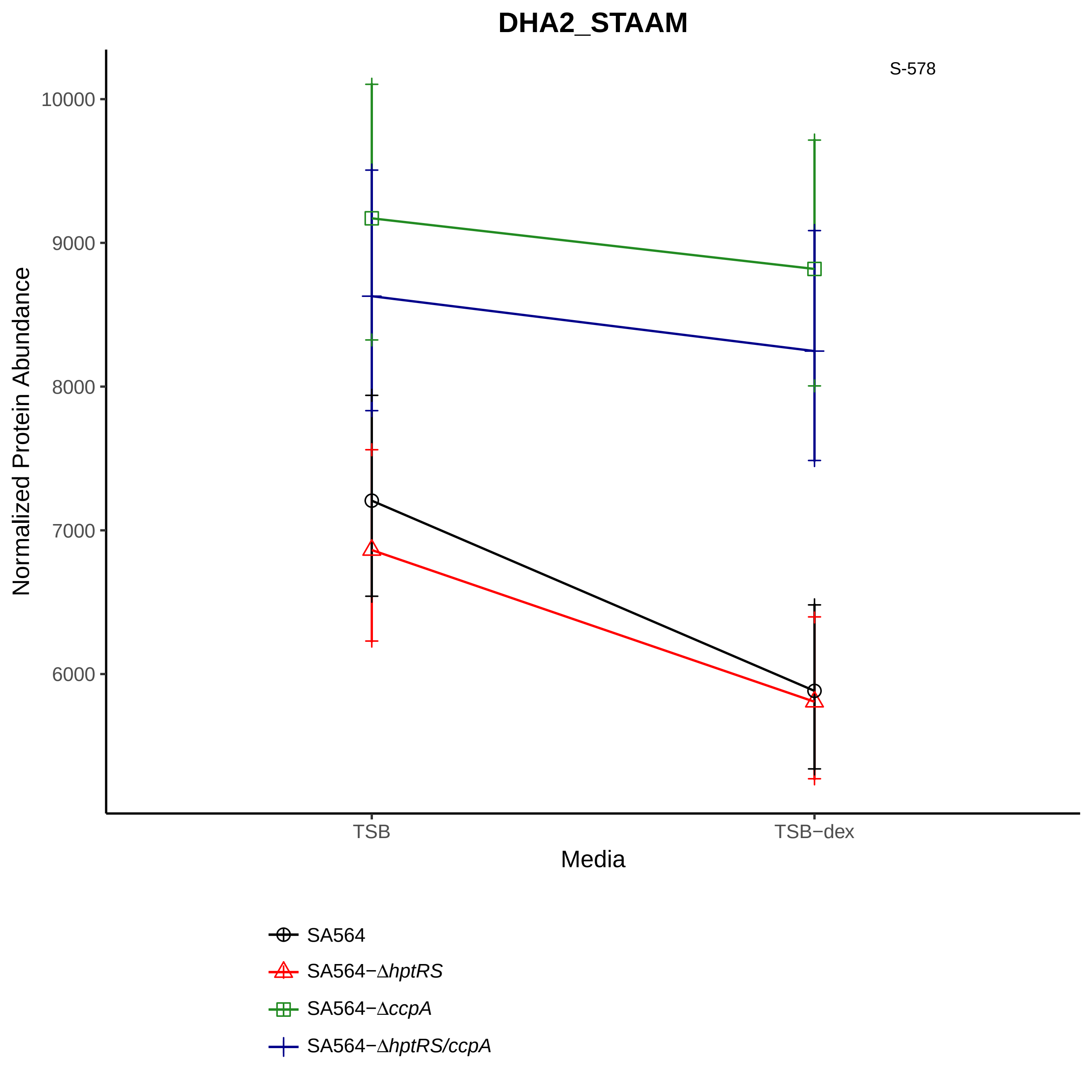
DHA2_STAAM
S-578
Normalized Protein Abundance
10000
9000
8000
7000
6000
TSB
TSB−dex
Media
SA564
SA564−ΔhptRS
SA564−ΔccpA
SA564−ΔhptRS/ccpA

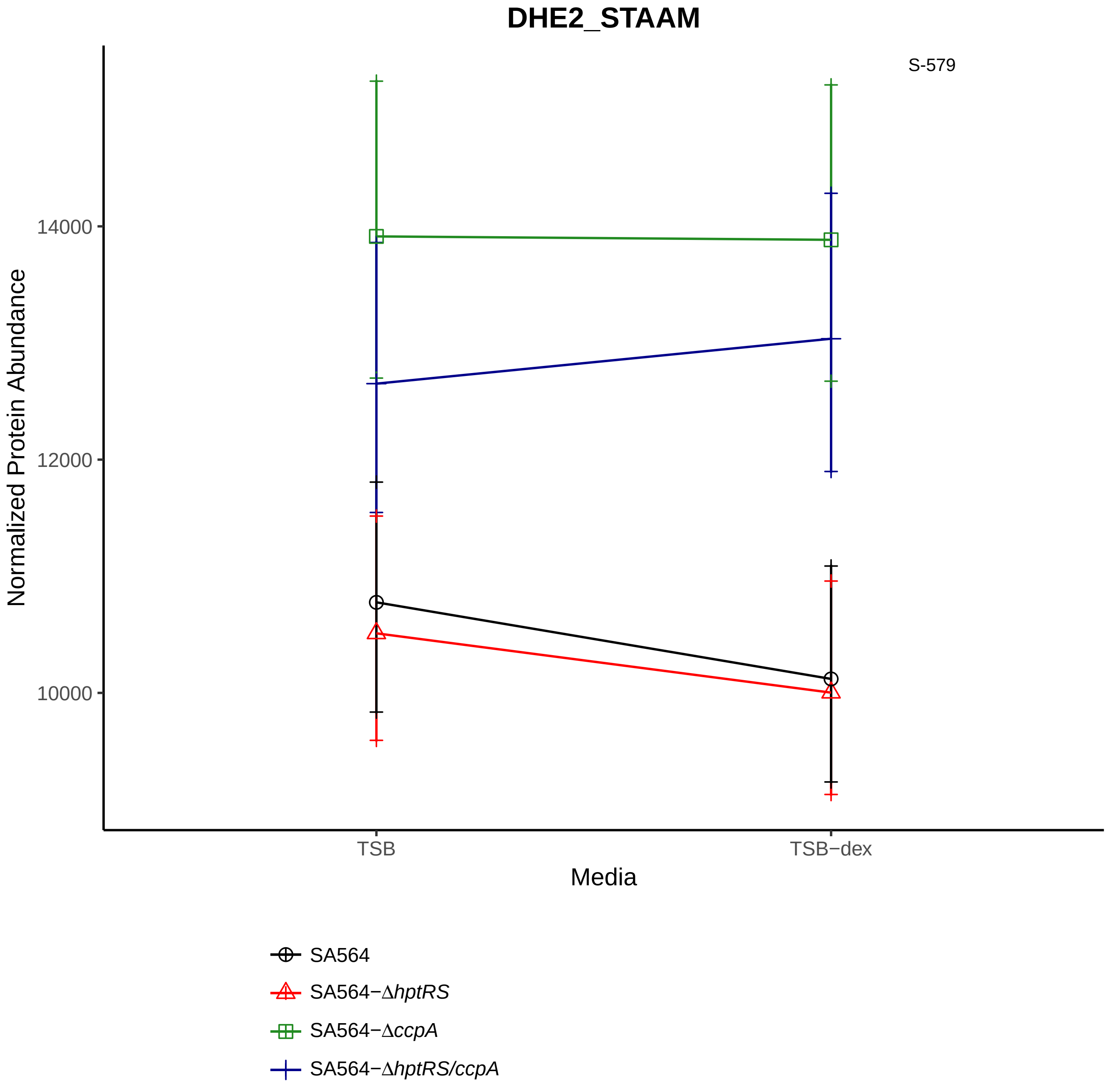

DHE2_STAAM
S-579
Normalized Protein Abundance
14000
12000
10000
TSB
TSB−dex
Media
SA564
SA564−Δ*hptRS*
SA564−Δ*ccpA*
SA564−Δ*hptRS/ccpA*

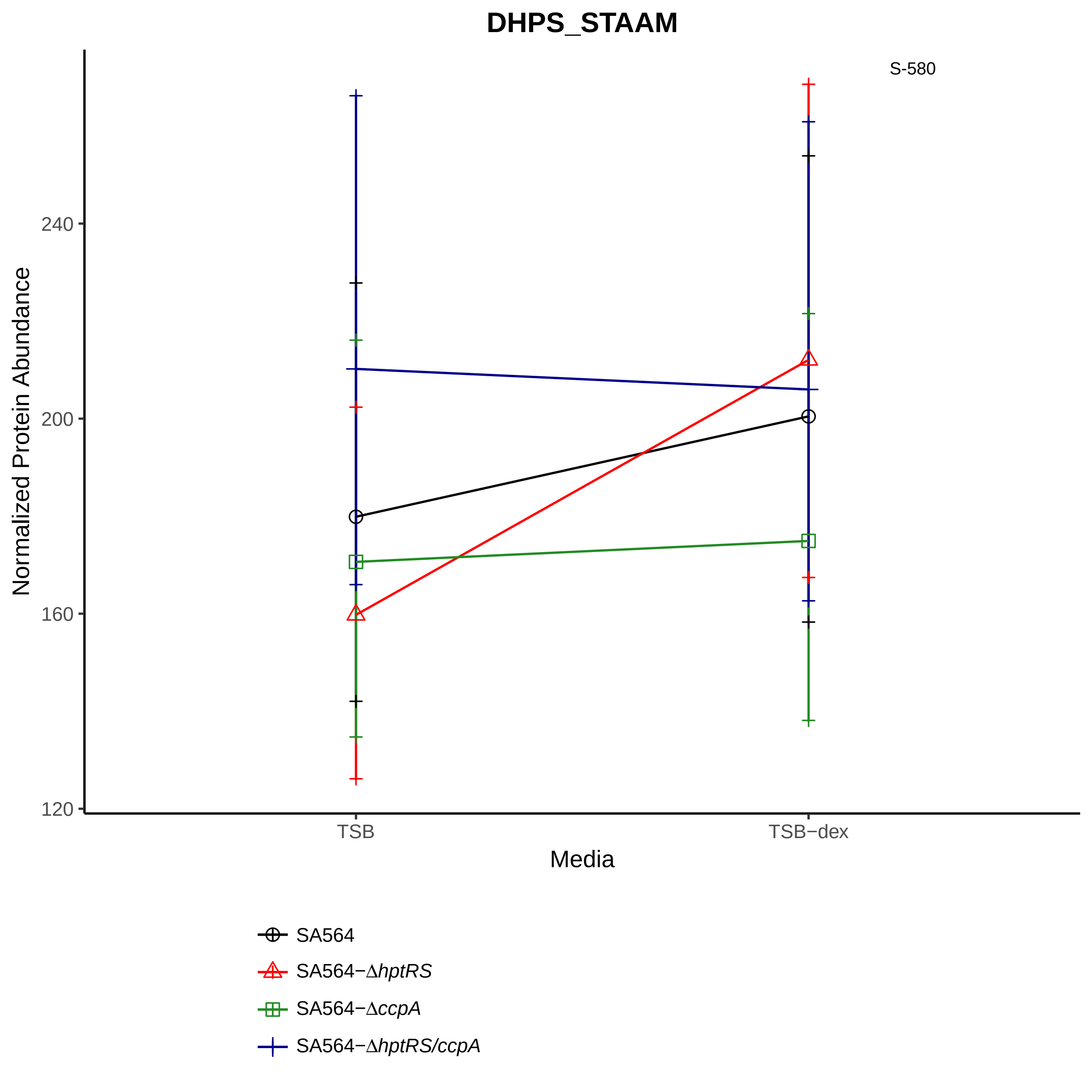
DHPS_STAAM
S-580
Normalized Protein Abundance
240
200
160
120
TSB
TSB−dex
Media
SA564
SA564−ΔhptRS
SA564−ΔccpA
SA564−ΔhptRS/ccpA

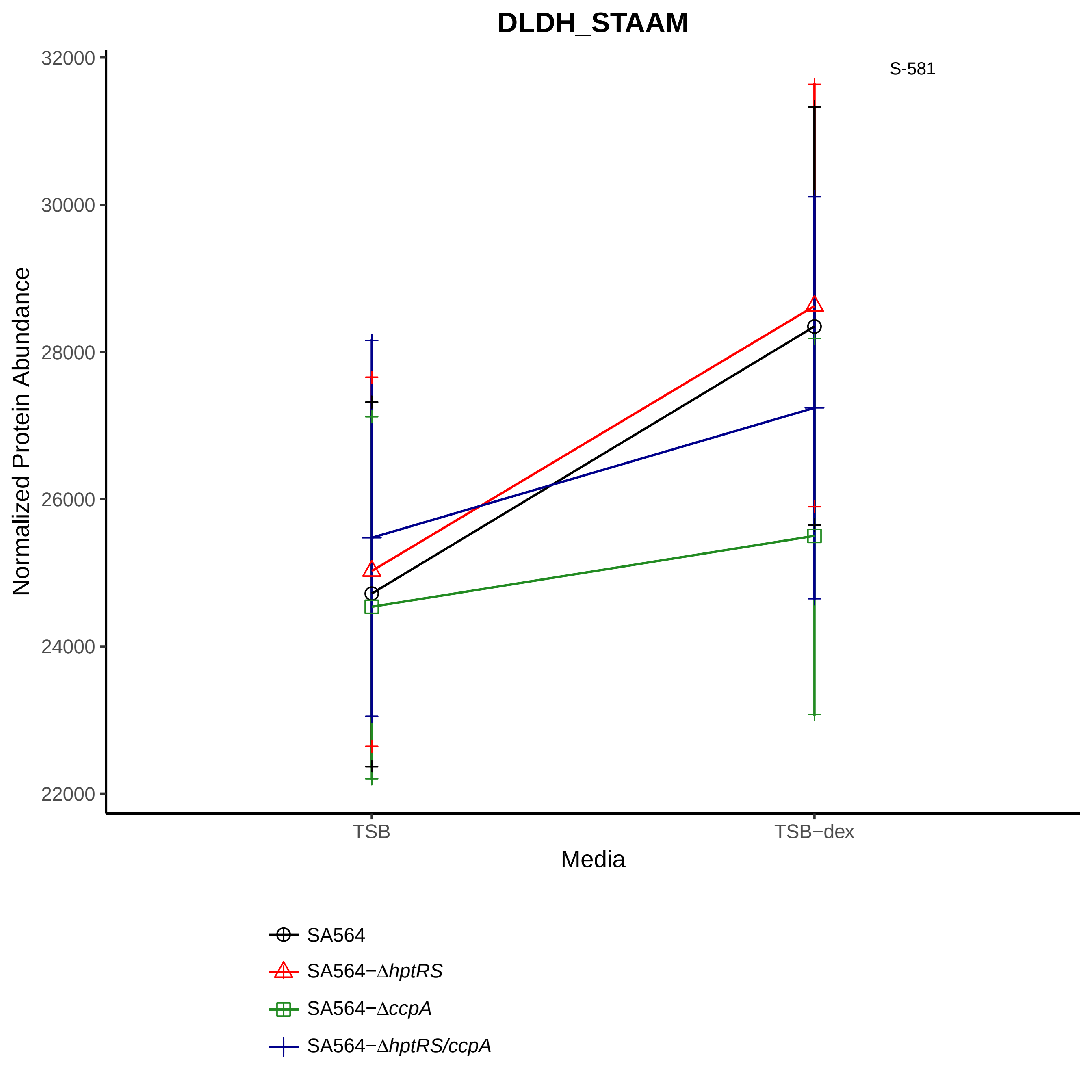

DLDH_STAAM
S-581
Normalized Protein Abundance
32000
30000
28000
26000
24000
22000
TSB
TSB−dex
Media
SA564
SA564−Δ*hptRS*
SA564−Δ*ccpA*
SA564−Δ*hptRS/ccpA*

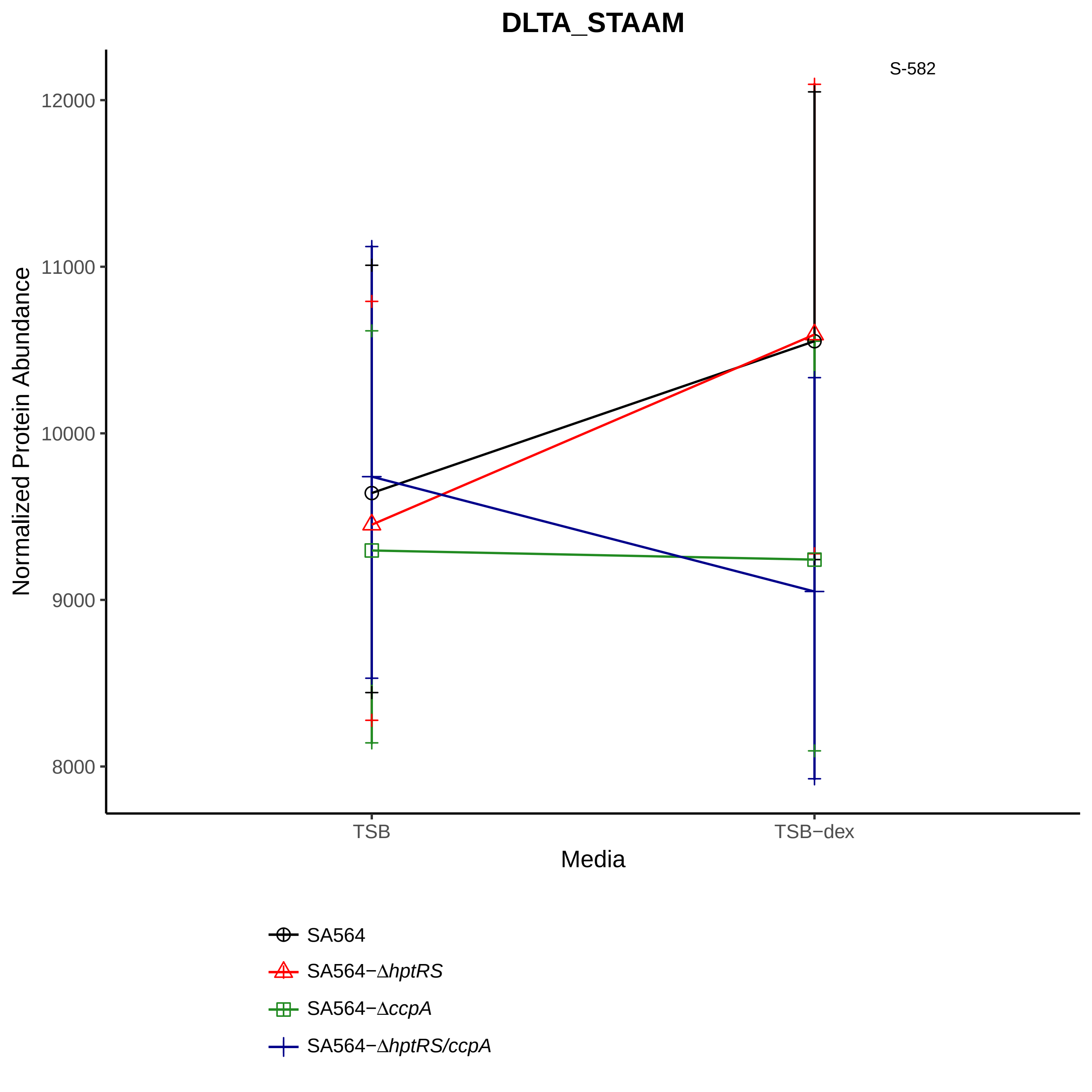

DLTA_STAAM
S-582
Normalized Protein Abundance
12000
11000
10000
9000
8000
TSB
TSB−dex
Media
SA564
SA564−ΔhptRS
SA564−ΔccpA
SA564−ΔhptRS/ccpA

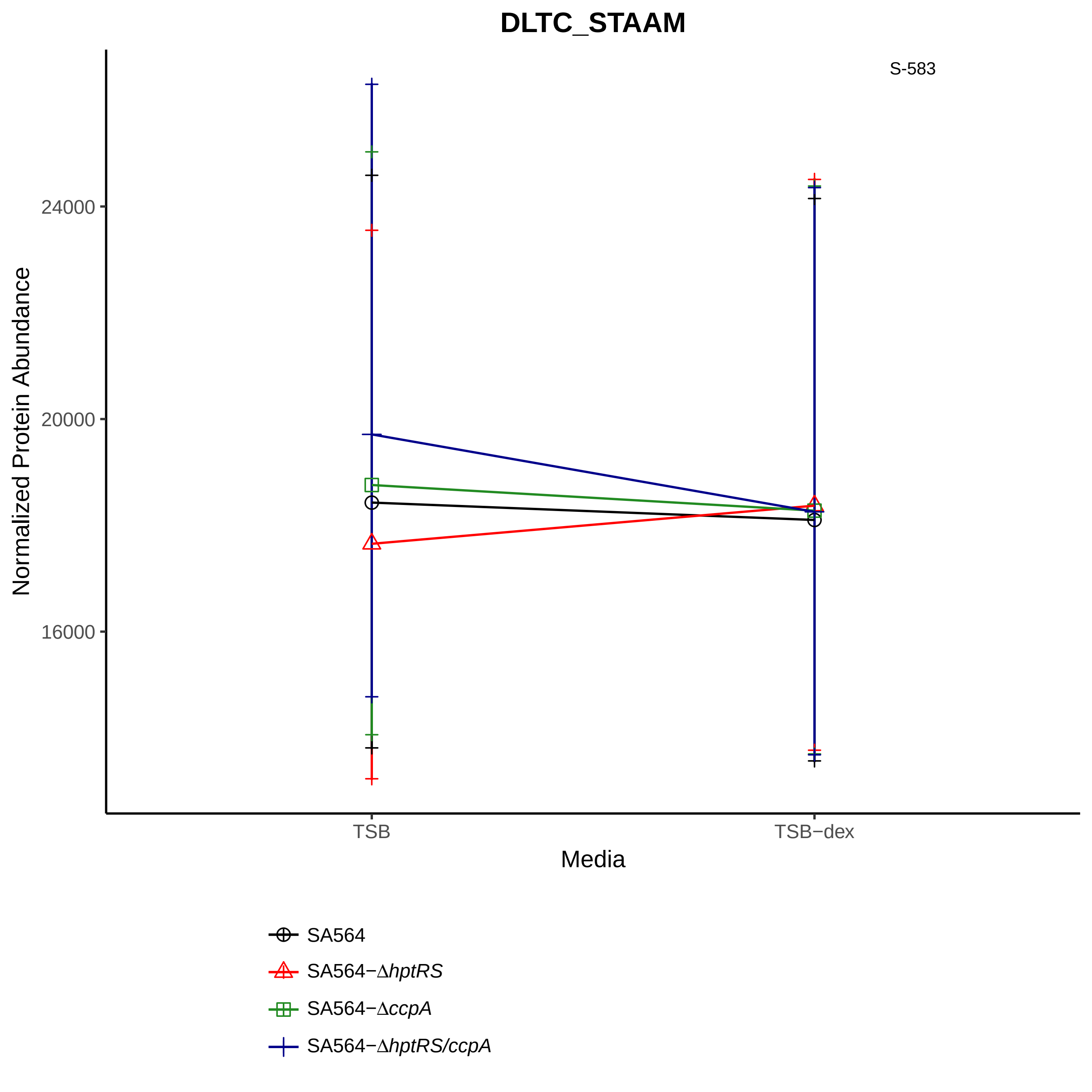
DLTC_STAAM
S-583
Normalized Protein Abundance
24000
20000
16000
TSB
TSB−dex
Media
SA564
SA564−ΔhptRS
SA564−ΔccpA
SA564−ΔhptRS/ccpA

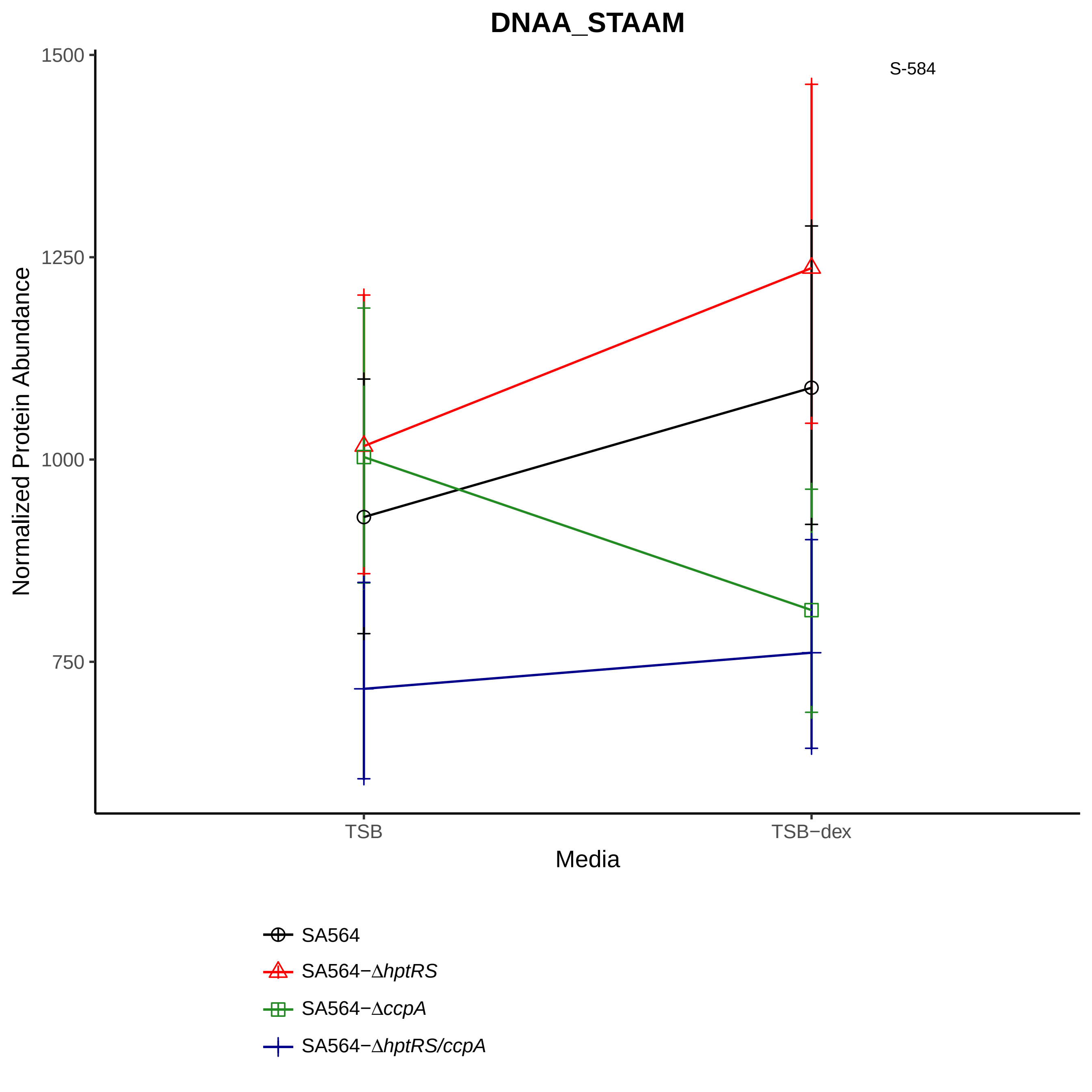

DNAA_STAAM
S-584
1500
1250
1000
750
Normalized Protein Abundance
TSB
TSB−dex
Media
SA564
SA564−Δ*hptRS*
SA564−Δ*ccpA*
SA564−Δ*hptRS/ccpA*

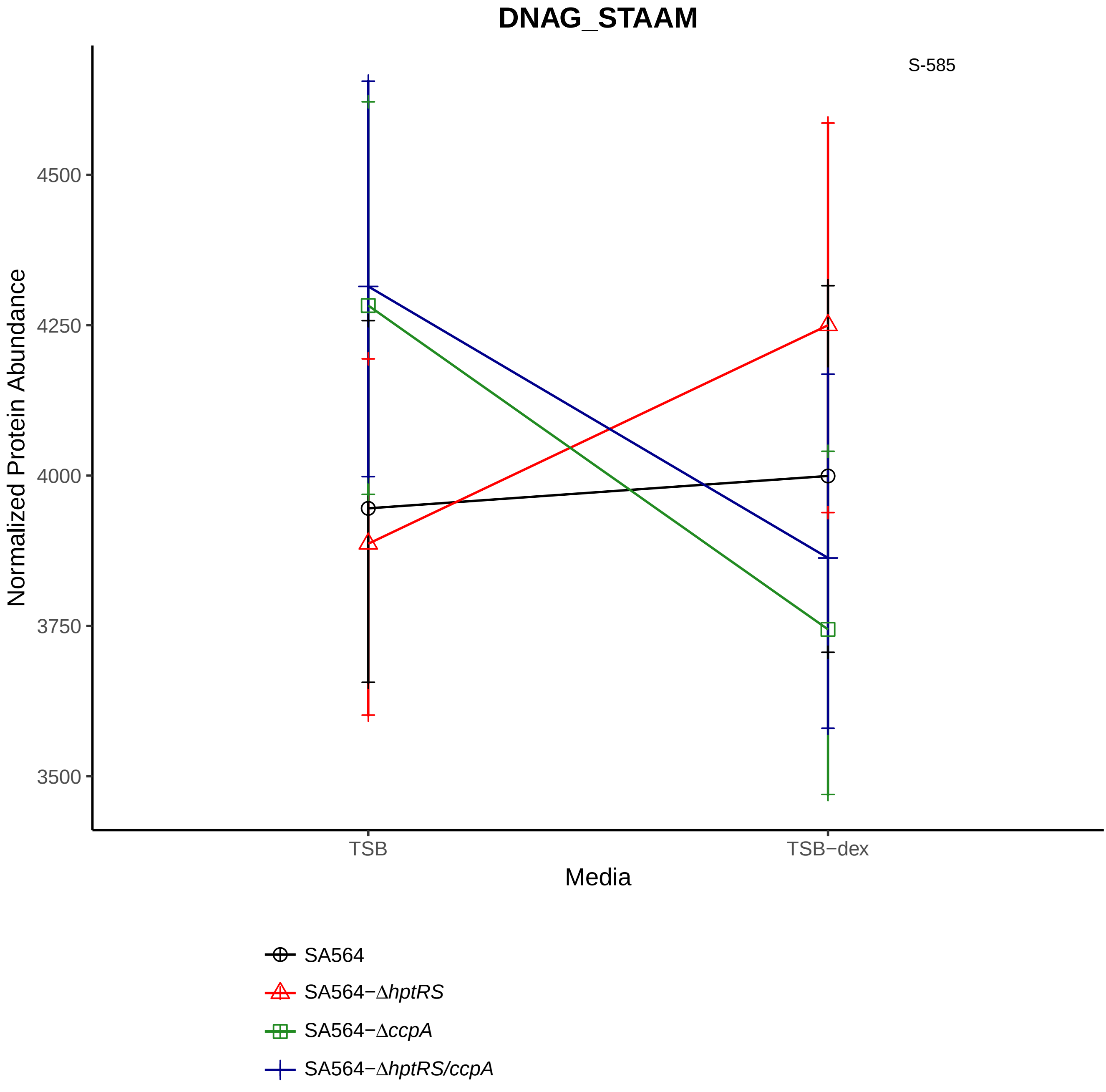

DNAG_STAAM
S-585
Normalized Protein Abundance
4500
4250
4000
3750
3500
TSB
TSB−dex
Media
SA564
SA564−Δ*hptRS*
SA564−Δ*ccpA*
SA564−Δ*hptRS/ccpA*

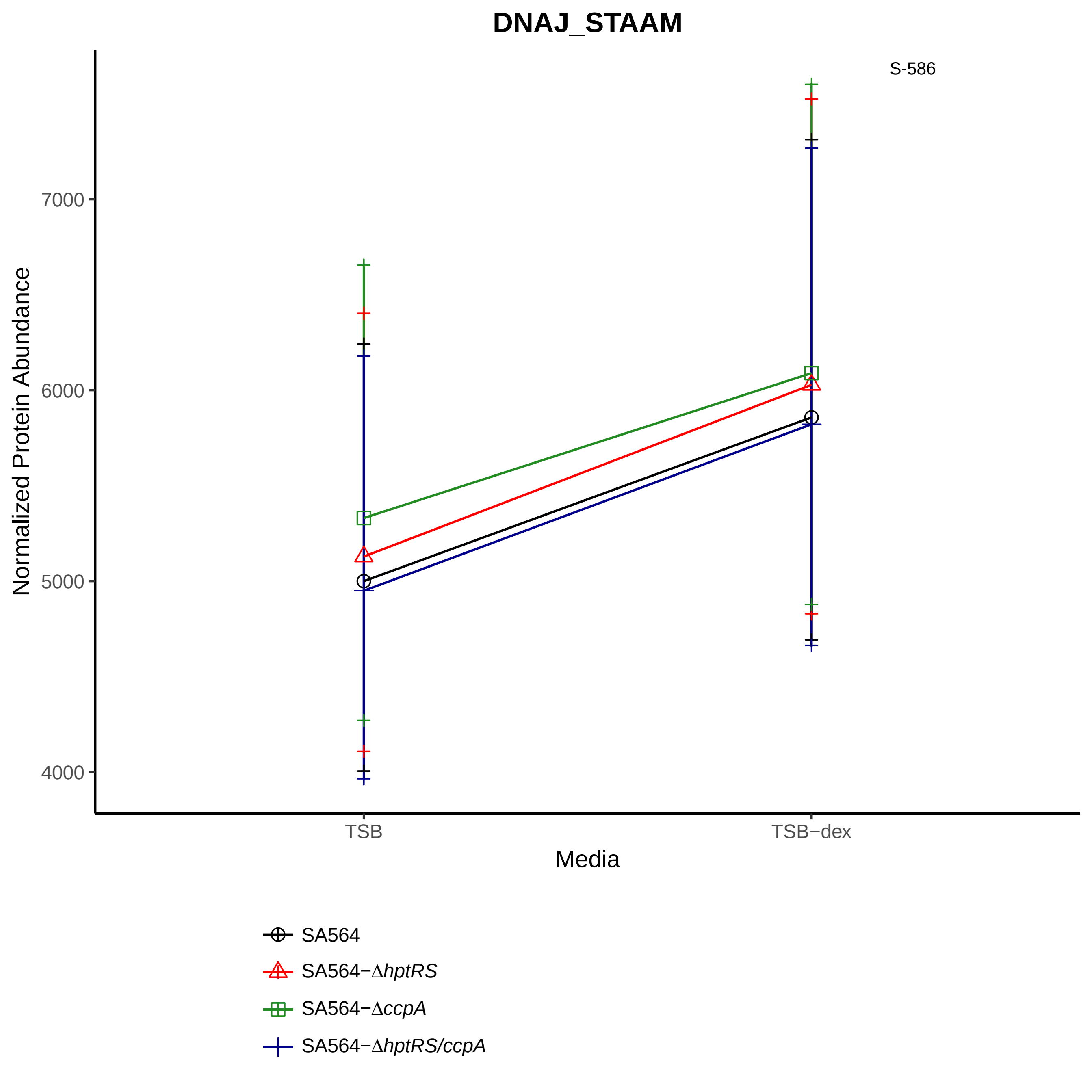

DNAJ_STAAM
S-586
Normalized Protein Abundance
7000
6000
5000
4000
TSB
TSB−dex
Media
SA564
SA564−Δ*hptRS*
SA564−Δ*ccpA*
SA564−Δ*hptRS/ccpA*

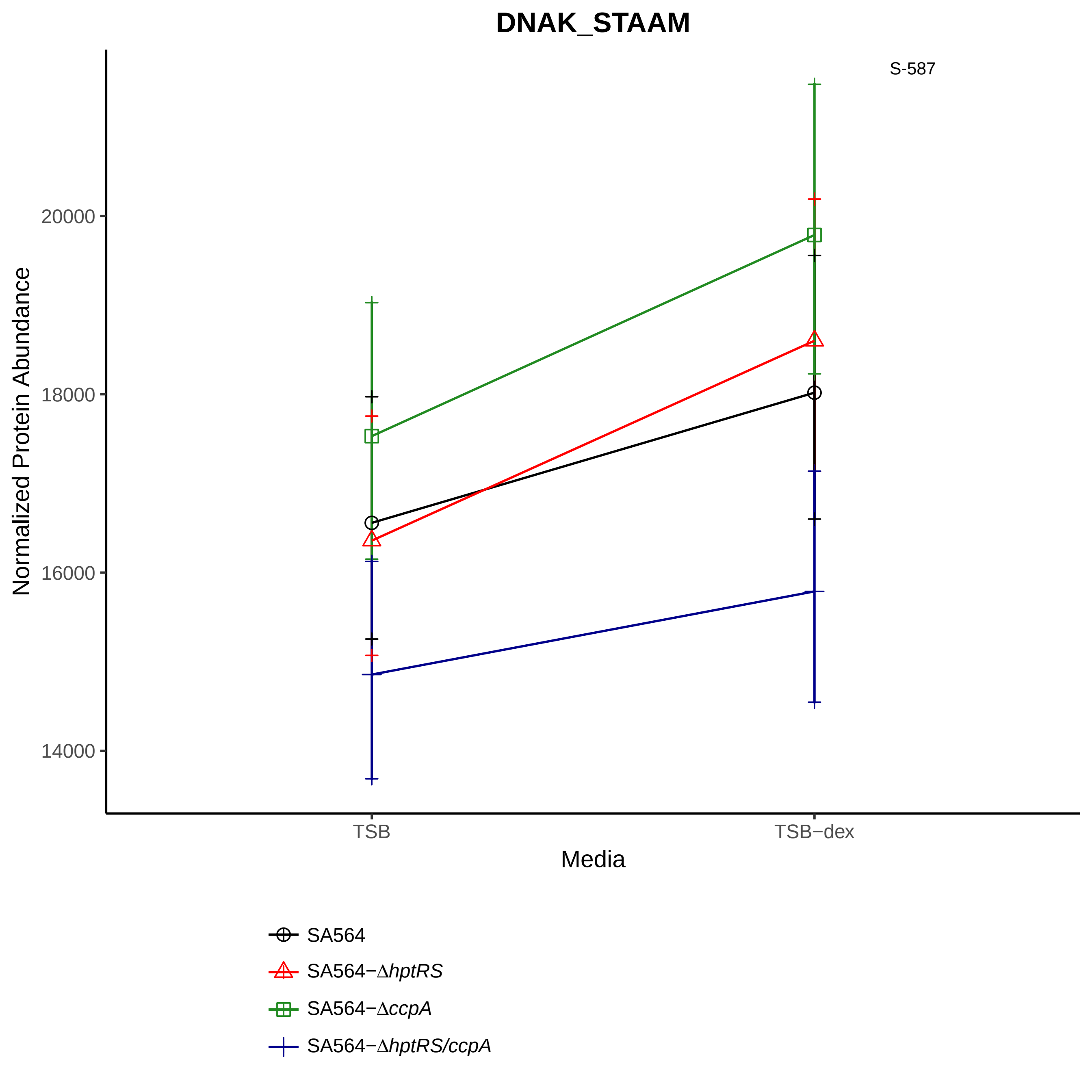

DNAK_STAAM
S-587
Normalized Protein Abundance
20000
18000
16000
14000
TSB
TSB−dex
Media
SA564
SA564−ΔhptRS
SA564−ΔccpA
SA564−ΔhptRS/ccpA

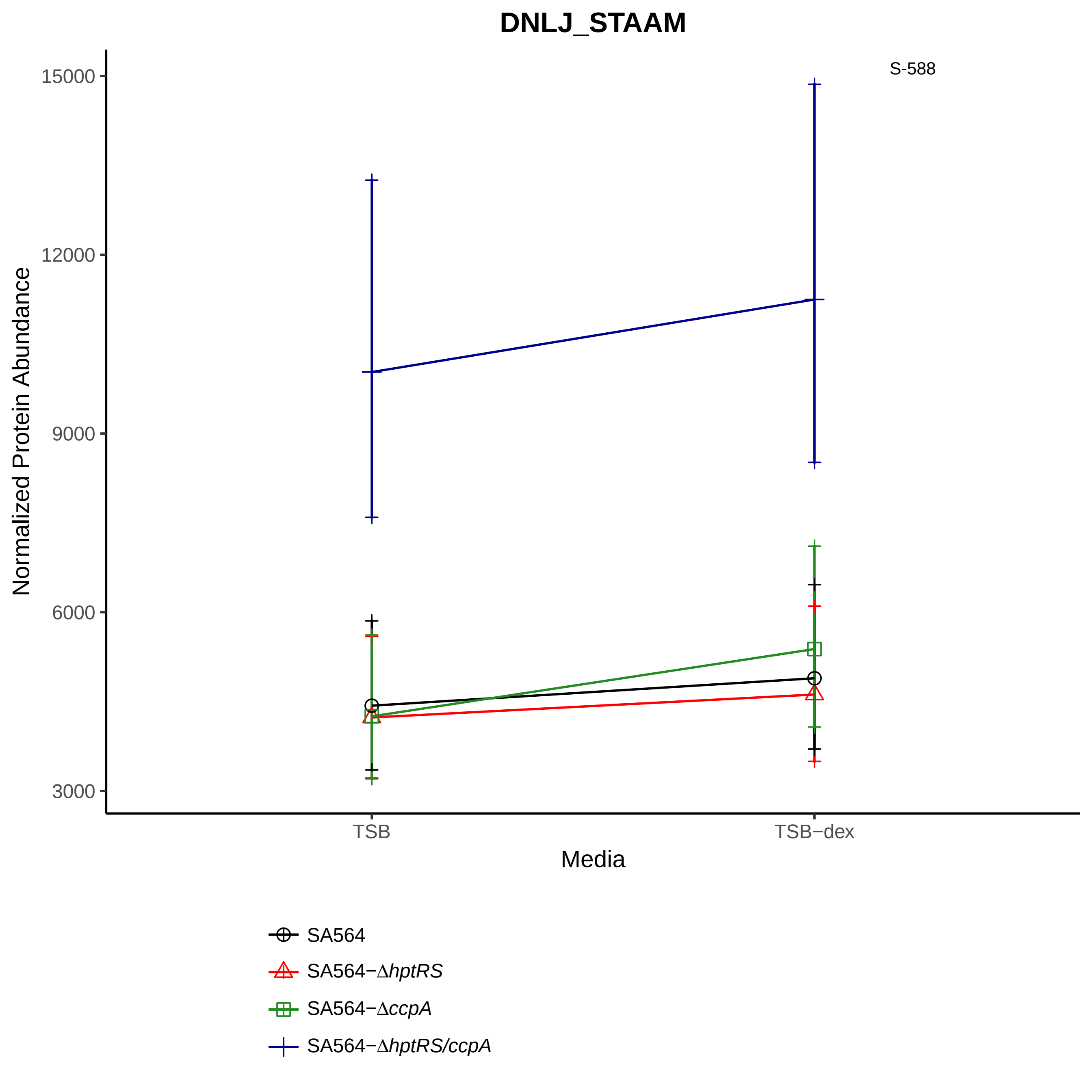

DNLJ_STAAM
S-588
Normalized Protein Abundance
15000
12000
9000
6000
3000
TSB
TSB−dex
Media
SA564
SA564−ΔhptRS
SA564−ΔccpA
SA564−ΔhptRS/ccpA

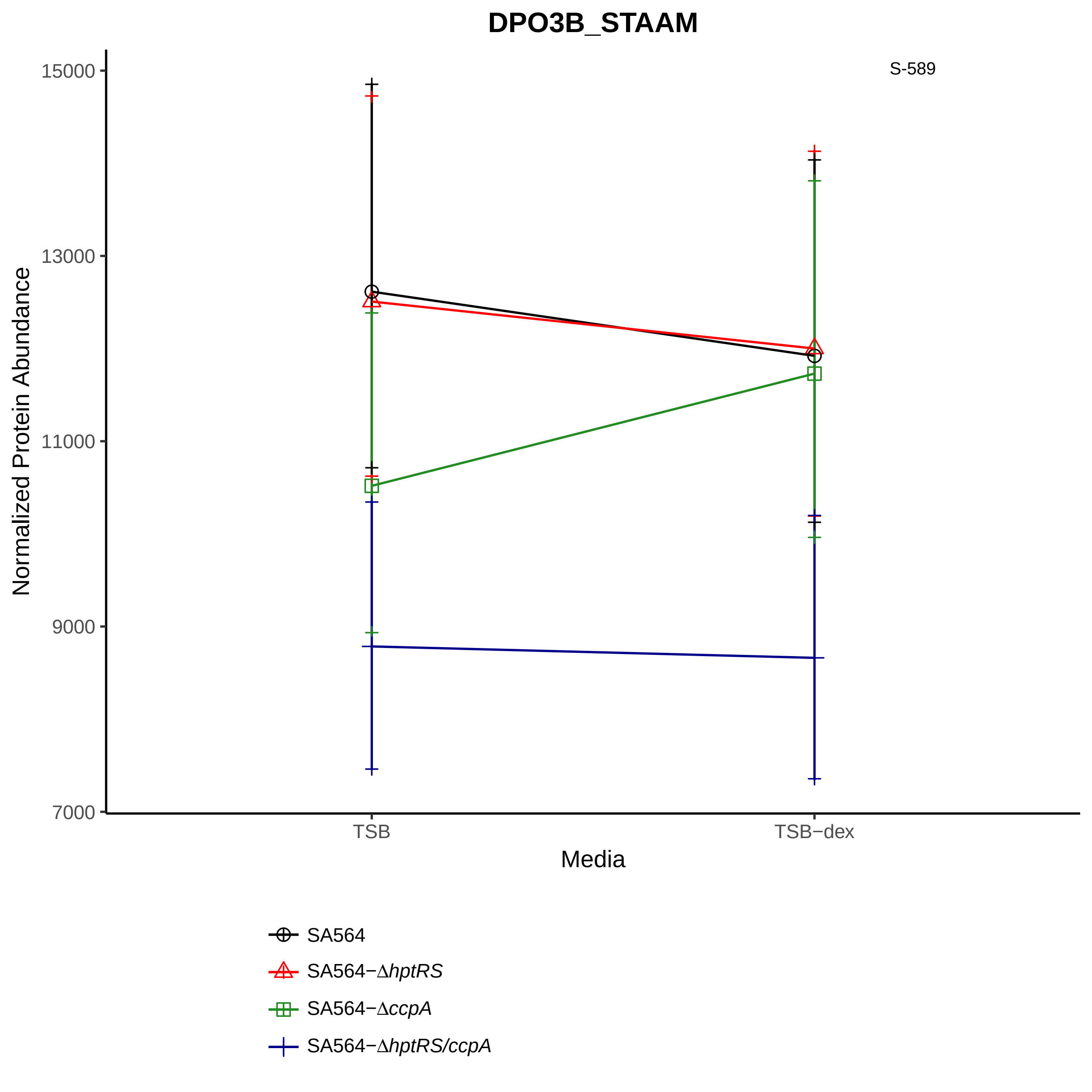

DPO3B_STAAM
S-589
15000
13000
11000
9000
7000
Normalized Protein Abundance
TSB
TSB−dex
Media
SA564
SA564−ΔhptRS
SA564−ΔccpA
SA564−ΔhptRS/ccpA

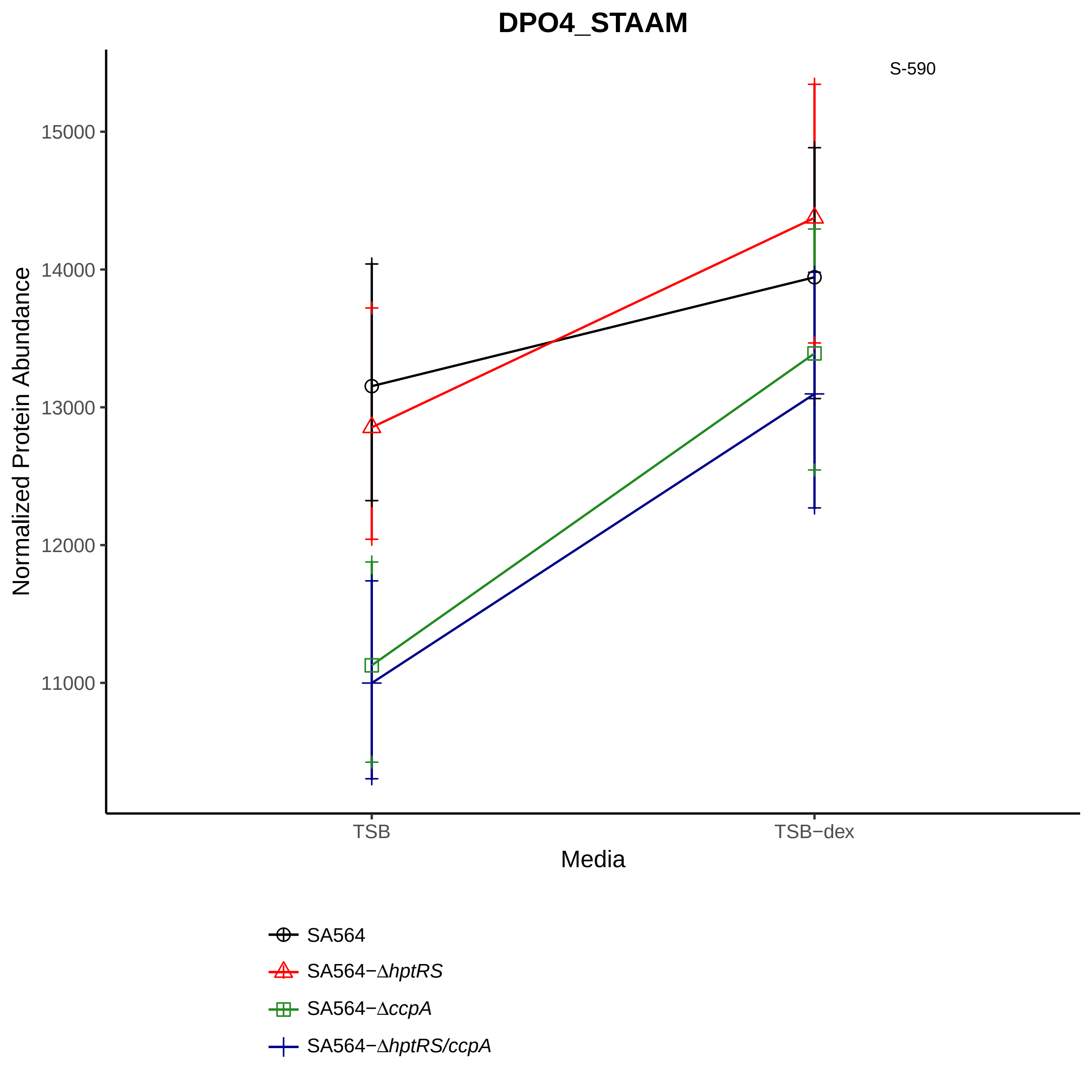

DPO4_STAAM
S-590
Normalized Protein Abundance
15000
14000
13000
12000
11000
TSB
TSB−dex
Media
SA564
SA564−Δ*hptRS*
SA564−Δ*ccpA*
SA564−Δ*hptRS/ccpA*

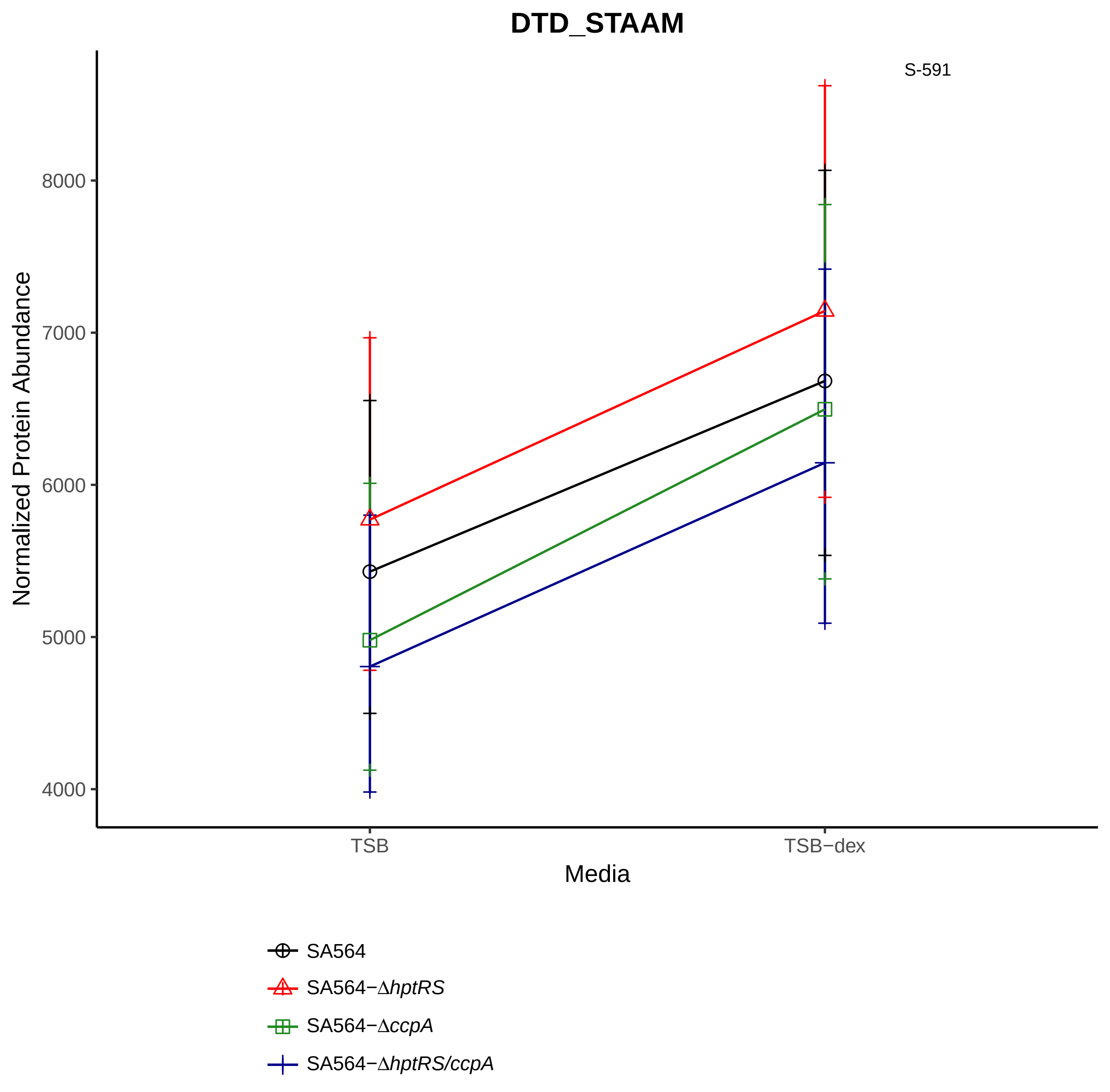

DTD_STAAM
S-591
Normalized Protein Abundance
8000
7000
6000
5000
4000
TSB
TSB−dex
Media
SA564
SA564−Δ*hptRS*
SA564−Δ*ccpA*
SA564−Δ*hptRS/ccpA*

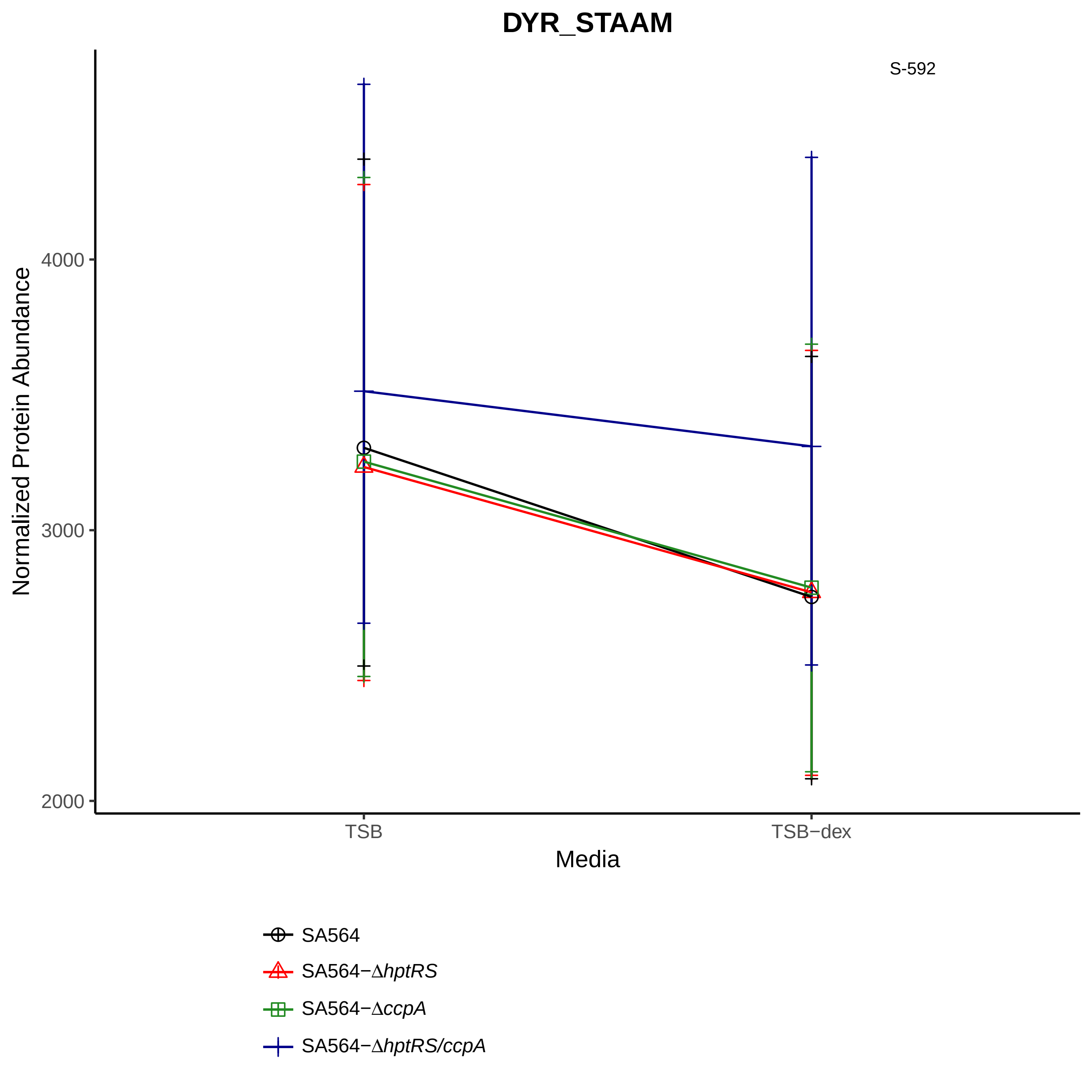

DYR_STAAM
S-592
Normalized Protein Abundance
4000
3000
2000
TSB
TSB−dex
Media
SA564
SA564−Δ*hptRS*
SA564−Δ*ccpA*
SA564−Δ*hptRS/ccpA*

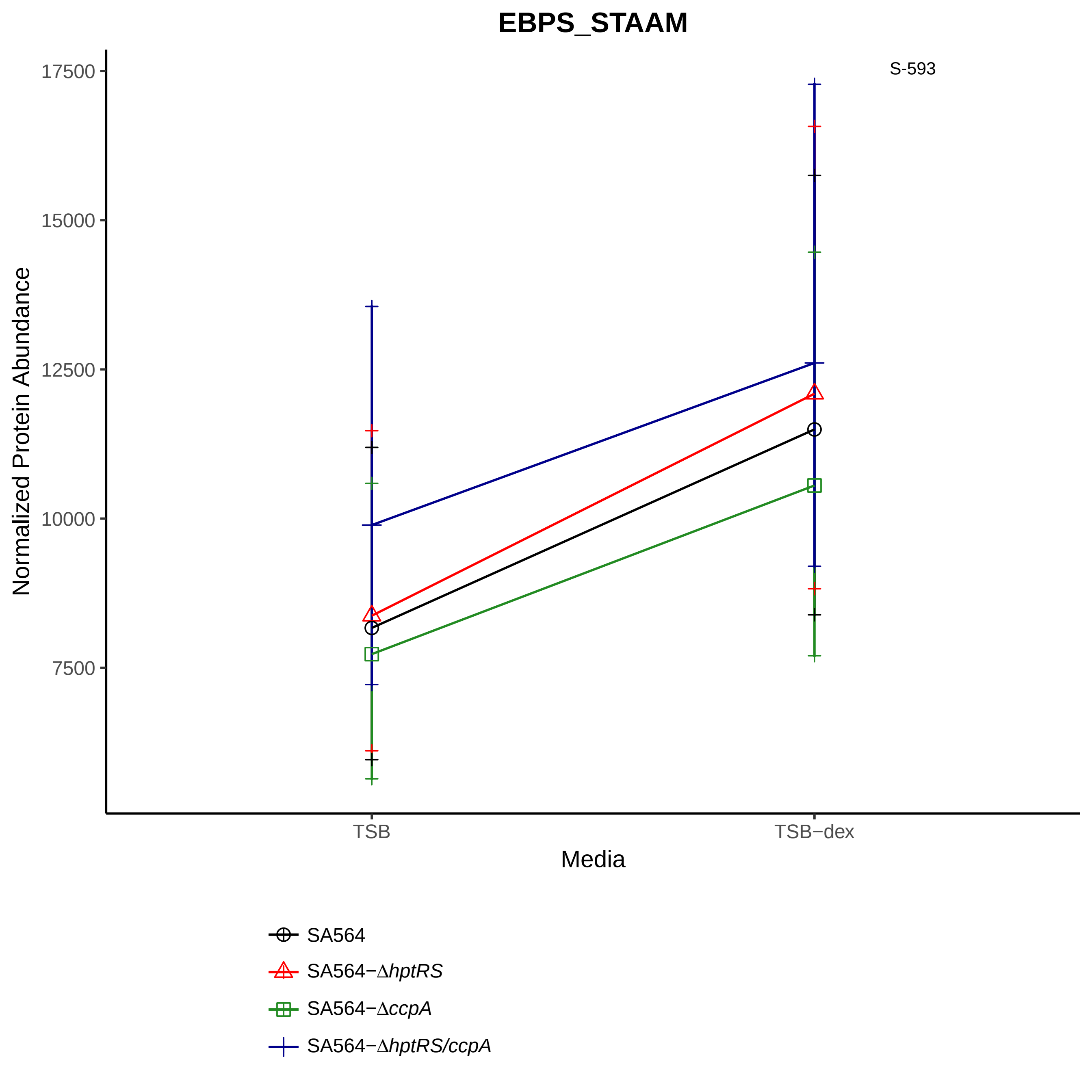

EBPS_STAAM
S-593
Normalized Protein Abundance
17500
15000
12500
10000
7500
TSB
TSB−dex
Media
SA564
SA564−Δ*hptRS*
SA564−Δ*ccpA*
SA564−Δ*hptRS/ccpA*

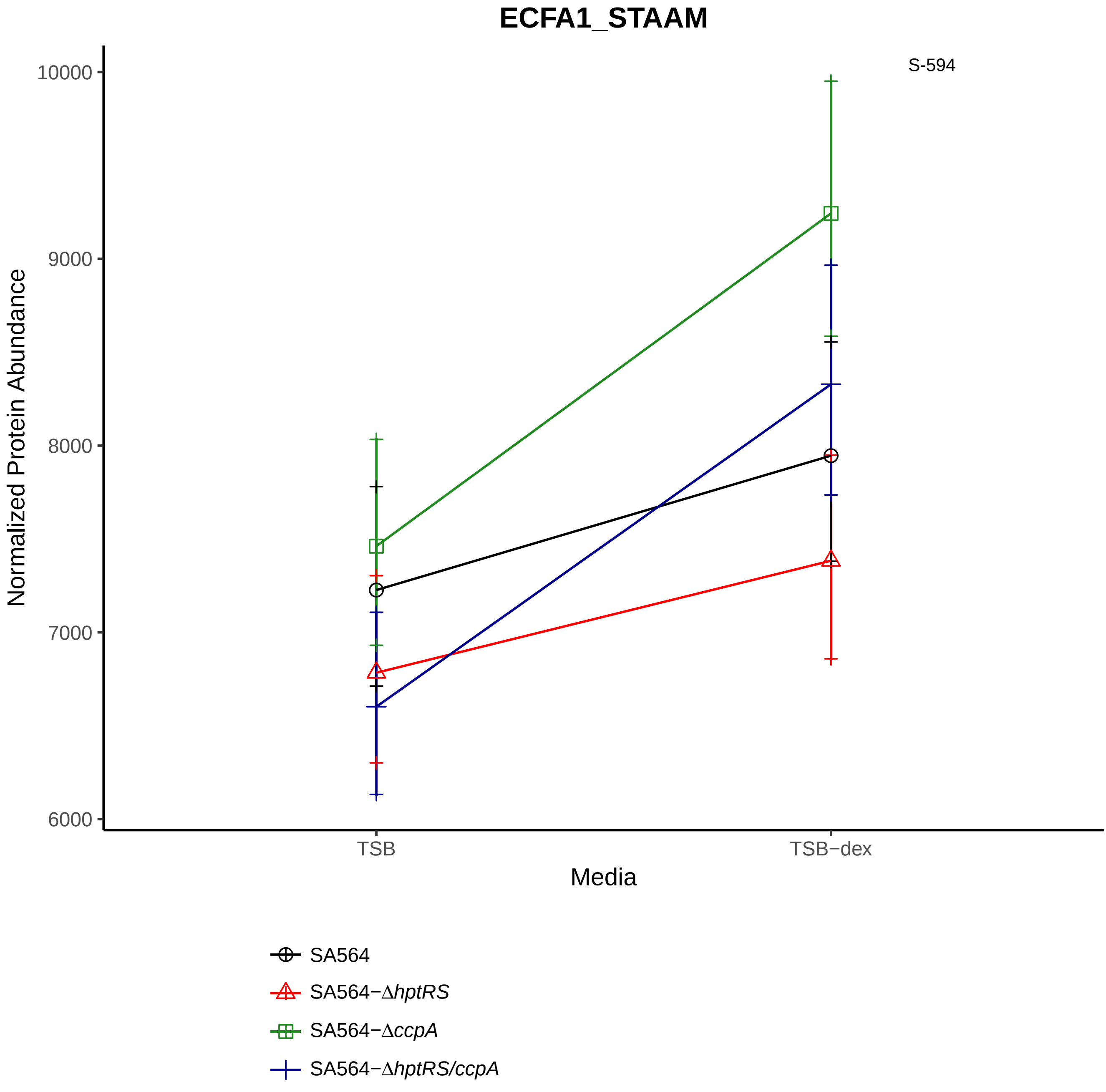

ECFA1_STAAM
S-594
10000
9000
8000
7000
6000
Normalized Protein Abundance
TSB
TSB−dex
Media
SA564
SA564−Δ*hptRS*
SA564−Δ*ccpA*
SA564−Δ*hptRS/ccpA*

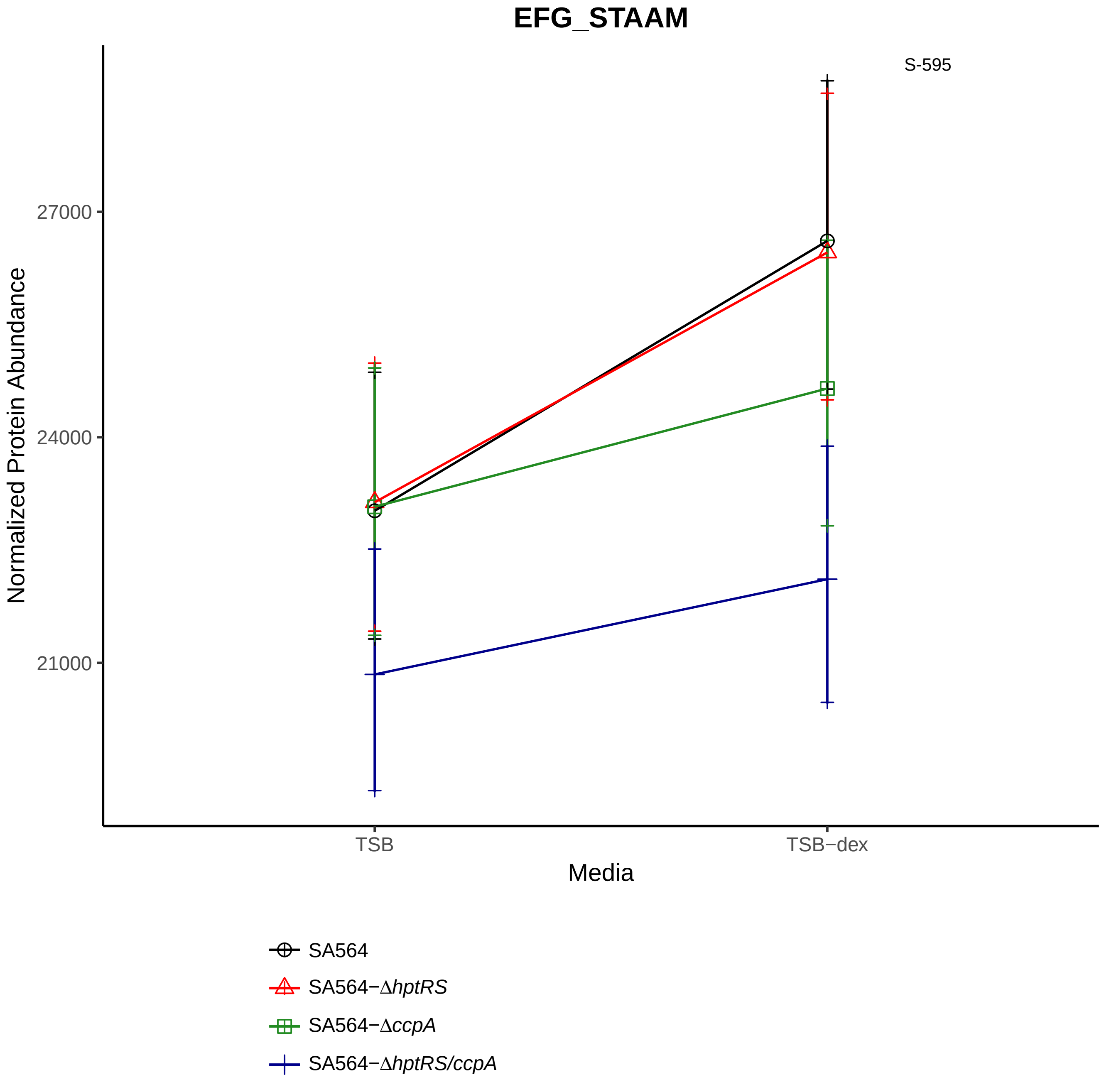

EFG_STAAM
S-595
Normalized Protein Abundance
27000
24000
21000
TSB
TSB−dex
Media
SA564
SA564−Δ*hptRS*
SA564−Δ*ccpA*
SA564−Δ*hptRS/ccpA*

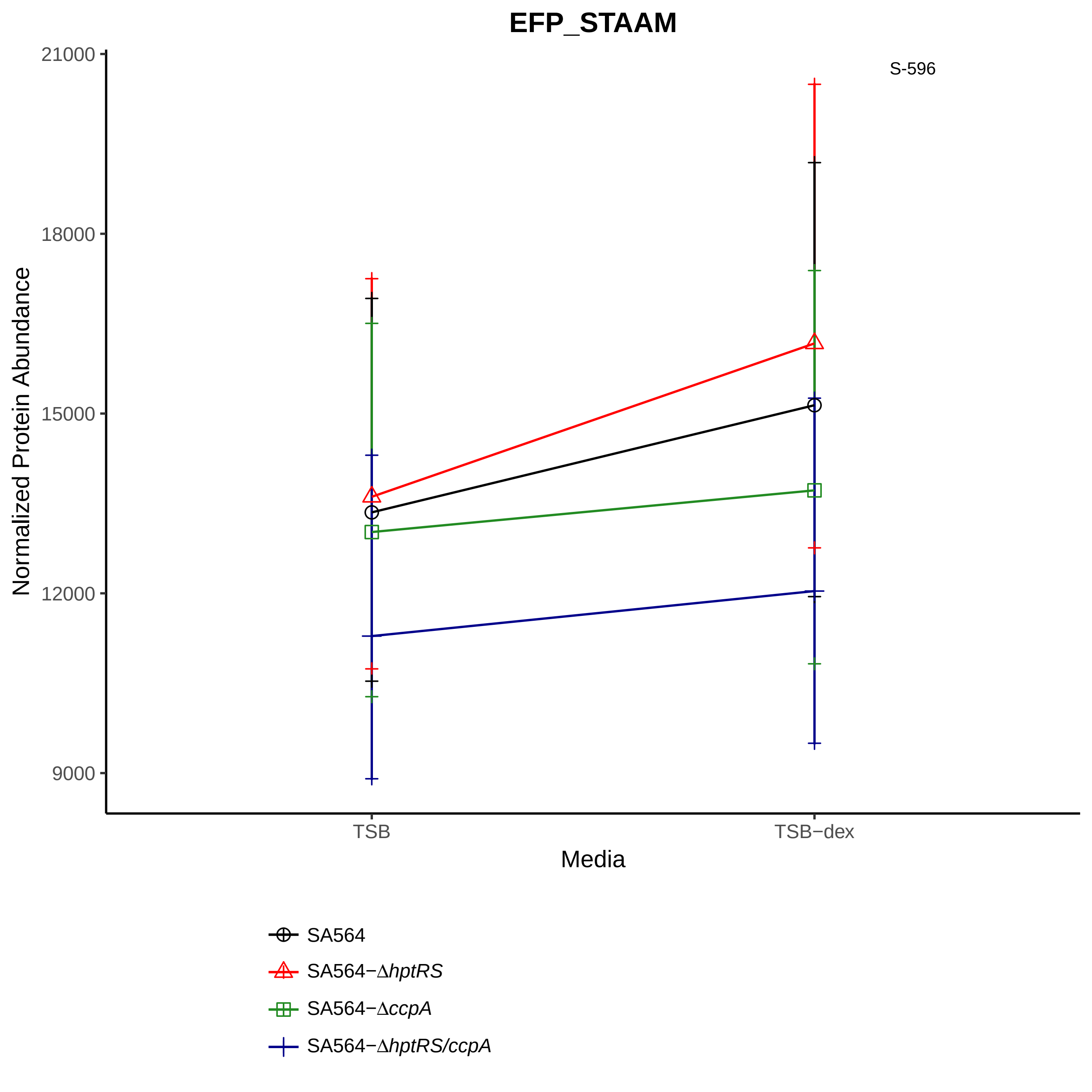
EFP_STAAM
S-596
Normalized Protein Abundance
21000
18000
15000
12000
9000
TSB
TSB−dex
Media
SA564
SA564−Δ*hptRS*
SA564−Δ*ccpA*
SA564−Δ*hptRS/ccpA*

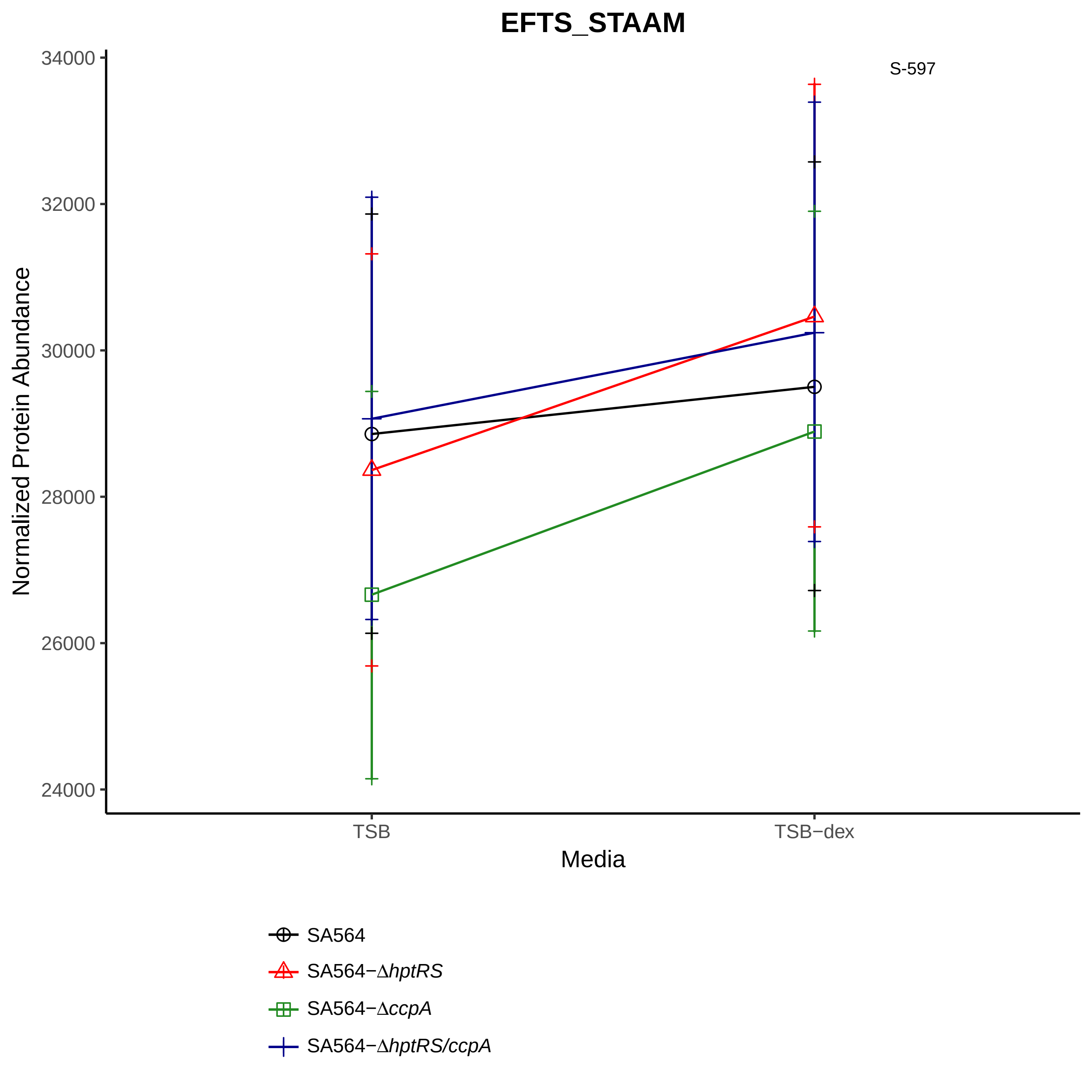
EFTS_STAAM
S-597
Normalized Protein Abundance
34000
32000
30000
28000
26000
24000
TSB
TSB−dex
Media
SA564
SA564−Δ*hptRS*
SA564−Δ*ccpA*
SA564−Δ*hptRS/ccpA*

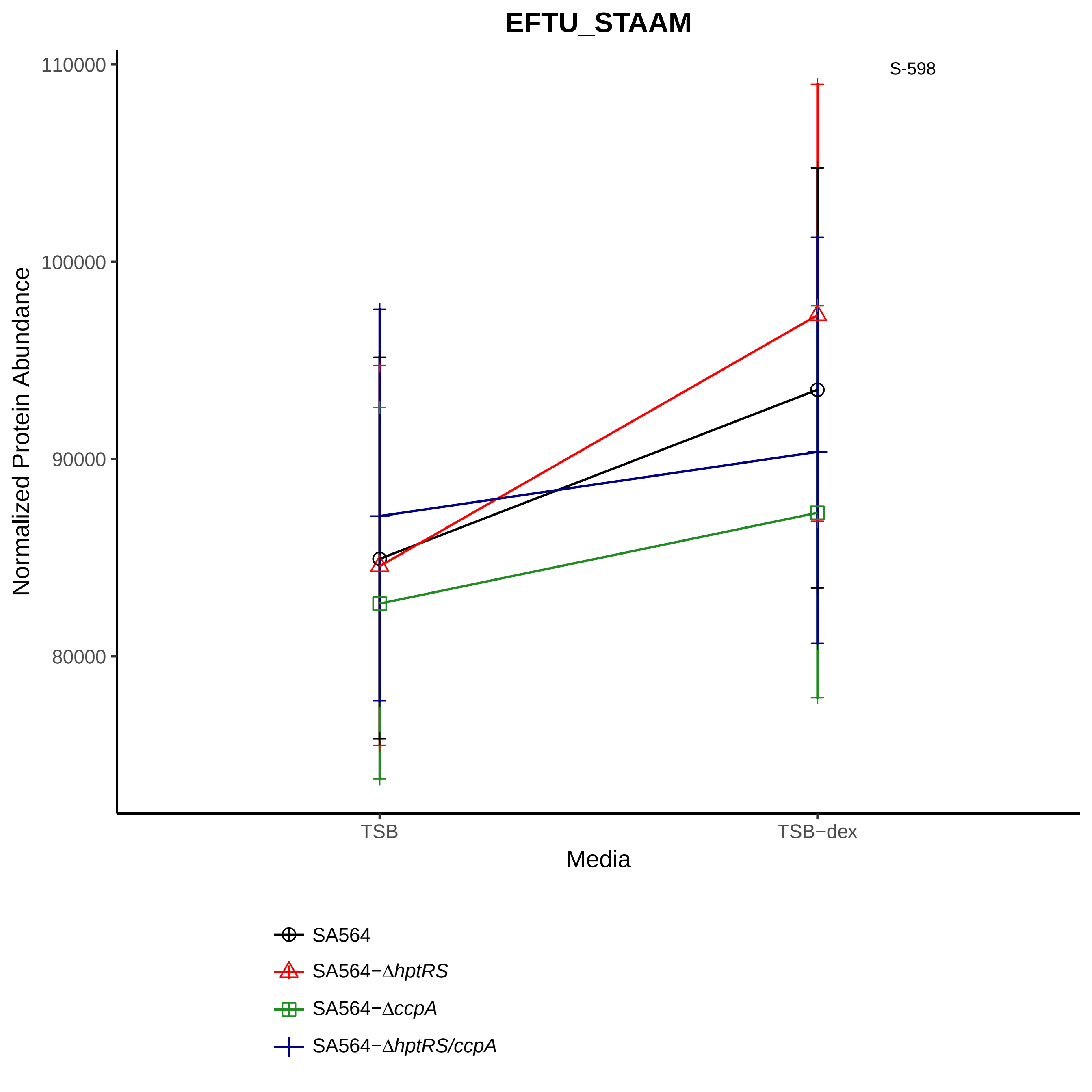
EFTU_STAAM
S-598
Normalized Protein Abundance
110000
100000
90000
80000
TSB
TSB−dex
Media
SA564
SA564−ΔhptRS
SA564−ΔccpA
SA564−ΔhptRS/ccpA

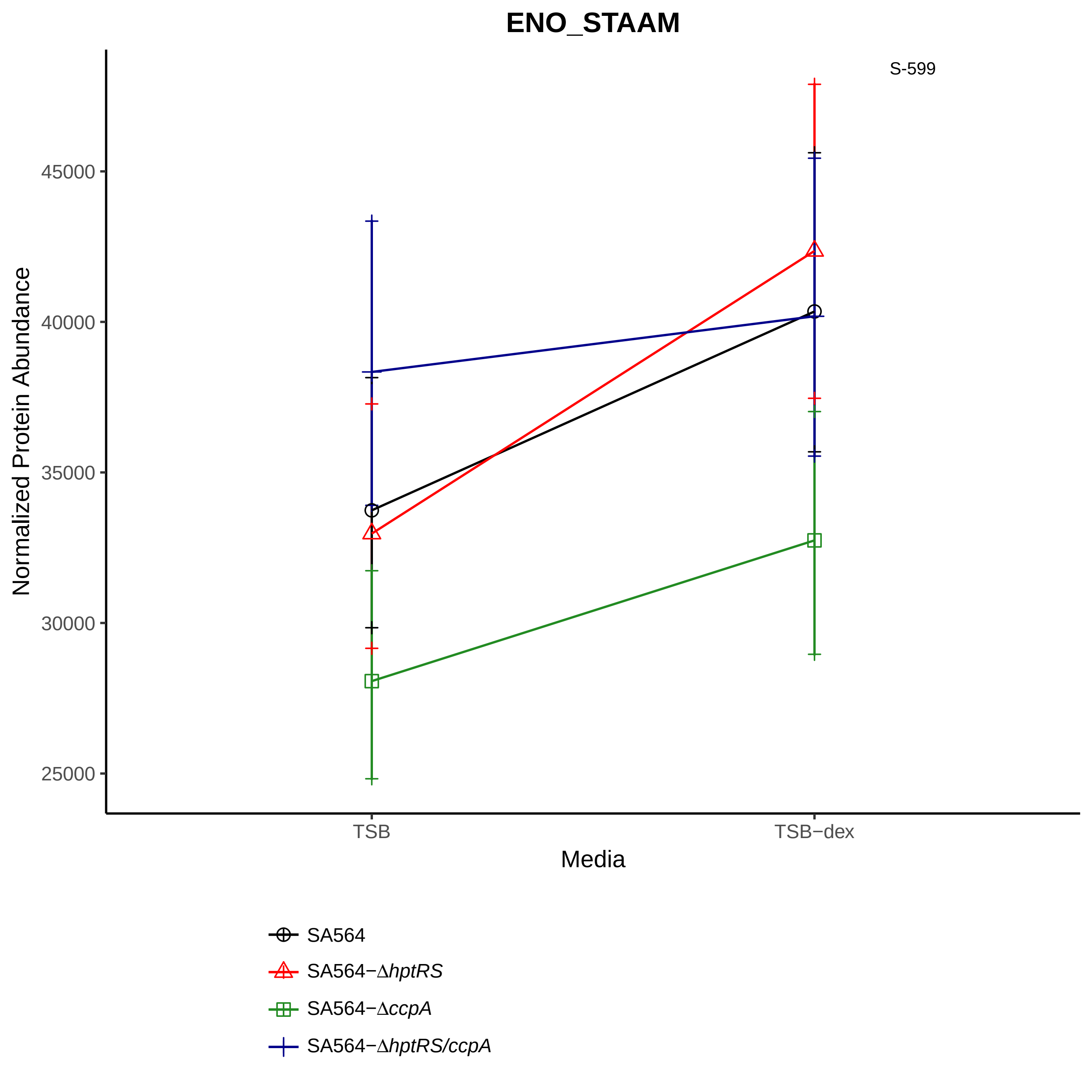
ENO_STAAM
S-599
Normalized Protein Abundance
45000
40000
35000
30000
25000
TSB
TSB−dex
Media
SA564
SA564−Δ*hptRS*
SA564−Δ*ccpA*
SA564−Δ*hptRS/ccpA*

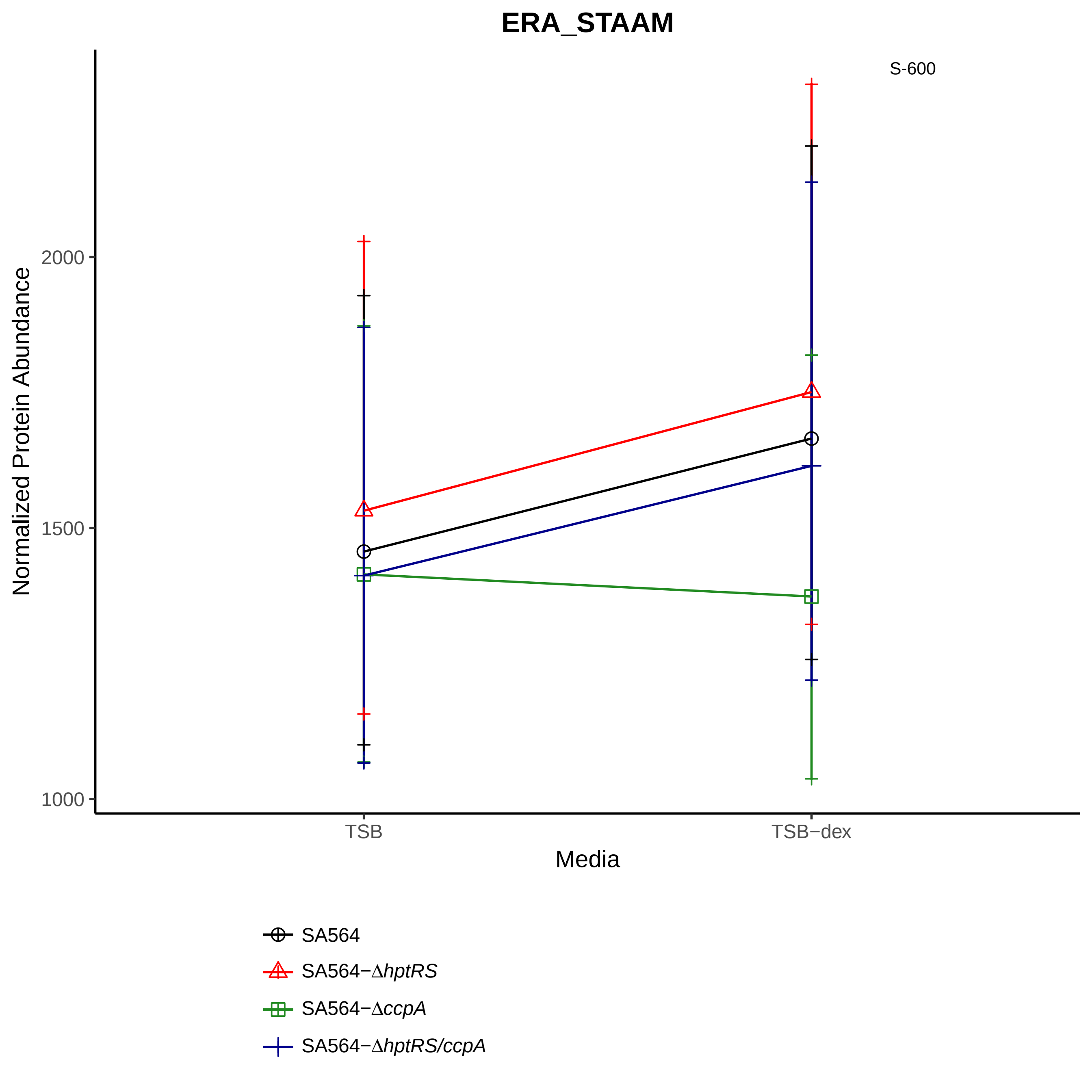

ERA_STAAM
S-600
Normalized Protein Abundance
2000
1500
1000
TSB
TSB−dex
Media
SA564
SA564−ΔhptRS
SA564−ΔccpA
SA564−ΔhptRS/ccpA

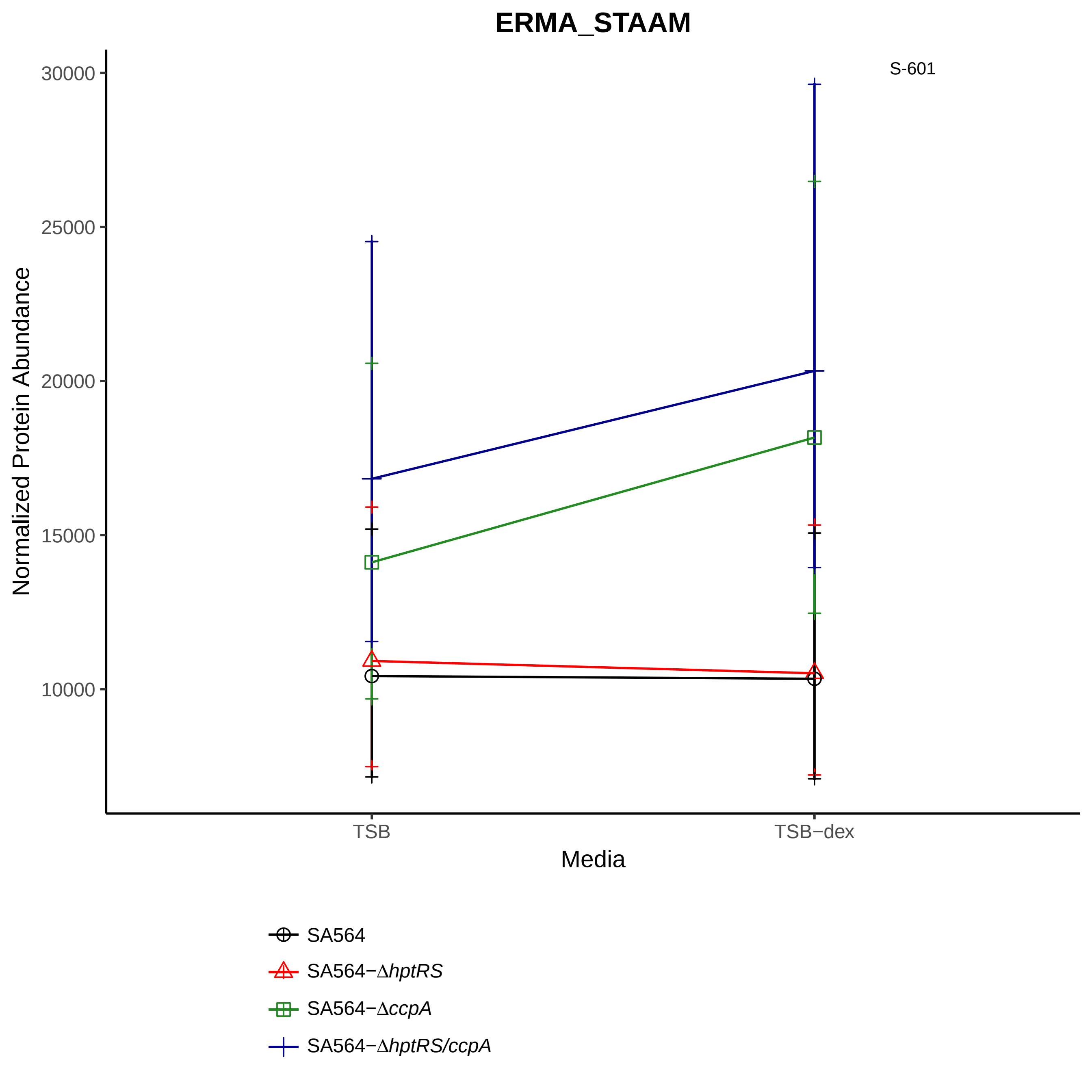

ERMA_STAAM
S-601
Normalized Protein Abundance
30000
25000
20000
15000
10000
TSB
TSB−dex
Media
SA564
SA564−Δ*hptRS*
SA564−Δ*ccpA*
SA564−Δ*hptRS/ccpA*

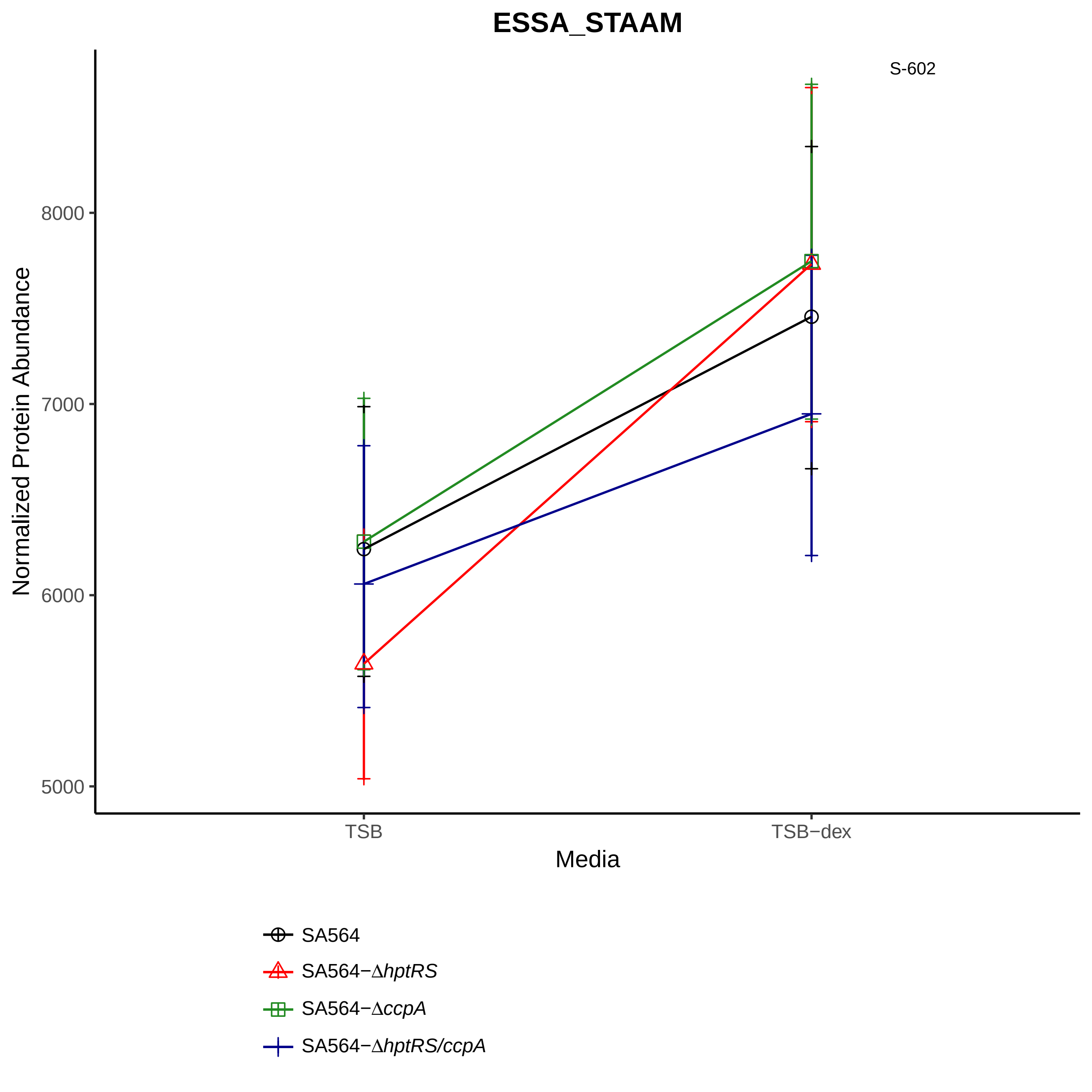
ESSA_STAAM
S-602
Normalized Protein Abundance
8000
7000
6000
5000
TSB
TSB−dex
Media
SA564
SA564−Δ*hptRS*
SA564−Δ*ccpA*
SA564−Δ*hptRS/ccpA*

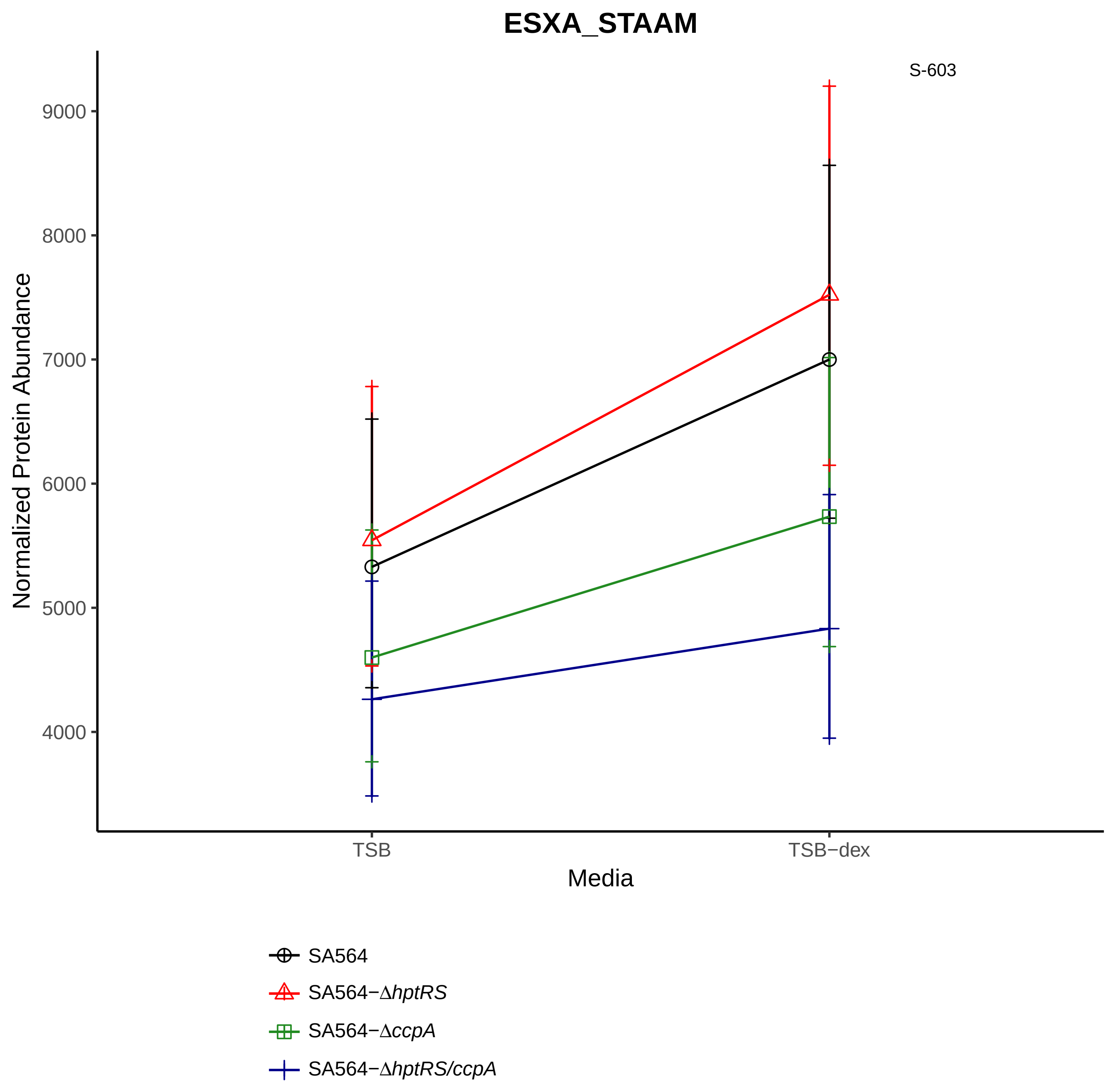

ESXA_STAAM
S-603
Normalized Protein Abundance
9000
8000
7000
6000
5000
4000
TSB
TSB−dex
Media
SA564
SA564−Δ*hptRS*
SA564−Δ*ccpA*
SA564−Δ*hptRS/ccpA*

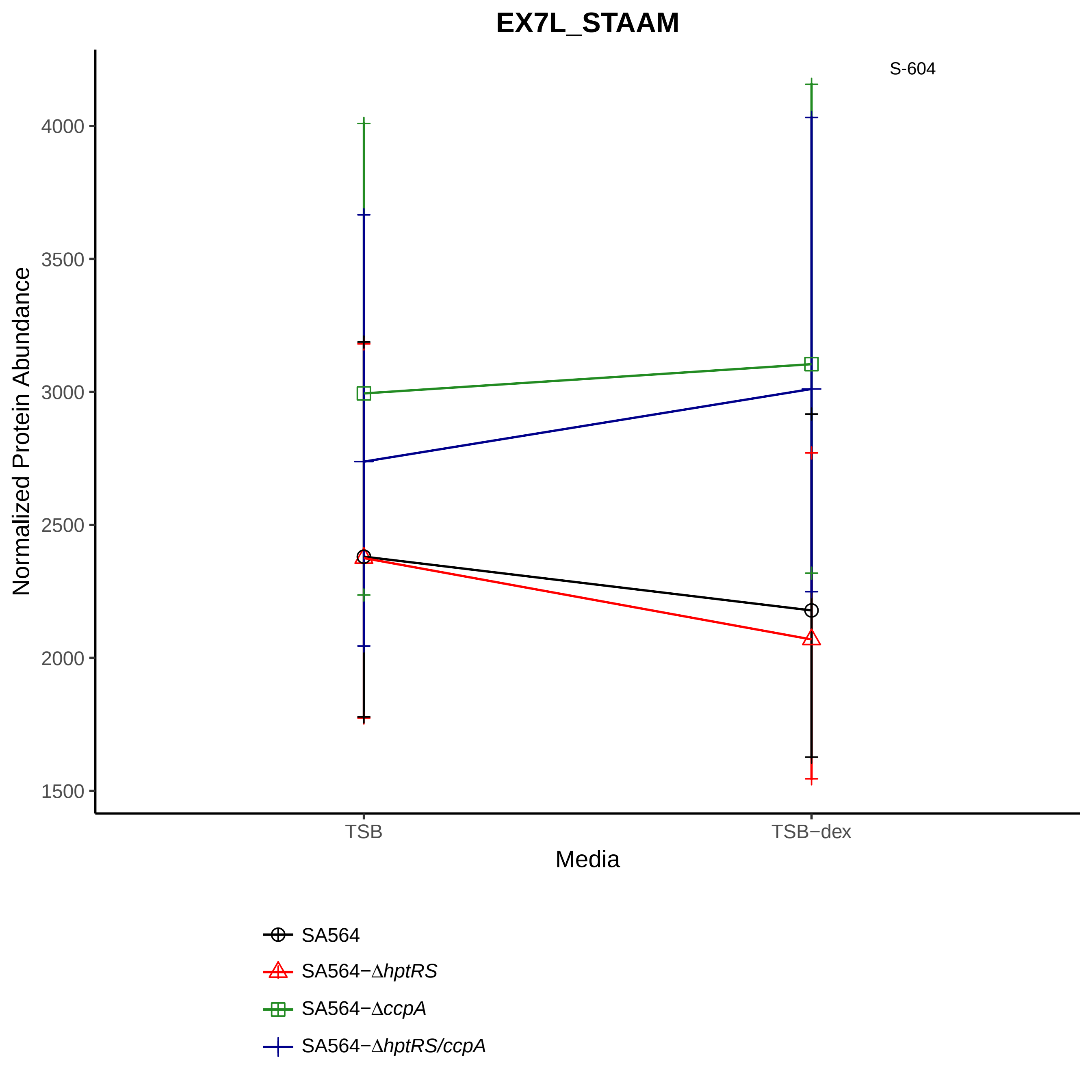

EX7L_STAAM
S-604
Normalized Protein Abundance
4000
3500
3000
2500
2000
1500
TSB
TSB−dex
Media
SA564
SA564−Δ*hptRS*
SA564−Δ*ccpA*
SA564−Δ*hptRS/ccpA*

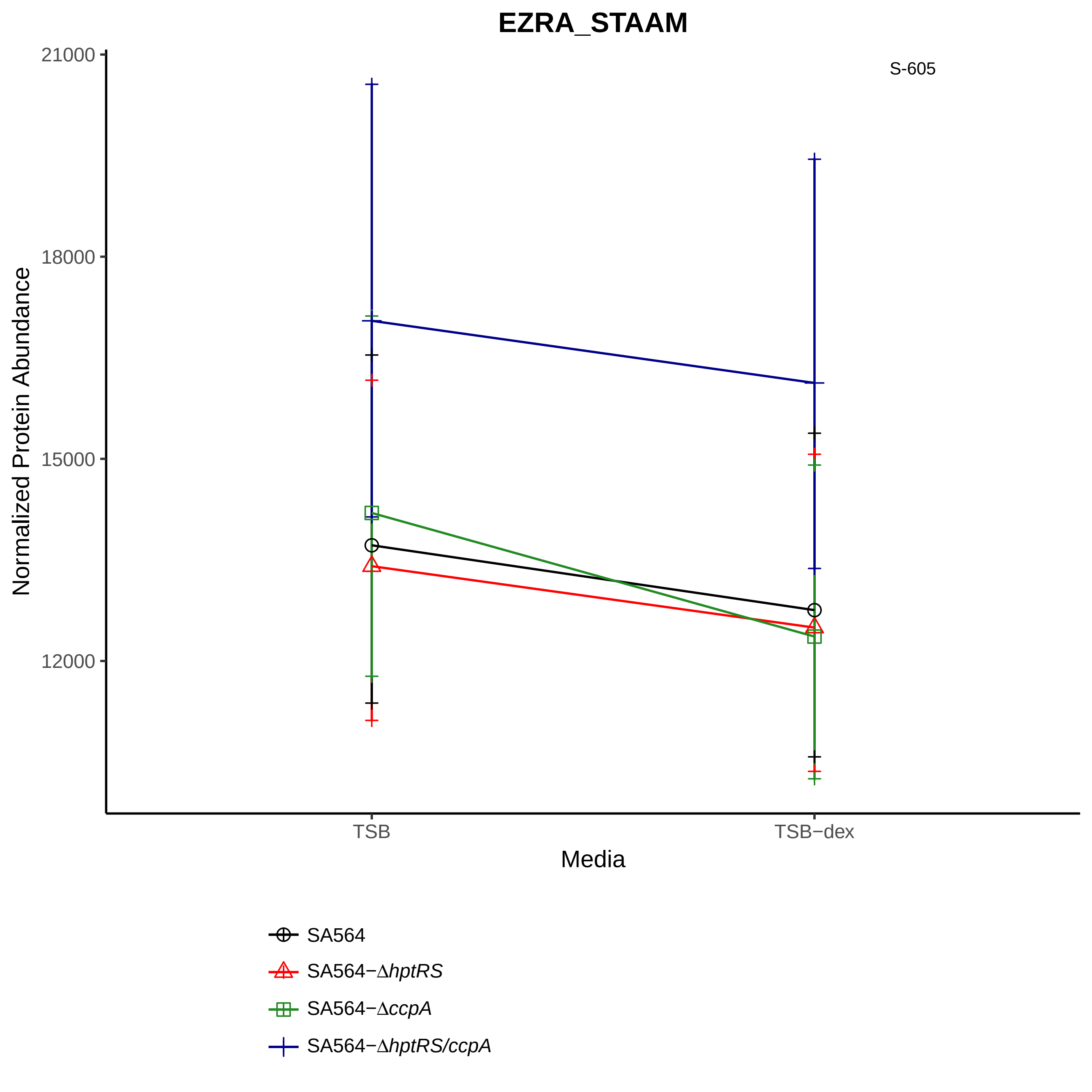

EZRA_STAAM
S-605
21000
18000
15000
12000
Normalized Protein Abundance
TSB
TSB−dex
Media
SA564
SA564−Δ*hptRS*
SA564−Δ*ccpA*
SA564−Δ*hptRS/ccpA*

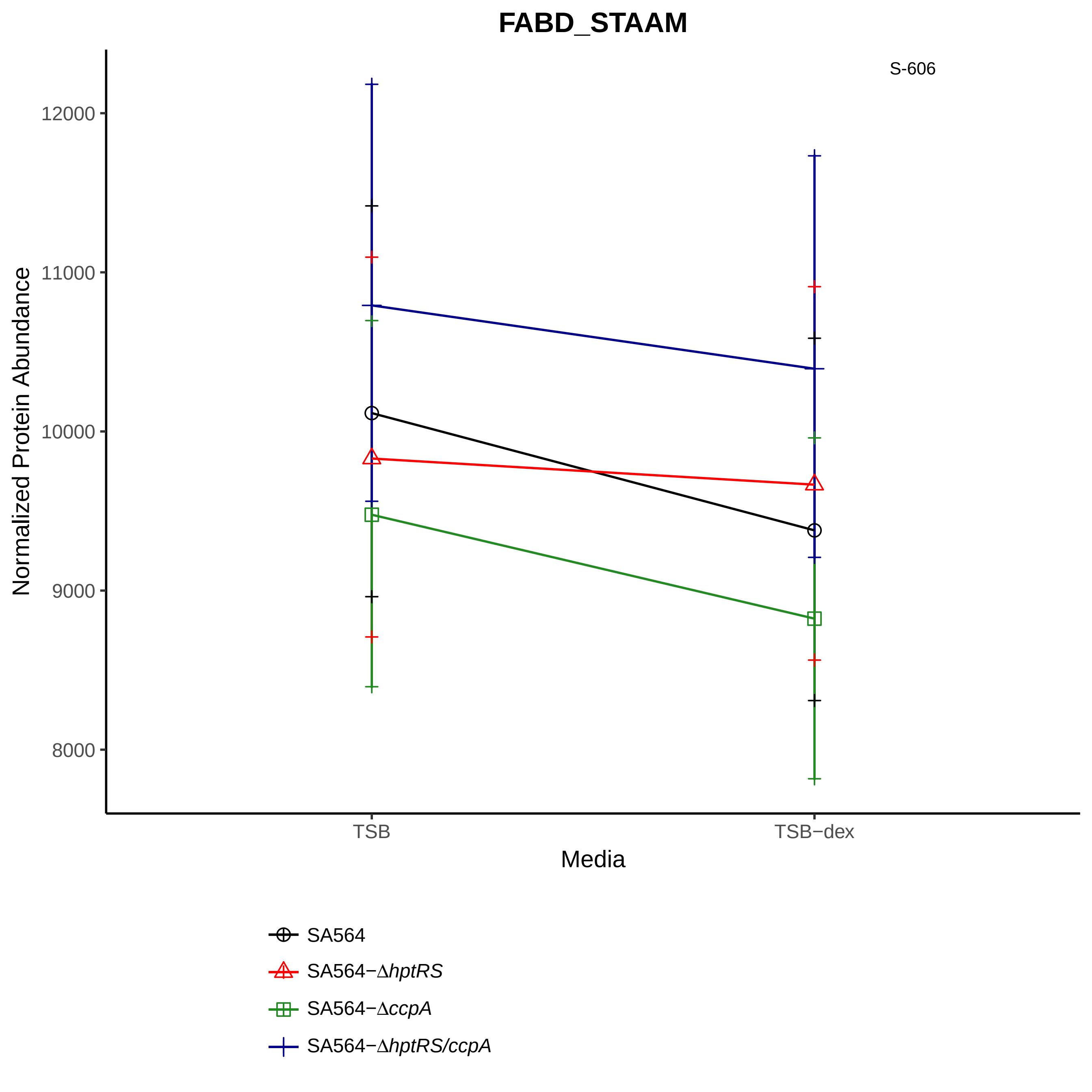

FABD_STAAM
S-606
Normalized Protein Abundance
12000
11000
10000
9000
8000
TSB
TSB−dex
Media
SA564
SA564−Δ*hptRS*
SA564−Δ*ccpA*
SA564−Δ*hptRS/ccpA*

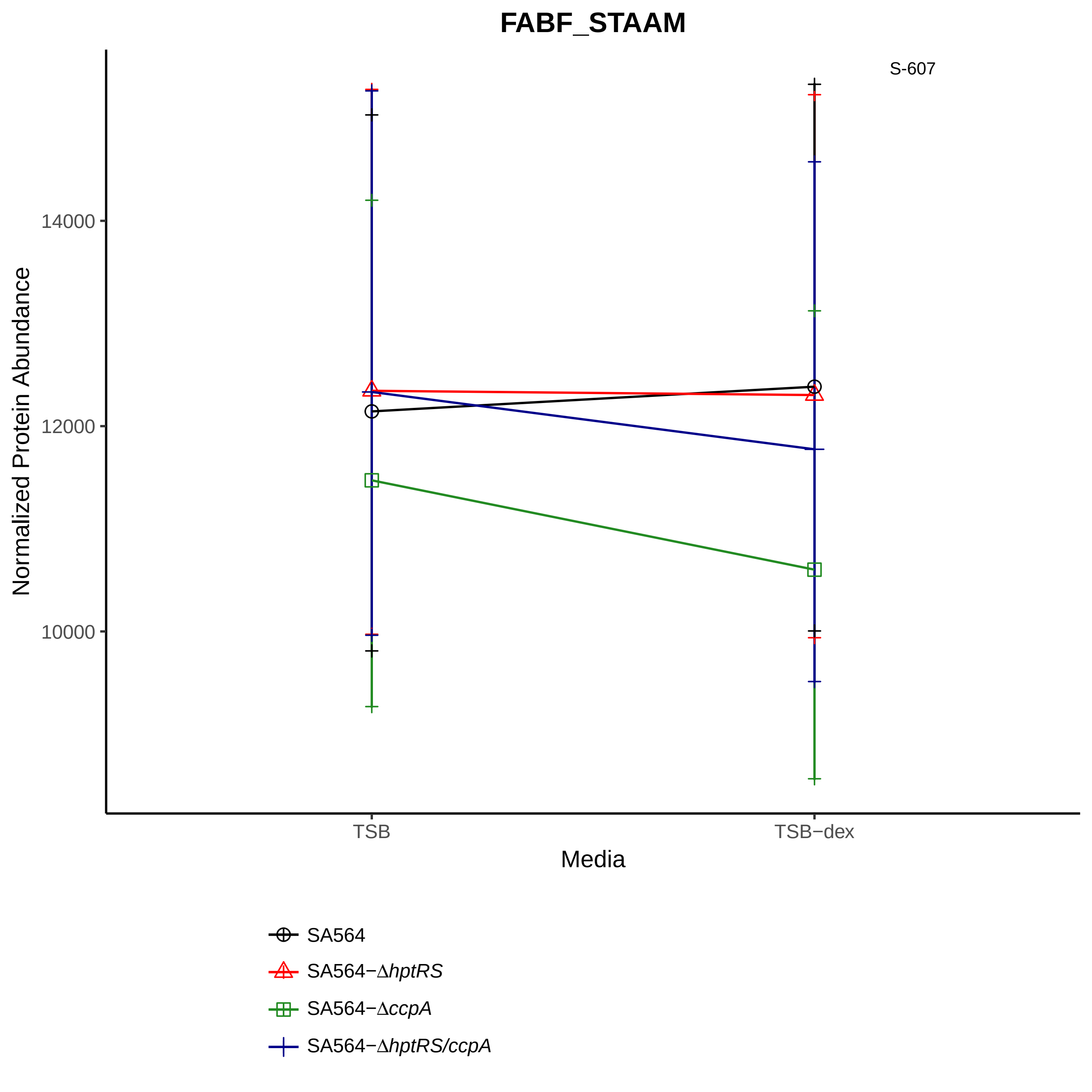
FABF_STAAM
S-607
Normalized Protein Abundance
14000
12000
10000
TSB
TSB−dex
Media
SA564
SA564−Δ*hptRS*
SA564−Δ*ccpA*
SA564−Δ*hptRS/ccpA*

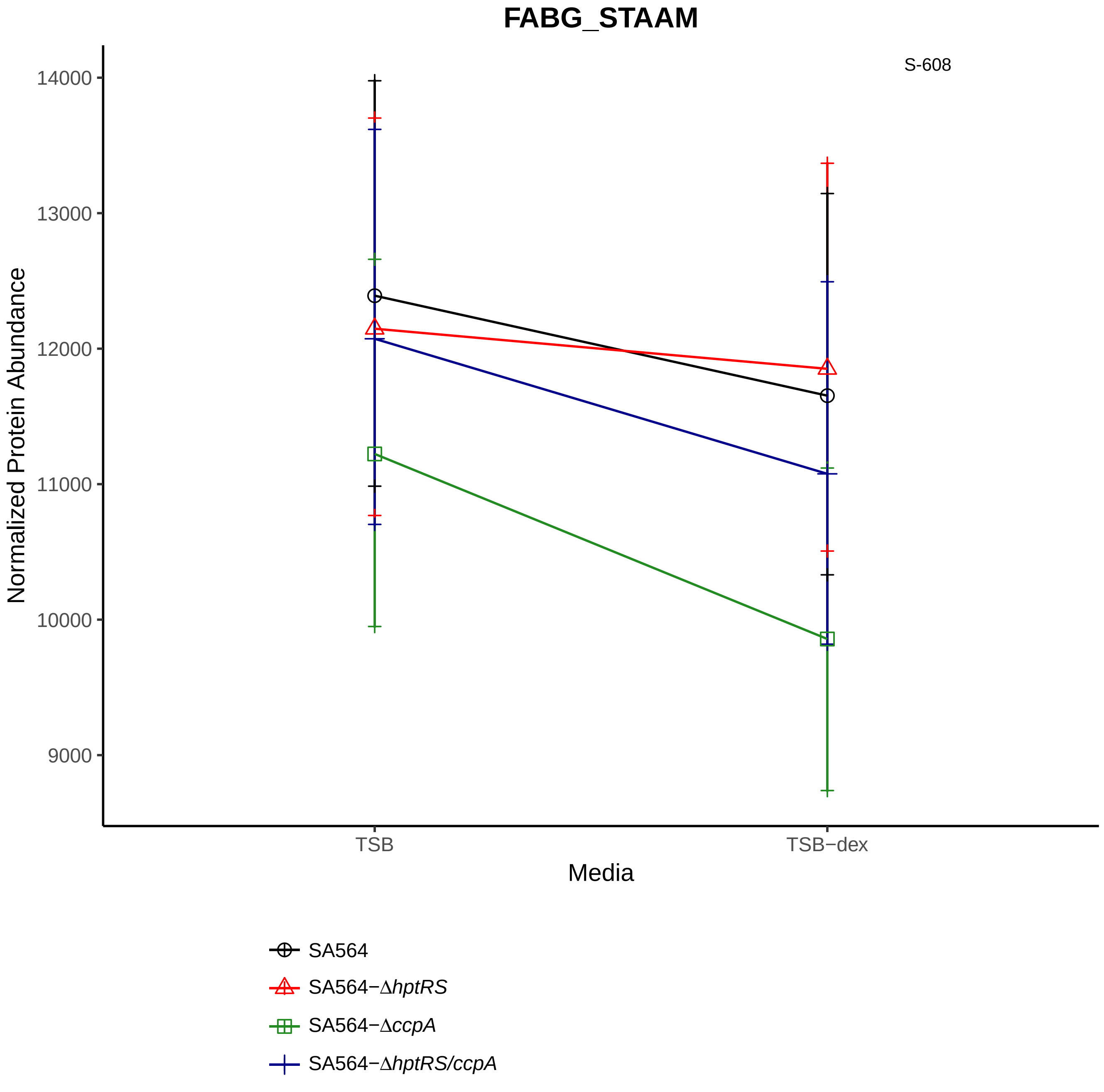

FABG_STAAM
S-608
Normalized Protein Abundance
14000
13000
12000
11000
10000
9000
TSB
TSB−dex
Media
SA564
SA564−ΔhptRS
SA564−ΔccpA
SA564−ΔhptRS/ccpA

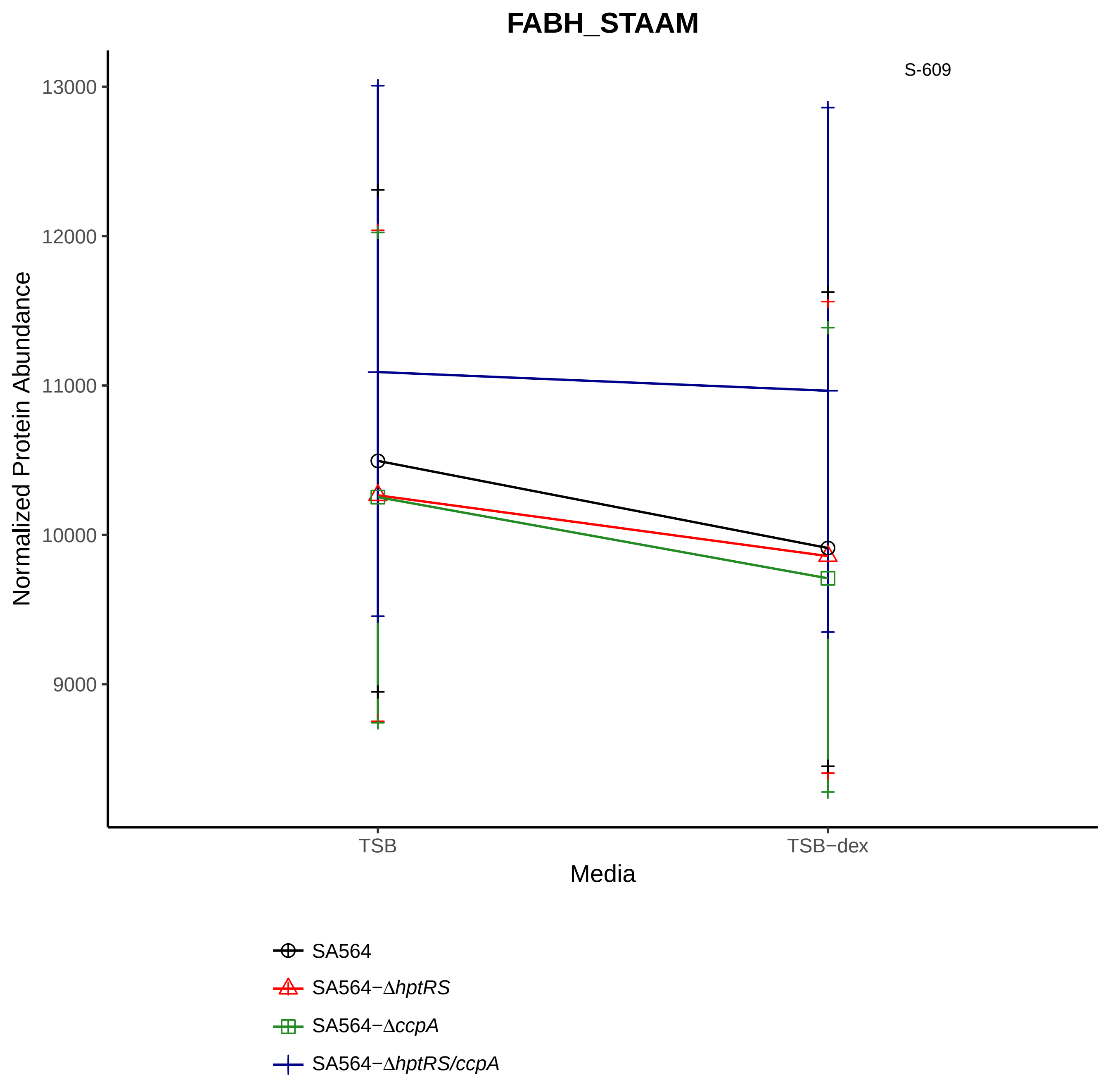

FABH_STAAM
S-609
Normalized Protein Abundance
13000
12000
11000
10000
9000
TSB
TSB−dex
Media
SA564
SA564−ΔhptRS
SA564−ΔccpA
SA564−ΔhptRS/ccpA

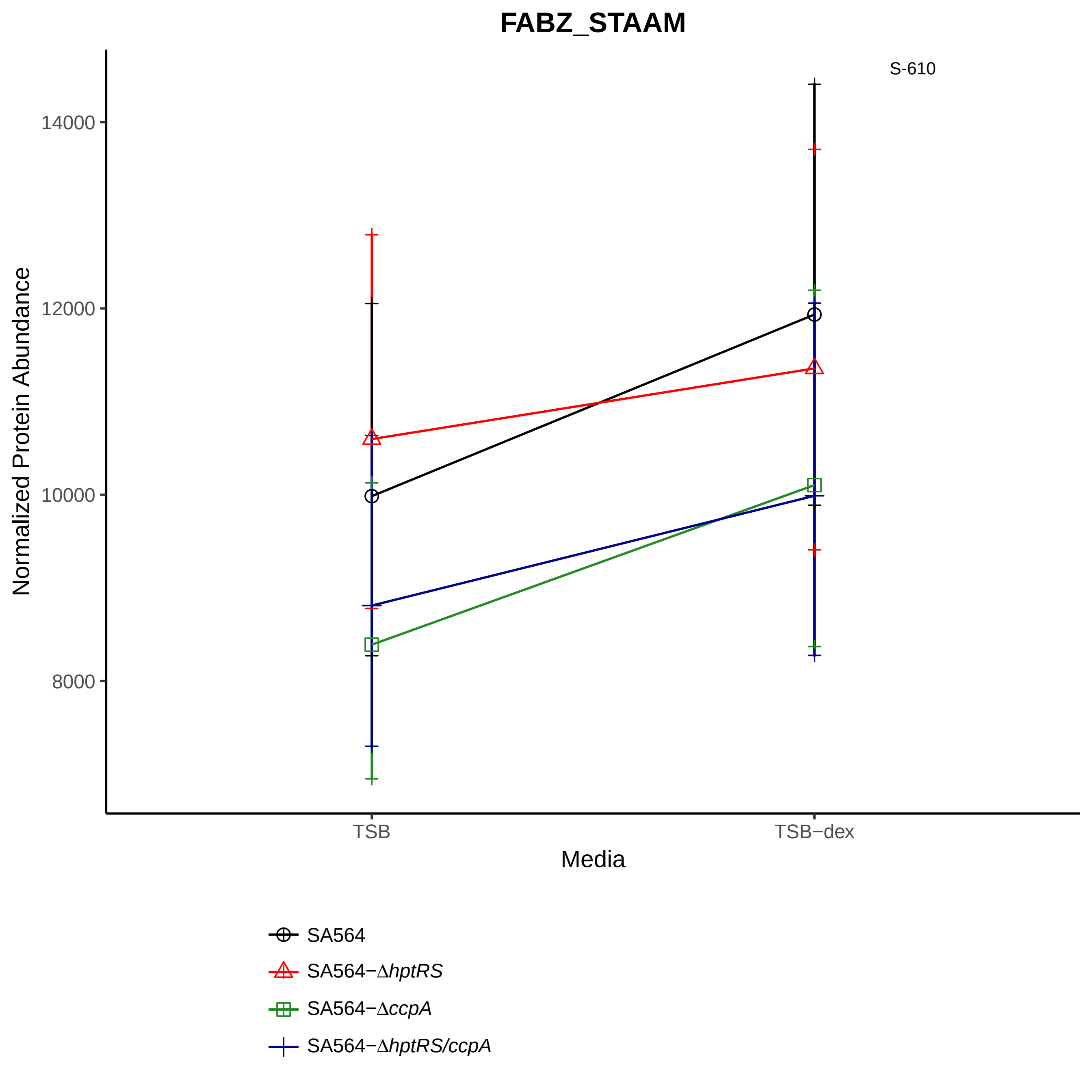

FABZ_STAAM
S-610
Normalized Protein Abundance
14000
12000
10000
8000
TSB
TSB−dex
Media
SA564
SA564−ΔhptRS
SA564−ΔccpA
SA564−ΔhptRS/ccpA

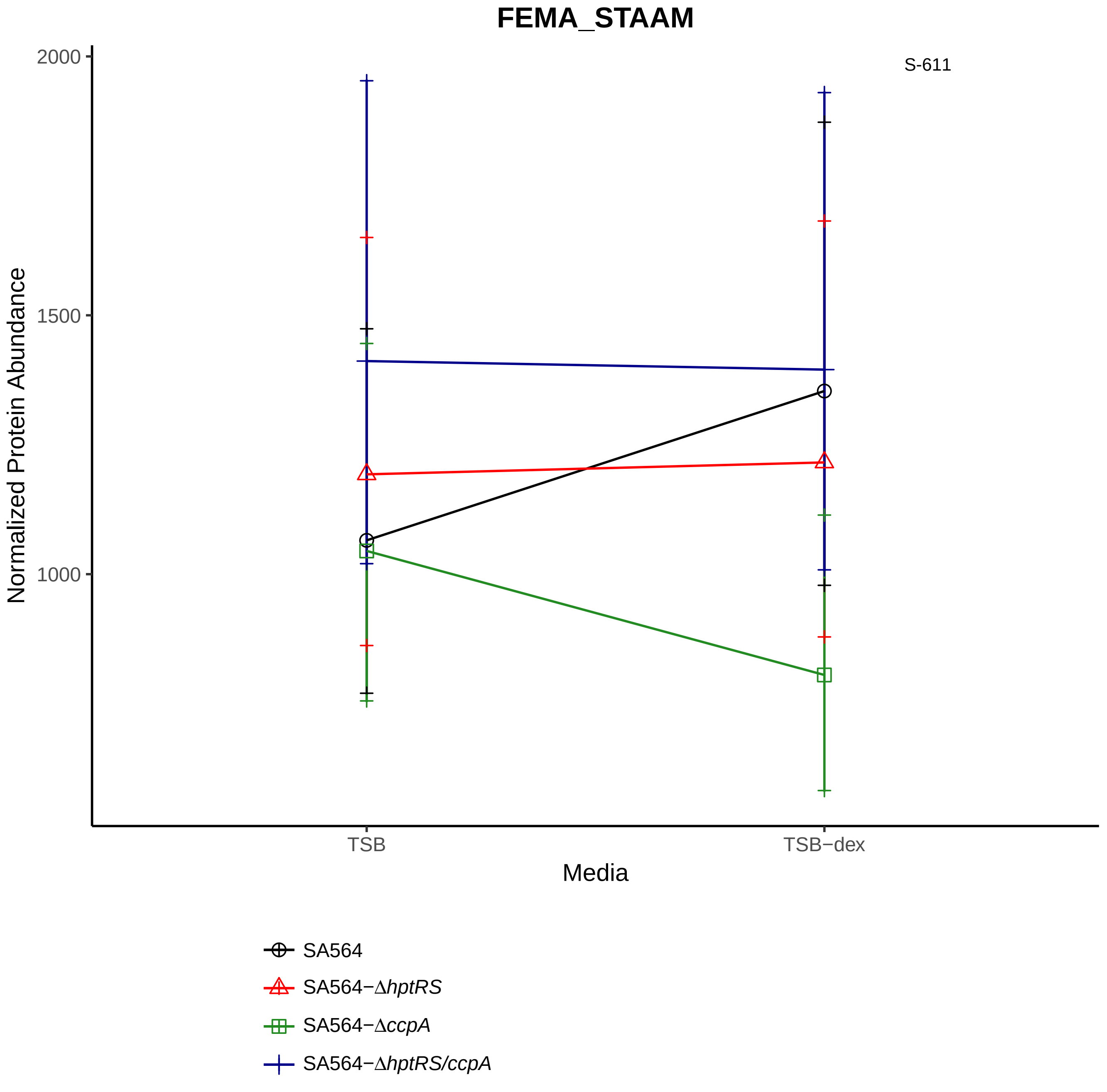

FEMA_STAAM
S-611
2000
1500
1000
Normalized Protein Abundance
TSB
TSB−dex
Media
SA564
SA564−Δ*hptRS*
SA564−Δ*ccpA*
SA564−Δ*hptRS/ccpA*

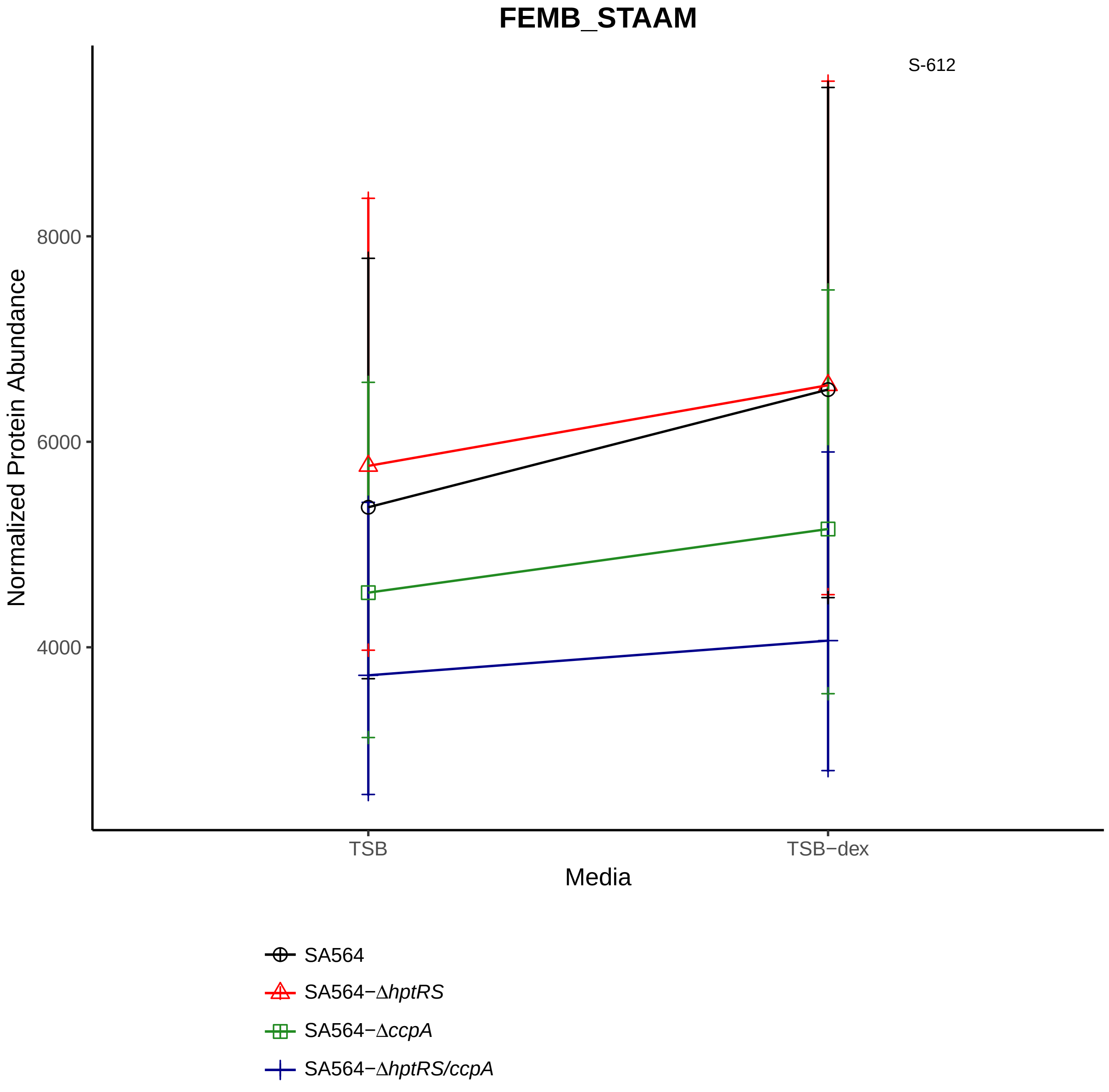

FEMB_STAAM
S-612
Normalized Protein Abundance
8000
6000
4000
TSB
TSB−dex
Media
SA564
SA564−ΔhptRS
SA564−ΔccpA
SA564−ΔhptRS/ccpA

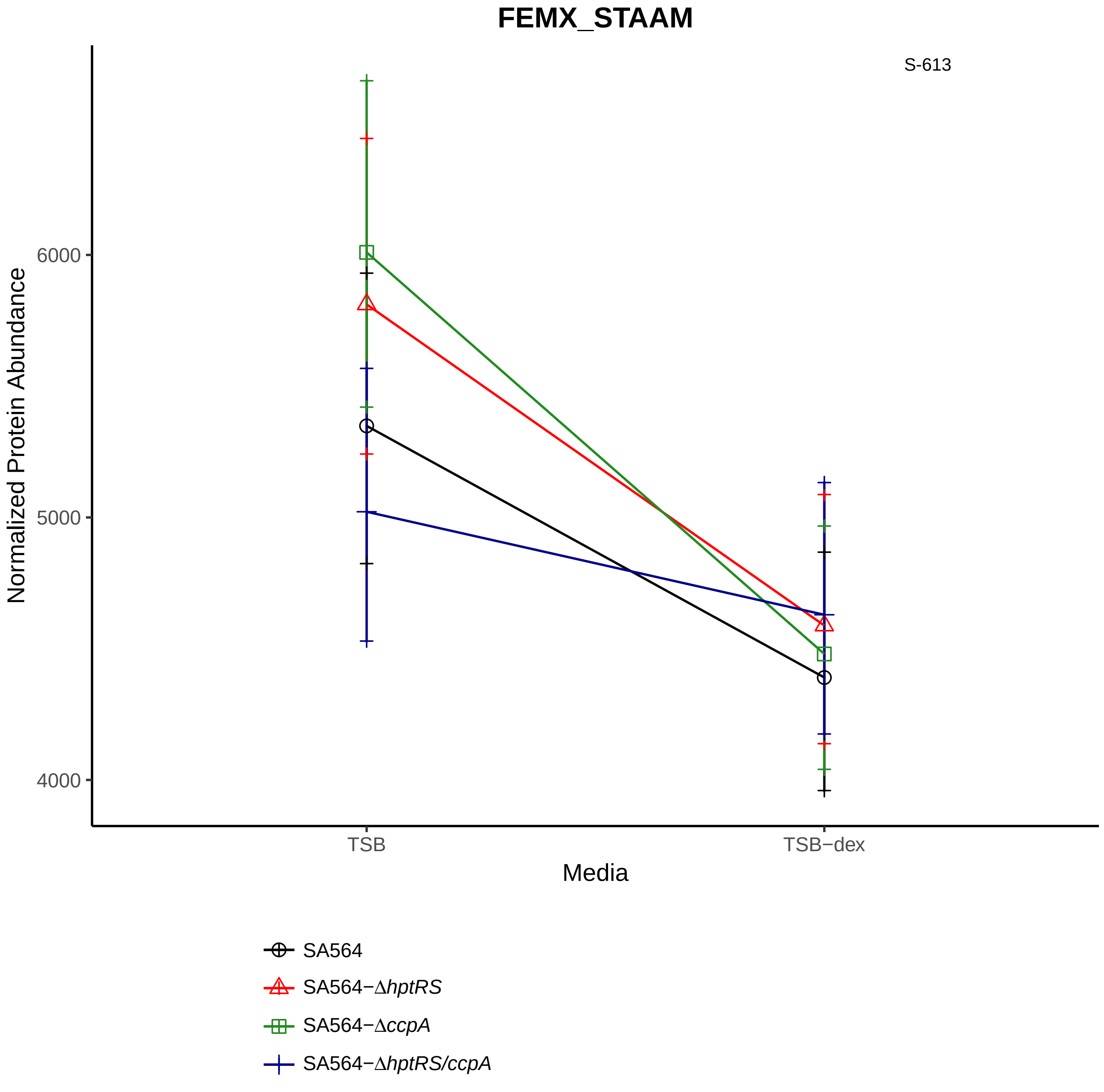
FEMX_STAAM
S-613
Normalized Protein Abundance
6000
5000
4000
TSB
TSB−dex
Media
SA564
SA564−Δ*hptRS*
SA564−Δ*ccpA*
SA564−Δ*hptRS/ccpA*

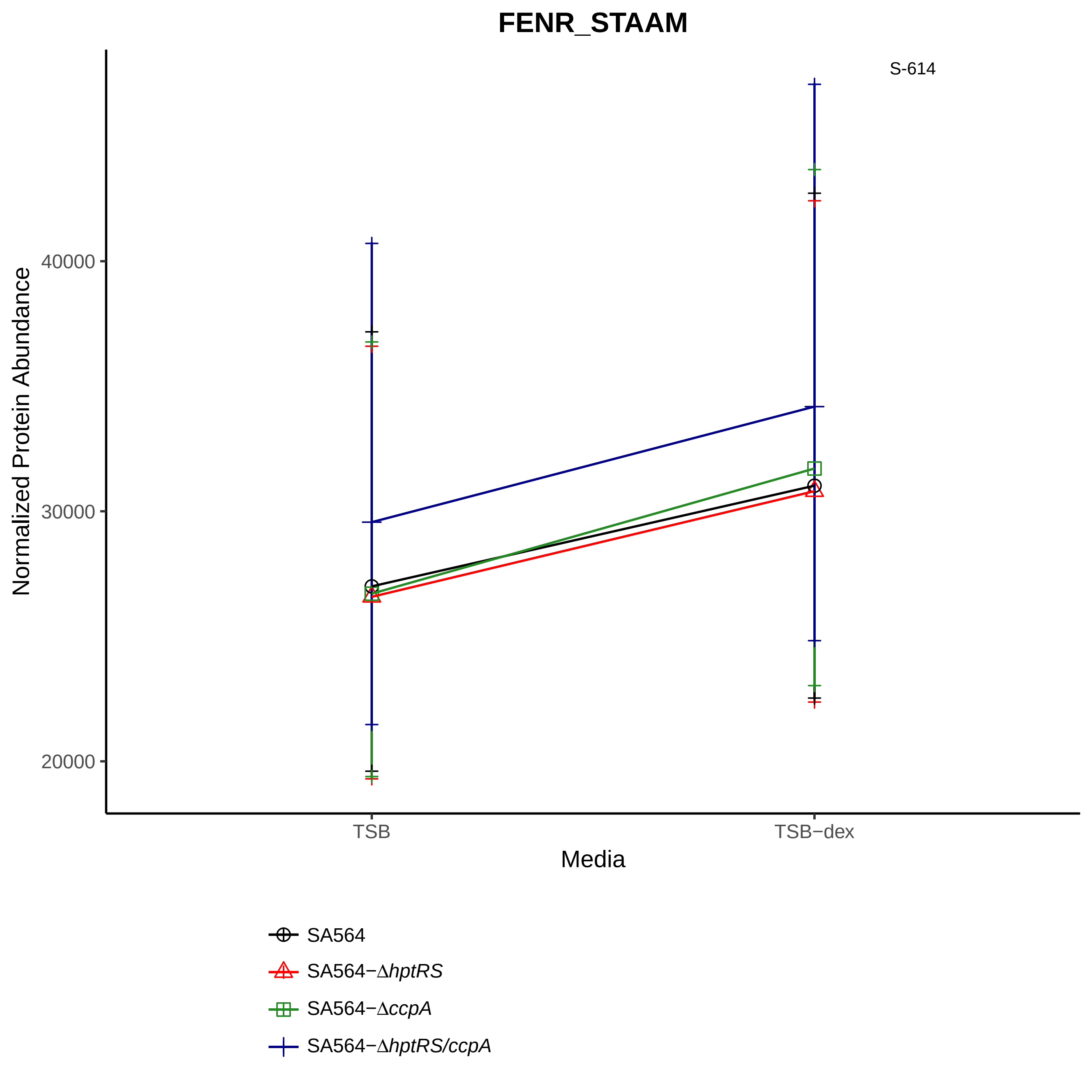
FENR_STAAM
S-614
Normalized Protein Abundance
40000
30000
20000
TSB
TSB−dex
Media
SA564
SA564−Δ*hptRS*
SA564−Δ*ccpA*
SA564−Δ*hptRS/ccpA*

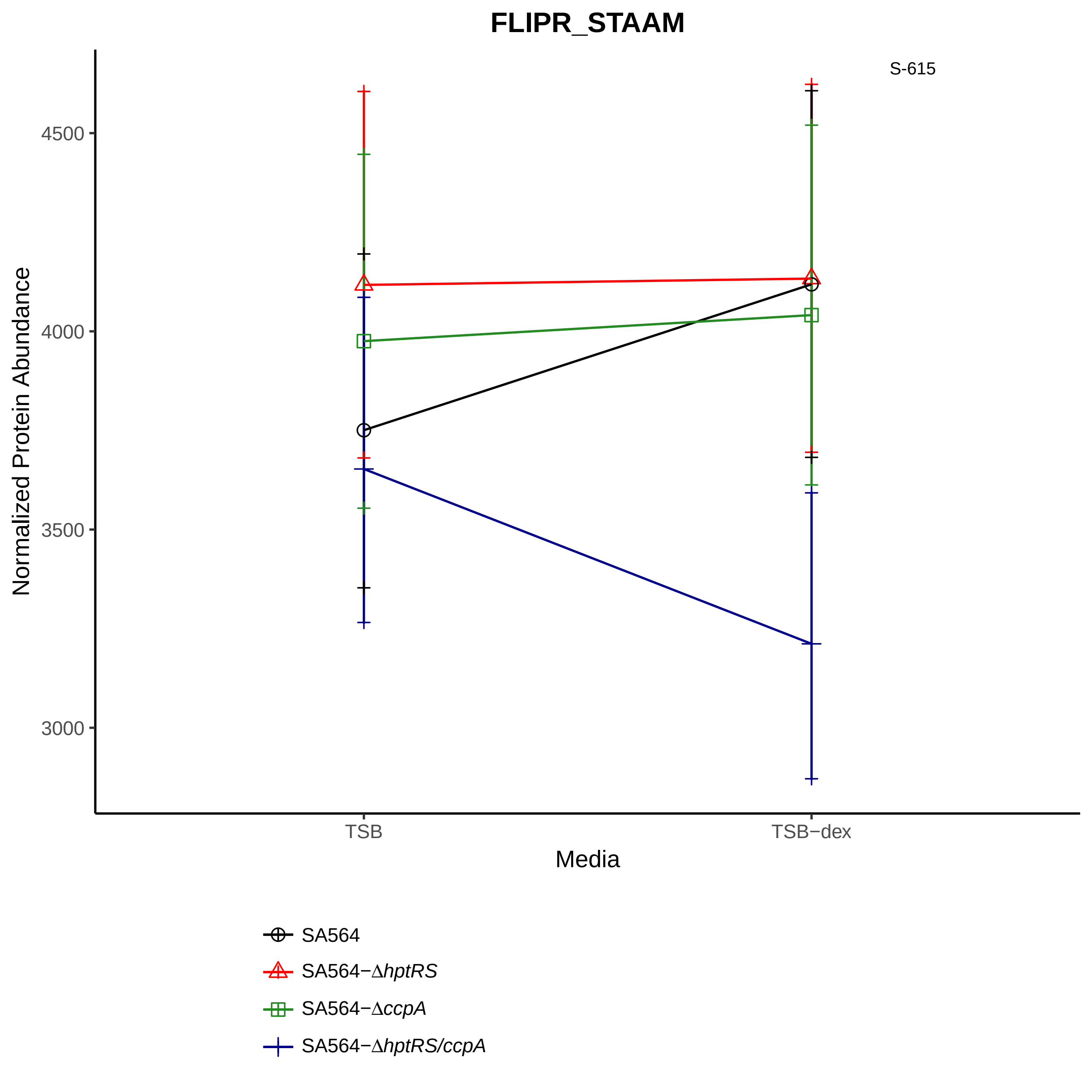

FLIPR_STAAM
S-615
4500
4000
3500
3000
Normalized Protein Abundance
TSB
TSB−dex
Media
SA564
SA564−Δ*hptRS*
SA564−Δ*ccpA*
SA564−Δ*hptRS/ccpA*

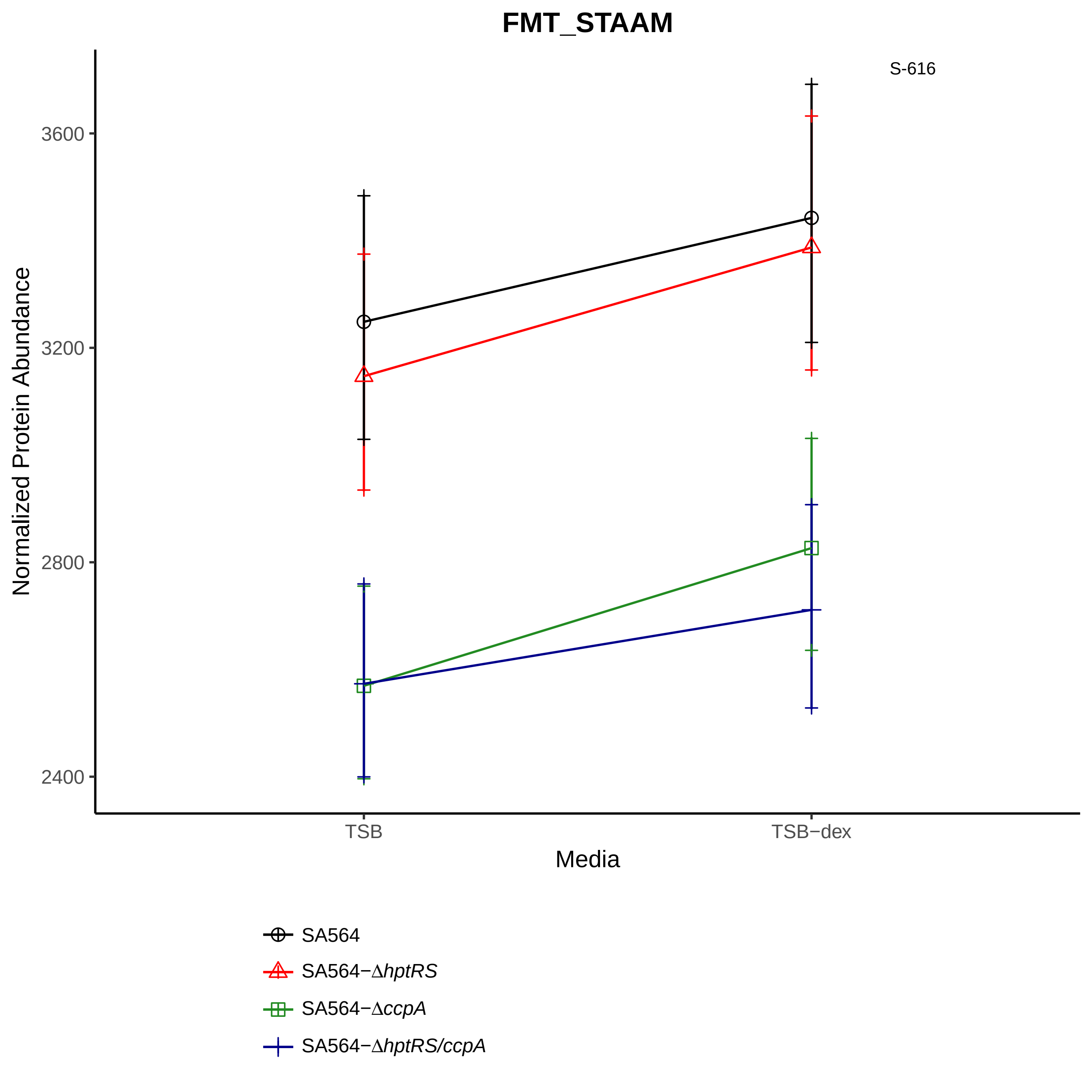

FMT_STAAM
S-616
Normalized Protein Abundance
3600
3200
2800
2400
TSB
TSB−dex
Media
SA564
SA564−Δ*hptRS*
SA564−Δ*ccpA*
SA564−Δ*hptRS/ccpA*

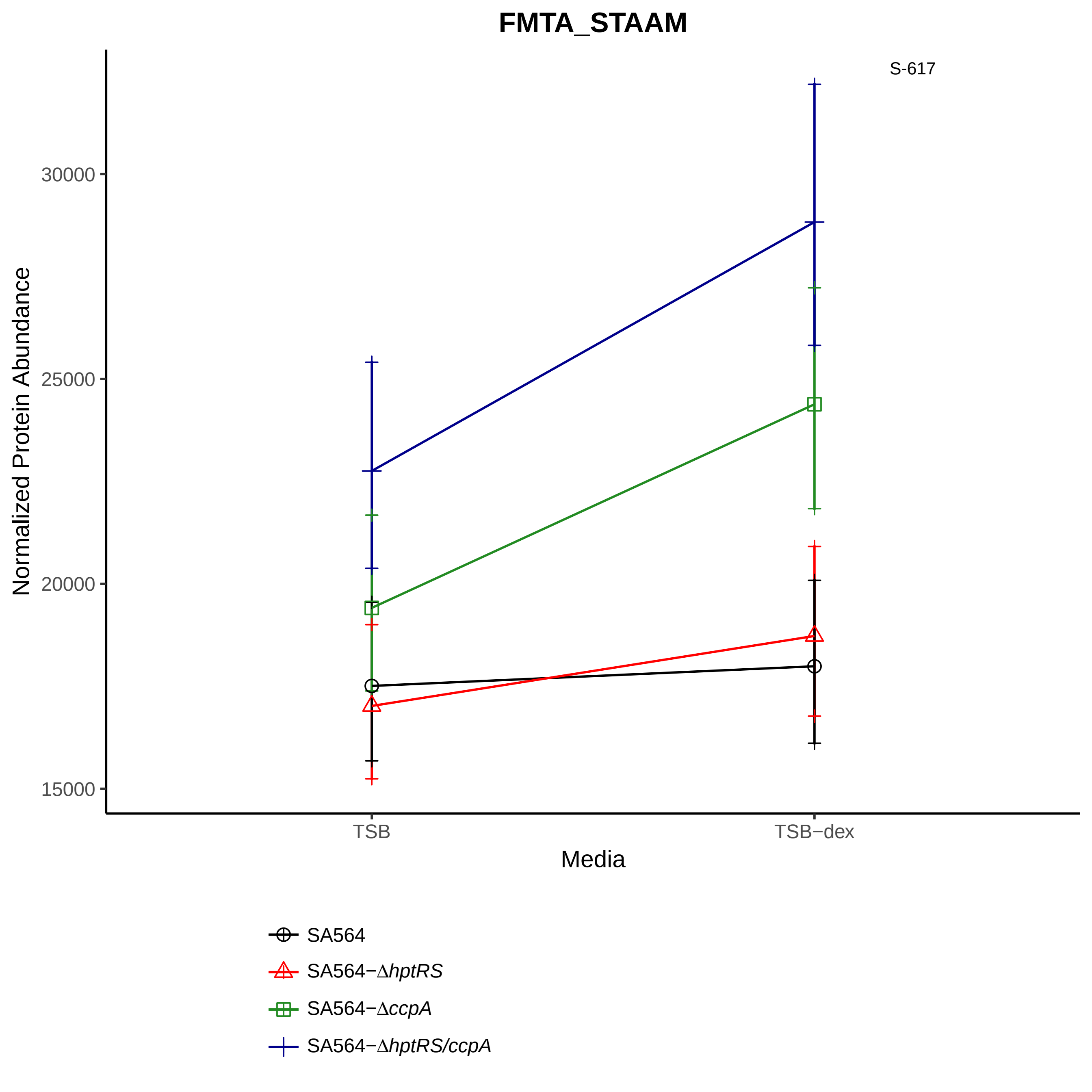

FMTA_STAAM
S-617
Normalized Protein Abundance
30000
25000
20000
15000
TSB
TSB−dex
Media
SA564
SA564−ΔhptRS
SA564−ΔccpA
SA564−ΔhptRS/ccpA

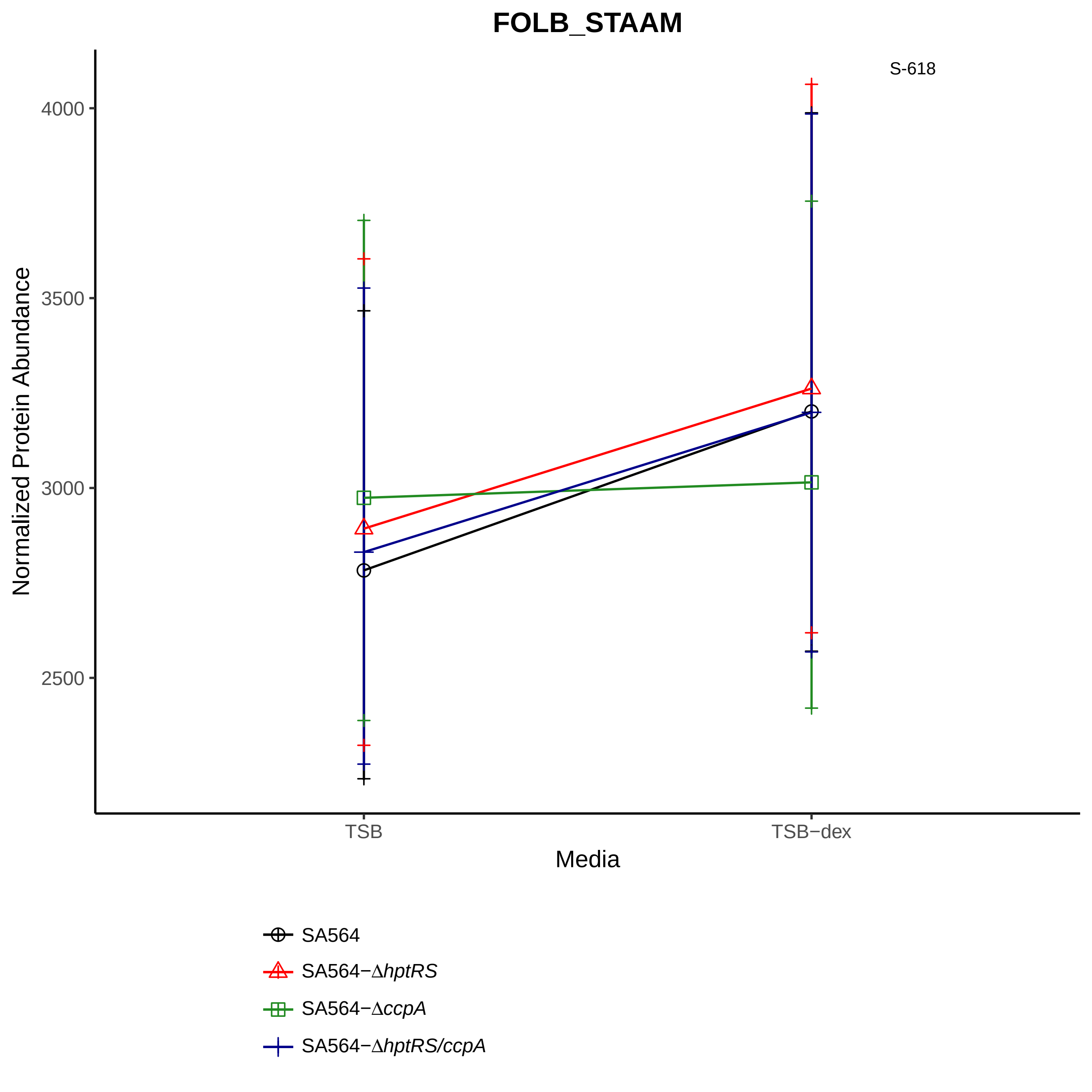
FOLB_STAAM
S-618
Normalized Protein Abundance
4000
3500
3000
2500
TSB
TSB−dex
Media
SA564
SA564−ΔhptRS
SA564−ΔccpA
SA564−ΔhptRS/ccpA

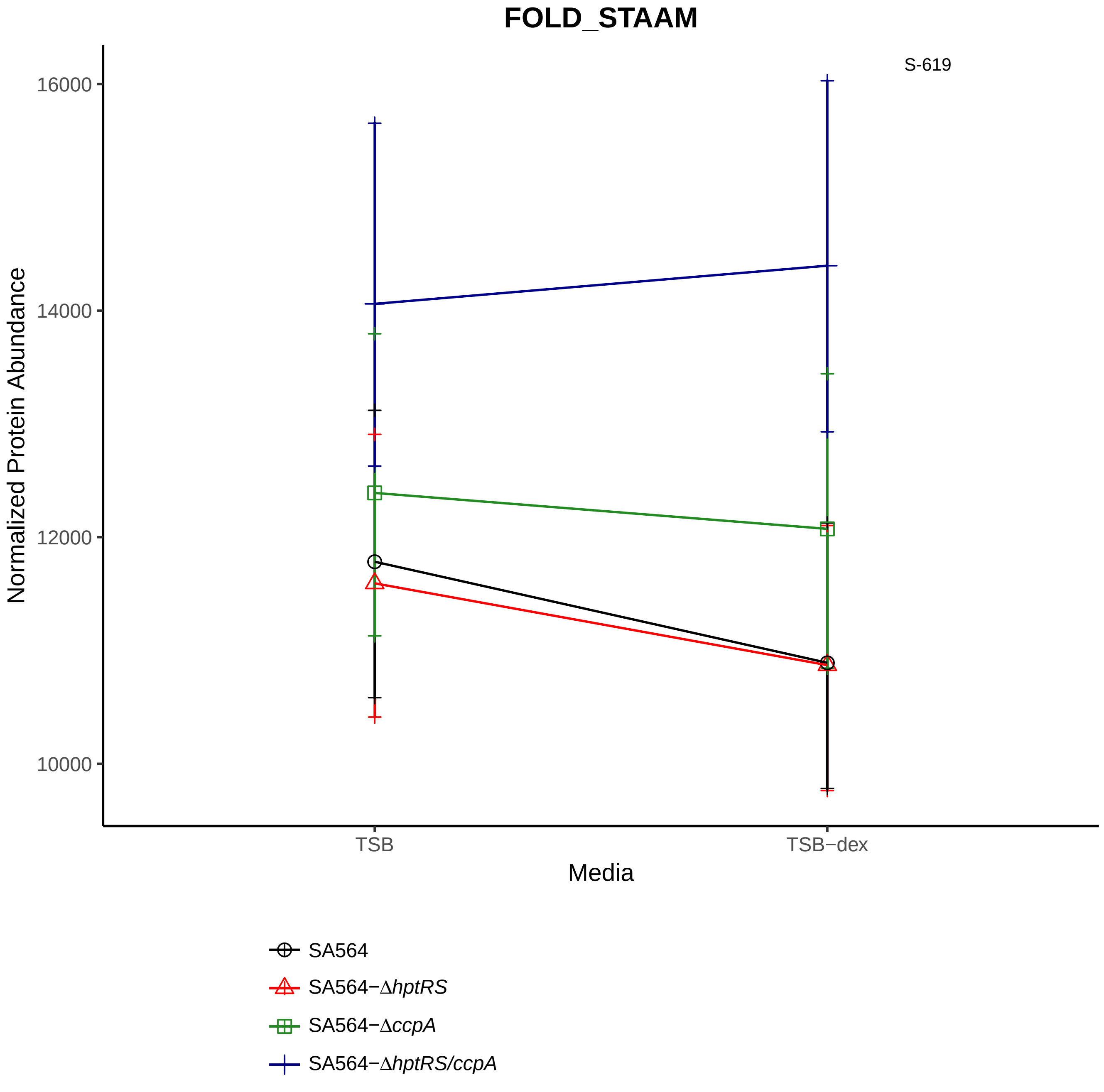
FOLD_STAAM
S-619
16000
14000
12000
10000
Normalized Protein Abundance
TSB
TSB−dex
Media
SA564
SA564−Δ*hptRS*
SA564−Δ*ccpA*
SA564−Δ*hptRS/ccpA*

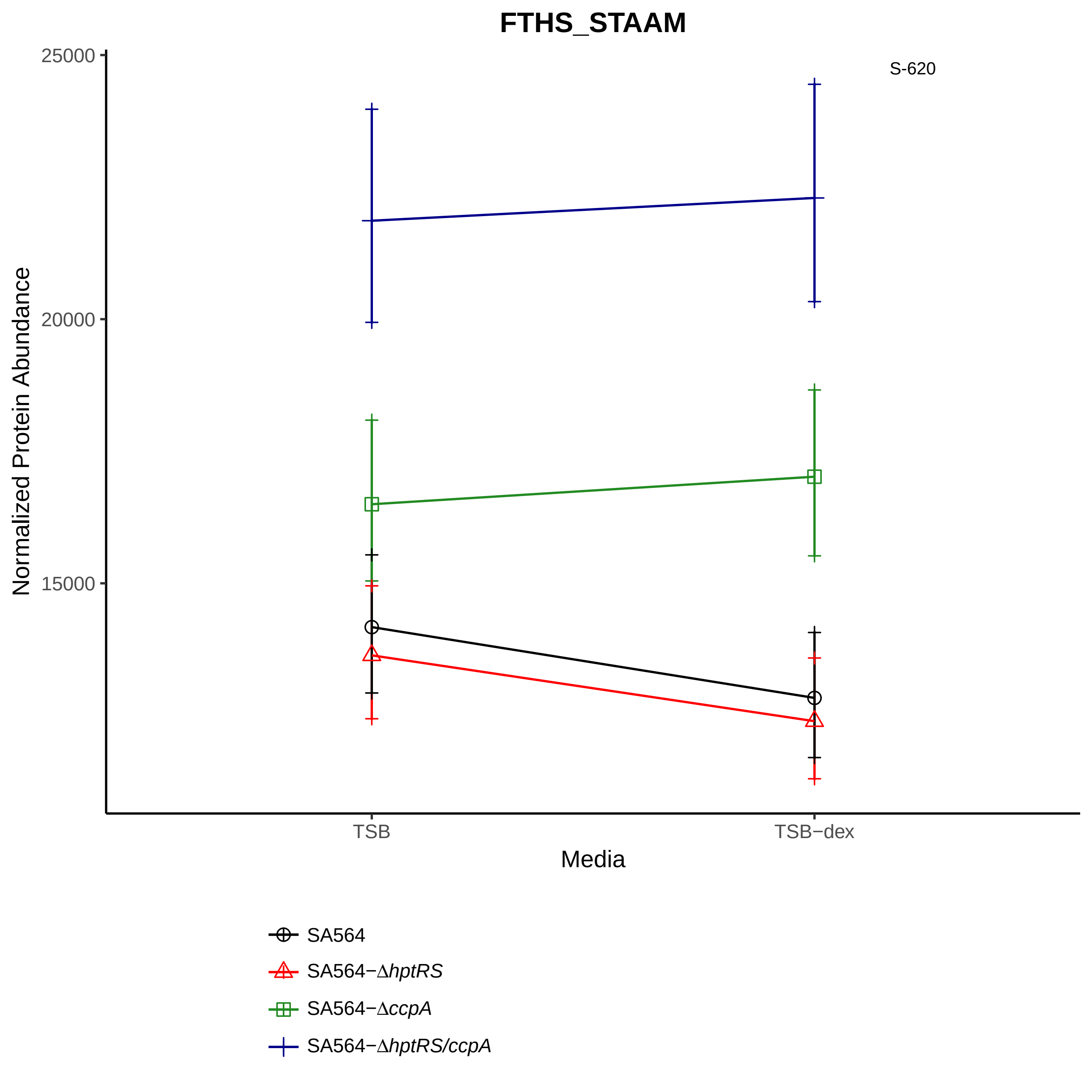

FTHS_STAAM
S-620
Normalized Protein Abundance
25000
20000
15000
TSB
TSB−dex
Media
SA564
SA564−Δ*hptRS*
SA564−Δ*ccpA*
SA564−Δ*hptRS/ccpA*

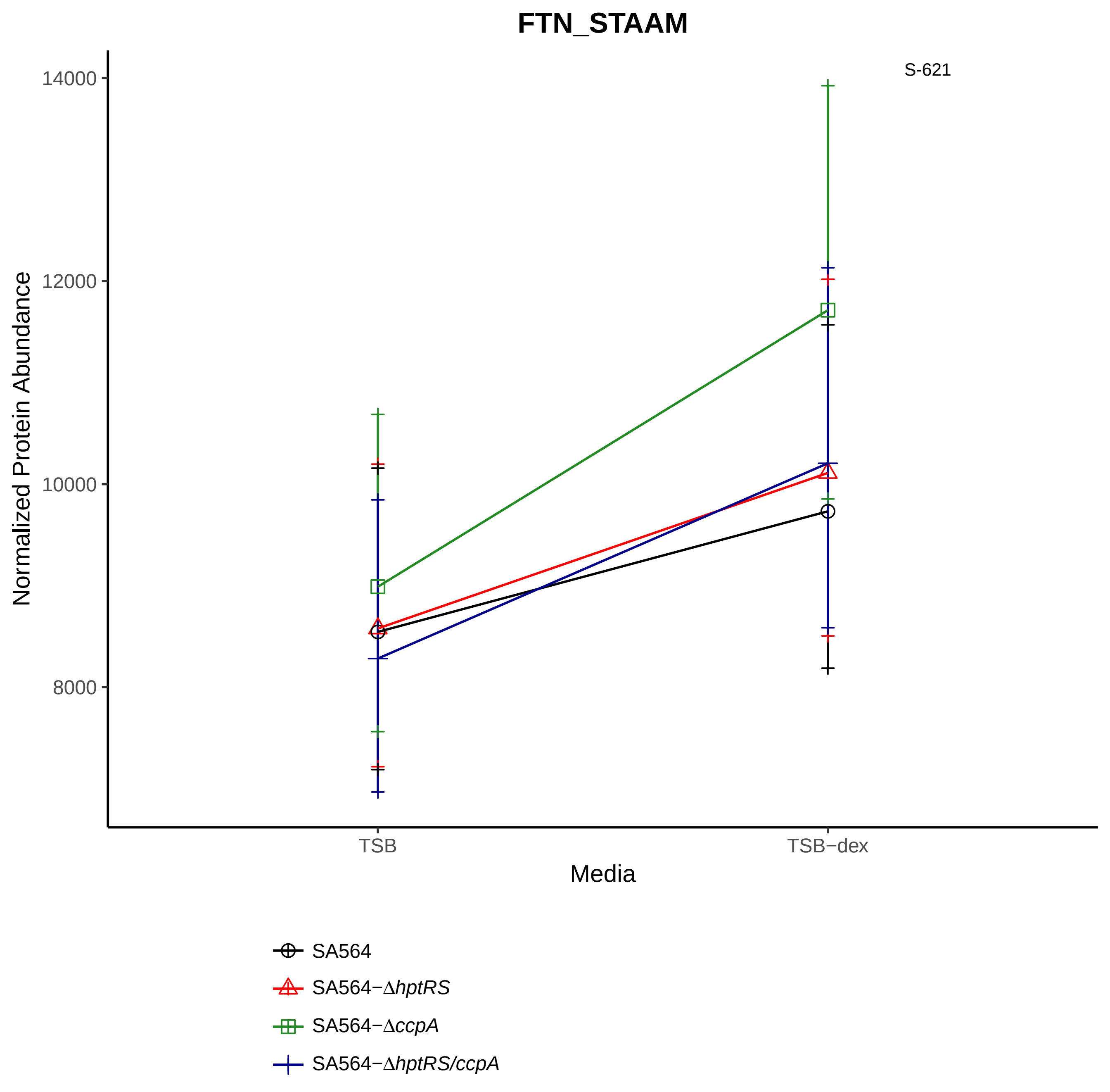
FTN_STAAM
S-621
Normalized Protein Abundance
14000
12000
10000
8000
TSB
TSB−dex
Media
SA564
SA564−ΔhptRS
SA564−ΔccpA
SA564−ΔhptRS/ccpA

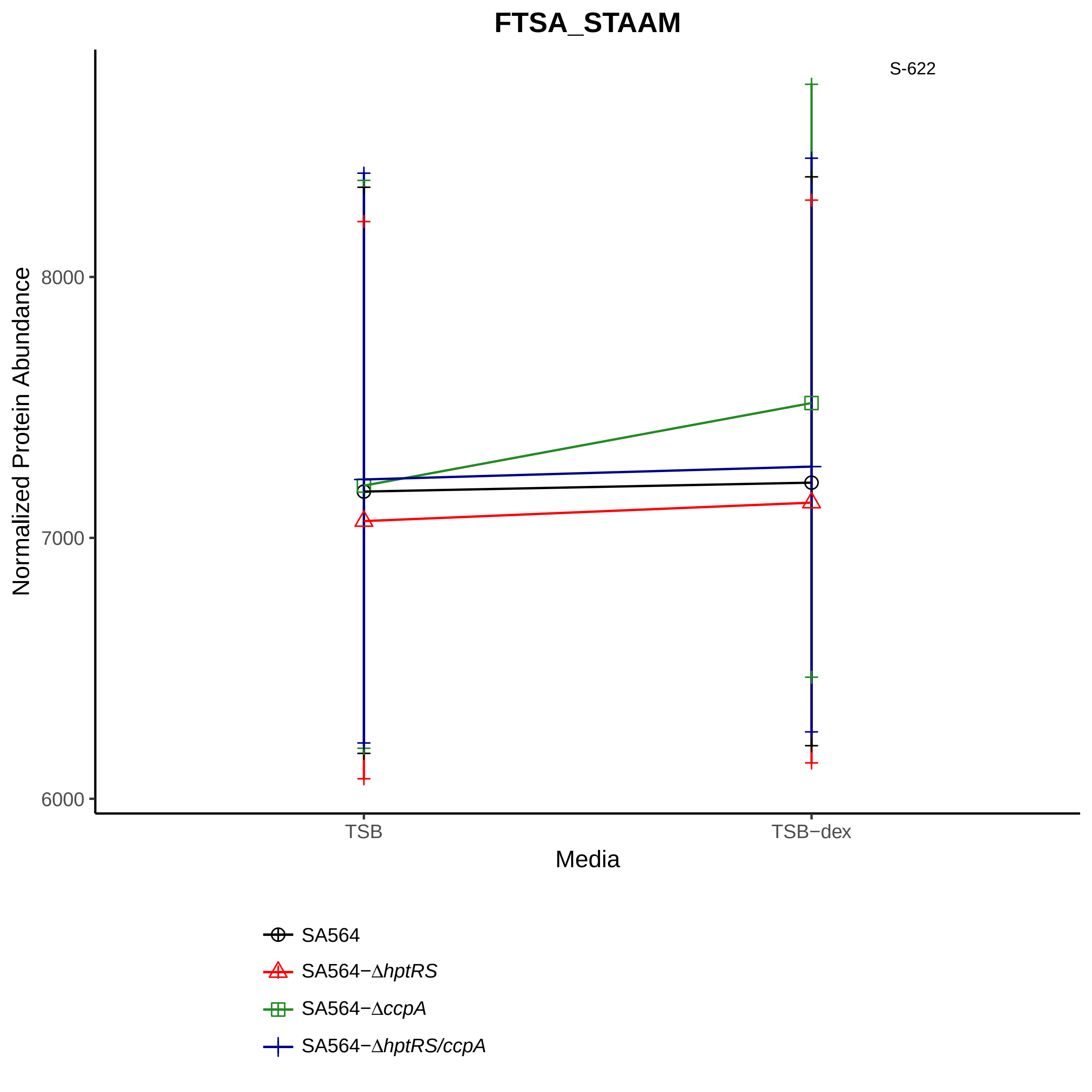

FTSA_STAAM
S-622
Normalized Protein Abundance
8000
7000
6000
TSB
TSB−dex
Media
SA564
SA564−ΔhptRS
SA564−ΔccpA
SA564−ΔhptRS/ccpA

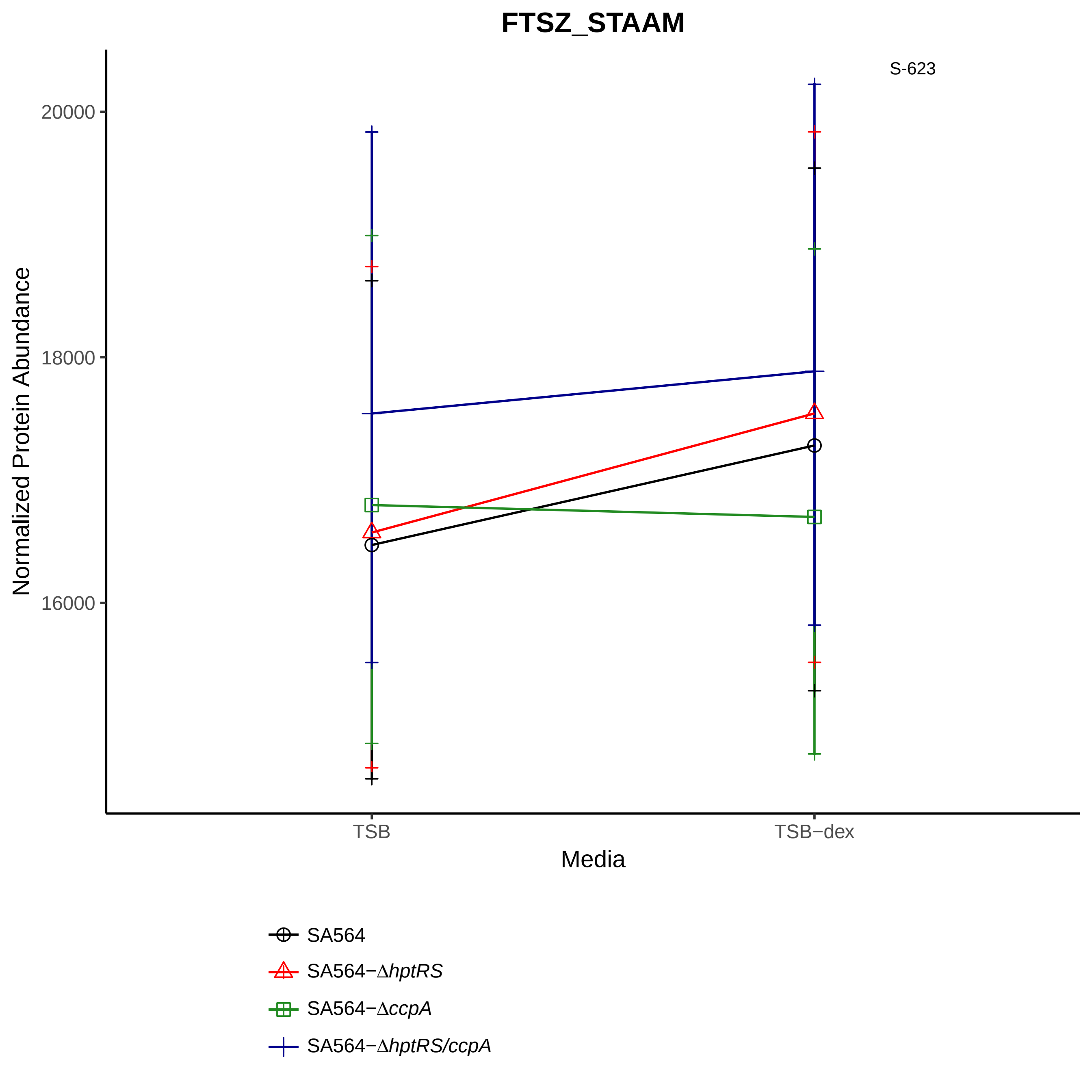
FTSZ_STAAM
S-623
20000
18000
16000
Normalized Protein Abundance
TSB
TSB−dex
Media
SA564
SA564−ΔhptRS
SA564−ΔccpA
SA564−ΔhptRS/ccpA

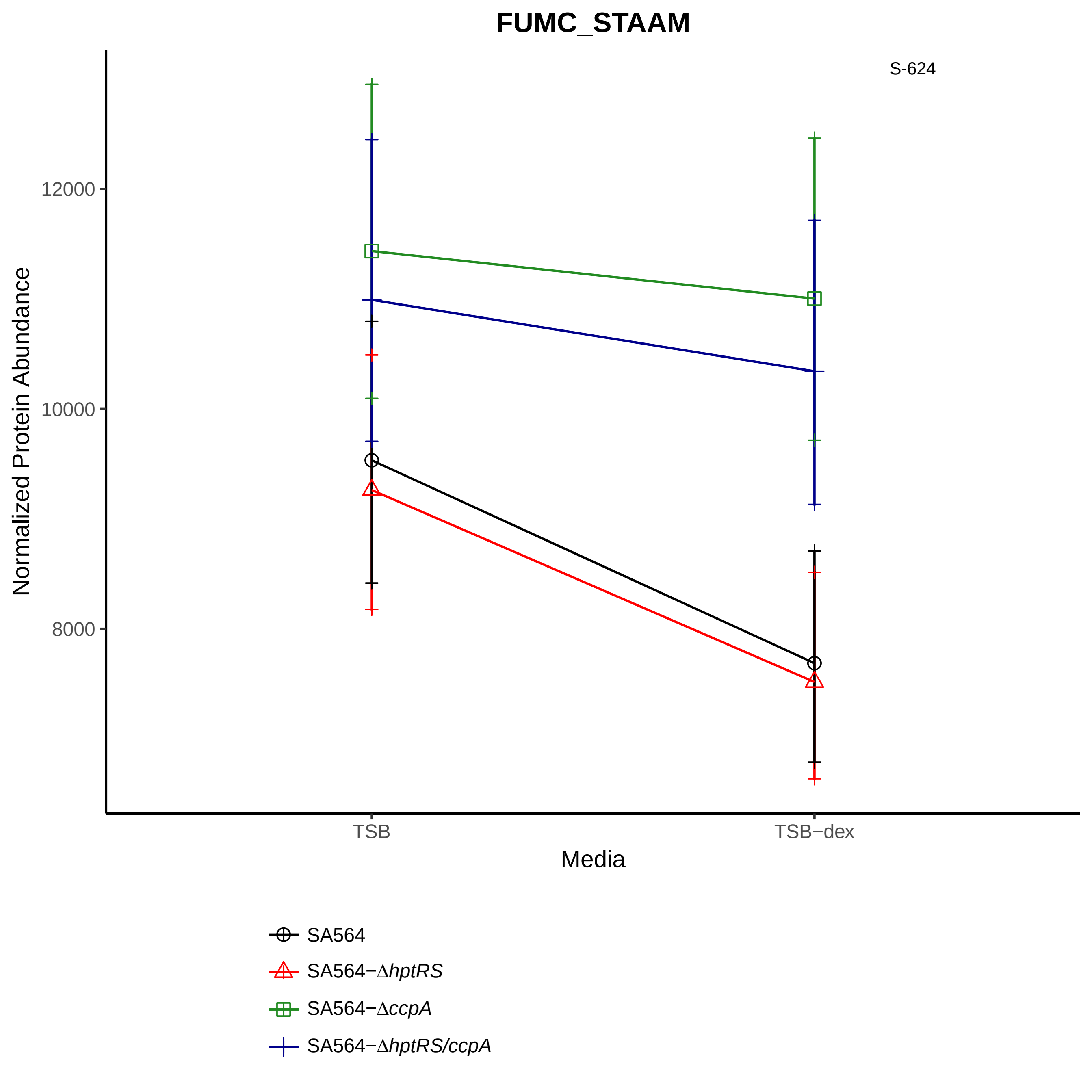
FUMC_STAAM
S-624
Normalized Protein Abundance
12000
10000
8000
TSB
TSB−dex
Media
SA564
SA564−Δ*hptRS*
SA564−Δ*ccpA*
SA564−Δ*hptRS/ccpA*

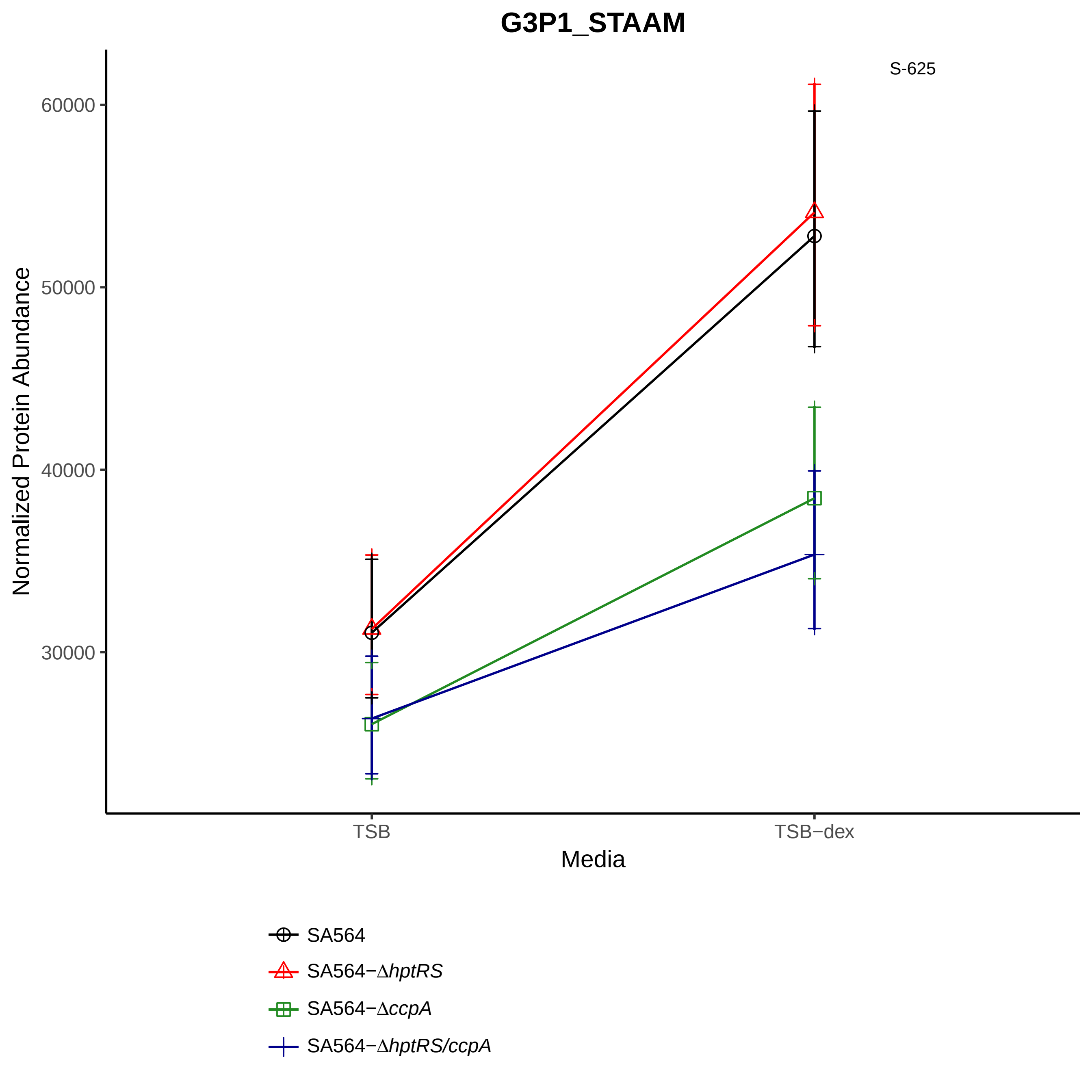

G3P1_STAAM
S-625
Normalized Protein Abundance
60000
50000
40000
30000
TSB
TSB−dex
Media
SA564
SA564−ΔhptRS
SA564−ΔccpA
SA564−ΔhptRS/ccpA

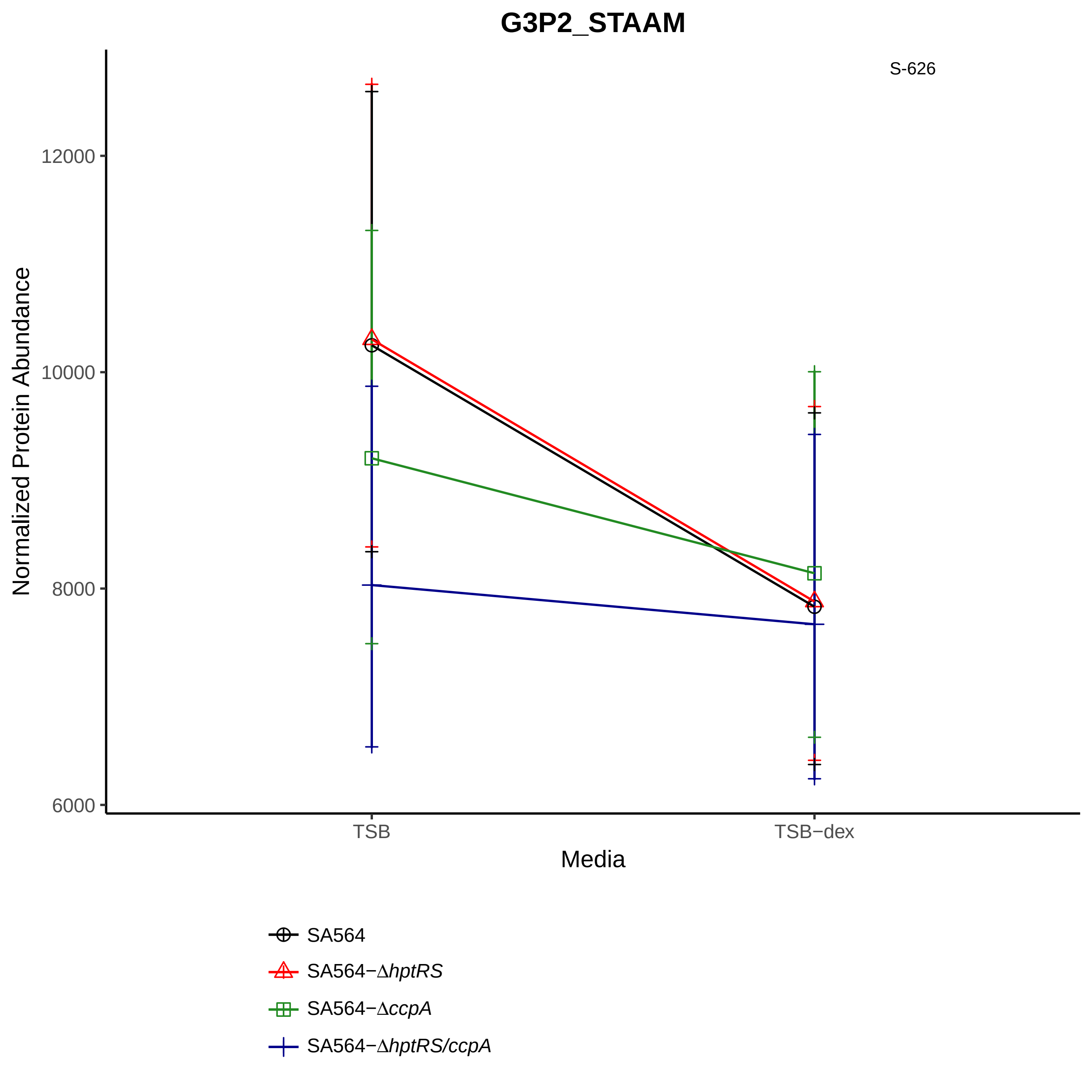

G3P2_STAAM
S-626
Normalized Protein Abundance
12000
10000
8000
6000
TSB
TSB−dex
Media
SA564
SA564−ΔhptRS
SA564−ΔccpA
SA564−ΔhptRS/ccpA

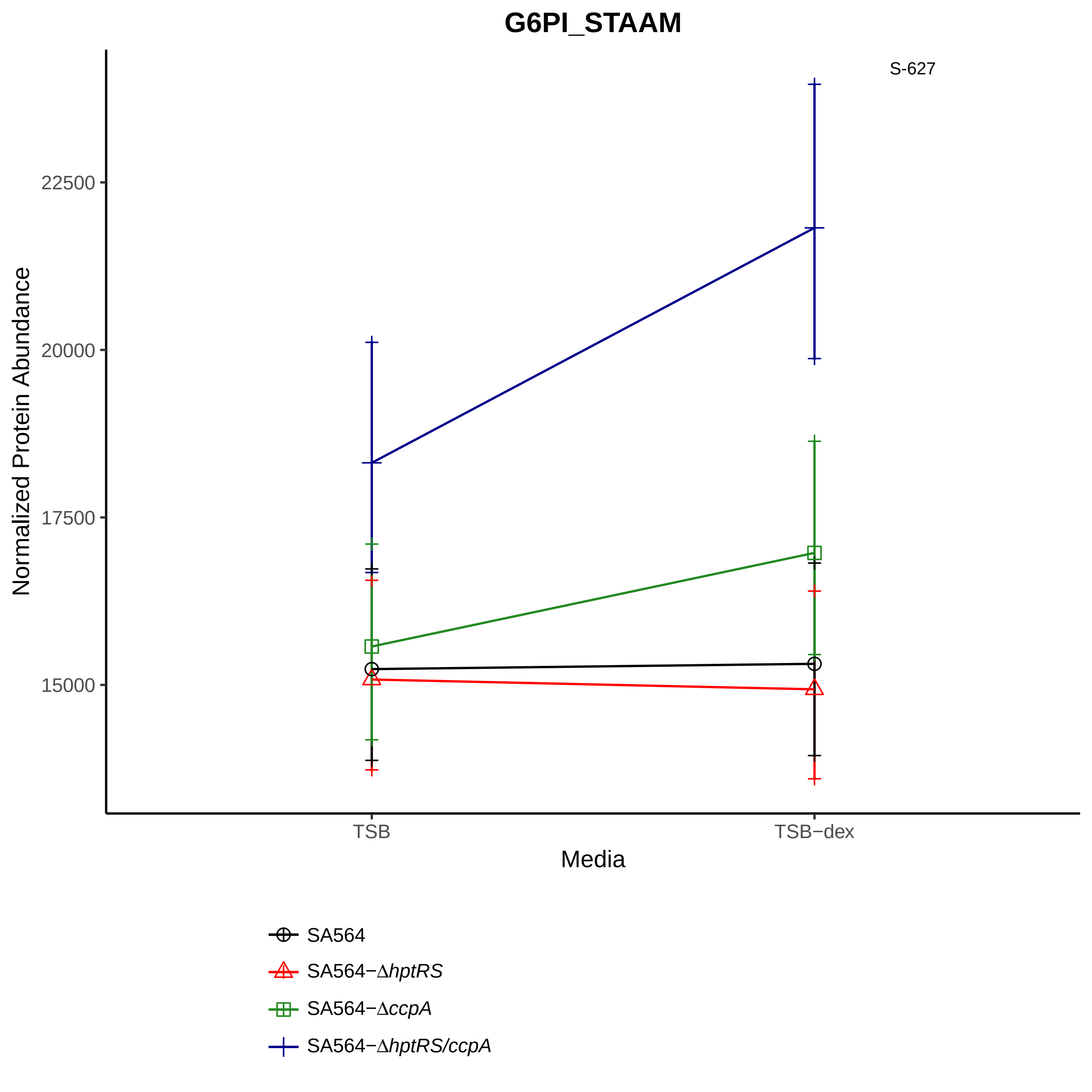

G6PI_STAAM
S-627
Normalized Protein Abundance
22500
20000
17500
15000
TSB
TSB−dex
Media
SA564
SA564−Δ*hptRS*
SA564−Δ*ccpA*
SA564−Δ*hptRS/ccpA*

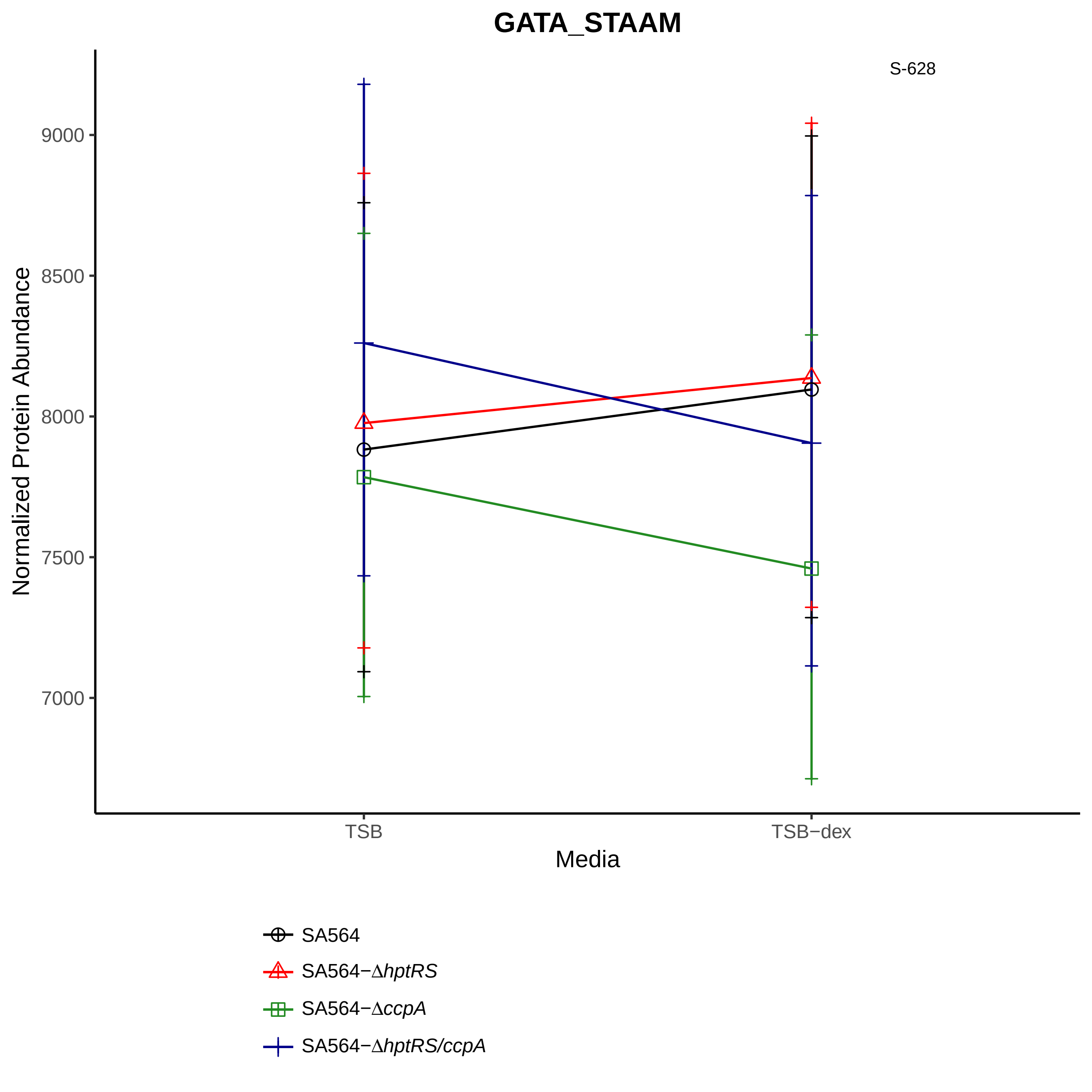

GATA_STAAM
S-628
Normalized Protein Abundance
9000
8500
8000
7500
7000
TSB
TSB−dex
Media
SA564
SA564−ΔhptRS
SA564−ΔccpA
SA564−ΔhptRS/ccpA

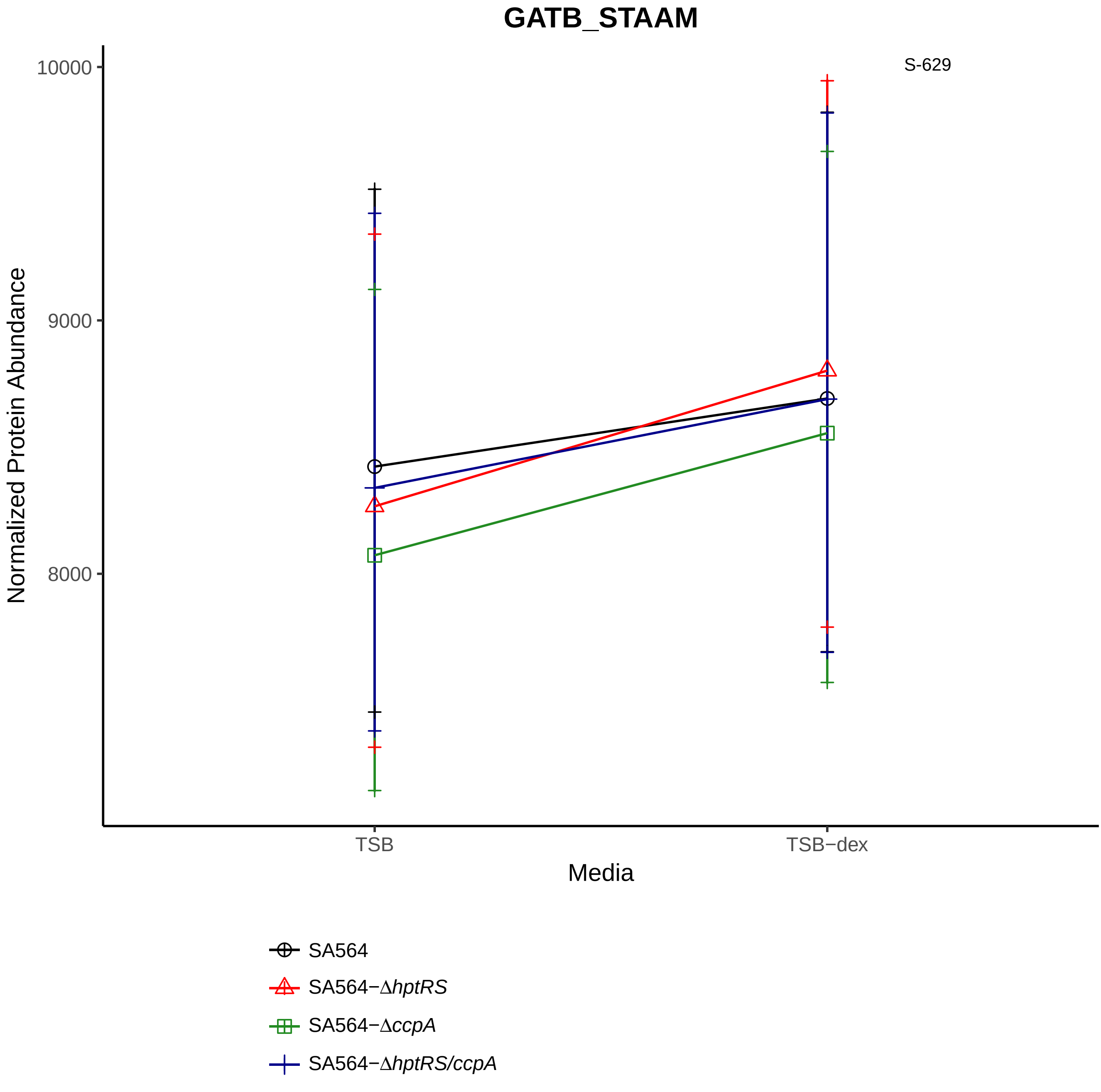

GATB_STAAM
S-629
Normalized Protein Abundance
10000
9000
8000
TSB
TSB−dex
Media
SA564
SA564−Δ*hptRS*
SA564−Δ*ccpA*
SA564−Δ*hptRS/ccpA*

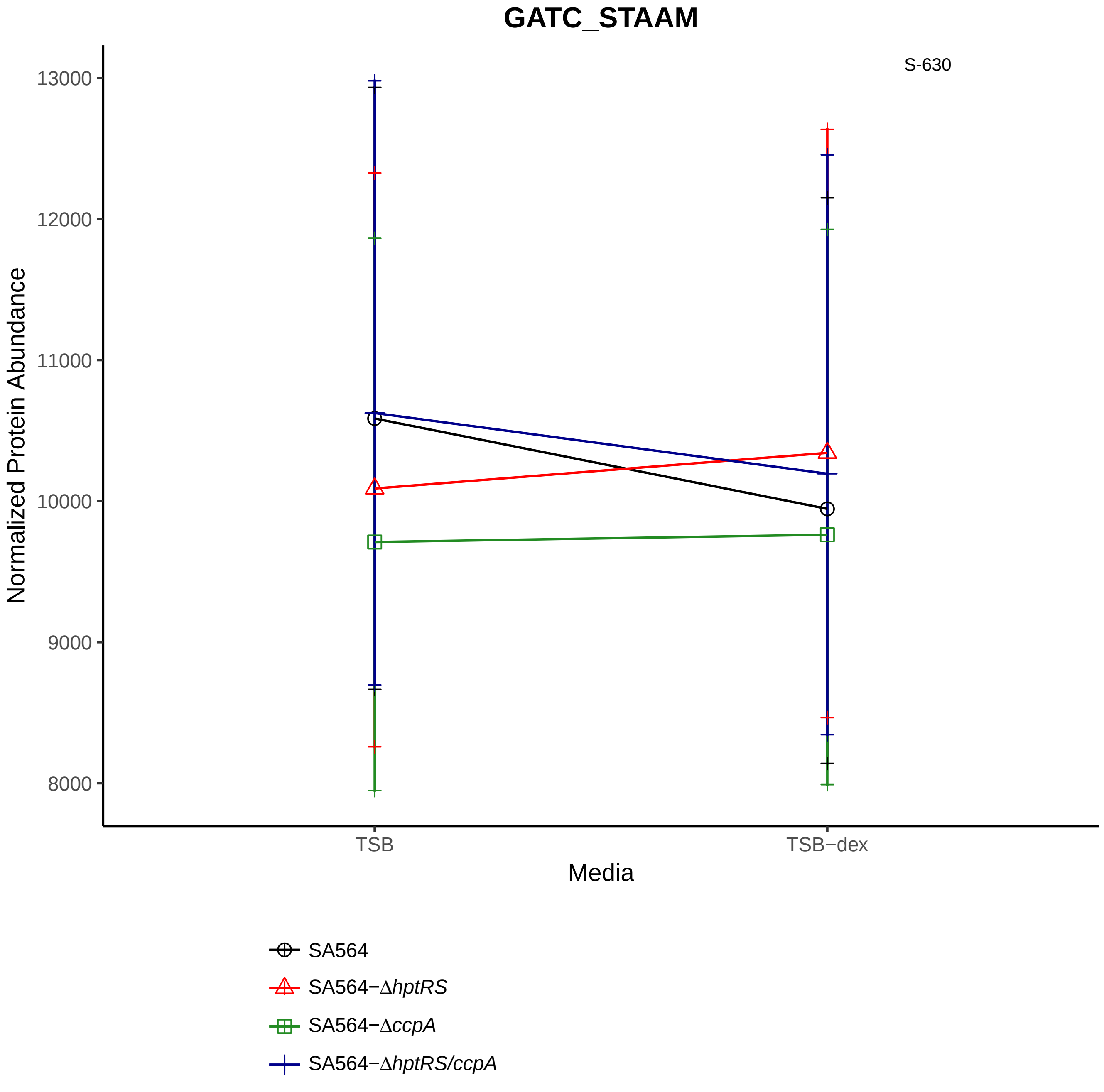
GATC_STAAM
S-630
Normalized Protein Abundance
13000
12000
11000
10000
9000
8000
TSB
TSB−dex
Media
SA564
SA564−Δ*hptRS*
SA564−Δ*ccpA*
SA564−Δ*hptRS/ccpA*

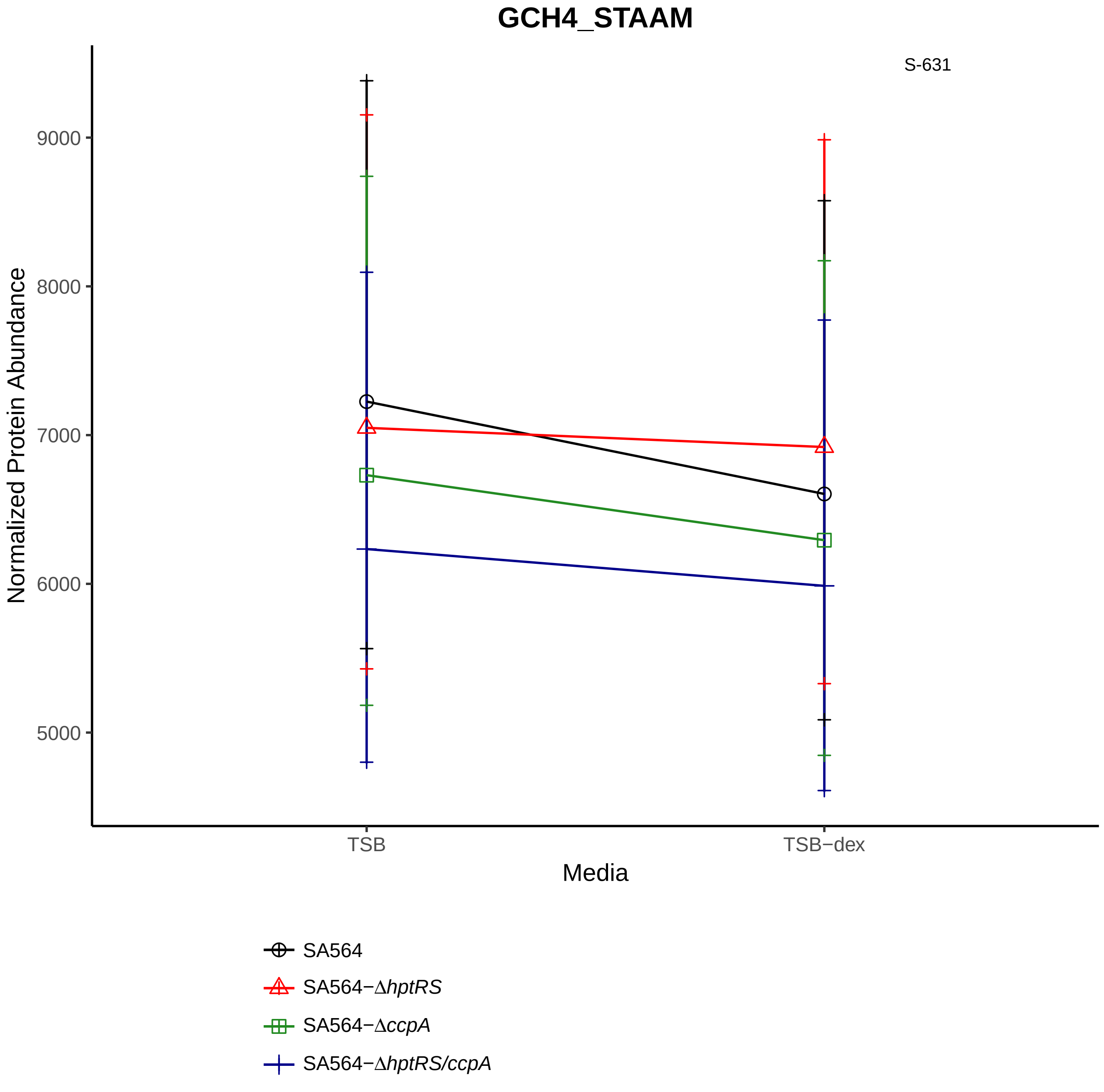

GCH4_STAAM
S-631
Normalized Protein Abundance
9000
8000
7000
6000
5000
TSB
TSB−dex
Media
SA564
SA564−Δ*hptRS*
SA564−Δ*ccpA*
SA564−Δ*hptRS/ccpA*

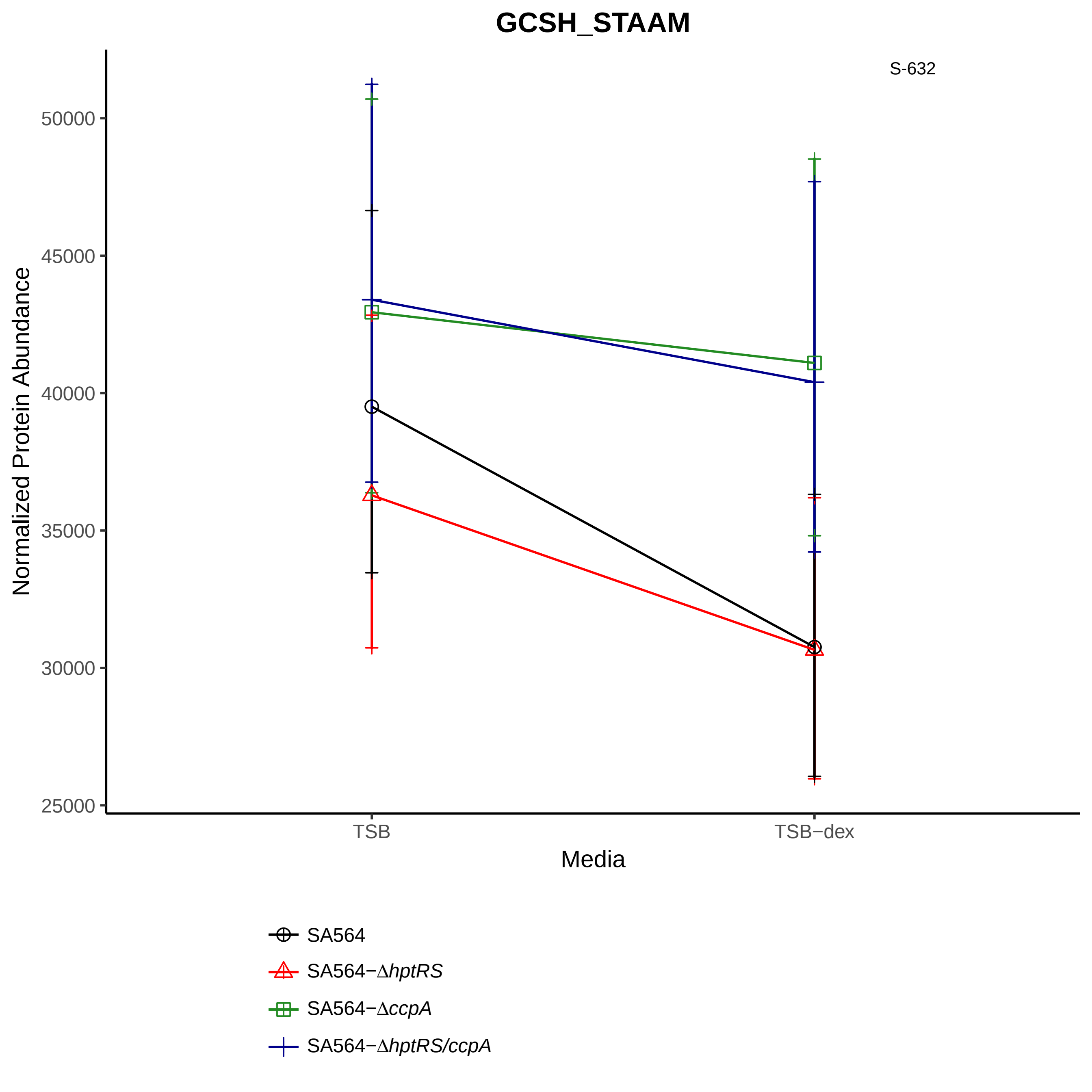

GCSH_STAAM
S-632
Normalized Protein Abundance
50000
45000
40000
35000
30000
25000
TSB
TSB−dex
Media
SA564
SA564−ΔhptRS
SA564−ΔccpA
SA564−ΔhptRS/ccpA

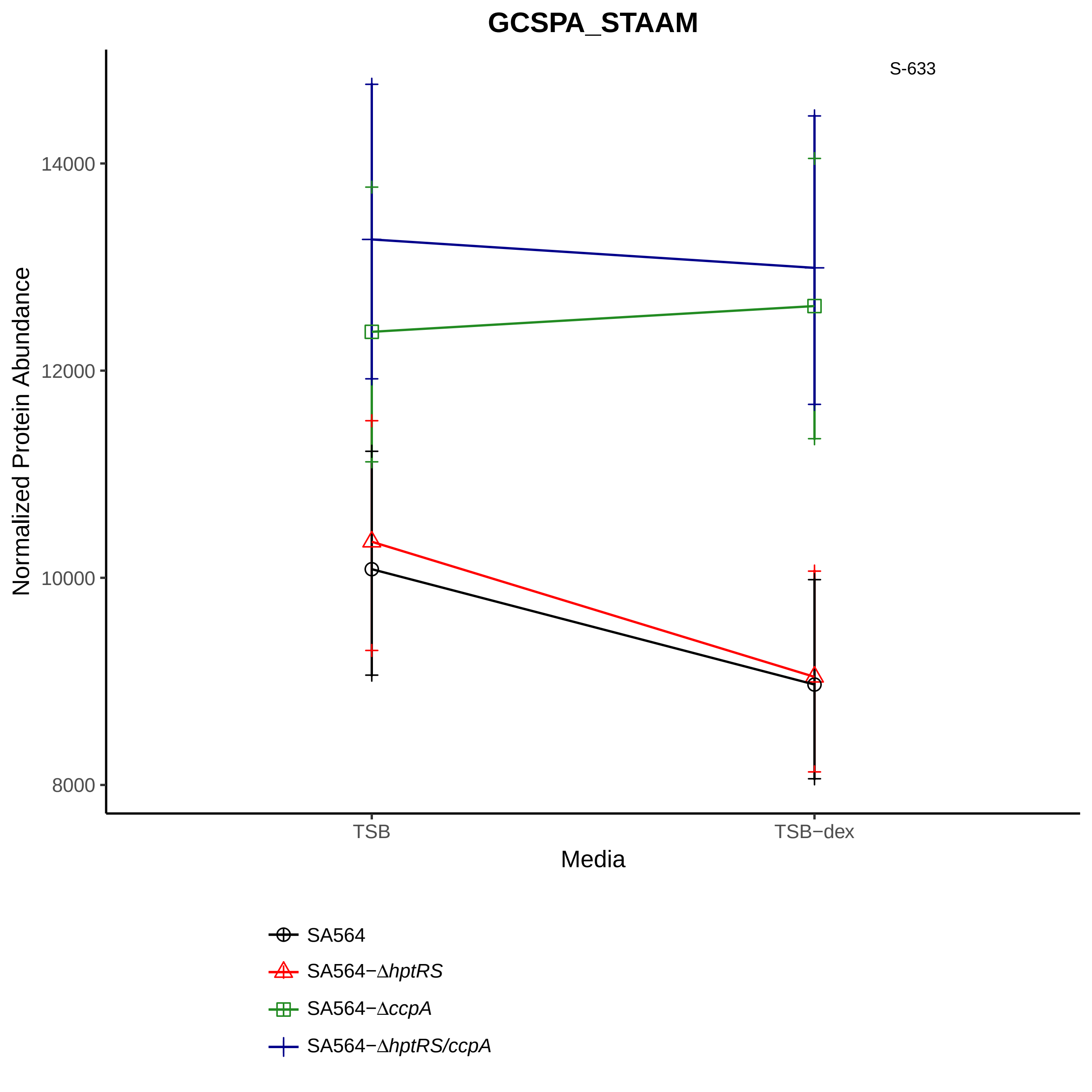

GCSPA_STAAM
S-633
Normalized Protein Abundance
14000
12000
10000
8000
TSB
TSB−dex
Media
SA564
SA564−ΔhptRS
SA564−ΔccpA
SA564−ΔhptRS/ccpA

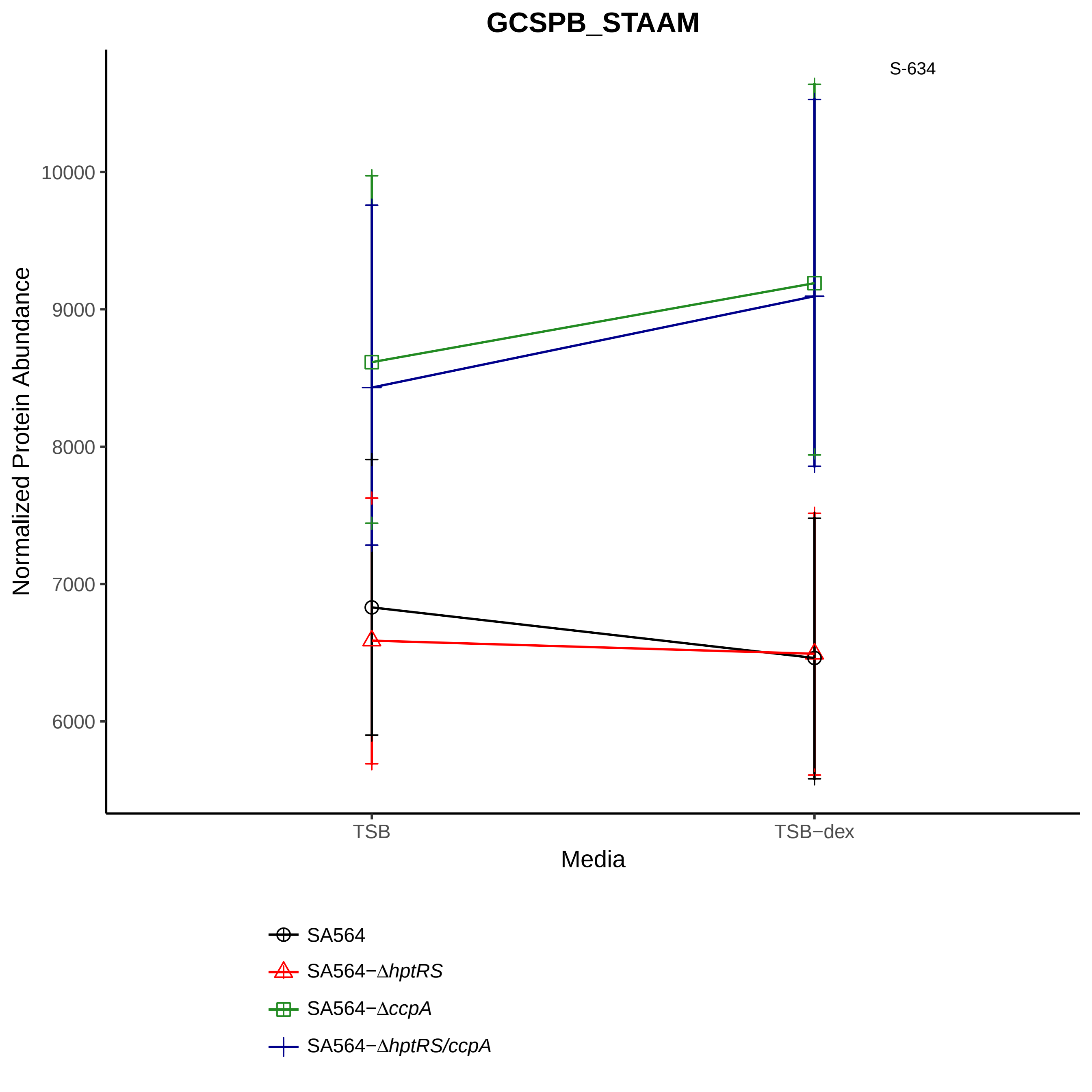

GCSPB_STAAM
S-634
Normalized Protein Abundance
10000
9000
8000
7000
6000
TSB
TSB−dex
Media
SA564
SA564−ΔhptRS
SA564−ΔccpA
SA564−ΔhptRS/ccpA

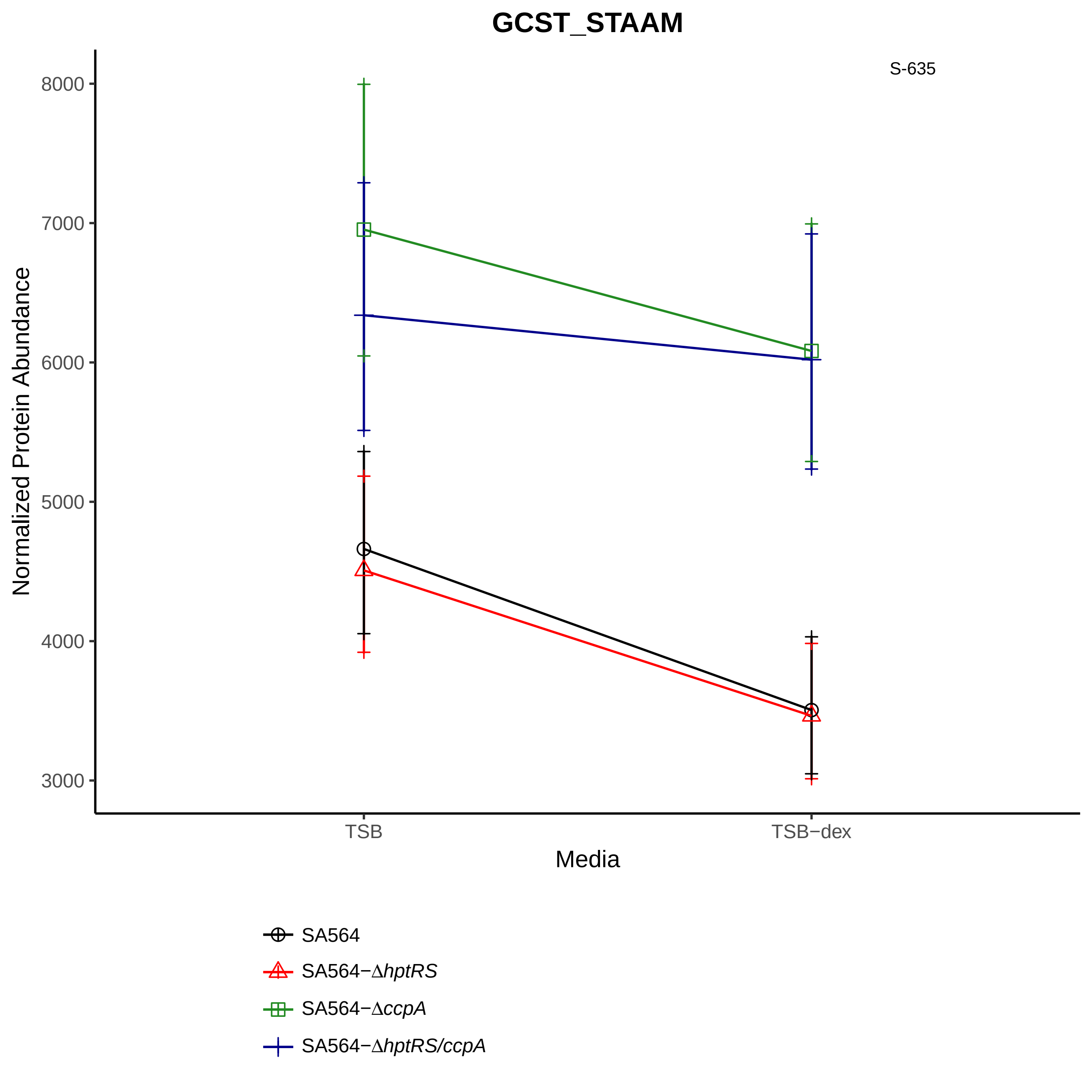

GCST_STAAM
S-635
Normalized Protein Abundance
8000
7000
6000
5000
4000
3000
TSB
TSB−dex
Media
SA564
SA564−Δ*hptRS*
SA564−Δ*ccpA*
SA564−Δ*hptRS/ccpA*

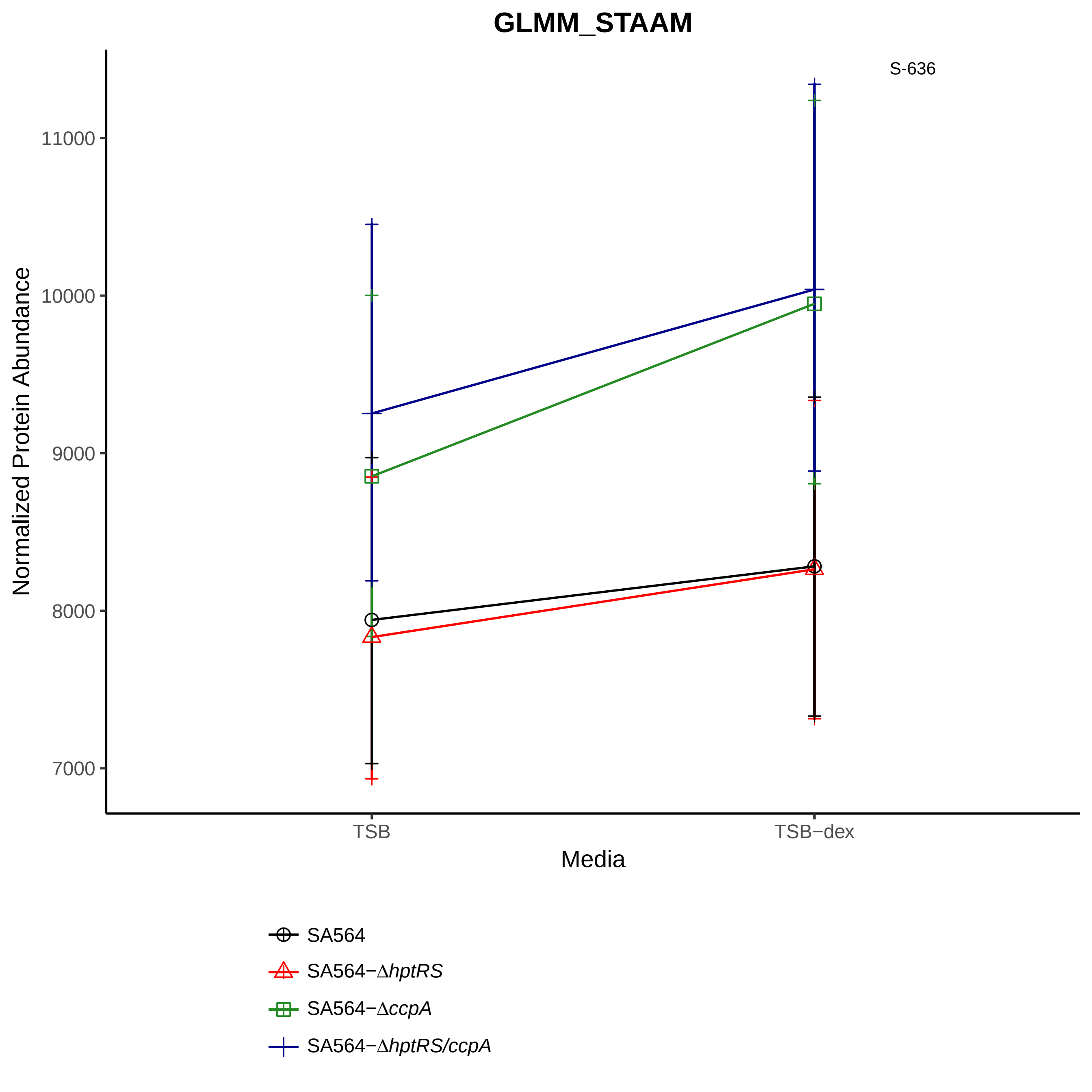
GLMM_STAAM
S-636
Normalized Protein Abundance
11000
10000
9000
8000
7000
TSB
TSB−dex
Media
SA564
SA564−Δ*hptRS*
SA564−Δ*ccpA*
SA564−Δ*hptRS/ccpA*

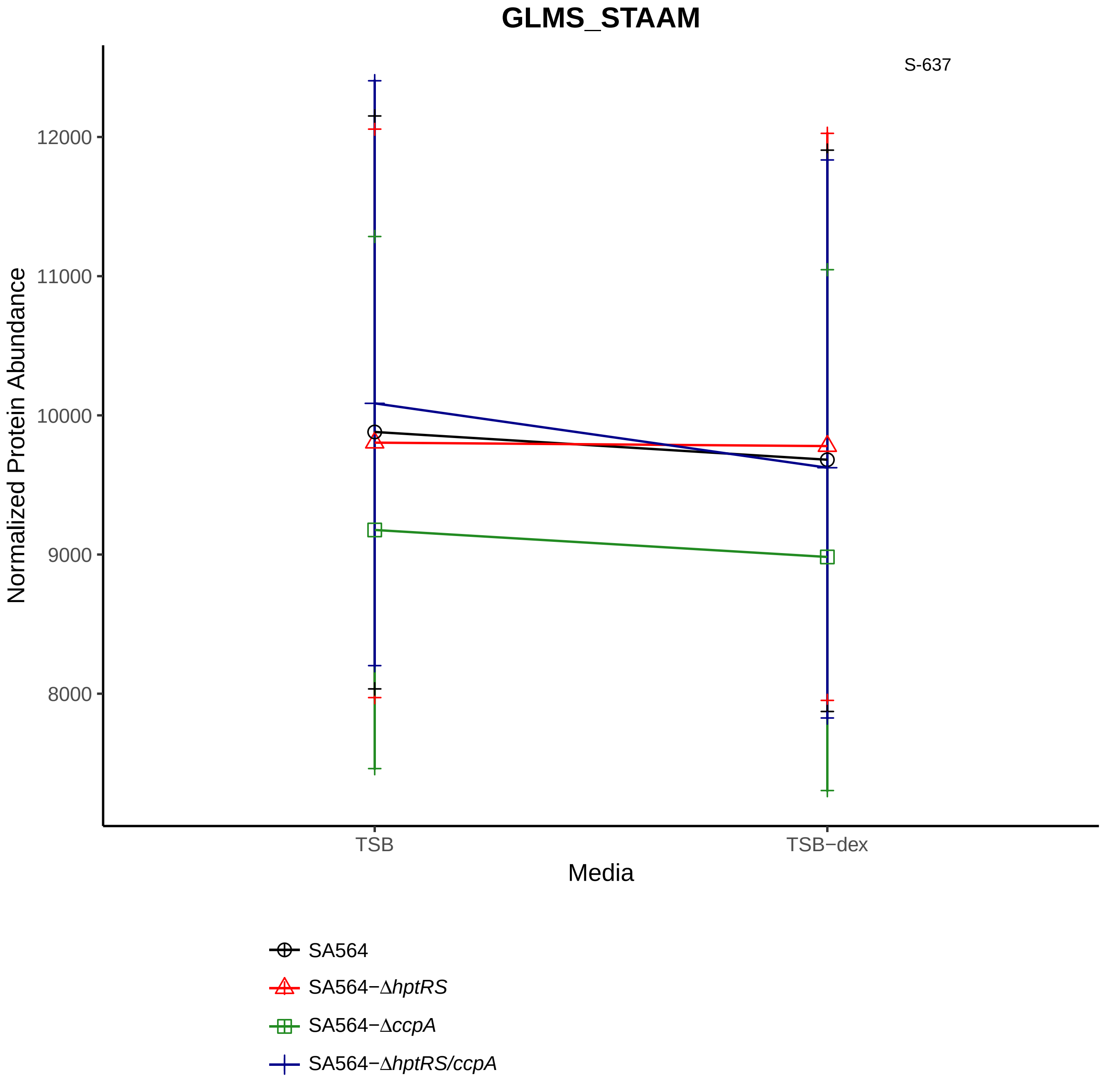

GLMS_STAAM
S-637
Normalized Protein Abundance
12000
11000
10000
9000
8000
TSB
TSB−dex
Media
SA564
SA564−Δ*hptRS*
SA564−Δ*ccpA*
SA564−Δ*hptRS/ccpA*

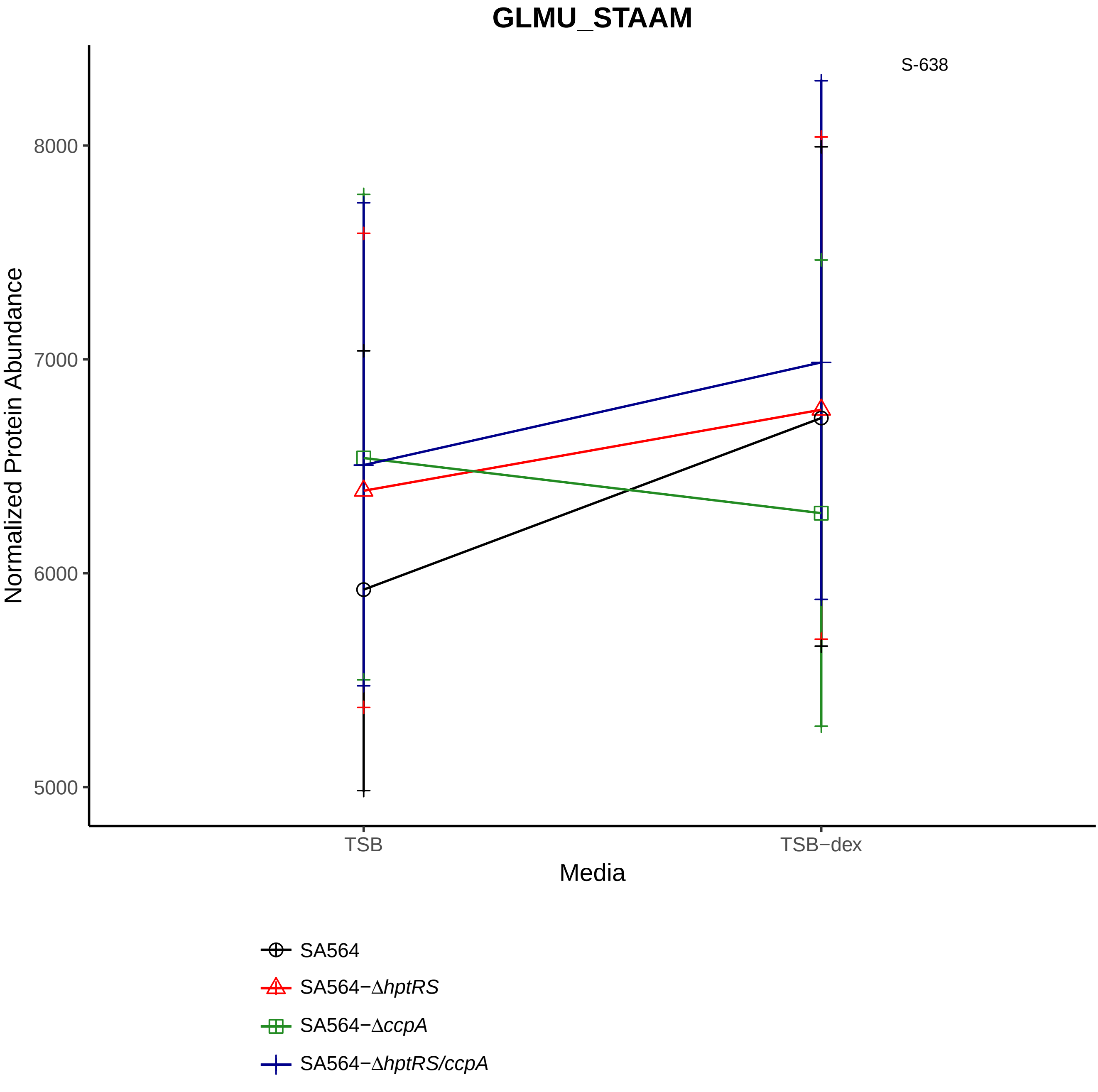

GLMU_STAAM
S-638
Normalized Protein Abundance
8000
7000
6000
5000
TSB
TSB−dex
Media
SA564
SA564−Δ*hptRS*
SA564−Δ*ccpA*
SA564−Δ*hptRS/ccpA*

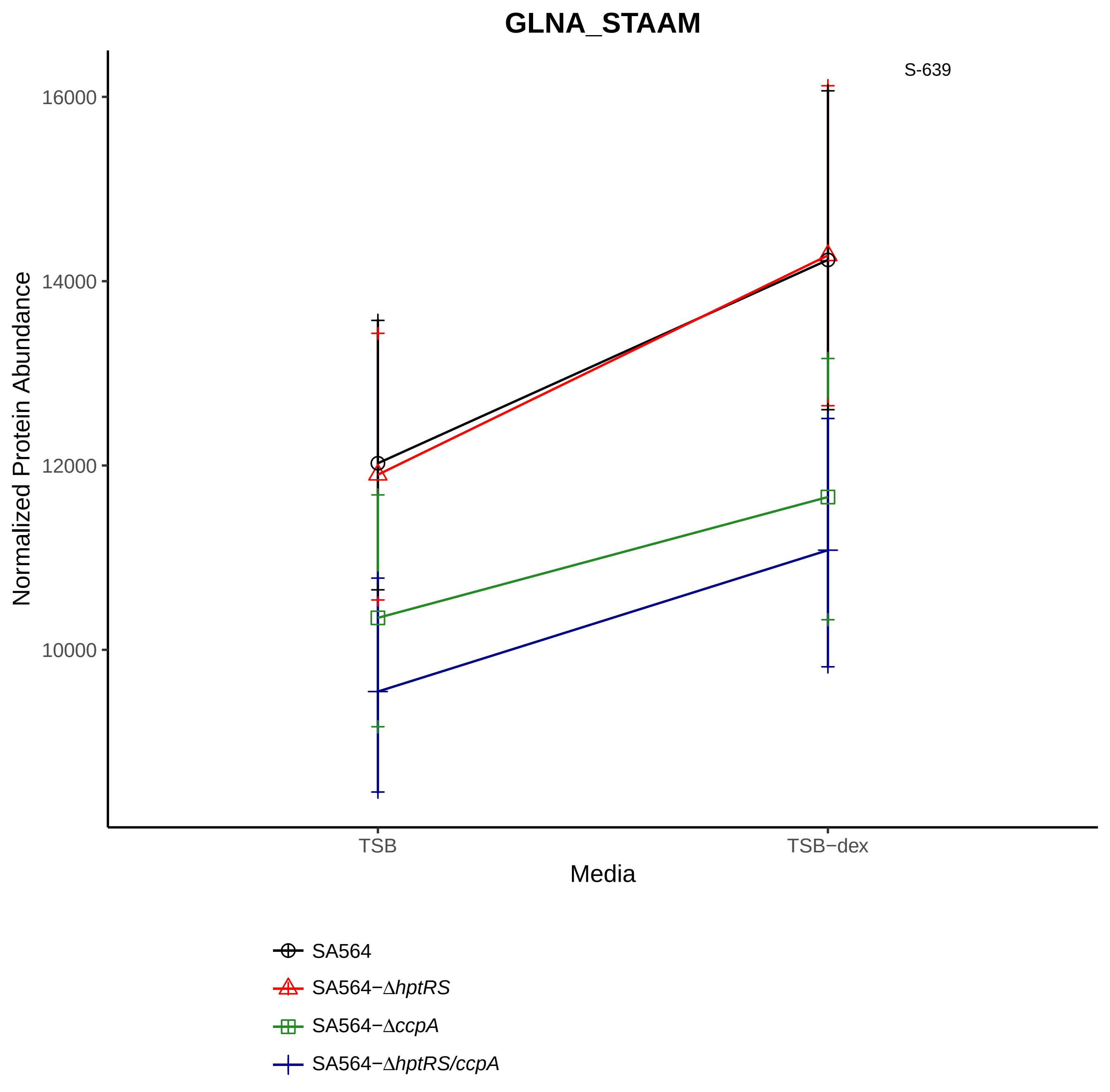

GLNA_STAAM
S-639
16000
14000
12000
10000
Normalized Protein Abundance
TSB
TSB−dex
Media
SA564
SA564−Δ*hptRS*
SA564−Δ*ccpA*
SA564−Δ*hptRS/ccpA*

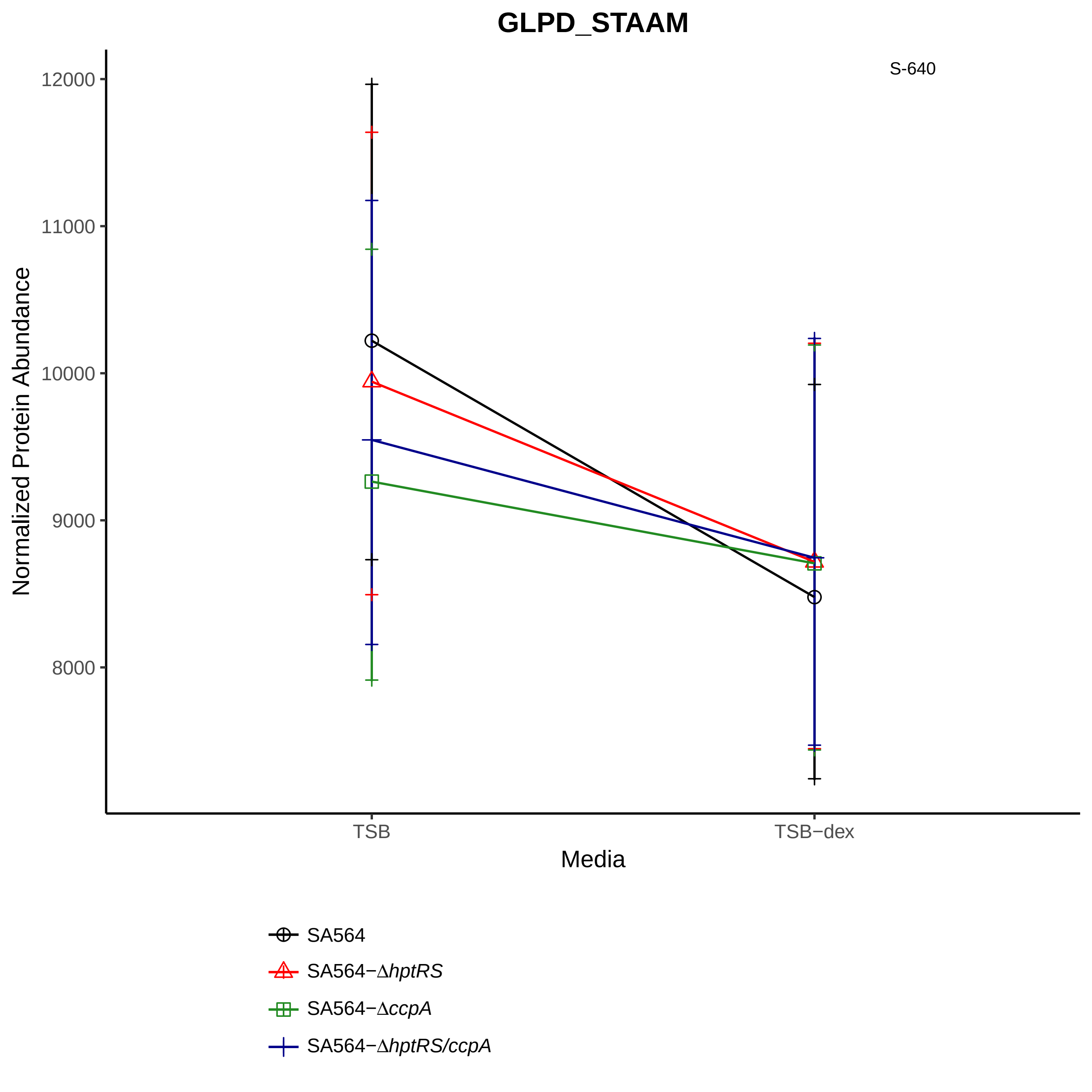

GLPD_STAAM
S-640
Normalized Protein Abundance
12000
11000
10000
9000
8000
TSB
TSB−dex
Media
SA564
SA564−ΔhptRS
SA564−ΔccpA
SA564−ΔhptRS/ccpA

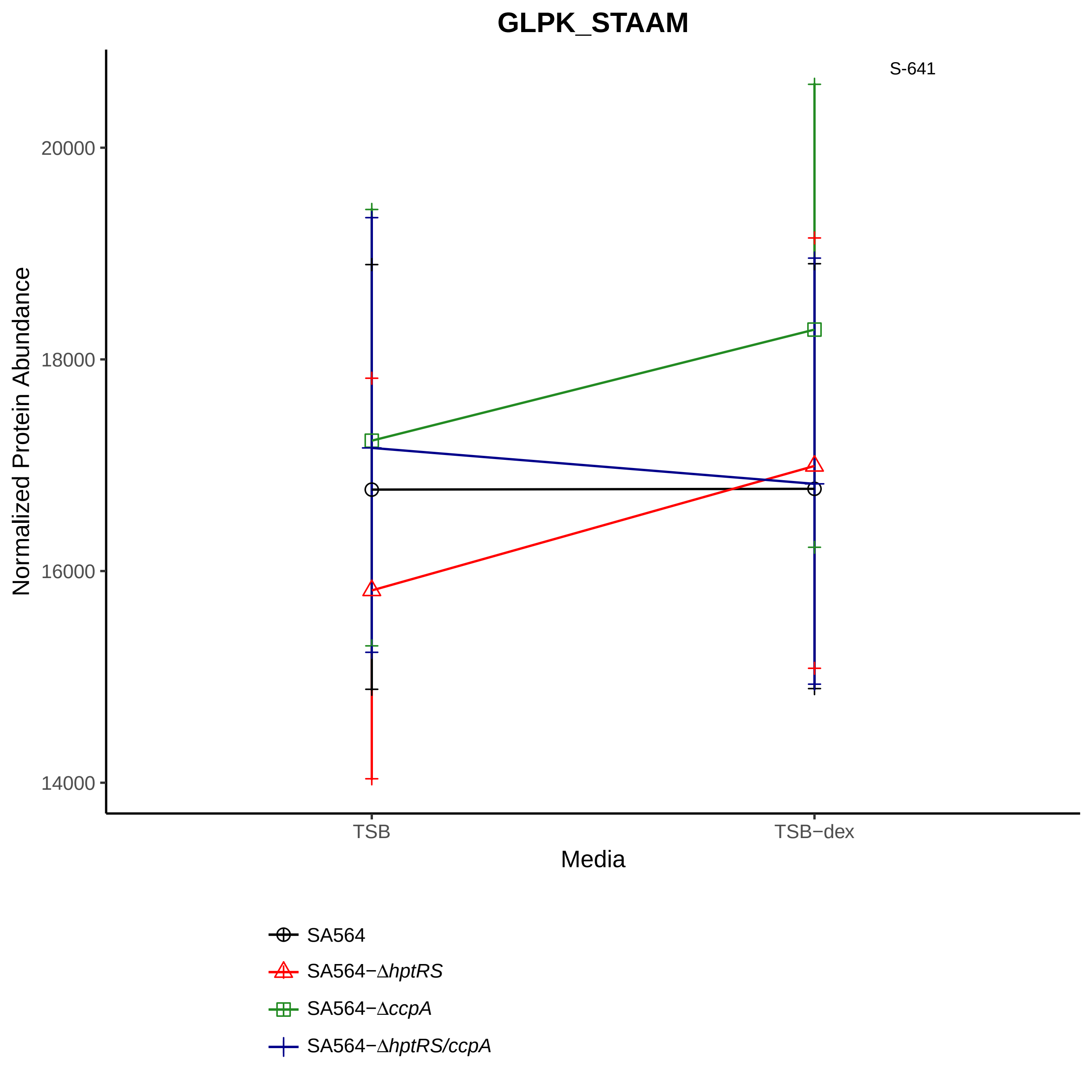
GLPK_STAAM
S-641
Normalized Protein Abundance
20000
18000
16000
14000
TSB
TSB−dex
Media
SA564
SA564−Δ*hptRS*
SA564−Δ*ccpA*
SA564−Δ*hptRS/ccpA*

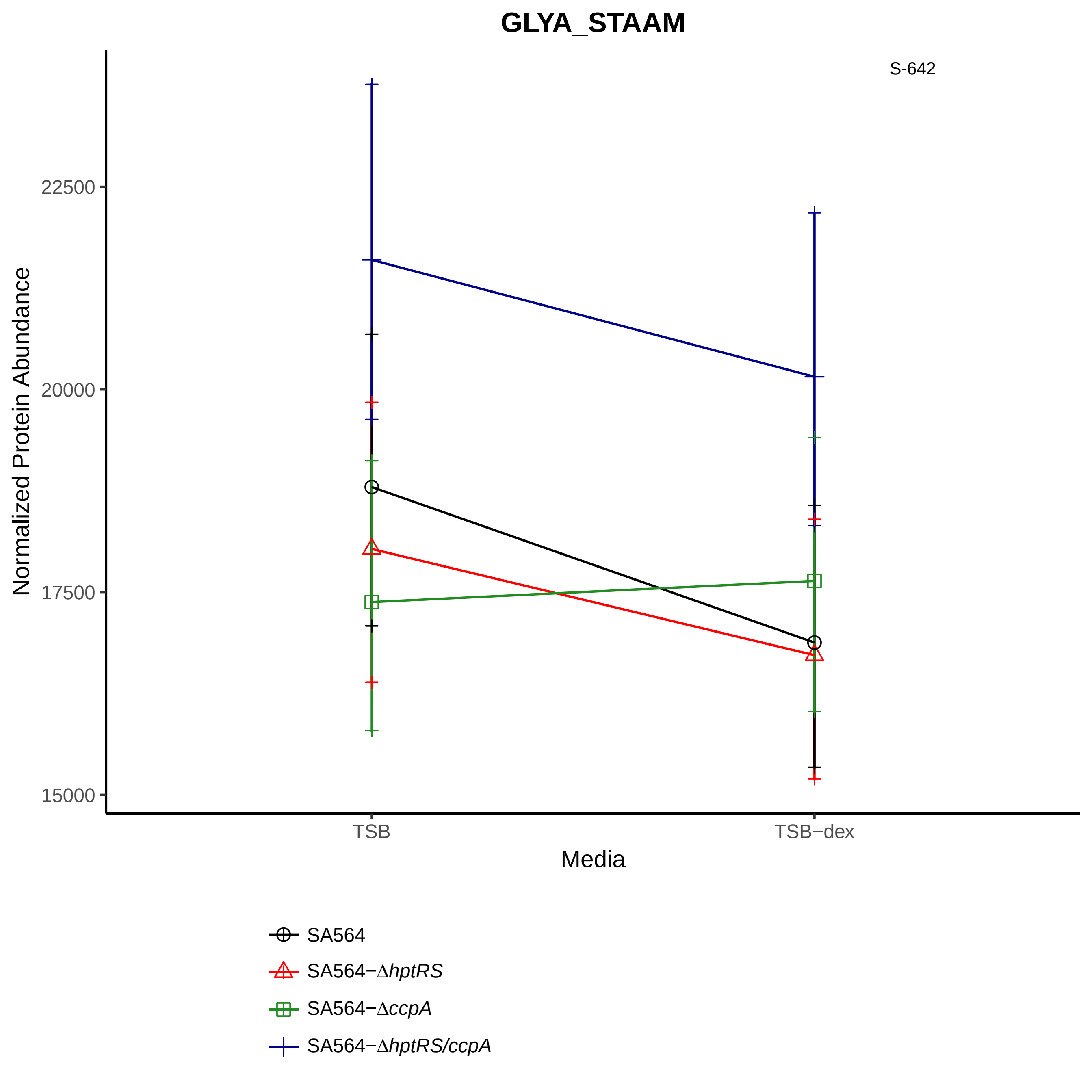

GLYA_STAAM
S-642
Normalized Protein Abundance
22500
20000
17500
15000
TSB
TSB−dex
Media
SA564
SA564−ΔhptRS
SA564−ΔccpA
SA564−ΔhptRS/ccpA

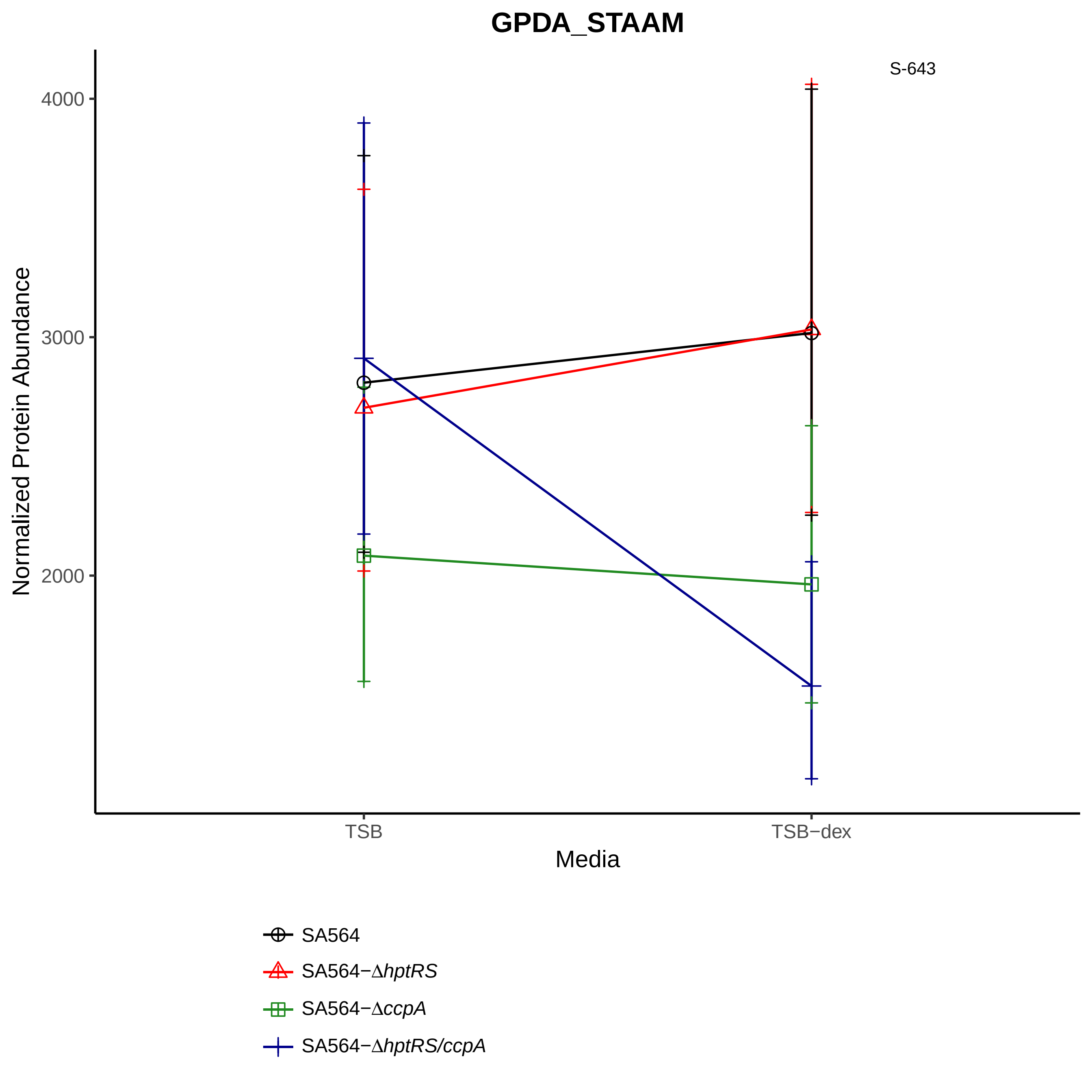

GPDA_STAAM
S-643
4000
3000
2000
Normalized Protein Abundance
TSB
TSB−dex
Media
SA564
SA564−Δ*hptRS*
SA564−Δ*ccpA*
SA564−Δ*hptRS/ccpA*

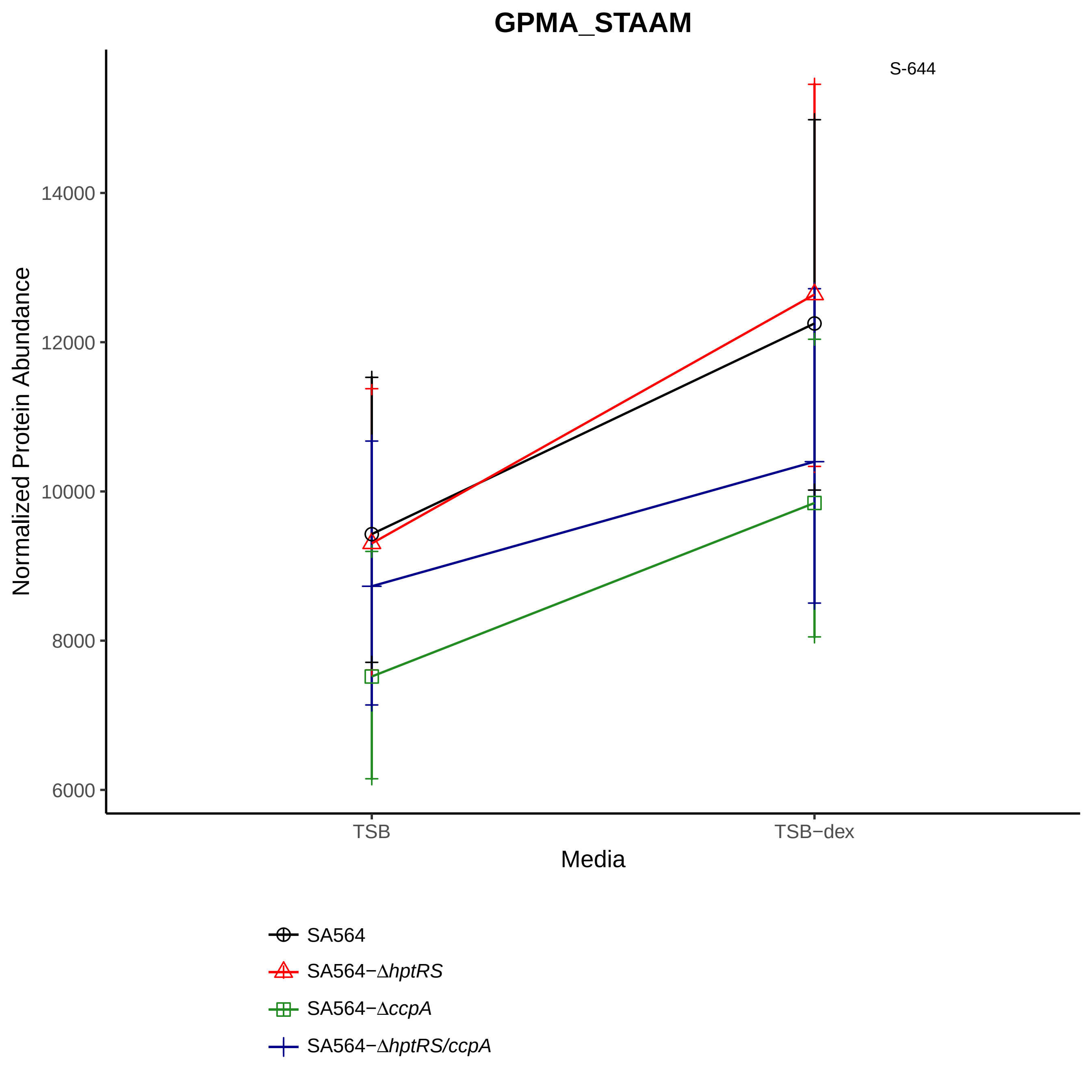

GPMA_STAAM
S-644
Normalized Protein Abundance
14000
12000
10000
8000
6000
TSB
TSB−dex
Media
SA564
SA564−Δ*hptRS*
SA564−Δ*ccpA*
SA564−Δ*hptRS/ccpA*

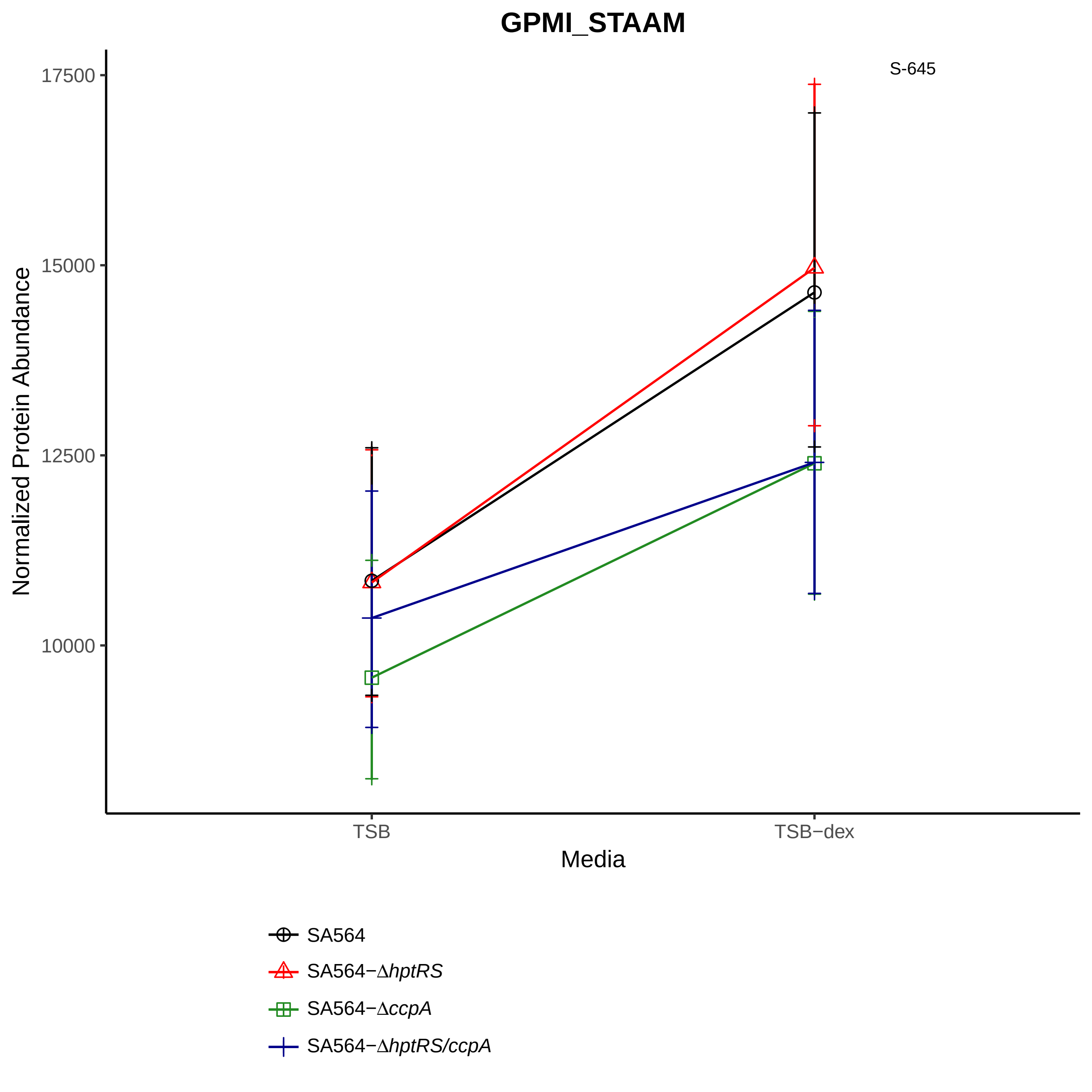

GPMI_STAAM
S-645
Normalized Protein Abundance
17500
15000
12500
10000
TSB
TSB−dex
Media
SA564
SA564−Δ*hptRS*
SA564−Δ*ccpA*
SA564−Δ*hptRS/ccpA*

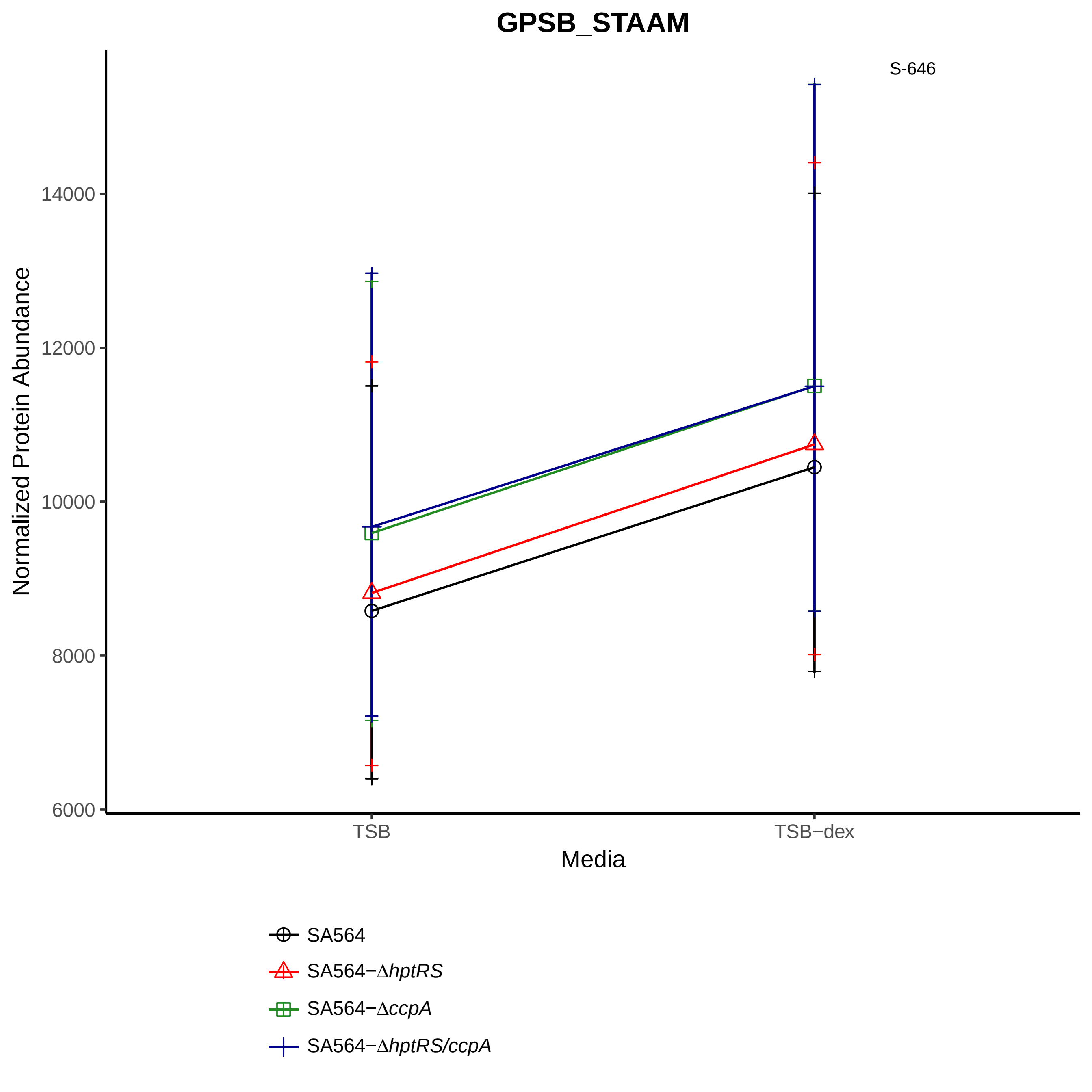

GPSB_STAAM
S-646
Normalized Protein Abundance
14000
12000
10000
8000
6000
TSB
TSB−dex
Media
SA564
SA564−Δ*hptRS*
SA564−Δ*ccpA*
SA564−Δ*hptRS/ccpA*

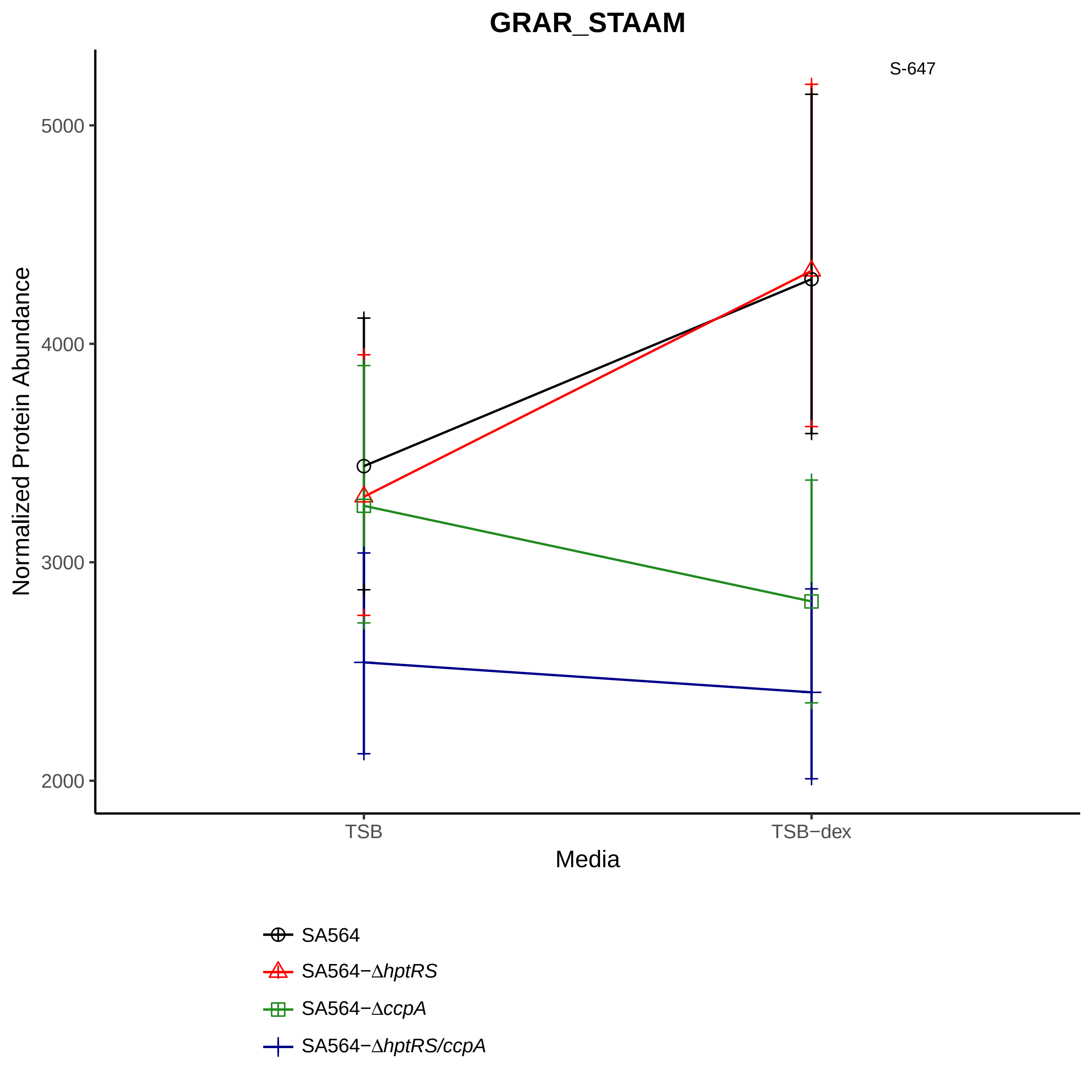

GRAR_STAAM
S-647
Normalized Protein Abundance
5000
4000
3000
2000
TSB
TSB−dex
Media
SA564
SA564−ΔhptRS
SA564−ΔccpA
SA564−ΔhptRS/ccpA

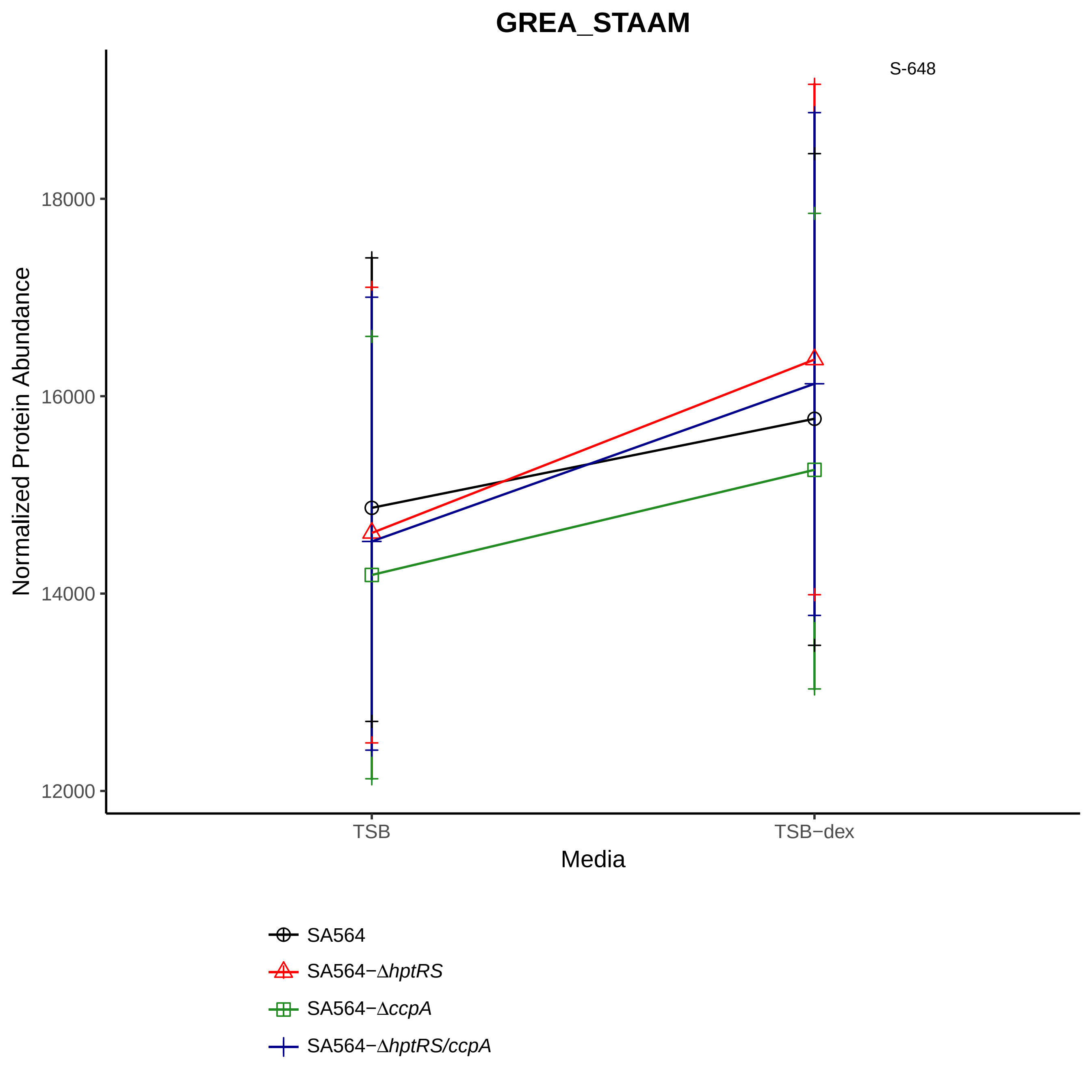
GREA_STAAM
S-648
Normalized Protein Abundance
18000
16000
14000
12000
TSB
TSB−dex
Media
SA564
SA564−Δ*hptRS*
SA564−Δ*ccpA*
SA564−Δ*hptRS/ccpA*

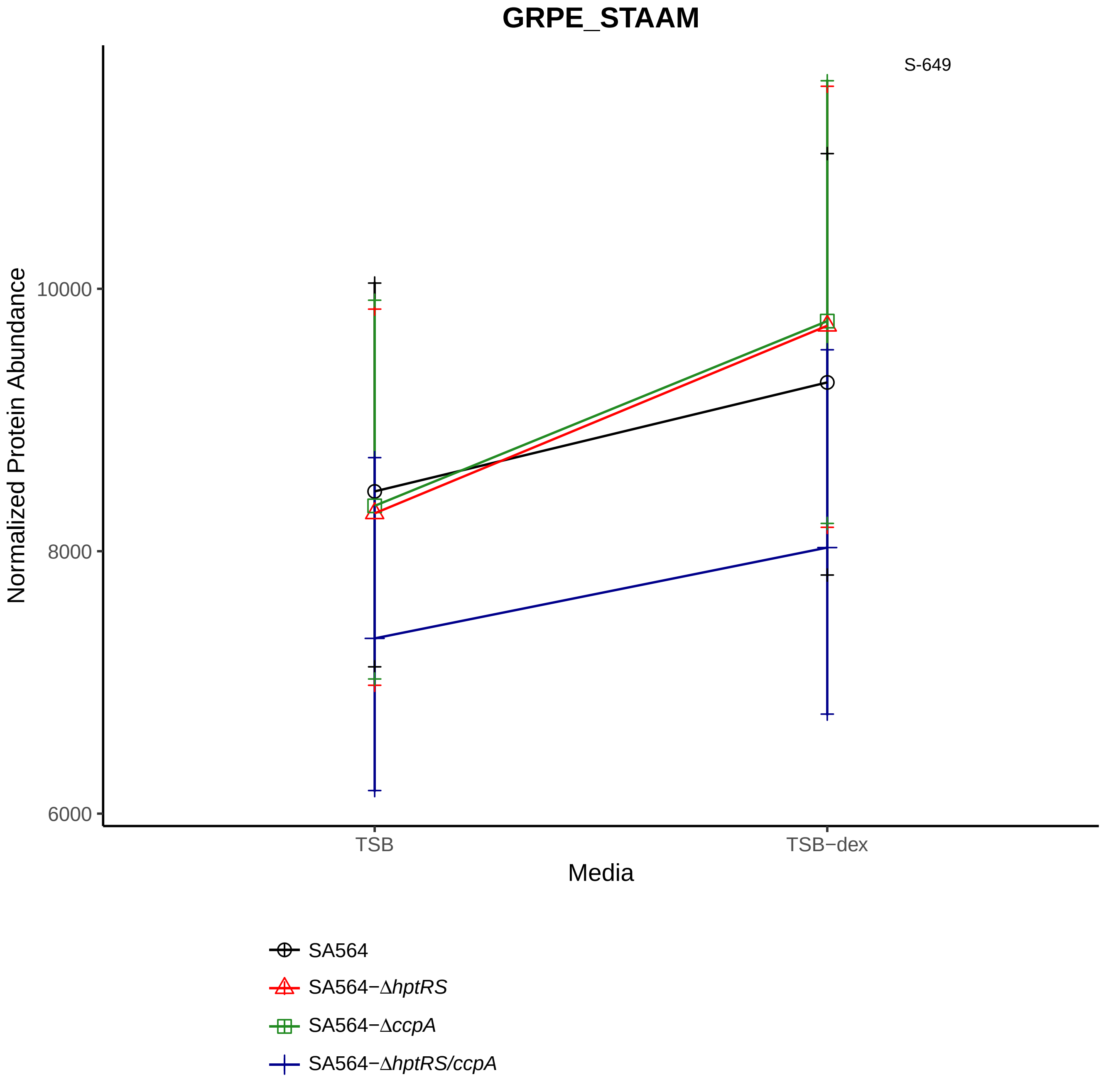

GRPE_STAAM
S-649
Normalized Protein Abundance
10000
8000
6000
TSB
TSB−dex
Media
SA564
SA564−Δ*hptRS*
SA564−Δ*ccpA*
SA564−Δ*hptRS/ccpA*

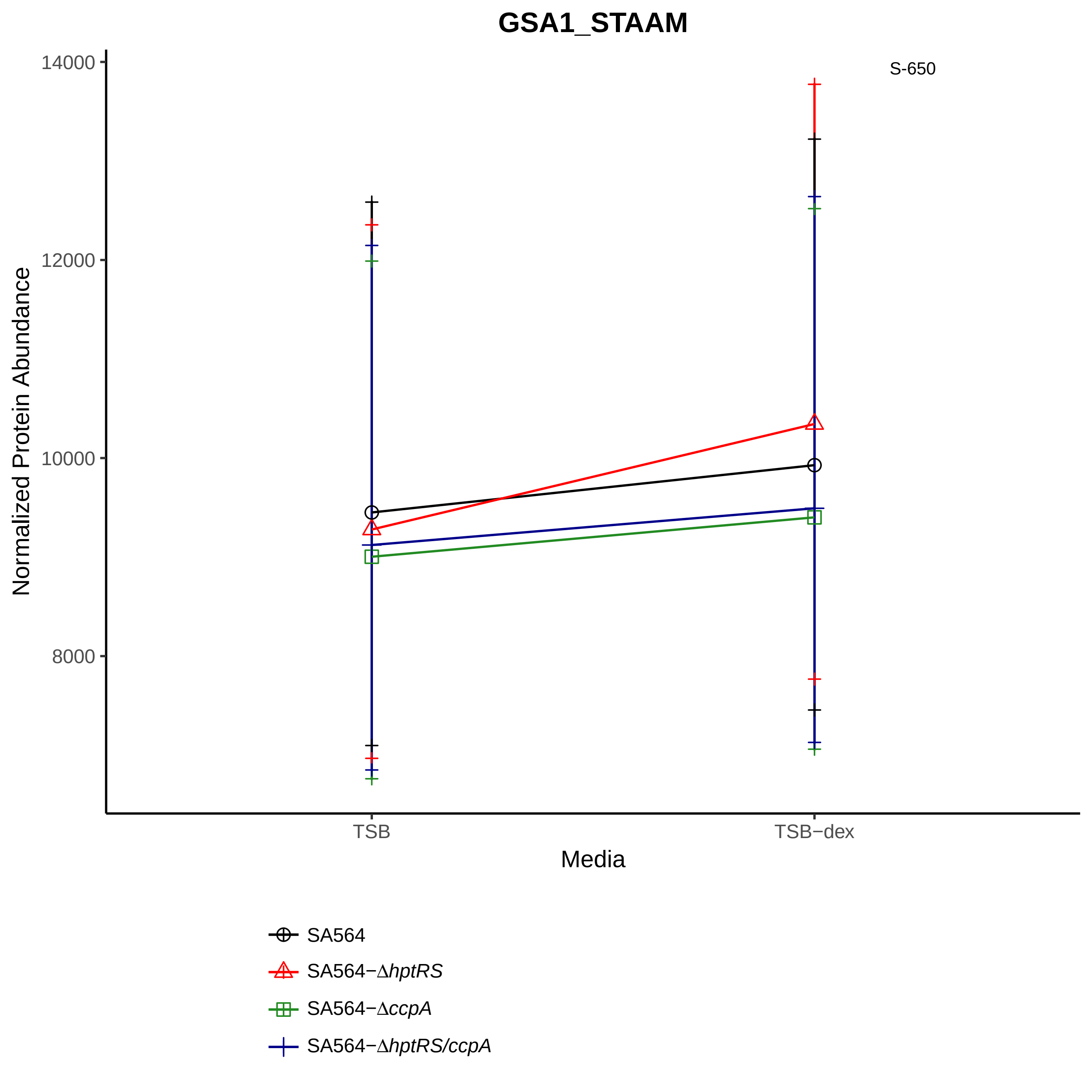
GSA1_STAAM
S-650
14000
12000
10000
8000
Normalized Protein Abundance
TSB
TSB−dex
Media
SA564
SA564−ΔhptRS
SA564−ΔccpA
SA564−ΔhptRS/ccpA

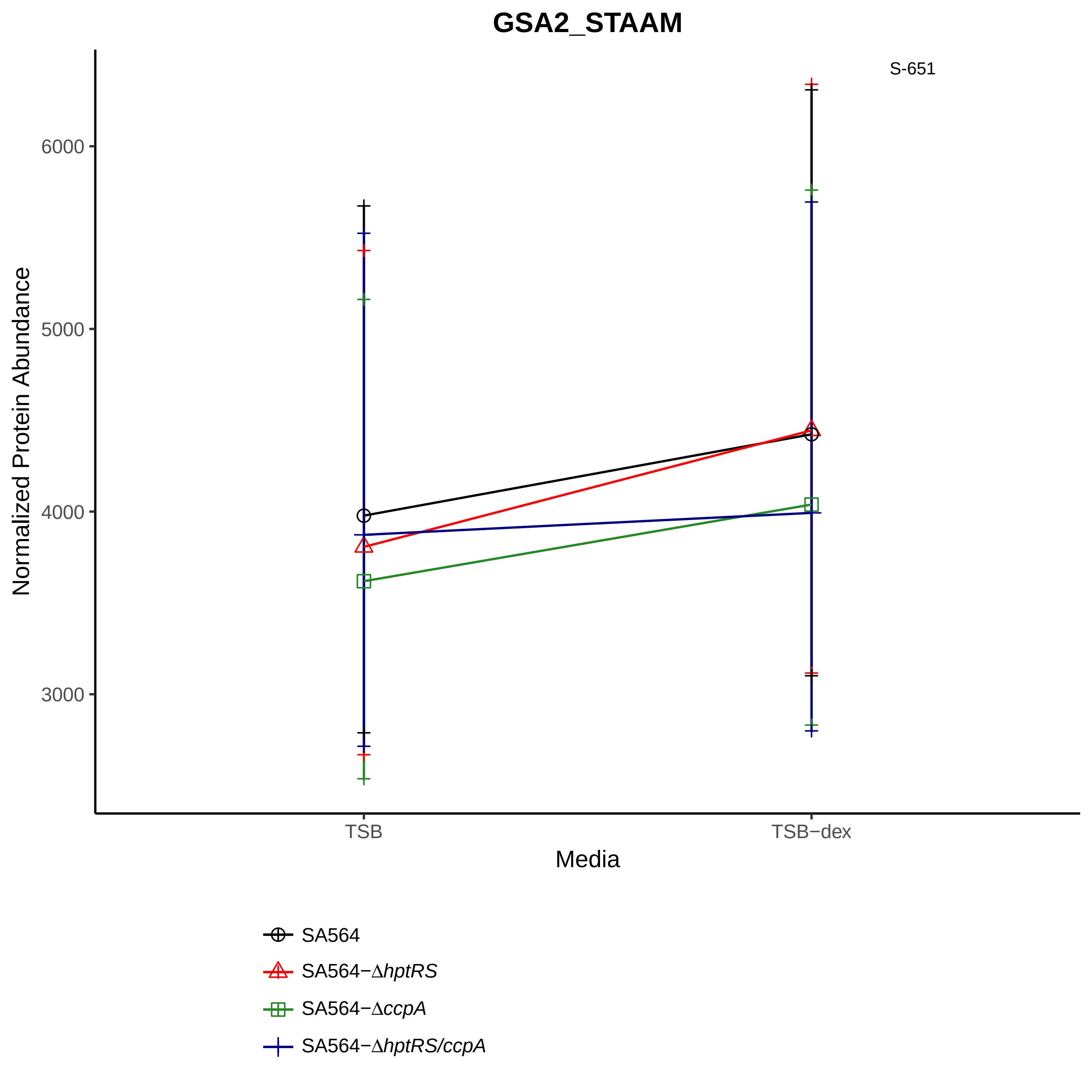
GSA2_STAAM
S-651
Normalized Protein Abundance
6000
5000
4000
3000
TSB
TSB−dex
Media
SA564
SA564−Δ*hptRS*
SA564−Δ*ccpA*
SA564−Δ*hptRS/ccpA*

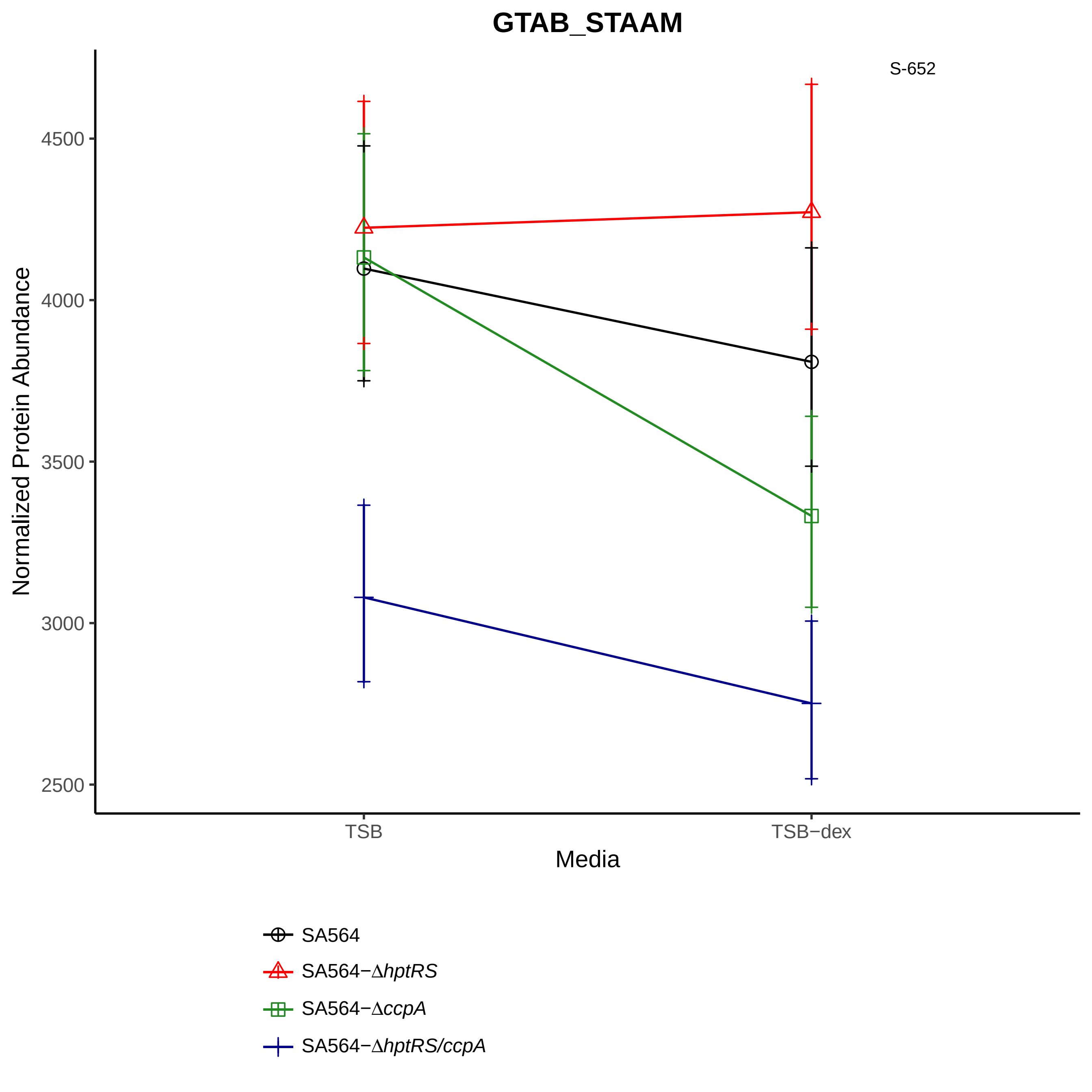

GTAB_STAAM
S-652
Normalized Protein Abundance
4500
4000
3500
3000
2500
TSB
TSB−dex
Media
SA564
SA564−Δ*hptRS*
SA564−Δ*ccpA*
SA564−Δ*hptRS/ccpA*

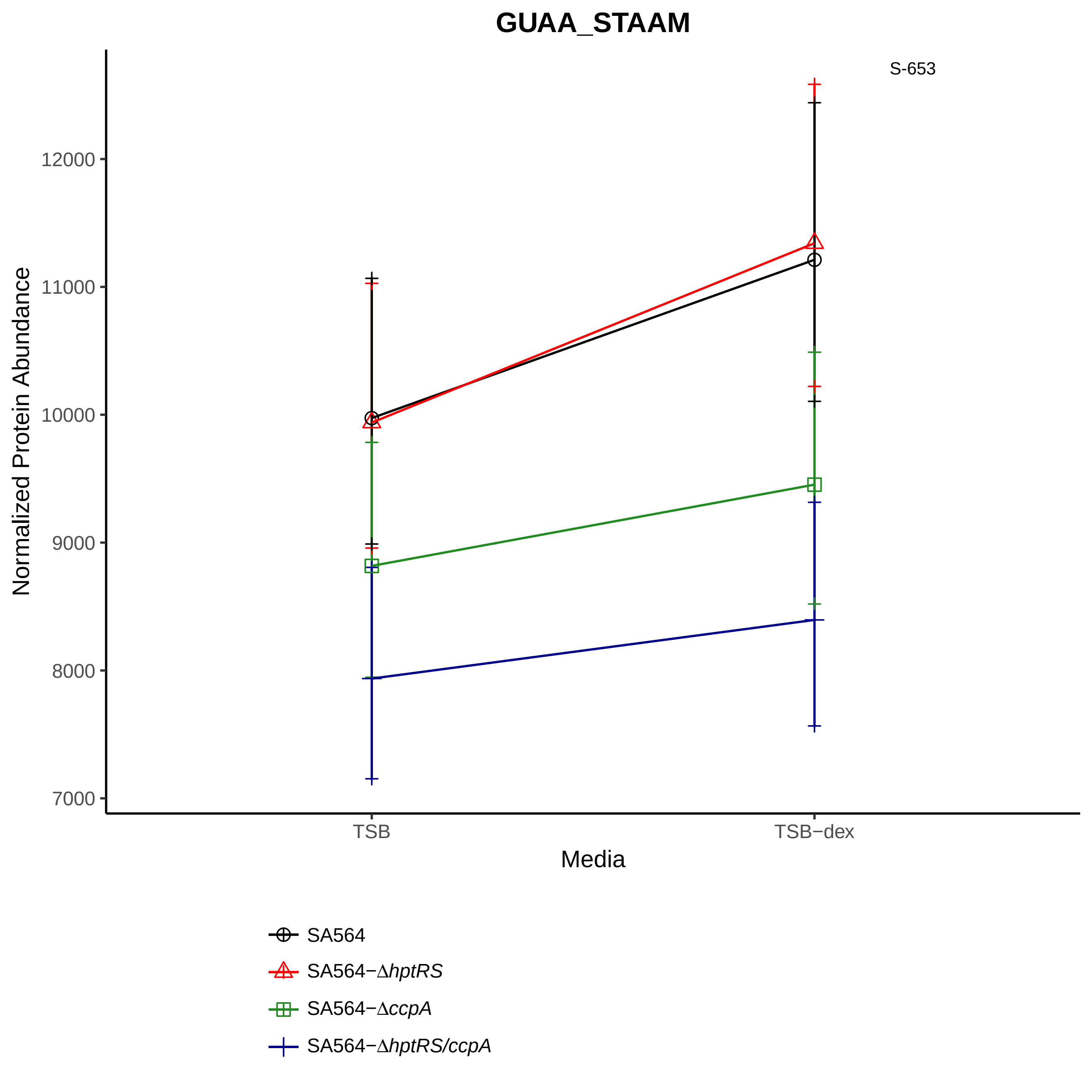

GUAA_STAAM
S-653
Normalized Protein Abundance
12000
11000
10000
9000
8000
7000
TSB
TSB−dex
Media
SA564
SA564−Δ*hptRS*
SA564−Δ*ccpA*
SA564−Δ*hptRS/ccpA*

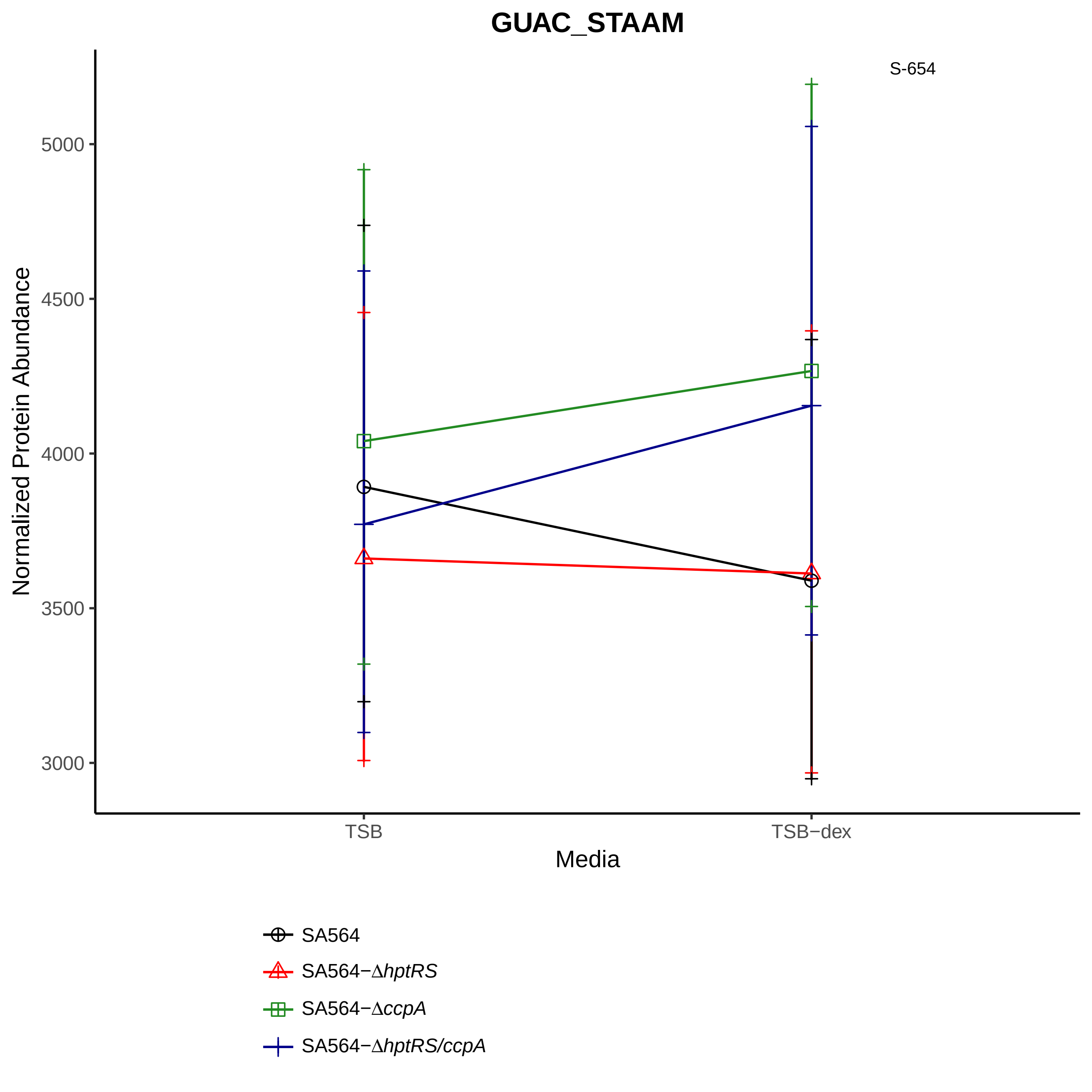
GUAC_STAAM
S-654
Normalized Protein Abundance
5000
4500
4000
3500
3000
TSB
TSB−dex
Media
SA564
SA564−ΔhptRS
SA564−ΔccpA
SA564−ΔhptRS/ccpA

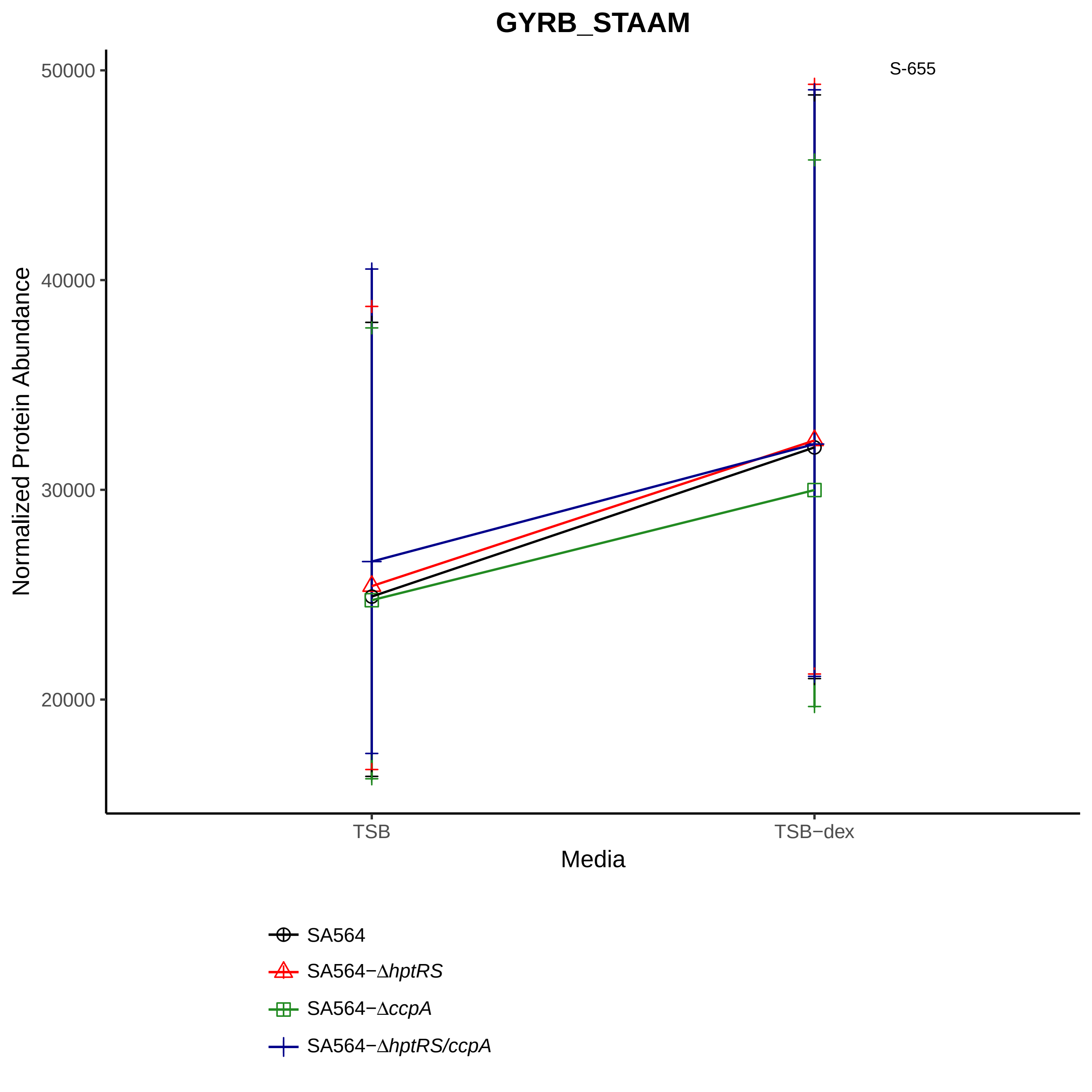
GYRB_STAAM
S-655
Normalized Protein Abundance
50000
40000
30000
20000
TSB
TSB−dex
Media
SA564
SA564−ΔhptRS
SA564−ΔccpA
SA564−ΔhptRS/ccpA

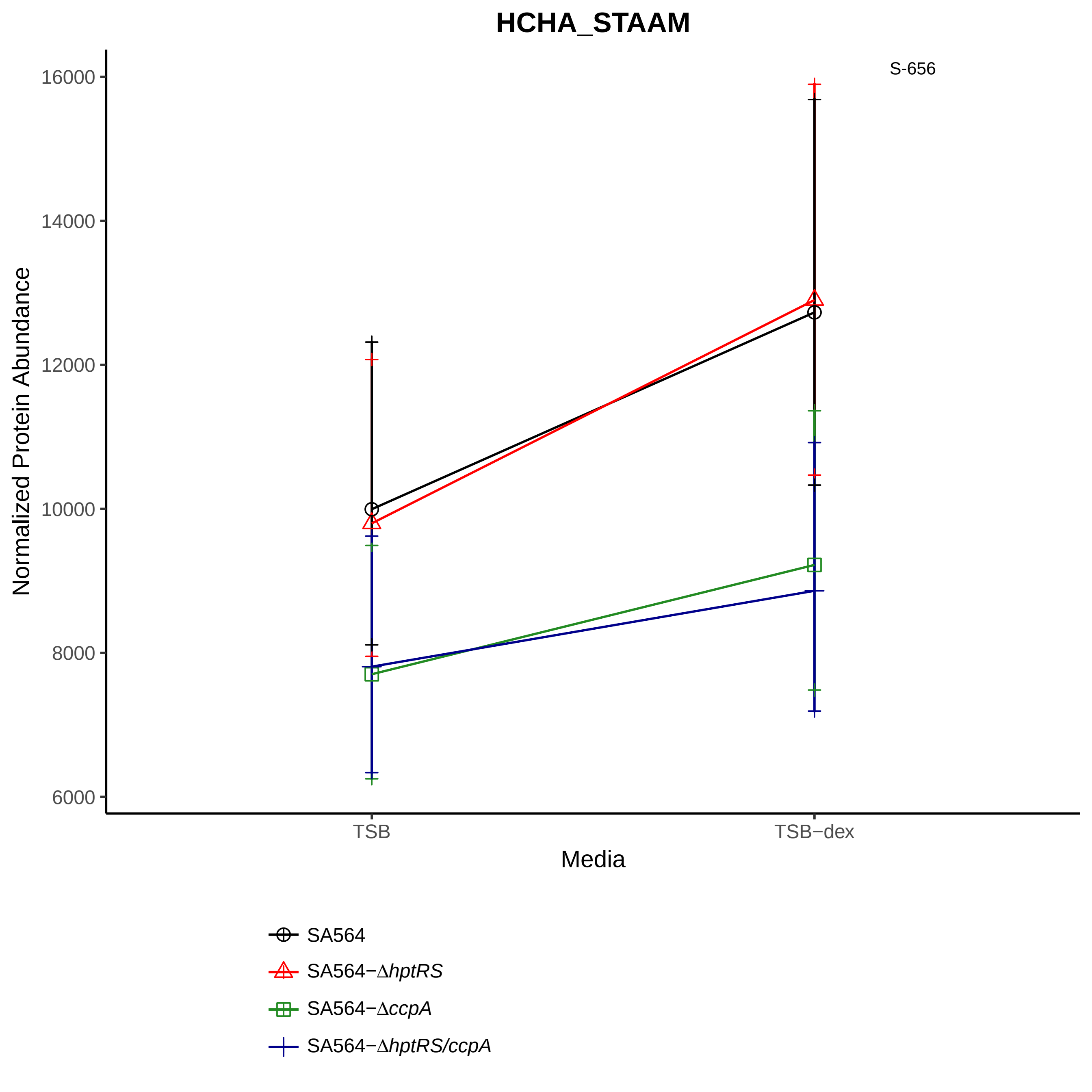

HCHA_STAAM
S-656
Normalized Protein Abundance
16000
14000
12000
10000
8000
6000
TSB
TSB−dex
Media
SA564
SA564−ΔhptRS
SA564−ΔccpA
SA564−ΔhptRS/ccpA

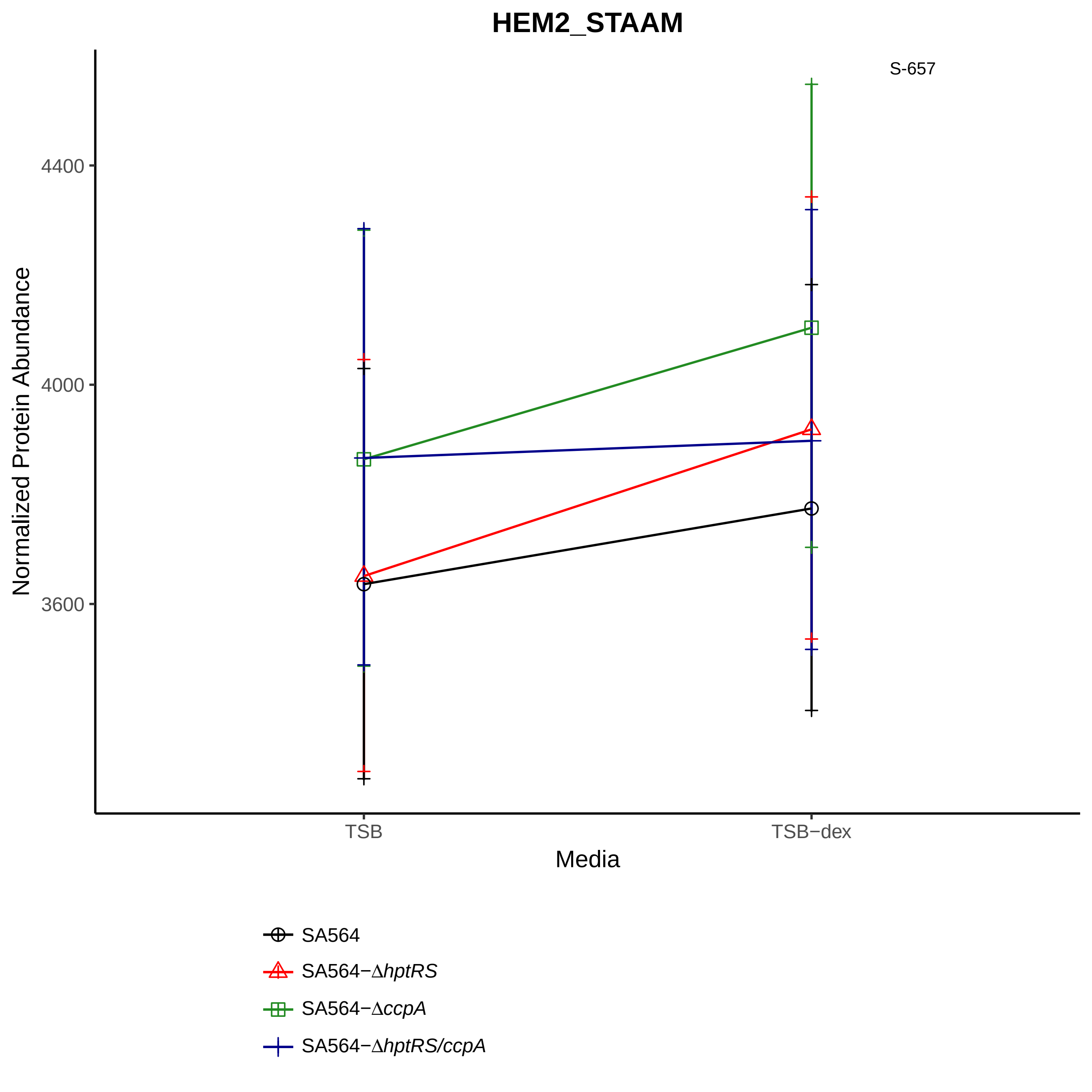

HEM2_STAAM
S-657
Normalized Protein Abundance
4400
4000
3600
TSB
TSB−dex
Media
SA564
SA564−Δ*hptRS*
SA564−Δ*ccpA*
SA564−Δ*hptRS/ccpA*

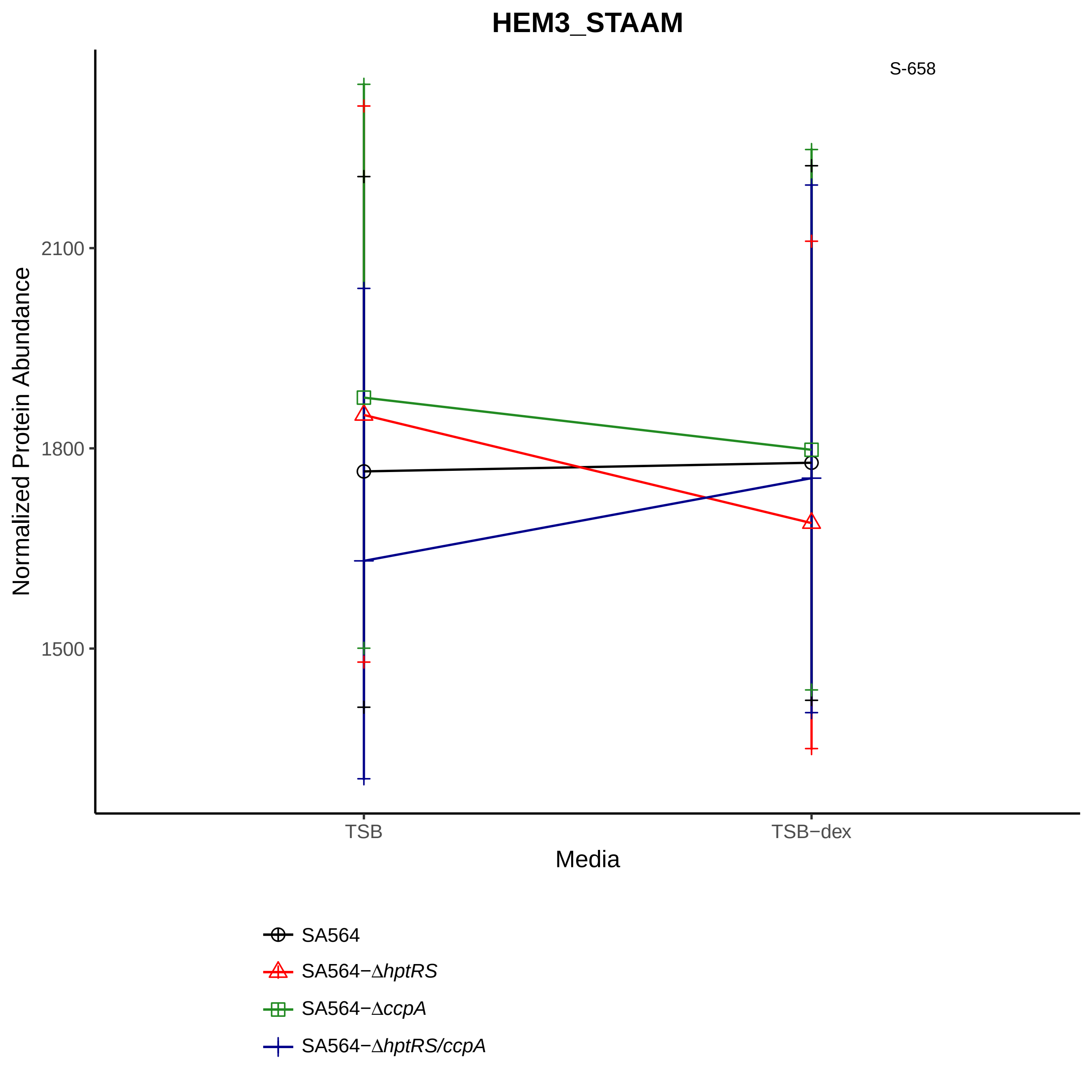

HEM3_STAAM
S-658
Normalized Protein Abundance
2100
1800
1500
TSB
TSB−dex
Media
SA564
SA564−ΔhptRS
SA564−ΔccpA
SA564−ΔhptRS/ccpA

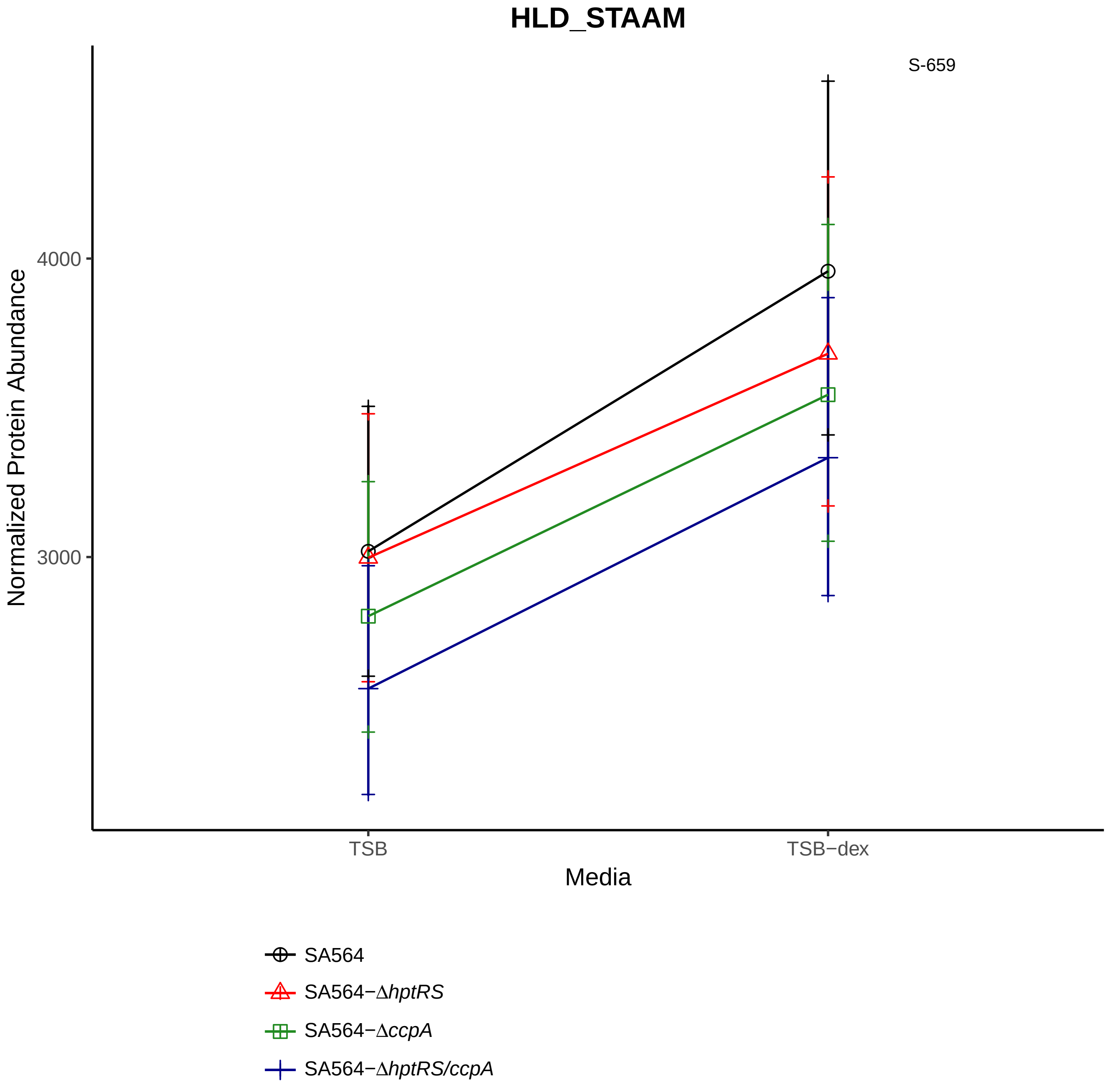

HLD_STAAM
S-659
4000
3000
Normalized Protein Abundance
TSB
TSB−dex
Media
SA564
SA564−Δ*hptRS*
SA564−Δ*ccpA*
SA564−Δ*hptRS/ccpA*

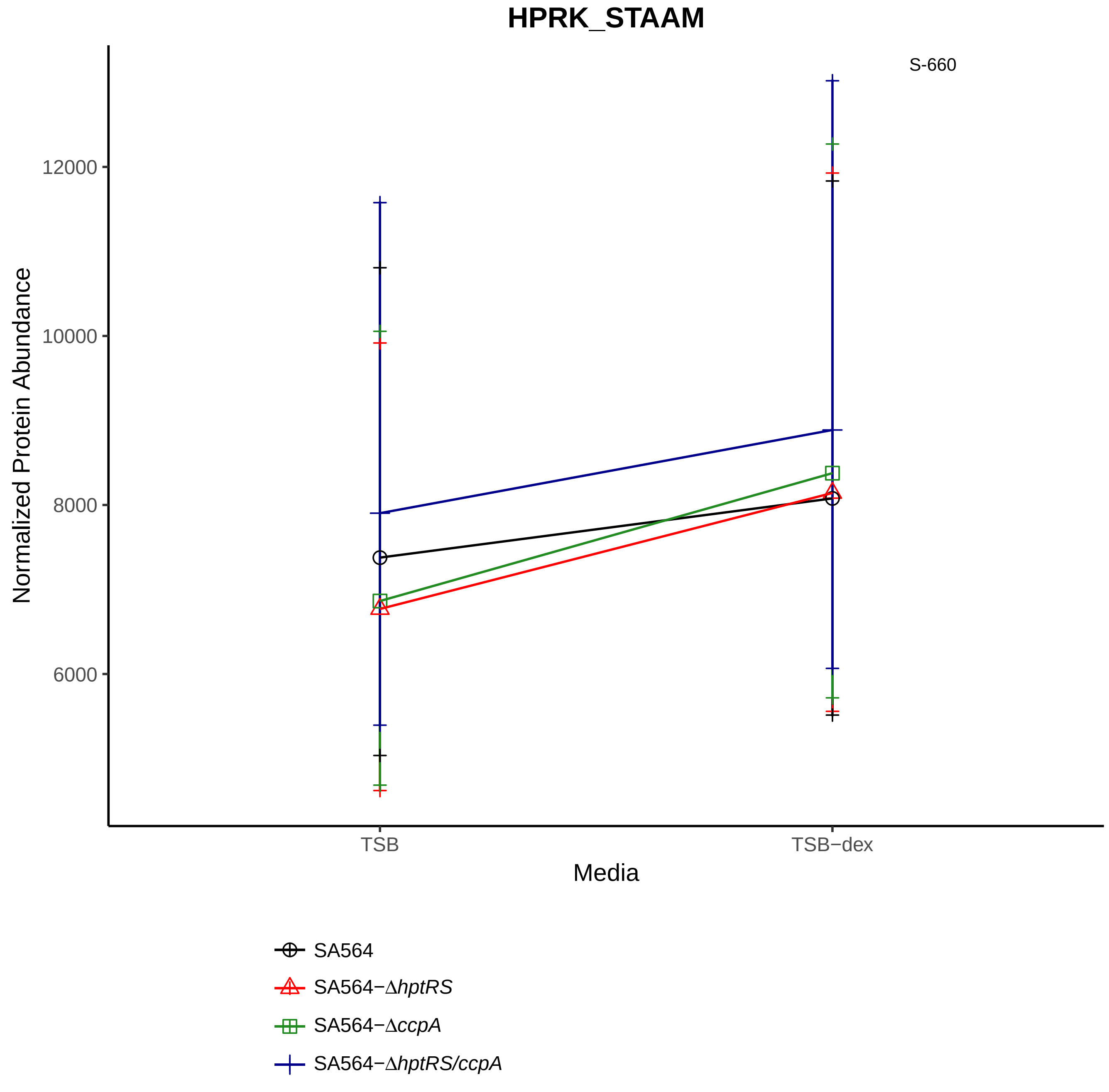

HPRK_STAAM
S-660
Normalized Protein Abundance
12000
10000
8000
6000
TSB
TSB−dex
Media
SA564
SA564−Δ*hptRS*
SA564−Δ*ccpA*
SA564−Δ*hptRS/ccpA*

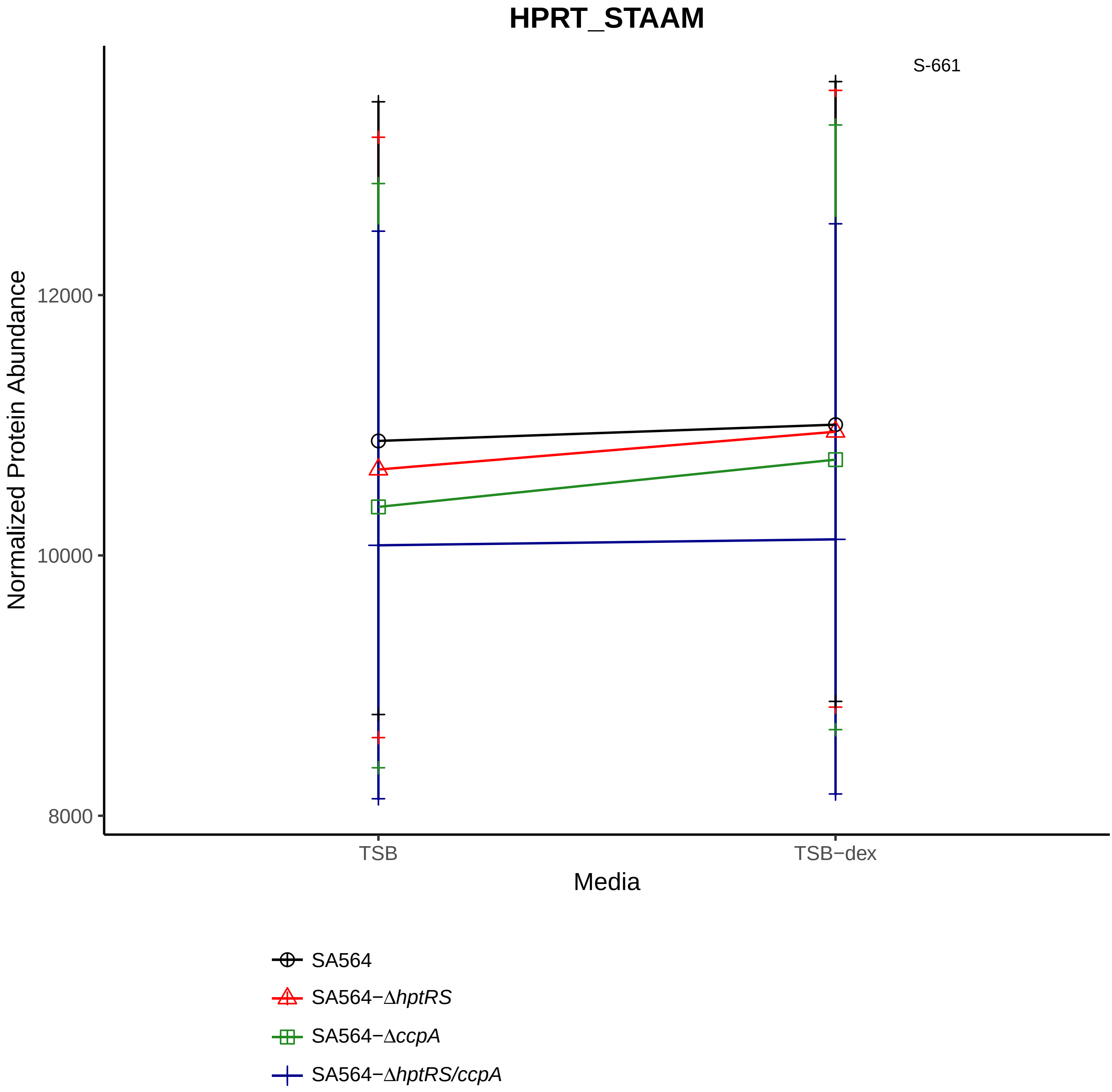

HPRT_STAAM
S-661
Normalized Protein Abundance
12000
10000
8000
TSB
TSB−dex
Media
SA564
SA564−ΔhptRS
SA564−ΔccpA
SA564−ΔhptRS/ccpA

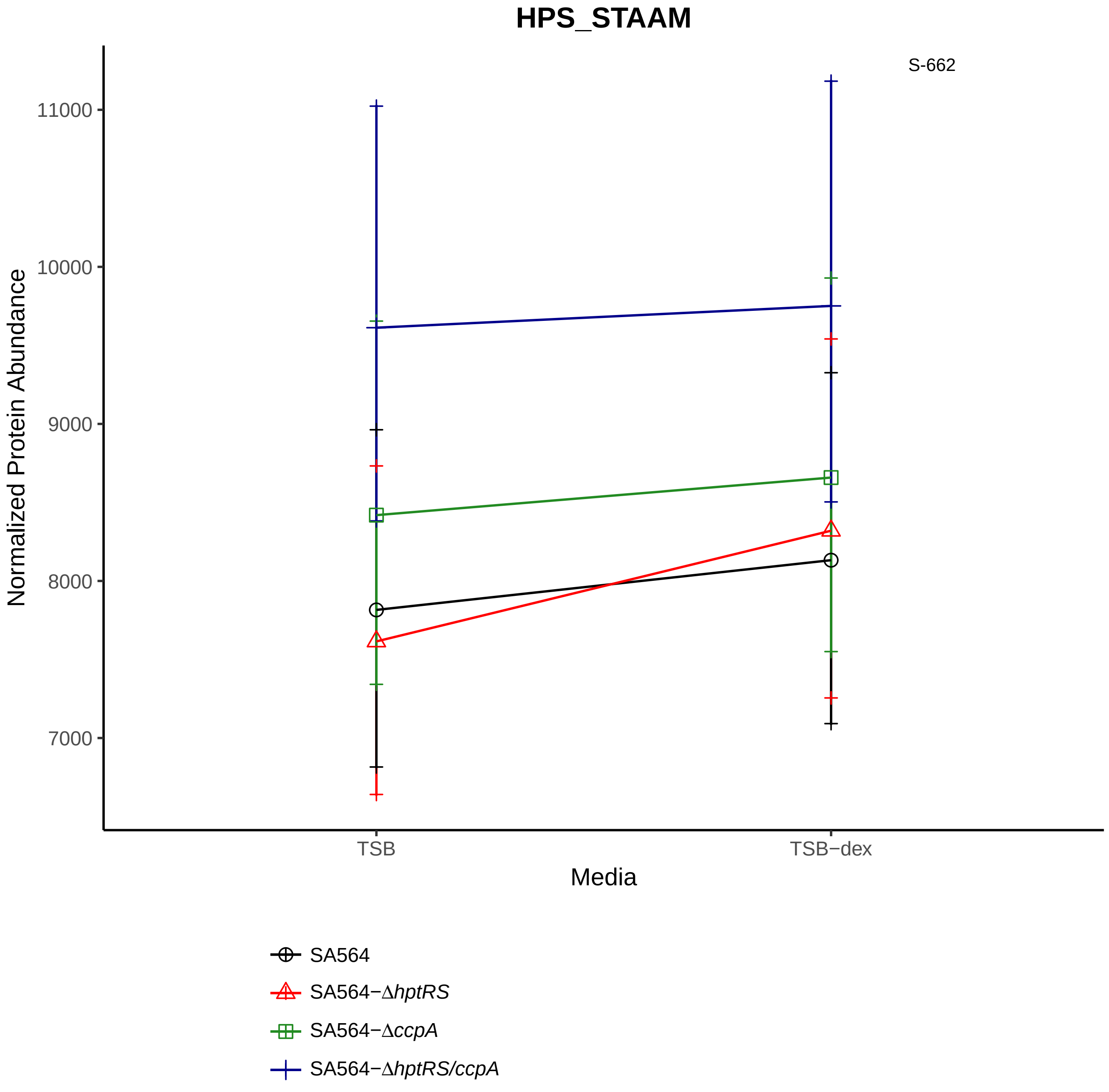
HPS_STAAM
S-662
Normalized Protein Abundance
11000
10000
9000
8000
7000
TSB
TSB−dex
Media
SA564
SA564−Δ*hptRS*
SA564−Δ*ccpA*
SA564−Δ*hptRS/ccpA*

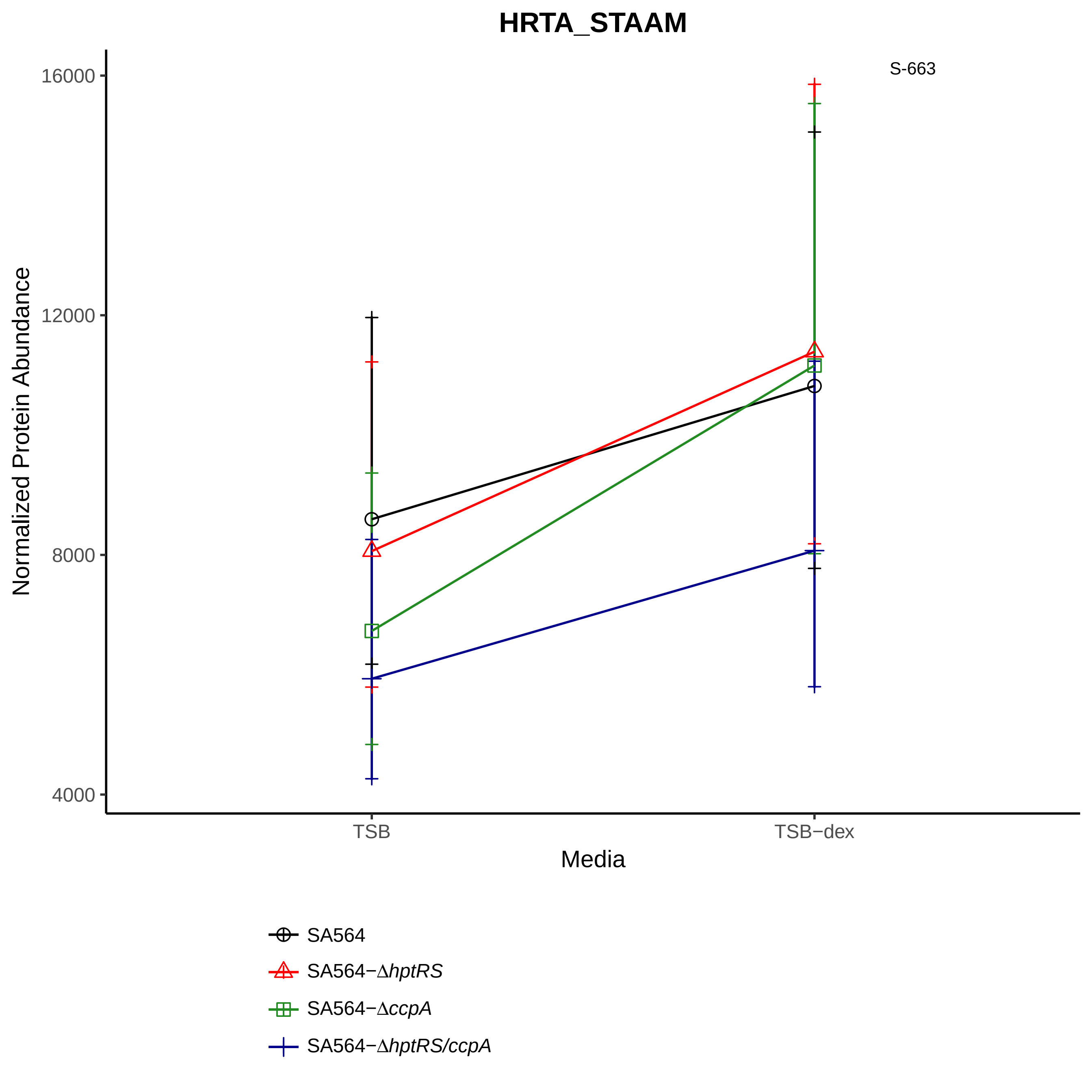

HRTA_STAAM
S-663
Normalized Protein Abundance
16000
12000
8000
4000
TSB
TSB−dex
Media
SA564
SA564−Δ*hptRS*
SA564−Δ*ccpA*
SA564−Δ*hptRS/ccpA*

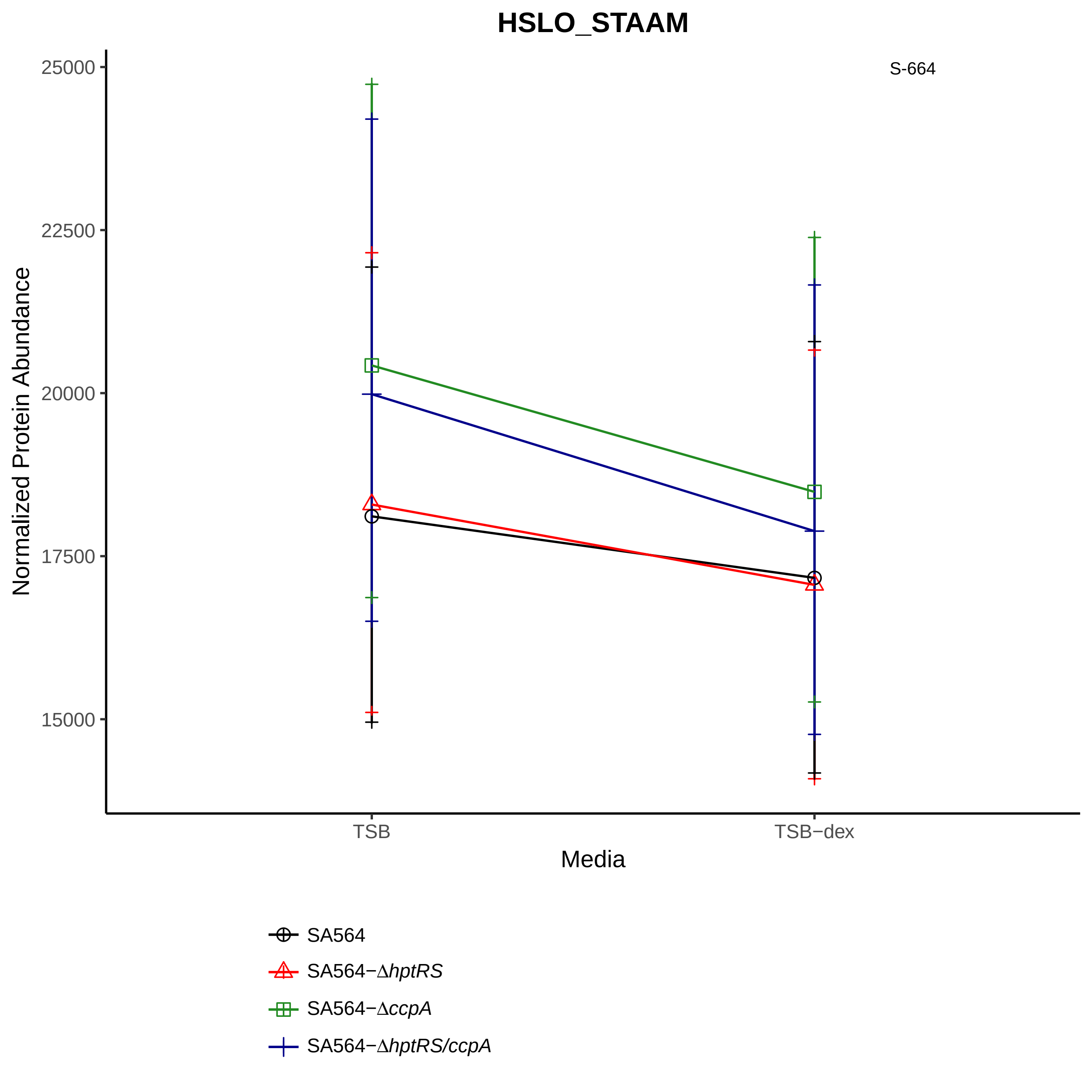

HSLO_STAAM
S-664
Normalized Protein Abundance
25000
22500
20000
17500
15000
TSB
TSB−dex
Media
SA564
SA564−Δ*hptRS*
SA564−Δ*ccpA*
SA564−Δ*hptRS/ccpA*

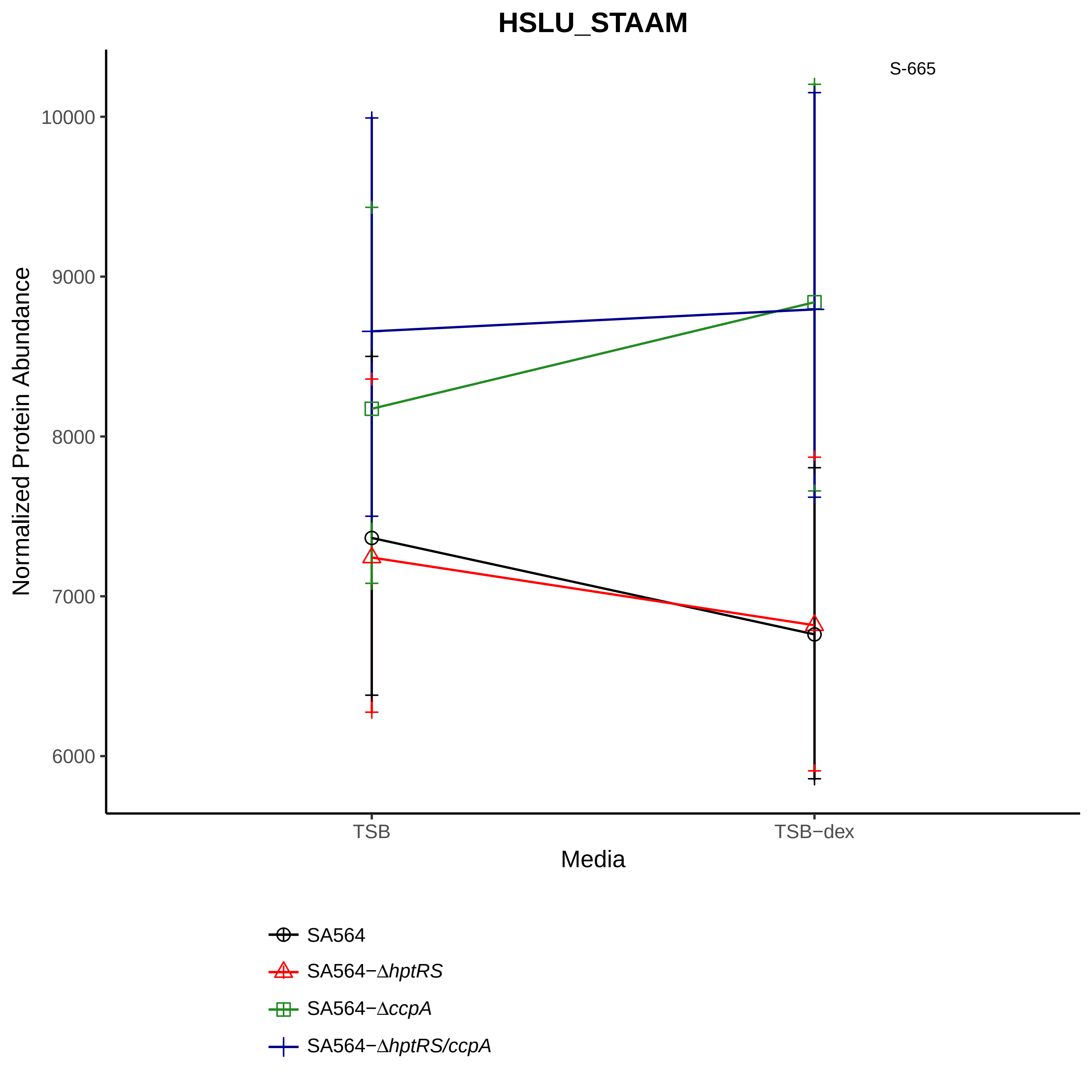

HSLU_STAAM
S-665
Normalized Protein Abundance
10000
9000
8000
7000
6000
TSB
TSB−dex
Media
SA564
SA564−Δ*hptRS*
SA564−Δ*ccpA*
SA564−Δ*hptRS/ccpA*

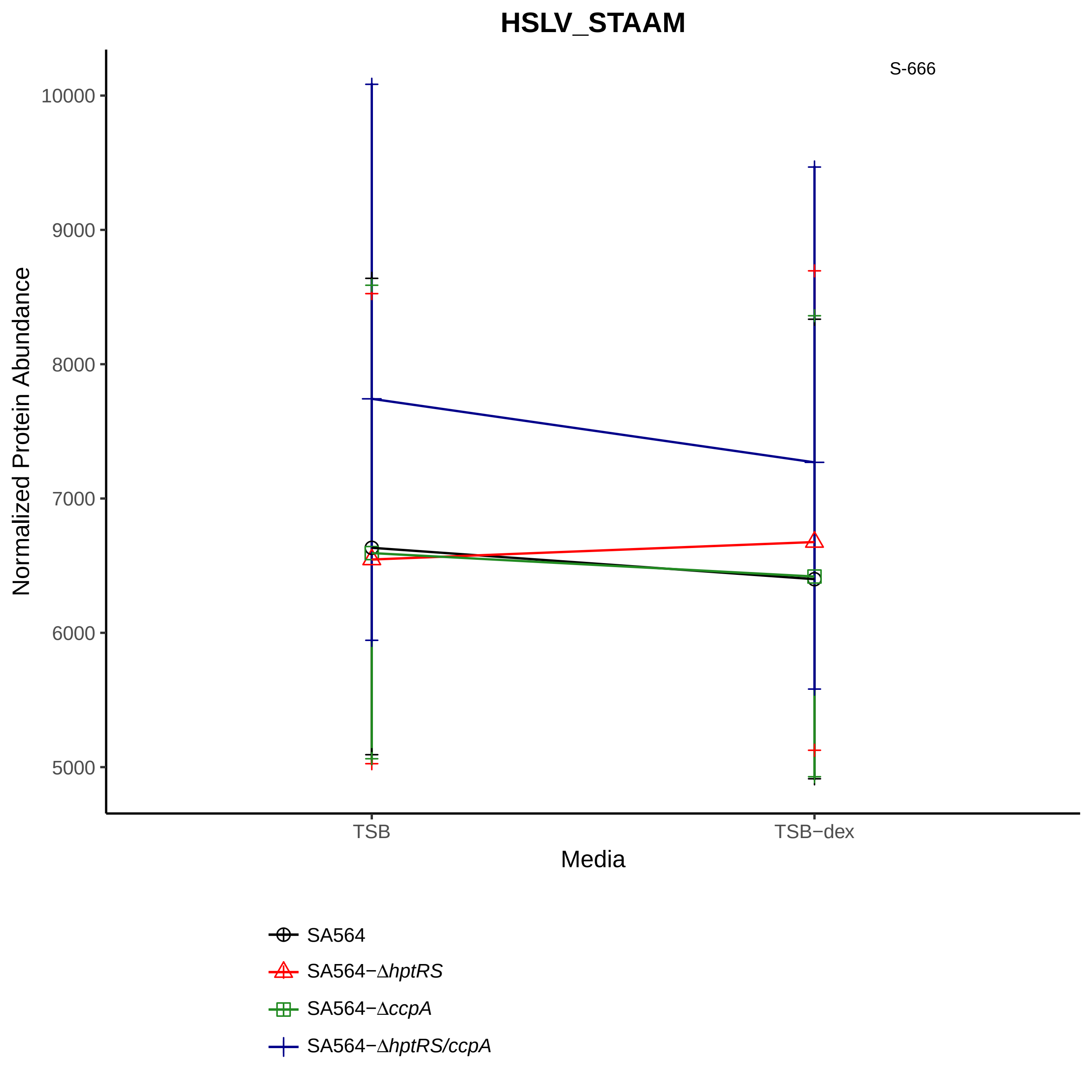

HSLV_STAAM
S-666
Normalized Protein Abundance
10000
9000
8000
7000
6000
5000
TSB
TSB−dex
Media
SA564
SA564−Δ*hptRS*
SA564−Δ*ccpA*
SA564−Δ*hptRS/ccpA*

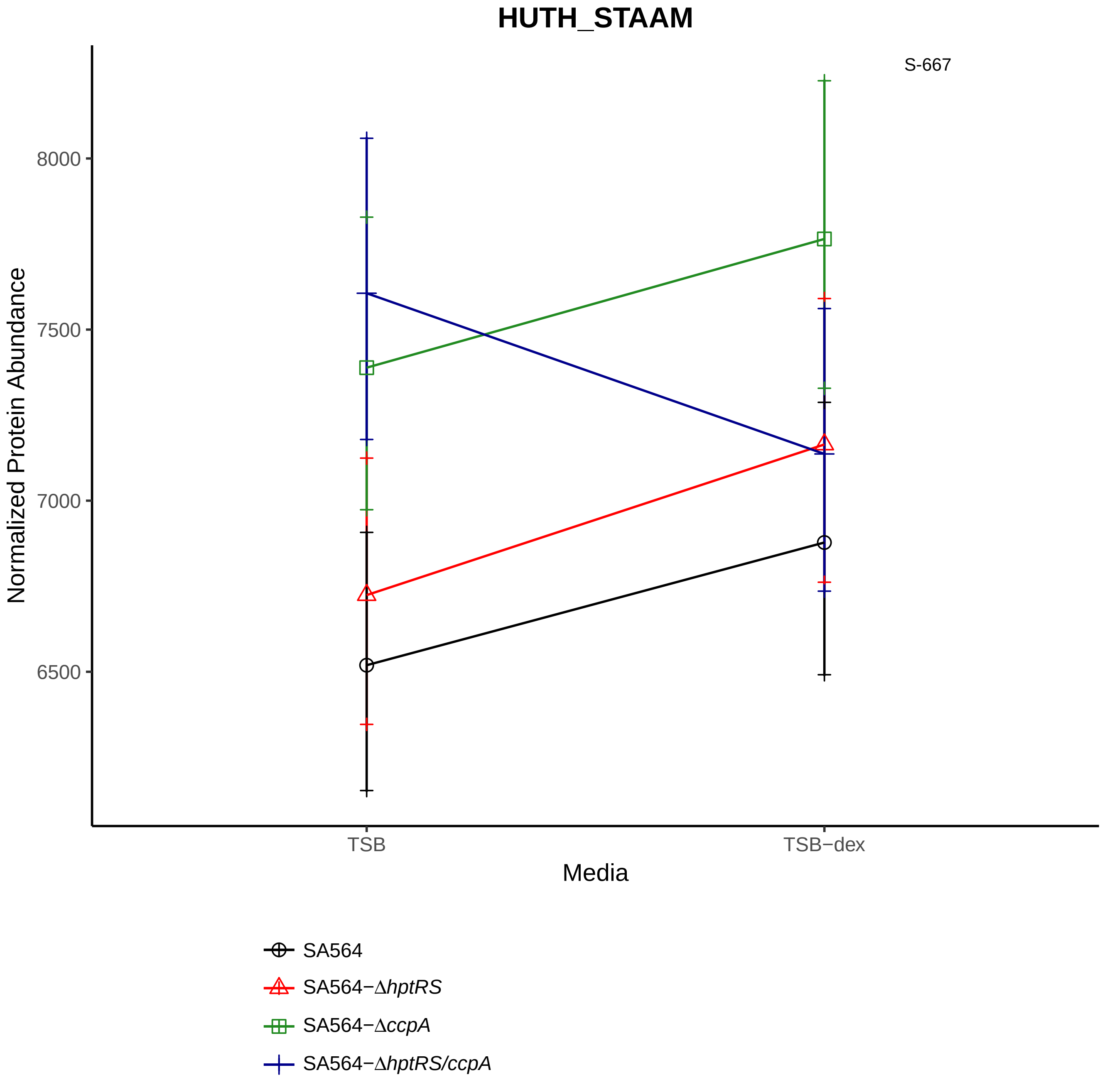

HUTH_STAAM
S-667
Normalized Protein Abundance
8000
7500
7000
6500
TSB
TSB−dex
Media
SA564
SA564−ΔhptRS
SA564−ΔccpA
SA564−ΔhptRS/ccpA

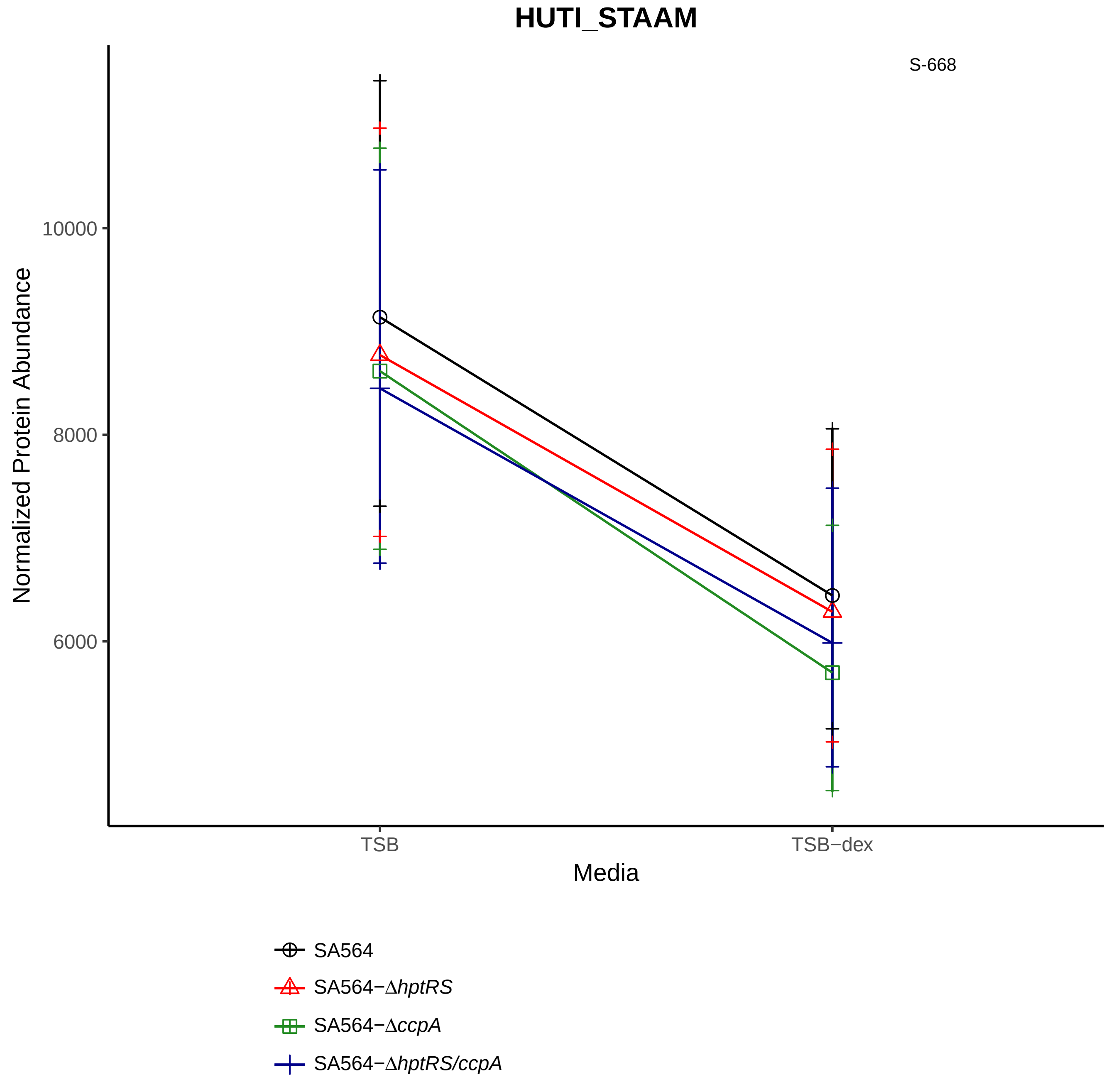

HUTI_STAAM
S-668
Normalized Protein Abundance
10000
8000
6000
TSB
TSB−dex
Media
SA564
SA564−Δ*hptRS*
SA564−Δ*ccpA*
SA564−Δ*hptRS/ccpA*

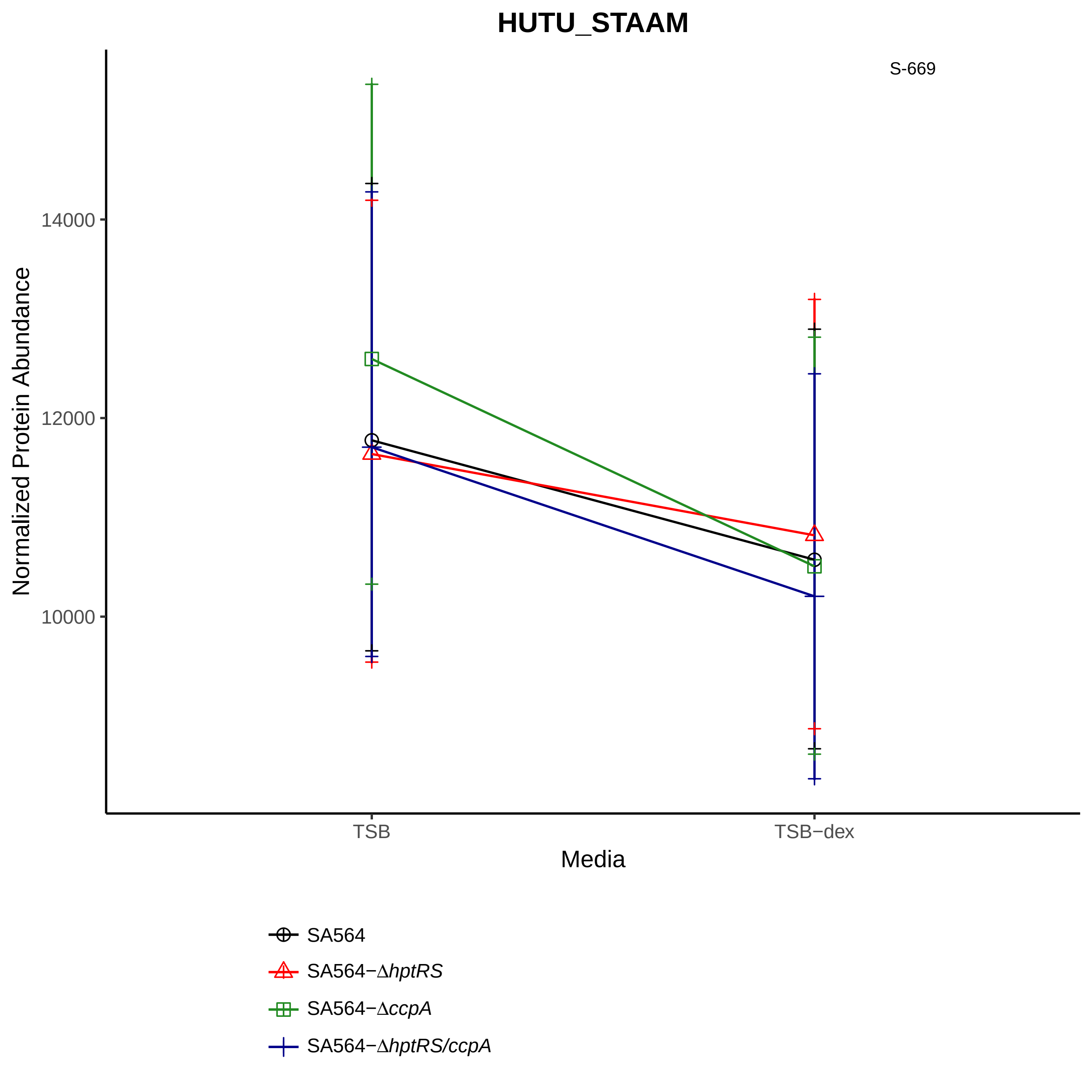

HUTU_STAAM
S-669
Normalized Protein Abundance
14000
12000
10000
TSB
TSB−dex
Media
SA564
SA564−Δ*hptRS*
SA564−Δ*ccpA*
SA564−Δ*hptRS/ccpA*

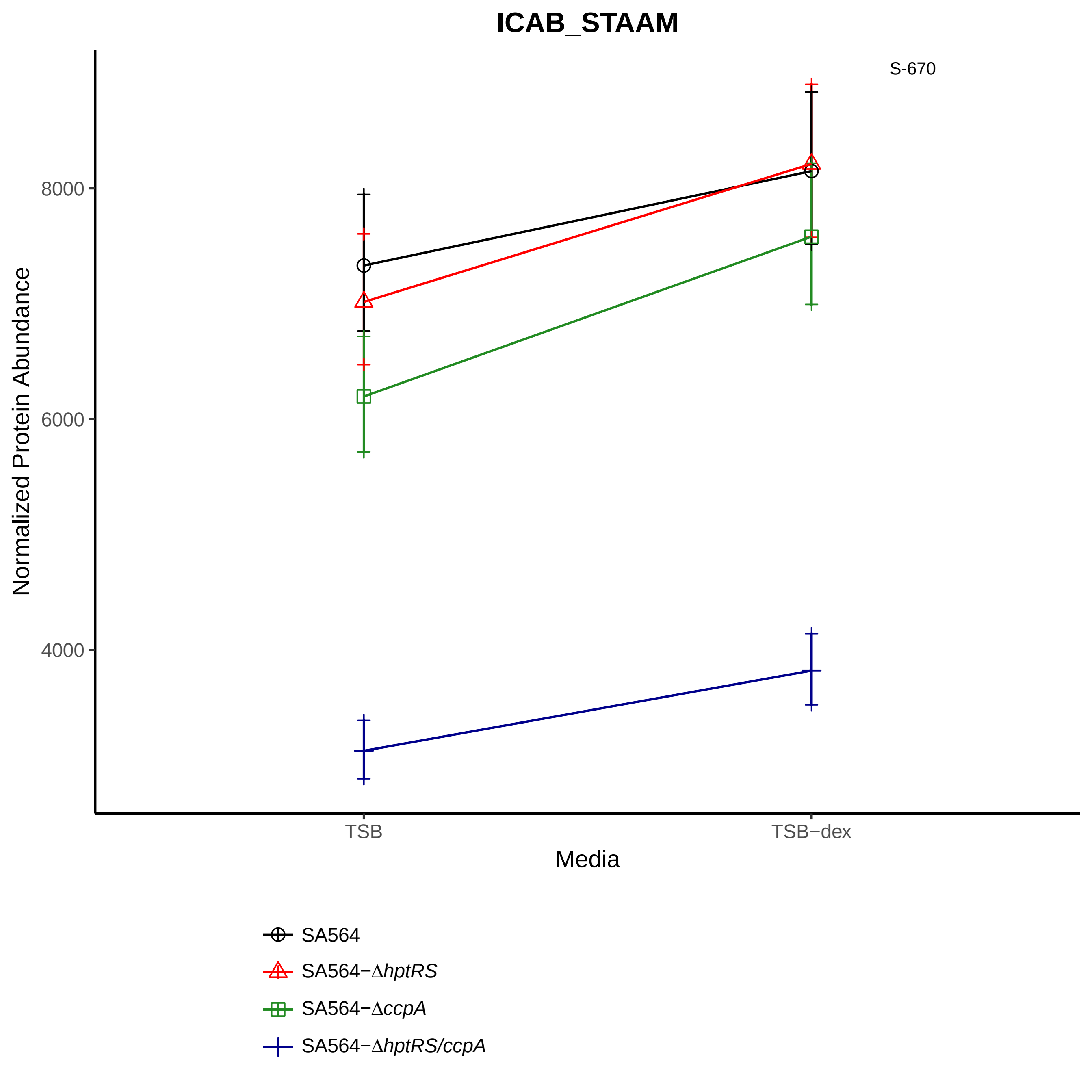
ICAB_STAAM
S-670
Normalized Protein Abundance
8000
6000
4000
TSB
TSB−dex
Media
SA564
SA564−ΔhptRS
SA564−ΔccpA
SA564−ΔhptRS/ccpA

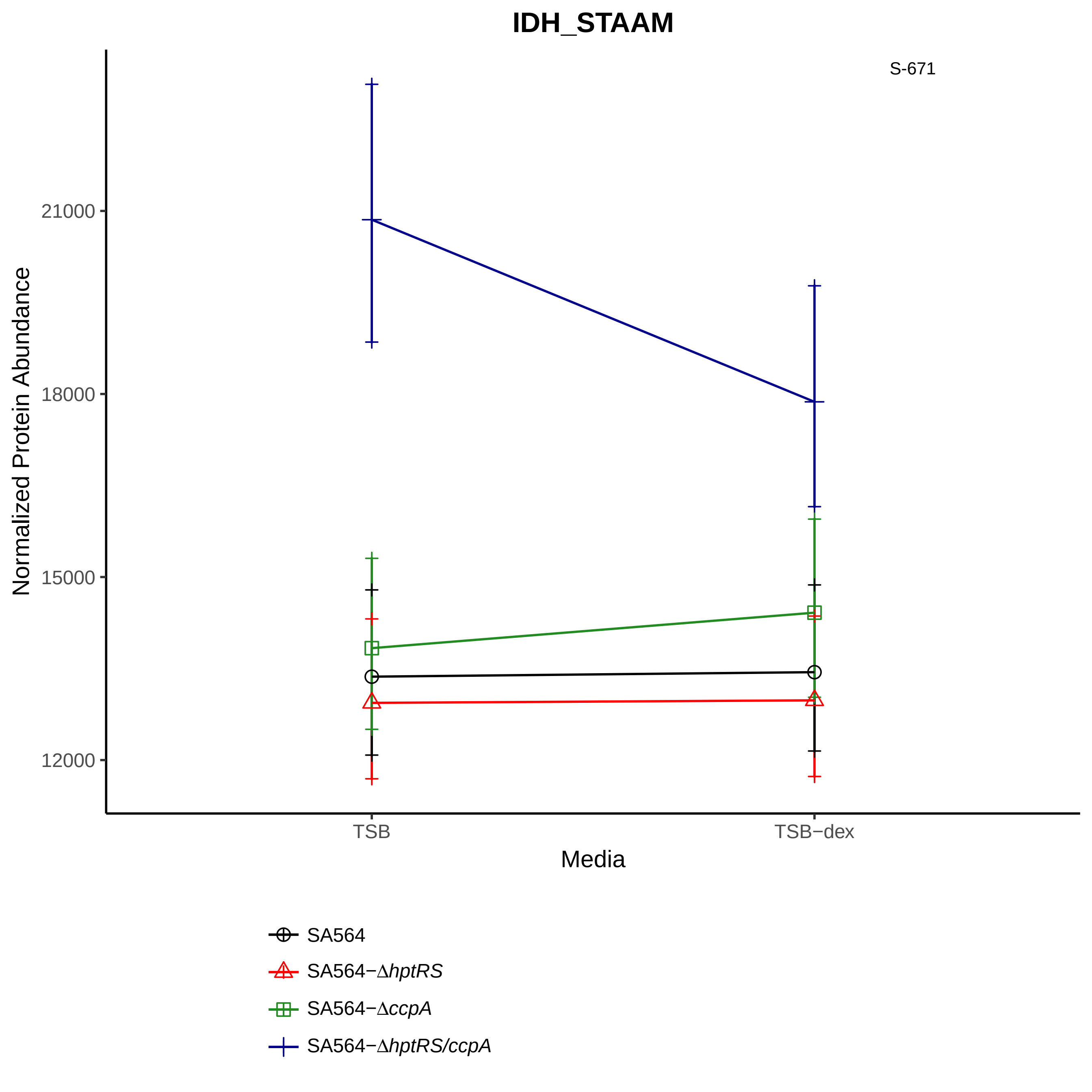

IDH_STAAM
S-671
Normalized Protein Abundance
21000
18000
15000
12000
TSB
TSB−dex
Media
SA564
SA564−Δ*hptRS*
SA564−Δ*ccpA*
SA564−Δ*hptRS/ccpA*

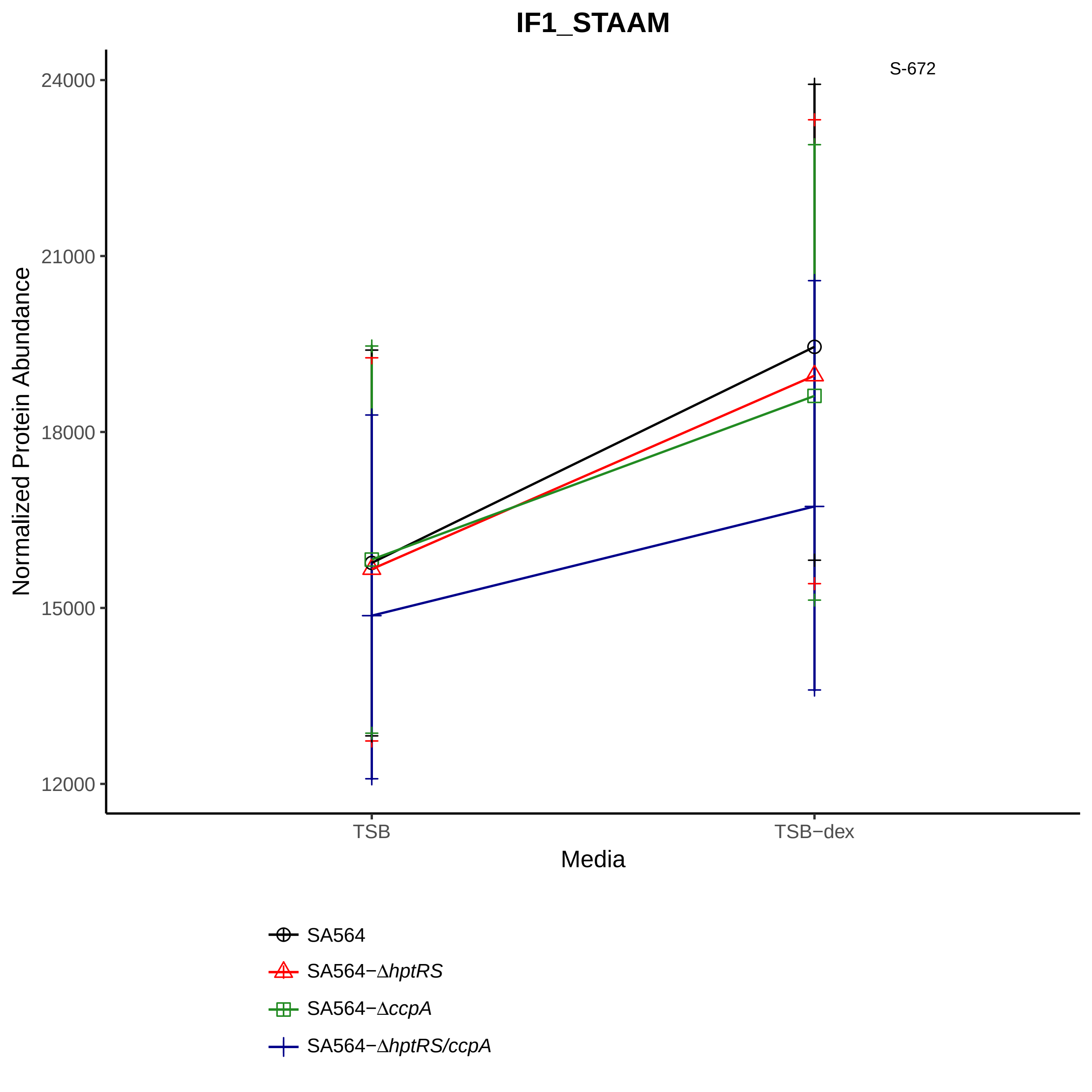

IF1_STAAM
S-672
Normalized Protein Abundance
24000
21000
18000
15000
12000
TSB
TSB−dex
Media
SA564
SA564−ΔhptRS
SA564−ΔccpA
SA564−ΔhptRS/ccpA

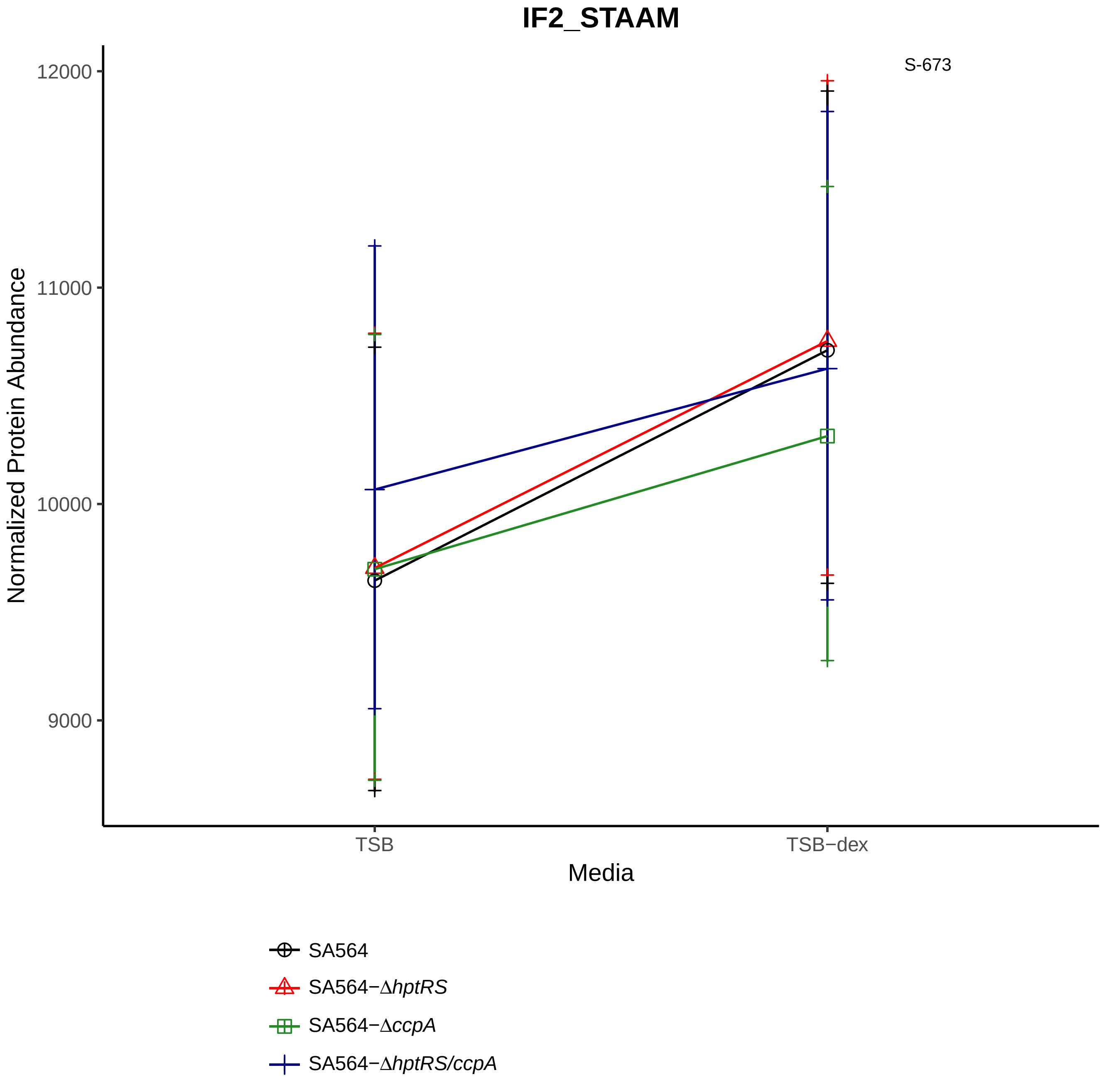

IF2_STAAM
S-673
12000
11000
10000
9000
Normalized Protein Abundance
TSB
TSB−dex
Media
SA564
SA564−Δ*hptRS*
SA564−Δ*ccpA*
SA564−Δ*hptRS/ccpA*

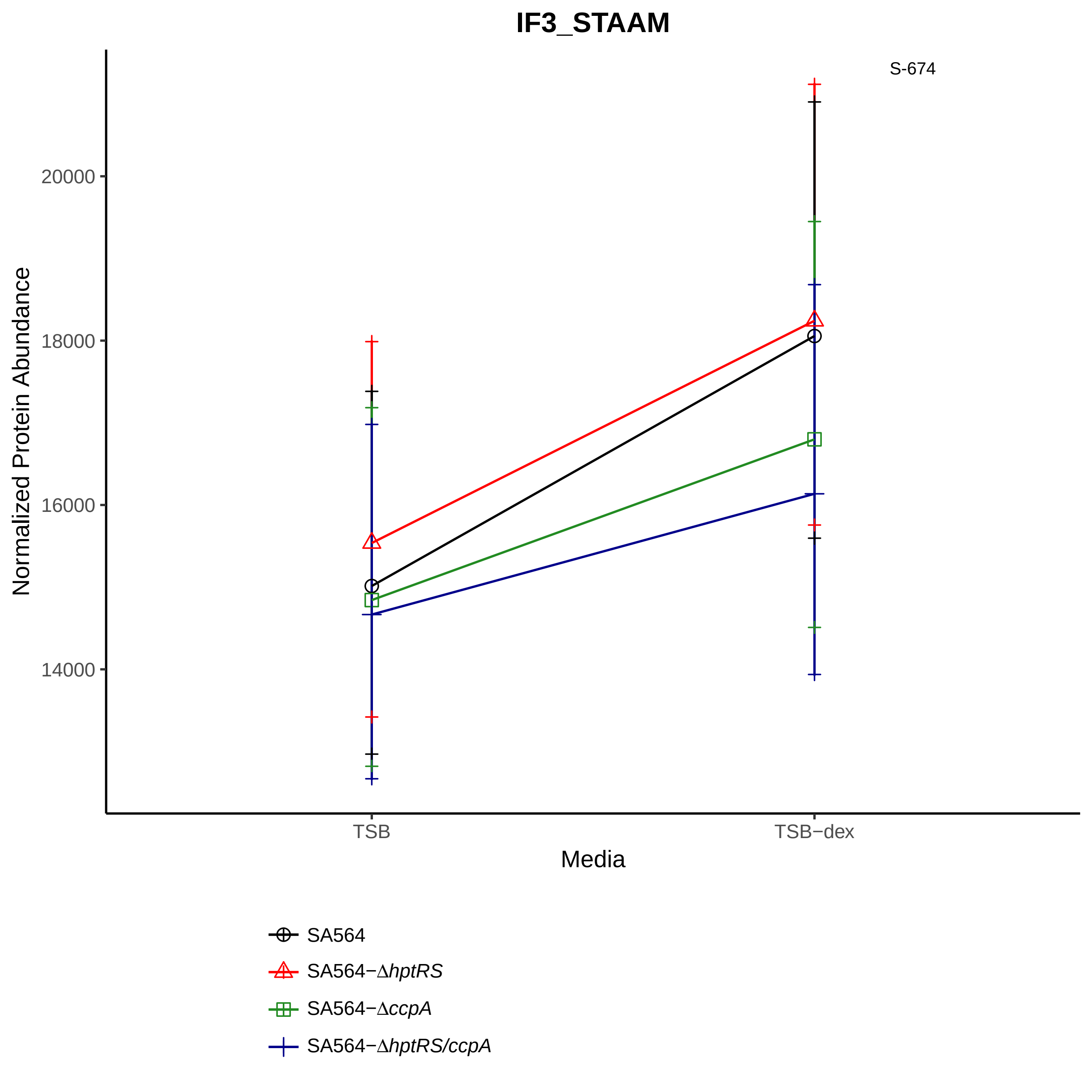

IF3_STAAM
S-674
20000
18000
16000
14000
Normalized Protein Abundance
TSB
TSB−dex
Media
SA564
SA564−Δ*hptRS*
SA564−Δ*ccpA*
SA564−Δ*hptRS/ccpA*

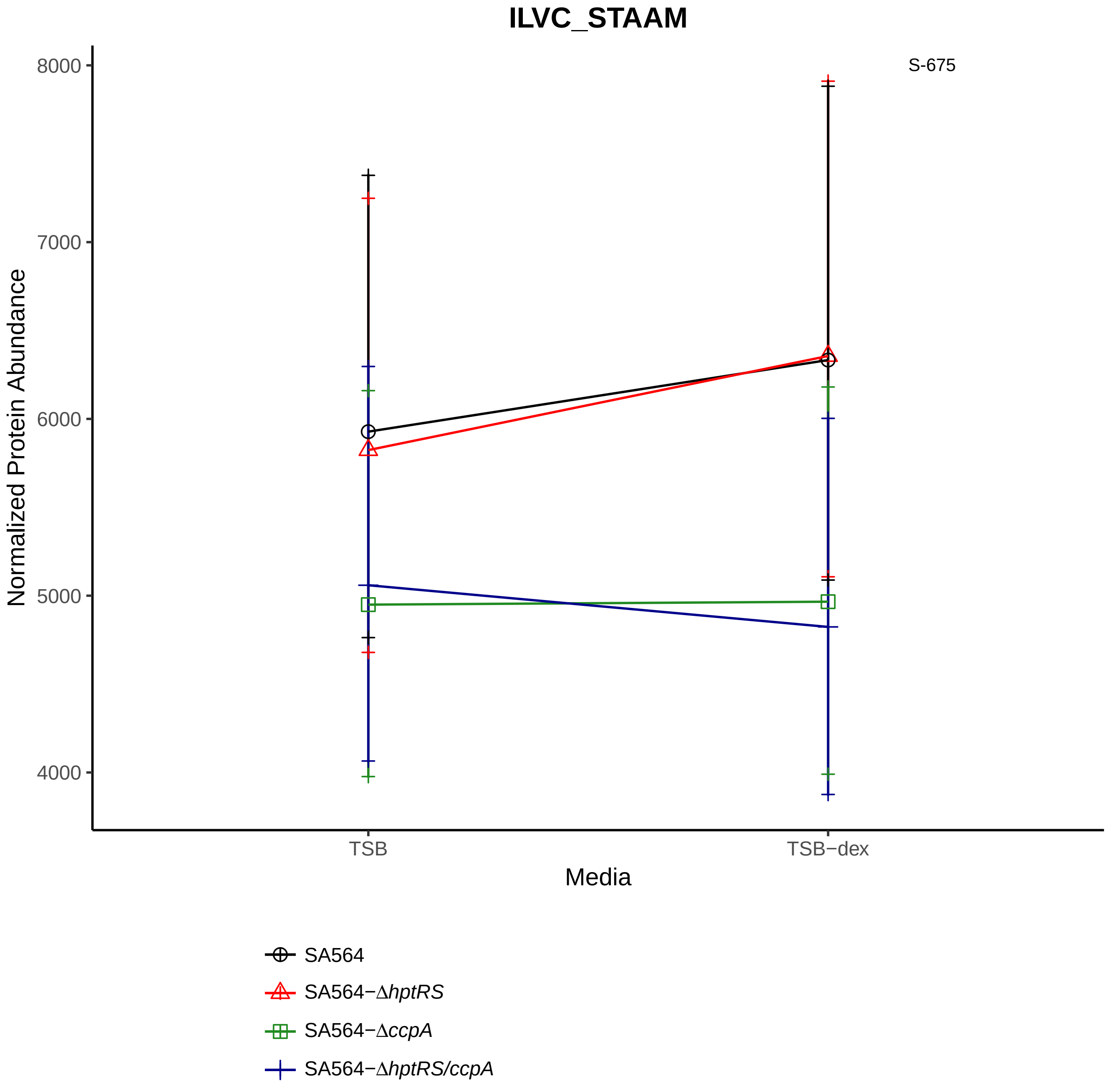

ILVC_STAAM
S-675
Normalized Protein Abundance
8000
7000
6000
5000
4000
TSB
TSB−dex
Media
SA564
SA564−ΔhptRS
SA564−ΔccpA
SA564−ΔhptRS/ccpA

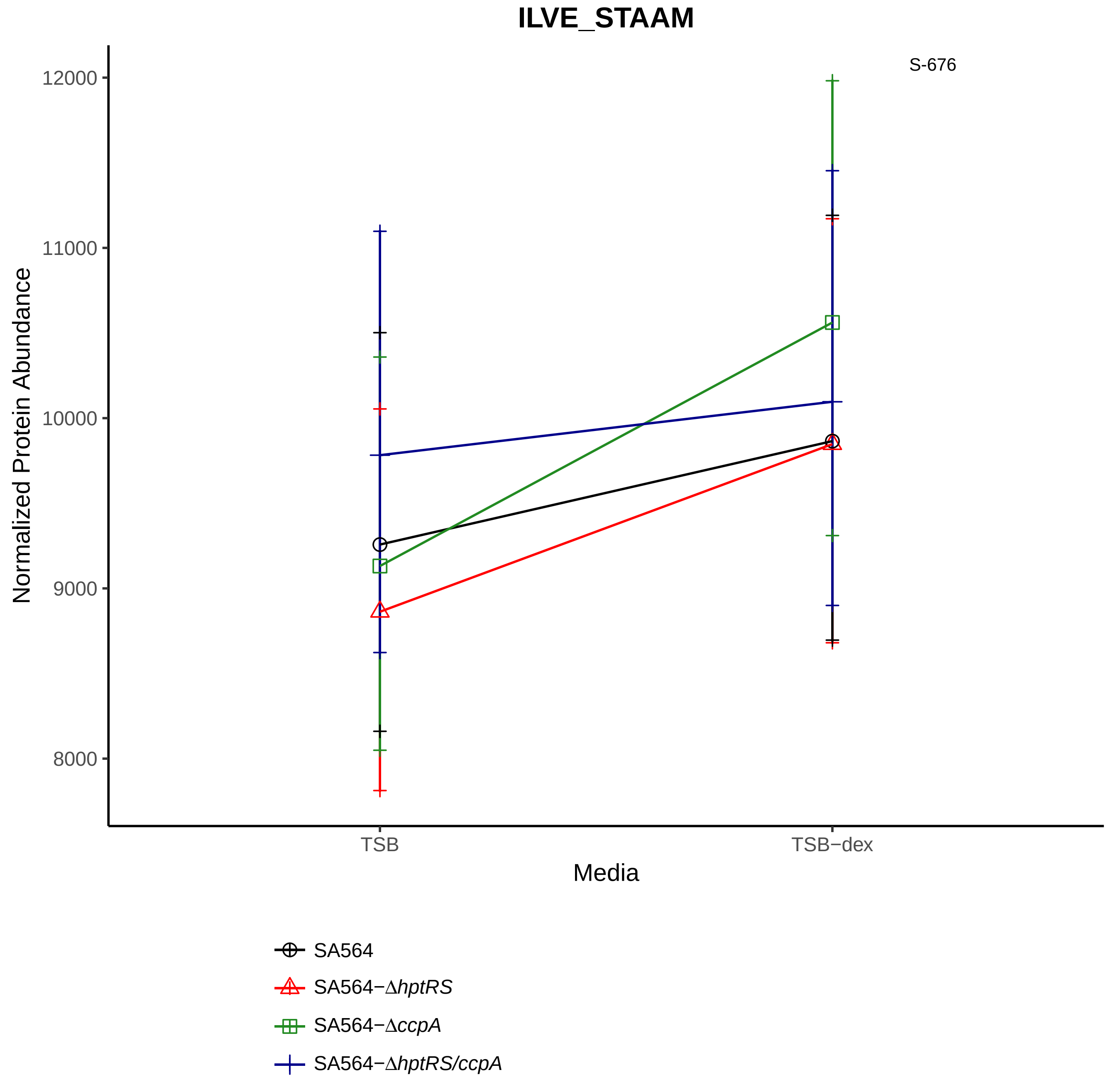

ILVE_STAAM
S-676
Normalized Protein Abundance
12000
11000
10000
9000
8000
TSB
TSB−dex
Media
SA564
SA564−Δ*hptRS*
SA564−Δ*ccpA*
SA564−Δ*hptRS/ccpA*

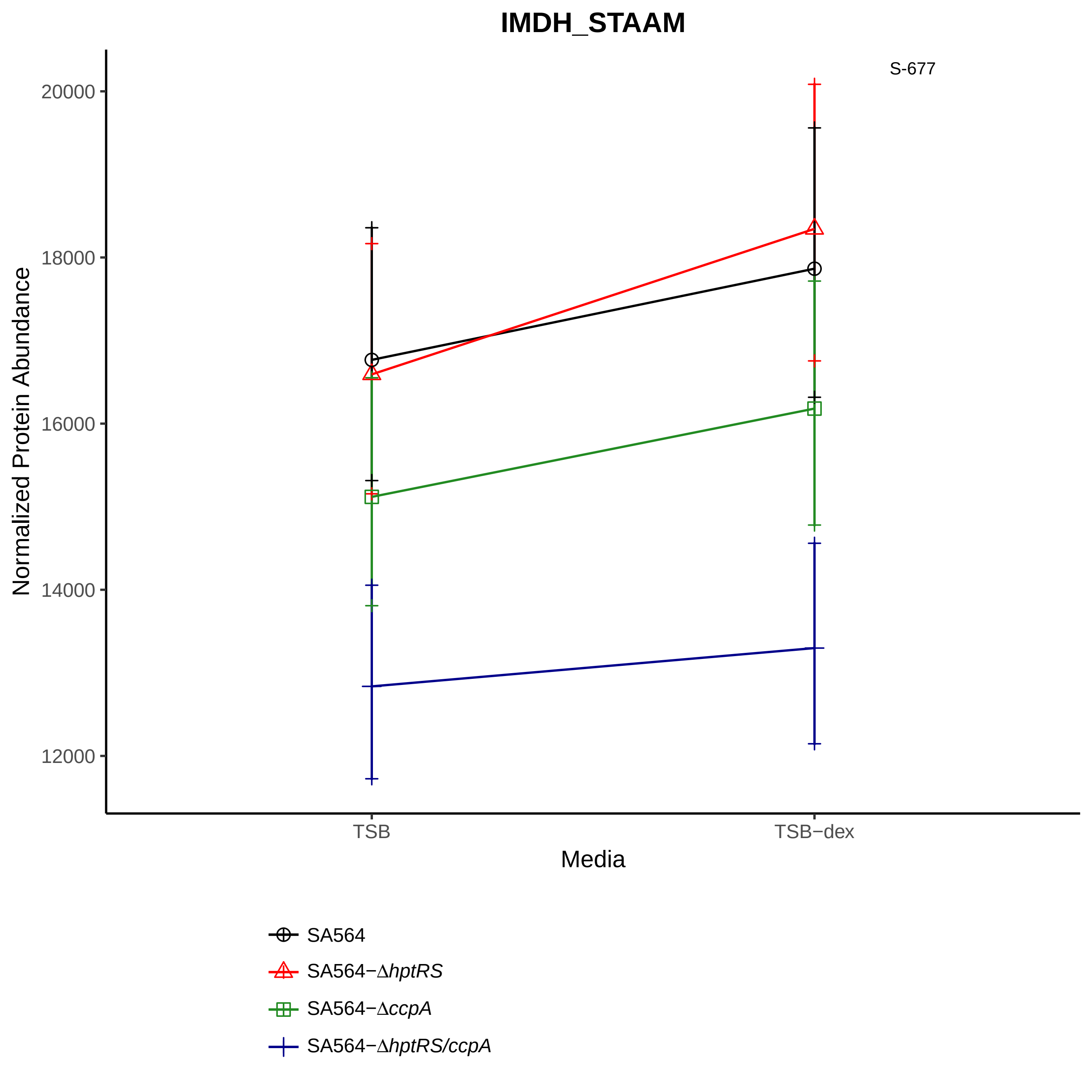

IMDH_STAAM
S-677
Normalized Protein Abundance
20000
18000
16000
14000
12000
TSB
TSB−dex
Media
SA564
SA564−Δ*hptRS*
SA564−Δ*ccpA*
SA564−Δ*hptRS/ccpA*

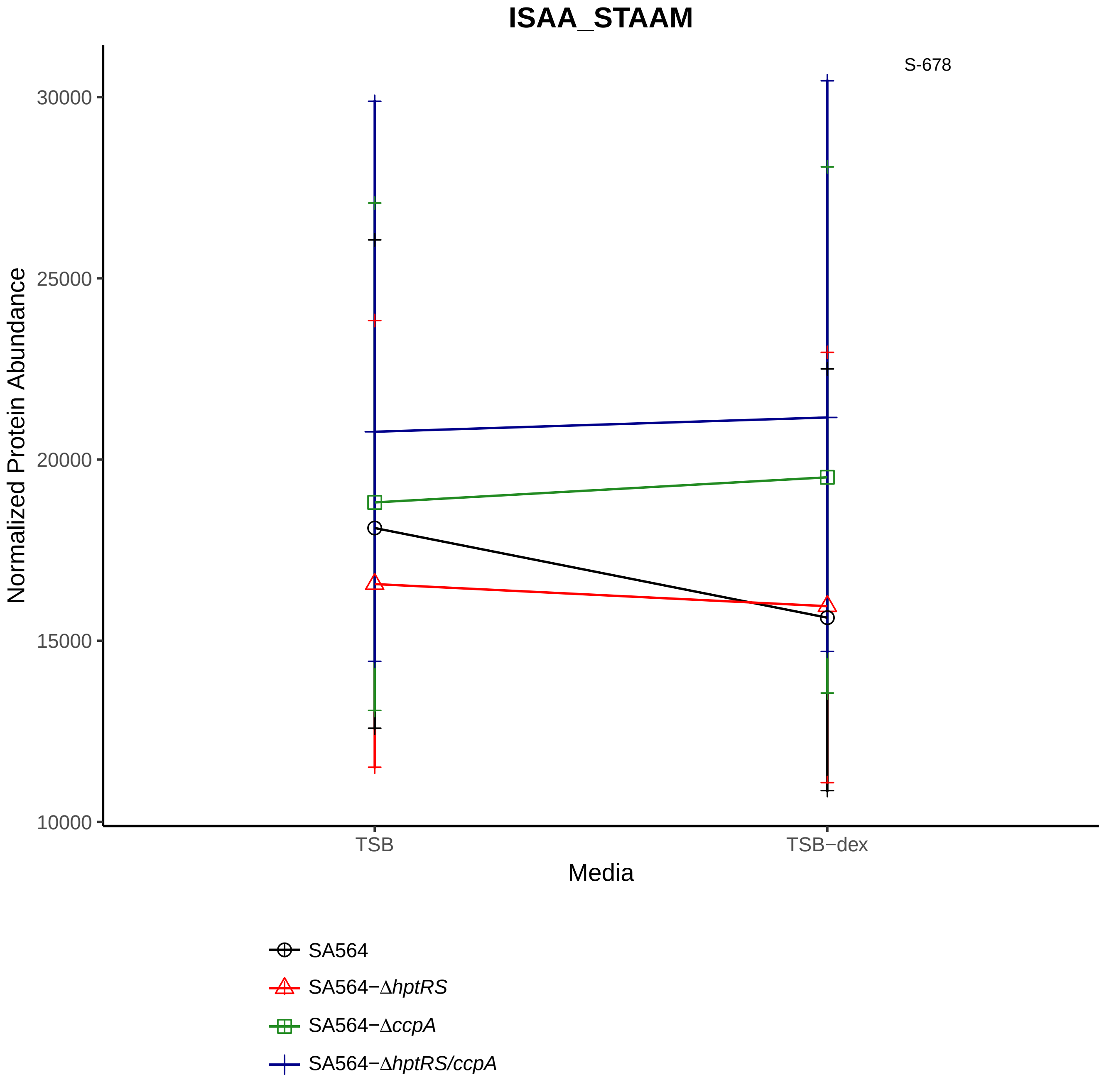

ISAA_STAAM
S-678
Normalized Protein Abundance
30000
25000
20000
15000
10000
TSB
TSB−dex
Media
SA564
SA564−Δ*hptRS*
SA564−Δ*ccpA*
SA564−Δ*hptRS/ccpA*

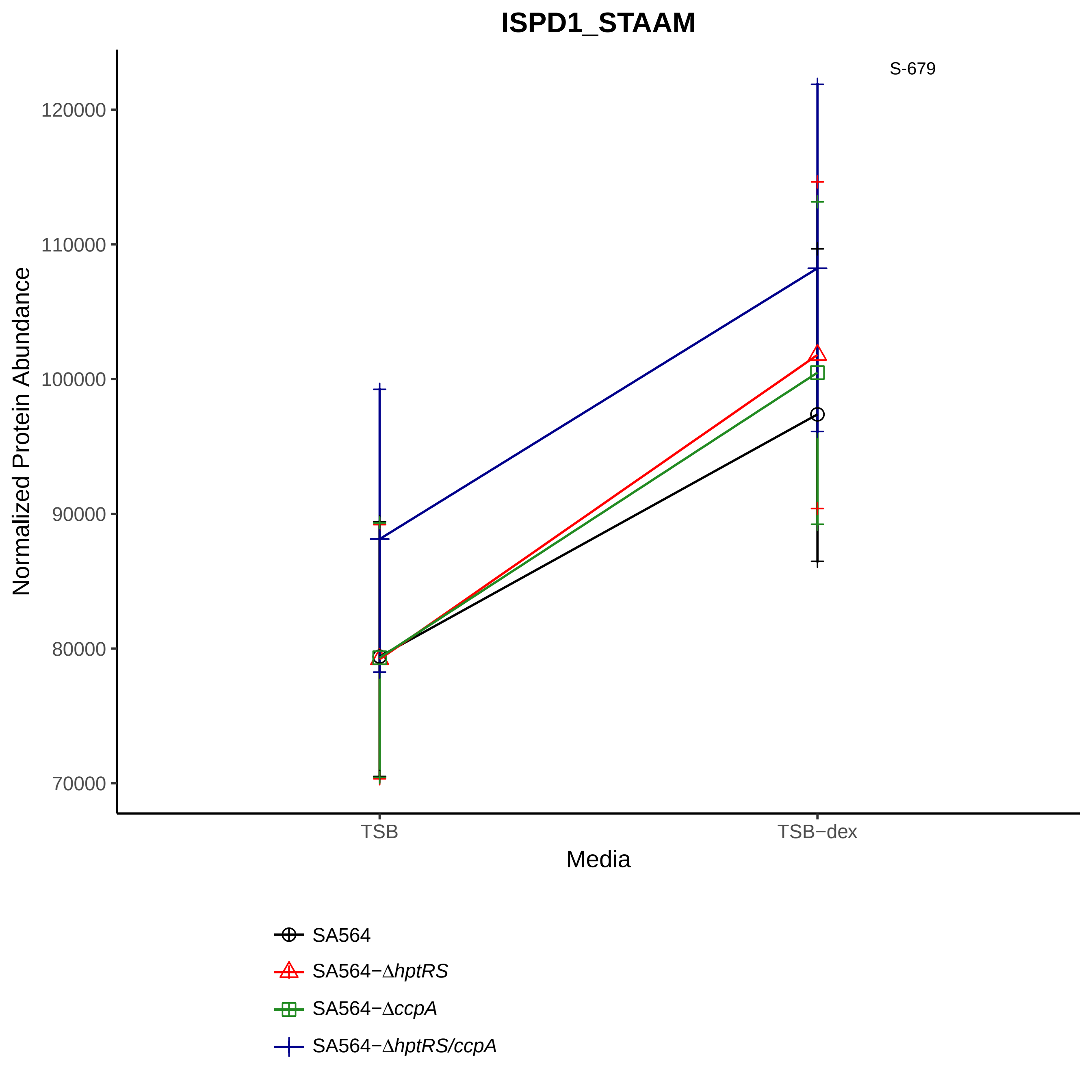
ISPD1_STAAM
S-679
Normalized Protein Abundance
120000
110000
100000
90000
80000
70000
TSB
TSB−dex
Media
SA564
SA564−ΔhptRS
SA564−ΔccpA
SA564−ΔhptRS/ccpA

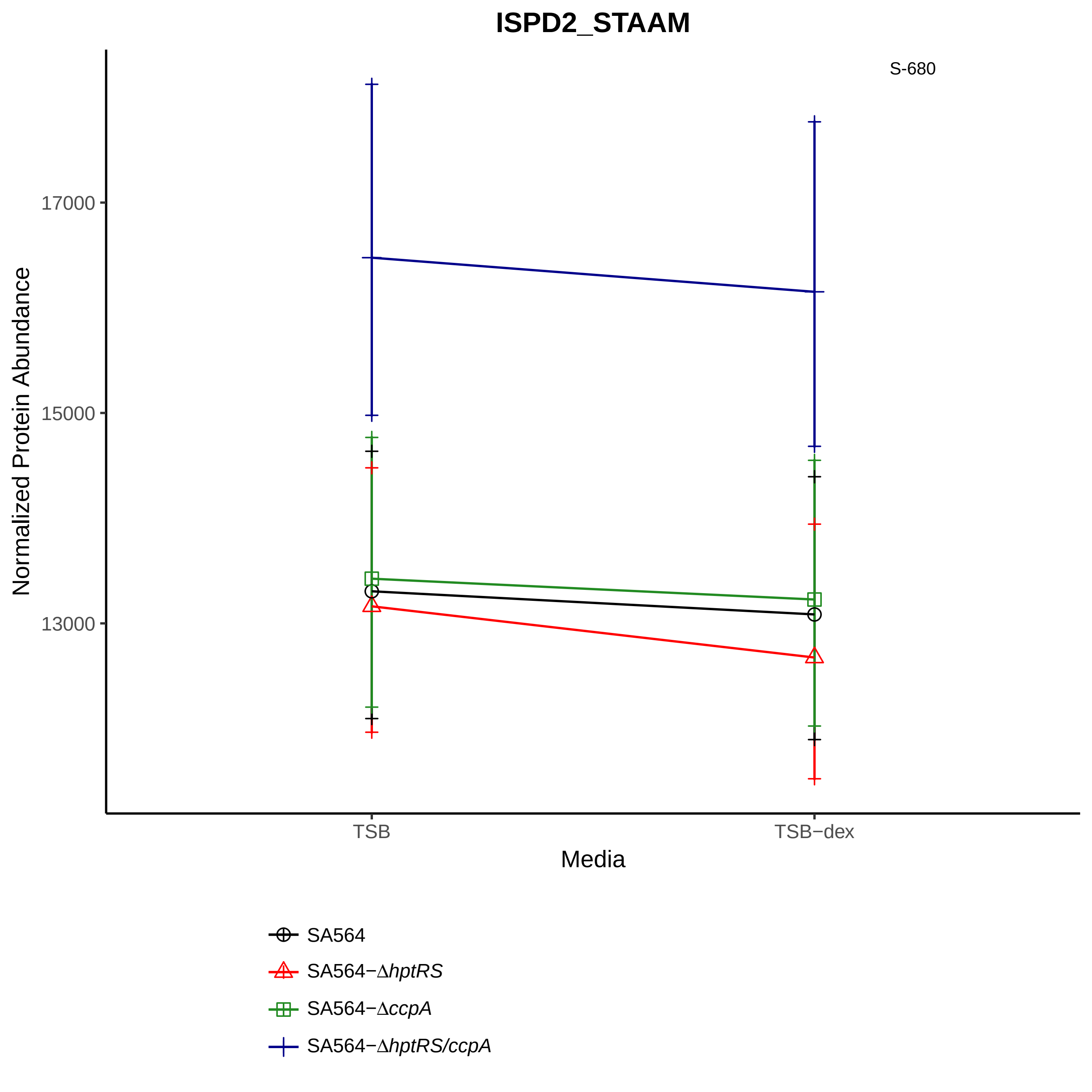

ISPD2_STAAM
S-680
17000
15000
13000
Normalized Protein Abundance
TSB
TSB−dex
Media
SA564
SA564−Δ*hptRS*
SA564−Δ*ccpA*
SA564−Δ*hptRS/ccpA*

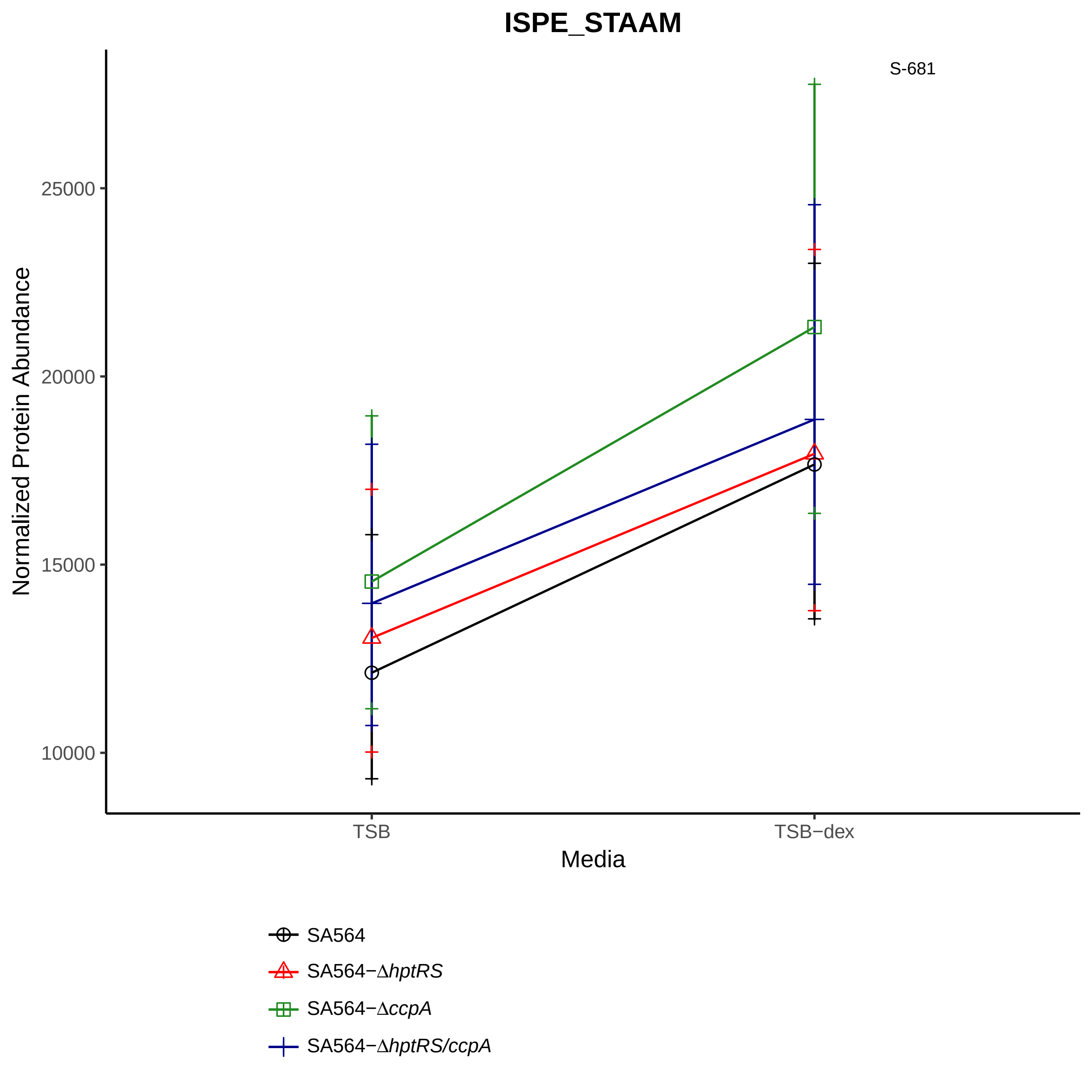

ISPE_STAAM
S-681
Normalized Protein Abundance
25000
20000
15000
10000
TSB
TSB−dex
Media
SA564
SA564−Δ*hptRS*
SA564−Δ*ccpA*
SA564−Δ*hptRS/ccpA*

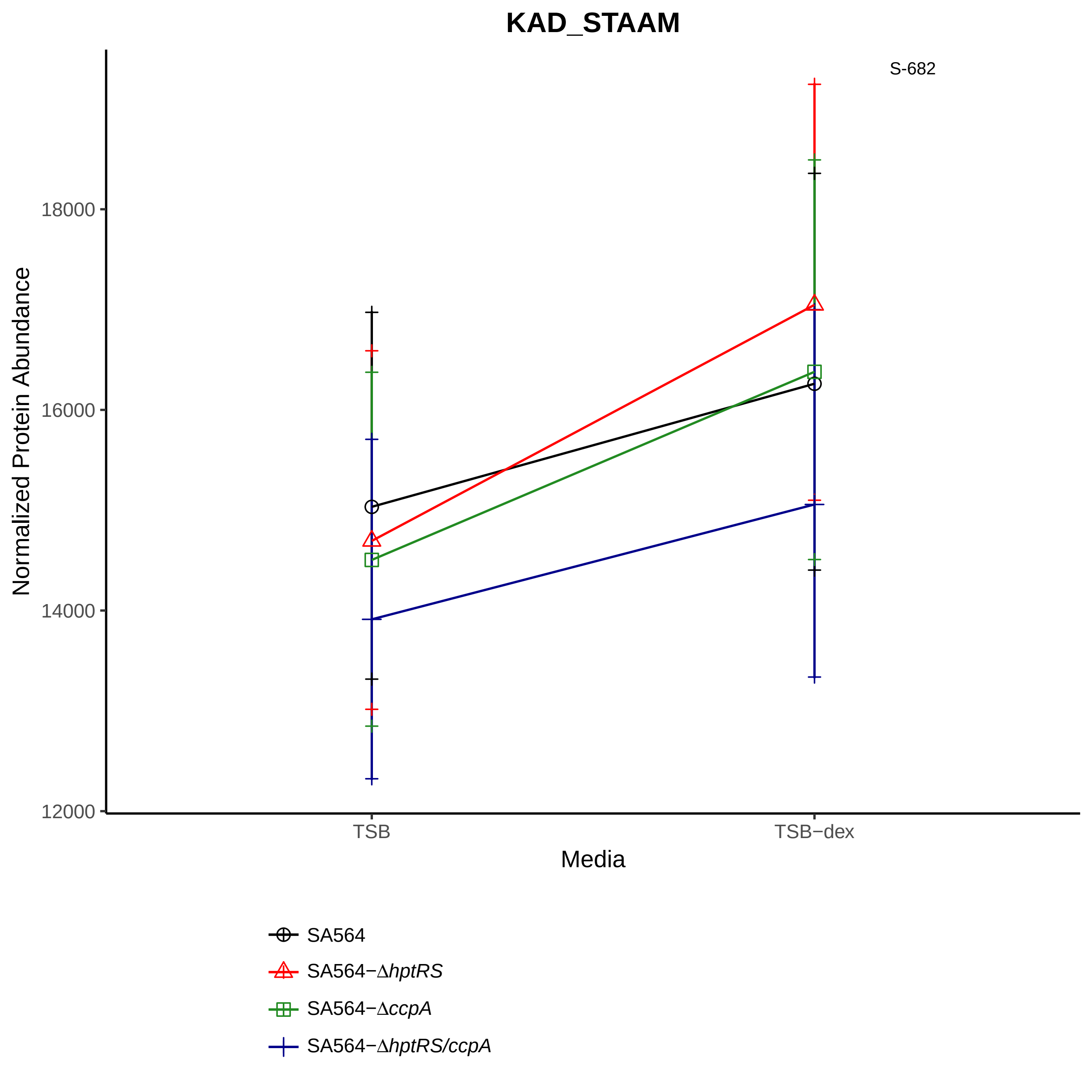
KAD_STAAM
S-682
Normalized Protein Abundance
18000
16000
14000
12000
TSB
TSB−dex
Media
SA564
SA564−Δ*hptRS*
SA564−Δ*ccpA*
SA564−Δ*hptRS/ccpA*

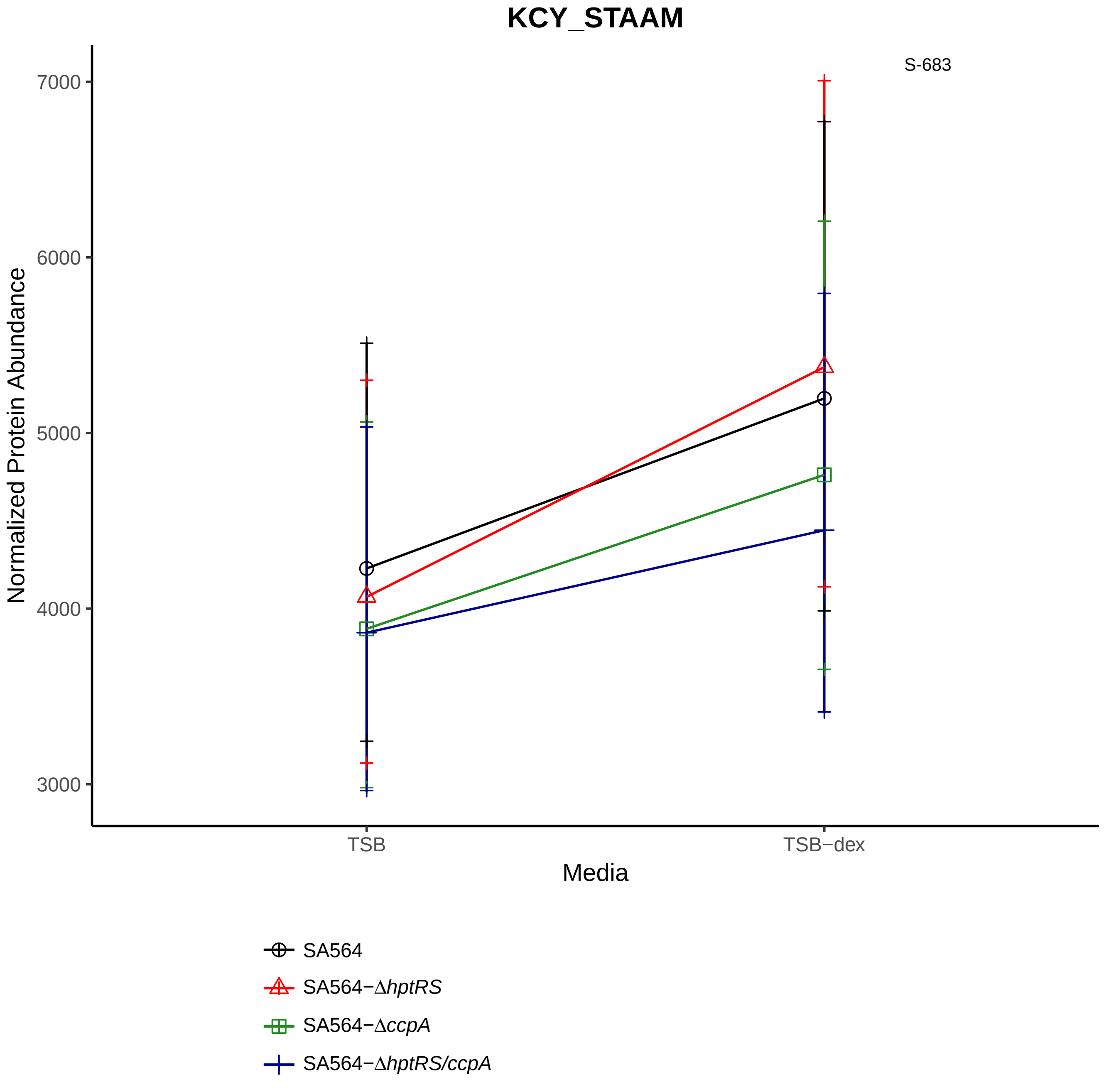

KCY_STAAM
S-683
Normalized Protein Abundance
7000
6000
5000
4000
3000
TSB
TSB−dex
Media
SA564
SA564−Δ*hptRS*
SA564−Δ*ccpA*
SA564−Δ*hptRS/ccpA*

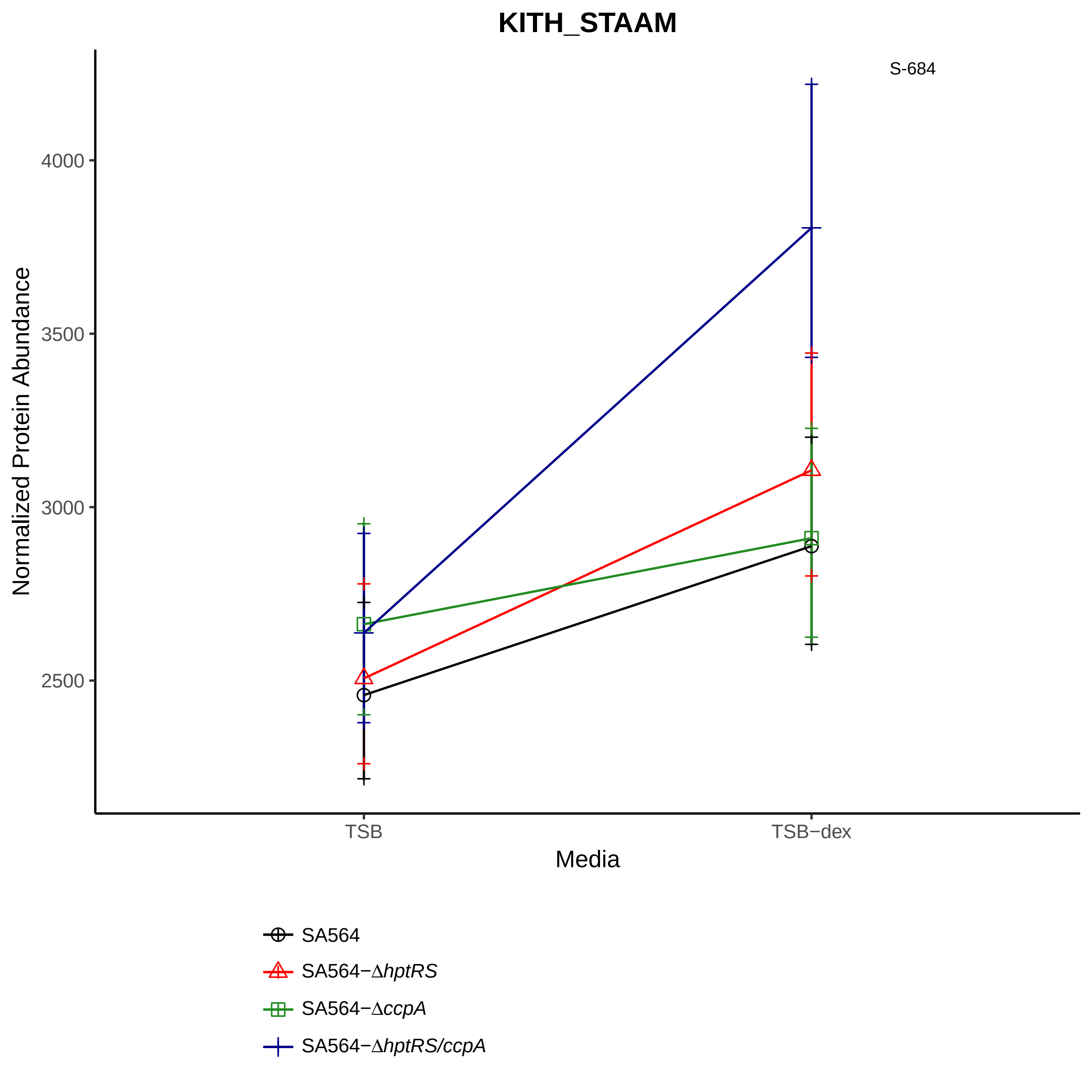

KITH_STAAM
S-684
Normalized Protein Abundance
4000
3500
3000
2500
TSB
TSB−dex
Media
SA564
SA564−ΔhptRS
SA564−ΔccpA
SA564−ΔhptRS/ccpA

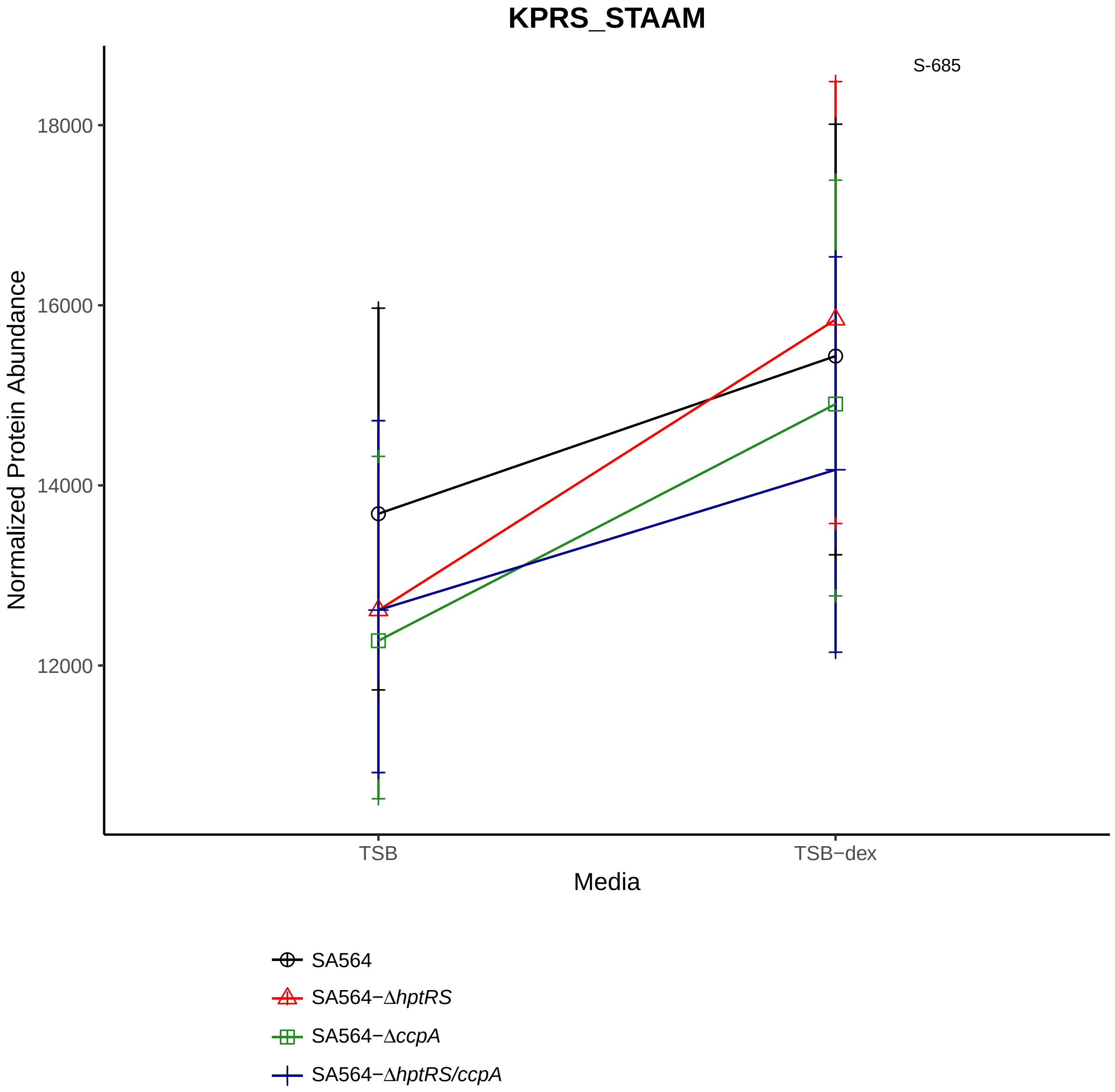
KPRS_STAAM
S-685
Normalized Protein Abundance
18000
16000
14000
12000
TSB
TSB−dex
Media
SA564
SA564−Δ*hptRS*
SA564−Δ*ccpA*
SA564−Δ*hptRS/ccpA*

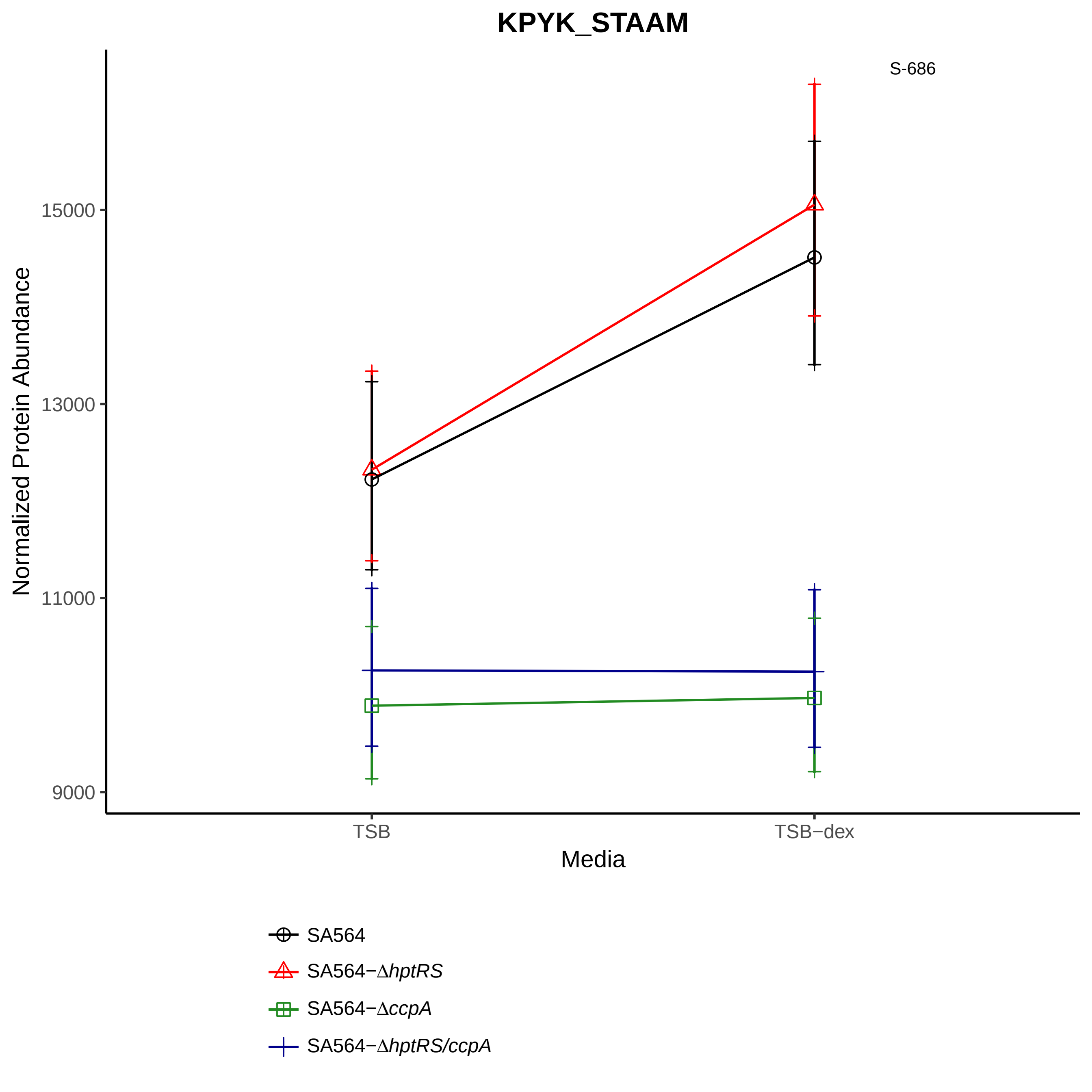

KPYK_STAAM
S-686
Normalized Protein Abundance
15000
13000
11000
9000
TSB
TSB−dex
Media
SA564
SA564−Δ*hptRS*
SA564−Δ*ccpA*
SA564−Δ*hptRS/ccpA*

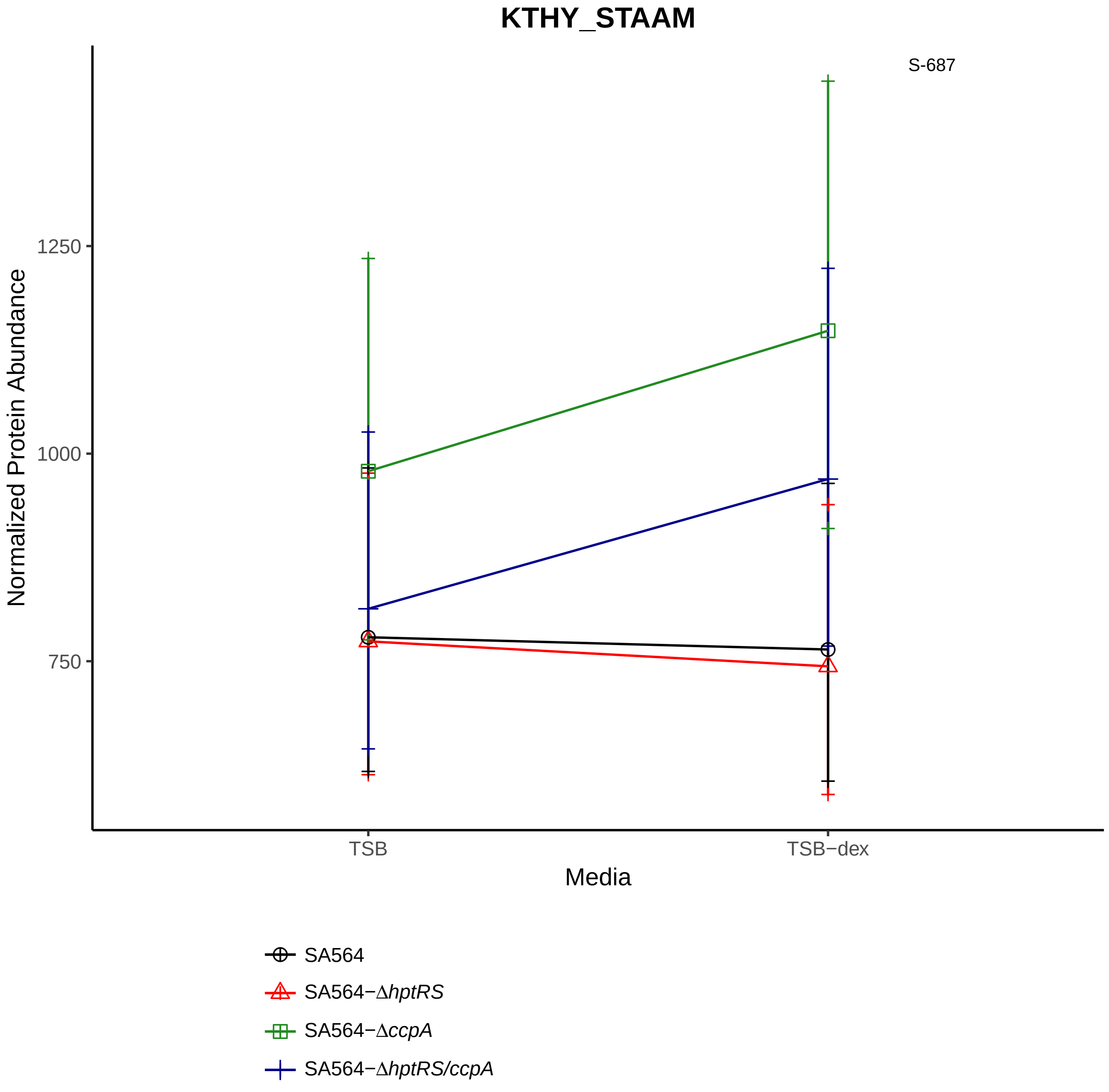

KTHY_STAAM
S-687
Normalized Protein Abundance
1250
1000
750
TSB
TSB−dex
Media
SA564
SA564−Δ*hptRS*
SA564−Δ*ccpA*
SA564−Δ*hptRS/ccpA*

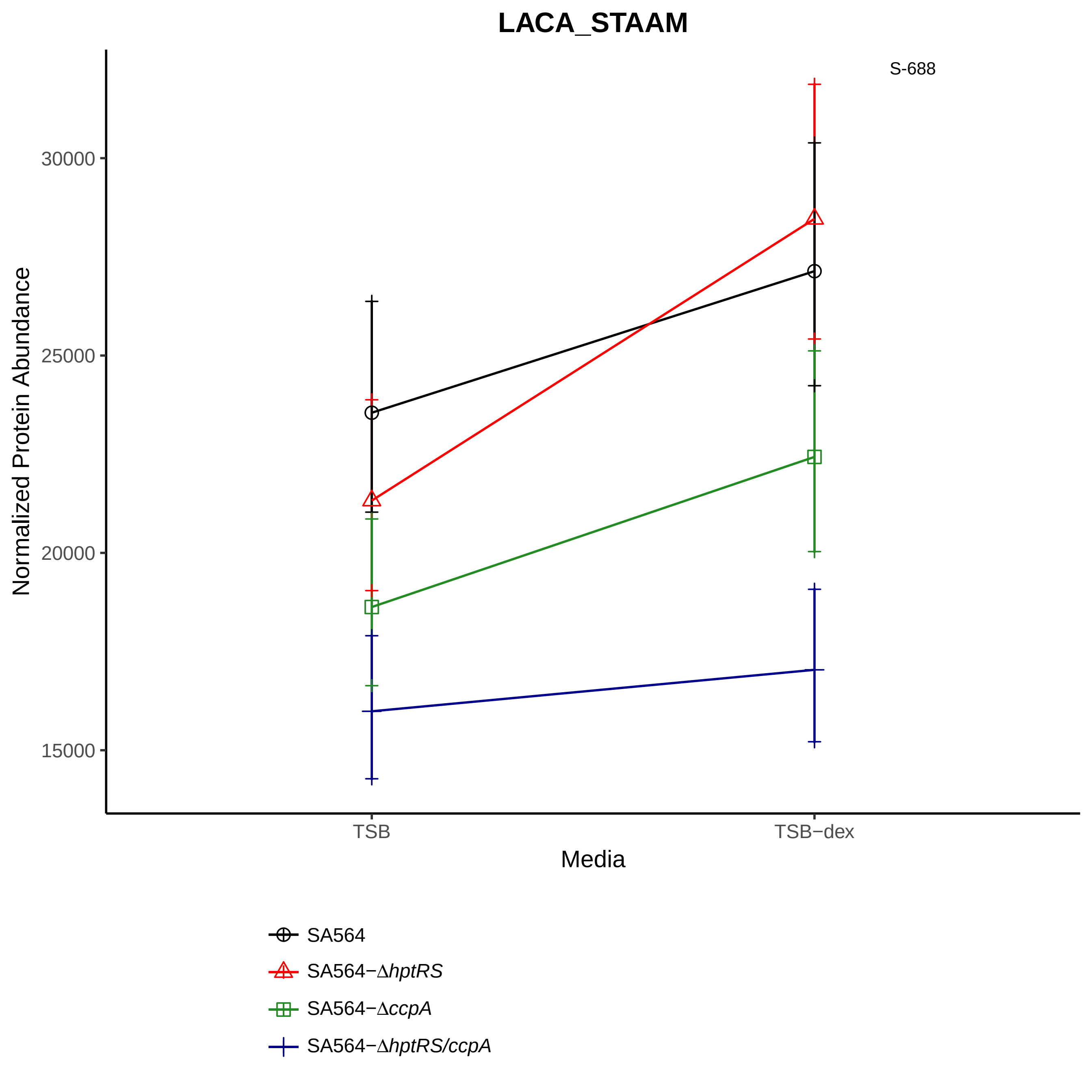
LACA_STAAM
S-688
Normalized Protein Abundance
30000
25000
20000
15000
TSB
TSB−dex
Media
SA564
SA564−Δ*hptRS*
SA564−Δ*ccpA*
SA564−Δ*hptRS/ccpA*

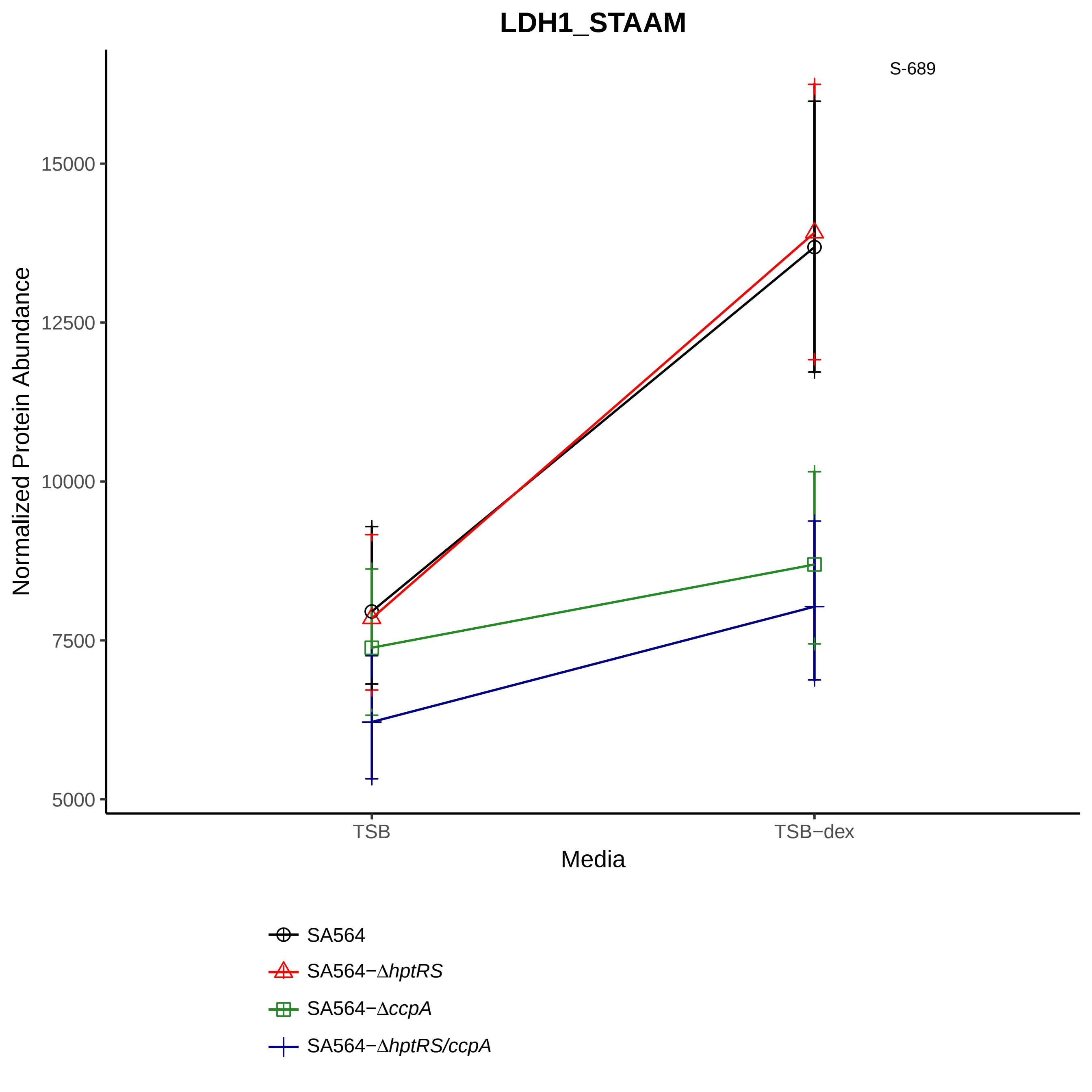
LDH1_STAAM
S-689
Normalized Protein Abundance
15000
12500
10000
7500
5000
TSB
TSB−dex
Media
SA564
SA564−Δ*hptRS*
SA564−Δ*ccpA*
SA564−Δ*hptRS/ccpA*

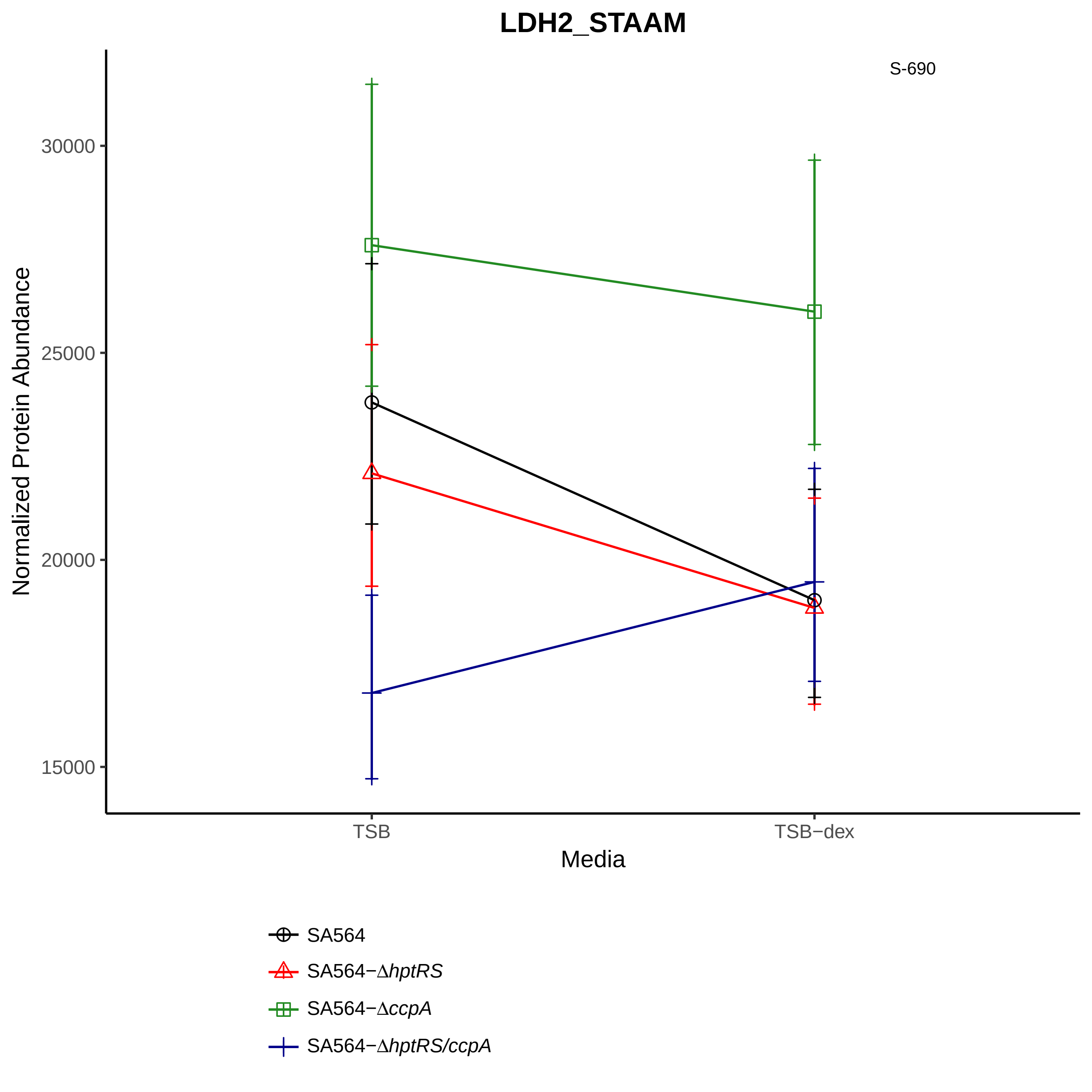

LDH2_STAAM
S-690
30000
25000
20000
15000
Normalized Protein Abundance
TSB
TSB−dex
Media
SA564
SA564−ΔhptRS
SA564−ΔccpA
SA564−ΔhptRS/ccpA

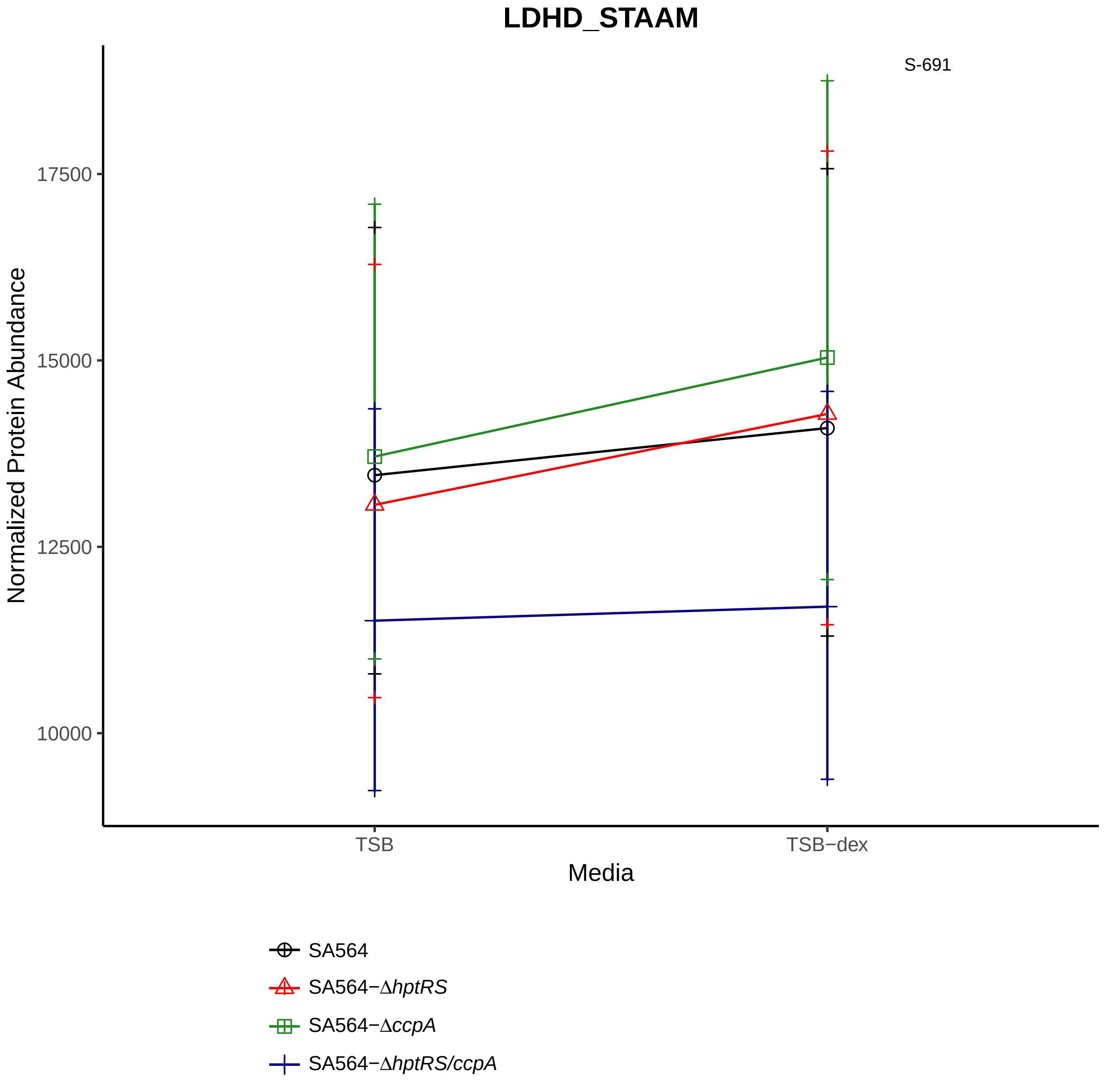

LDHD_STAAM
S-691
Normalized Protein Abundance
17500
15000
12500
10000
TSB
TSB−dex
Media
SA564
SA564−ΔhptRS
SA564−ΔccpA
SA564−ΔhptRS/ccpA

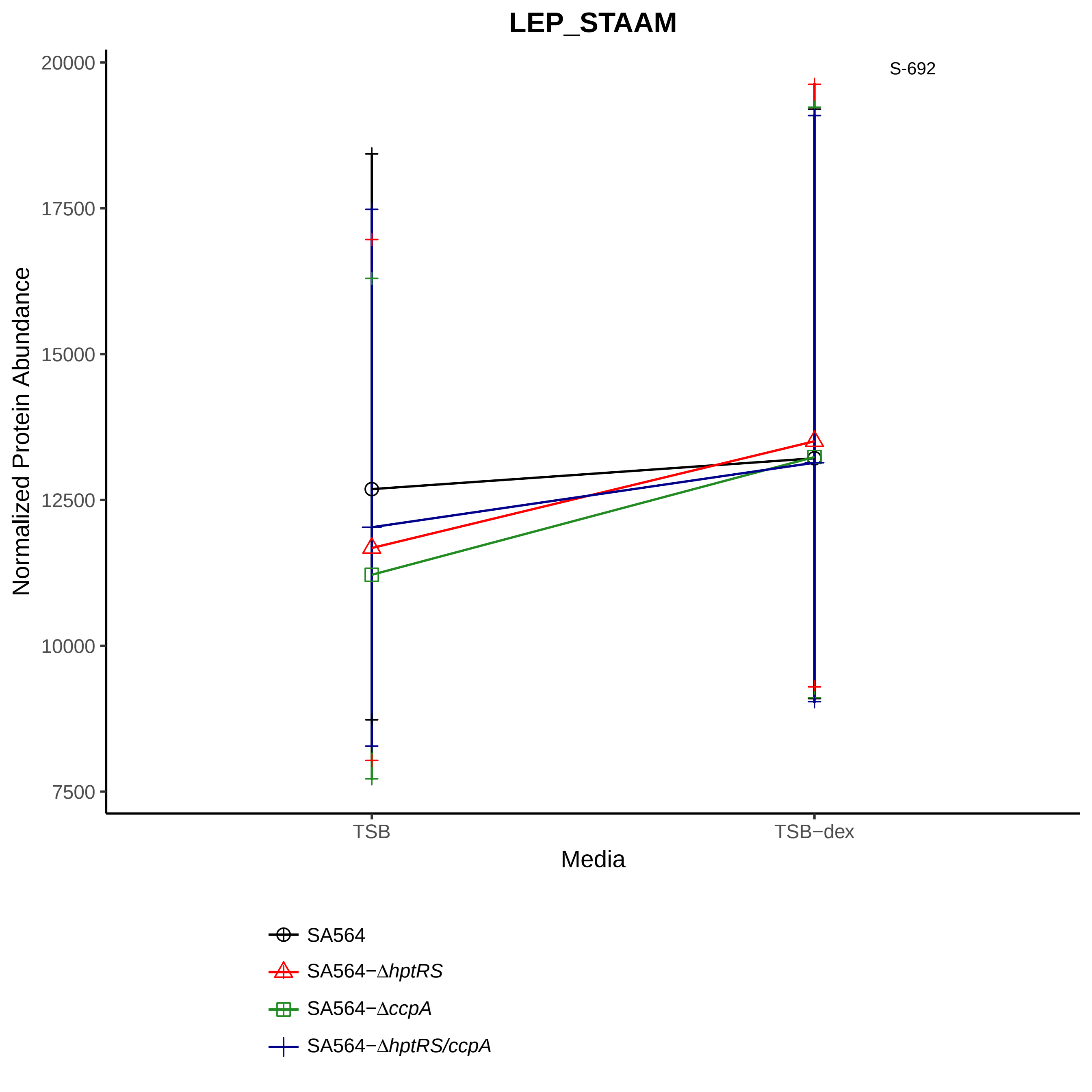

LEP_STAAM
S-692
Normalized Protein Abundance
20000
17500
15000
12500
10000
7500
TSB
TSB−dex
Media
SA564
SA564−Δ*hptRS*
SA564−Δ*ccpA*
SA564−Δ*hptRS/ccpA*

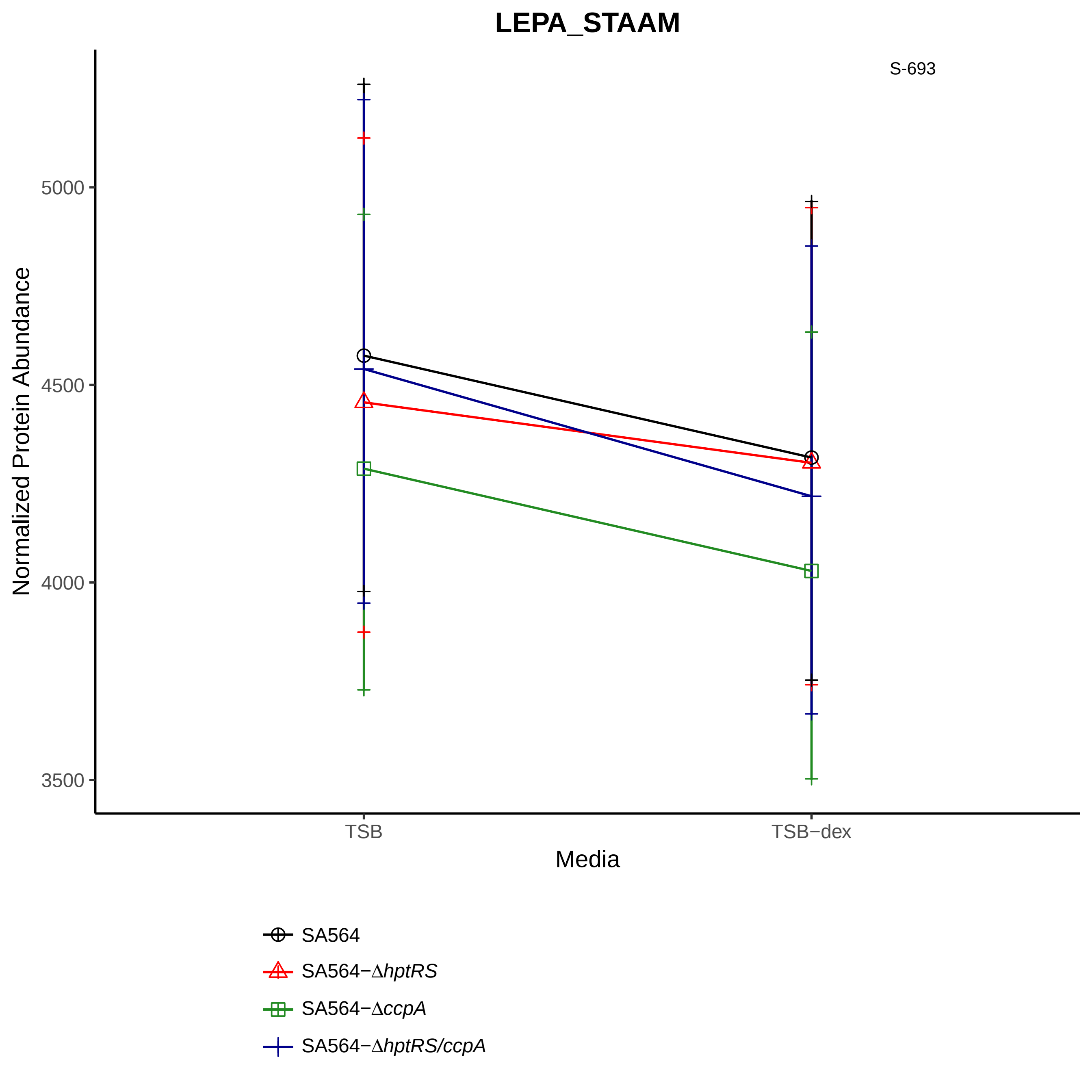
LEPA_STAAM
S-693
Normalized Protein Abundance
5000
4500
4000
3500
TSB
TSB−dex
Media
SA564
SA564−Δ*hptRS*
SA564−Δ*ccpA*
SA564−Δ*hptRS/ccpA*

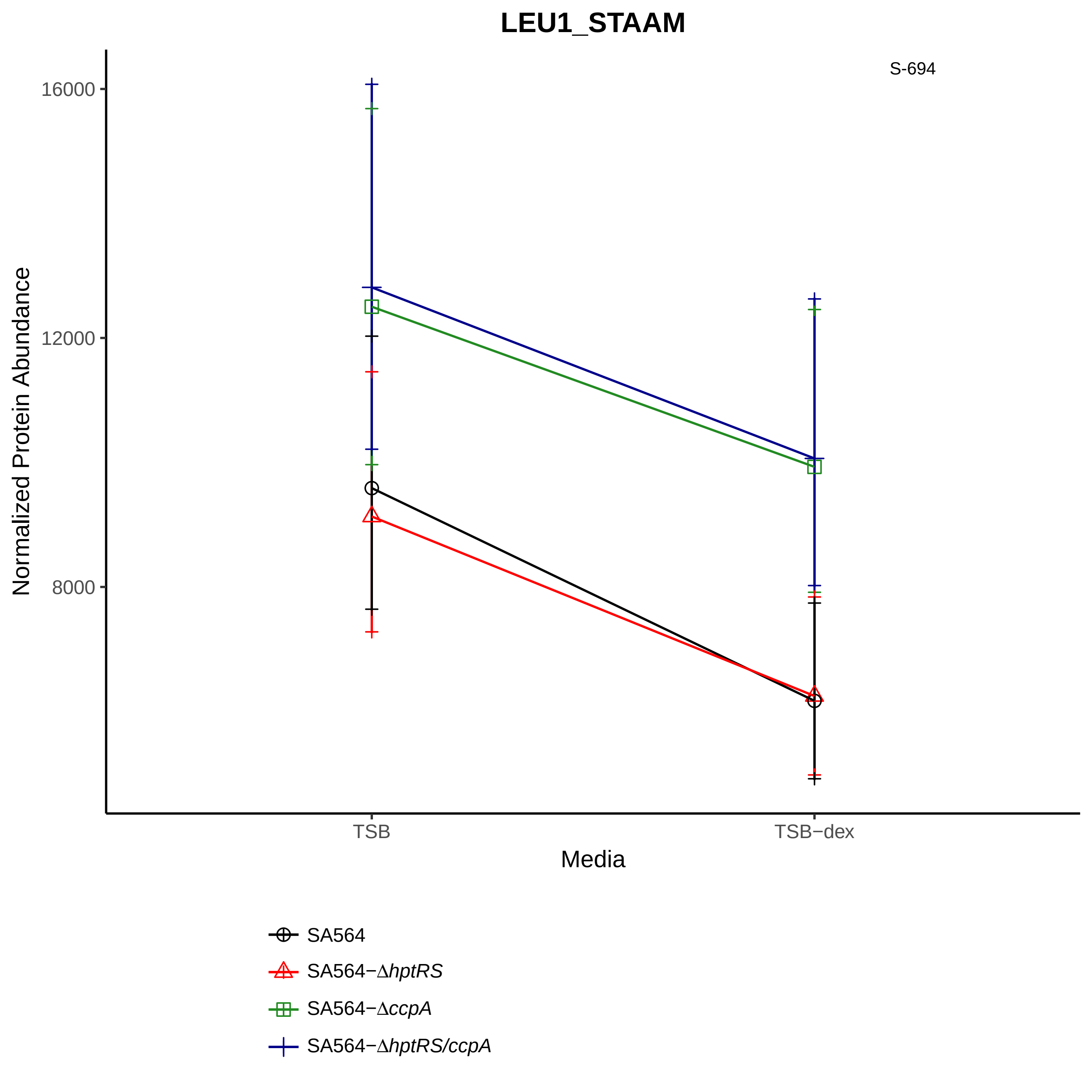

LEU1_STAAM
S-694
16000
12000
8000
Normalized Protein Abundance
TSB
TSB−dex
Media
SA564
SA564−Δ*hptRS*
SA564−Δ*ccpA*
SA564−Δ*hptRS/ccpA*

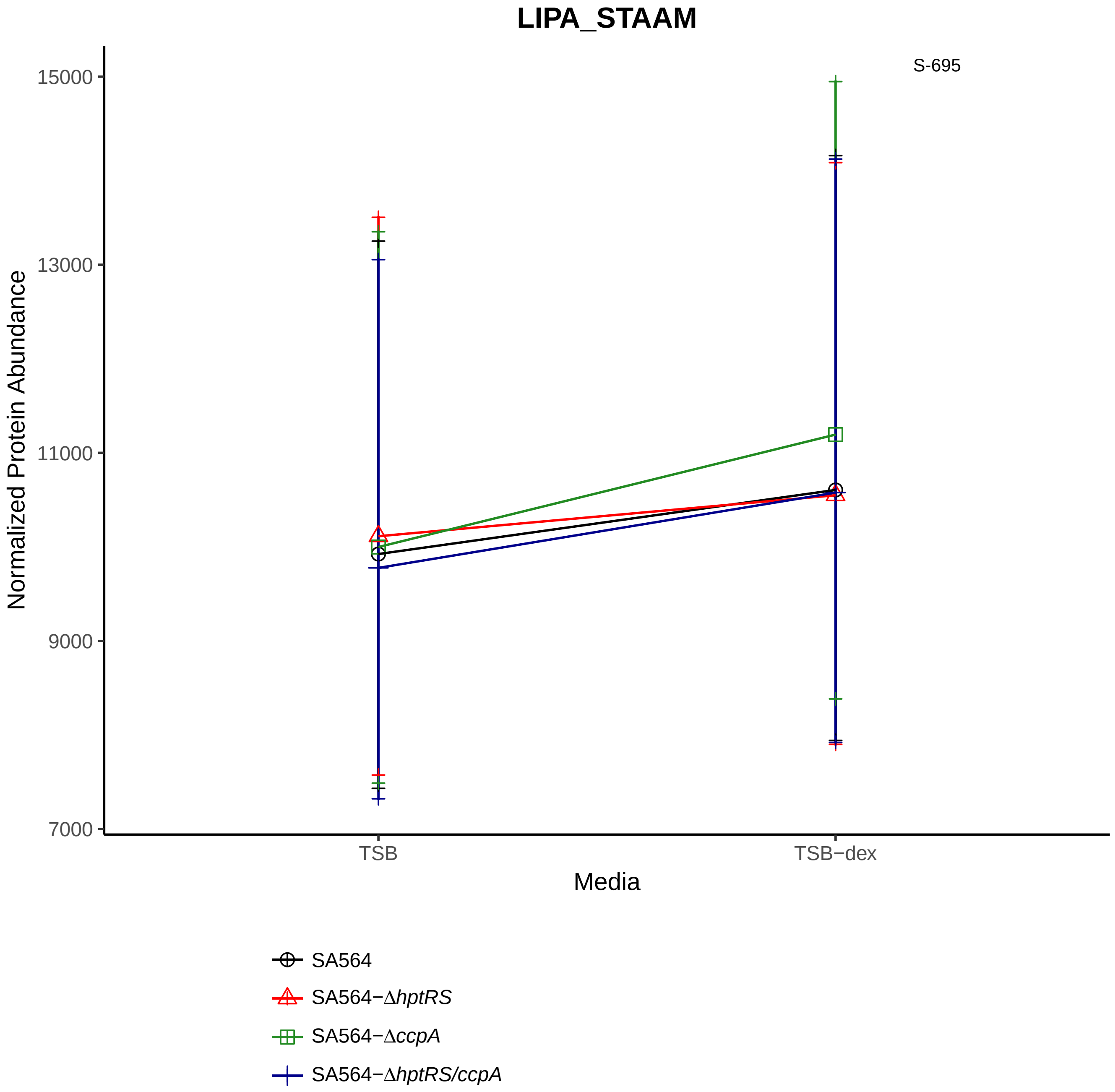

LIPA_STAAM
S-695
Normalized Protein Abundance
15000
13000
11000
9000
7000
TSB
TSB−dex
Media
SA564
SA564−Δ*hptRS*
SA564−Δ*ccpA*
SA564−Δ*hptRS/ccpA*

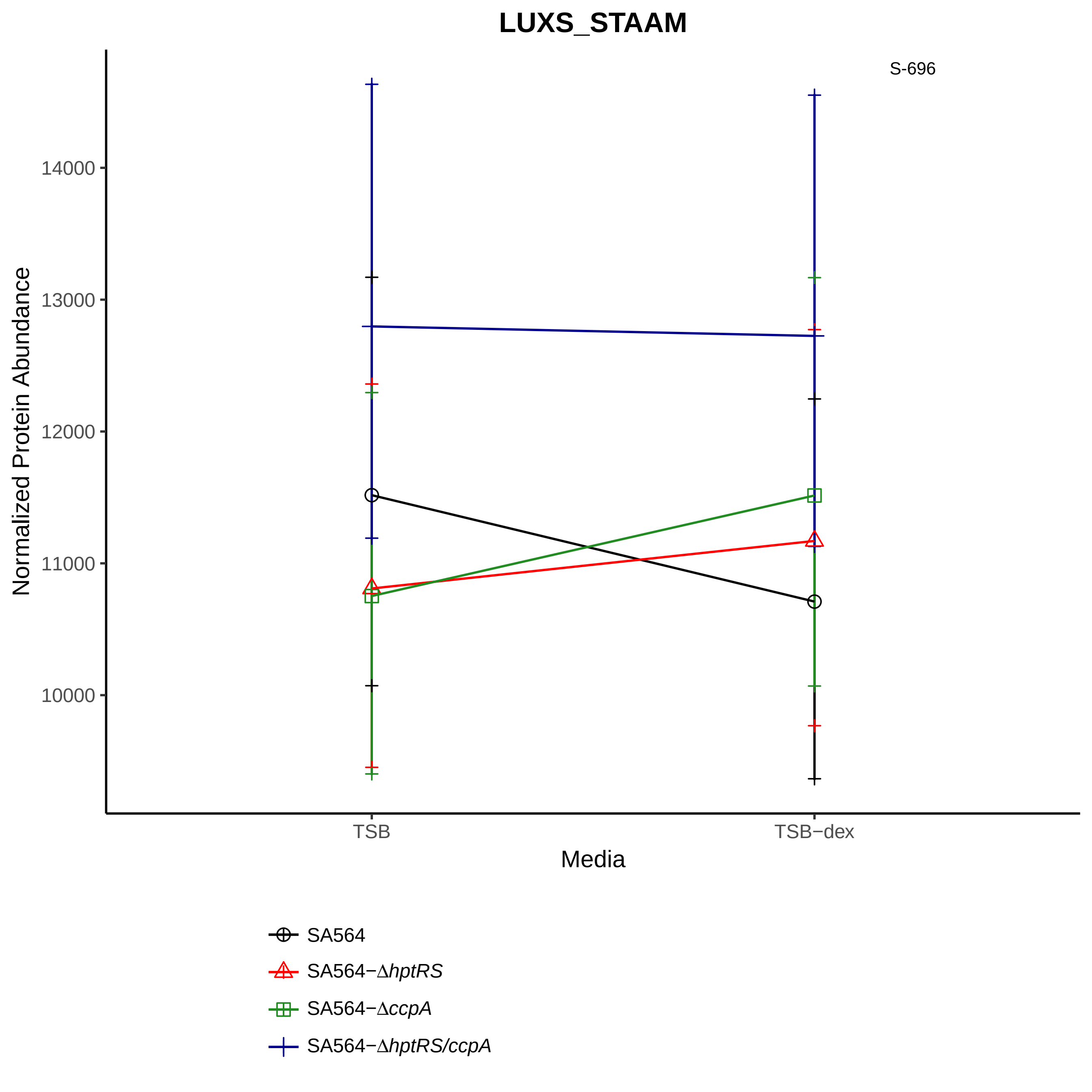

LUXS_STAAM
S-696
Normalized Protein Abundance
14000
13000
12000
11000
10000
TSB
TSB−dex
Media
SA564
SA564−ΔhptRS
SA564−ΔccpA
SA564−ΔhptRS/ccpA

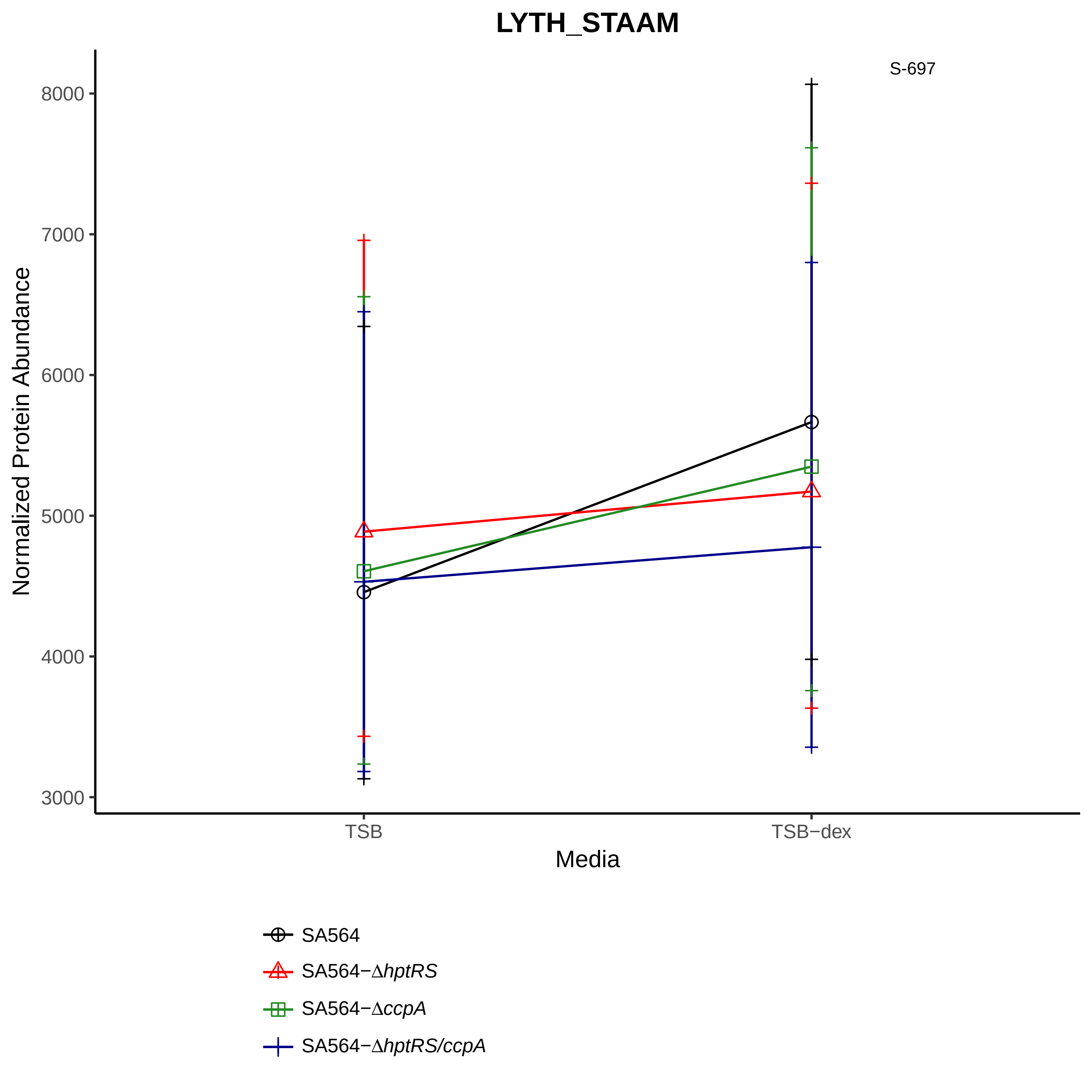

LYTH_STAAM
S-697
Normalized Protein Abundance
8000
7000
6000
5000
4000
3000
TSB
TSB−dex
Media
SA564
SA564−ΔhptRS
SA564−ΔccpA
SA564−ΔhptRS/ccpA

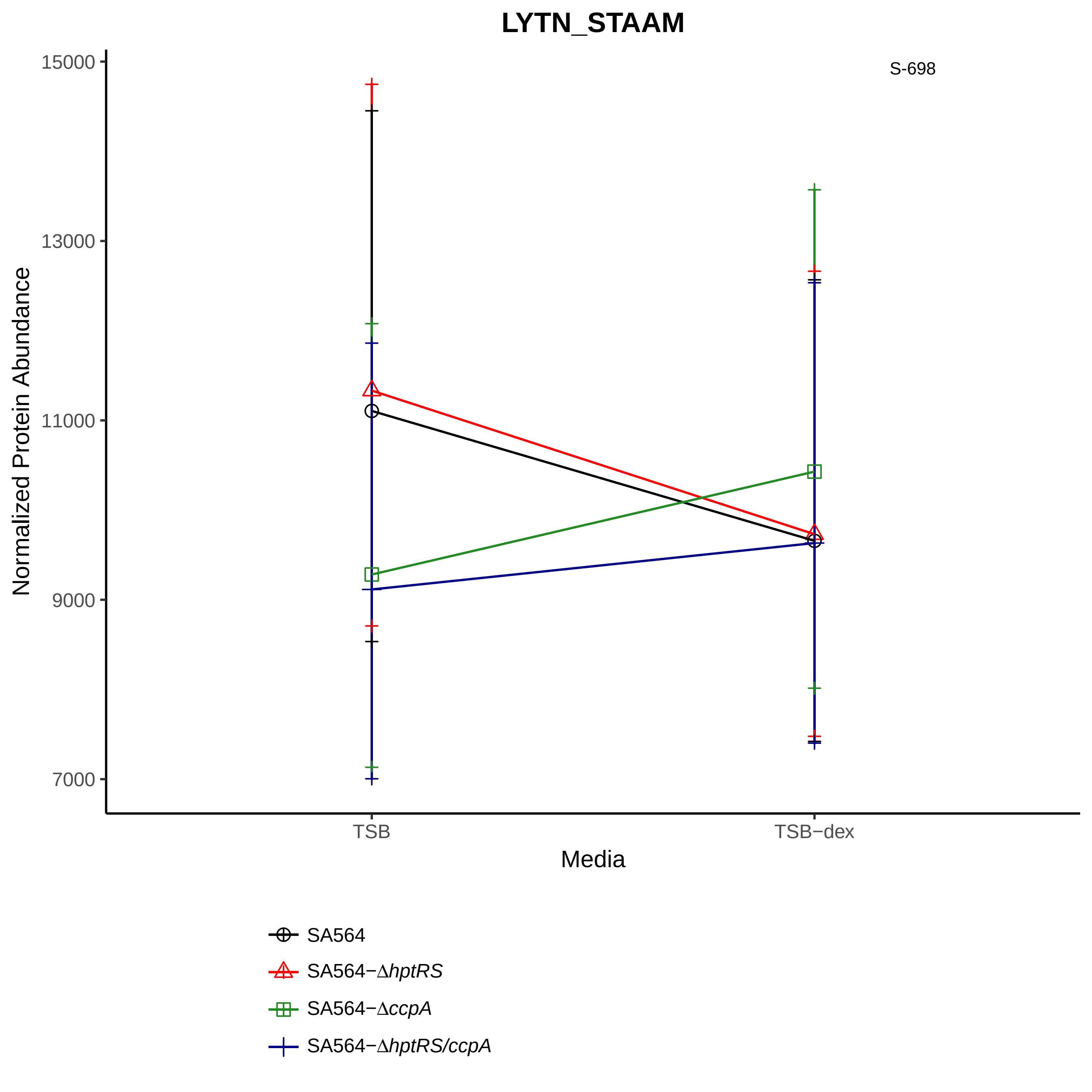

LYTN_STAAM
S-698
Normalized Protein Abundance
15000
13000
11000
9000
7000
TSB
TSB−dex
Media
SA564
SA564−Δ*hptRS*
SA564−Δ*ccpA*
SA564−Δ*hptRS/ccpA*

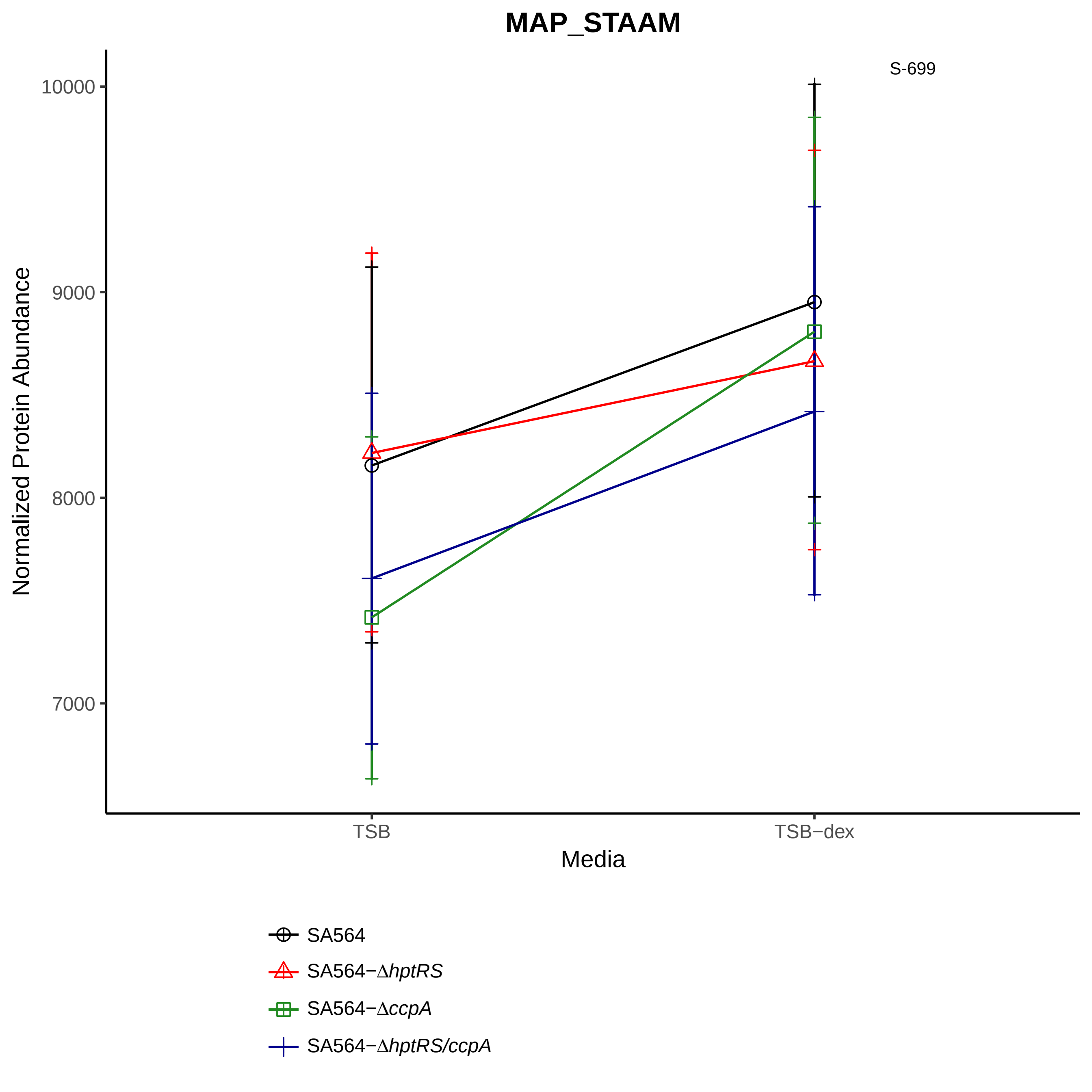

MAP_STAAM
S-699
Normalized Protein Abundance
10000
9000
8000
7000
TSB
TSB−dex
Media
SA564
SA564−Δ*hptRS*
SA564−Δ*ccpA*
SA564−Δ*hptRS/ccpA*

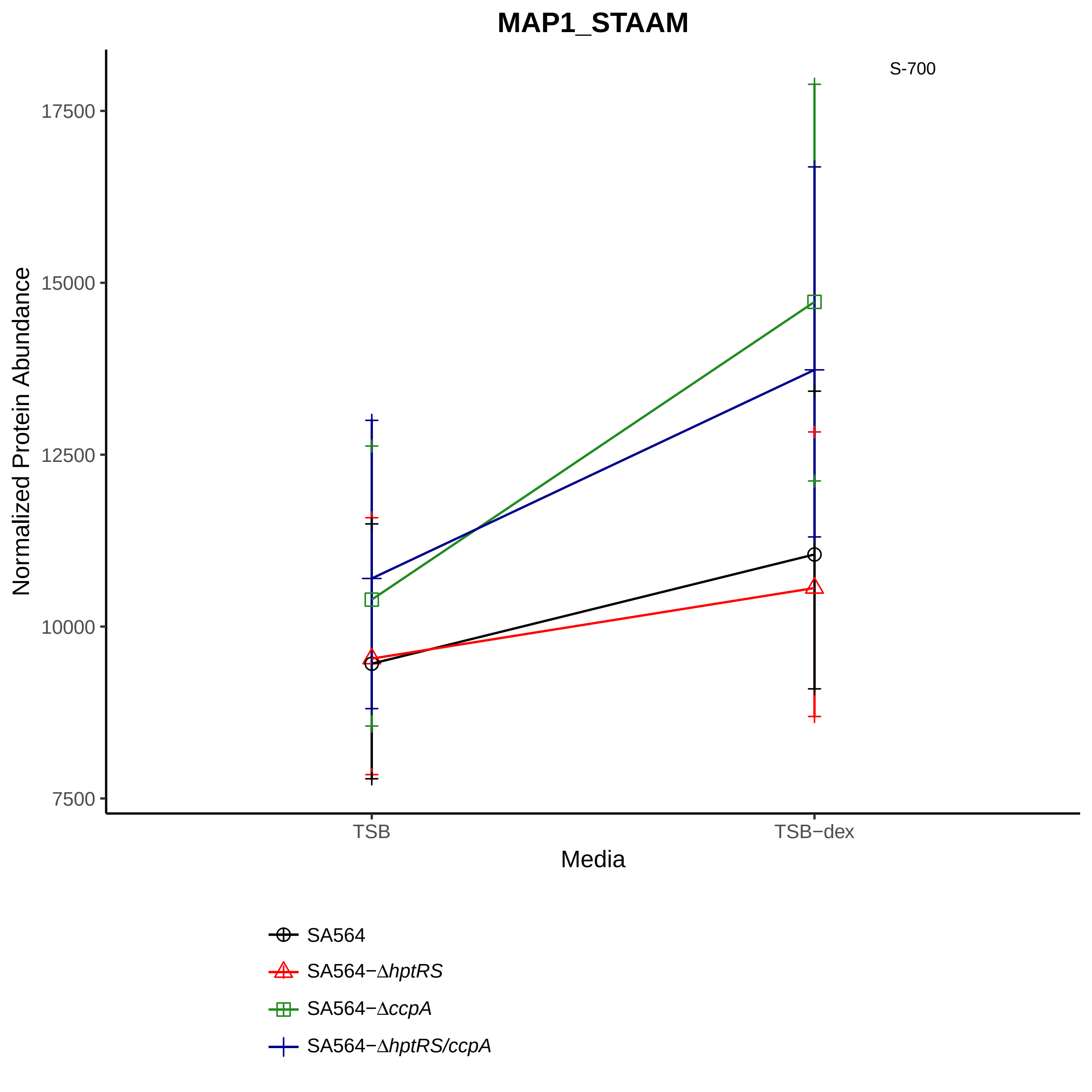

MAP1_STAAM
S-700
17500
15000
12500
10000
7500
Normalized Protein Abundance
TSB
TSB−dex
Media
SA564
SA564−Δ*hptRS*
SA564−Δ*ccpA*
SA564−Δ*hptRS/ccpA*

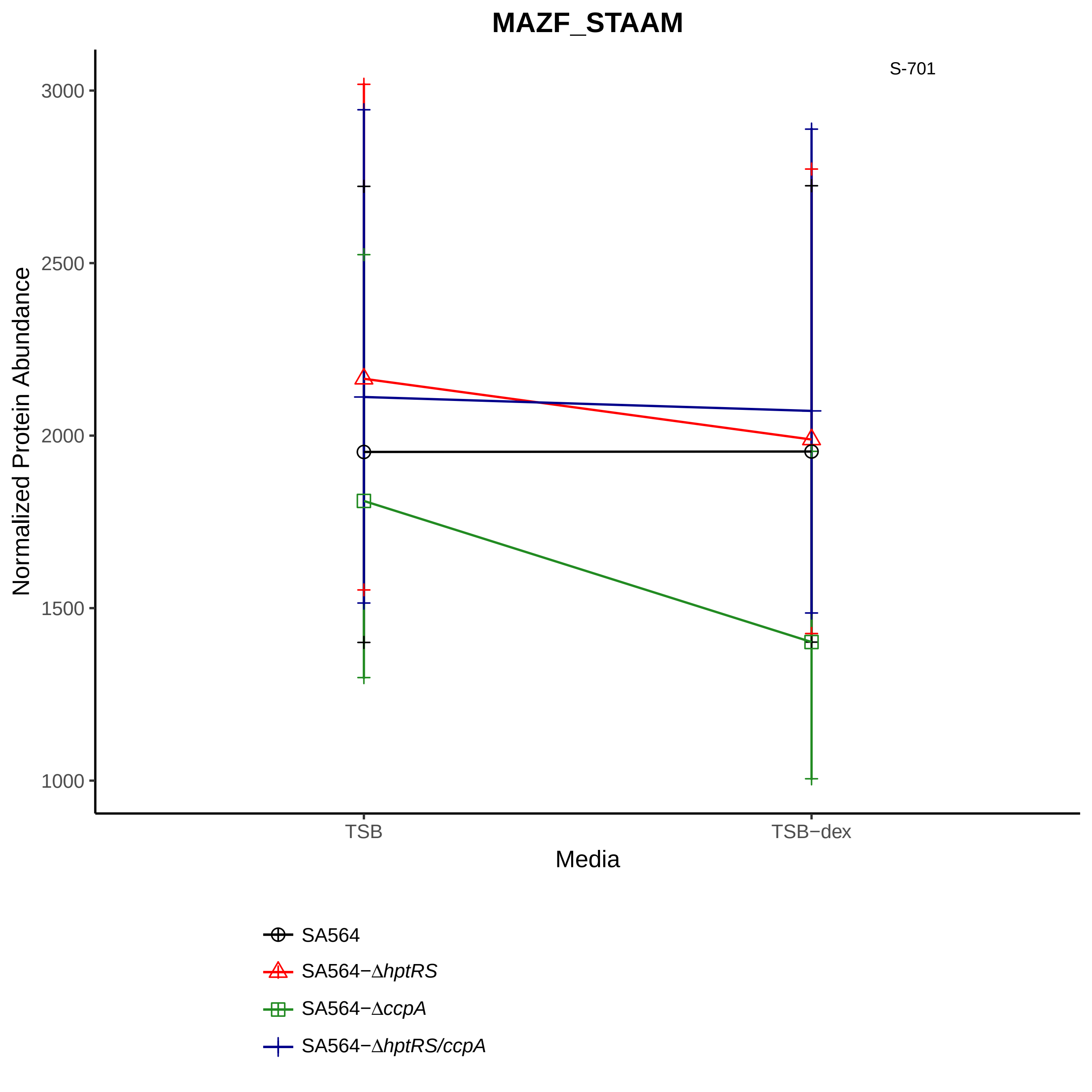

MAZF_STAAM
S-701
Normalized Protein Abundance
3000
2500
2000
1500
1000
TSB
TSB−dex
Media
SA564
SA564−Δ*hptRS*
SA564−Δ*ccpA*
SA564−Δ*hptRS/ccpA*

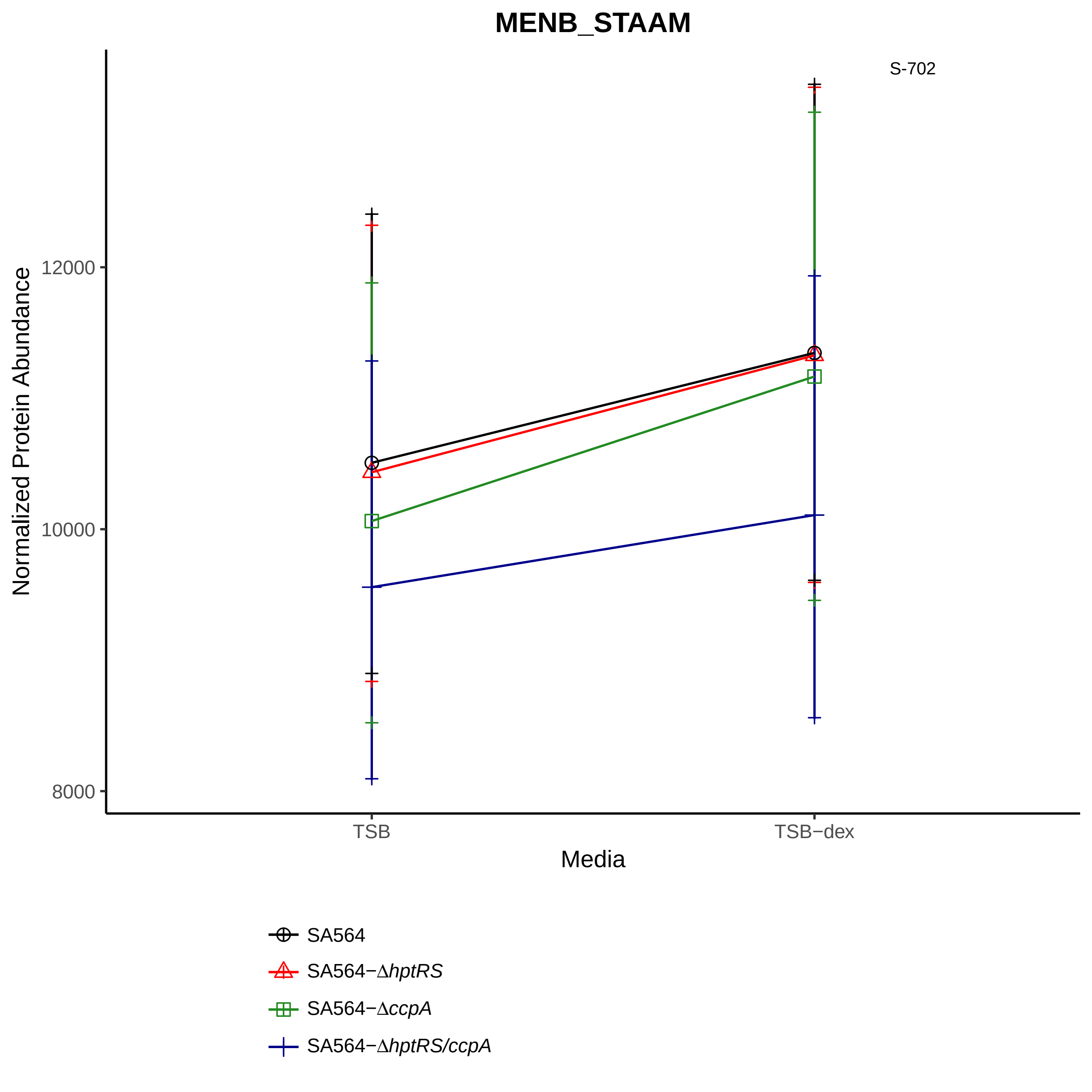

MENB_STAAM
S-702
Normalized Protein Abundance
12000
10000
8000
TSB
TSB−dex
Media
SA564
SA564−ΔhptRS
SA564−ΔccpA
SA564−ΔhptRS/ccpA

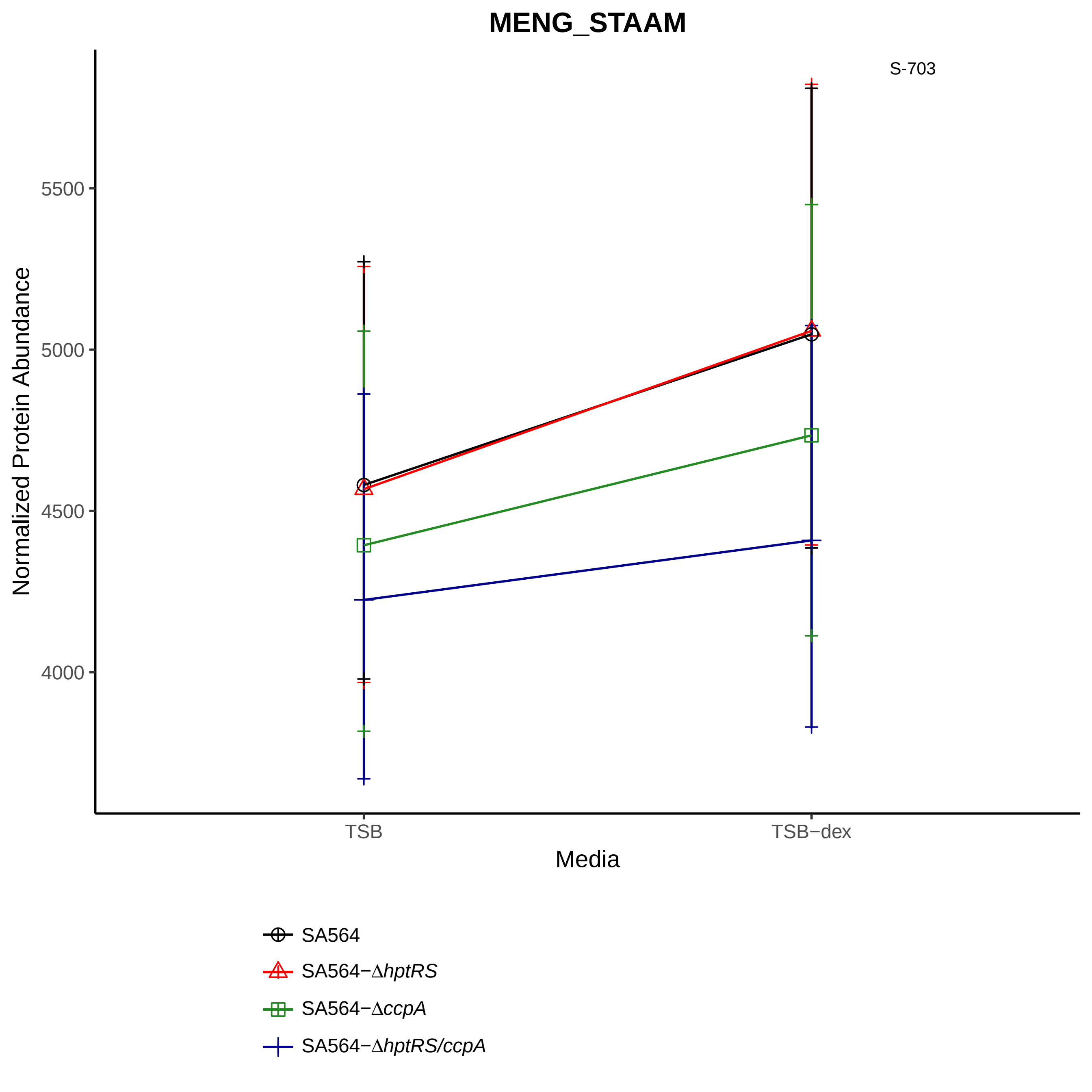

MENG_STAAM
S-703
Normalized Protein Abundance
5500
5000
4500
4000
TSB
TSB−dex
Media
SA564
SA564−Δ*hptRS*
SA564−Δ*ccpA*
SA564−Δ*hptRS/ccpA*

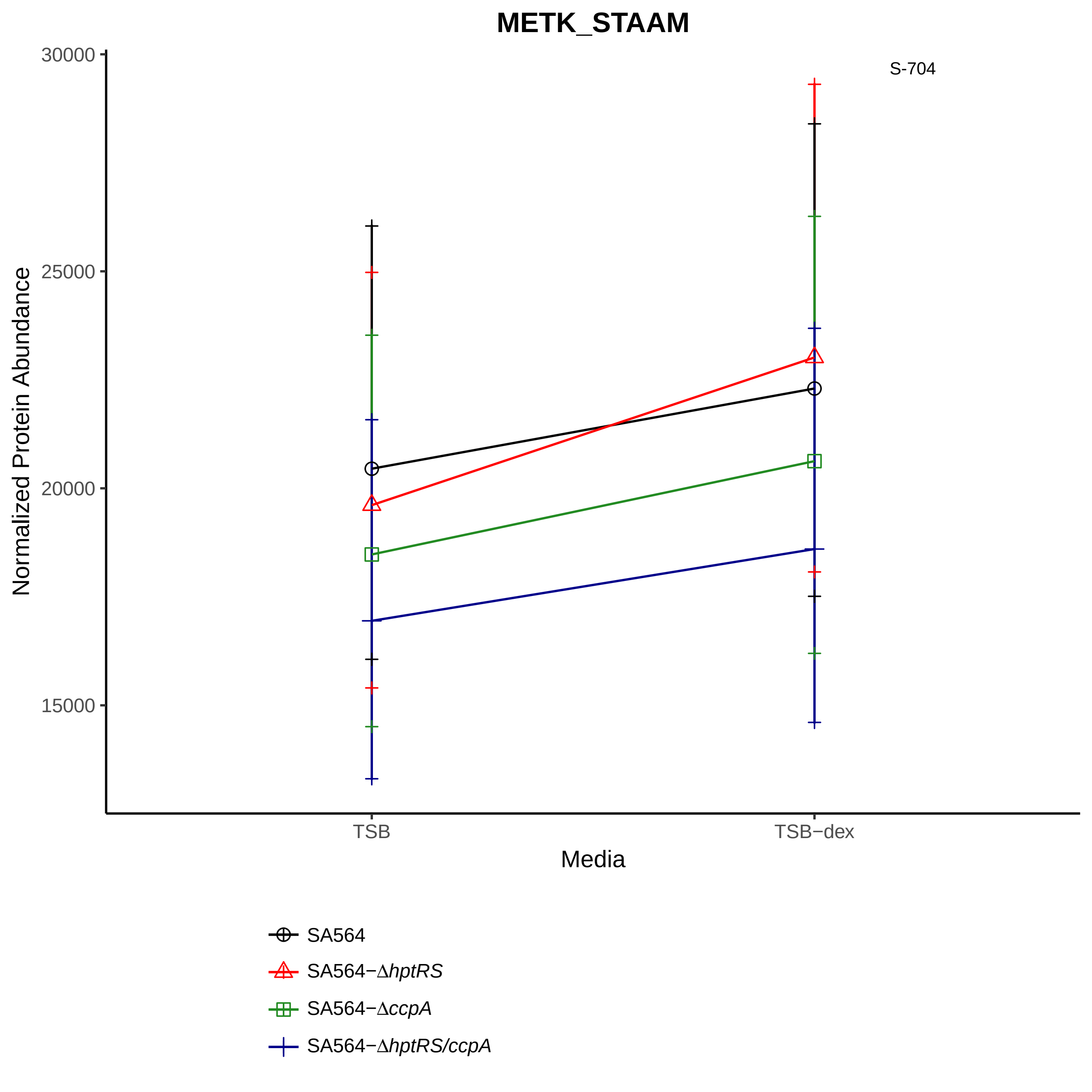
METK_STAAM
S-704
Normalized Protein Abundance
30000
25000
20000
15000
TSB
TSB−dex
Media
SA564
SA564−Δ*hptRS*
SA564−Δ*ccpA*
SA564−Δ*hptRS/ccpA*

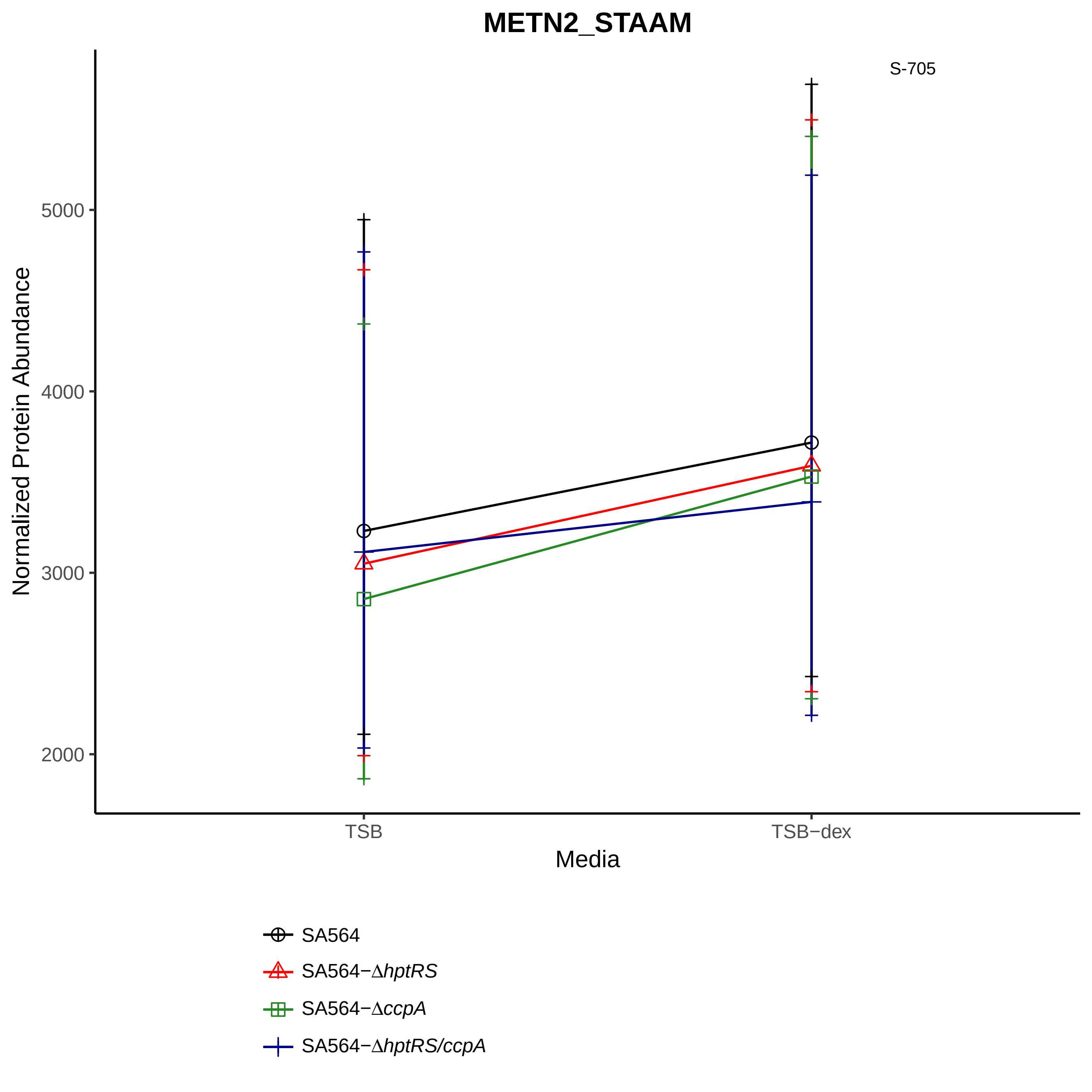

METN2_STAAM
S-705
Normalized Protein Abundance
5000
4000
3000
2000
TSB
TSB−dex
Media
SA564
SA564−Δ*hptRS*
SA564−Δ*ccpA*
SA564−Δ*hptRS/ccpA*

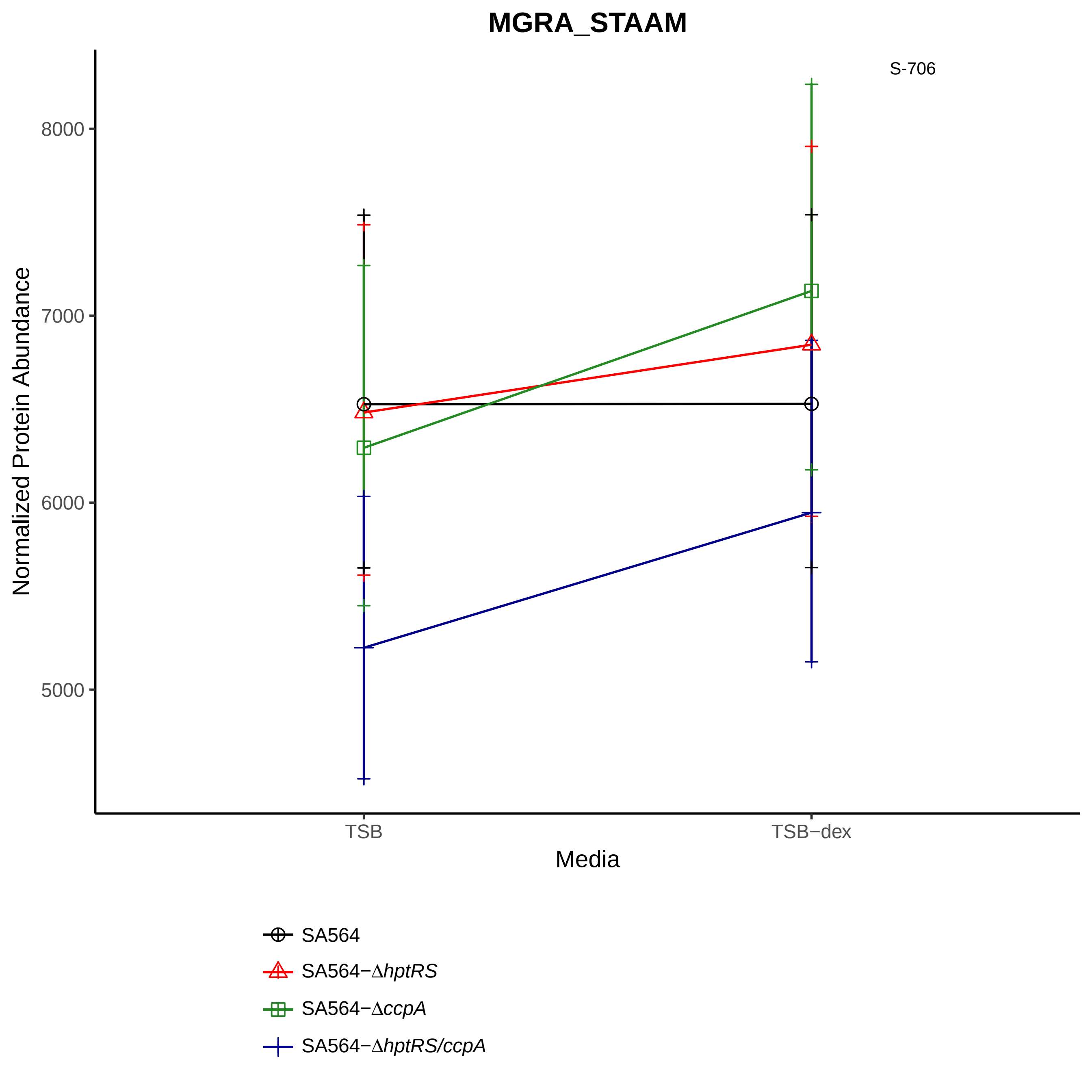

MGRA_STAAM
S-706
Normalized Protein Abundance
8000
7000
6000
5000
TSB
TSB−dex
Media
SA564
SA564−Δ*hptRS*
SA564−Δ*ccpA*
SA564−Δ*hptRS/ccpA*

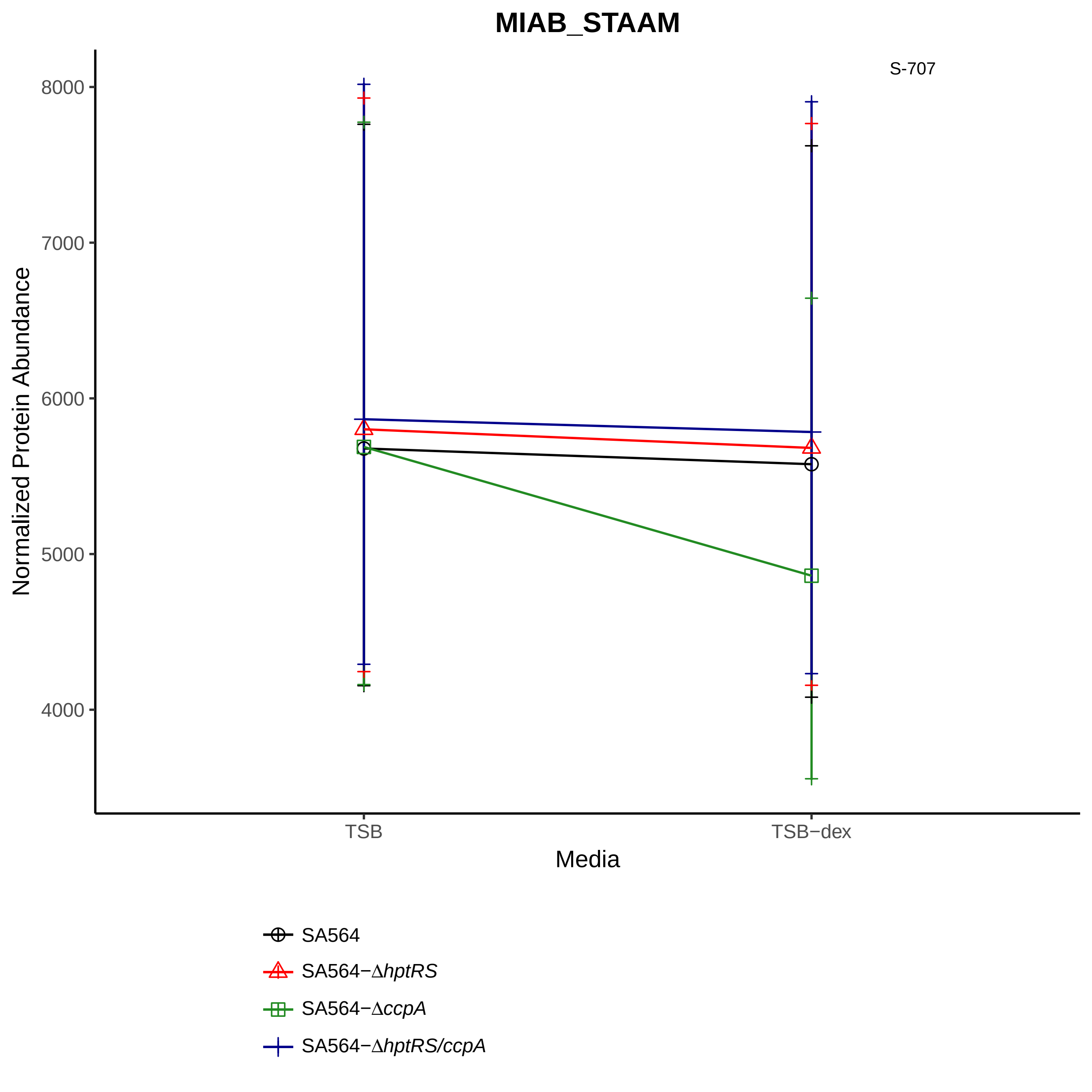
MIAB_STAAM
S-707
Normalized Protein Abundance
8000
7000
6000
5000
4000
TSB
TSB−dex
Media
SA564
SA564−ΔhptRS
SA564−ΔccpA
SA564−ΔhptRS/ccpA

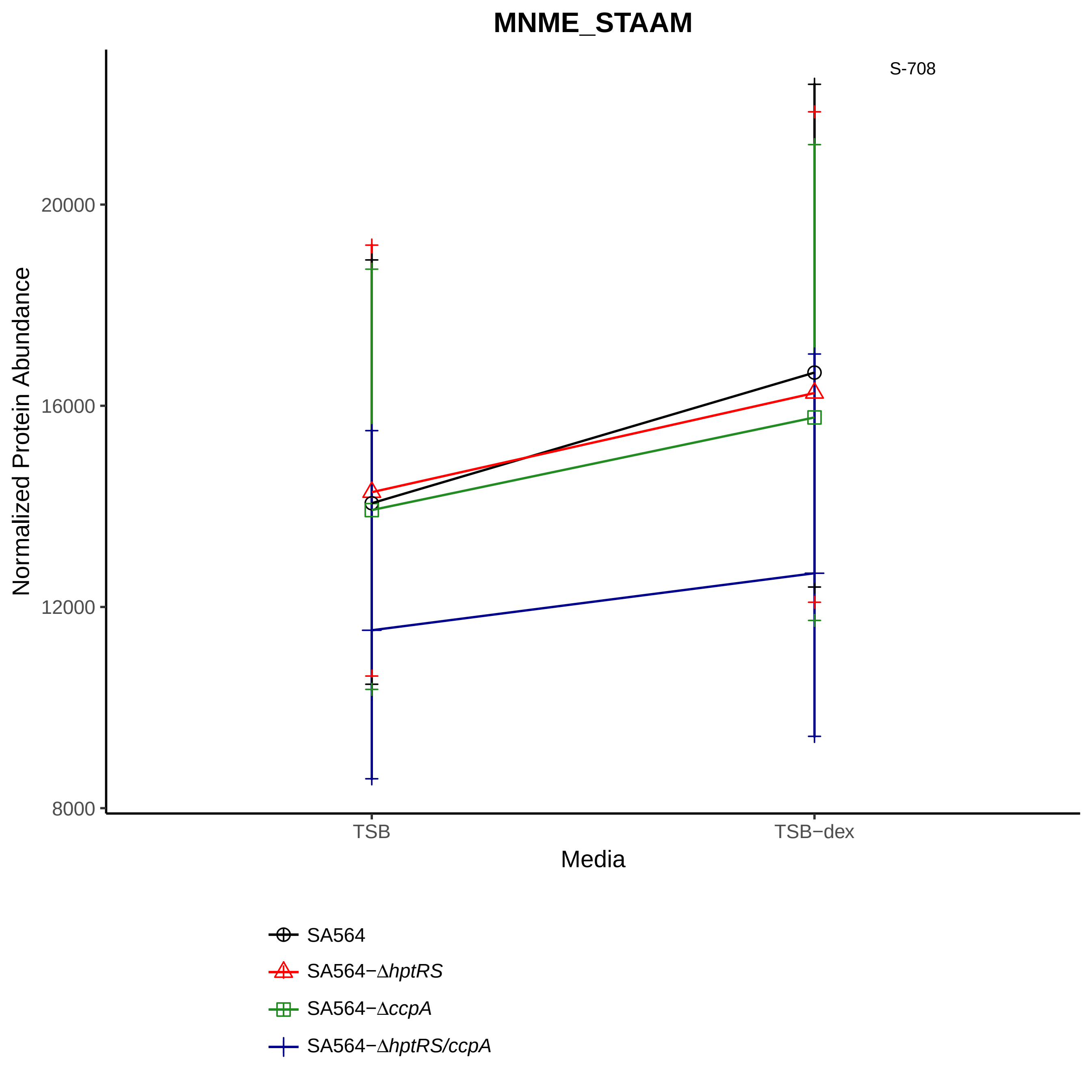

MNME_STAAM
S-708
Normalized Protein Abundance
20000
16000
12000
8000
TSB
TSB−dex
Media
SA564
SA564−Δ*hptRS*
SA564−Δ*ccpA*
SA564−Δ*hptRS/ccpA*

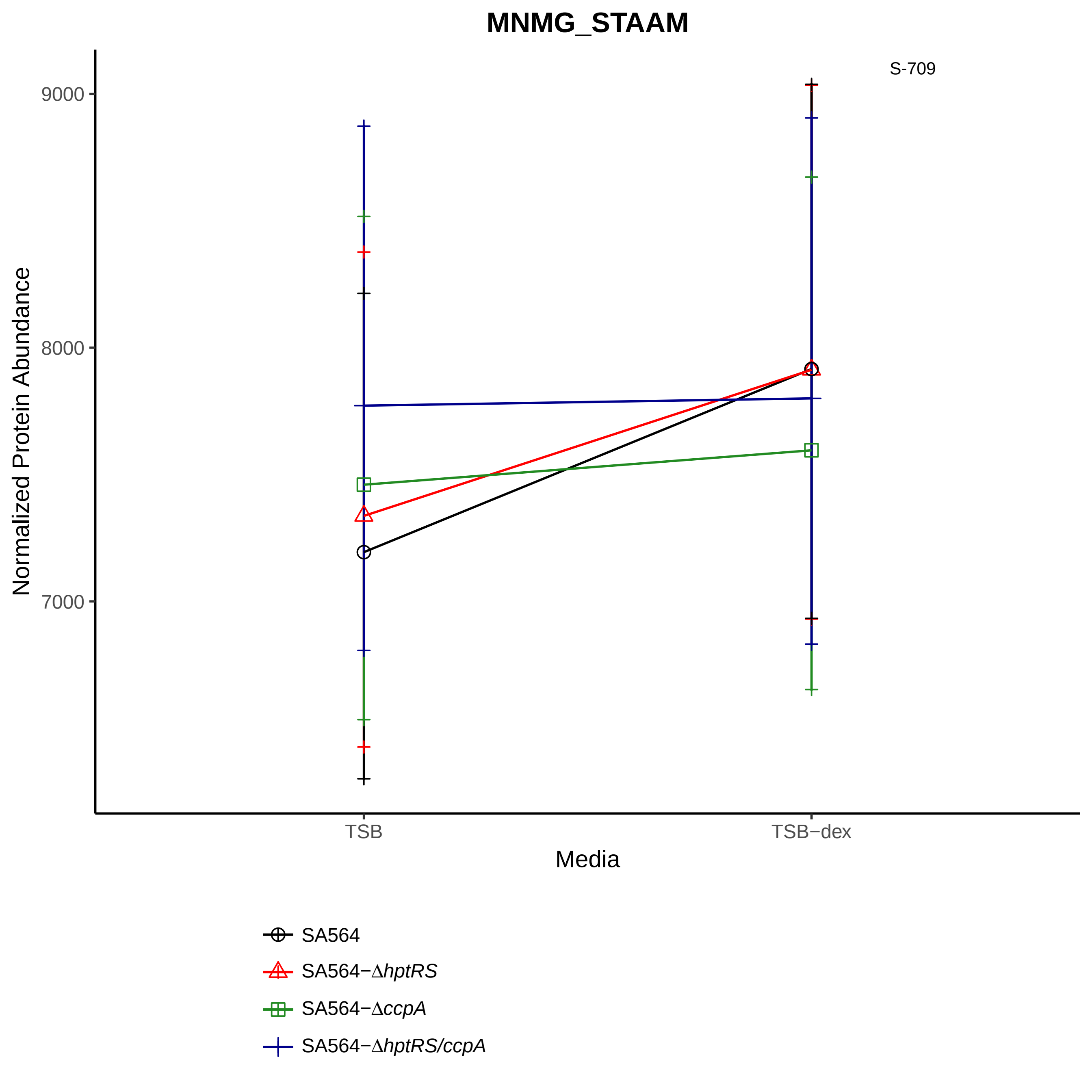
MNMG_STAAM
S-709
Normalized Protein Abundance
9000
8000
7000
TSB
TSB−dex
Media
SA564
SA564−Δ*hptRS*
SA564−Δ*ccpA*
SA564−Δ*hptRS/ccpA*

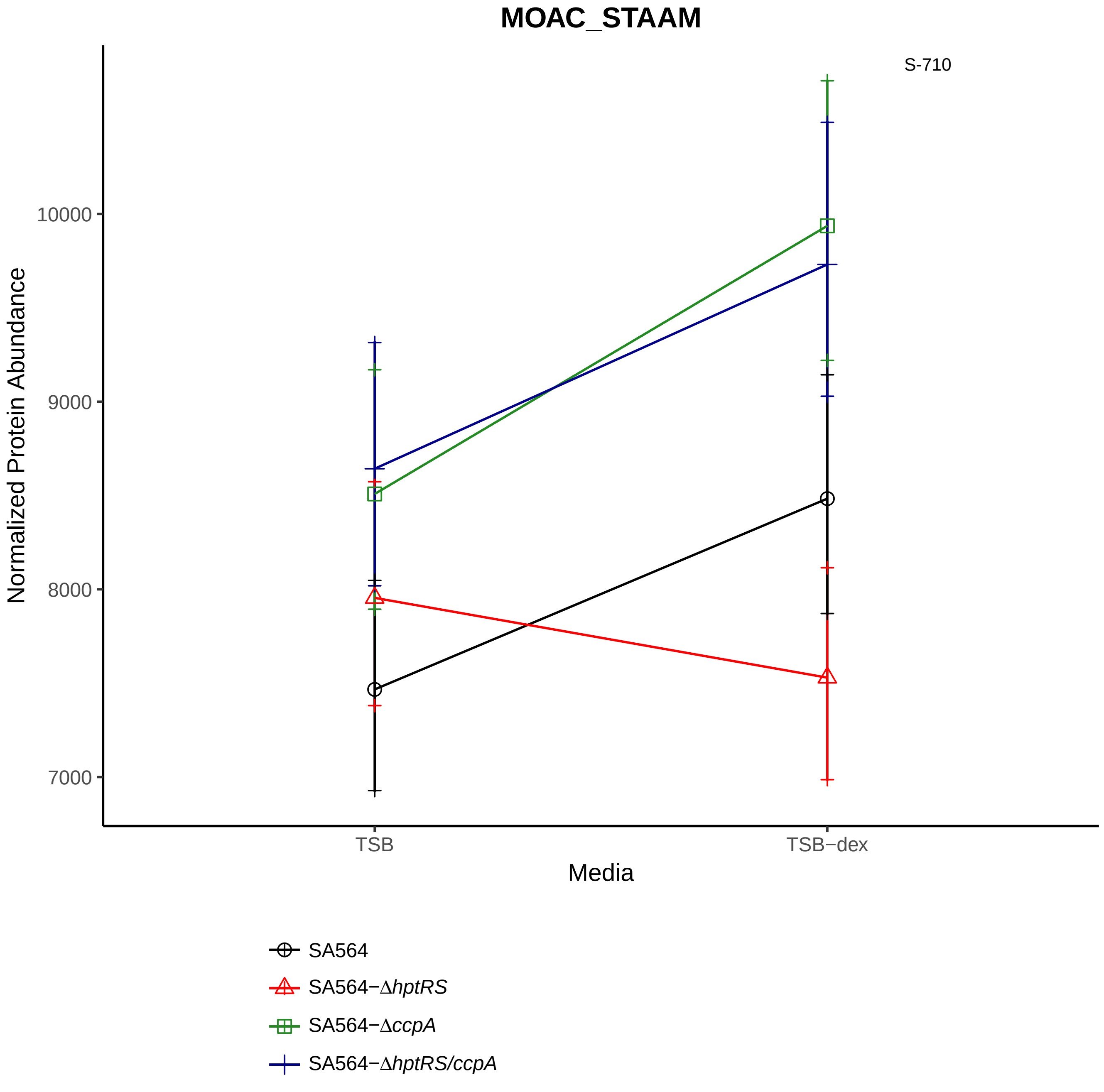
MOAC_STAAM
S-710
Normalized Protein Abundance
10000
9000
8000
7000
TSB
TSB−dex
Media
SA564
SA564−Δ*hptRS*
SA564−Δ*ccpA*
SA564−Δ*hptRS/ccpA*

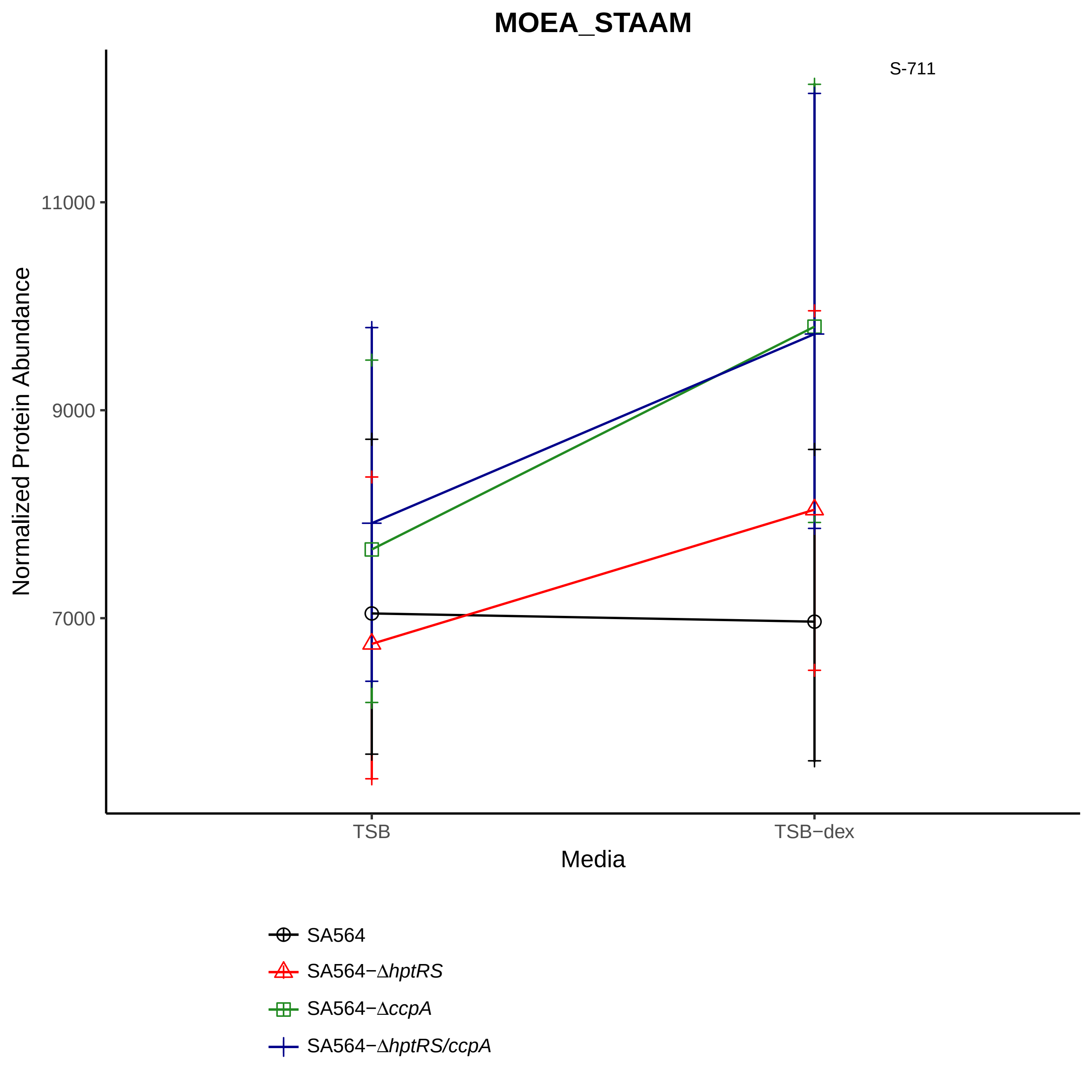

MOEA_STAAM
S-711
Normalized Protein Abundance
11000
9000
7000
TSB
TSB−dex
Media
SA564
SA564−Δ*hptRS*
SA564−Δ*ccpA*
SA564−Δ*hptRS/ccpA*

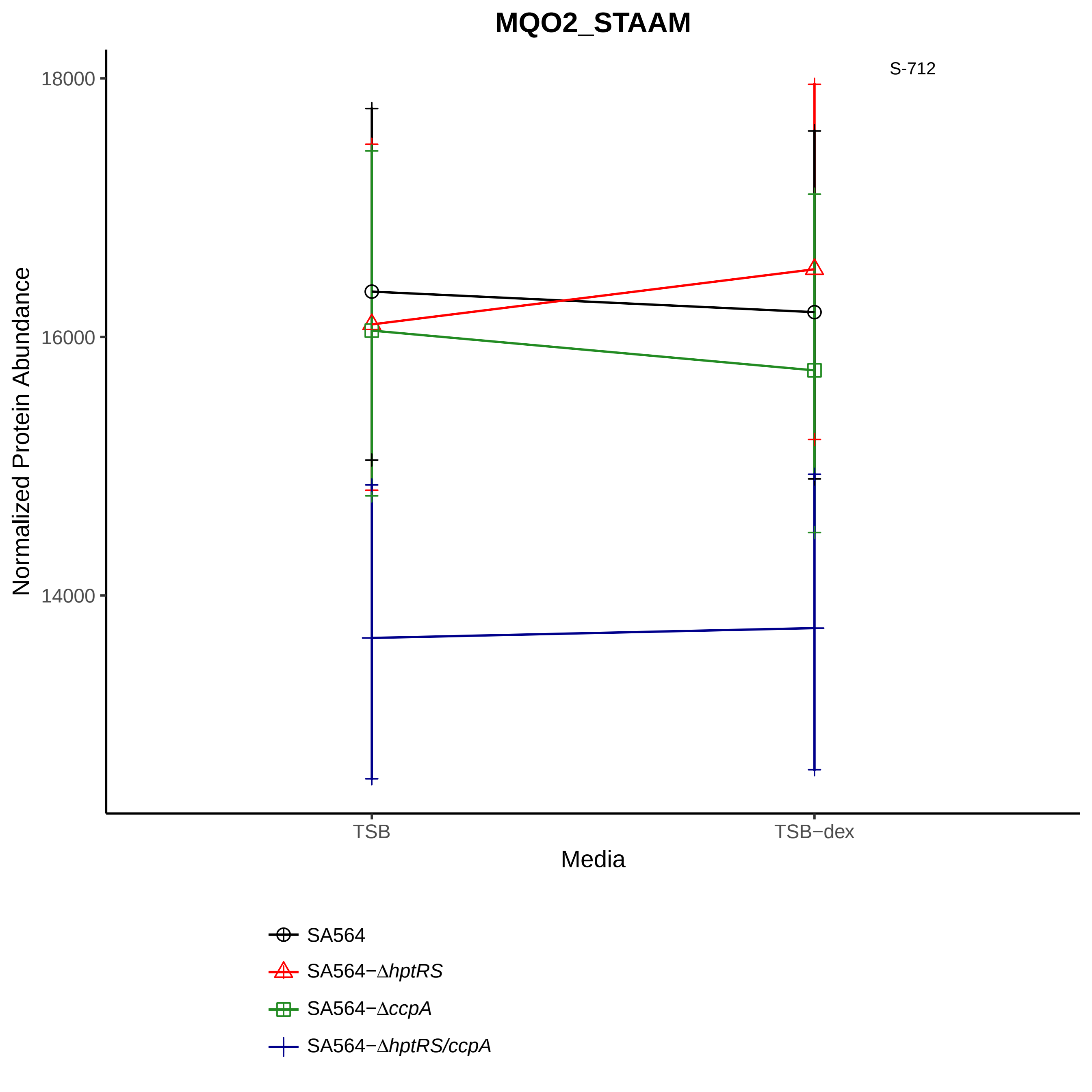

MQO2_STAAM
S-712
18000
16000
14000
Normalized Protein Abundance
TSB
TSB−dex
Media
SA564
SA564−Δ*hptRS*
SA564−Δ*ccpA*
SA564−Δ*hptRS/ccpA*

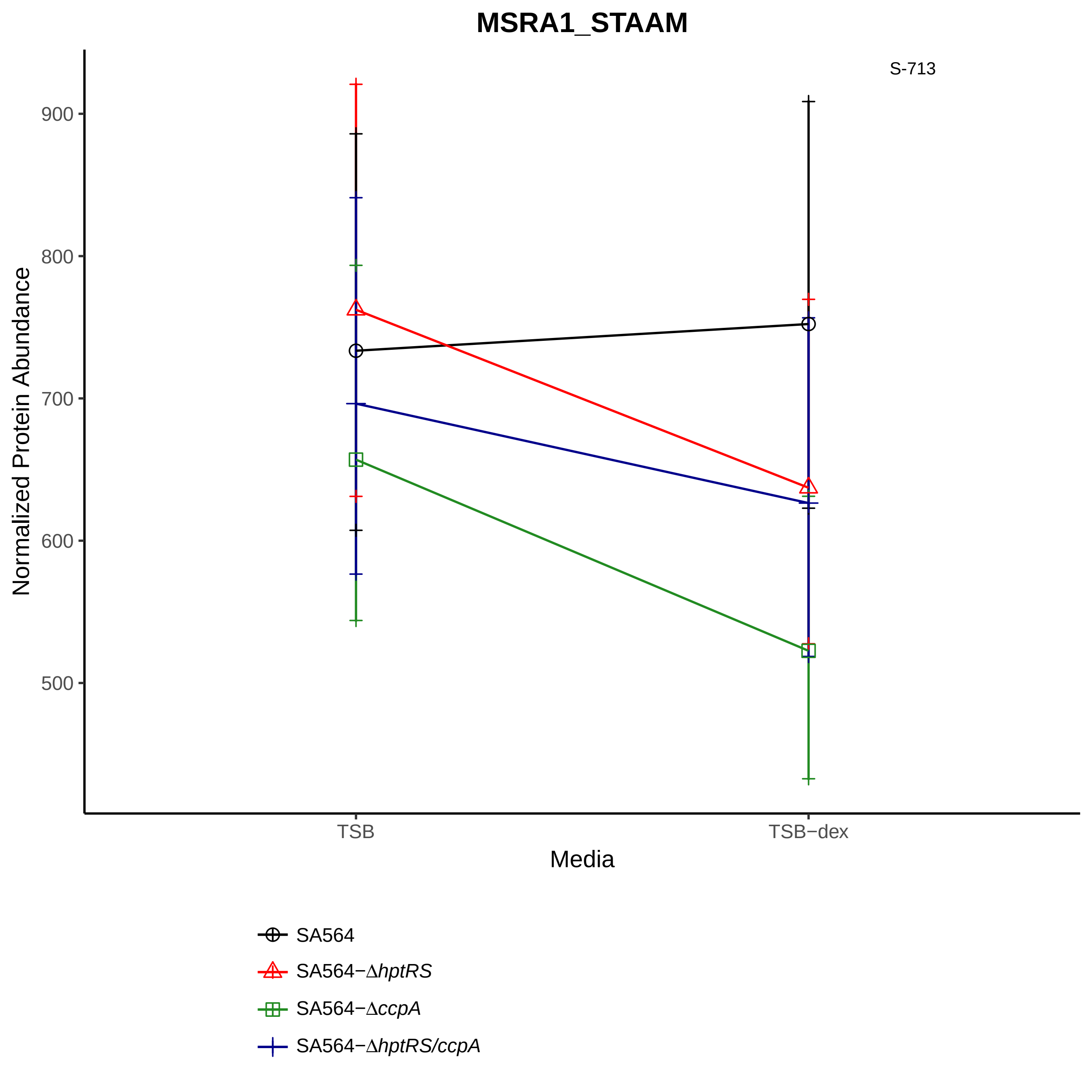

MSRA1_STAAM
S-713
900
800
700
600
500
Normalized Protein Abundance
TSB
TSB−dex
Media
SA564
SA564−Δ*hptRS*
SA564−Δ*ccpA*
SA564−Δ*hptRS/ccpA*

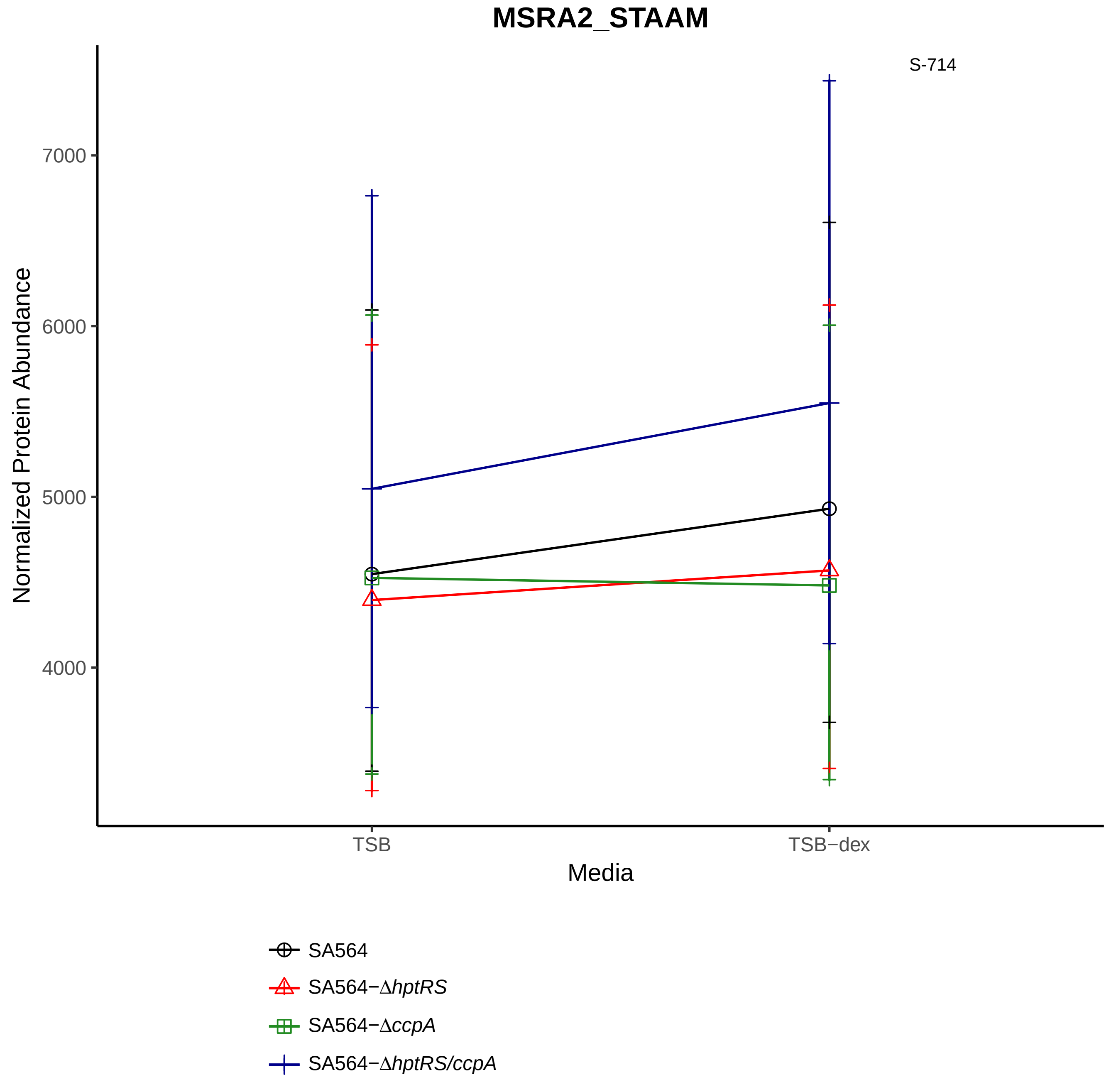

MSRA2_STAAM
S-714
Normalized Protein Abundance
7000
6000
5000
4000
TSB
TSB−dex
Media
SA564
SA564−Δ*hptRS*
SA564−Δ*ccpA*
SA564−Δ*hptRS/ccpA*

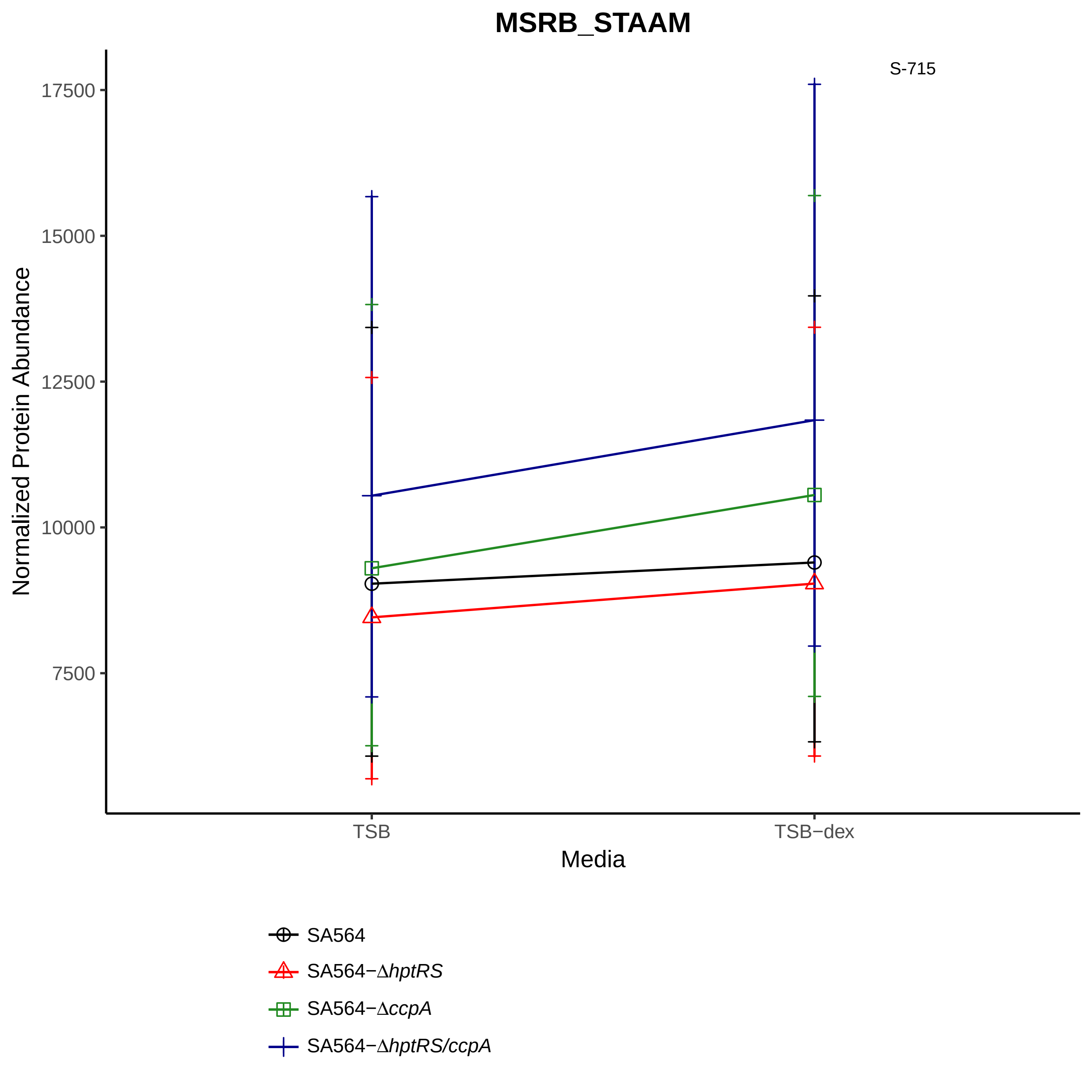

MSRB_STAAM
S-715
Normalized Protein Abundance
17500
15000
12500
10000
7500
TSB
TSB−dex
Media
SA564
SA564−ΔhptRS
SA564−ΔccpA
SA564−ΔhptRS/ccpA

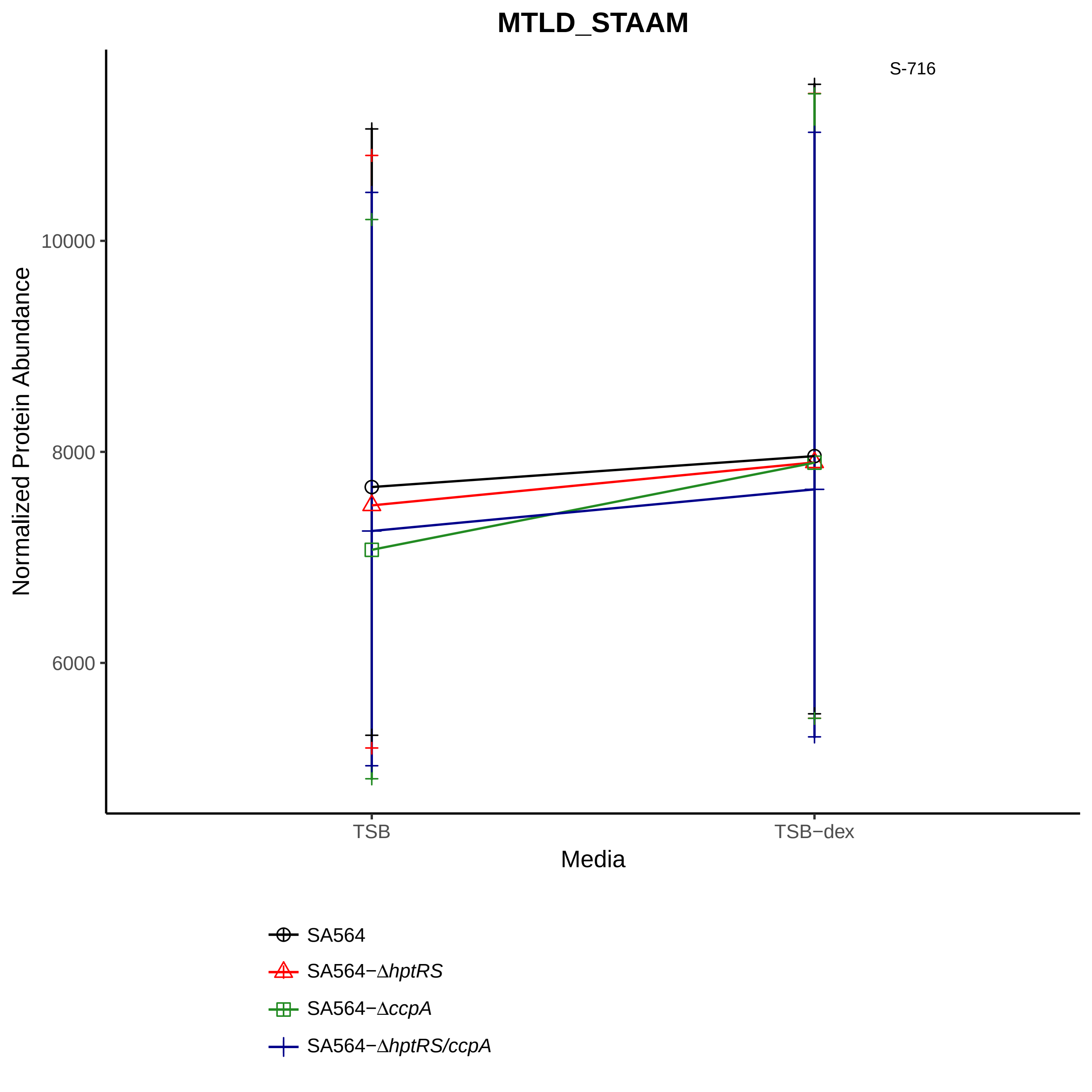
MTLD_STAAM
S-716
Normalized Protein Abundance
10000
8000
6000
TSB
TSB−dex
Media
SA564
SA564−Δ*hptRS*
SA564−Δ*ccpA*
SA564−Δ*hptRS/ccpA*

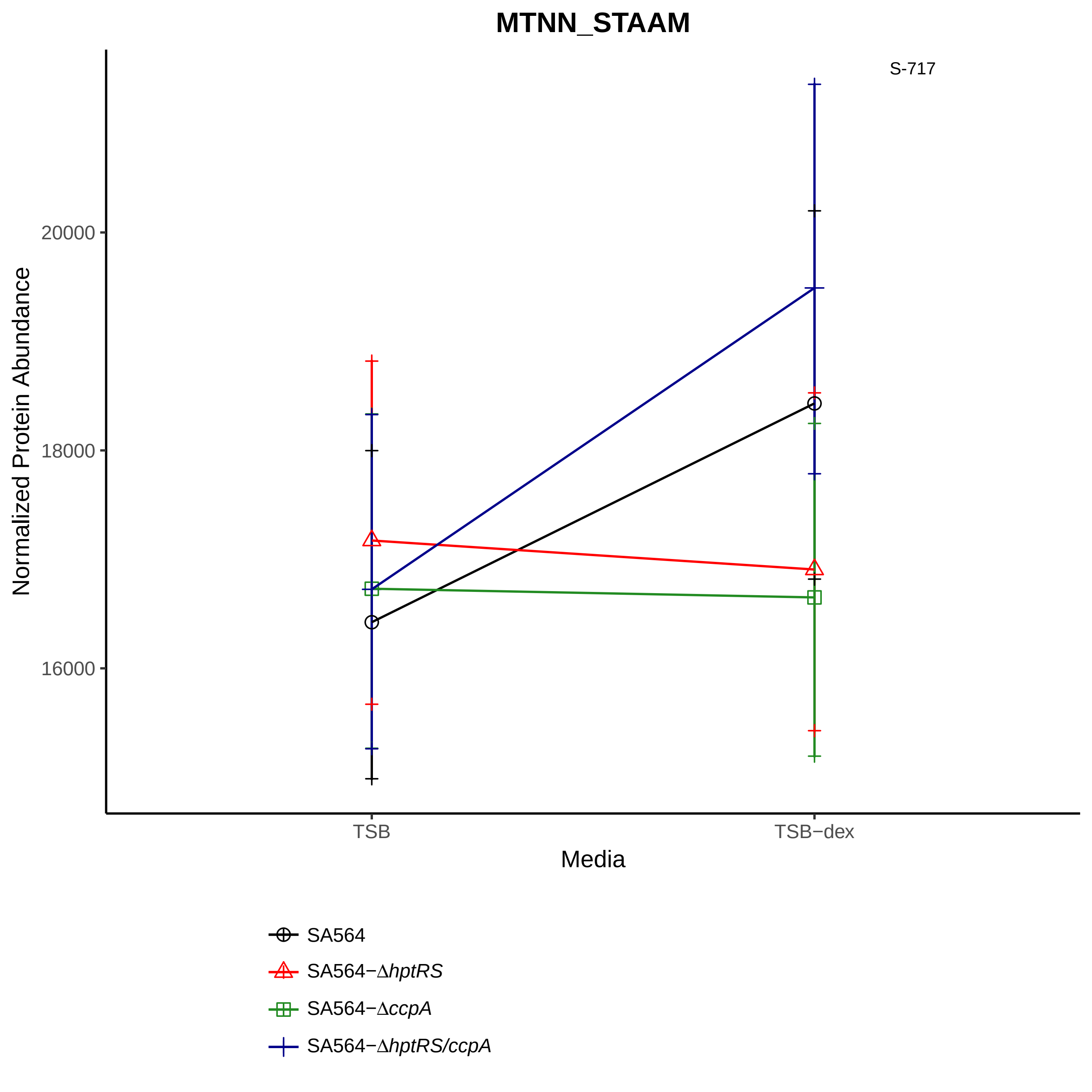

MTNN_STAAM
S-717
Normalized Protein Abundance
20000
18000
16000
TSB
TSB−dex
Media
SA564
SA564−ΔhptRS
SA564−ΔccpA
SA564−ΔhptRS/ccpA

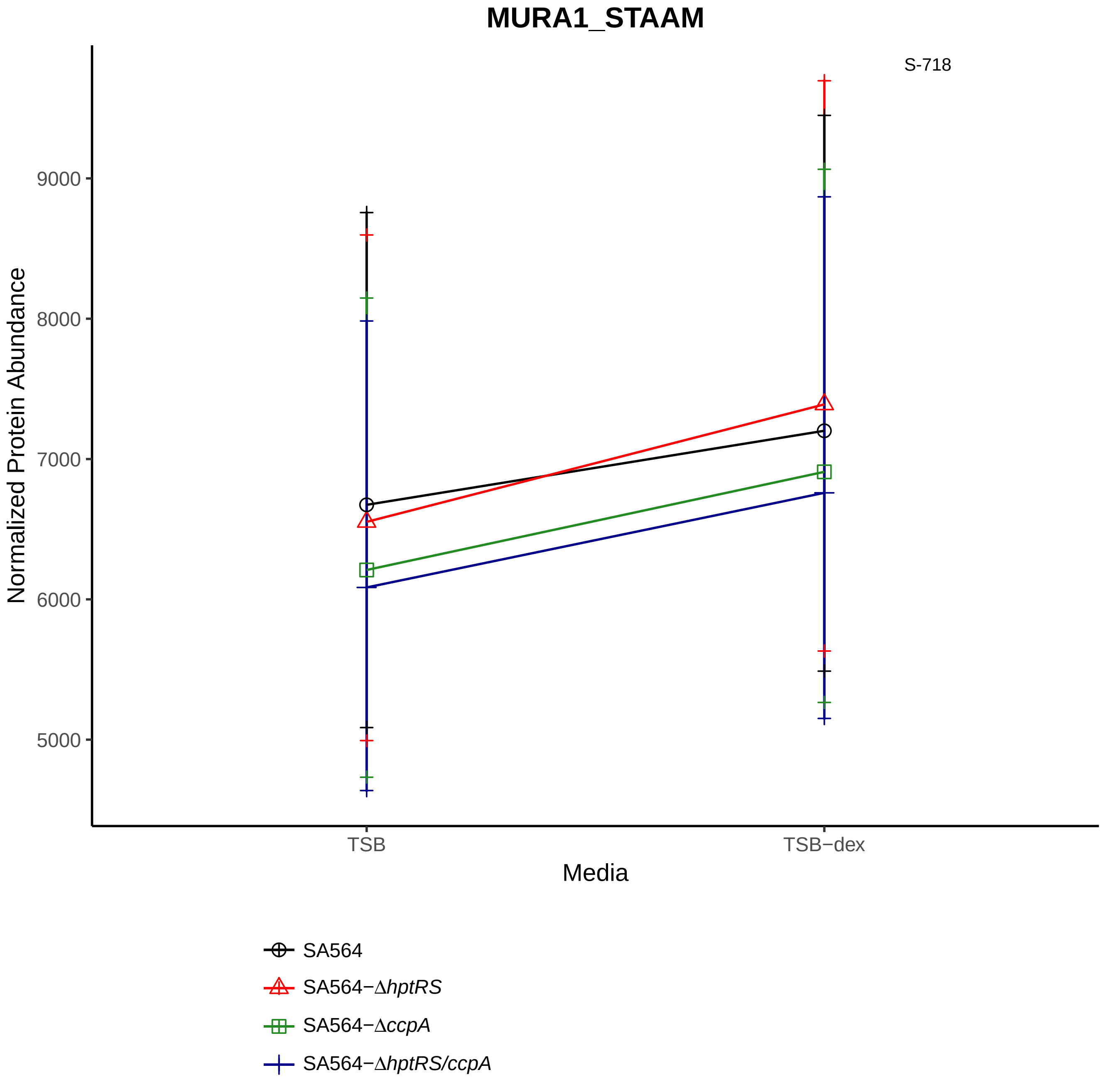
MURA1_STAAM
S-718
Normalized Protein Abundance
9000
8000
7000
6000
5000
TSB
TSB−dex
Media
SA564
SA564−Δ*hptRS*
SA564−Δ*ccpA*
SA564−Δ*hptRS/ccpA*

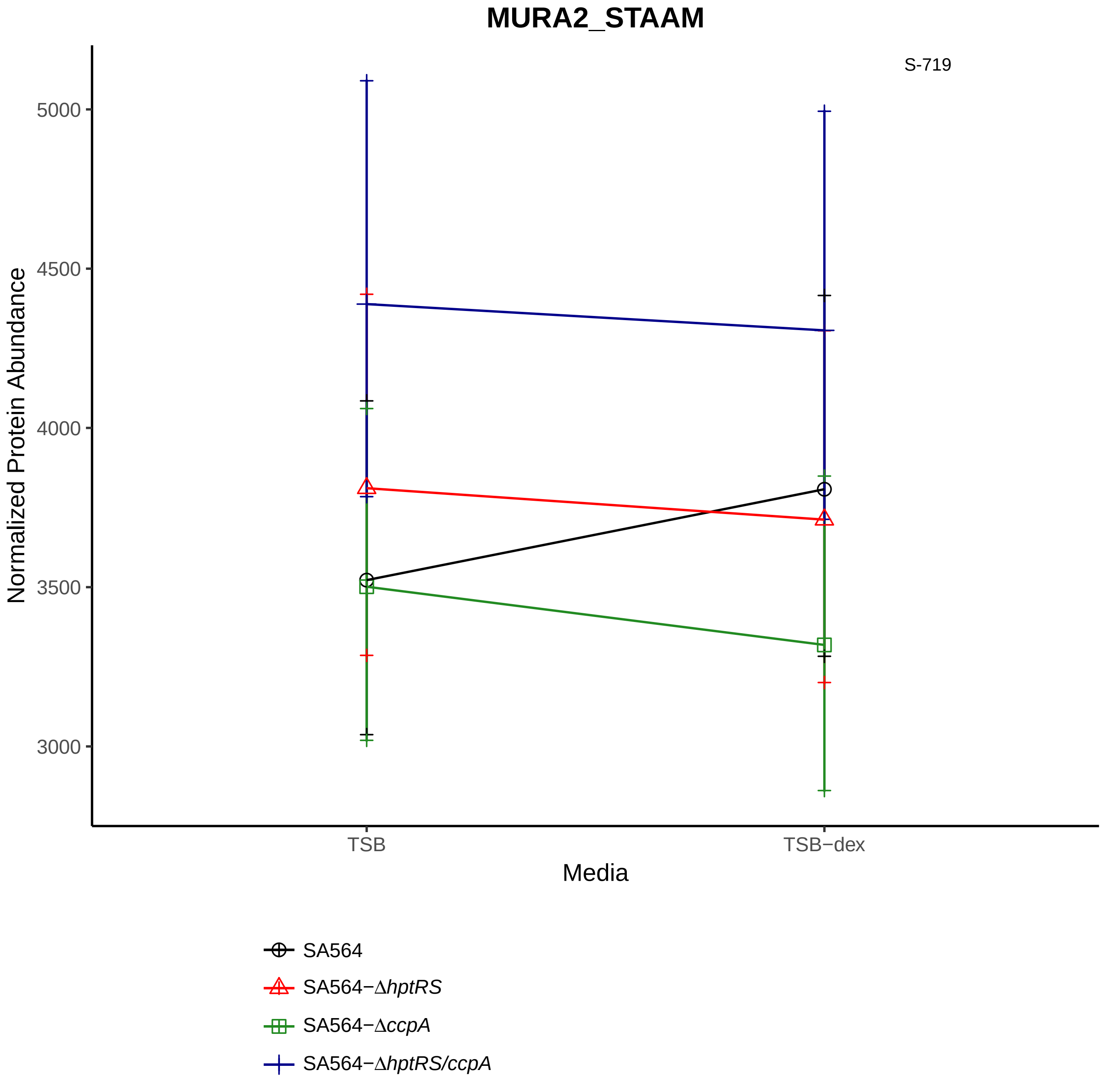

MURA2_STAAM
S-719
Normalized Protein Abundance
5000
4500
4000
3500
3000
TSB
TSB−dex
Media
SA564
SA564−Δ*hptRS*
SA564−Δ*ccpA*
SA564−Δ*hptRS/ccpA*

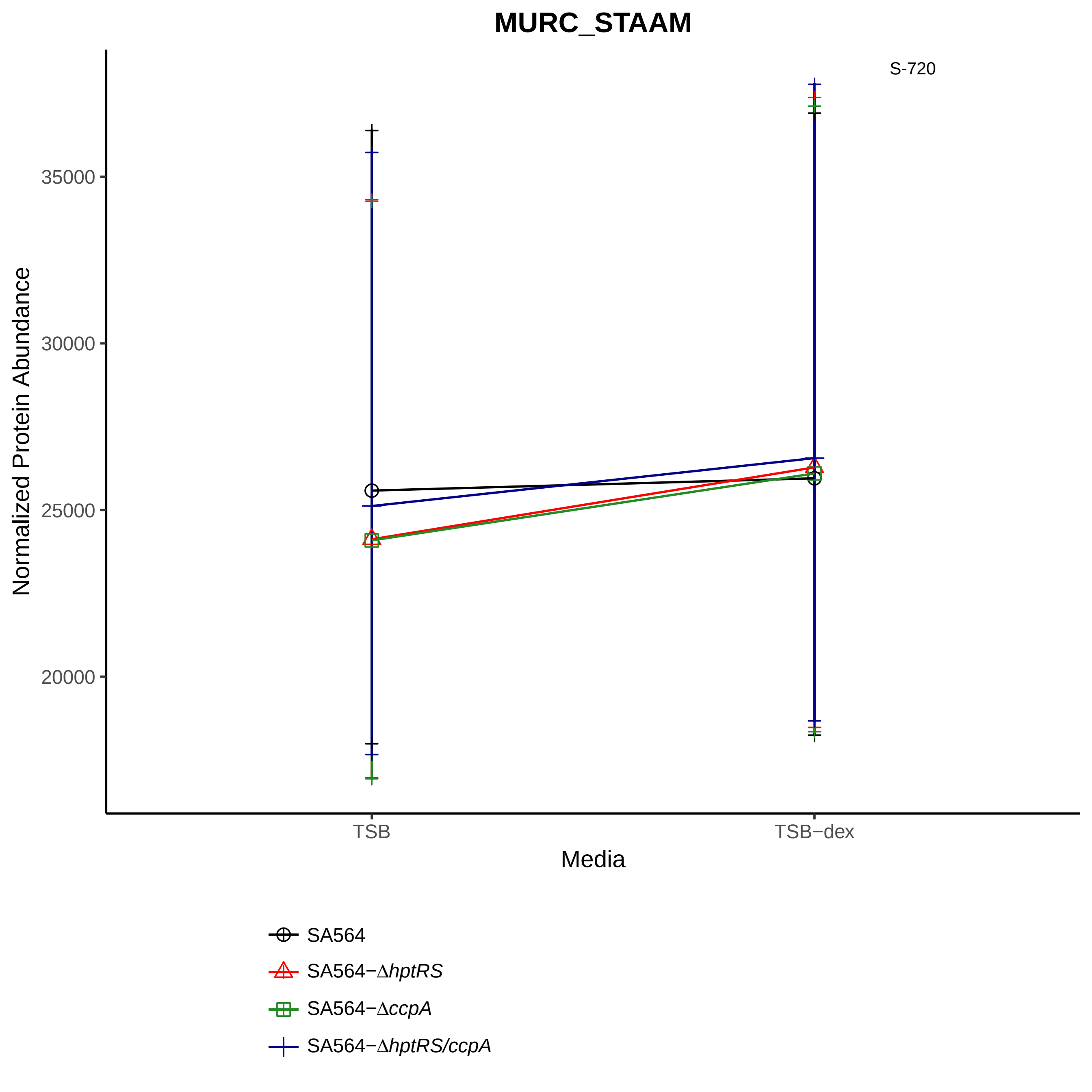
MURC_STAAM
S-720
Normalized Protein Abundance
35000
30000
25000
20000
TSB
TSB−dex
Media
SA564
SA564−Δ*hptRS*
SA564−Δ*ccpA*
SA564−Δ*hptRS/ccpA*

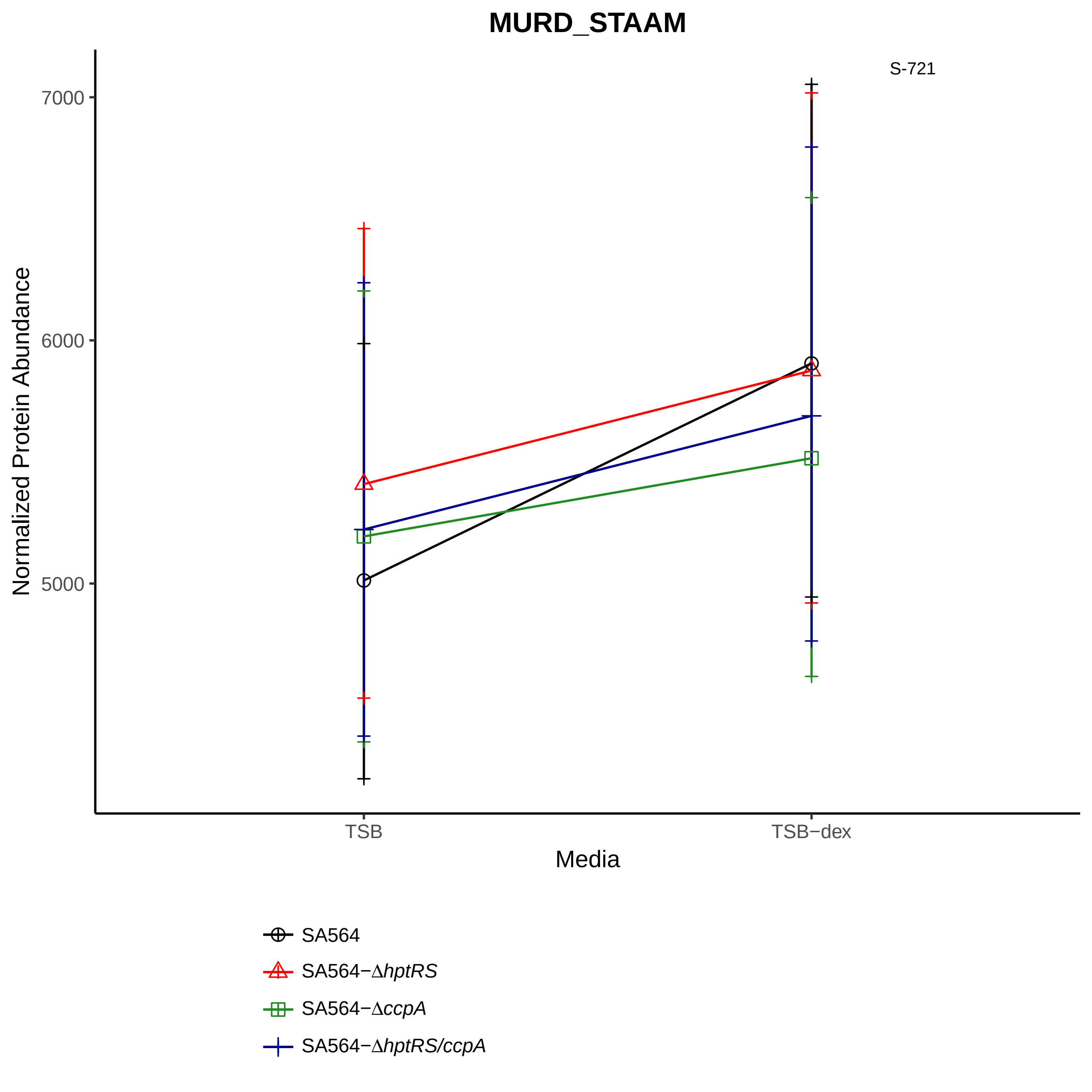

MURD_STAAM
S-721
7000
6000
5000
Normalized Protein Abundance
TSB
TSB−dex
Media
SA564
SA564−ΔhptRS
SA564−ΔccpA
SA564−ΔhptRS/ccpA

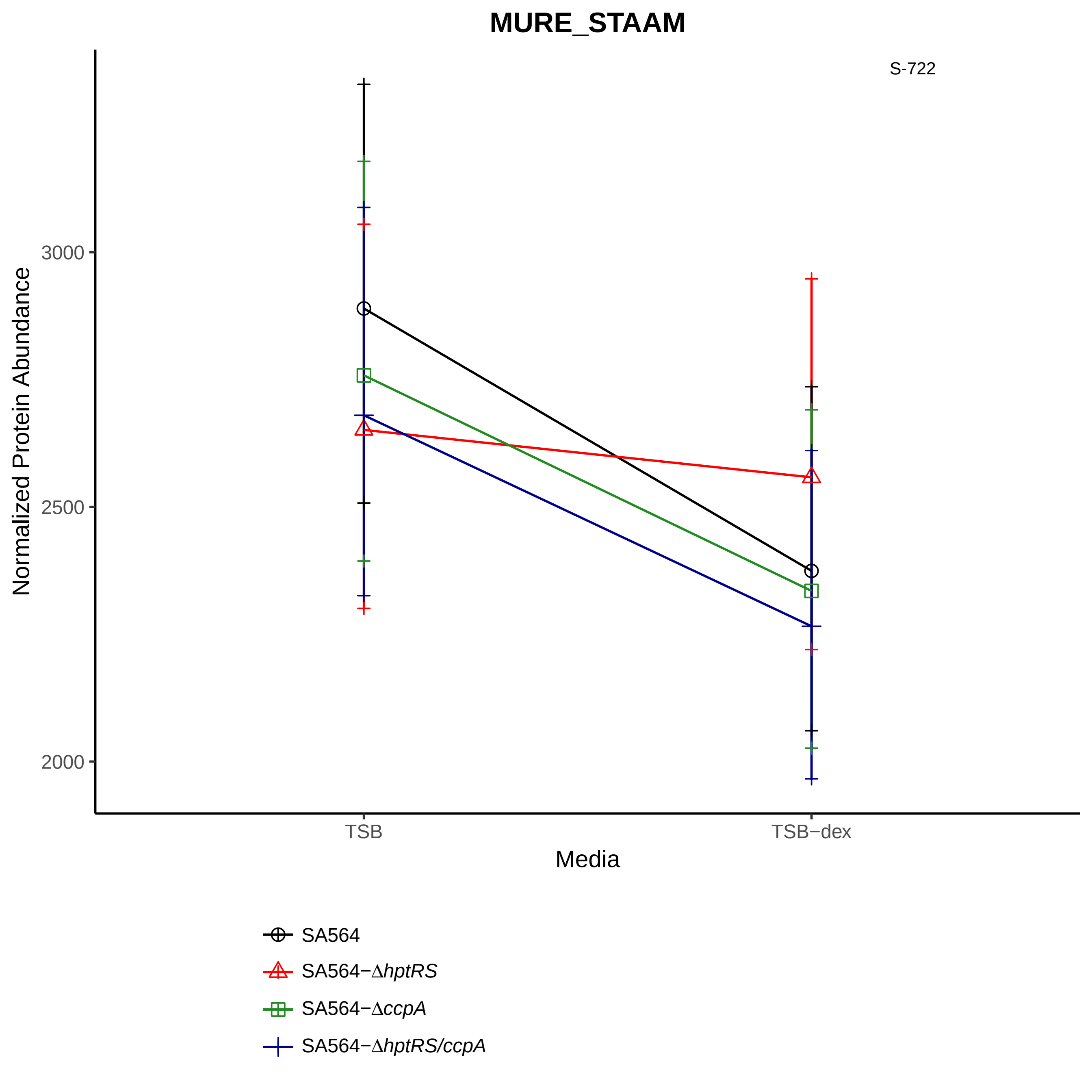

MURE_STAAM
S-722
Normalized Protein Abundance
3000
2500
2000
TSB
TSB−dex
Media
SA564
SA564−ΔhptRS
SA564−ΔccpA
SA564−ΔhptRS/ccpA

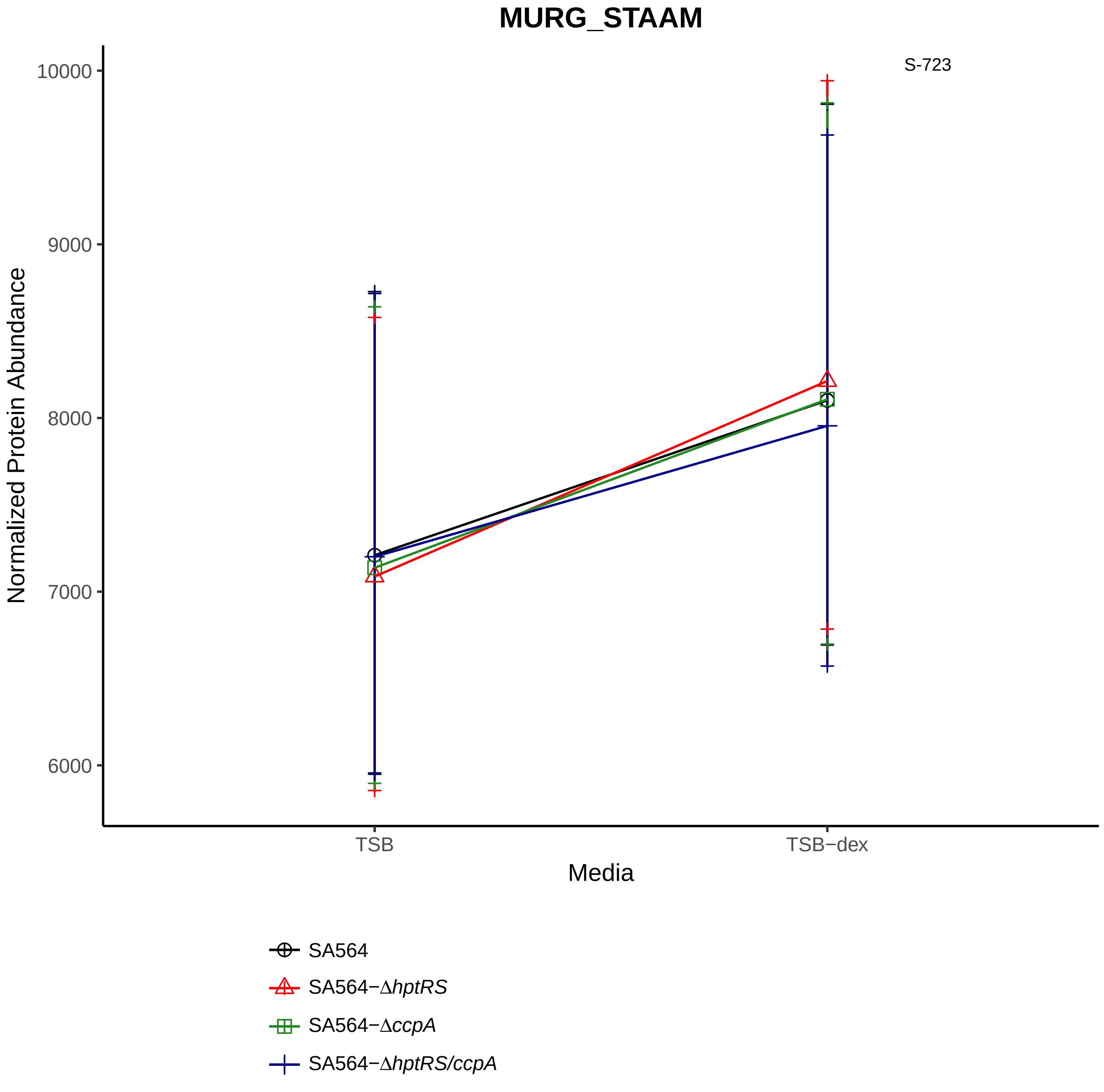
MURG_STAAM
S-723
Normalized Protein Abundance
10000
9000
8000
7000
6000
TSB
TSB−dex
Media
SA564
SA564−ΔhptRS
SA564−ΔccpA
SA564−ΔhptRS/ccpA

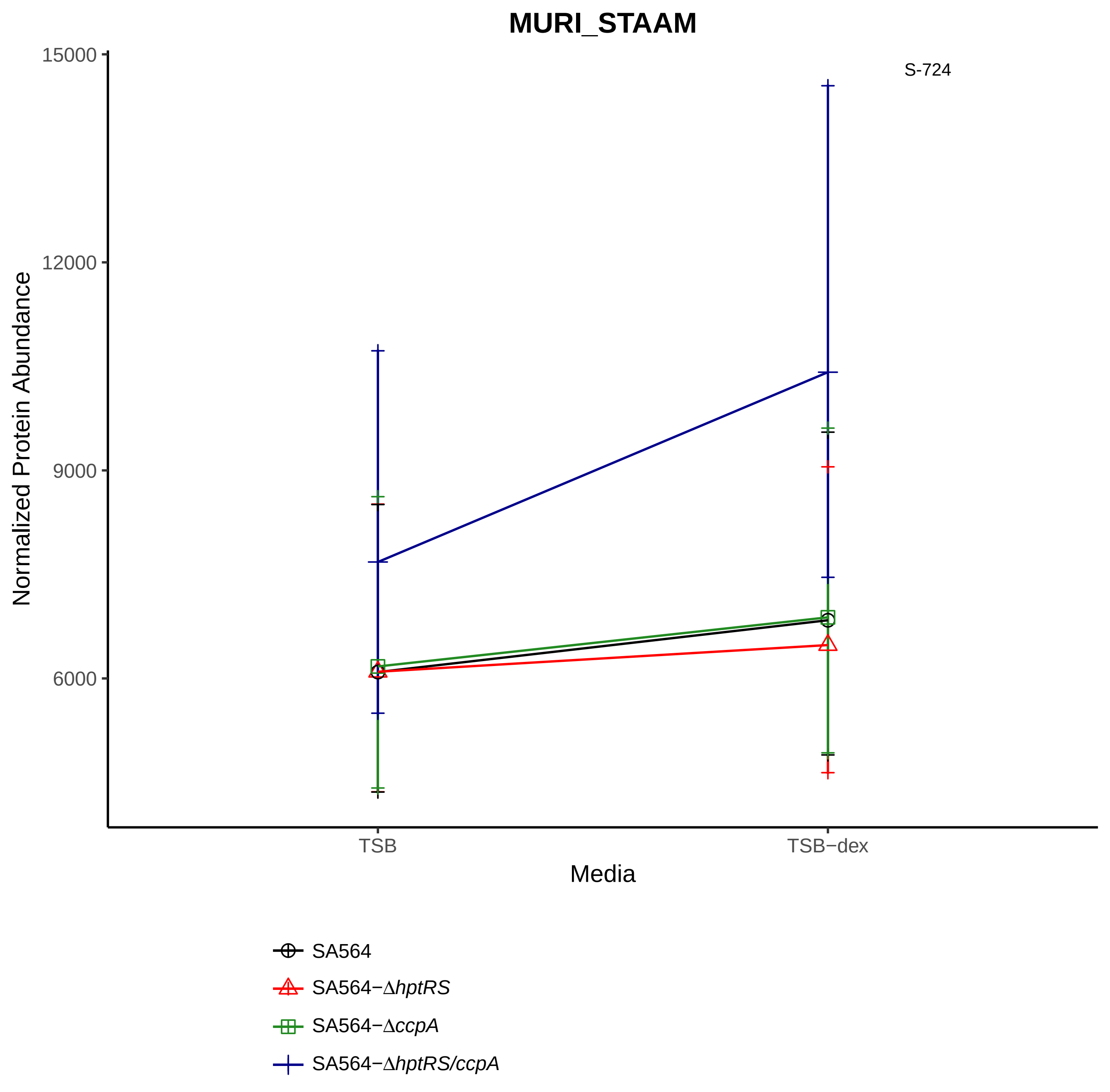

MURI_STAAM
S-724
15000
12000
9000
6000
Normalized Protein Abundance
TSB
TSB−dex
Media
SA564
SA564−ΔhptRS
SA564−ΔccpA
SA564−ΔhptRS/ccpA

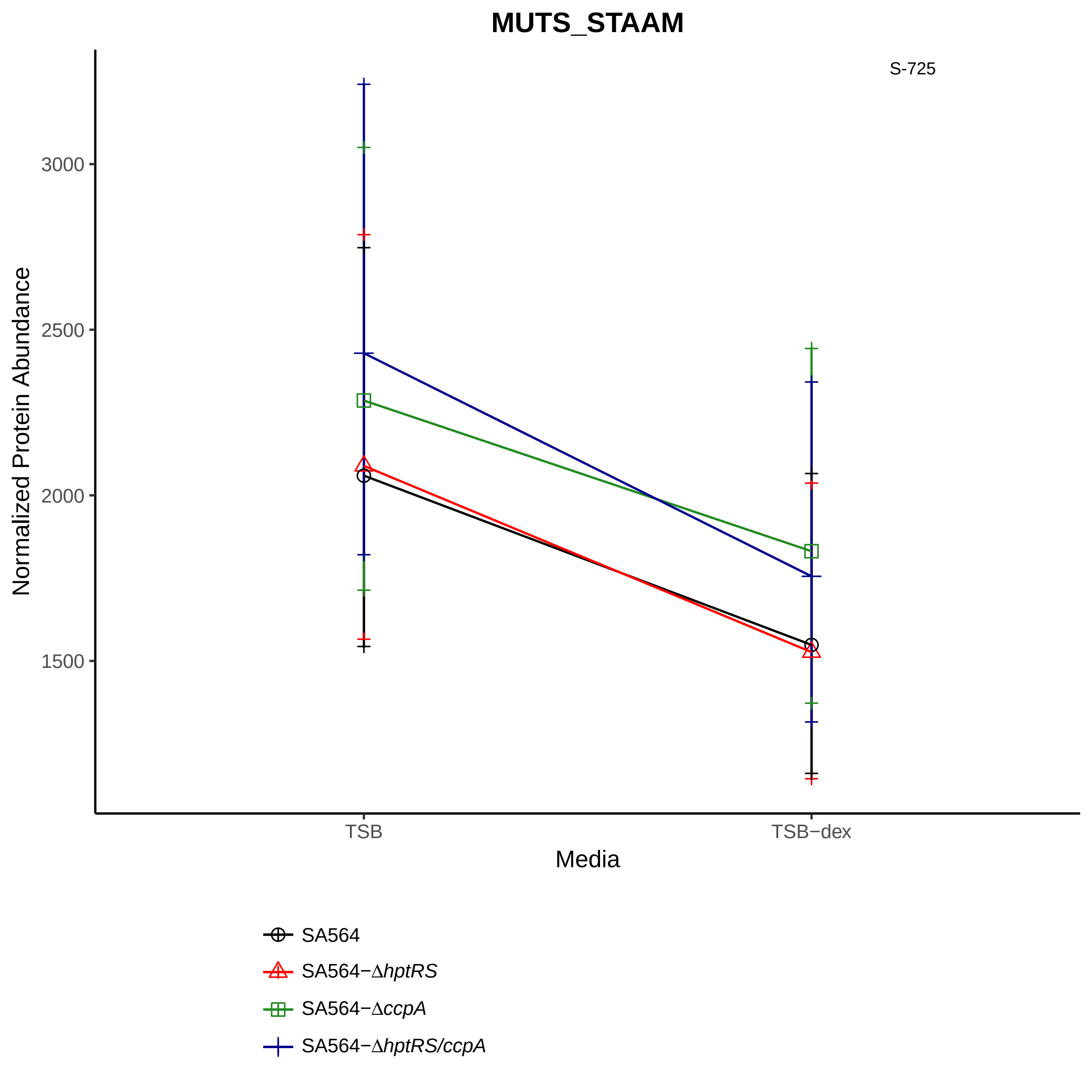

MUTS_STAAM
S-725
Normalized Protein Abundance
3000
2500
2000
1500
TSB
TSB−dex
Media
SA564
SA564−Δ*hptRS*
SA564−Δ*ccpA*
SA564−Δ*hptRS/ccpA*

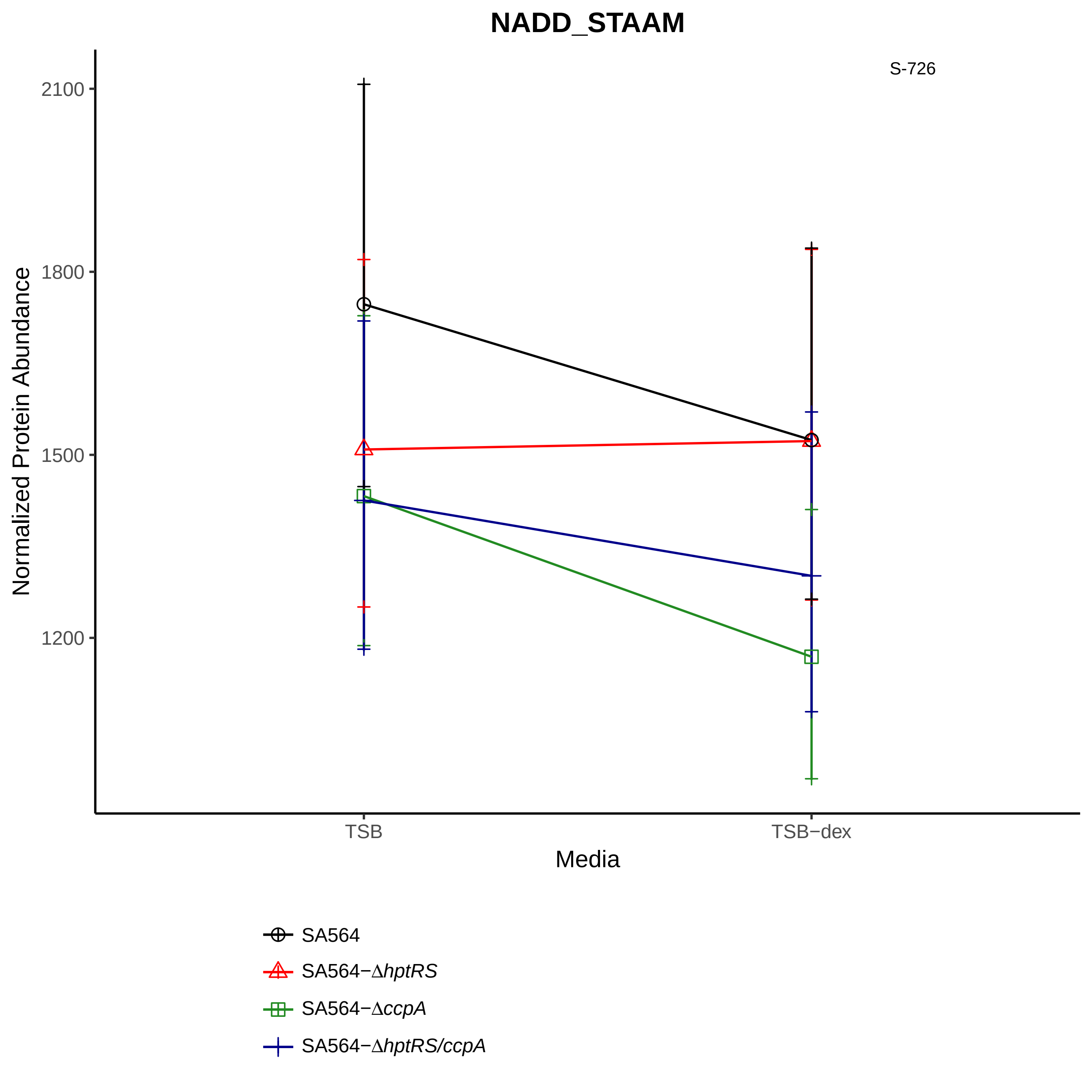

NADD_STAAM
S-726
Normalized Protein Abundance
2100
1800
1500
1200
TSB
TSB−dex
Media
SA564
SA564−Δ*hptRS*
SA564−Δ*ccpA*
SA564−Δ*hptRS/ccpA*

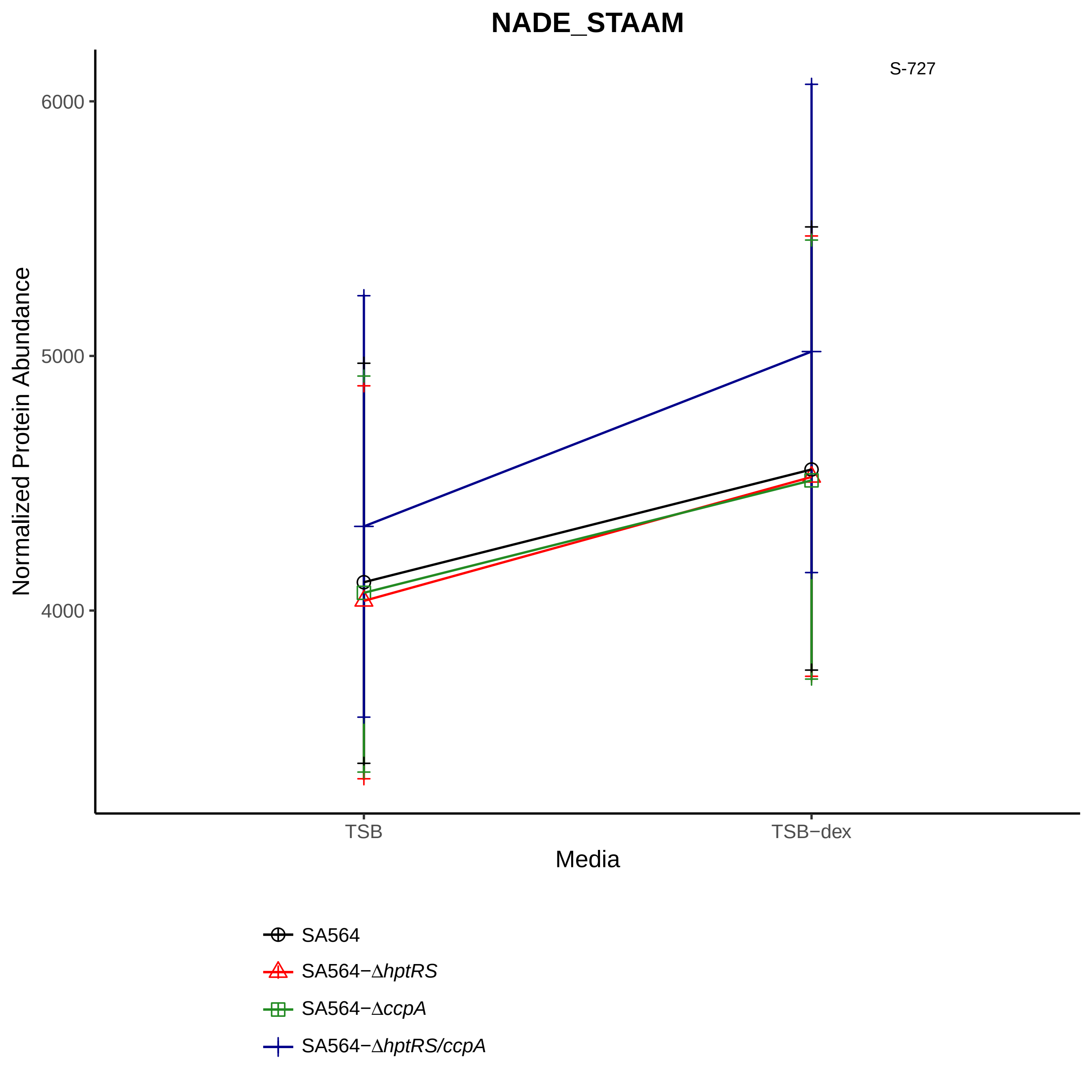

NADE_STAAM
S-727
6000
5000
4000
Normalized Protein Abundance
TSB
TSB−dex
Media
SA564
SA564−ΔhptRS
SA564−ΔccpA
SA564−ΔhptRS/ccpA

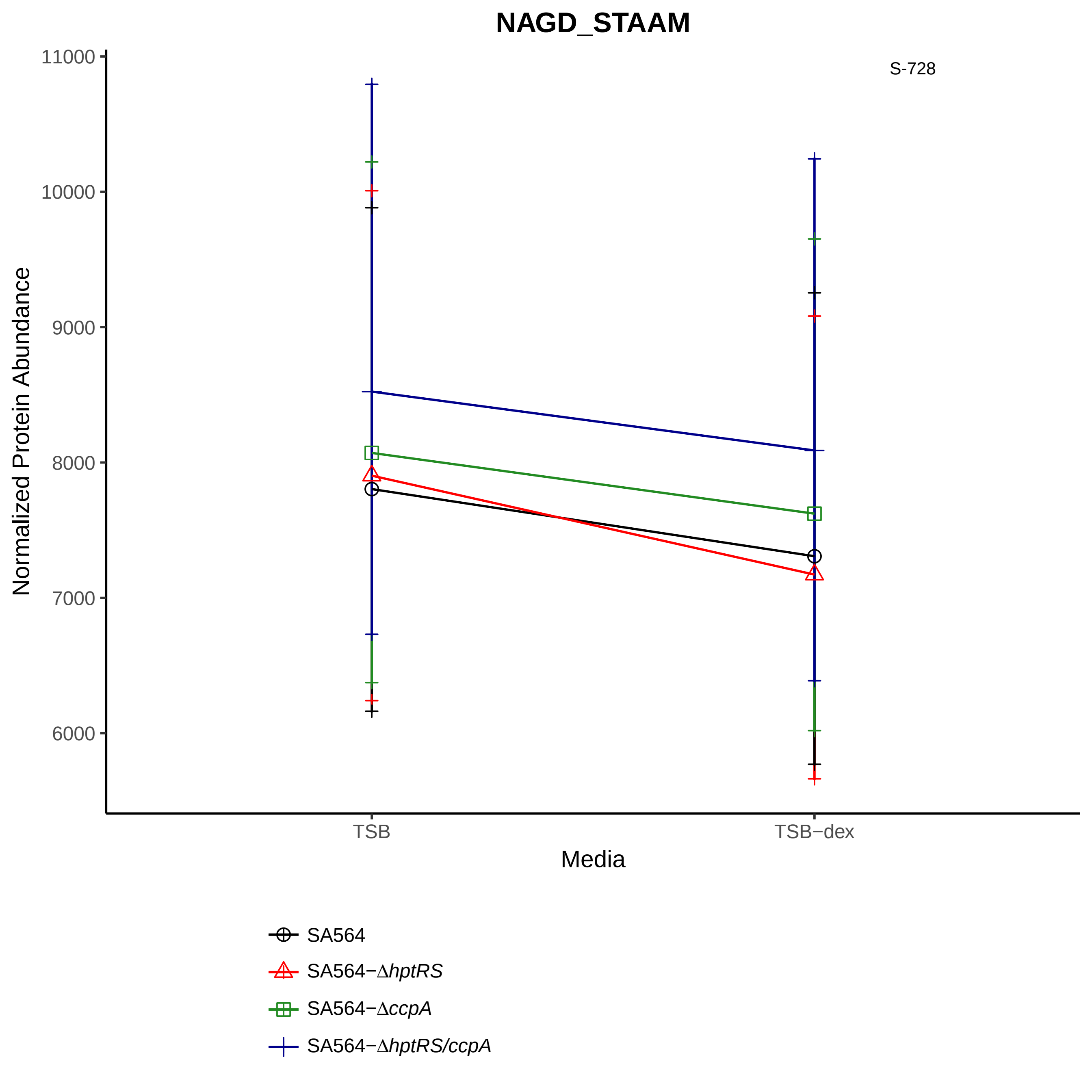
NAGD_STAAM
S-728
Normalized Protein Abundance
11000
10000
9000
8000
7000
6000
TSB
TSB−dex
Media
SA564
SA564−ΔhptRS
SA564−ΔccpA
SA564−ΔhptRS/ccpA

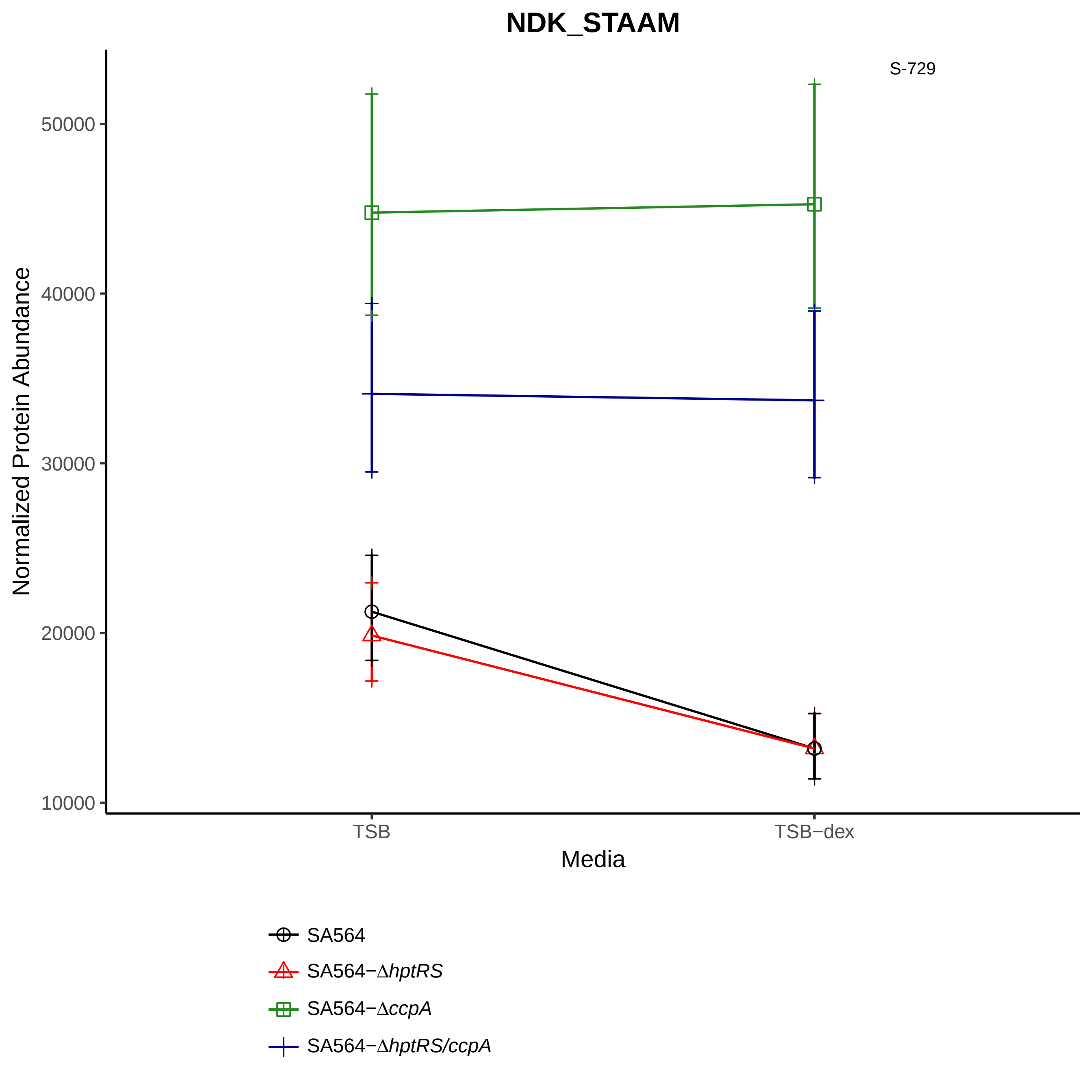
NDK_STAAM
S-729
Normalized Protein Abundance
50000
40000
30000
20000
10000
TSB
TSB−dex
Media
SA564
SA564−ΔhptRS
SA564−ΔccpA
SA564−ΔhptRS/ccpA

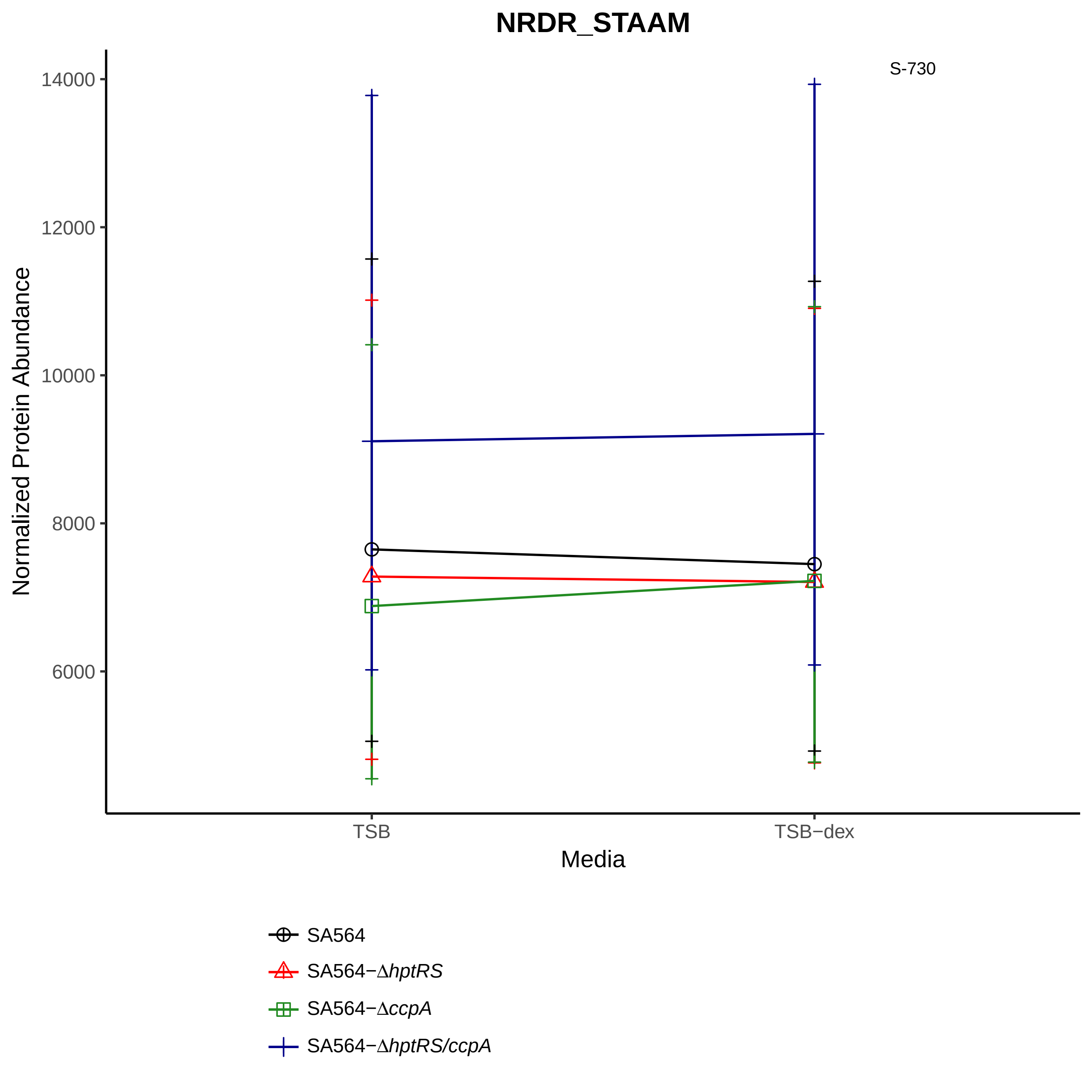

NRDR_STAAM
S-730
Normalized Protein Abundance
14000
12000
10000
8000
6000
TSB
TSB−dex
Media
SA564
SA564−ΔhptRS
SA564−ΔccpA
SA564−ΔhptRS/ccpA

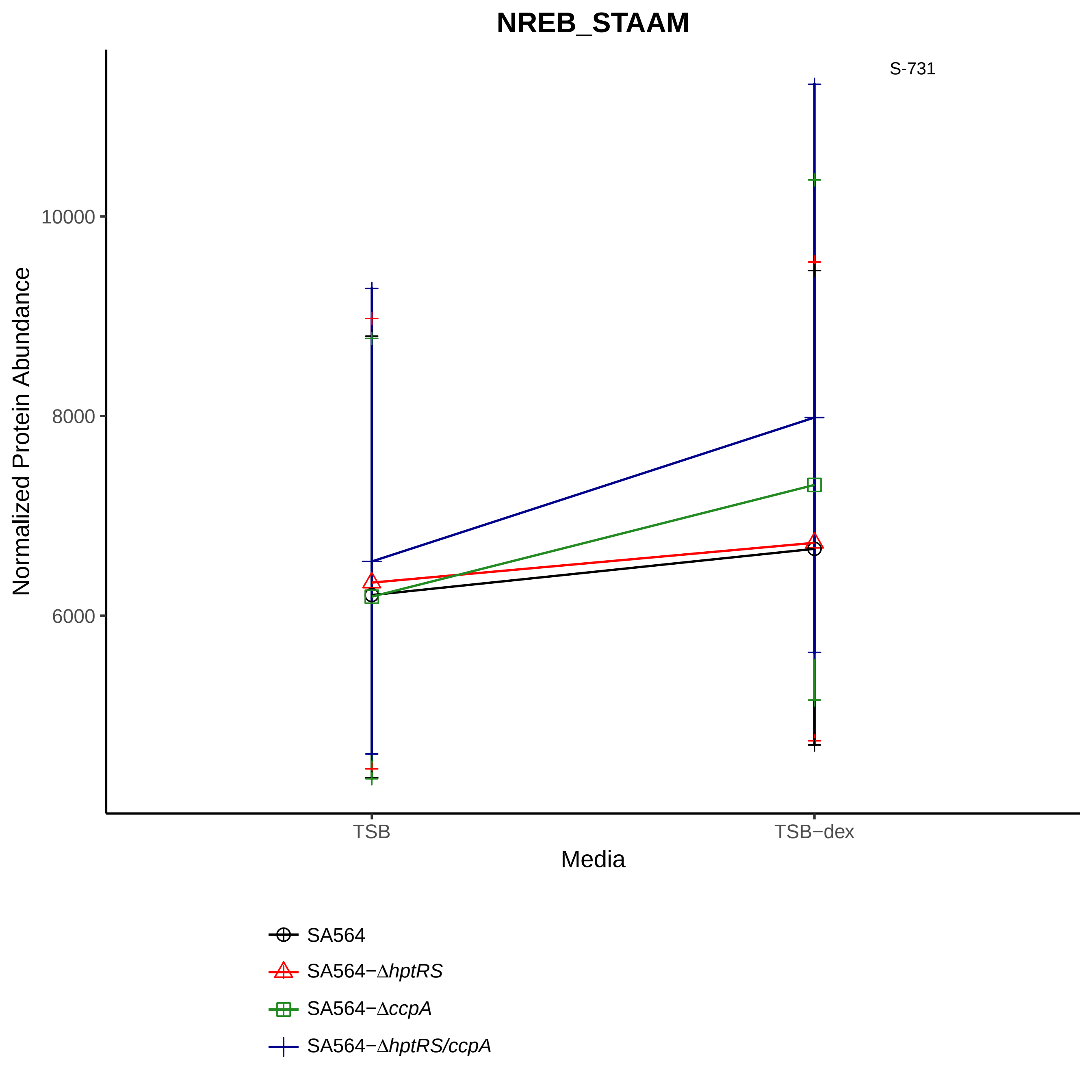

NREB_STAAM
S-731
Normalized Protein Abundance
10000
8000
6000
TSB
TSB−dex
Media
SA564
SA564−Δ*hptRS*
SA564−Δ*ccpA*
SA564−Δ*hptRS/ccpA*

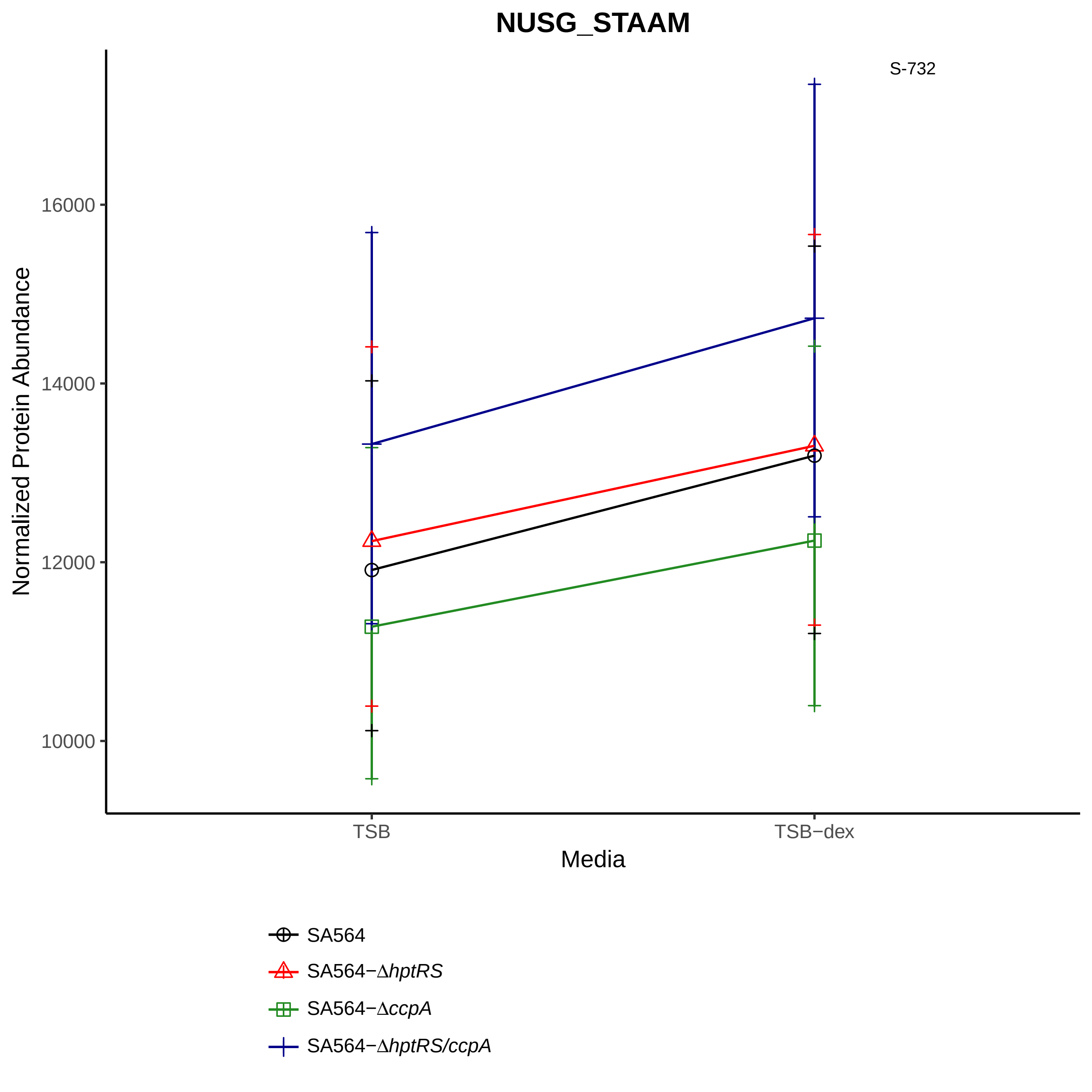
NUSG_STAAM
S-732
Normalized Protein Abundance
16000
14000
12000
10000
TSB
TSB−dex
Media
SA564
SA564−Δ*hptRS*
SA564−Δ*ccpA*
SA564−Δ*hptRS/ccpA*

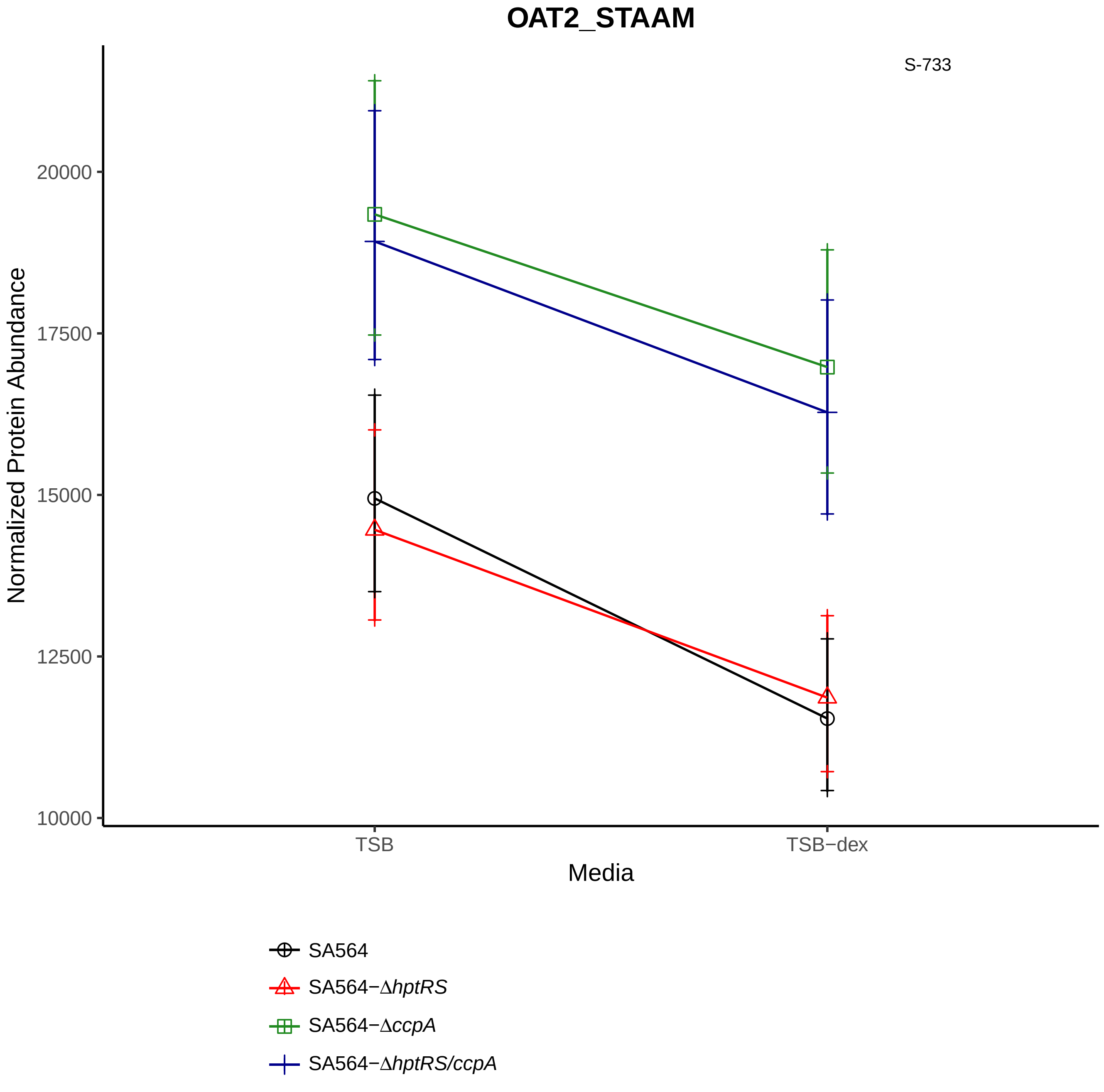

OAT2_STAAM
S-733
Normalized Protein Abundance
20000
17500
15000
12500
10000
TSB
TSB−dex
Media
SA564
SA564−Δ*hptRS*
SA564−Δ*ccpA*
SA564−Δ*hptRS/ccpA*

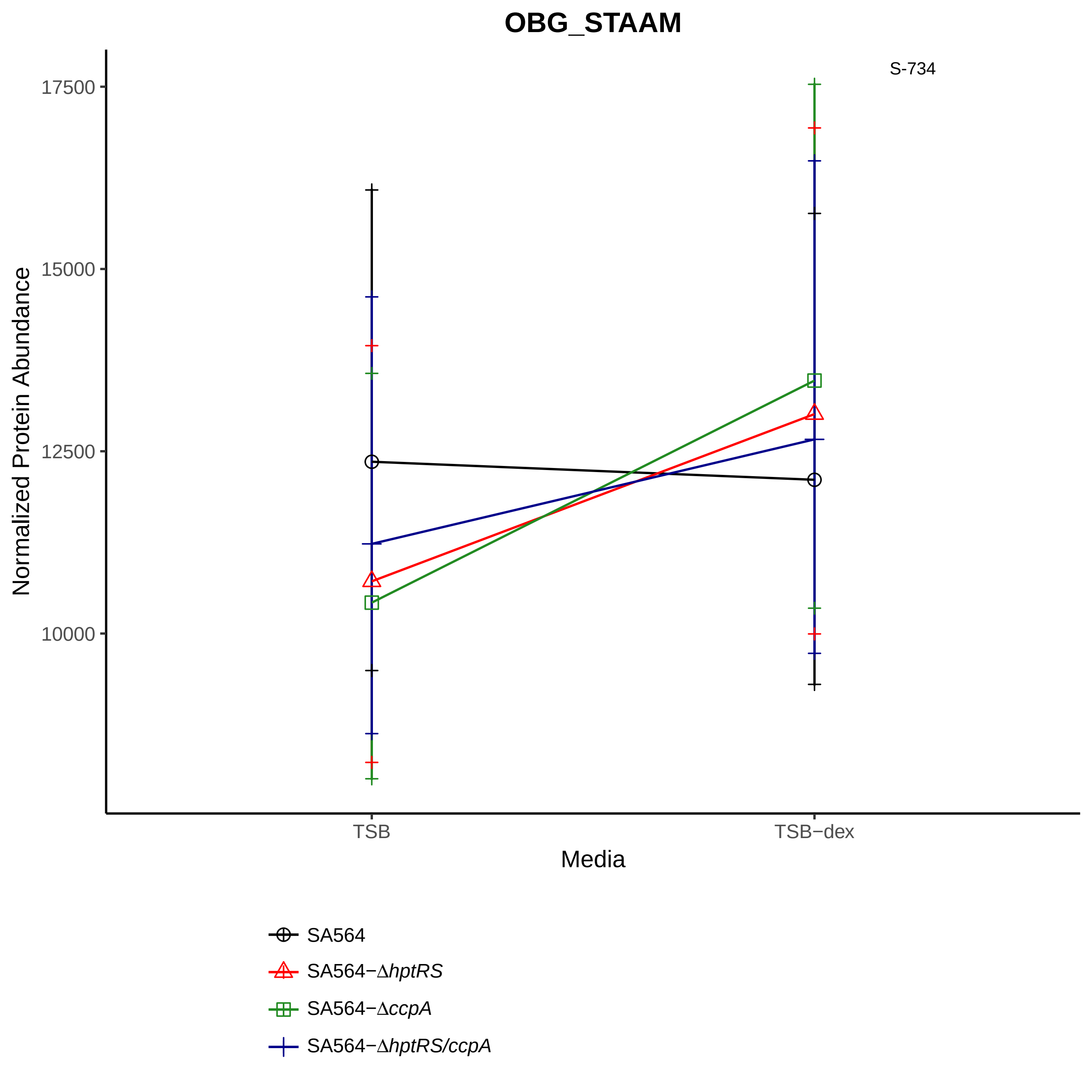

OBG_STAAM
S-734
17500
15000
12500
10000
Normalized Protein Abundance
TSB
TSB−dex
Media
SA564
SA564−Δ*hptRS*
SA564−Δ*ccpA*
SA564−Δ*hptRS/ccpA*

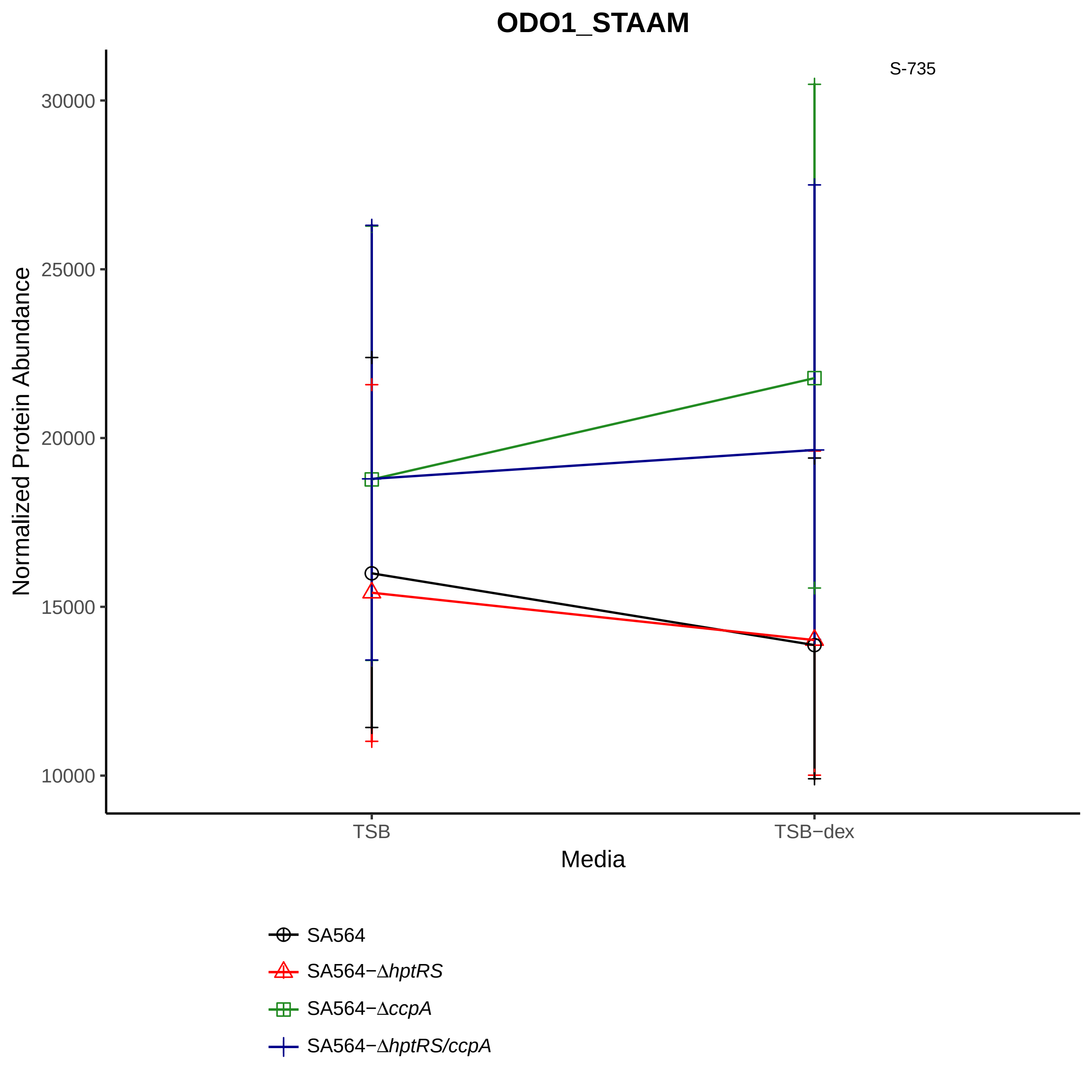

ODO1_STAAM
S-735
30000
25000
20000
15000
10000
Normalized Protein Abundance
TSB
TSB−dex
Media
SA564
SA564−Δ*hptRS*
SA564−Δ*ccpA*
SA564−Δ*hptRS/ccpA*

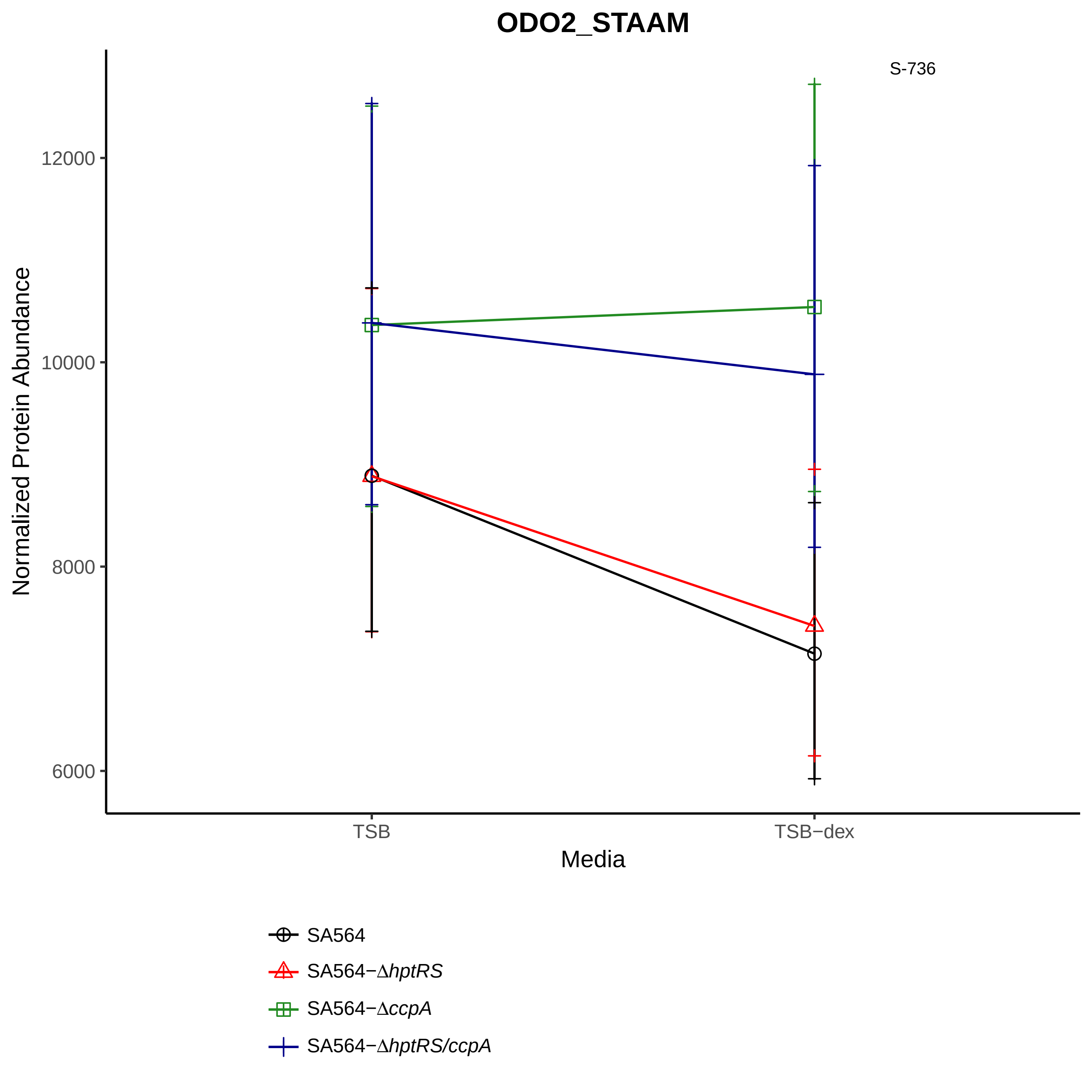

ODO2_STAAM
S-736
Normalized Protein Abundance
12000
10000
8000
6000
TSB
TSB−dex
Media
SA564
SA564−ΔhptRS
SA564−ΔccpA
SA564−ΔhptRS/ccpA

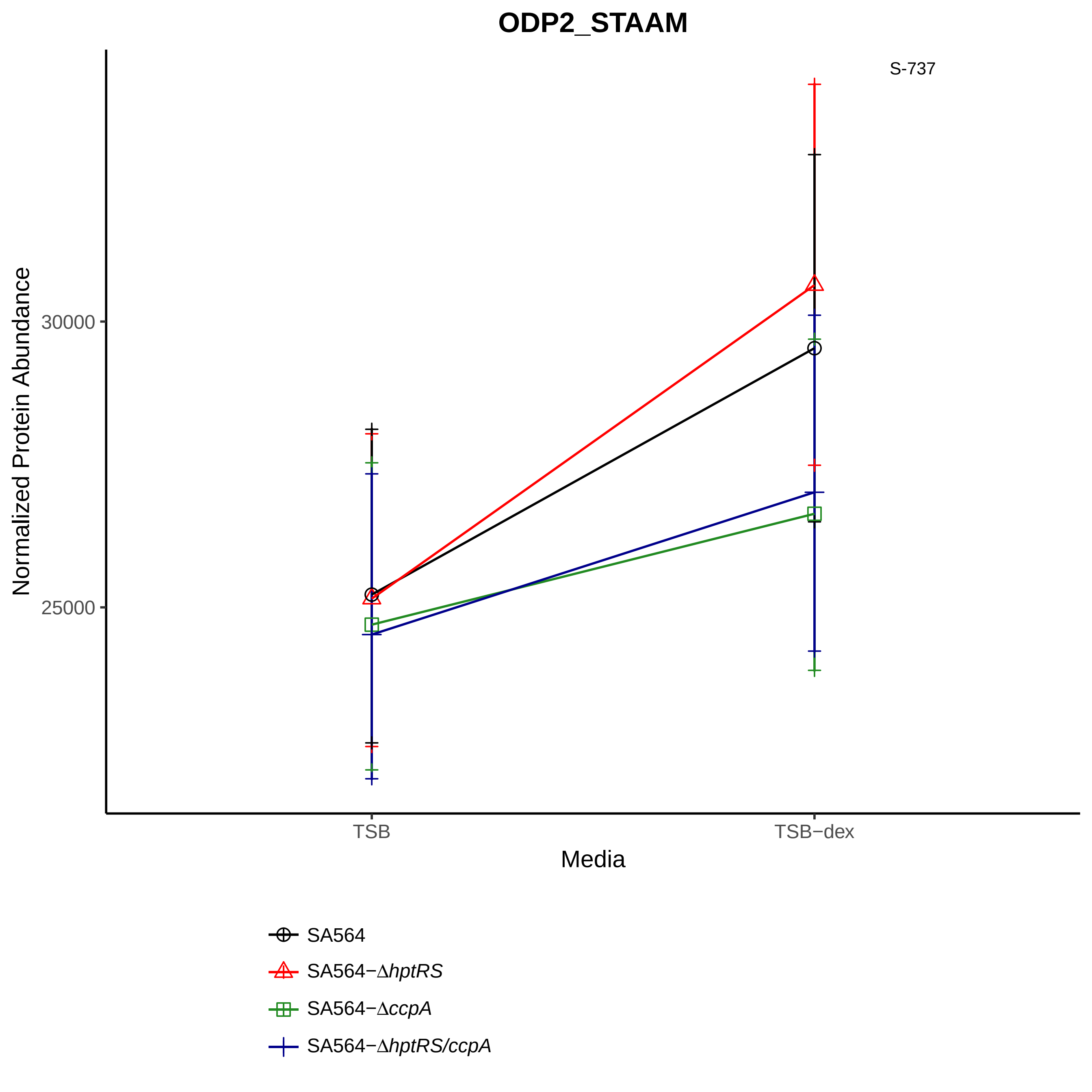

ODP2_STAAM
S-737
Normalized Protein Abundance
30000
25000
TSB
TSB−dex
Media
SA564
SA564−Δ*hptRS*
SA564−Δ*ccpA*
SA564−Δ*hptRS/ccpA*

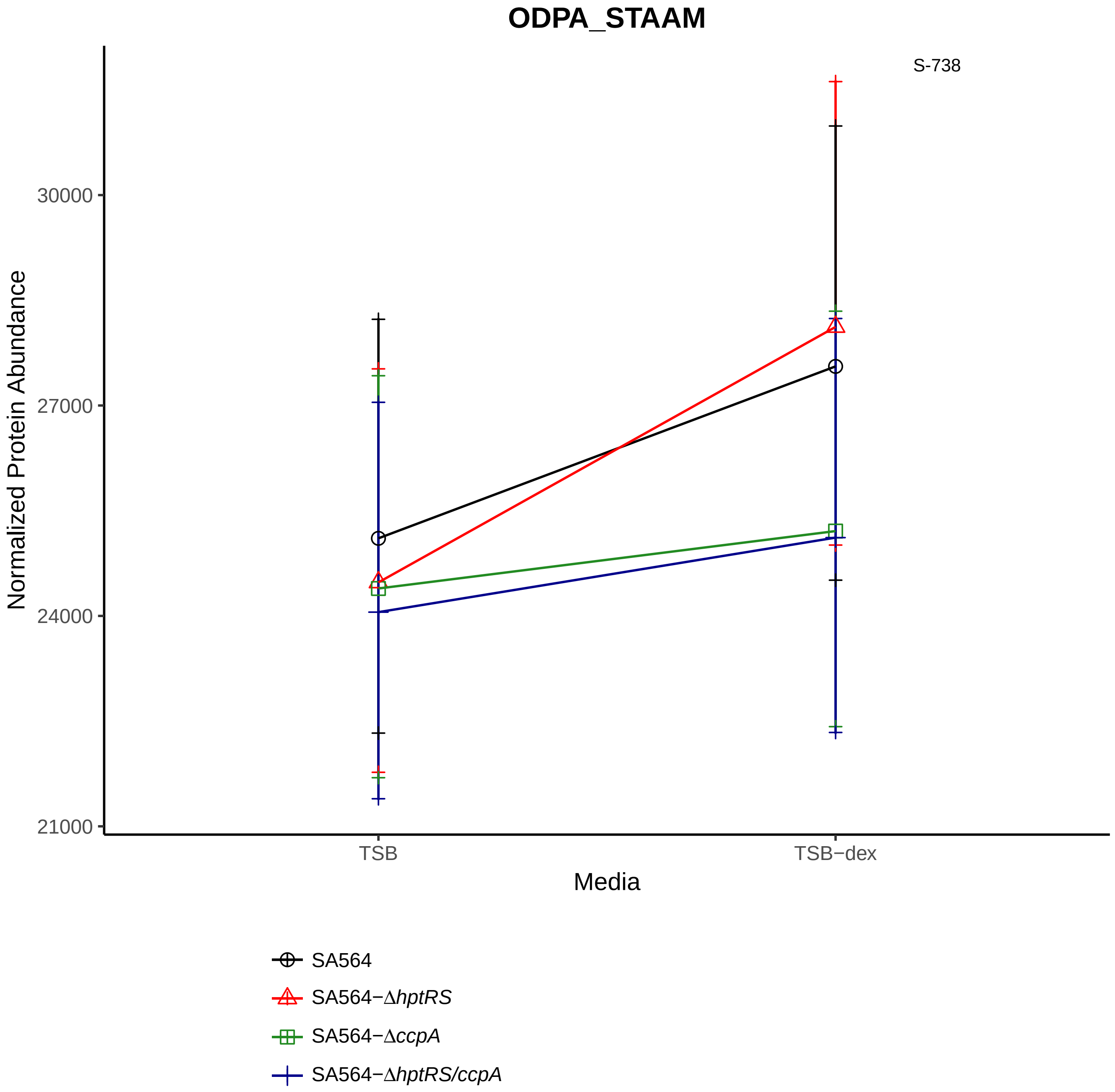

ODPA_STAAM
S-738
Normalized Protein Abundance
30000
27000
24000
21000
TSB
TSB−dex
Media
SA564
SA564−Δ*hptRS*
SA564−Δ*ccpA*
SA564−Δ*hptRS/ccpA*

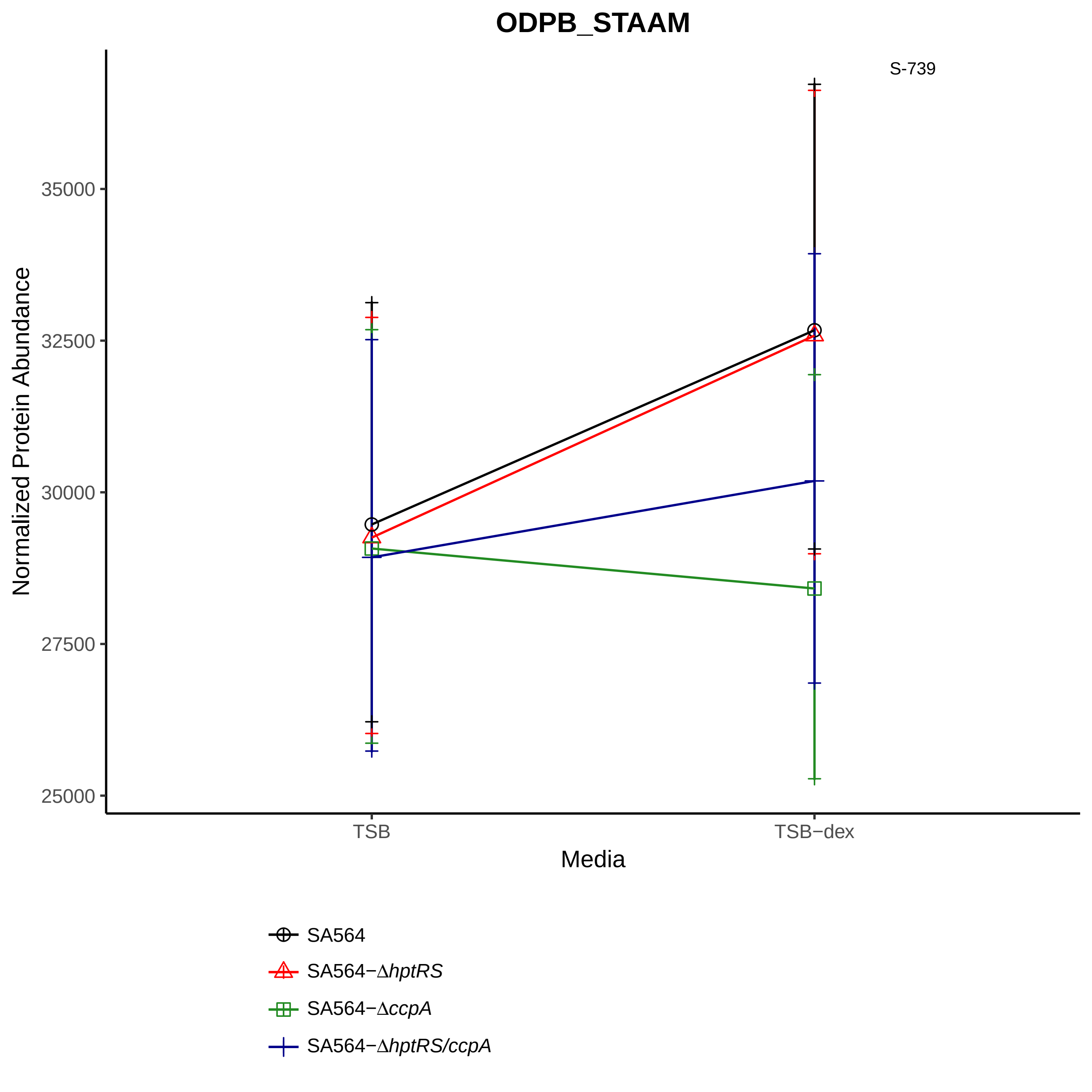

ODPB_STAAM
S-739
35000
32500
30000
27500
25000
Normalized Protein Abundance
TSB
TSB−dex
Media
SA564
SA564−Δ*hptRS*
SA564−Δ*ccpA*
SA564−Δ*hptRS/ccpA*

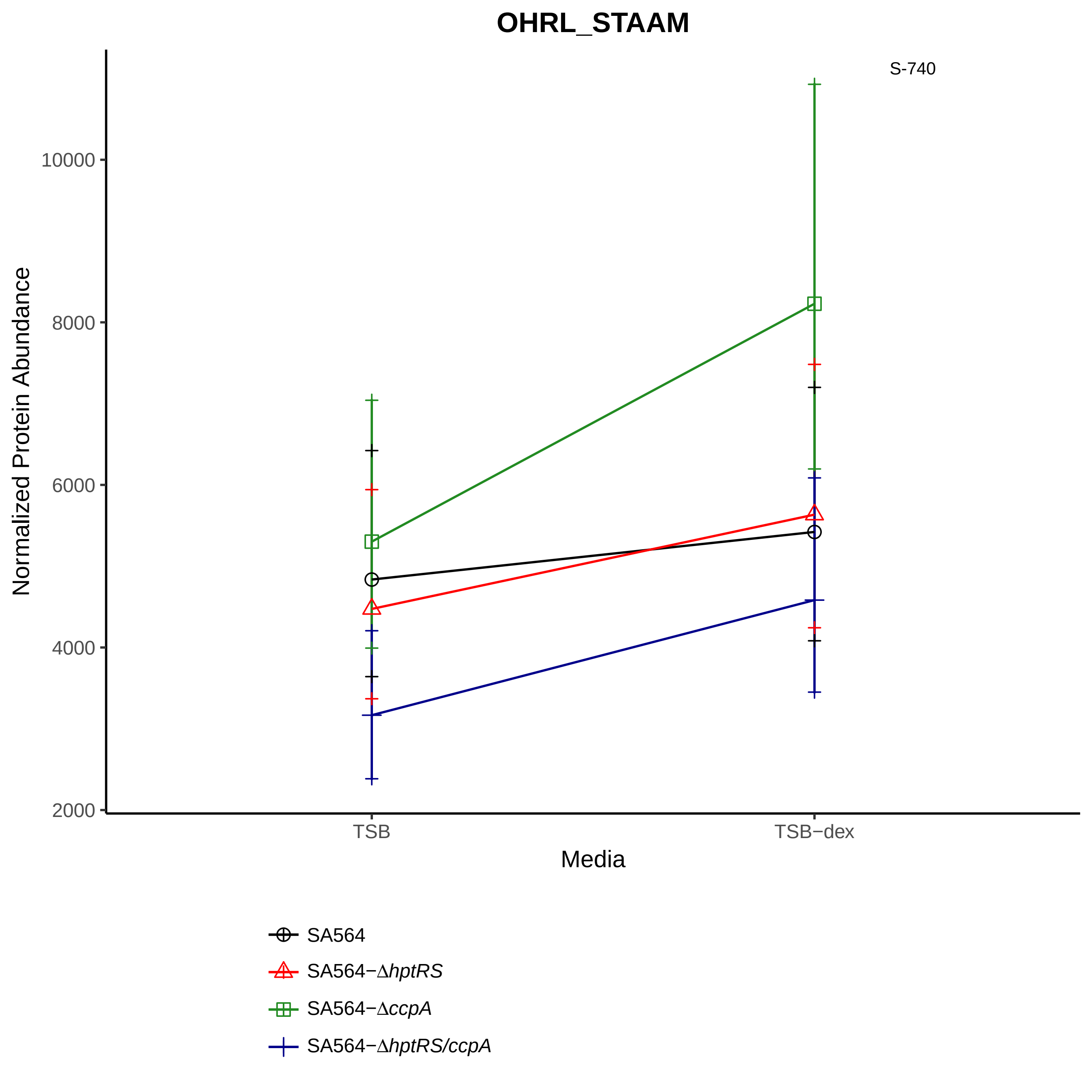
OHRL_STAAM
S-740
Normalized Protein Abundance
10000
8000
6000
4000
2000
TSB
TSB−dex
Media
SA564
SA564−Δ*hptRS*
SA564−Δ*ccpA*
SA564−Δ*hptRS/ccpA*

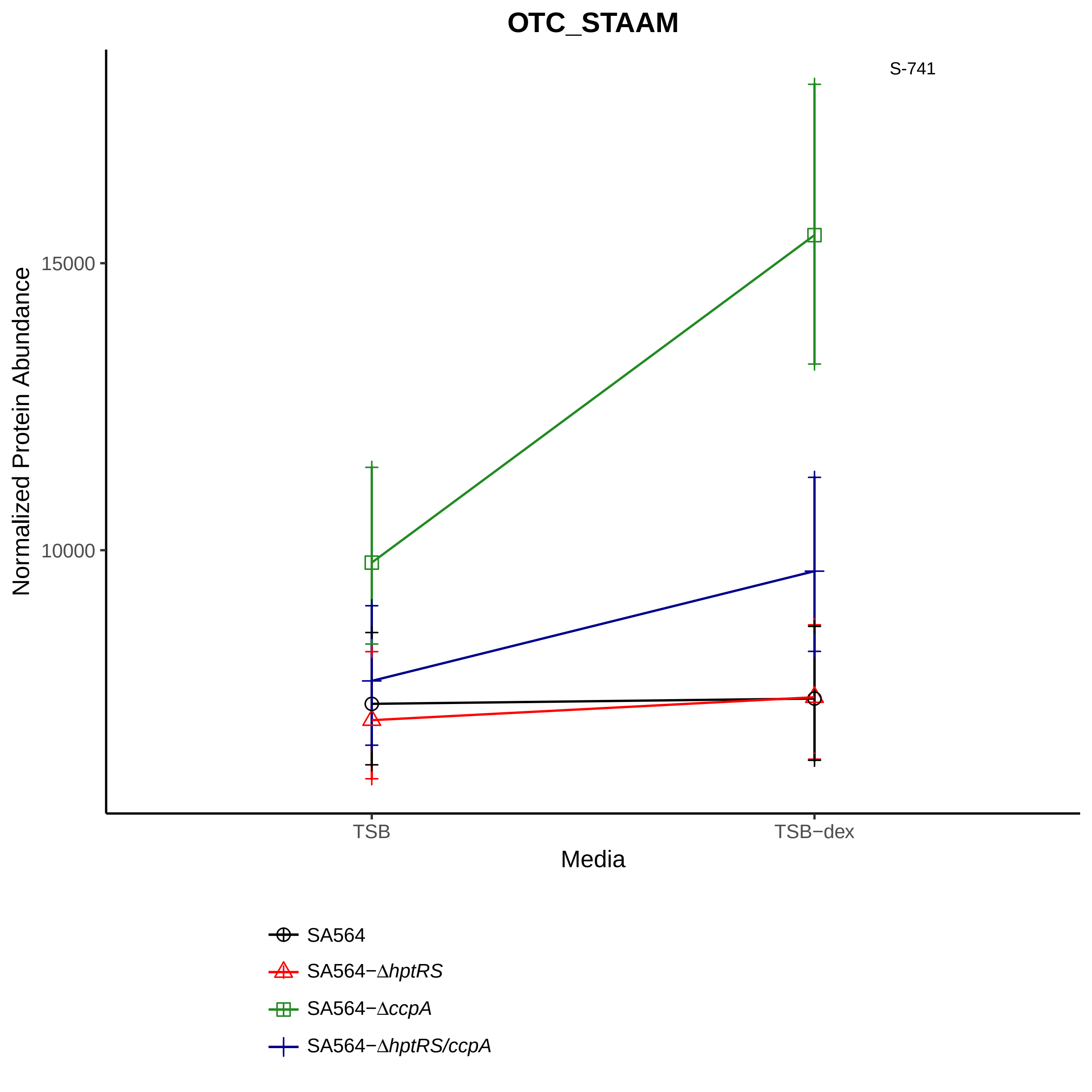

OTC_STAAM
S-741
Normalized Protein Abundance
15000
10000
TSB
TSB−dex
Media
SA564
SA564−ΔhptRS
SA564−ΔccpA
SA564−ΔhptRS/ccpA

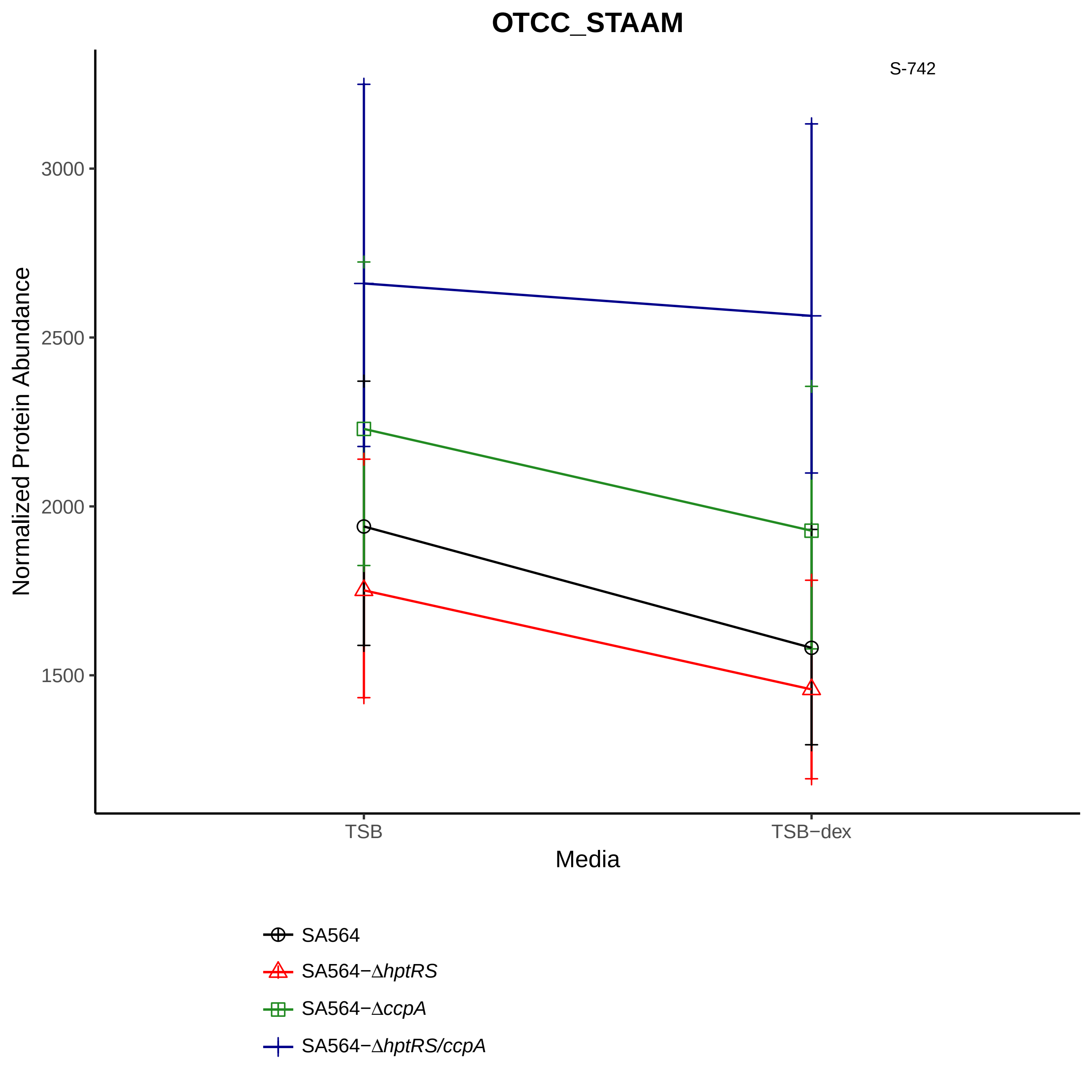

OTCC_STAAM
S-742
Normalized Protein Abundance
3000
2500
2000
1500
TSB
TSB−dex
Media
SA564
SA564−Δ*hptRS*
SA564−Δ*ccpA*
SA564−Δ*hptRS/ccpA*

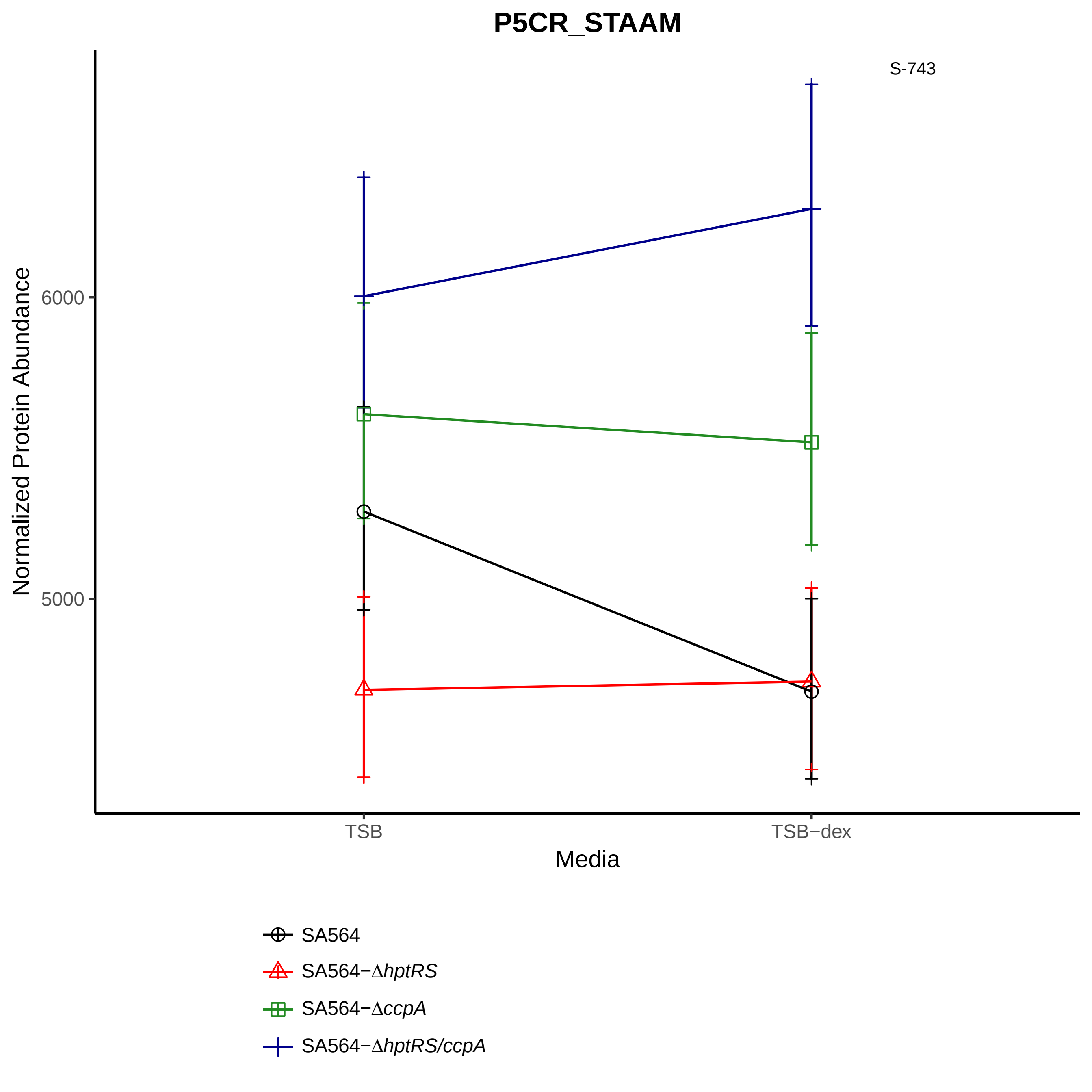

P5CR_STAAM
S-743
Normalized Protein Abundance
6000
5000
TSB
TSB−dex
Media
SA564
SA564−Δ*hptRS*
SA564−Δ*ccpA*
SA564−Δ*hptRS/ccpA*

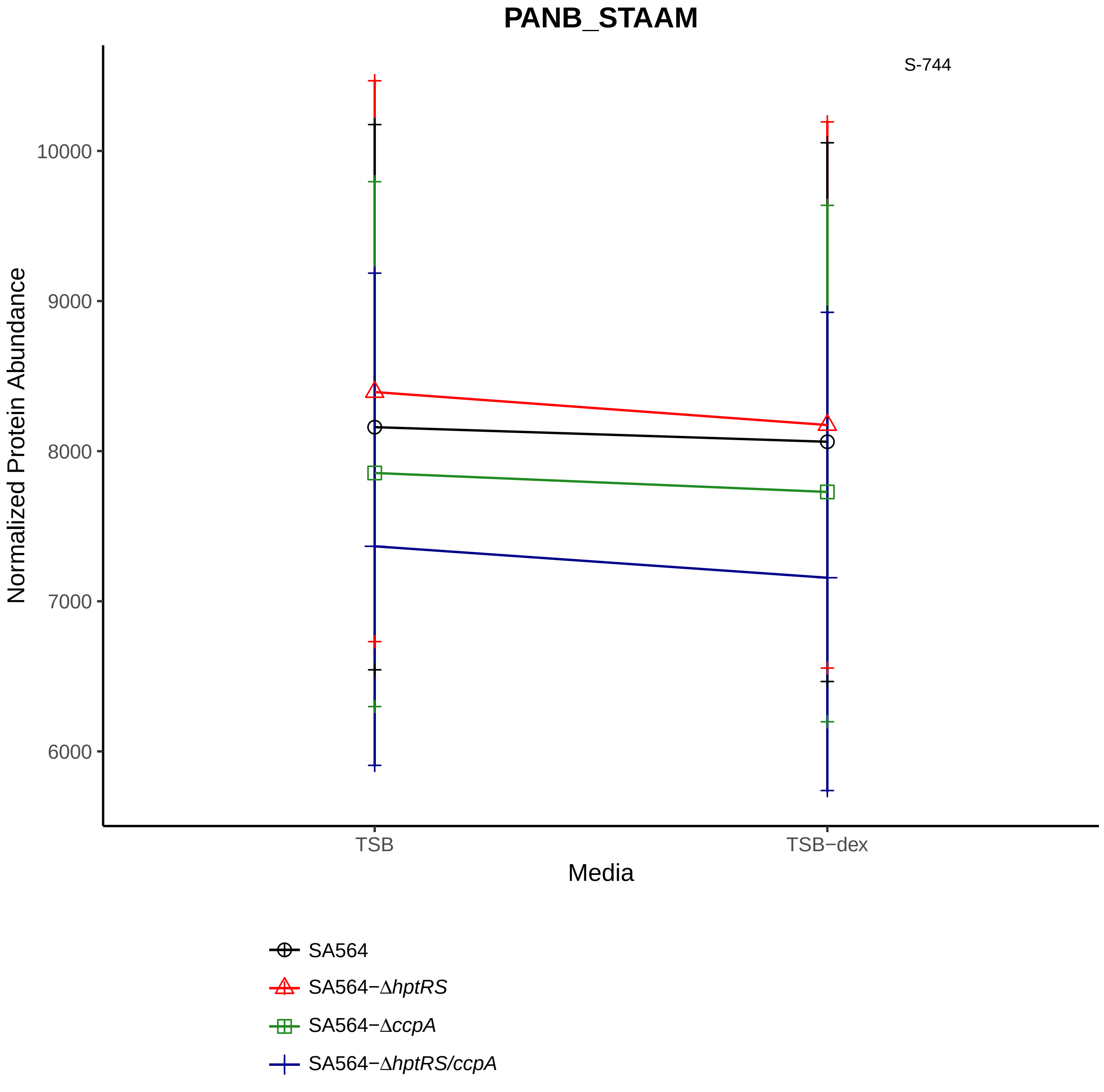

PANB_STAAM
S-744
Normalized Protein Abundance
10000
9000
8000
7000
6000
TSB
TSB−dex
Media
SA564
SA564−Δ*hptRS*
SA564−Δ*ccpA*
SA564−Δ*hptRS/ccpA*

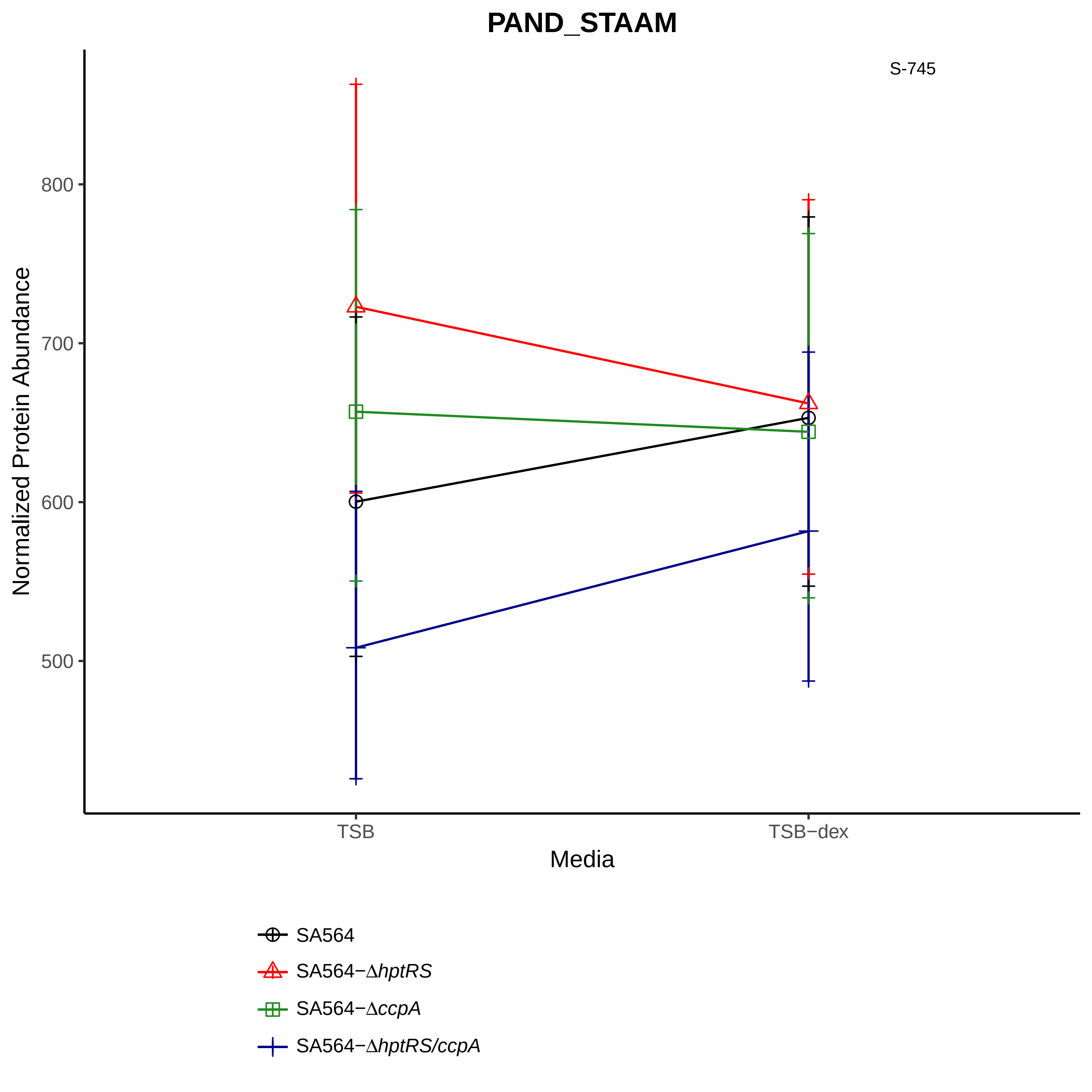

PAND_STAAM
S-745
Normalized Protein Abundance
800
700
600
500
TSB
TSB−dex
Media
SA564
SA564−Δ*hptRS*
SA564−Δ*ccpA*
SA564−Δ*hptRS/ccpA*

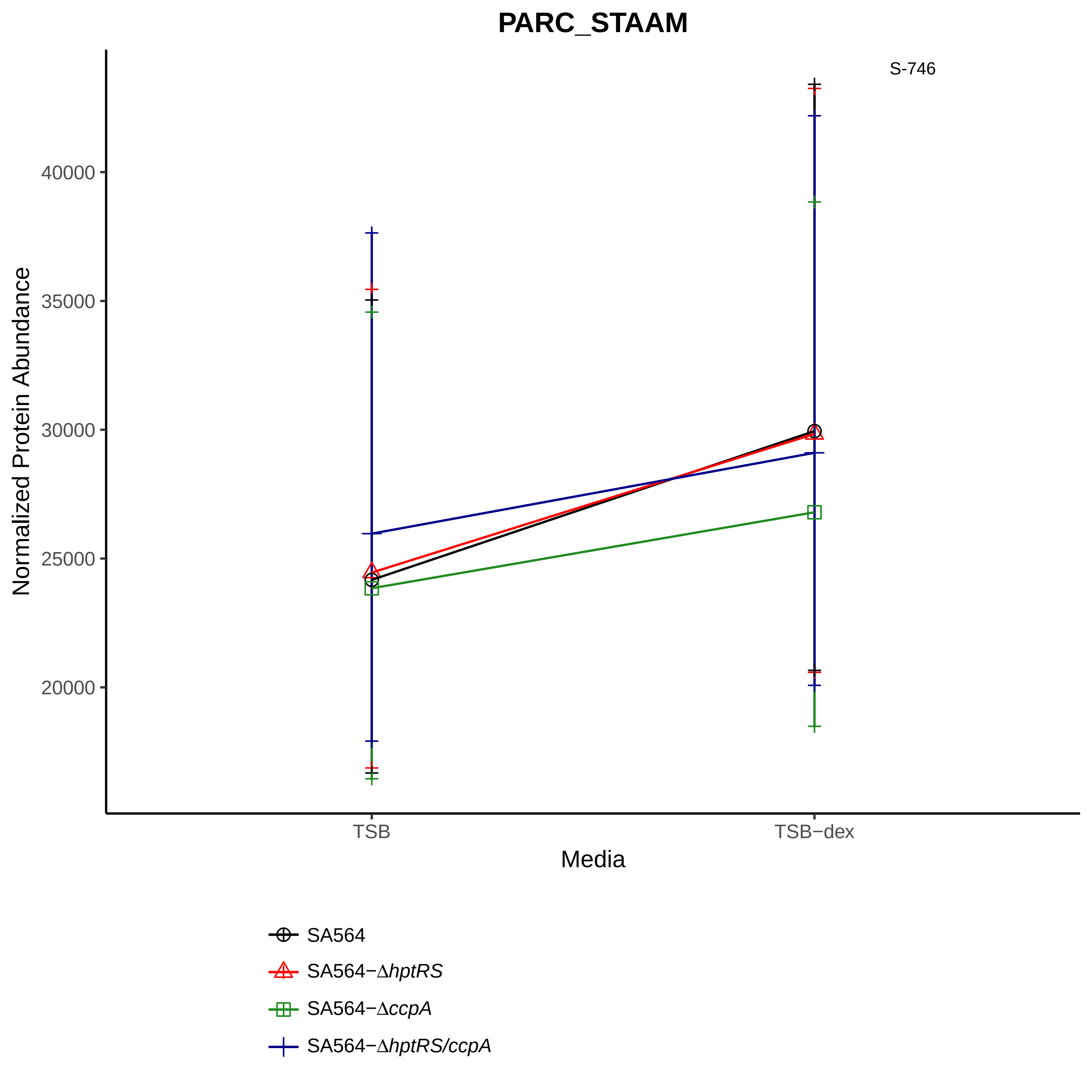

PARC_STAAM
S-746
Normalized Protein Abundance
40000
35000
30000
25000
20000
TSB
TSB−dex
Media
SA564
SA564−Δ*hptRS*
SA564−Δ*ccpA*
SA564−Δ*hptRS/ccpA*

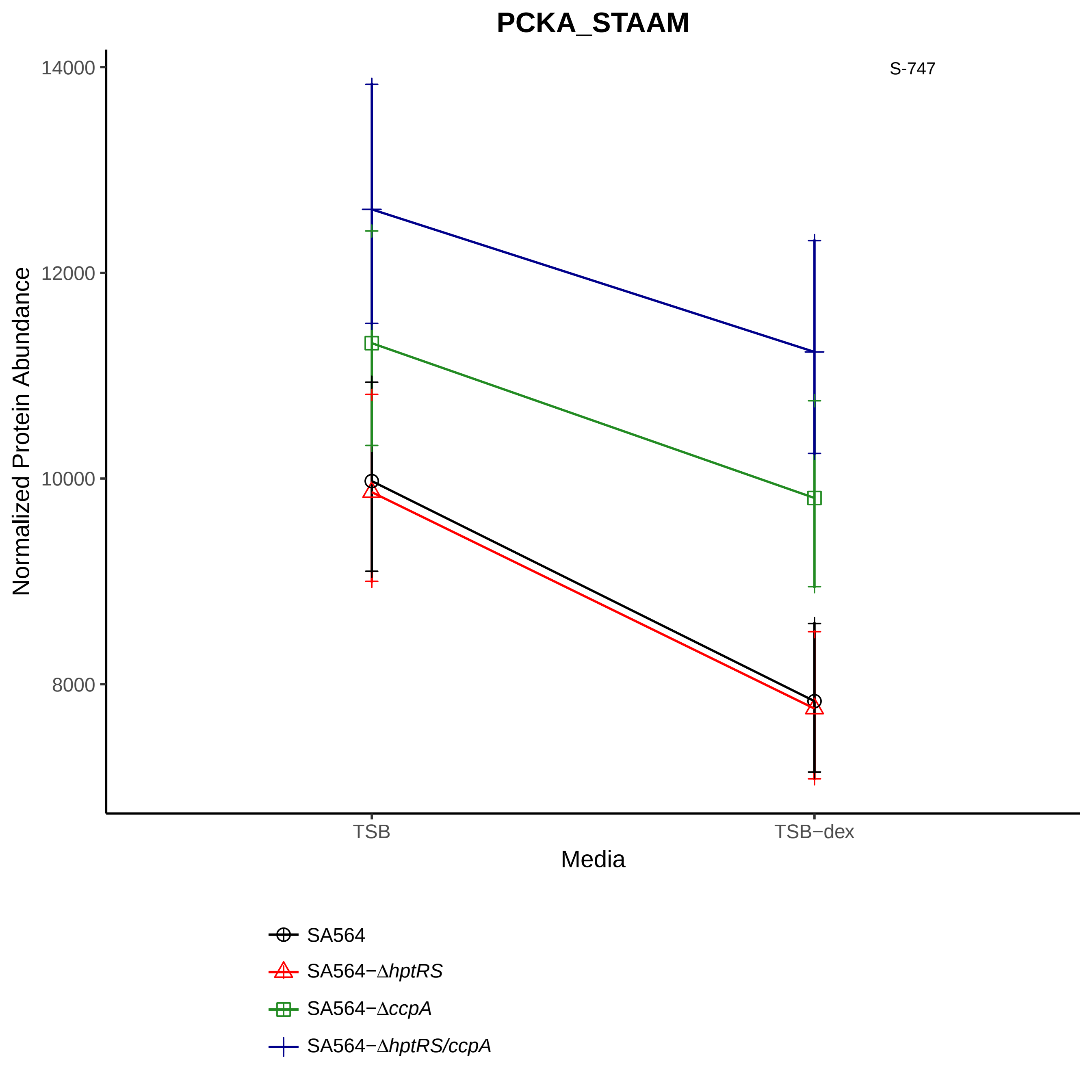
PCKA_STAAM
S-747
Normalized Protein Abundance
14000
12000
10000
8000
TSB
TSB−dex
Media
SA564
SA564−ΔhptRS
SA564−ΔccpA
SA564−ΔhptRS/ccpA

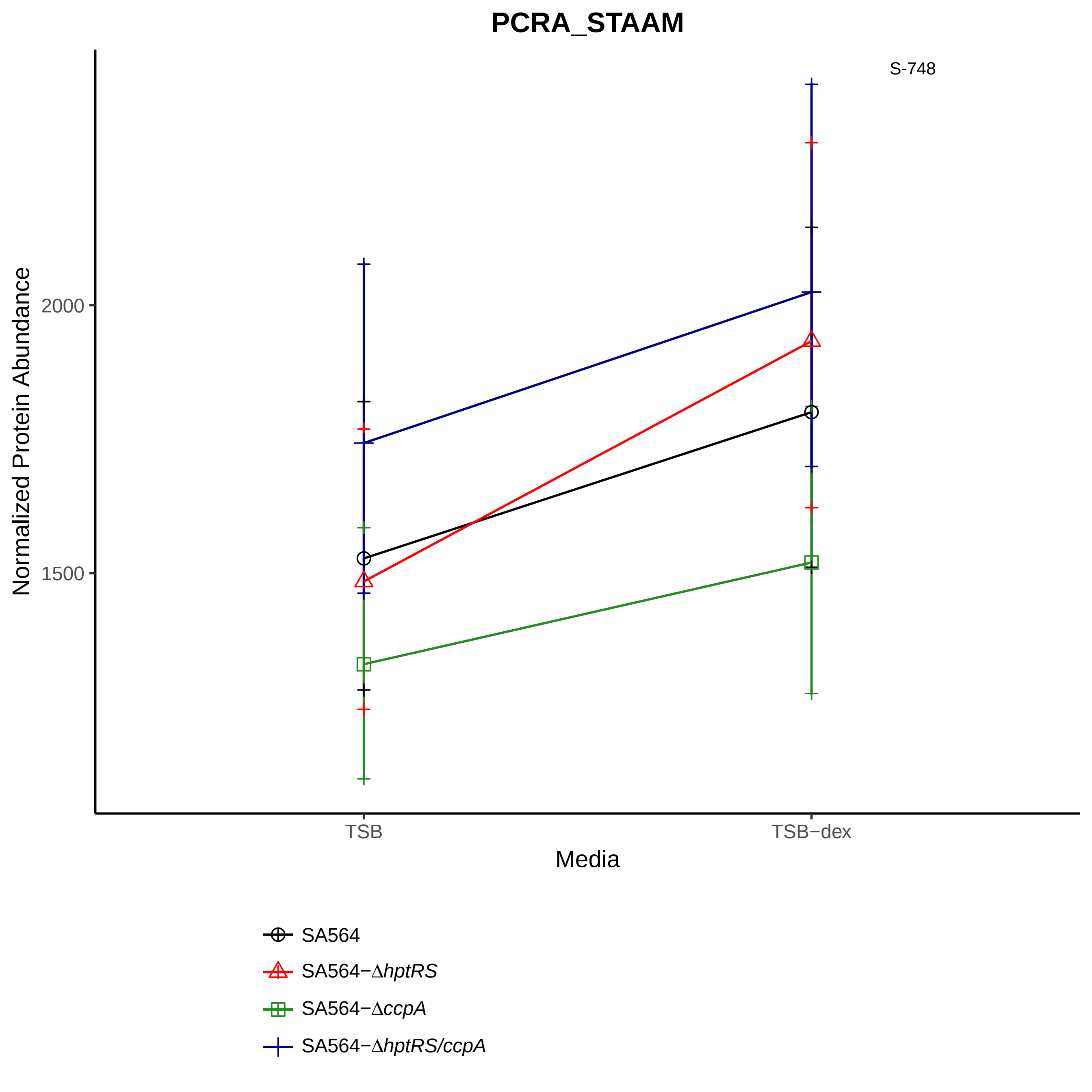

PCRA_STAAM
S-748
Normalized Protein Abundance
2000
1500
TSB
TSB−dex
Media
SA564
SA564−ΔhptRS
SA564−ΔccpA
SA564−ΔhptRS/ccpA

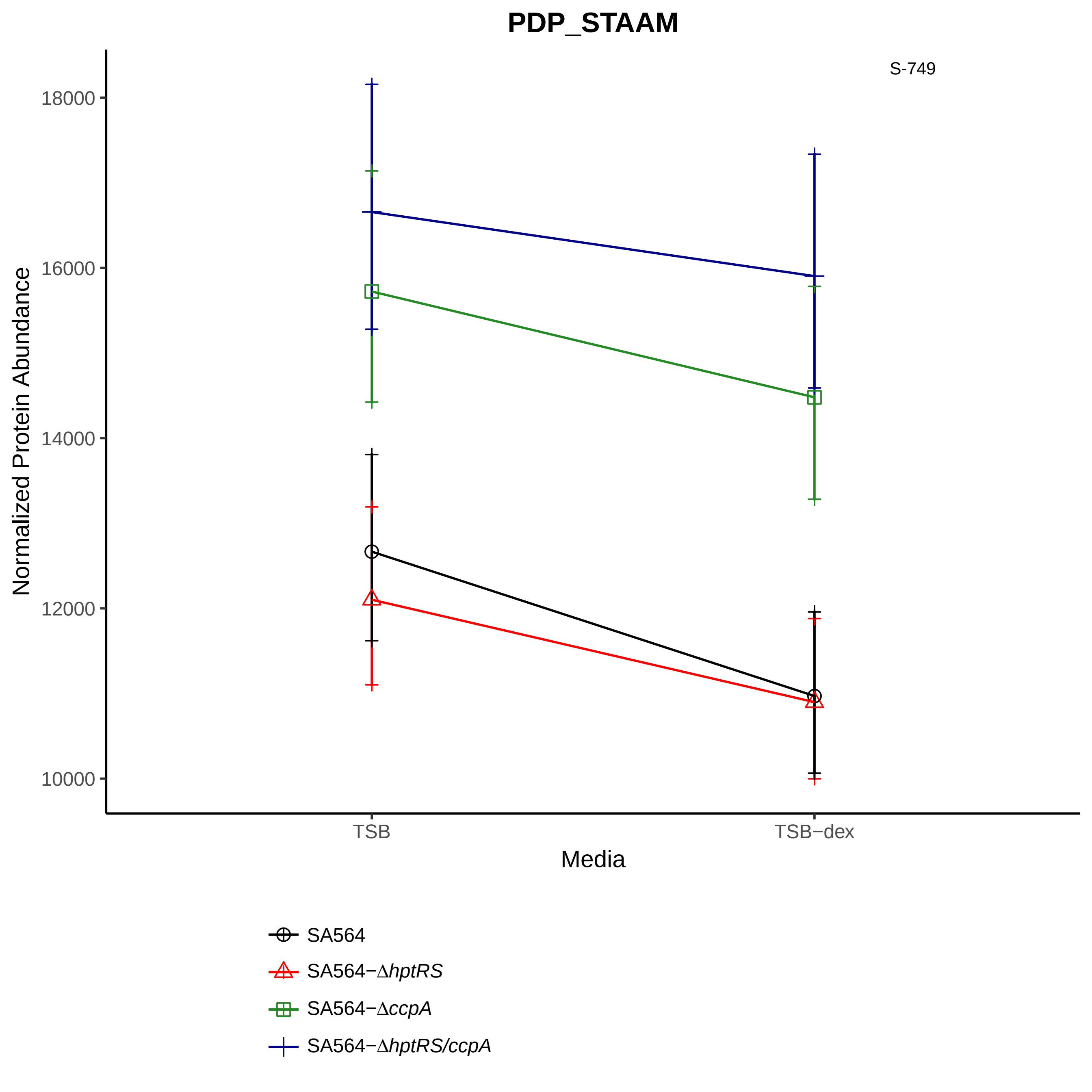

PDP_STAAM
S-749
Normalized Protein Abundance
18000
16000
14000
12000
10000
TSB
TSB−dex
Media
SA564
SA564−Δ*hptRS*
SA564−Δ*ccpA*
SA564−Δ*hptRS/ccpA*

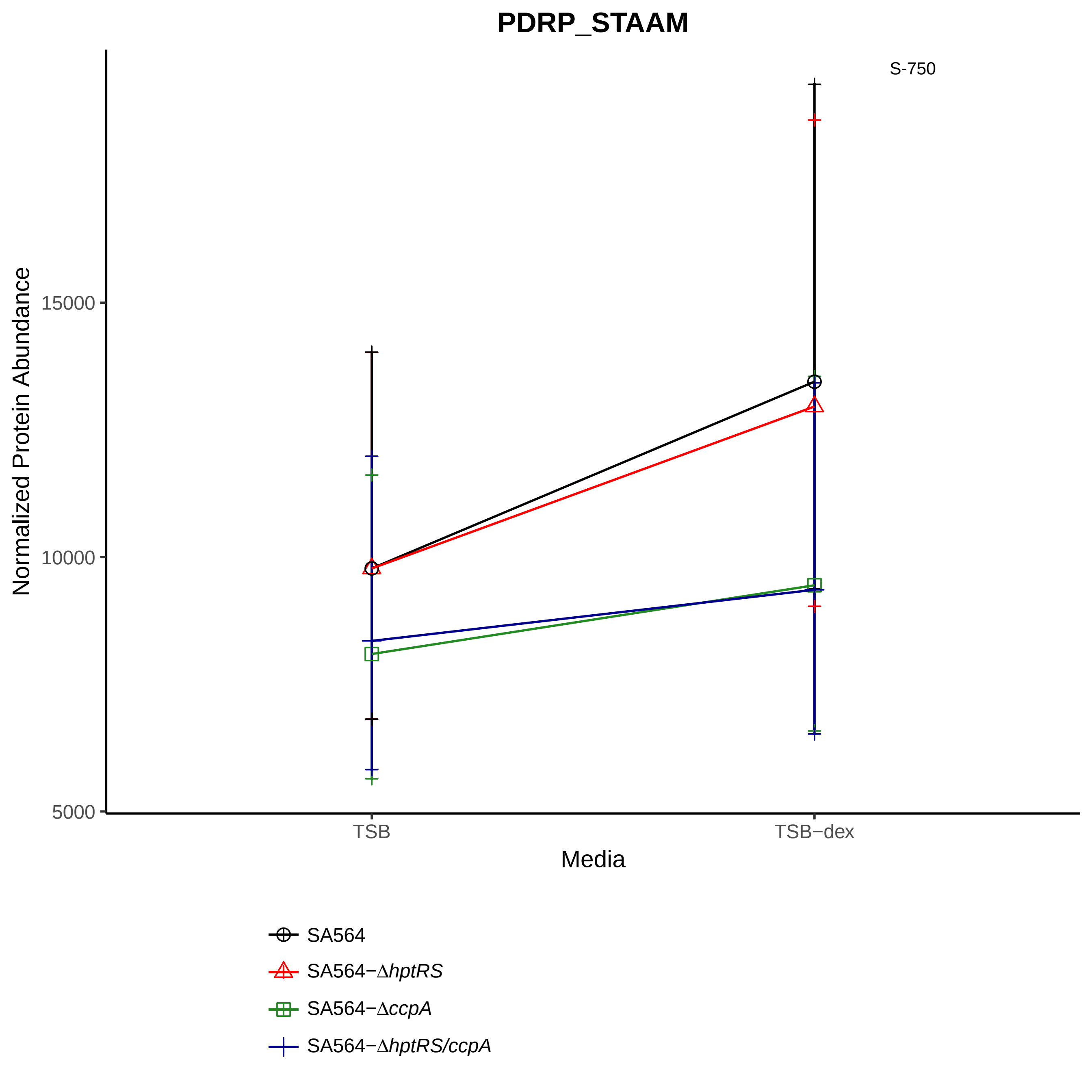

PDRP_STAAM
S-750
Normalized Protein Abundance
15000
10000
5000
TSB
TSB−dex
Media
SA564
SA564−Δ*hptRS*
SA564−Δ*ccpA*
SA564−Δ*hptRS/ccpA*

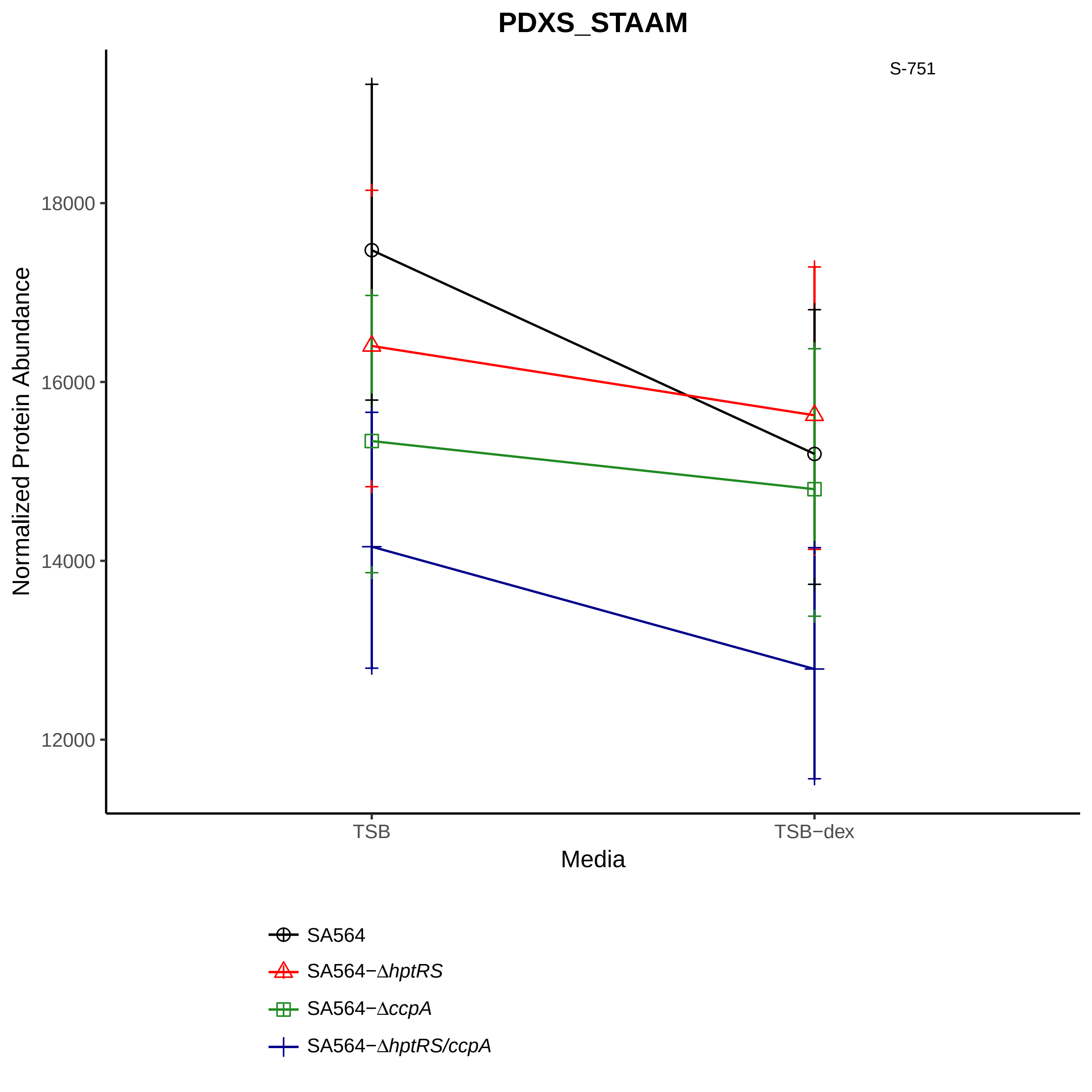
PDXS_STAAM
S-751
Normalized Protein Abundance
18000
16000
14000
12000
TSB
TSB−dex
Media
SA564
SA564−Δ*hptRS*
SA564−Δ*ccpA*
SA564−Δ*hptRS/ccpA*

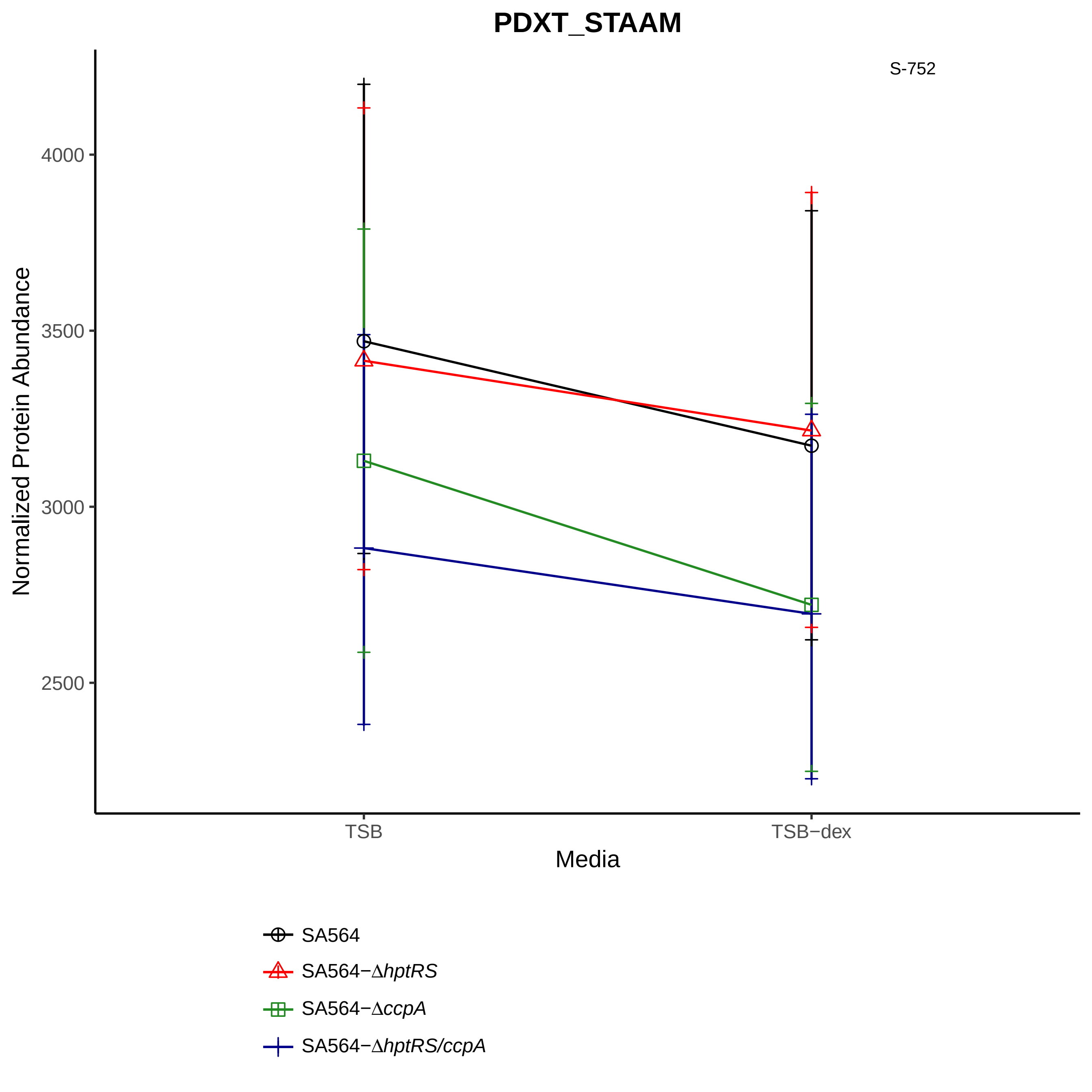

PDXT_STAAM
S-752
Normalized Protein Abundance
4000
3500
3000
2500
TSB
TSB−dex
Media
SA564
SA564−Δ*hptRS*
SA564−Δ*ccpA*
SA564−Δ*hptRS/ccpA*

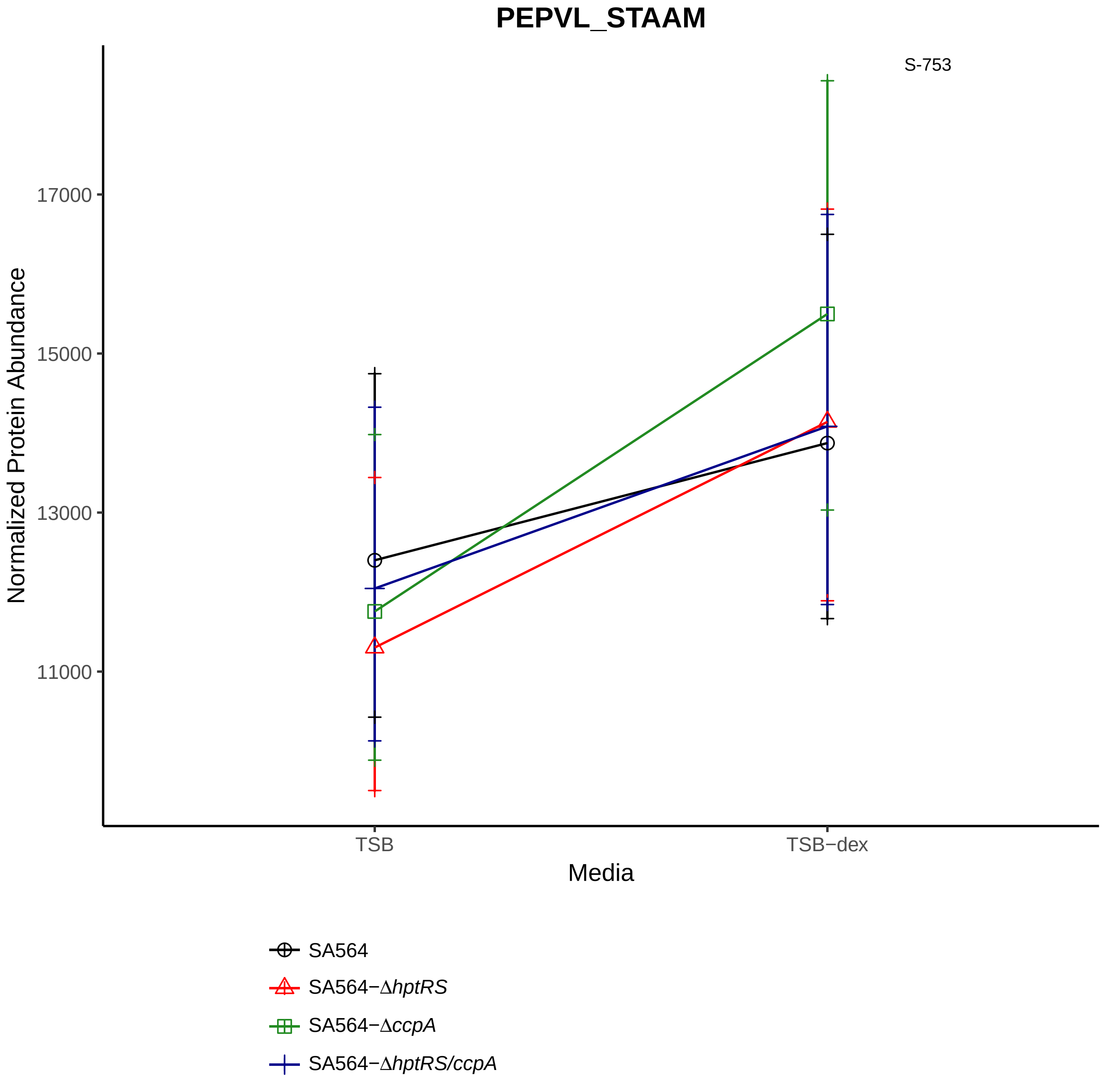

PEPVL_STAAM
S-753
Normalized Protein Abundance
17000
15000
13000
11000
TSB
TSB−dex
Media
SA564
SA564−Δ*hptRS*
SA564−Δ*ccpA*
SA564−Δ*hptRS/ccpA*

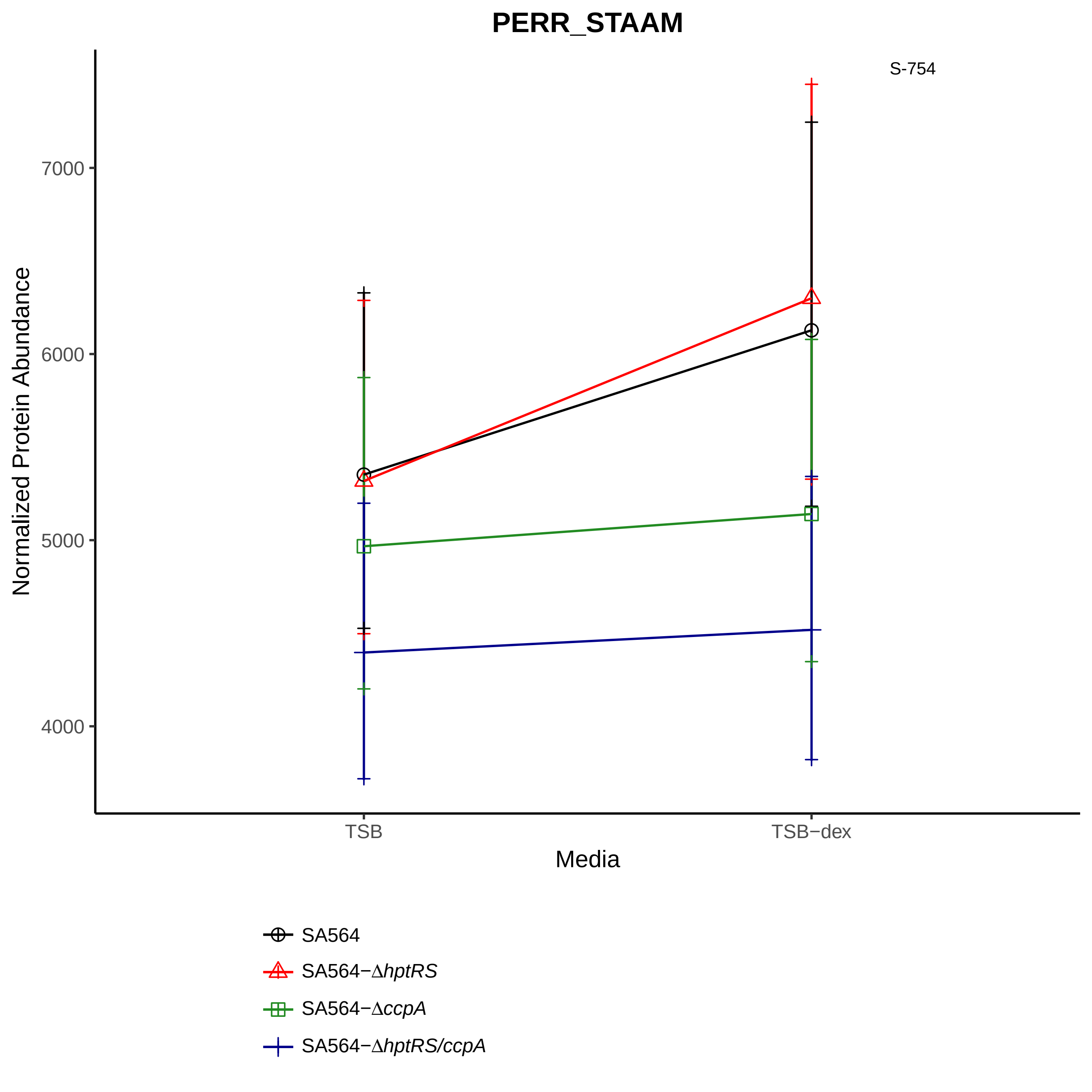

PERR_STAAM
S-754
Normalized Protein Abundance
7000
6000
5000
4000
TSB
TSB−dex
Media
SA564
SA564−Δ*hptRS*
SA564−Δ*ccpA*
SA564−Δ*hptRS/ccpA*

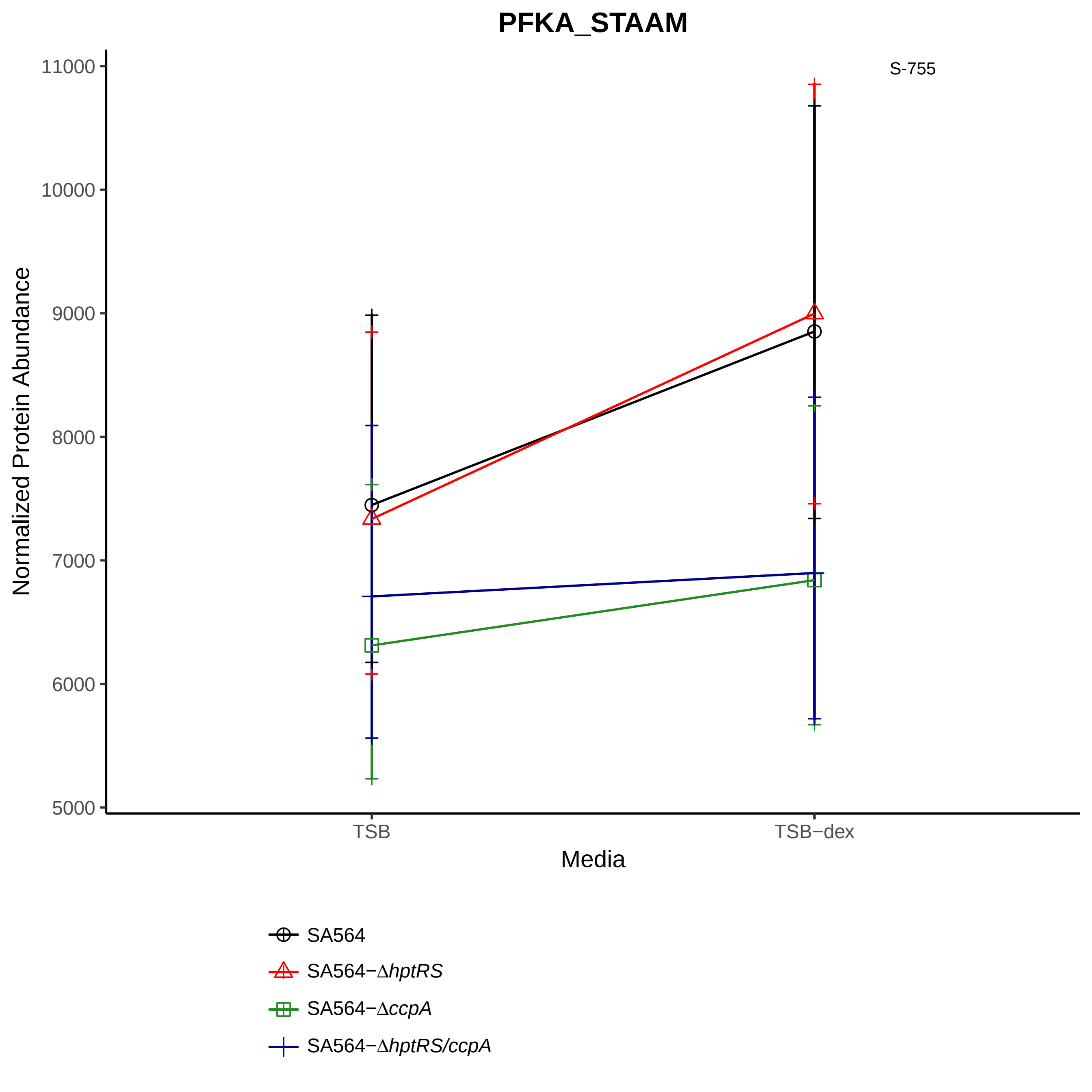
PFKA_STAAM
S-755
Normalized Protein Abundance
11000
10000
9000
8000
7000
6000
5000
TSB
TSB−dex
Media
SA564
SA564−Δ*hptRS*
SA564−Δ*ccpA*
SA564−Δ*hptRS/ccpA*

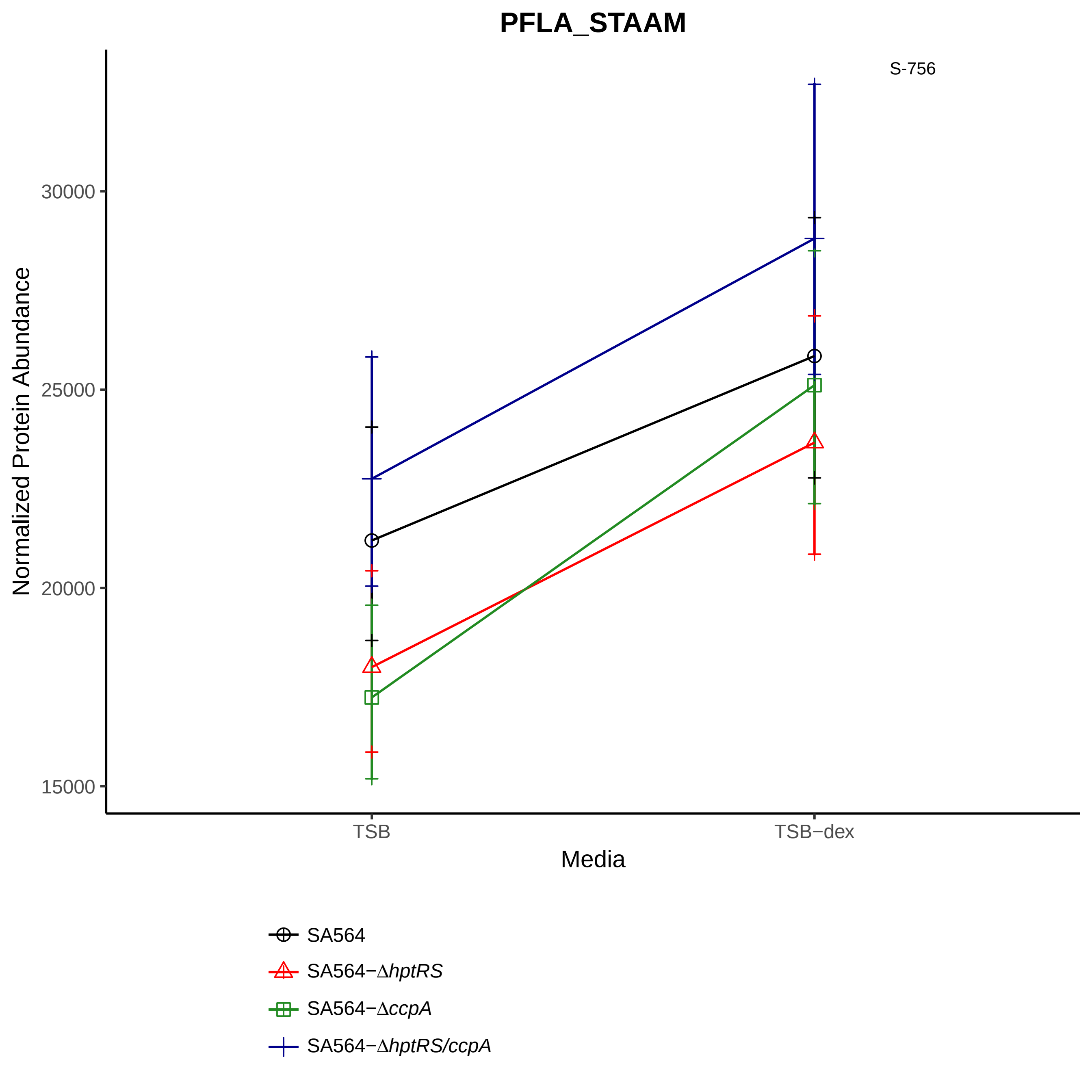

PFLA_STAAM
S-756
Normalized Protein Abundance
30000
25000
20000
15000
TSB
TSB−dex
Media
SA564
SA564−Δ*hptRS*
SA564−Δ*ccpA*
SA564−Δ*hptRS/ccpA*

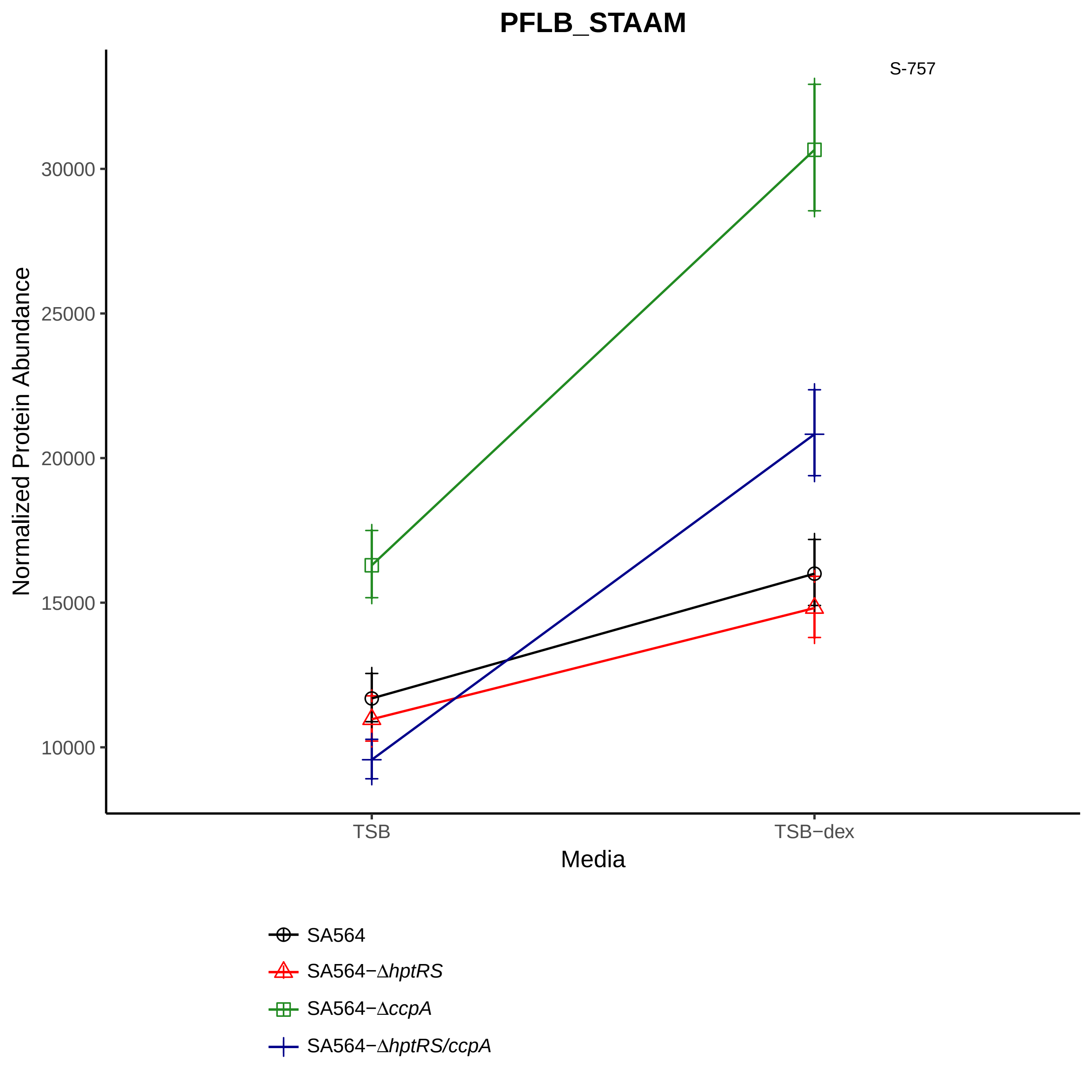

PFLB_STAAM
S-757
Normalized Protein Abundance
30000
25000
20000
15000
10000
TSB
TSB−dex
Media
SA564
SA564−Δ*hptRS*
SA564−Δ*ccpA*
SA564−Δ*hptRS/ccpA*

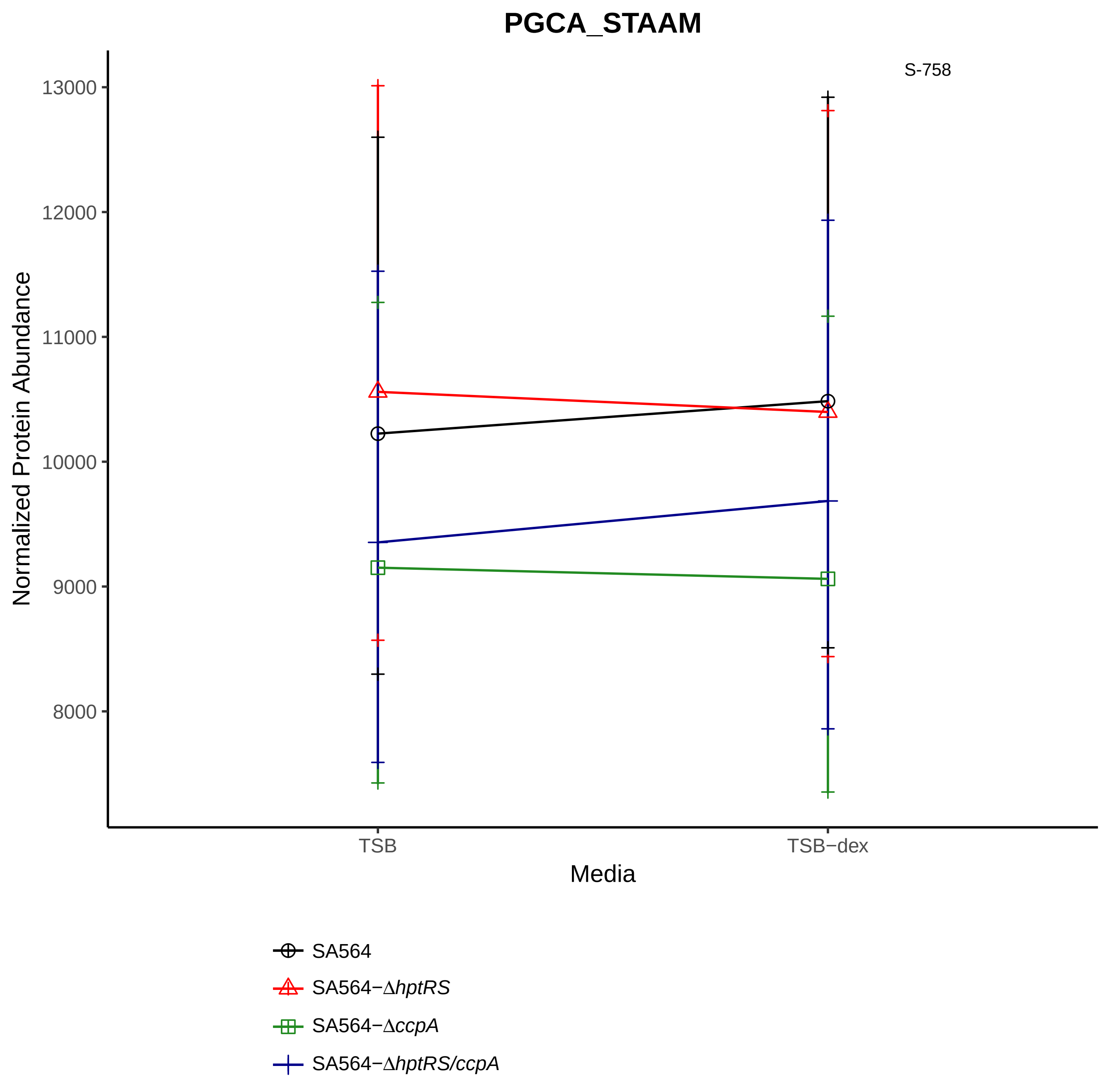

PGCA_STAAM
S-758
Normalized Protein Abundance
13000
12000
11000
10000
9000
8000
TSB
TSB−dex
Media
SA564
SA564−Δ*hptRS*
SA564−Δ*ccpA*
SA564−Δ*hptRS/ccpA*

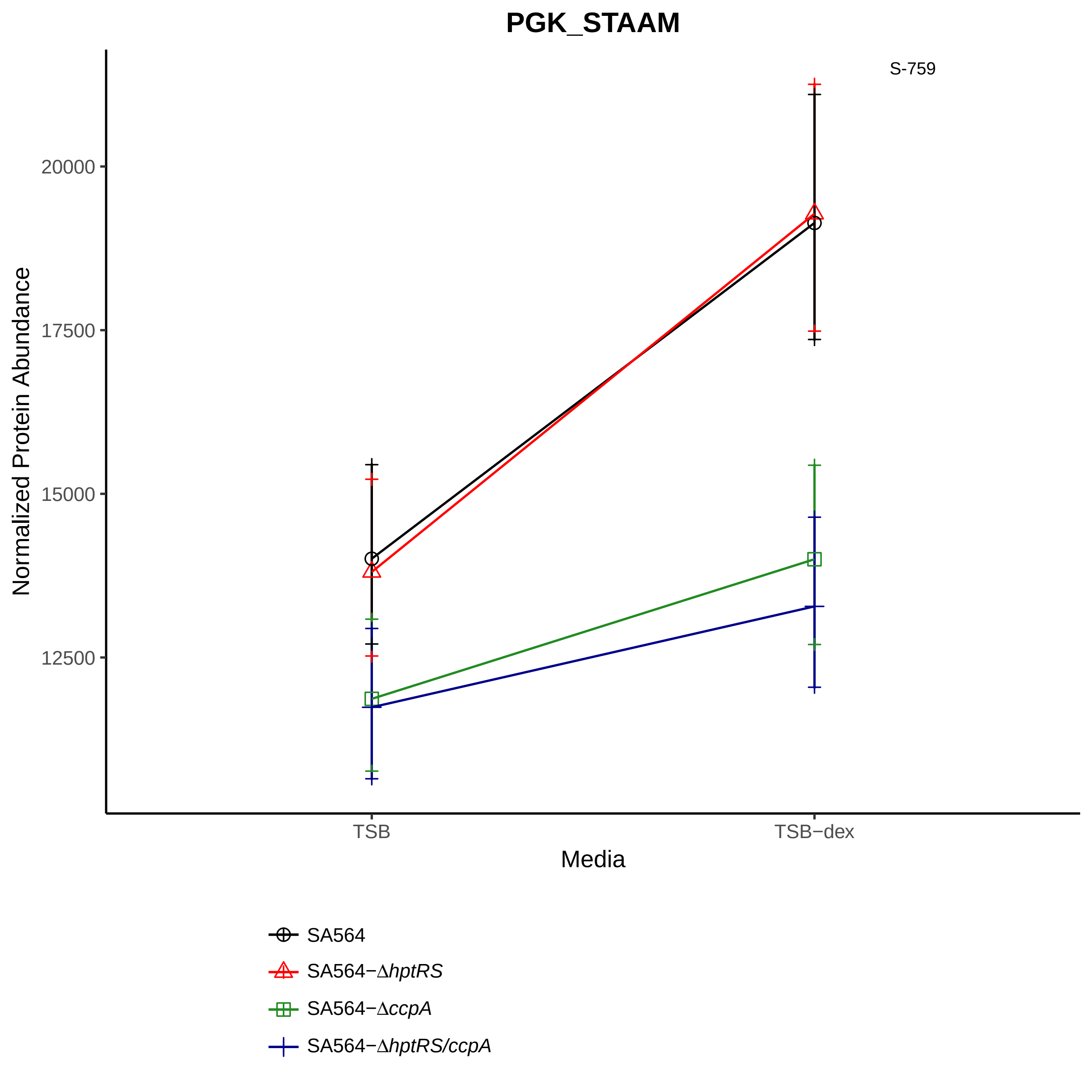
PGK_STAAM
S-759
Normalized Protein Abundance
20000
17500
15000
12500
TSB
TSB−dex
Media
SA564
SA564−Δ*hptRS*
SA564−Δ*ccpA*
SA564−Δ*hptRS/ccpA*

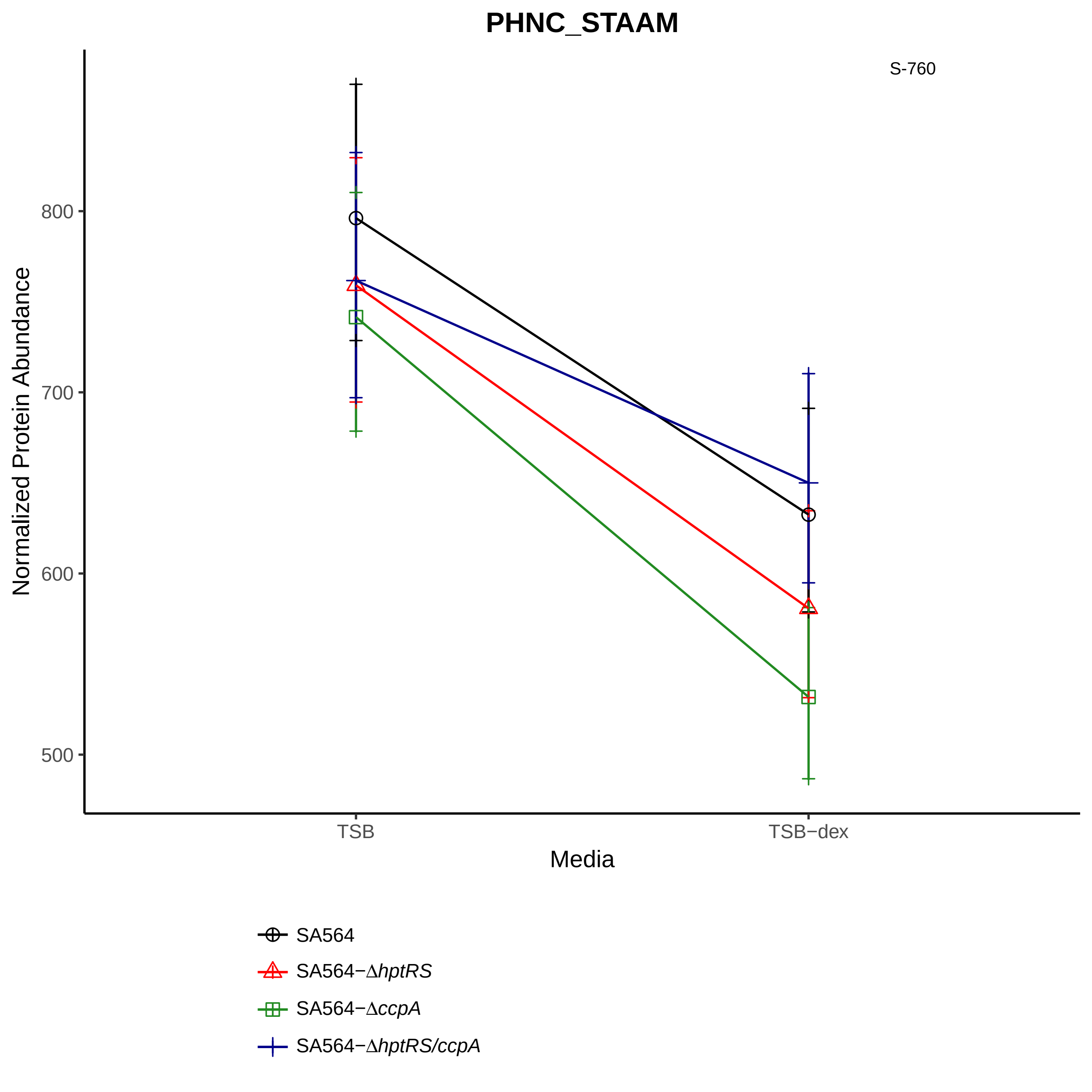

PHNC_STAAM
S-760
Normalized Protein Abundance
800
700
600
500
TSB
TSB−dex
Media
SA564
SA564−Δ*hptRS*
SA564−Δ*ccpA*
SA564−Δ*hptRS/ccpA*

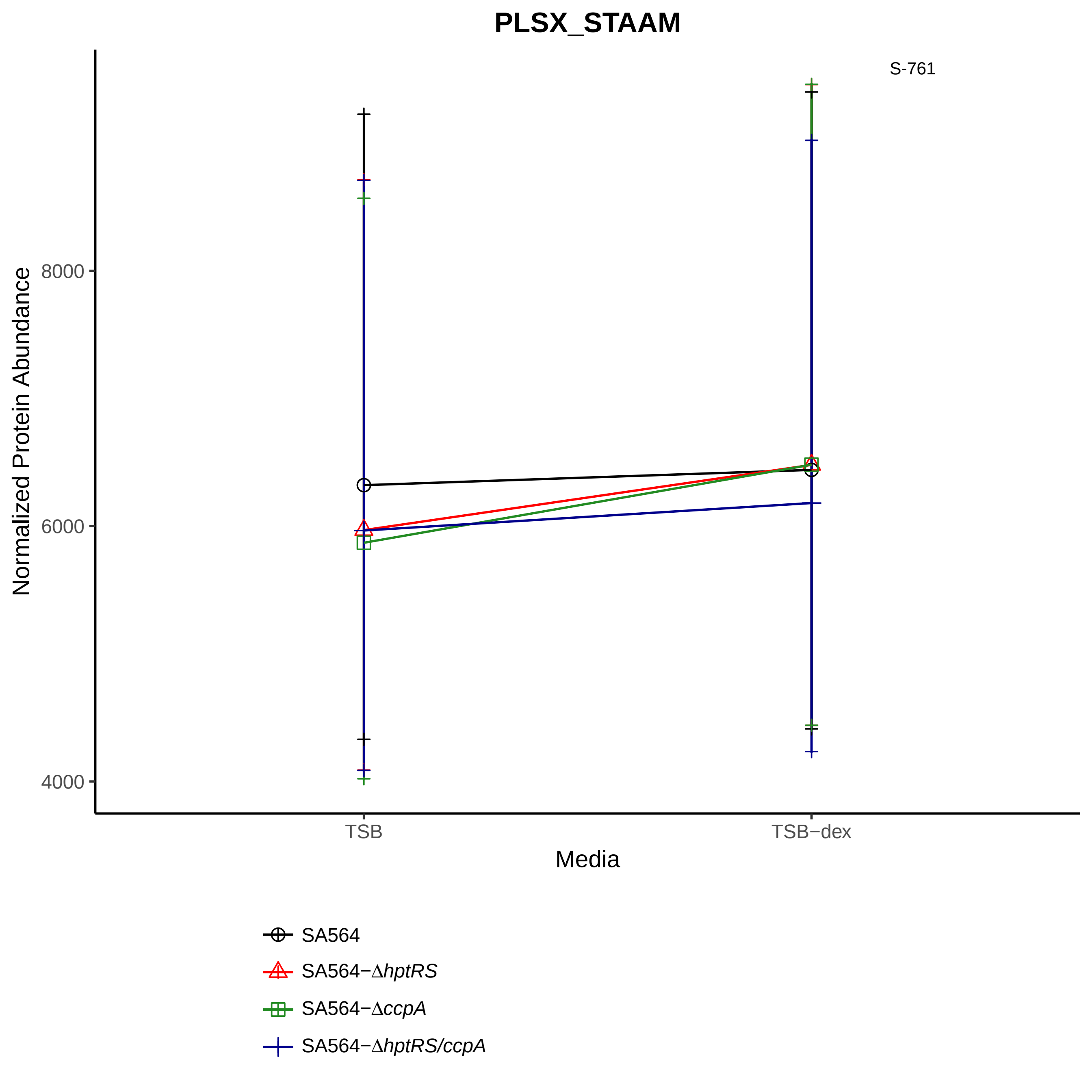

PLSX_STAAM
S-761
Normalized Protein Abundance
8000
6000
4000
TSB
TSB−dex
Media
SA564
SA564−Δ*hptRS*
SA564−Δ*ccpA*
SA564−Δ*hptRS/ccpA*

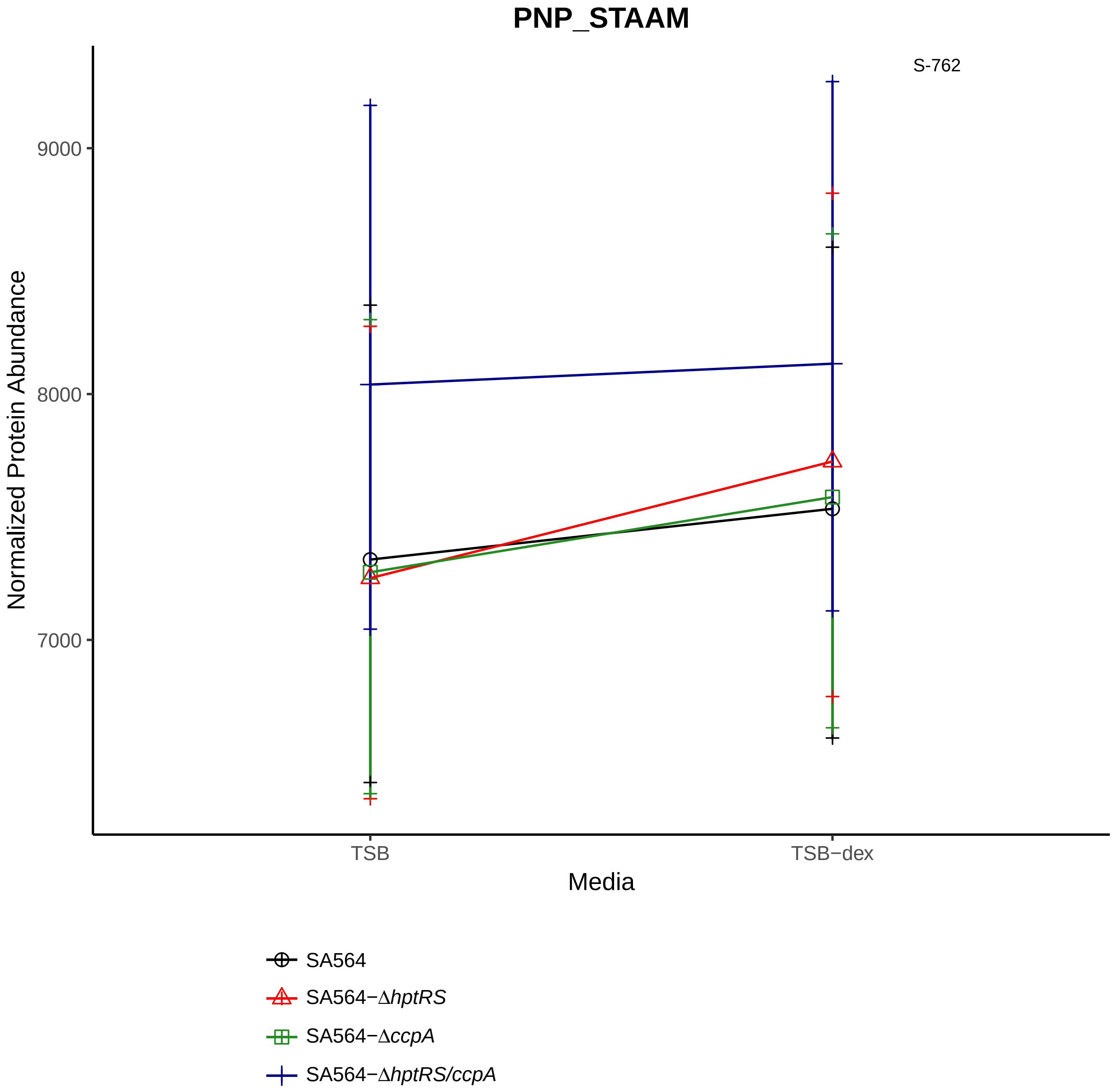

PNP_STAAM
S-762
9000
8000
7000
Normalized Protein Abundance
TSB
TSB−dex
Media
SA564
SA564−Δ*hptRS*
SA564−Δ*ccpA*
SA564−Δ*hptRS/ccpA*

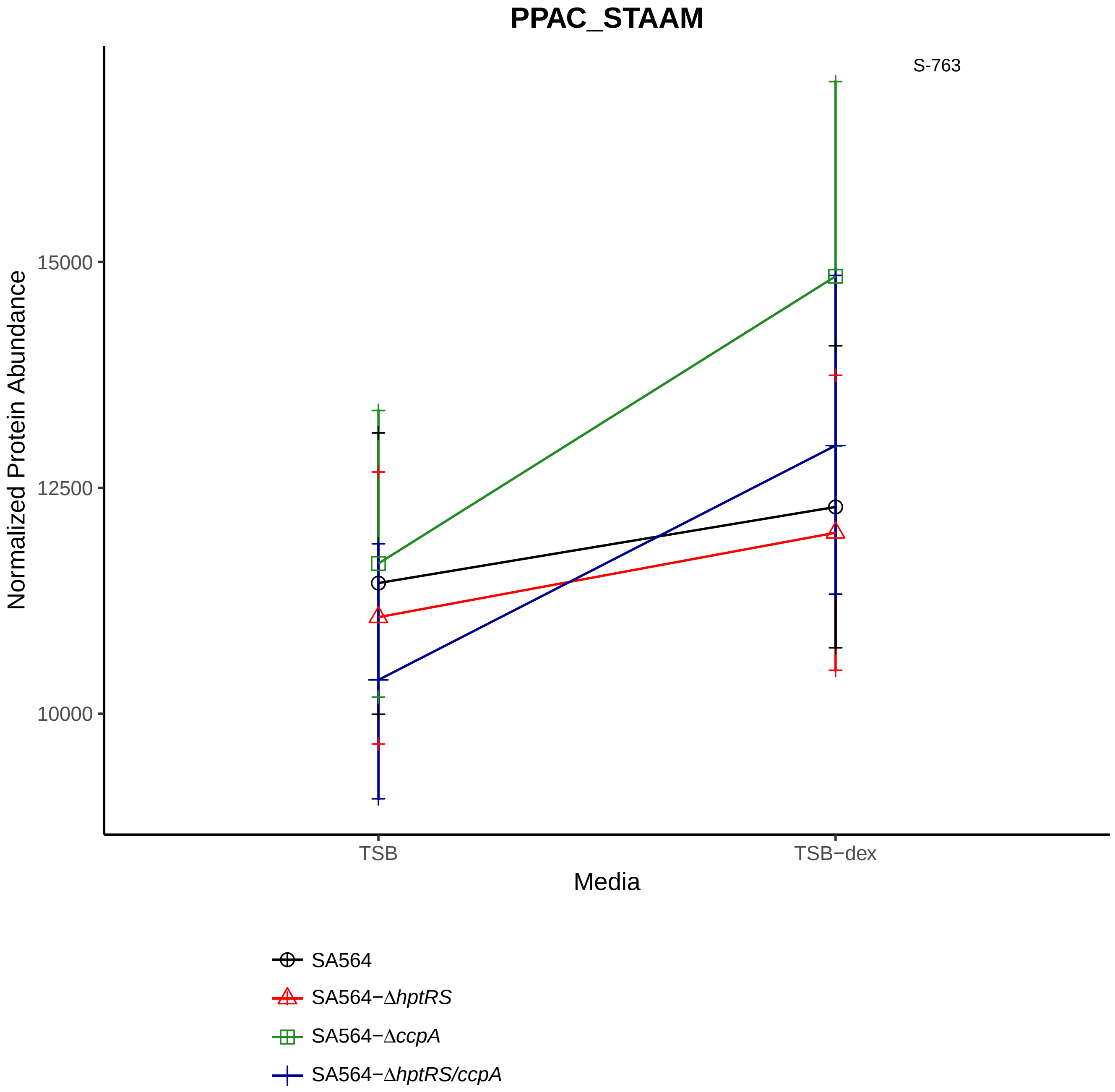
PPAC_STAAM
S-763
Normalized Protein Abundance
15000
12500
10000
TSB
TSB−dex
Media
SA564
SA564−Δ*hptRS*
SA564−Δ*ccpA*
SA564−Δ*hptRS/ccpA*

PPAT_STAAM

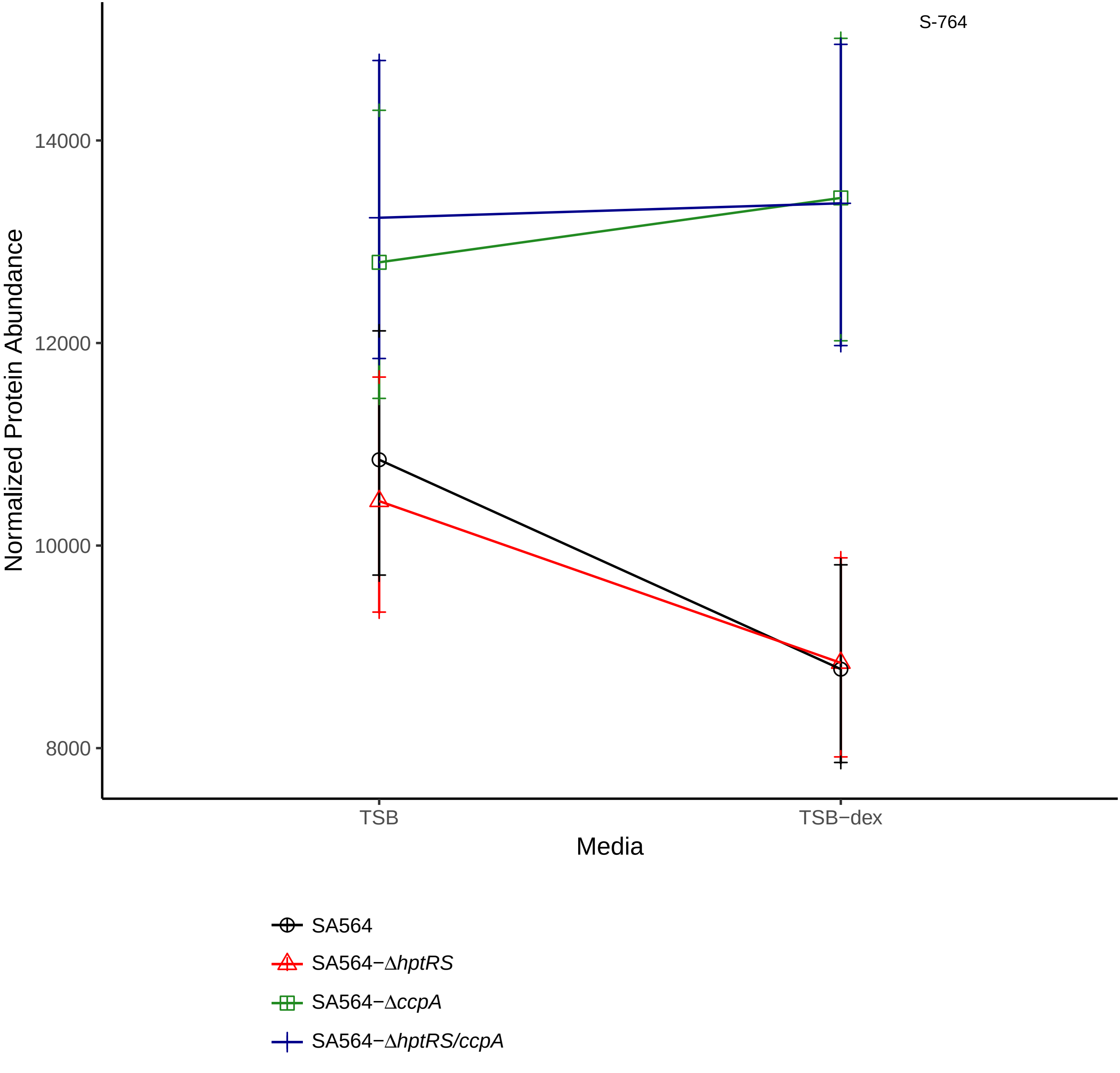

S-764
Normalized Protein Abundance
14000
12000
10000
8000
TSB
TSB−dex
Media
SA564
SA564−Δ*hptRS*
SA564−Δ*ccpA*
SA564−Δ*hptRS/ccpA*

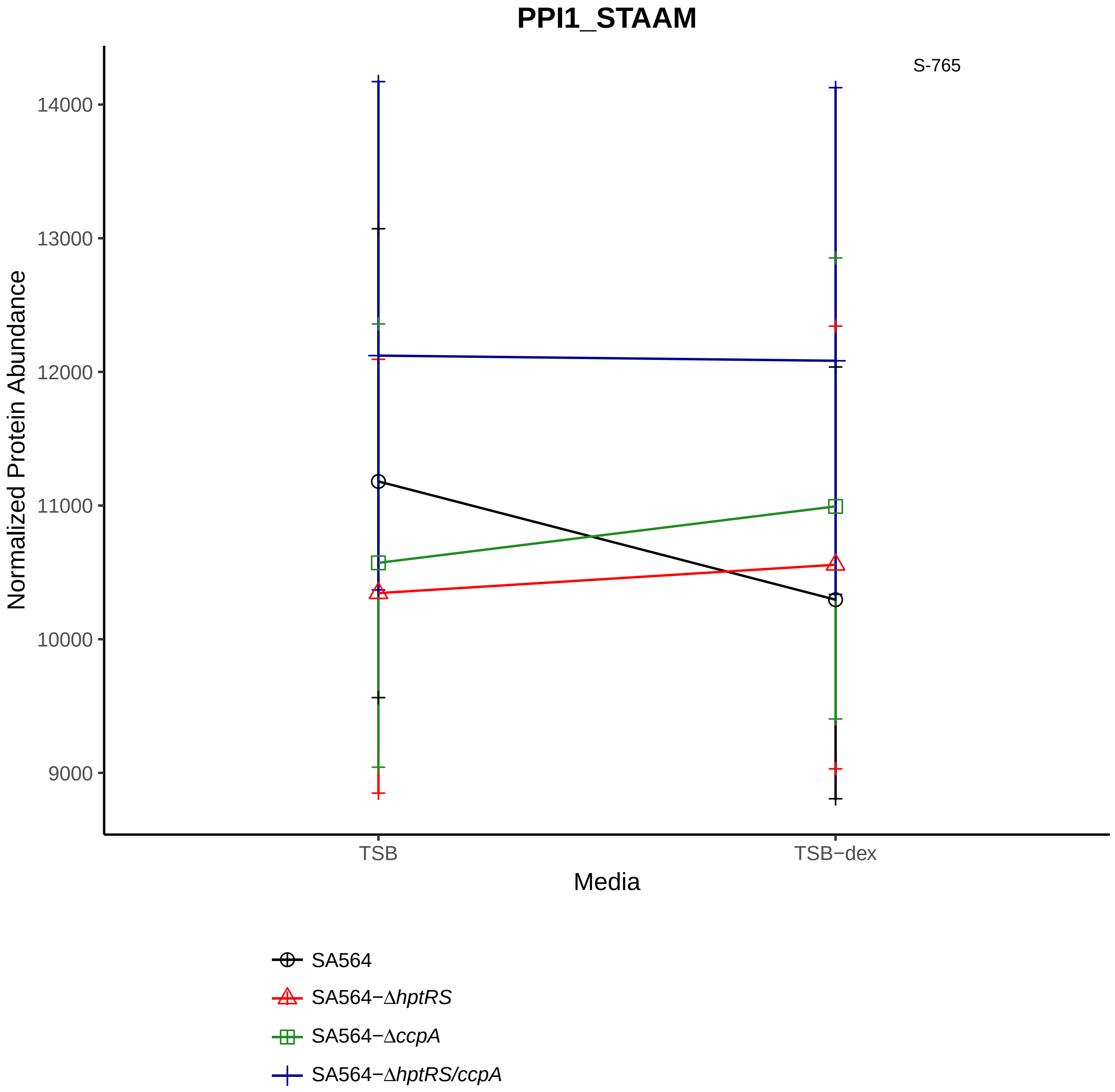

PPI1_STAAM
S-765
Normalized Protein Abundance
14000
13000
12000
11000
10000
9000
TSB
TSB−dex
Media
SA564
SA564−Δ*hptRS*
SA564−Δ*ccpA*
SA564−Δ*hptRS/ccpA*

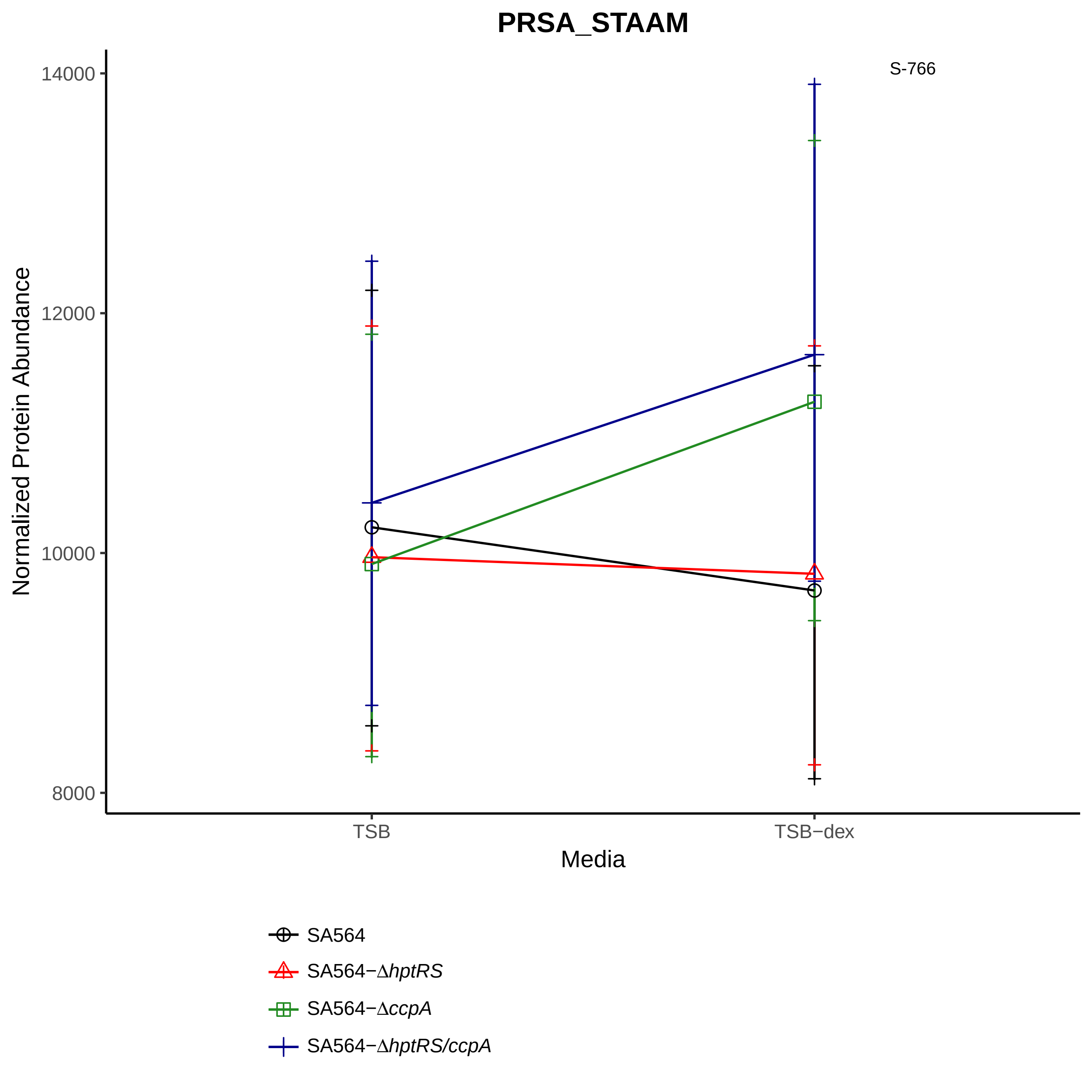

PRSA_STAAM
S-766
Normalized Protein Abundance
14000
12000
10000
8000
TSB
TSB−dex
Media
SA564
SA564−Δ*hptRS*
SA564−Δ*ccpA*
SA564−Δ*hptRS/ccpA*

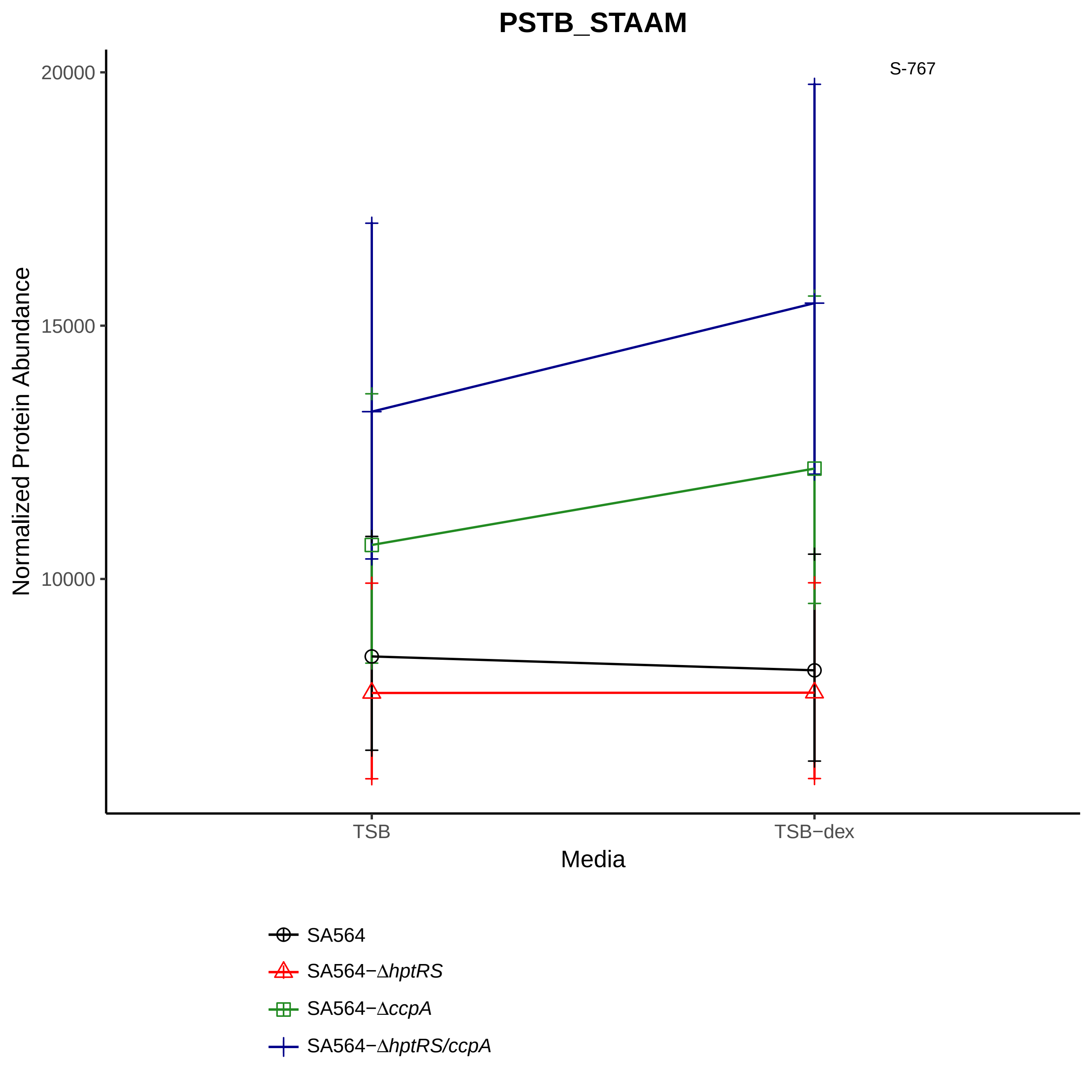

PSTB_STAAM
S-767
Normalized Protein Abundance
20000
15000
10000
TSB
TSB−dex
Media
SA564
SA564−ΔhptRS
SA564−ΔccpA
SA564−ΔhptRS/ccpA

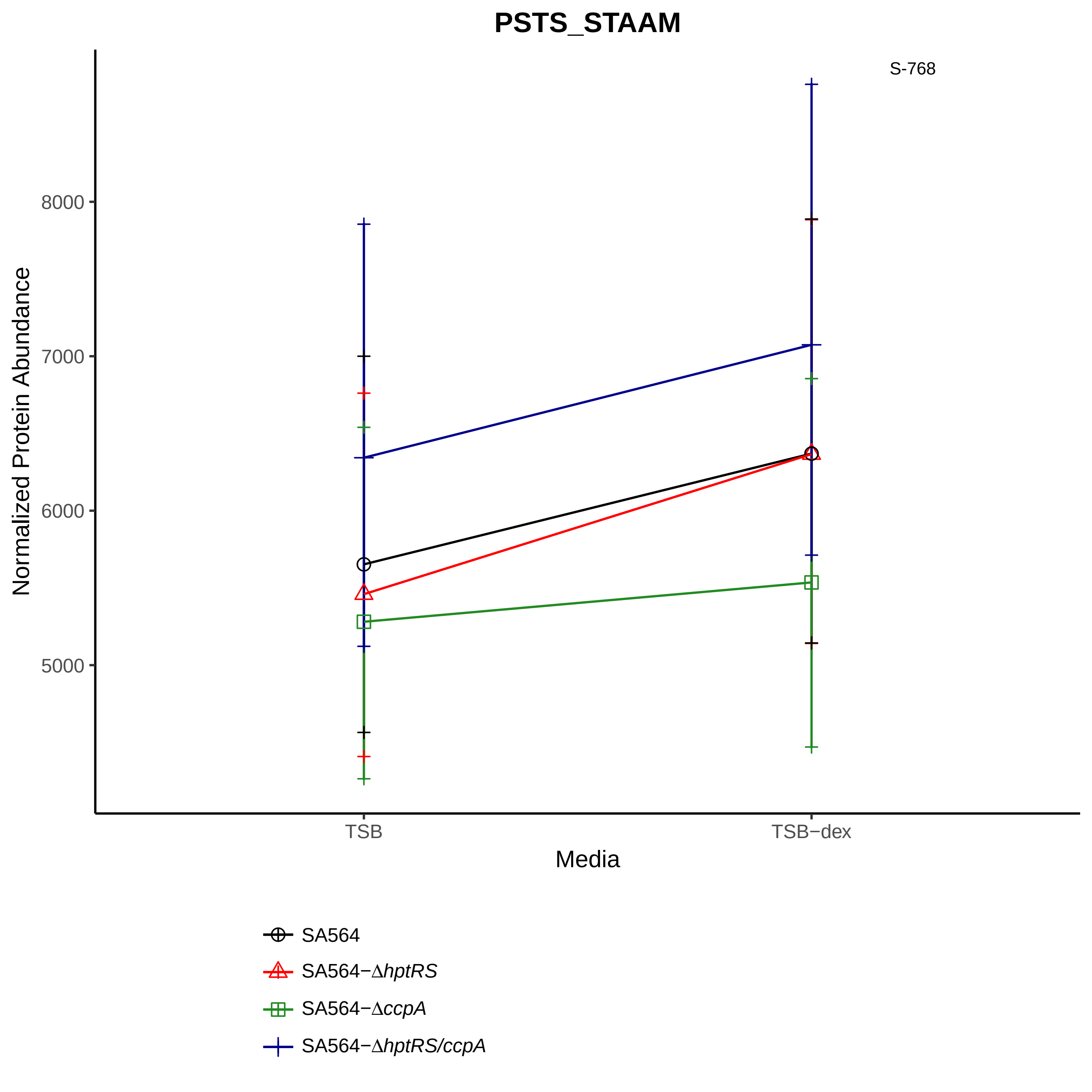
PSTS_STAAM
S-768
Normalized Protein Abundance
8000
7000
6000
5000
TSB
TSB−dex
Media
SA564
SA564−ΔhptRS
SA564−ΔccpA
SA564−ΔhptRS/ccpA

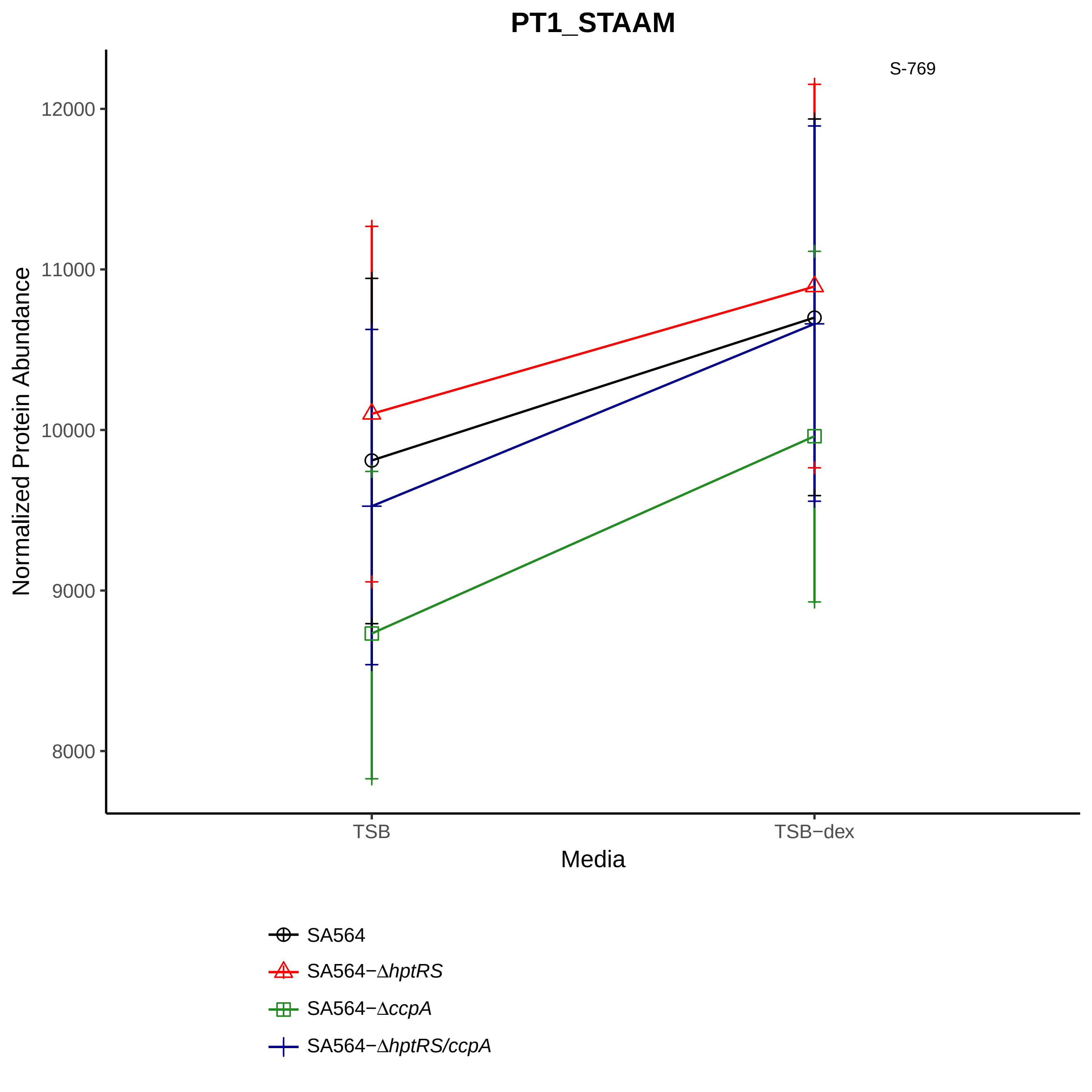
PT1_STAAM
S-769
Normalized Protein Abundance
12000
11000
10000
9000
8000
TSB
TSB−dex
Media
SA564
SA564−ΔhptRS
SA564−ΔccpA
SA564−ΔhptRS/ccpA

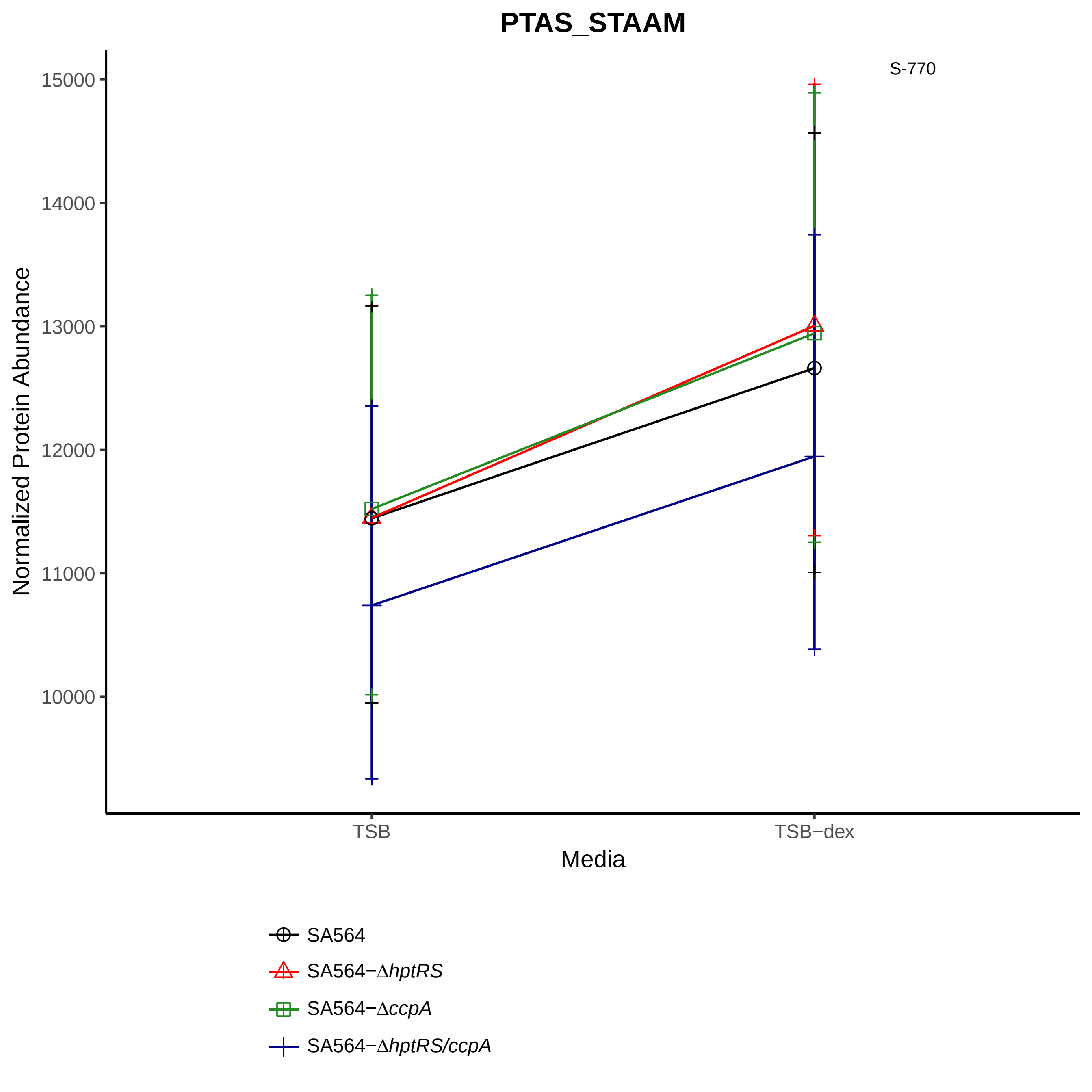

PTAS_STAAM
S-770
Normalized Protein Abundance
15000
14000
13000
12000
11000
10000
TSB
TSB−dex
Media
SA564
SA564−Δ*hptRS*
SA564−Δ*ccpA*
SA564−Δ*hptRS/ccpA*

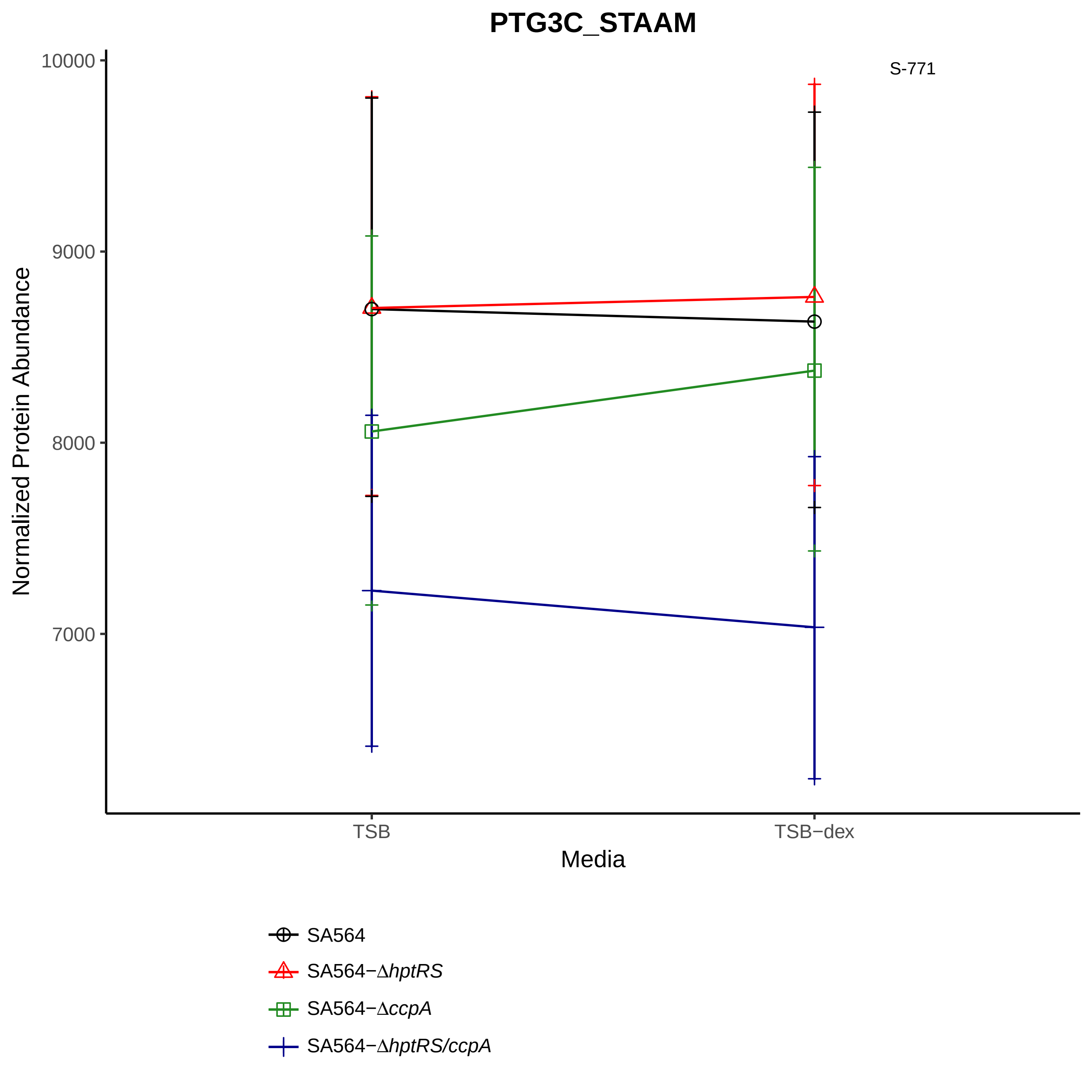

PTG3C_STAAM
S-771
Normalized Protein Abundance
10000
9000
8000
7000
TSB
TSB−dex
Media
SA564
SA564−Δ*hptRS*
SA564−Δ*ccpA*
SA564−Δ*hptRS/ccpA*

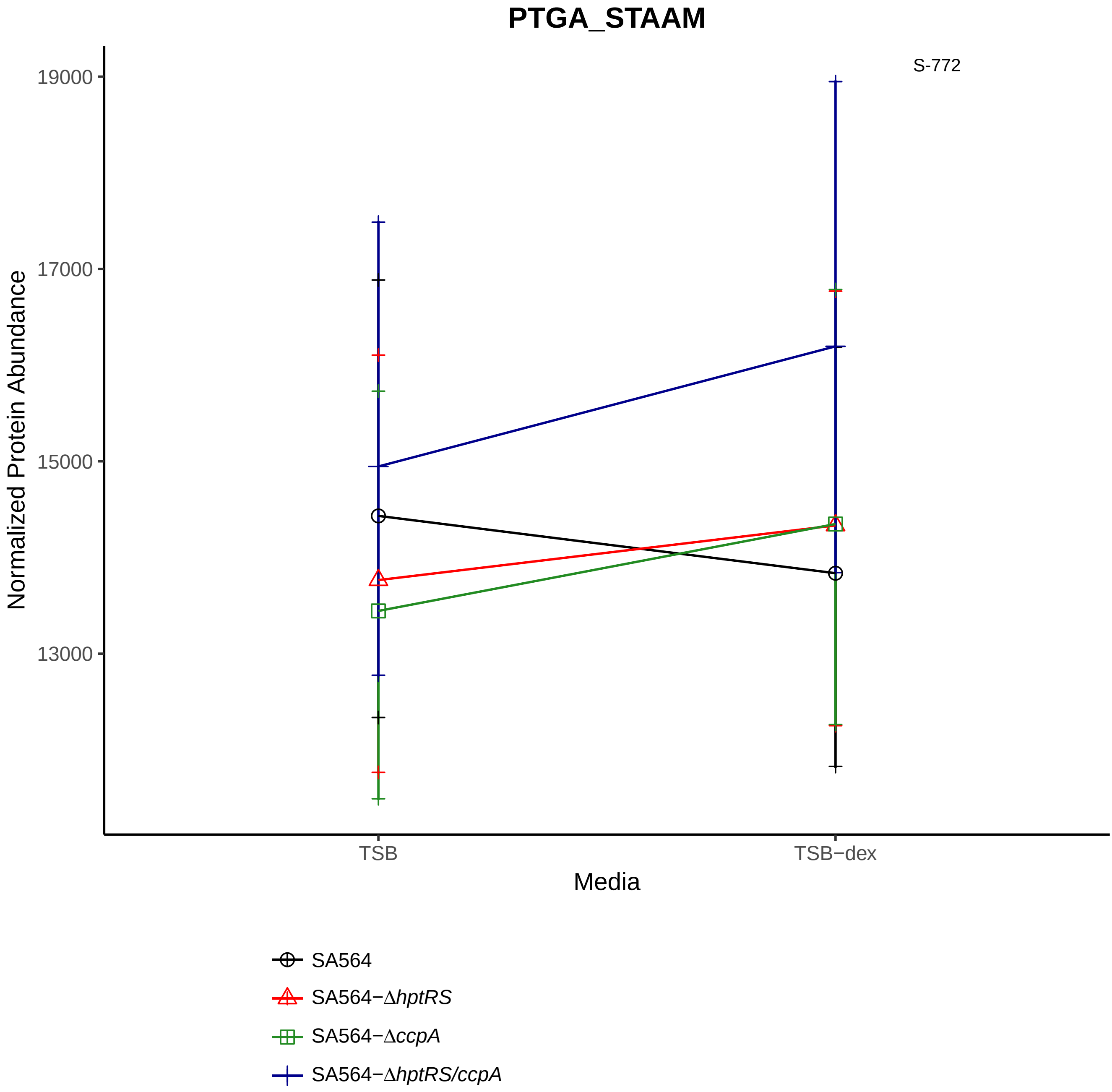

PTGA_STAAM
S-772
19000
17000
15000
13000
Normalized Protein Abundance
TSB
TSB−dex
Media
SA564
SA564−Δ*hptRS*
SA564−Δ*ccpA*
SA564−Δ*hptRS/ccpA*

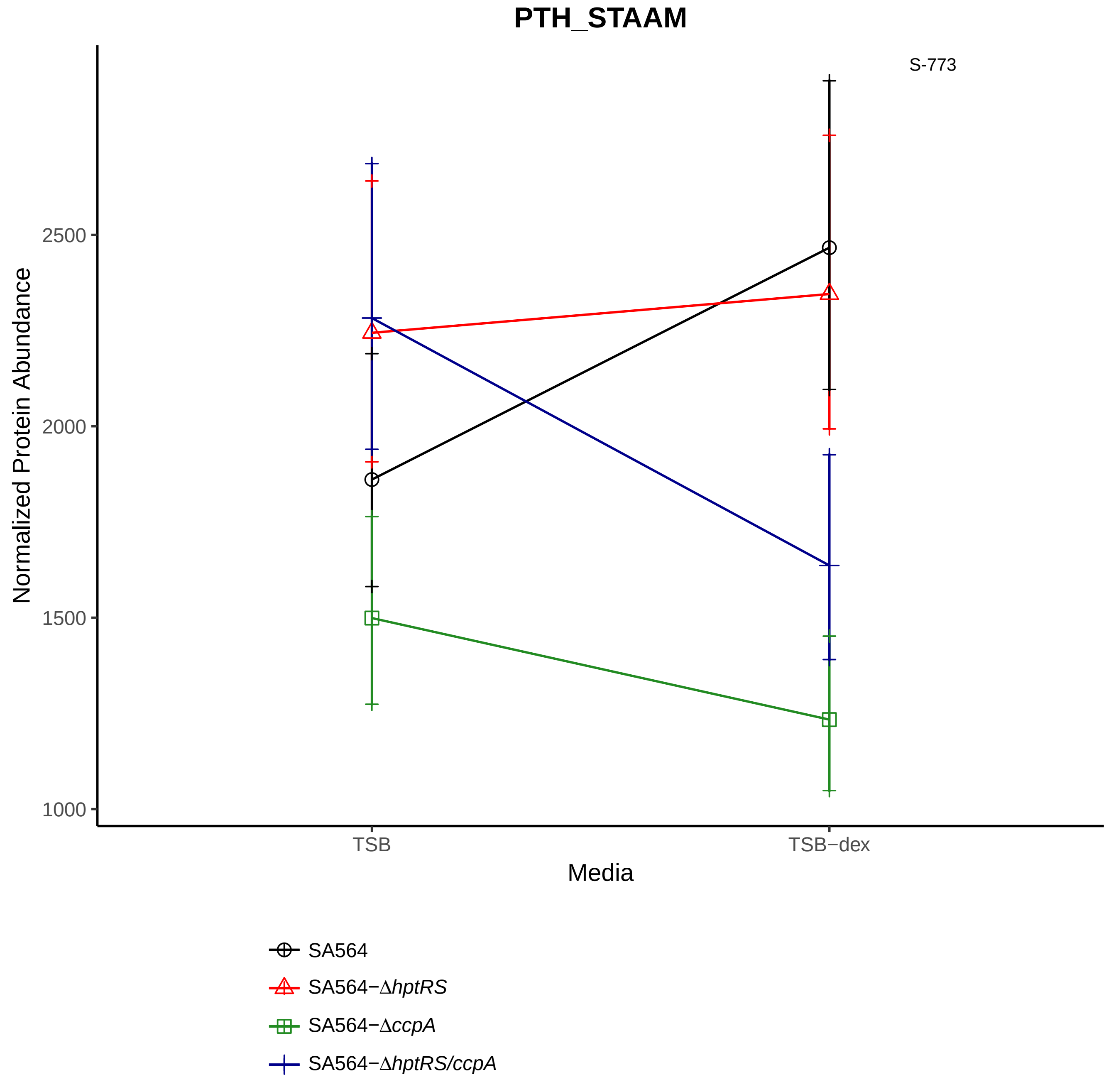

PTH_STAAM
S-773
Normalized Protein Abundance
2500
2000
1500
1000
TSB
TSB−dex
Media
SA564
SA564−Δ*hptRS*
SA564−Δ*ccpA*
SA564−Δ*hptRS/ccpA*

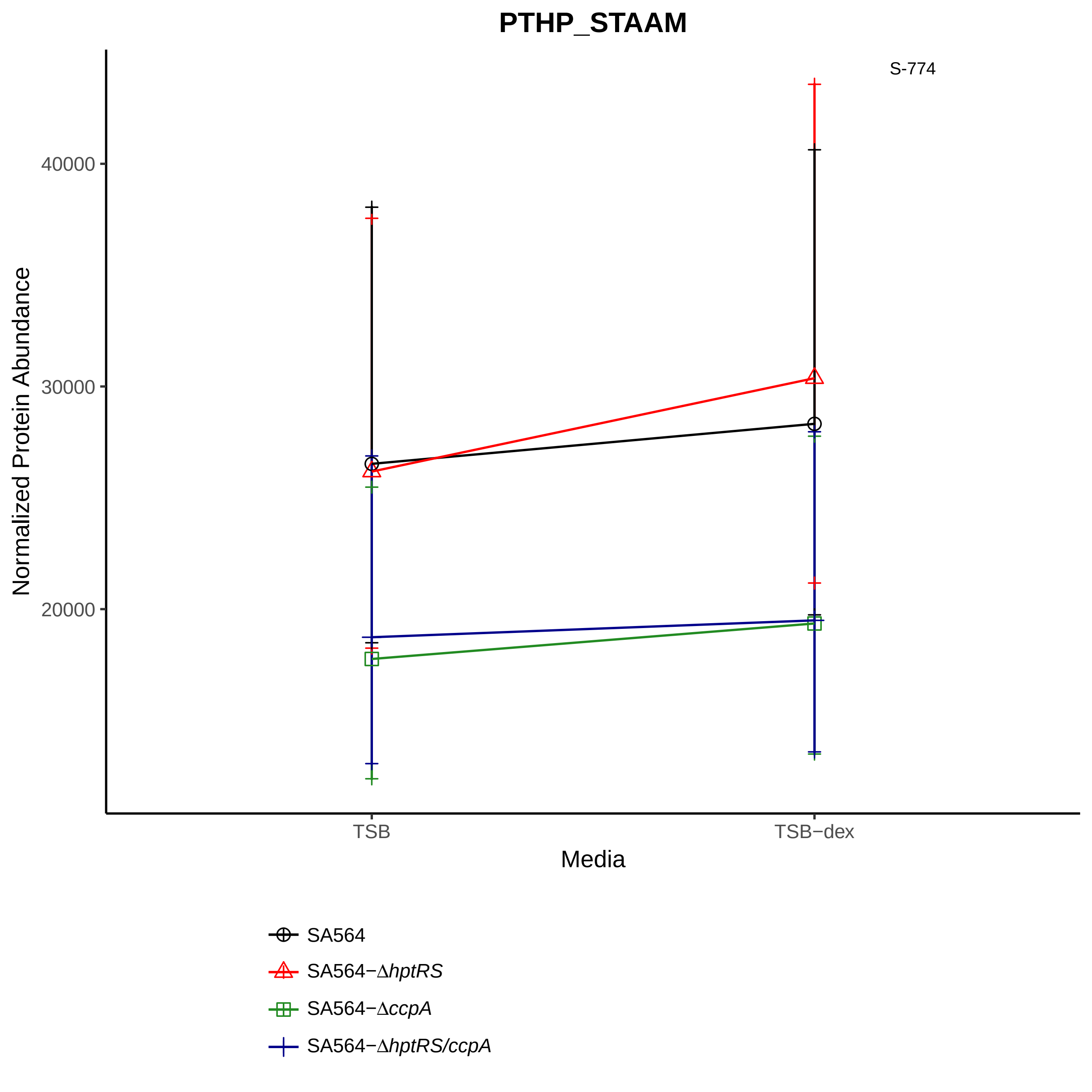

PTHP_STAAM
S-774
Normalized Protein Abundance
40000
30000
20000
TSB
TSB−dex
Media
SA564
SA564−Δ*hptRS*
SA564−Δ*ccpA*
SA564−Δ*hptRS/ccpA*

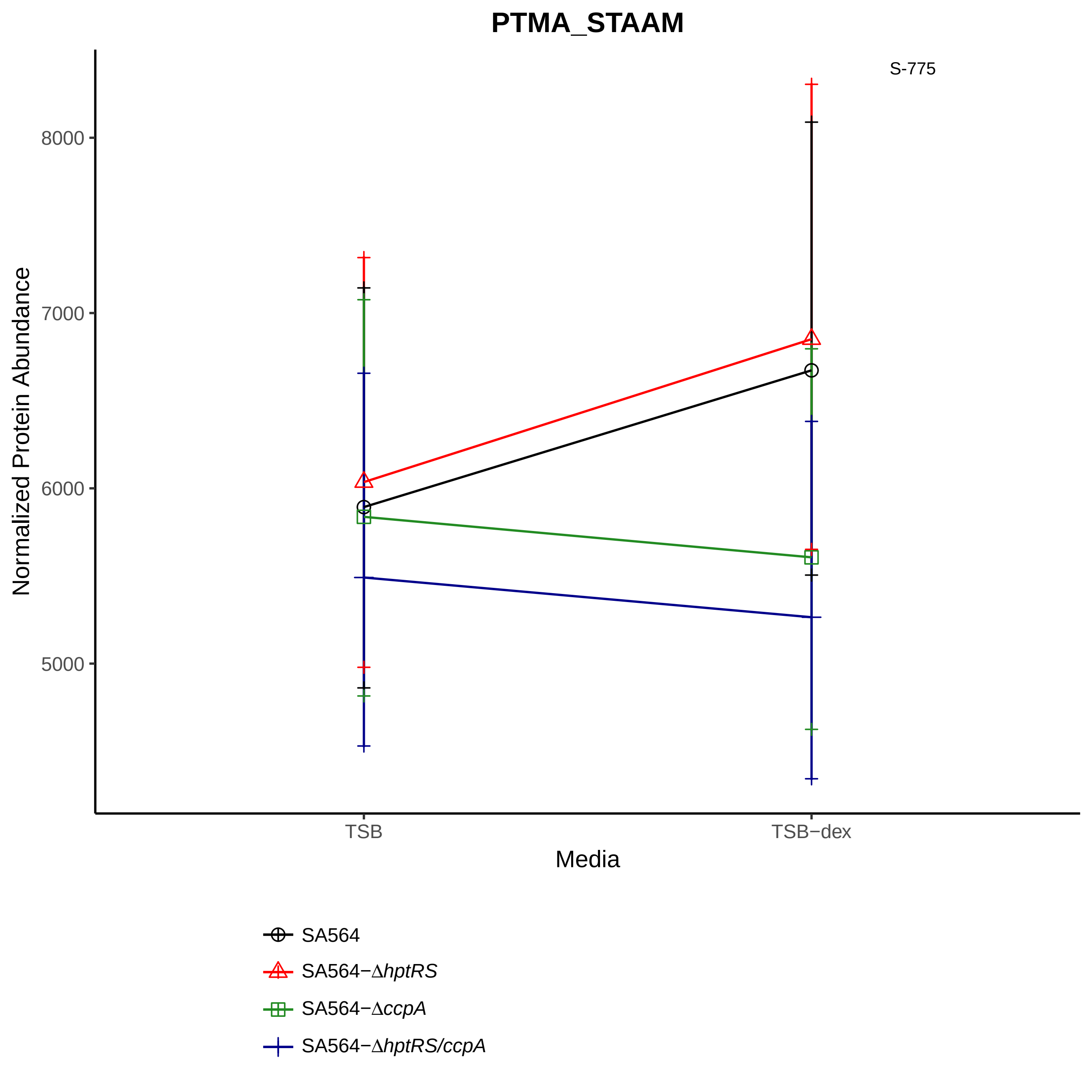

PTMA_STAAM
S-775
Normalized Protein Abundance
8000
7000
6000
5000
TSB
TSB−dex
Media
SA564
SA564−Δ*hptRS*
SA564−Δ*ccpA*
SA564−Δ*hptRS/ccpA*

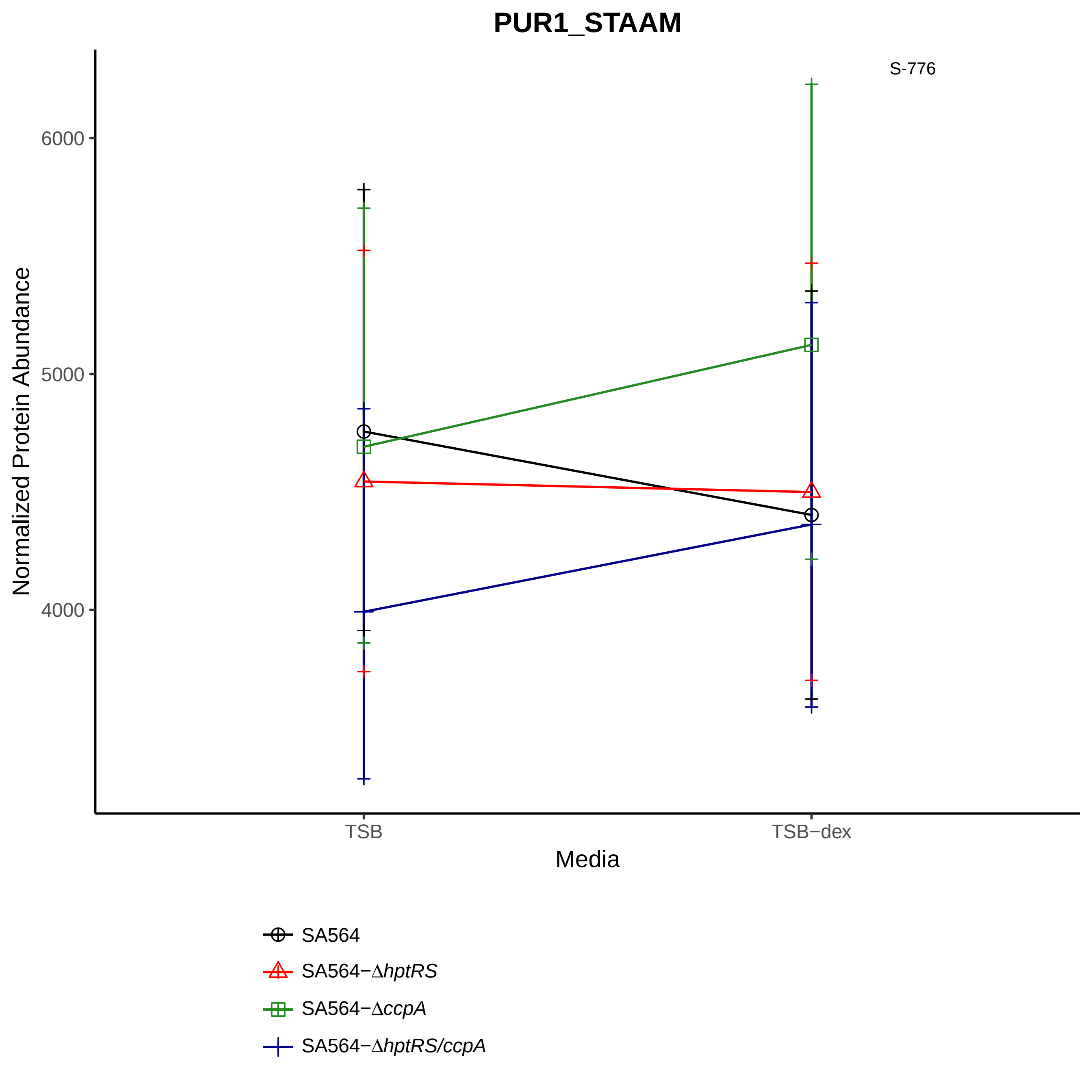
PUR1_STAAM
S-776
Normalized Protein Abundance
6000
5000
4000
TSB
TSB−dex
Media
SA564
SA564−Δ*hptRS*
SA564−Δ*ccpA*
SA564−Δ*hptRS/ccpA*

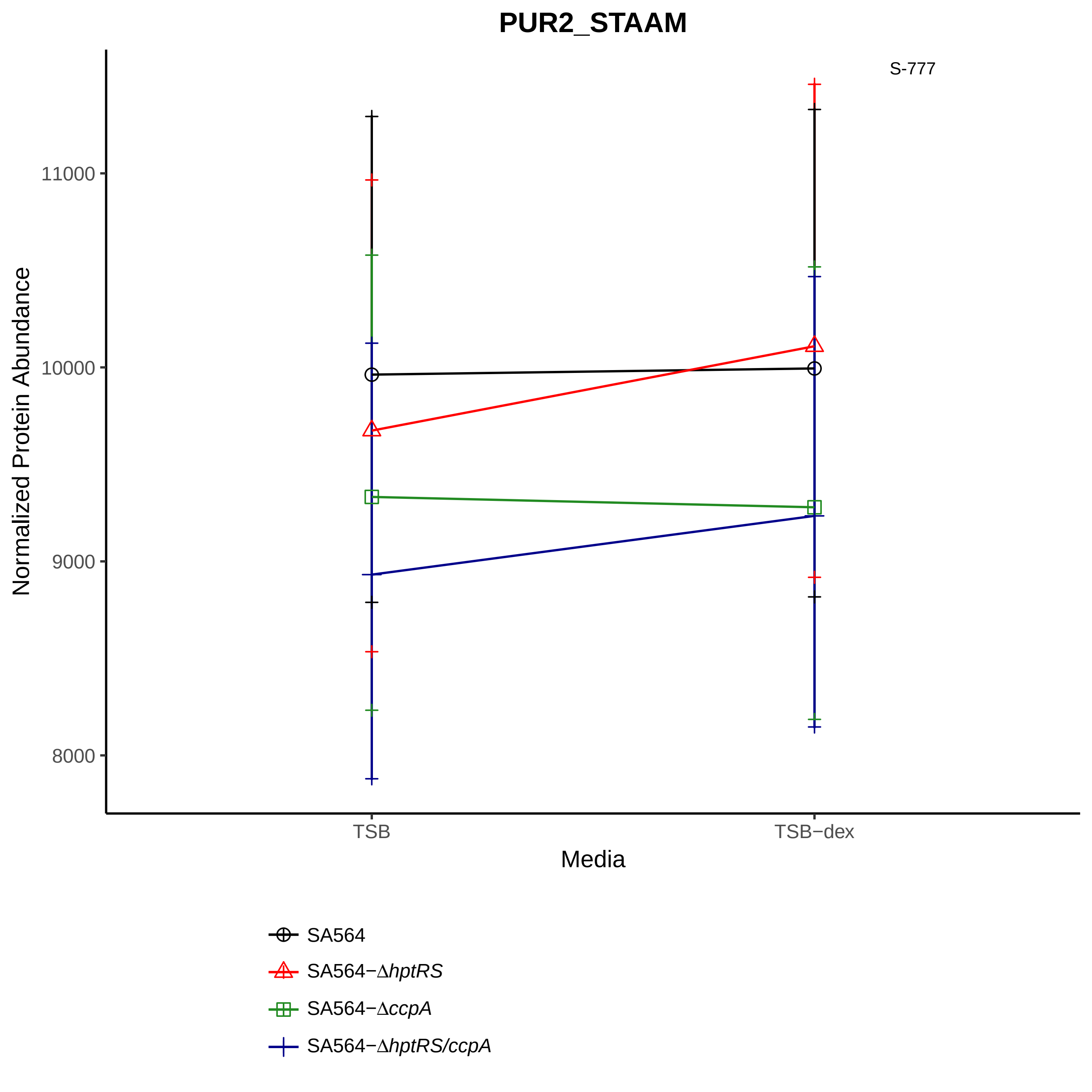

PUR2_STAAM
S-777
Normalized Protein Abundance
11000
10000
9000
8000
TSB
TSB−dex
Media
SA564
SA564−ΔhptRS
SA564−ΔccpA
SA564−ΔhptRS/ccpA

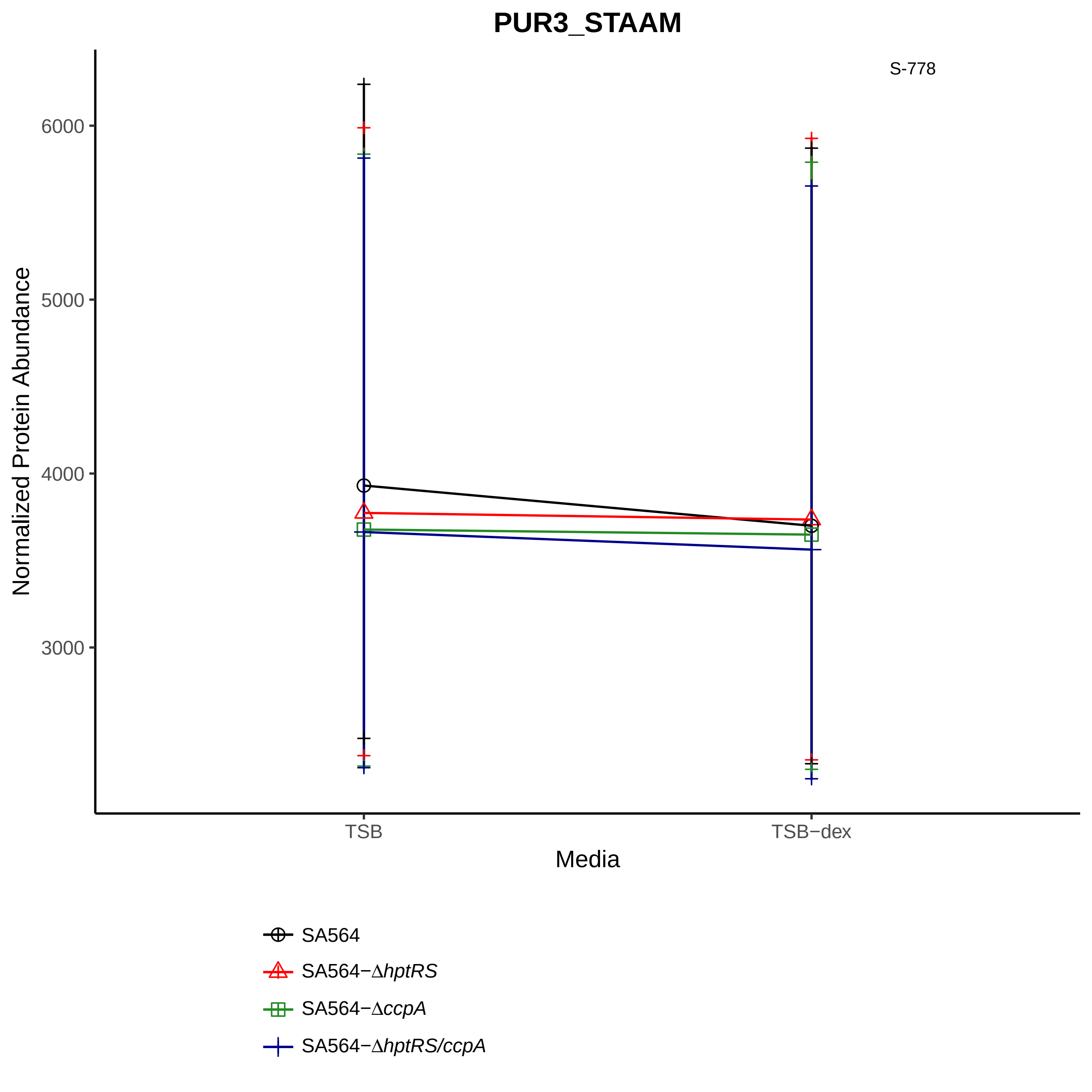
PUR3_STAAM
S-778
Normalized Protein Abundance
6000
5000
4000
3000
TSB
TSB−dex
Media
SA564
SA564−ΔhptRS
SA564−ΔccpA
SA564−ΔhptRS/ccpA

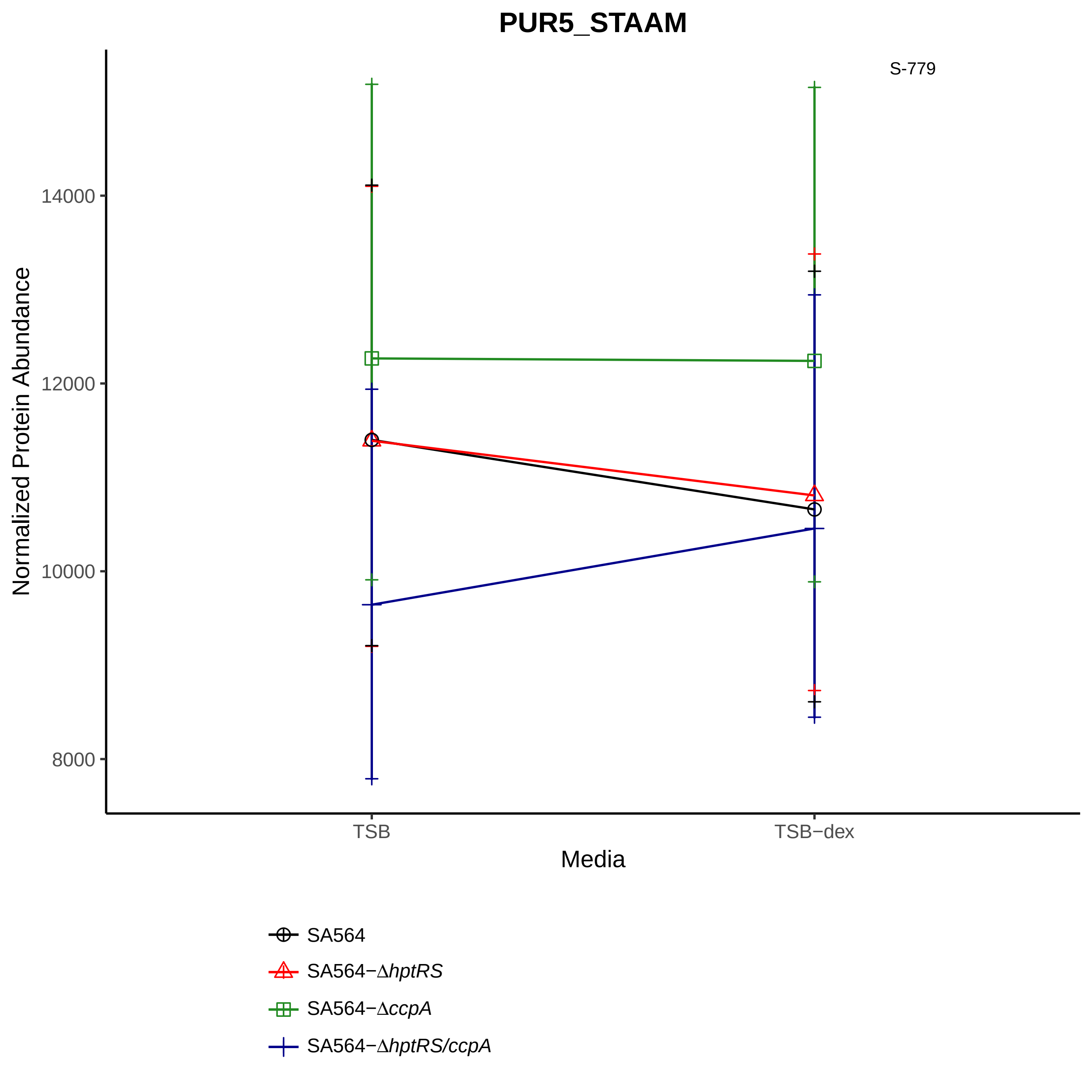

PUR5_STAAM
S-779
Normalized Protein Abundance
14000
12000
10000
8000
TSB
TSB−dex
Media
SA564
SA564−ΔhptRS
SA564−ΔccpA
SA564−ΔhptRS/ccpA

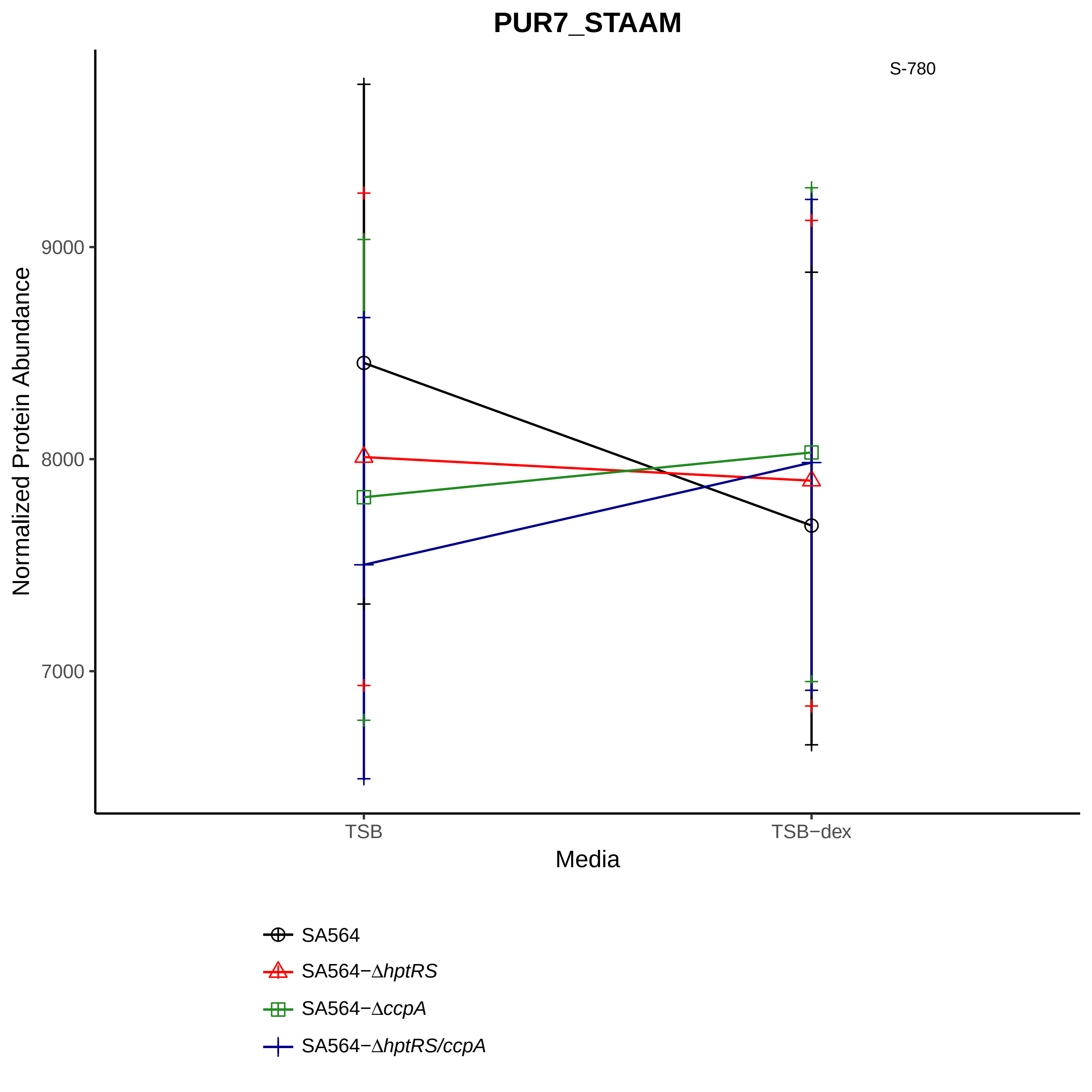

PUR7_STAAM
S-780
Normalized Protein Abundance
9000
8000
7000
TSB
TSB−dex
Media
SA564
SA564−Δ*hptRS*
SA564−Δ*ccpA*
SA564−Δ*hptRS/ccpA*

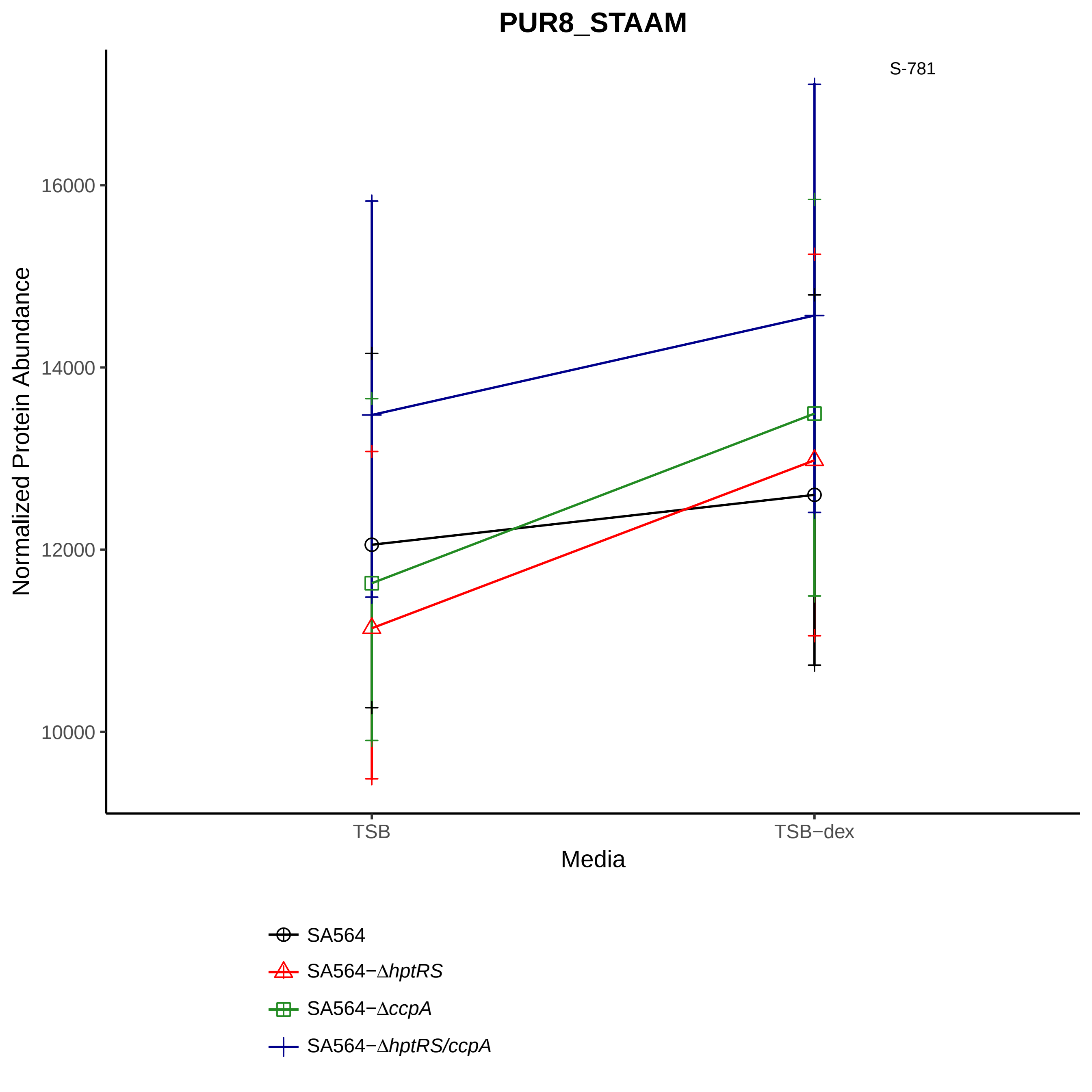
PUR8_STAAM
S-781
Normalized Protein Abundance
16000
14000
12000
10000
TSB
TSB−dex
Media
SA564
SA564−Δ*hptRS*
SA564−Δ*ccpA*
SA564−Δ*hptRS/ccpA*

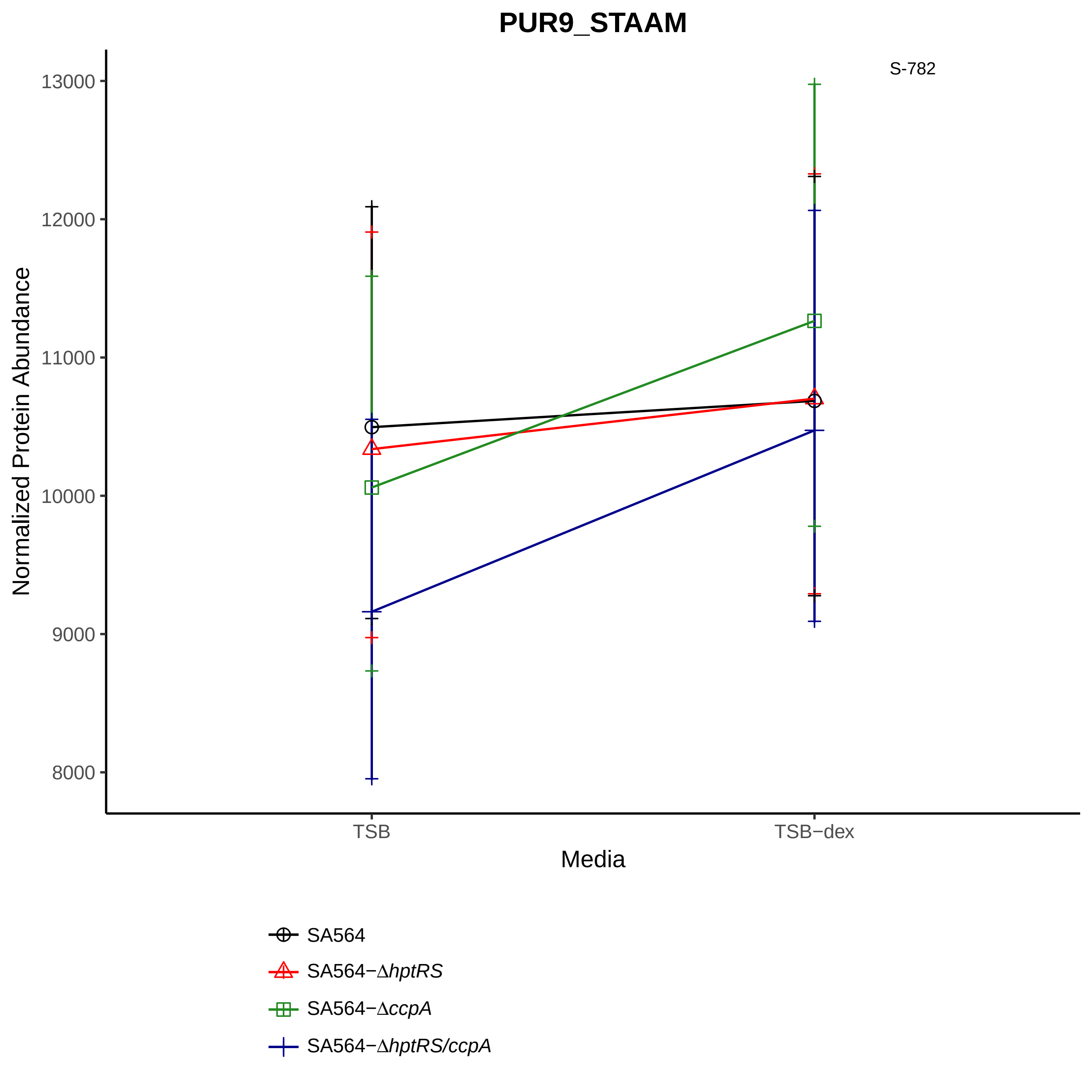

PUR9_STAAM
S-782
Normalized Protein Abundance
13000
12000
11000
10000
9000
8000
TSB
TSB−dex
Media
SA564
SA564−Δ*hptRS*
SA564−Δ*ccpA*
SA564−Δ*hptRS/ccpA*

PURA_STAAM

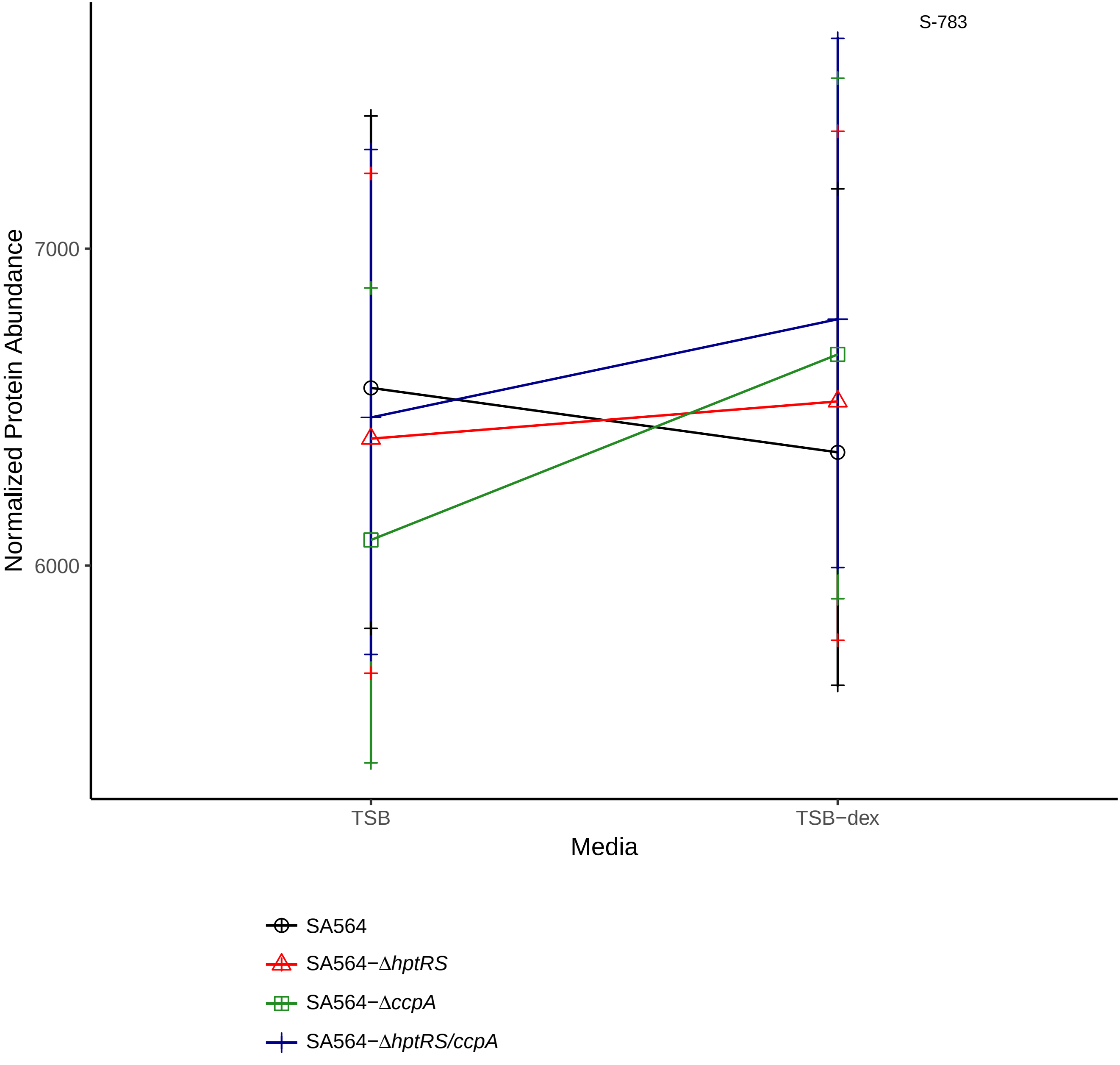

S-783
Normalized Protein Abundance
7000
6000
TSB
TSB−dex
Media
SA564
SA564−Δ*hptRS*
SA564−Δ*ccpA*
SA564−Δ*hptRS/ccpA*

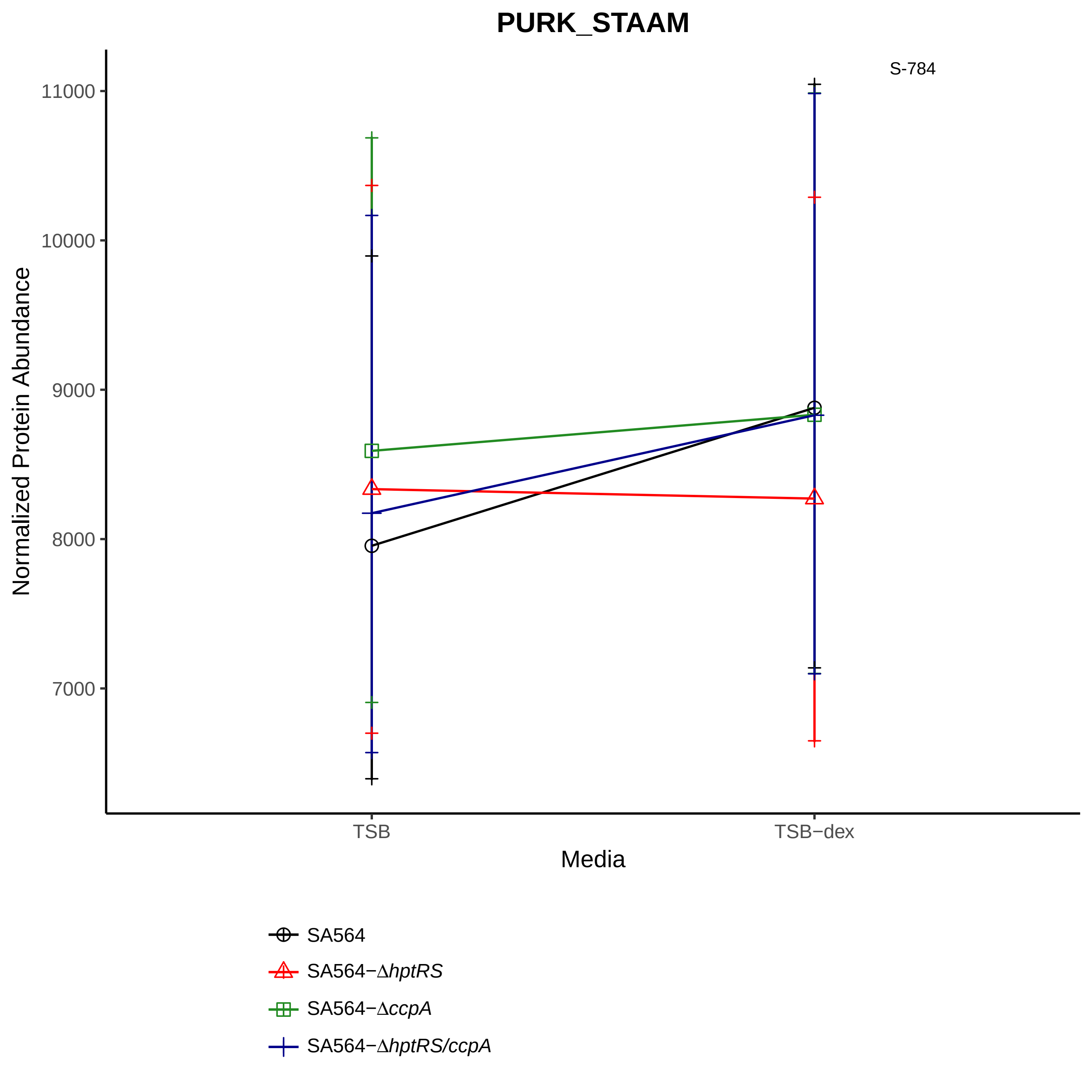

PURK_STAAM
S-784
Normalized Protein Abundance
11000
10000
9000
8000
7000
TSB
TSB−dex
Media
SA564
SA564−Δ*hptRS*
SA564−Δ*ccpA*
SA564−Δ*hptRS/ccpA*

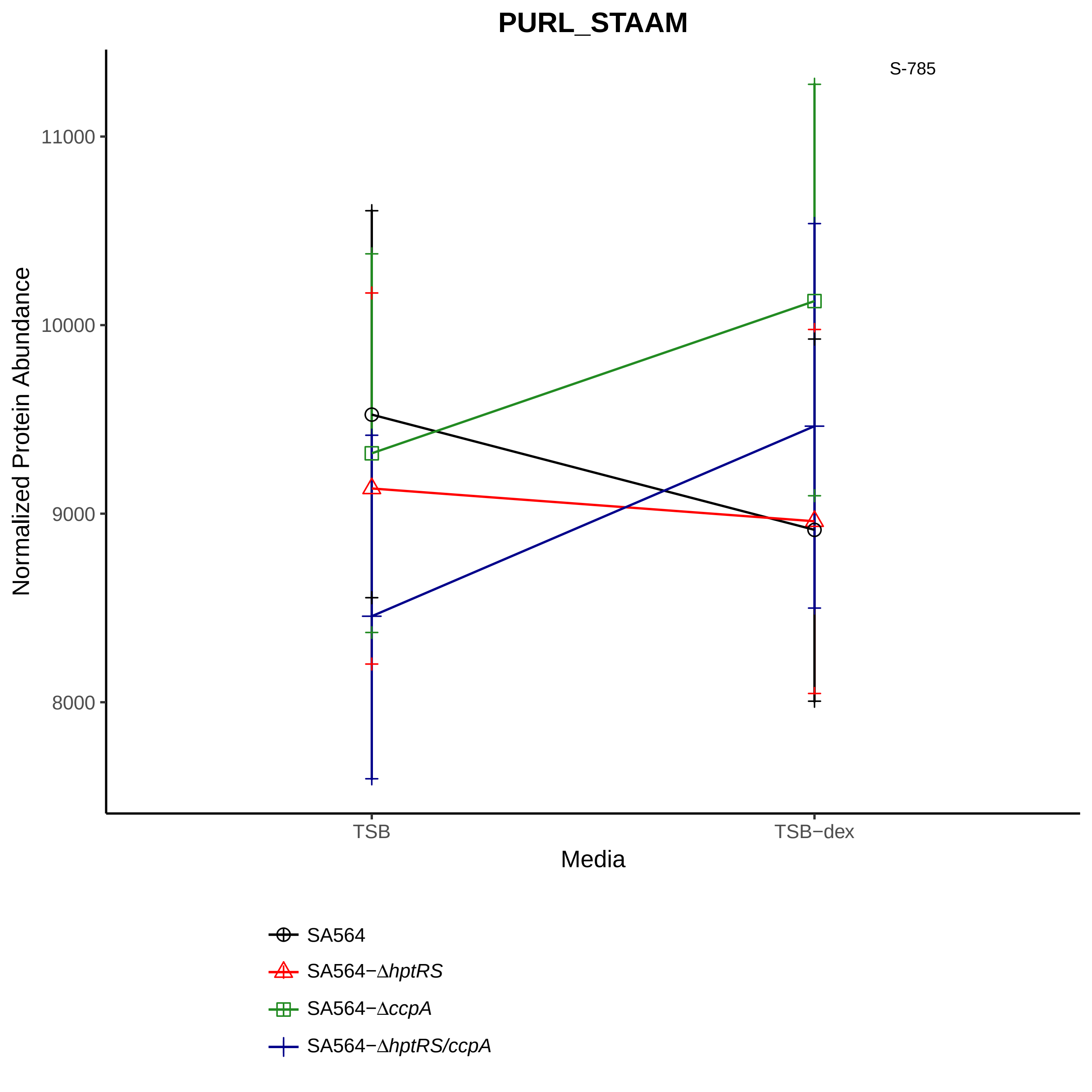
PURL_STAAM
S-785
Normalized Protein Abundance
11000
10000
9000
8000
TSB
TSB−dex
Media
SA564
SA564−Δ*hptRS*
SA564−Δ*ccpA*
SA564−Δ*hptRS/ccpA*

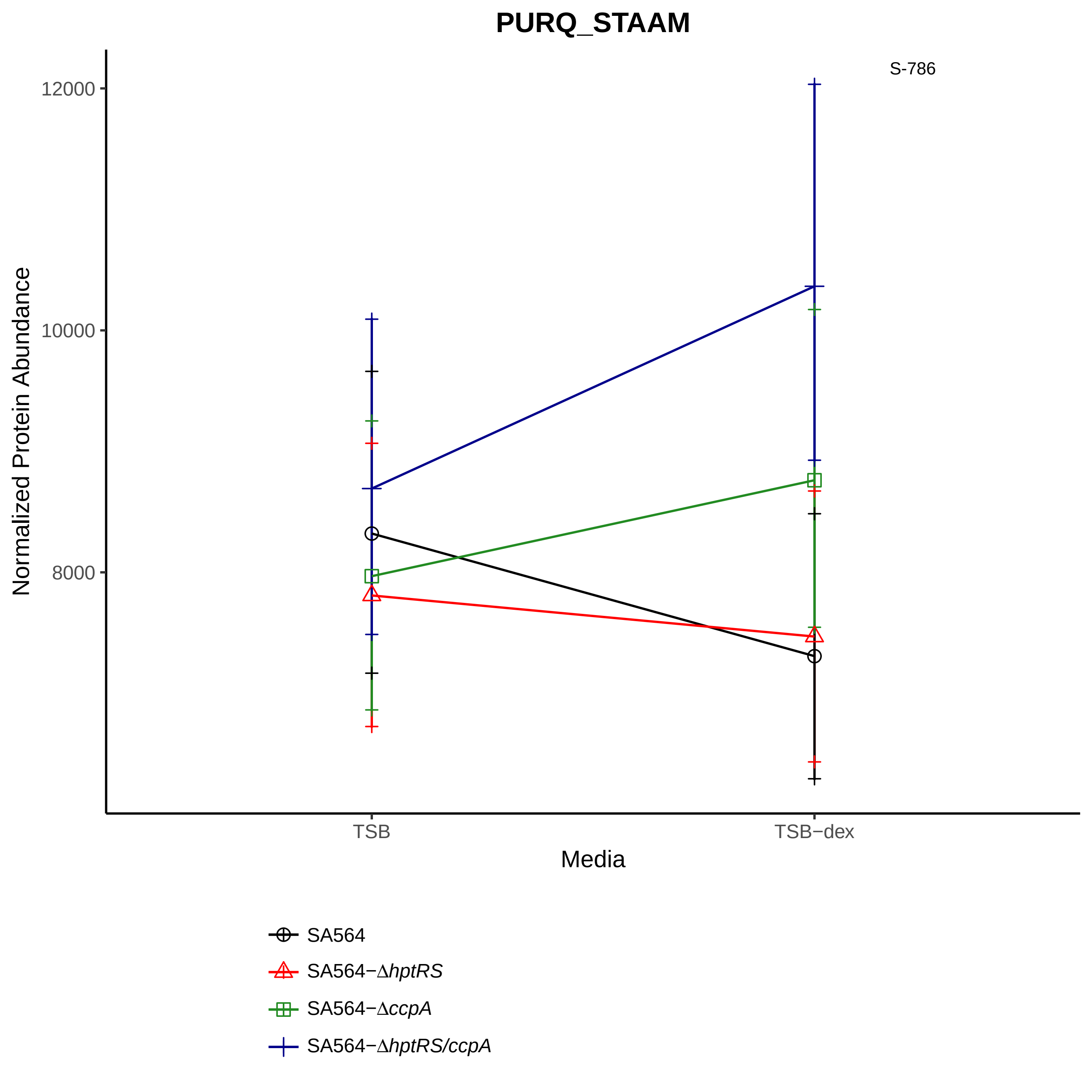

PURQ_STAAM
S-786
12000
10000
8000
Normalized Protein Abundance
TSB
TSB−dex
Media
SA564
SA564−Δ*hptRS*
SA564−Δ*ccpA*
SA564−Δ*hptRS/ccpA*

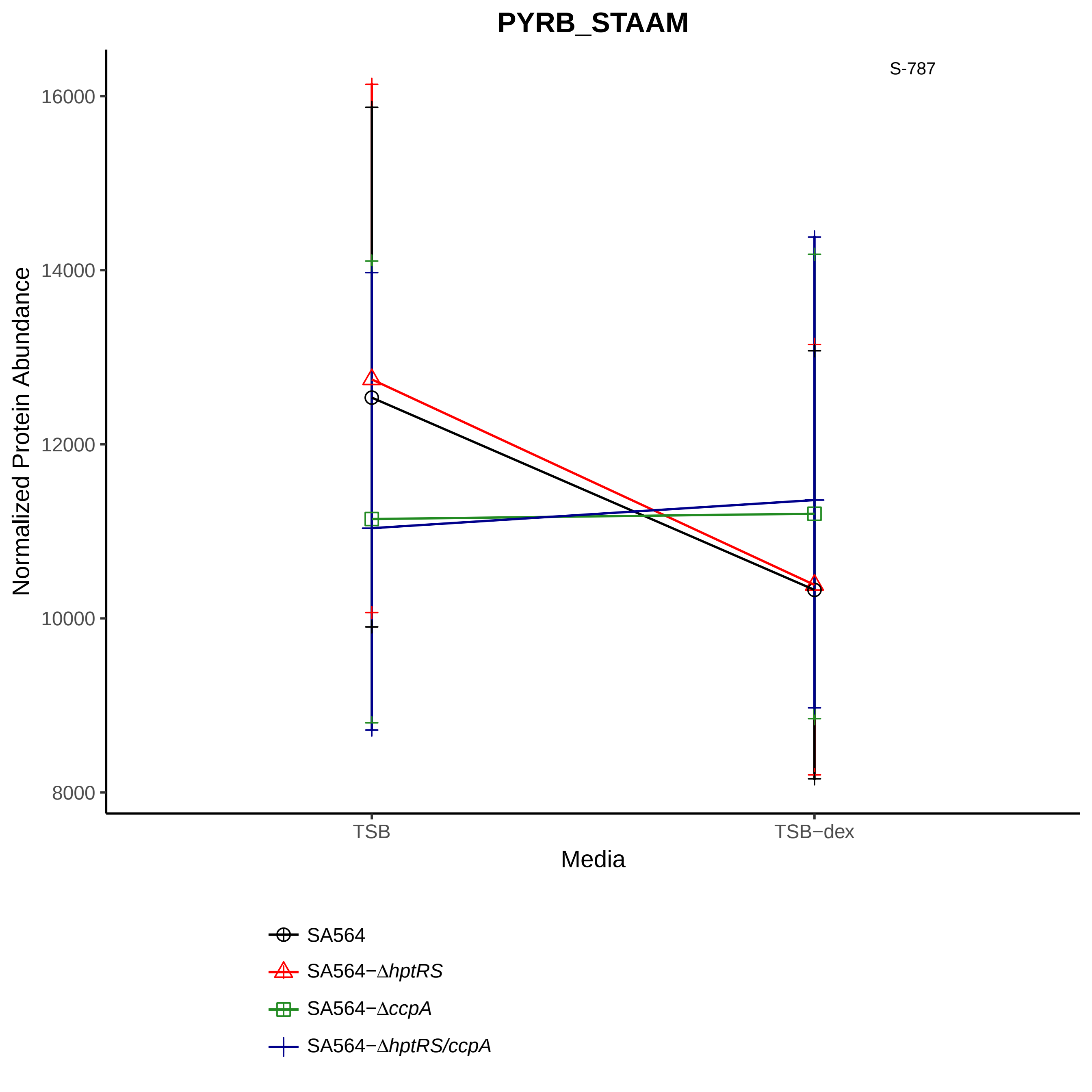

PYRB_STAAM
S-787
Normalized Protein Abundance
16000
14000
12000
10000
8000
TSB
TSB−dex
Media
SA564
SA564−ΔhptRS
SA564−ΔccpA
SA564−ΔhptRS/ccpA

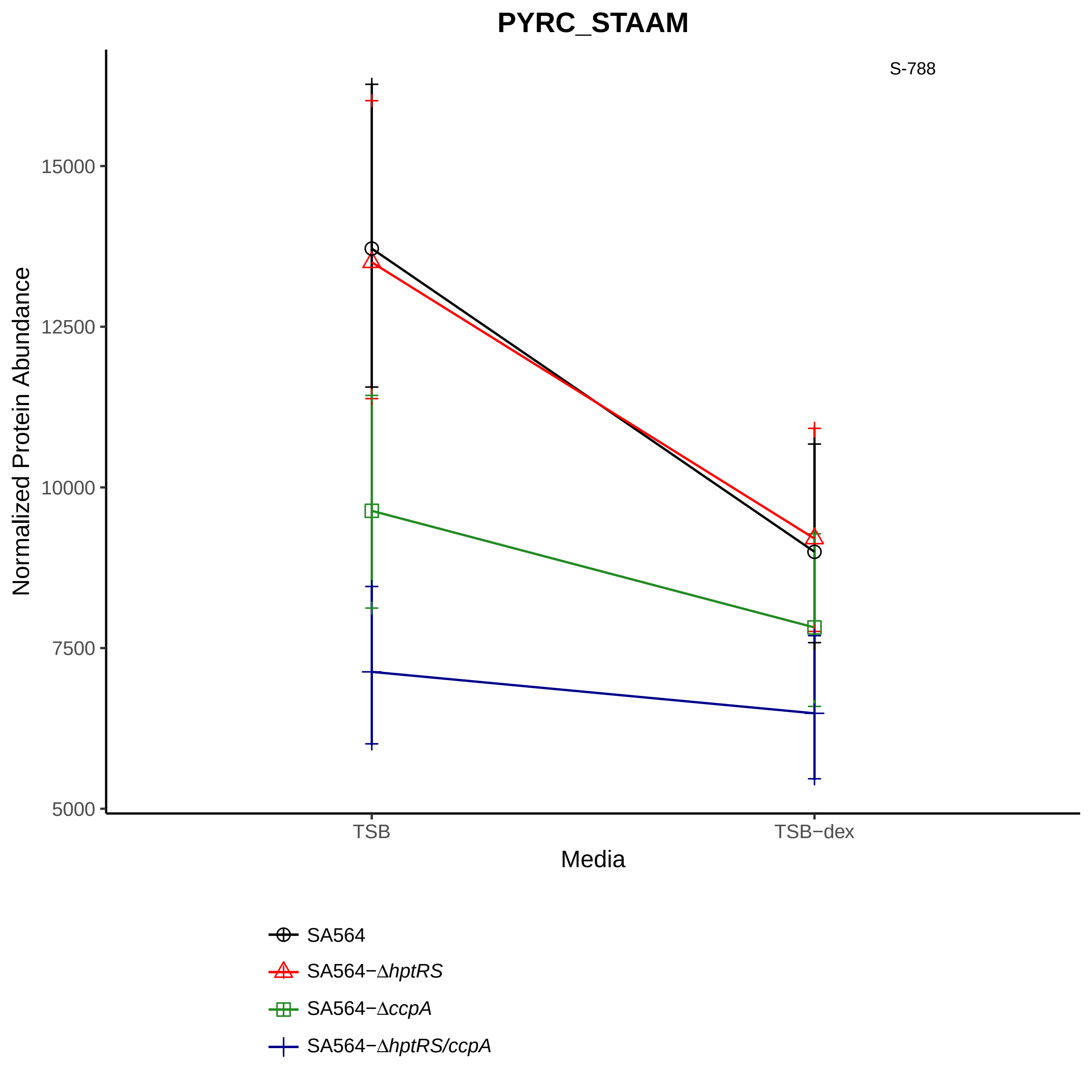

PYRC_STAAM
S-788
Normalized Protein Abundance
15000
12500
10000
7500
5000
TSB
TSB−dex
Media
SA564
SA564−Δ*hptRS*
SA564−Δ*ccpA*
SA564−Δ*hptRS/ccpA*

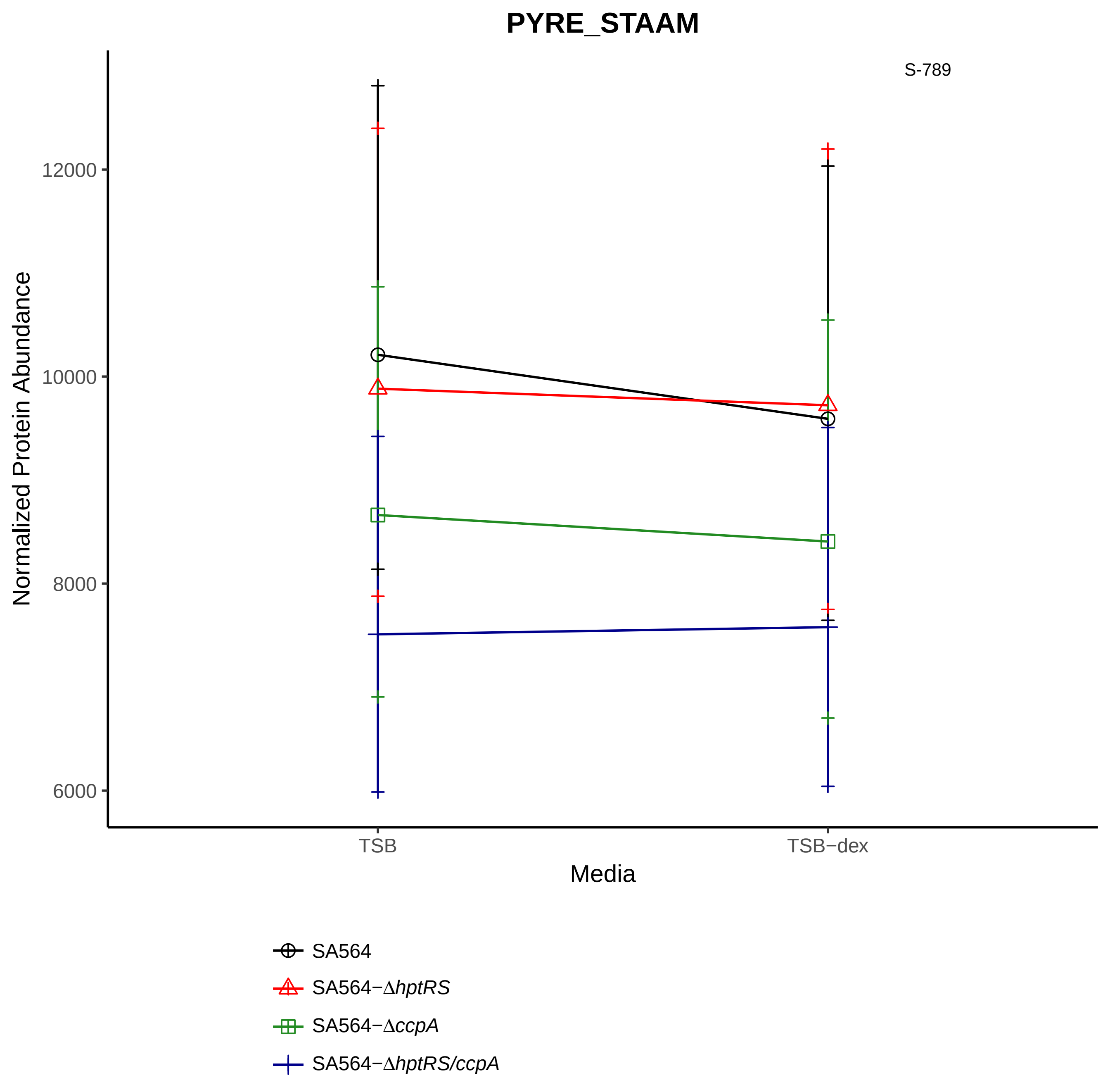

PYRE_STAAM
S-789
Normalized Protein Abundance
12000
10000
8000
6000
TSB
TSB−dex
Media
SA564
SA564−Δ*hptRS*
SA564−Δ*ccpA*
SA564−Δ*hptRS/ccpA*

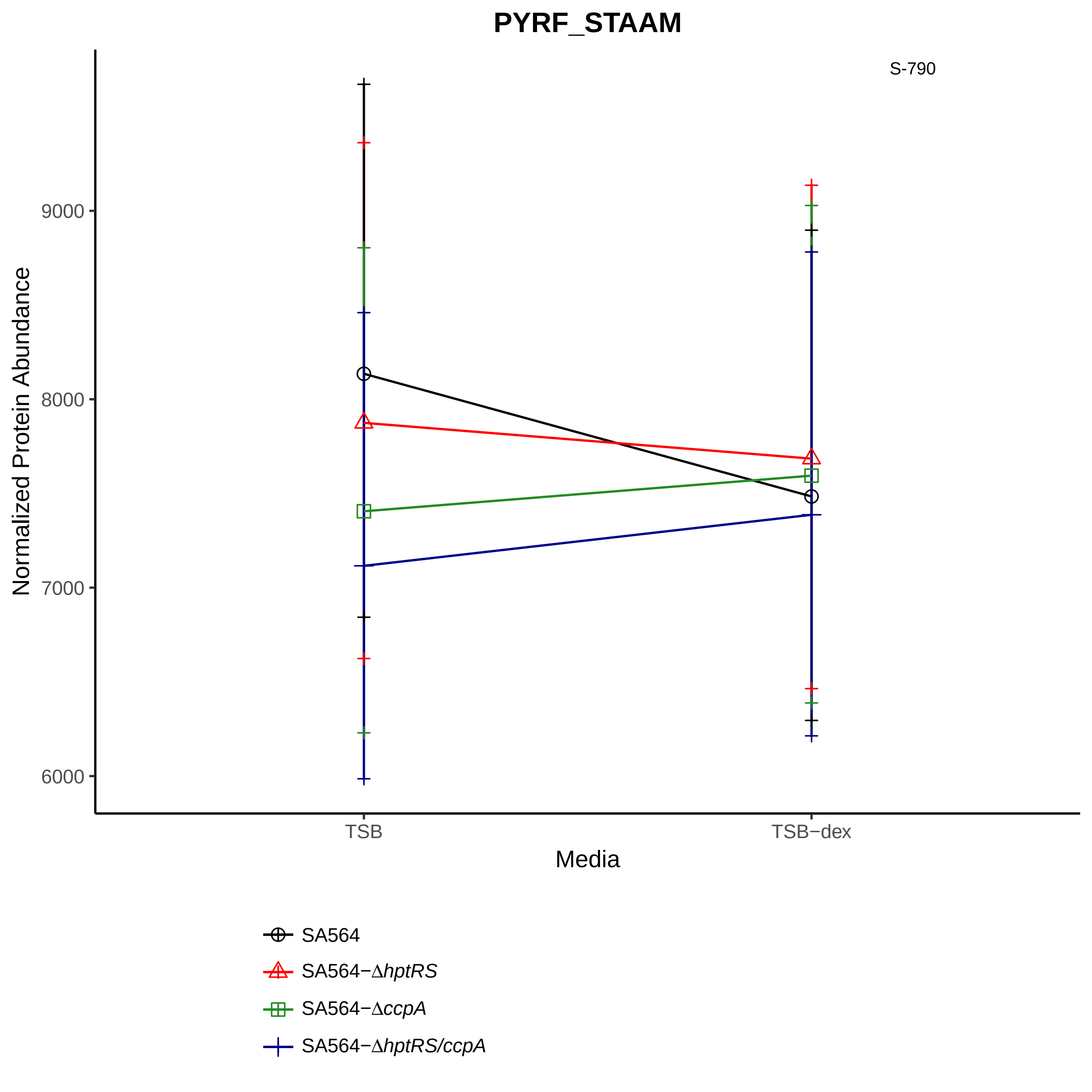

PYRF_STAAM
S-790
Normalized Protein Abundance
9000
8000
7000
6000
TSB
TSB−dex
Media
SA564
SA564−Δ*hptRS*
SA564−Δ*ccpA*
SA564−Δ*hptRS/ccpA*

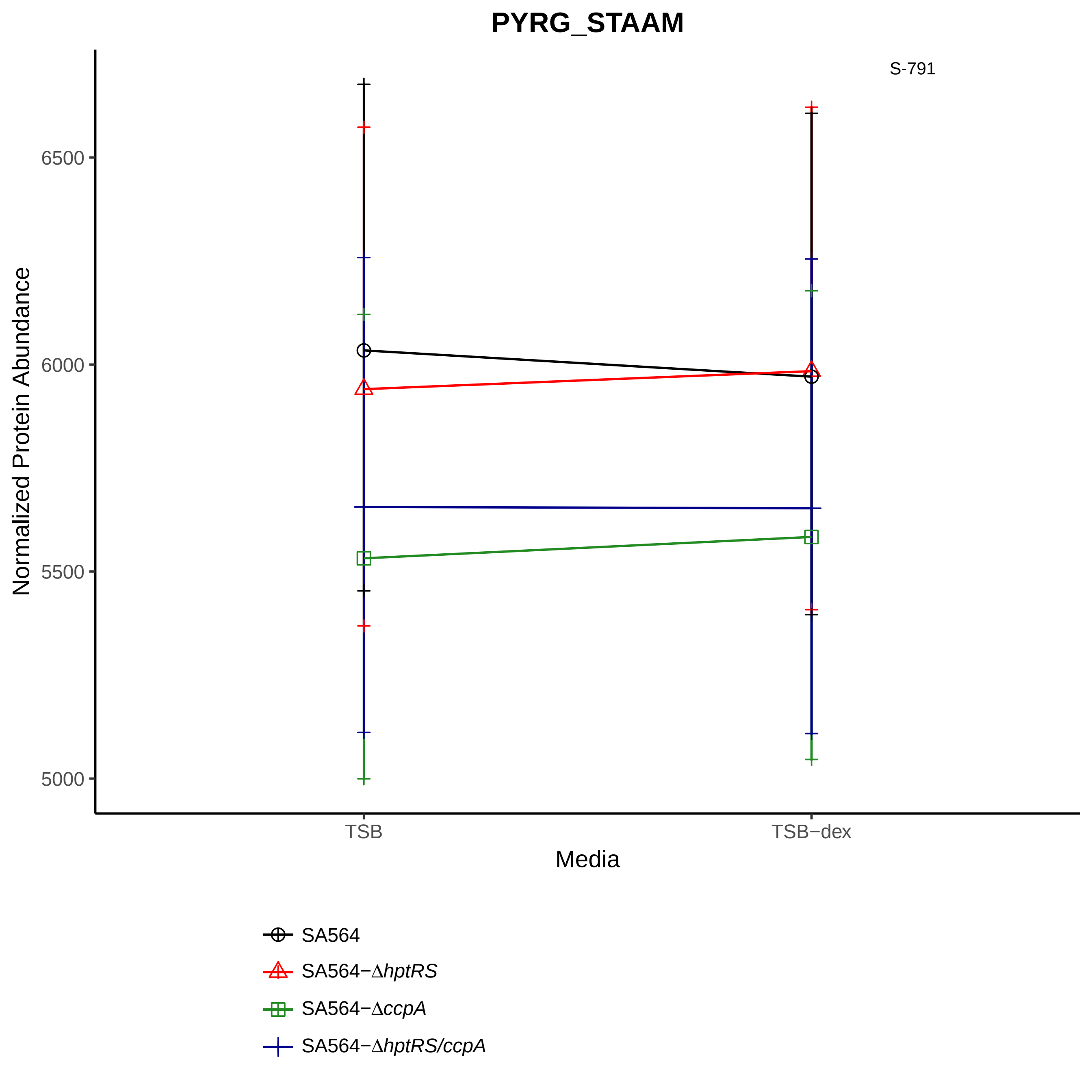

PYRG_STAAM
S-791
Normalized Protein Abundance
6500
6000
5500
5000
TSB
TSB−dex
Media
SA564
SA564−ΔhptRS
SA564−ΔccpA
SA564−ΔhptRS/ccpA

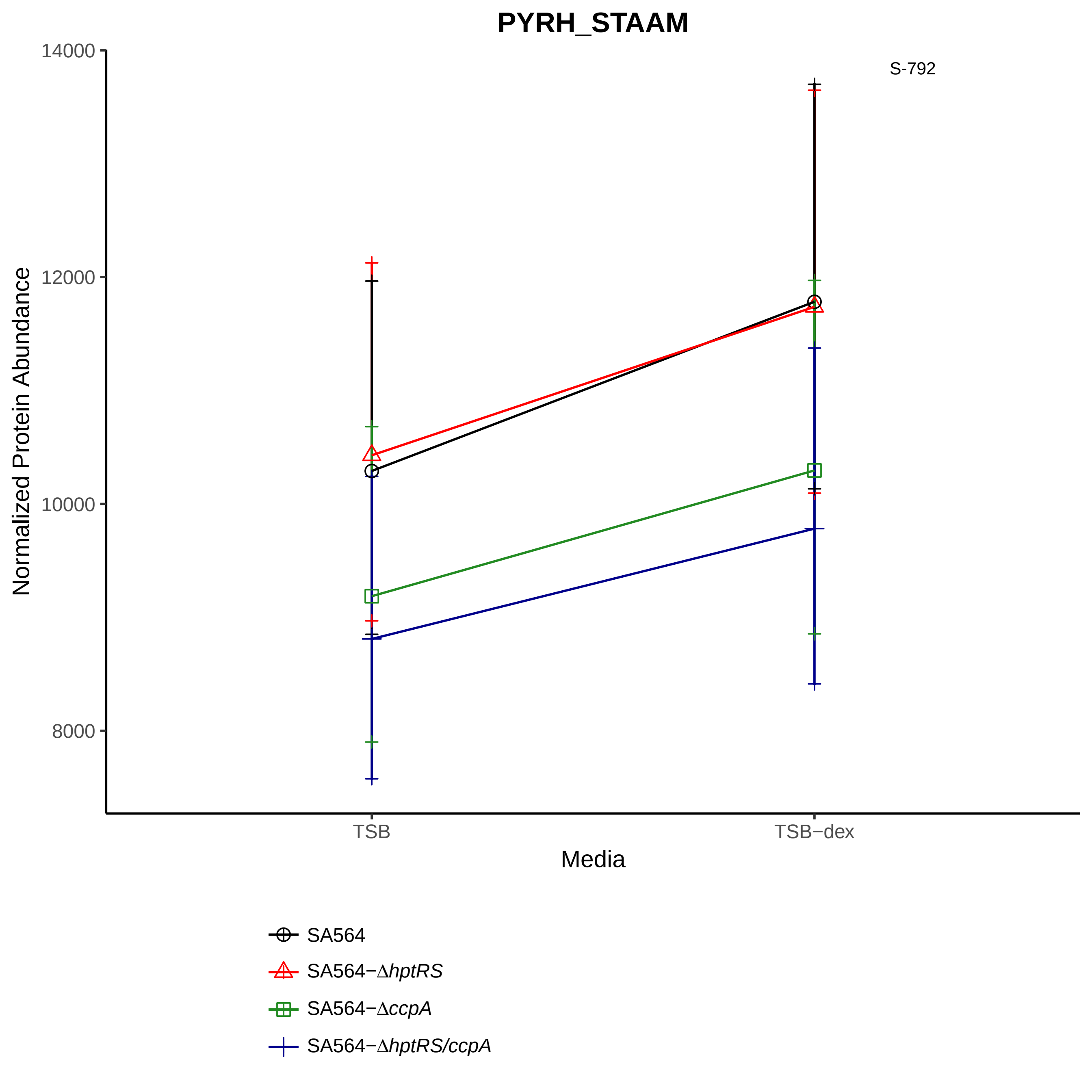
PYRH_STAAM
S-792
Normalized Protein Abundance
14000
12000
10000
8000
TSB
TSB−dex
Media
SA564
SA564−ΔhptRS
SA564−ΔccpA
SA564−ΔhptRS/ccpA

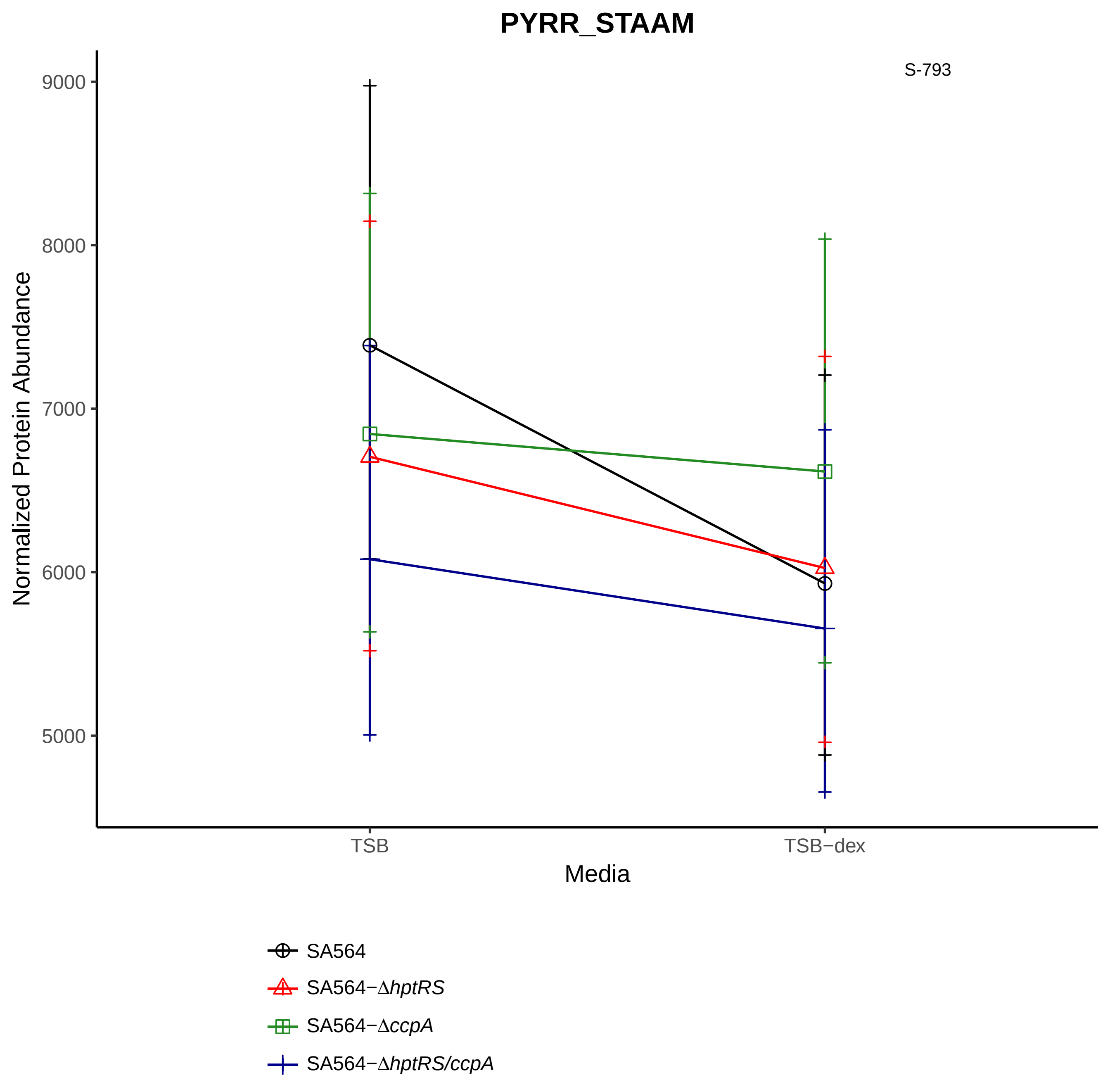
PYRR_STAAM
S-793
Normalized Protein Abundance
9000
8000
7000
6000
5000
TSB
TSB−dex
Media
SA564
SA564−ΔhptRS
SA564−ΔccpA
SA564−ΔhptRS/ccpA

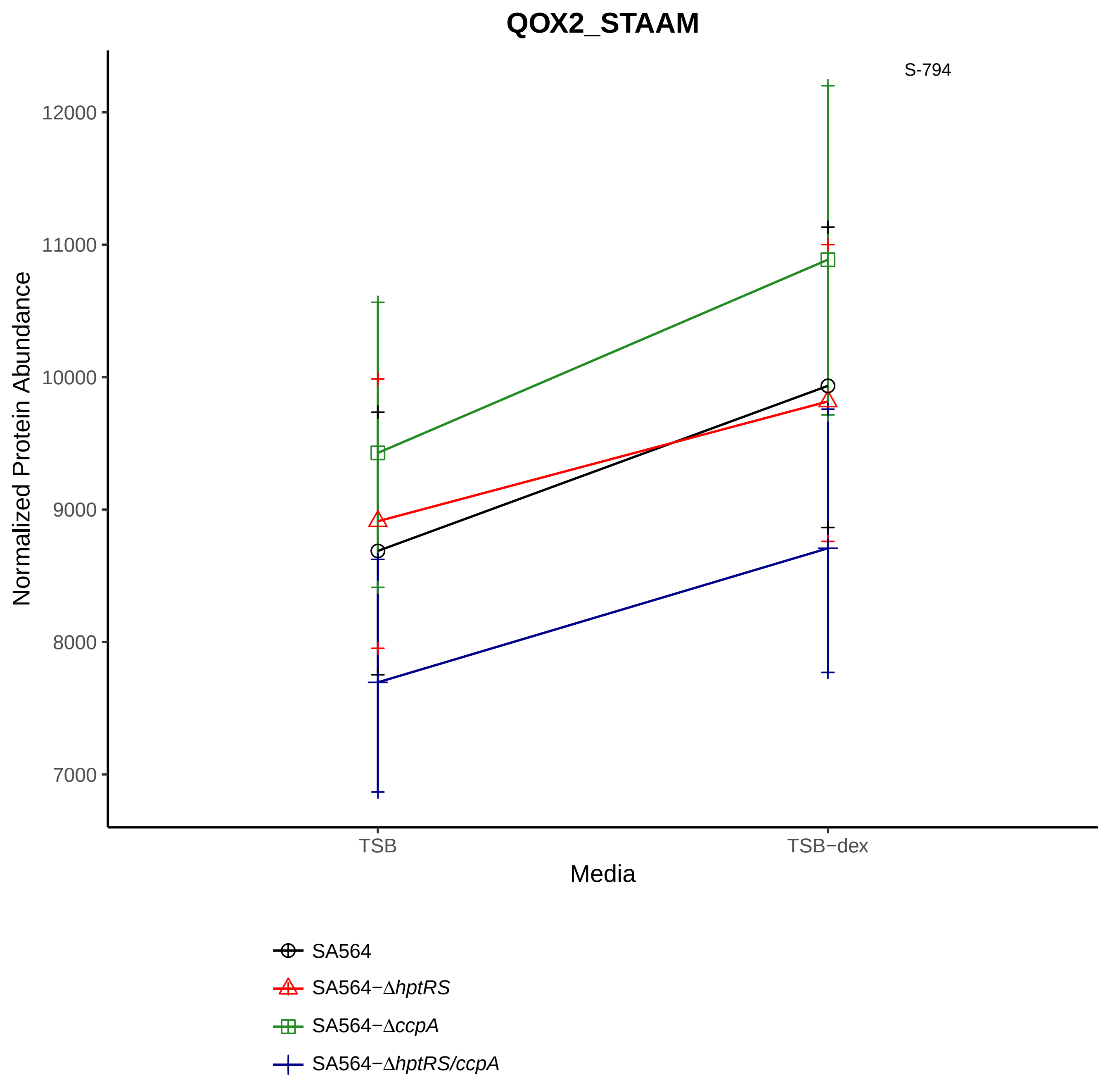

QOX2_STAAM
S-794
Normalized Protein Abundance
12000
11000
10000
9000
8000
7000
TSB
TSB−dex
Media
SA564
SA564−ΔhptRS
SA564−ΔccpA
SA564−ΔhptRS/ccpA

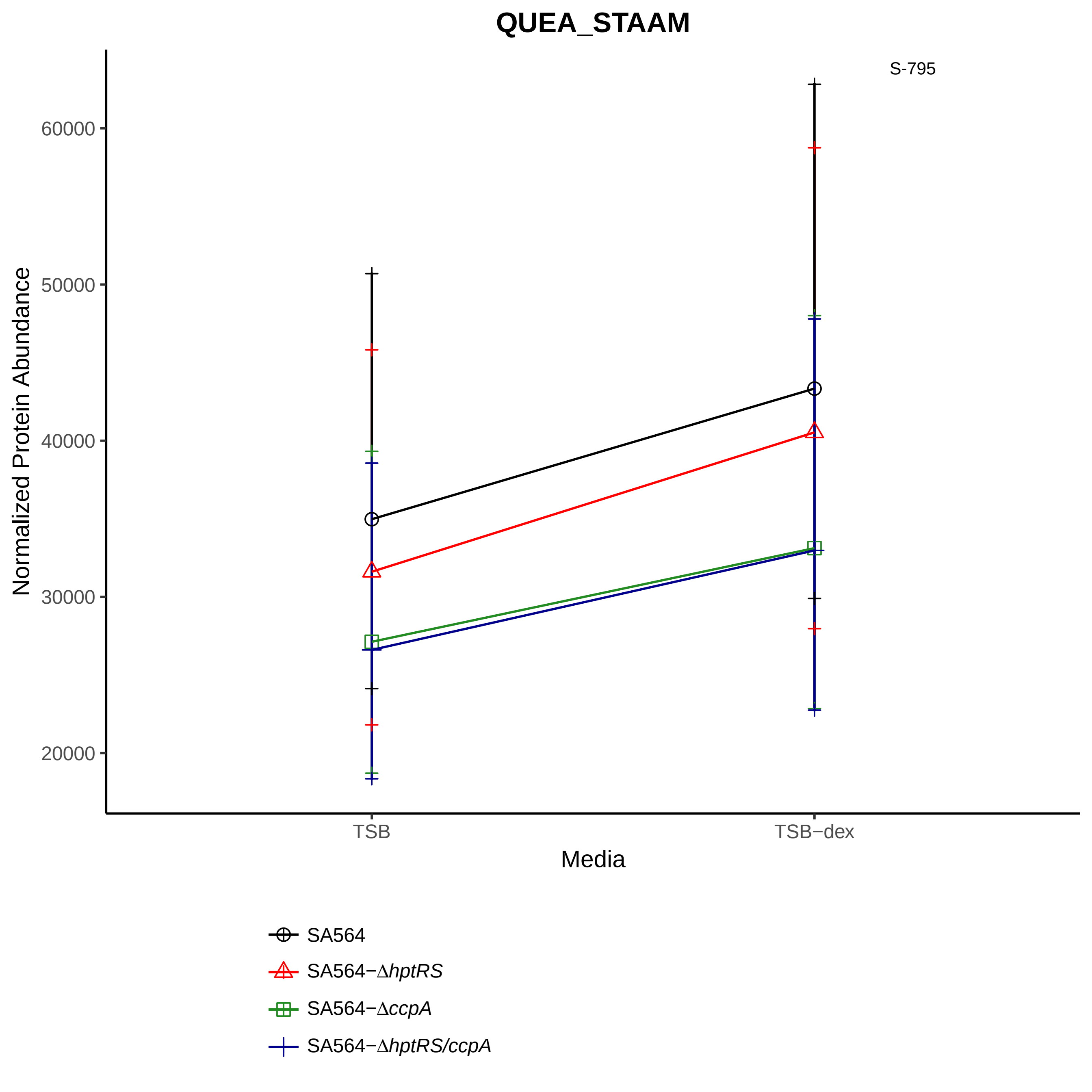

QUEA_STAAM
S-795
Normalized Protein Abundance
60000
50000
40000
30000
20000
TSB
TSB−dex
Media
SA564
SA564−ΔhptRS
SA564−ΔccpA
SA564−ΔhptRS/ccpA

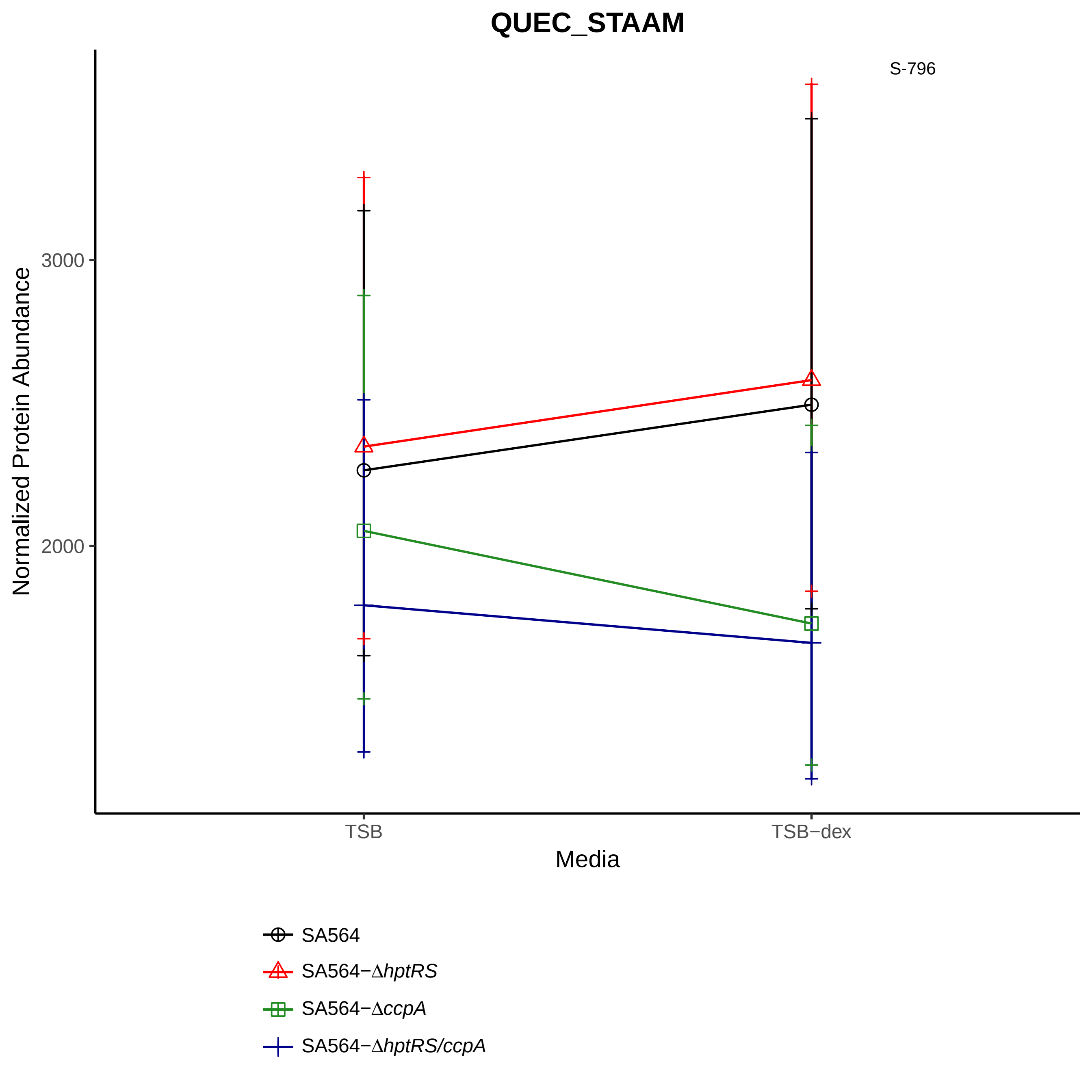

QUEC_STAAM
S-796
Normalized Protein Abundance
3000
2000
TSB
TSB−dex
Media
SA564
SA564−ΔhptRS
SA564−ΔccpA
SA564−ΔhptRS/ccpA

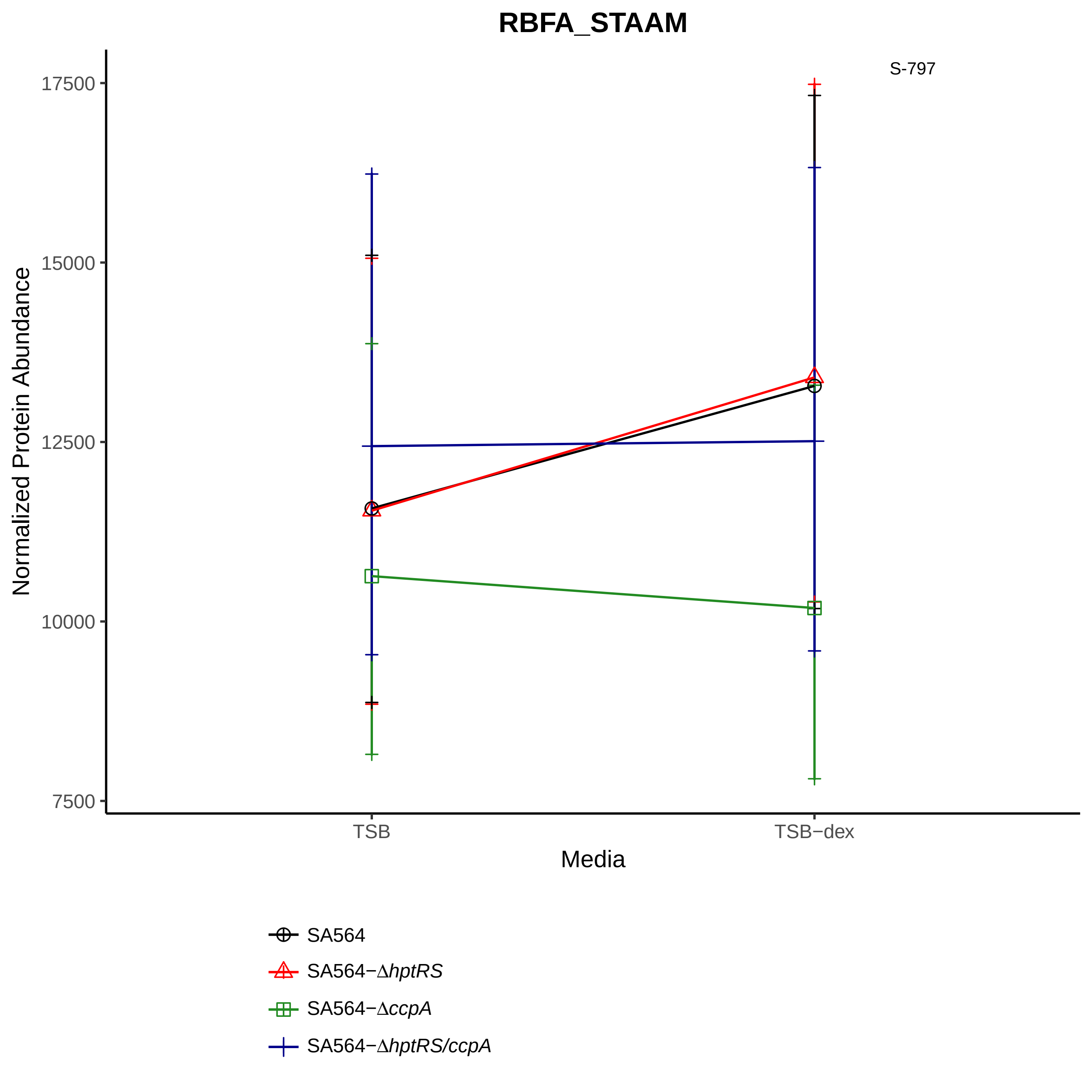
RBFA_STAAM
S-797
Normalized Protein Abundance
17500
15000
12500
10000
7500
TSB
TSB−dex
Media
SA564
SA564−Δ*hptRS*
SA564−Δ*ccpA*
SA564−Δ*hptRS/ccpA*

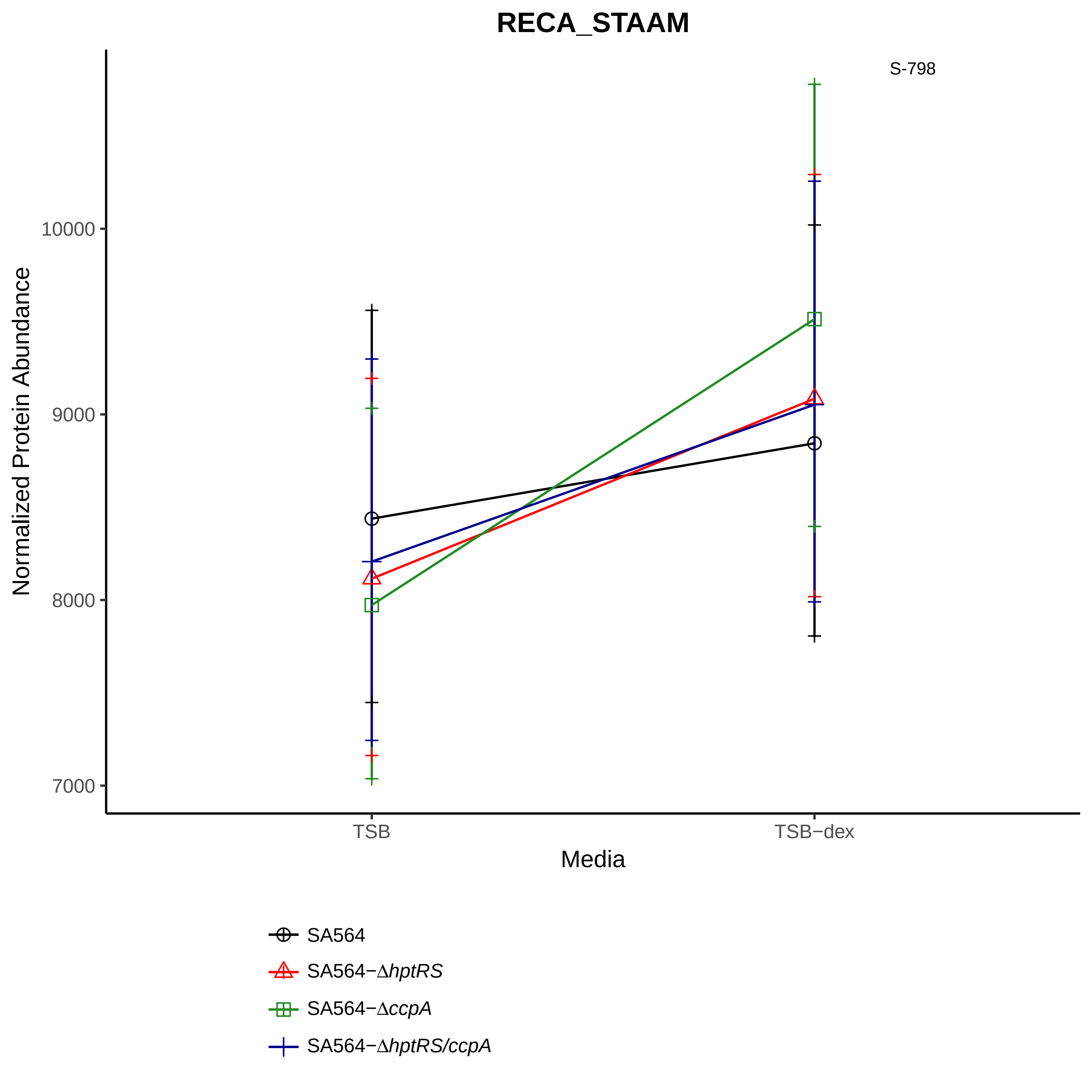
RECA_STAAM
S-798
Normalized Protein Abundance
10000
9000
8000
7000
TSB
TSB−dex
Media
SA564
SA564−ΔhptRS
SA564−ΔccpA
SA564−ΔhptRS/ccpA

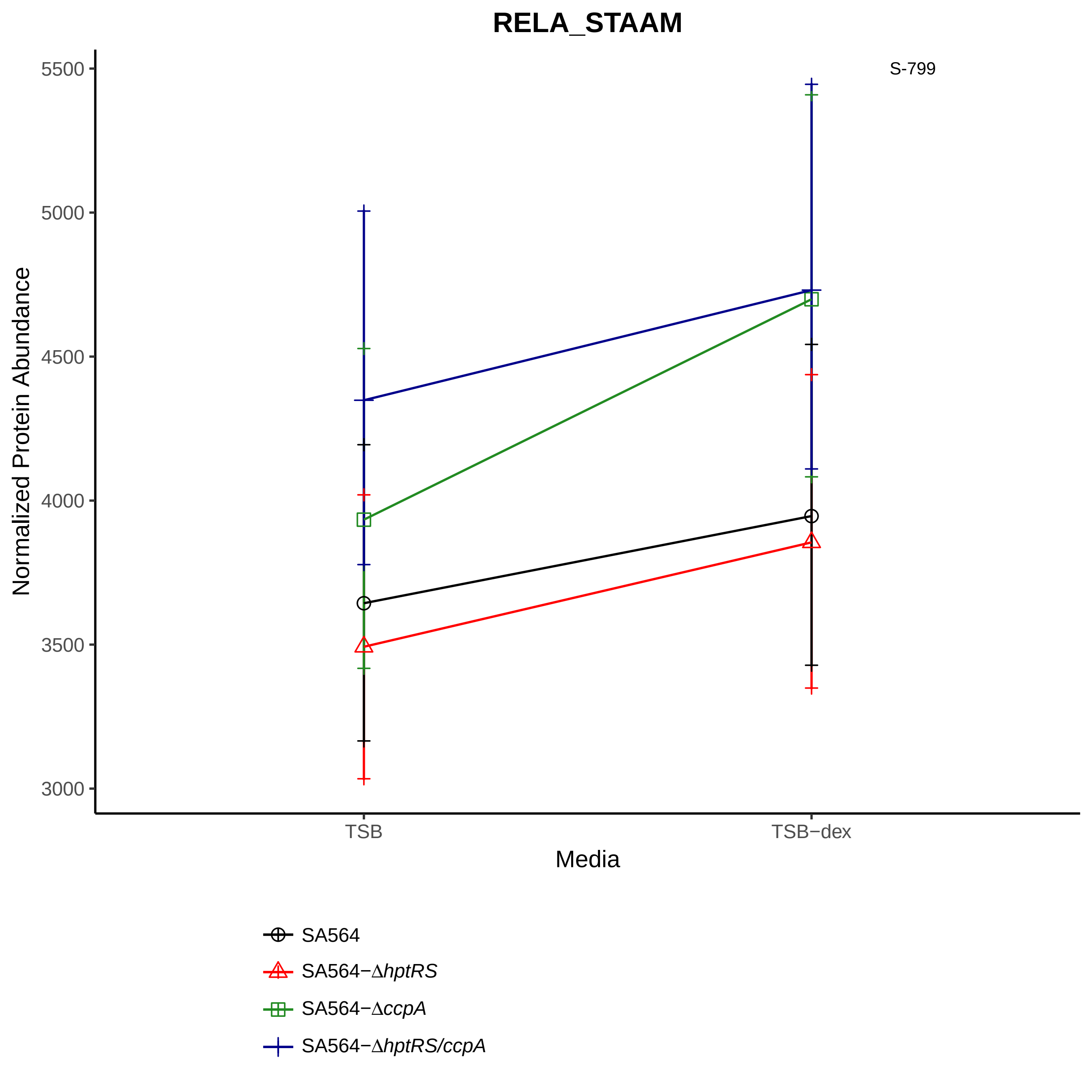

RELA_STAAM
S-799
Normalized Protein Abundance
5500
5000
4500
4000
3500
3000
TSB
TSB−dex
Media
SA564
SA564−Δ*hptRS*
SA564−Δ*ccpA*
SA564−Δ*hptRS/ccpA*

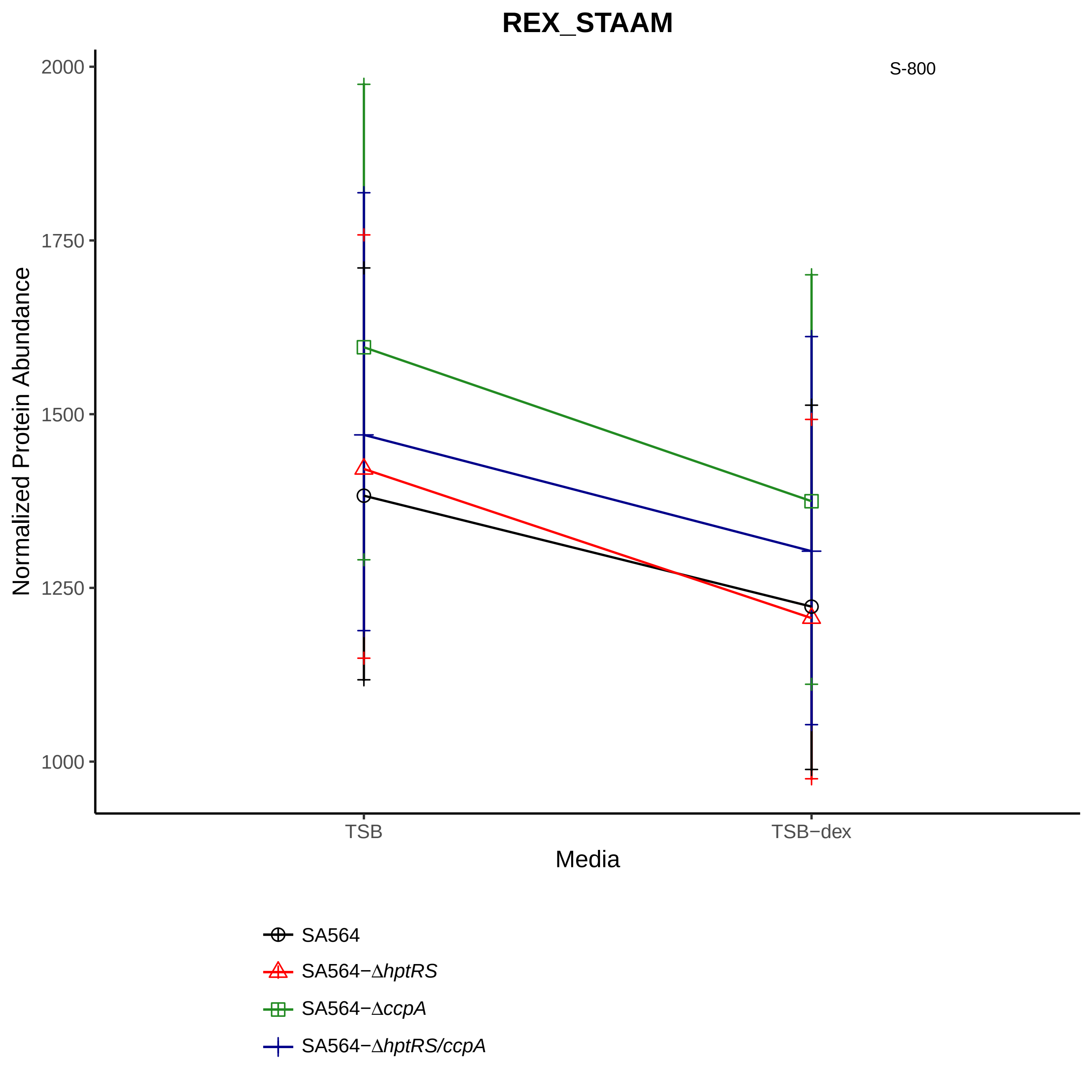
REX_STAAM
S-800
Normalized Protein Abundance
2000
1750
1500
1250
1000
TSB
TSB−dex
Media
SA564
SA564−ΔhptRS
SA564−ΔccpA
SA564−ΔhptRS/ccpA

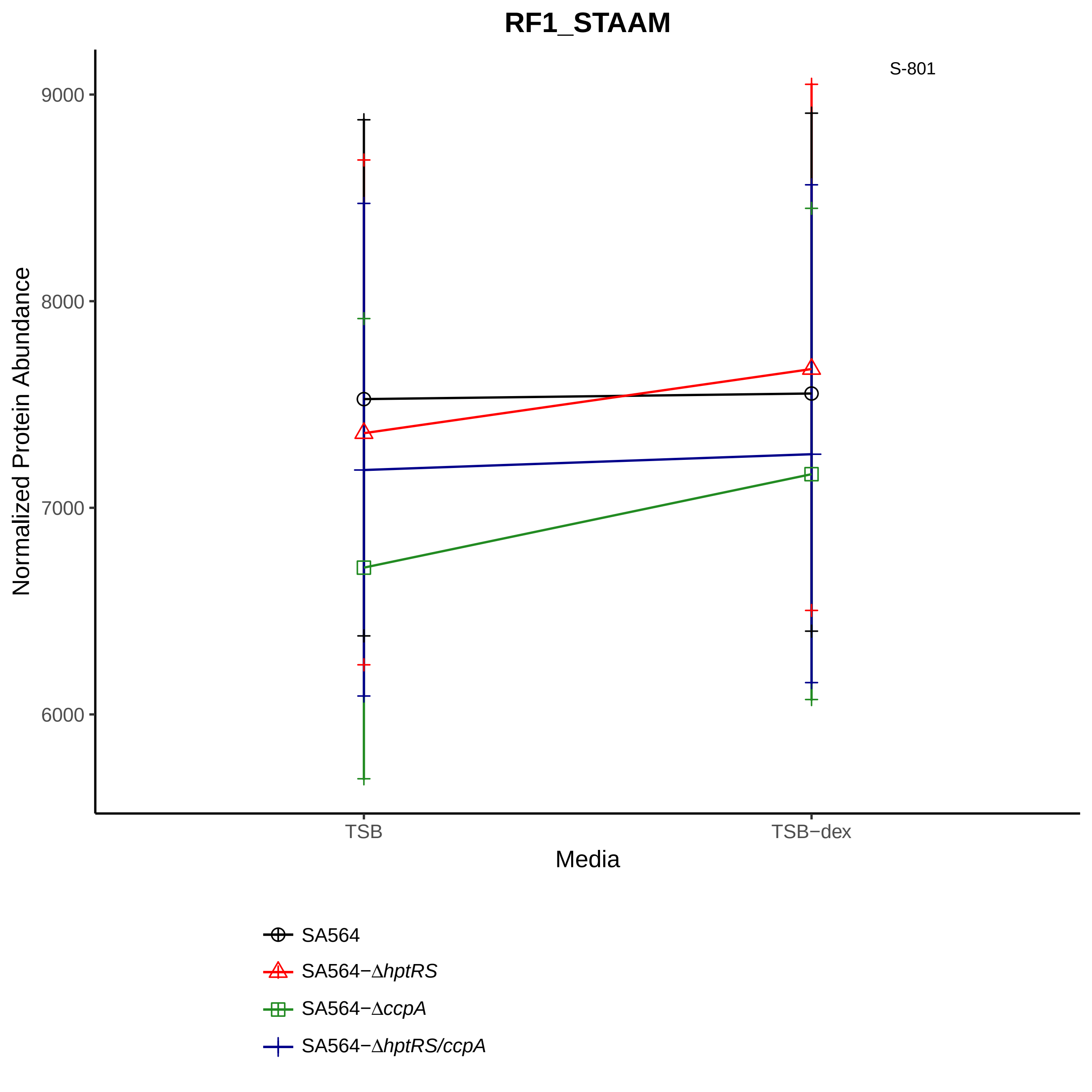
RF1_STAAM
S-801
Normalized Protein Abundance
9000
8000
7000
6000
TSB
TSB−dex
Media
SA564
SA564−Δ*hptRS*
SA564−Δ*ccpA*
SA564−Δ*hptRS/ccpA*

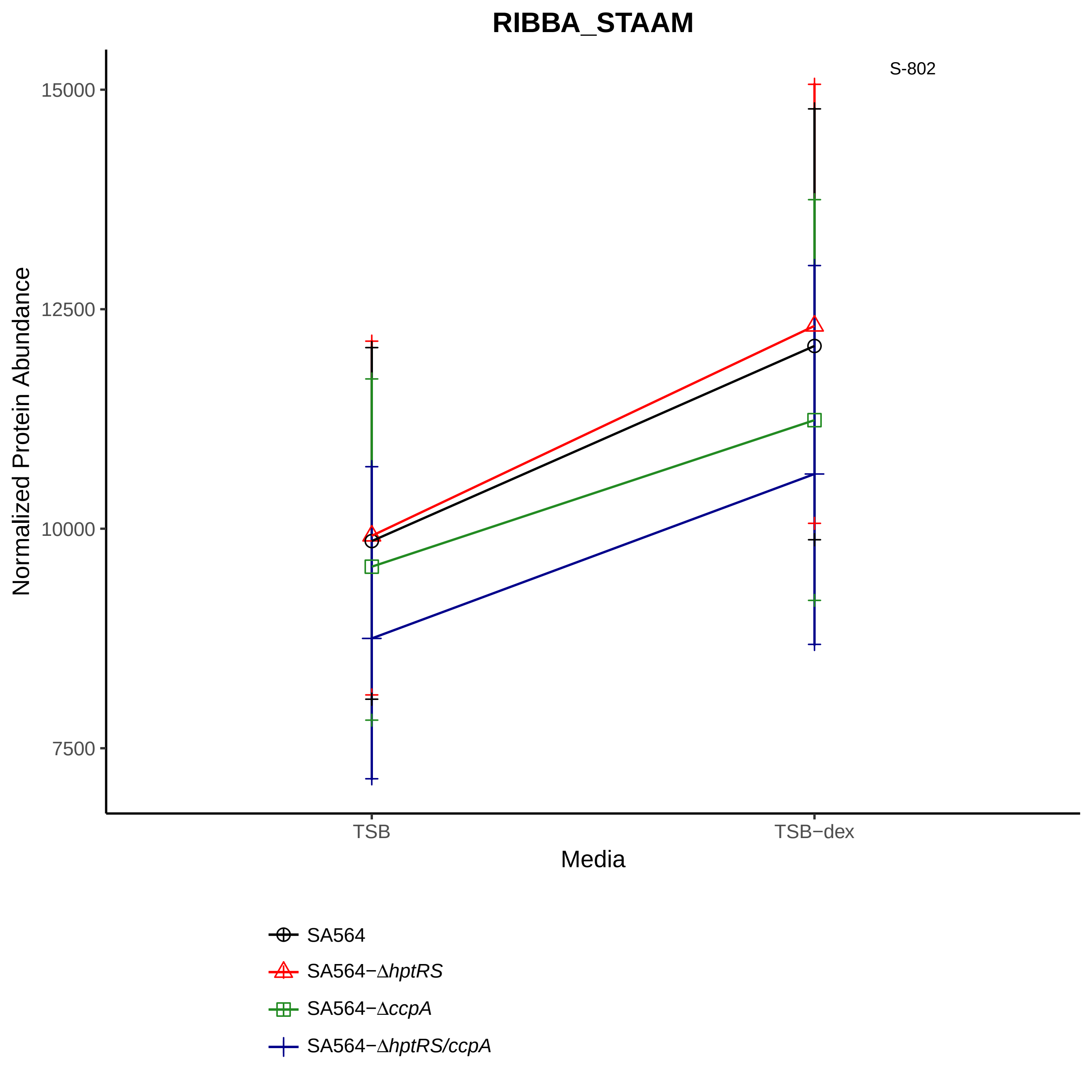
RIBBA_STAAM
S-802
Normalized Protein Abundance
15000
12500
10000
7500
TSB
TSB−dex
Media
SA564
SA564−ΔhptRS
SA564−ΔccpA
SA564−ΔhptRS/ccpA

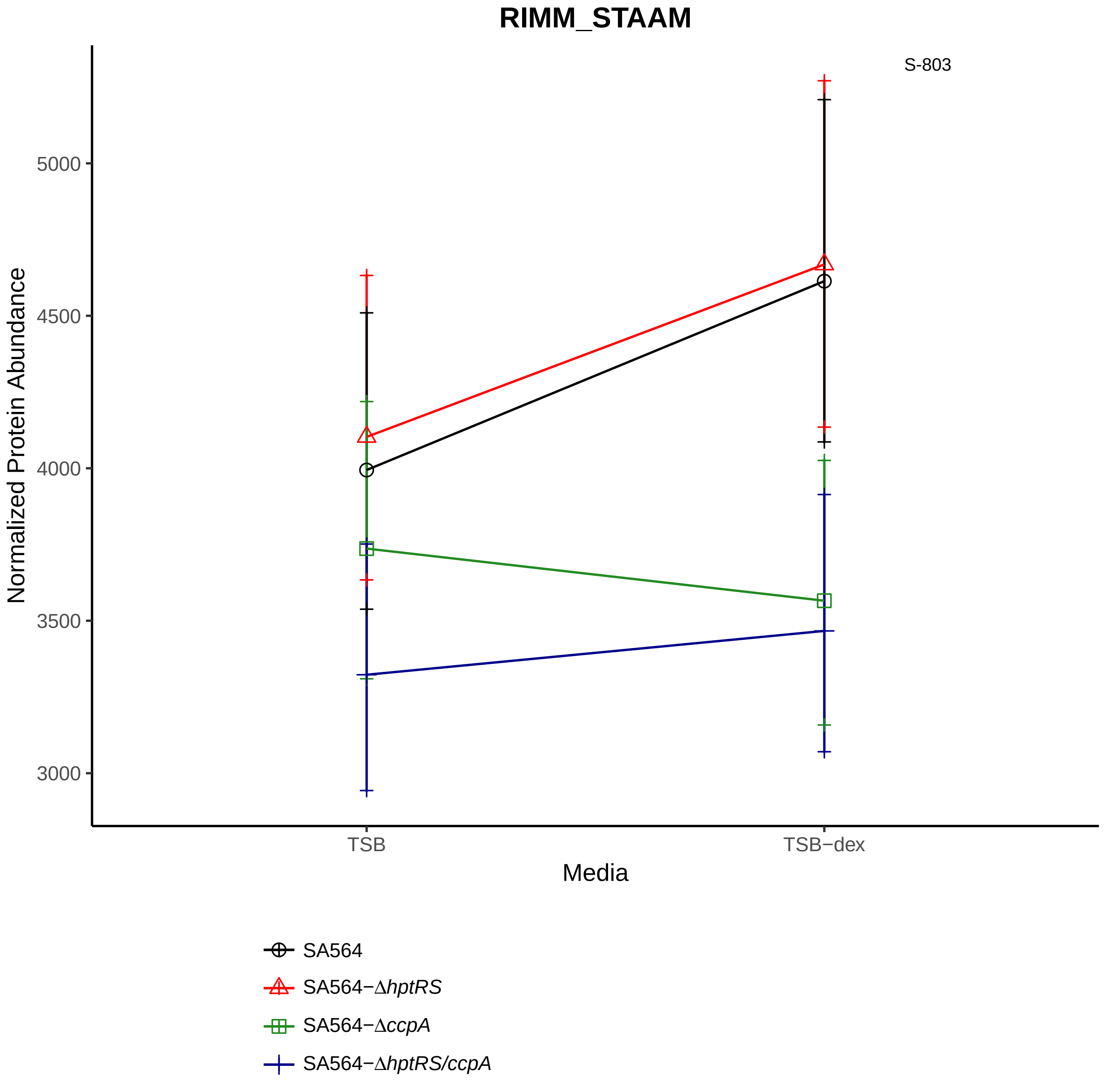

RIMM_STAAM
S-803
Normalized Protein Abundance
5000
4500
4000
3500
3000
TSB
TSB−dex
Media
SA564
SA564−Δ*hptRS*
SA564−Δ*ccpA*
SA564−Δ*hptRS/ccpA*

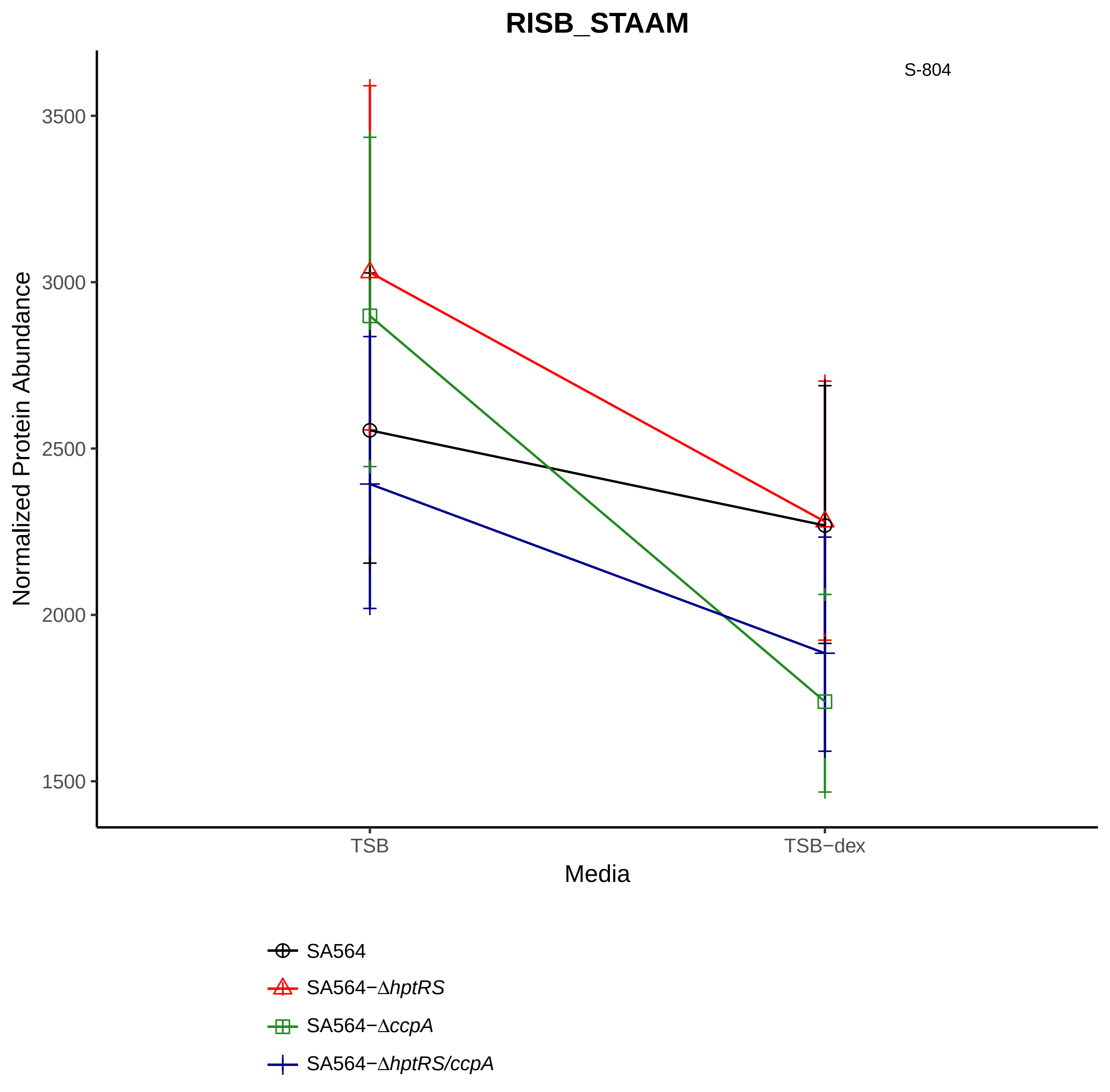

RISB_STAAM
S-804
3500
3000
2500
2000
1500
Normalized Protein Abundance
TSB
TSB−dex
Media
SA564
SA564−ΔhptRS
SA564−ΔccpA
SA564−ΔhptRS/ccpA

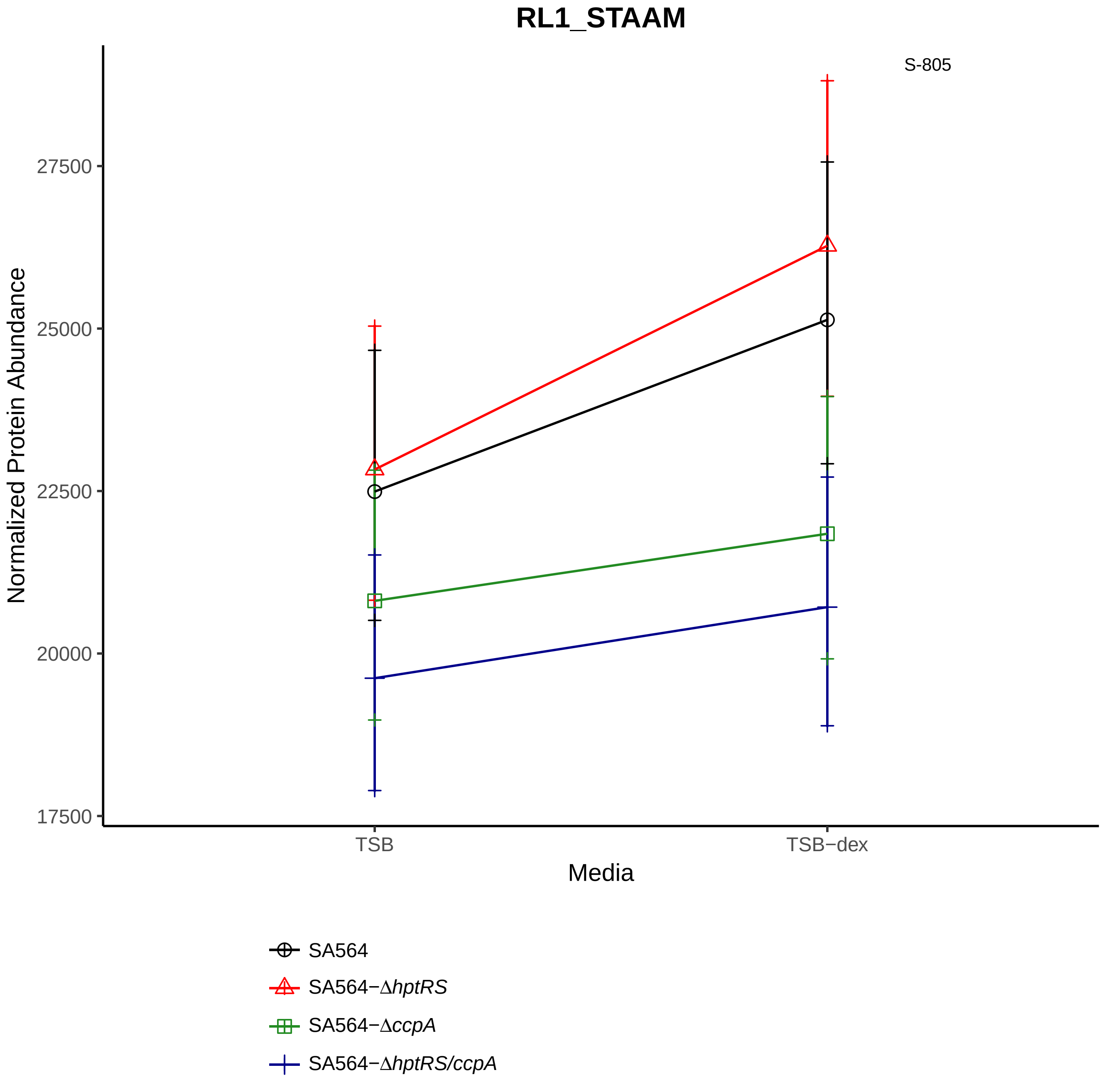

RL1_STAAM
S-805
Normalized Protein Abundance
27500
25000
22500
20000
17500
TSB
TSB−dex
Media
SA564
SA564−Δ*hptRS*
SA564−Δ*ccpA*
SA564−Δ*hptRS/ccpA*

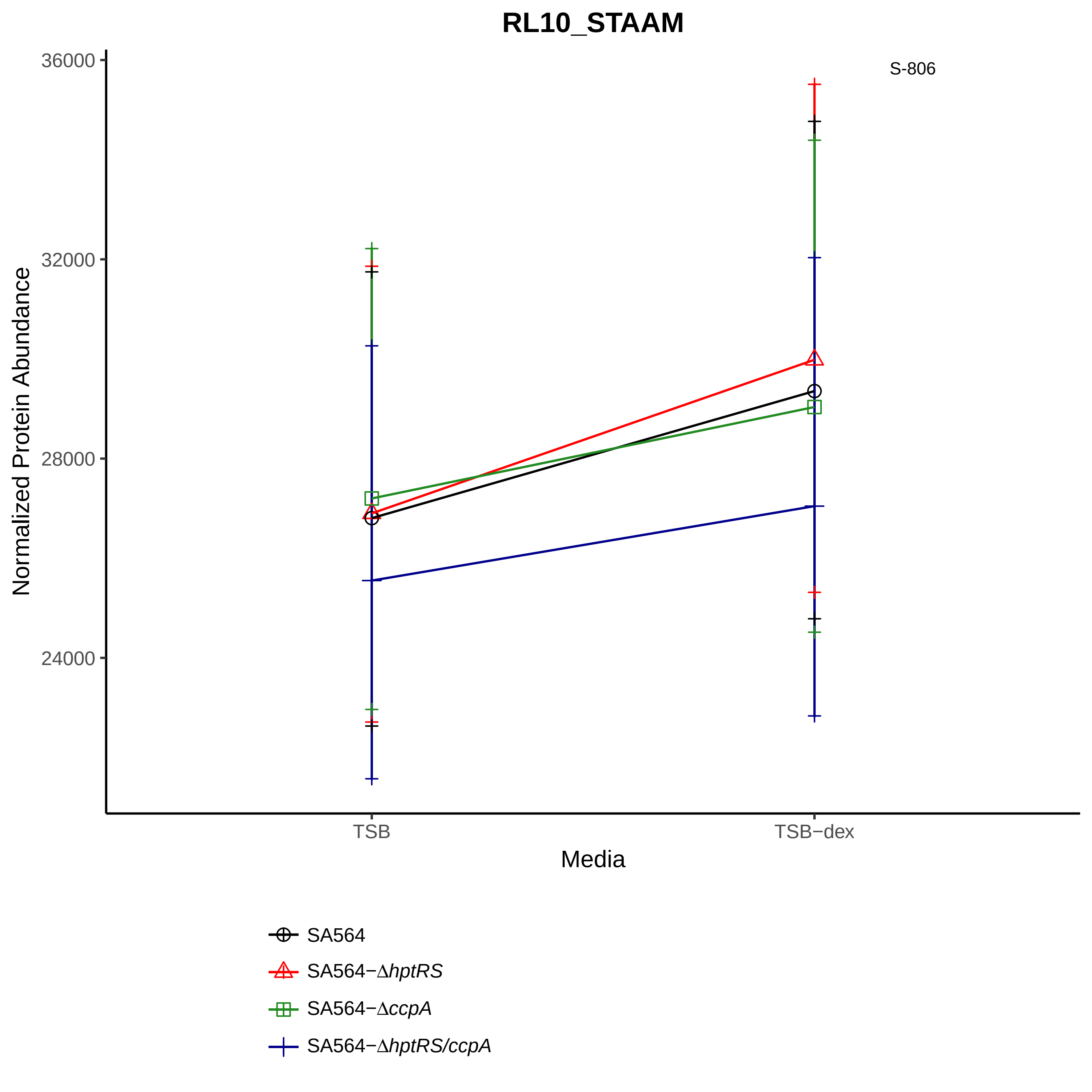

RL10_STAAM
S-806
Normalized Protein Abundance
36000
32000
28000
24000
TSB
TSB−dex
Media
SA564
SA564−Δ*hptRS*
SA564−Δ*ccpA*
SA564−Δ*hptRS/ccpA*

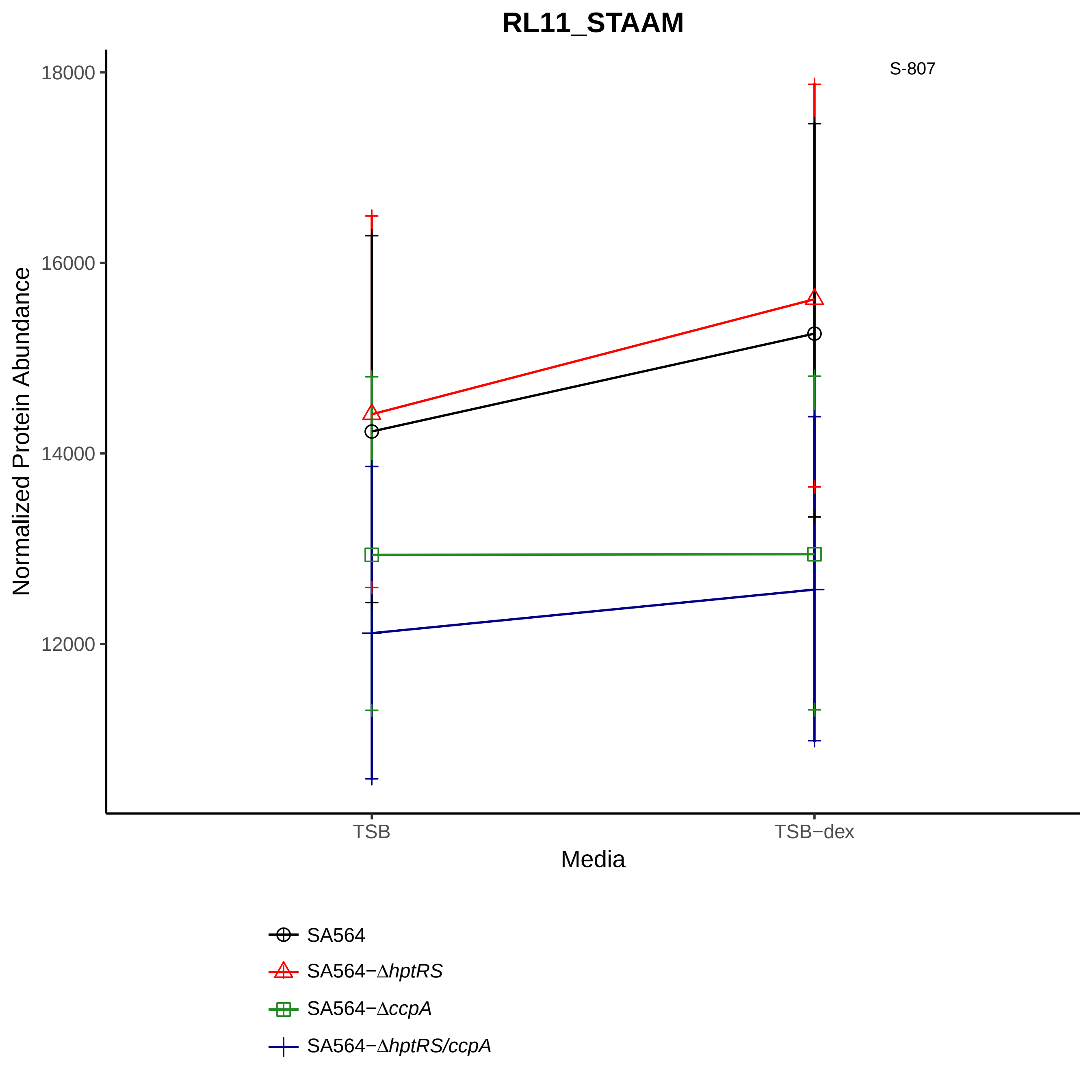

RL11_STAAM
S-807
Normalized Protein Abundance
18000
16000
14000
12000
TSB
TSB−dex
Media
SA564
SA564−Δ*hptRS*
SA564−Δ*ccpA*
SA564−Δ*hptRS/ccpA*

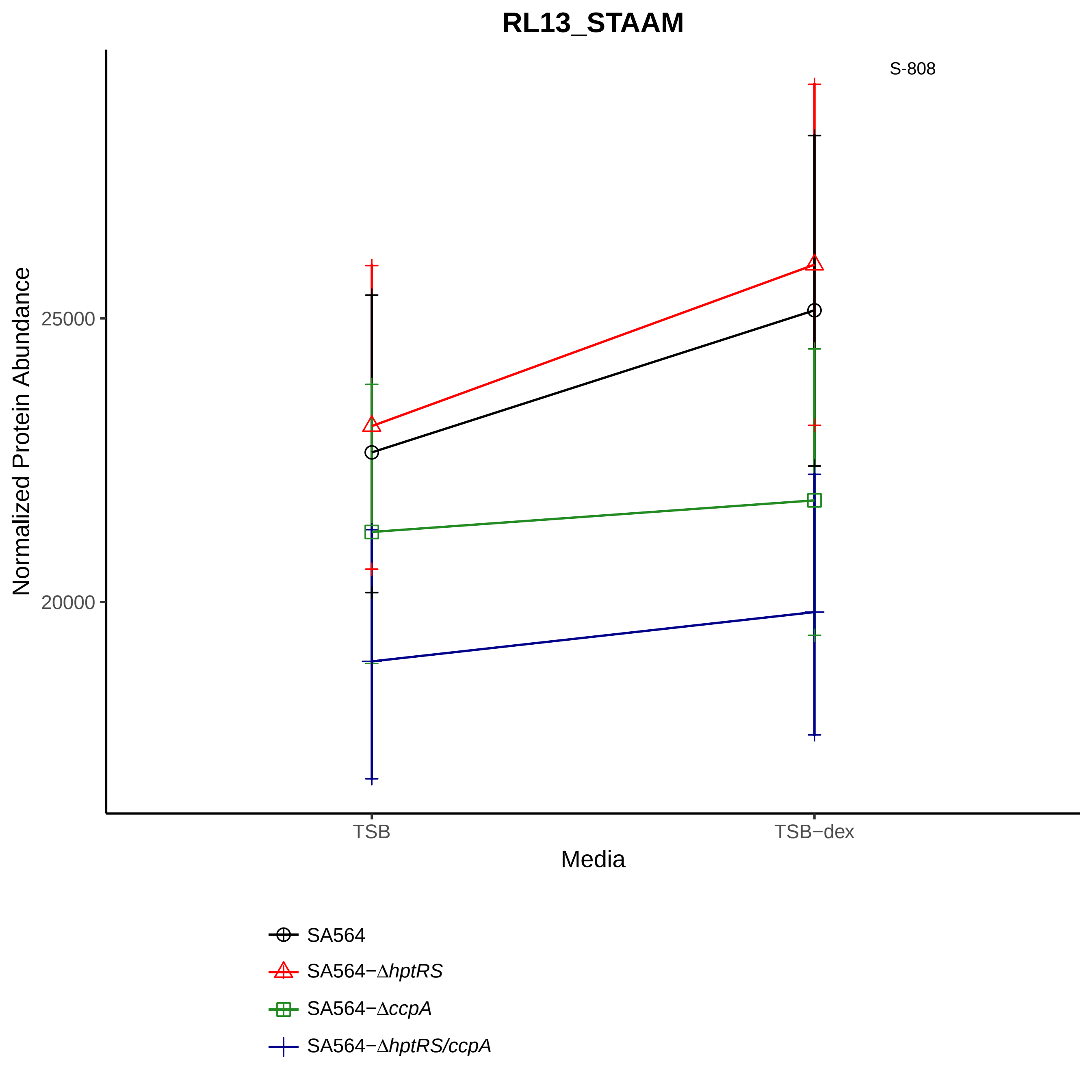

RL13_STAAM
S-808
Normalized Protein Abundance
25000
20000
TSB
TSB−dex
Media
SA564
SA564−Δ*hptRS*
SA564−Δ*ccpA*
SA564−Δ*hptRS/ccpA*

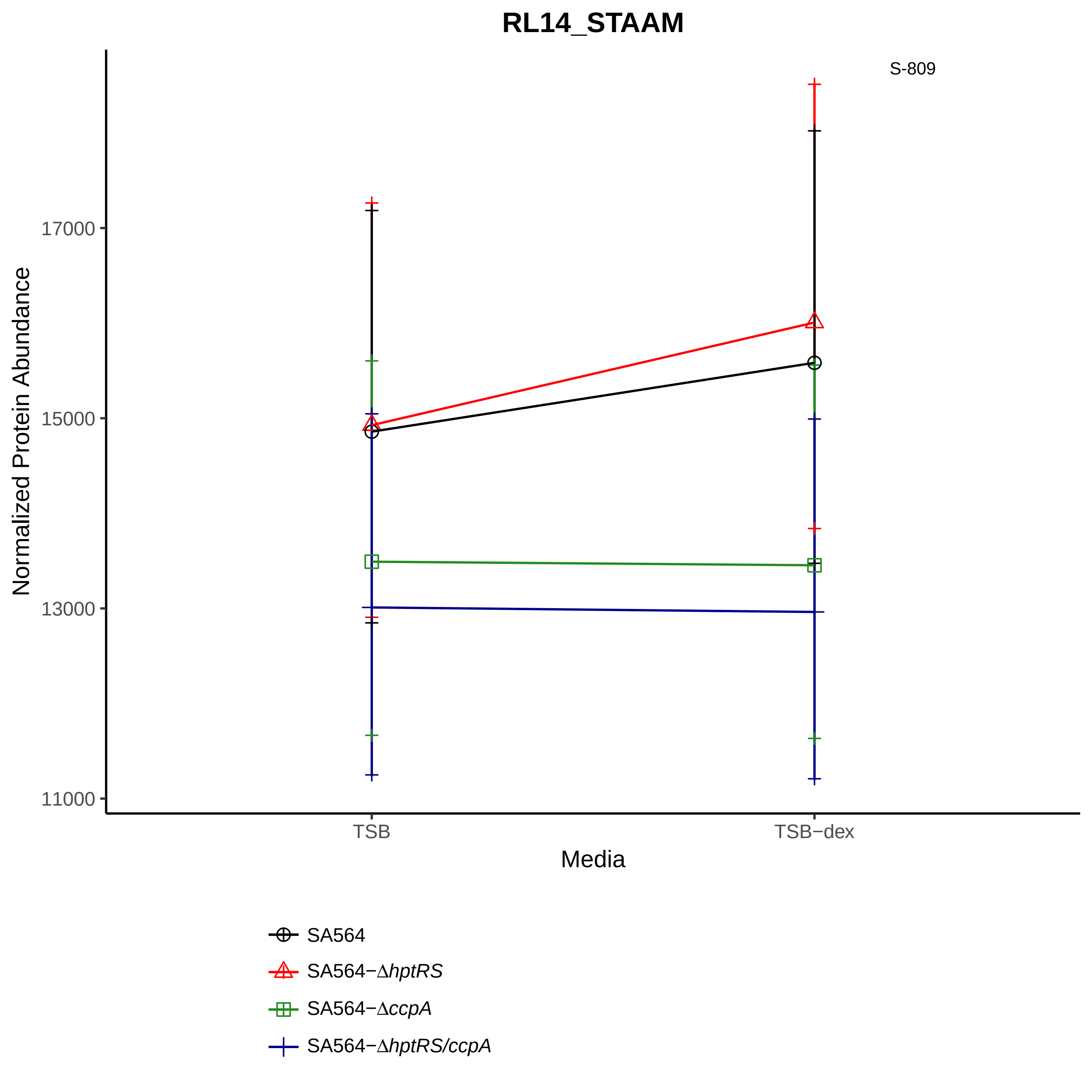
RL14_STAAM
S-809
Normalized Protein Abundance
17000
15000
13000
11000
TSB
TSB−dex
Media
SA564
SA564−Δ*hptRS*
SA564−Δ*ccpA*
SA564−Δ*hptRS/ccpA*

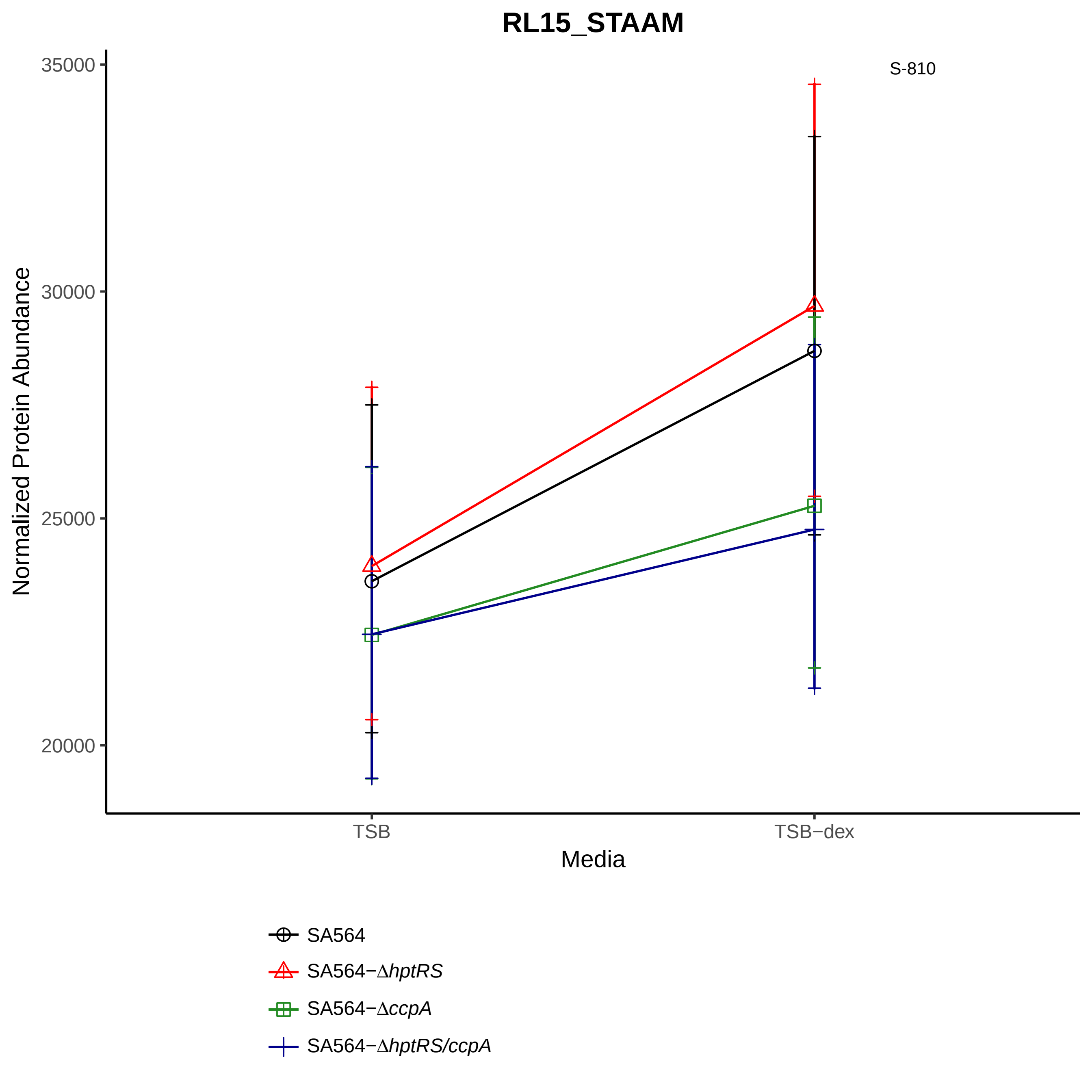

RL15_STAAM
S-810
Normalized Protein Abundance
35000
30000
25000
20000
TSB
TSB−dex
Media
SA564
SA564−Δ*hptRS*
SA564−Δ*ccpA*
SA564−Δ*hptRS/ccpA*

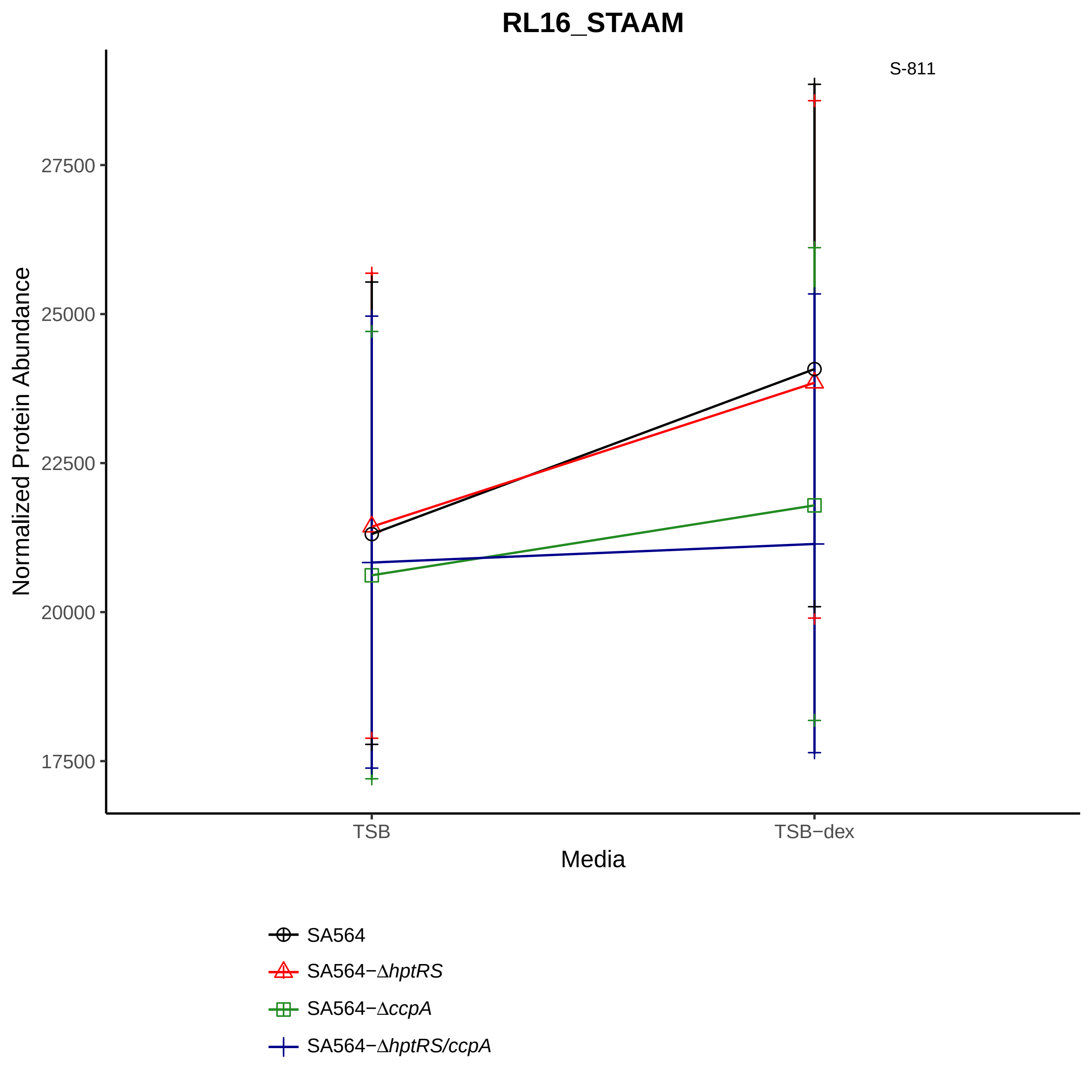
RL16_STAAM
S-811
Normalized Protein Abundance
27500
25000
22500
20000
17500
TSB
TSB−dex
Media
SA564
SA564−ΔhptRS
SA564−ΔccpA
SA564−ΔhptRS/ccpA

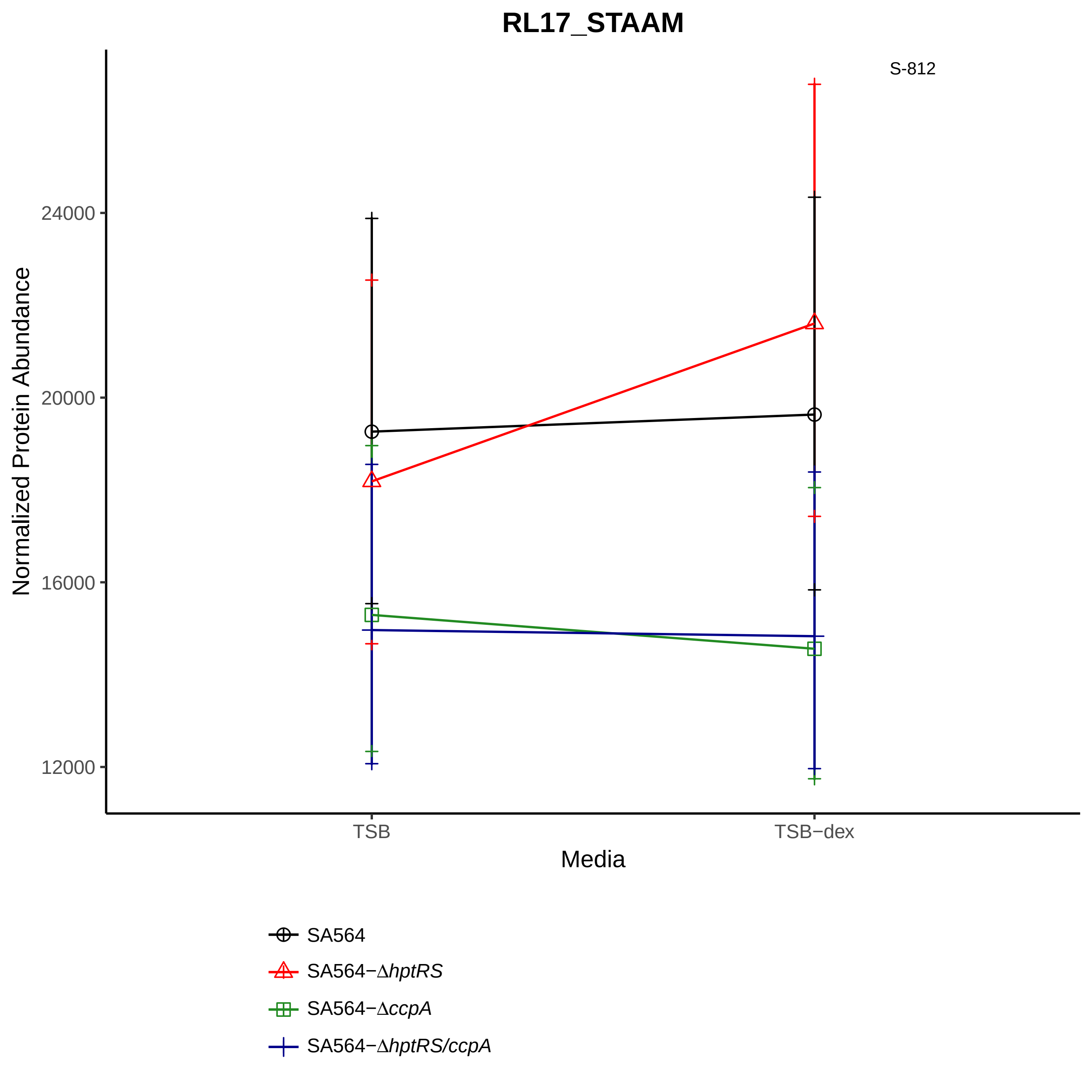

RL17_STAAM
S-812
Normalized Protein Abundance
24000
20000
16000
12000
TSB
TSB−dex
Media
SA564
SA564−Δ*hptRS*
SA564−Δ*ccpA*
SA564−Δ*hptRS/ccpA*

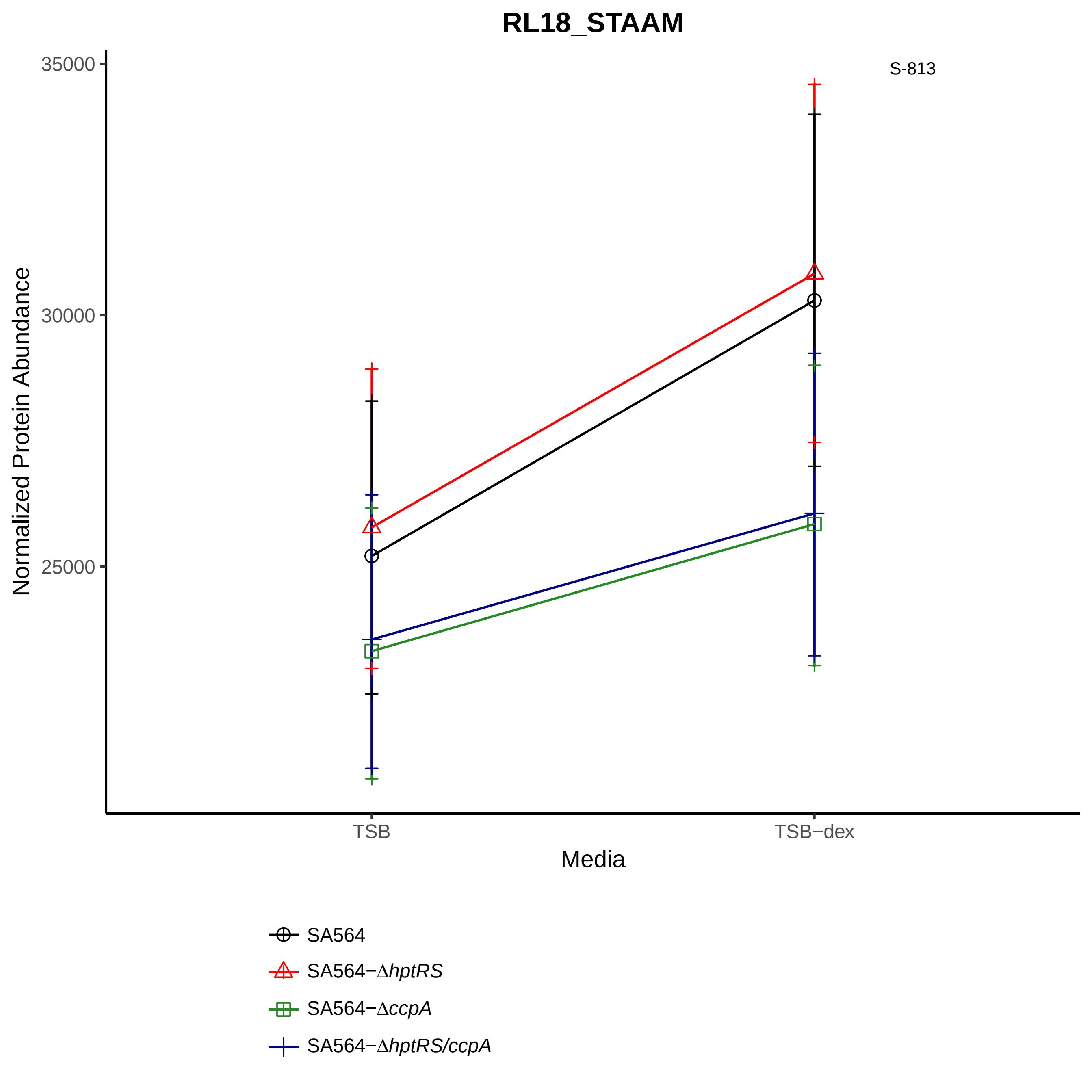

RL18_STAAM
S-813
35000
30000
25000
Normalized Protein Abundance
TSB
TSB−dex
Media
SA564
SA564−ΔhptRS
SA564−ΔccpA
SA564−ΔhptRS/ccpA

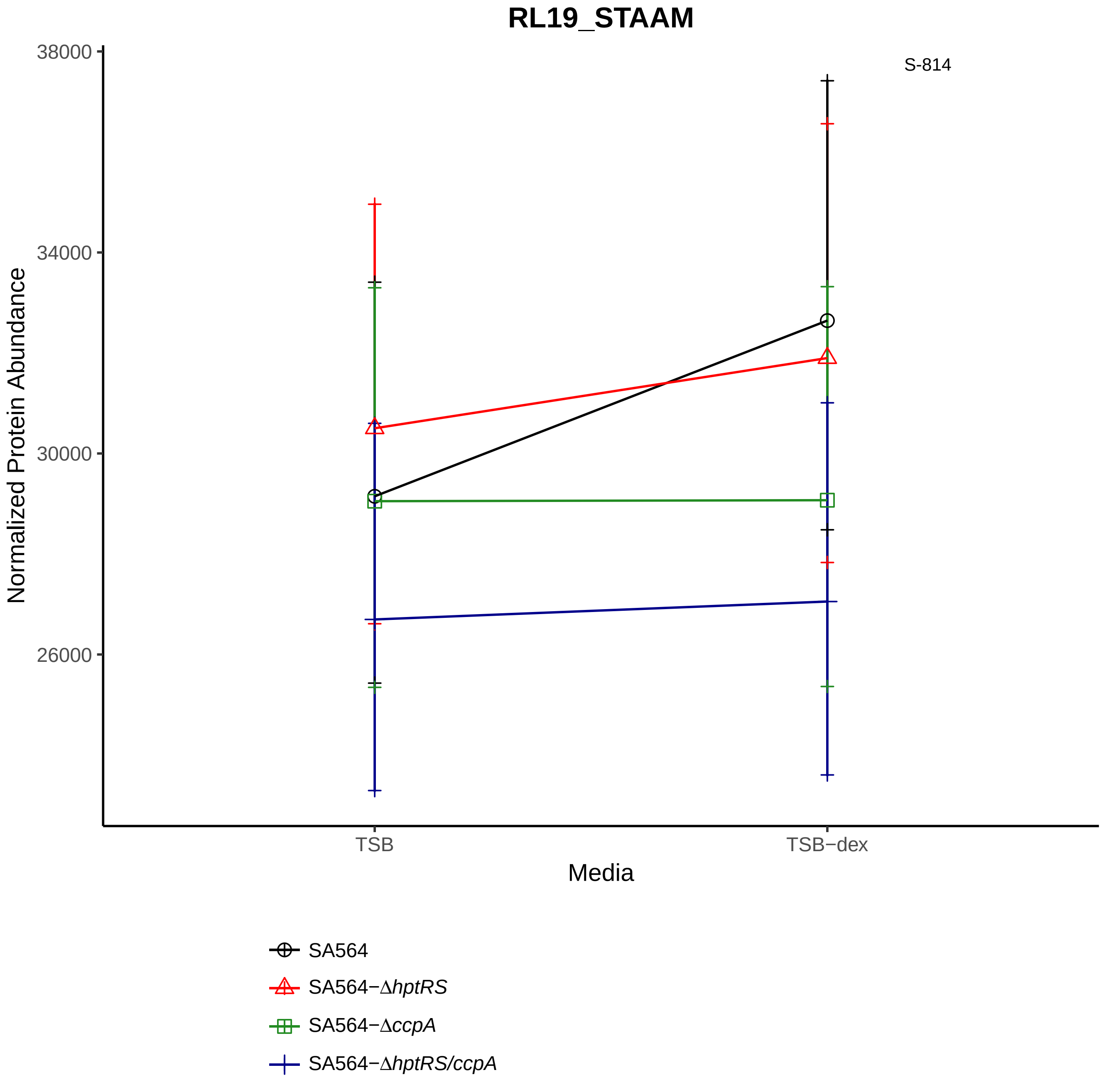
RL19_STAAM
S-814
Normalized Protein Abundance
38000
34000
30000
26000
TSB
TSB−dex
Media
SA564
SA564−ΔhptRS
SA564−ΔccpA
SA564−ΔhptRS/ccpA

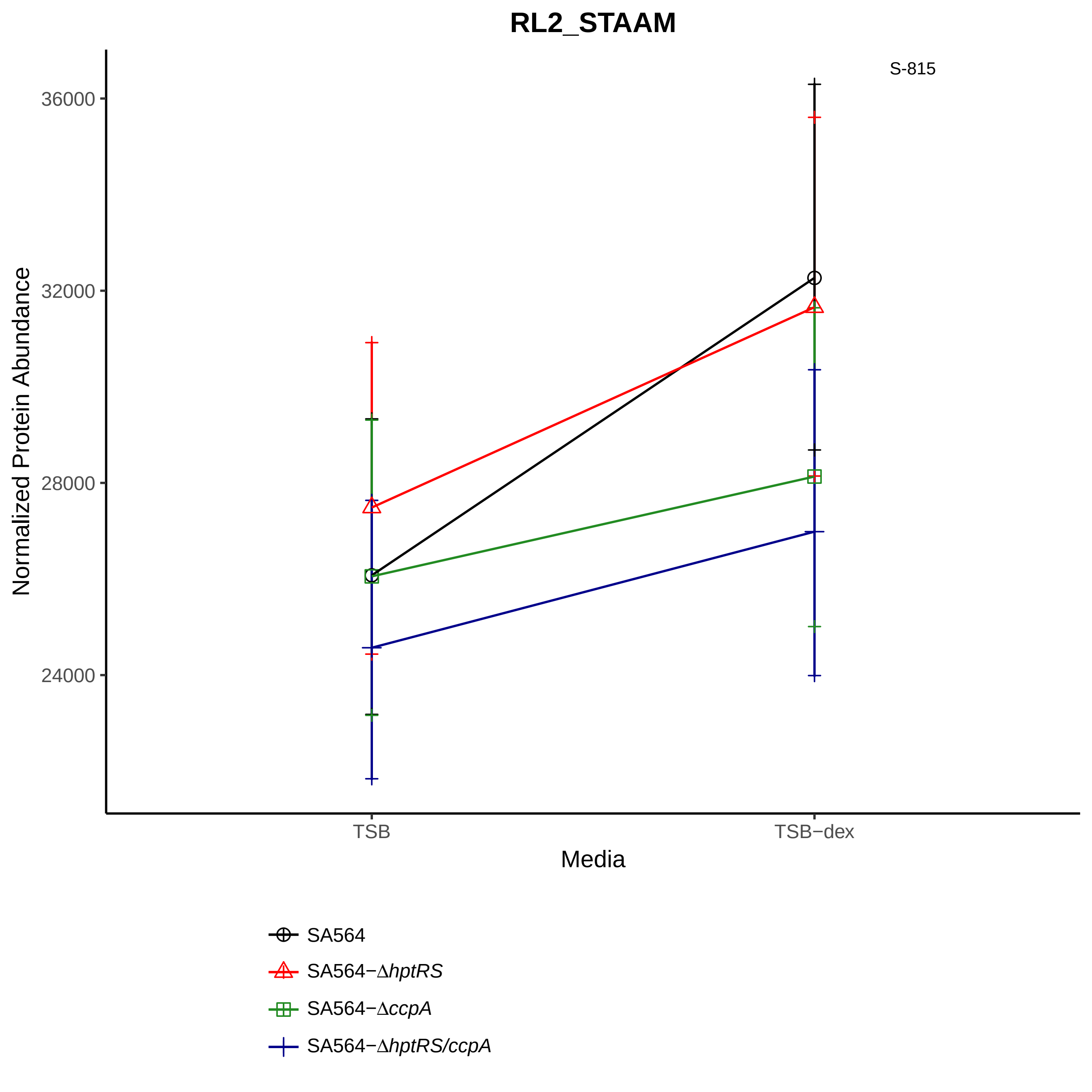

RL2_STAAM
S-815
36000
32000
28000
24000
Normalized Protein Abundance
TSB
TSB−dex
Media
SA564
SA564−Δ*hptRS*
SA564−Δ*ccpA*
SA564−Δ*hptRS/ccpA*

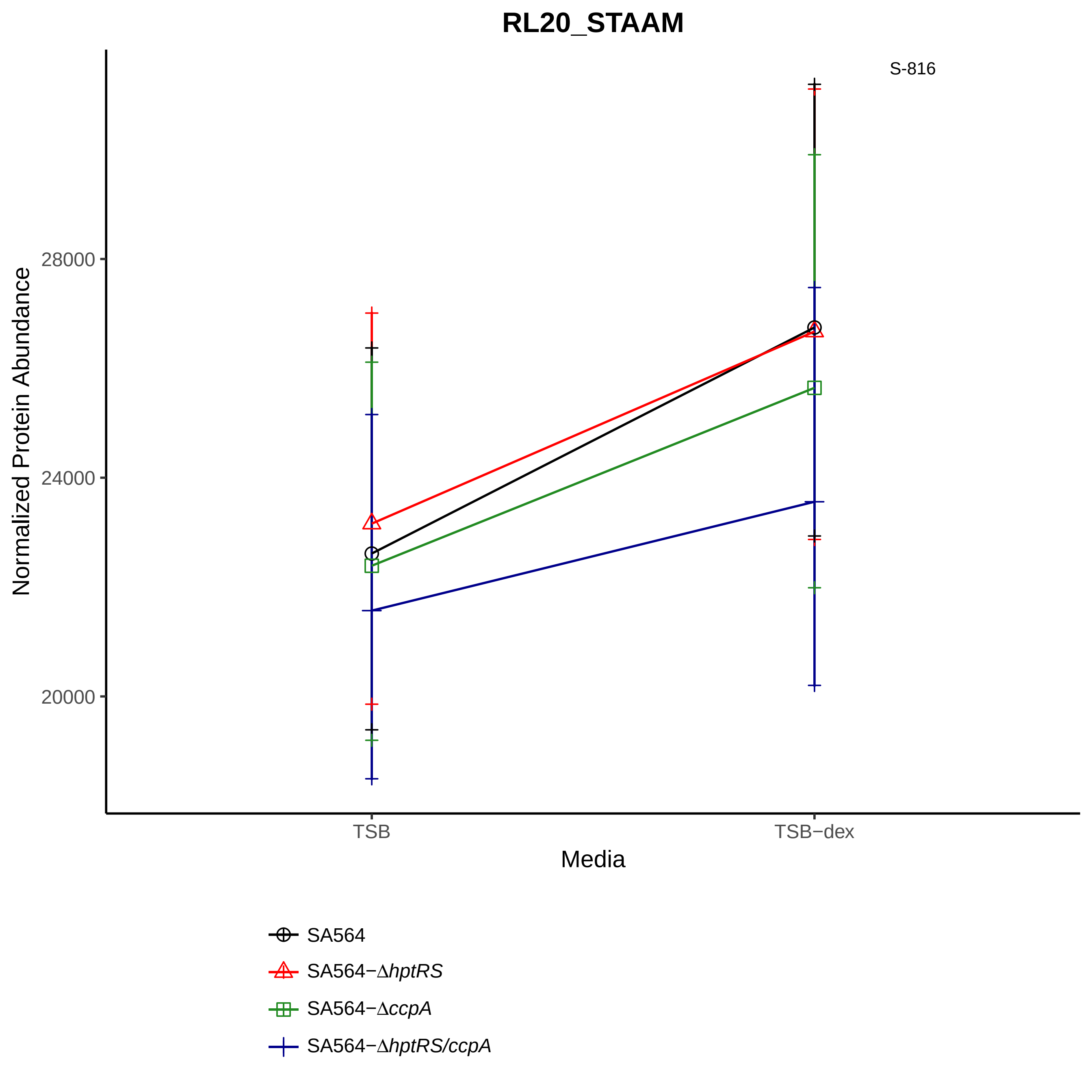

RL20_STAAM
S-816
Normalized Protein Abundance
28000
24000
20000
TSB
TSB−dex
Media
SA564
SA564−ΔhptRS
SA564−ΔccpA
SA564−ΔhptRS/ccpA

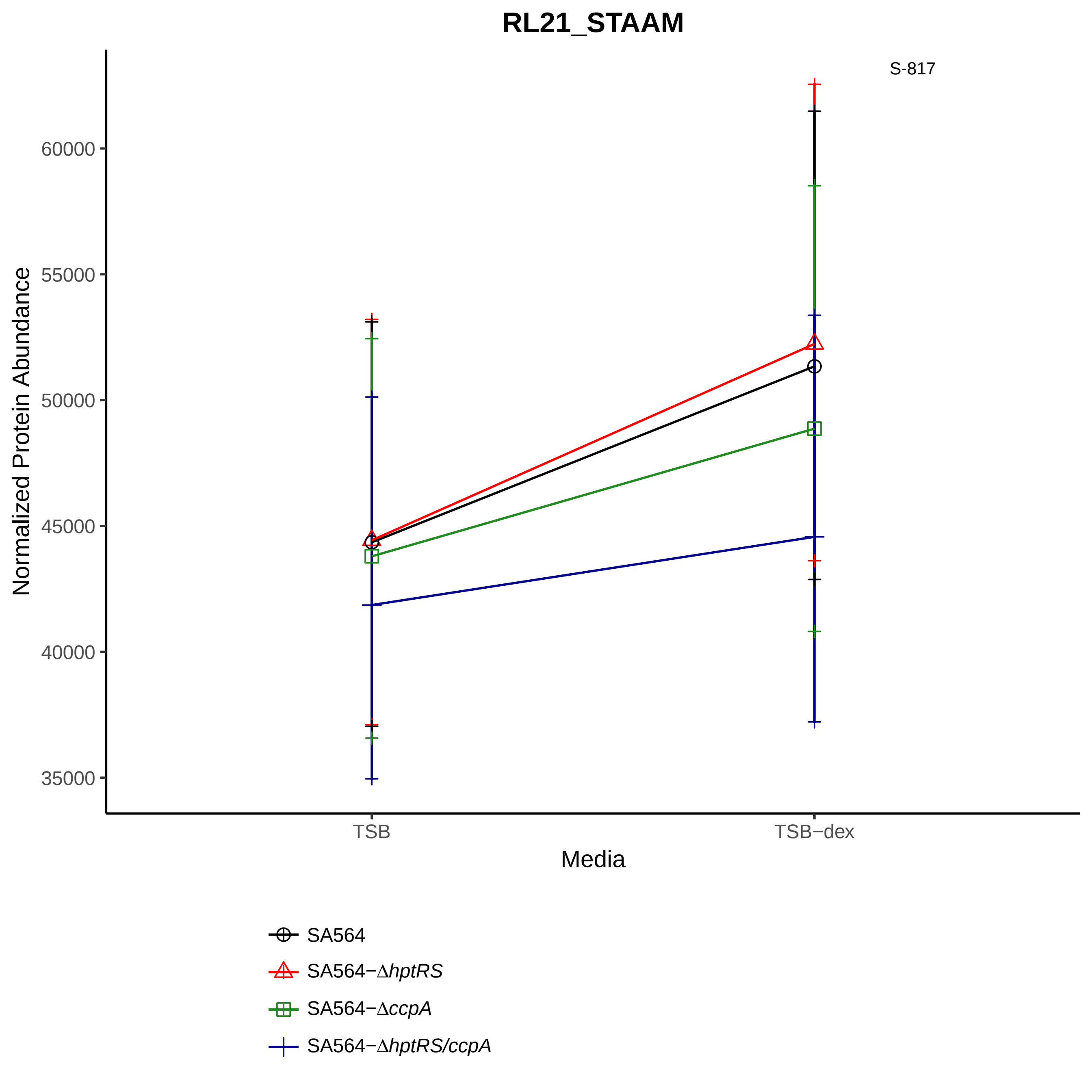

RL21_STAAM
S-817
Normalized Protein Abundance
60000
55000
50000
45000
40000
35000
TSB
TSB−dex
Media
SA564
SA564−Δ*hptRS*
SA564−Δ*ccpA*
SA564−Δ*hptRS/ccpA*

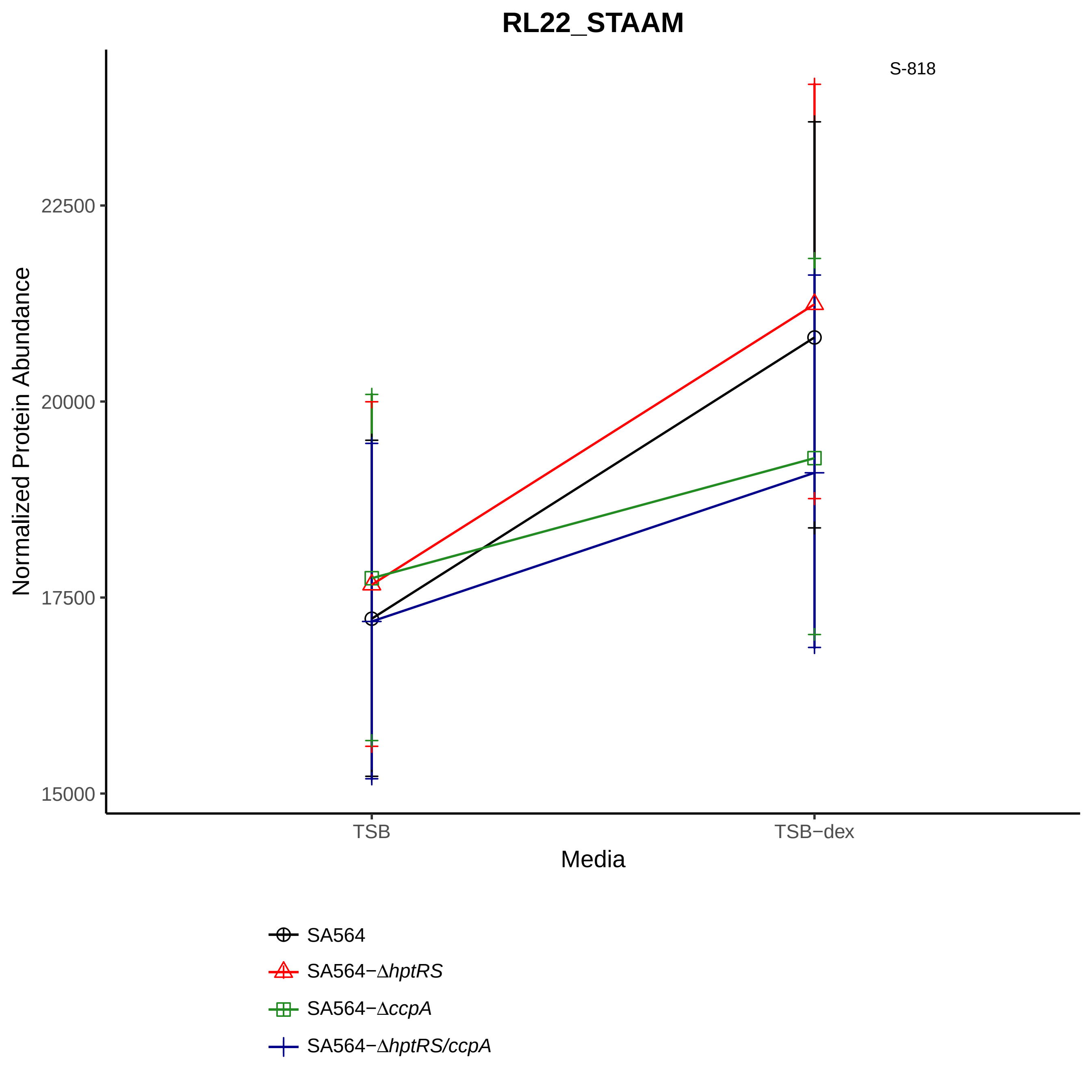

RL22_STAAM
S-818
Normalized Protein Abundance
22500
20000
17500
15000
TSB
TSB−dex
Media
SA564
SA564−ΔhptRS
SA564−ΔccpA
SA564−ΔhptRS/ccpA

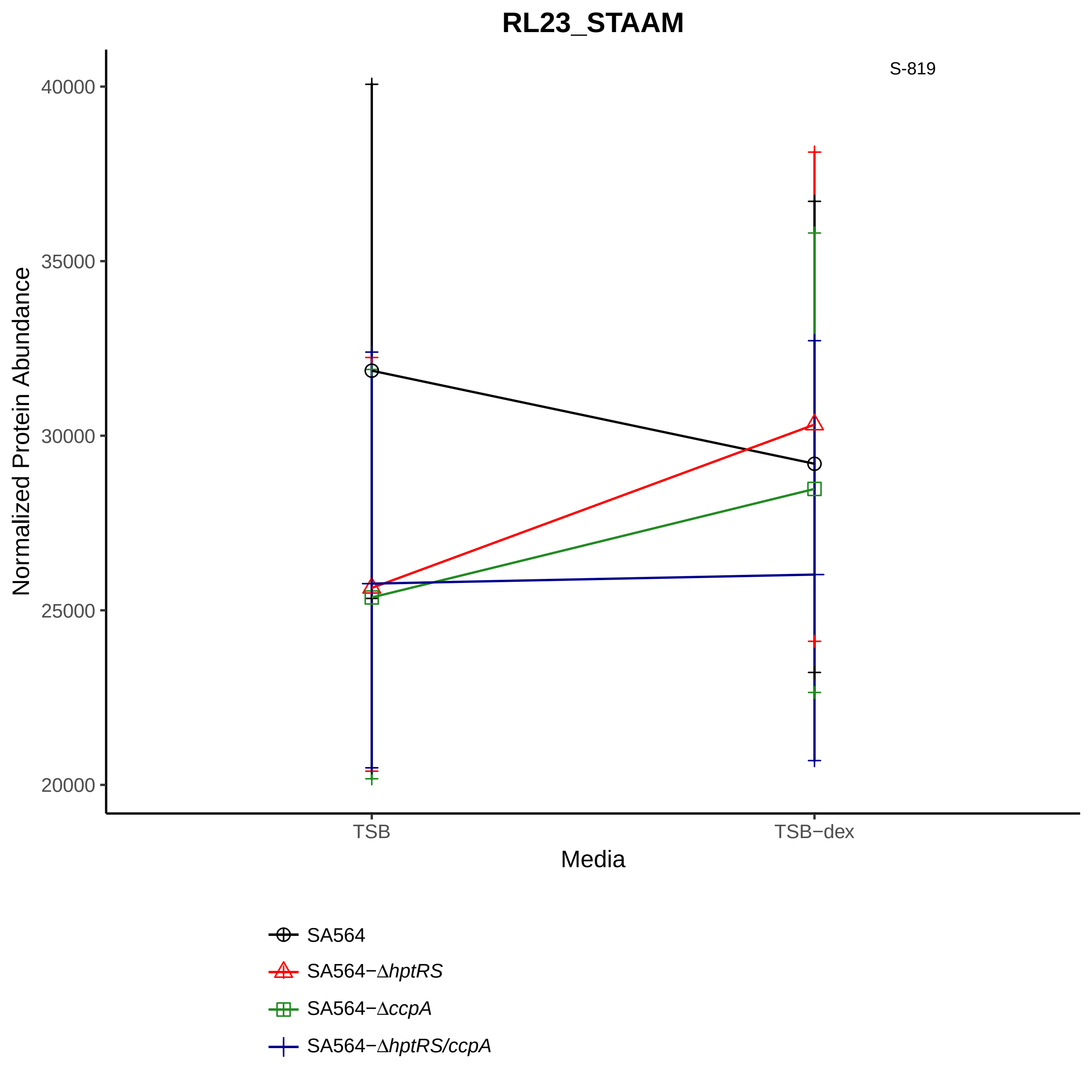
RL23_STAAM
S-819
Normalized Protein Abundance
40000
35000
30000
25000
20000
TSB
TSB−dex
Media
SA564
SA564−Δ*hptRS*
SA564−Δ*ccpA*
SA564−Δ*hptRS/ccpA*

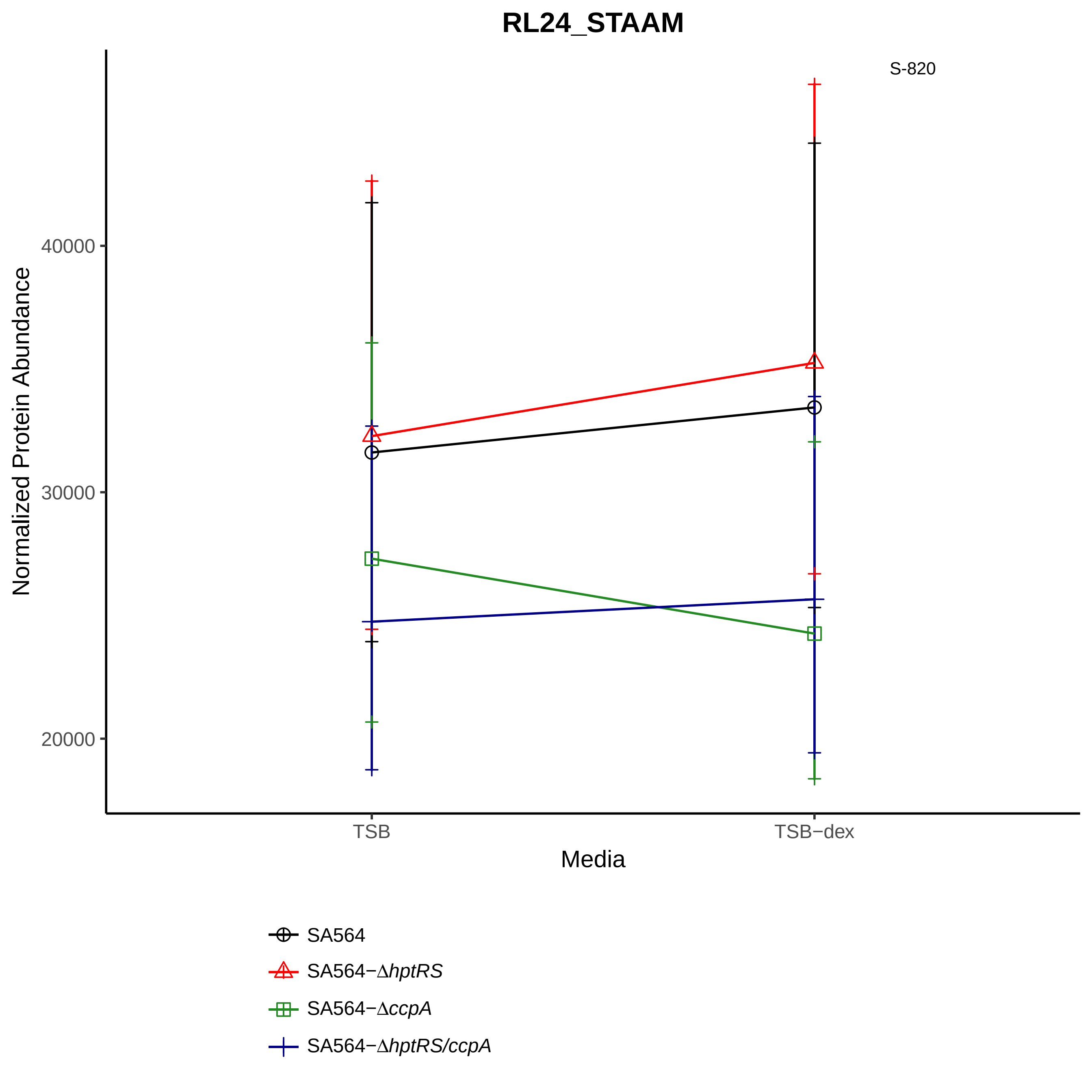

RL24_STAAM
S-820
Normalized Protein Abundance
40000
30000
20000
TSB
TSB−dex
Media
SA564
SA564−Δ*hptRS*
SA564−Δ*ccpA*
SA564−Δ*hptRS/ccpA*

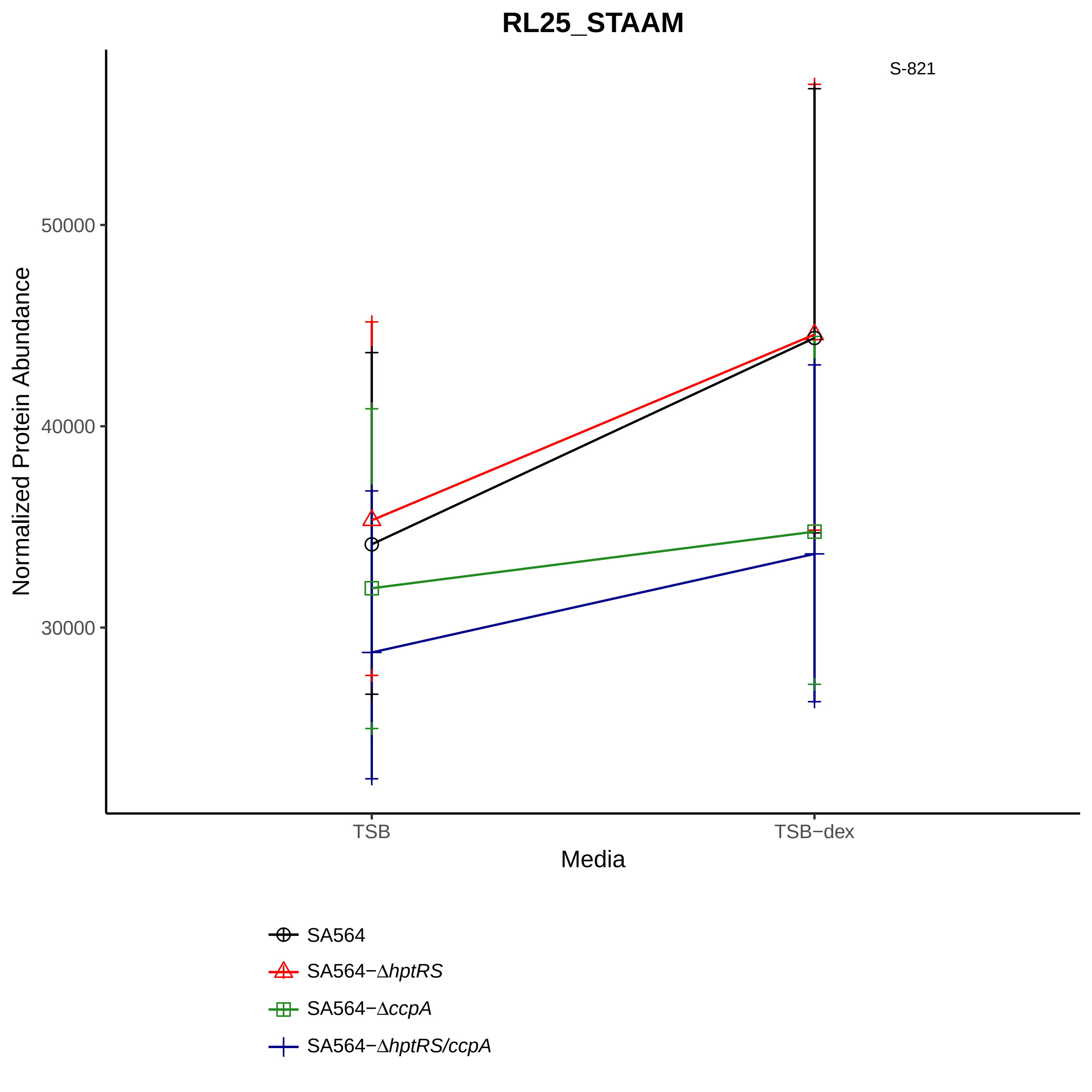

RL25_STAAM
S-821
Normalized Protein Abundance
50000
40000
30000
TSB
TSB−dex
Media
SA564
SA564−ΔhptRS
SA564−ΔccpA
SA564−ΔhptRS/ccpA

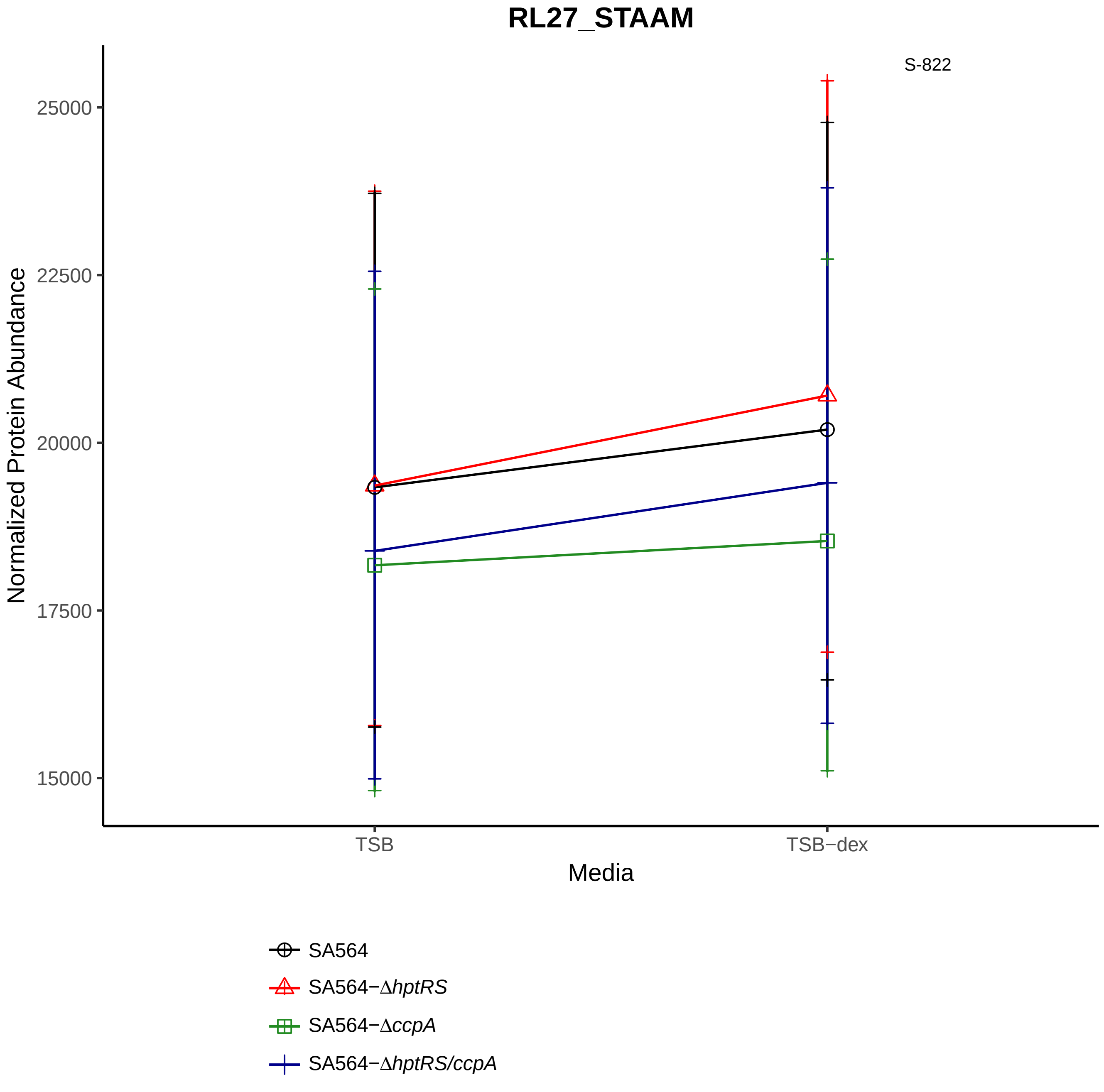
RL27_STAAM
S-822
Normalized Protein Abundance
25000
22500
20000
17500
15000
TSB
TSB−dex
Media
SA564
SA564−Δ*hptRS*
SA564−Δ*ccpA*
SA564−Δ*hptRS/ccpA*

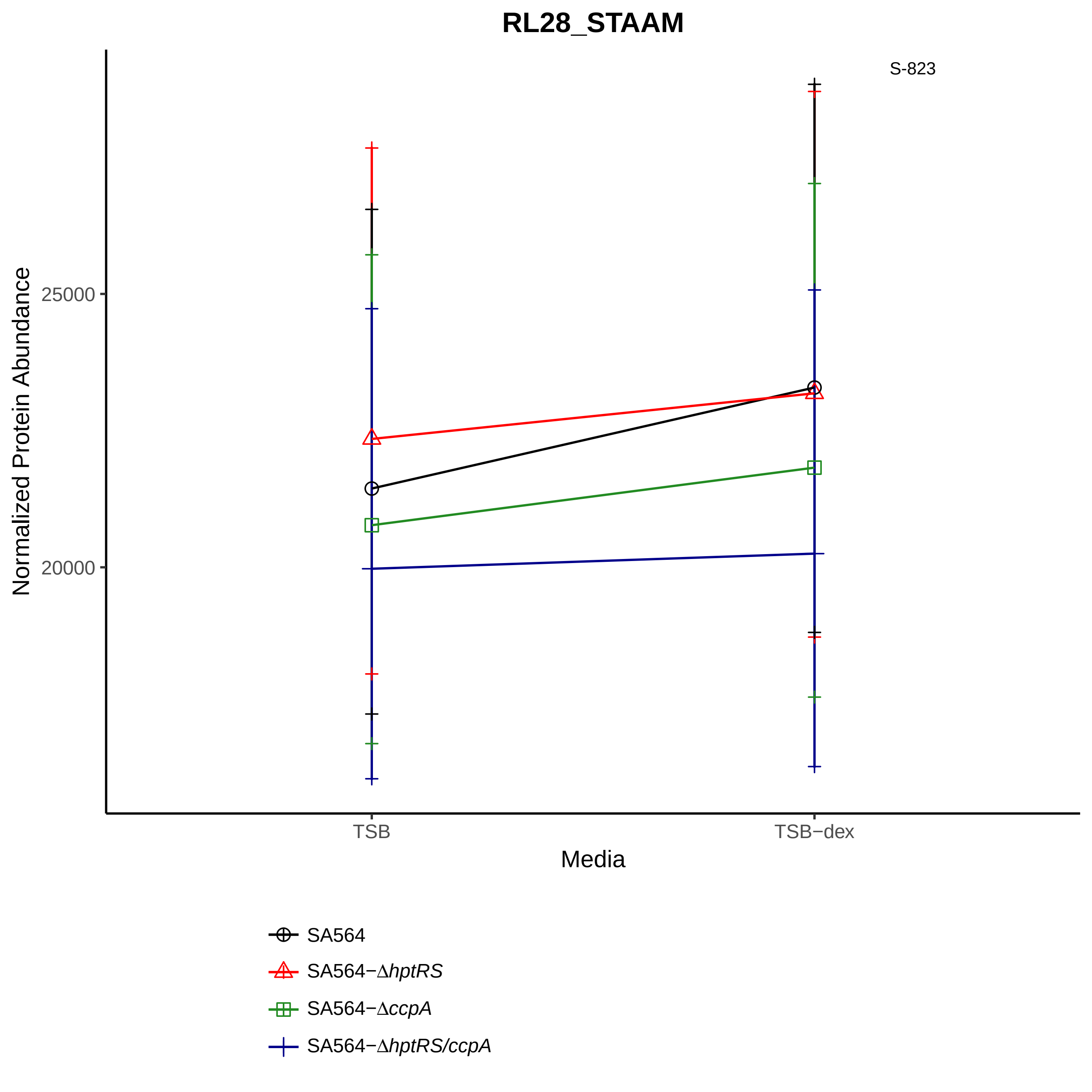
RL28_STAAM
S-823
Normalized Protein Abundance
25000
20000
TSB
TSB−dex
Media
SA564
SA564−ΔhptRS
SA564−ΔccpA
SA564−ΔhptRS/ccpA

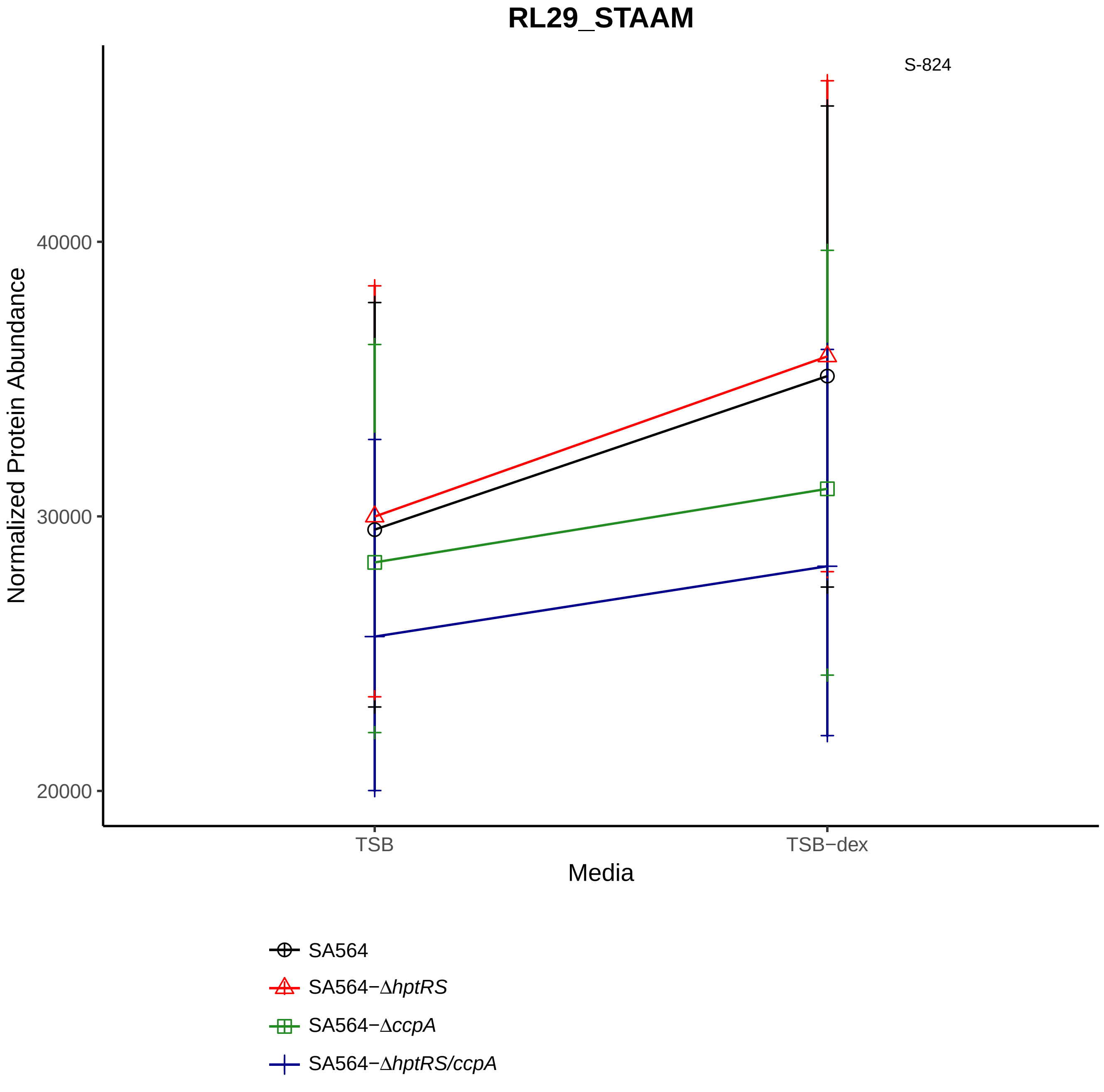

RL29_STAAM
S-824
Normalized Protein Abundance
40000
30000
20000
TSB
TSB−dex
Media
SA564
SA564−Δ*hptRS*
SA564−Δ*ccpA*
SA564−Δ*hptRS/ccpA*

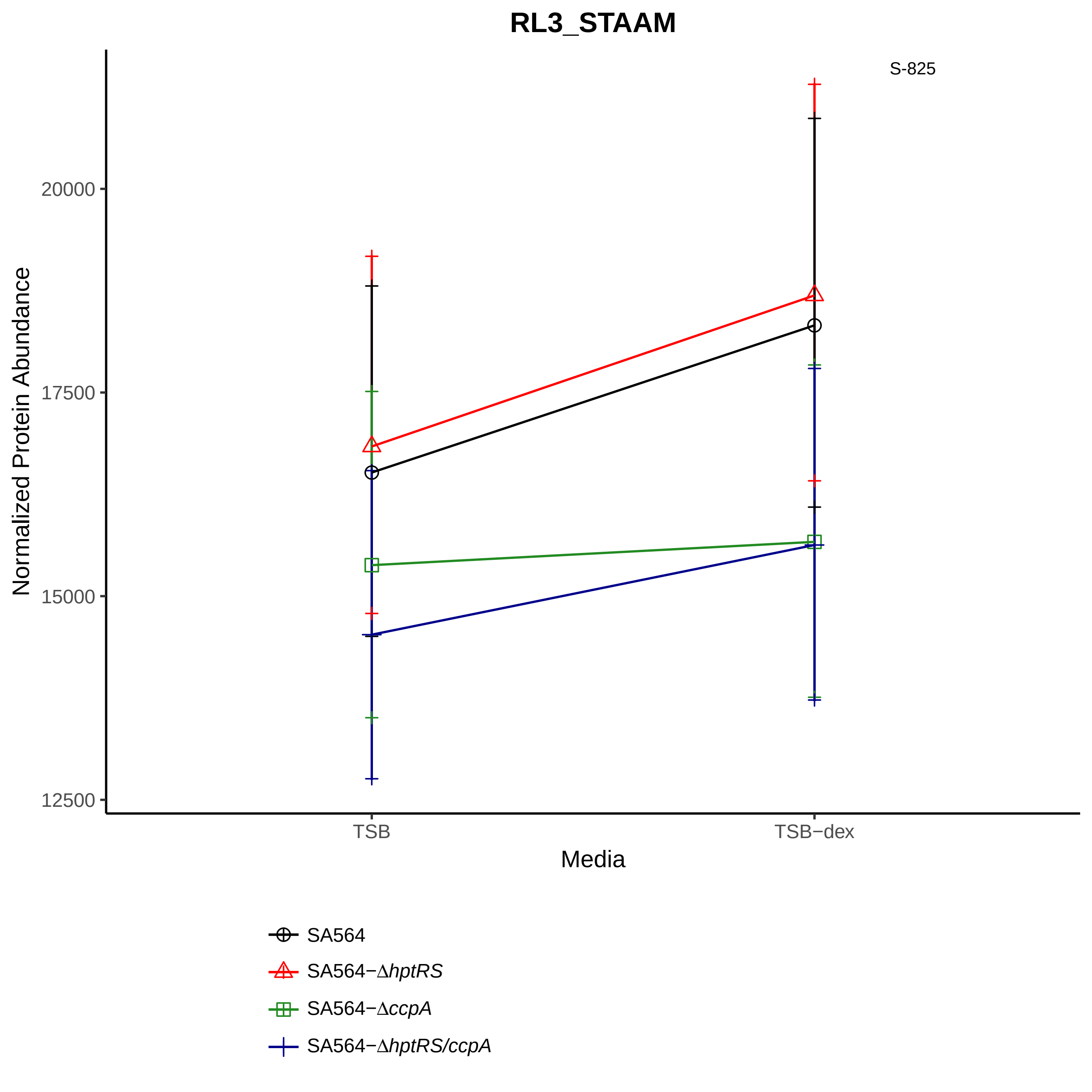
RL3_STAAM
S-825
Normalized Protein Abundance
20000
17500
15000
12500
TSB
TSB−dex
Media
SA564
SA564−ΔhptRS
SA564−ΔccpA
SA564−ΔhptRS/ccpA

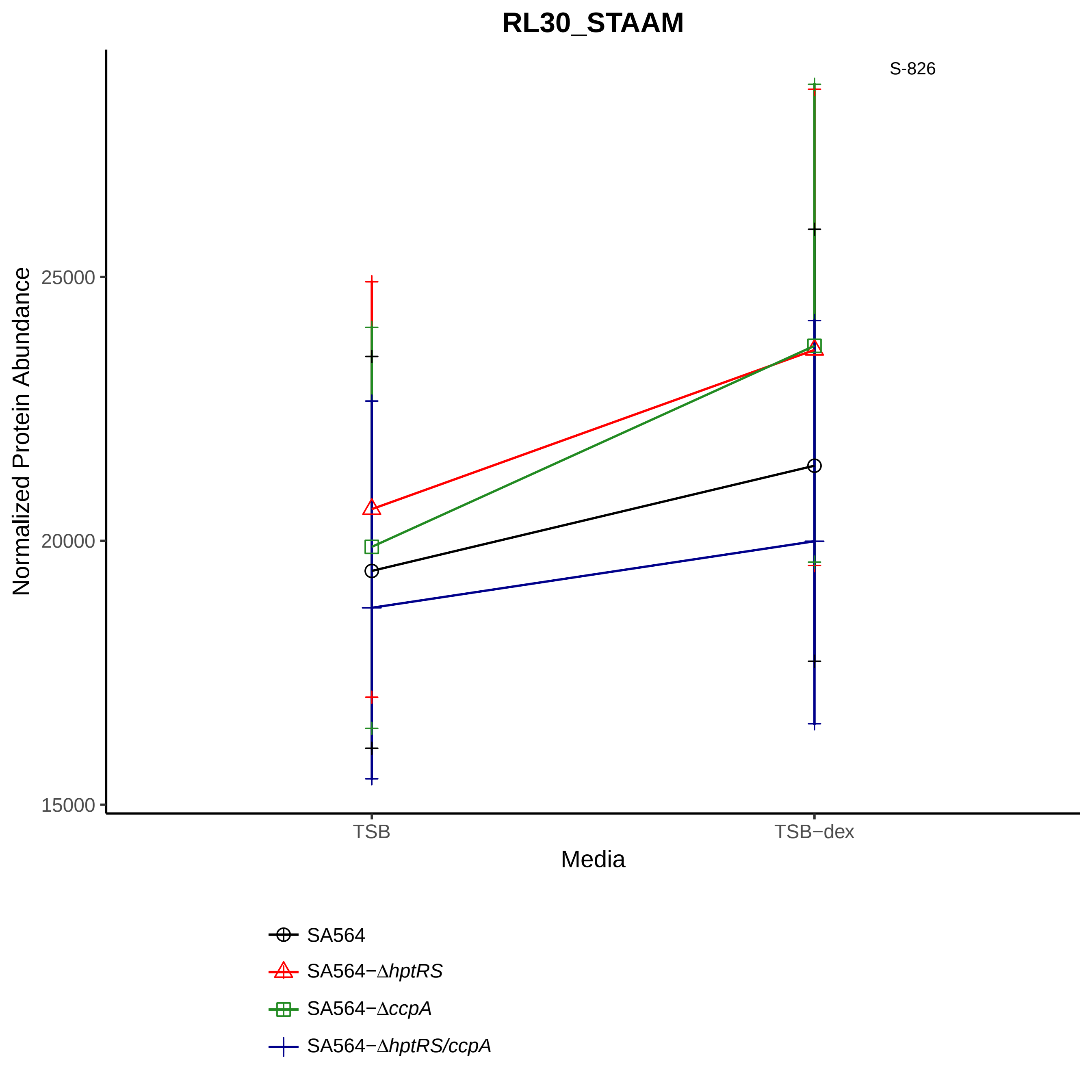
RL30_STAAM
S-826
Normalized Protein Abundance
25000
20000
15000
TSB
TSB−dex
Media
SA564
SA564−Δ*hptRS*
SA564−Δ*ccpA*
SA564−Δ*hptRS/ccpA*

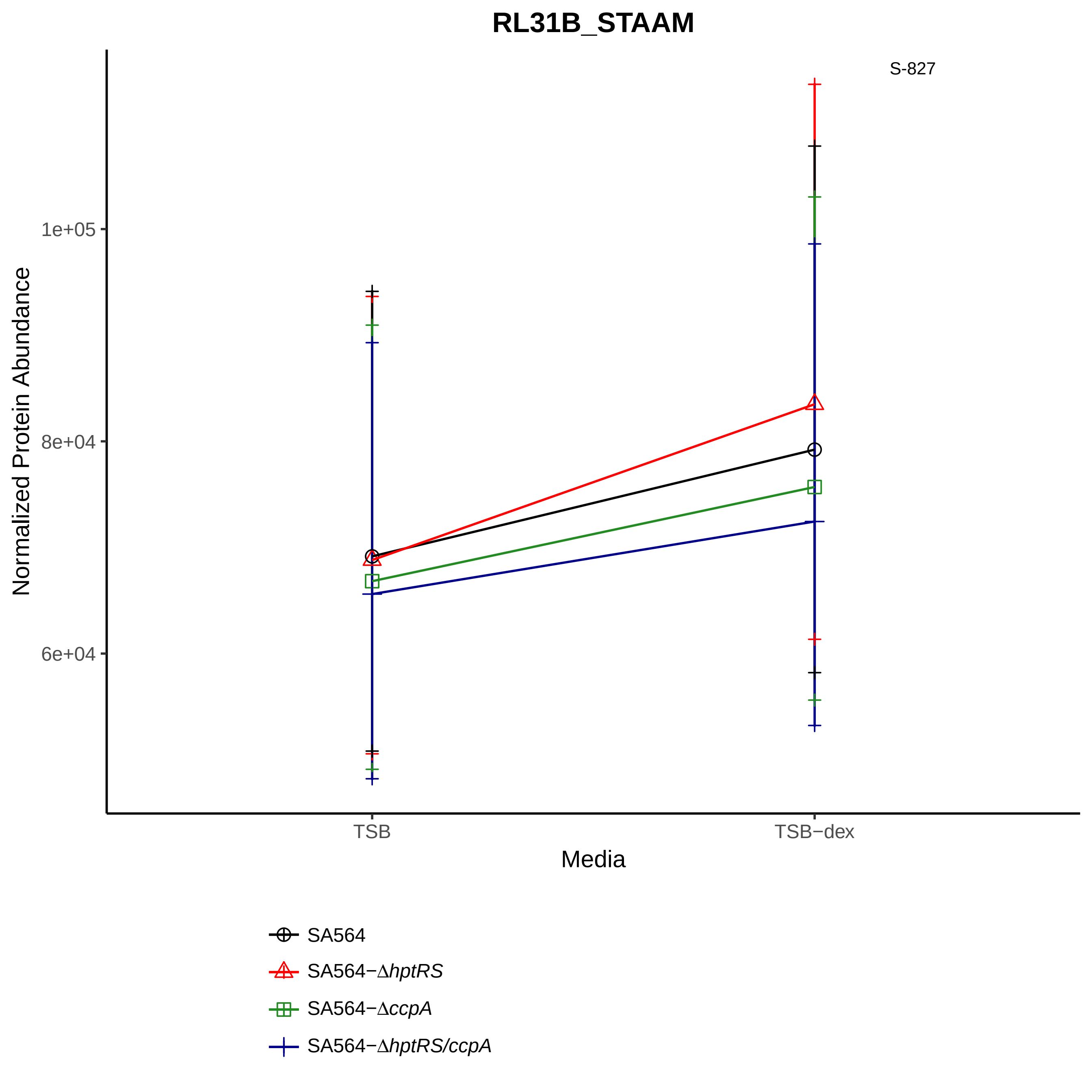
RL31B_STAAM
S-827
Normalized Protein Abundance
1e+05
8e+04
6e+04
TSB
TSB−dex
Media
SA564
SA564−ΔhptRS
SA564−ΔccpA
SA564−ΔhptRS/ccpA

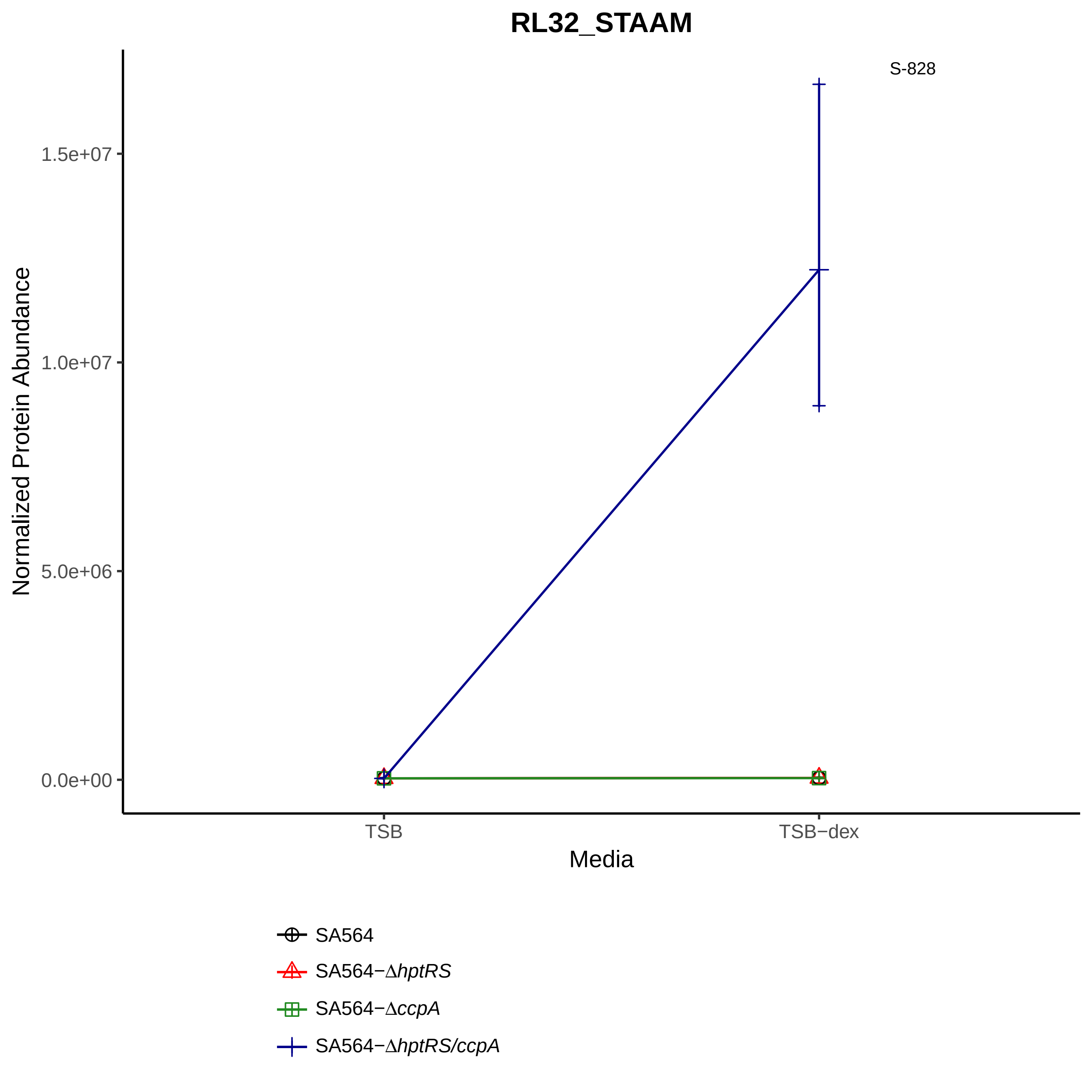

RL32_STAAM
S-828
Normalized Protein Abundance
1.5e+07
1.0e+07
5.0e+06
0.0e+00
TSB
TSB−dex
Media
SA564
SA564−Δ*hptRS*
SA564−Δ*ccpA*
SA564−Δ*hptRS/ccpA*

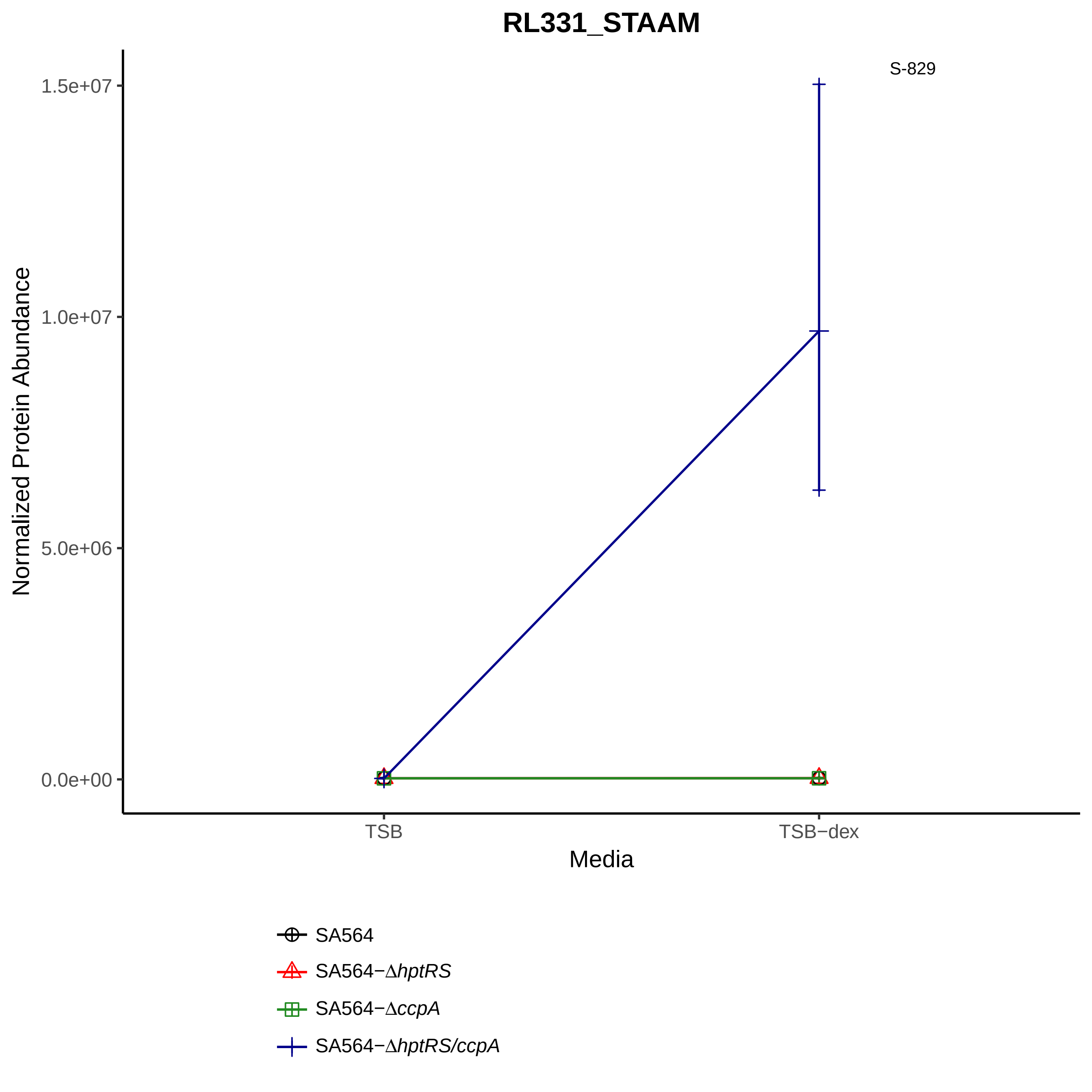

RL331_STAAM
S-829
Normalized Protein Abundance
1.5e+07
1.0e+07
5.0e+06
0.0e+00
TSB
TSB−dex
Media
SA564
SA564−ΔhptRS
SA564−ΔccpA
SA564−ΔhptRS/ccpA

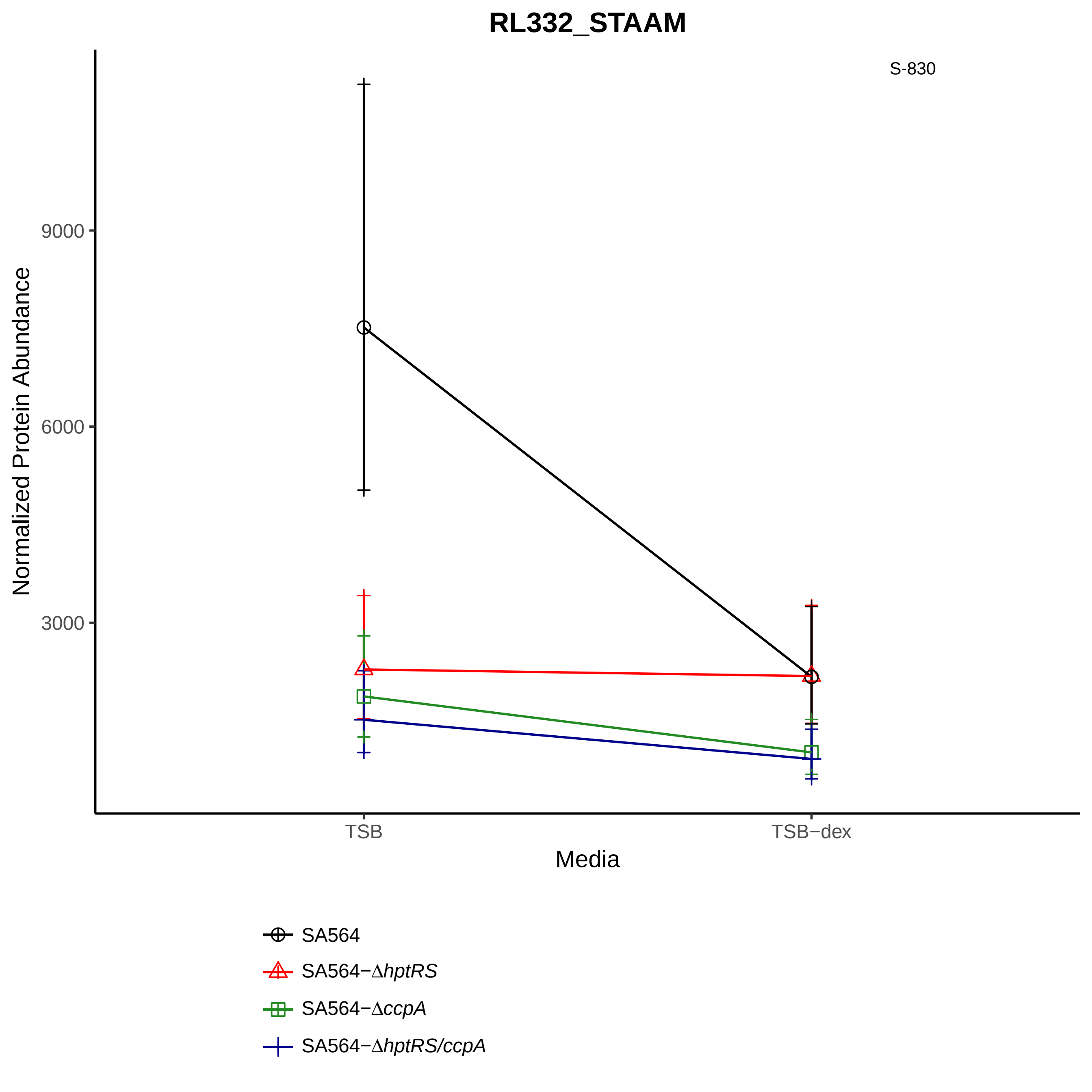
RL332_STAAM
S-830
Normalized Protein Abundance
9000
6000
3000
TSB
TSB−dex
Media
SA564
SA564−Δ*hptRS*
SA564−Δ*ccpA*
SA564−Δ*hptRS/ccpA*

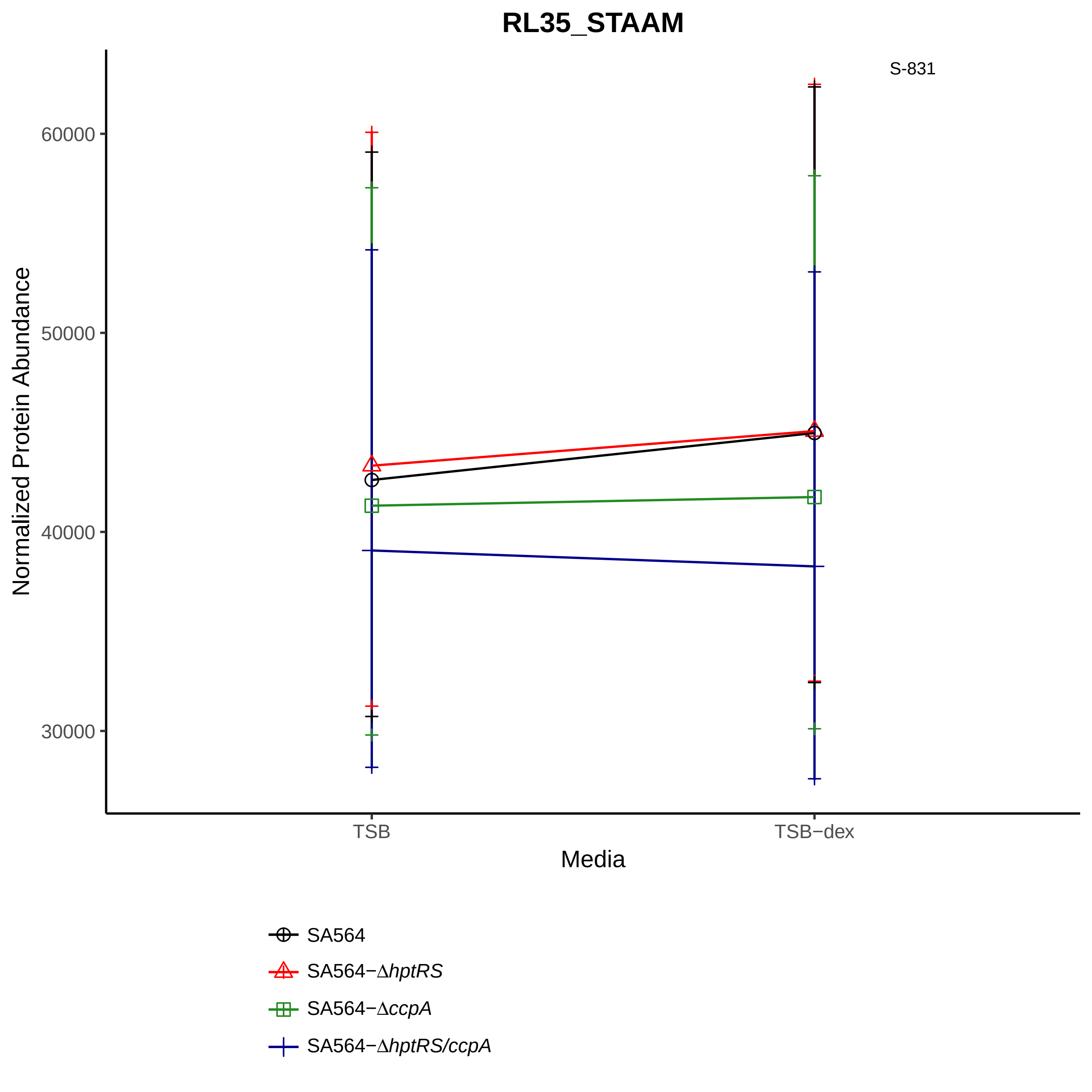

RL35_STAAM
S-831
Normalized Protein Abundance
60000
50000
40000
30000
TSB
TSB−dex
Media
SA564
SA564−Δ*hptRS*
SA564−Δ*ccpA*
SA564−Δ*hptRS/ccpA*

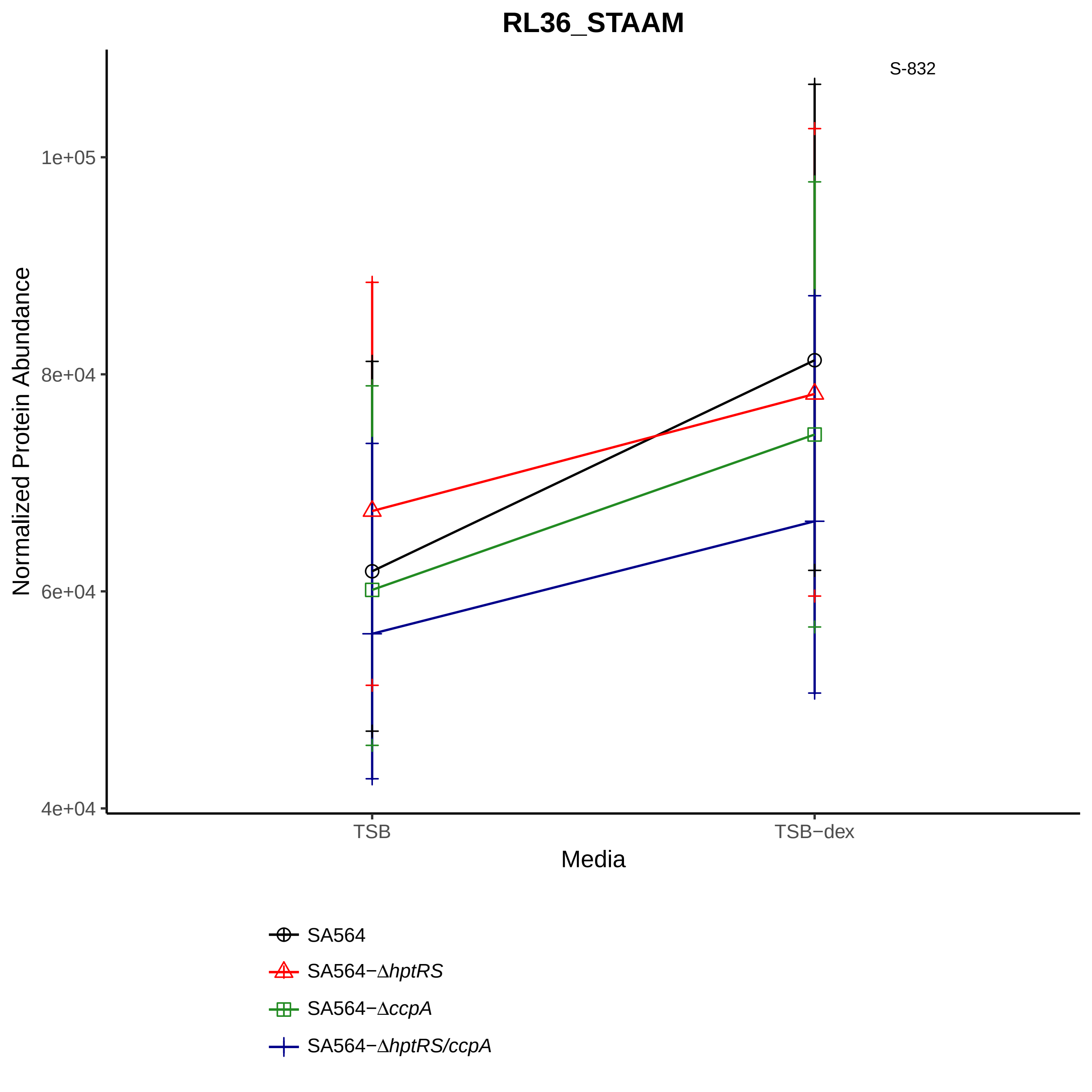

RL36_STAAM
S-832
Normalized Protein Abundance
1e+05
8e+04
6e+04
4e+04
TSB
TSB−dex
Media
SA564
SA564−Δ*hptRS*
SA564−Δ*ccpA*
SA564−Δ*hptRS/ccpA*

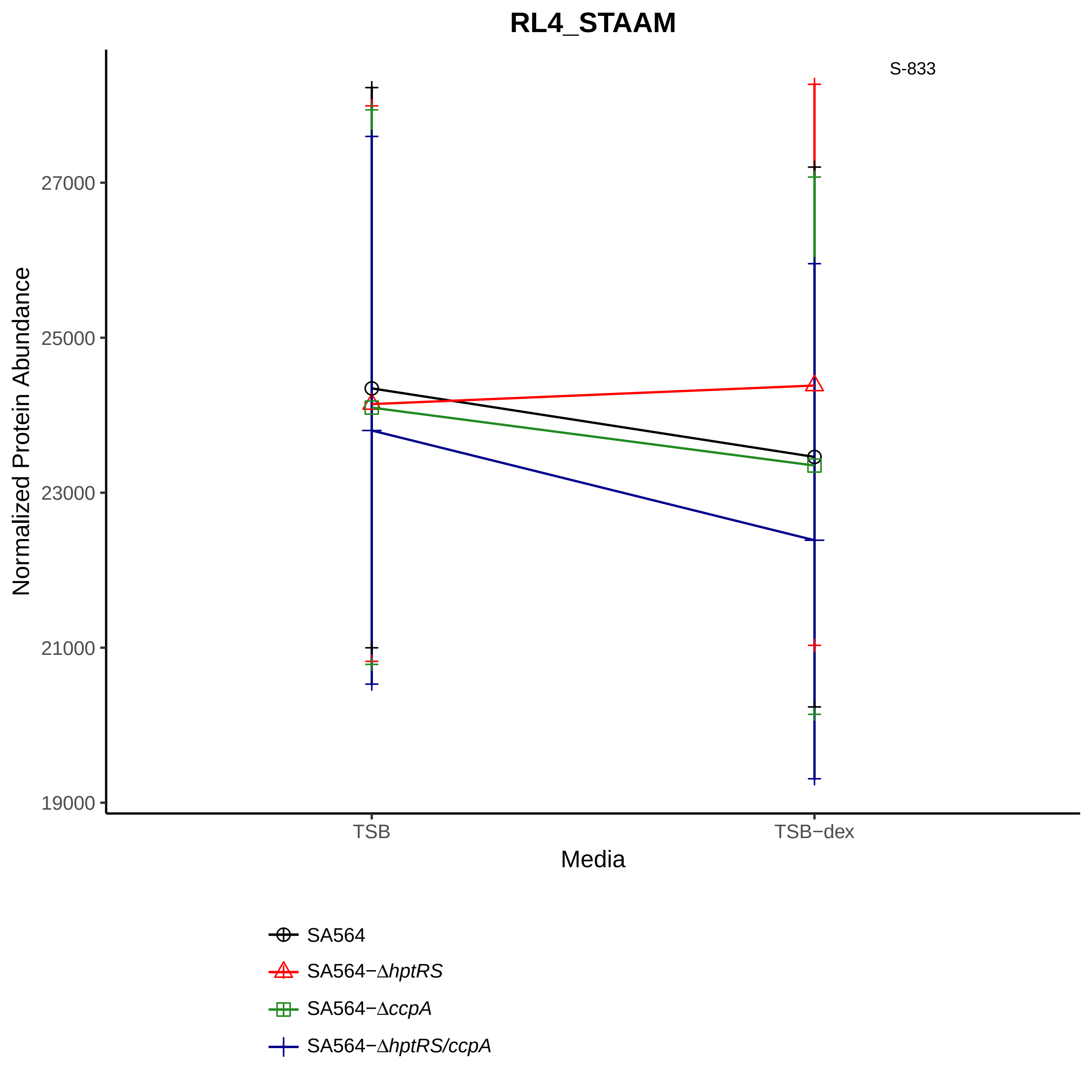

RL4_STAAM
S-833
Normalized Protein Abundance
19000
21000
23000
25000
27000
TSB
TSB−dex
Media
SA564
SA564−ΔhptRS
SA564−ΔccpA
SA564−ΔhptRS/ccpA

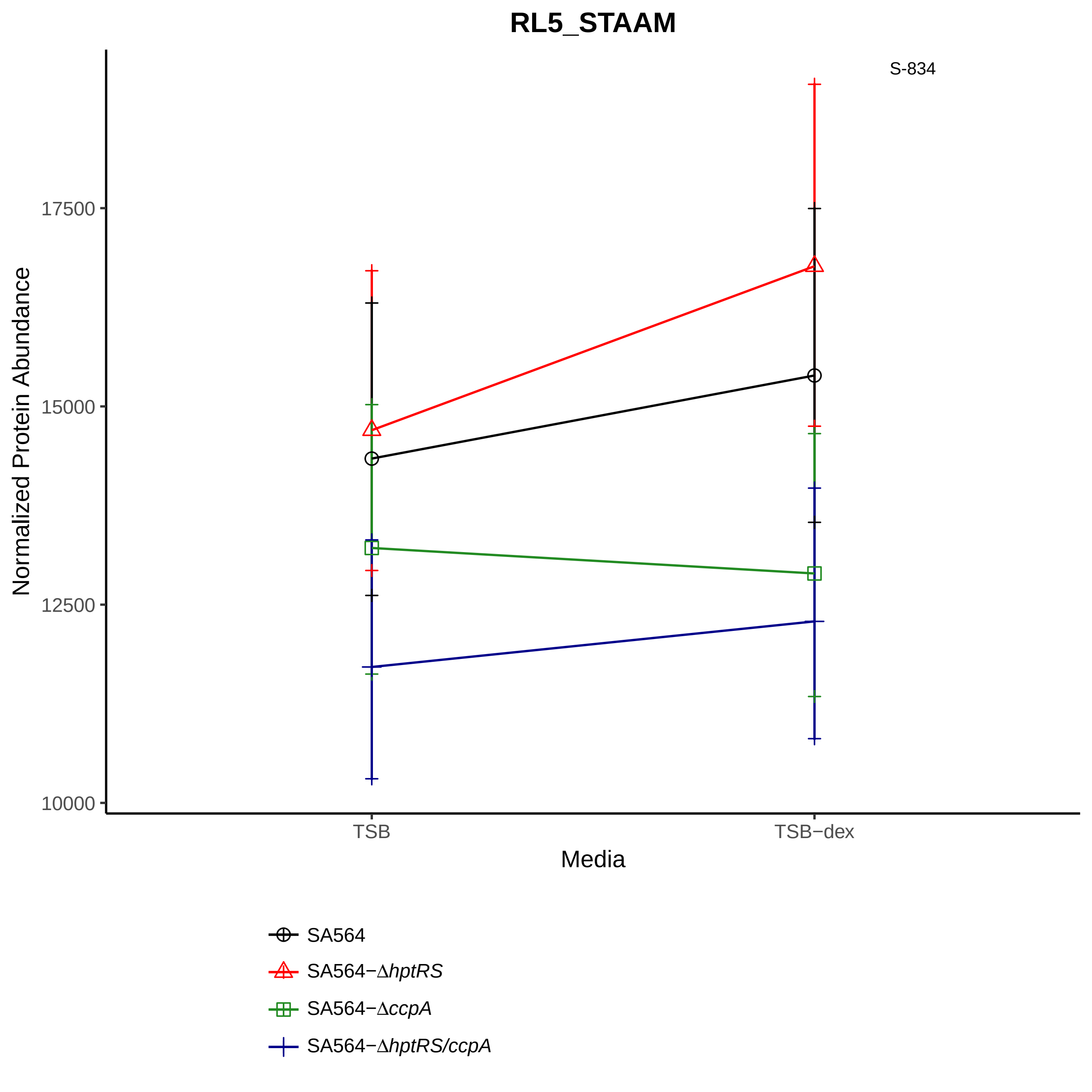

RL5_STAAM
S-834
Normalized Protein Abundance
17500
15000
12500
10000
TSB
TSB−dex
Media
SA564
SA564−Δ*hptRS*
SA564−Δ*ccpA*
SA564−Δ*hptRS/ccpA*

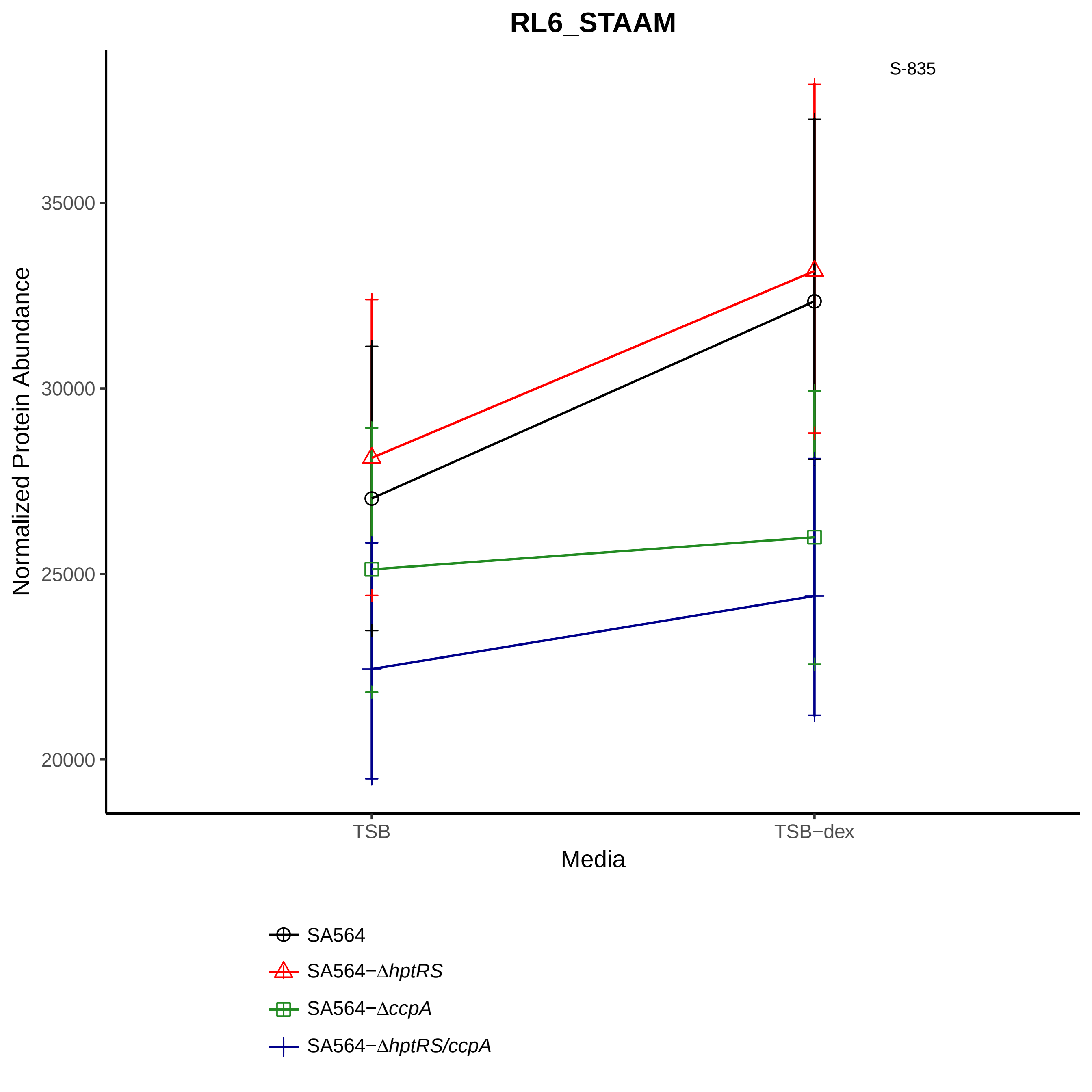

RL6_STAAM
S-835
35000
30000
25000
20000
Normalized Protein Abundance
TSB
TSB−dex
Media
SA564
SA564−Δ*hptRS*
SA564−Δ*ccpA*
SA564−Δ*hptRS/ccpA*

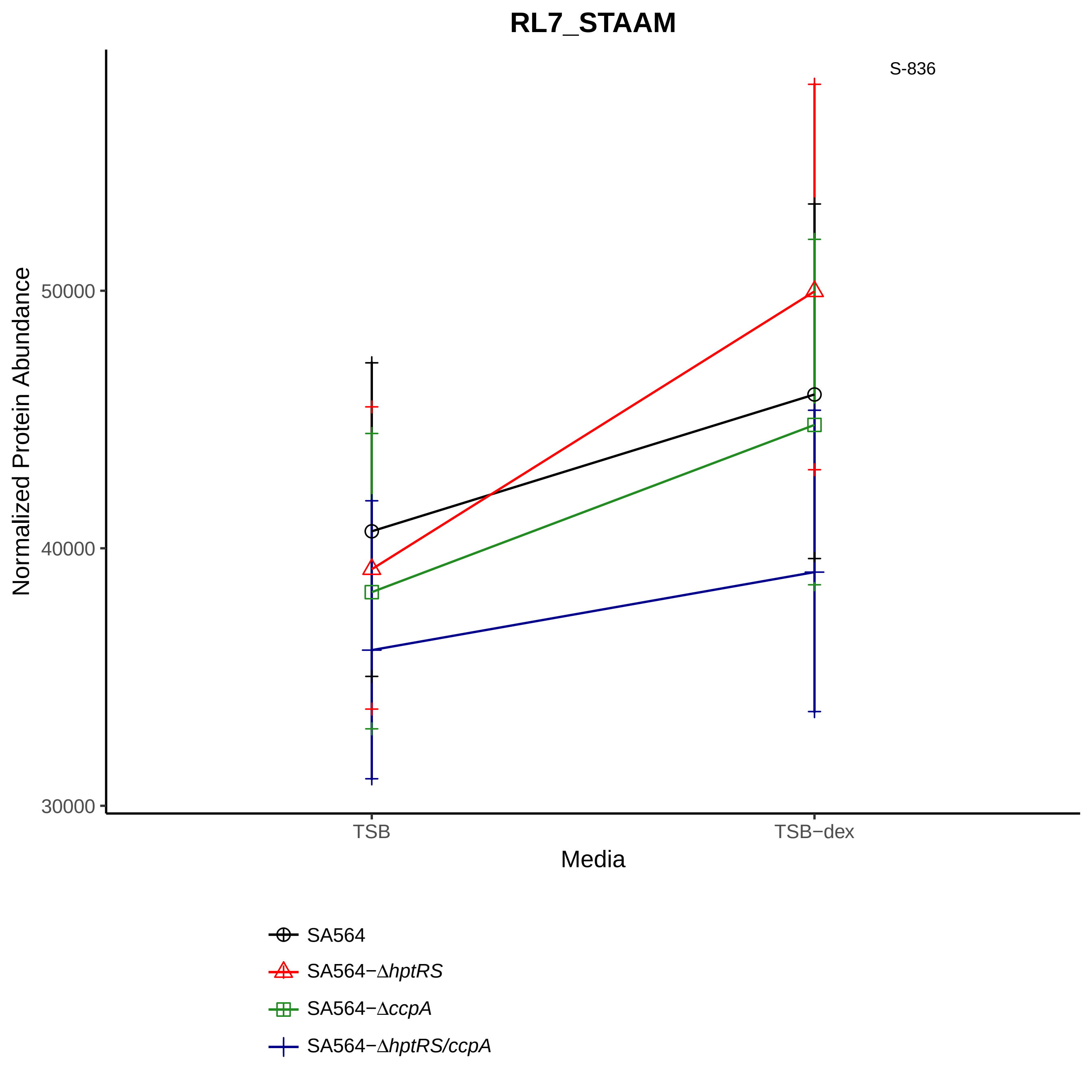

RL7_STAAM
S-836
Normalized Protein Abundance
50000
40000
30000
TSB
TSB−dex
Media
SA564
SA564−ΔhptRS
SA564−ΔccpA
SA564−ΔhptRS/ccpA

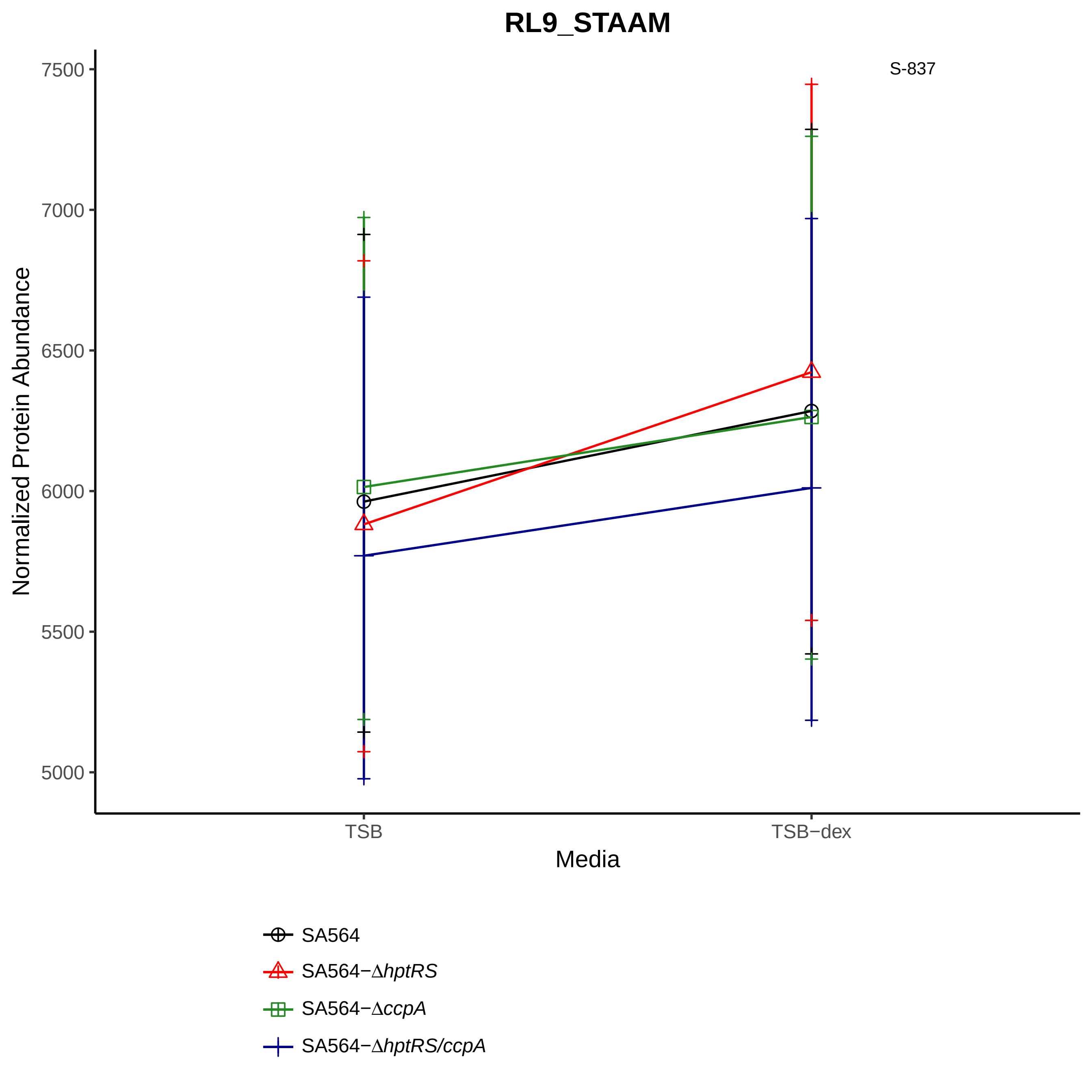
RL9_STAAM
S-837
Normalized Protein Abundance
7500
7000
6500
6000
5500
5000
TSB
TSB−dex
Media
SA564
SA564−ΔhptRS
SA564−ΔccpA
SA564−ΔhptRS/ccpA

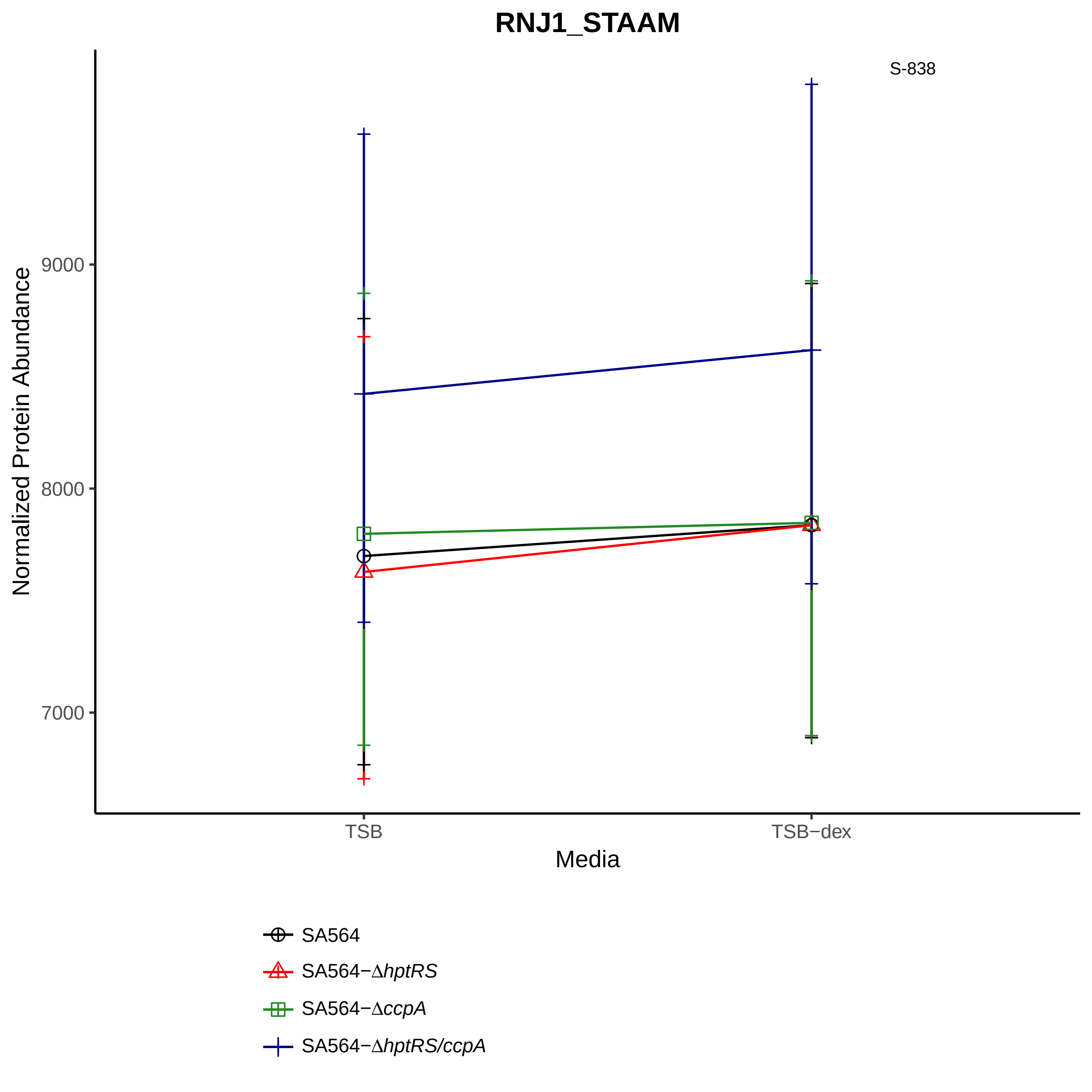

RNJ1_STAAM
S-838
Normalized Protein Abundance
9000
8000
7000
TSB
TSB−dex
Media
SA564
SA564−Δ*hptRS*
SA564−Δ*ccpA*
SA564−Δ*hptRS/ccpA*

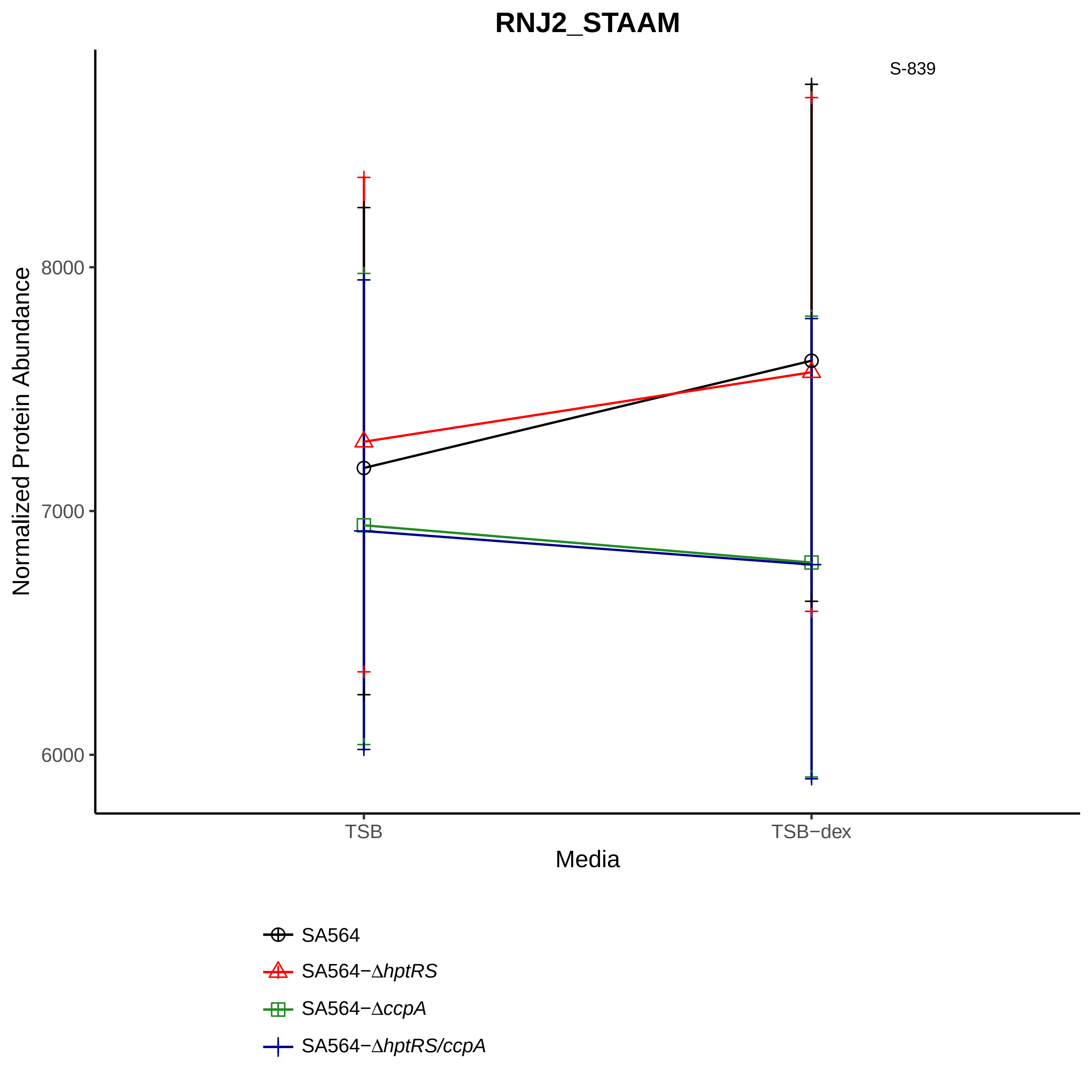

RNJ2_STAAM
S-839
Normalized Protein Abundance
8000
7000
6000
TSB
TSB−dex
Media
SA564
SA564−Δ*hptRS*
SA564−Δ*ccpA*
SA564−Δ*hptRS/ccpA*

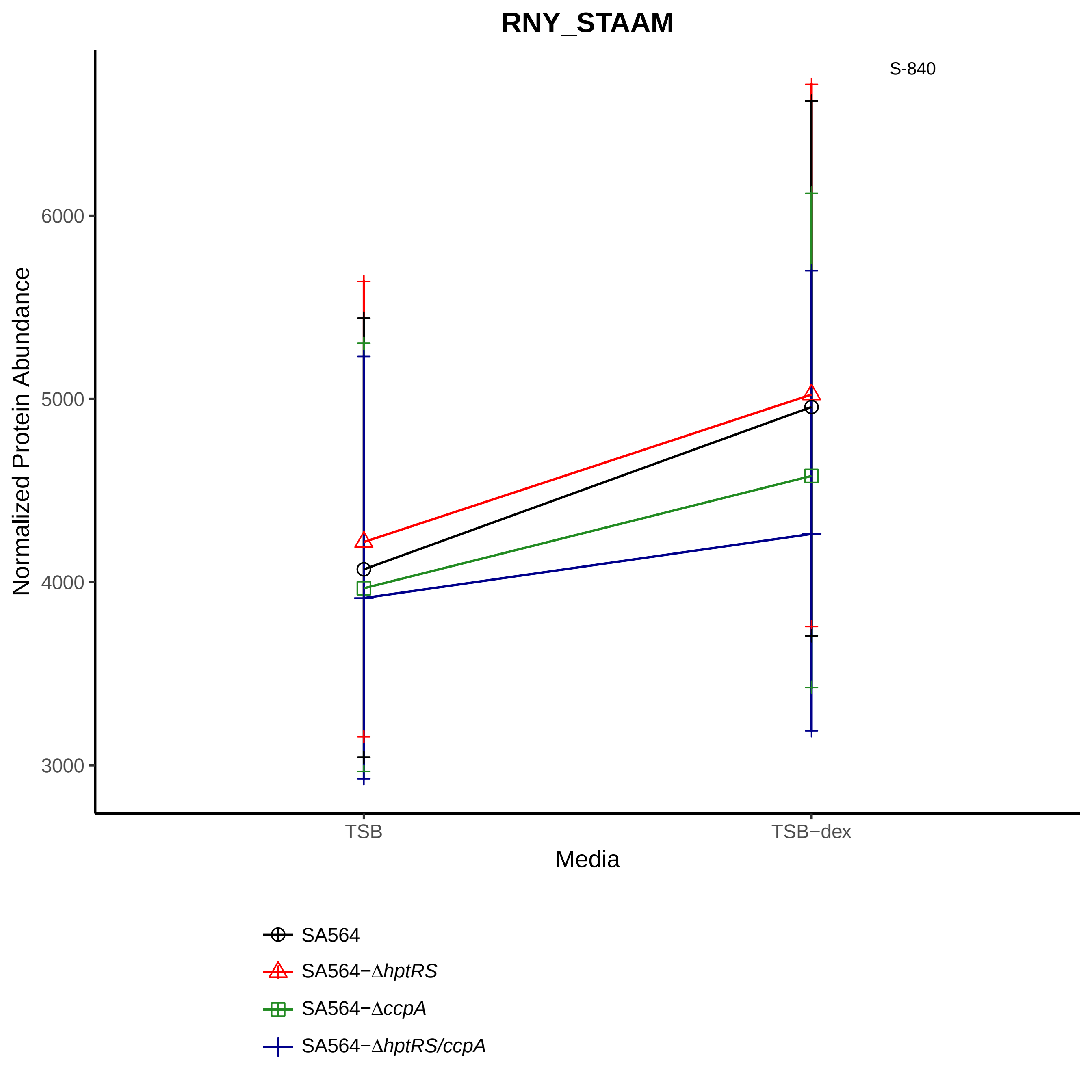

RNY_STAAM
S-840
Normalized Protein Abundance
6000
5000
4000
3000
TSB
TSB−dex
Media
SA564
SA564−Δ*hptRS*
SA564−Δ*ccpA*
SA564−Δ*hptRS/ccpA*

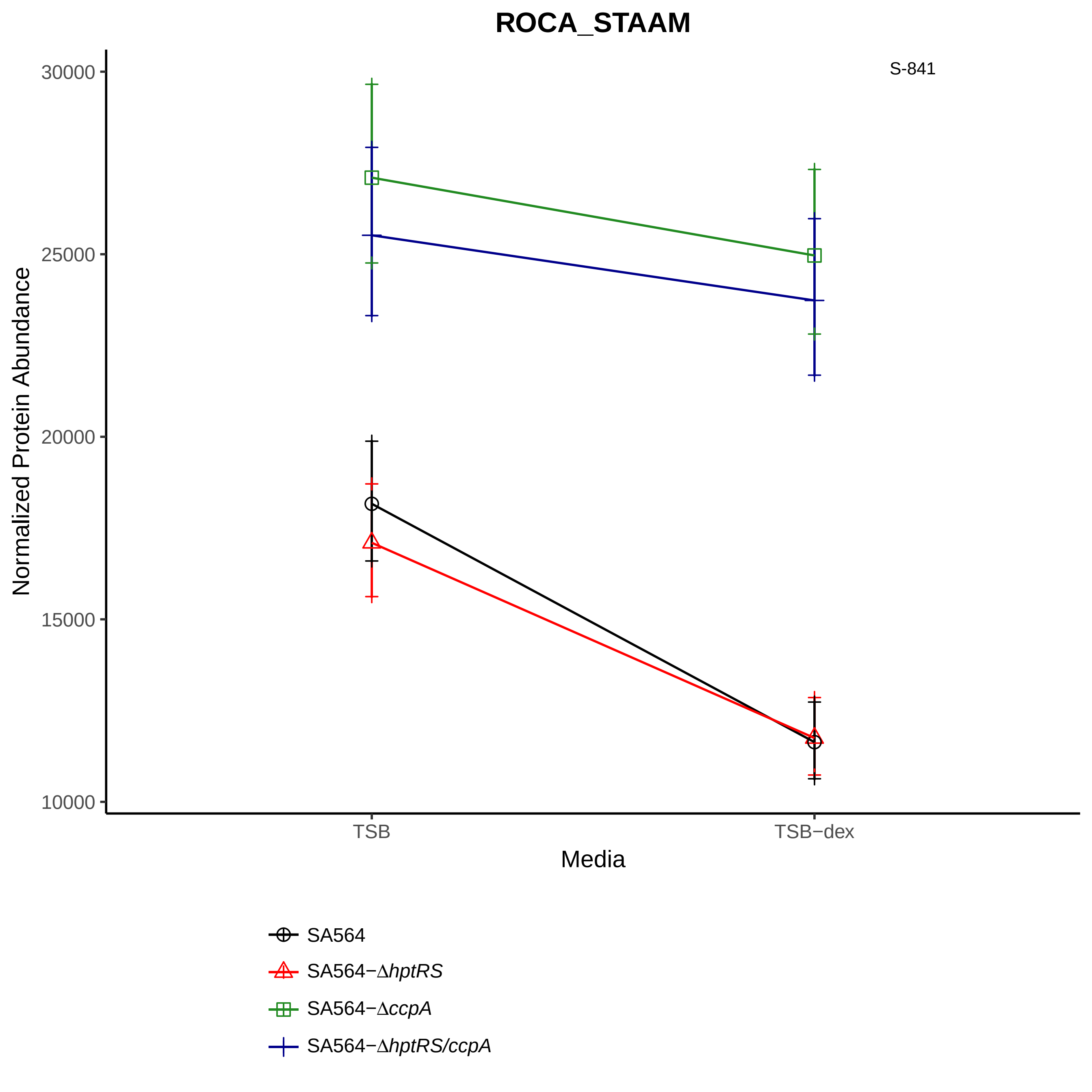

ROCA_STAAM
S-841
30000
25000
20000
15000
10000
Normalized Protein Abundance
TSB
TSB−dex
Media
SA564
SA564−Δ*hptRS*
SA564−Δ*ccpA*
SA564−Δ*hptRS/ccpA*

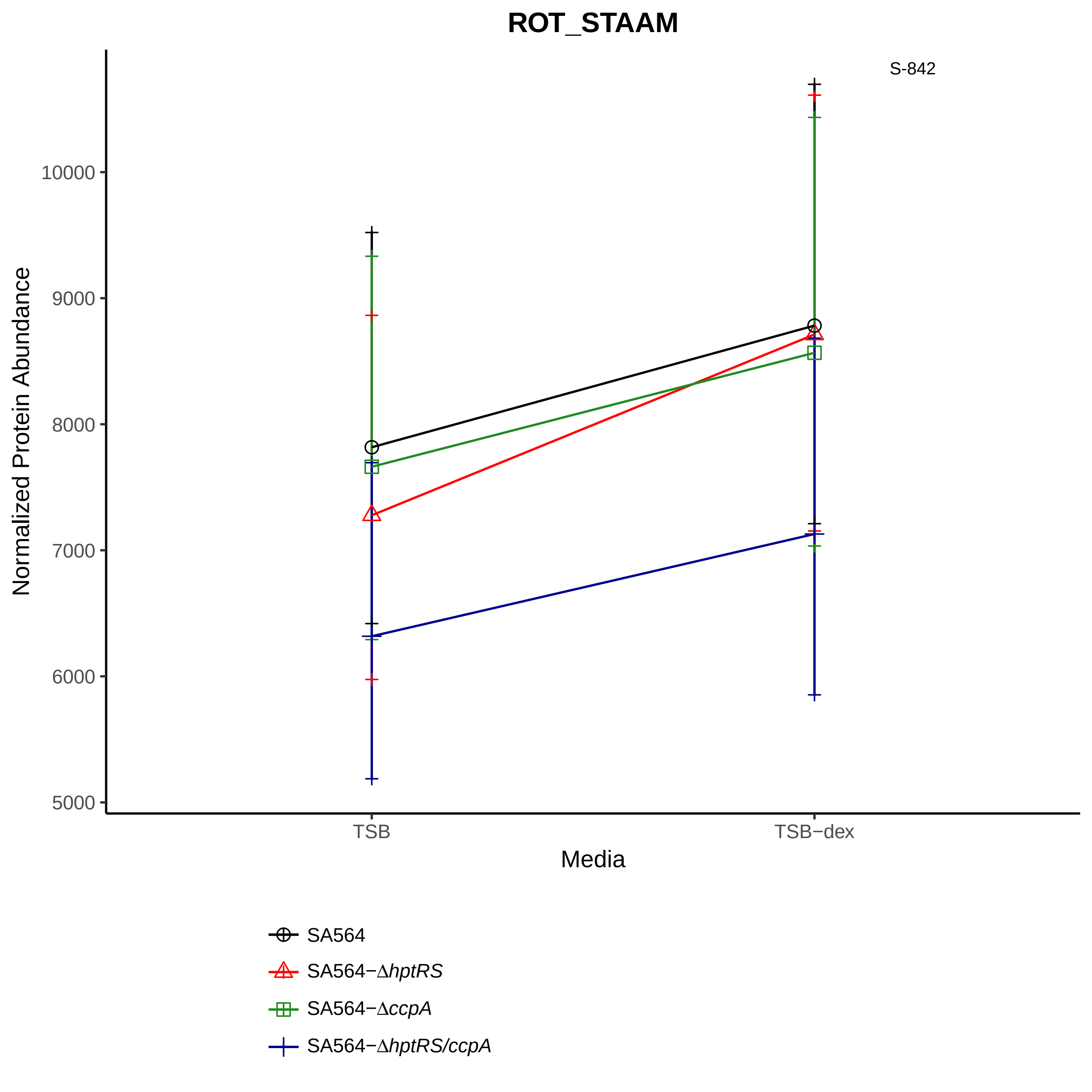

ROT_STAAM
S-842
10000
9000
8000
7000
6000
5000
Normalized Protein Abundance
TSB
TSB−dex
Media
SA564
SA564−Δ*hptRS*
SA564−Δ*ccpA*
SA564−Δ*hptRS/ccpA*

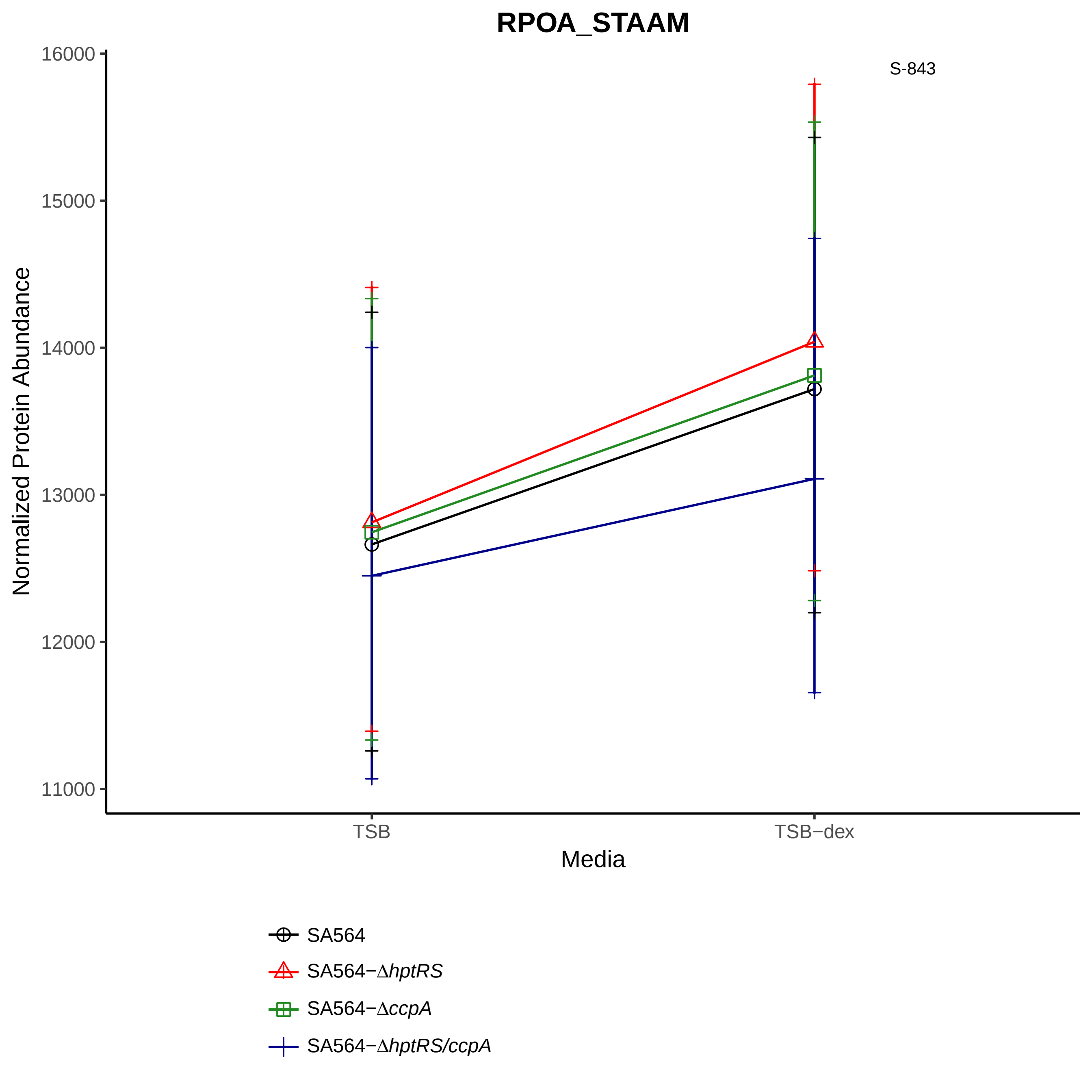

RPOA_STAAM
S-843
16000
15000
14000
13000
12000
11000
Normalized Protein Abundance
TSB
TSB−dex
Media
SA564
SA564−Δ*hptRS*
SA564−Δ*ccpA*
SA564−Δ*hptRS/ccpA*

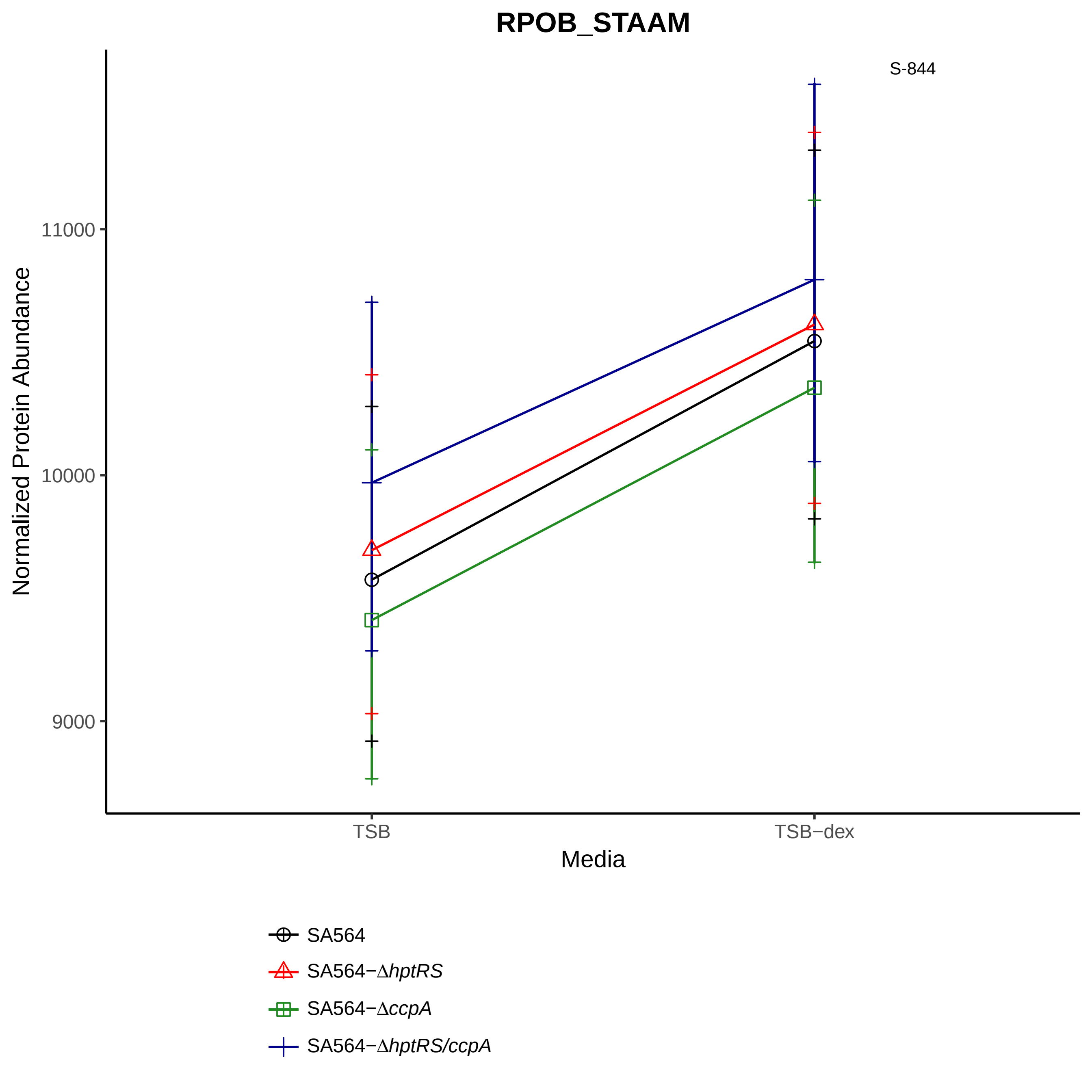

RPOB_STAAM
S-844
Normalized Protein Abundance
11000
10000
9000
TSB
TSB−dex
Media
SA564
SA564−Δ*hptRS*
SA564−Δ*ccpA*
SA564−Δ*hptRS/ccpA*

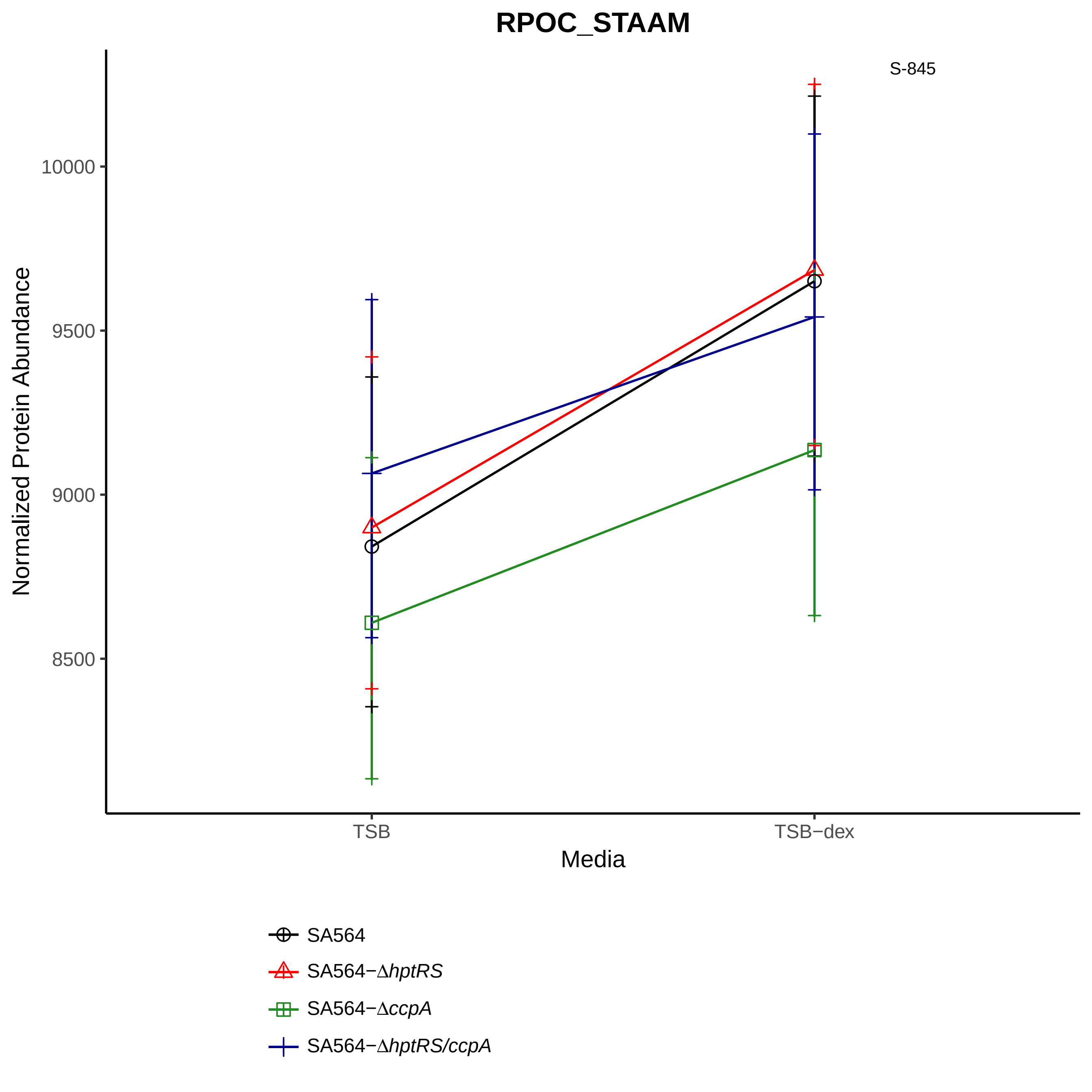
RPOC_STAAM
S-845
Normalized Protein Abundance
10000
9500
9000
8500
TSB
TSB−dex
Media
SA564
SA564−Δ*hptRS*
SA564−Δ*ccpA*
SA564−Δ*hptRS/ccpA*

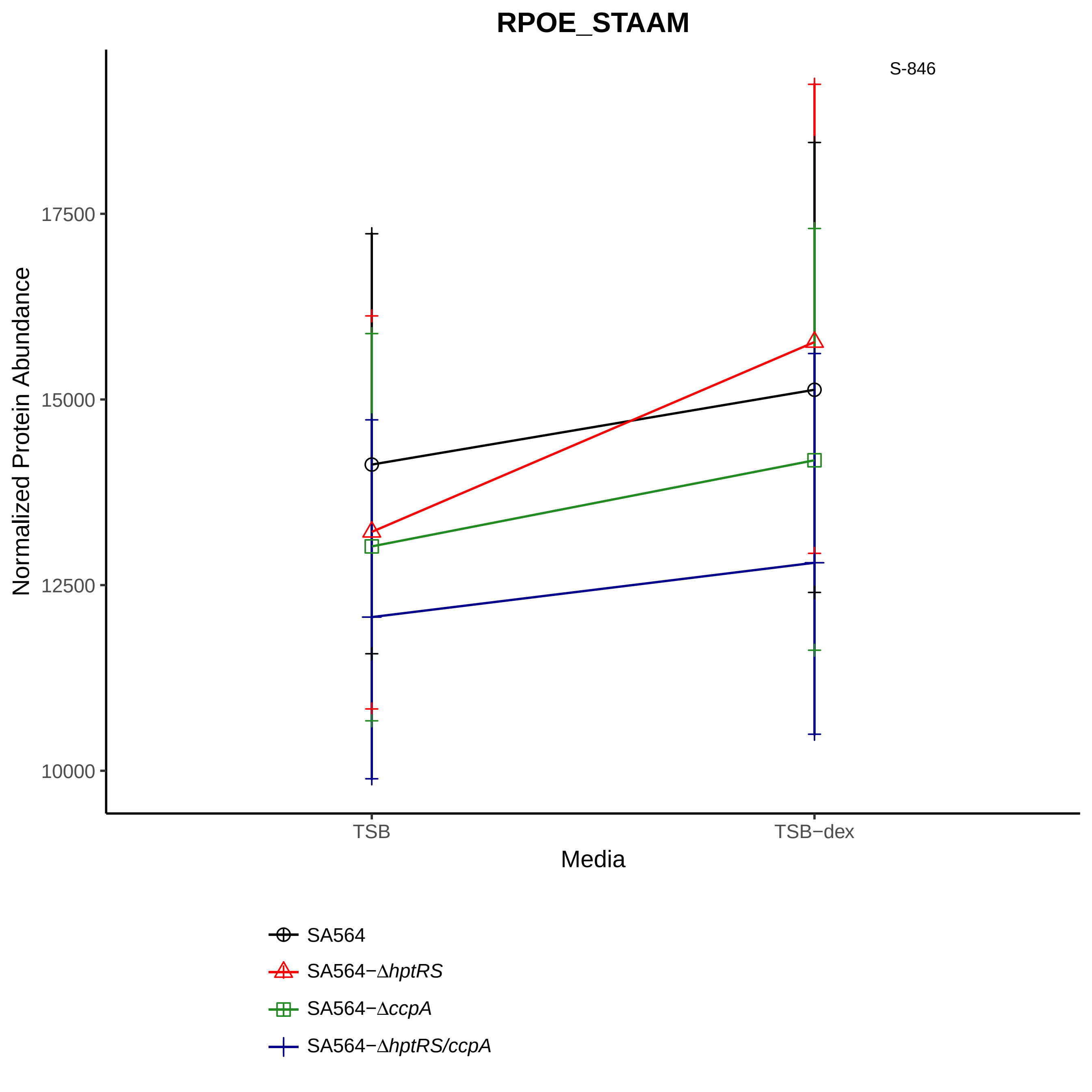

RPOE_STAAM
S-846
Normalized Protein Abundance
17500
15000
12500
10000
TSB
TSB−dex
Media
SA564
SA564−Δ*hptRS*
SA564−Δ*ccpA*
SA564−Δ*hptRS/ccpA*

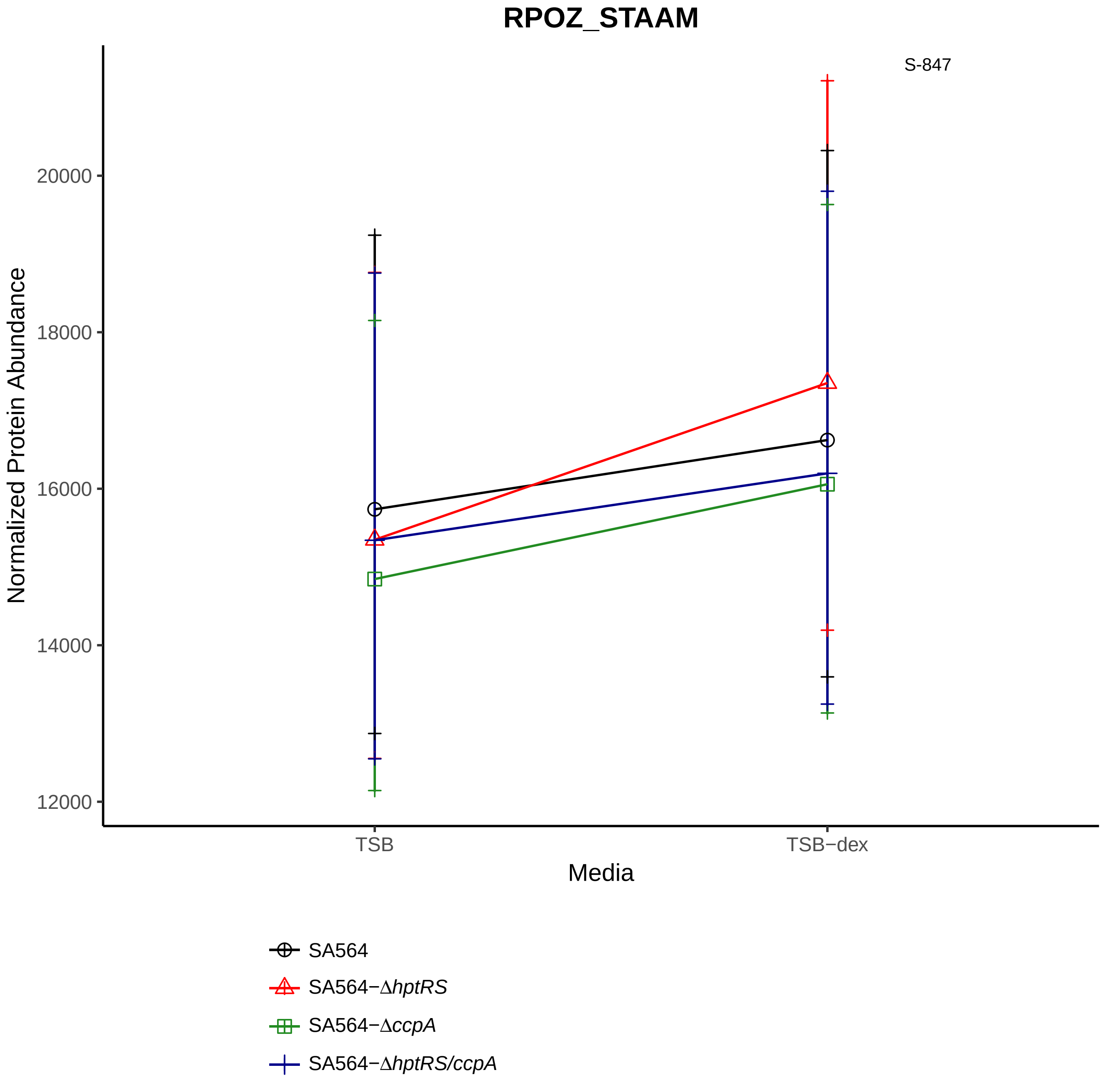

RPOZ_STAAM
S-847
Normalized Protein Abundance
20000
18000
16000
14000
12000
TSB
TSB−dex
Media
SA564
SA564−Δ*hptRS*
SA564−Δ*ccpA*
SA564−Δ*hptRS/ccpA*

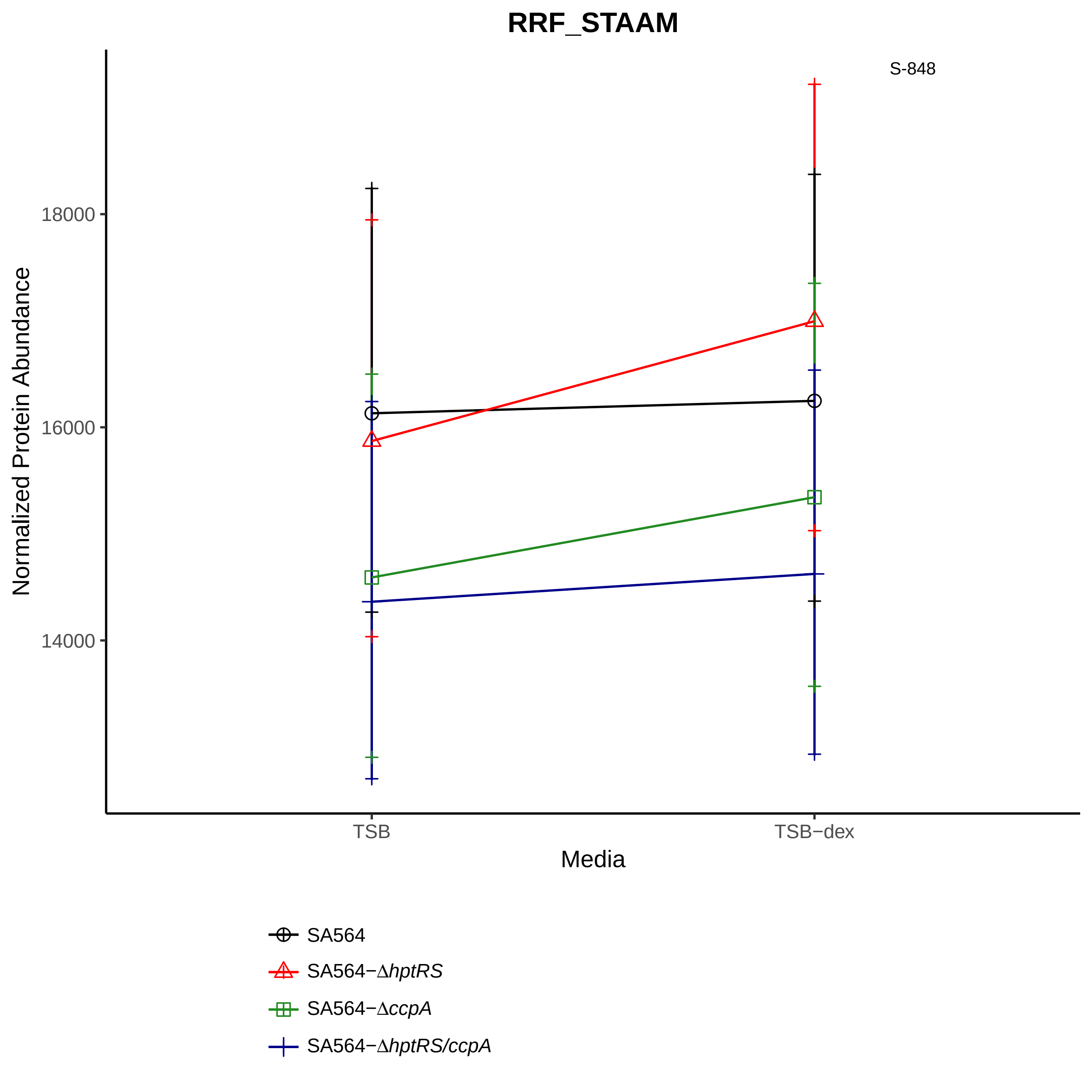
RRF_STAAM
S-848
Normalized Protein Abundance
18000
16000
14000
TSB
TSB−dex
Media
SA564
SA564−ΔhptRS
SA564−ΔccpA
SA564−ΔhptRS/ccpA

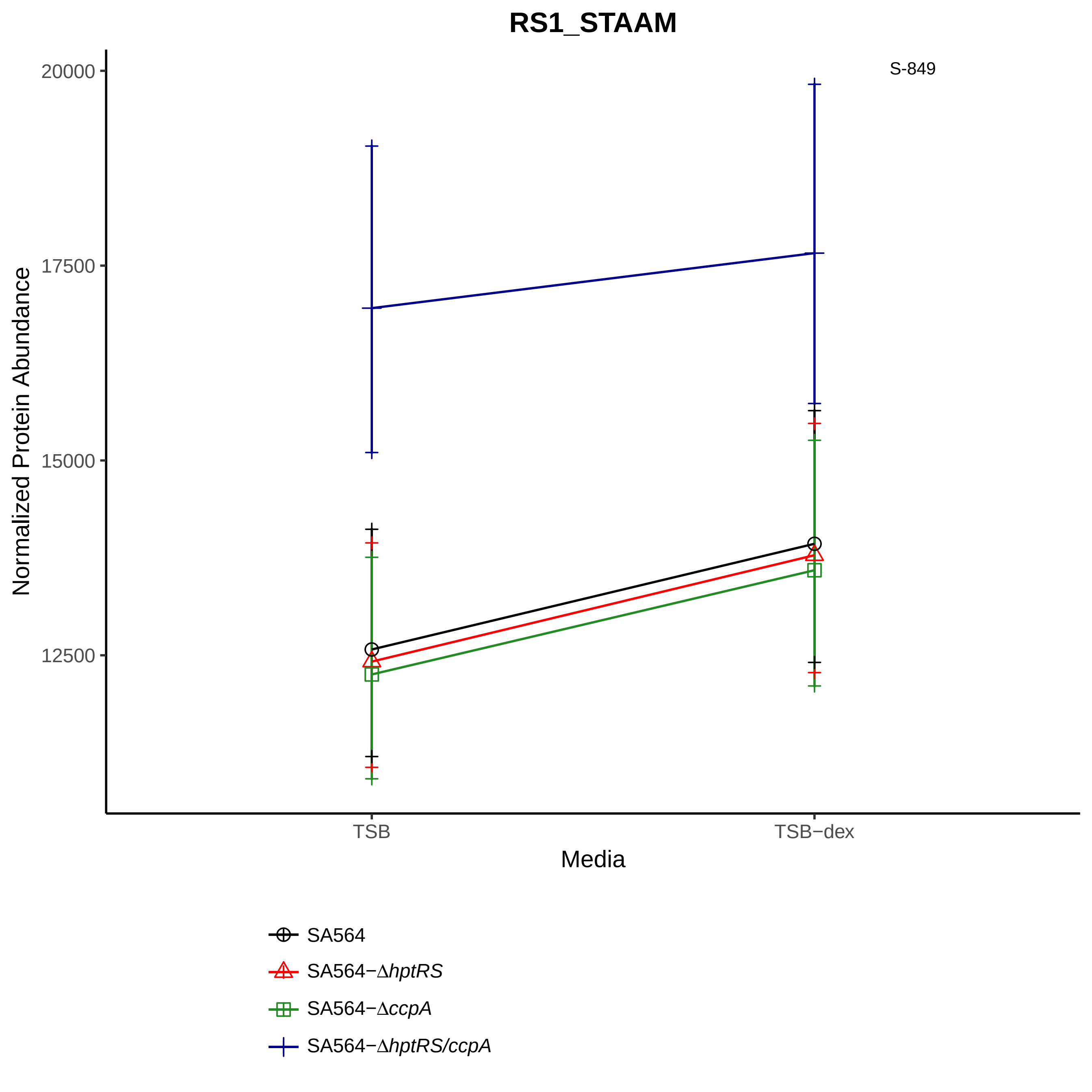

RS1_STAAM
S-849
Normalized Protein Abundance
20000
17500
15000
12500
TSB
TSB−dex
Media
SA564
SA564−ΔhptRS
SA564−ΔccpA
SA564−ΔhptRS/ccpA

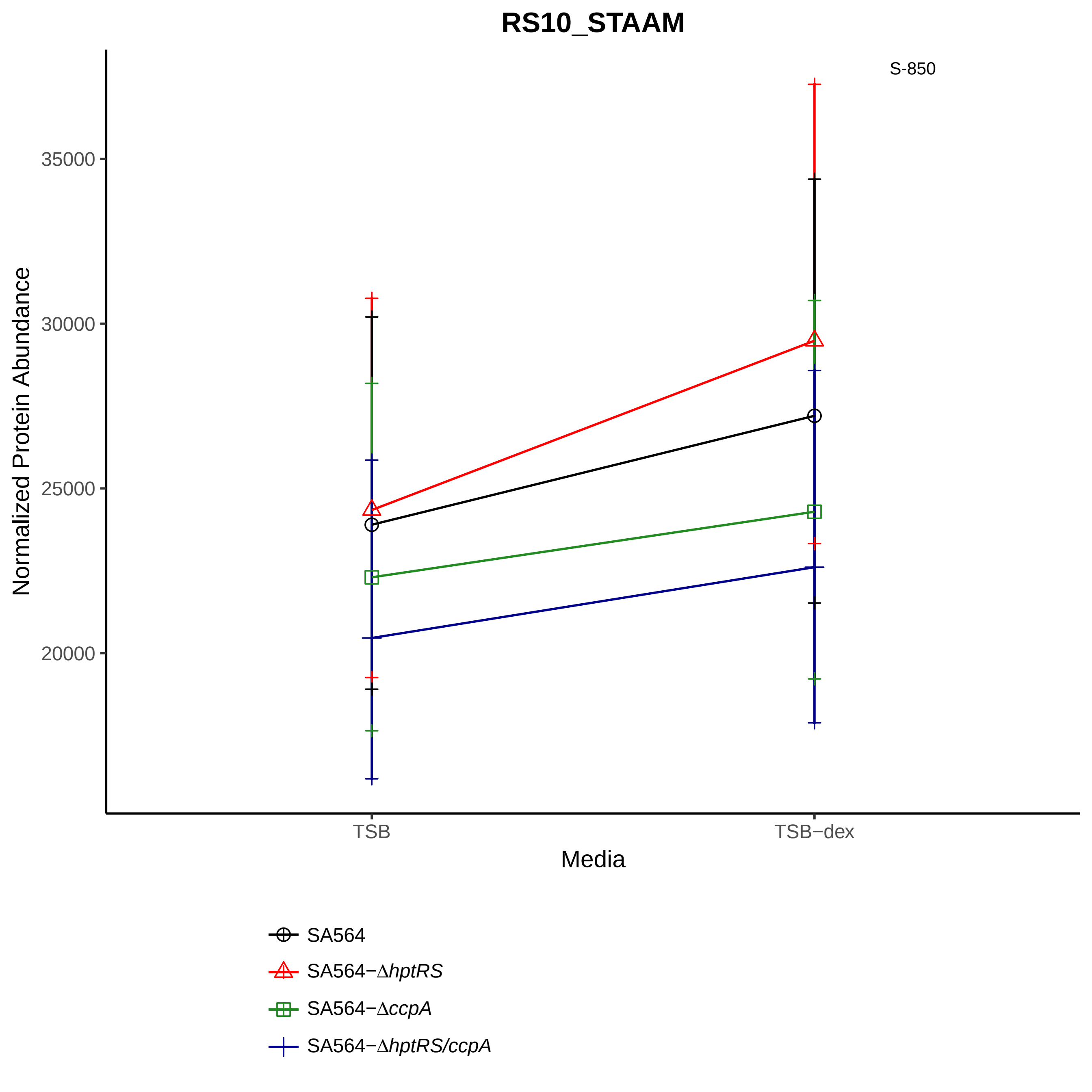
RS10_STAAM
S-850
Normalized Protein Abundance
35000
30000
25000
20000
TSB
TSB−dex
Media
SA564
SA564−ΔhptRS
SA564−ΔccpA
SA564−ΔhptRS/ccpA

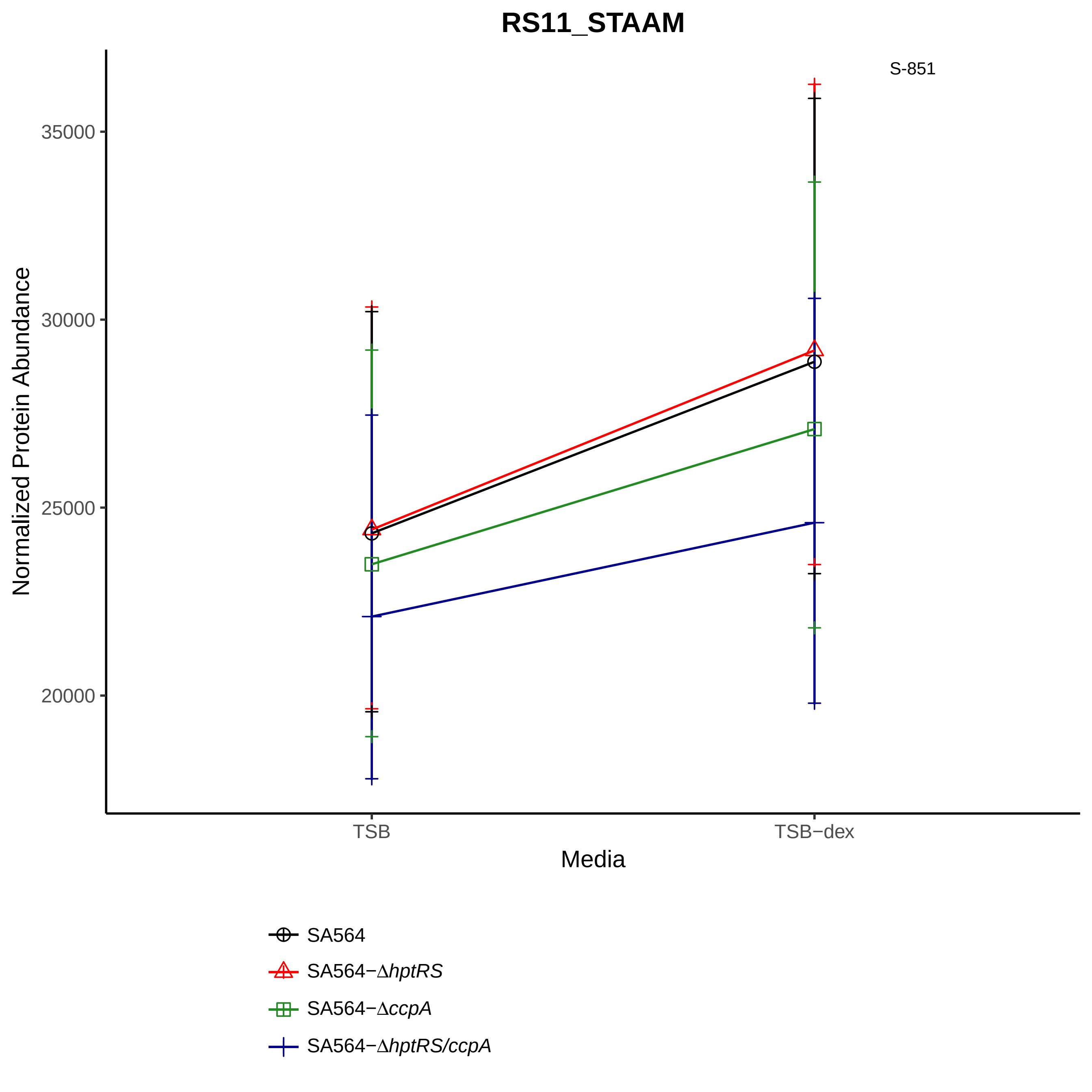

RS11_STAAM
S-851
Normalized Protein Abundance
35000
30000
25000
20000
TSB
TSB−dex
Media
SA564
SA564−ΔhptRS
SA564−ΔccpA
SA564−ΔhptRS/ccpA

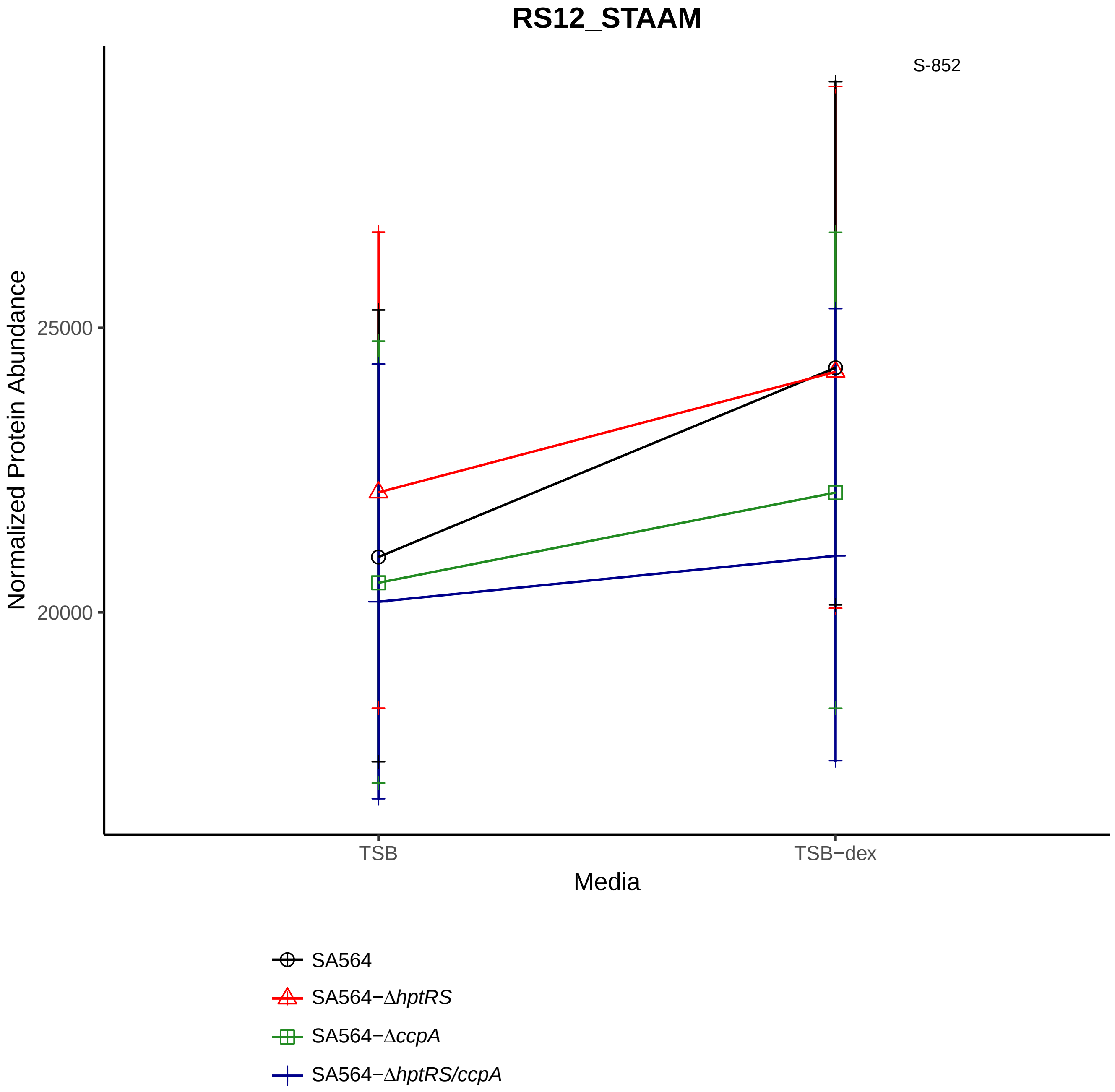

RS12_STAAM
S-852
Normalized Protein Abundance
25000
20000
TSB
TSB−dex
Media
SA564
SA564−Δ*hptRS*
SA564−Δ*ccpA*
SA564−Δ*hptRS/ccpA*

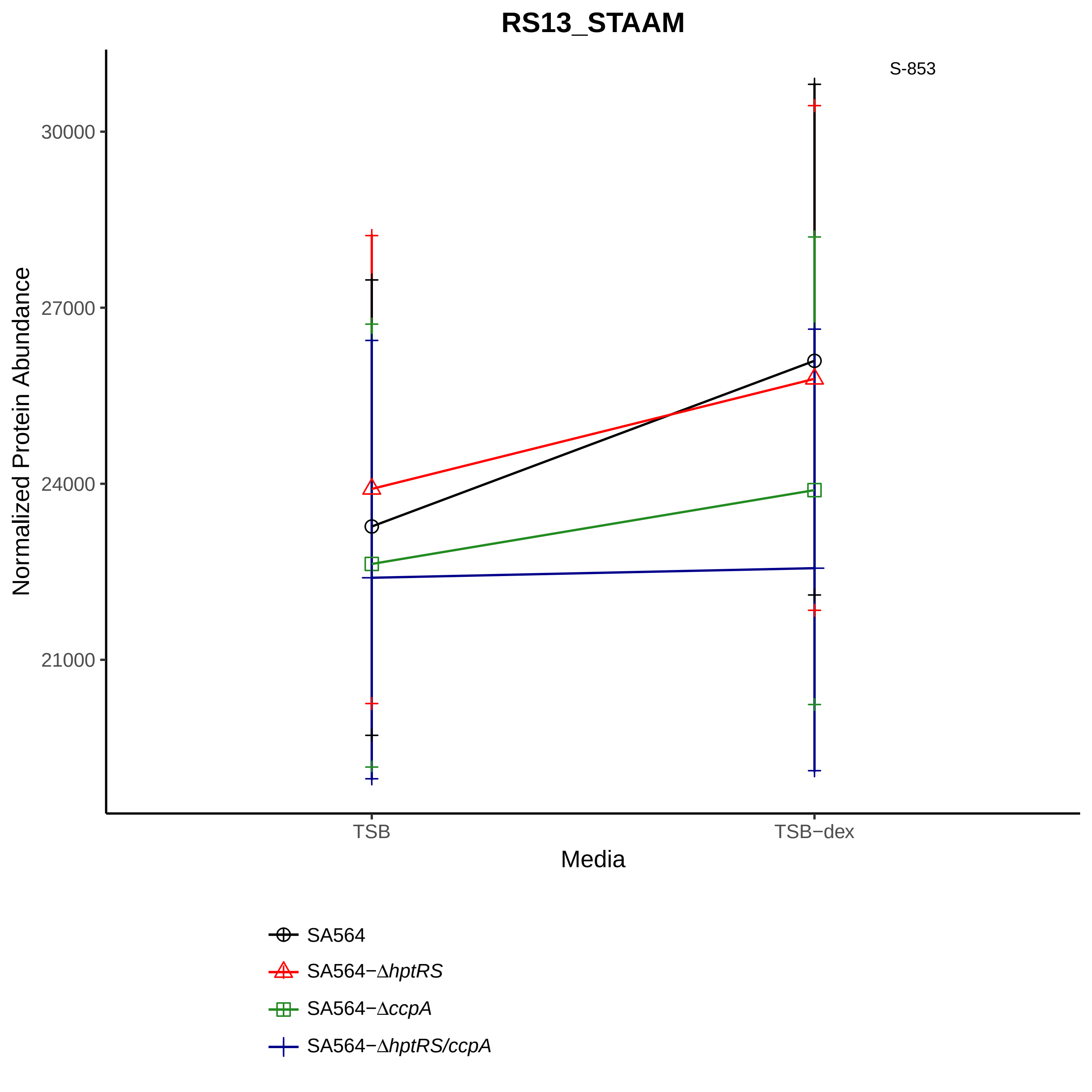

RS13_STAAM
S-853
30000
27000
24000
21000
Normalized Protein Abundance
TSB
TSB−dex
Media
SA564
SA564−Δ*hptRS*
SA564−Δ*ccpA*
SA564−Δ*hptRS/ccpA*

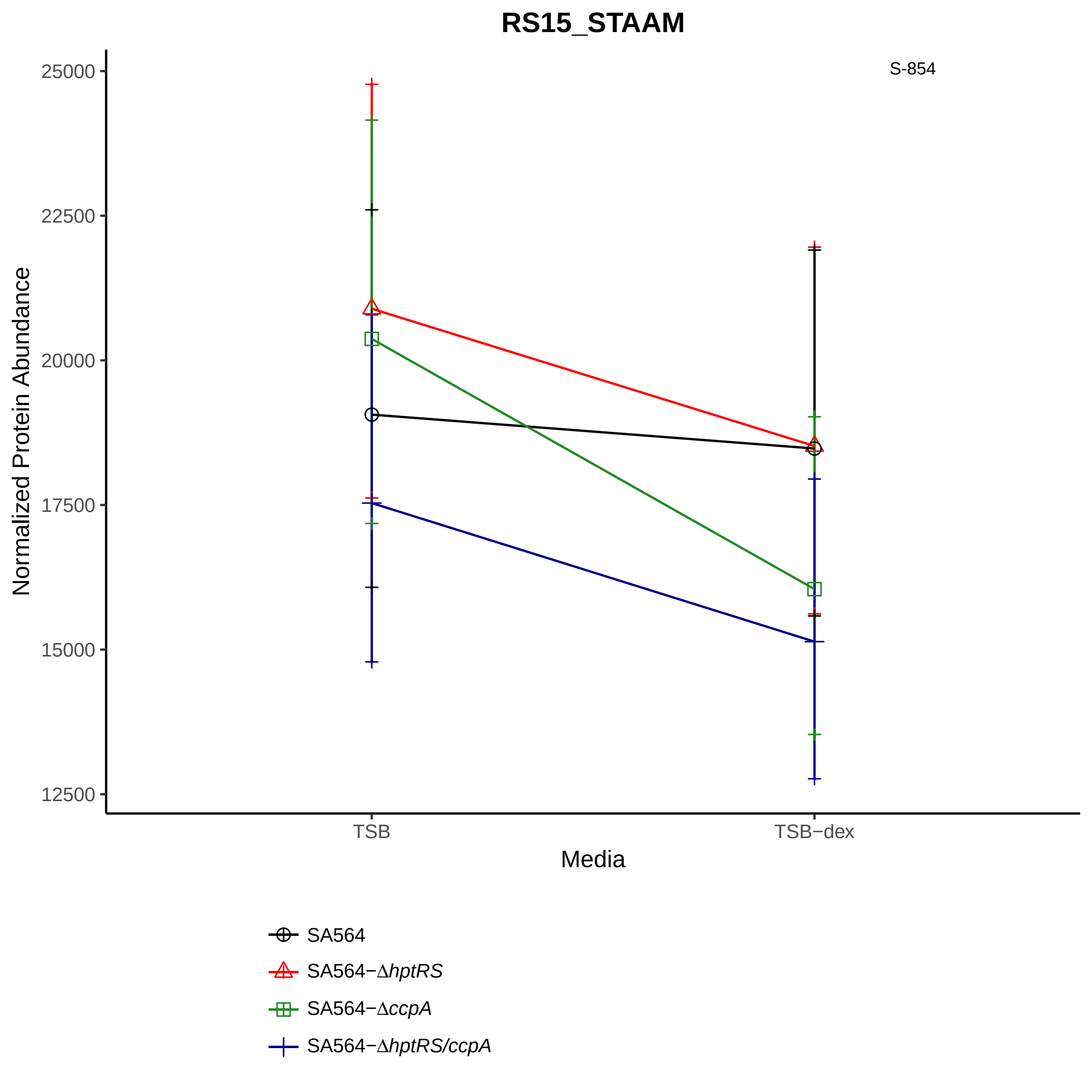

RS15_STAAM
S-854
Normalized Protein Abundance
25000
22500
20000
17500
15000
12500
TSB
TSB−dex
Media
SA564
SA564−ΔhptRS
SA564−ΔccpA
SA564−ΔhptRS/ccpA

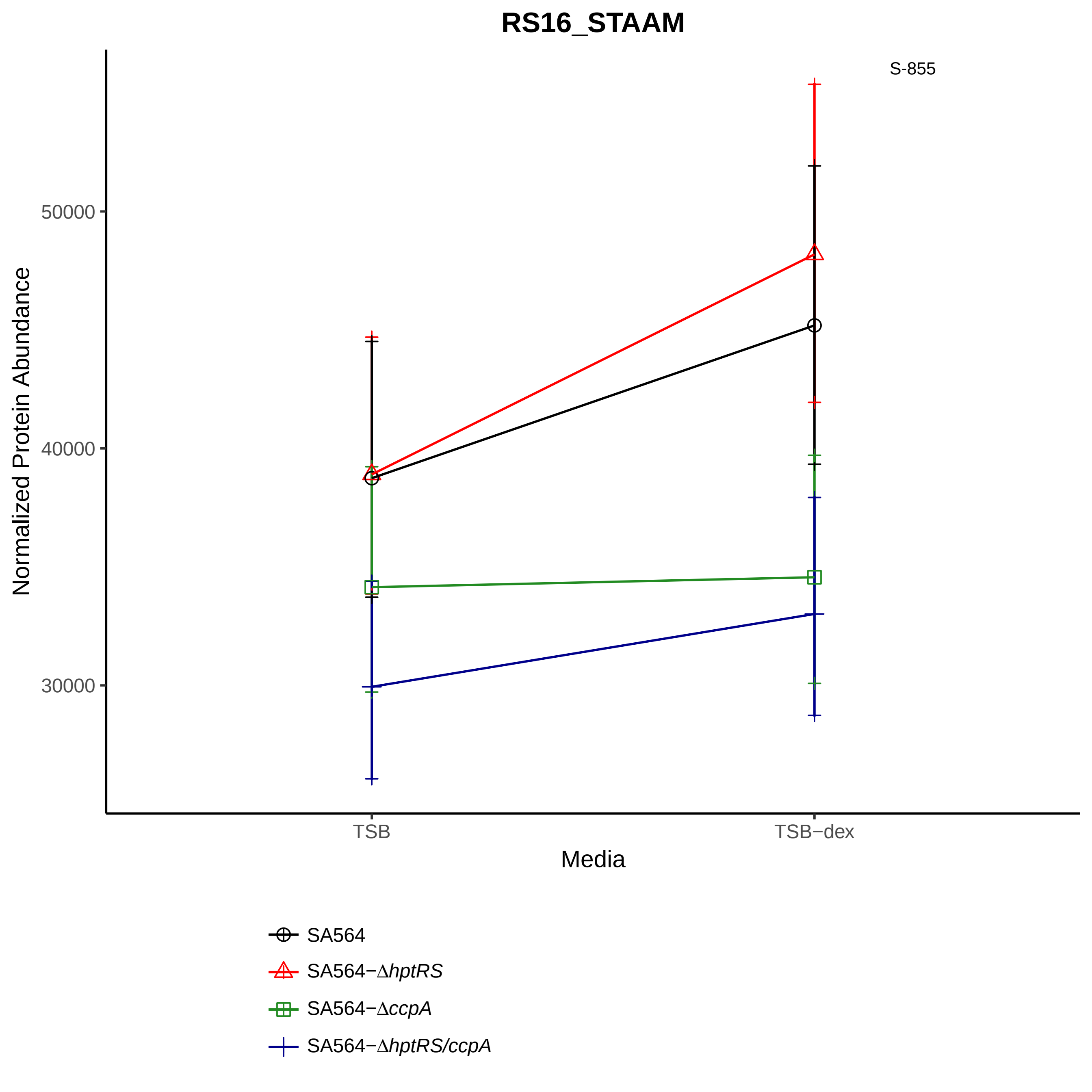

RS16_STAAM
S-855
Normalized Protein Abundance
50000
40000
30000
TSB
TSB−dex
Media
SA564
SA564−ΔhptRS
SA564−ΔccpA
SA564−ΔhptRS/ccpA

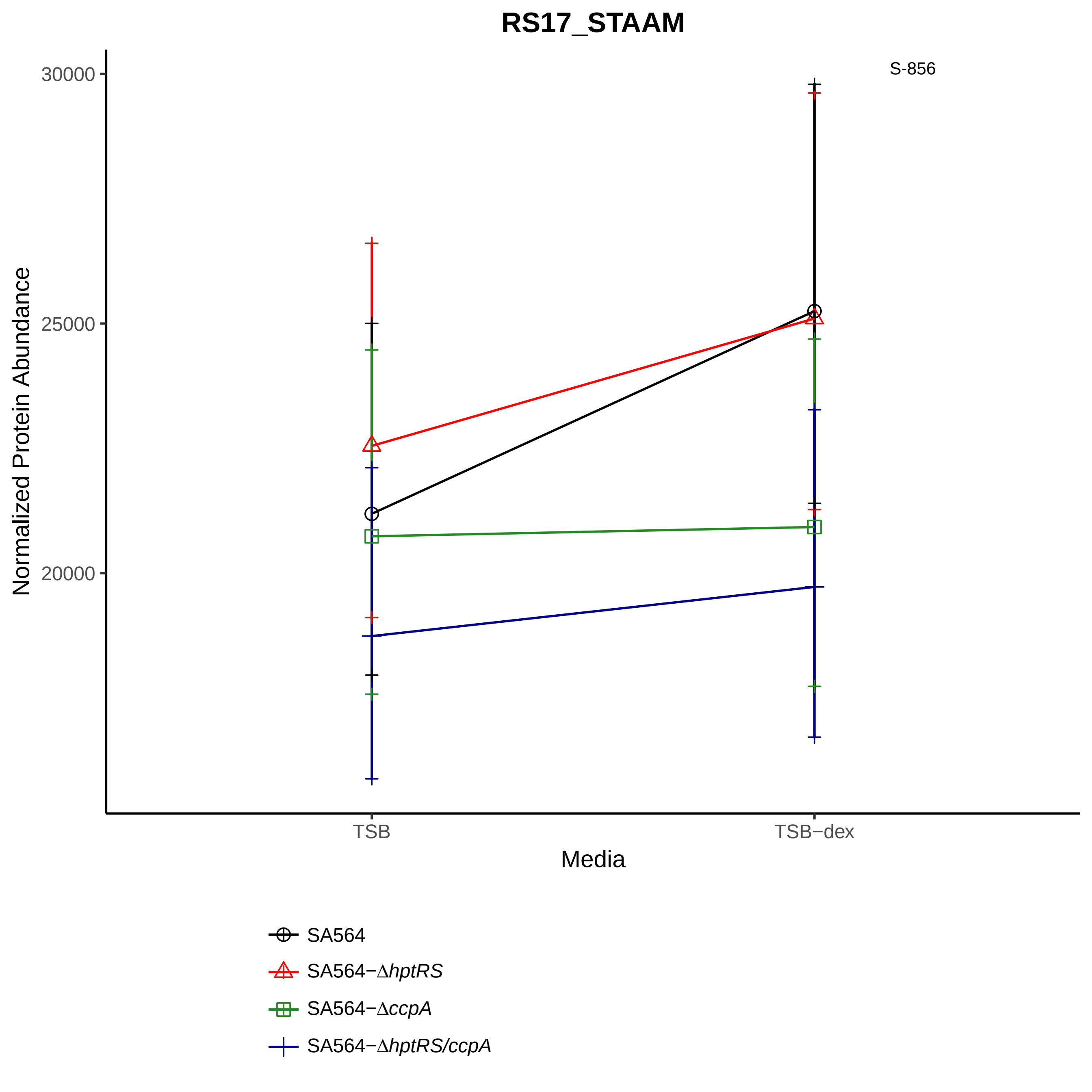
RS17_STAAM
S-856
30000
25000
20000
Normalized Protein Abundance
TSB
TSB−dex
Media
SA564
SA564−ΔhptRS
SA564−ΔccpA
SA564−ΔhptRS/ccpA

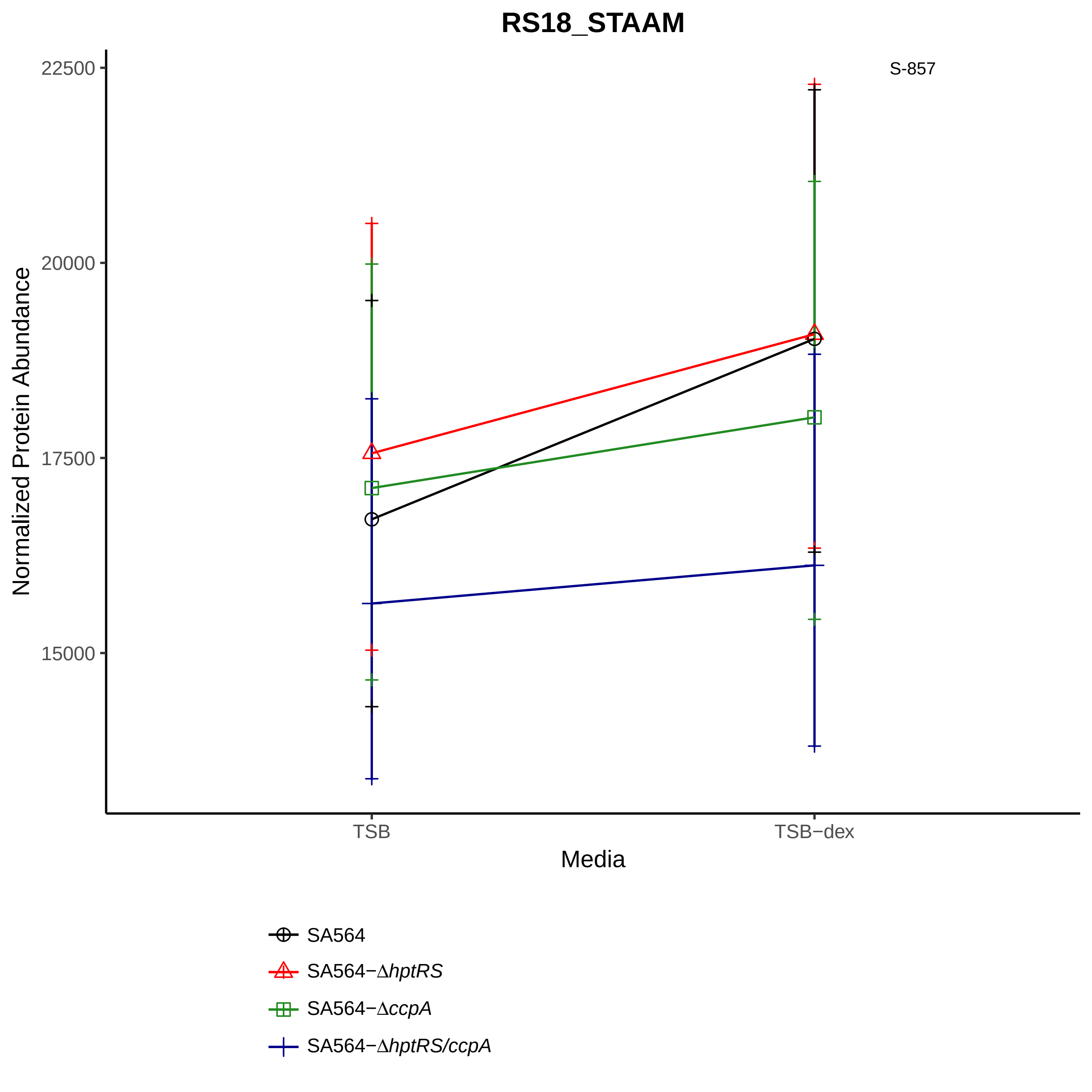

RS18_STAAM
S-857
22500
20000
17500
15000
Normalized Protein Abundance
TSB
TSB−dex
Media
SA564
SA564−Δ*hptRS*
SA564−Δ*ccpA*
SA564−Δ*hptRS/ccpA*

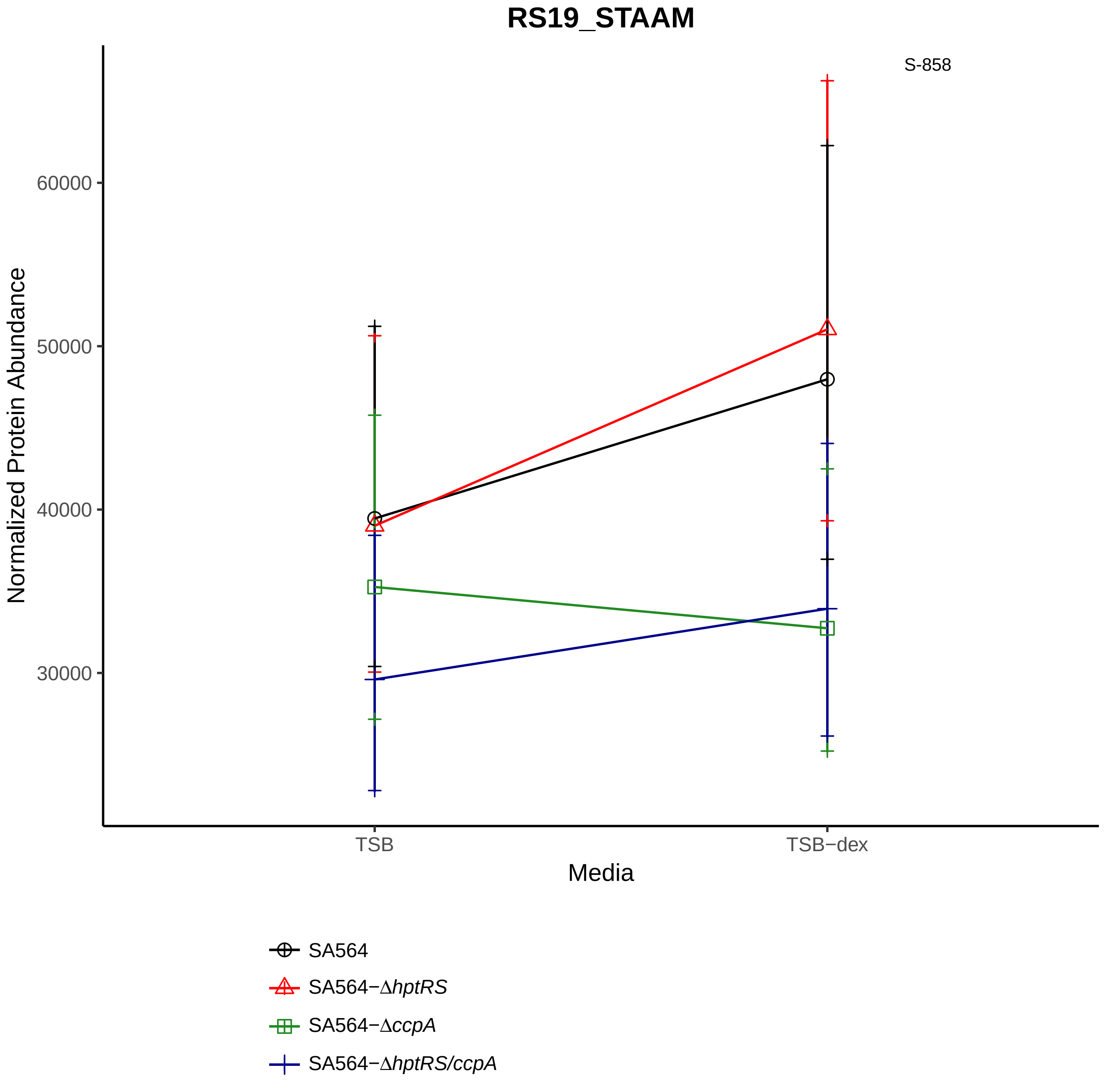
RS19_STAAM
S-858
60000
50000
40000
30000
Normalized Protein Abundance
TSB
TSB−dex
Media
SA564
SA564−Δ*hptRS*
SA564−Δ*ccpA*
SA564−Δ*hptRS/ccpA*

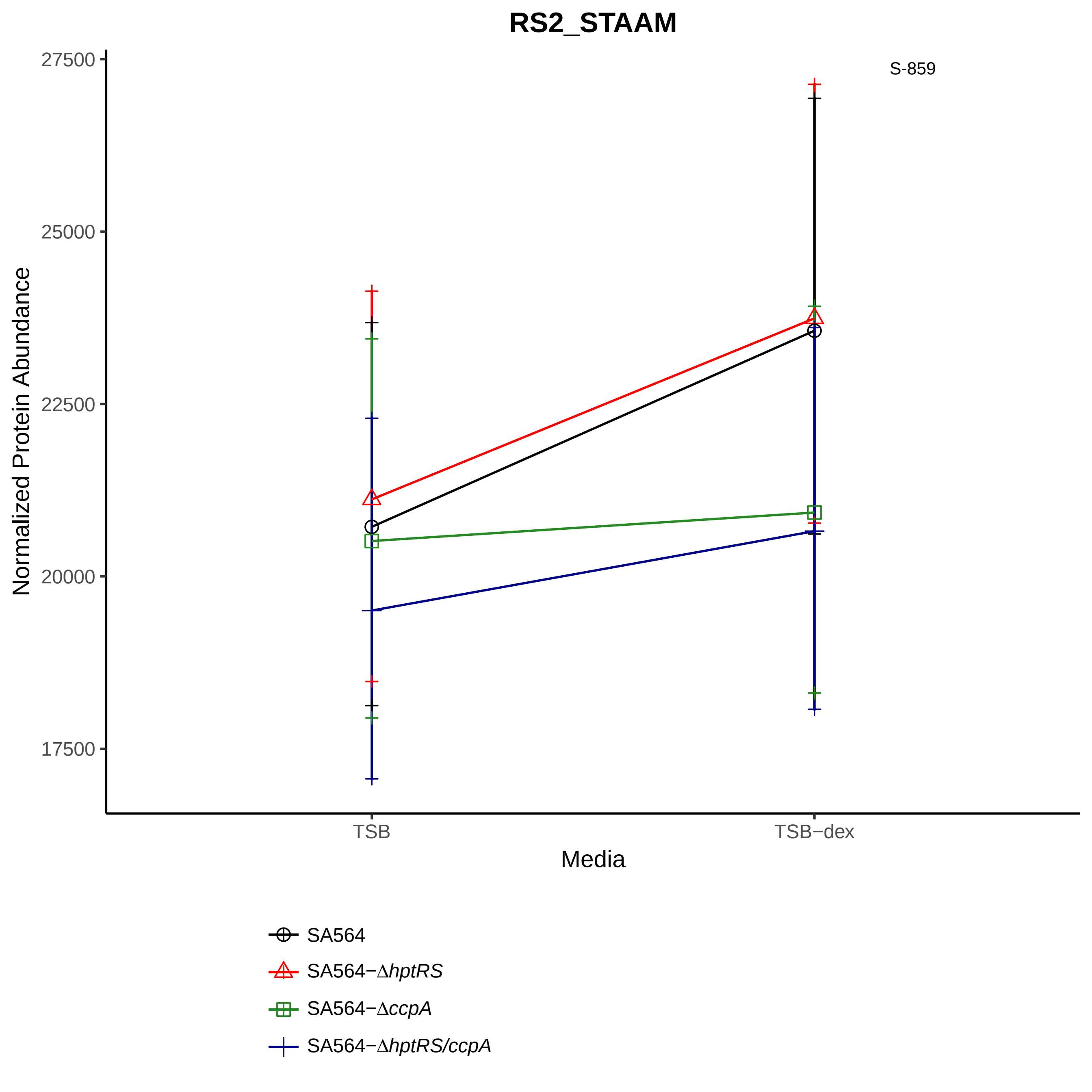
RS2_STAAM
S-859
Normalized Protein Abundance
27500
25000
22500
20000
17500
TSB
TSB−dex
Media
SA564
SA564−ΔhptRS
SA564−ΔccpA
SA564−ΔhptRS/ccpA

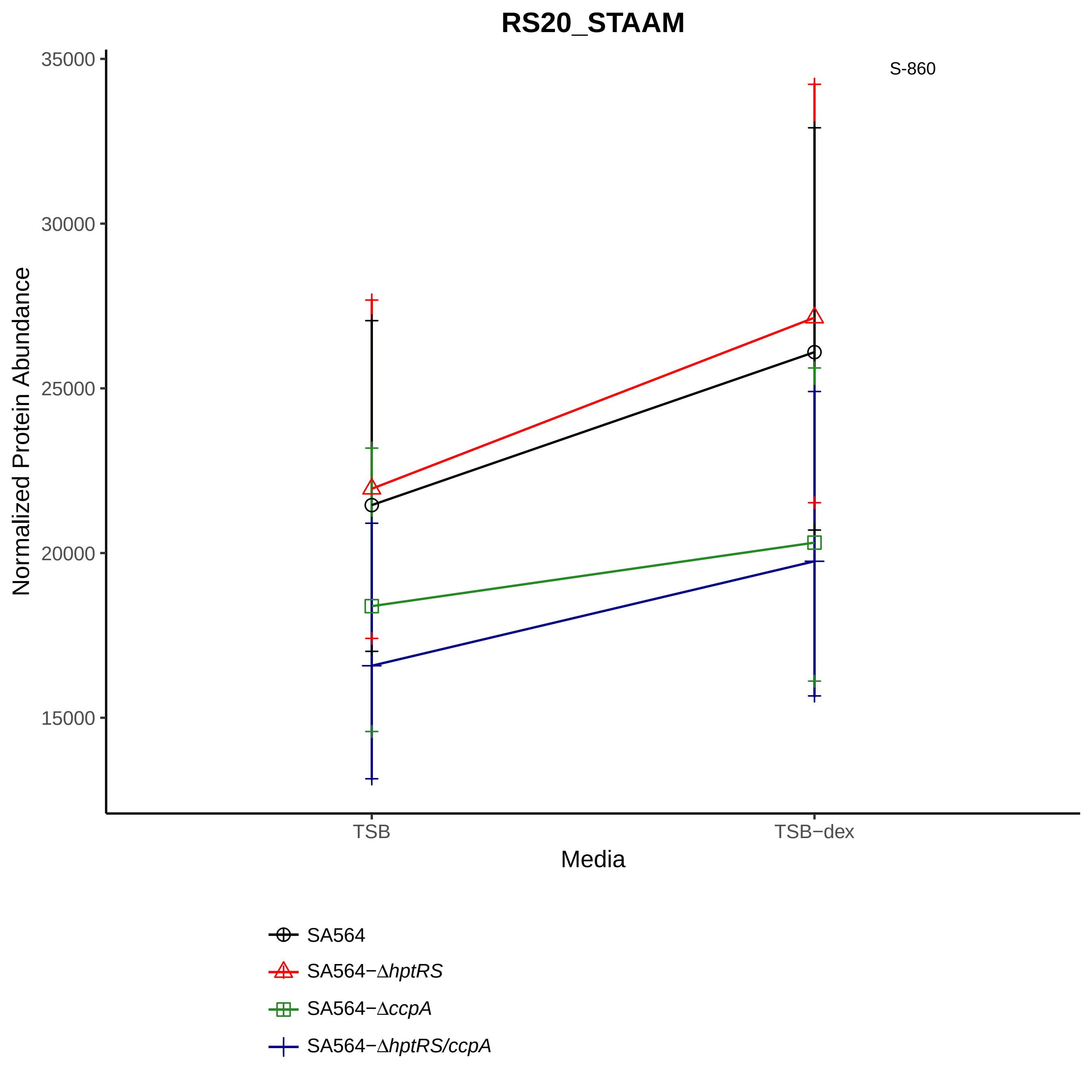

RS20_STAAM
S-860
Normalized Protein Abundance
35000
30000
25000
20000
15000
TSB
TSB−dex
Media
SA564
SA564−Δ*hptRS*
SA564−Δ*ccpA*
SA564−Δ*hptRS/ccpA*

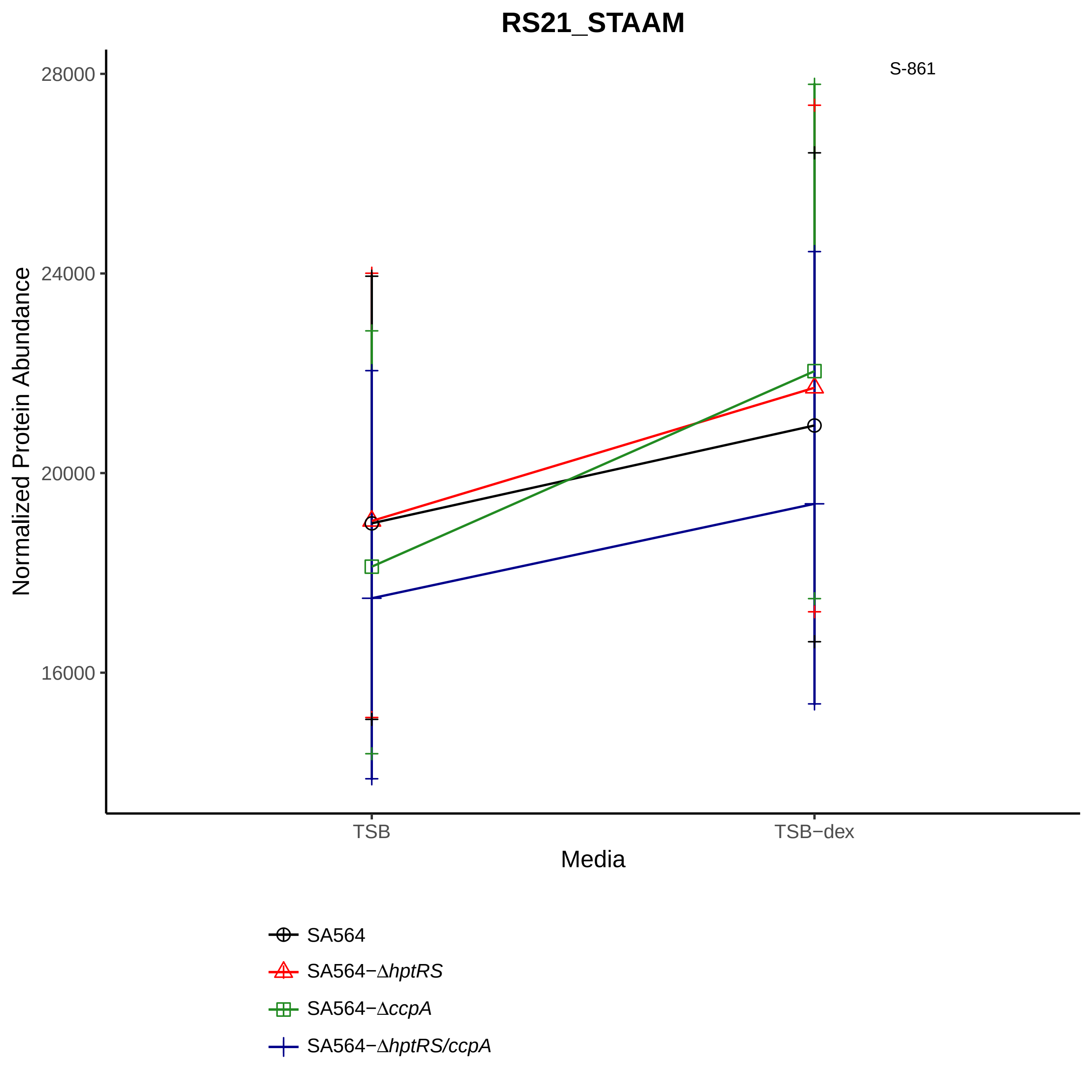

RS21_STAAM
S-861
28000
24000
20000
16000
Normalized Protein Abundance
TSB
TSB−dex
Media
SA564
SA564−ΔhptRS
SA564−ΔccpA
SA564−ΔhptRS/ccpA

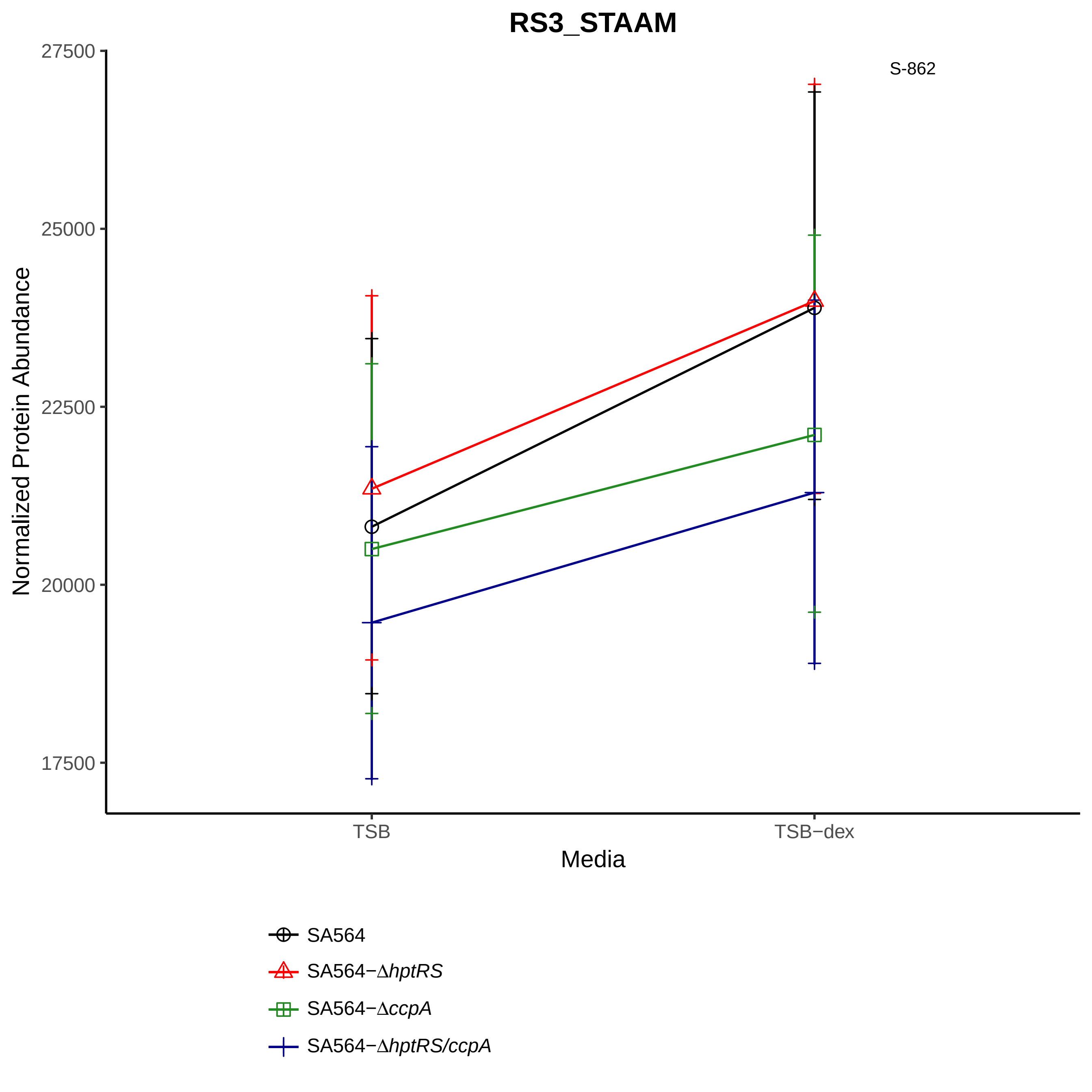

RS3_STAAM
S-862
27500
25000
22500
20000
17500
Normalized Protein Abundance
TSB
TSB−dex
Media
SA564
SA564−Δ*hptRS*
SA564−Δ*ccpA*
SA564−Δ*hptRS/ccpA*

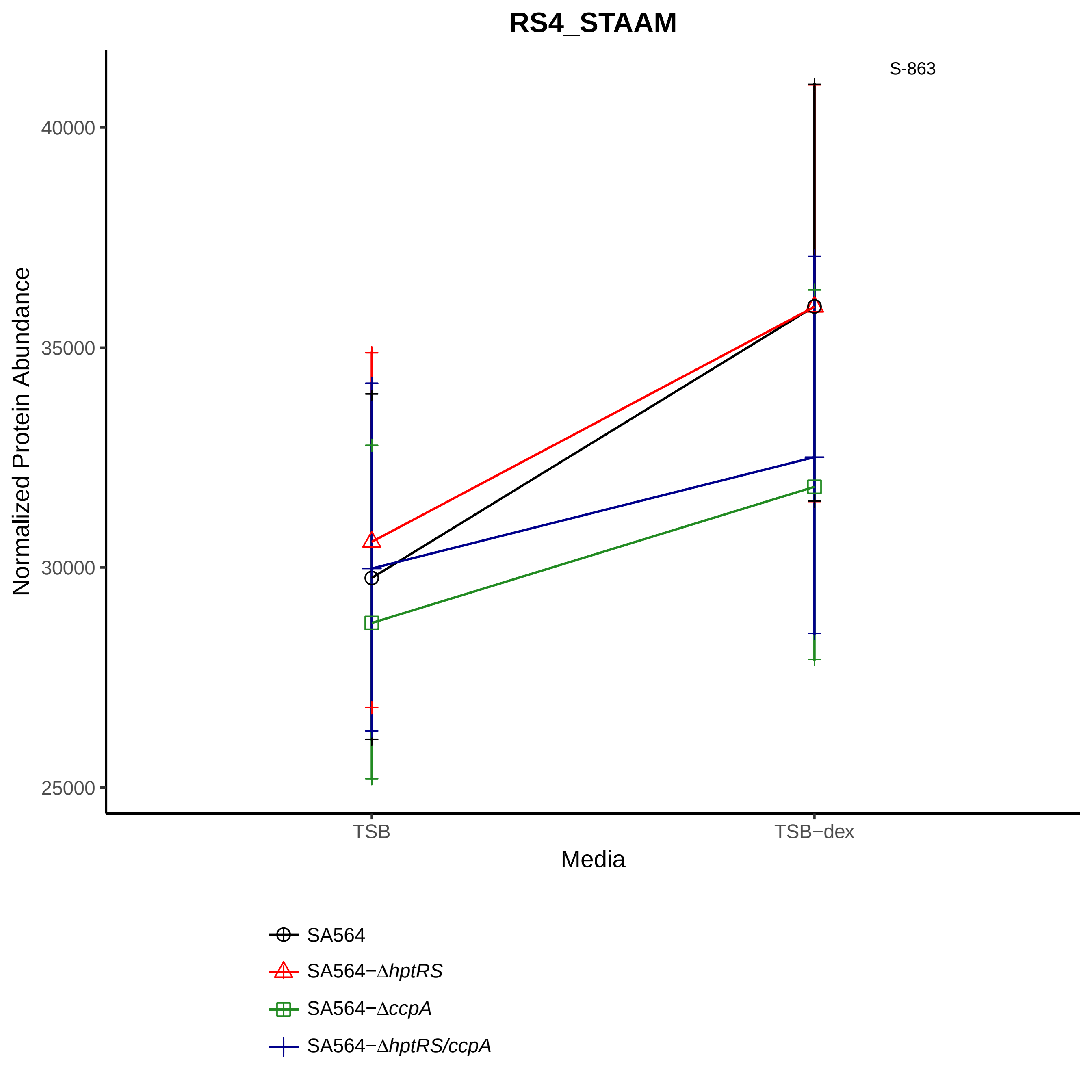

RS4_STAAM
S-863
40000
35000
30000
25000
Normalized Protein Abundance
TSB
TSB−dex
Media
SA564
SA564−ΔhptRS
SA564−ΔccpA
SA564−ΔhptRS/ccpA

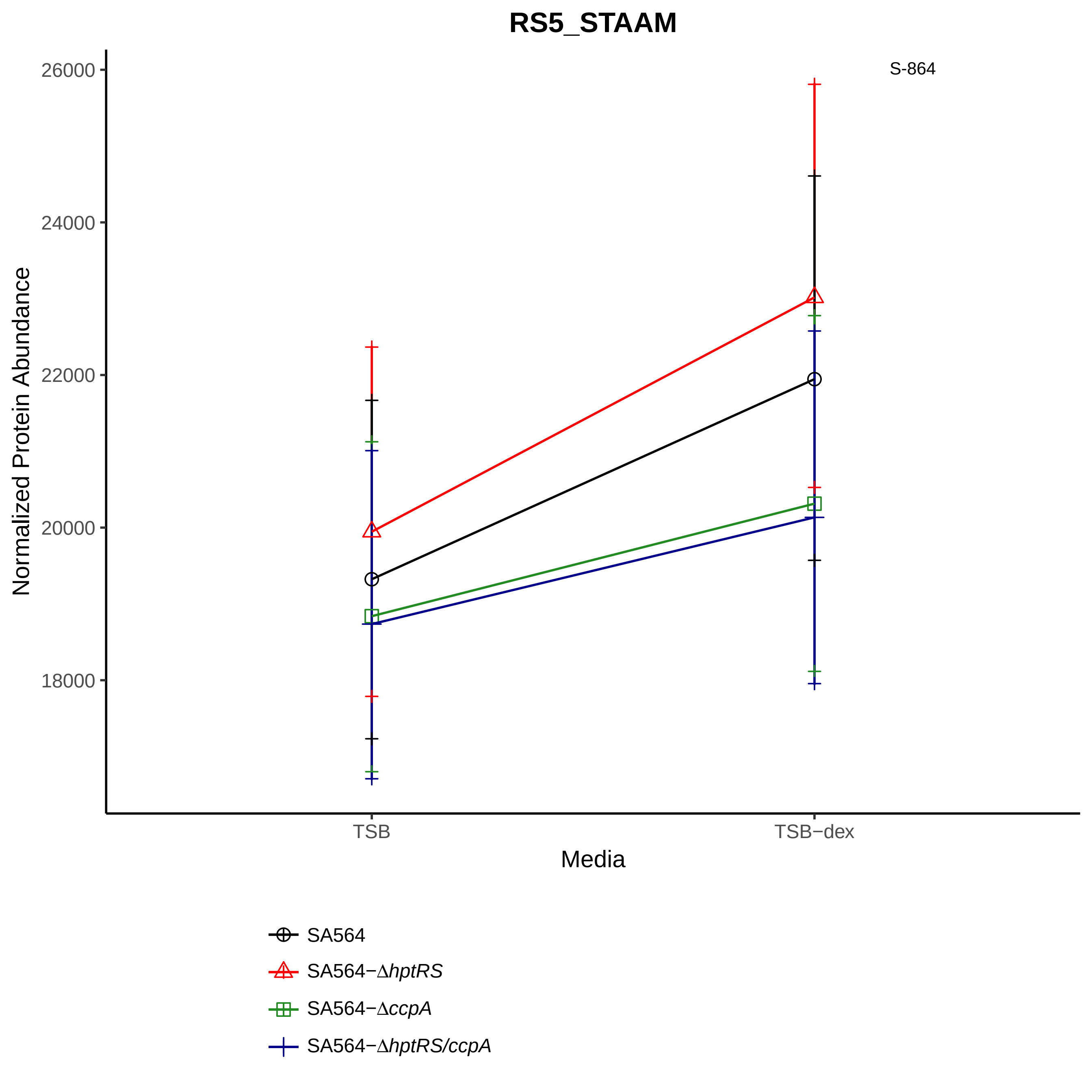

RS5_STAAM
S-864
Normalized Protein Abundance
26000
24000
22000
20000
18000
TSB
TSB−dex
Media
SA564
SA564−Δ*hptRS*
SA564−Δ*ccpA*
SA564−Δ*hptRS/ccpA*

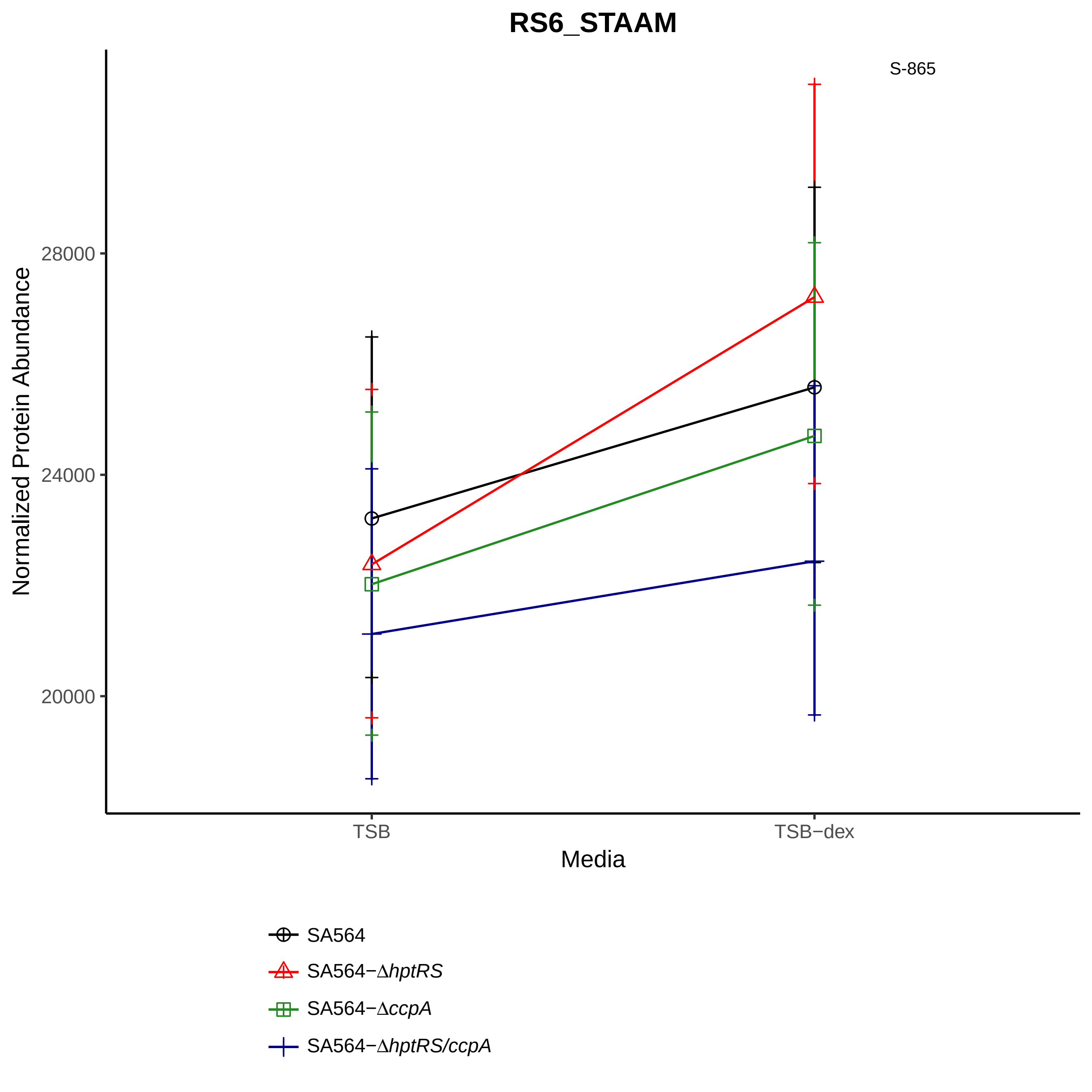

RS6_STAAM
S-865
Normalized Protein Abundance
28000
24000
20000
TSB
TSB−dex
Media
SA564
SA564−Δ*hptRS*
SA564−Δ*ccpA*
SA564−Δ*hptRS/ccpA*

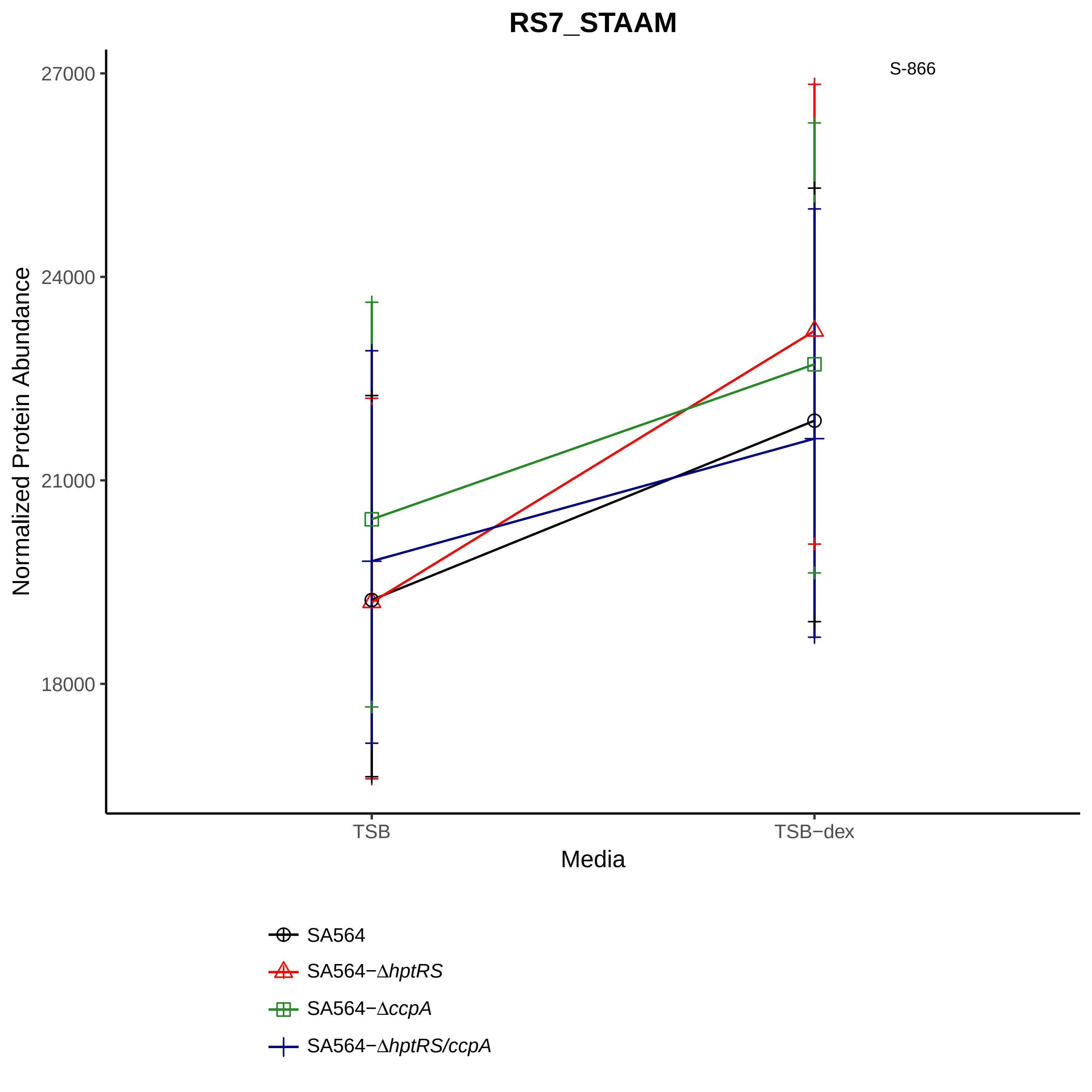

RS7_STAAM
S-866
27000
24000
21000
18000
Normalized Protein Abundance
TSB
TSB−dex
Media
SA564
SA564−Δ*hptRS*
SA564−Δ*ccpA*
SA564−Δ*hptRS/ccpA*

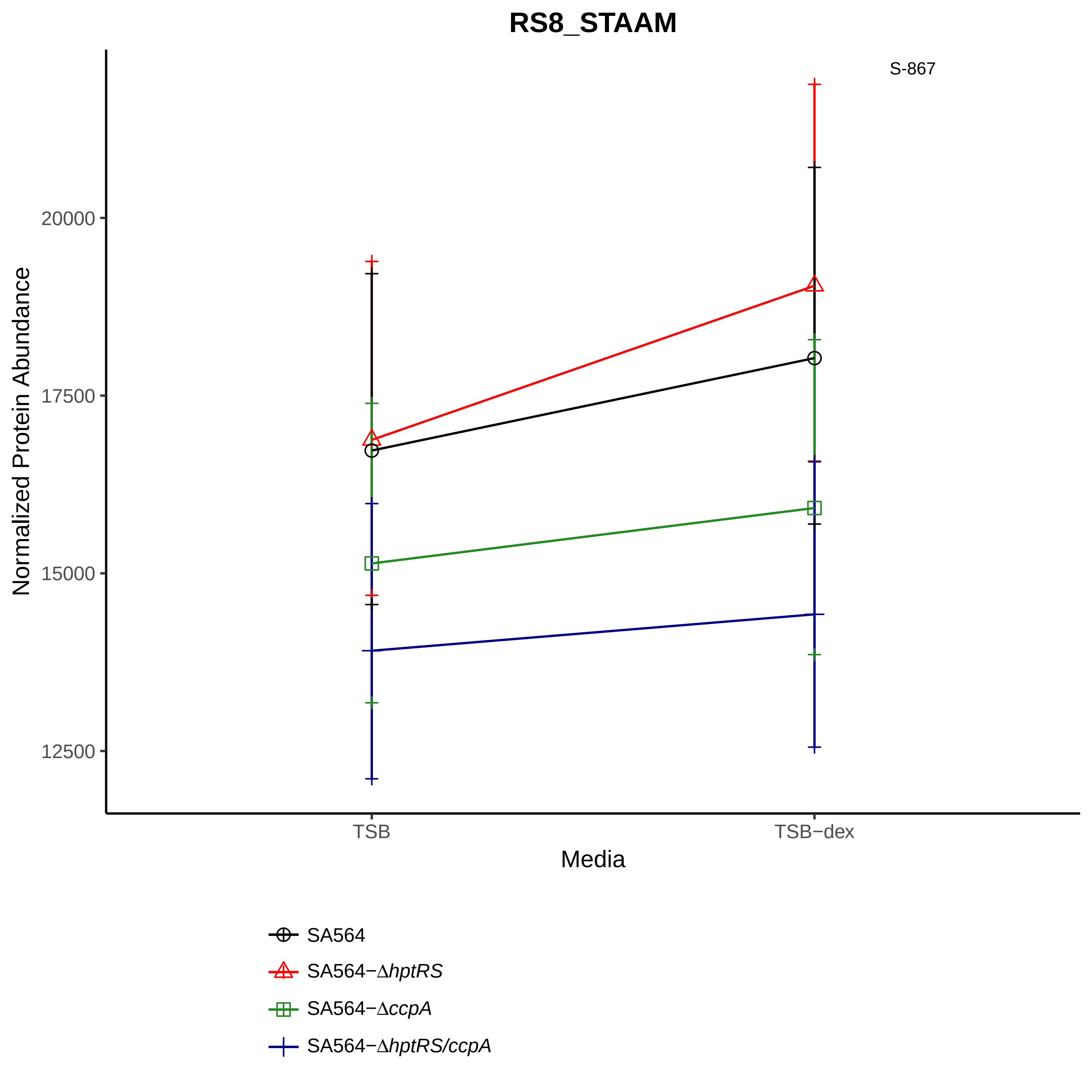

RS8_STAAM
S-867
Normalized Protein Abundance
20000
17500
15000
12500
TSB
TSB−dex
Media
SA564
SA564−ΔhptRS
SA564−ΔccpA
SA564−ΔhptRS/ccpA

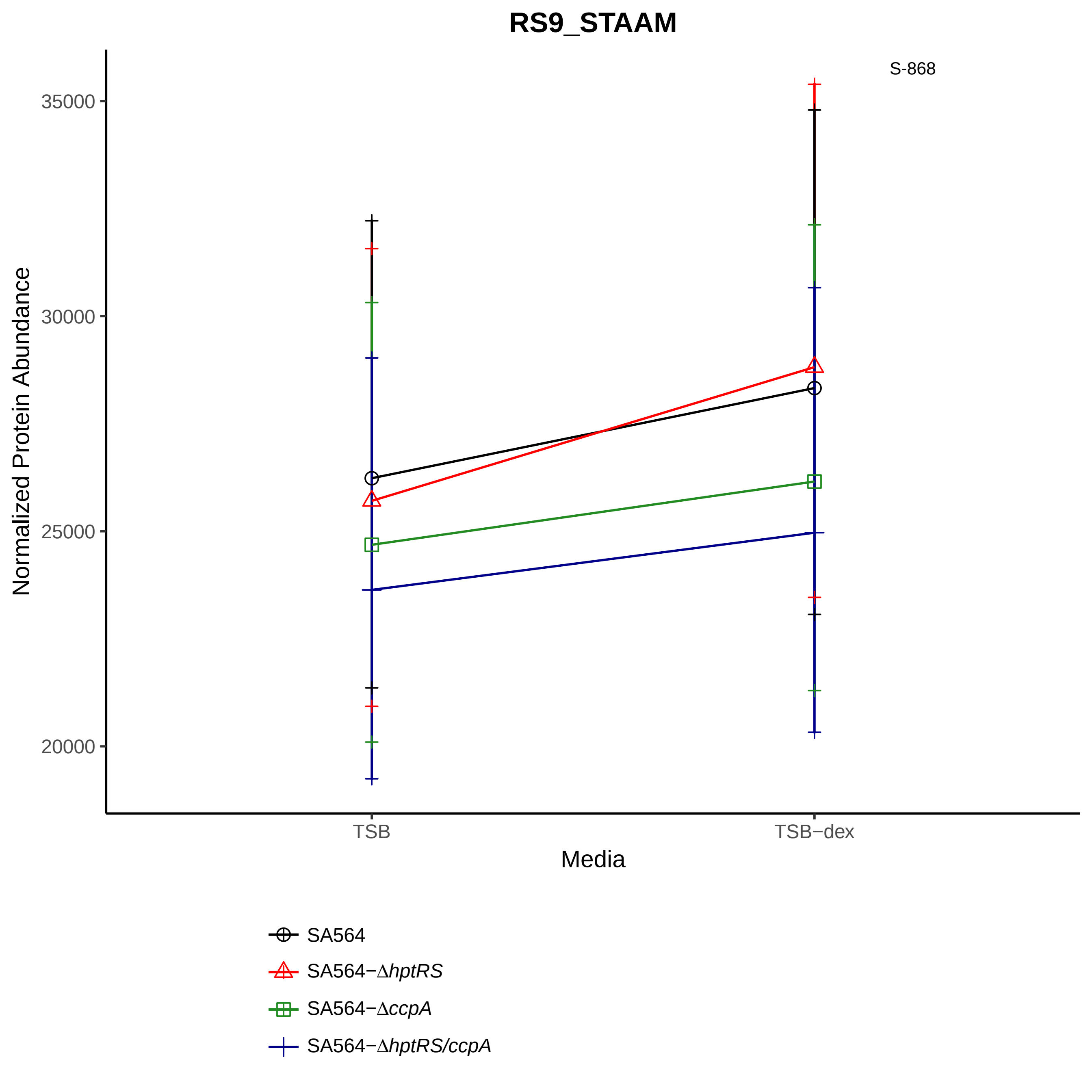

RS9_STAAM
S-868
Normalized Protein Abundance
35000
30000
25000
20000
TSB
TSB−dex
Media
SA564
SA564−Δ*hptRS*
SA564−Δ*ccpA*
SA564−Δ*hptRS/ccpA*

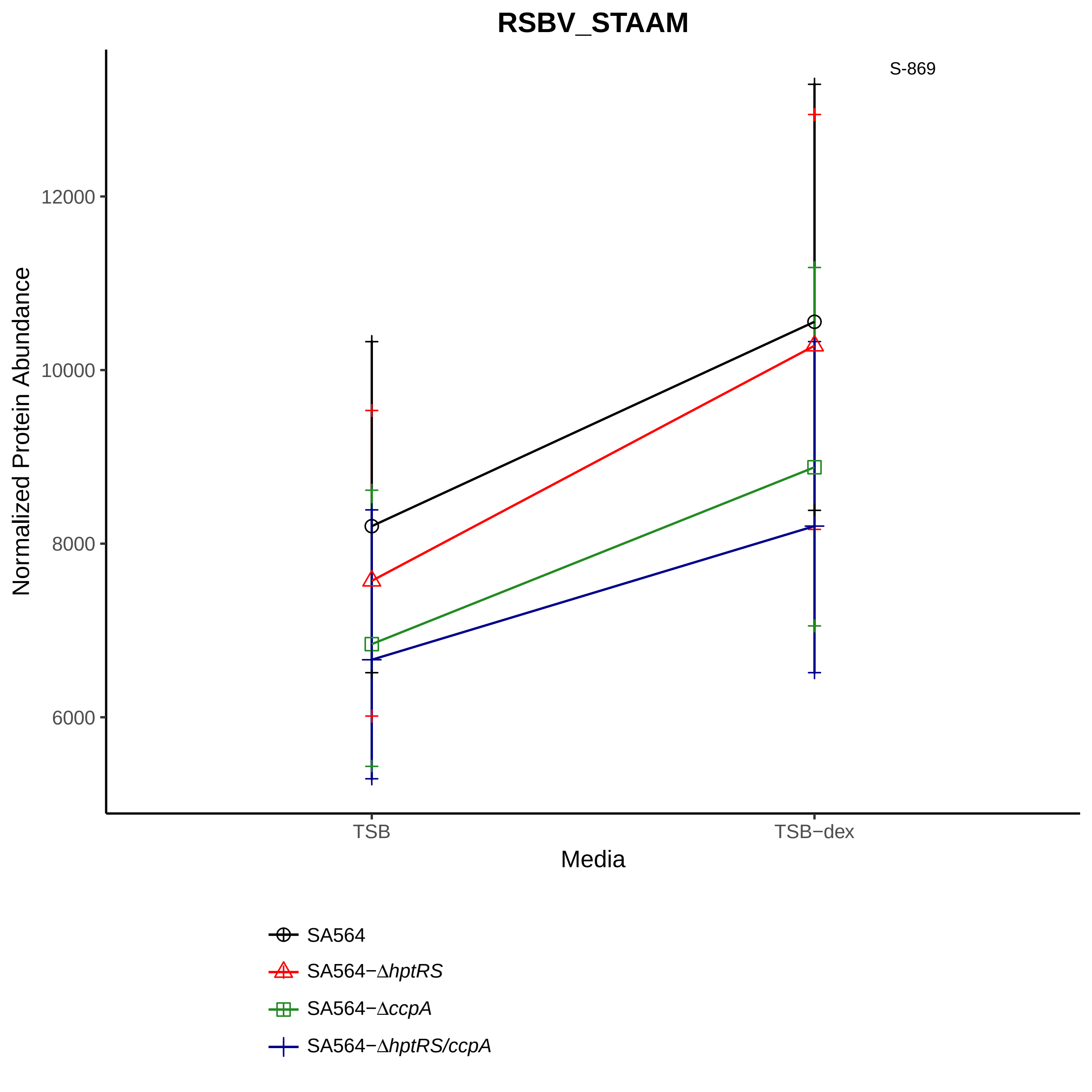

RSBV_STAAM
S-869
Normalized Protein Abundance
12000
10000
8000
6000
TSB
TSB−dex
Media
SA564
SA564−Δ*hptRS*
SA564−Δ*ccpA*
SA564−Δ*hptRS/ccpA*

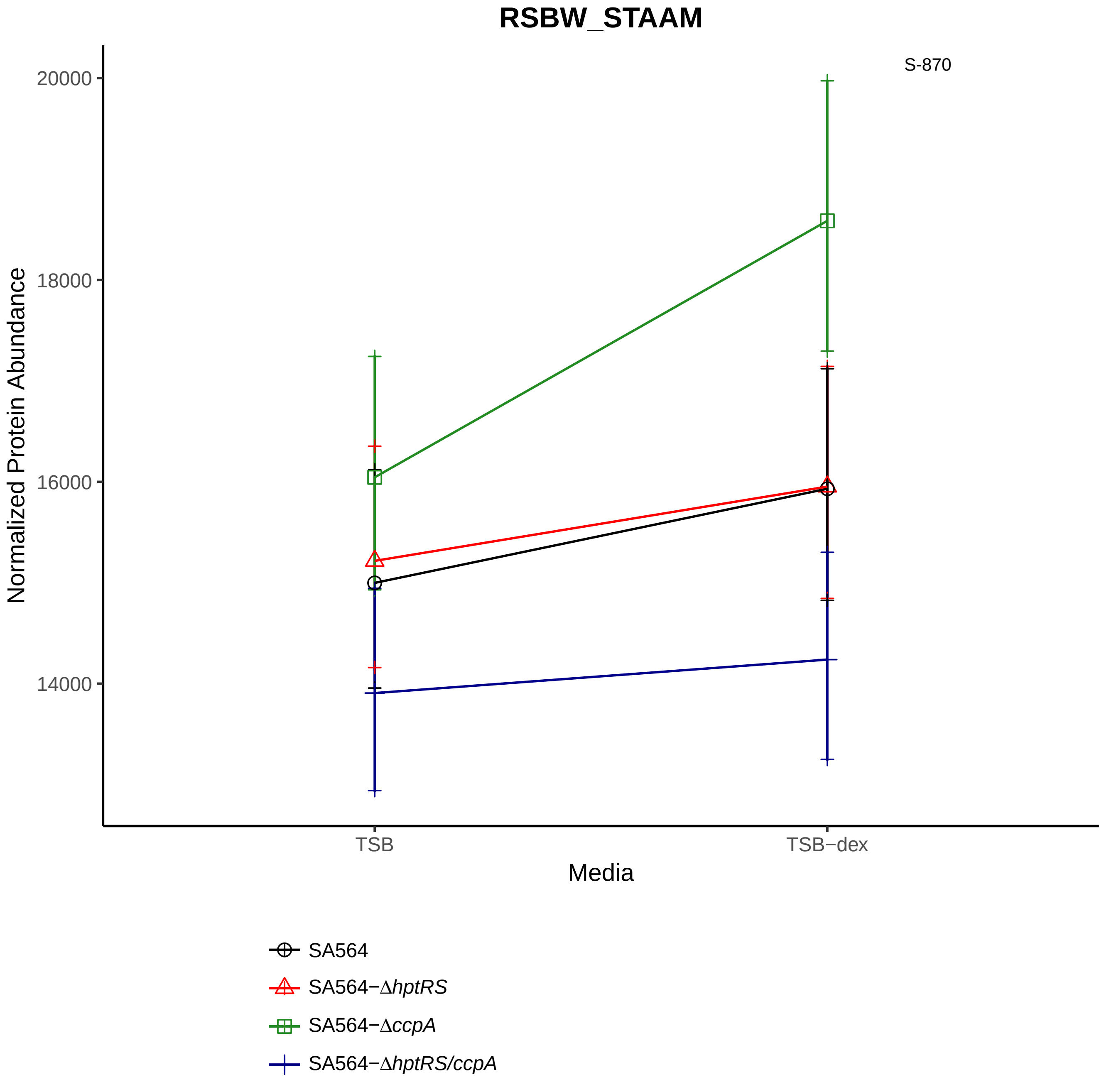
RSBW_STAAM
S-870
20000
18000
16000
14000
Normalized Protein Abundance
TSB
TSB−dex
Media
SA564
SA564−Δ*hptRS*
SA564−Δ*ccpA*
SA564−Δ*hptRS/ccpA*

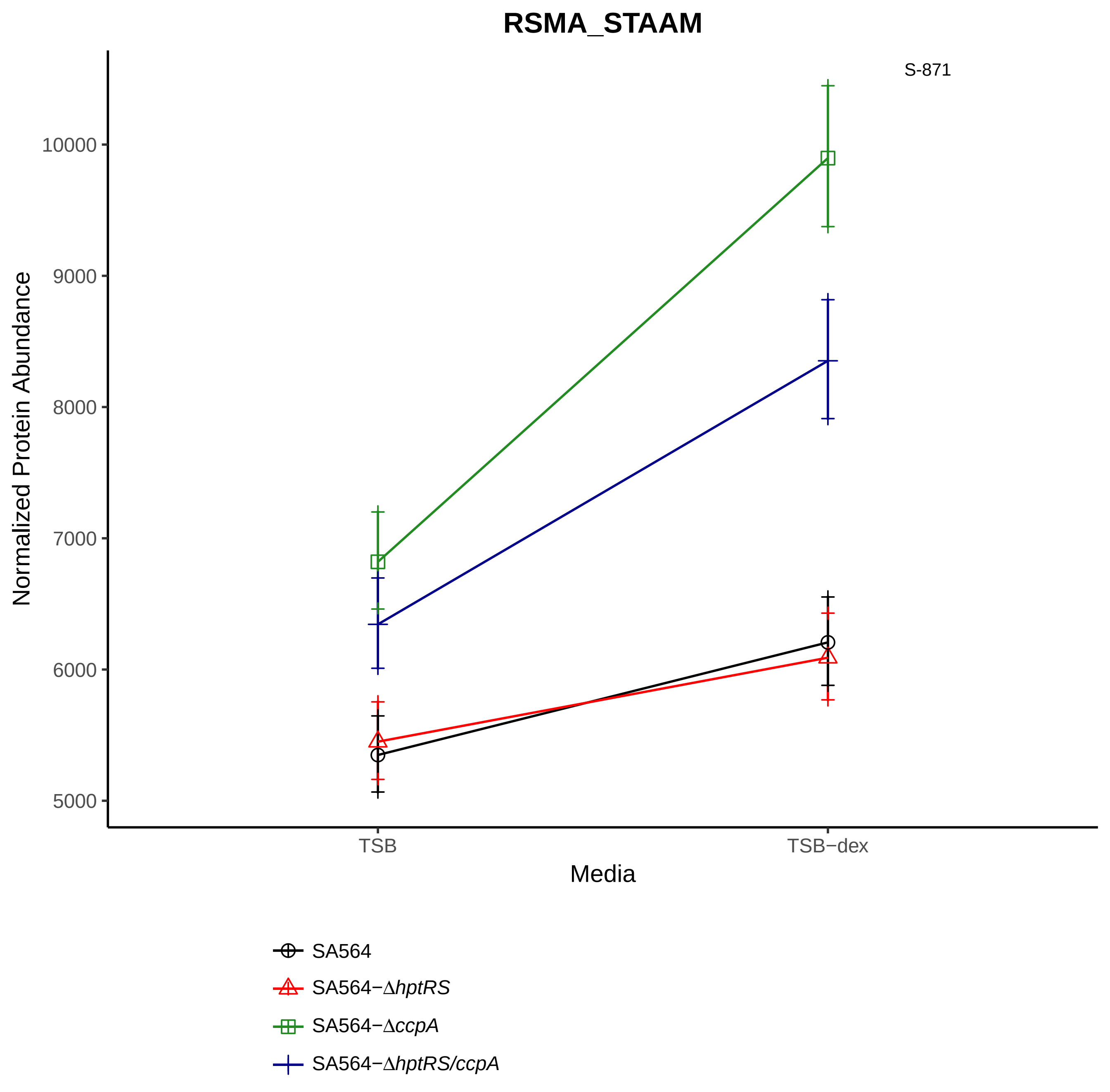
RSMA_STAAM
S-871
Normalized Protein Abundance
10000
9000
8000
7000
6000
5000
TSB
TSB−dex
Media
SA564
SA564−Δ*hptRS*
SA564−Δ*ccpA*
SA564−Δ*hptRS/ccpA*

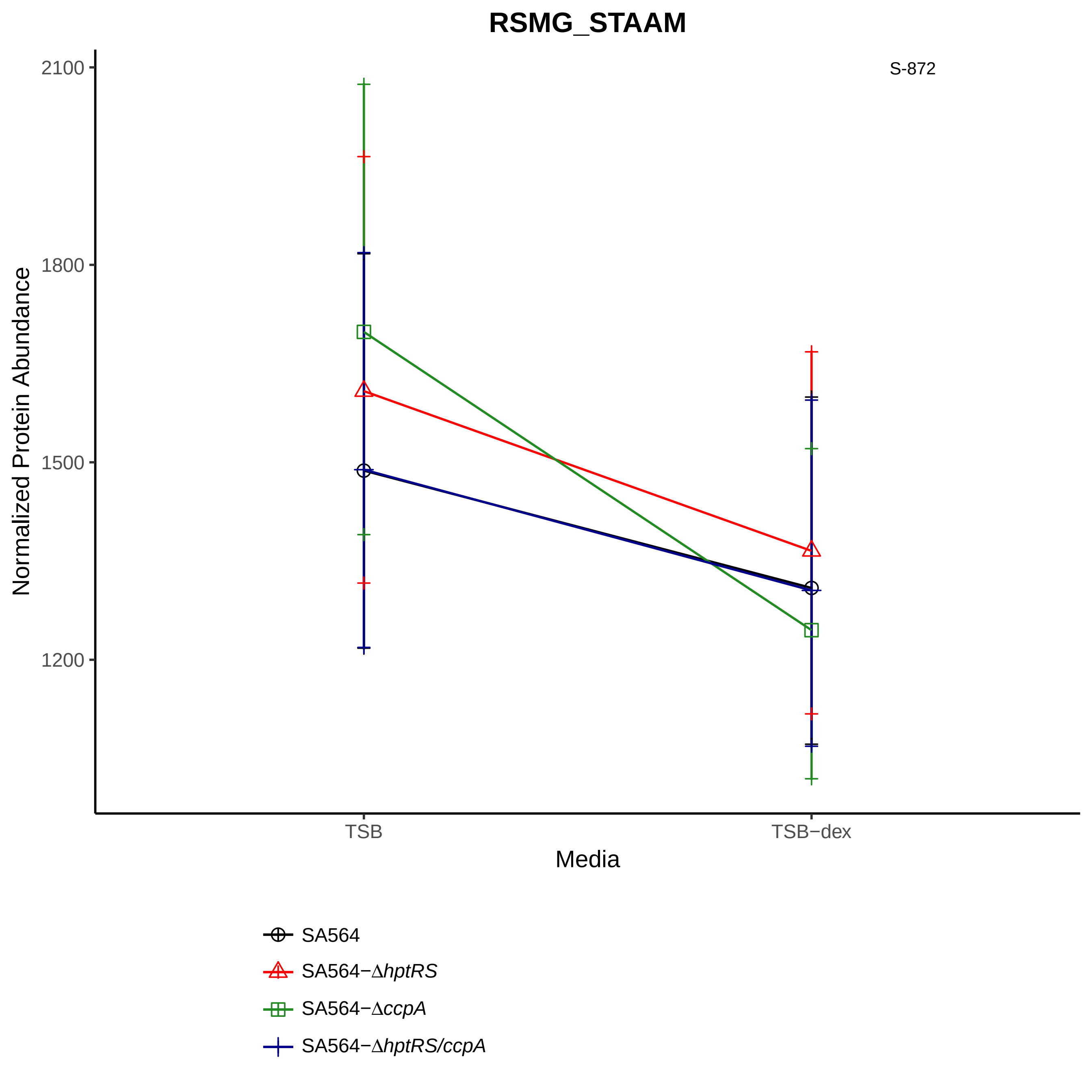

RSMG_STAAM
S-872
Normalized Protein Abundance
2100
1800
1500
1200
TSB
TSB−dex
Media
SA564
SA564−Δ*hptRS*
SA564−Δ*ccpA*
SA564−Δ*hptRS/ccpA*

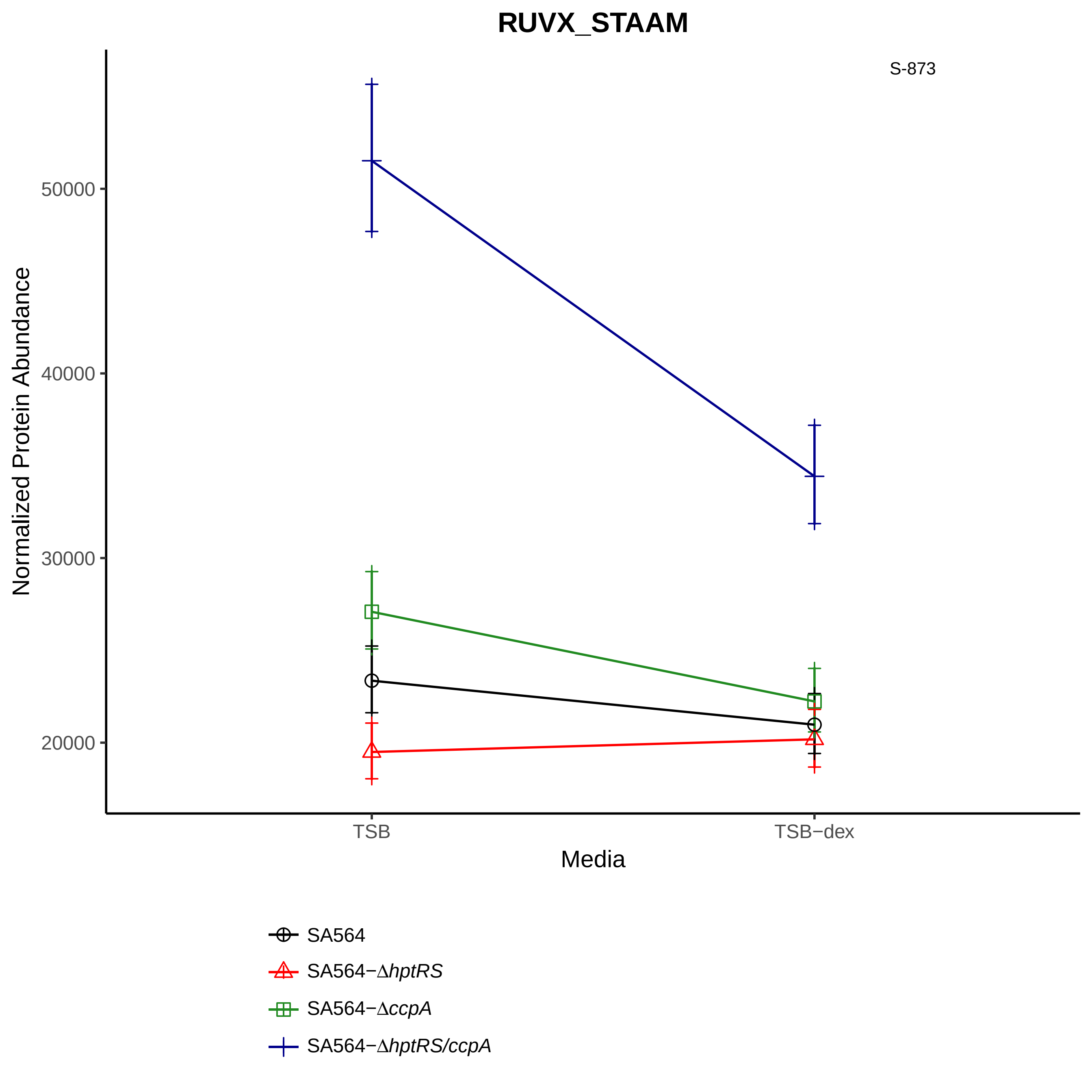

RUVX_STAAM
S-873
Normalized Protein Abundance
50000
40000
30000
20000
TSB
TSB−dex
Media
SA564
SA564−ΔhptRS
SA564−ΔccpA
SA564−ΔhptRS/ccpA

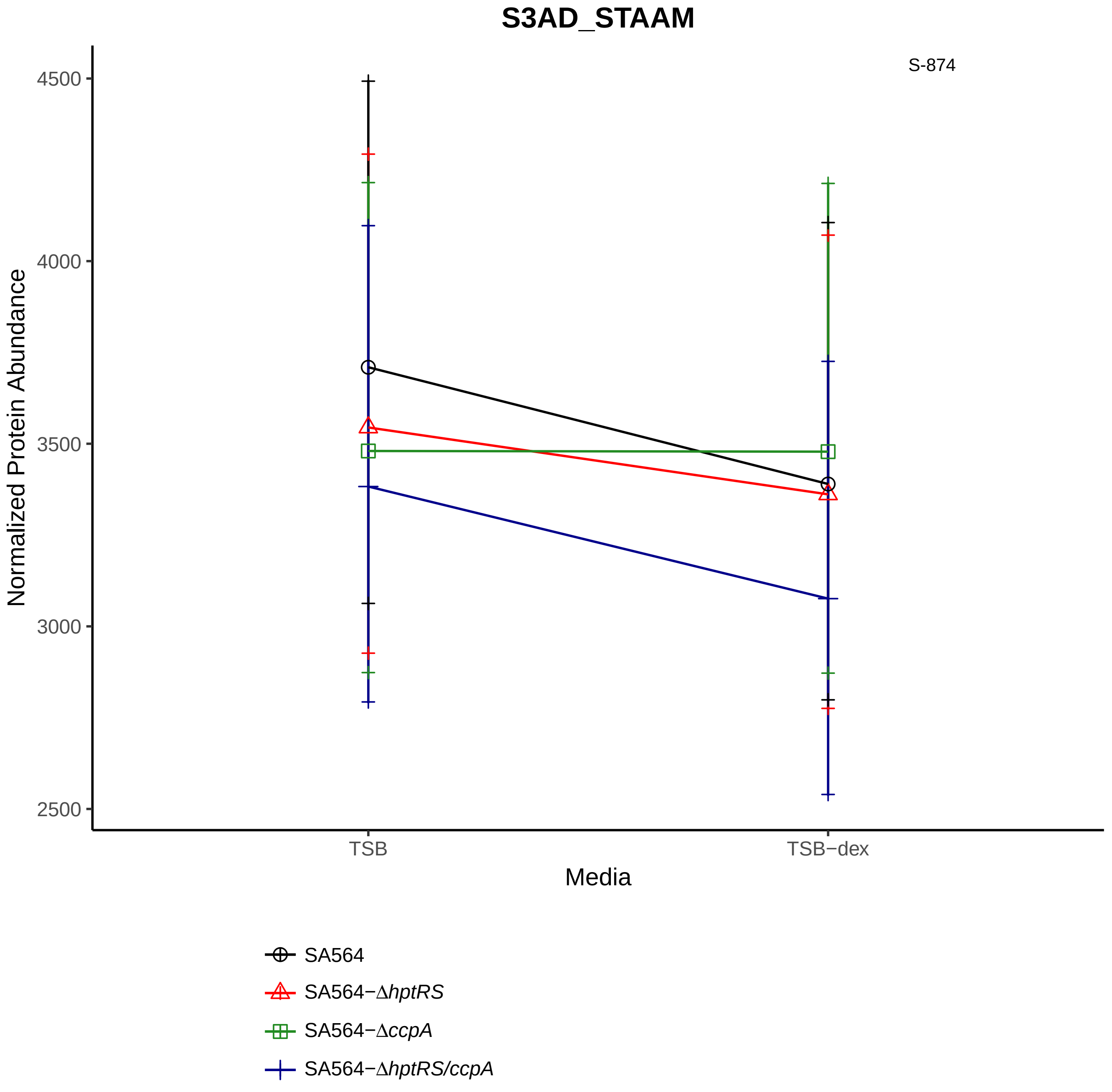

S3AD_STAAM
S-874
4500
4000
3500
3000
2500
Normalized Protein Abundance
TSB
TSB−dex
Media
SA564
SA564−Δ*hptRS*
SA564−Δ*ccpA*
SA564−Δ*hptRS/ccpA*

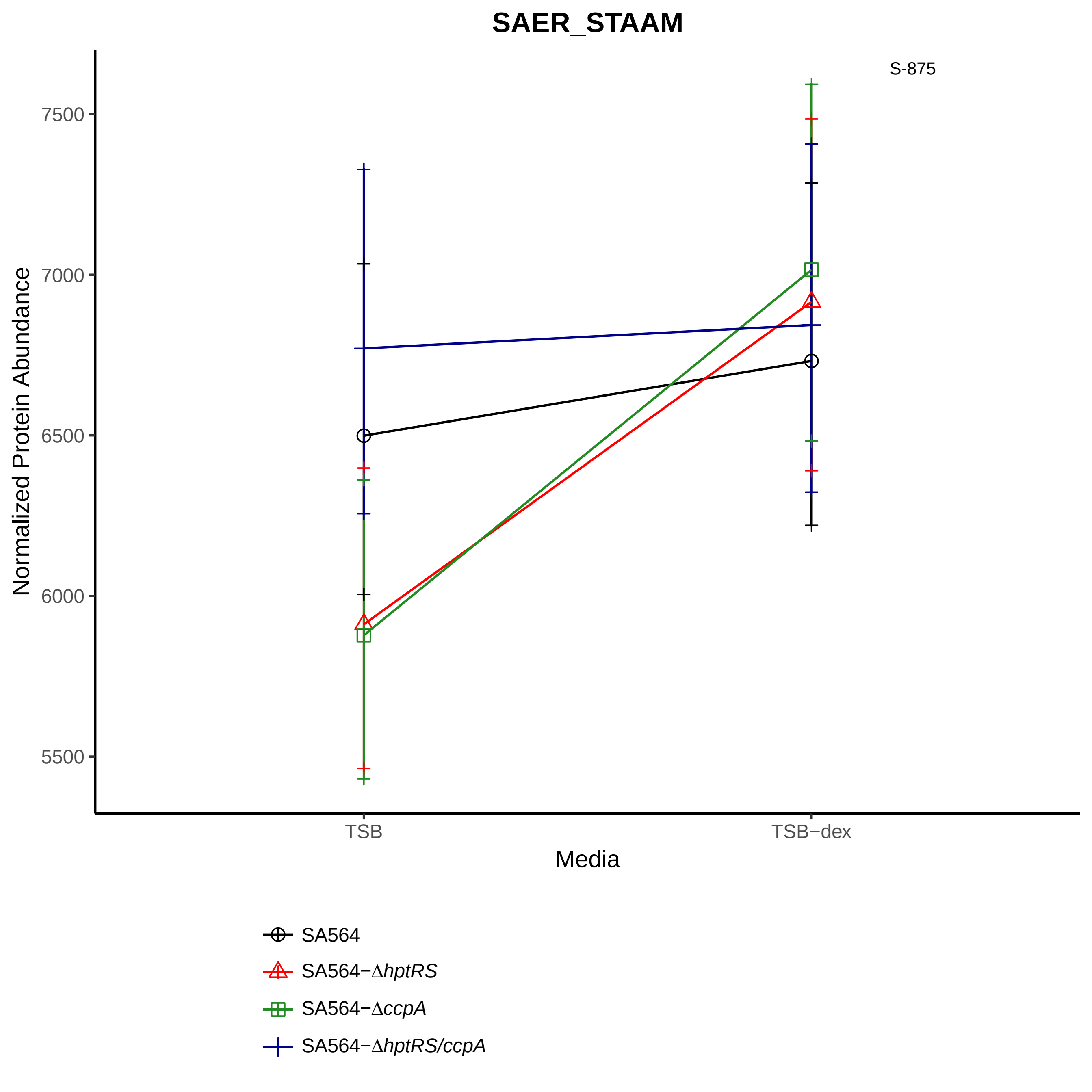

SAER_STAAM
S-875
Normalized Protein Abundance
7500
7000
6500
6000
5500
TSB
TSB−dex
Media
SA564
SA564−ΔhptRS
SA564−ΔccpA
SA564−ΔhptRS/ccpA

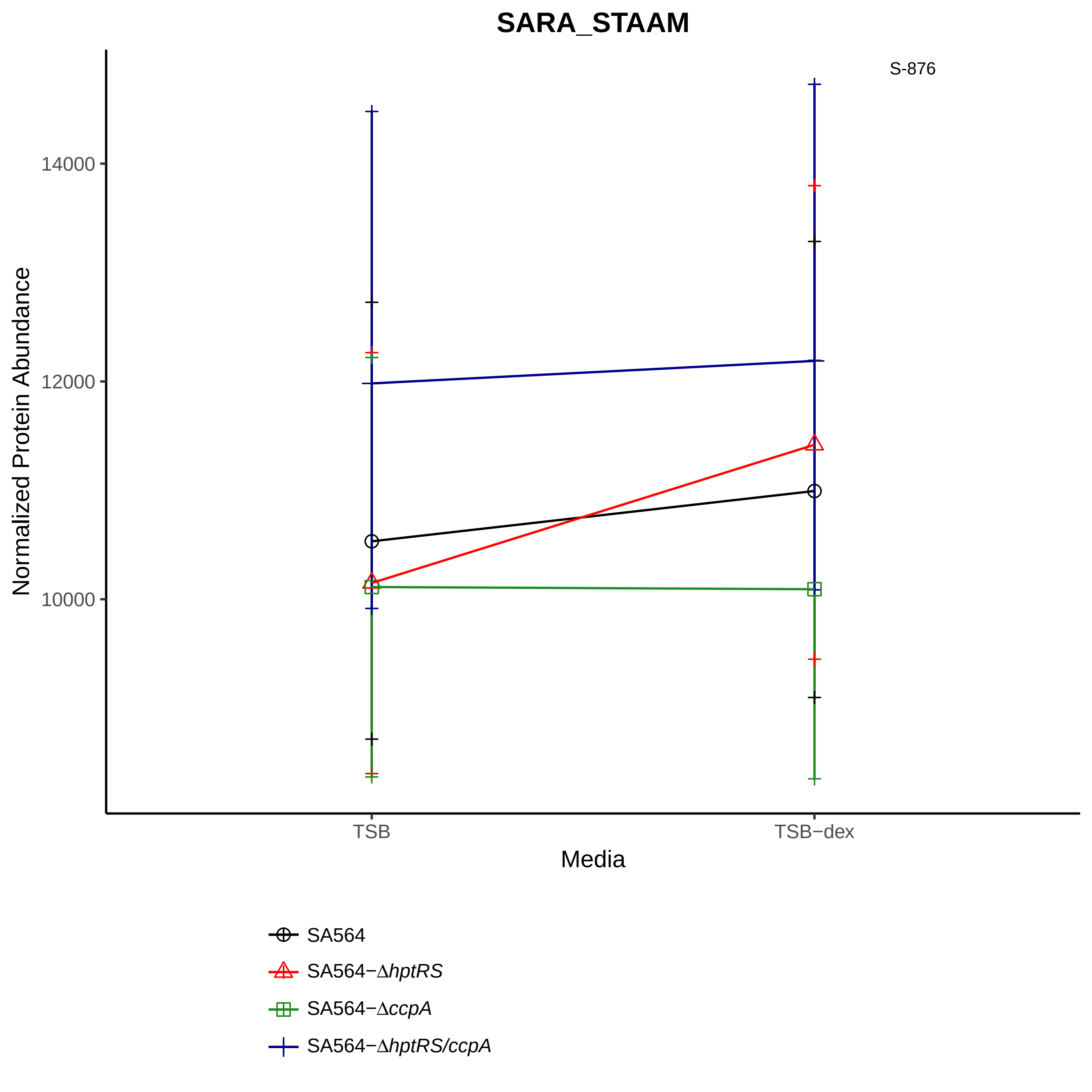
SARA_STAAM
S-876
14000
12000
10000
Normalized Protein Abundance
TSB
TSB−dex
Media
SA564
SA564−ΔhptRS
SA564−ΔccpA
SA564−ΔhptRS/ccpA

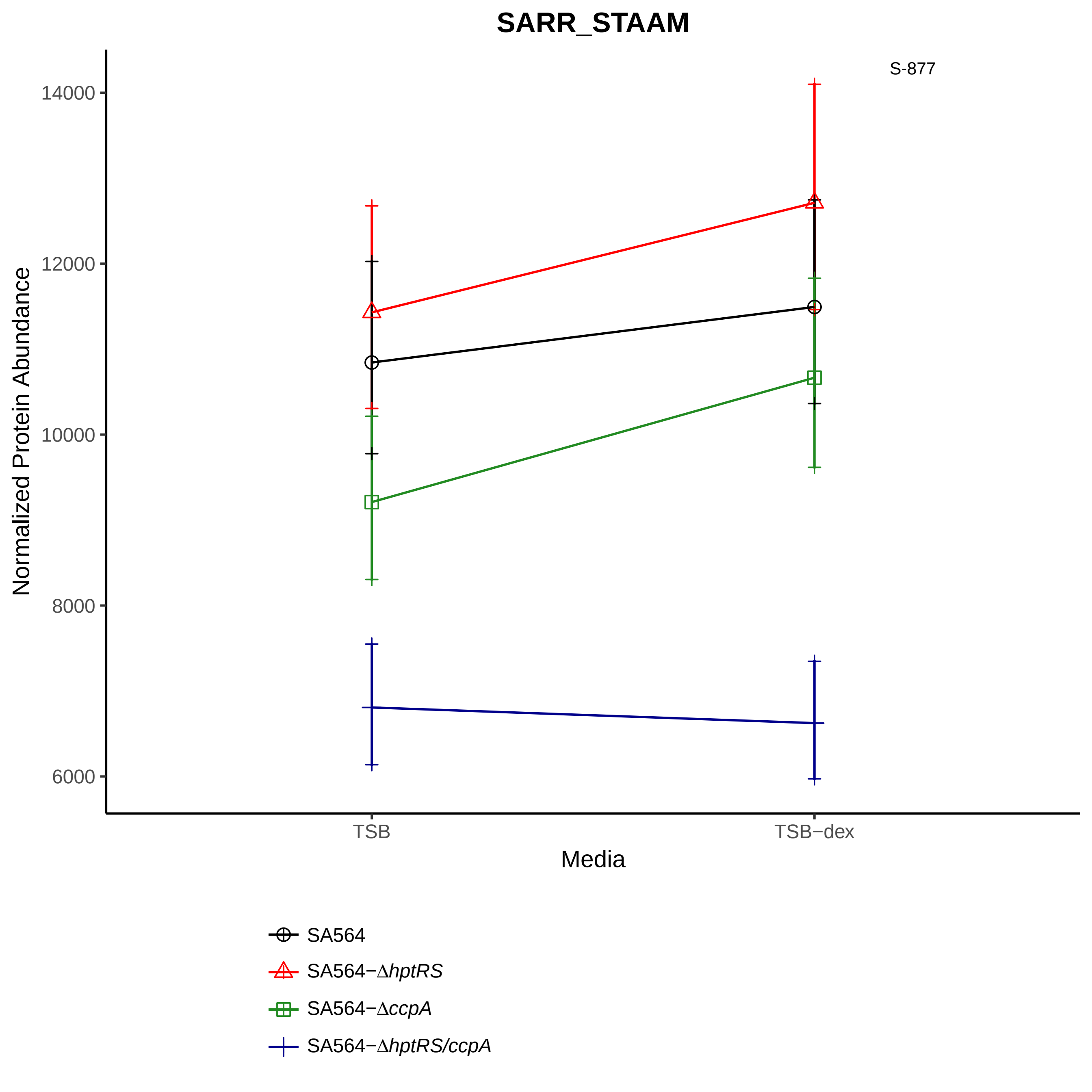
SARR_STAAM
S-877
Normalized Protein Abundance
14000
12000
10000
8000
6000
TSB
TSB−dex
Media
SA564
SA564−Δ*hptRS*
SA564−Δ*ccpA*
SA564−Δ*hptRS/ccpA*

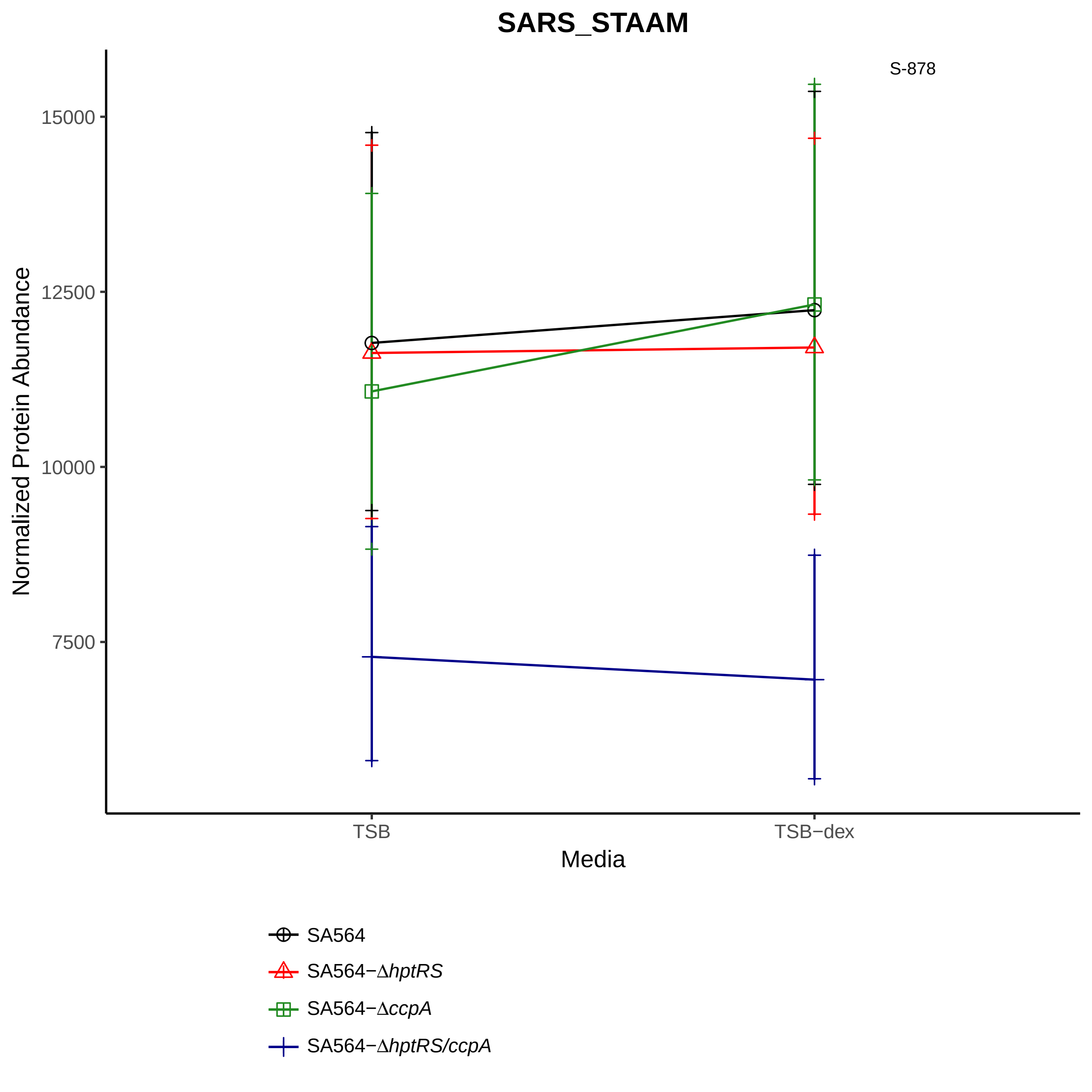

SARS_STAAM
S-878
15000
12500
10000
7500
Normalized Protein Abundance
TSB
TSB−dex
Media
SA564
SA564−ΔhptRS
SA564−ΔccpA
SA564−ΔhptRS/ccpA

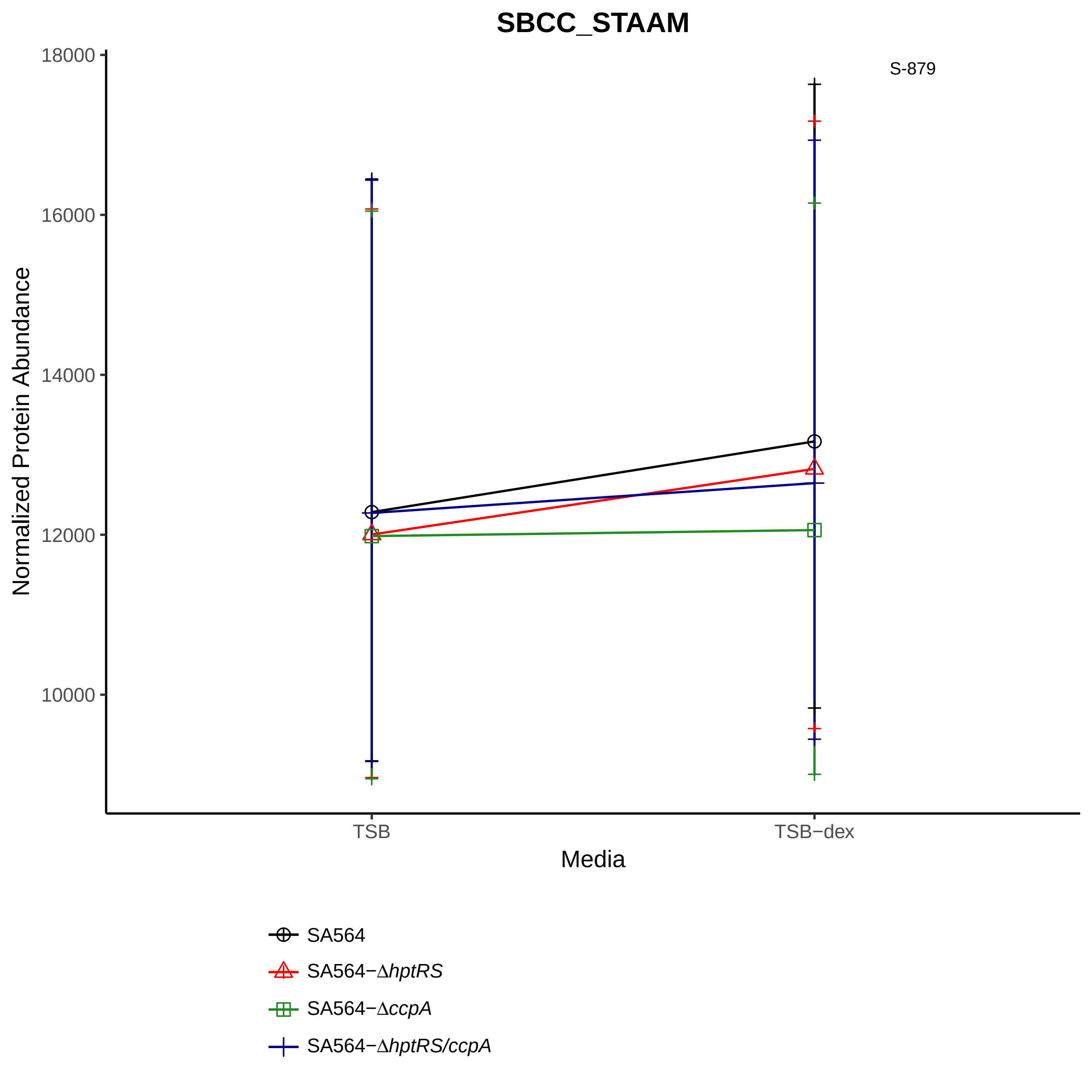

SBCC_STAAM
S-879
Normalized Protein Abundance
18000
16000
14000
12000
10000
TSB
TSB−dex
Media
SA564
SA564−ΔhptRS
SA564−ΔccpA
SA564−ΔhptRS/ccpA

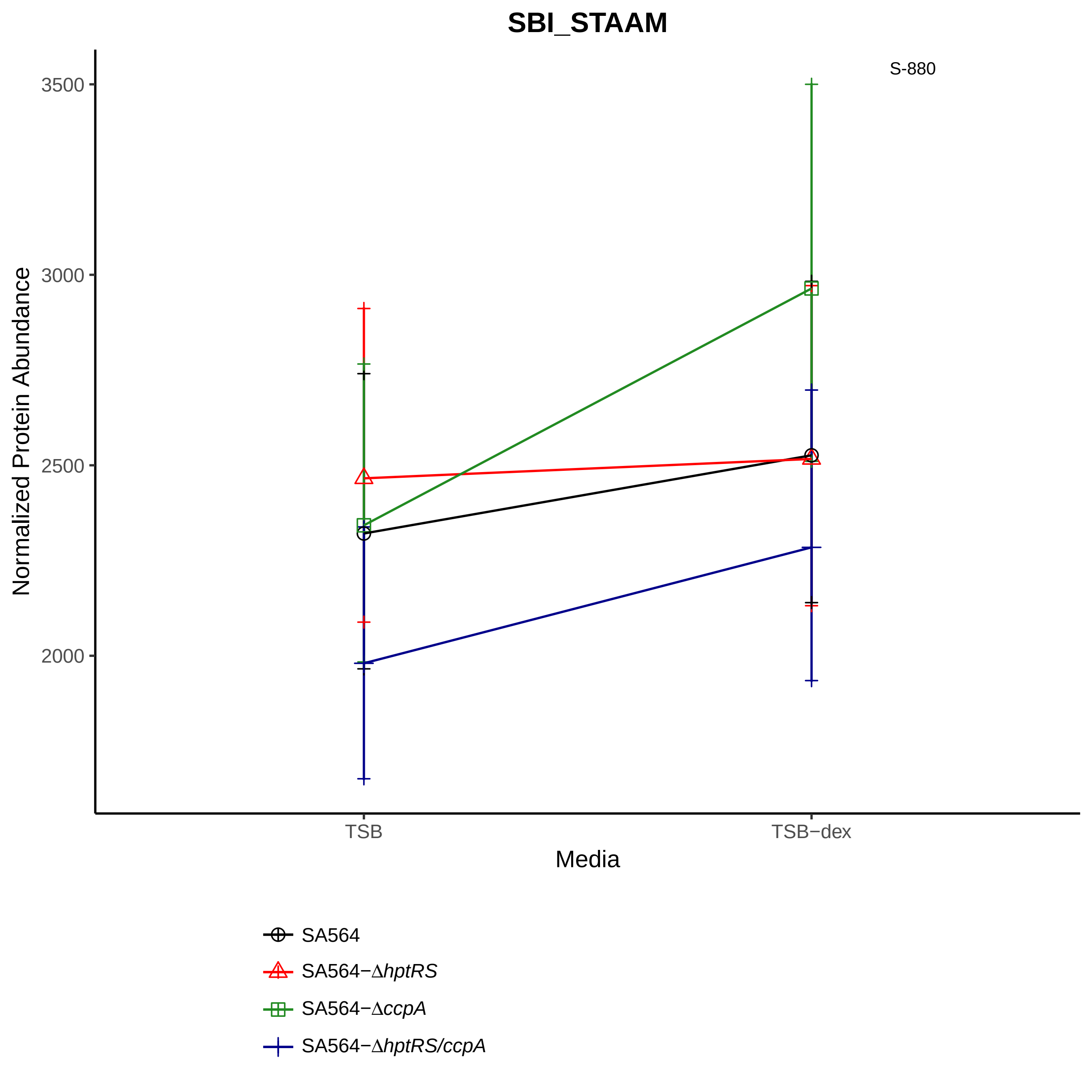

SBI_STAAM
S-880
3500
3000
2500
2000
Normalized Protein Abundance
TSB
TSB−dex
Media
SA564
SA564−Δ*hptRS*
SA564−Δ*ccpA*
SA564−Δ*hptRS/ccpA*

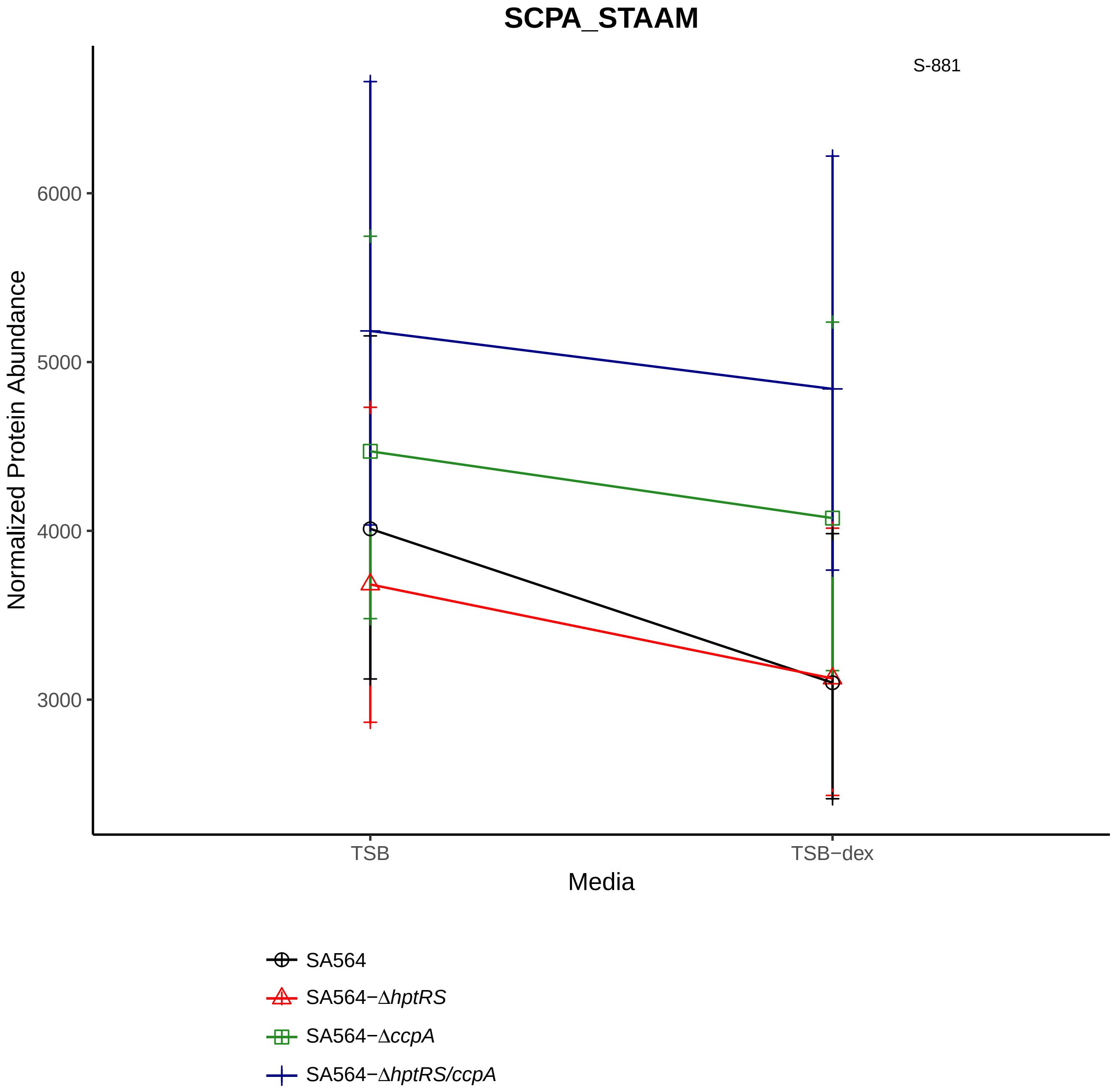

SCPA_STAAM
S-881
Normalized Protein Abundance
6000
5000
4000
3000
TSB
TSB−dex
Media
SA564
SA564−Δ*hptRS*
SA564−Δ*ccpA*
SA564−Δ*hptRS/ccpA*

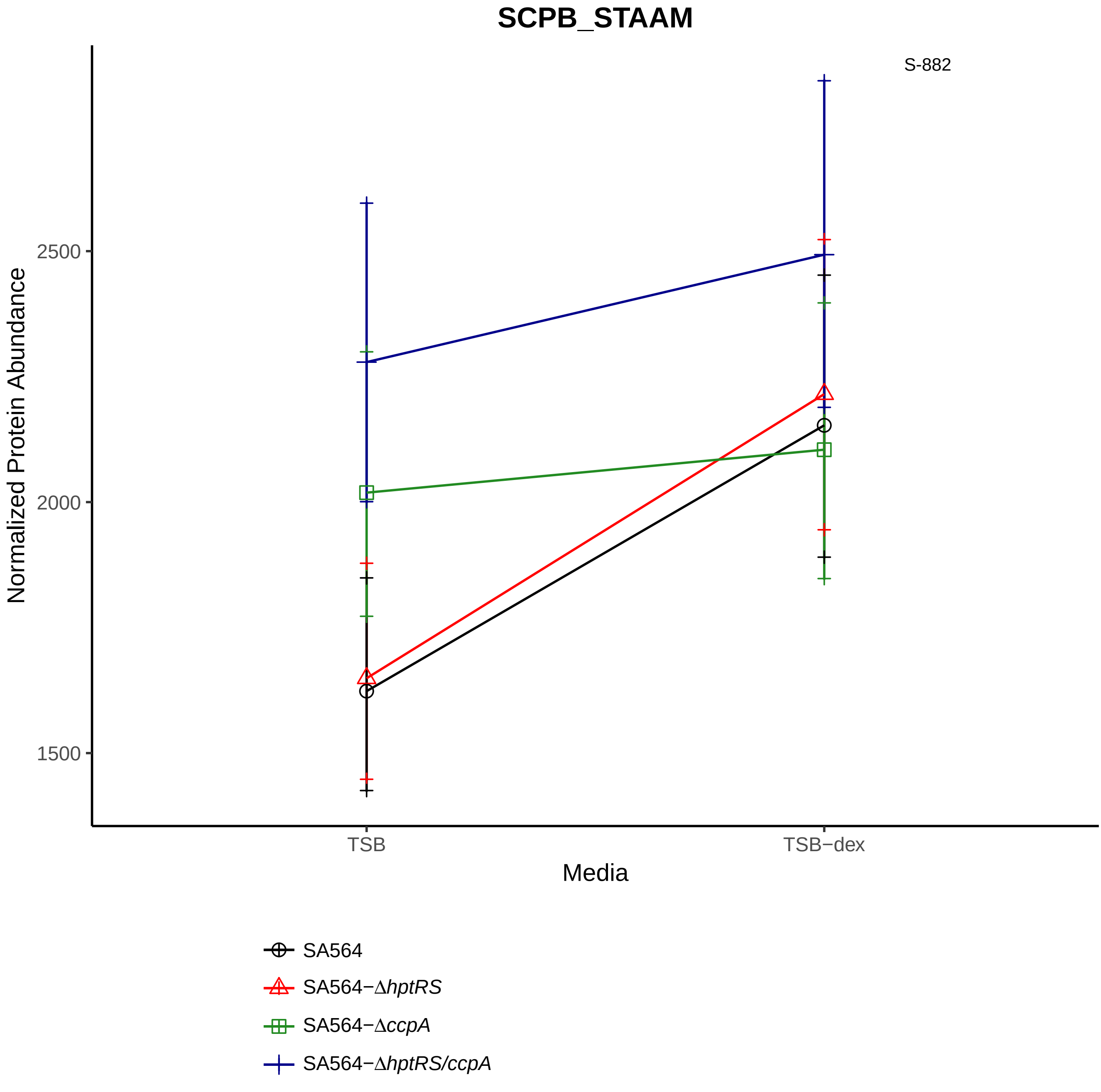

SCPB_STAAM
S-882
Normalized Protein Abundance
2500
2000
1500
TSB
TSB−dex
Media
SA564
SA564−Δ*hptRS*
SA564−Δ*ccpA*
SA564−Δ*hptRS/ccpA*

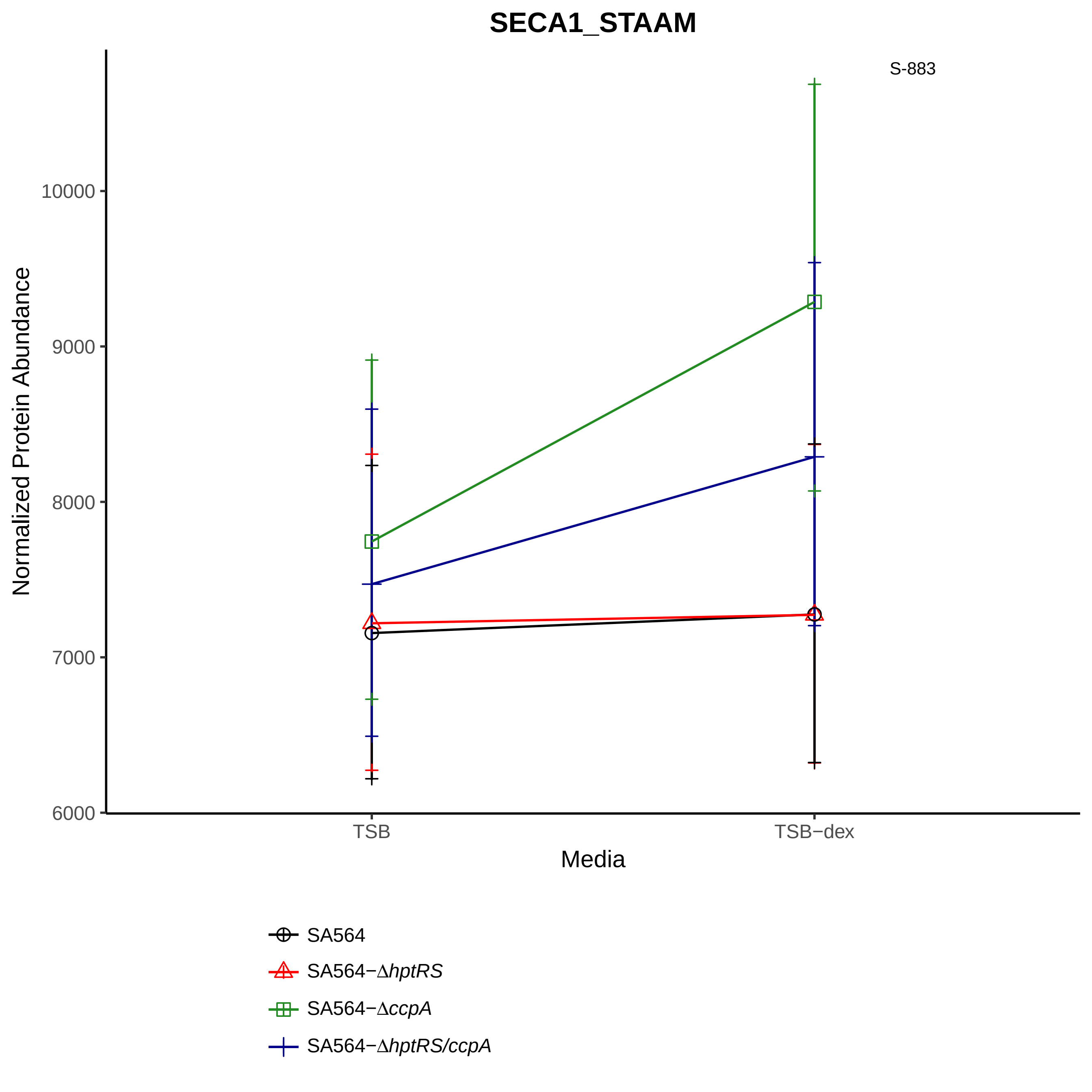

SECA1_STAAM
S-883
Normalized Protein Abundance
10000
9000
8000
7000
6000
TSB
TSB−dex
Media
SA564
SA564−Δ*hptRS*
SA564−Δ*ccpA*
SA564−Δ*hptRS/ccpA*

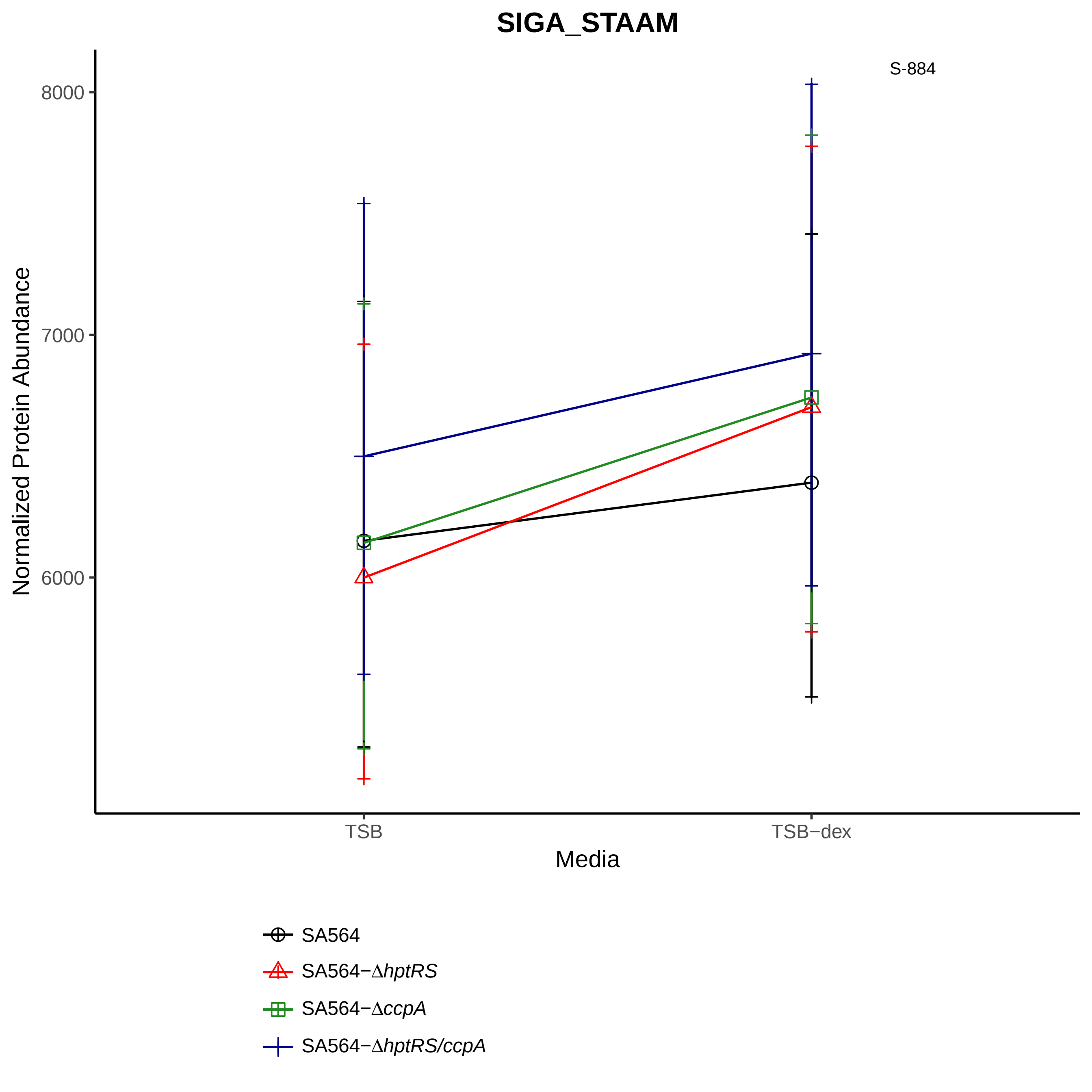

SIGA_STAAM
S-884
8000
7000
6000
Normalized Protein Abundance
TSB
TSB−dex
Media
SA564
SA564−Δ*hptRS*
SA564−Δ*ccpA*
SA564−Δ*hptRS/ccpA*

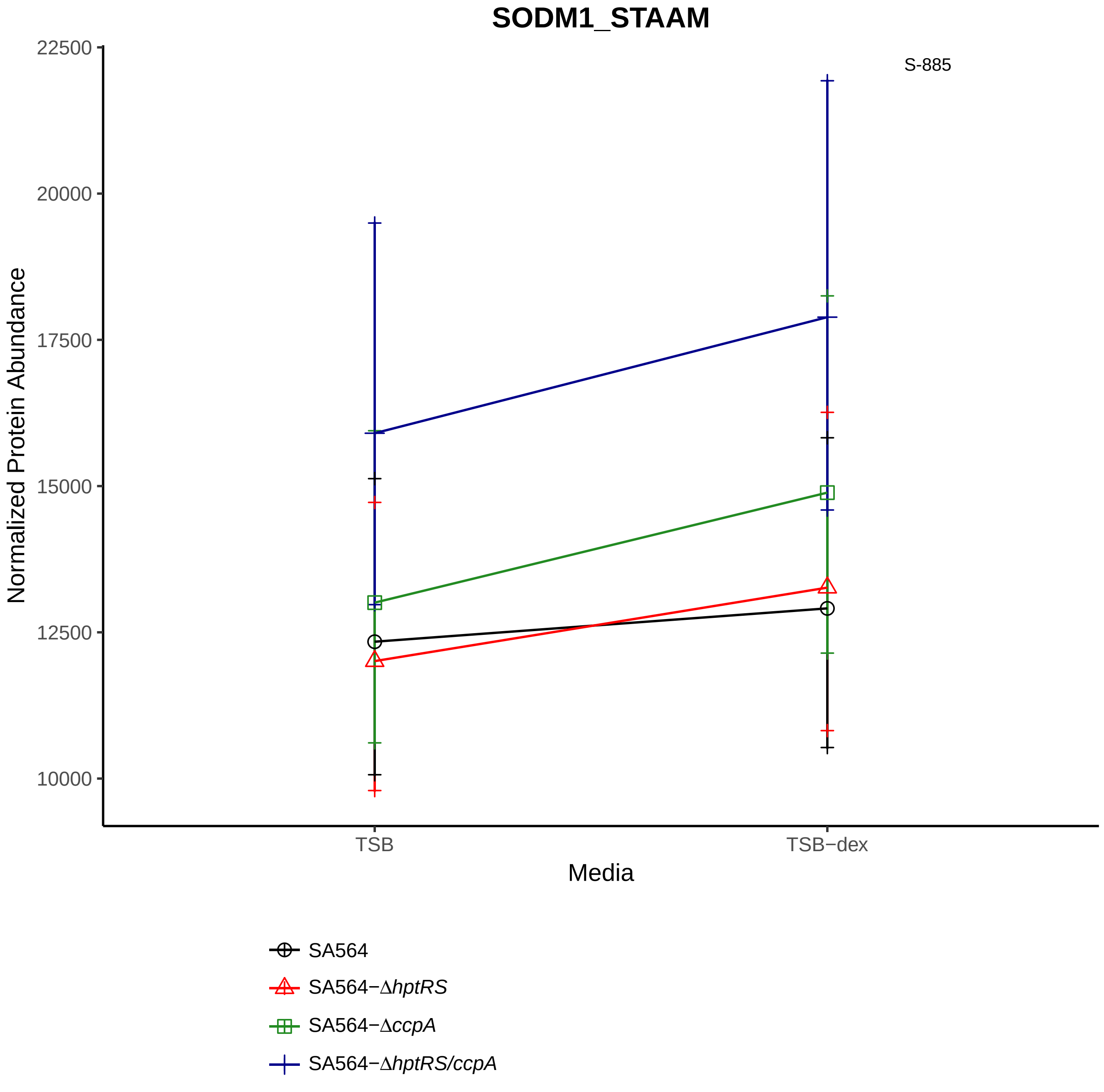
SODM1_STAAM
S-885
Normalized Protein Abundance
22500
20000
17500
15000
12500
10000
TSB
TSB−dex
Media
SA564
SA564−Δ*hptRS*
SA564−Δ*ccpA*
SA564−Δ*hptRS/ccpA*

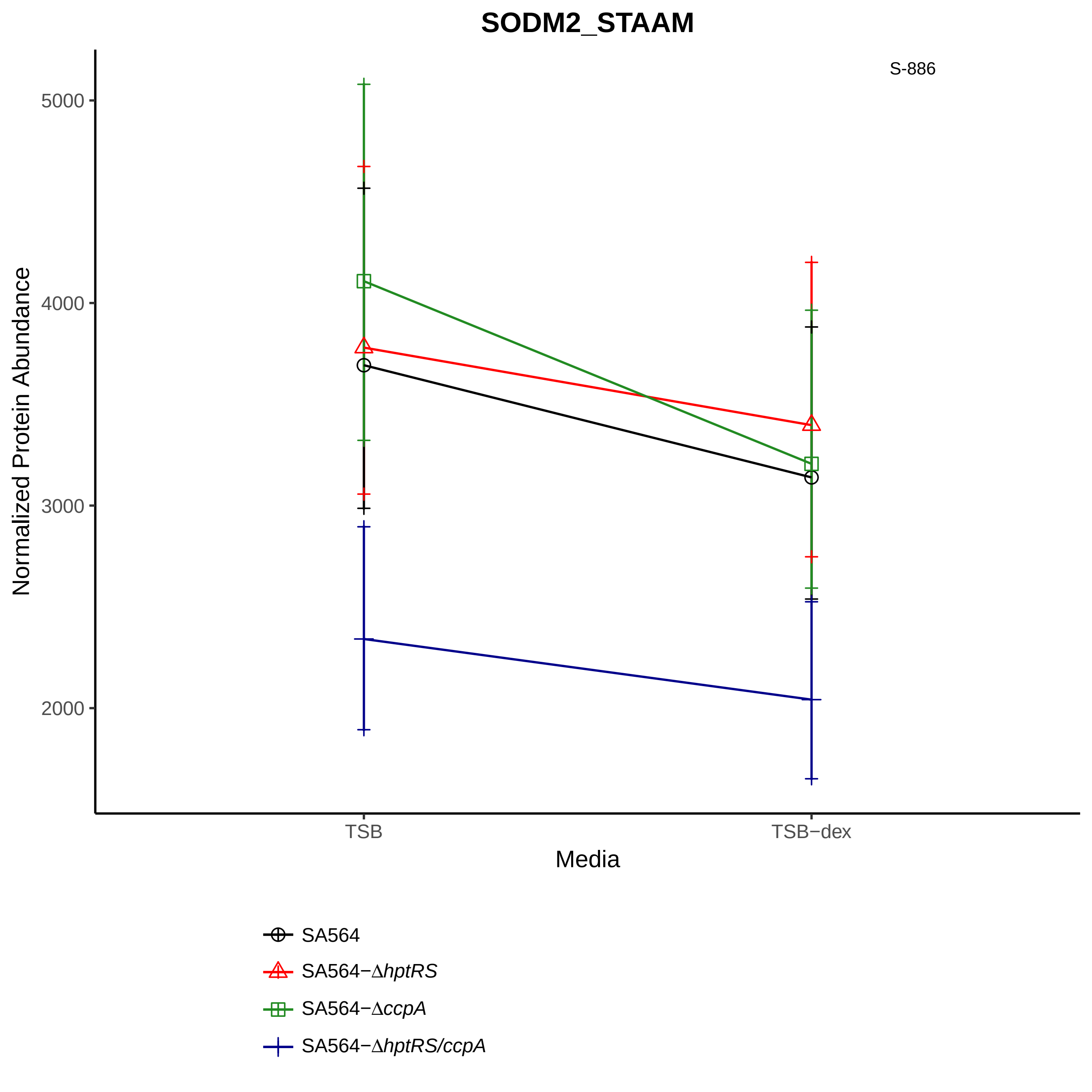

SODM2_STAAM
S-886
Normalized Protein Abundance
5000
4000
3000
2000
TSB
TSB−dex
Media
SA564
SA564−Δ*hptRS*
SA564−Δ*ccpA*
SA564−Δ*hptRS/ccpA*

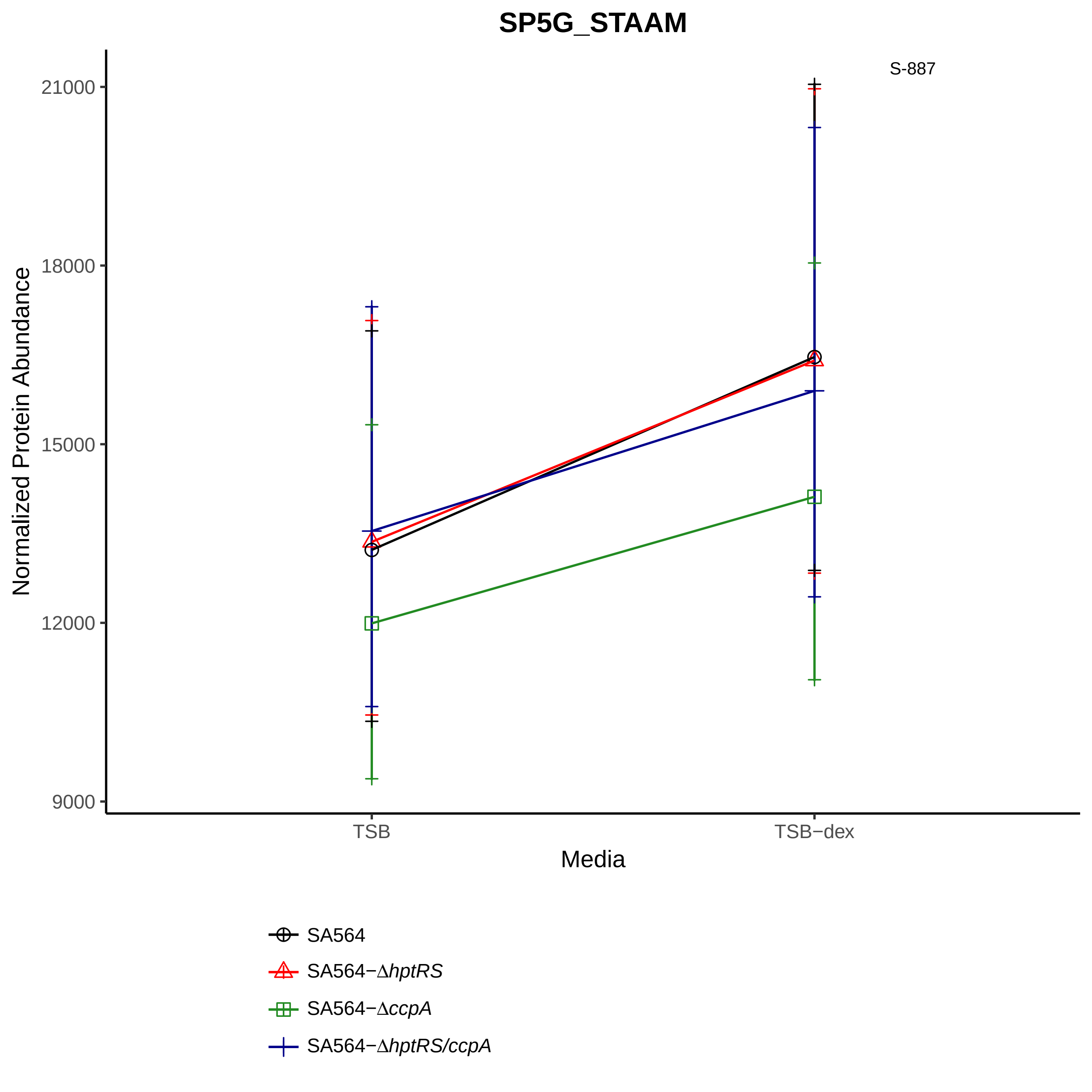

SP5G_STAAM
S-887
Normalized Protein Abundance
21000
18000
15000
12000
9000
TSB
TSB−dex
Media
SA564
SA564−Δ*hptRS*
SA564−Δ*ccpA*
SA564−Δ*hptRS/ccpA*

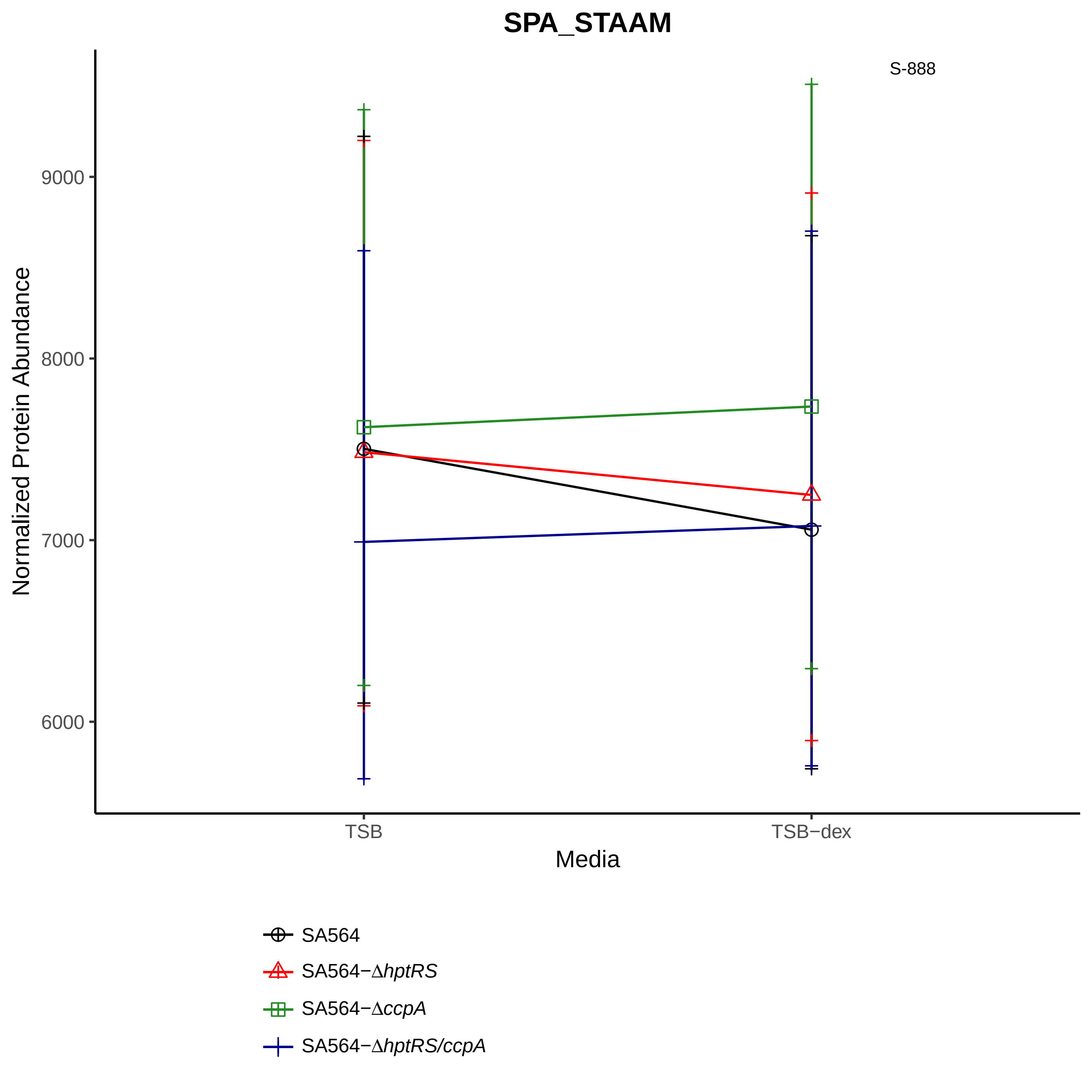

SPA_STAAM
S-888
Normalized Protein Abundance
9000
8000
7000
6000
TSB
TSB−dex
Media
SA564
SA564−ΔhptRS
SA564−ΔccpA
SA564−ΔhptRS/ccpA

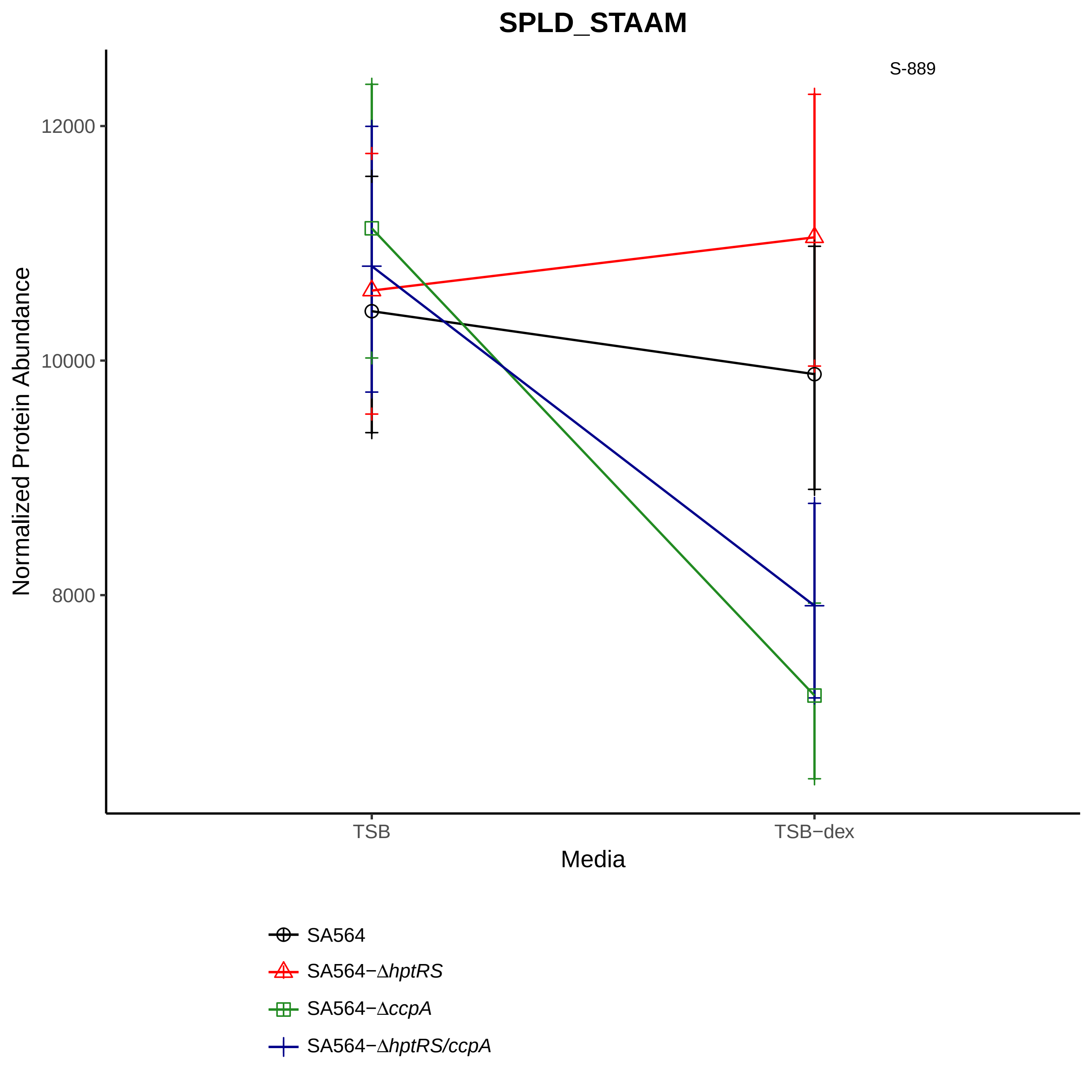

SPLD_STAAM
S-889
12000
10000
8000
Normalized Protein Abundance
TSB
TSB−dex
Media
SA564
SA564−Δ*hptRS*
SA564−Δ*ccpA*
SA564−Δ*hptRS/ccpA*

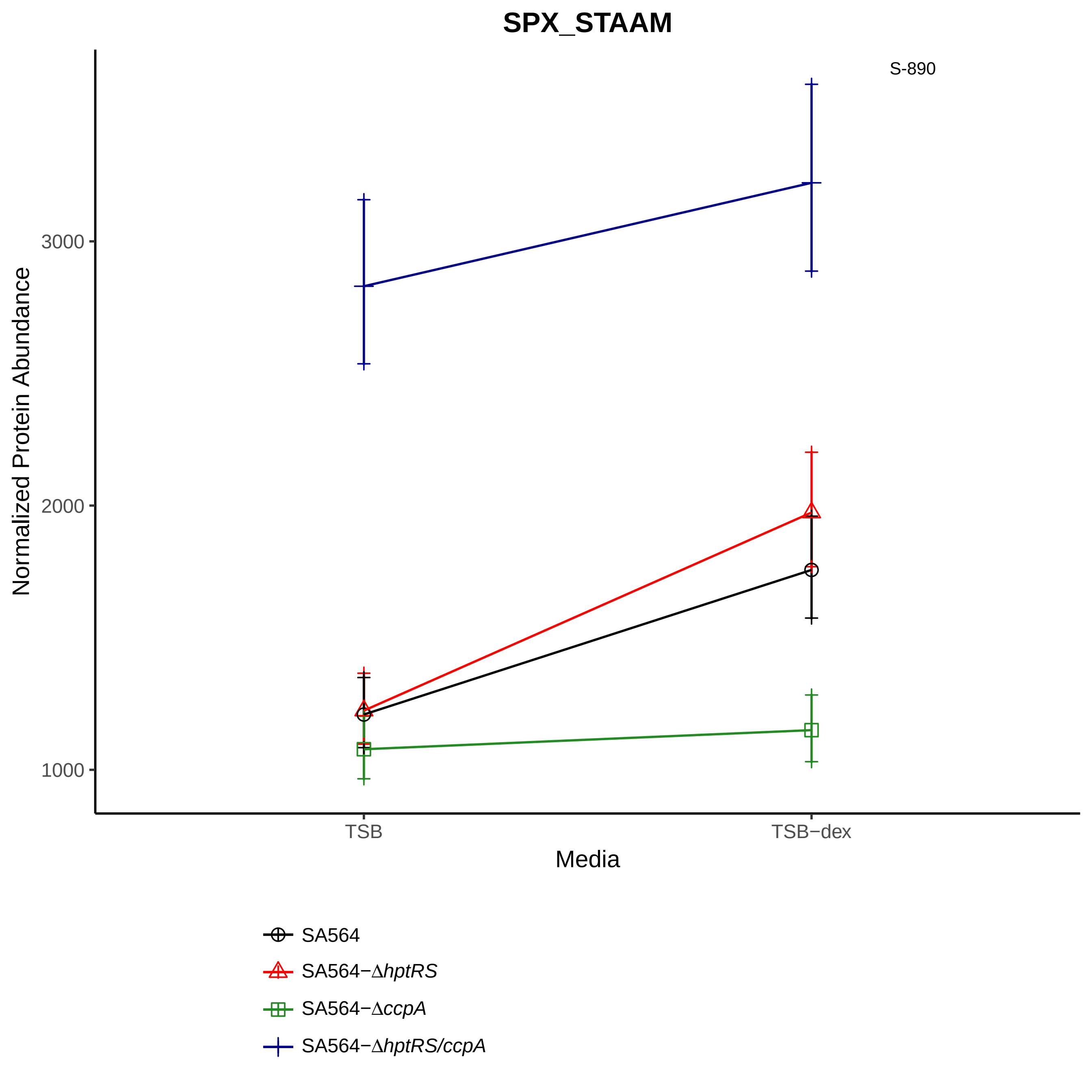

SPX_STAAM
S-890
Normalized Protein Abundance
3000
2000
1000
TSB
TSB−dex
Media
SA564
SA564−Δ*hptRS*
SA564−Δ*ccpA*
SA564−Δ*hptRS/ccpA*

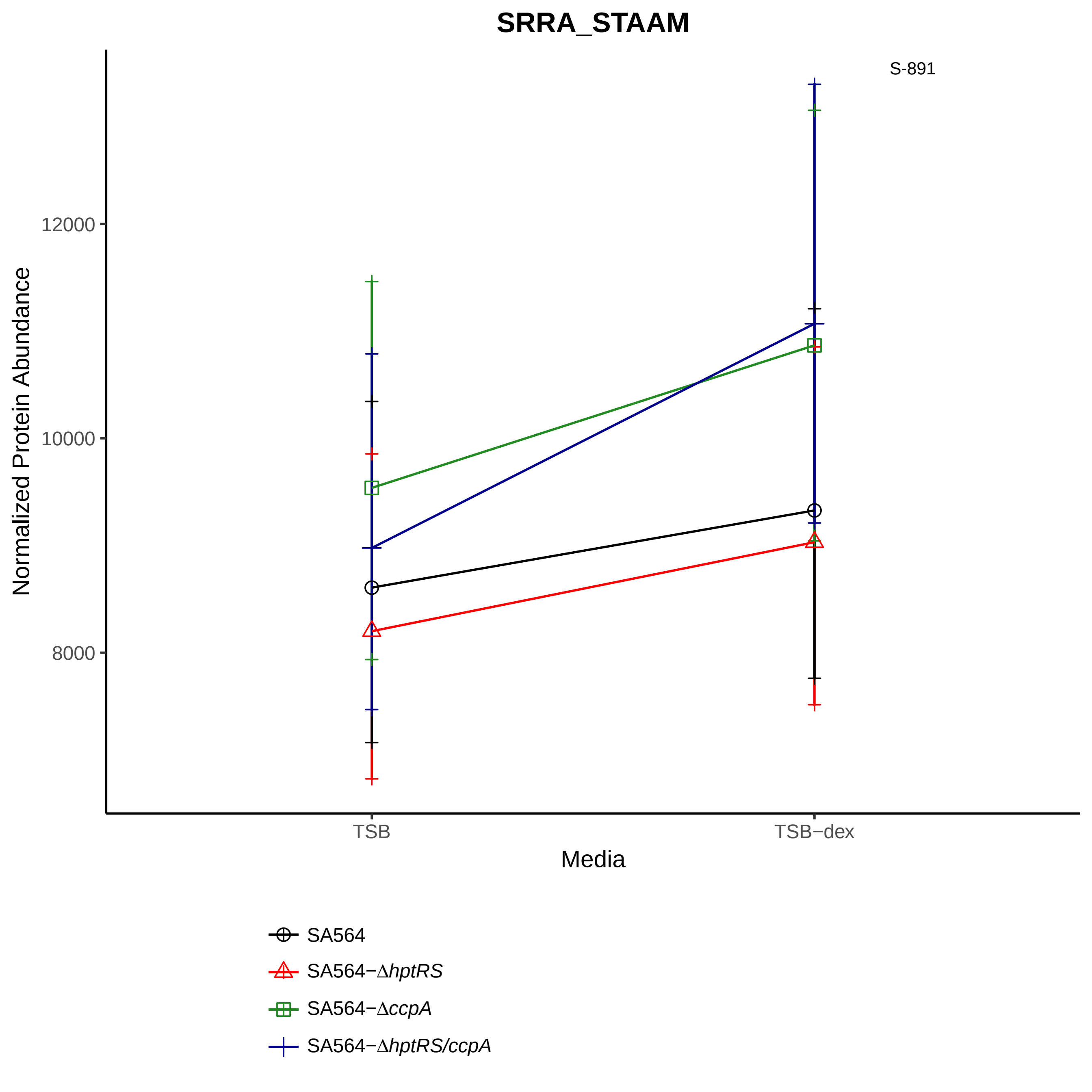

SRRA_STAAM
S-891
Normalized Protein Abundance
12000
10000
8000
TSB
TSB−dex
Media
SA564
SA564−ΔhptRS
SA564−ΔccpA
SA564−ΔhptRS/ccpA

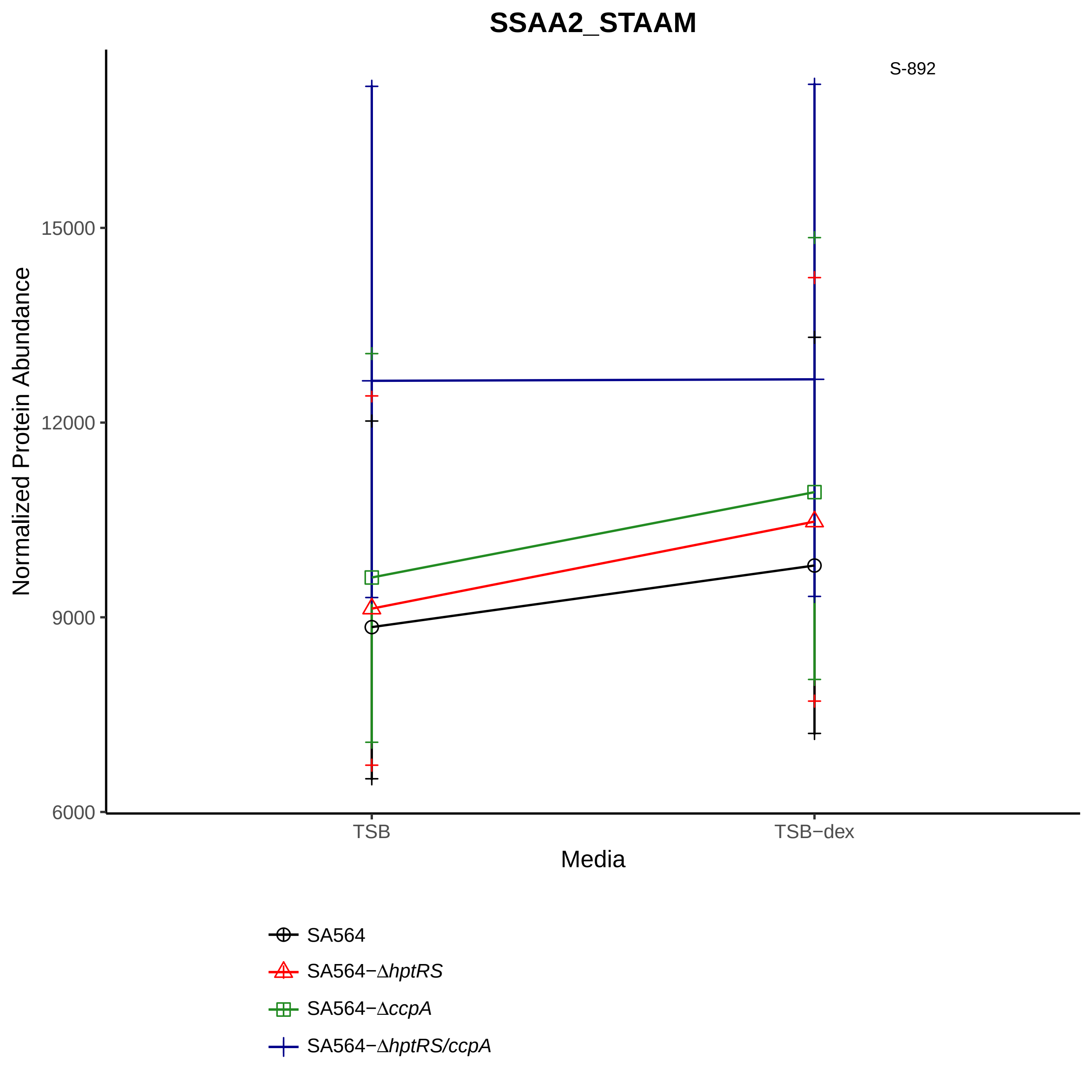

SSAA2_STAAM
S-892
Normalized Protein Abundance
15000
12000
9000
6000
TSB
TSB−dex
Media
SA564
SA564−Δ*hptRS*
SA564−Δ*ccpA*
SA564−Δ*hptRS/ccpA*

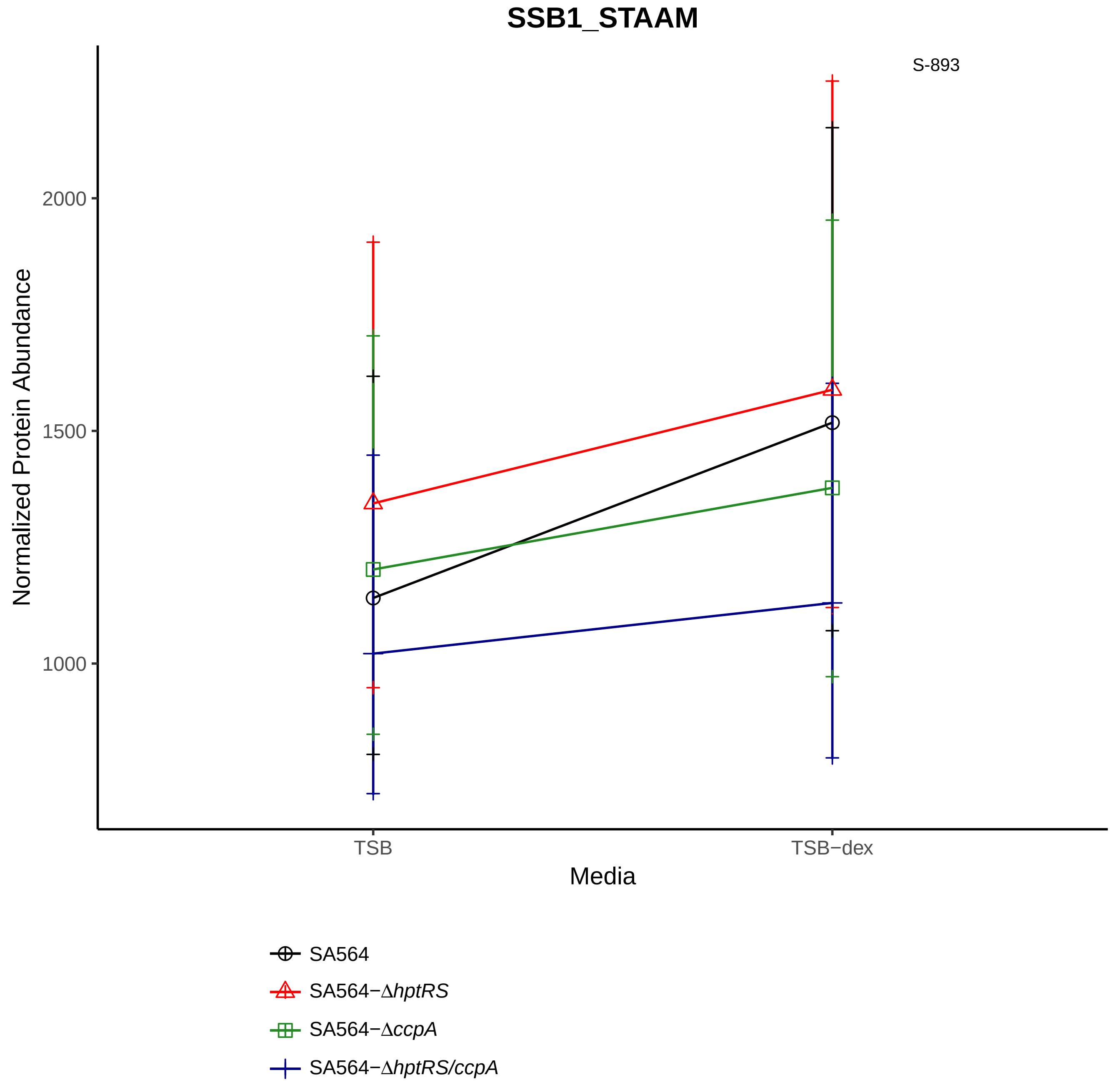
SSB1_STAAM
S-893
Normalized Protein Abundance
2000
1500
1000
TSB
TSB−dex
Media
SA564
SA564−ΔhptRS
SA564−ΔccpA
SA564−ΔhptRS/ccpA

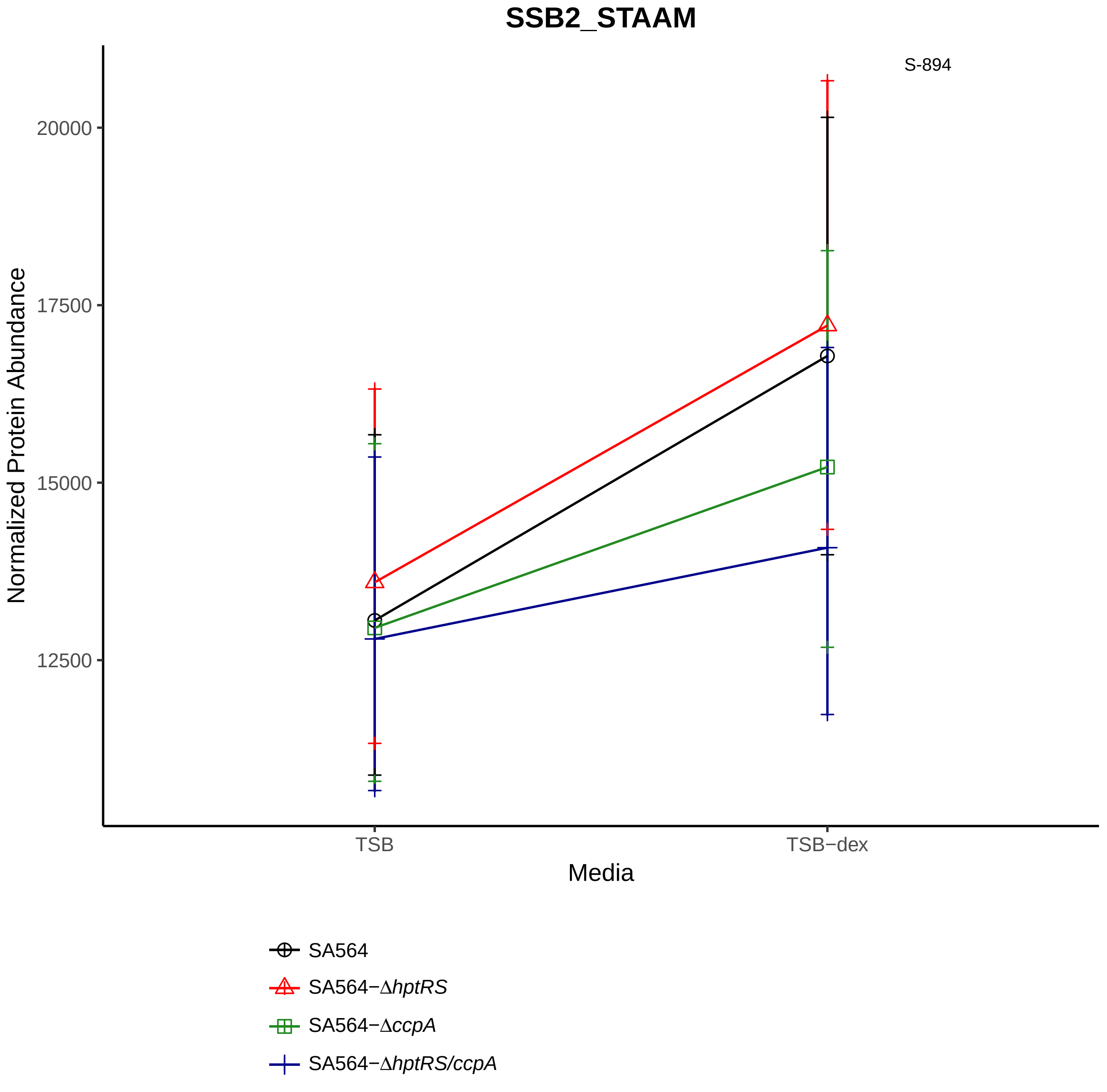

SSB2_STAAM
S-894
Normalized Protein Abundance
20000
17500
15000
12500
TSB
TSB−dex
Media
SA564
SA564−Δ*hptRS*
SA564−Δ*ccpA*
SA564−Δ*hptRS/ccpA*

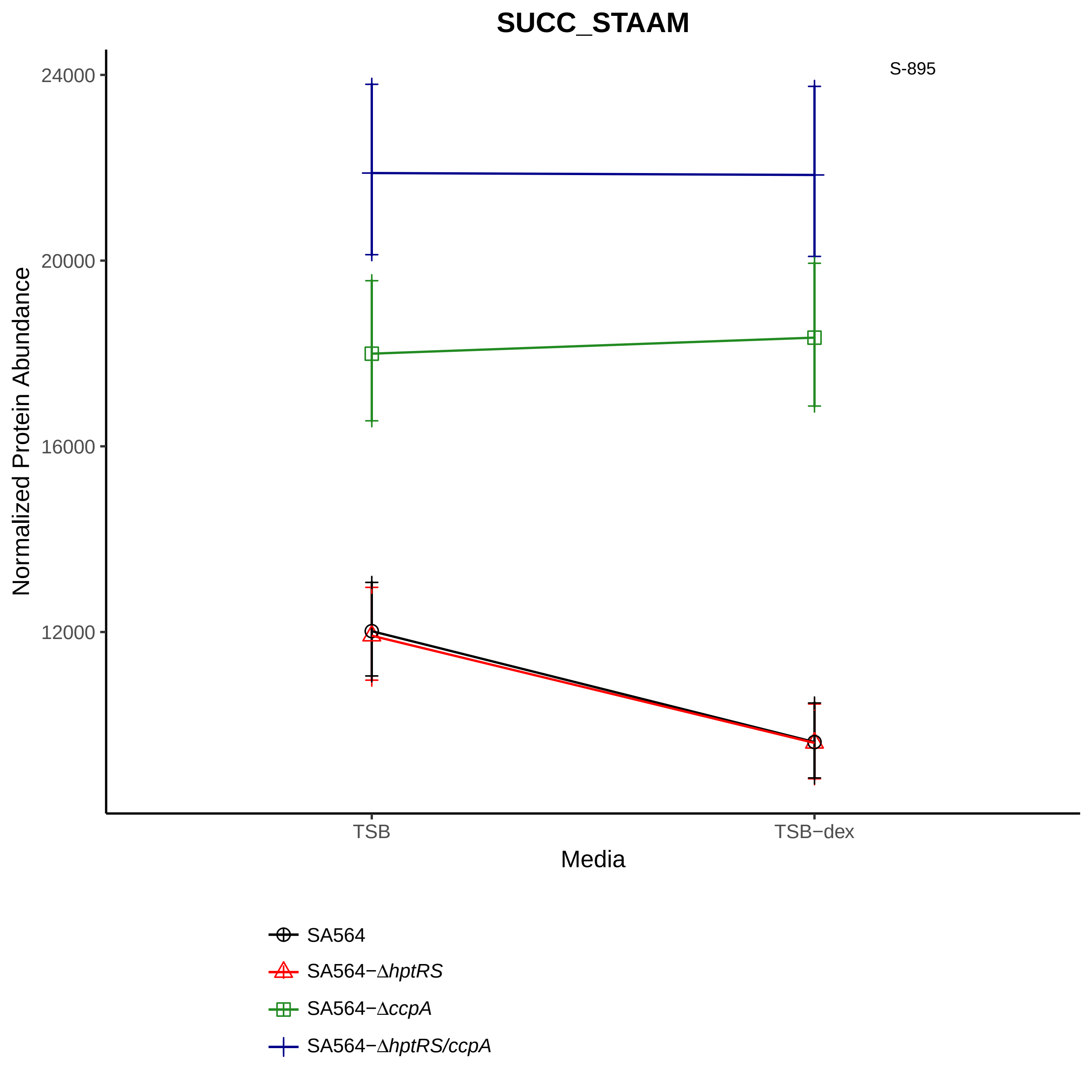

SUCC_STAAM
S-895
24000
20000
16000
12000
Normalized Protein Abundance
TSB
TSB−dex
Media
SA564
SA564−Δ*hptRS*
SA564−Δ*ccpA*
SA564−Δ*hptRS/ccpA*

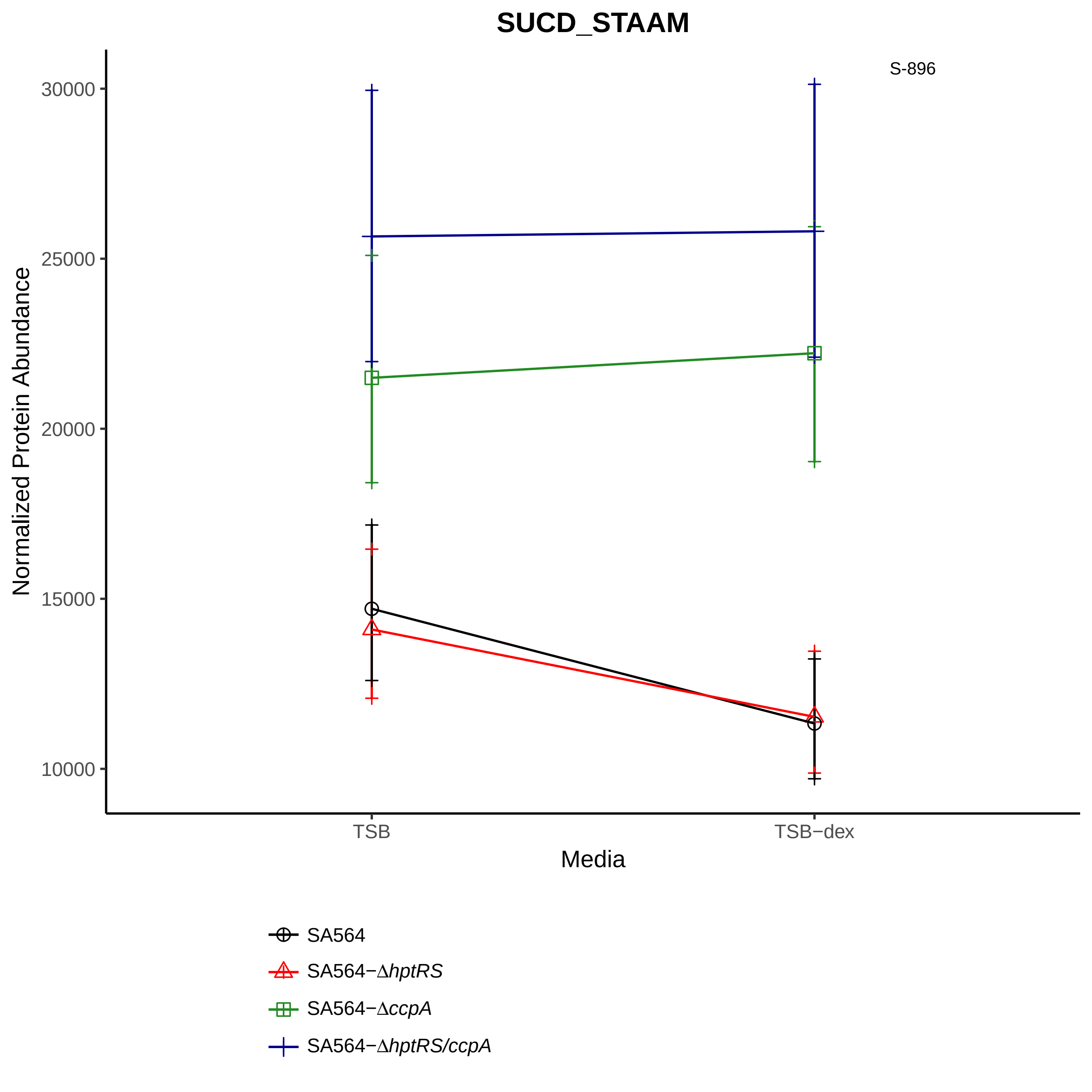

SUCD_STAAM
S-896
Normalized Protein Abundance
30000
25000
20000
15000
10000
TSB
TSB−dex
Media
SA564
SA564−Δ*hptRS*
SA564−Δ*ccpA*
SA564−Δ*hptRS/ccpA*

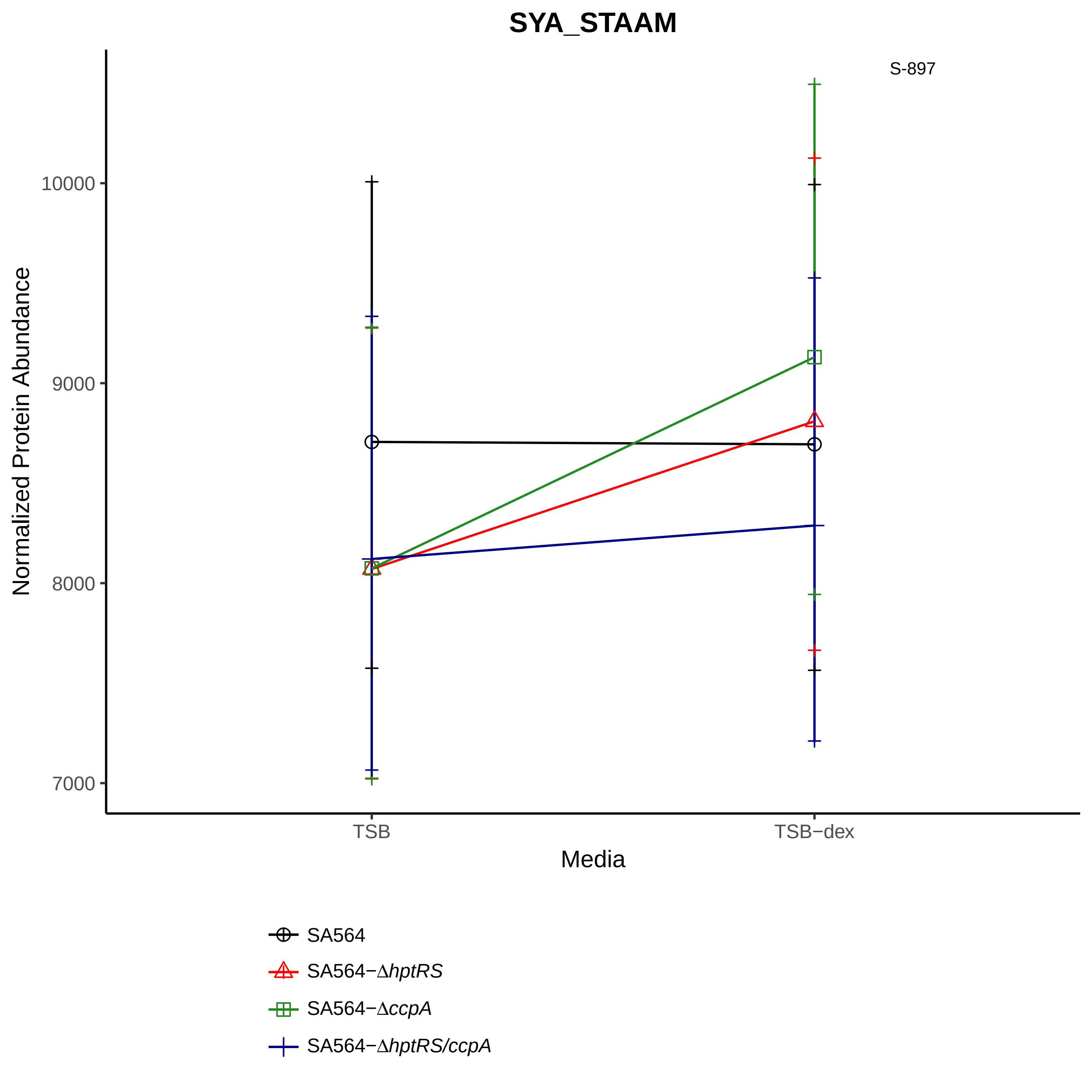

SYA_STAAM
S-897
Normalized Protein Abundance
10000
9000
8000
7000
TSB
TSB−dex
Media
SA564
SA564−Δ*hptRS*
SA564−Δ*ccpA*
SA564−Δ*hptRS/ccpA*

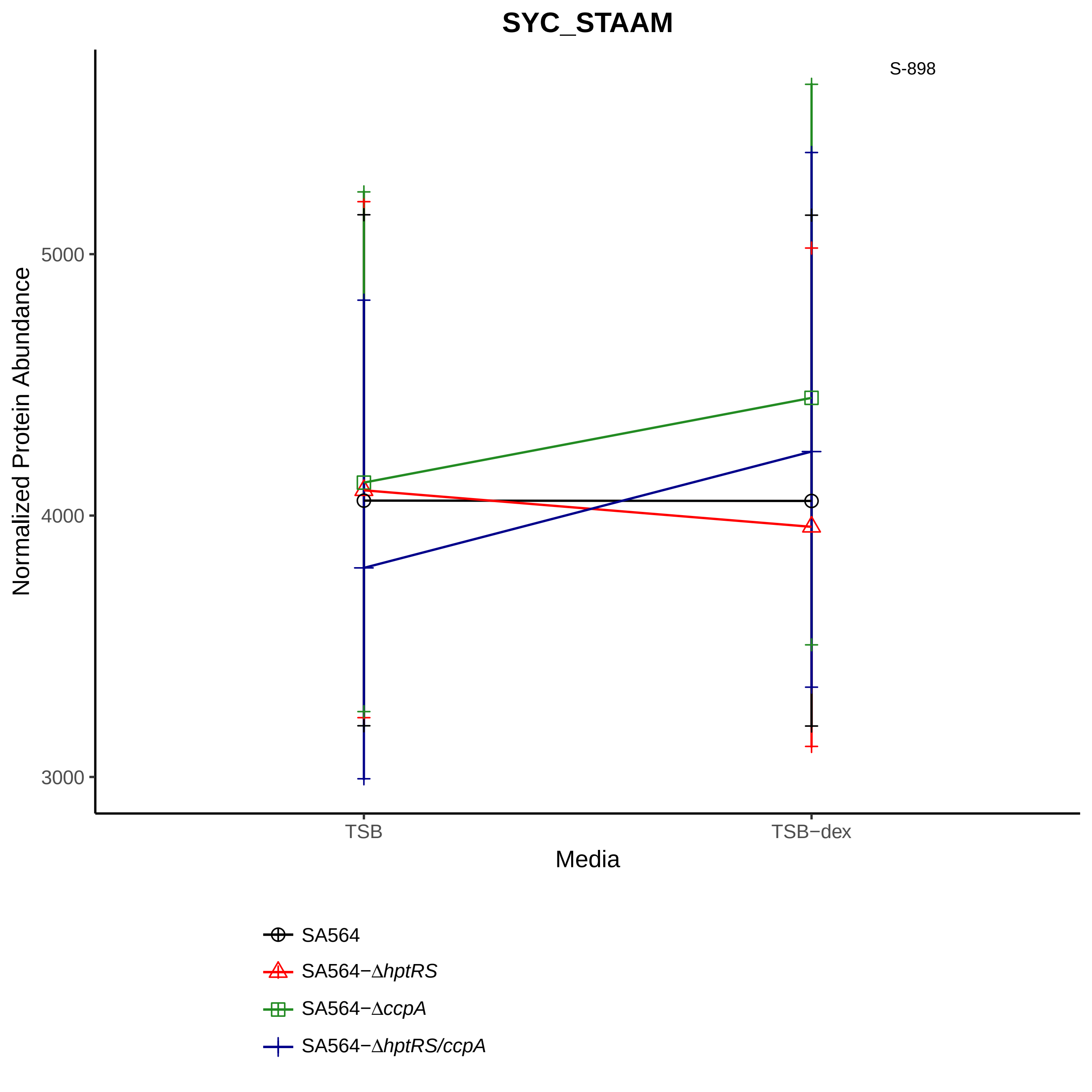

SYC_STAAM
S-898
Normalized Protein Abundance
5000
4000
3000
TSB
TSB−dex
Media
SA564
SA564−Δ*hptRS*
SA564−Δ*ccpA*
SA564−Δ*hptRS/ccpA*

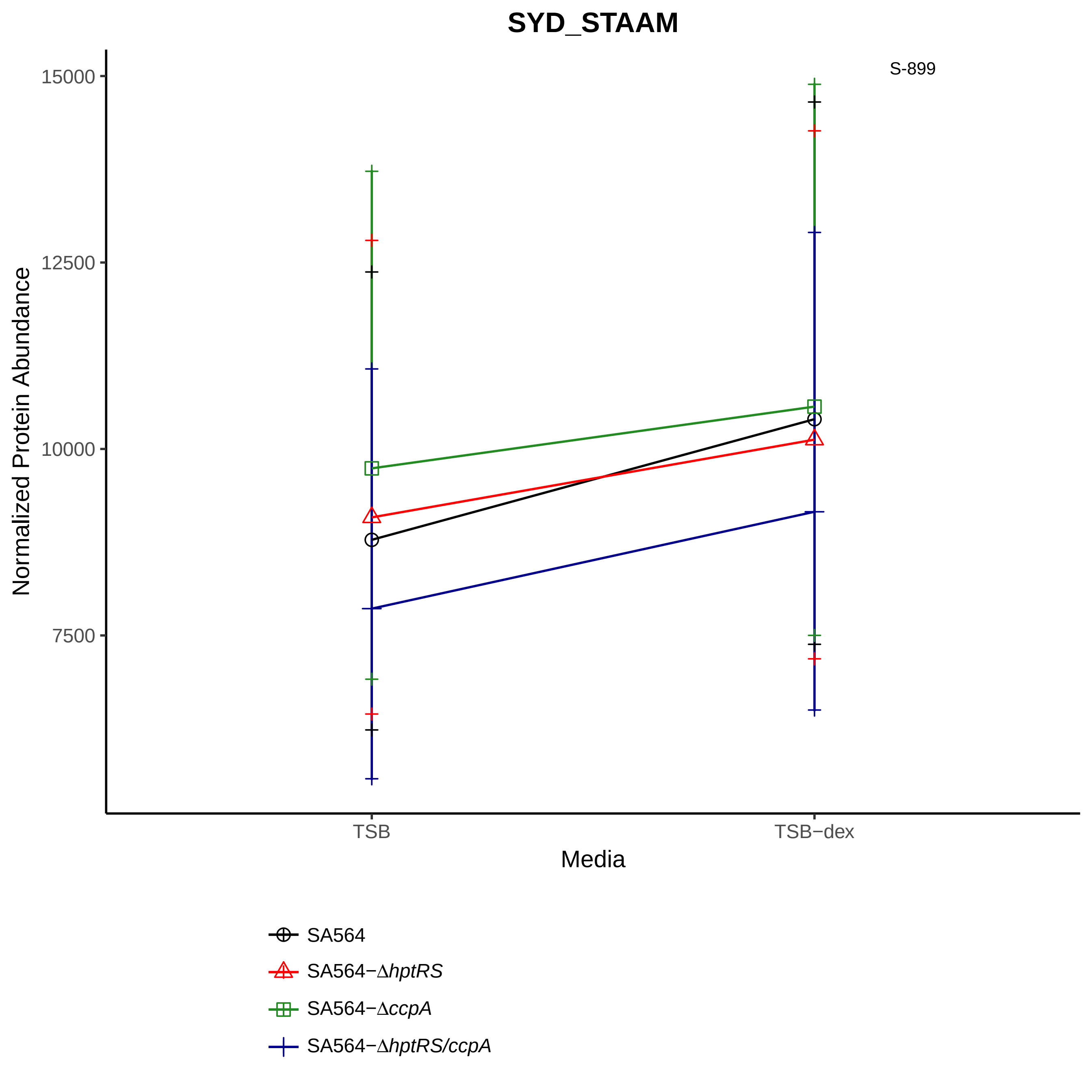
SYD_STAAM
S-899
Normalized Protein Abundance
15000
12500
10000
7500
TSB
TSB−dex
Media
SA564
SA564−Δ*hptRS*
SA564−Δ*ccpA*
SA564−Δ*hptRS/ccpA*

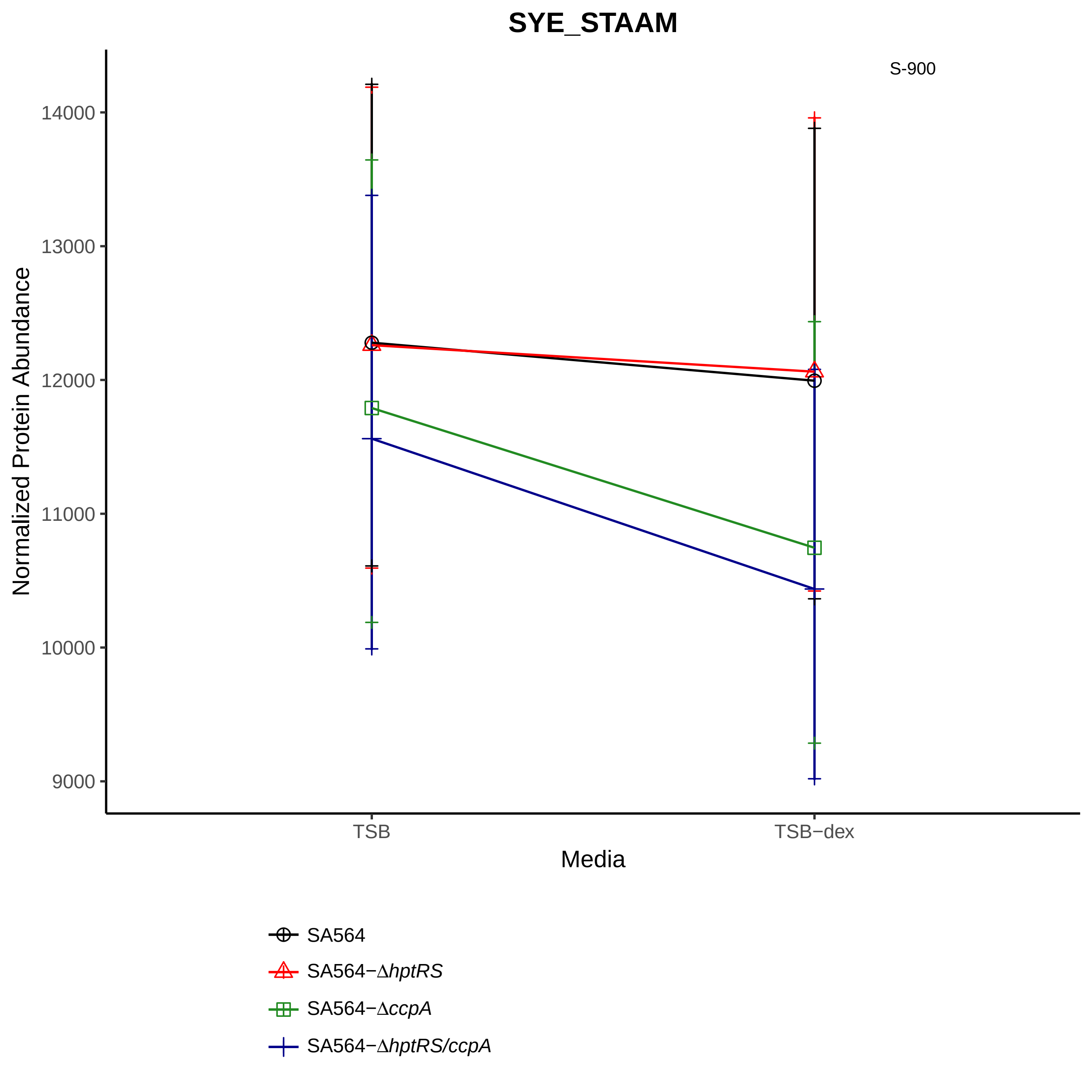

SYE_STAAM
S-900
Normalized Protein Abundance
14000
13000
12000
11000
10000
9000
TSB
TSB−dex
Media
SA564
SA564−ΔhptRS
SA564−ΔccpA
SA564−ΔhptRS/ccpA

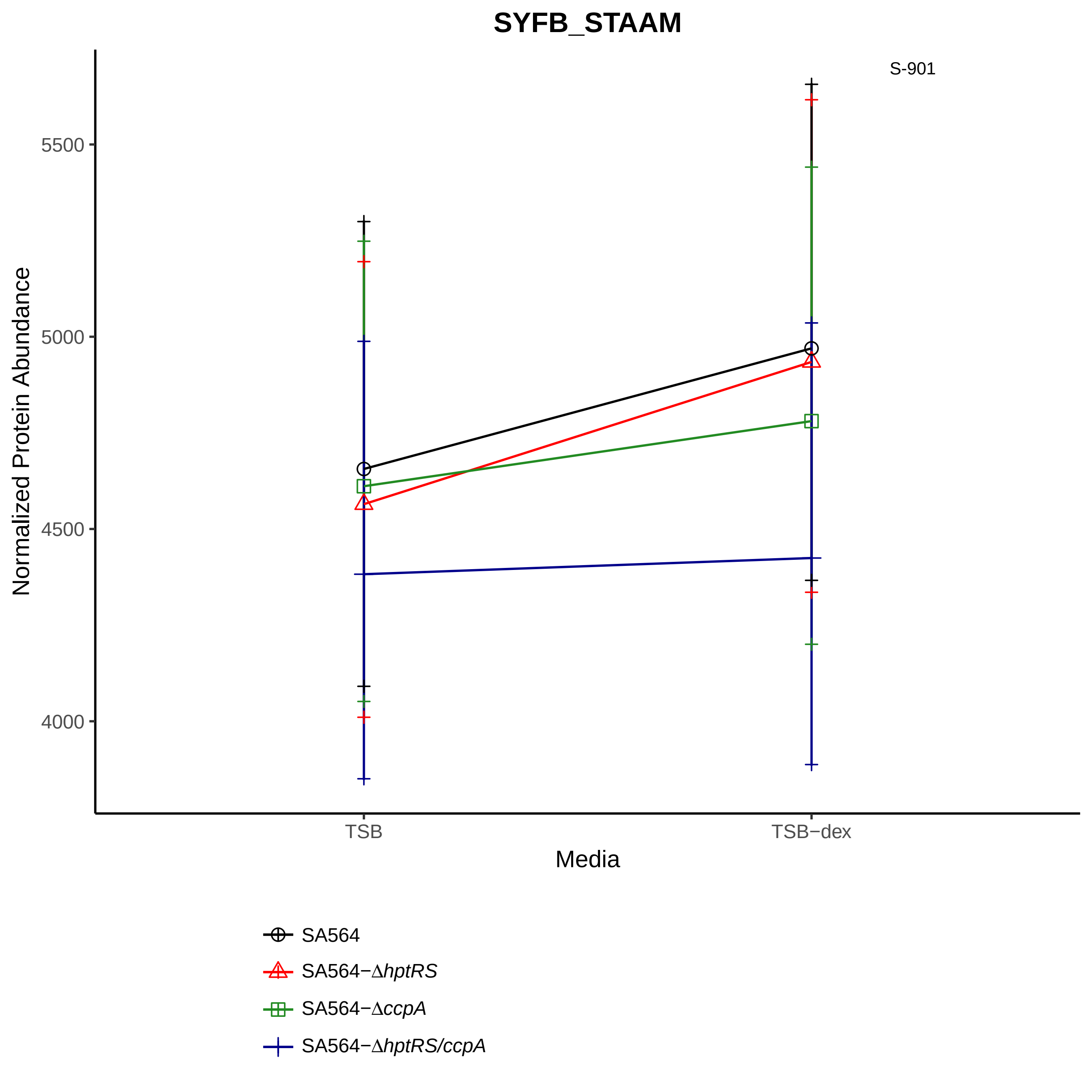

SYFB_STAAM
S-901
Normalized Protein Abundance
5500
5000
4500
4000
TSB
TSB−dex
Media
SA564
SA564−ΔhptRS
SA564−ΔccpA
SA564−ΔhptRS/ccpA

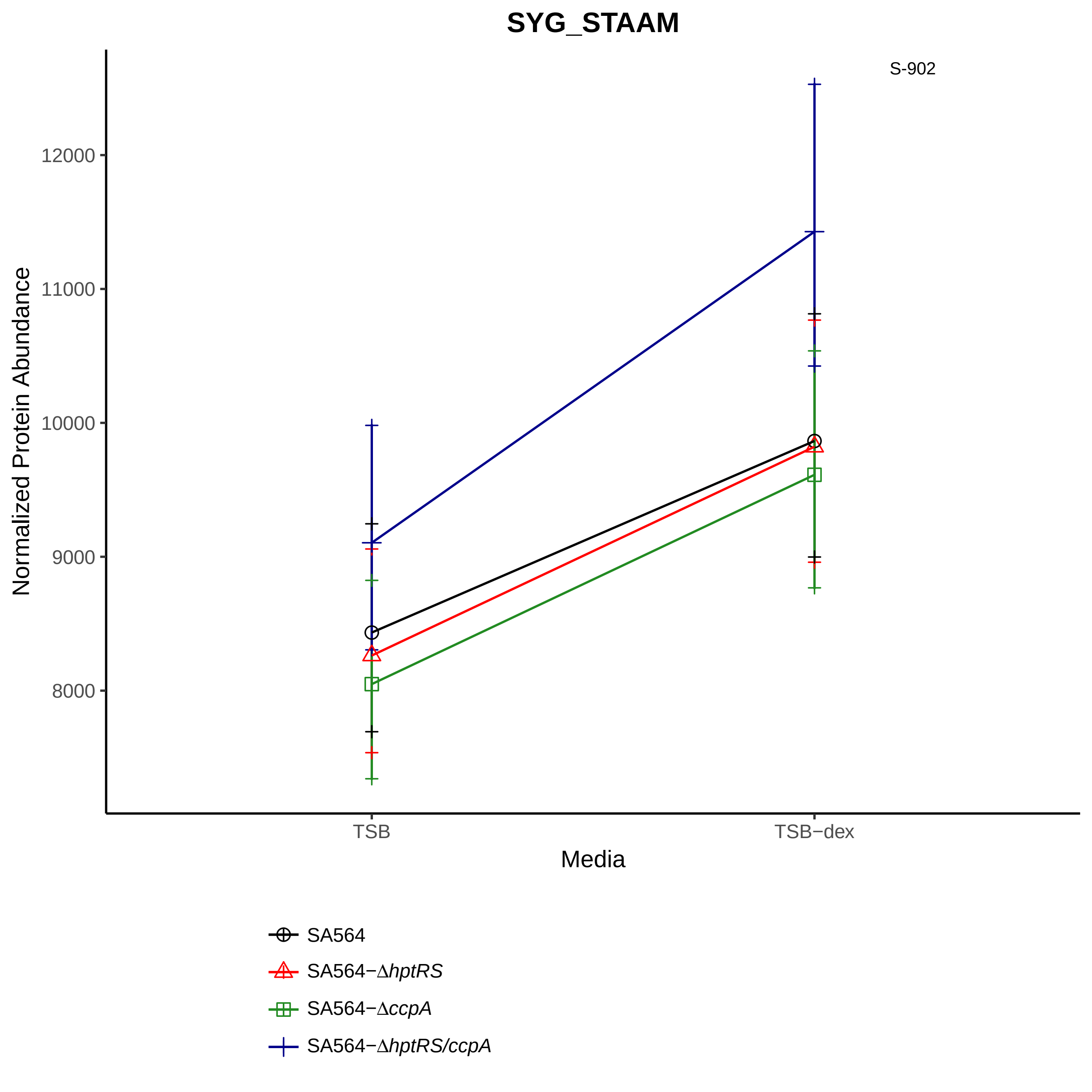

SYG_STAAM
S-902
Normalized Protein Abundance
12000
11000
10000
9000
8000
TSB
TSB−dex
Media
SA564
SA564−Δ*hptRS*
SA564−Δ*ccpA*
SA564−Δ*hptRS/ccpA*

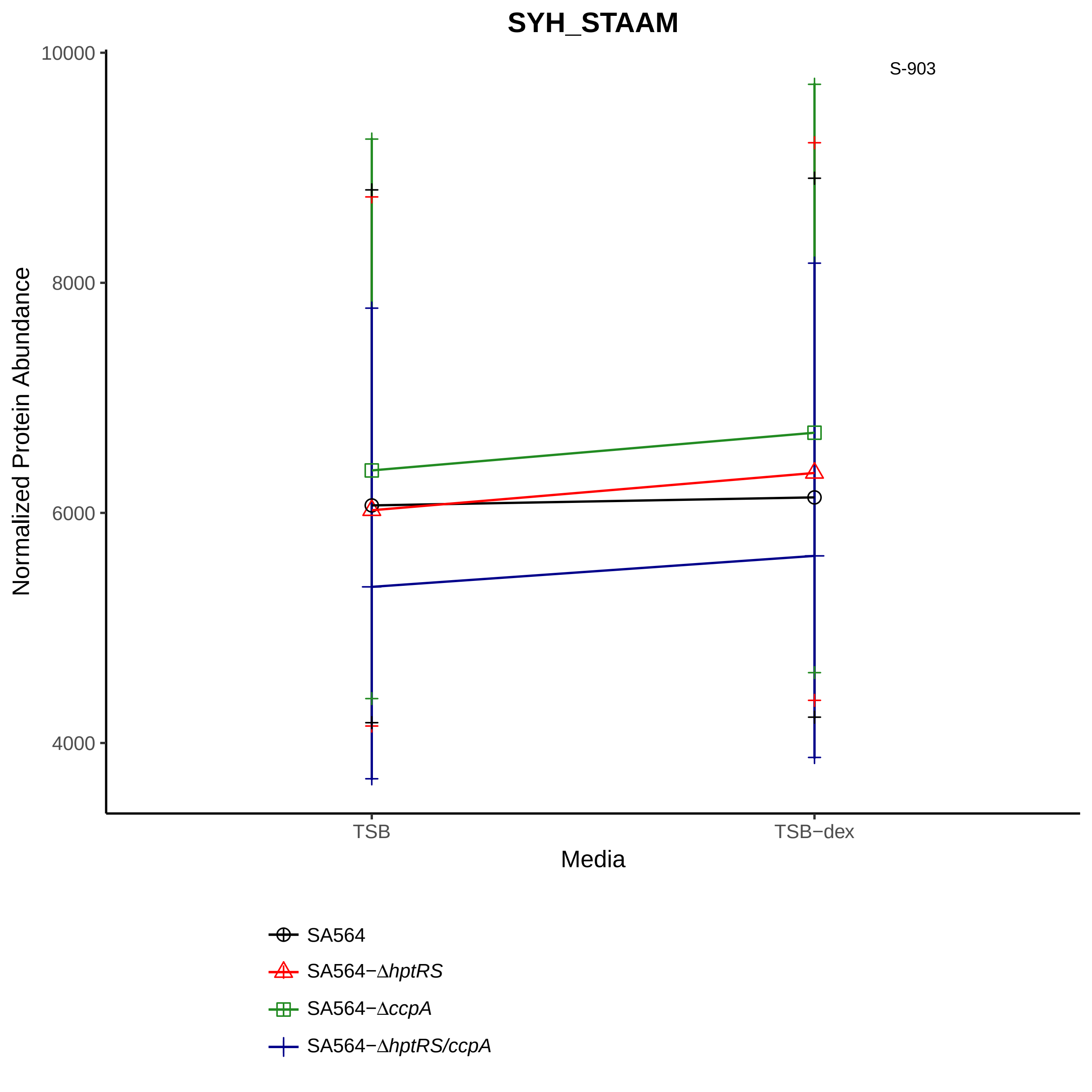

SYH_STAAM
S-903
Normalized Protein Abundance
10000
8000
6000
4000
TSB
TSB−dex
Media
SA564
SA564−ΔhptRS
SA564−ΔccpA
SA564−ΔhptRS/ccpA

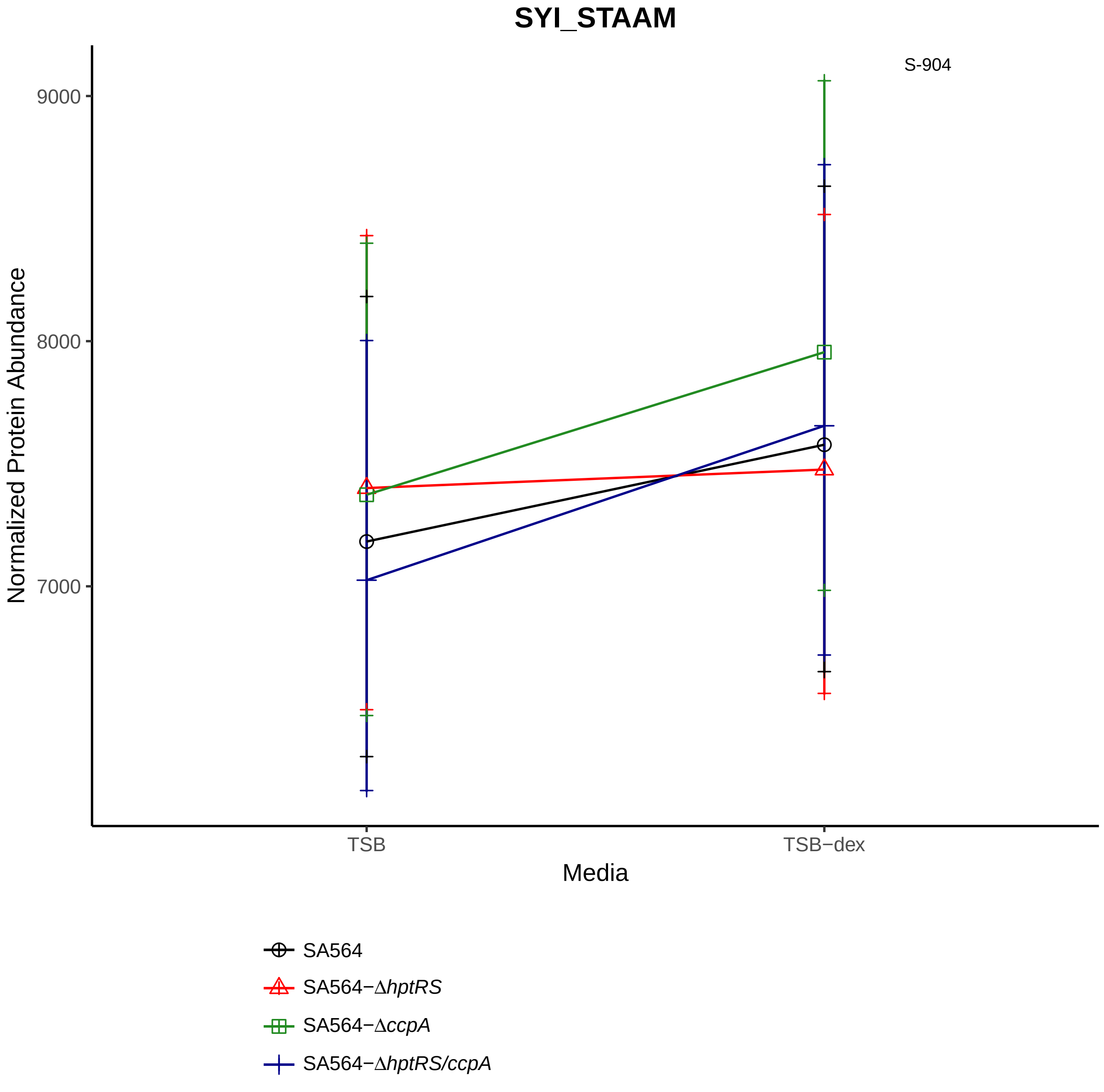
SYI_STAAM
S-904
Normalized Protein Abundance
9000
8000
7000
TSB
TSB−dex
Media
SA564
SA564−ΔhptRS
SA564−ΔccpA
SA564−ΔhptRS/ccpA

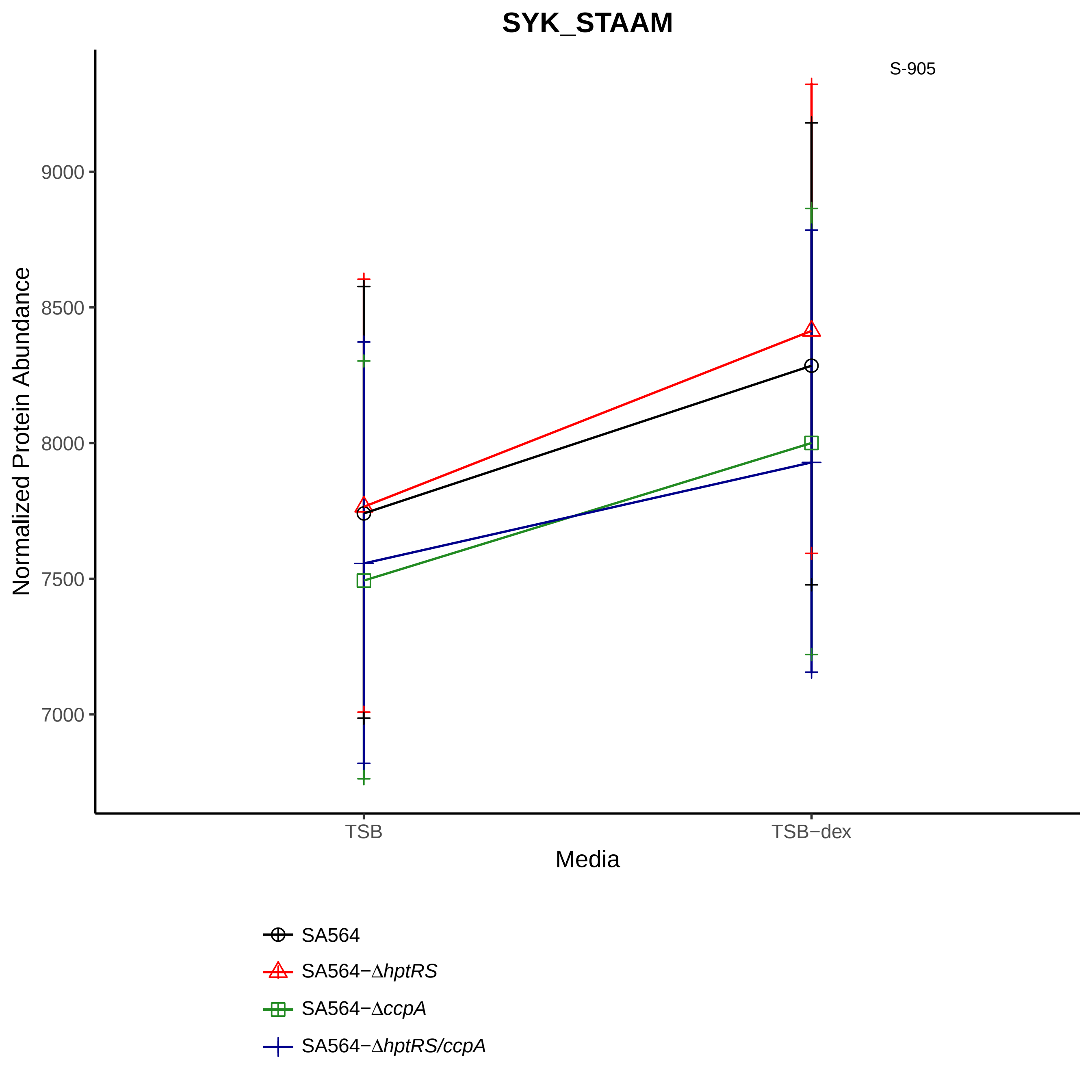

SYK_STAAM
S-905
Normalized Protein Abundance
9000
8500
8000
7500
7000
TSB
TSB−dex
Media
SA564
SA564−Δ*hptRS*
SA564−Δ*ccpA*
SA564−Δ*hptRS/ccpA*

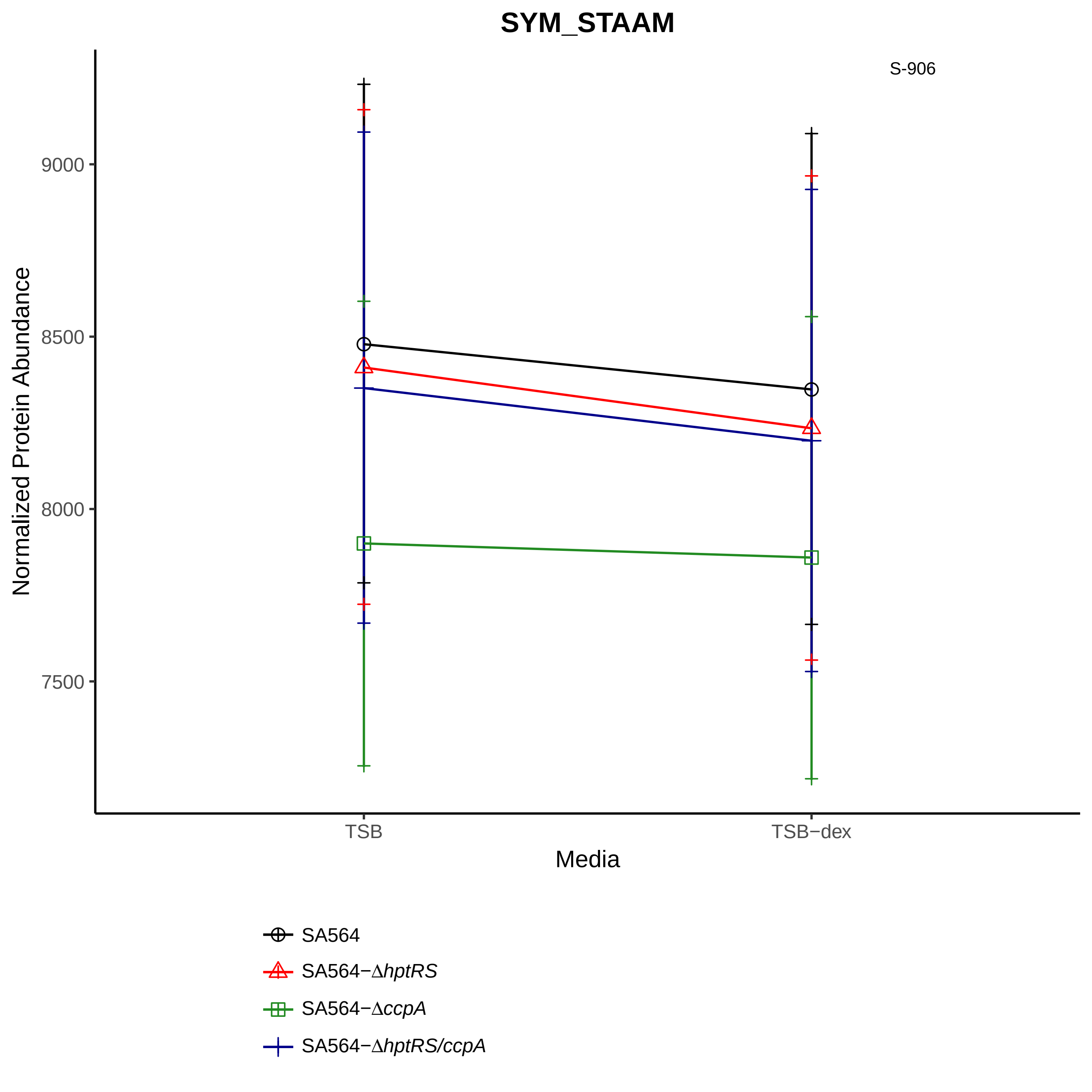

SYM_STAAM
S-906
Normalized Protein Abundance
9000
8500
8000
7500
TSB
TSB−dex
Media
SA564
SA564−ΔhptRS
SA564−ΔccpA
SA564−ΔhptRS/ccpA

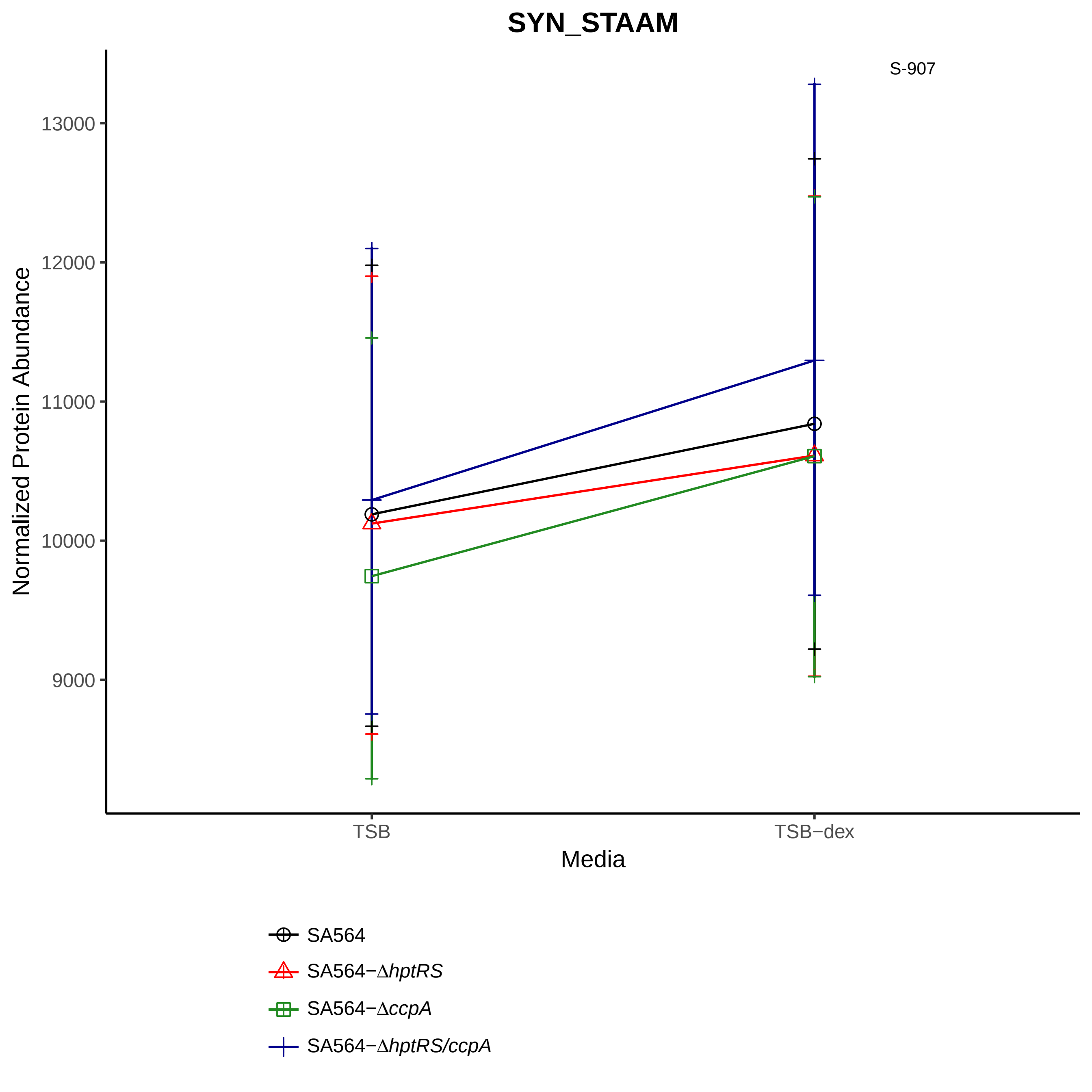

SYN_STAAM
S-907
Normalized Protein Abundance
13000
12000
11000
10000
9000
TSB
TSB−dex
Media
SA564
SA564−Δ*hptRS*
SA564−Δ*ccpA*
SA564−Δ*hptRS/ccpA*

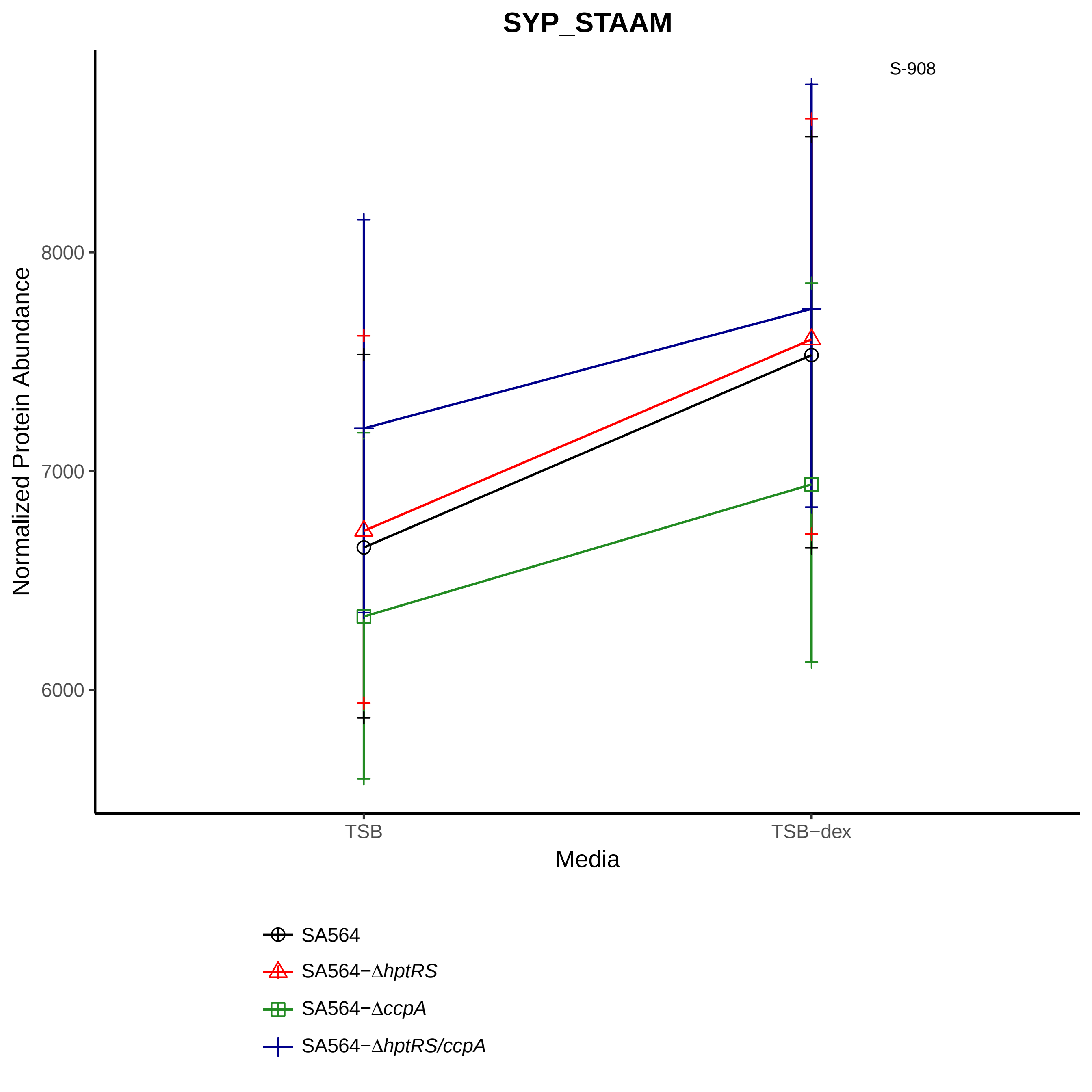

SYP_STAAM
S-908
Normalized Protein Abundance
8000
7000
6000
TSB
TSB−dex
Media
SA564
SA564−ΔhptRS
SA564−ΔccpA
SA564−ΔhptRS/ccpA

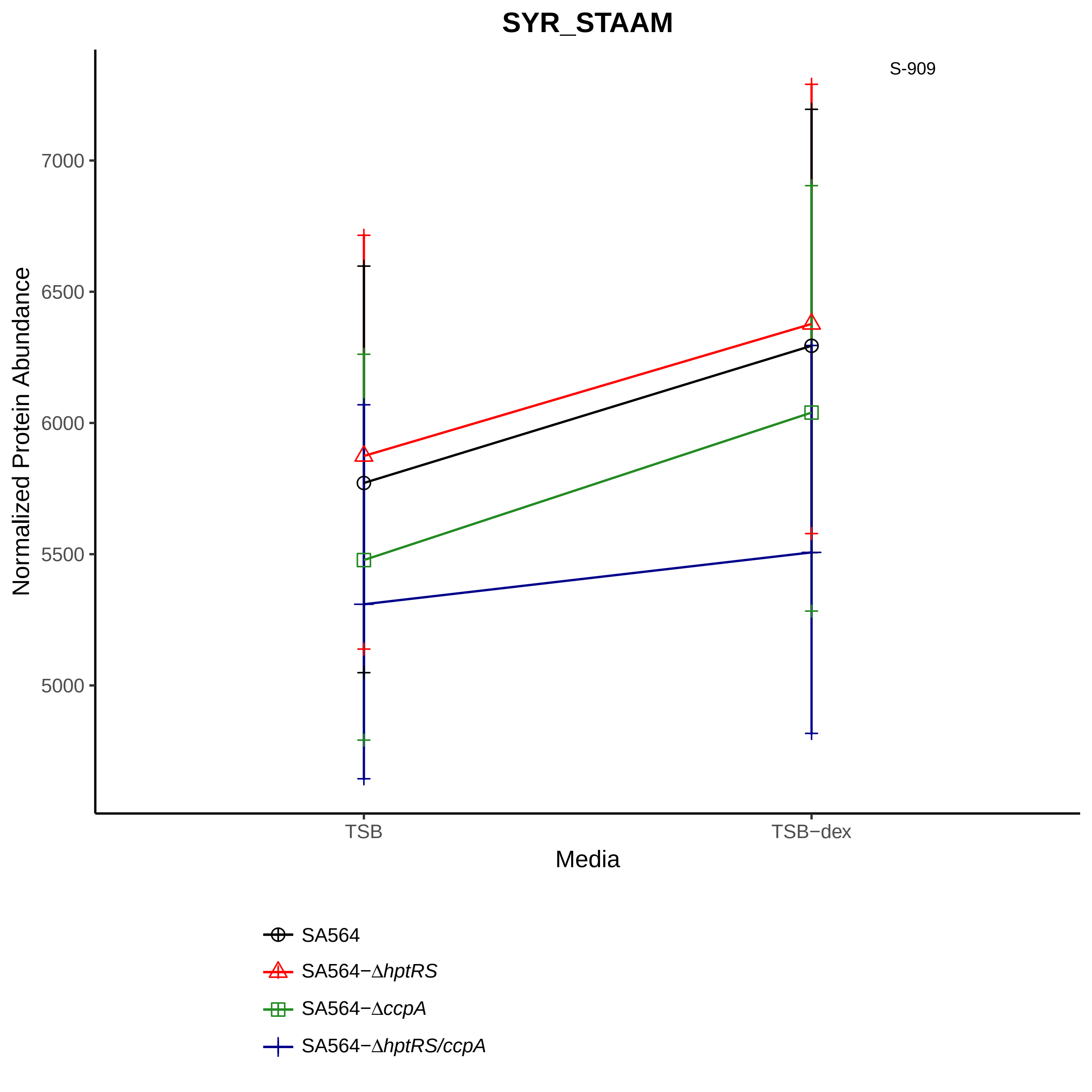
SYR_STAAM
S-909
Normalized Protein Abundance
7000
6500
6000
5500
5000
TSB
TSB−dex
Media
SA564
SA564−Δ*hptRS*
SA564−Δ*ccpA*
SA564−Δ*hptRS/ccpA*

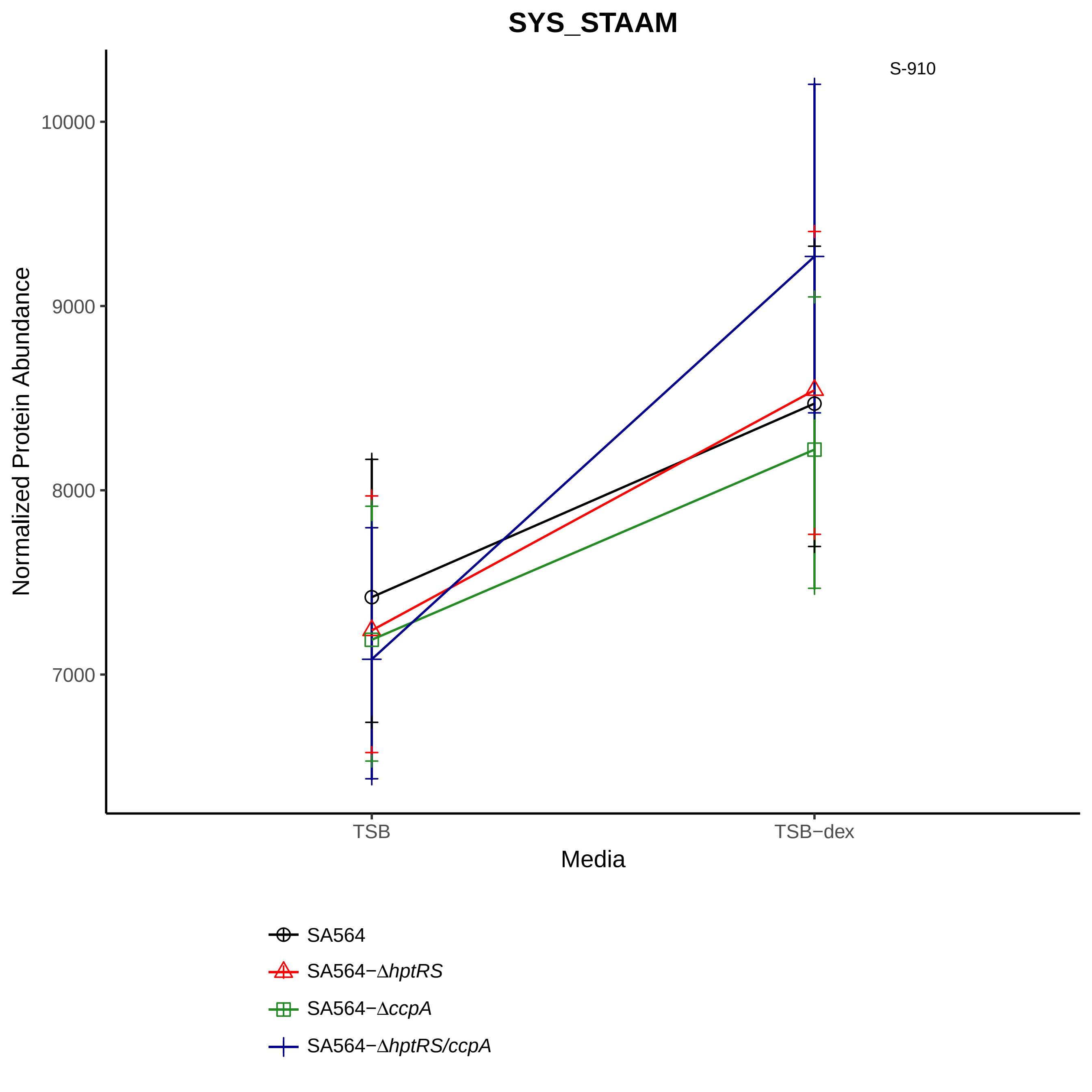
SYS_STAAM
S-910
Normalized Protein Abundance
10000
9000
8000
7000
TSB
TSB−dex
Media
SA564
SA564−ΔhptRS
SA564−ΔccpA
SA564−ΔhptRS/ccpA

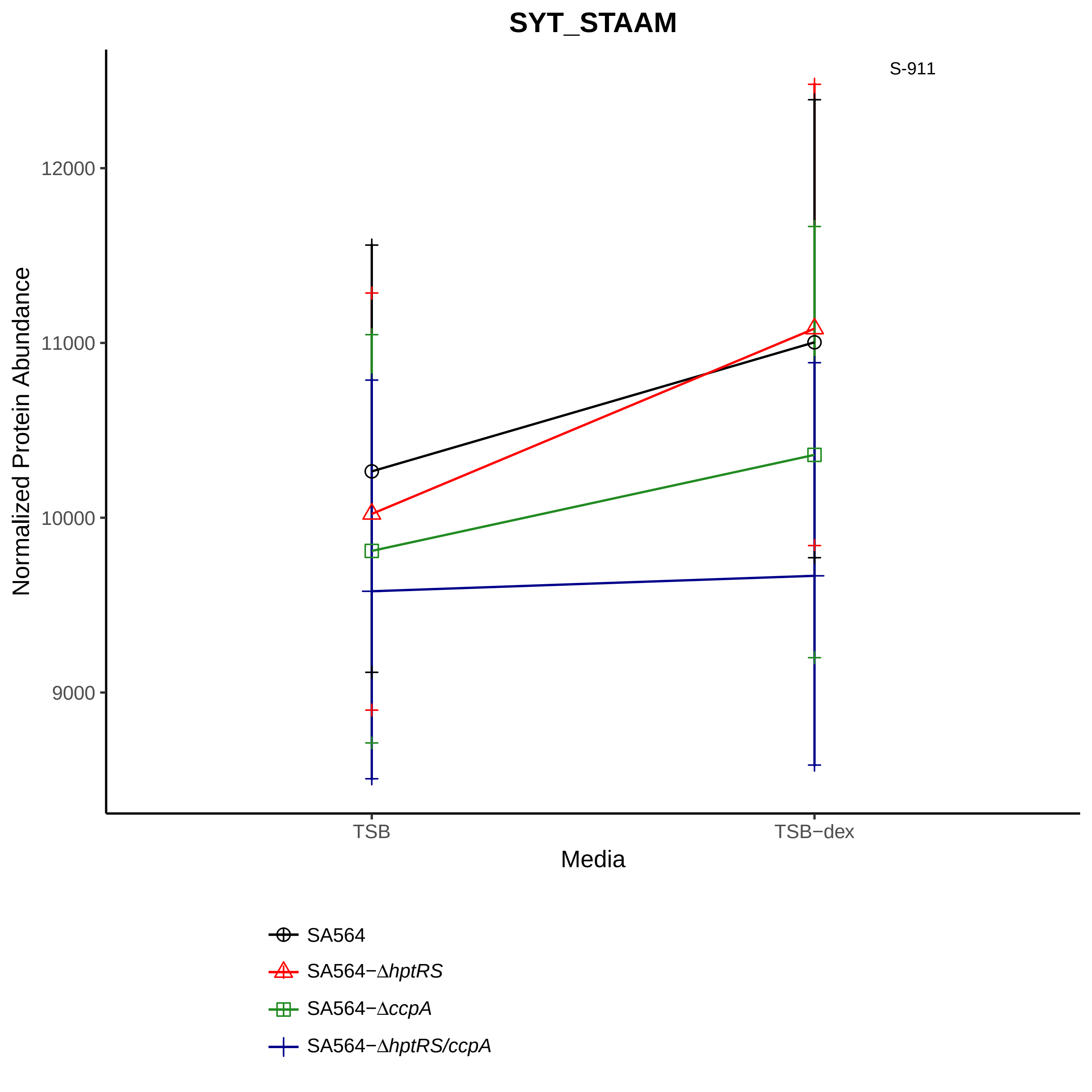

SYT_STAAM
S-911
Normalized Protein Abundance
12000
11000
10000
9000
TSB
TSB−dex
Media
SA564
SA564−Δ*hptRS*
SA564−Δ*ccpA*
SA564−Δ*hptRS/ccpA*

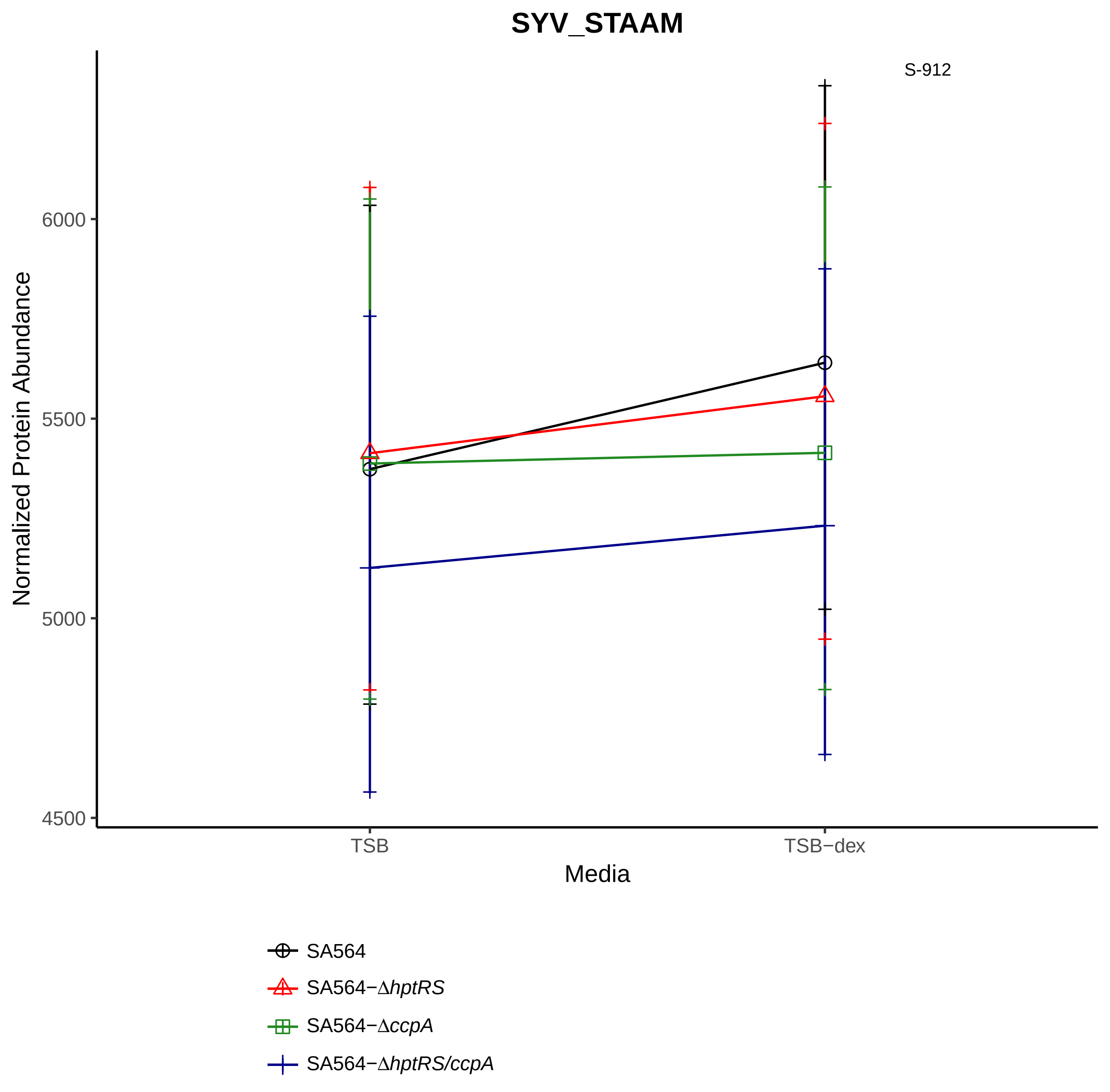

SYV_STAAM
S-912
Normalized Protein Abundance
6000
5500
5000
4500
TSB
TSB−dex
Media
SA564
SA564−Δ*hptRS*
SA564−Δ*ccpA*
SA564−Δ*hptRS/ccpA*

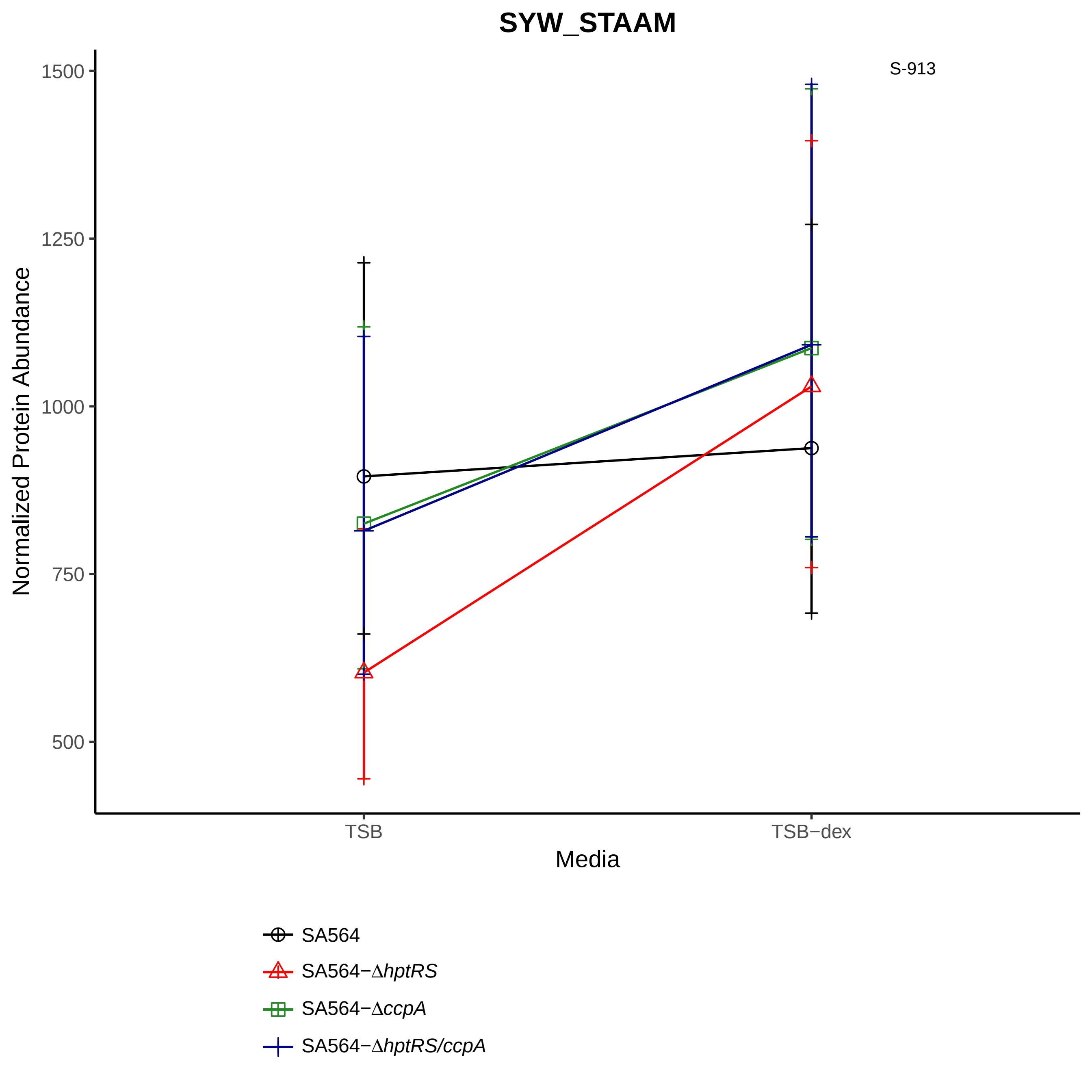

SYW_STAAM
S-913
Normalized Protein Abundance
1500
1250
1000
750
500
TSB
TSB−dex
Media
SA564
SA564−Δ*hptRS*
SA564−Δ*ccpA*
SA564−Δ*hptRS/ccpA*

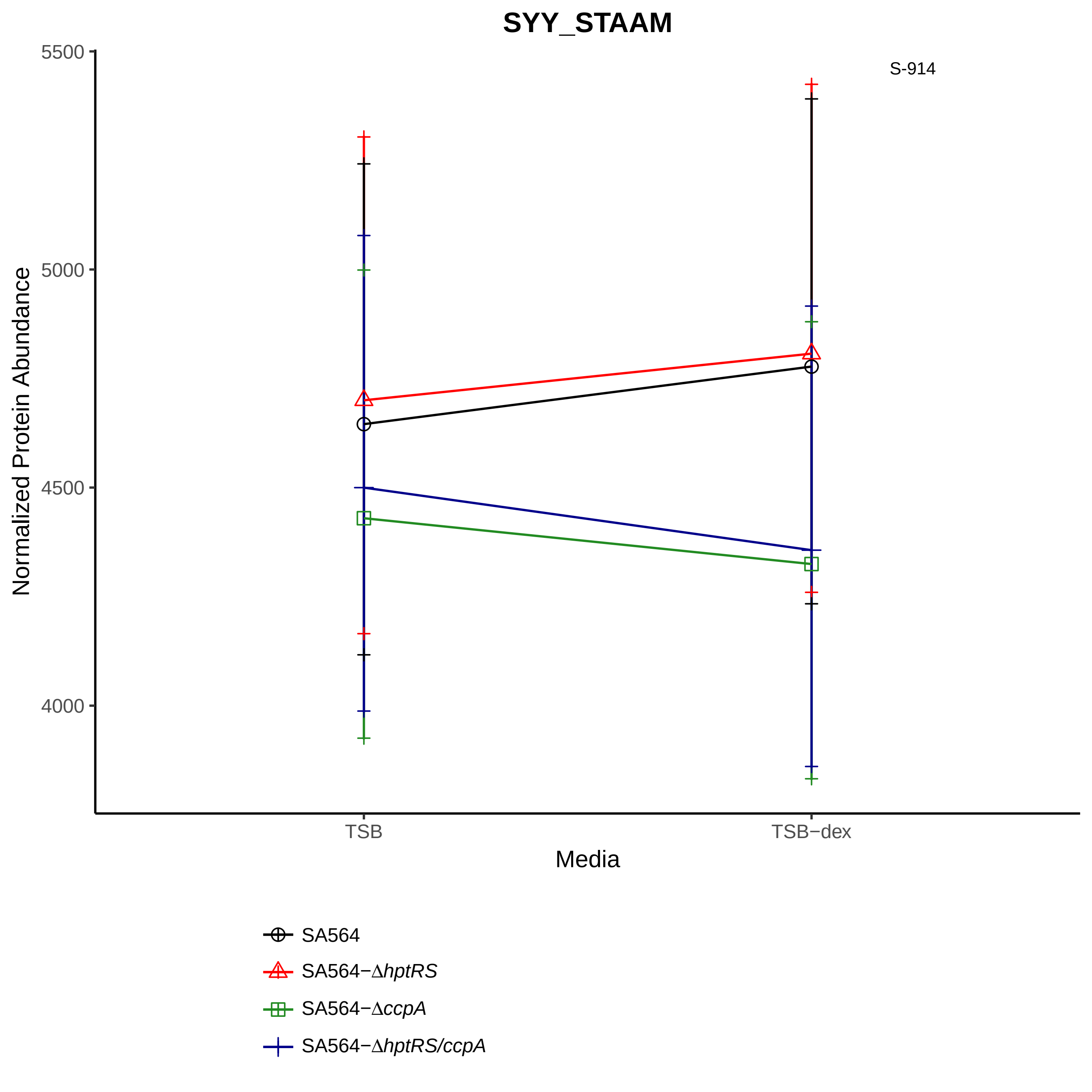

SYY_STAAM
S-914
5500
5000
4500
4000
Normalized Protein Abundance
TSB
TSB−dex
Media
SA564
SA564−Δ*hptRS*
SA564−Δ*ccpA*
SA564−Δ*hptRS/ccpA*

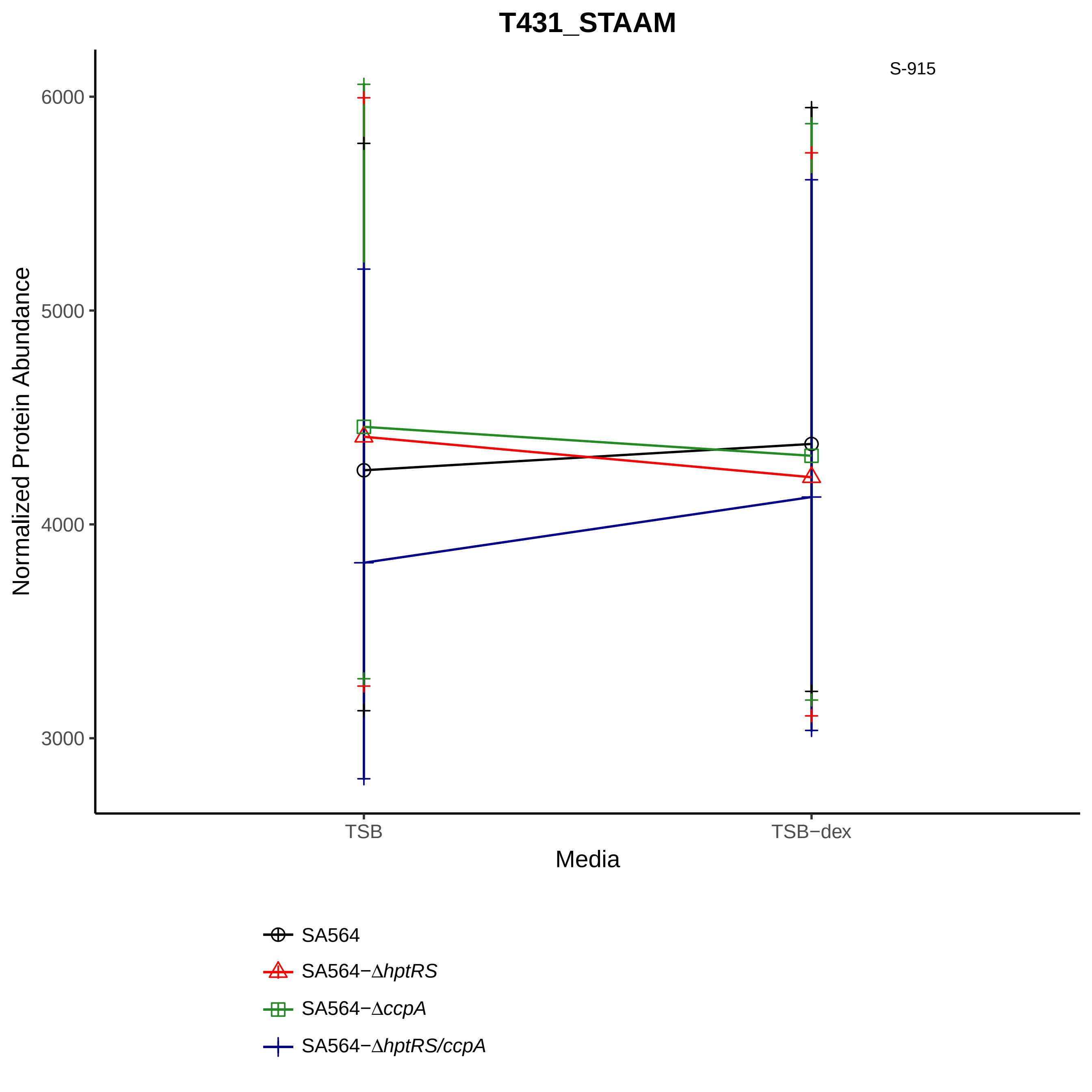

T431_STAAM
S-915
Normalized Protein Abundance
6000
5000
4000
3000
TSB
TSB−dex
Media
SA564
SA564−Δ*hptRS*
SA564−Δ*ccpA*
SA564−Δ*hptRS/ccpA*

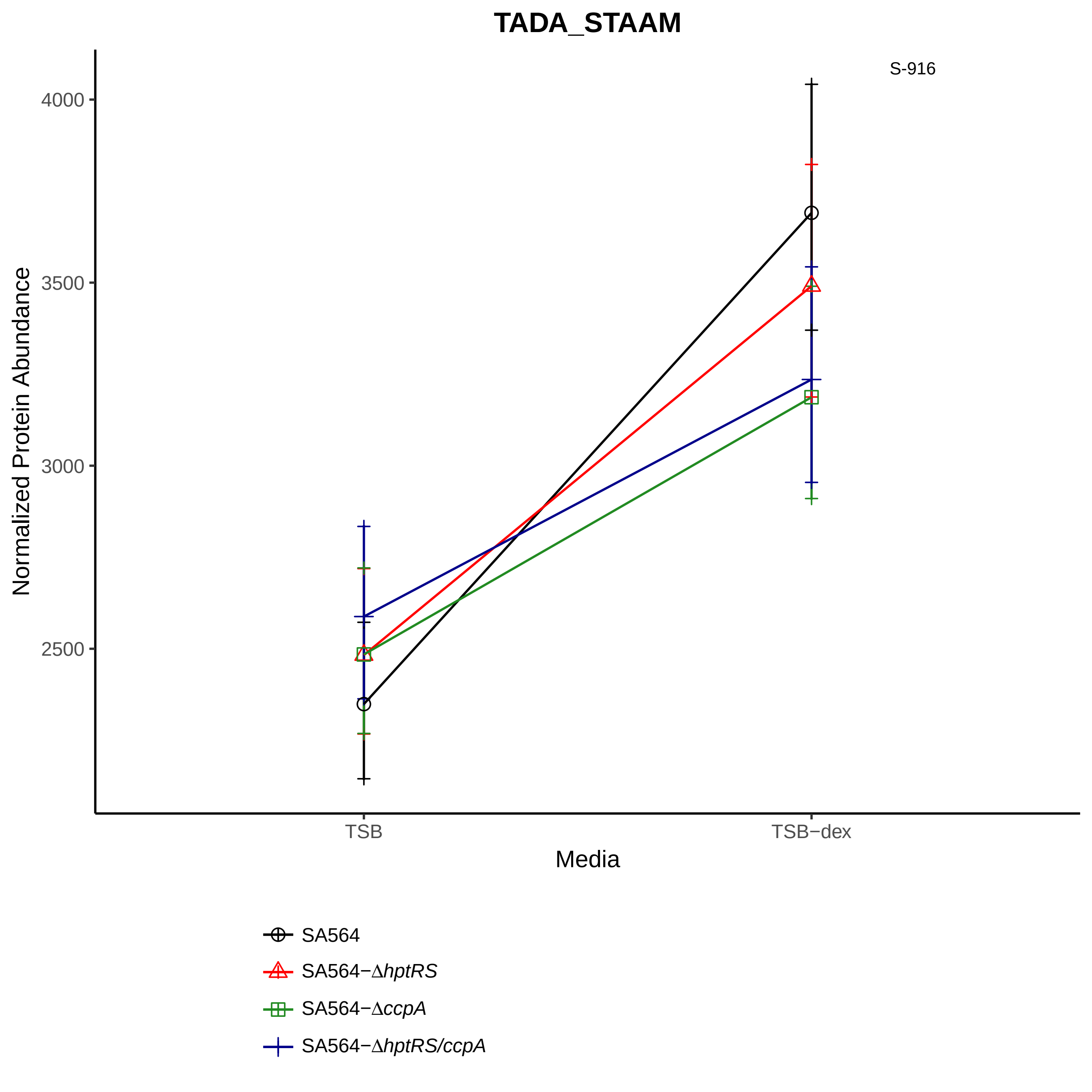

TADA_STAAM
S-916
Normalized Protein Abundance
4000
3500
3000
2500
TSB
TSB−dex
Media
SA564
SA564−ΔhptRS
SA564−ΔccpA
SA564−ΔhptRS/ccpA

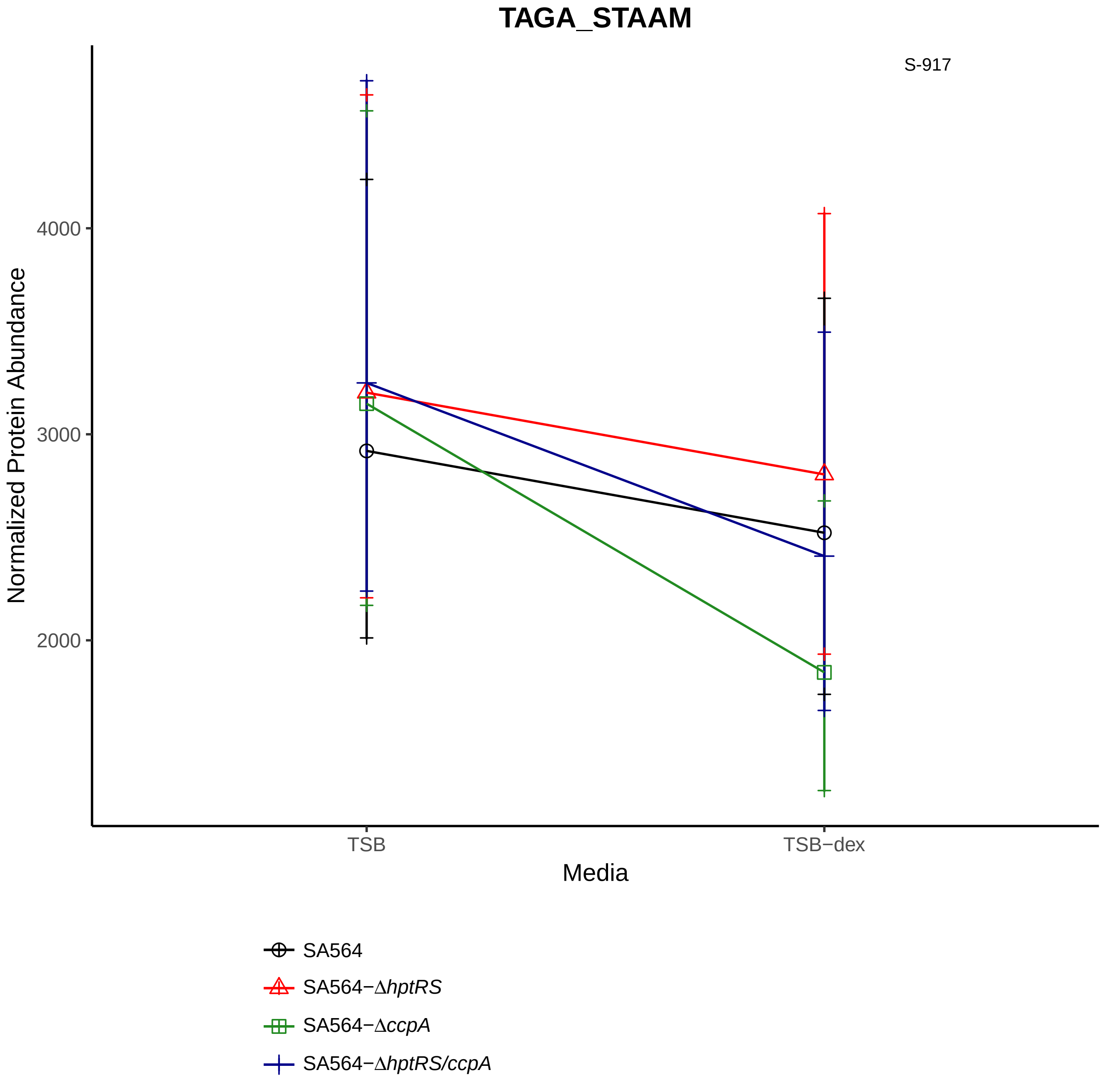

TAGA_STAAM
S-917
Normalized Protein Abundance
4000
3000
2000
TSB
TSB−dex
Media
SA564
SA564−Δ*hptRS*
SA564−Δ*ccpA*
SA564−Δ*hptRS/ccpA*

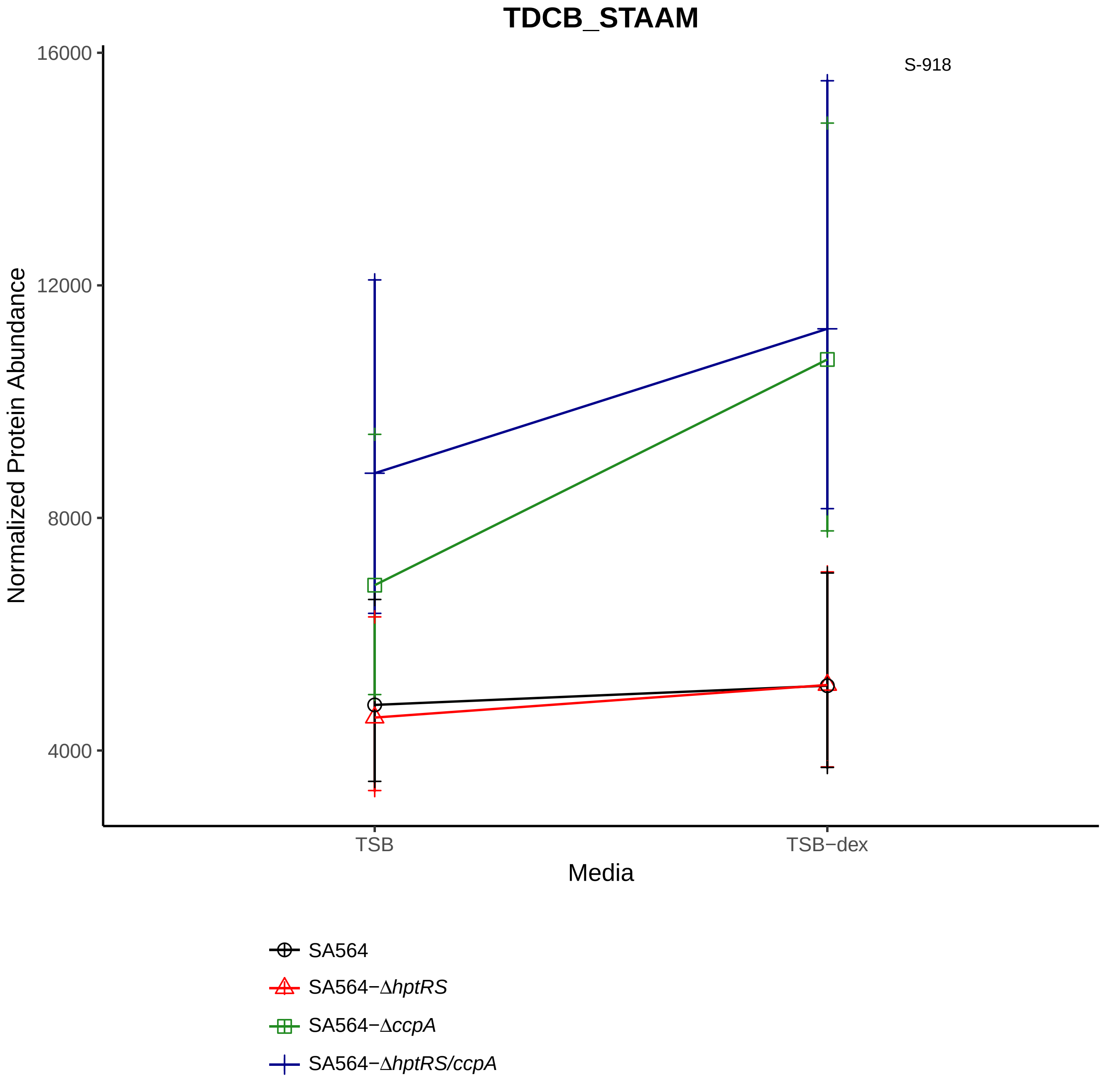
TDCB_STAAM
S-918
Normalized Protein Abundance
16000
12000
8000
4000
TSB
TSB−dex
Media
SA564
SA564−Δ*hptRS*
SA564−Δ*ccpA*
SA564−Δ*hptRS/ccpA*

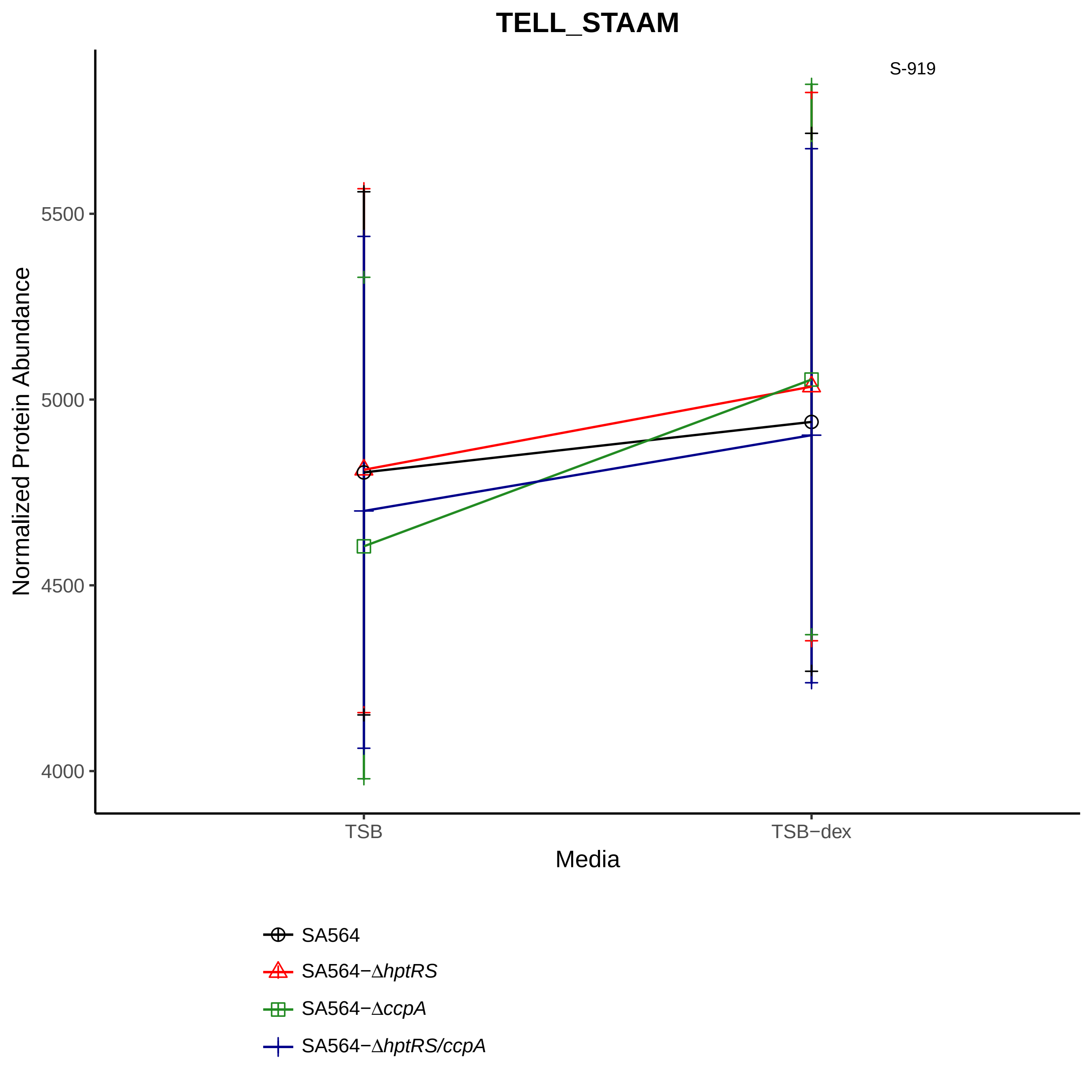

TELL_STAAM
S-919
Normalized Protein Abundance
5500
5000
4500
4000
TSB
TSB−dex
Media
SA564
SA564−Δ*hptRS*
SA564−Δ*ccpA*
SA564−Δ*hptRS/ccpA*

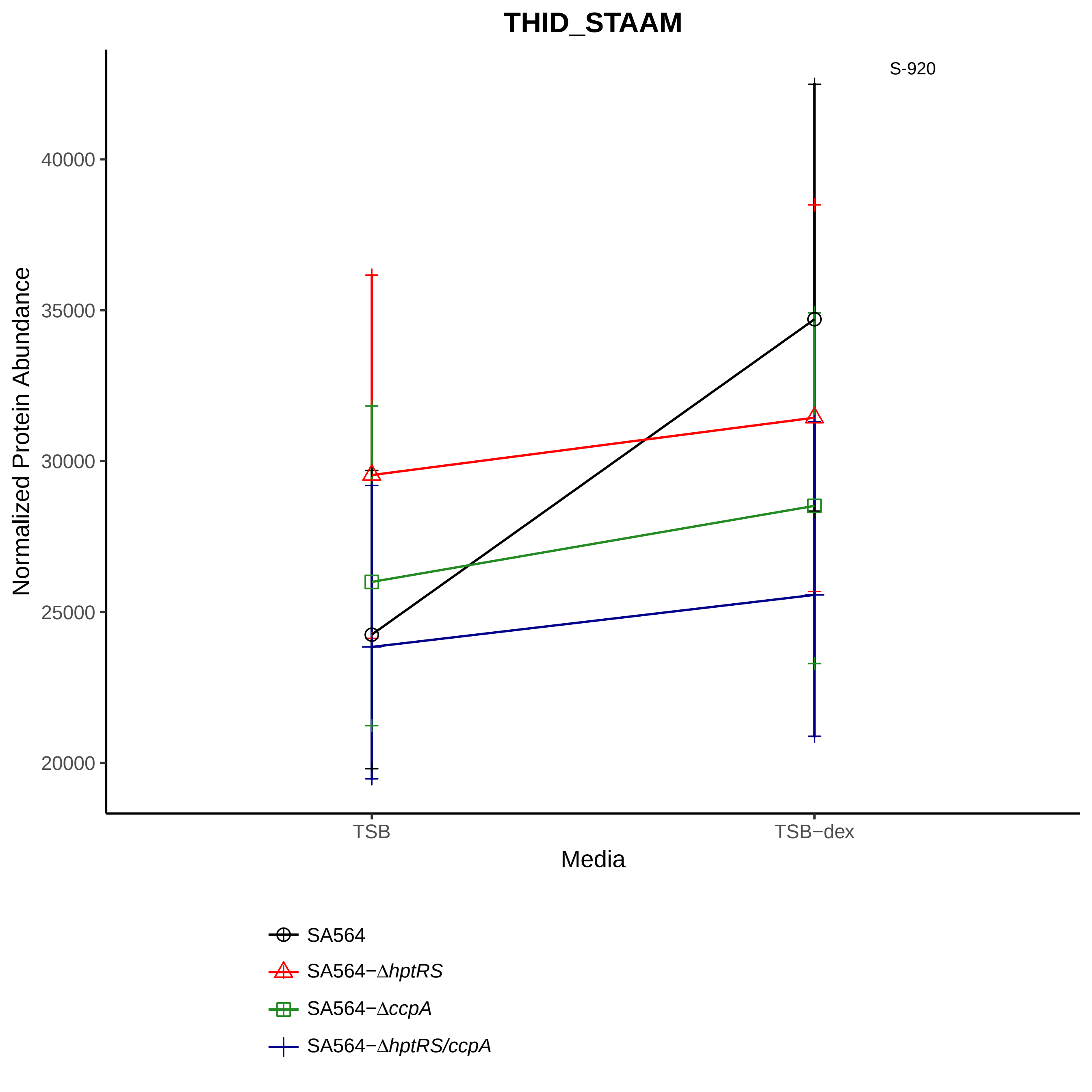
THID_STAAM
S-920
Normalized Protein Abundance
40000
35000
30000
25000
20000
TSB
TSB−dex
Media
SA564
SA564−Δ*hptRS*
SA564−Δ*ccpA*
SA564−Δ*hptRS/ccpA*

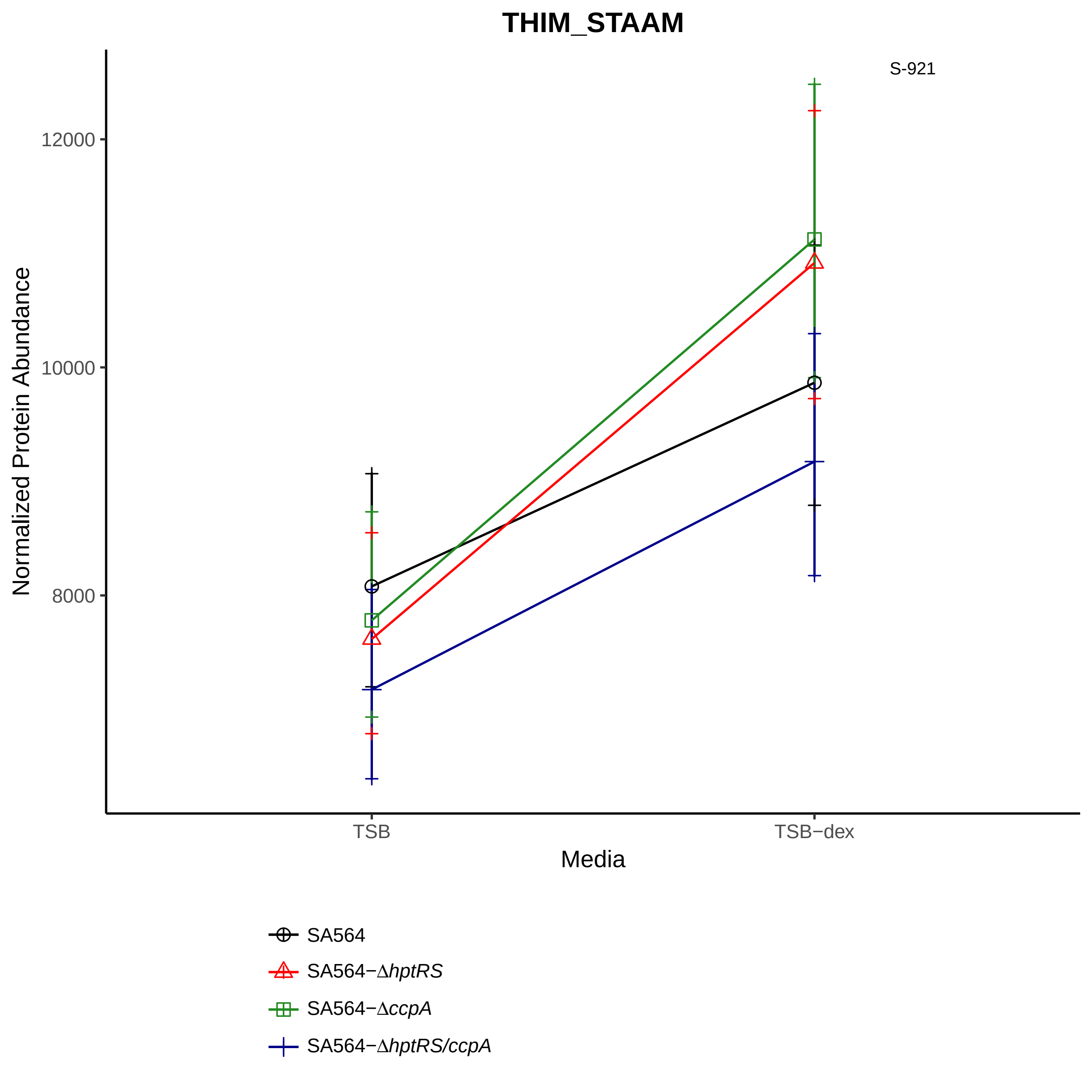
THIM_STAAM
S-921
Normalized Protein Abundance
12000
10000
8000
TSB
TSB−dex
Media
SA564
SA564−ΔhptRS
SA564−ΔccpA
SA564−ΔhptRS/ccpA

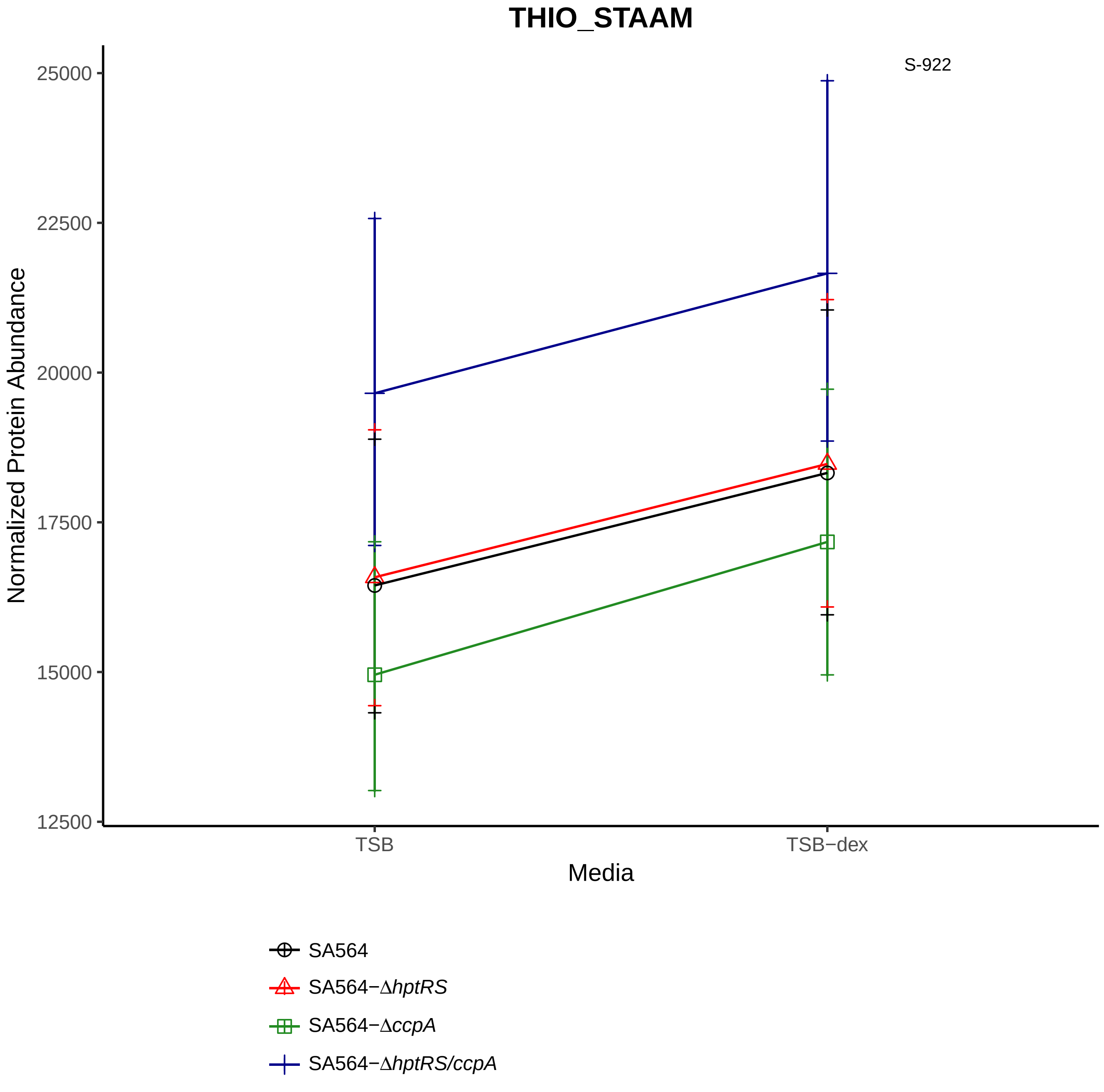
THIO_STAAM
S-922
Normalized Protein Abundance
25000
22500
20000
17500
15000
12500
TSB
TSB−dex
Media
SA564
SA564−Δ*hptRS*
SA564−Δ*ccpA*
SA564−Δ*hptRS/ccpA*

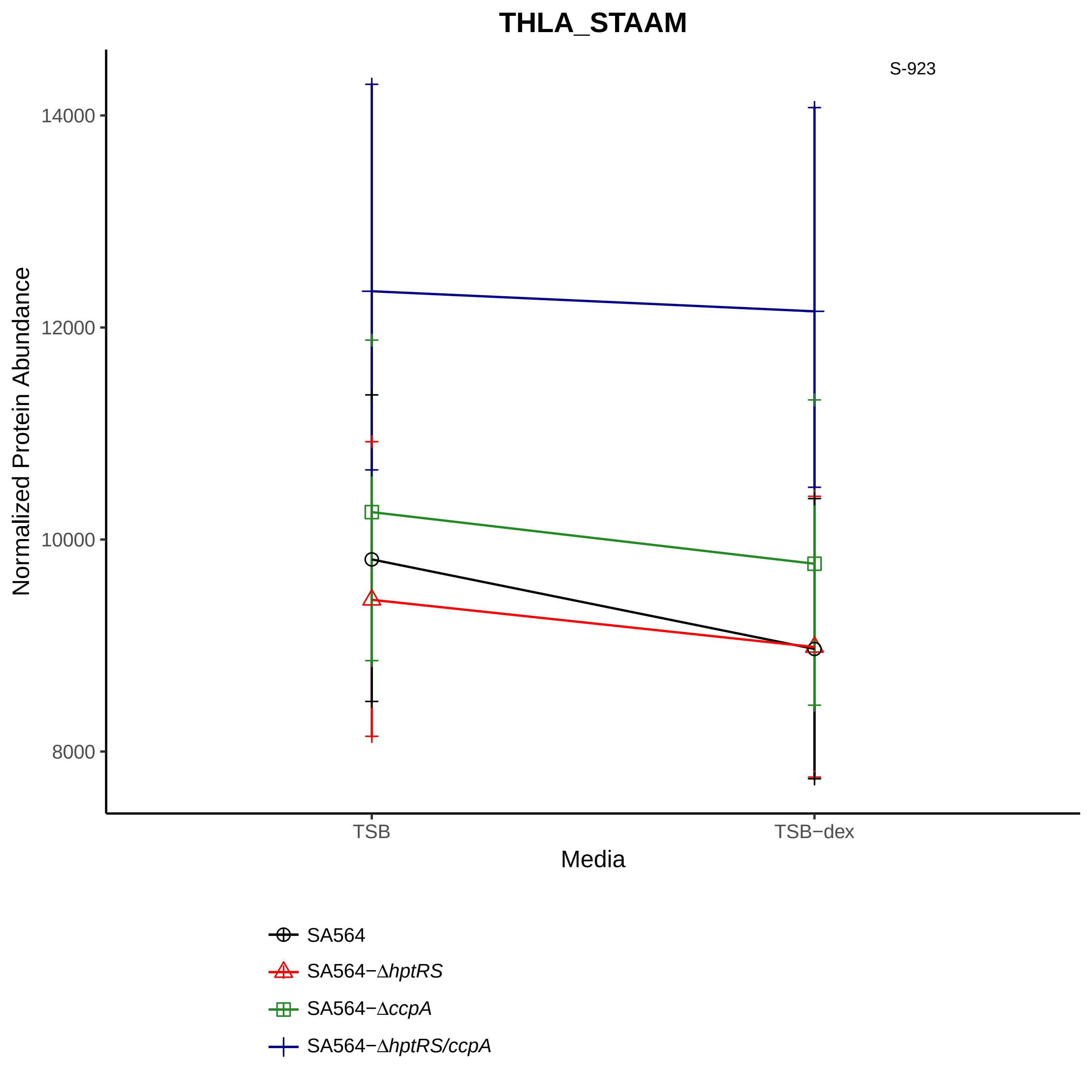

THLA_STAAM
S-923
Normalized Protein Abundance
14000
12000
10000
8000
TSB
TSB−dex
Media
SA564
SA564−ΔhptRS
SA564−ΔccpA
SA564−ΔhptRS/ccpA

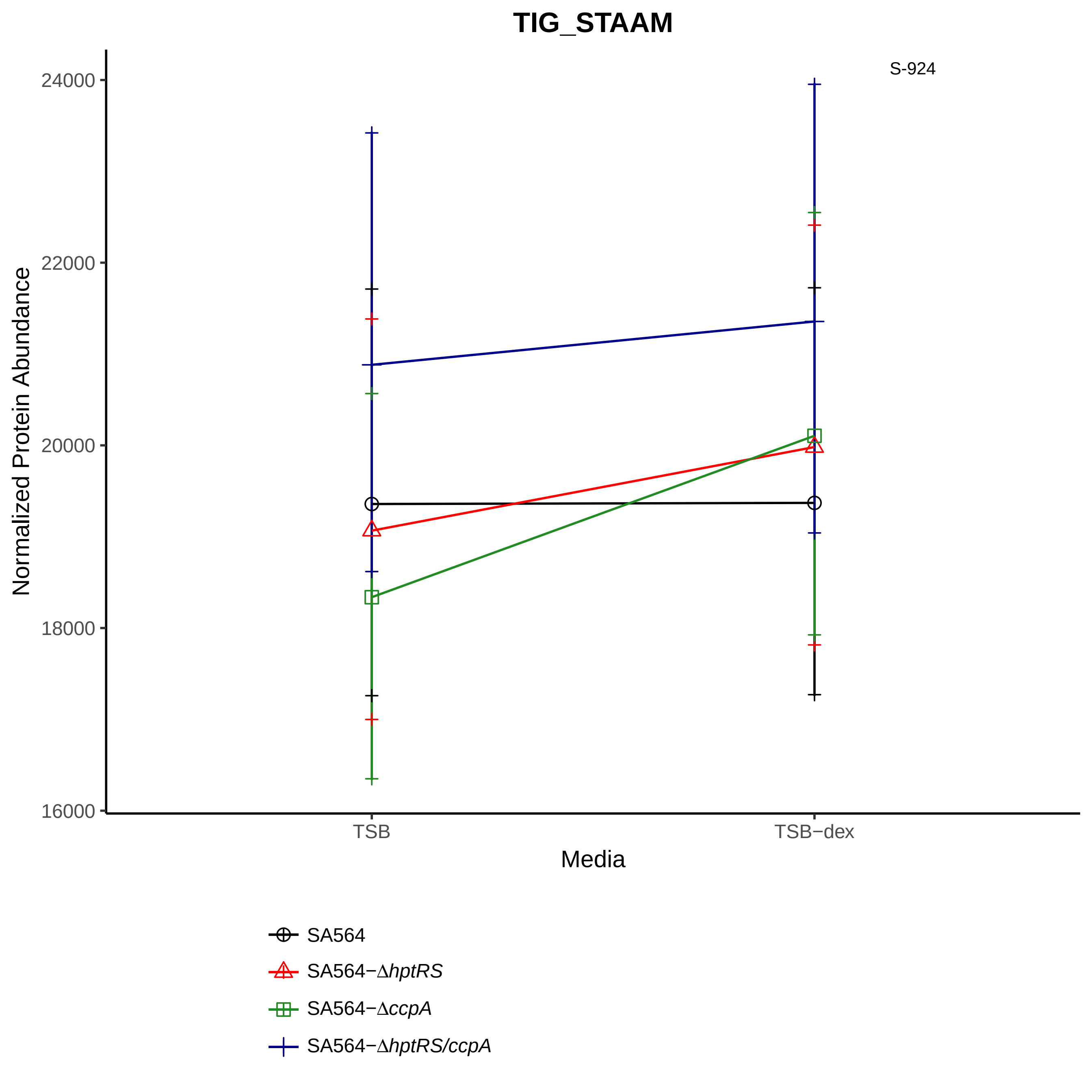

TIG_STAAM
S-924
Normalized Protein Abundance
24000
22000
20000
18000
16000
TSB
TSB−dex
Media
SA564
SA564−Δ*hptRS*
SA564−Δ*ccpA*
SA564−Δ*hptRS/ccpA*

TKT_STAAM

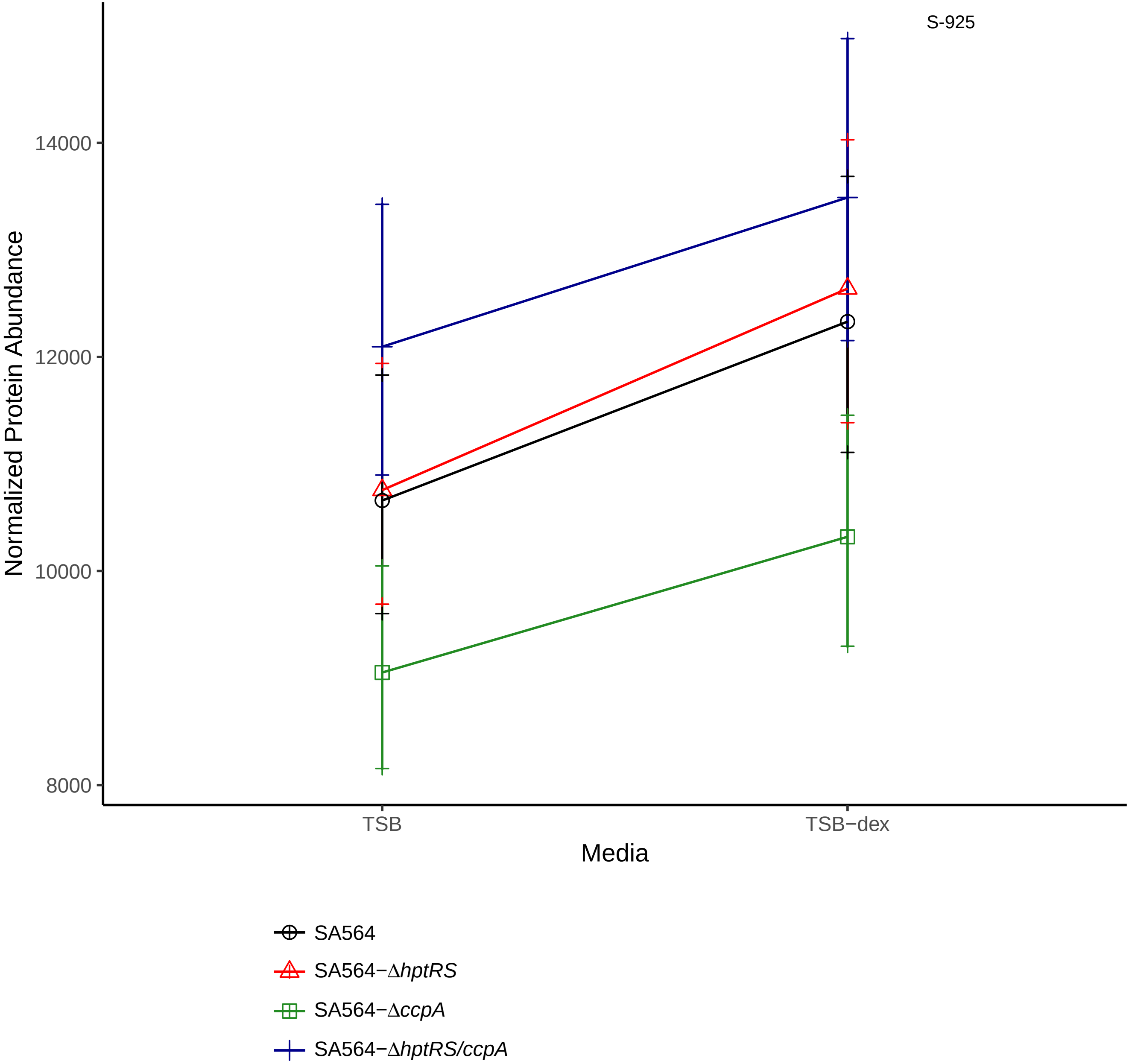

S-925
14000
12000
10000
8000
Normalized Protein Abundance
TSB
TSB−dex
Media
SA564
SA564−Δ*hptRS*
SA564−Δ*ccpA*
SA564−Δ*hptRS/ccpA*

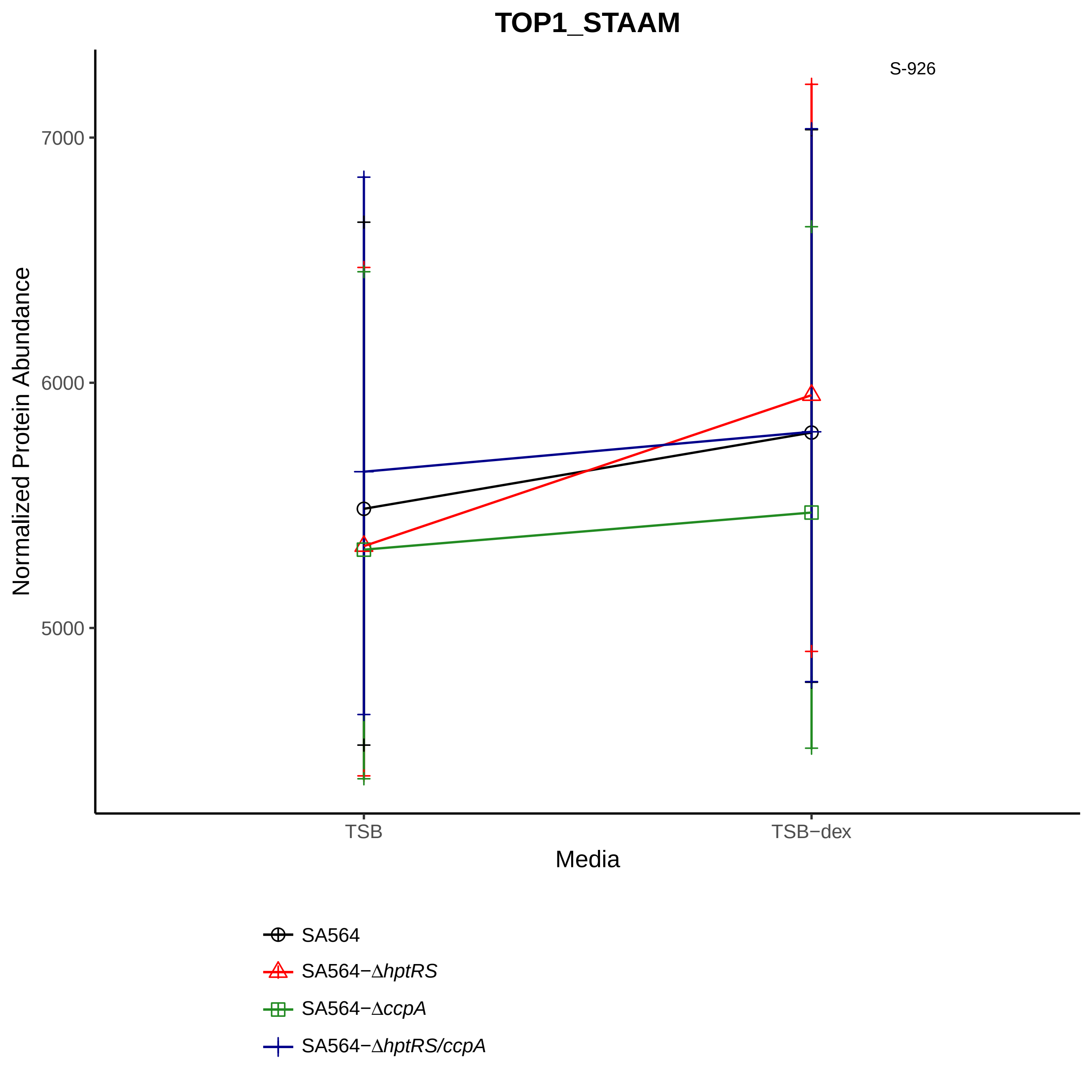

TOP1_STAAM
S-926
7000
6000
5000
Normalized Protein Abundance
TSB
TSB−dex
Media
SA564
SA564−ΔhptRS
SA564−ΔccpA
SA564−ΔhptRS/ccpA

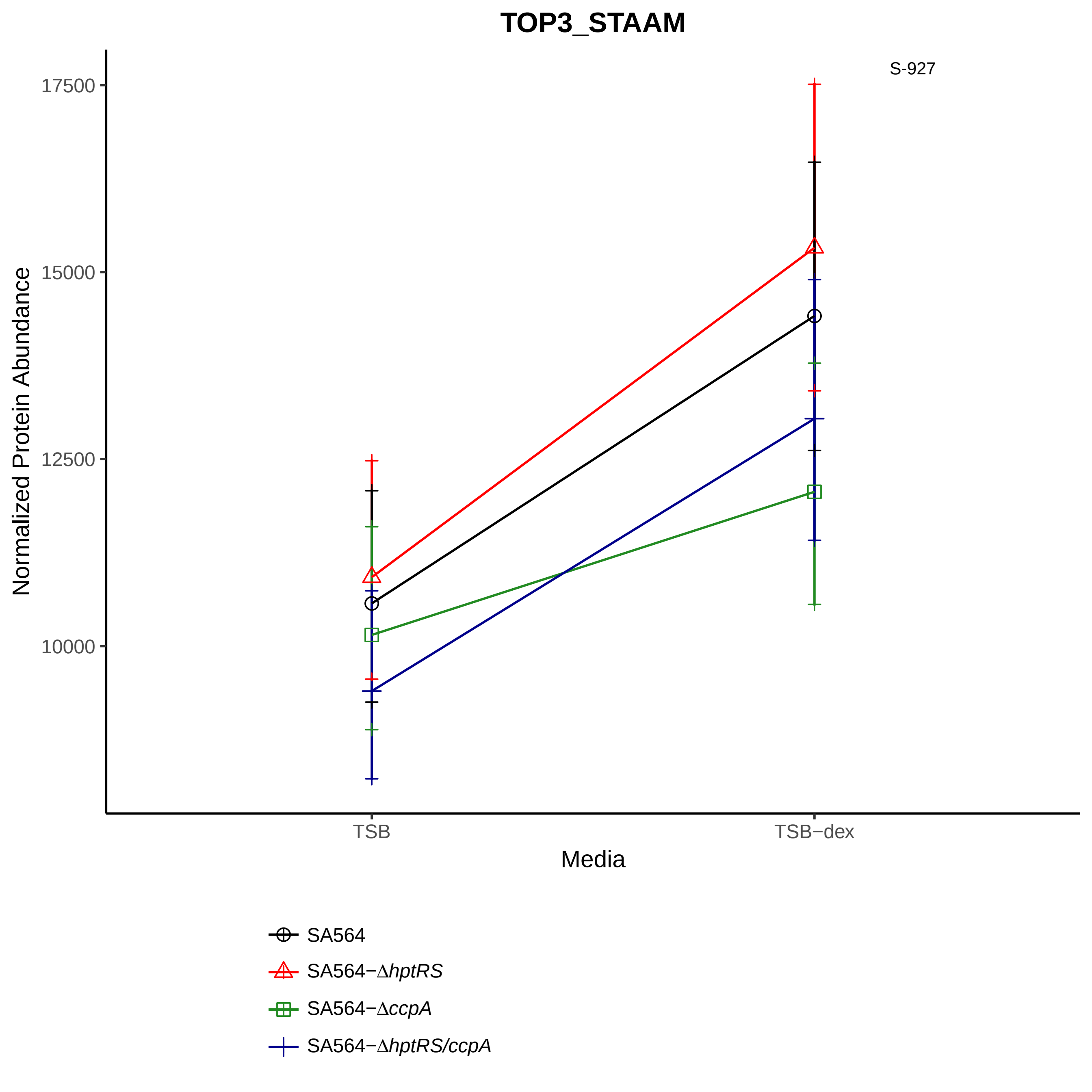

TOP3_STAAM
S-927
Normalized Protein Abundance
17500
15000
12500
10000
TSB
TSB−dex
Media
SA564
SA564−Δ*hptRS*
SA564−Δ*ccpA*
SA564−Δ*hptRS/ccpA*

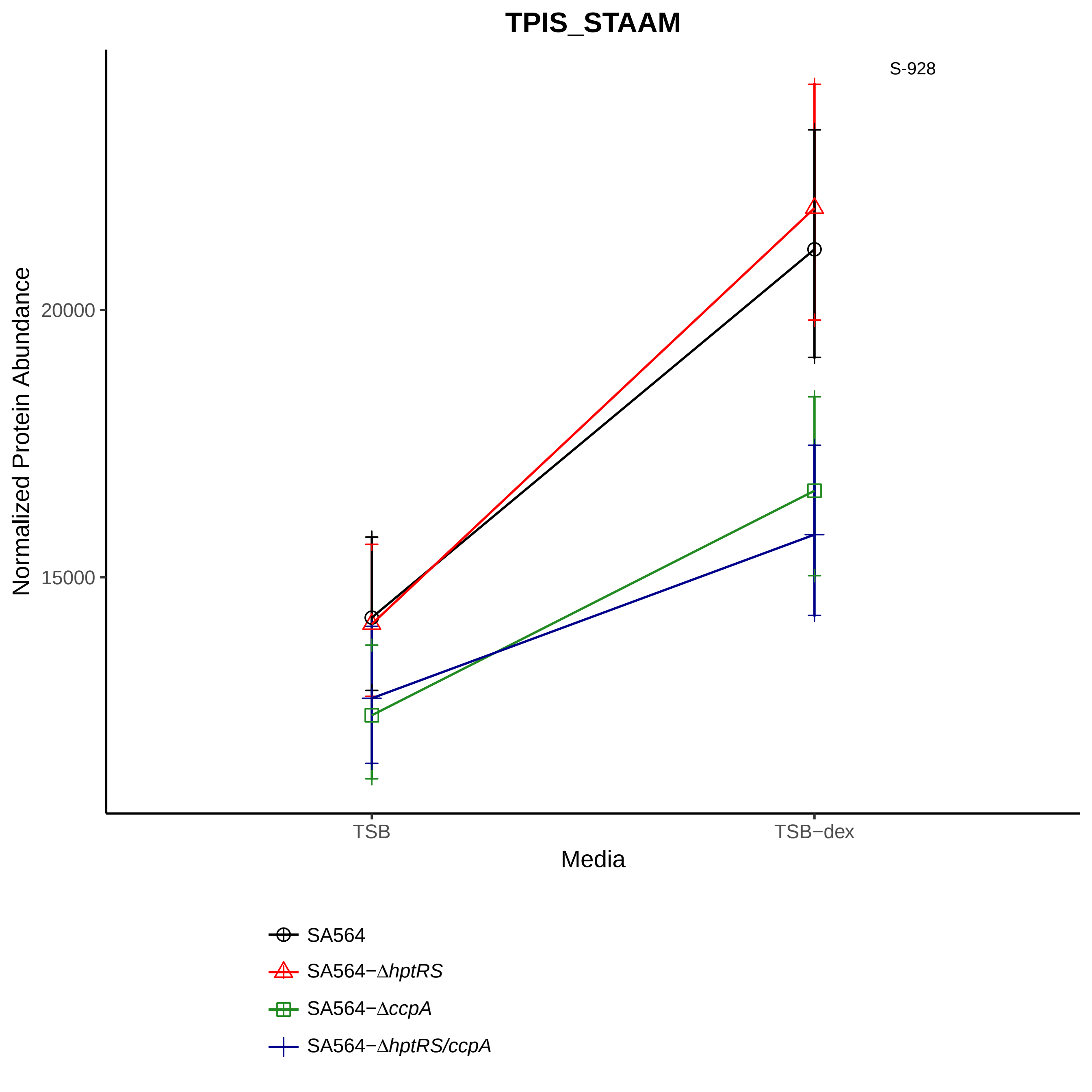

TPIS_STAAM
S-928
Normalized Protein Abundance
20000
15000
TSB
TSB−dex
Media
SA564
SA564−ΔhptRS
SA564−ΔccpA
SA564−ΔhptRS/ccpA

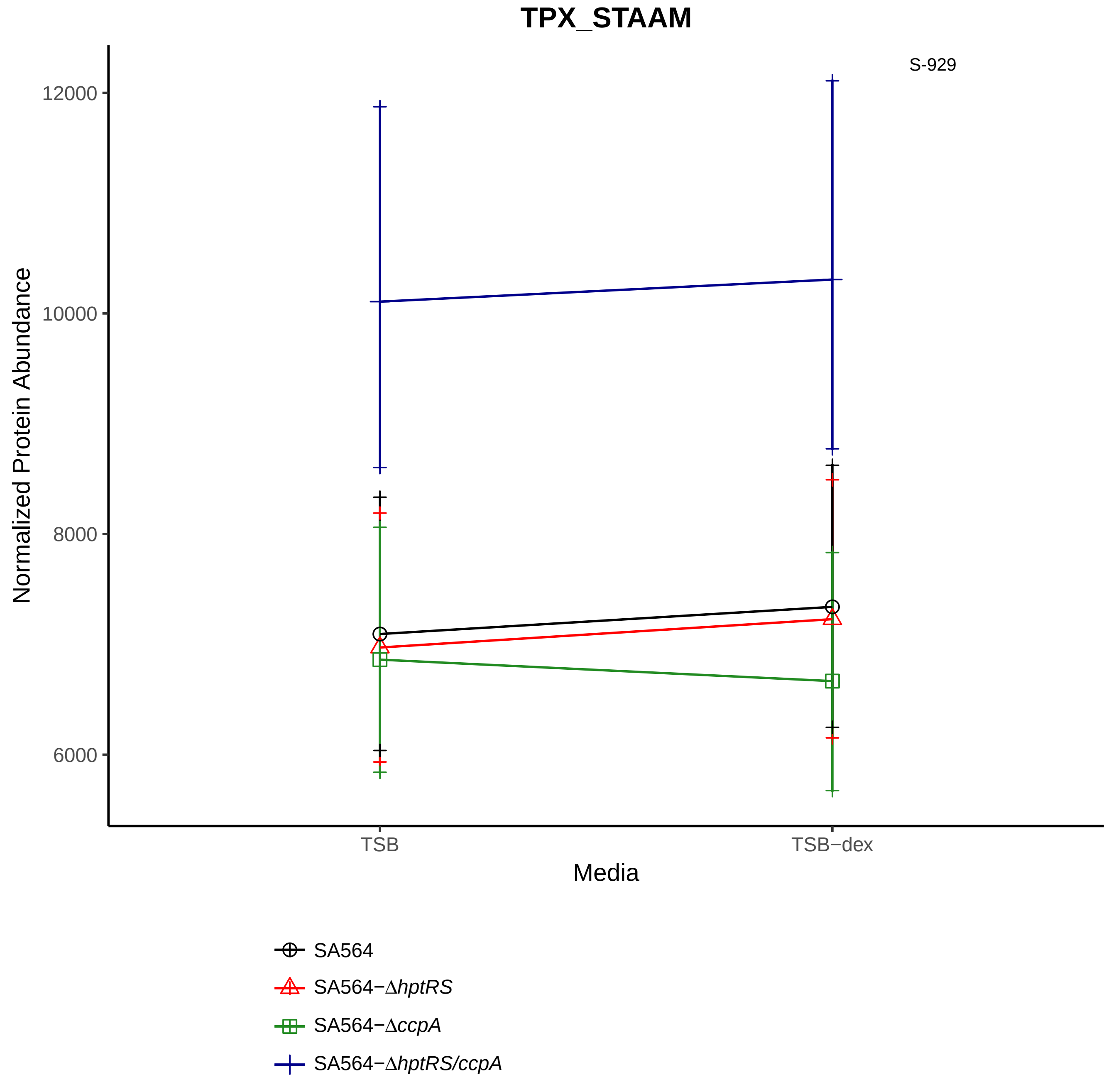

TPX_STAAM
S-929
Normalized Protein Abundance
12000
10000
8000
6000
TSB
TSB−dex
Media
SA564
SA564−ΔhptRS
SA564−ΔccpA
SA564−ΔhptRS/ccpA

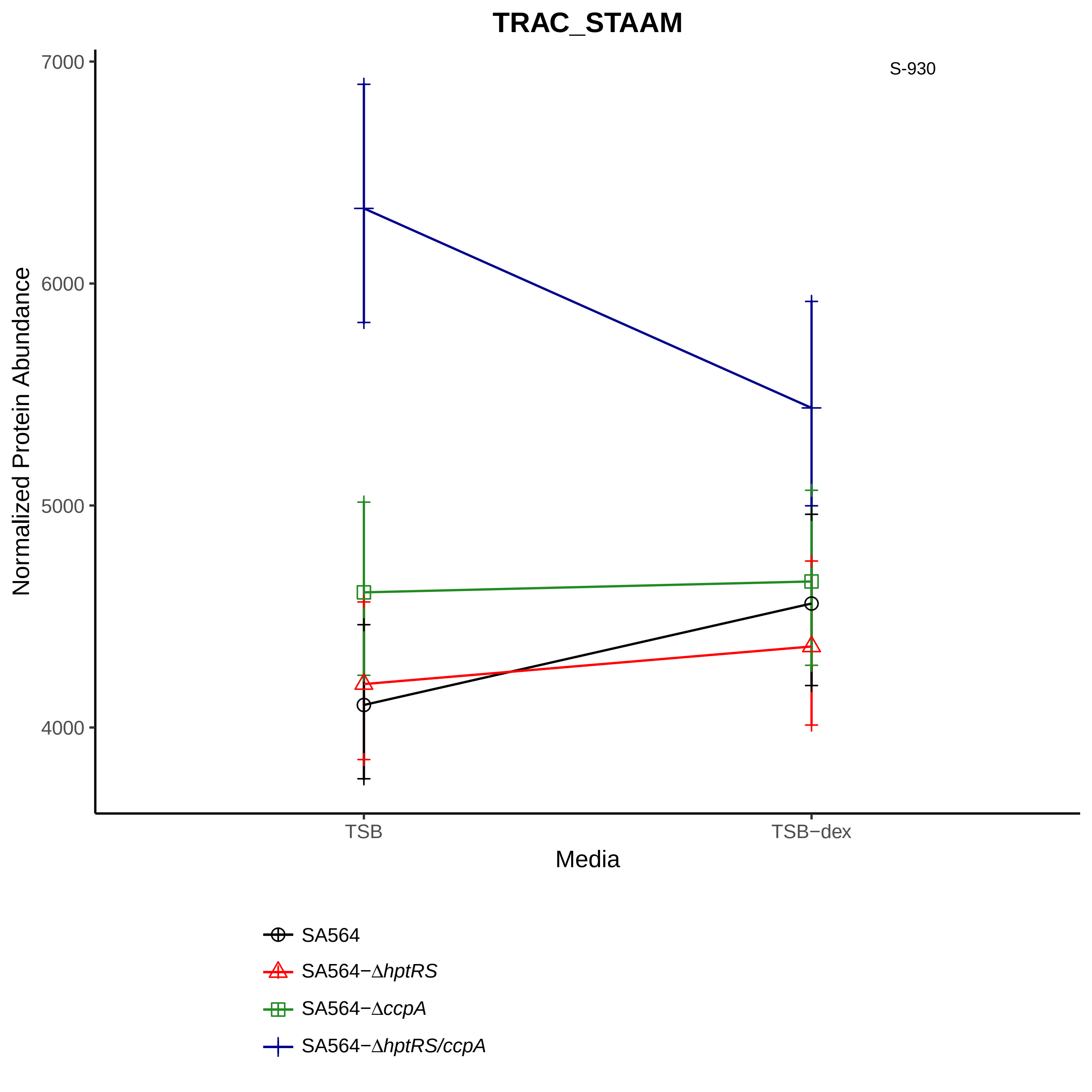

TRAC_STAAM
S-930
Normalized Protein Abundance
7000
6000
5000
4000
TSB
TSB−dex
Media
SA564
SA564−Δ*hptRS*
SA564−Δ*ccpA*
SA564−Δ*hptRS/ccpA*

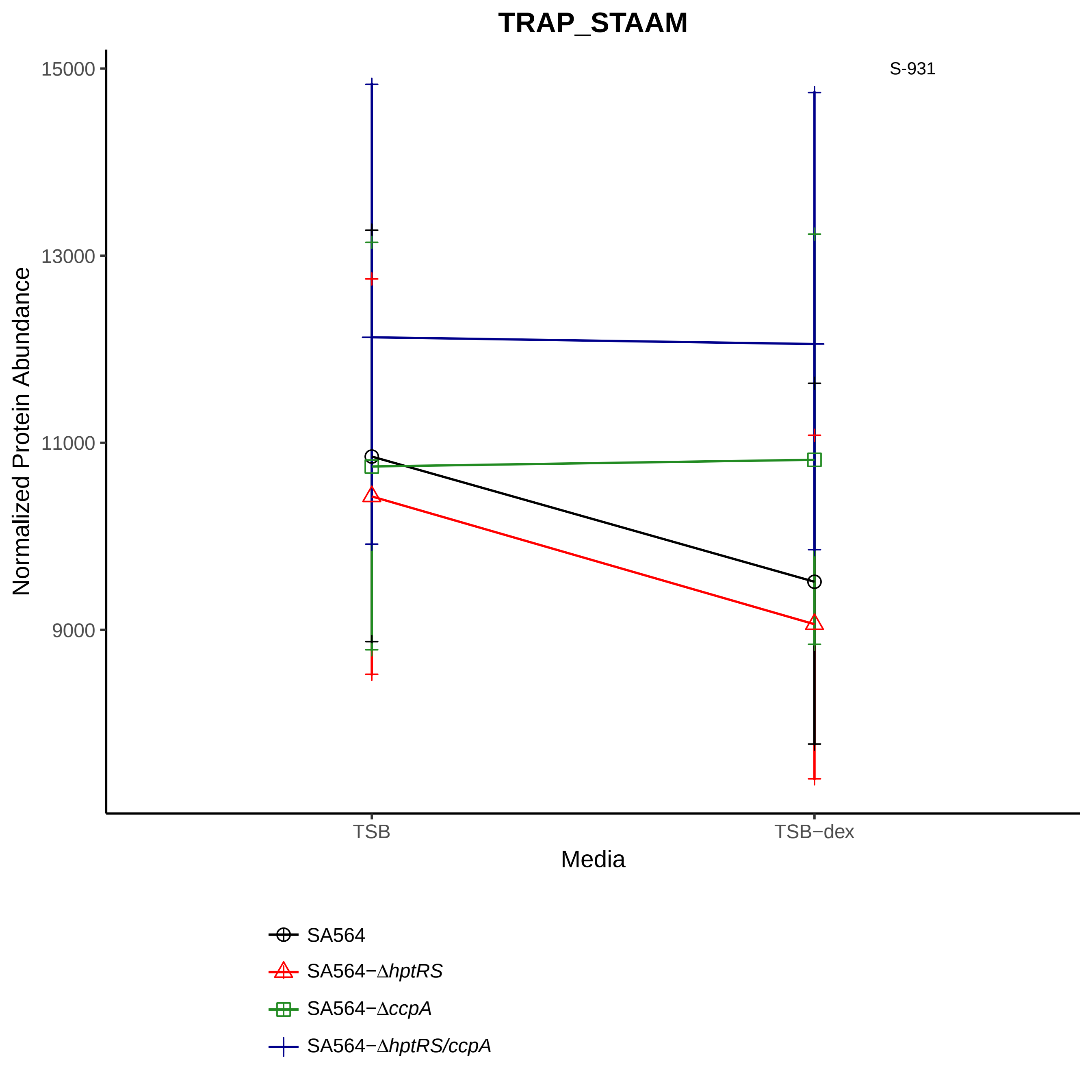
TRAP_STAAM
S-931
15000
13000
11000
9000
Normalized Protein Abundance
TSB
TSB−dex
Media
SA564
SA564−Δ*hptRS*
SA564−Δ*ccpA*
SA564−Δ*hptRS/ccpA*

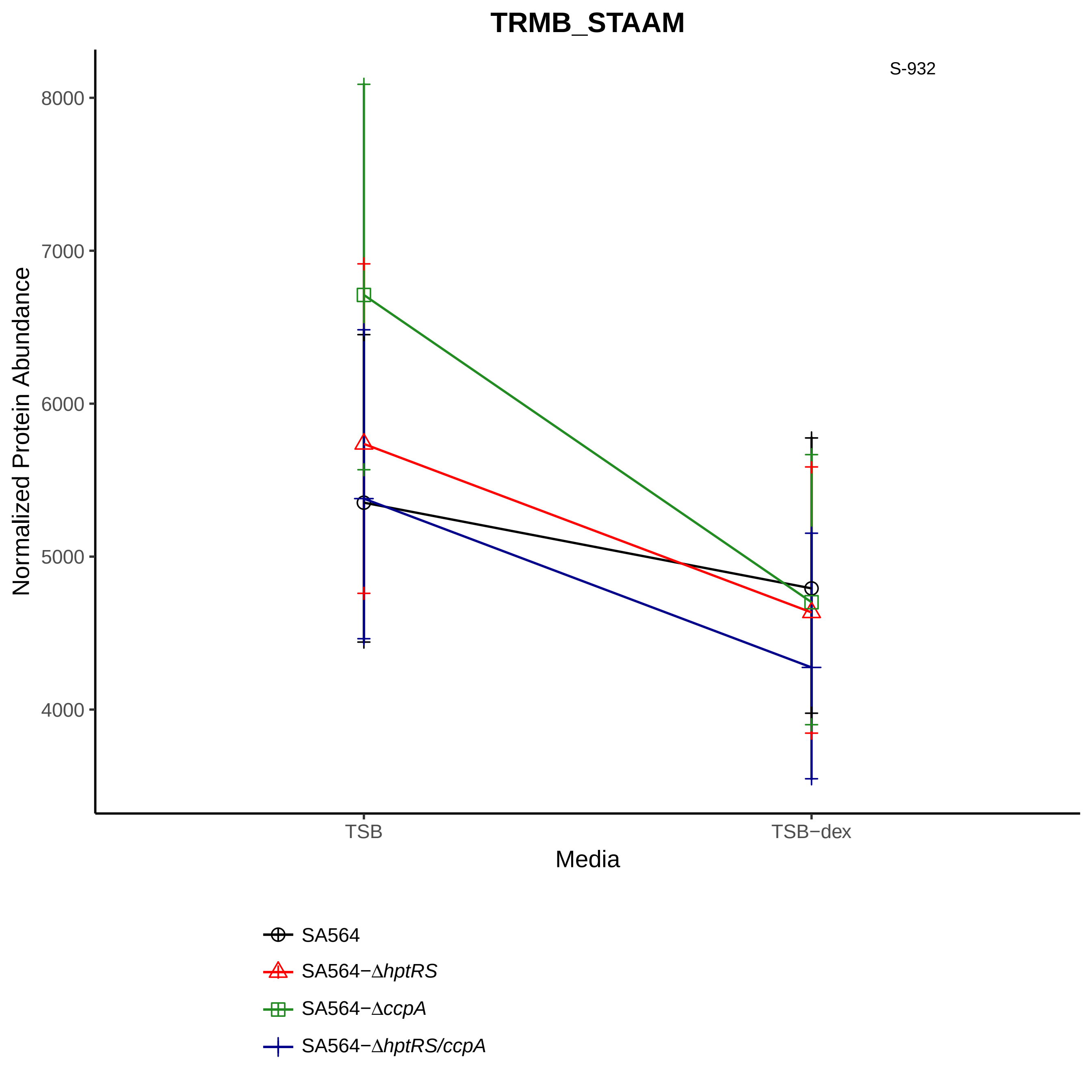
TRMB_STAAM
S-932
Normalized Protein Abundance
8000
7000
6000
5000
4000
TSB
TSB−dex
Media
SA564
SA564−ΔhptRS
SA564−ΔccpA
SA564−ΔhptRS/ccpA

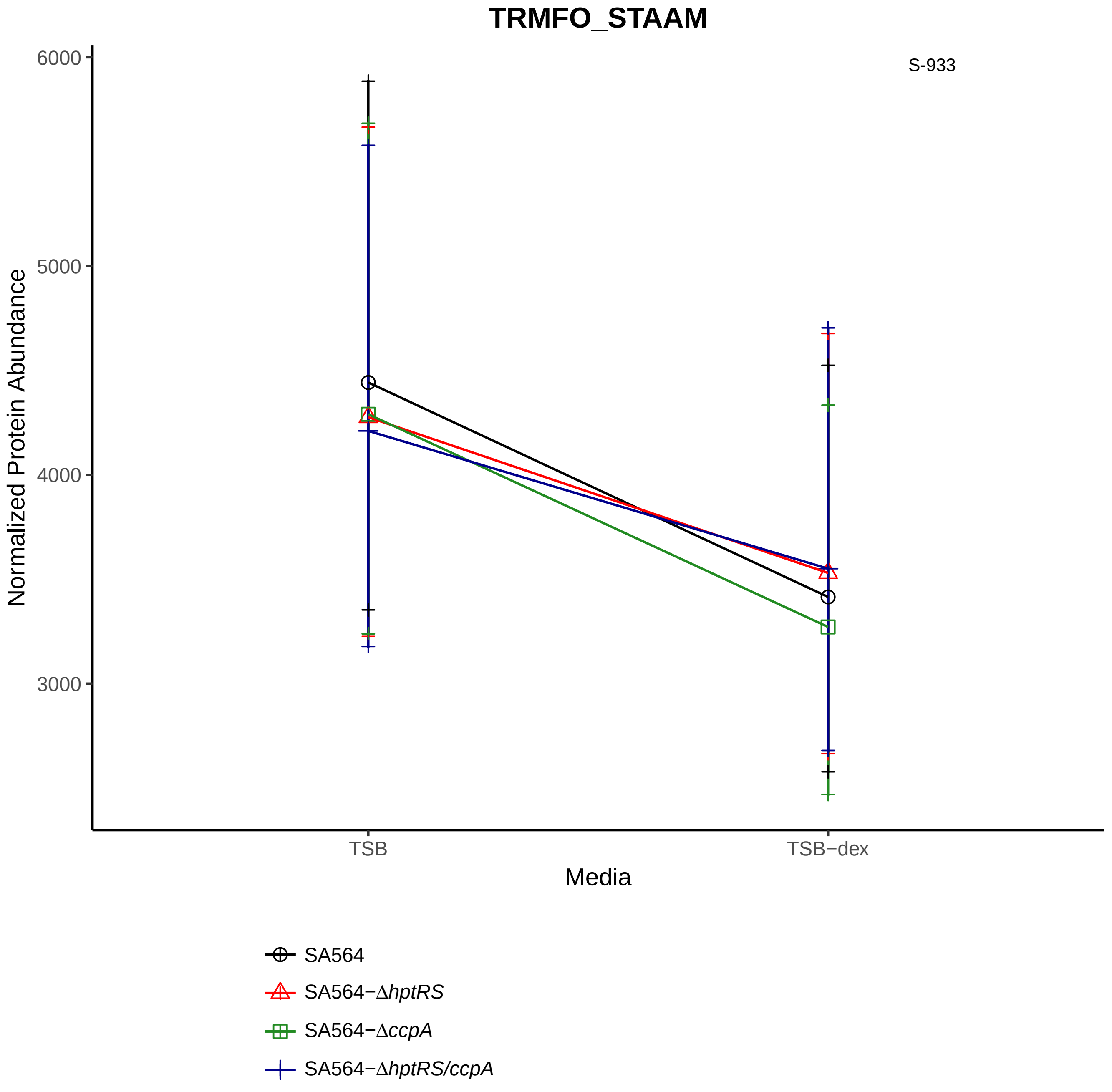
TRMFO_STAAM
S-933
Normalized Protein Abundance
6000
5000
4000
3000
TSB
TSB−dex
Media
SA564
SA564−ΔhptRS
SA564−ΔccpA
SA564−ΔhptRS/ccpA

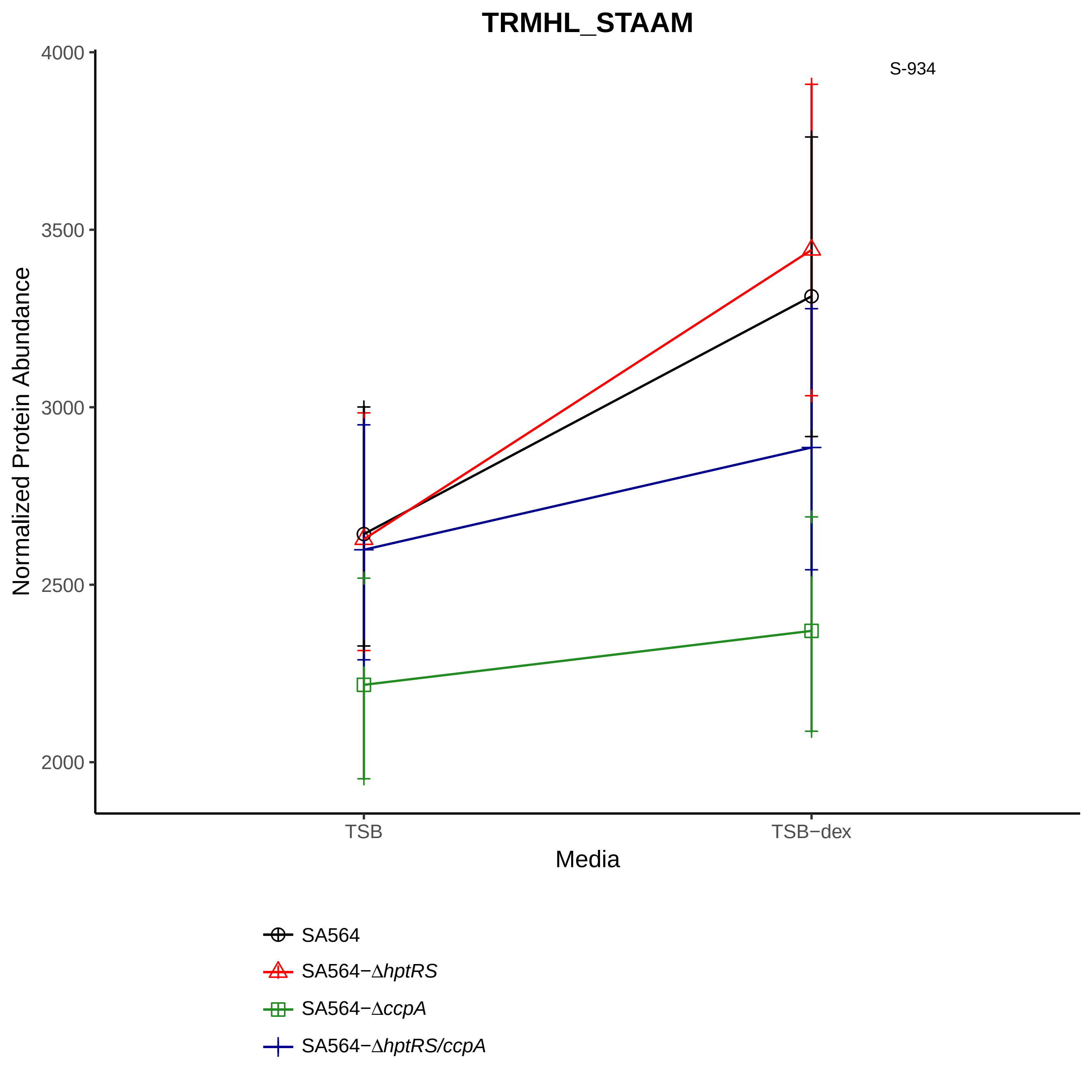

TRMHL_STAAM
S-934
4000
3500
3000
2500
2000
Normalized Protein Abundance
TSB
TSB−dex
Media
SA564
SA564−Δ*hptRS*
SA564−Δ*ccpA*
SA564−Δ*hptRS/ccpA*

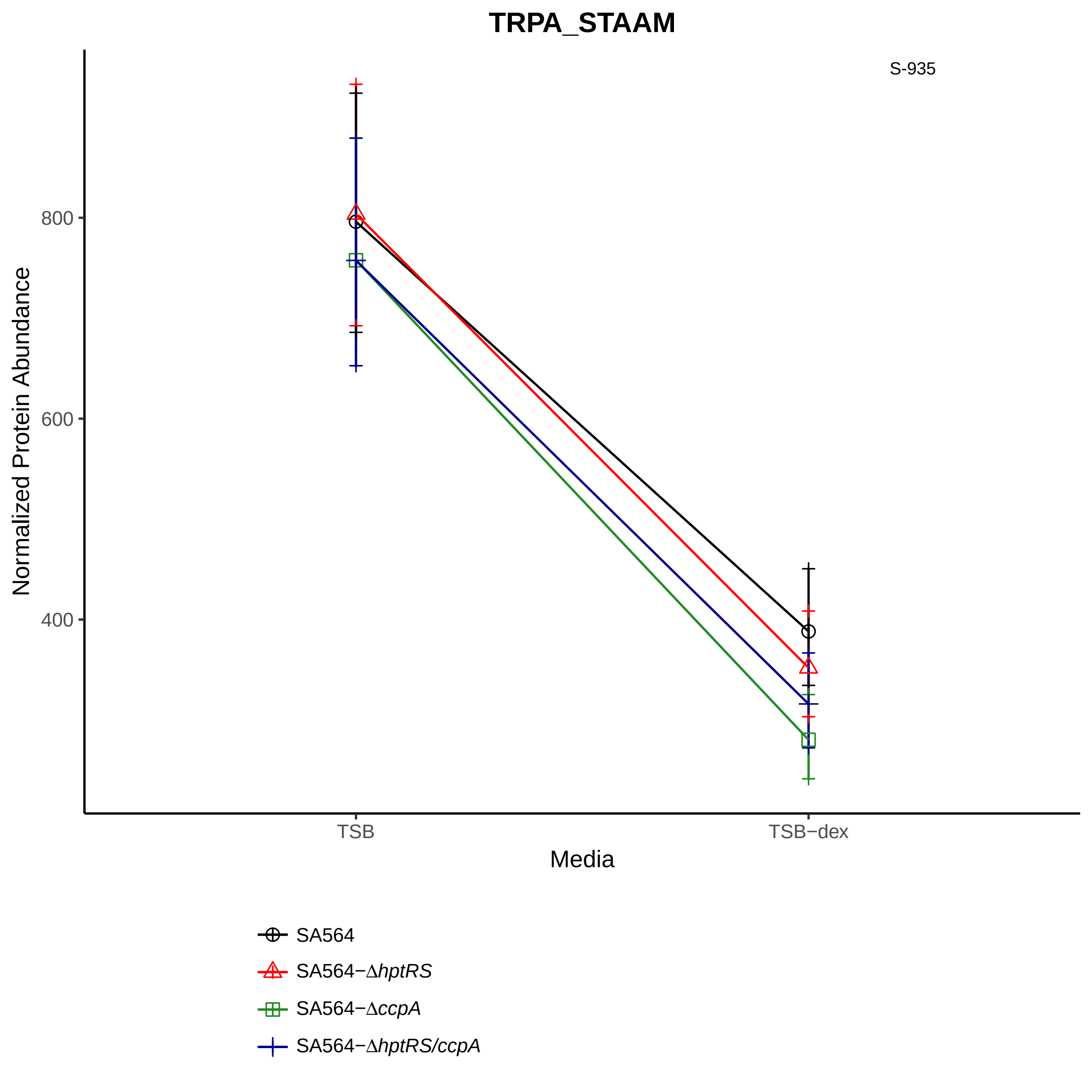

TRPA_STAAM
S-935
Normalized Protein Abundance
800
600
400
TSB
TSB−dex
Media
SA564
SA564−ΔhptRS
SA564−ΔccpA
SA564−ΔhptRS/ccpA

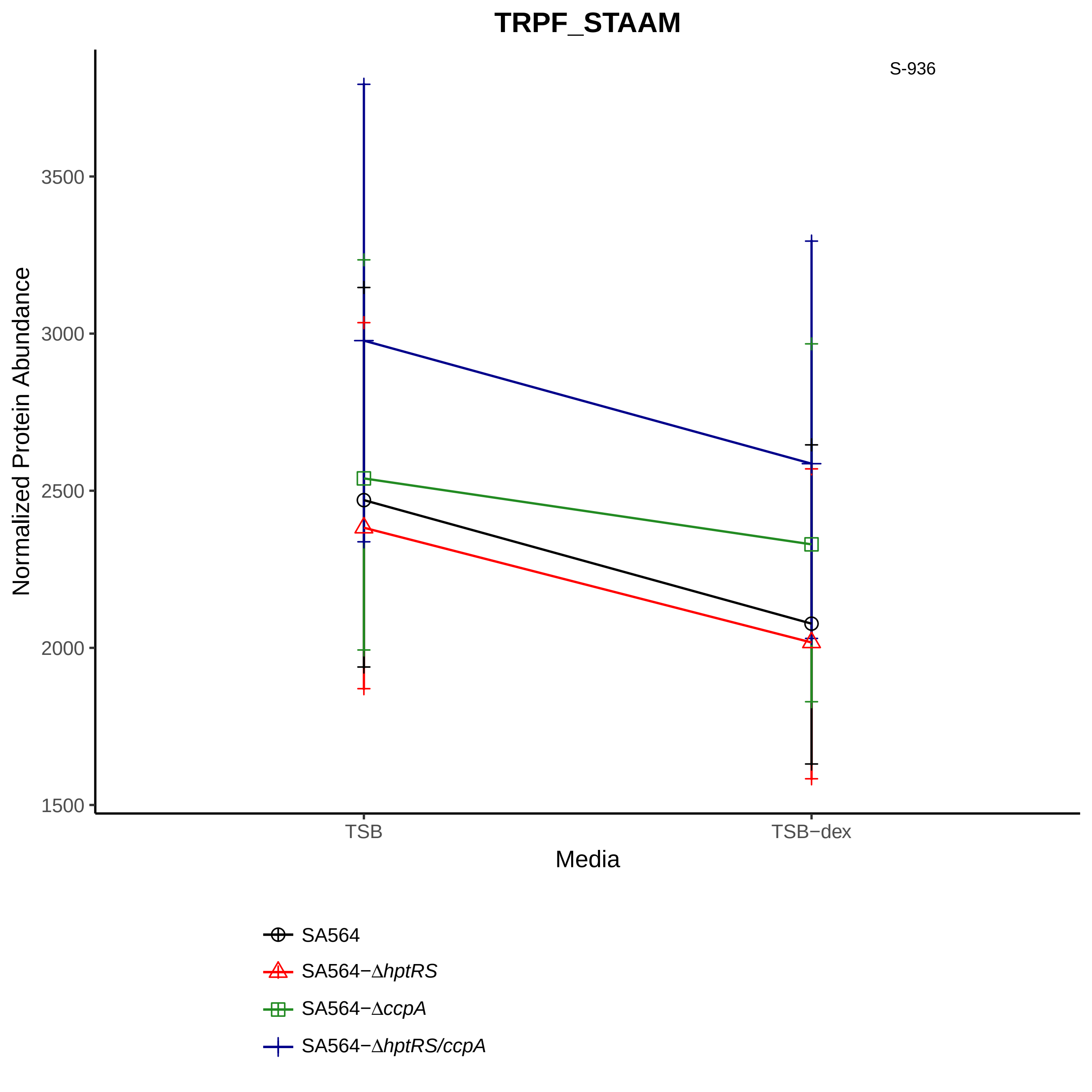
TRPF_STAAM
S-936
Normalized Protein Abundance
3500
3000
2500
2000
1500
TSB
TSB−dex
Media
SA564
SA564−Δ*hptRS*
SA564−Δ*ccpA*
SA564−Δ*hptRS/ccpA*

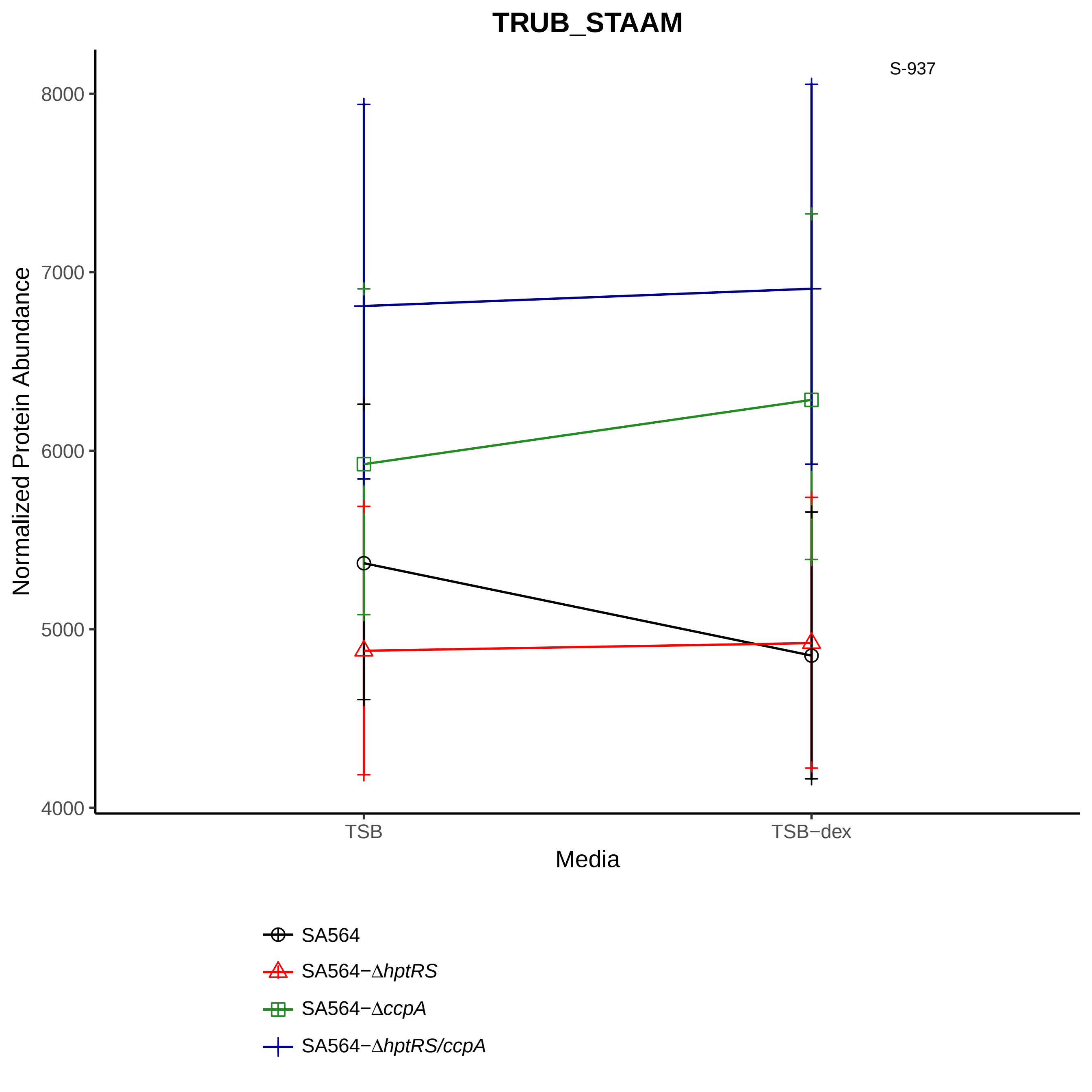

TRUB_STAAM
S-937
Normalized Protein Abundance
8000
7000
6000
5000
4000
TSB
TSB−dex
Media
SA564
SA564−ΔhptRS
SA564−ΔccpA
SA564−ΔhptRS/ccpA

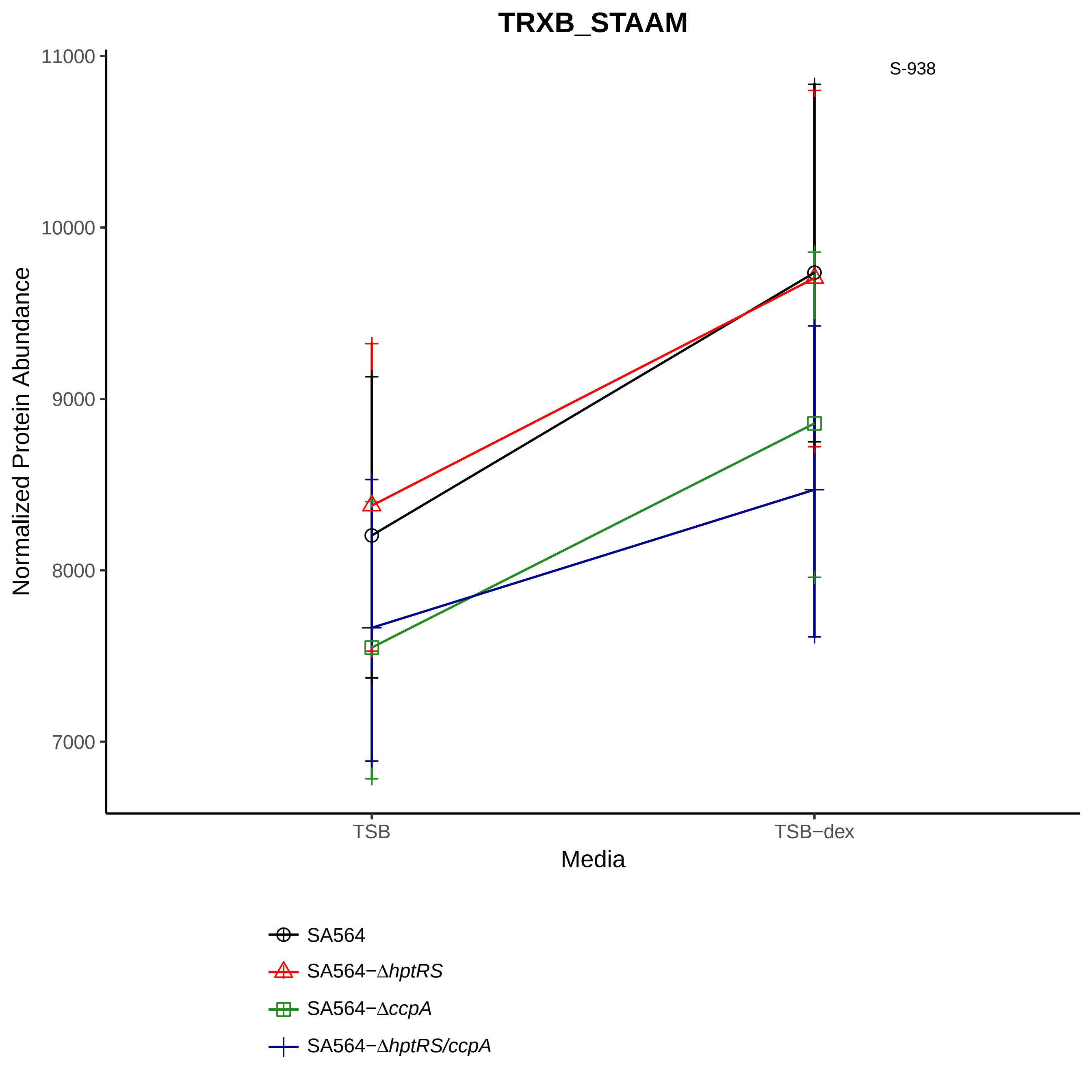

TRXB_STAAM
S-938
Normalized Protein Abundance
11000
10000
9000
8000
7000
TSB
TSB−dex
Media
SA564
SA564−ΔhptRS
SA564−ΔccpA
SA564−ΔhptRS/ccpA

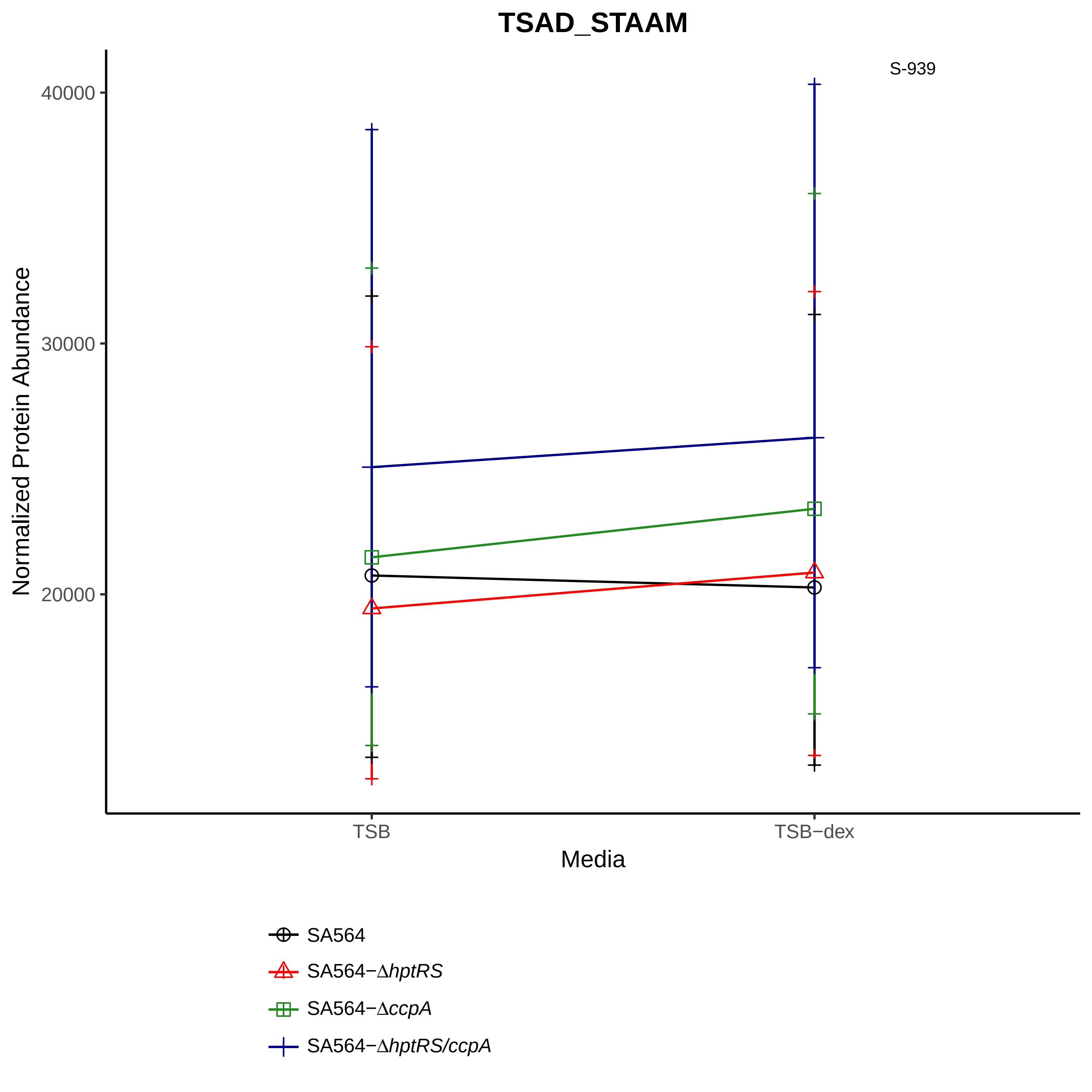

TSAD_STAAM
S-939
40000
30000
20000
Normalized Protein Abundance
TSB
TSB−dex
Media
SA564
SA564−Δ*hptRS*
SA564−Δ*ccpA*
SA564−Δ*hptRS/ccpA*

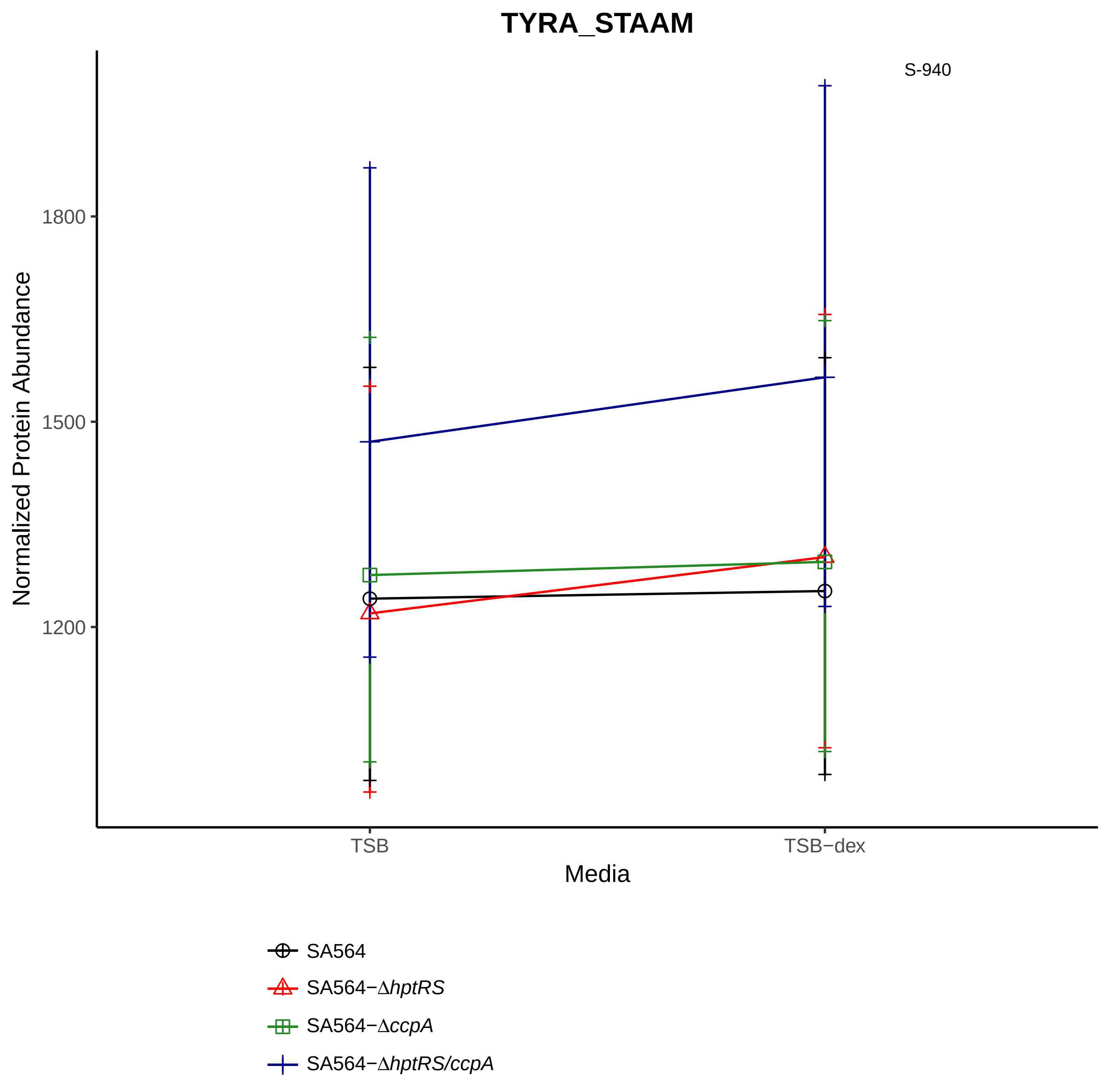
TYRA_STAAM
S-940
Normalized Protein Abundance
1800
1500
1200
TSB
TSB−dex
Media
SA564
SA564−Δ*hptRS*
SA564−Δ*ccpA*
SA564−Δ*hptRS/ccpA*

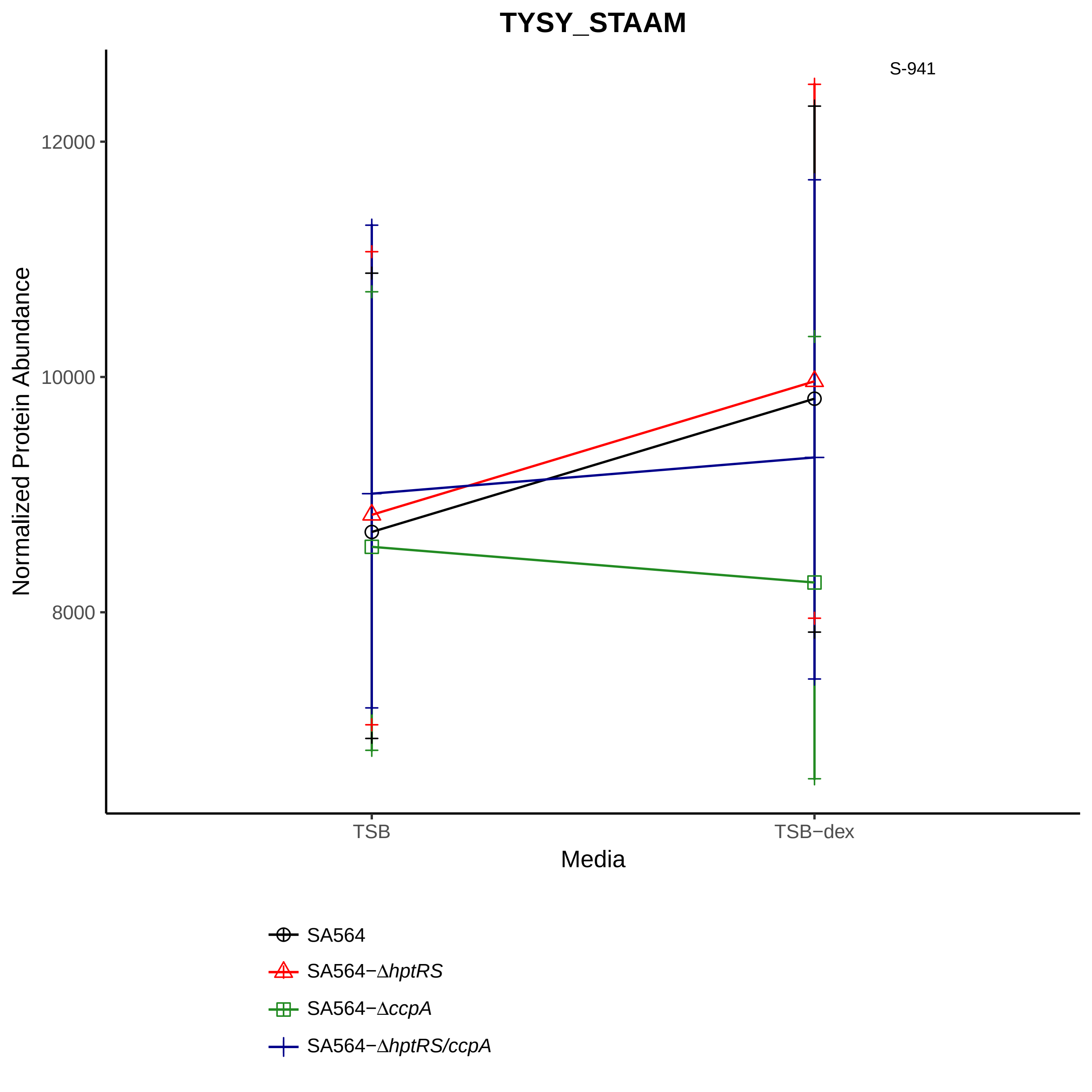

TYSY_STAAM
S-941
Normalized Protein Abundance
12000
10000
8000
TSB
TSB−dex
Media
SA564
SA564−Δ*hptRS*
SA564−Δ*ccpA*
SA564−Δ*hptRS/ccpA*

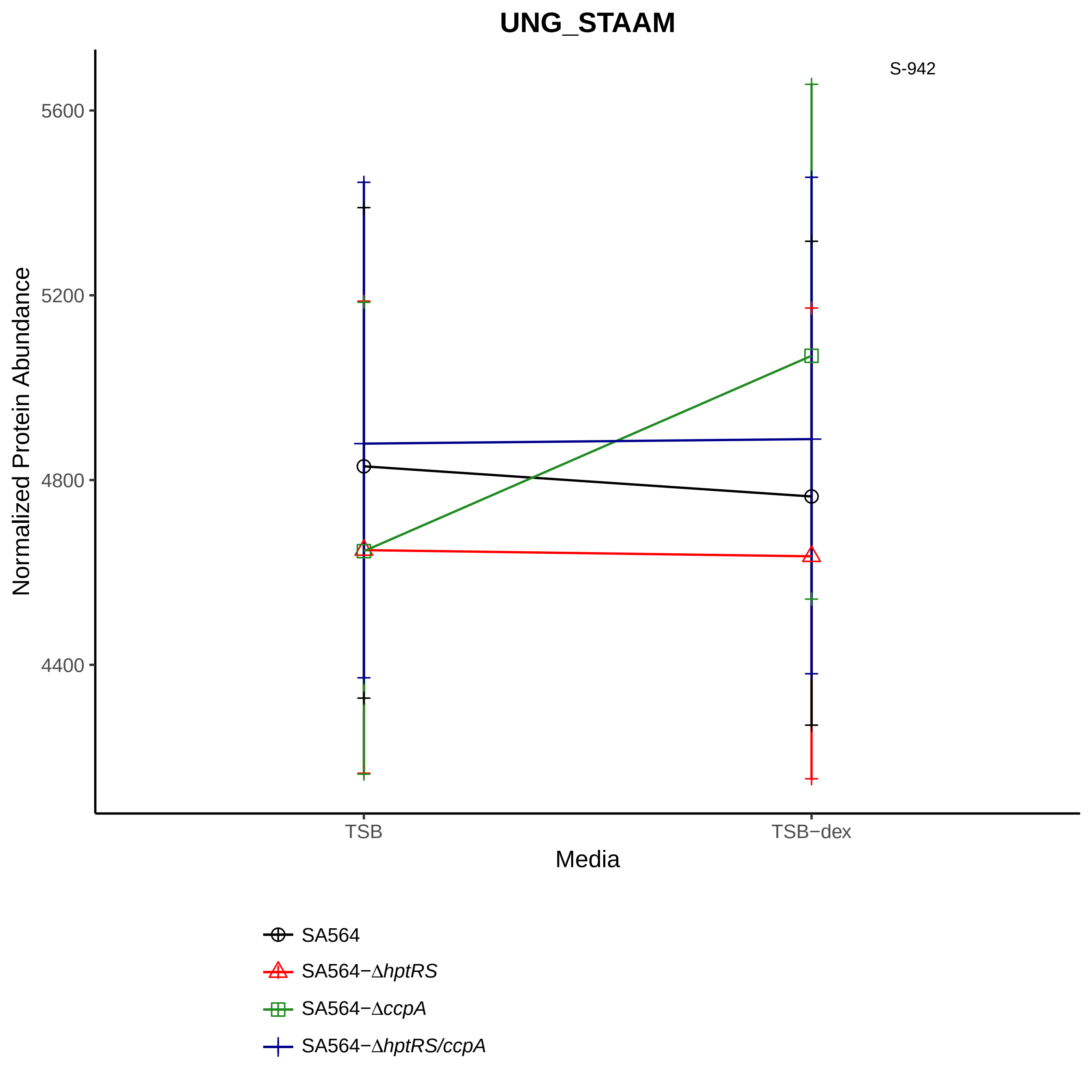

UNG_STAAM
S-942
Normalized Protein Abundance
5600
5200
4800
4400
TSB
TSB−dex
Media
SA564
SA564−ΔhptRS
SA564−ΔccpA
SA564−ΔhptRS/ccpA

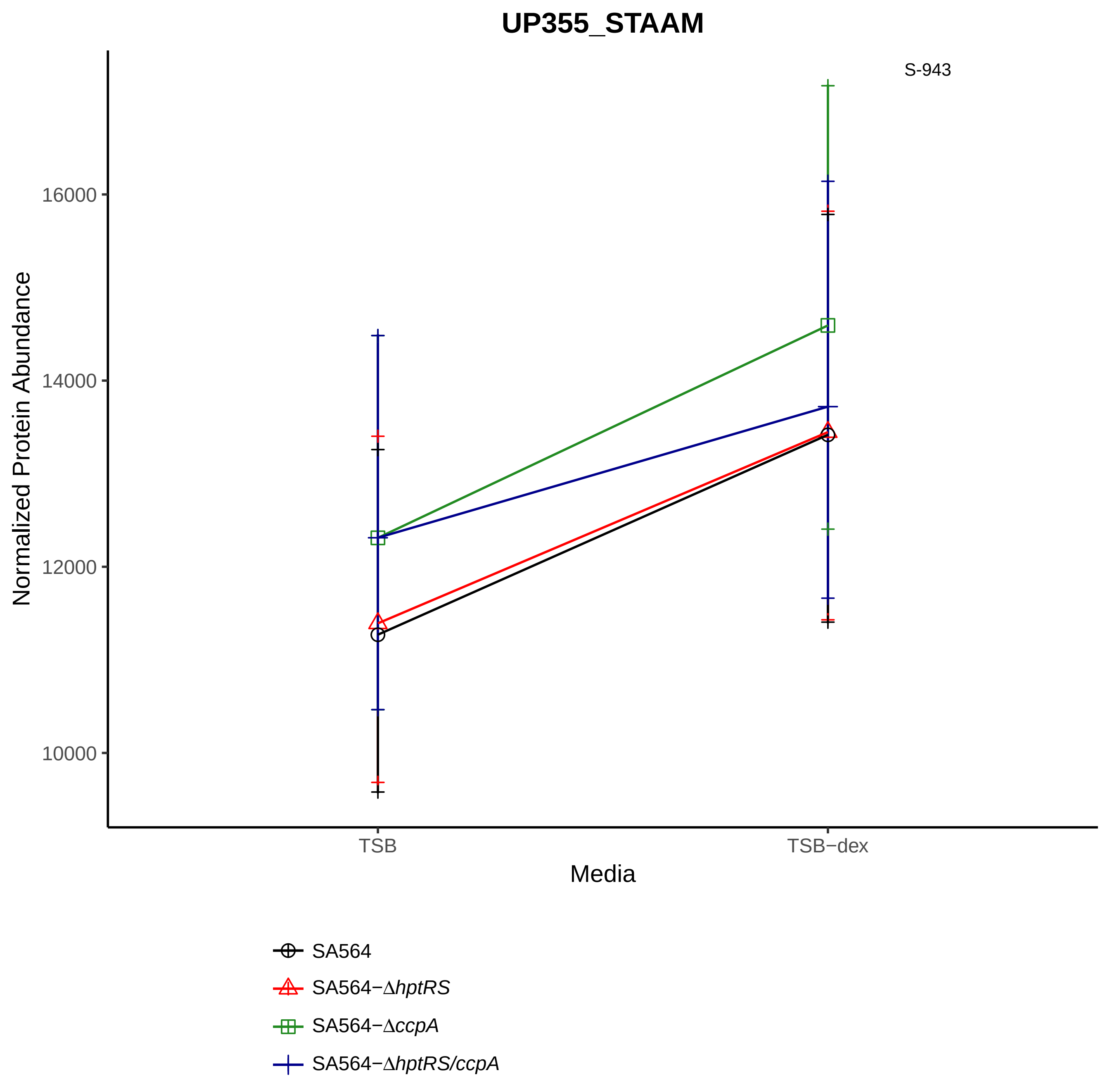

UP355_STAAM
S-943
Normalized Protein Abundance
16000
14000
12000
10000
TSB
TSB−dex
Media
SA564
SA564−ΔhptRS
SA564−ΔccpA
SA564−ΔhptRS/ccpA

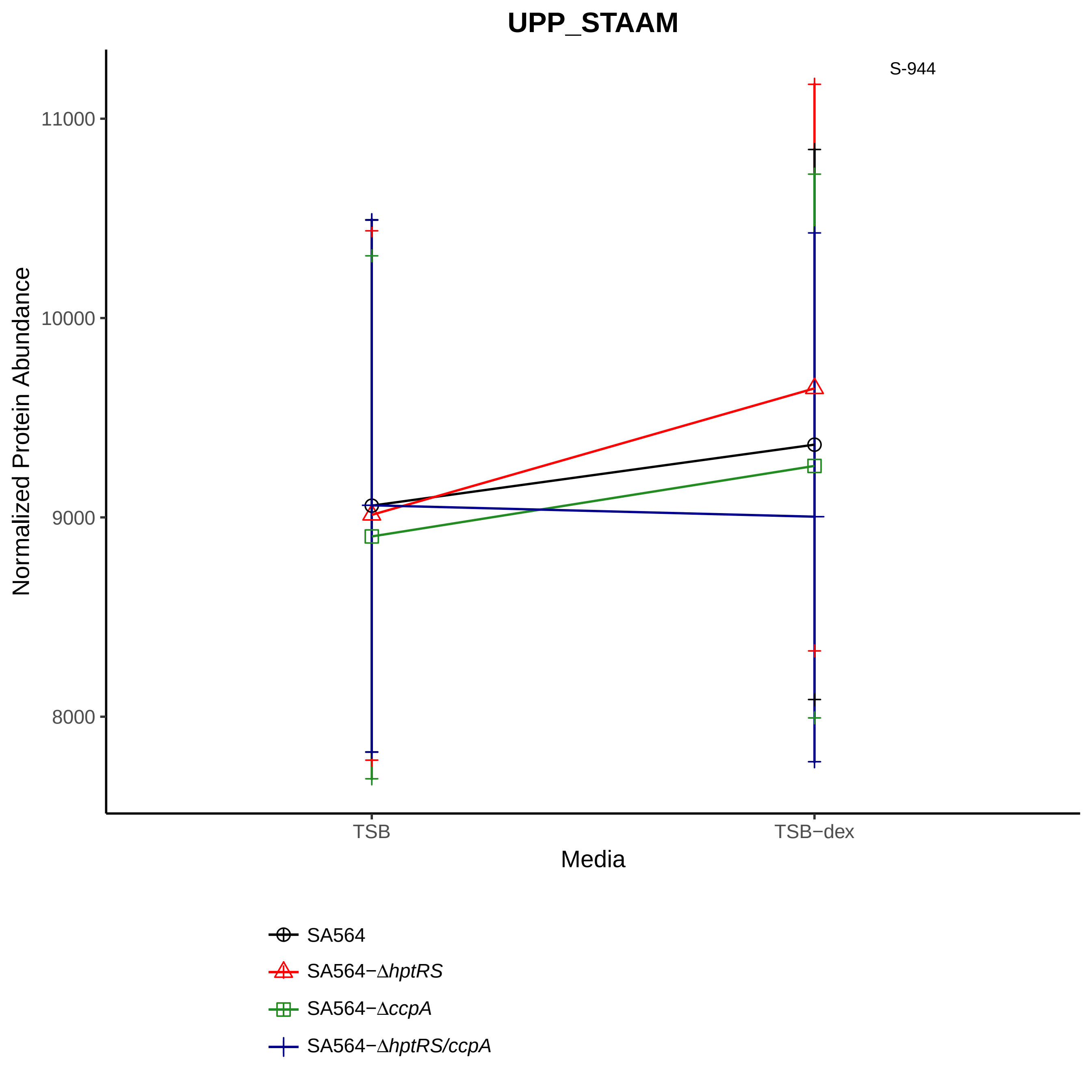

UPP_STAAM
S-944
Normalized Protein Abundance
11000
10000
9000
8000
TSB
TSB−dex
Media
SA564
SA564−Δ*hptRS*
SA564−Δ*ccpA*
SA564−Δ*hptRS/ccpA*

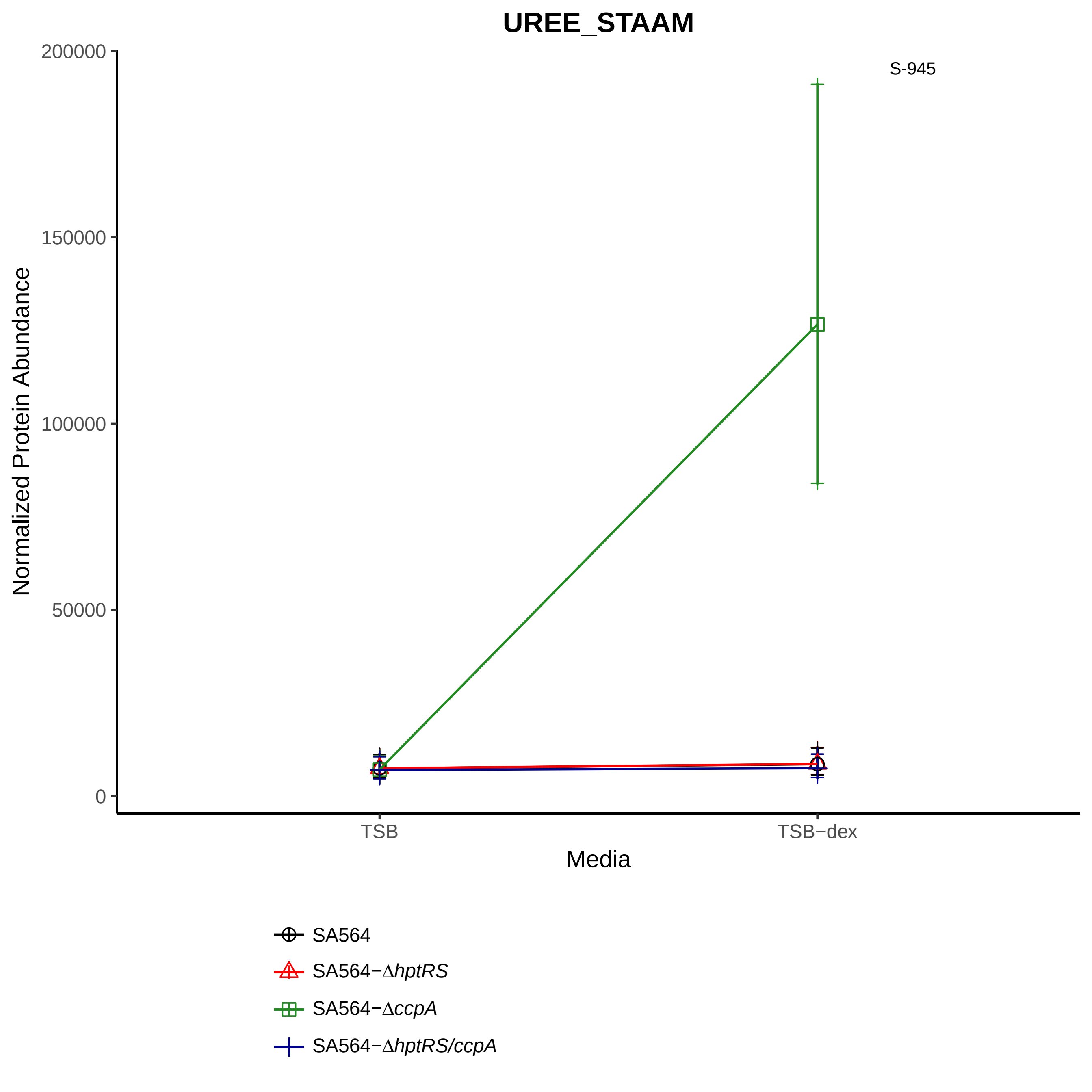
UREE_STAAM
S-945
Normalized Protein Abundance
200000
150000
100000
50000
0
TSB
TSB−dex
Media
SA564
SA564−Δ*hptRS*
SA564−Δ*ccpA*
SA564−Δ*hptRS/ccpA*

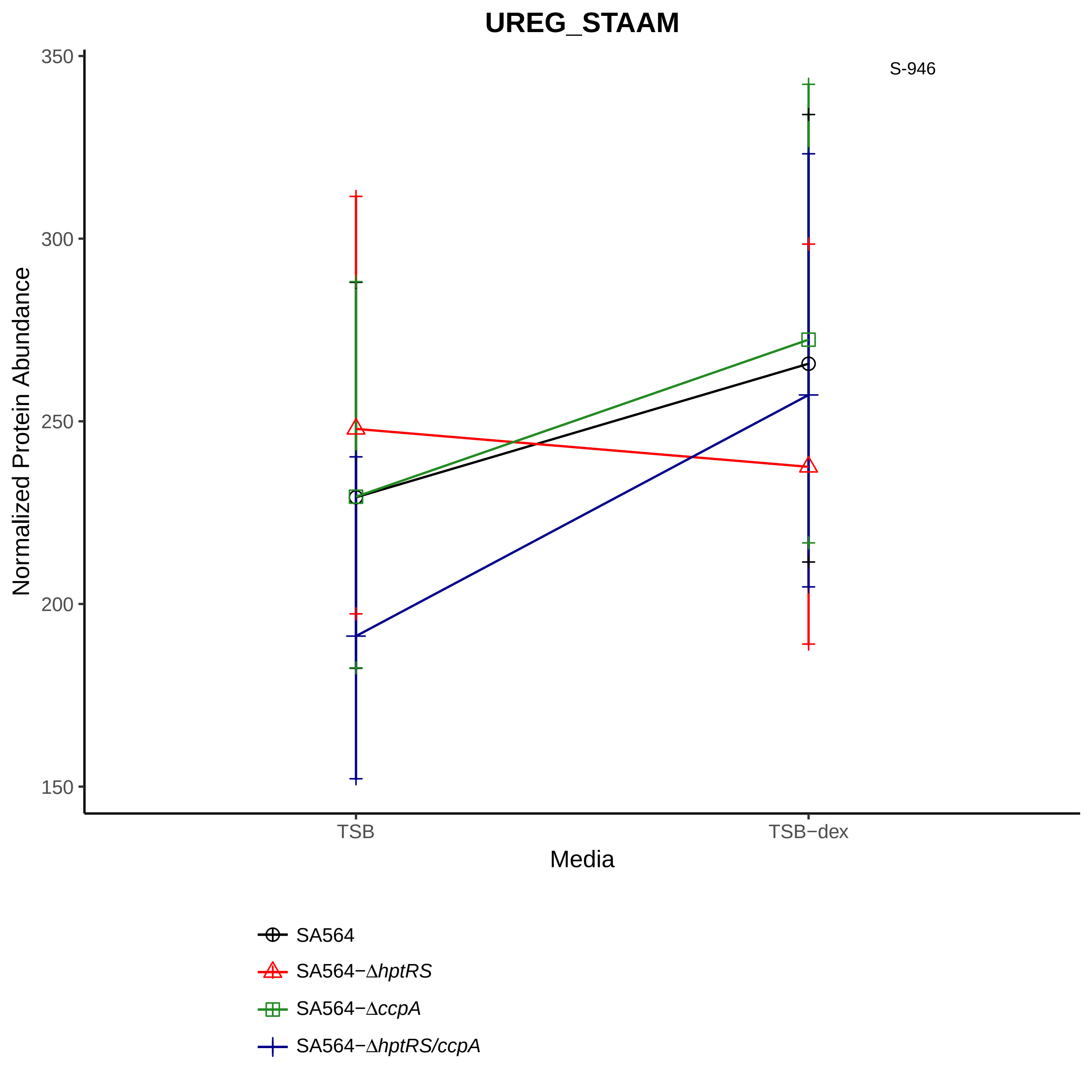
UREG_STAAM
S-946
Normalized Protein Abundance
350
300
250
200
150
TSB
TSB−dex
Media
SA564
SA564−Δ*hptRS*
SA564−Δ*ccpA*
SA564−Δ*hptRS/ccpA*

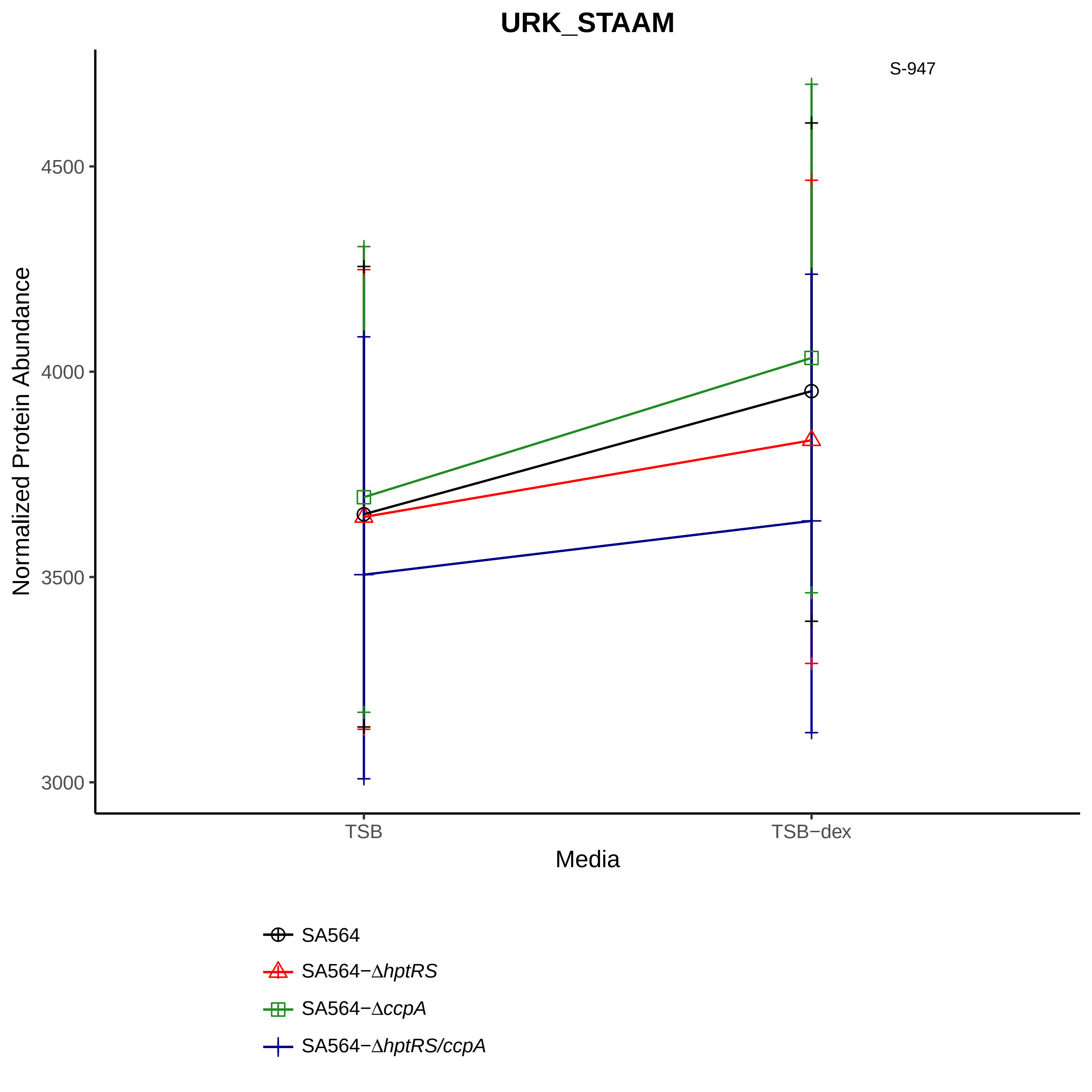
URK_STAAM
S-947
Normalized Protein Abundance
4500
4000
3500
3000
TSB
TSB−dex
Media
SA564
SA564−ΔhptRS
SA564−ΔccpA
SA564−ΔhptRS/ccpA

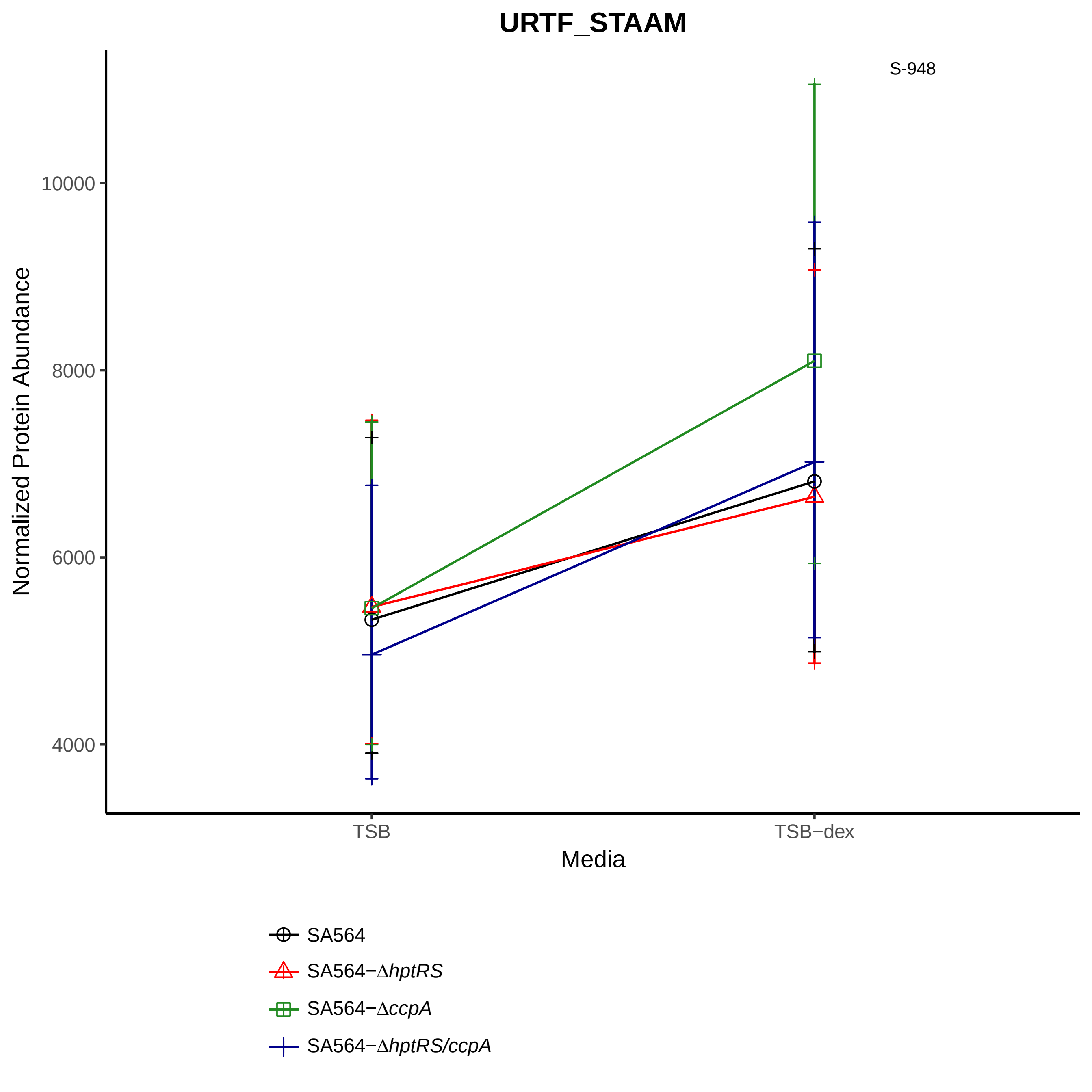

URTF_STAAM
S-948
Normalized Protein Abundance
10000
8000
6000
4000
TSB
TSB−dex
Media
SA564
SA564−Δ*hptRS*
SA564−Δ*ccpA*
SA564−Δ*hptRS/ccpA*

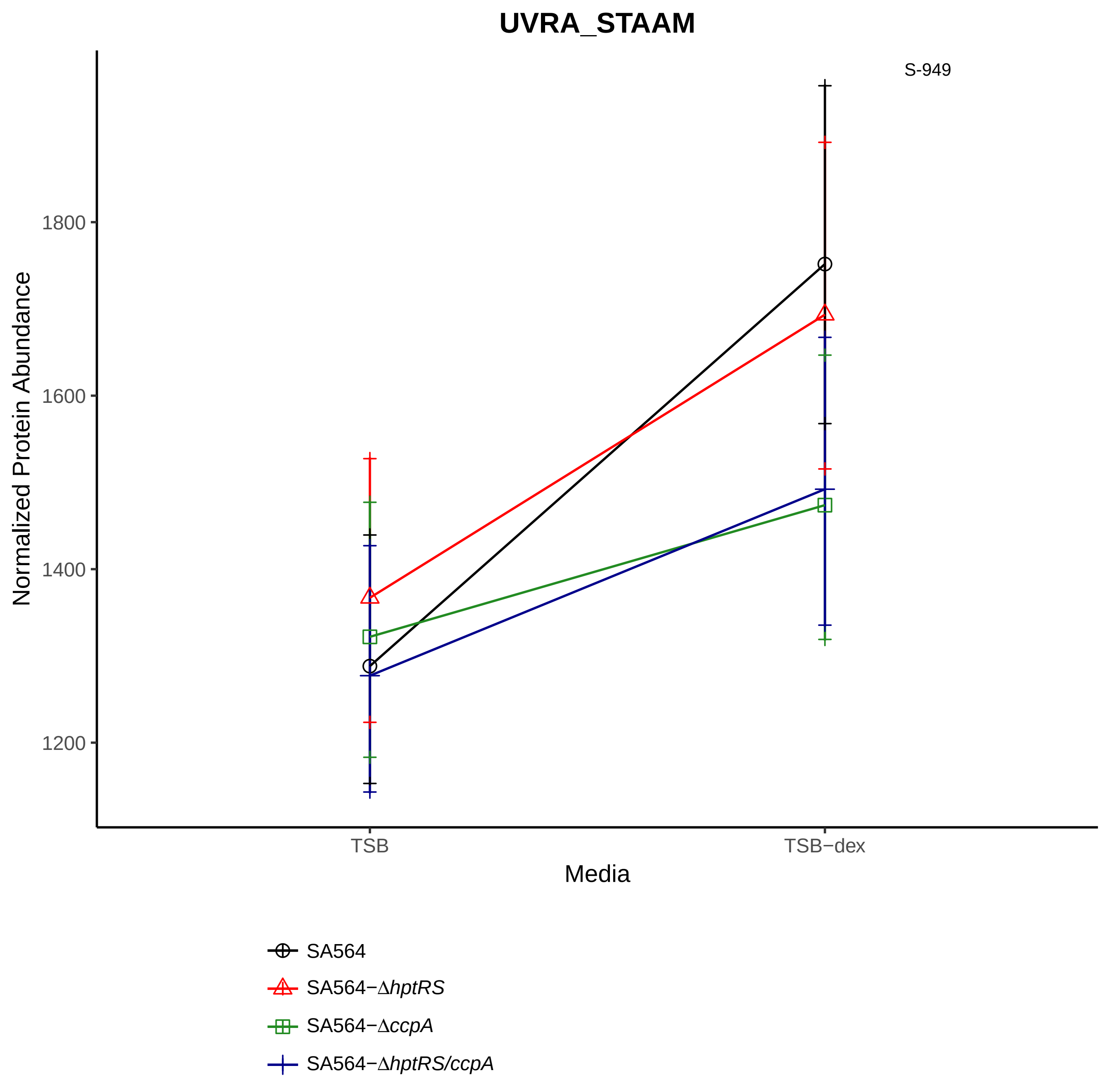
UVRA_STAAM
S-949
Normalized Protein Abundance
1800
1600
1400
1200
TSB
TSB−dex
Media
SA564
SA564−Δ*hptRS*
SA564−Δ*ccpA*
SA564−Δ*hptRS/ccpA*

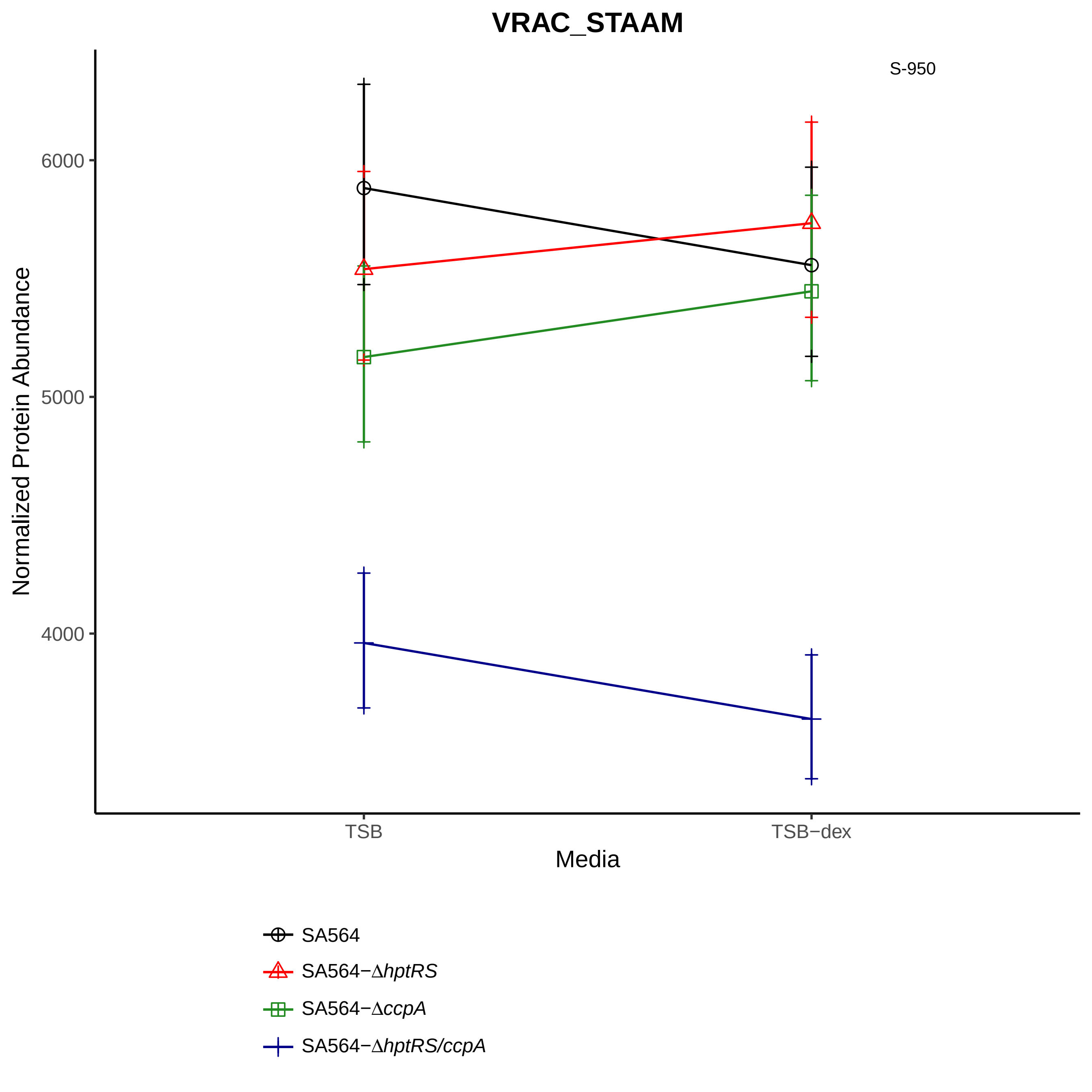

VRAC_STAAM
S-950
Normalized Protein Abundance
6000
5000
4000
TSB
TSB−dex
Media
SA564
SA564−Δ*hptRS*
SA564−Δ*ccpA*
SA564−Δ*hptRS/ccpA*

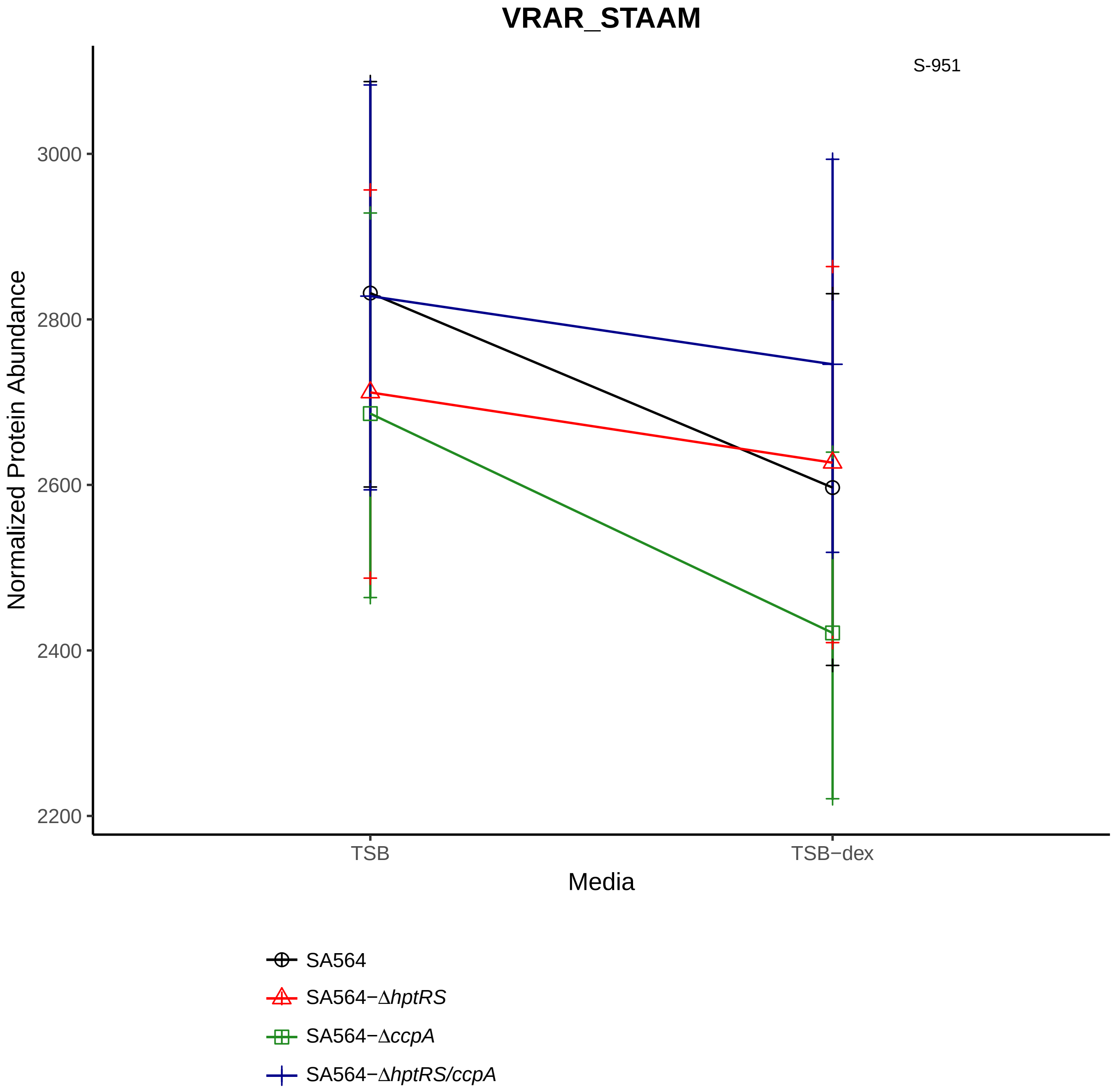

VRAR_STAAM
S-951
Normalized Protein Abundance
3000
2800
2600
2400
2200
TSB
TSB−dex
Media
SA564
SA564−Δ*hptRS*
SA564−Δ*ccpA*
SA564−Δ*hptRS/ccpA*

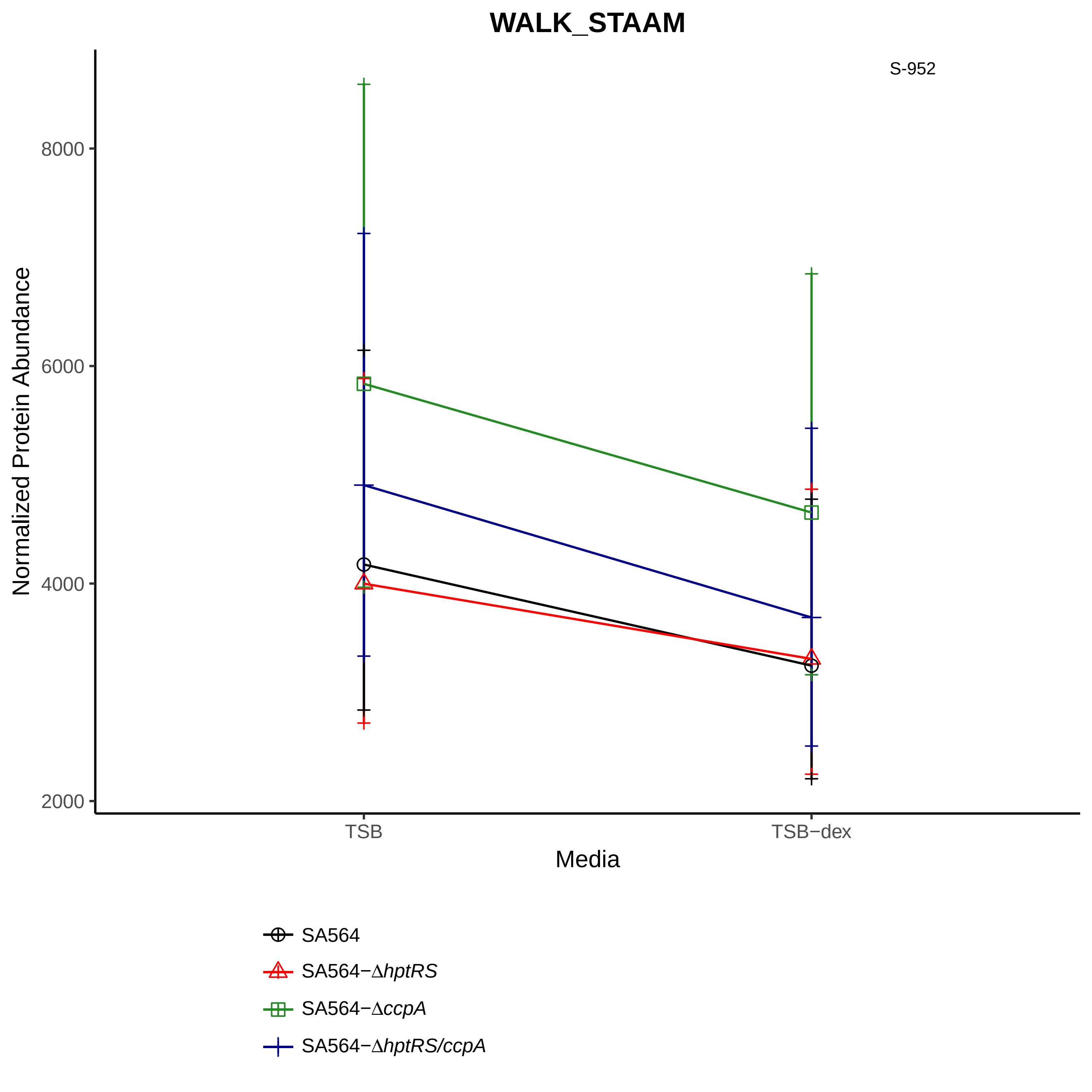

WALK_STAAM
S-952
Normalized Protein Abundance
8000
6000
4000
2000
TSB
TSB−dex
Media
SA564
SA564−Δ*hptRS*
SA564−Δ*ccpA*
SA564−Δ*hptRS/ccpA*

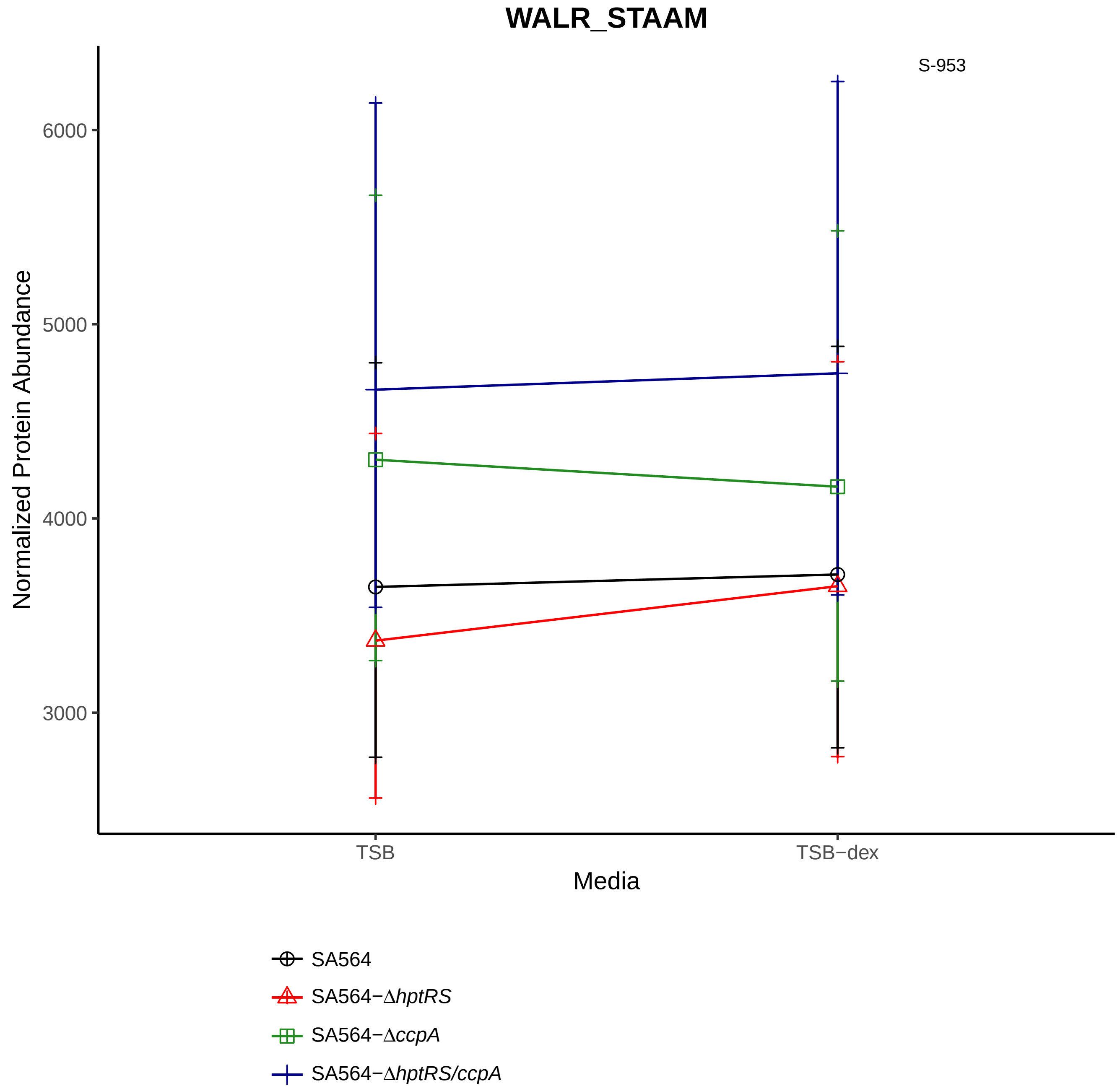

WALR_STAAM
S-953
6000
5000
4000
3000
Normalized Protein Abundance
TSB
TSB−dex
Media
SA564
SA564−ΔhptRS
SA564−ΔccpA
SA564−ΔhptRS/ccpA

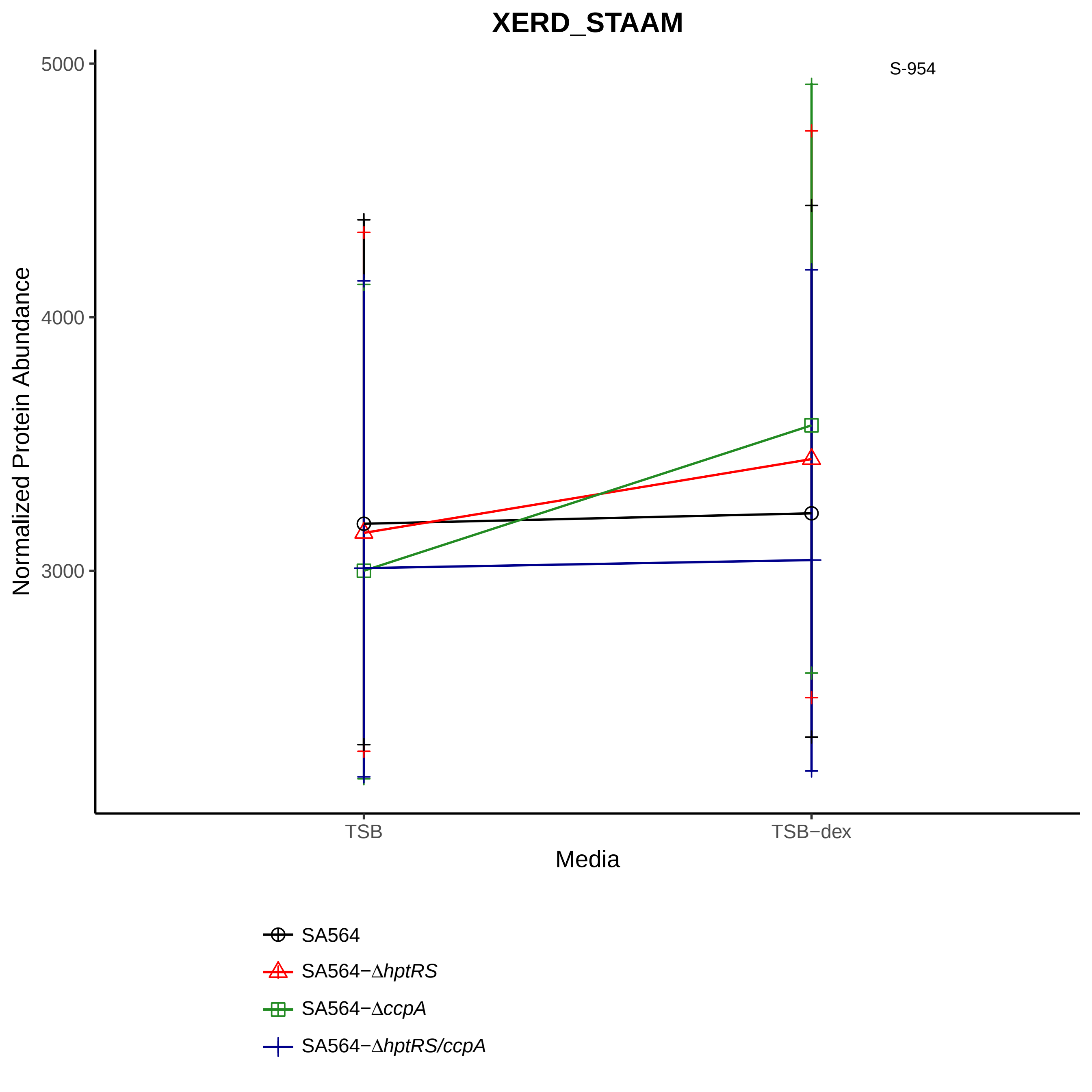

XERD_STAAM
S-954
5000
4000
3000
Normalized Protein Abundance
TSB
TSB−dex
Media
SA564
SA564−Δ*hptRS*
SA564−Δ*ccpA*
SA564−Δ*hptRS/ccpA*

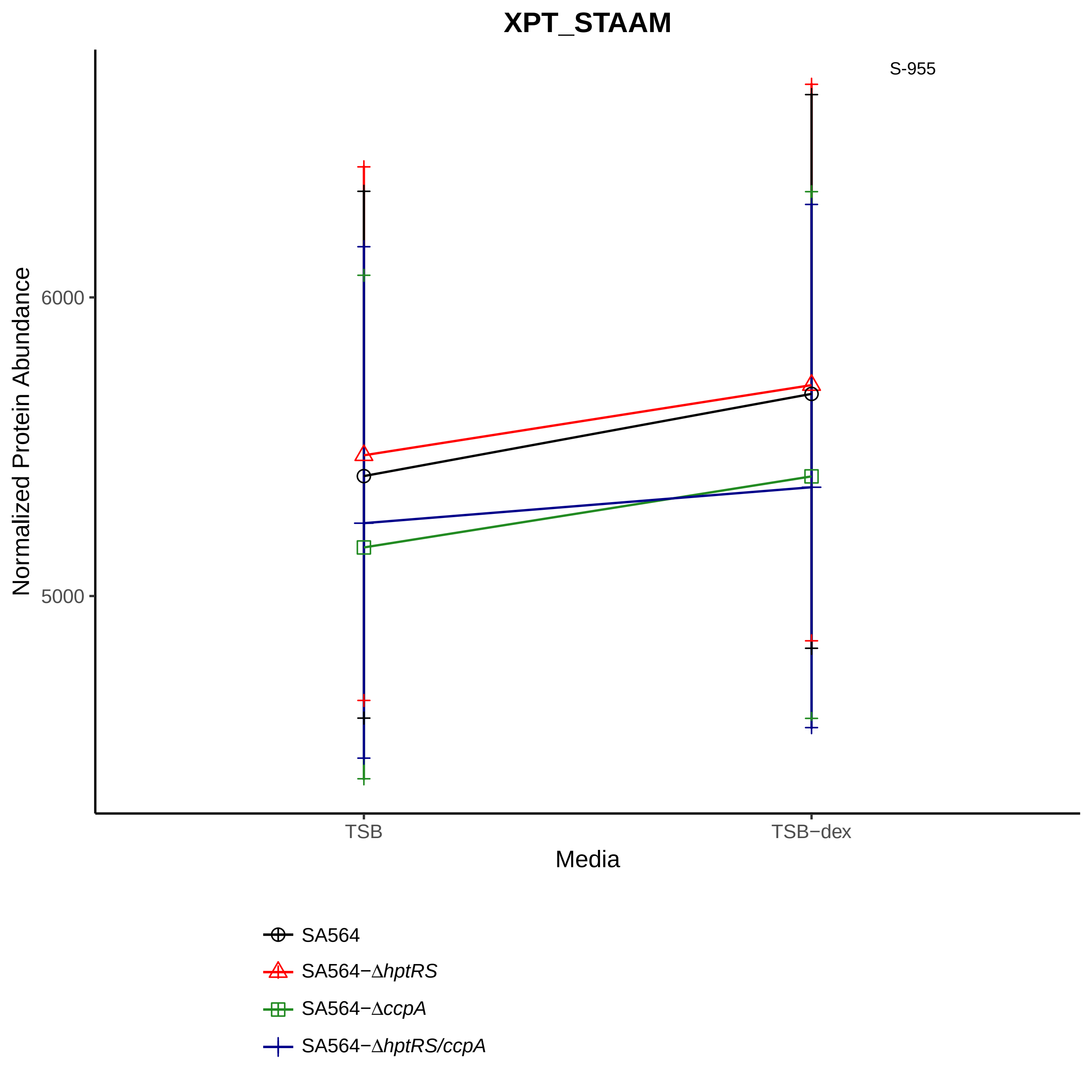

XPT_STAAM
S-955
Normalized Protein Abundance
6000
5000
TSB
TSB−dex
Media
SA564
SA564−ΔhptRS
SA564−ΔccpA
SA564−ΔhptRS/ccpA

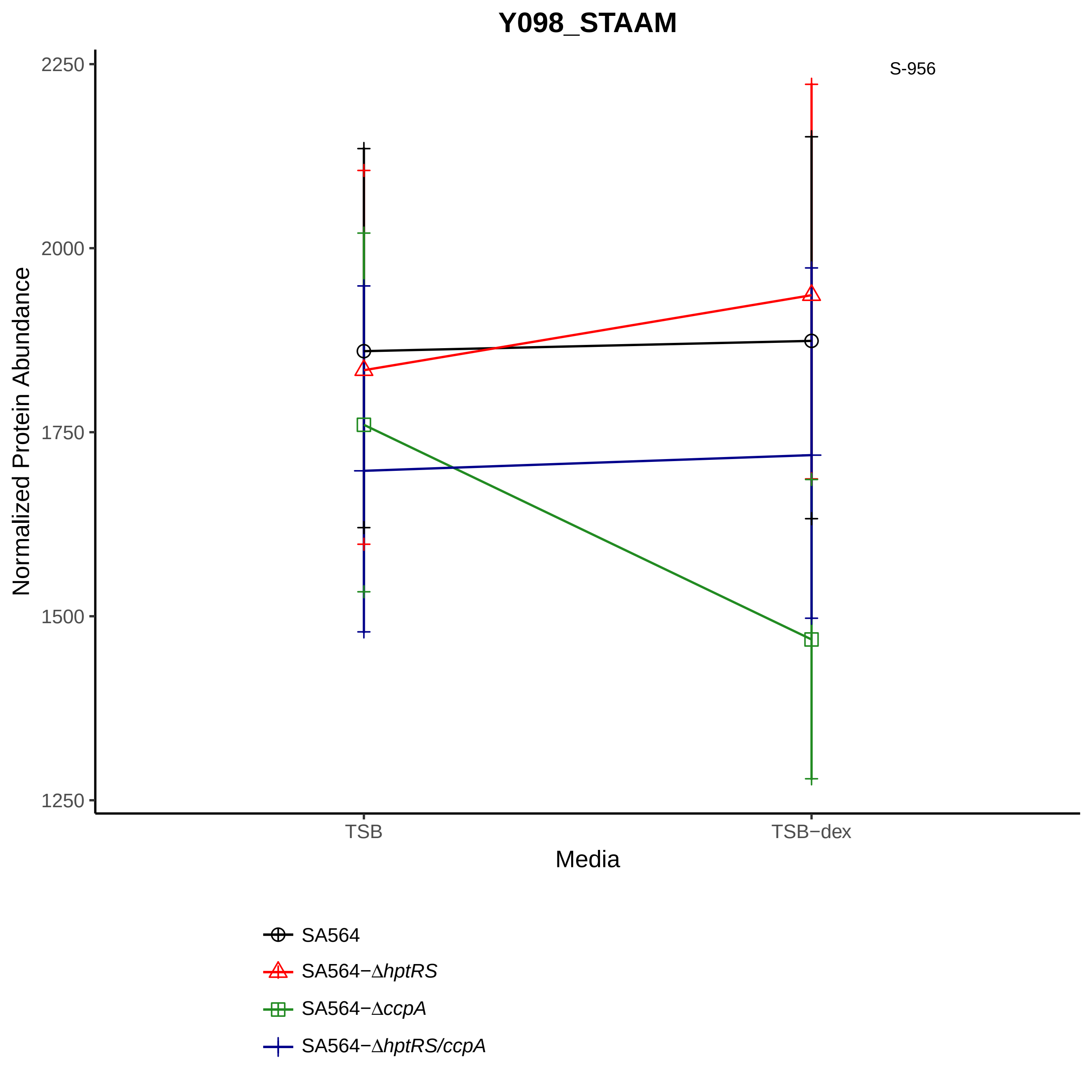

Y098_STAAM
S-956
Normalized Protein Abundance
2250
2000
1750
1500
1250
TSB
TSB−dex
Media
SA564
SA564−Δ*hptRS*
SA564−Δ*ccpA*
SA564−Δ*hptRS/ccpA*

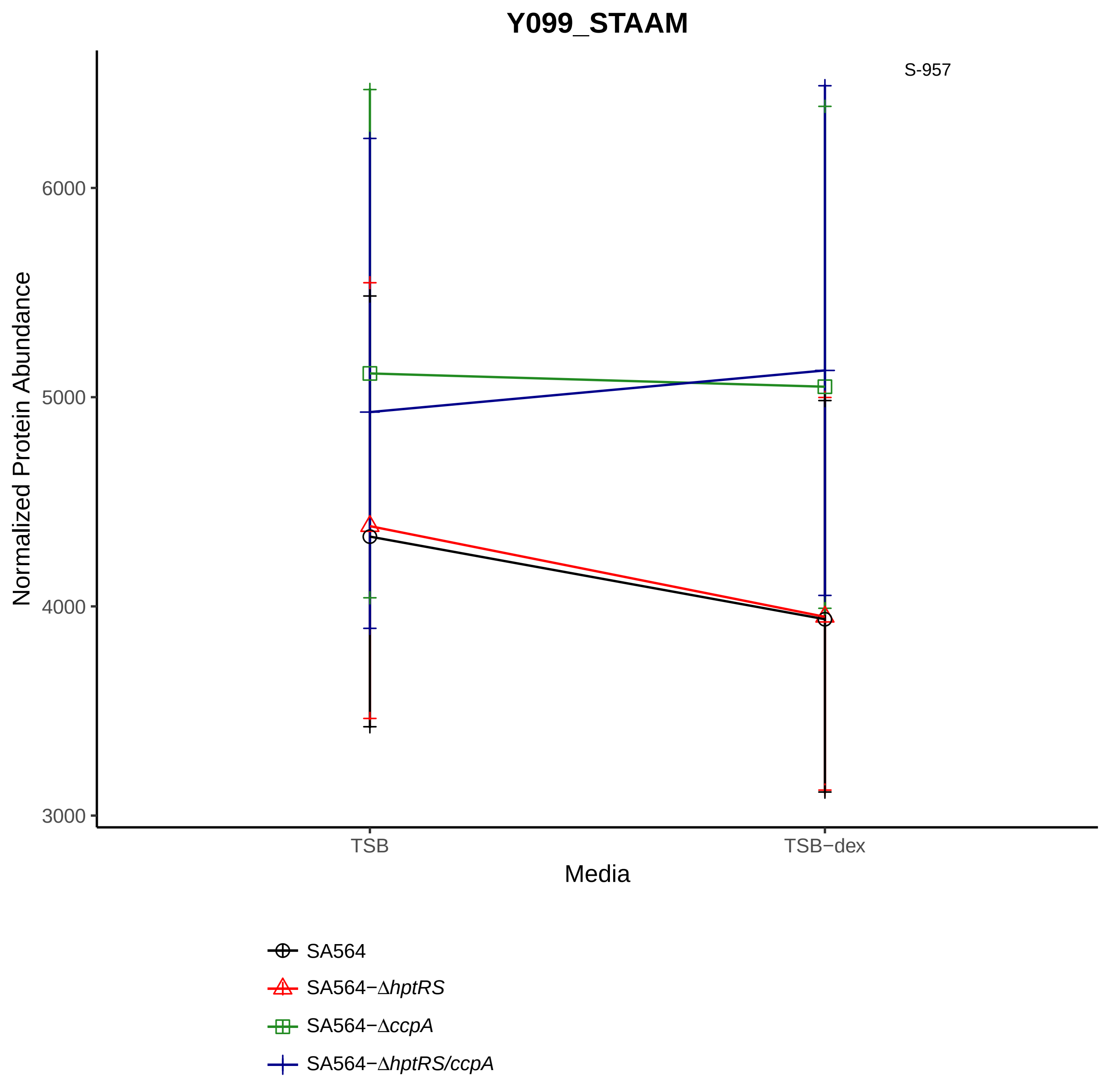

Y099_STAAM
S-957
Normalized Protein Abundance
6000
5000
4000
3000
TSB
TSB−dex
Media
SA564
SA564−ΔhptRS
SA564−ΔccpA
SA564−ΔhptRS/ccpA

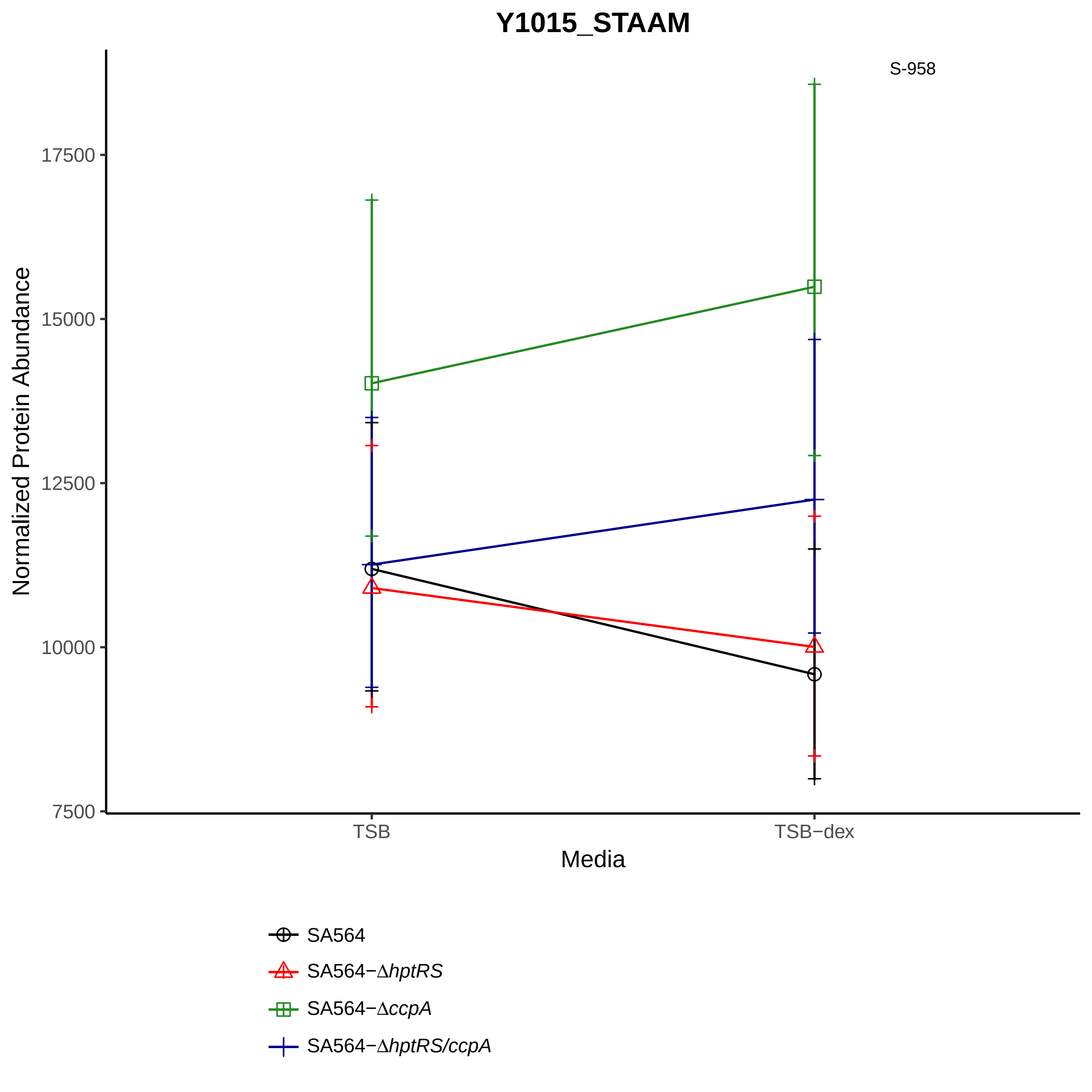

Y1015_STAAM
S-958
Normalized Protein Abundance
17500
15000
12500
10000
7500
TSB
TSB−dex
Media
SA564
SA564−Δ*hptRS*
SA564−Δ*ccpA*
SA564−Δ*hptRS/ccpA*

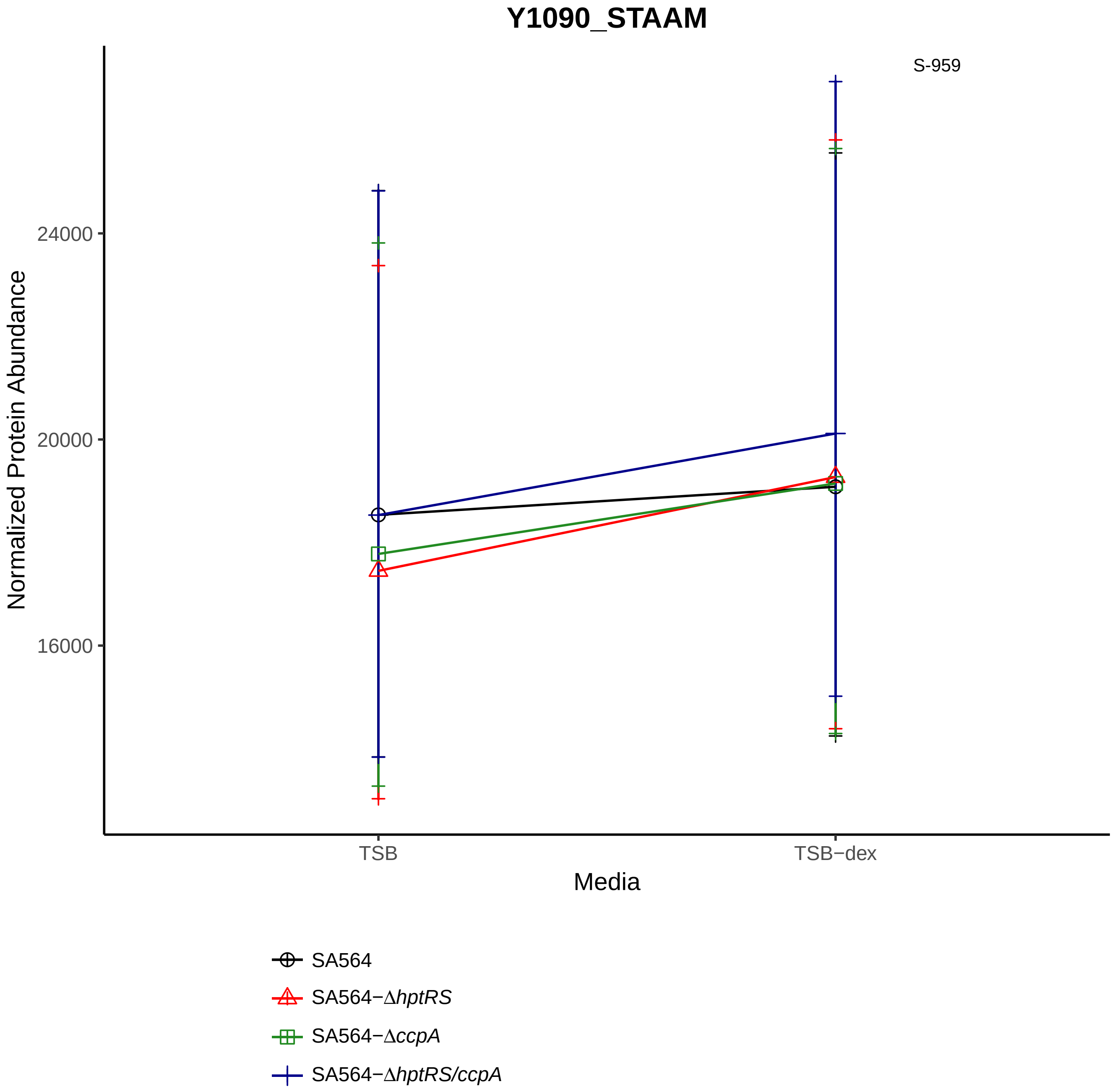
Y1090_STAAM
S-959
Normalized Protein Abundance
24000
20000
16000
TSB
TSB−dex
Media
SA564
SA564−ΔhptRS
SA564−ΔccpA
SA564−ΔhptRS/ccpA

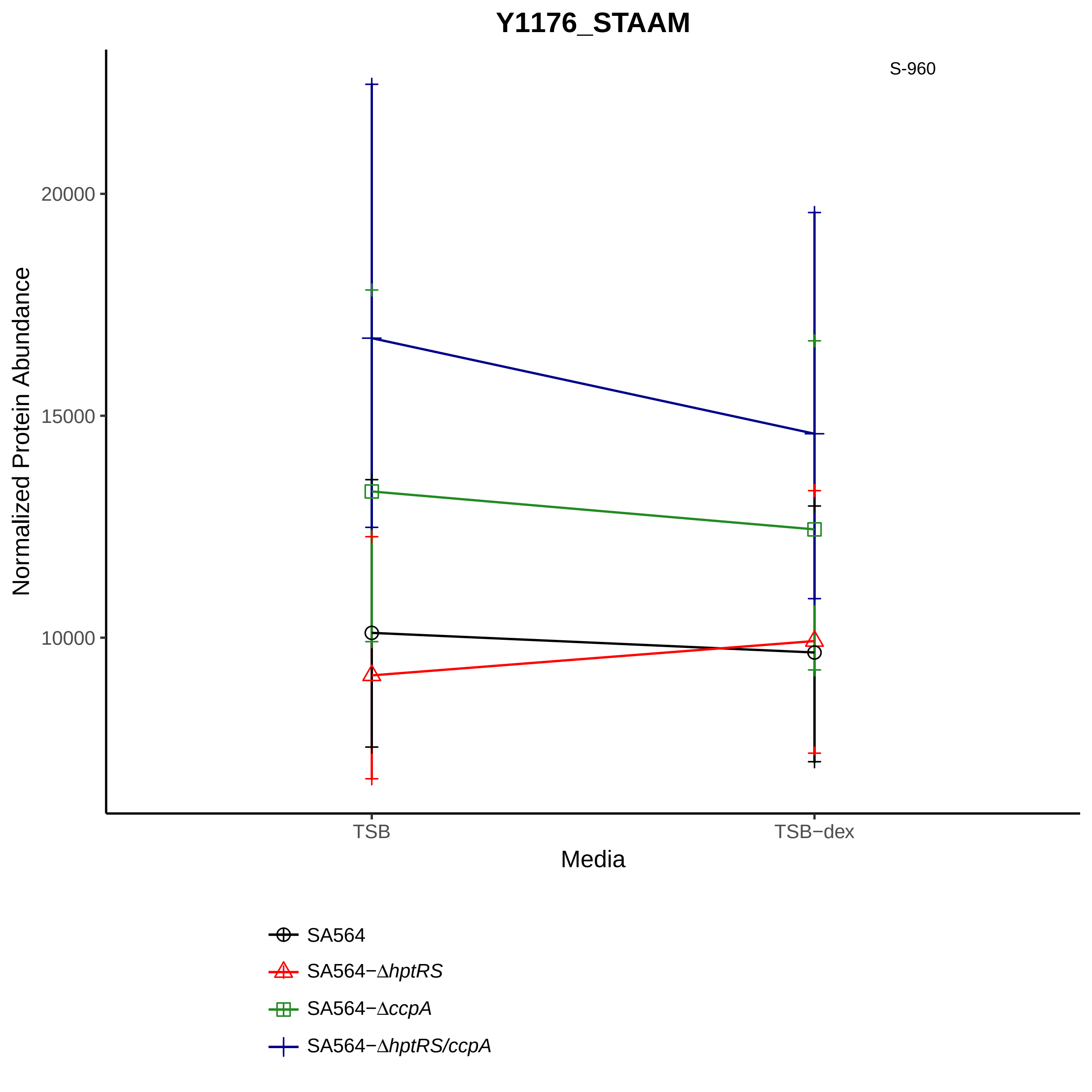

Y1176_STAAM
S-960
Normalized Protein Abundance
20000
15000
10000
TSB
TSB−dex
Media
SA564
SA564−ΔhptRS
SA564−ΔccpA
SA564−ΔhptRS/ccpA

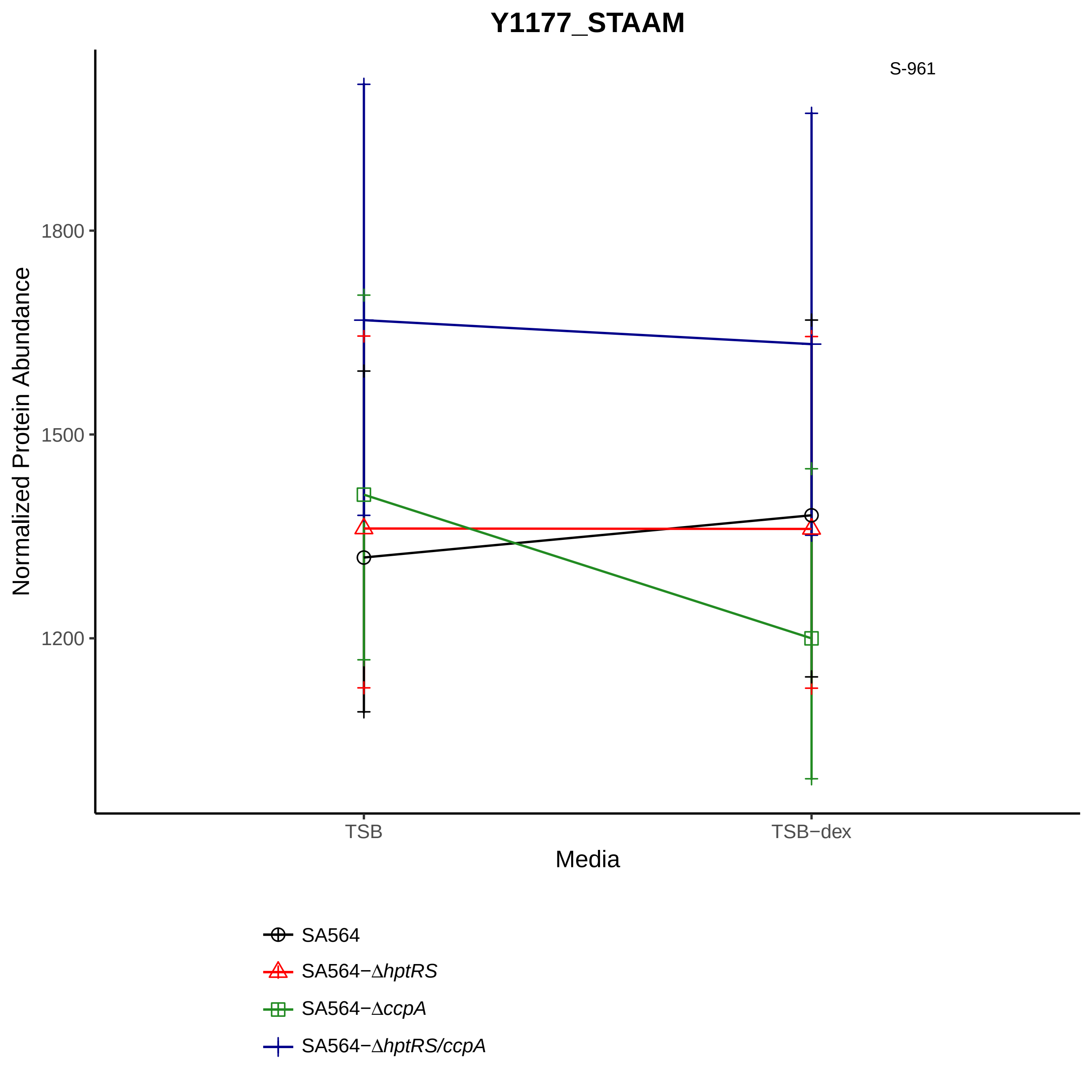

Y1177_STAAM
S-961
Normalized Protein Abundance
1800
1500
1200
TSB
TSB−dex
Media
SA564
SA564−Δ*hptRS*
SA564−Δ*ccpA*
SA564−Δ*hptRS/ccpA*

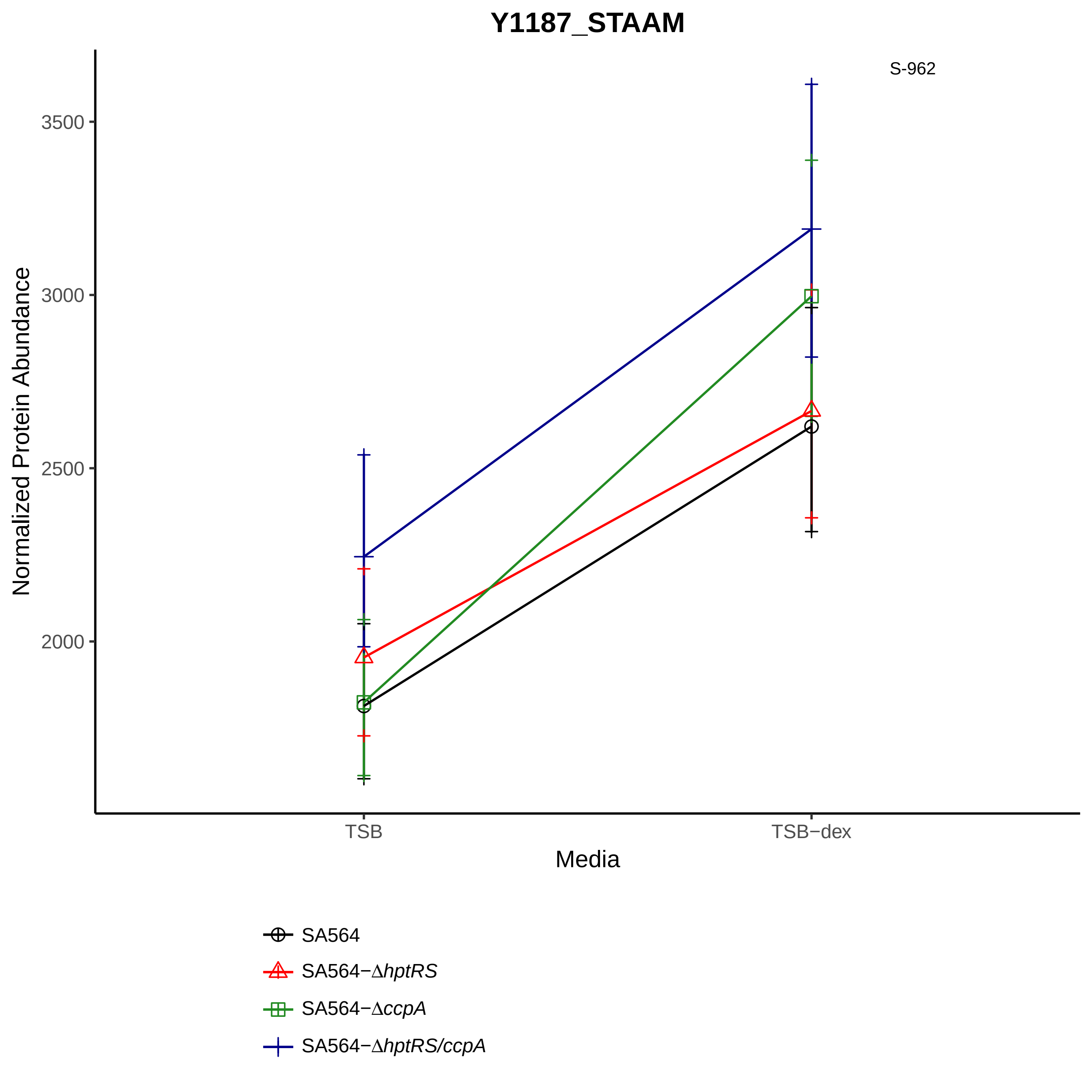

Y1187_STAAM
S-962
3500
3000
2500
2000
Normalized Protein Abundance
TSB
TSB−dex
Media
SA564
SA564−ΔhptRS
SA564−ΔccpA
SA564−ΔhptRS/ccpA

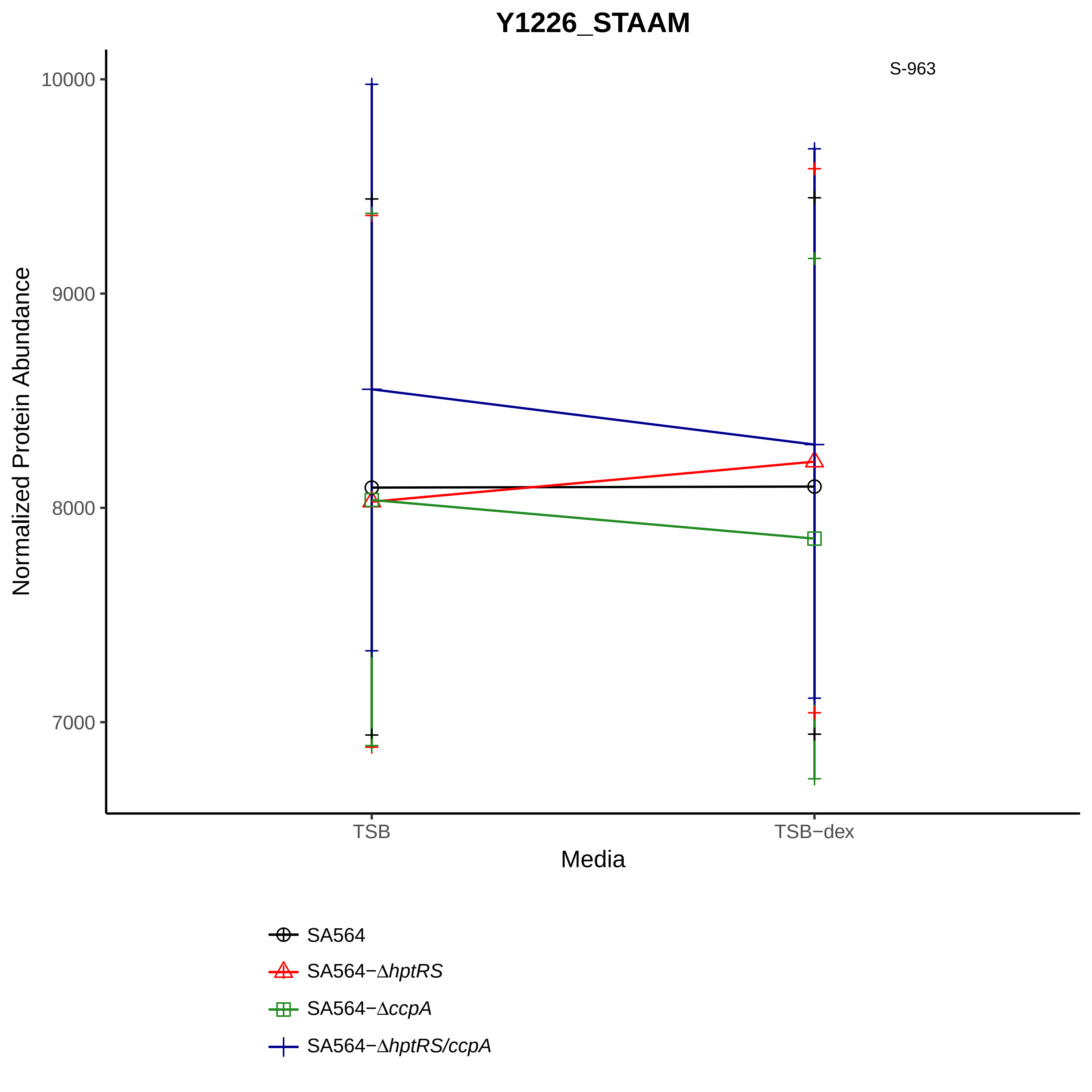

Y1226_STAAM
S-963
Normalized Protein Abundance
10000
9000
8000
7000
TSB
TSB−dex
Media
SA564
SA564−ΔhptRS
SA564−ΔccpA
SA564−ΔhptRS/ccpA

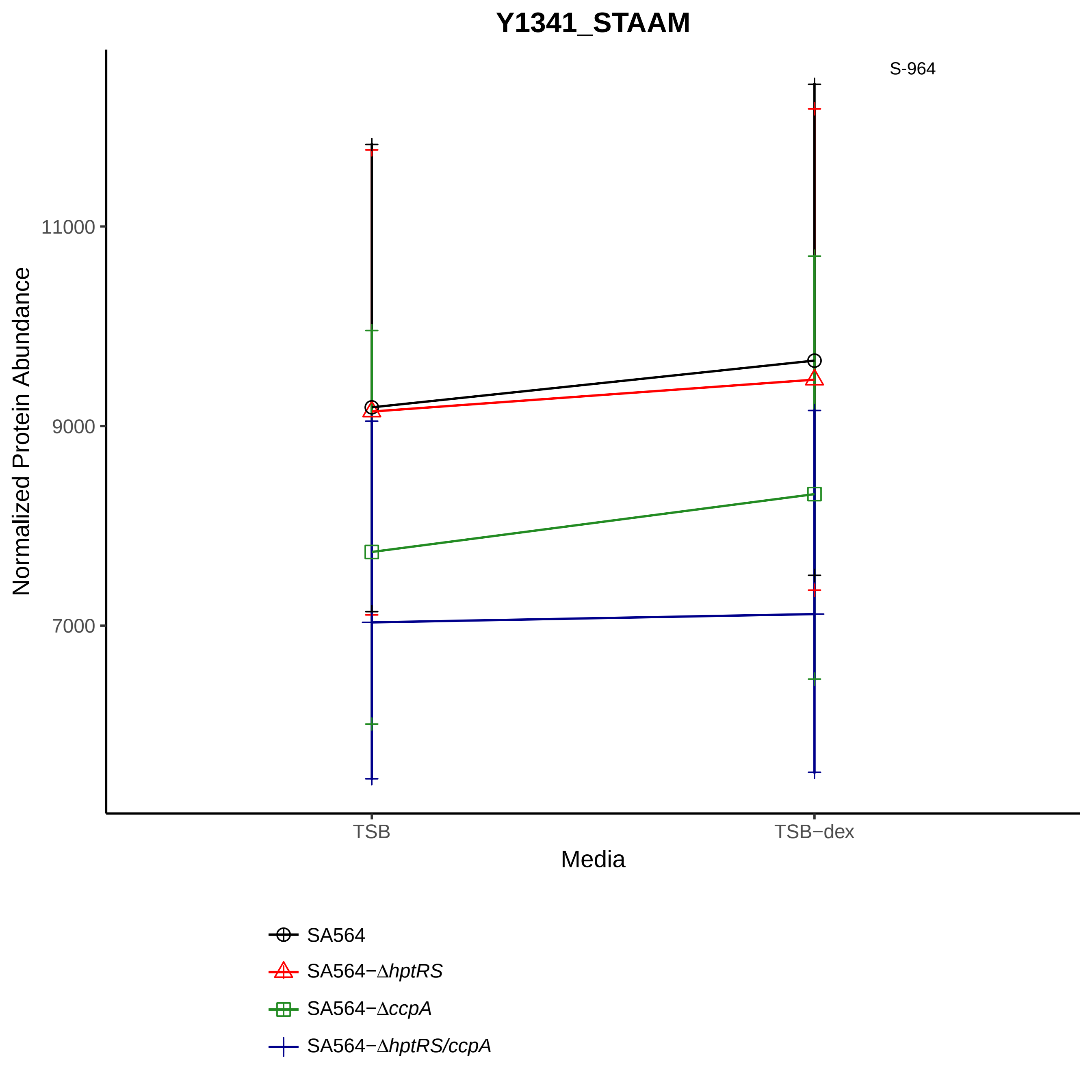

Y1341_STAAM
S-964
Normalized Protein Abundance
11000
9000
7000
TSB
TSB−dex
Media
SA564
SA564−Δ*hptRS*
SA564−Δ*ccpA*
SA564−Δ*hptRS/ccpA*

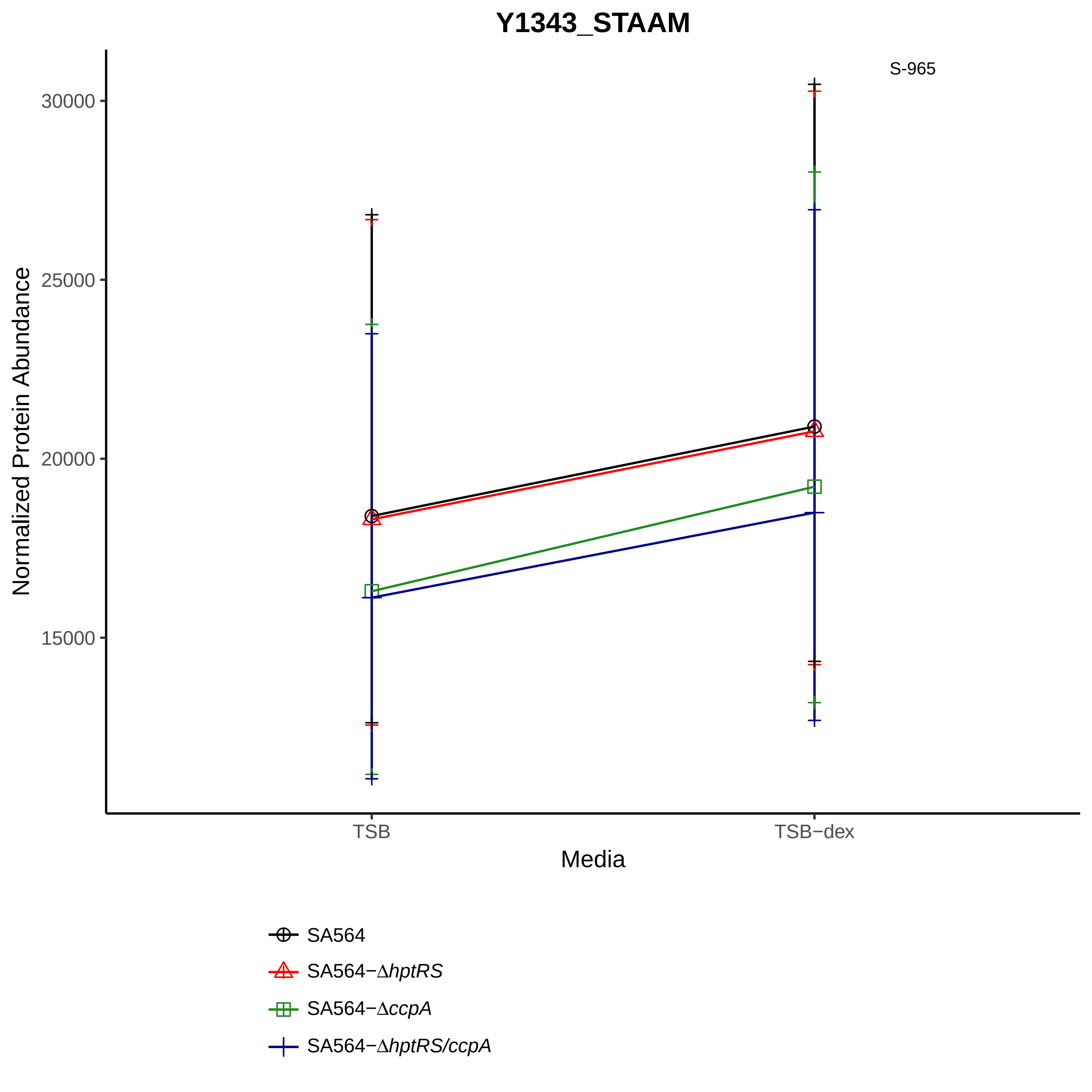
Y1343_STAAM
S-965
Normalized Protein Abundance
30000
25000
20000
15000
TSB
TSB−dex
Media
SA564
SA564−Δ*hptRS*
SA564−Δ*ccpA*
SA564−Δ*hptRS/ccpA*

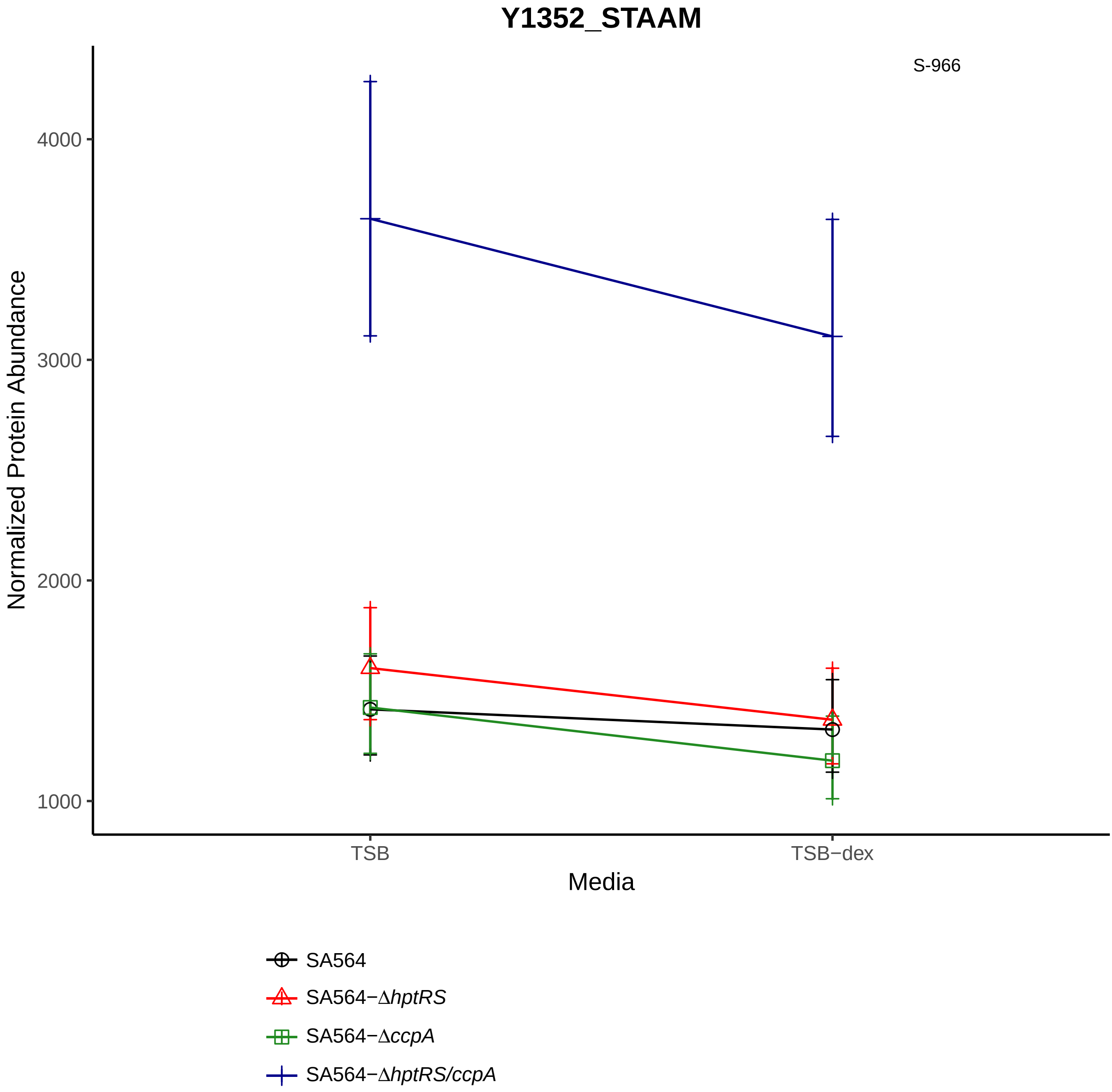

Y1352_STAAM
S-966
Normalized Protein Abundance
4000
3000
2000
1000
TSB
TSB−dex
Media
SA564
SA564−ΔhptRS
SA564−ΔccpA
SA564−ΔhptRS/ccpA

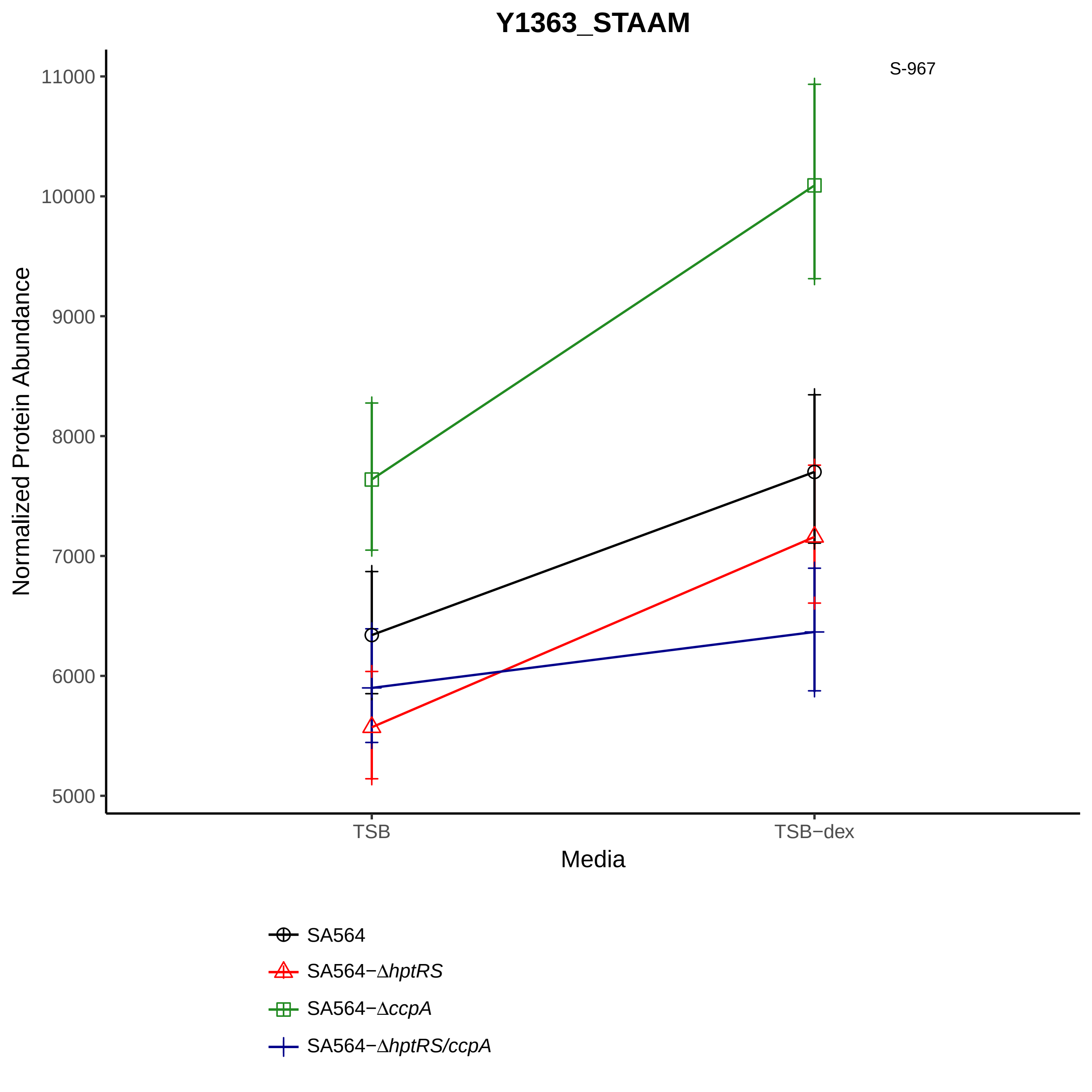

Y1363_STAAM
S-967
Normalized Protein Abundance
11000
10000
9000
8000
7000
6000
5000
TSB
TSB−dex
Media
SA564
SA564−Δ*hptRS*
SA564−Δ*ccpA*
SA564−Δ*hptRS/ccpA*

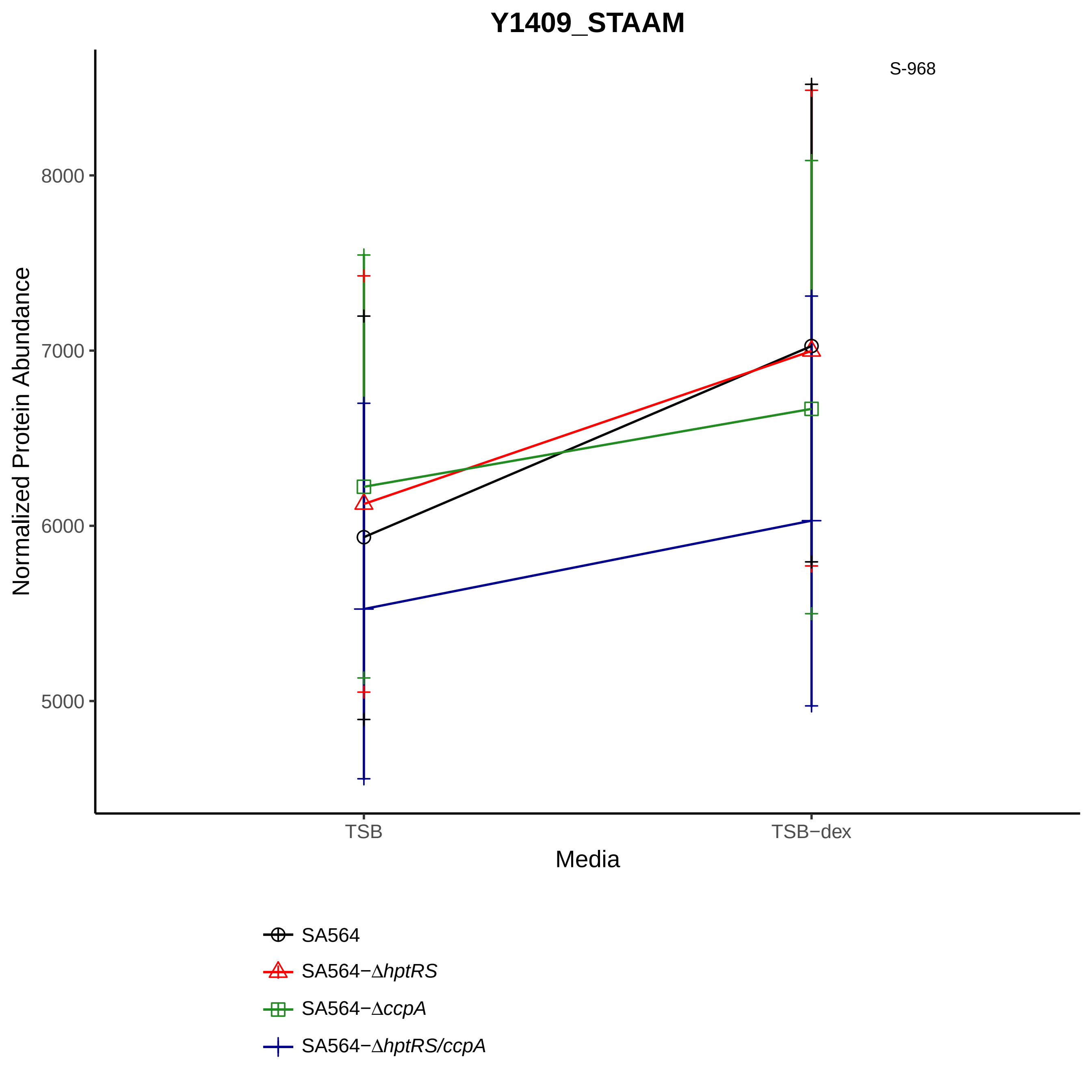
Y1409_STAAM
S-968
Normalized Protein Abundance
8000
7000
6000
5000
TSB
TSB−dex
Media
SA564
SA564−Δ*hptRS*
SA564−Δ*ccpA*
SA564−Δ*hptRS/ccpA*

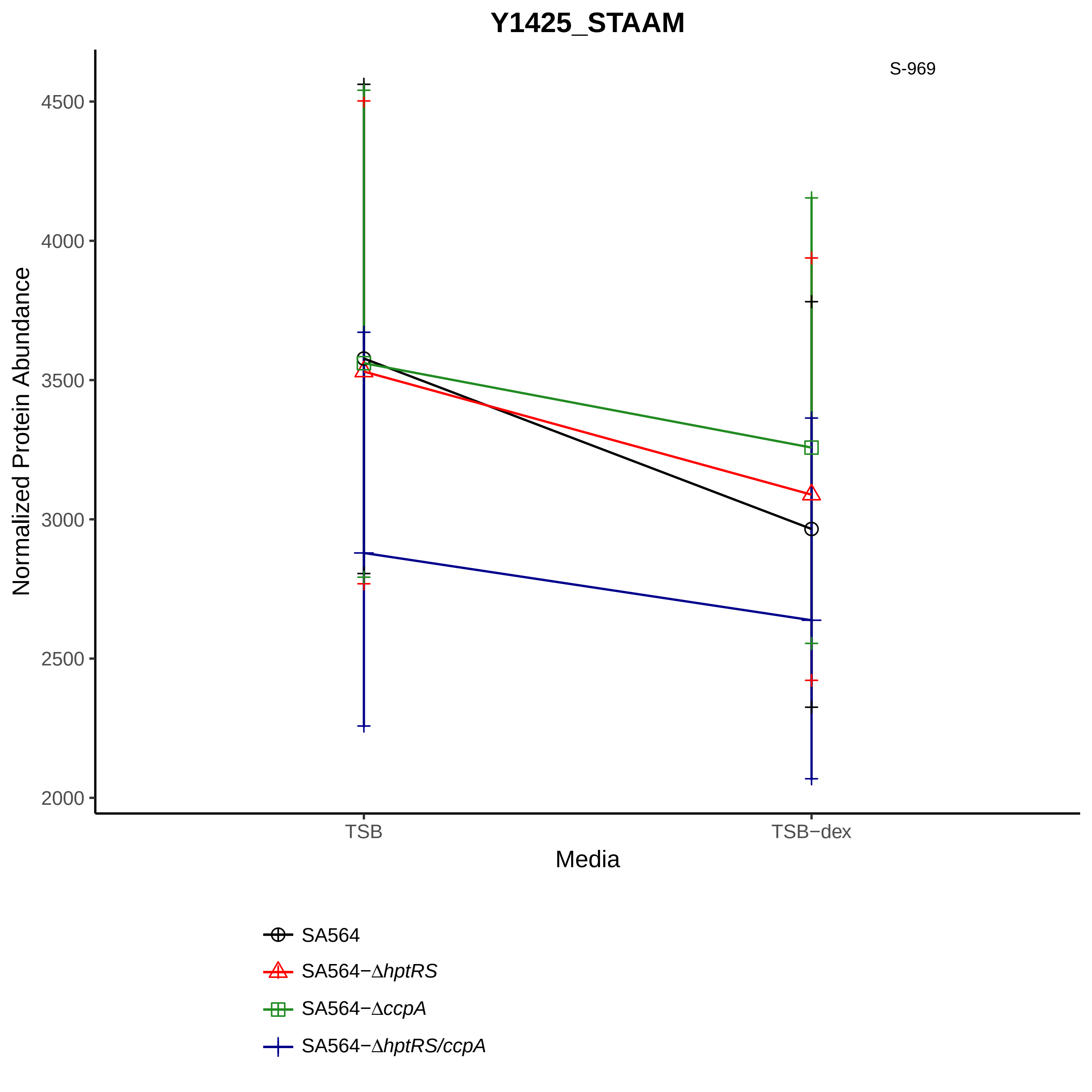
Y1425_STAAM
S-969
Normalized Protein Abundance
4500
4000
3500
3000
2500
2000
TSB
TSB−dex
Media
SA564
SA564−ΔhptRS
SA564−ΔccpA
SA564−ΔhptRS/ccpA

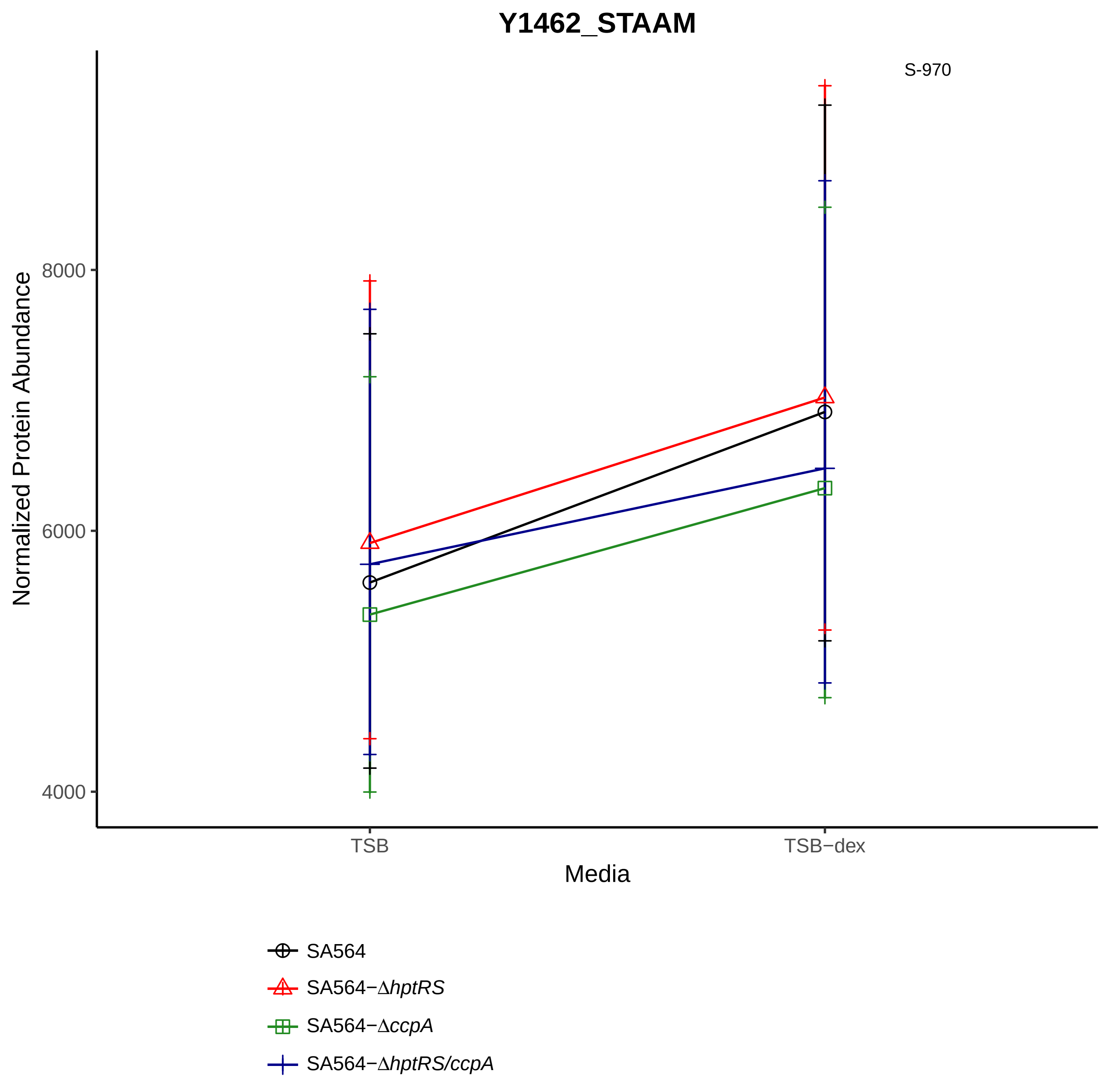

Y1462_STAAM
S-970
Normalized Protein Abundance
8000
6000
4000
TSB
TSB−dex
Media
SA564
SA564−ΔhptRS
SA564−ΔccpA
SA564−ΔhptRS/ccpA

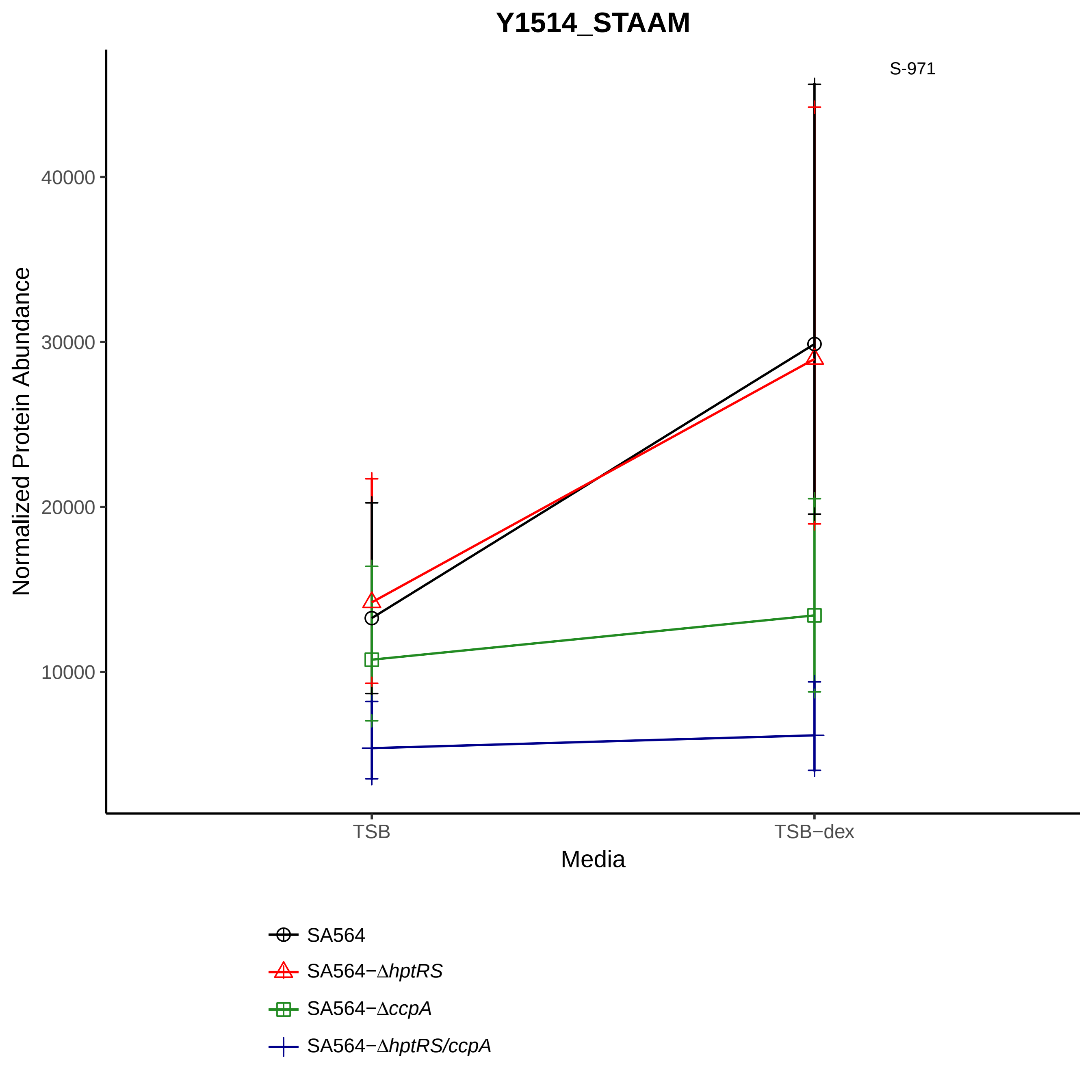

Y1514_STAAM
S-971
Normalized Protein Abundance
40000
30000
20000
10000
TSB
TSB−dex
Media
SA564
SA564−Δ*hptRS*
SA564−Δ*ccpA*
SA564−Δ*hptRS/ccpA*

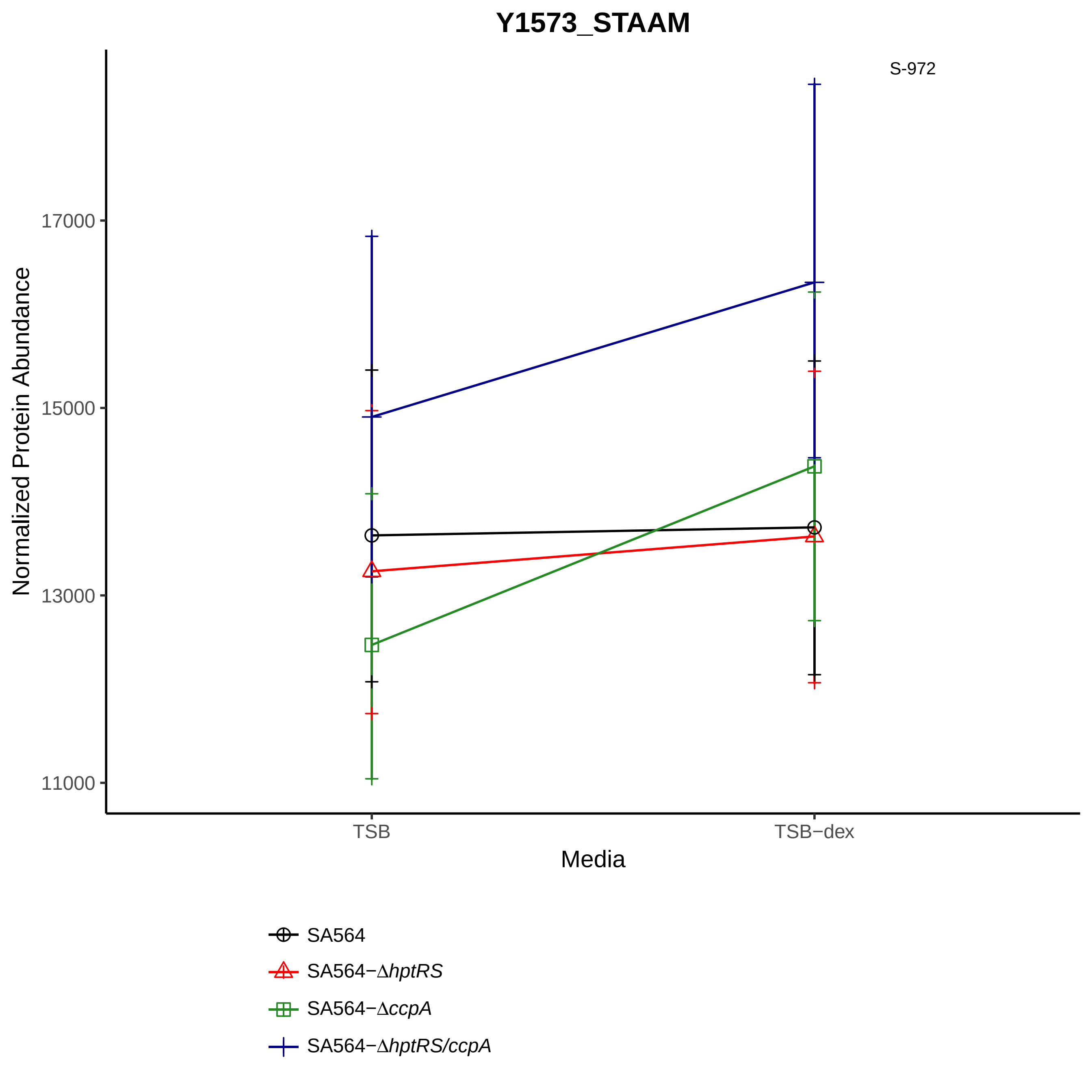

Y1573_STAAM
S-972
Normalized Protein Abundance
17000
15000
13000
11000
TSB
TSB−dex
Media
SA564
SA564−Δ*hptRS*
SA564−Δ*ccpA*
SA564−Δ*hptRS/ccpA*

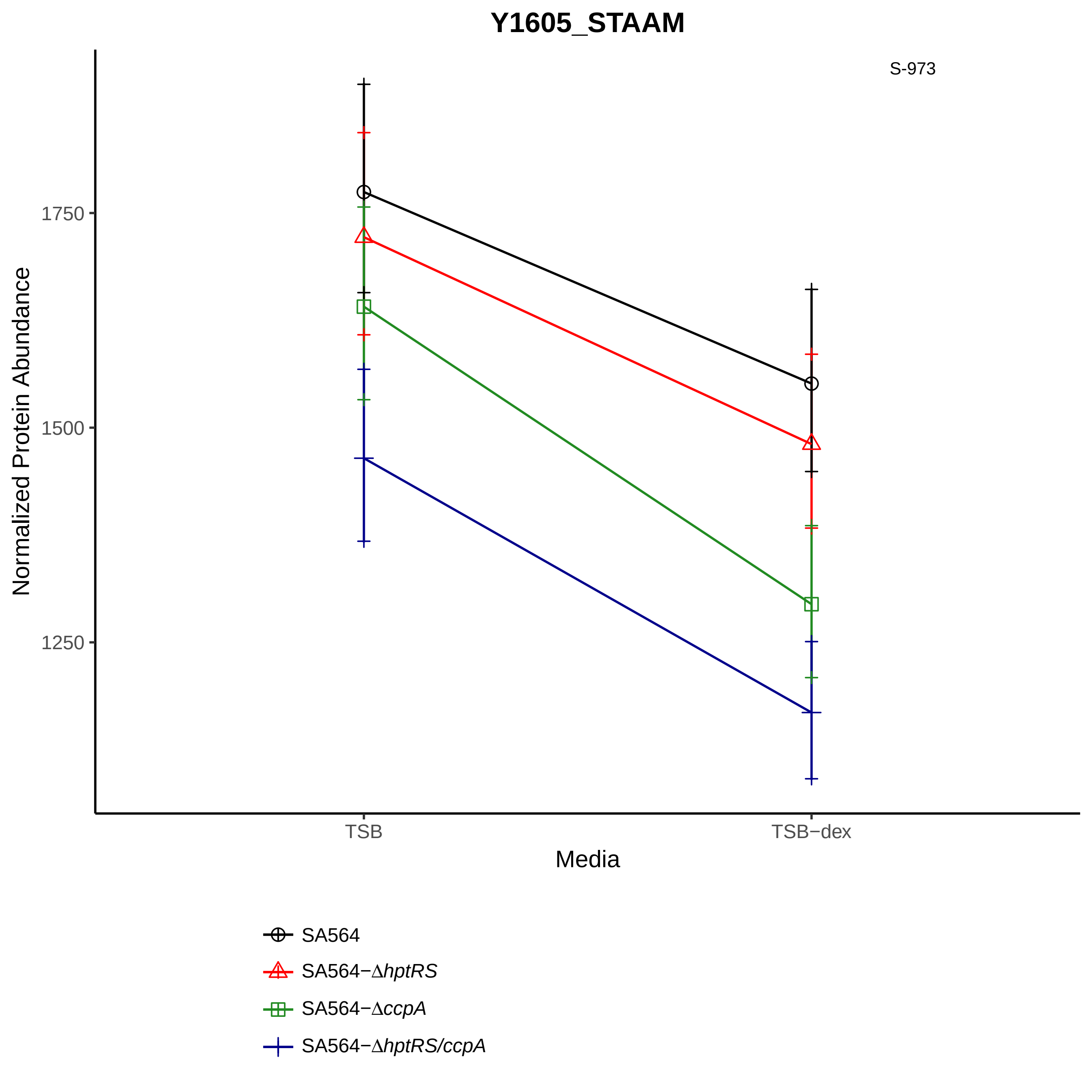

Y1605_STAAM
S-973
Normalized Protein Abundance
1750
1500
1250
TSB
TSB−dex
Media
SA564
SA564−Δ*hptRS*
SA564−Δ*ccpA*
SA564−Δ*hptRS/ccpA*

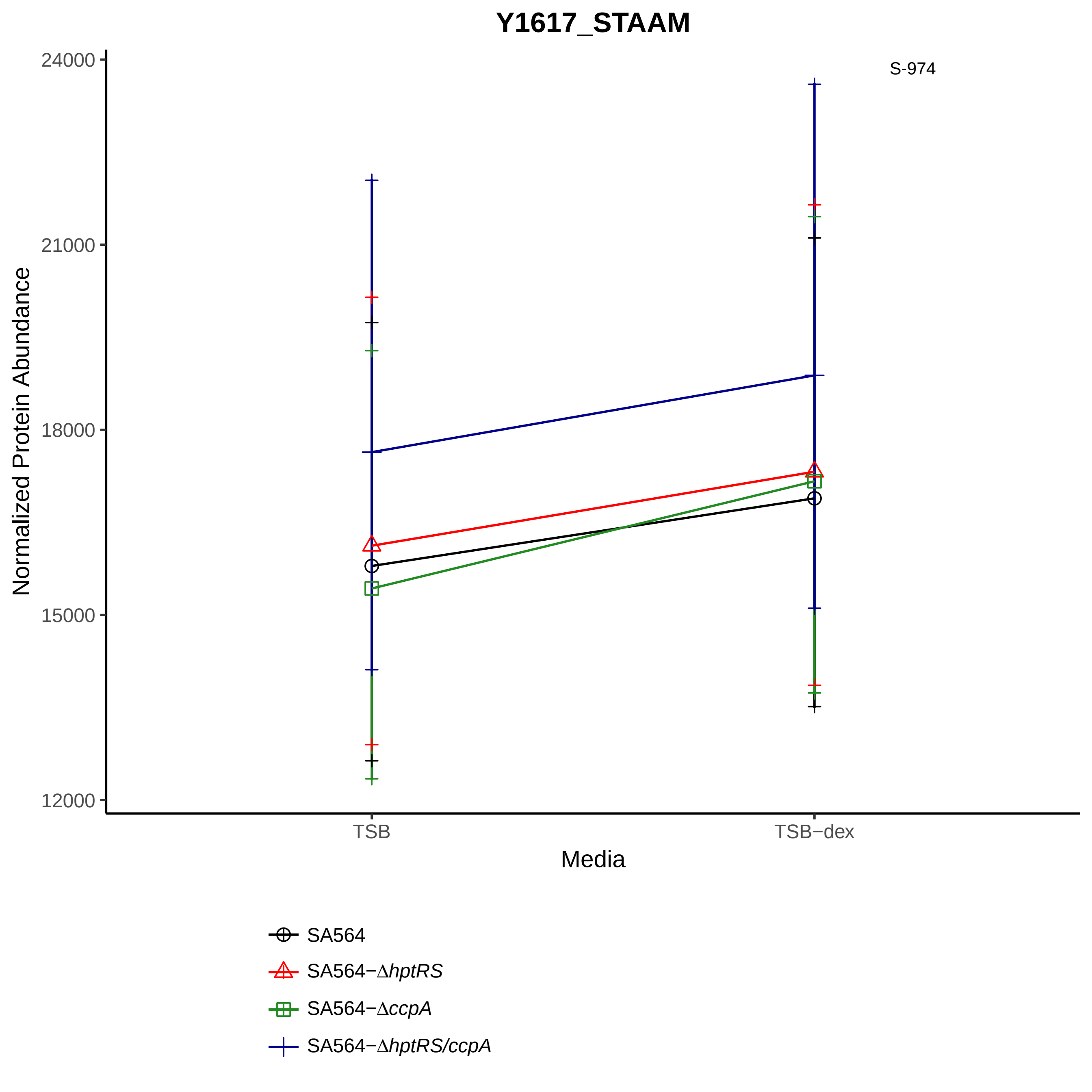
Y1617_STAAM
S-974
Normalized Protein Abundance
24000
21000
18000
15000
12000
TSB
TSB−dex
Media
SA564
SA564−Δ*hptRS*
SA564−Δ*ccpA*
SA564−Δ*hptRS/ccpA*

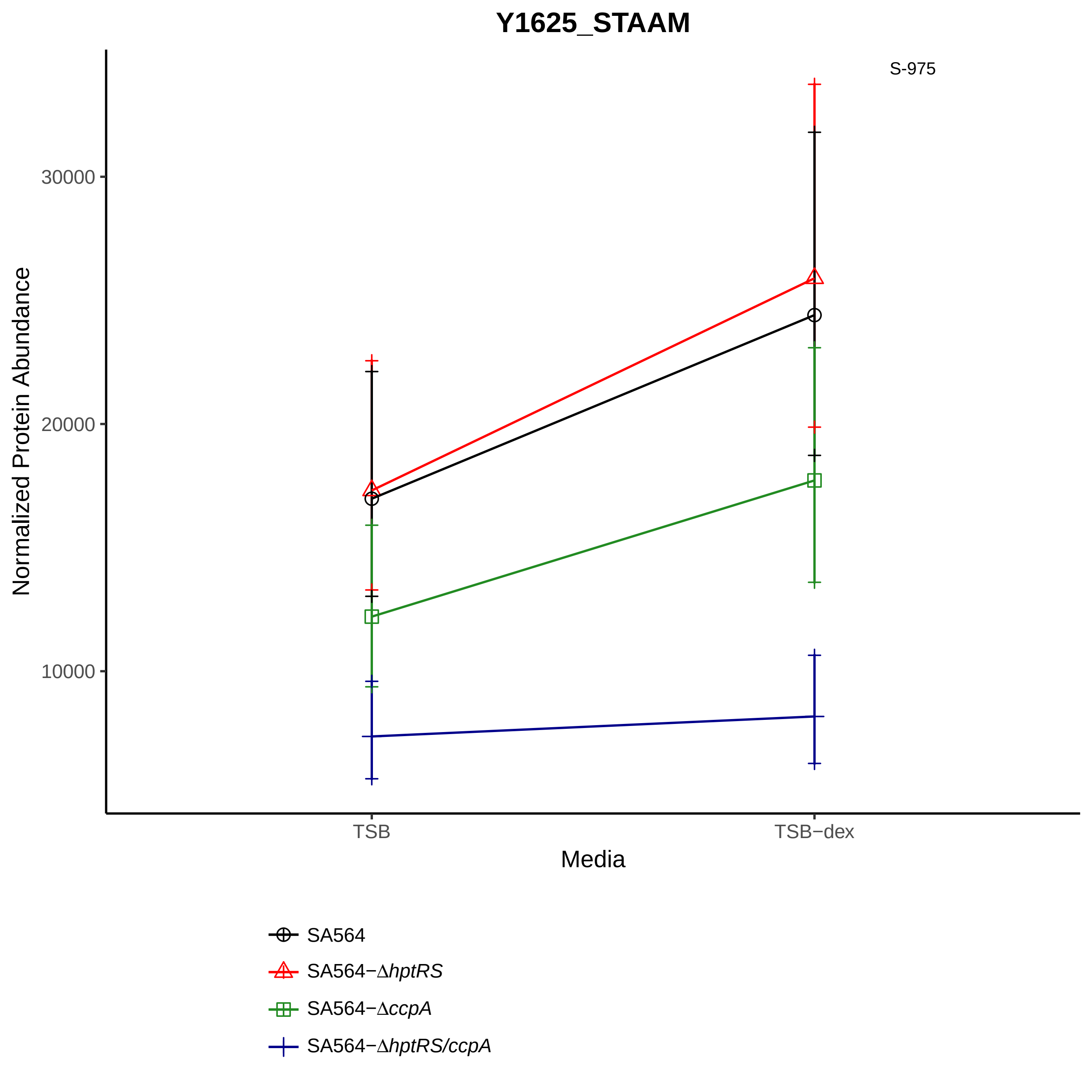

Y1625_STAAM
S-975
30000
20000
10000
Normalized Protein Abundance
TSB
TSB−dex
Media
SA564
SA564−Δ*hptRS*
SA564−Δ*ccpA*
SA564−Δ*hptRS/ccpA*

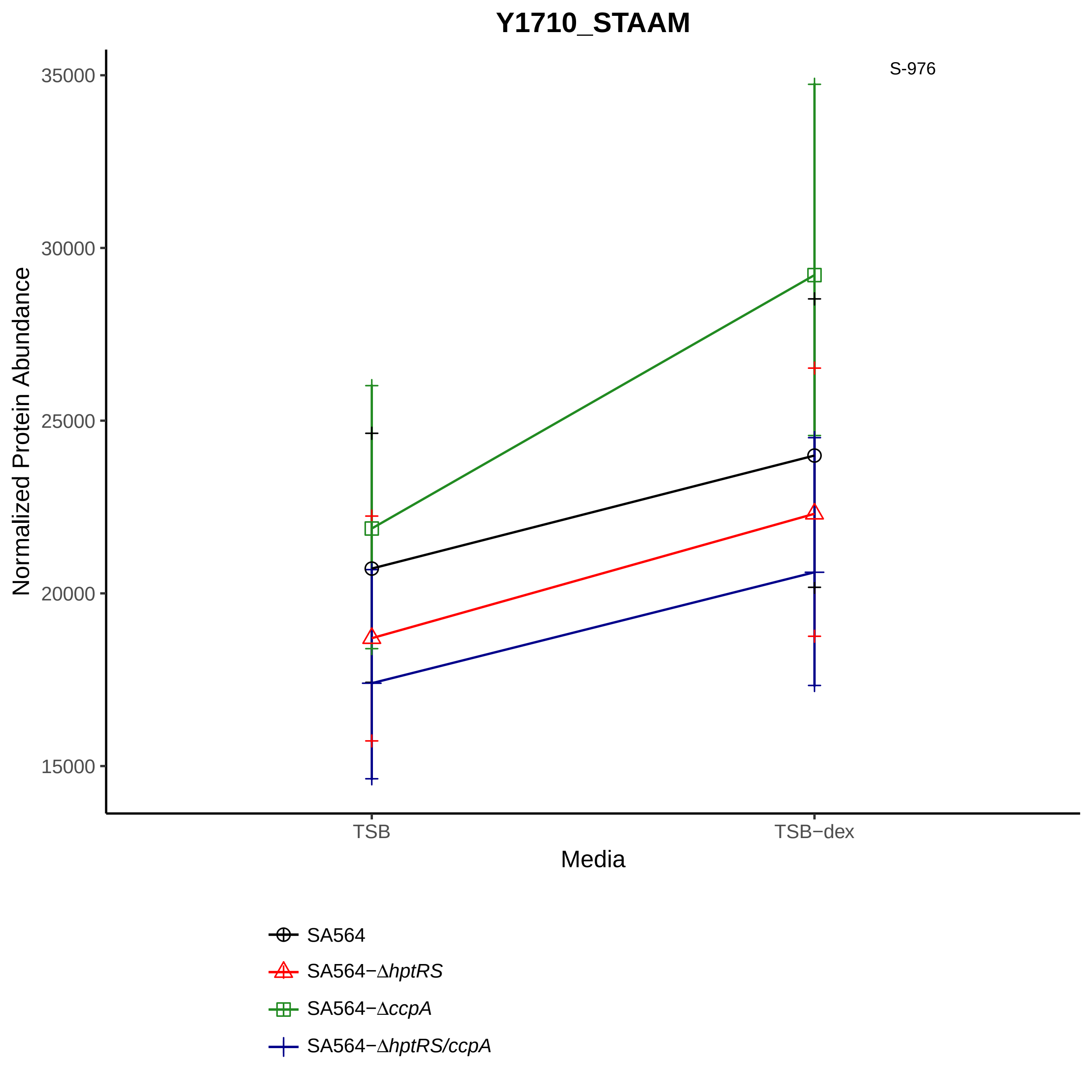

Y1710_STAAM
S-976
Normalized Protein Abundance
35000
30000
25000
20000
15000
TSB
TSB−dex
Media
SA564
SA564−Δ*hptRS*
SA564−Δ*ccpA*
SA564−Δ*hptRS/ccpA*

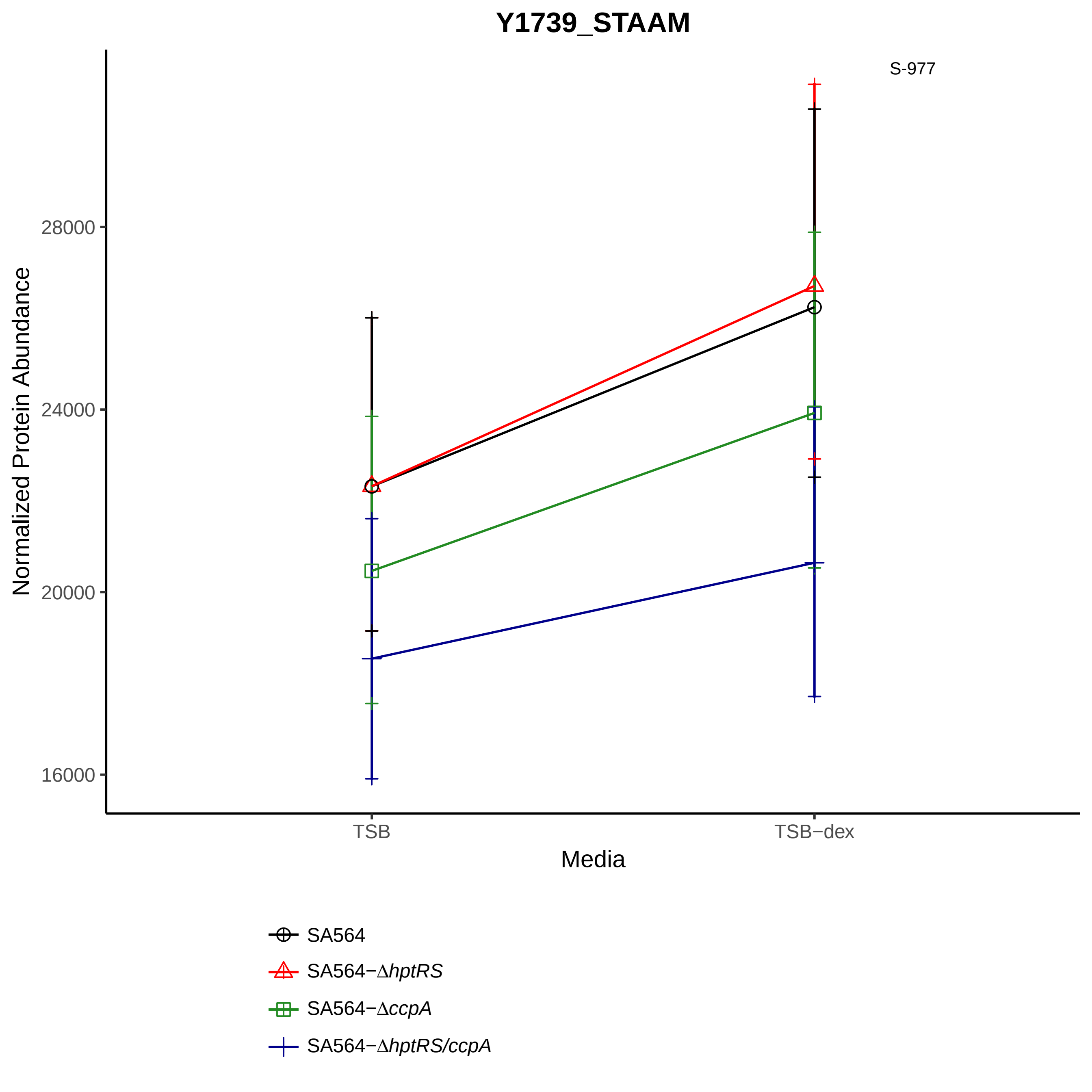

Y1739_STAAM
S-977
Normalized Protein Abundance
28000
24000
20000
16000
TSB
TSB−dex
Media
SA564
SA564−ΔhptRS
SA564−ΔccpA
SA564−ΔhptRS/ccpA

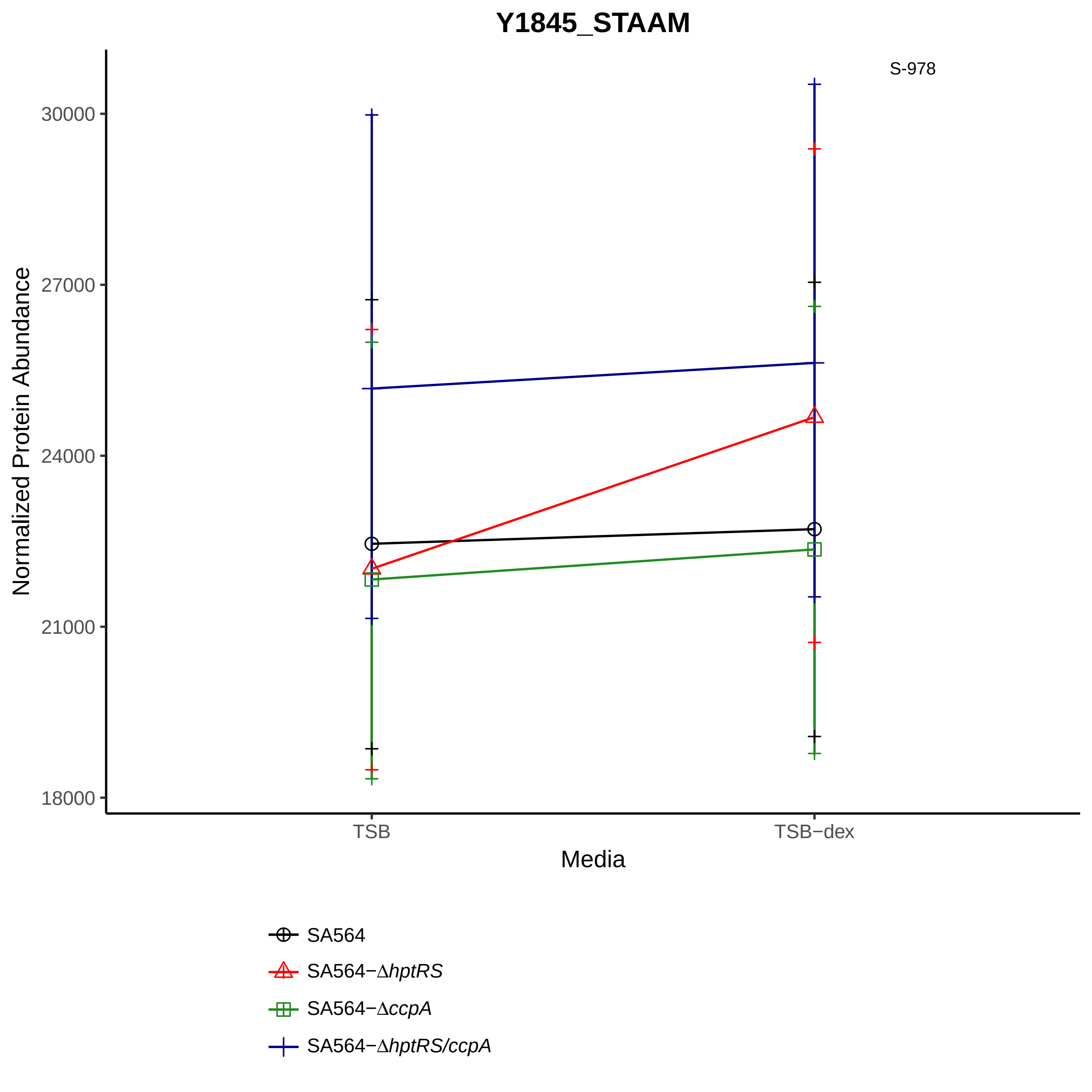

Y1845_STAAM
S-978
30000
27000
24000
21000
18000
Normalized Protein Abundance
TSB
TSB−dex
Media
SA564
SA564−ΔhptRS
SA564−ΔccpA
SA564−ΔhptRS/ccpA

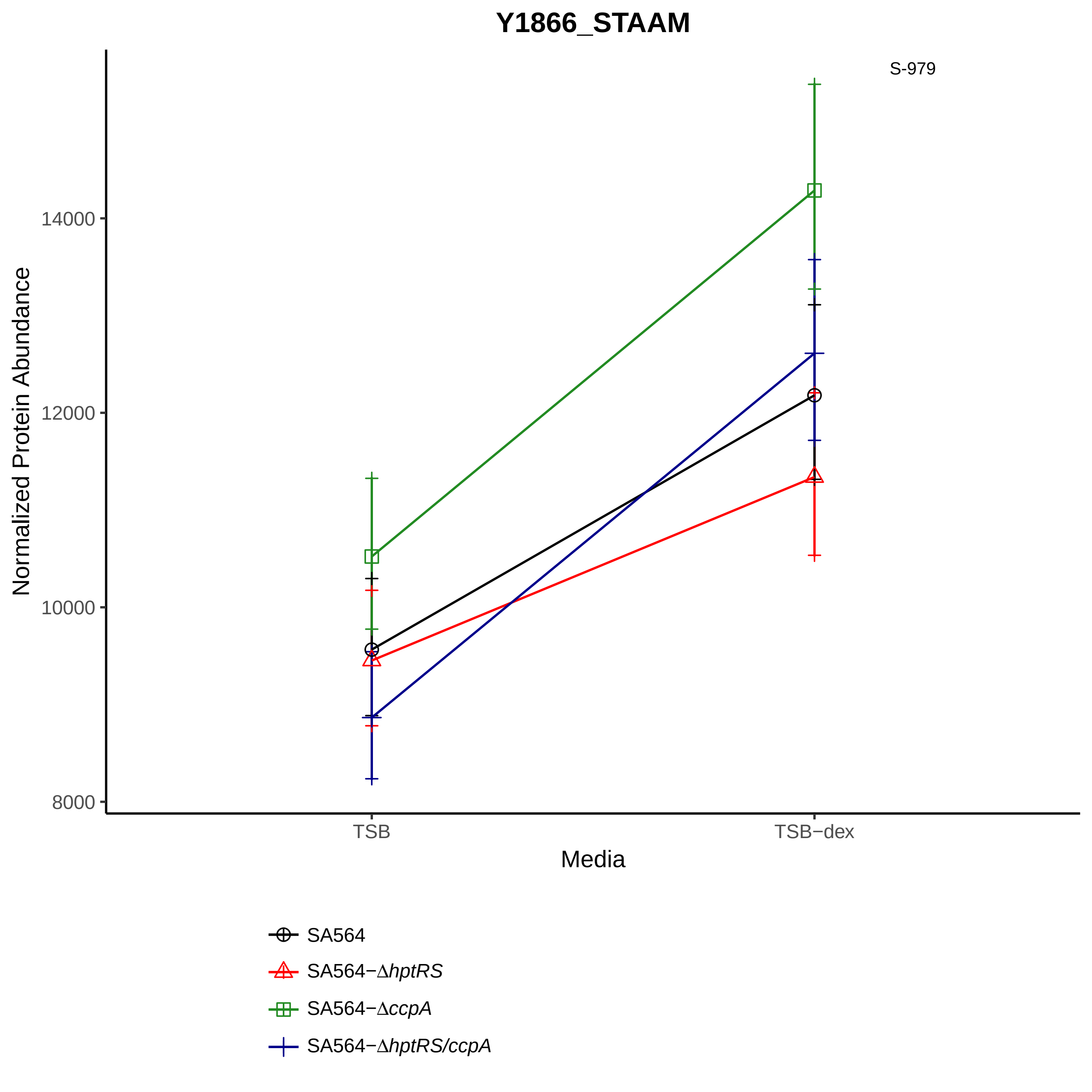
Y1866_STAAM
S-979
Normalized Protein Abundance
14000
12000
10000
8000
TSB
TSB−dex
Media
SA564
SA564−ΔhptRS
SA564−ΔccpA
SA564−ΔhptRS/ccpA

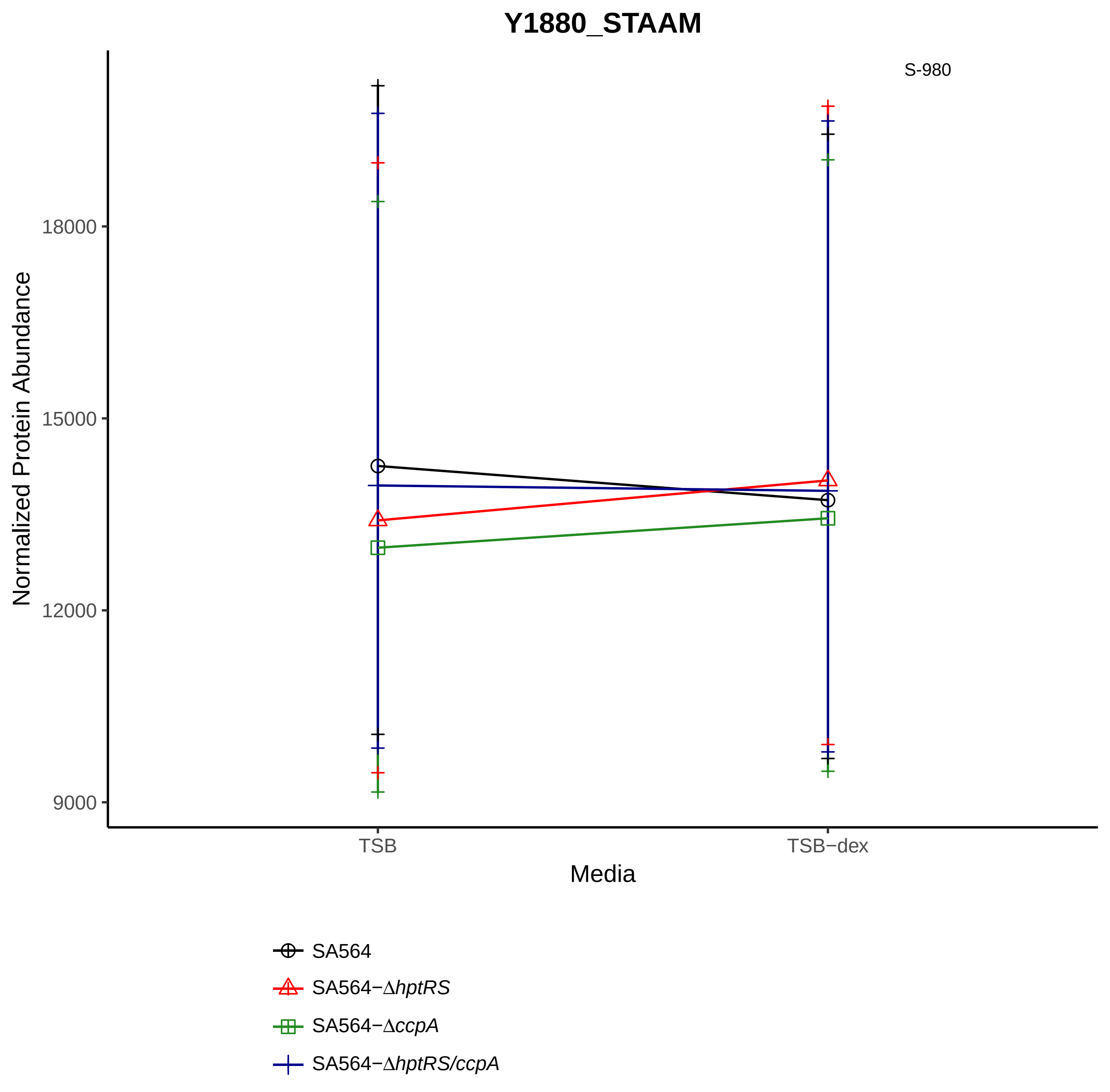

Y1880_STAAM
S-980
Normalized Protein Abundance
18000
15000
12000
9000
TSB
TSB−dex
Media
SA564
SA564−Δ*hptRS*
SA564−Δ*ccpA*
SA564−Δ*hptRS/ccpA*

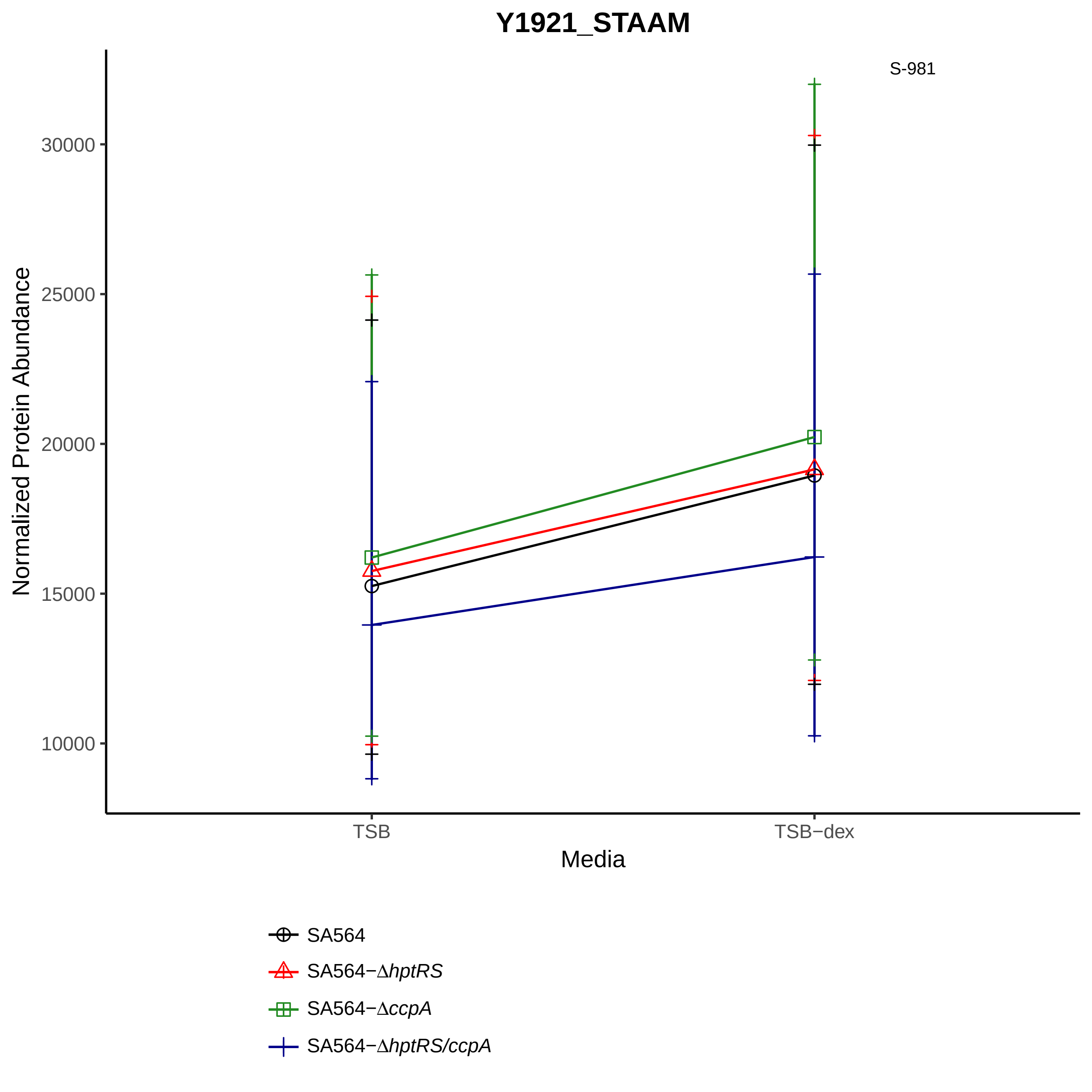

Y1921_STAAM
S-981
Normalized Protein Abundance
30000
25000
20000
15000
10000
TSB
TSB−dex
Media
SA564
SA564−Δ*hptRS*
SA564−Δ*ccpA*
SA564−Δ*hptRS/ccpA*

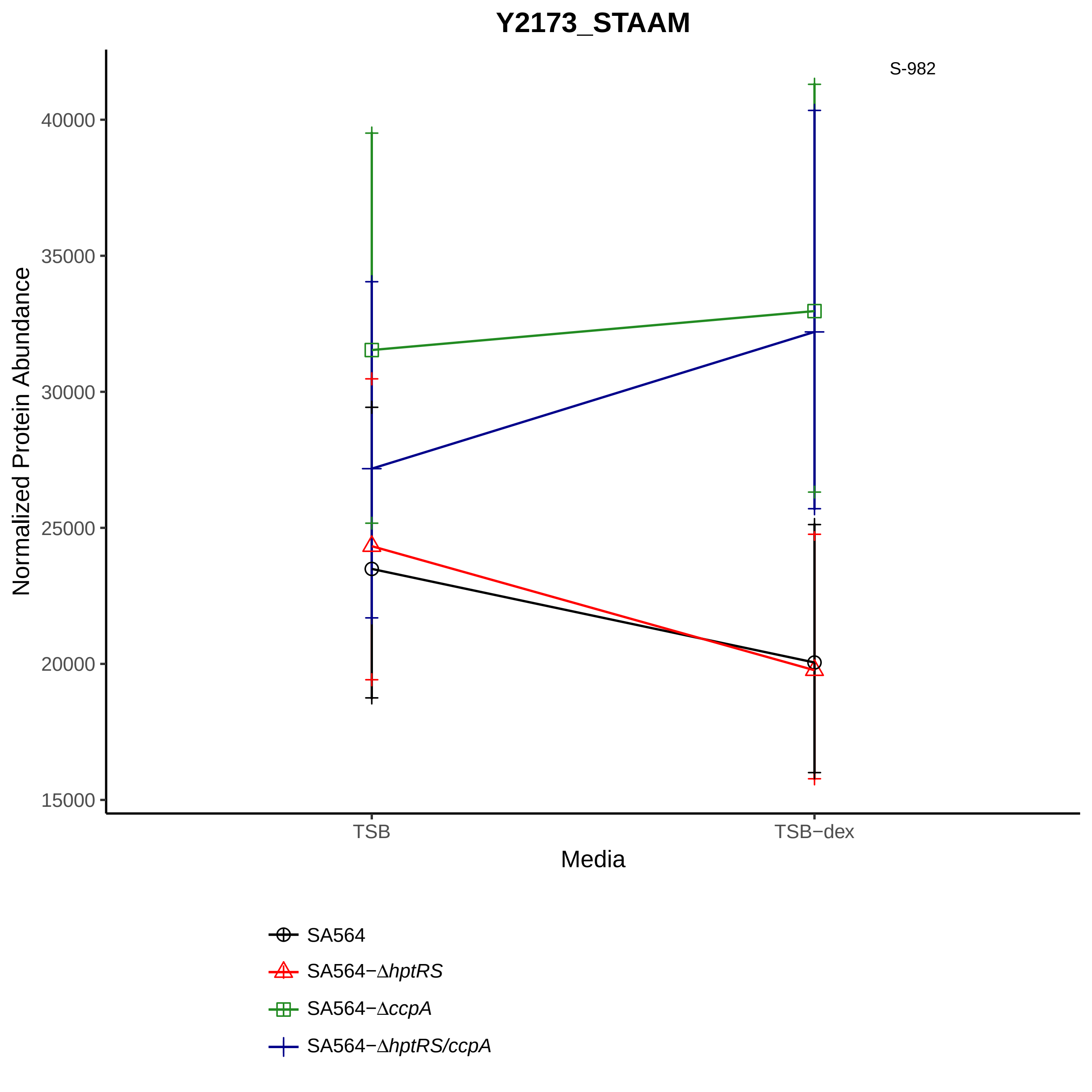
Y2173_STAAM
S-982
Normalized Protein Abundance
40000
35000
30000
25000
20000
15000
TSB
TSB−dex
Media
SA564
SA564−Δ*hptRS*
SA564−Δ*ccpA*
SA564−Δ*hptRS/ccpA*

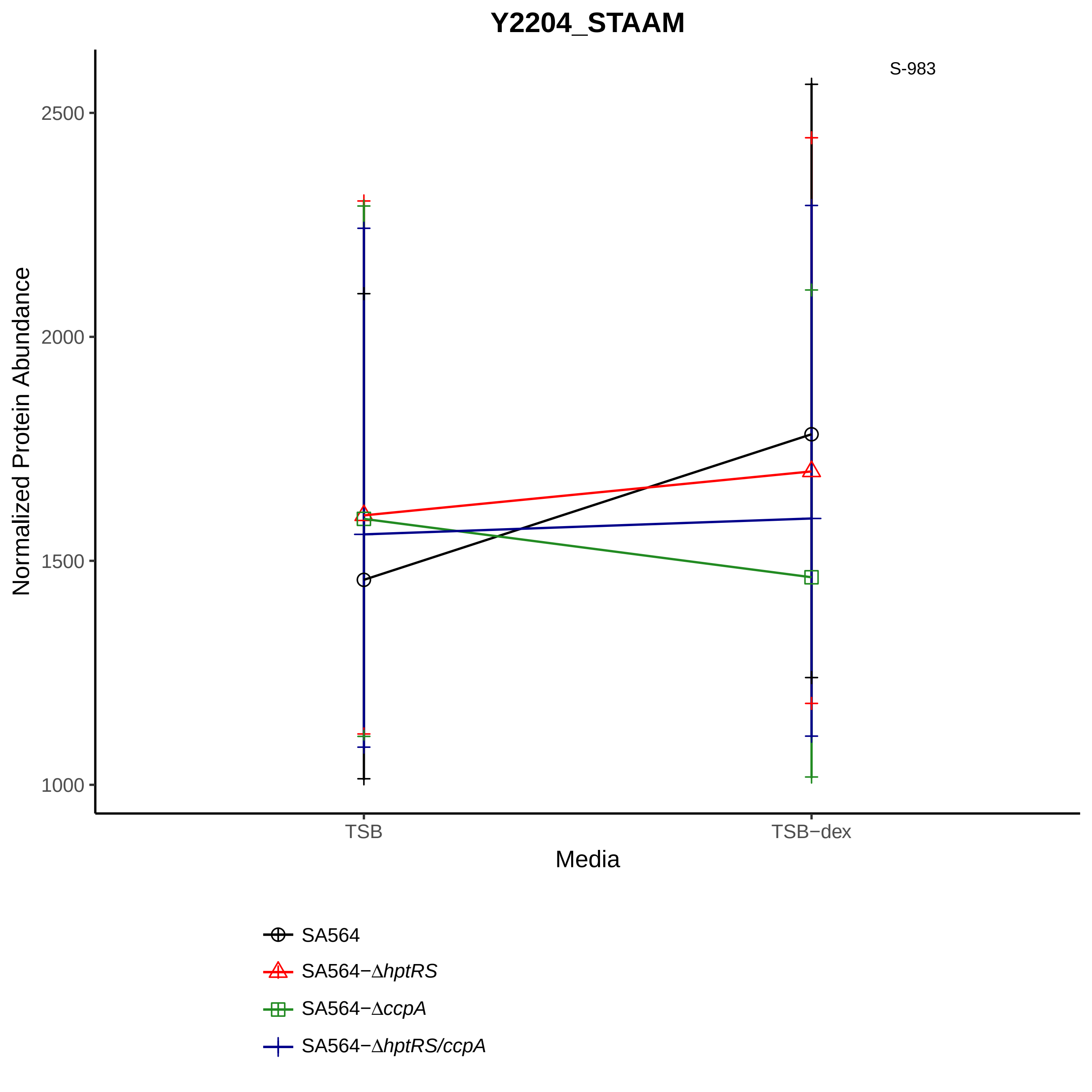
Y2204_STAAM
S-983
Normalized Protein Abundance
2500
2000
1500
1000
TSB
TSB−dex
Media
SA564
SA564−ΔhptRS
SA564−ΔccpA
SA564−ΔhptRS/ccpA

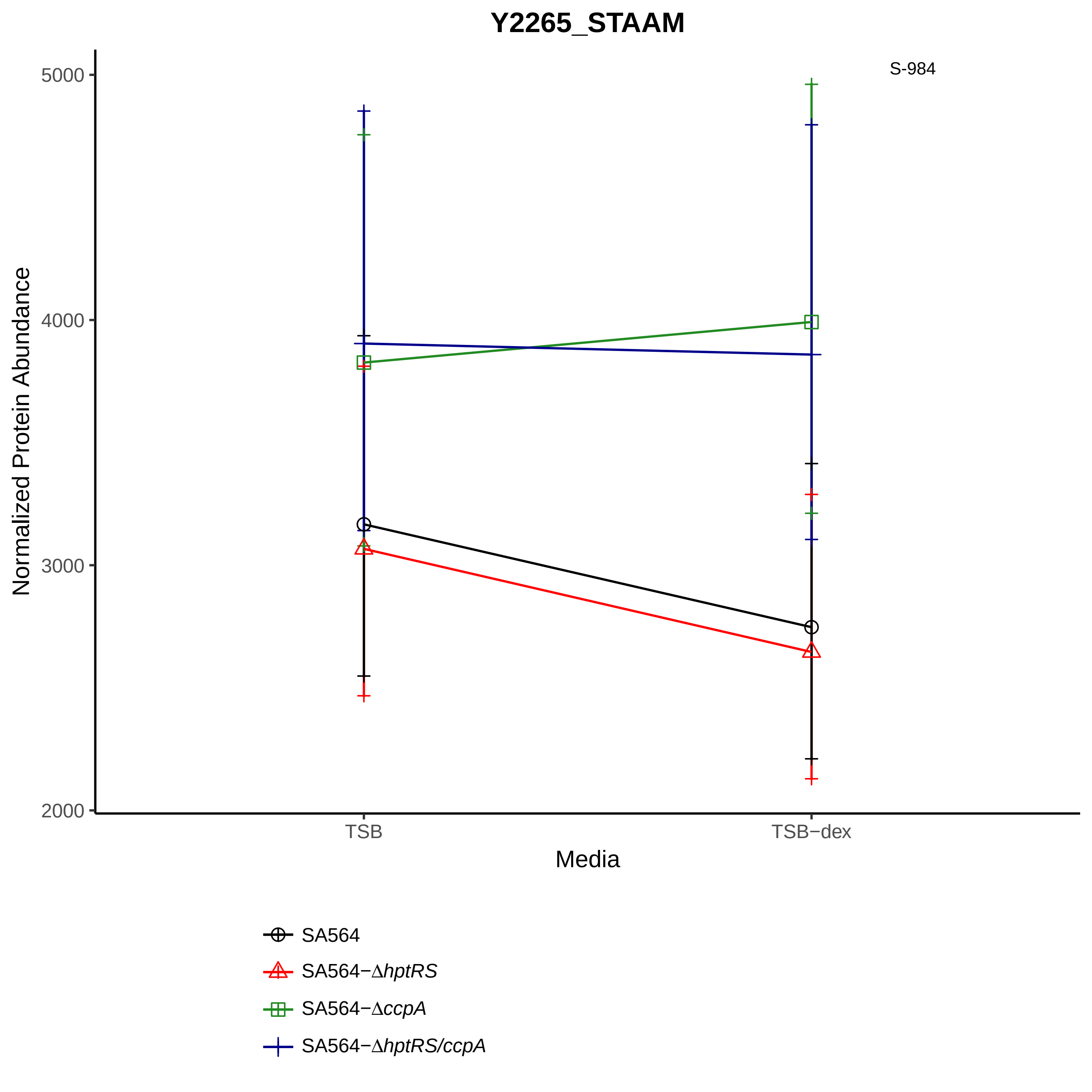

Y2265_STAAM
S-984
5000
4000
3000
2000
Normalized Protein Abundance
TSB
TSB−dex
Media
SA564
SA564−ΔhptRS
SA564−ΔccpA
SA564−ΔhptRS/ccpA

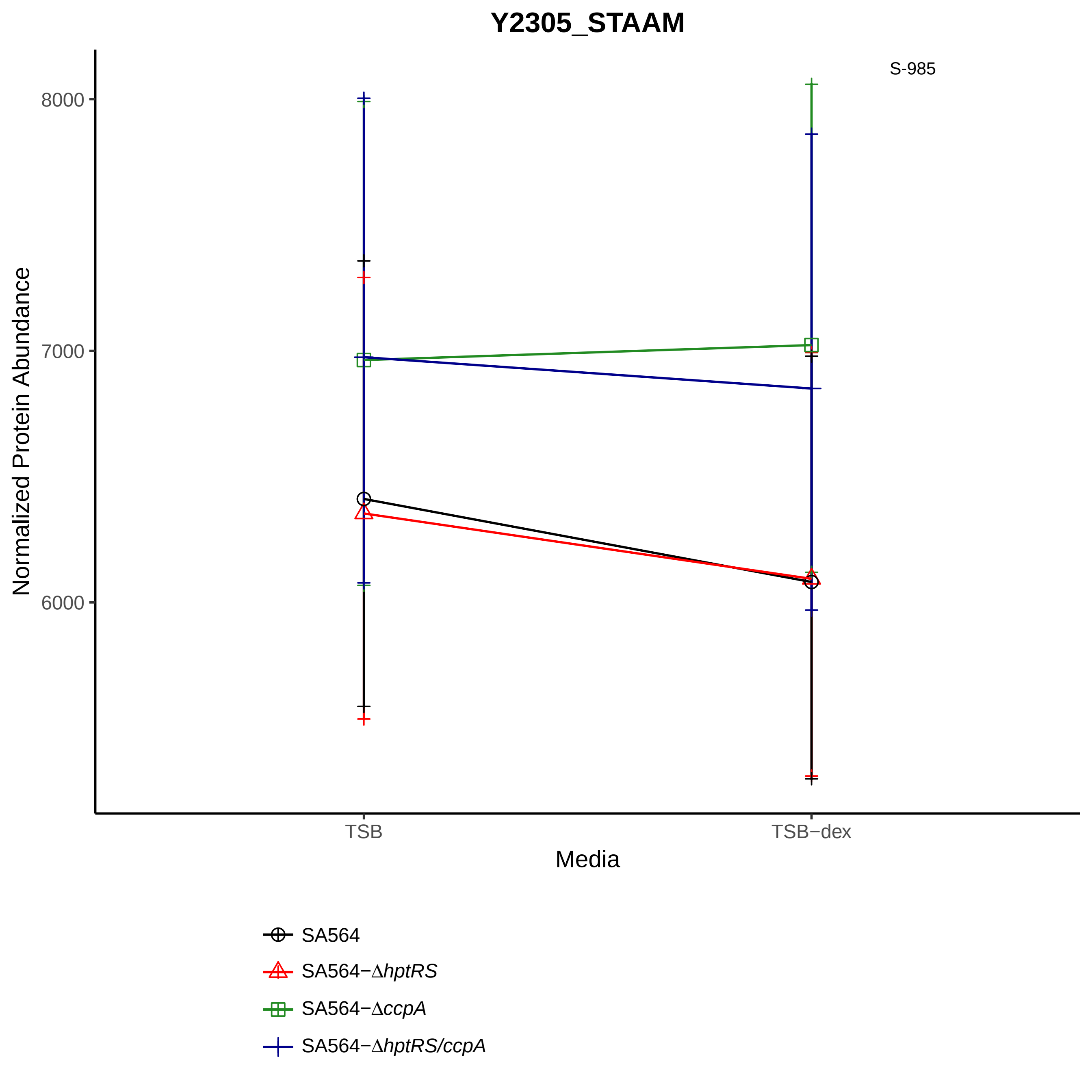
Y2305_STAAM
S-985
8000
7000
6000
Normalized Protein Abundance
TSB
TSB−dex
Media
SA564
SA564−Δ*hptRS*
SA564−Δ*ccpA*
SA564−Δ*hptRS/ccpA*

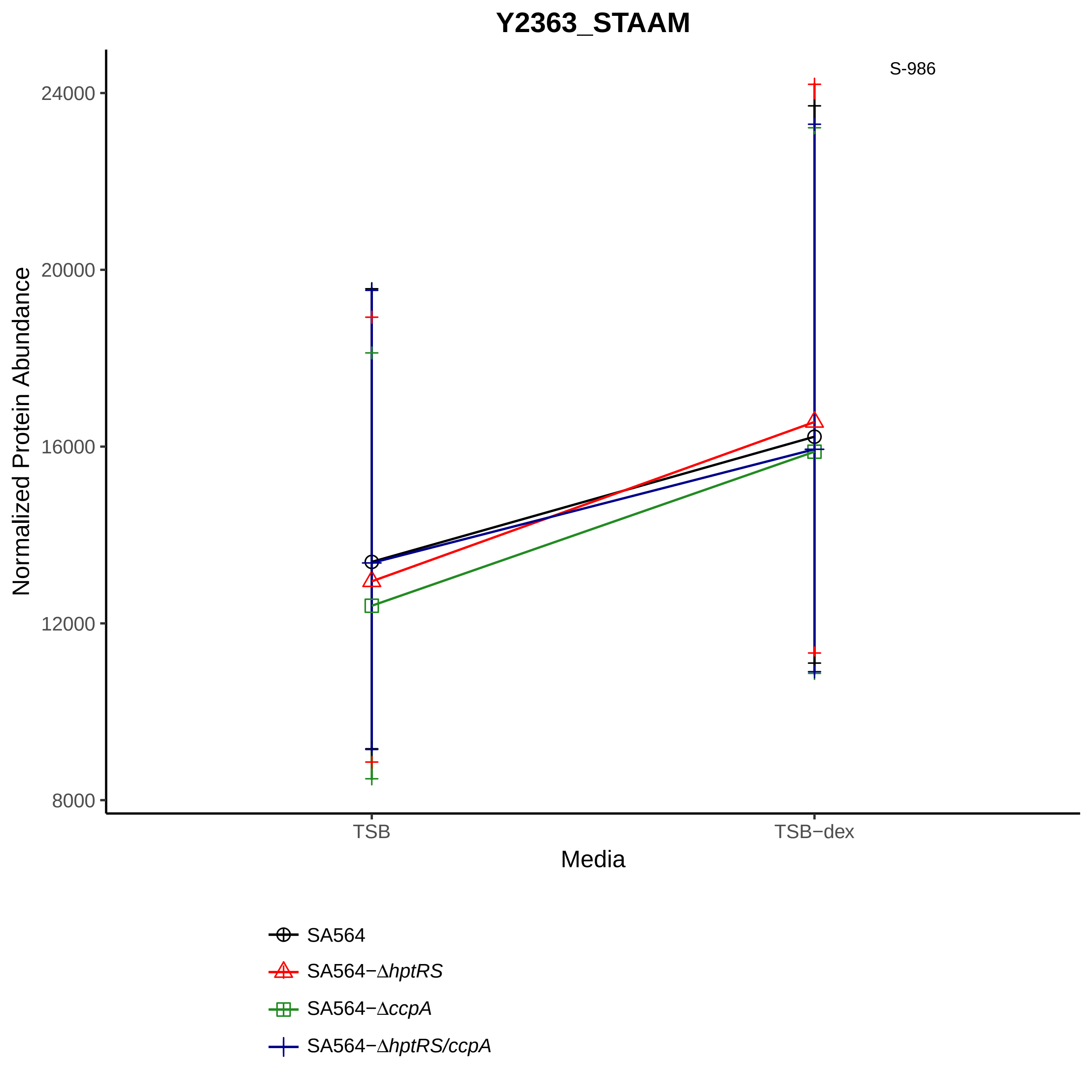

Y2363_STAAM
S-986
24000
20000
16000
12000
8000
Normalized Protein Abundance
TSB
TSB−dex
Media
SA564
SA564−Δ*hptRS*
SA564−Δ*ccpA*
SA564−Δ*hptRS/ccpA*

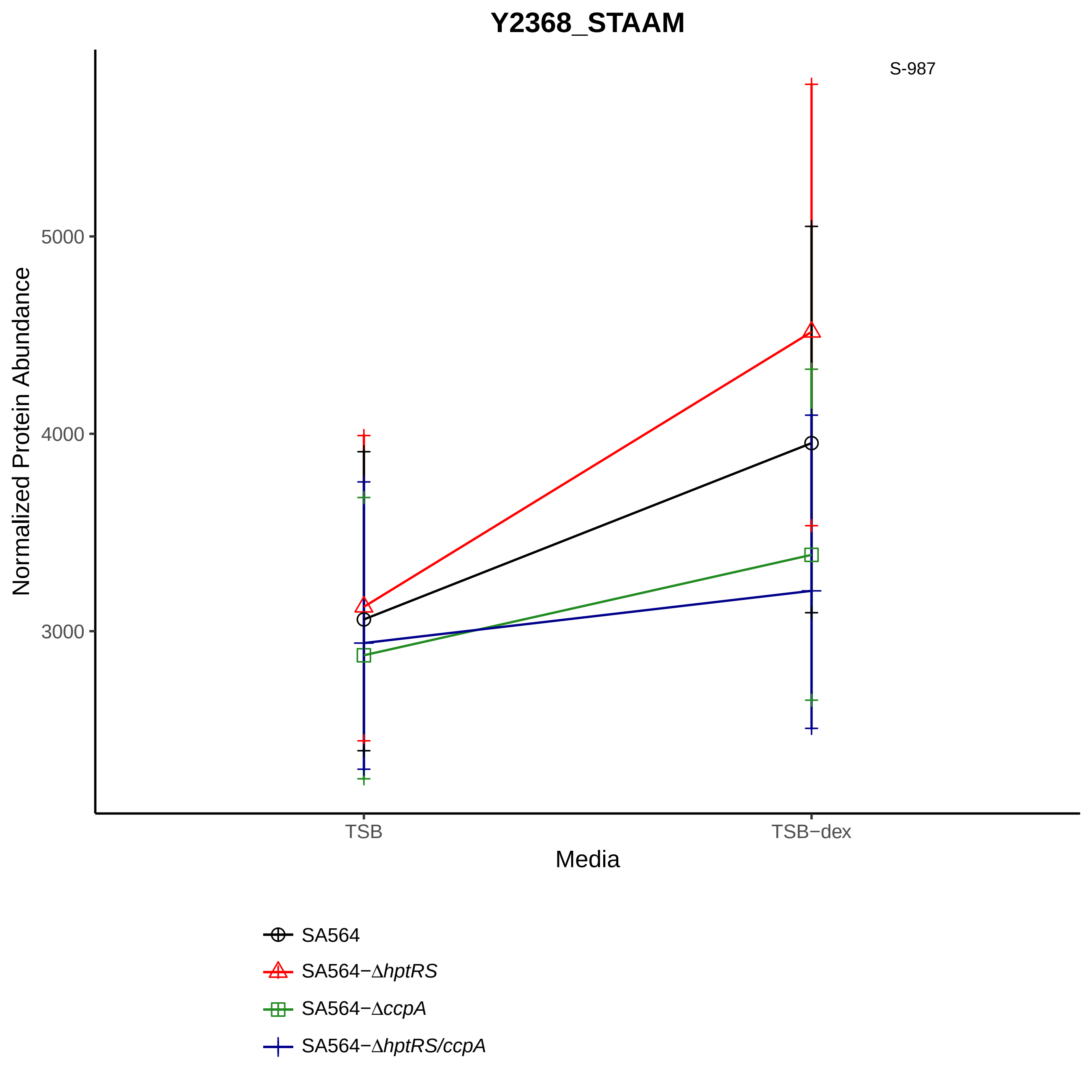

Y2368_STAAM
S-987
Normalized Protein Abundance
5000
4000
3000
TSB
TSB−dex
Media
SA564
SA564−ΔhptRS
SA564−ΔccpA
SA564−ΔhptRS/ccpA

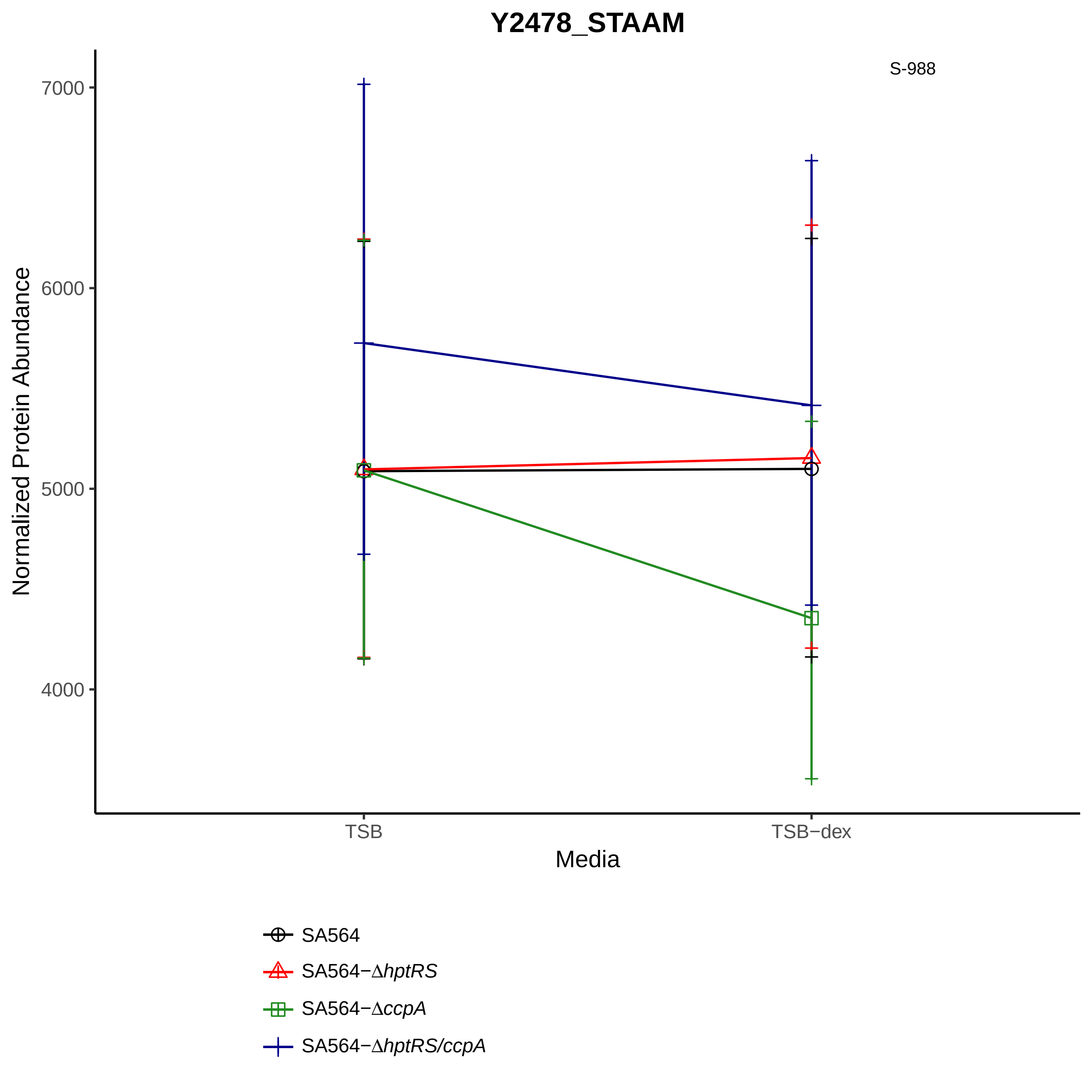

Y2478_STAAM
S-988
Normalized Protein Abundance
7000
6000
5000
4000
TSB
TSB−dex
Media
SA564
SA564−ΔhptRS
SA564−ΔccpA
SA564−ΔhptRS/ccpA

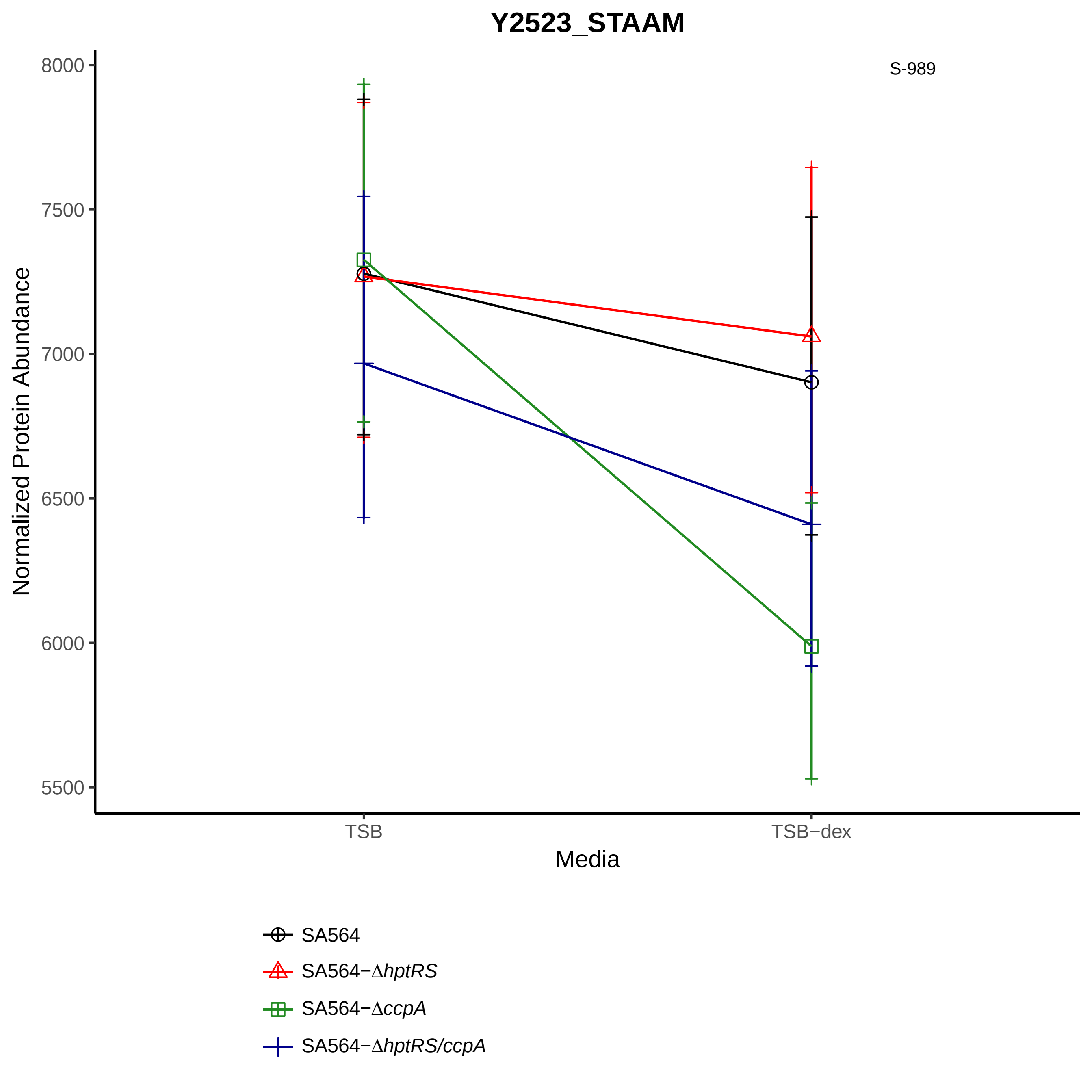

Y2523_STAAM
S-989
Normalized Protein Abundance
8000
7500
7000
6500
6000
5500
TSB
TSB−dex
Media
SA564
SA564−Δ*hptRS*
SA564−Δ*ccpA*
SA564−Δ*hptRS/ccpA*

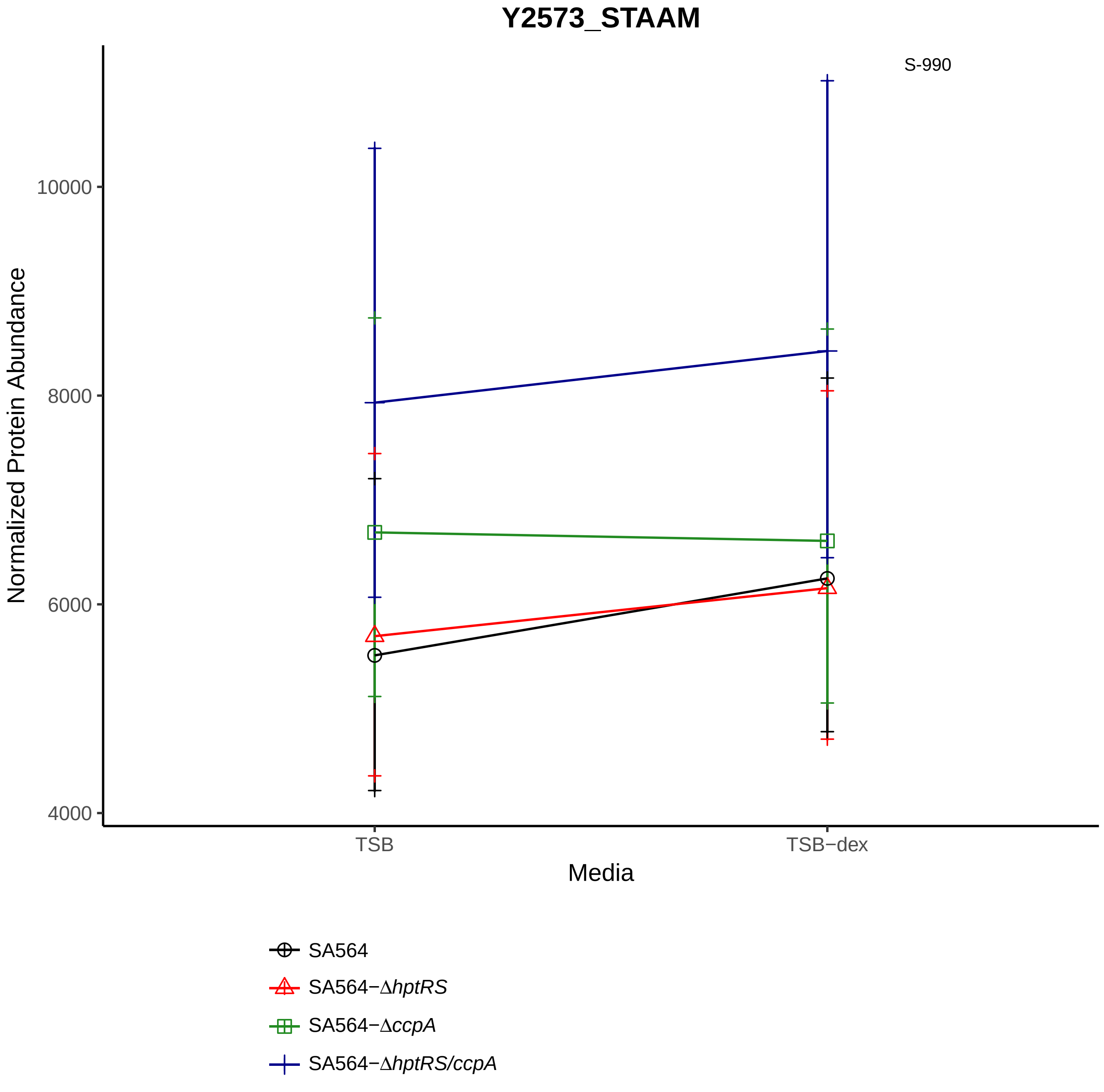

Y2573_STAAM
S-990
Normalized Protein Abundance
10000
8000
6000
4000
TSB
TSB−dex
Media
SA564
SA564−Δ*hptRS*
SA564−Δ*ccpA*
SA564−Δ*hptRS/ccpA*

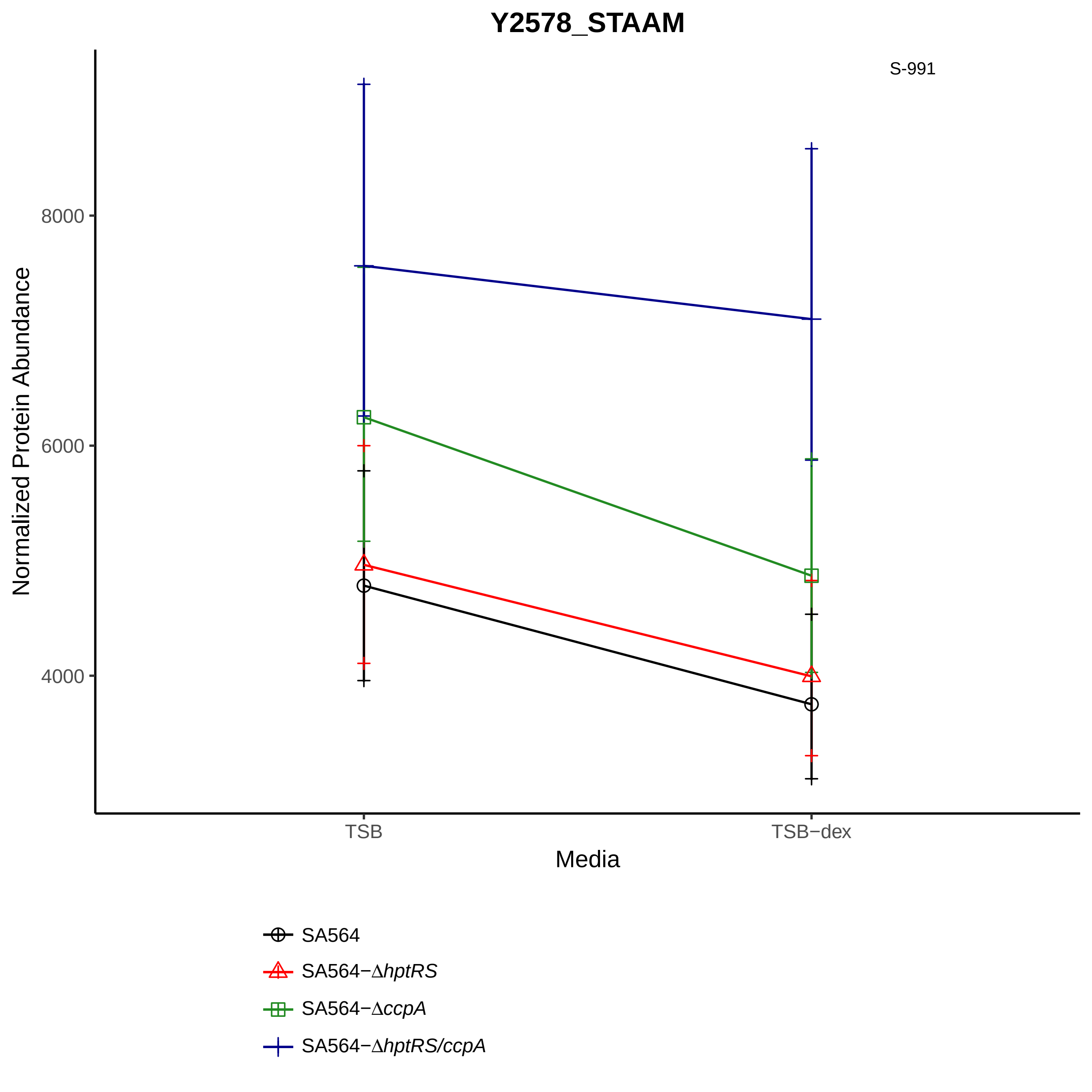

Y2578_STAAM
S-991
Normalized Protein Abundance
8000
6000
4000
TSB
TSB−dex
Media
SA564
SA564−Δ*hptRS*
SA564−Δ*ccpA*
SA564−Δ*hptRS/ccpA*

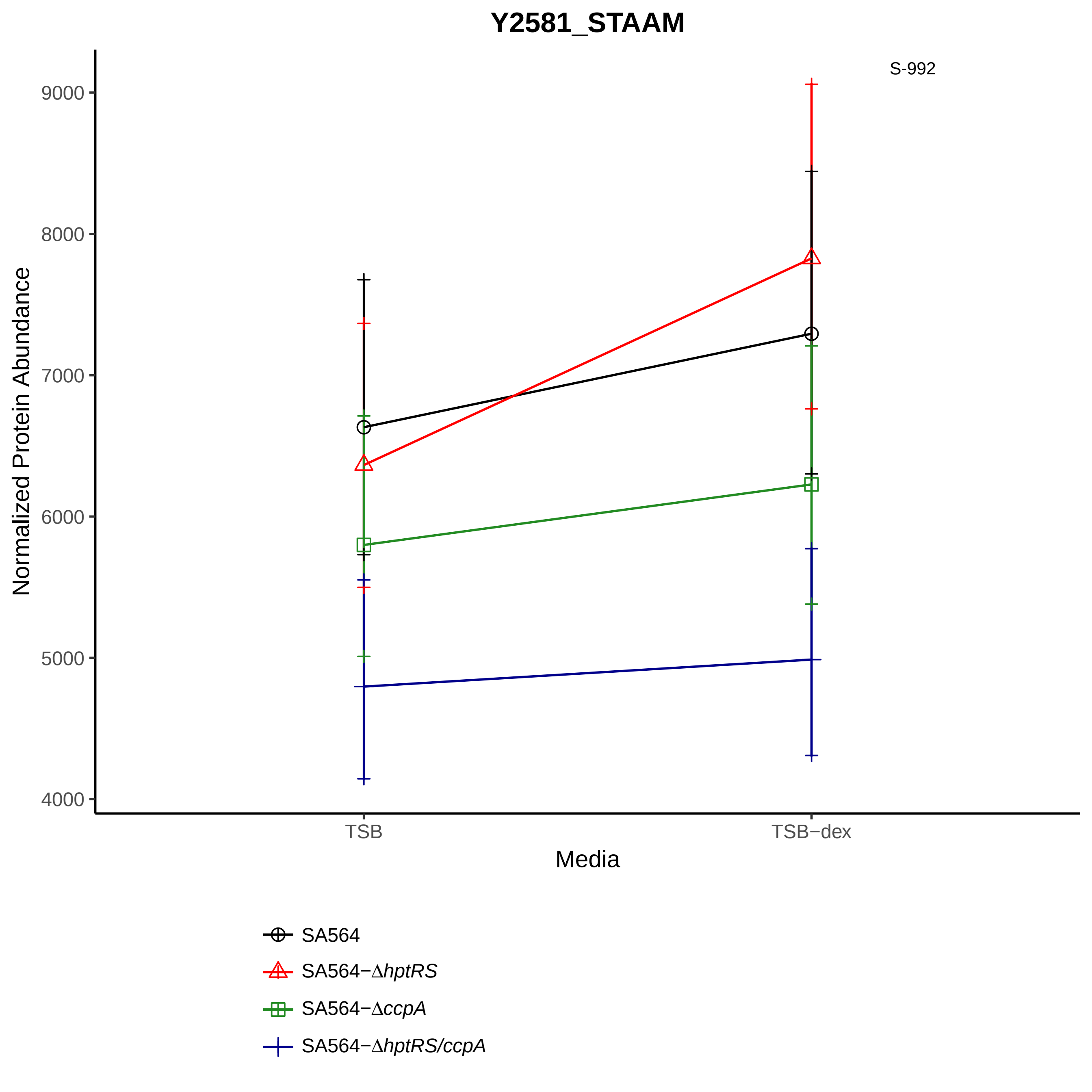

Y2581_STAAM
S-992
Normalized Protein Abundance
9000
8000
7000
6000
5000
4000
TSB
TSB−dex
Media
SA564
SA564−Δ*hptRS*
SA564−Δ*ccpA*
SA564−Δ*hptRS/ccpA*

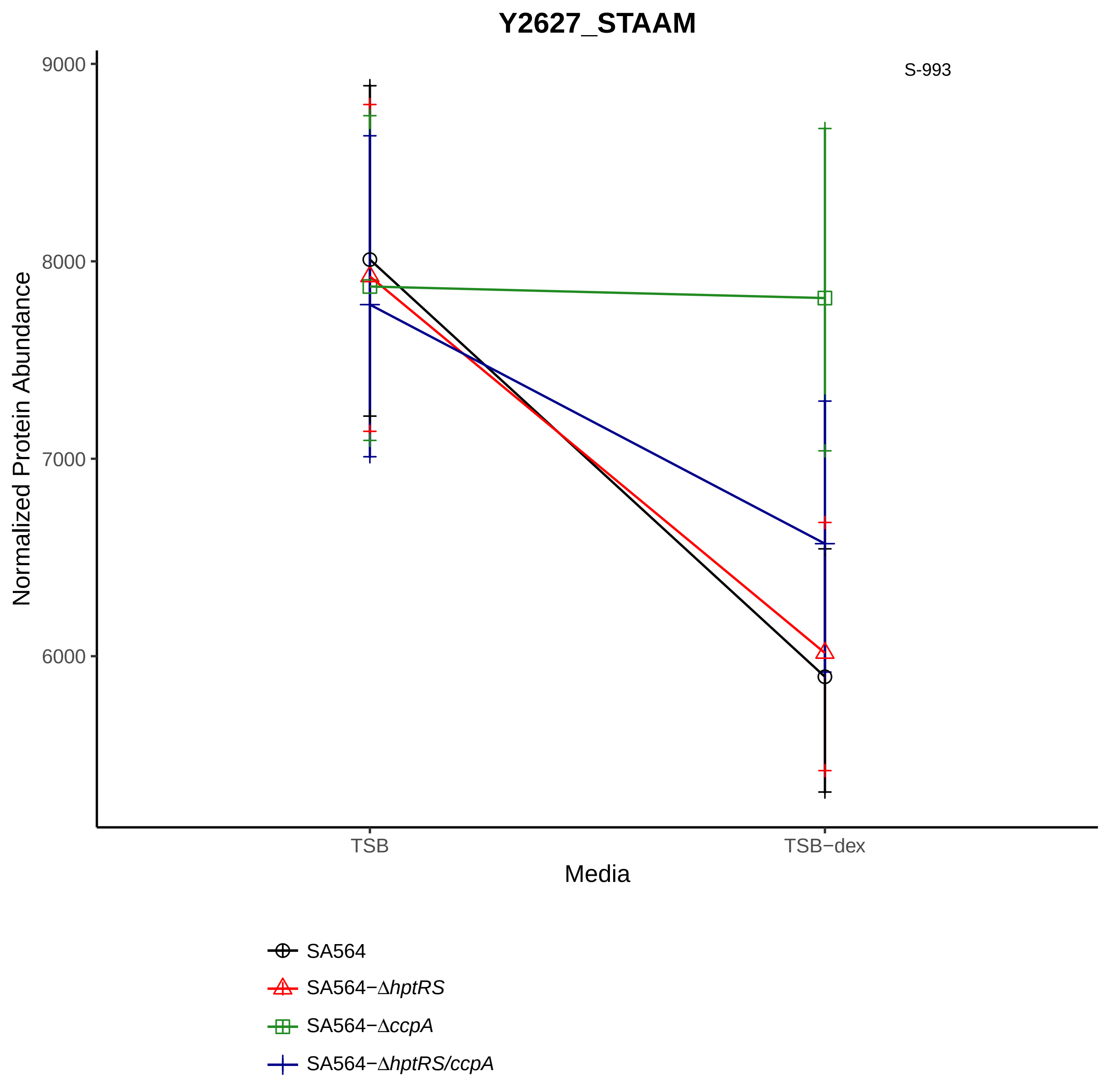

Y2627_STAAM
S-993
9000
8000
7000
6000
Normalized Protein Abundance
TSB
TSB−dex
Media
SA564
SA564−Δ*hptRS*
SA564−Δ*ccpA*
SA564−Δ*hptRS/ccpA*

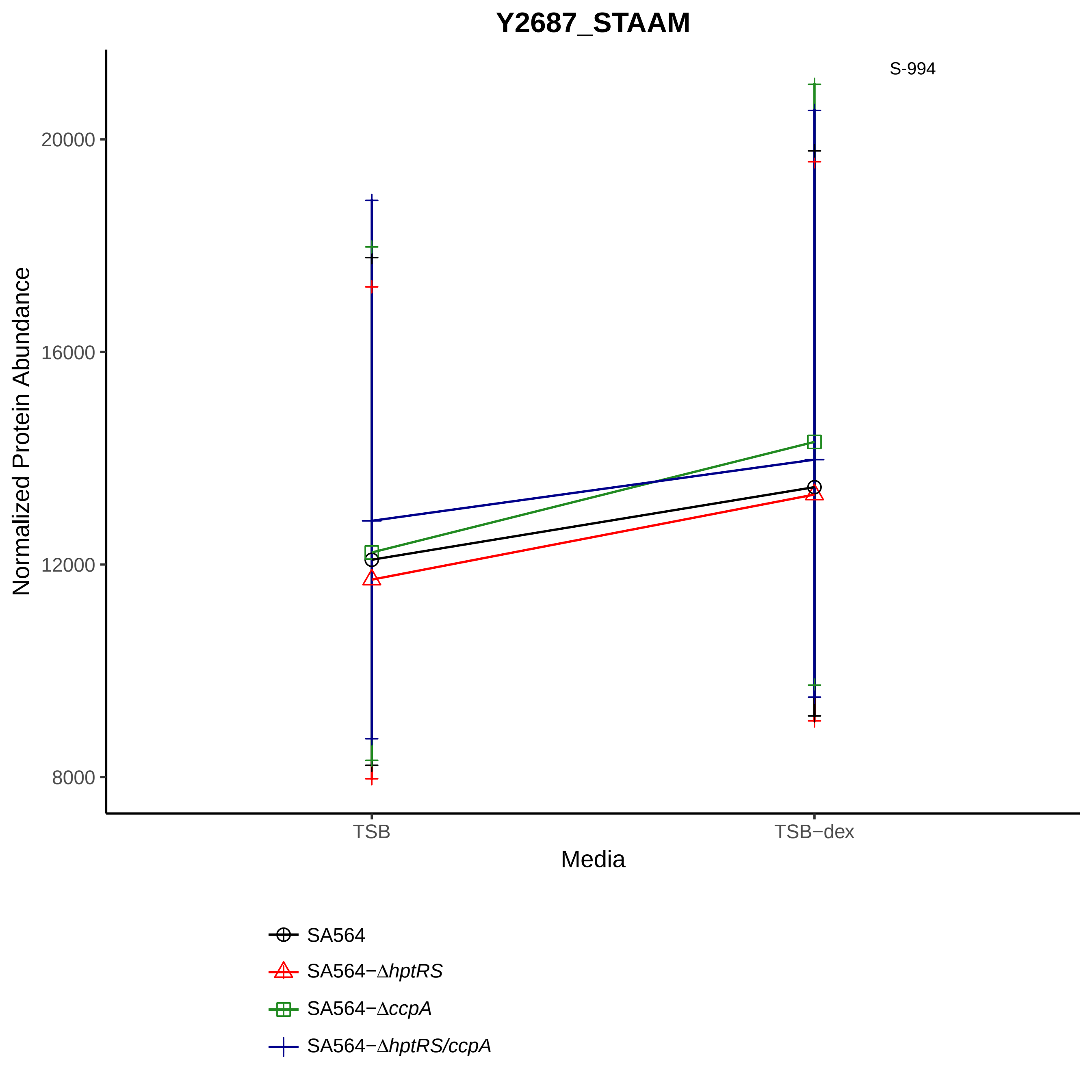

Y2687_STAAM
S-994
Normalized Protein Abundance
20000
16000
12000
8000
TSB
TSB−dex
Media
SA564
SA564−Δ*hptRS*
SA564−Δ*ccpA*
SA564−Δ*hptRS/ccpA*

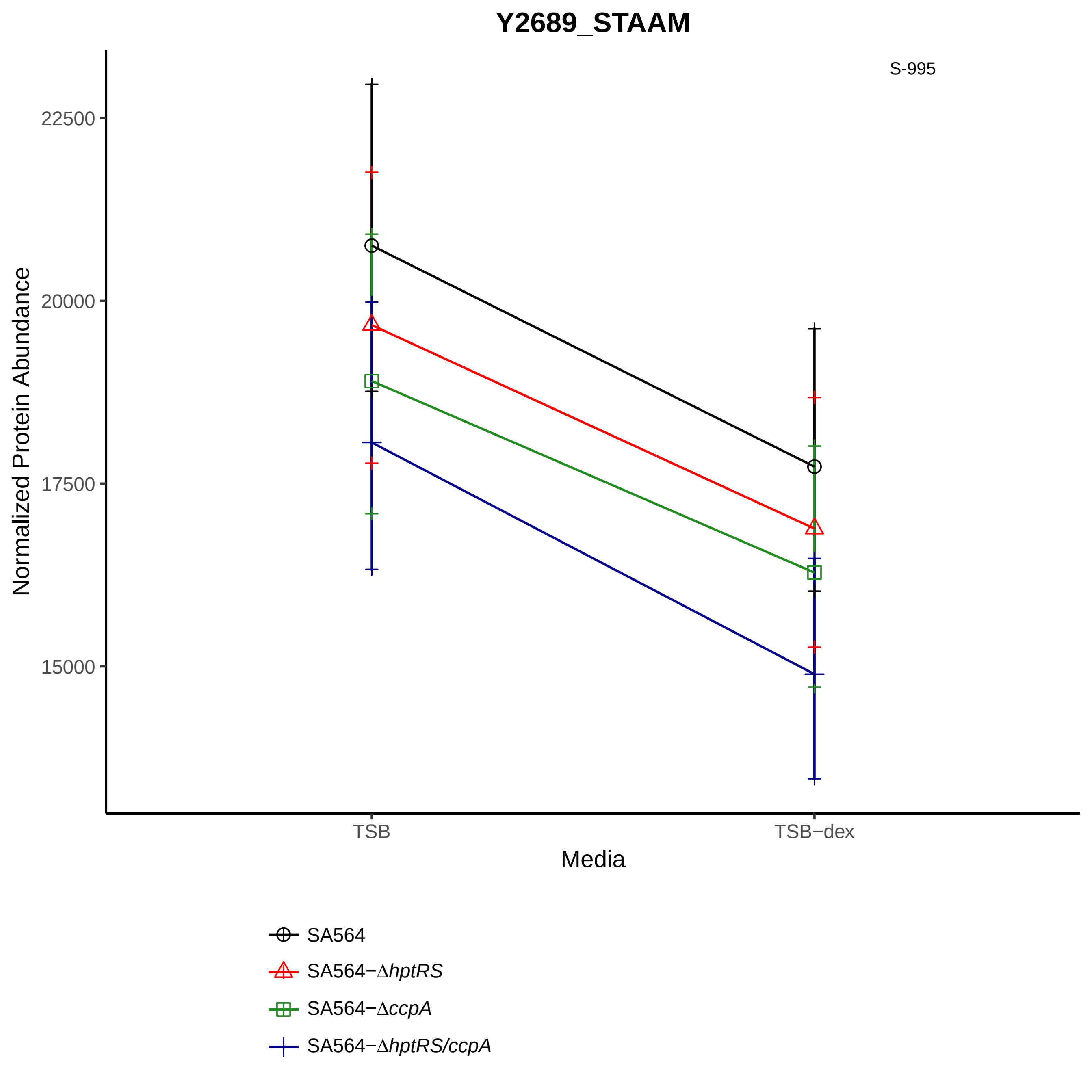

Y2689_STAAM
S-995
Normalized Protein Abundance
22500
20000
17500
15000
TSB
TSB−dex
Media
SA564
SA564−Δ*hptRS*
SA564−Δ*ccpA*
SA564−Δ*hptRS/ccpA*

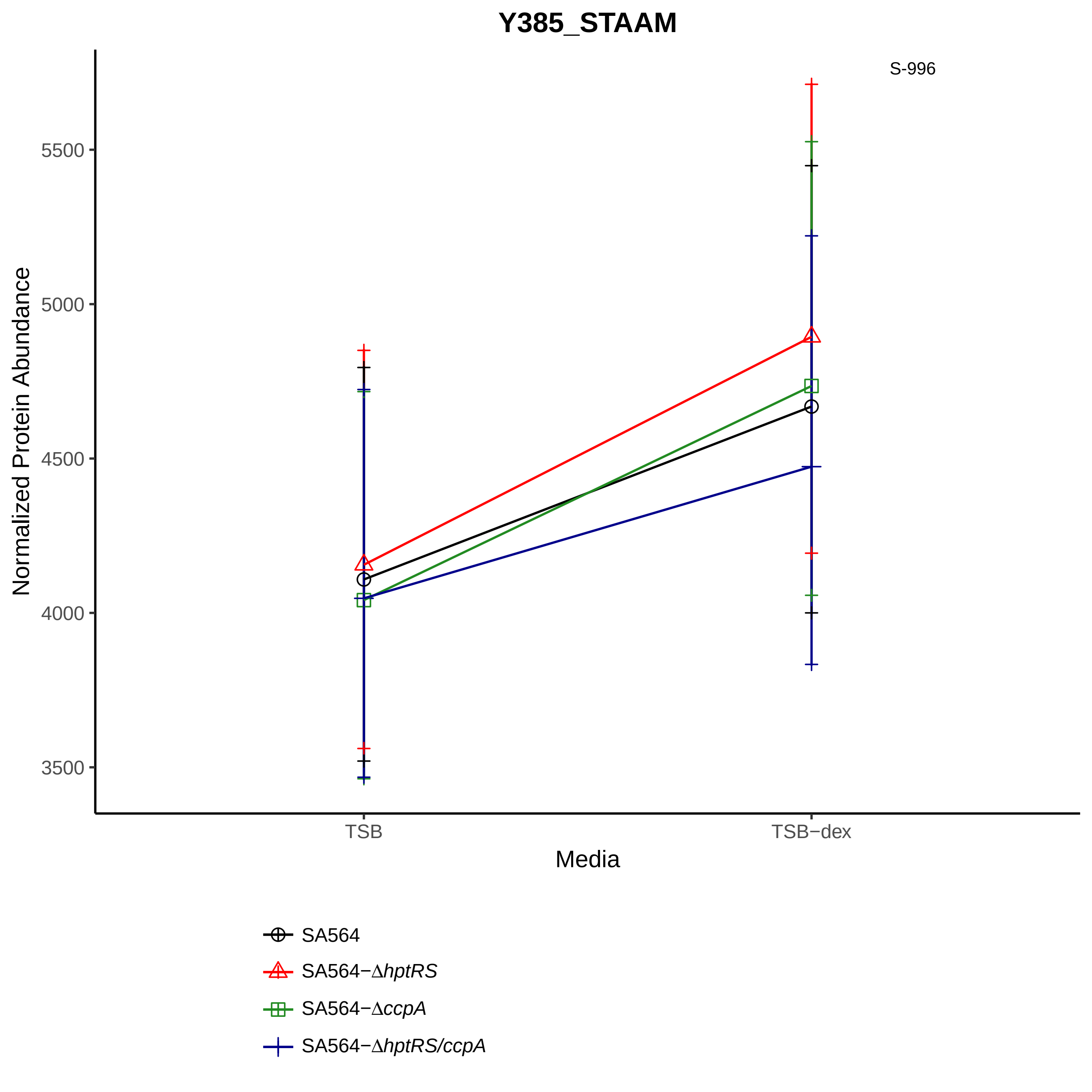

Y385_STAAM
S-996
Normalized Protein Abundance
5500
5000
4500
4000
3500
TSB
TSB−dex
Media
SA564
SA564−ΔhptRS
SA564−ΔccpA
SA564−ΔhptRS/ccpA

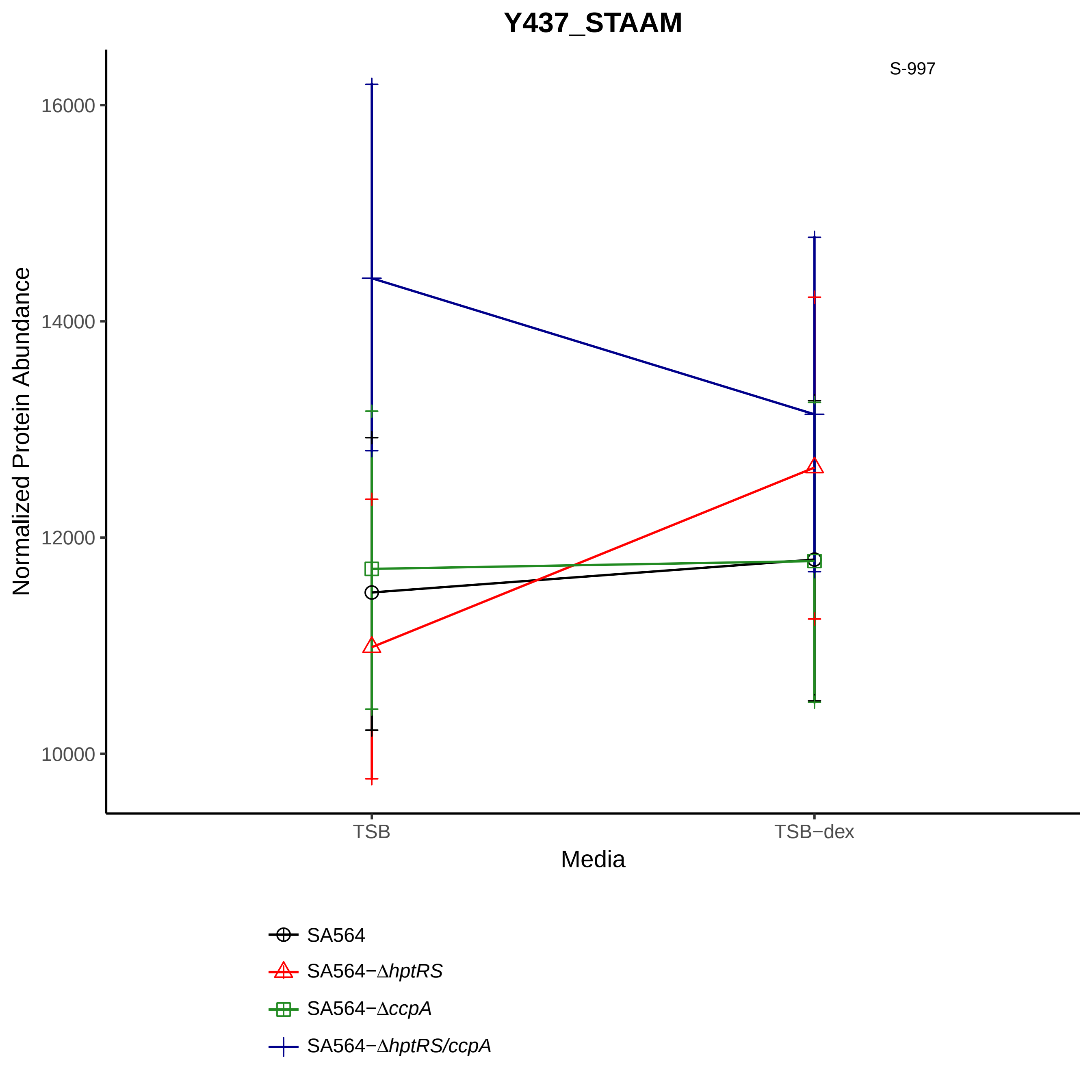

Y437_STAAM
S-997
Normalized Protein Abundance
16000
14000
12000
10000
TSB
TSB−dex
Media
SA564
SA564−Δ*hptRS*
SA564−Δ*ccpA*
SA564−Δ*hptRS/ccpA*

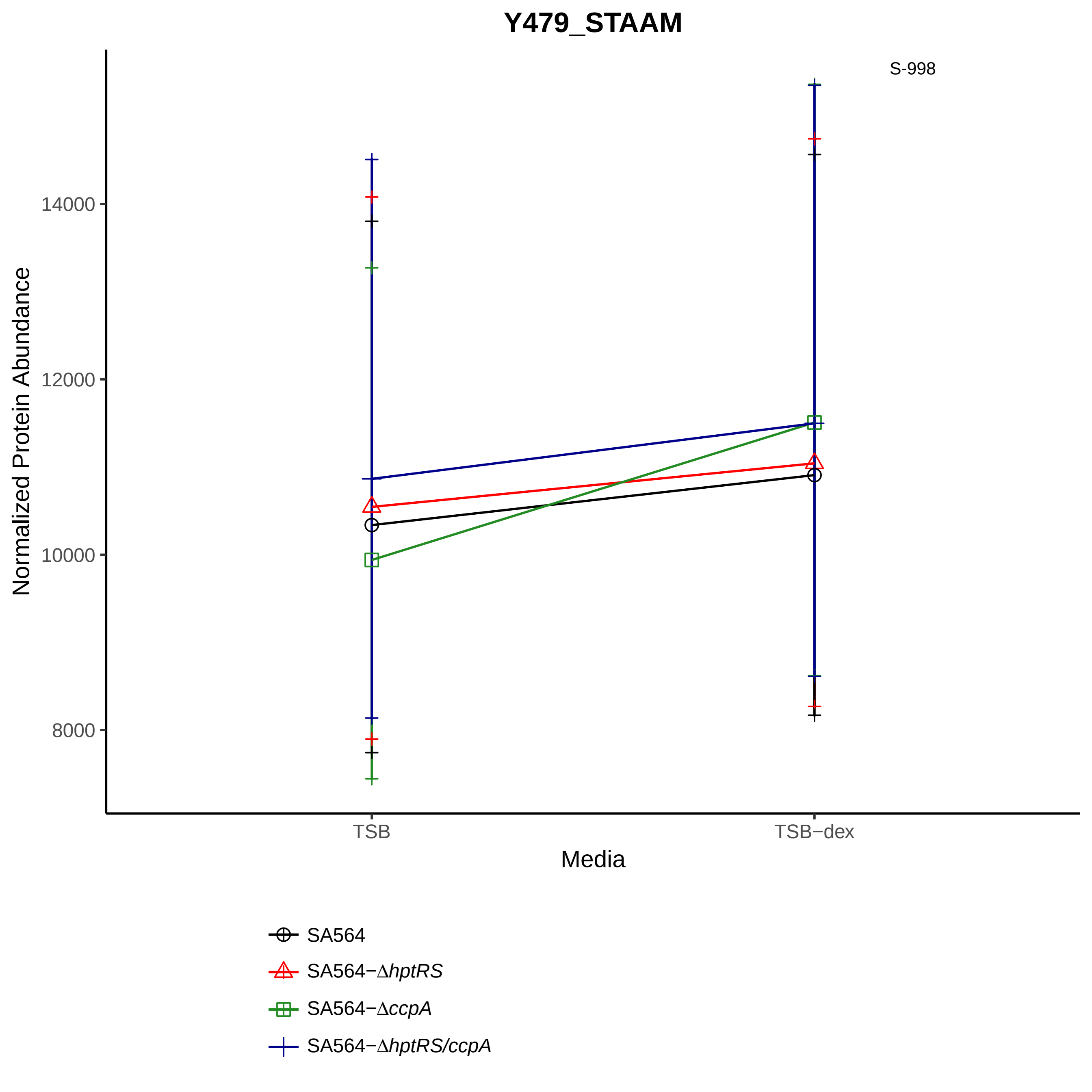
Y479_STAAM
S-998
Normalized Protein Abundance
14000
12000
10000
8000
TSB
TSB−dex
Media
SA564
SA564−Δ*hptRS*
SA564−Δ*ccpA*
SA564−Δ*hptRS/ccpA*

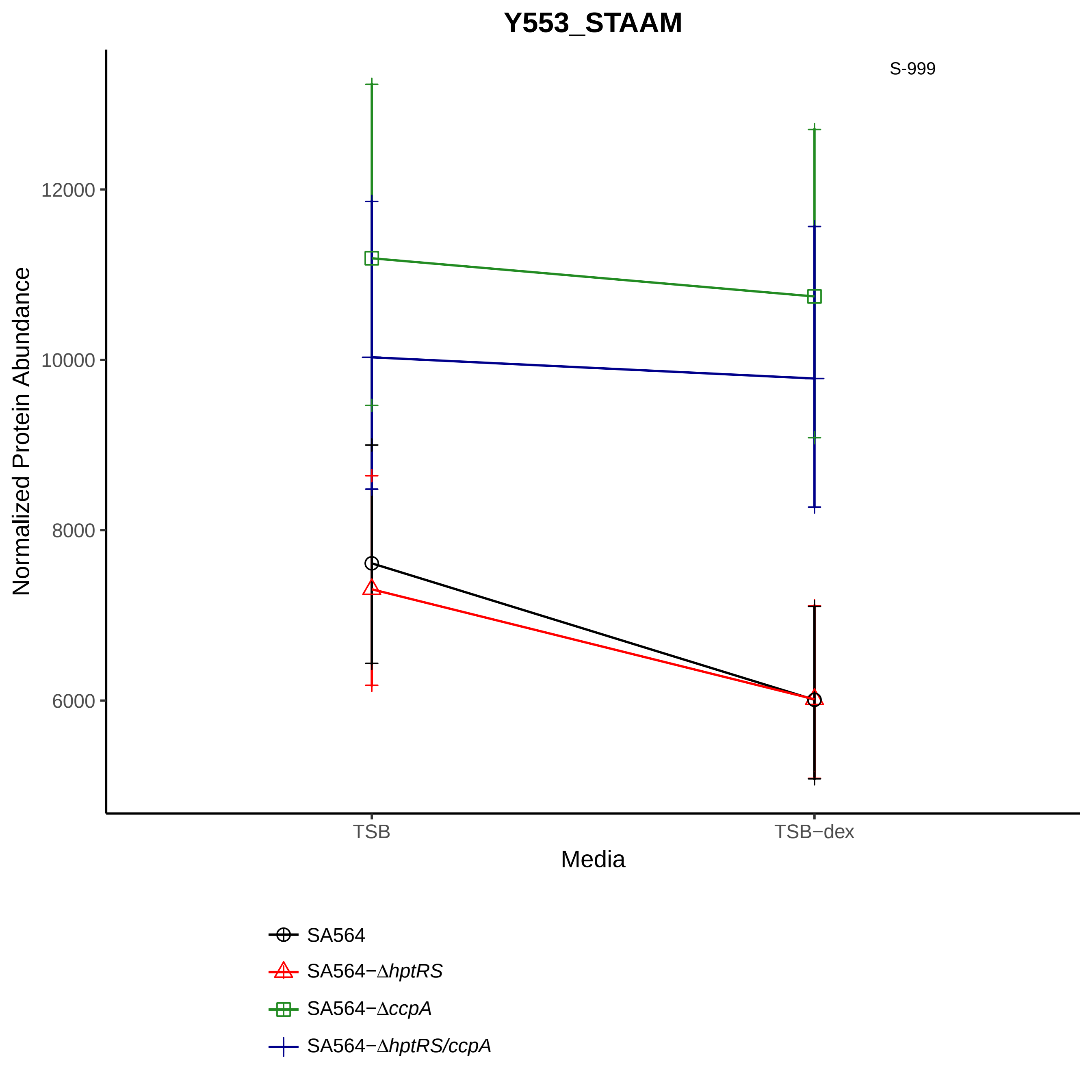

Y553_STAAM
S-999
Normalized Protein Abundance
12000
10000
8000
6000
TSB
TSB−dex
Media
SA564
SA564−Δ*hptRS*
SA564−Δ*ccpA*
SA564−Δ*hptRS/ccpA*

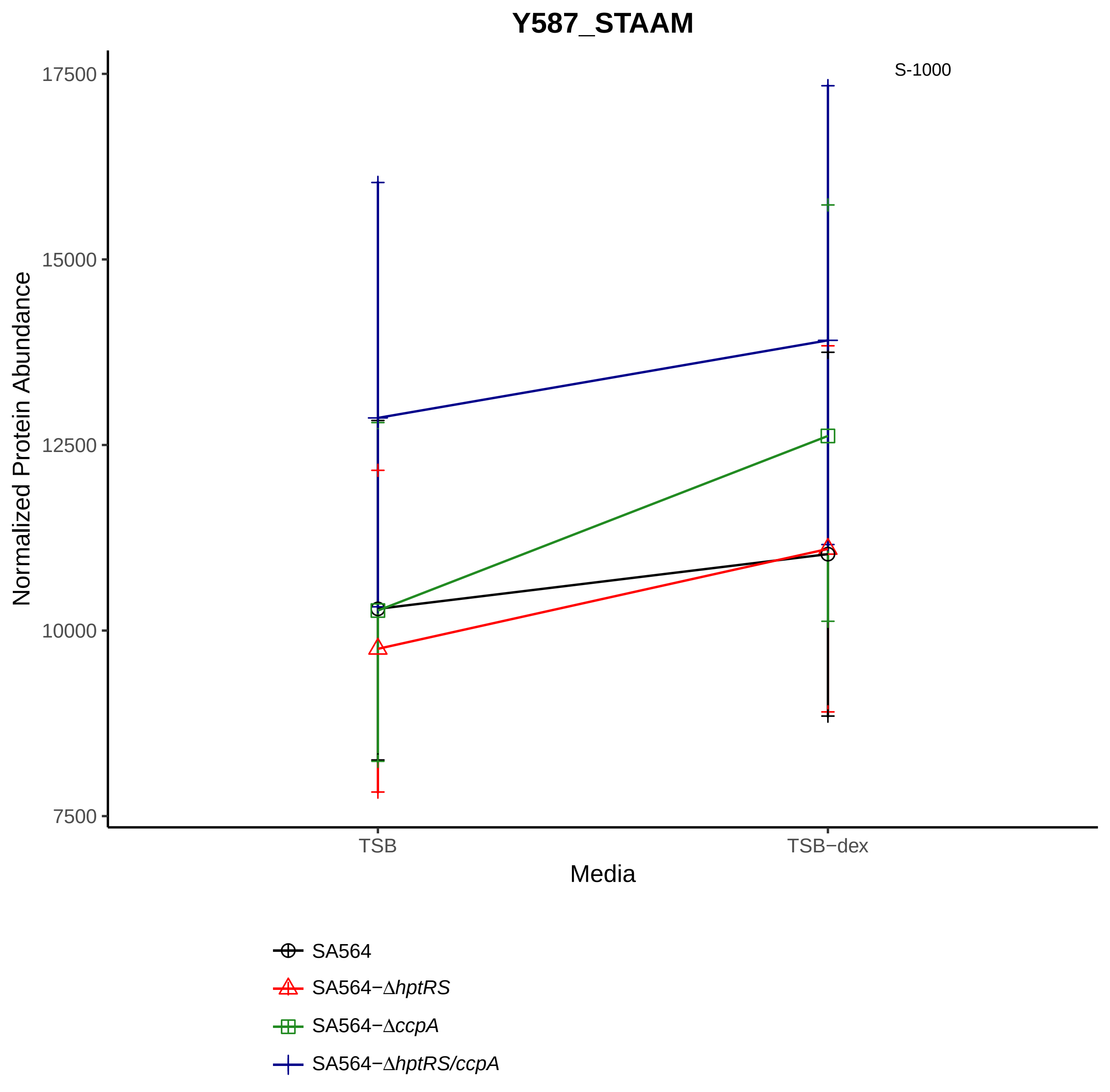
Y587_STAAM
S-1000
Normalized Protein Abundance
17500
15000
12500
10000
7500
TSB
TSB−dex
Media
SA564
SA564−ΔhptRS
SA564−ΔccpA
SA564−ΔhptRS/ccpA

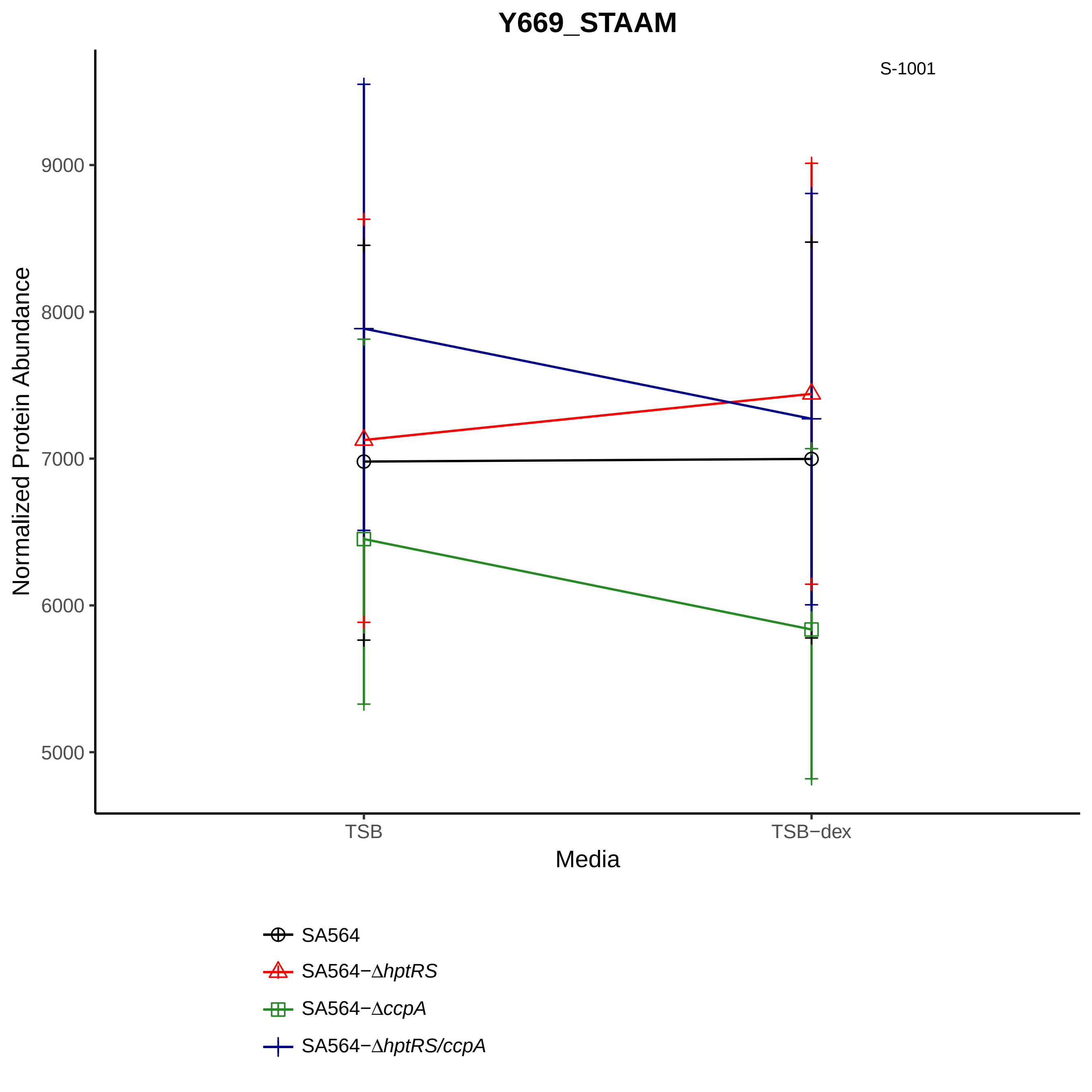

Y669_STAAM
S-1001
Normalized Protein Abundance
9000
8000
7000
6000
5000
TSB
TSB−dex
Media
SA564
SA564−Δ*hptRS*
SA564−Δ*ccpA*
SA564−Δ*hptRS/ccpA*

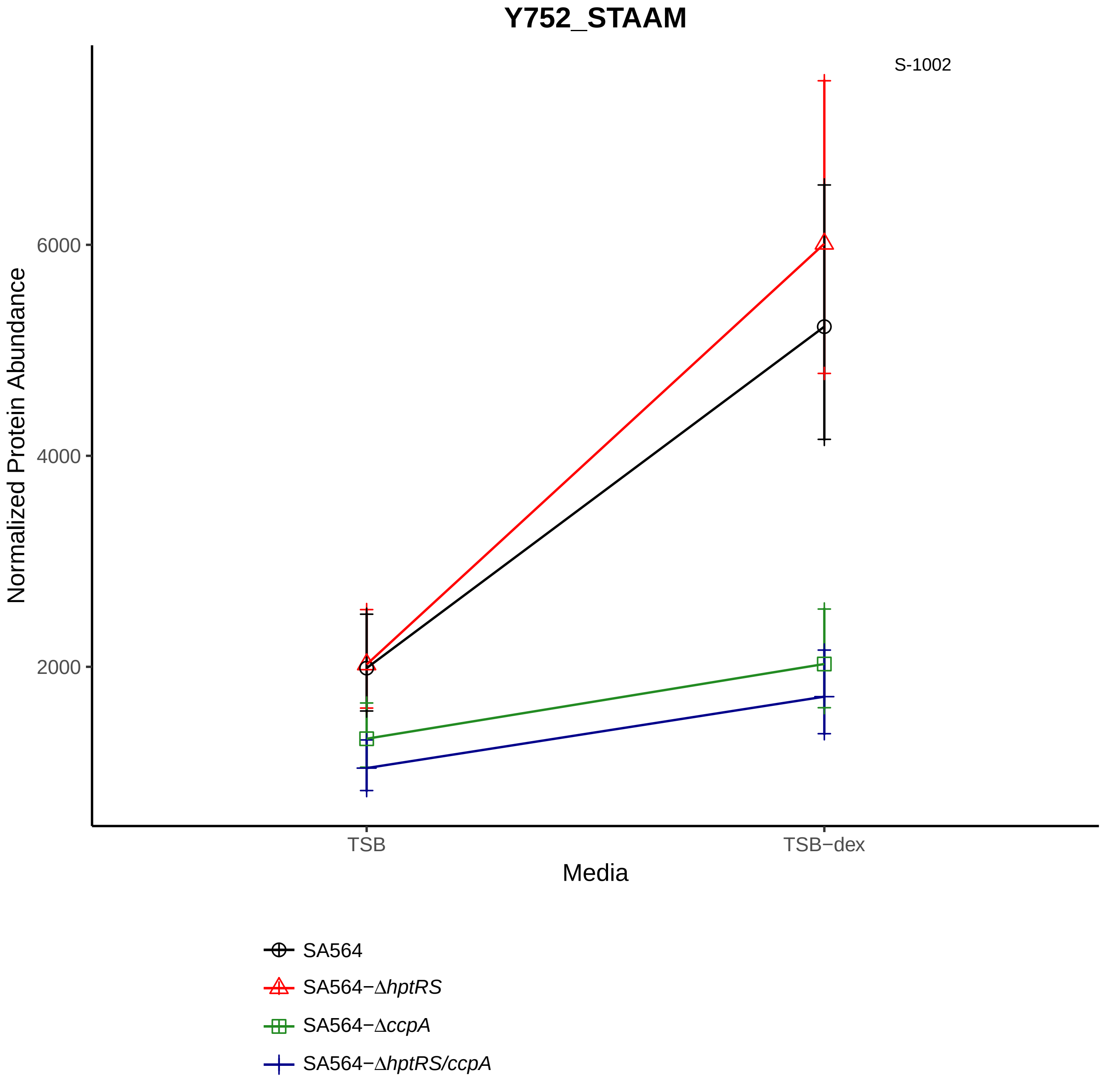
Y752_STAAM
S-1002
Normalized Protein Abundance
6000
4000
2000
TSB
TSB−dex
Media
SA564
SA564−Δ*hptRS*
SA564−Δ*ccpA*
SA564−Δ*hptRS/ccpA*

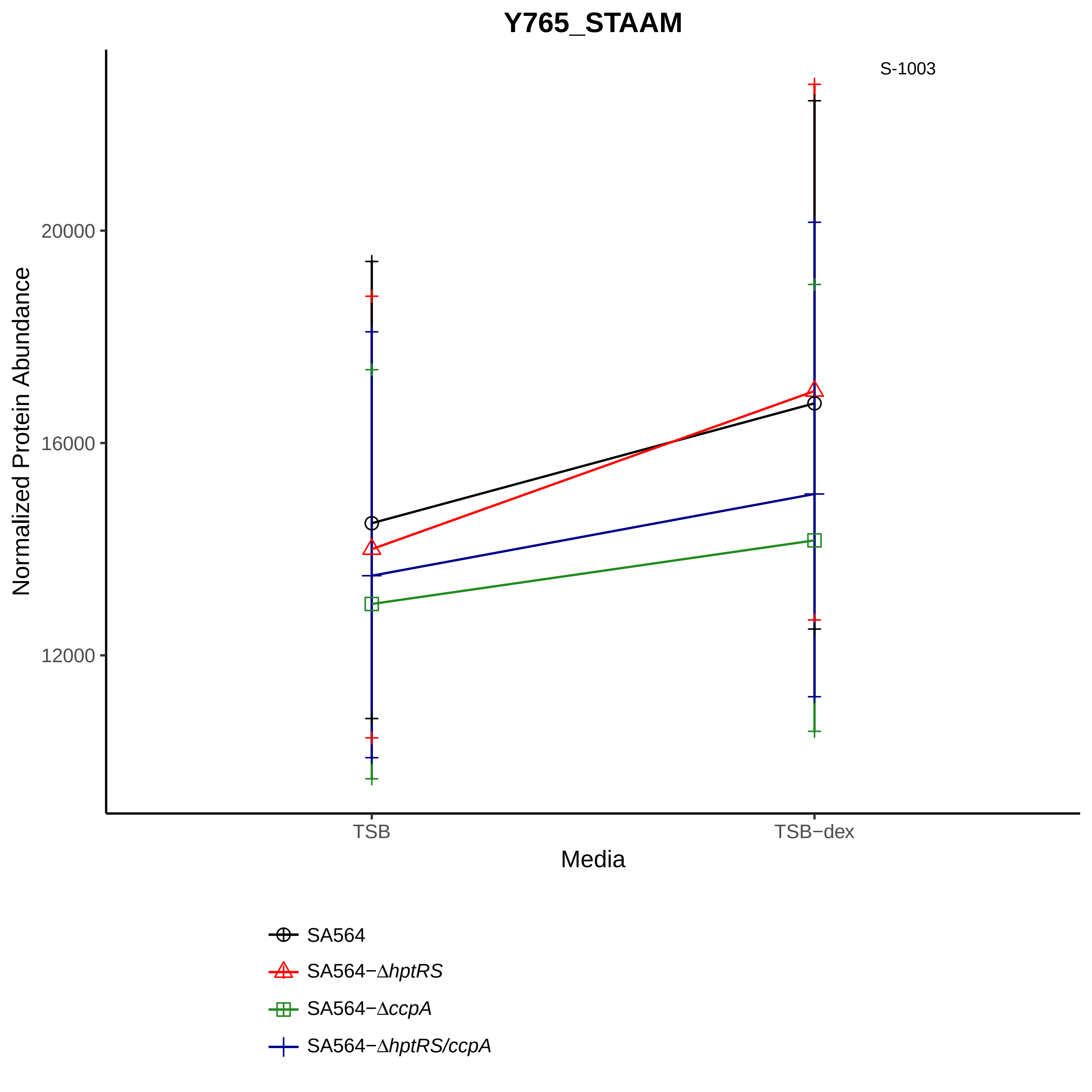

Y765_STAAM
S-1003
Normalized Protein Abundance
20000
16000
12000
TSB
TSB−dex
Media
SA564
SA564−ΔhptRS
SA564−ΔccpA
SA564−ΔhptRS/ccpA

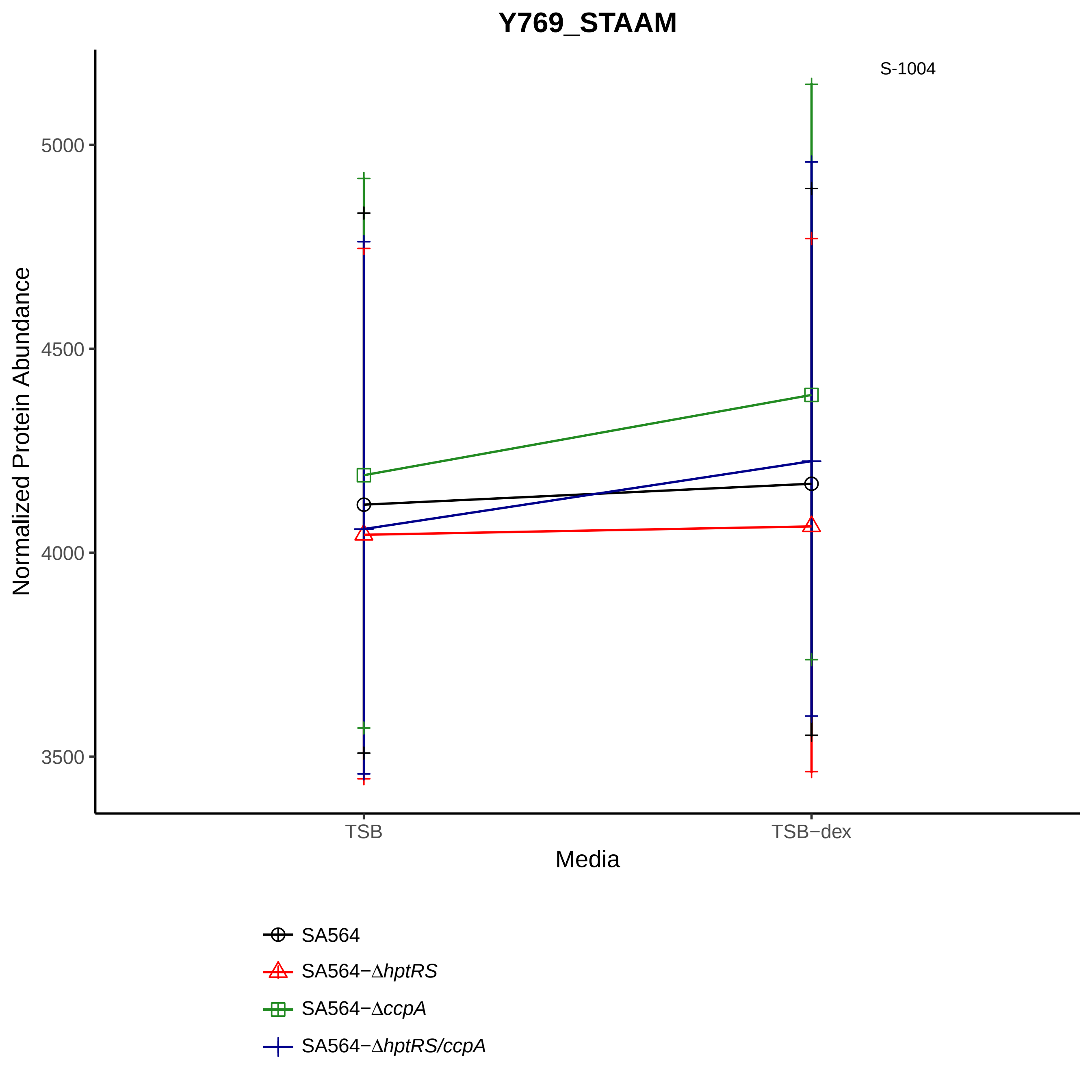

Y769_STAAM
S-1004
Normalized Protein Abundance
5000
4500
4000
3500
TSB
TSB−dex
Media
SA564
SA564−ΔhptRS
SA564−ΔccpA
SA564−ΔhptRS/ccpA

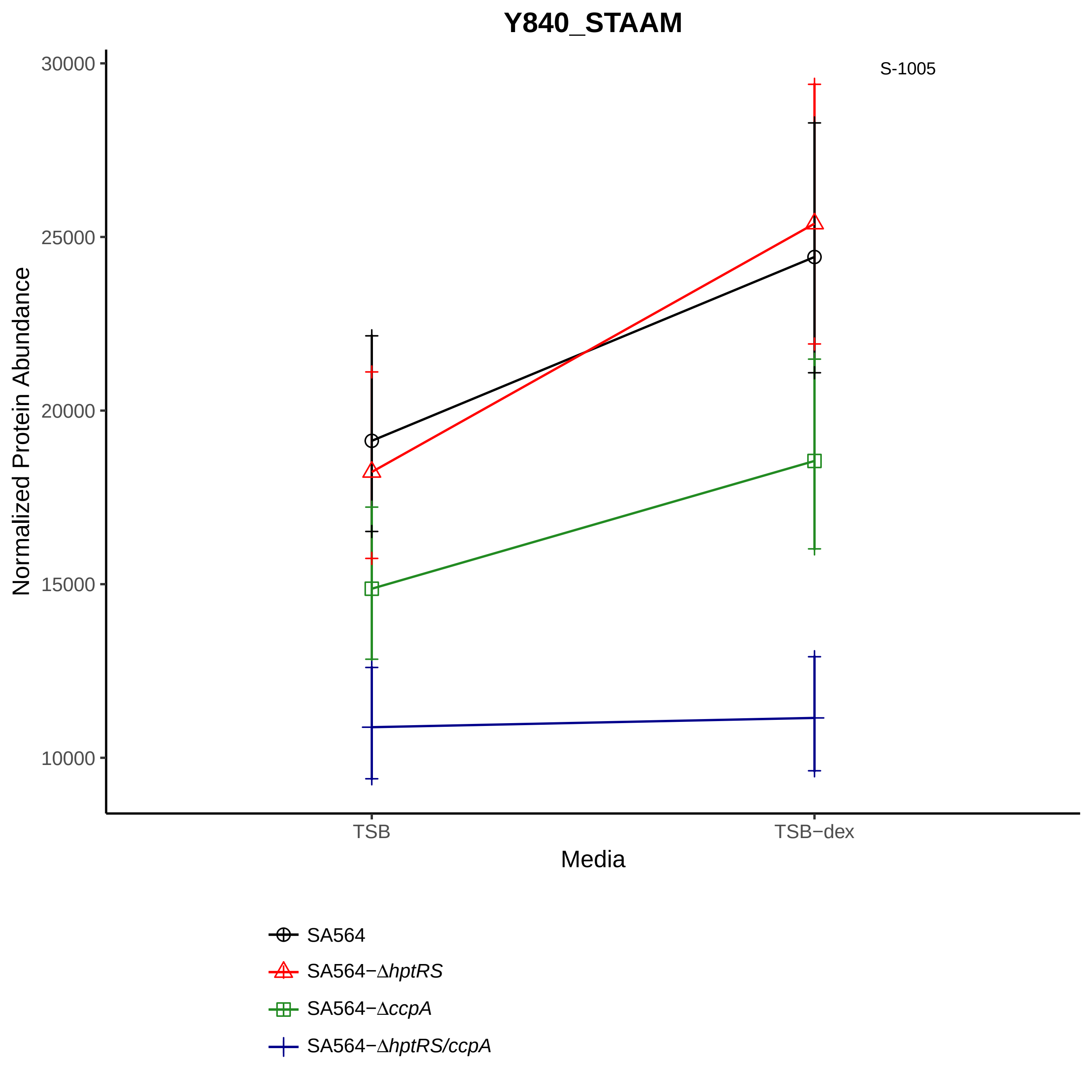

Y840_STAAM
S-1005
Normalized Protein Abundance
30000
25000
20000
15000
10000
TSB
TSB−dex
Media
SA564
SA564−Δ*hptRS*
SA564−Δ*ccpA*
SA564−Δ*hptRS/ccpA*

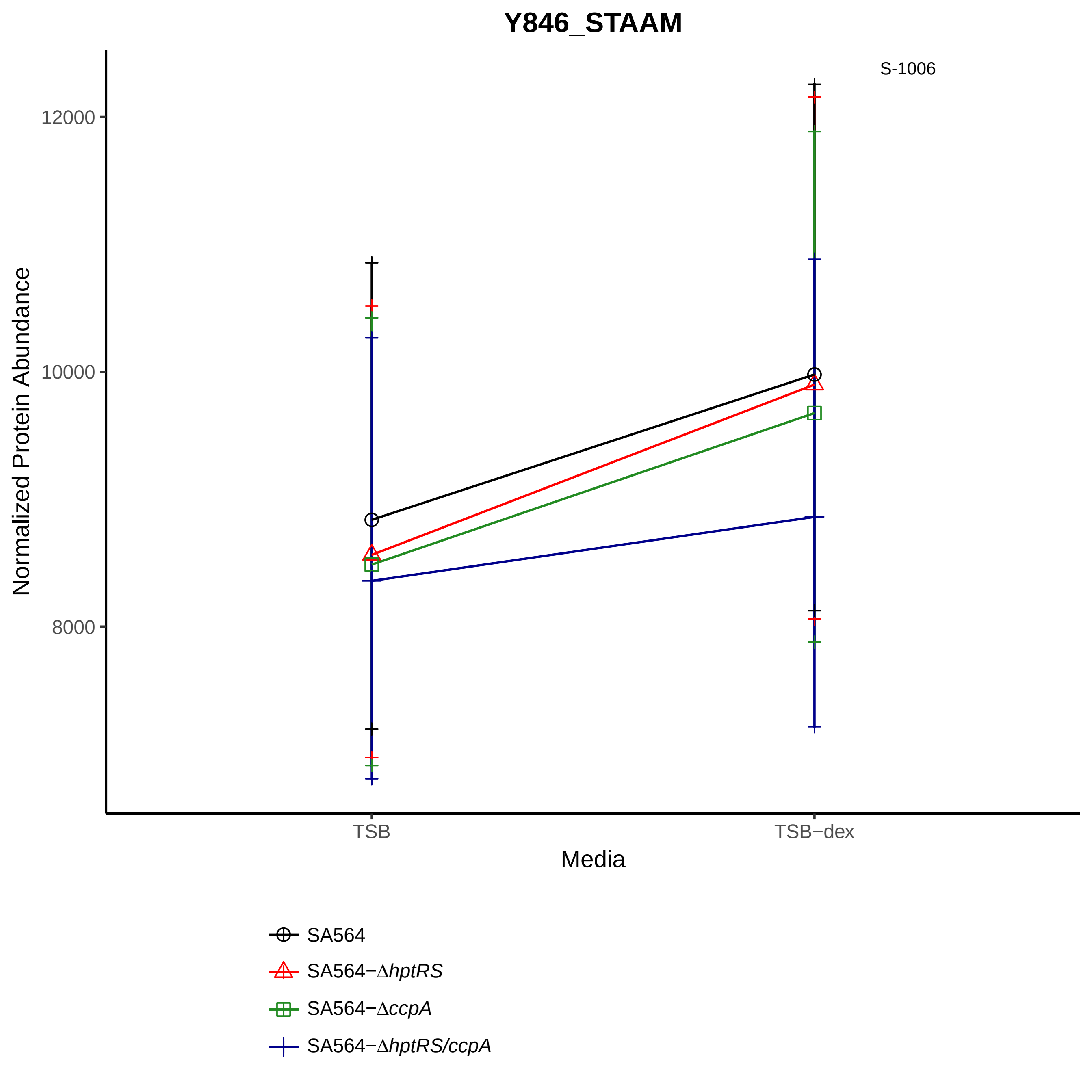

Y846_STAAM
S-1006
12000
10000
8000
Normalized Protein Abundance
TSB
TSB−dex
Media
SA564
SA564−Δ*hptRS*
SA564−Δ*ccpA*
SA564−Δ*hptRS/ccpA*

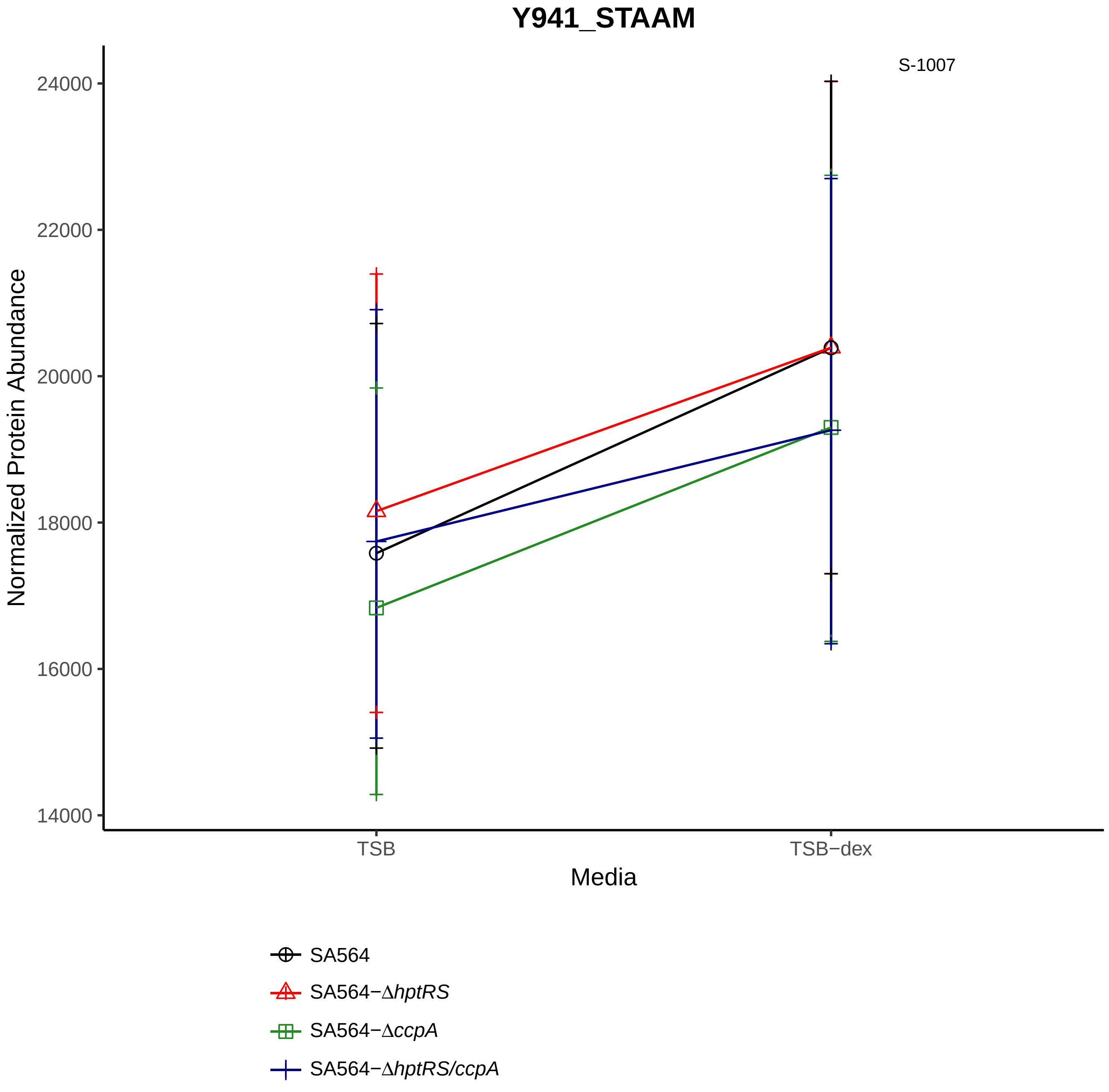

Y941_STAAM
S-1007
Normalized Protein Abundance
24000
22000
20000
18000
16000
14000
TSB
TSB−dex
Media
SA564
SA564−ΔhptRS
SA564−ΔccpA
SA564−ΔhptRS/ccpA

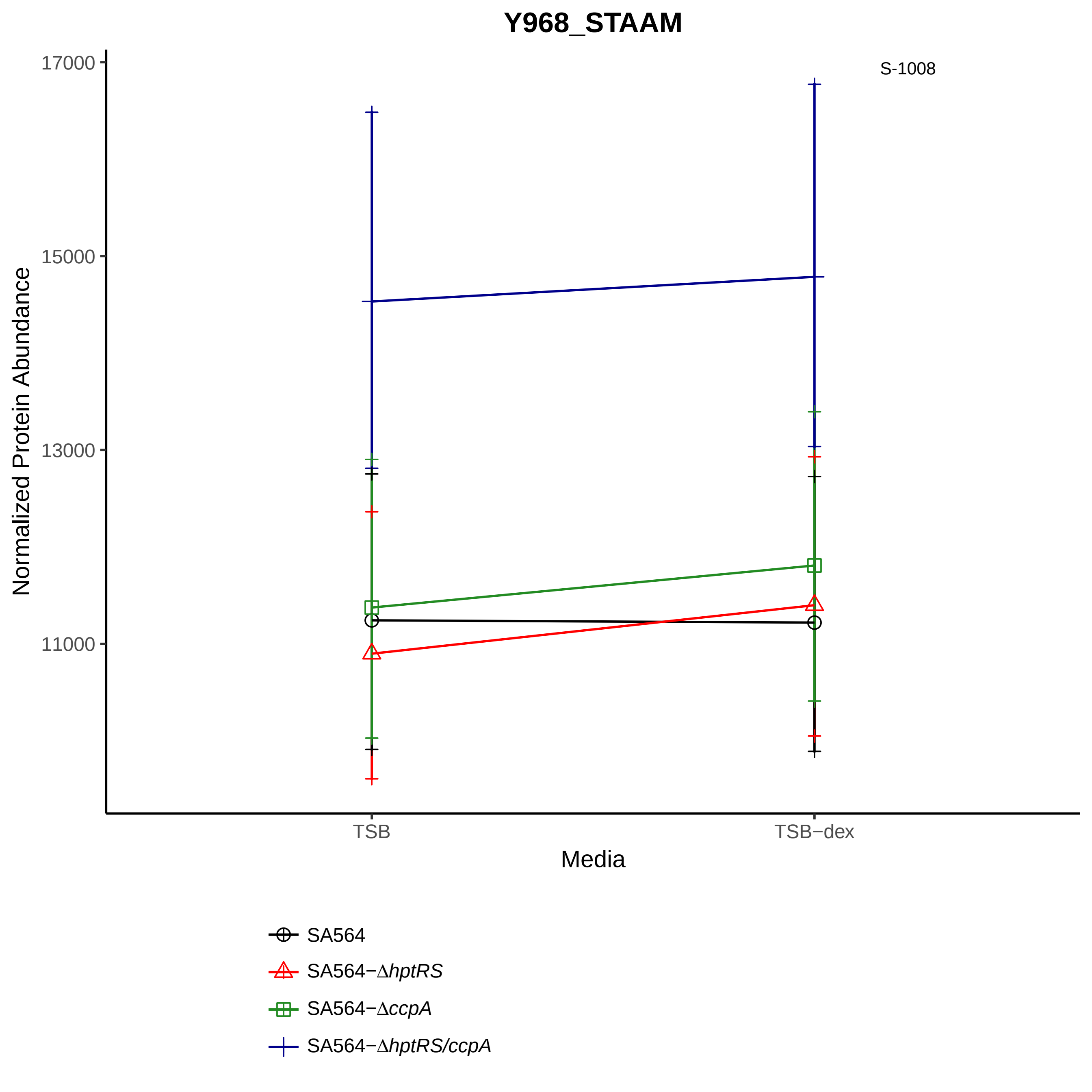

Y968_STAAM
S-1008
Normalized Protein Abundance
17000
15000
13000
11000
TSB
TSB−dex
Media
SA564
SA564−ΔhptRS
SA564−ΔccpA
SA564−ΔhptRS/ccpA

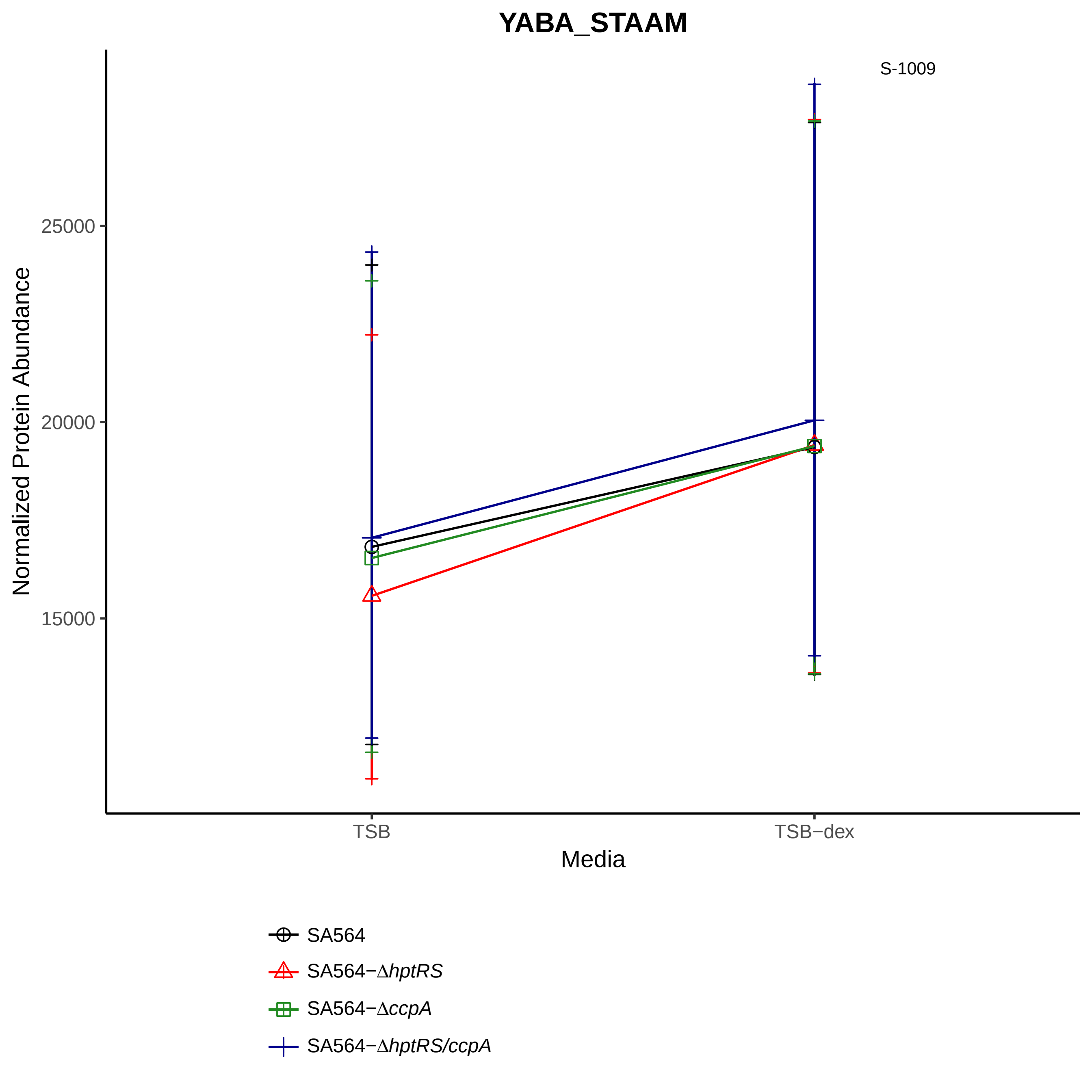
YABA_STAAM
S-1009
Normalized Protein Abundance
25000
20000
15000
TSB
TSB−dex
Media
SA564
SA564−ΔhptRS
SA564−ΔccpA
SA564−ΔhptRS/ccpA

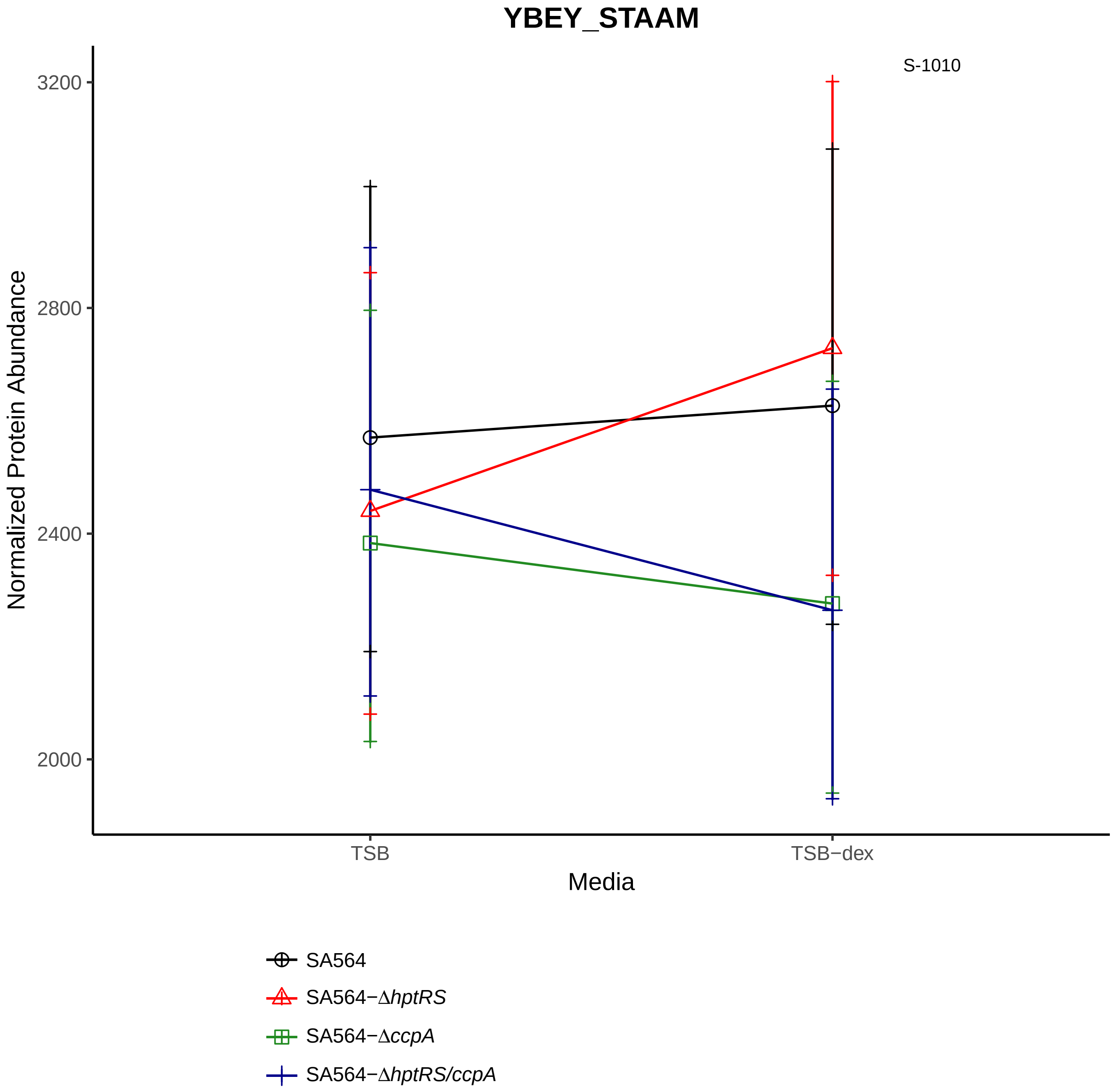

YBEY_STAAM
S-1010
Normalized Protein Abundance
3200
2800
2400
2000
TSB
TSB−dex
Media
SA564
SA564−Δ*hptRS*
SA564−Δ*ccpA*
SA564−Δ*hptRS/ccpA*

Figure H.

2-way analysis: s x h | m

Pages: S1011-S1512

The normalized protein abundance, lower 95% confidence interval, and upper 95% confidence interval were plotted for each protein. These plots were analyzed if a 3-way interaction was rejected and 2-way interaction was identified between strain and hour.

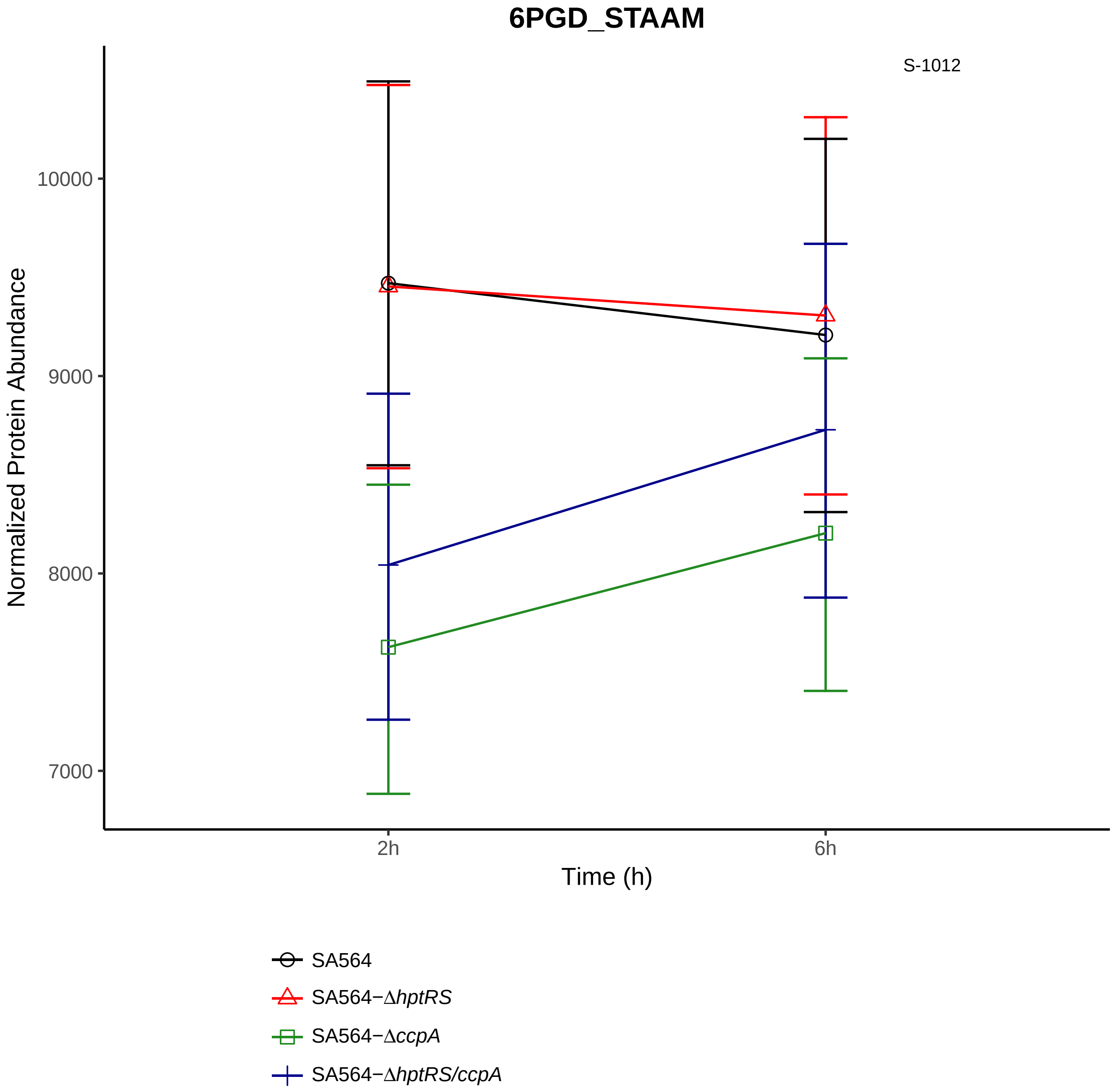

6PGD_STAAM
S-1012
Normalized Protein Abundance
10000
9000
8000
7000
2h
6h
Time (h)
SA564
SA564−Δ*hptRS*
SA564−Δ*ccpA*
SA564−Δ*hptRS/ccpA*

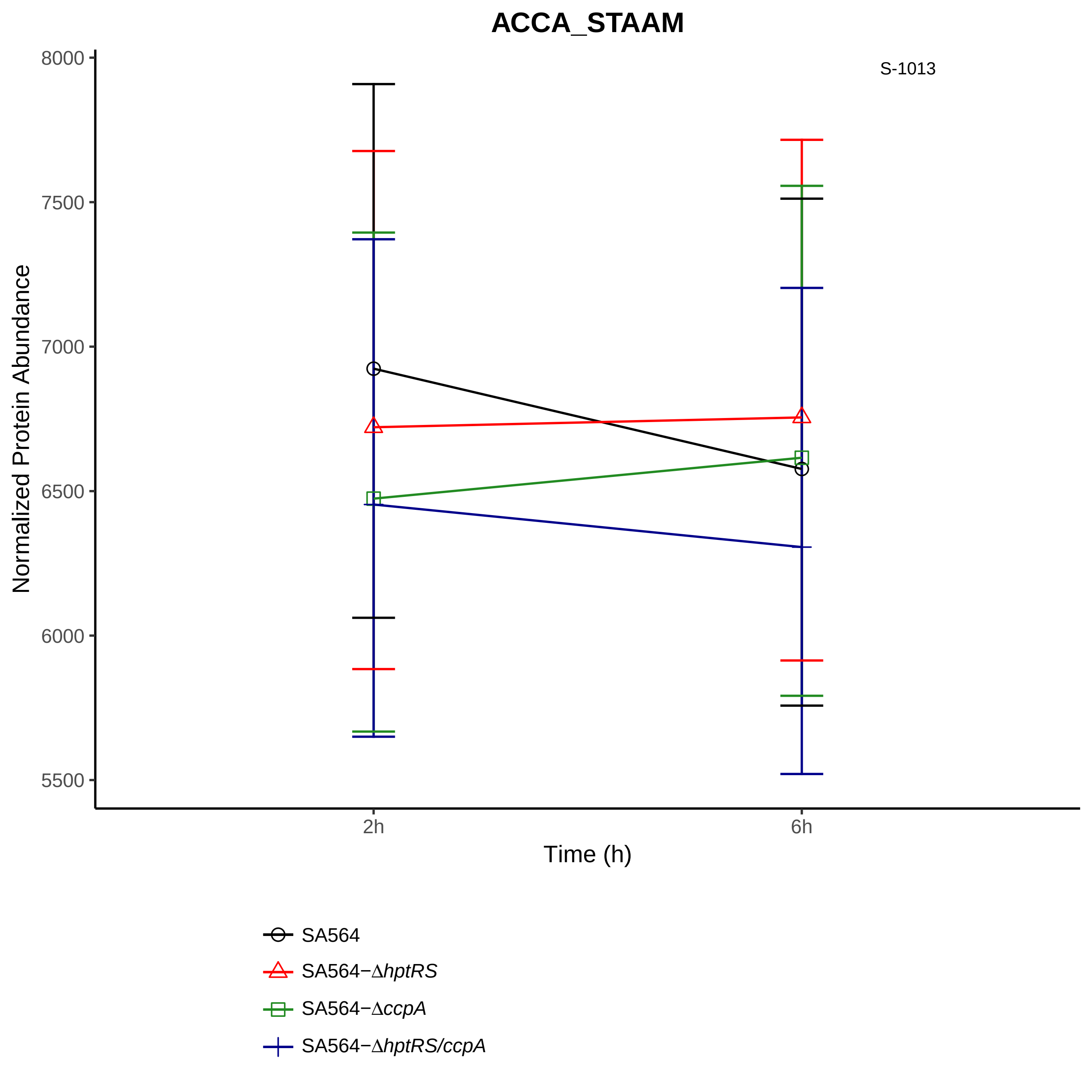

ACCA_STAAM
S-1013
Normalized Protein Abundance
8000
7500
7000
6500
6000
5500
2h
6h
Time (h)
SA564
SA564−Δ*hptRS*
SA564−Δ*ccpA*
SA564−Δ*hptRS/ccpA*

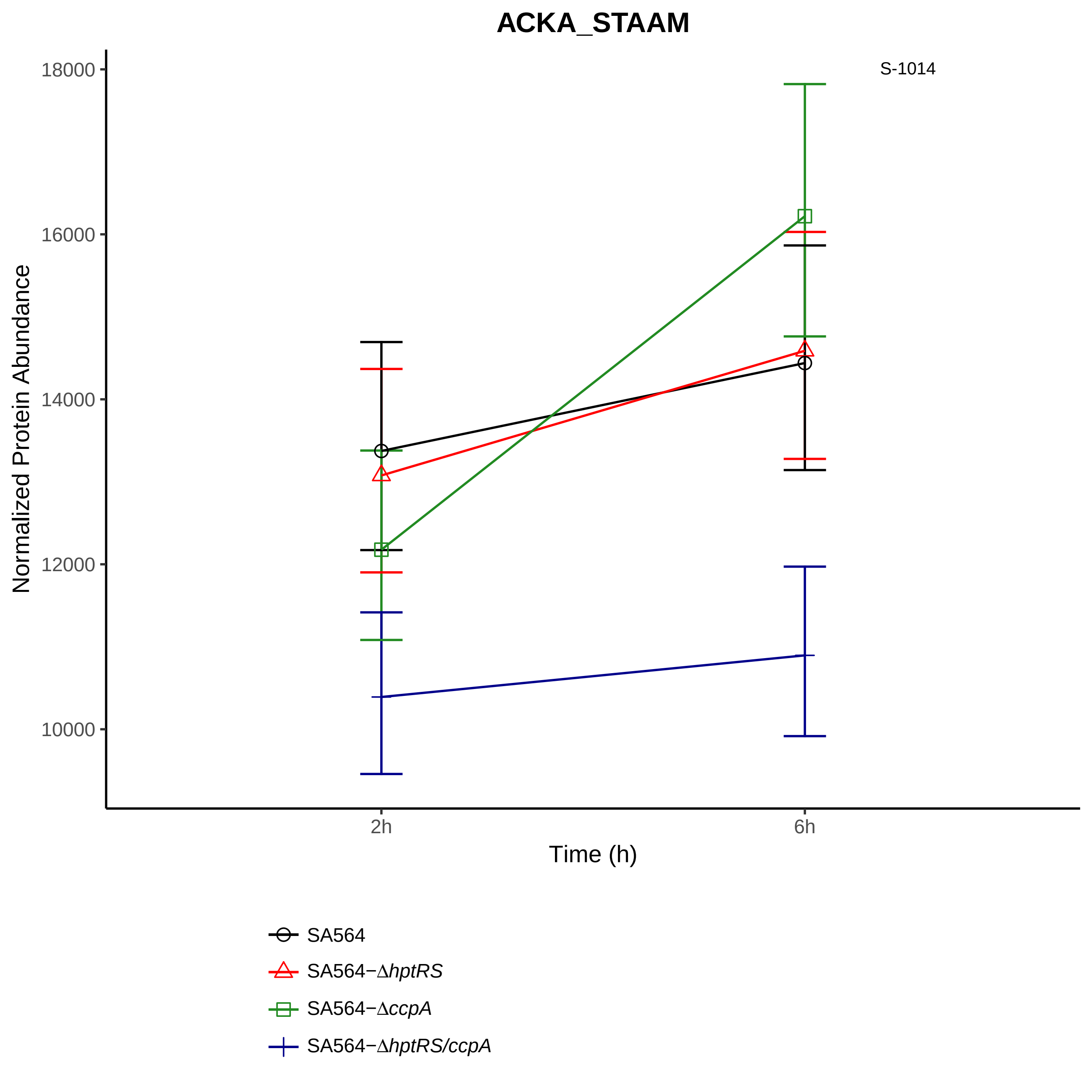
ACKA_STAAM
S-1014
Normalized Protein Abundance
18000
16000
14000
12000
10000
2h
6h
Time (h)
SA564
SA564−Δ*hptRS*
SA564−Δ*ccpA*
SA564−Δ*hptRS/ccpA*

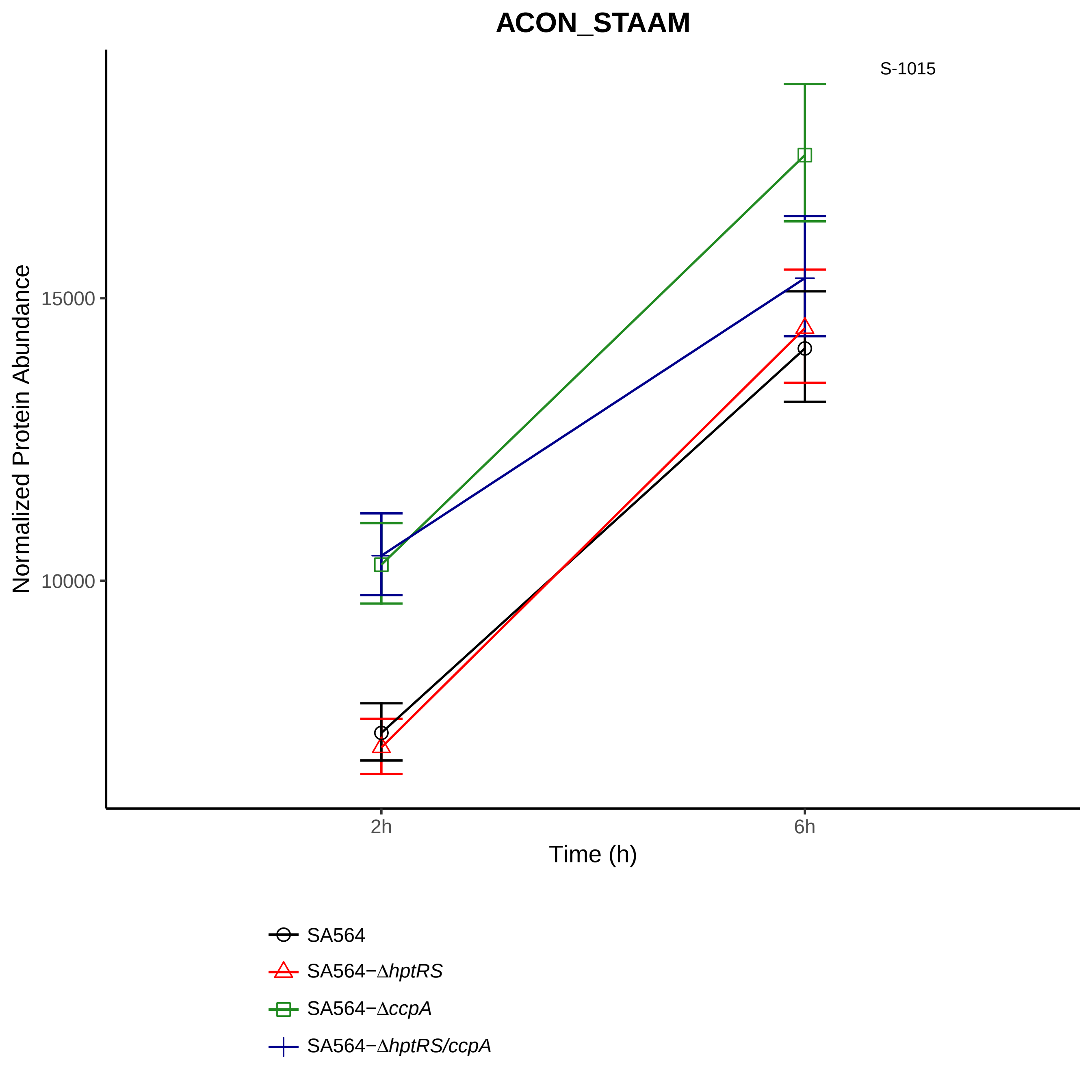

ACON_STAAM
S-1015
Normalized Protein Abundance
15000
10000
2h
6h
Time (h)
SA564
SA564−Δ*hptRS*
SA564−Δ*ccpA*
SA564−Δ*hptRS/ccpA*

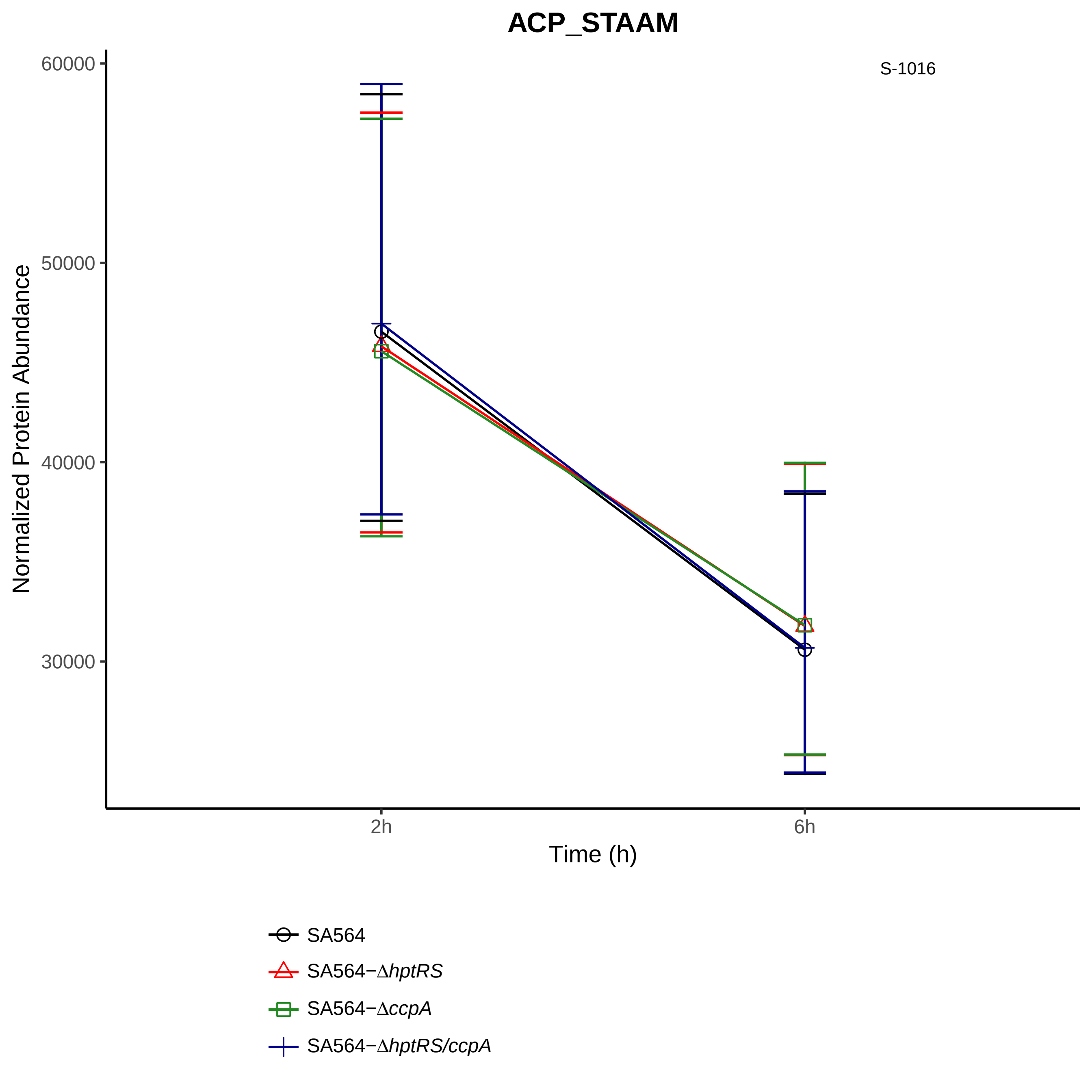

ACP_STAAM
S-1016
60000
50000
40000
30000
Normalized Protein Abundance
2h
6h
Time (h)
SA564
SA564−Δ*hptRS*
SA564−Δ*ccpA*
SA564−Δ*hptRS/ccpA*

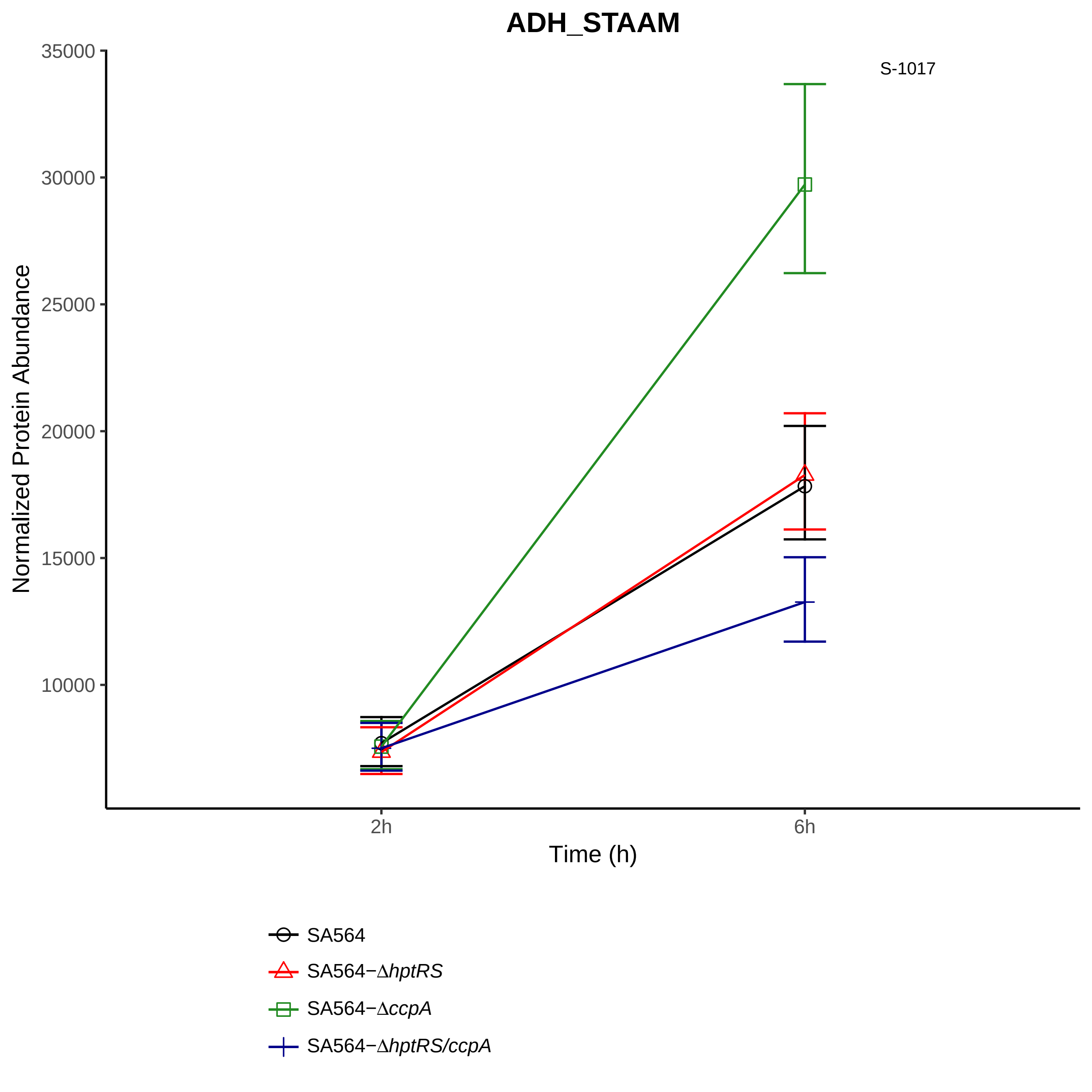

ADH_STAAM
S-1017
35000
30000
25000
20000
15000
10000
Normalized Protein Abundance
2h
6h
Time (h)
SA564
SA564−Δ*hptRS*
SA564−Δ*ccpA*
SA564−Δ*hptRS/ccpA*

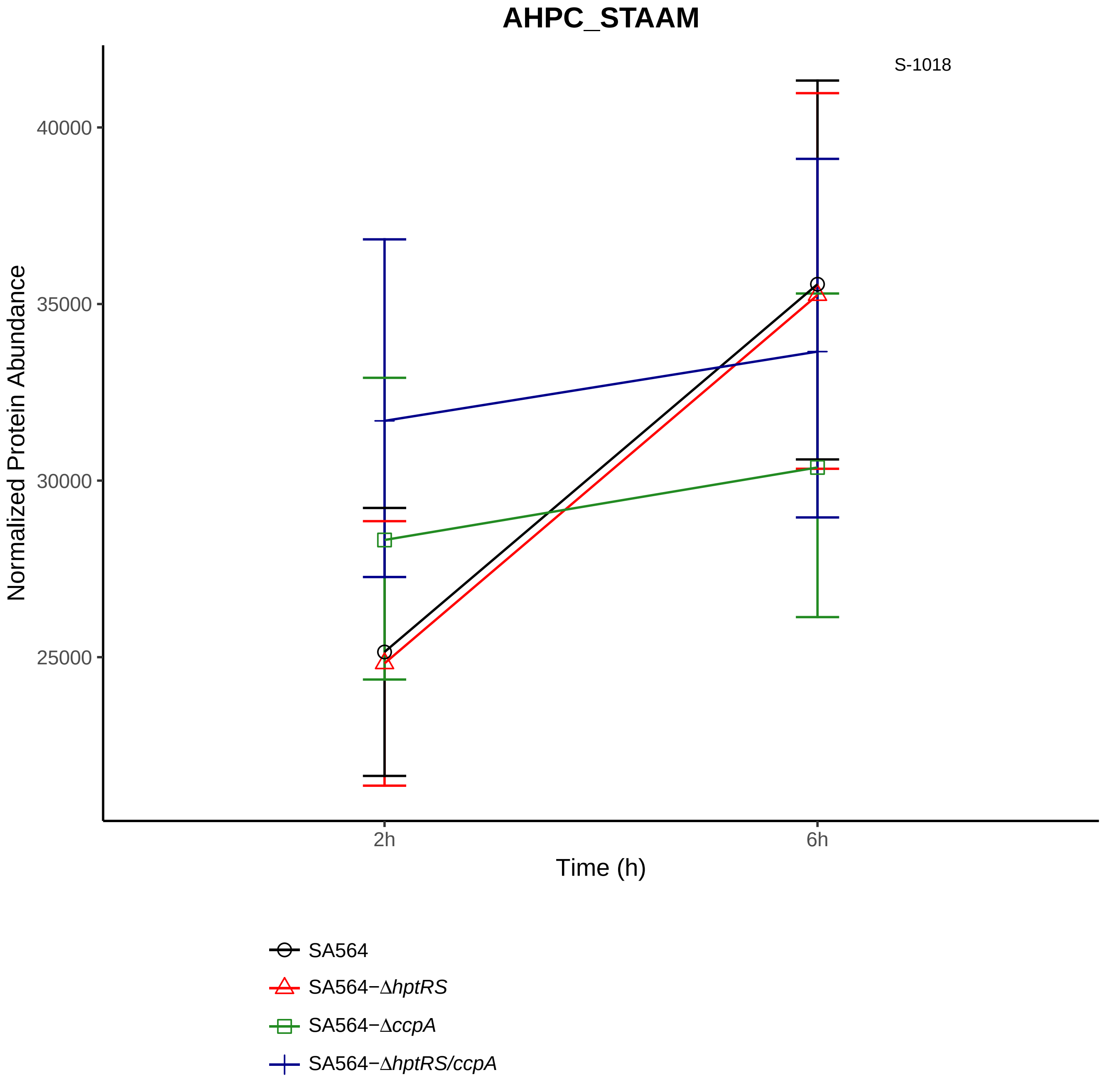

AHPC_STAAM
S-1018
Normalized Protein Abundance
40000
35000
30000
25000
2h
6h
Time (h)
SA564
SA564−Δ*hptRS*
SA564−Δ*ccpA*
SA564−Δ*hptRS/ccpA*

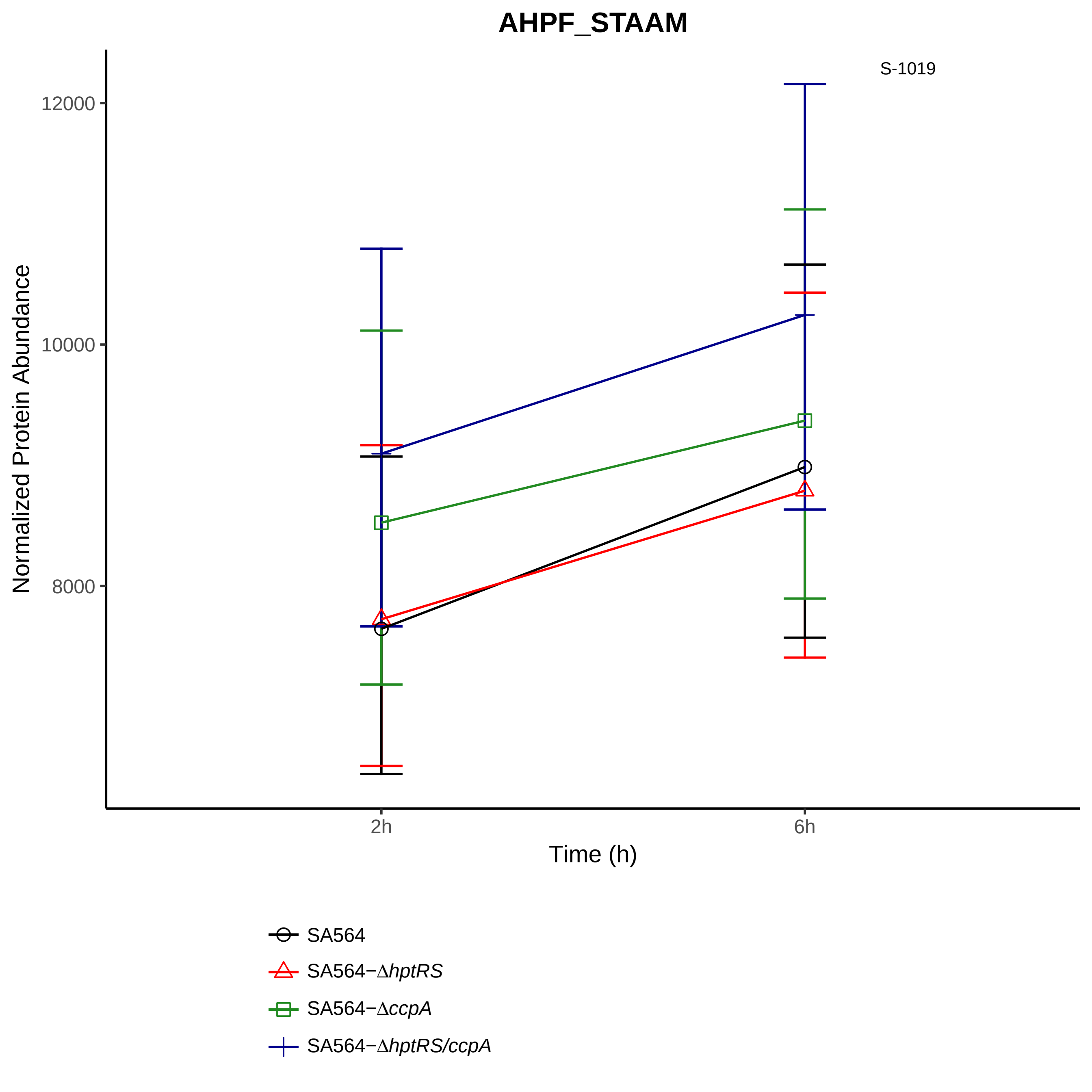

AHPF_STAAM
S-1019
12000
10000
8000
Normalized Protein Abundance
2h
6h
Time (h)
SA564
SA564−Δ*hptRS*
SA564−Δ*ccpA*
SA564−Δ*hptRS/ccpA*

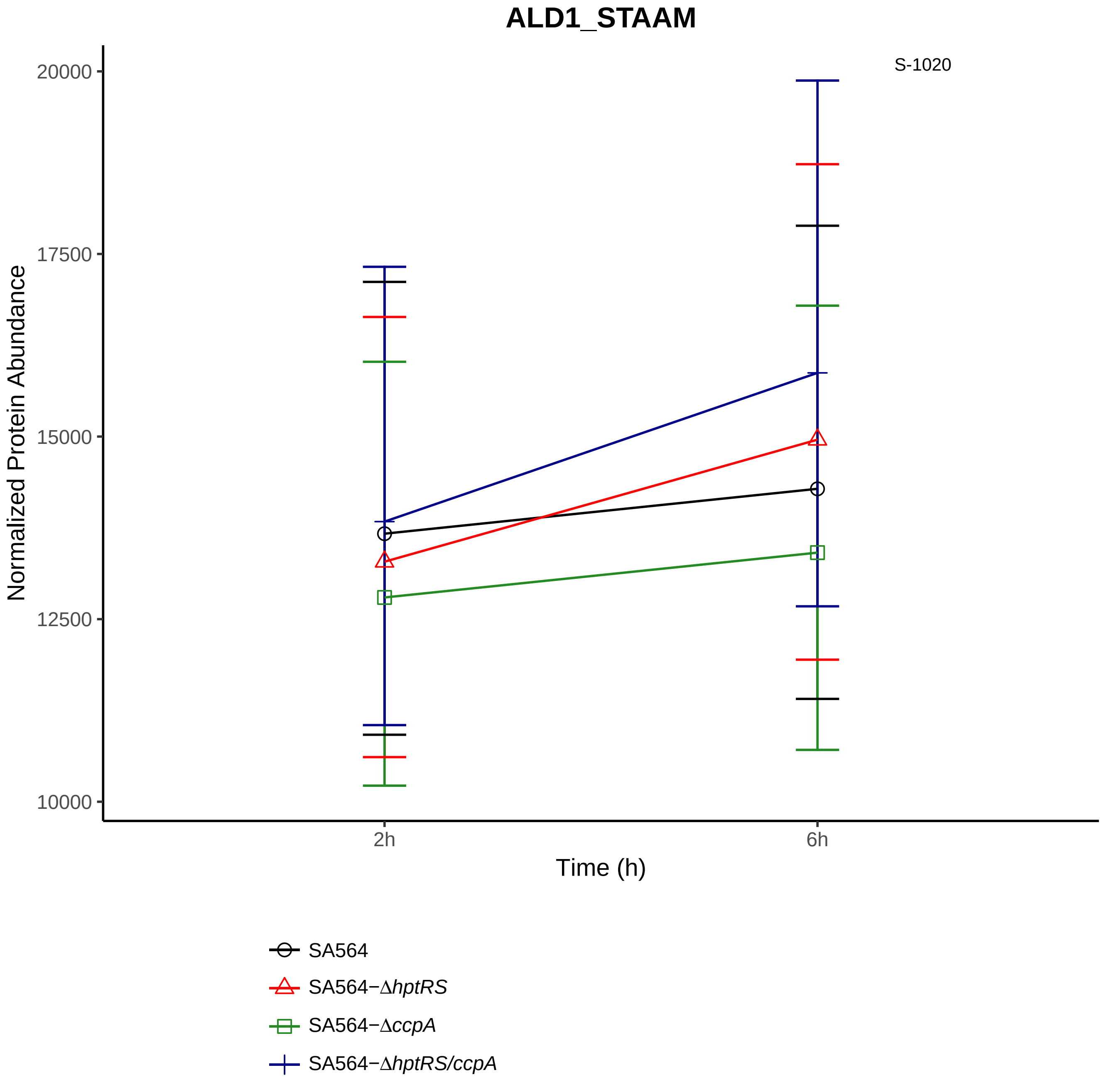
ALD1_STAAM
S-1020
Normalized Protein Abundance
20000
17500
15000
12500
10000
2h
6h
Time (h)
SA564
SA564−Δ*hptRS*
SA564−Δ*ccpA*
SA564−Δ*hptRS/ccpA*

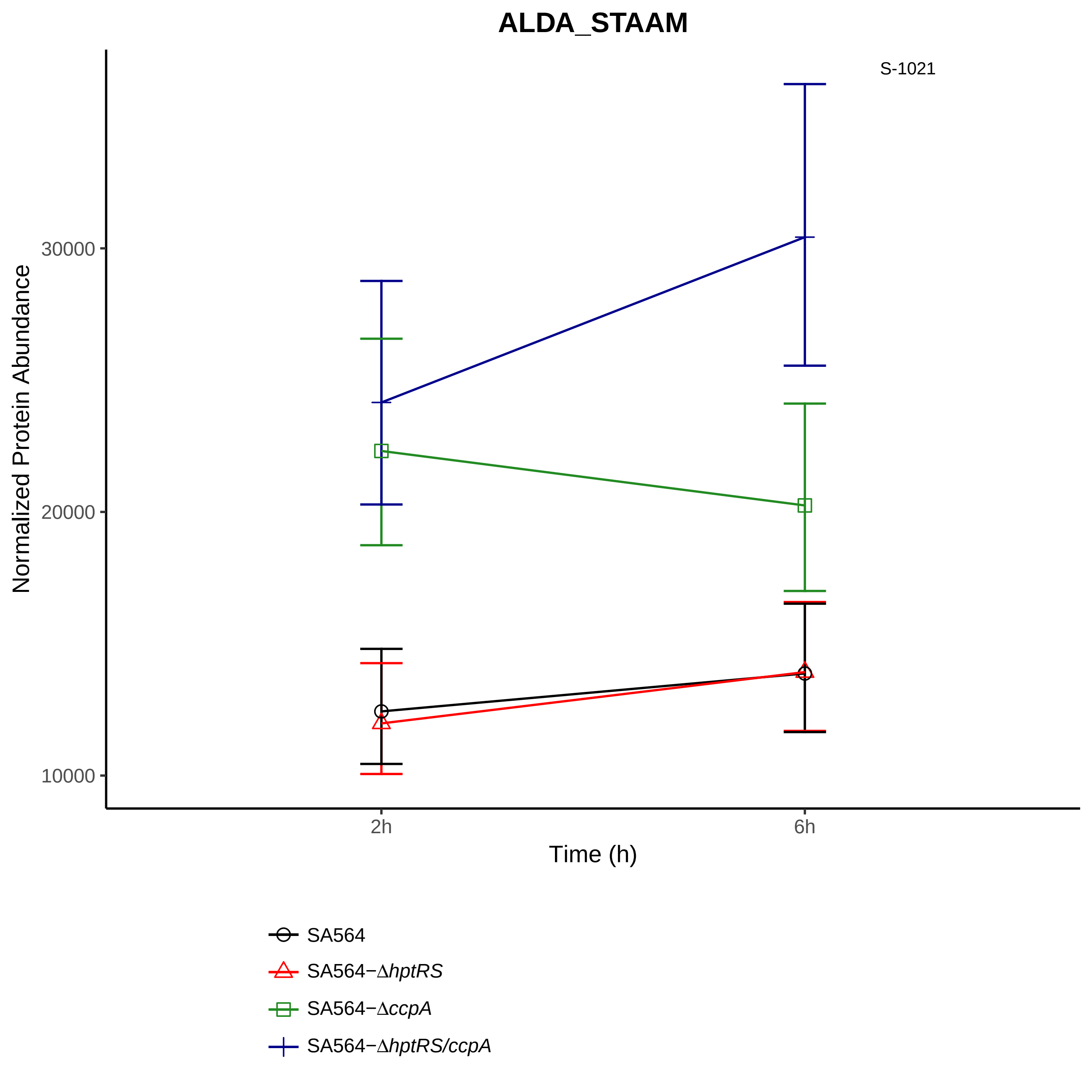

ALDA_STAAM
S-1021
Normalized Protein Abundance
30000
20000
10000
2h
6h
Time (h)
SA564
SA564−Δ*hptRS*
SA564−Δ*ccpA*
SA564−Δ*hptRS/ccpA*

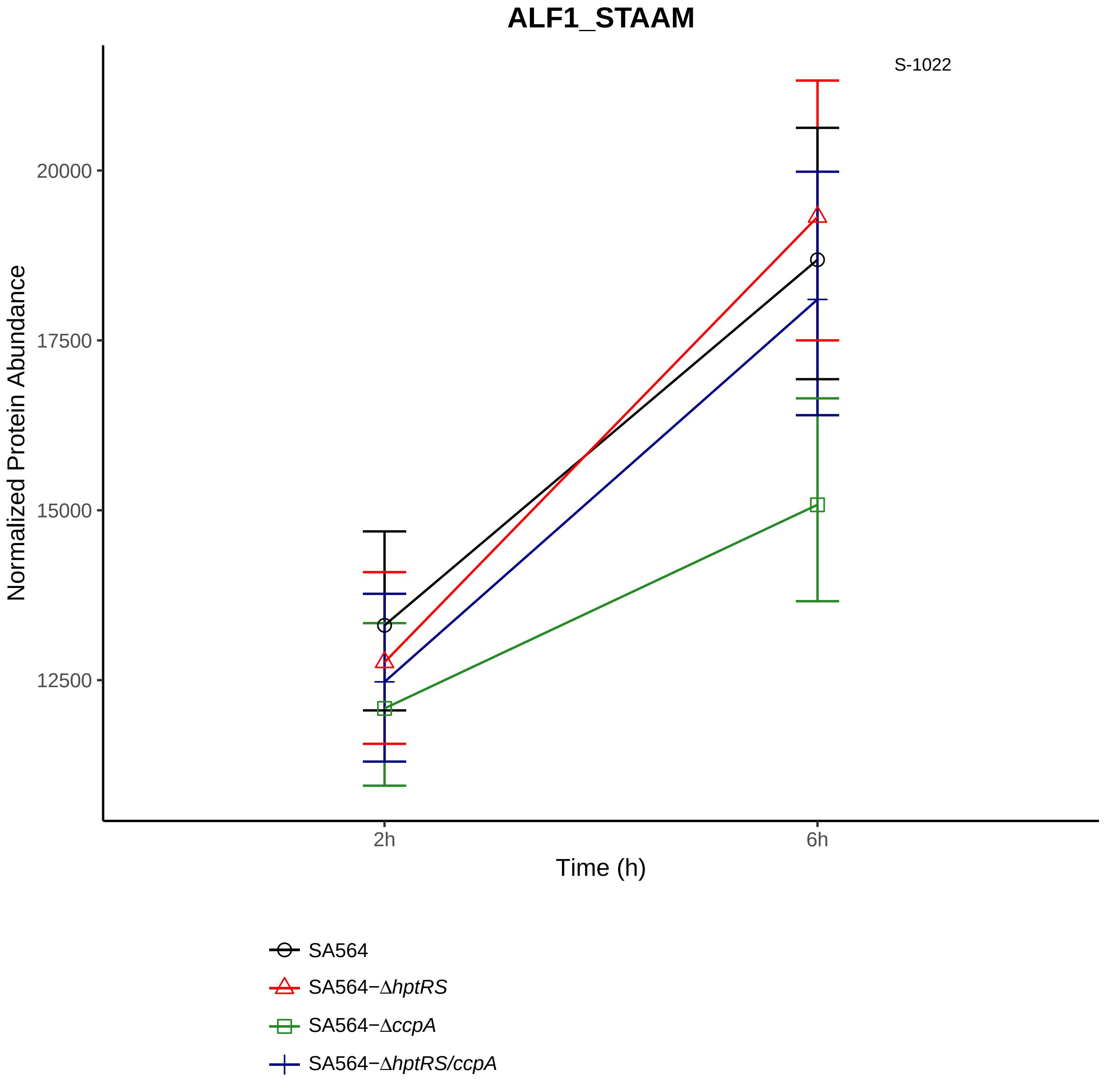

ALF1_STAAM
S-1022
Normalized Protein Abundance
20000
17500
15000
12500
2h
6h
Time (h)
SA564
SA564−Δ*hptRS*
SA564−Δ*ccpA*
SA564−Δ*hptRS/ccpA*

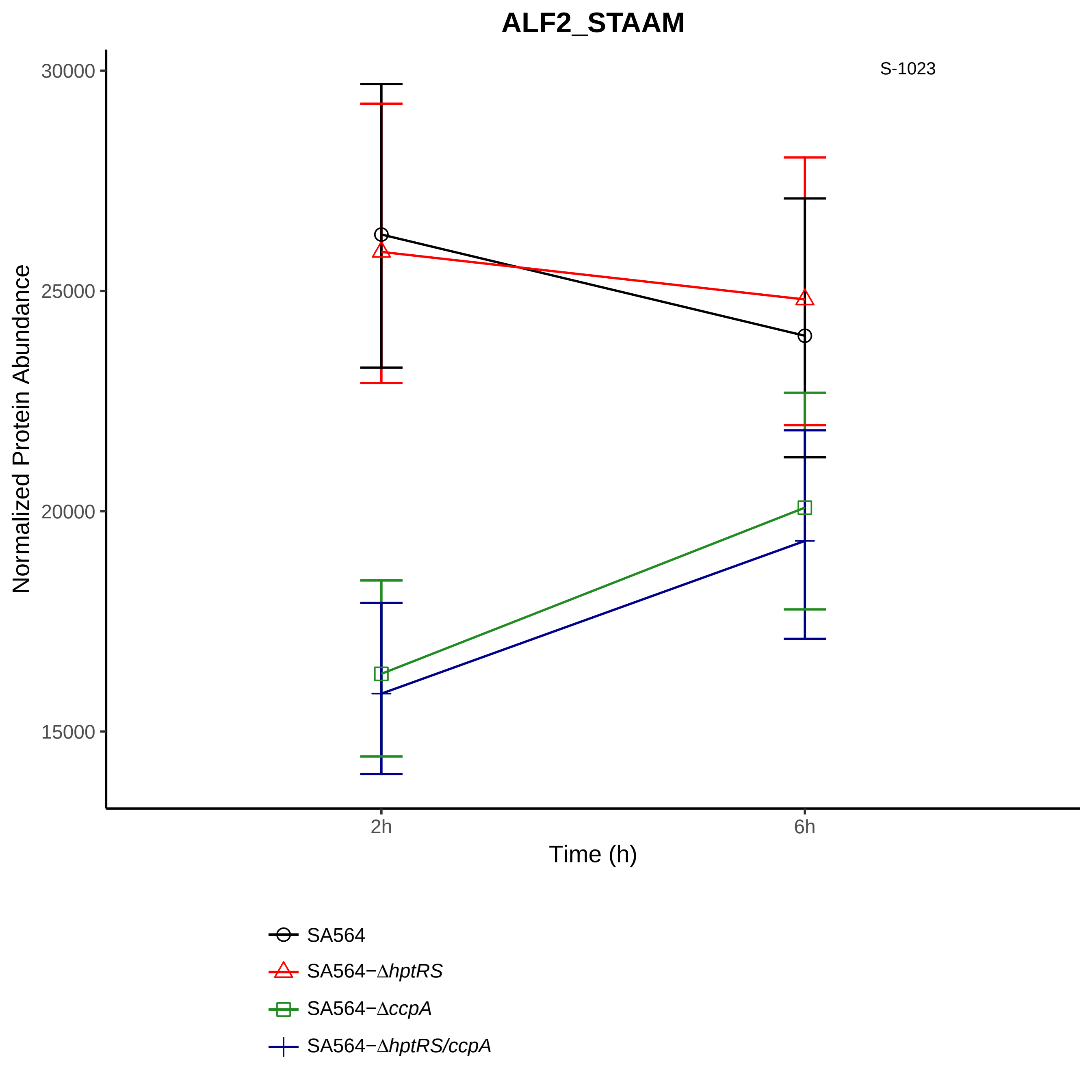

ALF2_STAAM
S-1023
Normalized Protein Abundance
30000
25000
20000
15000
2h
6h
Time (h)
SA564
SA564−ΔhptRS
SA564−ΔccpA
SA564−ΔhptRS/ccpA

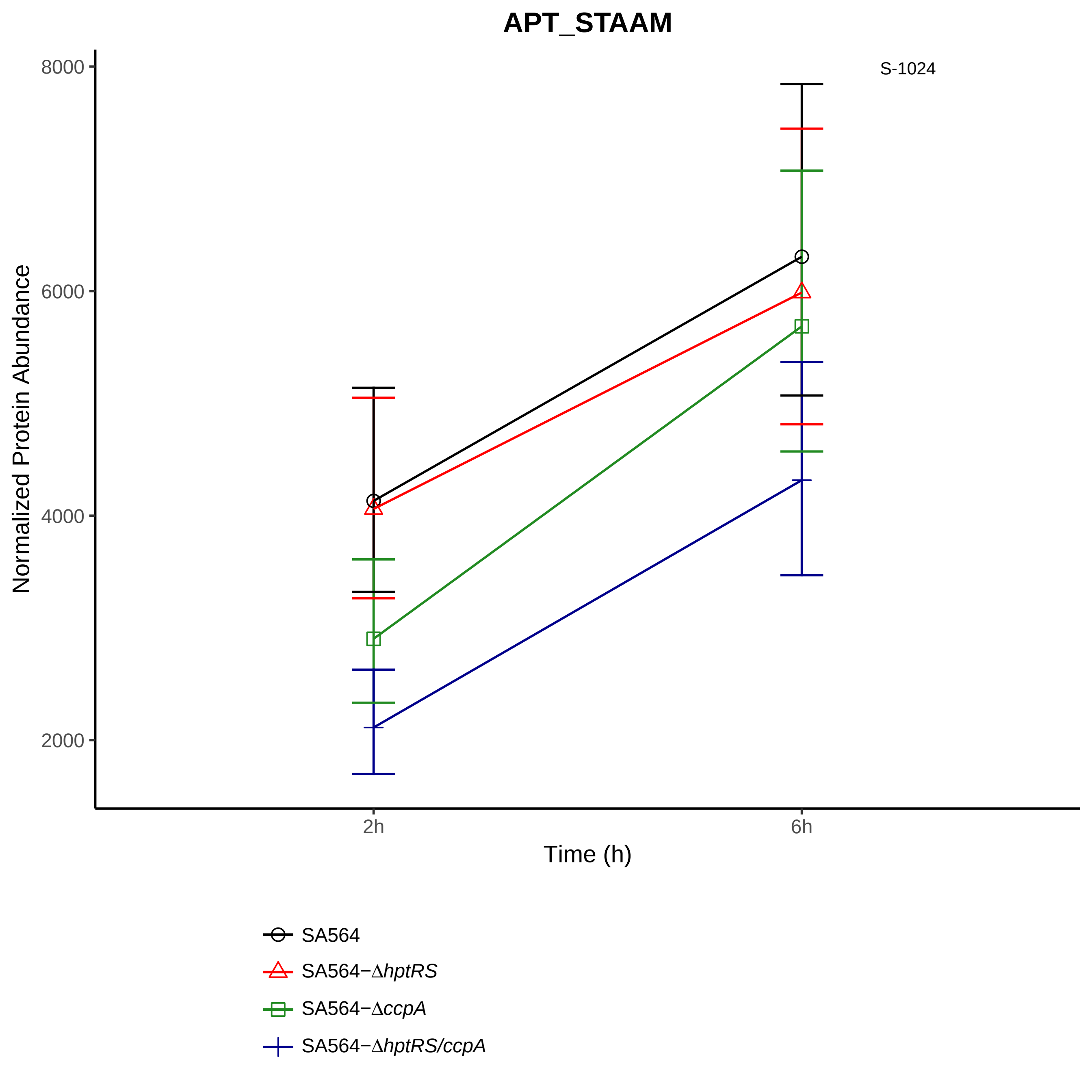

APT_STAAM
S-1024
Normalized Protein Abundance
8000
6000
4000
2000
2h
6h
Time (h)
SA564
SA564−Δ*hptRS*
SA564−Δ*ccpA*
SA564−Δ*hptRS/ccpA*

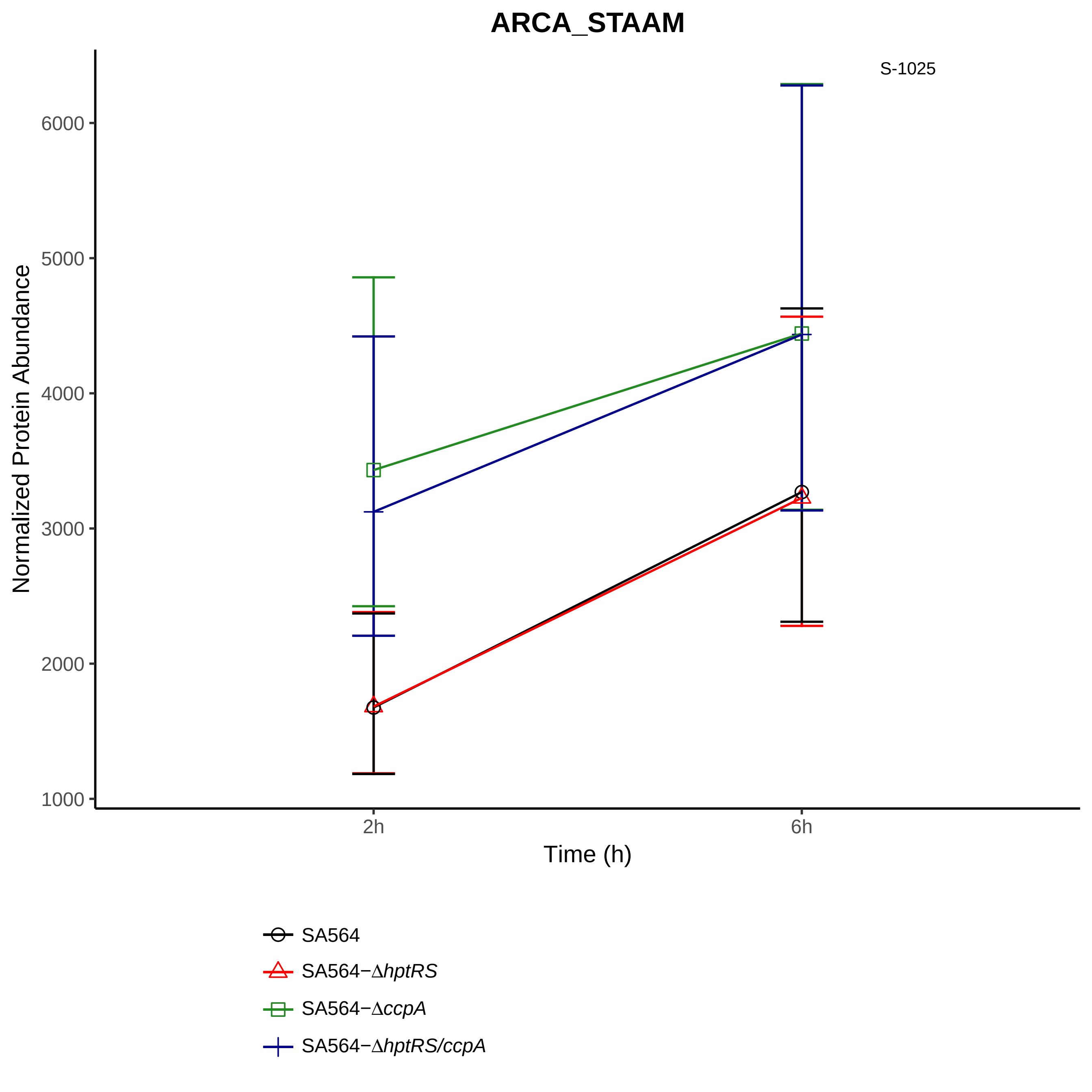

ARCA_STAAM
S-1025
Normalized Protein Abundance
1000
2000
3000
4000
5000
6000
2h
6h
Time (h)
SA564
SA564−ΔhptRS
SA564−ΔccpA
SA564−ΔhptRS/ccpA

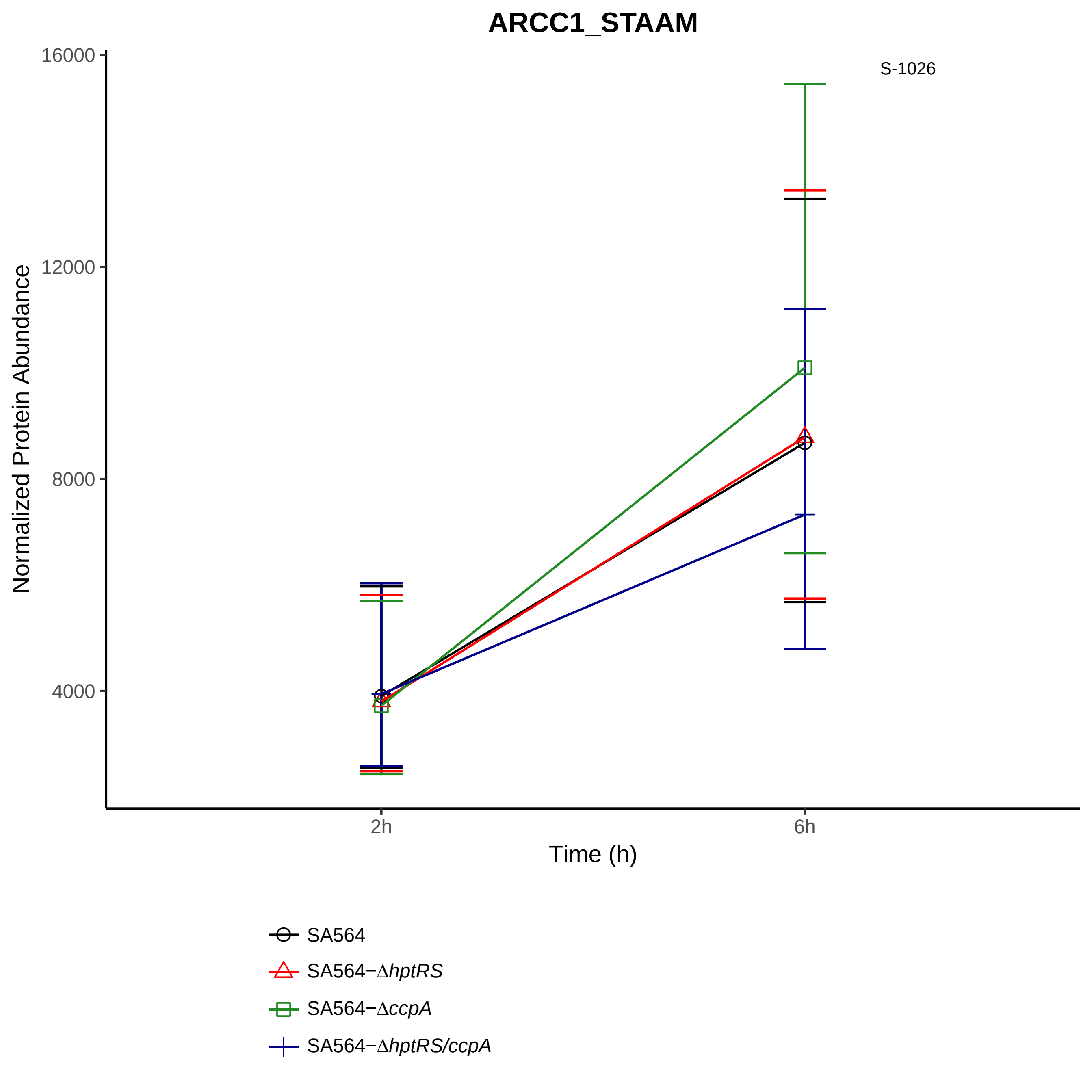

ARCC1_STAAM
S-1026
16000
12000
8000
4000
Normalized Protein Abundance
2h
6h
Time (h)
SA564
SA564−Δ*hptRS*
SA564−Δ*ccpA*
SA564−Δ*hptRS/ccpA*

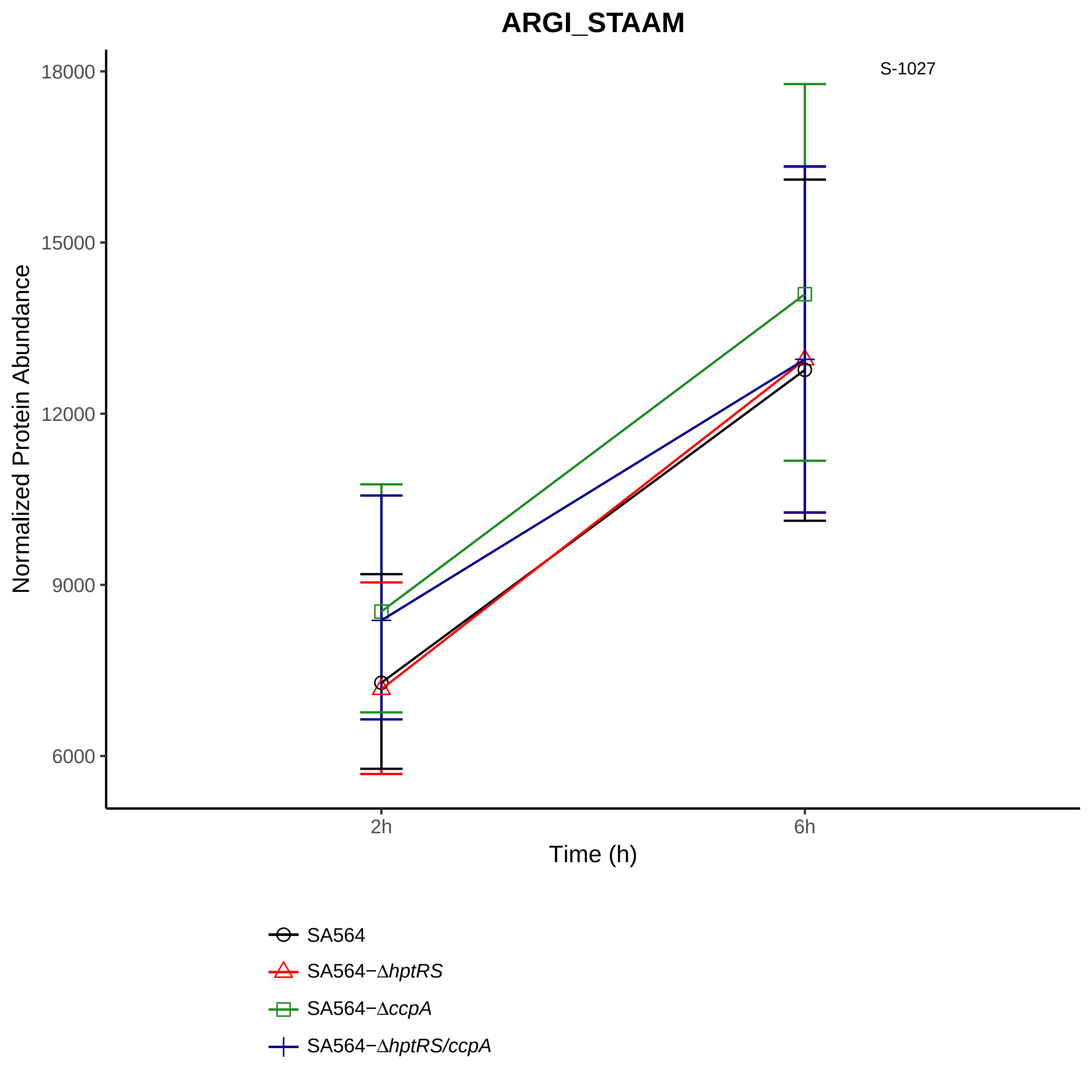

ARGI_STAAM
S-1027
Normalized Protein Abundance
18000
15000
12000
9000
6000
2h
6h
Time (h)
SA564
SA564−Δ*hptRS*
SA564−Δ*ccpA*
SA564−Δ*hptRS/ccpA*

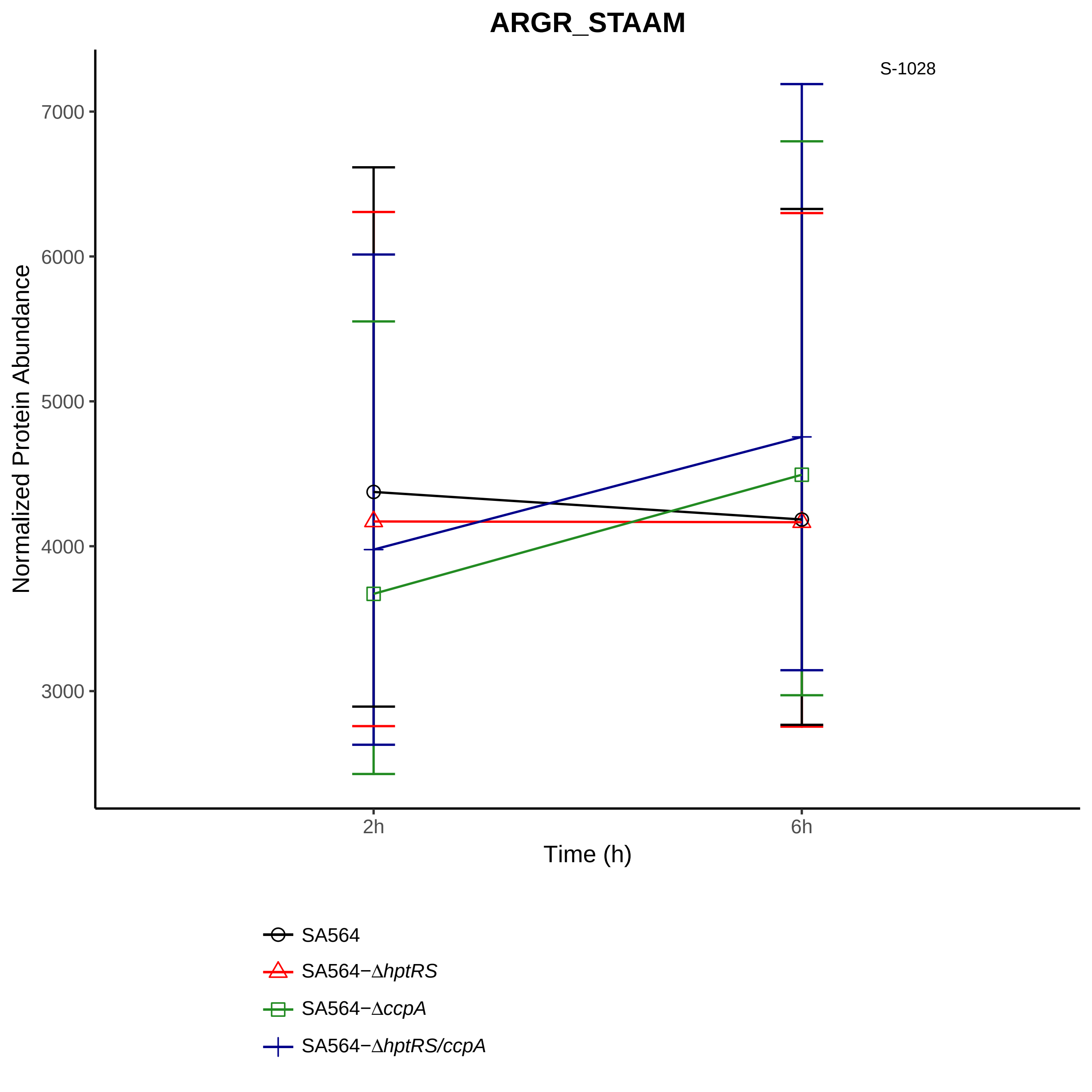

ARGR_STAAM
S-1028
Normalized Protein Abundance
7000
6000
5000
4000
3000
2h
6h
Time (h)
SA564
SA564−Δ*hptRS*
SA564−Δ*ccpA*
SA564−Δ*hptRS/ccpA*

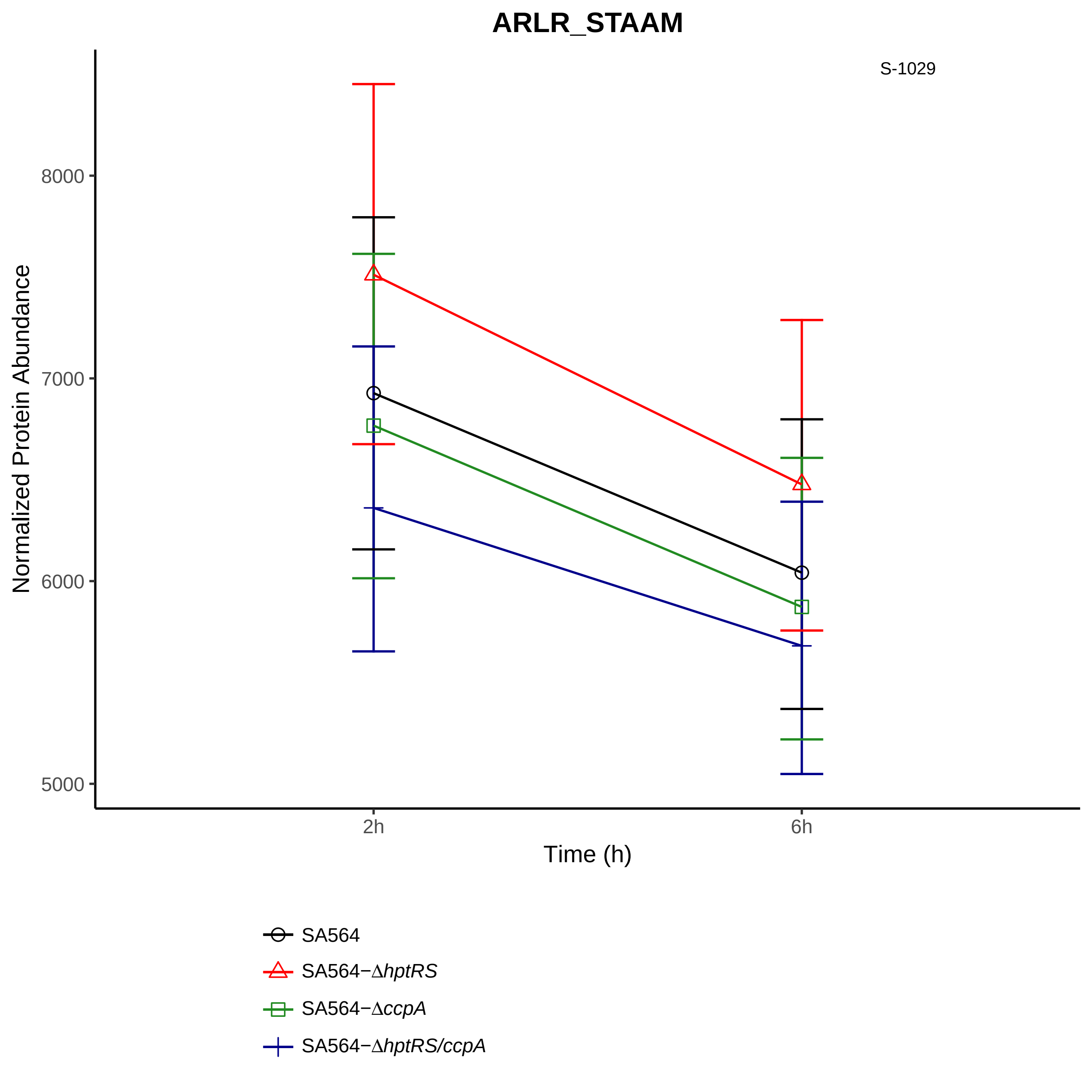

ARLR_STAAM
S-1029
Normalized Protein Abundance
8000
7000
6000
5000
2h
6h
Time (h)
SA564
SA564−Δ*hptRS*
SA564−Δ*ccpA*
SA564−Δ*hptRS/ccpA*

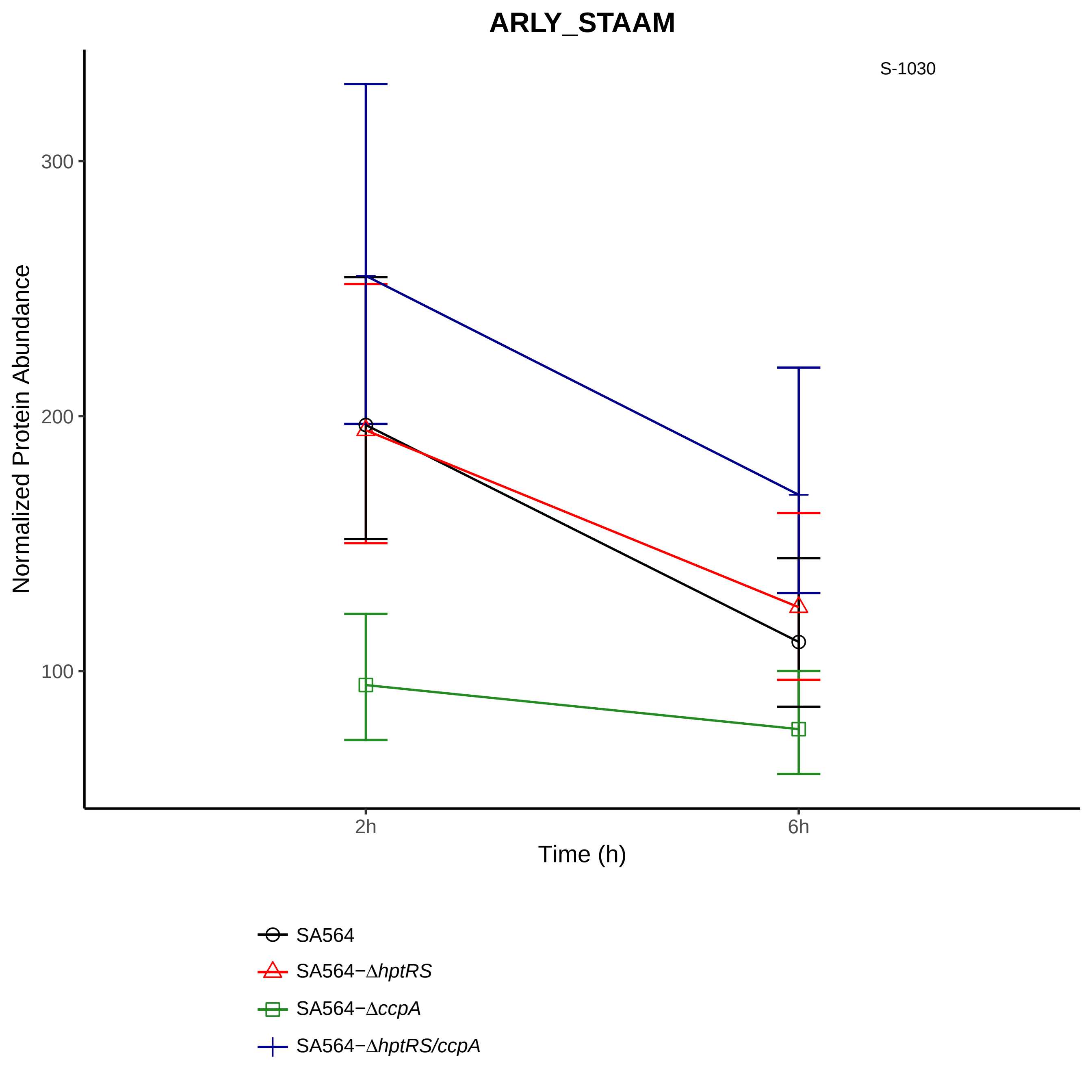

ARLY_STAAM
S-1030
300
200
100
Normalized Protein Abundance
2h
6h
Time (h)
SA564
SA564−Δ*hptRS*
SA564−Δ*ccpA*
SA564−Δ*hptRS/ccpA*

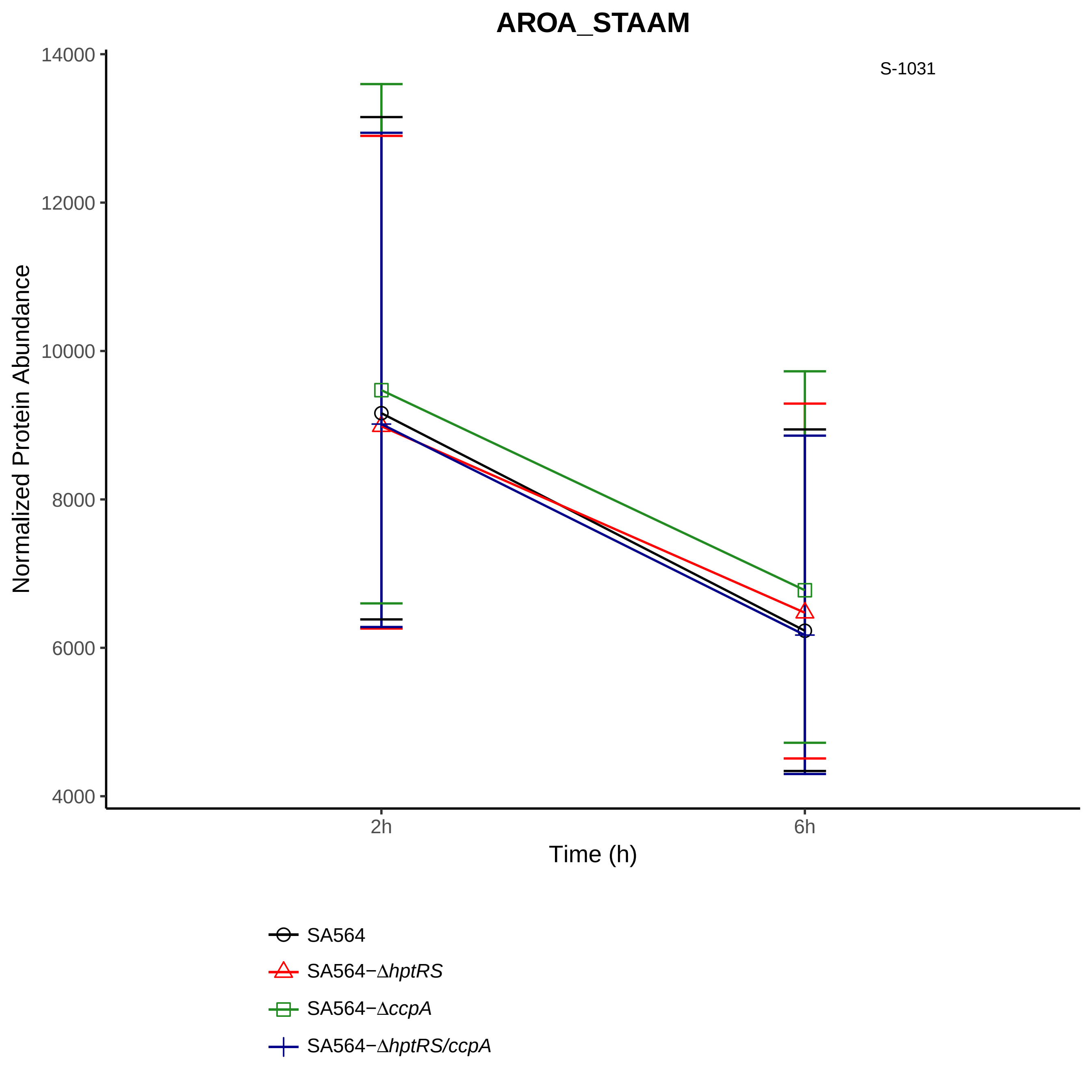

AROA_STAAM
S-1031
14000
12000
10000
8000
6000
4000
Normalized Protein Abundance
2h
6h
Time (h)
SA564
SA564−Δ*hptRS*
SA564−Δ*ccpA*
SA564−Δ*hptRS/ccpA*

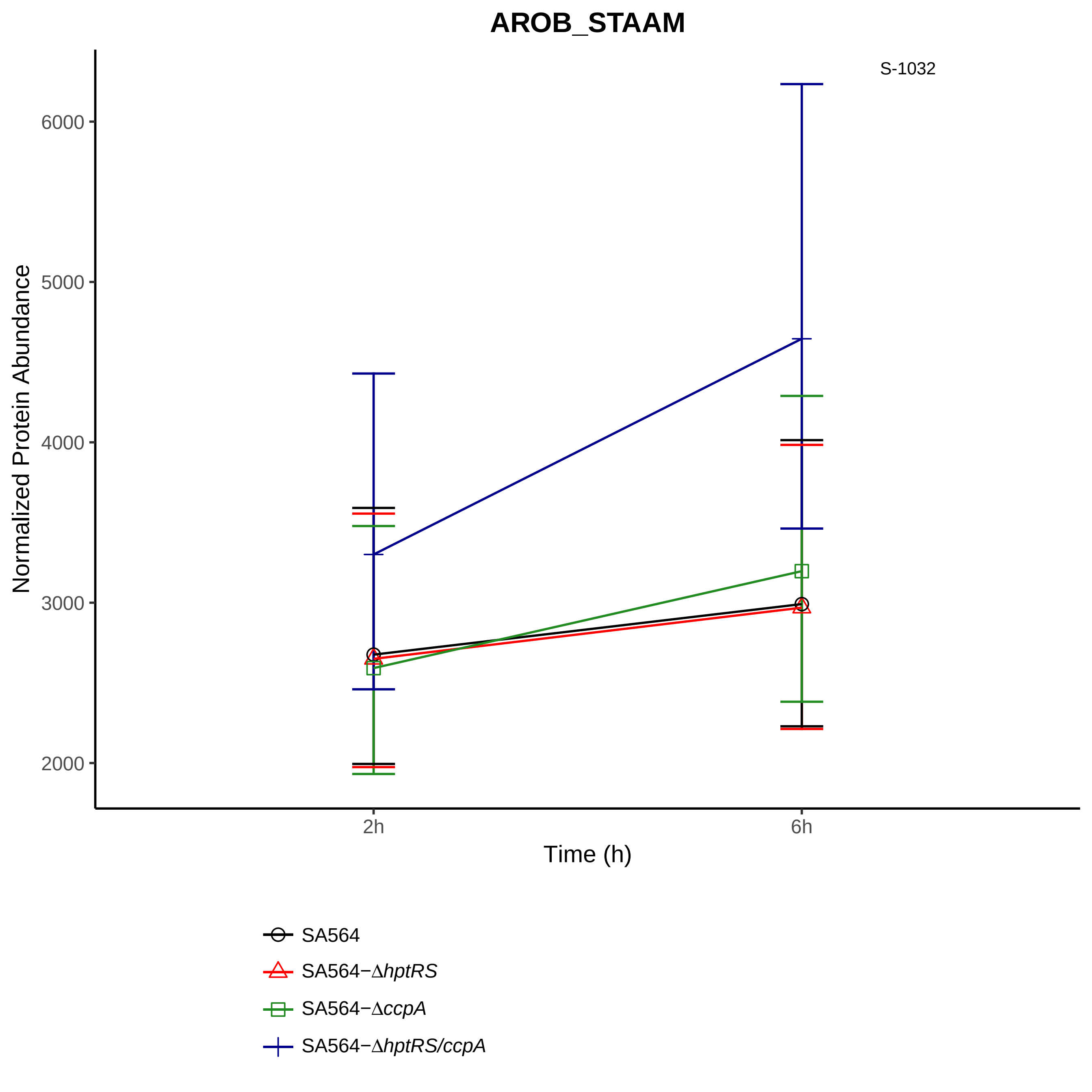

AROB_STAAM
S-1032
Normalized Protein Abundance
6000
5000
4000
3000
2000
2h
6h
Time (h)
SA564
SA564−Δ*hptRS*
SA564−Δ*ccpA*
SA564−Δ*hptRS/ccpA*

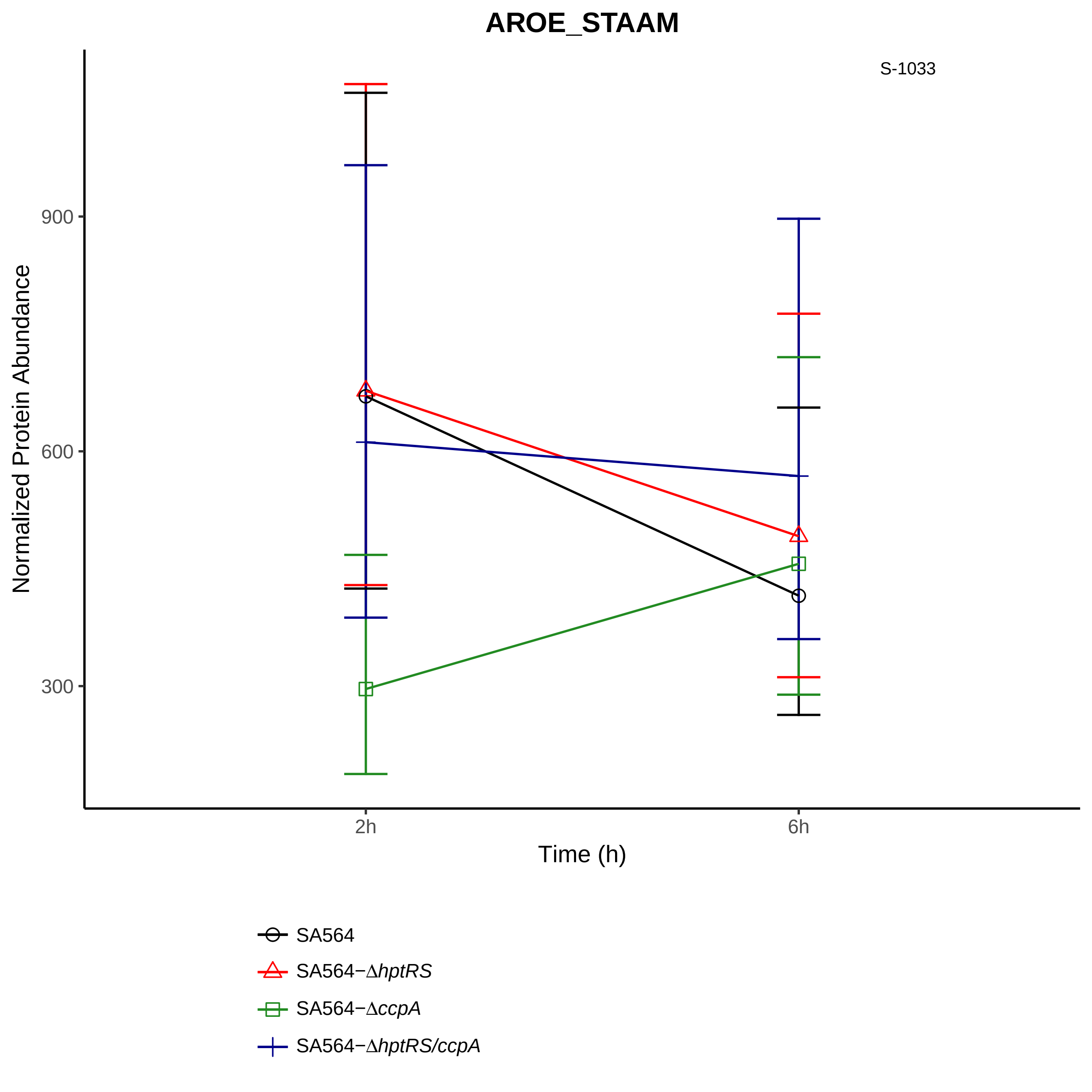

AROE_STAAM
S-1033
Normalized Protein Abundance
900
600
300
2h
6h
Time (h)
SA564
SA564−ΔhptRS
SA564−ΔccpA
SA564−ΔhptRS/ccpA

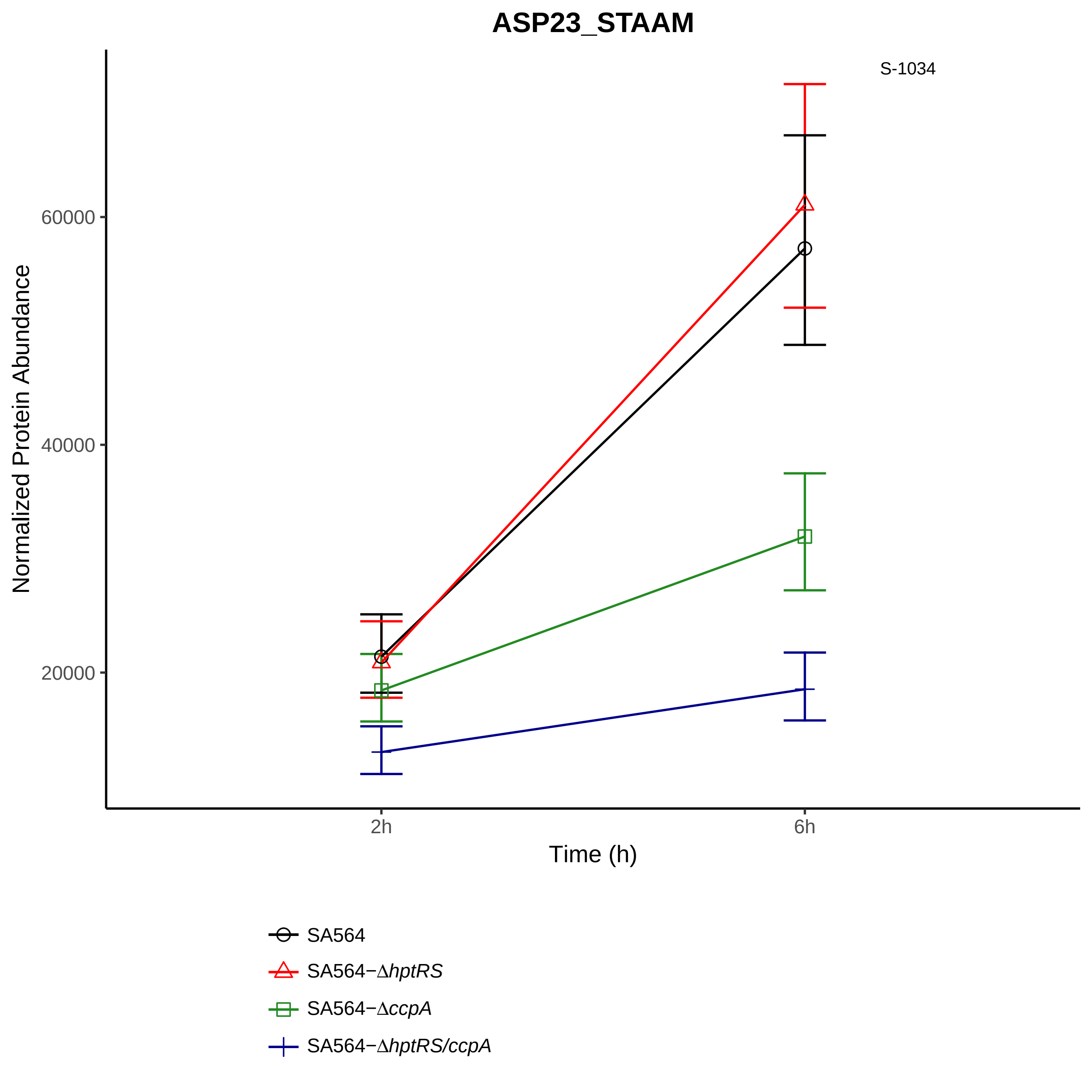
ASP23_STAAM
S-1034
Normalized Protein Abundance
60000
40000
20000
2h
6h
Time (h)
SA564
SA564−ΔhptRS
SA564−ΔccpA
SA564−ΔhptRS/ccpA

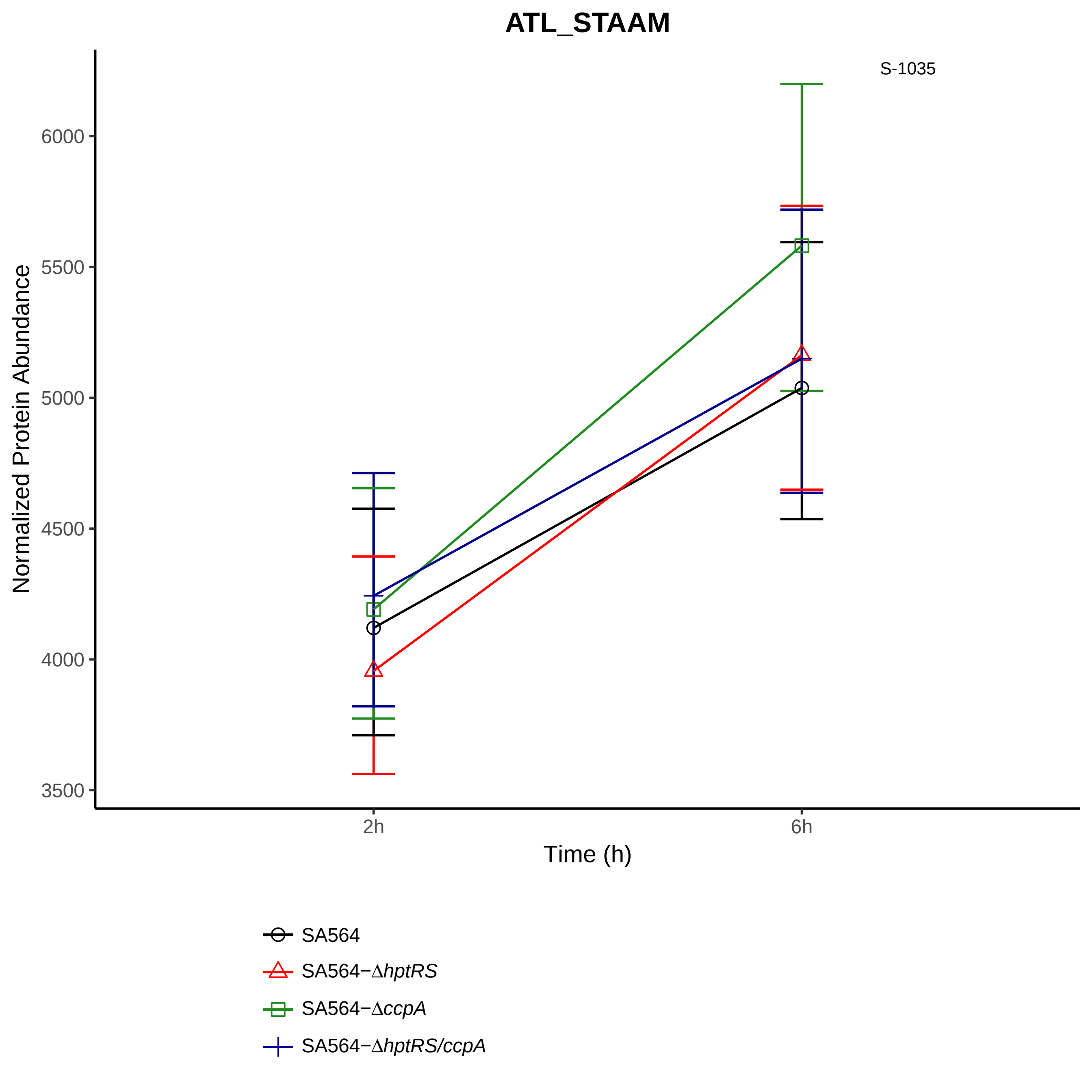

ATL_STAAM
S-1035
Normalized Protein Abundance
6000
5500
5000
4500
4000
3500
2h
6h
Time (h)
SA564
SA564−Δ*hptRS*
SA564−Δ*ccpA*
SA564−Δ*hptRS/ccpA*

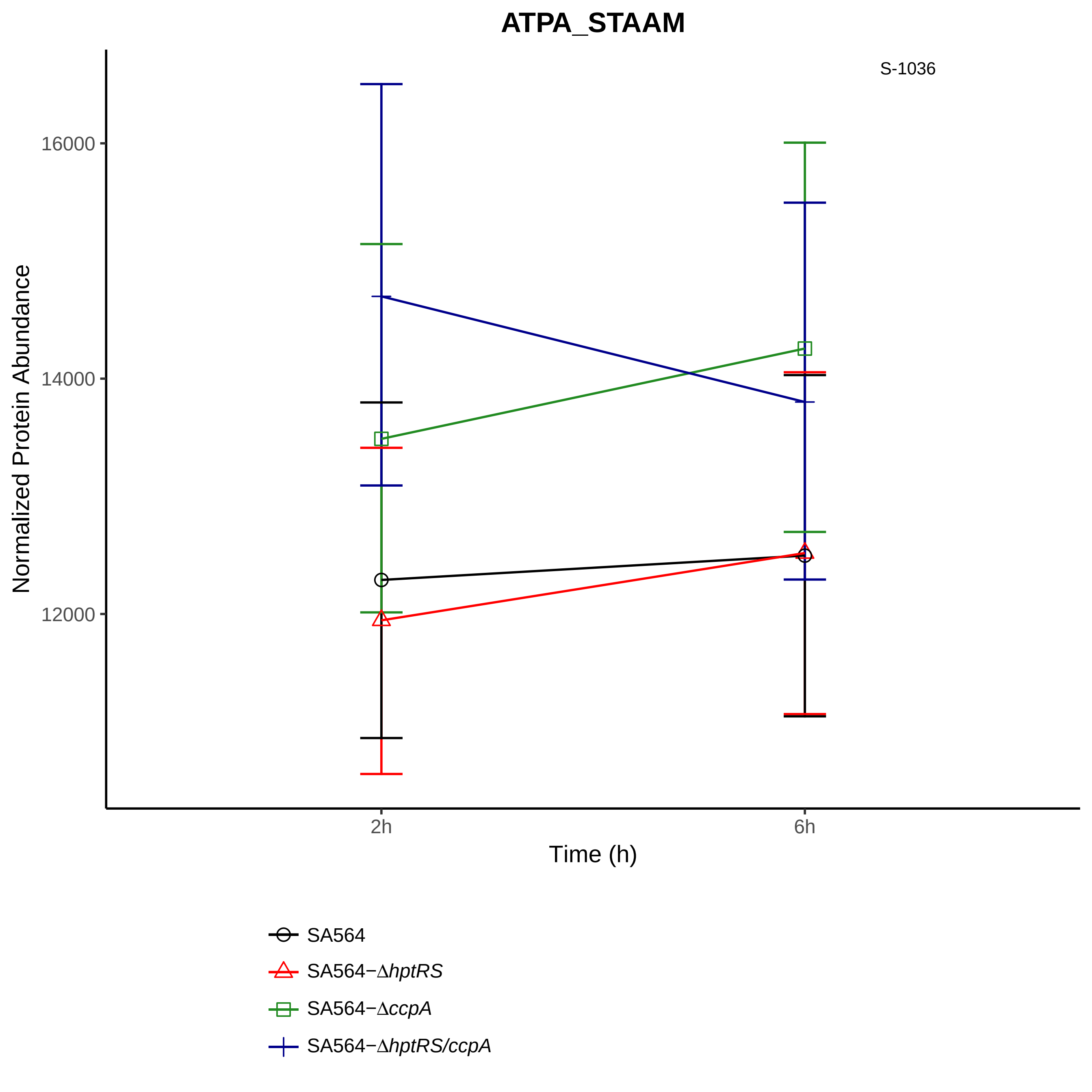
ATPA_STAAM
S-1036
Normalized Protein Abundance
16000
14000
12000
2h
6h
Time (h)
SA564
SA564−ΔhptRS
SA564−ΔccpA
SA564−ΔhptRS/ccpA

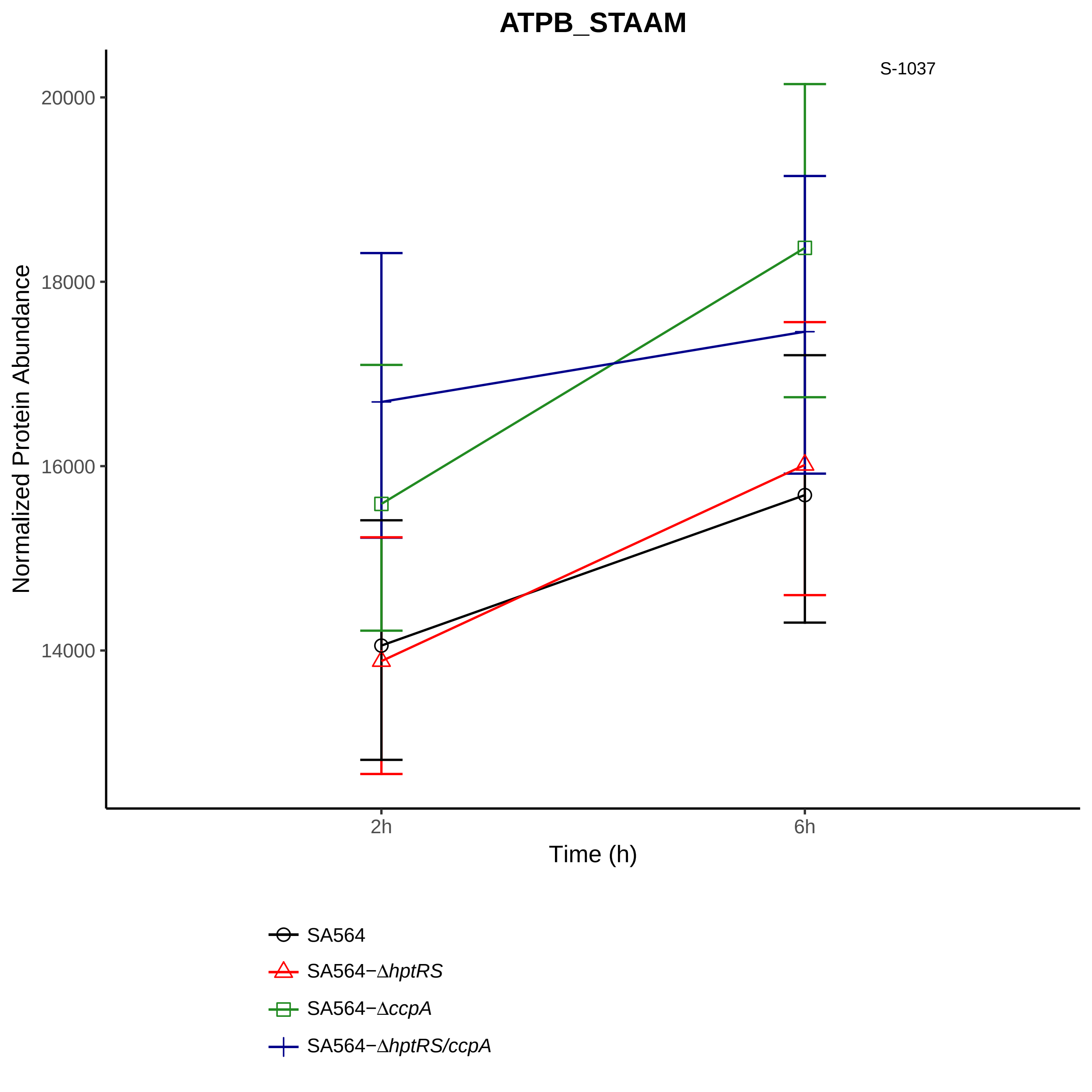
ATPB_STAAM
S-1037
20000
18000
16000
14000
Normalized Protein Abundance
2h
6h
Time (h)
SA564
SA564−Δ*hptRS*
SA564−Δ*ccpA*
SA564−Δ*hptRS/ccpA*

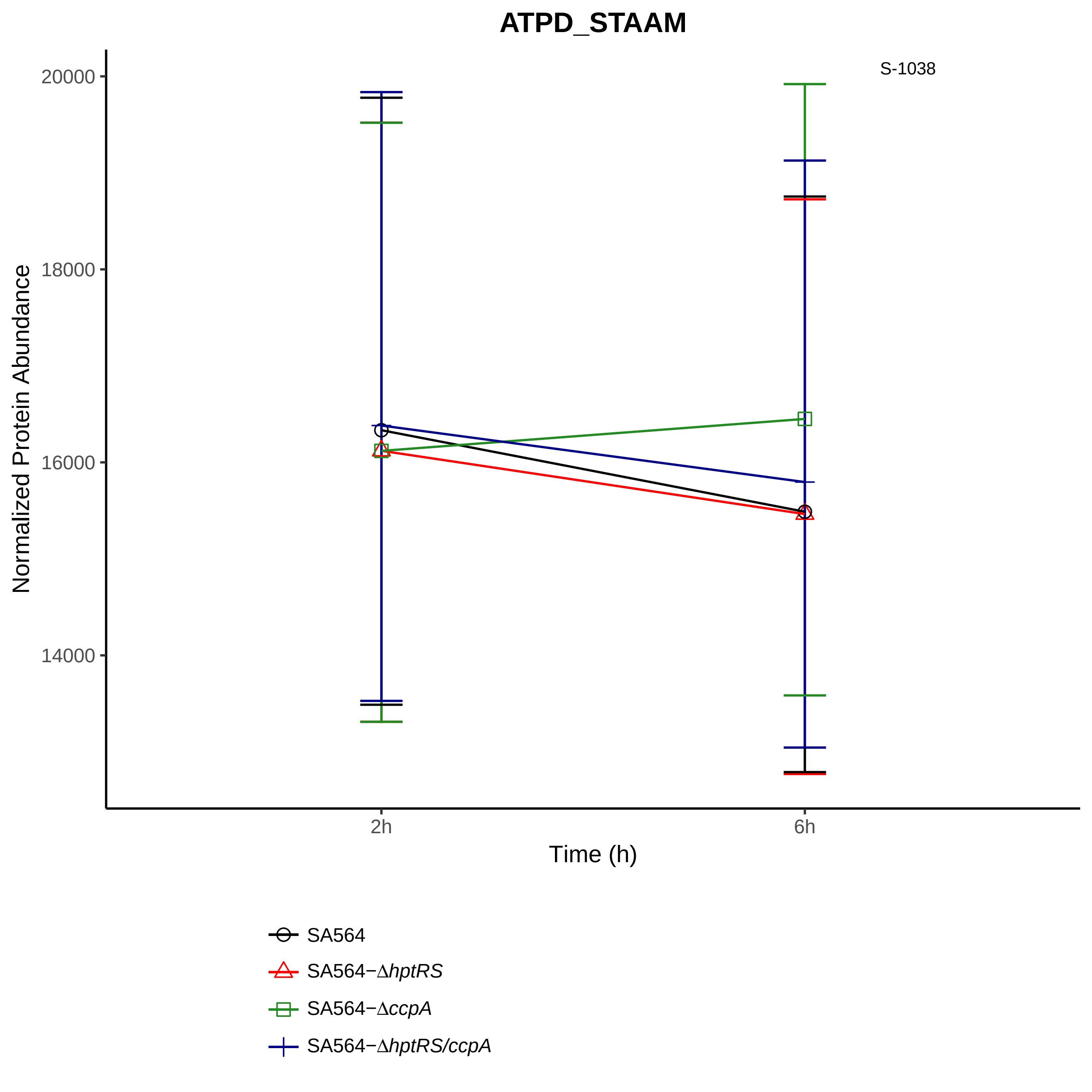

ATPD_STAAM
S-1038
20000
18000
16000
14000
Normalized Protein Abundance
2h
6h
Time (h)
SA564
SA564−Δ*hptRS*
SA564−Δ*ccpA*
SA564−Δ*hptRS/ccpA*

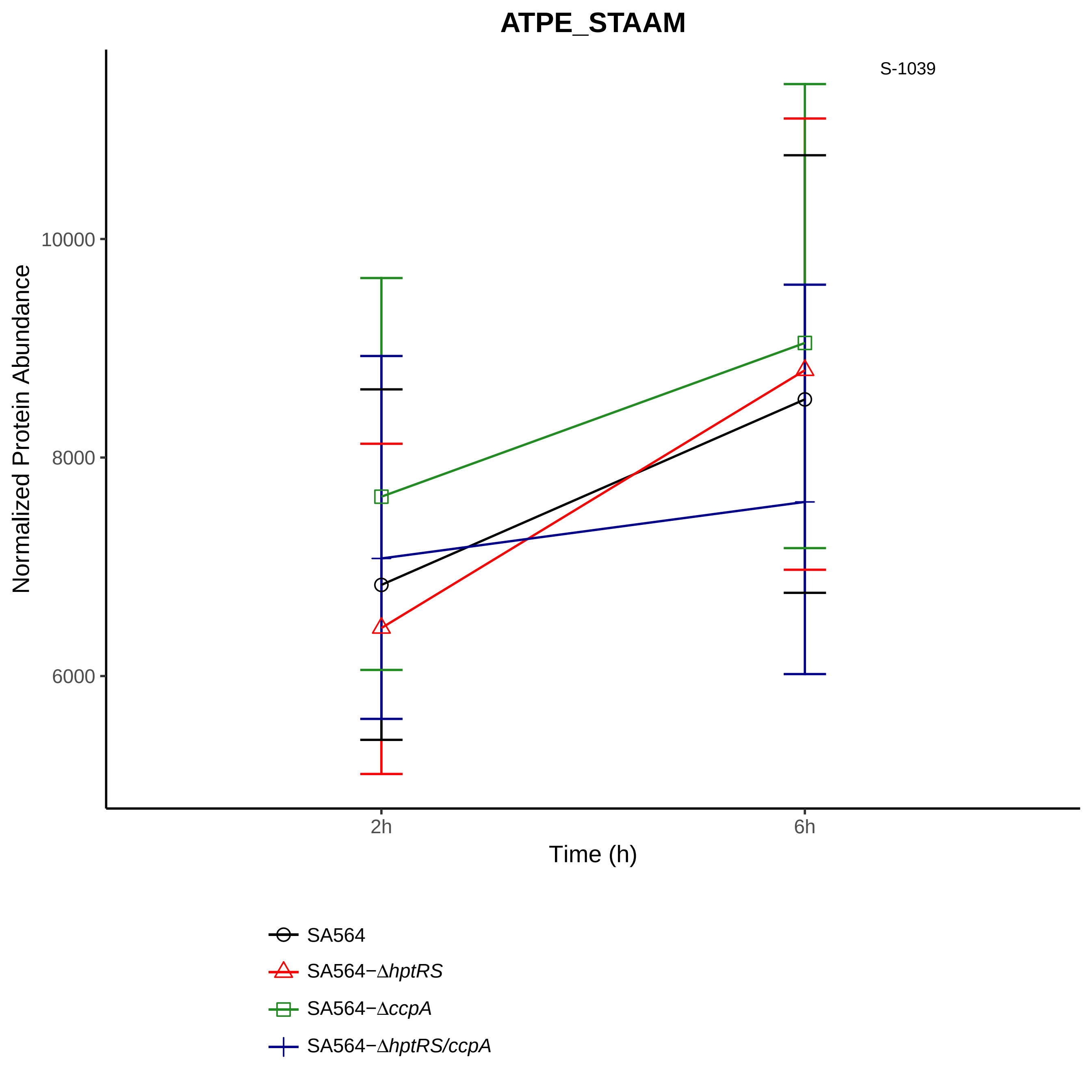
ATPE_STAAM
S-1039
Normalized Protein Abundance
10000
8000
6000
2h
6h
Time (h)
SA564
SA564−Δ*hptRS*
SA564−Δ*ccpA*
SA564−Δ*hptRS/ccpA*

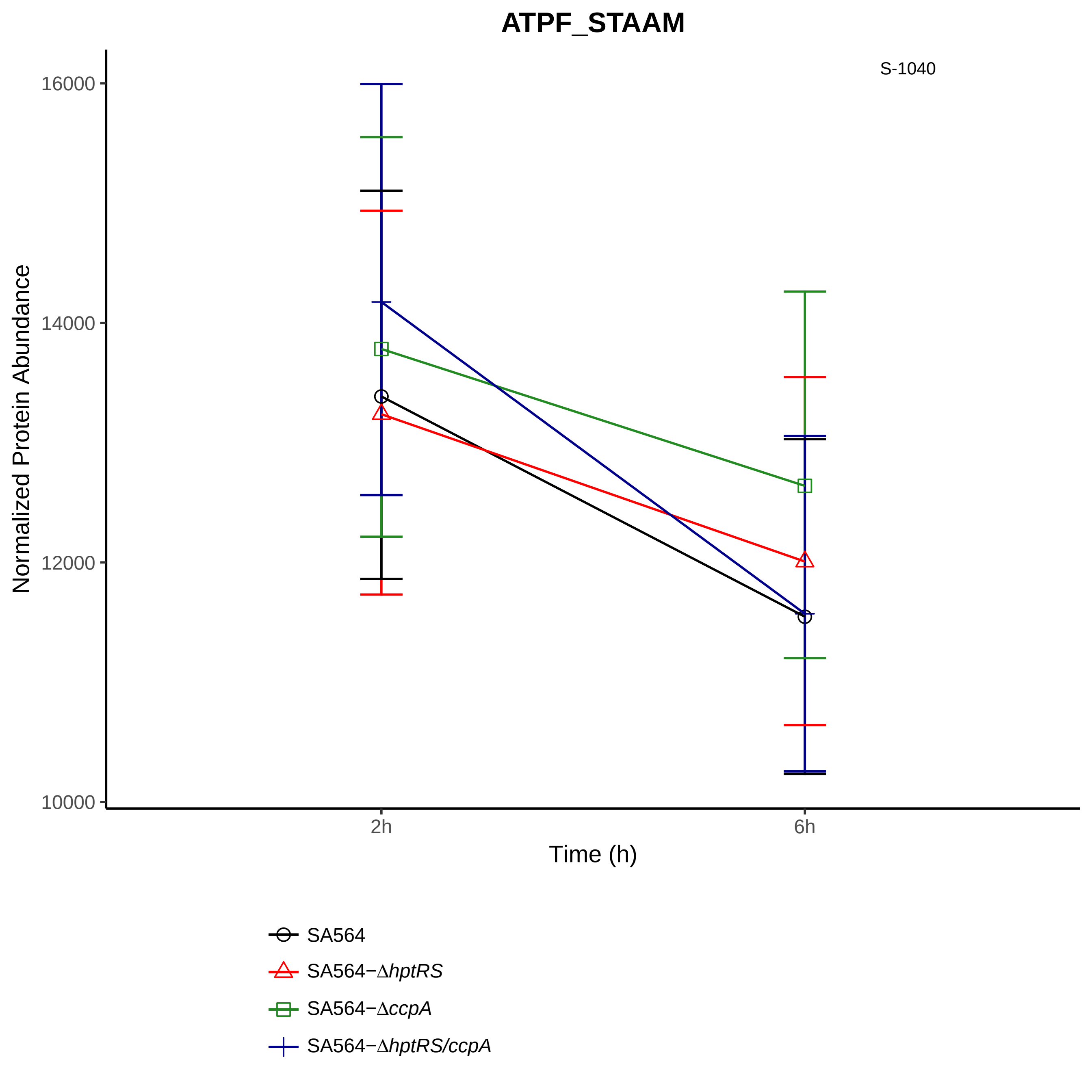

ATPF_STAAM
S-1040
16000
14000
12000
10000
Normalized Protein Abundance
2h
6h
Time (h)
SA564
SA564−Δ*hptRS*
SA564−Δ*ccpA*
SA564−Δ*hptRS/ccpA*

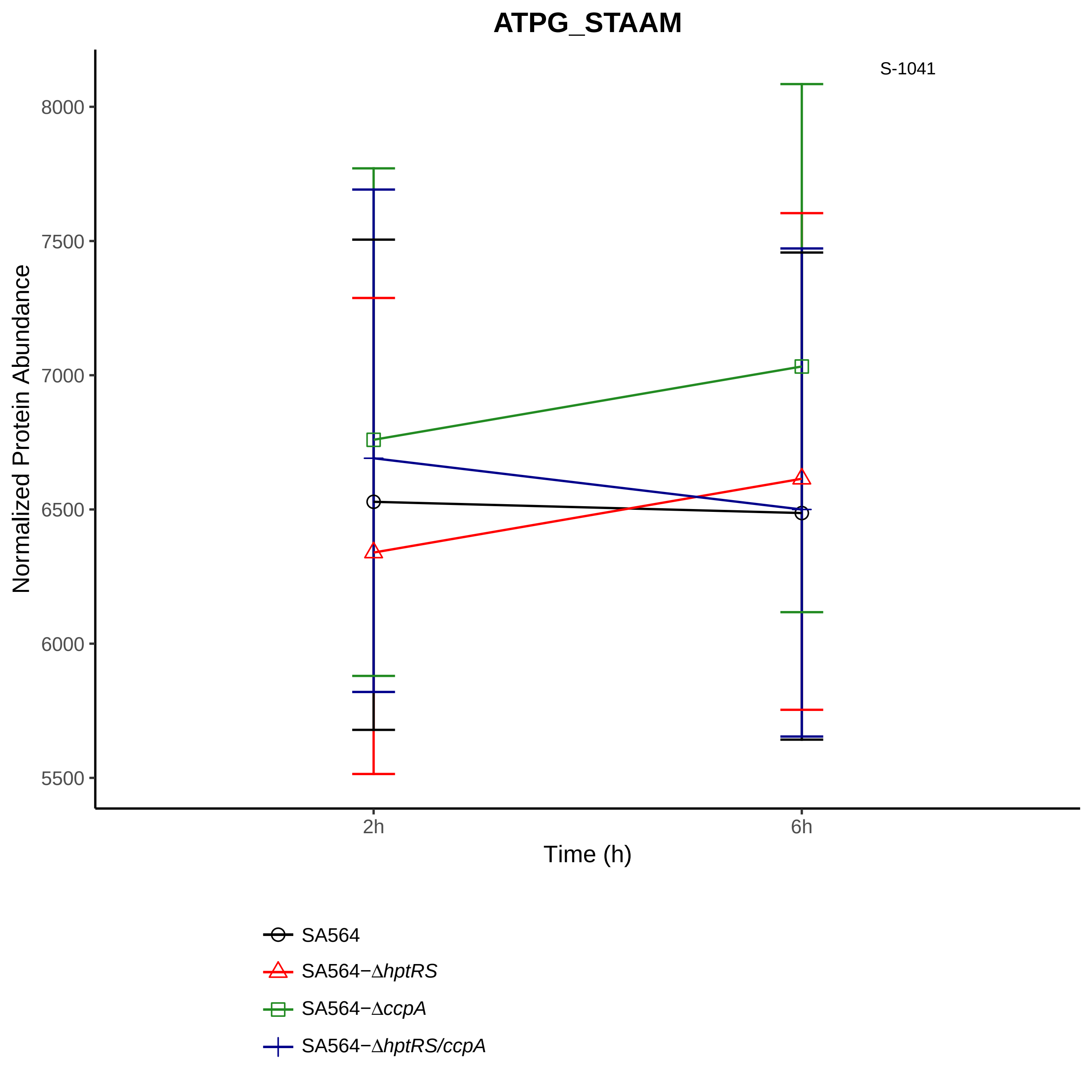
ATPG_STAAM
S-1041
Normalized Protein Abundance
8000
7500
7000
6500
6000
5500
2h
6h
Time (h)
SA564
SA564−Δ*hptRS*
SA564−Δ*ccpA*
SA564−Δ*hptRS/ccpA*

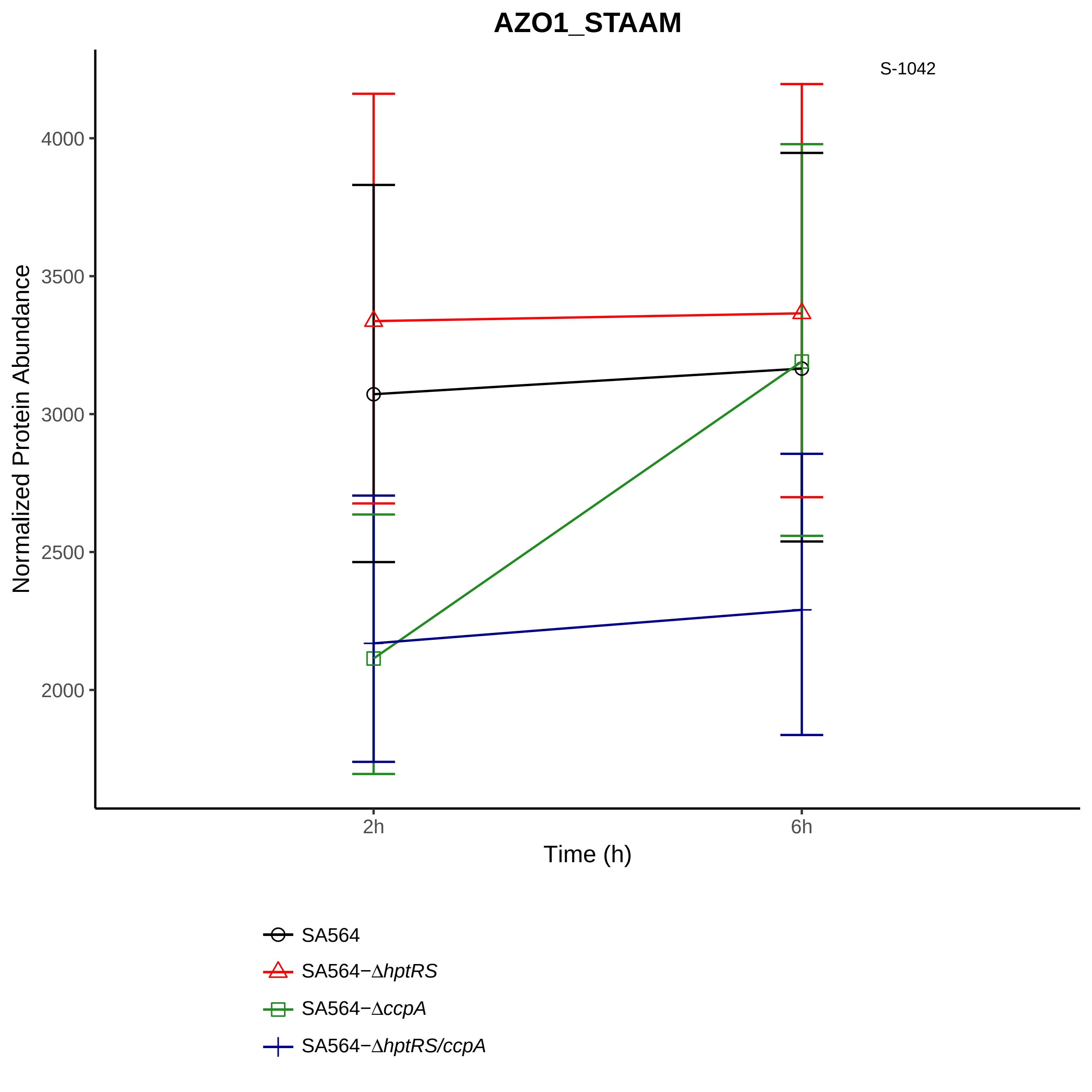

AZO1_STAAM
S-1042
Normalized Protein Abundance
2000
2500
3000
3500
4000
2h
6h
Time (h)
SA564
SA564−Δ*hptRS*
SA564−Δ*ccpA*
SA564−Δ*hptRS/ccpA*

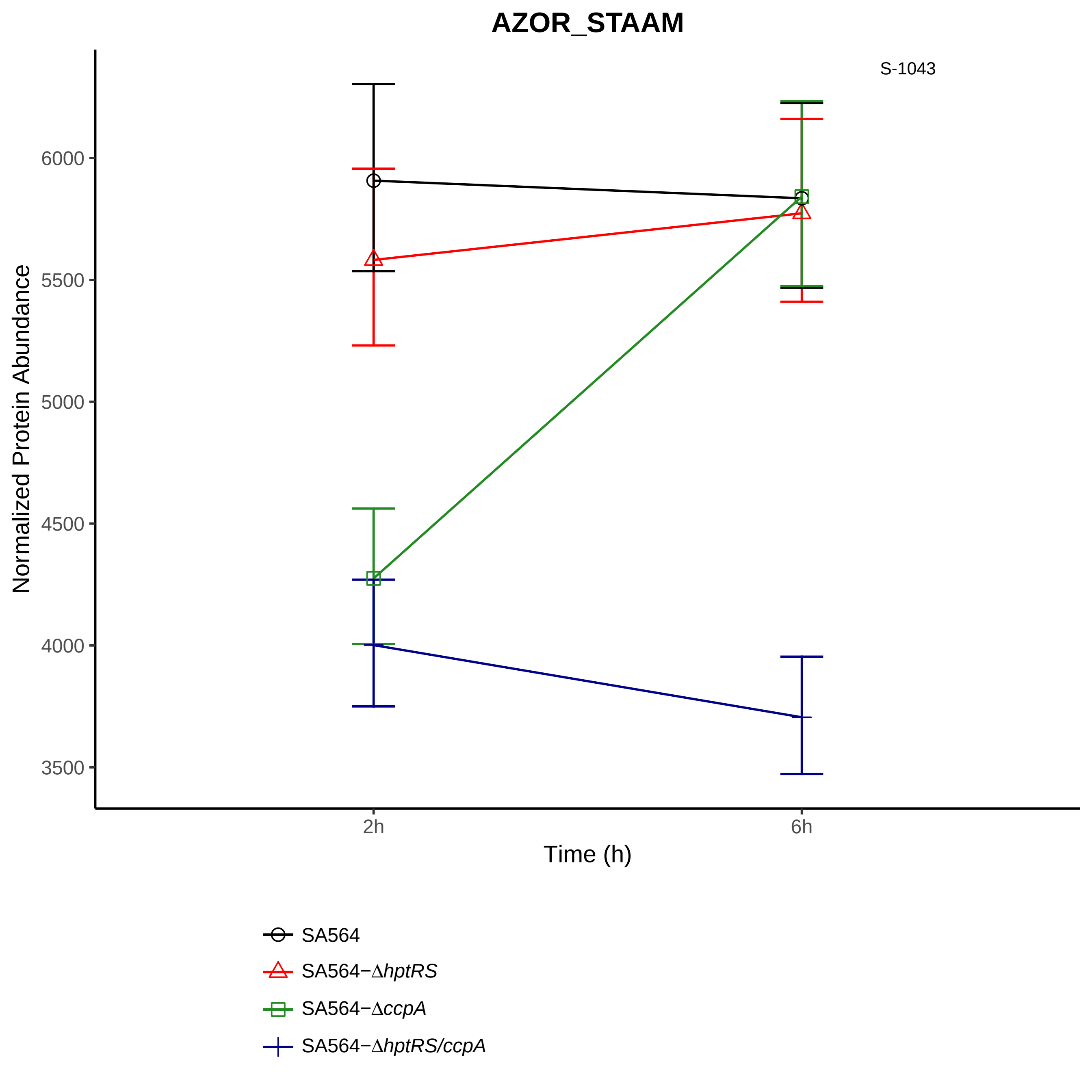
AZOR_STAAM
S-1043
Normalized Protein Abundance
6000
5500
5000
4500
4000
3500
2h
6h
Time (h)
SA564
SA564−Δ*hptRS*
SA564−Δ*ccpA*
SA564−Δ*hptRS/ccpA*

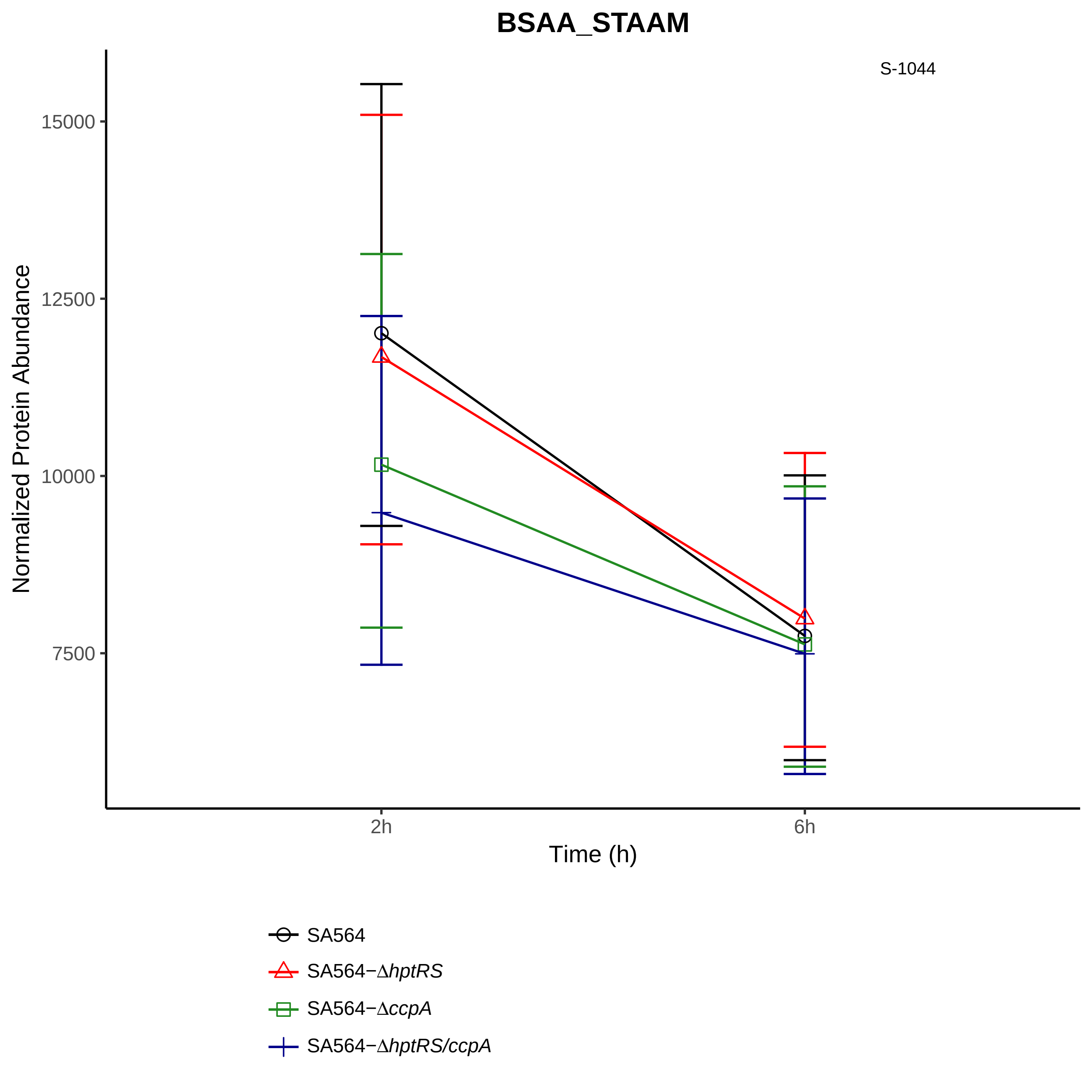

BSAA_STAAM
S-1044
15000
12500
10000
7500
Normalized Protein Abundance
2h
6h
Time (h)
SA564
SA564−Δ*hptRS*
SA564−Δ*ccpA*
SA564−Δ*hptRS/ccpA*

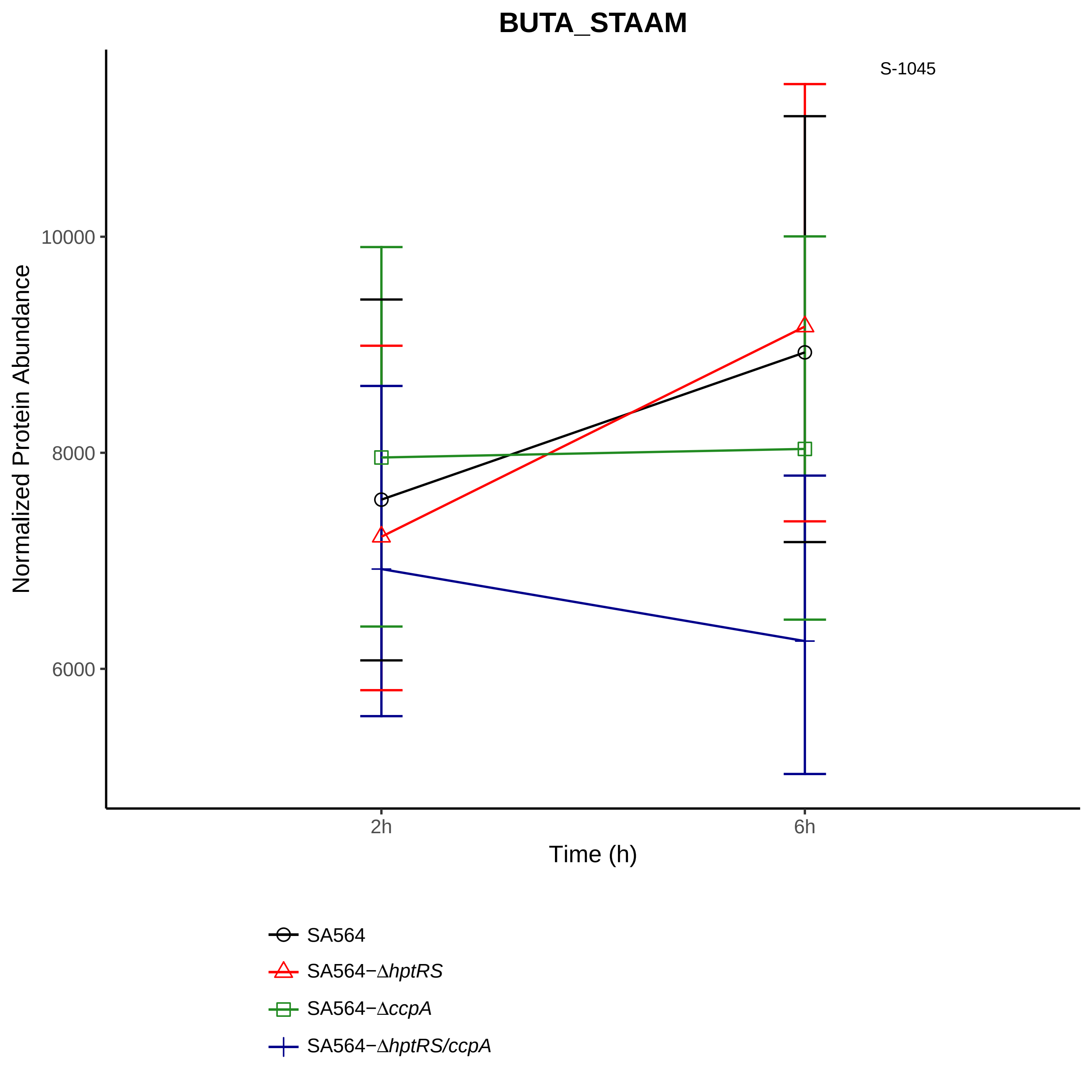

BUTA_STAAM
S-1045
Normalized Protein Abundance
10000
8000
6000
2h
6h
Time (h)
SA564
SA564−ΔhptRS
SA564−ΔccpA
SA564−ΔhptRS/ccpA

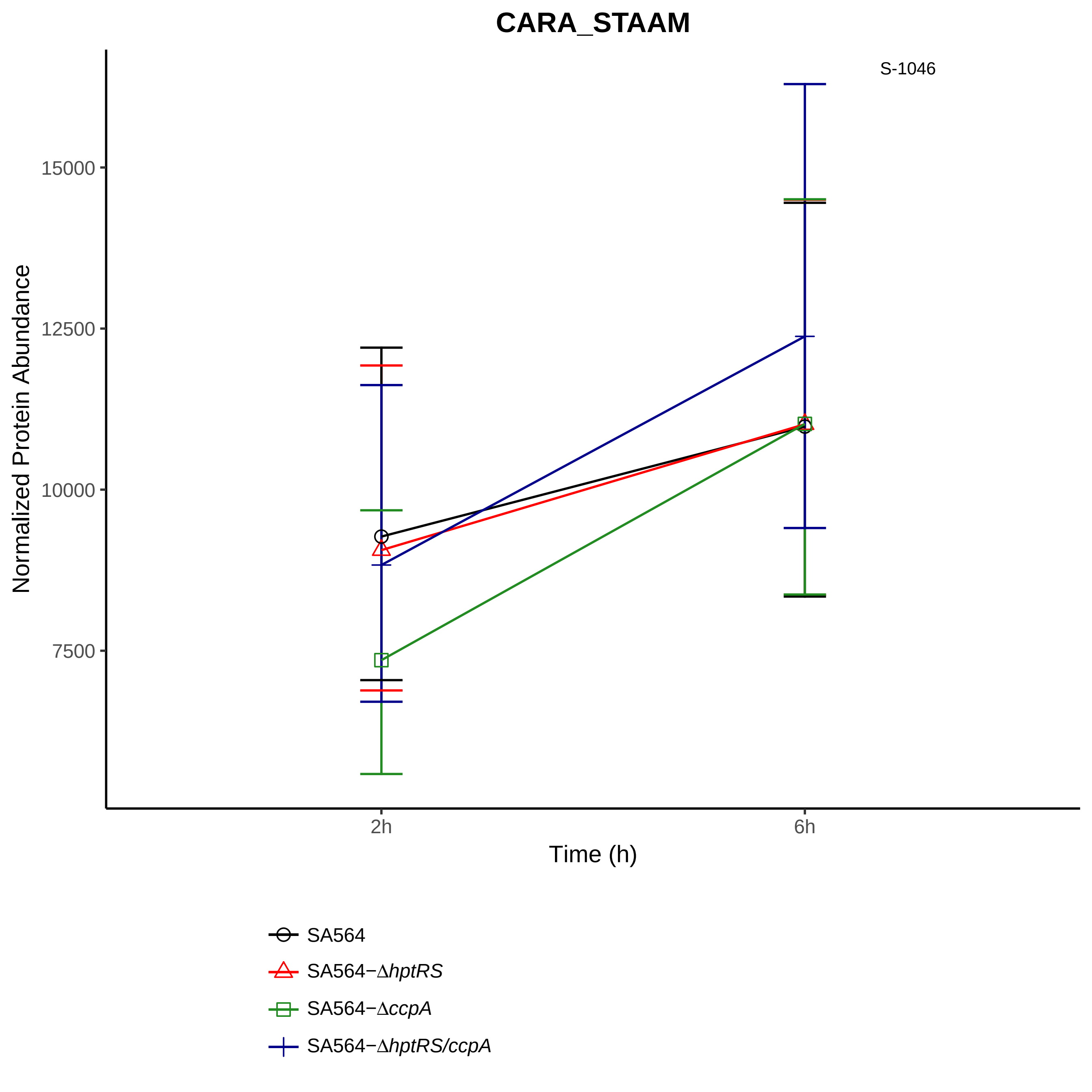

CARA_STAAM
S-1046
Normalized Protein Abundance
15000
12500
10000
7500
2h
6h
Time (h)
SA564
SA564−Δ*hptRS*
SA564−Δ*ccpA*
SA564−Δ*hptRS/ccpA*

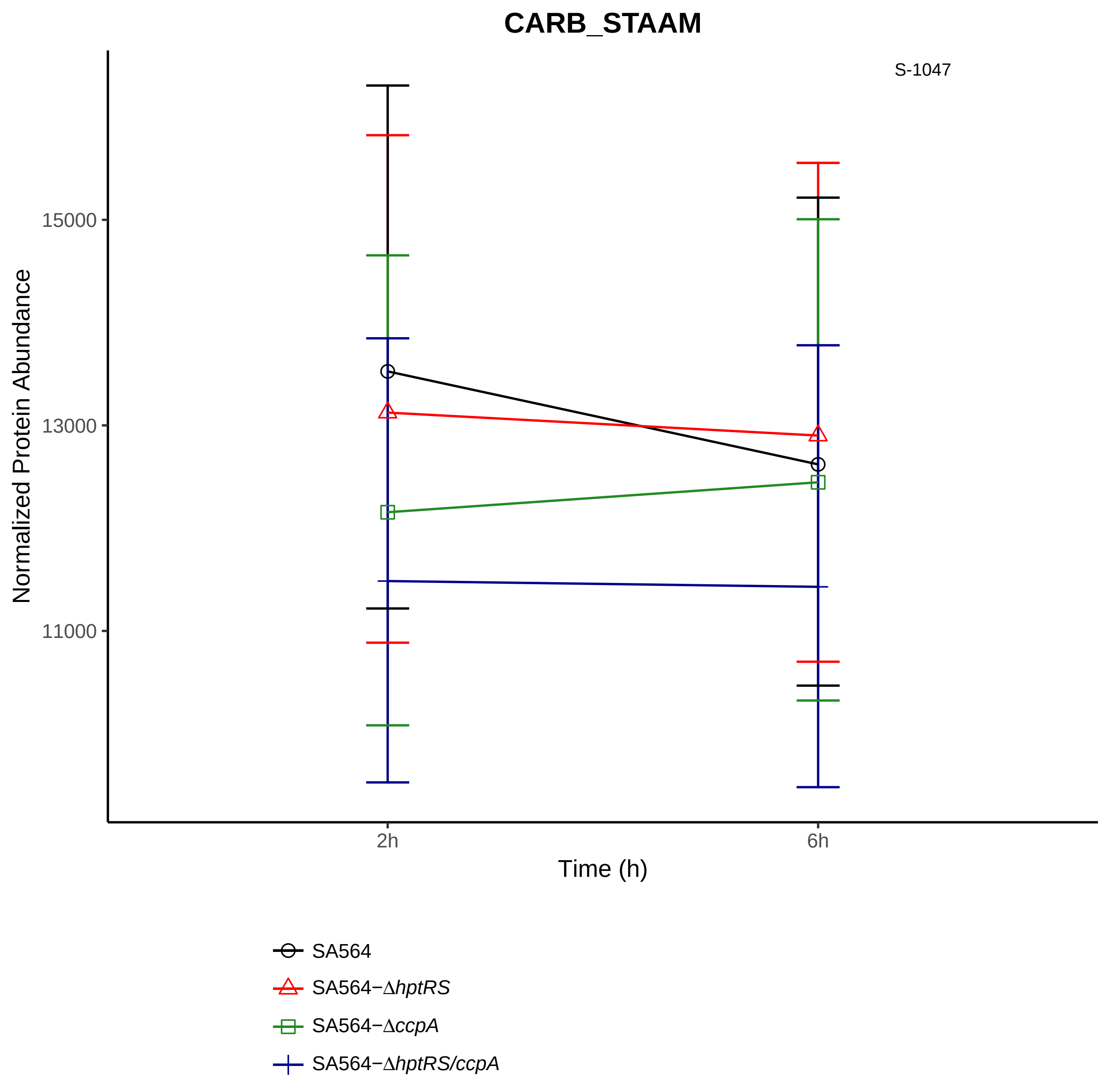

CARB_STAAM
S-1047
Normalized Protein Abundance
15000
13000
11000
2h
6h
Time (h)
SA564
SA564−Δ*hptRS*
SA564−Δ*ccpA*
SA564−Δ*hptRS/ccpA*

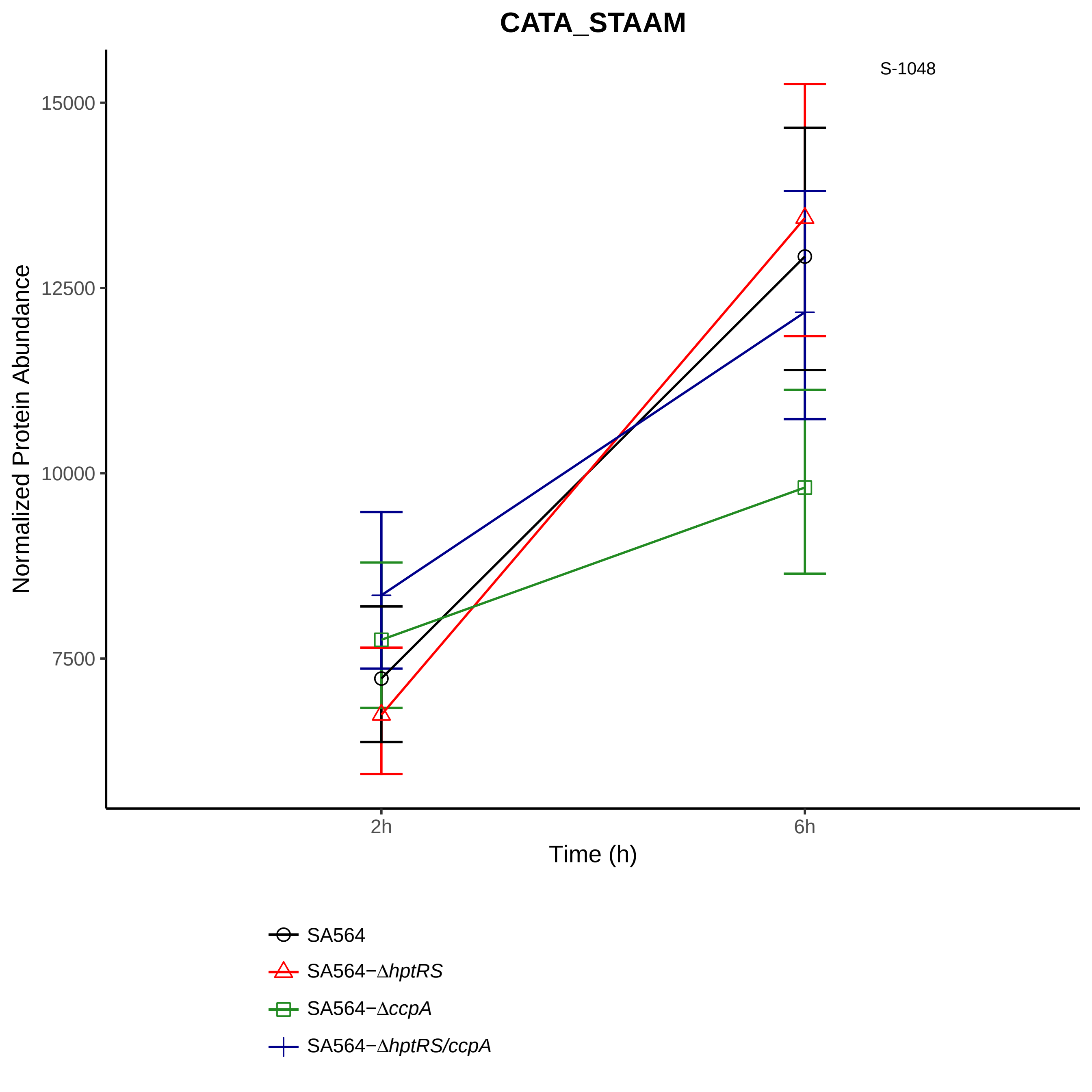

CATA_STAAM
S-1048
15000
12500
10000
7500
Normalized Protein Abundance
2h
6h
Time (h)
SA564
SA564−Δ*hptRS*
SA564−Δ*ccpA*
SA564−Δ*hptRS/ccpA*

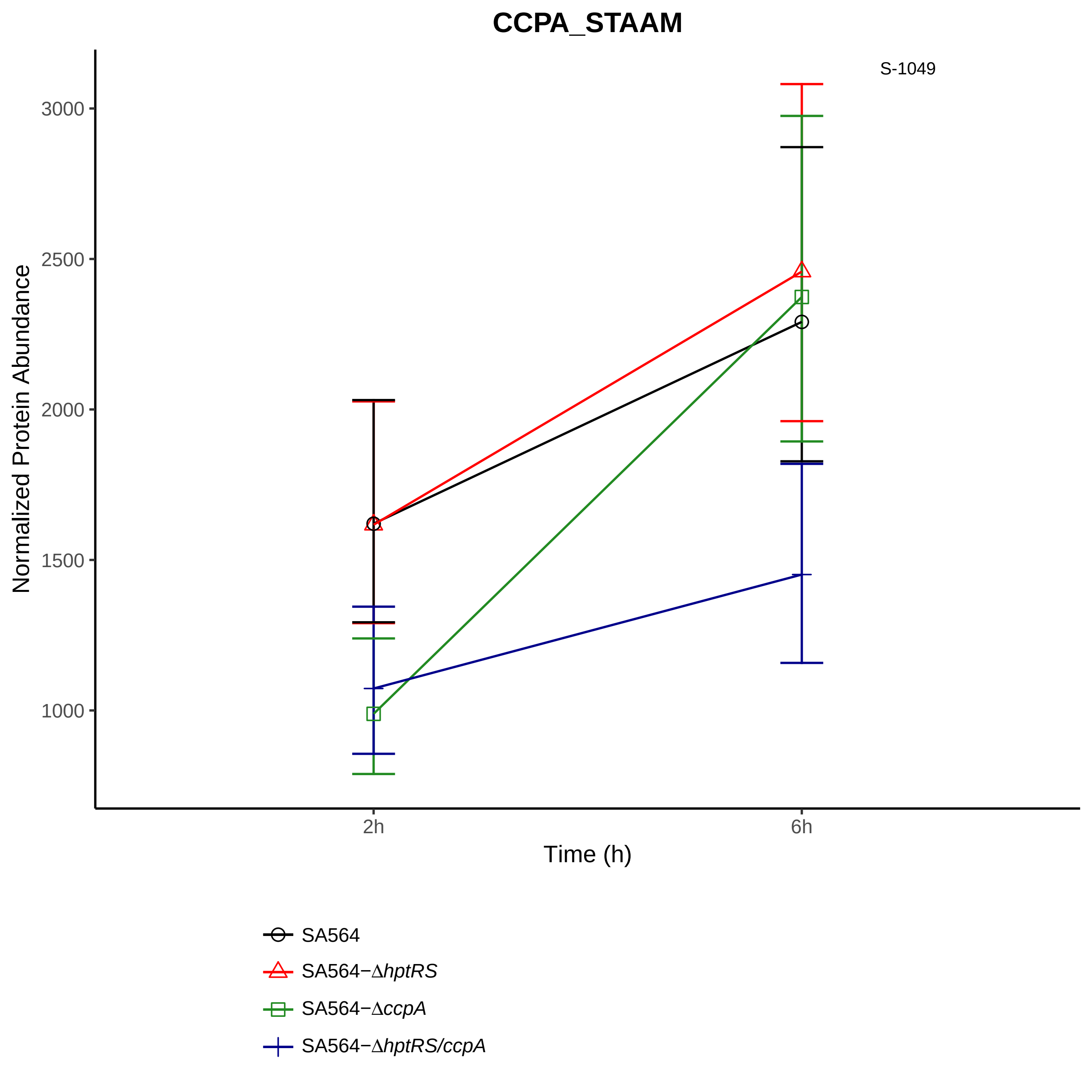
CCPA_STAAM
S-1049
3000
2500
2000
1500
1000
Normalized Protein Abundance
2h
6h
Time (h)
SA564
SA564−Δ*hptRS*
SA564−Δ*ccpA*
SA564−Δ*hptRS/ccpA*

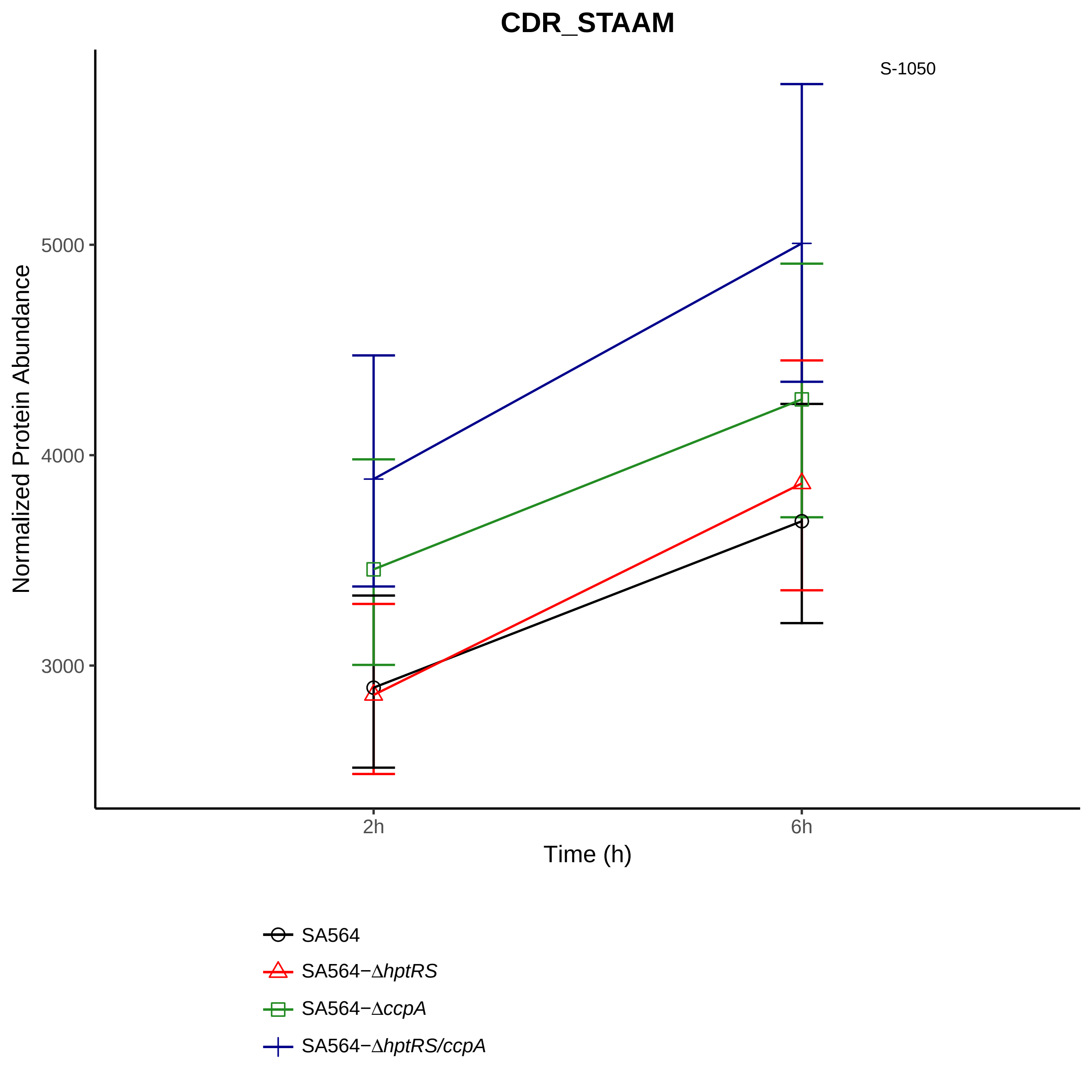

CDR_STAAM
S-1050
Normalized Protein Abundance
5000
4000
3000
2h
6h
Time (h)
SA564
SA564−ΔhptRS
SA564−ΔccpA
SA564−ΔhptRS/ccpA

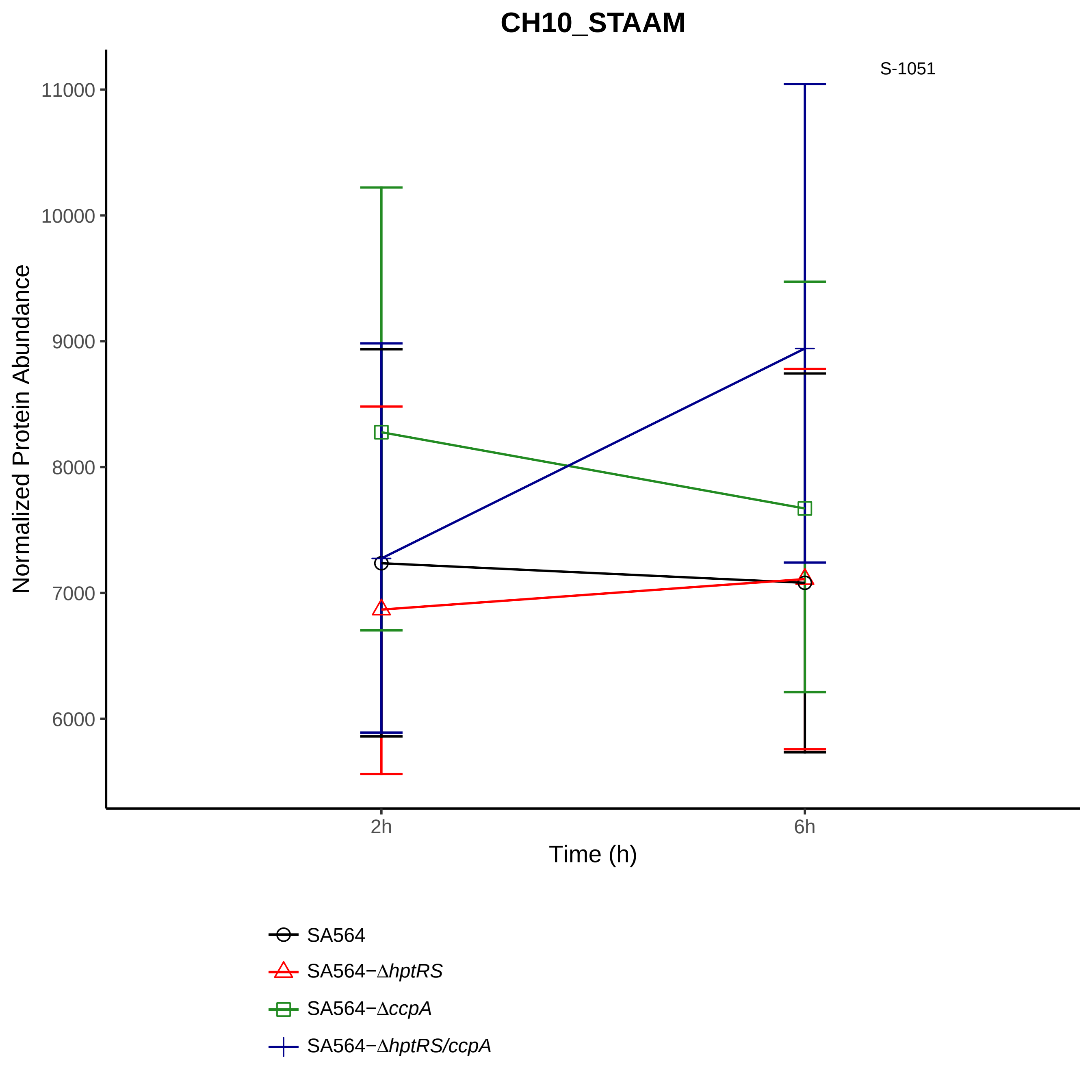

CH10_STAAM
S-1051
Normalized Protein Abundance
11000
10000
9000
8000
7000
6000
2h
6h
Time (h)
SA564
SA564−Δ*hptRS*
SA564−Δ*ccpA*
SA564−Δ*hptRS/ccpA*

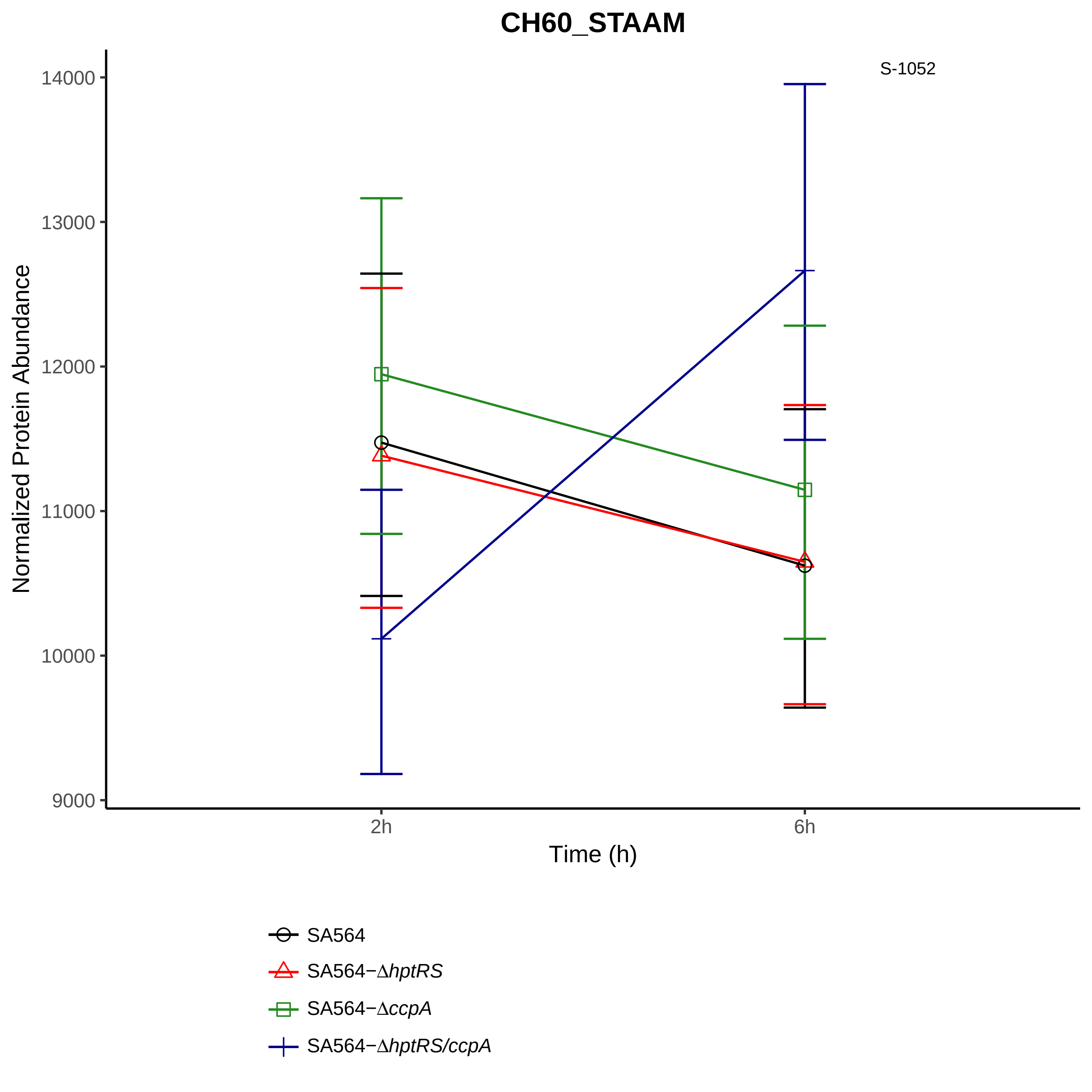

CH60_STAAM
S-1052
Normalized Protein Abundance
14000
13000
12000
11000
10000
9000
2h
6h
Time (h)
SA564
SA564−Δ*hptRS*
SA564−Δ*ccpA*
SA564−Δ*hptRS/ccpA*

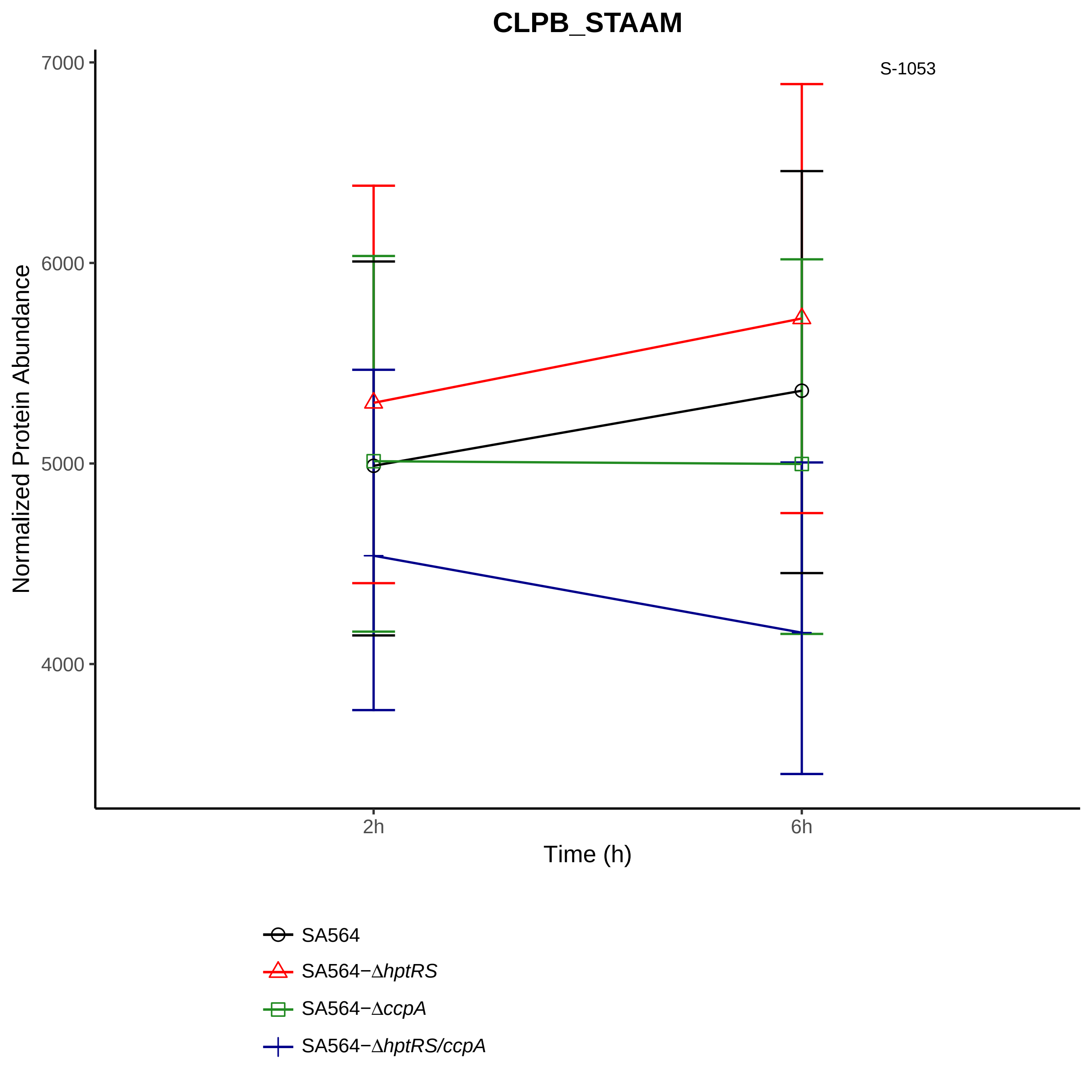
CLPB_STAAM
S-1053
Normalized Protein Abundance
7000
6000
5000
4000
2h
6h
Time (h)
SA564
SA564−Δ*hptRS*
SA564−Δ*ccpA*
SA564−Δ*hptRS/ccpA*

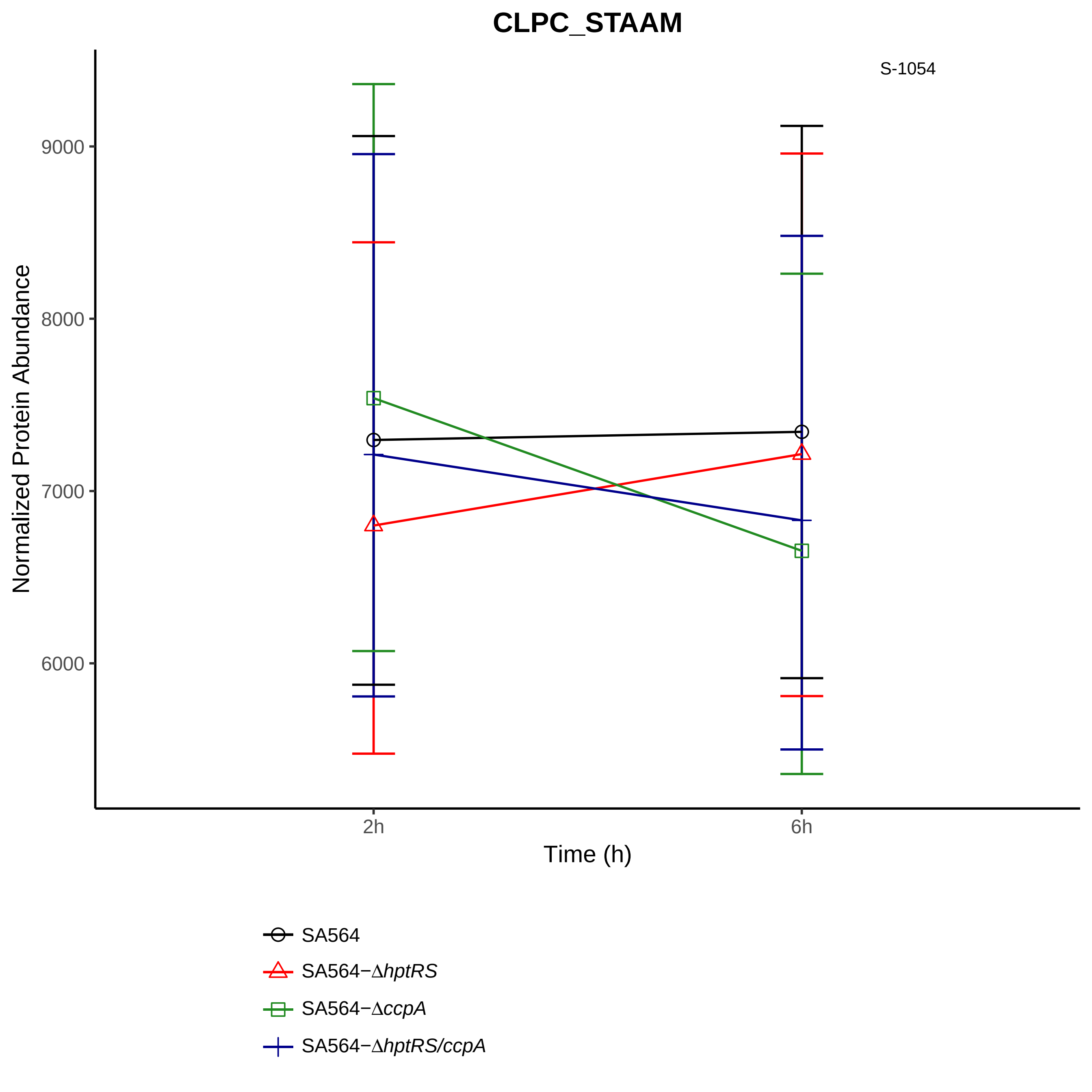
CLPC_STAAM
S-1054
Normalized Protein Abundance
9000
8000
7000
6000
2h
6h
Time (h)
SA564
SA564−ΔhptRS
SA564−ΔccpA
SA564−ΔhptRS/ccpA

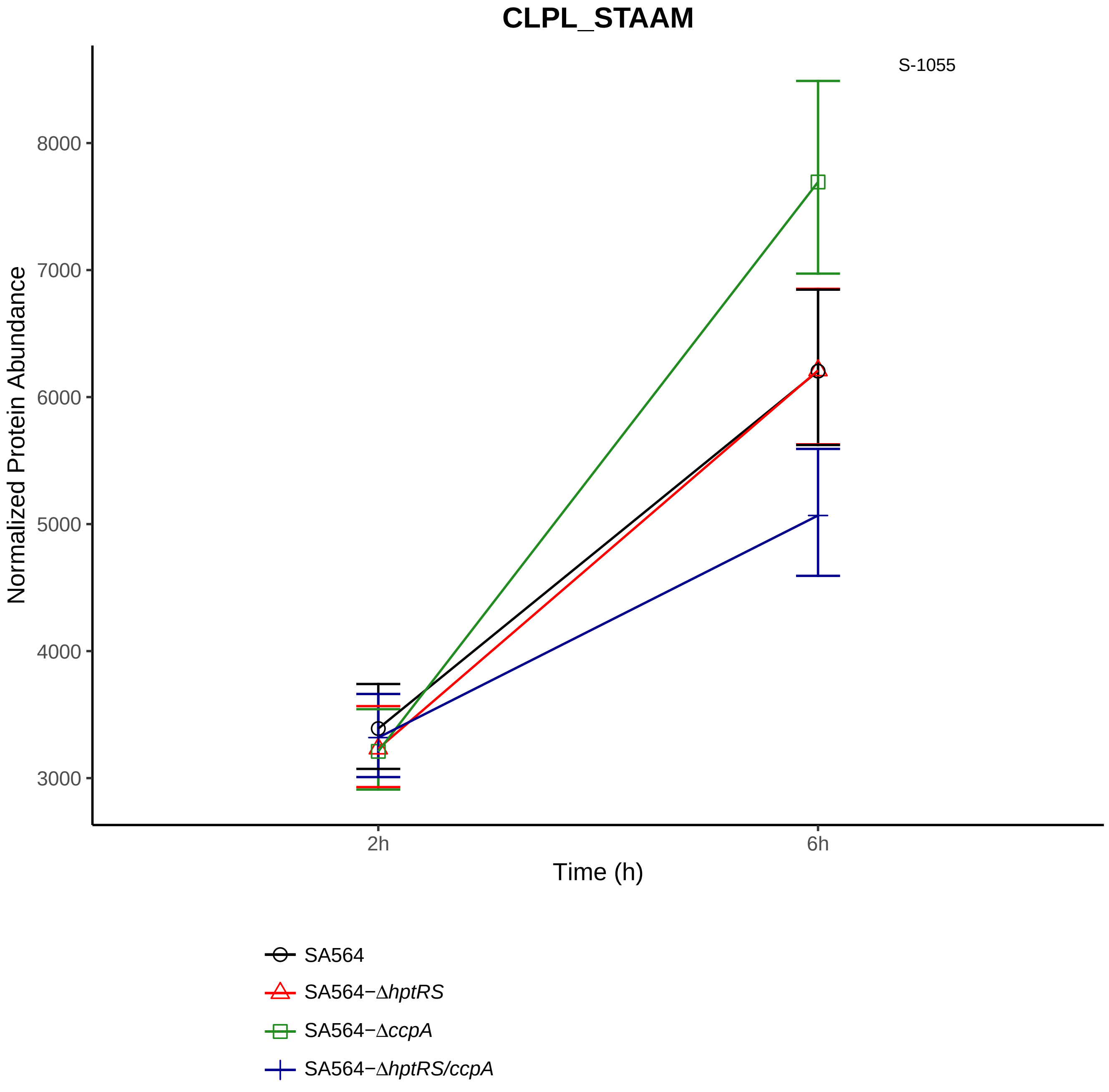

CLPL_STAAM
S-1055
Normalized Protein Abundance
3000
4000
5000
6000
7000
8000
2h
6h
Time (h)
SA564
SA564−Δ*hptRS*
SA564−Δ*ccpA*
SA564−Δ*hptRS/ccpA*

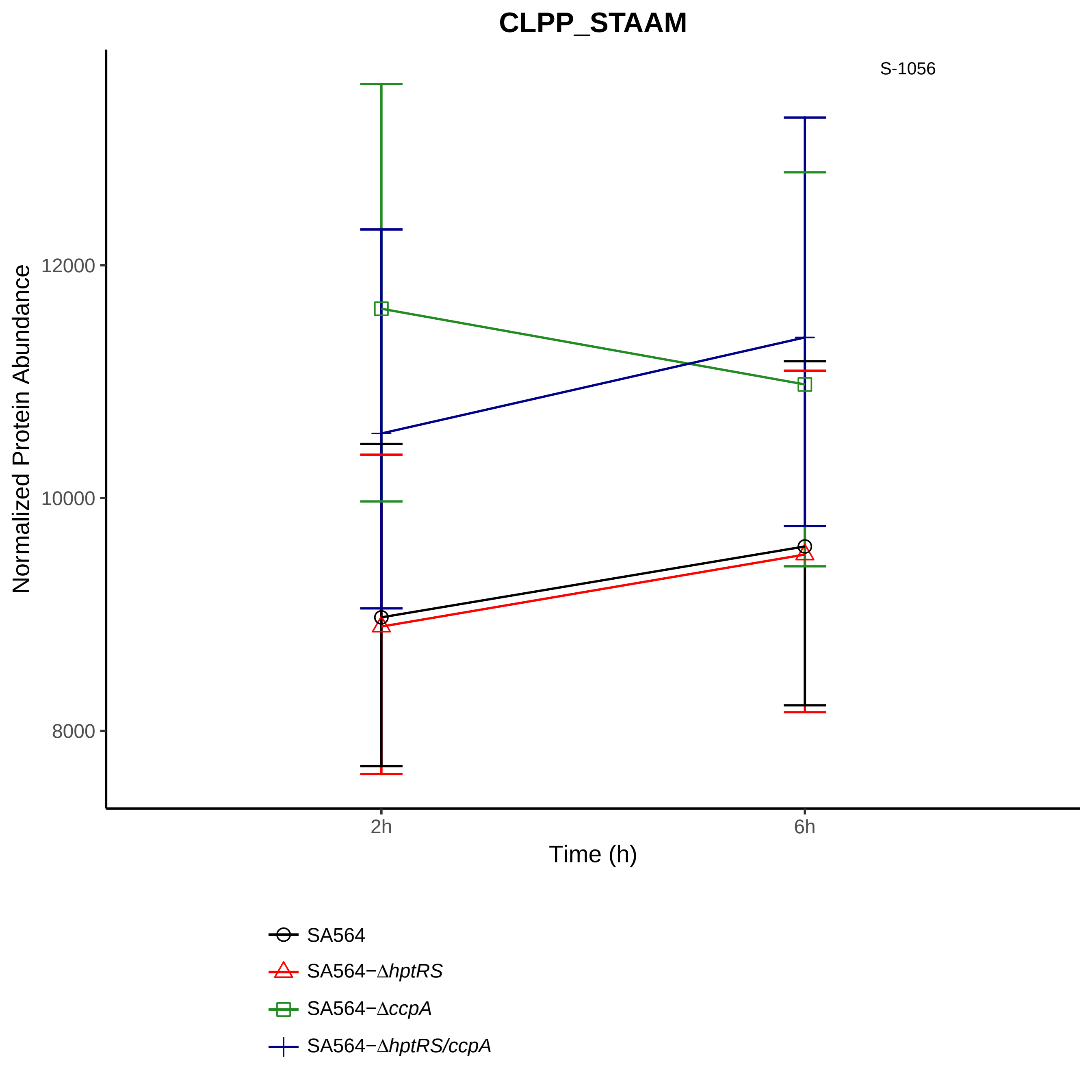
CLPP_STAAM
S-1056
Normalized Protein Abundance
12000
10000
8000
2h
6h
Time (h)
SA564
SA564−Δ*hptRS*
SA564−Δ*ccpA*
SA564−Δ*hptRS/ccpA*

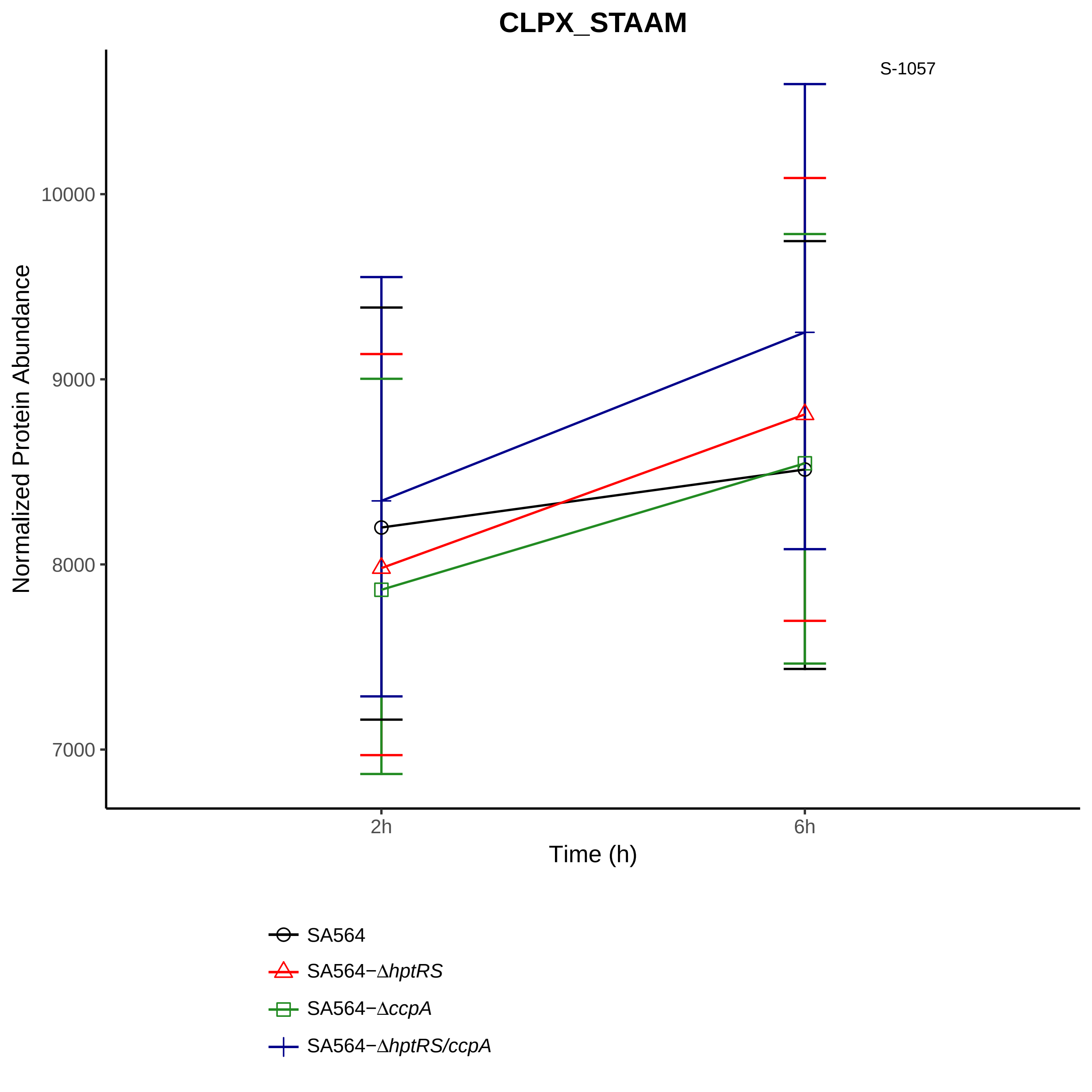

CLPX_STAAM
S-1057
Normalized Protein Abundance
10000
9000
8000
7000
2h
6h
Time (h)
SA564
SA564−Δ*hptRS*
SA564−Δ*ccpA*
SA564−Δ*hptRS/ccpA*

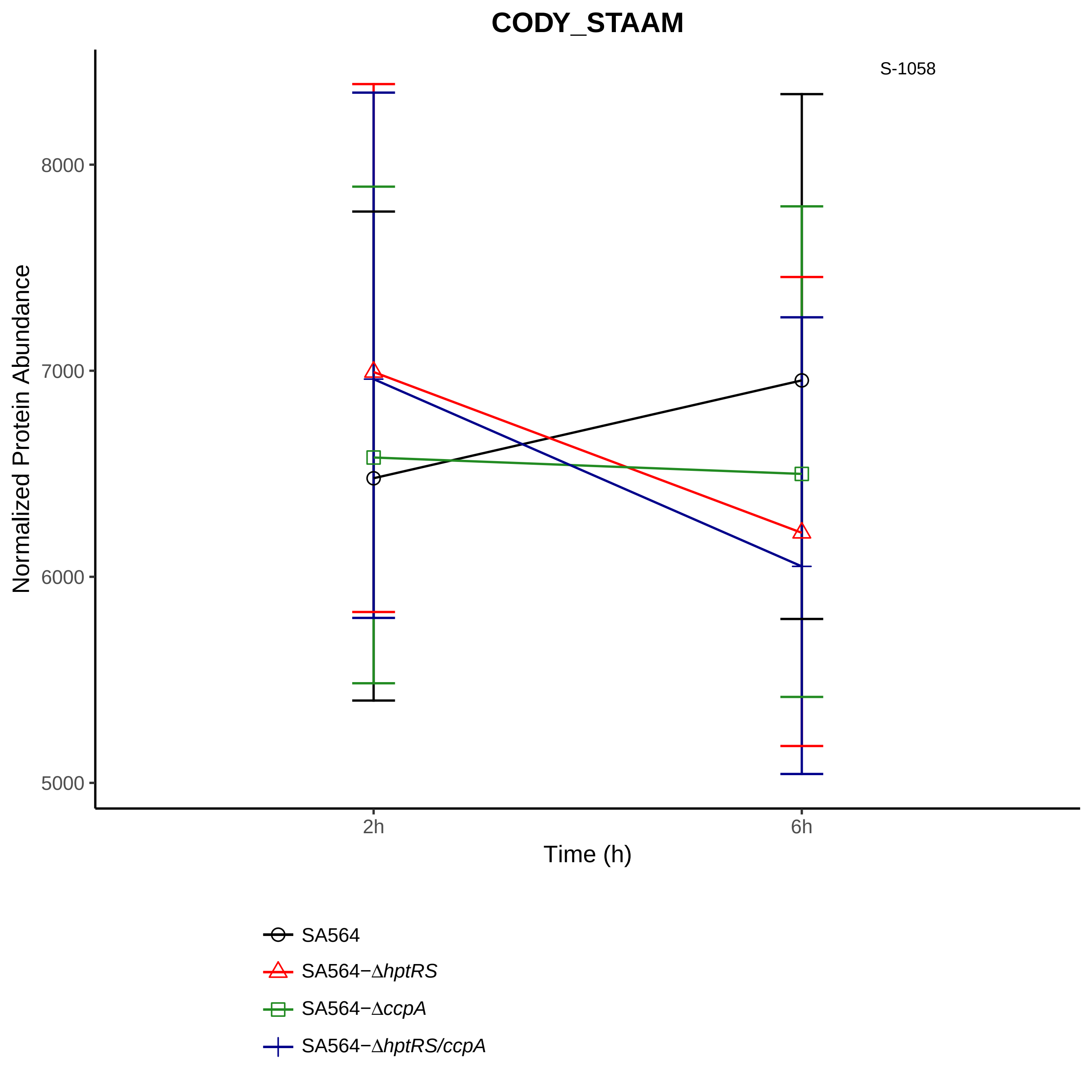

CODY_STAAM
S-1058
Normalized Protein Abundance
8000
7000
6000
5000
2h
6h
Time (h)
SA564
SA564−Δ*hptRS*
SA564−Δ*ccpA*
SA564−Δ*hptRS/ccpA*

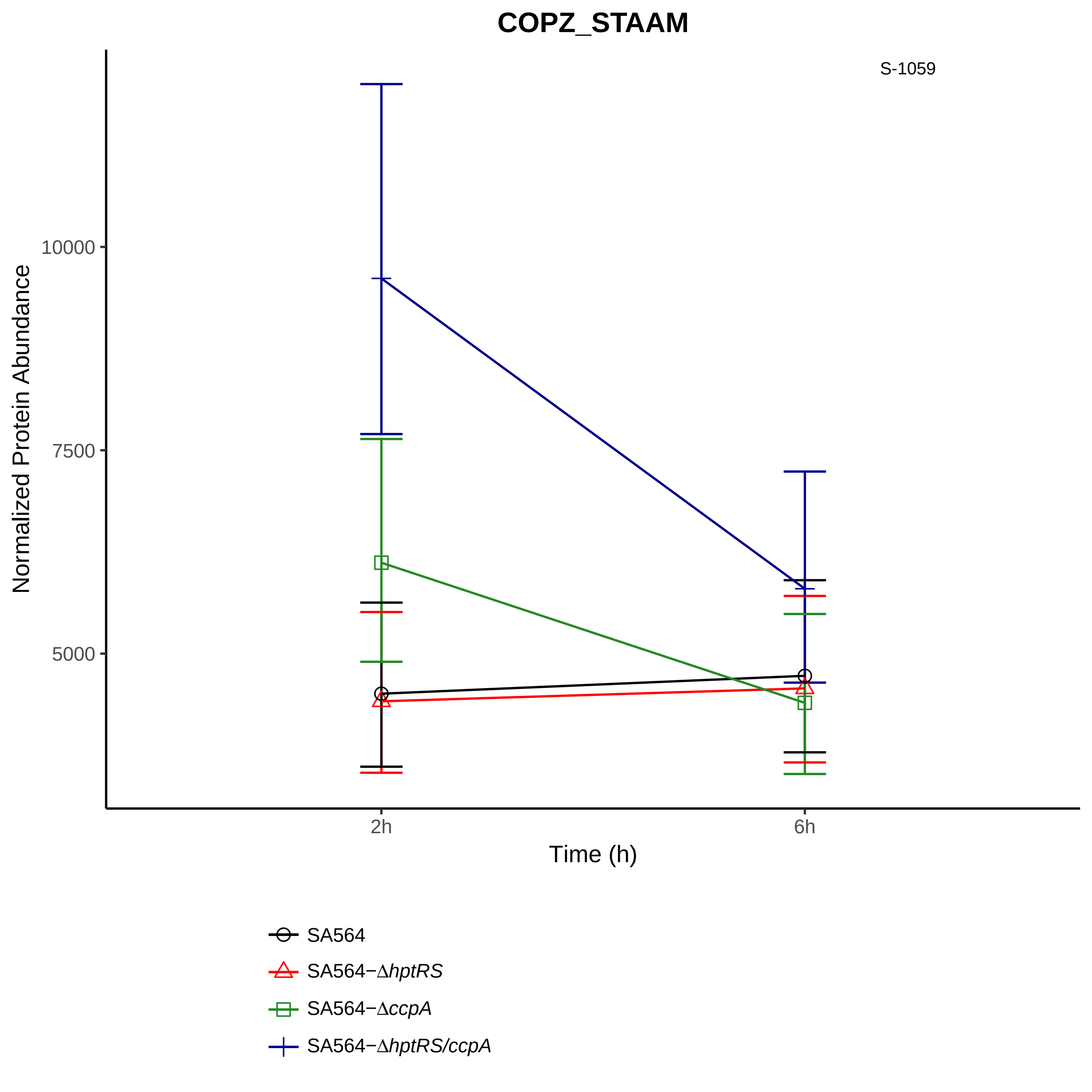
COPZ_STAAM
S-1059
Normalized Protein Abundance
10000
7500
5000
2h
6h
Time (h)
SA564
SA564−ΔhptRS
SA564−ΔccpA
SA564−ΔhptRS/ccpA

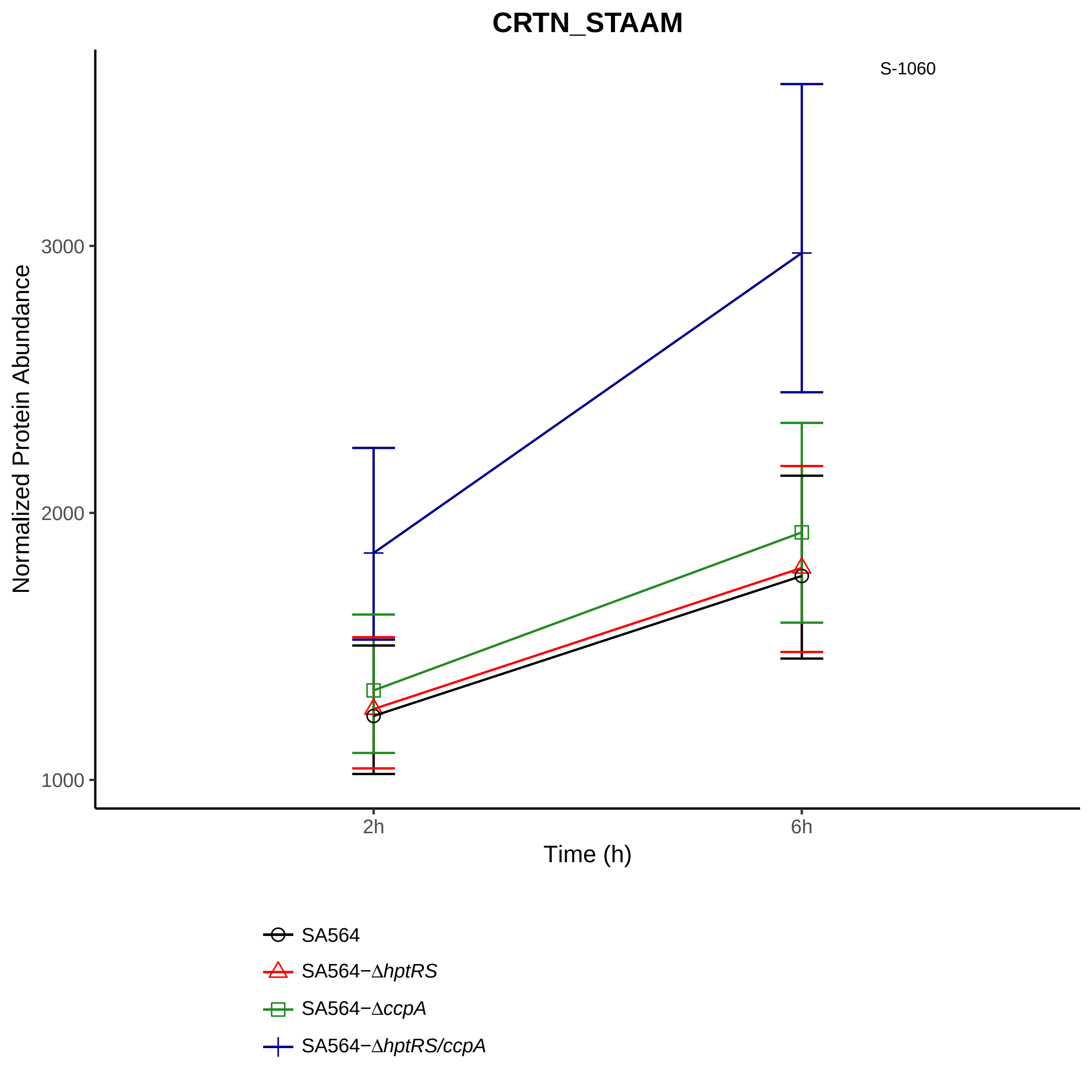

CRTN_STAAM
S-1060
Normalized Protein Abundance
3000
2000
1000
2h
6h
Time (h)
SA564
SA564−Δ*hptRS*
SA564−Δ*ccpA*
SA564−Δ*hptRS/ccpA*

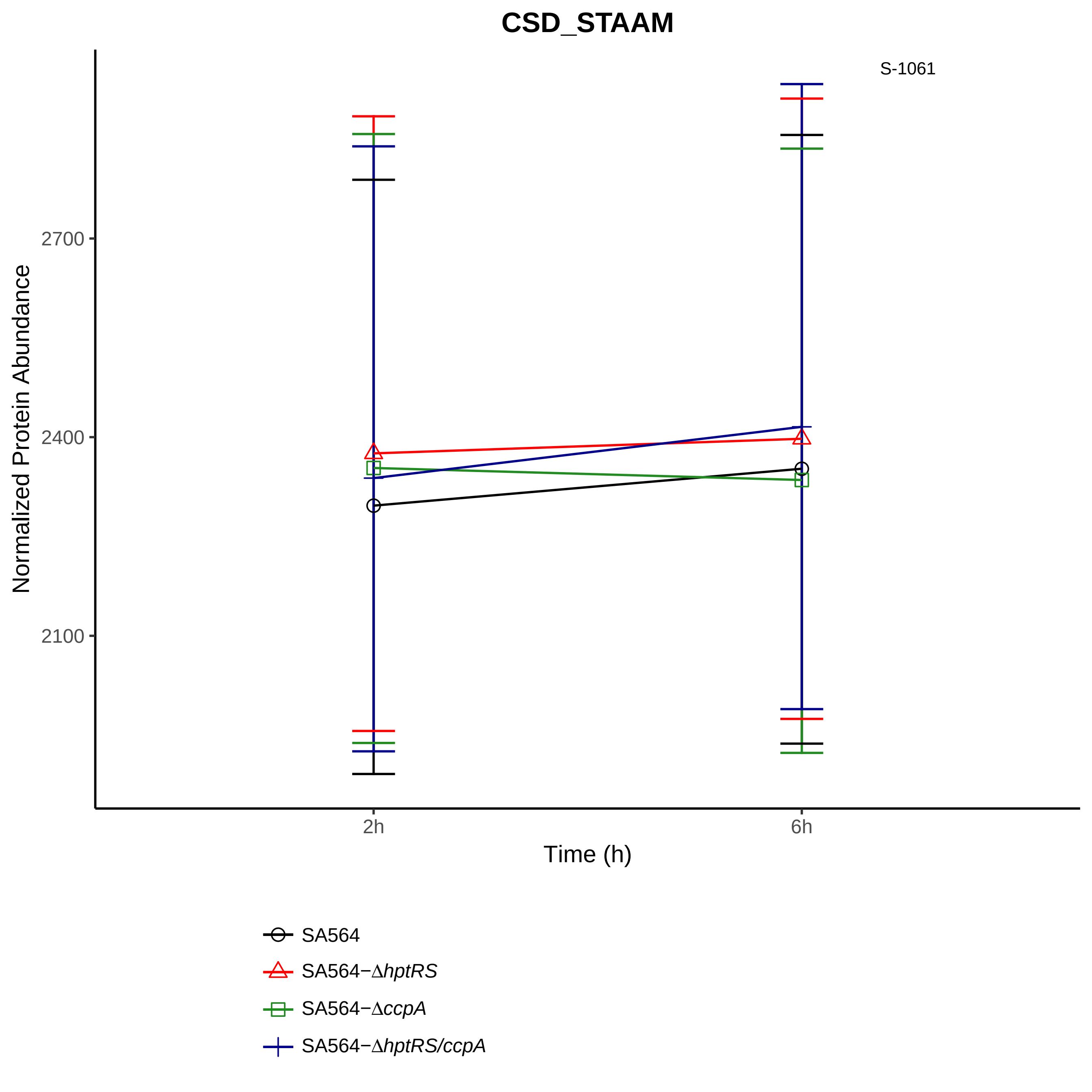

CSD_STAAM
S-1061
Normalized Protein Abundance
2700
2400
2100
2h
6h
Time (h)
SA564
SA564−Δ*hptRS*
SA564−Δ*ccpA*
SA564−Δ*hptRS/ccpA*

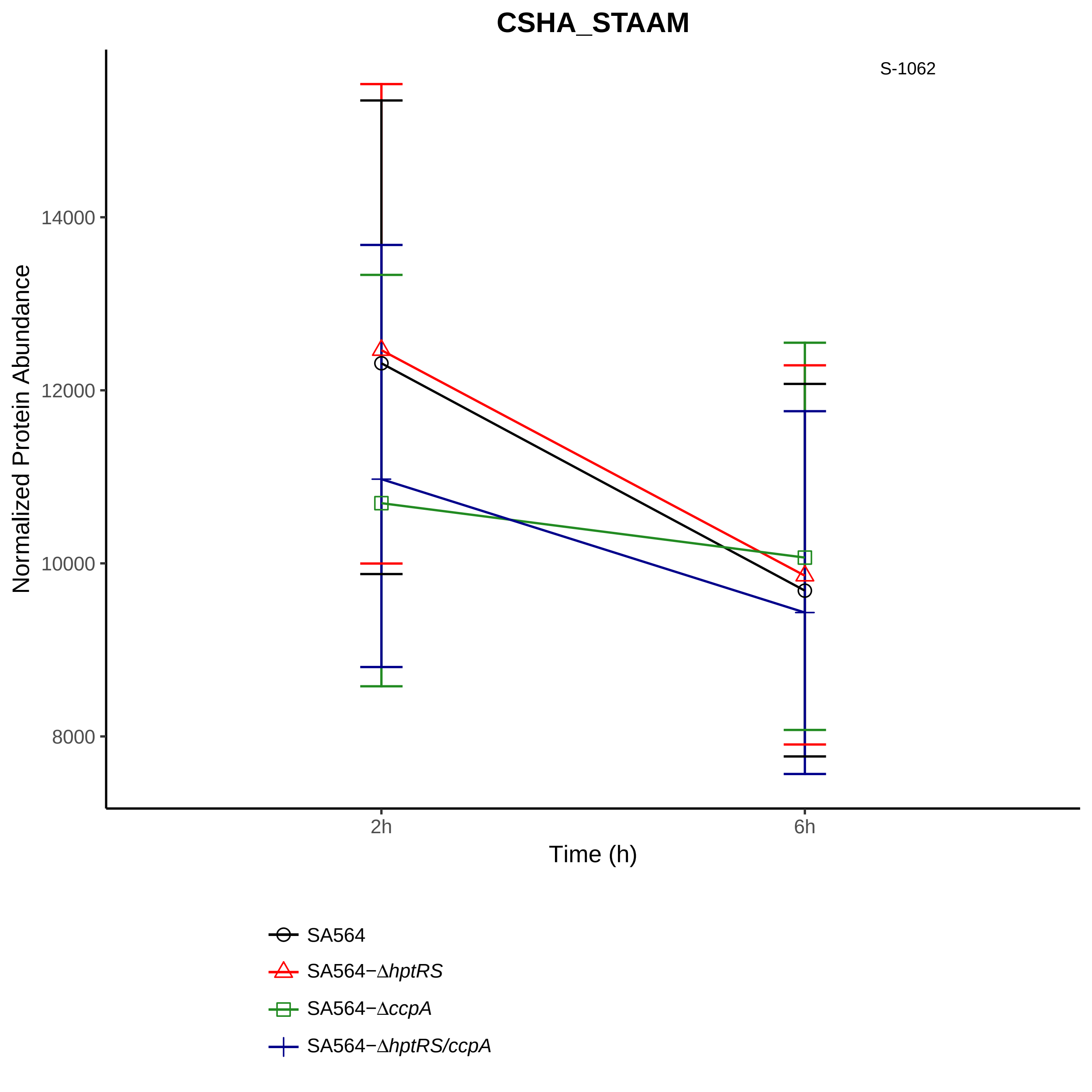

CSHA_STAAM
S-1062
Normalized Protein Abundance
14000
12000
10000
8000
2h
6h
Time (h)
SA564
SA564−Δ*hptRS*
SA564−Δ*ccpA*
SA564−Δ*hptRS/ccpA*

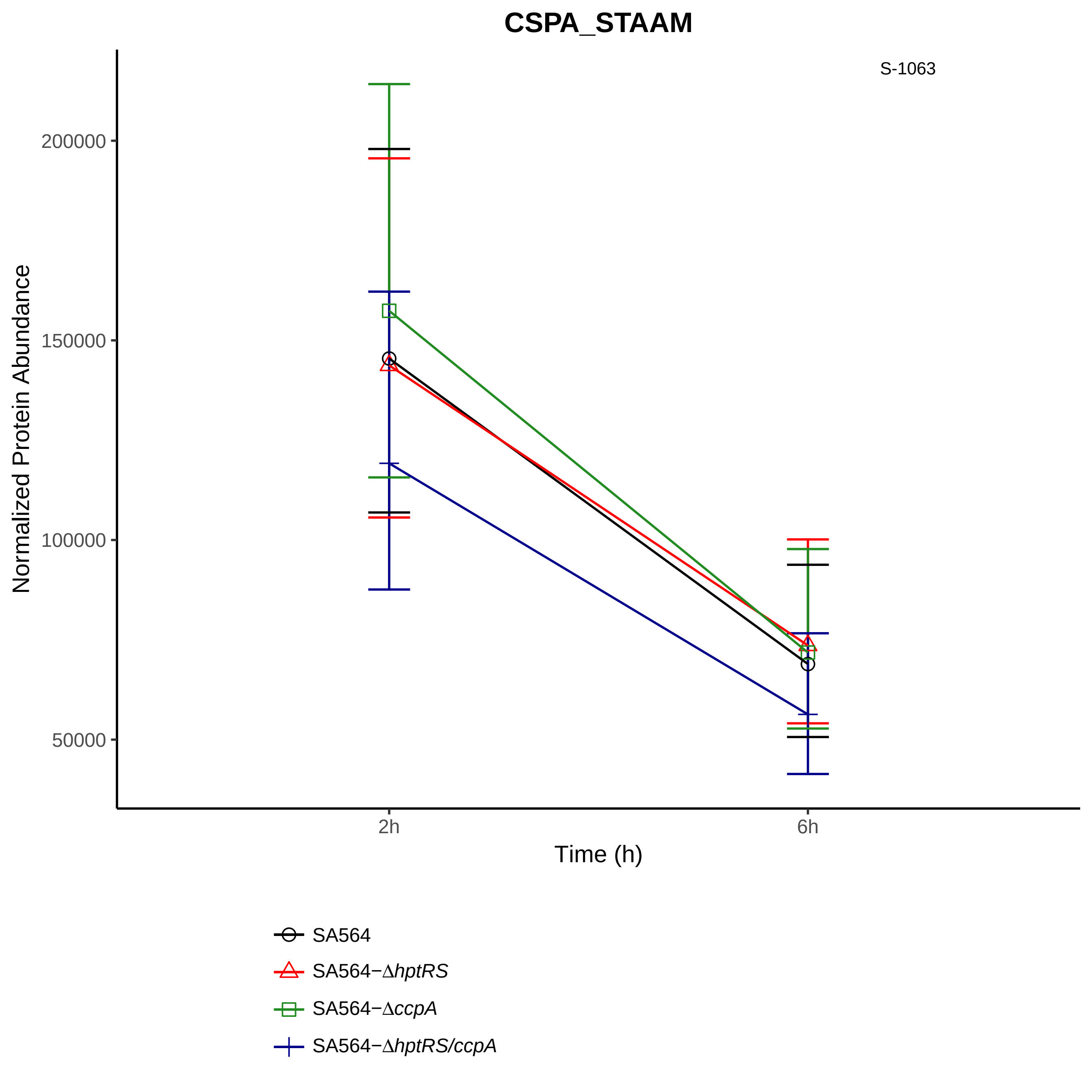

CSPA_STAAM
S-1063
Normalized Protein Abundance
200000
150000
100000
50000
2h
6h
Time (h)
SA564
SA564−ΔhptRS
SA564−ΔccpA
SA564−ΔhptRS/ccpA

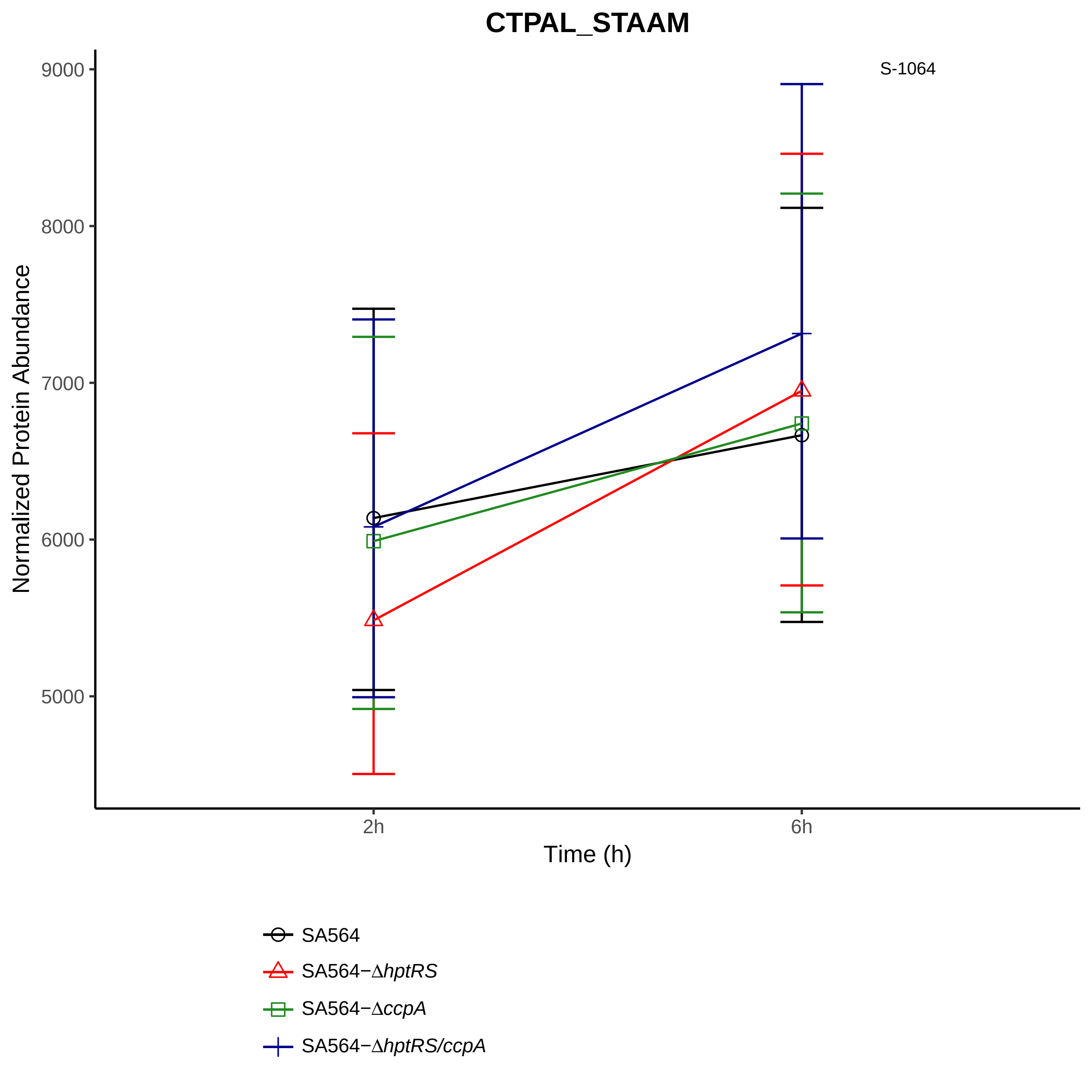

CTPAL_STAAM
S-1064
Normalized Protein Abundance
9000
8000
7000
6000
5000
2h
6h
Time (h)
SA564
SA564−Δ*hptRS*
SA564−Δ*ccpA*
SA564−Δ*hptRS/ccpA*

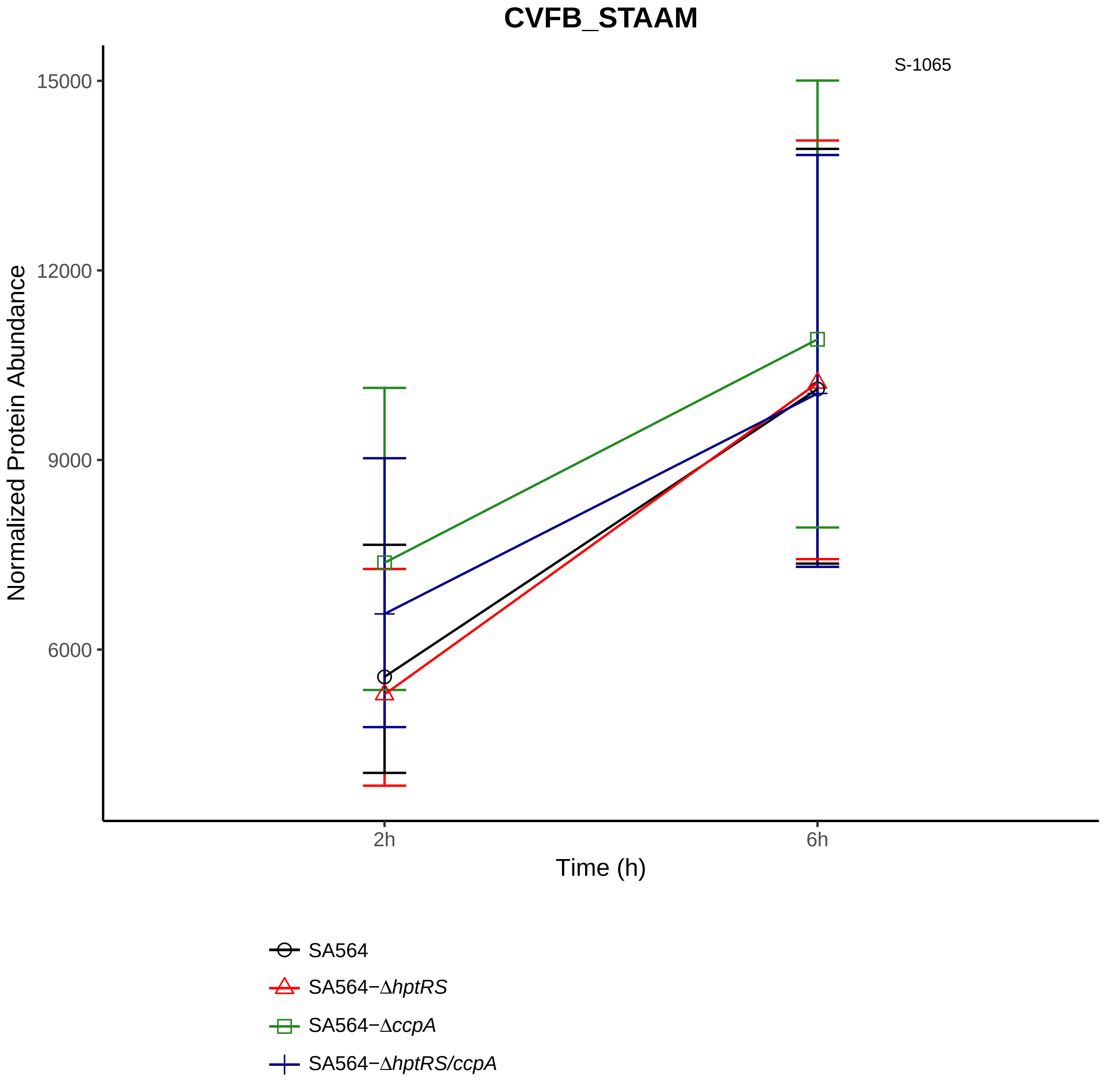
CVFB_STAAM
S-1065
Normalized Protein Abundance
15000
12000
9000
6000
2h
6h
Time (h)
SA564
SA564−ΔhptRS
SA564−ΔccpA
SA564−ΔhptRS/ccpA

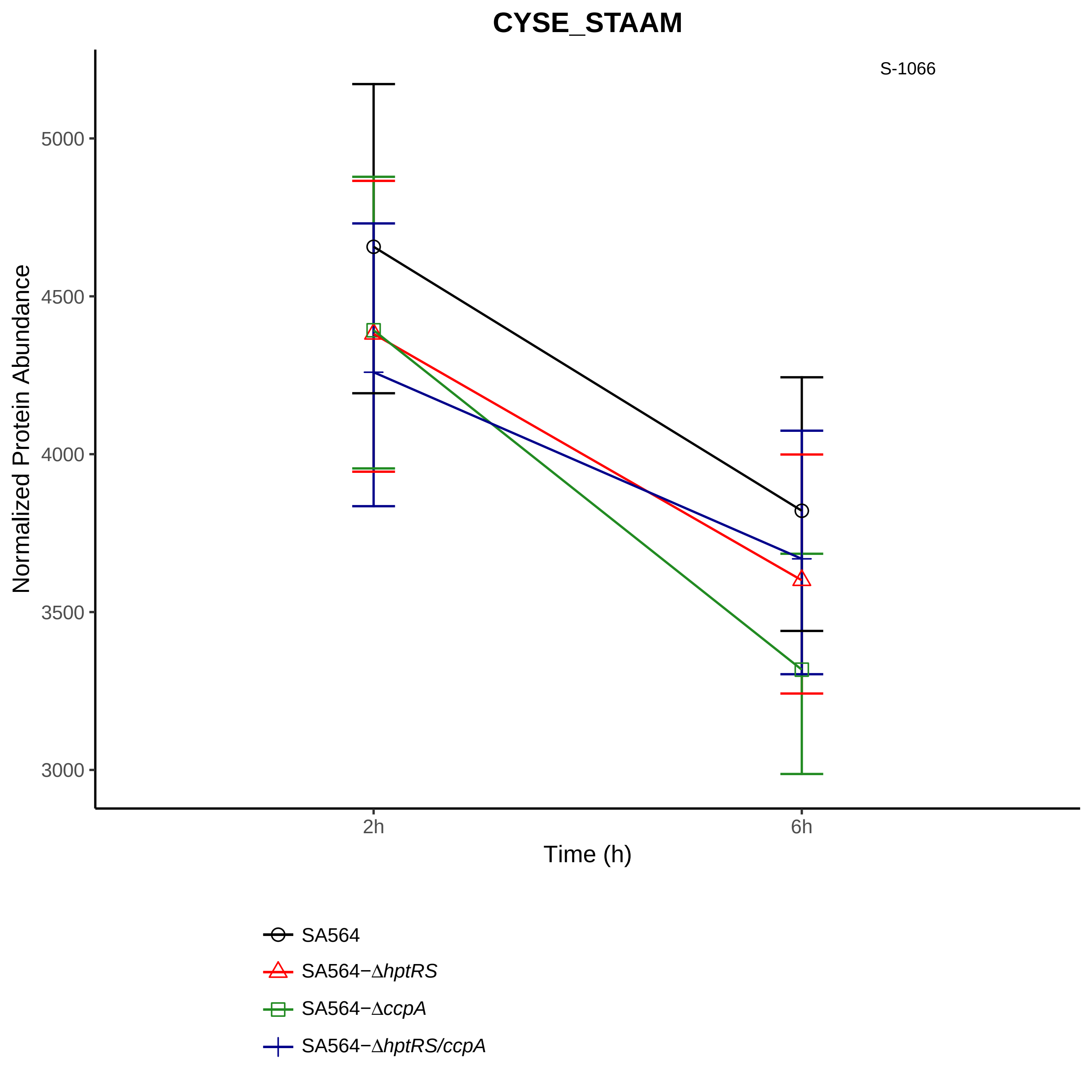
CYSE_STAAM
S-1066
Normalized Protein Abundance
5000
4500
4000
3500
3000
2h
6h
Time (h)
SA564
SA564−ΔhptRS
SA564−ΔccpA
SA564−ΔhptRS/ccpA

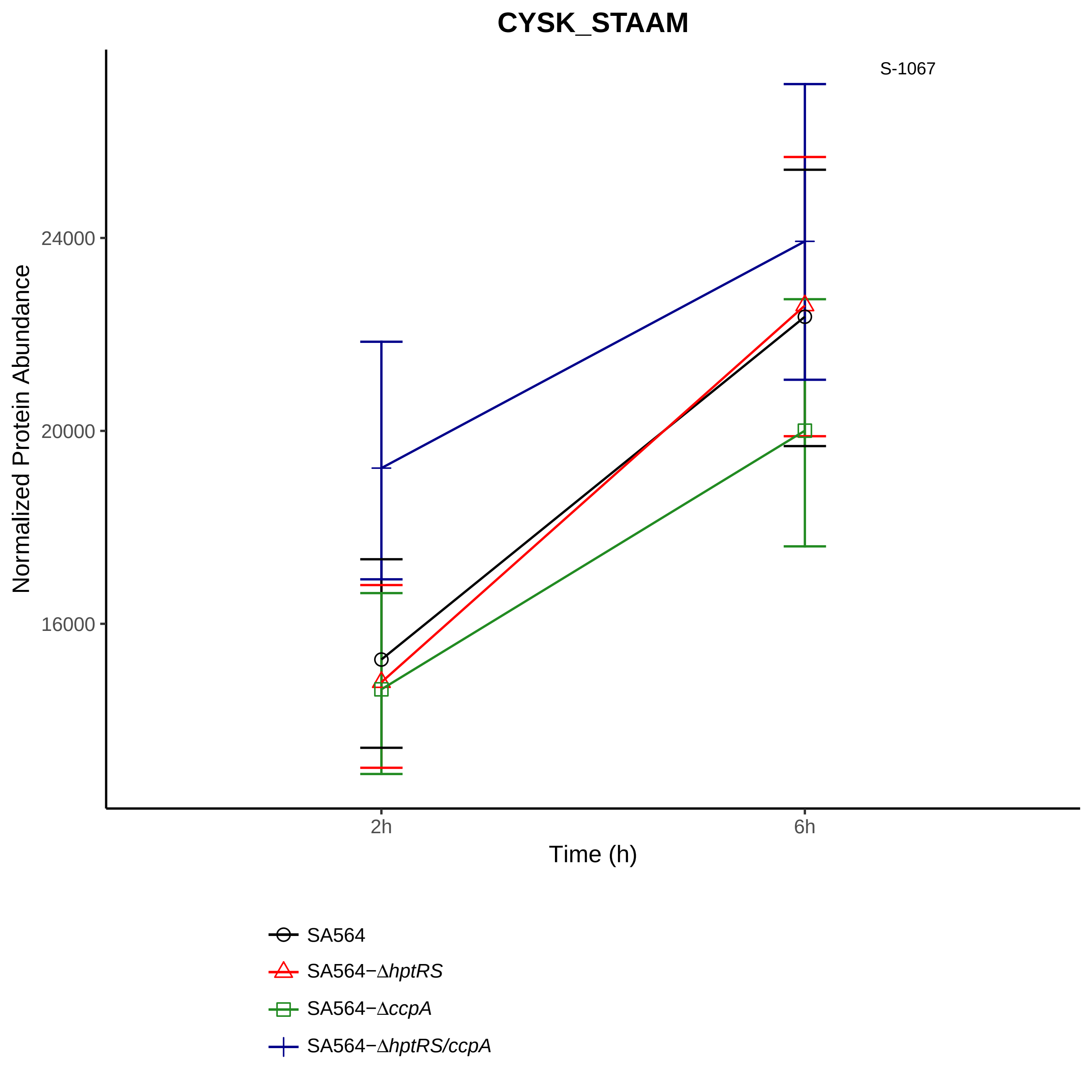

CYSK_STAAM
S-1067
Normalized Protein Abundance
24000
20000
16000
2h
6h
Time (h)
SA564
SA564−ΔhptRS
SA564−ΔccpA
SA564−ΔhptRS/ccpA

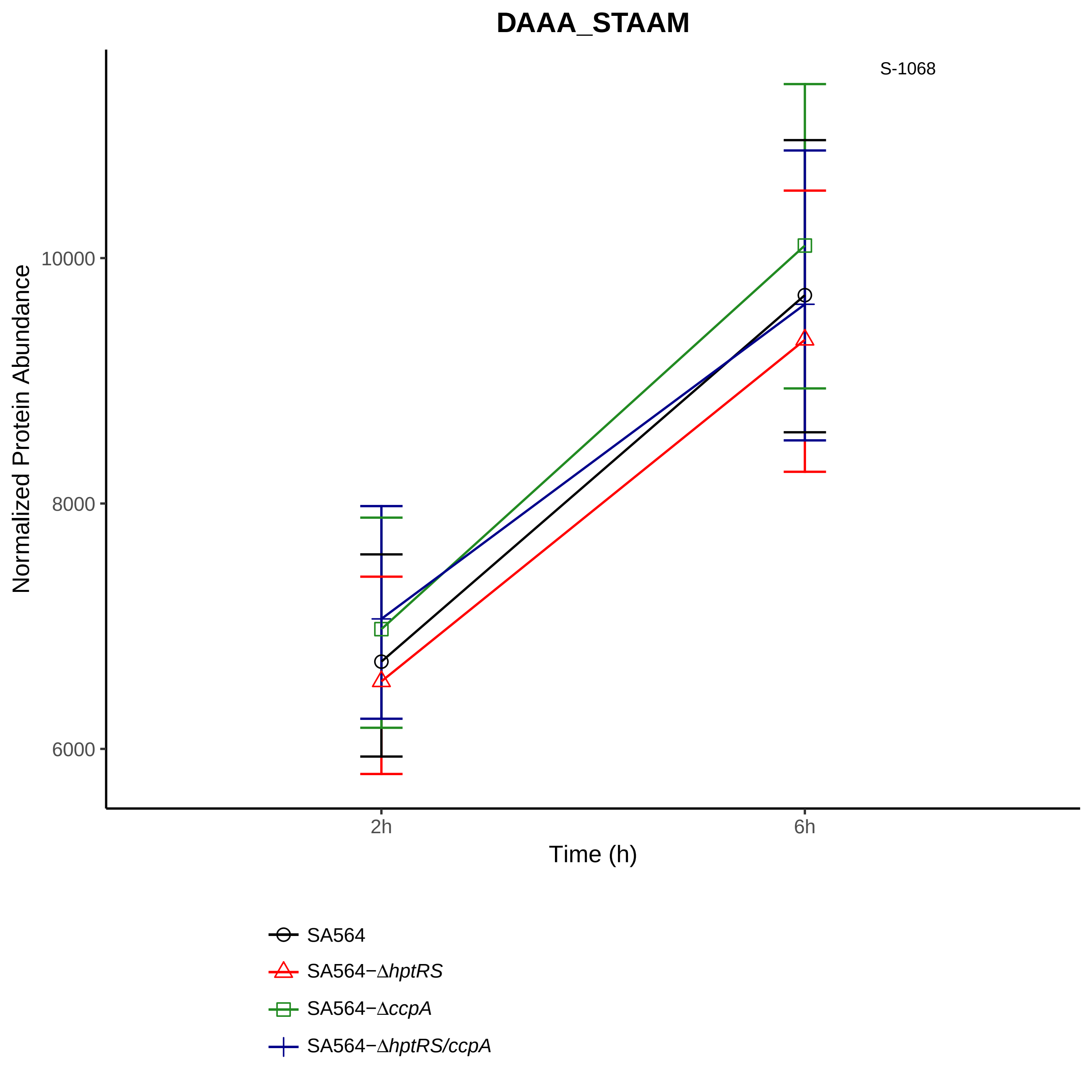
DAAA_STAAM
S-1068
Normalized Protein Abundance
10000
8000
6000
2h
6h
Time (h)
SA564
SA564−ΔhptRS
SA564−ΔccpA
SA564−ΔhptRS/ccpA

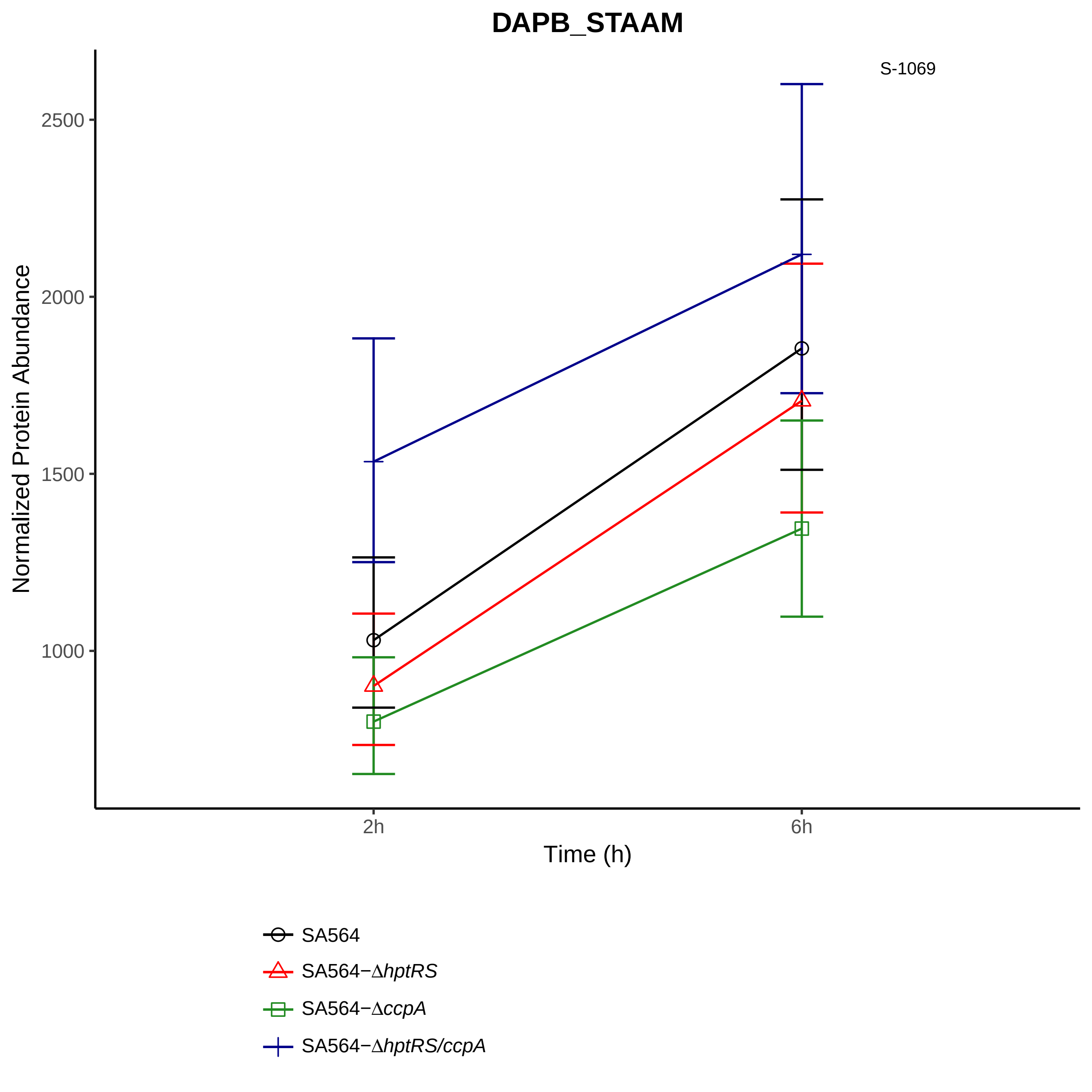
DAPB_STAAM
S-1069
Normalized Protein Abundance
2500
2000
1500
1000
2h
6h
Time (h)
SA564
SA564−Δ*hptRS*
SA564−Δ*ccpA*
SA564−Δ*hptRS/ccpA*

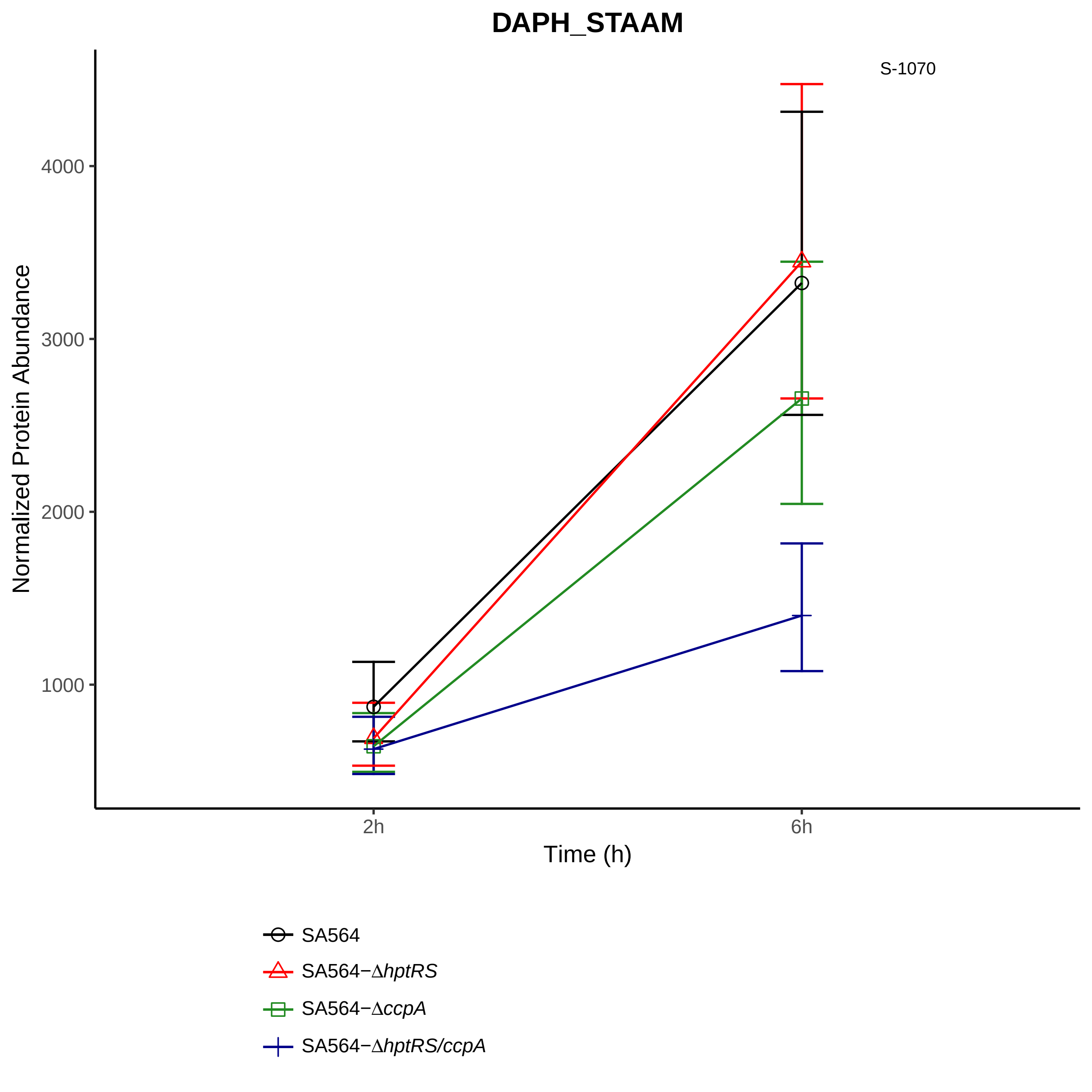

DAPH_STAAM
S-1070
Normalized Protein Abundance
4000
3000
2000
1000
2h
6h
Time (h)
SA564
SA564−Δ*hptRS*
SA564−Δ*ccpA*
SA564−Δ*hptRS/ccpA*

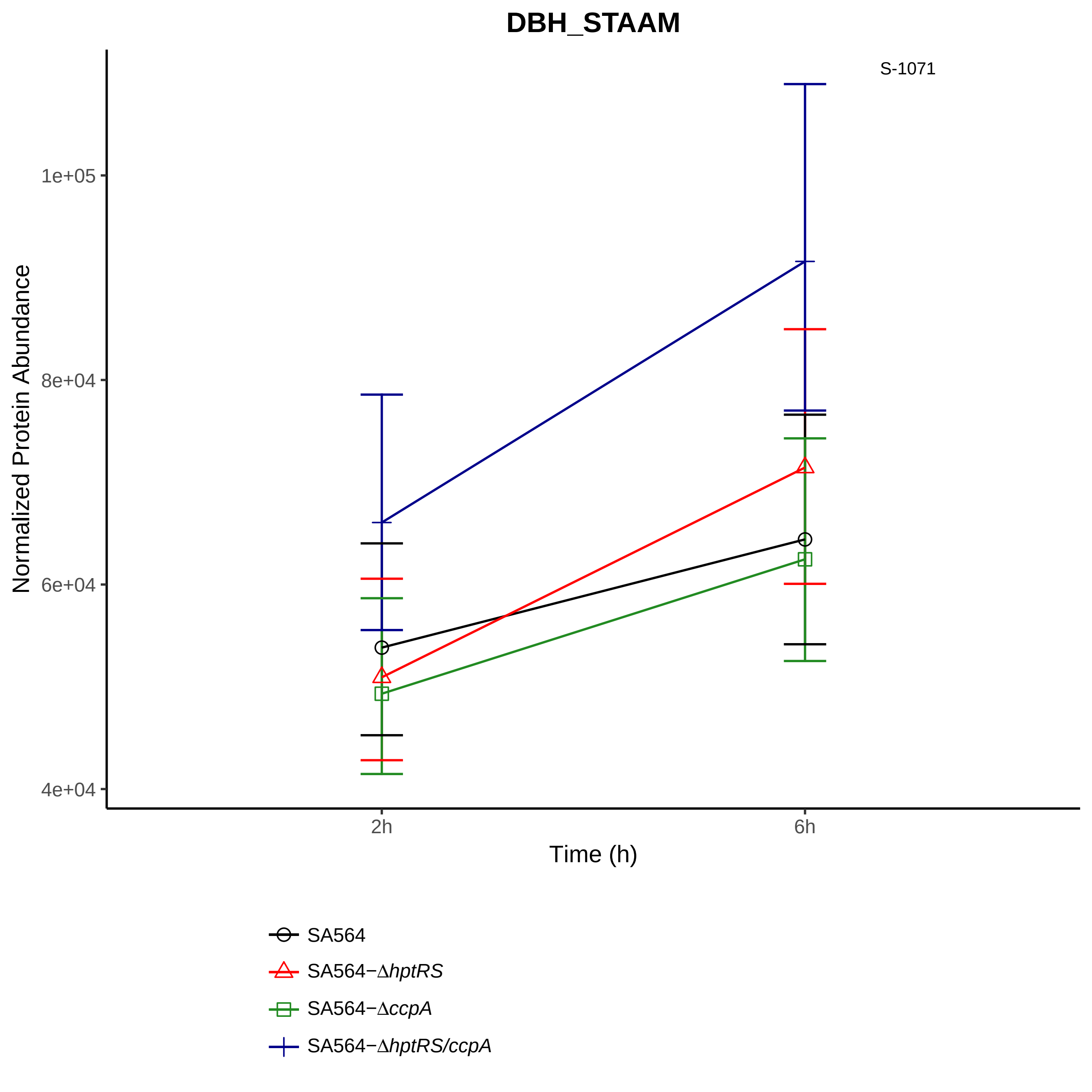
DBH_STAAM
S-1071
Normalized Protein Abundance
1e+05
8e+04
6e+04
4e+04
2h
6h
Time (h)
SA564
SA564−ΔhptRS
SA564−ΔccpA
SA564−ΔhptRS/ccpA

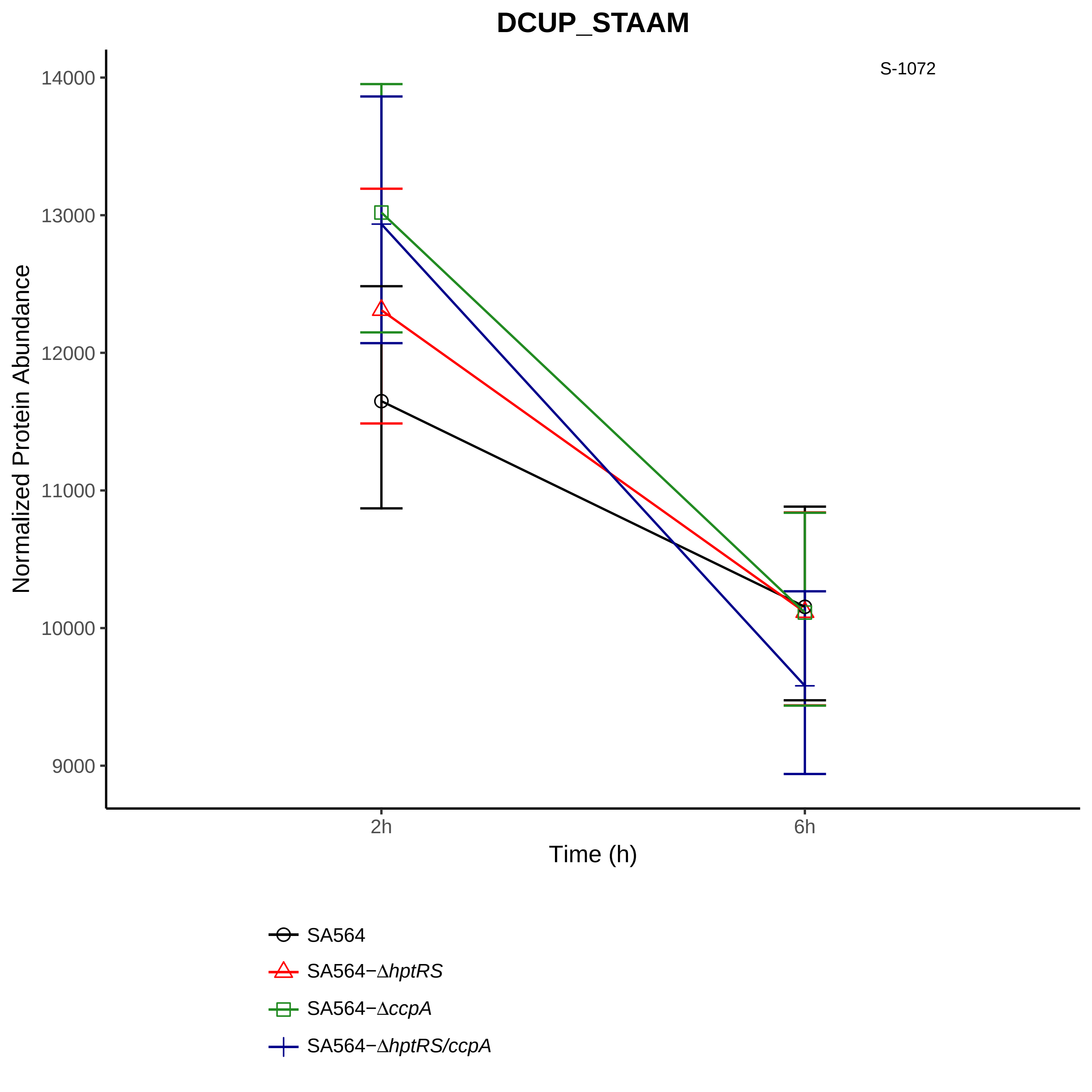
DCUP_STAAM
S-1072
Normalized Protein Abundance
14000
13000
12000
11000
10000
9000
2h
6h
Time (h)
SA564
SA564−ΔhptRS
SA564−ΔccpA
SA564−ΔhptRS/ccpA

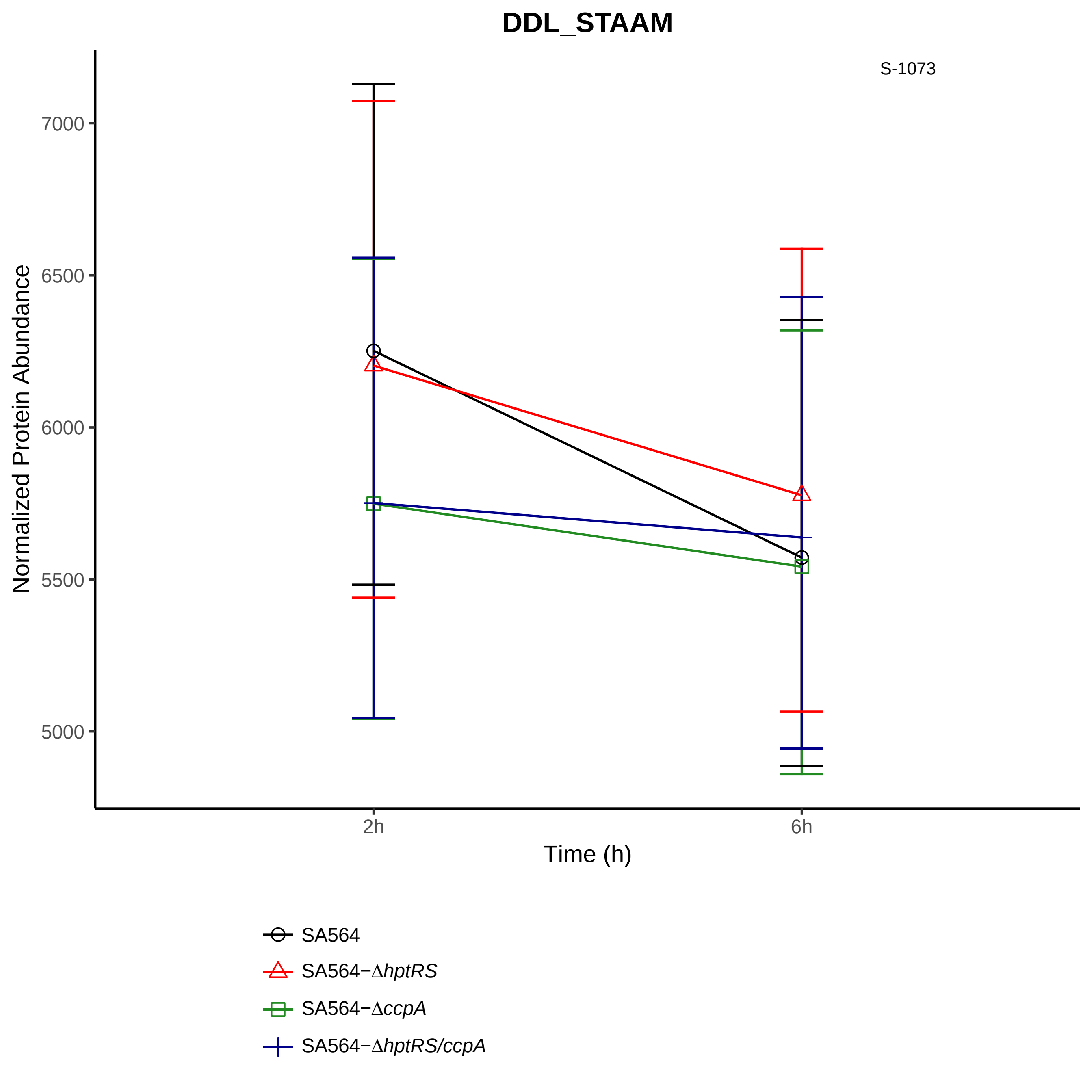

DDL_STAAM
S-1073
Normalized Protein Abundance
7000
6500
6000
5500
5000
2h
6h
Time (h)
SA564
SA564−Δ*hptRS*
SA564−Δ*ccpA*
SA564−Δ*hptRS/ccpA*

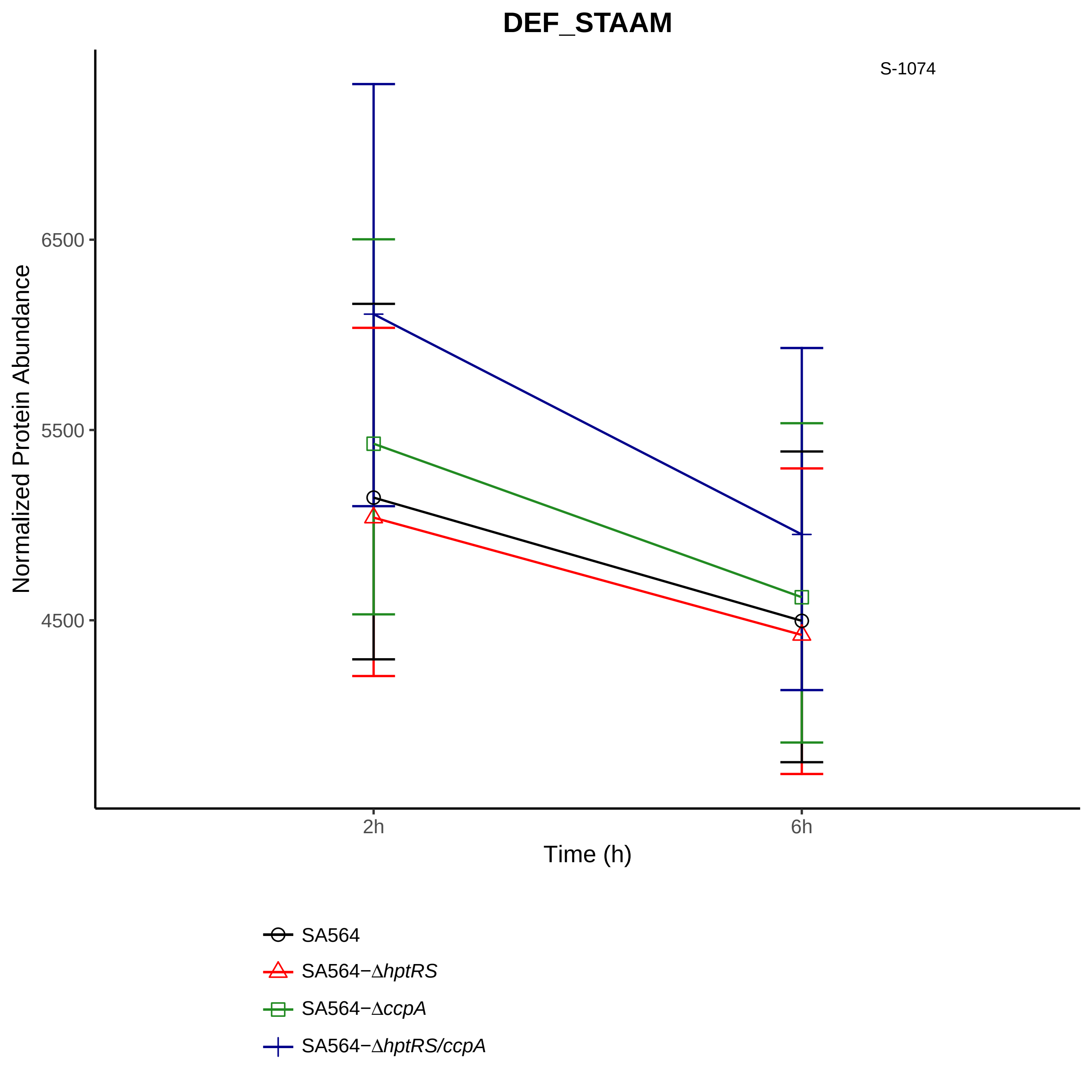
DEF_STAAM
S-1074
Normalized Protein Abundance
6500
5500
4500
2h
6h
Time (h)
SA564
SA564−ΔhptRS
SA564−ΔccpA
SA564−ΔhptRS/ccpA

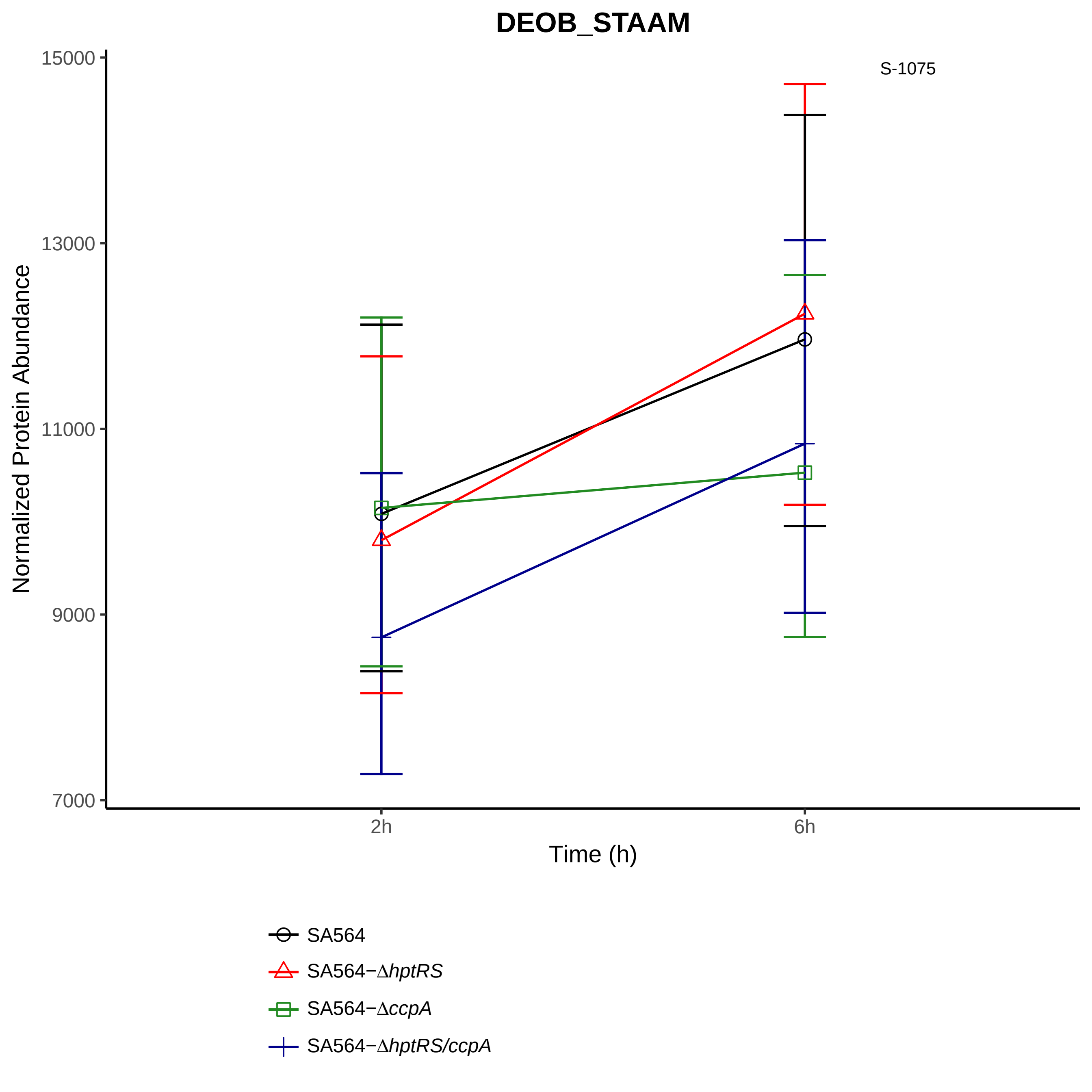
DEOB_STAAM
S-1075
15000
13000
11000
9000
7000
Normalized Protein Abundance
2h
6h
Time (h)
SA564
SA564−ΔhptRS
SA564−ΔccpA
SA564−ΔhptRS/ccpA

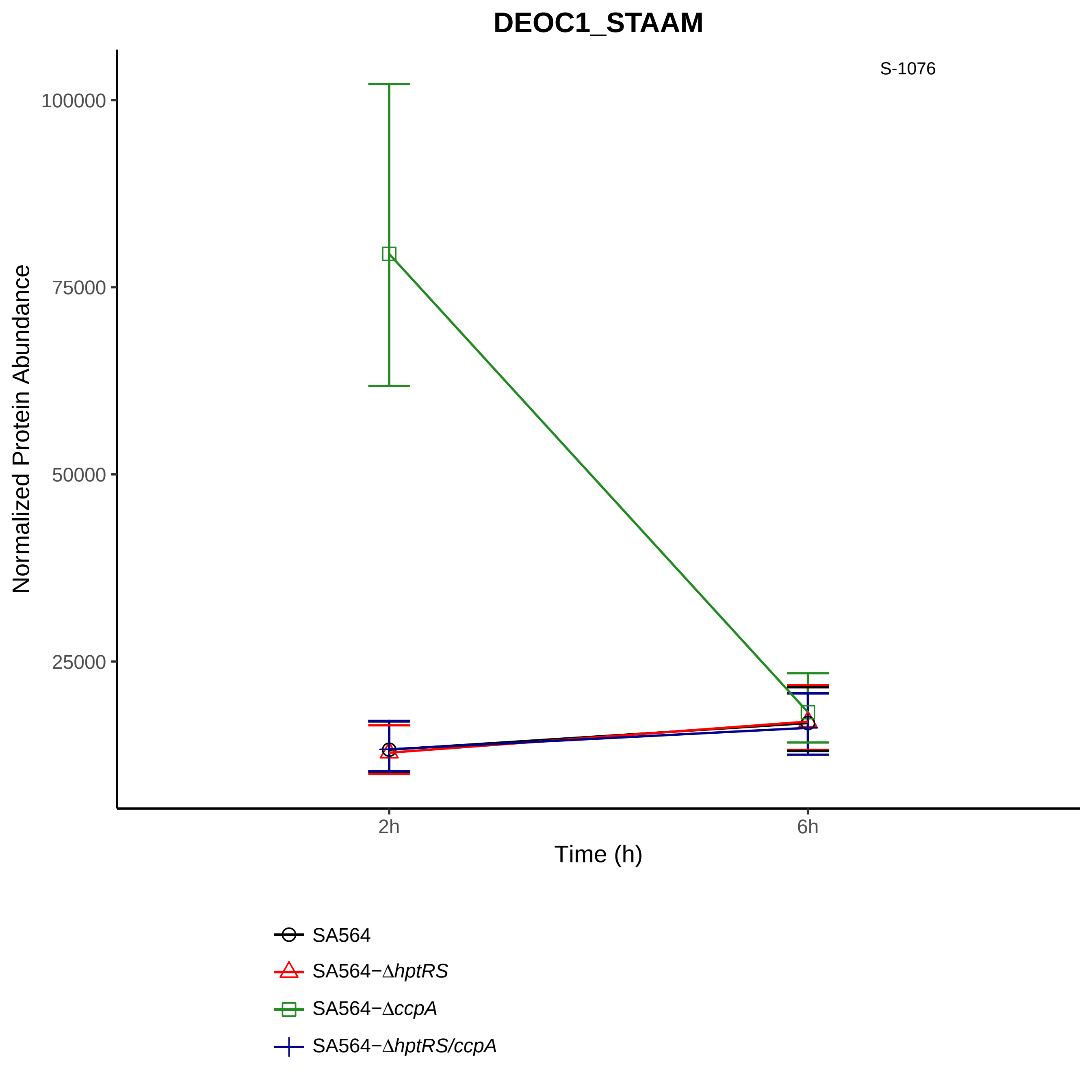
DEOC1_STAAM
S-1076
Normalized Protein Abundance
100000
75000
50000
25000
2h
6h
Time (h)
SA564
SA564−Δ*hptRS*
SA564−Δ*ccpA*
SA564−Δ*hptRS/ccpA*

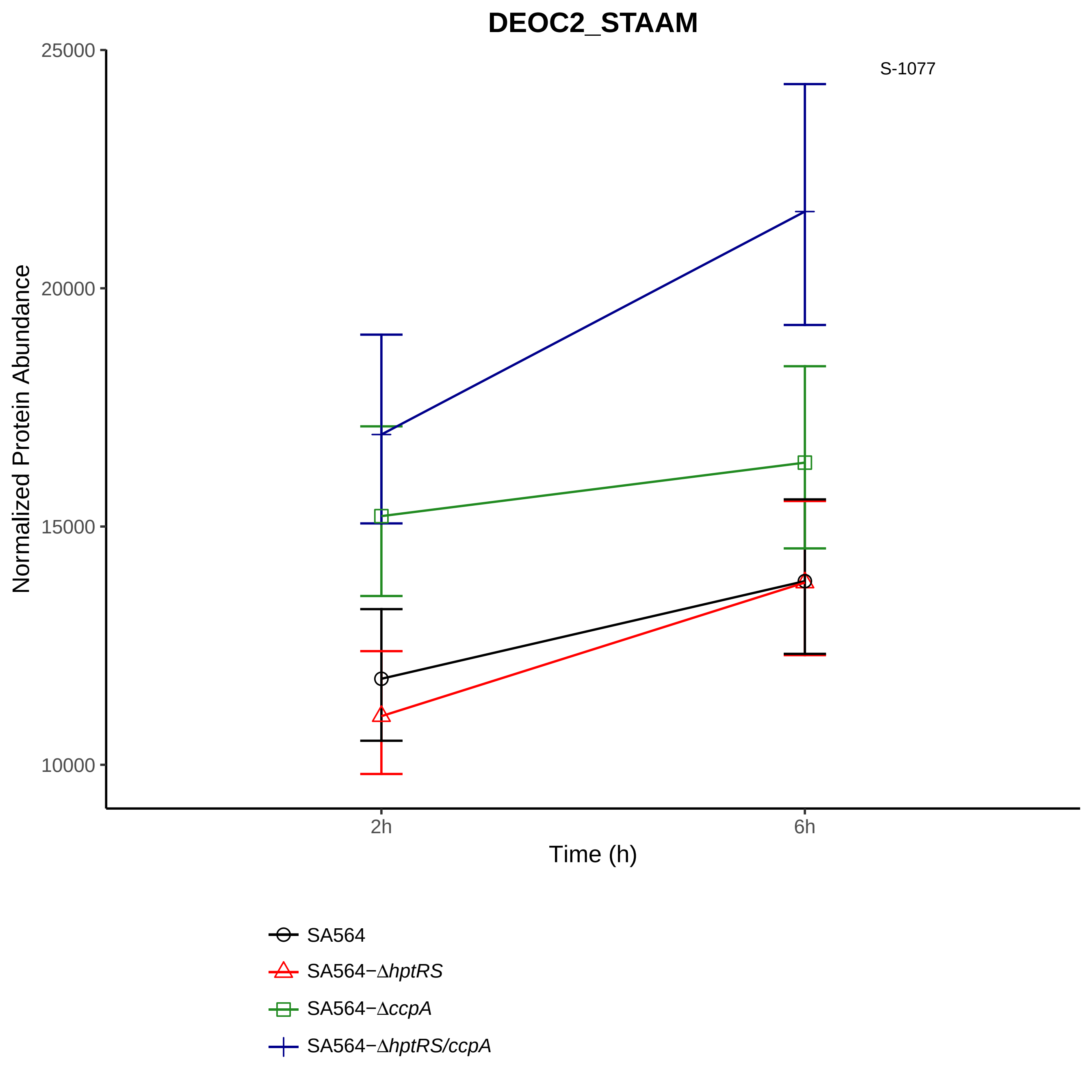

DEOC2_STAAM
S-1077
25000
20000
15000
10000
Normalized Protein Abundance
2h
6h
Time (h)
SA564
SA564−ΔhptRS
SA564−ΔccpA
SA564−ΔhptRS/ccpA

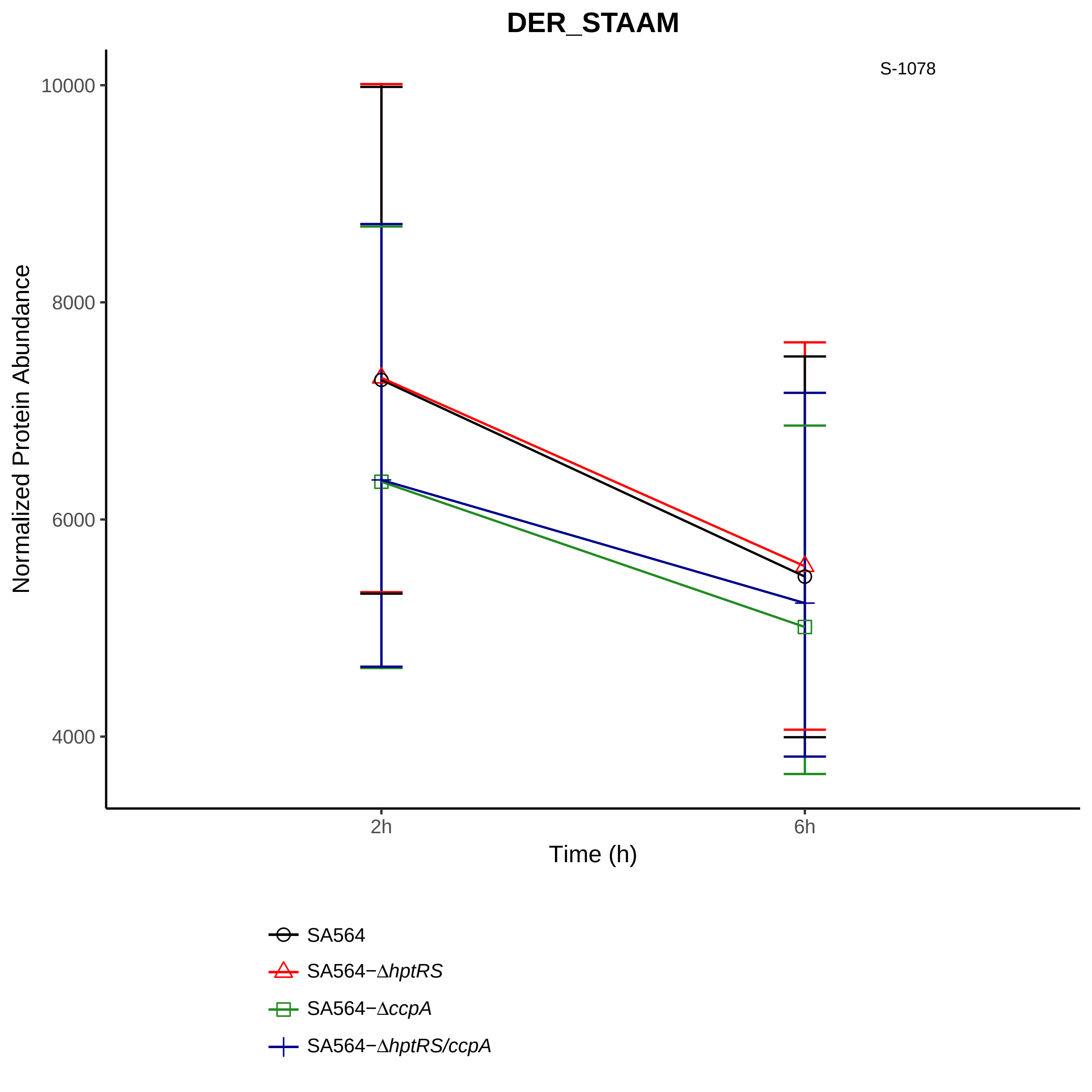

DER_STAAM
S-1078
Normalized Protein Abundance
10000
8000
6000
4000
2h
6h
Time (h)
SA564
SA564−Δ*hptRS*
SA564−Δ*ccpA*
SA564−Δ*hptRS/ccpA*

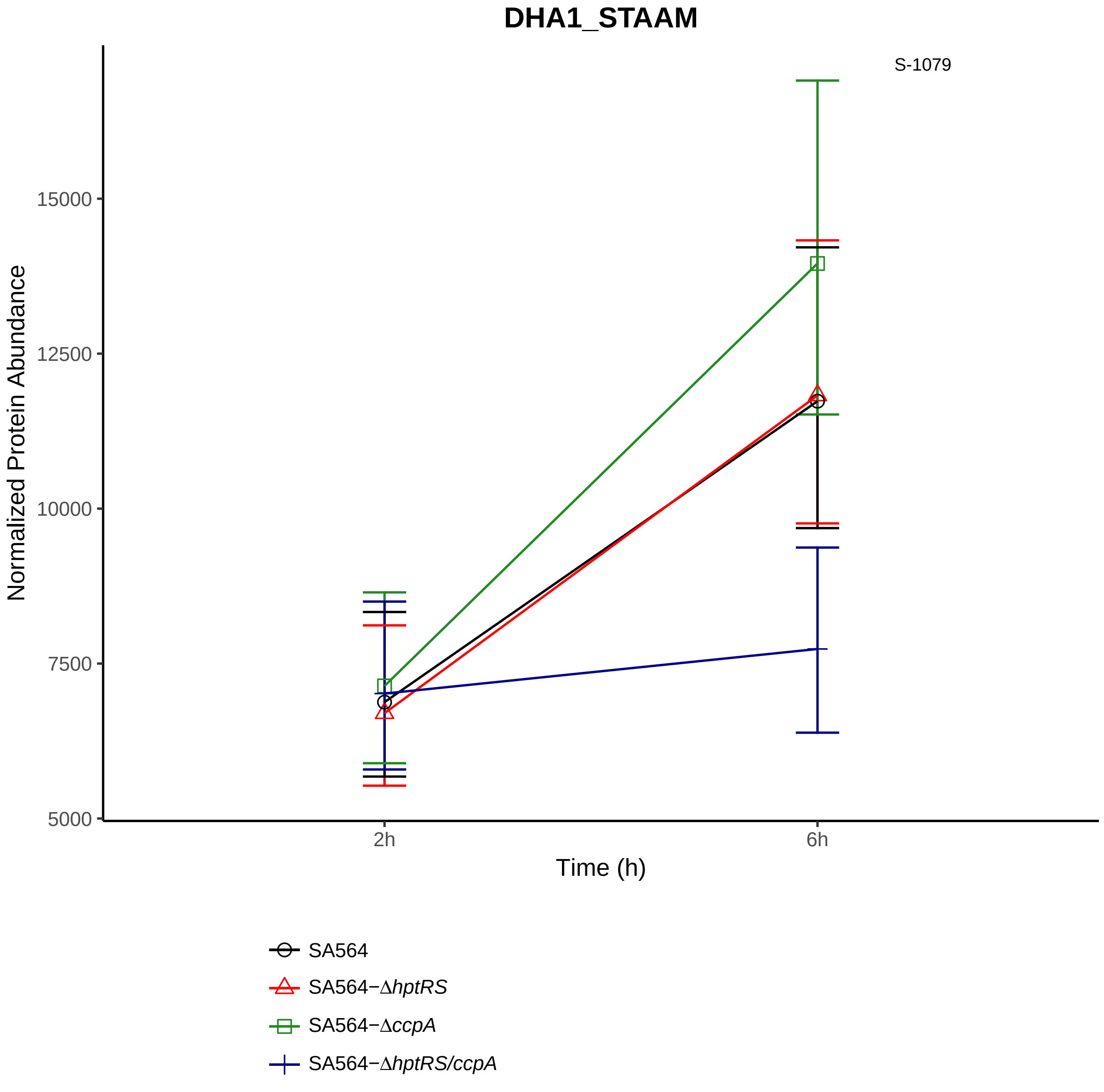

DHA1_STAAM
S-1079
Normalized Protein Abundance
15000
12500
10000
7500
5000
2h
6h
Time (h)
SA564
SA564−Δ*hptRS*
SA564−Δ*ccpA*
SA564−Δ*hptRS/ccpA*

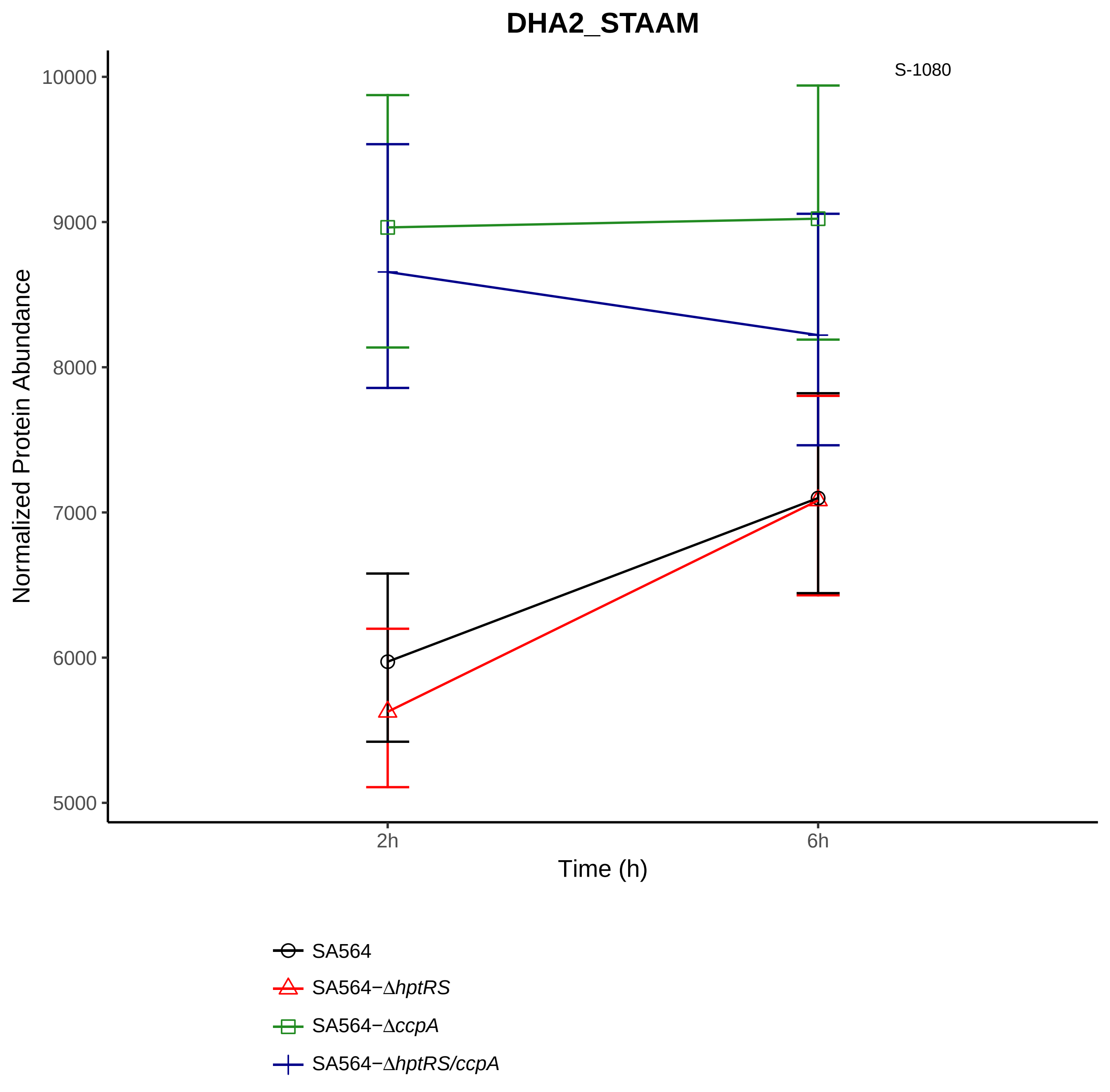

DHA2_STAAM
S-1080
Normalized Protein Abundance
10000
9000
8000
7000
6000
5000
2h
6h
Time (h)
SA564
SA564−Δ*hptRS*
SA564−Δ*ccpA*
SA564−Δ*hptRS/ccpA*

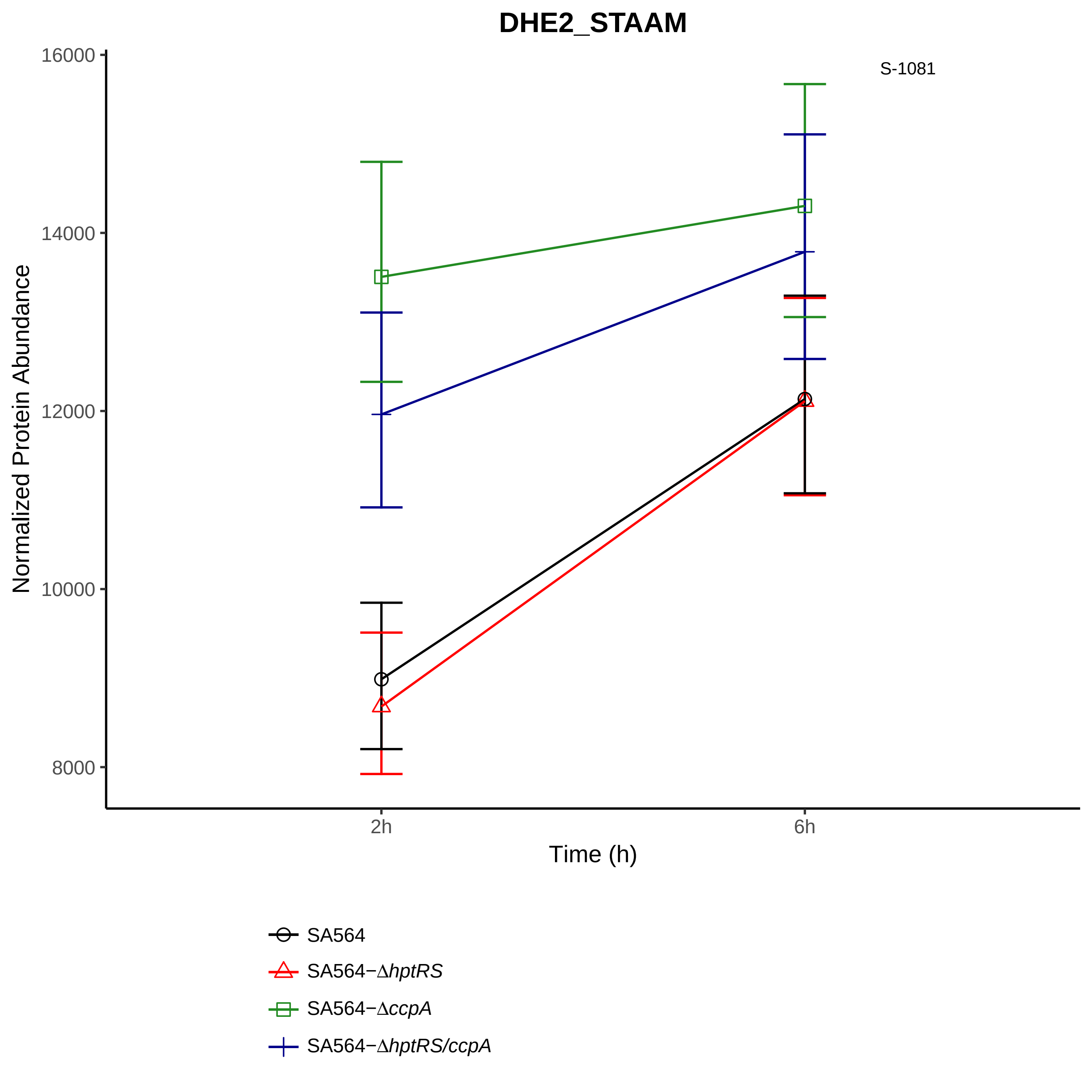

DHE2_STAAM
S-1081
Normalized Protein Abundance
16000
14000
12000
10000
8000
2h
6h
Time (h)
SA564
SA564−Δ*hptRS*
SA564−Δ*ccpA*
SA564−Δ*hptRS/ccpA*

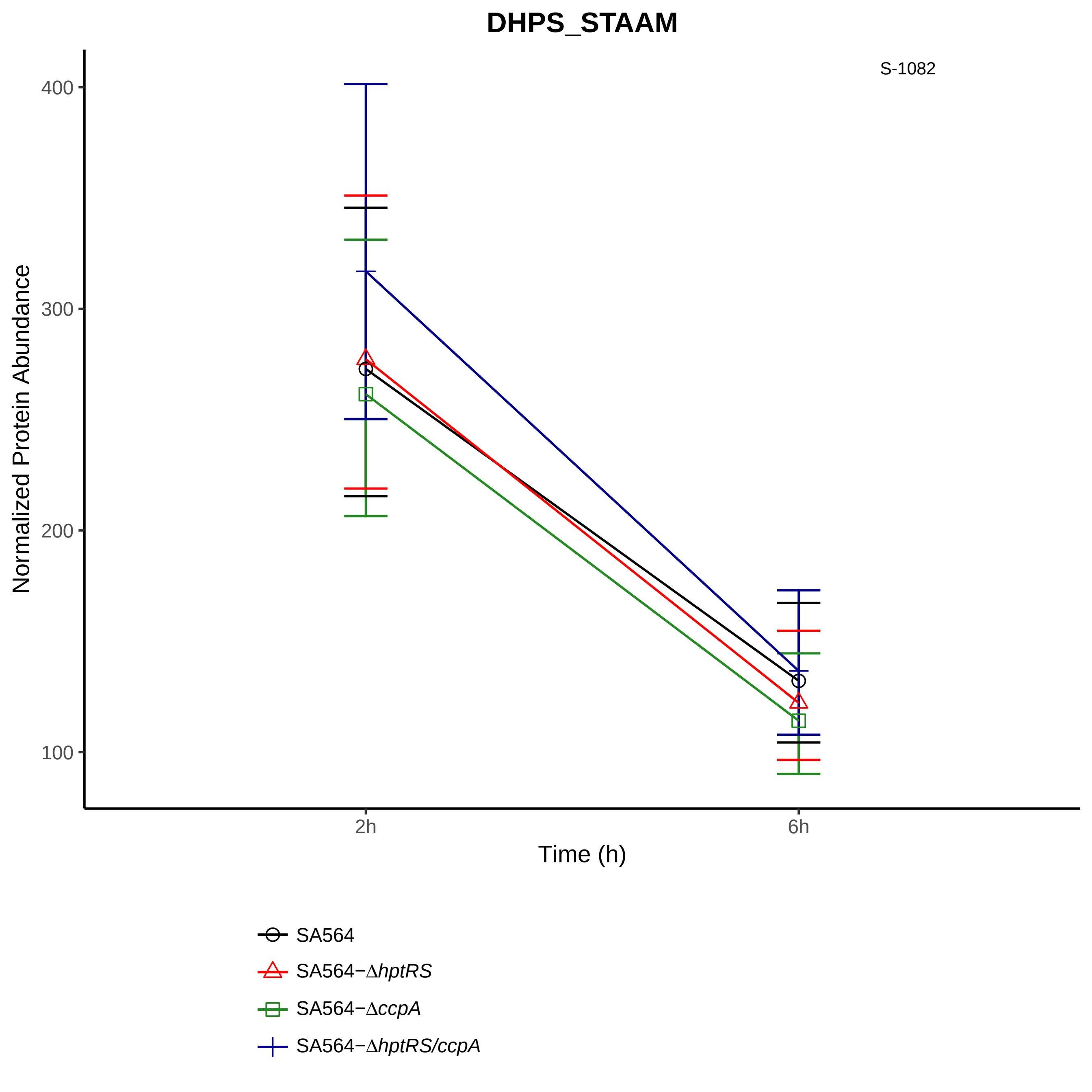

DHPS_STAAM
S-1082
Normalized Protein Abundance
400
300
200
100
2h
6h
Time (h)
SA564
SA564−Δ*hptRS*
SA564−Δ*ccpA*
SA564−Δ*hptRS/ccpA*

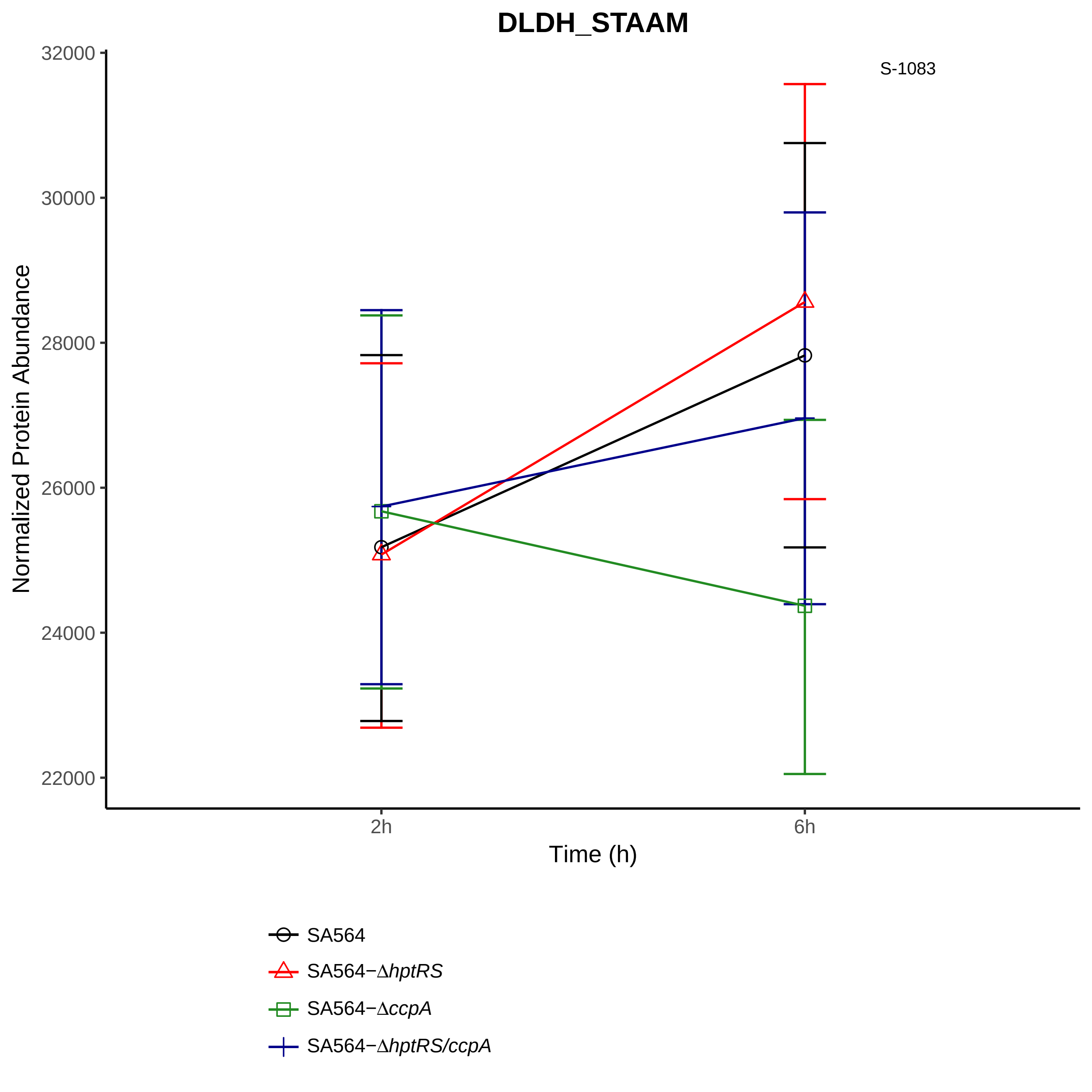

DLDH_STAAM
S-1083
Normalized Protein Abundance
32000
30000
28000
26000
24000
22000
2h
6h
Time (h)
SA564
SA564−Δ*hptRS*
SA564−Δ*ccpA*
SA564−Δ*hptRS/ccpA*

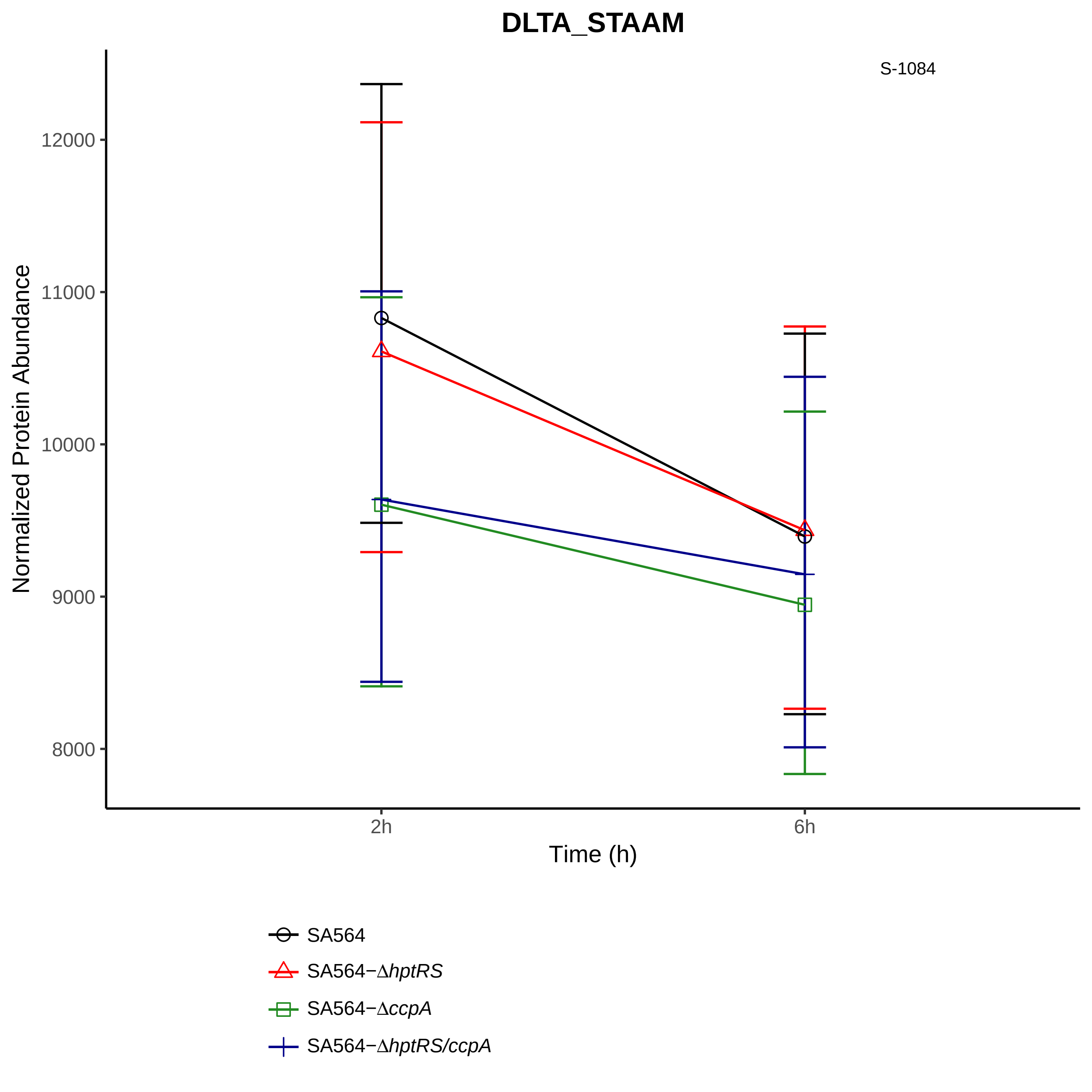
DLTA_STAAM
S-1084
Normalized Protein Abundance
12000
11000
10000
9000
8000
2h
6h
Time (h)
SA564
SA564−Δ*hptRS*
SA564−Δ*ccpA*
SA564−Δ*hptRS/ccpA*

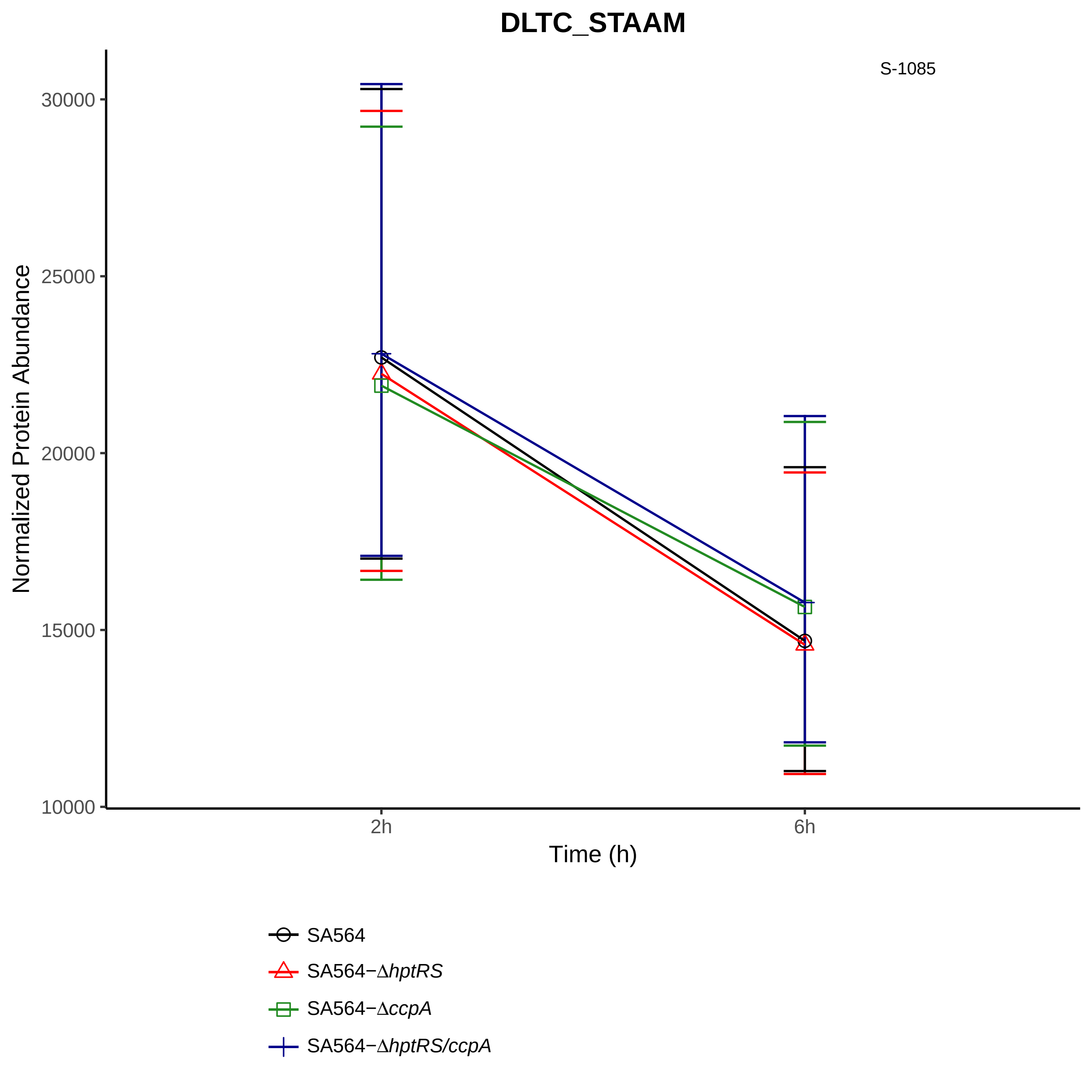
DLTC_STAAM
S-1085
Normalized Protein Abundance
30000
25000
20000
15000
10000
2h
6h
Time (h)
SA564
SA564−Δ*hptRS*
SA564−Δ*ccpA*
SA564−Δ*hptRS/ccpA*

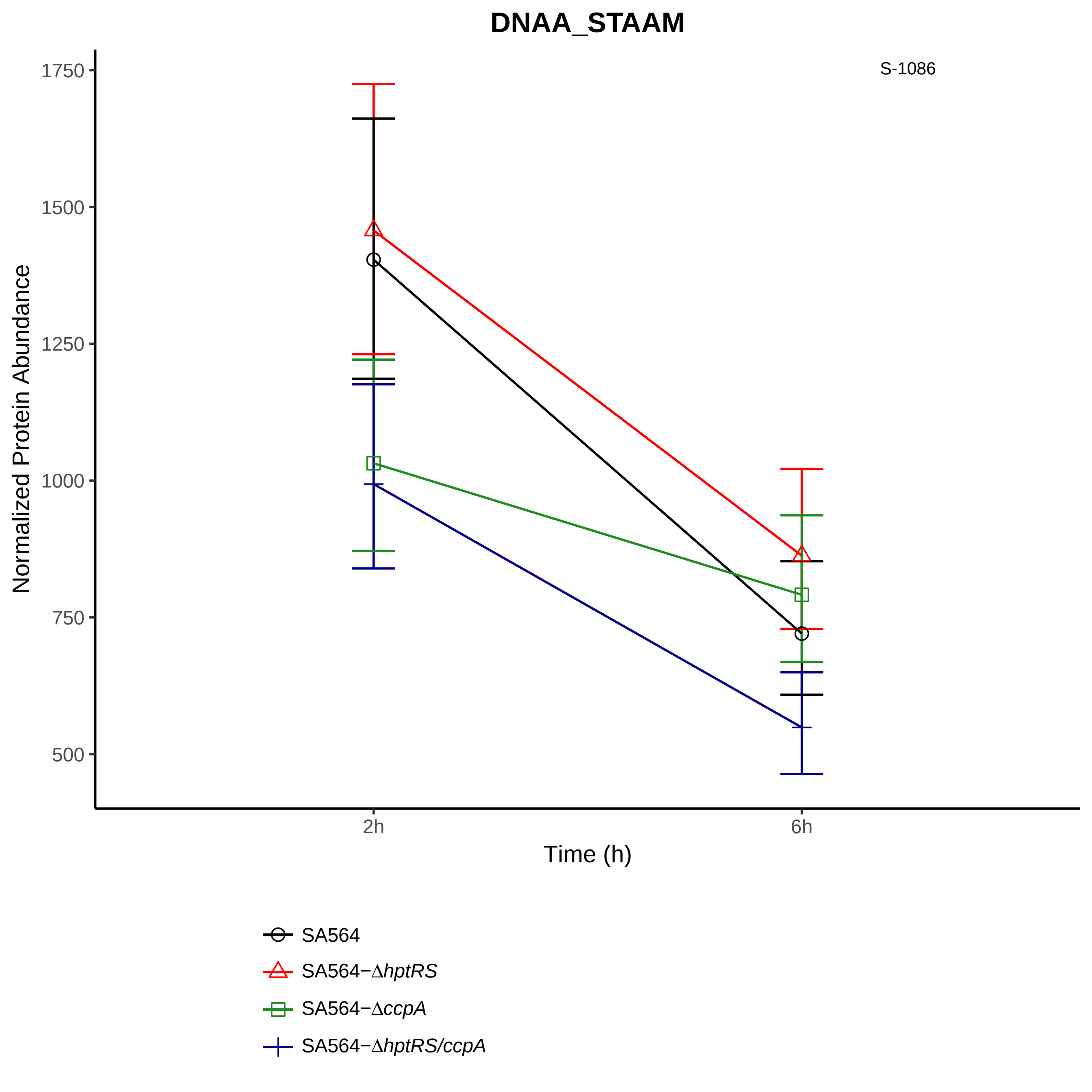

DNAA_STAAM
S-1086
Normalized Protein Abundance
1750
1500
1250
1000
750
500
2h
6h
Time (h)
SA564
SA564−Δ*hptRS*
SA564−Δ*ccpA*
SA564−Δ*hptRS/ccpA*

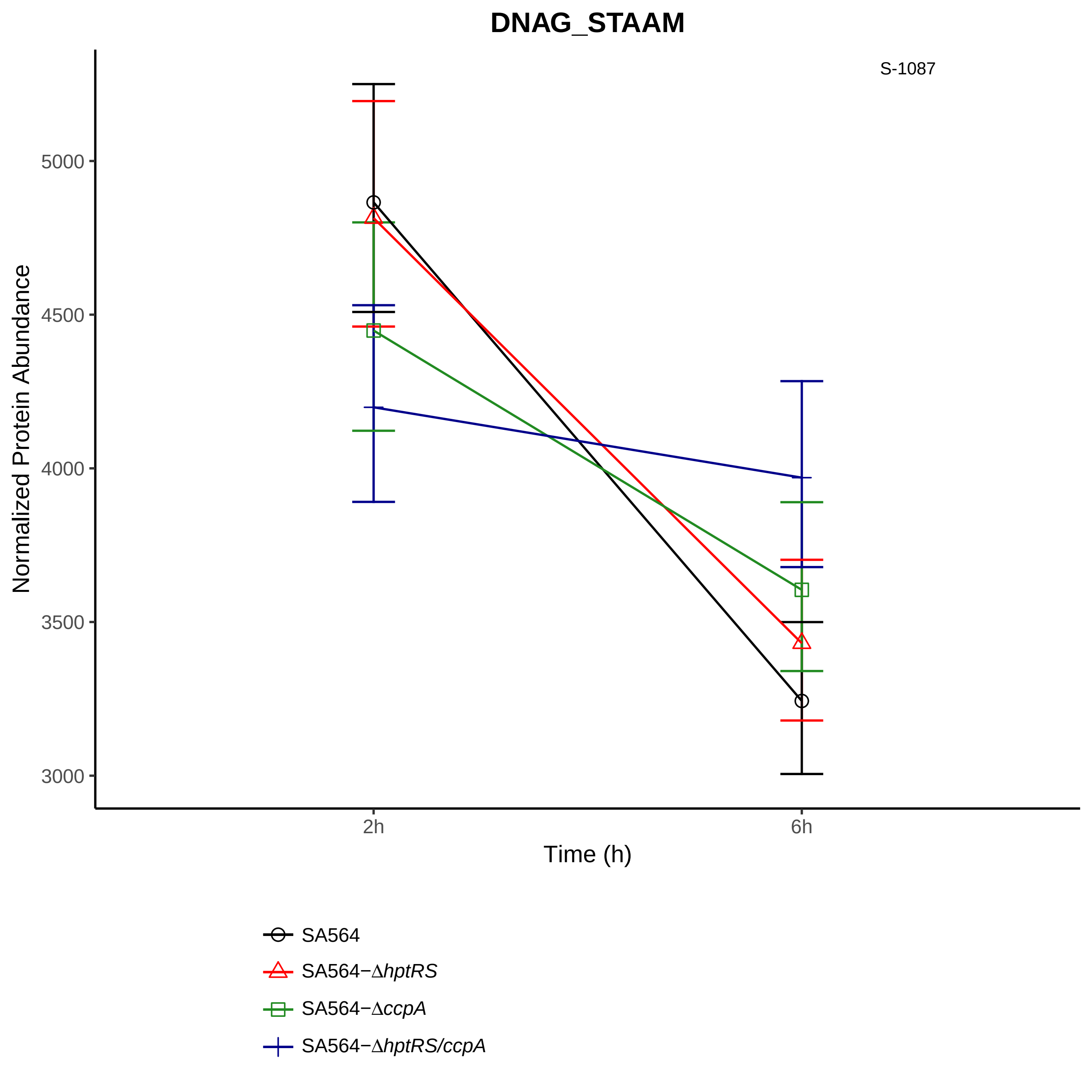

DNAG_STAAM
S-1087
5000
4500
4000
3500
3000
Normalized Protein Abundance
2h
6h
Time (h)
SA564
SA564−Δ*hptRS*
SA564−Δ*ccpA*
SA564−Δ*hptRS/ccpA*

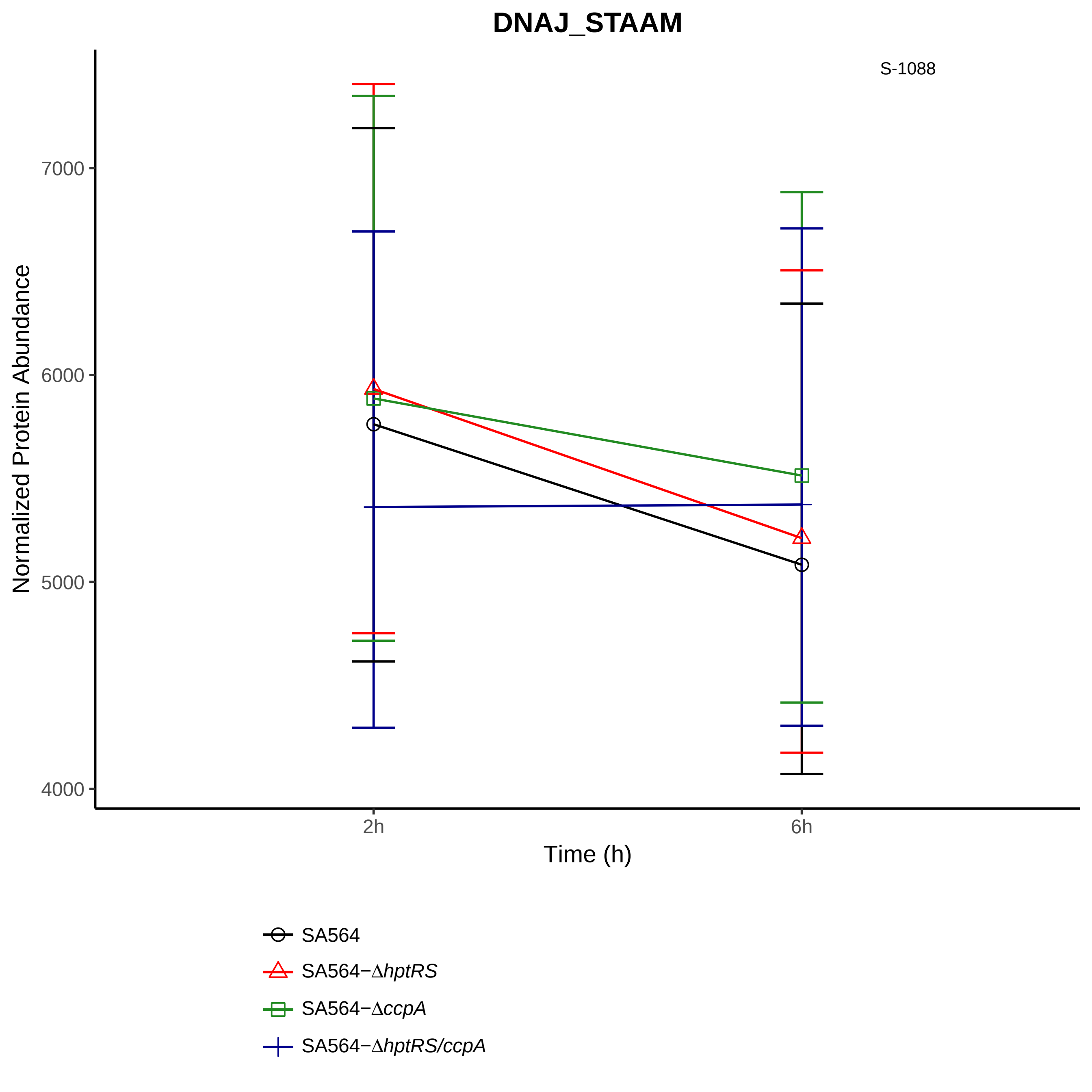

DNAJ_STAAM
S-1088
Normalized Protein Abundance
7000
6000
5000
4000
2h
6h
Time (h)
SA564
SA564−ΔhptRS
SA564−ΔccpA
SA564−ΔhptRS/ccpA

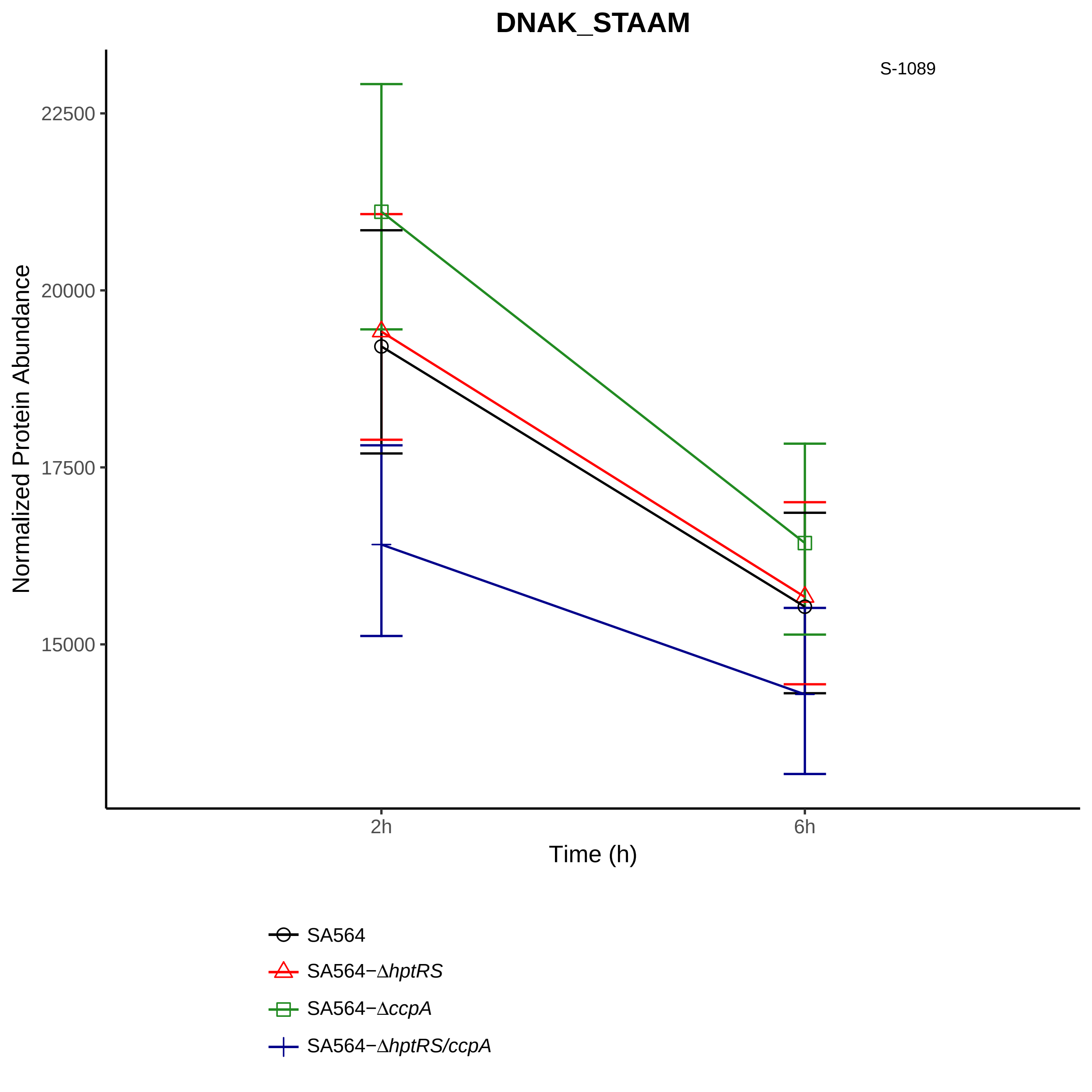

DNAK_STAAM
S-1089
Normalized Protein Abundance
22500
20000
17500
15000
2h
6h
Time (h)
SA564
SA564−Δ*hptRS*
SA564−Δ*ccpA*
SA564−Δ*hptRS/ccpA*

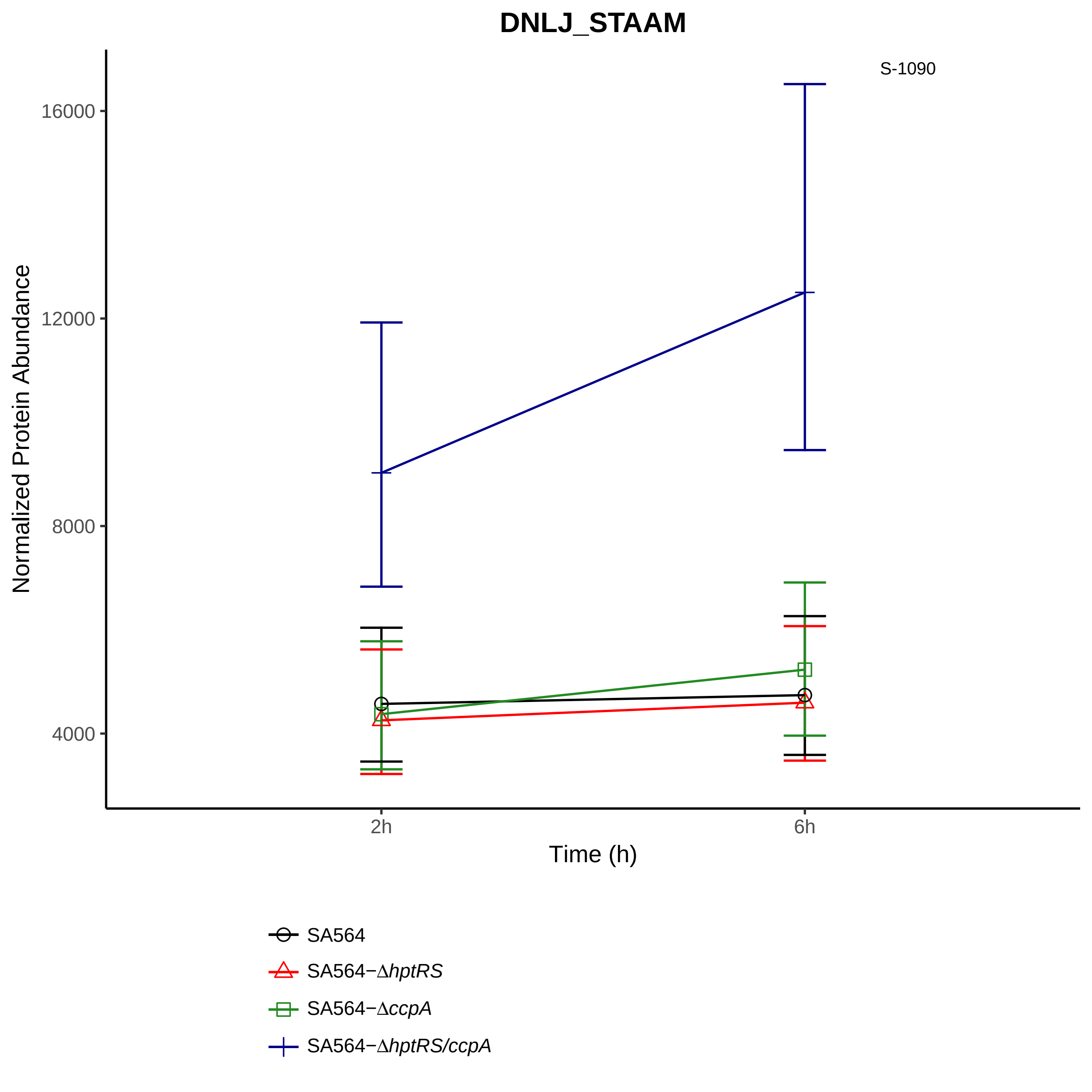
DNLJ_STAAM
S-1090
Normalized Protein Abundance
16000
12000
8000
4000
2h
6h
Time (h)
SA564
SA564−ΔhptRS
SA564−ΔccpA
SA564−ΔhptRS/ccpA

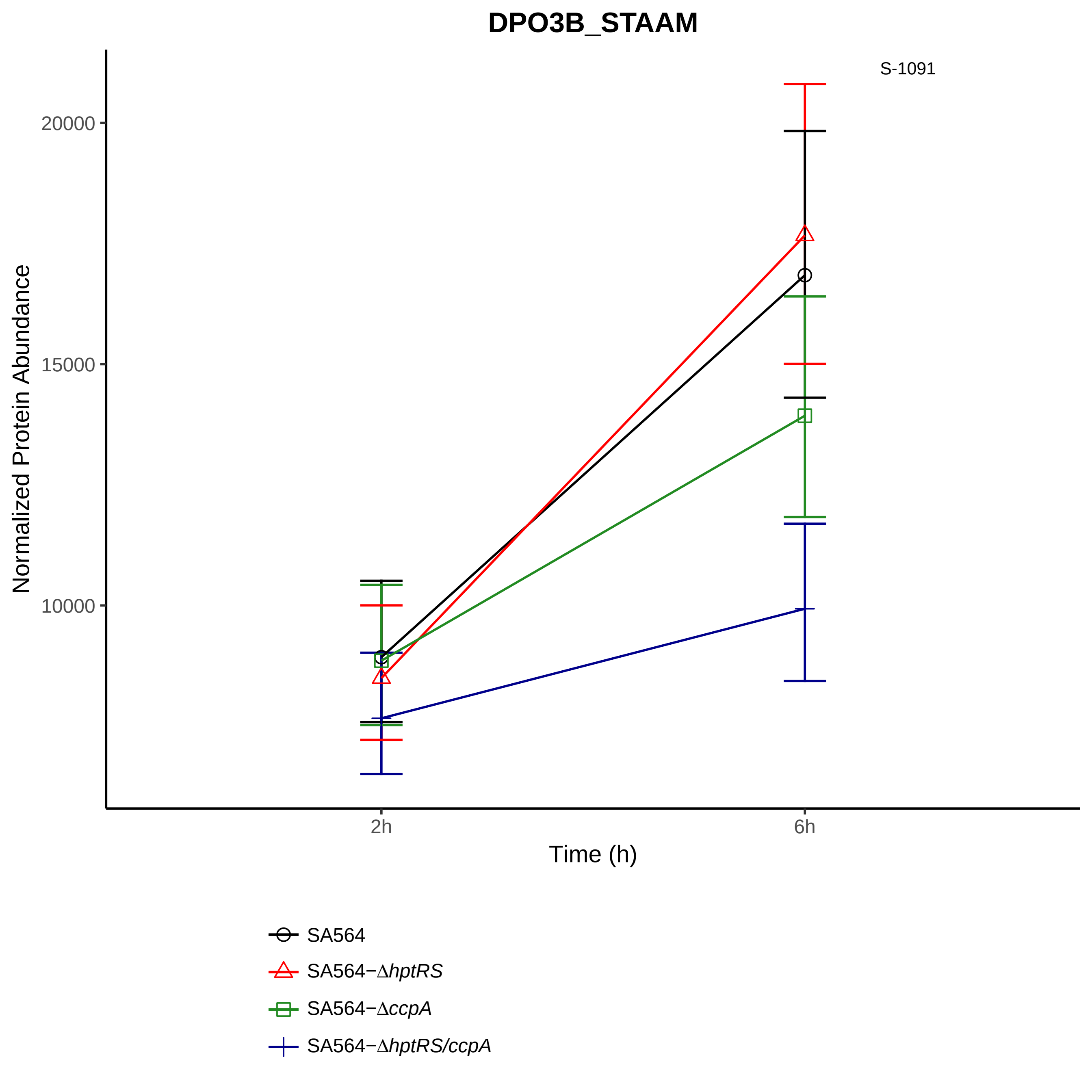
DPO3B_STAAM
S-1091
Normalized Protein Abundance
20000
15000
10000
2h
6h
Time (h)
SA564
SA564−ΔhptRS
SA564−ΔccpA
SA564−ΔhptRS/ccpA

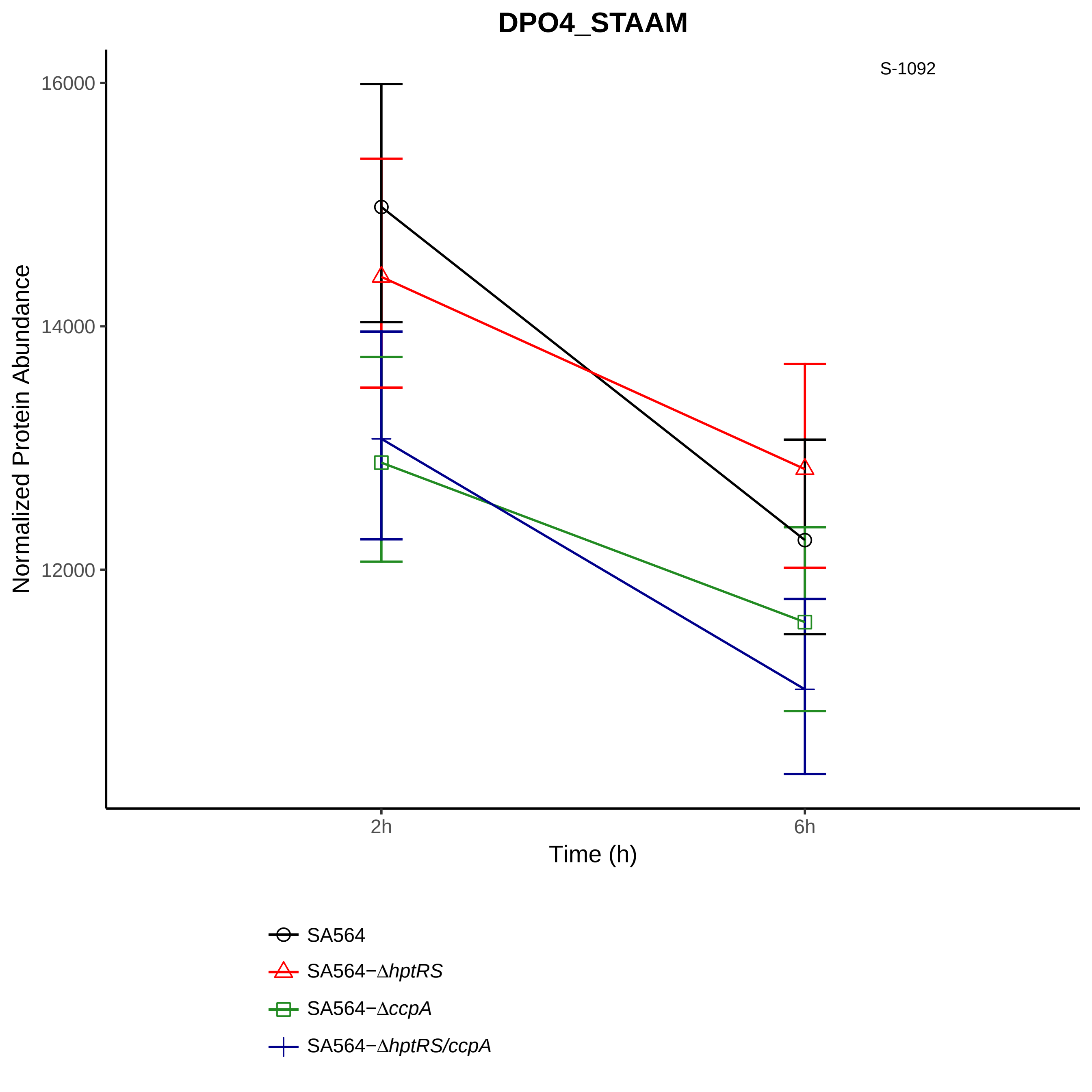

DPO4_STAAM
S-1092
16000
14000
12000
Normalized Protein Abundance
2h
6h
Time (h)
SA564
SA564−Δ*hptRS*
SA564−Δ*ccpA*
SA564−Δ*hptRS/ccpA*

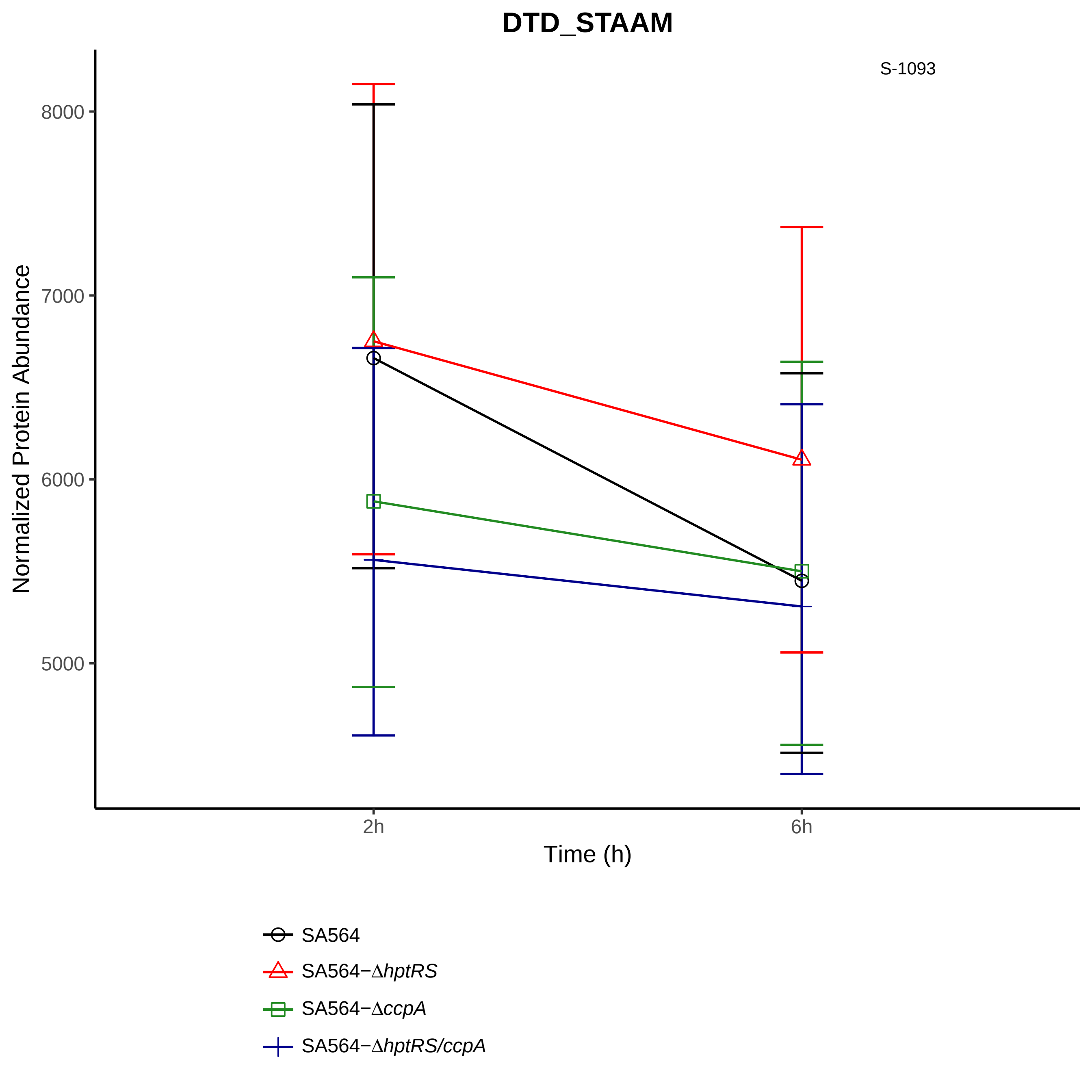

DTD_STAAM
S-1093
8000
7000
6000
5000
Normalized Protein Abundance
2h
6h
Time (h)
SA564
SA564−Δ*hptRS*
SA564−Δ*ccpA*
SA564−Δ*hptRS/ccpA*

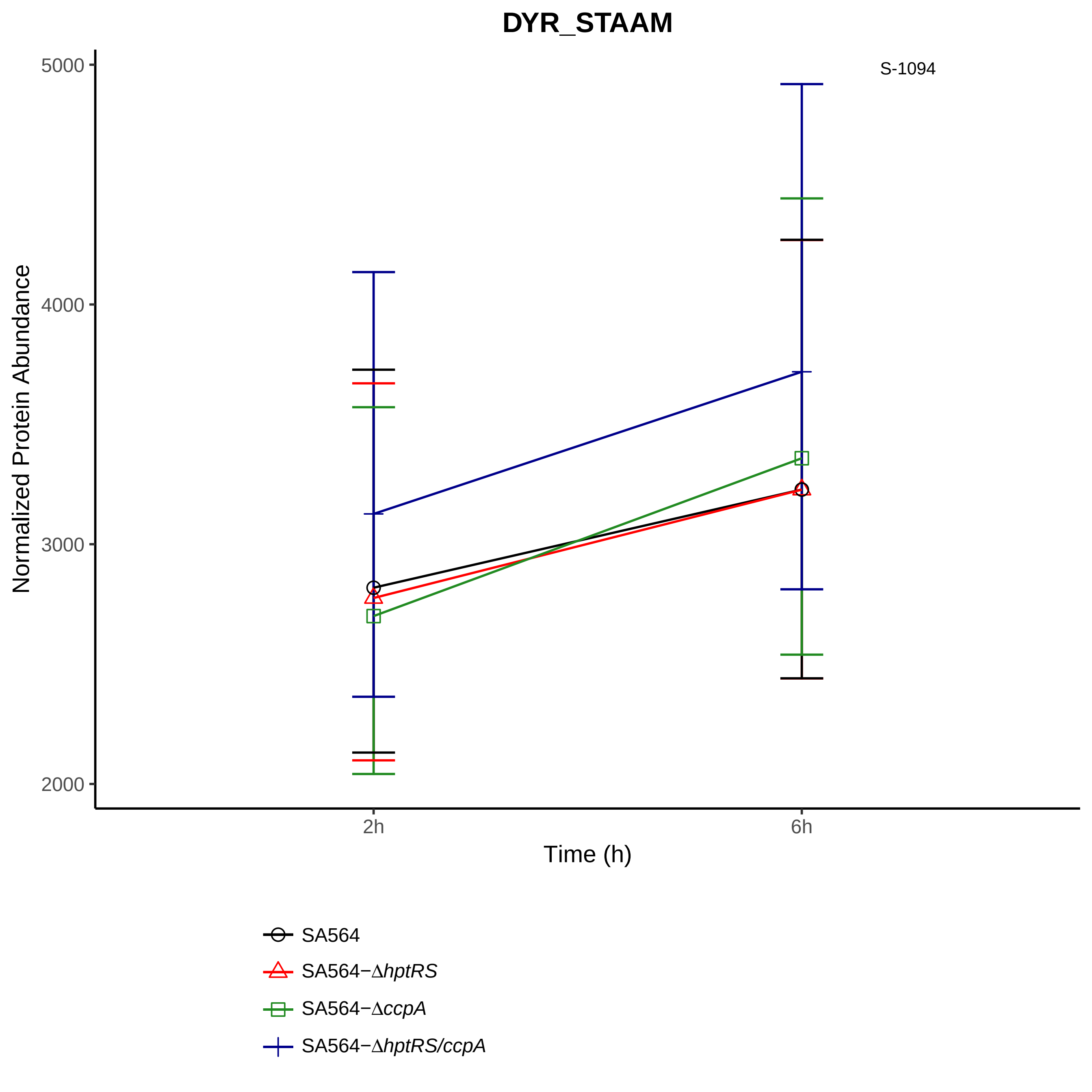

DYR_STAAM
S-1094
5000
4000
3000
2000
Normalized Protein Abundance
2h
6h
Time (h)
SA564
SA564−ΔhptRS
SA564−ΔccpA
SA564−ΔhptRS/ccpA

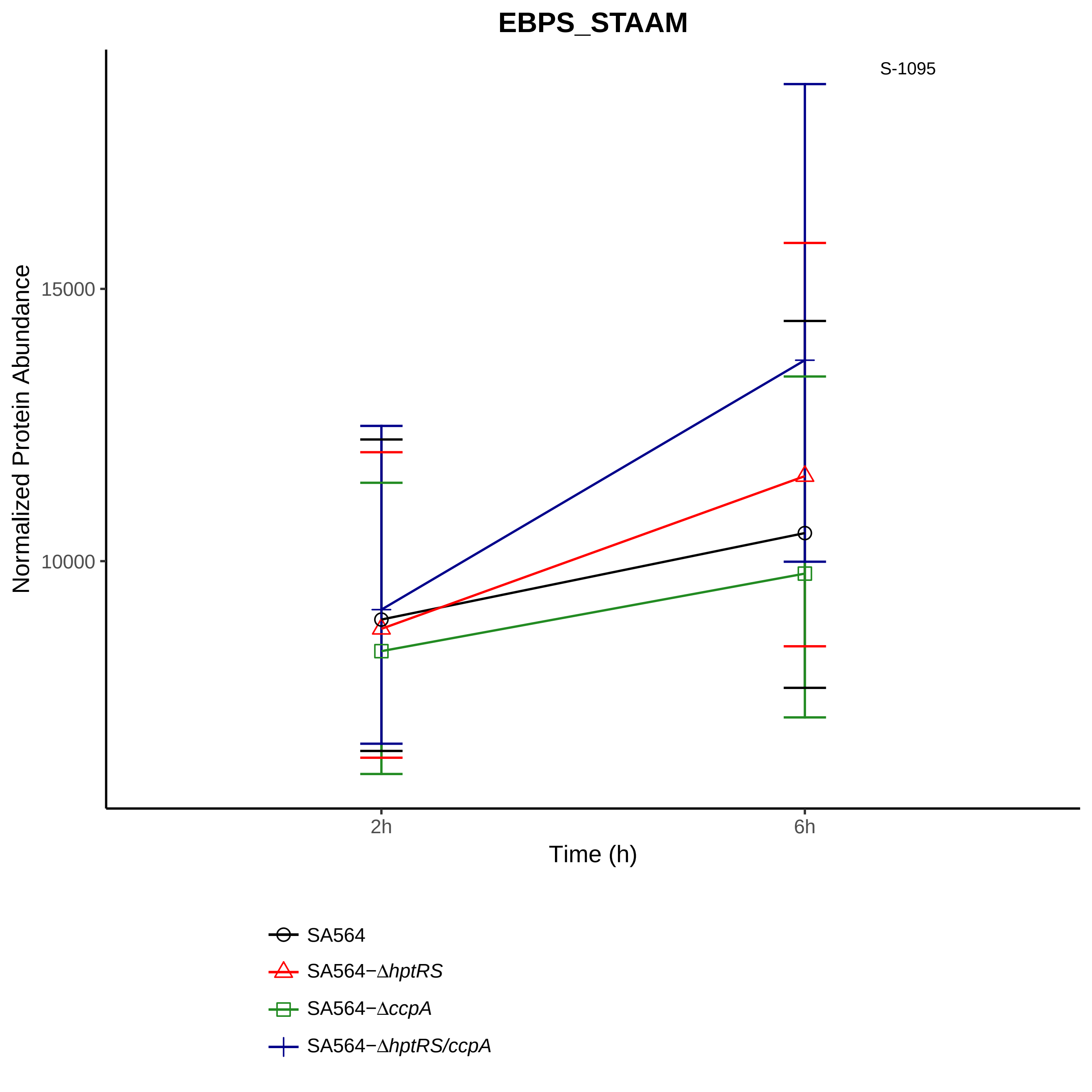

EBPS_STAAM
S-1095
15000
10000
Normalized Protein Abundance
2h
6h
Time (h)
SA564
SA564−Δ*hptRS*
SA564−Δ*ccpA*
SA564−Δ*hptRS/ccpA*

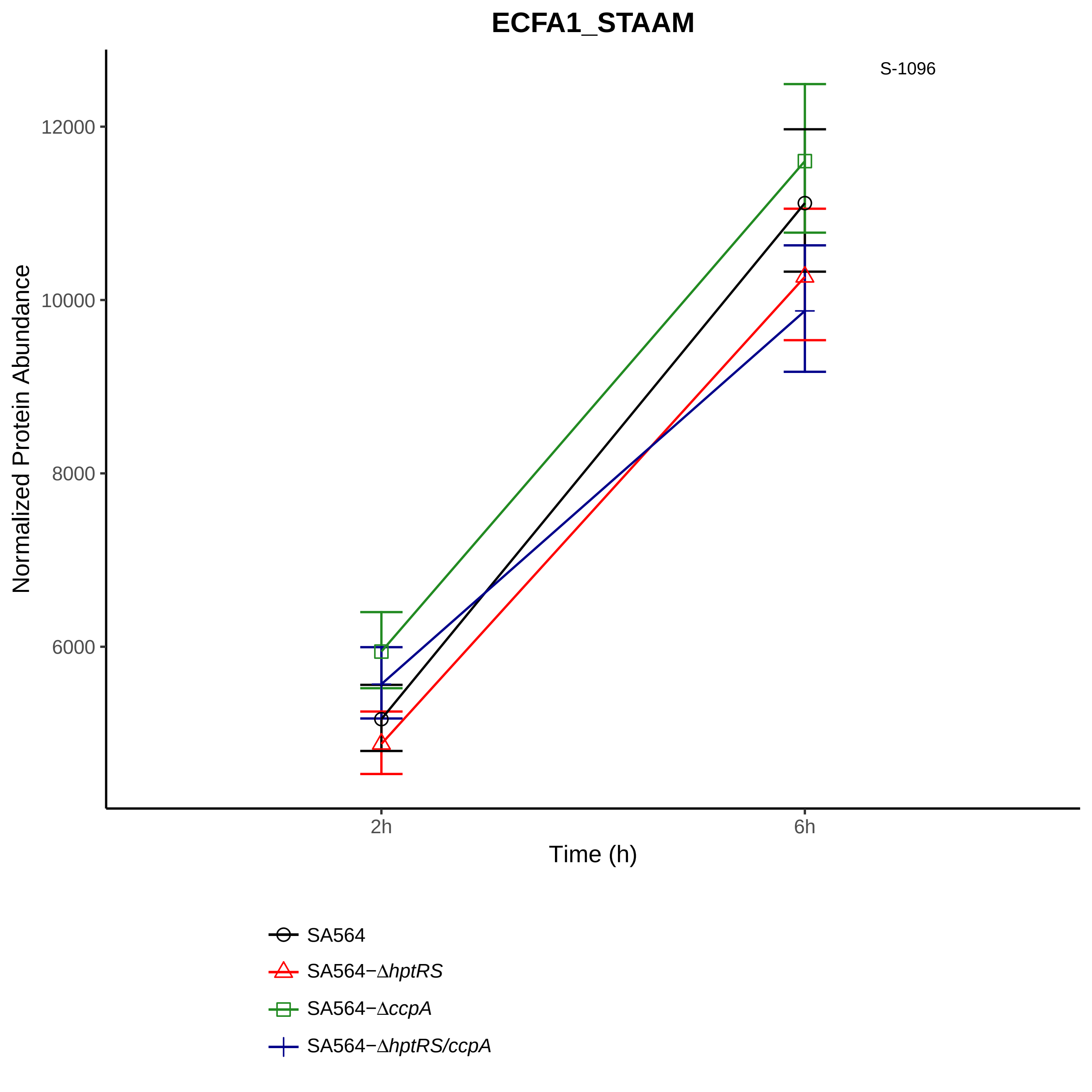
ECFA1_STAAM
S-1096
Normalized Protein Abundance
12000
10000
8000
6000
2h
6h
Time (h)
SA564
SA564−ΔhptRS
SA564−ΔccpA
SA564−ΔhptRS/ccpA

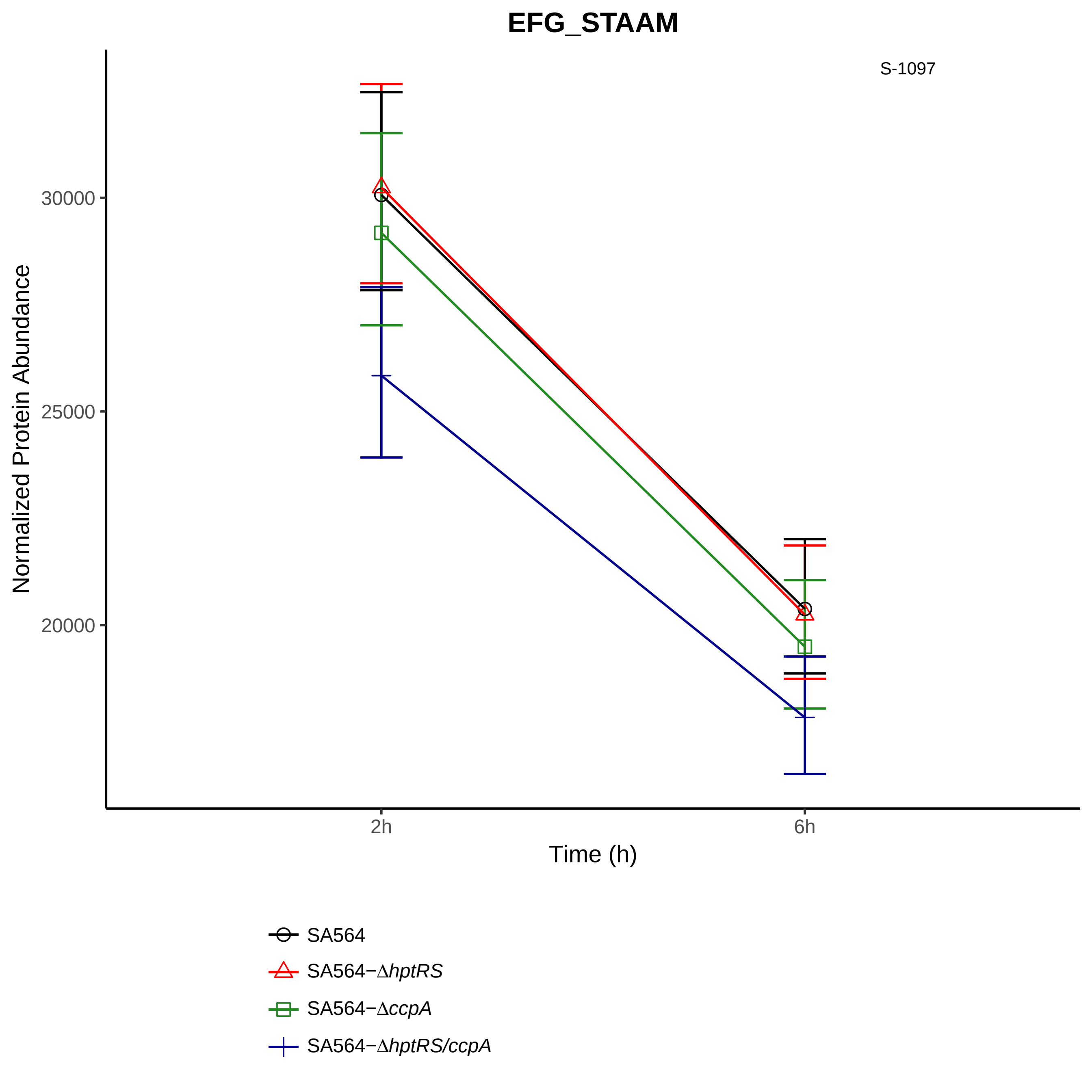

EFG_STAAM
S-1097
30000
25000
20000
Normalized Protein Abundance
2h
6h
Time (h)
SA564
SA564−Δ*hptRS*
SA564−Δ*ccpA*
SA564−Δ*hptRS/ccpA*

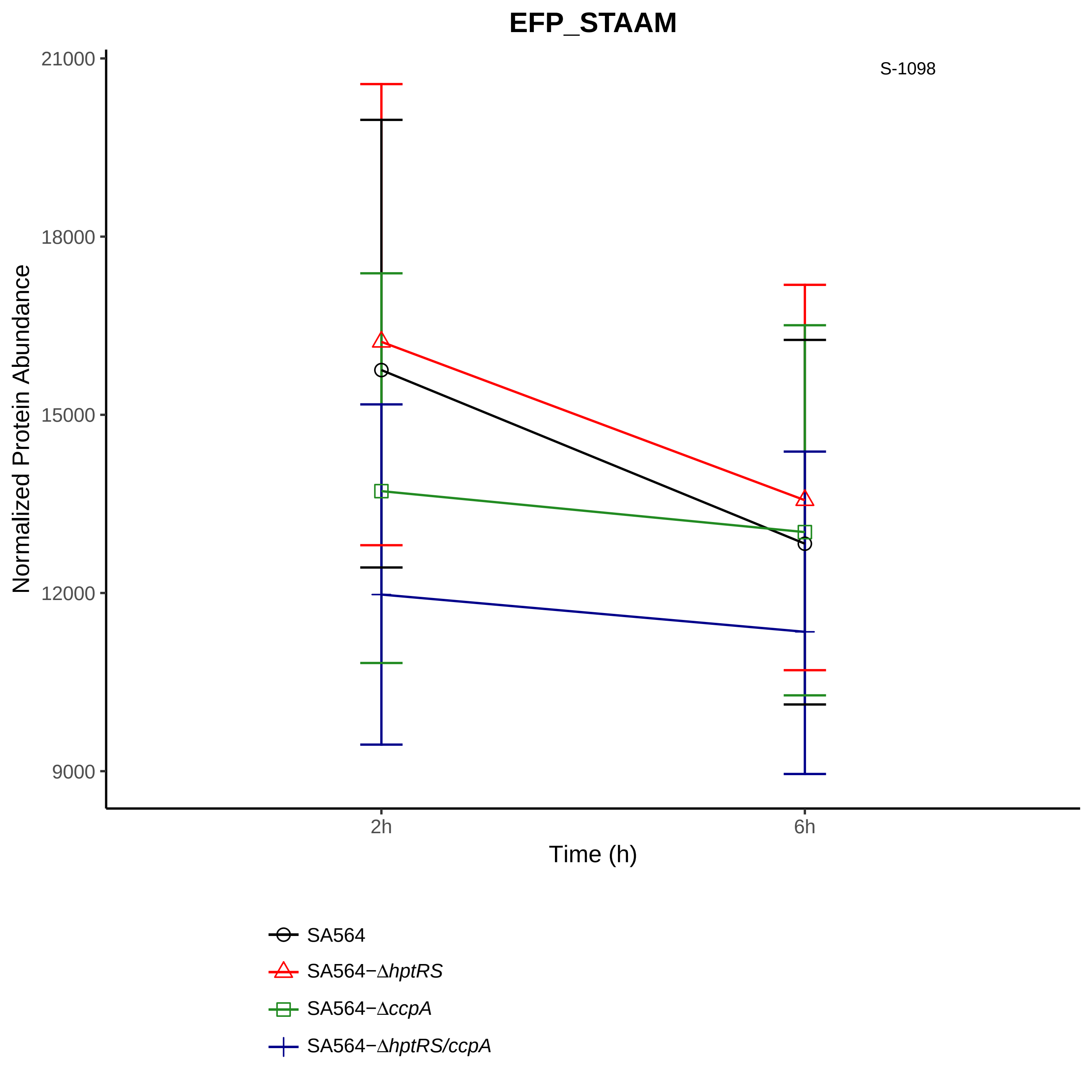

EFP_STAAM
S-1098
Normalized Protein Abundance
21000
18000
15000
12000
9000
2h
6h
Time (h)
SA564
SA564−ΔhptRS
SA564−ΔccpA
SA564−ΔhptRS/ccpA

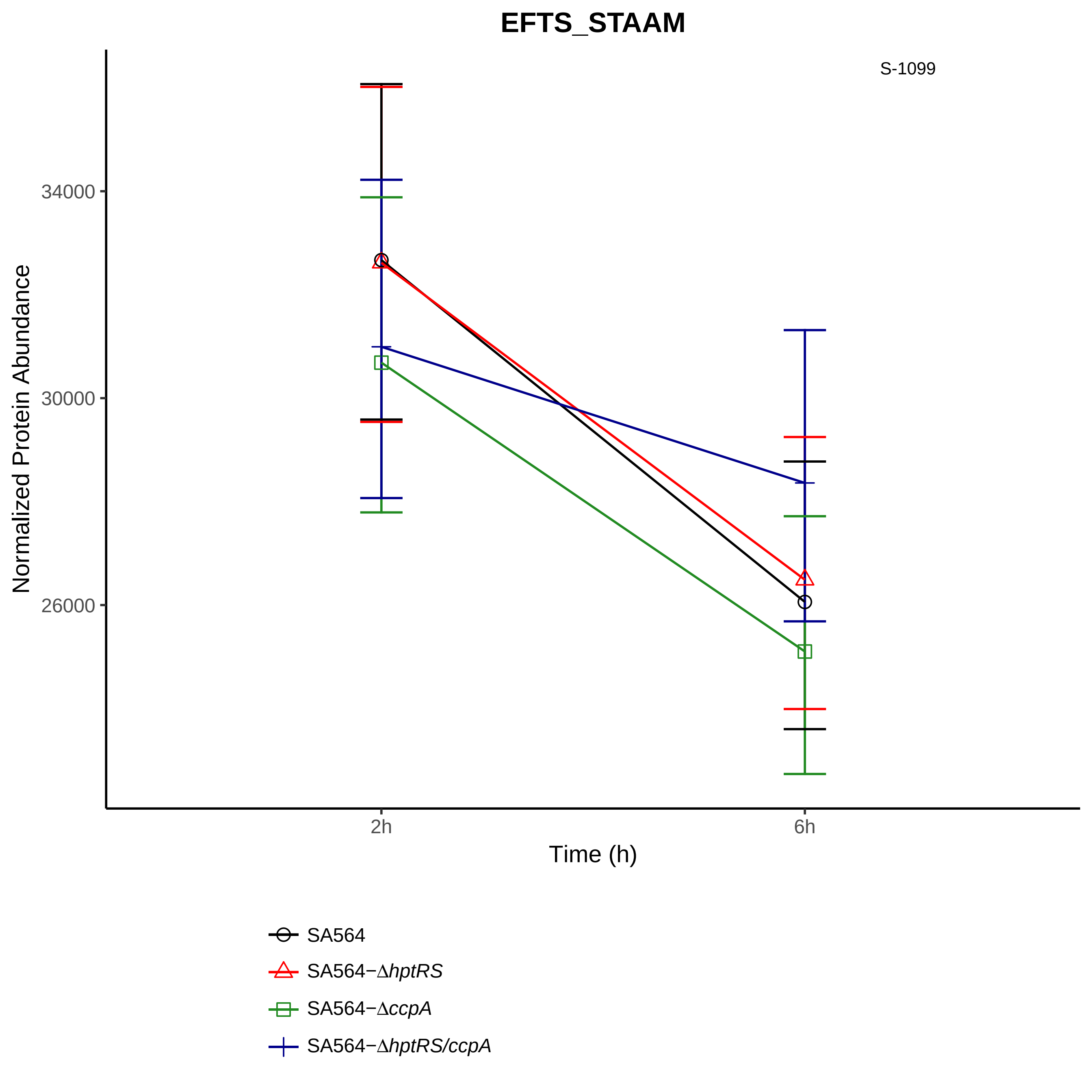

EFTS_STAAM
S-1099
Normalized Protein Abundance
34000
30000
26000
2h
6h
Time (h)
SA564
SA564−Δ*hptRS*
SA564−Δ*ccpA*
SA564−Δ*hptRS/ccpA*

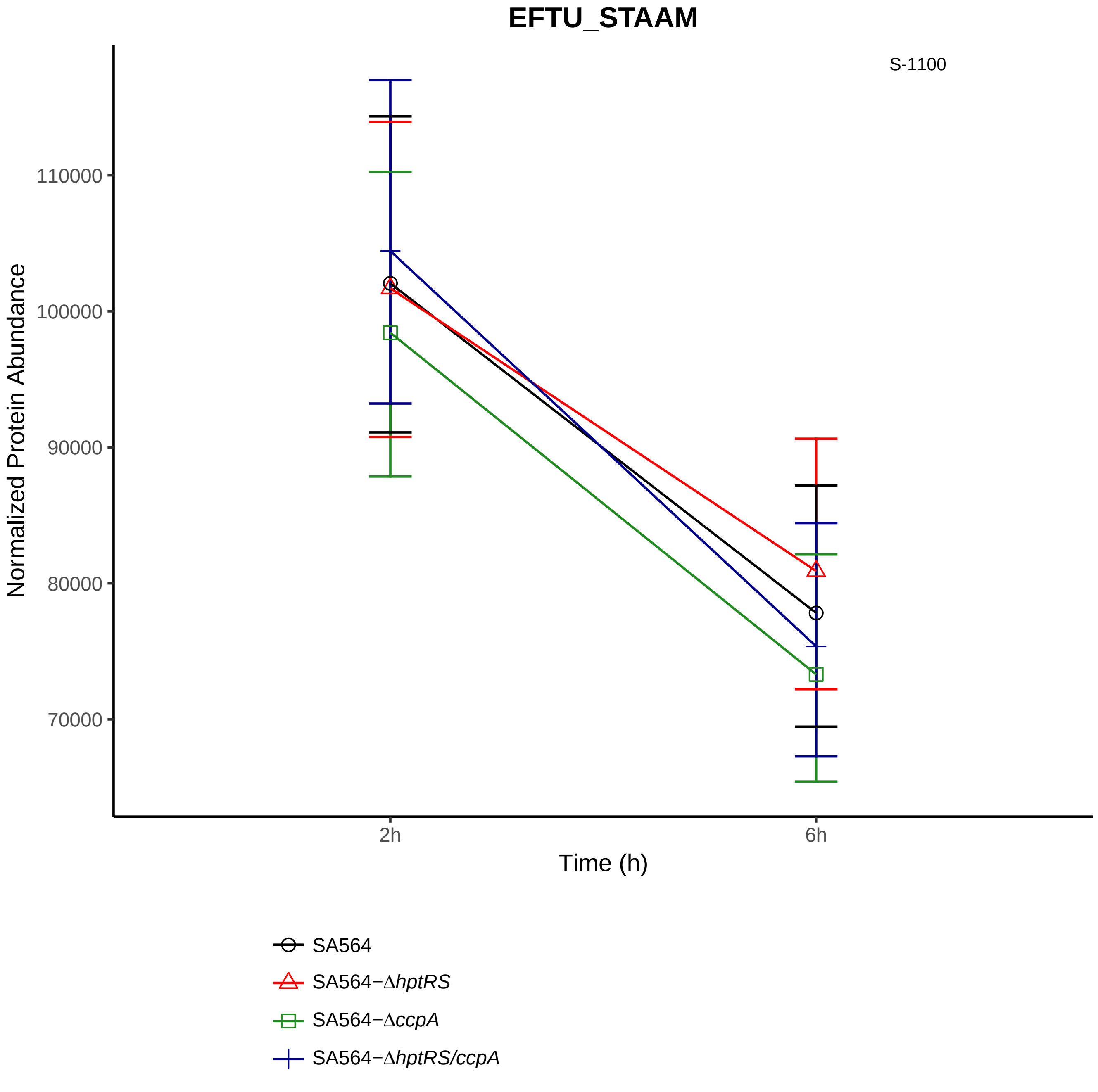
EFTU_STAAM
S-1100
Normalized Protein Abundance
110000
100000
90000
80000
70000
2h
6h
Time (h)
SA564
SA564−Δ*hptRS*
SA564−Δ*ccpA*
SA564−Δ*hptRS/ccpA*

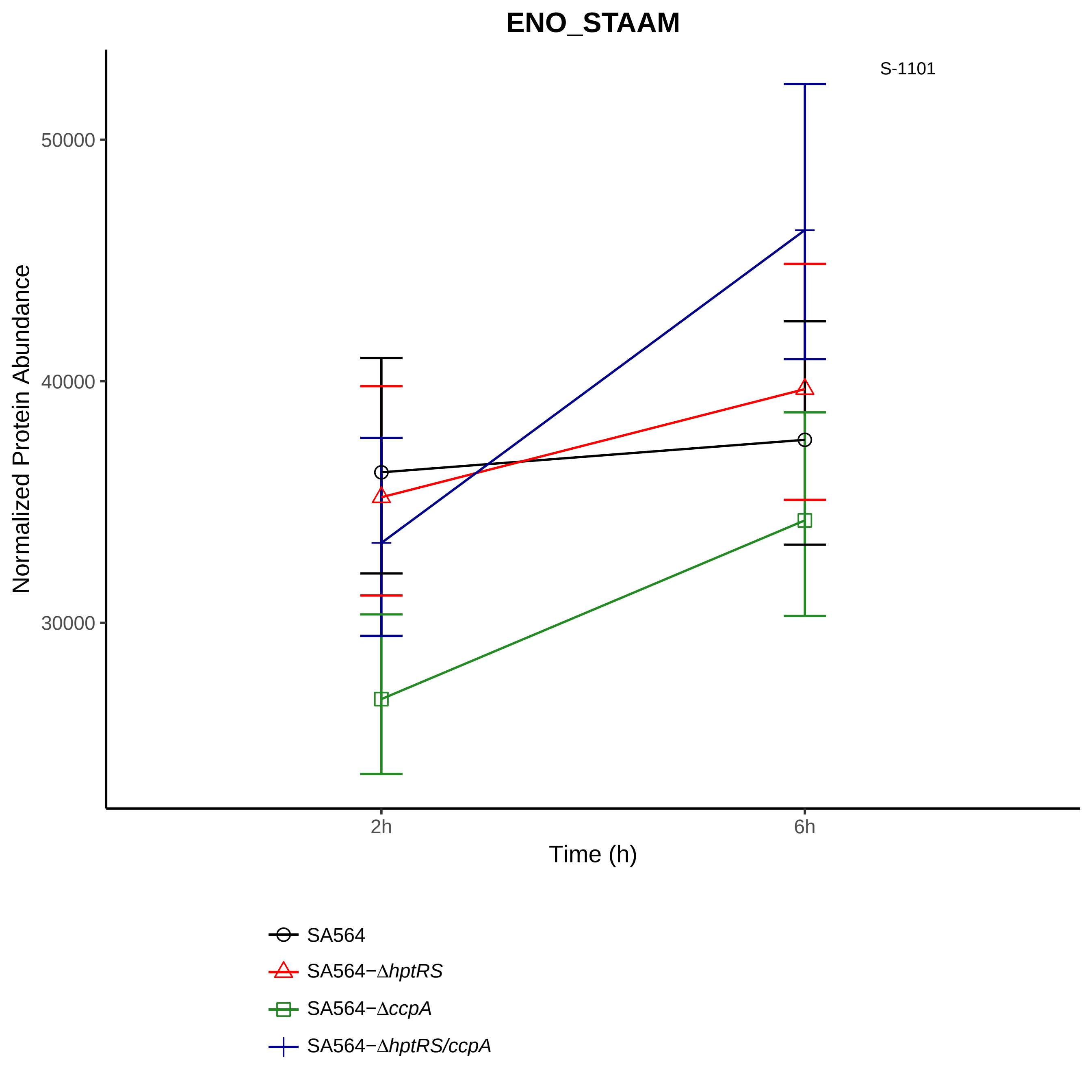

ENO_STAAM
S-1101
Normalized Protein Abundance
50000
40000
30000
2h
6h
Time (h)
SA564
SA564−Δ*hptRS*
SA564−Δ*ccpA*
SA564−Δ*hptRS/ccpA*

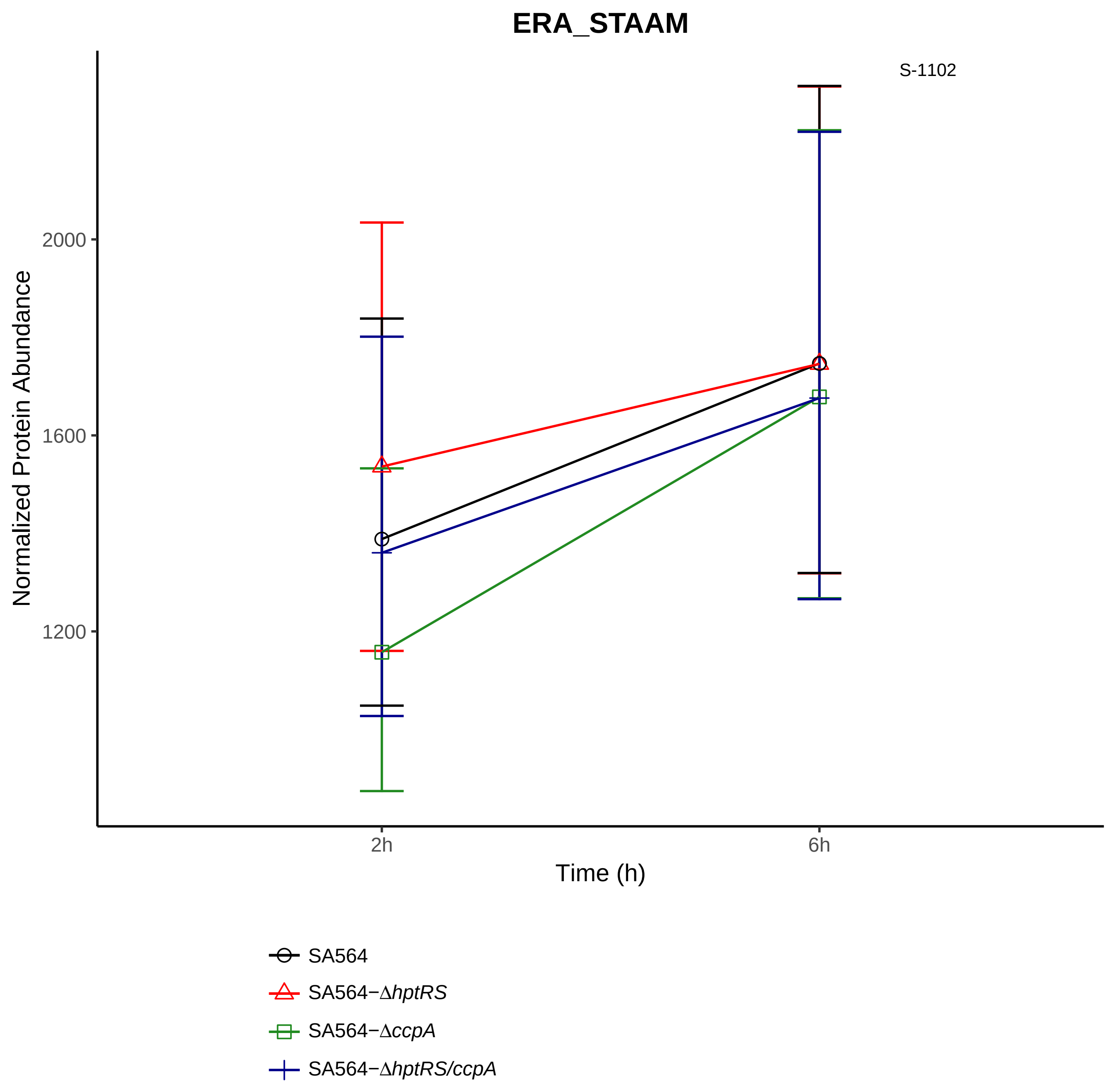

ERA_STAAM
S-1102
Normalized Protein Abundance
2000
1600
1200
2h
6h
Time (h)
SA564
SA564−Δ*hptRS*
SA564−Δ*ccpA*
SA564−Δ*hptRS/ccpA*

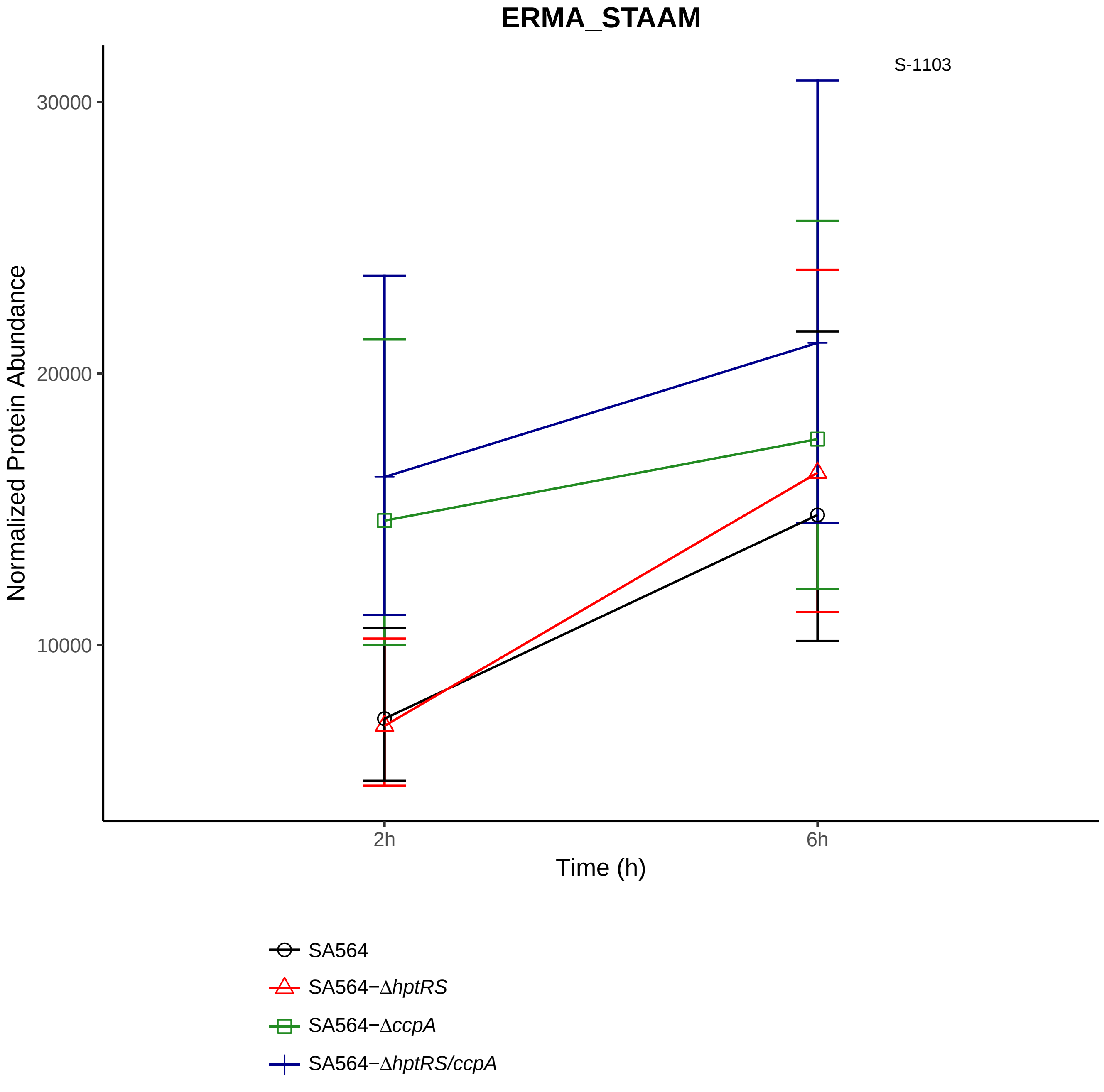

ERMA_STAAM
S-1103
30000
20000
10000
Normalized Protein Abundance
2h
6h
Time (h)
SA564
SA564−Δ*hptRS*
SA564−Δ*ccpA*
SA564−Δ*hptRS/ccpA*

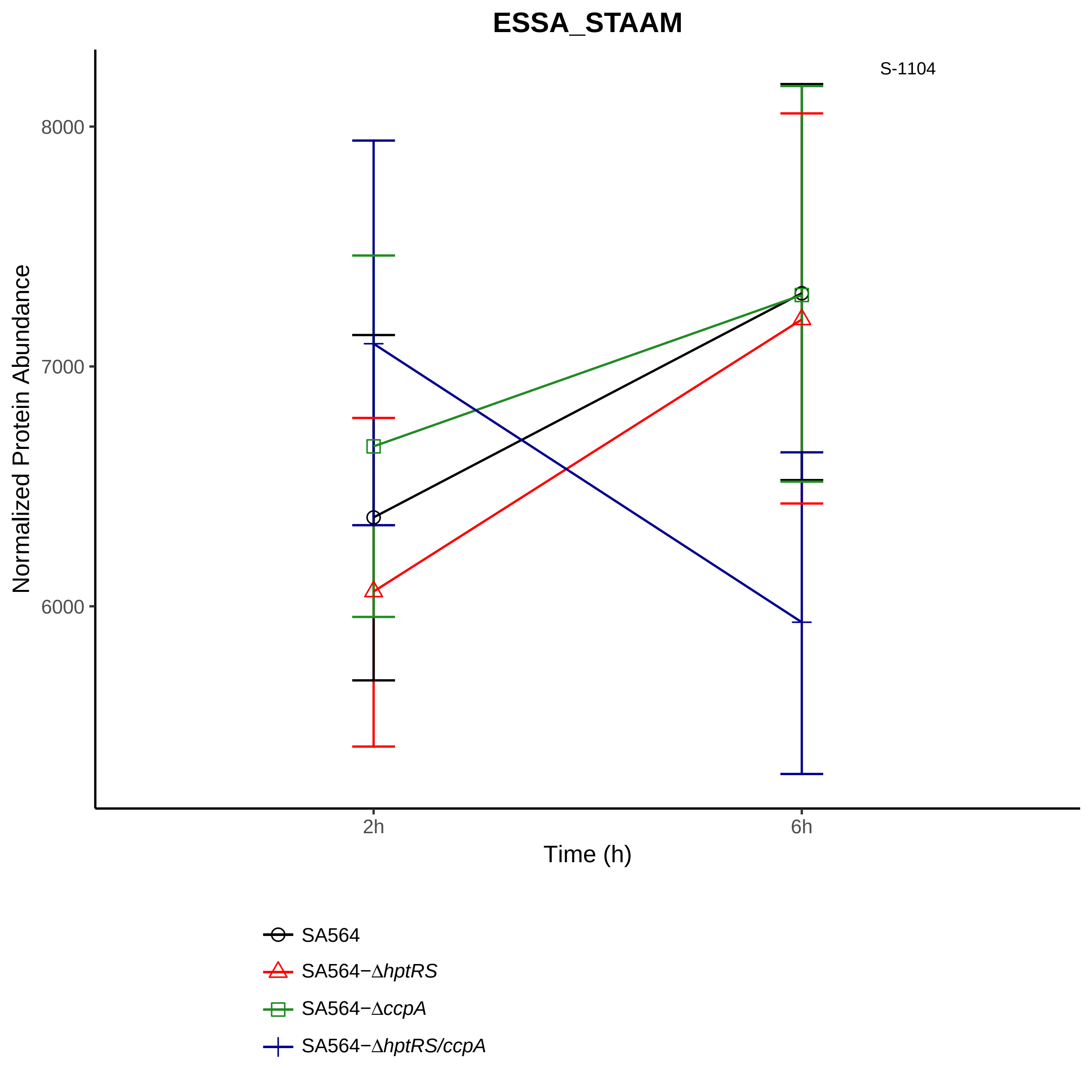

ESSA_STAAM
S-1104
8000
7000
6000
Normalized Protein Abundance
2h
6h
Time (h)
SA564
SA564−Δ*hptRS*
SA564−Δ*ccpA*
SA564−Δ*hptRS/ccpA*

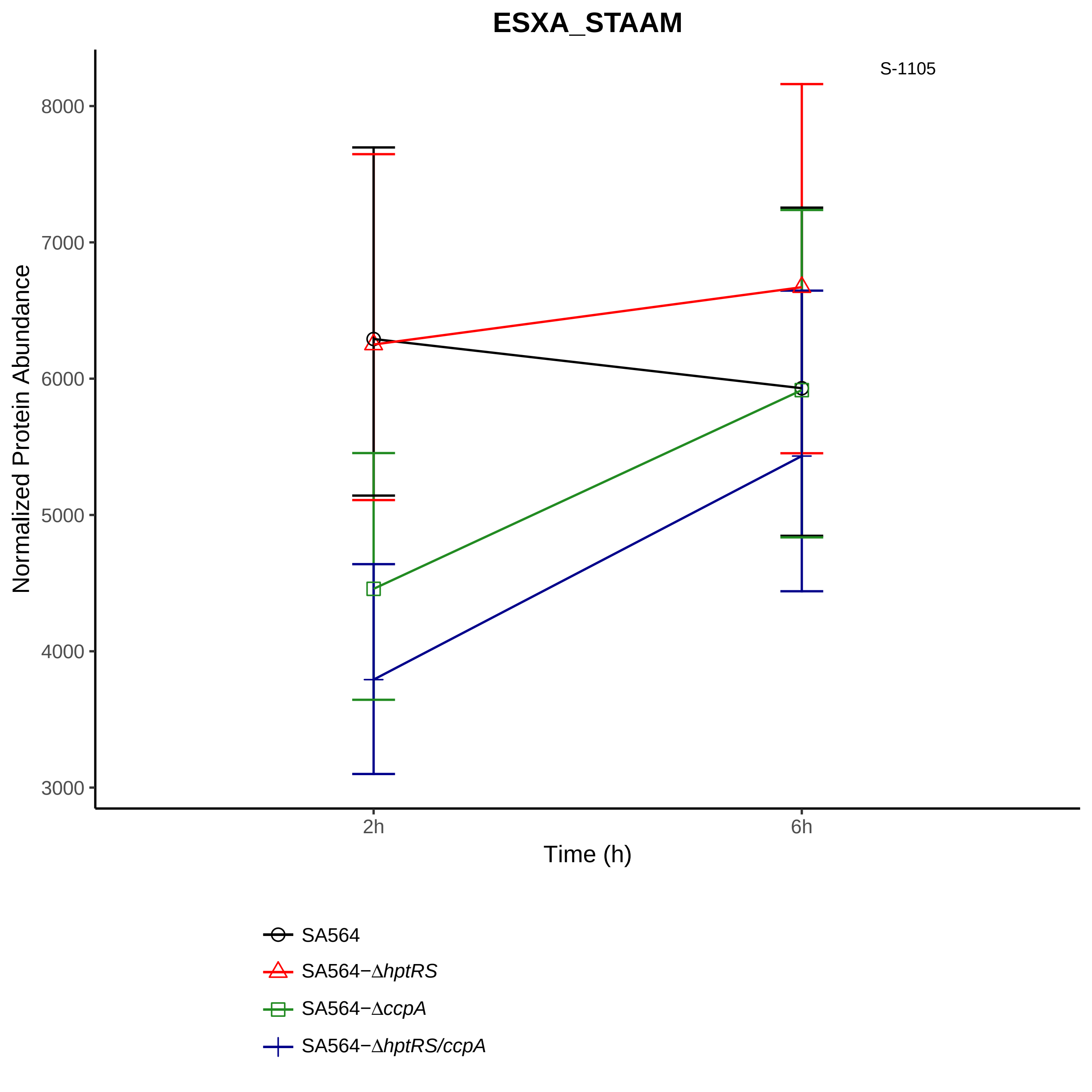

ESXA_STAAM
S-1105
Normalized Protein Abundance
8000
7000
6000
5000
4000
3000
2h
6h
Time (h)
SA564
SA564−Δ*hptRS*
SA564−Δ*ccpA*
SA564−Δ*hptRS/ccpA*

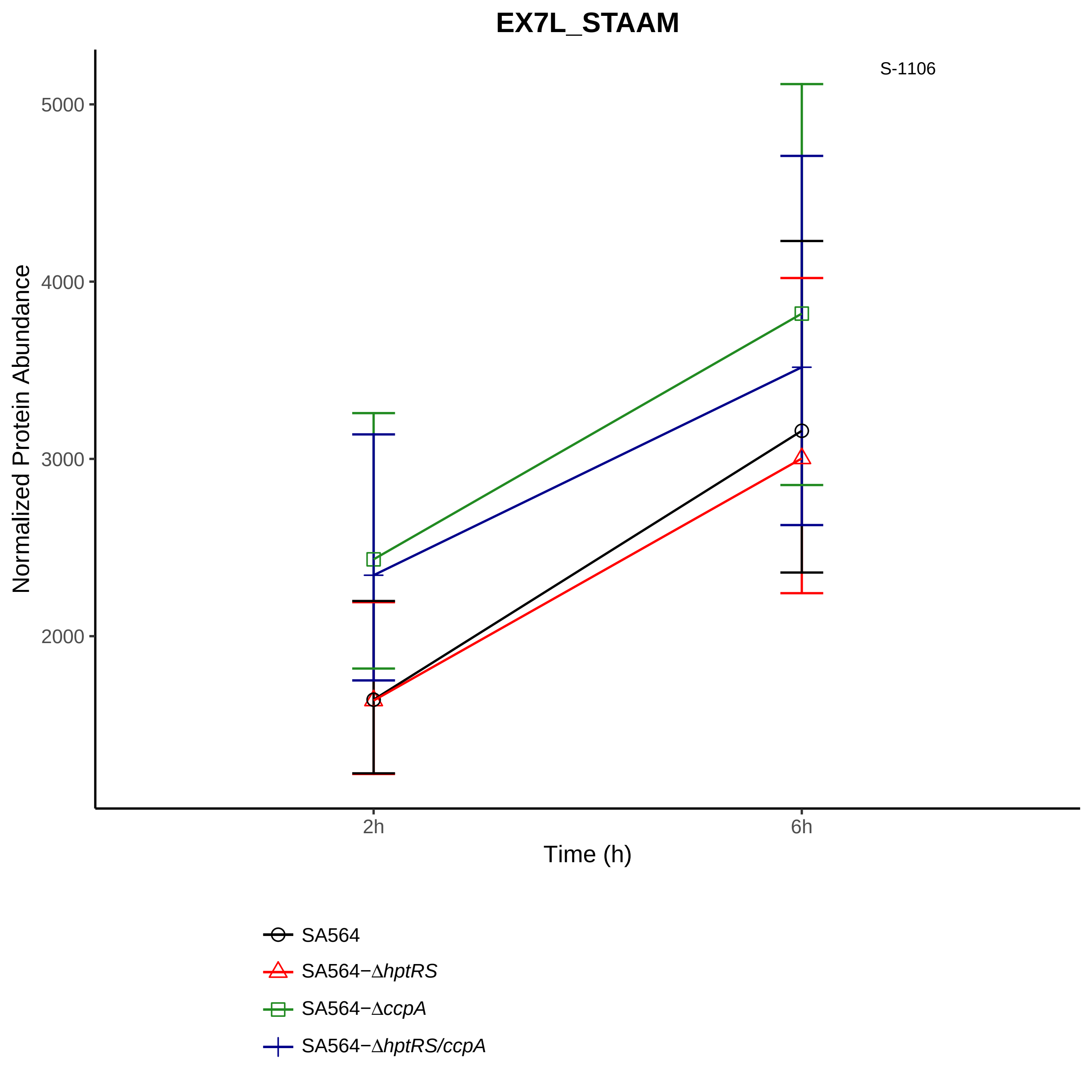

EX7L_STAAM
S-1106
5000
4000
3000
2000
Normalized Protein Abundance
2h
6h
Time (h)
SA564
SA564−ΔhptRS
SA564−ΔccpA
SA564−ΔhptRS/ccpA

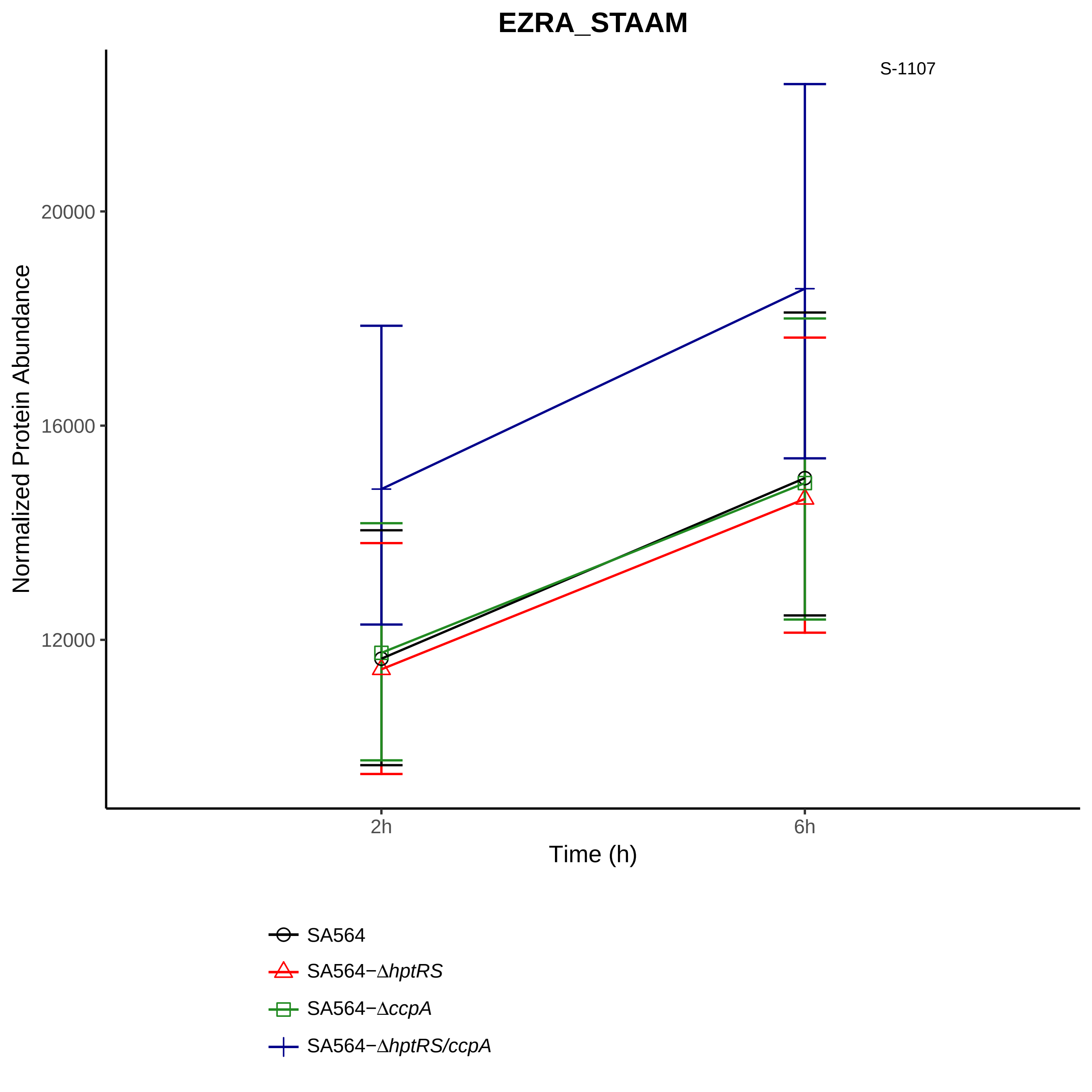

EZRA_STAAM
S-1107
Normalized Protein Abundance
20000
16000
12000
2h
6h
Time (h)
SA564
SA564−Δ*hptRS*
SA564−Δ*ccpA*
SA564−Δ*hptRS/ccpA*

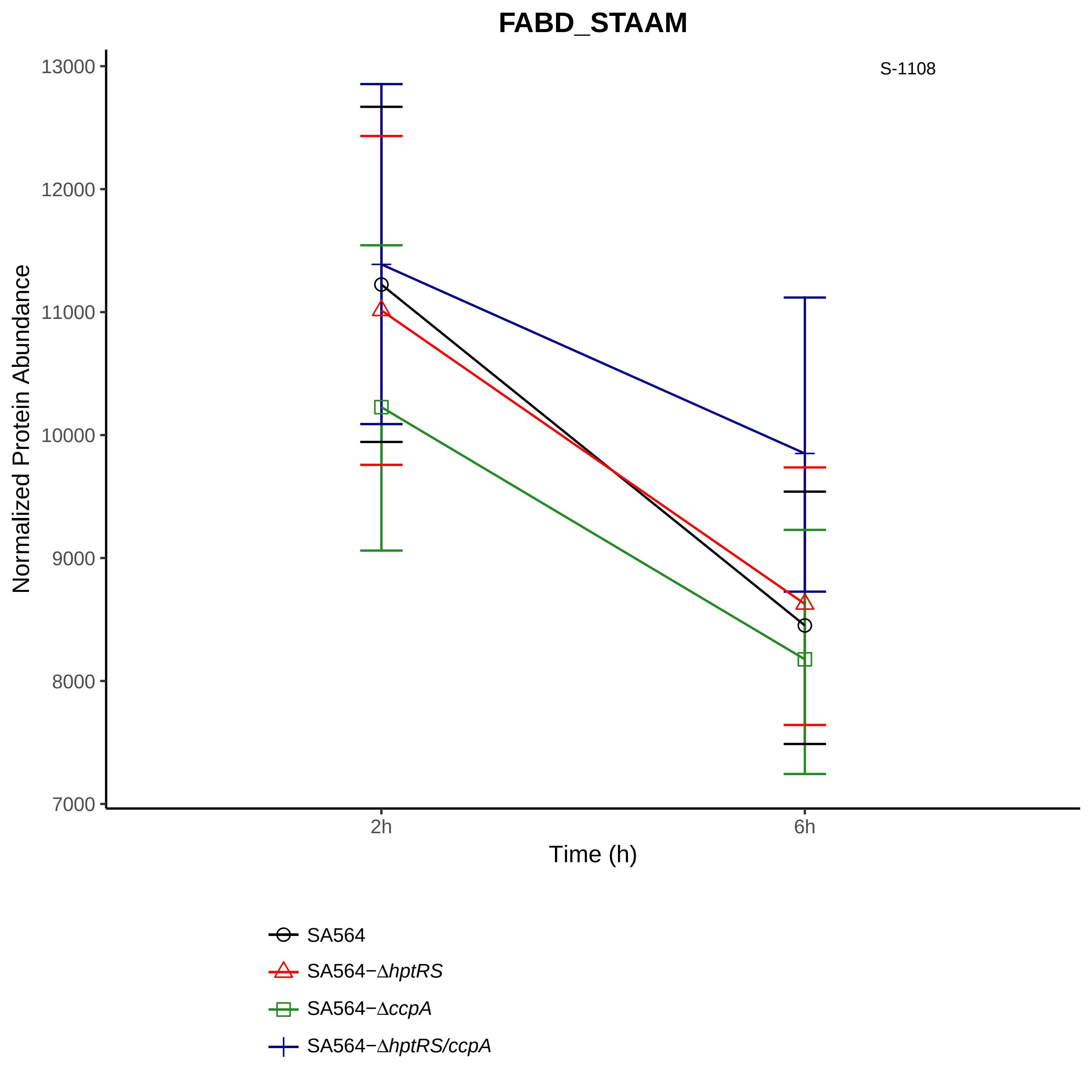
FABD_STAAM
S-1108
Normalized Protein Abundance
13000
12000
11000
10000
9000
8000
7000
2h
6h
Time (h)
SA564
SA564−Δ*hptRS*
SA564−Δ*ccpA*
SA564−Δ*hptRS/ccpA*

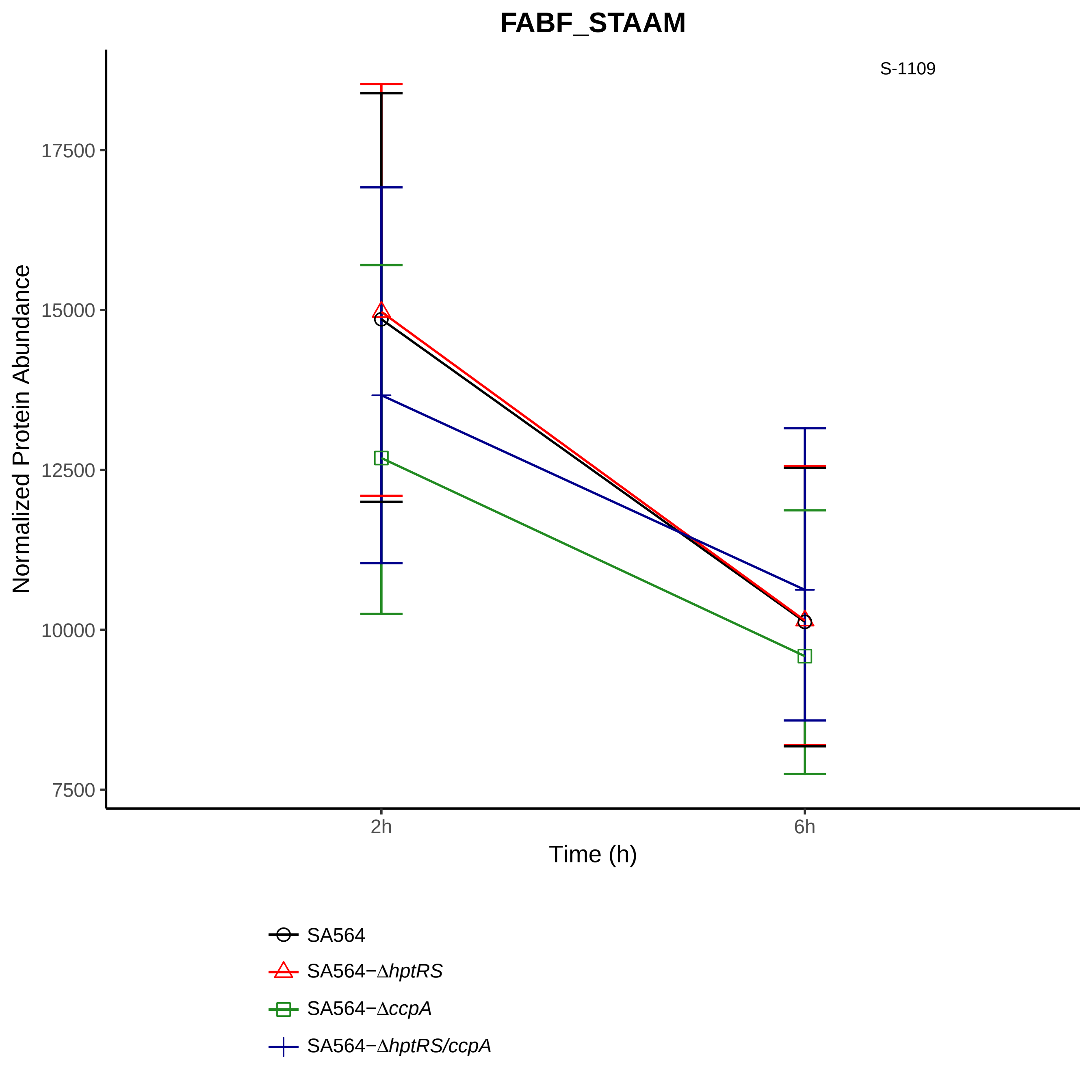

FABF_STAAM
S-1109
Normalized Protein Abundance
17500
15000
12500
10000
7500
2h
6h
Time (h)
SA564
SA564−Δ*hptRS*
SA564−Δ*ccpA*
SA564−Δ*hptRS/ccpA*

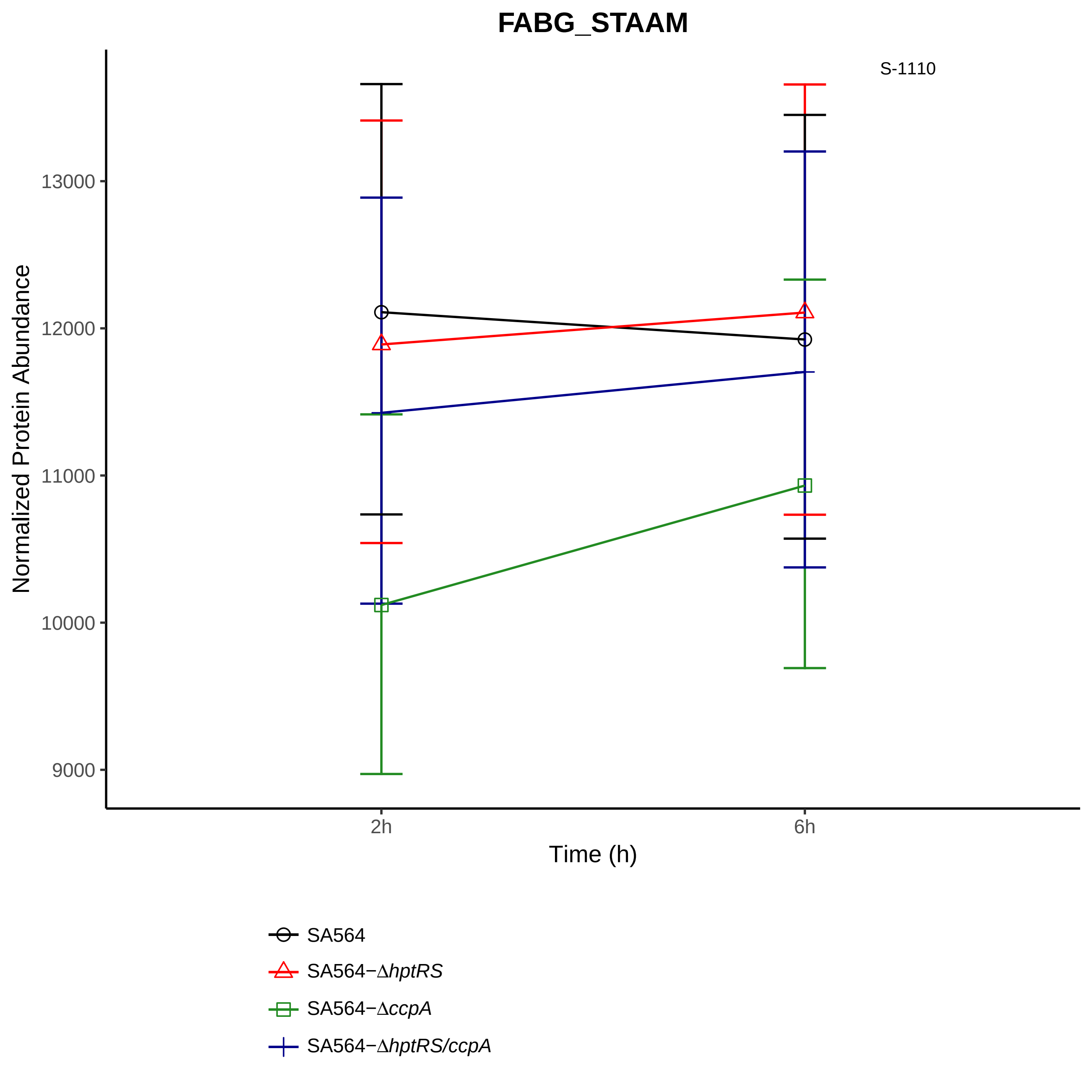

FABG_STAAM
S-1110
Normalized Protein Abundance
13000
12000
11000
10000
9000
2h
6h
Time (h)
SA564
SA564−Δ*hptRS*
SA564−Δ*ccpA*
SA564−Δ*hptRS/ccpA*

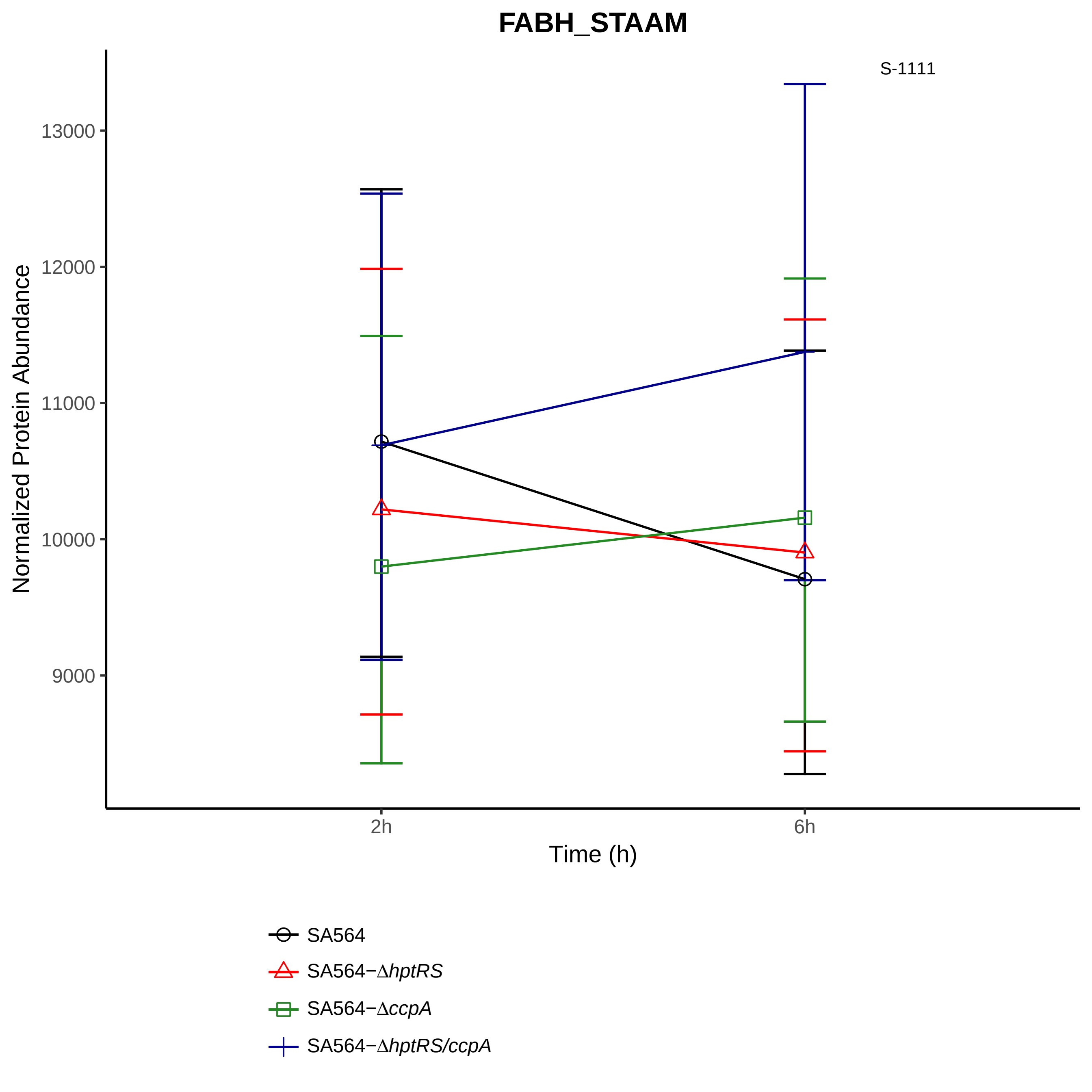

FABH_STAAM
S-1111
Normalized Protein Abundance
13000
12000
11000
10000
9000
2h
6h
Time (h)
SA564
SA564−Δ*hptRS*
SA564−Δ*ccpA*
SA564−Δ*hptRS/ccpA*

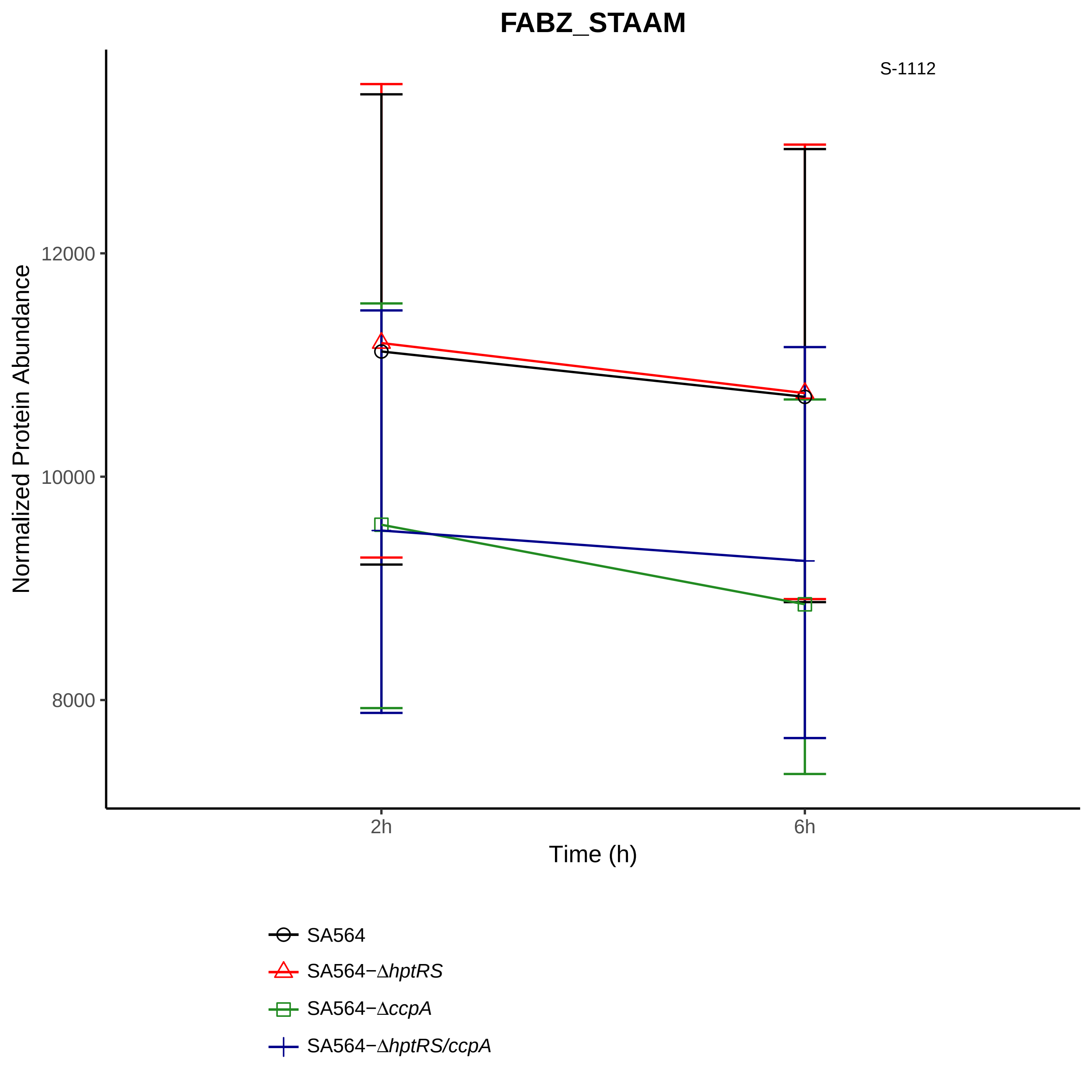

FABZ_STAAM
S-1112
Normalized Protein Abundance
12000
10000
8000
2h
6h
Time (h)
SA564
SA564−Δ*hptRS*
SA564−Δ*ccpA*
SA564−Δ*hptRS/ccpA*

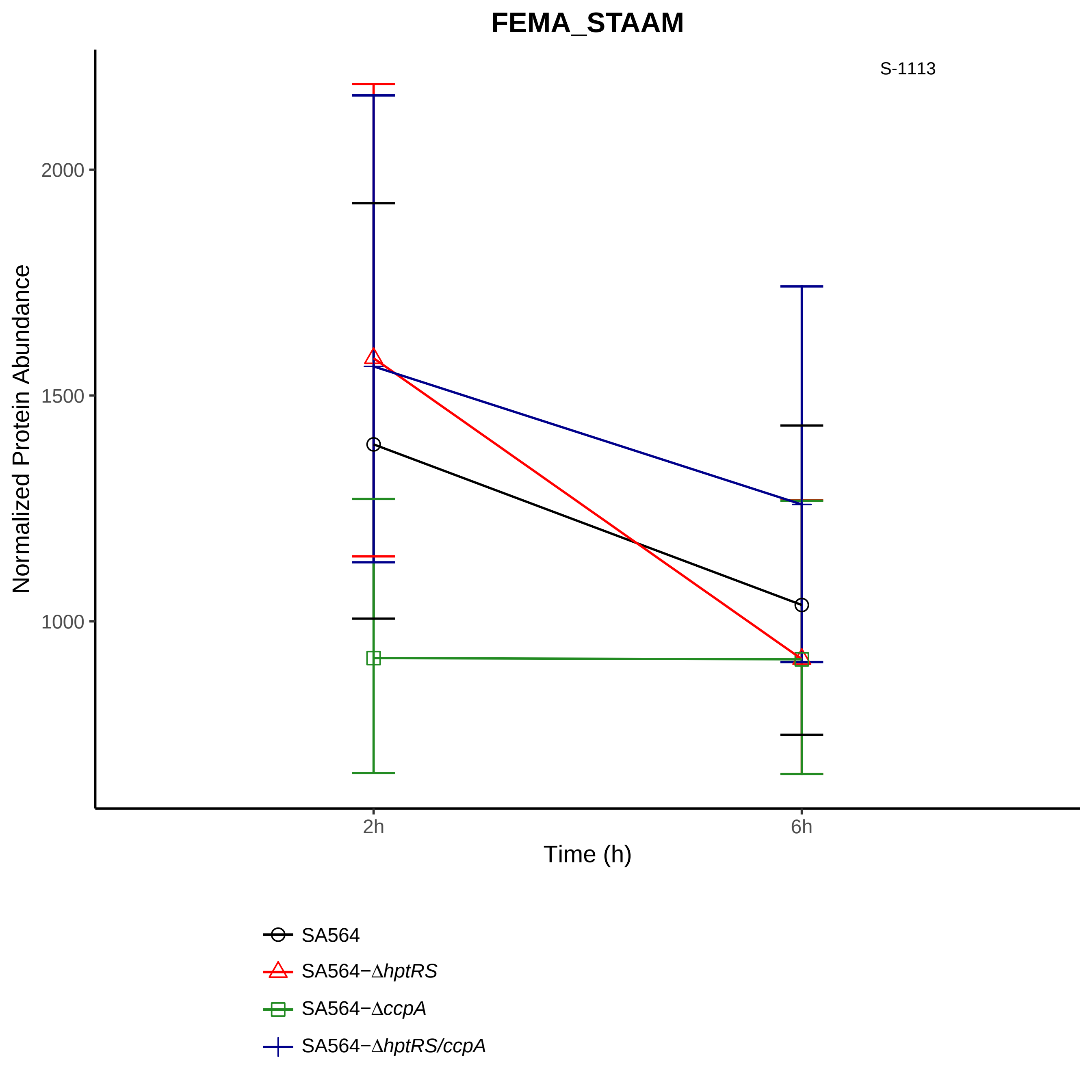

FEMA_STAAM
S-1113
Normalized Protein Abundance
2000
1500
1000
2h
6h
Time (h)
SA564
SA564−Δ*hptRS*
SA564−Δ*ccpA*
SA564−Δ*hptRS/ccpA*

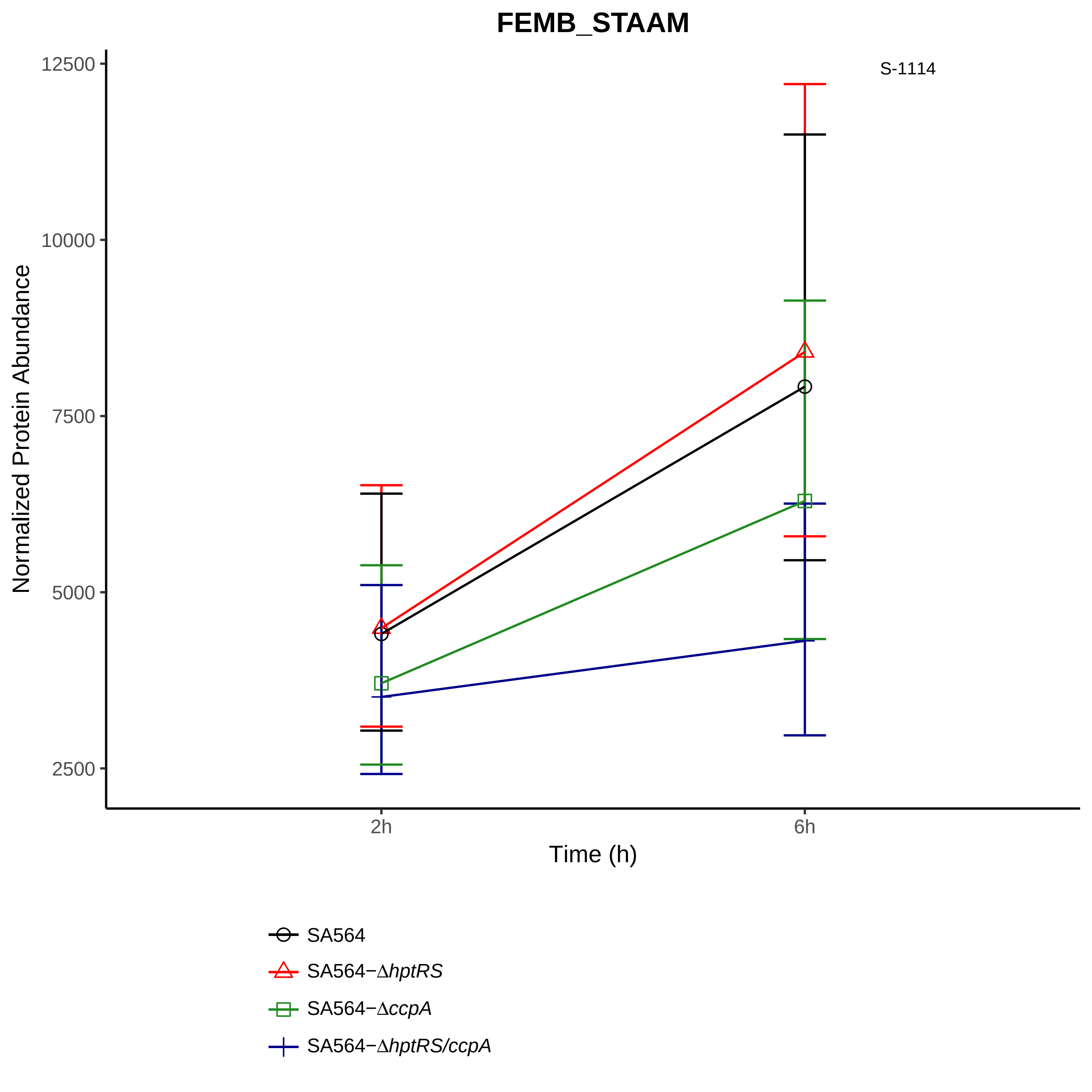

FEMB_STAAM
S-1114
Normalized Protein Abundance
12500
10000
7500
5000
2500
2h
6h
Time (h)
SA564
SA564−Δ*hptRS*
SA564−Δ*ccpA*
SA564−Δ*hptRS/ccpA*

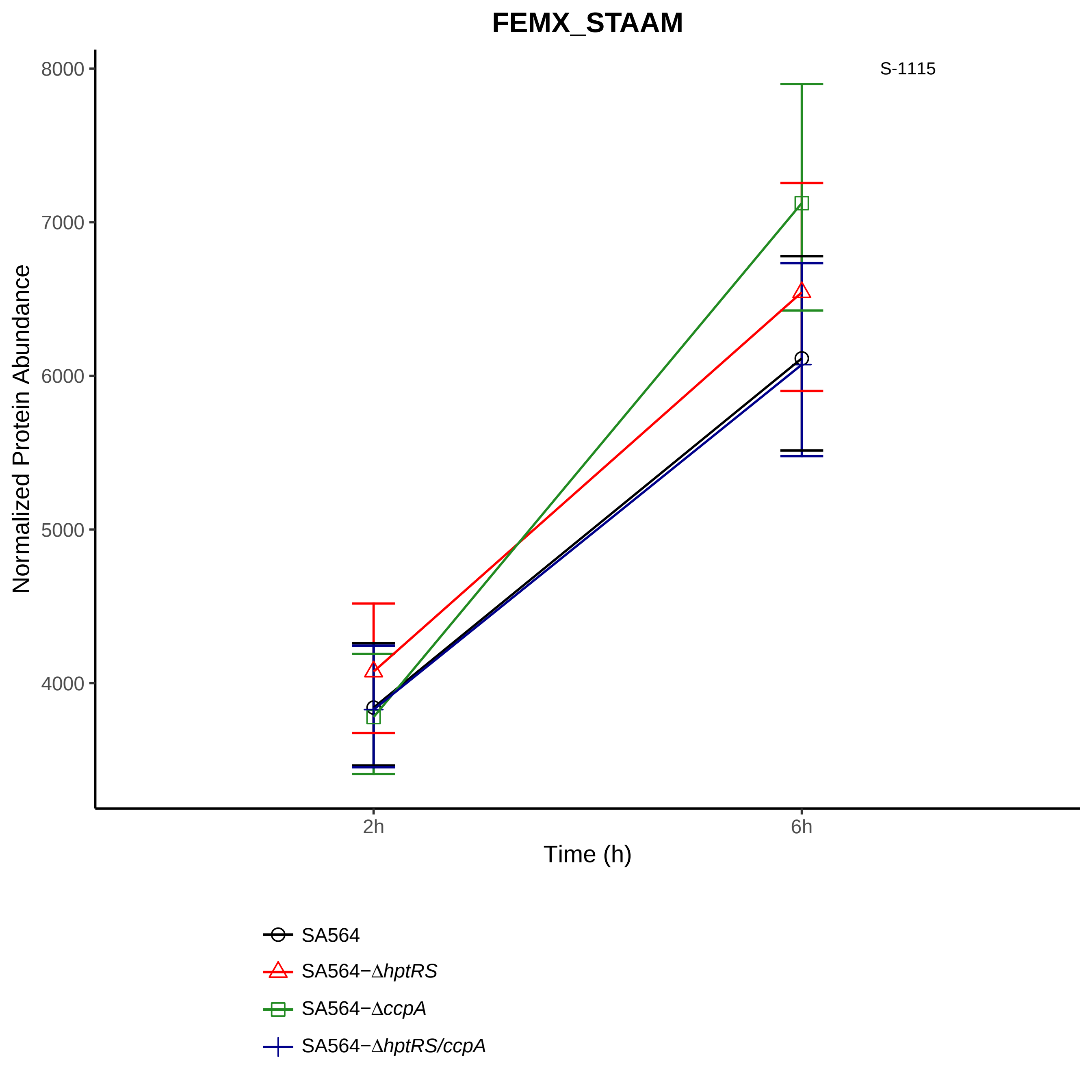

FEMX_STAAM
S-1115
8000
7000
6000
5000
4000
Normalized Protein Abundance
2h
6h
Time (h)
SA564
SA564−Δ*hptRS*
SA564−Δ*ccpA*
SA564−Δ*hptRS/ccpA*

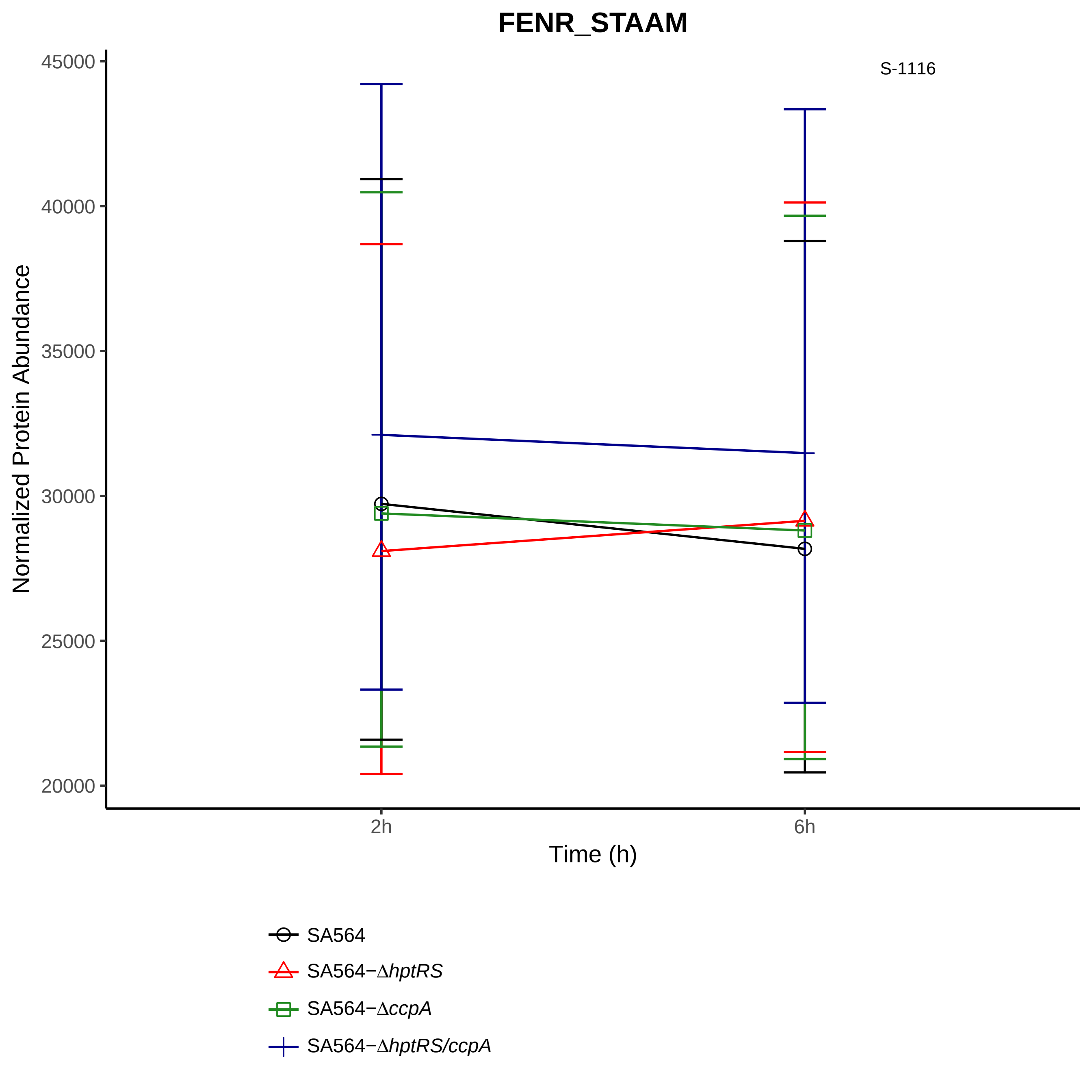

FENR_STAAM
S-1116
Normalized Protein Abundance
45000
40000
35000
30000
25000
20000
2h
6h
Time (h)
SA564
SA564−Δ*hptRS*
SA564−Δ*ccpA*
SA564−Δ*hptRS/ccpA*

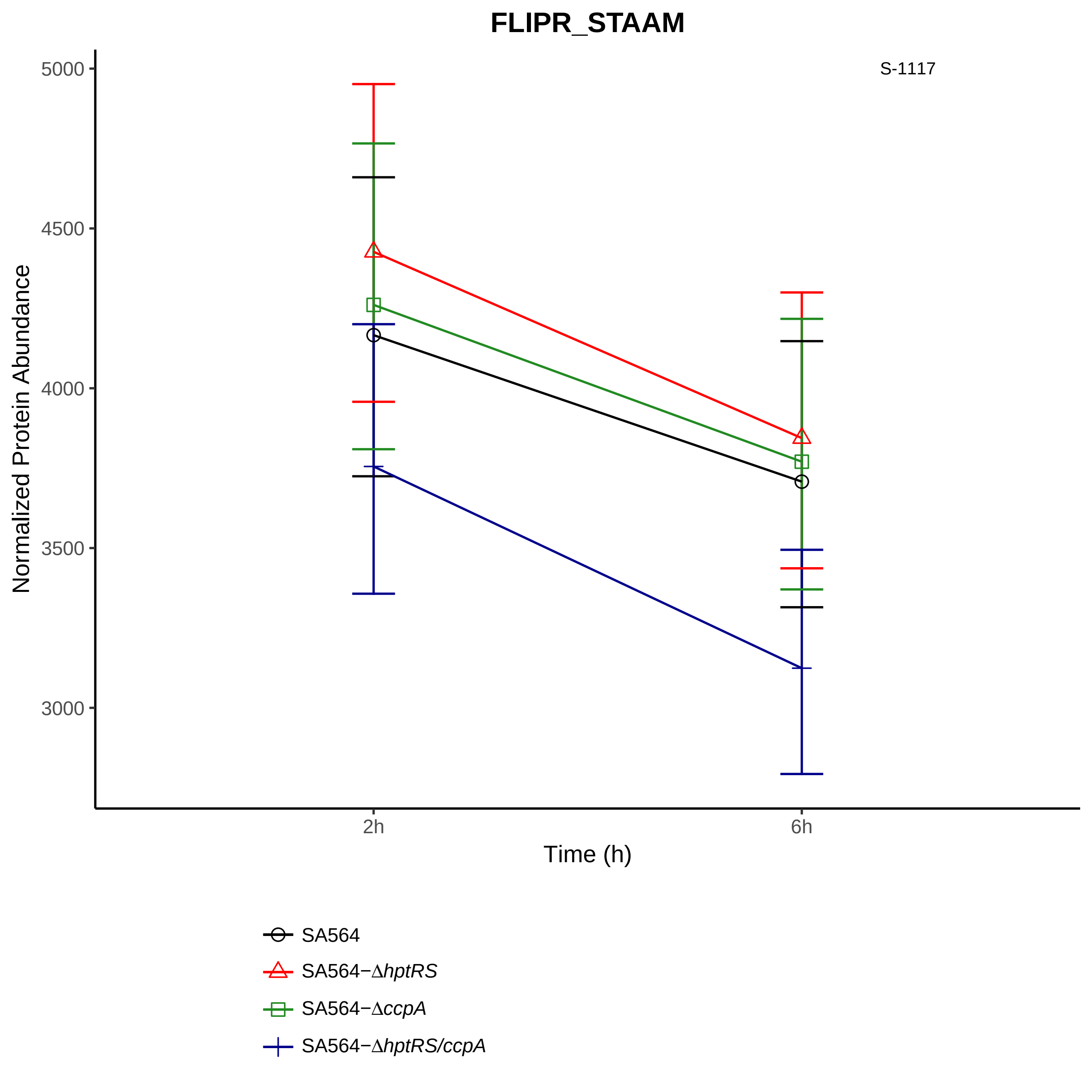
FLIPR_STAAM
S-1117
Normalized Protein Abundance
5000
4500
4000
3500
3000
2h
6h
Time (h)
SA564
SA564−Δ*hptRS*
SA564−Δ*ccpA*
SA564−Δ*hptRS/ccpA*

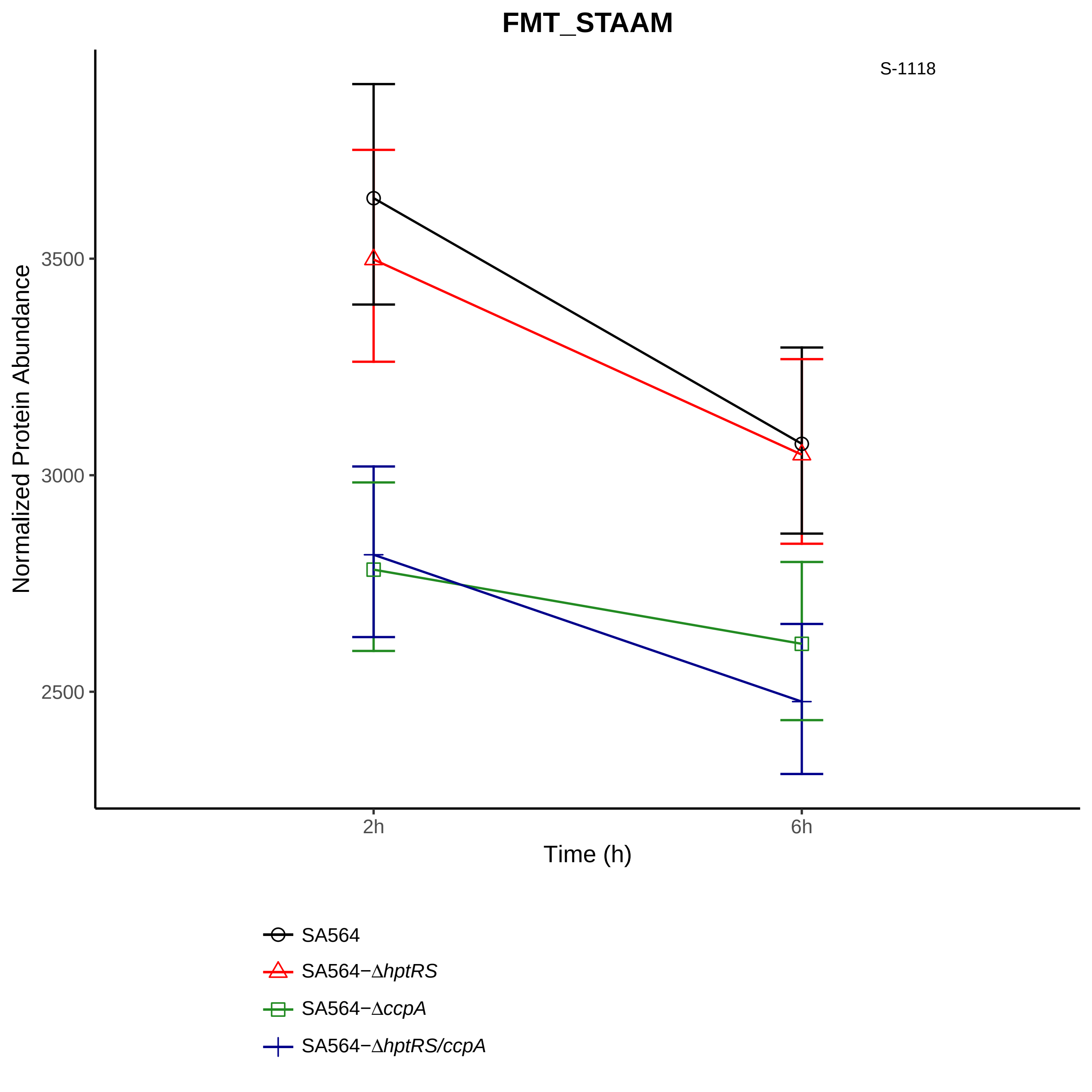

FMT_STAAM
S-1118
Normalized Protein Abundance
3500
3000
2500
2h
6h
Time (h)
SA564
SA564−Δ*hptRS*
SA564−Δ*ccpA*
SA564−Δ*hptRS/ccpA*

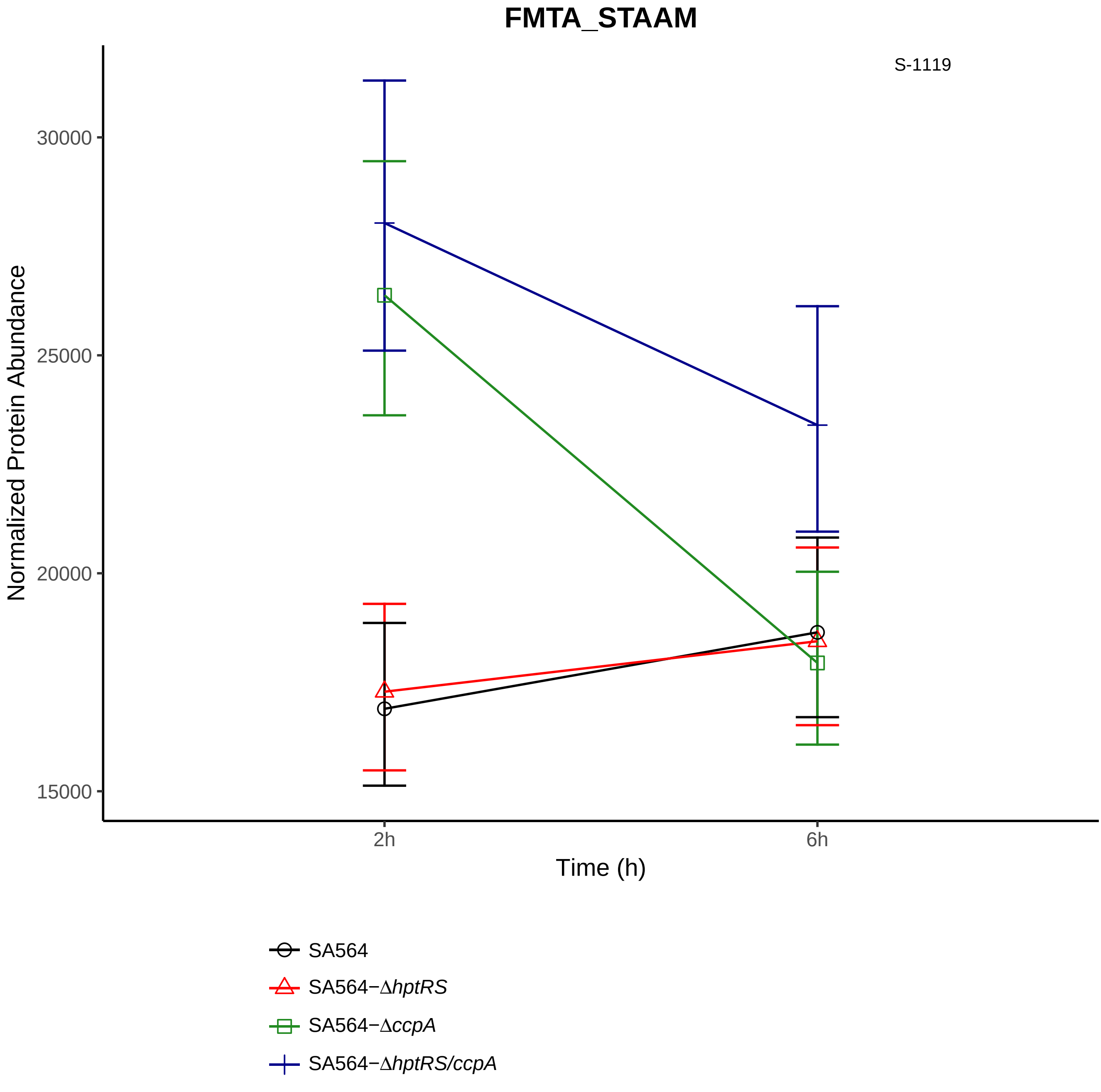

FMTA_STAAM
S-1119
Normalized Protein Abundance
30000
25000
20000
15000
2h
6h
Time (h)
SA564
SA564−Δ*hptRS*
SA564−Δ*ccpA*
SA564−Δ*hptRS/ccpA*

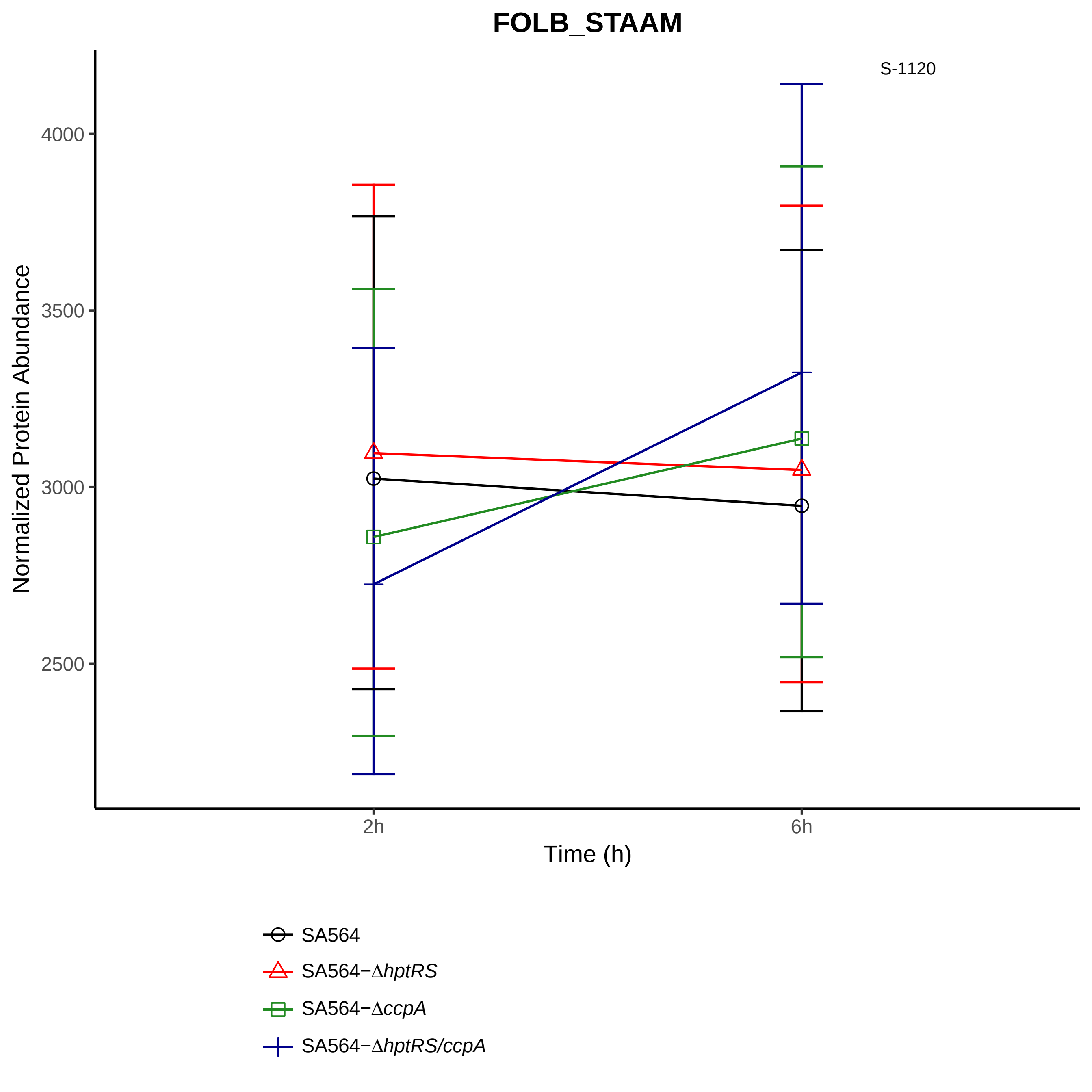
FOLB_STAAM
S-1120
Normalized Protein Abundance
4000
3500
3000
2500
2h
6h
Time (h)
SA564
SA564−Δ*hptRS*
SA564−Δ*ccpA*
SA564−Δ*hptRS/ccpA*

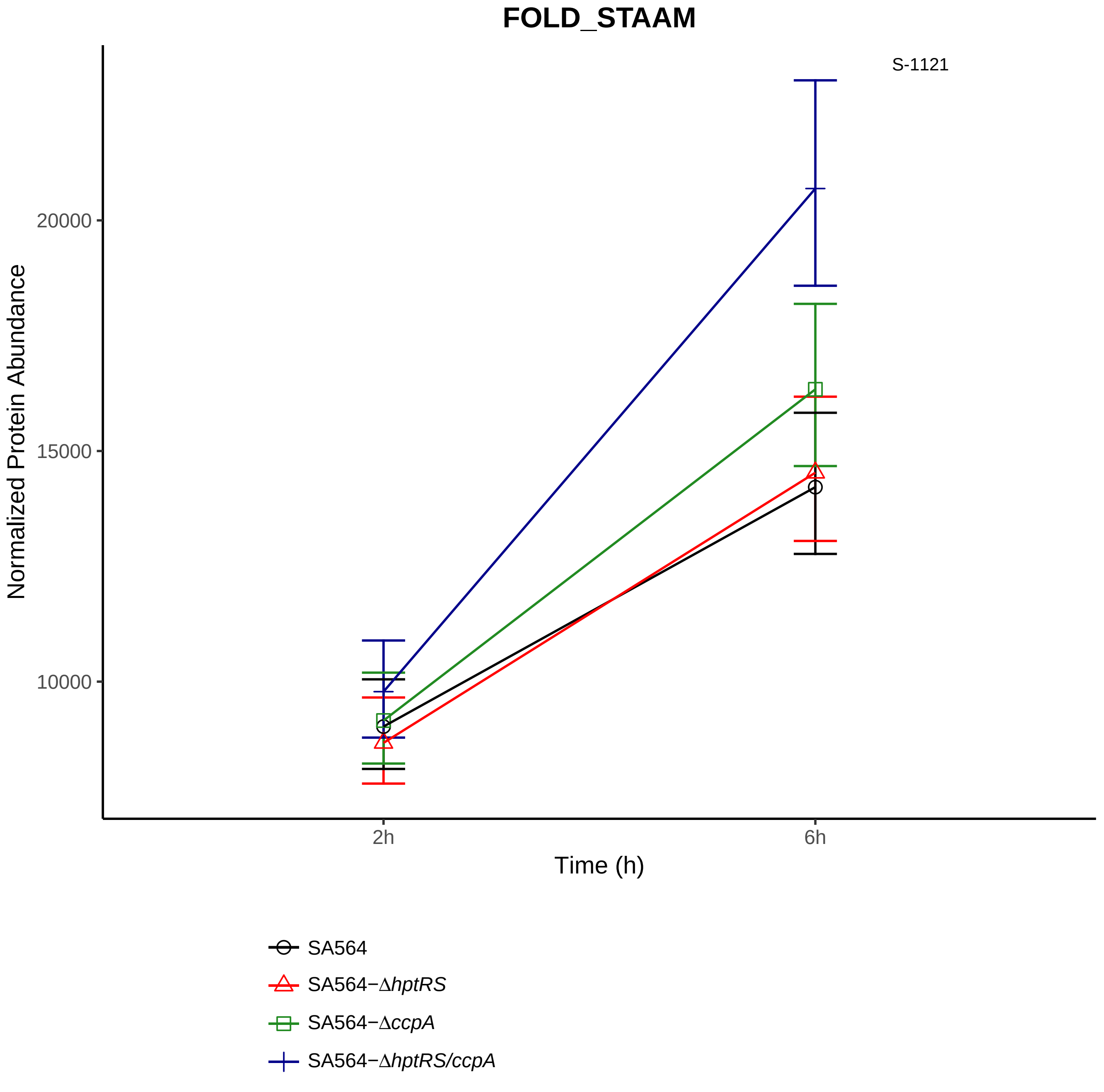
FOLD_STAAM
S-1121
Normalized Protein Abundance
20000
15000
10000
2h
6h
Time (h)
SA564
SA564−Δ*hptRS*
SA564−Δ*ccpA*
SA564−Δ*hptRS/ccpA*

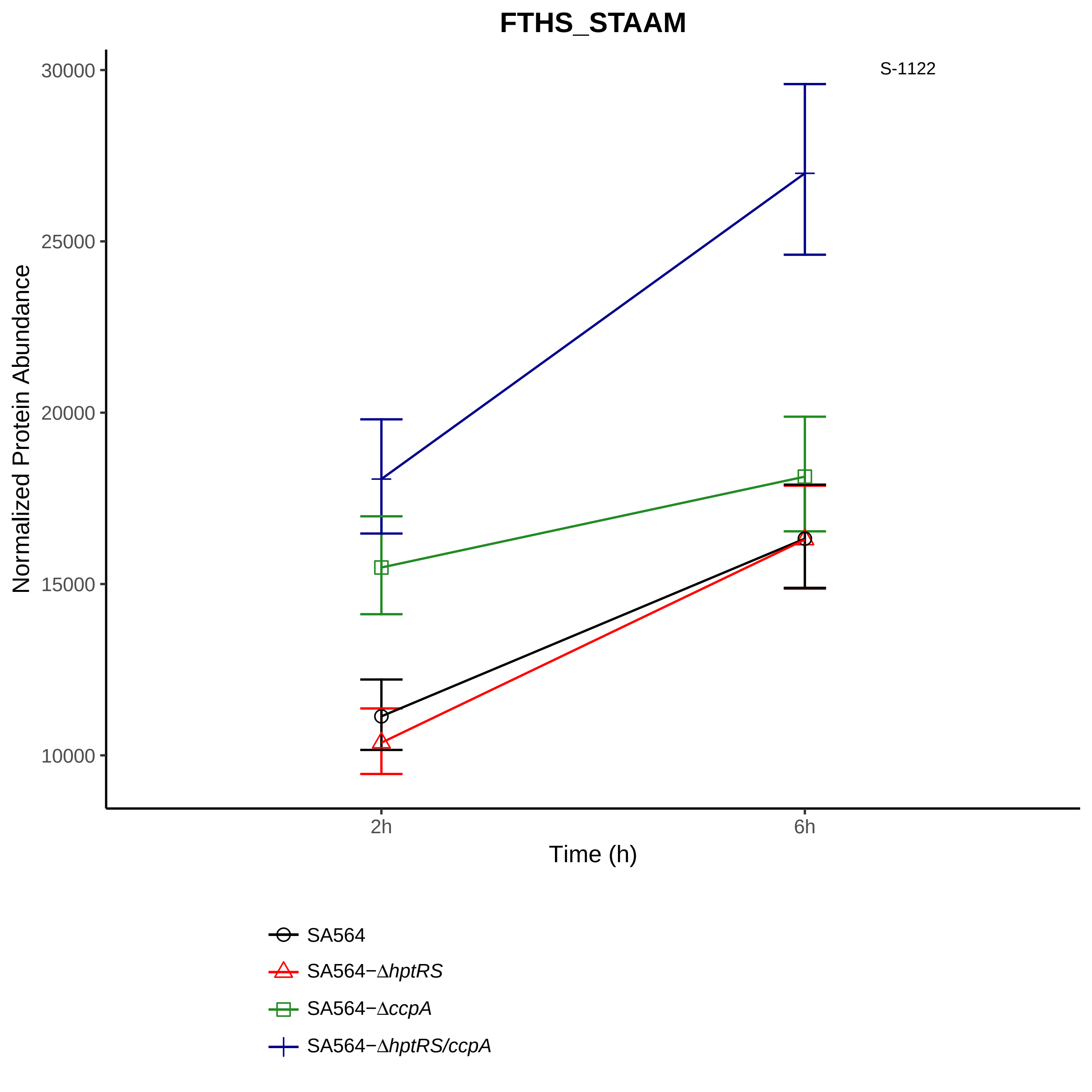

FTHS_STAAM
S-1122
Normalized Protein Abundance
30000
25000
20000
15000
10000
2h
6h
Time (h)
SA564
SA564−Δ*hptRS*
SA564−Δ*ccpA*
SA564−Δ*hptRS/ccpA*

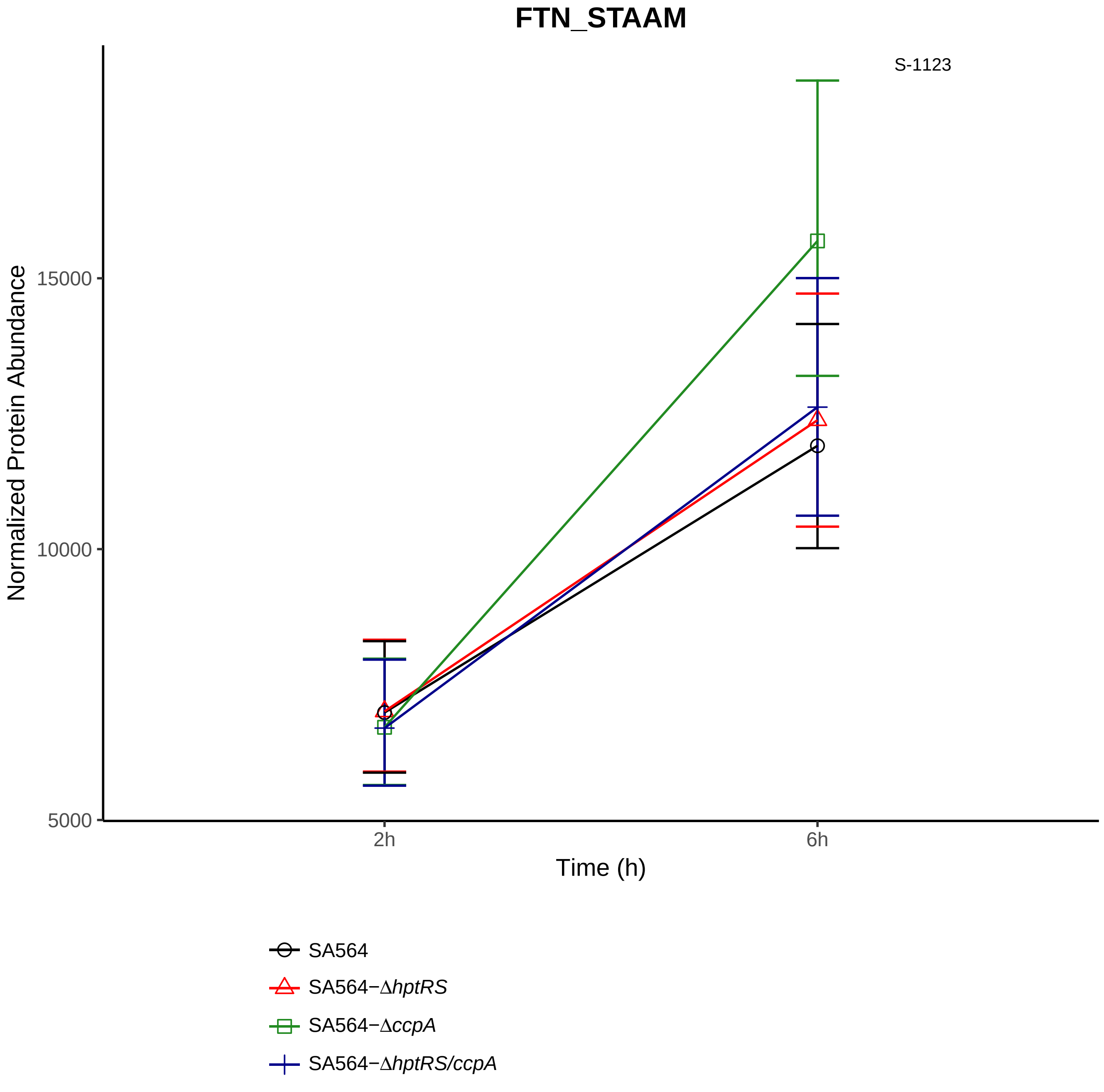
FTN_STAAM
S-1123
Normalized Protein Abundance
15000
10000
5000
2h
6h
Time (h)
SA564
SA564−Δ*hptRS*
SA564−Δ*ccpA*
SA564−Δ*hptRS/ccpA*

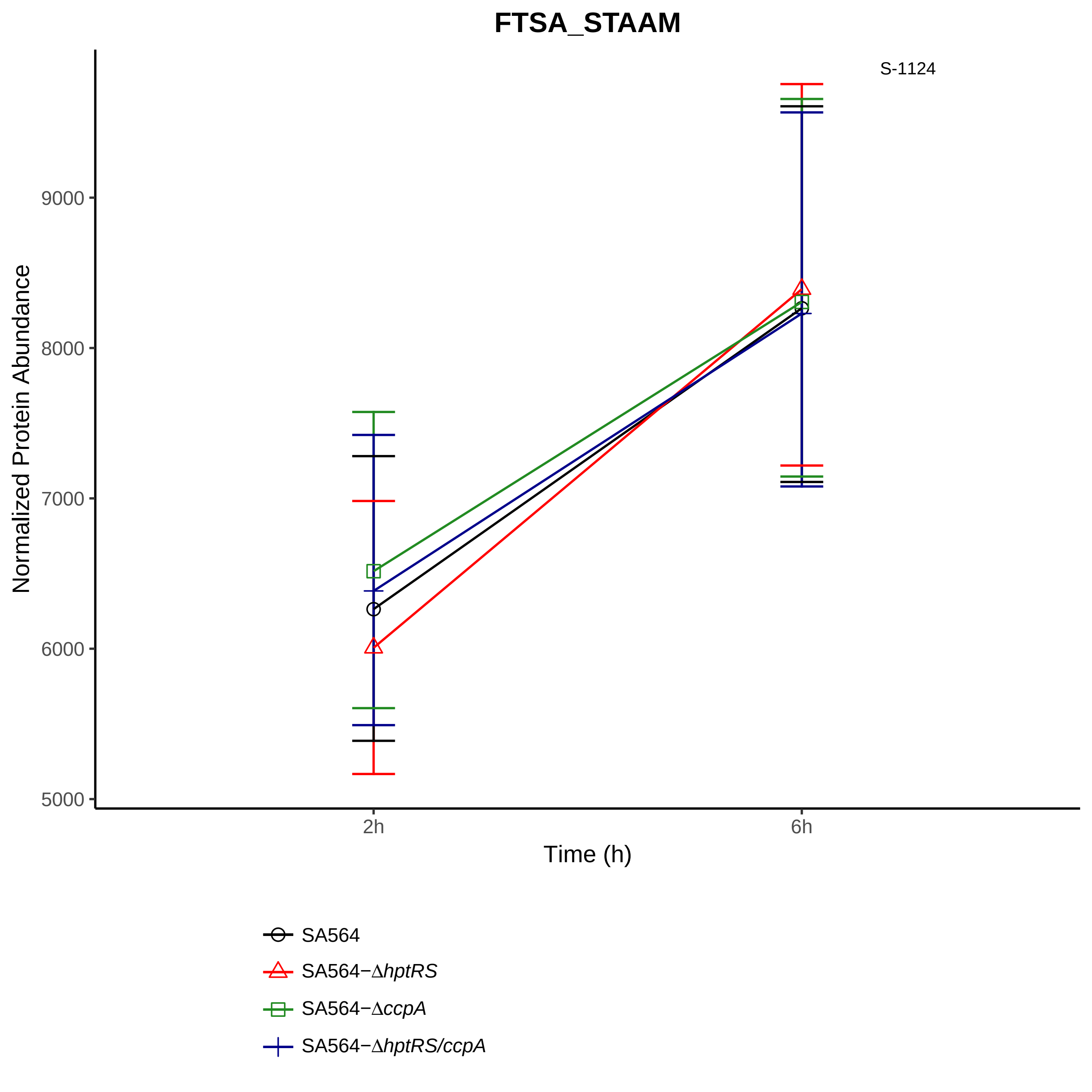

FTSA_STAAM
S-1124
Normalized Protein Abundance
5000
6000
7000
8000
9000
2h
6h
Time (h)
SA564
SA564−Δ*hptRS*
SA564−Δ*ccpA*
SA564−Δ*hptRS/ccpA*

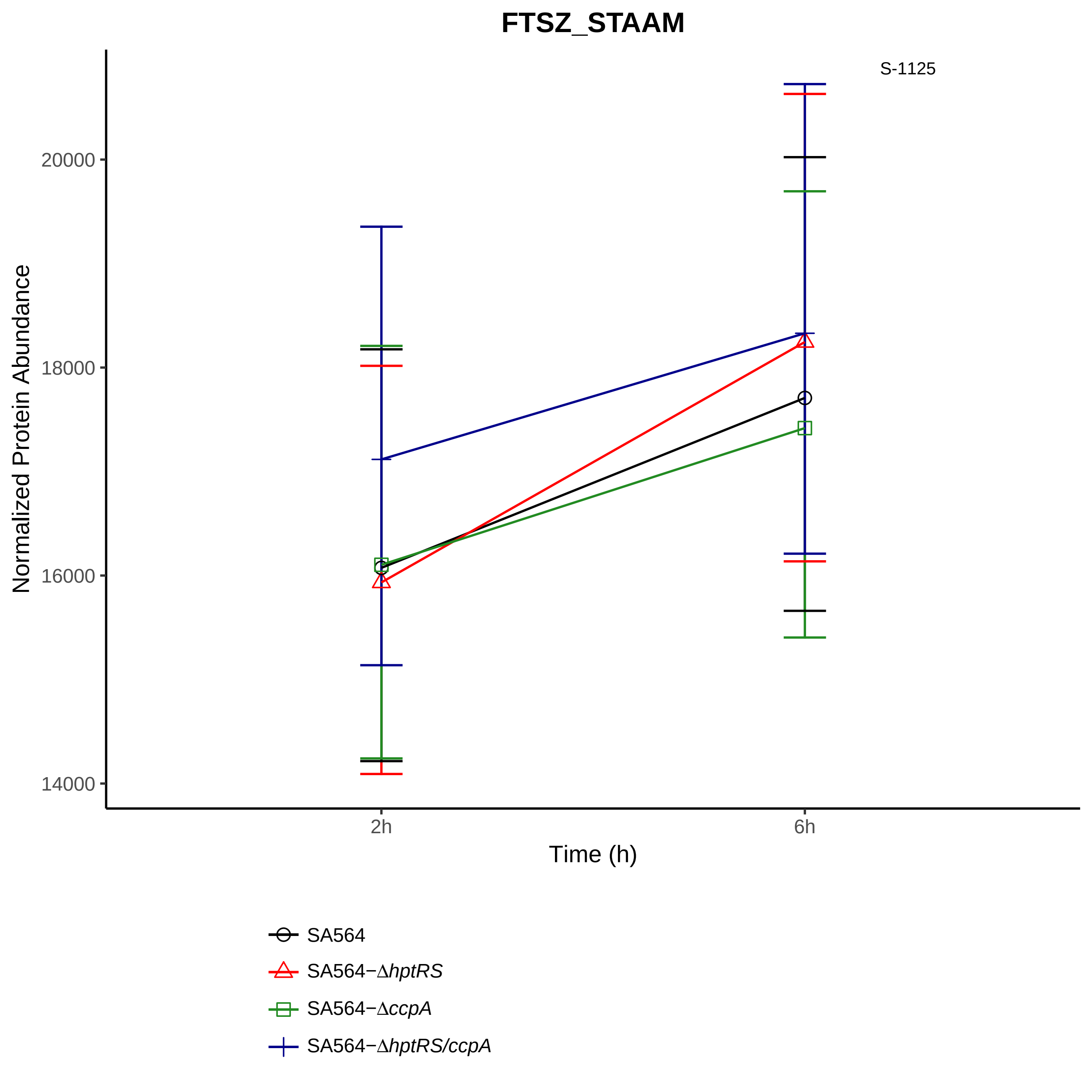

FTSZ_STAAM
S-1125
Normalized Protein Abundance
20000
18000
16000
14000
2h
6h
Time (h)
SA564
SA564−Δ*hptRS*
SA564−Δ*ccpA*
SA564−Δ*hptRS/ccpA*

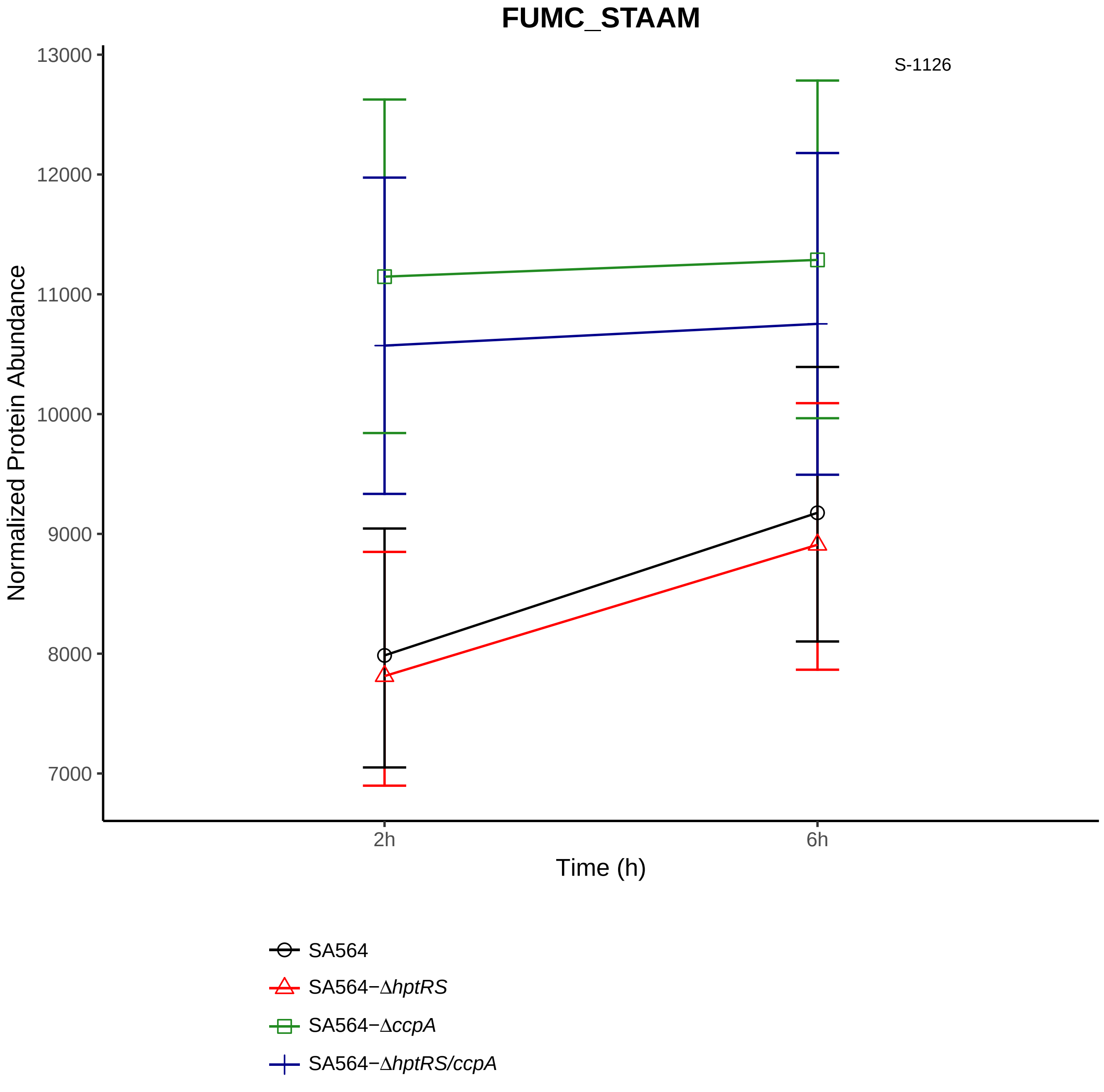

FUMC_STAAM
S-1126
Normalized Protein Abundance
13000
12000
11000
10000
9000
8000
7000
2h
6h
Time (h)
SA564
SA564−ΔhptRS
SA564−ΔccpA
SA564−ΔhptRS/ccpA

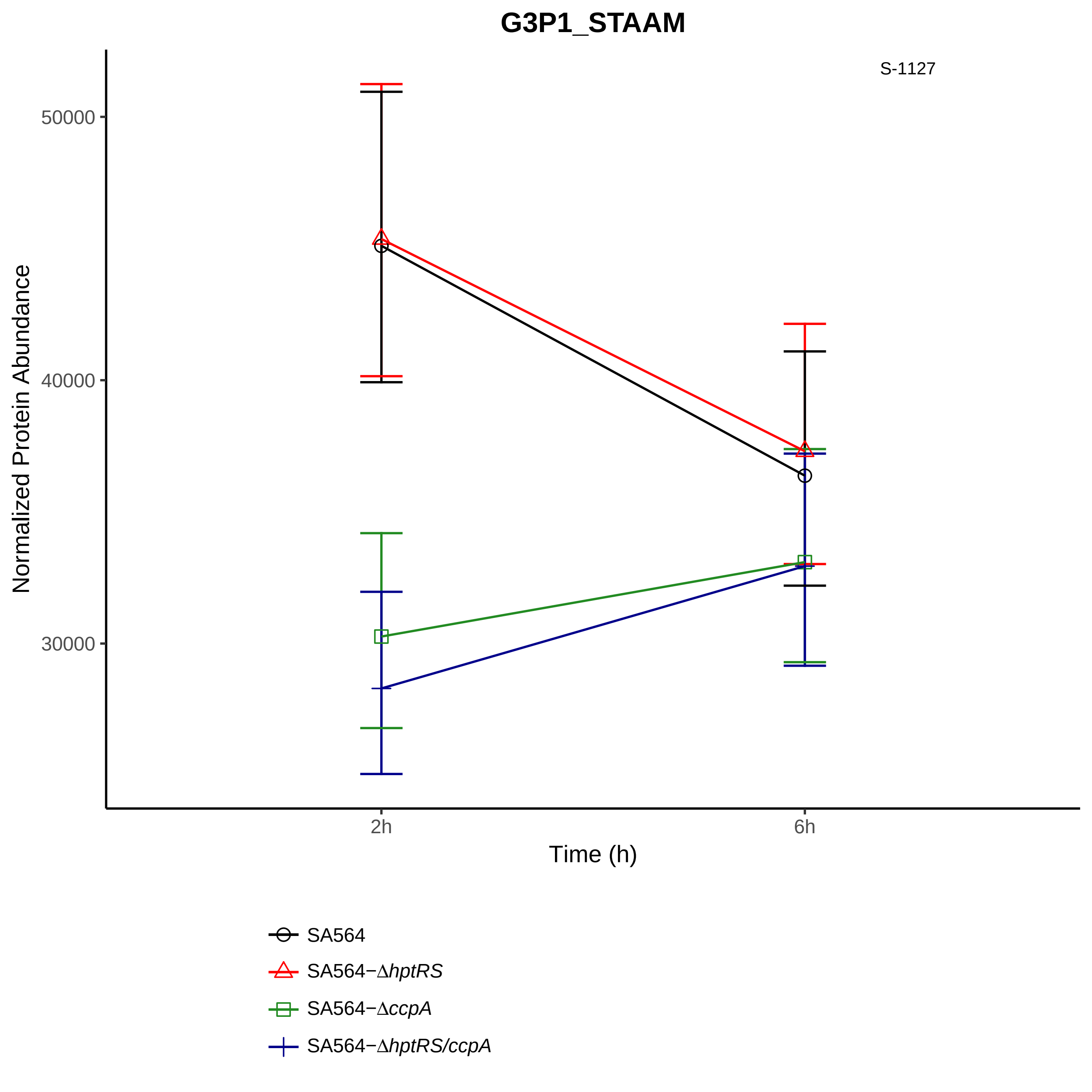

G3P1_STAAM
S-1127
50000
40000
30000
Normalized Protein Abundance
2h
6h
Time (h)
SA564
SA564−Δ*hptRS*
SA564−Δ*ccpA*
SA564−Δ*hptRS/ccpA*

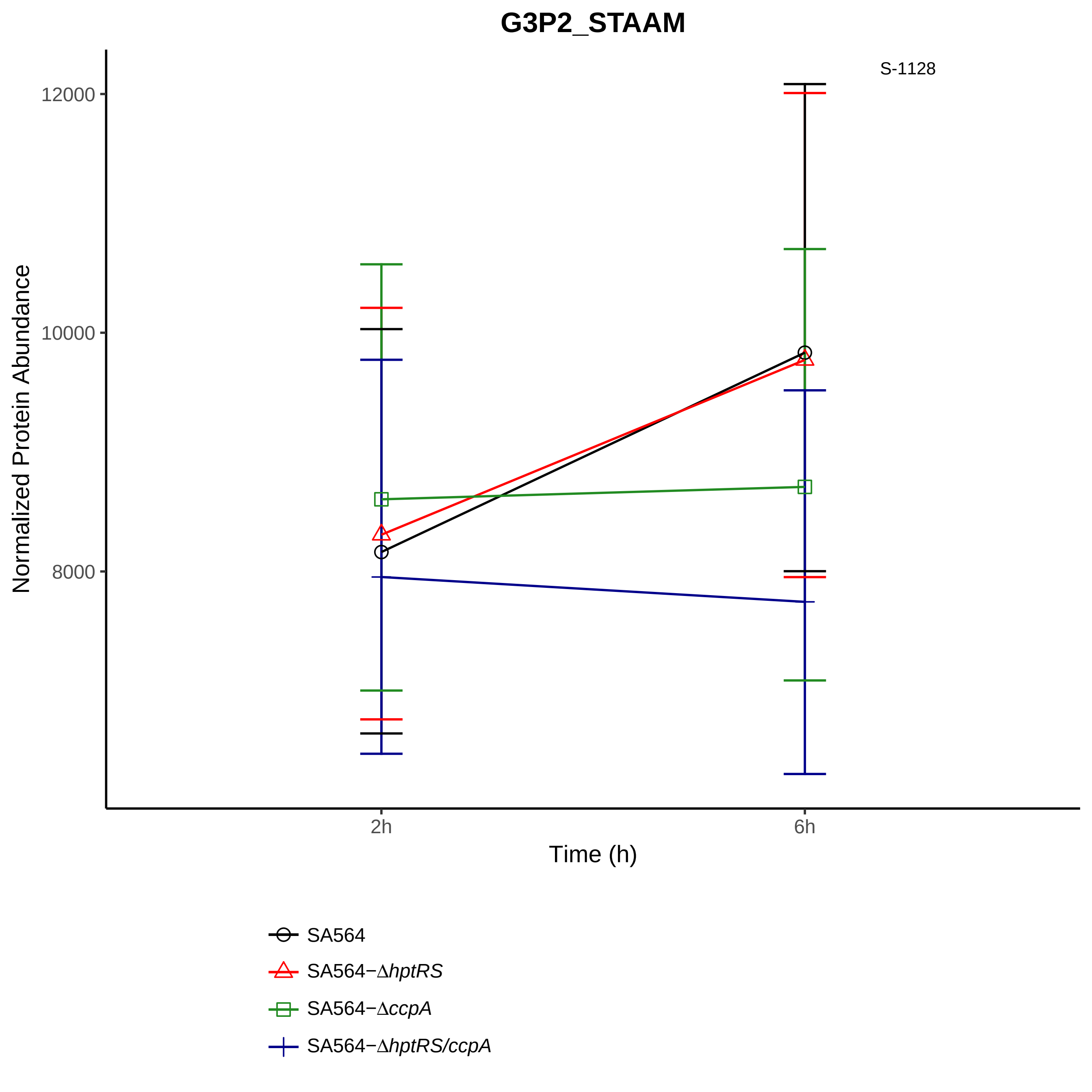
G3P2_STAAM
S-1128
12000
10000
8000
Normalized Protein Abundance
2h
6h
Time (h)
SA564
SA564−Δ*hptRS*
SA564−Δ*ccpA*
SA564−Δ*hptRS/ccpA*

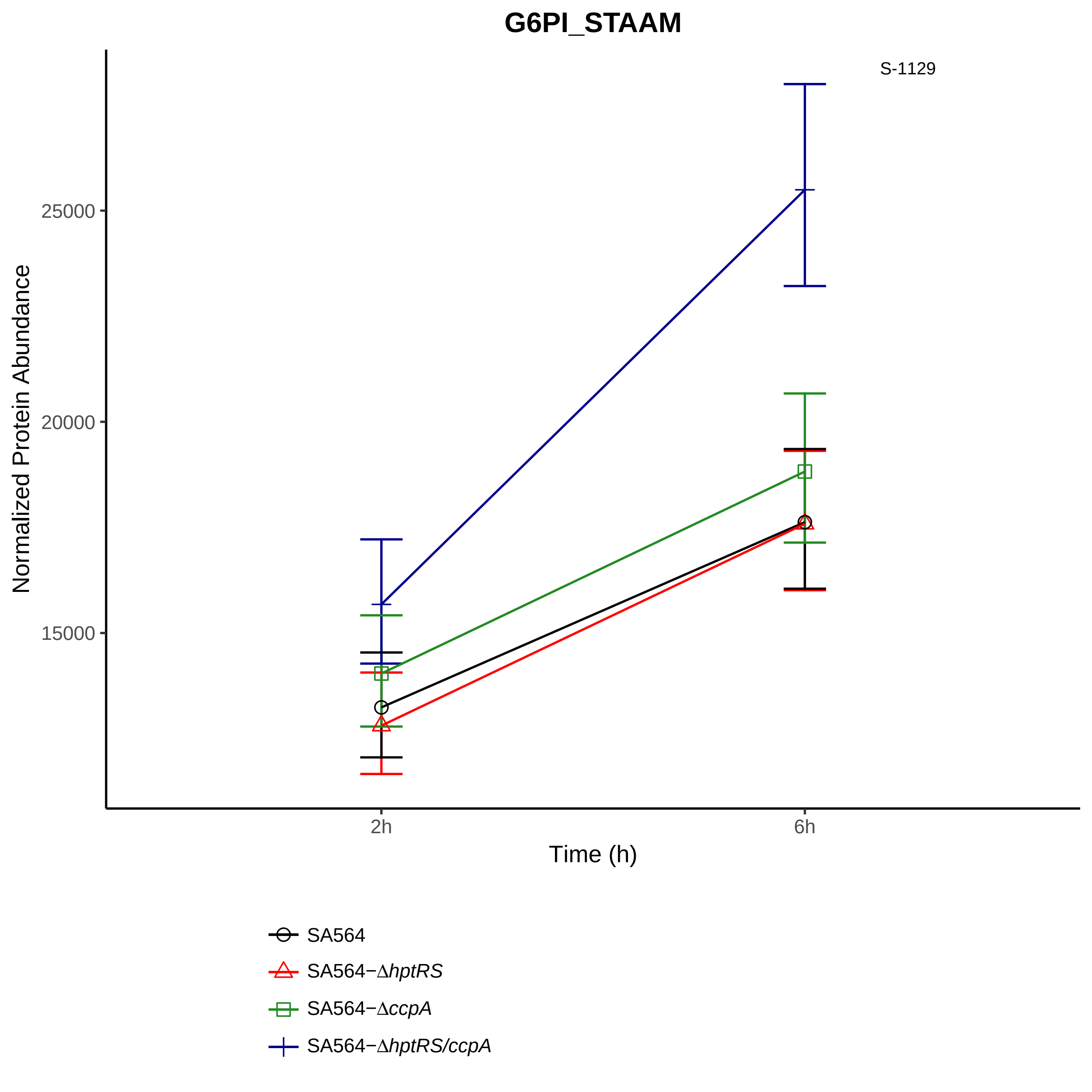
G6PI_STAAM
S-1129
25000
20000
15000
Normalized Protein Abundance
2h
6h
Time (h)
SA564
SA564−ΔhptRS
SA564−ΔccpA
SA564−ΔhptRS/ccpA

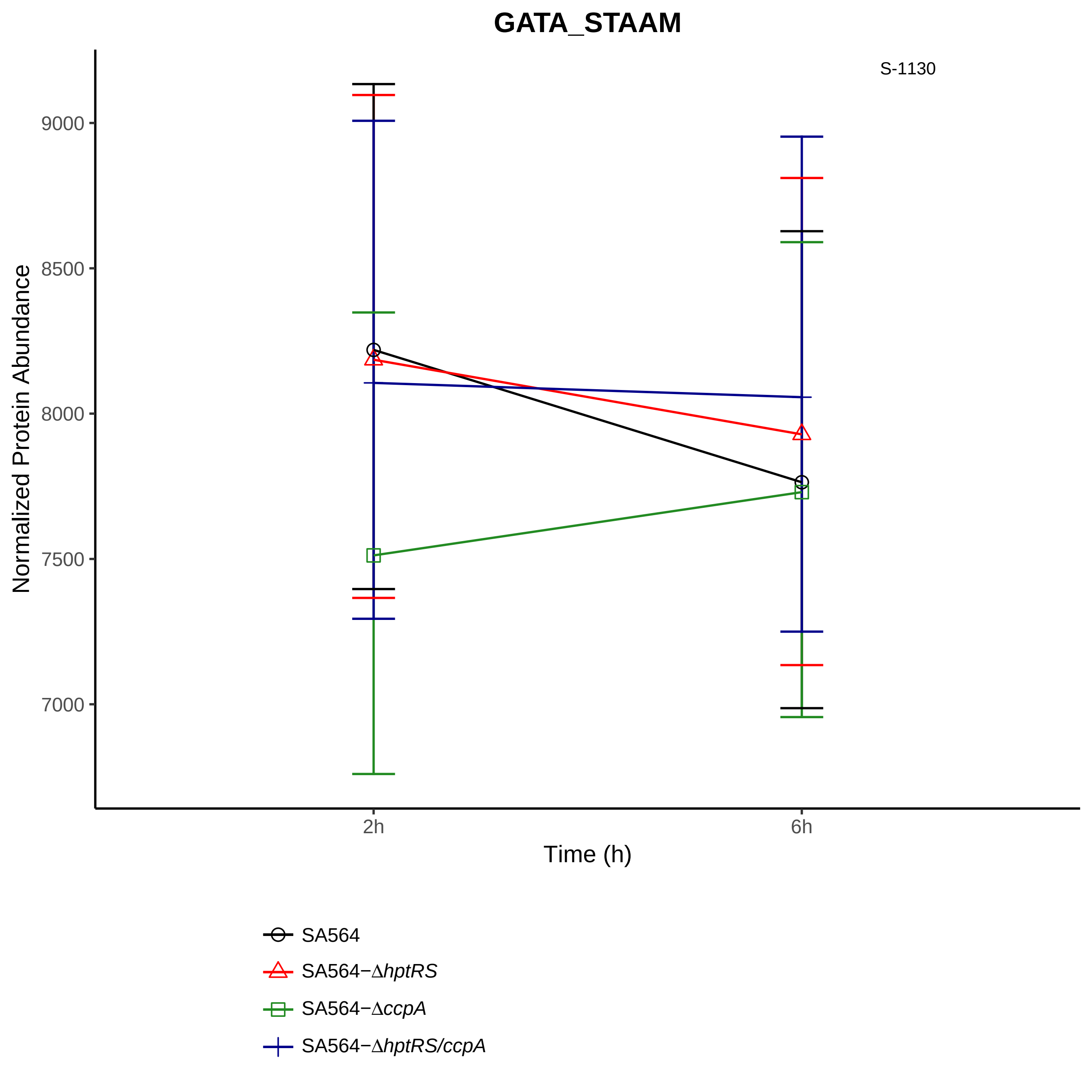

GATA_STAAM
S-1130
Normalized Protein Abundance
9000
8500
8000
7500
7000
2h
6h
Time (h)
SA564
SA564−Δ*hptRS*
SA564−Δ*ccpA*
SA564−Δ*hptRS/ccpA*

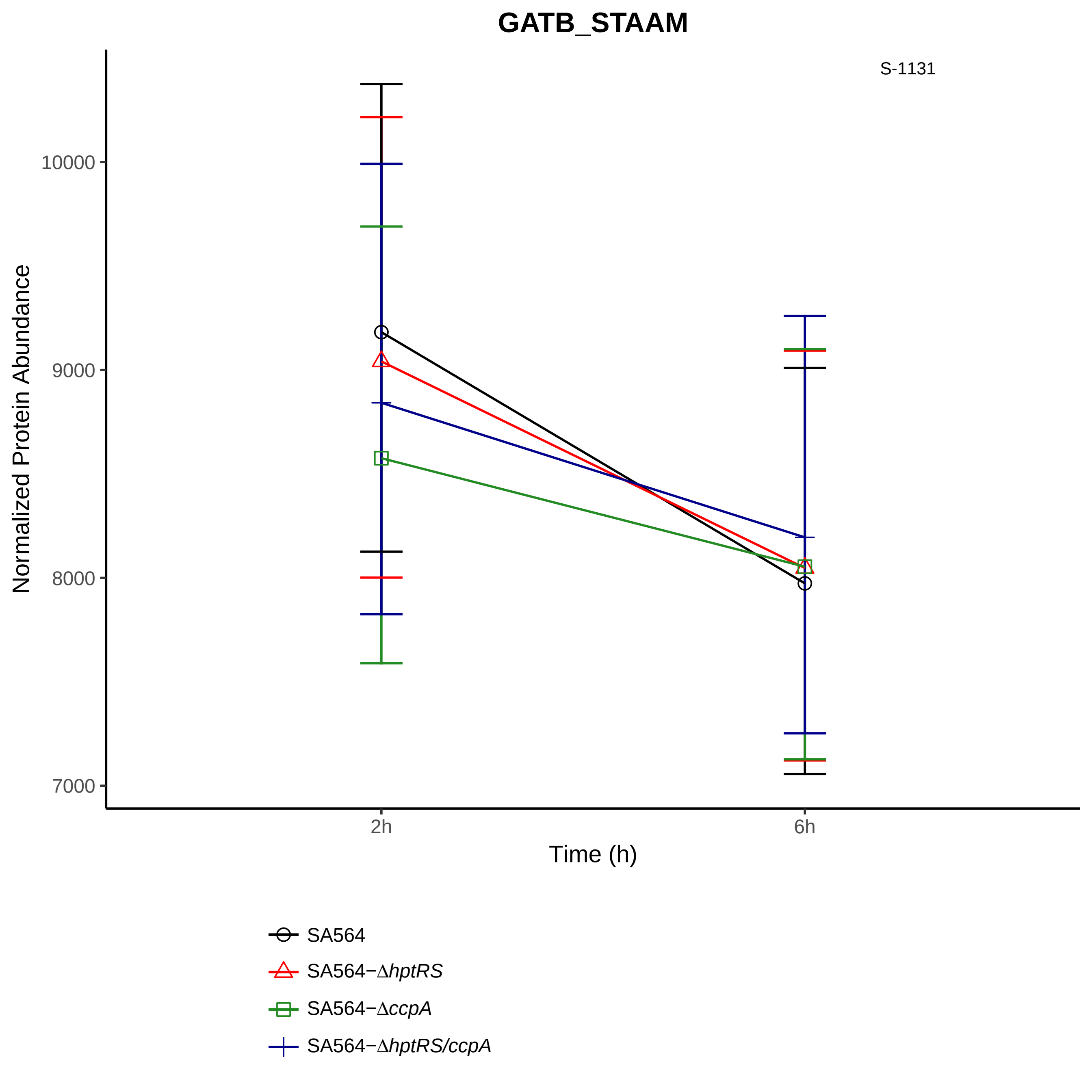
GATB_STAAM
S-1131
Normalized Protein Abundance
10000
9000
8000
7000
2h
6h
Time (h)
SA564
SA564−ΔhptRS
SA564−ΔccpA
SA564−ΔhptRS/ccpA

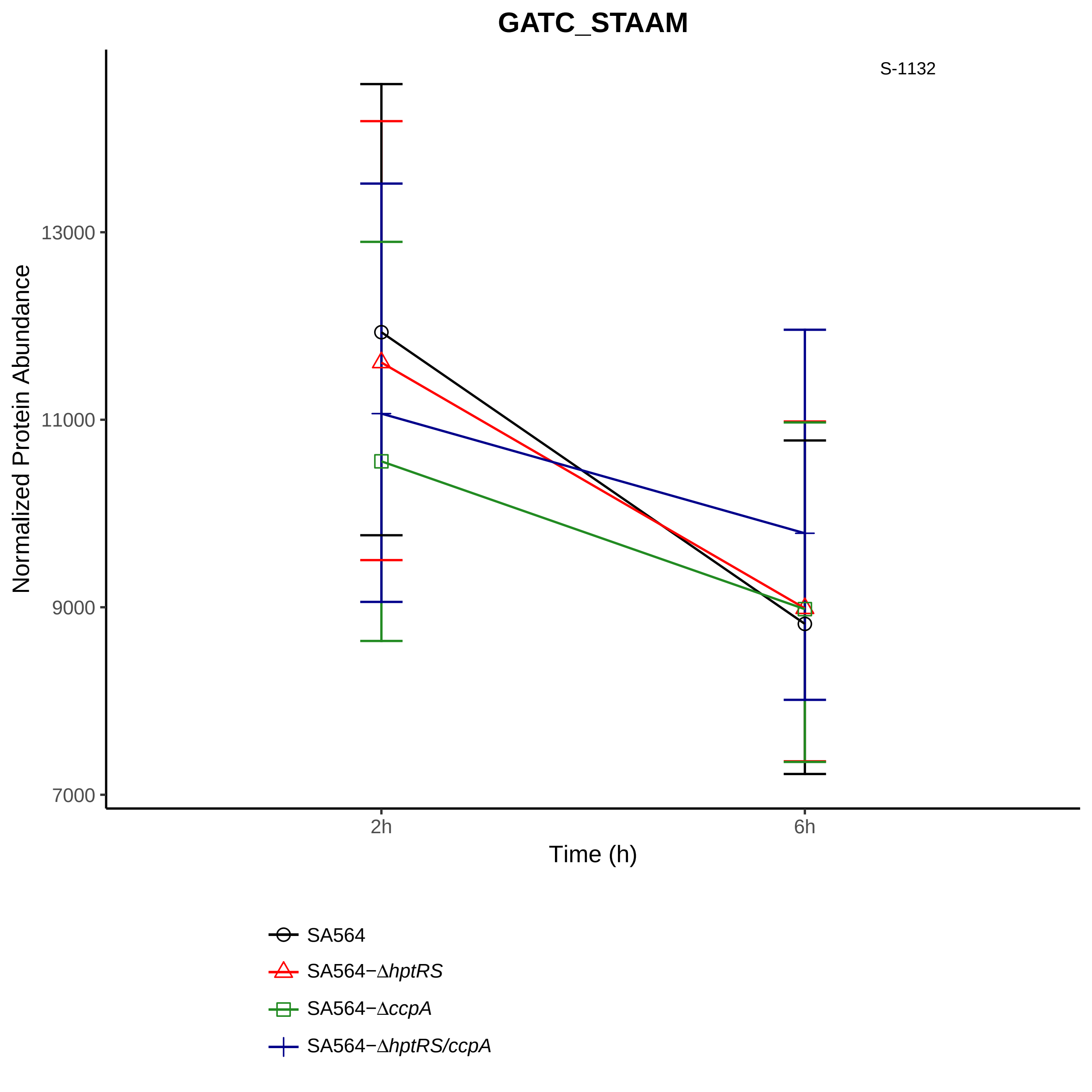

GATC_STAAM
S-1132
Normalized Protein Abundance
13000
11000
9000
7000
2h
6h
Time (h)
SA564
SA564−Δ*hptRS*
SA564−Δ*ccpA*
SA564−Δ*hptRS/ccpA*

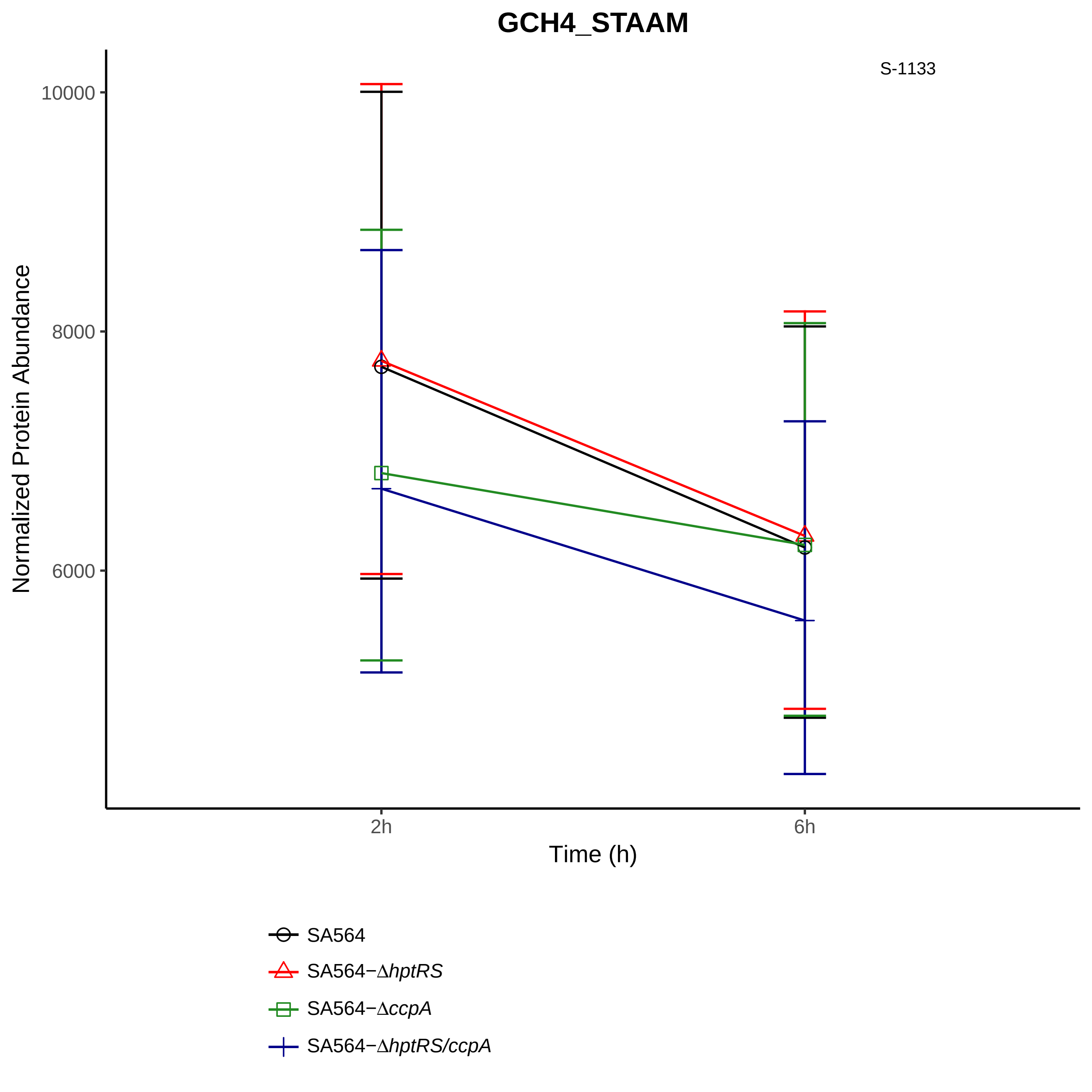

GCH4_STAAM
S-1133
Normalized Protein Abundance
10000
8000
6000
2h
6h
Time (h)
SA564
SA564−Δ*hptRS*
SA564−Δ*ccpA*
SA564−Δ*hptRS/ccpA*

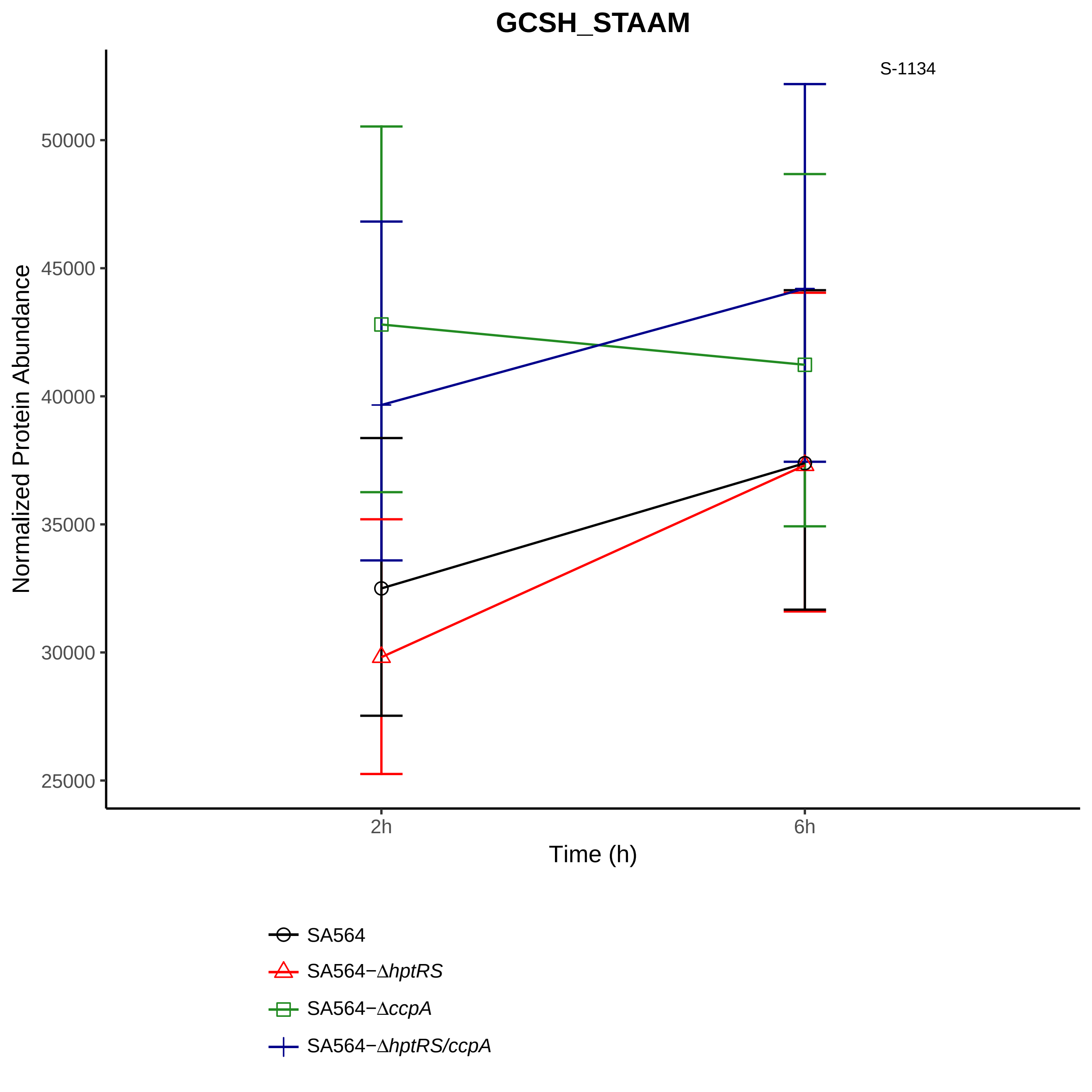
GCSH_STAAM
S-1134
Normalized Protein Abundance
50000
45000
40000
35000
30000
25000
2h
6h
Time (h)
SA564
SA564−ΔhptRS
SA564−ΔccpA
SA564−ΔhptRS/ccpA

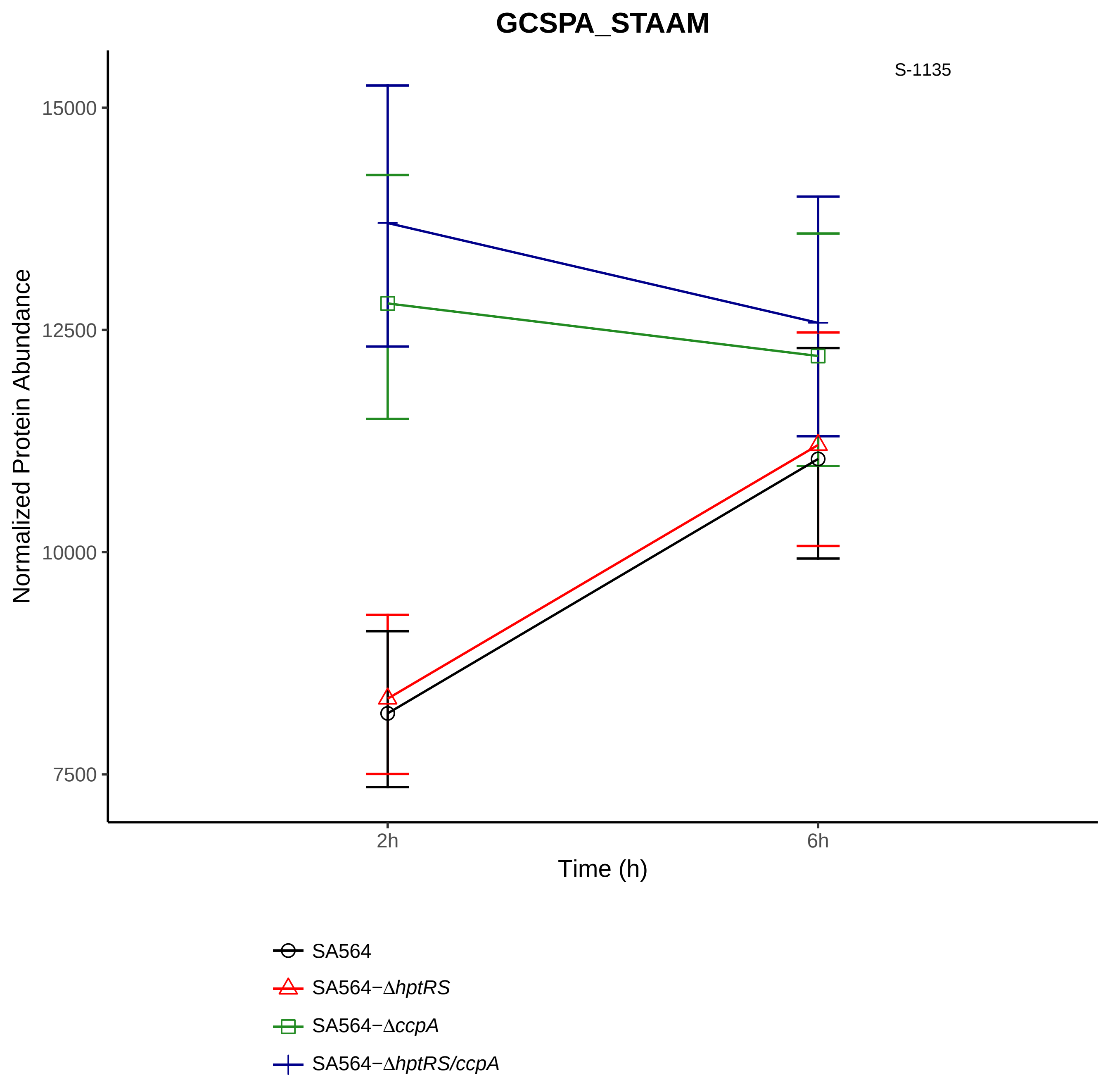
GCSPA_STAAM
S-1135
Normalized Protein Abundance
15000
12500
10000
7500
2h
6h
Time (h)
SA564
SA564−Δ*hptRS*
SA564−Δ*ccpA*
SA564−Δ*hptRS/ccpA*

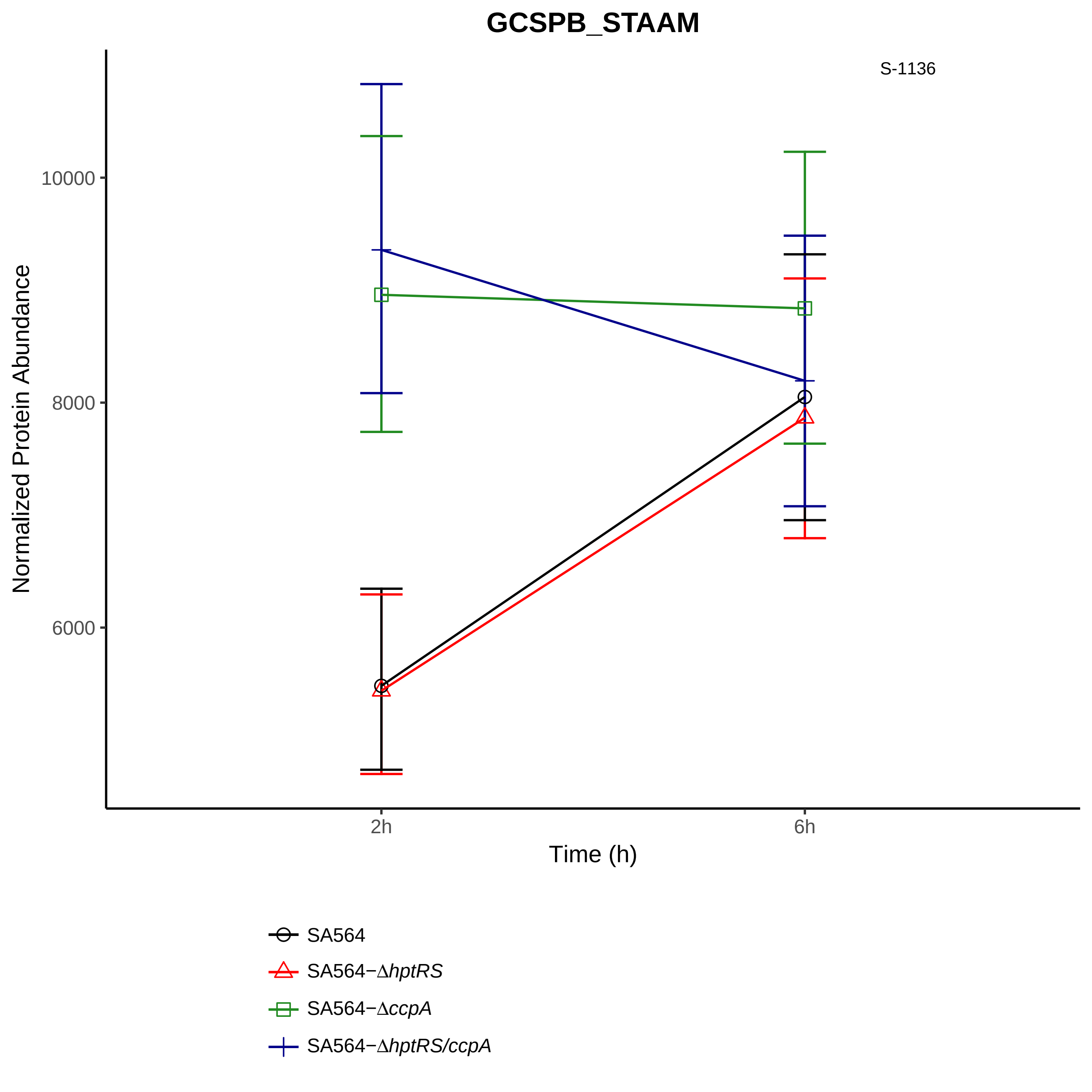
GCSPB_STAAM
S-1136
Normalized Protein Abundance
10000
8000
6000
2h
6h
Time (h)
SA564
SA564−ΔhptRS
SA564−ΔccpA
SA564−ΔhptRS/ccpA

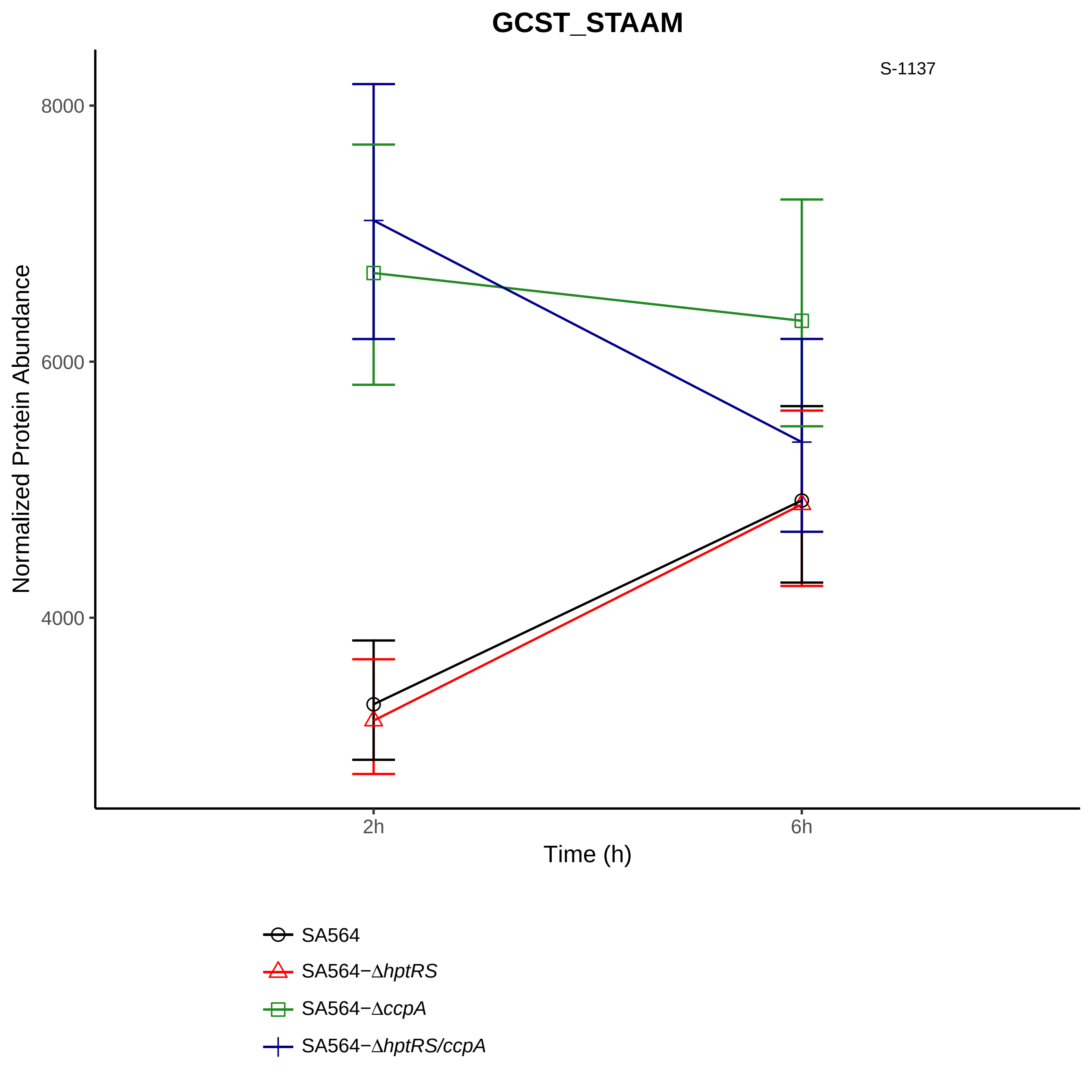

GCST_STAAM
S-1137
8000
6000
4000
Normalized Protein Abundance
2h
6h
Time (h)
SA564
SA564−Δ*hptRS*
SA564−Δ*ccpA*
SA564−Δ*hptRS/ccpA*

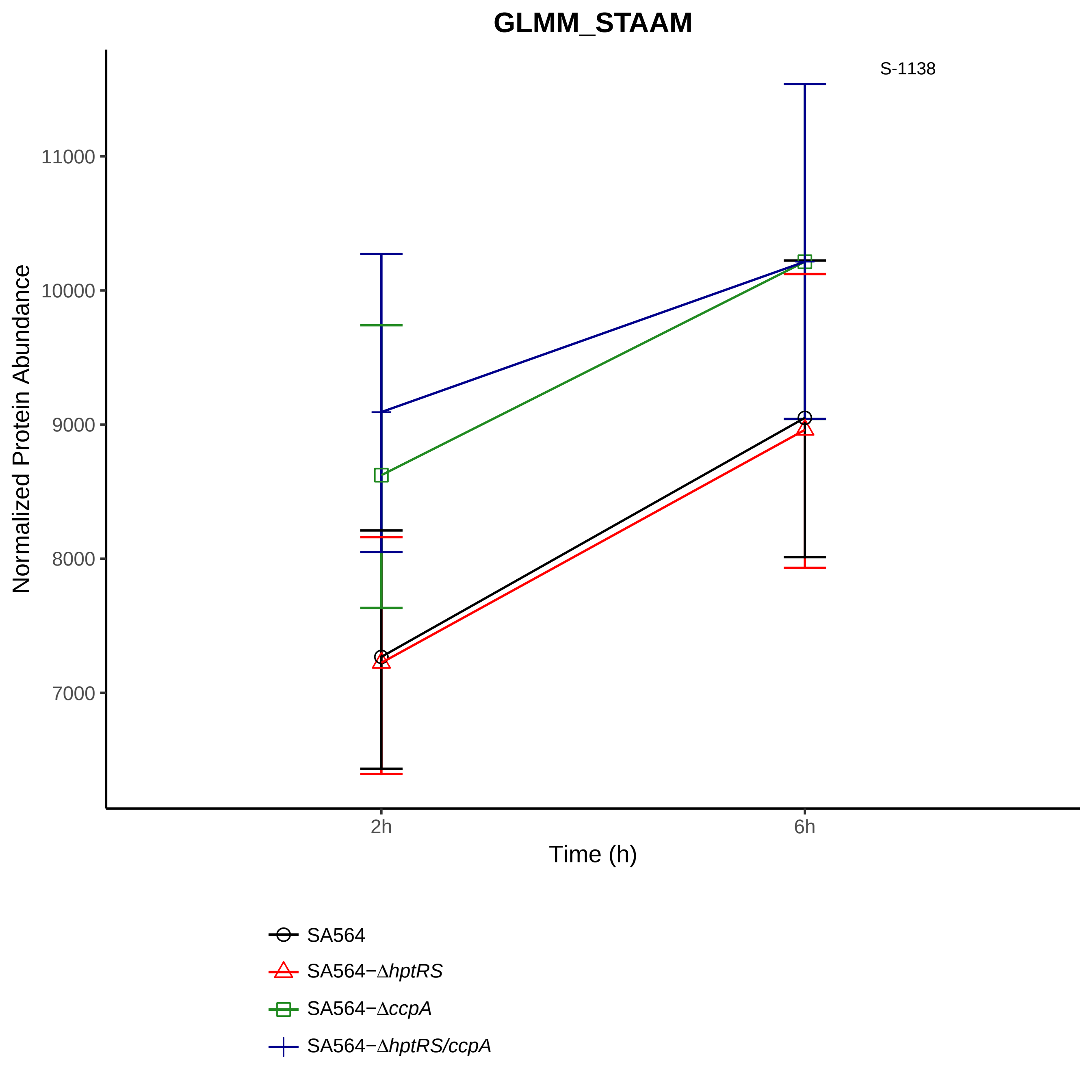

GLMM_STAAM
S-1138
Normalized Protein Abundance
11000
10000
9000
8000
7000
2h
6h
Time (h)
SA564
SA564−Δ*hptRS*
SA564−Δ*ccpA*
SA564−Δ*hptRS/ccpA*

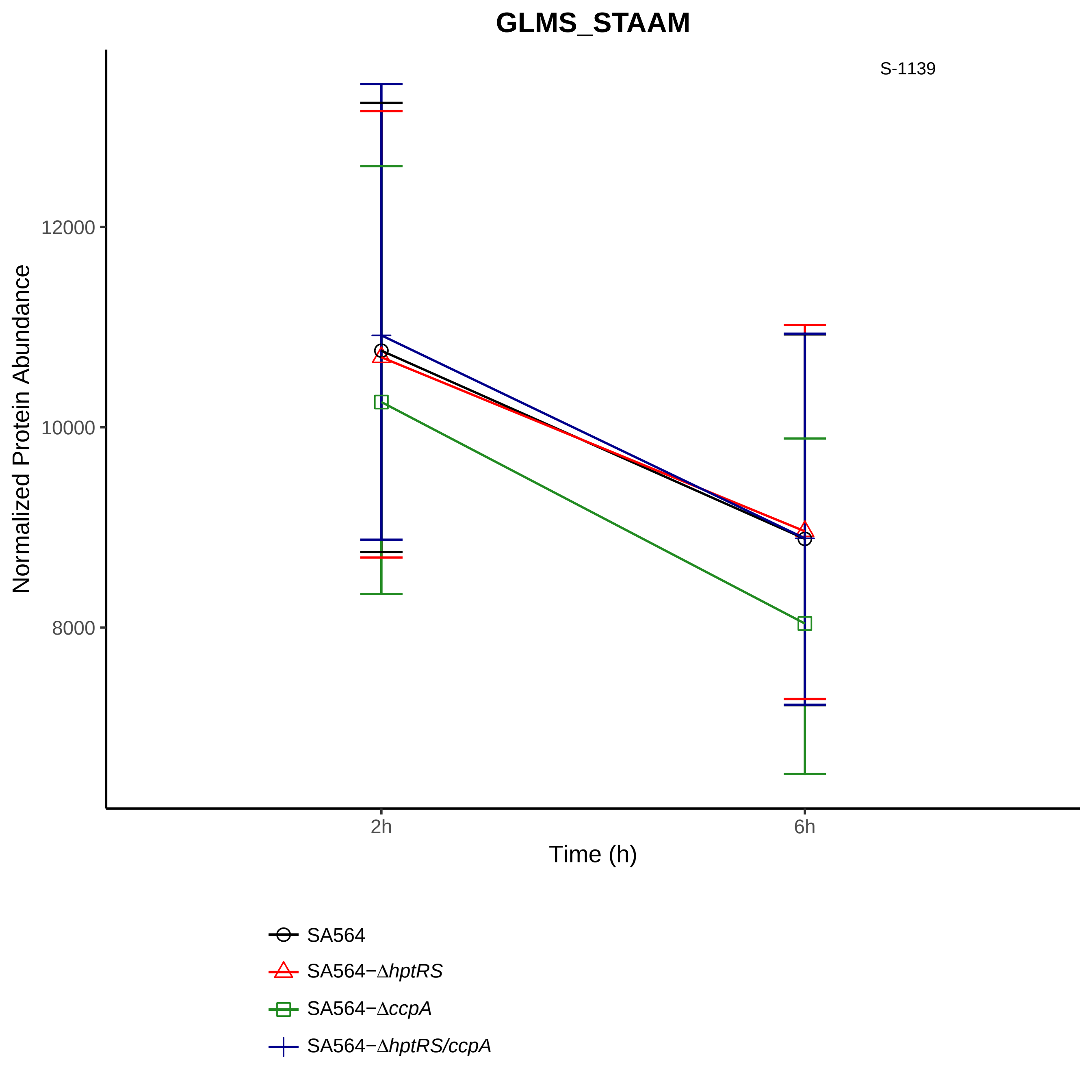

GLMS_STAAM
S-1139
Normalized Protein Abundance
12000
10000
8000
2h
6h
Time (h)
SA564
SA564−Δ*hptRS*
SA564−Δ*ccpA*
SA564−Δ*hptRS/ccpA*

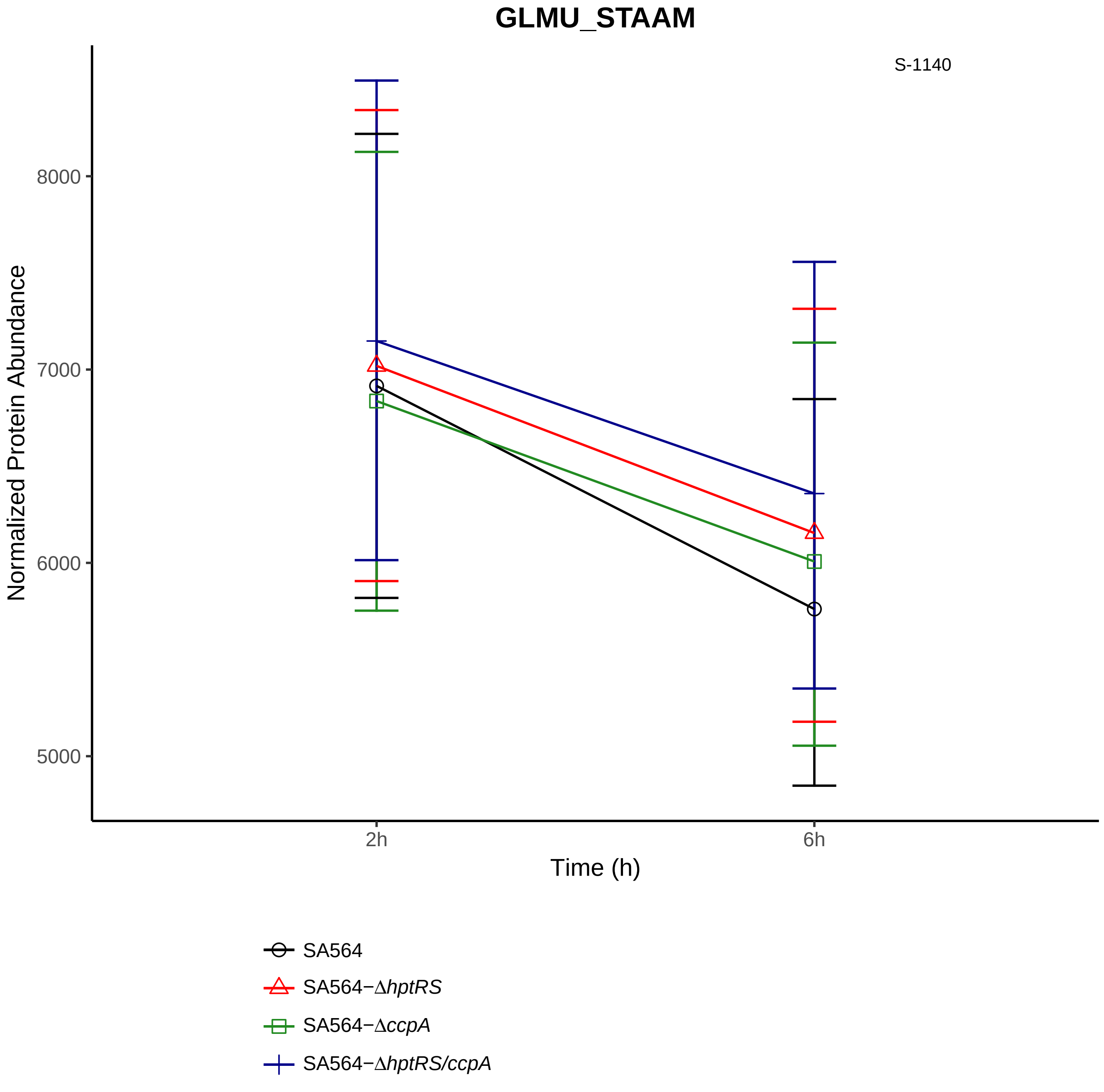
GLMU_STAAM
S-1140
Normalized Protein Abundance
8000
7000
6000
5000
2h
6h
Time (h)
SA564
SA564−Δ*hptRS*
SA564−Δ*ccpA*
SA564−Δ*hptRS/ccpA*

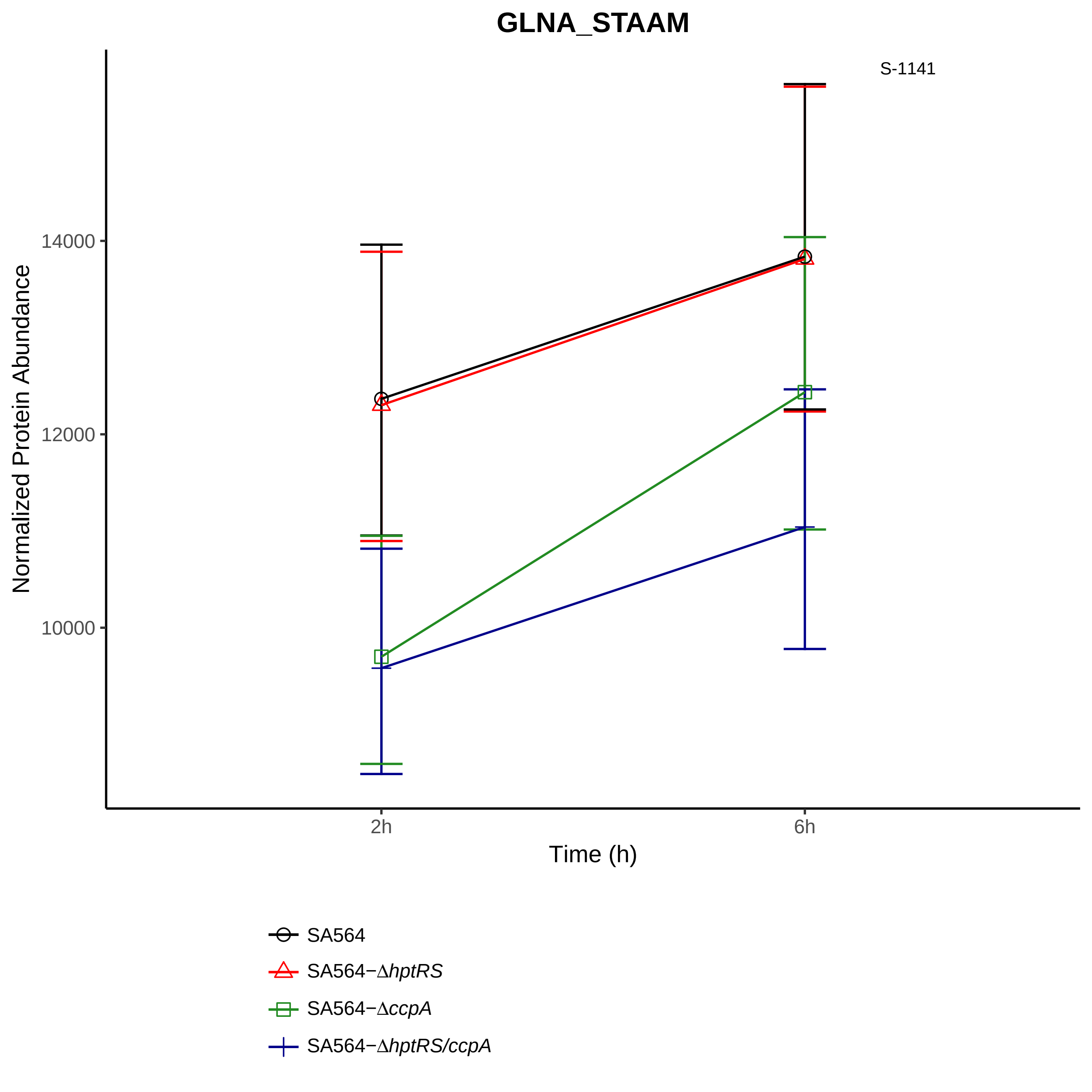

GLNA_STAAM
S-1141
Normalized Protein Abundance
14000
12000
10000
2h
6h
Time (h)
SA564
SA564−Δ*hptRS*
SA564−Δ*ccpA*
SA564−Δ*hptRS/ccpA*

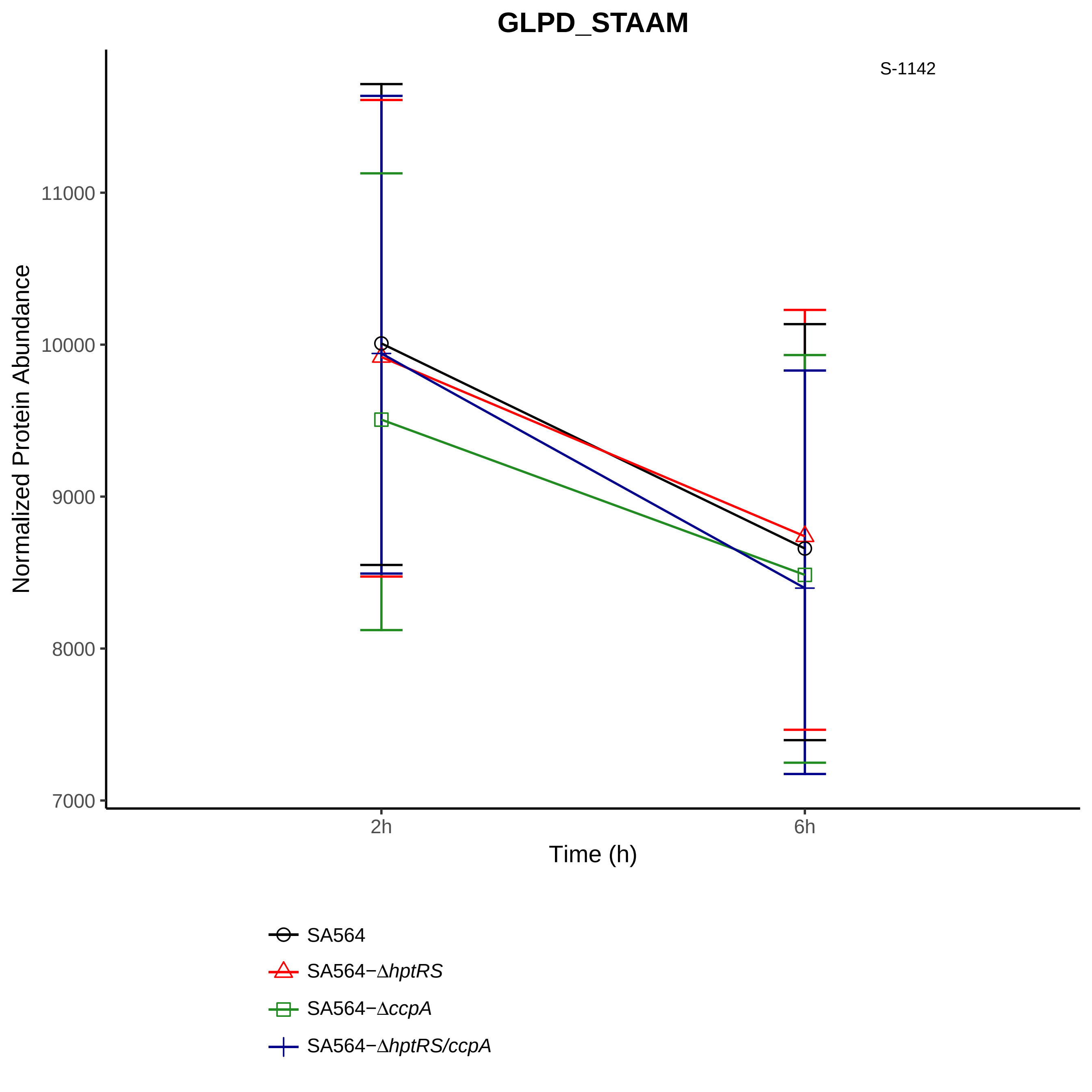

GLPD_STAAM
S-1142
Normalized Protein Abundance
11000
10000
9000
8000
7000
2h
6h
Time (h)
SA564
SA564−Δ*hptRS*
SA564−Δ*ccpA*
SA564−Δ*hptRS/ccpA*

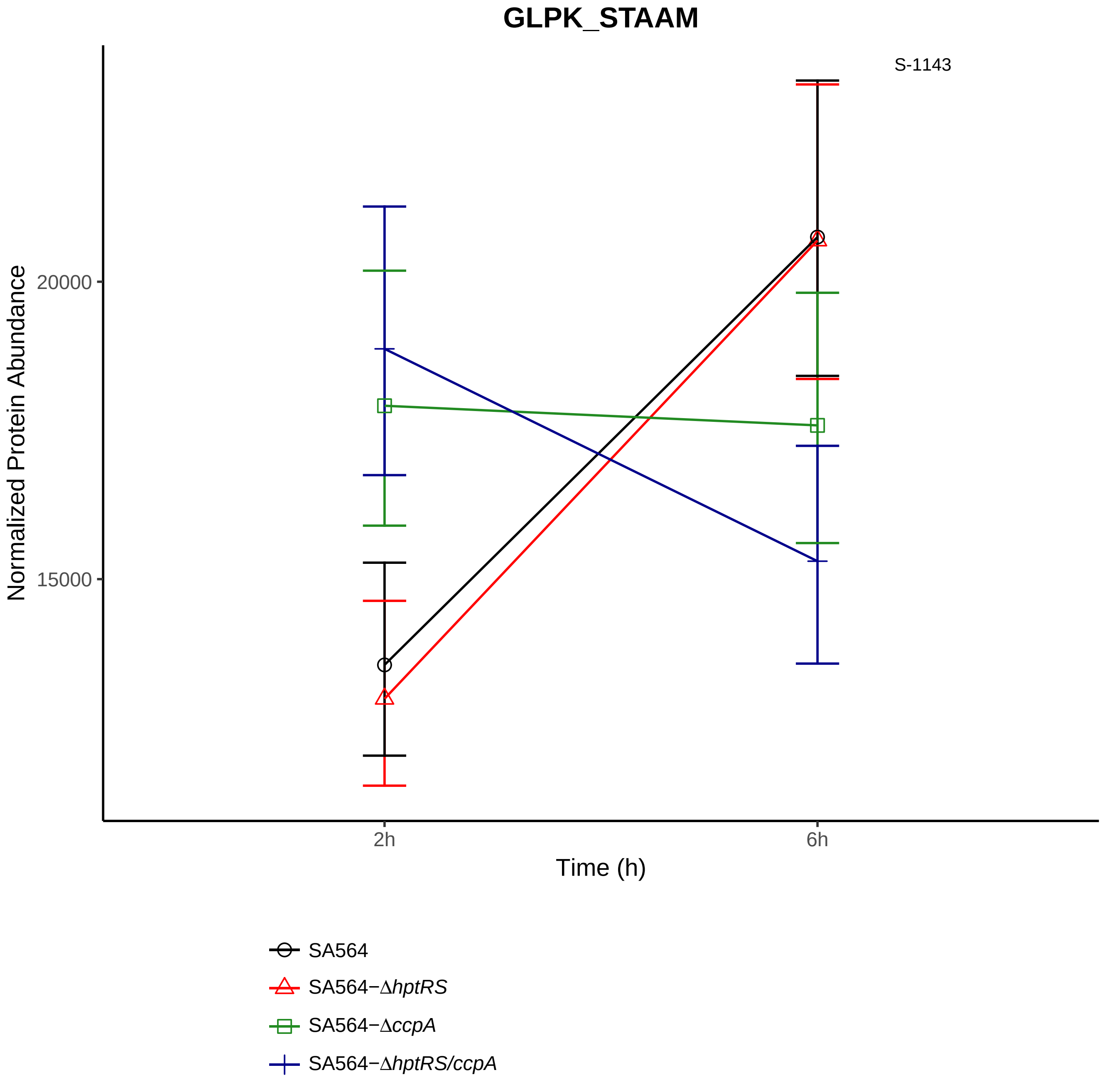
GLPK_STAAM
S-1143
Normalized Protein Abundance
20000
15000
2h
6h
Time (h)
SA564
SA564−Δ*hptRS*
SA564−Δ*ccpA*
SA564−Δ*hptRS/ccpA*

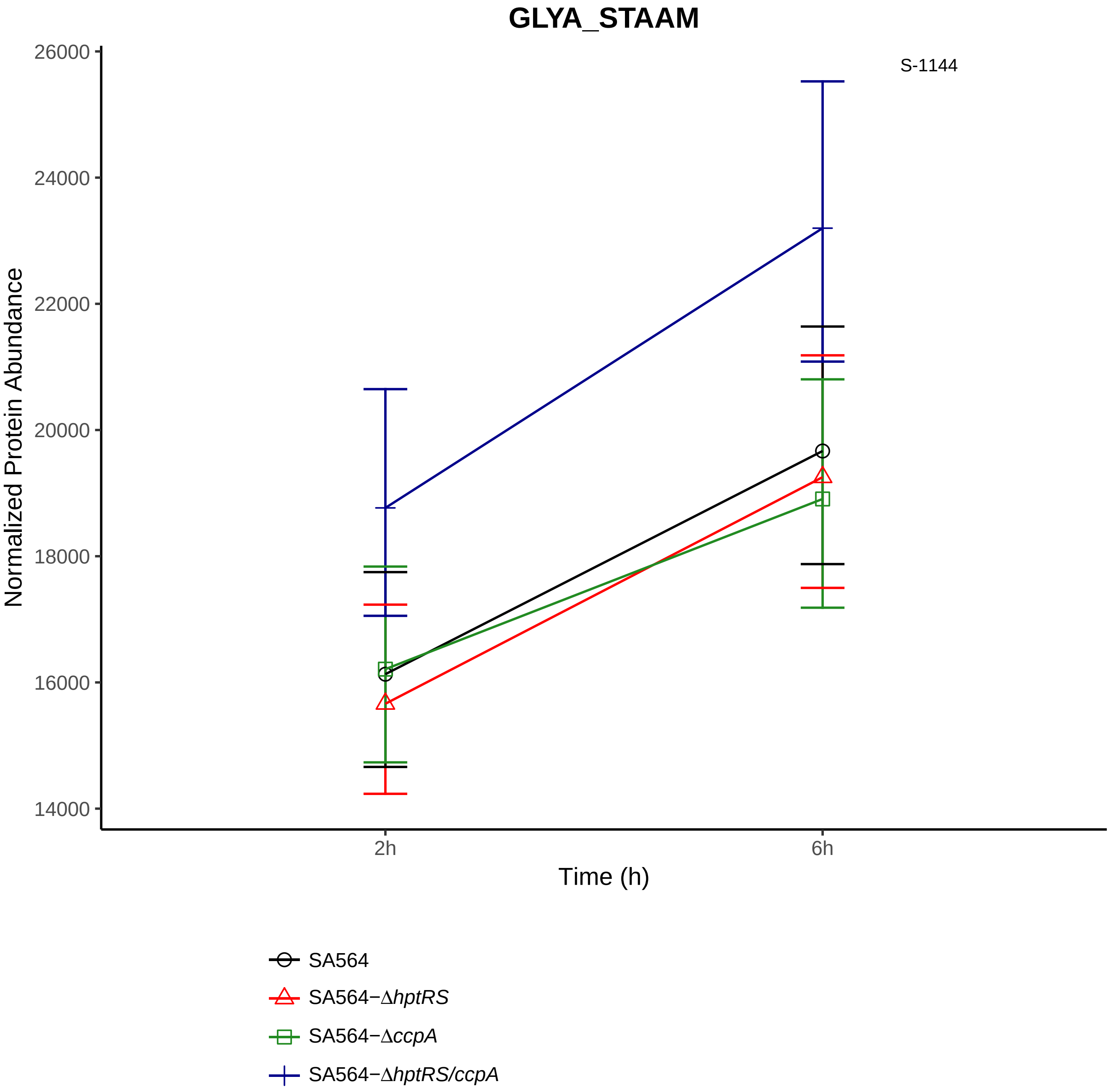
GLYA_STAAM
S-1144
Normalized Protein Abundance
26000
24000
22000
20000
18000
16000
14000
2h
6h
Time (h)
SA564
SA564−Δ*hptRS*
SA564−Δ*ccpA*
SA564−Δ*hptRS/ccpA*

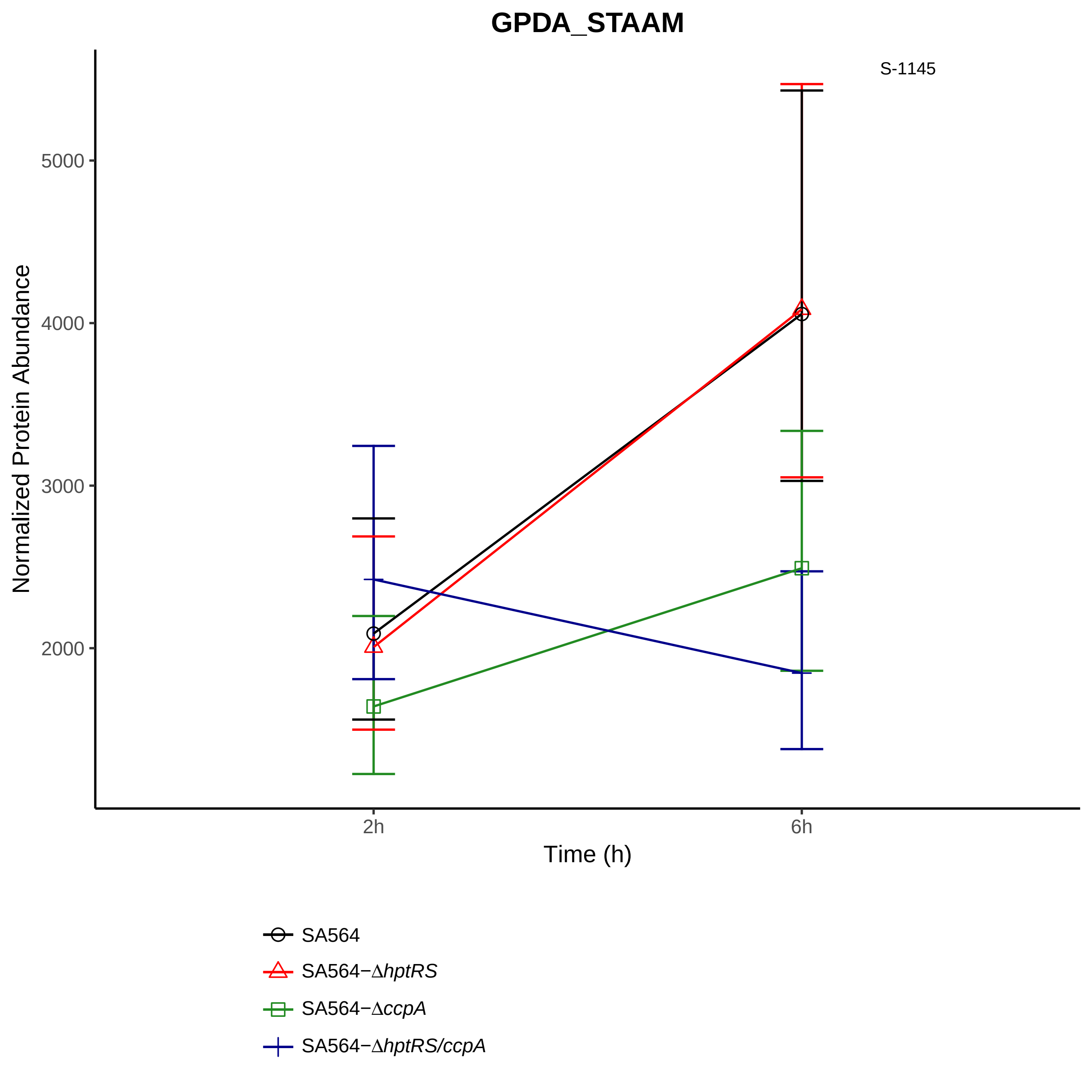

GPDA_STAAM
S-1145
Normalized Protein Abundance
5000
4000
3000
2000
2h
6h
Time (h)
SA564
SA564−Δ*hptRS*
SA564−Δ*ccpA*
SA564−Δ*hptRS/ccpA*

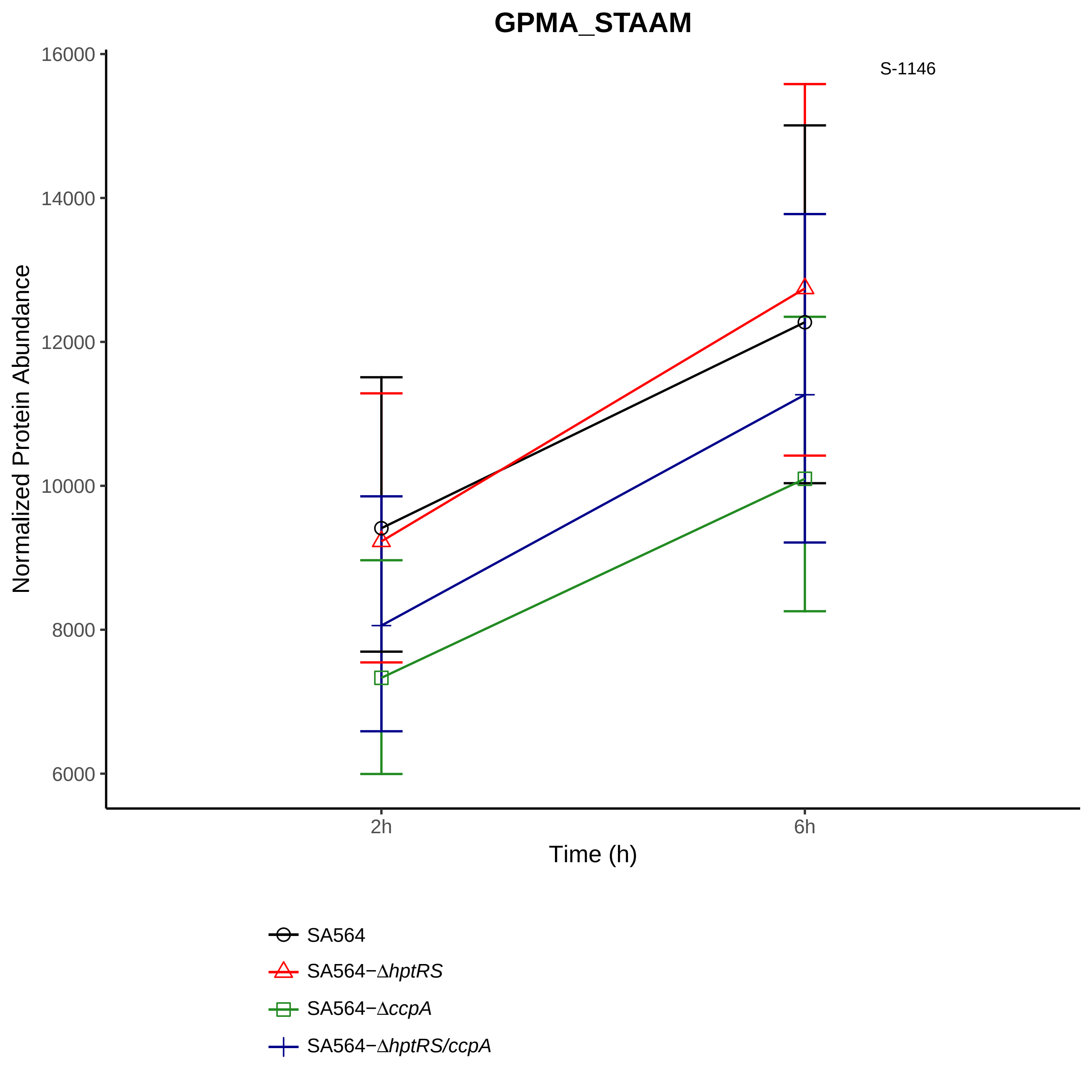
GPMA_STAAM
S-1146
Normalized Protein Abundance
16000
14000
12000
10000
8000
6000
2h
6h
Time (h)
SA564
SA564−ΔhptRS
SA564−ΔccpA
SA564−ΔhptRS/ccpA

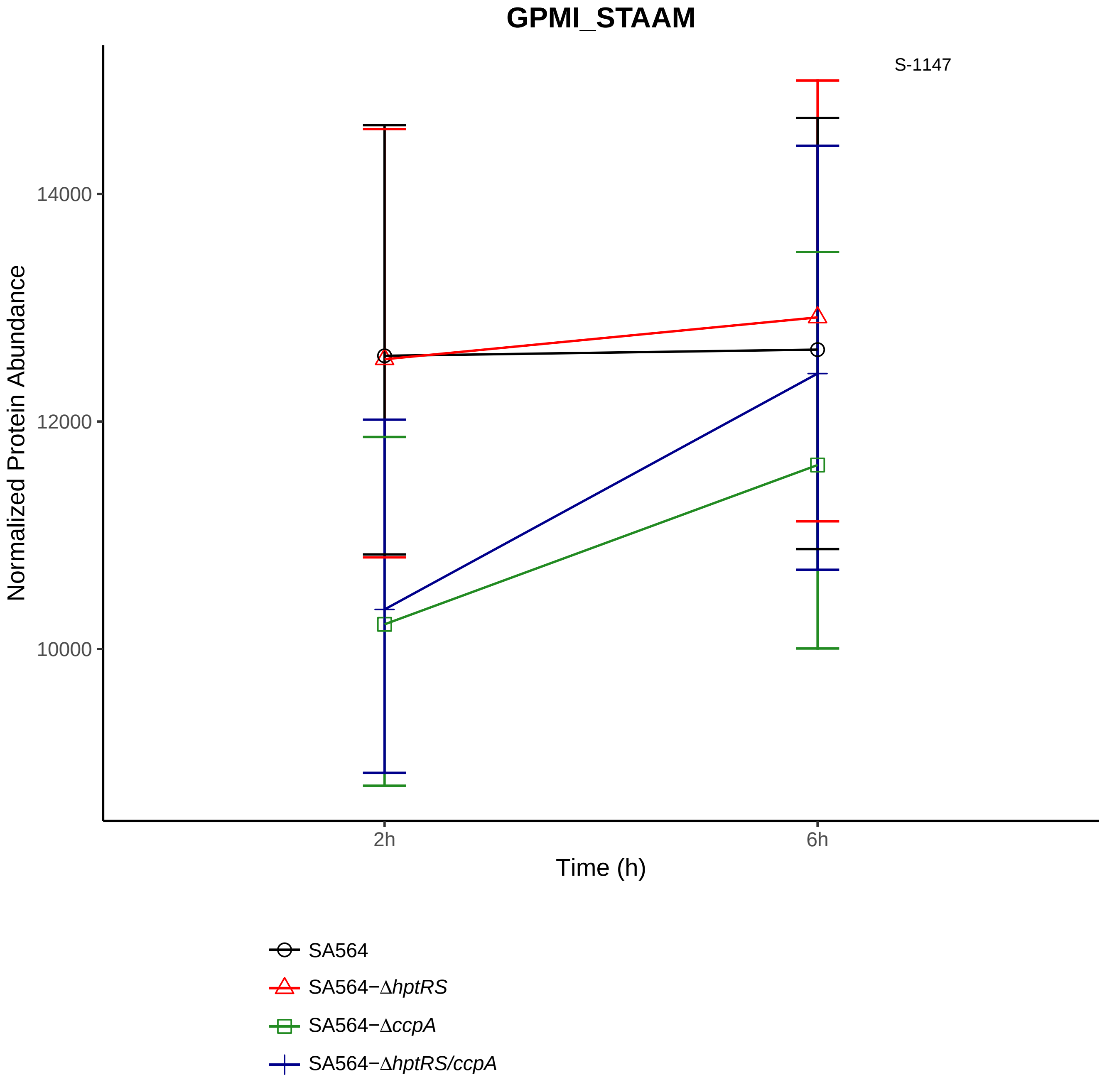
GPMI_STAAM
S-1147
Normalized Protein Abundance
14000
12000
10000
2h
6h
Time (h)
SA564
SA564−Δ*hptRS*
SA564−Δ*ccpA*
SA564−Δ*hptRS/ccpA*

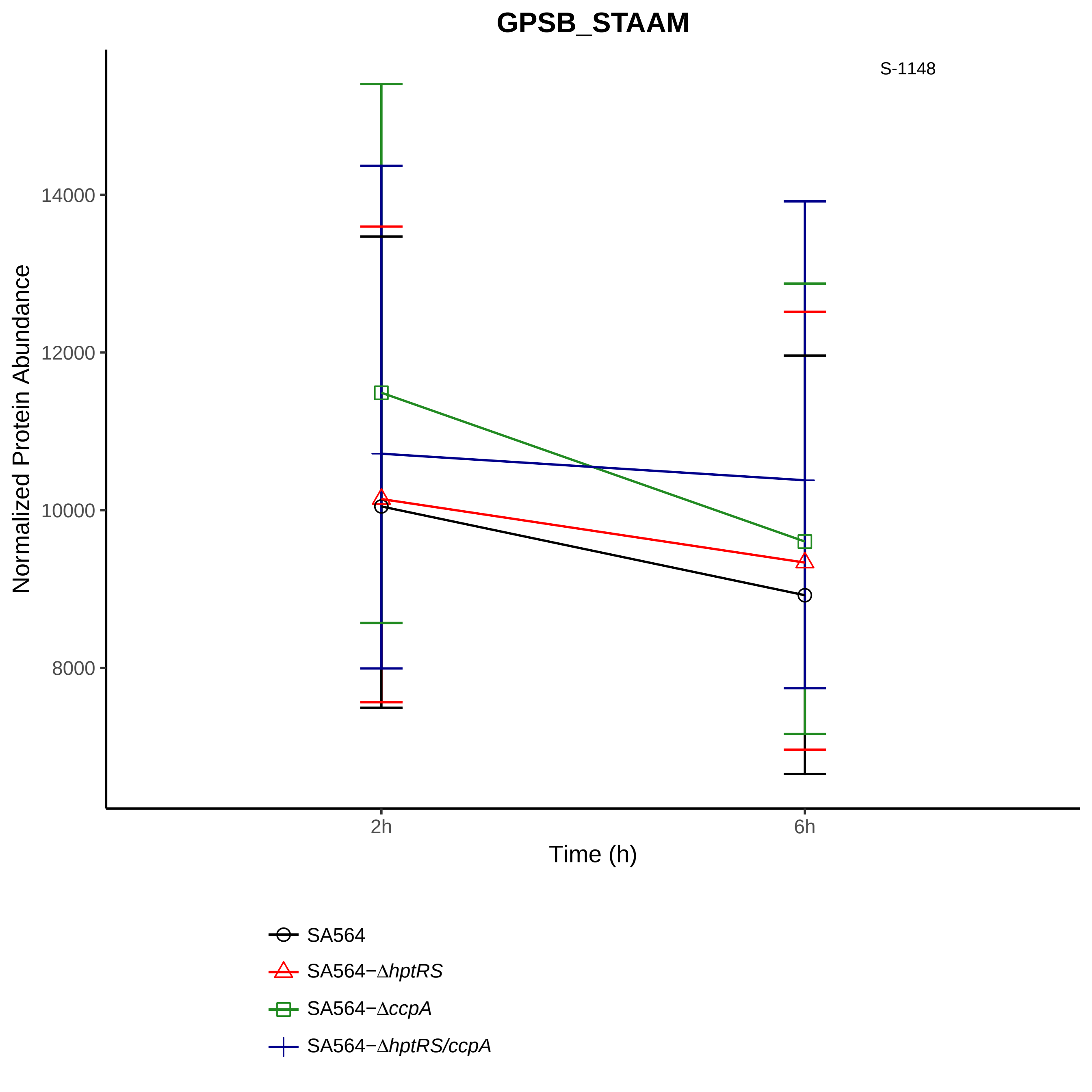

GPSB_STAAM
S-1148
Normalized Protein Abundance
14000
12000
10000
8000
2h
6h
Time (h)
SA564
SA564−Δ*hptRS*
SA564−Δ*ccpA*
SA564−Δ*hptRS/ccpA*

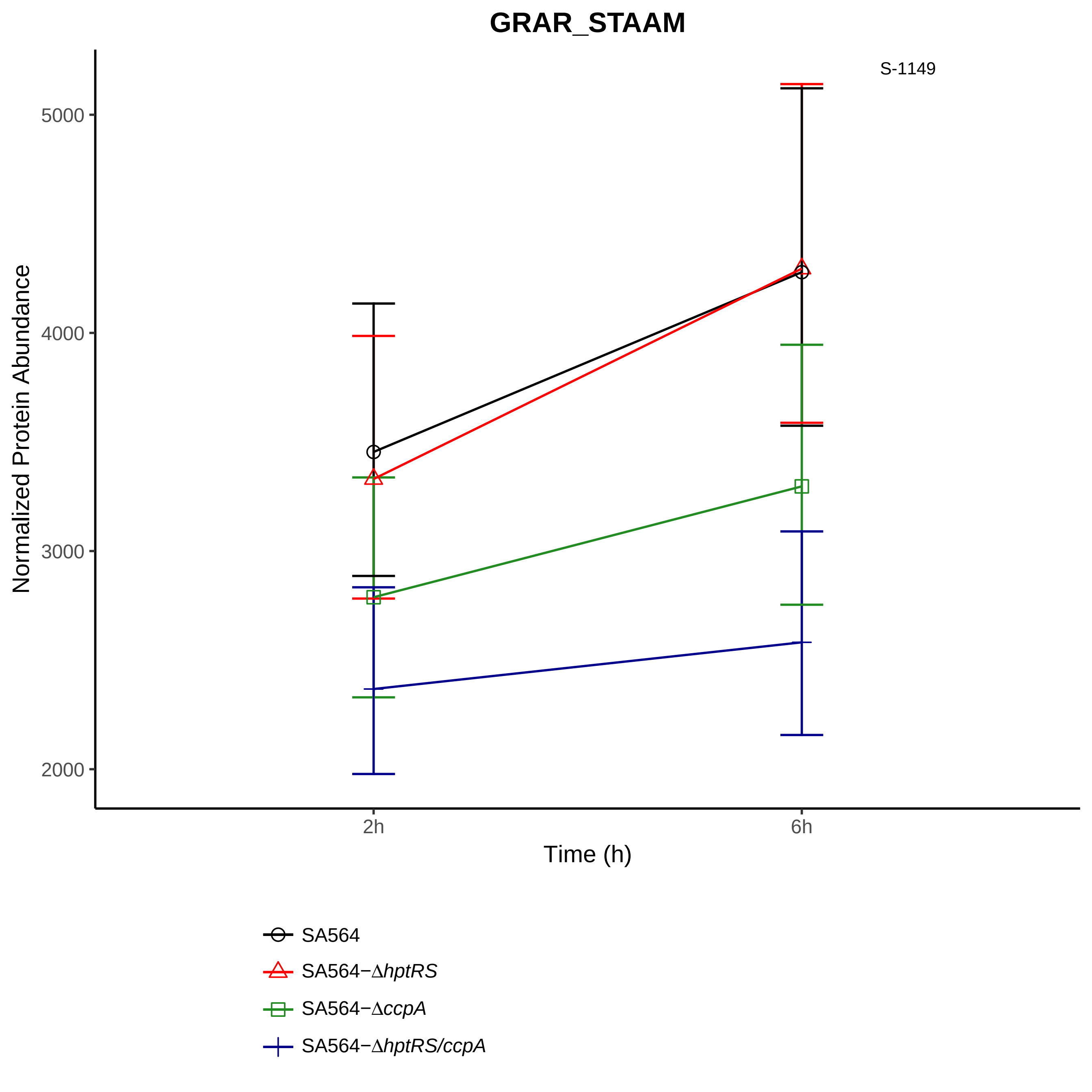
GRAR_STAAM
S-1149
Normalized Protein Abundance
5000
4000
3000
2000
2h
6h
Time (h)
SA564
SA564−Δ*hptRS*
SA564−Δ*ccpA*
SA564−Δ*hptRS/ccpA*

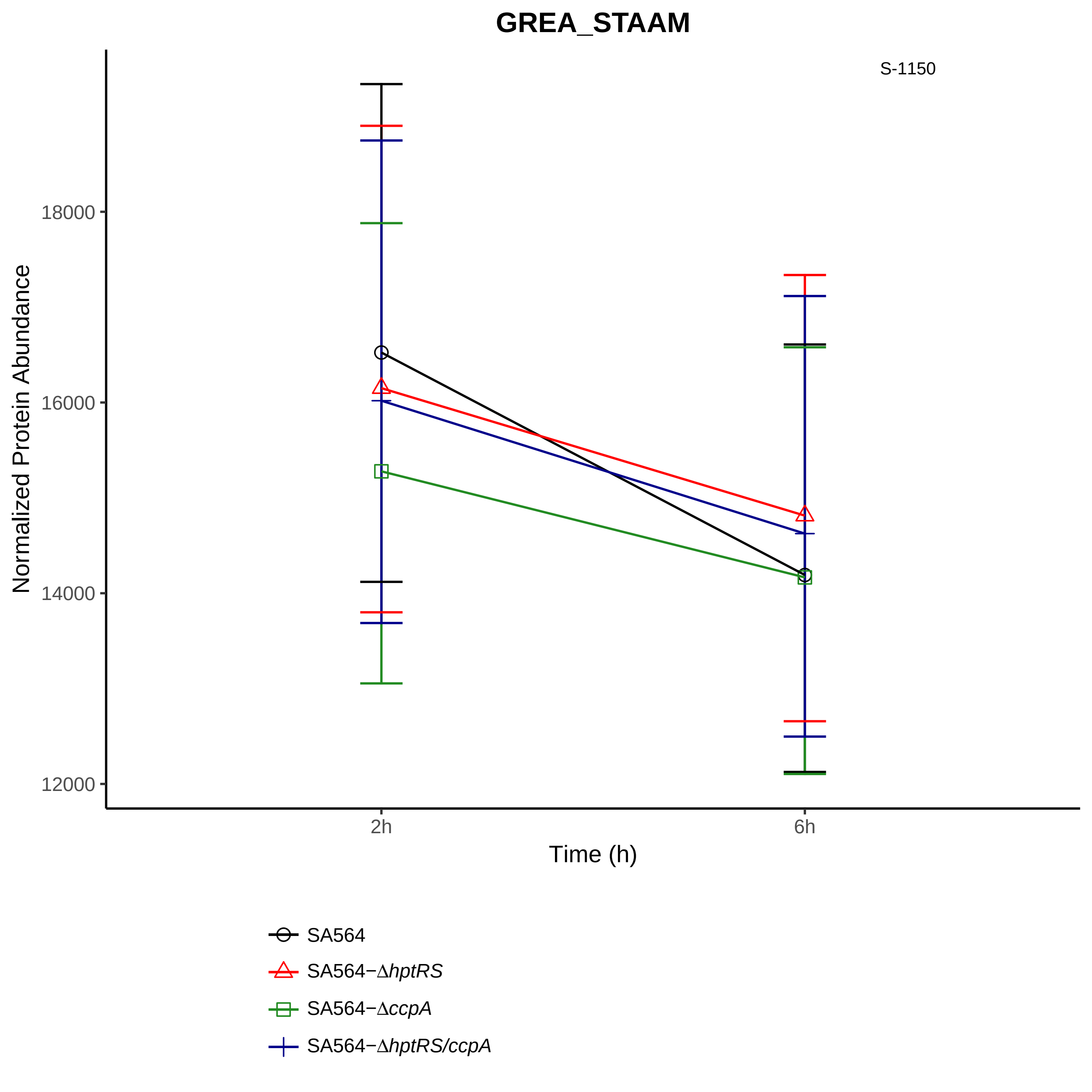

GREA_STAAM
S-1150
Normalized Protein Abundance
18000
16000
14000
12000
2h
6h
Time (h)
SA564
SA564−Δ*hptRS*
SA564−Δ*ccpA*
SA564−Δ*hptRS/ccpA*

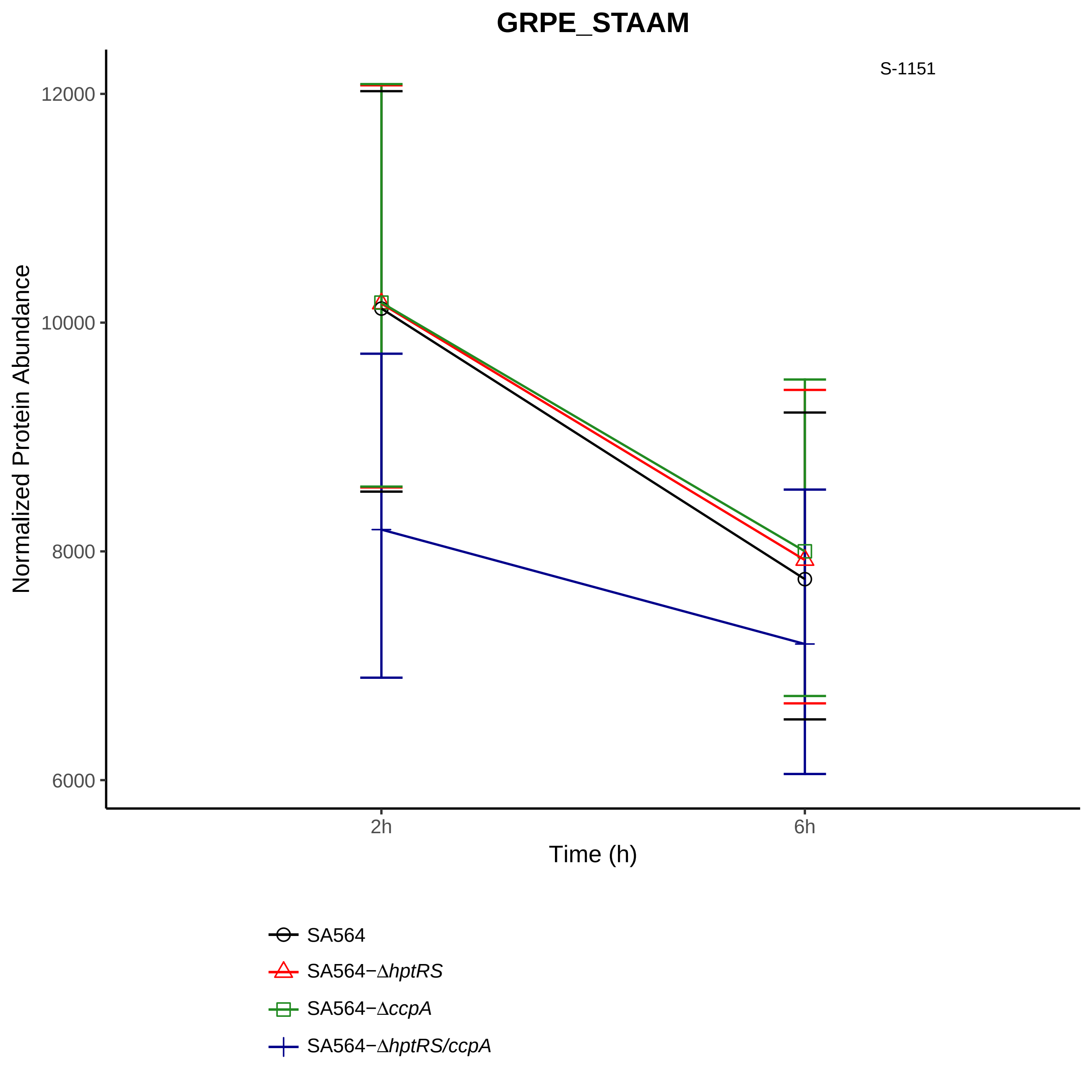
GRPE_STAAM
S-1151
Normalized Protein Abundance
12000
10000
8000
6000
2h
6h
Time (h)
SA564
SA564−ΔhptRS
SA564−ΔccpA
SA564−ΔhptRS/ccpA

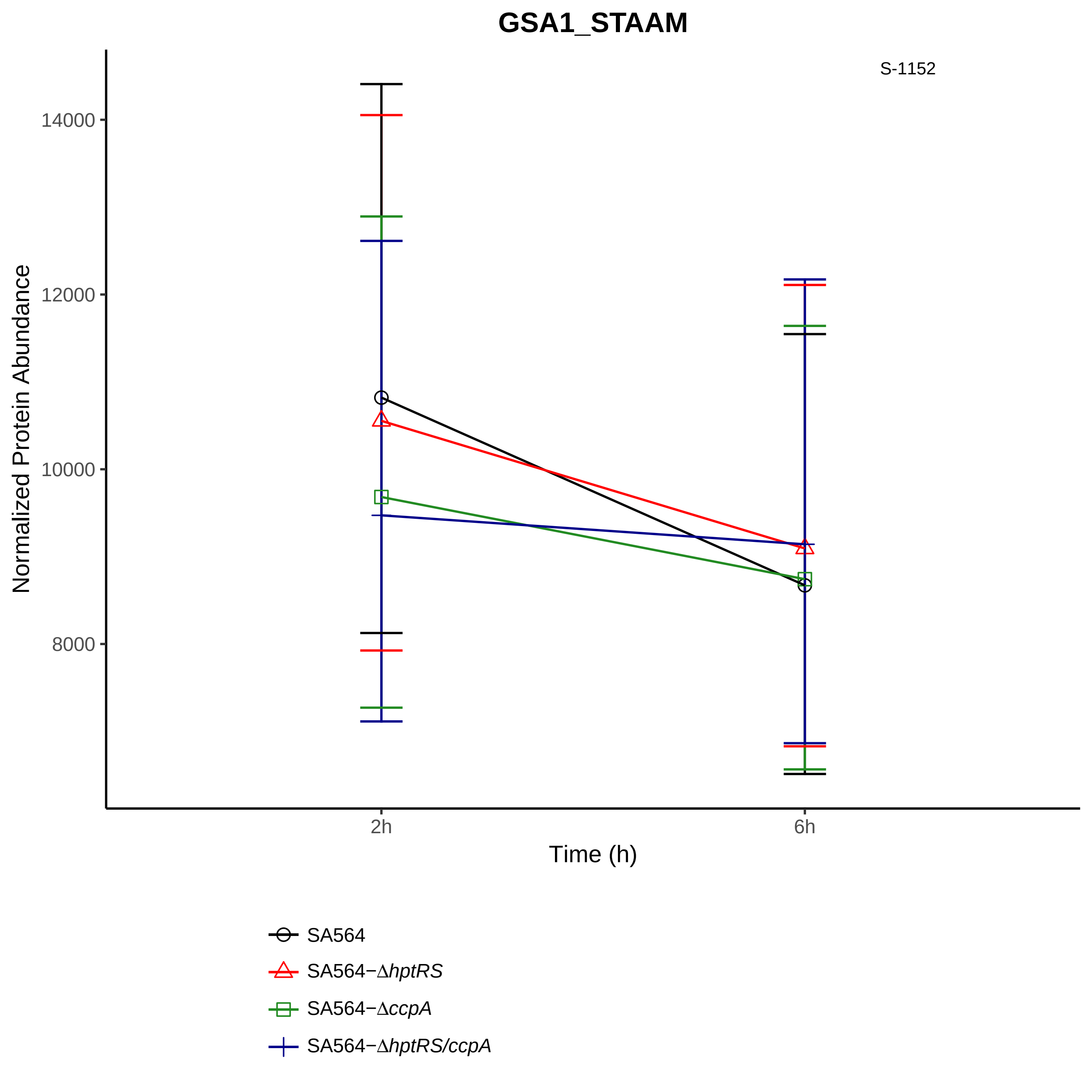

GSA1_STAAM
S-1152
14000
12000
10000
8000
Normalized Protein Abundance
2h
6h
Time (h)
SA564
SA564−ΔhptRS
SA564−ΔccpA
SA564−ΔhptRS/ccpA

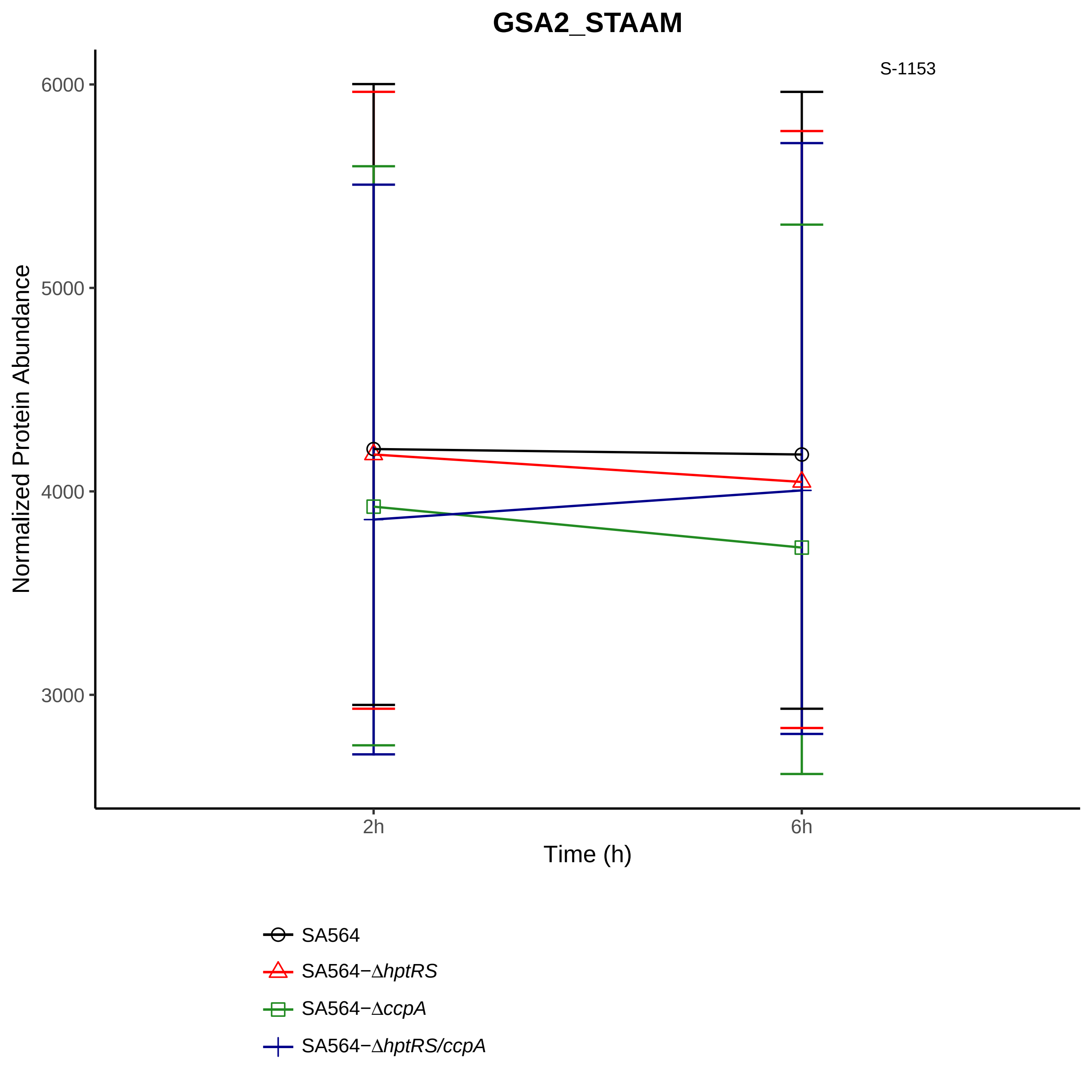

GSA2_STAAM
S-1153
Normalized Protein Abundance
6000
5000
4000
3000
2h
6h
Time (h)
SA564
SA564−Δ*hptRS*
SA564−Δ*ccpA*
SA564−Δ*hptRS/ccpA*

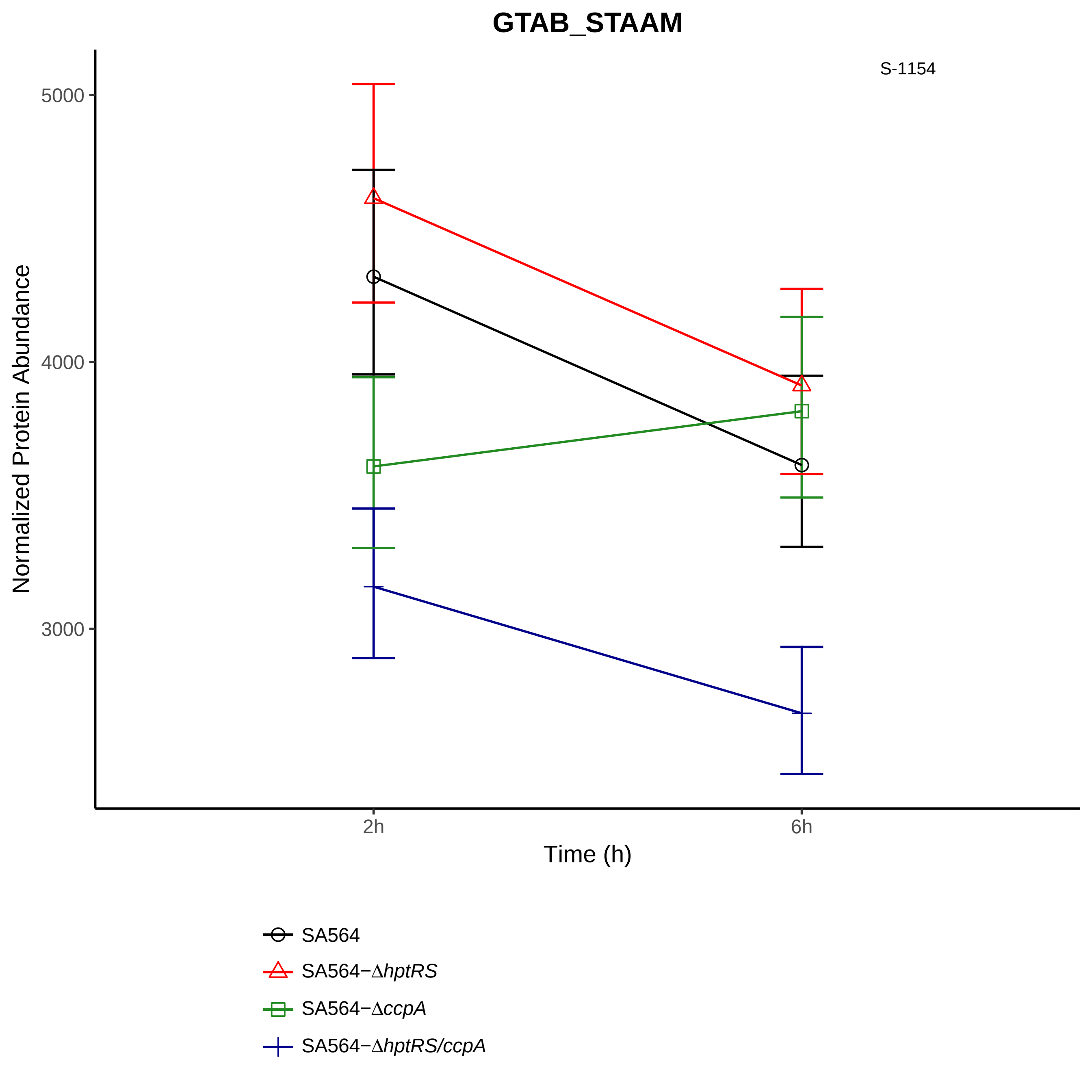

GTAB_STAAM
S-1154
5000
4000
3000
Normalized Protein Abundance
2h
6h
Time (h)
SA564
SA564−Δ*hptRS*
SA564−Δ*ccpA*
SA564−Δ*hptRS/ccpA*

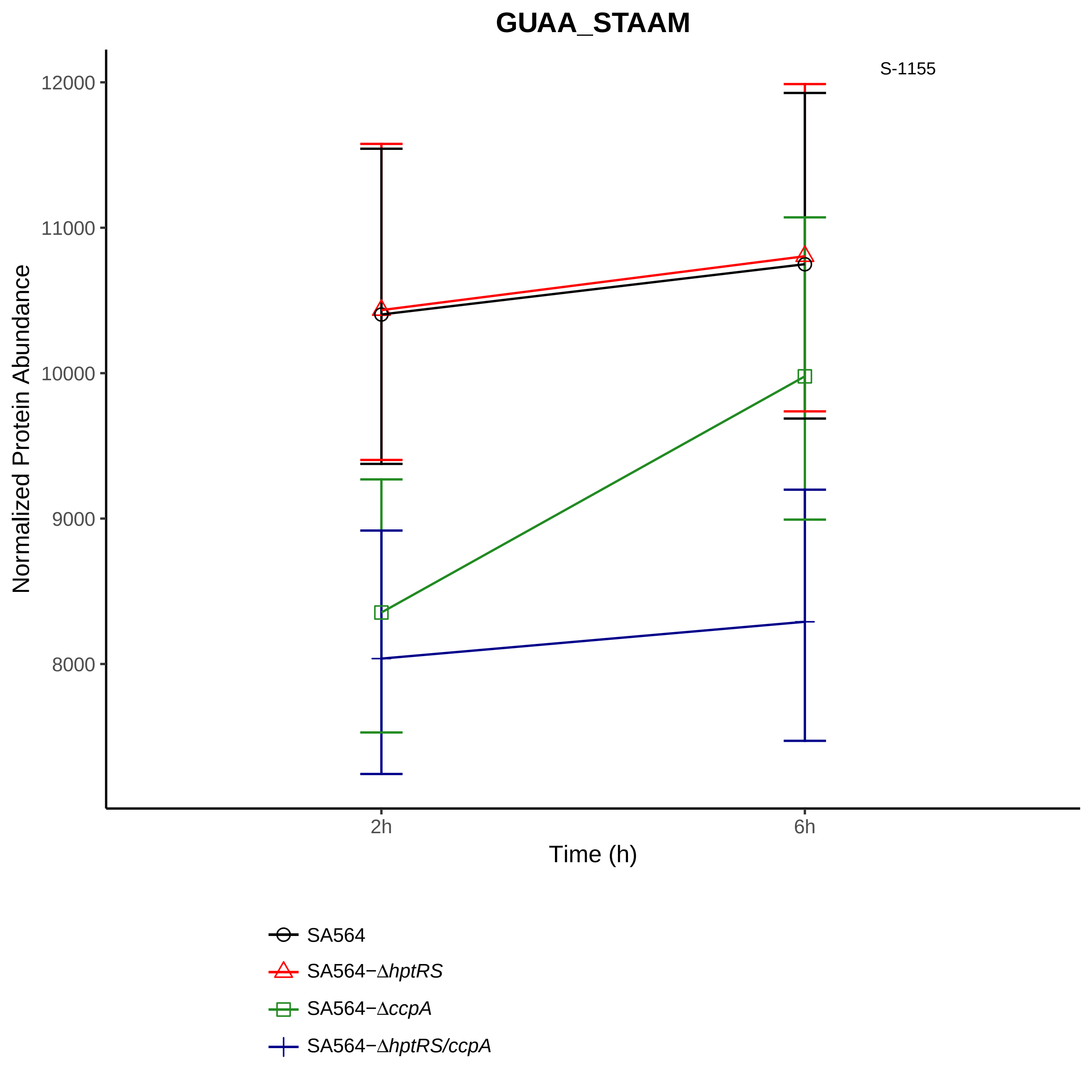
GUAA_STAAM
S-1155
Normalized Protein Abundance
12000
11000
10000
9000
8000
2h
6h
Time (h)
SA564
SA564−ΔhptRS
SA564−ΔccpA
SA564−ΔhptRS/ccpA

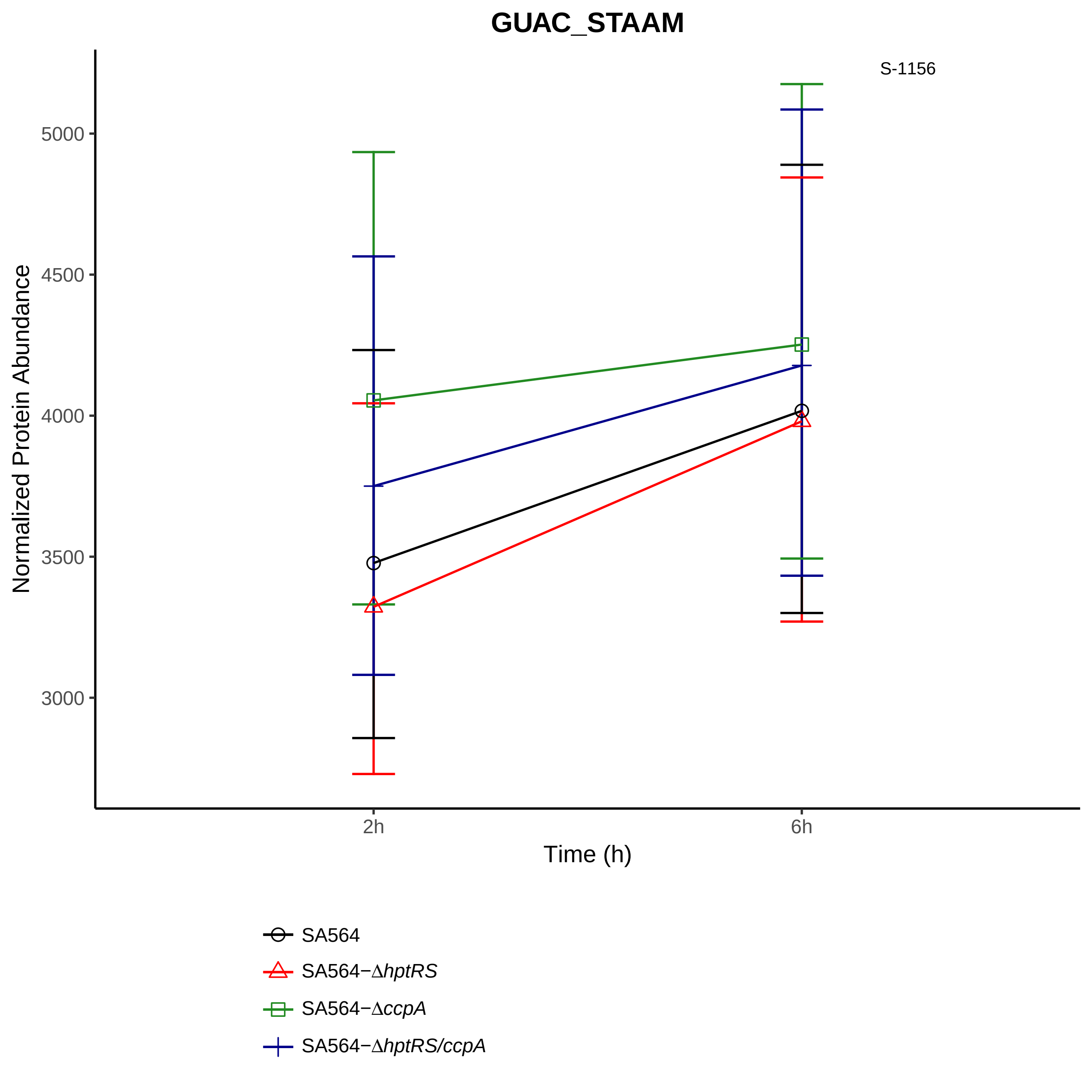
GUAC_STAAM
S-1156
Normalized Protein Abundance
5000
4500
4000
3500
3000
2h
6h
Time (h)
SA564
SA564−ΔhptRS
SA564−ΔccpA
SA564−ΔhptRS/ccpA

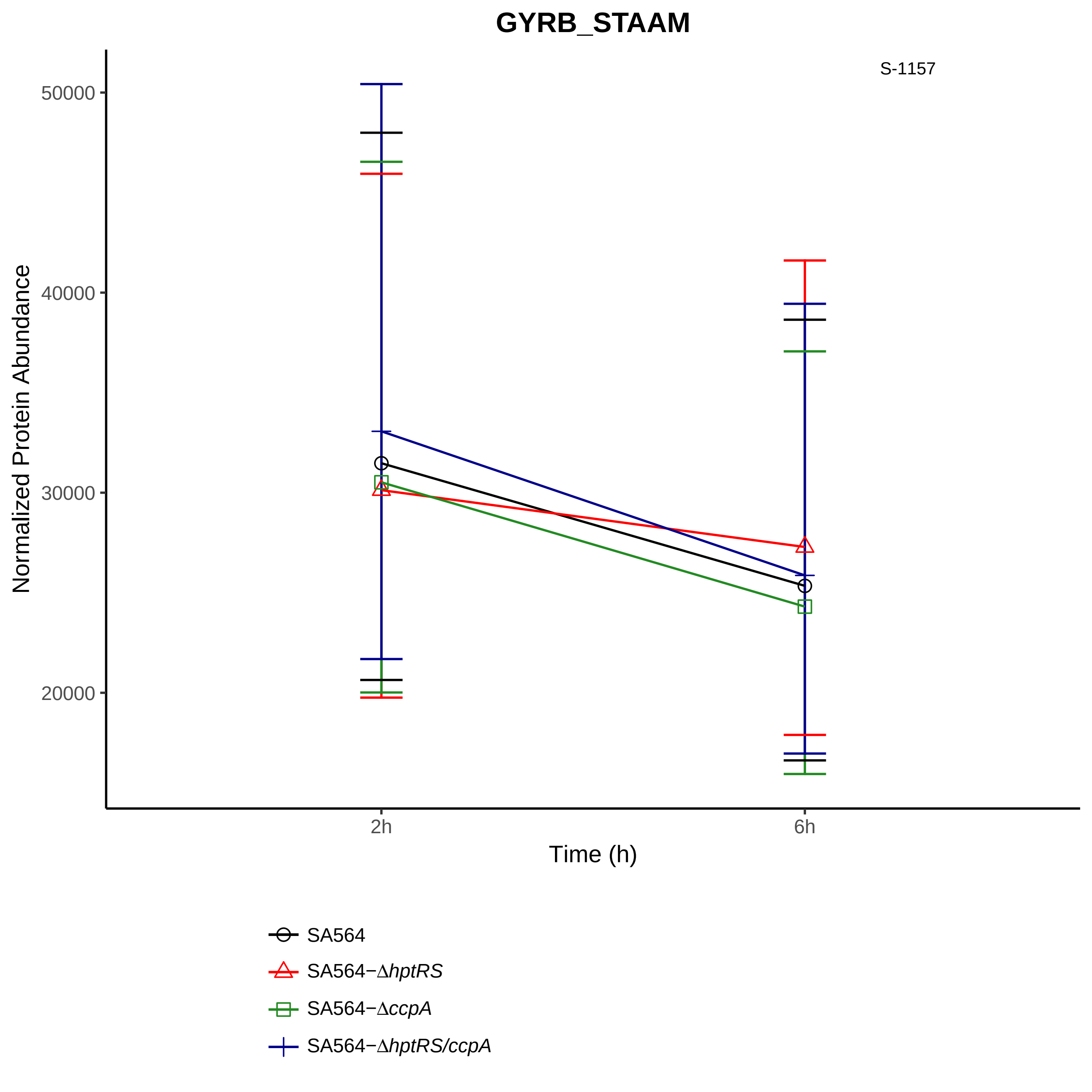

GYRB_STAAM
S-1157
Normalized Protein Abundance
50000
40000
30000
20000
2h
6h
Time (h)
SA564
SA564−Δ*hptRS*
SA564−Δ*ccpA*
SA564−Δ*hptRS/ccpA*

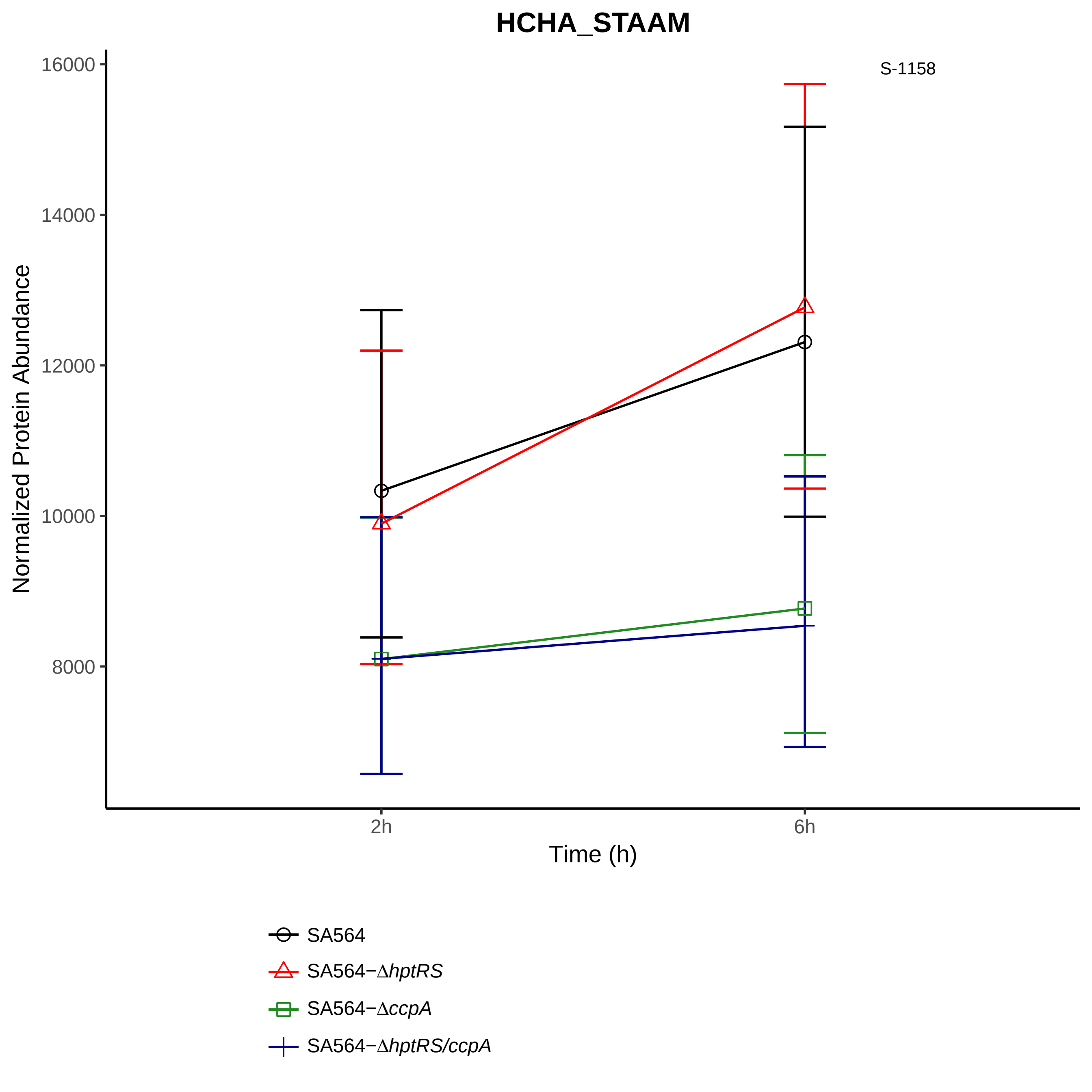

HCHA_STAAM
S-1158
16000
14000
12000
10000
8000
Normalized Protein Abundance
2h
6h
Time (h)
SA564
SA564−Δ*hptRS*
SA564−Δ*ccpA*
SA564−Δ*hptRS/ccpA*

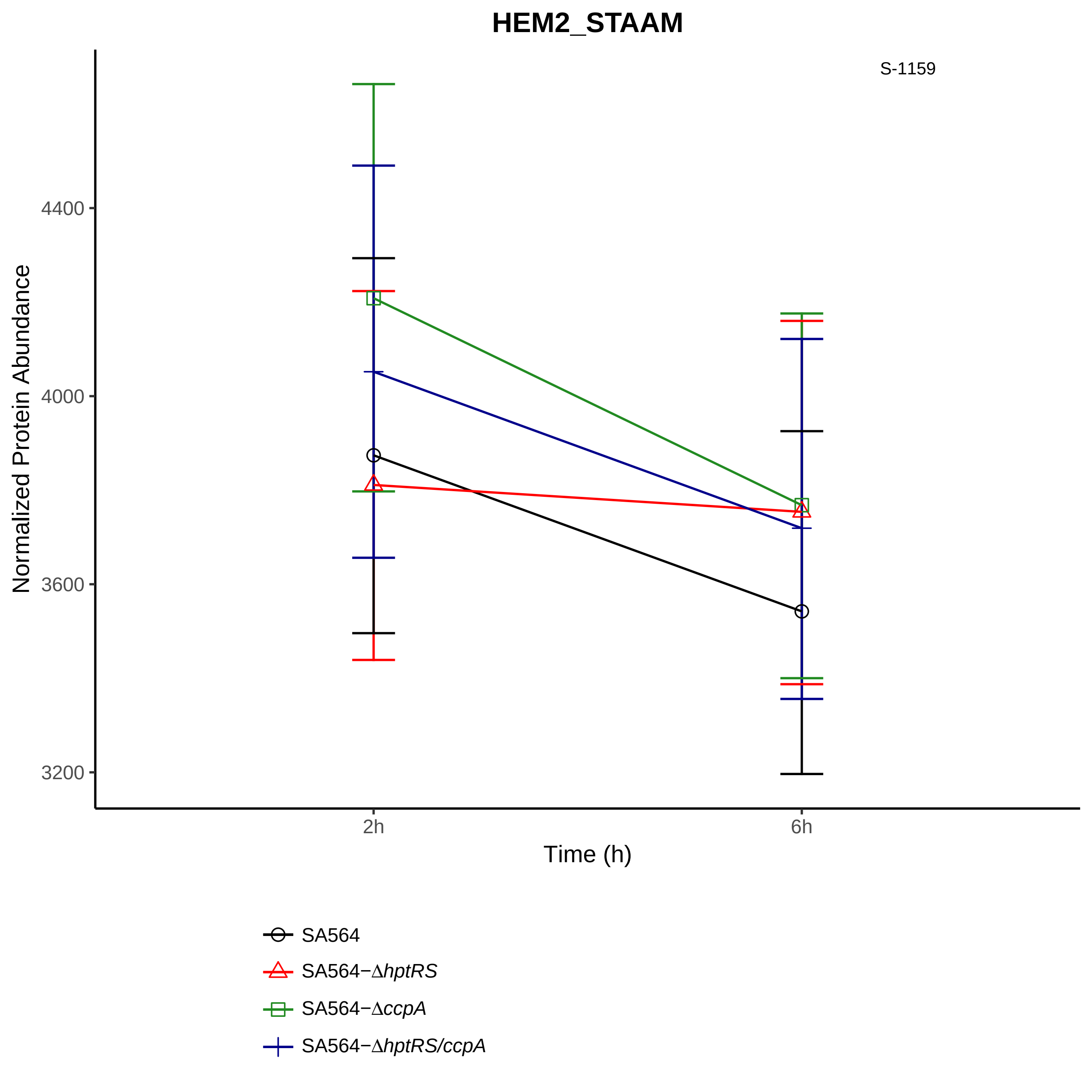
HEM2_STAAM
S-1159
Normalized Protein Abundance
4400
4000
3600
3200
2h
6h
Time (h)
SA564
SA564−ΔhptRS
SA564−ΔccpA
SA564−ΔhptRS/ccpA

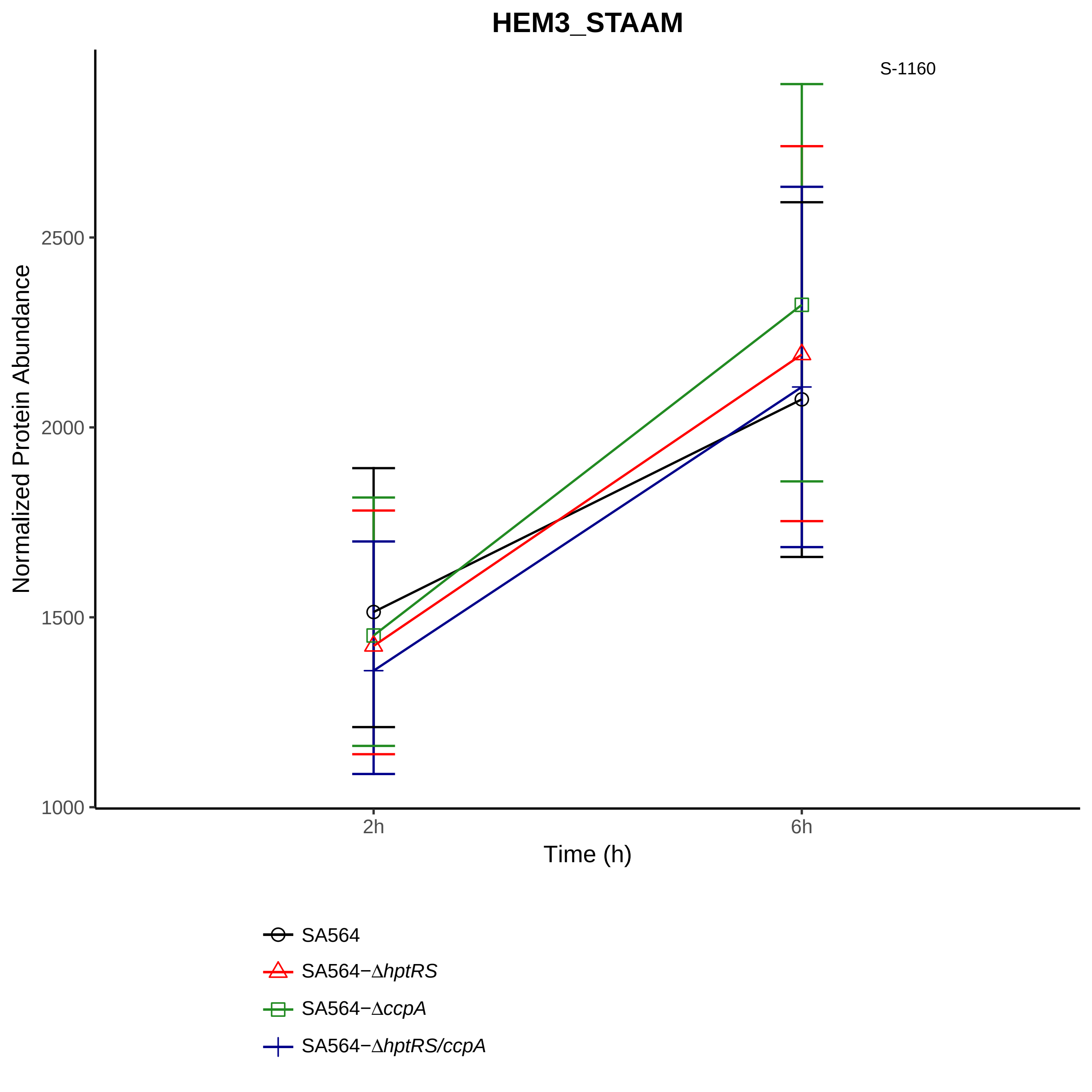
HEM3_STAAM
S-1160
Normalized Protein Abundance
2500
2000
1500
1000
2h
6h
Time (h)
SA564
SA564−Δ*hptRS*
SA564−Δ*ccpA*
SA564−Δ*hptRS/ccpA*

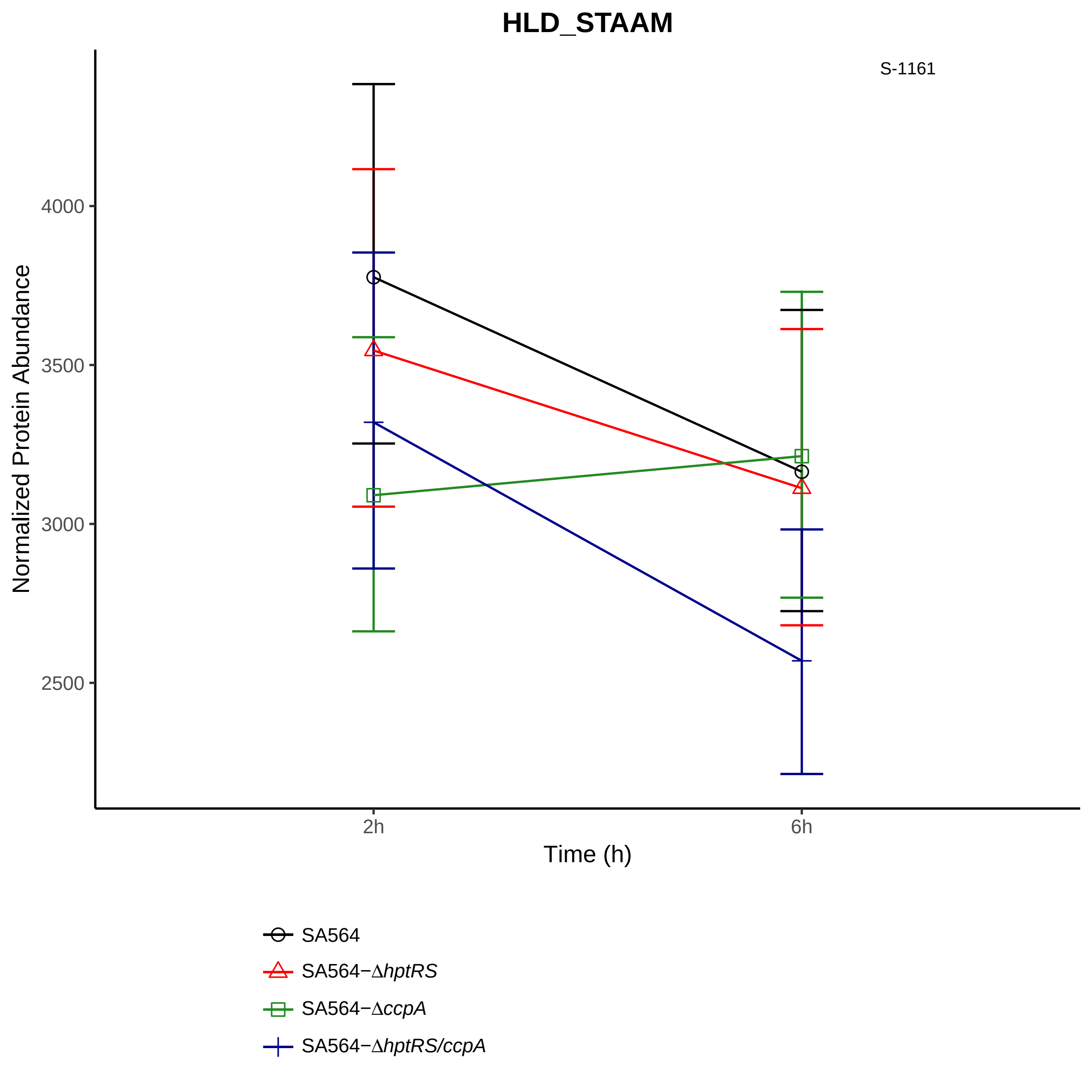
HLD_STAAM
S-1161
Normalized Protein Abundance
4000
3500
3000
2500
2h
6h
Time (h)
SA564
SA564−Δ*hptRS*
SA564−Δ*ccpA*
SA564−Δ*hptRS/ccpA*

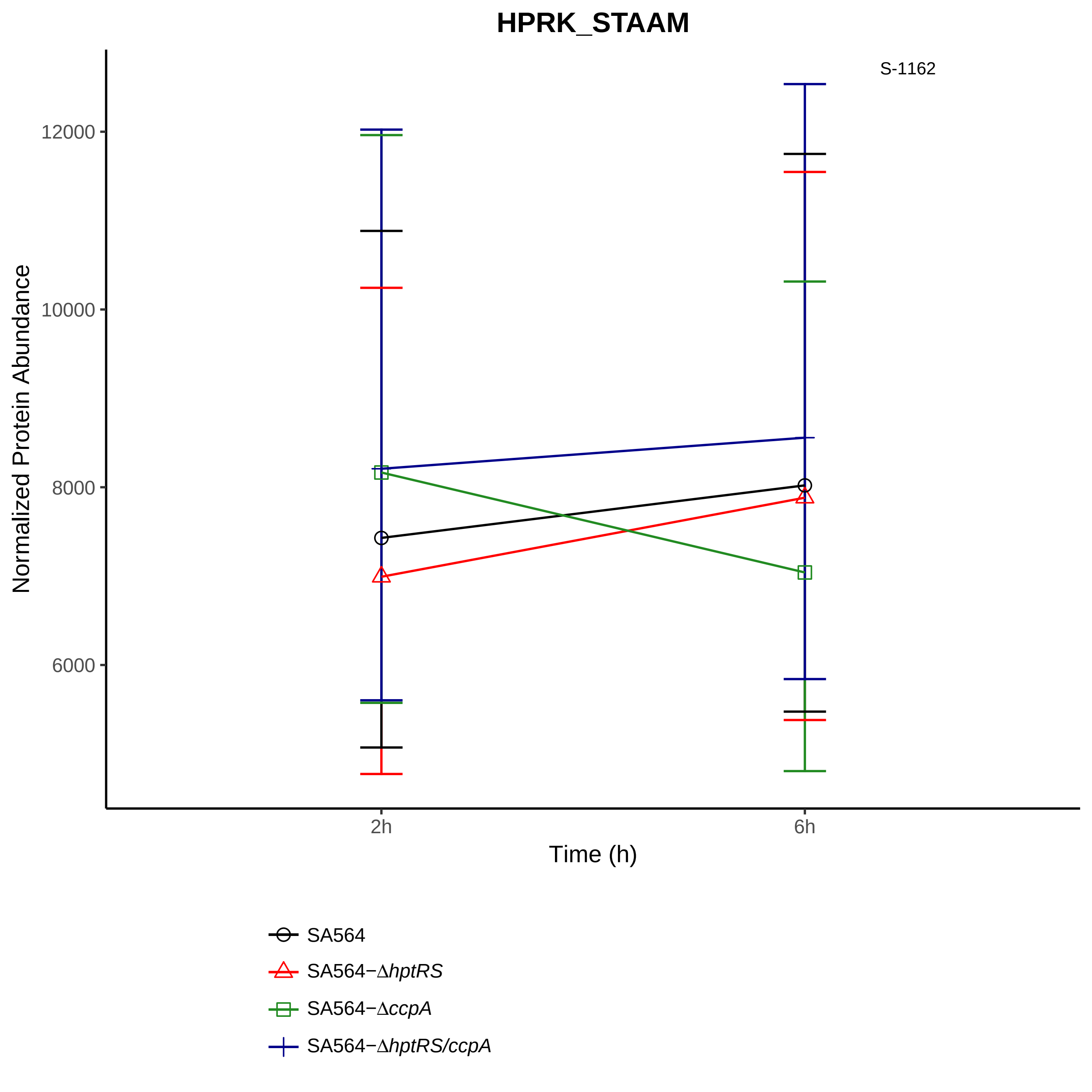

HPRK_STAAM
S-1162
Normalized Protein Abundance
12000
10000
8000
6000
2h
6h
Time (h)
SA564
SA564−Δ*hptRS*
SA564−Δ*ccpA*
SA564−Δ*hptRS/ccpA*

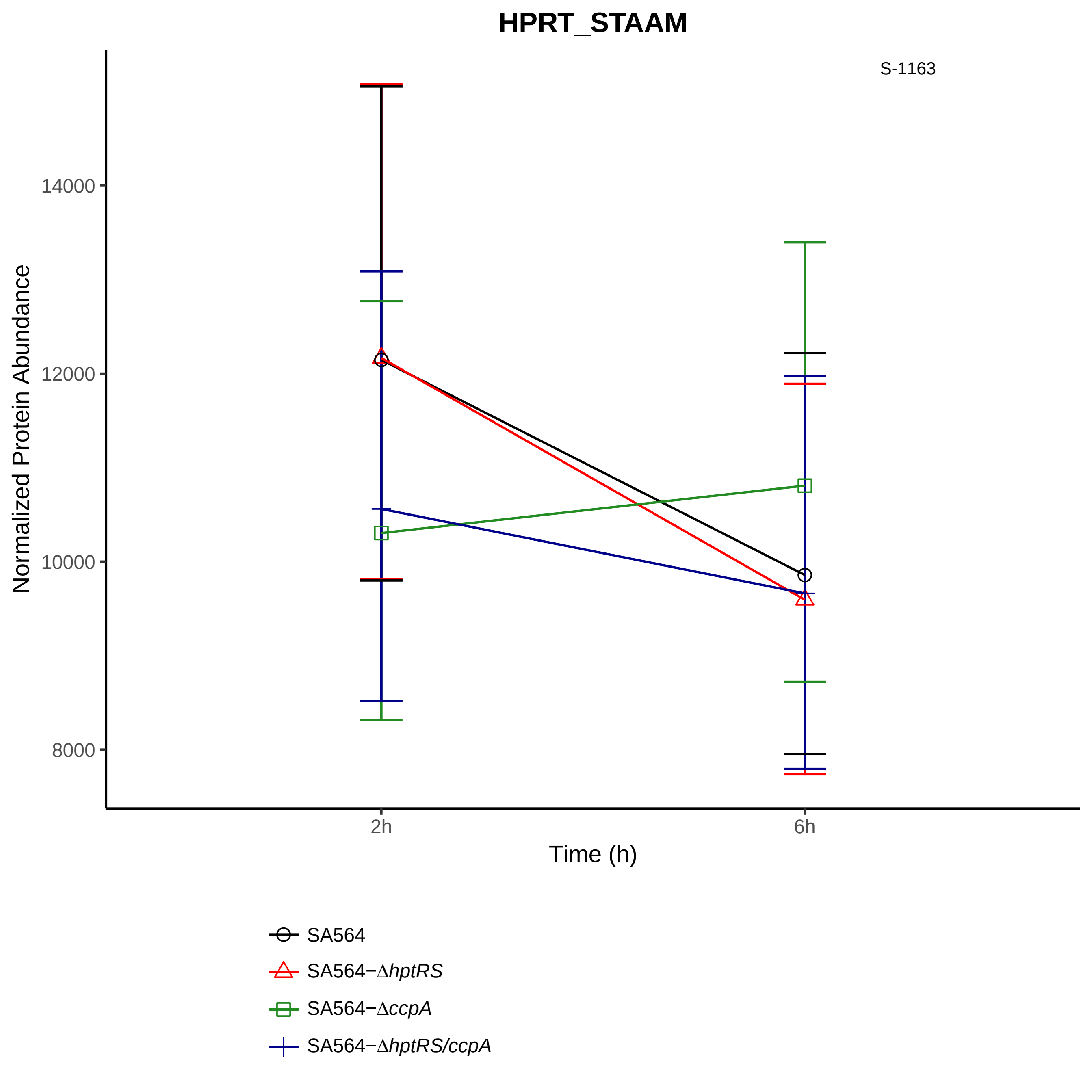

HPRT_STAAM
S-1163
Normalized Protein Abundance
14000
12000
10000
8000
2h
6h
Time (h)
SA564
SA564−Δ*hptRS*
SA564−Δ*ccpA*
SA564−Δ*hptRS/ccpA*

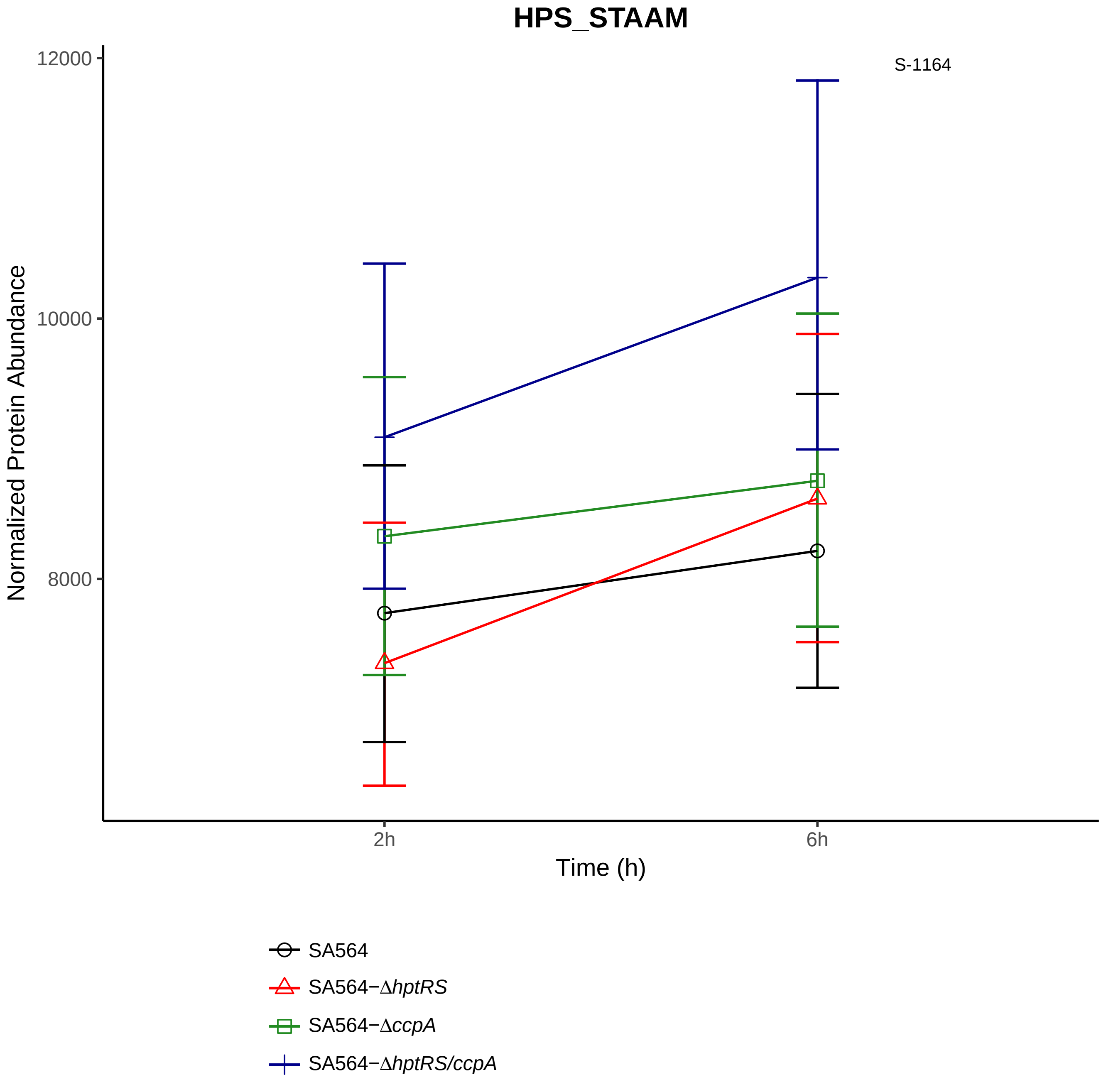

HPS_STAAM
S-1164
12000
10000
8000
Normalized Protein Abundance
2h
6h
Time (h)
SA564
SA564−Δ*hptRS*
SA564−Δ*ccpA*
SA564−Δ*hptRS/ccpA*

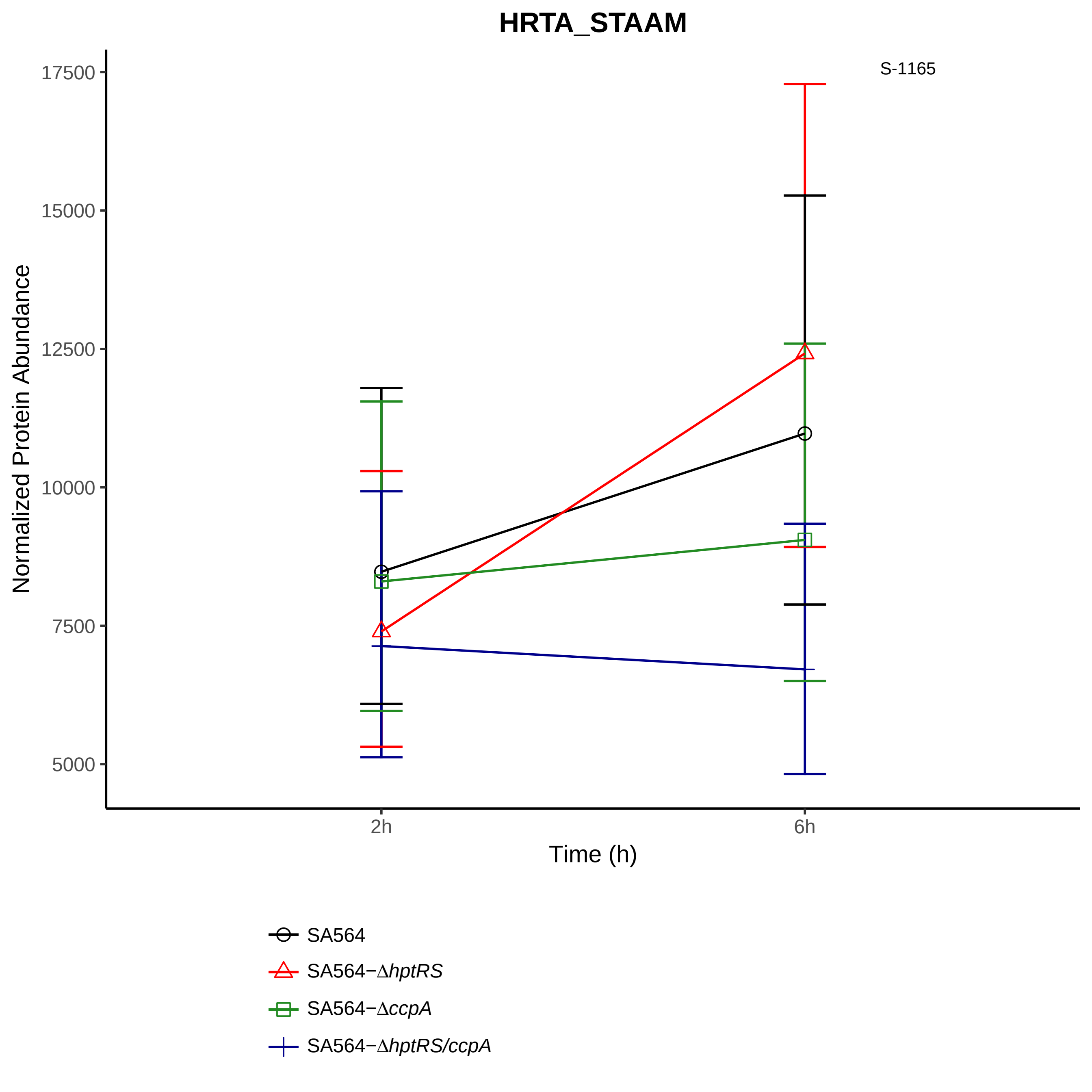

HRTA_STAAM
S-1165
Normalized Protein Abundance
17500
15000
12500
10000
7500
5000
2h
6h
Time (h)
SA564
SA564−Δ*hptRS*
SA564−Δ*ccpA*
SA564−Δ*hptRS/ccpA*

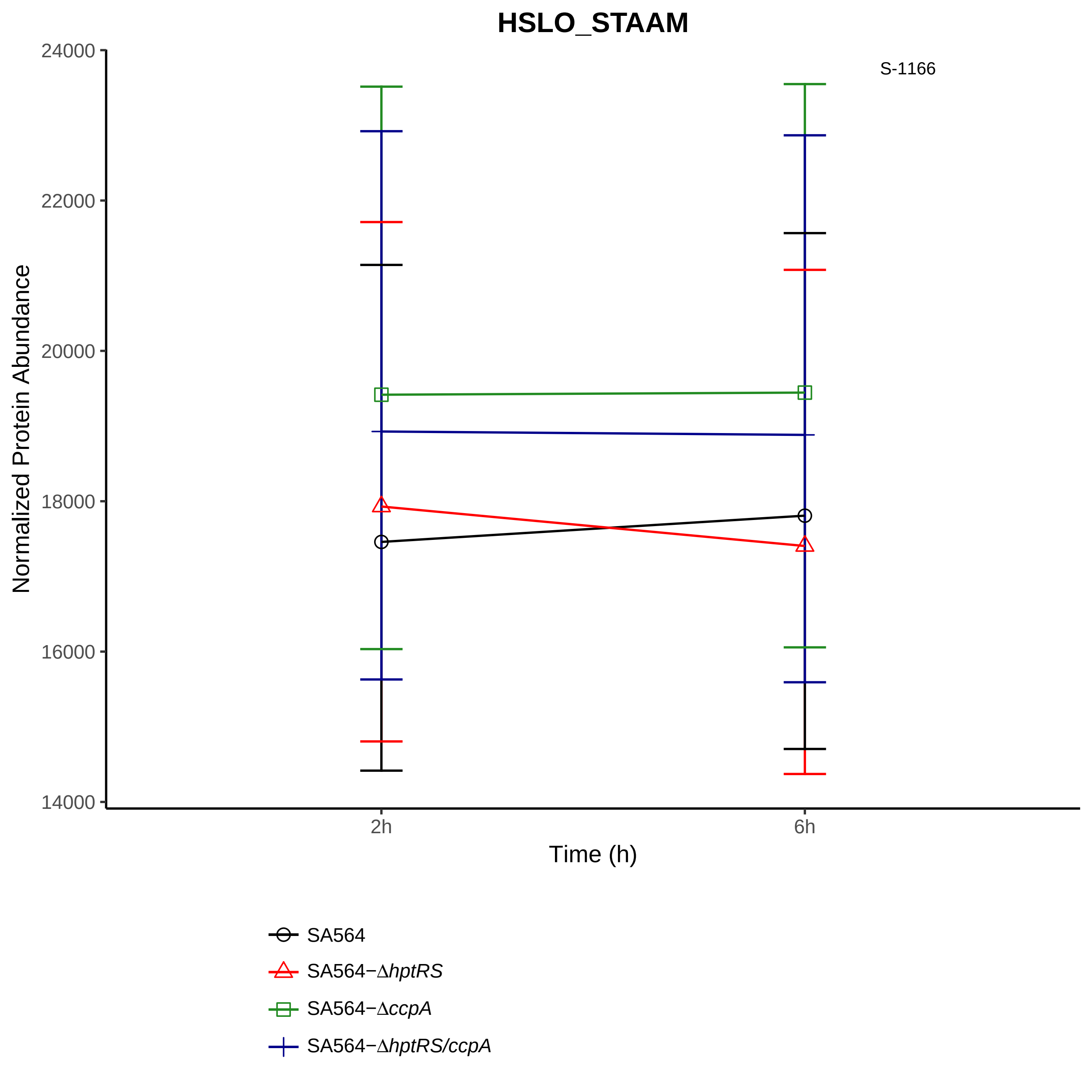

HSLO_STAAM
S-1166
24000
22000
20000
18000
16000
14000
Normalized Protein Abundance
2h
6h
Time (h)
SA564
SA564−Δ*hptRS*
SA564−Δ*ccpA*
SA564−Δ*hptRS/ccpA*

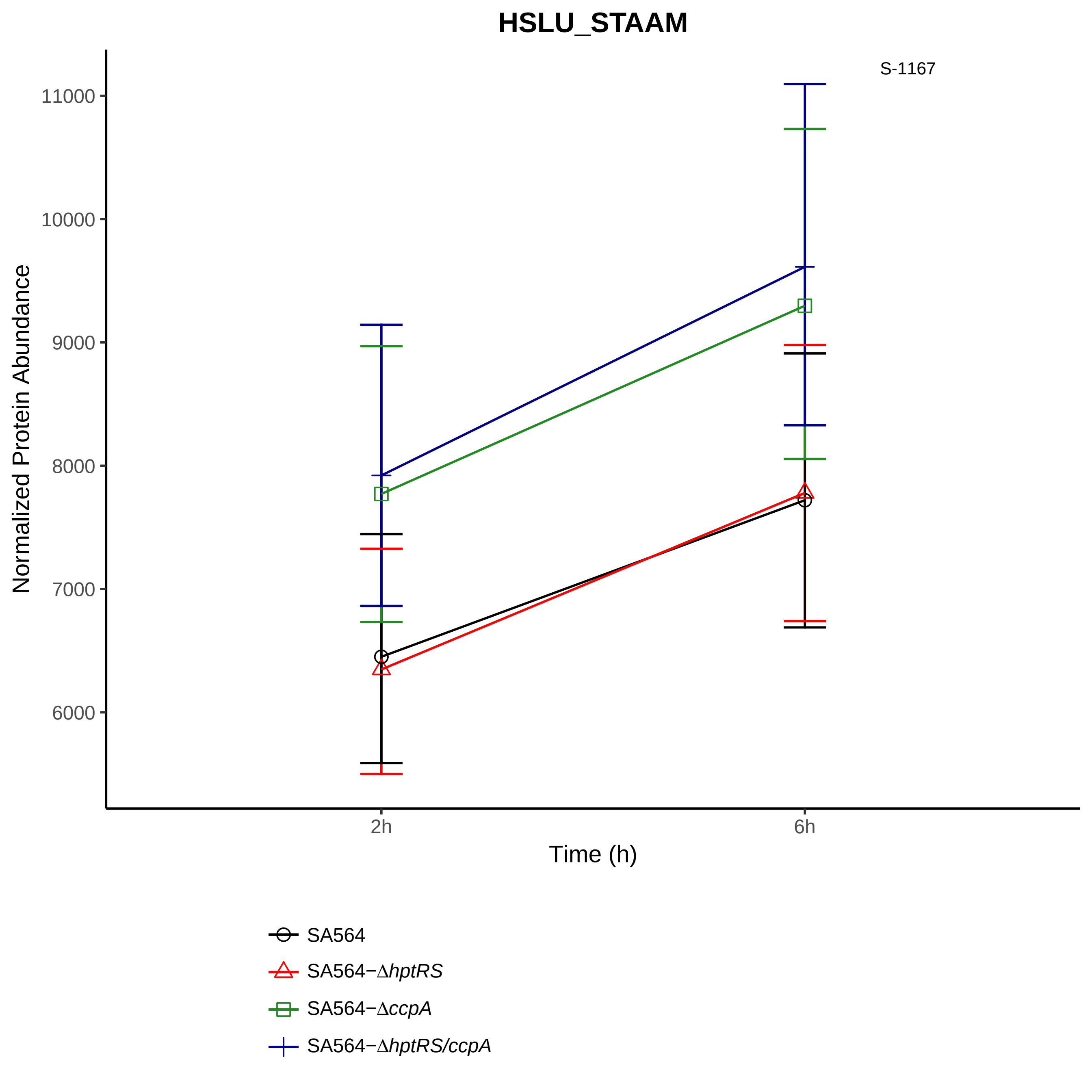
HSLU_STAAM
S-1167
Normalized Protein Abundance
11000
10000
9000
8000
7000
6000
2h
6h
Time (h)
SA564
SA564−ΔhptRS
SA564−ΔccpA
SA564−ΔhptRS/ccpA

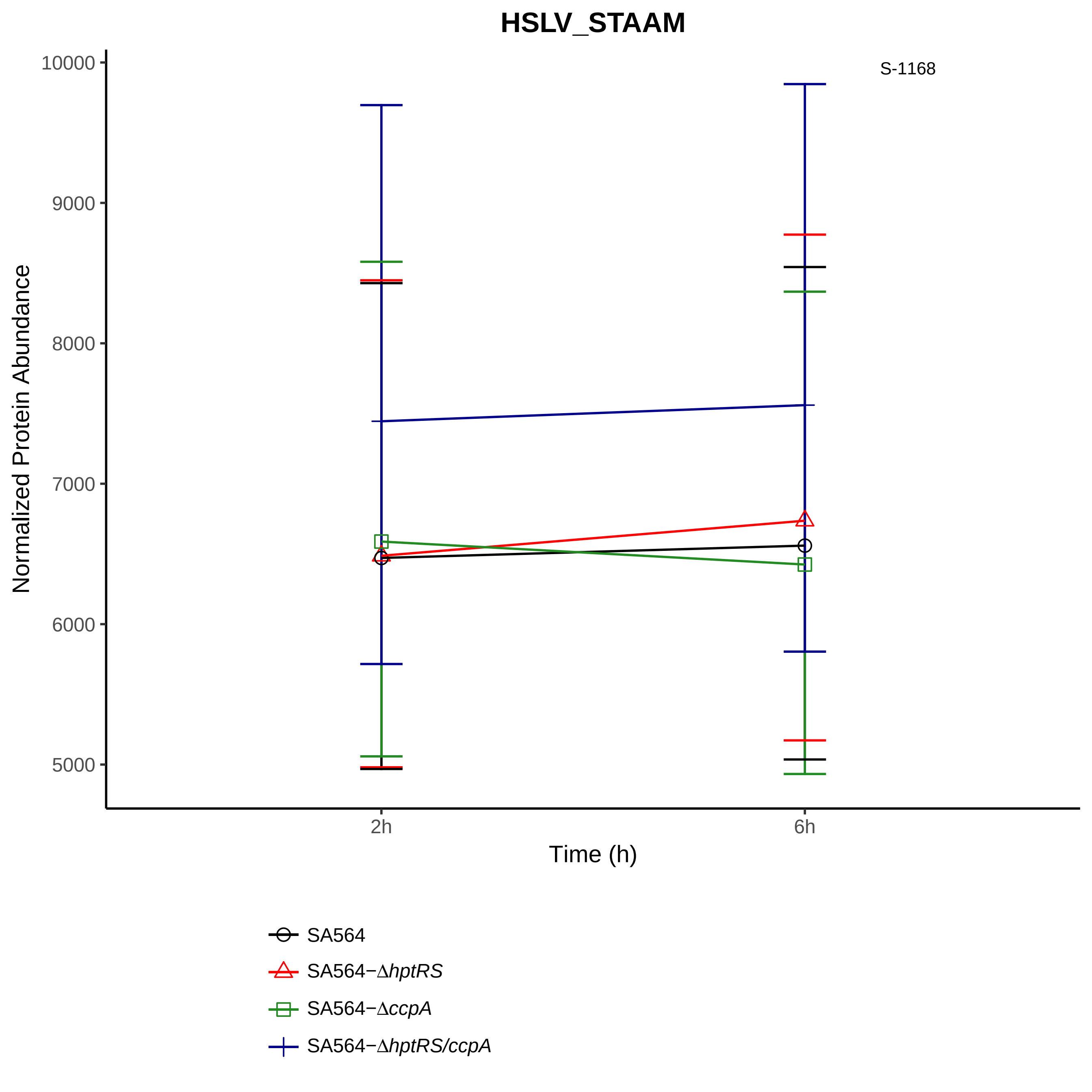

HSLV_STAAM
S-1168
Normalized Protein Abundance
10000
9000
8000
7000
6000
5000
2h
6h
Time (h)
SA564
SA564−Δ*hptRS*
SA564−Δ*ccpA*
SA564−Δ*hptRS/ccpA*

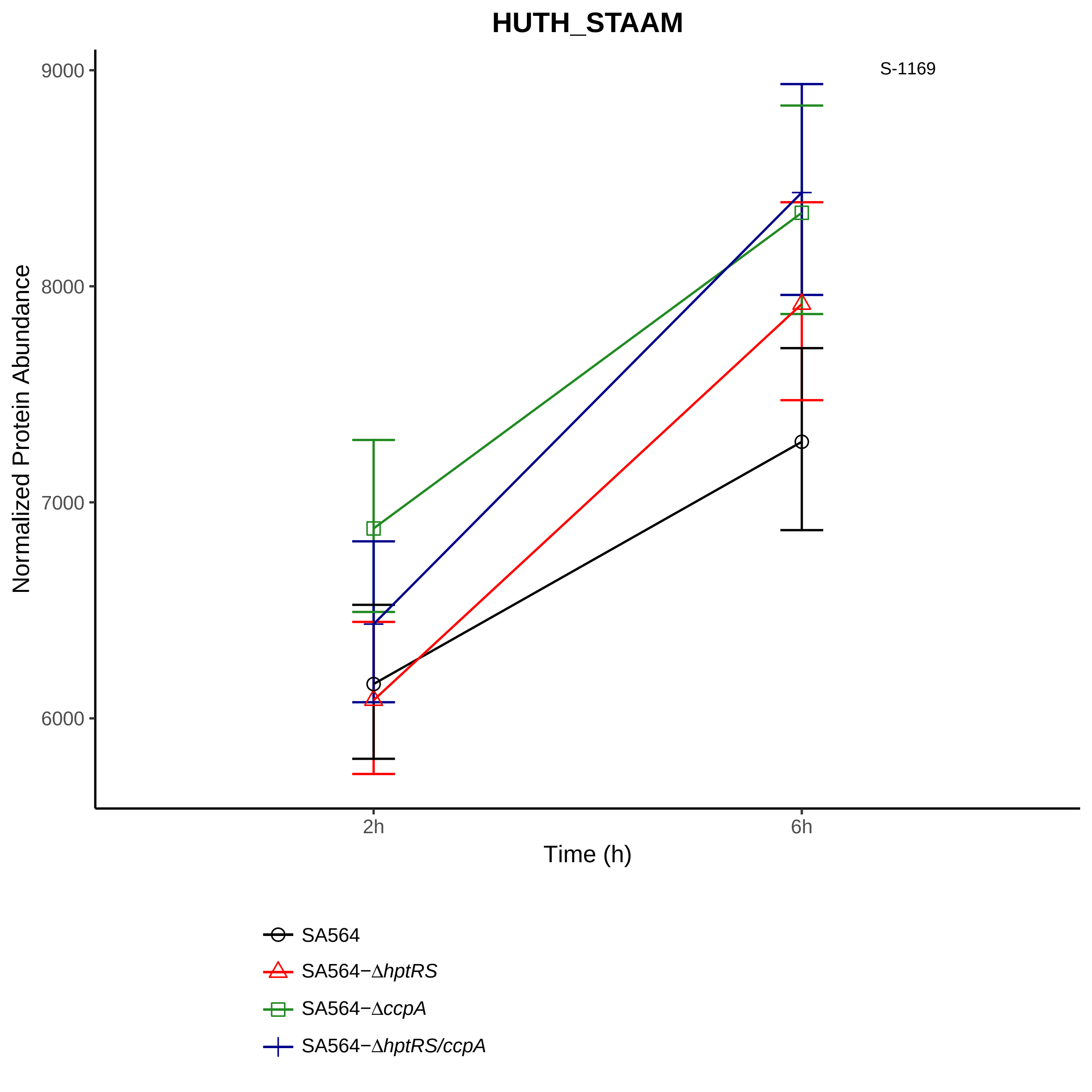
HUTH_STAAM
S-1169
9000
8000
7000
6000
Normalized Protein Abundance
2h
6h
Time (h)
SA564
SA564−Δ*hptRS*
SA564−Δ*ccpA*
SA564−Δ*hptRS/ccpA*

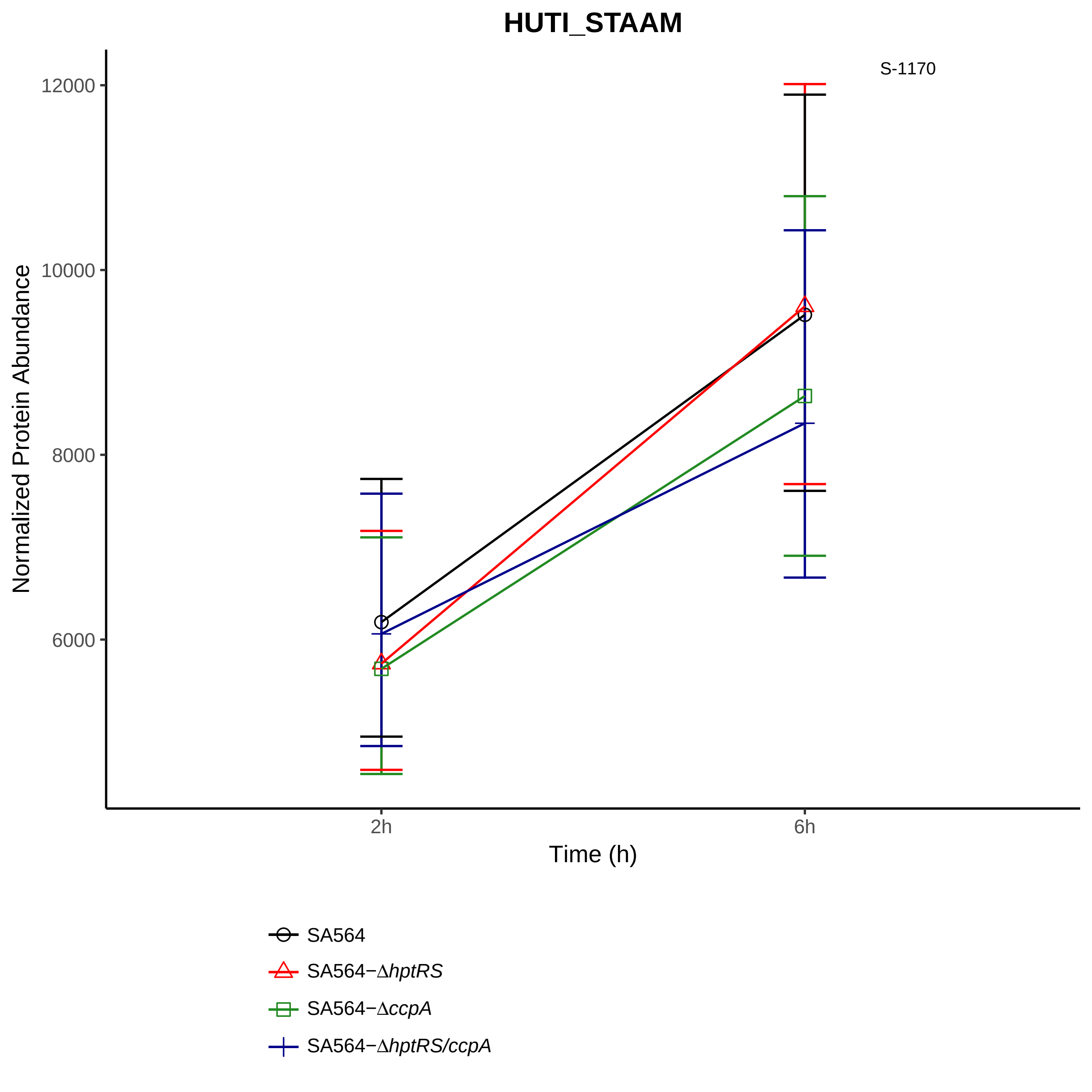

HUTI_STAAM
S-1170
12000
10000
8000
6000
Normalized Protein Abundance
2h
6h
Time (h)
SA564
SA564−Δ*hptRS*
SA564−Δ*ccpA*
SA564−Δ*hptRS/ccpA*

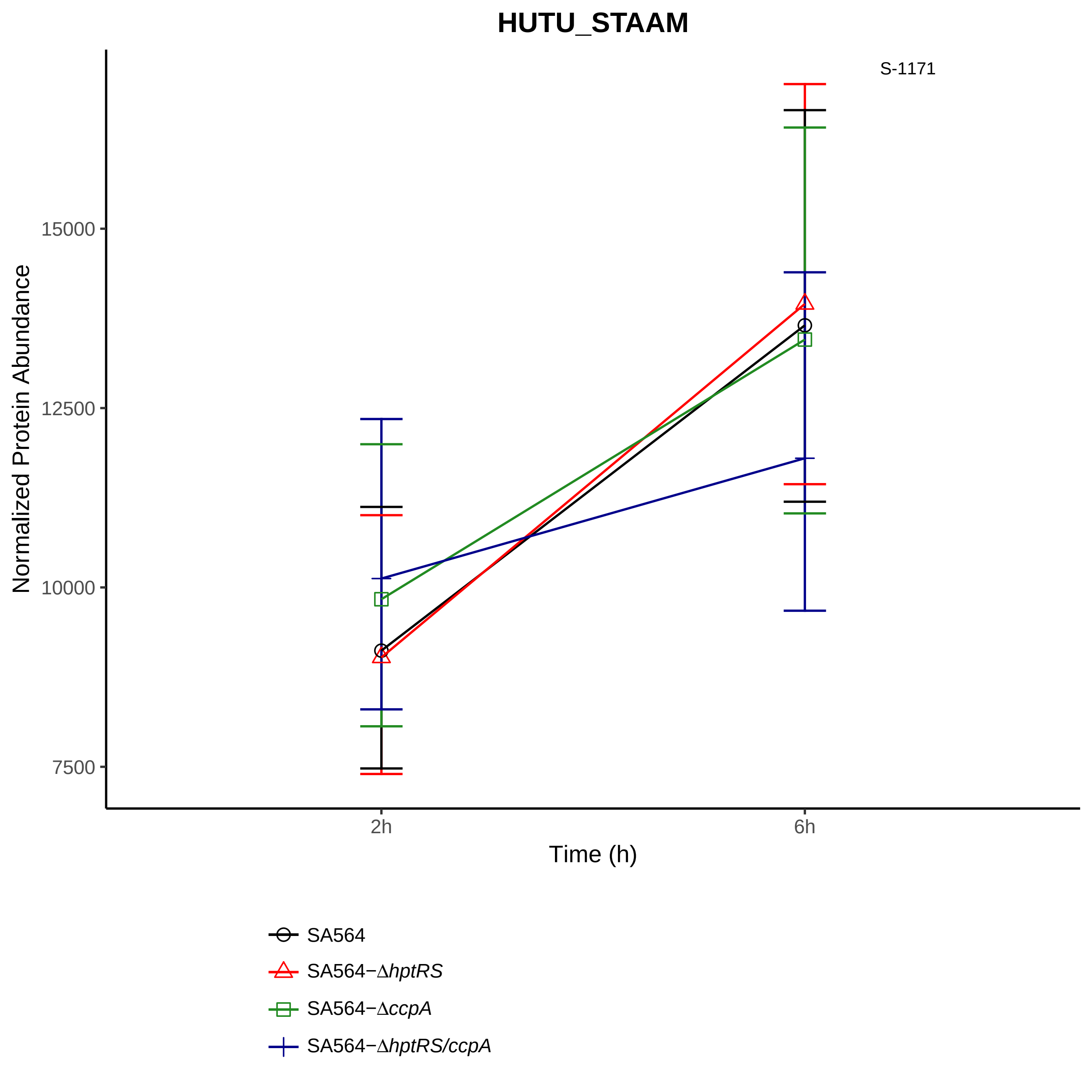

HUTU_STAAM
S-1171
Normalized Protein Abundance
15000
12500
10000
7500
2h
6h
Time (h)
SA564
SA564−Δ*hptRS*
SA564−Δ*ccpA*
SA564−Δ*hptRS/ccpA*

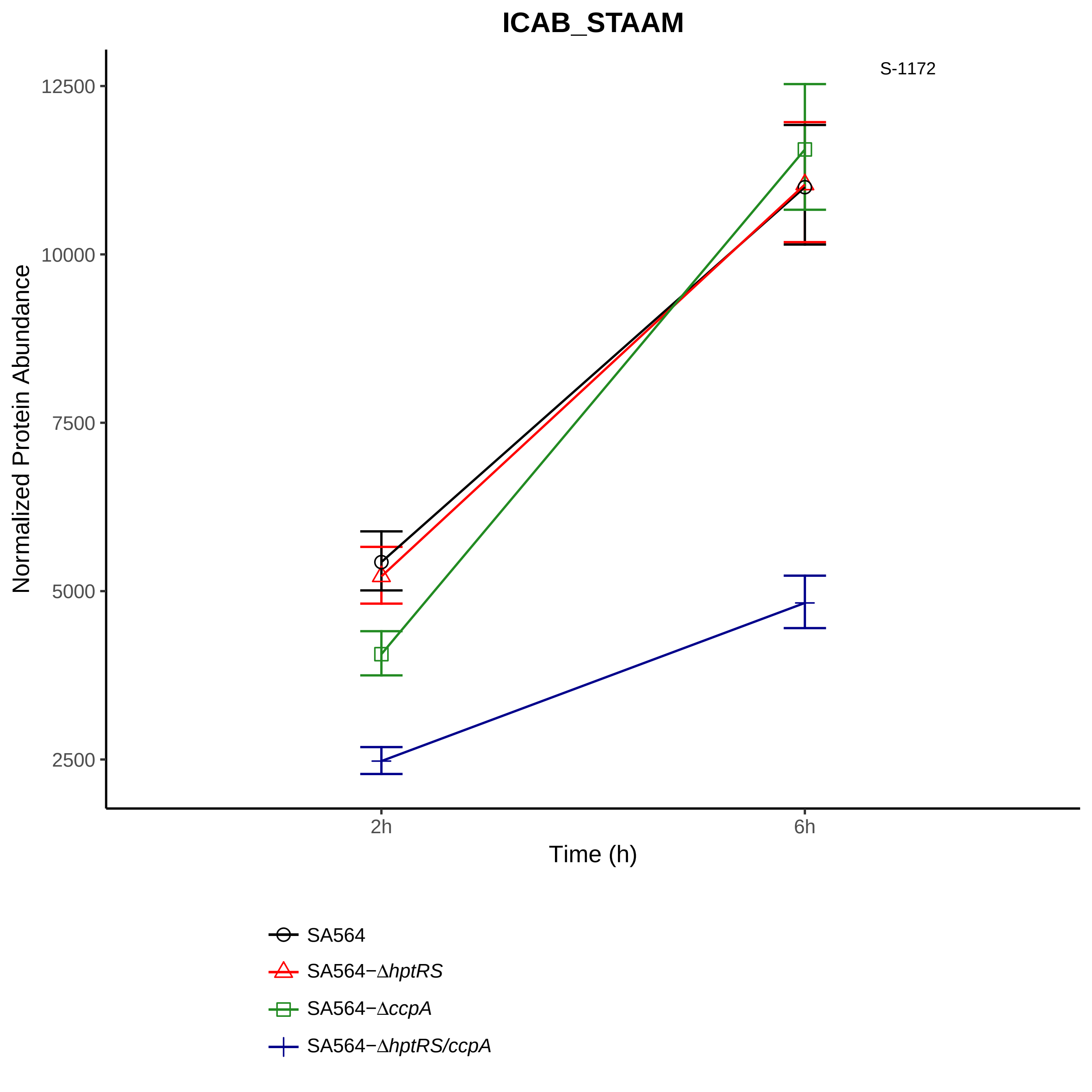
ICAB_STAAM
S-1172
Normalized Protein Abundance
12500
10000
7500
5000
2500
2h
6h
Time (h)
SA564
SA564−Δ*hptRS*
SA564−Δ*ccpA*
SA564−Δ*hptRS/ccpA*

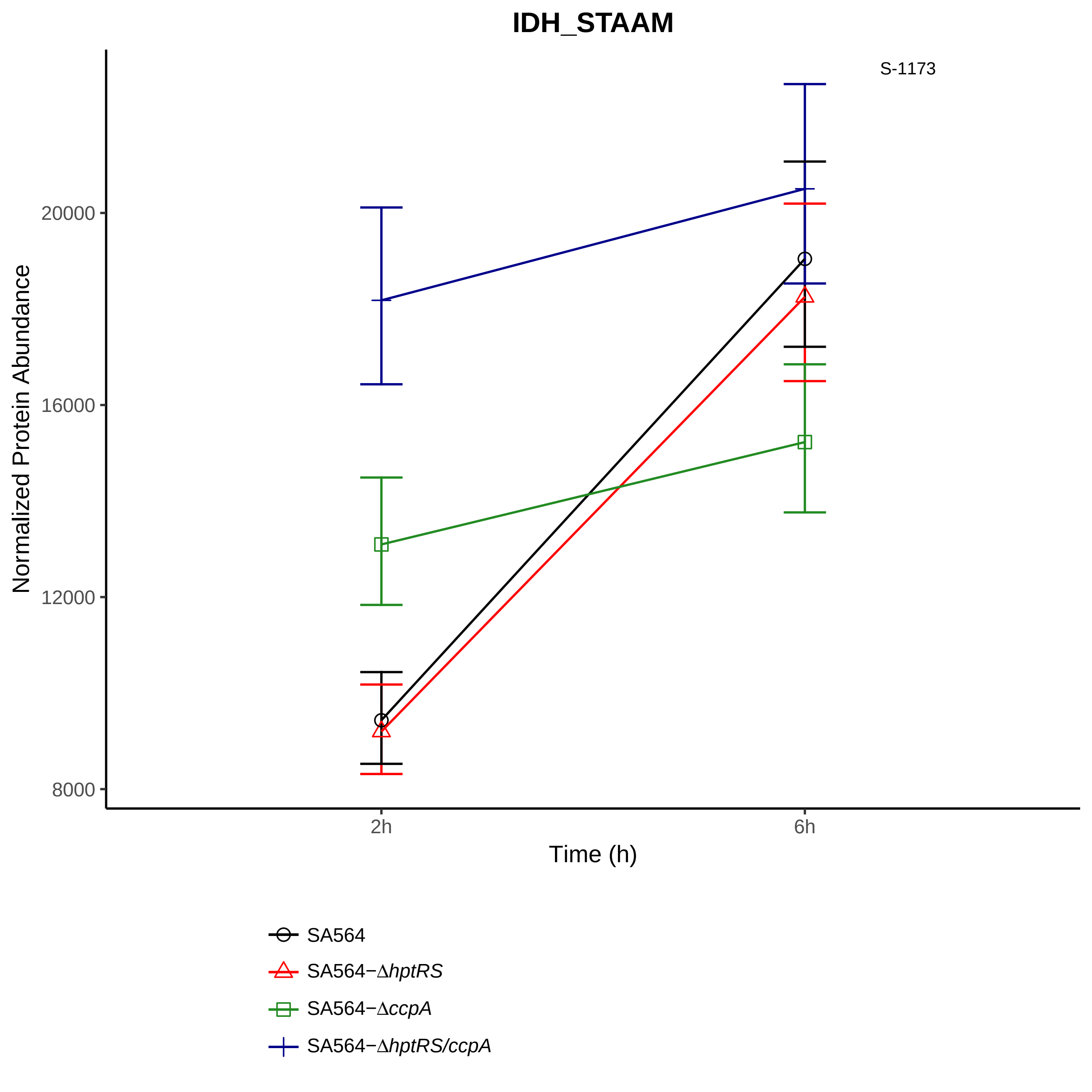
IDH_STAAM
S-1173
Normalized Protein Abundance
20000
16000
12000
8000
2h
6h
Time (h)
SA564
SA564−Δ*hptRS*
SA564−Δ*ccpA*
SA564−Δ*hptRS/ccpA*

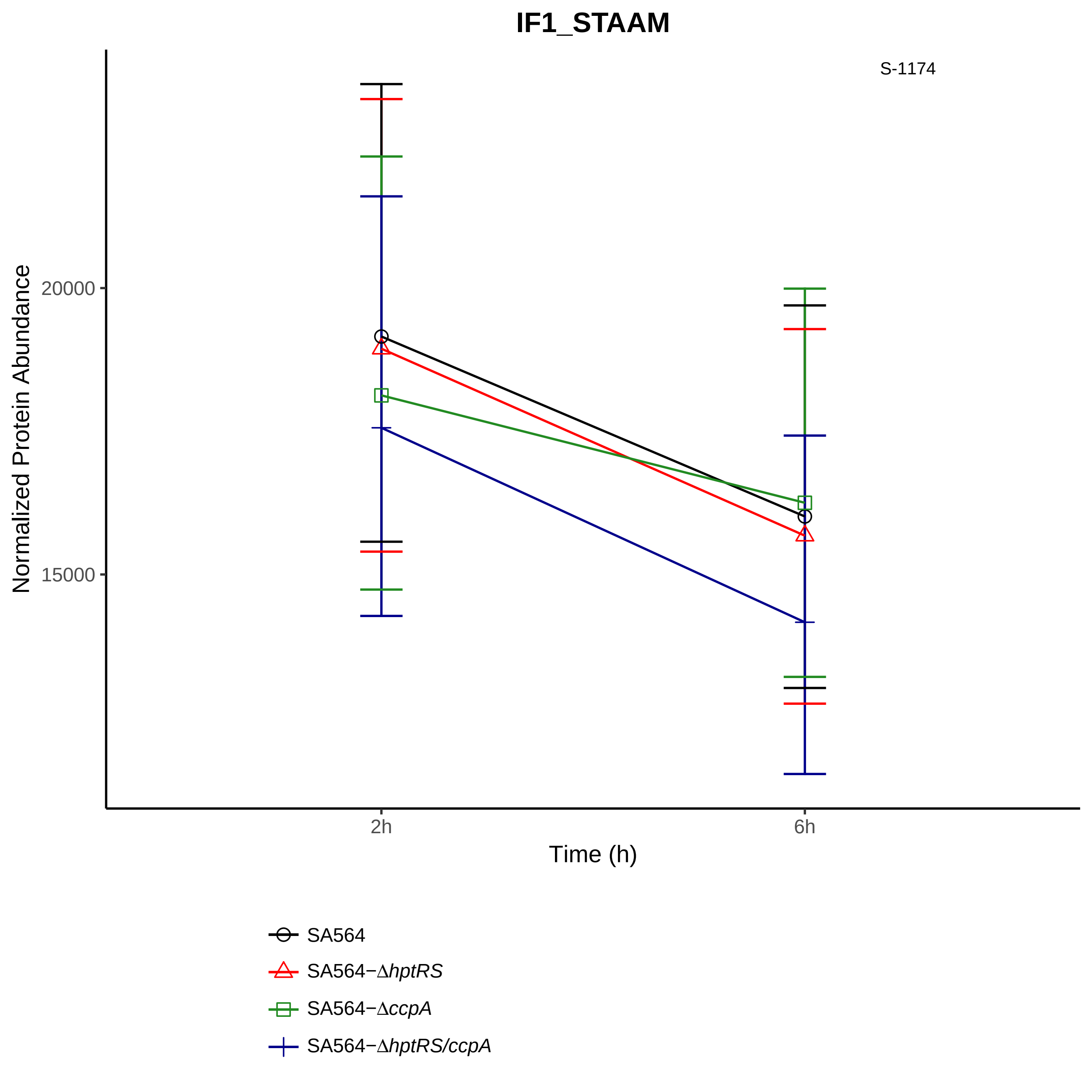
IF1_STAAM
S-1174
Normalized Protein Abundance
20000
15000
2h
6h
Time (h)
SA564
SA564−ΔhptRS
SA564−ΔccpA
SA564−ΔhptRS/ccpA

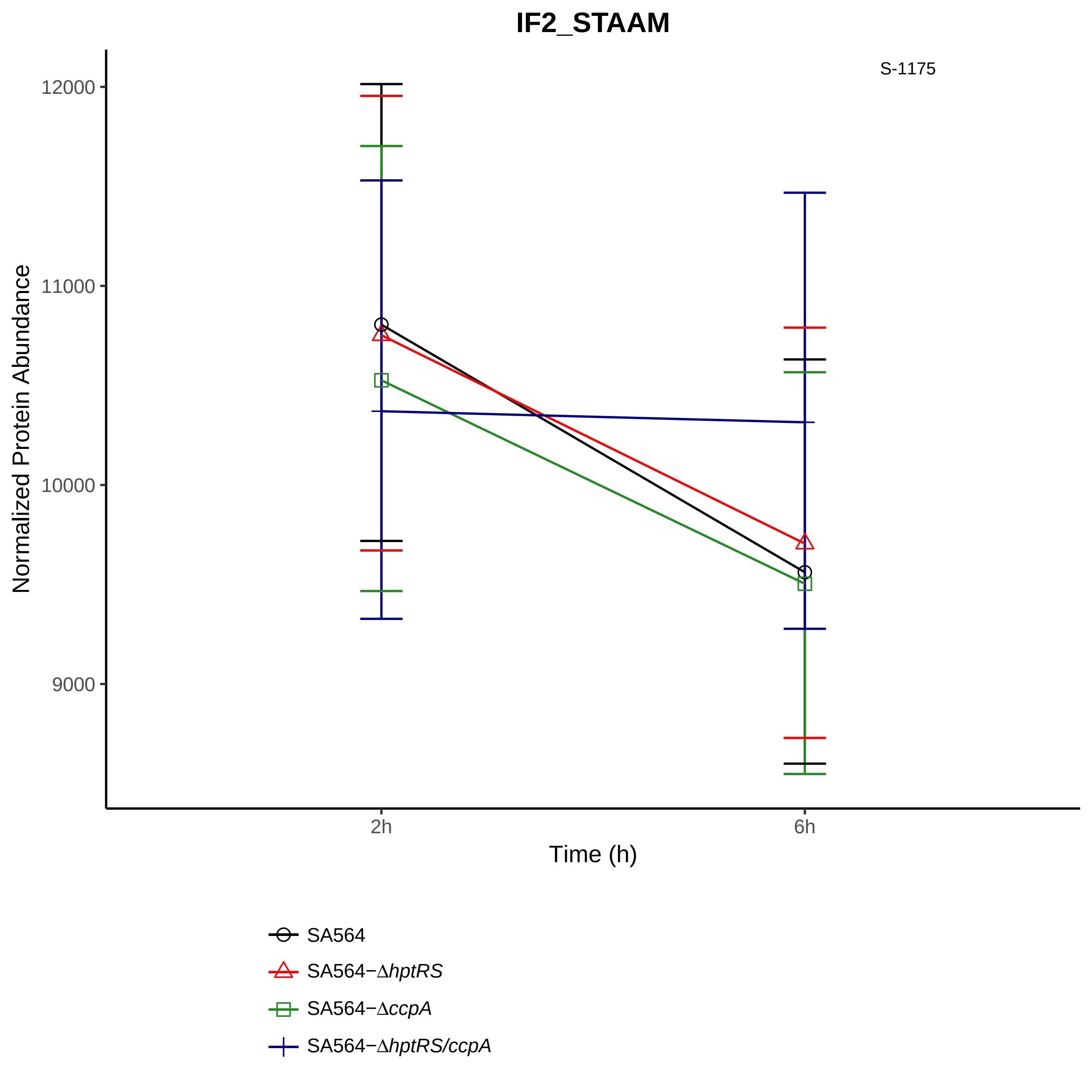

IF2_STAAM
S-1175
Normalized Protein Abundance
12000
11000
10000
9000
2h
6h
Time (h)
SA564
SA564−Δ*hptRS*
SA564−Δ*ccpA*
SA564−Δ*hptRS/ccpA*

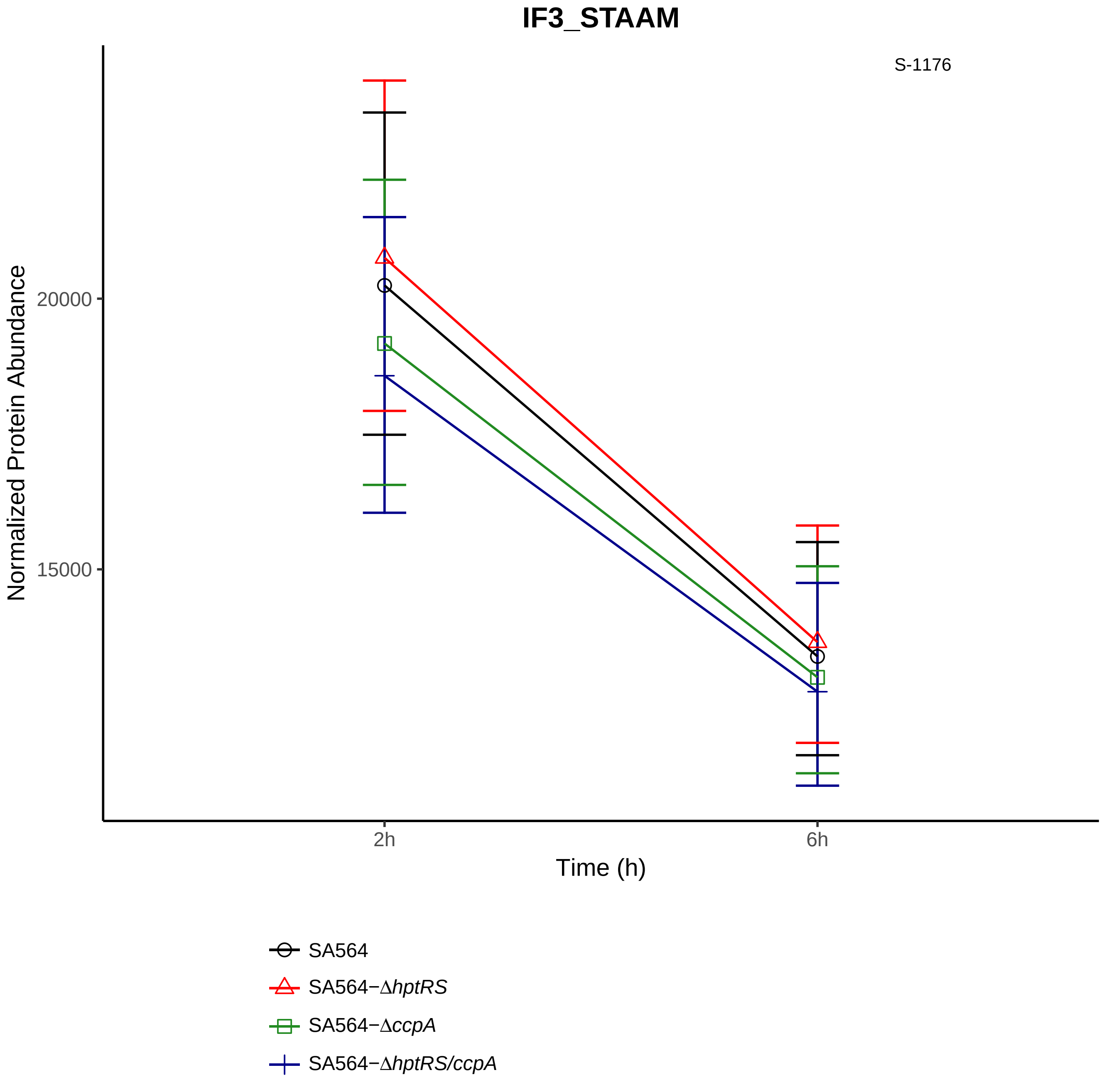

IF3_STAAM
S-1176
Normalized Protein Abundance
20000
15000
2h
6h
Time (h)
SA564
SA564−ΔhptRS
SA564−ΔccpA
SA564−ΔhptRS/ccpA

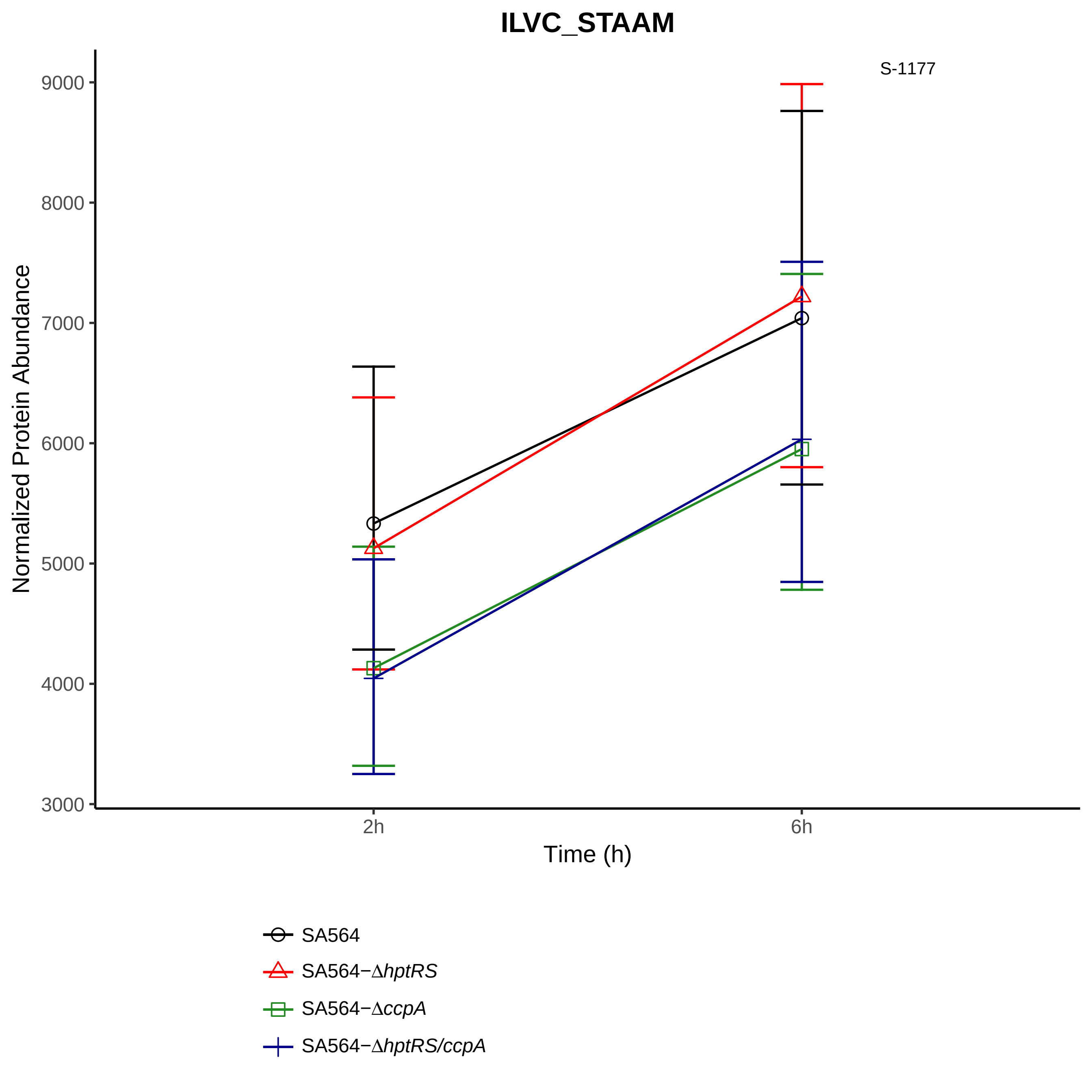

ILVC_STAAM
S-1177
Normalized Protein Abundance
9000
8000
7000
6000
5000
4000
3000
2h
6h
Time (h)
SA564
SA564−Δ*hptRS*
SA564−Δ*ccpA*
SA564−Δ*hptRS/ccpA*

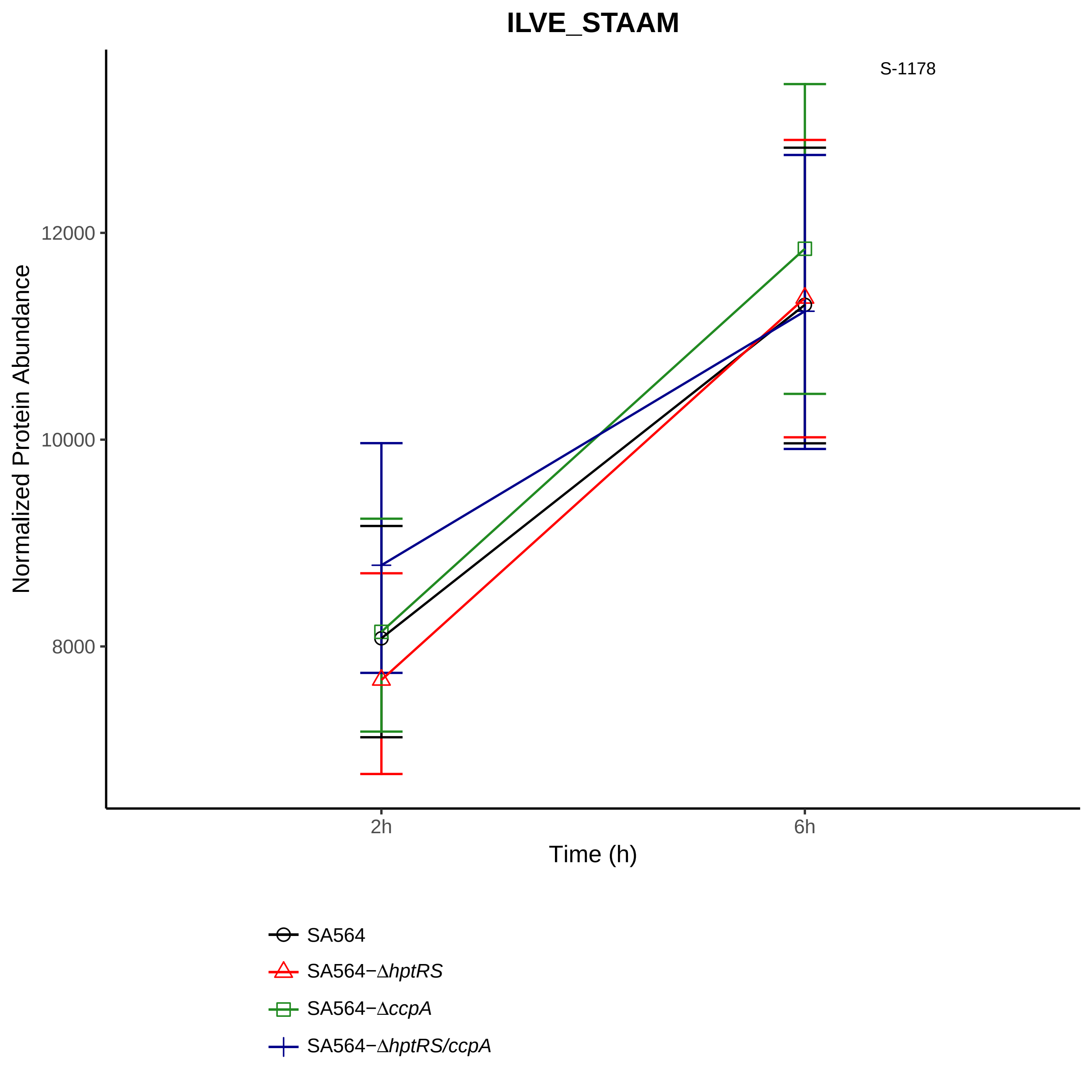

ILVE_STAAM
S-1178
Normalized Protein Abundance
12000
10000
8000
2h
6h
Time (h)
SA564
SA564−Δ*hptRS*
SA564−Δ*ccpA*
SA564−Δ*hptRS/ccpA*

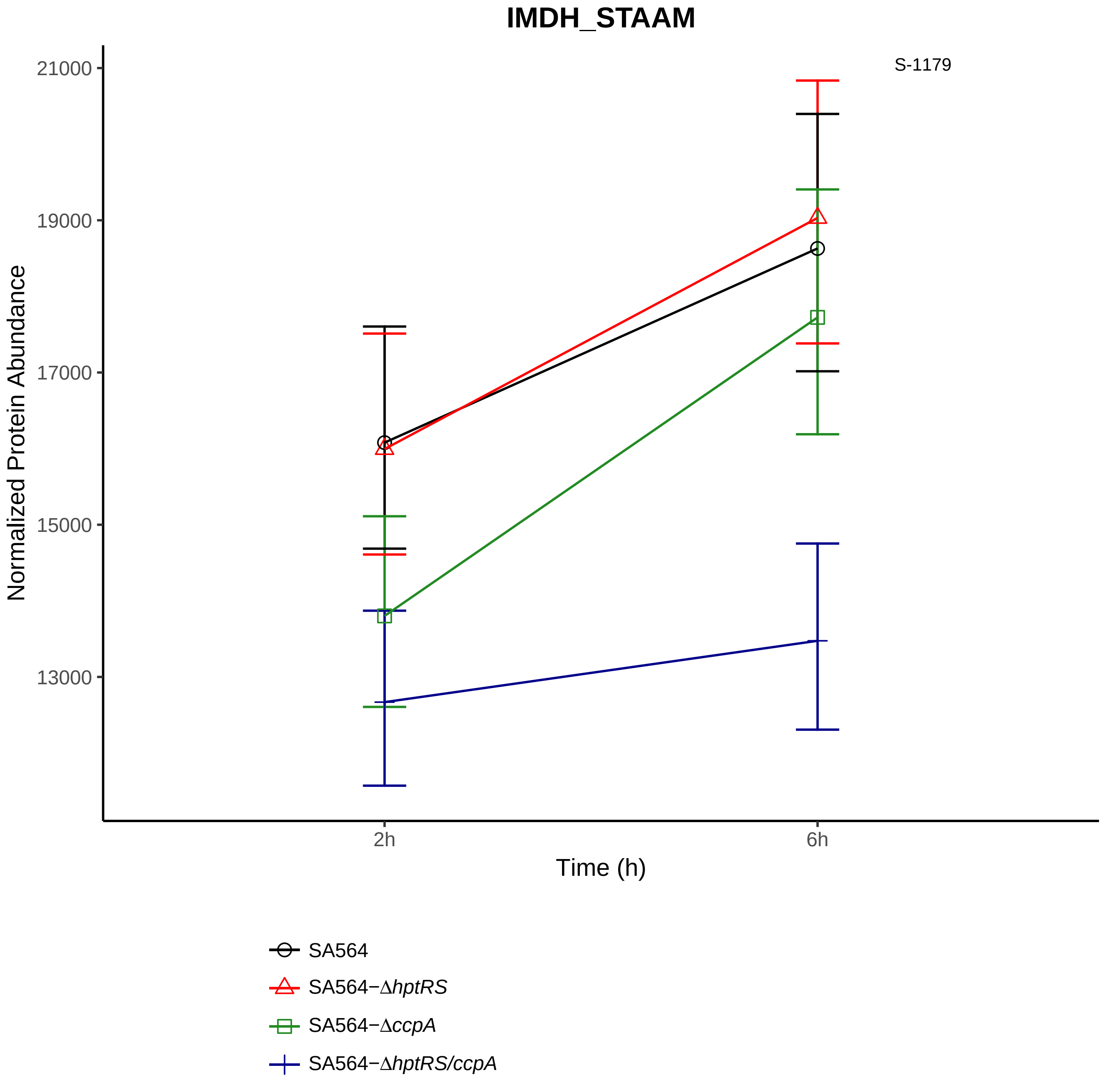

IMDH_STAAM
S-1179
Normalized Protein Abundance
21000
19000
17000
15000
13000
2h
6h
Time (h)
SA564
SA564−Δ*hptRS*
SA564−Δ*ccpA*
SA564−Δ*hptRS/ccpA*

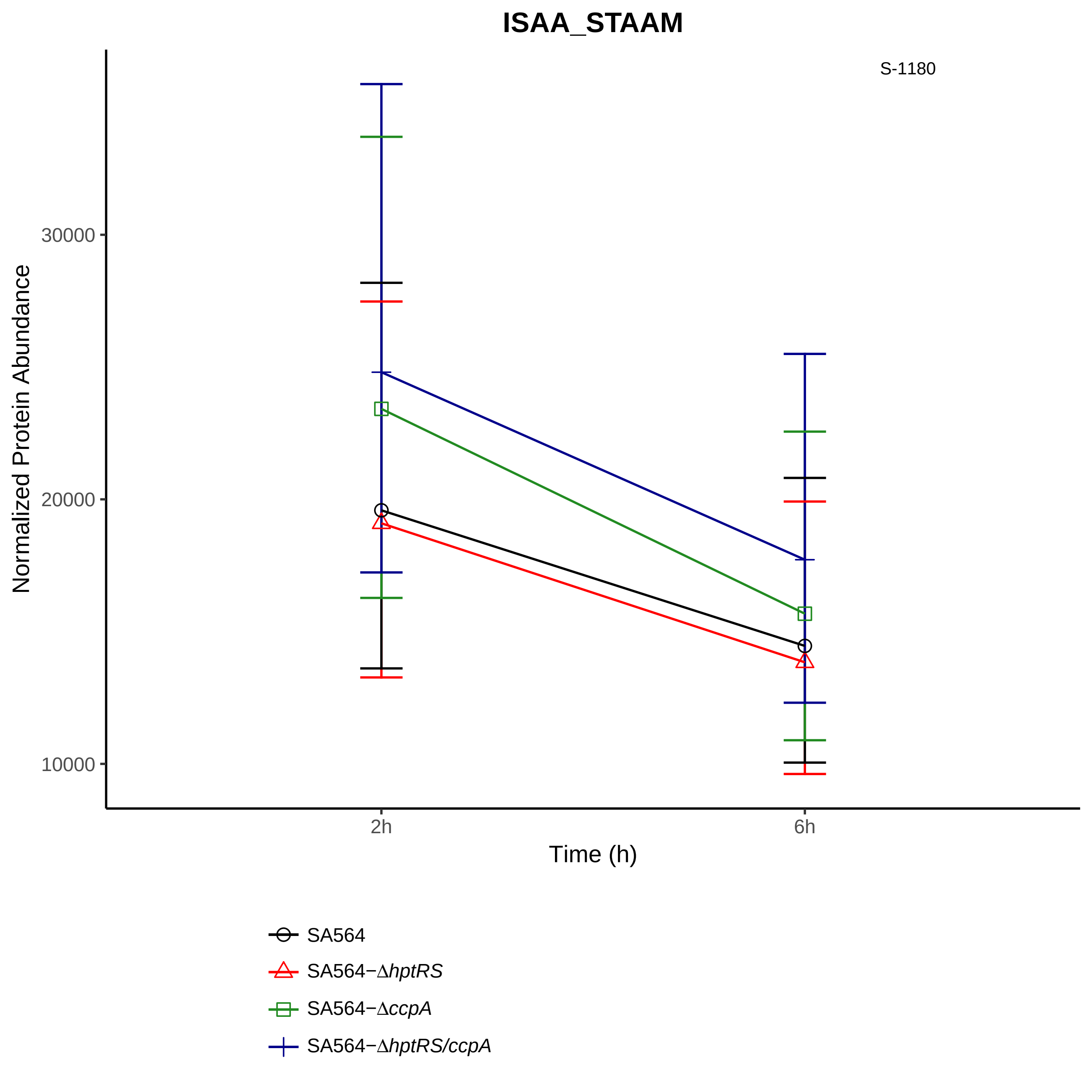

ISAA_STAAM
S-1180
Normalized Protein Abundance
30000
20000
10000
2h
6h
Time (h)
SA564
SA564−Δ*hptRS*
SA564−Δ*ccpA*
SA564−Δ*hptRS/ccpA*

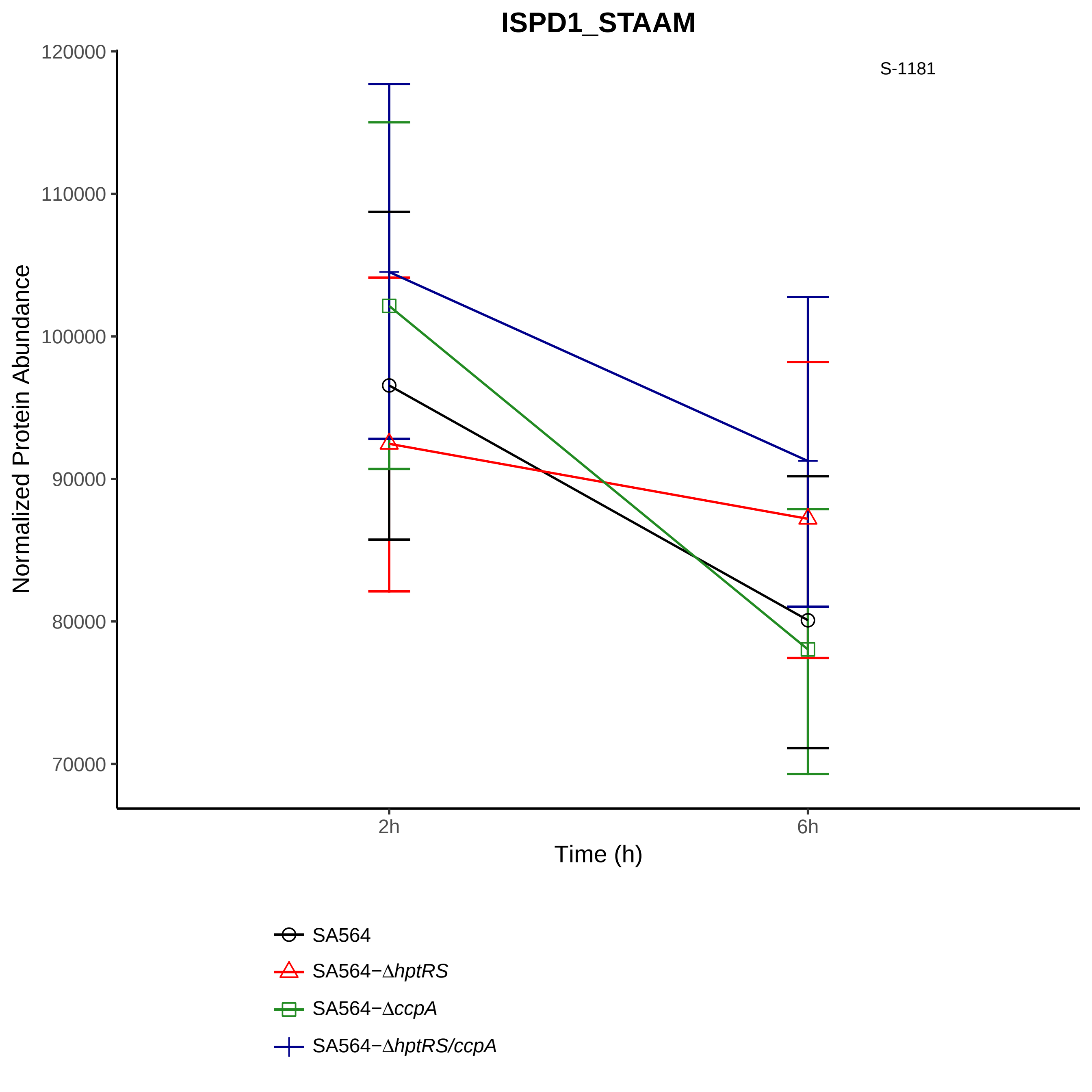

ISPD1_STAAM
S-1181
Normalized Protein Abundance
120000
110000
100000
90000
80000
70000
2h
6h
Time (h)
SA564
SA564−ΔhptRS
SA564−ΔccpA
SA564−ΔhptRS/ccpA

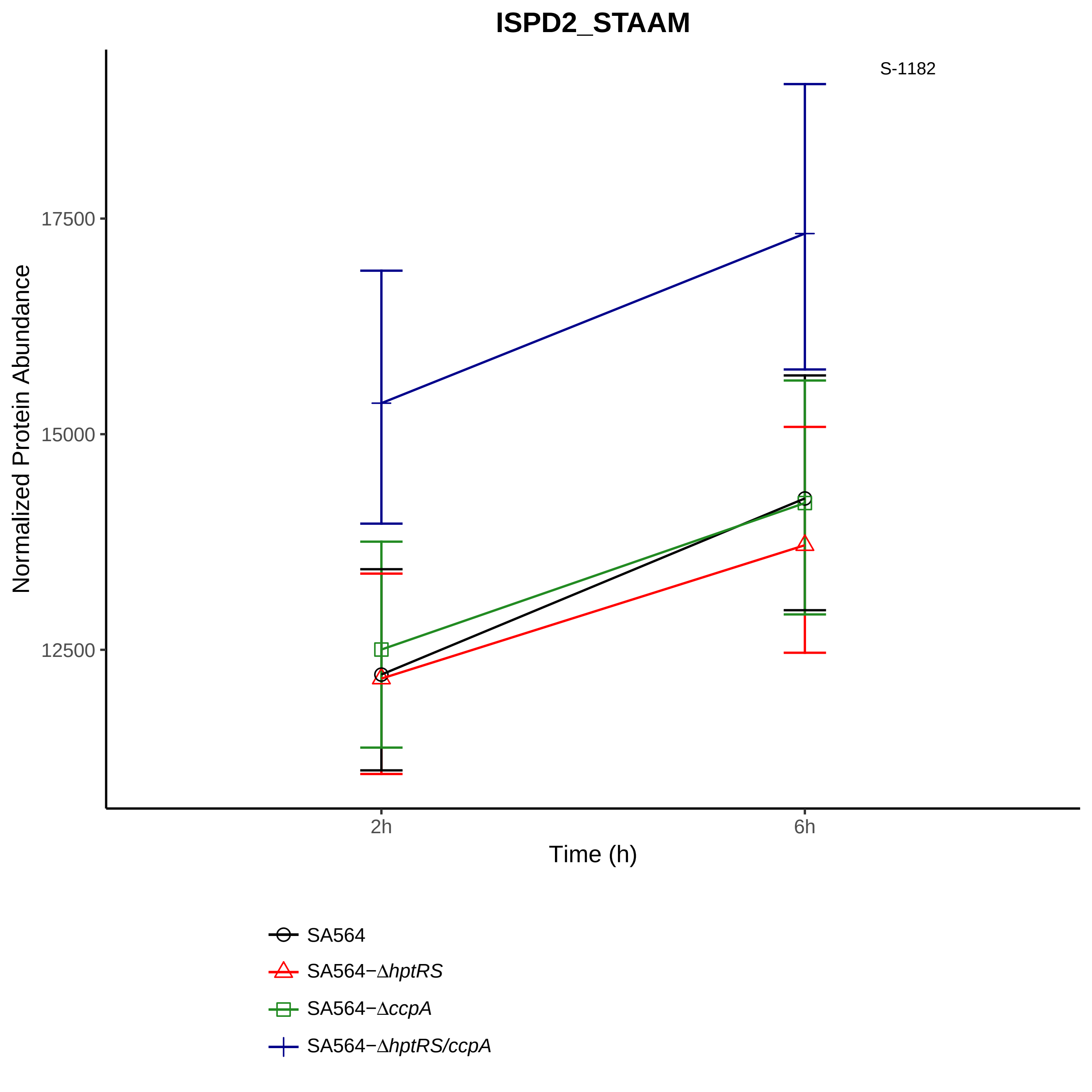

ISPD2_STAAM
S-1182
Normalized Protein Abundance
17500
15000
12500
2h
6h
Time (h)
SA564
SA564−Δ*hptRS*
SA564−Δ*ccpA*
SA564−Δ*hptRS/ccpA*

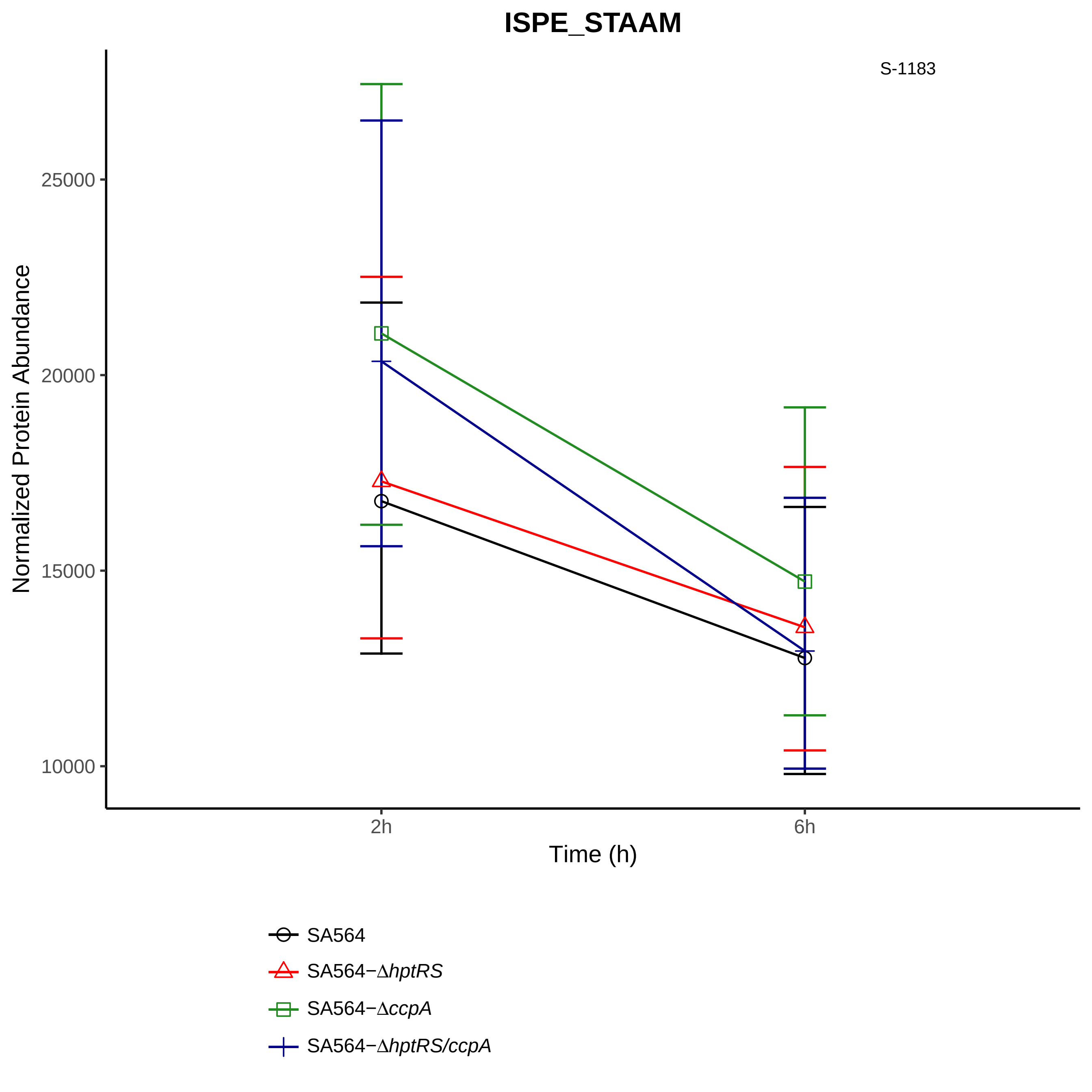

ISPE_STAAM
S-1183
Normalized Protein Abundance
25000
20000
15000
10000
2h
6h
Time (h)
SA564
SA564−Δ*hptRS*
SA564−Δ*ccpA*
SA564−Δ*hptRS/ccpA*

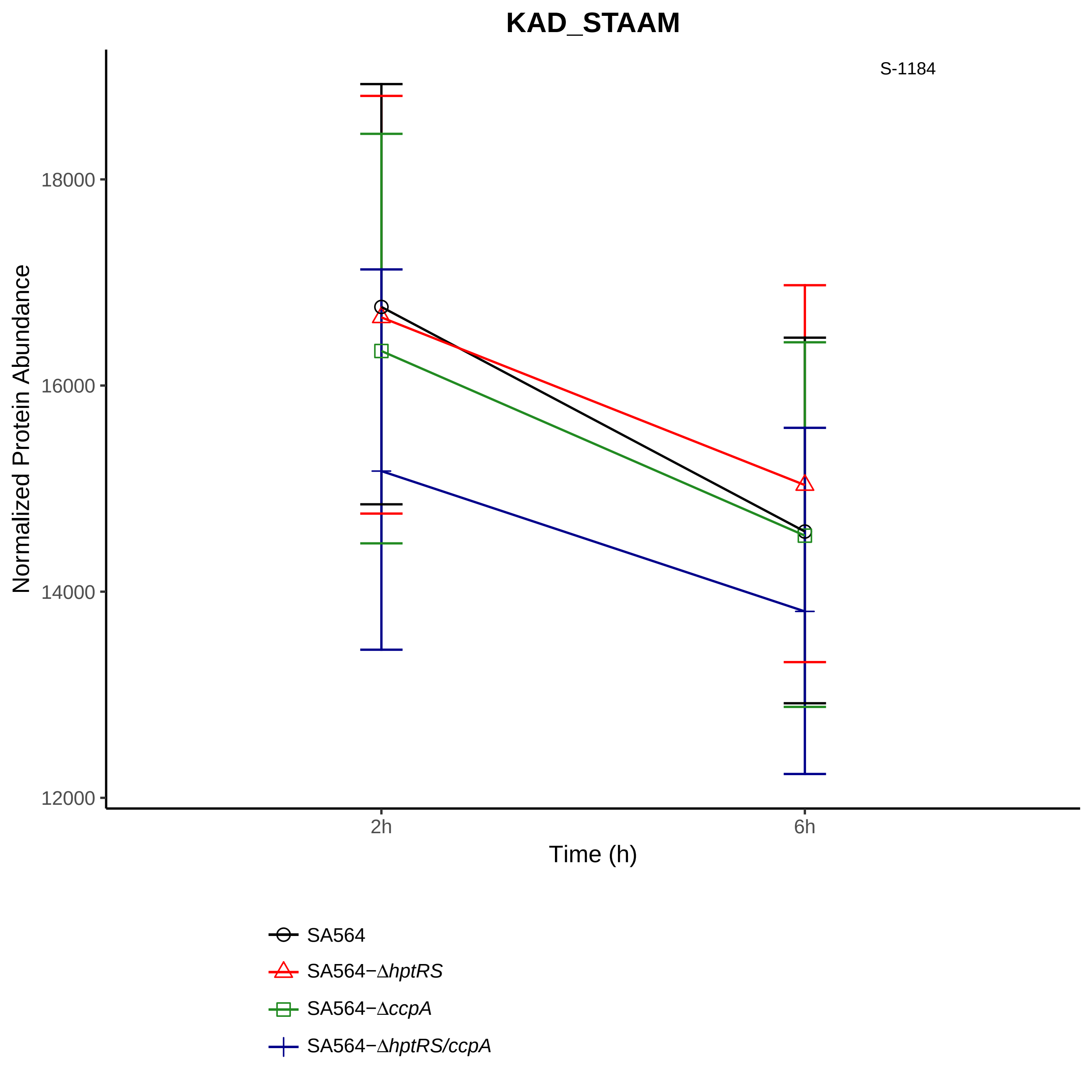

KAD_STAAM
S-1184
Normalized Protein Abundance
18000
16000
14000
12000
2h
6h
Time (h)
SA564
SA564−ΔhptRS
SA564−ΔccpA
SA564−ΔhptRS/ccpA

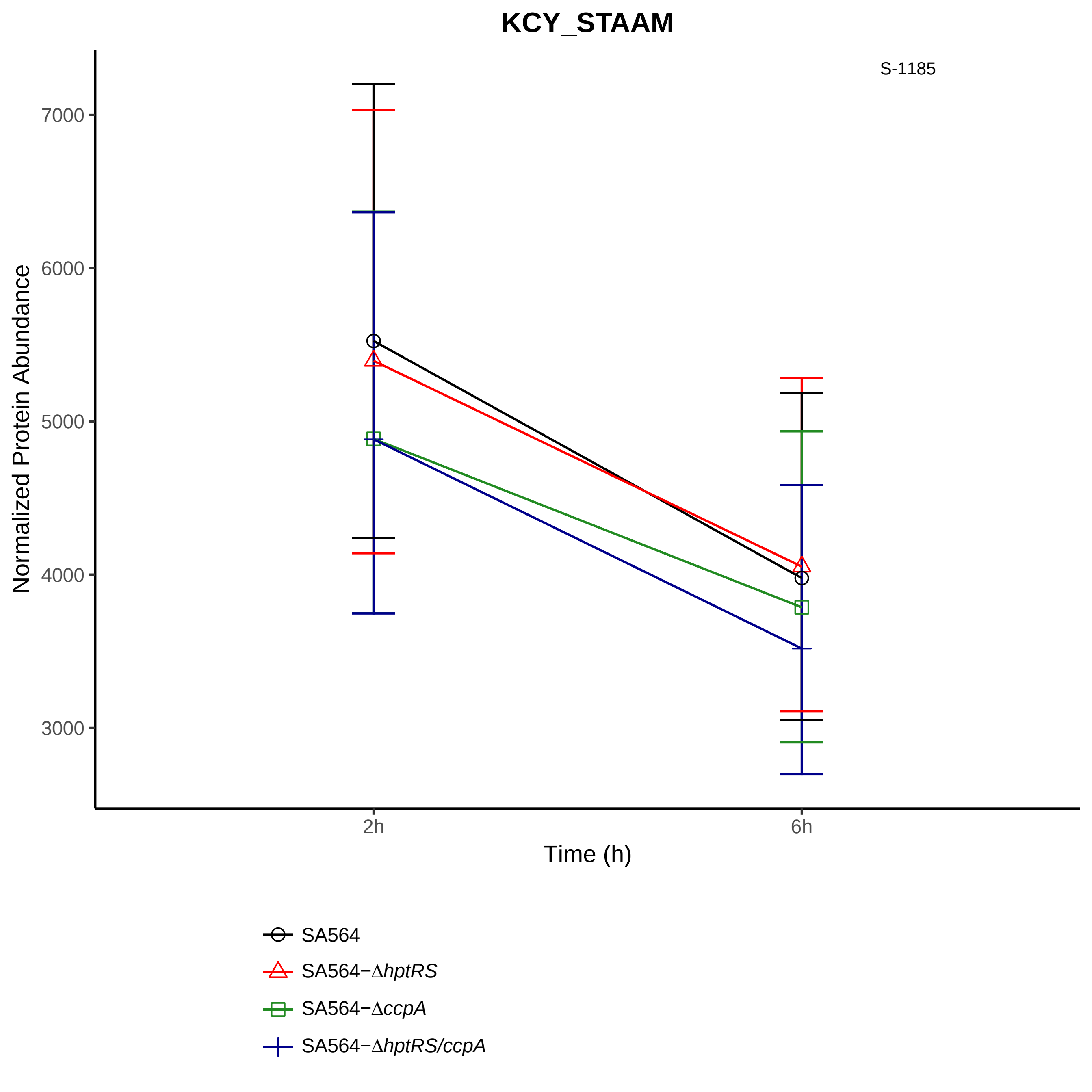

KCY_STAAM
S-1185
Normalized Protein Abundance
7000
6000
5000
4000
3000
2h
6h
Time (h)
SA564
SA564−Δ*hptRS*
SA564−Δ*ccpA*
SA564−Δ*hptRS/ccpA*

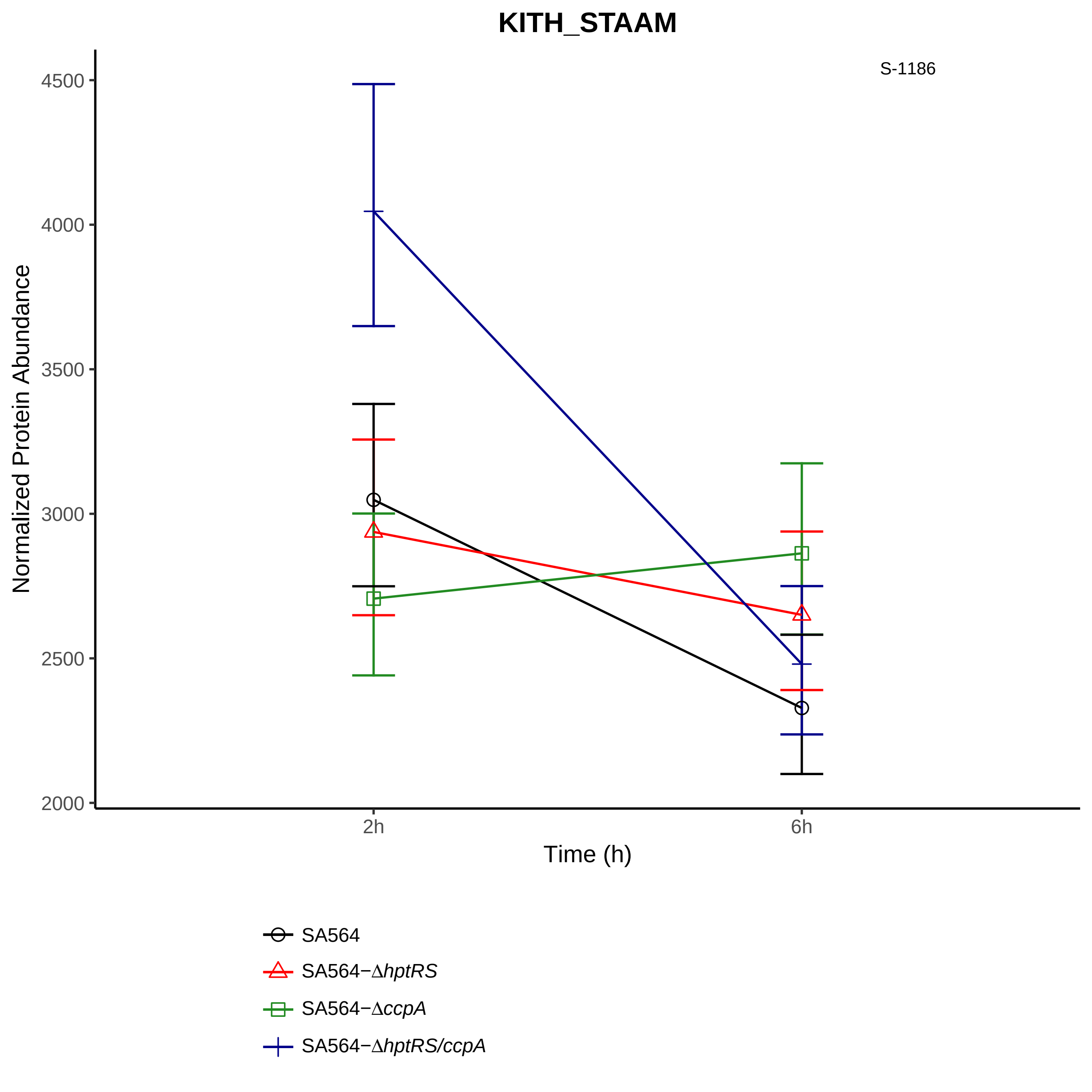

KITH_STAAM
S-1186
Normalized Protein Abundance
4500
4000
3500
3000
2500
2000
2h
6h
Time (h)
SA564
SA564−Δ*hptRS*
SA564−Δ*ccpA*
SA564−Δ*hptRS/ccpA*

KPRS_STAAM

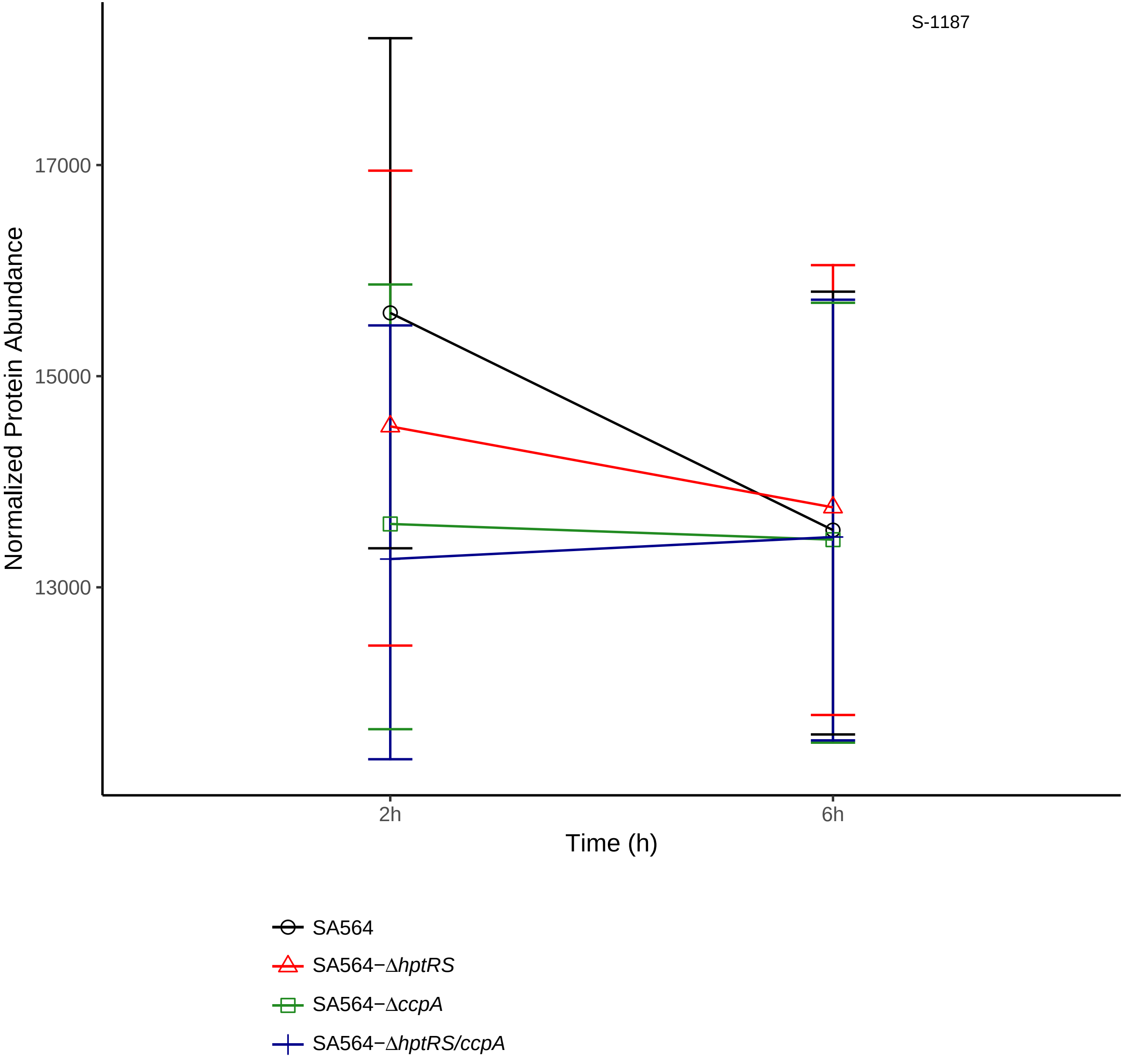

S-1187
Normalized Protein Abundance
17000
15000
13000
2h
6h
Time (h)
SA564
SA564−Δ*hptRS*
SA564−Δ*ccpA*
SA564−Δ*hptRS/ccpA*

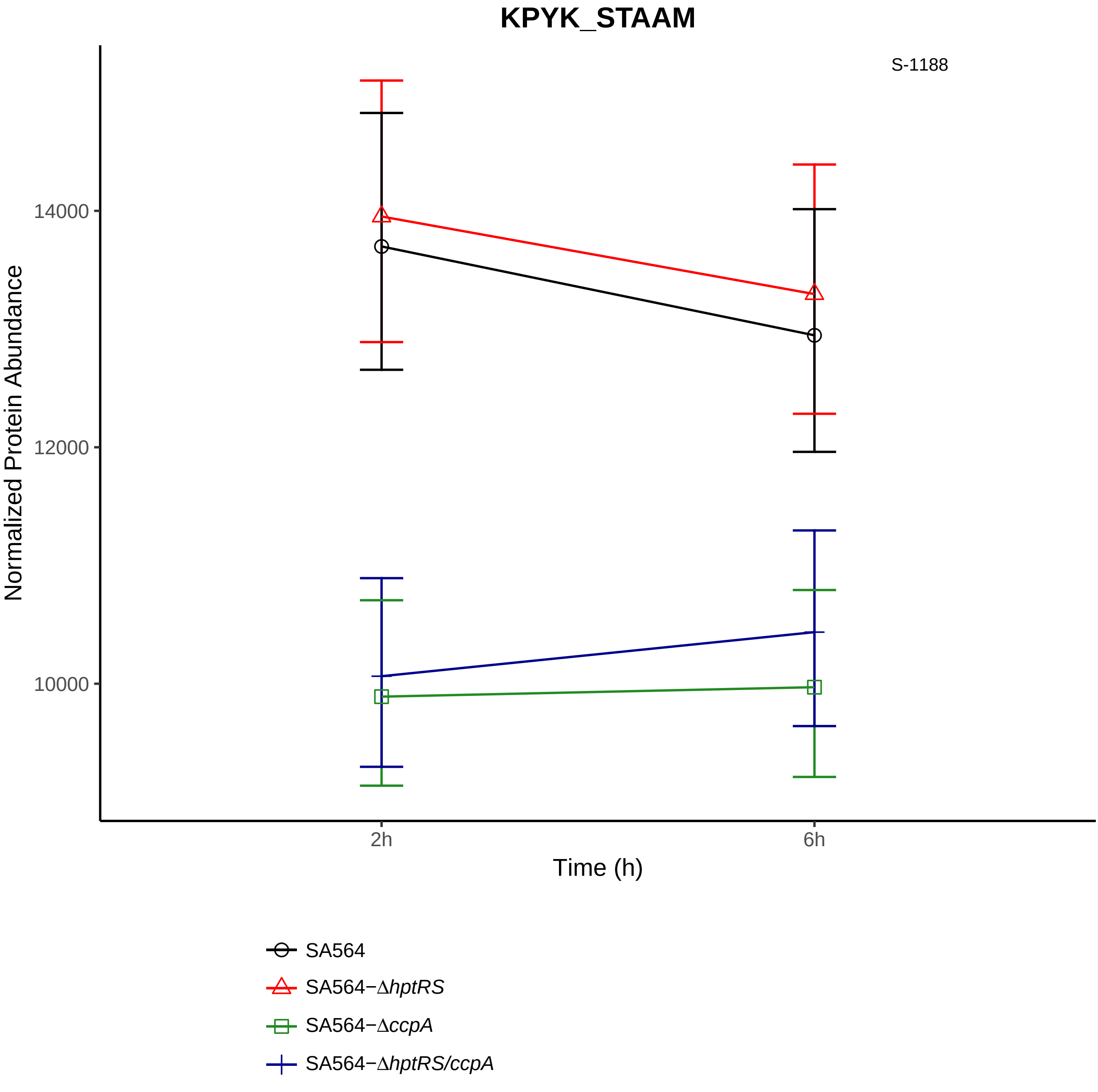

KPYK_STAAM
S-1188
Normalized Protein Abundance
14000
12000
10000
2h
6h
Time (h)
SA564
SA564−Δ*hptRS*
SA564−Δ*ccpA*
SA564−Δ*hptRS/ccpA*

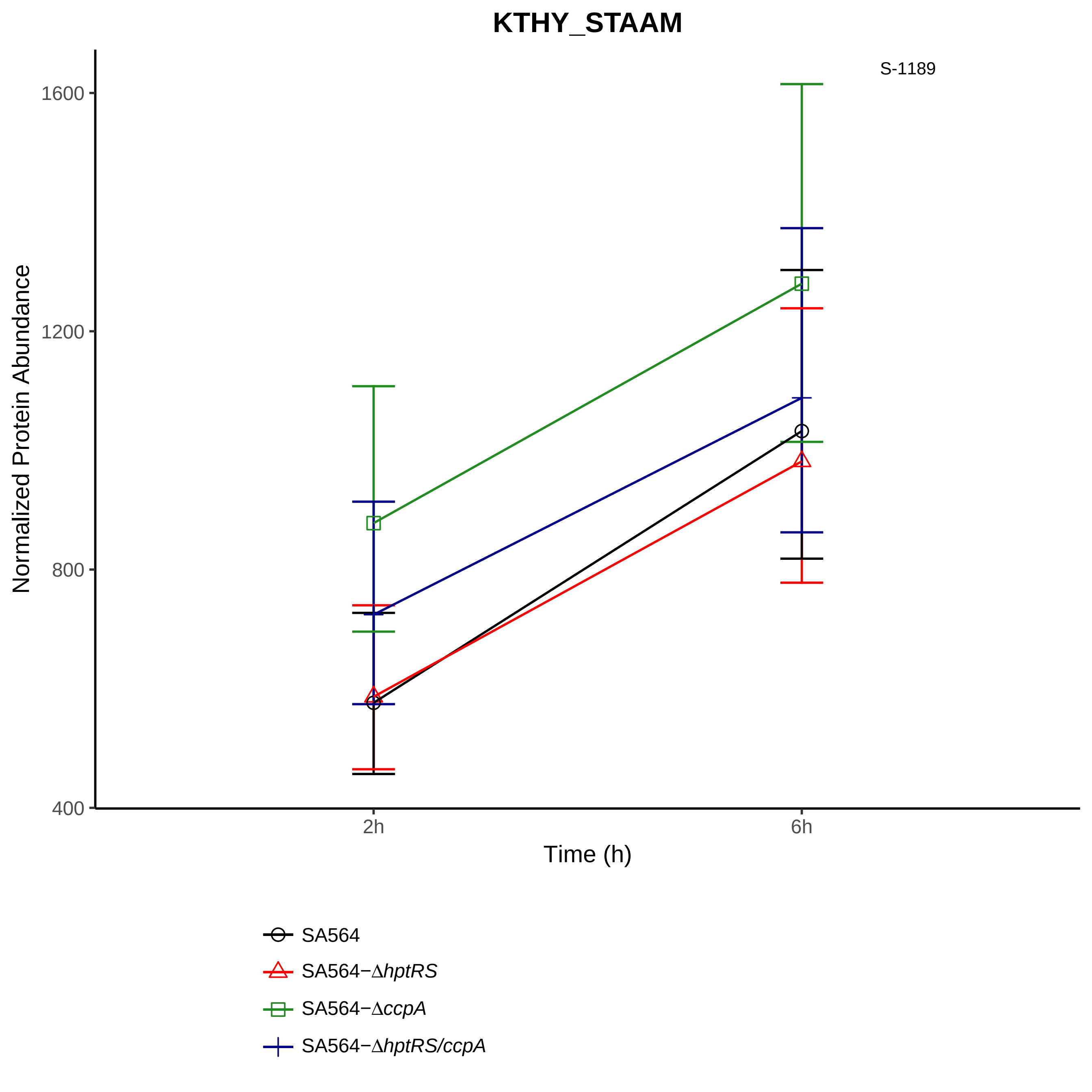

KTHY_STAAM
S-1189
1600
1200
800
400
Normalized Protein Abundance
2h
6h
Time (h)
SA564
SA564−Δ*hptRS*
SA564−Δ*ccpA*
SA564−Δ*hptRS/ccpA*

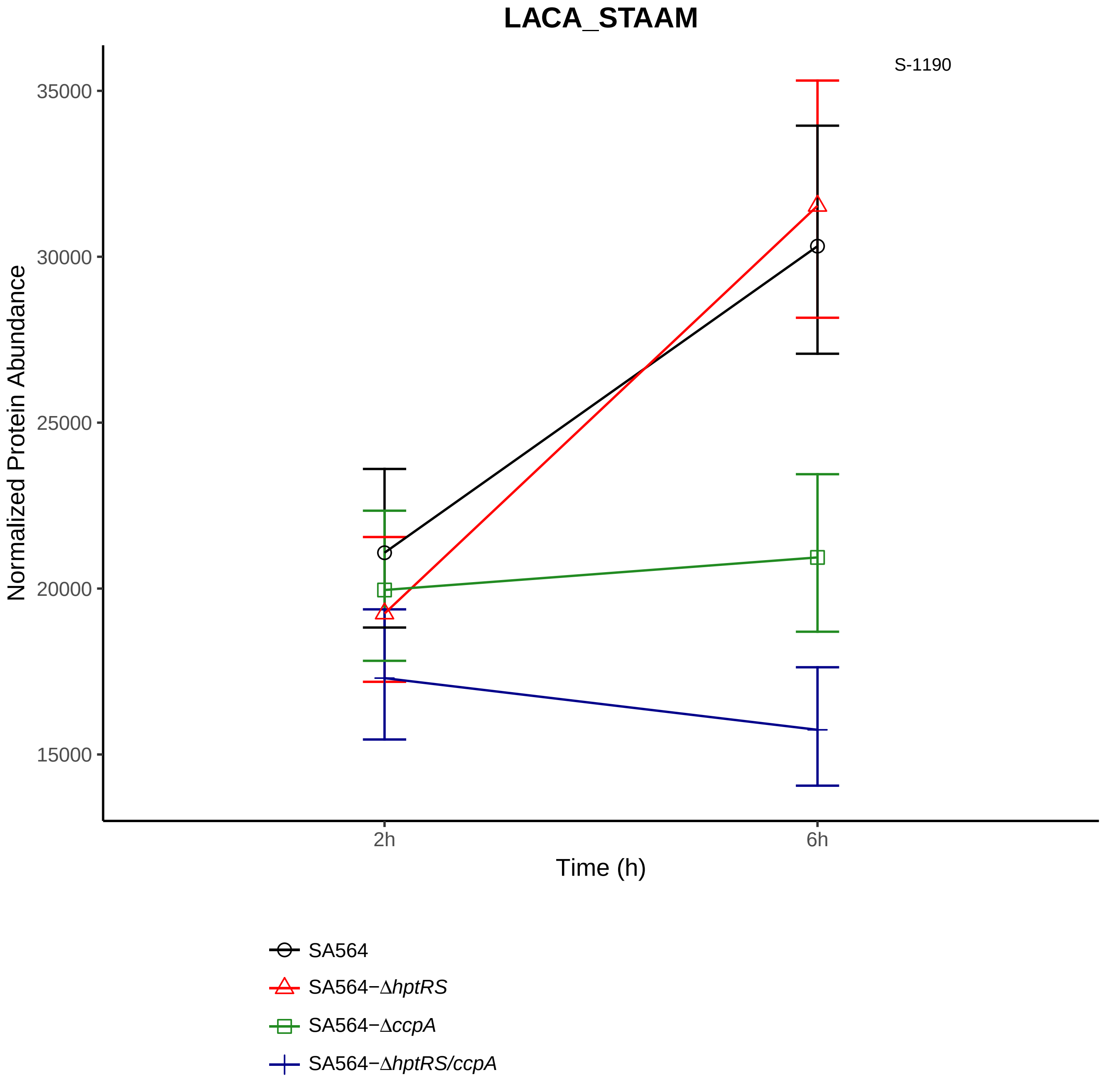

LACA_STAAM
S-1190
35000
30000
25000
20000
15000
Normalized Protein Abundance
2h
6h
Time (h)
SA564
SA564−Δ*hptRS*
SA564−Δ*ccpA*
SA564−Δ*hptRS/ccpA*

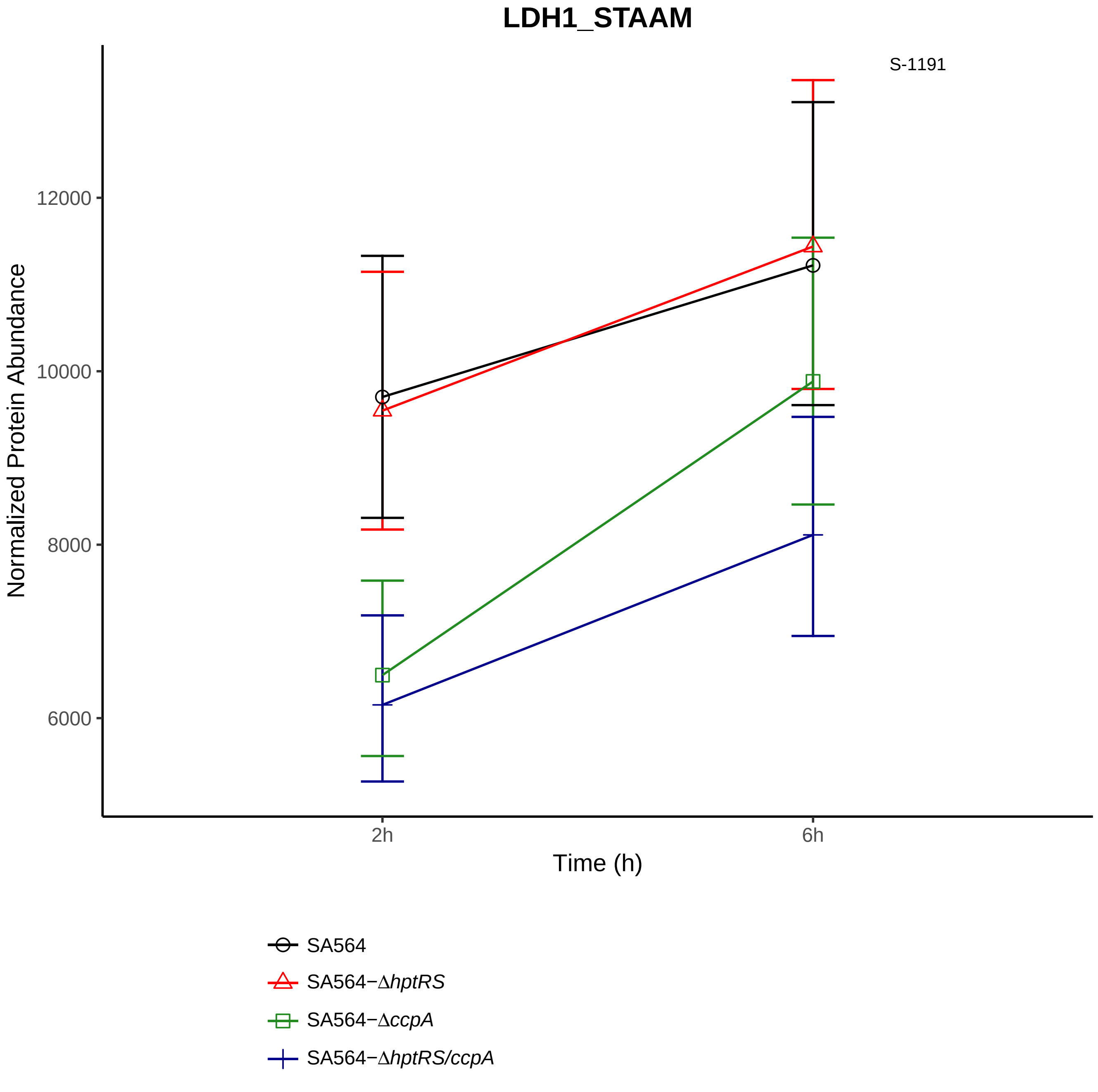

LDH1_STAAM
S-1191
Normalized Protein Abundance
12000
10000
8000
6000
2h
6h
Time (h)
SA564
SA564−Δ*hptRS*
SA564−Δ*ccpA*
SA564−Δ*hptRS/ccpA*

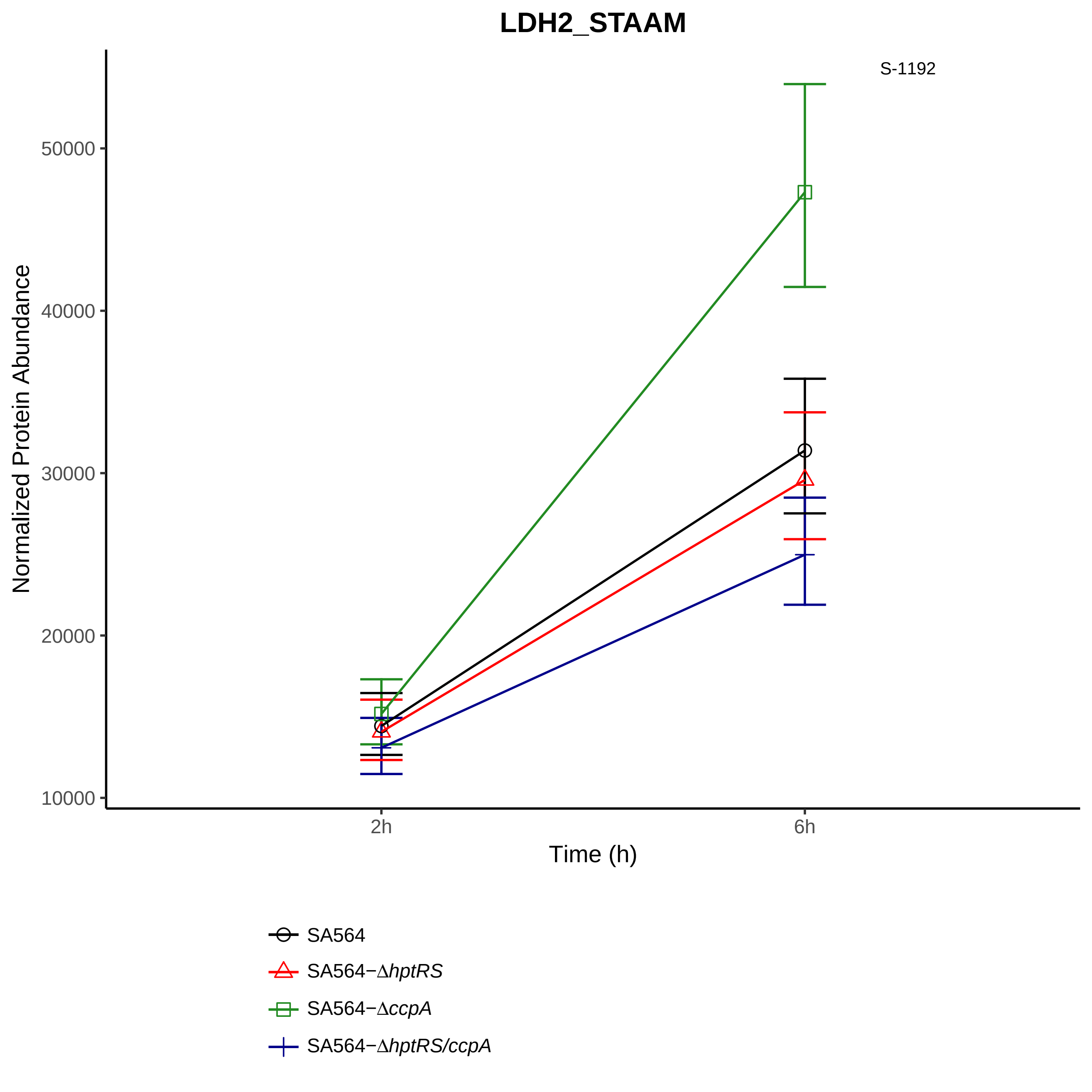

LDH2_STAAM
S-1192
Normalized Protein Abundance
50000
40000
30000
20000
10000
2h
6h
Time (h)
SA564
SA564−Δ*hptRS*
SA564−Δ*ccpA*
SA564−Δ*hptRS/ccpA*

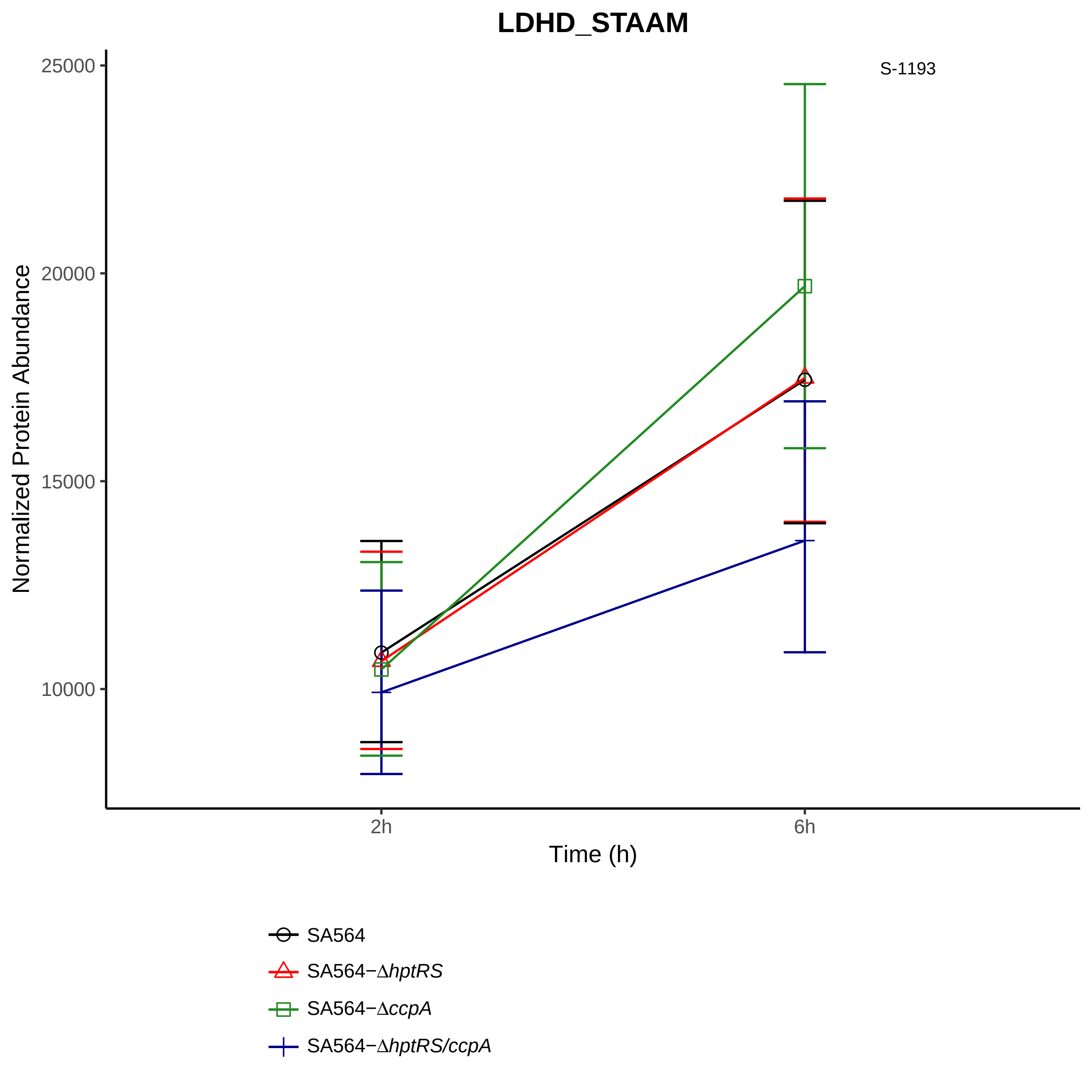

LDHD_STAAM
S-1193
Normalized Protein Abundance
25000
20000
15000
10000
2h
6h
Time (h)
SA564
SA564−Δ*hptRS*
SA564−Δ*ccpA*
SA564−Δ*hptRS/ccpA*

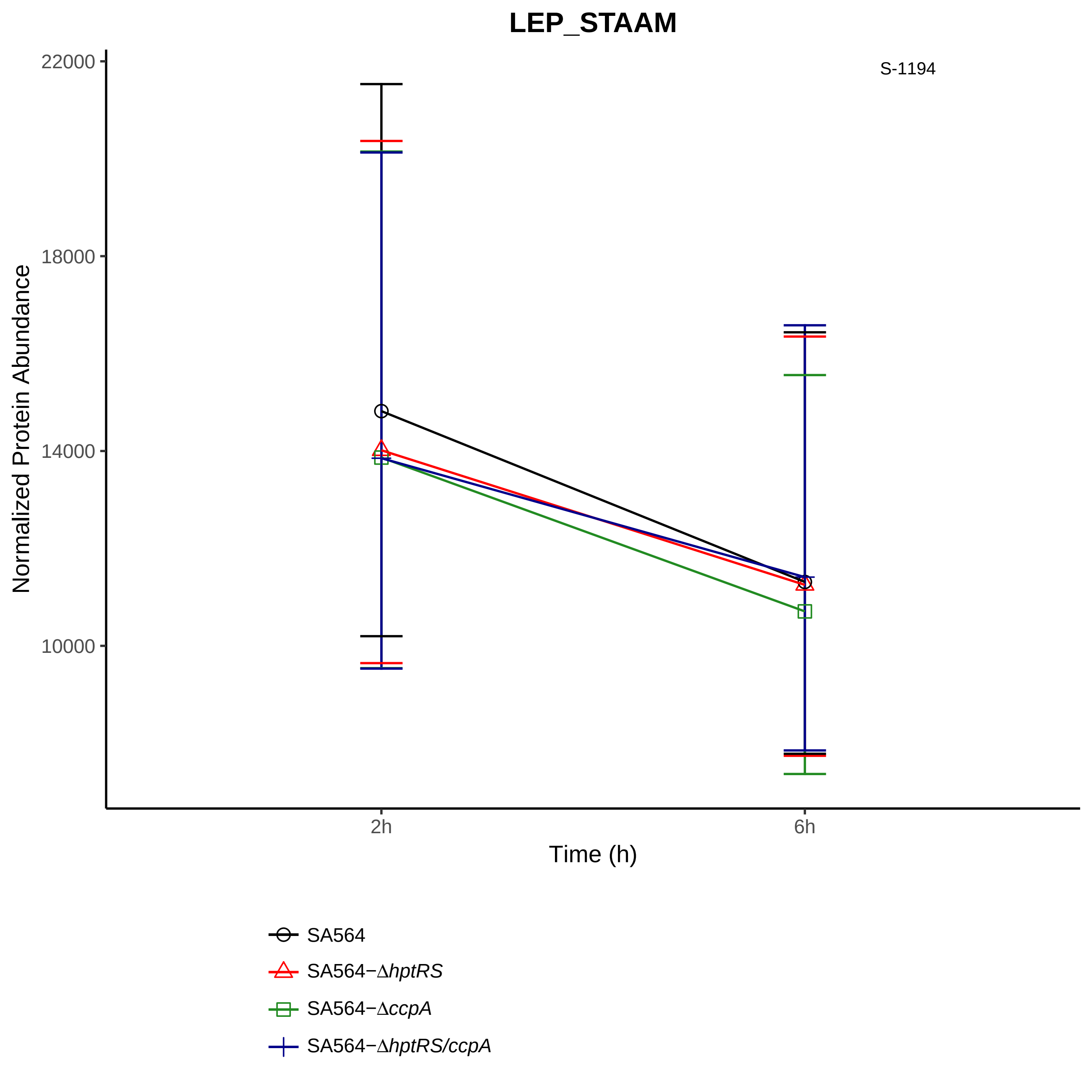

LEP_STAAM
S-1194
22000
18000
14000
10000
Normalized Protein Abundance
2h
6h
Time (h)
SA564
SA564−Δ*hptRS*
SA564−Δ*ccpA*
SA564−Δ*hptRS/ccpA*

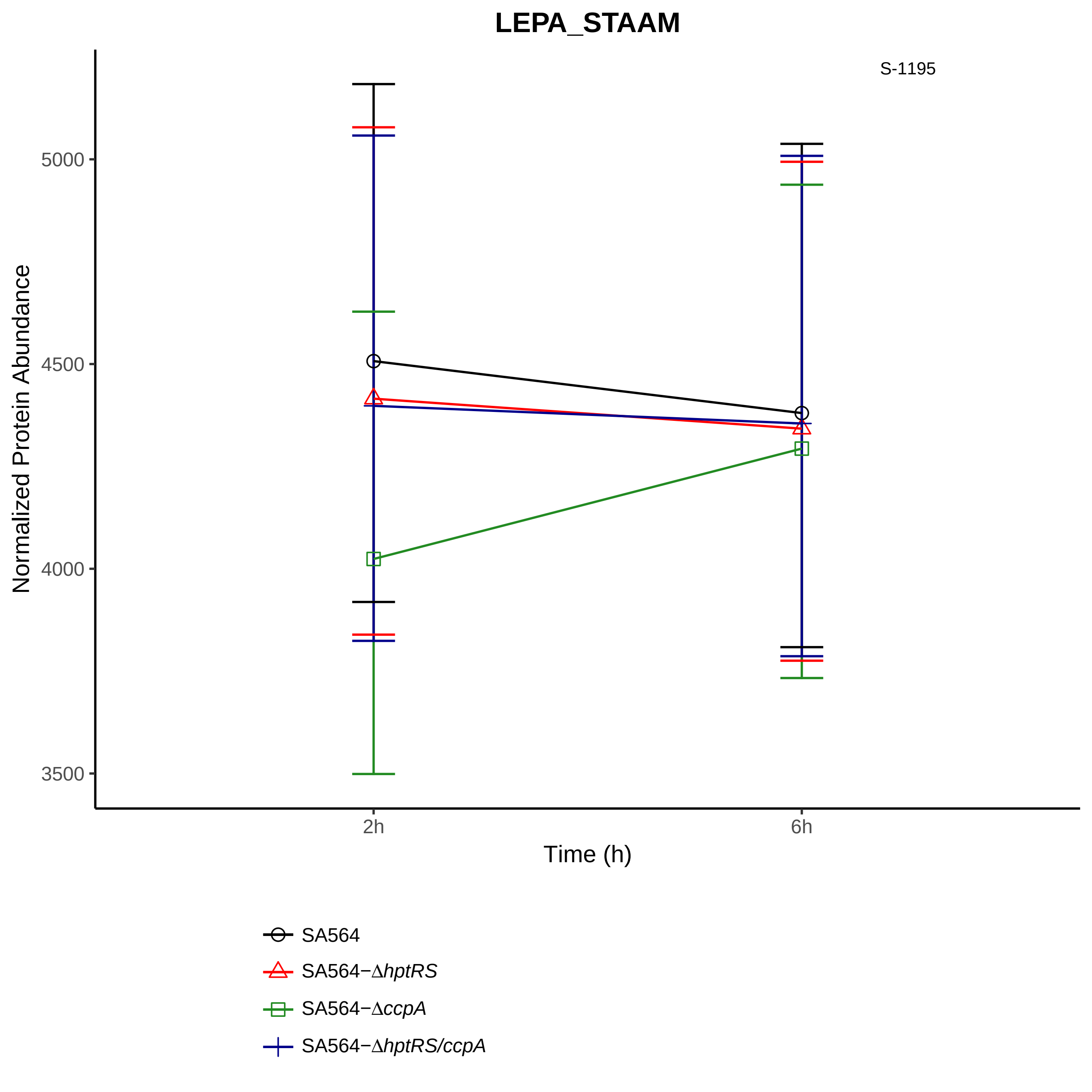

LEPA_STAAM
S-1195
Normalized Protein Abundance
5000
4500
4000
3500
2h
6h
Time (h)
SA564
SA564−Δ*hptRS*
SA564−Δ*ccpA*
SA564−Δ*hptRS/ccpA*

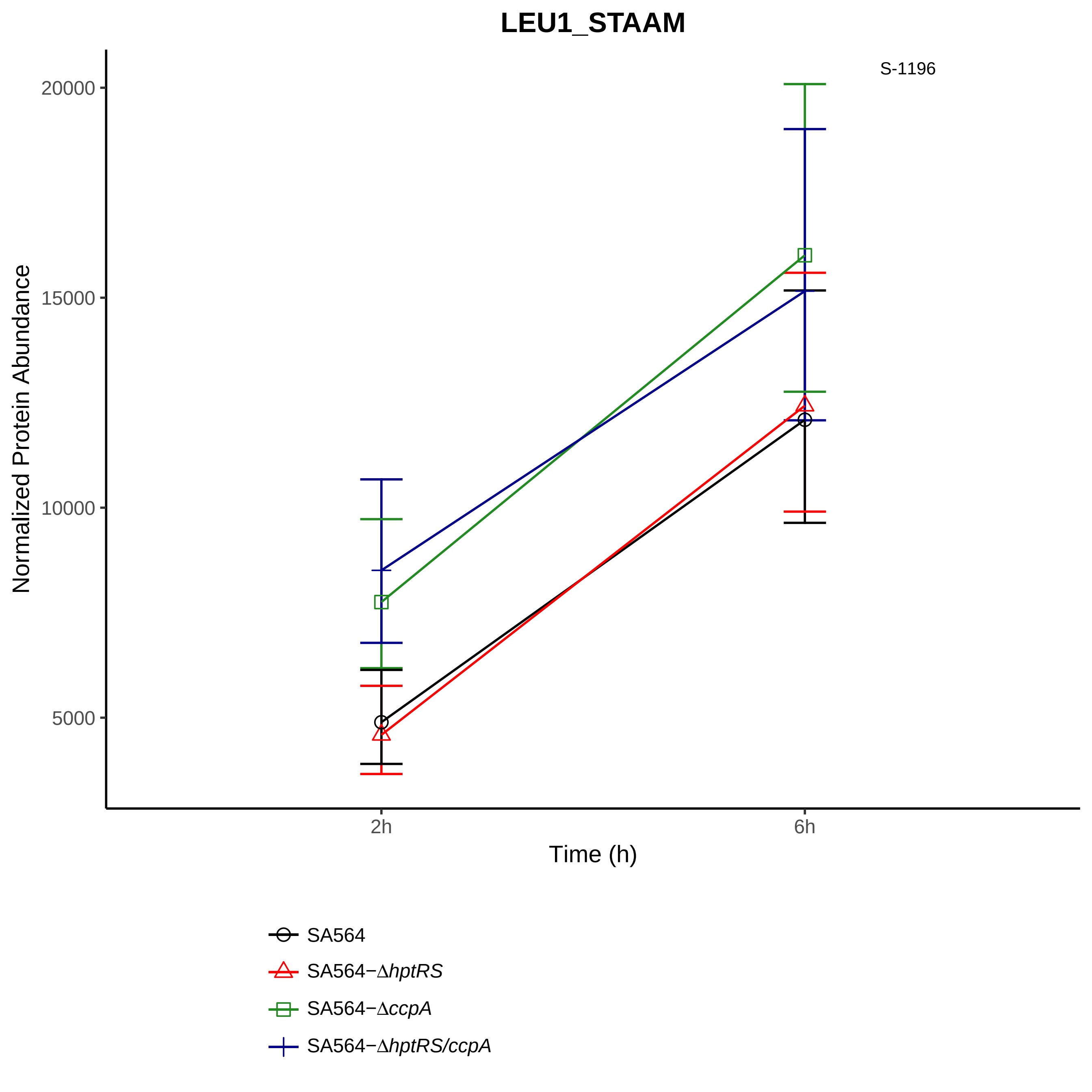

LEU1_STAAM
S-1196
Normalized Protein Abundance
20000
15000
10000
5000
2h
6h
Time (h)
SA564
SA564−Δ*hptRS*
SA564−Δ*ccpA*
SA564−Δ*hptRS/ccpA*

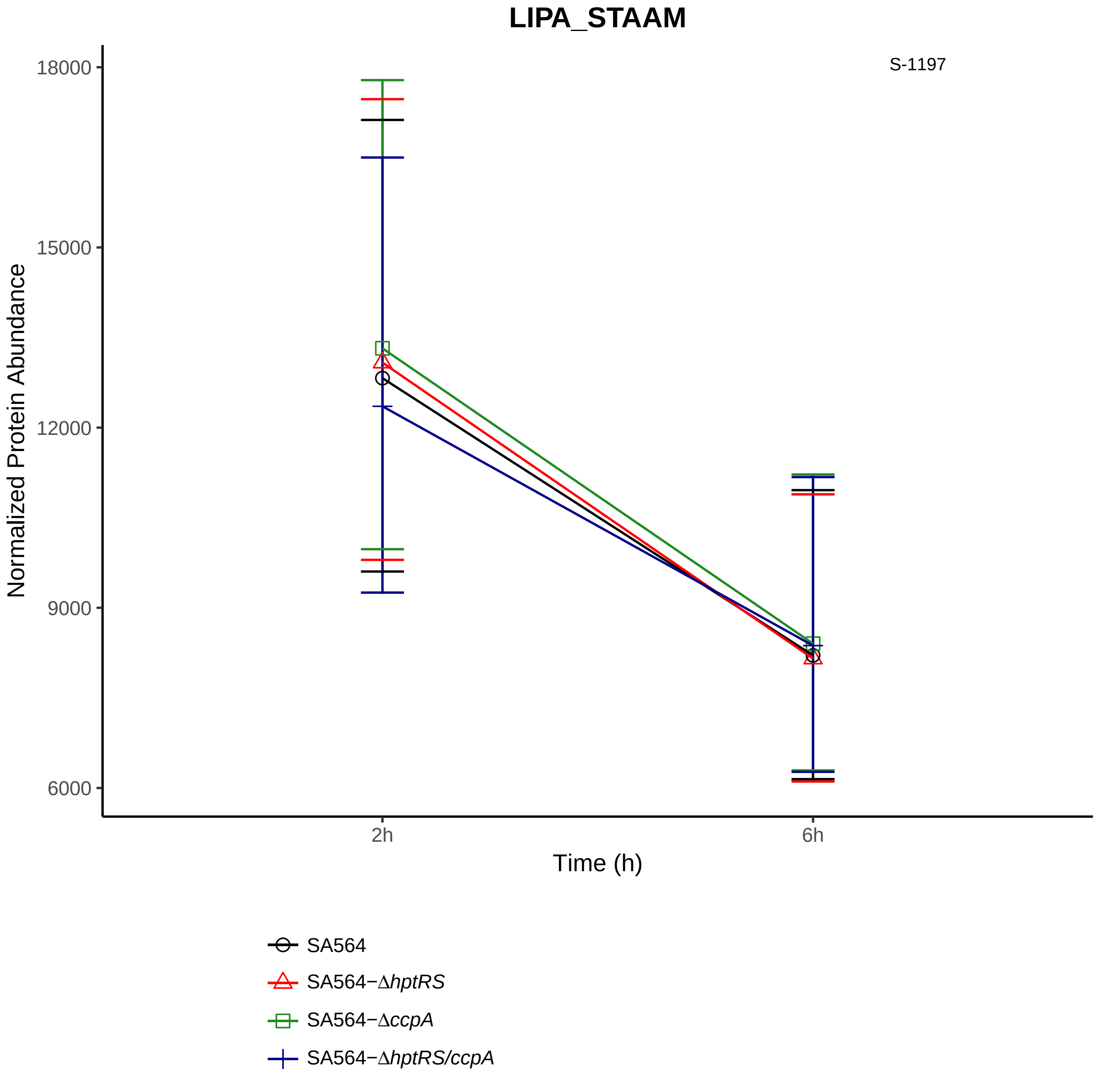

LIPA_STAAM
S-1197
18000
15000
12000
9000
6000
Normalized Protein Abundance
2h
6h
Time (h)
SA564
SA564−Δ*hptRS*
SA564−Δ*ccpA*
SA564−Δ*hptRS/ccpA*

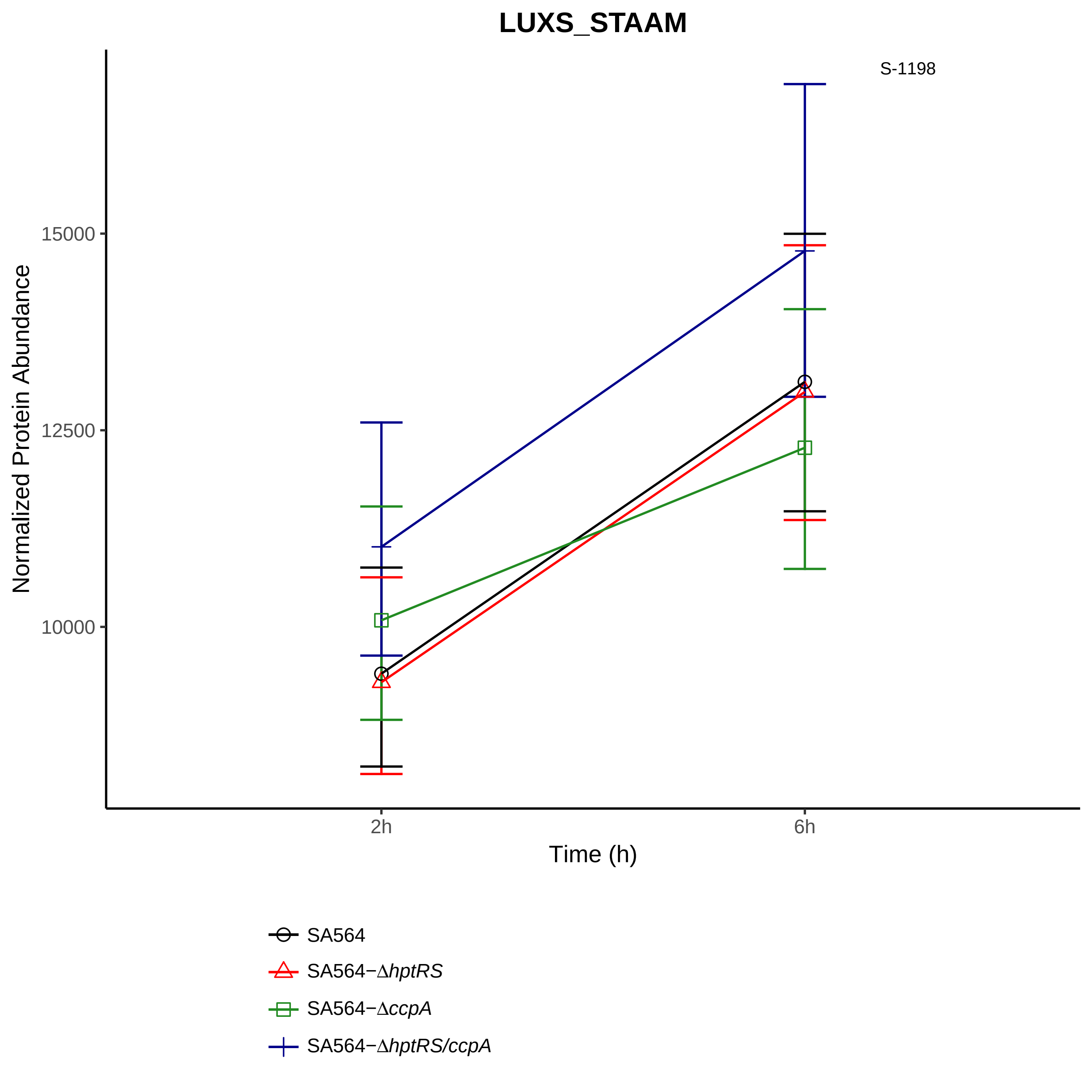

LUXS_STAAM
S-1198
15000
12500
10000
Normalized Protein Abundance
2h
6h
Time (h)
SA564
SA564−Δ*hptRS*
SA564−Δ*ccpA*
SA564−Δ*hptRS/ccpA*

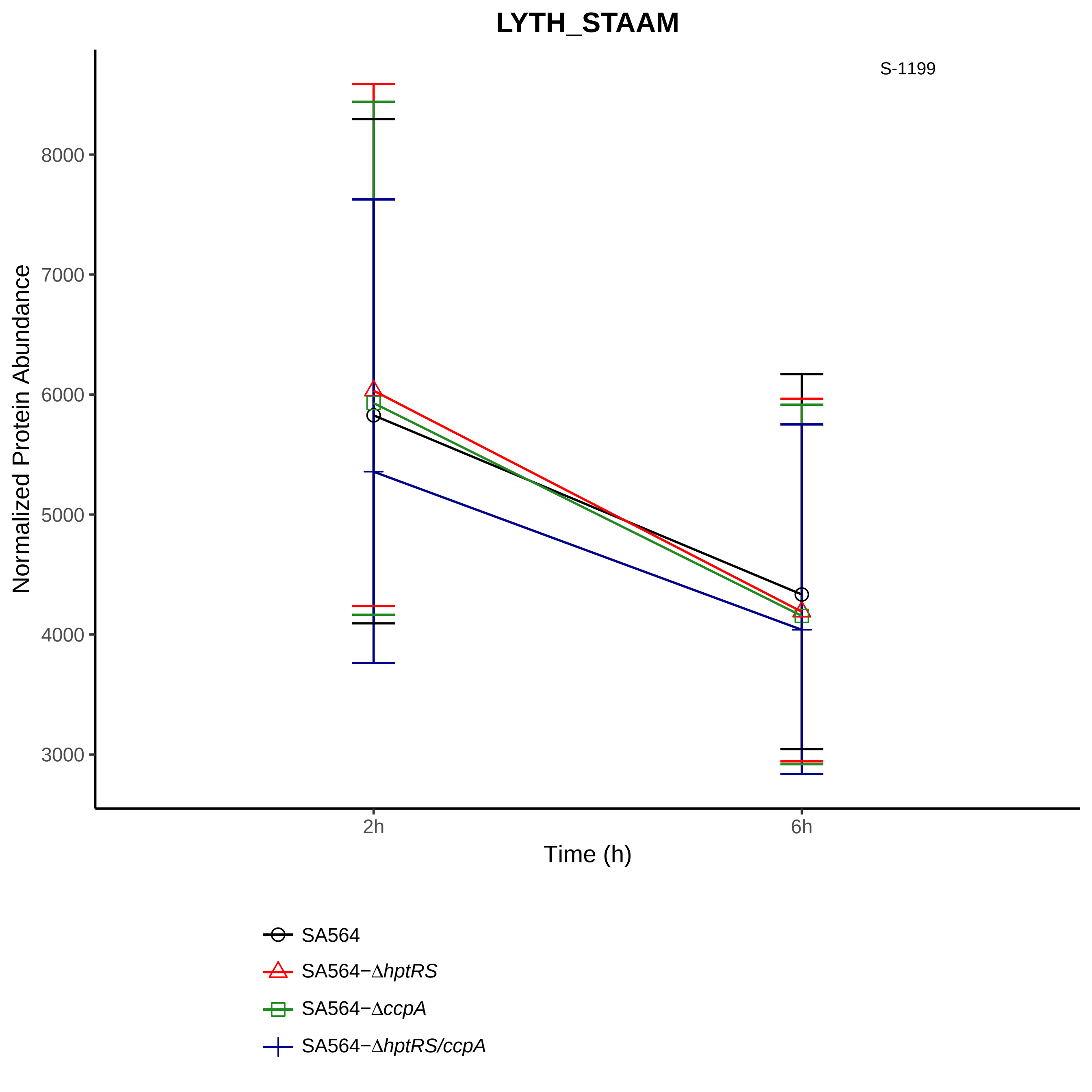
LYTH_STAAM
S-1199
Normalized Protein Abundance
8000
7000
6000
5000
4000
3000
2h
6h
Time (h)
SA564
SA564−Δ*hptRS*
SA564−Δ*ccpA*
SA564−Δ*hptRS/ccpA*

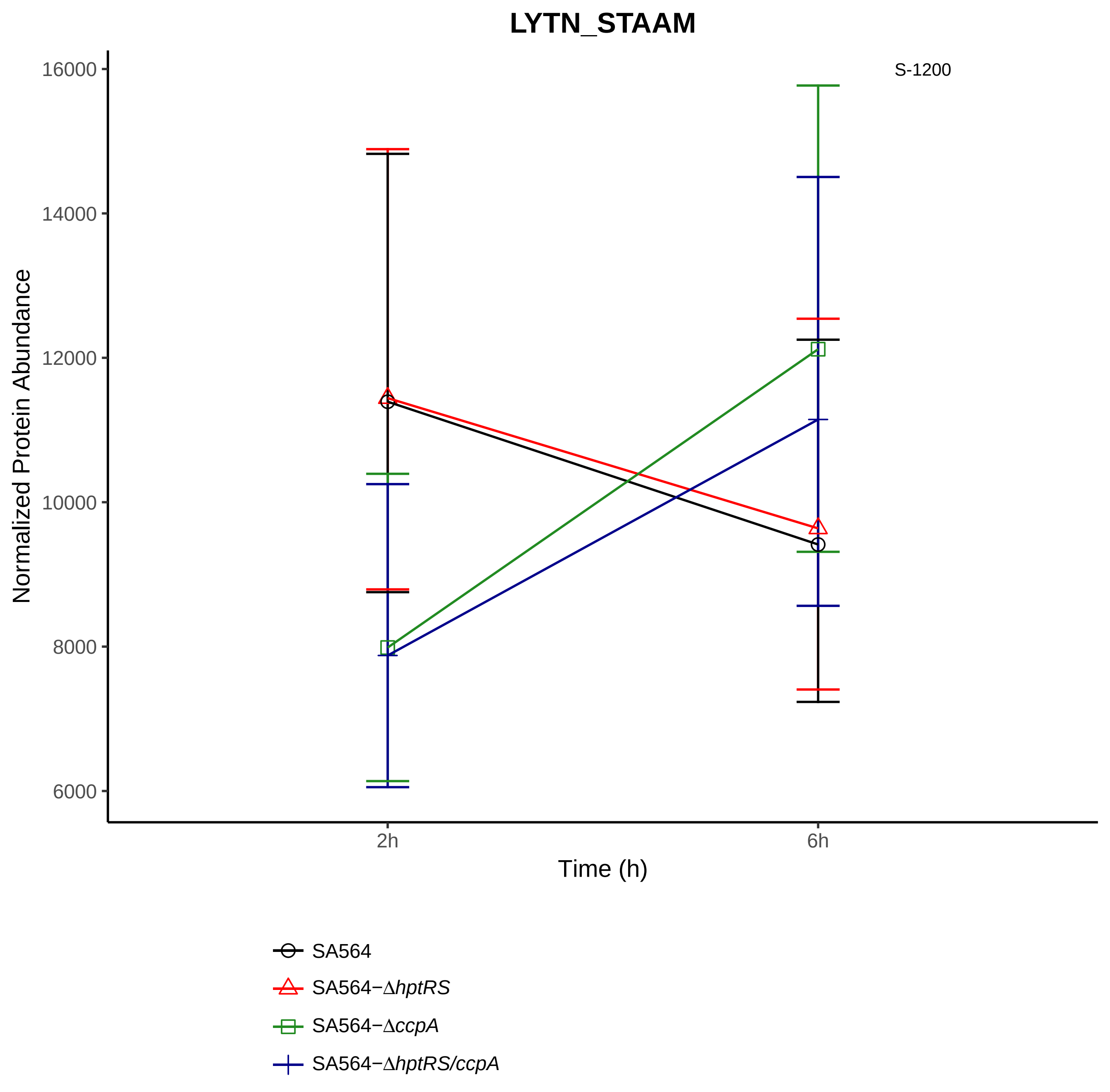

LYTN_STAAM
S-1200
16000
14000
12000
10000
8000
6000
Normalized Protein Abundance
2h
6h
Time (h)
SA564
SA564−Δ*hptRS*
SA564−Δ*ccpA*
SA564−Δ*hptRS/ccpA*

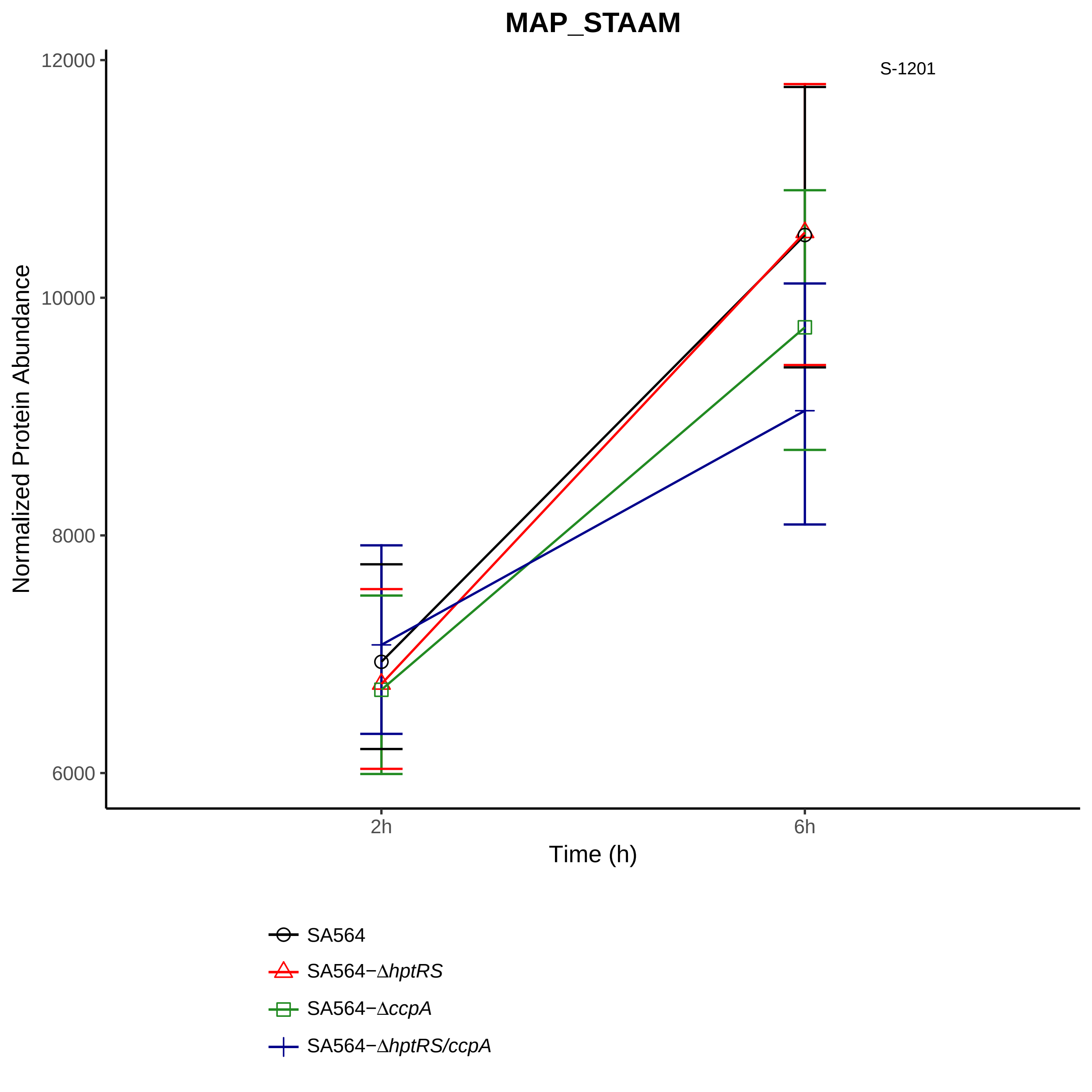

MAP_STAAM
S-1201
Normalized Protein Abundance
12000
10000
8000
6000
2h
6h
Time (h)
SA564
SA564−Δ*hptRS*
SA564−Δ*ccpA*
SA564−Δ*hptRS/ccpA*

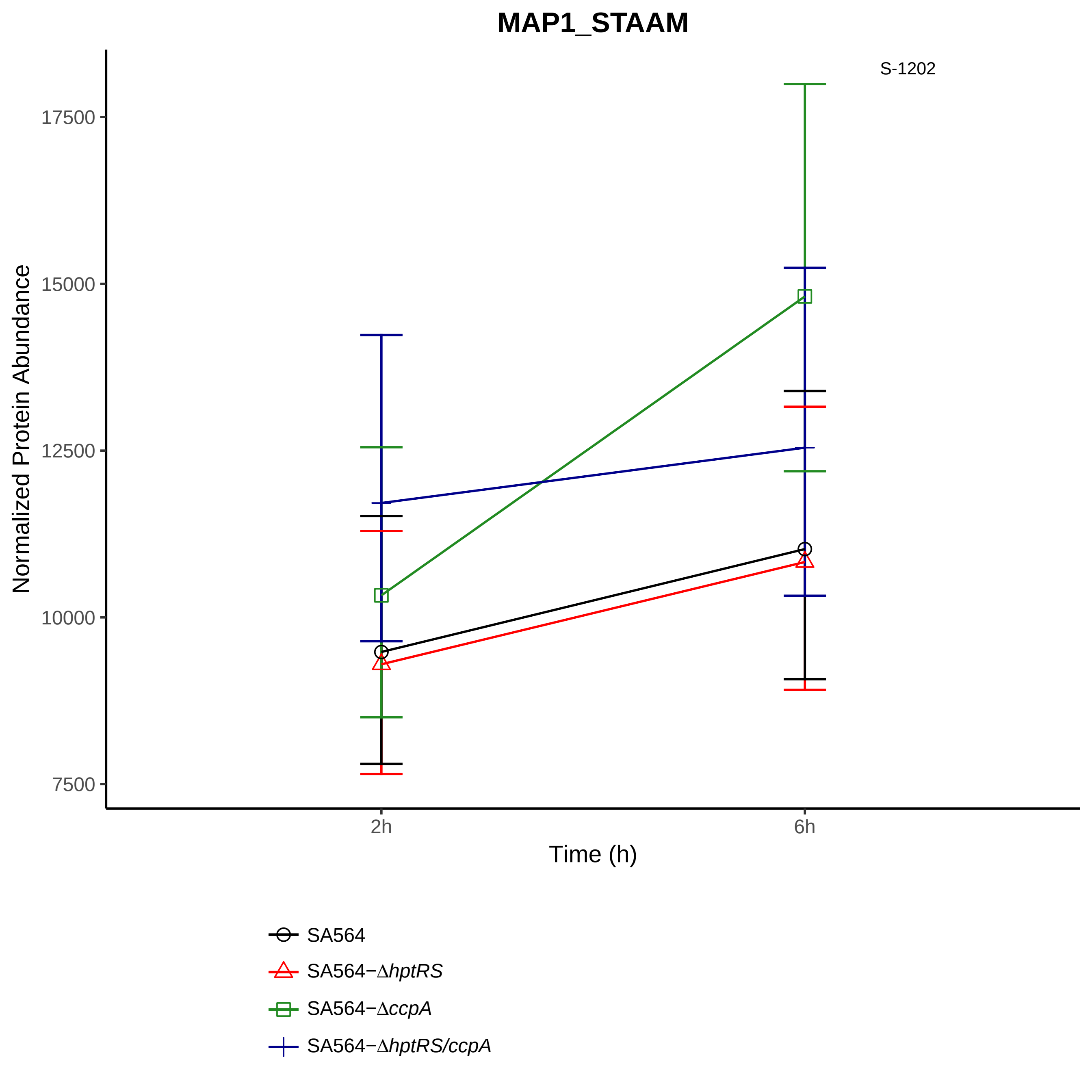

MAP1_STAAM
S-1202
17500
15000
12500
10000
7500
Normalized Protein Abundance
2h
6h
Time (h)
SA564
SA564−Δ*hptRS*
SA564−Δ*ccpA*
SA564−Δ*hptRS/ccpA*

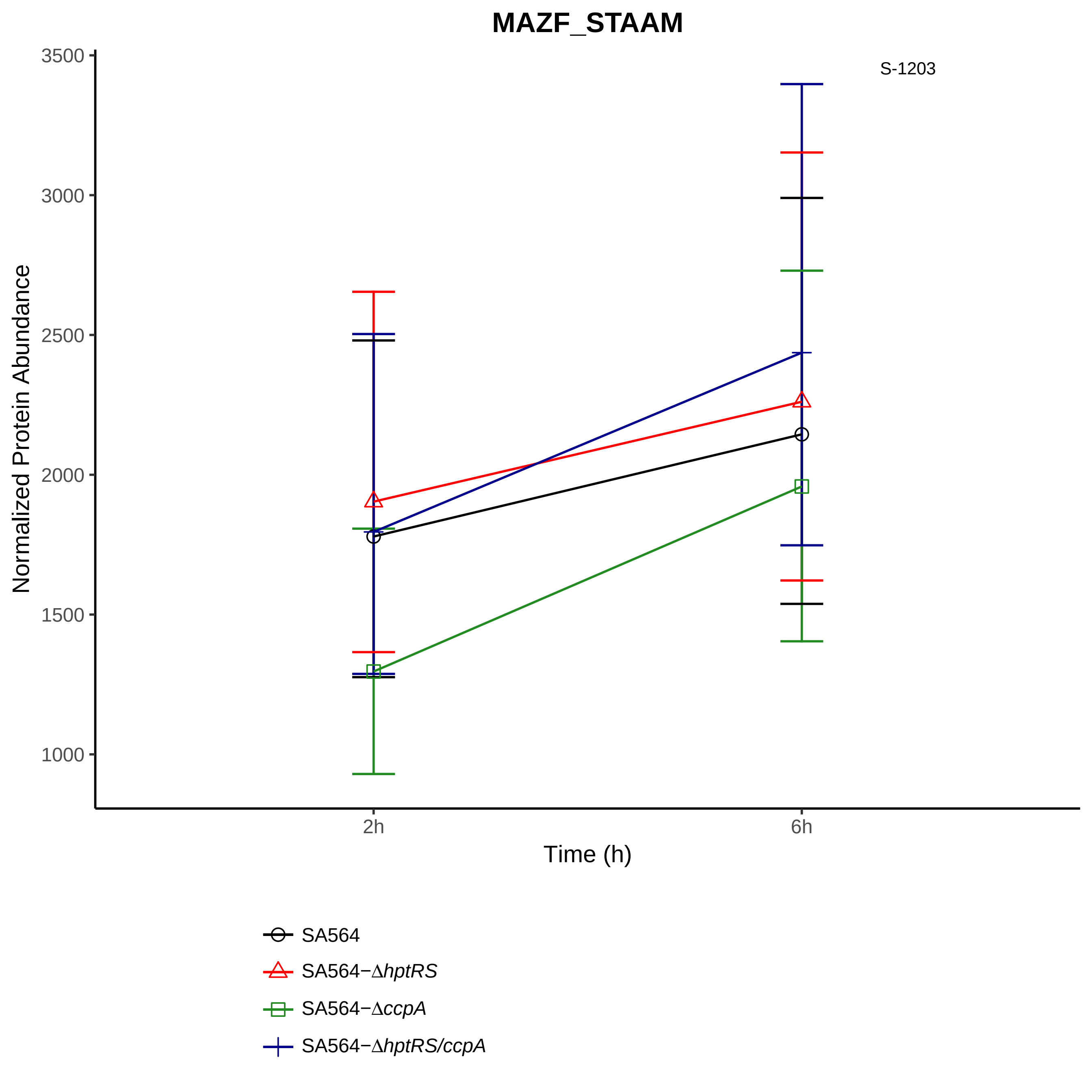

MAZF_STAAM
S-1203
Normalized Protein Abundance
3500
3000
2500
2000
1500
1000
2h
6h
Time (h)
SA564
SA564−ΔhptRS
SA564−ΔccpA
SA564−ΔhptRS/ccpA

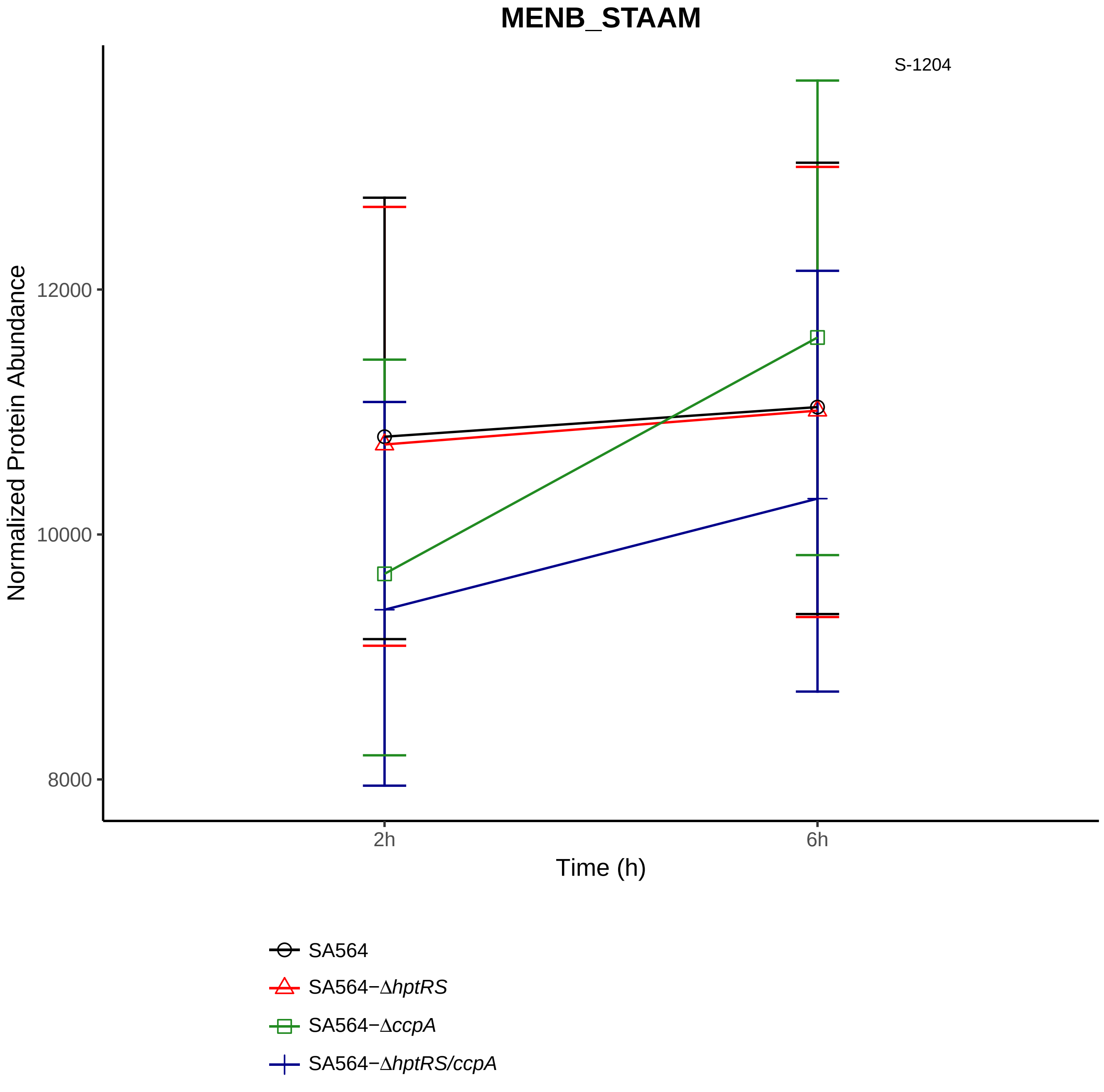
MENB_STAAM
S-1204
Normalized Protein Abundance
12000
10000
8000
2h
6h
Time (h)
SA564
SA564−Δ*hptRS*
SA564−Δ*ccpA*
SA564−Δ*hptRS/ccpA*

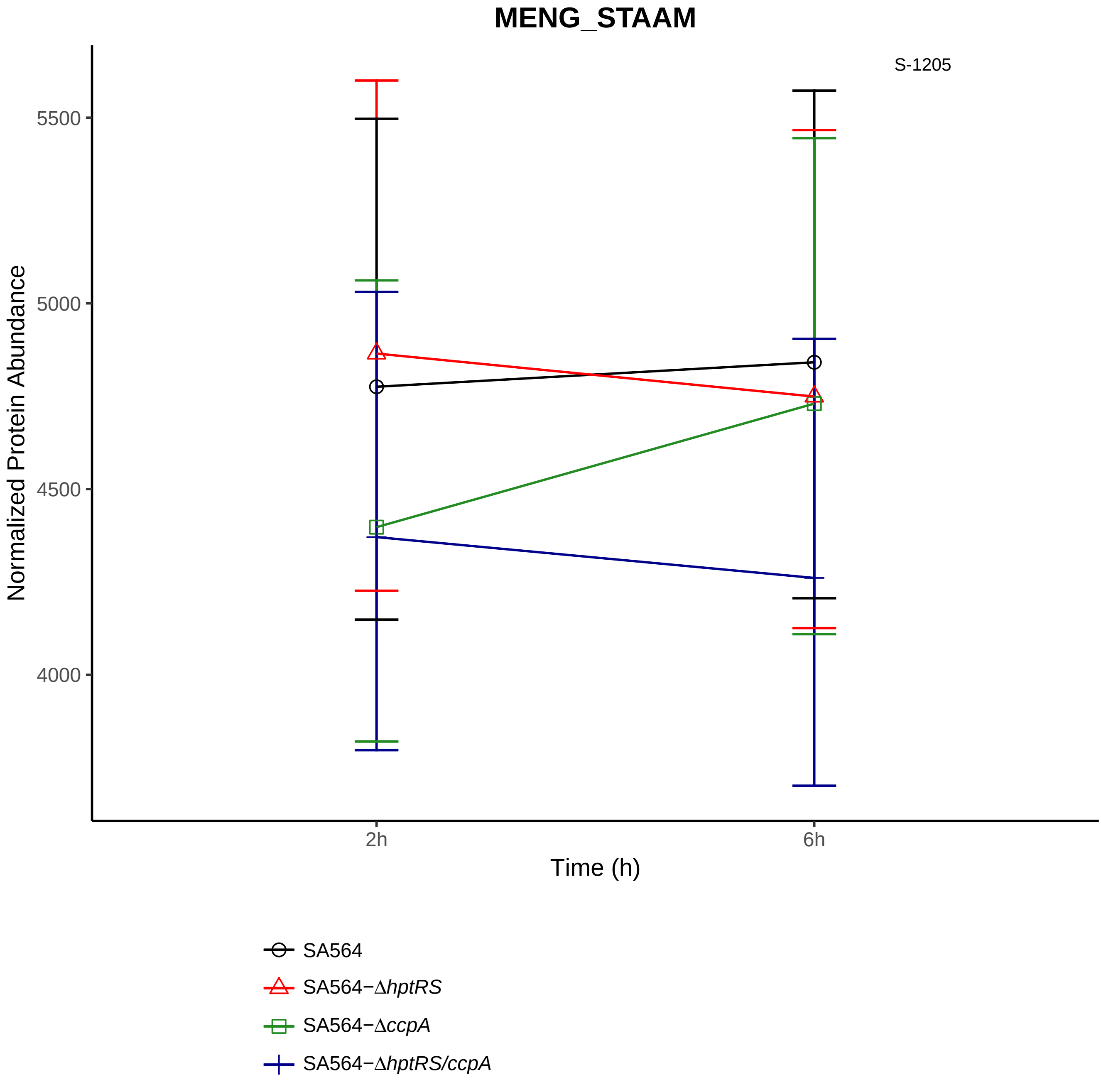

MENG_STAAM
S-1205
Normalized Protein Abundance
5500
5000
4500
4000
2h
6h
Time (h)
SA564
SA564−ΔhptRS
SA564−ΔccpA
SA564−ΔhptRS/ccpA

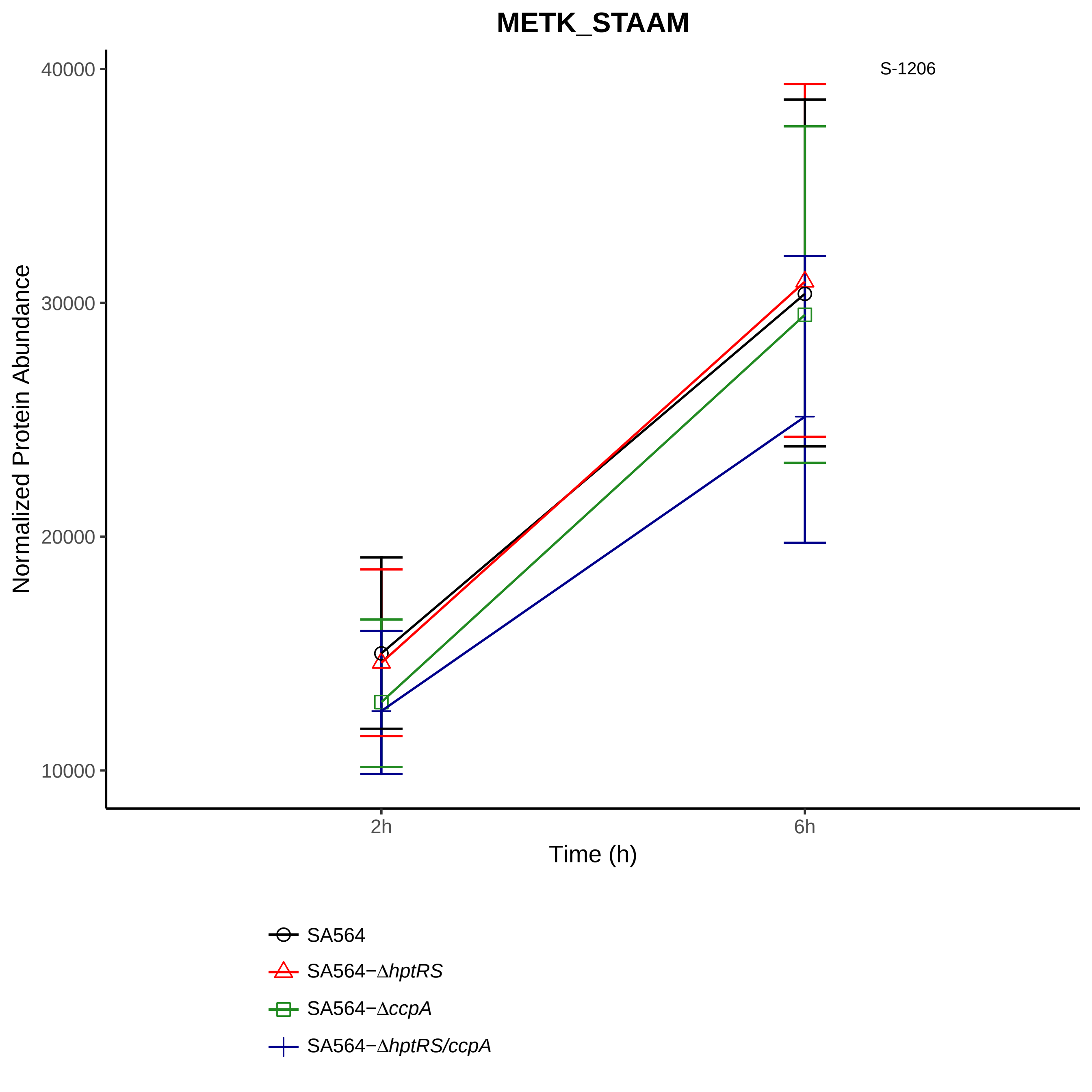

METK_STAAM
S-1206
Normalized Protein Abundance
40000
30000
20000
10000
2h
6h
Time (h)
SA564
SA564−Δ*hptRS*
SA564−Δ*ccpA*
SA564−Δ*hptRS/ccpA*

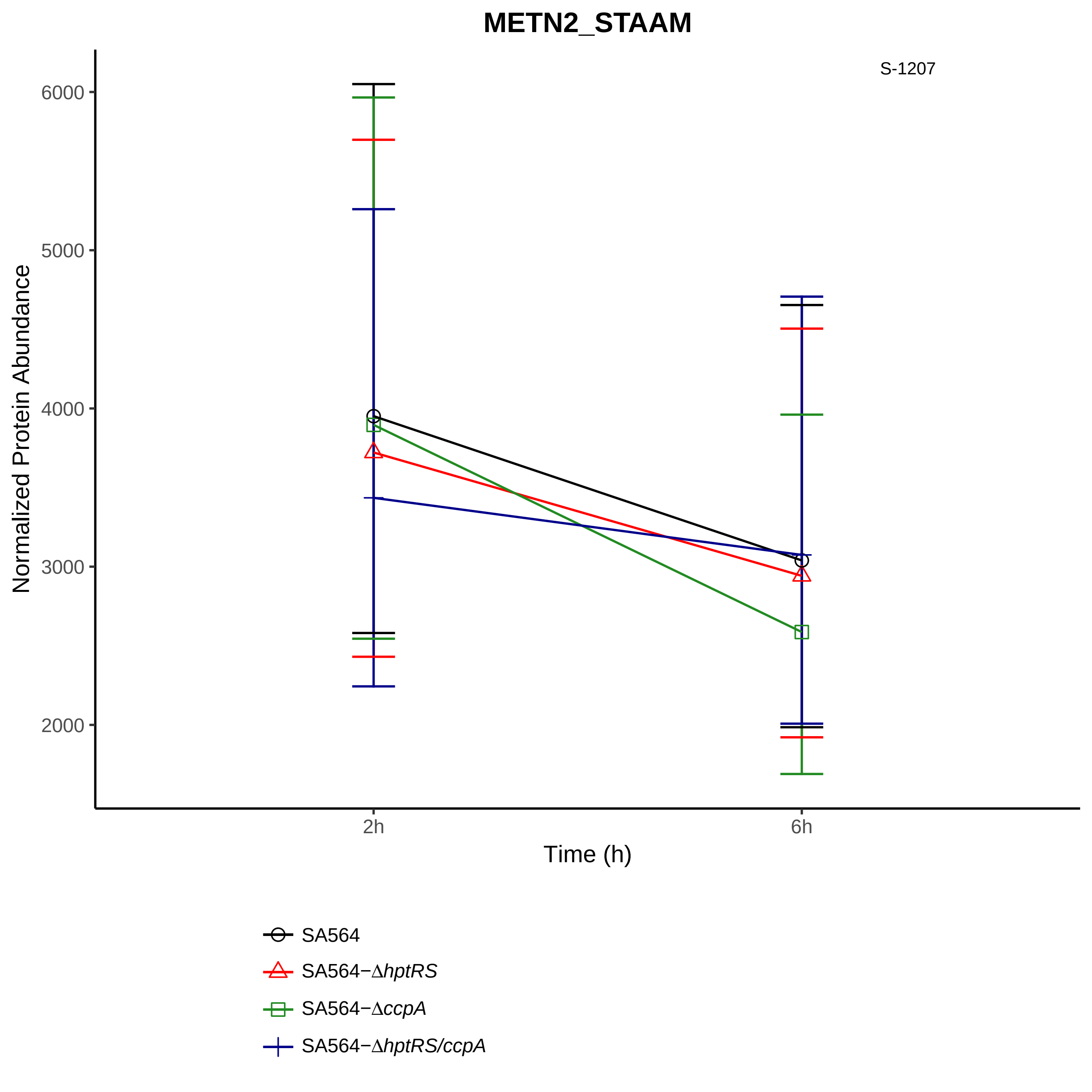

METN2_STAAM
S-1207
Normalized Protein Abundance
6000
5000
4000
3000
2000
2h
6h
Time (h)
SA564
SA564−Δ*hptRS*
SA564−Δ*ccpA*
SA564−Δ*hptRS/ccpA*

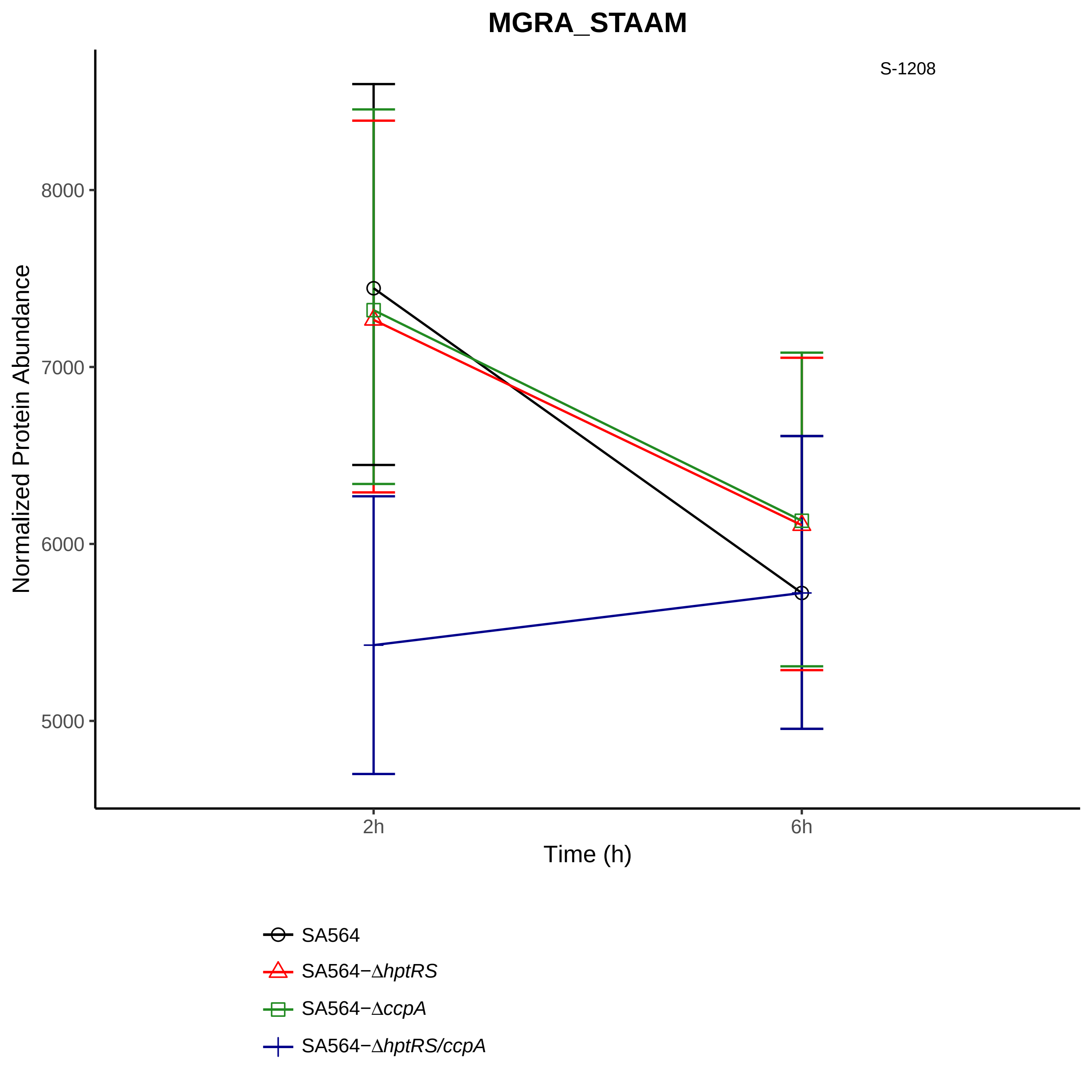

MGRA_STAAM
S-1208
Normalized Protein Abundance
8000
7000
6000
5000
2h
6h
Time (h)
SA564
SA564−Δ*hptRS*
SA564−Δ*ccpA*
SA564−Δ*hptRS/ccpA*

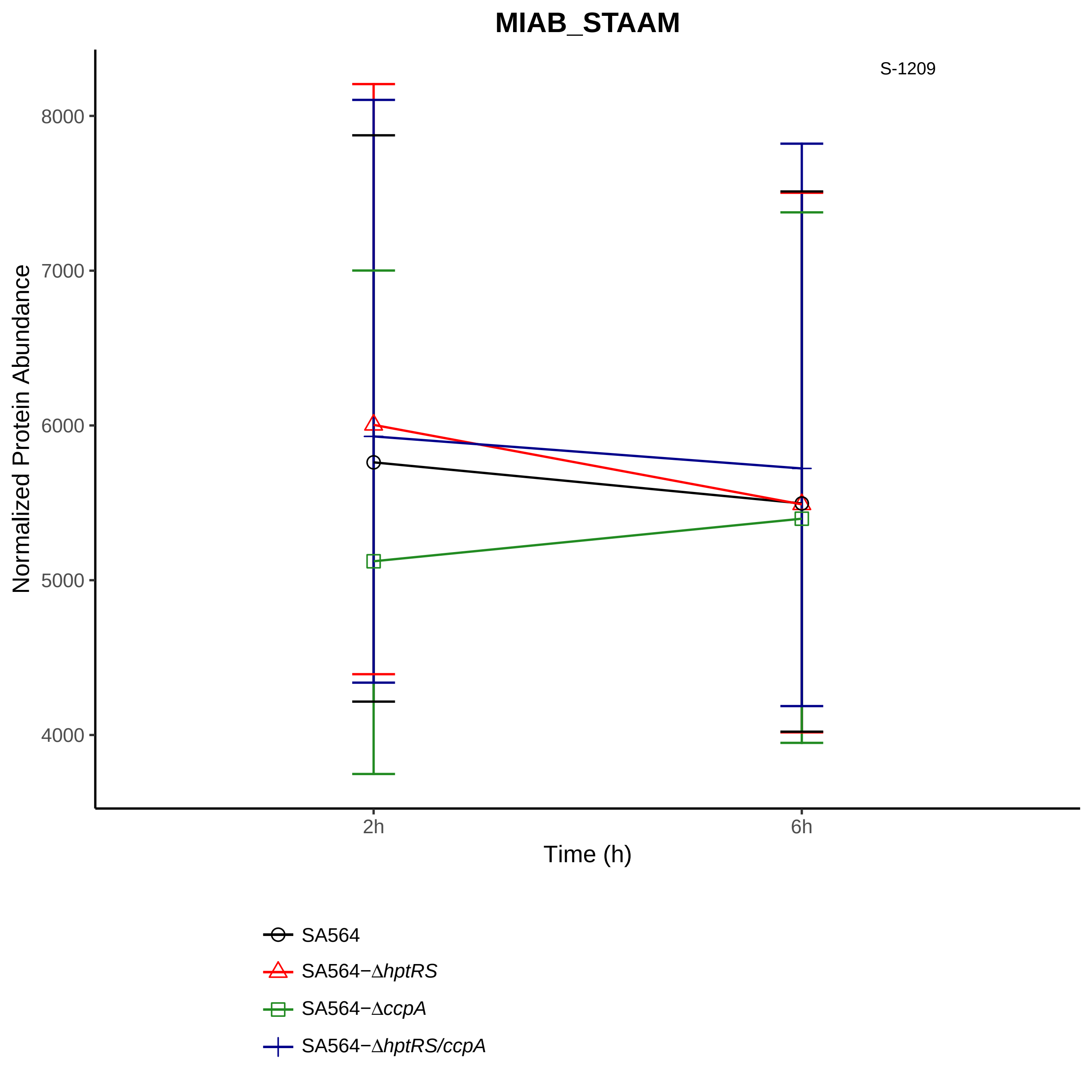

MIAB_STAAM
S-1209
Normalized Protein Abundance
8000
7000
6000
5000
4000
2h
6h
Time (h)
SA564
SA564−ΔhptRS
SA564−ΔccpA
SA564−ΔhptRS/ccpA

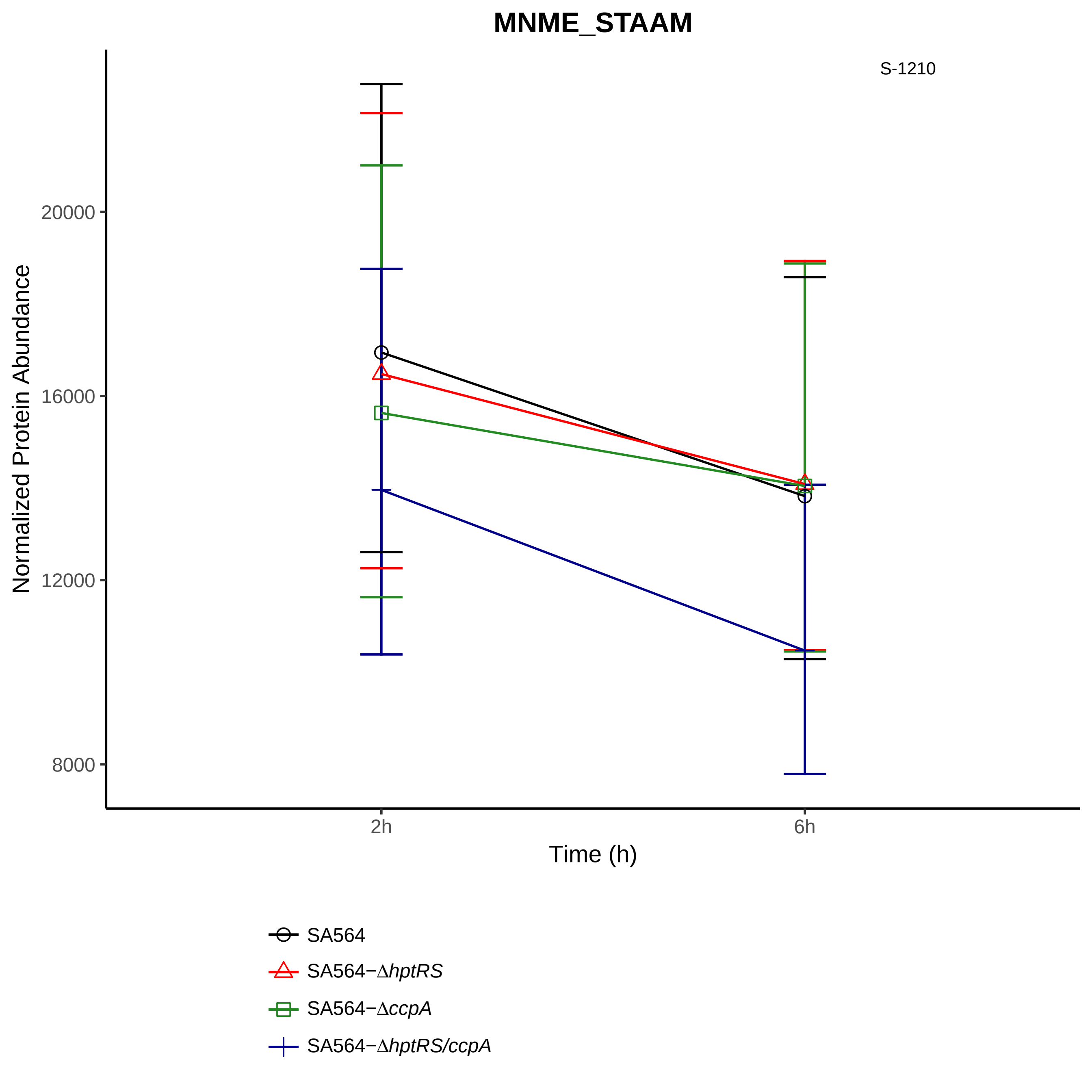
MNME_STAAM
S-1210
Normalized Protein Abundance
20000
16000
12000
8000
2h
6h
Time (h)
SA564
SA564−Δ*hptRS*
SA564−Δ*ccpA*
SA564−Δ*hptRS/ccpA*

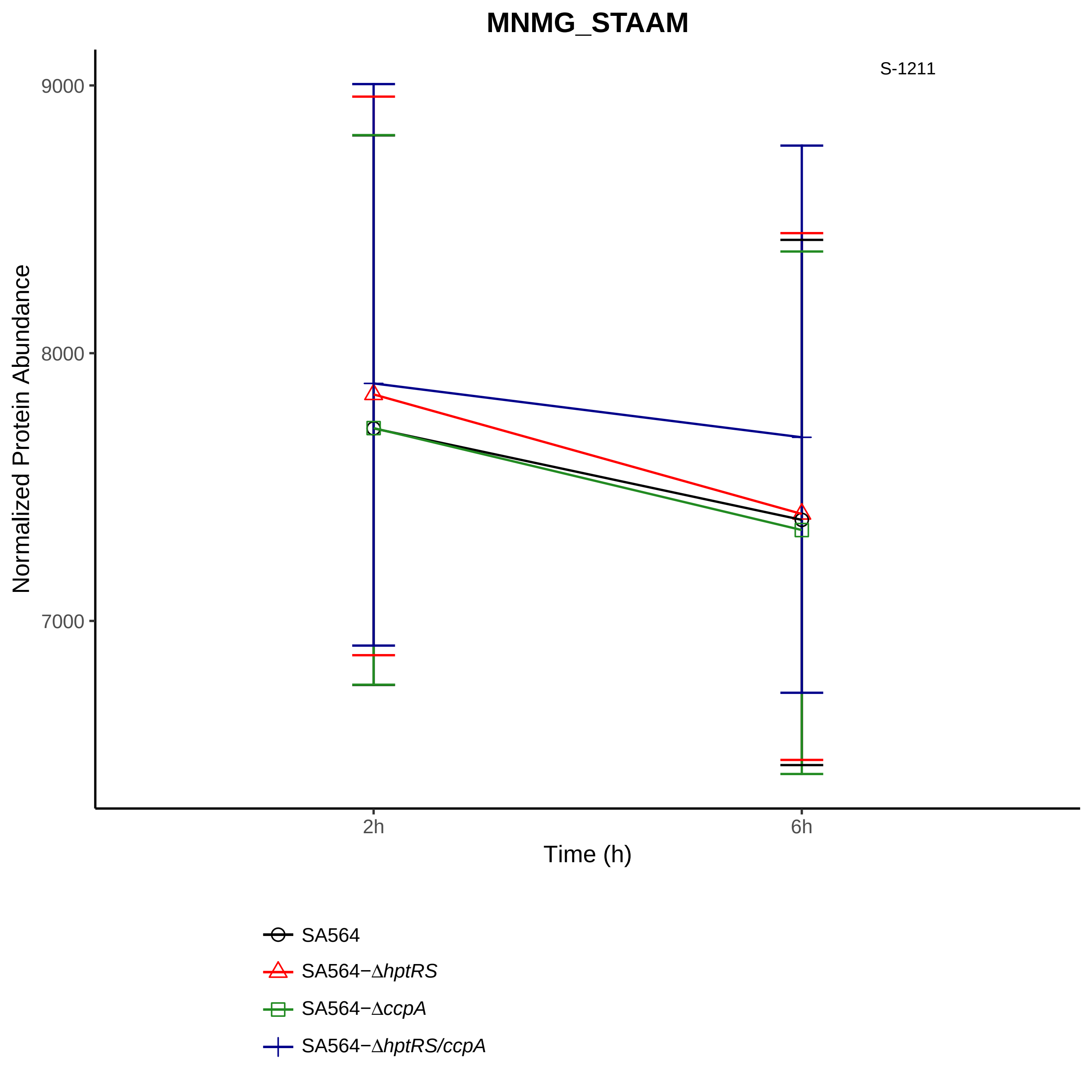

MNMG_STAAM
S-1211
9000
8000
7000
Normalized Protein Abundance
2h
6h
Time (h)
SA564
SA564−Δ*hptRS*
SA564−Δ*ccpA*
SA564−Δ*hptRS/ccpA*

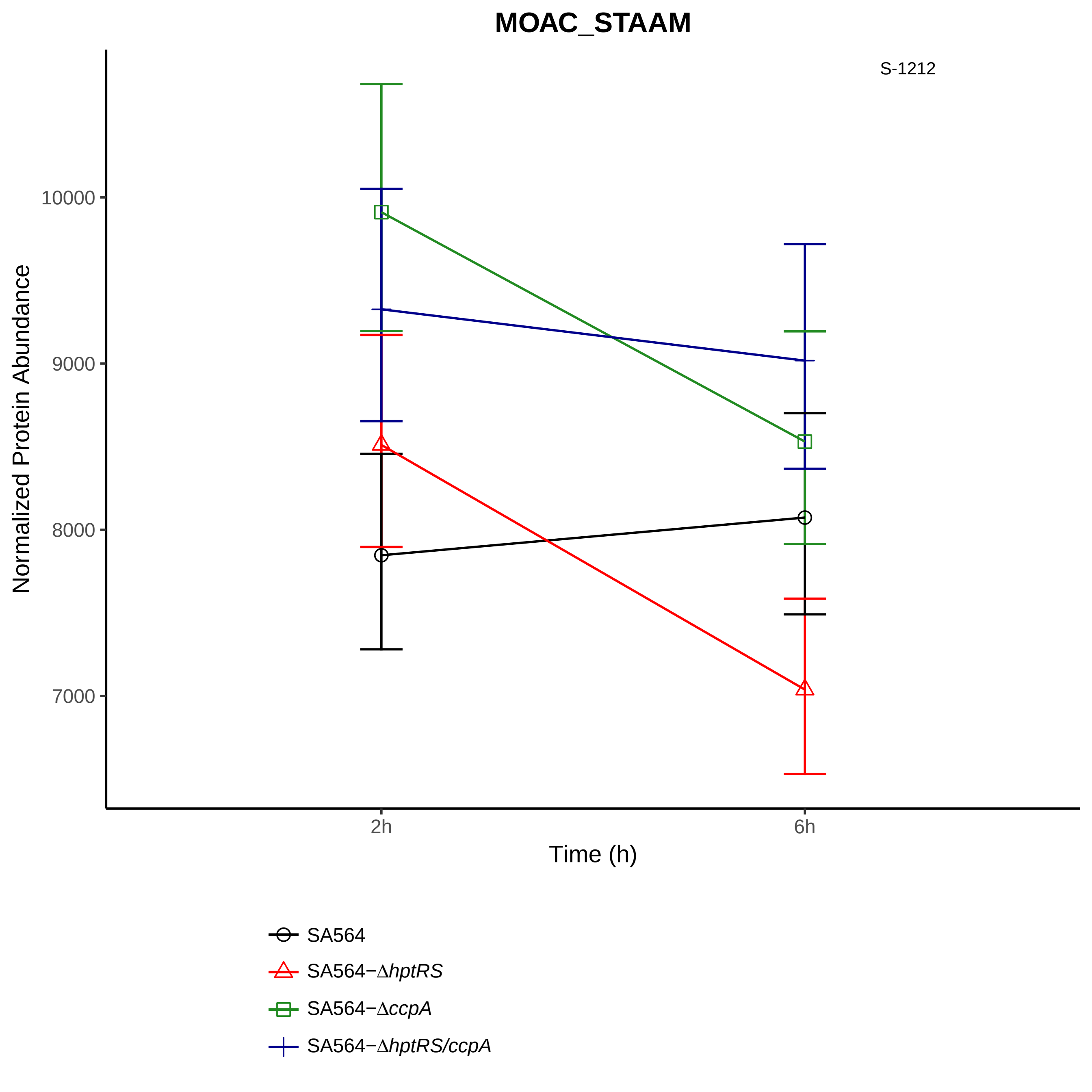

MOAC_STAAM
S-1212
Normalized Protein Abundance
10000
9000
8000
7000
2h
6h
Time (h)
SA564
SA564−Δ*hptRS*
SA564−Δ*ccpA*
SA564−Δ*hptRS/ccpA*

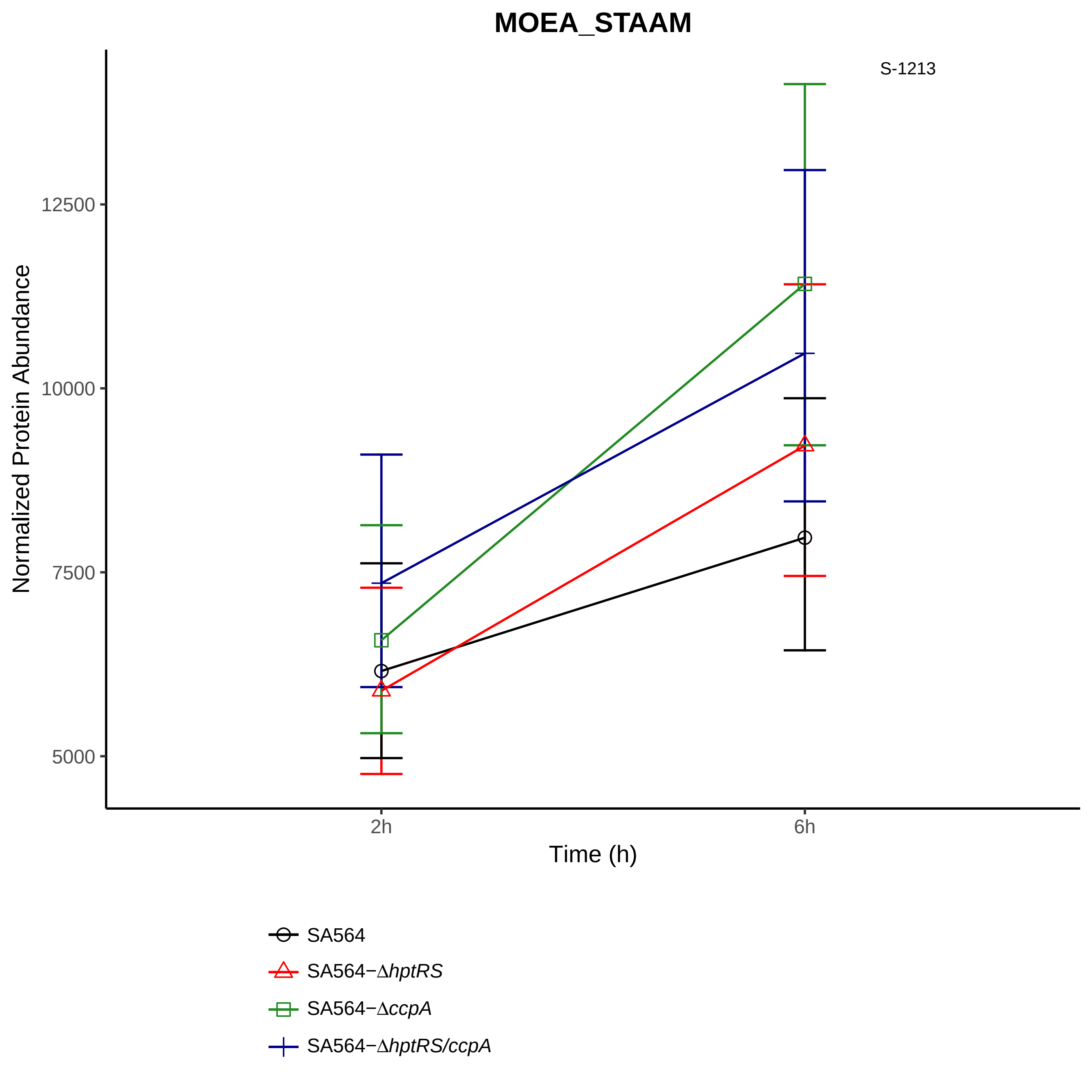
MOEA_STAAM
S-1213
Normalized Protein Abundance
12500
10000
7500
5000
2h
6h
Time (h)
SA564
SA564−ΔhptRS
SA564−ΔccpA
SA564−ΔhptRS/ccpA

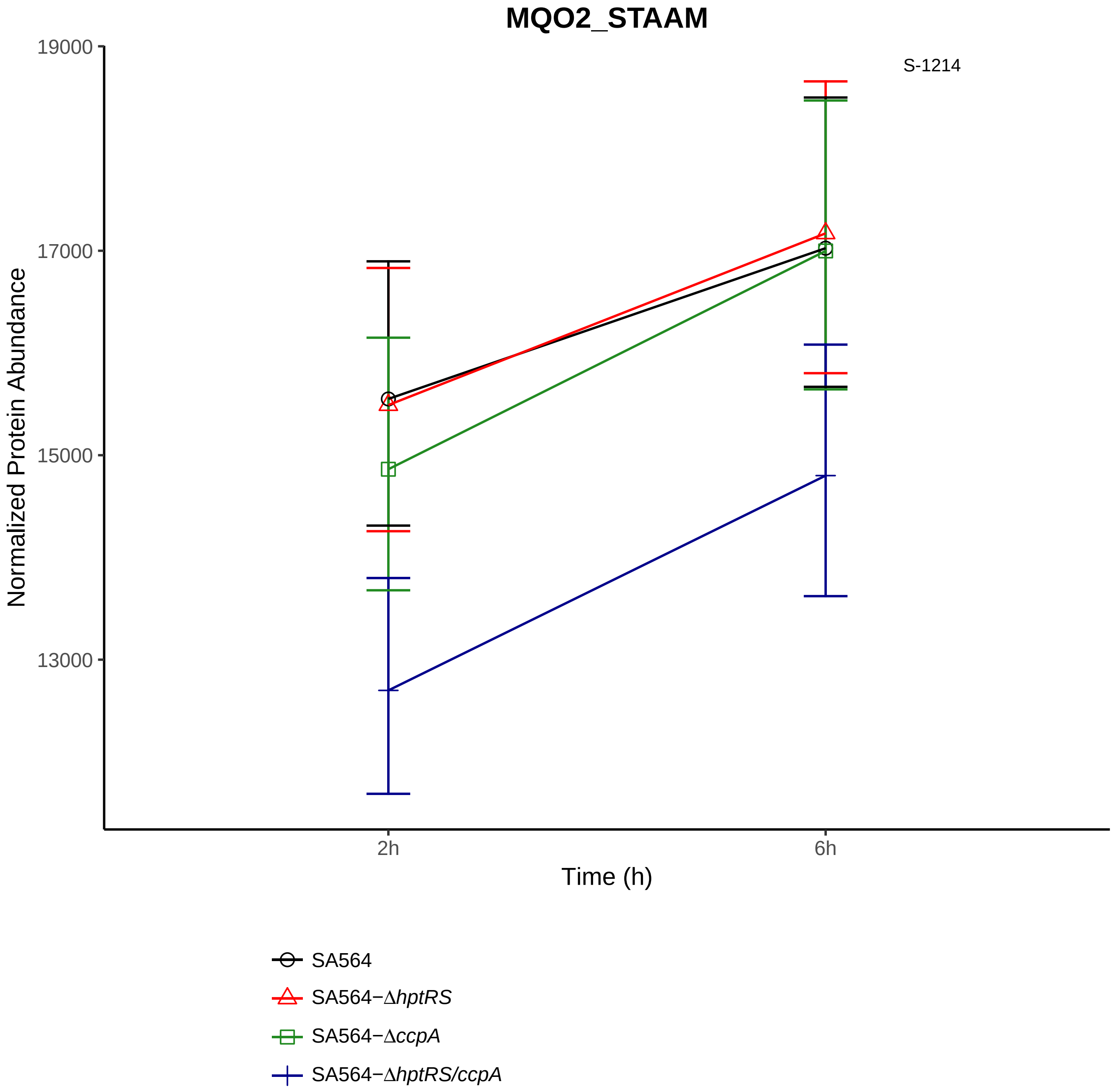

MQO2_STAAM
S-1214
19000
17000
15000
13000
Normalized Protein Abundance
2h
6h
Time (h)
SA564
SA564−Δ*hptRS*
SA564−Δ*ccpA*
SA564−Δ*hptRS/ccpA*

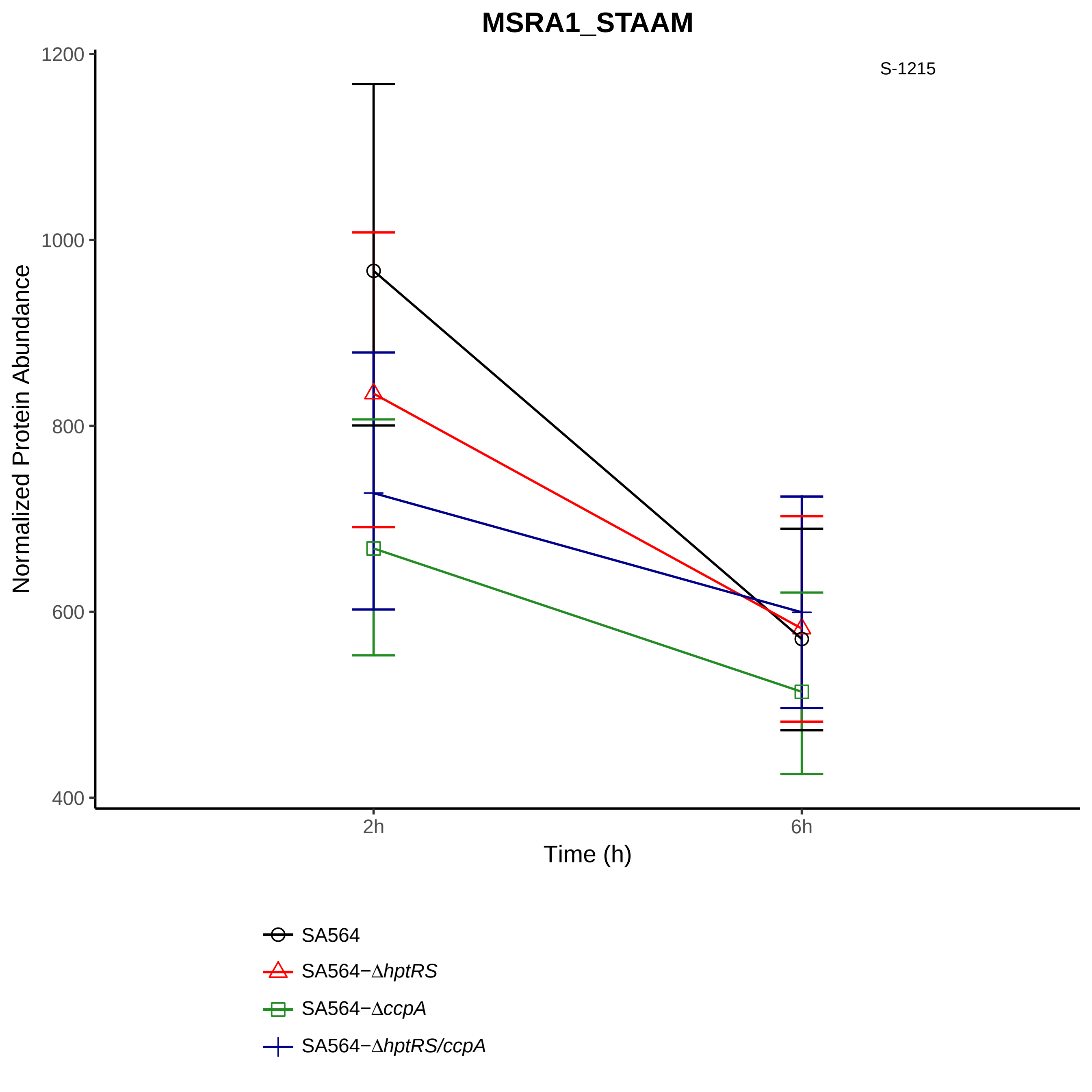
MSRA1_STAAM
S-1215
Normalized Protein Abundance
1200
1000
800
600
400
2h
6h
Time (h)
SA564
SA564−Δ*hptRS*
SA564−Δ*ccpA*
SA564−Δ*hptRS/ccpA*

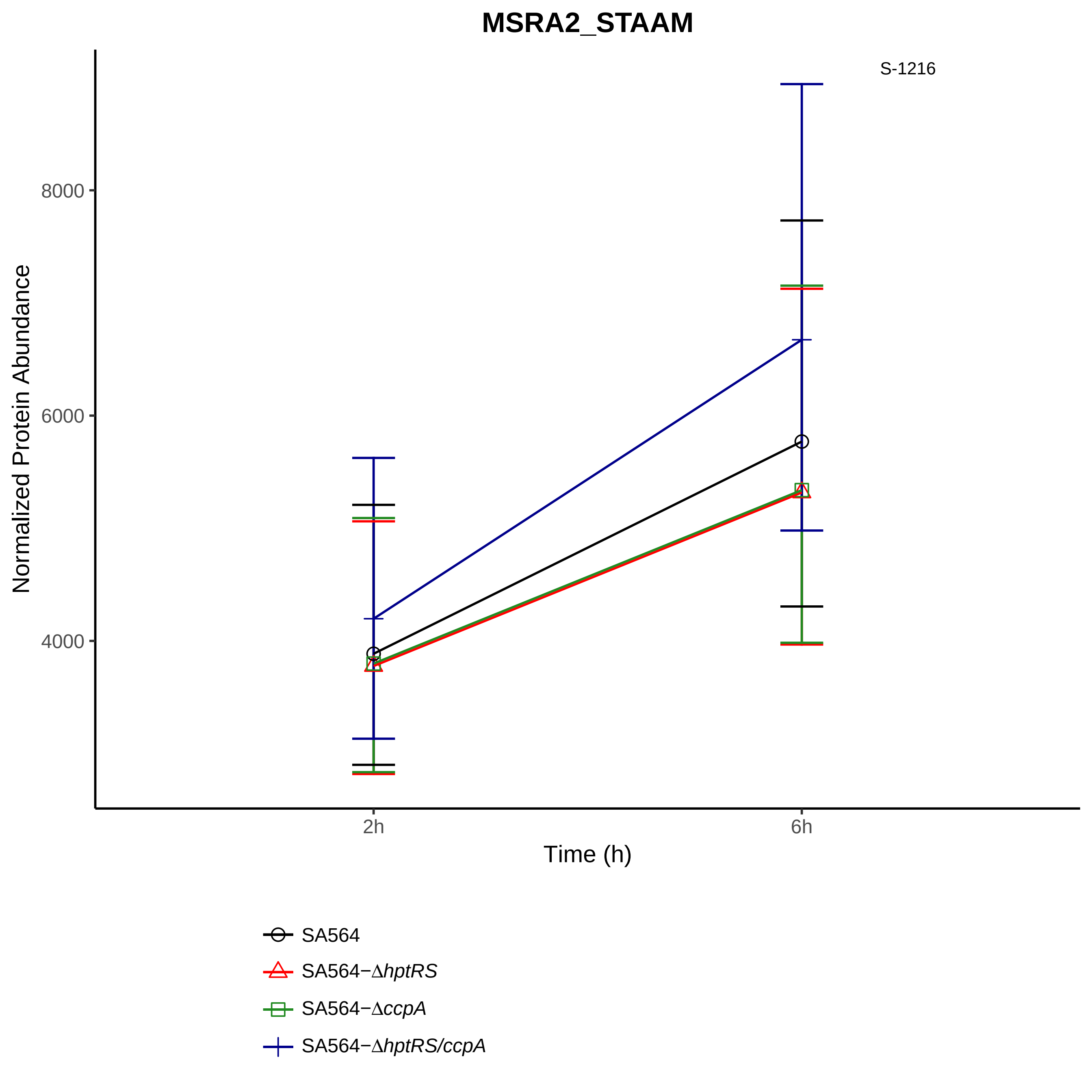

MSRA2_STAAM
S-1216
Normalized Protein Abundance
8000
6000
4000
2h
6h
Time (h)
SA564
SA564−Δ*hptRS*
SA564−Δ*ccpA*
SA564−Δ*hptRS/ccpA*

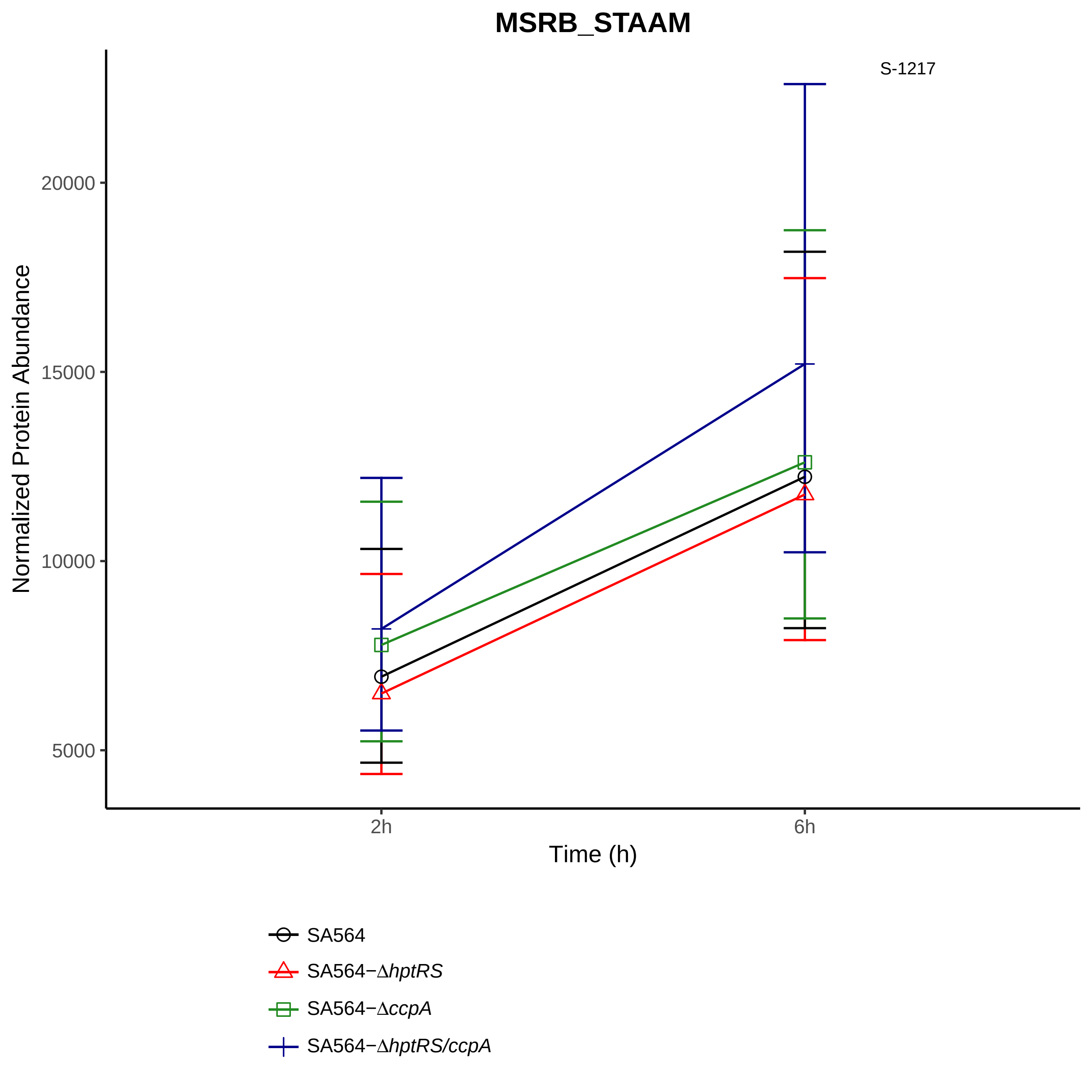

MSRB_STAAM
S-1217
Normalized Protein Abundance
20000
15000
10000
5000
2h
6h
Time (h)
SA564
SA564−Δ*hptRS*
SA564−Δ*ccpA*
SA564−Δ*hptRS/ccpA*

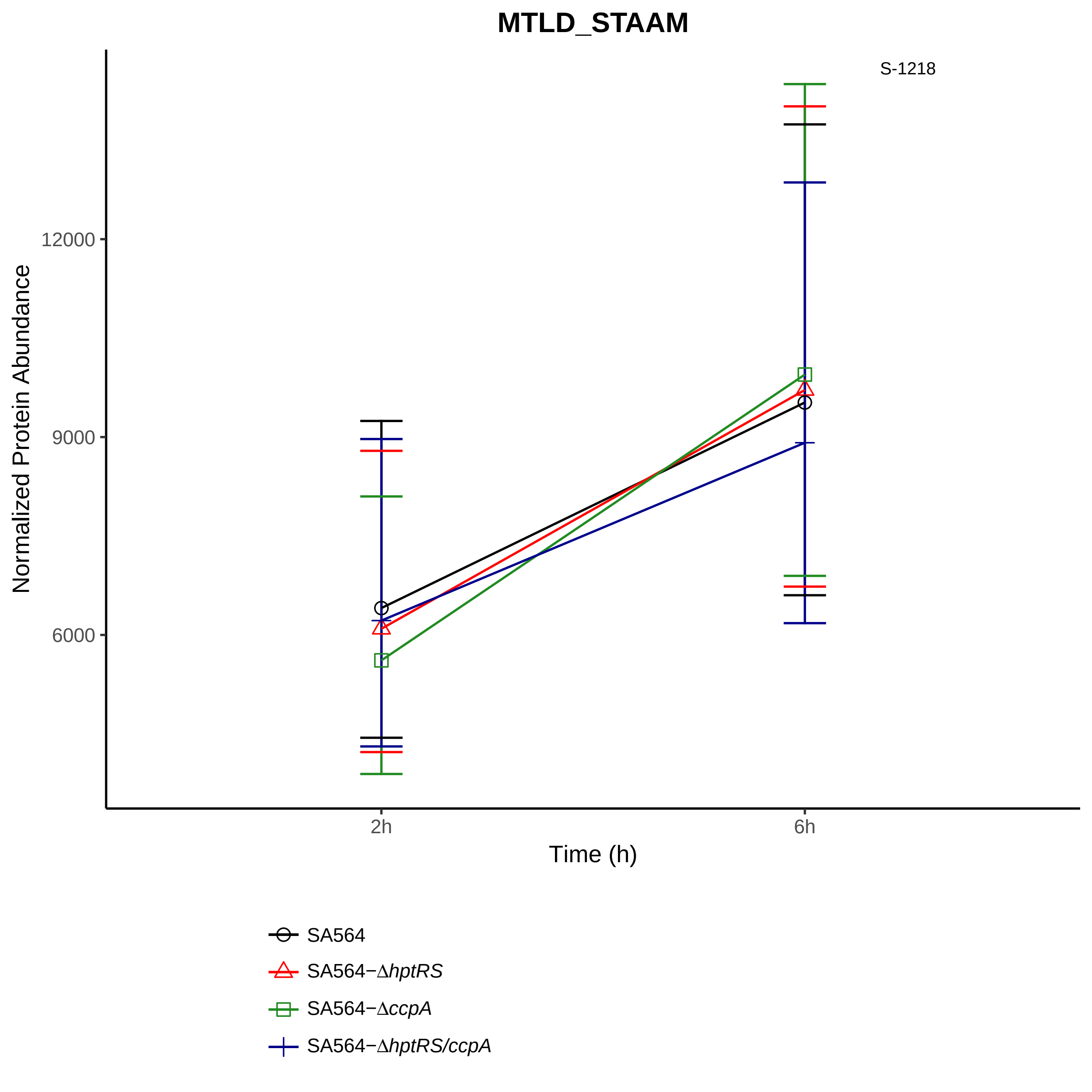

MTLD_STAAM
S-1218
Normalized Protein Abundance
12000
9000
6000
2h
6h
Time (h)
SA564
SA564−Δ*hptRS*
SA564−Δ*ccpA*
SA564−Δ*hptRS/ccpA*

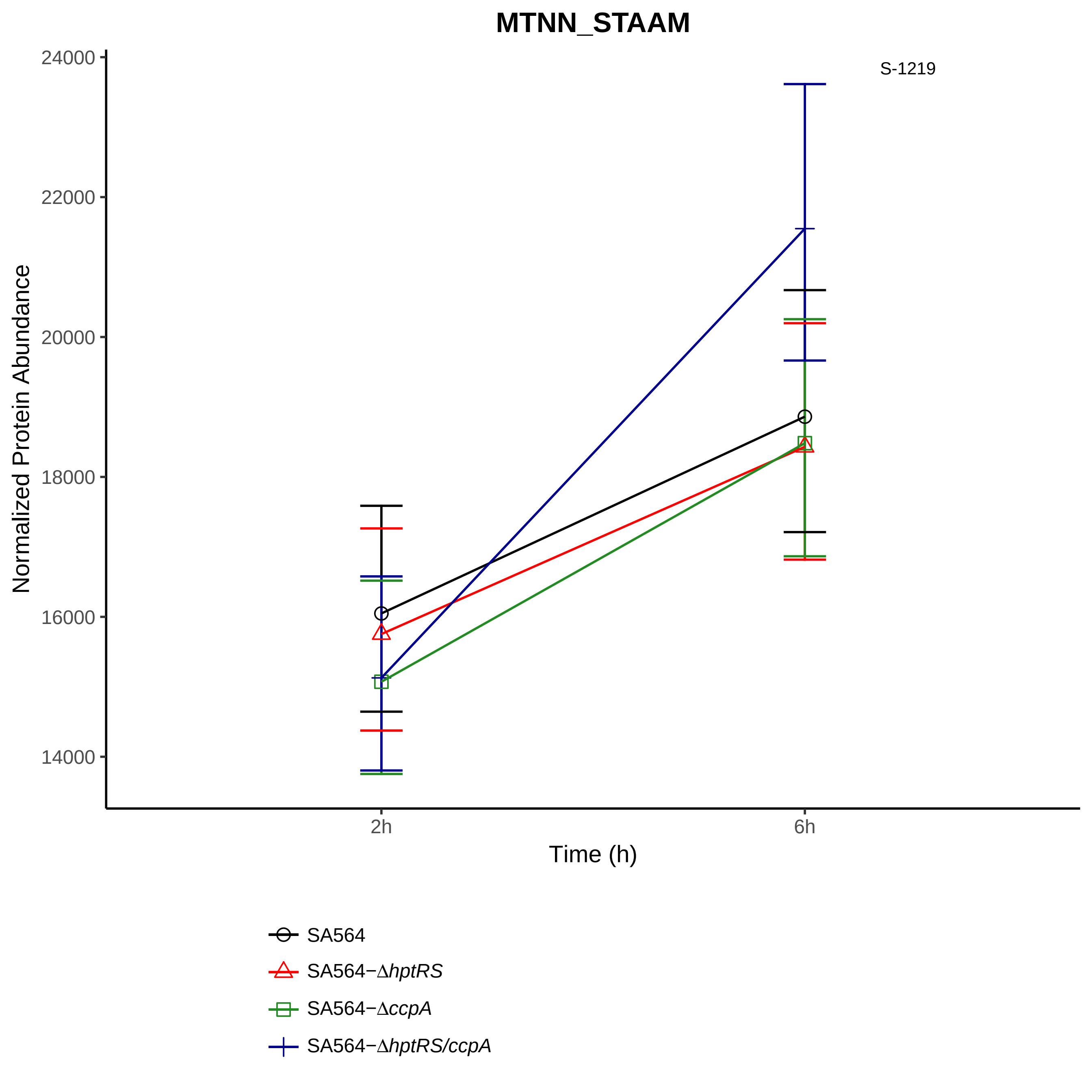

MTNN_STAAM
S-1219
Normalized Protein Abundance
24000
22000
20000
18000
16000
14000
2h
6h
Time (h)
SA564
SA564−Δ*hptRS*
SA564−Δ*ccpA*
SA564−Δ*hptRS/ccpA*

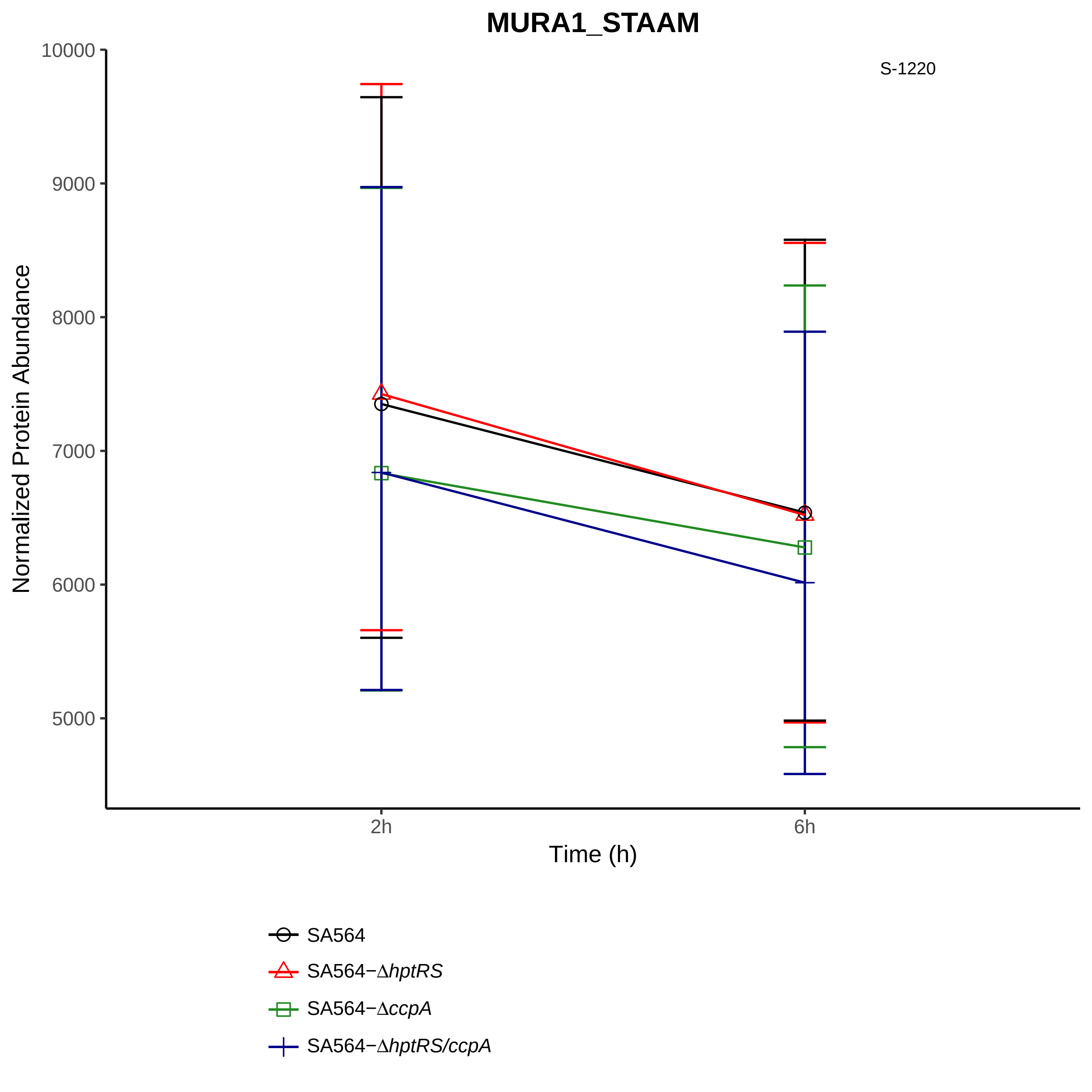

MURA1_STAAM
S-1220
Normalized Protein Abundance
10000
9000
8000
7000
6000
5000
2h
6h
Time (h)
SA564
SA564−Δ*hptRS*
SA564−Δ*ccpA*
SA564−Δ*hptRS/ccpA*

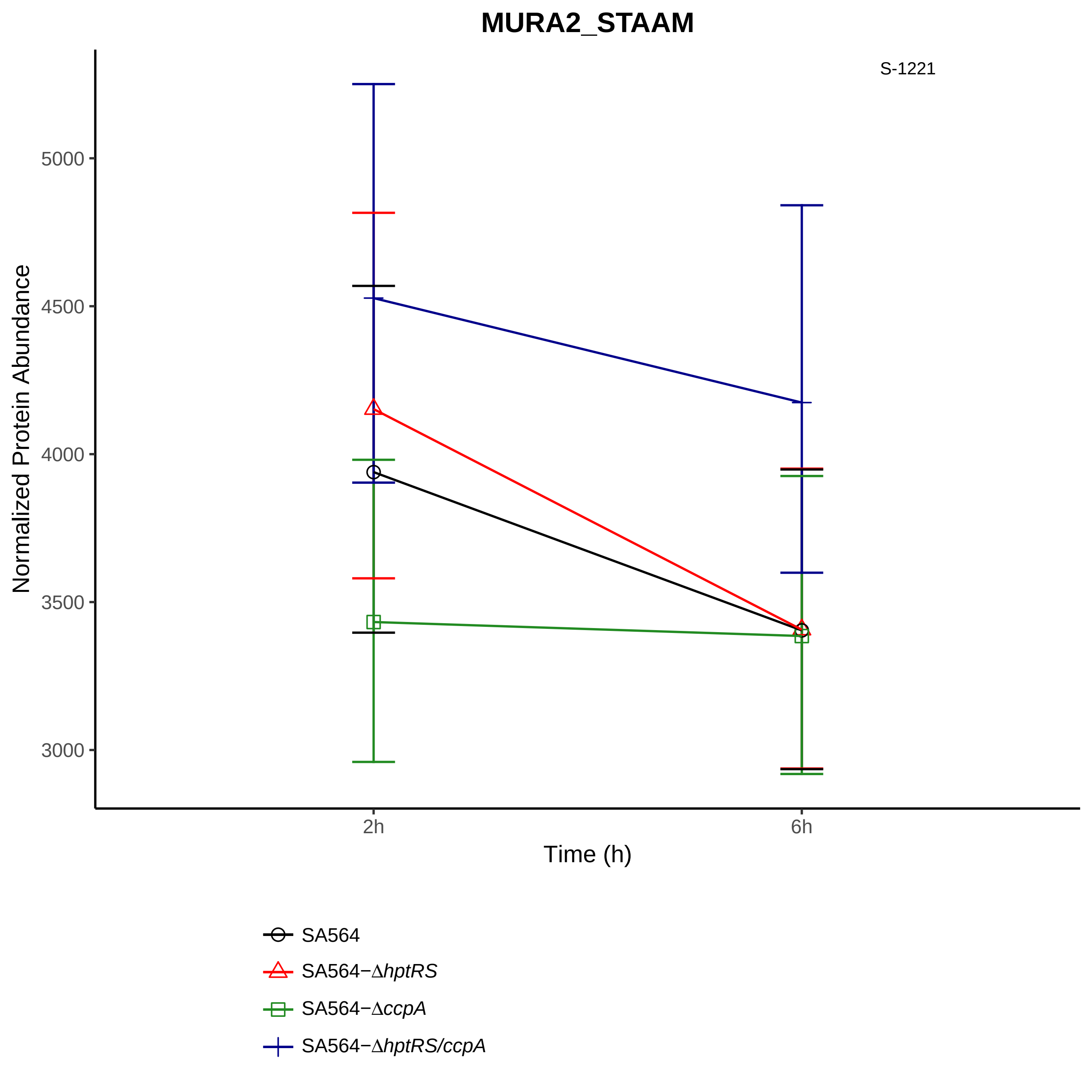

MURA2_STAAM
S-1221
Normalized Protein Abundance
5000
4500
4000
3500
3000
2h
6h
Time (h)
SA564
SA564−ΔhptRS
SA564−ΔccpA
SA564−ΔhptRS/ccpA

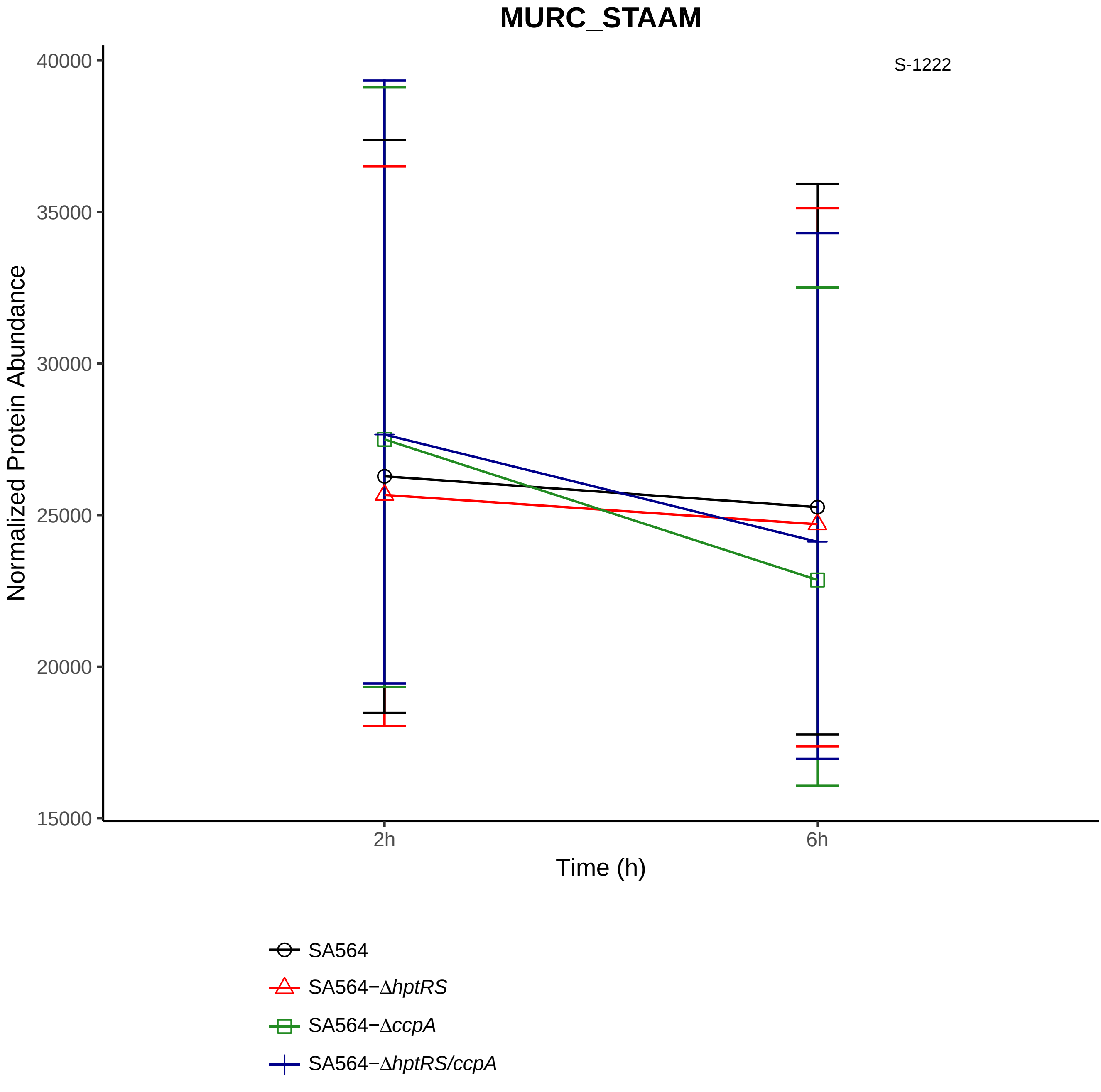

MURC_STAAM
S-1222
Normalized Protein Abundance
40000
35000
30000
25000
20000
15000
2h
6h
Time (h)
SA564
SA564−Δ*hptRS*
SA564−Δ*ccpA*
SA564−Δ*hptRS/ccpA*

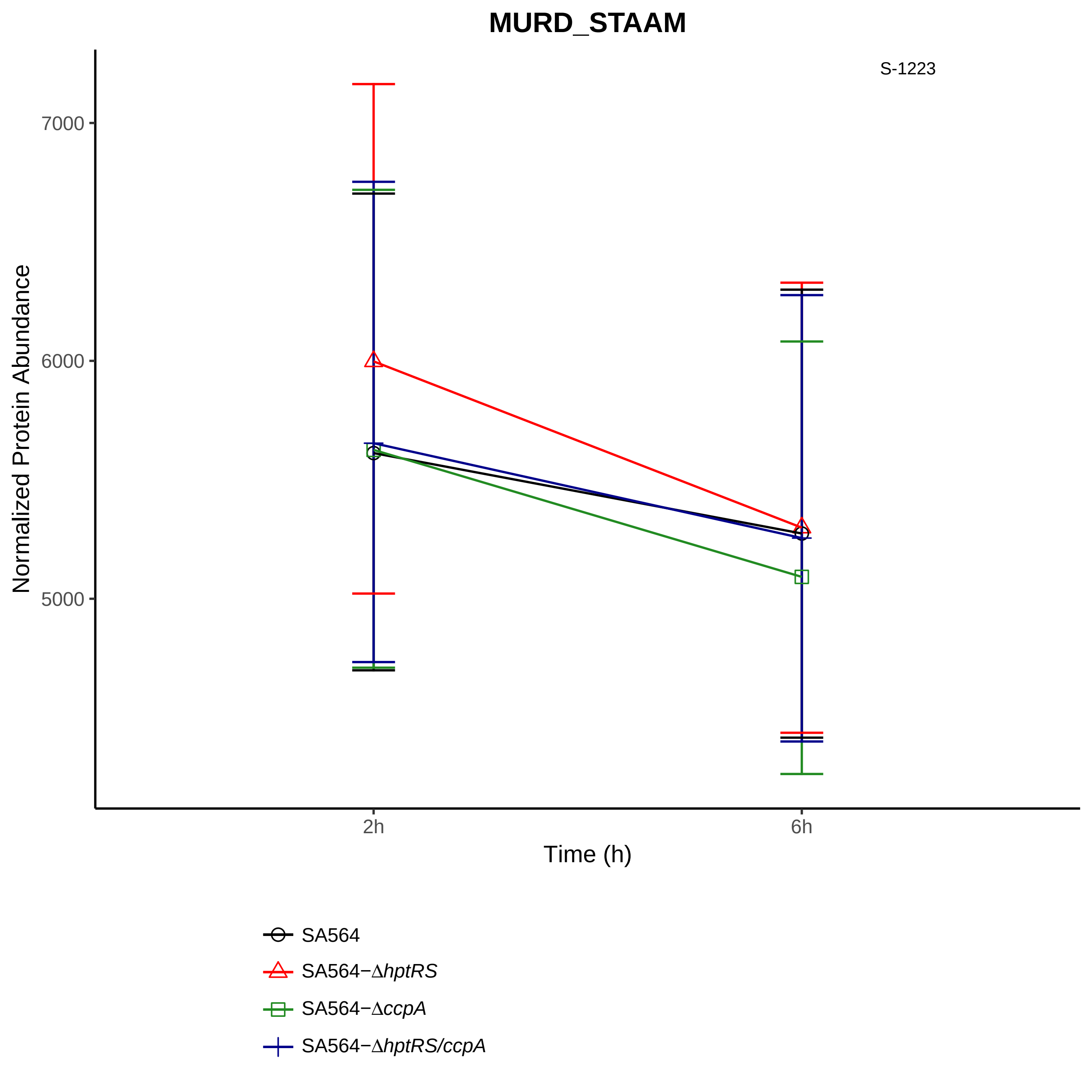
MURD_STAAM
S-1223
Normalized Protein Abundance
7000
6000
5000
2h
6h
Time (h)
SA564
SA564−Δ*hptRS*
SA564−Δ*ccpA*
SA564−Δ*hptRS/ccpA*

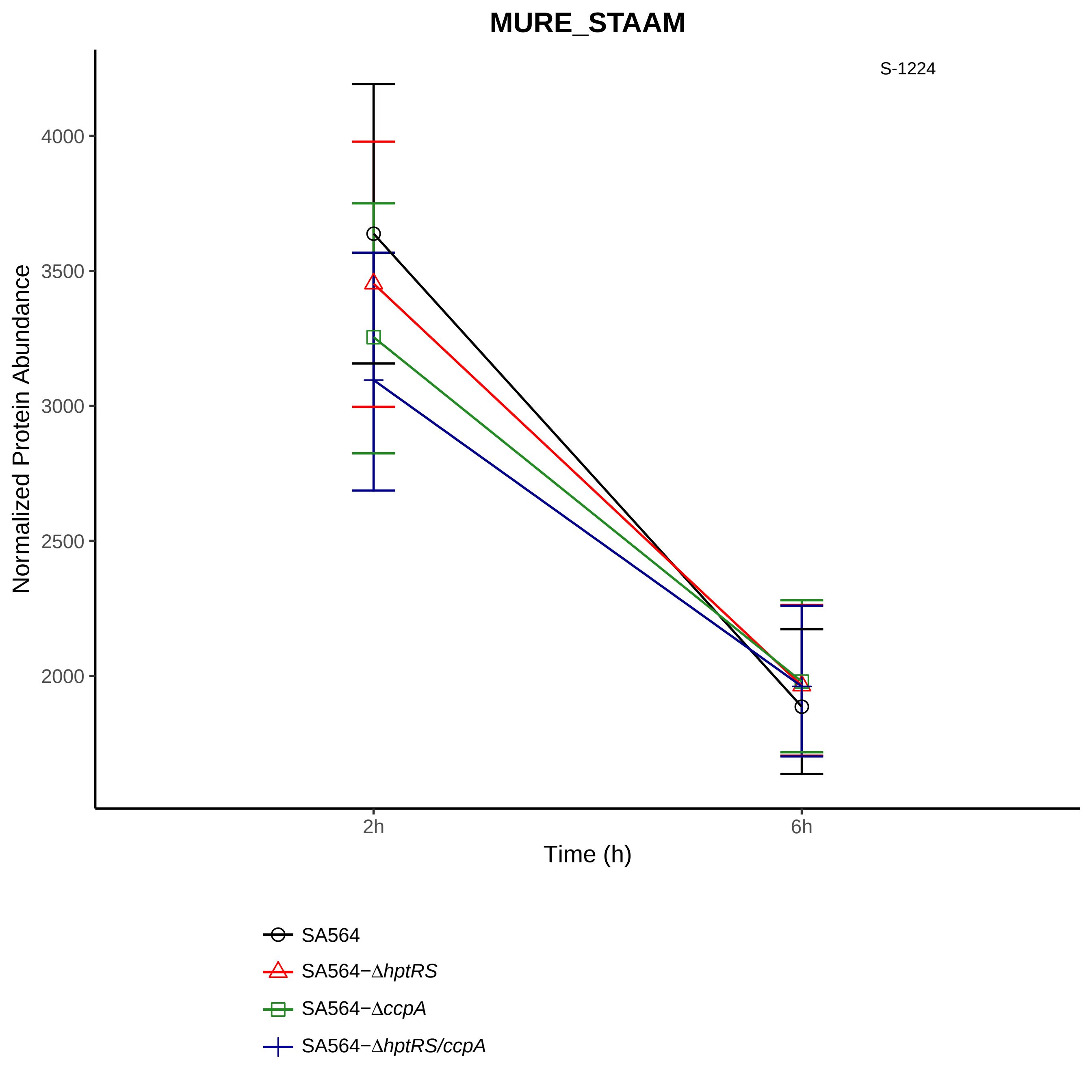

MURE_STAAM
S-1224
Normalized Protein Abundance
4000
3500
3000
2500
2000
2h
6h
Time (h)
SA564
SA564−Δ*hptRS*
SA564−Δ*ccpA*
SA564−Δ*hptRS/ccpA*

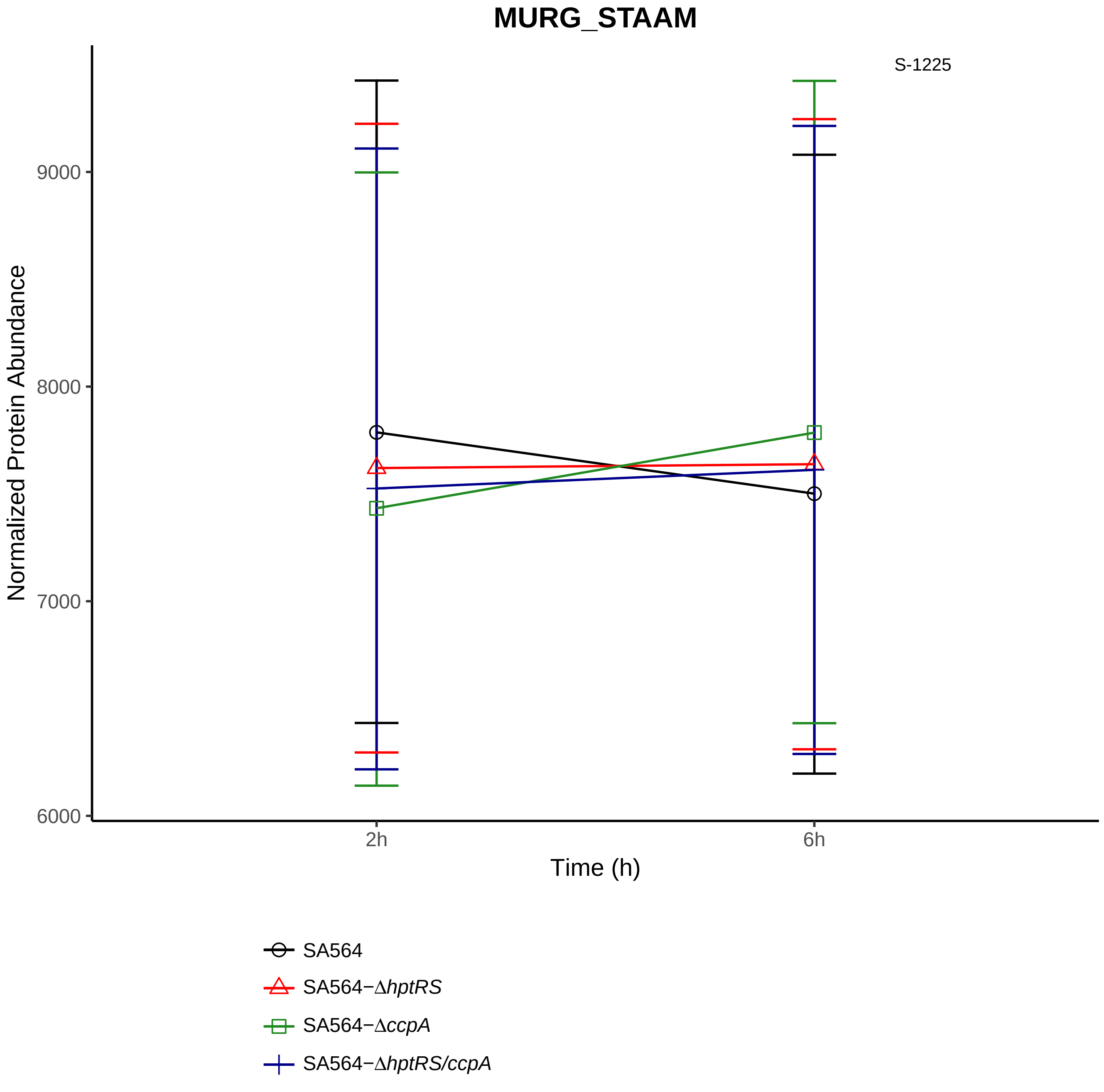

MURG_STAAM
S-1225
Normalized Protein Abundance
9000
8000
7000
6000
2h
6h
Time (h)
SA564
SA564−ΔhptRS
SA564−ΔccpA
SA564−ΔhptRS/ccpA

MURI_STAAM

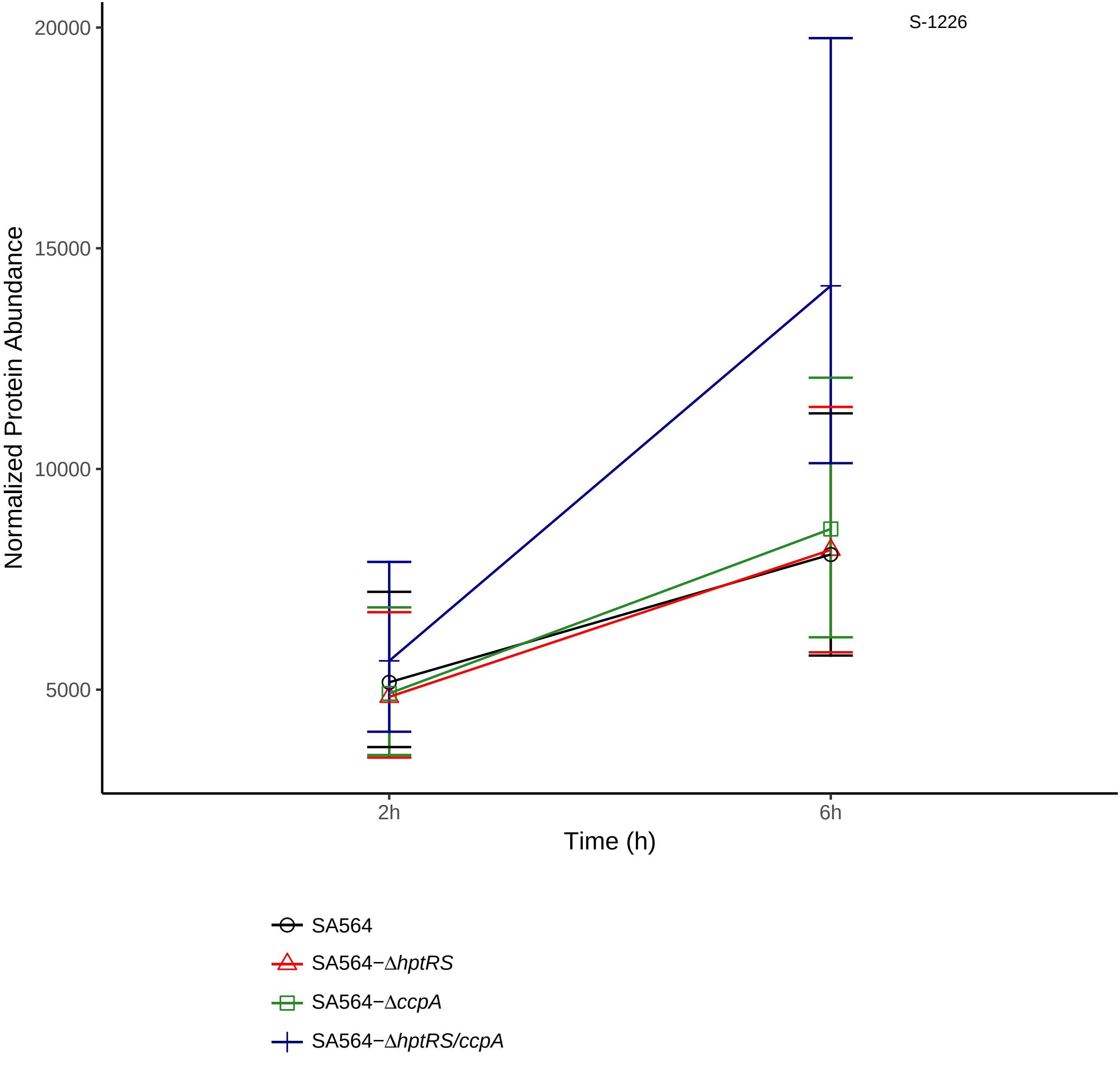

S-1226
Normalized Protein Abundance
20000
15000
10000
5000
2h
6h
Time (h)
SA564
SA564−Δ*hptRS*
SA564−Δ*ccpA*
SA564−Δ*hptRS/ccpA*

MUTS_STAAM

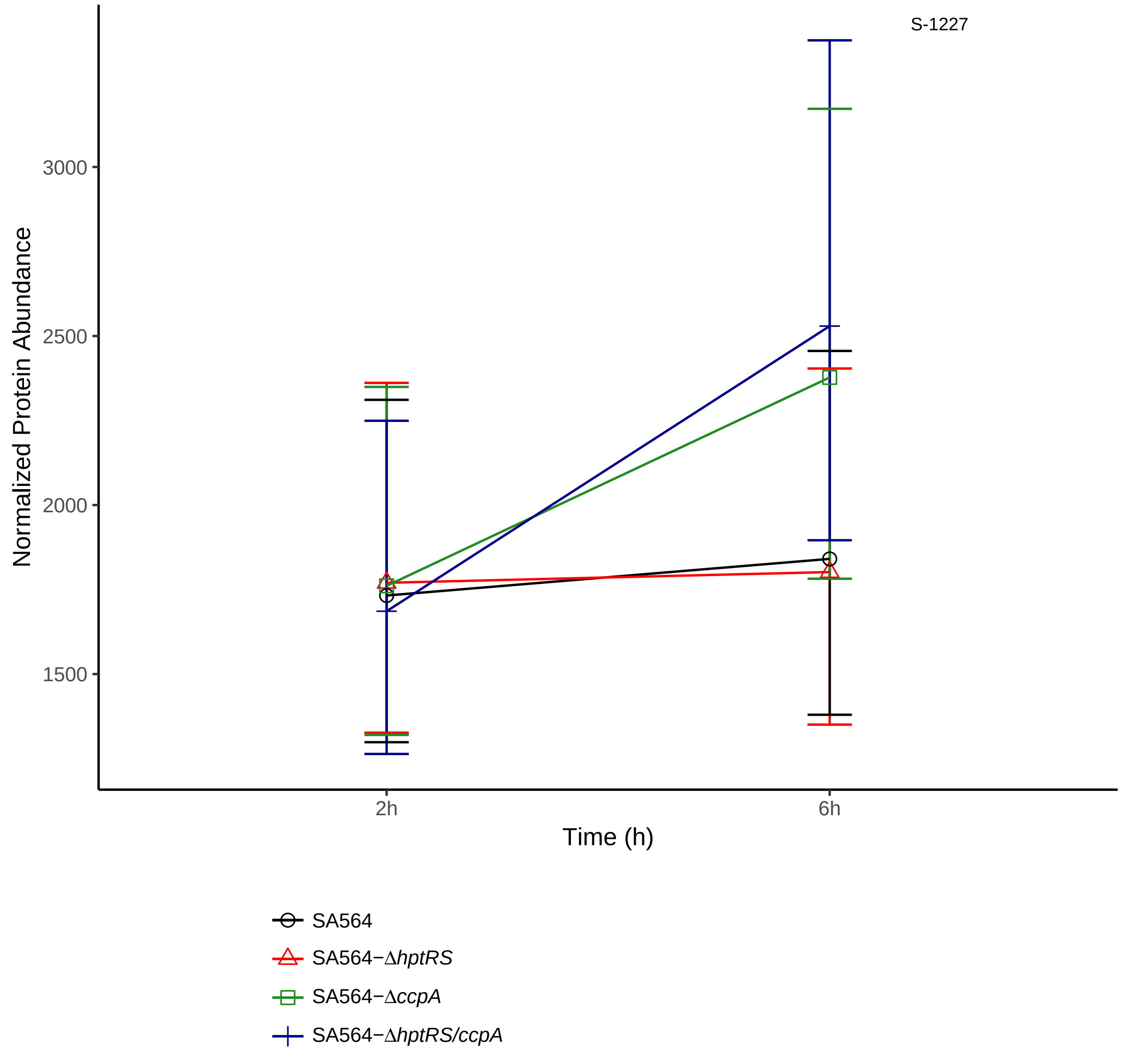

S-1227
Normalized Protein Abundance
3000
2500
2000
1500
2h
6h
Time (h)
SA564
SA564−Δ*hptRS*
SA564−Δ*ccpA*
SA564−Δ*hptRS/ccpA*

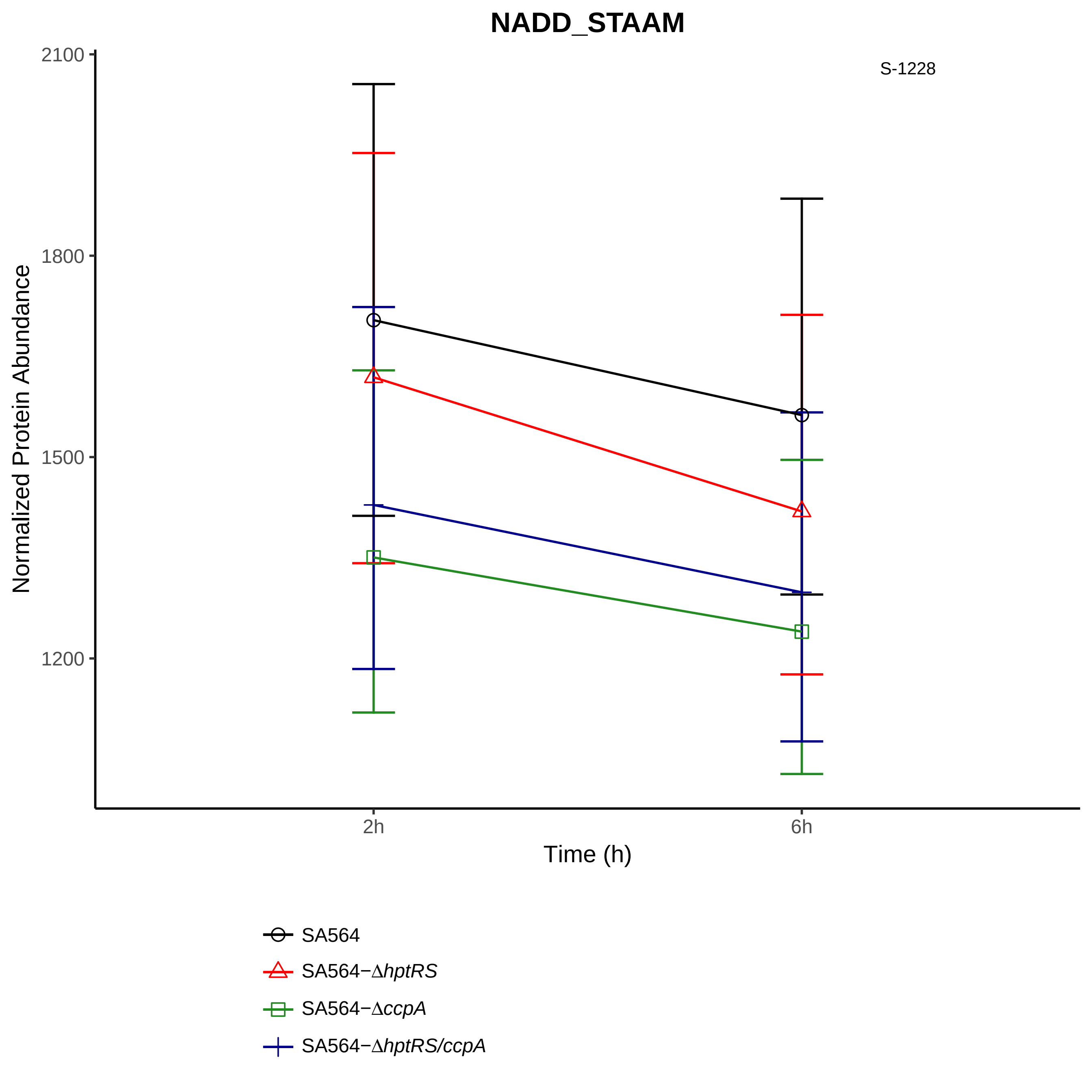

NADD_STAAM
S-1228
2100
1800
1500
1200
Normalized Protein Abundance
2h
6h
Time (h)
SA564
SA564−ΔhptRS
SA564−ΔccpA
SA564−ΔhptRS/ccpA

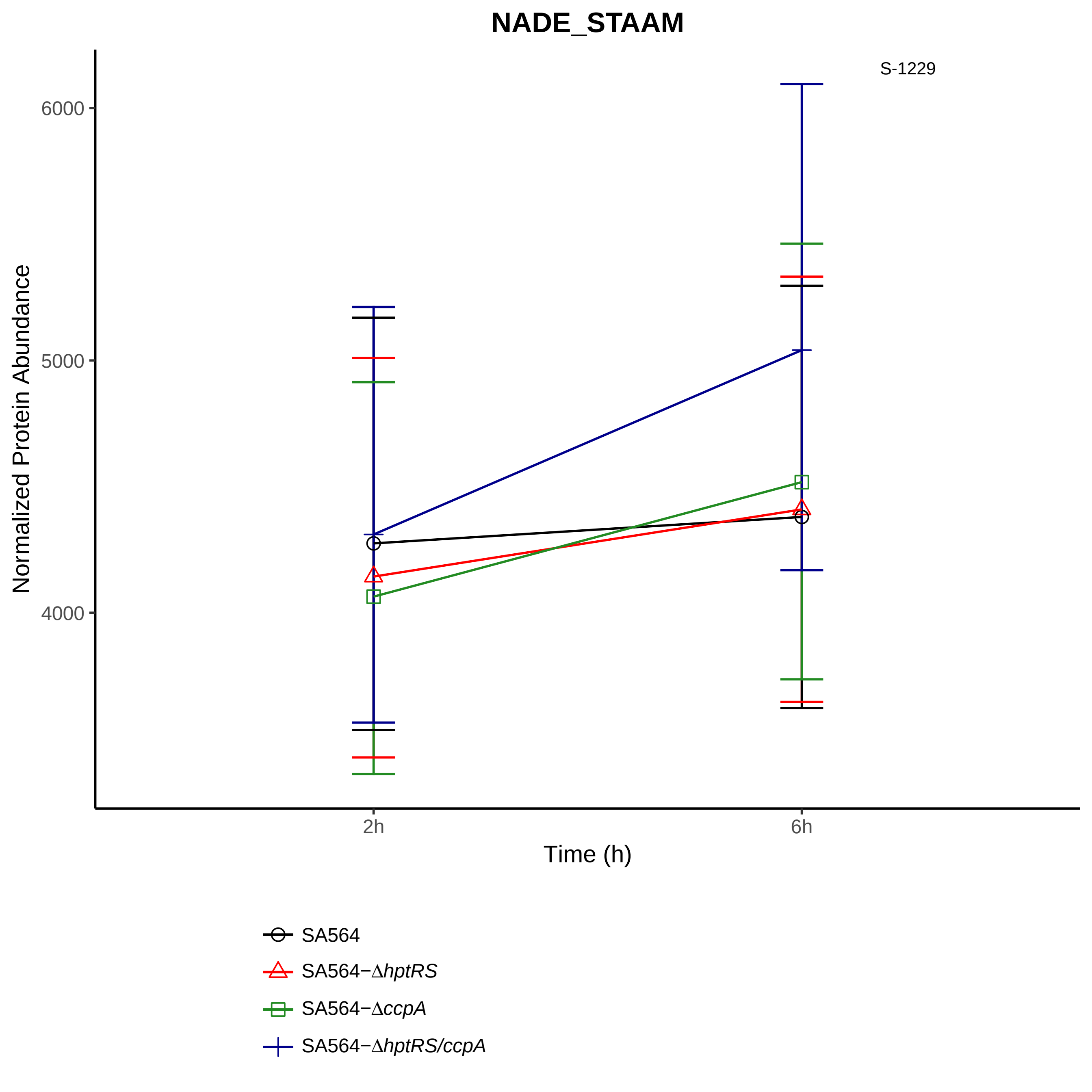

NADE_STAAM
S-1229
6000
5000
4000
Normalized Protein Abundance
2h
6h
Time (h)
SA564
SA564−Δ*hptRS*
SA564−Δ*ccpA*
SA564−Δ*hptRS/ccpA*

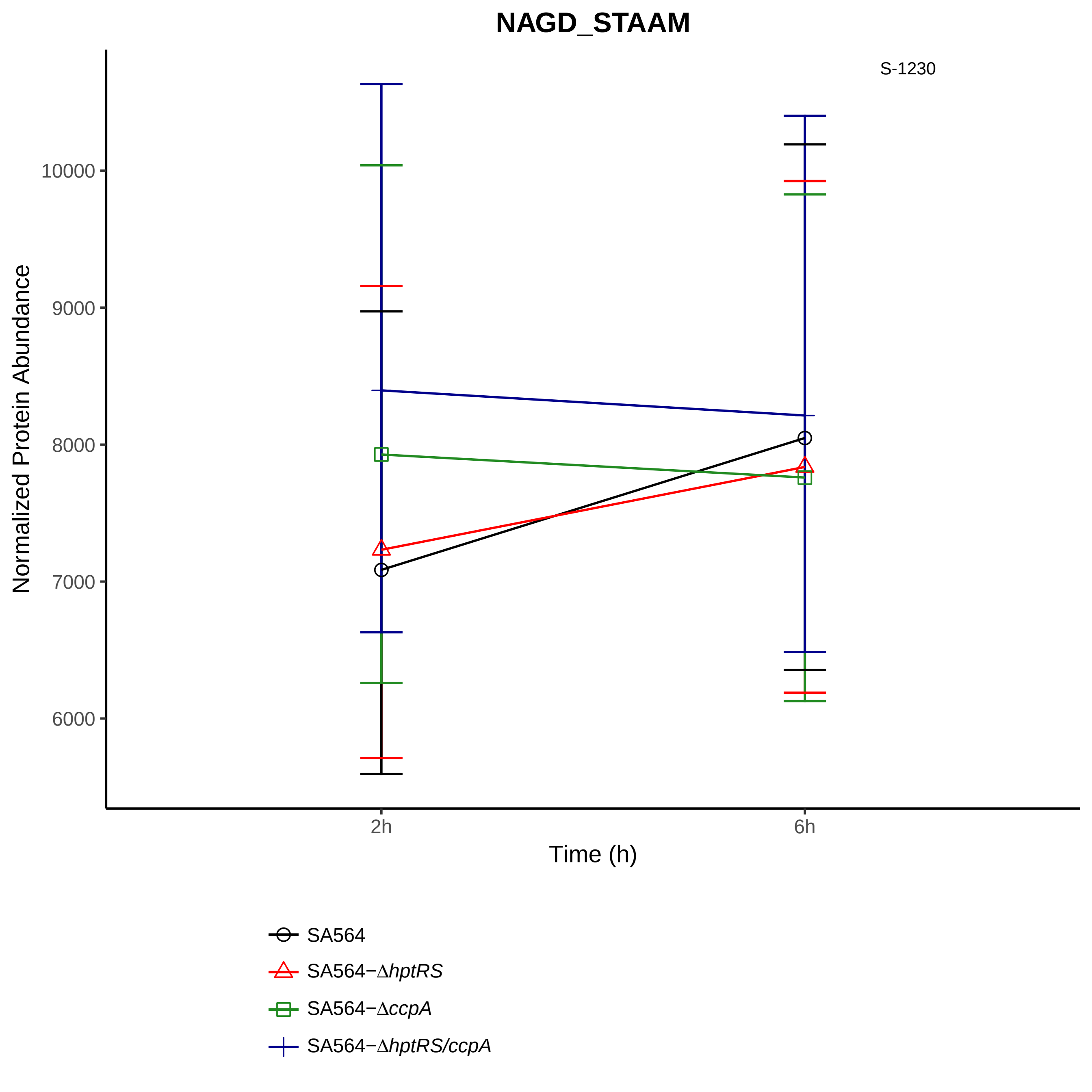

NAGD_STAAM
S-1230
Normalized Protein Abundance
10000
9000
8000
7000
6000
2h
6h
Time (h)
SA564
SA564−Δ*hptRS*
SA564−Δ*ccpA*
SA564−Δ*hptRS/ccpA*

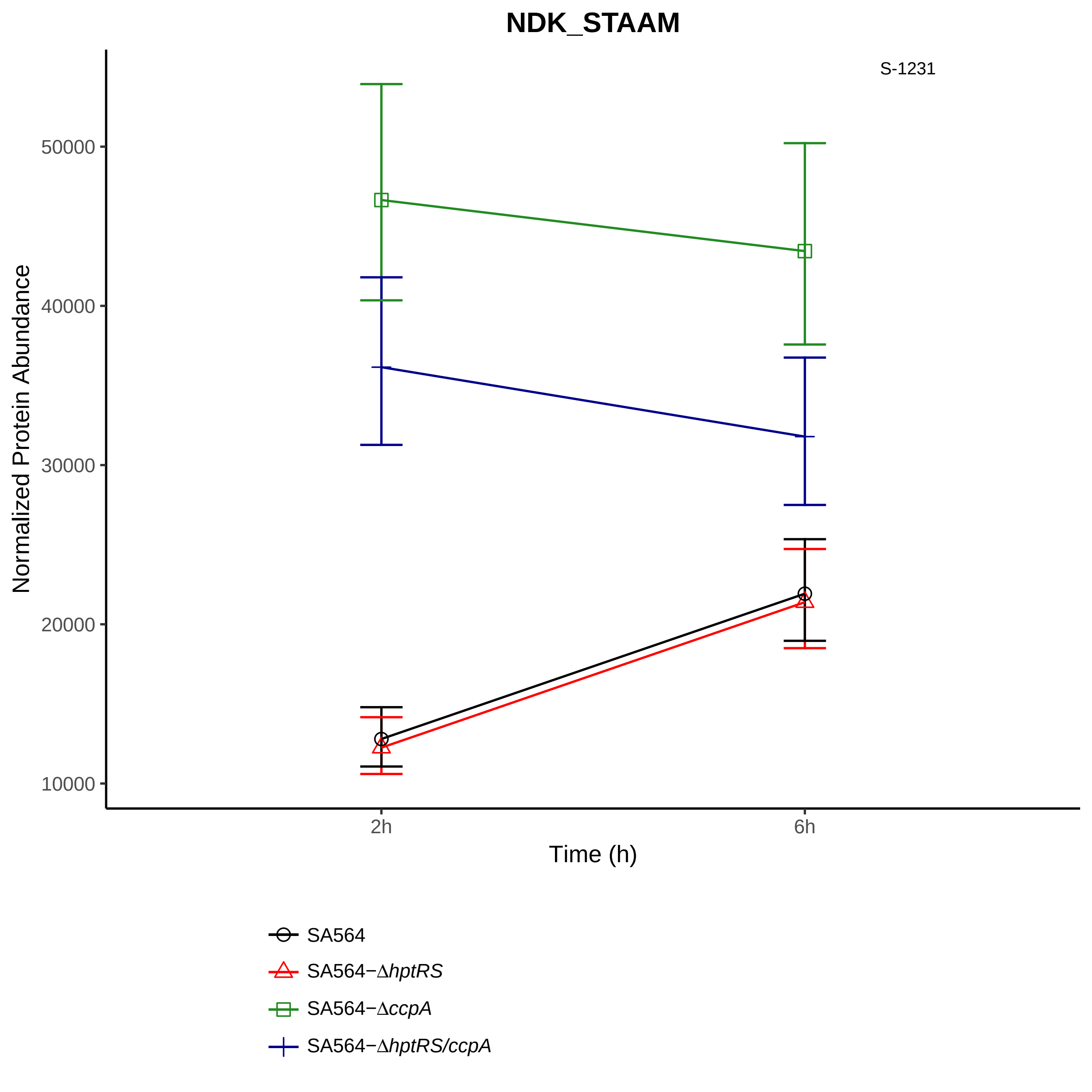

NDK_STAAM
S-1231
Normalized Protein Abundance
50000
40000
30000
20000
10000
2h
6h
Time (h)
SA564
SA564−Δ*hptRS*
SA564−Δ*ccpA*
SA564−Δ*hptRS/ccpA*

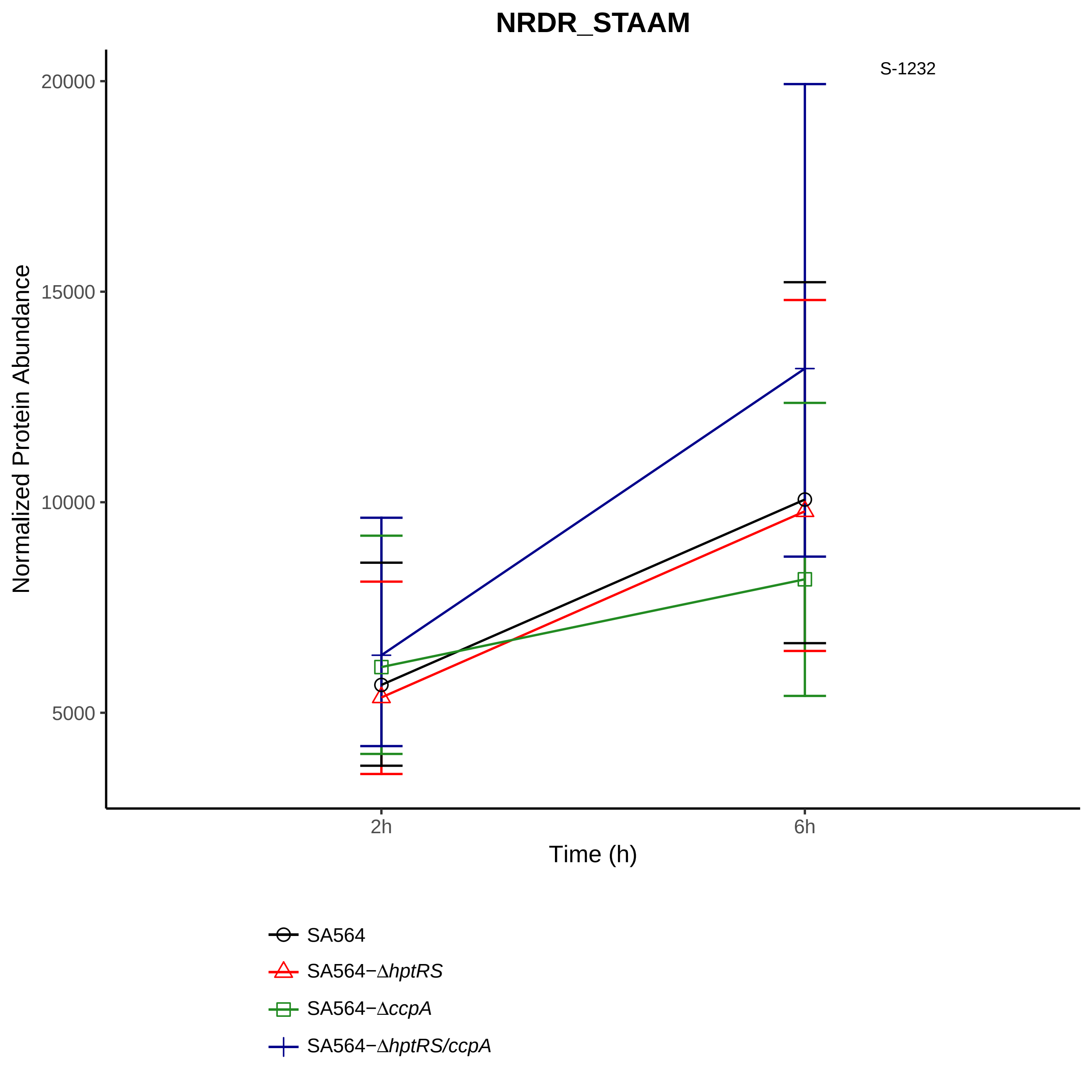

NRDR_STAAM
S-1232
Normalized Protein Abundance
20000
15000
10000
5000
2h
6h
Time (h)
SA564
SA564−ΔhptRS
SA564−ΔccpA
SA564−ΔhptRS/ccpA

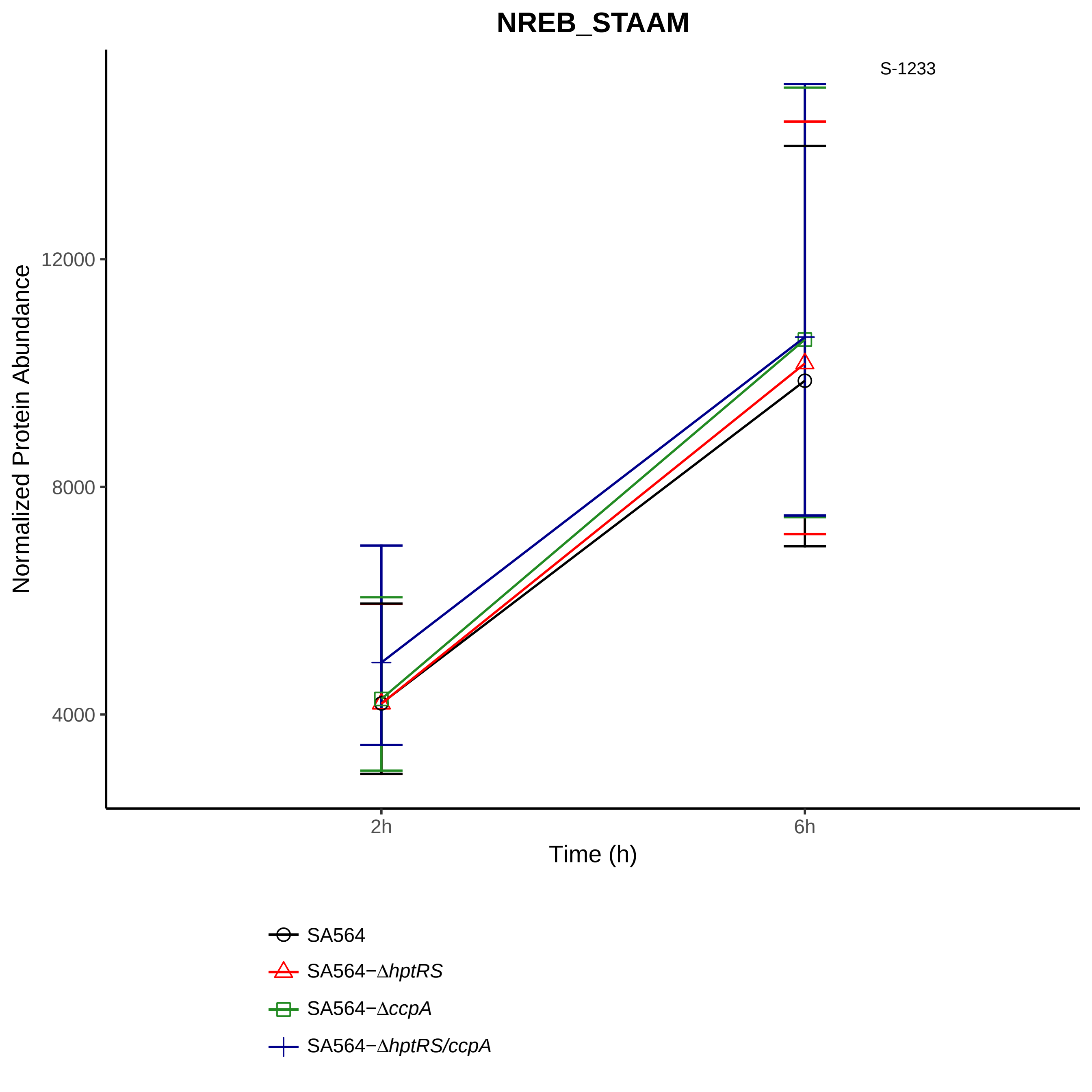

NREB_STAAM
S-1233
Normalized Protein Abundance
12000
8000
4000
2h
6h
Time (h)
SA564
SA564−Δ*hptRS*
SA564−Δ*ccpA*
SA564−Δ*hptRS/ccpA*

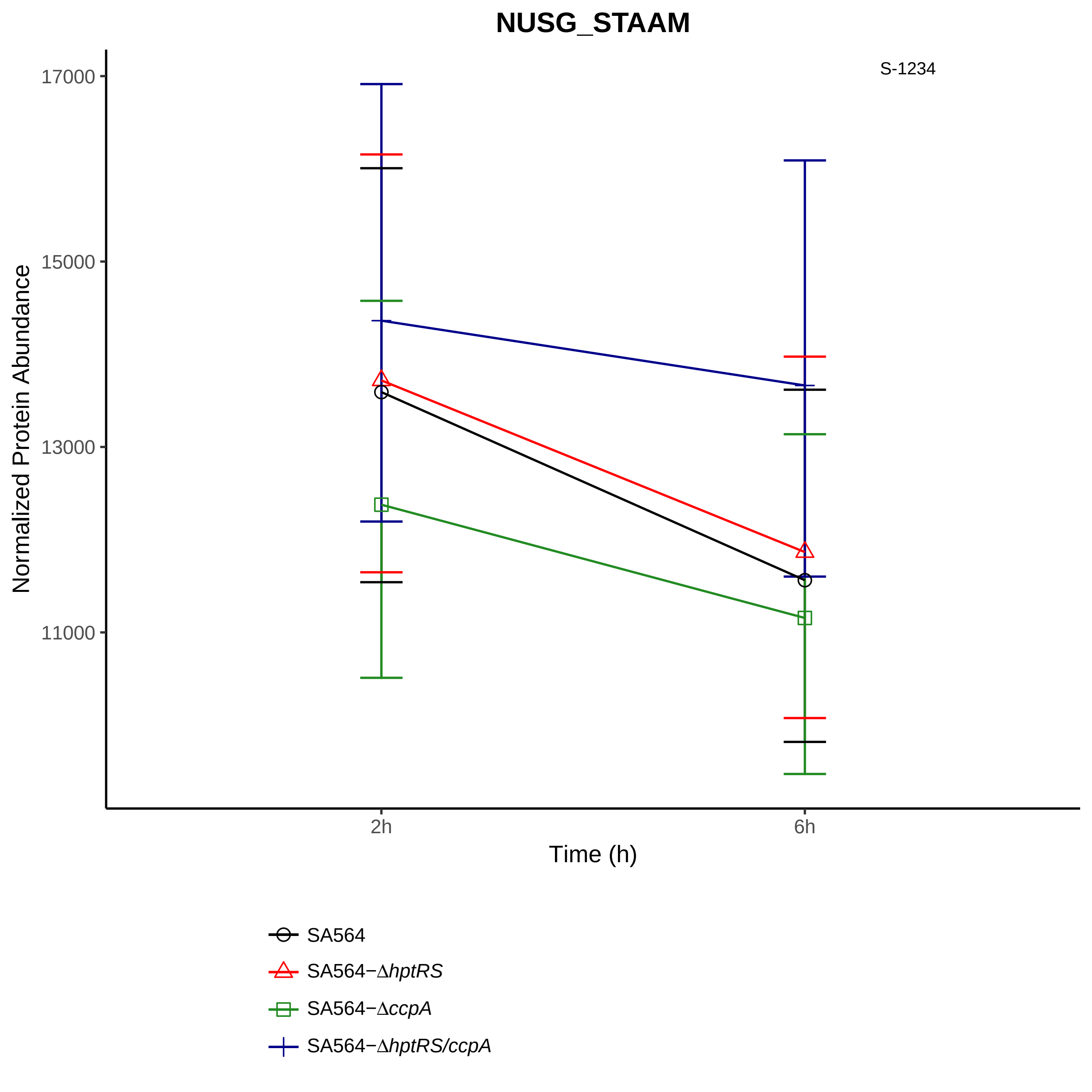

NUSG_STAAM
S-1234
17000
15000
13000
11000
Normalized Protein Abundance
2h
6h
Time (h)
SA564
SA564−Δ*hptRS*
SA564−Δ*ccpA*
SA564−Δ*hptRS/ccpA*

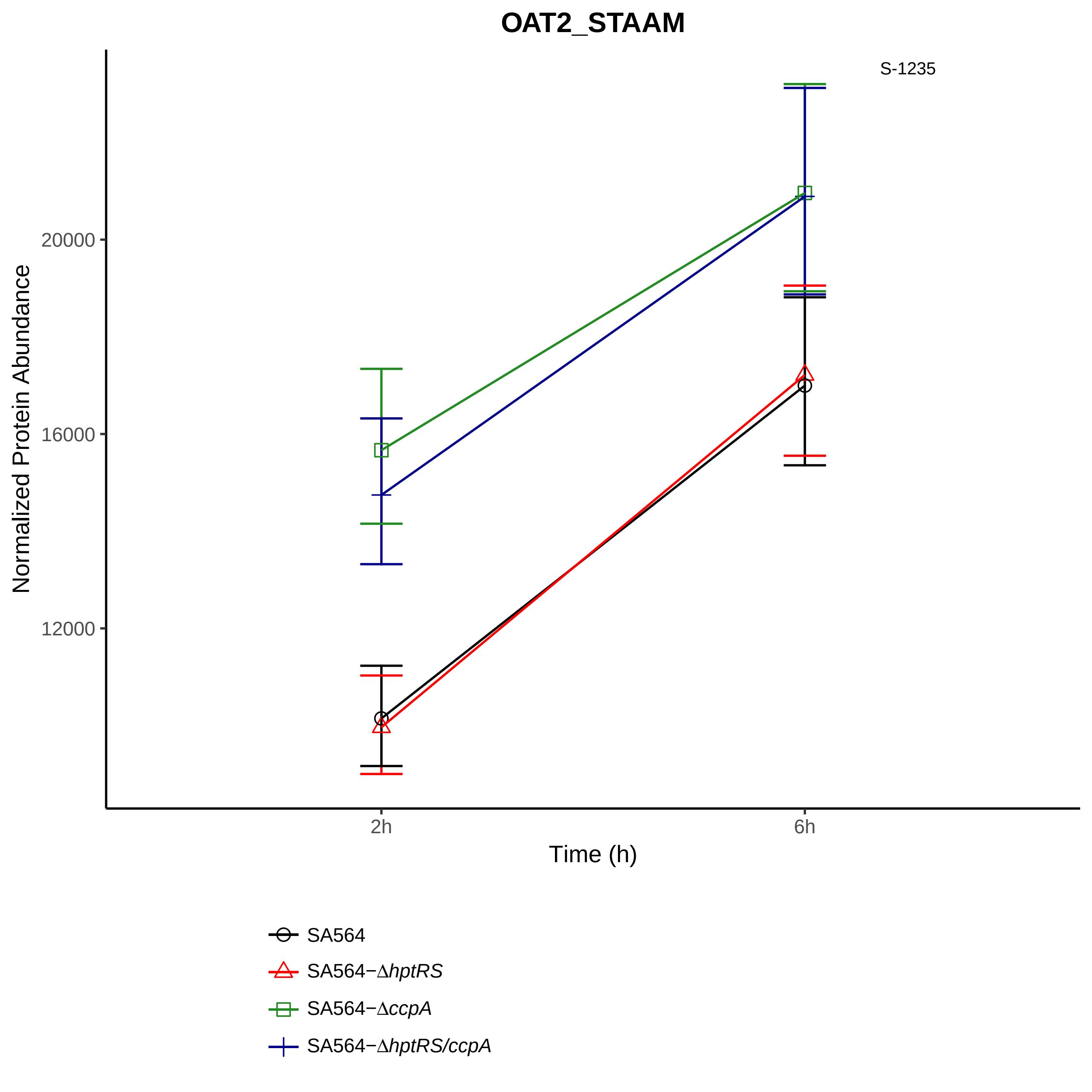

OAT2_STAAM
S-1235
20000
16000
12000
Normalized Protein Abundance
2h
6h
Time (h)
SA564
SA564−Δ*hptRS*
SA564−Δ*ccpA*
SA564−Δ*hptRS/ccpA*

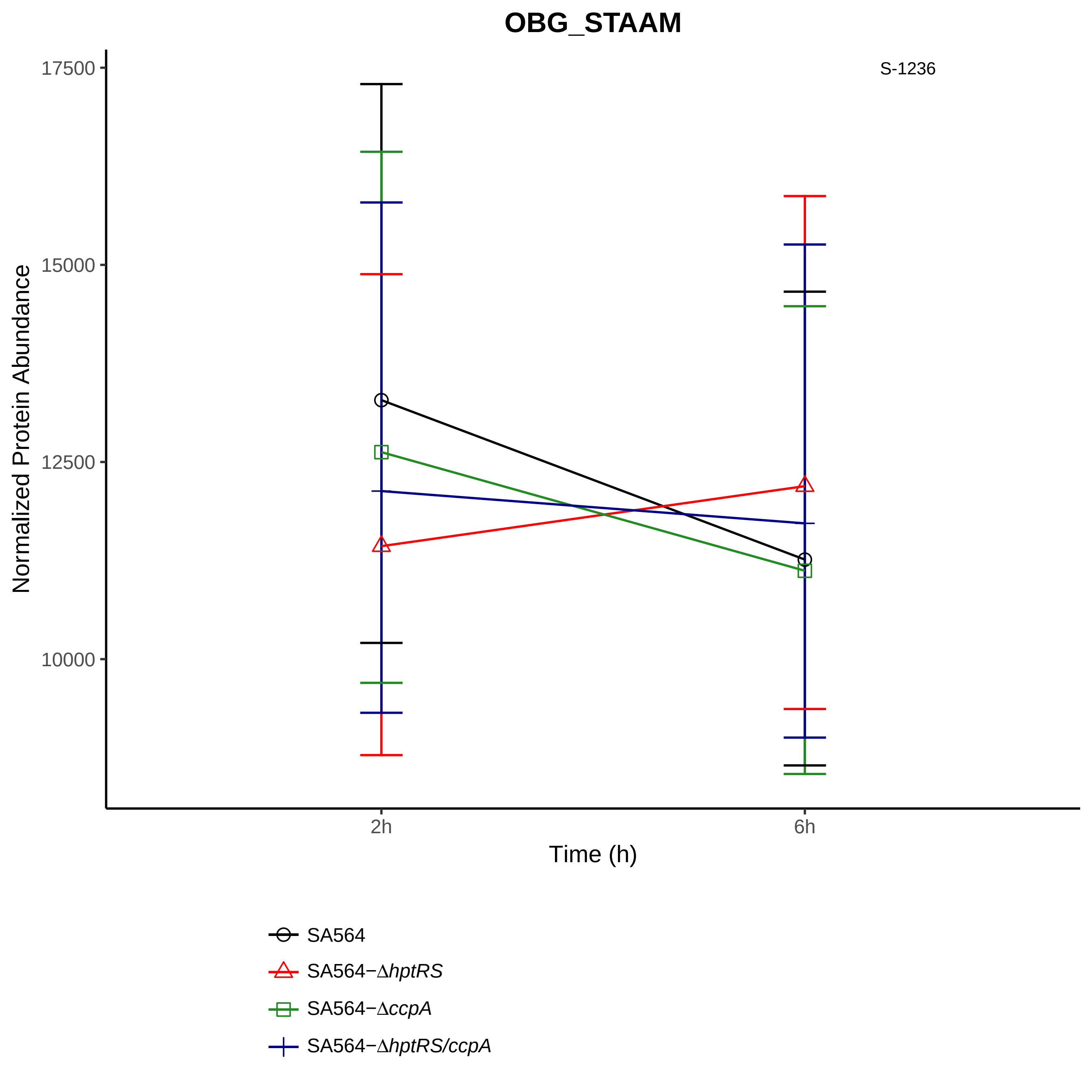

OBG_STAAM
S-1236
17500
15000
12500
10000
Normalized Protein Abundance
2h
6h
Time (h)
SA564
SA564−Δ*hptRS*
SA564−Δ*ccpA*
SA564−Δ*hptRS/ccpA*

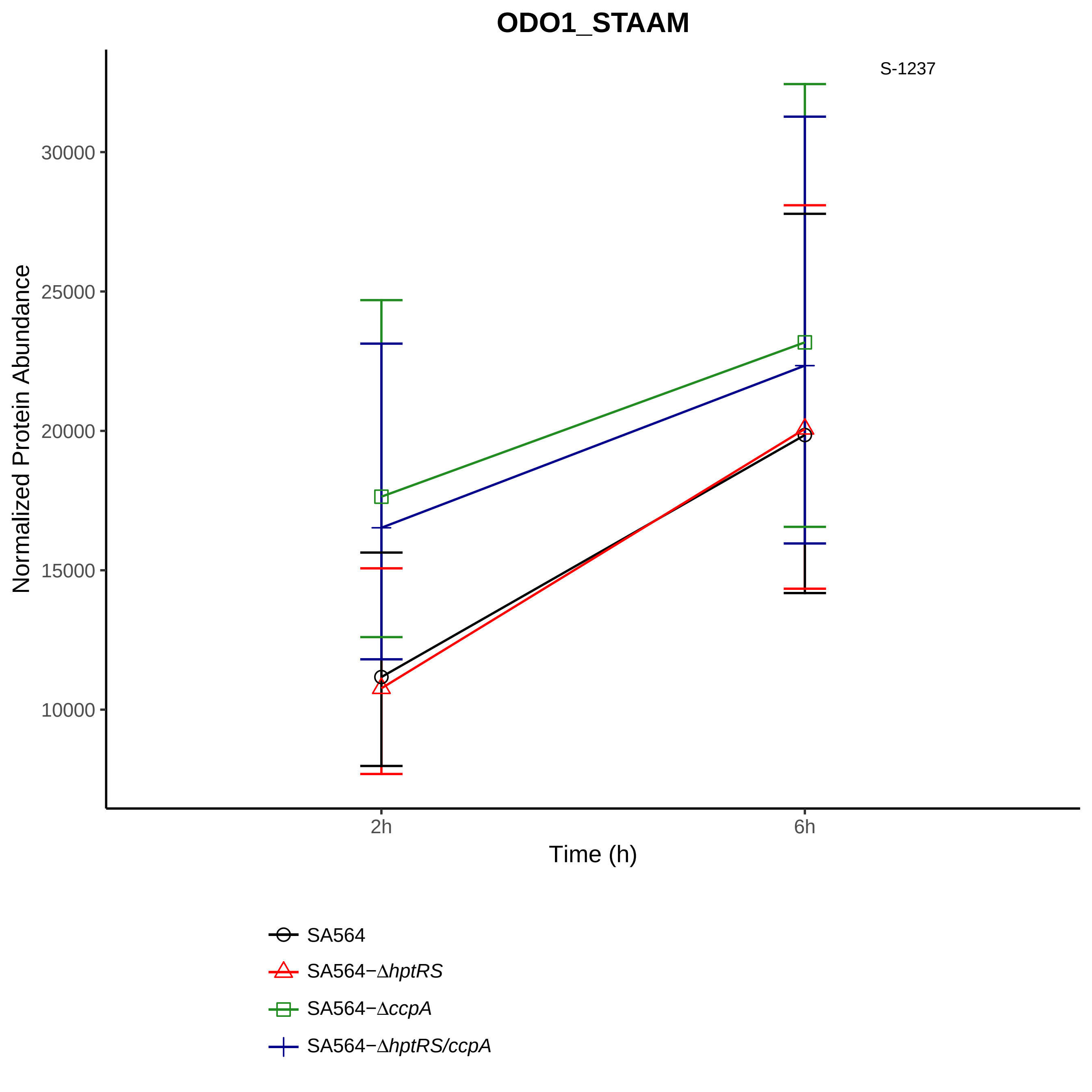

ODO1_STAAM
S-1237
Normalized Protein Abundance
30000
25000
20000
15000
10000
2h
6h
Time (h)
SA564
SA564−Δ*hptRS*
SA564−Δ*ccpA*
SA564−Δ*hptRS/ccpA*

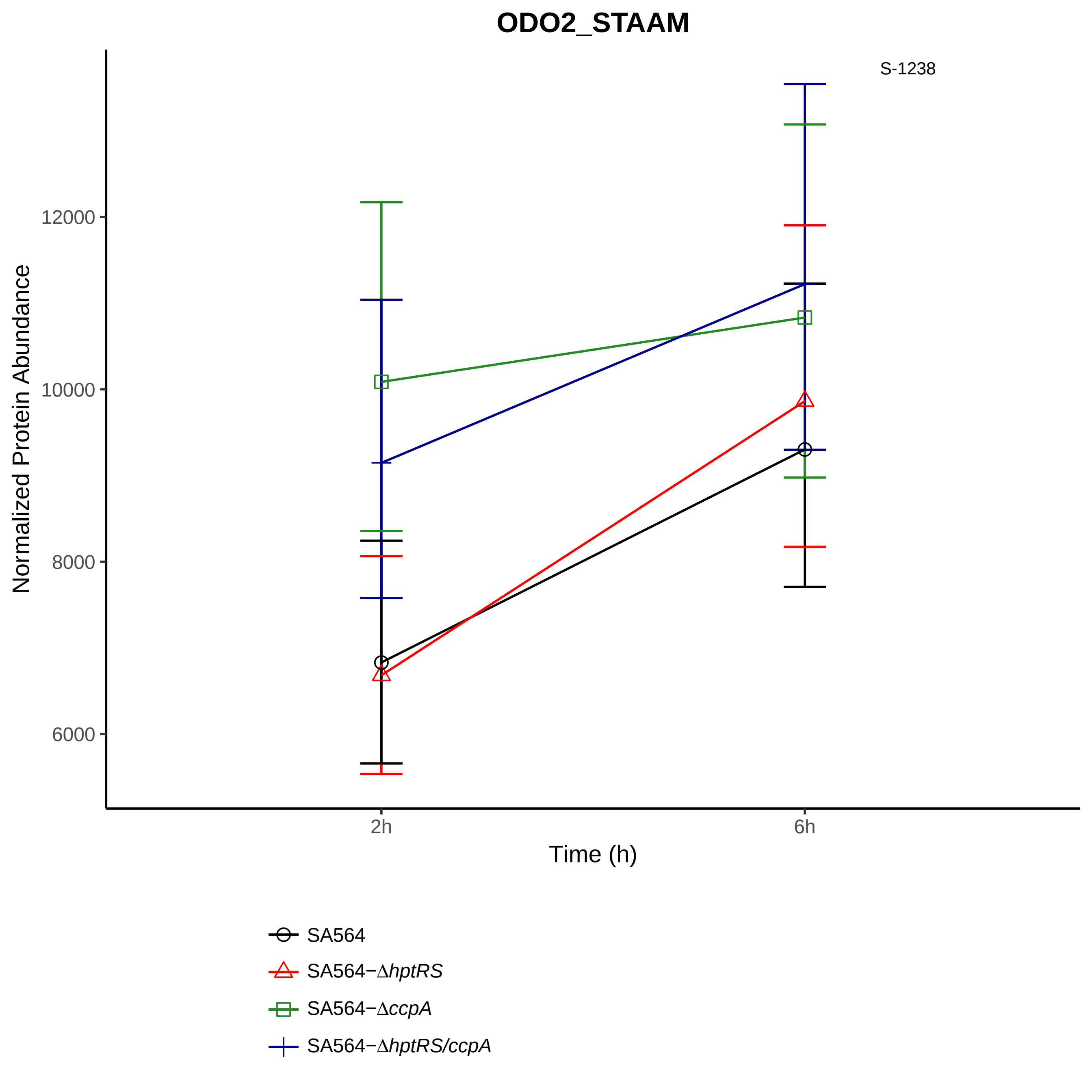

ODO2_STAAM
S-1238
Normalized Protein Abundance
12000
10000
8000
6000
2h
6h
Time (h)
SA564
SA564−ΔhptRS
SA564−ΔccpA
SA564−ΔhptRS/ccpA

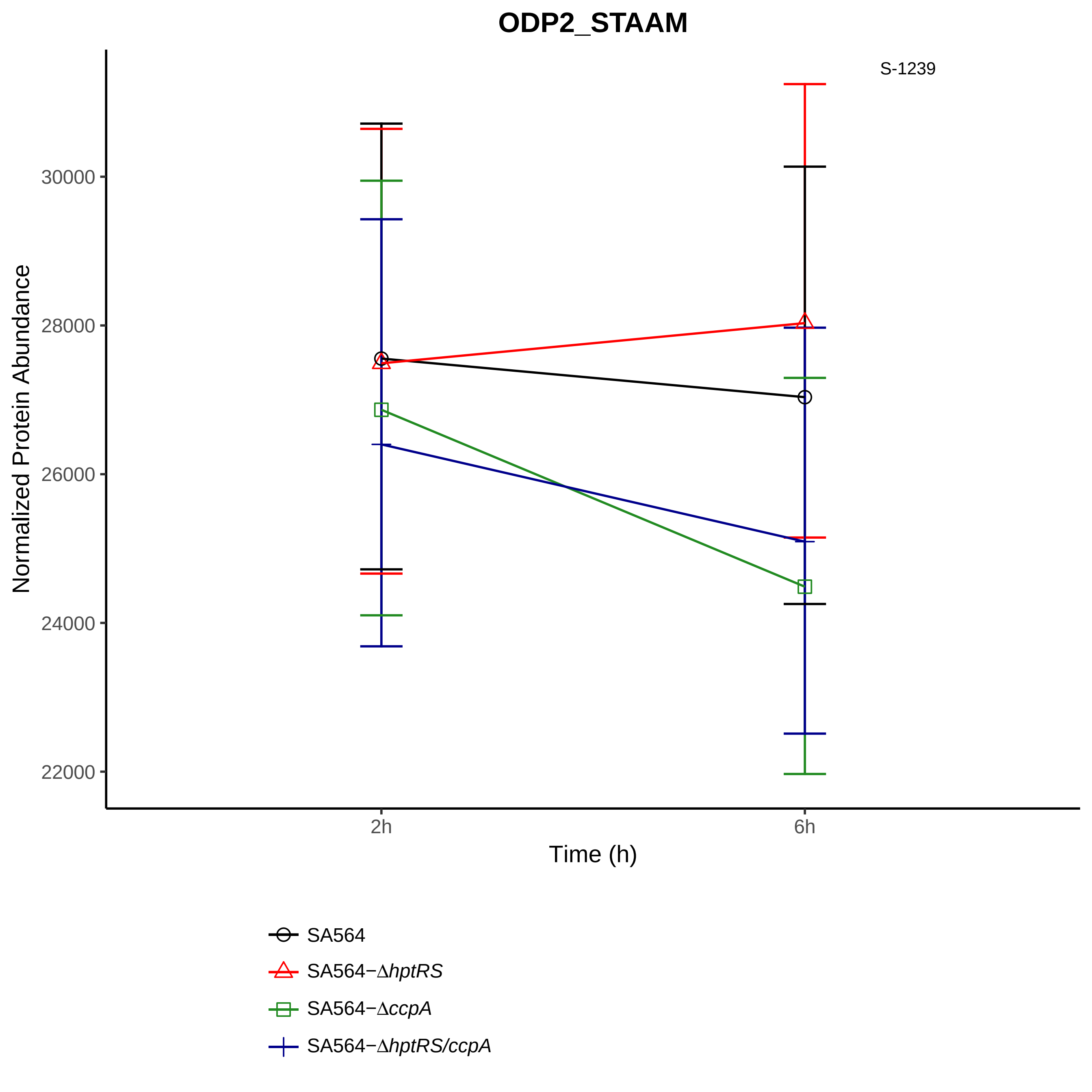

ODP2_STAAM
S-1239
Normalized Protein Abundance
30000
28000
26000
24000
22000
2h
6h
Time (h)
SA564
SA564−Δ*hptRS*
SA564−Δ*ccpA*
SA564−Δ*hptRS/ccpA*

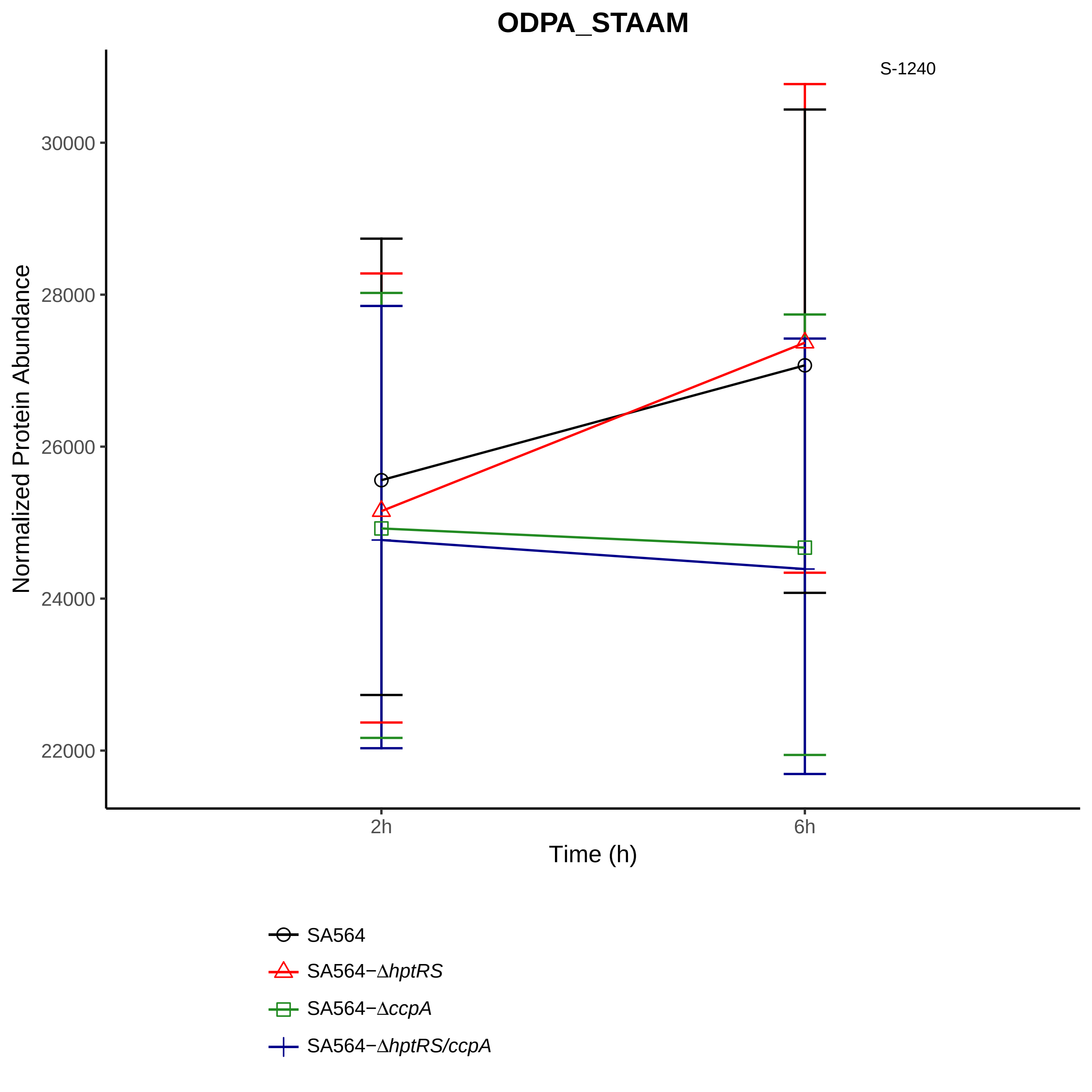

ODPA_STAAM
S-1240
30000
28000
26000
24000
22000
Normalized Protein Abundance
2h
6h
Time (h)
SA564
SA564−Δ*hptRS*
SA564−Δ*ccpA*
SA564−Δ*hptRS/ccpA*

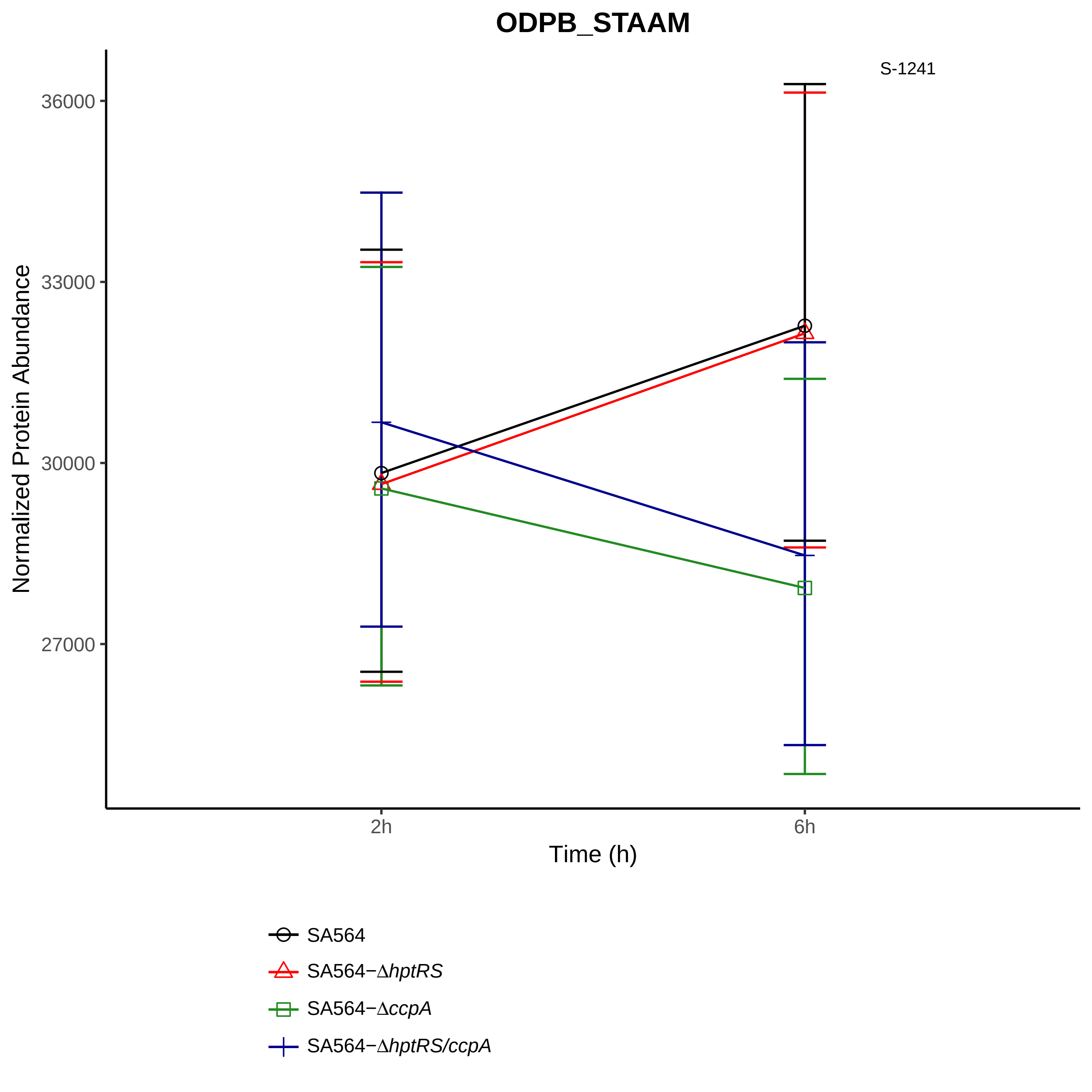
ODPB_STAAM
S-1241
Normalized Protein Abundance
36000
33000
30000
27000
2h
6h
Time (h)
SA564
SA564−ΔhptRS
SA564−ΔccpA
SA564−ΔhptRS/ccpA

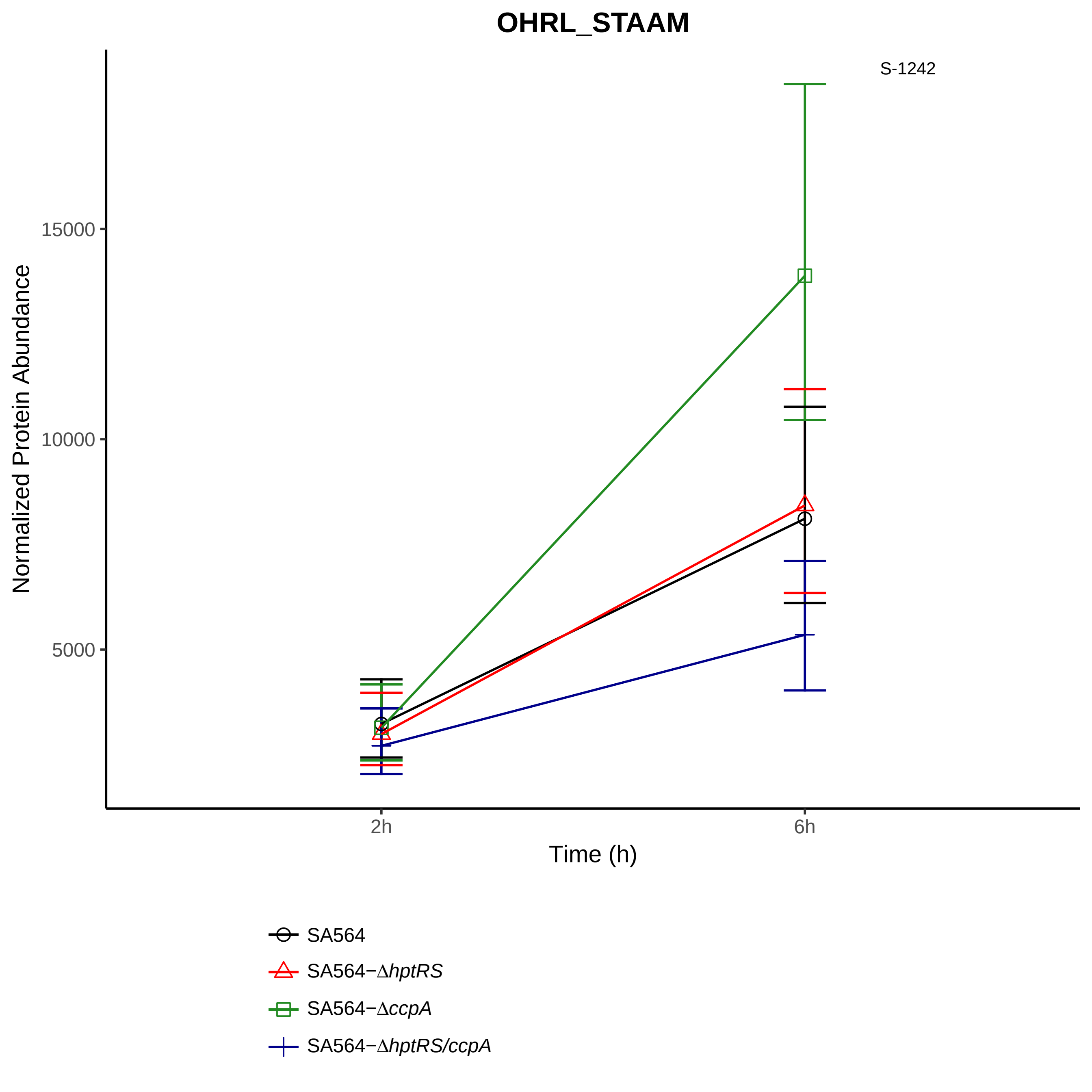

OHRL_STAAM
S-1242
Normalized Protein Abundance
15000
10000
5000
2h
6h
Time (h)
SA564
SA564−Δ*hptRS*
SA564−Δ*ccpA*
SA564−Δ*hptRS/ccpA*

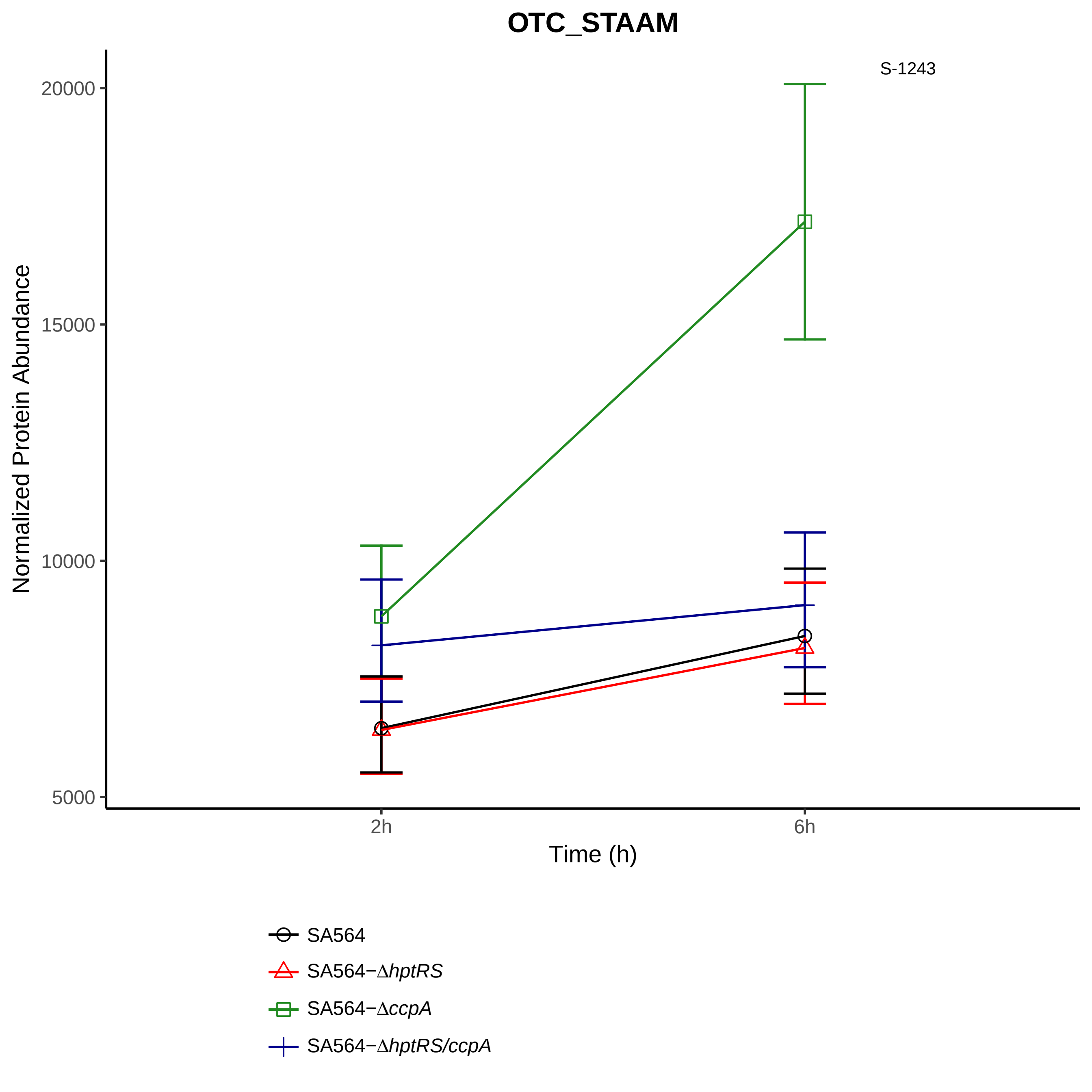
OTC_STAAM
S-1243
Normalized Protein Abundance
20000
15000
10000
5000
2h
6h
Time (h)
SA564
SA564−Δ*hptRS*
SA564−Δ*ccpA*
SA564−Δ*hptRS/ccpA*

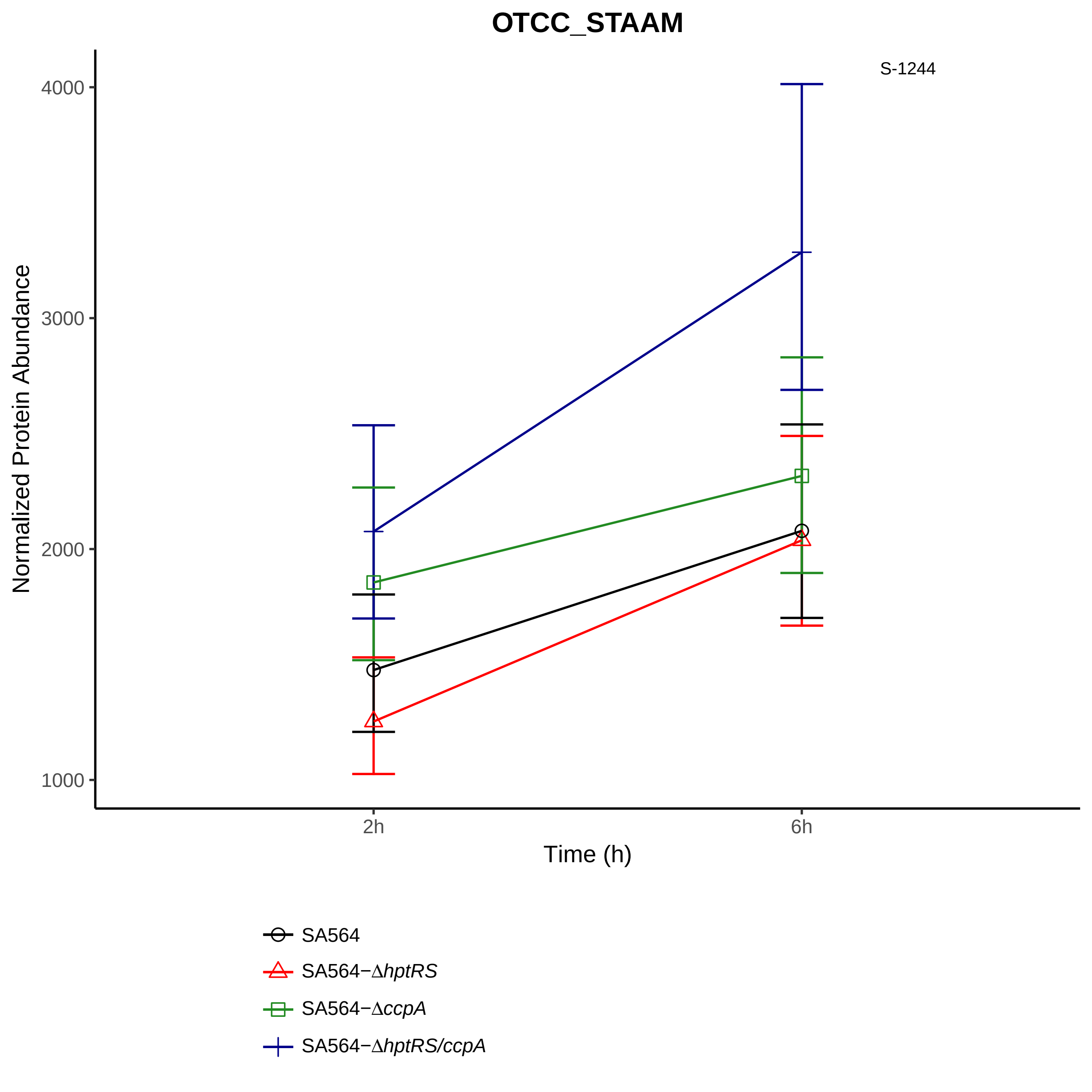

OTCC_STAAM
S-1244
Normalized Protein Abundance
4000
3000
2000
1000
2h
6h
Time (h)
SA564
SA564−Δ*hptRS*
SA564−Δ*ccpA*
SA564−Δ*hptRS/ccpA*

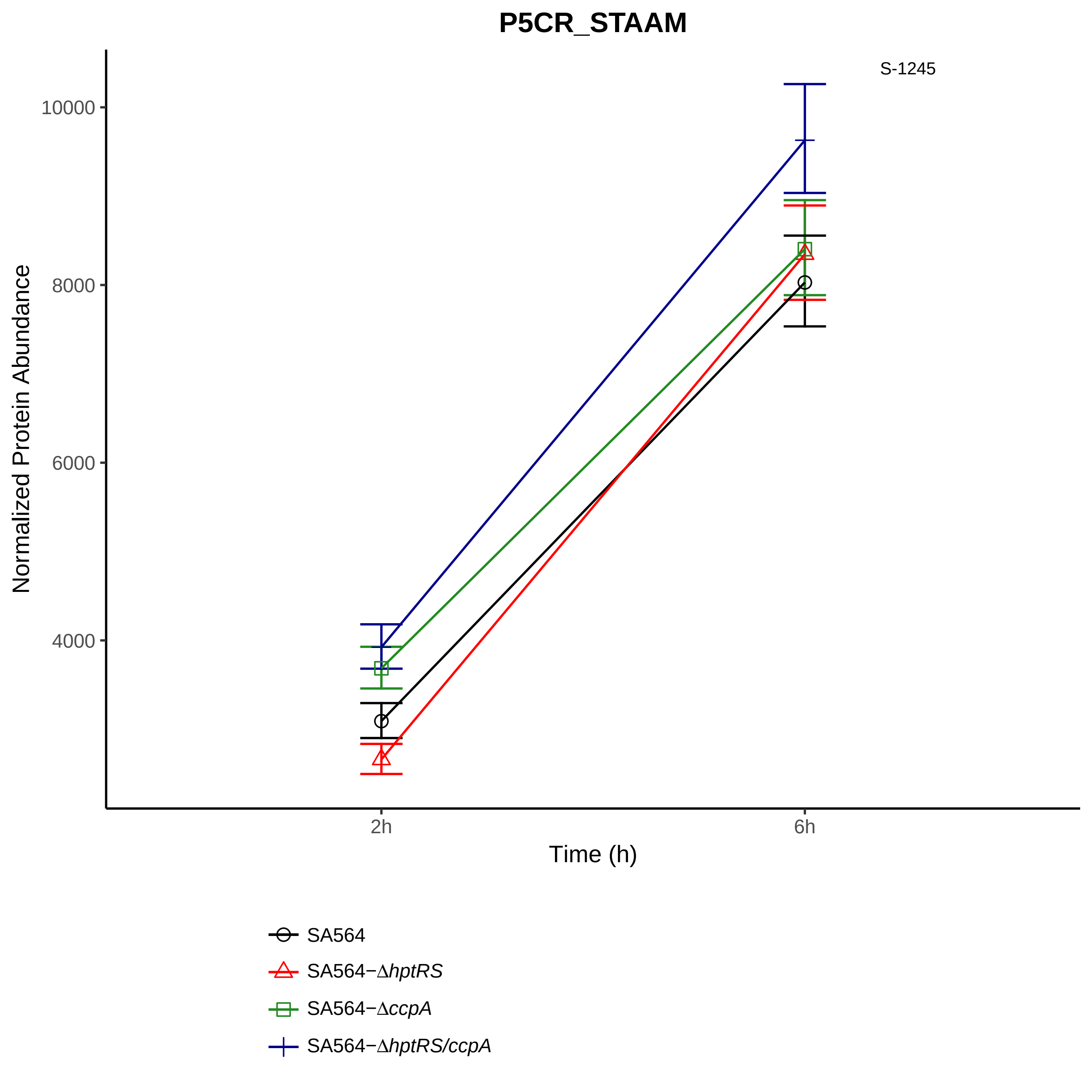

P5CR_STAAM
S-1245
Normalized Protein Abundance
10000
8000
6000
4000
2h
6h
Time (h)
SA564
SA564−Δ*hptRS*
SA564−Δ*ccpA*
SA564−Δ*hptRS/ccpA*

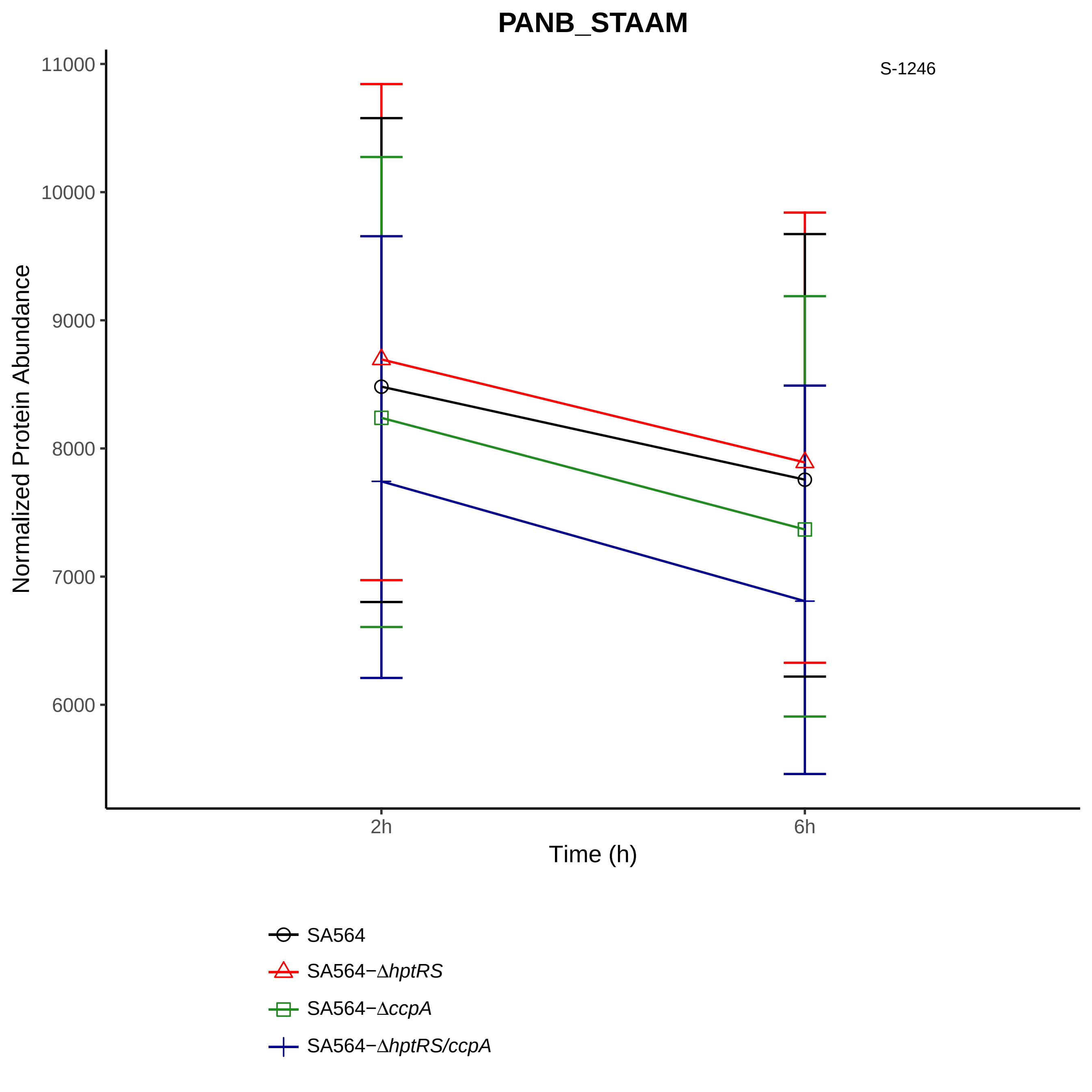
PANB_STAAM
S-1246
Normalized Protein Abundance
11000
10000
9000
8000
7000
6000
2h
6h
Time (h)
SA564
SA564−Δ*hptRS*
SA564−Δ*ccpA*
SA564−Δ*hptRS/ccpA*

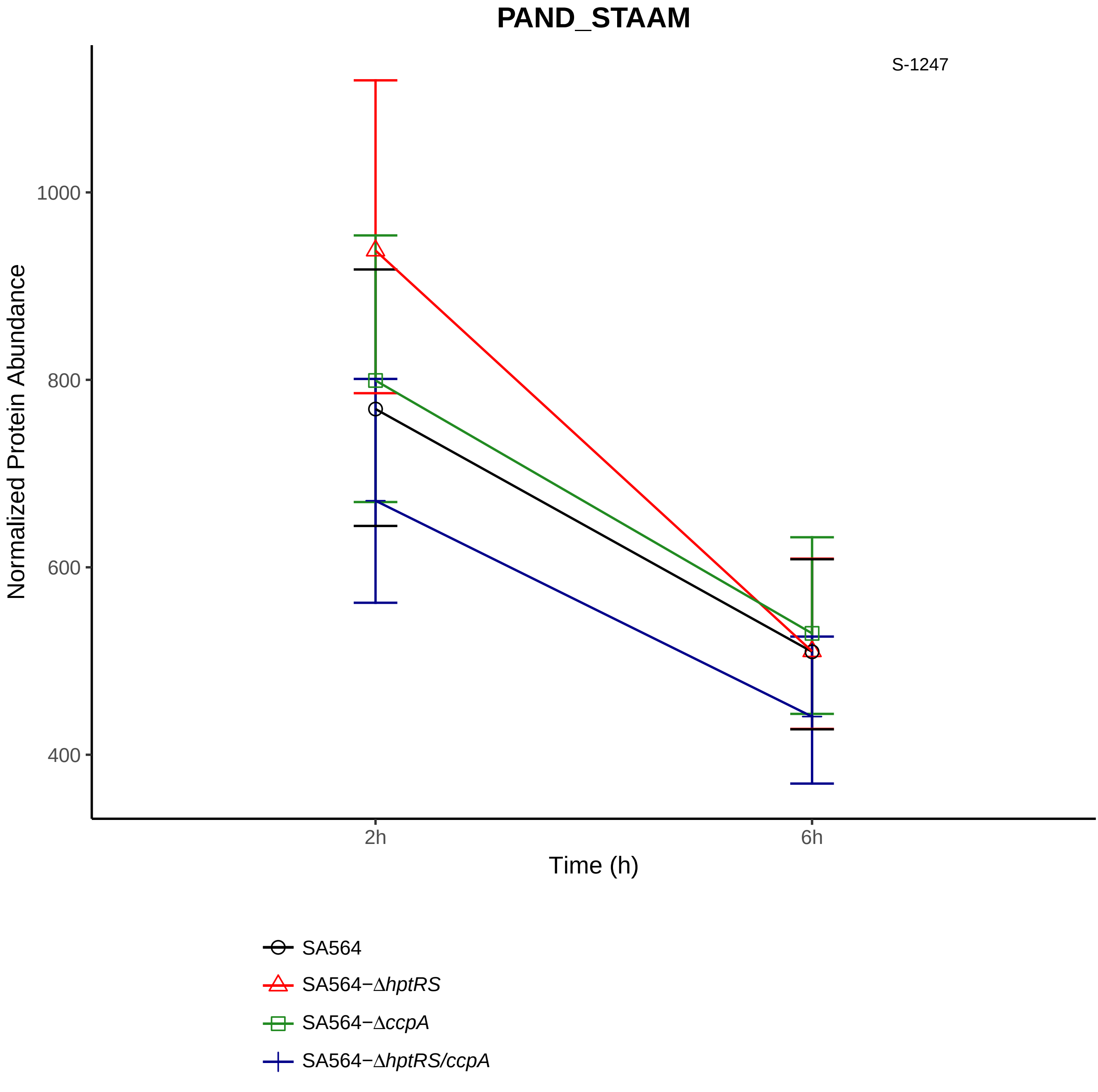

PAND_STAAM
S-1247
Normalized Protein Abundance
1000
800
600
400
2h
6h
Time (h)
SA564
SA564−ΔhptRS
SA564−ΔccpA
SA564−ΔhptRS/ccpA

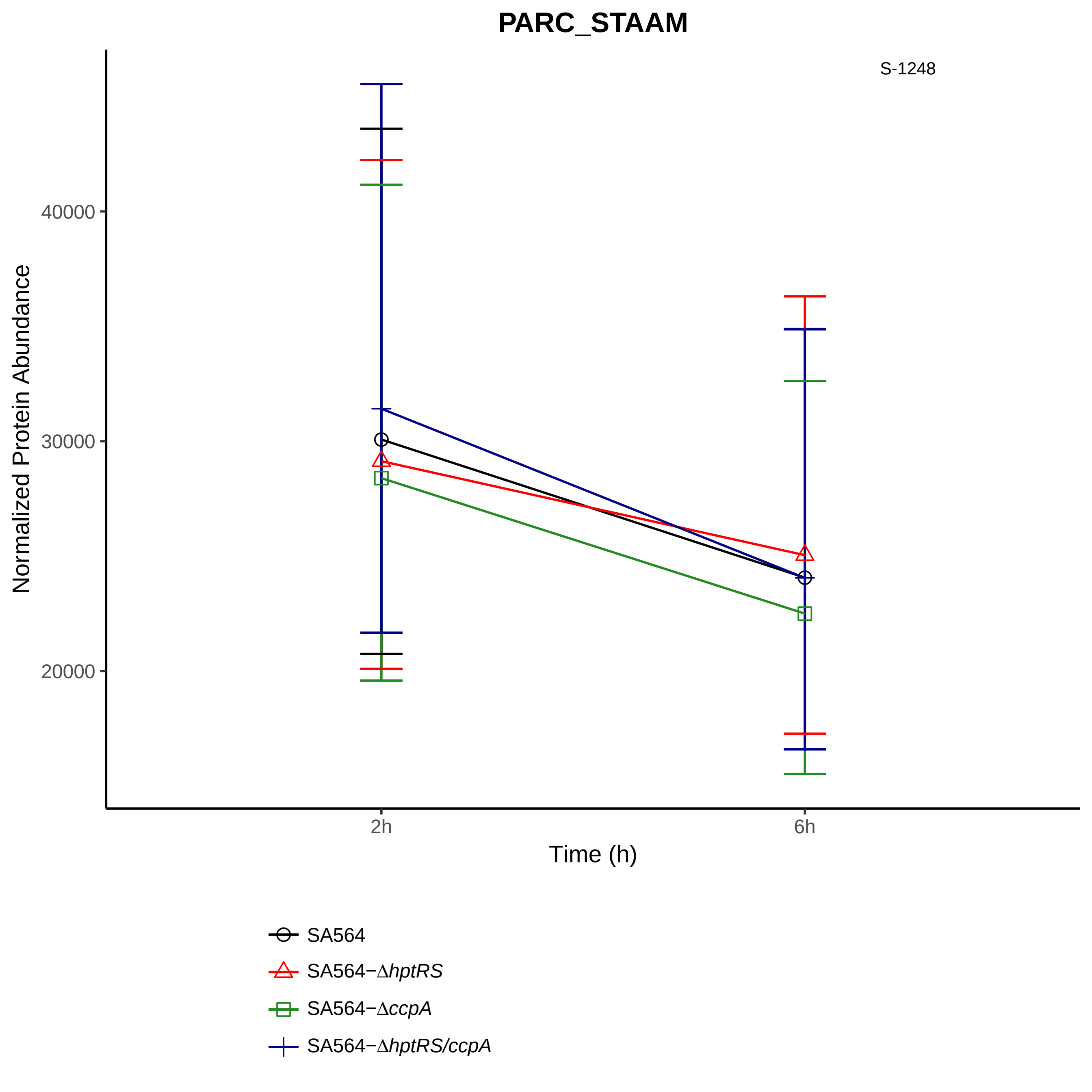

PARC_STAAM
S-1248
Normalized Protein Abundance
40000
30000
20000
2h
6h
Time (h)
SA564
SA564−ΔhptRS
SA564−ΔccpA
SA564−ΔhptRS/ccpA

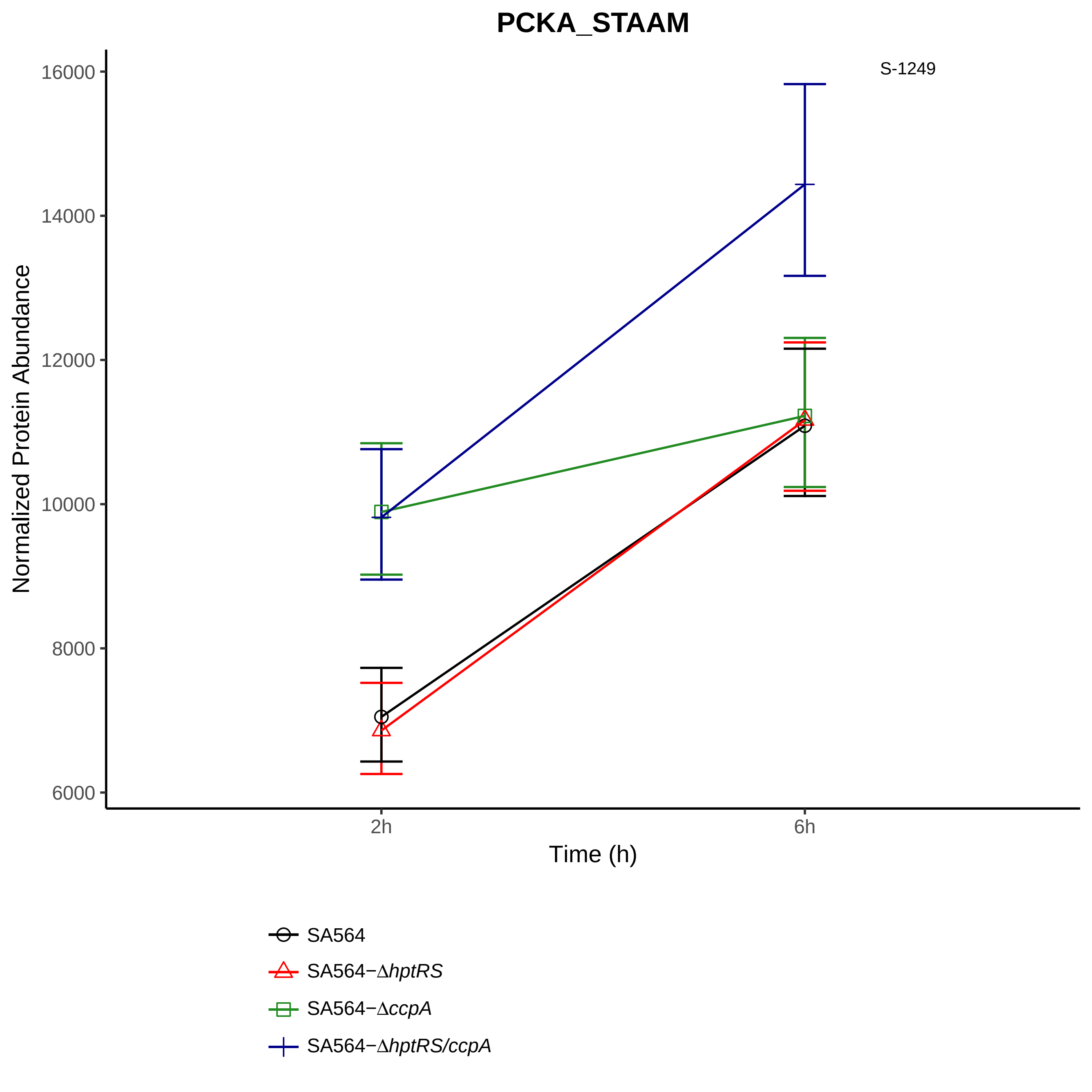

PCKA_STAAM
S-1249
Normalized Protein Abundance
16000
14000
12000
10000
8000
6000
2h
6h
Time (h)
SA564
SA564−ΔhptRS
SA564−ΔccpA
SA564−ΔhptRS/ccpA

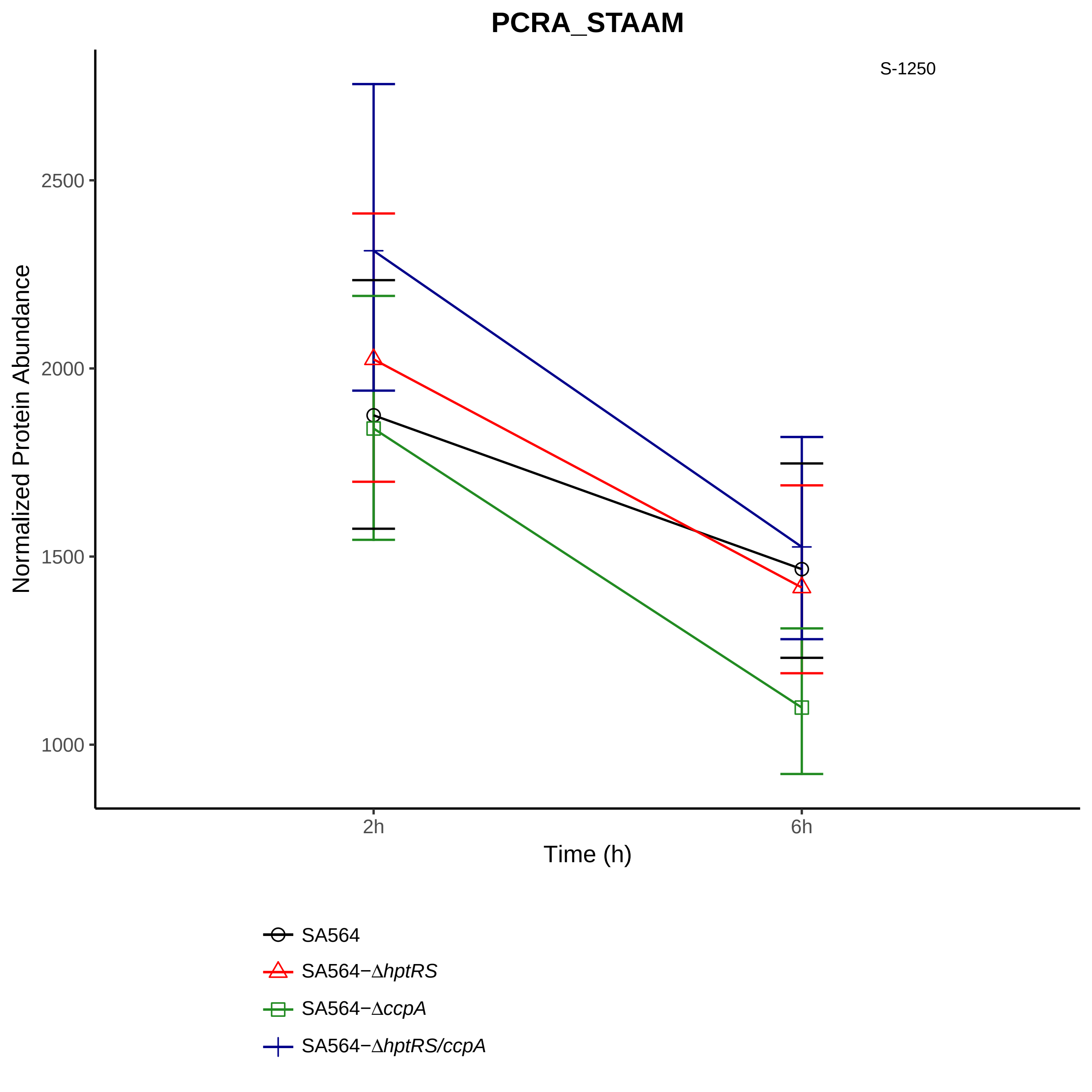

PCRA_STAAM
S-1250
Normalized Protein Abundance
2500
2000
1500
1000
2h
6h
Time (h)
SA564
SA564−Δ*hptRS*
SA564−Δ*ccpA*
SA564−Δ*hptRS/ccpA*

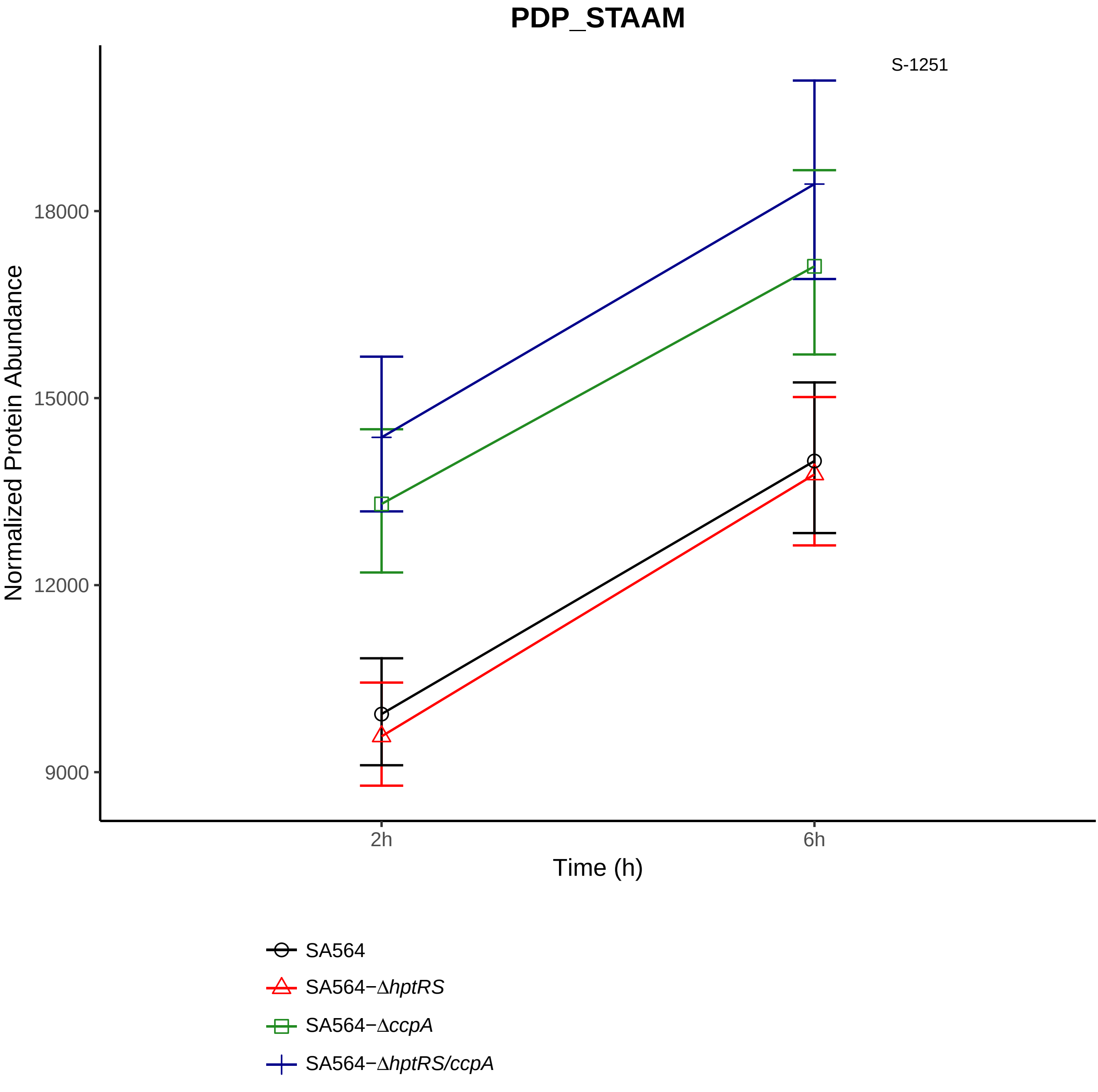
PDP_STAAM
S-1251
Normalized Protein Abundance
18000
15000
12000
9000
2h
6h
Time (h)
SA564
SA564−Δ*hptRS*
SA564−Δ*ccpA*
SA564−Δ*hptRS/ccpA*

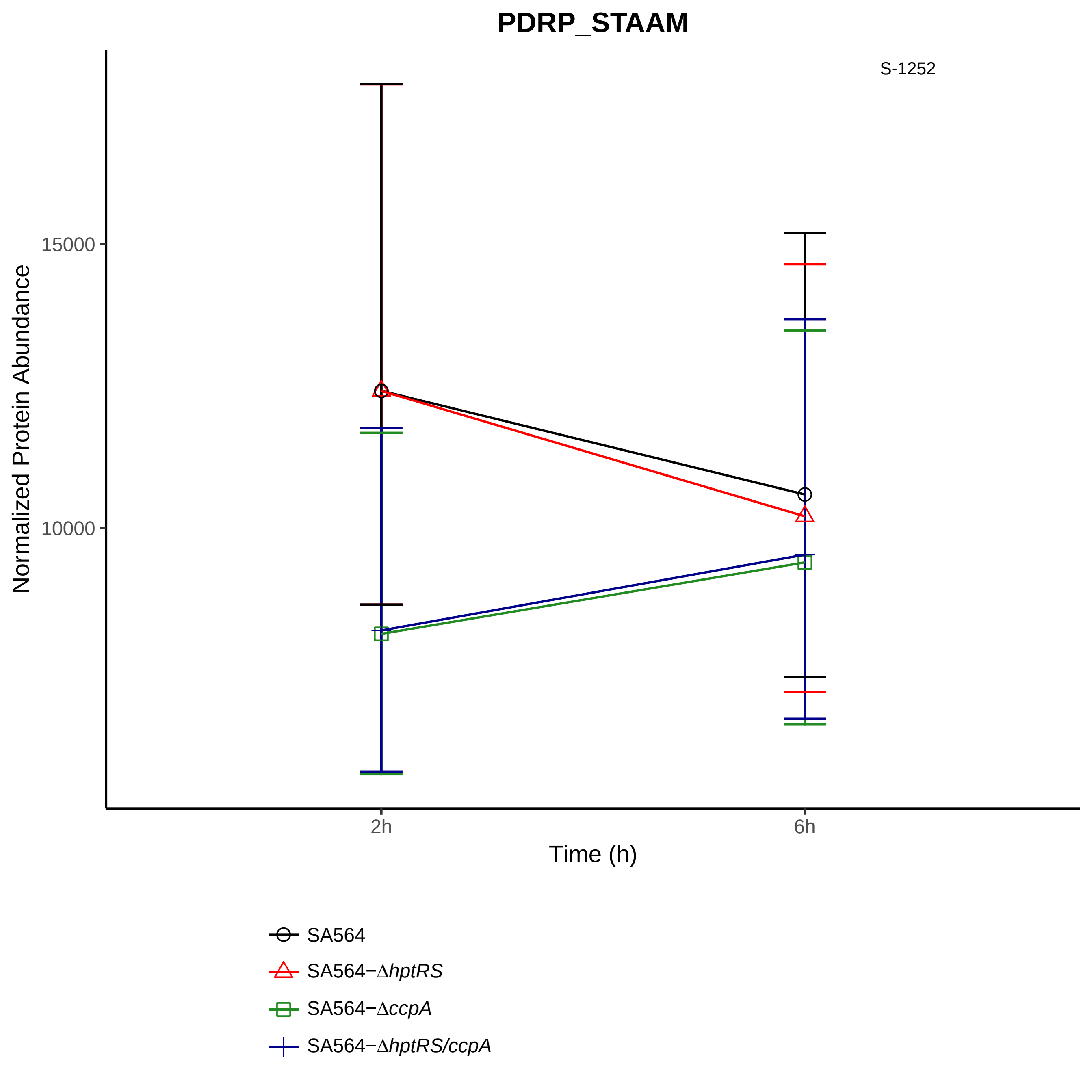

PDRP_STAAM
S-1252
Normalized Protein Abundance
15000
10000
2h
6h
Time (h)
SA564
SA564−Δ*hptRS*
SA564−Δ*ccpA*
SA564−Δ*hptRS/ccpA*

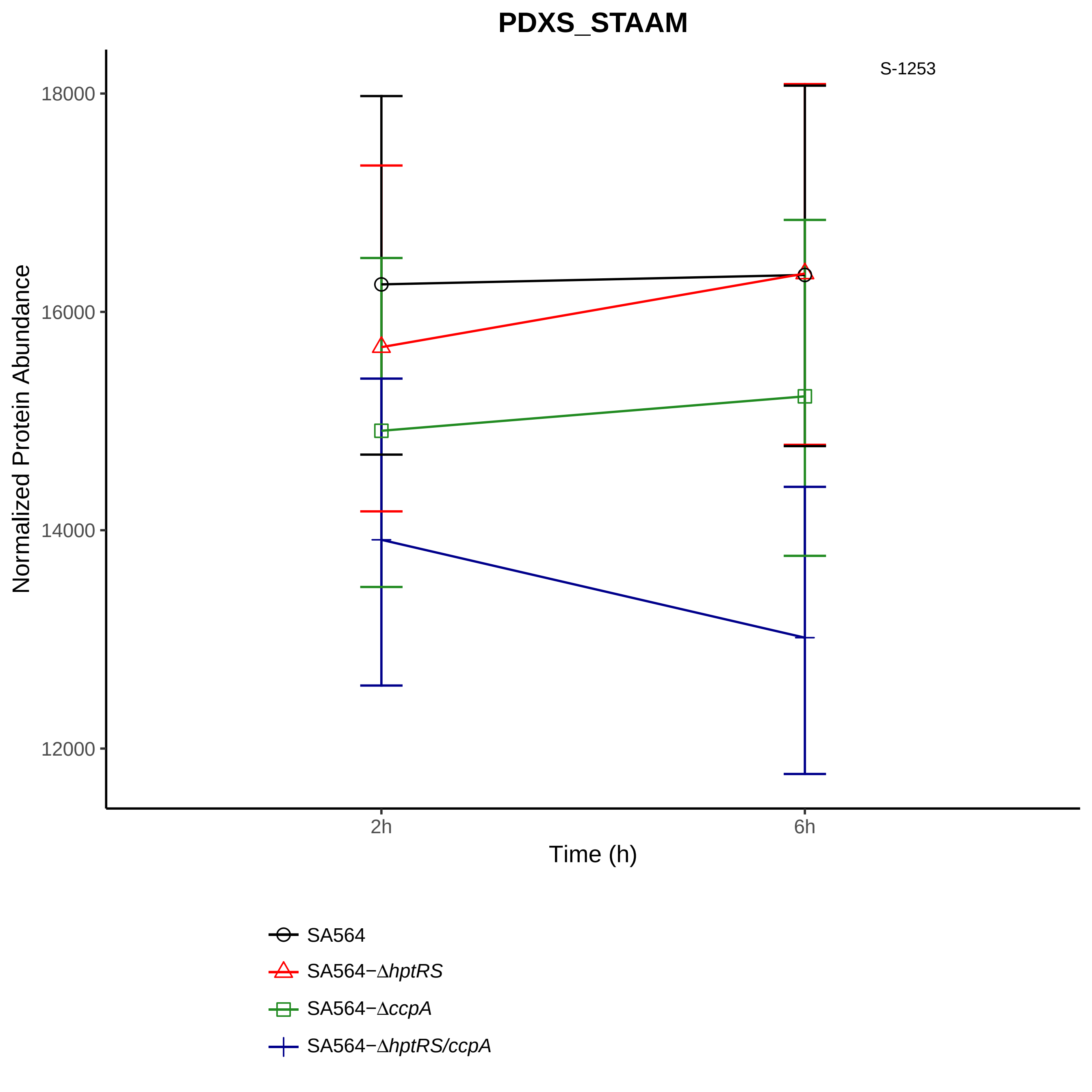

PDXS_STAAM
S-1253
18000
16000
14000
12000
Normalized Protein Abundance
2h
6h
Time (h)
SA564
SA564−ΔhptRS
SA564−ΔccpA
SA564−ΔhptRS/ccpA

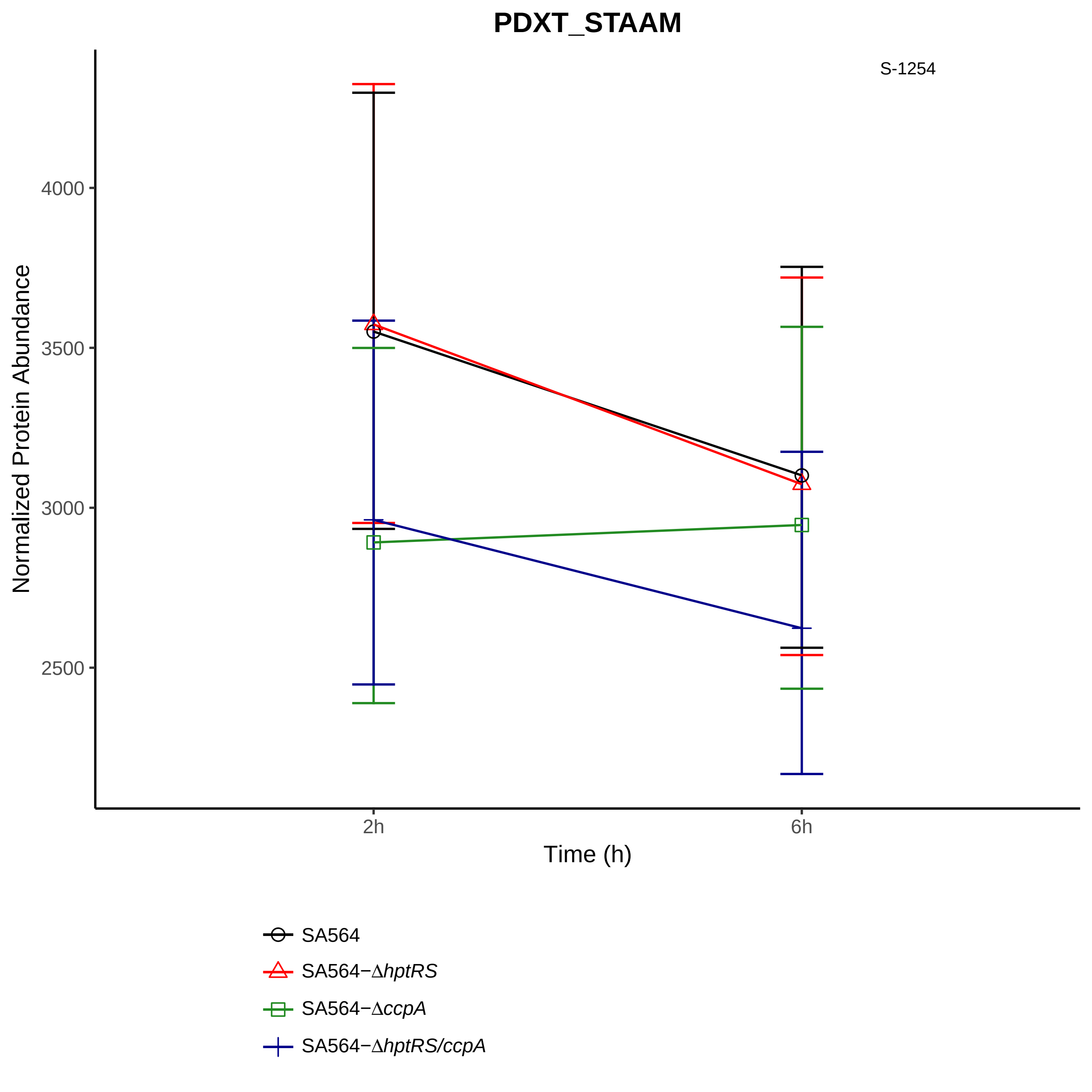

PDXT_STAAM
S-1254
Normalized Protein Abundance
4000
3500
3000
2500
2h
6h
Time (h)
SA564
SA564−Δ*hptRS*
SA564−Δ*ccpA*
SA564−Δ*hptRS/ccpA*

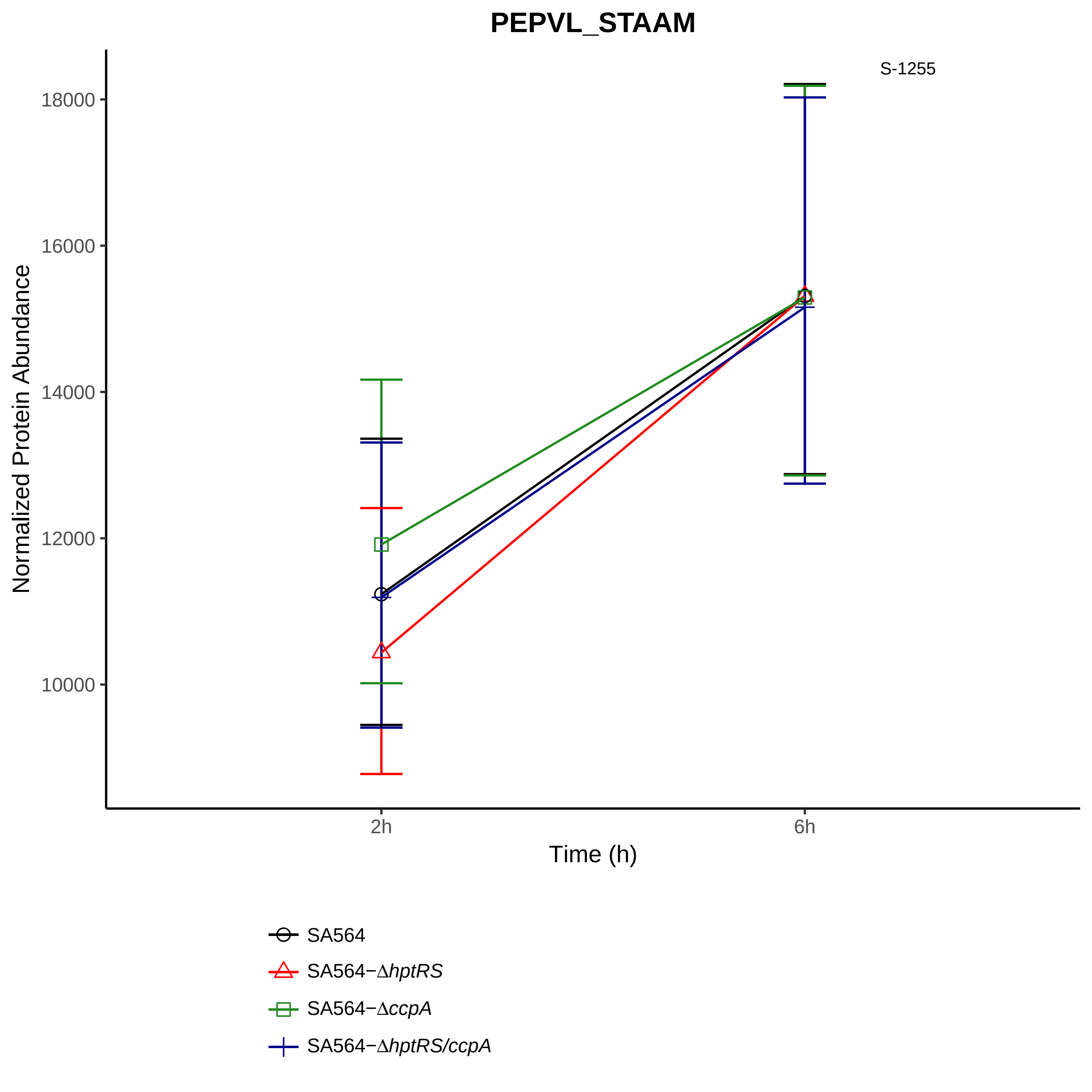

PEPVL_STAAM
S-1255
Normalized Protein Abundance
18000
16000
14000
12000
10000
2h
6h
Time (h)
SA564
SA564−Δ*hptRS*
SA564−Δ*ccpA*
SA564−Δ*hptRS/ccpA*

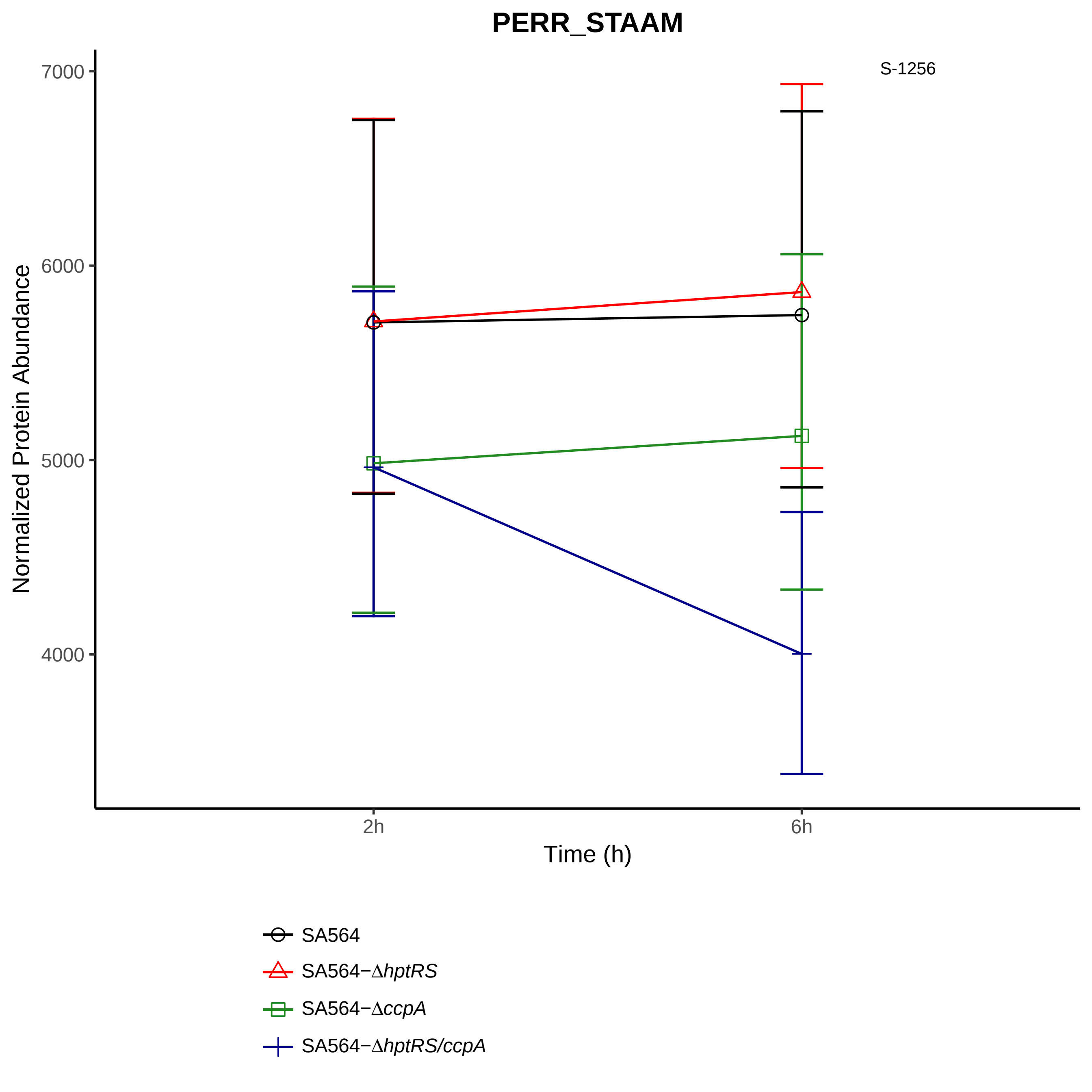

PERR_STAAM
S-1256
7000
6000
5000
4000
Normalized Protein Abundance
2h
6h
Time (h)
SA564
SA564−Δ*hptRS*
SA564−Δ*ccpA*
SA564−Δ*hptRS/ccpA*

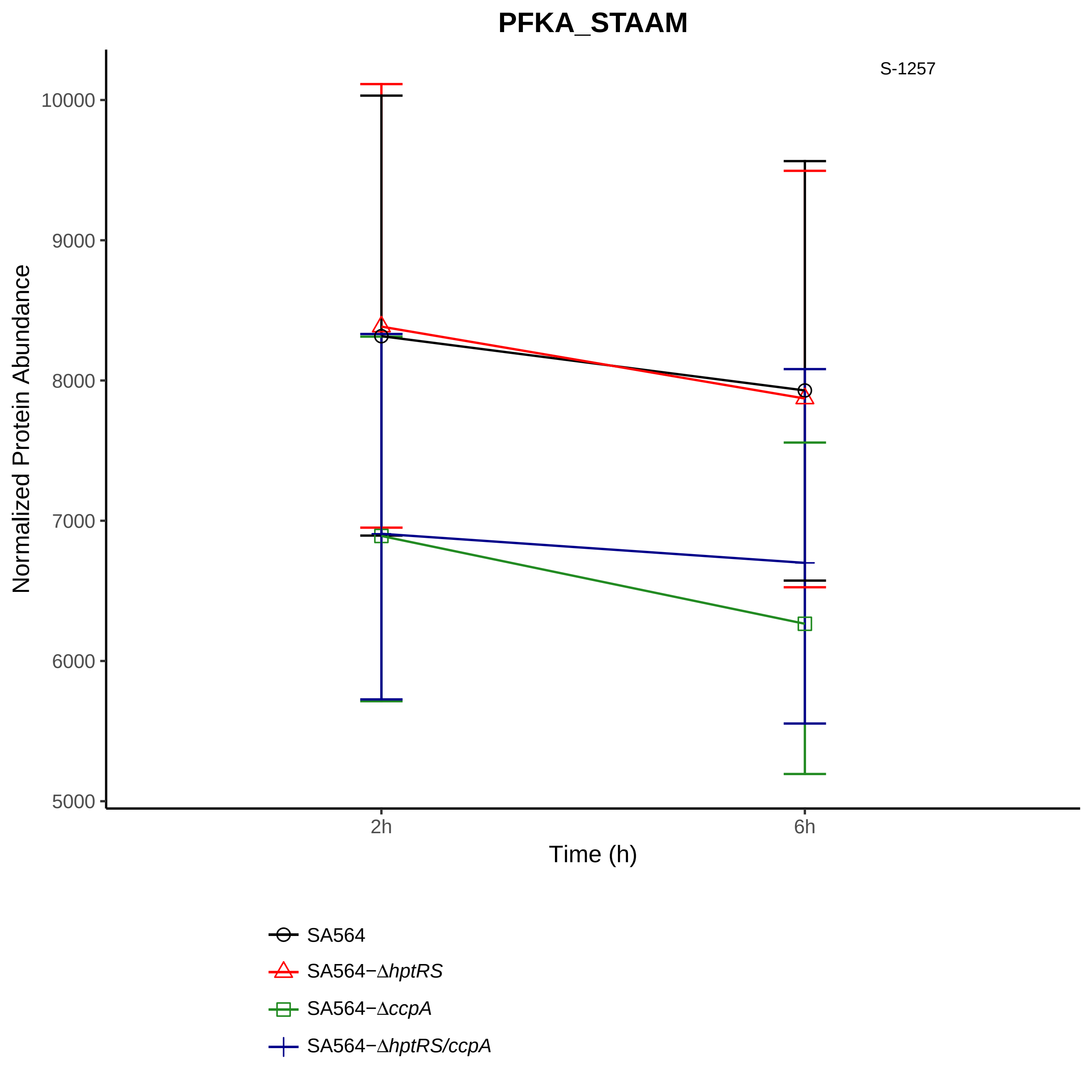

PFKA_STAAM
S-1257
Normalized Protein Abundance
10000
9000
8000
7000
6000
5000
2h
6h
Time (h)
SA564
SA564−Δ*hptRS*
SA564−Δ*ccpA*
SA564−Δ*hptRS/ccpA*

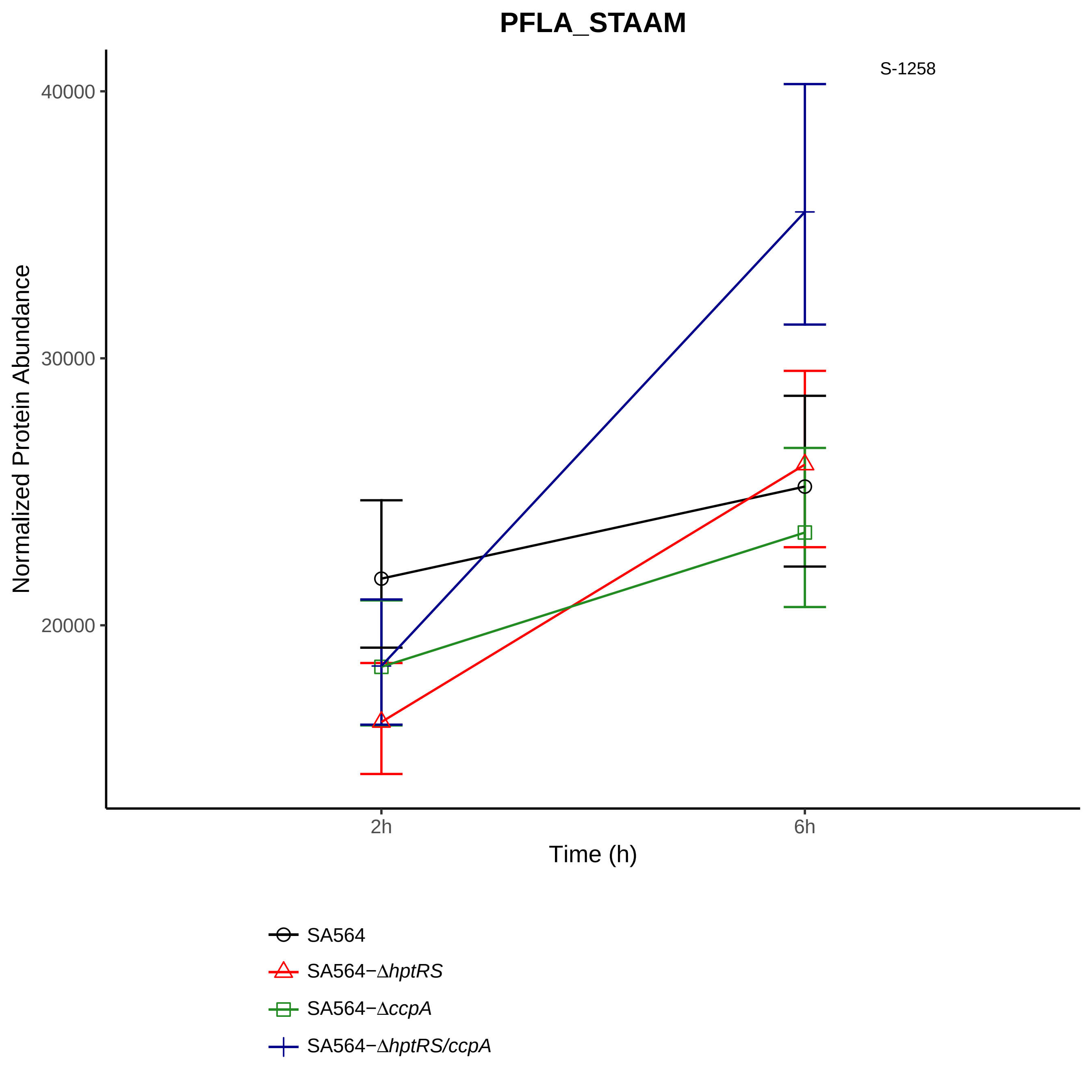
PFLA_STAAM
S-1258
40000
30000
20000
Normalized Protein Abundance
2h
6h
Time (h)
SA564
SA564−Δ*hptRS*
SA564−Δ*ccpA*
SA564−Δ*hptRS/ccpA*

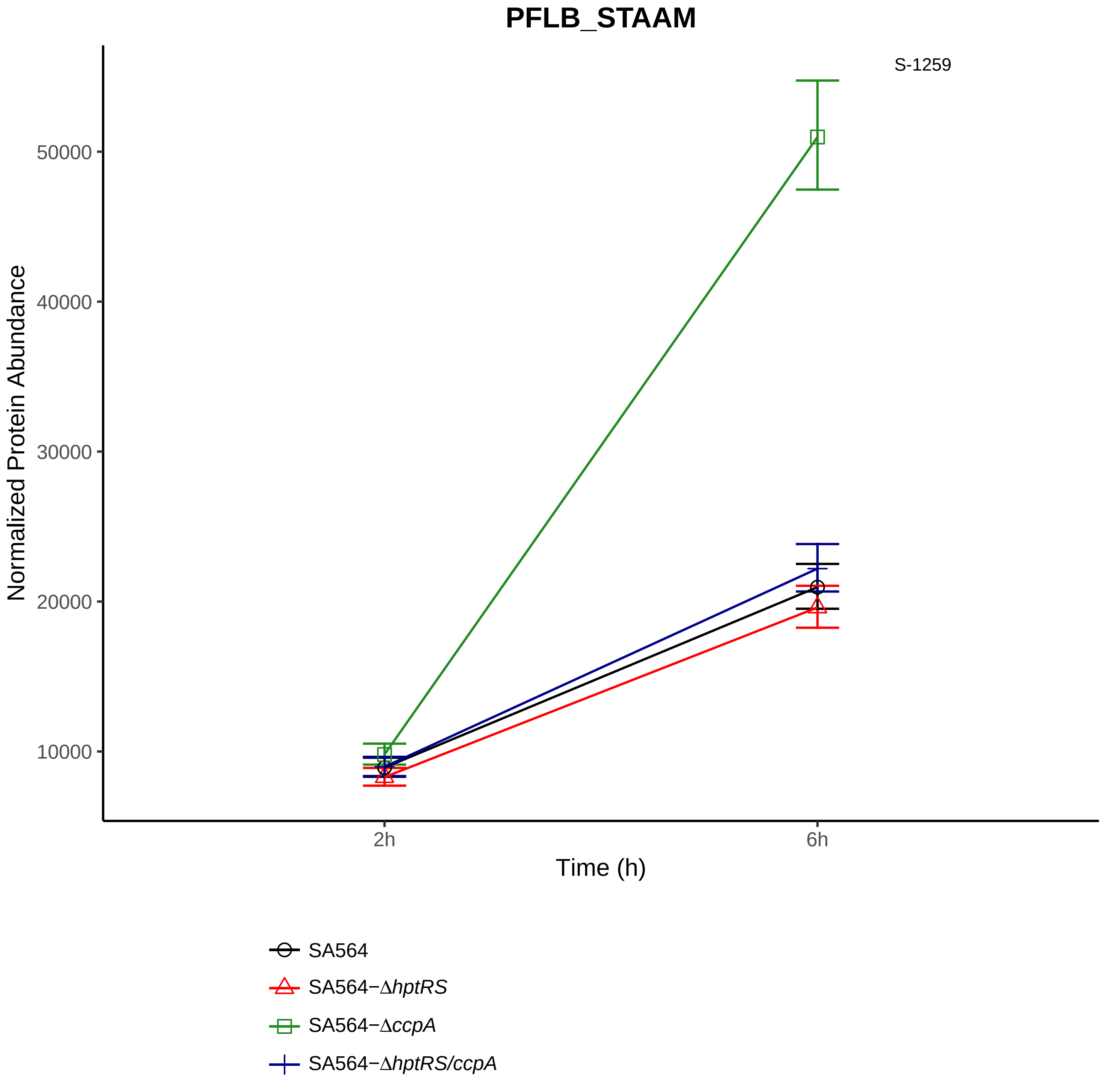

PFLB_STAAM
S-1259
Normalized Protein Abundance
50000
40000
30000
20000
10000
2h
6h
Time (h)
SA564
SA564−Δ*hptRS*
SA564−Δ*ccpA*
SA564−Δ*hptRS/ccpA*

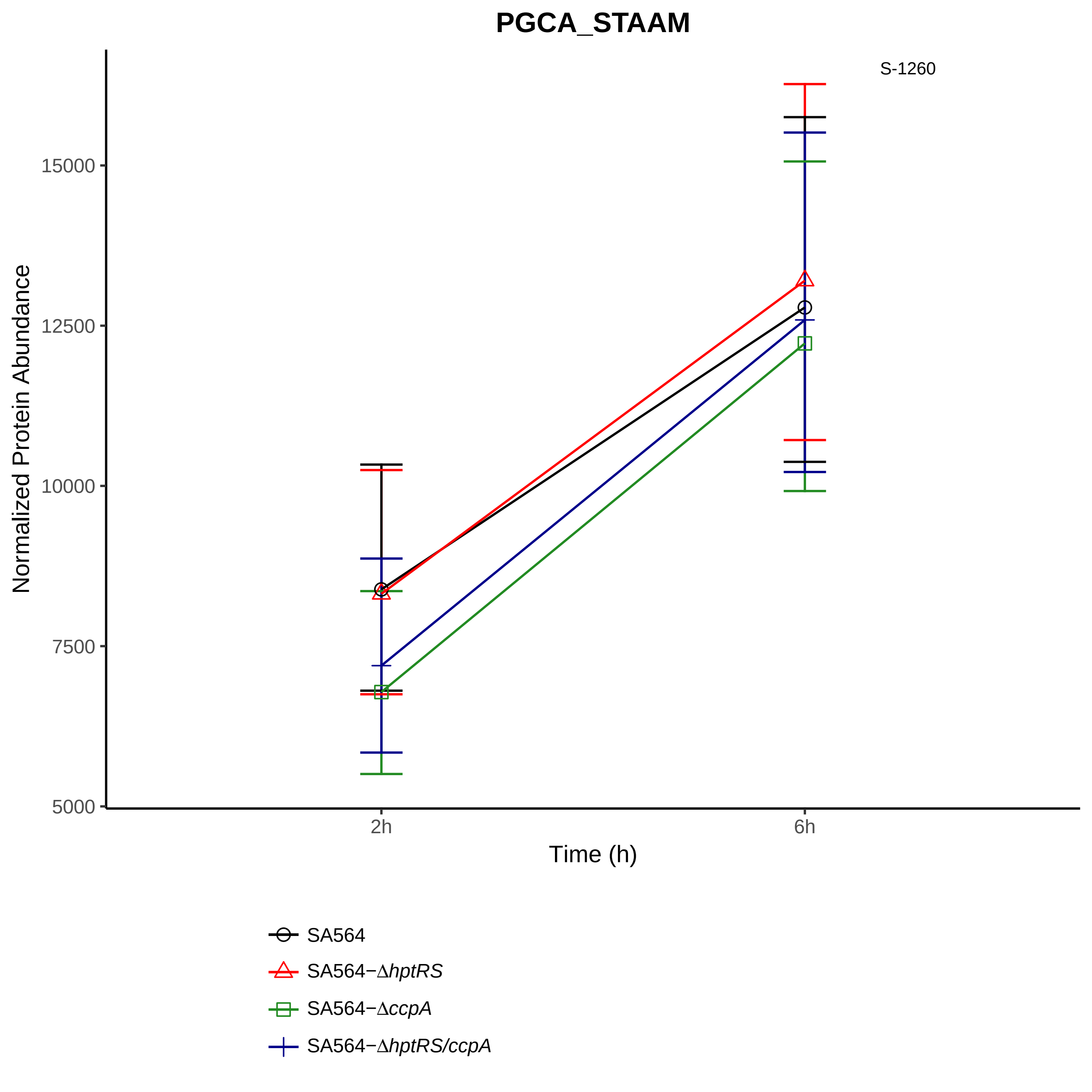
PGCA_STAAM
S-1260
Normalized Protein Abundance
15000
12500
10000
7500
5000
2h
6h
Time (h)
SA564
SA564−Δ*hptRS*
SA564−Δ*ccpA*
SA564−Δ*hptRS/ccpA*

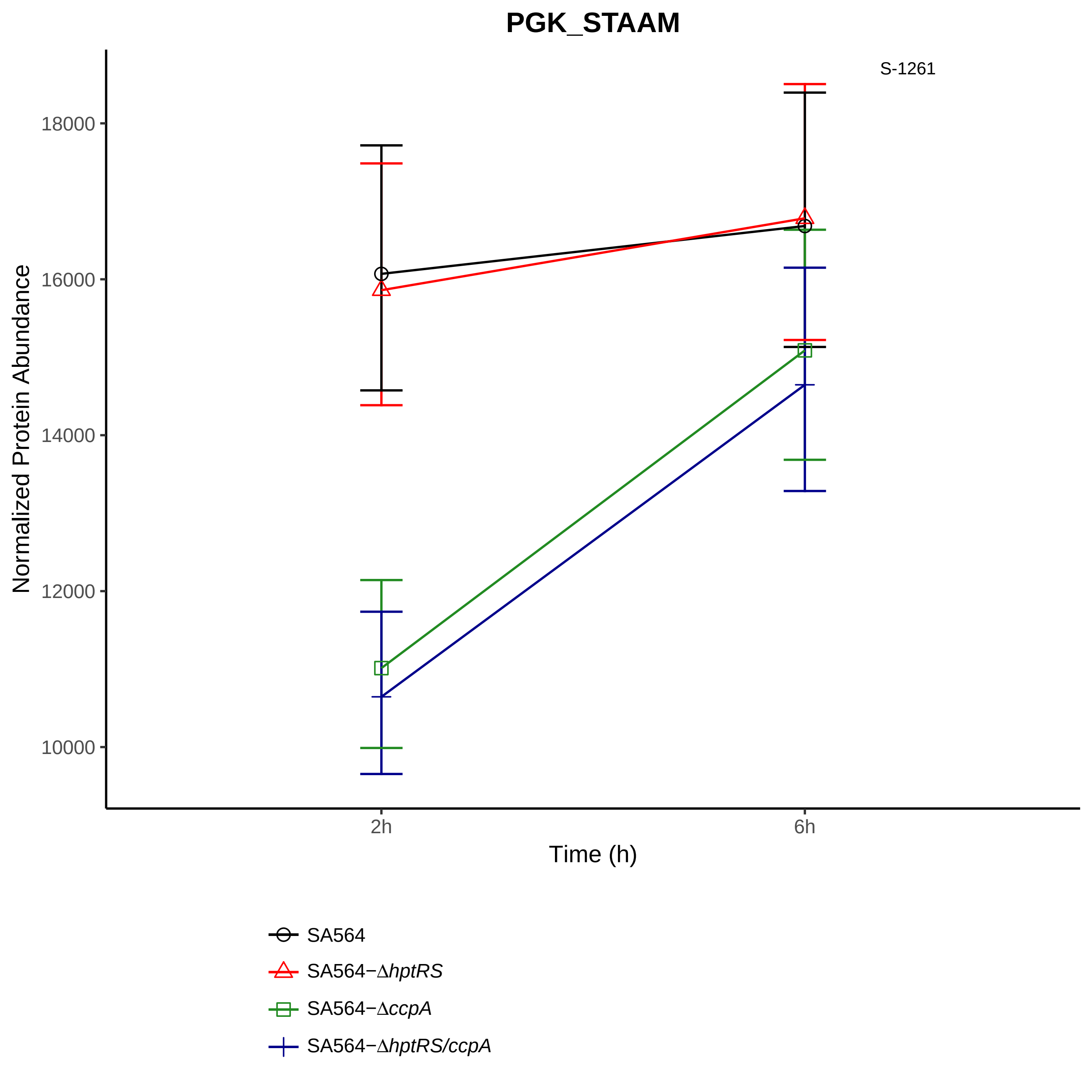

PGK_STAAM
S-1261
18000
16000
14000
12000
10000
Normalized Protein Abundance
2h
6h
Time (h)
SA564
SA564−Δ*hptRS*
SA564−Δ*ccpA*
SA564−Δ*hptRS/ccpA*

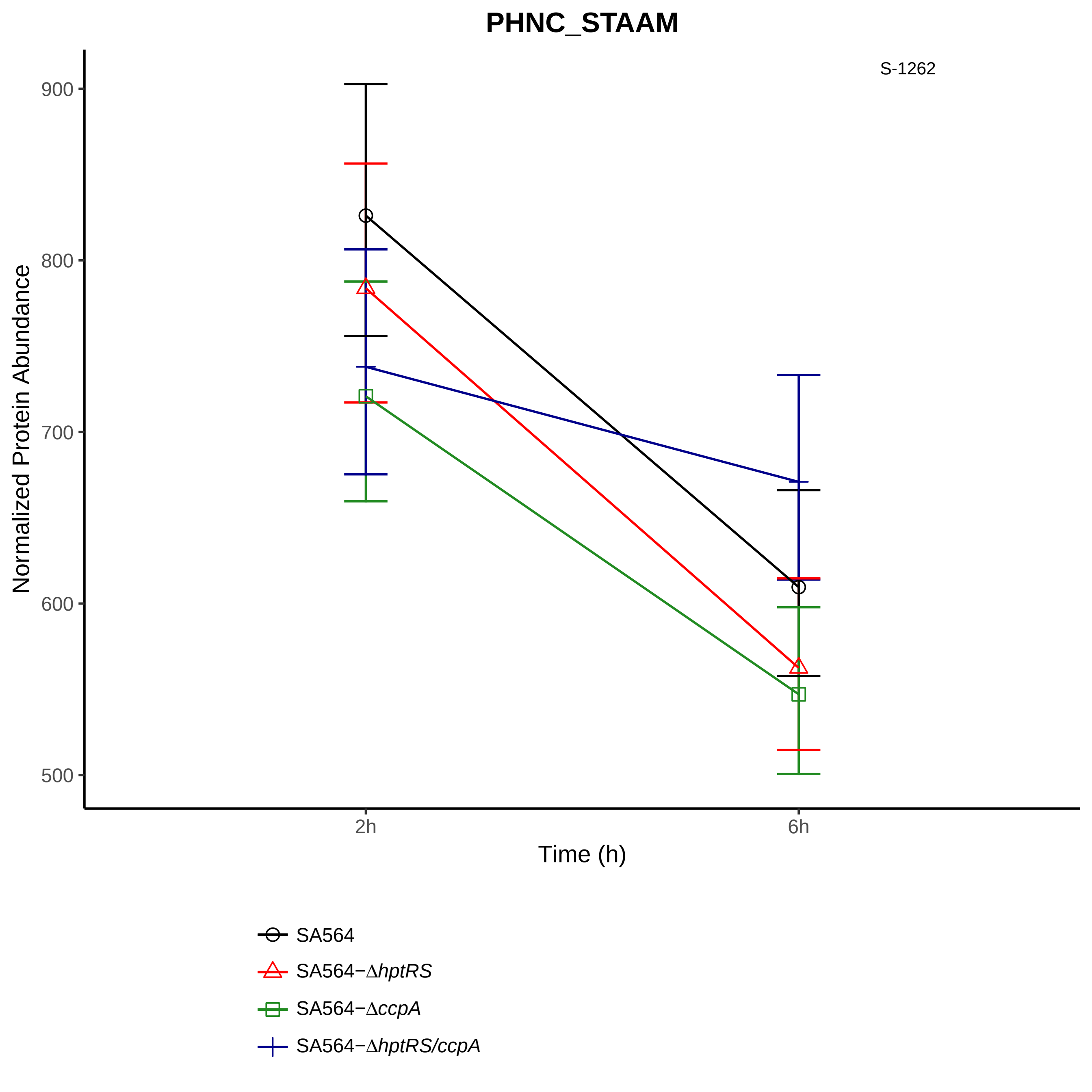

PHNC_STAAM
S-1262
Normalized Protein Abundance
900
800
700
600
500
2h
6h
Time (h)
SA564
SA564−ΔhptRS
SA564−ΔccpA
SA564−ΔhptRS/ccpA

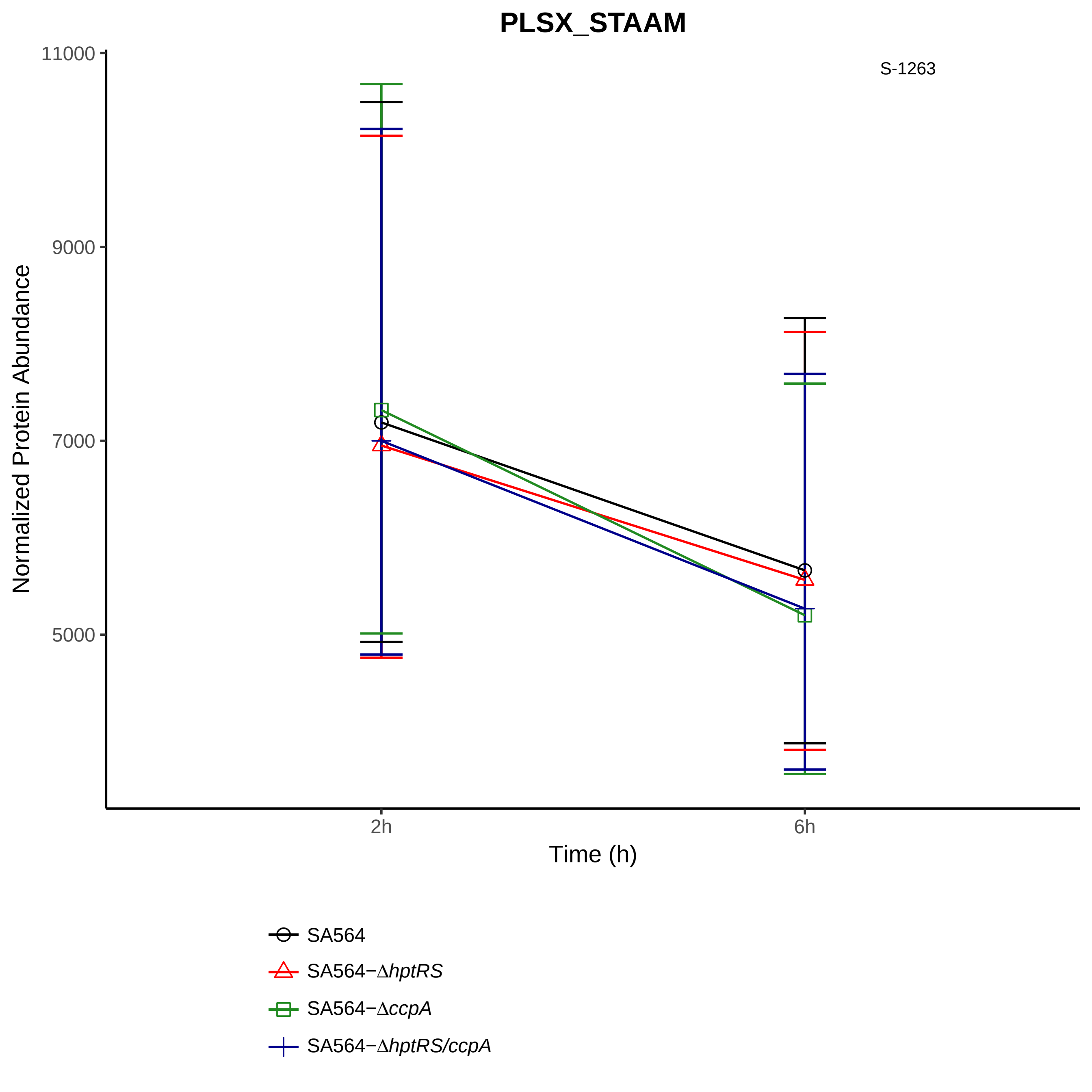

PLSX_STAAM
S-1263
11000
9000
7000
5000
Normalized Protein Abundance
2h
6h
Time (h)
SA564
SA564−Δ*hptRS*
SA564−Δ*ccpA*
SA564−Δ*hptRS/ccpA*

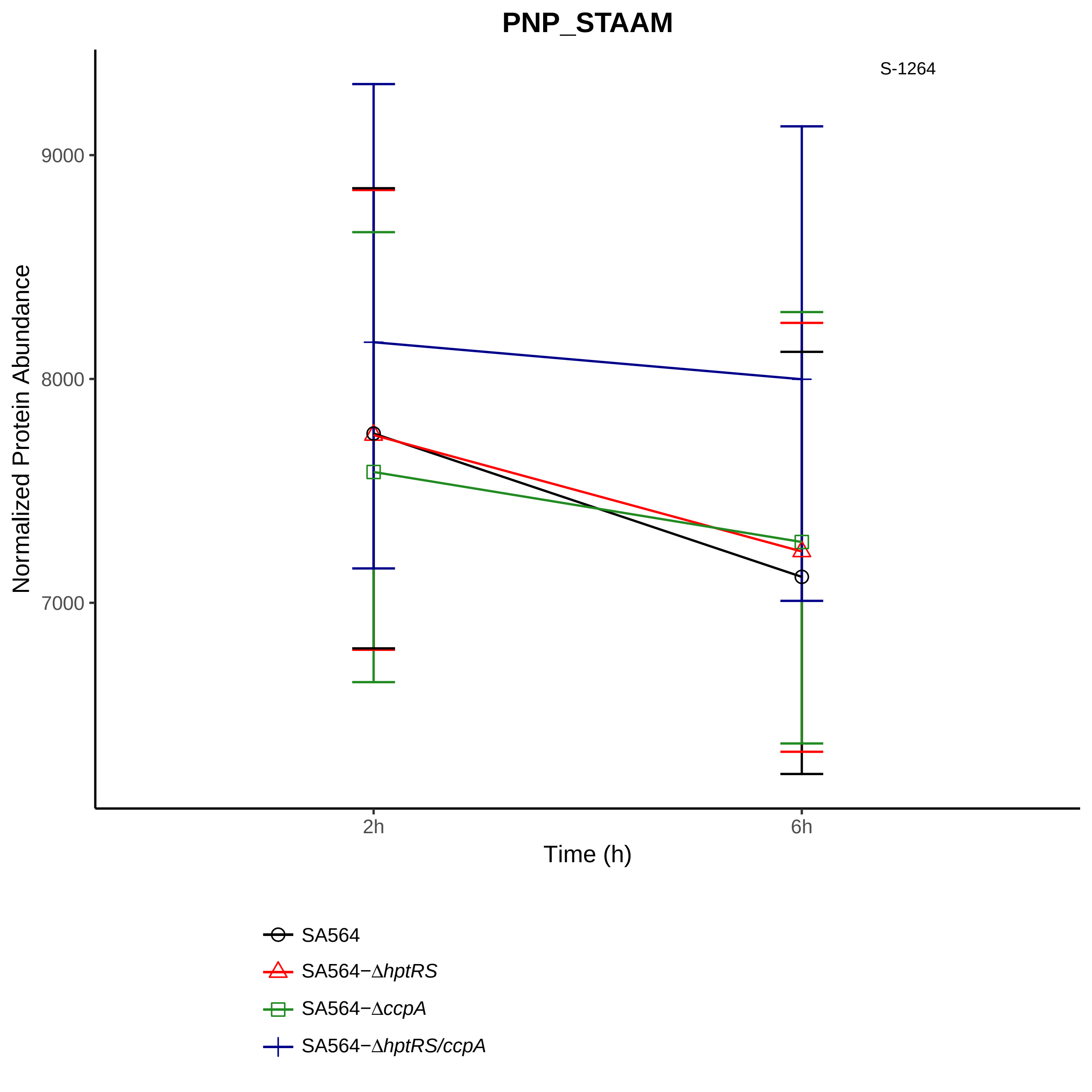

PNP_STAAM
S-1264
Normalized Protein Abundance
9000
8000
7000
2h
6h
Time (h)
SA564
SA564−ΔhptRS
SA564−ΔccpA
SA564−ΔhptRS/ccpA

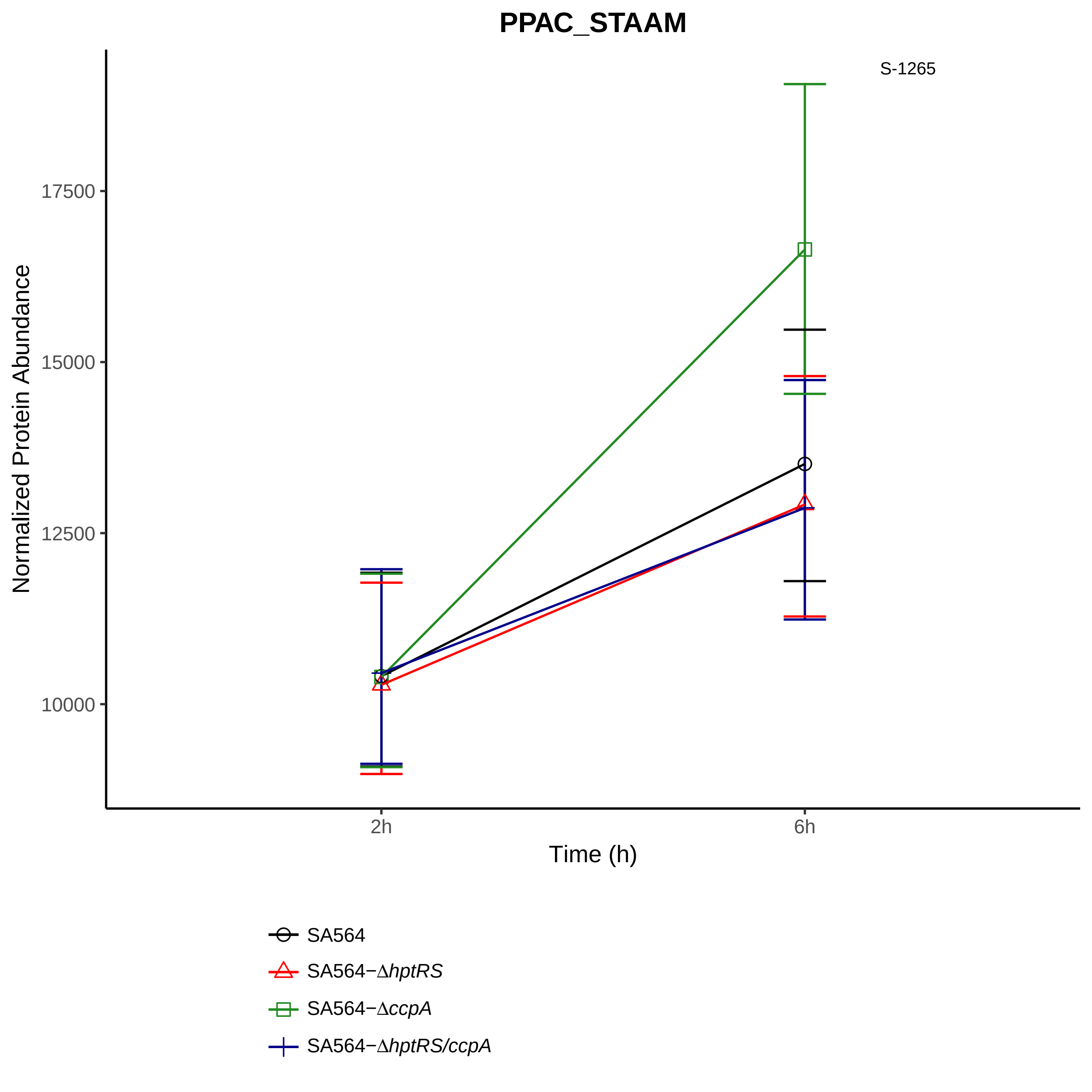

PPAC_STAAM
S-1265
17500
15000
12500
10000
Normalized Protein Abundance
2h
6h
Time (h)
SA564
SA564−ΔhptRS
SA564−ΔccpA
SA564−ΔhptRS/ccpA

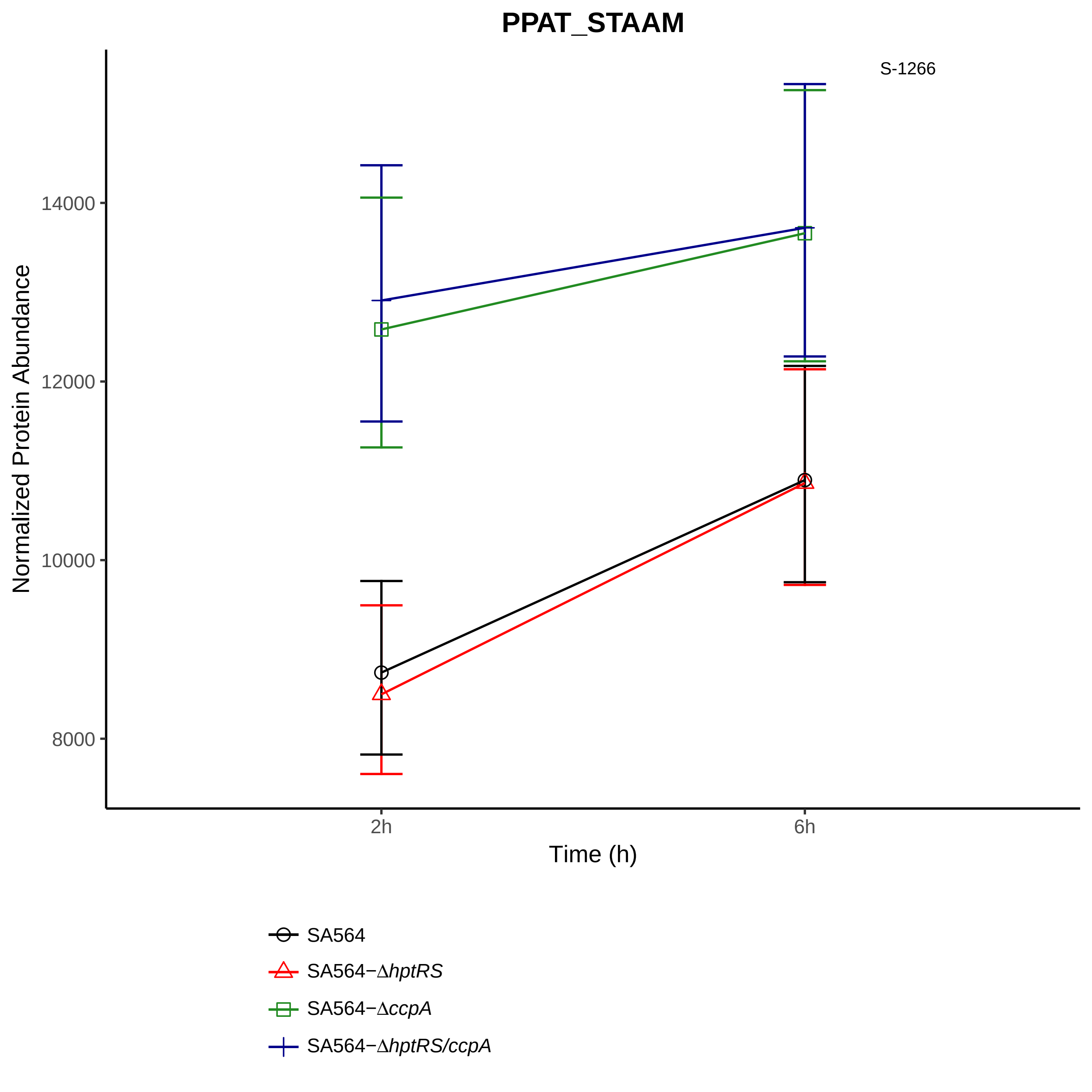

PPAT_STAAM
S-1266
Normalized Protein Abundance
14000
12000
10000
8000
2h
6h
Time (h)
SA564
SA564−ΔhptRS
SA564−ΔccpA
SA564−ΔhptRS/ccpA

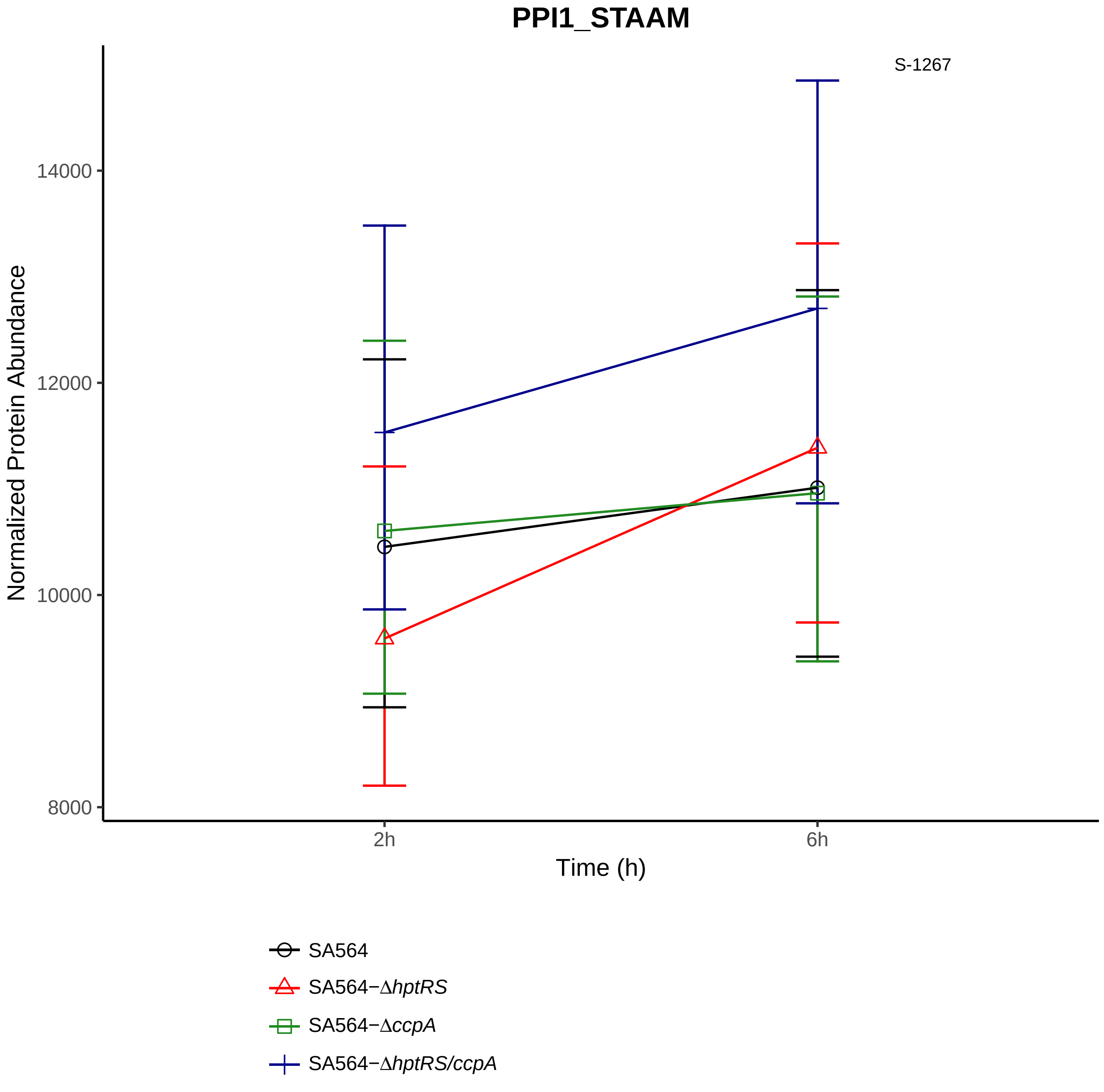
PPI1_STAAM
S-1267
Normalized Protein Abundance
14000
12000
10000
8000
2h
6h
Time (h)
SA564
SA564−Δ*hptRS*
SA564−Δ*ccpA*
SA564−Δ*hptRS/ccpA*

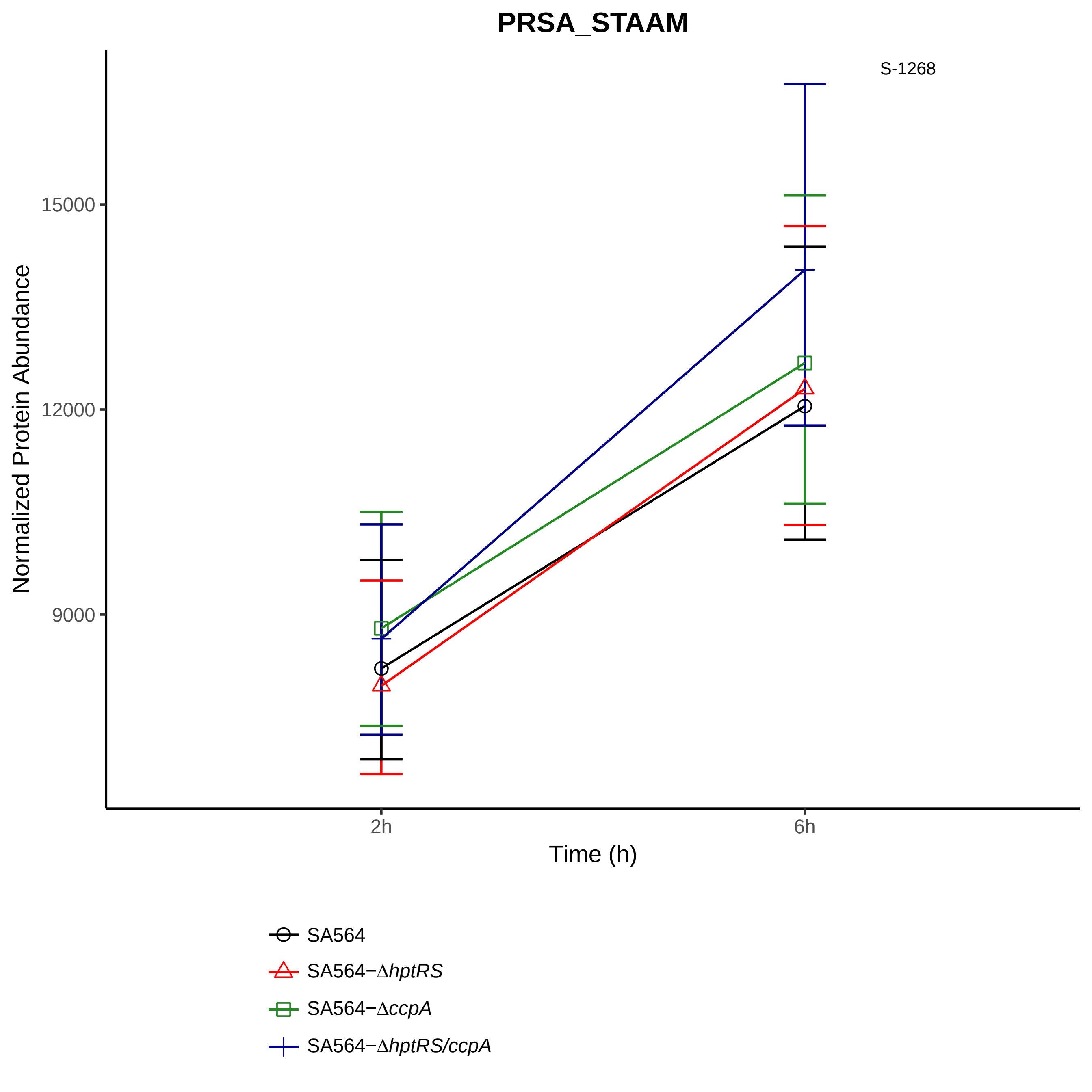
PRSA_STAAM
S-1268
Normalized Protein Abundance
15000
12000
9000
2h
6h
Time (h)
SA564
SA564−ΔhptRS
SA564−ΔccpA
SA564−ΔhptRS/ccpA

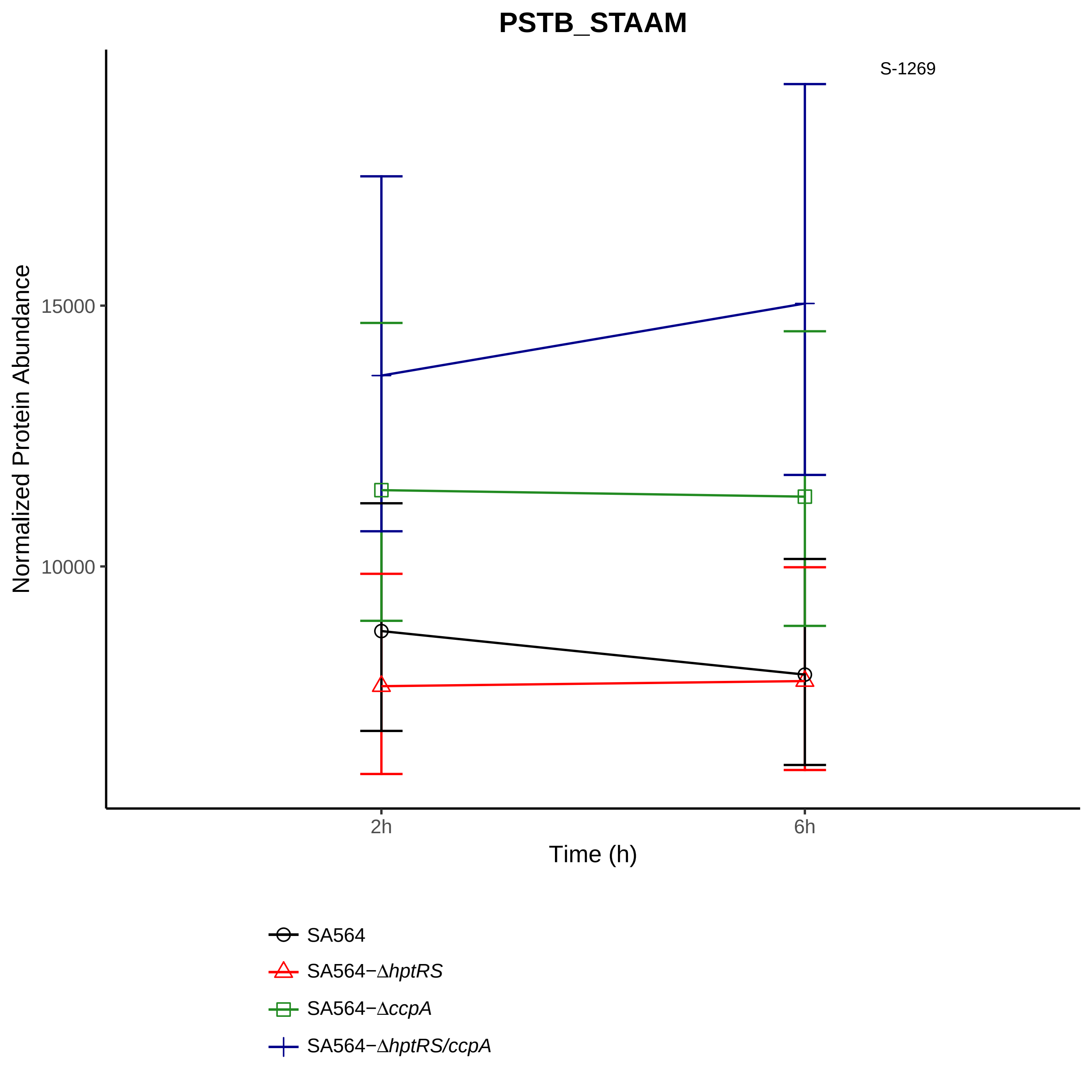
PSTB_STAAM
S-1269
Normalized Protein Abundance
15000
10000
2h
6h
Time (h)
SA564
SA564−Δ*hptRS*
SA564−Δ*ccpA*
SA564−Δ*hptRS/ccpA*

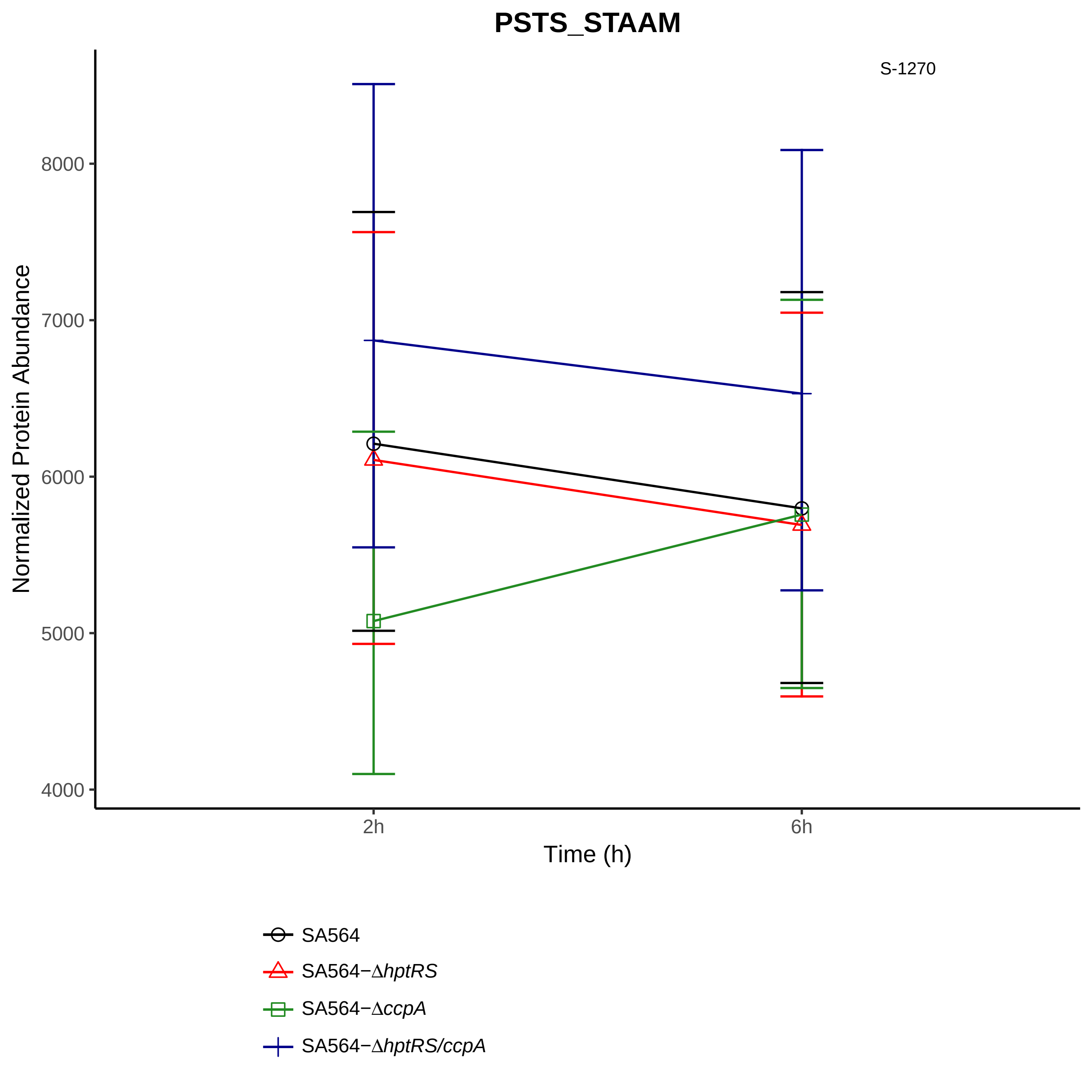

PSTS_STAAM
S-1270
Normalized Protein Abundance
8000
7000
6000
5000
4000
2h
6h
Time (h)
SA564
SA564−Δ*hptRS*
SA564−Δ*ccpA*
SA564−Δ*hptRS/ccpA*

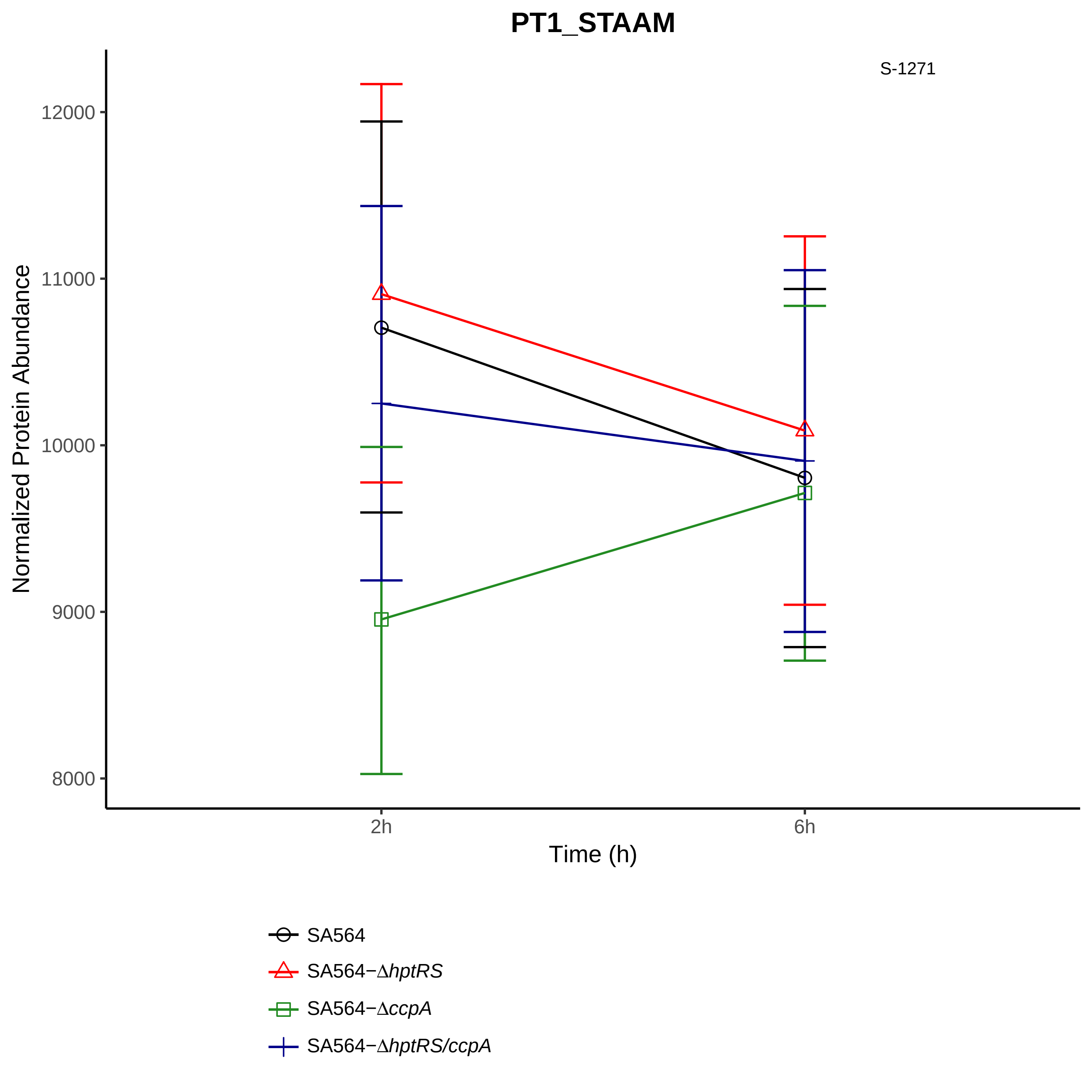
PT1_STAAM
S-1271
Normalized Protein Abundance
12000
11000
10000
9000
8000
2h
6h
Time (h)
SA564
SA564−Δ*hptRS*
SA564−Δ*ccpA*
SA564−Δ*hptRS/ccpA*

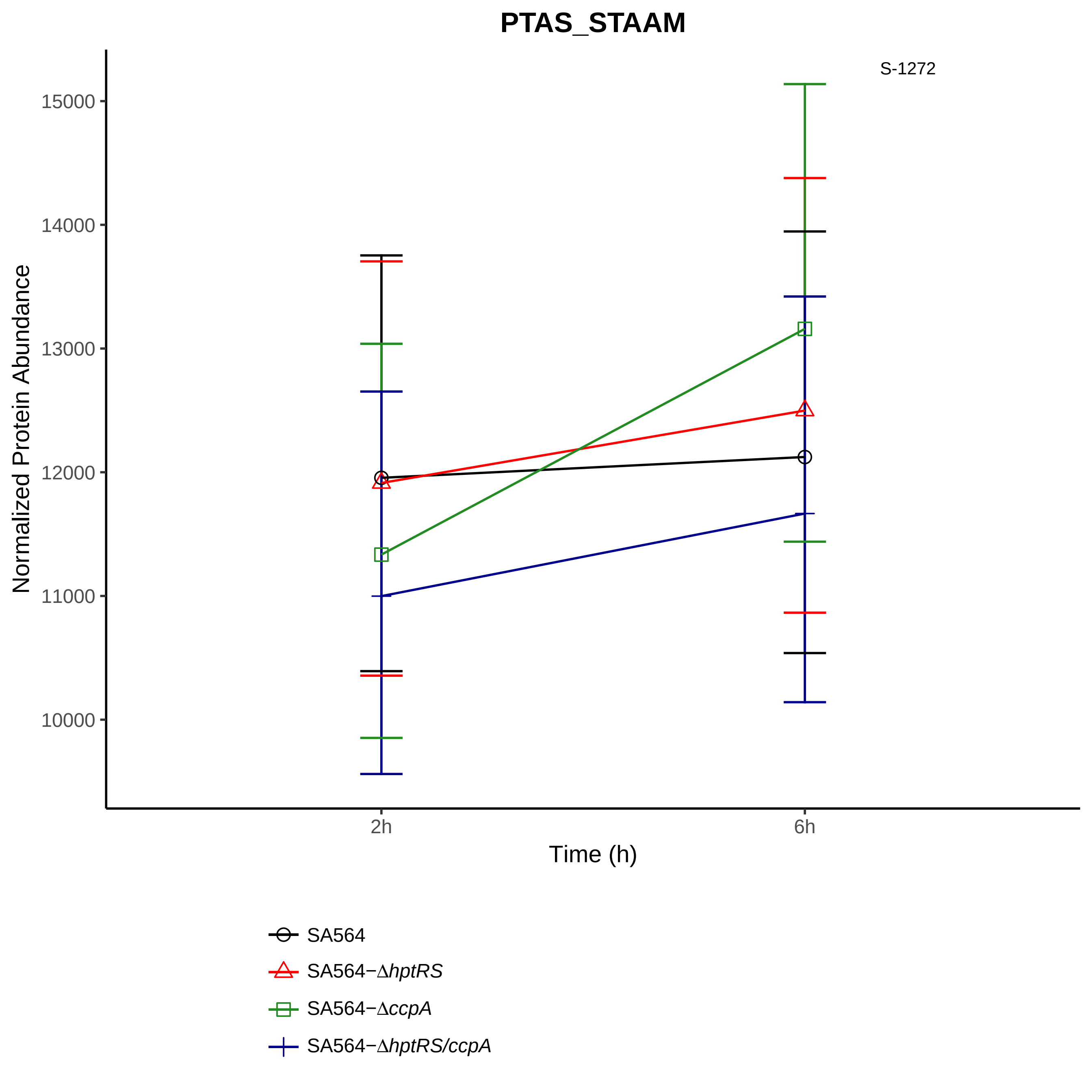

PTAS_STAAM
S-1272
Normalized Protein Abundance
15000
14000
13000
12000
11000
10000
2h
6h
Time (h)
SA564
SA564−ΔhptRS
SA564−ΔccpA
SA564−ΔhptRS/ccpA

PTG3C_STAAM

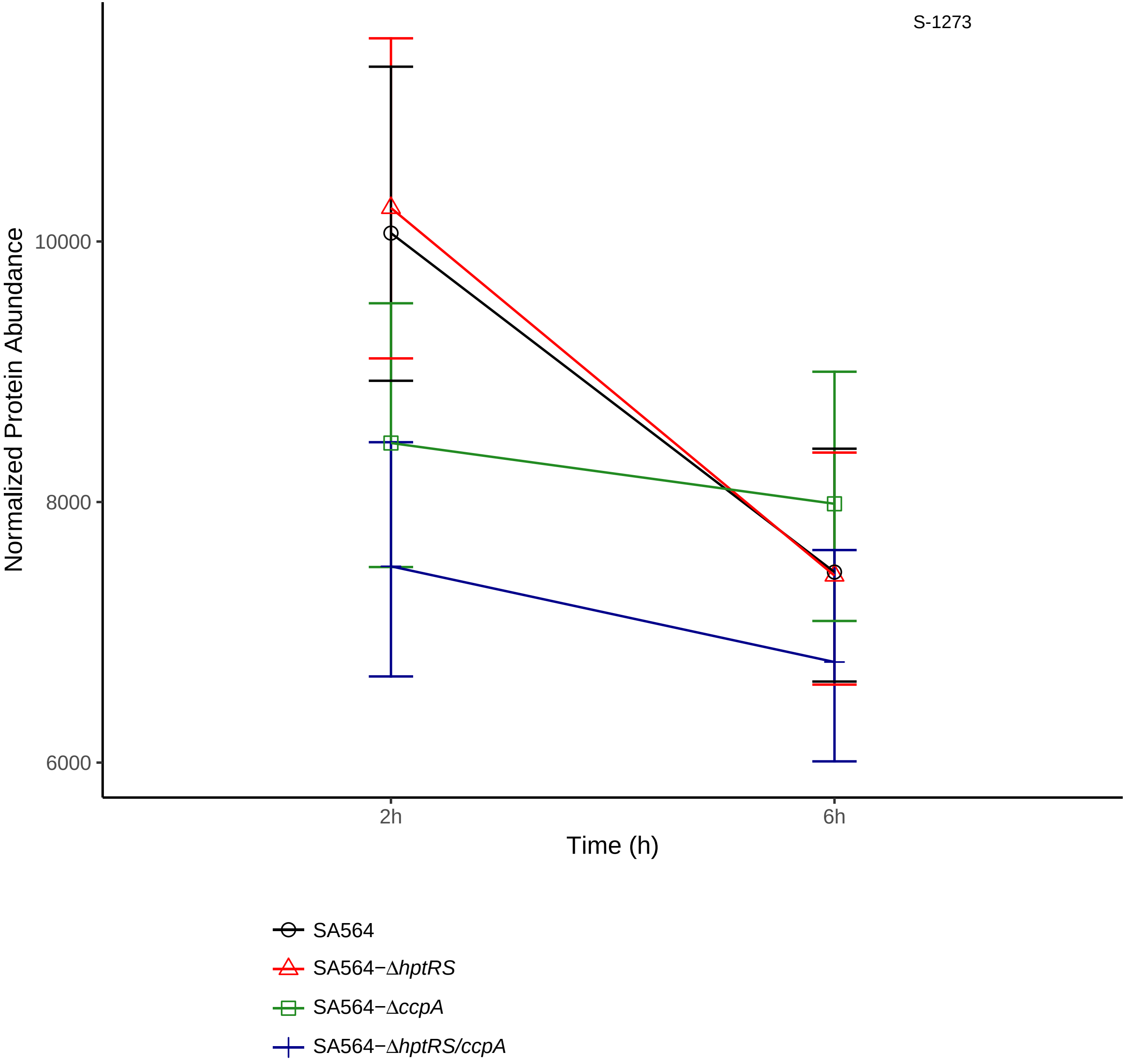

S-1273
Normalized Protein Abundance
10000
8000
6000
2h
6h
Time (h)
SA564
SA564−Δ*hptRS*
SA564−Δ*ccpA*
SA564−Δ*hptRS/ccpA*

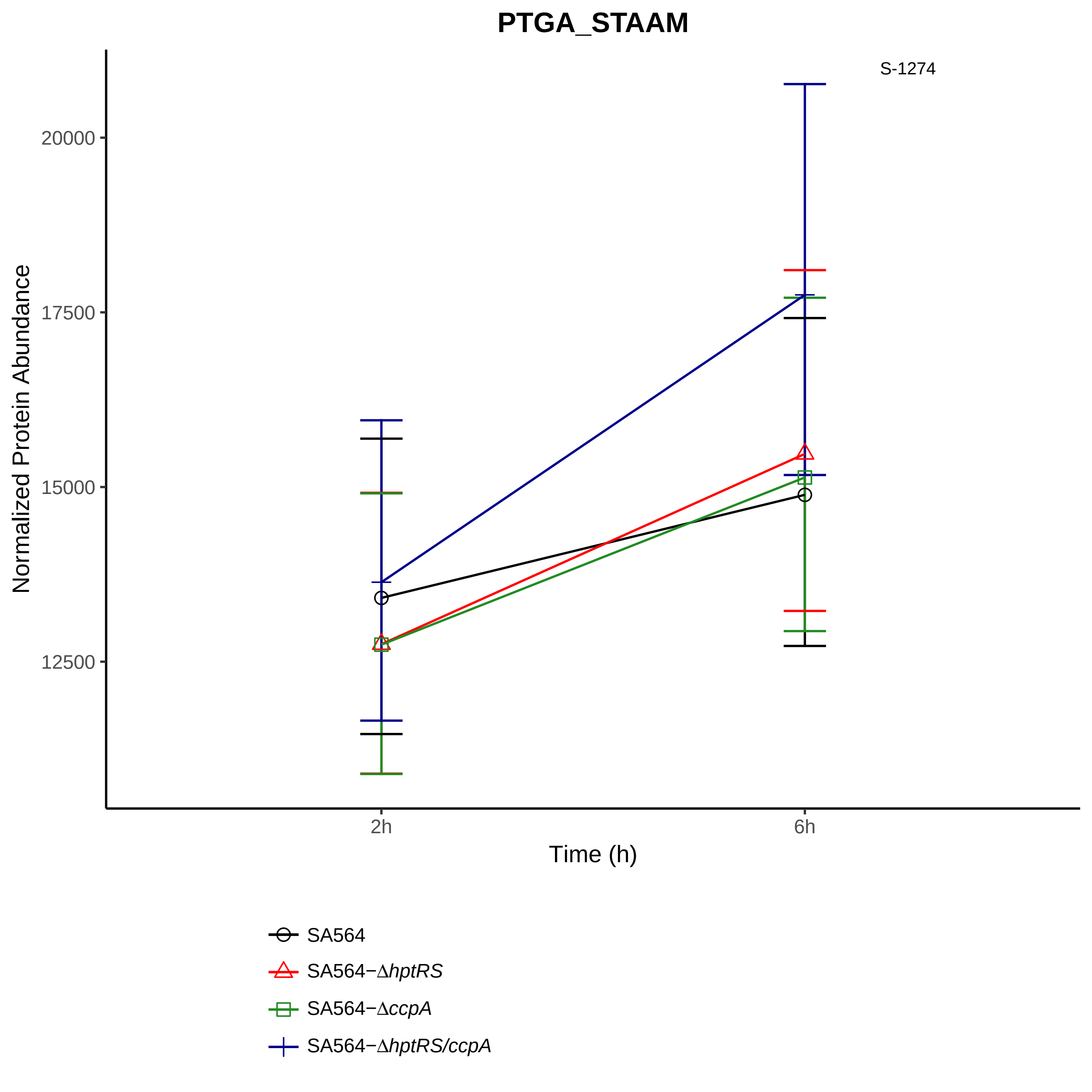
PTGA_STAAM
S-1274
Normalized Protein Abundance
20000
17500
15000
12500
2h
6h
Time (h)
SA564
SA564−Δ*hptRS*
SA564−Δ*ccpA*
SA564−Δ*hptRS/ccpA*

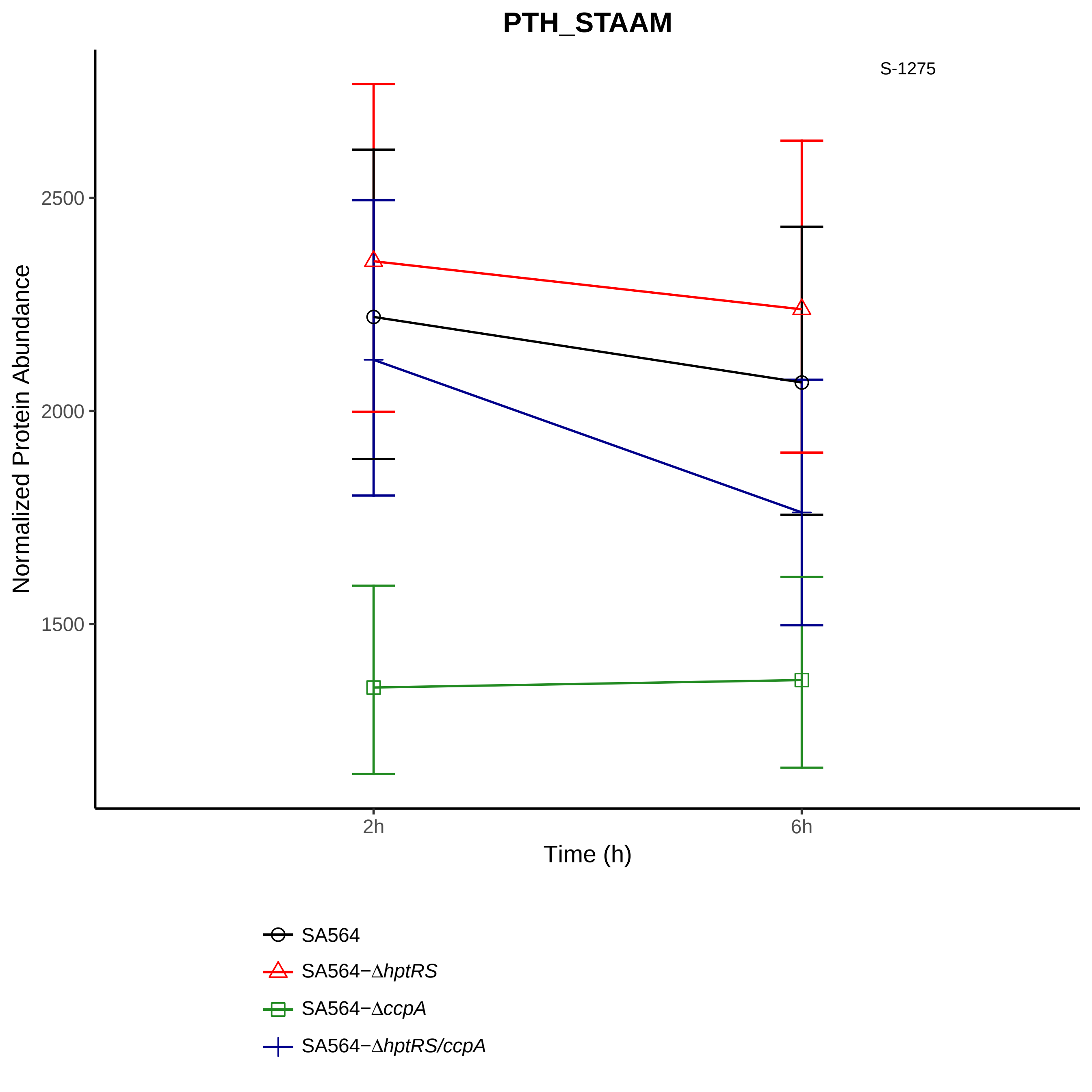

PTH_STAAM
S-1275
2500
2000
1500
Normalized Protein Abundance
2h
6h
Time (h)
SA564
SA564−ΔhptRS
SA564−ΔccpA
SA564−ΔhptRS/ccpA

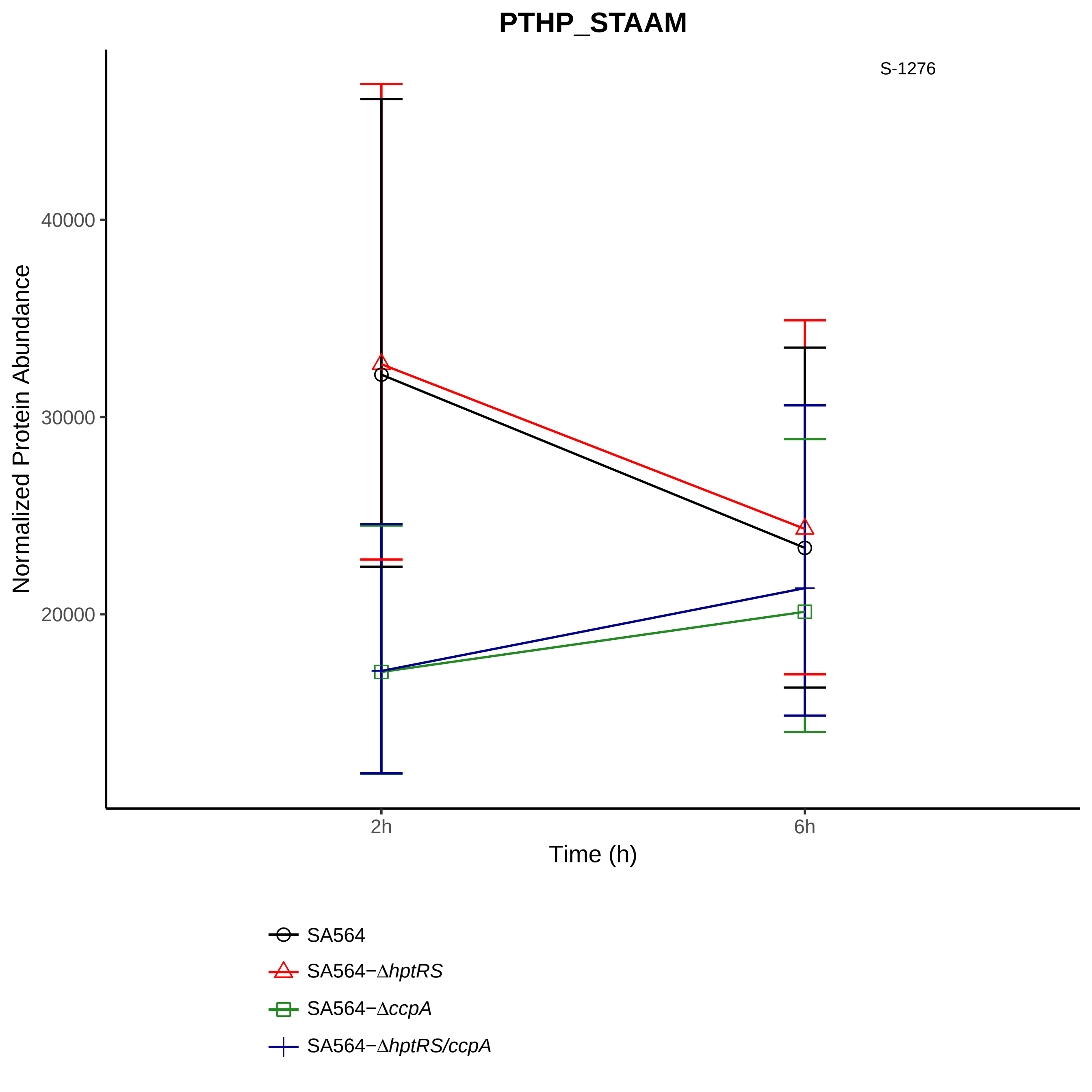

PTHP_STAAM
S-1276
Normalized Protein Abundance
40000
30000
20000
2h
6h
Time (h)
SA564
SA564−Δ*hptRS*
SA564−Δ*ccpA*
SA564−Δ*hptRS/ccpA*

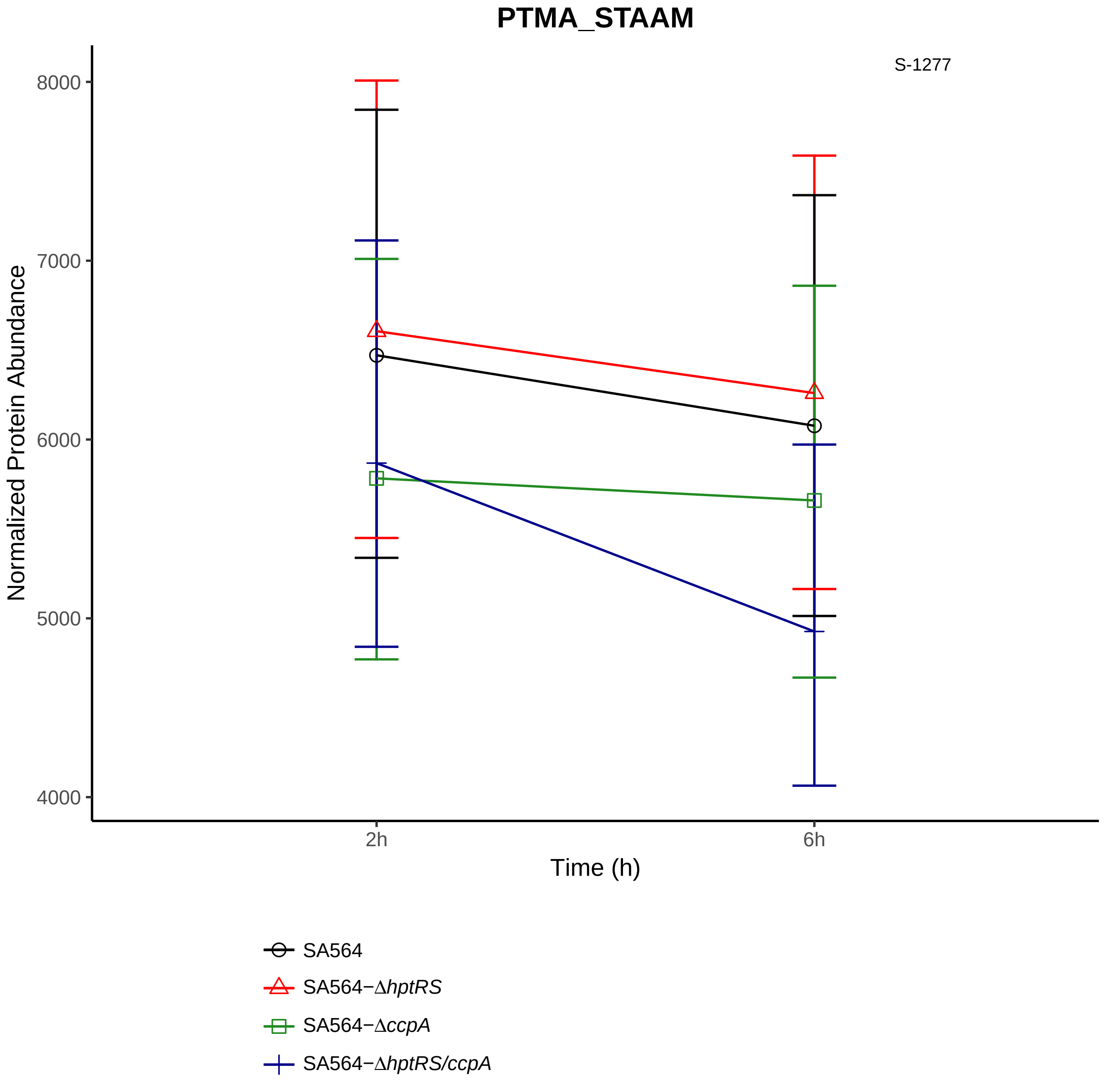

PTMA_STAAM
S-1277
Normalized Protein Abundance
8000
7000
6000
5000
4000
2h
6h
Time (h)
SA564
SA564−Δ*hptRS*
SA564−Δ*ccpA*
SA564−Δ*hptRS/ccpA*

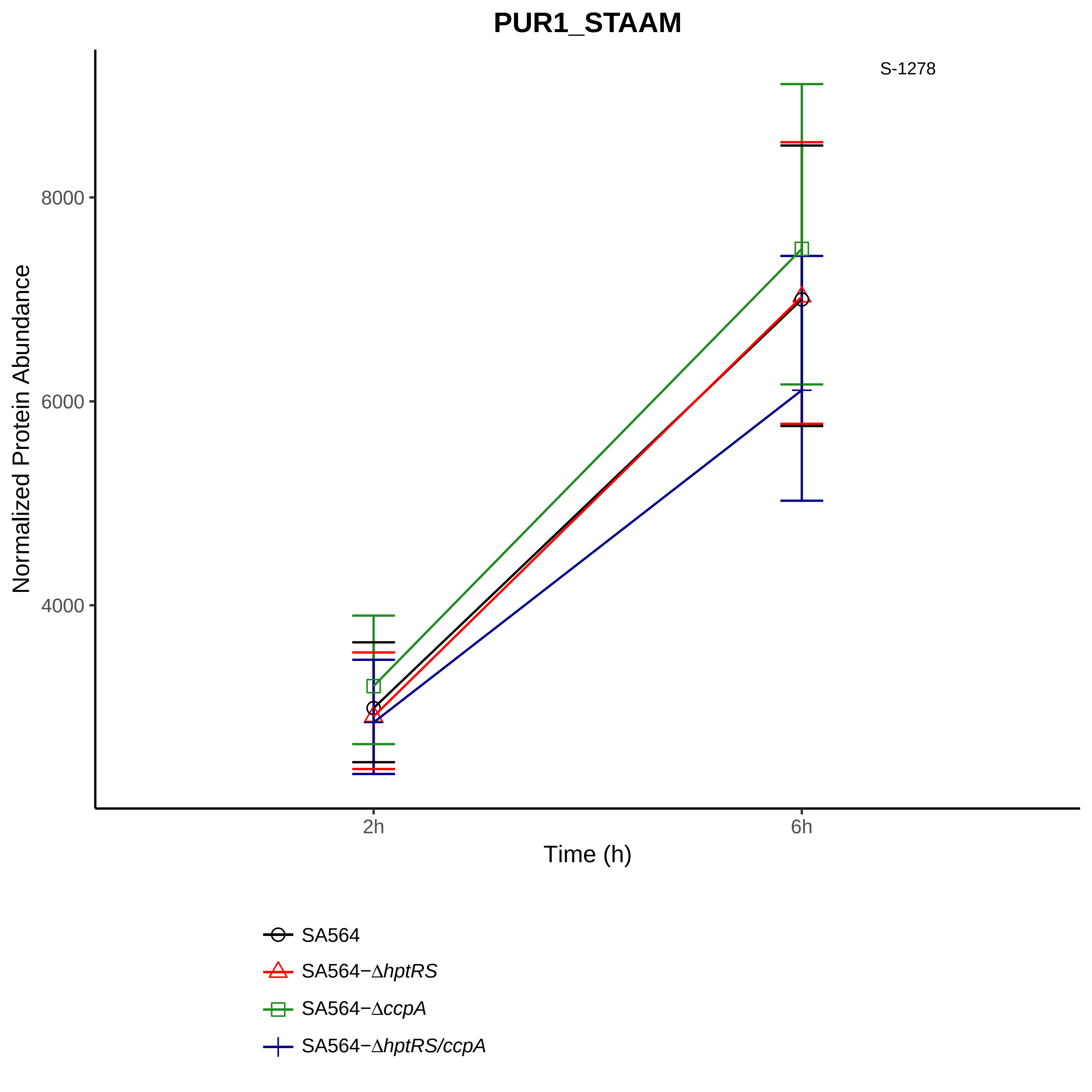

PUR1_STAAM
S-1278
Normalized Protein Abundance
8000
6000
4000
2h
6h
Time (h)
SA564
SA564−Δ*hptRS*
SA564−Δ*ccpA*
SA564−Δ*hptRS/ccpA*

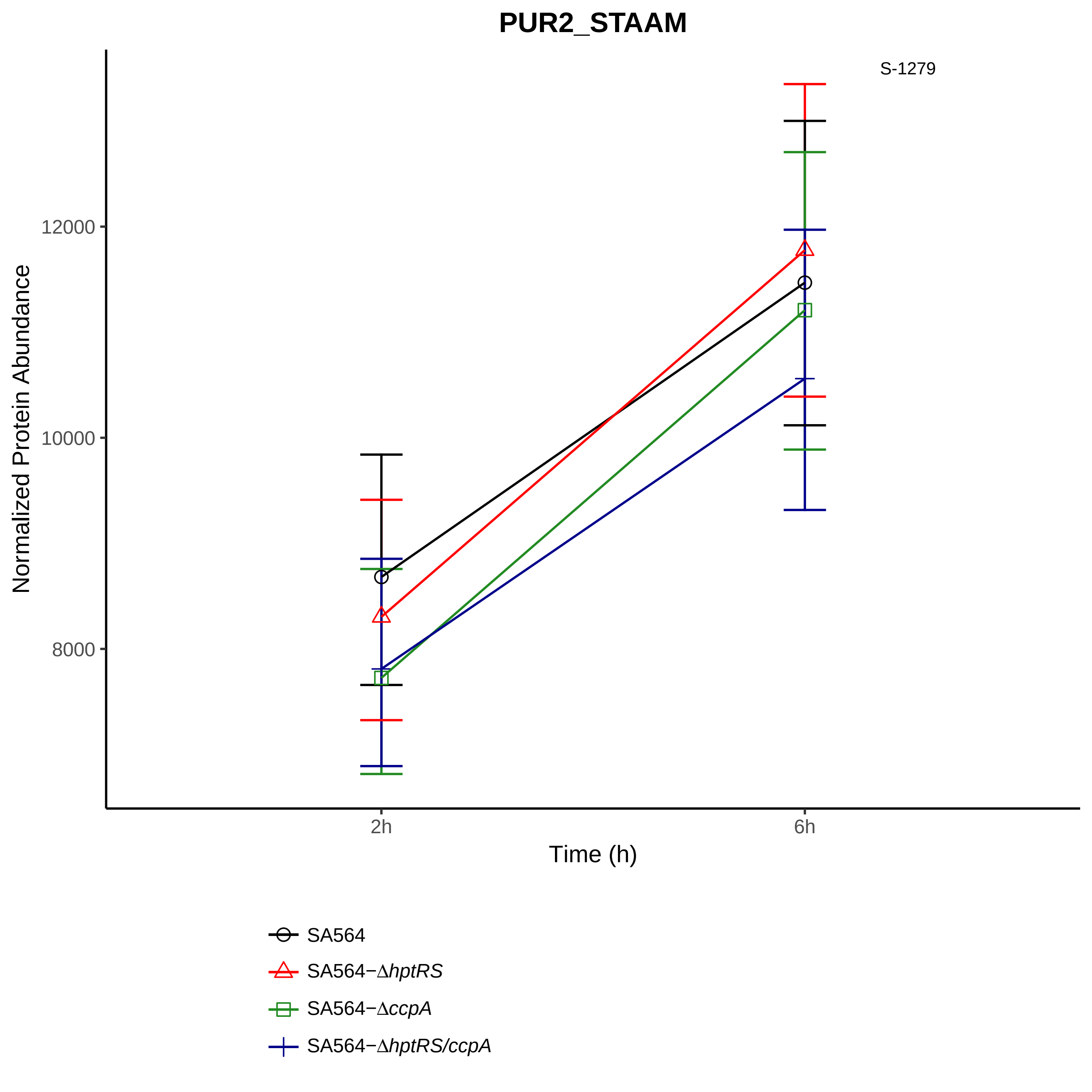

PUR2_STAAM
S-1279
Normalized Protein Abundance
12000
10000
8000
2h
6h
Time (h)
SA564
SA564−ΔhptRS
SA564−ΔccpA
SA564−ΔhptRS/ccpA

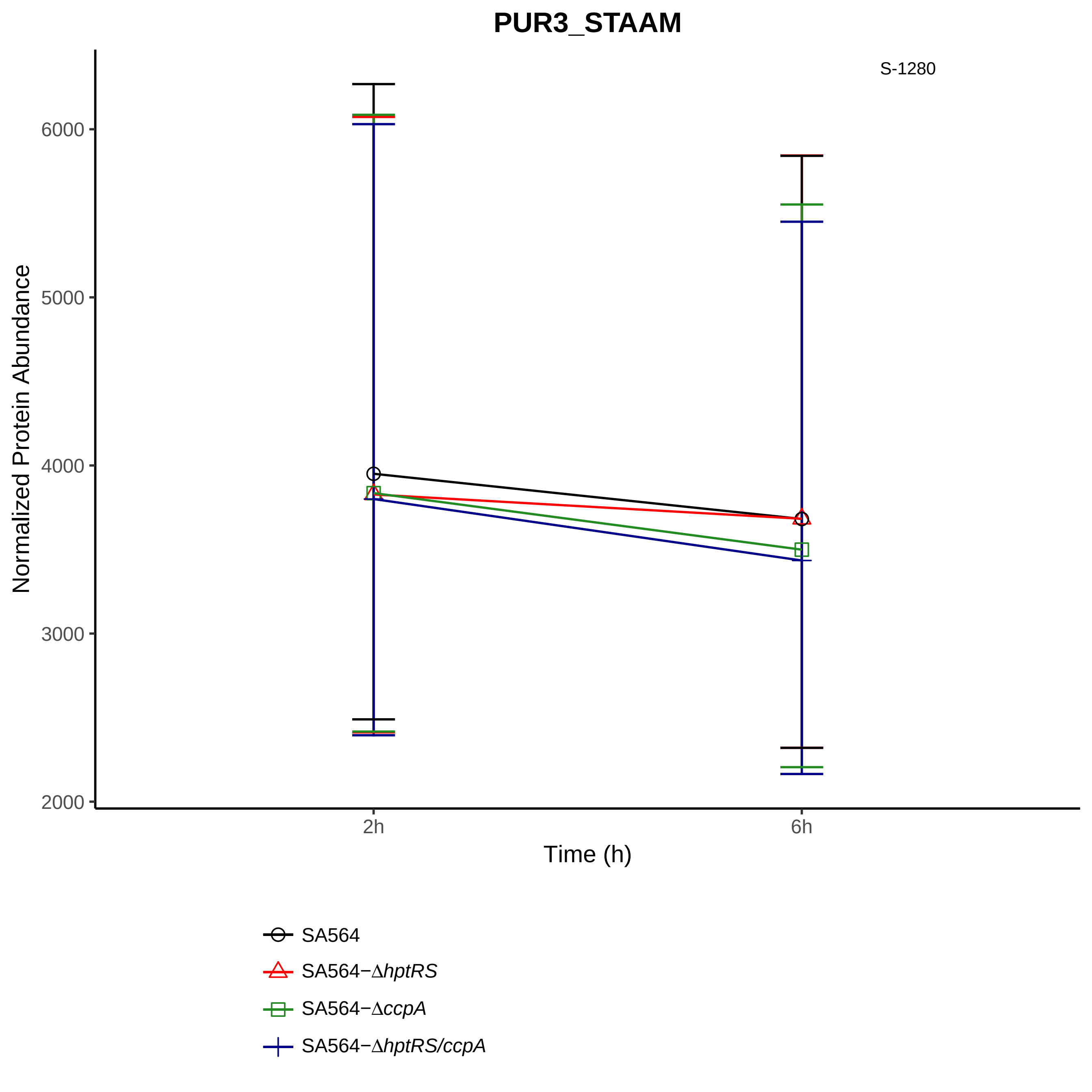

PUR3_STAAM
S-1280
Normalized Protein Abundance
6000
5000
4000
3000
2000
2h
6h
Time (h)
SA564
SA564−Δ*hptRS*
SA564−Δ*ccpA*
SA564−Δ*hptRS/ccpA*

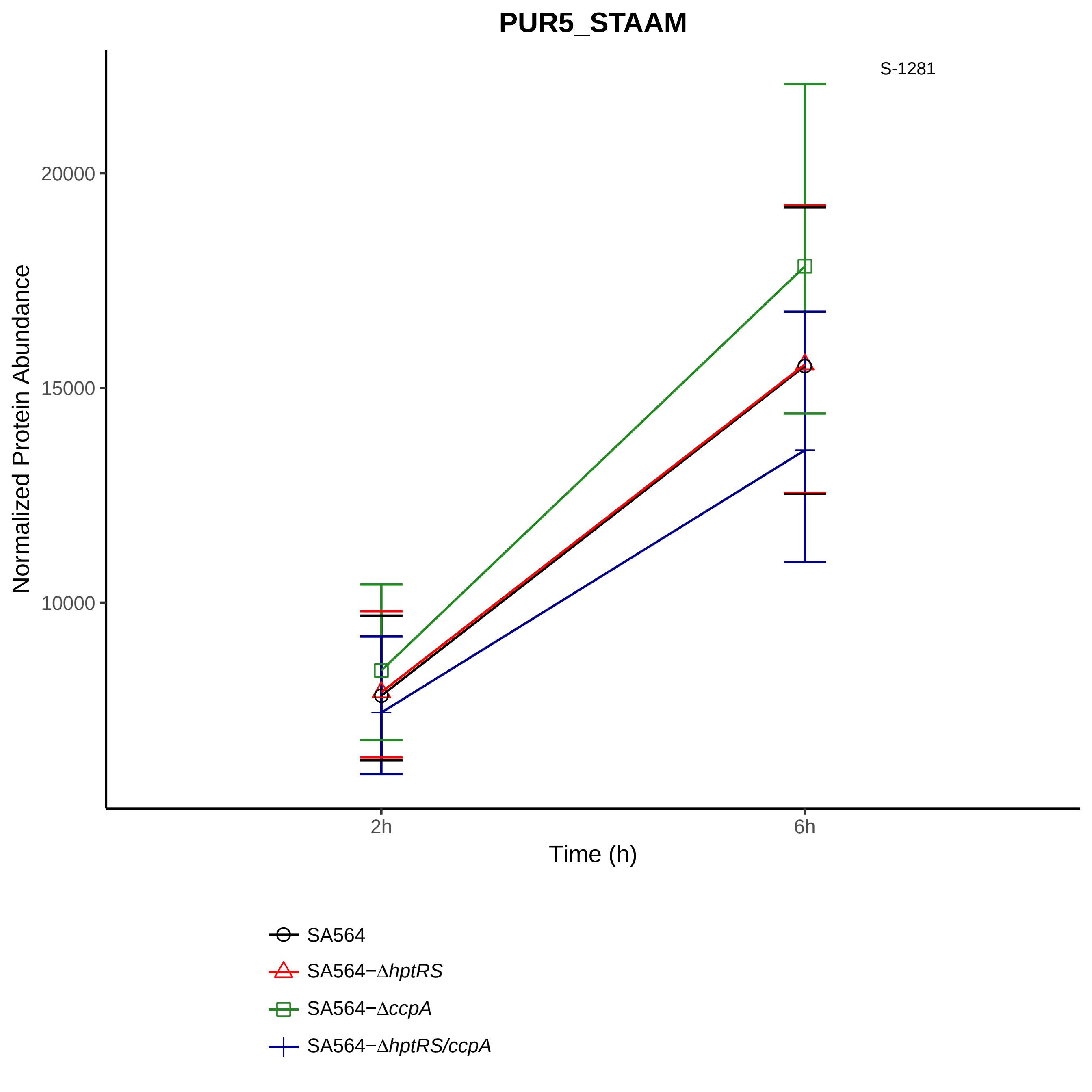
PUR5_STAAM
S-1281
Normalized Protein Abundance
20000
15000
10000
2h
6h
Time (h)
SA564
SA564−ΔhptRS
SA564−ΔccpA
SA564−ΔhptRS/ccpA

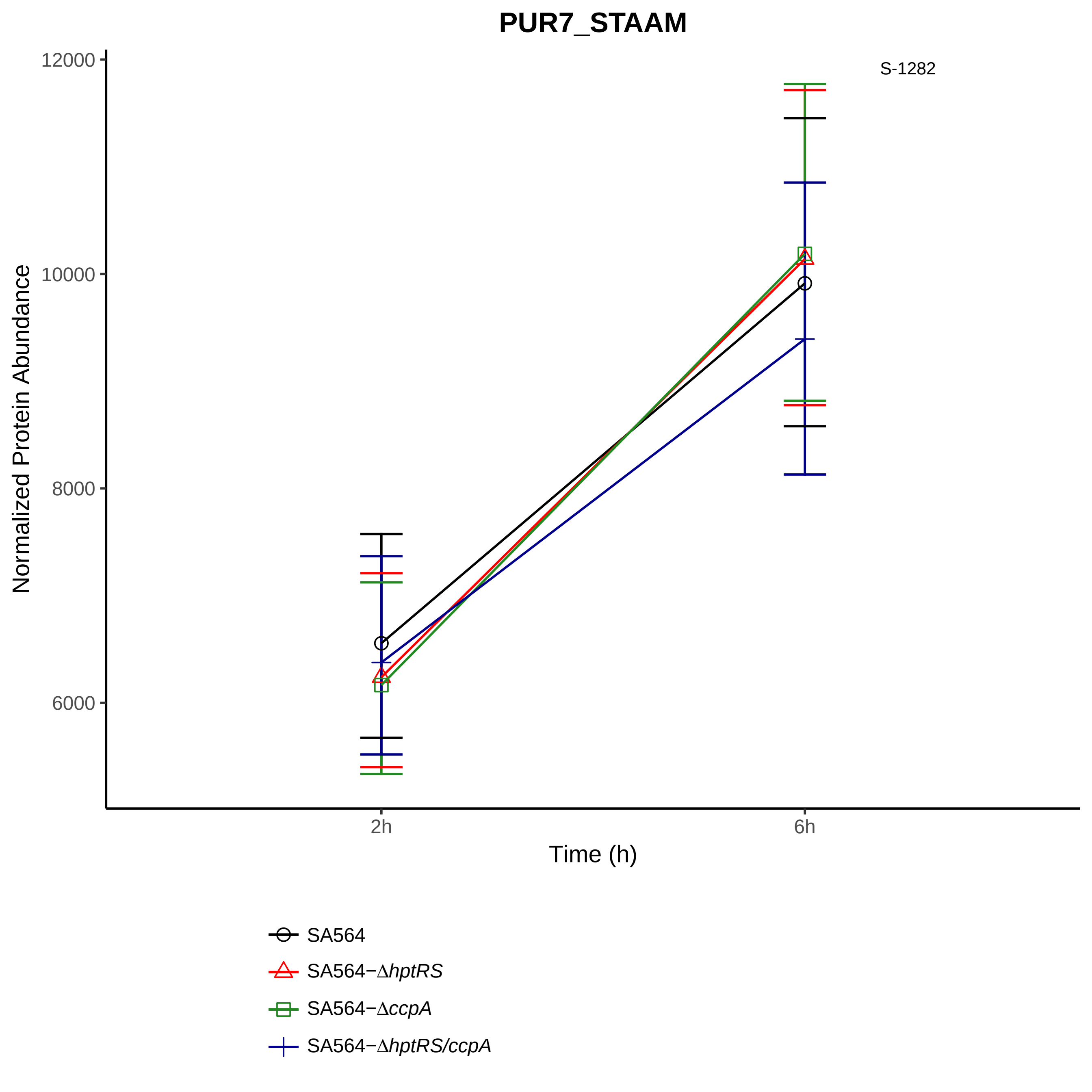

PUR7_STAAM
S-1282
Normalized Protein Abundance
12000
10000
8000
6000
2h
6h
Time (h)
SA564
SA564−Δ*hptRS*
SA564−Δ*ccpA*
SA564−Δ*hptRS/ccpA*

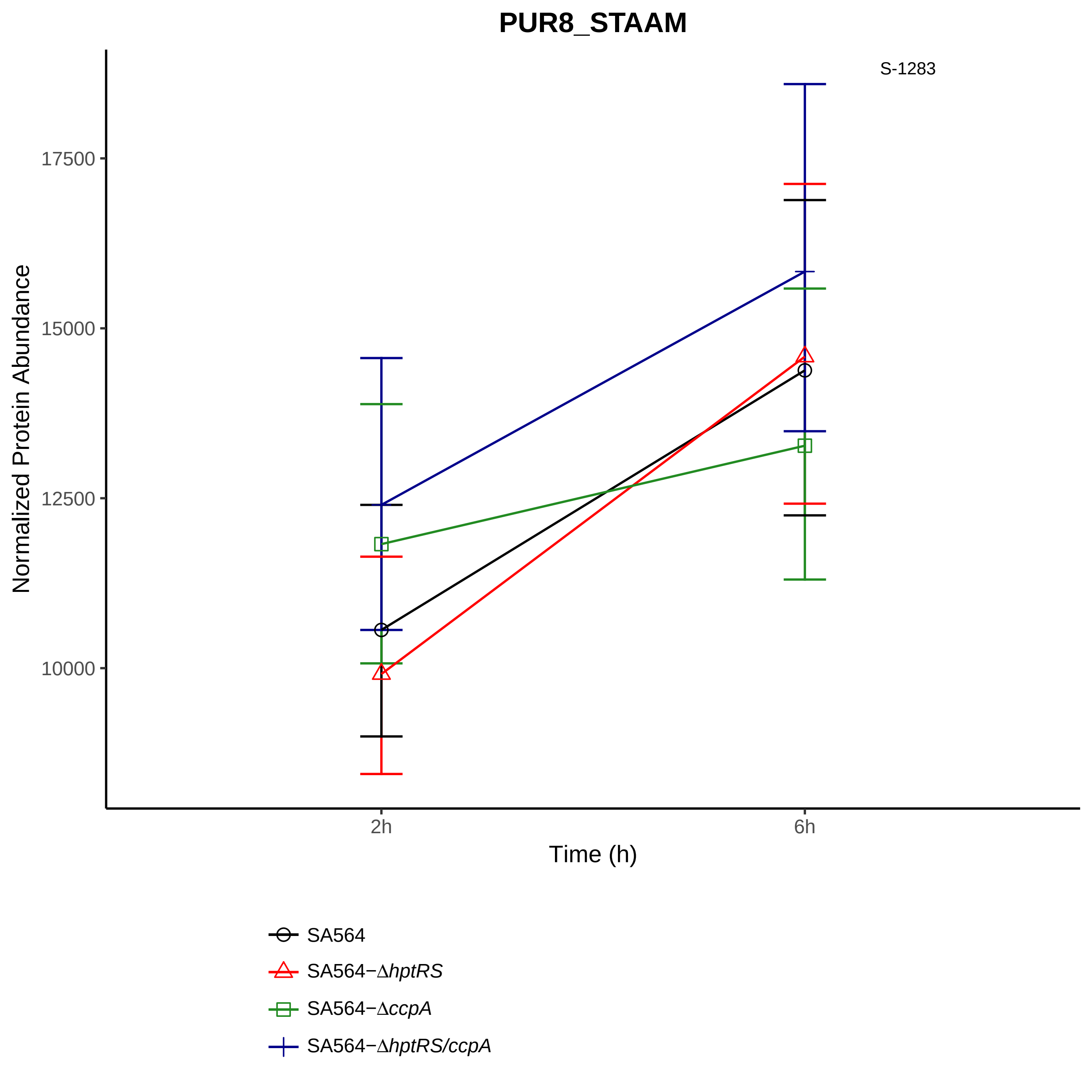

PUR8_STAAM
S-1283
Normalized Protein Abundance
17500
15000
12500
10000
2h
6h
Time (h)
SA564
SA564−Δ*hptRS*
SA564−Δ*ccpA*
SA564−Δ*hptRS/ccpA*

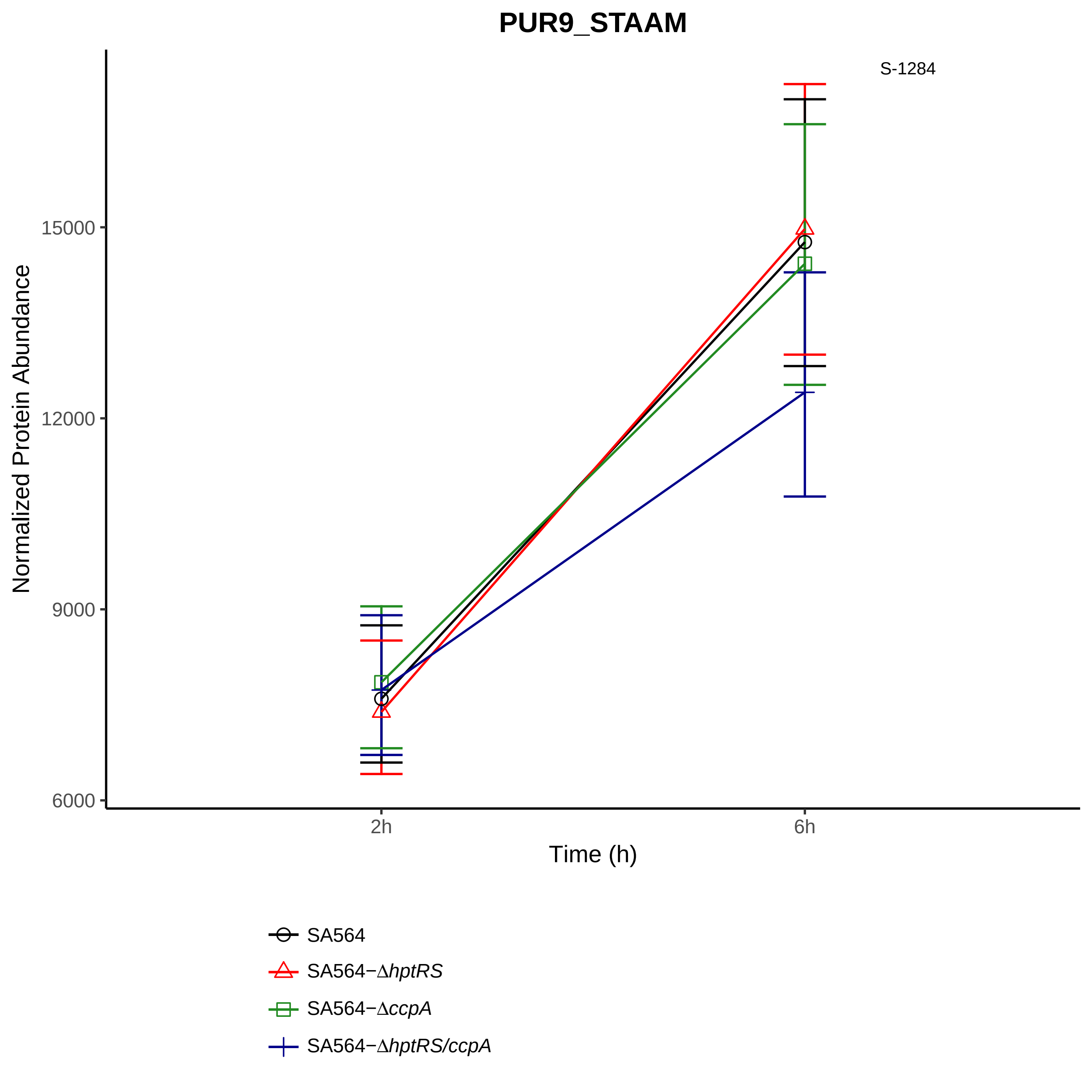
PUR9_STAAM
S-1284
Normalized Protein Abundance
15000
12000
9000
6000
2h
6h
Time (h)
SA564
SA564−ΔhptRS
SA564−ΔccpA
SA564−ΔhptRS/ccpA

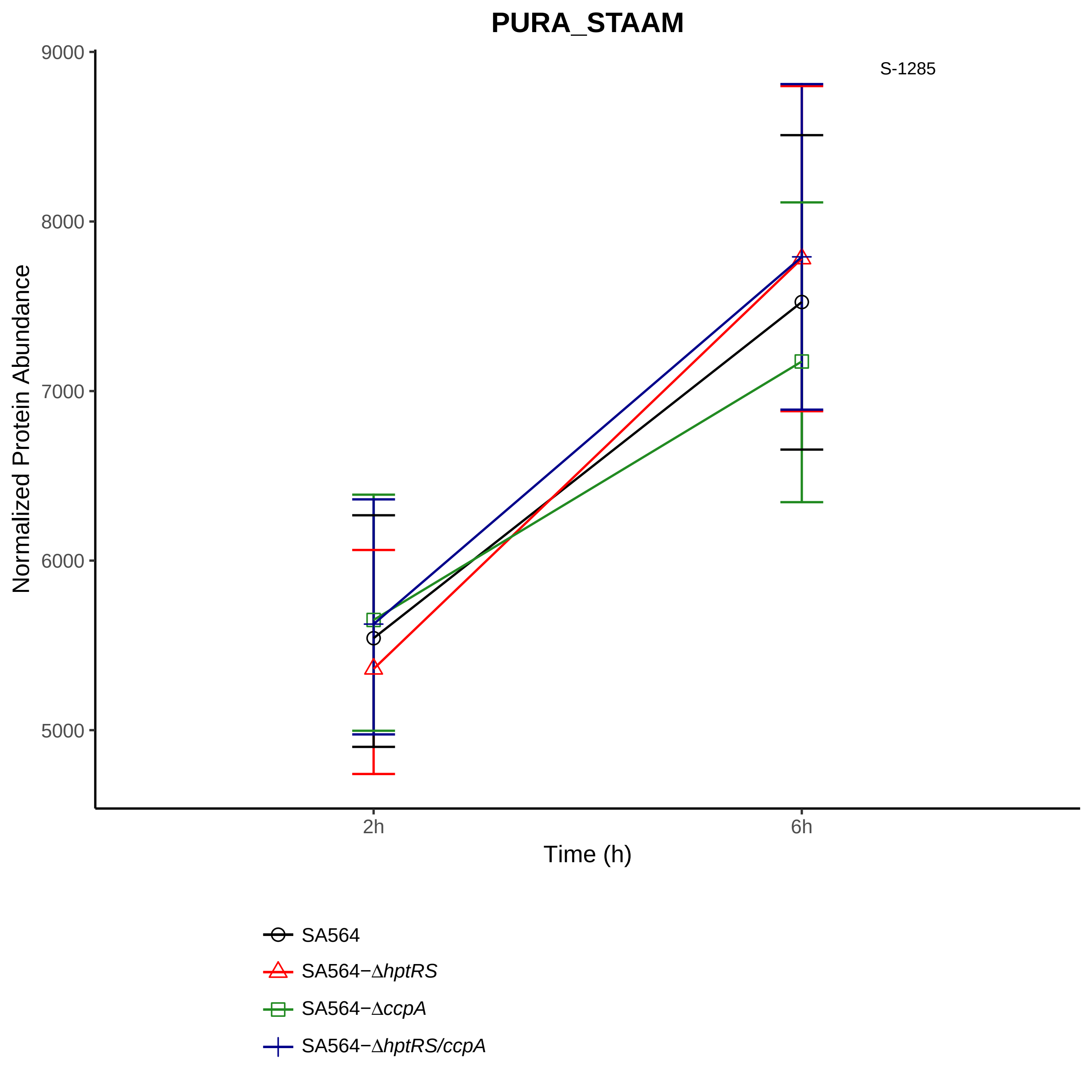
PURA_STAAM
S-1285
Normalized Protein Abundance
9000
8000
7000
6000
5000
2h
6h
Time (h)
SA564
SA564−Δ*hptRS*
SA564−Δ*ccpA*
SA564−Δ*hptRS/ccpA*

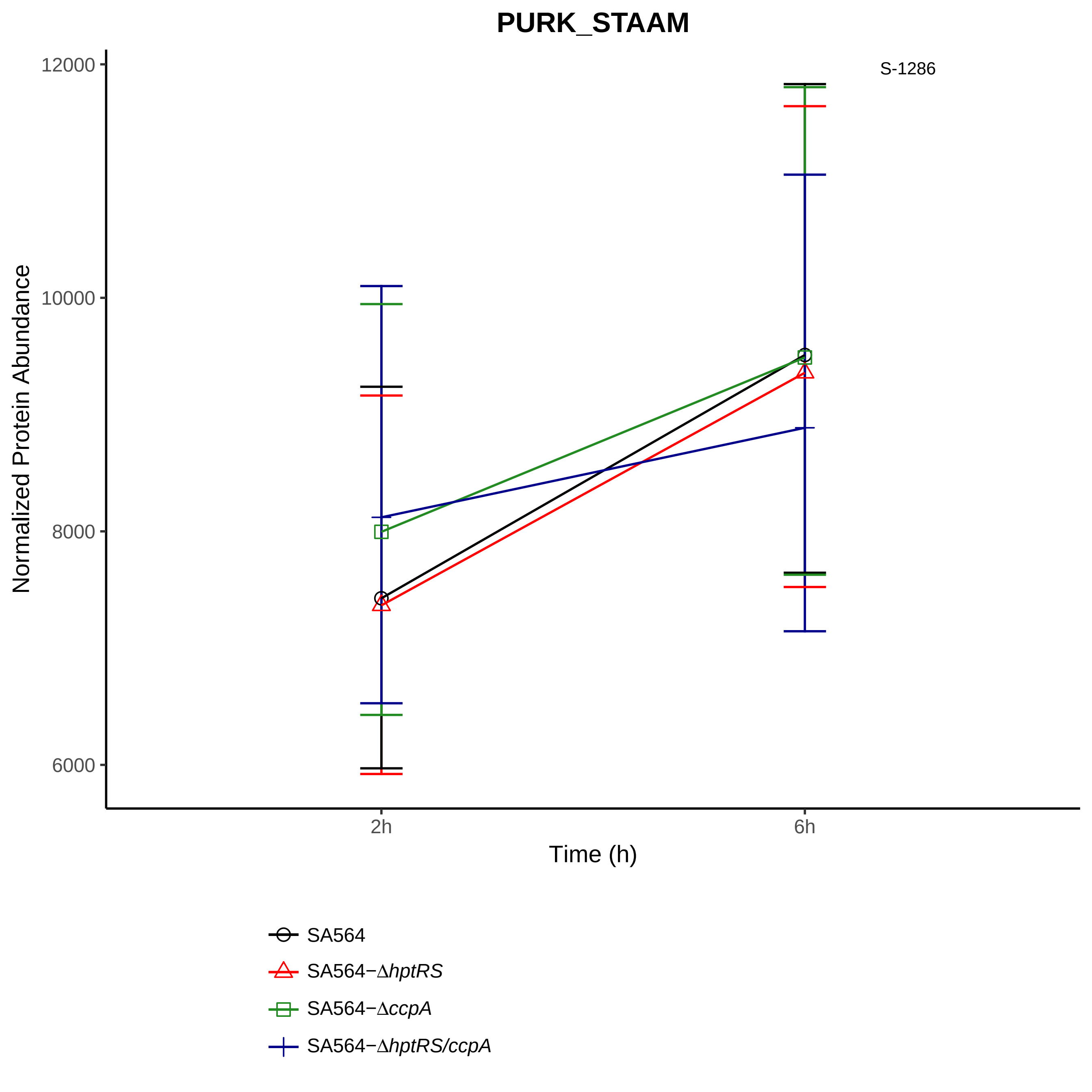
PURK_STAAM
S-1286
Normalized Protein Abundance
12000
10000
8000
6000
2h
6h
Time (h)
SA564
SA564−Δ*hptRS*
SA564−Δ*ccpA*
SA564−Δ*hptRS/ccpA*

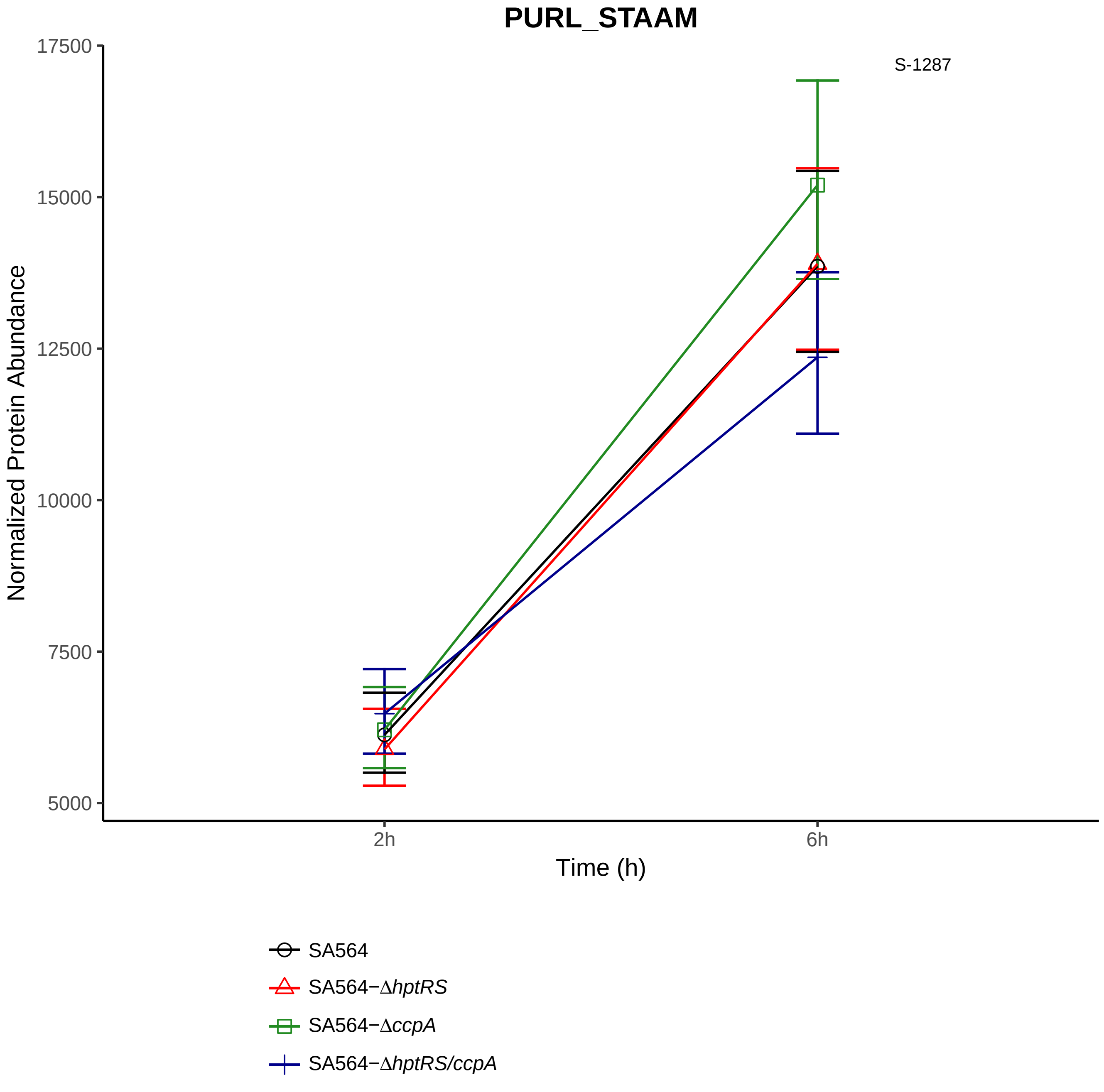

PURL_STAAM
S-1287
17500
15000
12500
10000
7500
5000
Normalized Protein Abundance
2h
6h
Time (h)
SA564
SA564−ΔhptRS
SA564−ΔccpA
SA564−ΔhptRS/ccpA

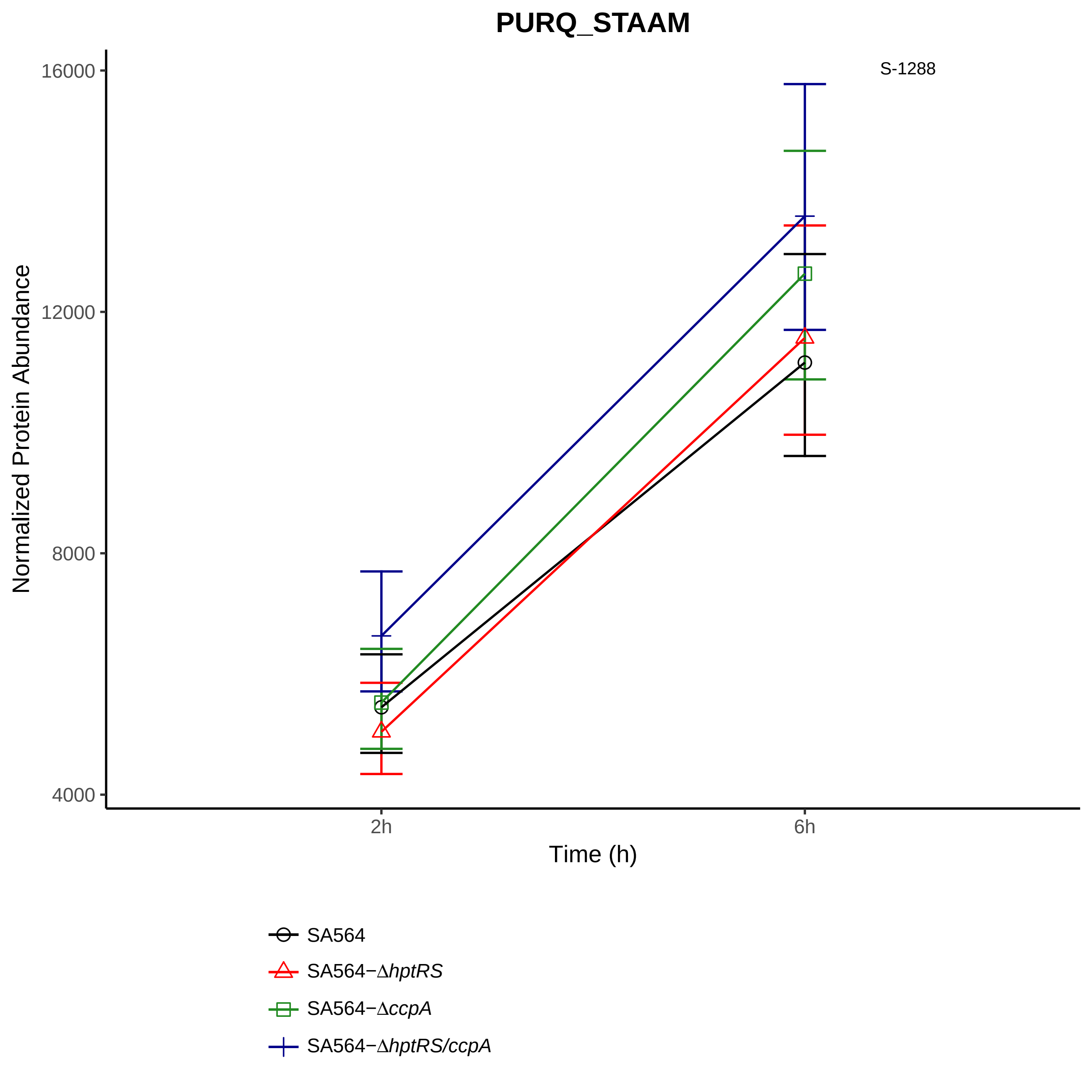

PURQ_STAAM
S-1288
16000
12000
8000
4000
Normalized Protein Abundance
2h
6h
Time (h)
SA564
SA564−Δ*hptRS*
SA564−Δ*ccpA*
SA564−Δ*hptRS/ccpA*

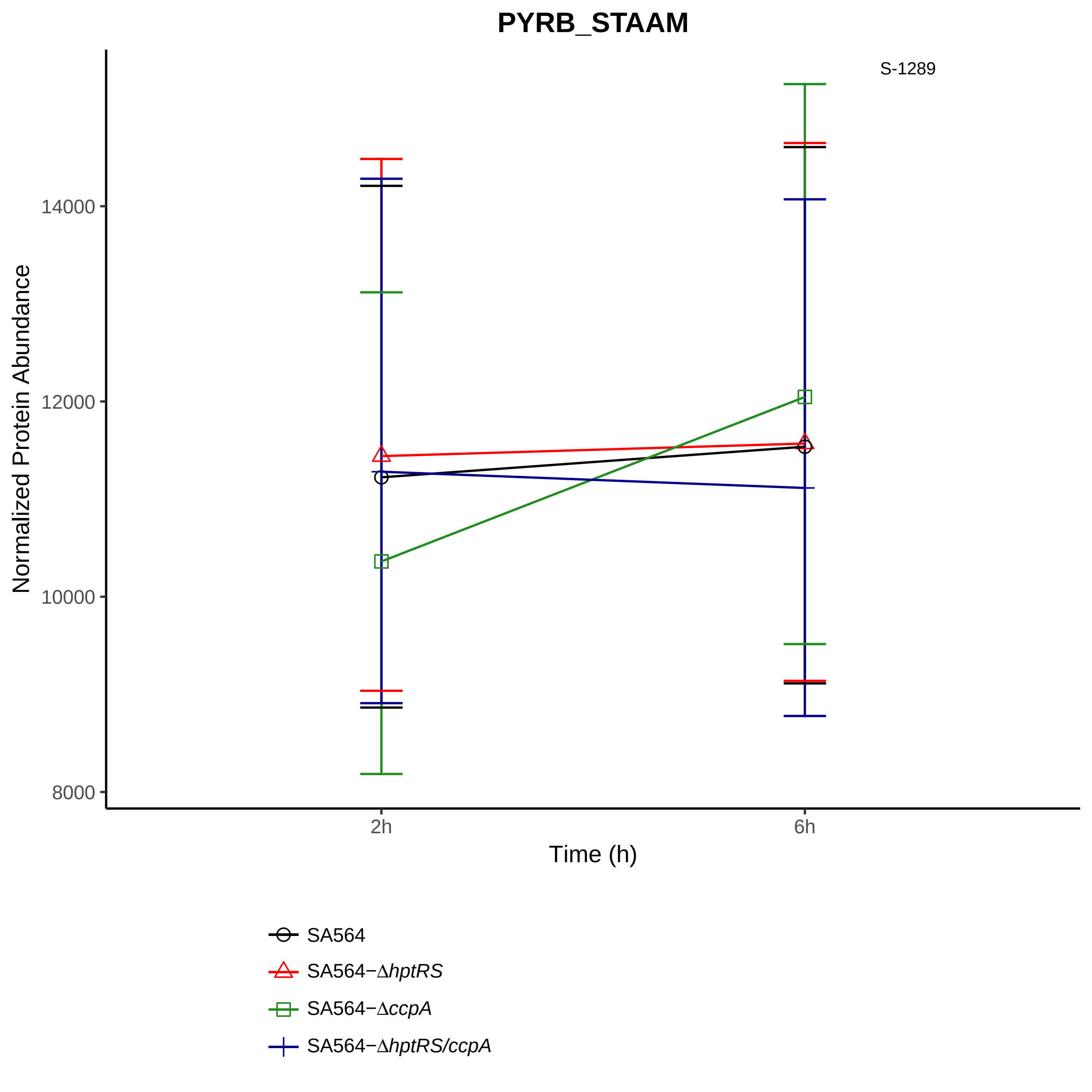

PYRB_STAAM
S-1289
Normalized Protein Abundance
14000
12000
10000
8000
2h
6h
Time (h)
SA564
SA564−Δ*hptRS*
SA564−Δ*ccpA*
SA564−Δ*hptRS/ccpA*

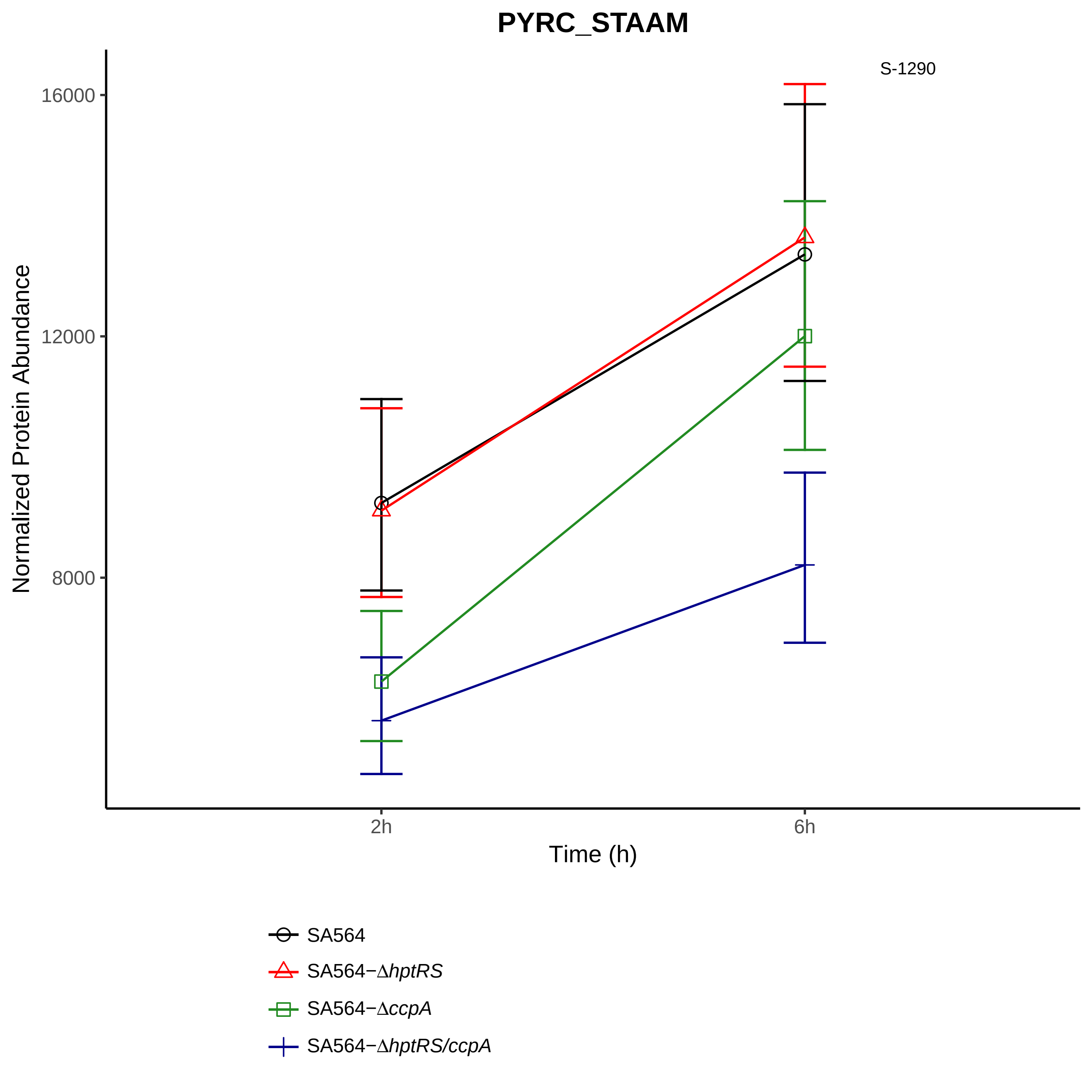

PYRC_STAAM
S-1290
16000
12000
8000
Normalized Protein Abundance
2h
6h
Time (h)
SA564
SA564−Δ*hptRS*
SA564−Δ*ccpA*
SA564−Δ*hptRS/ccpA*

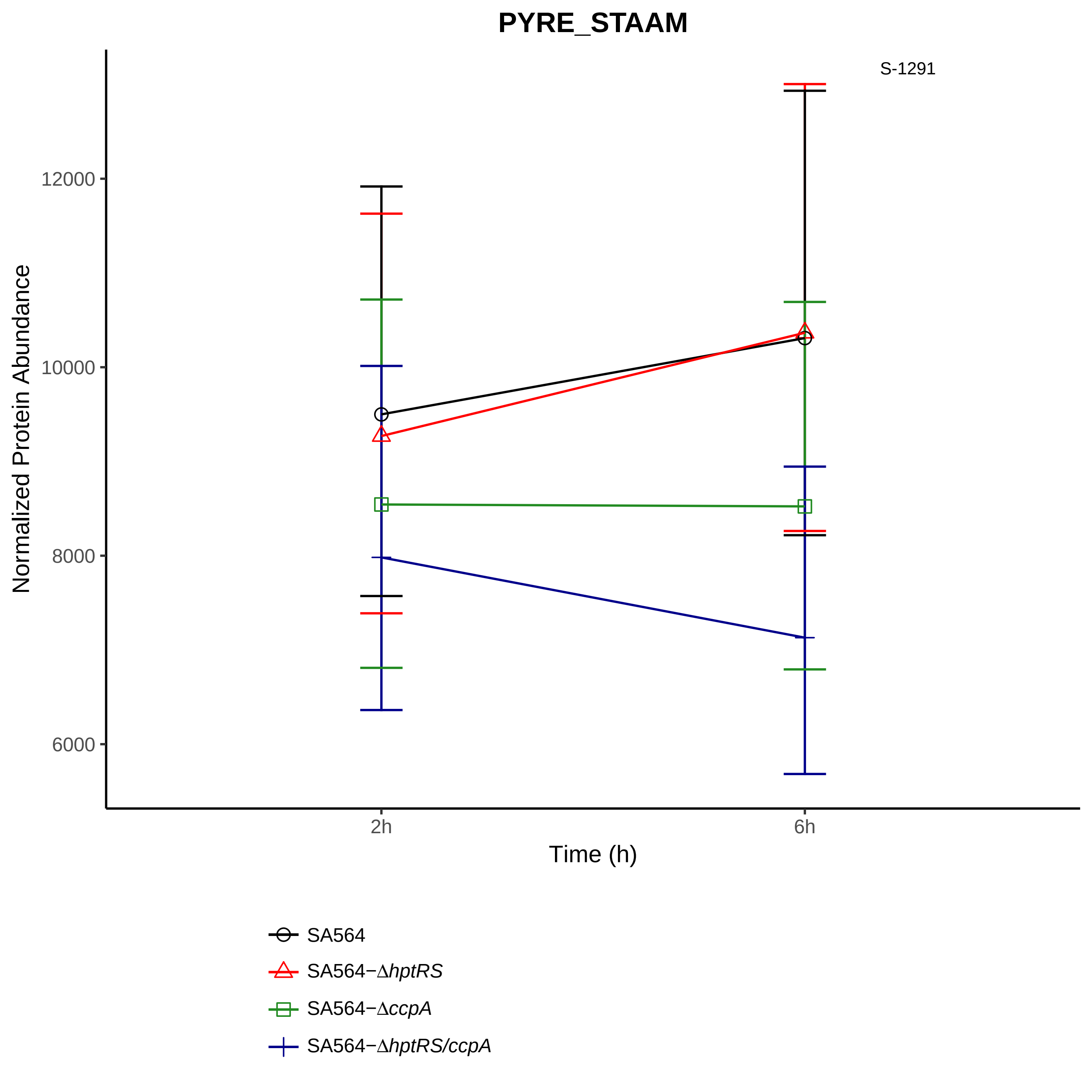

PYRE_STAAM
S-1291
Normalized Protein Abundance
12000
10000
8000
6000
2h
6h
Time (h)
SA564
SA564−ΔhptRS
SA564−ΔccpA
SA564−ΔhptRS/ccpA

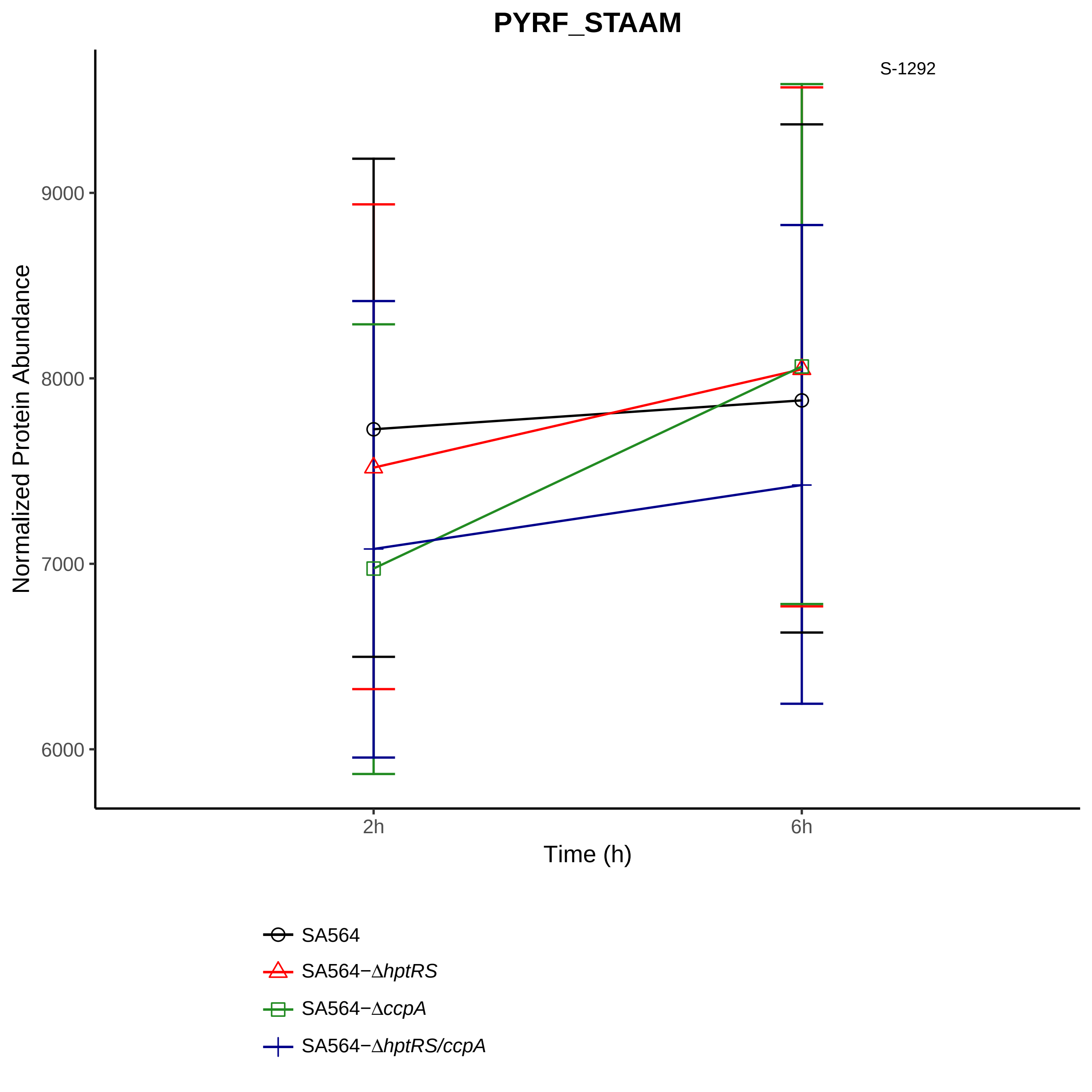

PYRF_STAAM
S-1292
Normalized Protein Abundance
9000
8000
7000
6000
2h
6h
Time (h)
SA564
SA564−ΔhptRS
SA564−ΔccpA
SA564−ΔhptRS/ccpA

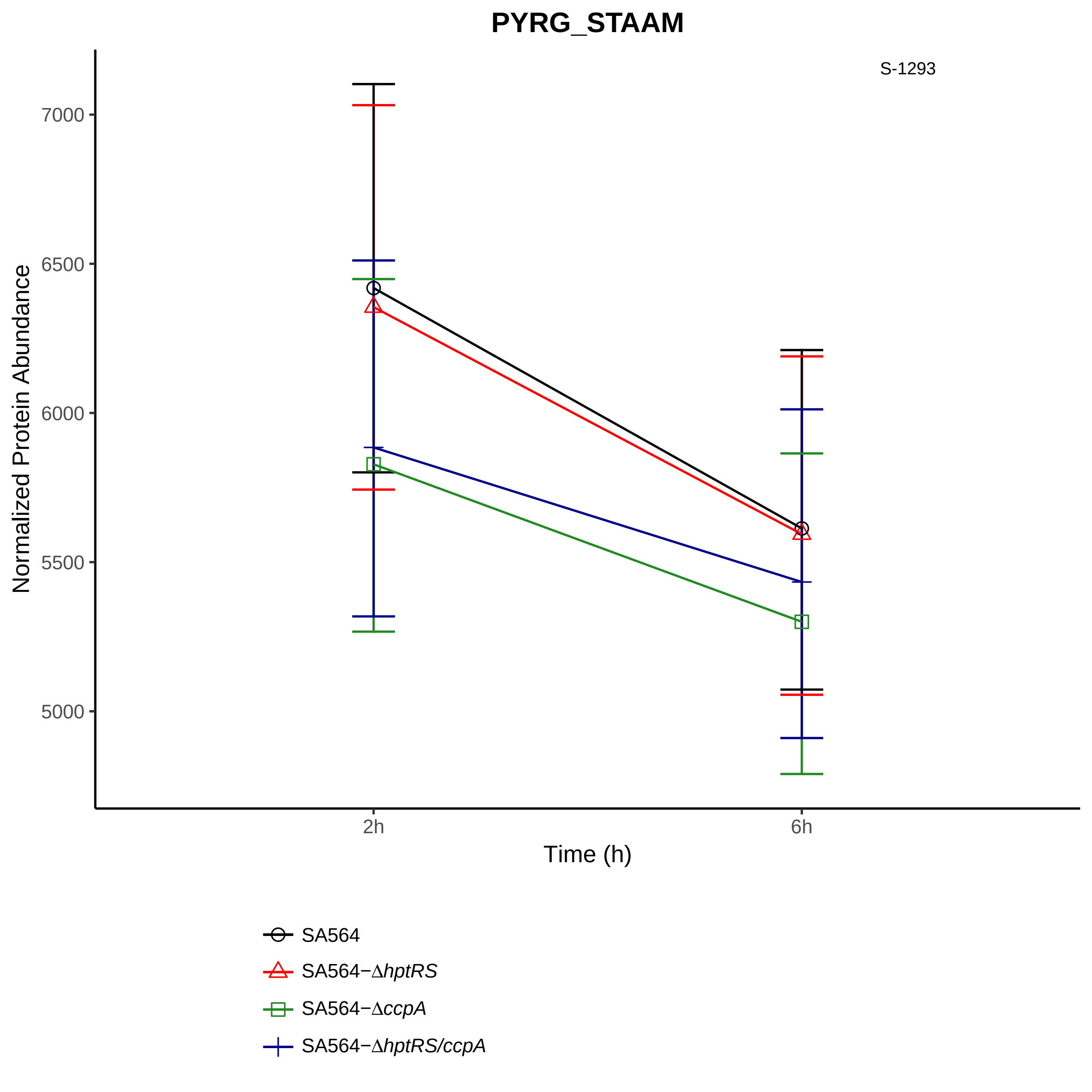
PYRG_STAAM
S-1293
Normalized Protein Abundance
7000
6500
6000
5500
5000
2h
6h
Time (h)
SA564
SA564−Δ*hptRS*
SA564−Δ*ccpA*
SA564−Δ*hptRS/ccpA*

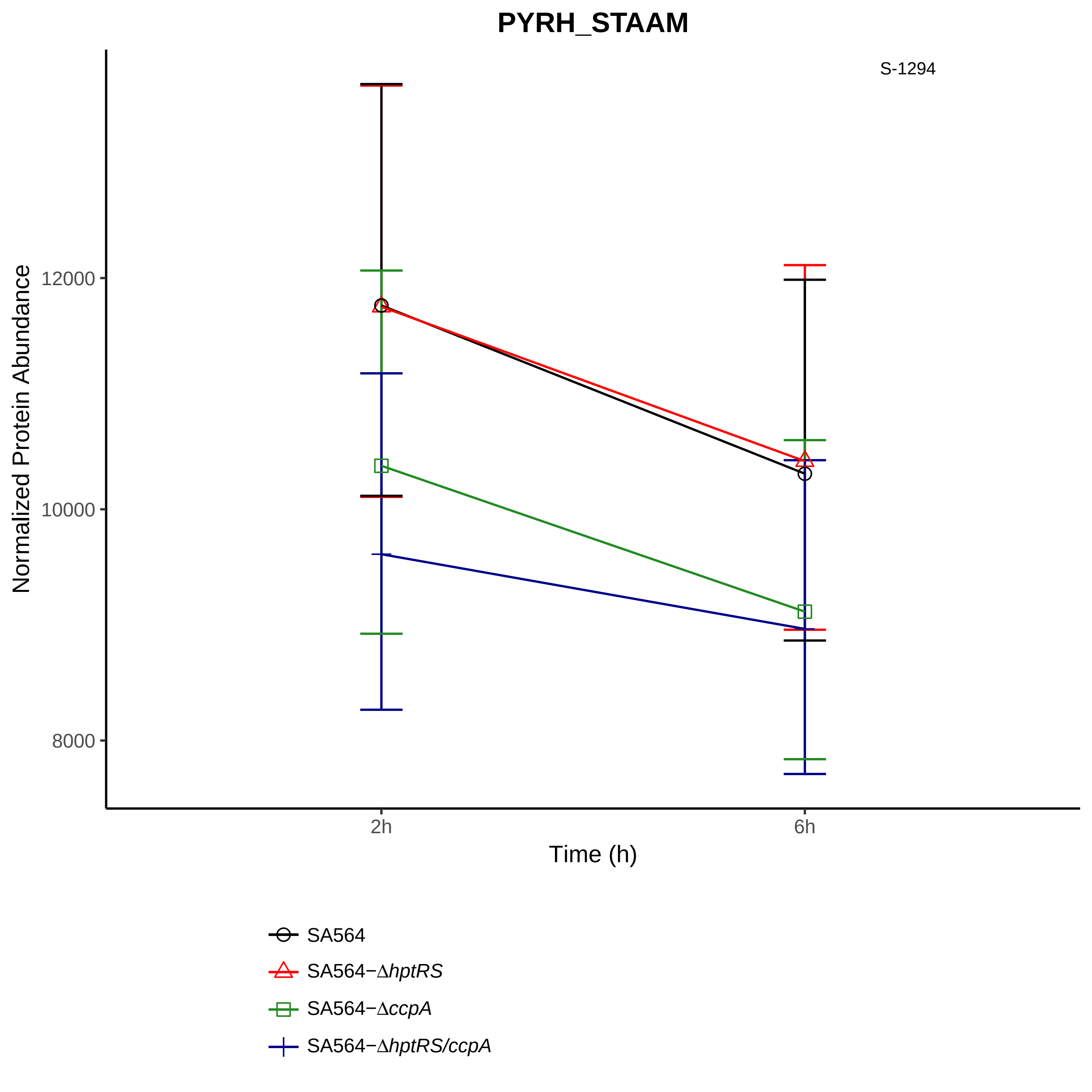

PYRH_STAAM
S-1294
Normalized Protein Abundance
12000
10000
8000
2h
6h
Time (h)
SA564
SA564−Δ*hptRS*
SA564−Δ*ccpA*
SA564−Δ*hptRS/ccpA*

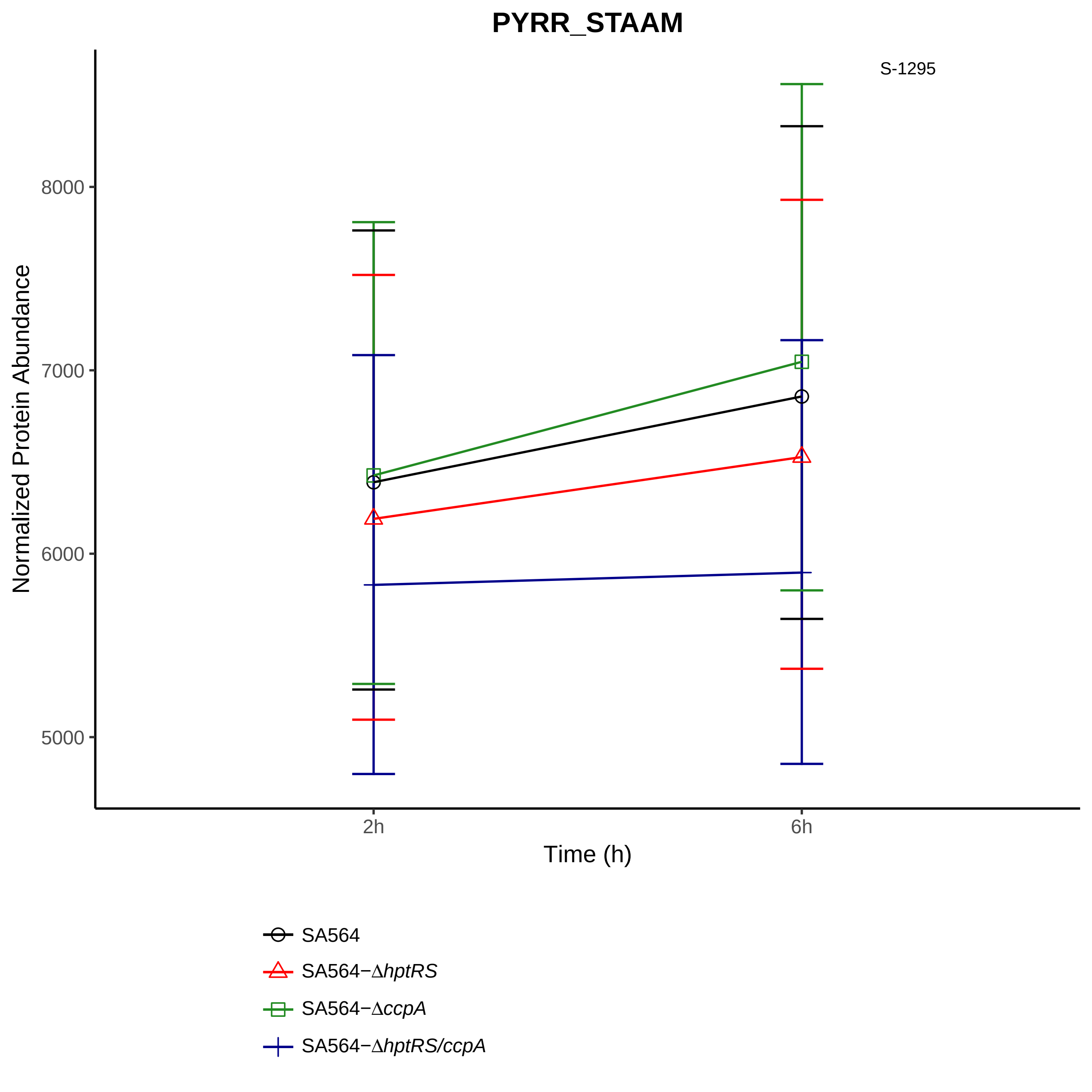

PYRR_STAAM
S-1295
Normalized Protein Abundance
8000
7000
6000
5000
2h
6h
Time (h)
SA564
SA564−Δ*hptRS*
SA564−Δ*ccpA*
SA564−Δ*hptRS/ccpA*

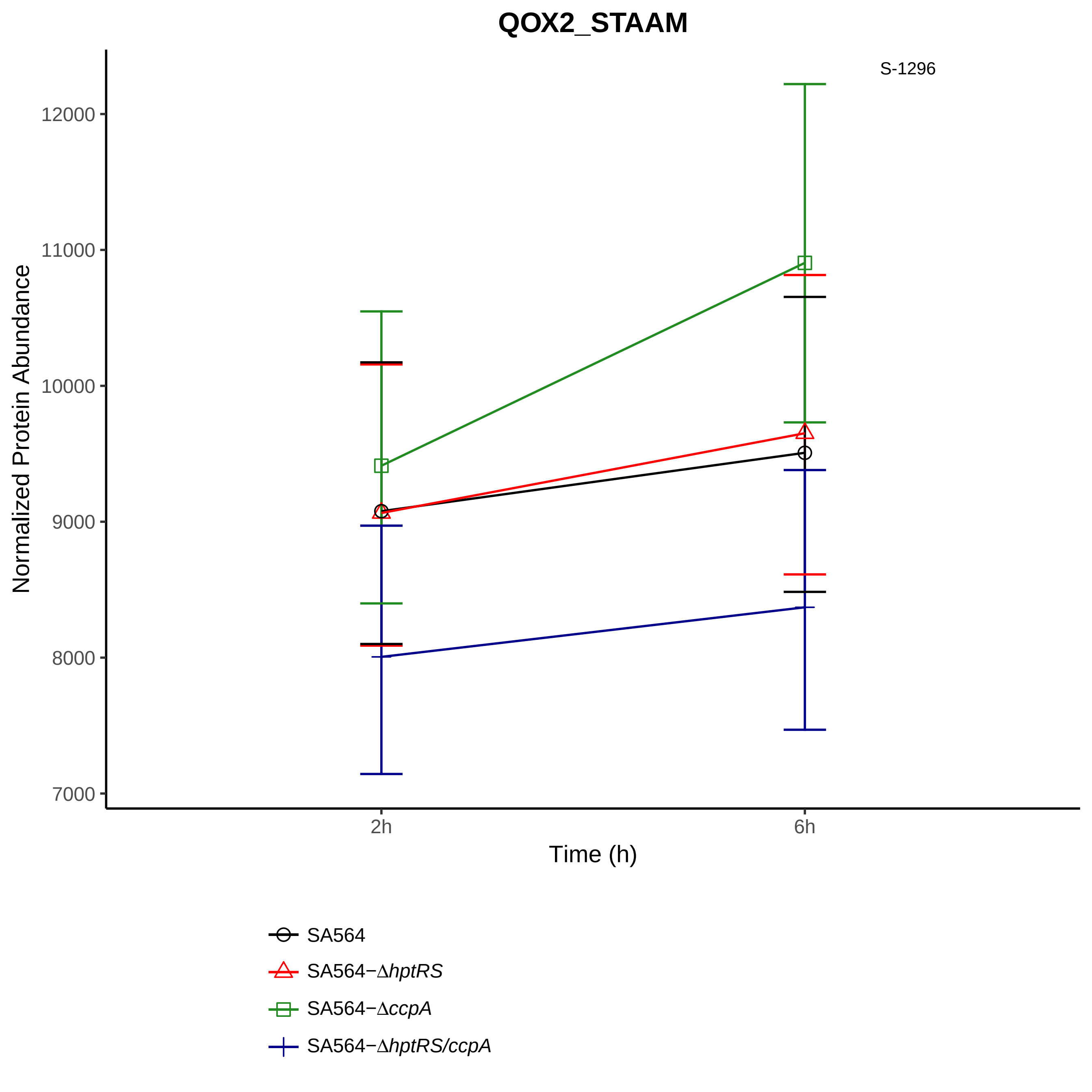

QOX2_STAAM
S-1296
Normalized Protein Abundance
12000
11000
10000
9000
8000
7000
2h
6h
Time (h)
SA564
SA564−Δ*hptRS*
SA564−Δ*ccpA*
SA564−Δ*hptRS/ccpA*

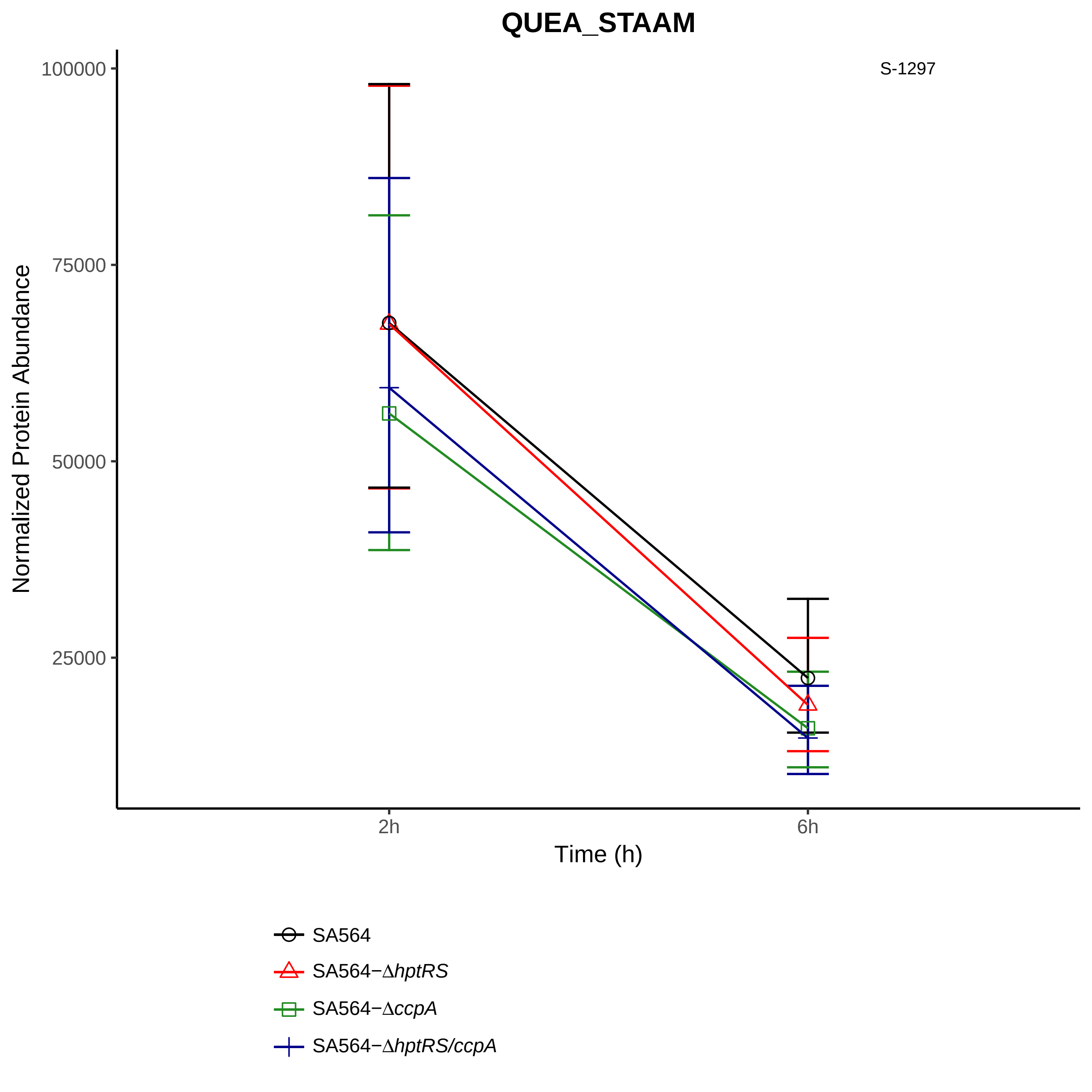
QUEA_STAAM
S-1297
Normalized Protein Abundance
100000
75000
50000
25000
2h
6h
Time (h)
SA564
SA564−ΔhptRS
SA564−ΔccpA
SA564−ΔhptRS/ccpA

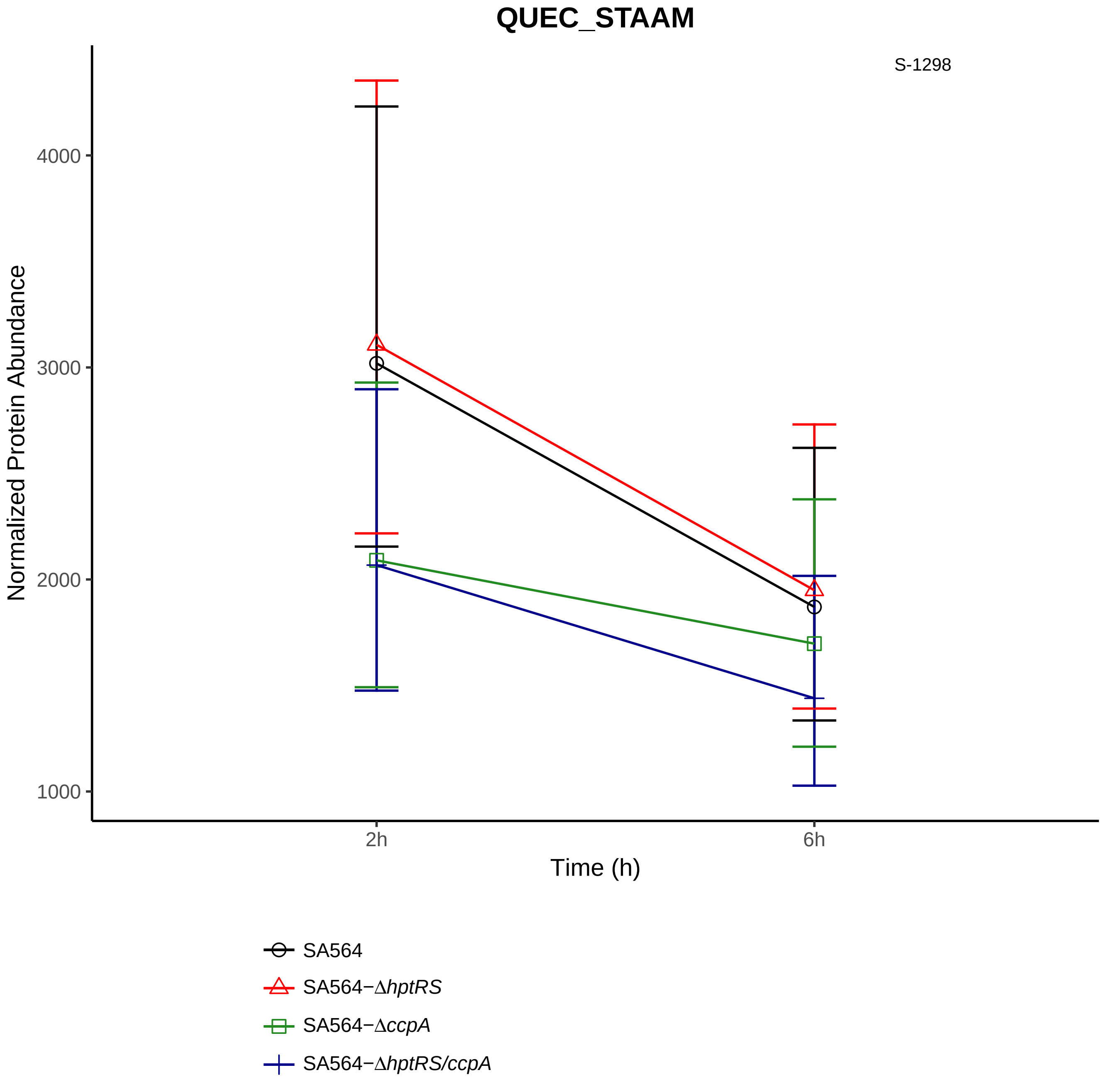

QUEC_STAAM
S-1298
Normalized Protein Abundance
4000
3000
2000
1000
2h
6h
Time (h)
SA564
SA564−Δ*hptRS*
SA564−Δ*ccpA*
SA564−Δ*hptRS/ccpA*

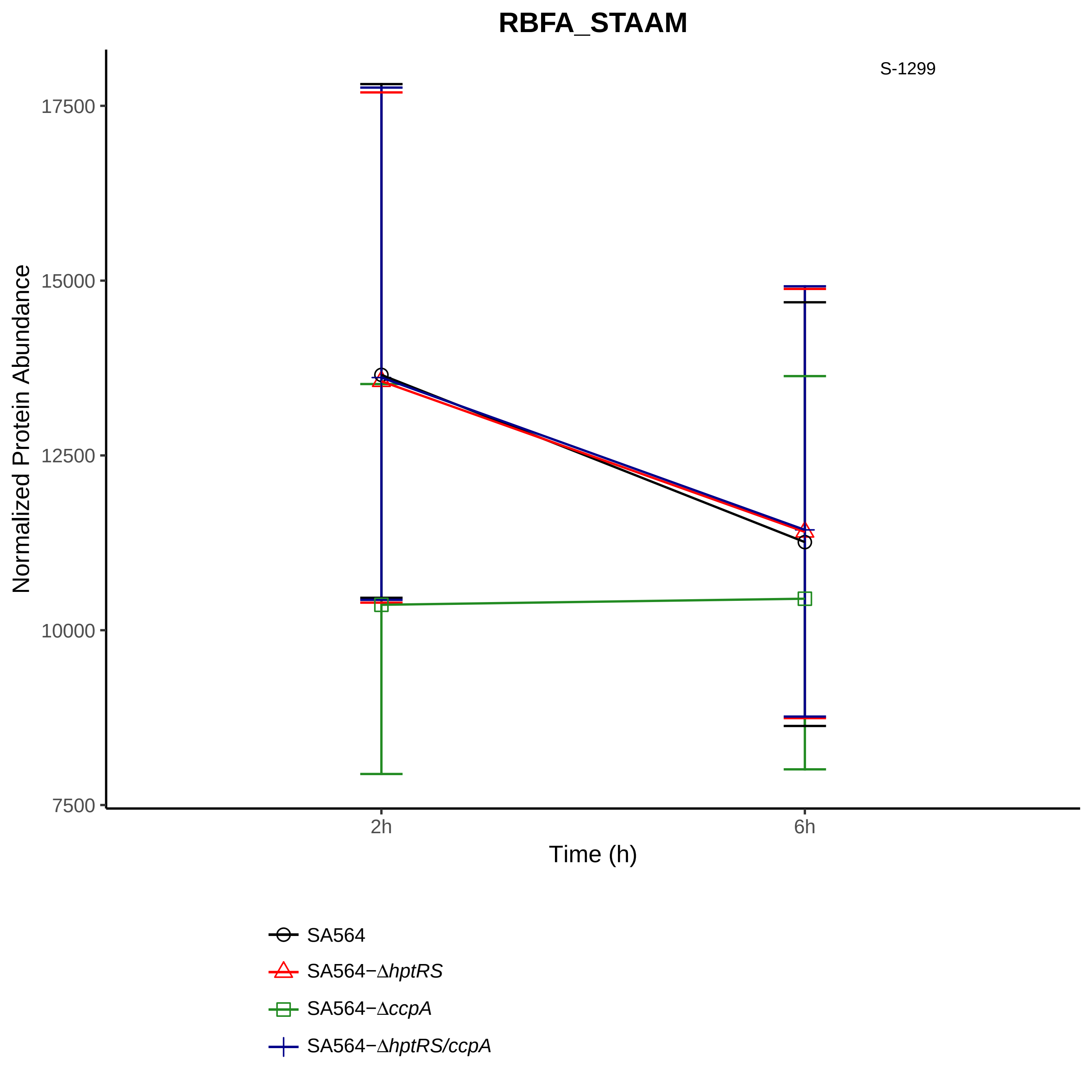

RBFA_STAAM
S-1299
17500
15000
12500
10000
7500
Normalized Protein Abundance
2h
6h
Time (h)
SA564
SA564−Δ*hptRS*
SA564−Δ*ccpA*
SA564−Δ*hptRS/ccpA*

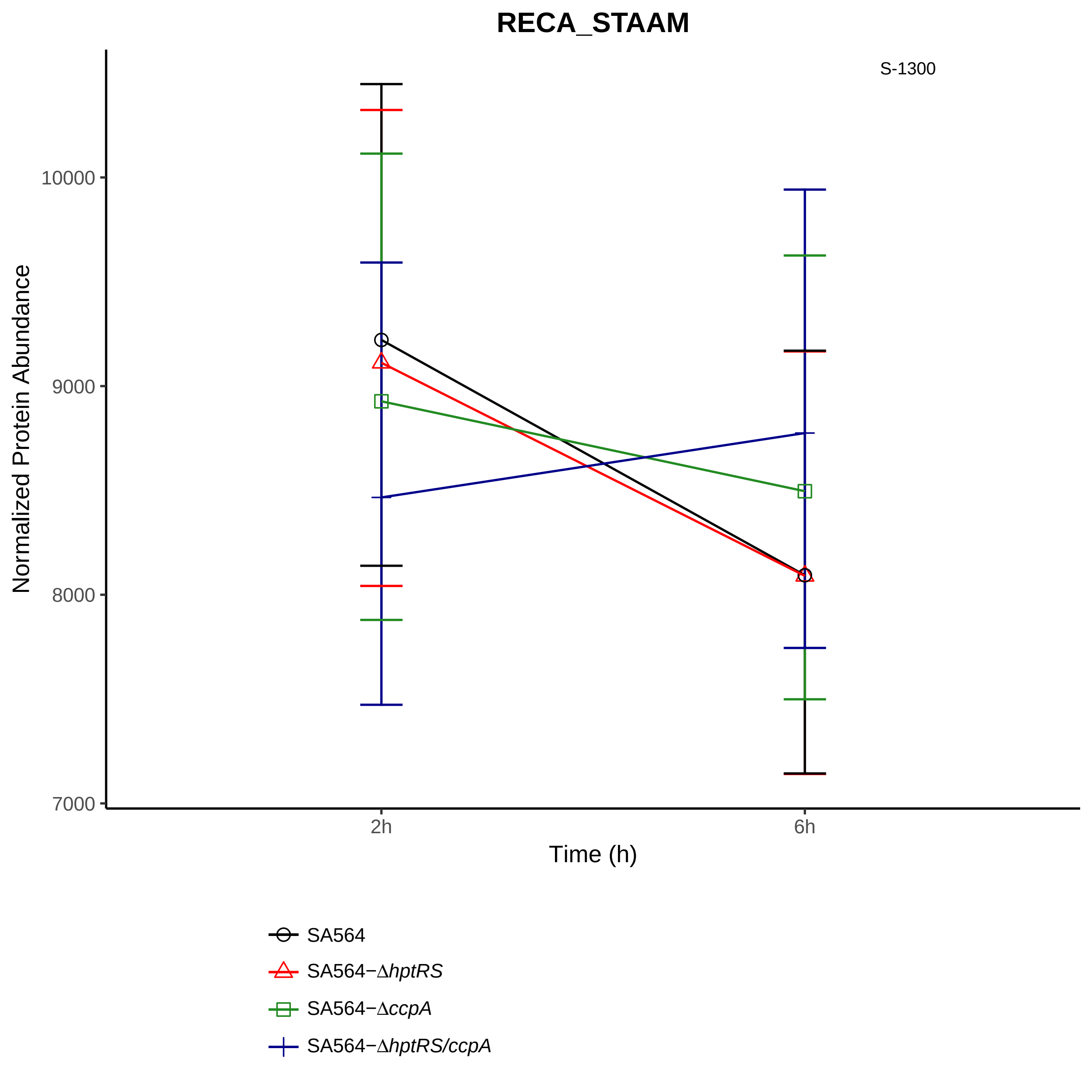

RECA_STAAM
S-1300
Normalized Protein Abundance
10000
9000
8000
7000
2h
6h
Time (h)
SA564
SA564−Δ*hptRS*
SA564−Δ*ccpA*
SA564−Δ*hptRS/ccpA*

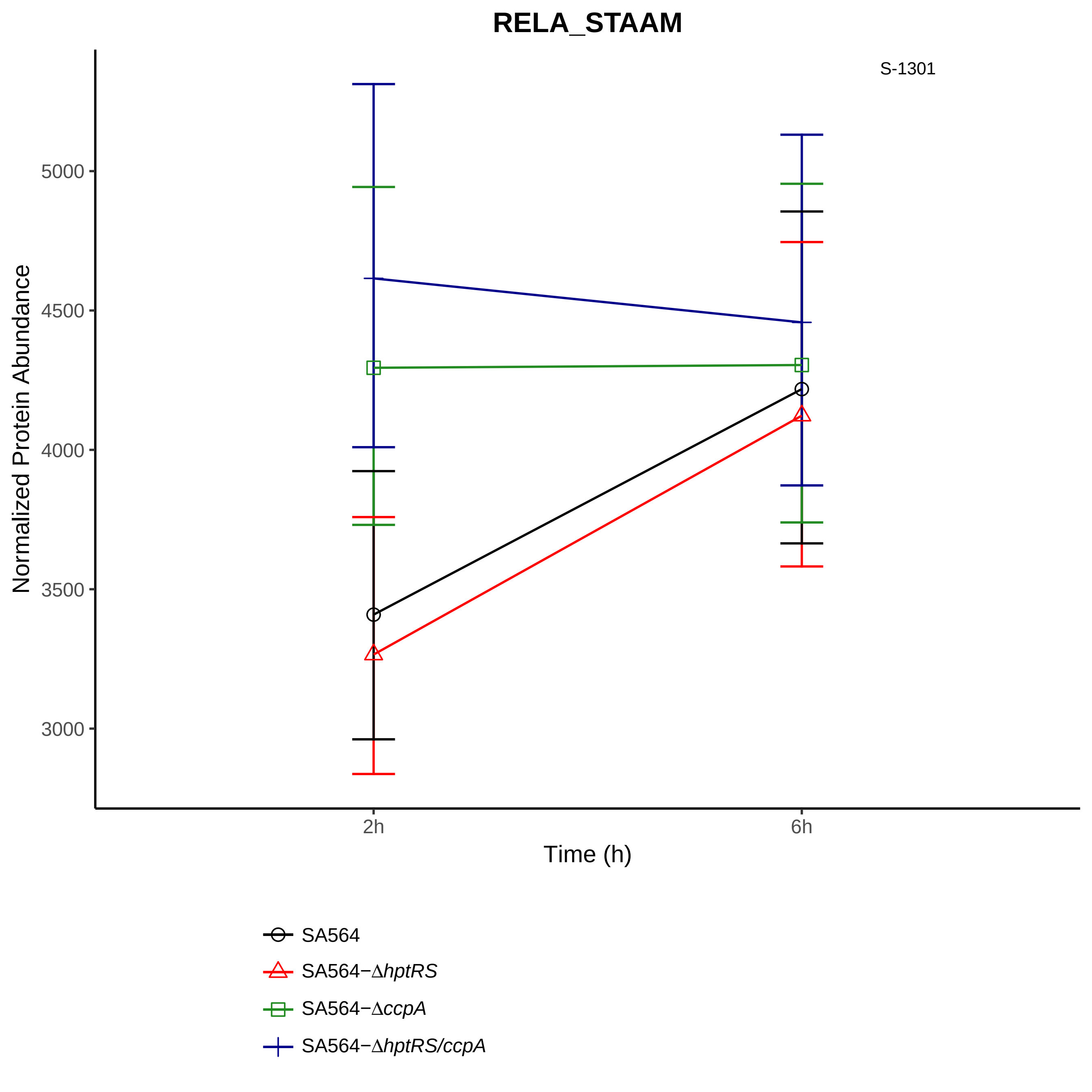

RELA_STAAM
S-1301
Normalized Protein Abundance
5000
4500
4000
3500
3000
2h
6h
Time (h)
SA564
SA564−Δ*hptRS*
SA564−Δ*ccpA*
SA564−Δ*hptRS/ccpA*

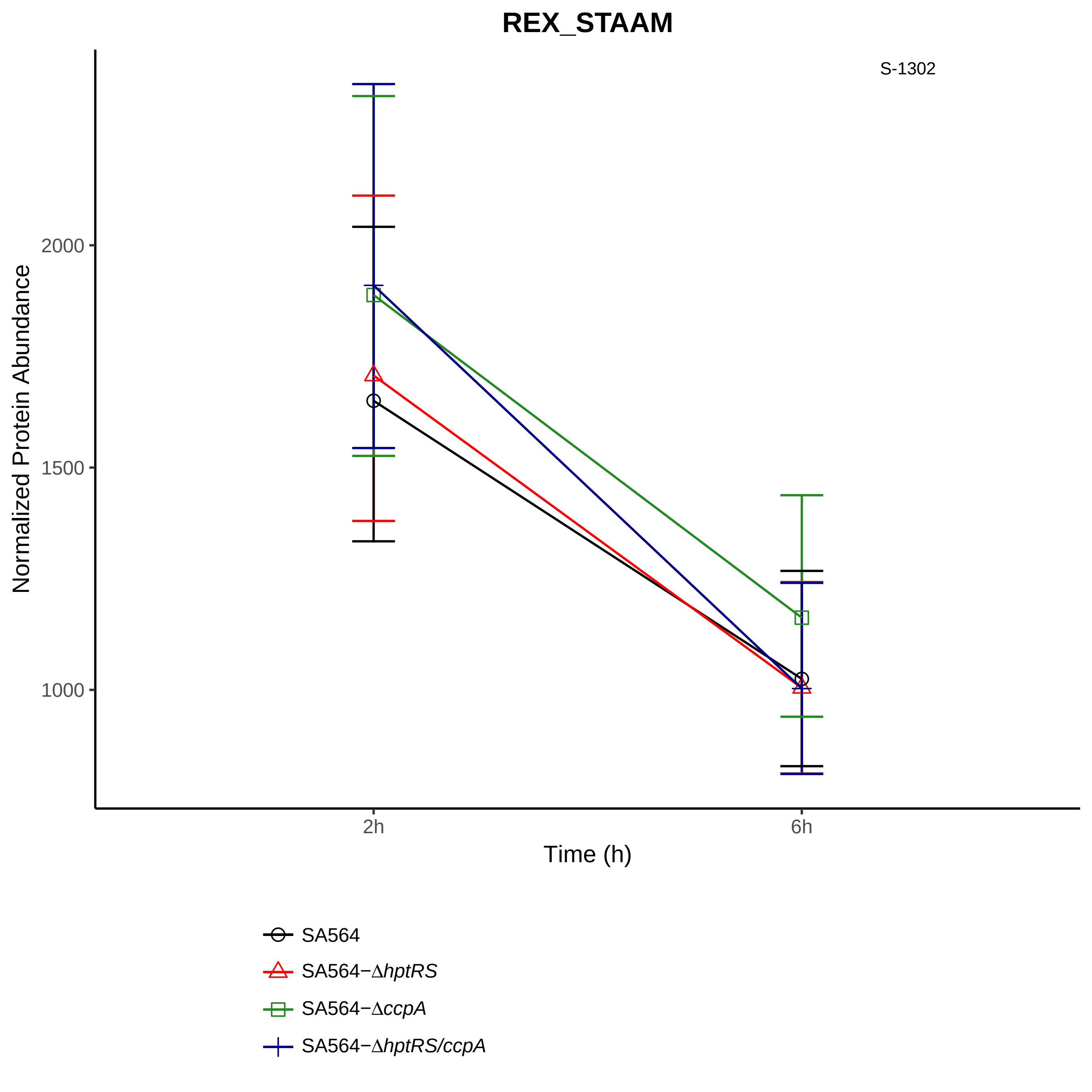

REX_STAAM
S-1302
Normalized Protein Abundance
2000
1500
1000
2h
6h
Time (h)
SA564
SA564−Δ*hptRS*
SA564−Δ*ccpA*
SA564−Δ*hptRS/ccpA*

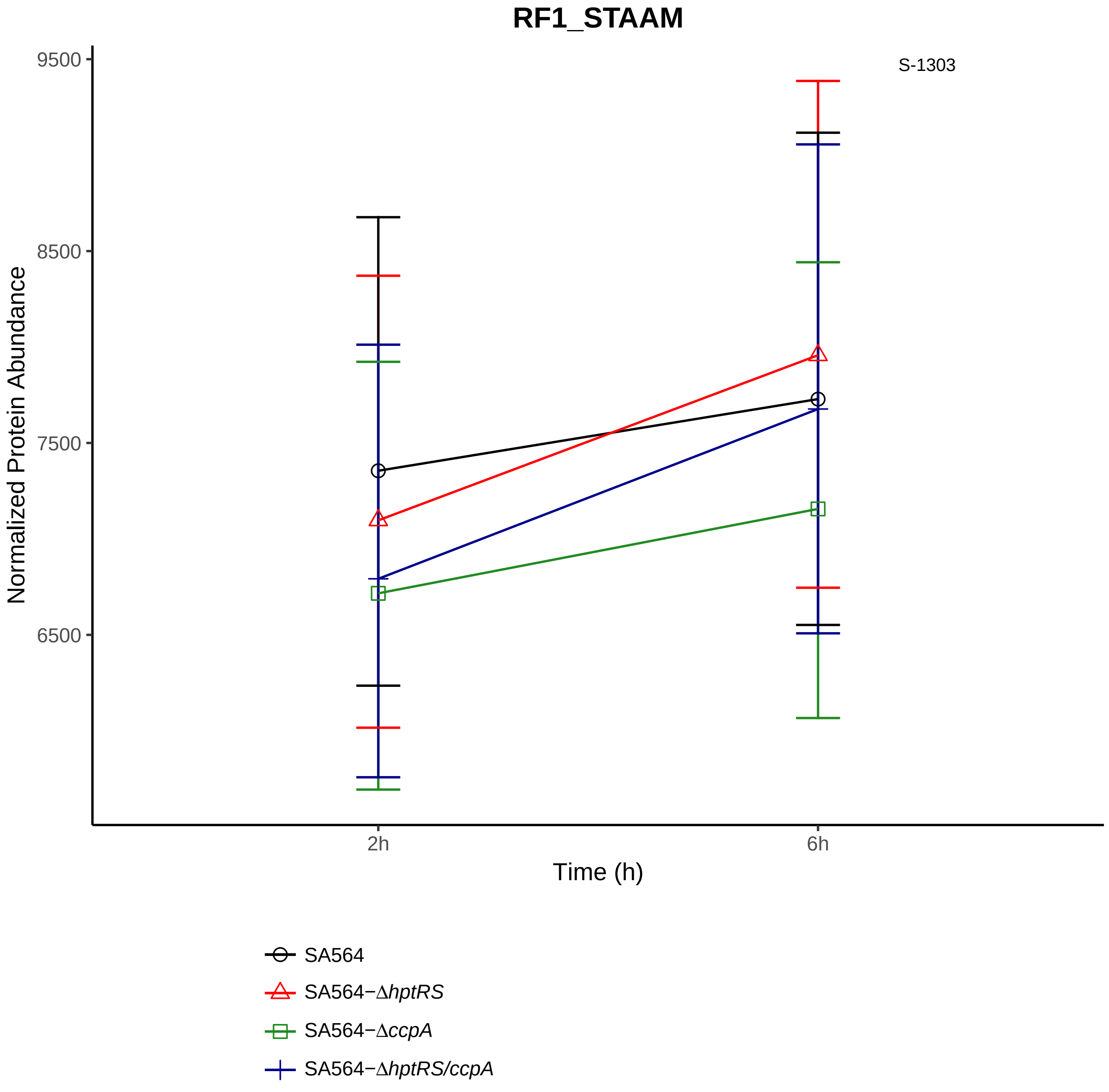
RF1_STAAM
S-1303
9500
8500
7500
6500
Normalized Protein Abundance
2h
6h
Time (h)
SA564
SA564−Δ*hptRS*
SA564−Δ*ccpA*
SA564−Δ*hptRS/ccpA*

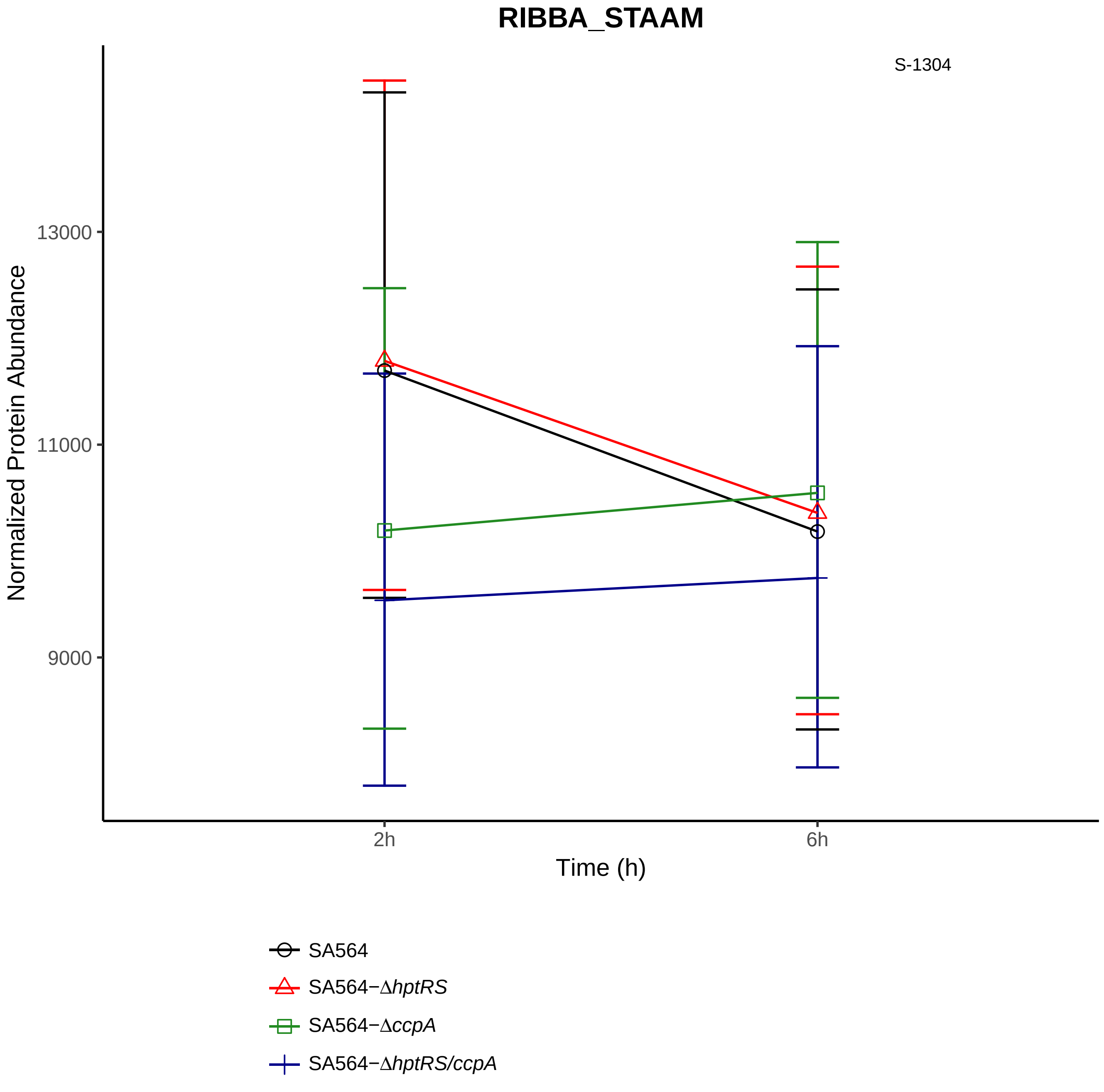

RIBBA_STAAM
S-1304
Normalized Protein Abundance
13000
11000
9000
2h
6h
Time (h)
SA564
SA564−Δ*hptRS*
SA564−Δ*ccpA*
SA564−Δ*hptRS/ccpA*

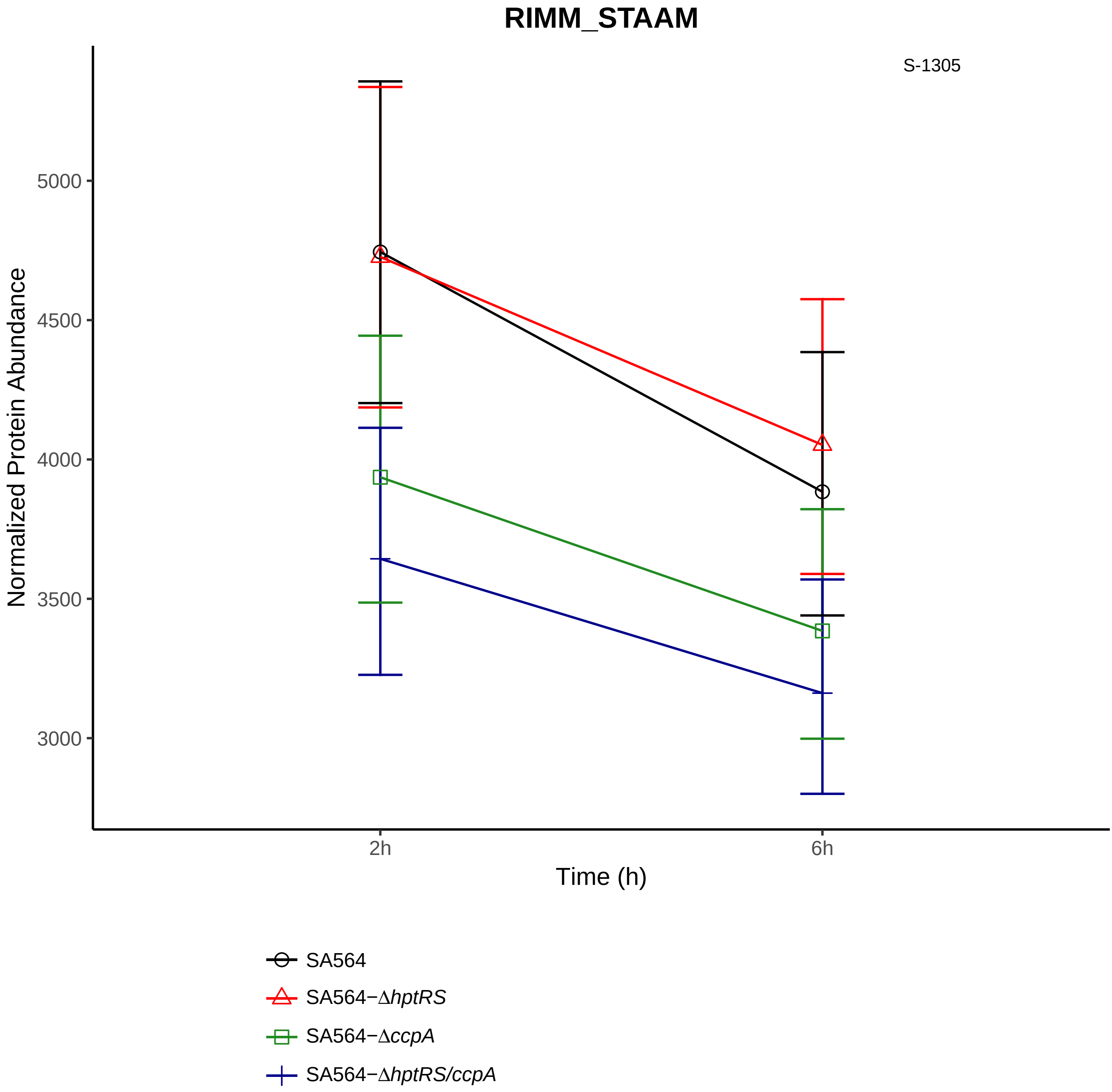

RIMM_STAAM
S-1305
Normalized Protein Abundance
5000
4500
4000
3500
3000
2h
6h
Time (h)
SA564
SA564−Δ*hptRS*
SA564−Δ*ccpA*
SA564−Δ*hptRS/ccpA*

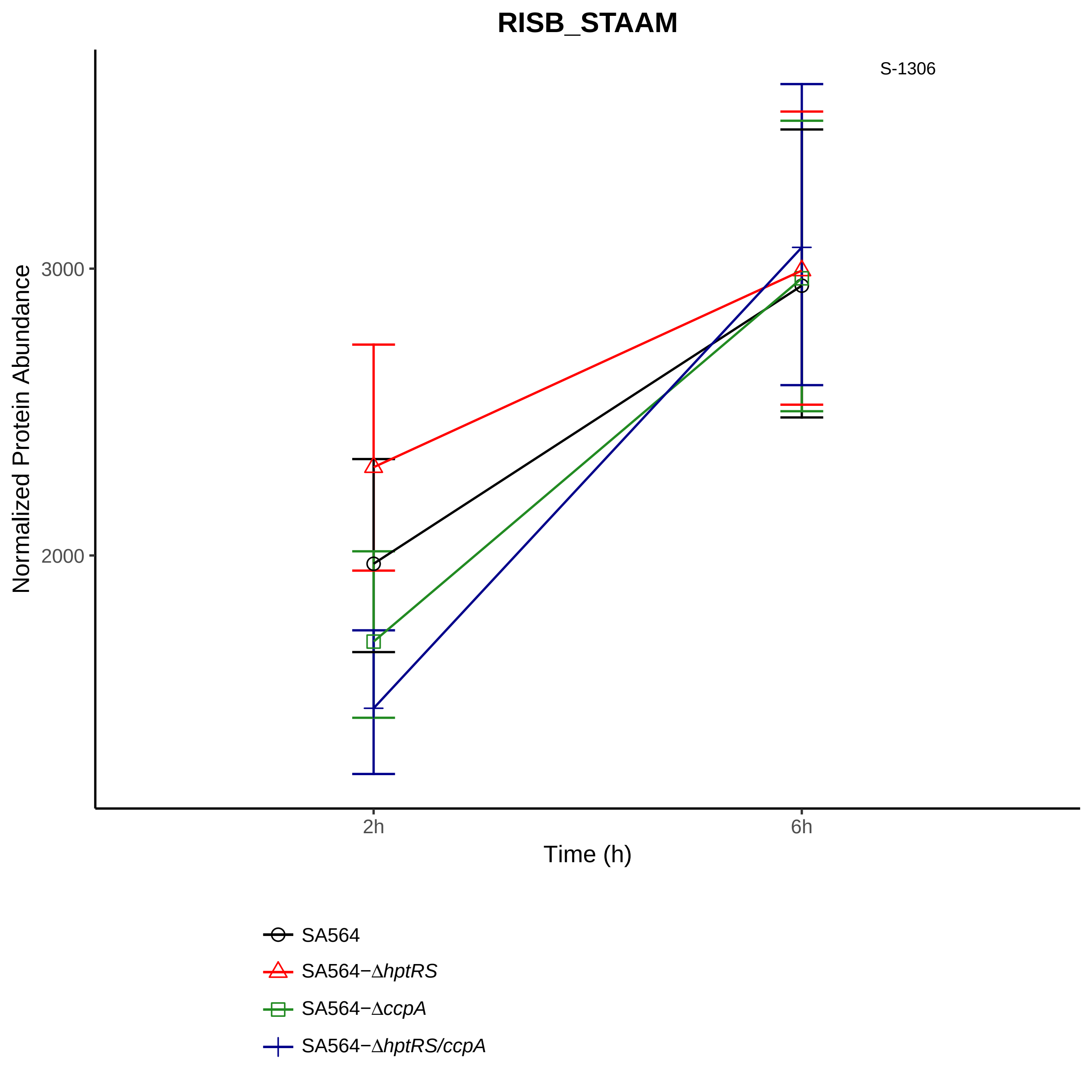

RISB_STAAM
S-1306
Normalized Protein Abundance
3000
2000
2h
6h
Time (h)
SA564
SA564−Δ*hptRS*
SA564−Δ*ccpA*
SA564−Δ*hptRS/ccpA*

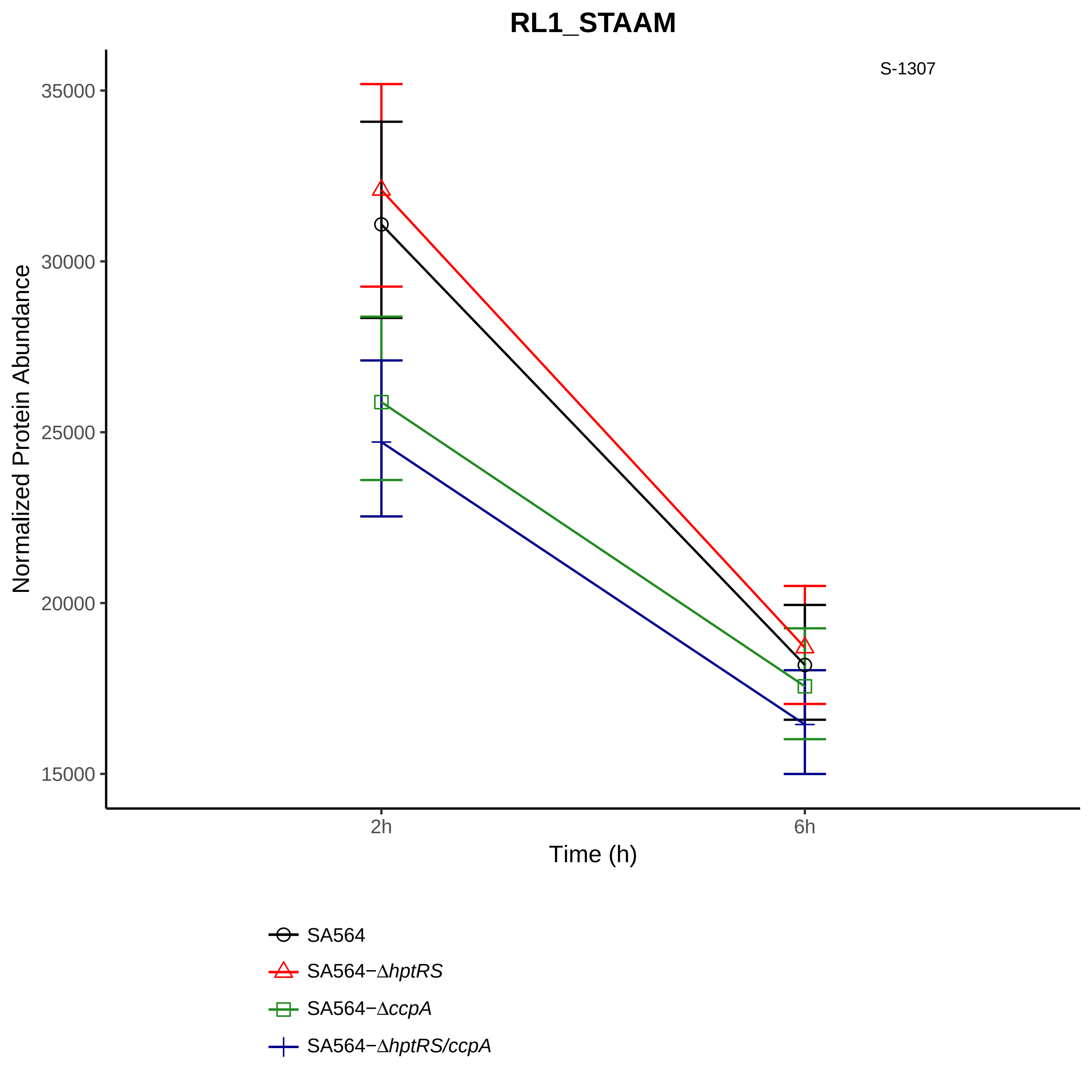
RL1_STAAM
S-1307
Normalized Protein Abundance
35000
30000
25000
20000
15000
2h
6h
Time (h)
SA564
SA564−Δ*hptRS*
SA564−Δ*ccpA*
SA564−Δ*hptRS/ccpA*

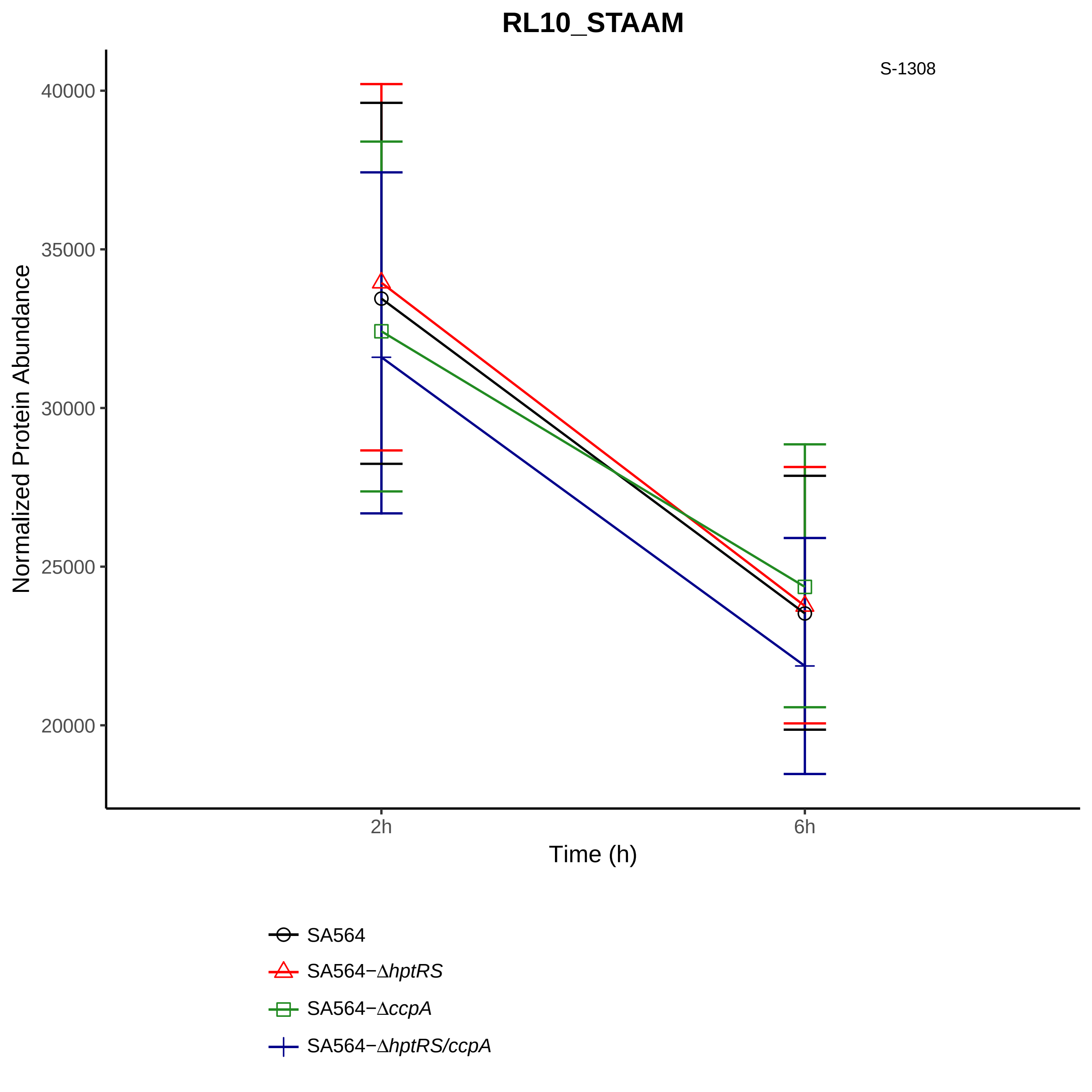
RL10_STAAM
S-1308
Normalized Protein Abundance
40000
35000
30000
25000
20000
2h
6h
Time (h)
SA564
SA564−Δ*hptRS*
SA564−Δ*ccpA*
SA564−Δ*hptRS/ccpA*

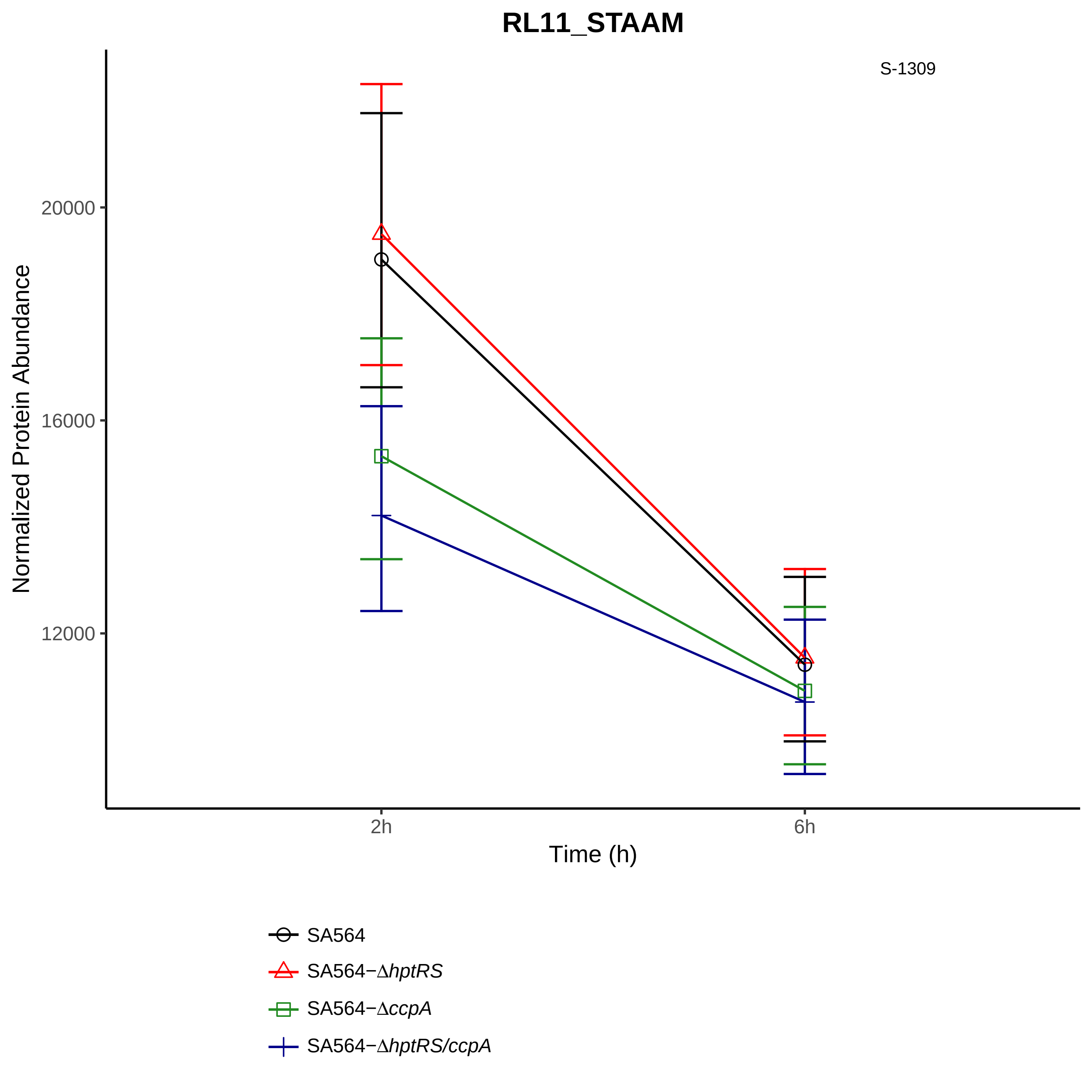

RL11_STAAM
S-1309
20000
16000
12000
Normalized Protein Abundance
2h
6h
Time (h)
SA564
SA564−Δ*hptRS*
SA564−Δ*ccpA*
SA564−Δ*hptRS/ccpA*

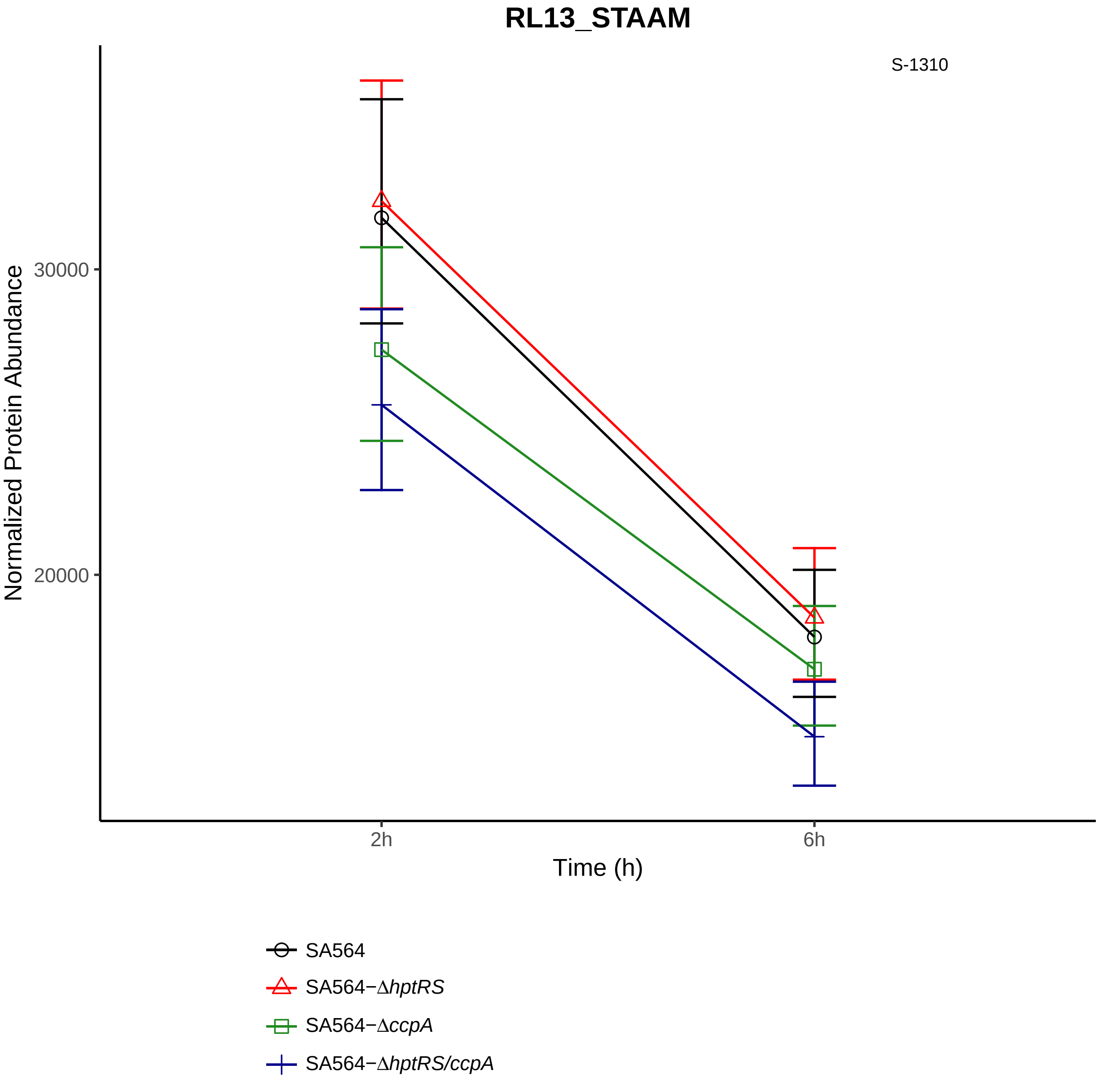
RL13_STAAM
S-1310
Normalized Protein Abundance
30000
20000
2h
6h
Time (h)
SA564
SA564−ΔhptRS
SA564−ΔccpA
SA564−ΔhptRS/ccpA

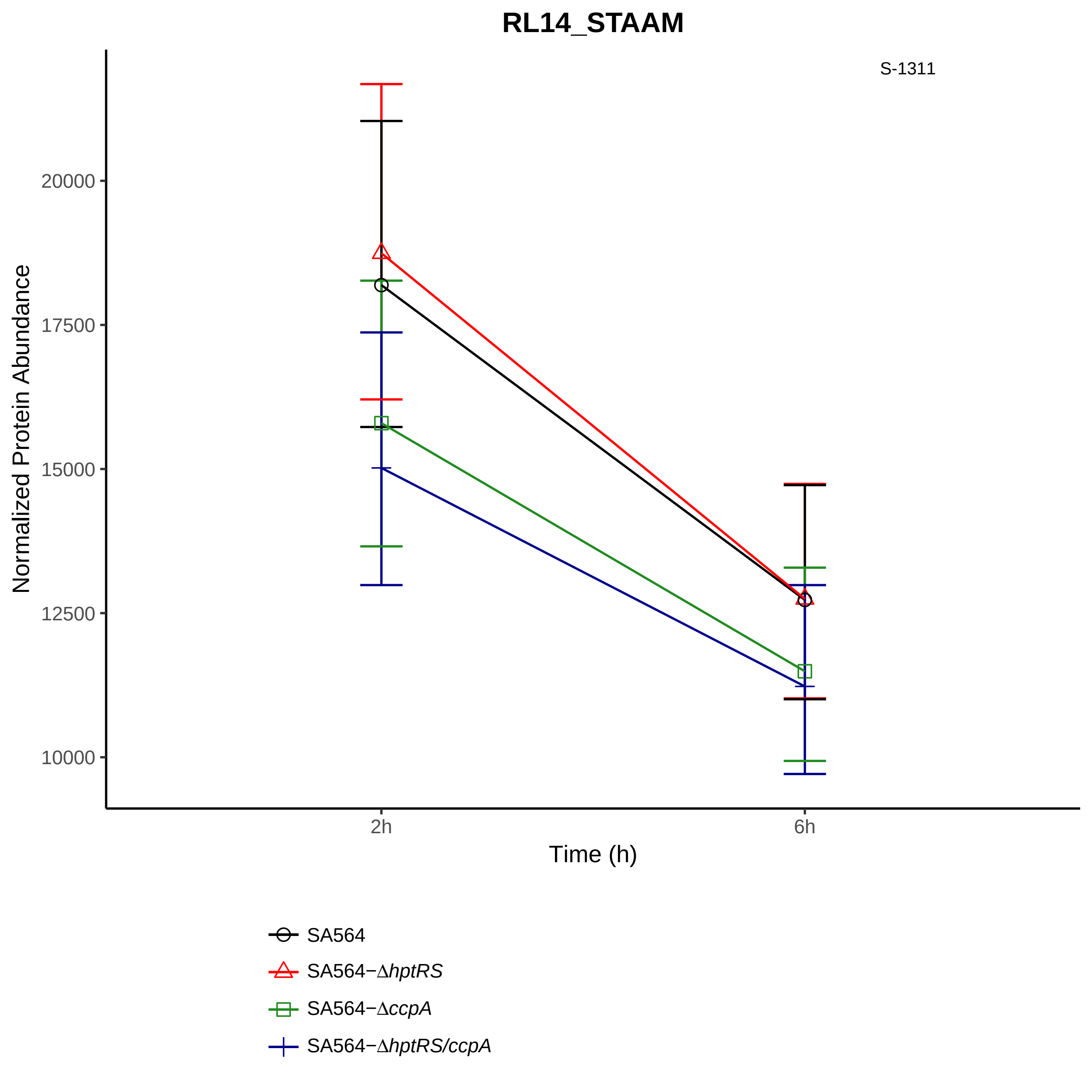

RL14_STAAM
S-1311
Normalized Protein Abundance
20000
17500
15000
12500
10000
2h
6h
Time (h)
SA564
SA564−ΔhptRS
SA564−ΔccpA
SA564−ΔhptRS/ccpA

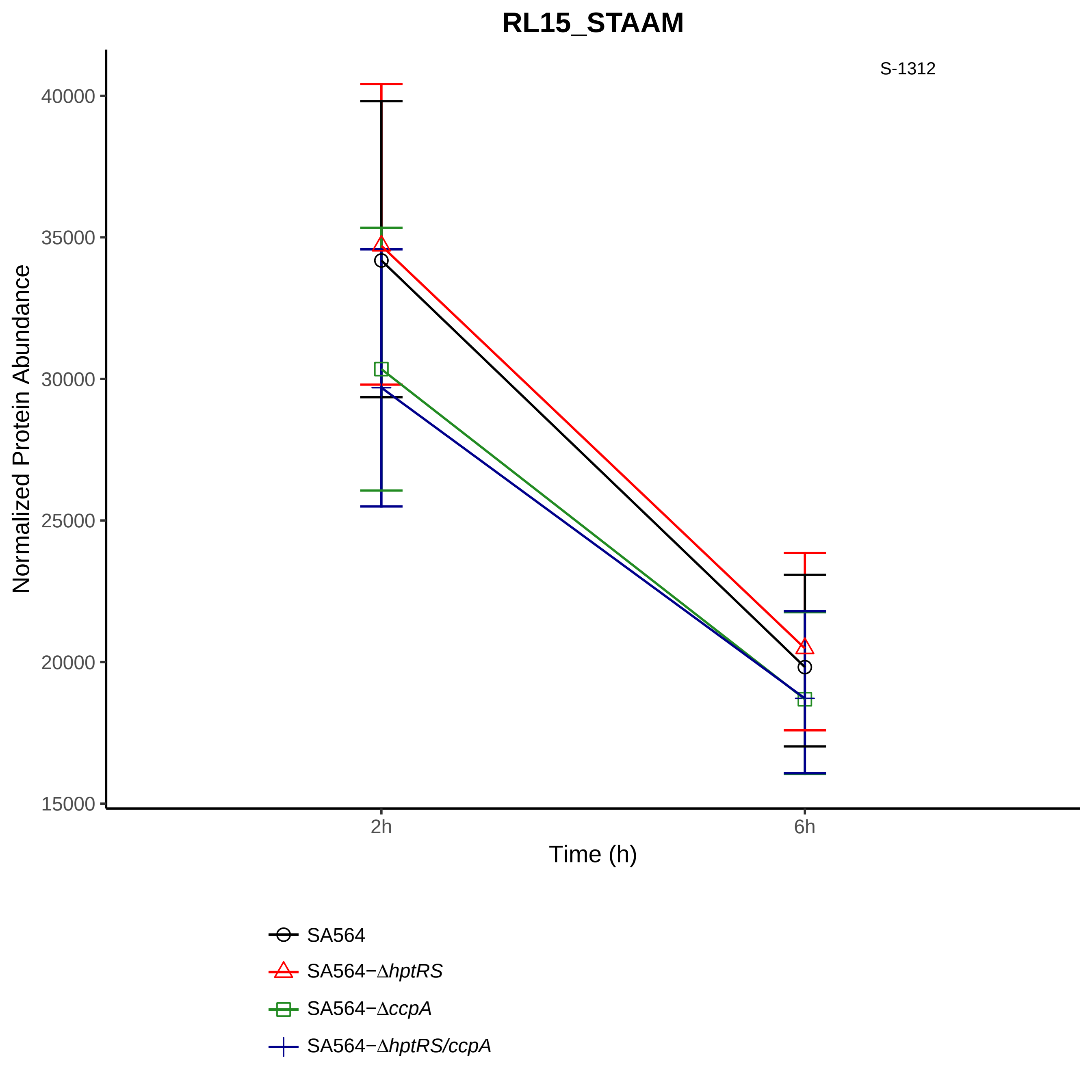

RL15_STAAM
S-1312
Normalized Protein Abundance
40000
35000
30000
25000
20000
15000
2h
6h
Time (h)
SA564
SA564−ΔhptRS
SA564−ΔccpA
SA564−ΔhptRS/ccpA

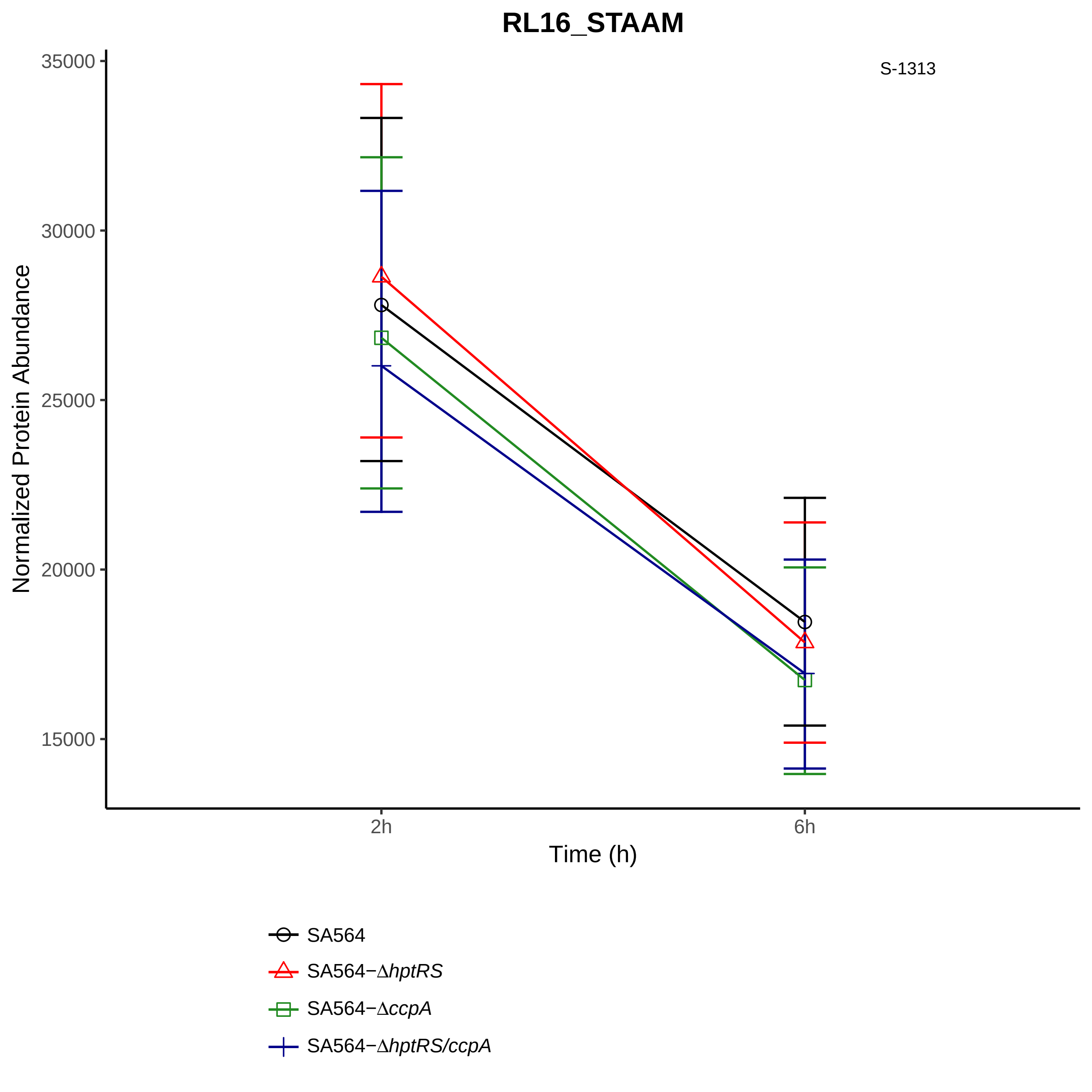
RL16_STAAM
S-1313
Normalized Protein Abundance
35000
30000
25000
20000
15000
2h
6h
Time (h)
SA564
SA564−Δ*hptRS*
SA564−Δ*ccpA*
SA564−Δ*hptRS/ccpA*

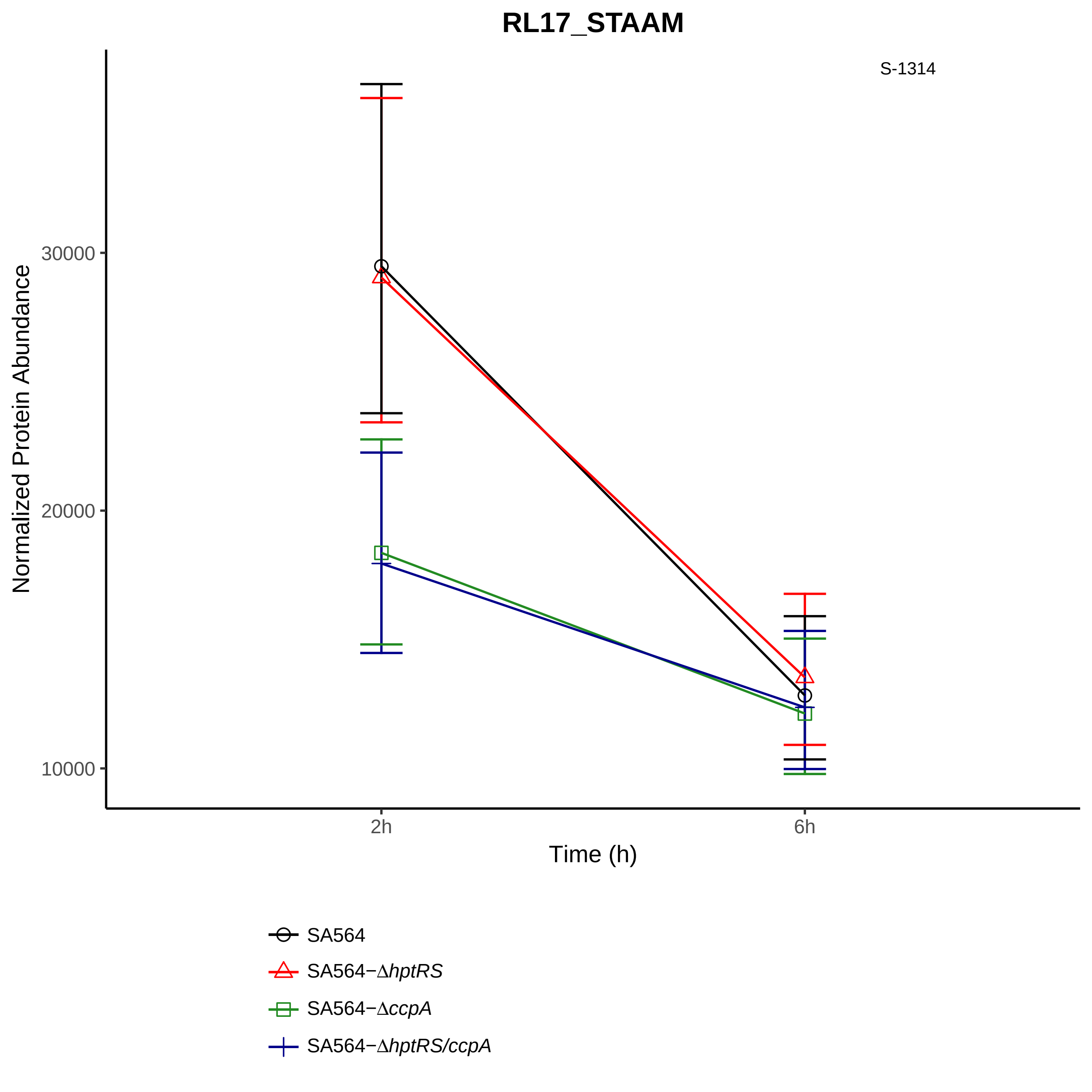

RL17_STAAM
S-1314
Normalized Protein Abundance
30000
20000
10000
2h
6h
Time (h)
SA564
SA564−Δ*hptRS*
SA564−Δ*ccpA*
SA564−Δ*hptRS/ccpA*

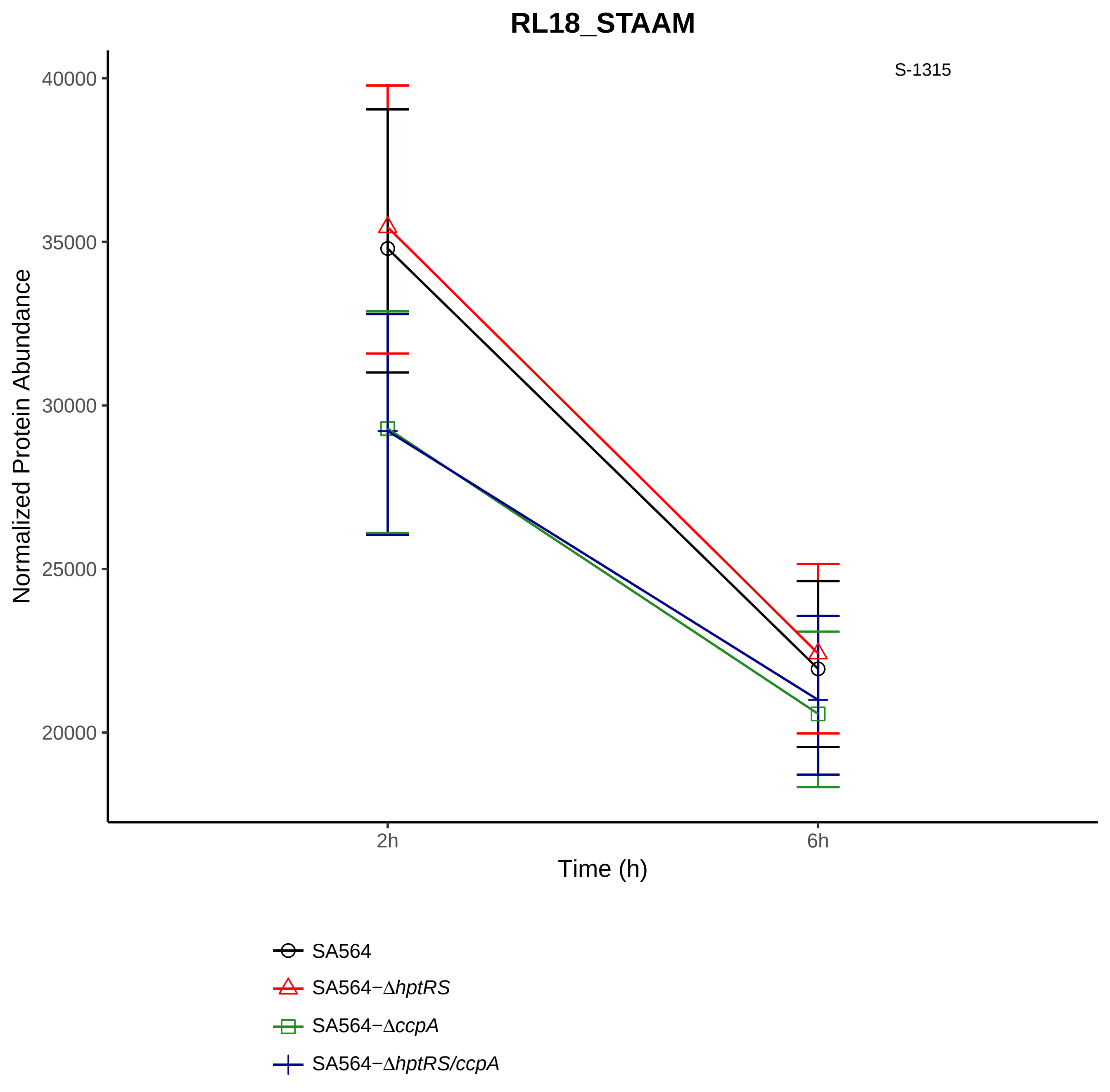

RL18_STAAM
S-1315
40000
35000
30000
25000
20000
Normalized Protein Abundance
2h
6h
Time (h)
SA564
SA564−Δ*hptRS*
SA564−Δ*ccpA*
SA564−Δ*hptRS/ccpA*

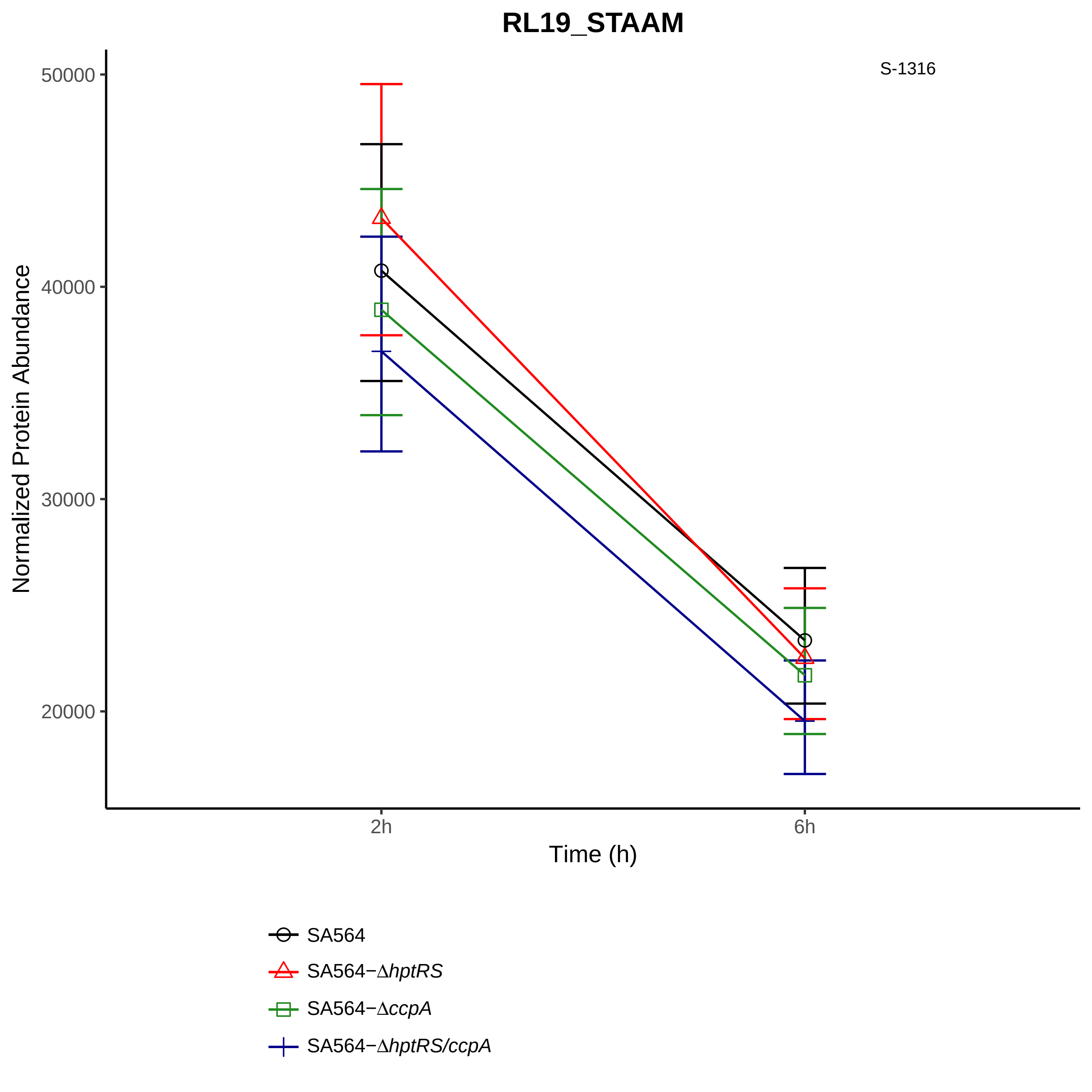

RL19_STAAM
S-1316
Normalized Protein Abundance
50000
40000
30000
20000
2h
6h
Time (h)
SA564
SA564−ΔhptRS
SA564−ΔccpA
SA564−ΔhptRS/ccpA

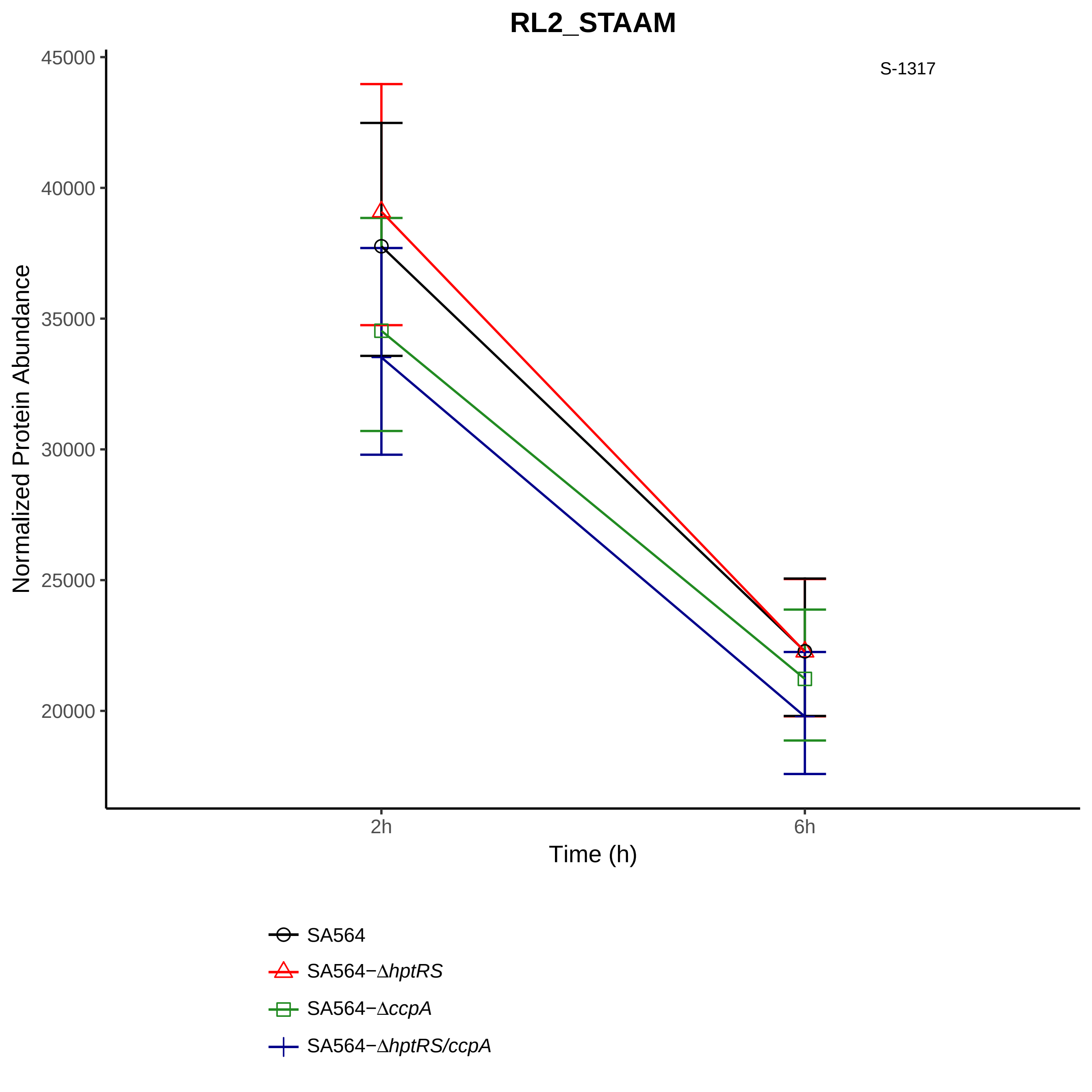

RL2_STAAM
S-1317
Normalized Protein Abundance
45000
40000
35000
30000
25000
20000
2h
6h
Time (h)
SA564
SA564−Δ*hptRS*
SA564−Δ*ccpA*
SA564−Δ*hptRS/ccpA*

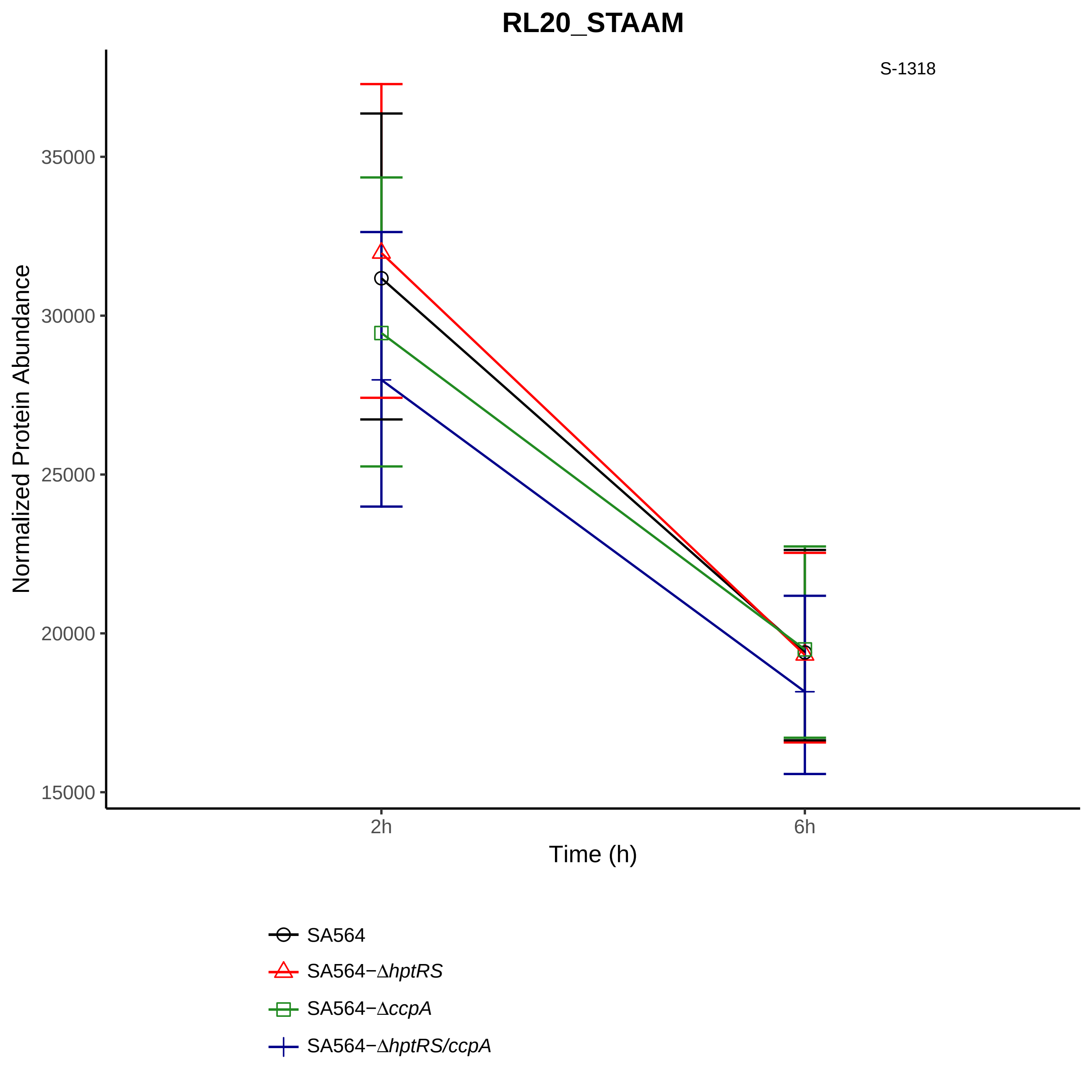

RL20_STAAM
S-1318
Normalized Protein Abundance
35000
30000
25000
20000
15000
2h
6h
Time (h)
SA564
SA564−Δ*hptRS*
SA564−Δ*ccpA*
SA564−Δ*hptRS/ccpA*

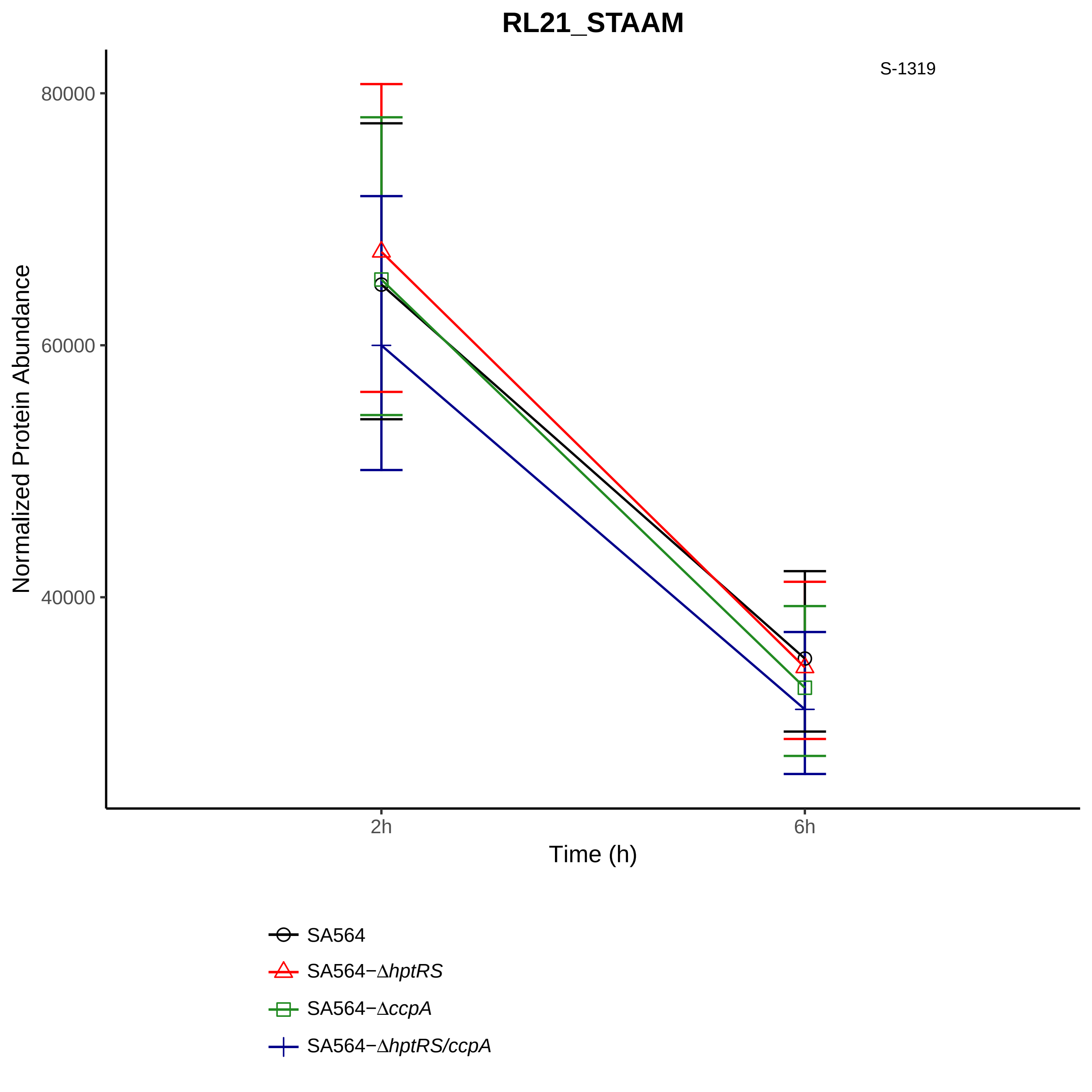
RL21_STAAM
S-1319
80000
60000
40000
Normalized Protein Abundance
2h
6h
Time (h)
SA564
SA564−ΔhptRS
SA564−ΔccpA
SA564−ΔhptRS/ccpA

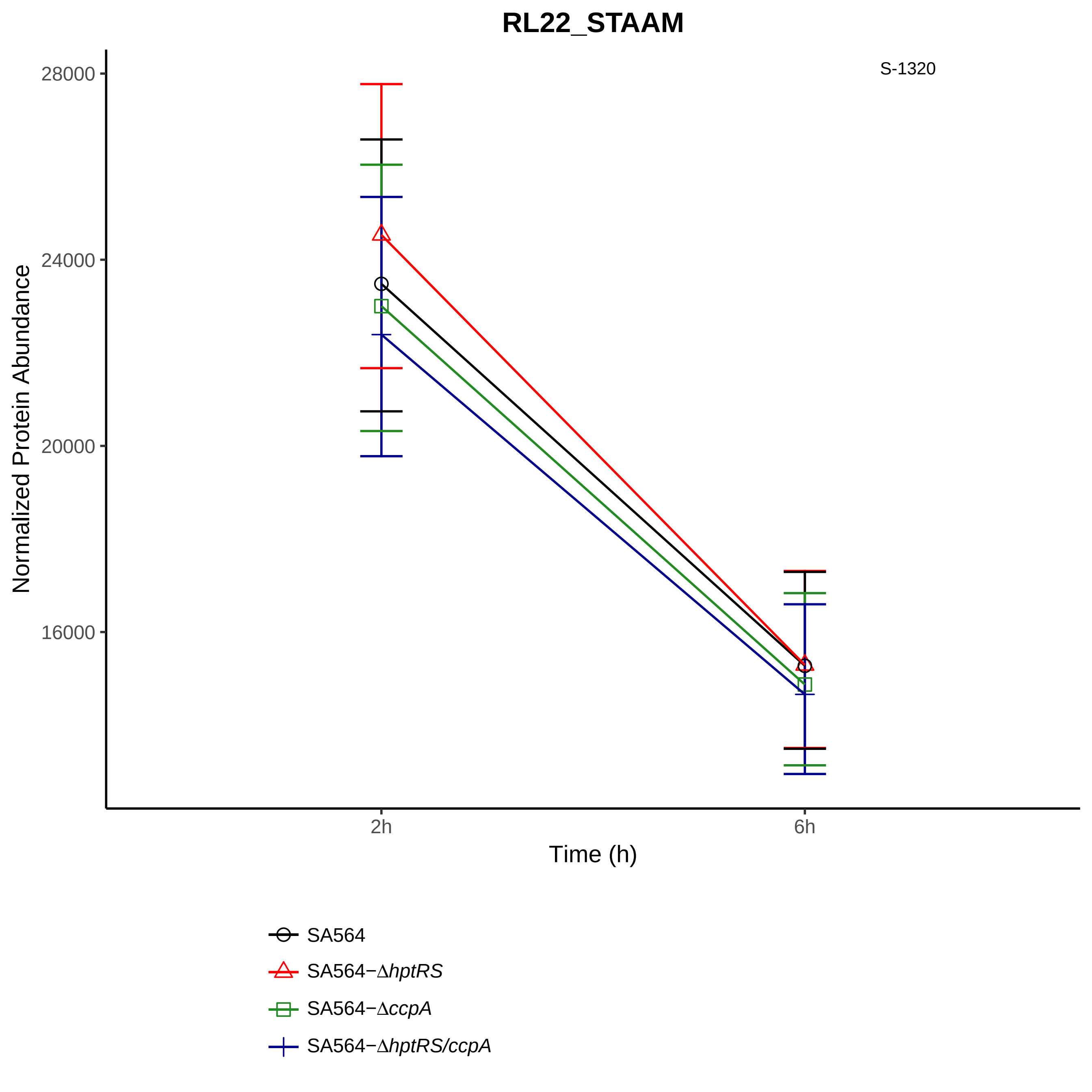
RL22_STAAM
S-1320
Normalized Protein Abundance
28000
24000
20000
16000
2h
6h
Time (h)
SA564
SA564−Δ*hptRS*
SA564−Δ*ccpA*
SA564−Δ*hptRS/ccpA*

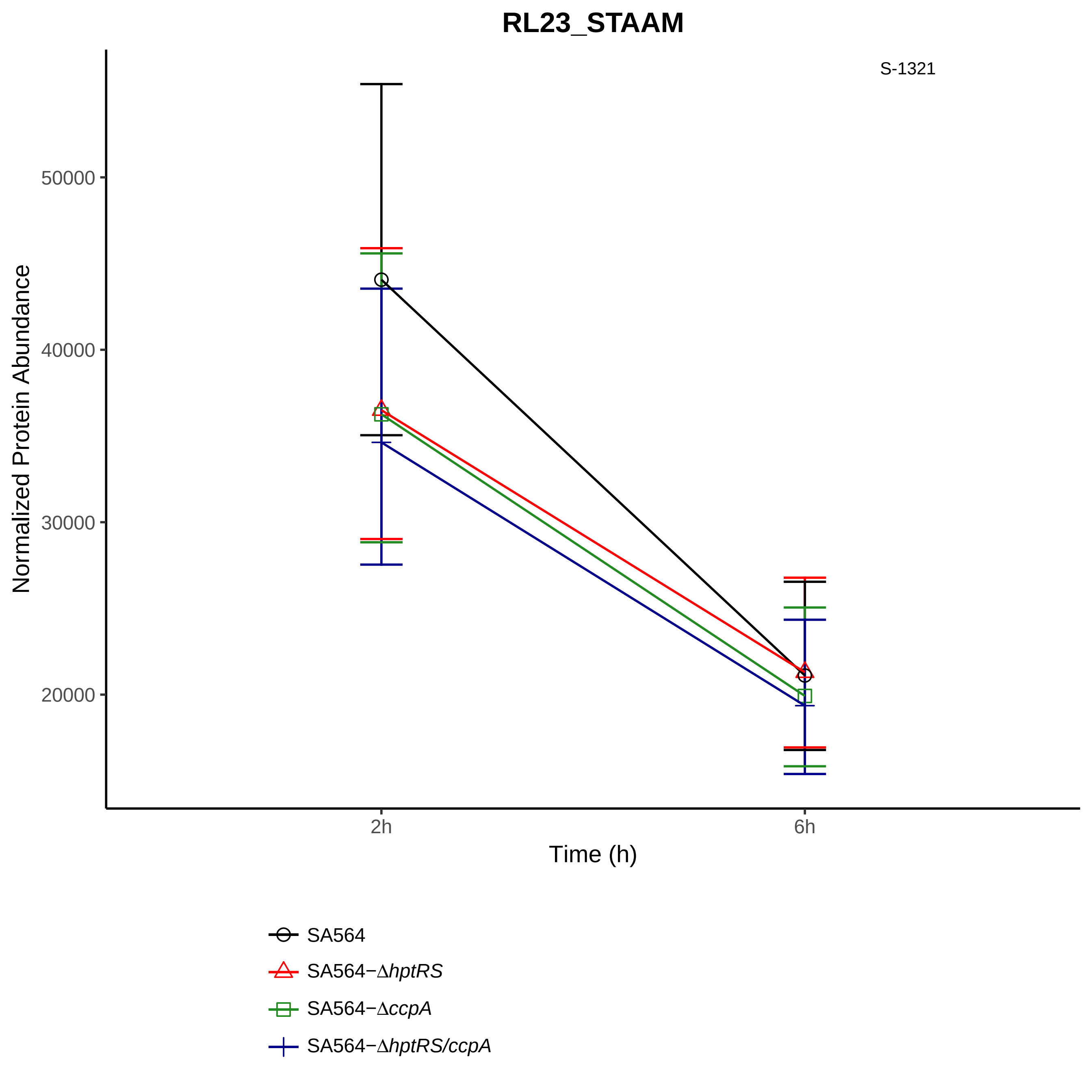

RL23_STAAM
S-1321
Normalized Protein Abundance
50000
40000
30000
20000
2h
6h
Time (h)
SA564
SA564−Δ*hptRS*
SA564−Δ*ccpA*
SA564−Δ*hptRS/ccpA*

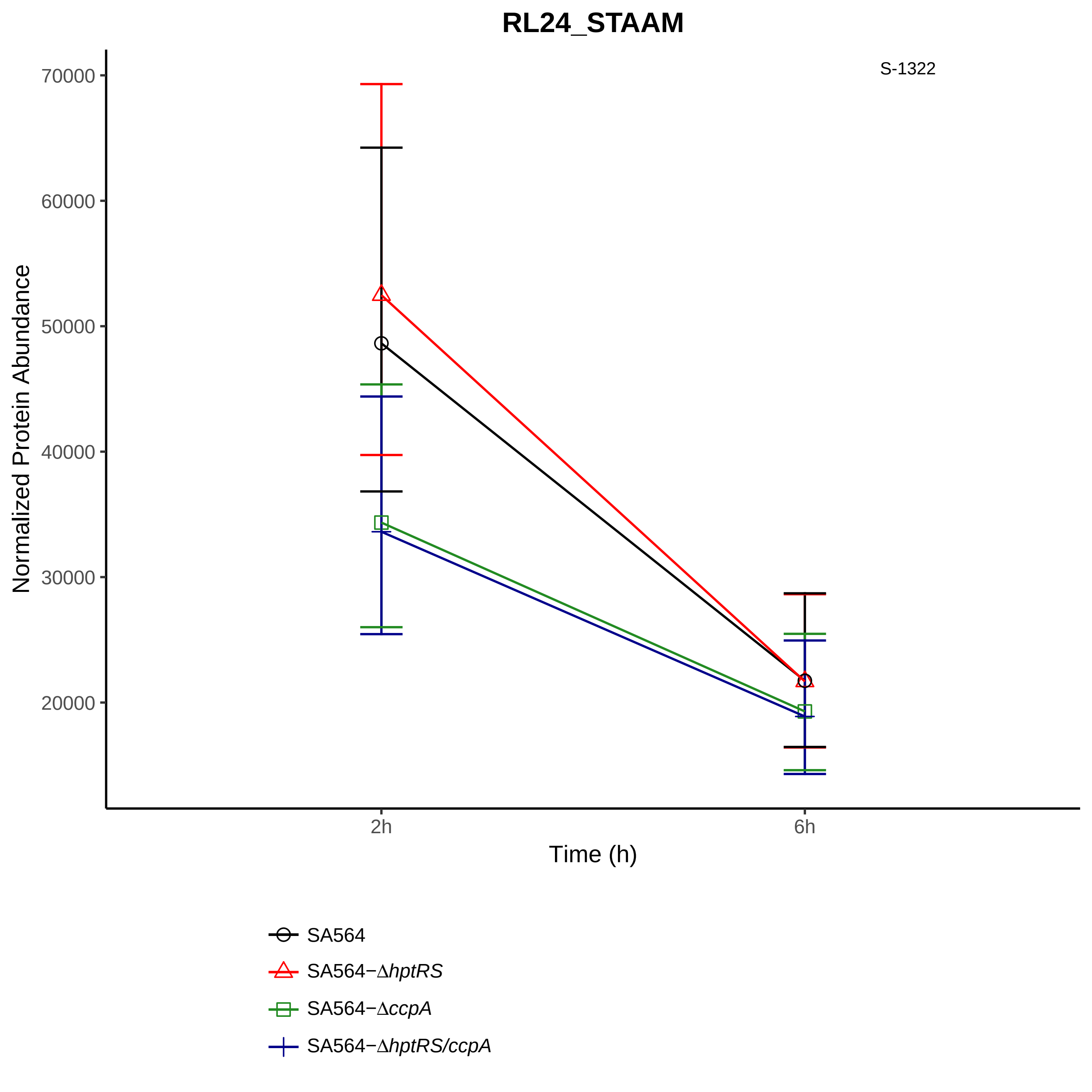

RL24_STAAM
S-1322
Normalized Protein Abundance
70000
60000
50000
40000
30000
20000
2h
6h
Time (h)
SA564
SA564−Δ*hptRS*
SA564−Δ*ccpA*
SA564−Δ*hptRS/ccpA*

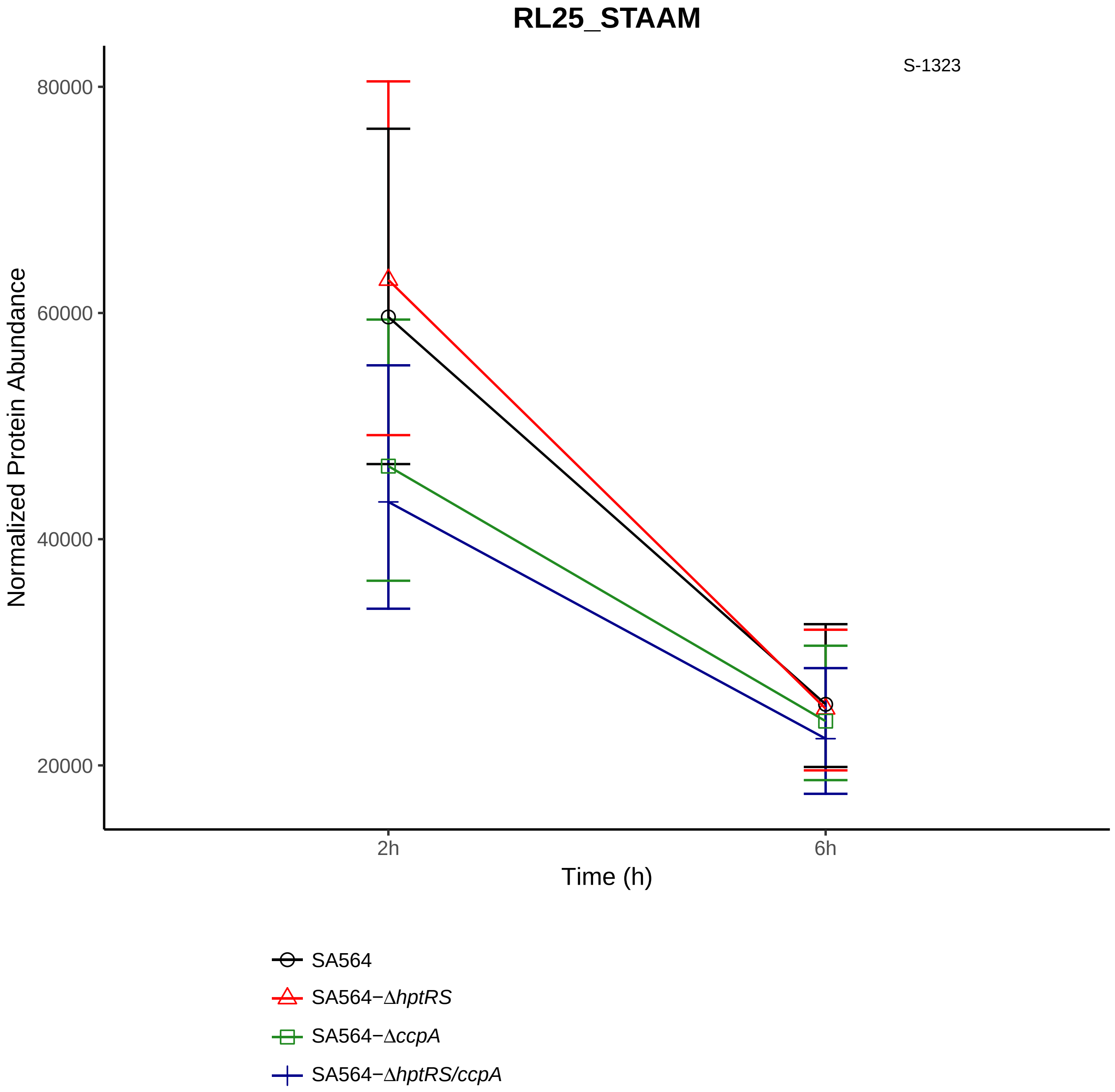

RL25_STAAM
S-1323
Normalized Protein Abundance
80000
60000
40000
20000
2h
6h
Time (h)
SA564
SA564−Δ*hptRS*
SA564−Δ*ccpA*
SA564−Δ*hptRS/ccpA*

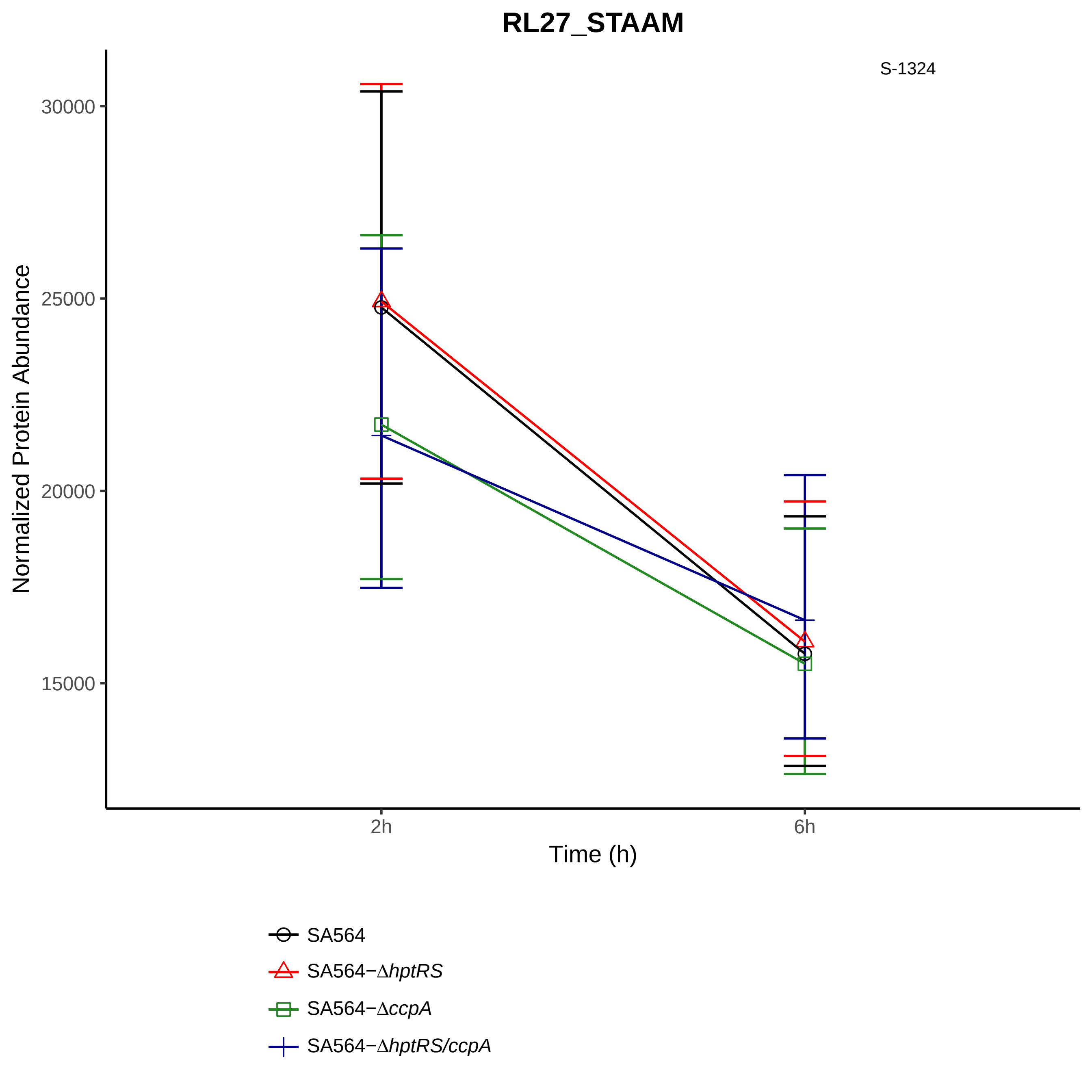

RL27_STAAM
S-1324
Normalized Protein Abundance
30000
25000
20000
15000
2h
6h
Time (h)
SA564
SA564−Δ*hptRS*
SA564−Δ*ccpA*
SA564−Δ*hptRS/ccpA*

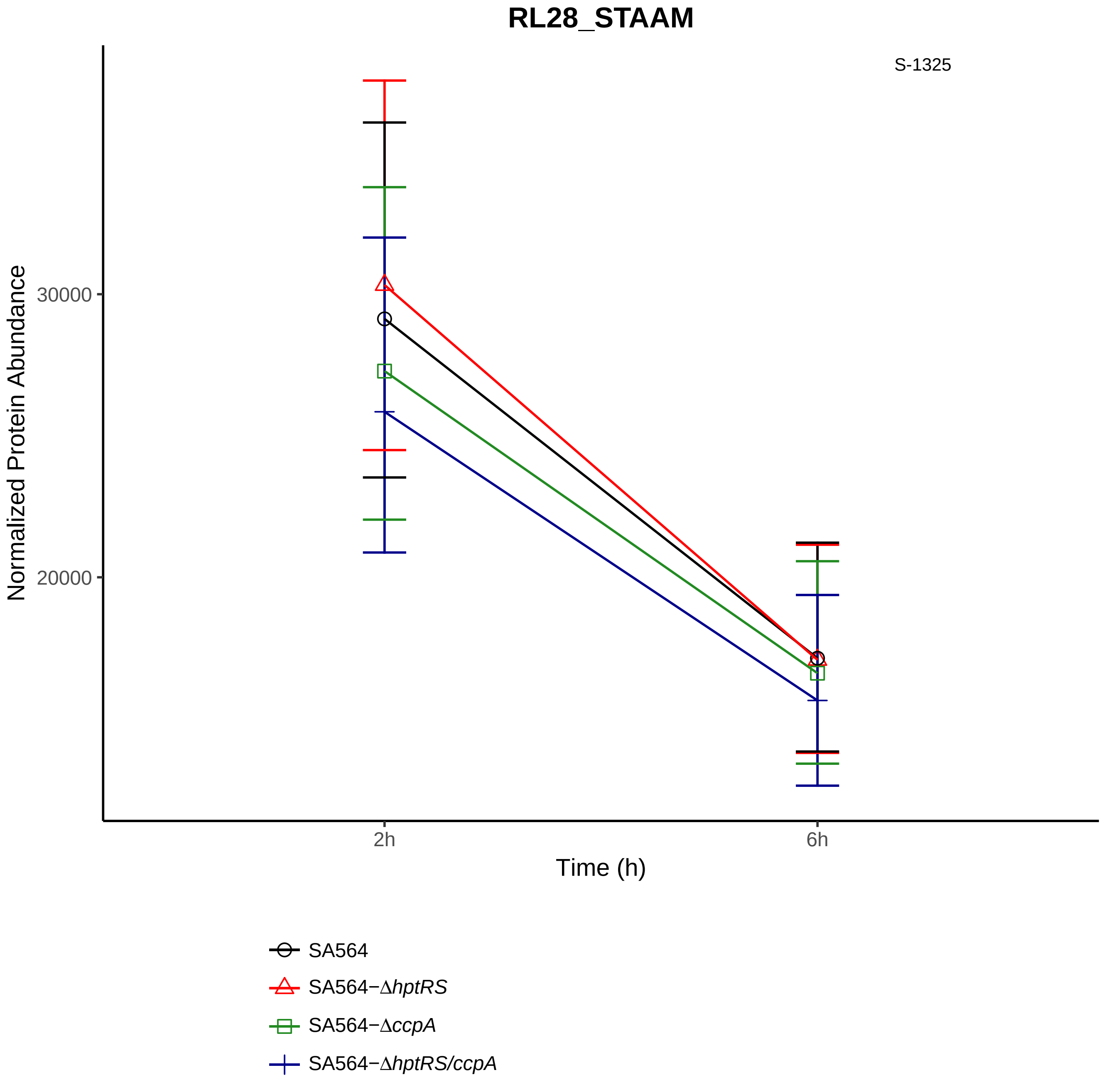

RL28_STAAM
S-1325
Normalized Protein Abundance
30000
20000
2h
6h
Time (h)
SA564
SA564−ΔhptRS
SA564−ΔccpA
SA564−ΔhptRS/ccpA

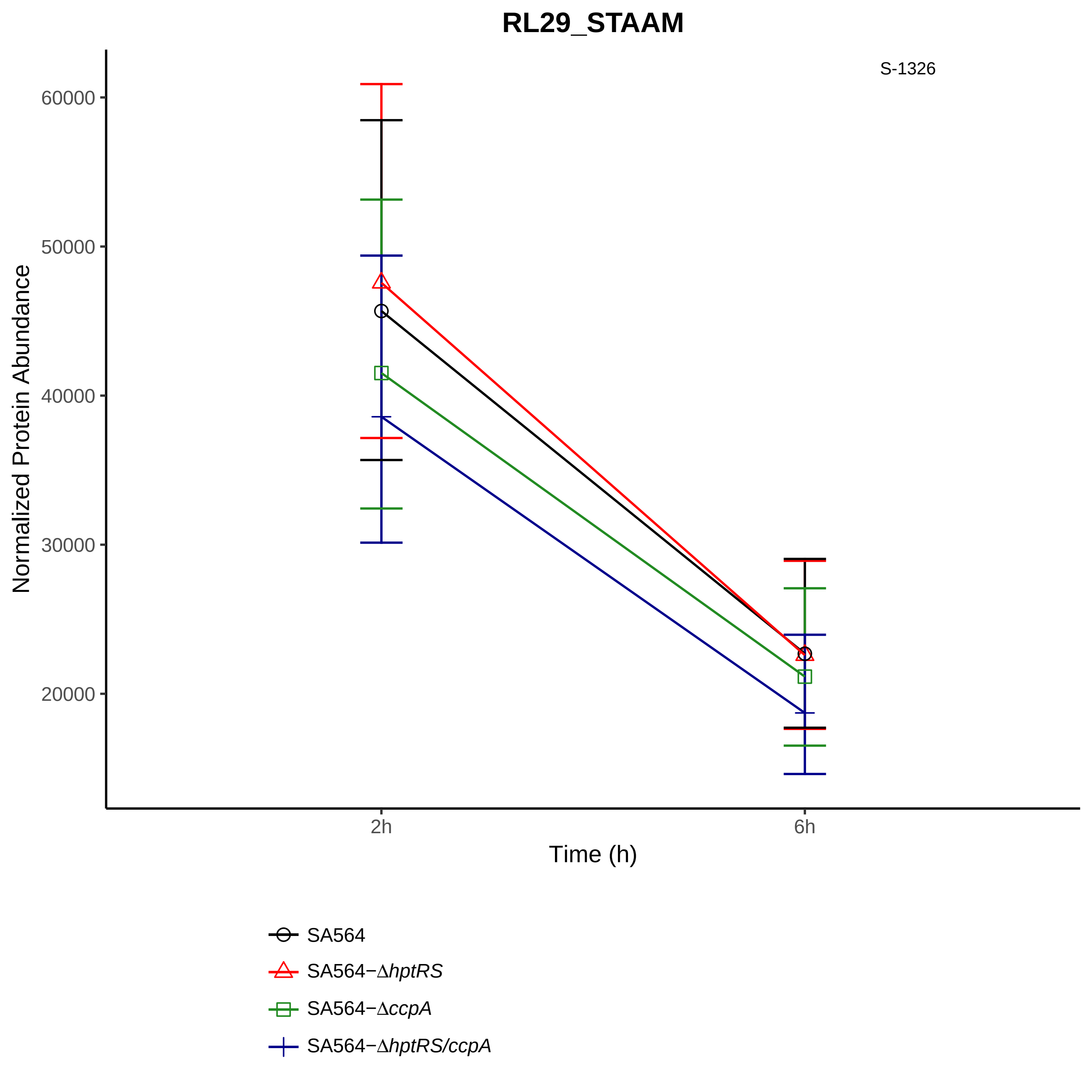

RL29_STAAM
S-1326
Normalized Protein Abundance
60000
50000
40000
30000
20000
2h
6h
Time (h)
SA564
SA564−Δ*hptRS*
SA564−Δ*ccpA*
SA564−Δ*hptRS/ccpA*

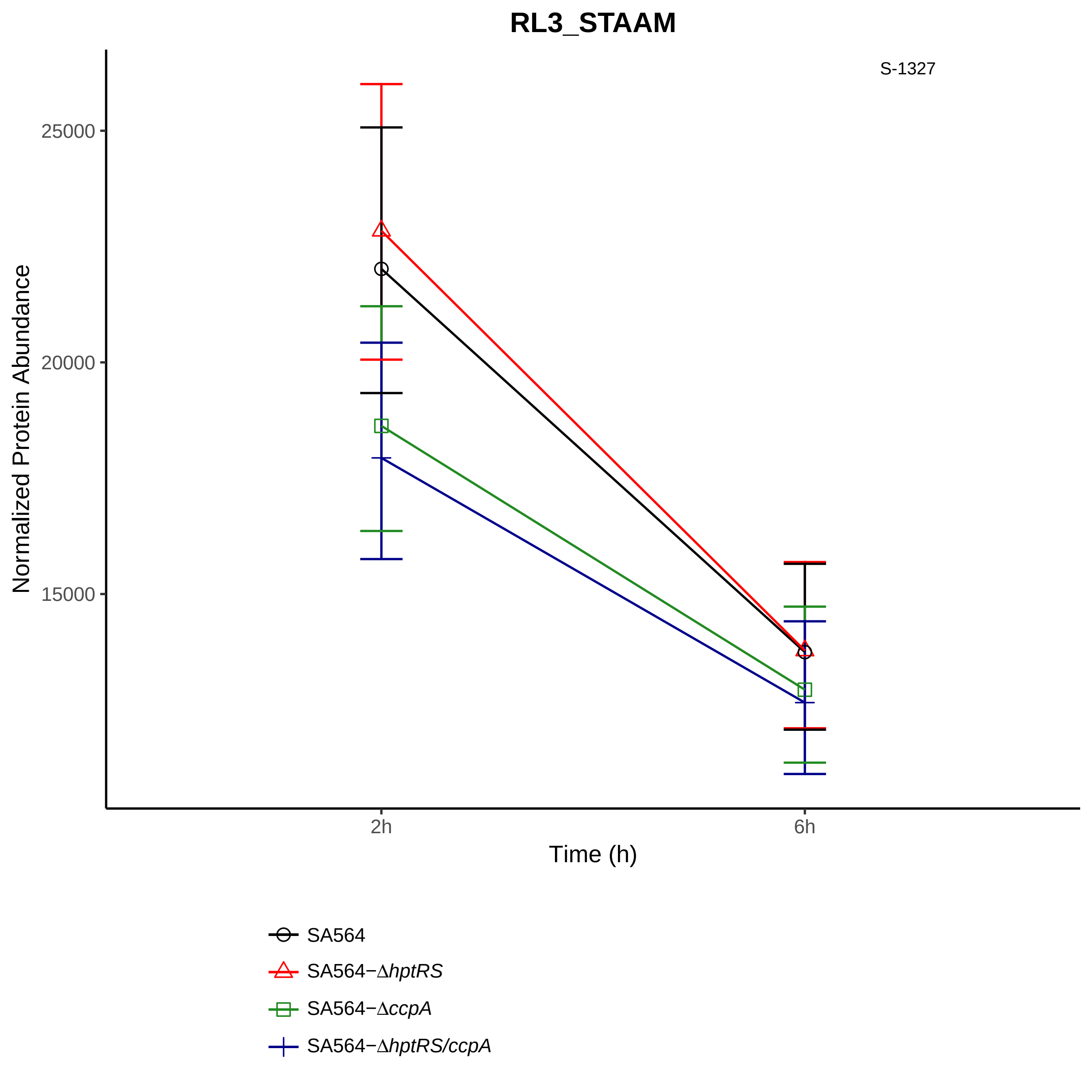
RL3_STAAM
S-1327
Normalized Protein Abundance
25000
20000
15000
2h
6h
Time (h)
SA564
SA564−Δ*hptRS*
SA564−Δ*ccpA*
SA564−Δ*hptRS/ccpA*

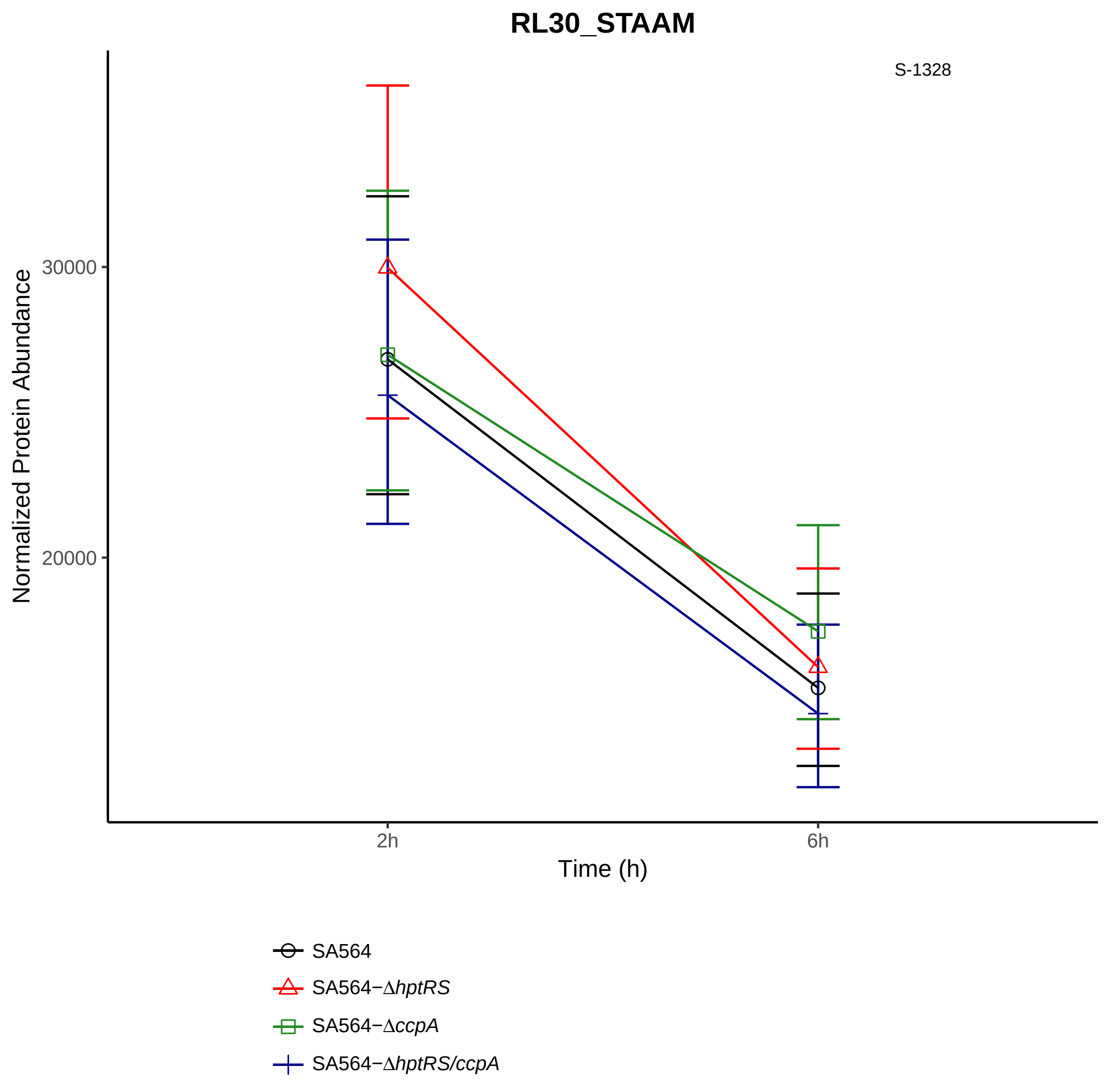

RL30_STAAM
S-1328
30000
20000
Normalized Protein Abundance
2h
6h
Time (h)
SA564
SA564−Δ*hptRS*
SA564−Δ*ccpA*
SA564−Δ*hptRS/ccpA*

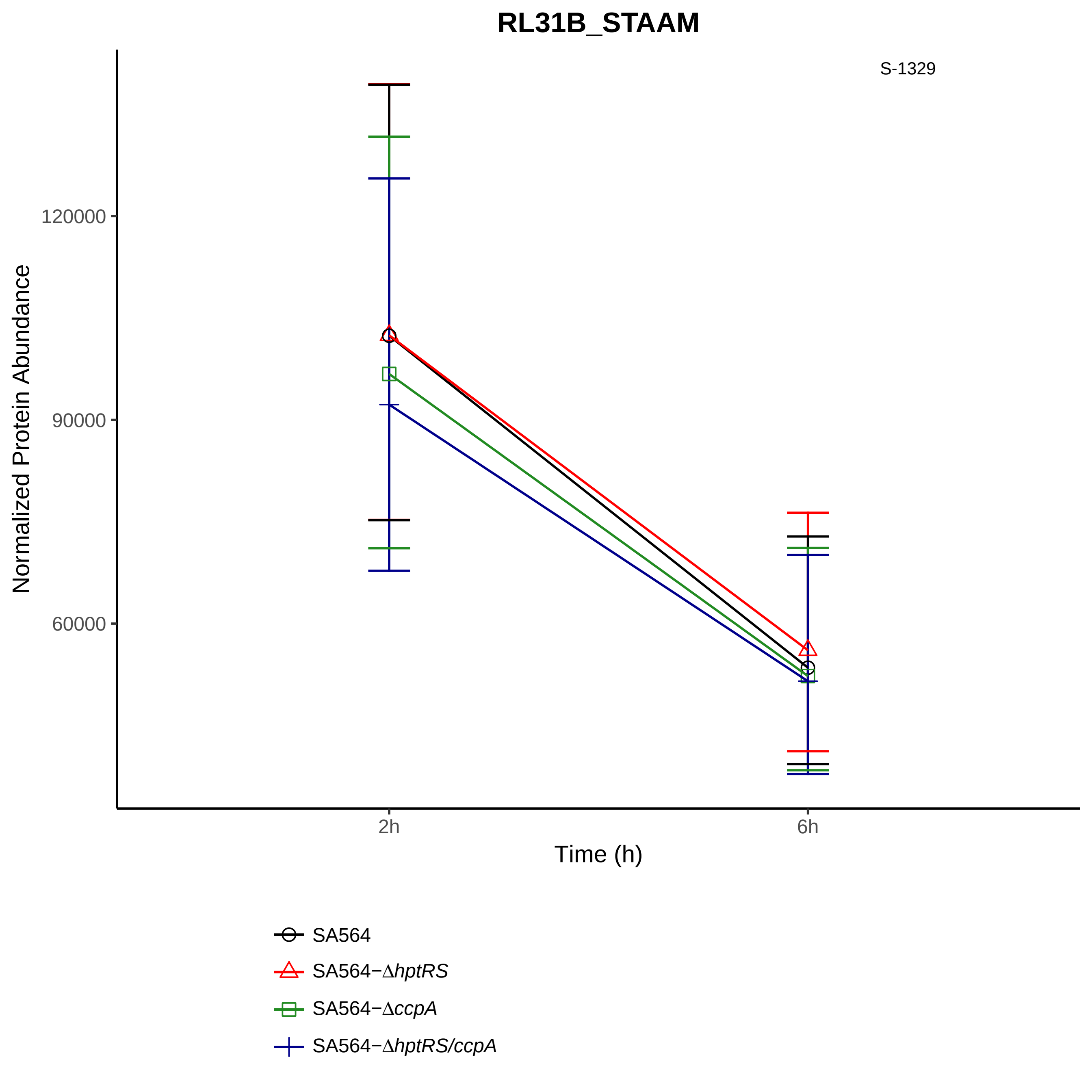

RL31B_STAAM
S-1329
Normalized Protein Abundance
120000
90000
60000
2h
6h
Time (h)
SA564
SA564−Δ*hptRS*
SA564−Δ*ccpA*
SA564−Δ*hptRS/ccpA*

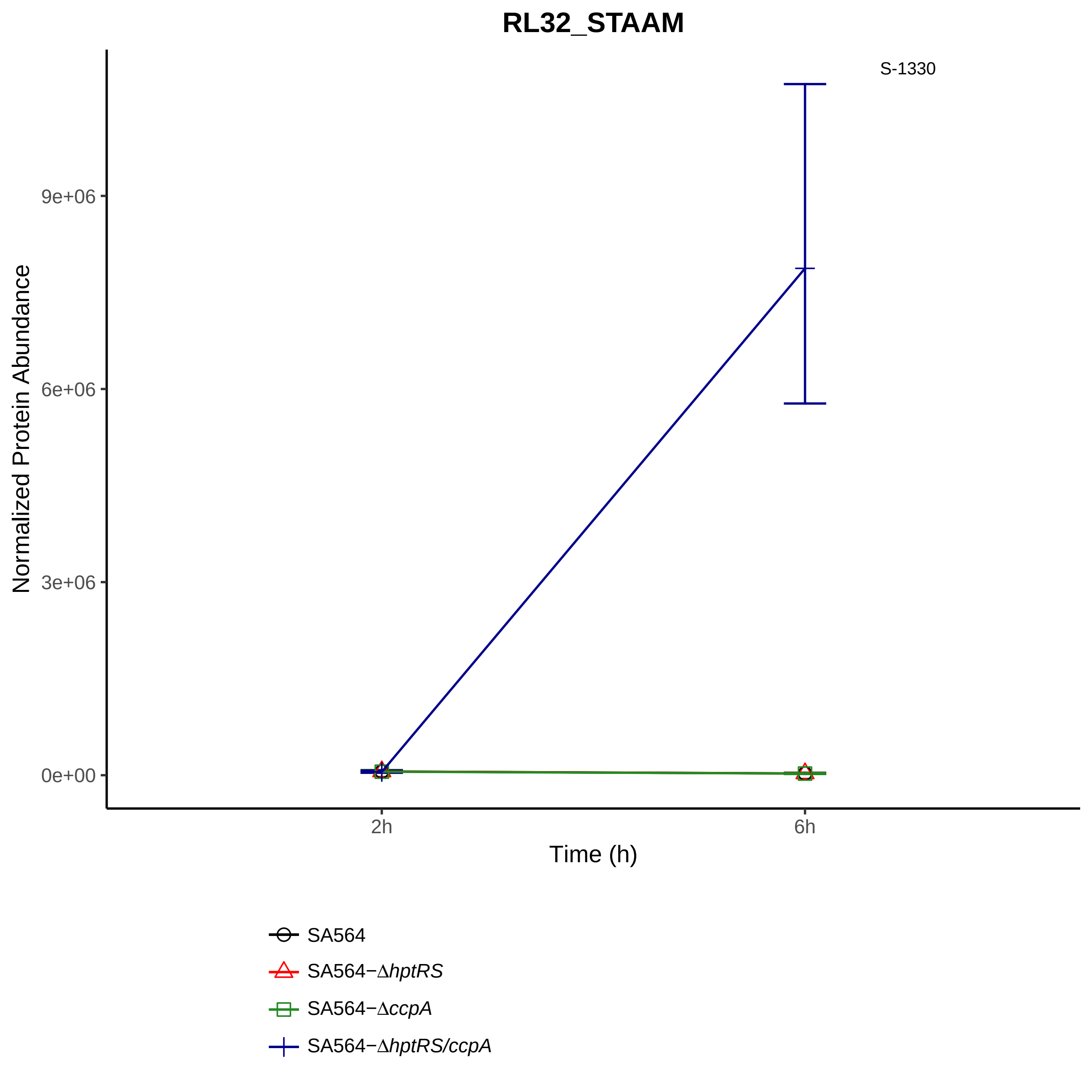

RL32_STAAM
S-1330
Normalized Protein Abundance
9e+06
6e+06
3e+06
0e+00
2h
6h
Time (h)
SA564
SA564−Δ*hptRS*
SA564−Δ*ccpA*
SA564−Δ*hptRS/ccpA*

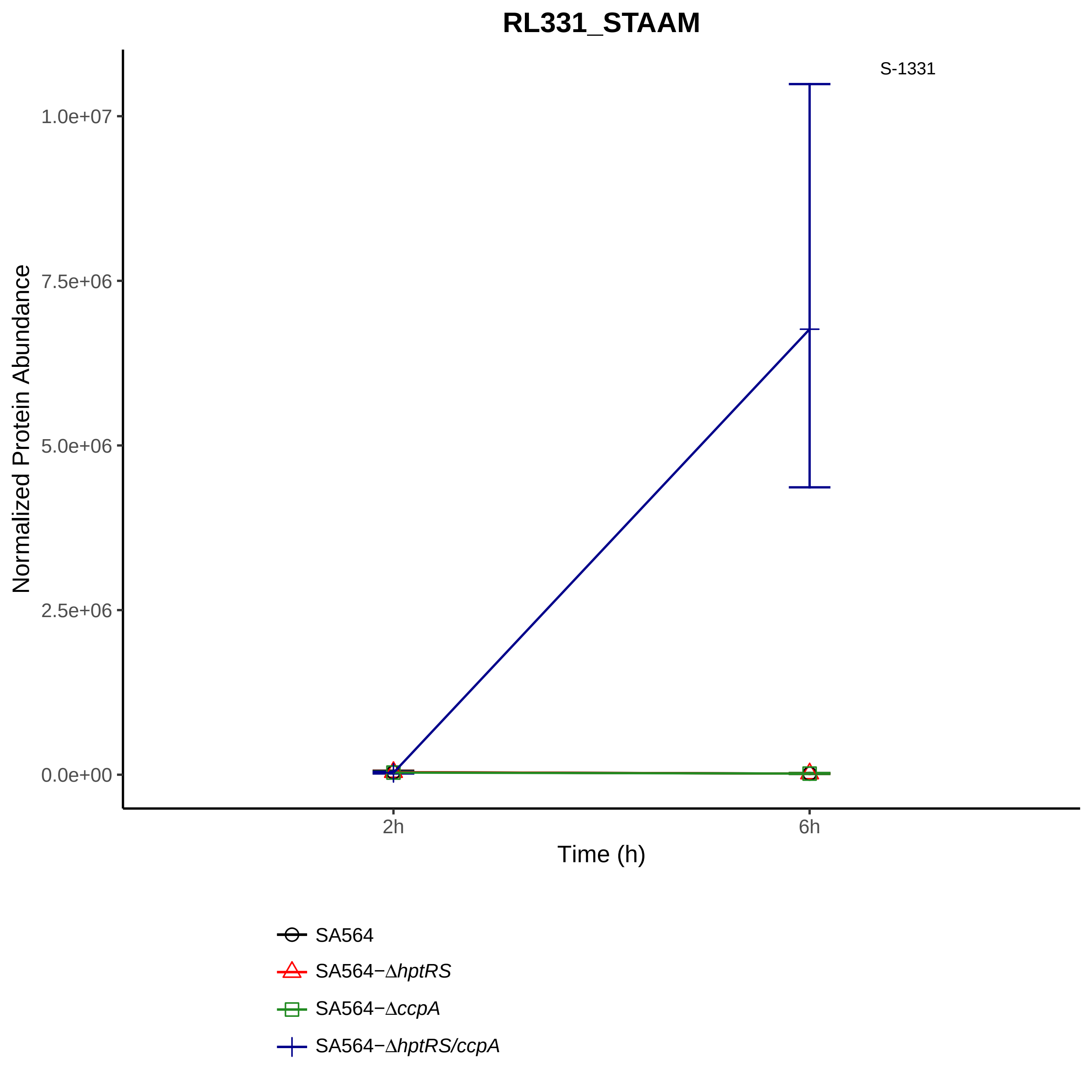

RL331_STAAM
S-1331
1.0e+07
7.5e+06
5.0e+06
2.5e+06
0.0e+00
Normalized Protein Abundance
2h
6h
Time (h)
SA564
SA564−ΔhptRS
SA564−ΔccpA
SA564−ΔhptRS/ccpA

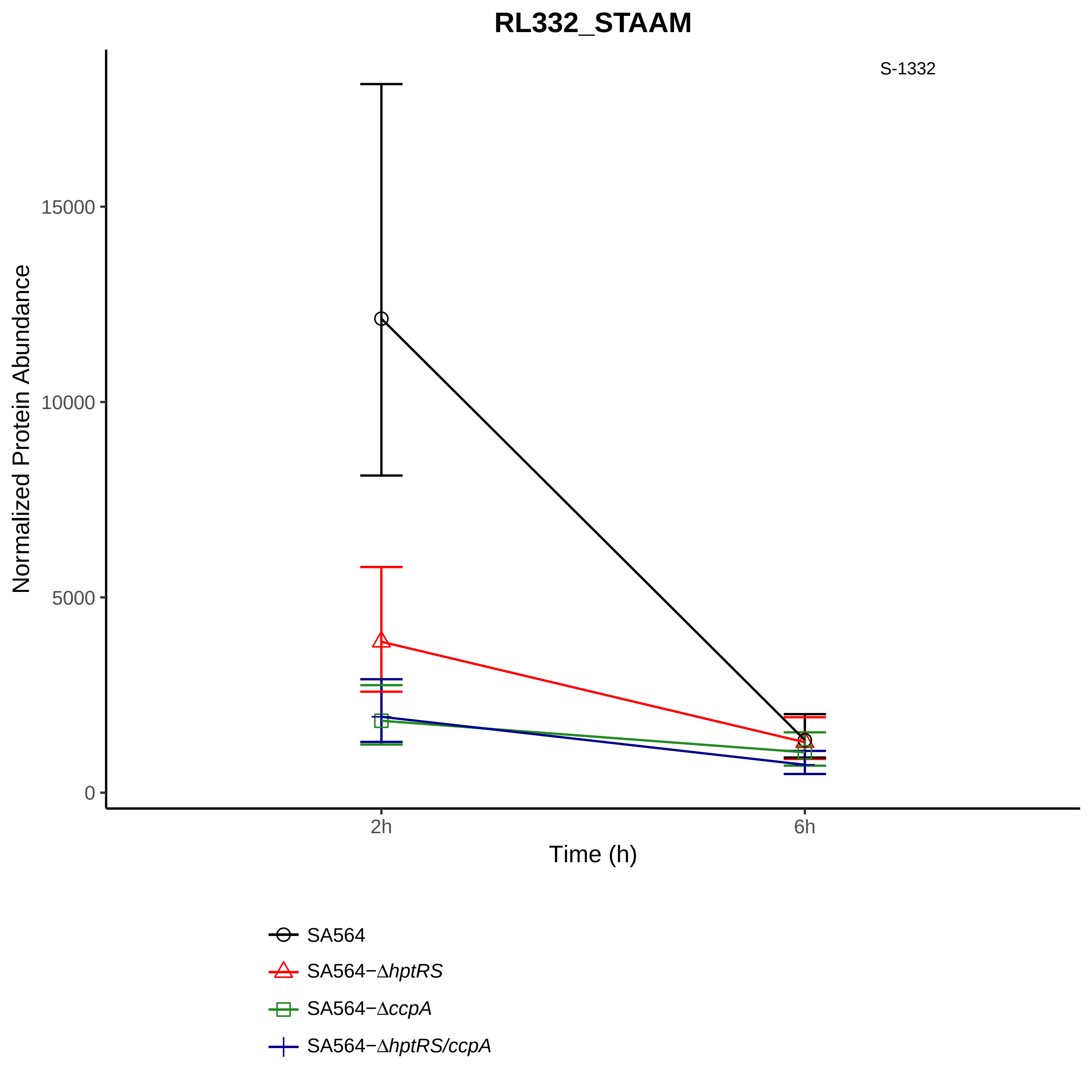

RL332_STAAM
S-1332
Normalized Protein Abundance
15000
10000
5000
0
2h
6h
Time (h)
SA564
SA564−Δ*hptRS*
SA564−Δ*ccpA*
SA564−Δ*hptRS/ccpA*

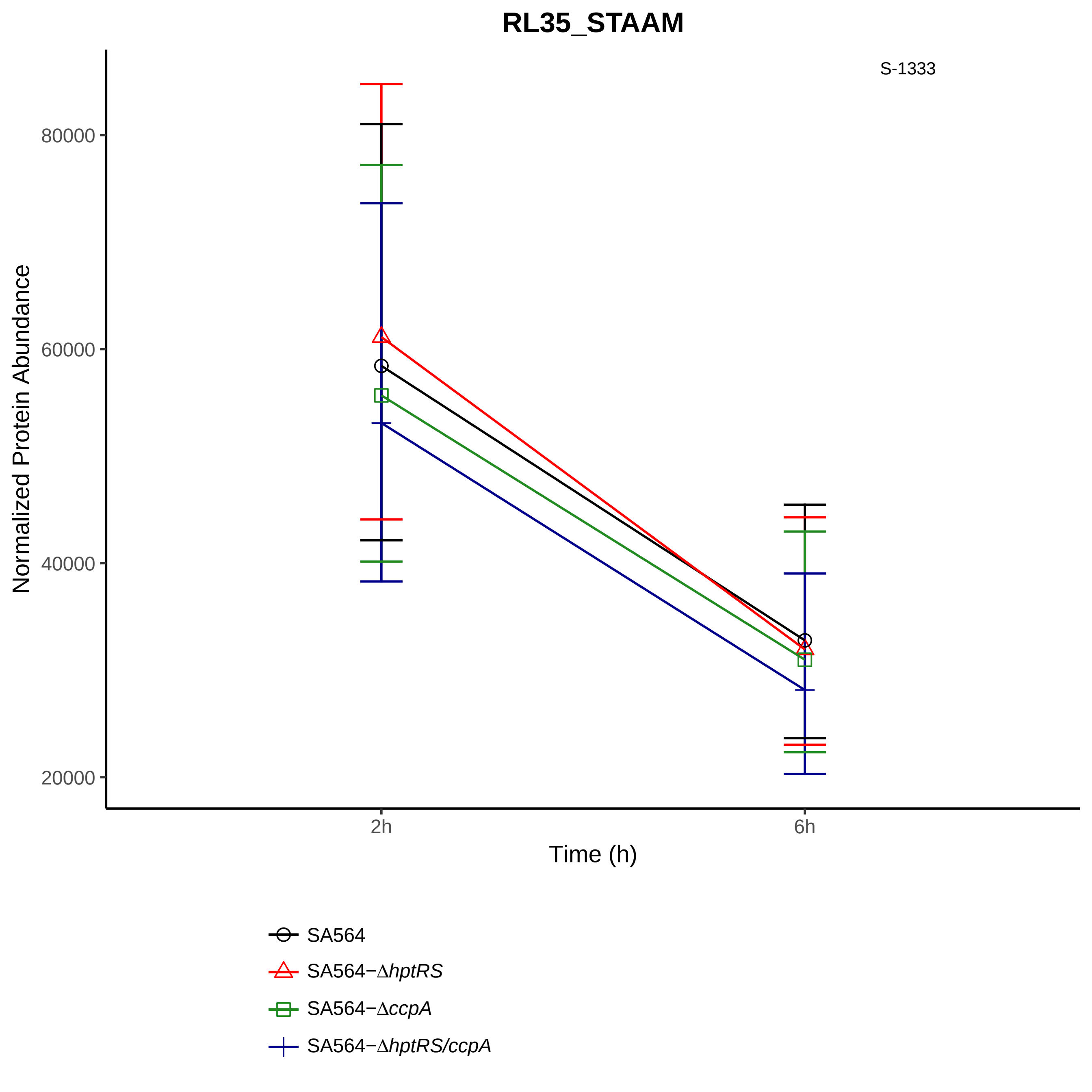

RL35_STAAM
S-1333
Normalized Protein Abundance
80000
60000
40000
20000
2h
6h
Time (h)
SA564
SA564−Δ*hptRS*
SA564−Δ*ccpA*
SA564−Δ*hptRS/ccpA*

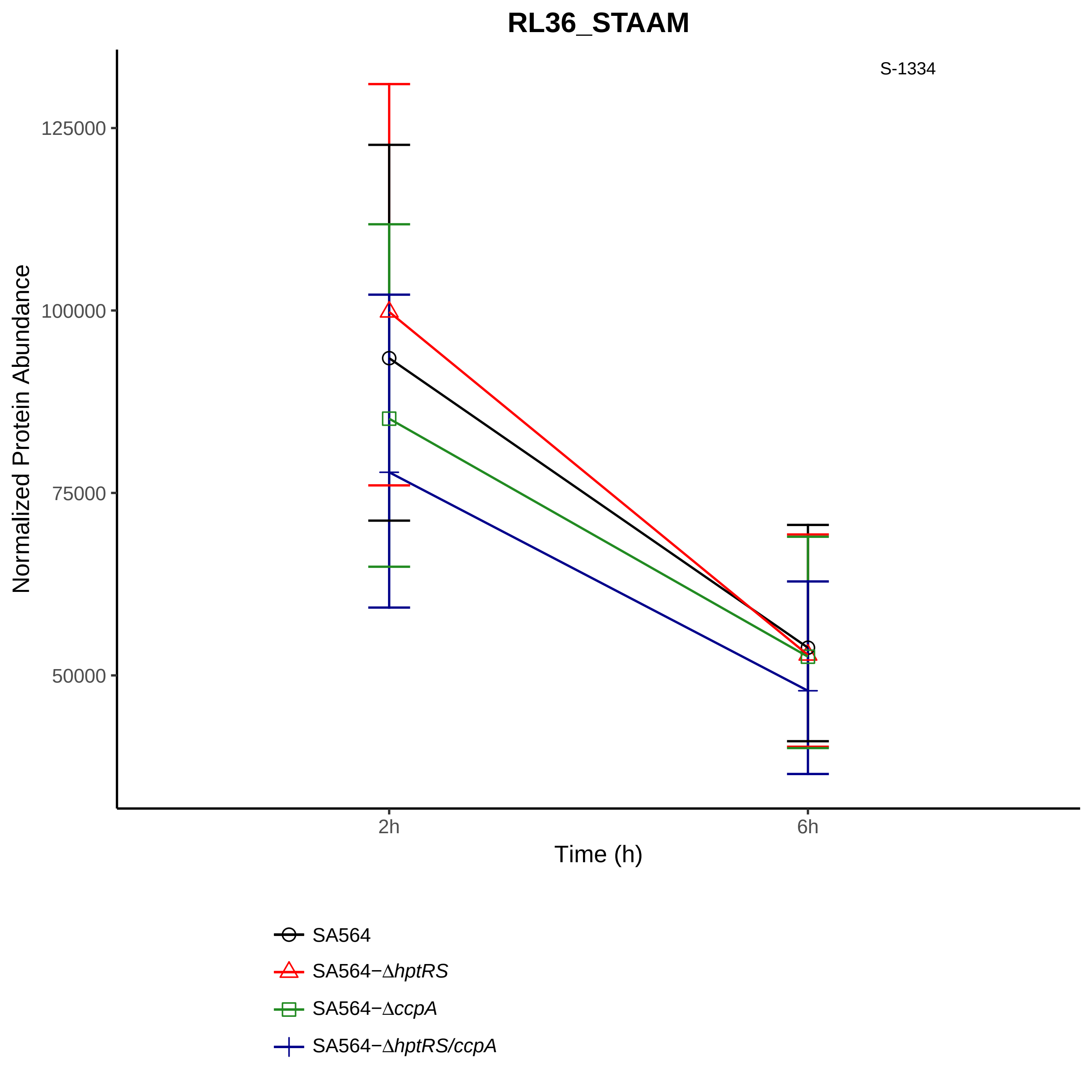

RL36_STAAM
S-1334
Normalized Protein Abundance
125000
100000
75000
50000
2h
6h
Time (h)
SA564
SA564−Δ*hptRS*
SA564−Δ*ccpA*
SA564−Δ*hptRS/ccpA*

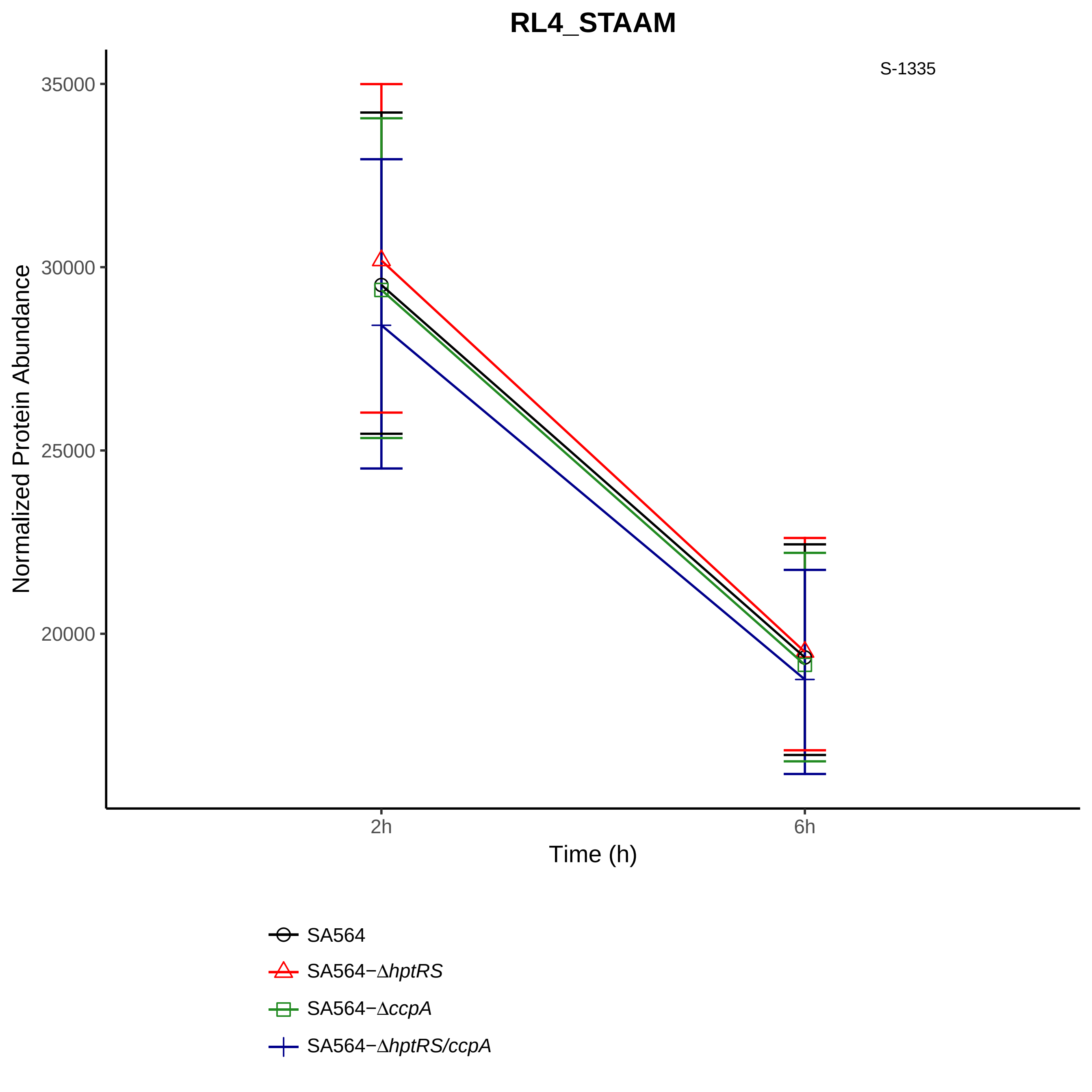

RL4_STAAM
S-1335
35000
30000
25000
20000
Normalized Protein Abundance
2h
6h
Time (h)
SA564
SA564−Δ*hptRS*
SA564−Δ*ccpA*
SA564−Δ*hptRS/ccpA*

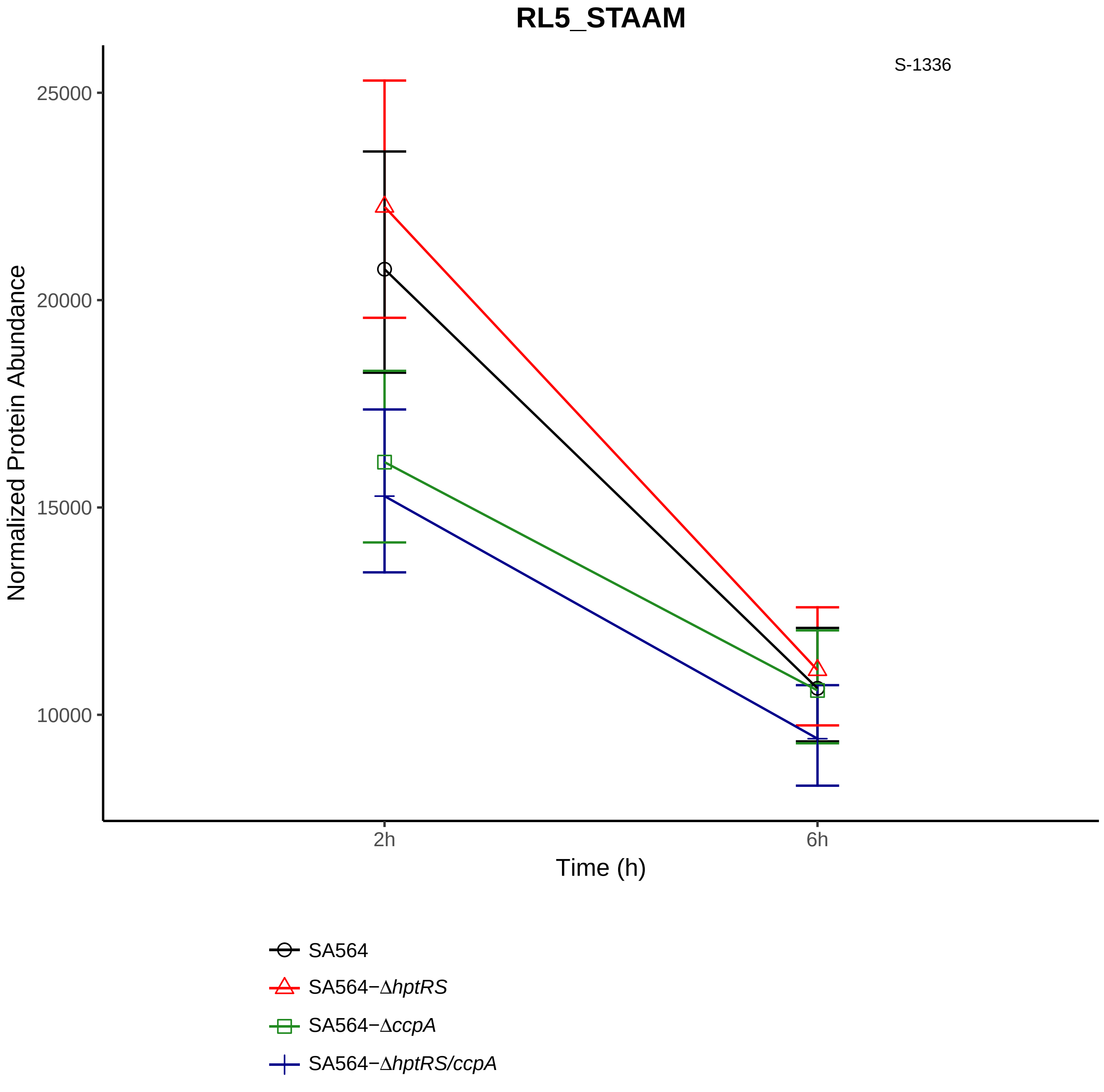

RL5_STAAM
S-1336
Normalized Protein Abundance
25000
20000
15000
10000
2h
6h
Time (h)
SA564
SA564−Δ*hptRS*
SA564−Δ*ccpA*
SA564−Δ*hptRS/ccpA*

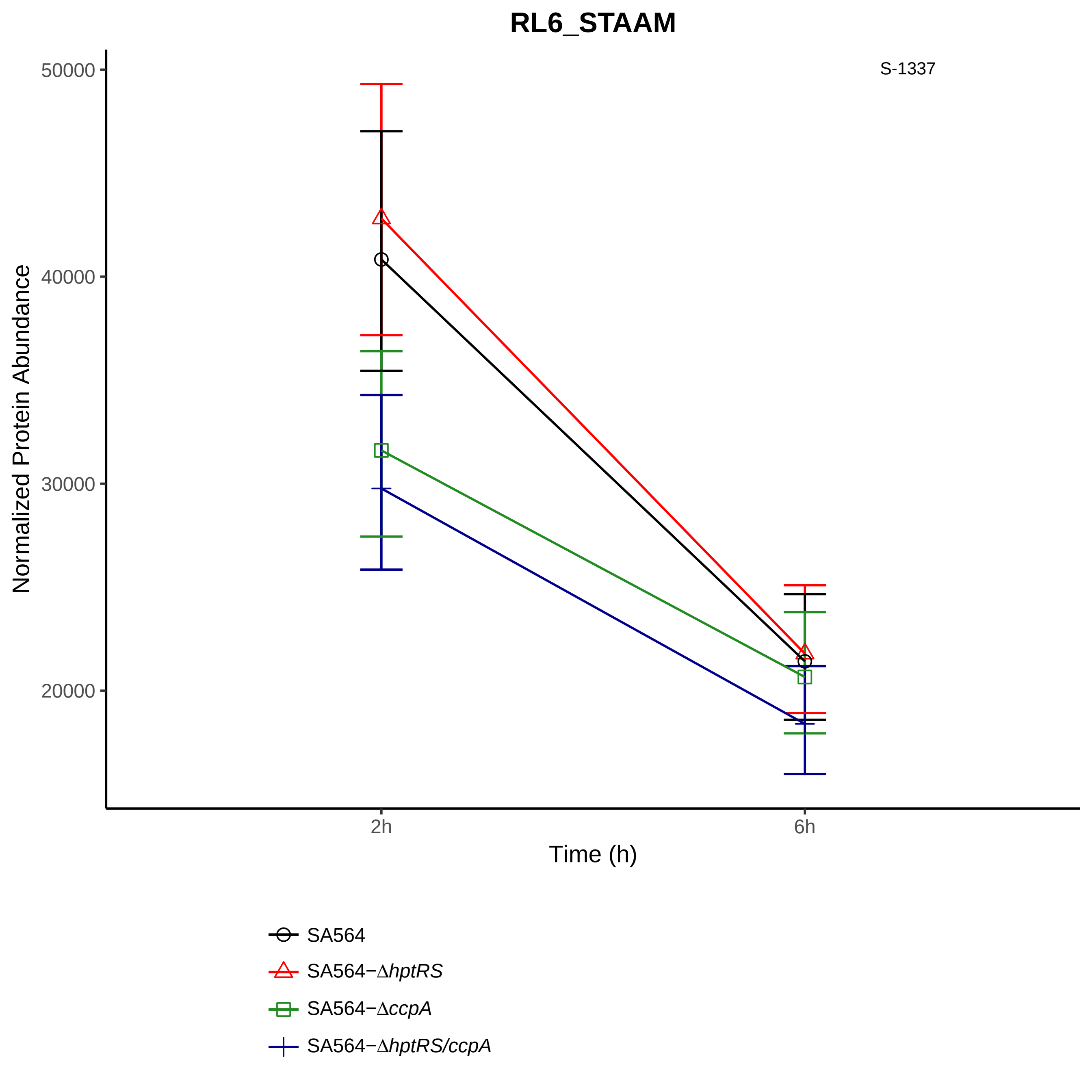

RL6_STAAM
S-1337
Normalized Protein Abundance
50000
40000
30000
20000
2h
6h
Time (h)
SA564
SA564−Δ*hptRS*
SA564−Δ*ccpA*
SA564−Δ*hptRS/ccpA*

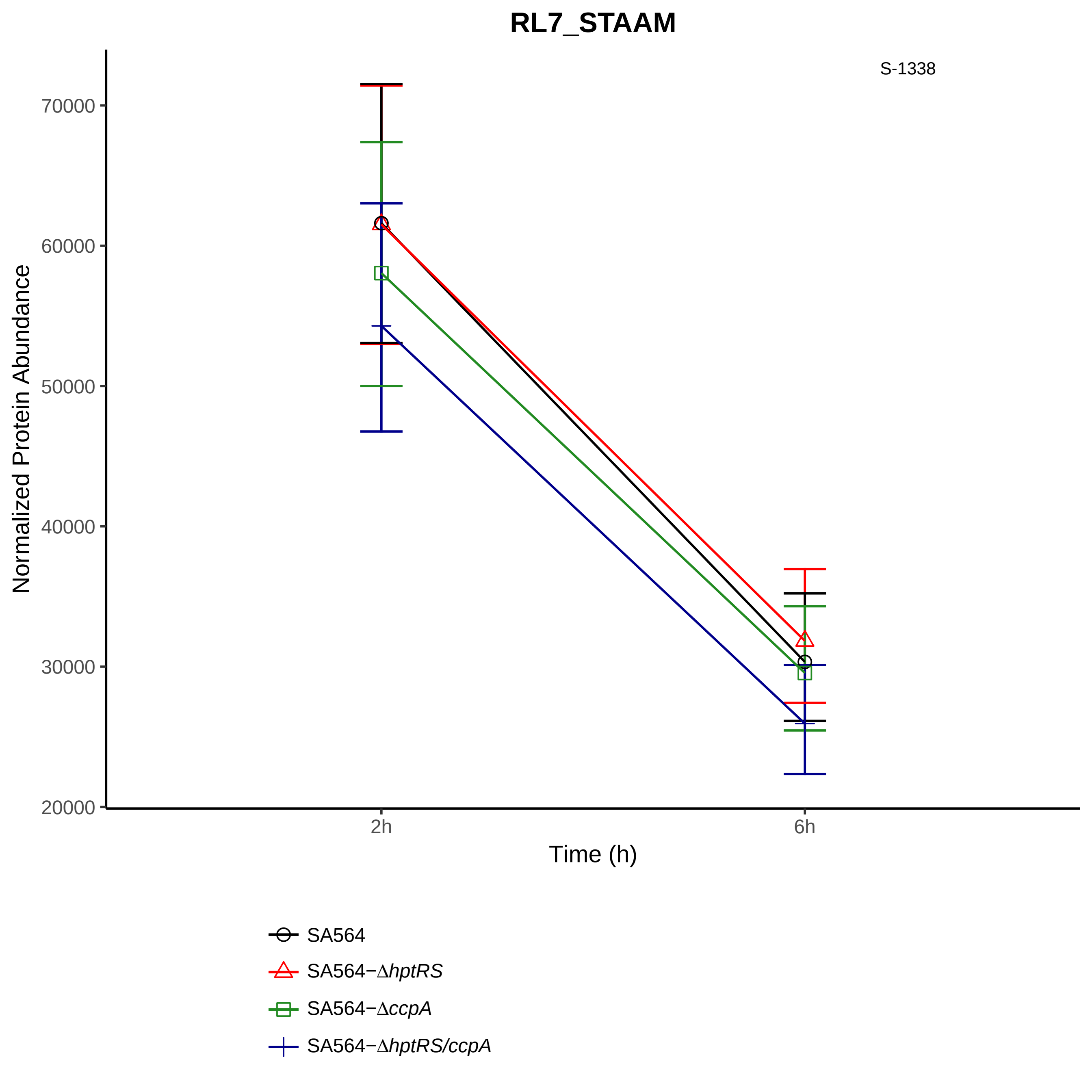

RL7_STAAM
S-1338
Normalized Protein Abundance
70000
60000
50000
40000
30000
20000
2h
6h
Time (h)
SA564
SA564−Δ*hptRS*
SA564−Δ*ccpA*
SA564−Δ*hptRS/ccpA*

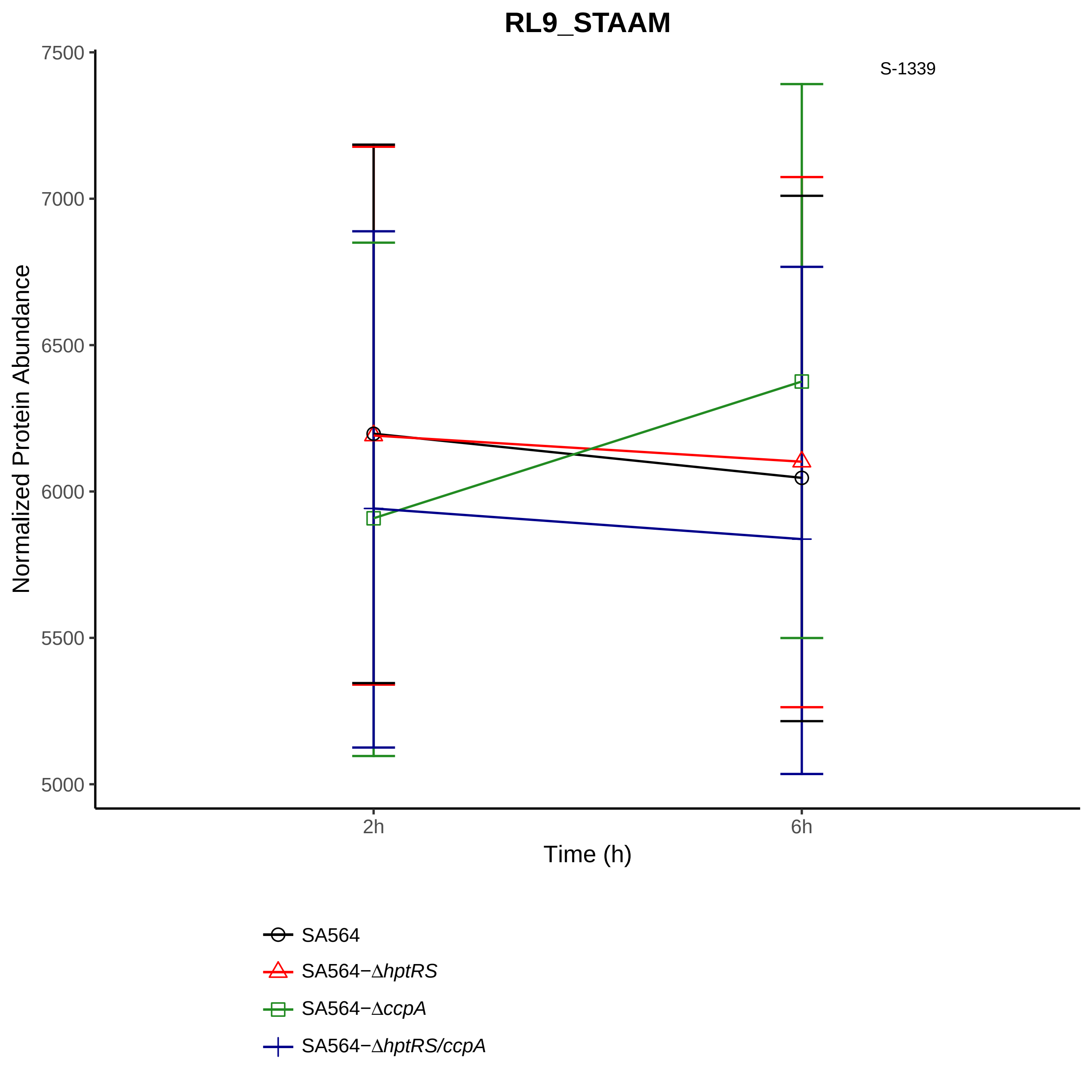

RL9_STAAM
S-1339
Normalized Protein Abundance
7500
7000
6500
6000
5500
5000
2h
6h
Time (h)
SA564
SA564−Δ*hptRS*
SA564−Δ*ccpA*
SA564−Δ*hptRS/ccpA*

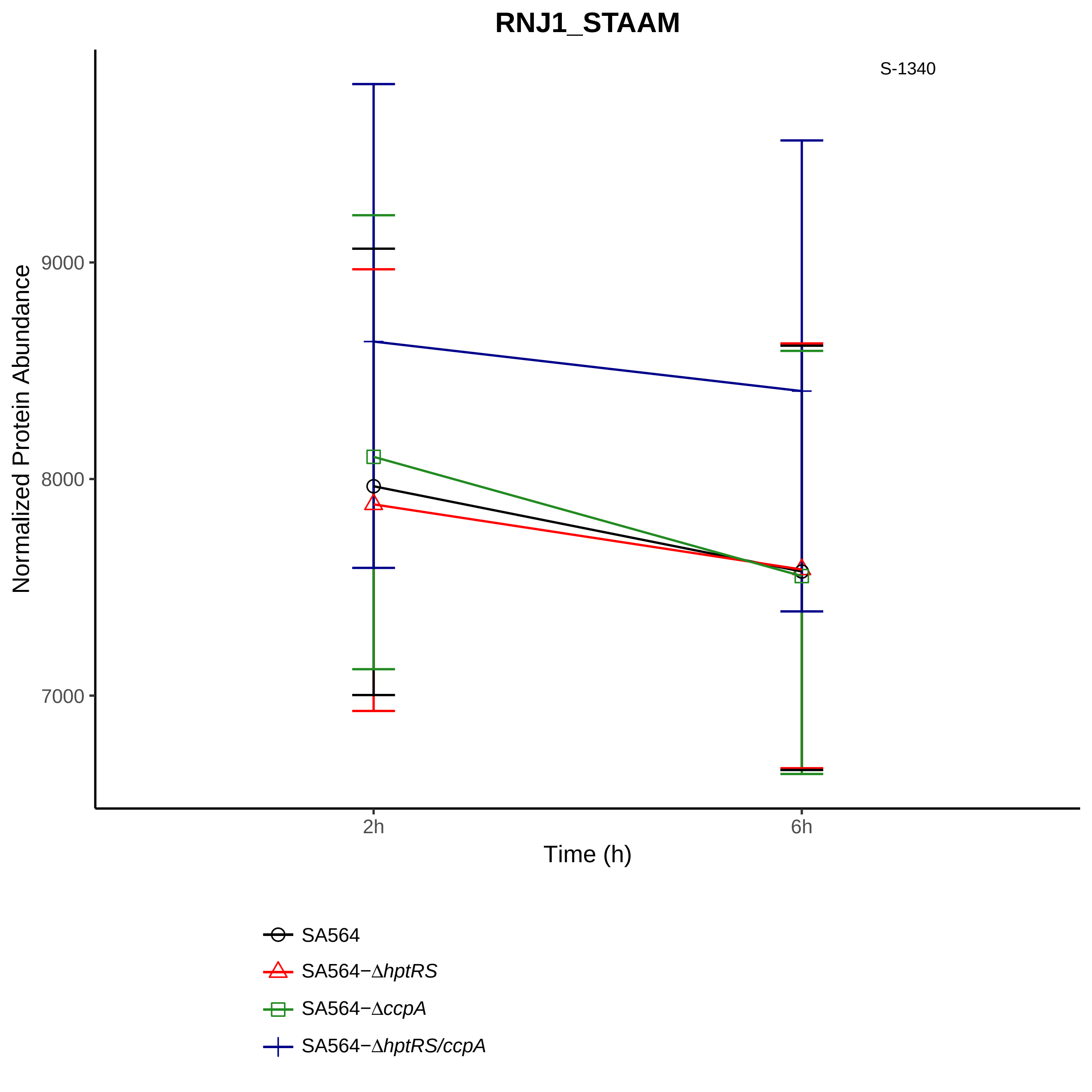
RNJ1_STAAM
S-1340
Normalized Protein Abundance
9000
8000
7000
2h
6h
Time (h)
SA564
SA564−ΔhptRS
SA564−ΔccpA
SA564−ΔhptRS/ccpA

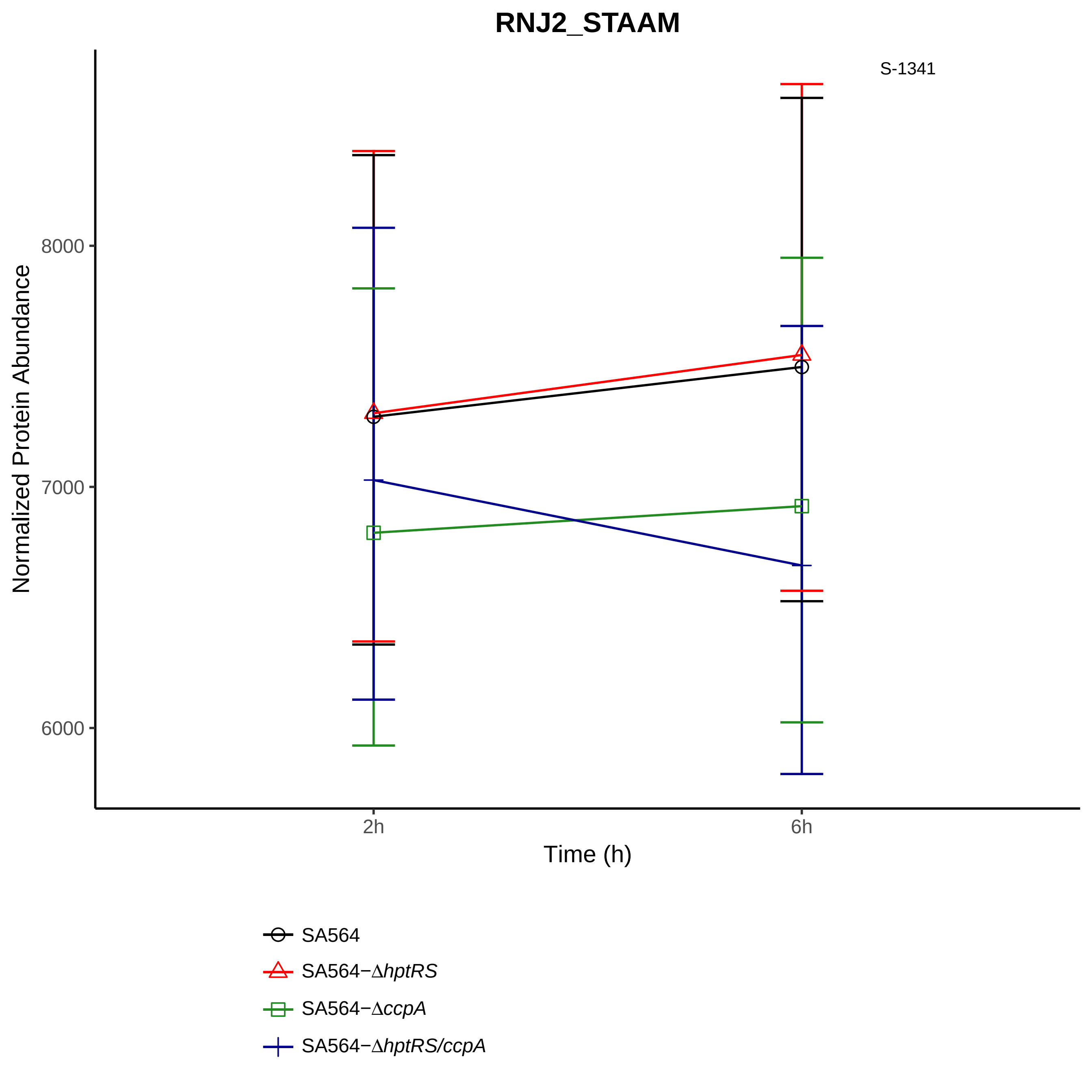

RNJ2_STAAM
S-1341
Normalized Protein Abundance
8000
7000
6000
2h
6h
Time (h)
SA564
SA564−Δ*hptRS*
SA564−Δ*ccpA*
SA564−Δ*hptRS/ccpA*

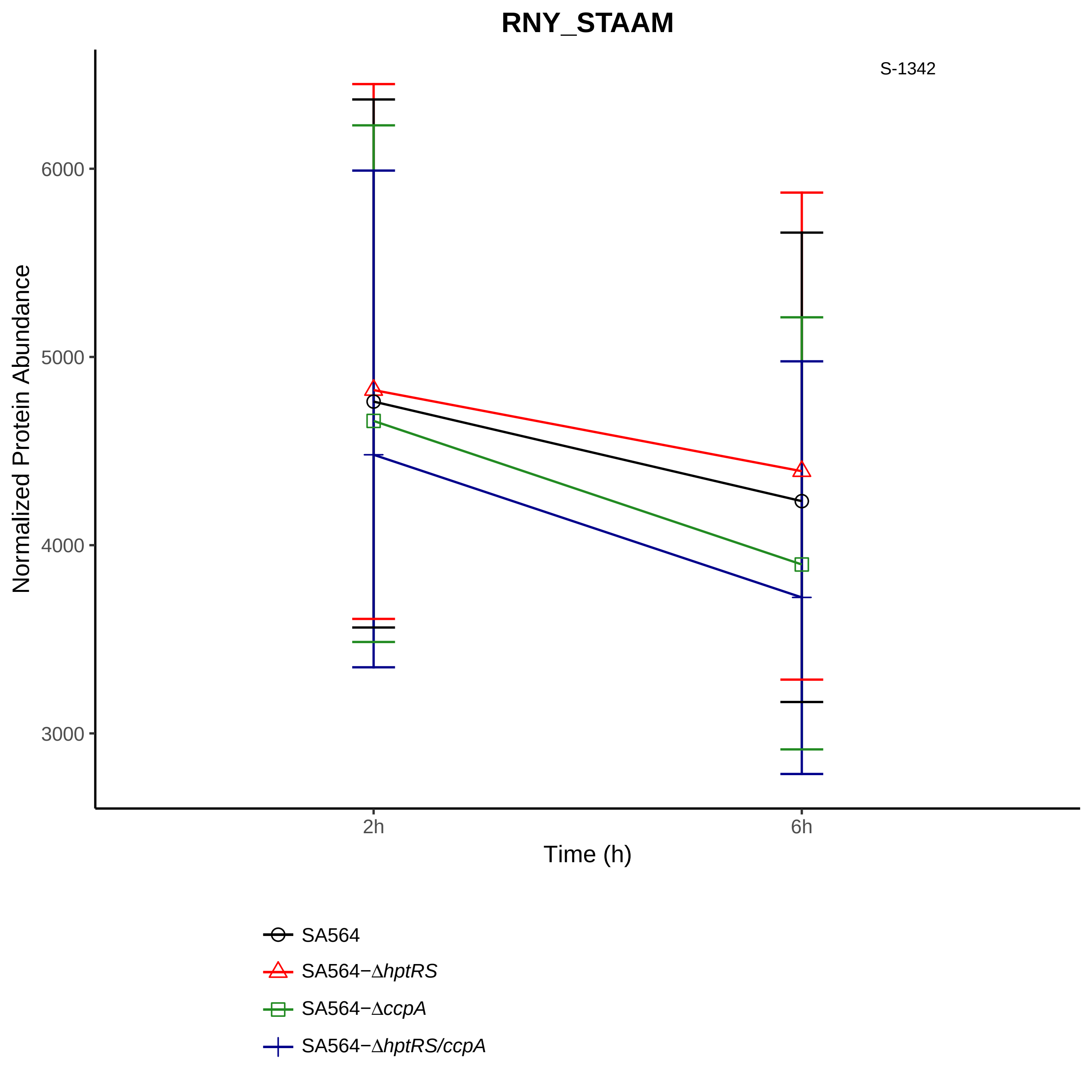

RNY_STAAM
S-1342
Normalized Protein Abundance
6000
5000
4000
3000
2h
6h
Time (h)
SA564
SA564−ΔhptRS
SA564−ΔccpA
SA564−ΔhptRS/ccpA

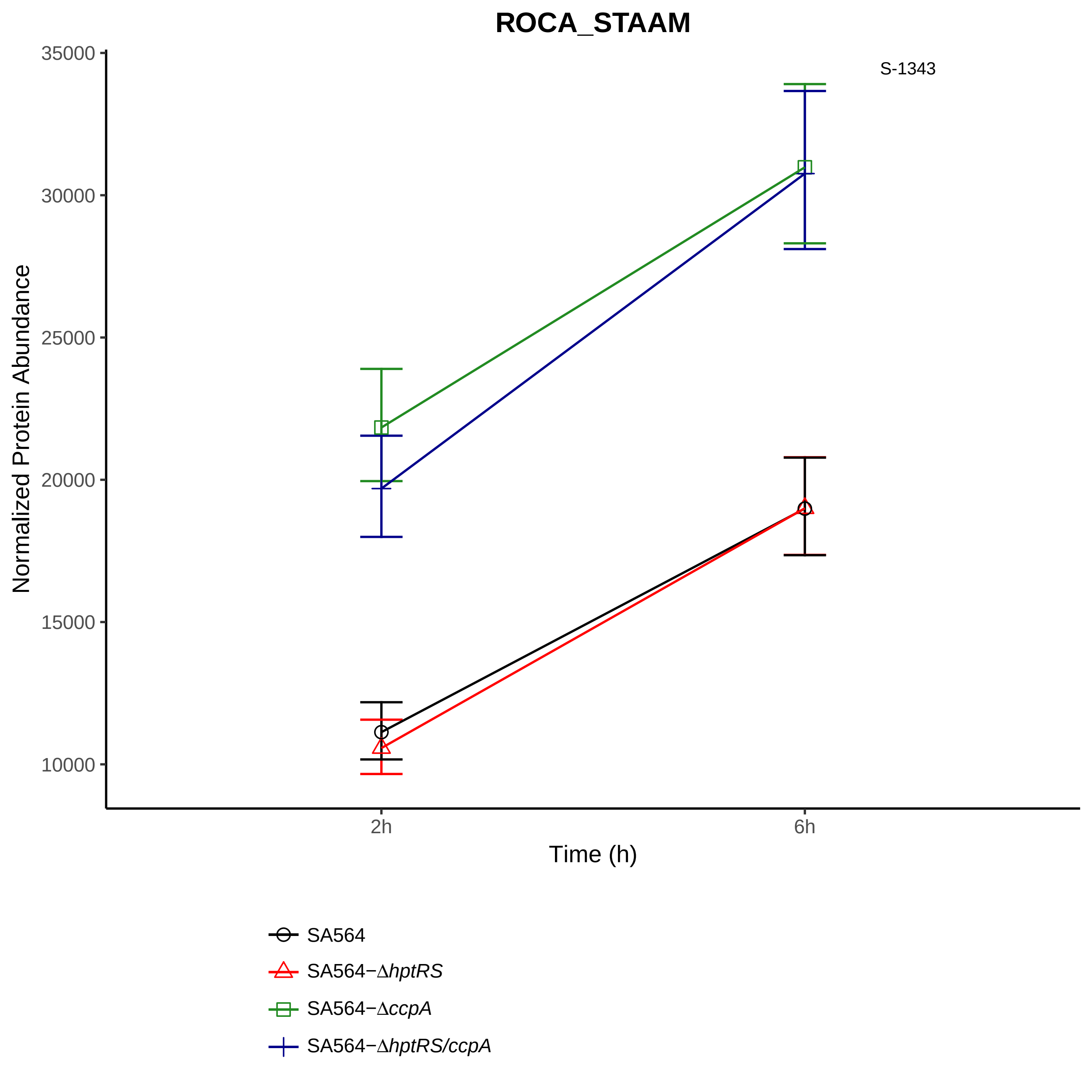
ROCA_STAAM
S-1343
Normalized Protein Abundance
35000
30000
25000
20000
15000
10000
2h
6h
Time (h)
SA564
SA564−Δ*hptRS*
SA564−Δ*ccpA*
SA564−Δ*hptRS/ccpA*

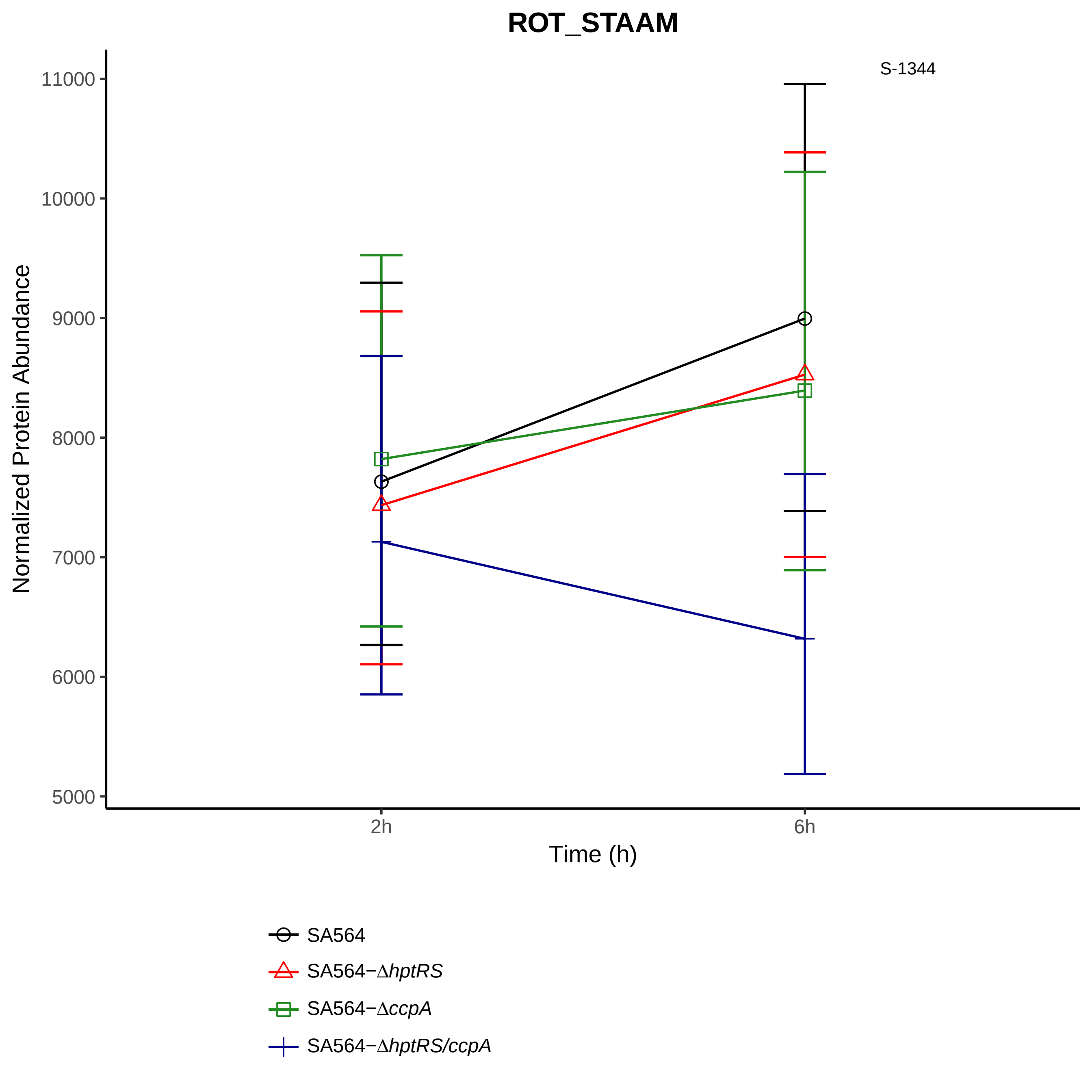

ROT_STAAM
S-1344
Normalized Protein Abundance
11000
10000
9000
8000
7000
6000
5000
2h
6h
Time (h)
SA564
SA564−Δ*hptRS*
SA564−Δ*ccpA*
SA564−Δ*hptRS/ccpA*

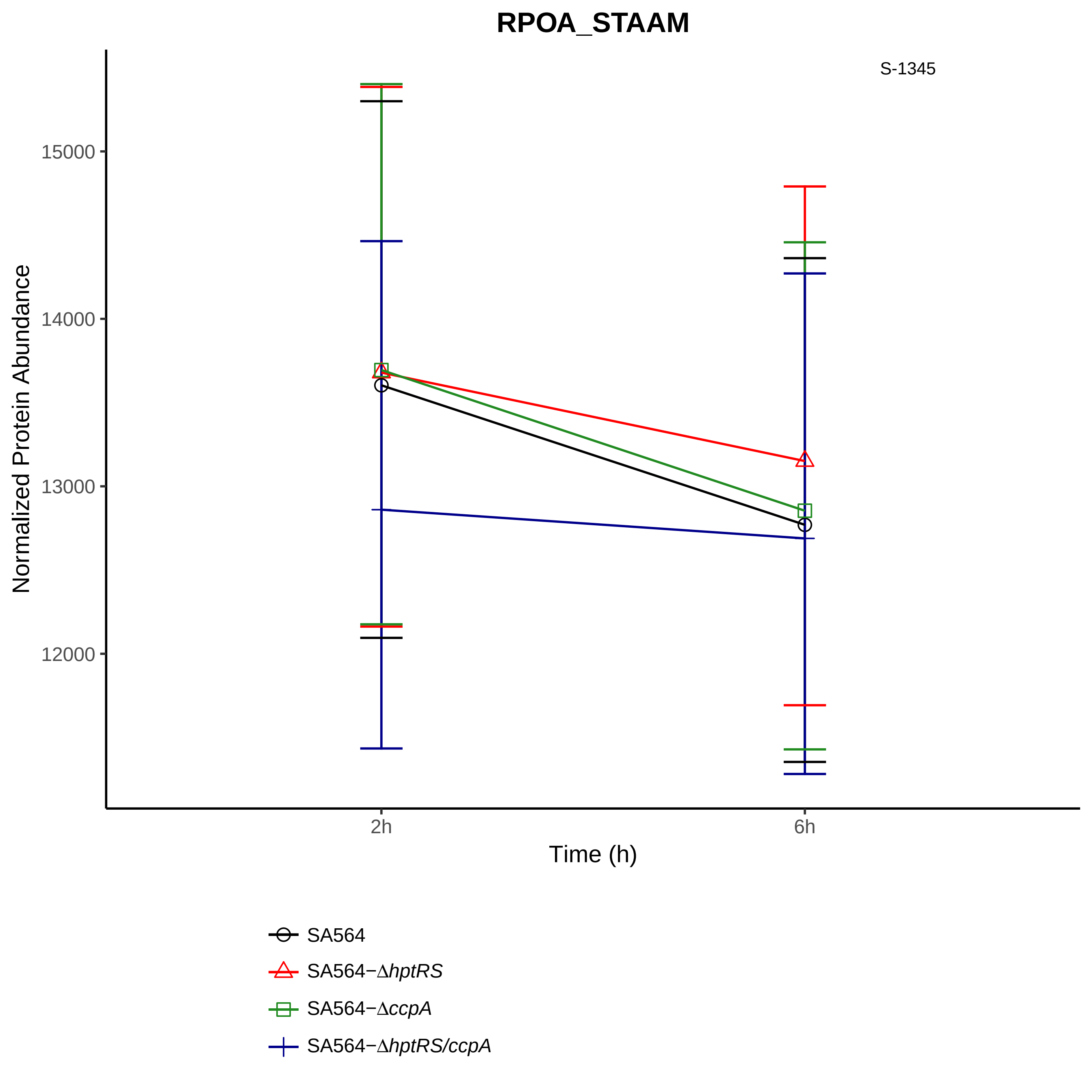

RPOA_STAAM
S-1345
Normalized Protein Abundance
15000
14000
13000
12000
2h
6h
Time (h)
SA564
SA564−Δ*hptRS*
SA564−Δ*ccpA*
SA564−Δ*hptRS/ccpA*

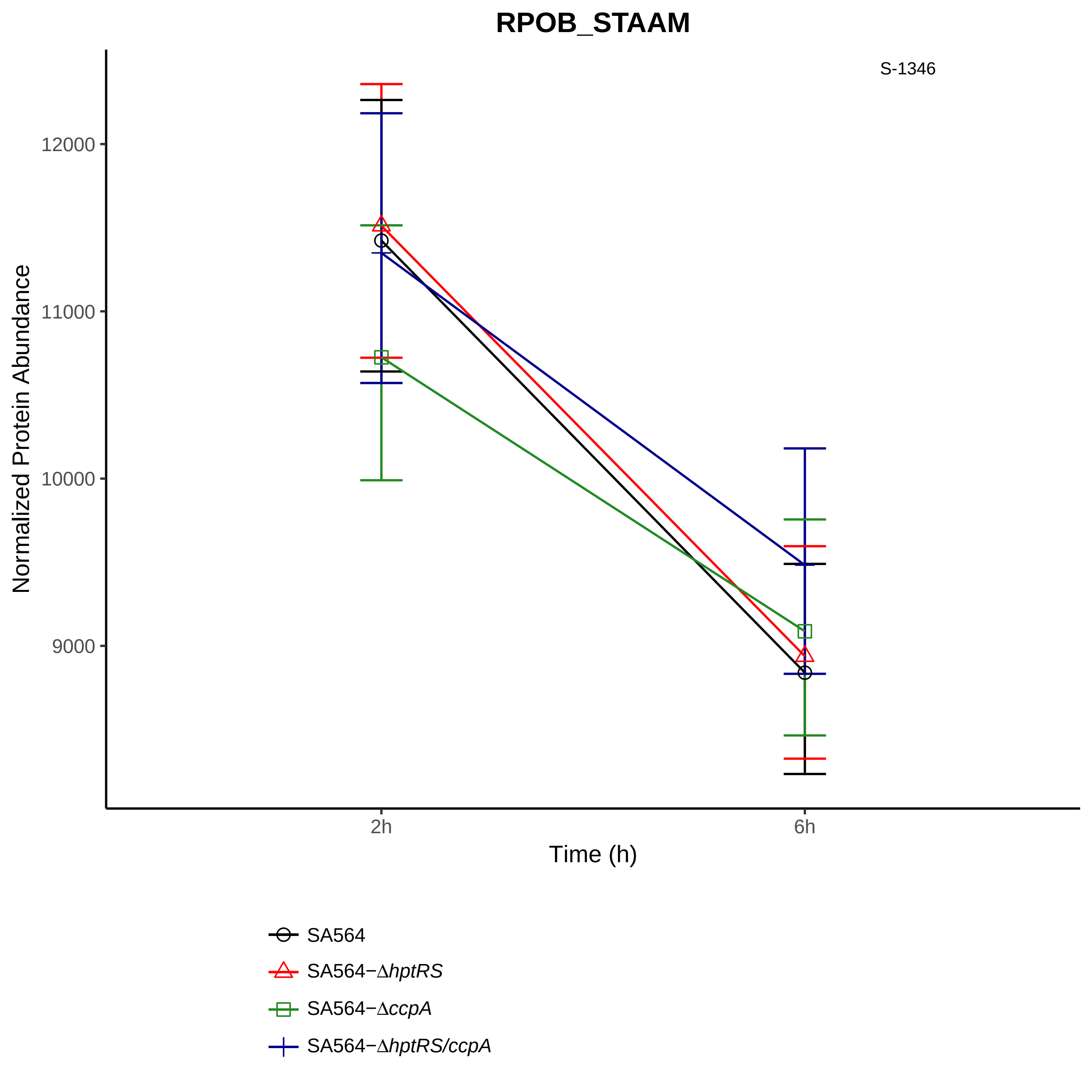

RPOB_STAAM
S-1346
Normalized Protein Abundance
12000
11000
10000
9000
2h
6h
Time (h)
SA564
SA564−Δ*hptRS*
SA564−Δ*ccpA*
SA564−Δ*hptRS/ccpA*

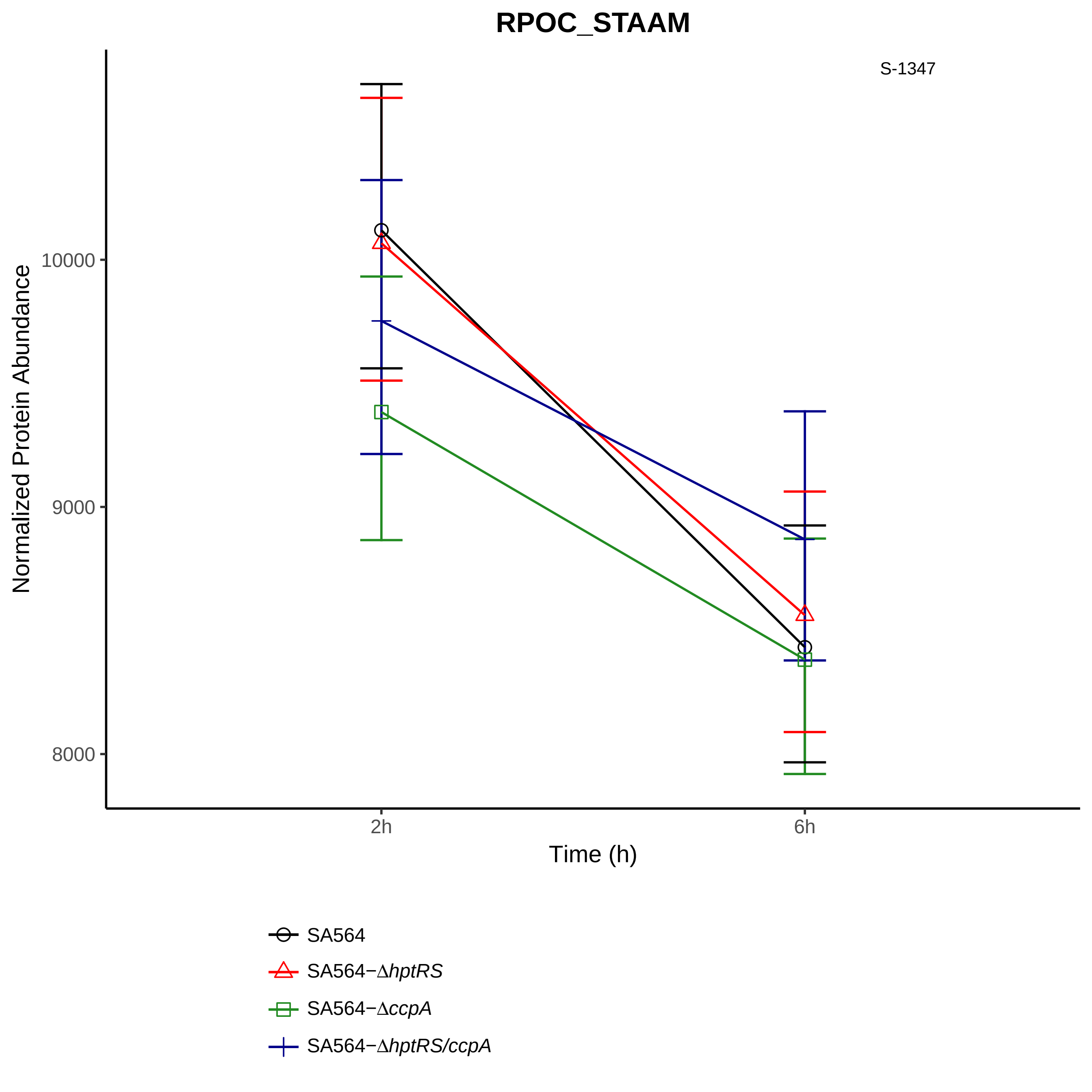
RPOC_STAAM
S-1347
Normalized Protein Abundance
10000
9000
8000
2h
6h
Time (h)
SA564
SA564−ΔhptRS
SA564−ΔccpA
SA564−ΔhptRS/ccpA

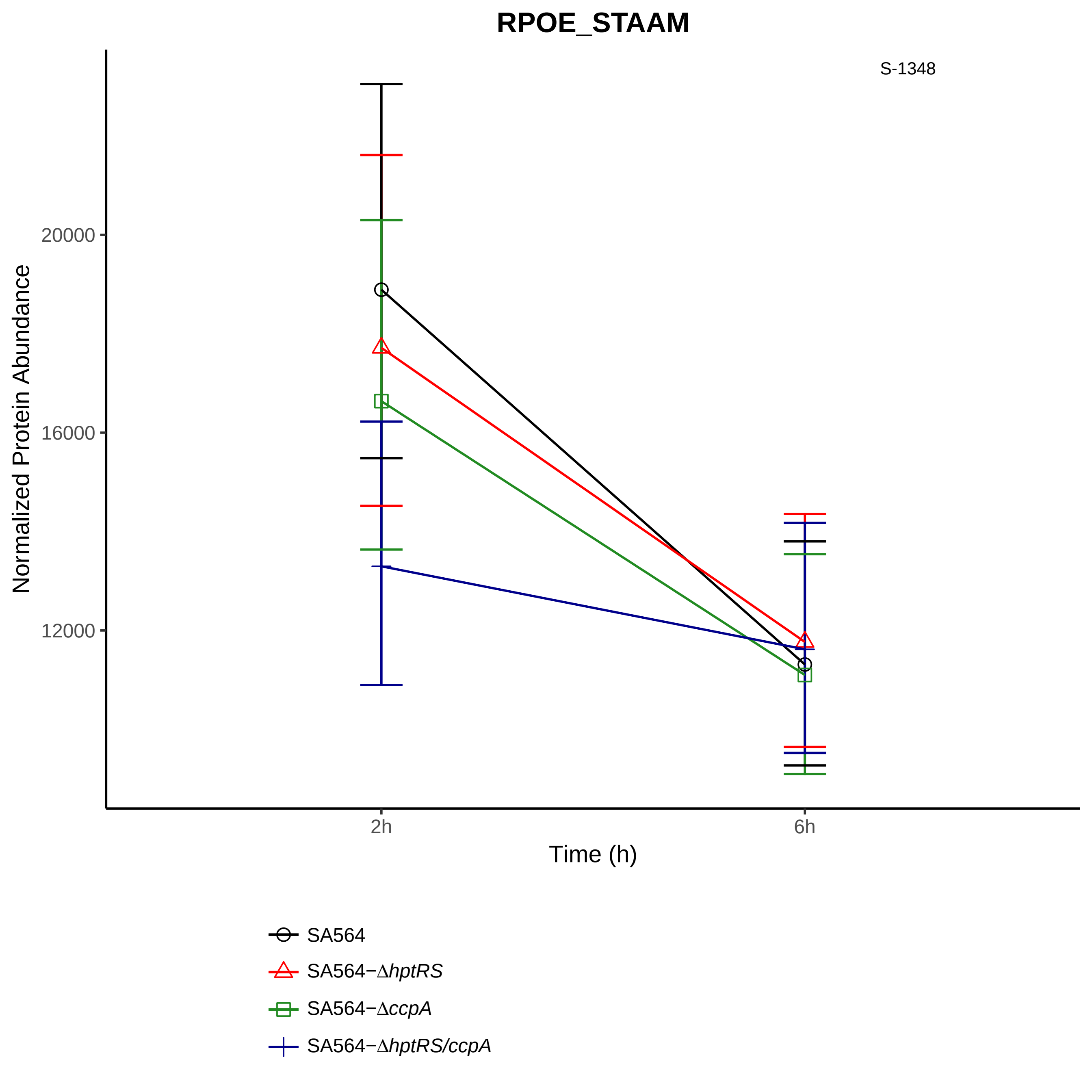

RPOE_STAAM
S-1348
Normalized Protein Abundance
20000
16000
12000
2h
6h
Time (h)
SA564
SA564−Δ*hptRS*
SA564−Δ*ccpA*
SA564−Δ*hptRS/ccpA*

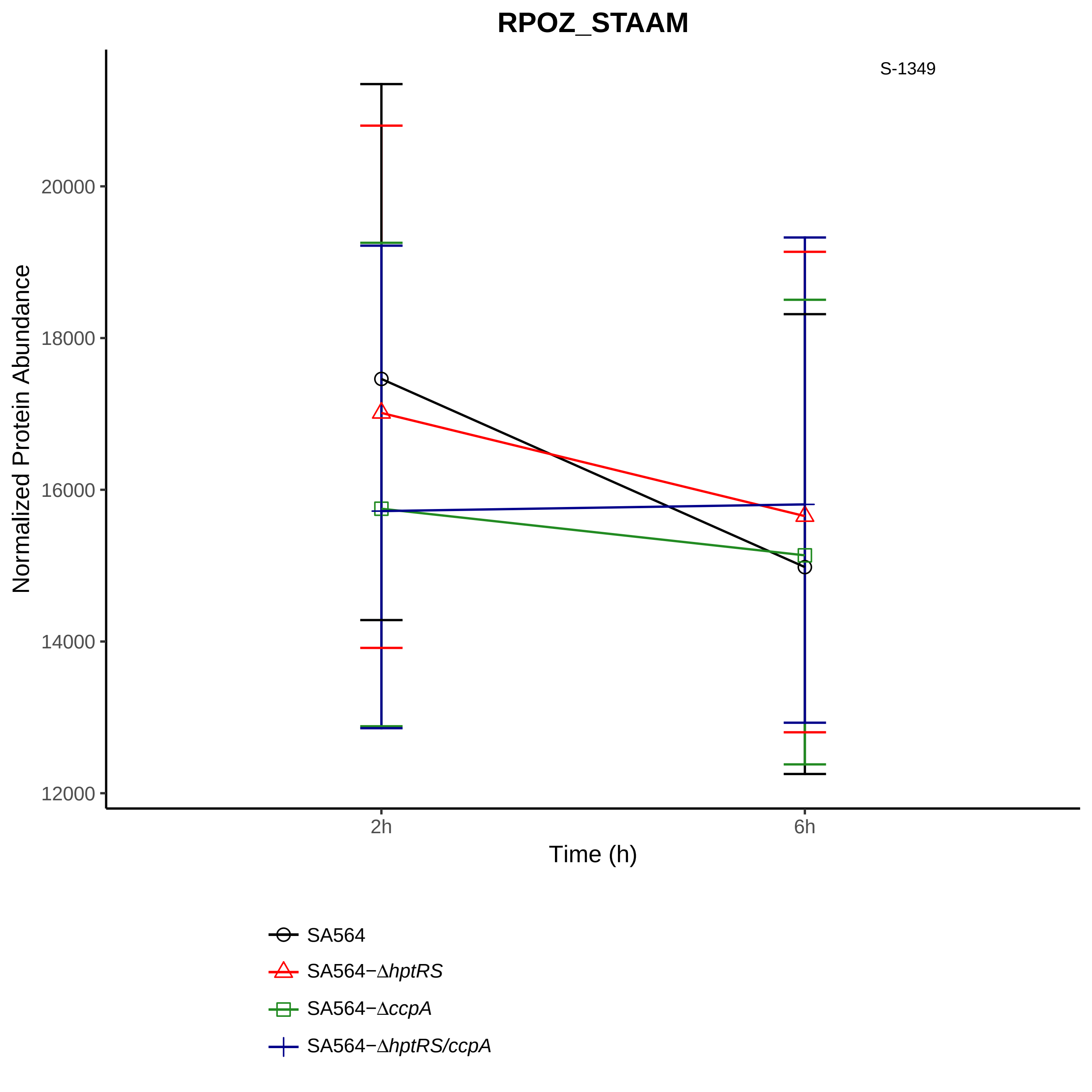

RPOZ_STAAM
S-1349
Normalized Protein Abundance
20000
18000
16000
14000
12000
2h
6h
Time (h)
SA564
SA564−Δ*hptRS*
SA564−Δ*ccpA*
SA564−Δ*hptRS/ccpA*

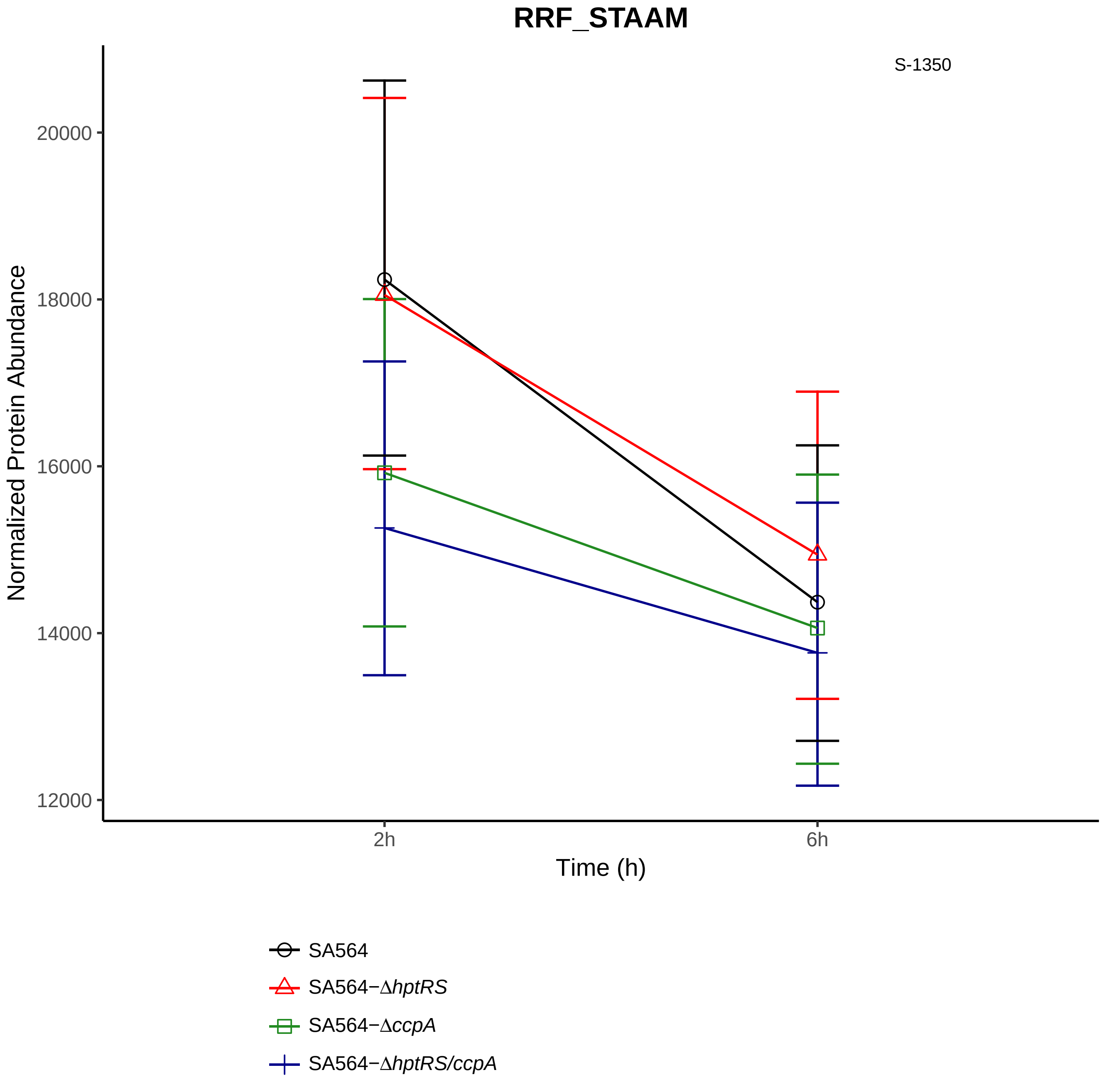

RRF_STAAM
S-1350
Normalized Protein Abundance
20000
18000
16000
14000
12000
2h
6h
Time (h)
SA564
SA564−ΔhptRS
SA564−ΔccpA
SA564−ΔhptRS/ccpA

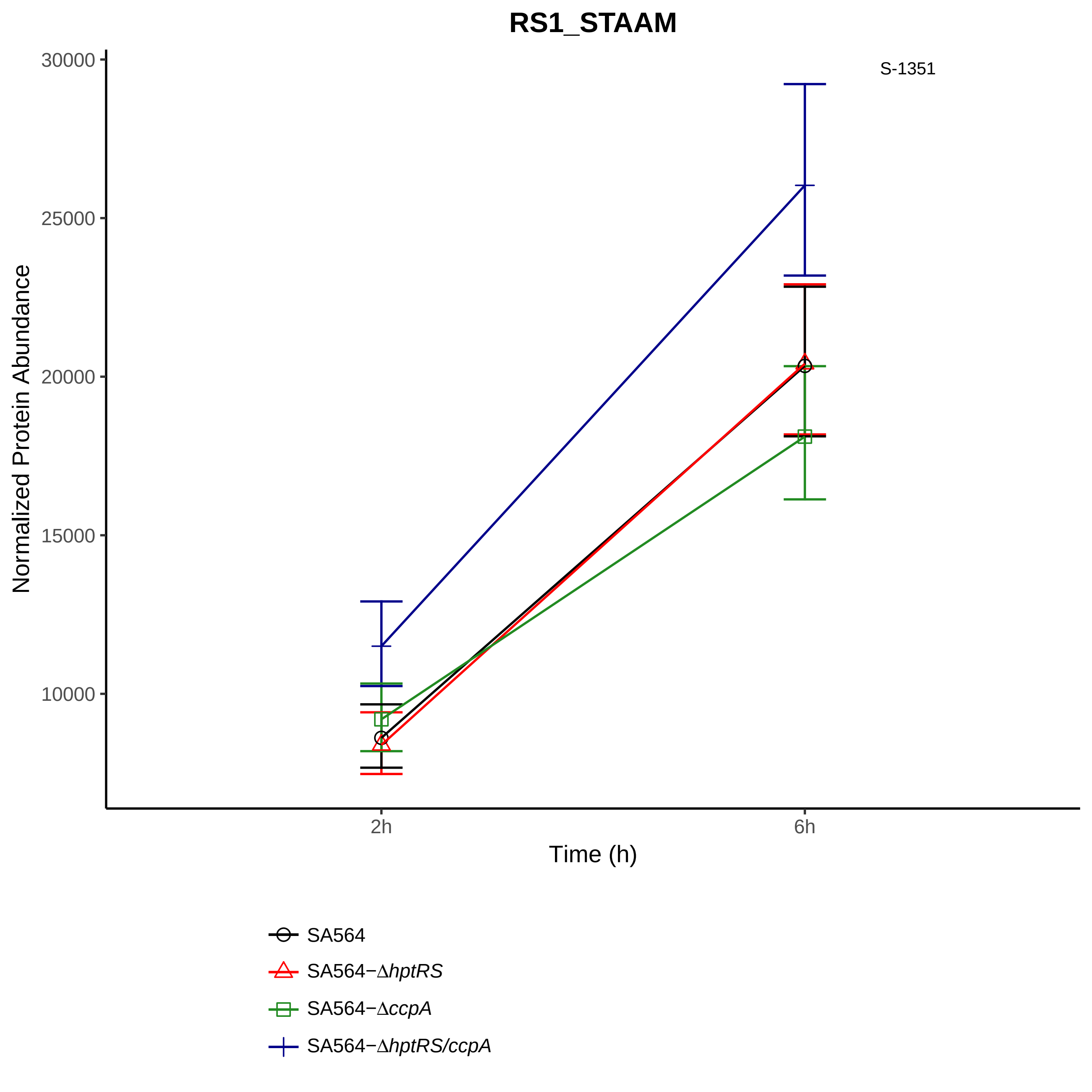

RS1_STAAM
S-1351
Normalized Protein Abundance
30000
25000
20000
15000
10000
2h
6h
Time (h)
SA564
SA564−Δ*hptRS*
SA564−Δ*ccpA*
SA564−Δ*hptRS/ccpA*

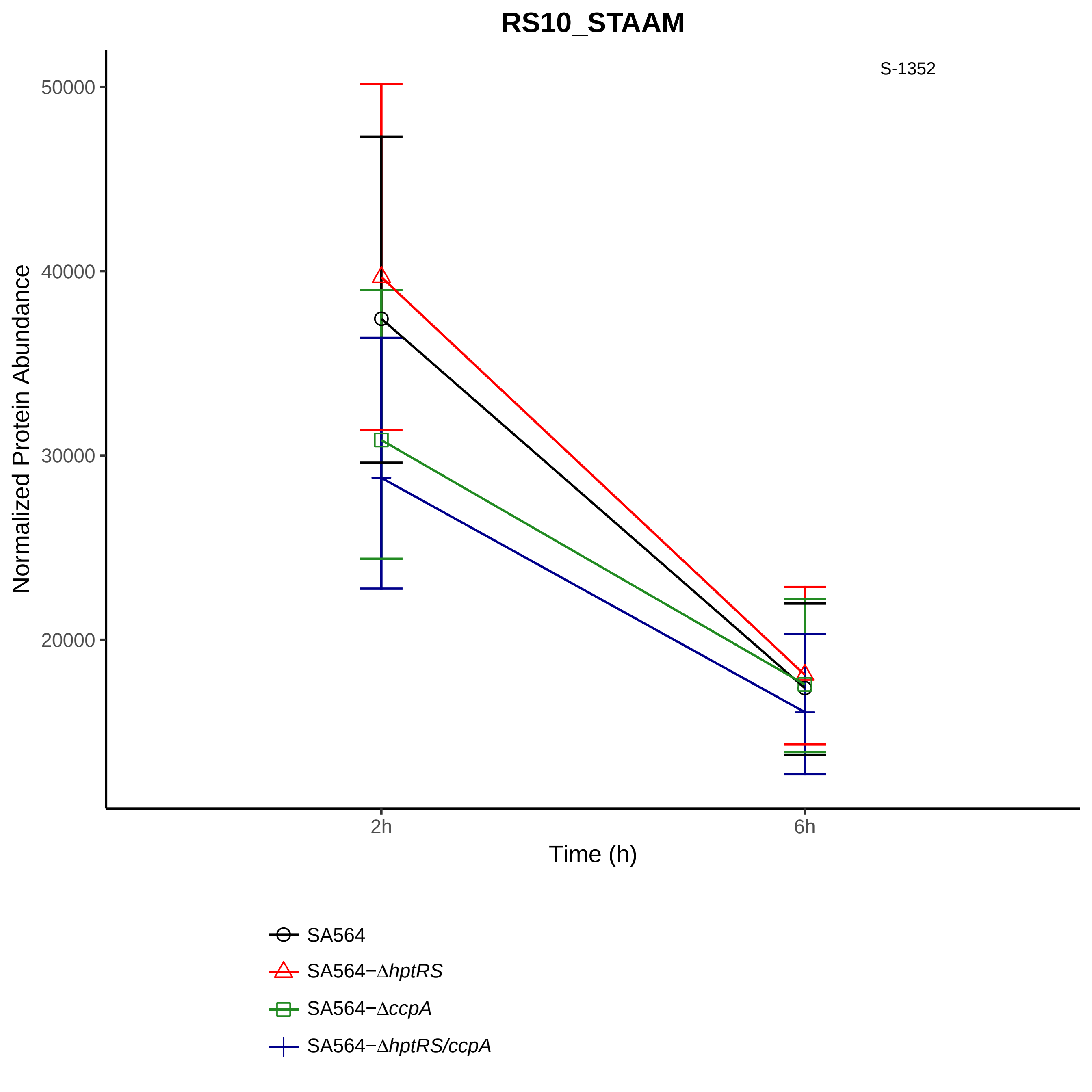

RS10_STAAM
S-1352
50000
40000
30000
20000
Normalized Protein Abundance
2h
6h
Time (h)
SA564
SA564−Δ*hptRS*
SA564−Δ*ccpA*
SA564−Δ*hptRS/ccpA*

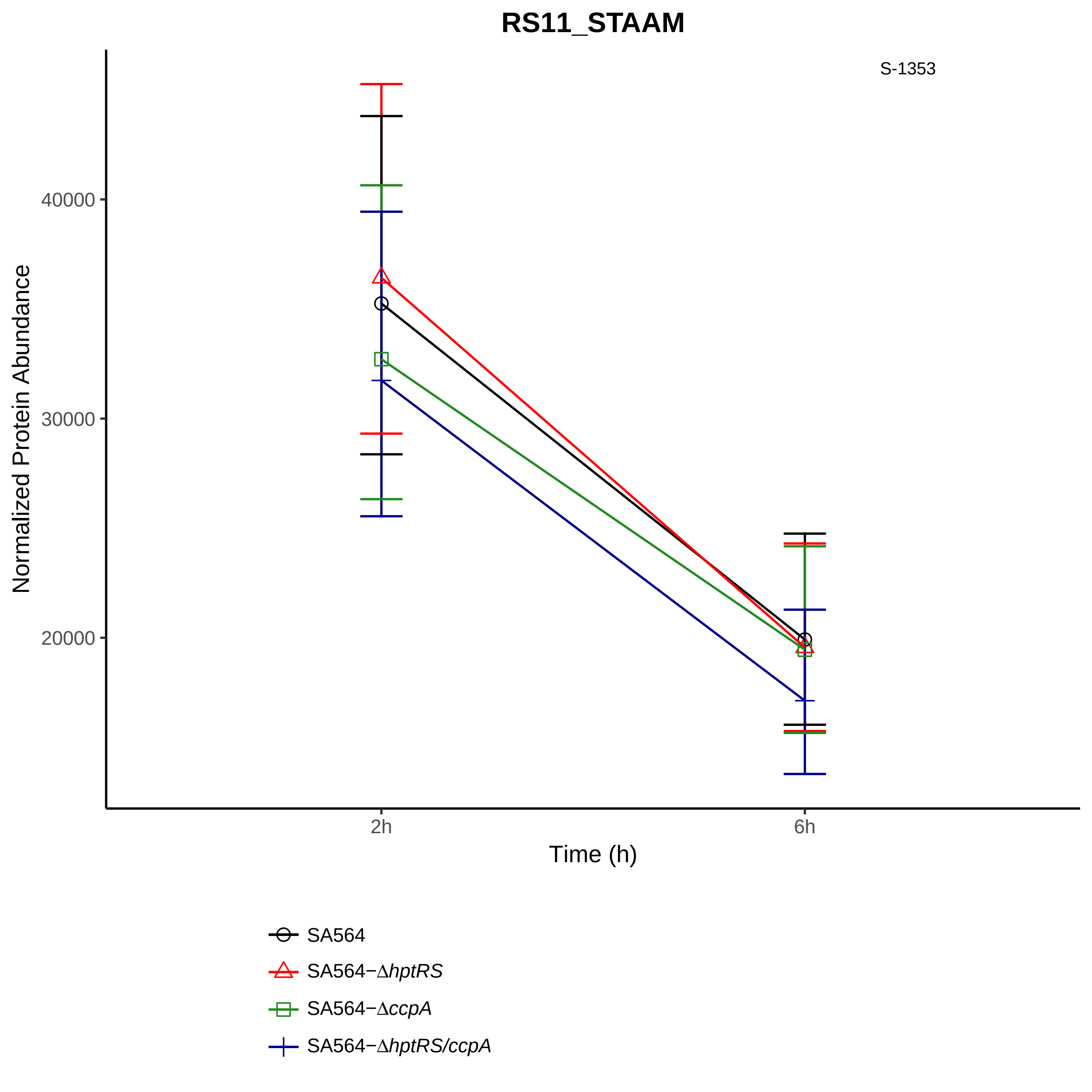
RS11_STAAM
S-1353
Normalized Protein Abundance
40000
30000
20000
2h
6h
Time (h)
SA564
SA564−Δ*hptRS*
SA564−Δ*ccpA*
SA564−Δ*hptRS/ccpA*

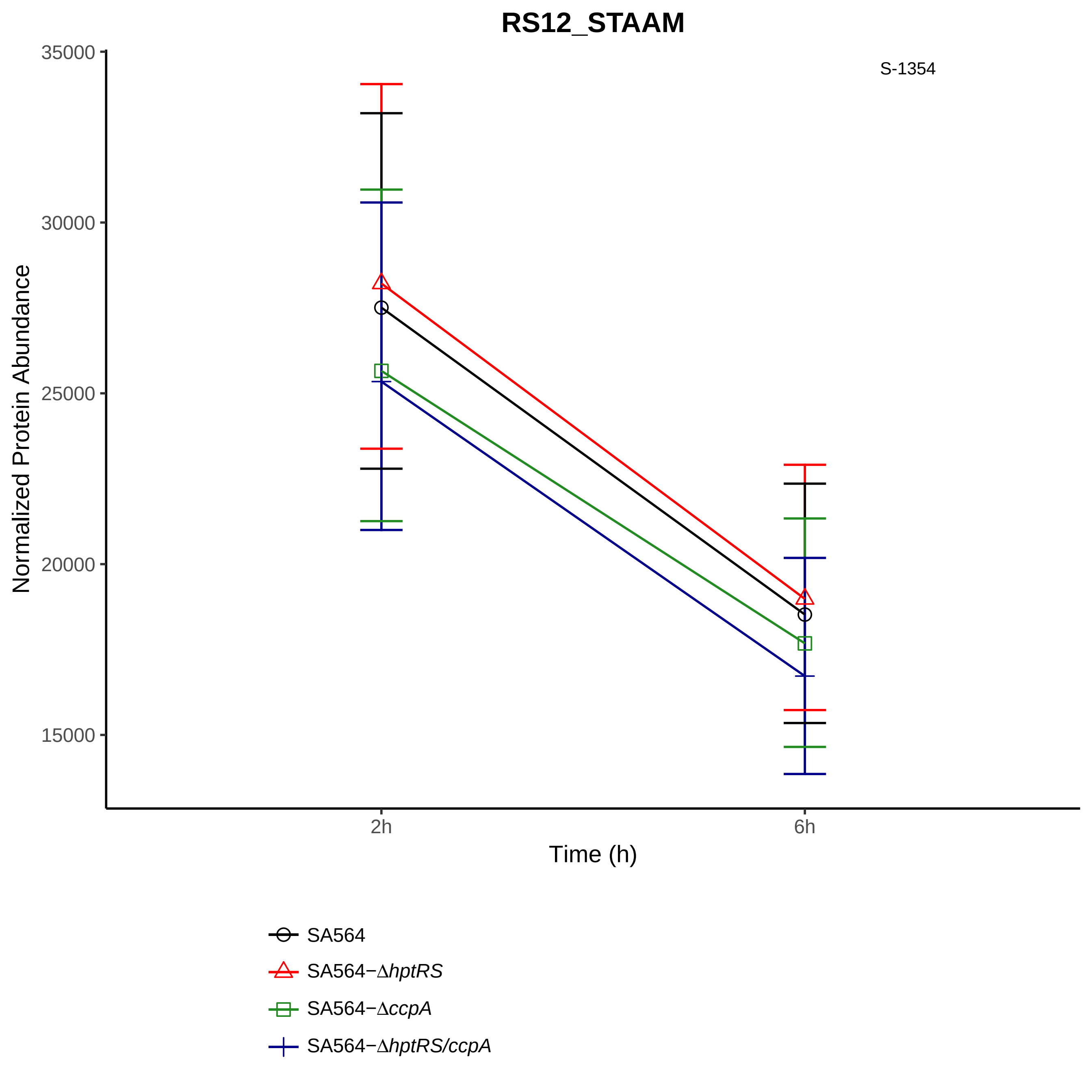

RS12_STAAM
S-1354
Normalized Protein Abundance
35000
30000
25000
20000
15000
2h
6h
Time (h)
SA564
SA564−Δ*hptRS*
SA564−Δ*ccpA*
SA564−Δ*hptRS/ccpA*

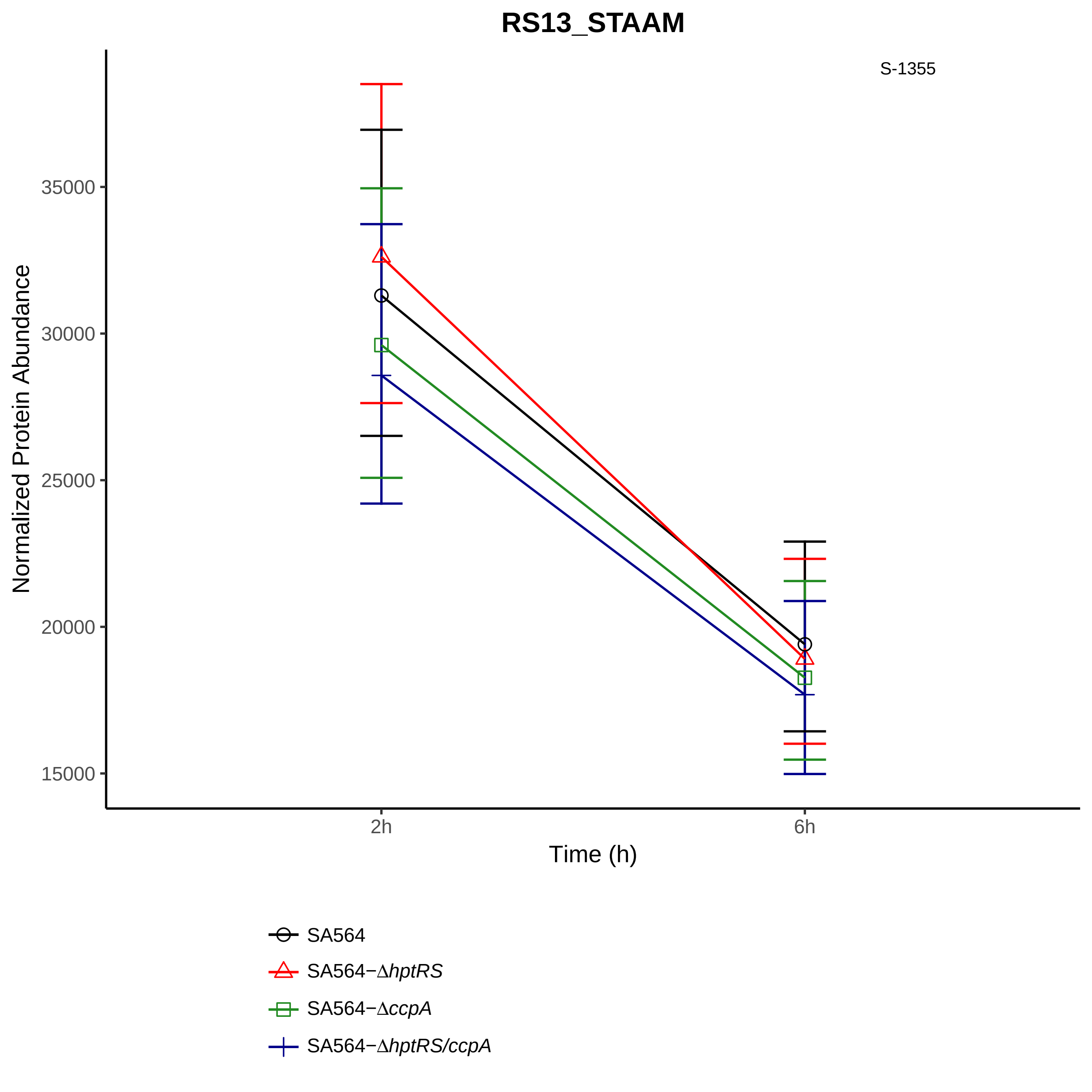

RS13_STAAM
S-1355
Normalized Protein Abundance
35000
30000
25000
20000
15000
2h
6h
Time (h)
SA564
SA564−Δ*hptRS*
SA564−Δ*ccpA*
SA564−Δ*hptRS/ccpA*

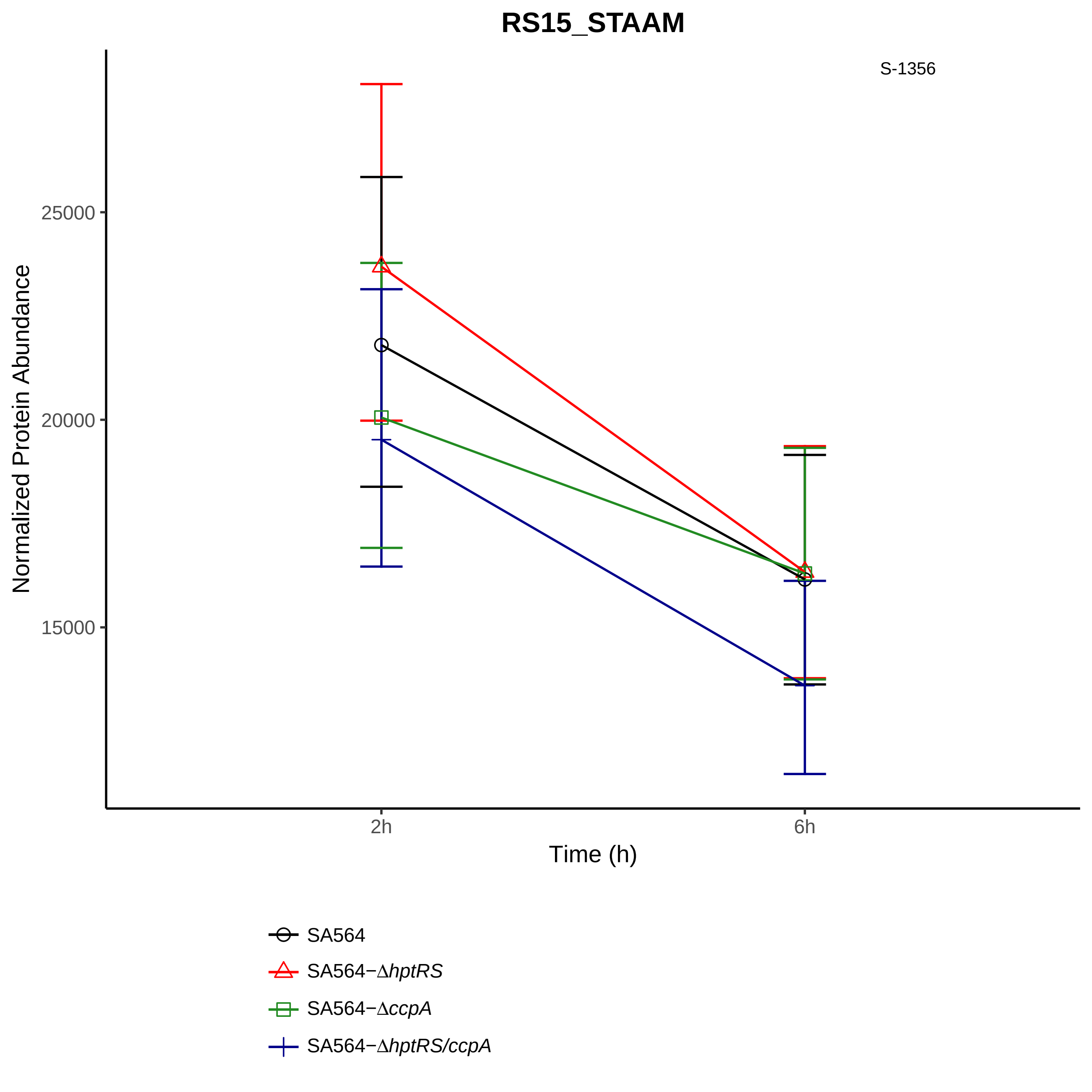

RS15_STAAM
S-1356
Normalized Protein Abundance
25000
20000
15000
2h
6h
Time (h)
SA564
SA564−Δ*hptRS*
SA564−Δ*ccpA*
SA564−Δ*hptRS/ccpA*

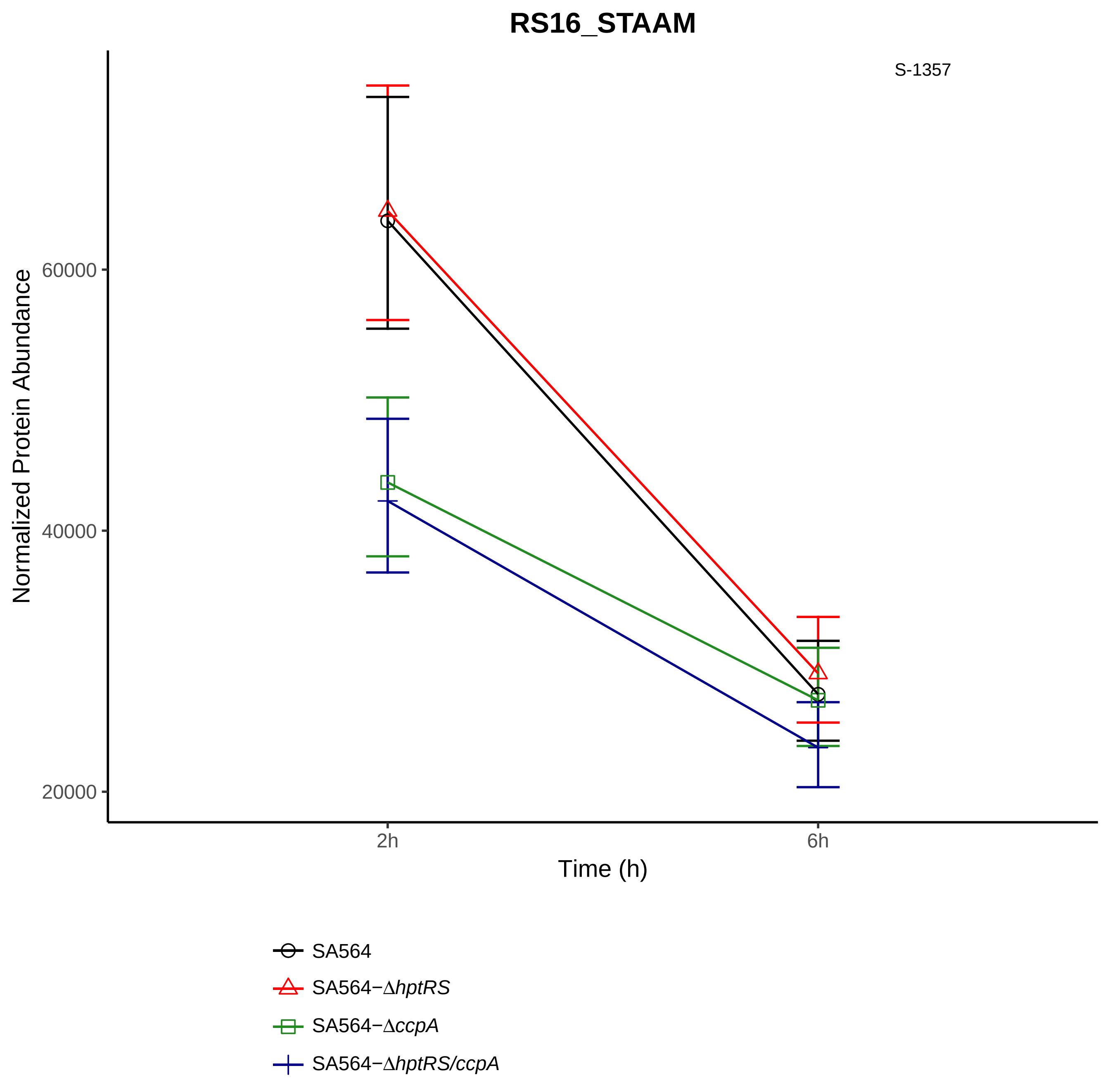

RS16_STAAM
S-1357
Normalized Protein Abundance
60000
40000
20000
2h
6h
Time (h)
SA564
SA564−ΔhptRS
SA564−ΔccpA
SA564−ΔhptRS/ccpA

RS17_STAAM

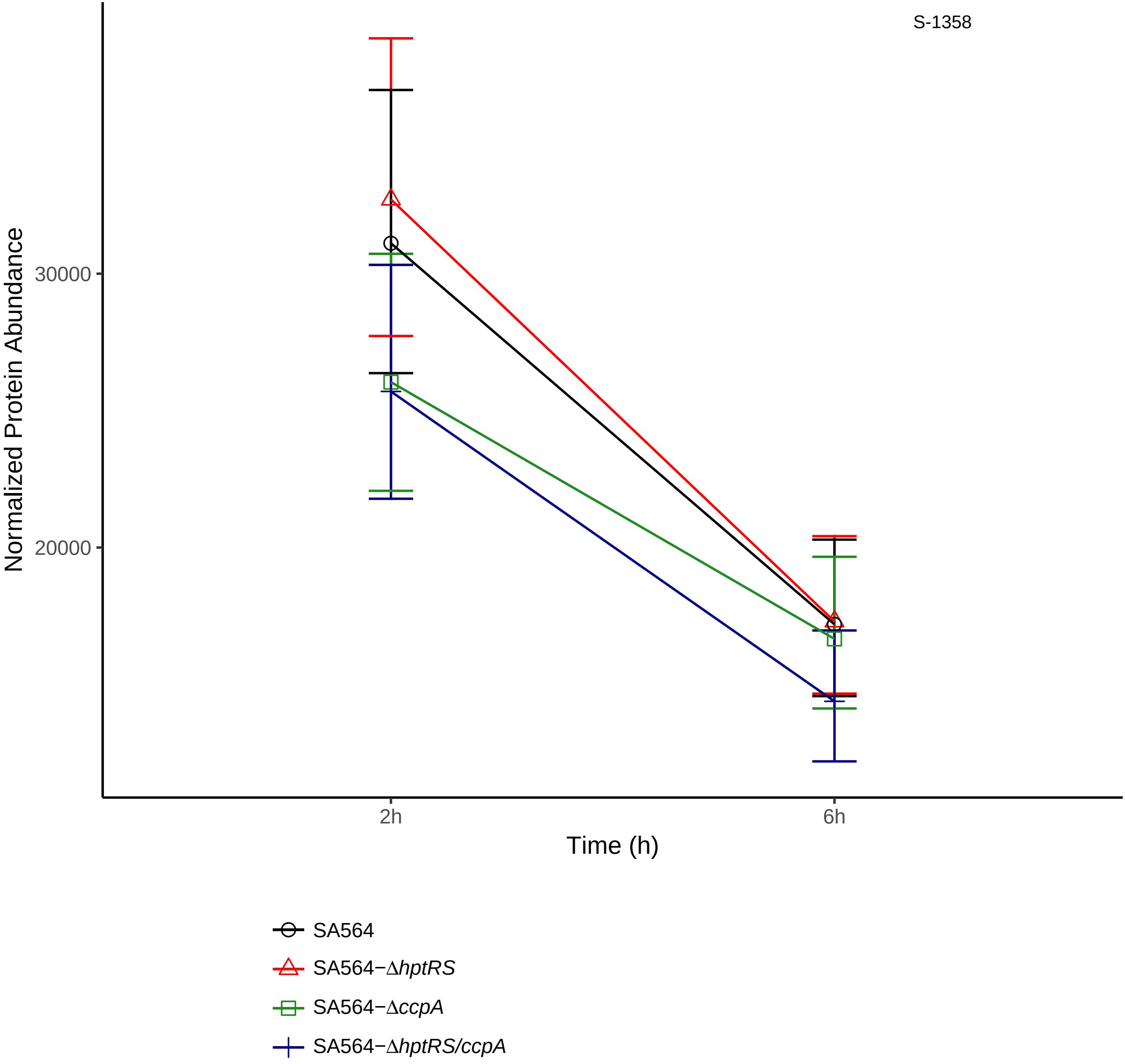

S-1358
Normalized Protein Abundance
30000
20000
2h
6h
Time (h)
SA564
SA564−Δ*hptRS*
SA564−Δ*ccpA*
SA564−Δ*hptRS/ccpA*

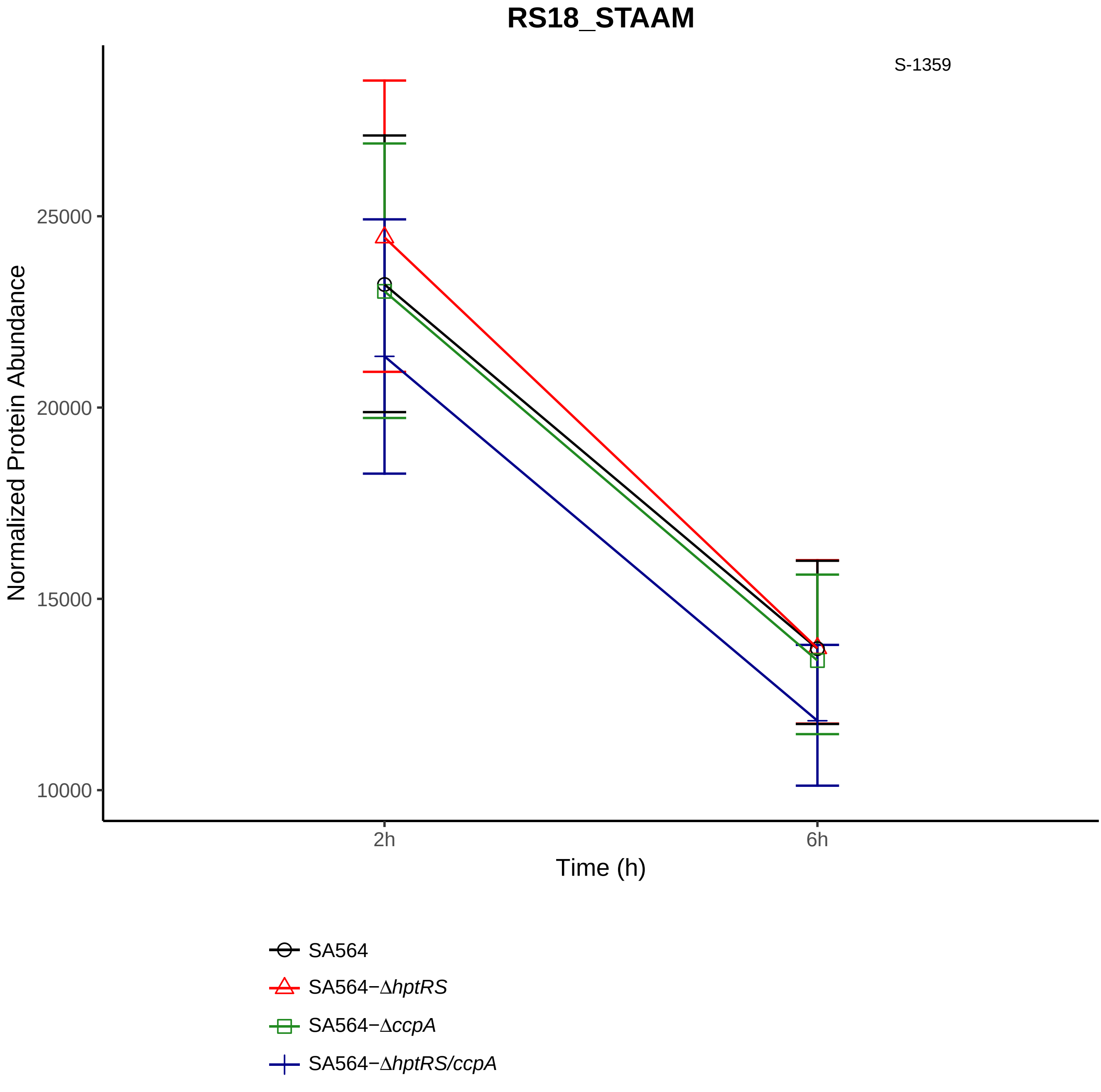

RS18_STAAM
S-1359
Normalized Protein Abundance
25000
20000
15000
10000
2h
6h
Time (h)
SA564
SA564−Δ*hptRS*
SA564−Δ*ccpA*
SA564−Δ*hptRS/ccpA*

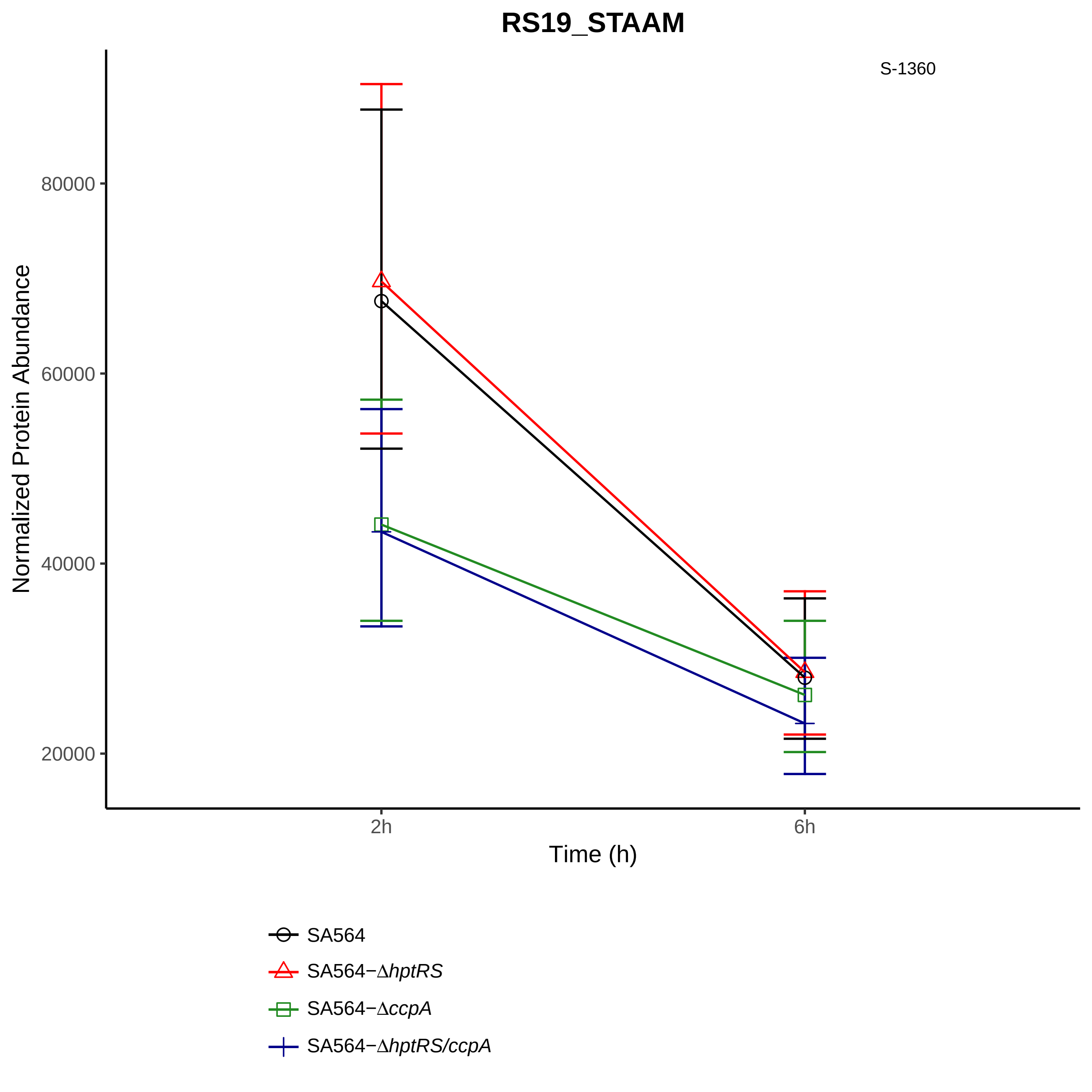

RS19_STAAM
S-1360
Normalized Protein Abundance
80000
60000
40000
20000
2h
6h
Time (h)
SA564
SA564−Δ*hptRS*
SA564−Δ*ccpA*
SA564−Δ*hptRS/ccpA*

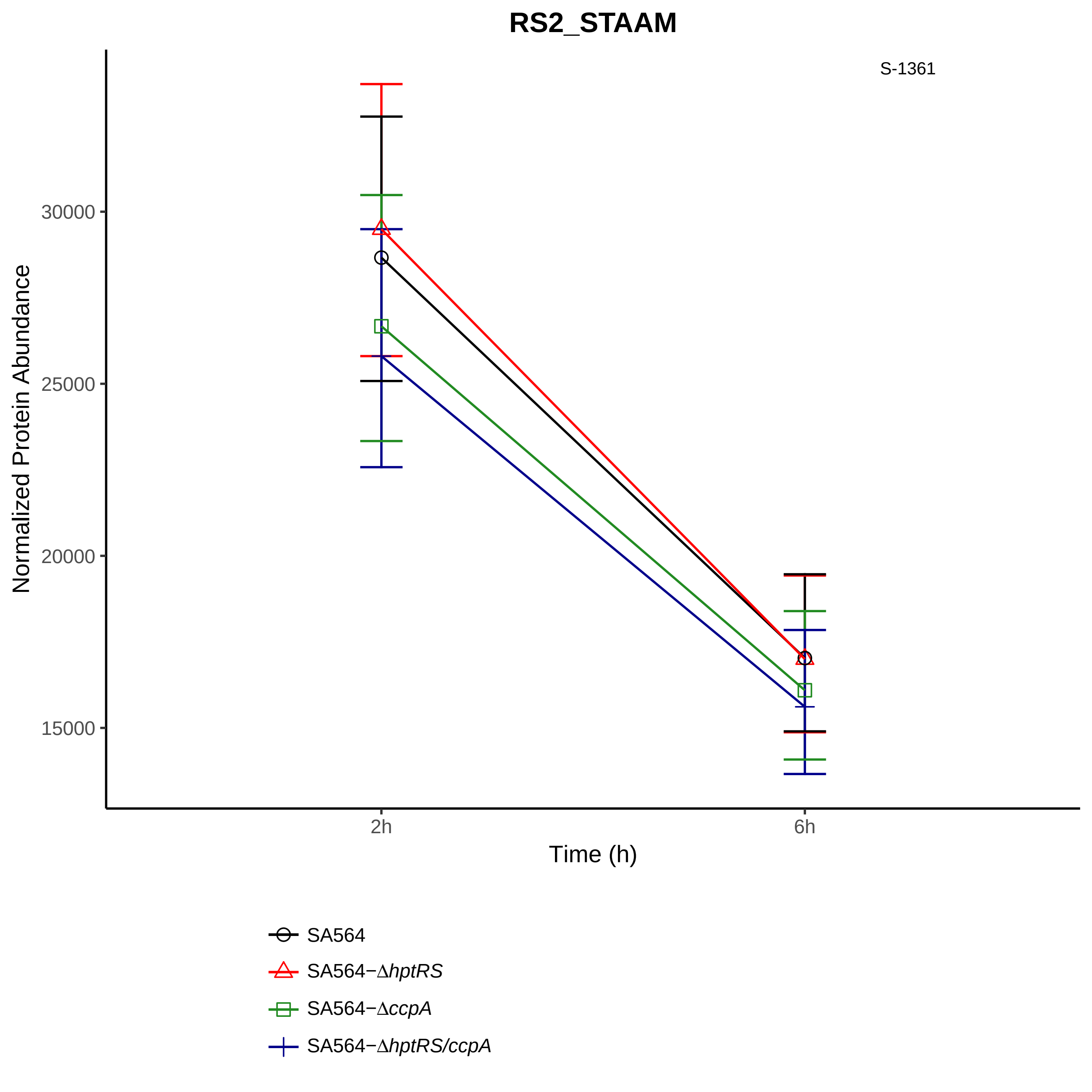

RS2_STAAM
S-1361
Normalized Protein Abundance
30000
25000
20000
15000
2h
6h
Time (h)
SA564
SA564−Δ*hptRS*
SA564−Δ*ccpA*
SA564−Δ*hptRS/ccpA*

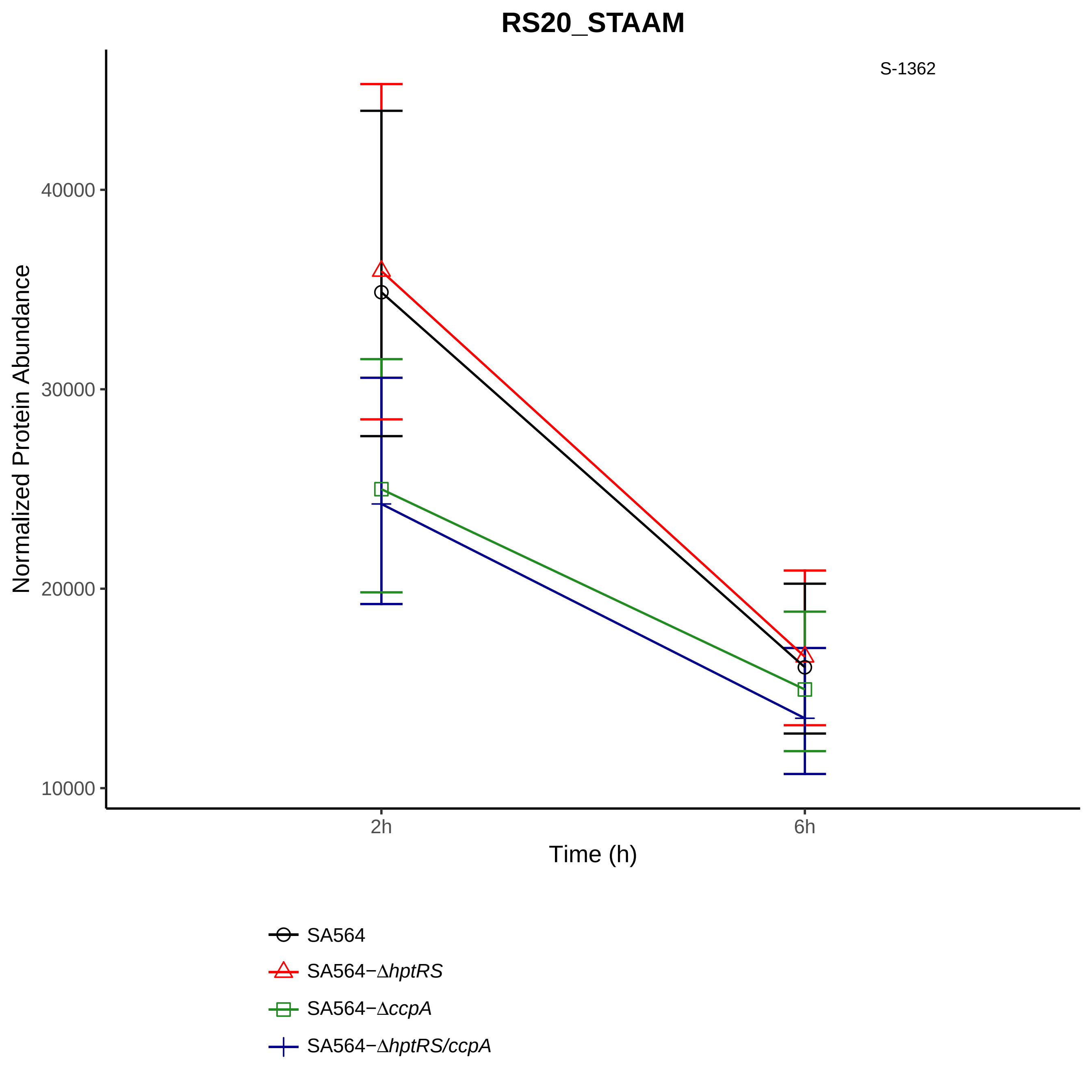

RS20_STAAM
S-1362
Normalized Protein Abundance
40000
30000
20000
10000
2h
6h
Time (h)
SA564
SA564−Δ*hptRS*
SA564−Δ*ccpA*
SA564−Δ*hptRS/ccpA*

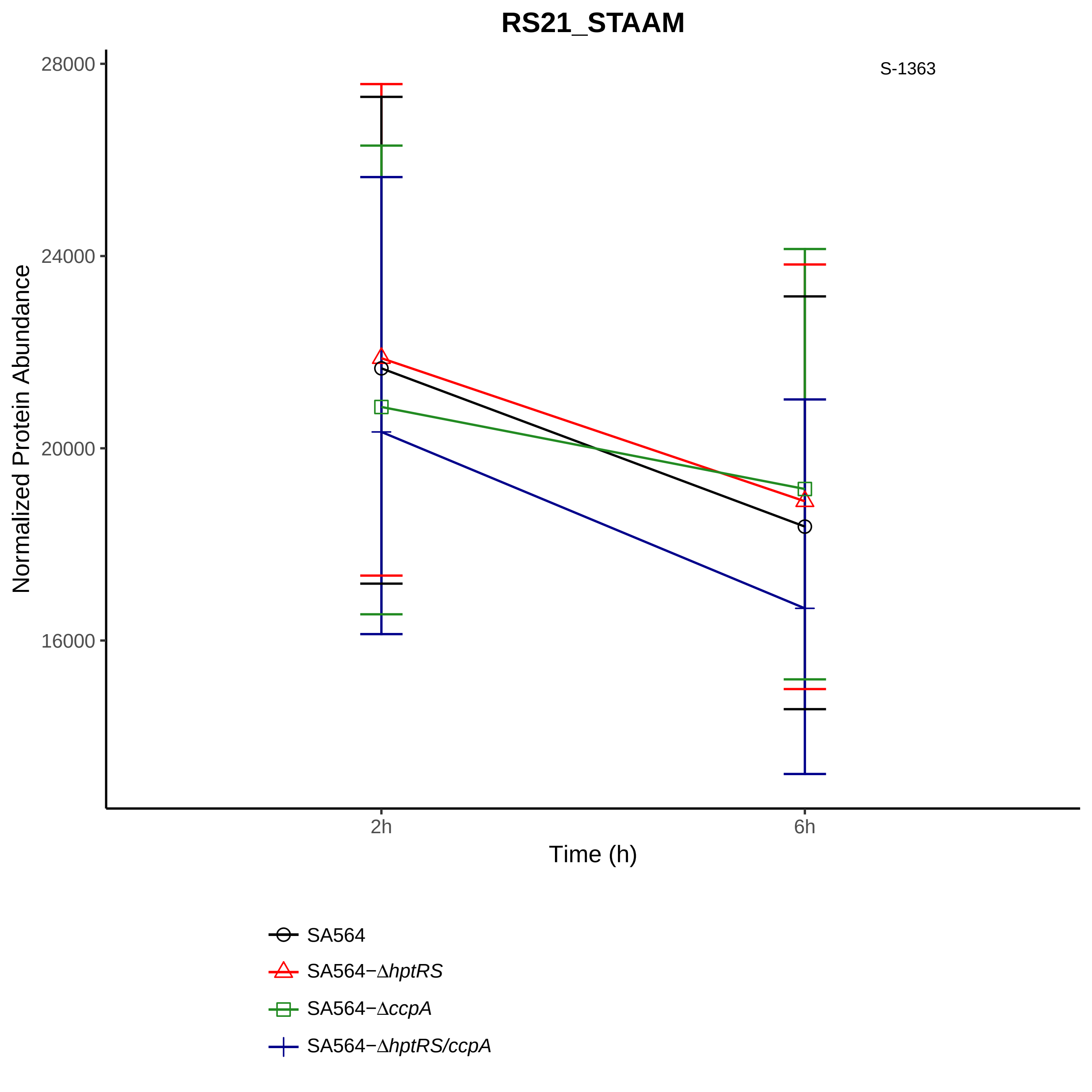
RS21_STAAM
S-1363
Normalized Protein Abundance
28000
24000
20000
16000
2h
6h
Time (h)
SA564
SA564−Δ*hptRS*
SA564−Δ*ccpA*
SA564−Δ*hptRS/ccpA*

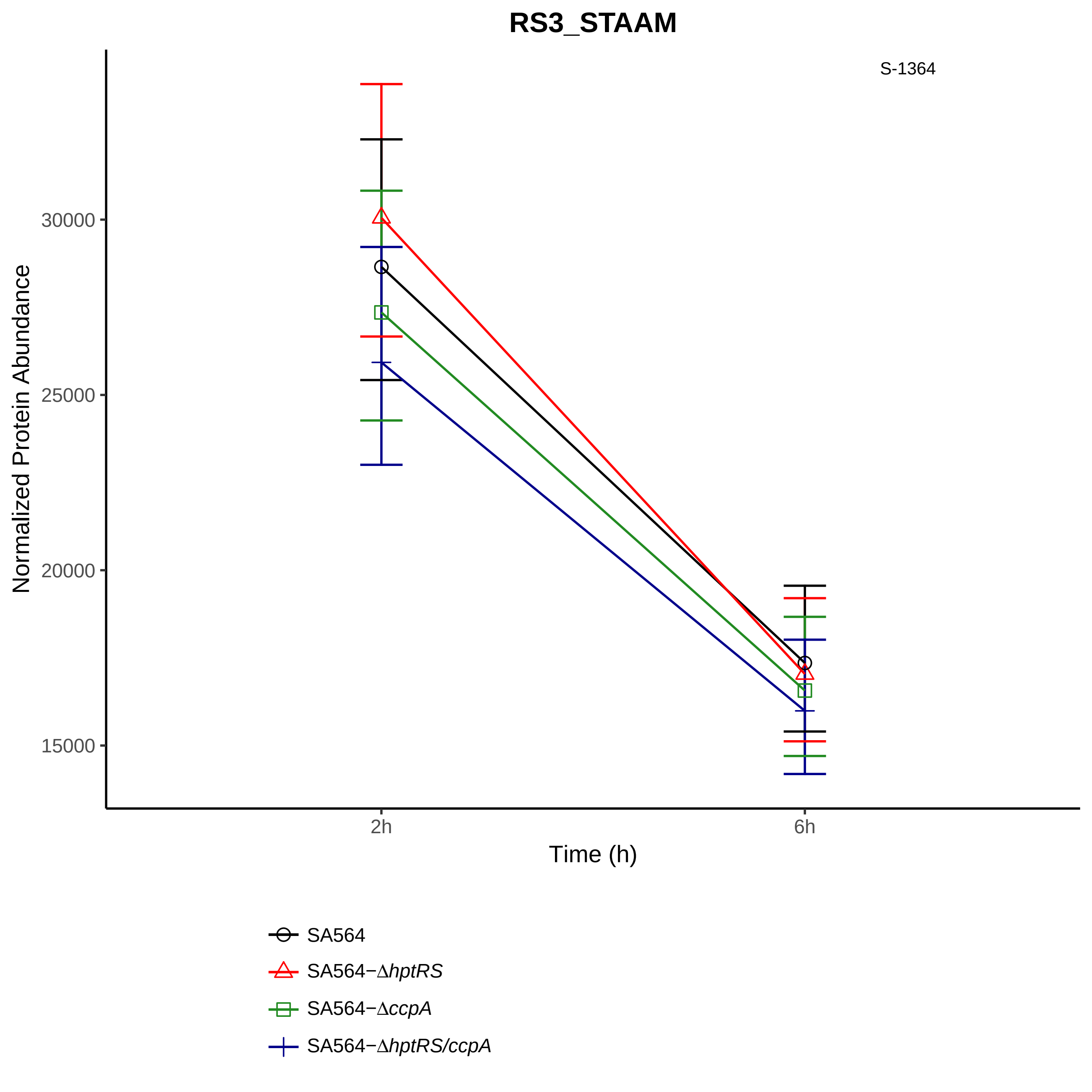

RS3_STAAM
S-1364
Normalized Protein Abundance
30000
25000
20000
15000
2h
6h
Time (h)
SA564
SA564−Δ*hptRS*
SA564−Δ*ccpA*
SA564−Δ*hptRS/ccpA*

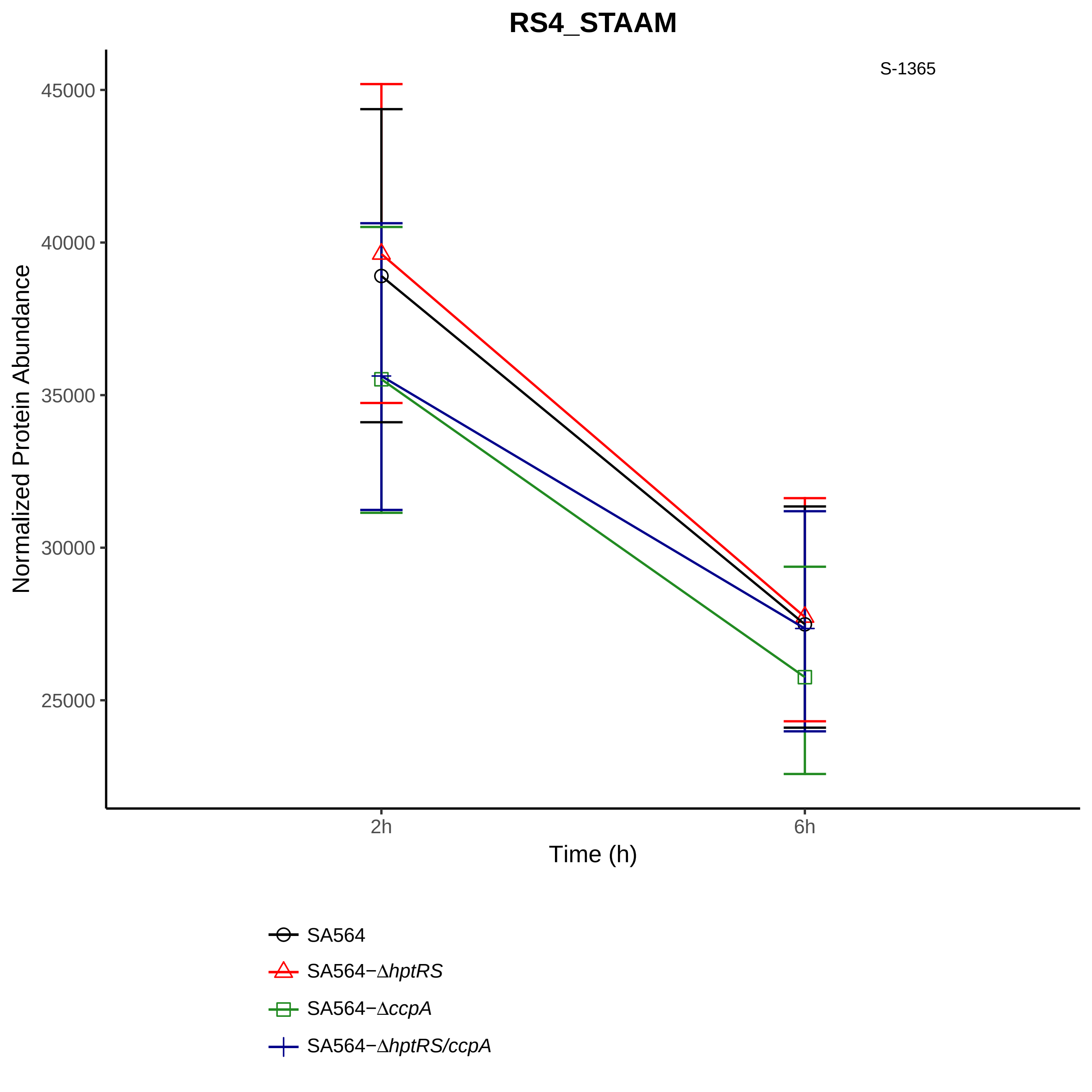
RS4_STAAM
S-1365
Normalized Protein Abundance
45000
40000
35000
30000
25000
2h
6h
Time (h)
SA564
SA564−Δ*hptRS*
SA564−Δ*ccpA*
SA564−Δ*hptRS/ccpA*

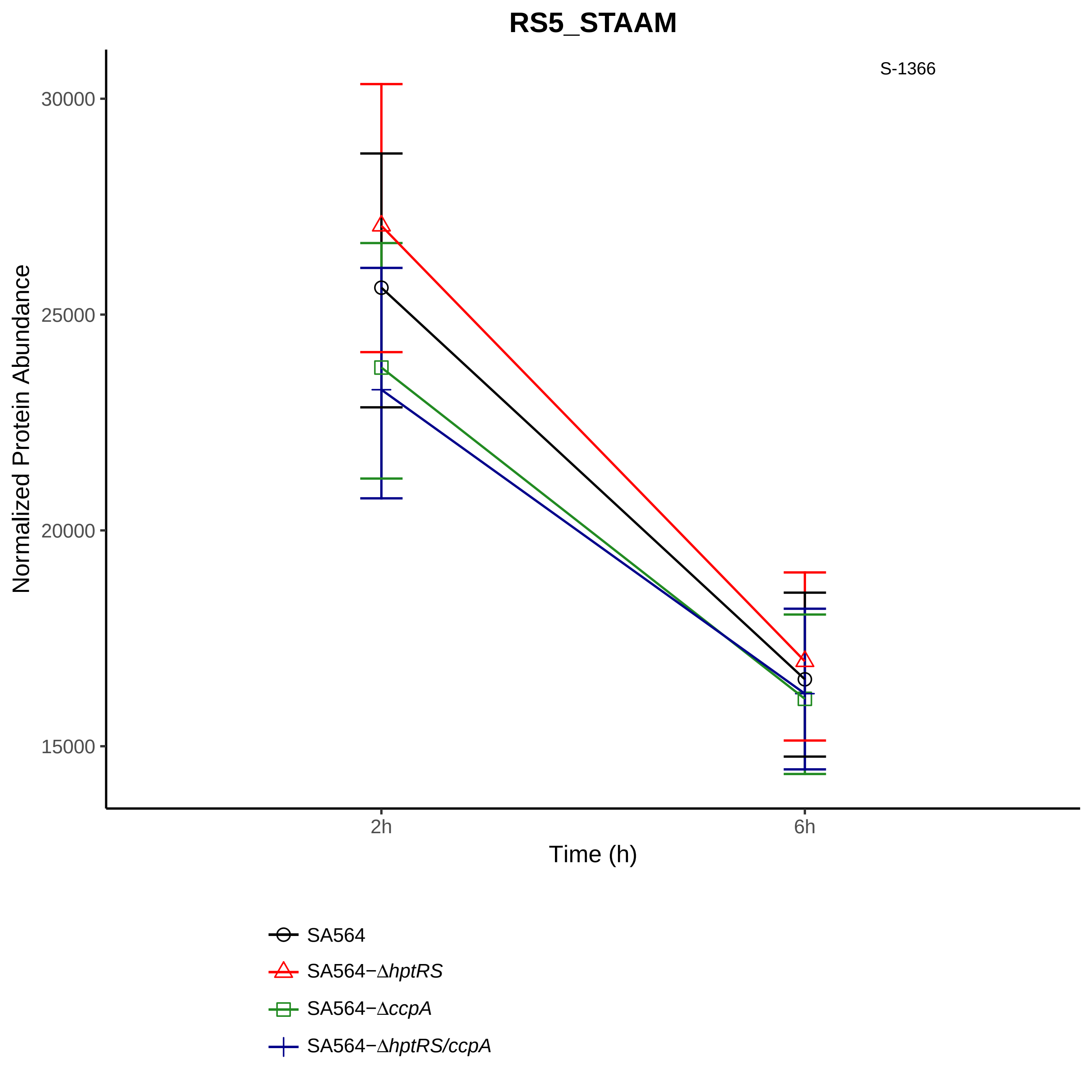
RS5_STAAM
S-1366
Normalized Protein Abundance
30000
25000
20000
15000
2h
6h
Time (h)
SA564
SA564−Δ*hptRS*
SA564−Δ*ccpA*
SA564−Δ*hptRS/ccpA*

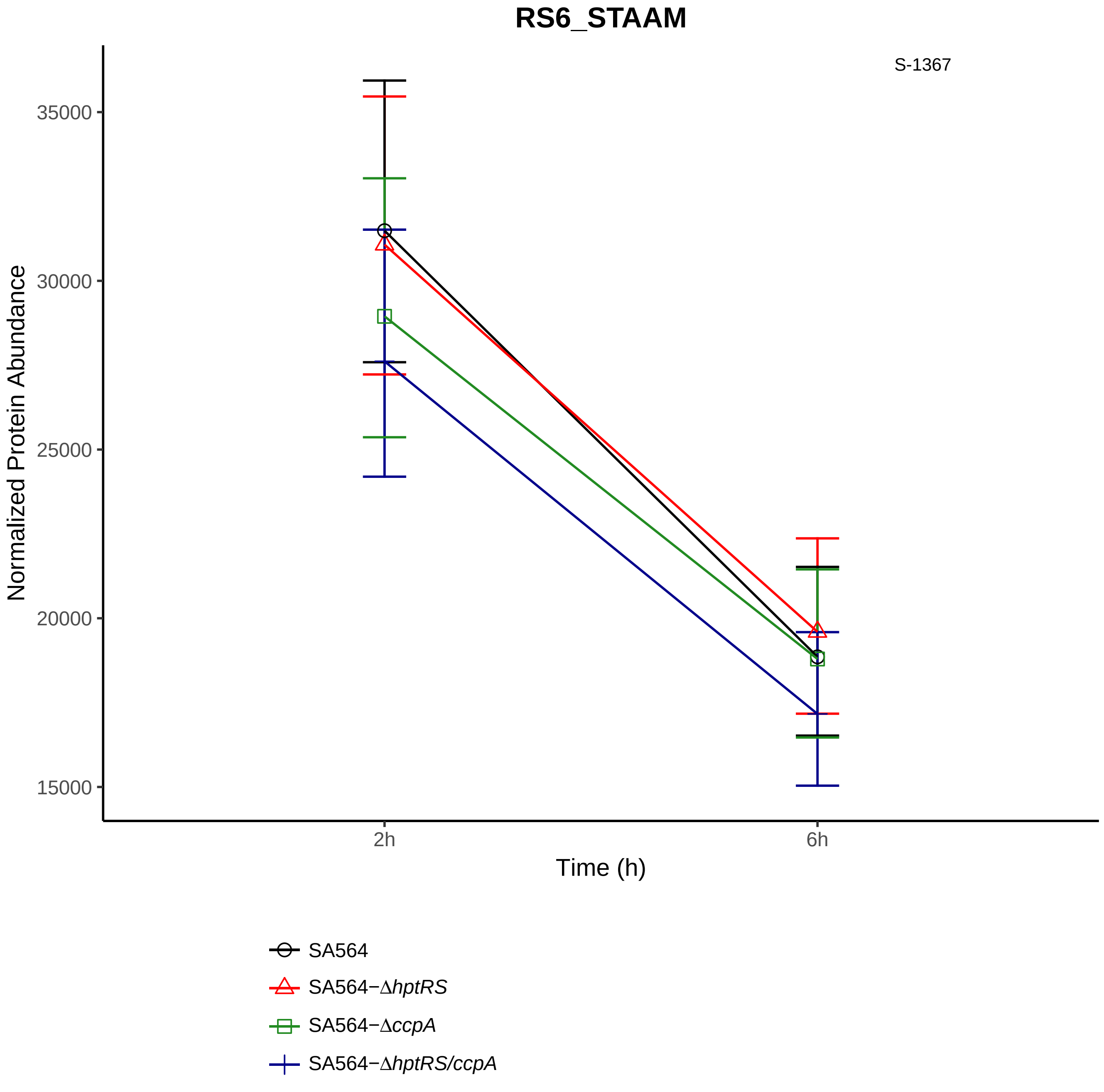
RS6_STAAM
S-1367
Normalized Protein Abundance
35000
30000
25000
20000
15000
2h
6h
Time (h)
SA564
SA564−Δ*hptRS*
SA564−Δ*ccpA*
SA564−Δ*hptRS/ccpA*

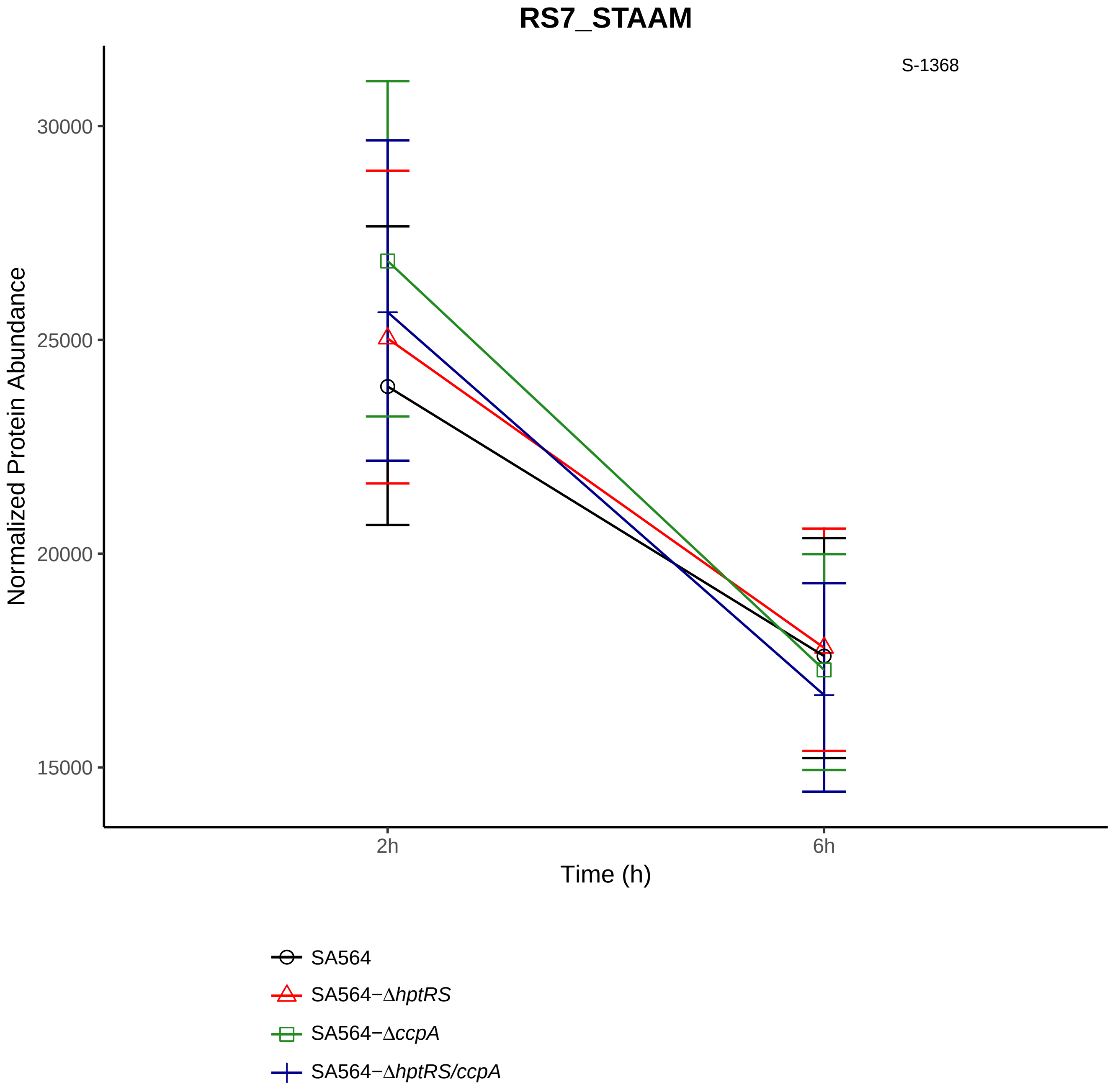

RS7_STAAM
S-1368
30000
25000
20000
15000
Normalized Protein Abundance
2h
6h
Time (h)
SA564
SA564−ΔhptRS
SA564−ΔccpA
SA564−ΔhptRS/ccpA

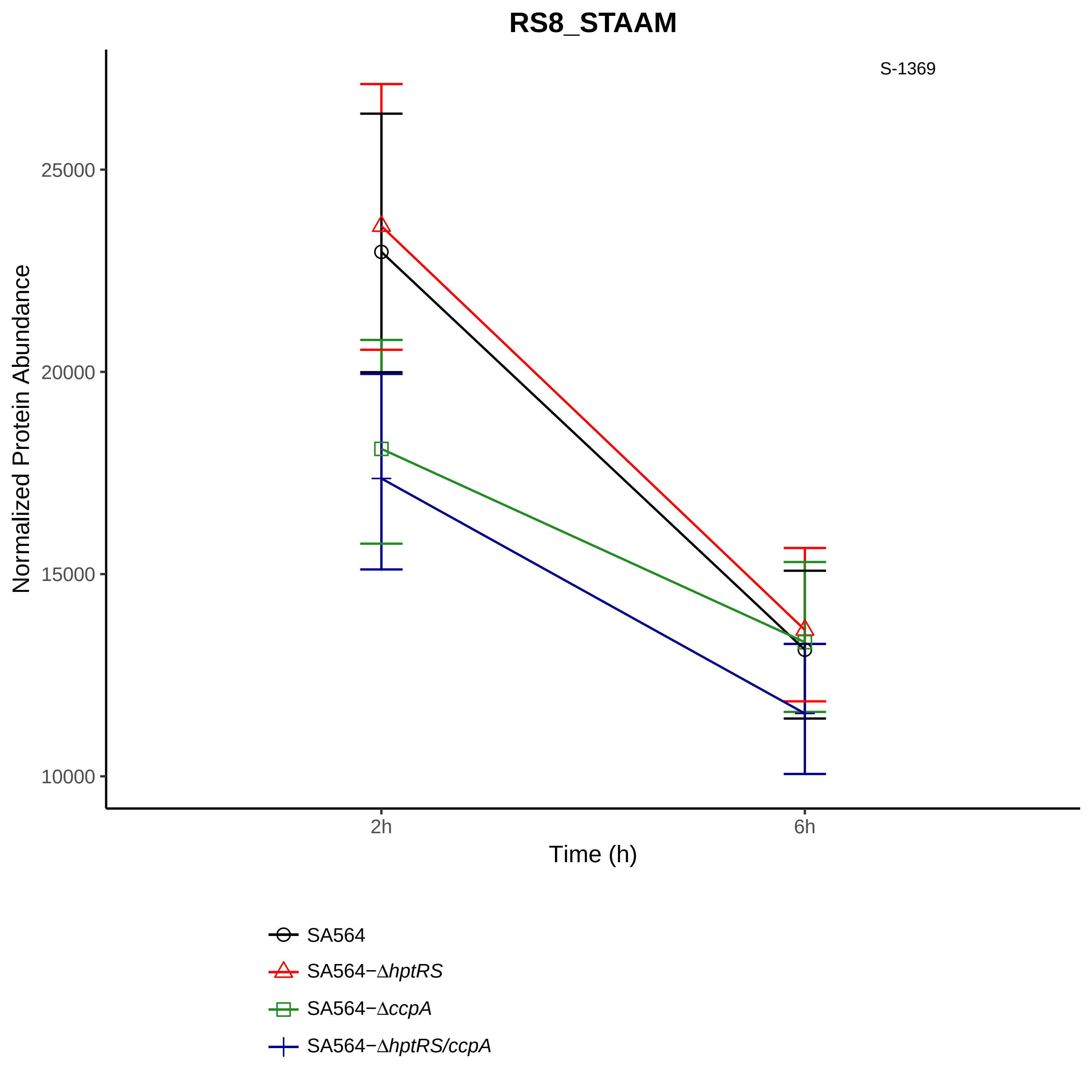

RS8_STAAM
S-1369
Normalized Protein Abundance
25000
20000
15000
10000
2h
6h
Time (h)
SA564
SA564−Δ*hptRS*
SA564−Δ*ccpA*
SA564−Δ*hptRS/ccpA*

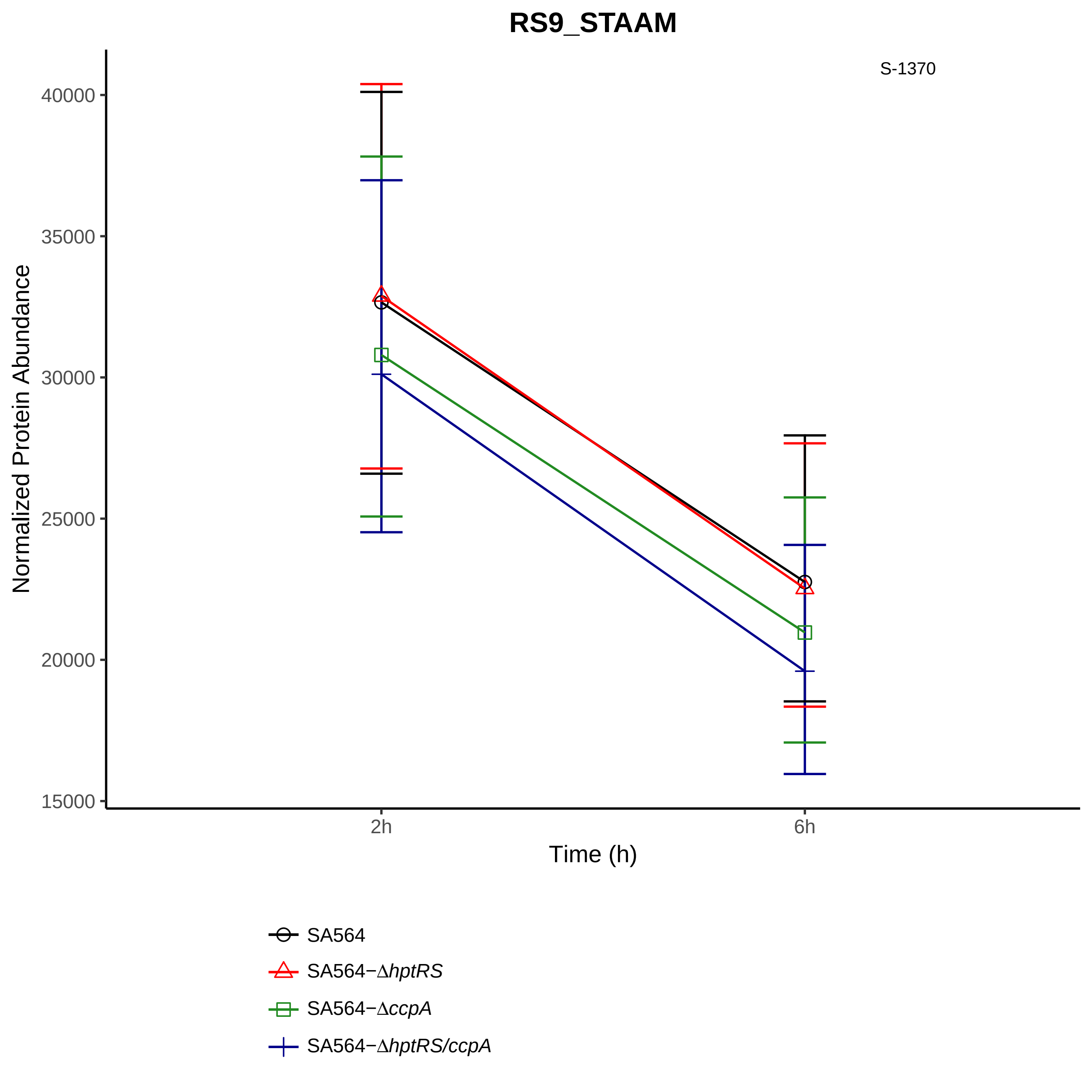
RS9_STAAM
S-1370
Normalized Protein Abundance
40000
35000
30000
25000
20000
15000
2h
6h
Time (h)
SA564
SA564−Δ*hptRS*
SA564−Δ*ccpA*
SA564−Δ*hptRS/ccpA*

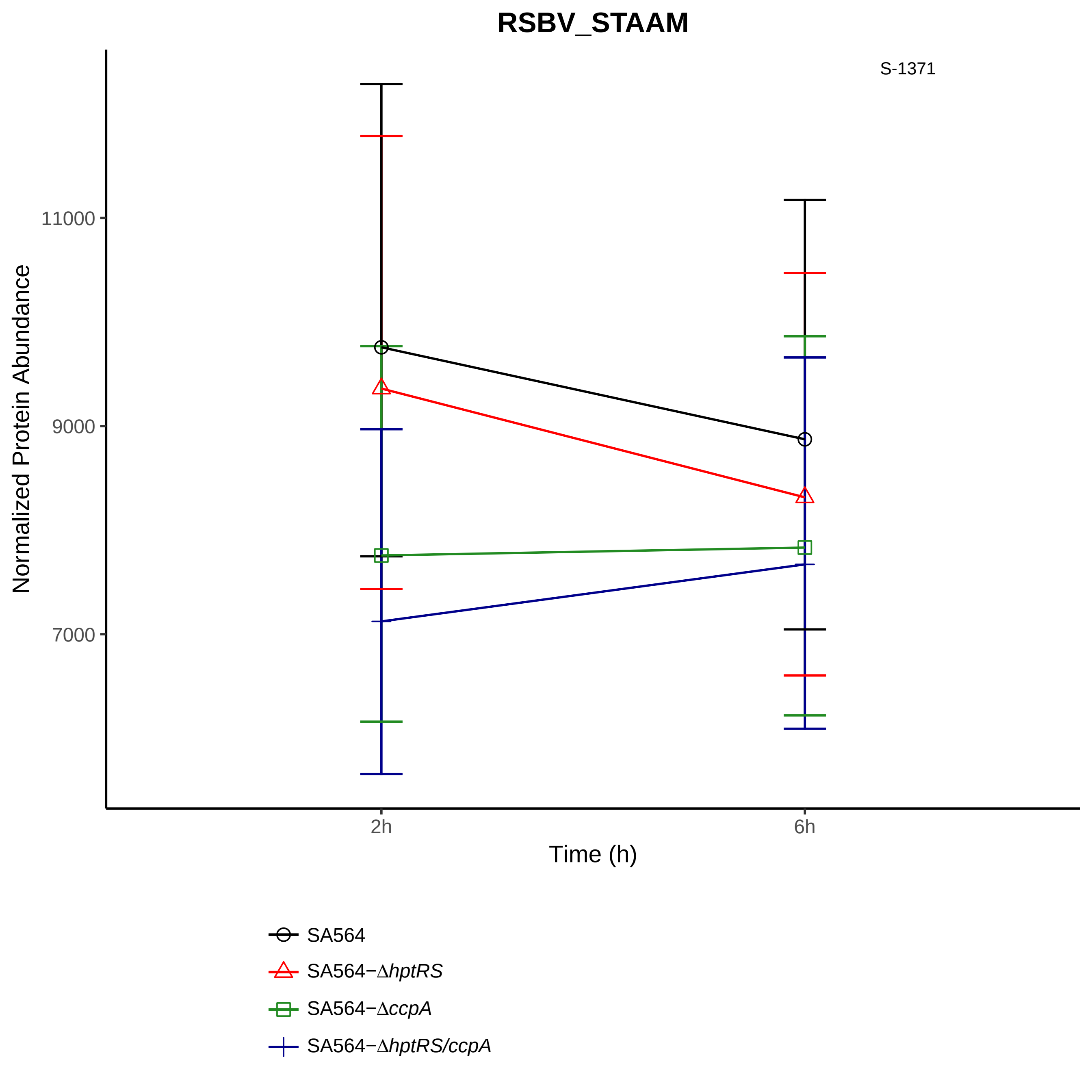
RSBV_STAAM
S-1371
Normalized Protein Abundance
11000
9000
7000
2h
6h
Time (h)
SA564
SA564−Δ*hptRS*
SA564−Δ*ccpA*
SA564−Δ*hptRS/ccpA*

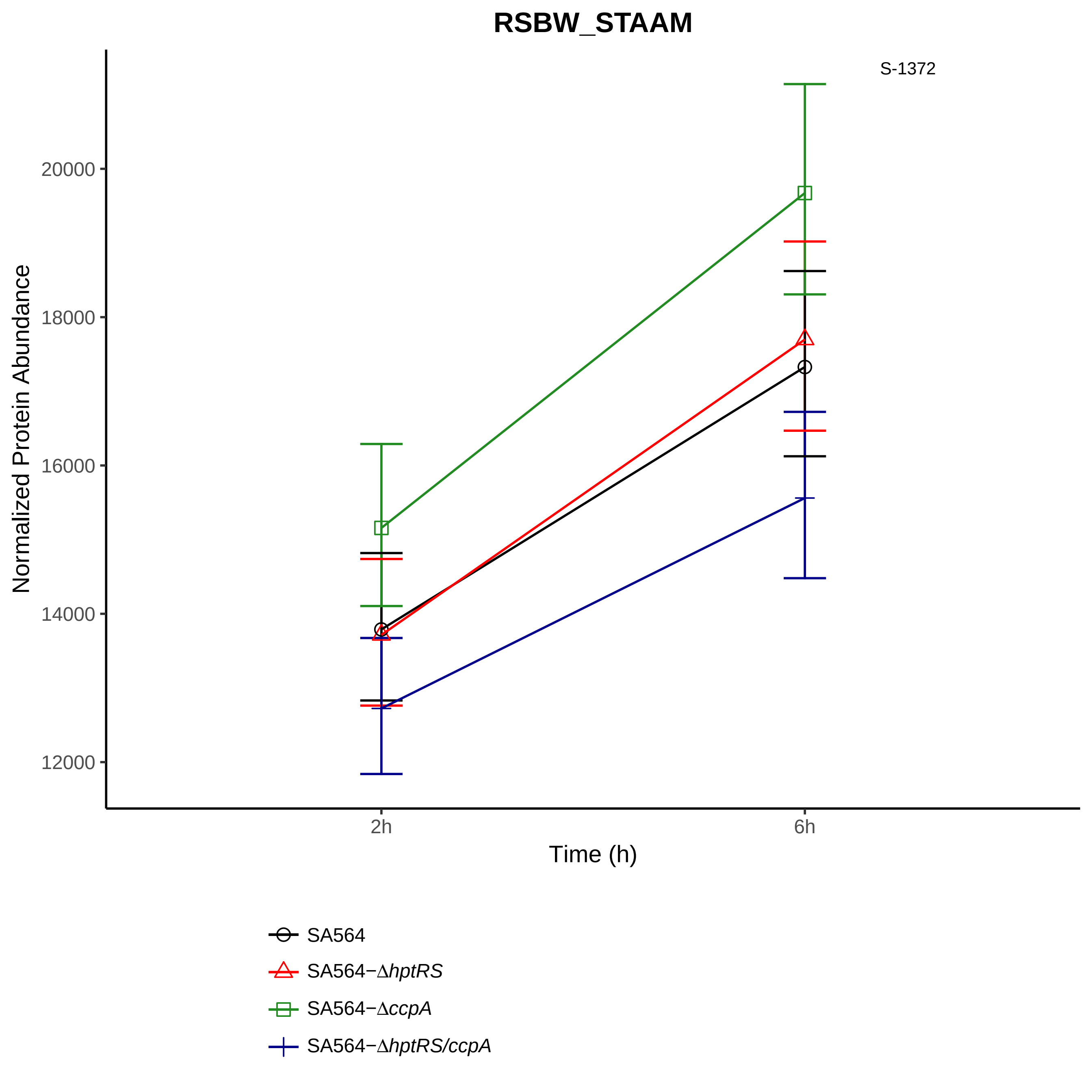

RSBW_STAAM
S-1372
Normalized Protein Abundance
20000
18000
16000
14000
12000
2h
6h
Time (h)
SA564
SA564−Δ*hptRS*
SA564−Δ*ccpA*
SA564−Δ*hptRS/ccpA*

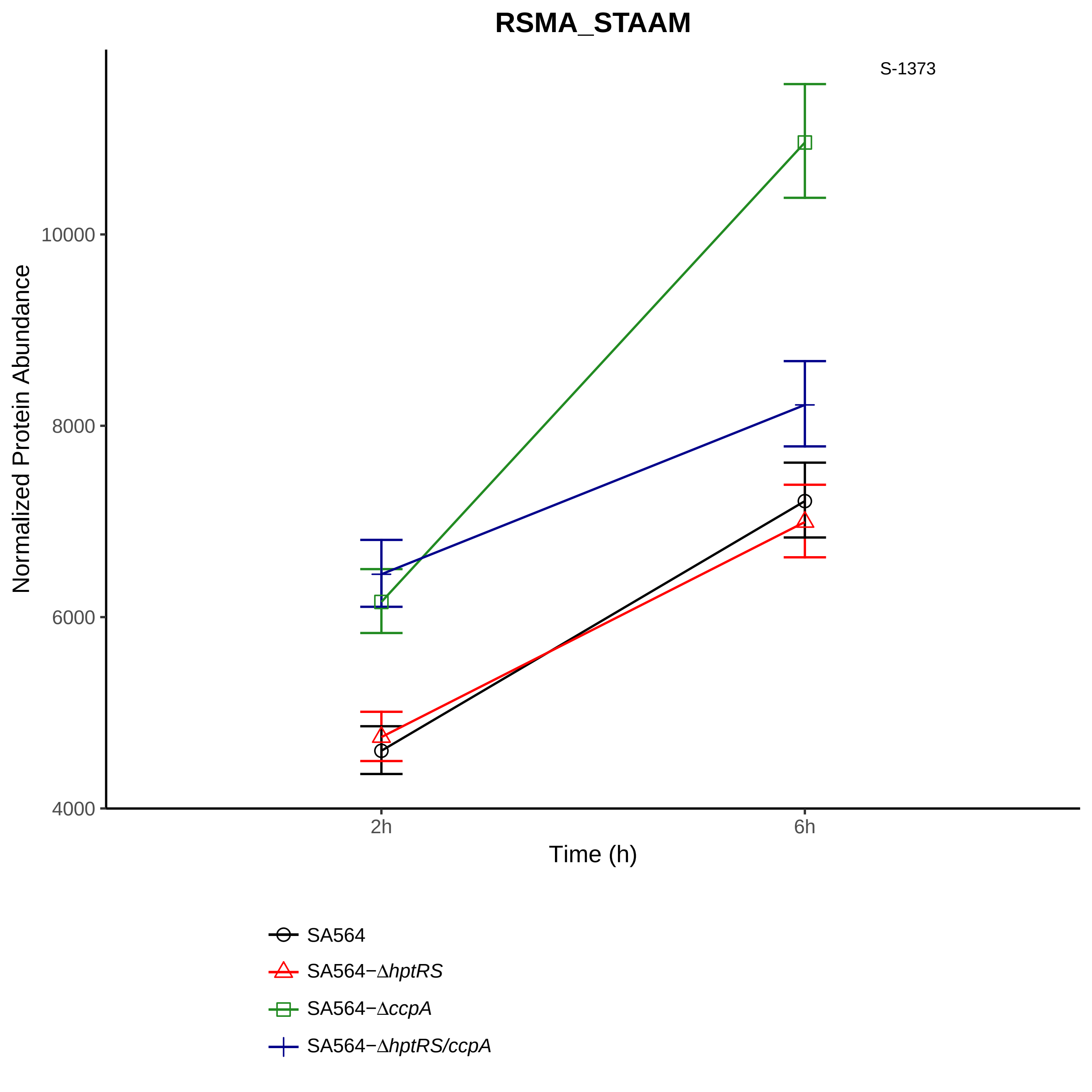

RSMA_STAAM
S-1373
Normalized Protein Abundance
10000
8000
6000
4000
2h
6h
Time (h)
SA564
SA564−Δ*hptRS*
SA564−Δ*ccpA*
SA564−Δ*hptRS/ccpA*

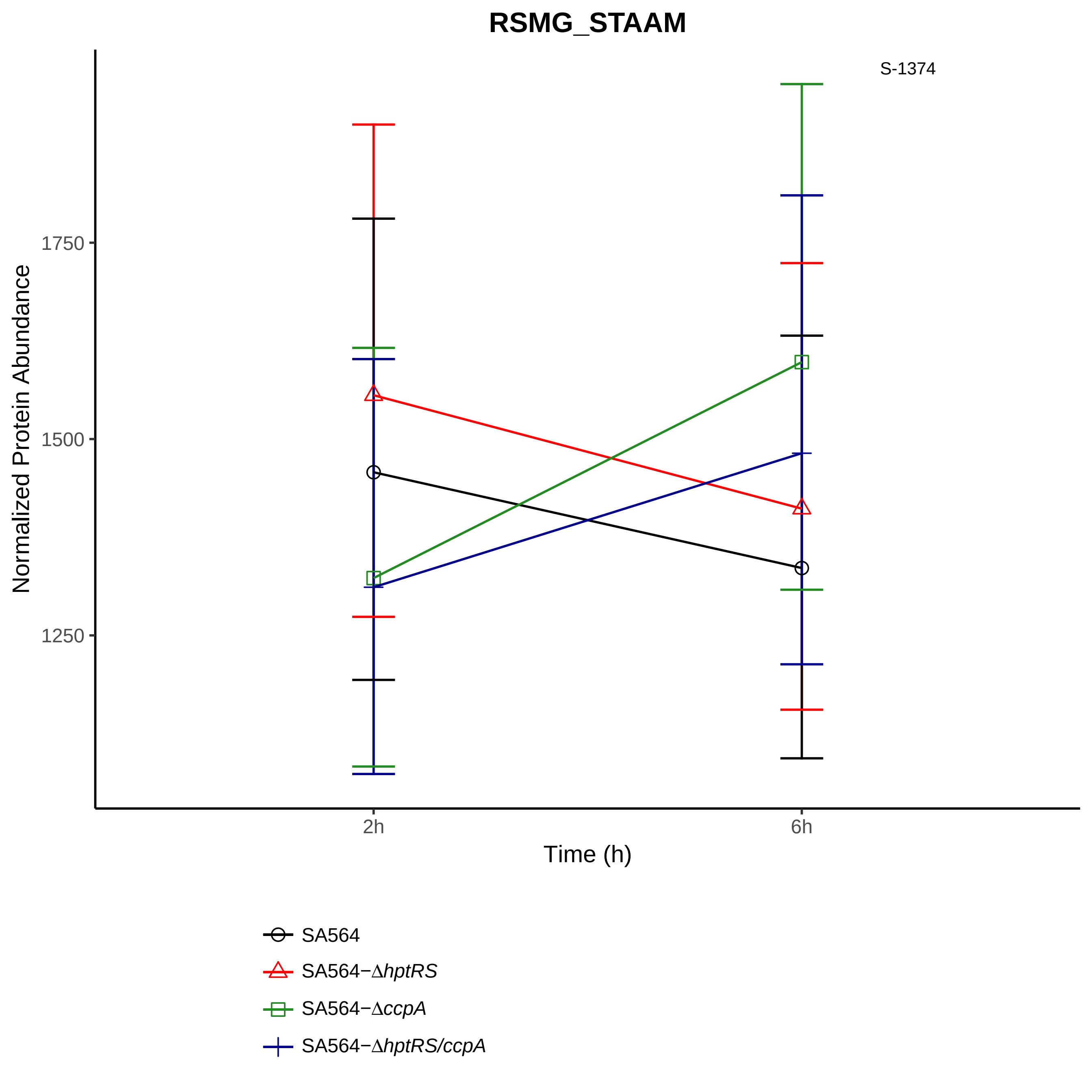

RSMG_STAAM
S-1374
Normalized Protein Abundance
1750
1500
1250
2h
6h
Time (h)
SA564
SA564−ΔhptRS
SA564−ΔccpA
SA564−ΔhptRS/ccpA

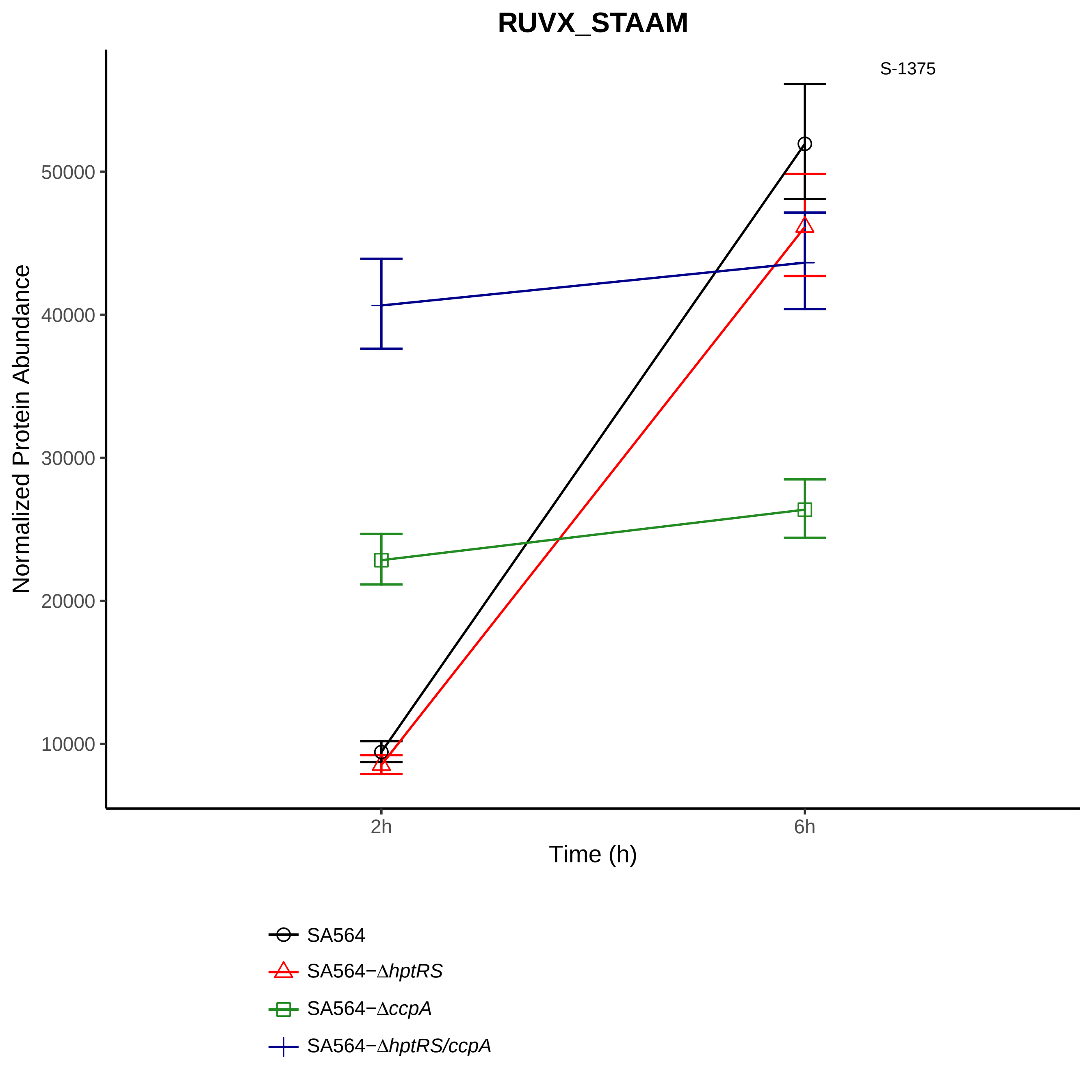

RUVX_STAAM
S-1375
Normalized Protein Abundance
50000
40000
30000
20000
10000
2h
6h
Time (h)
SA564
SA564−Δ*hptRS*
SA564−Δ*ccpA*
SA564−Δ*hptRS/ccpA*

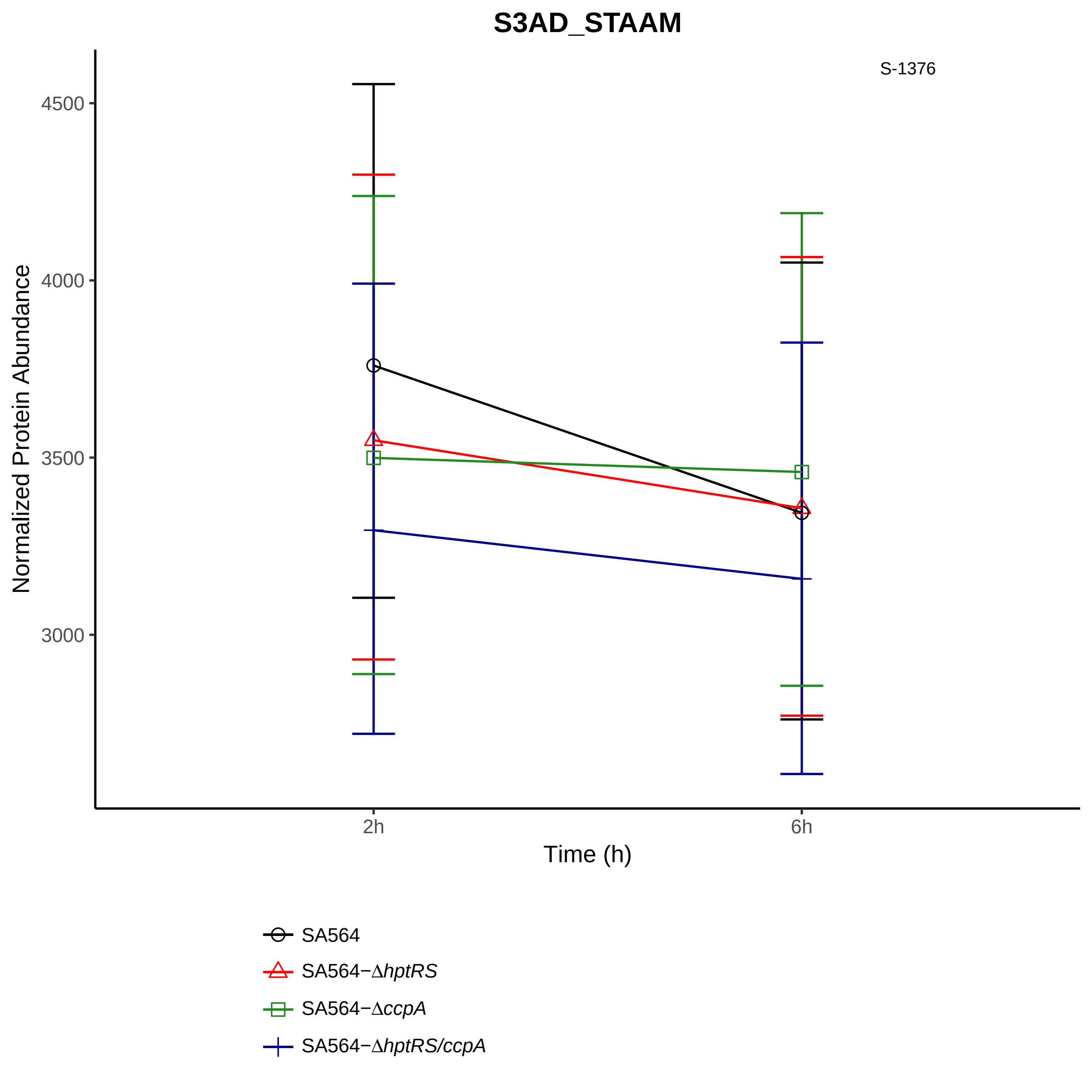

S3AD_STAAM
S-1376
Normalized Protein Abundance
4500
4000
3500
3000
2h
6h
Time (h)
SA564
SA564−Δ*hptRS*
SA564−Δ*ccpA*
SA564−Δ*hptRS/ccpA*

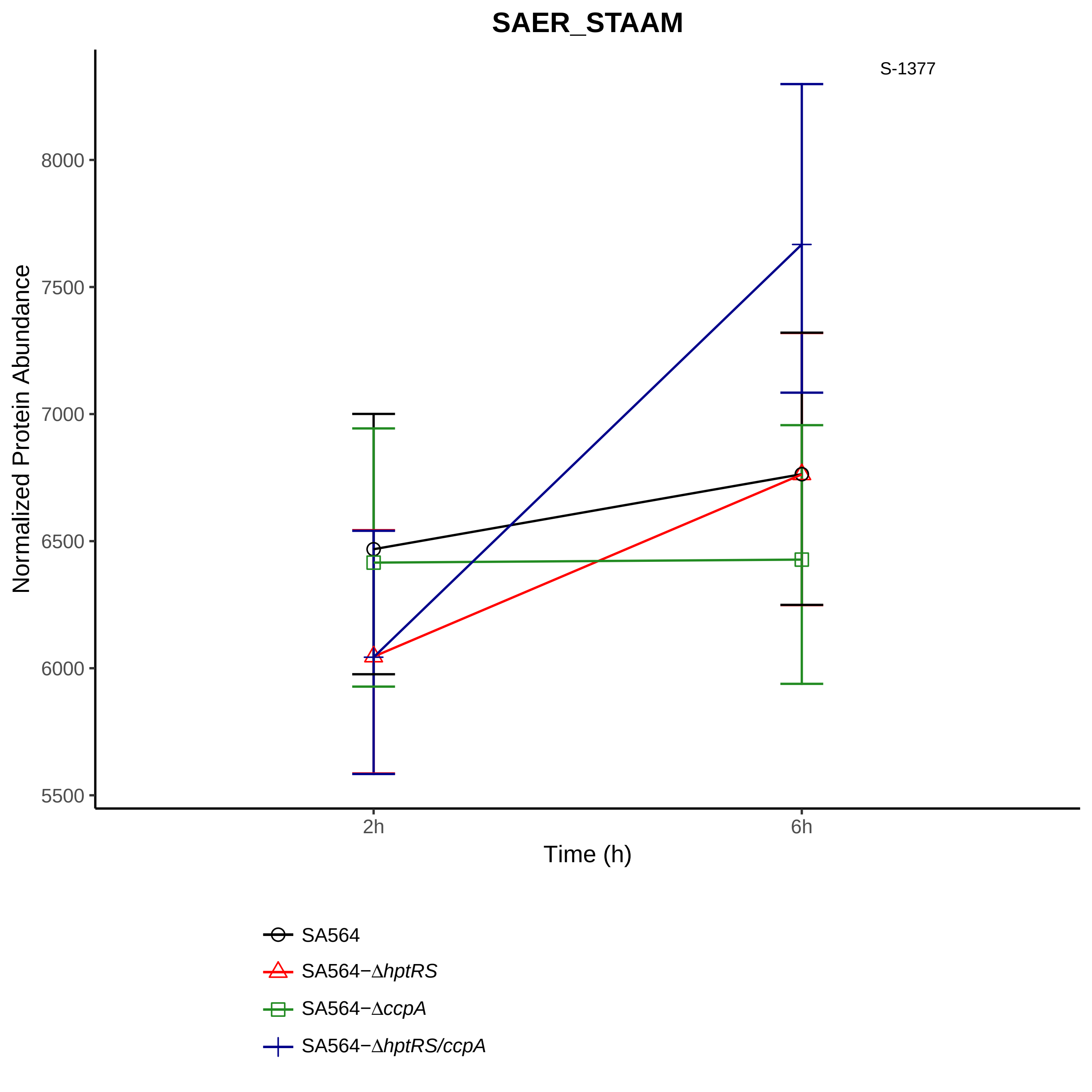

SAER_STAAM
S-1377
Normalized Protein Abundance
8000
7500
7000
6500
6000
5500
2h
6h
Time (h)
SA564
SA564−Δ*hptRS*
SA564−Δ*ccpA*
SA564−Δ*hptRS/ccpA*

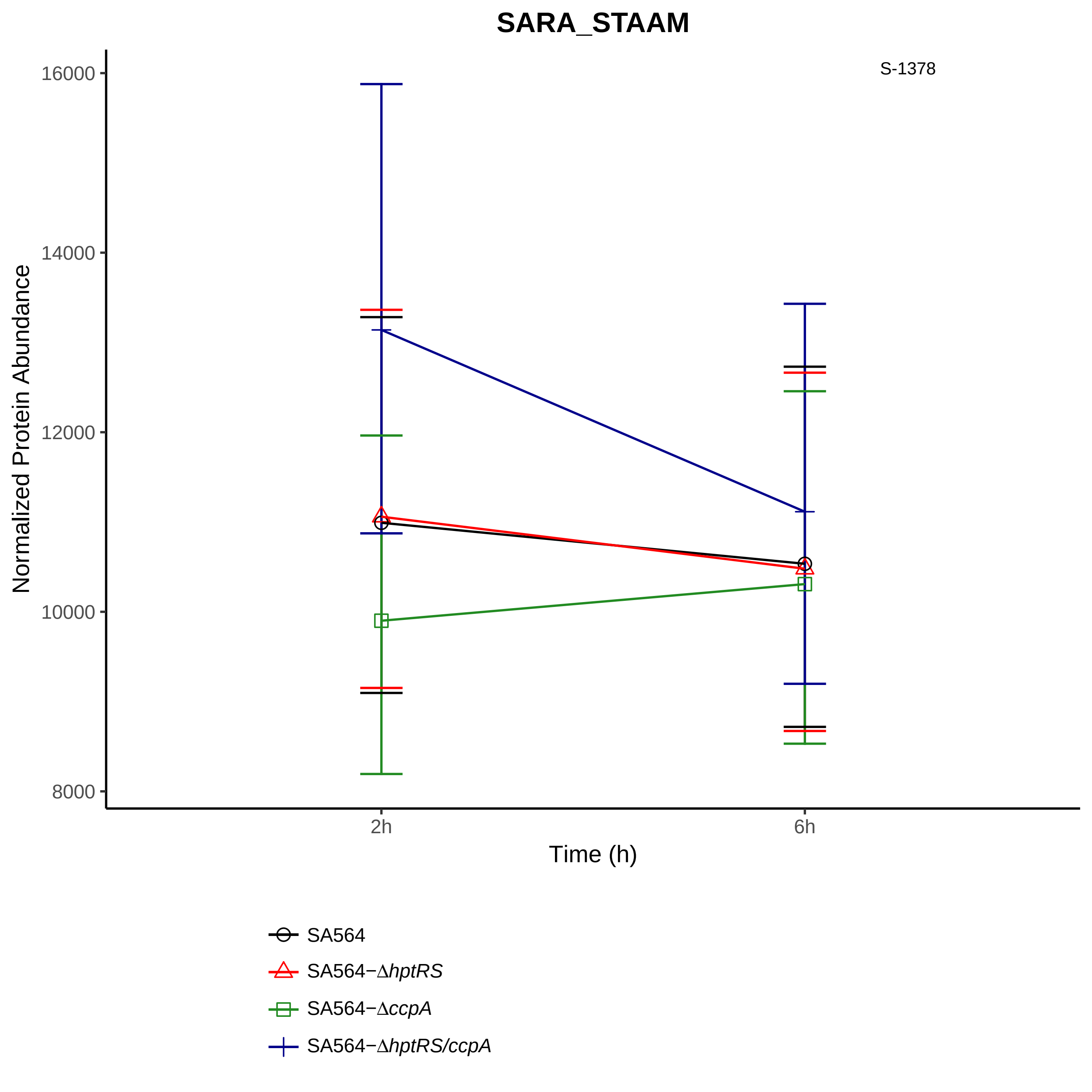

SARA_STAAM
S-1378
Normalized Protein Abundance
16000
14000
12000
10000
8000
2h
6h
Time (h)
SA564
SA564−Δ*hptRS*
SA564−Δ*ccpA*
SA564−Δ*hptRS/ccpA*

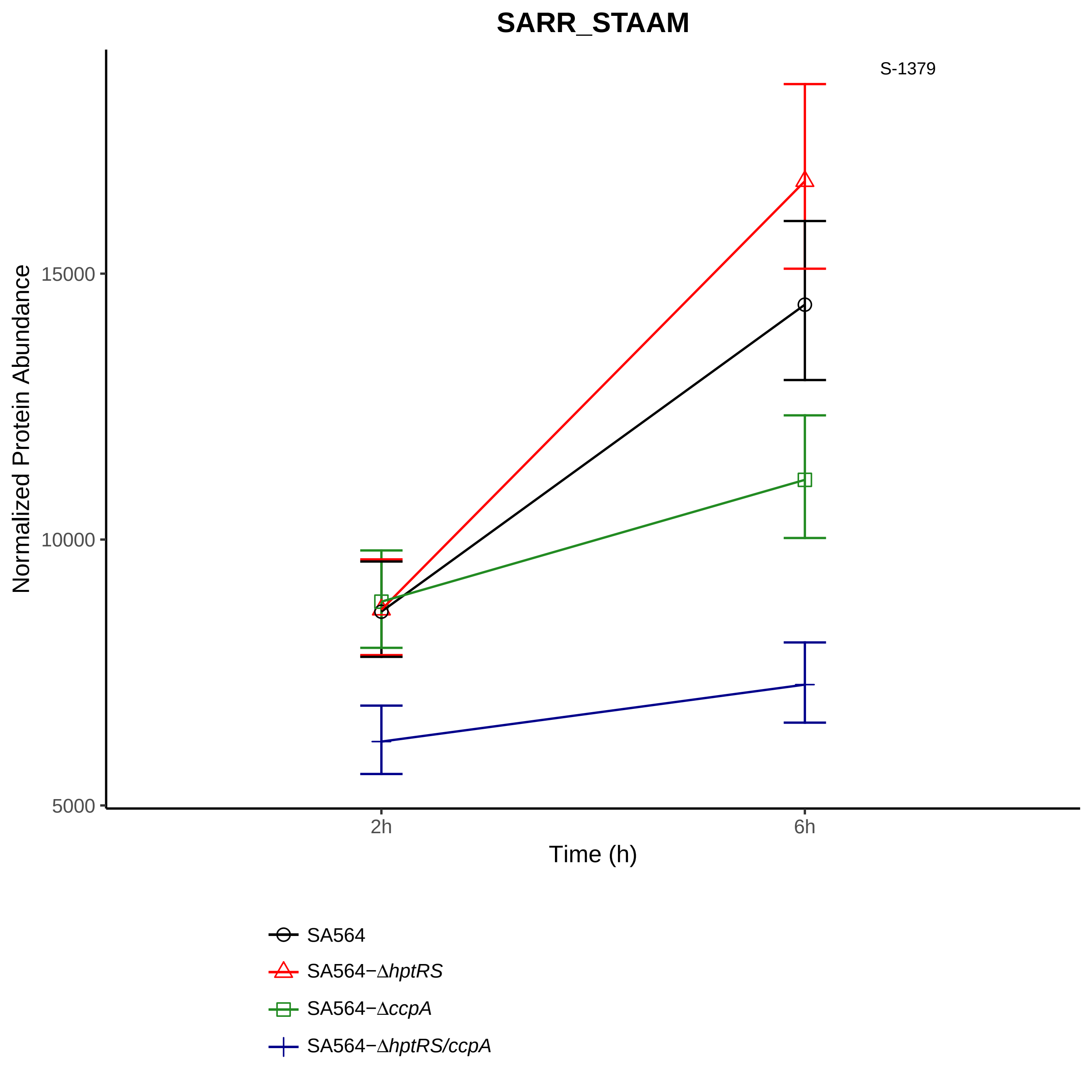

SARR_STAAM
S-1379
Normalized Protein Abundance
15000
10000
5000
2h
6h
Time (h)
SA564
SA564−Δ*hptRS*
SA564−Δ*ccpA*
SA564−Δ*hptRS/ccpA*

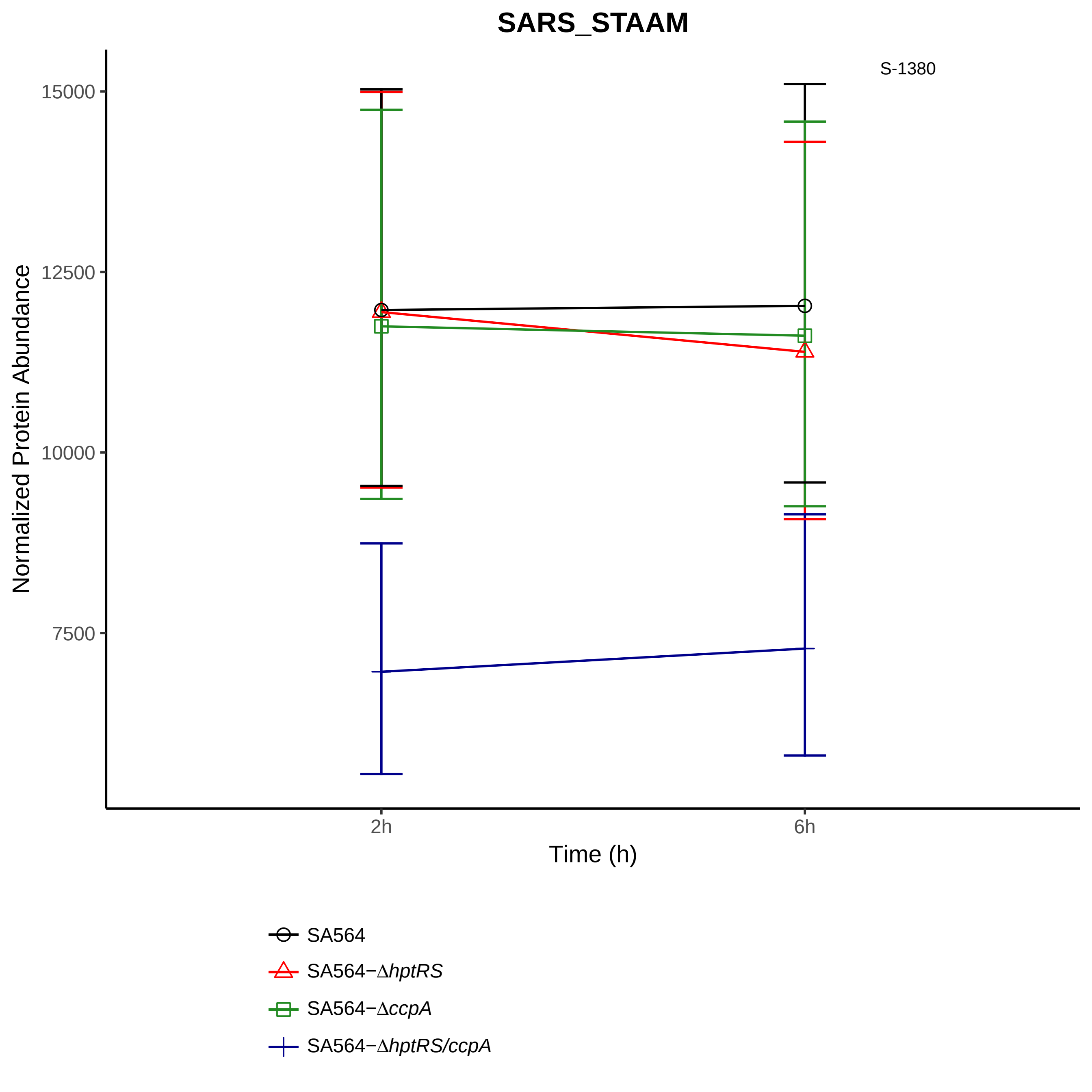

SARS_STAAM
S-1380
15000
12500
10000
7500
Normalized Protein Abundance
2h
6h
Time (h)
SA564
SA564−ΔhptRS
SA564−ΔccpA
SA564−ΔhptRS/ccpA

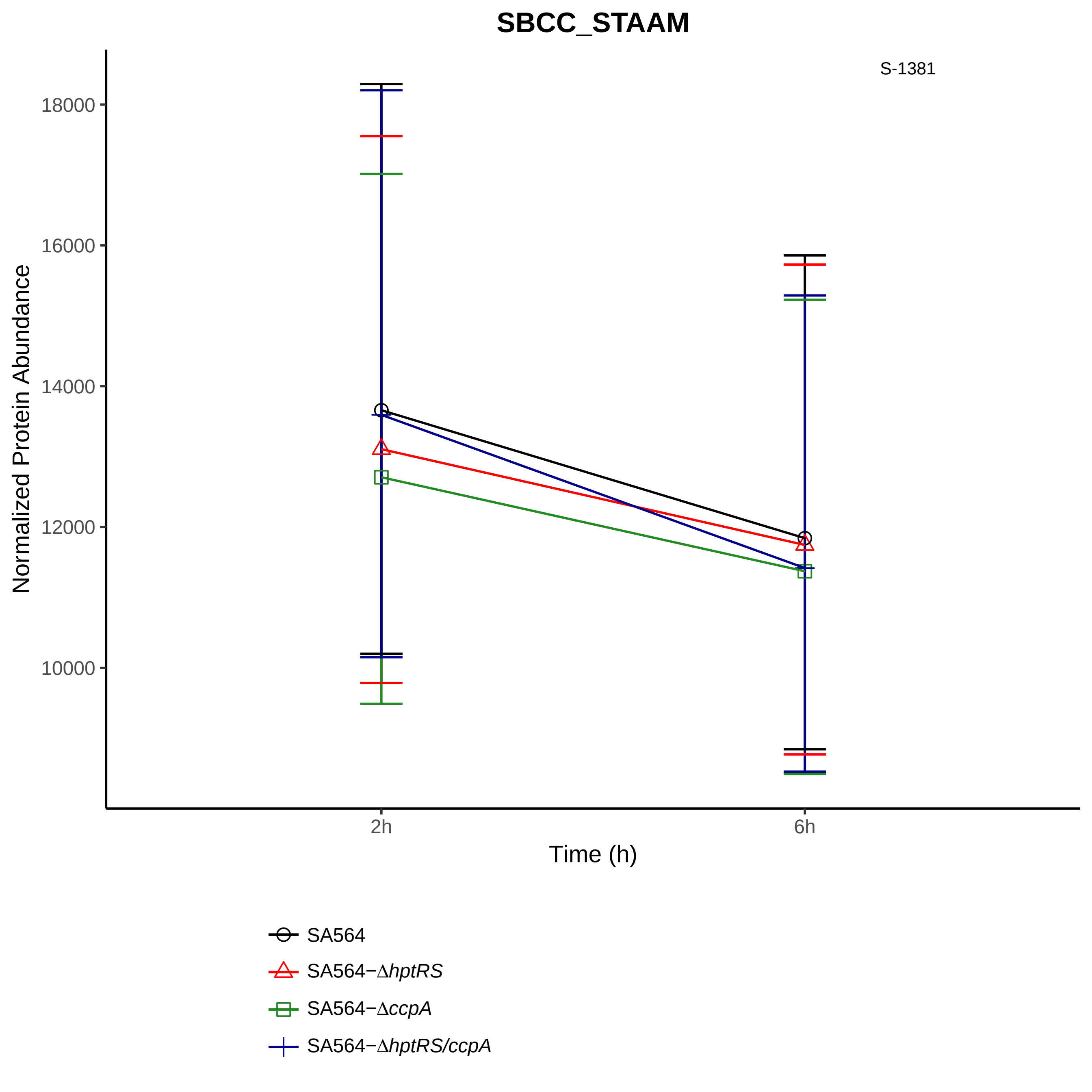

SBCC_STAAM
S-1381
Normalized Protein Abundance
18000
16000
14000
12000
10000
2h
6h
Time (h)
SA564
SA564−Δ*hptRS*
SA564−Δ*ccpA*
SA564−Δ*hptRS/ccpA*

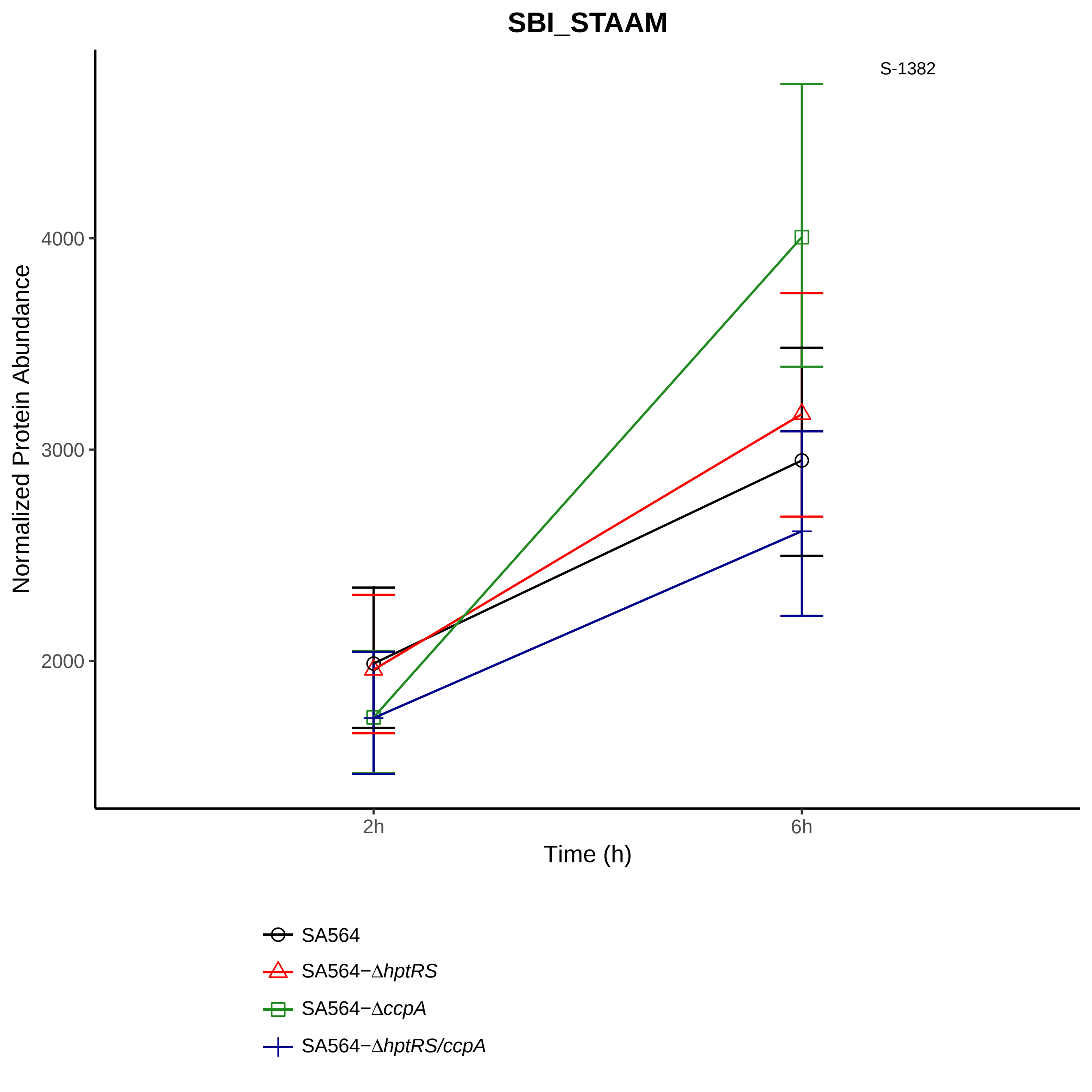

SBI_STAAM
S-1382
Normalized Protein Abundance
4000
3000
2000
2h
6h
Time (h)
SA564
SA564−Δ*hptRS*
SA564−Δ*ccpA*
SA564−Δ*hptRS/ccpA*

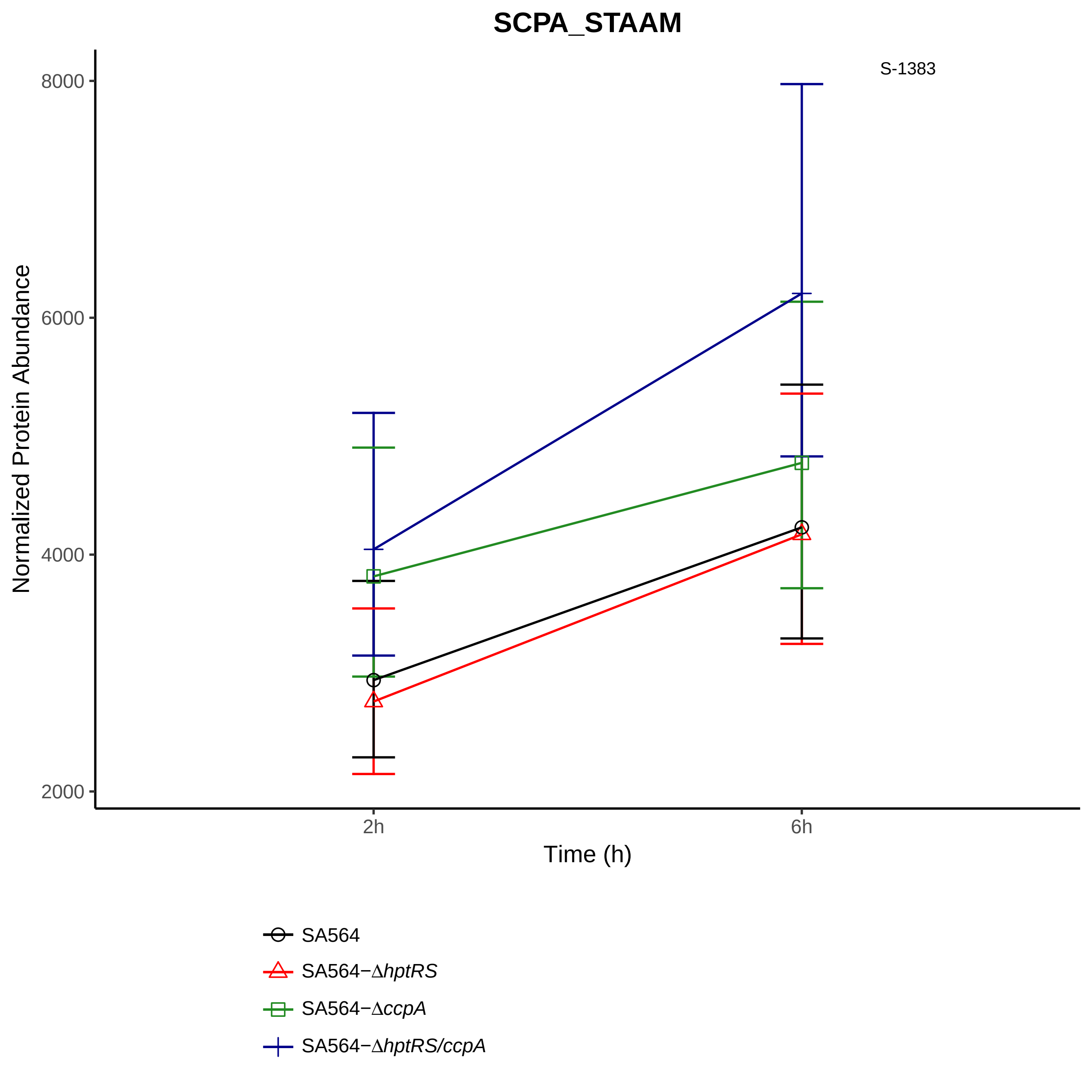
SCPA_STAAM
S-1383
8000
6000
4000
2000
Normalized Protein Abundance
2h
6h
Time (h)
SA564
SA564−ΔhptRS
SA564−ΔccpA
SA564−ΔhptRS/ccpA

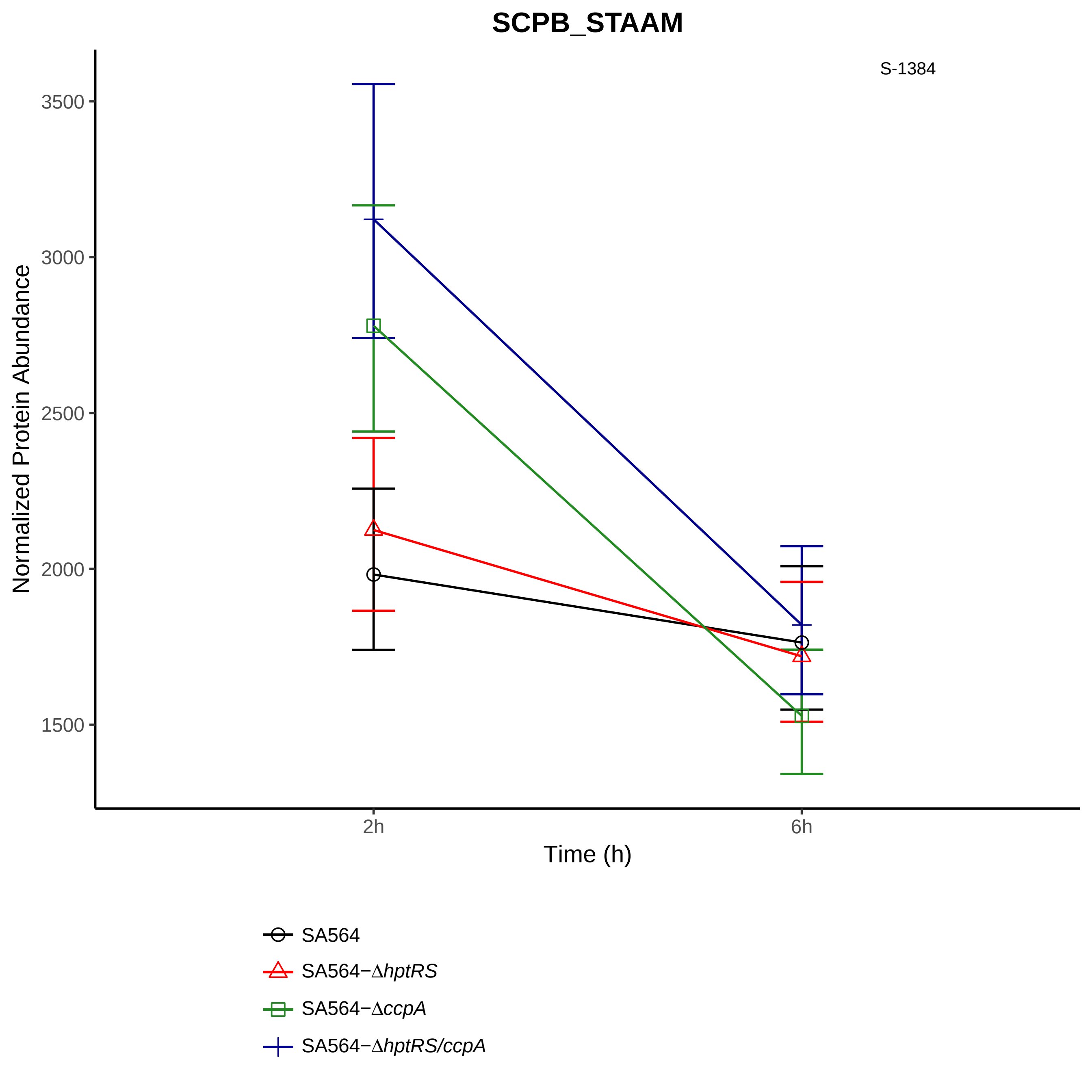

SCPB_STAAM
S-1384
Normalized Protein Abundance
3500
3000
2500
2000
1500
2h
6h
Time (h)
SA564
SA564−Δ*hptRS*
SA564−Δ*ccpA*
SA564−Δ*hptRS/ccpA*

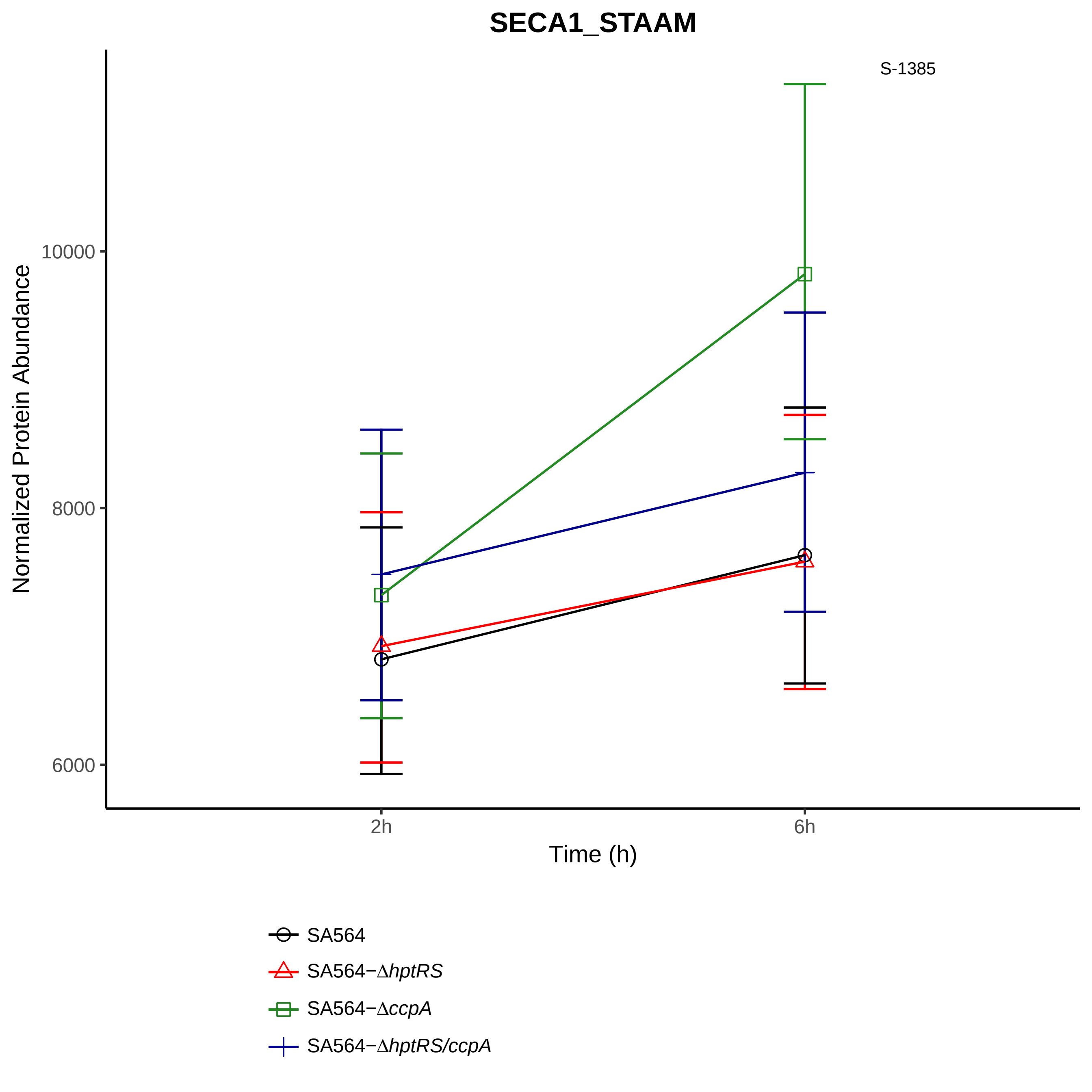
SECA1_STAAM
S-1385
Normalized Protein Abundance
10000
8000
6000
2h
6h
Time (h)
SA564
SA564−Δ*hptRS*
SA564−Δ*ccpA*
SA564−Δ*hptRS/ccpA*

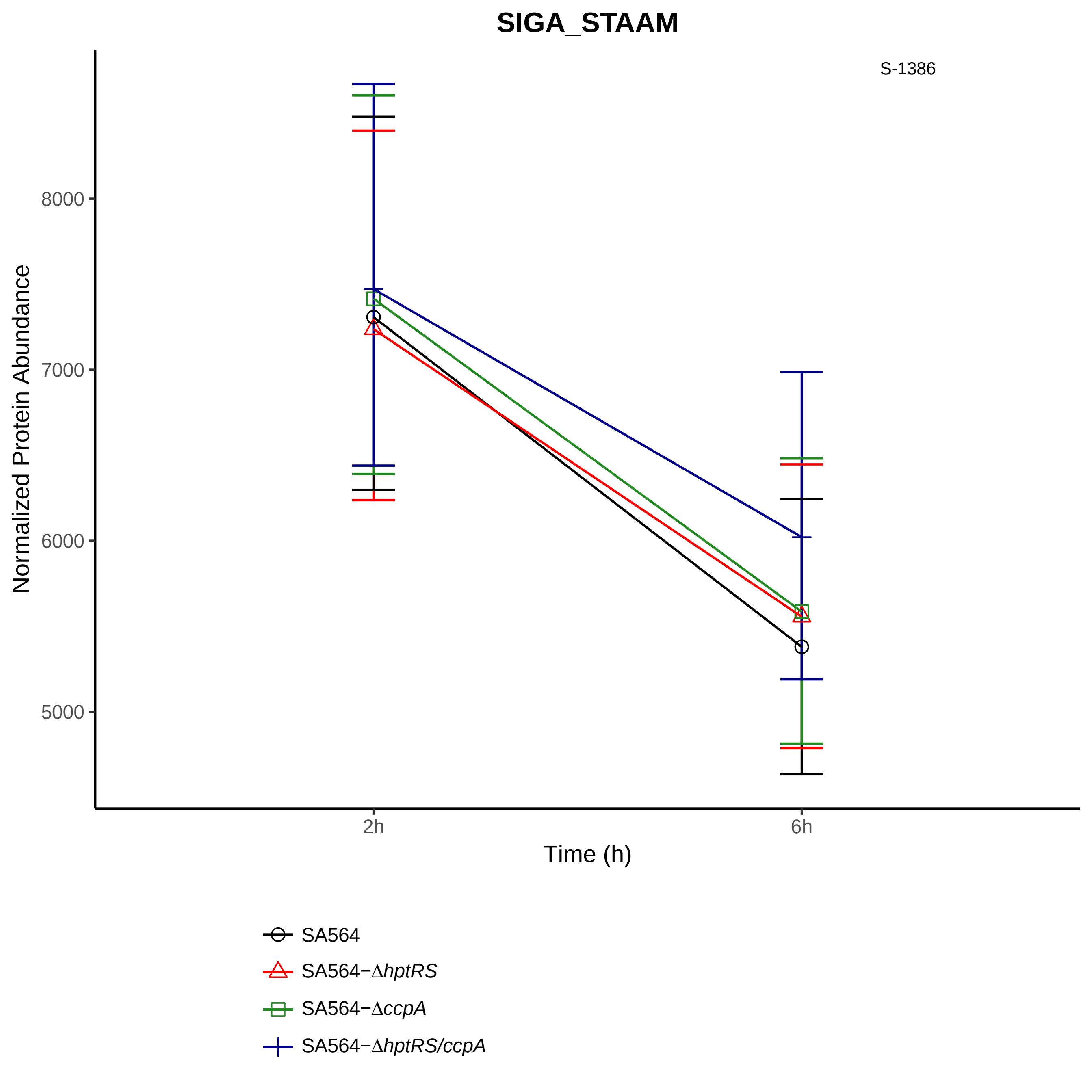
SIGA_STAAM
S-1386
Normalized Protein Abundance
8000
7000
6000
5000
2h
6h
Time (h)
SA564
SA564−ΔhptRS
SA564−ΔccpA
SA564−ΔhptRS/ccpA

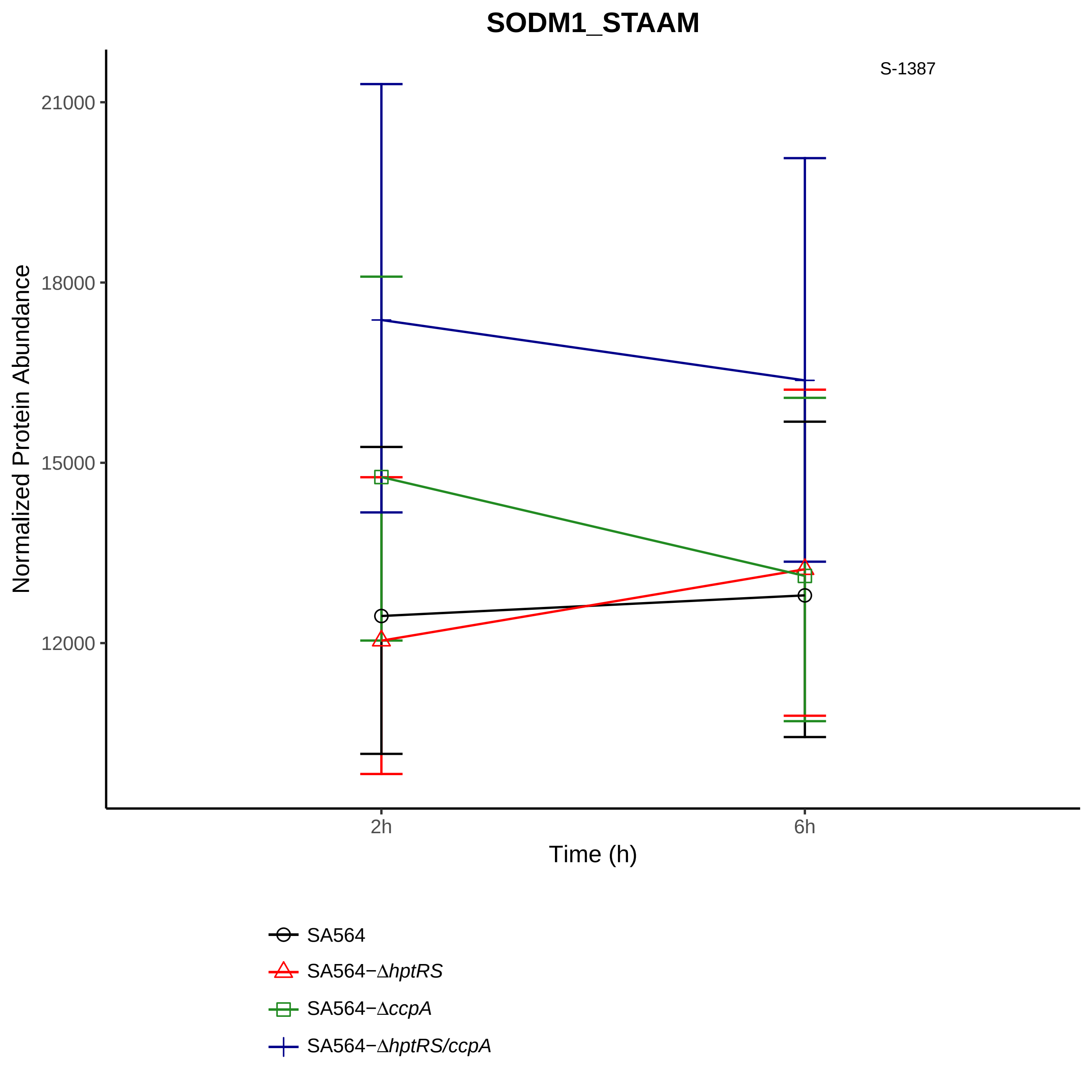

SODM1_STAAM
S-1387
Normalized Protein Abundance
21000
18000
15000
12000
2h
6h
Time (h)
SA564
SA564−Δ*hptRS*
SA564−Δ*ccpA*
SA564−Δ*hptRS/ccpA*

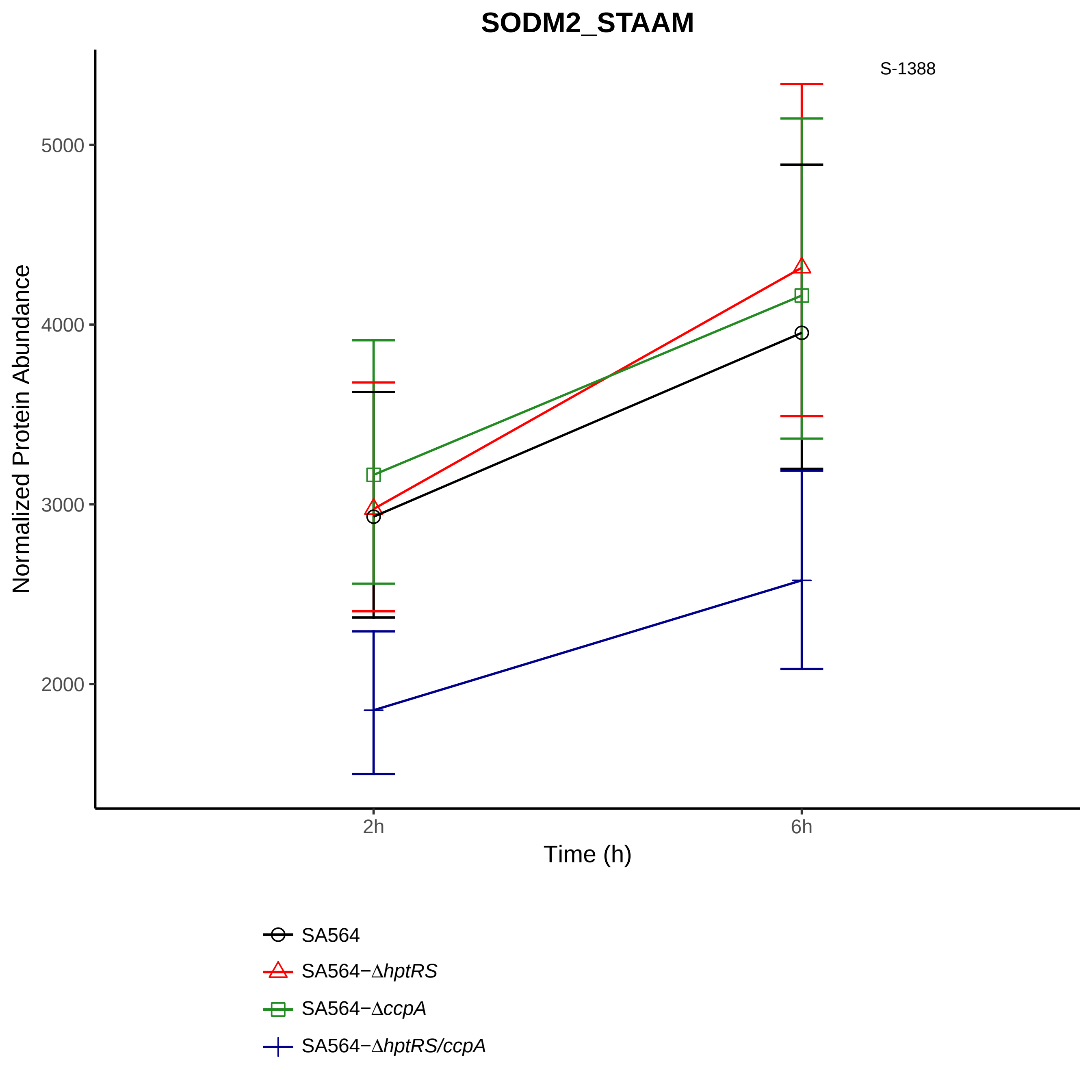

SODM2_STAAM
S-1388
Normalized Protein Abundance
5000
4000
3000
2000
2h
6h
Time (h)
SA564
SA564−Δ*hptRS*
SA564−Δ*ccpA*
SA564−Δ*hptRS/ccpA*

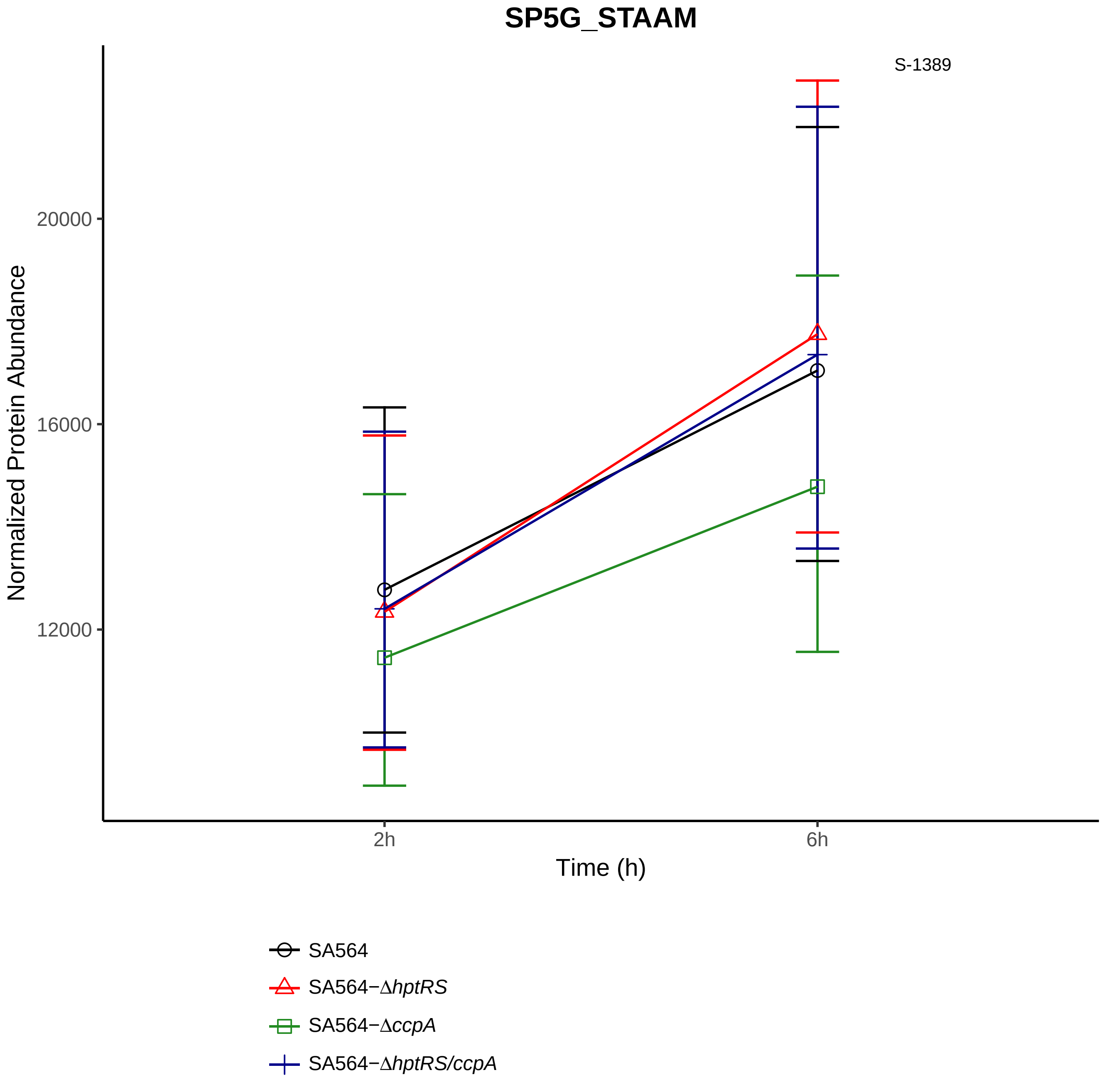

SP5G_STAAM
S-1389
20000
16000
12000
Normalized Protein Abundance
2h
6h
Time (h)
SA564
SA564−ΔhptRS
SA564−ΔccpA
SA564−ΔhptRS/ccpA

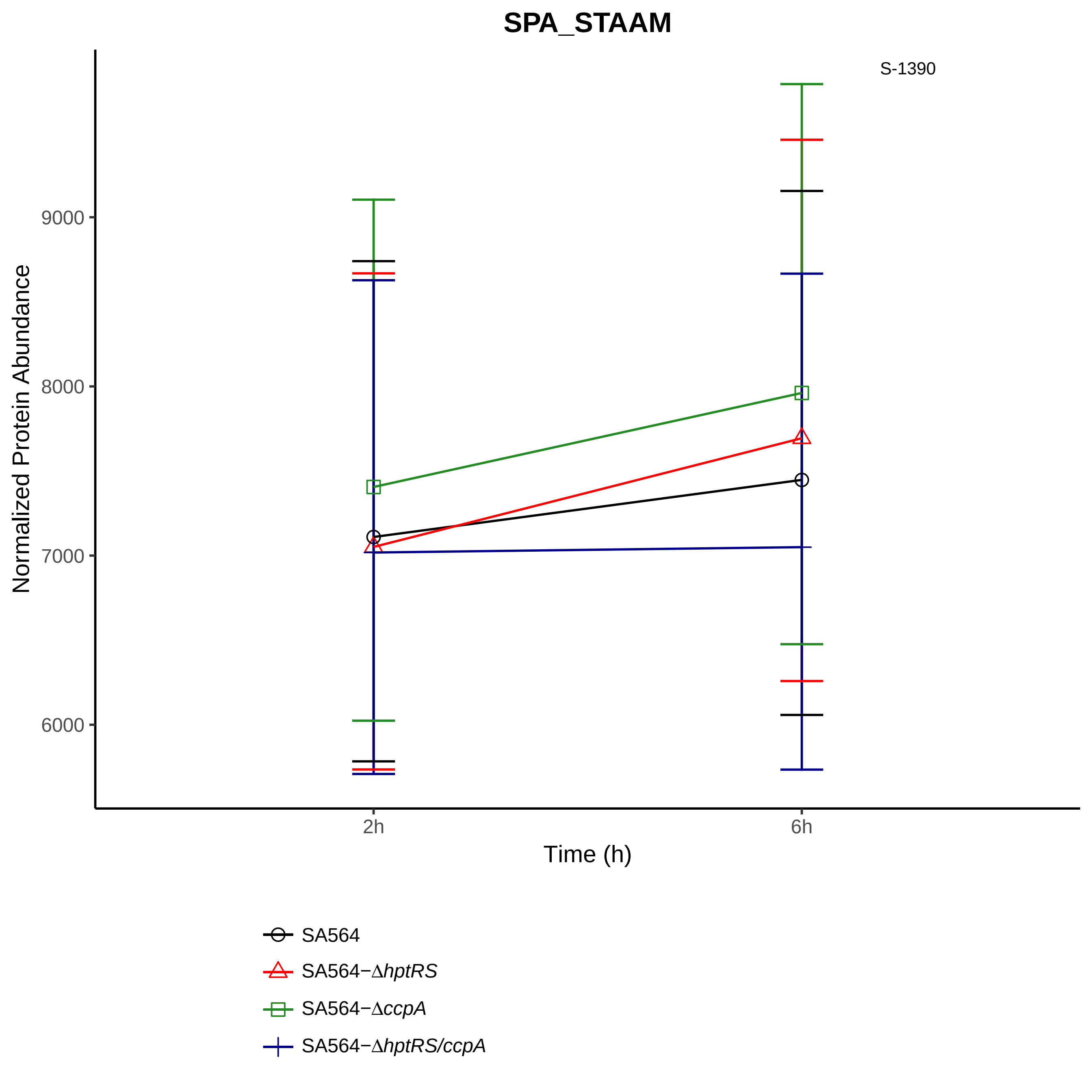

SPA_STAAM
S-1390
Normalized Protein Abundance
9000
8000
7000
6000
2h
6h
Time (h)
SA564
SA564−Δ*hptRS*
SA564−Δ*ccpA*
SA564−Δ*hptRS/ccpA*

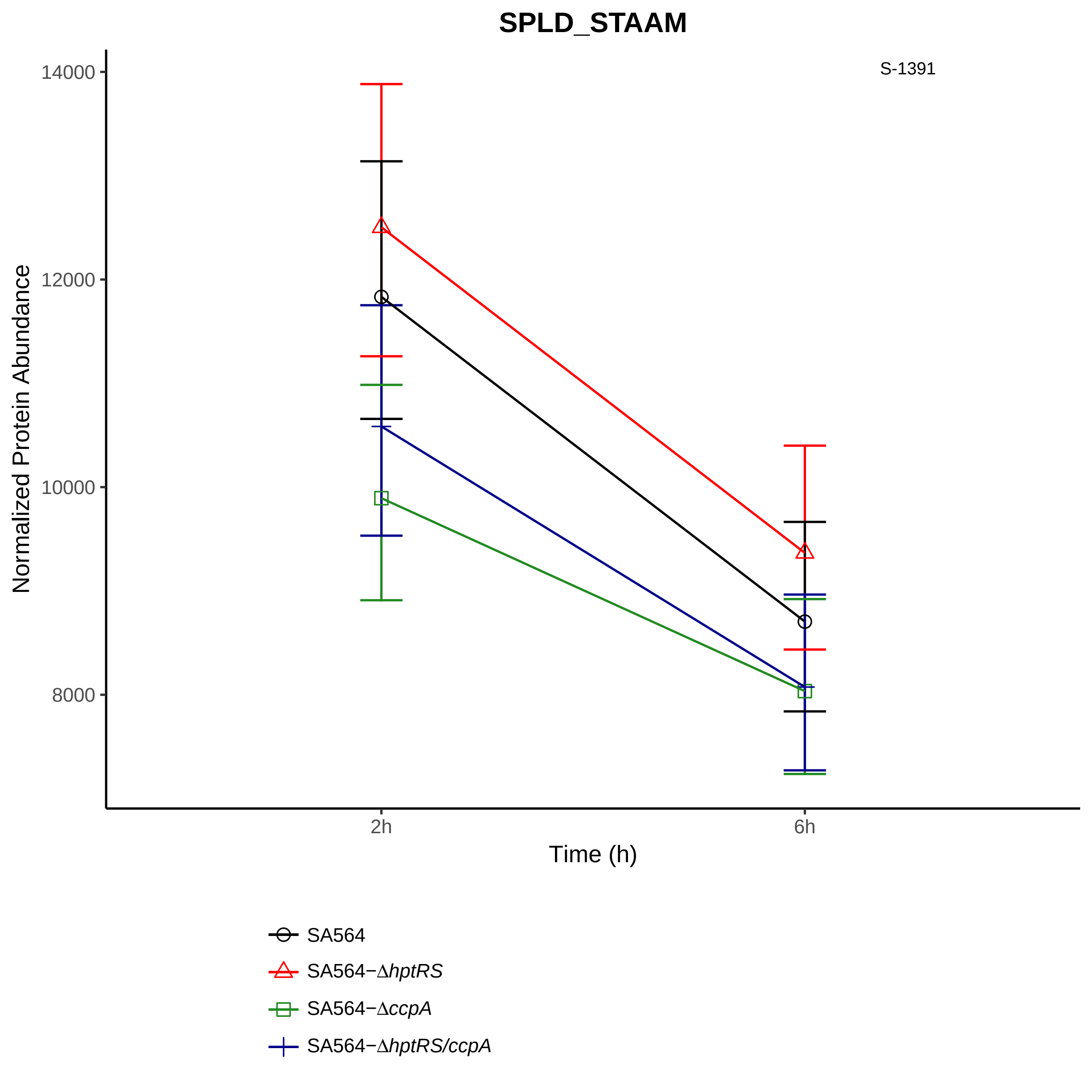

SPLD_STAAM
S-1391
Normalized Protein Abundance
14000
12000
10000
8000
2h
6h
Time (h)
SA564
SA564−Δ*hptRS*
SA564−Δ*ccpA*
SA564−Δ*hptRS/ccpA*

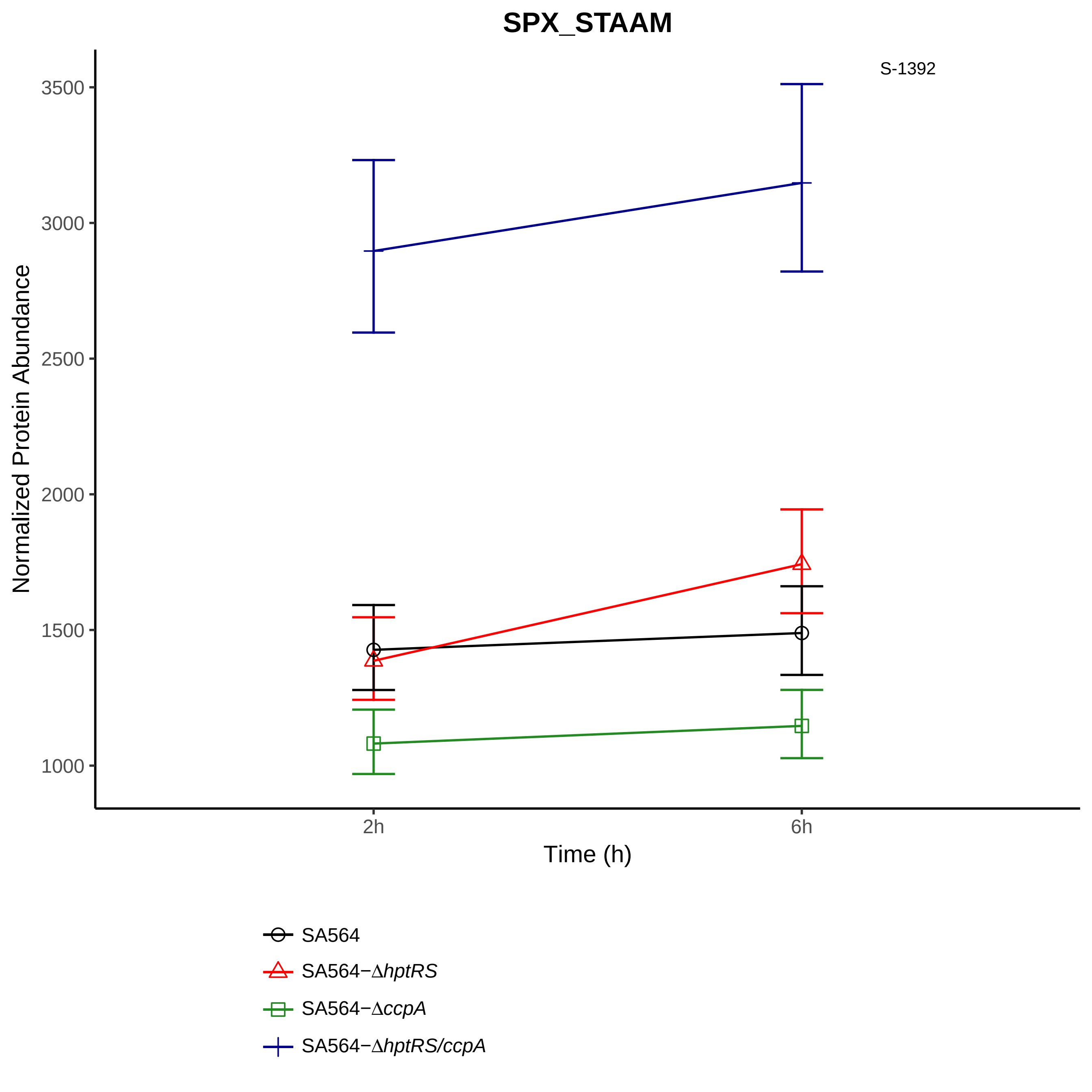

SPX_STAAM
S-1392
Normalized Protein Abundance
3500
3000
2500
2000
1500
1000
2h
6h
Time (h)
SA564
SA564−ΔhptRS
SA564−ΔccpA
SA564−ΔhptRS/ccpA

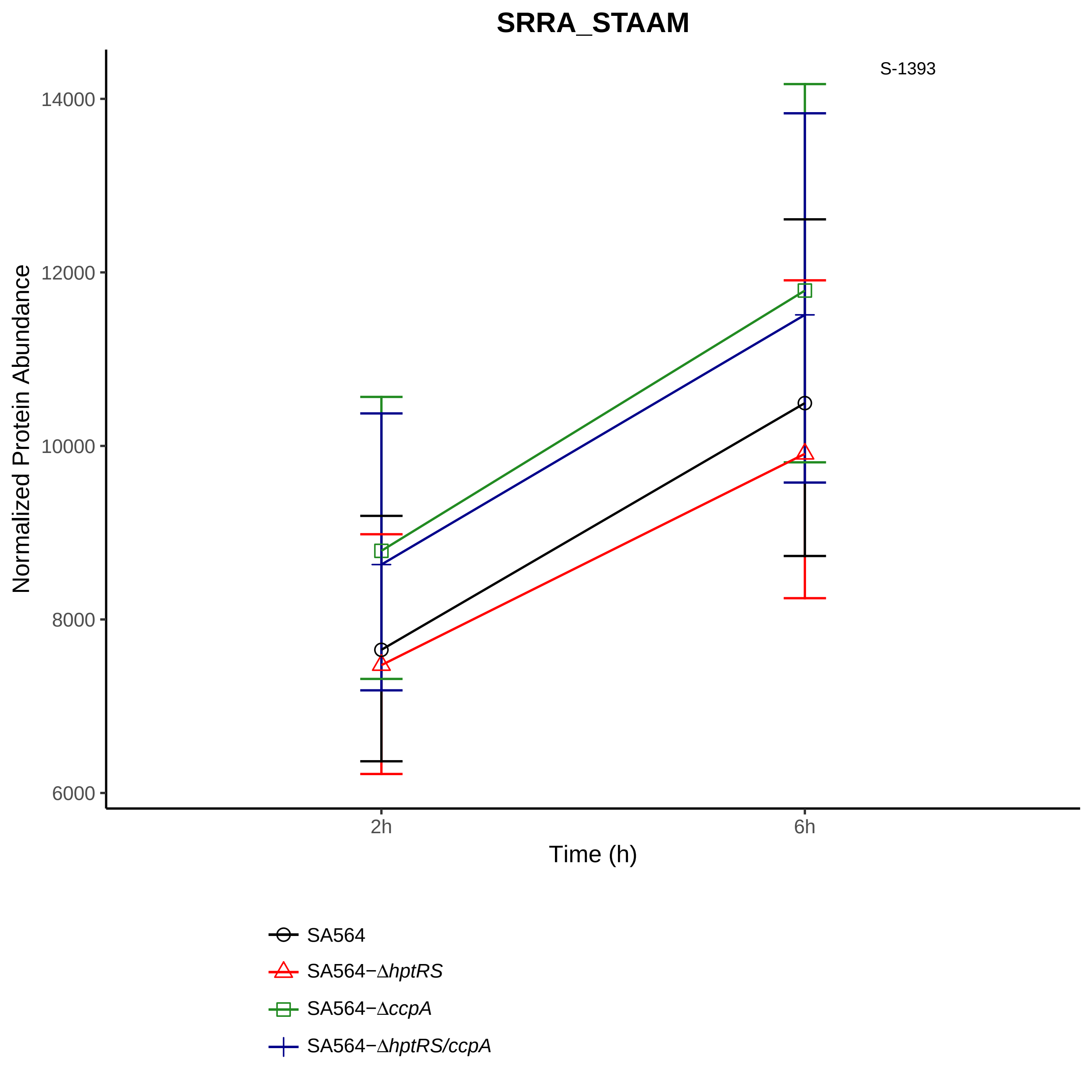

SRRA_STAAM
S-1393
14000
12000
10000
8000
6000
Normalized Protein Abundance
2h
6h
Time (h)
SA564
SA564−ΔhptRS
SA564−ΔccpA
SA564−ΔhptRS/ccpA

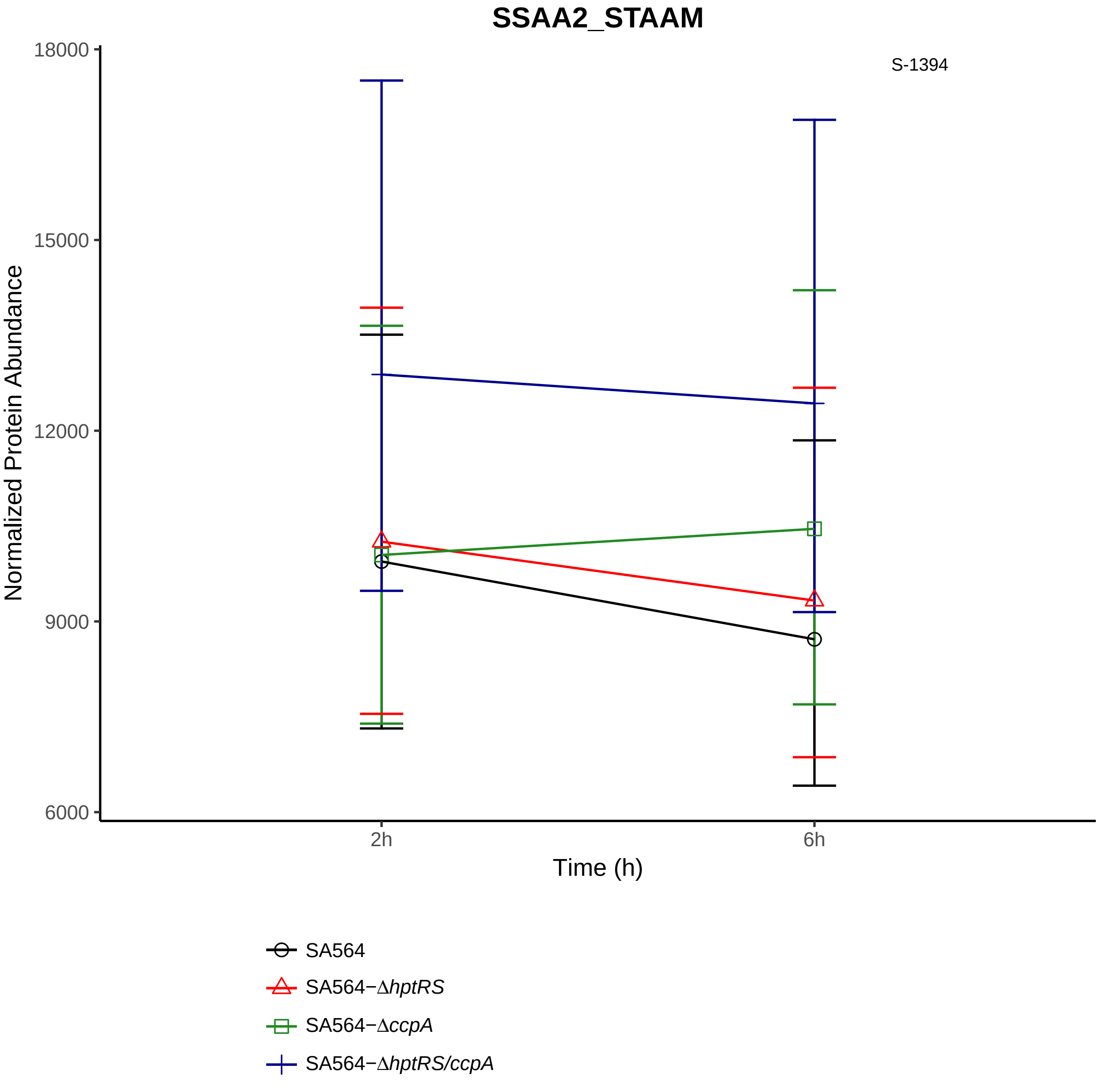

SSAA2_STAAM
S-1394
18000
15000
12000
9000
6000
Normalized Protein Abundance
2h
6h
Time (h)
SA564
SA564−Δ*hptRS*
SA564−Δ*ccpA*
SA564−Δ*hptRS/ccpA*

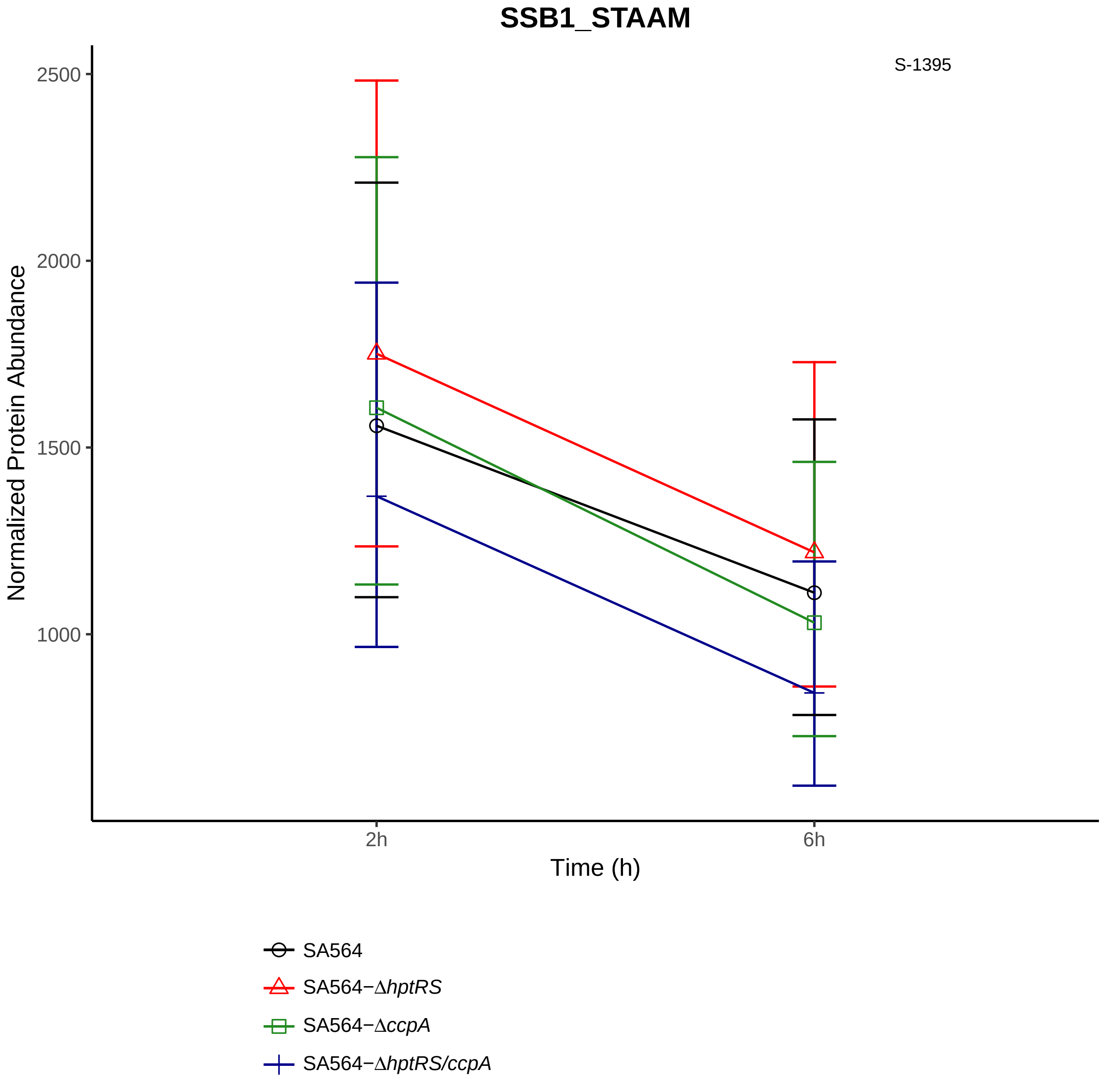

SSB1_STAAM
S-1395
Normalized Protein Abundance
2500
2000
1500
1000
2h
6h
Time (h)
SA564
SA564−Δ*hptRS*
SA564−Δ*ccpA*
SA564−Δ*hptRS/ccpA*

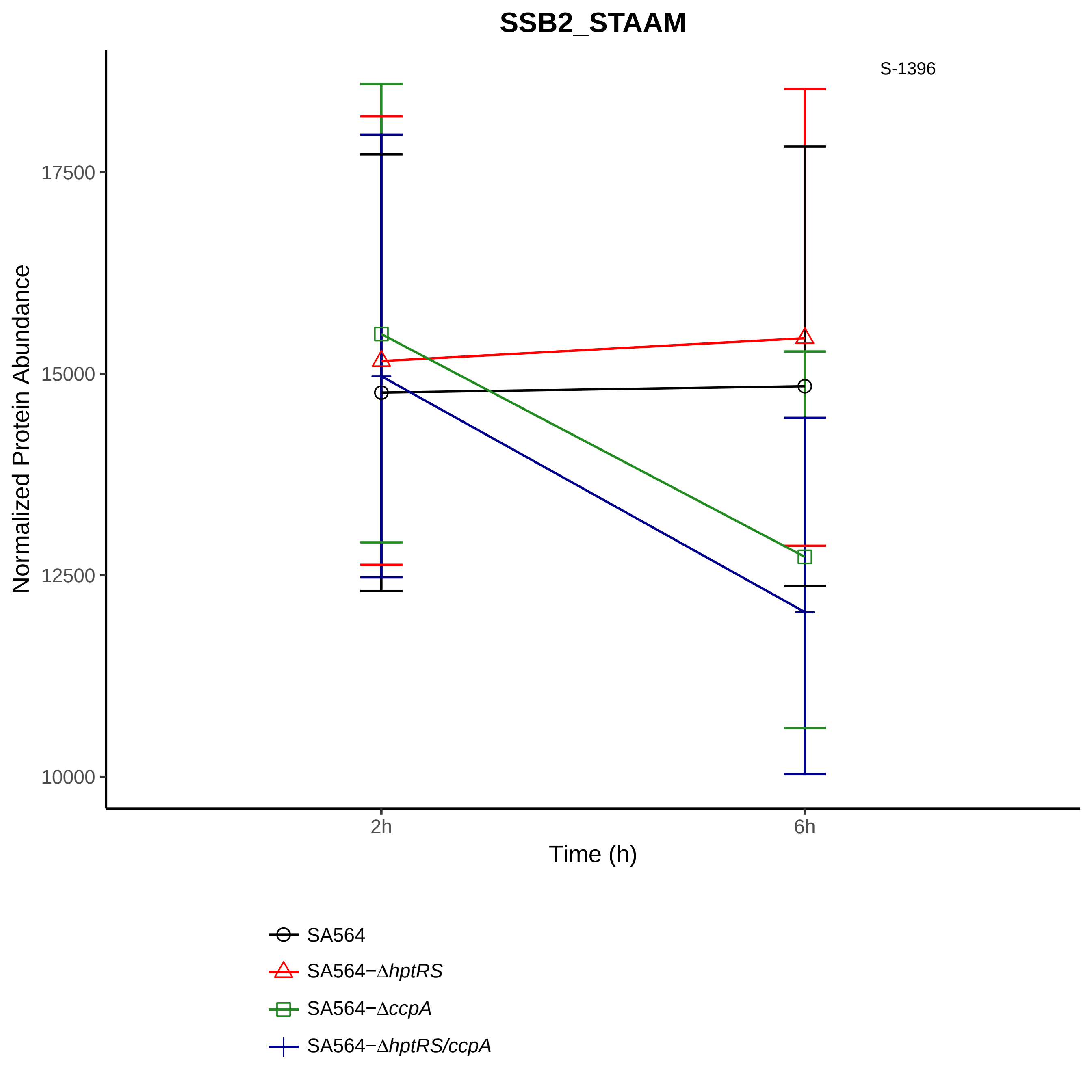
SSB2_STAAM
S-1396
Normalized Protein Abundance
17500
15000
12500
10000
2h
6h
Time (h)
SA564
SA564−ΔhptRS
SA564−ΔccpA
SA564−ΔhptRS/ccpA

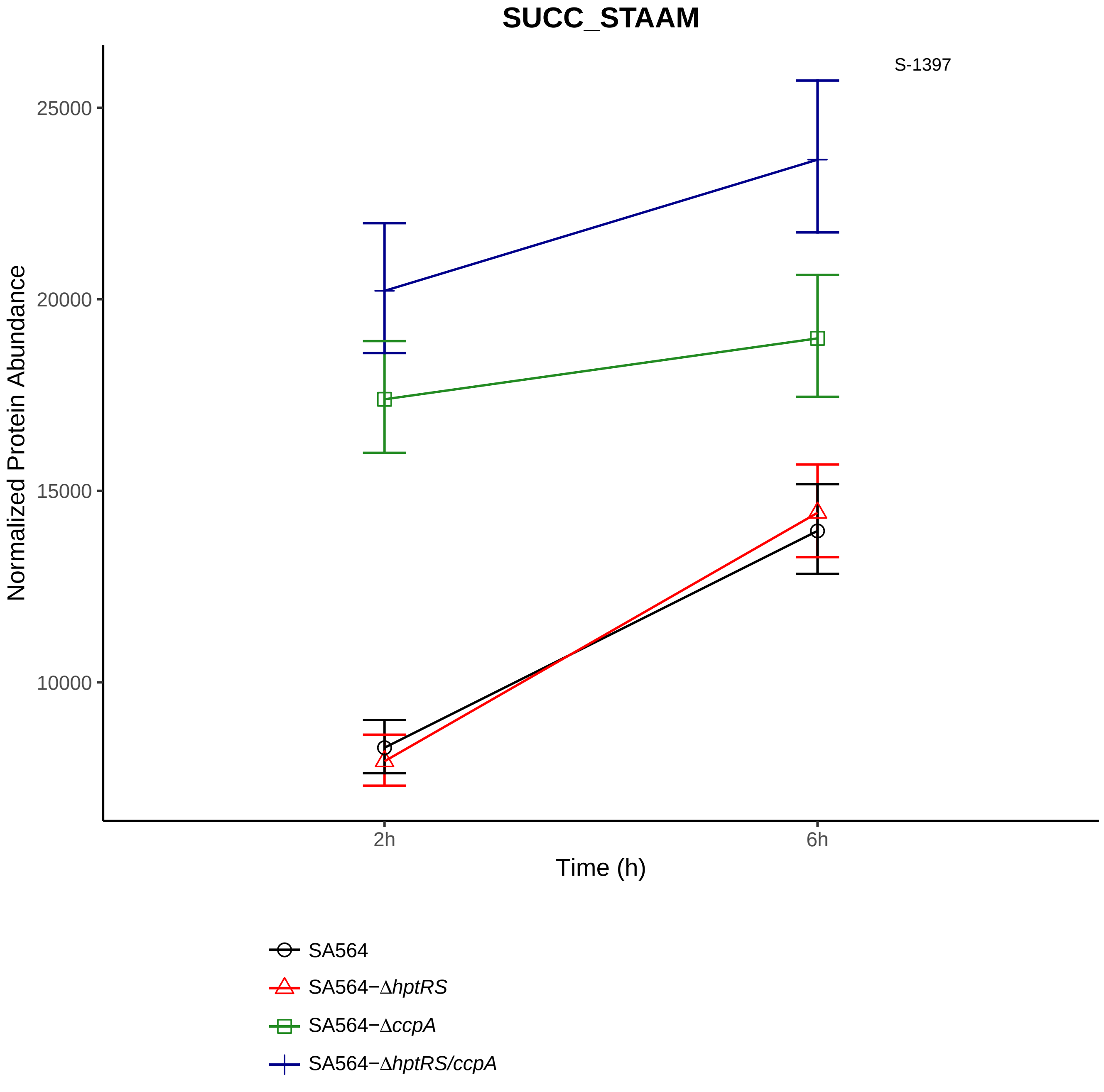

SUCC_STAAM
S-1397
Normalized Protein Abundance
25000
20000
15000
10000
2h
6h
Time (h)
SA564
SA564−Δ*hptRS*
SA564−Δ*ccpA*
SA564−Δ*hptRS/ccpA*

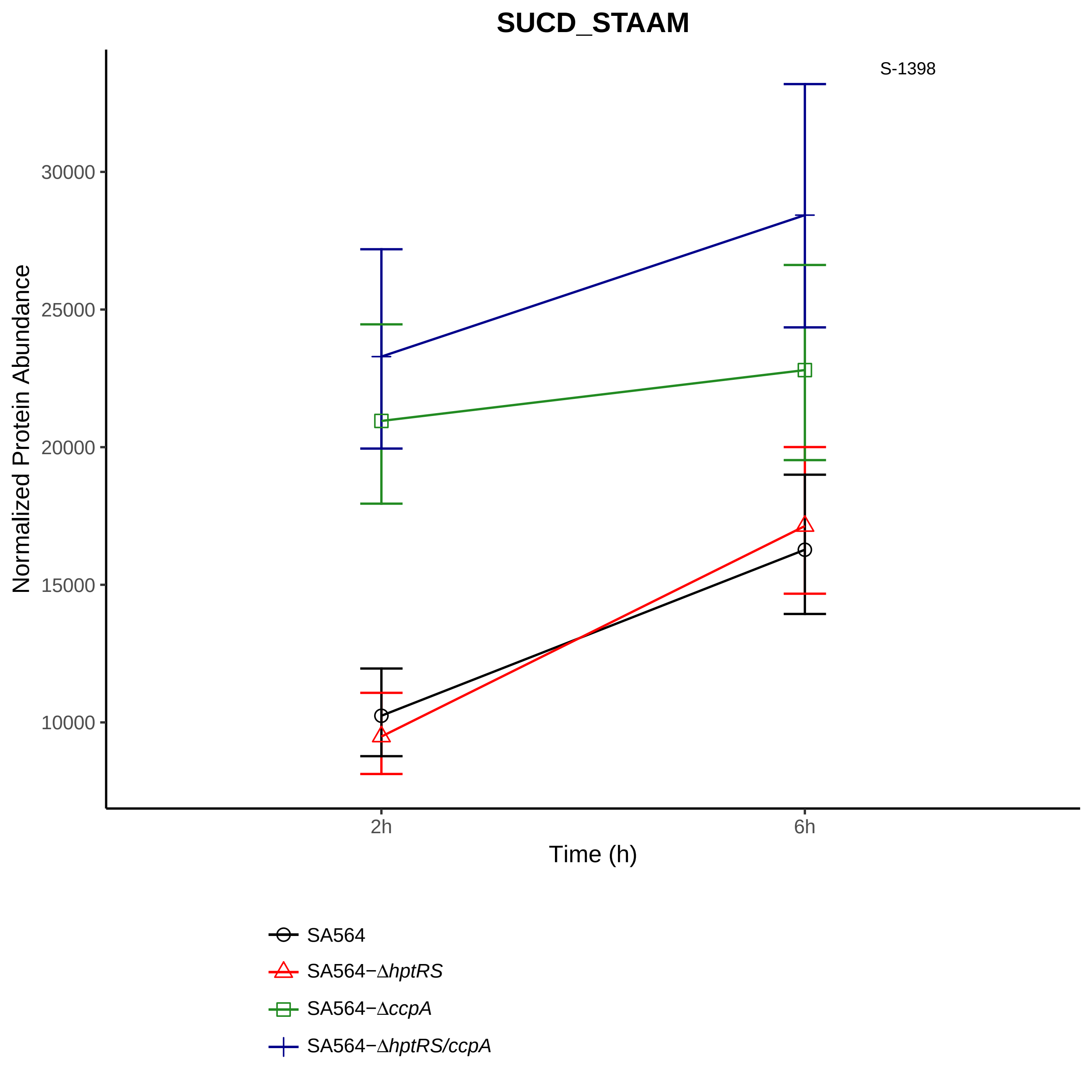
SUCD_STAAM
S-1398
Normalized Protein Abundance
30000
25000
20000
15000
10000
2h
6h
Time (h)
SA564
SA564−Δ*hptRS*
SA564−Δ*ccpA*
SA564−Δ*hptRS/ccpA*

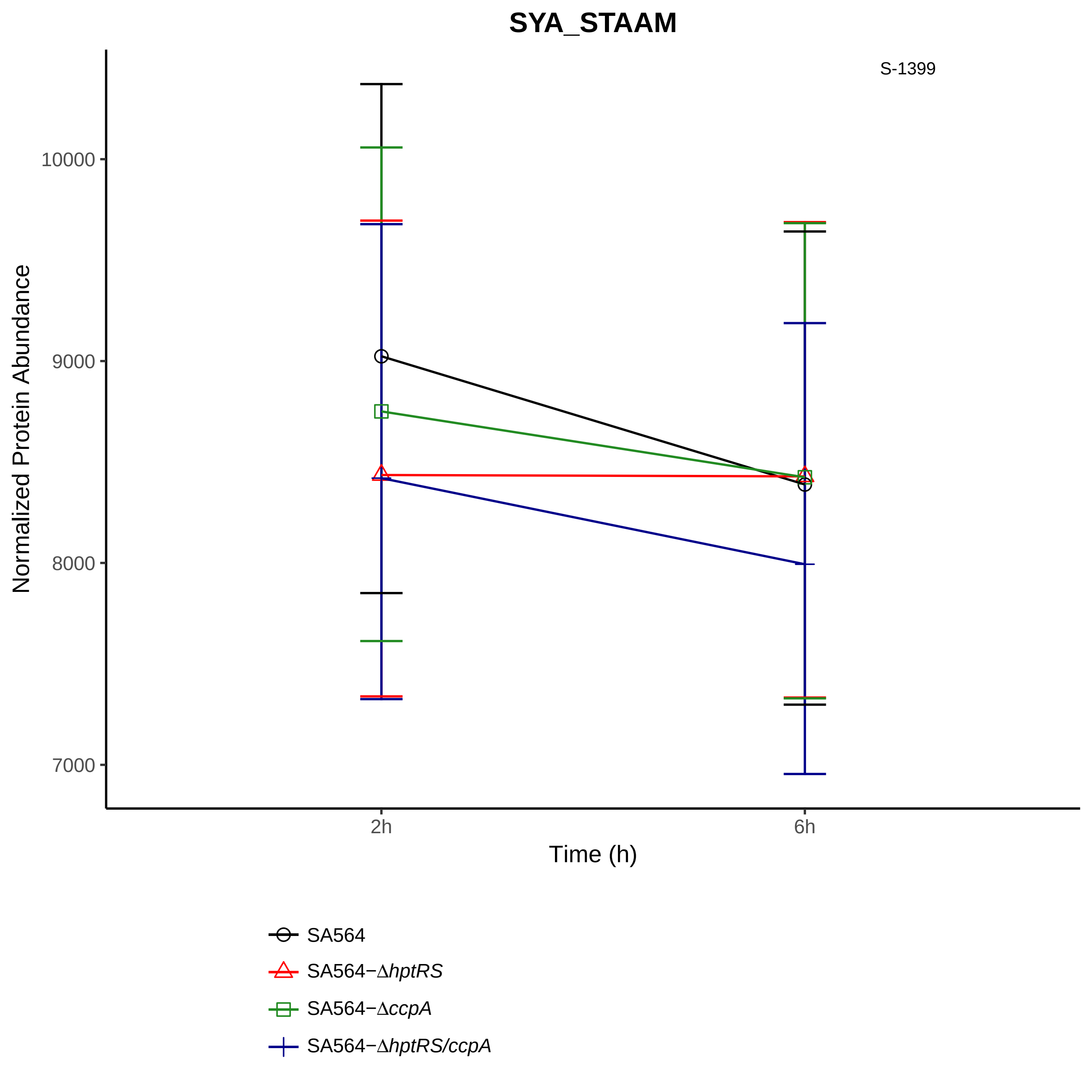

SYA_STAAM
S-1399
Normalized Protein Abundance
10000
9000
8000
7000
2h
6h
Time (h)
SA564
SA564−Δ*hptRS*
SA564−Δ*ccpA*
SA564−Δ*hptRS/ccpA*

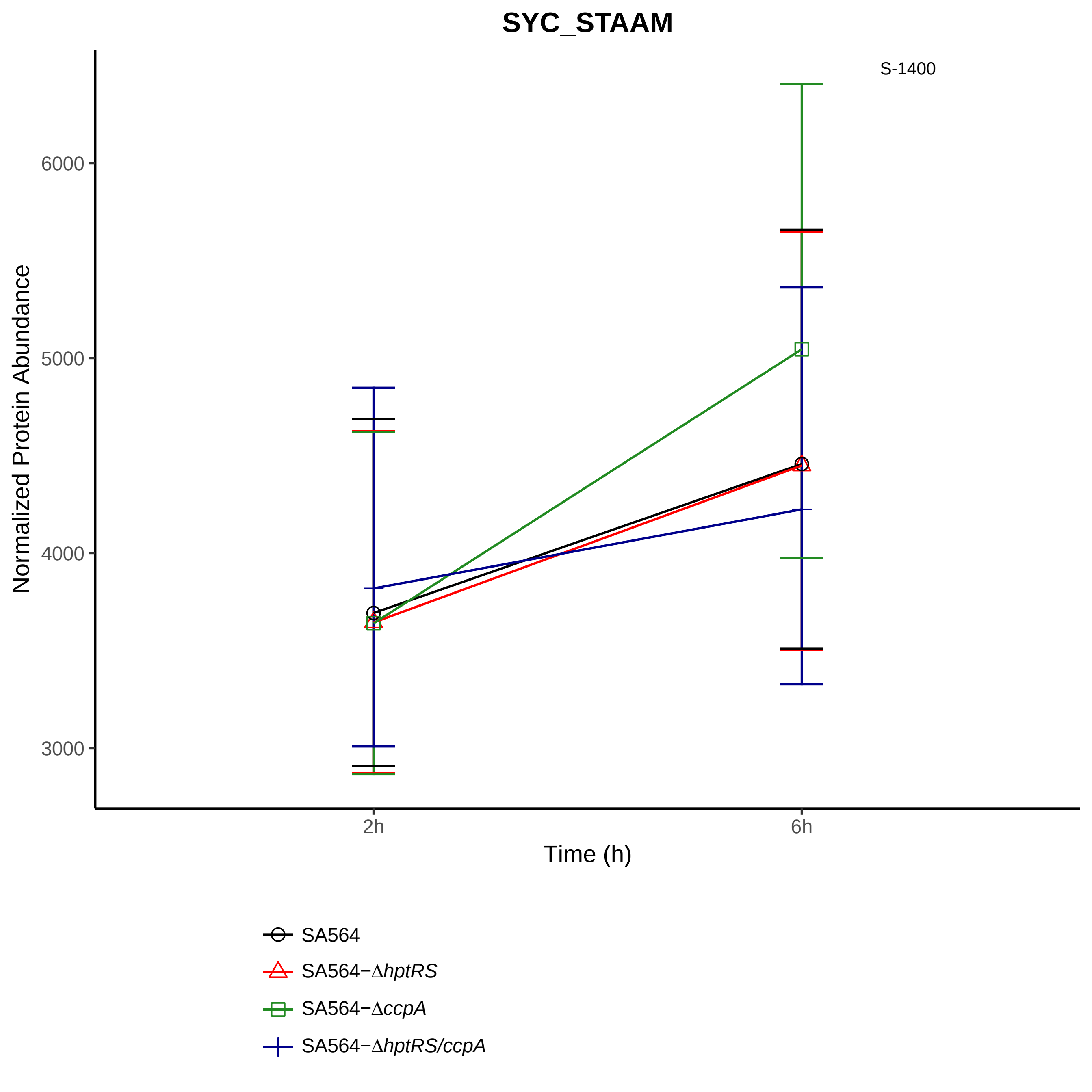
SYC_STAAM
S-1400
Normalized Protein Abundance
6000
5000
4000
3000
2h
6h
Time (h)
SA564
SA564−Δ*hptRS*
SA564−Δ*ccpA*
SA564−Δ*hptRS/ccpA*

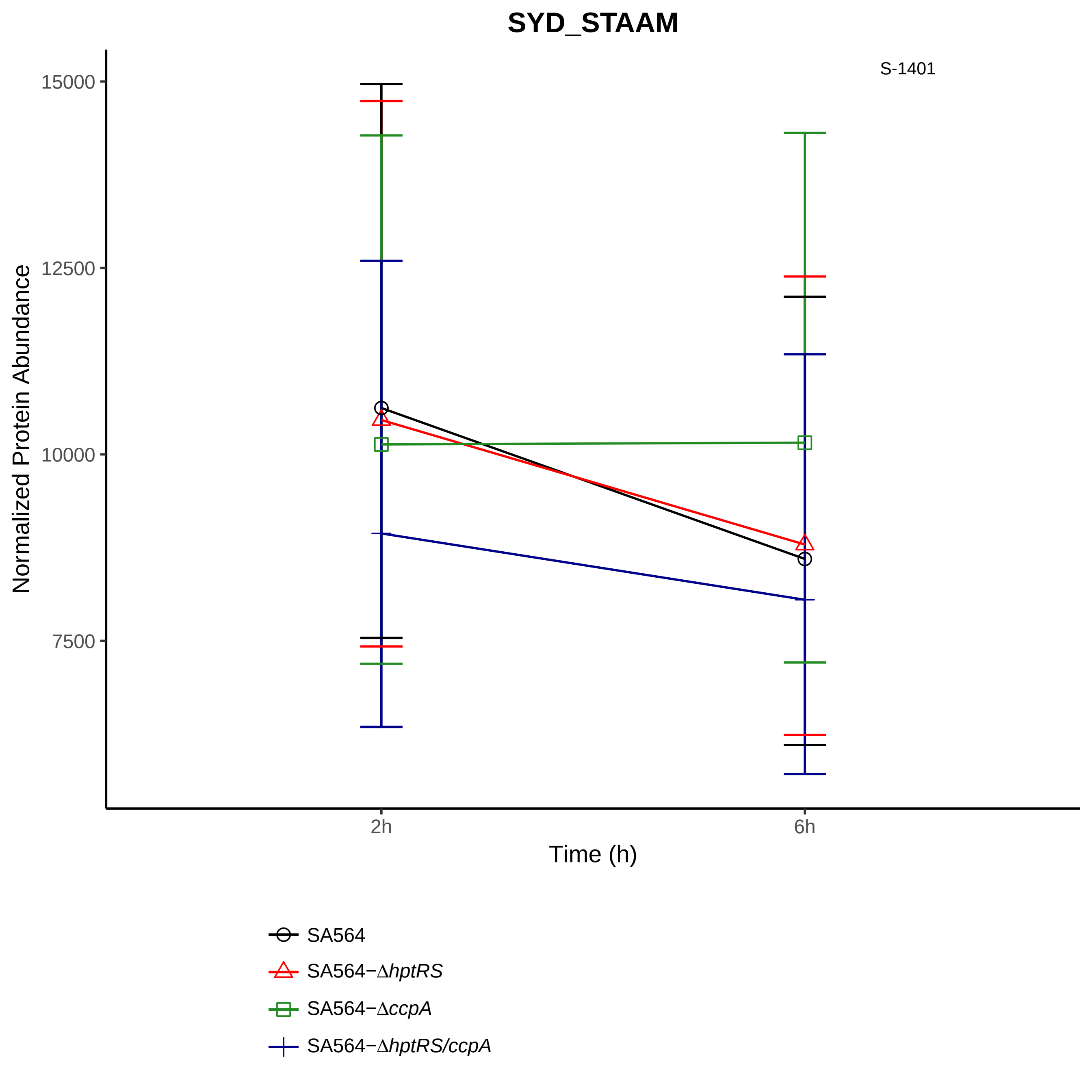

SYD_STAAM
S-1401
Normalized Protein Abundance
15000
12500
10000
7500
2h
6h
Time (h)
SA564
SA564−Δ*hptRS*
SA564−Δ*ccpA*
SA564−Δ*hptRS/ccpA*

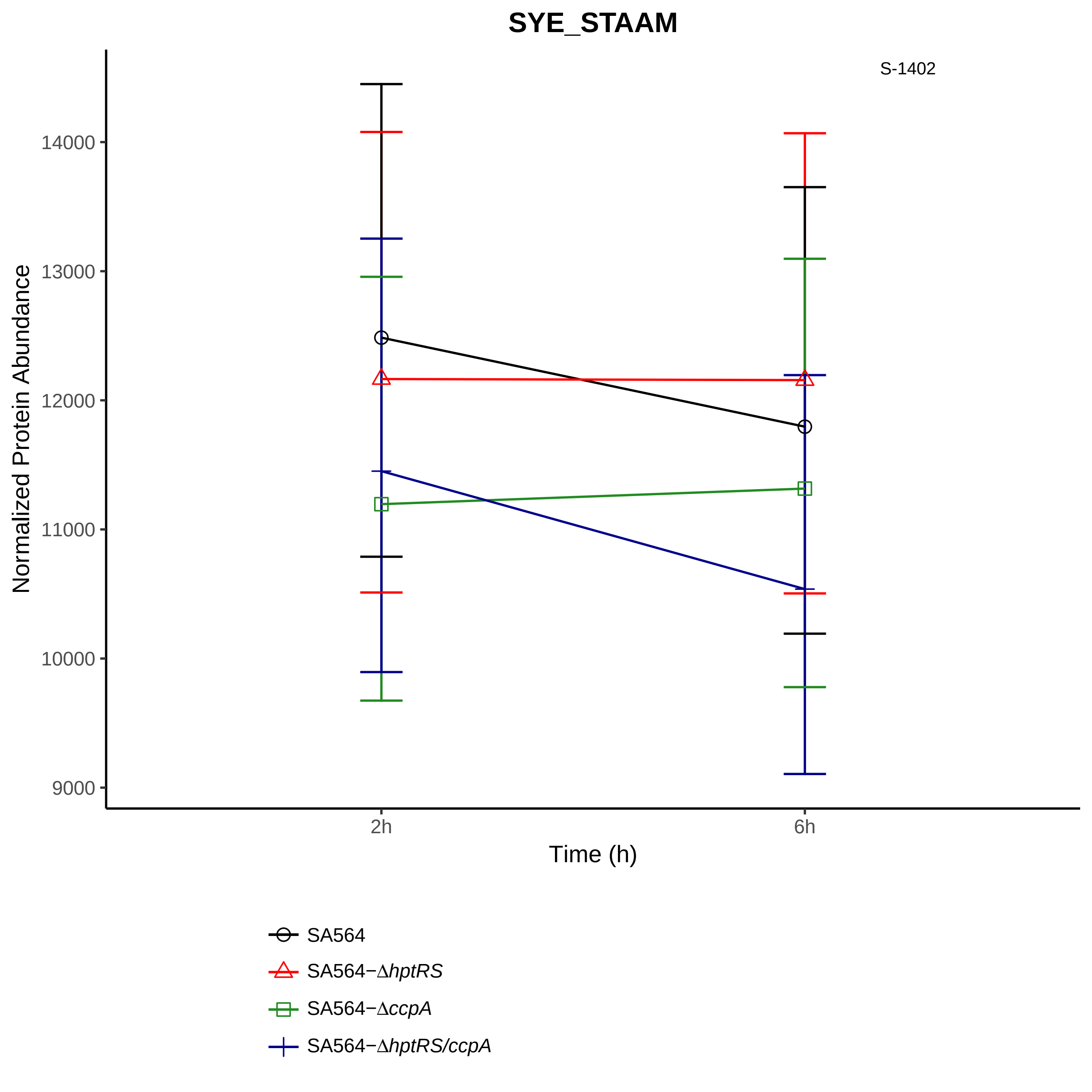

SYE_STAAM
S-1402
Normalized Protein Abundance
14000
13000
12000
11000
10000
9000
2h
6h
Time (h)
SA564
SA564−Δ*hptRS*
SA564−Δ*ccpA*
SA564−Δ*hptRS/ccpA*

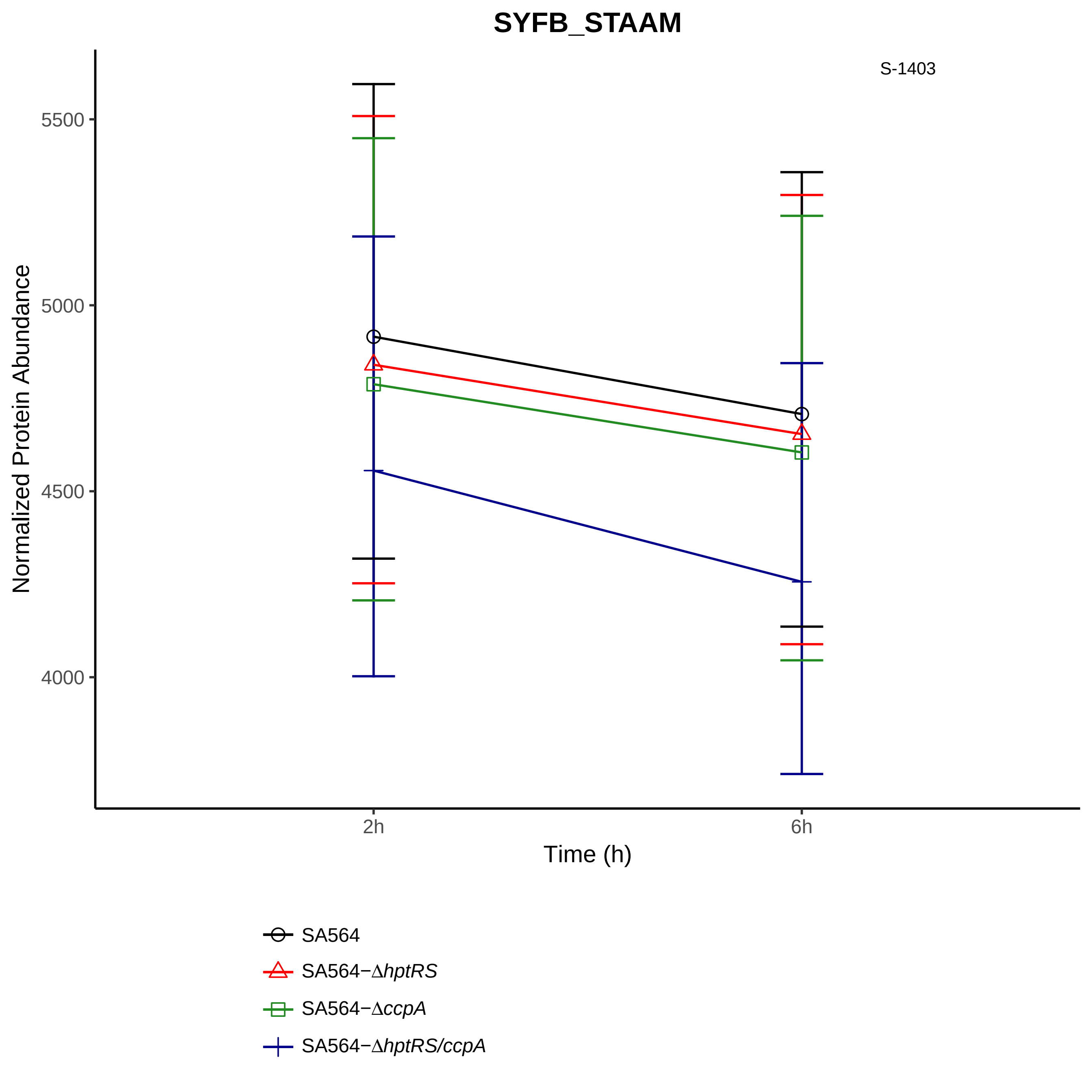

SYFB_STAAM
S-1403
Normalized Protein Abundance
5500
5000
4500
4000
2h
6h
Time (h)
SA564
SA564−Δ*hptRS*
SA564−Δ*ccpA*
SA564−Δ*hptRS/ccpA*

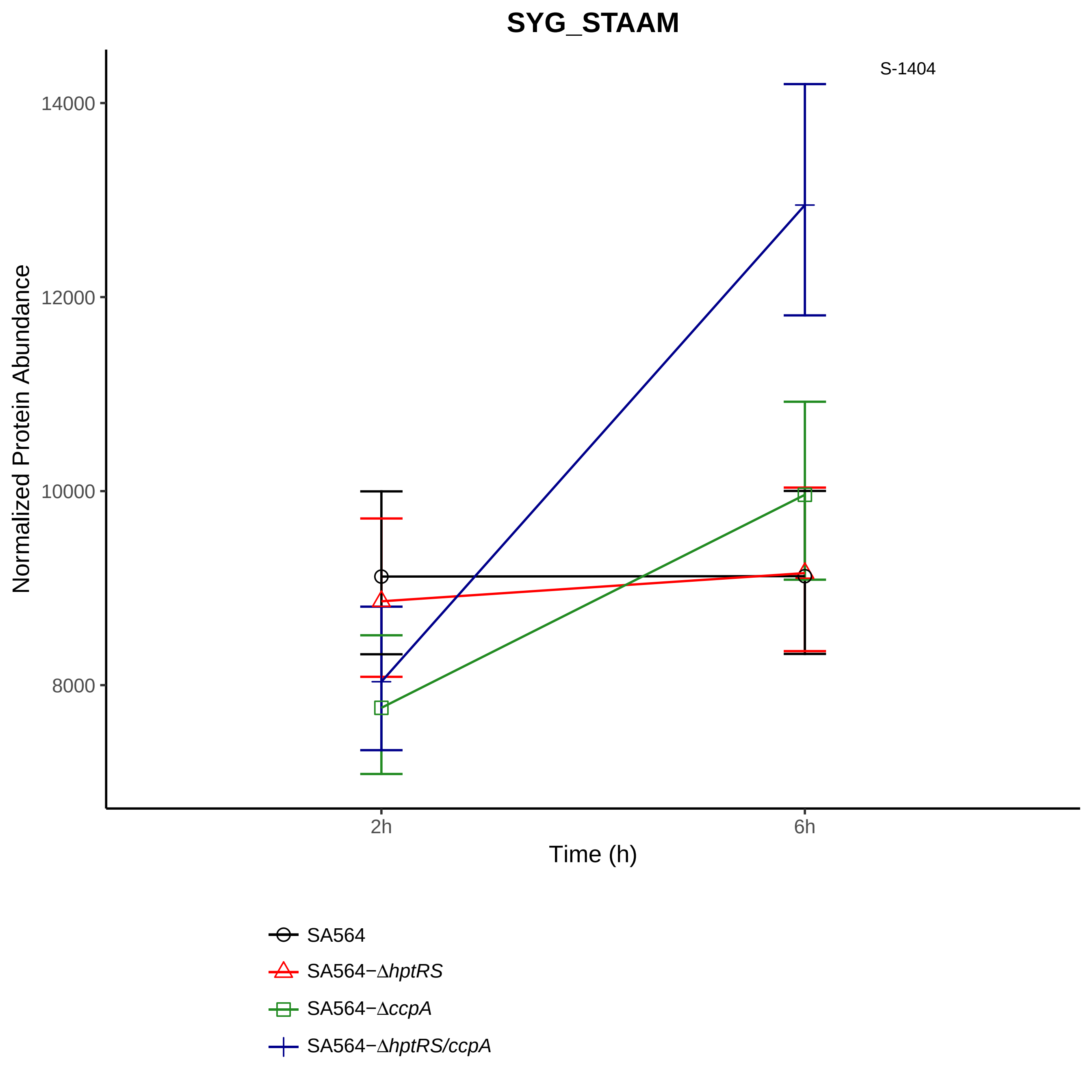

SYG_STAAM
S-1404
14000
12000
10000
8000
Normalized Protein Abundance
2h
6h
Time (h)
SA564
SA564−Δ*hptRS*
SA564−Δ*ccpA*
SA564−Δ*hptRS/ccpA*

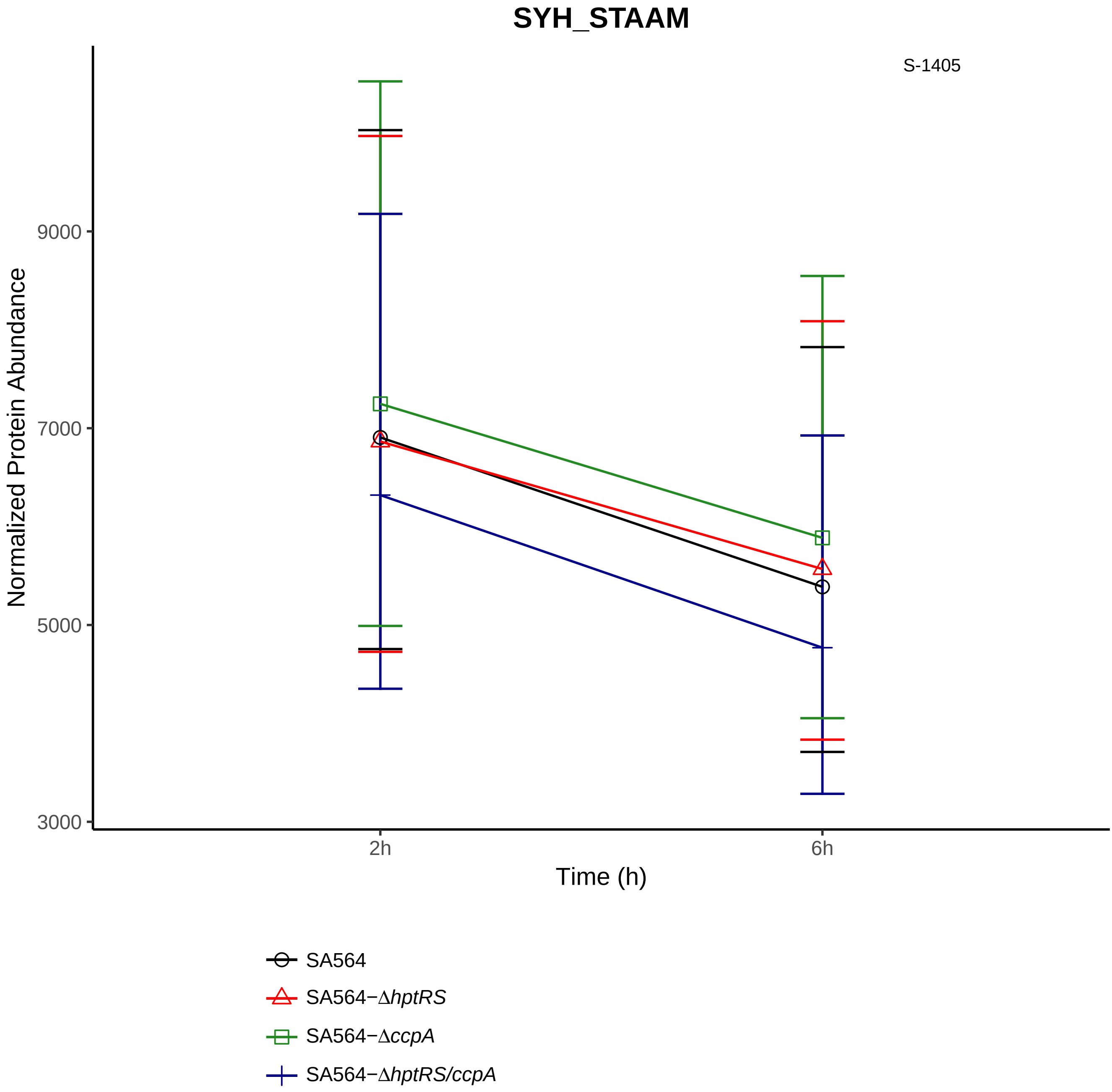

SYH_STAAM
S-1405
Normalized Protein Abundance
9000
7000
5000
3000
2h
6h
Time (h)
SA564
SA564−Δ*hptRS*
SA564−Δ*ccpA*
SA564−Δ*hptRS/ccpA*

SYI_STAAM

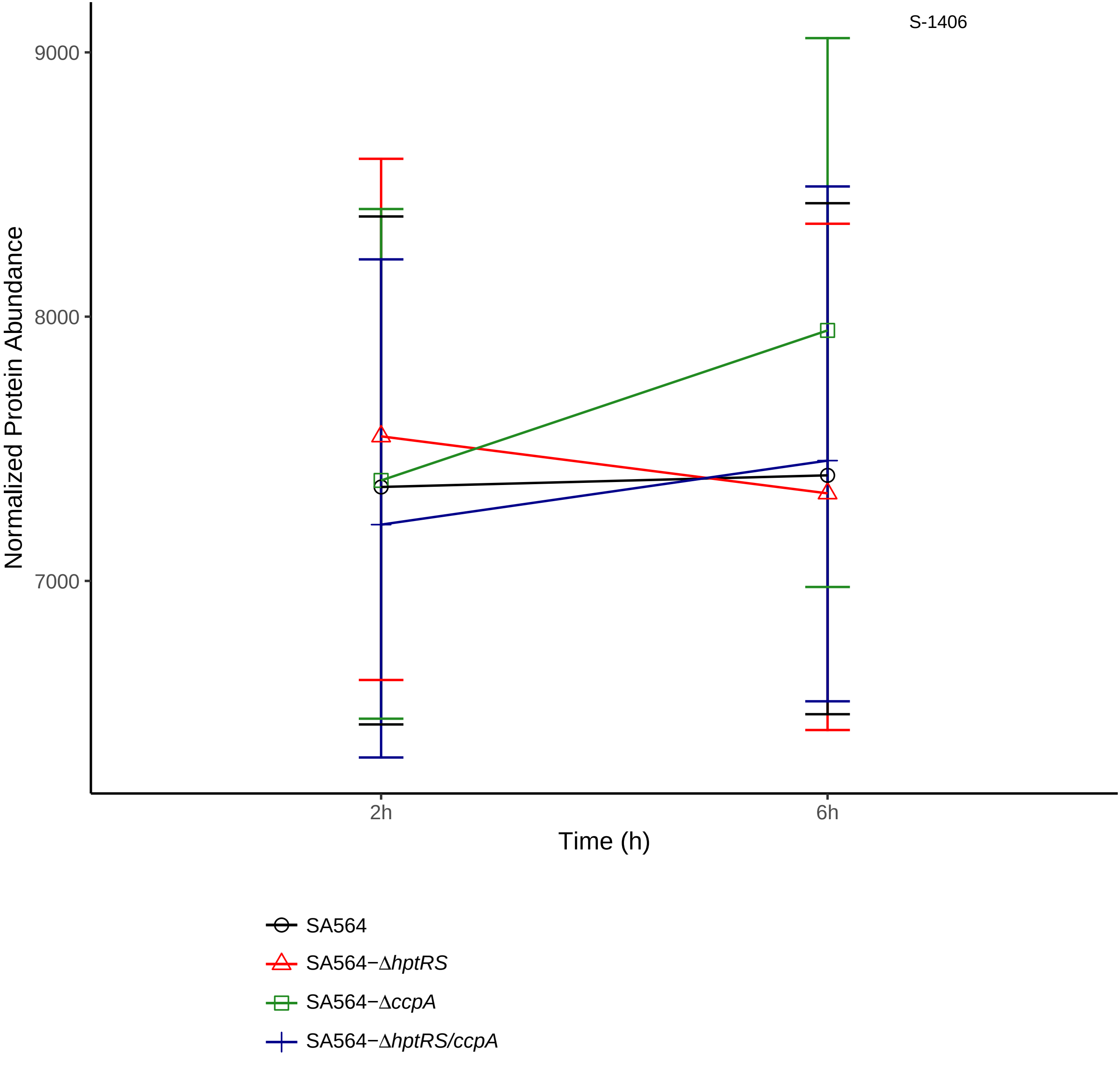

S-1406
9000
8000
7000
Normalized Protein Abundance
2h
6h
Time (h)
SA564
SA564−Δ*hptRS*
SA564−Δ*ccpA*
SA564−Δ*hptRS/ccpA*

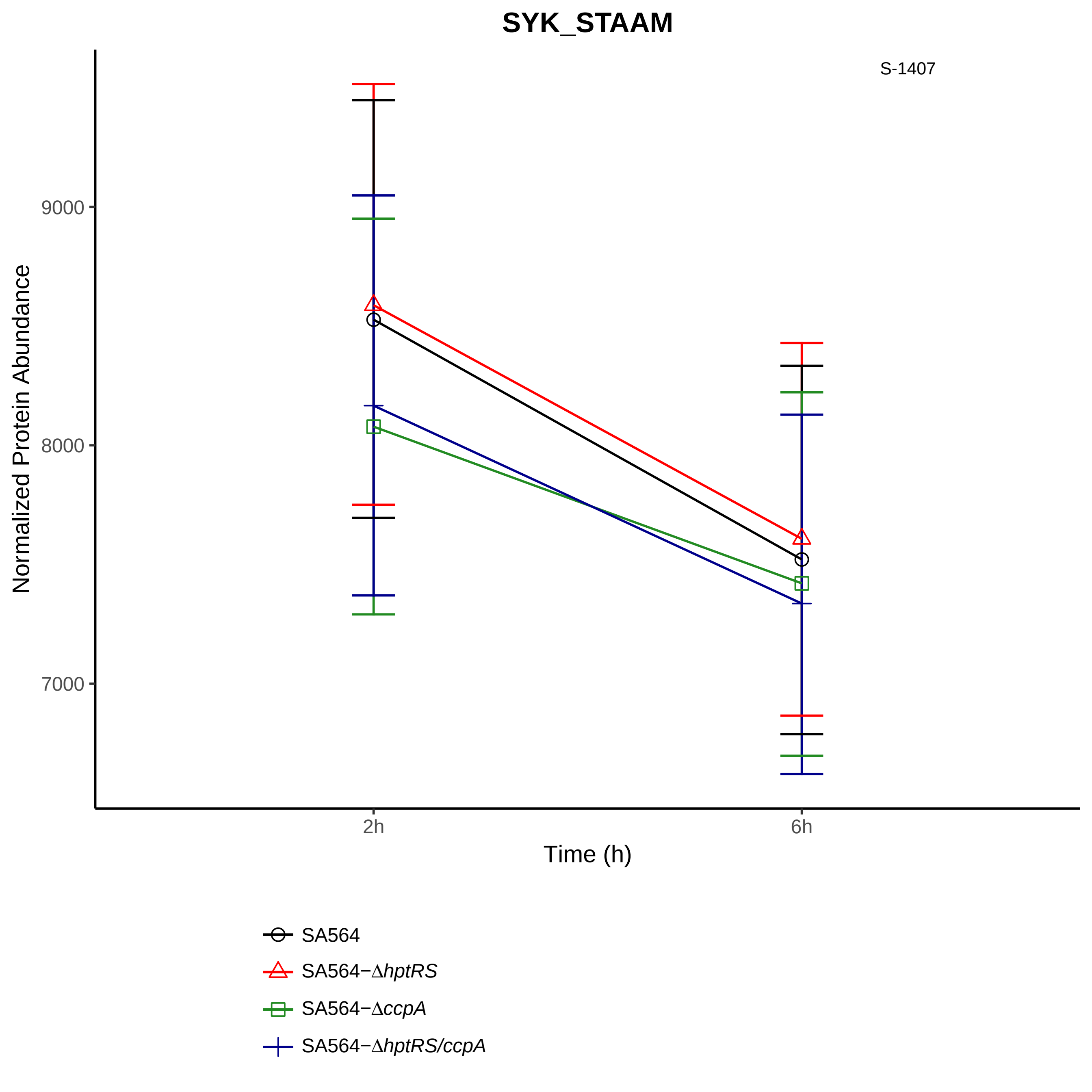

SYK_STAAM
S-1407
Normalized Protein Abundance
9000
8000
7000
2h
6h
Time (h)
SA564
SA564−Δ*hptRS*
SA564−Δ*ccpA*
SA564−Δ*hptRS/ccpA*

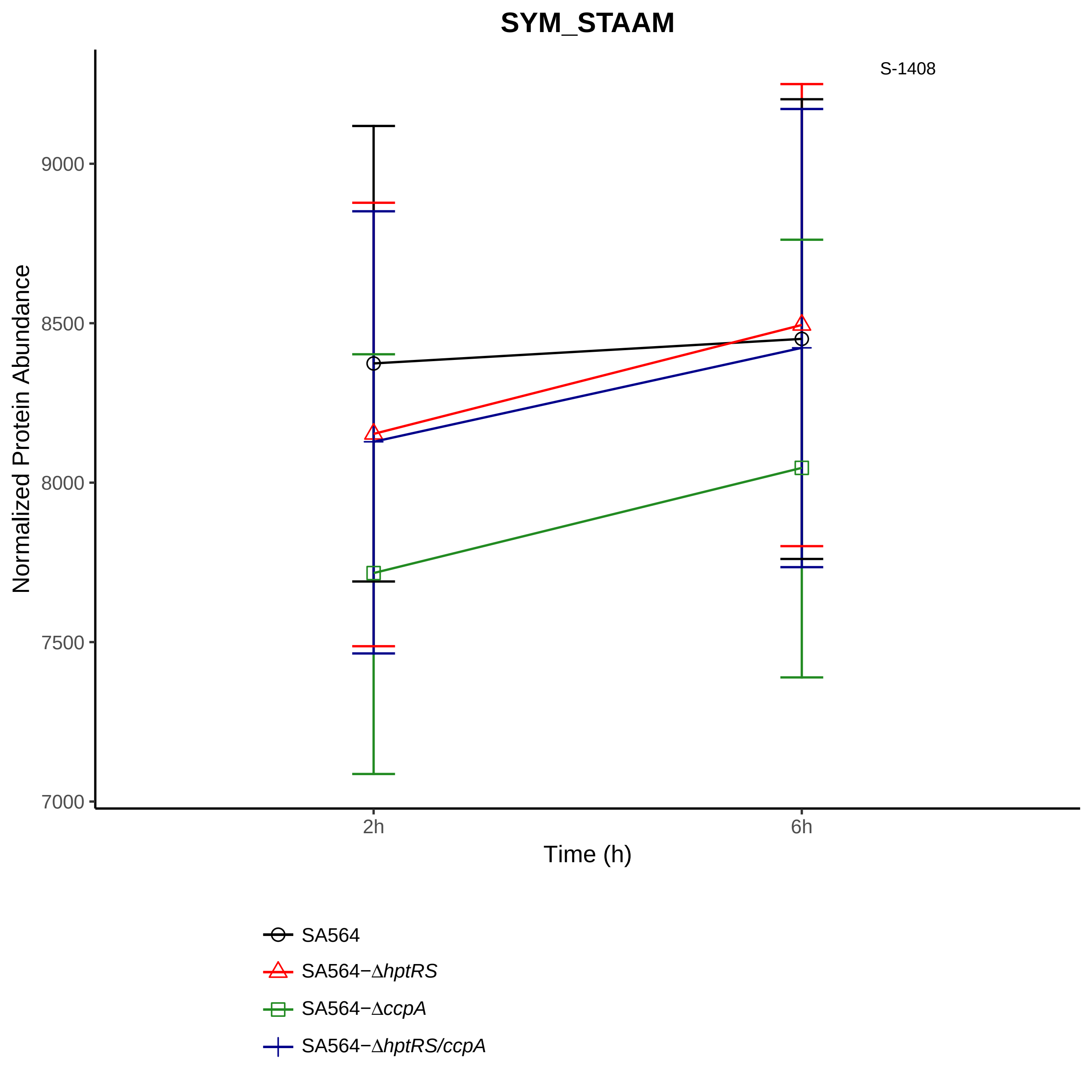

SYM_STAAM
S-1408
Normalized Protein Abundance
9000
8500
8000
7500
7000
2h
6h
Time (h)
SA564
SA564−Δ*hptRS*
SA564−Δ*ccpA*
SA564−Δ*hptRS/ccpA*

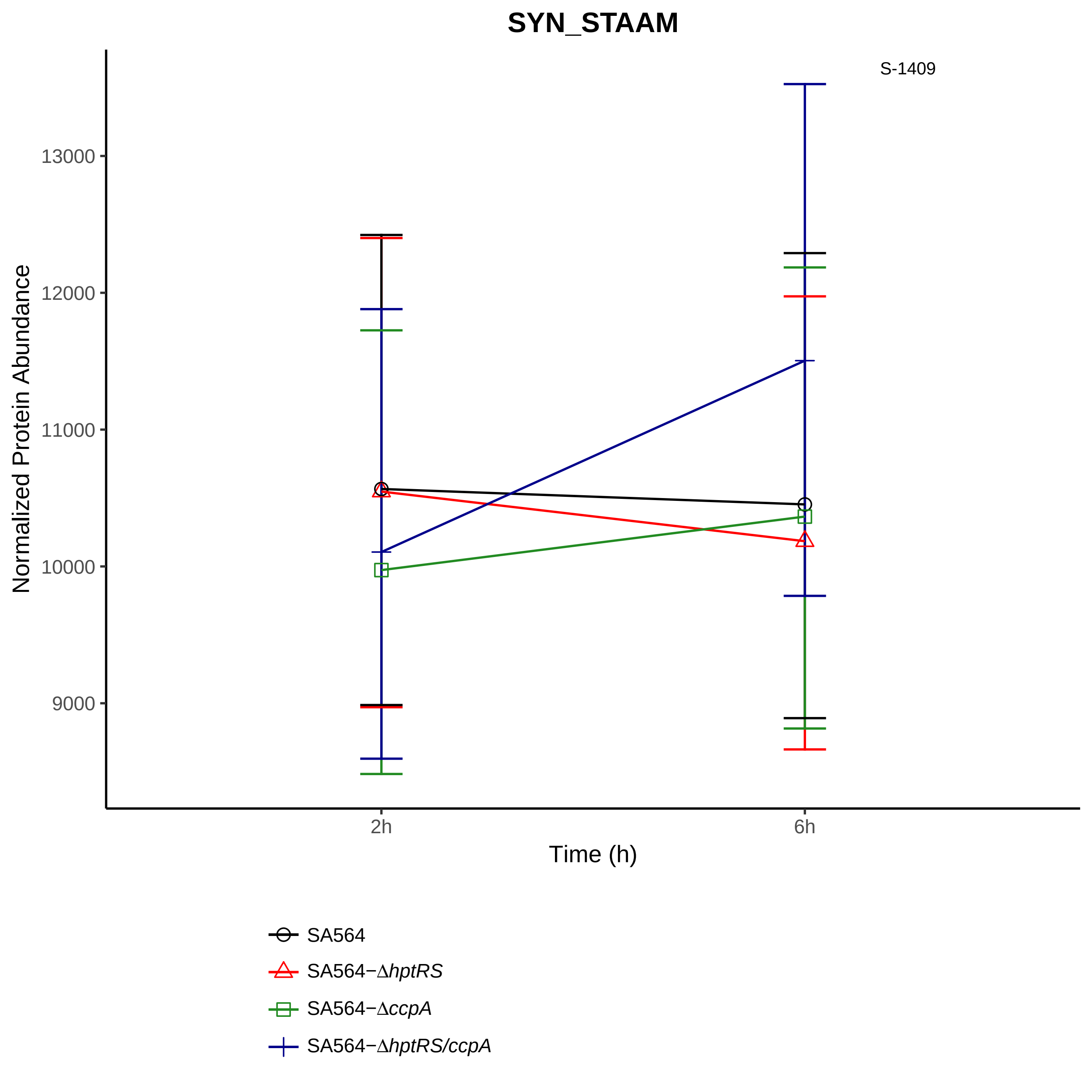

SYN_STAAM
S-1409
Normalized Protein Abundance
13000
12000
11000
10000
9000
2h
6h
Time (h)
SA564
SA564−Δ*hptRS*
SA564−Δ*ccpA*
SA564−Δ*hptRS/ccpA*

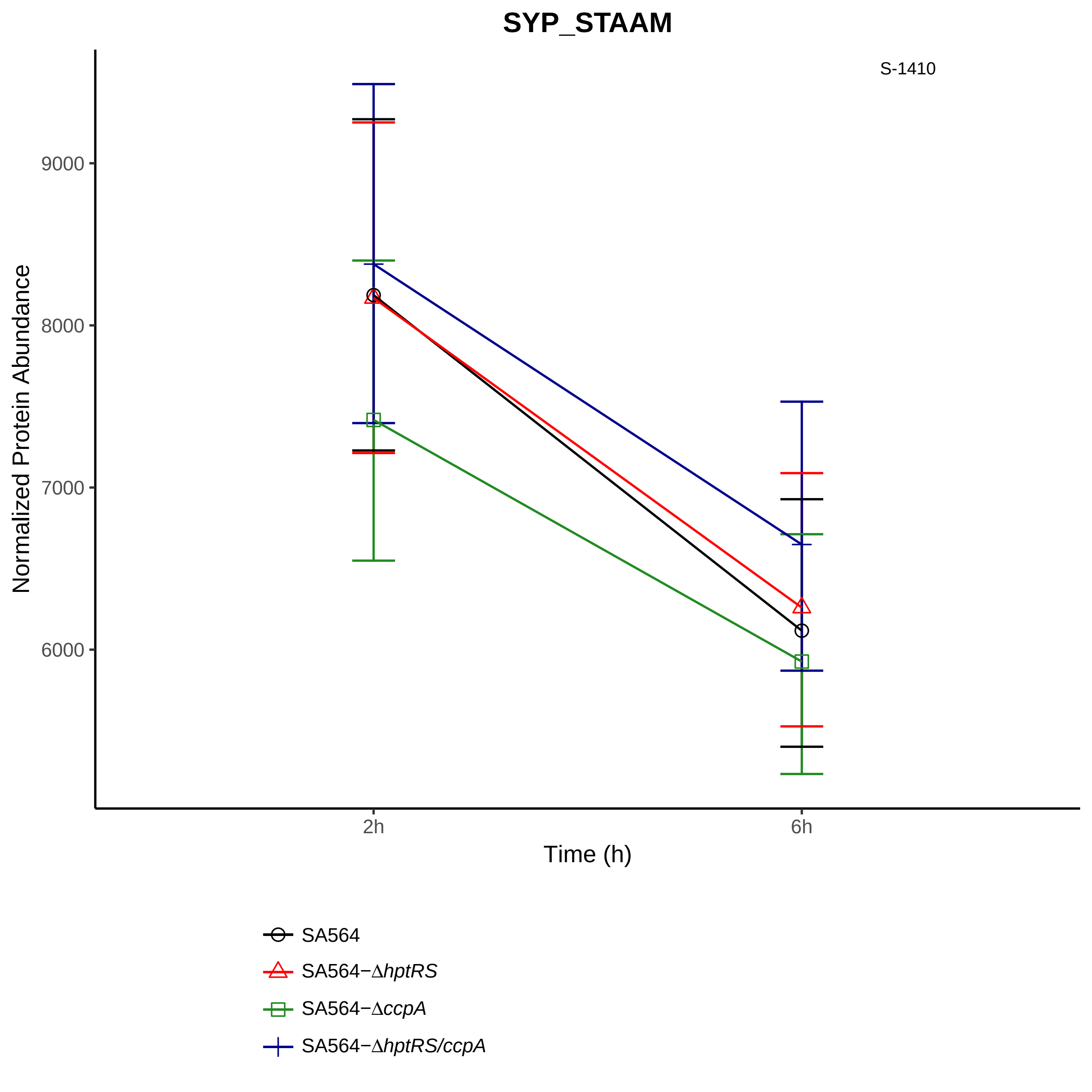
SYP_STAAM
S-1410
Normalized Protein Abundance
9000
8000
7000
6000
2h
6h
Time (h)
SA564
SA564−ΔhptRS
SA564−ΔccpA
SA564−ΔhptRS/ccpA

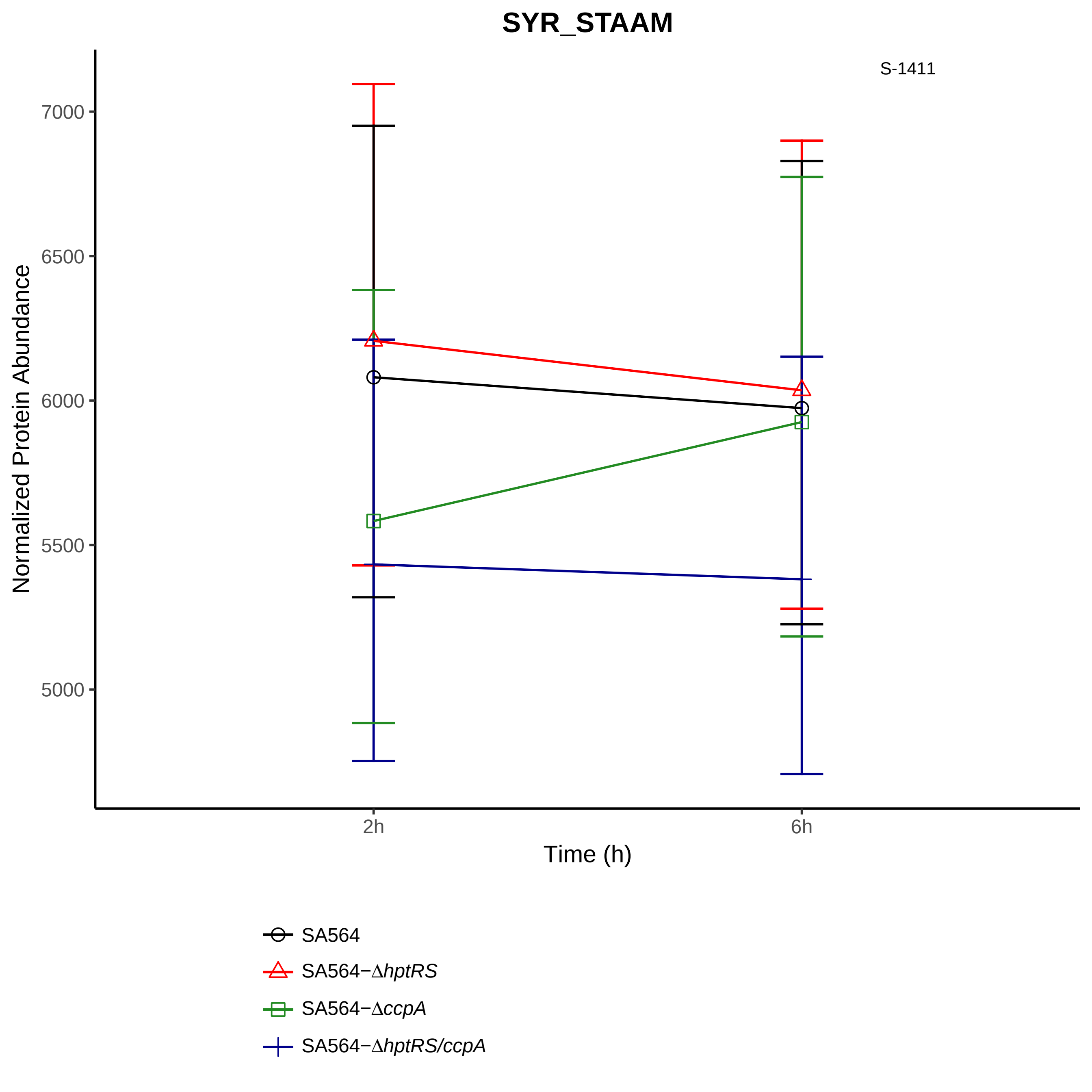
SYR_STAAM
S-1411
Normalized Protein Abundance
7000
6500
6000
5500
5000
2h
6h
Time (h)
SA564
SA564−ΔhptRS
SA564−ΔccpA
SA564−ΔhptRS/ccpA

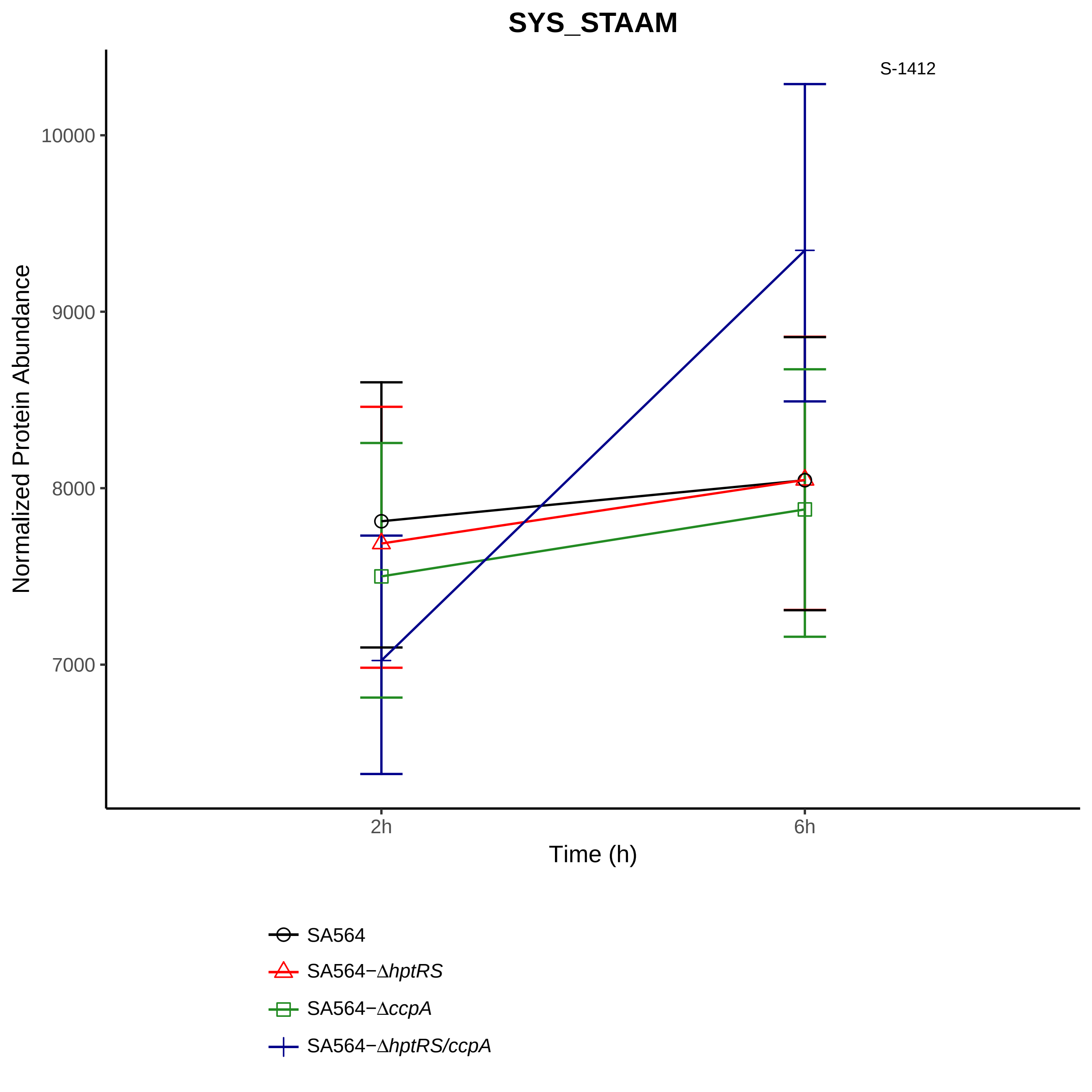

SYS_STAAM
S-1412
Normalized Protein Abundance
10000
9000
8000
7000
2h
6h
Time (h)
SA564
SA564−Δ*hptRS*
SA564−Δ*ccpA*
SA564−Δ*hptRS/ccpA*

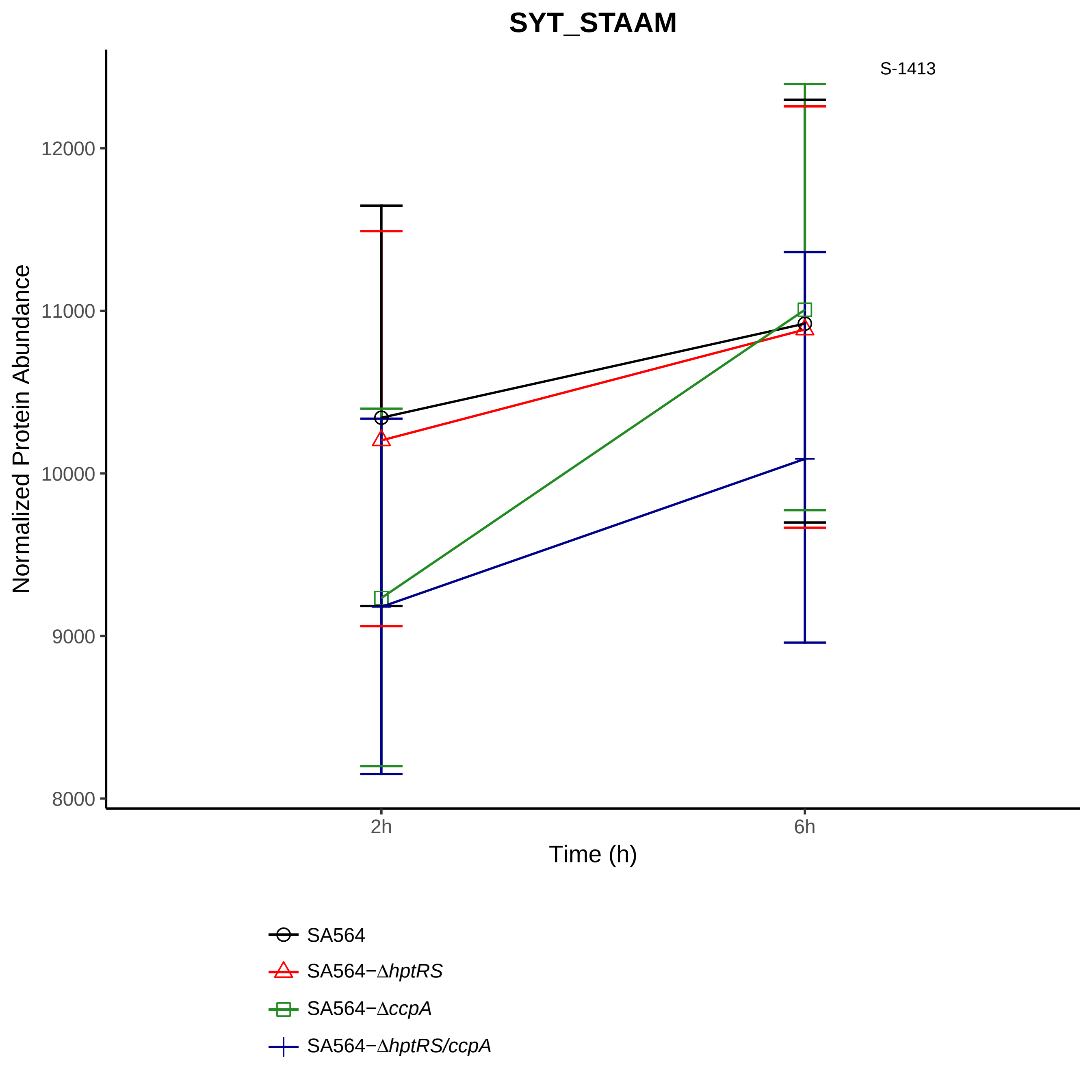

SYT_STAAM
S-1413
12000
11000
10000
9000
8000
Normalized Protein Abundance
2h
6h
Time (h)
SA564
SA564−Δ*hptRS*
SA564−Δ*ccpA*
SA564−Δ*hptRS/ccpA*

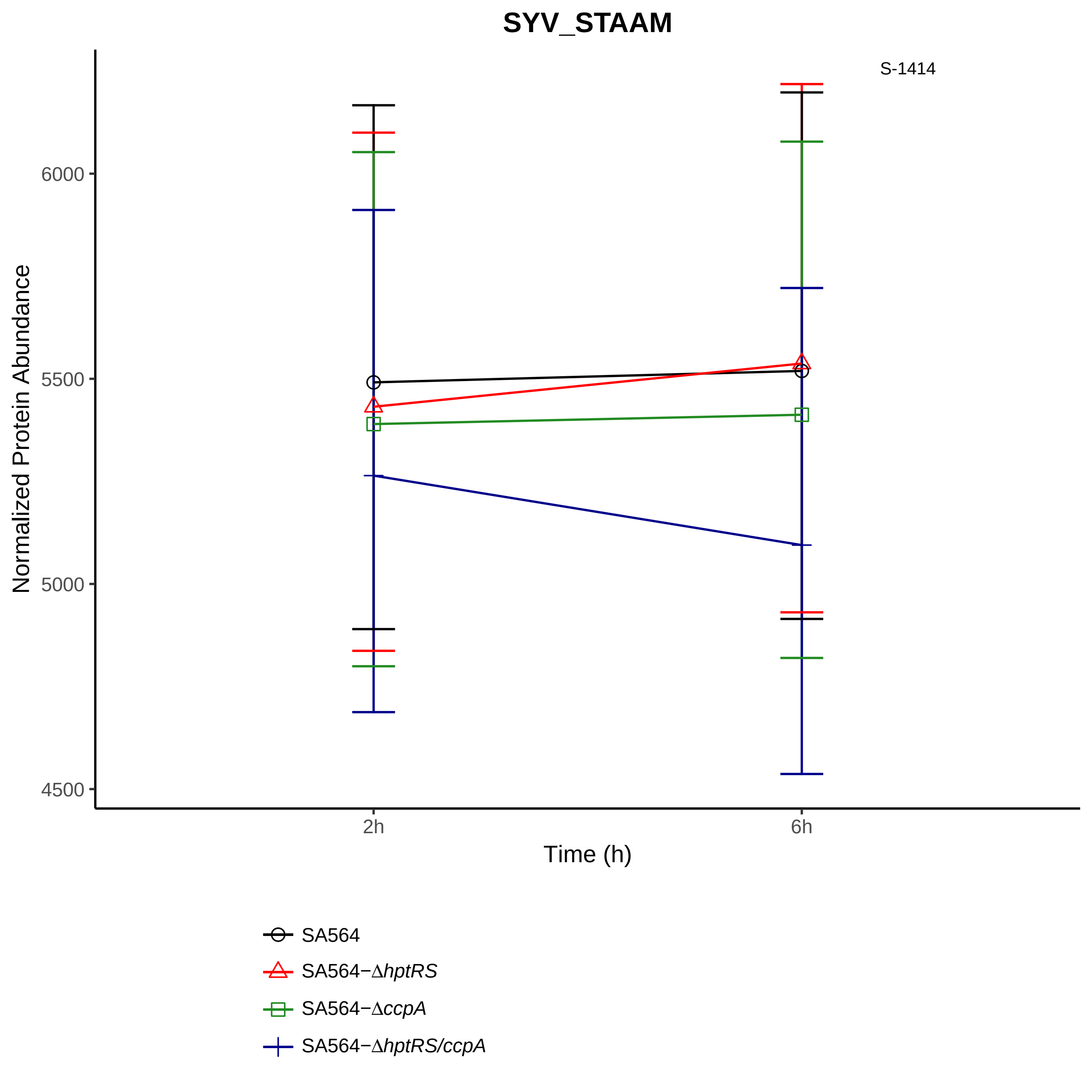

SYV_STAAM
S-1414
Normalized Protein Abundance
6000
5500
5000
4500
2h
6h
Time (h)
SA564
SA564−Δ*hptRS*
SA564−Δ*ccpA*
SA564−Δ*hptRS/ccpA*

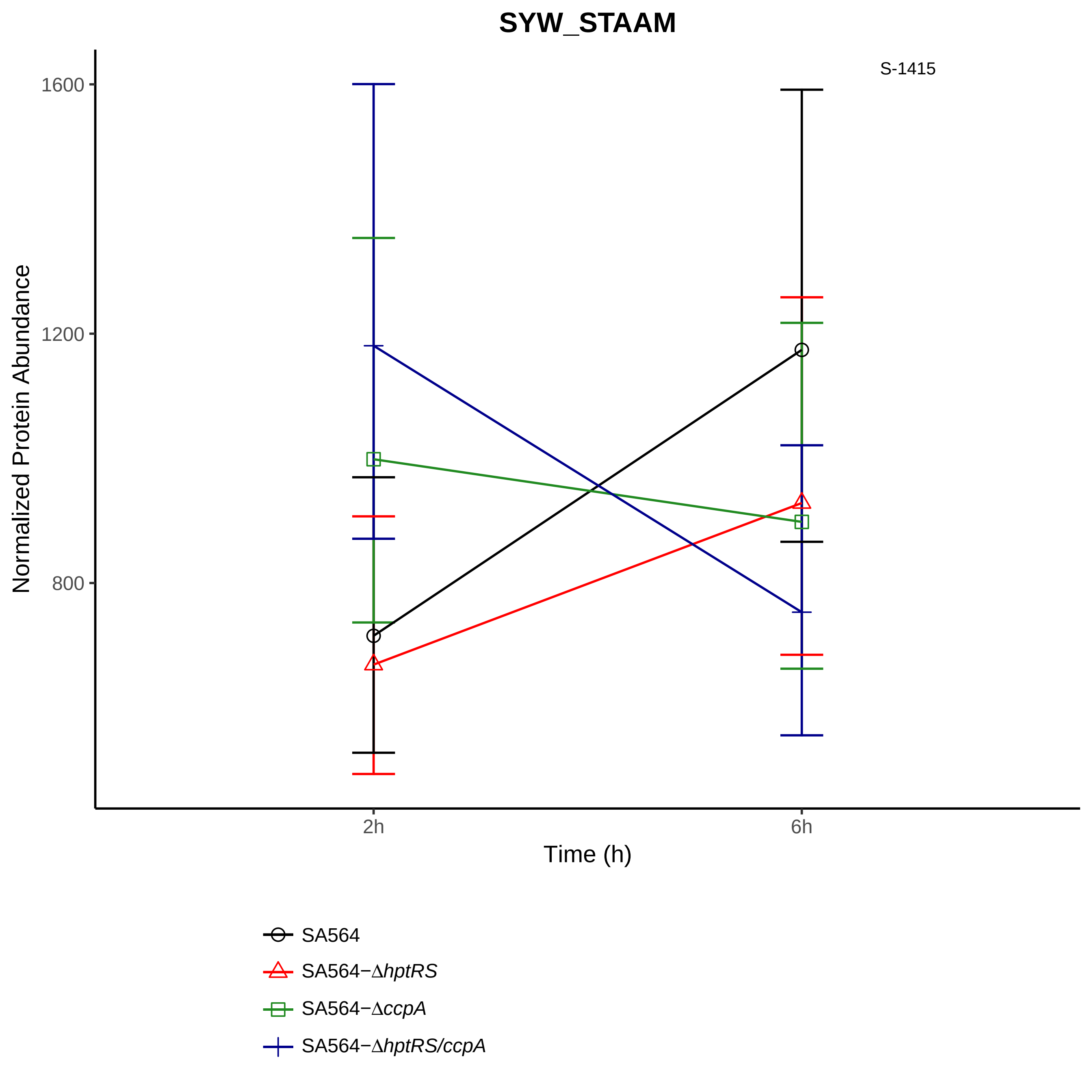

SYW_STAAM
S-1415
1600
1200
800
Normalized Protein Abundance
2h
6h
Time (h)
SA564
SA564−Δ*hptRS*
SA564−Δ*ccpA*
SA564−Δ*hptRS/ccpA*

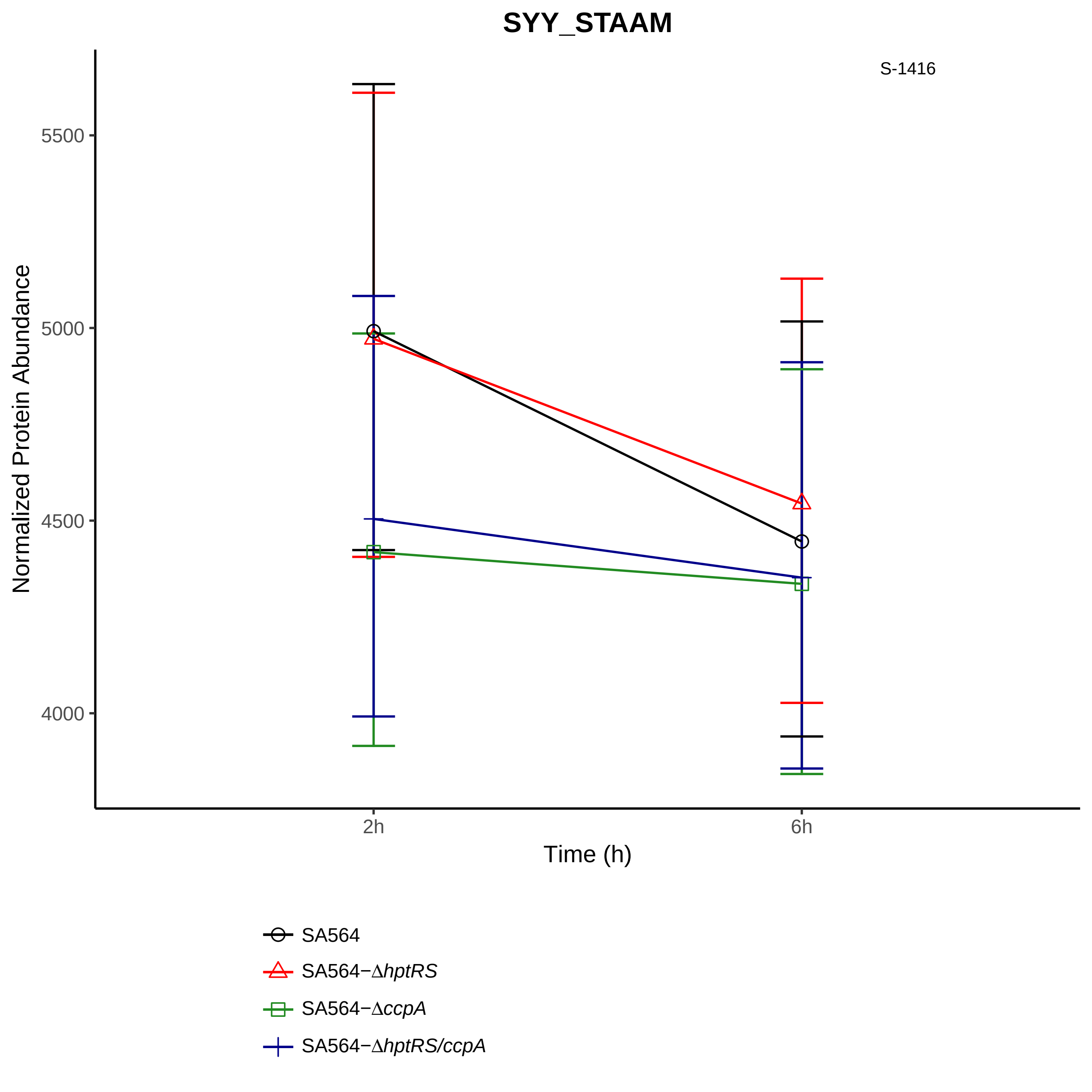
SYY_STAAM
S-1416
Normalized Protein Abundance
5500
5000
4500
4000
2h
6h
Time (h)
SA564
SA564−Δ*hptRS*
SA564−Δ*ccpA*
SA564−Δ*hptRS/ccpA*

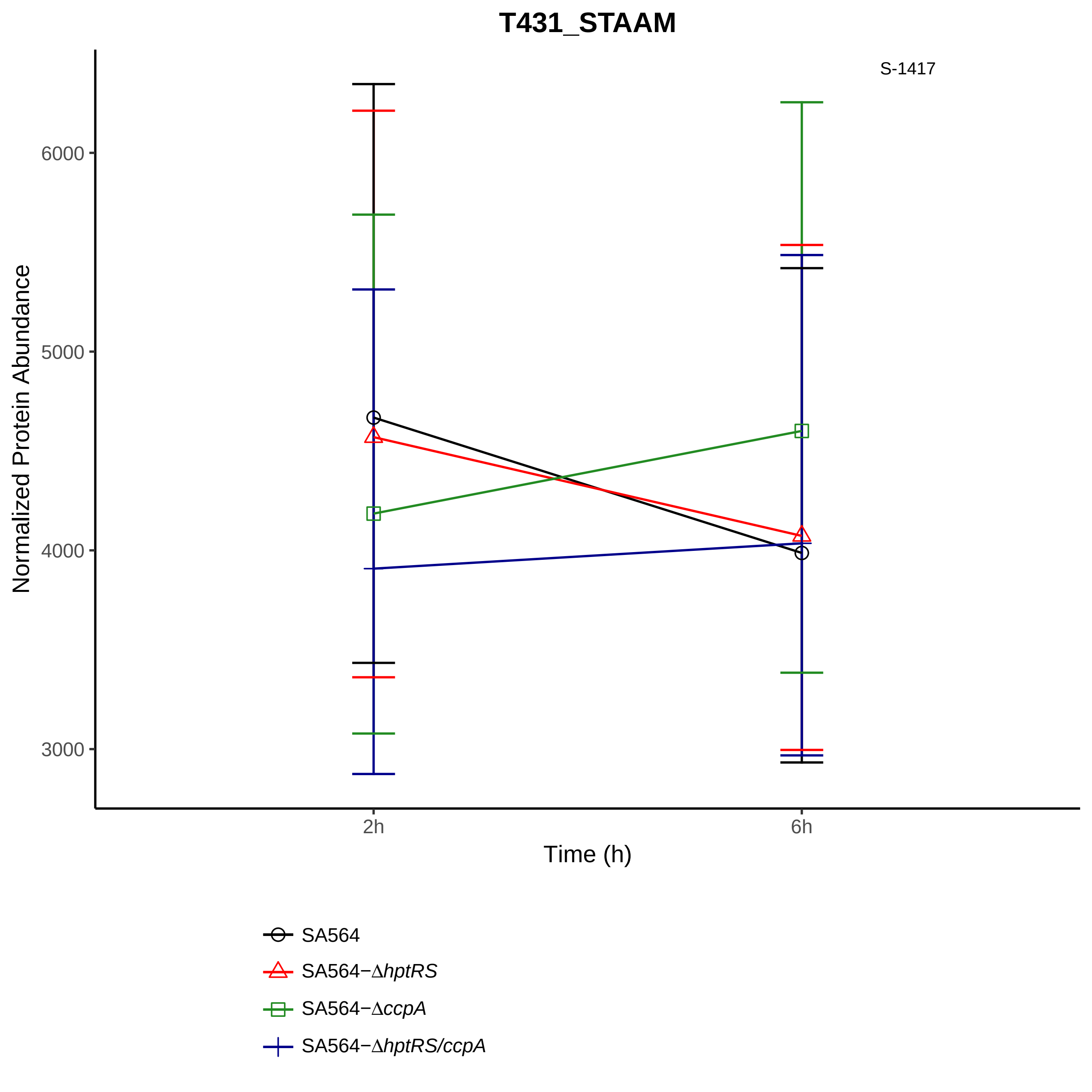

T431_STAAM
S-1417
Normalized Protein Abundance
6000
5000
4000
3000
2h
6h
Time (h)
SA564
SA564−Δ*hptRS*
SA564−Δ*ccpA*
SA564−Δ*hptRS/ccpA*

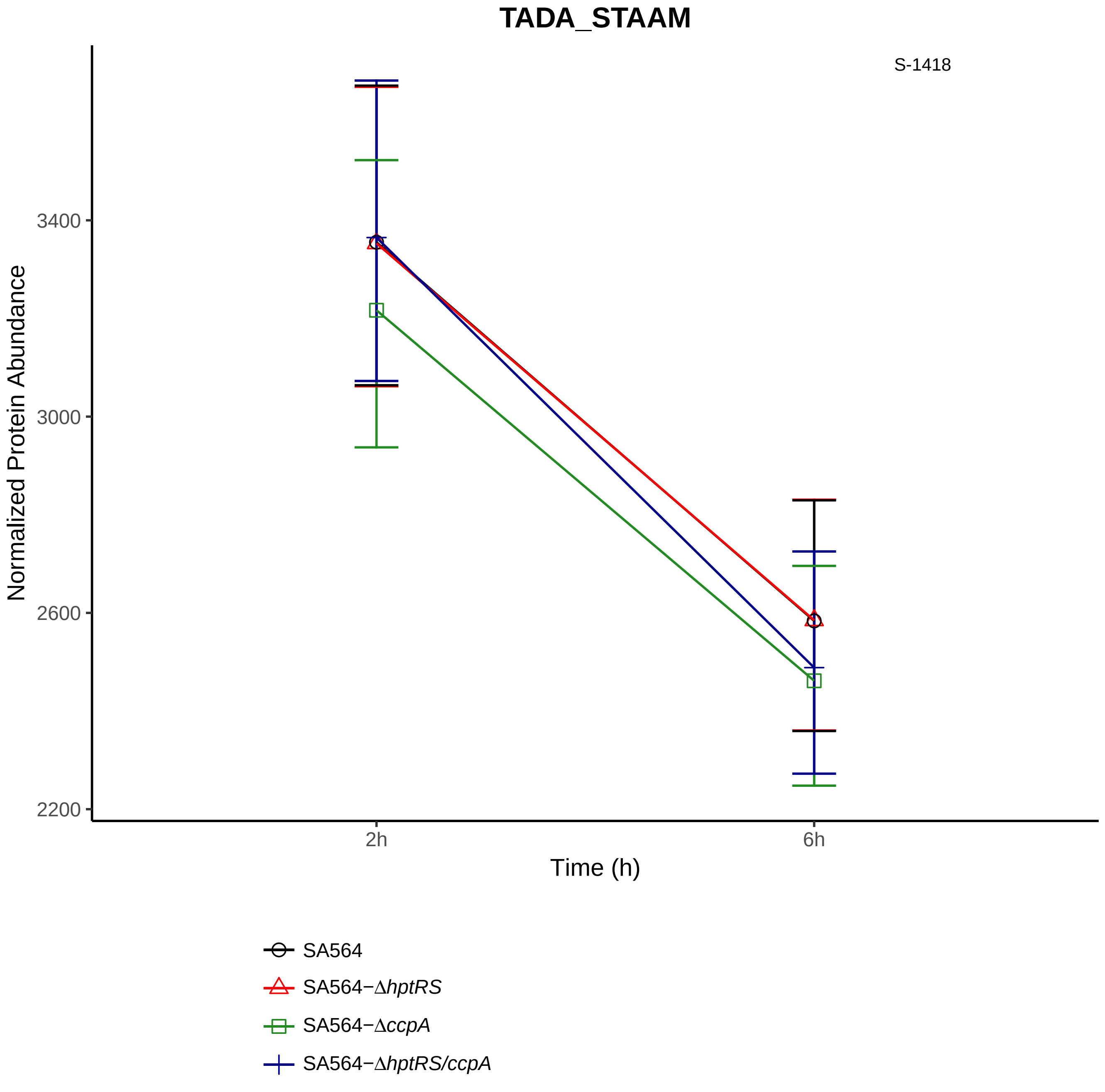

TADA_STAAM
S-1418
Normalized Protein Abundance
3400
3000
2600
2200
2h
6h
Time (h)
SA564
SA564−Δ*hptRS*
SA564−Δ*ccpA*
SA564−Δ*hptRS/ccpA*

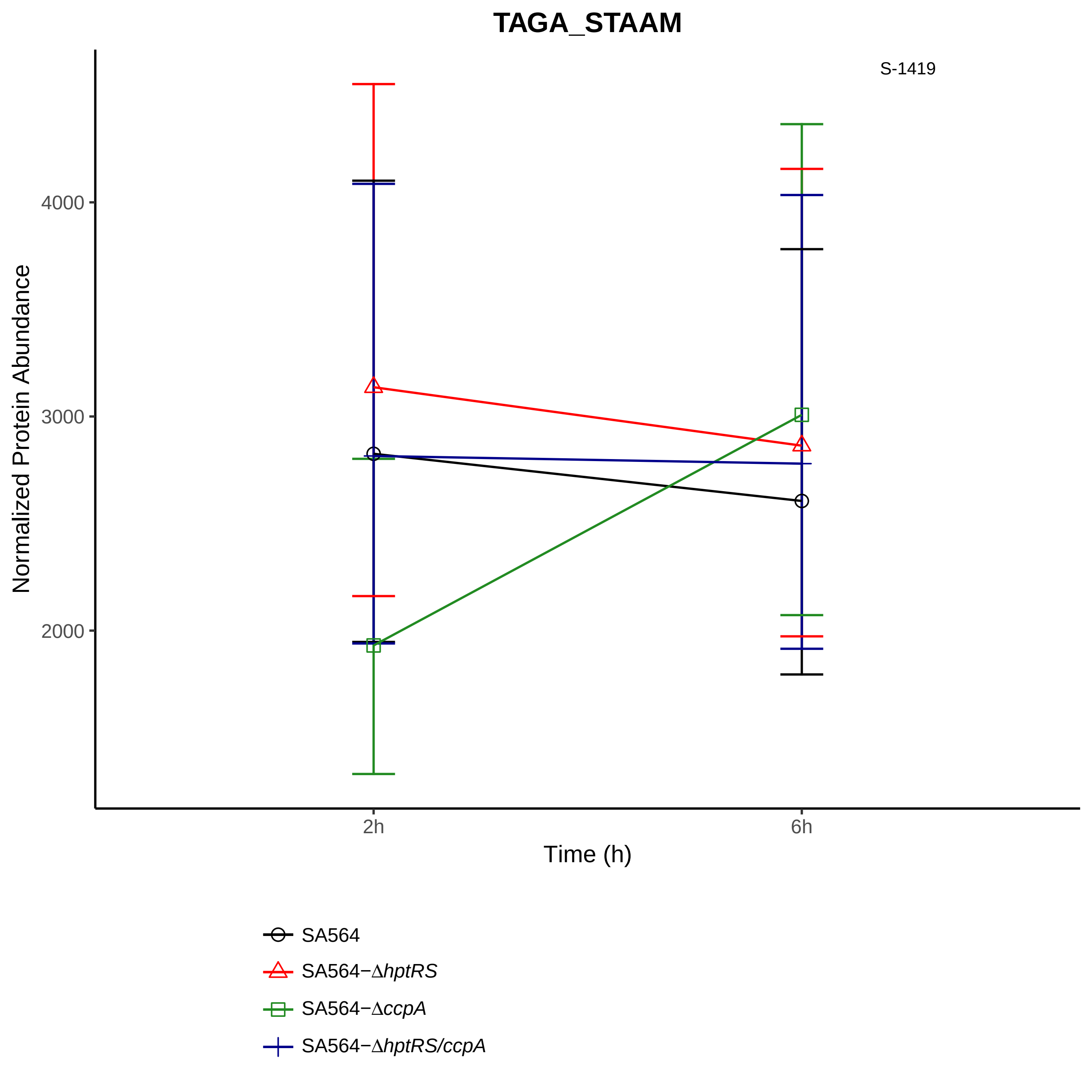

TAGA_STAAM
S-1419
Normalized Protein Abundance
2000
3000
4000
2h
6h
Time (h)
SA564
SA564−Δ*hptRS*
SA564−Δ*ccpA*
SA564−Δ*hptRS/ccpA*

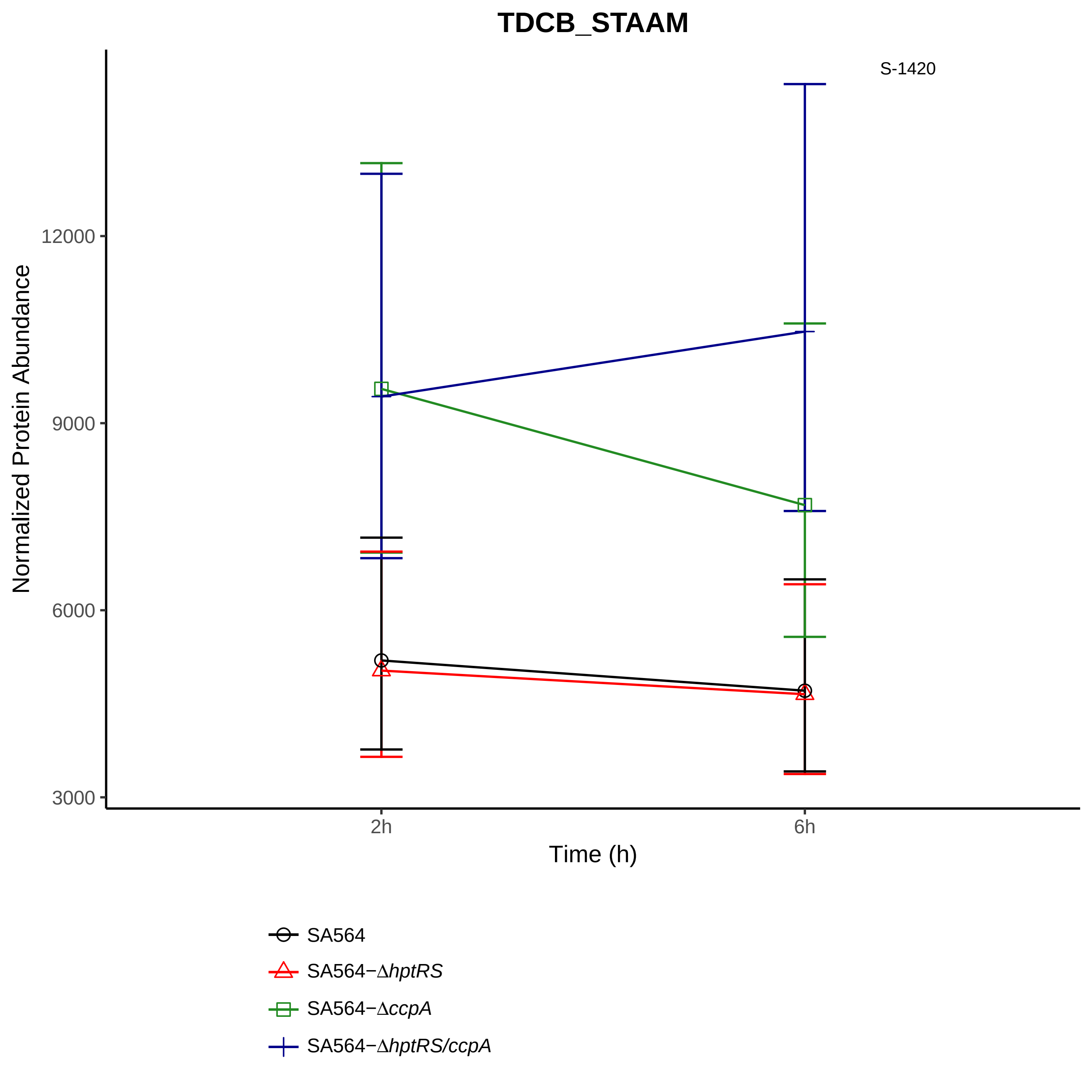
TDCB_STAAM
S-1420
Normalized Protein Abundance
12000
9000
6000
3000
2h
6h
Time (h)
SA564
SA564−ΔhptRS
SA564−ΔccpA
SA564−ΔhptRS/ccpA

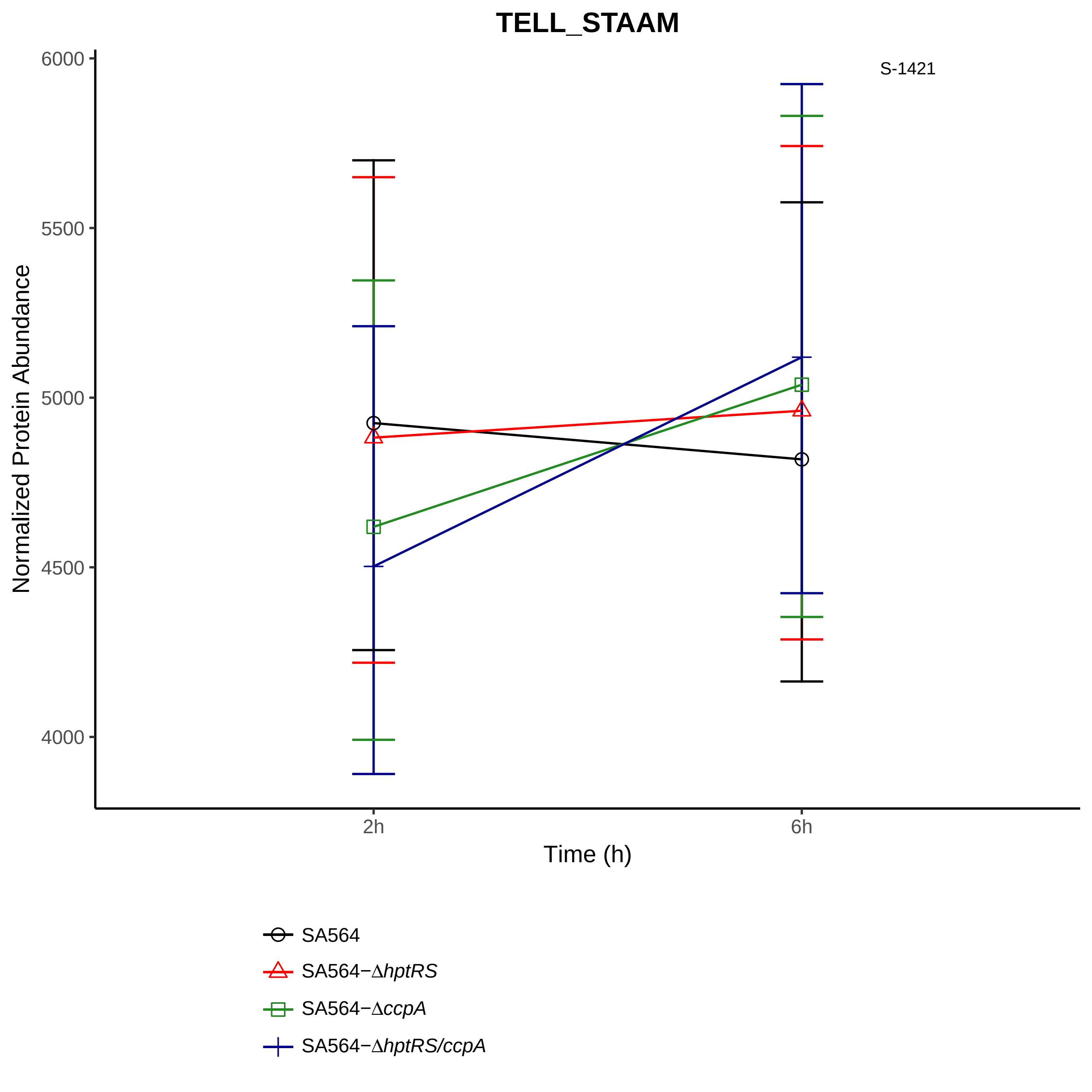

TELL_STAAM
S-1421
Normalized Protein Abundance
6000
5500
5000
4500
4000
2h
6h
Time (h)
SA564
SA564−Δ*hptRS*
SA564−Δ*ccpA*
SA564−Δ*hptRS/ccpA*

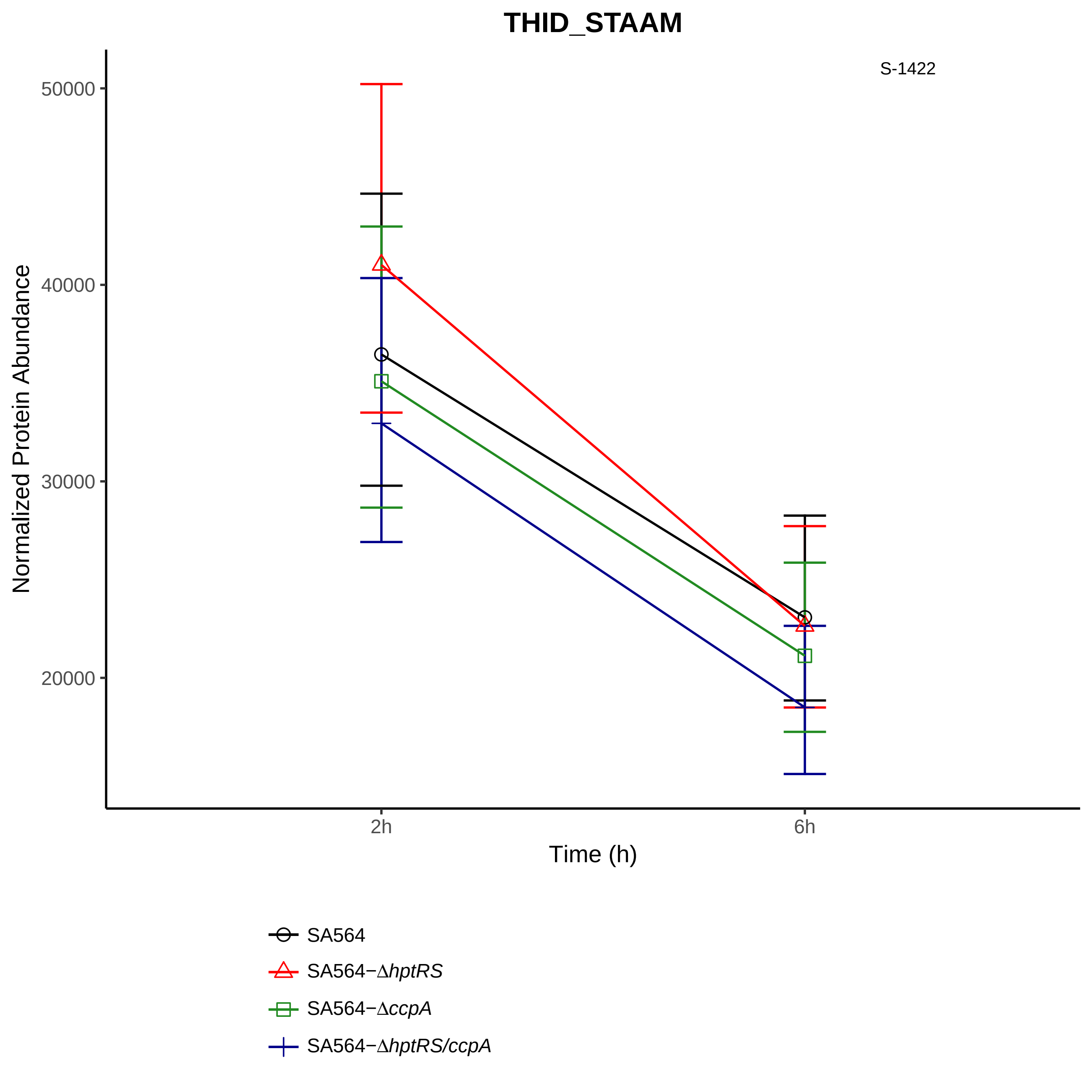

THID_STAAM
S-1422
Normalized Protein Abundance
50000
40000
30000
20000
2h
6h
Time (h)
SA564
SA564−Δ*hptRS*
SA564−Δ*ccpA*
SA564−Δ*hptRS/ccpA*

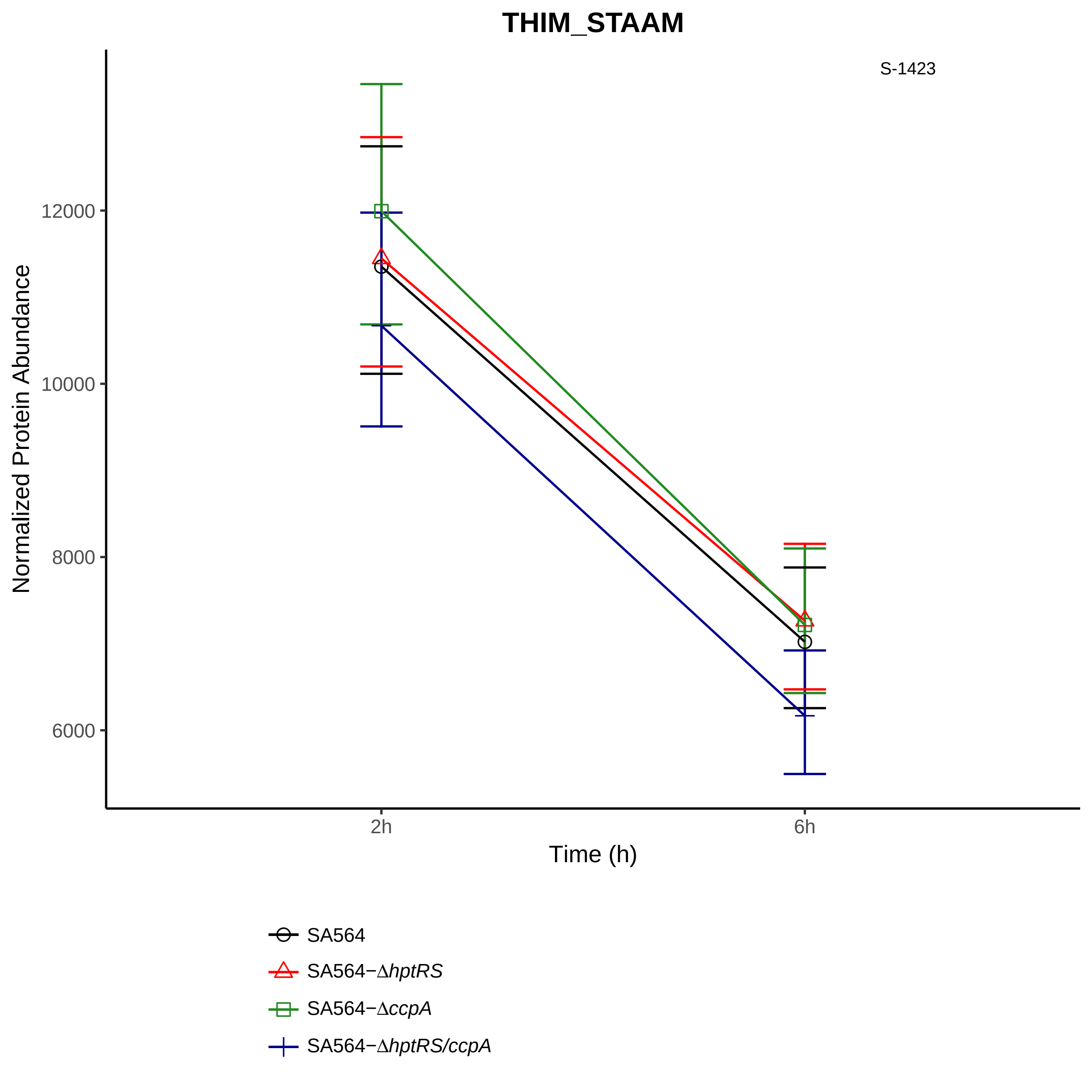

THIM_STAAM
S-1423
Normalized Protein Abundance
12000
10000
8000
6000
2h
6h
Time (h)
SA564
SA564−Δ*hptRS*
SA564−Δ*ccpA*
SA564−Δ*hptRS/ccpA*

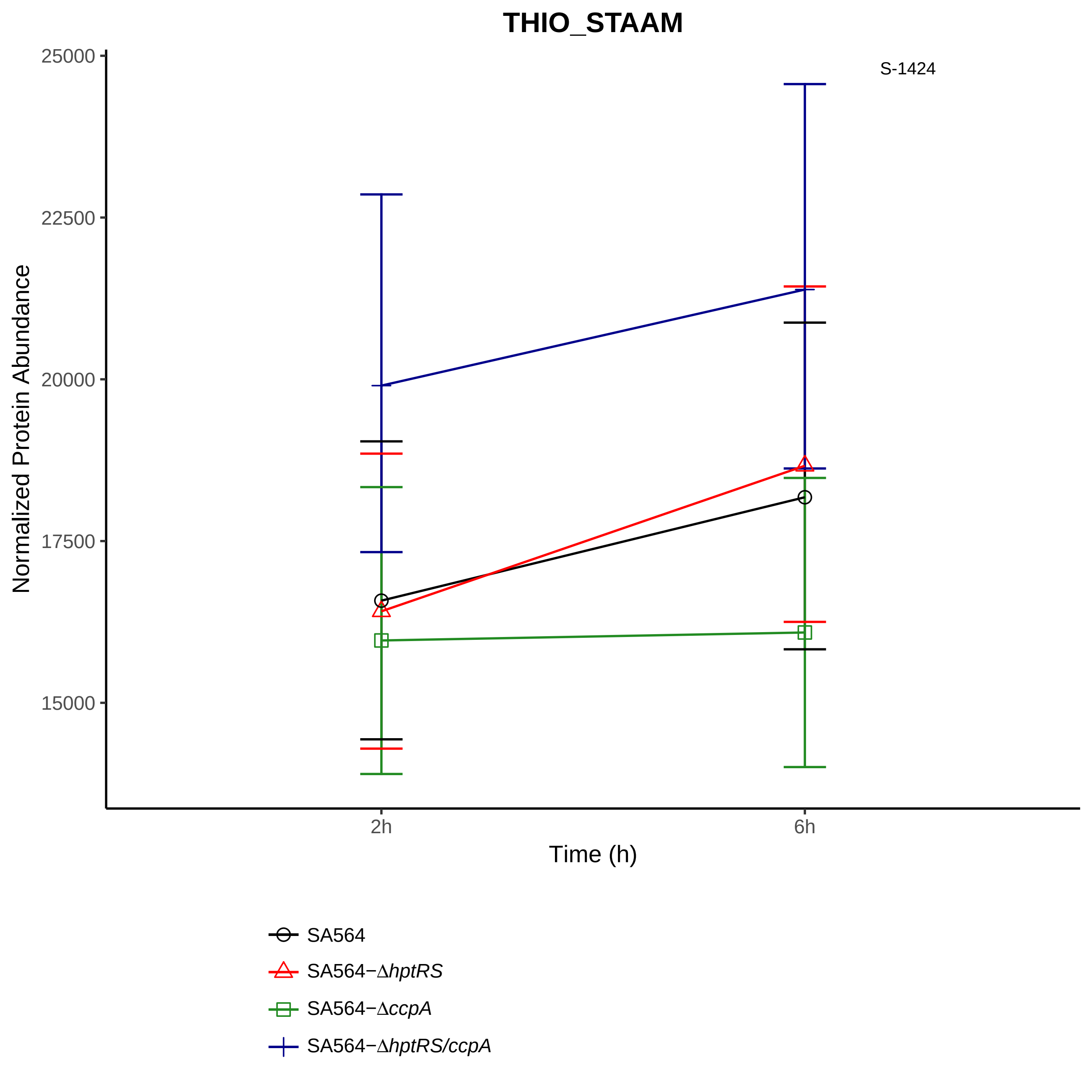

THIO_STAAM
S-1424
25000
22500
20000
17500
15000
Normalized Protein Abundance
2h
6h
Time (h)
SA564
SA564−Δ*hptRS*
SA564−Δ*ccpA*
SA564−Δ*hptRS/ccpA*

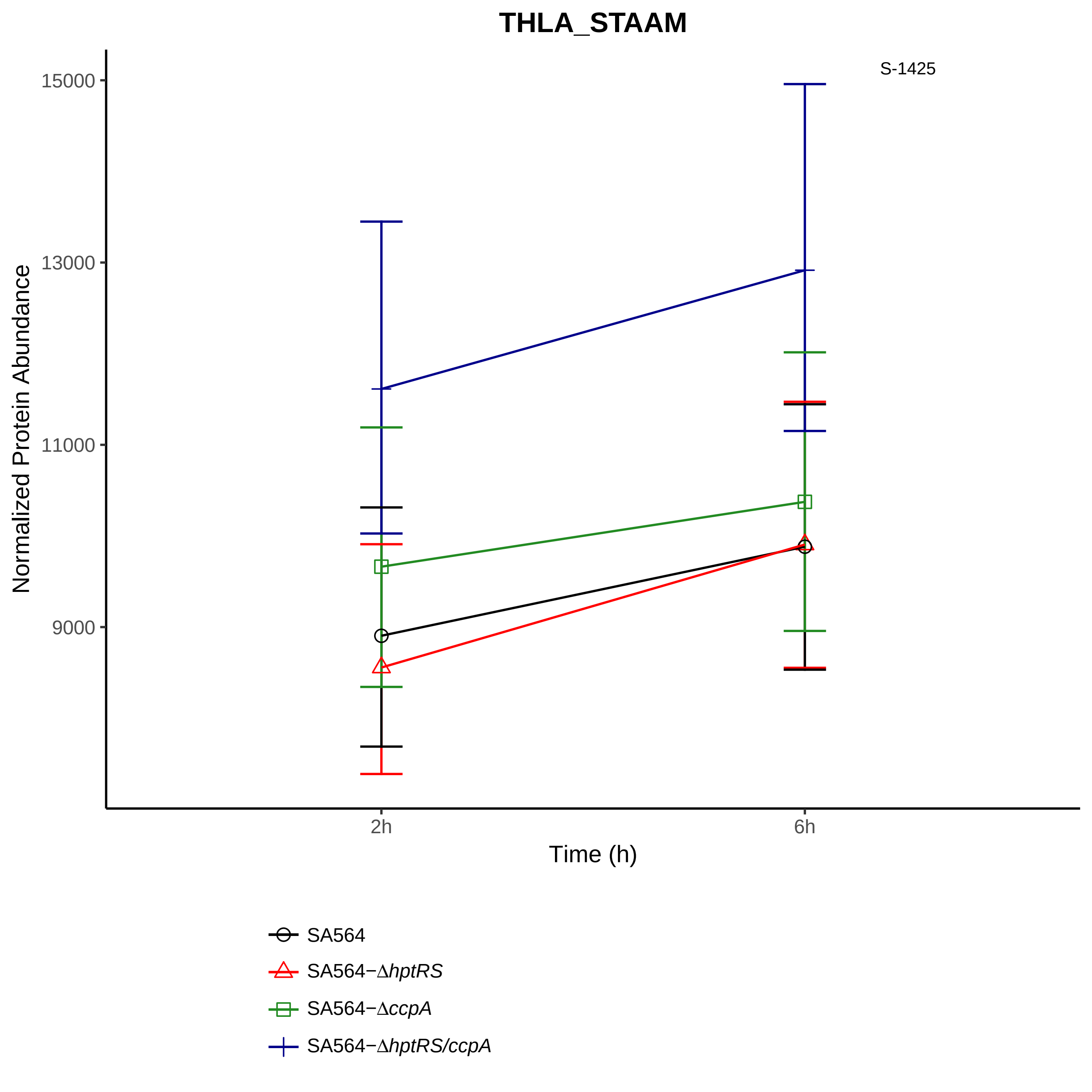

THLA_STAAM
S-1425
15000
13000
11000
9000
Normalized Protein Abundance
2h
6h
Time (h)
SA564
SA564−ΔhptRS
SA564−ΔccpA
SA564−ΔhptRS/ccpA

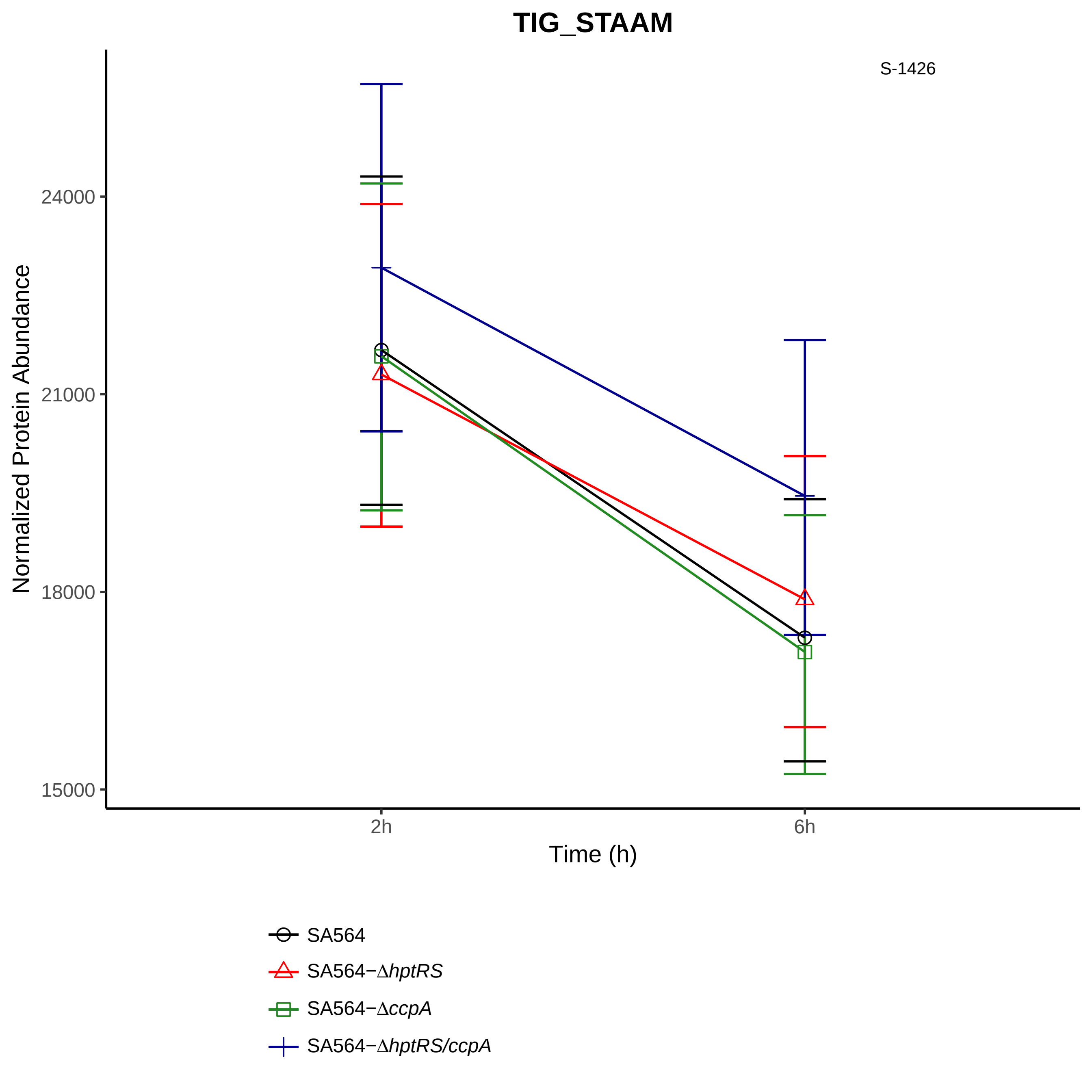

TIG_STAAM
S-1426
Normalized Protein Abundance
24000
21000
18000
15000
2h
6h
Time (h)
SA564
SA564−ΔhptRS
SA564−ΔccpA
SA564−ΔhptRS/ccpA

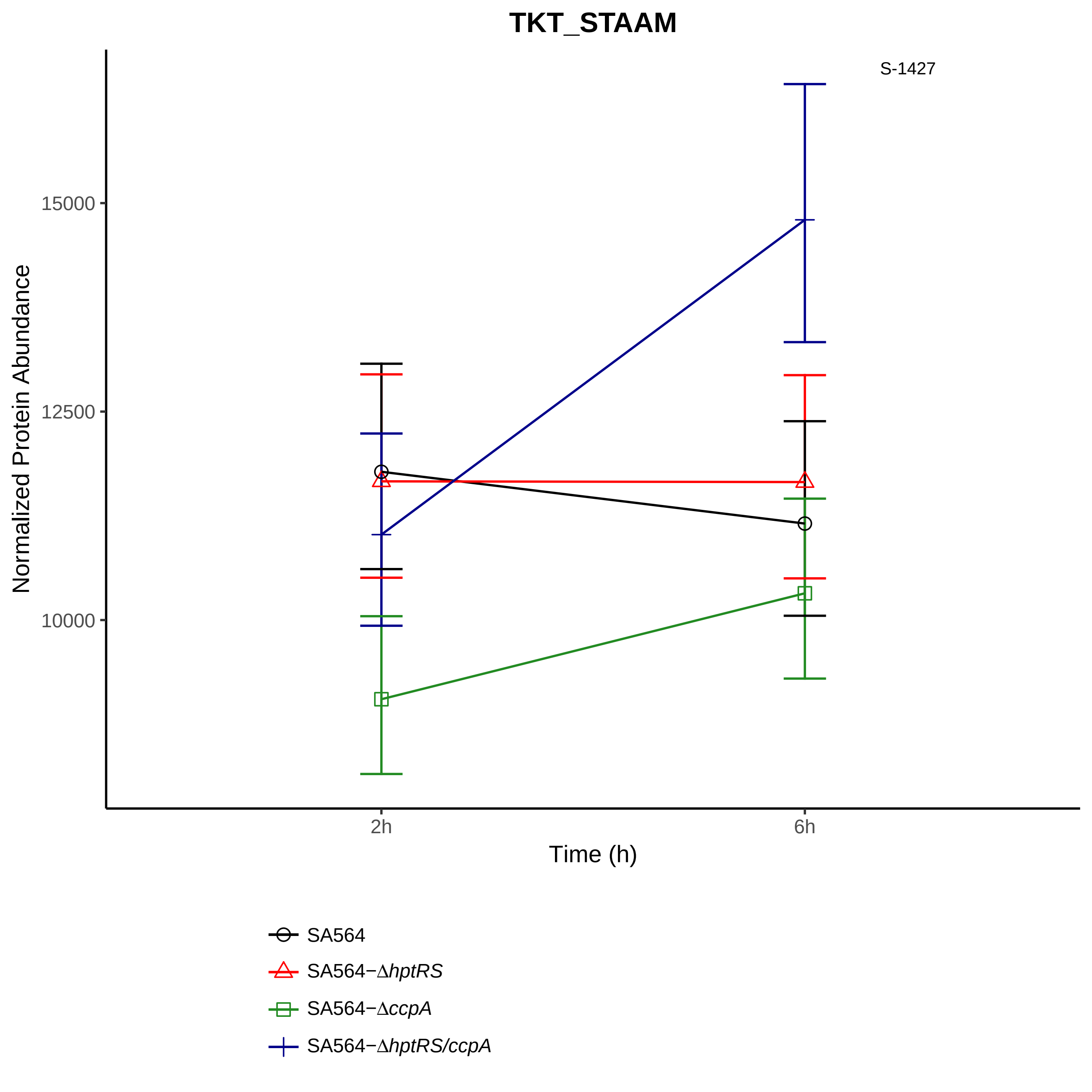

TKT_STAAM
S-1427
Normalized Protein Abundance
15000
12500
10000
2h
6h
Time (h)
SA564
SA564−Δ*hptRS*
SA564−Δ*ccpA*
SA564−Δ*hptRS/ccpA*

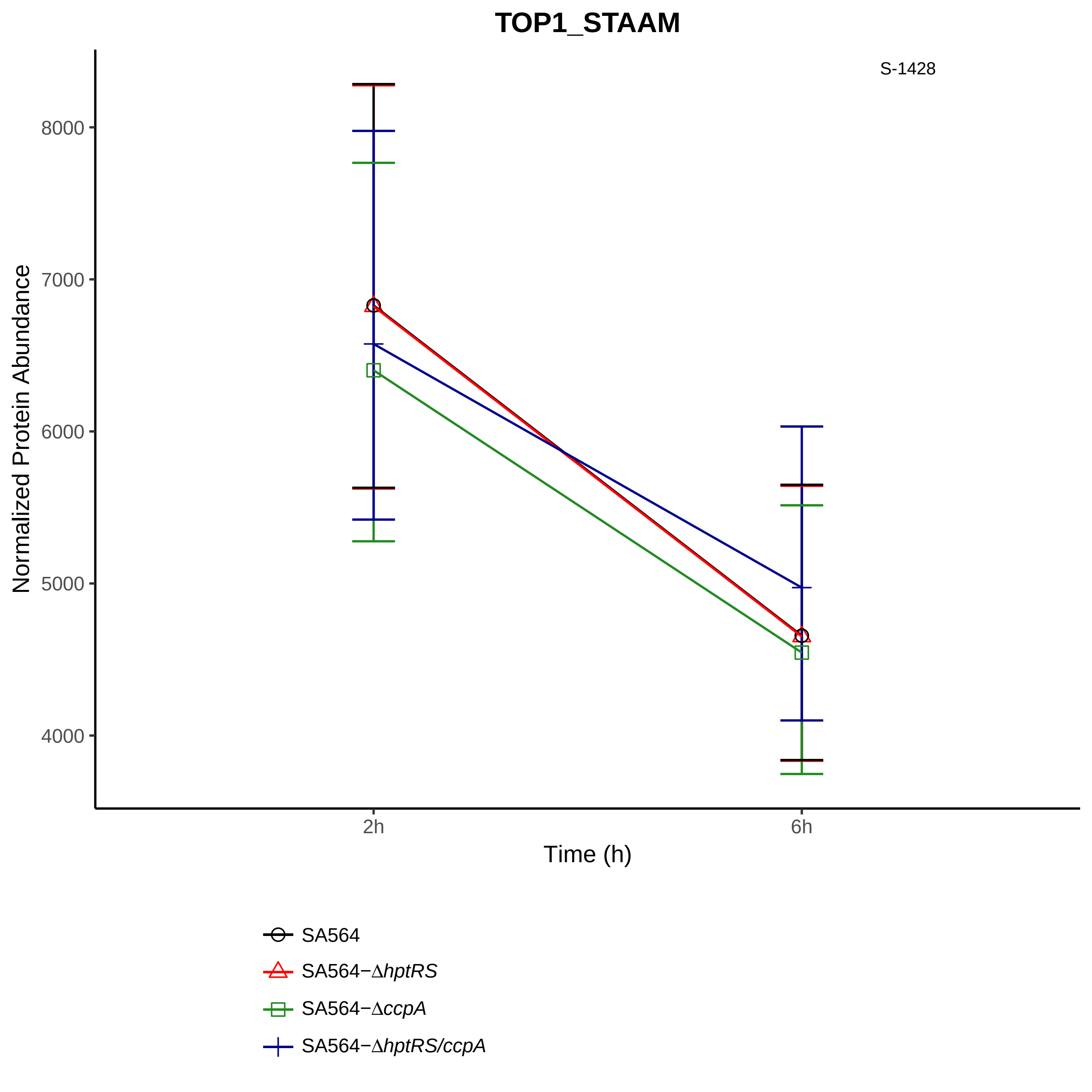

TOP1_STAAM
S-1428
Normalized Protein Abundance
8000
7000
6000
5000
4000
2h
6h
Time (h)
SA564
SA564−Δ*hptRS*
SA564−Δ*ccpA*
SA564−Δ*hptRS/ccpA*

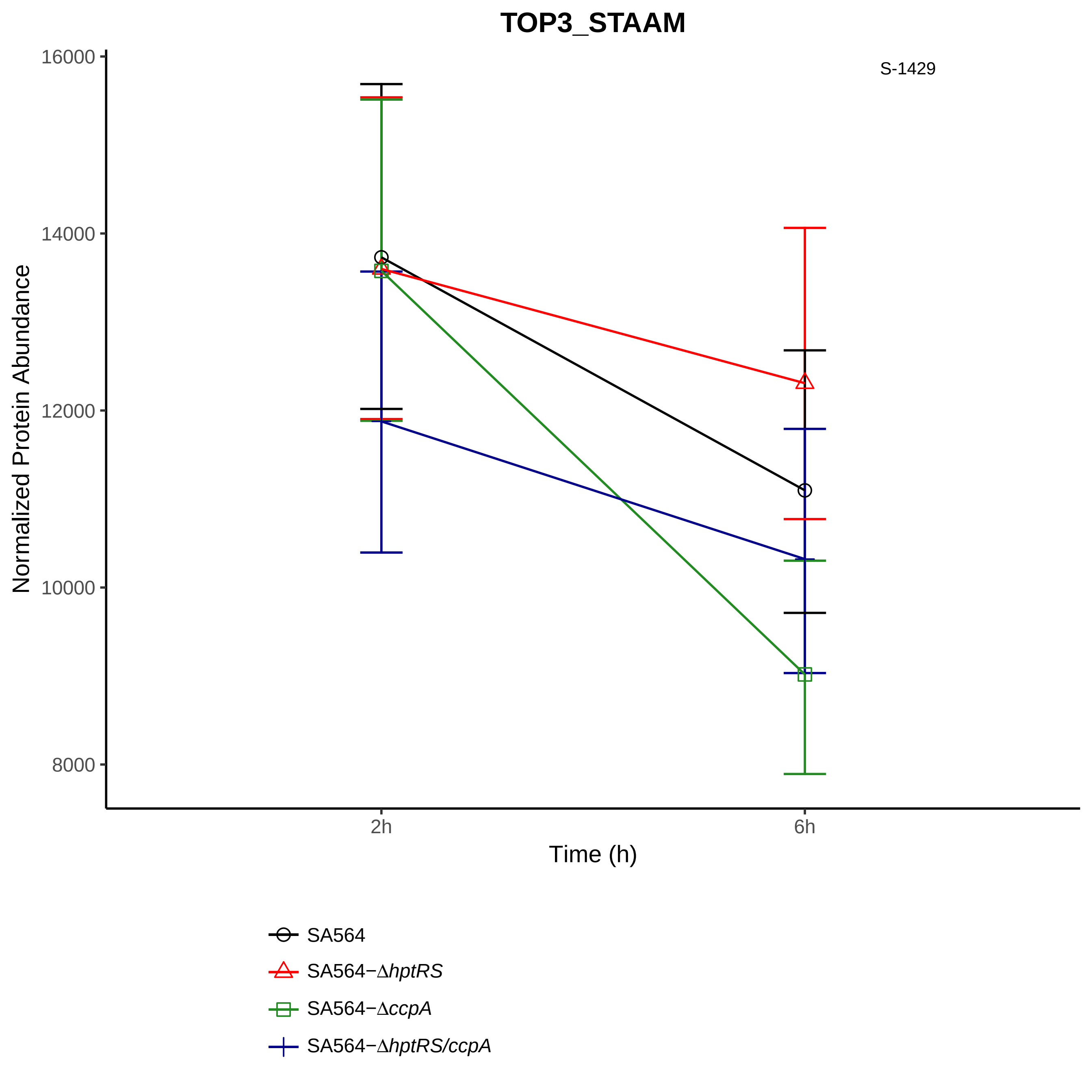

TOP3_STAAM
S-1429
Normalized Protein Abundance
16000
14000
12000
10000
8000
2h
6h
Time (h)
SA564
SA564−Δ*hptRS*
SA564−Δ*ccpA*
SA564−Δ*hptRS/ccpA*

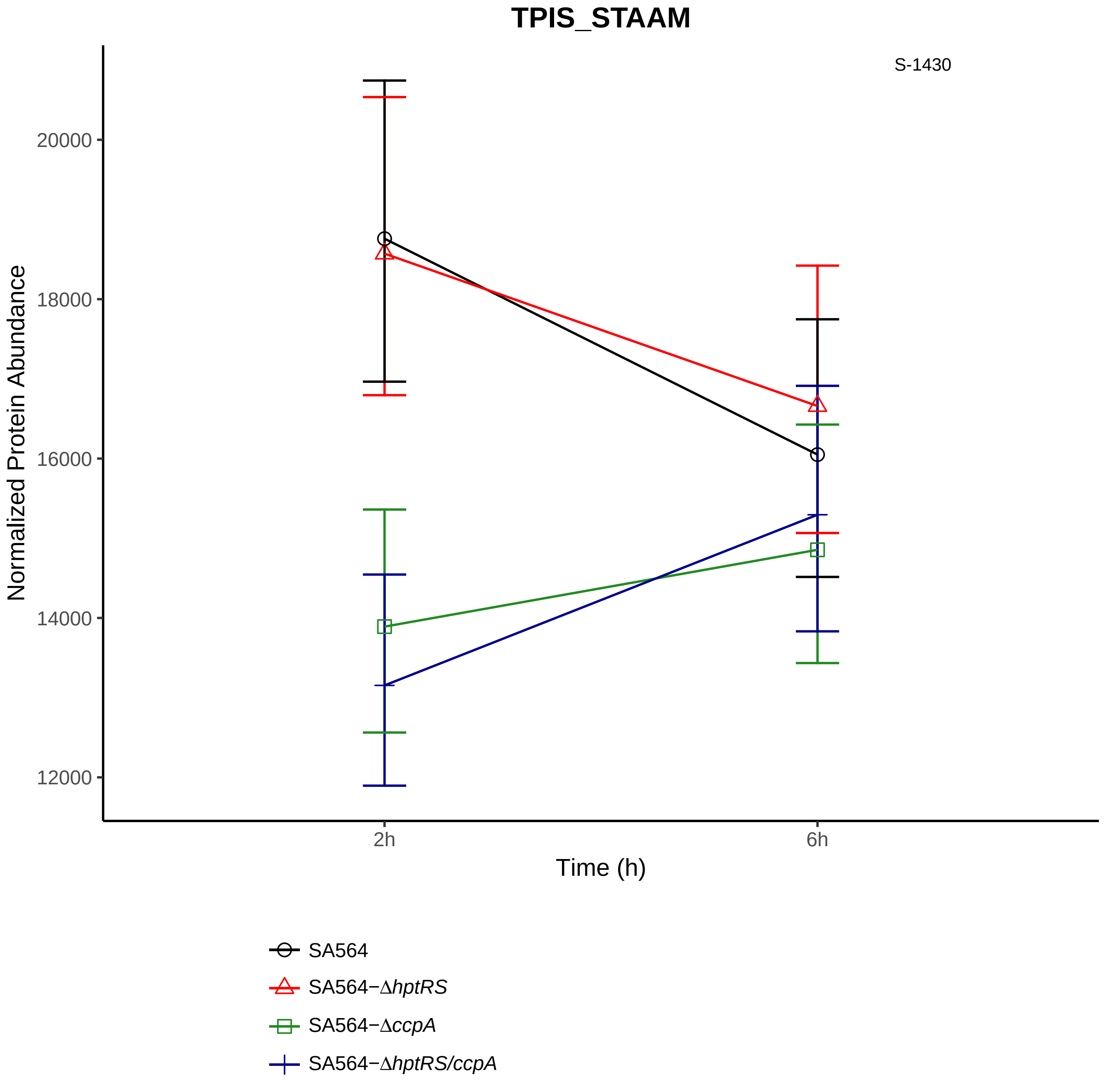

TPIS_STAAM
S-1430
Normalized Protein Abundance
20000
18000
16000
14000
12000
2h
6h
Time (h)
SA564
SA564−Δ*hptRS*
SA564−Δ*ccpA*
SA564−Δ*hptRS/ccpA*

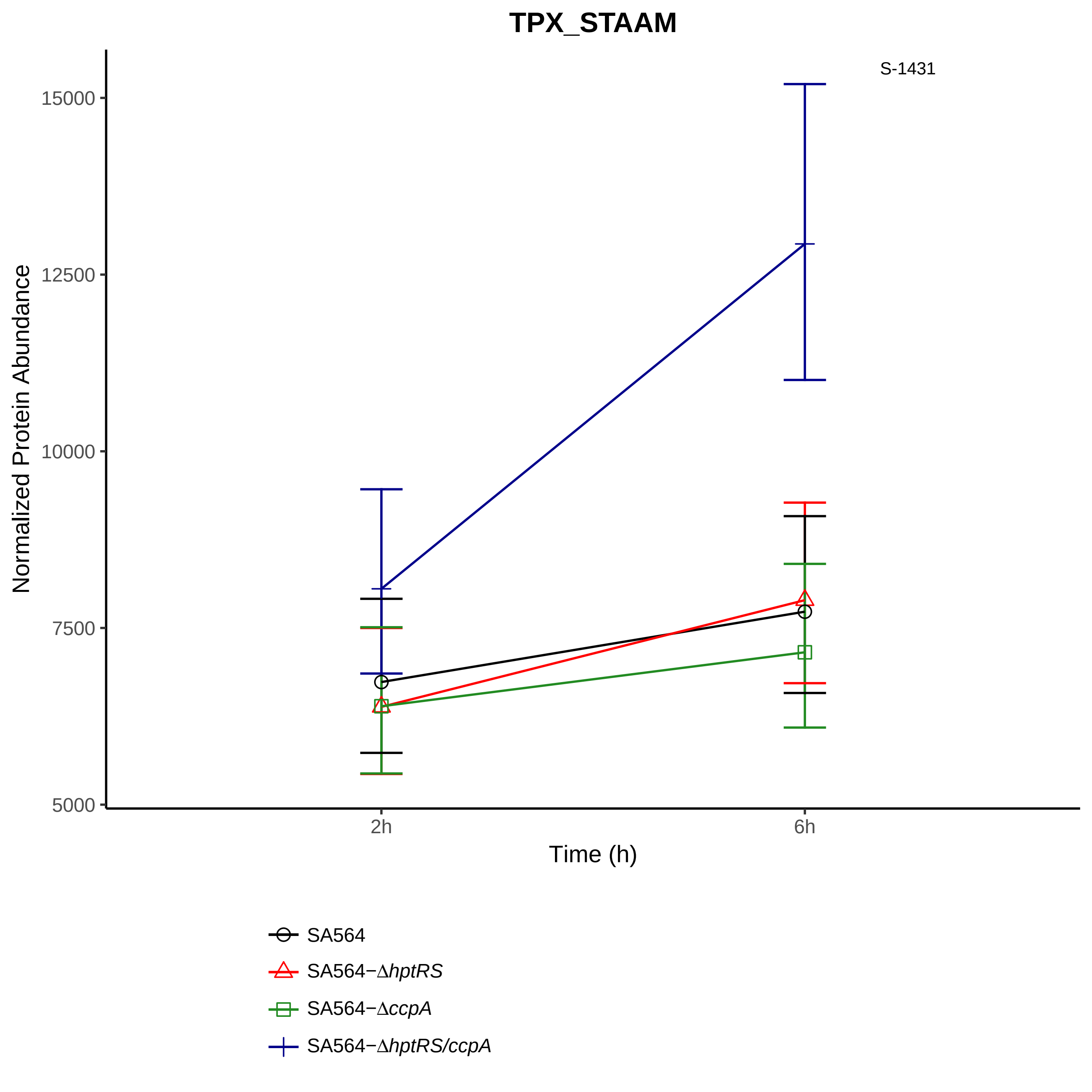
TPX_STAAM
S-1431
Normalized Protein Abundance
15000
12500
10000
7500
5000
2h
6h
Time (h)
SA564
SA564−Δ*hptRS*
SA564−Δ*ccpA*
SA564−Δ*hptRS/ccpA*

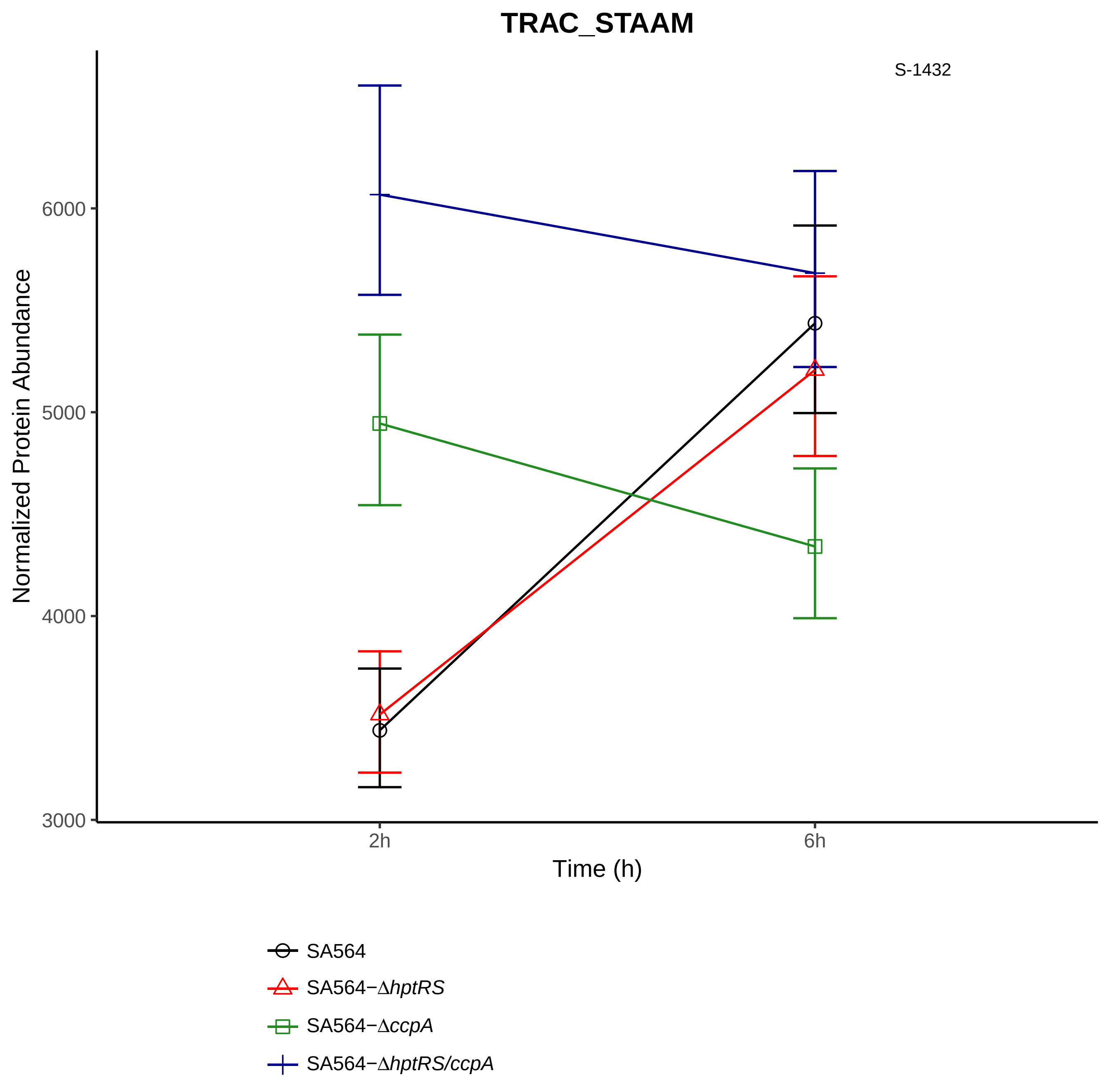
TRAC_STAAM
S-1432
Normalized Protein Abundance
6000
5000
4000
3000
2h
6h
Time (h)
SA564
SA564−Δ*hptRS*
SA564−Δ*ccpA*
SA564−Δ*hptRS/ccpA*

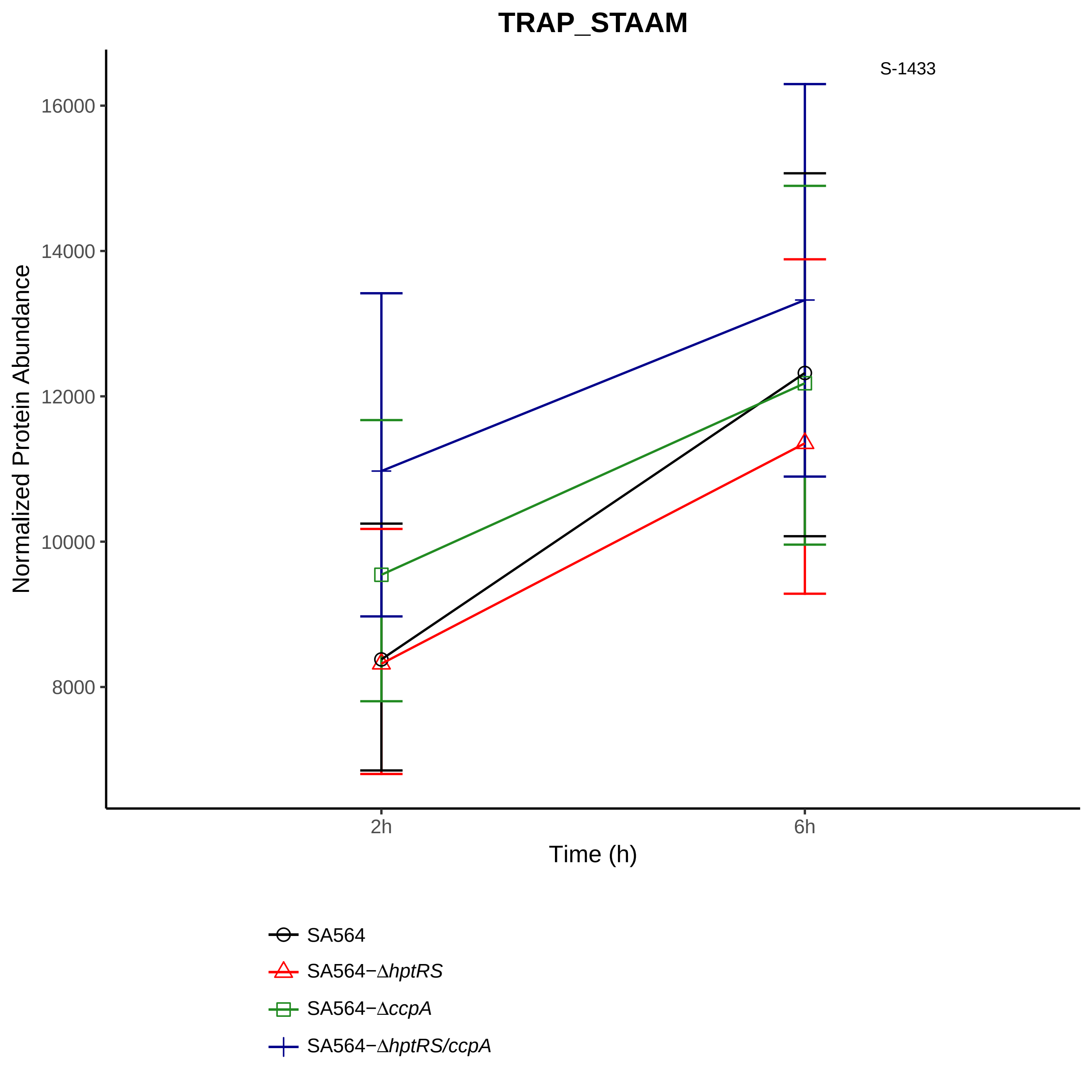

TRAP_STAAM
S-1433
Normalized Protein Abundance
16000
14000
12000
10000
8000
2h
6h
Time (h)
SA564
SA564−Δ*hptRS*
SA564−Δ*ccpA*
SA564−Δ*hptRS/ccpA*

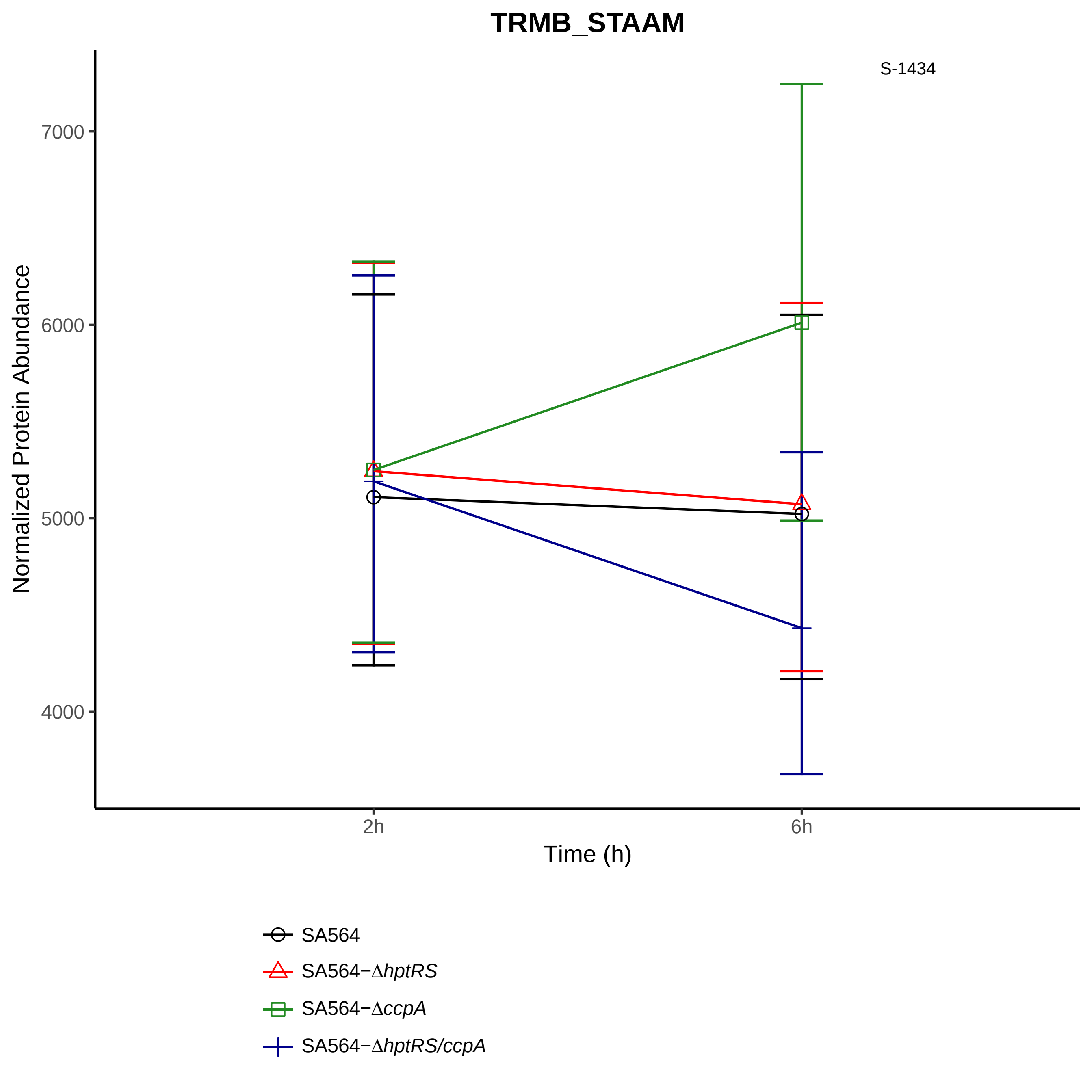

TRMB_STAAM
S-1434
7000
6000
5000
4000
Normalized Protein Abundance
2h
6h
Time (h)
SA564
SA564−Δ*hptRS*
SA564−Δ*ccpA*
SA564−Δ*hptRS/ccpA*

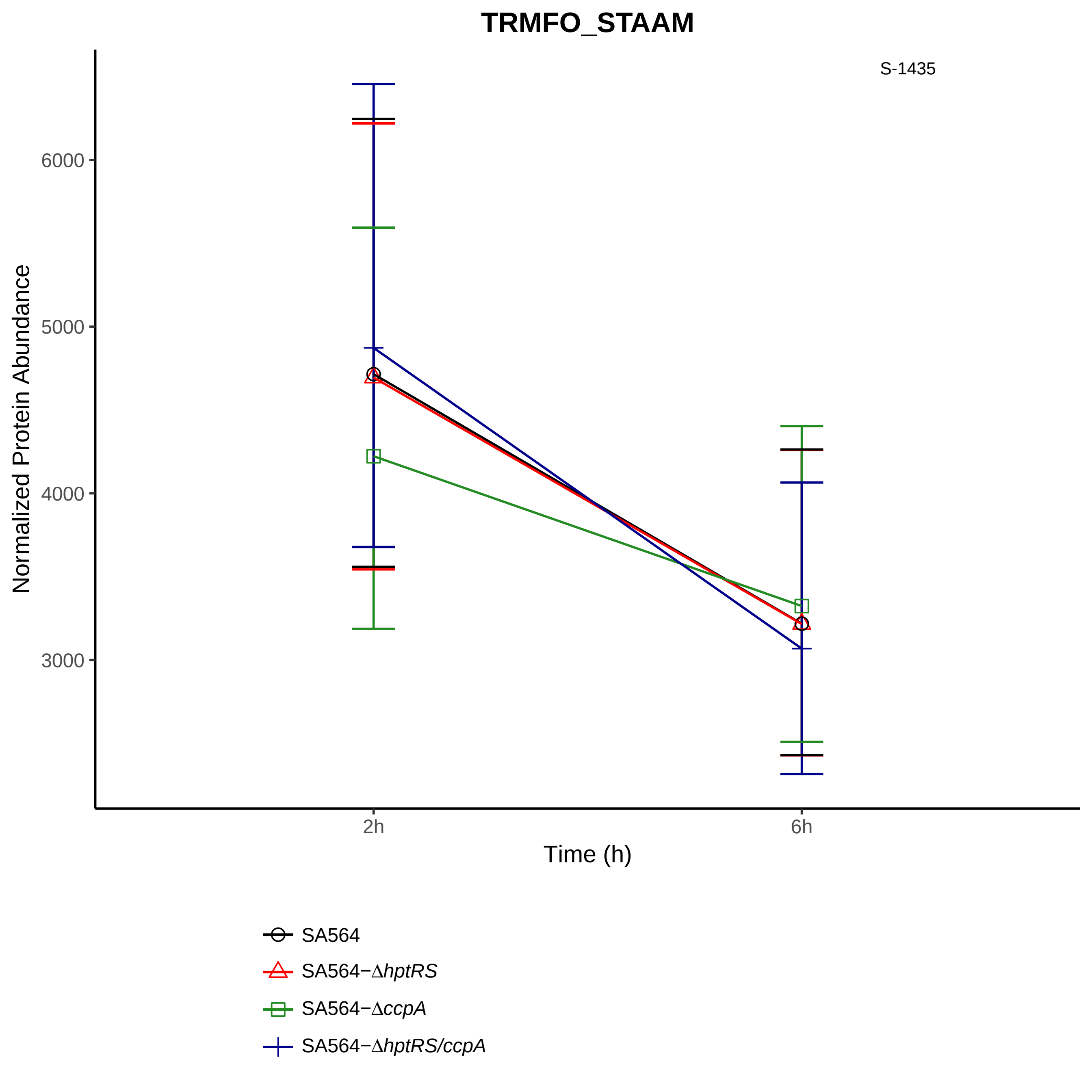

TRMFO_STAAM
S-1435
Normalized Protein Abundance
6000
5000
4000
3000
2h
6h
Time (h)
SA564
SA564−Δ*hptRS*
SA564−Δ*ccpA*
SA564−Δ*hptRS/ccpA*

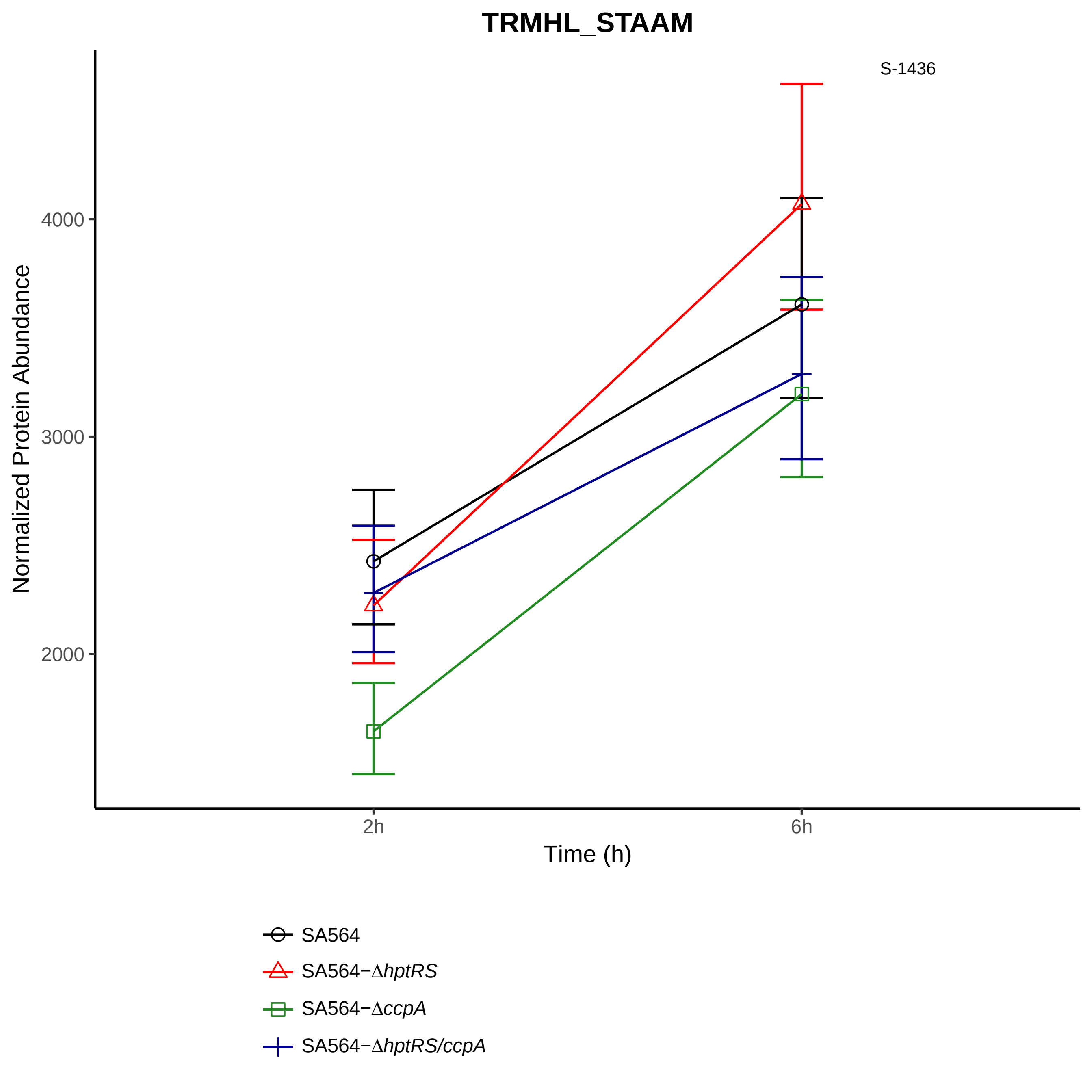

TRMHL_STAAM
S-1436
Normalized Protein Abundance
4000
3000
2000
2h
6h
Time (h)
SA564
SA564−Δ*hptRS*
SA564−Δ*ccpA*
SA564−Δ*hptRS/ccpA*

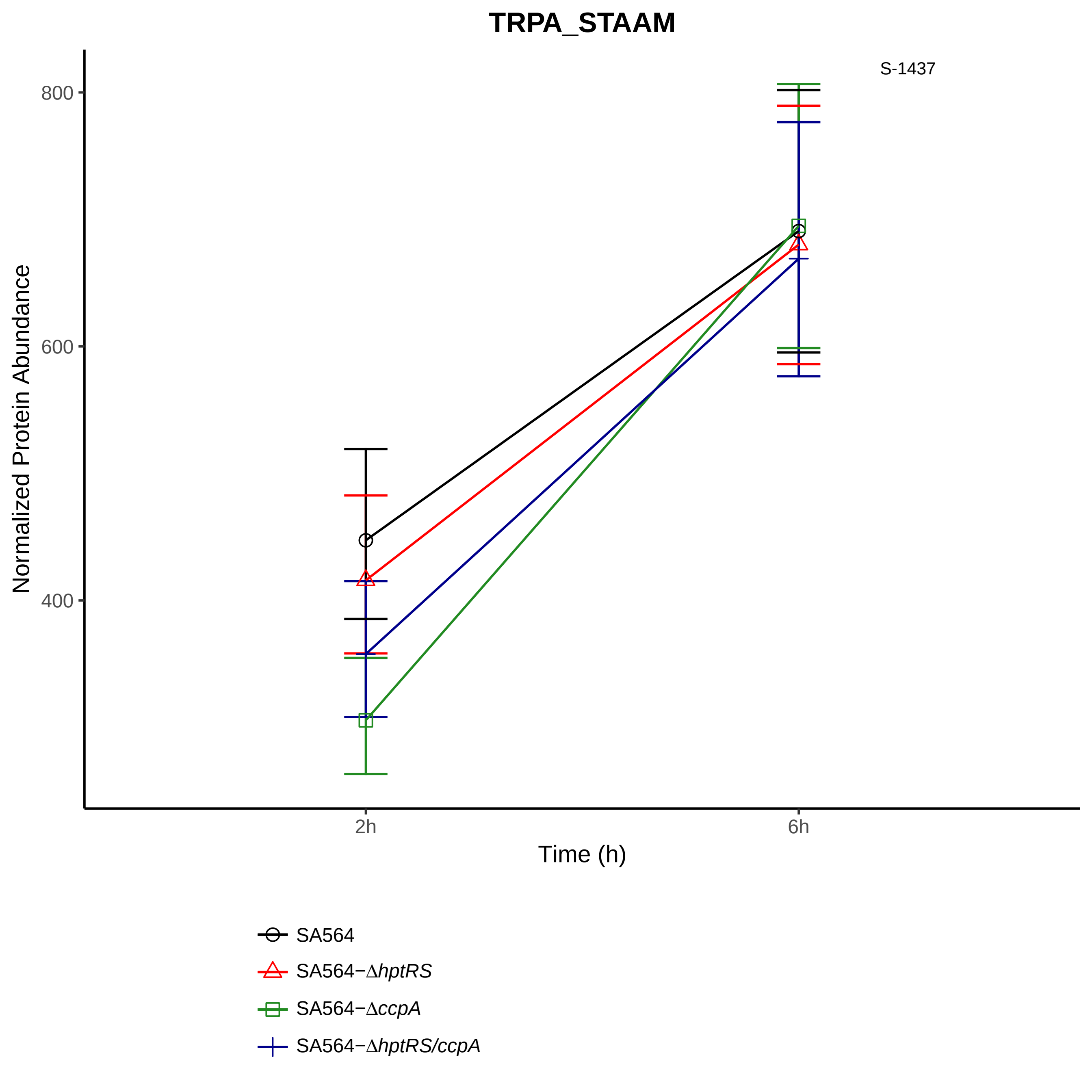

TRPA_STAAM
S-1437
800
600
400
Normalized Protein Abundance
2h
6h
Time (h)
SA564
SA564−Δ*hptRS*
SA564−Δ*ccpA*
SA564−Δ*hptRS/ccpA*

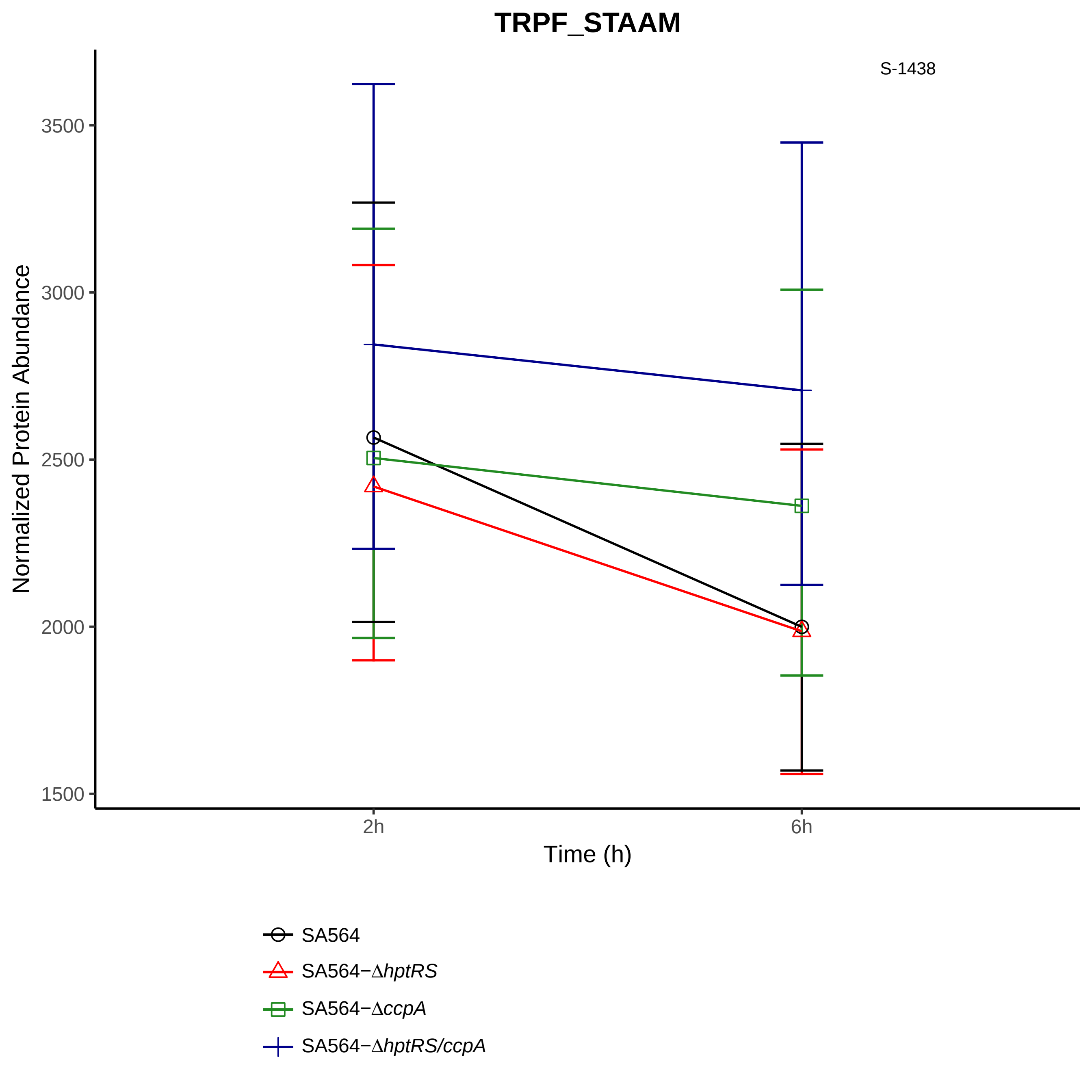

TRPF_STAAM
S-1438
Normalized Protein Abundance
3500
3000
2500
2000
1500
2h
6h
Time (h)
SA564
SA564−Δ*hptRS*
SA564−Δ*ccpA*
SA564−Δ*hptRS/ccpA*

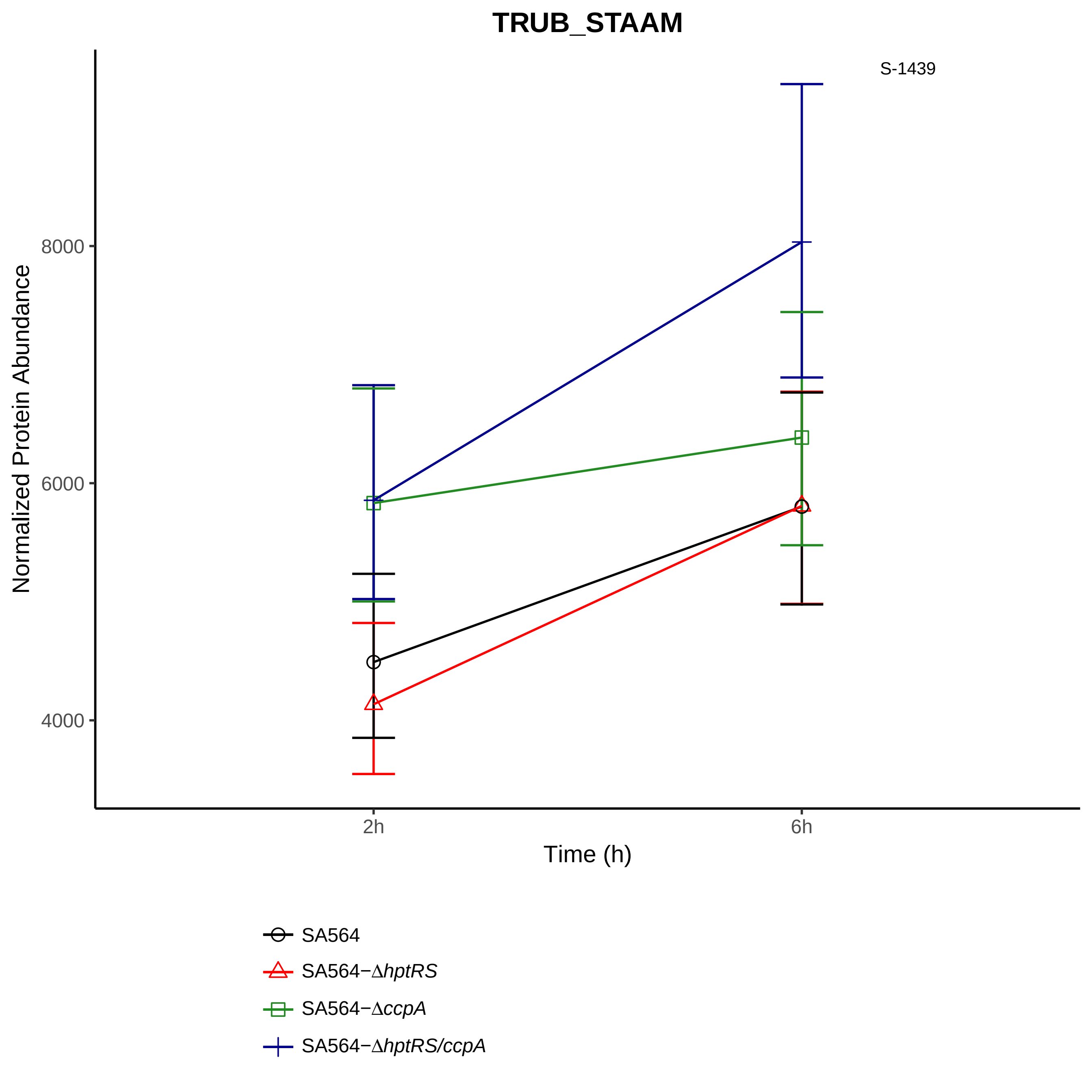

TRUB_STAAM
S-1439
Normalized Protein Abundance
8000
6000
4000
2h
6h
Time (h)
SA564
SA564−Δ*hptRS*
SA564−Δ*ccpA*
SA564−Δ*hptRS/ccpA*

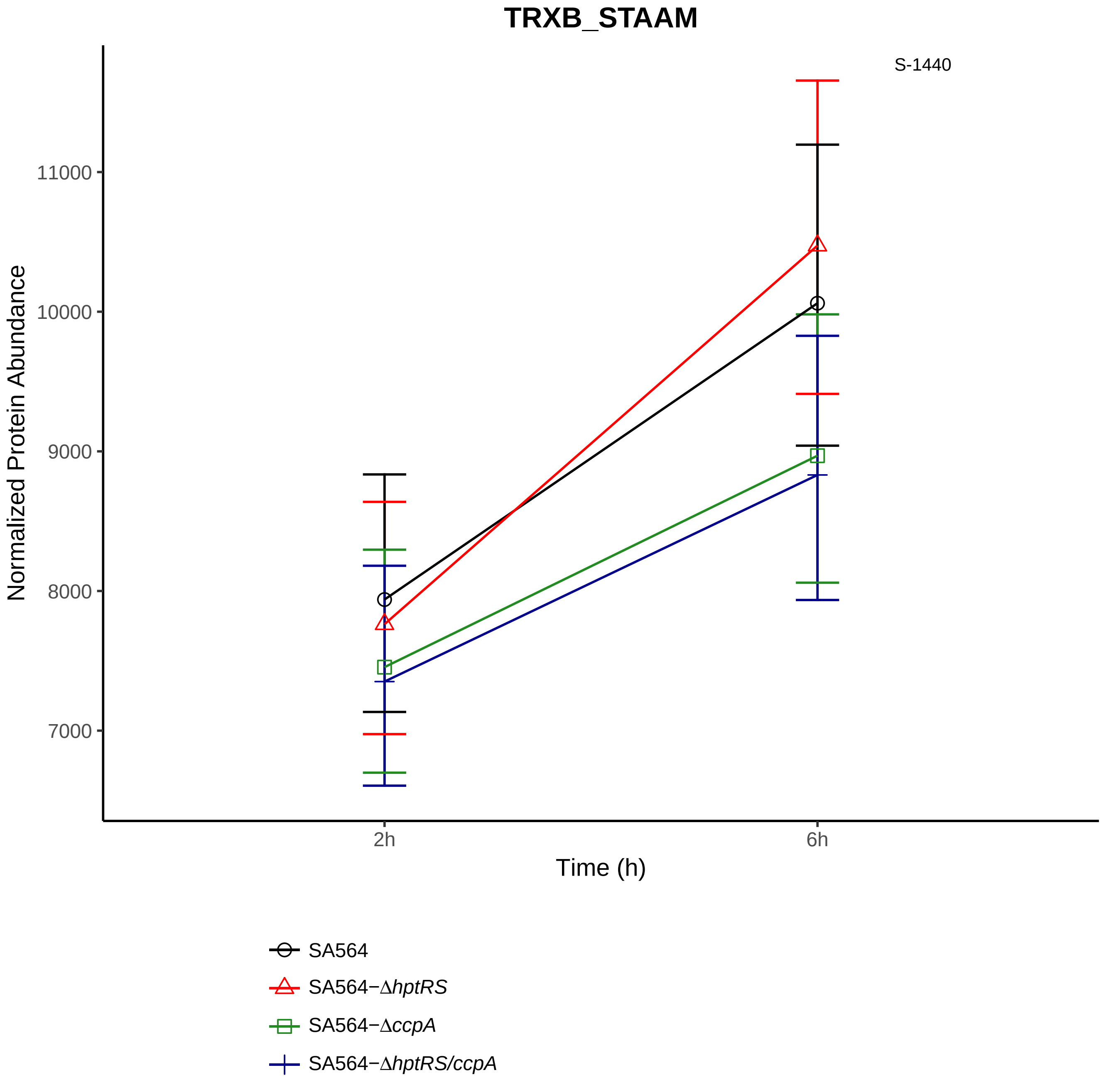
TRXB_STAAM
S-1440
Normalized Protein Abundance
11000
10000
9000
8000
7000
2h
6h
Time (h)
SA564
SA564−Δ*hptRS*
SA564−Δ*ccpA*
SA564−Δ*hptRS/ccpA*

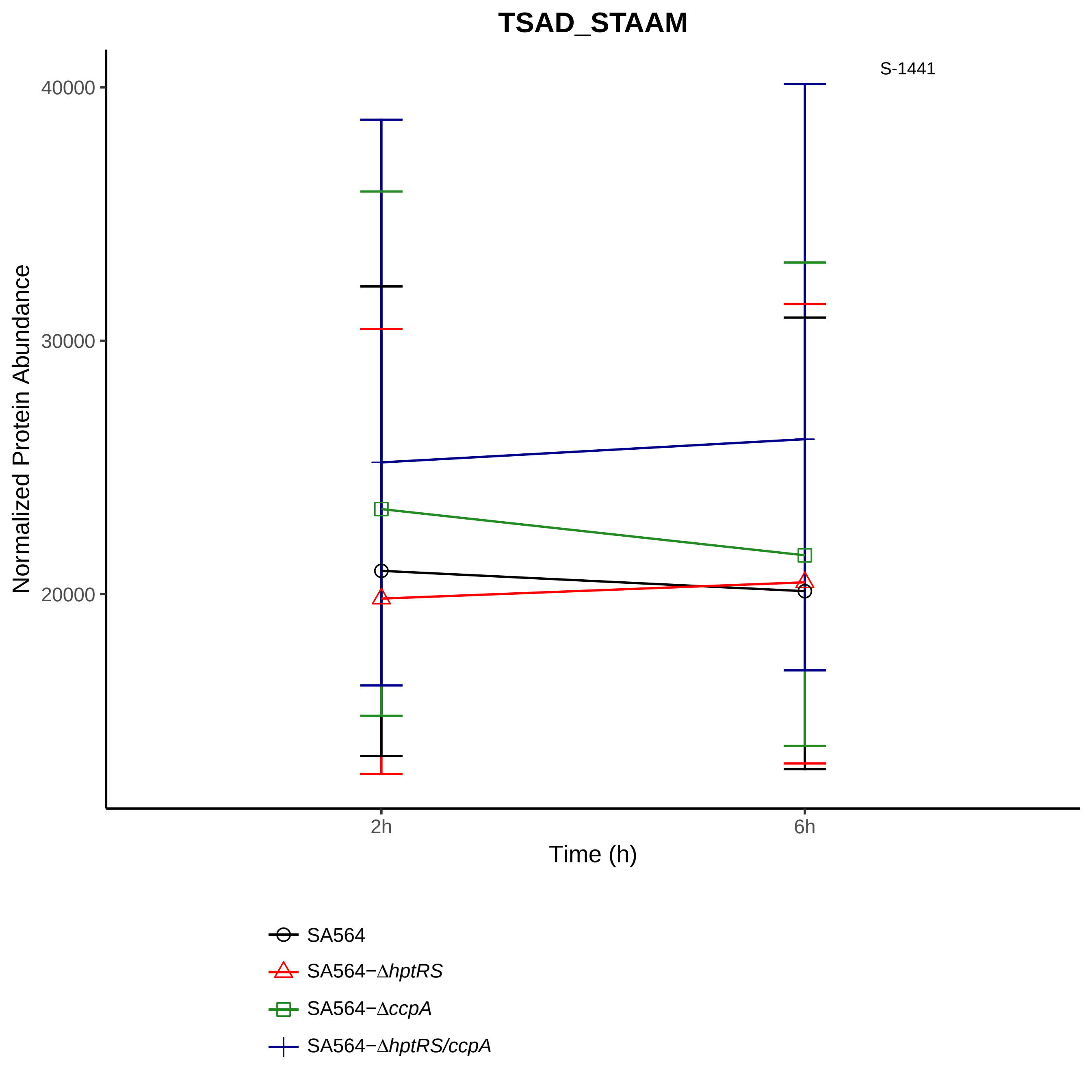

TSAD_STAAM
S-1441
40000
30000
20000
Normalized Protein Abundance
2h
6h
Time (h)
SA564
SA564−ΔhptRS
SA564−ΔccpA
SA564−ΔhptRS/ccpA

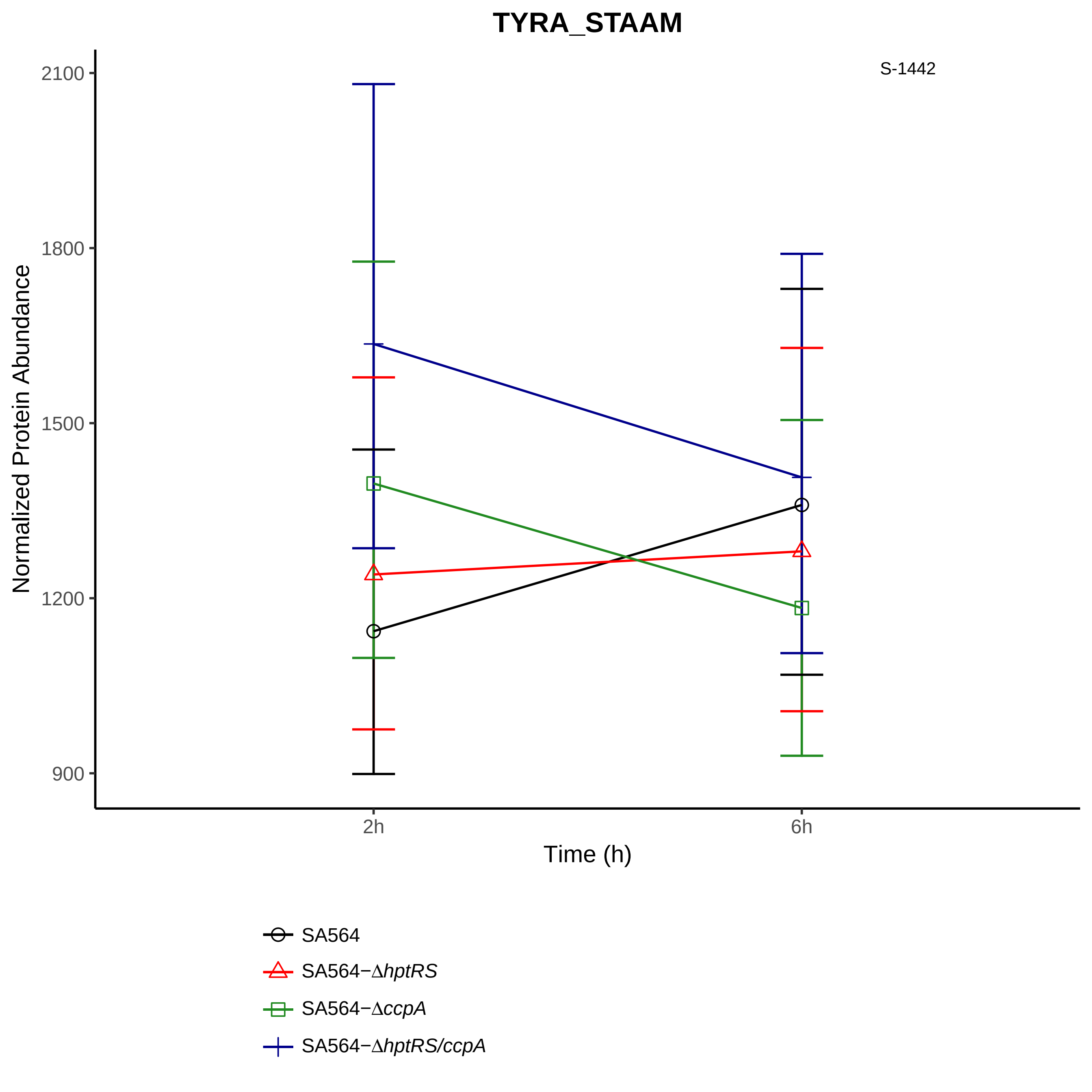

TYRA_STAAM
S-1442
Normalized Protein Abundance
2100
1800
1500
1200
900
2h
6h
Time (h)
SA564
SA564−ΔhptRS
SA564−ΔccpA
SA564−ΔhptRS/ccpA

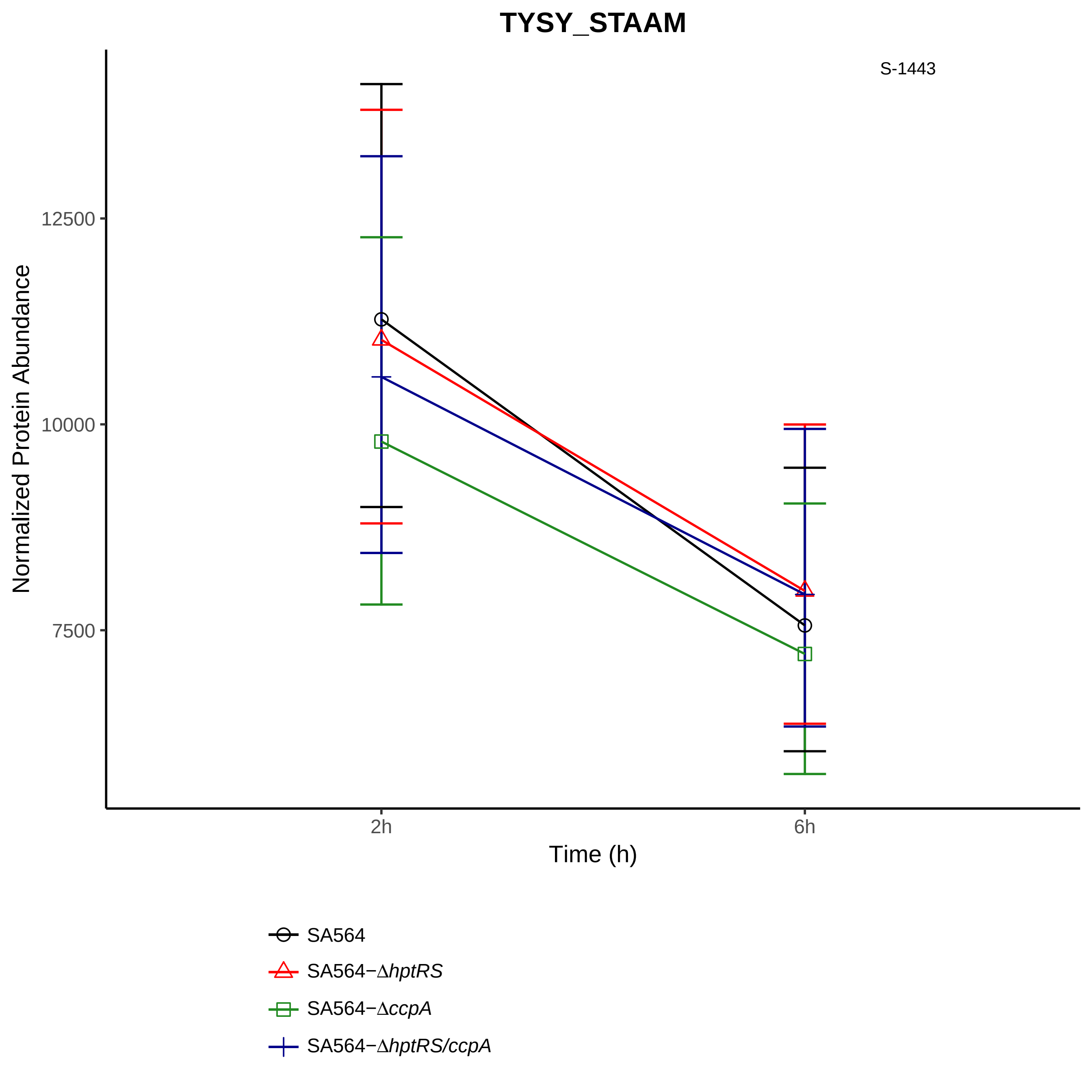

TYSY_STAAM
S-1443
Normalized Protein Abundance
12500
10000
7500
2h
6h
Time (h)
SA564
SA564−Δ*hptRS*
SA564−Δ*ccpA*
SA564−Δ*hptRS/ccpA*

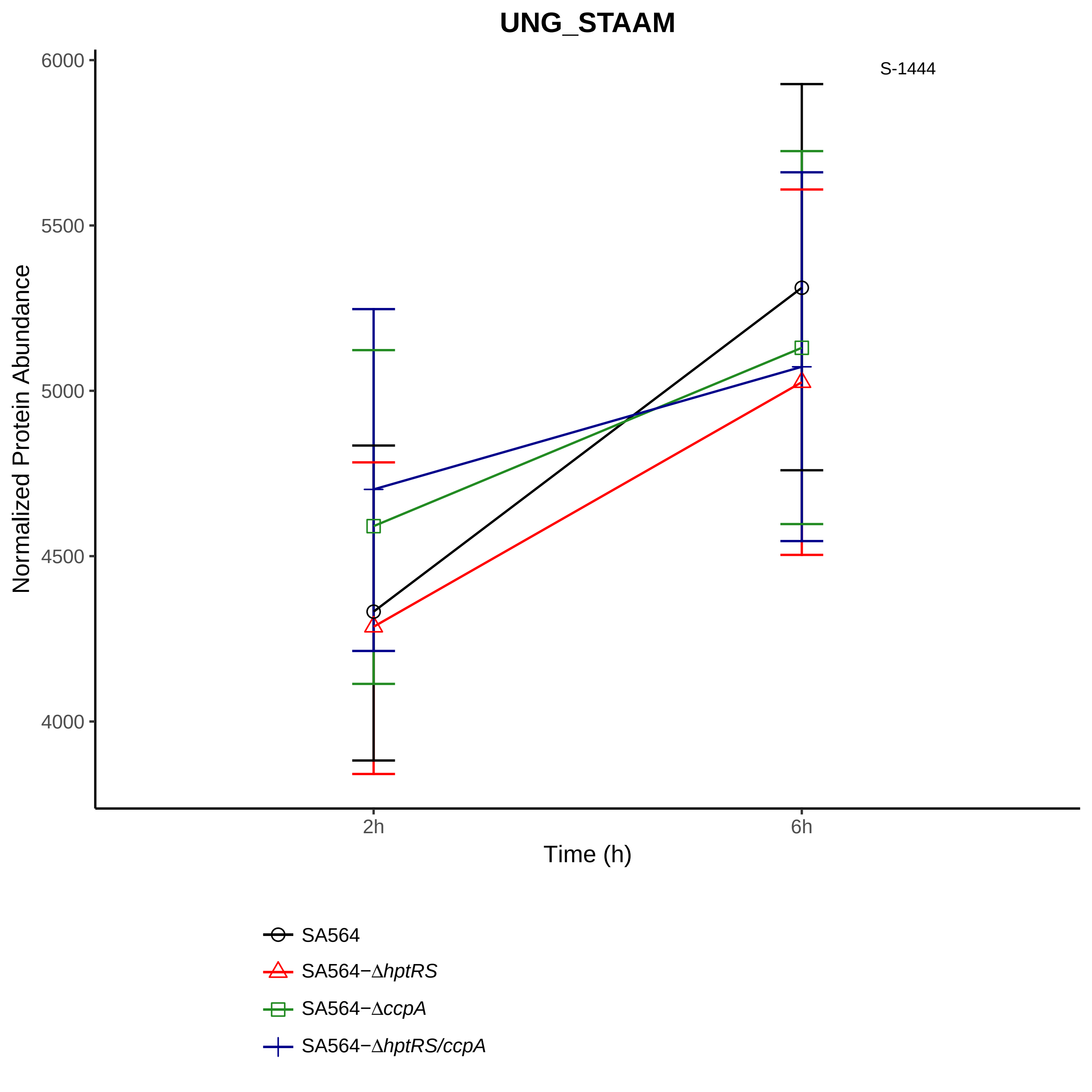

UNG_STAAM
S-1444
Normalized Protein Abundance
6000
5500
5000
4500
4000
2h
6h
Time (h)
SA564
SA564−Δ*hptRS*
SA564−Δ*ccpA*
SA564−Δ*hptRS/ccpA*

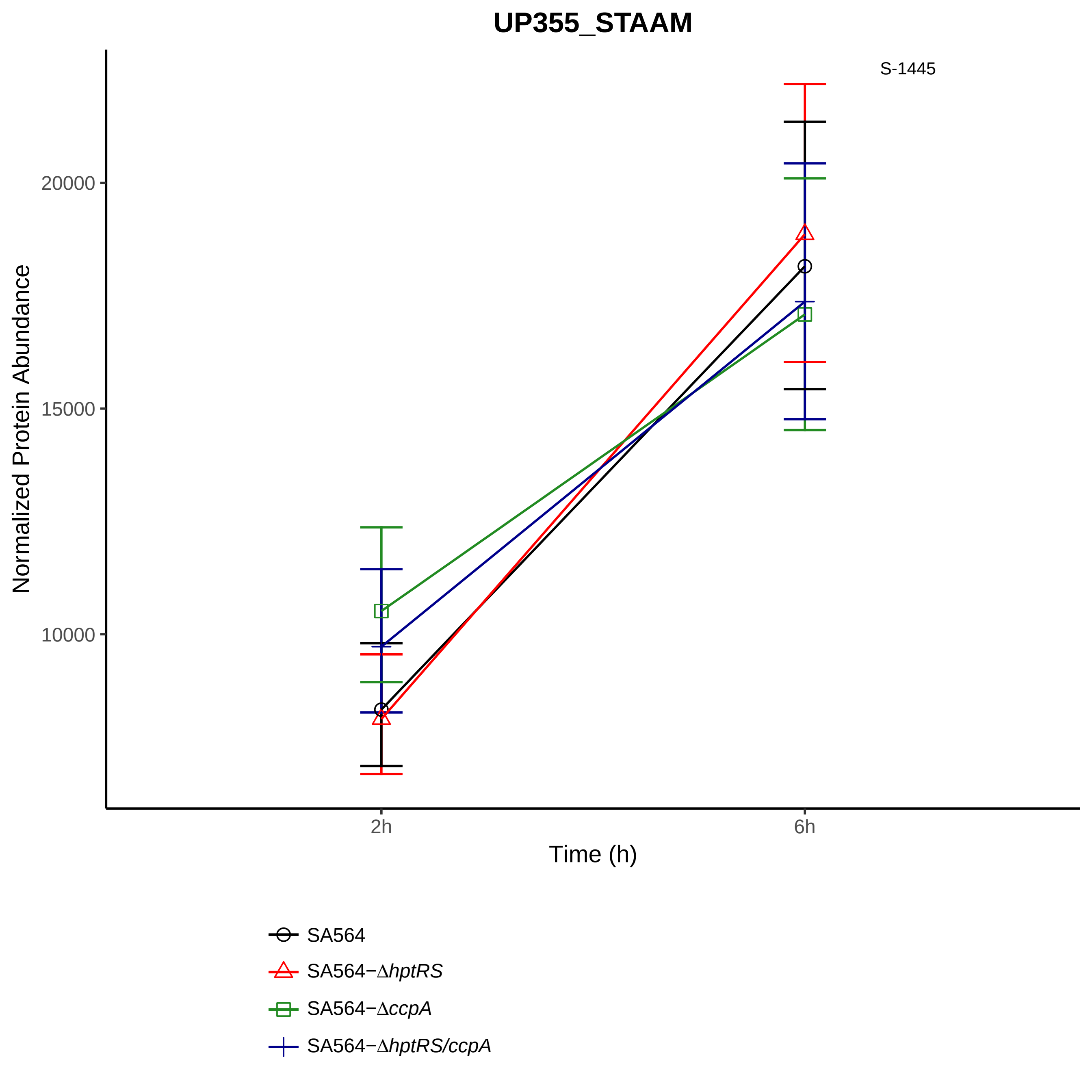

UP355_STAAM
S-1445
Normalized Protein Abundance
20000
15000
10000
2h
6h
Time (h)
SA564
SA564−Δ*hptRS*
SA564−Δ*ccpA*
SA564−Δ*hptRS/ccpA*

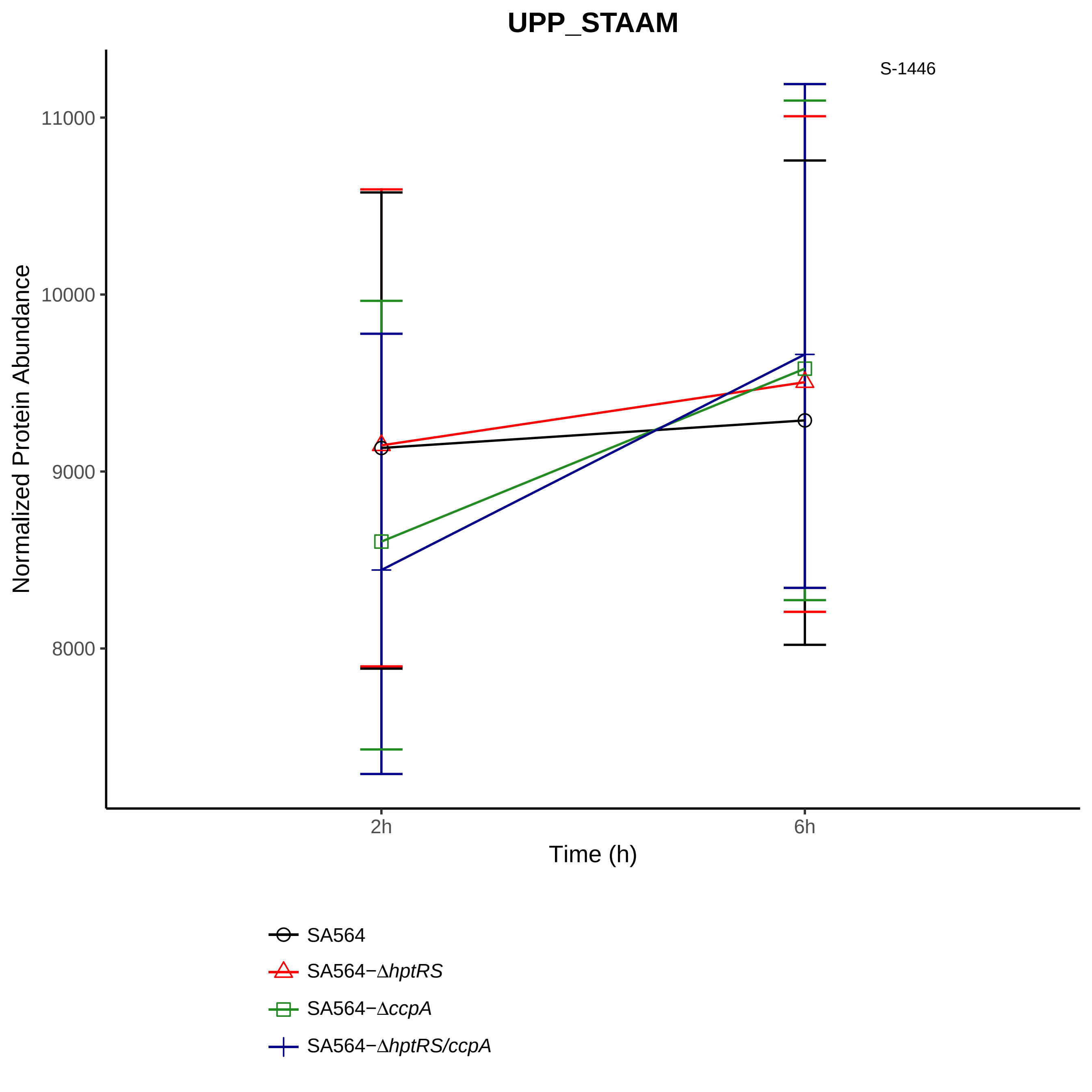

UPP_STAAM
S-1446
Normalized Protein Abundance
11000
10000
9000
8000
2h
6h
Time (h)
SA564
SA564−Δ*hptRS*
SA564−Δ*ccpA*
SA564−Δ*hptRS/ccpA*

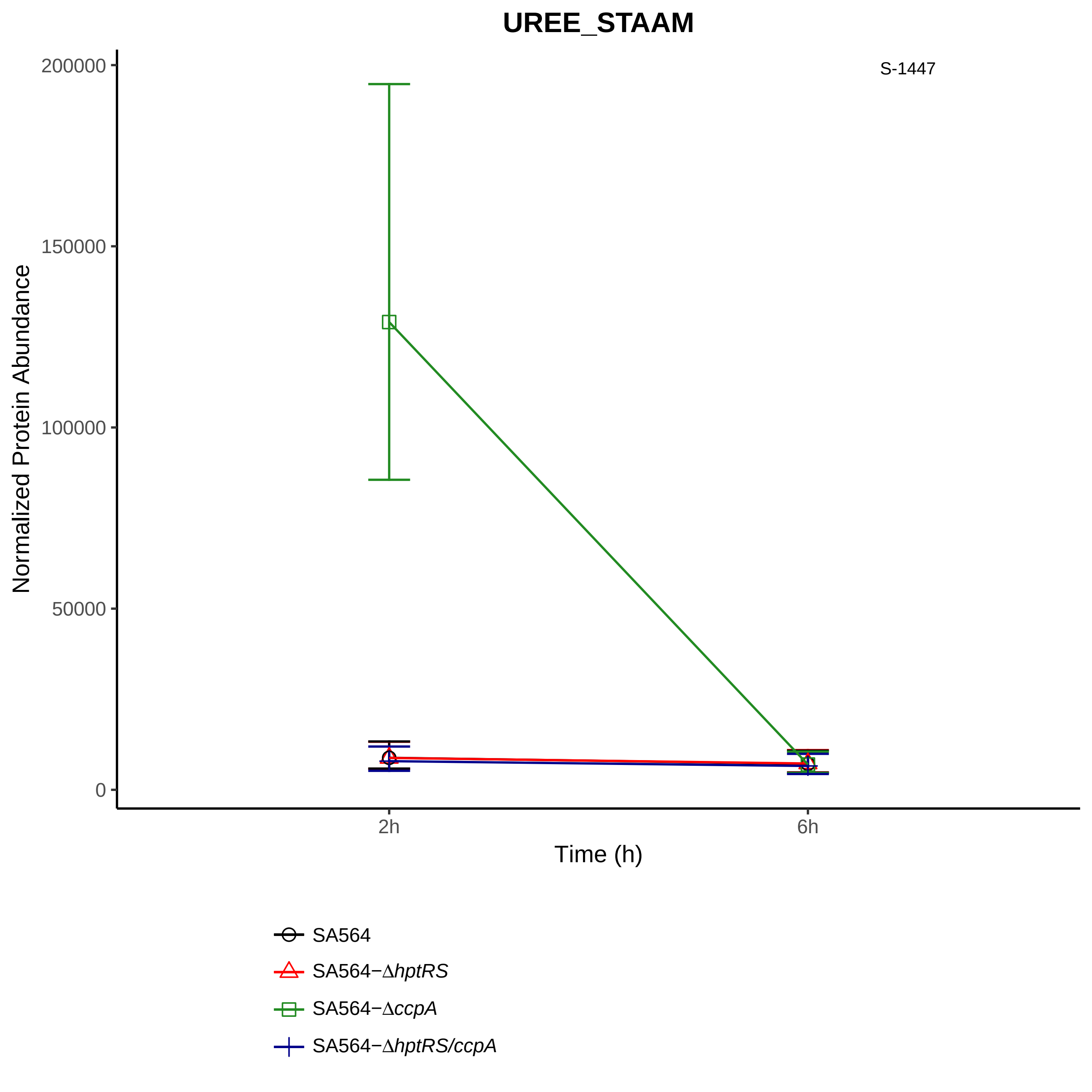

UREE_STAAM
S-1447
Normalized Protein Abundance
200000
150000
100000
50000
0
2h
6h
Time (h)
SA564
SA564−ΔhptRS
SA564−ΔccpA
SA564−ΔhptRS/ccpA

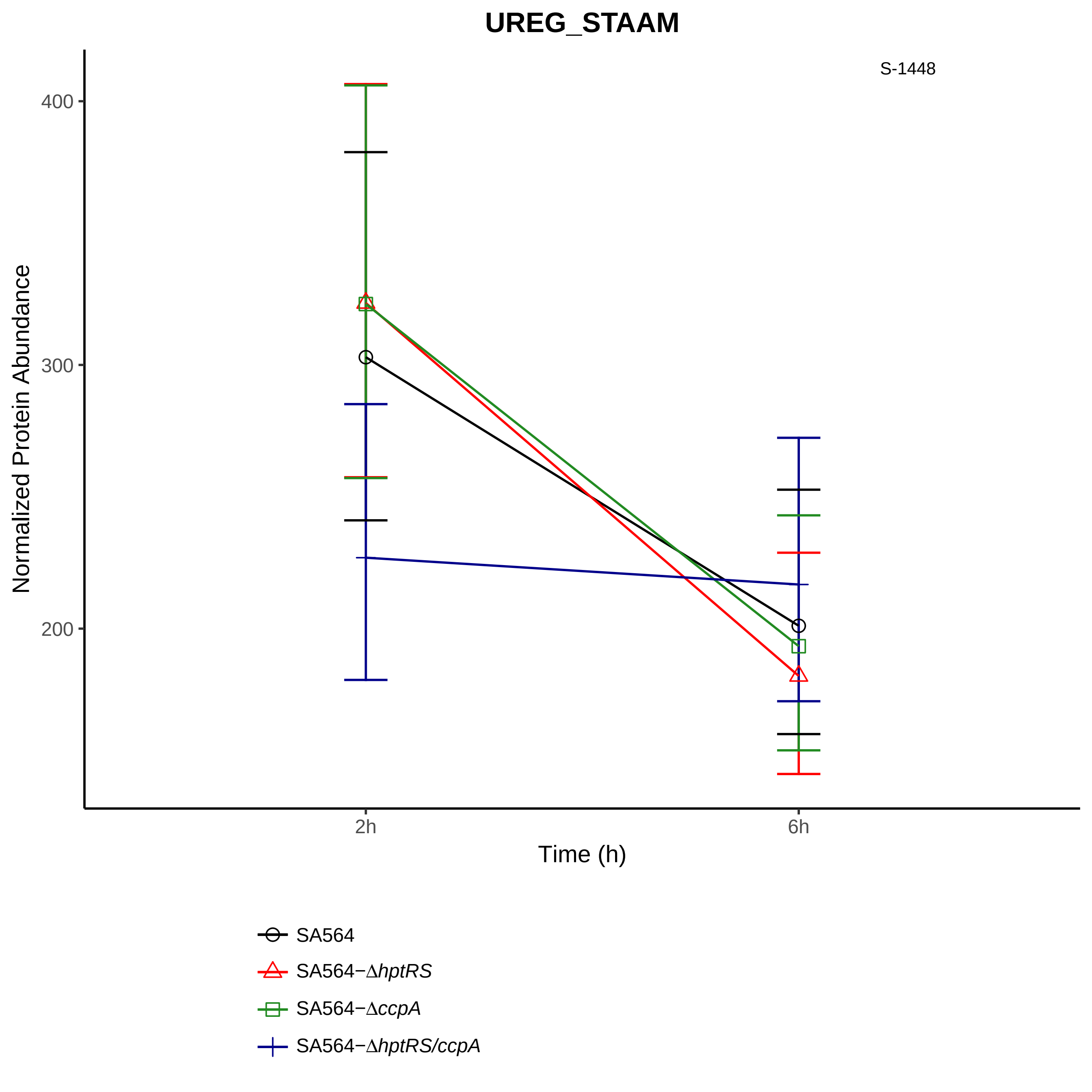

UREG_STAAM
S-1448
400
300
200
Normalized Protein Abundance
2h
6h
Time (h)
SA564
SA564−ΔhptRS
SA564−ΔccpA
SA564−ΔhptRS/ccpA

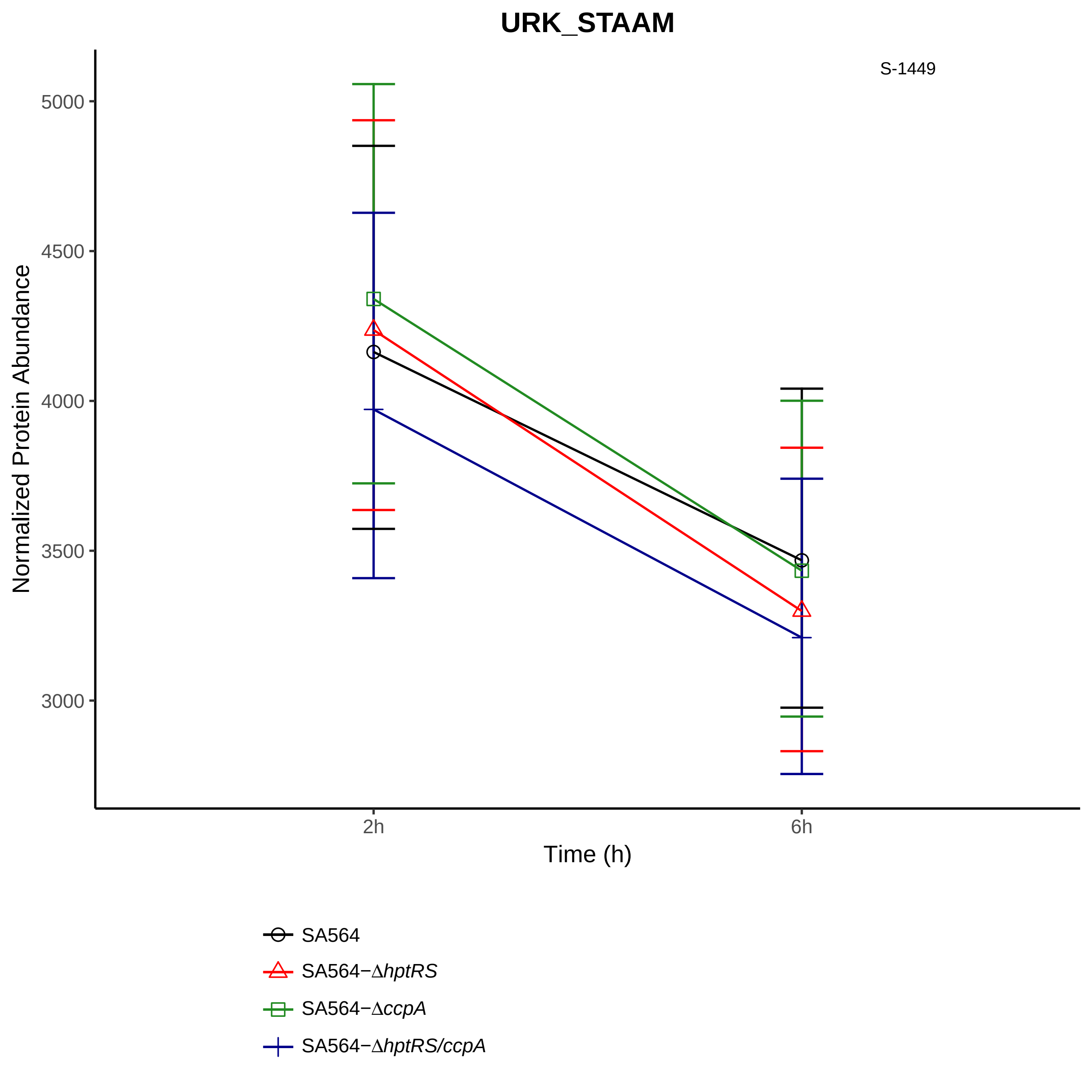
URK_STAAM
S-1449
Normalized Protein Abundance
5000
4500
4000
3500
3000
2h
6h
Time (h)
SA564
SA564−ΔhptRS
SA564−ΔccpA
SA564−ΔhptRS/ccpA

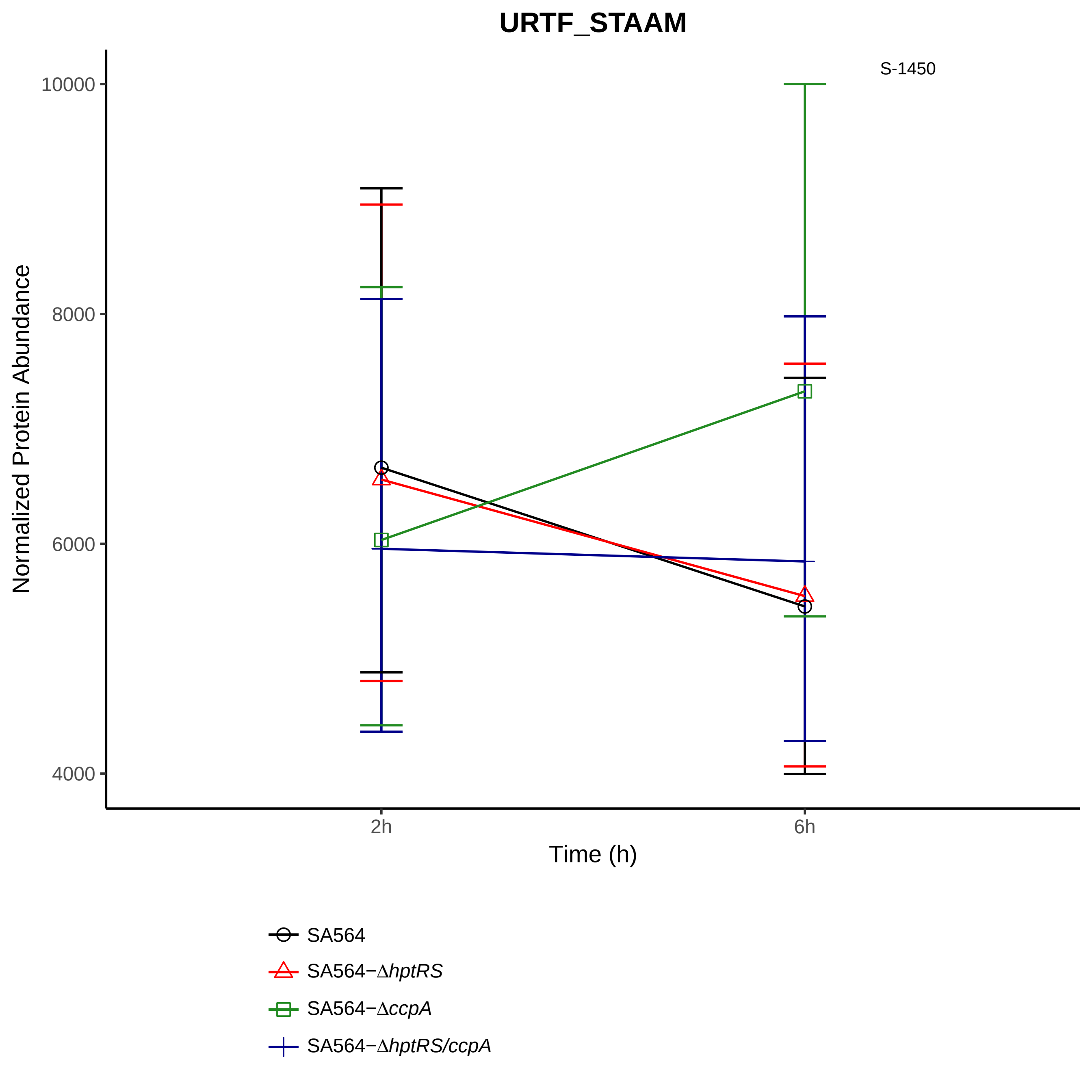

URTF_STAAM
S-1450
Normalized Protein Abundance
10000
8000
6000
4000
2h
6h
Time (h)
SA564
SA564−ΔhptRS
SA564−ΔccpA
SA564−ΔhptRS/ccpA

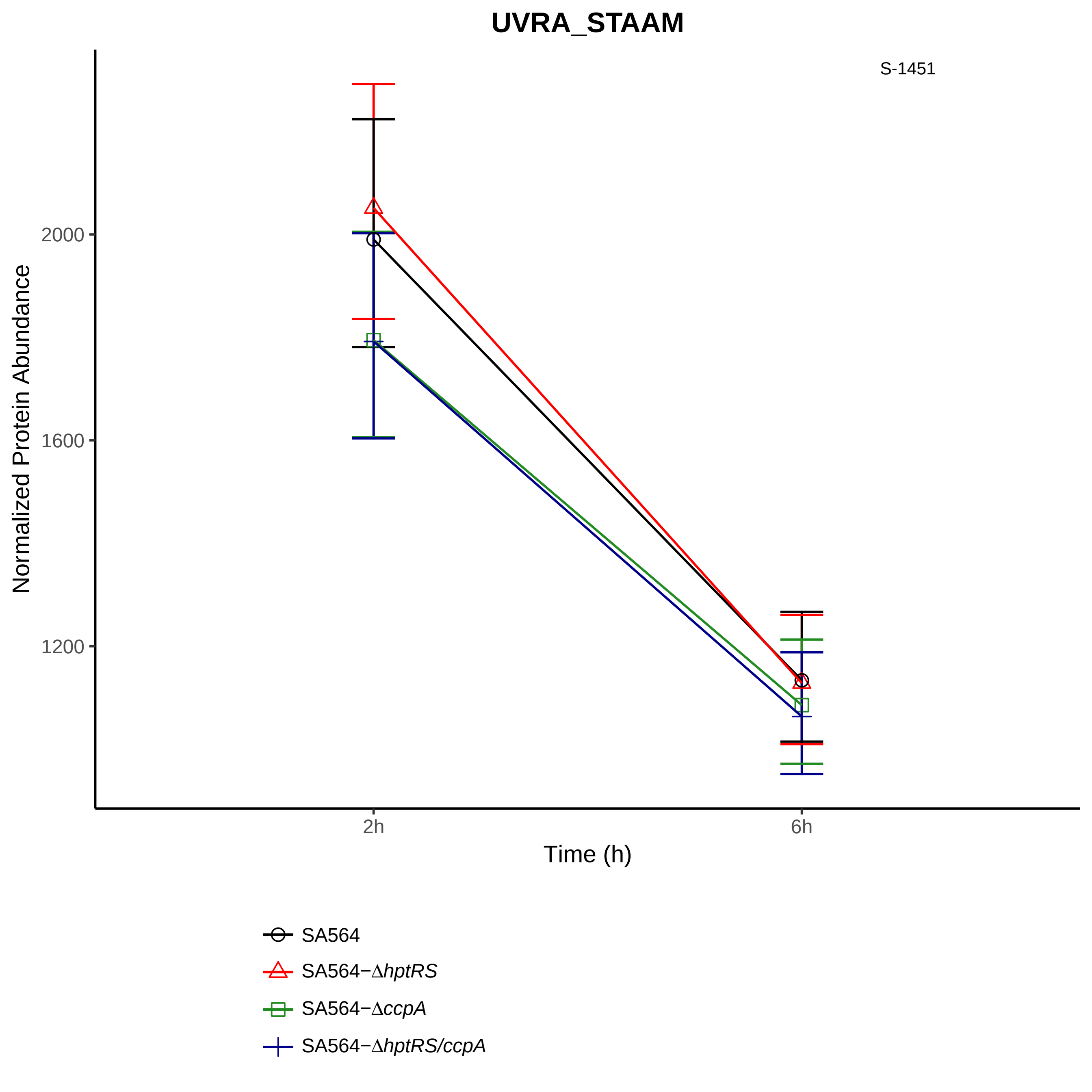

UVRA_STAAM
S-1451
Normalized Protein Abundance
2000
1600
1200
2h
6h
Time (h)
SA564
SA564−ΔhptRS
SA564−ΔccpA
SA564−ΔhptRS/ccpA

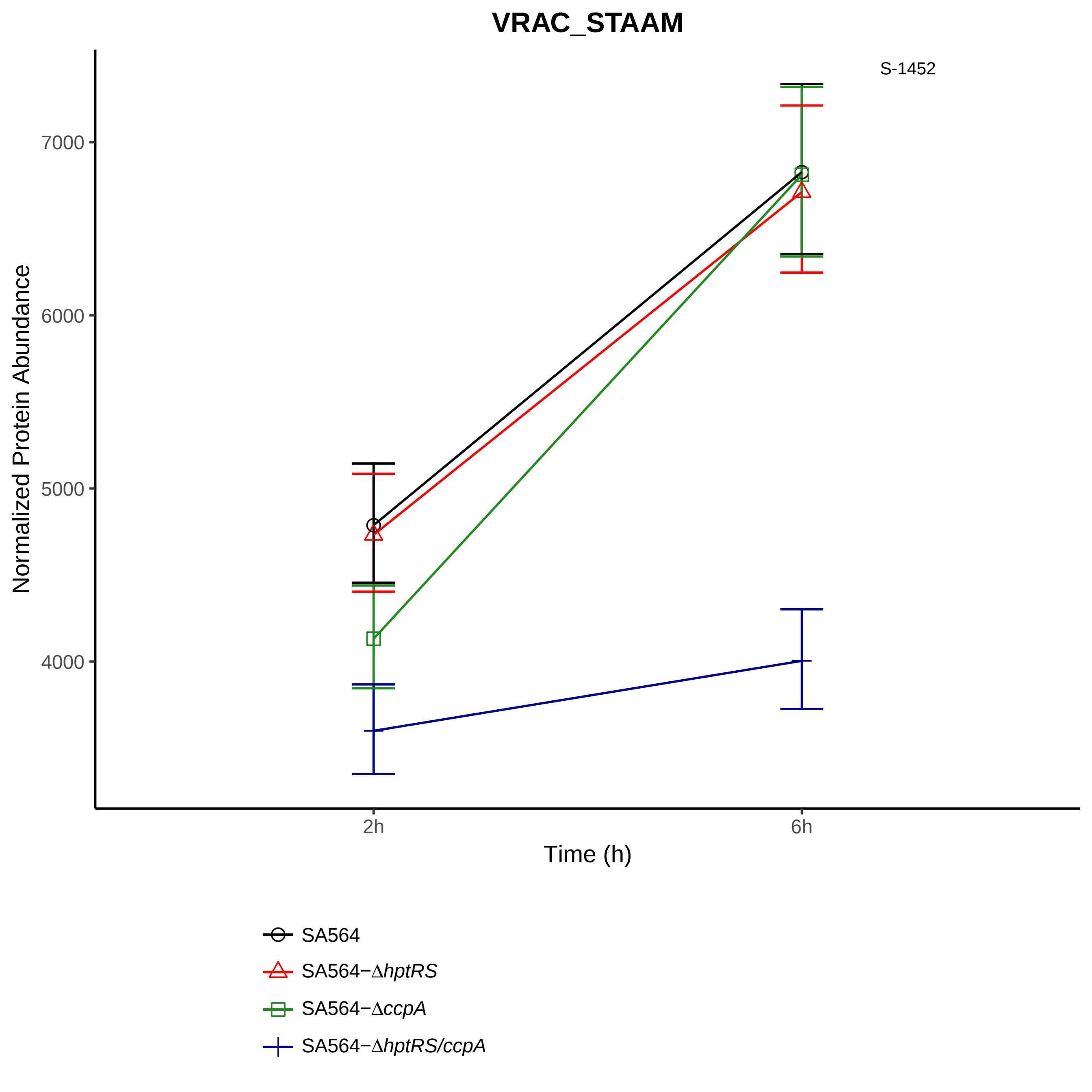
VRAC_STAAM
S-1452
Normalized Protein Abundance
7000
6000
5000
4000
2h
6h
Time (h)
SA564
SA564−ΔhptRS
SA564−ΔccpA
SA564−ΔhptRS/ccpA

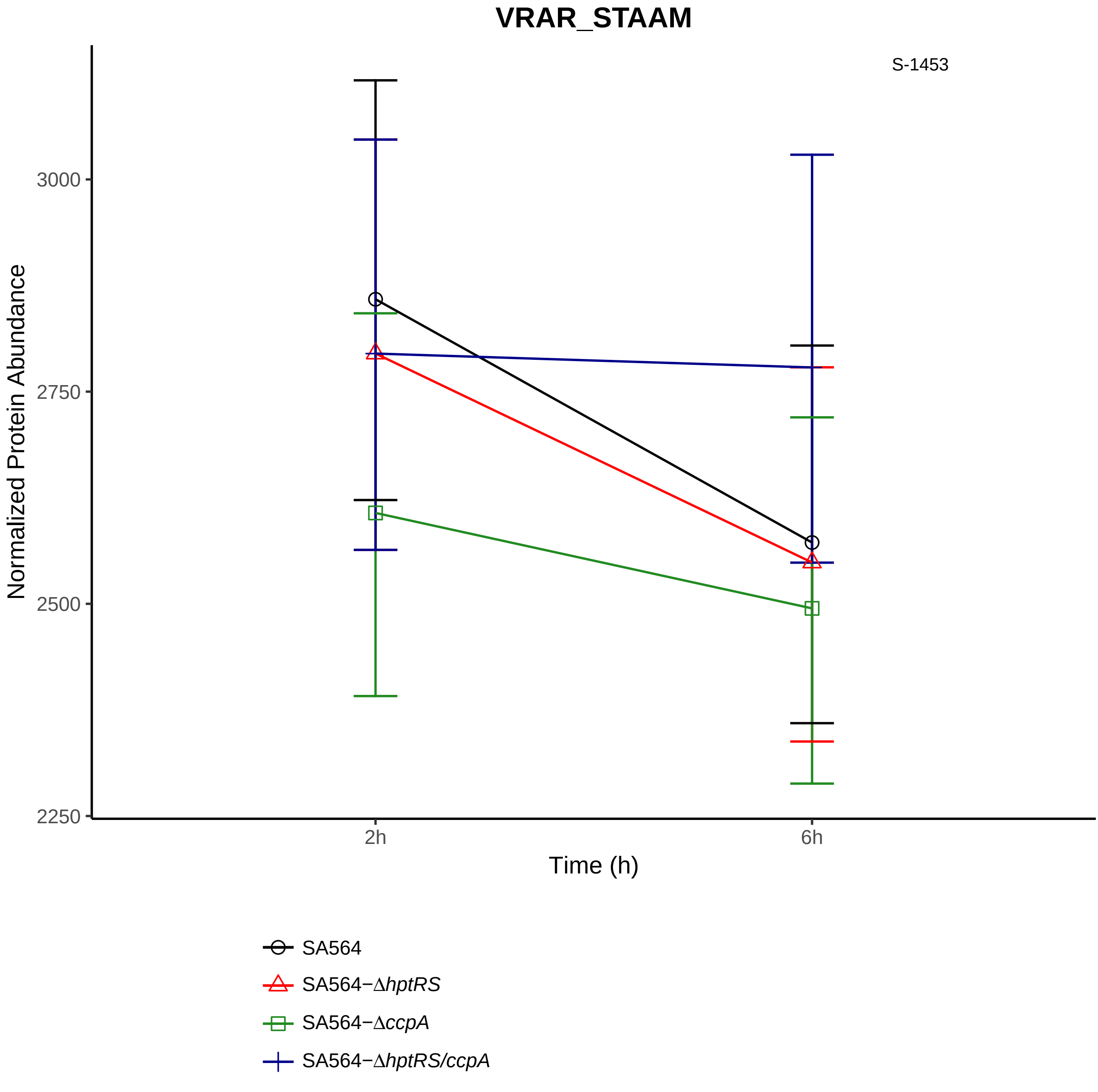

VRAR_STAAM
S-1453
Normalized Protein Abundance
3000
2750
2500
2250
2h
6h
Time (h)
SA564
SA564−Δ*hptRS*
SA564−Δ*ccpA*
SA564−Δ*hptRS/ccpA*

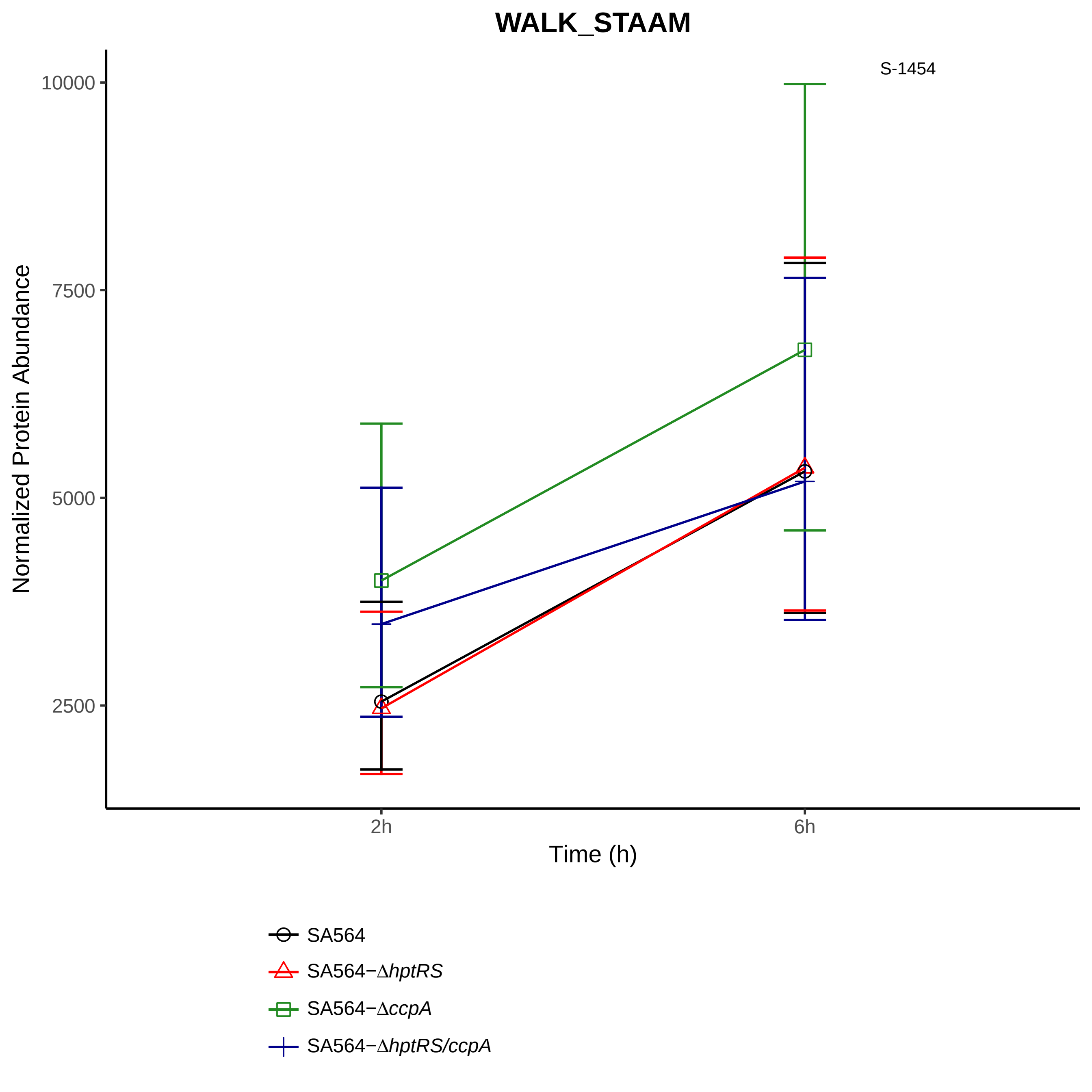
WALK_STAAM
S-1454
Normalized Protein Abundance
10000
7500
5000
2500
2h
6h
Time (h)
SA564
SA564−ΔhptRS
SA564−ΔccpA
SA564−ΔhptRS/ccpA

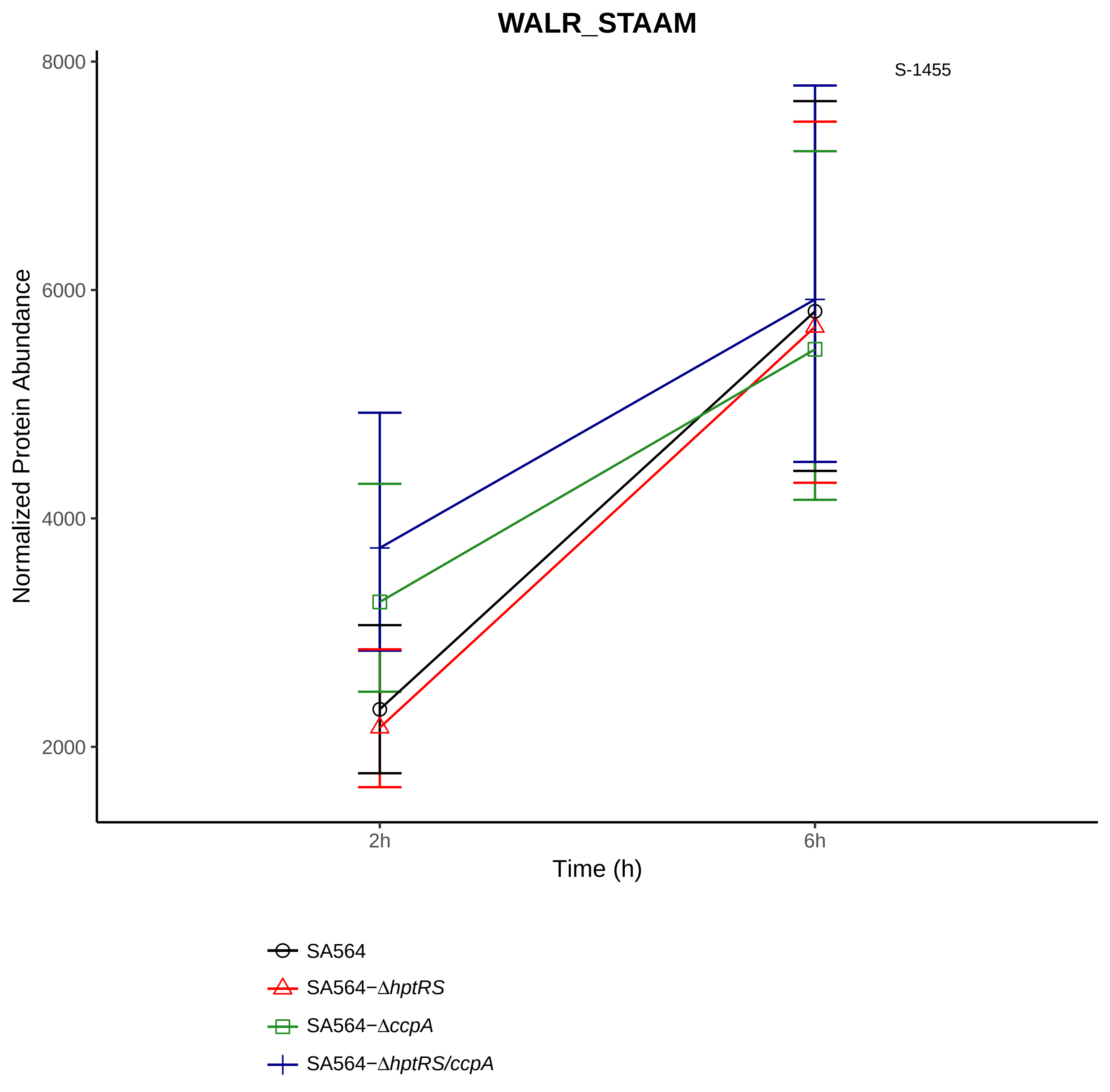

WALR_STAAM
S-1455
8000
6000
4000
2000
Normalized Protein Abundance
2h
6h
Time (h)
SA564
SA564−Δ*hptRS*
SA564−Δ*ccpA*
SA564−Δ*hptRS/ccpA*

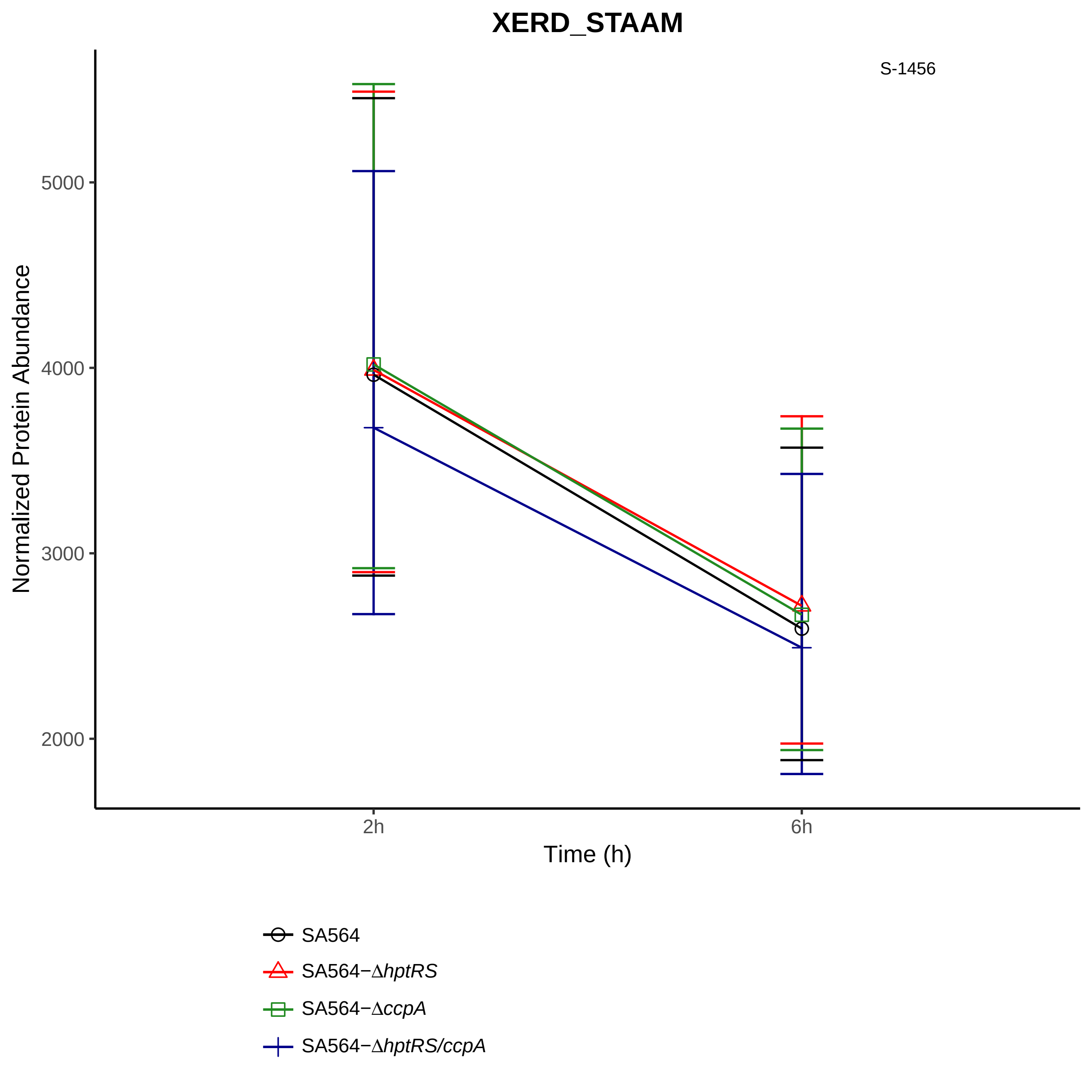

XERD_STAAM
S-1456
Normalized Protein Abundance
5000
4000
3000
2000
2h
6h
Time (h)
SA564
SA564−Δ*hptRS*
SA564−Δ*ccpA*
SA564−Δ*hptRS/ccpA*

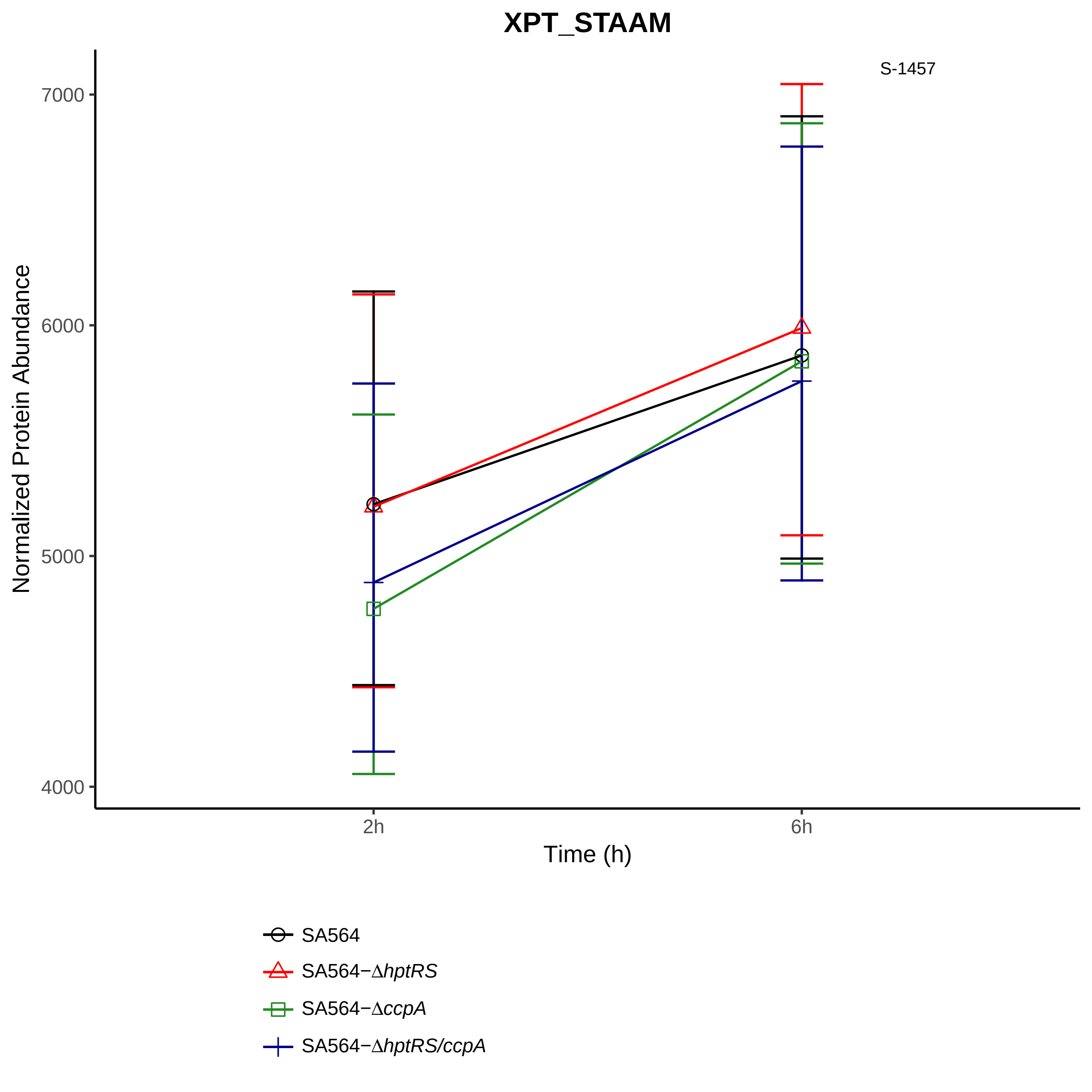
XPT_STAAM
S-1457
7000
6000
5000
4000
Normalized Protein Abundance
2h
6h
Time (h)
SA564
SA564−Δ*hptRS*
SA564−Δ*ccpA*
SA564−Δ*hptRS/ccpA*

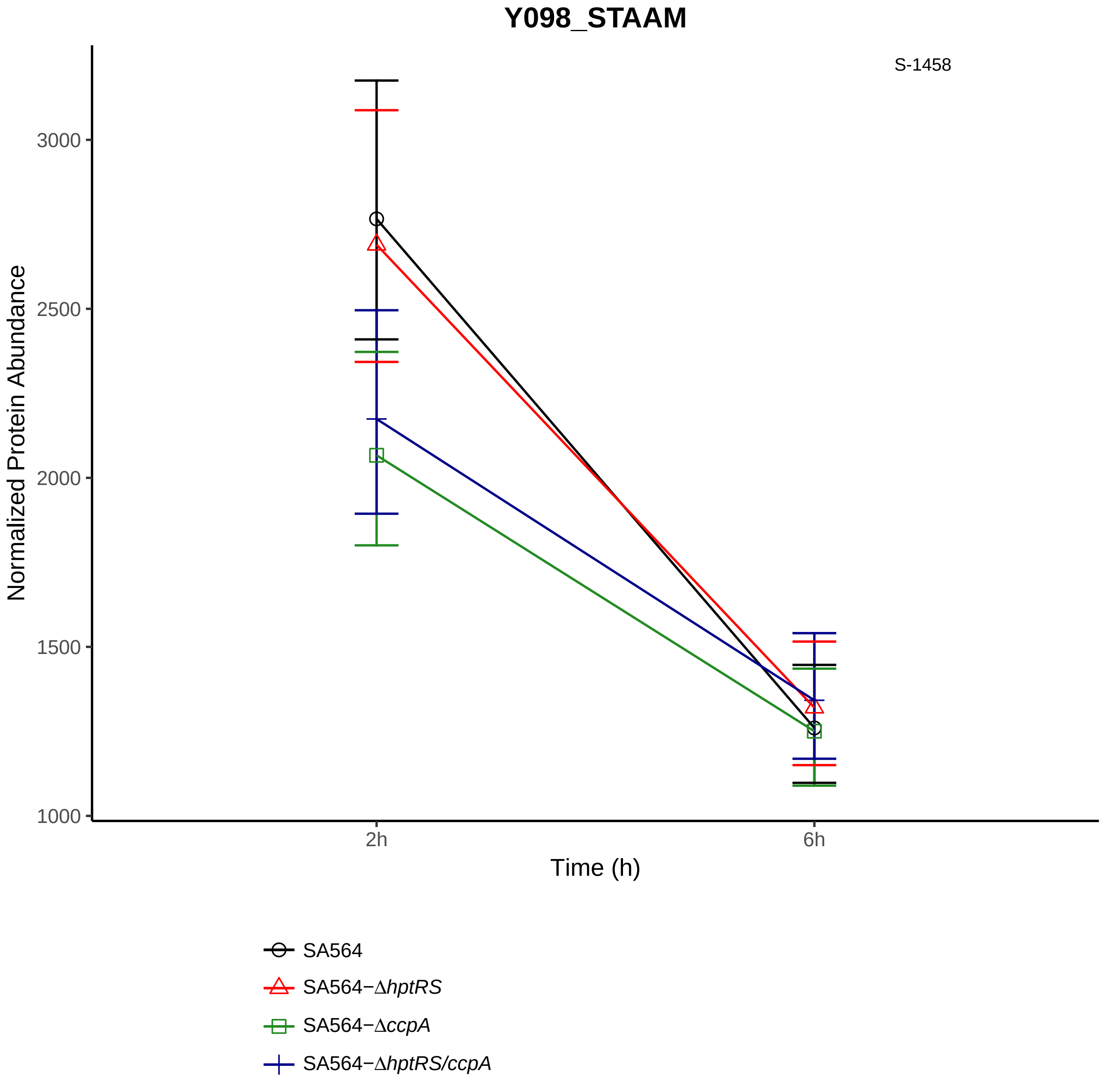
Y098_STAAM
S-1458
Normalized Protein Abundance
3000
2500
2000
1500
1000
2h
6h
Time (h)
SA564
SA564−Δ*hptRS*
SA564−Δ*ccpA*
SA564−Δ*hptRS/ccpA*

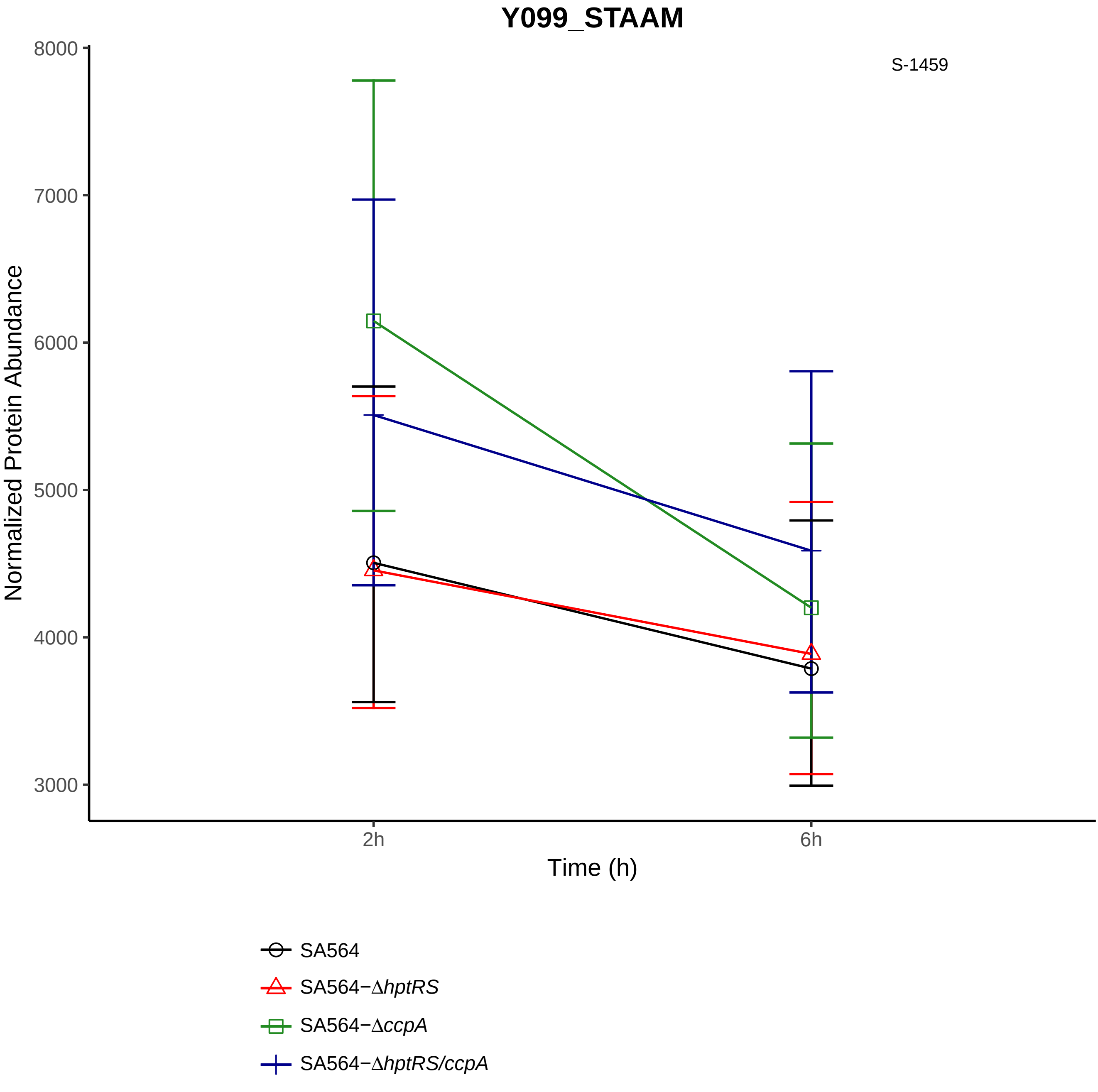

Y099_STAAM
S-1459
Normalized Protein Abundance
8000
7000
6000
5000
4000
3000
2h
6h
Time (h)
SA564
SA564−Δ*hptRS*
SA564−Δ*ccpA*
SA564−Δ*hptRS/ccpA*

Y1015_STAAM

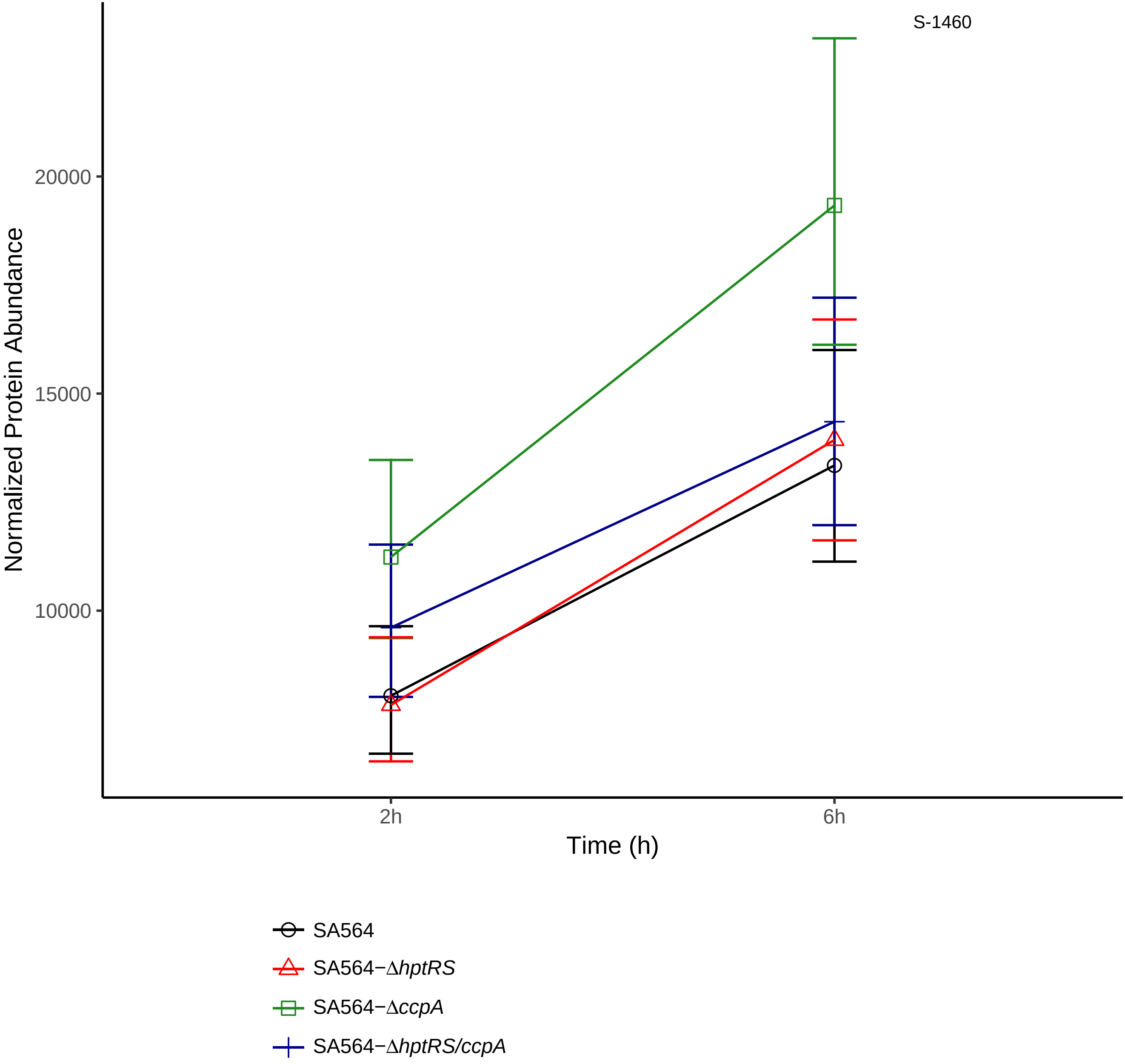

S-1460
Normalized Protein Abundance
20000
15000
10000
2h
6h
Time (h)
SA564
SA564−Δ*hptRS*
SA564−Δ*ccpA*
SA564−Δ*hptRS/ccpA*

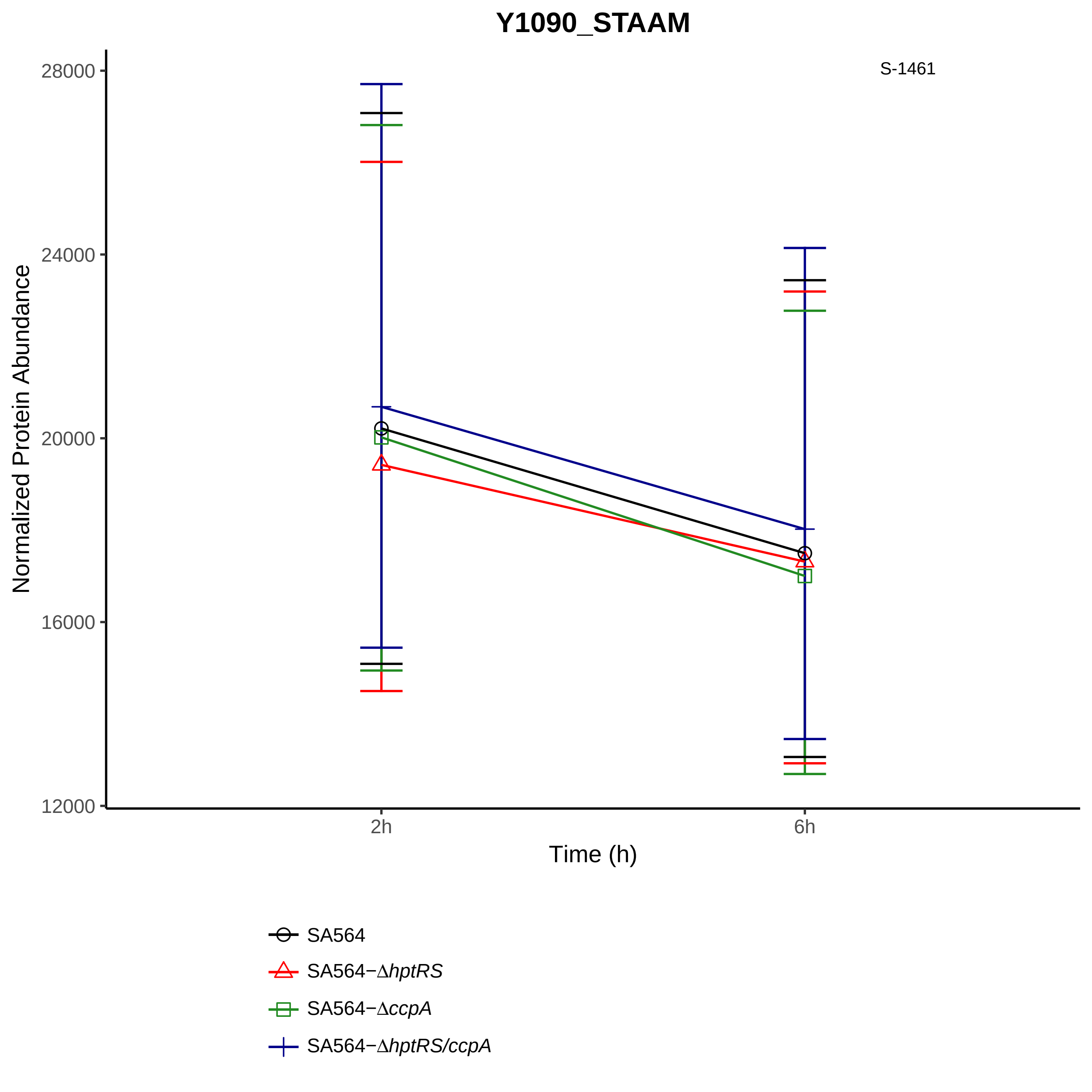

Y1090_STAAM
S-1461
Normalized Protein Abundance
28000
24000
20000
16000
12000
2h
6h
Time (h)
SA564
SA564−Δ*hptRS*
SA564−Δ*ccpA*
SA564−Δ*hptRS/ccpA*

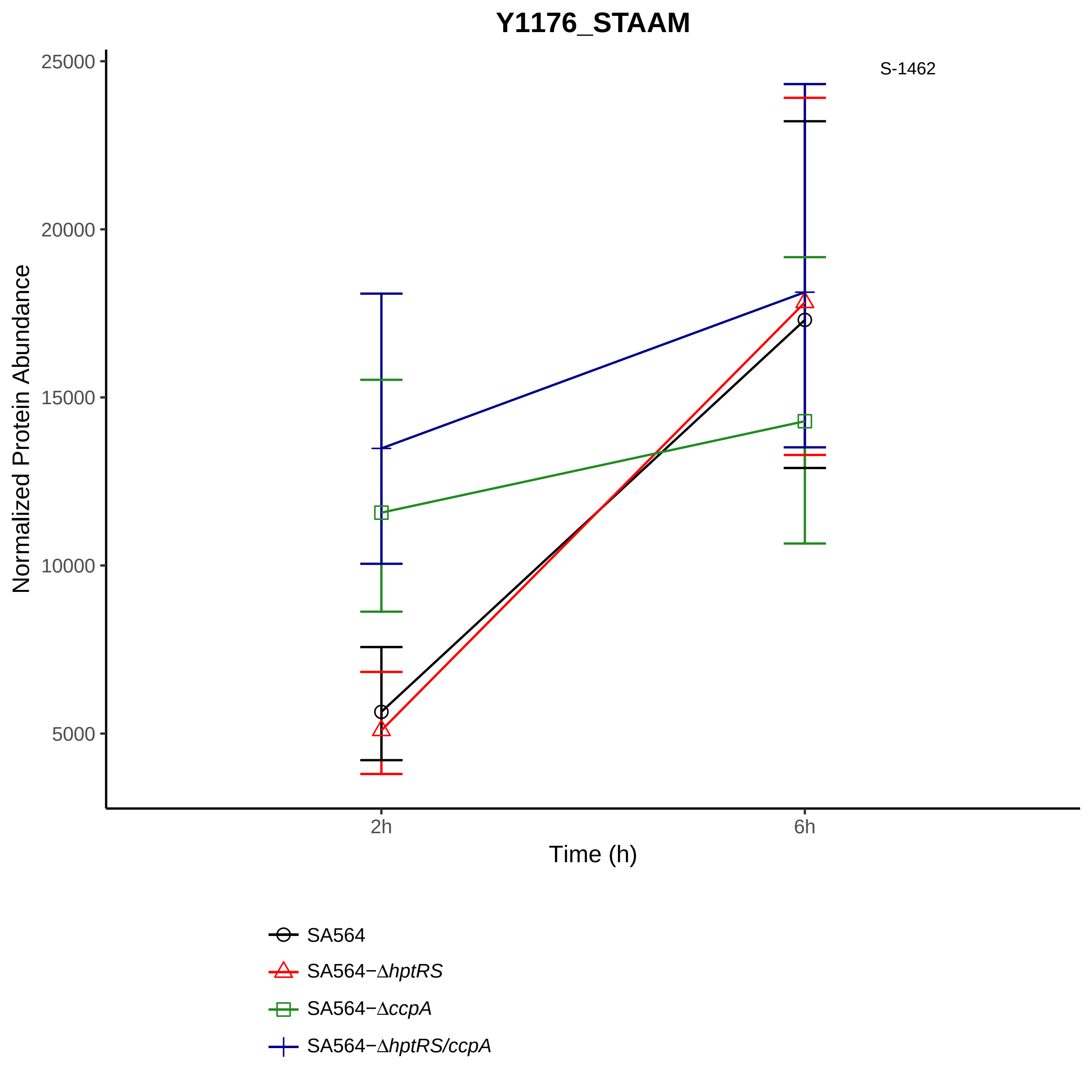
Y1176_STAAM
S-1462
Normalized Protein Abundance
25000
20000
15000
10000
5000
2h
6h
Time (h)
SA564
SA564−Δ*hptRS*
SA564−Δ*ccpA*
SA564−Δ*hptRS/ccpA*

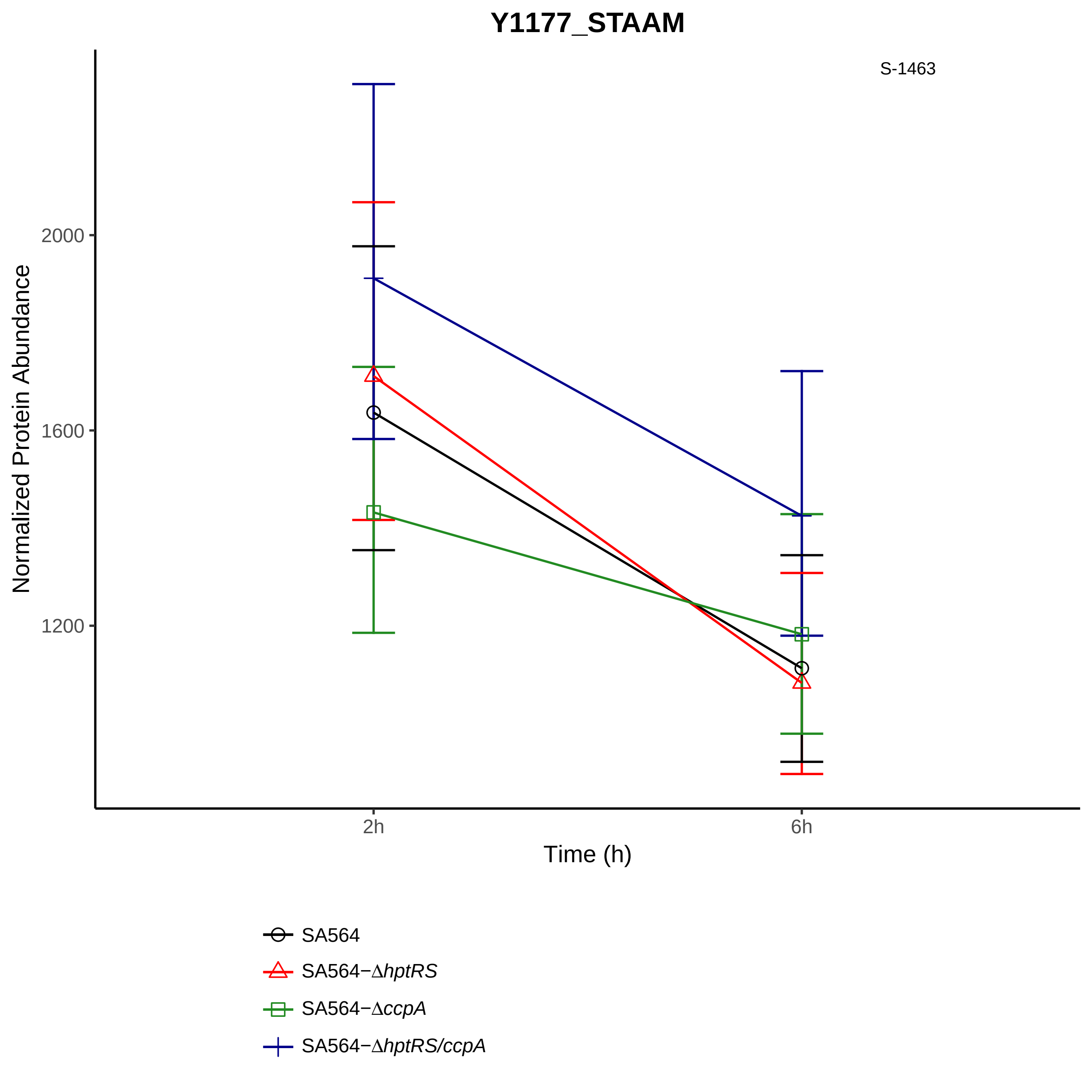

Y1177_STAAM
S-1463
Normalized Protein Abundance
2000
1600
1200
2h
6h
Time (h)
SA564
SA564−Δ*hptRS*
SA564−Δ*ccpA*
SA564−Δ*hptRS/ccpA*

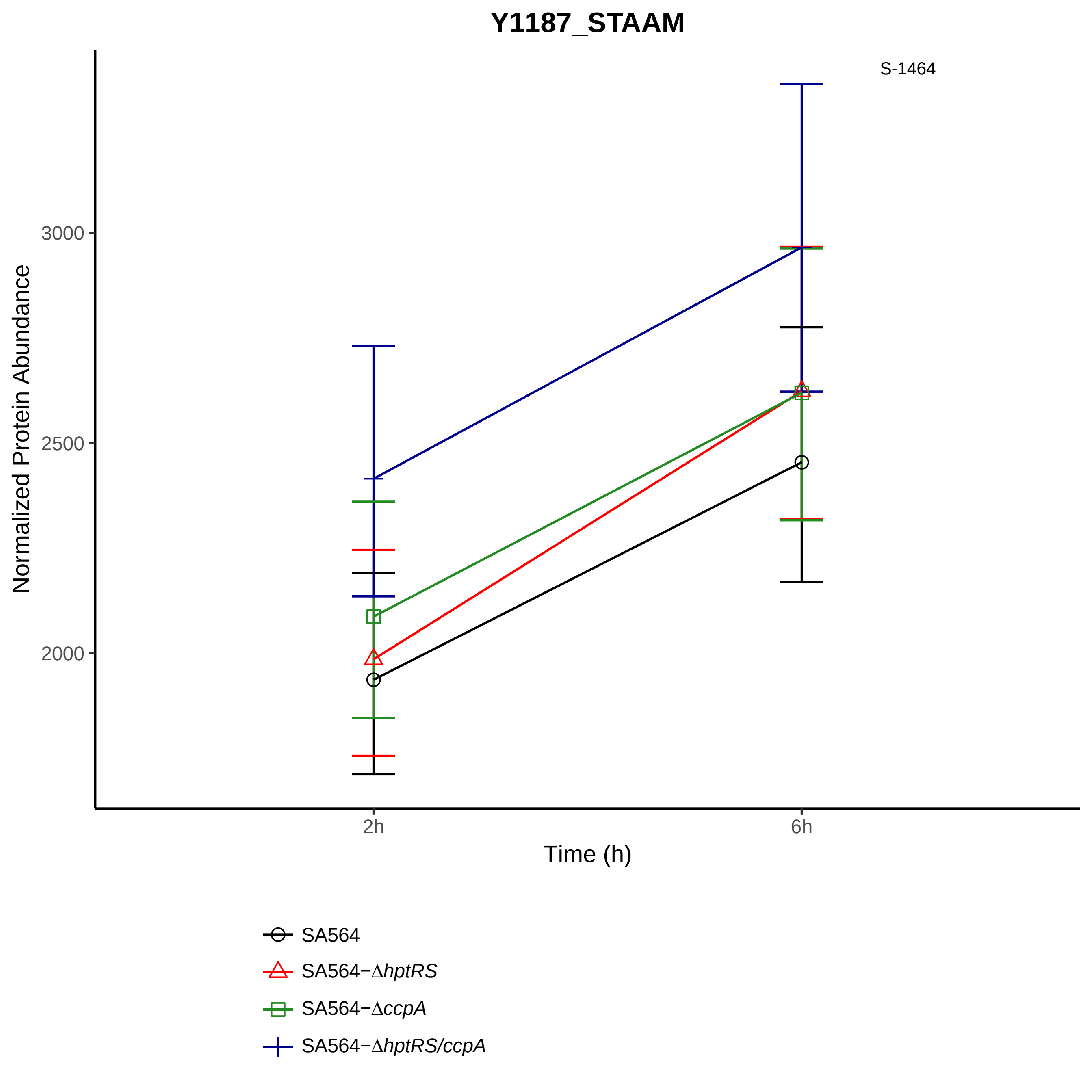
Y1187_STAAM
S-1464
Normalized Protein Abundance
3000
2500
2000
2h
6h
Time (h)
SA564
SA564−ΔhptRS
SA564−ΔccpA
SA564−ΔhptRS/ccpA

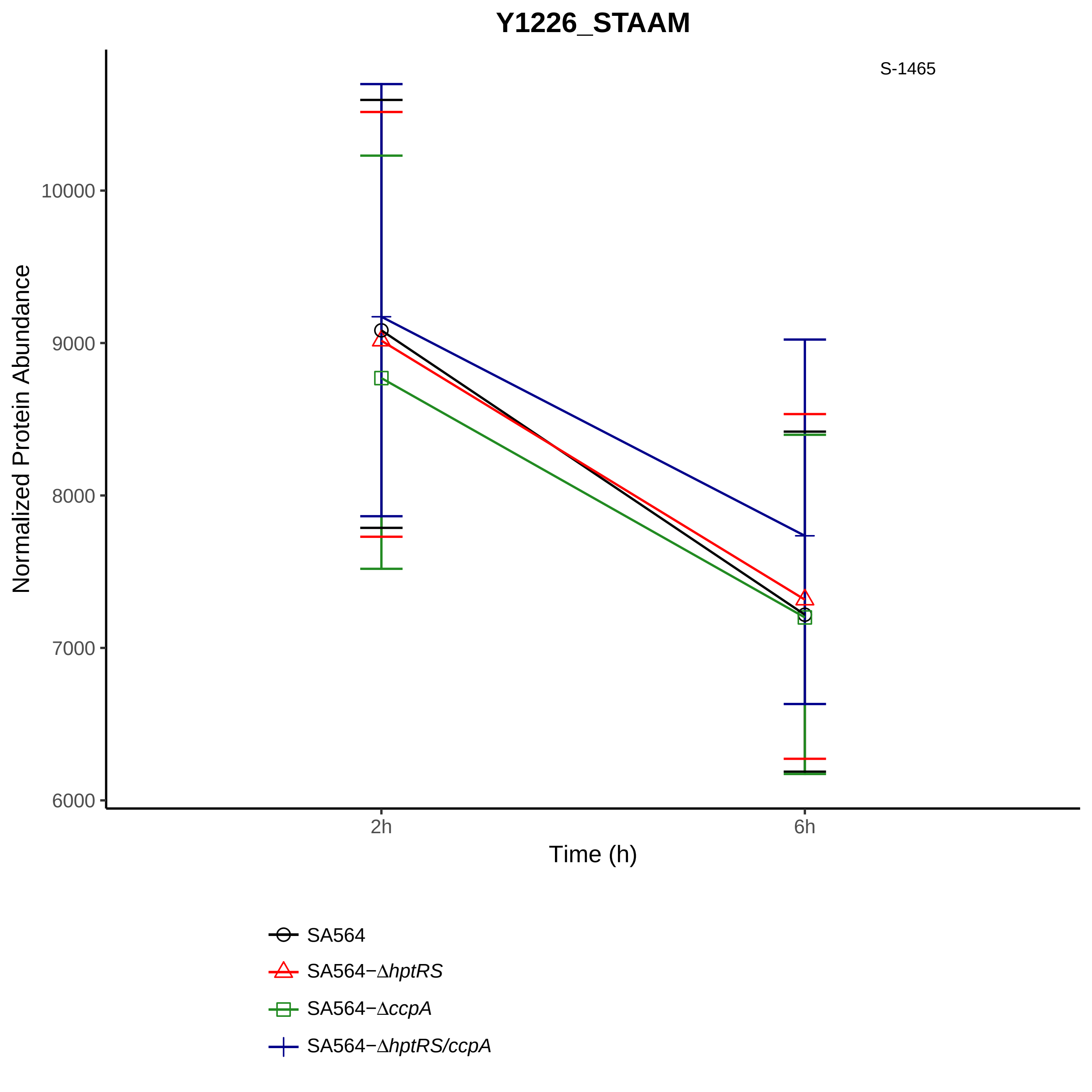

Y1226_STAAM
S-1465
Normalized Protein Abundance
10000
9000
8000
7000
6000
2h
6h
Time (h)
SA564
SA564−Δ*hptRS*
SA564−Δ*ccpA*
SA564−Δ*hptRS/ccpA*

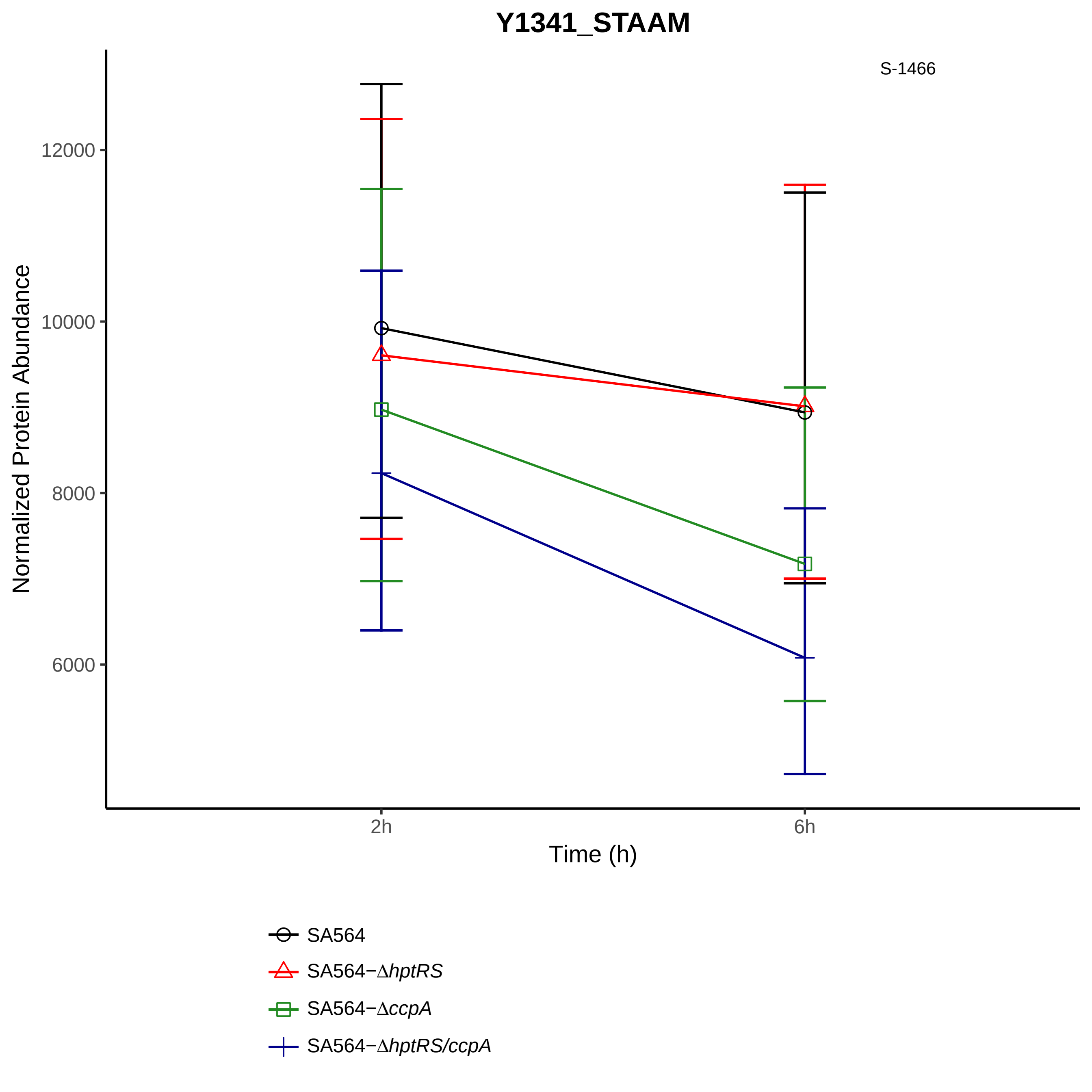

Y1341_STAAM
S-1466
Normalized Protein Abundance
12000
10000
8000
6000
2h
6h
Time (h)
SA564
SA564−Δ*hptRS*
SA564−Δ*ccpA*
SA564−Δ*hptRS/ccpA*

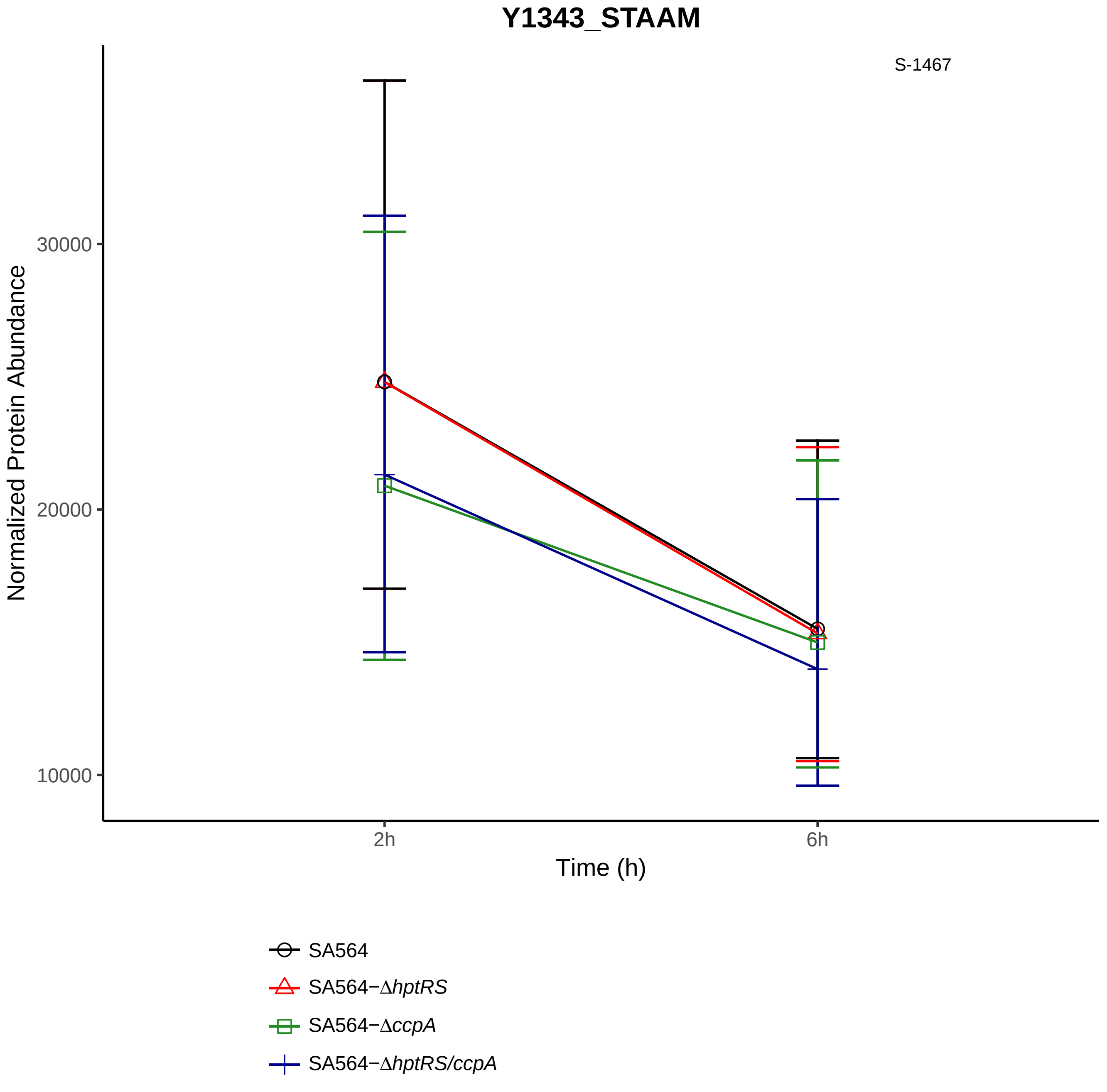
Y1343_STAAM
S-1467
Normalized Protein Abundance
30000
20000
10000
2h
6h
Time (h)
SA564
SA564−Δ*hptRS*
SA564−Δ*ccpA*
SA564−Δ*hptRS/ccpA*

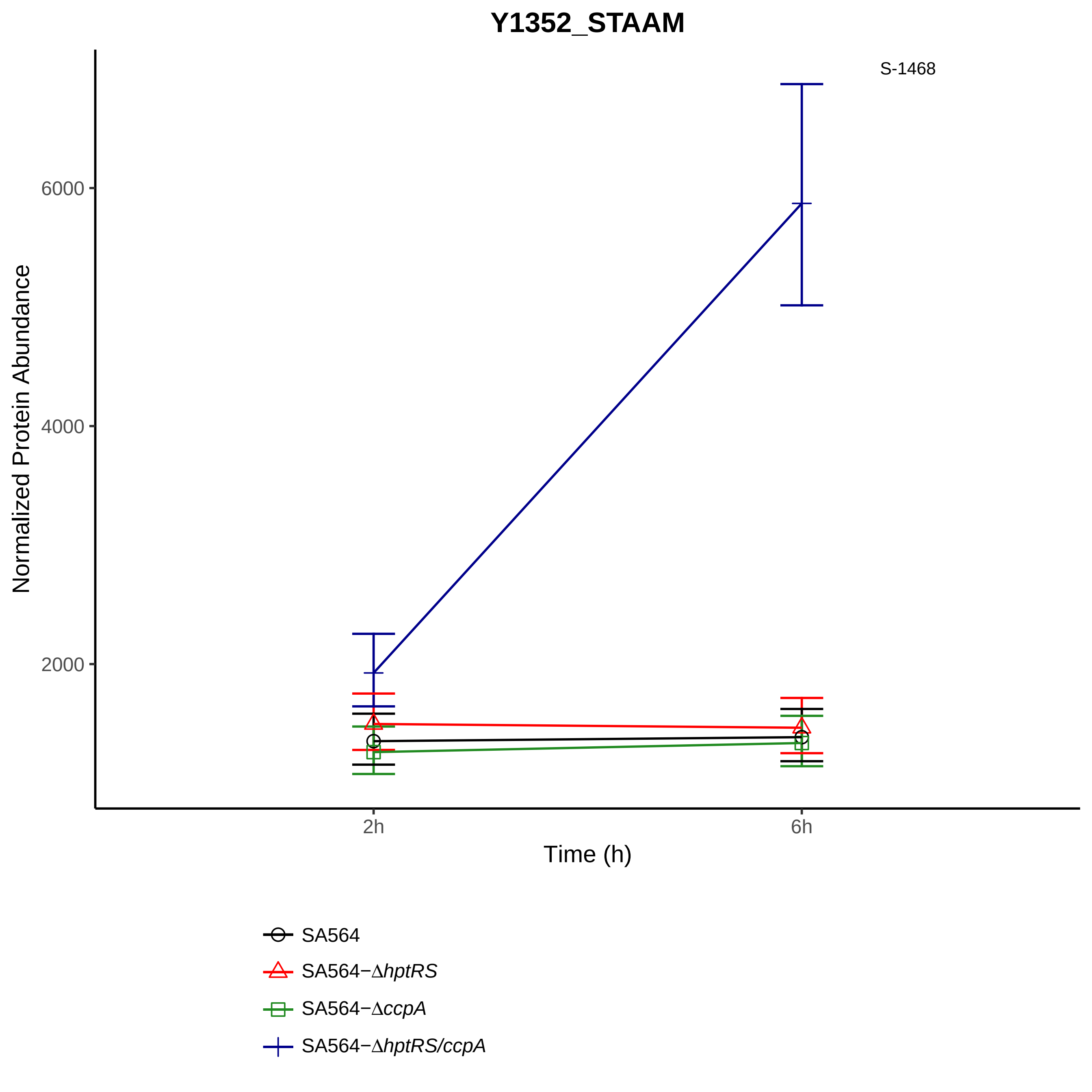
Y1352_STAAM
S-1468
6000
4000
2000
Normalized Protein Abundance
2h
6h
Time (h)
SA564
SA564−Δ*hptRS*
SA564−Δ*ccpA*
SA564−Δ*hptRS/ccpA*

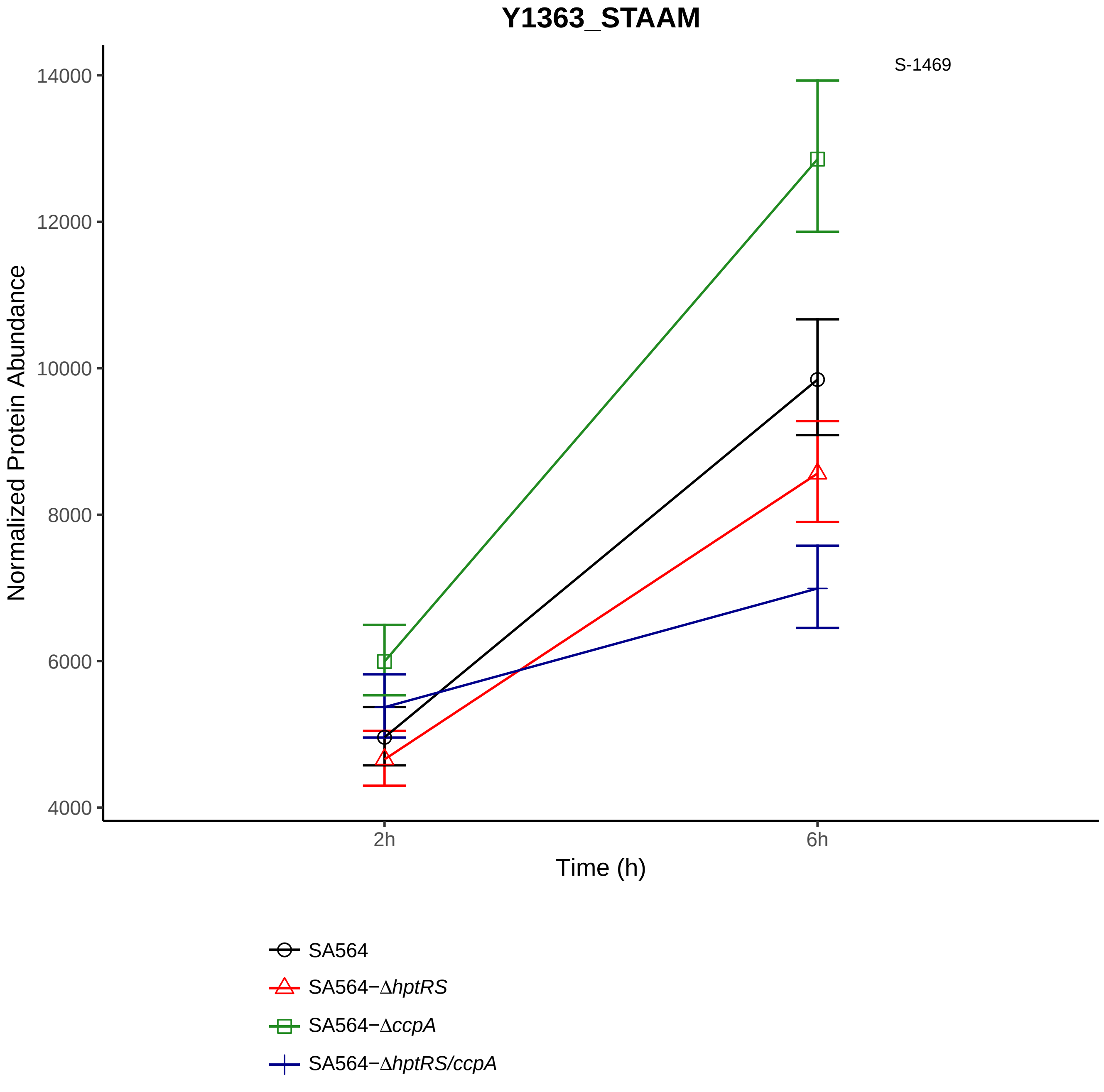

Y1363_STAAM
S-1469
14000
12000
10000
8000
6000
4000
Normalized Protein Abundance
2h
6h
Time (h)
SA564
SA564−ΔhptRS
SA564−ΔccpA
SA564−ΔhptRS/ccpA

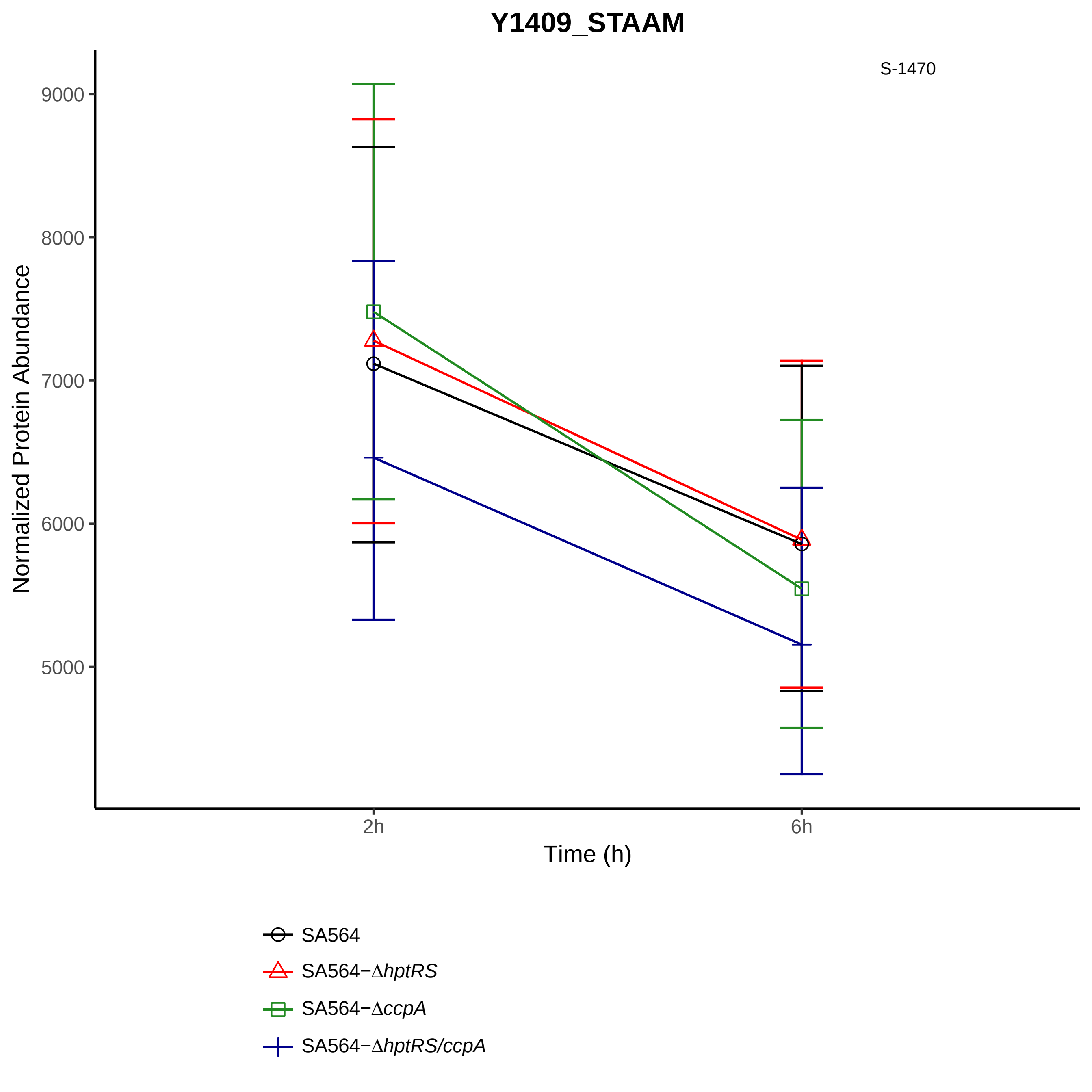

Y1409_STAAM
S-1470
Normalized Protein Abundance
9000
8000
7000
6000
5000
2h
6h
Time (h)
SA564
SA564−Δ*hptRS*
SA564−Δ*ccpA*
SA564−Δ*hptRS/ccpA*

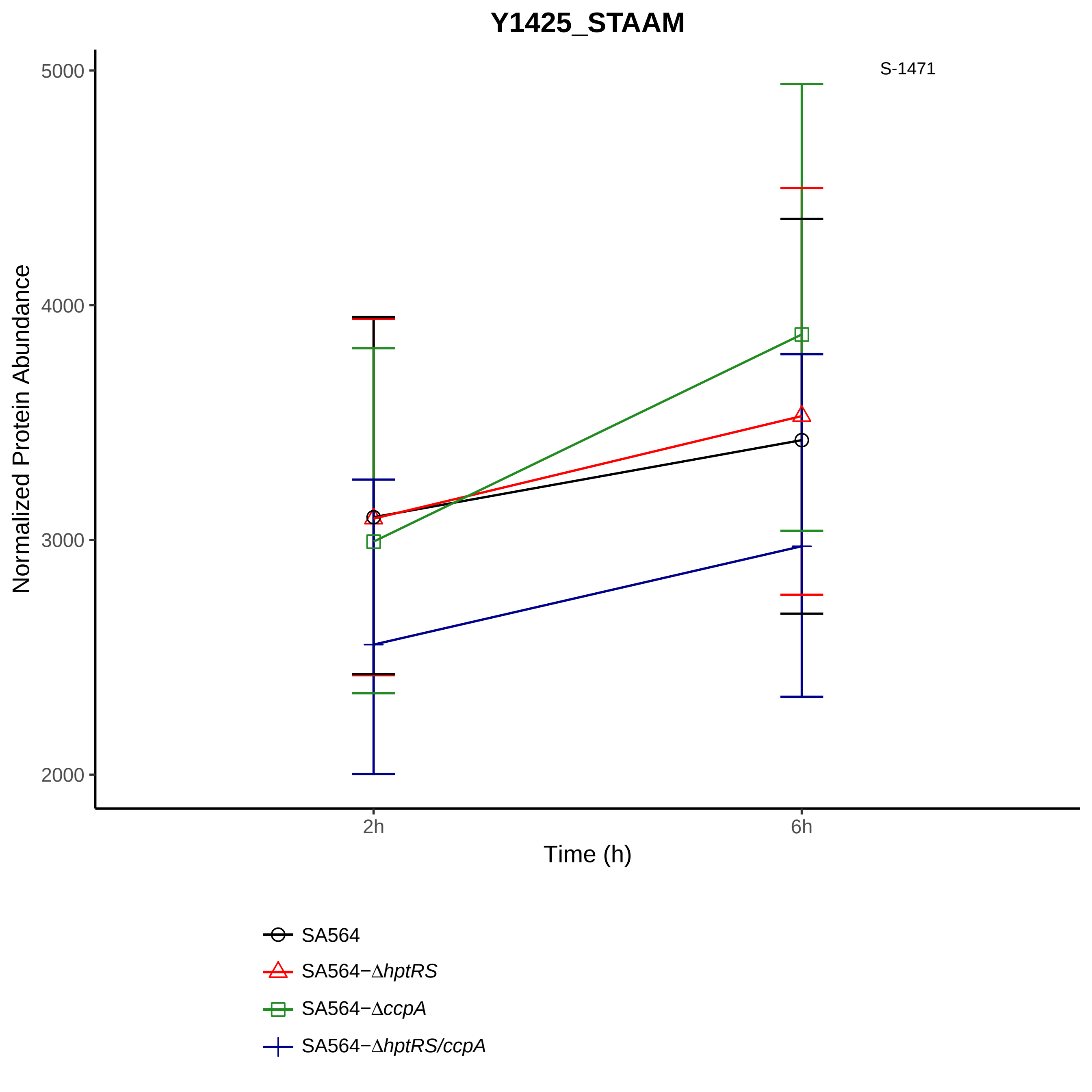

Y1425_STAAM
S-1471
Normalized Protein Abundance
5000
4000
3000
2000
2h
6h
Time (h)
SA564
SA564−ΔhptRS
SA564−ΔccpA
SA564−ΔhptRS/ccpA

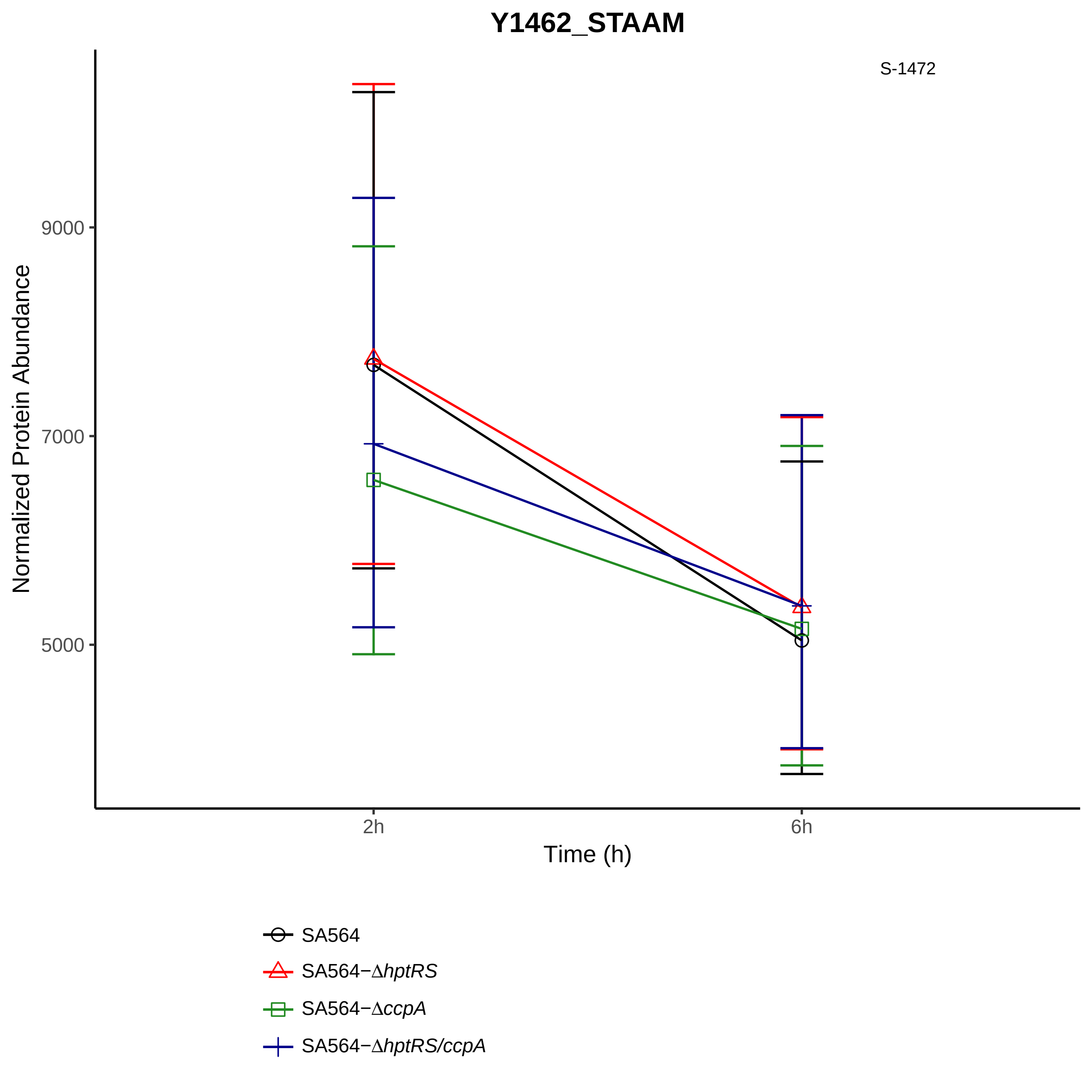

Y1462_STAAM
S-1472
Normalized Protein Abundance
9000
7000
5000
2h
6h
Time (h)
SA564
SA564−Δ*hptRS*
SA564−Δ*ccpA*
SA564−Δ*hptRS/ccpA*

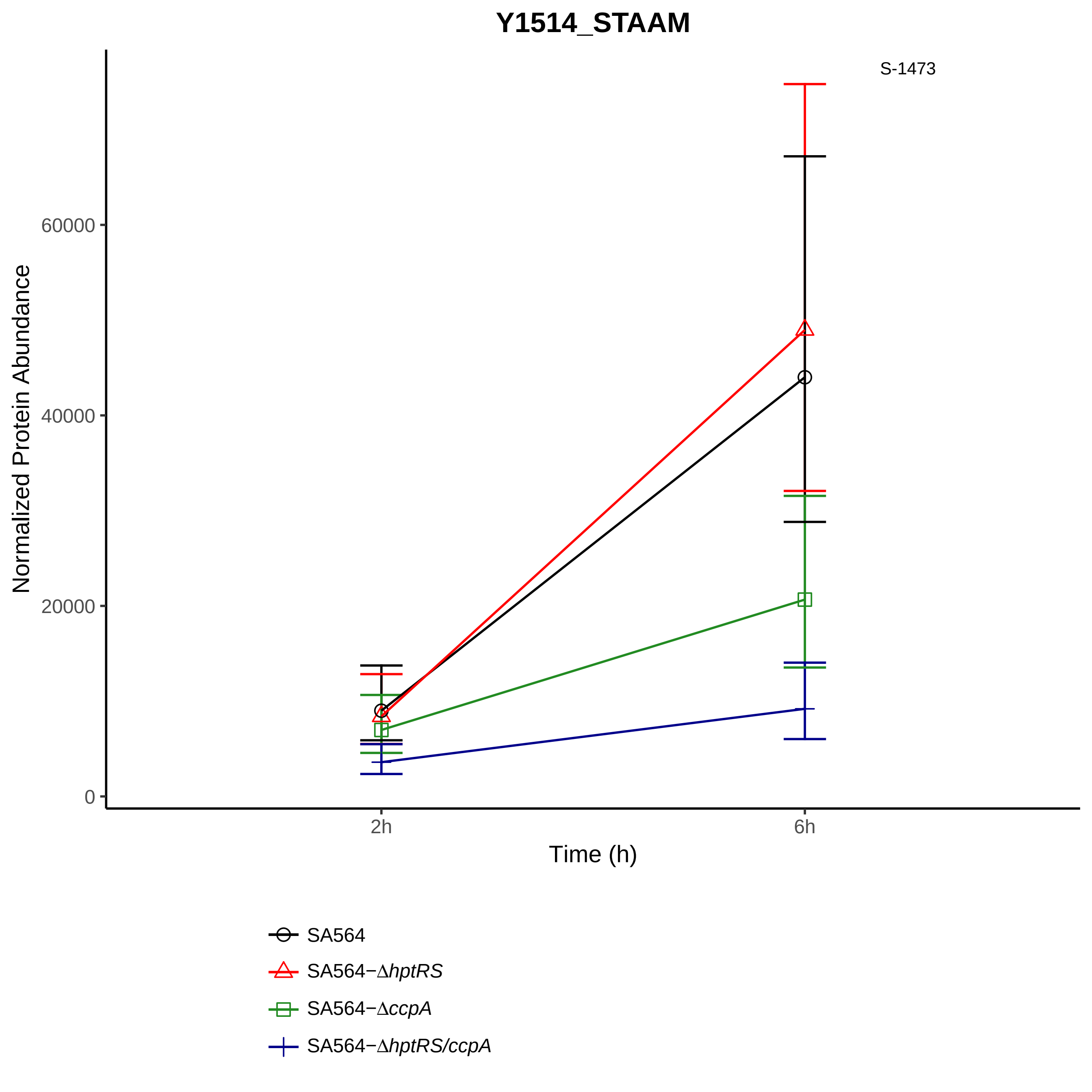
Y1514_STAAM
S-1473
Normalized Protein Abundance
60000
40000
20000
0
2h
6h
Time (h)
SA564
SA564−Δ*hptRS*
SA564−Δ*ccpA*
SA564−Δ*hptRS/ccpA*

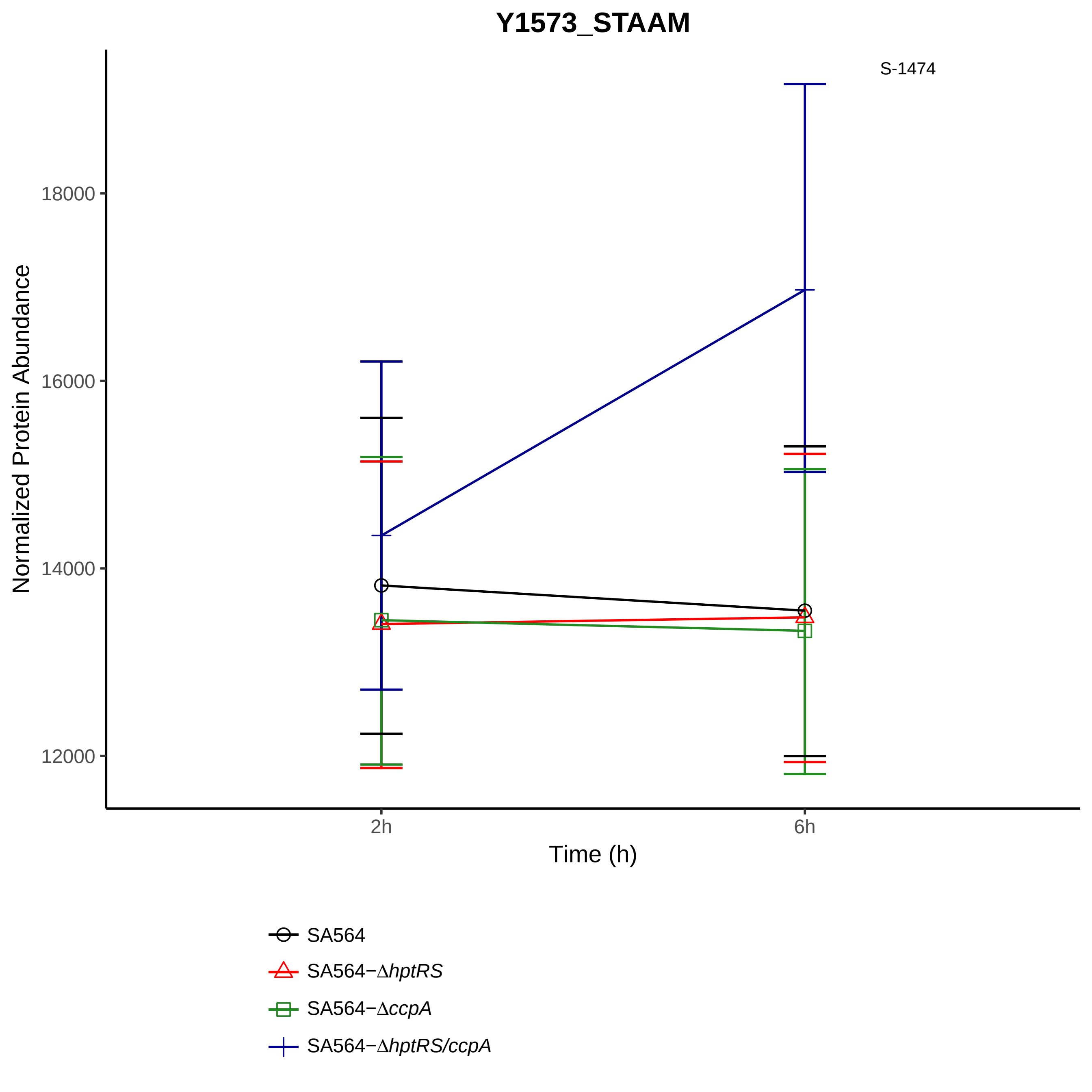
Y1573_STAAM
S-1474
Normalized Protein Abundance
18000
16000
14000
12000
2h
6h
Time (h)
SA564
SA564−Δ*hptRS*
SA564−Δ*ccpA*
SA564−Δ*hptRS/ccpA*

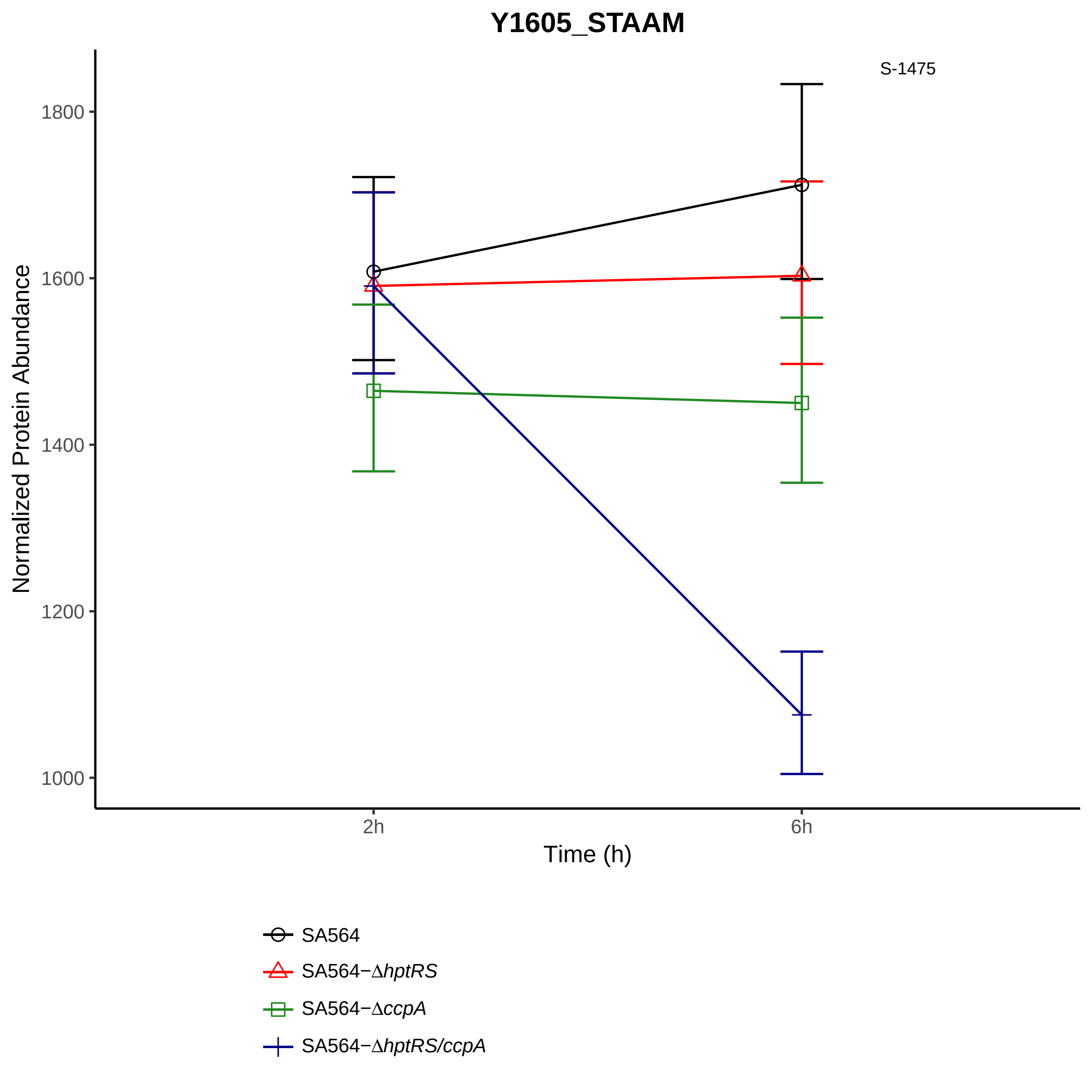

Y1605_STAAM
S-1475
Normalized Protein Abundance
1800
1600
1400
1200
1000
2h
6h
Time (h)
SA564
SA564−ΔhptRS
SA564−ΔccpA
SA564−ΔhptRS/ccpA

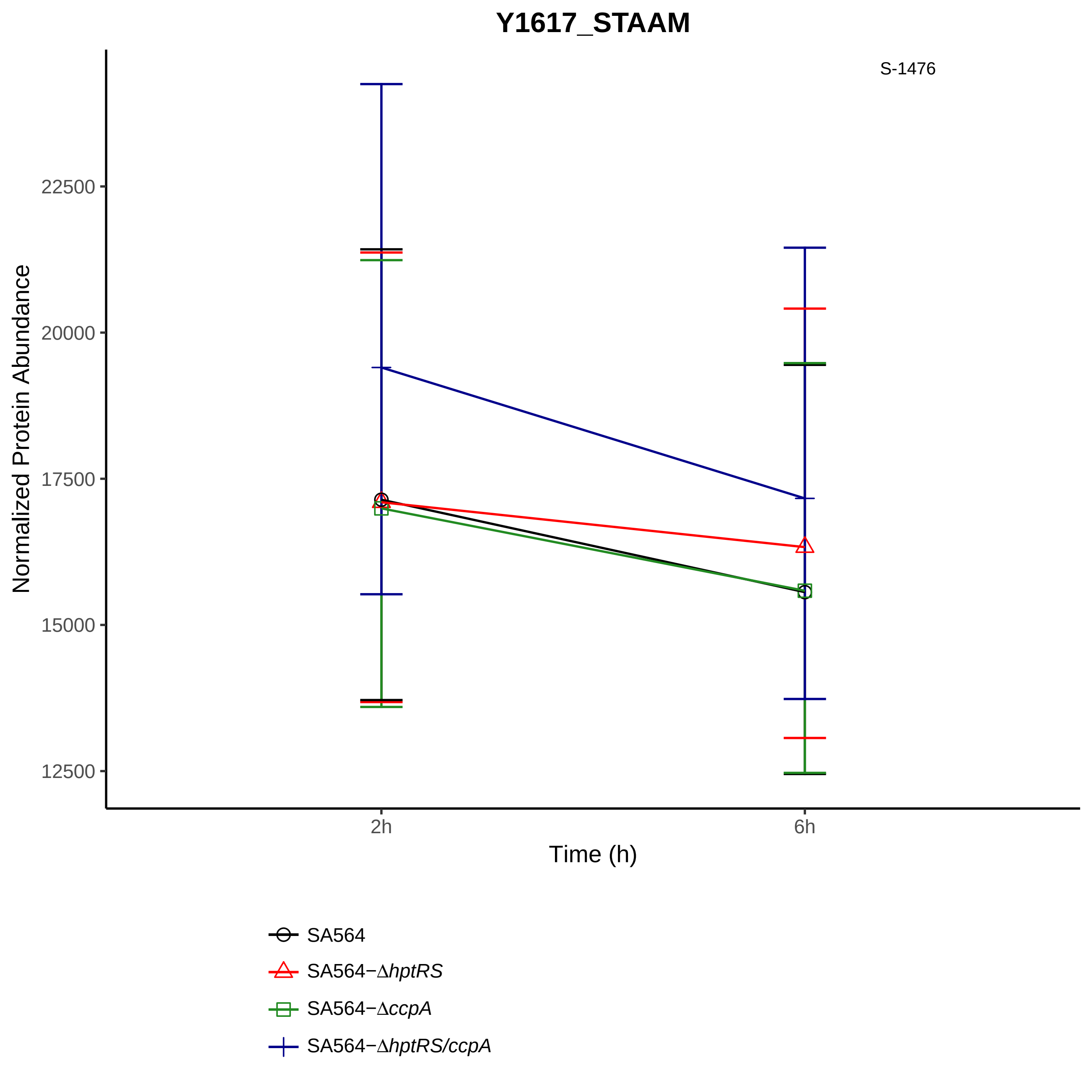

Y1617_STAAM
S-1476
Normalized Protein Abundance
22500
20000
17500
15000
12500
2h
6h
Time (h)
SA564
SA564−ΔhptRS
SA564−ΔccpA
SA564−ΔhptRS/ccpA

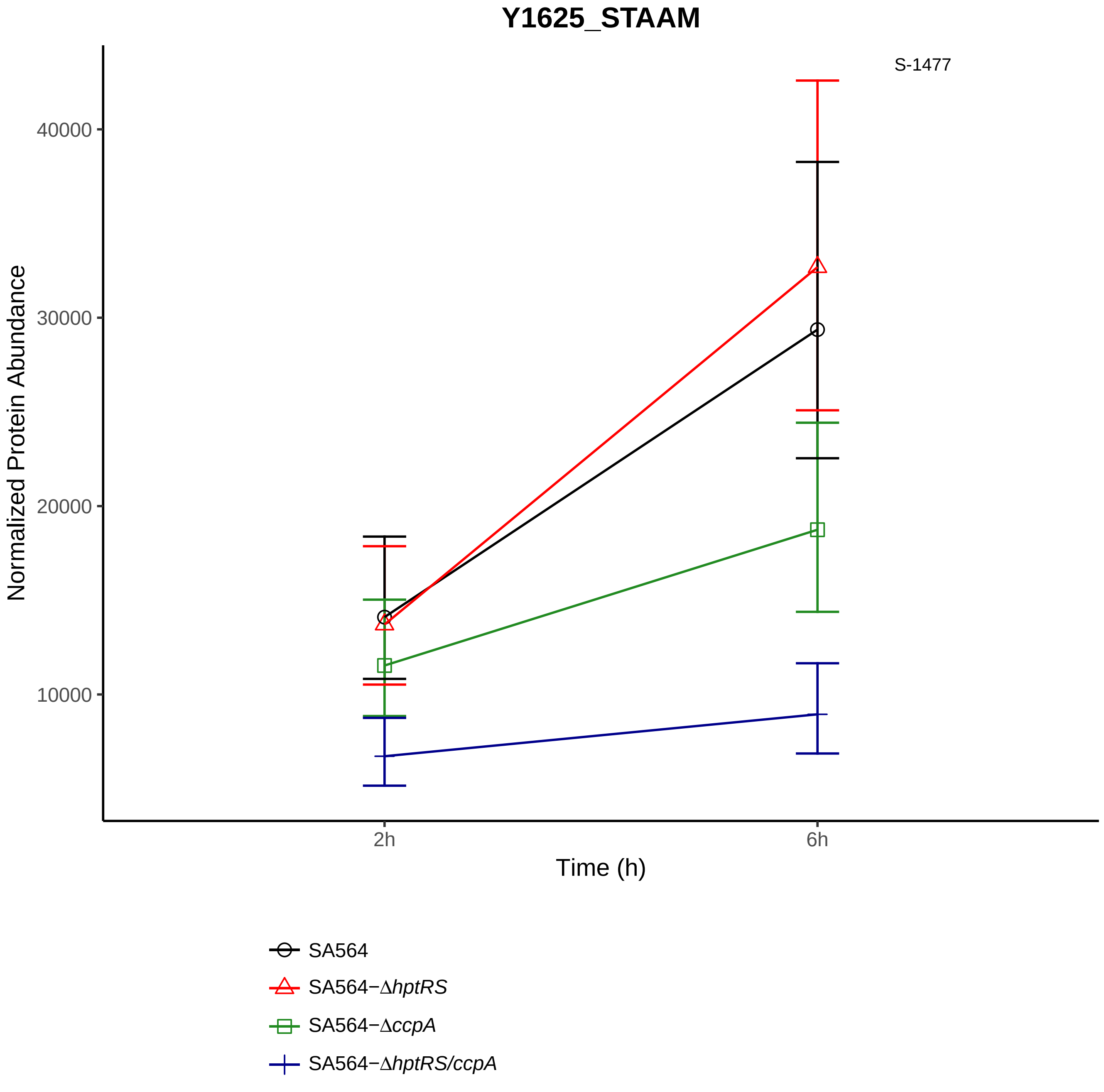
Y1625_STAAM
S-1477
Normalized Protein Abundance
40000
30000
20000
10000
2h
6h
Time (h)
SA564
SA564−ΔhptRS
SA564−ΔccpA
SA564−ΔhptRS/ccpA

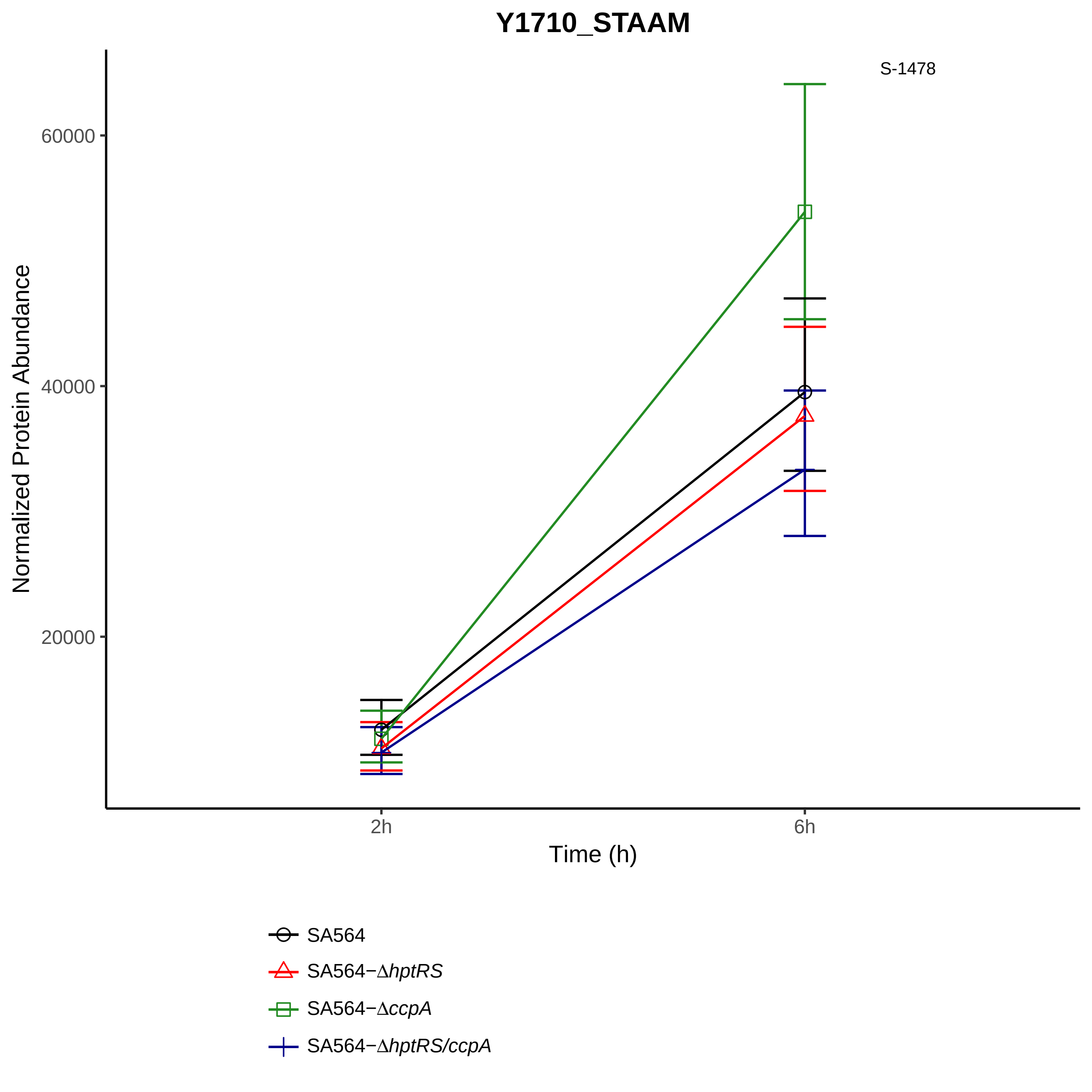
Y1710_STAAM
S-1478
Normalized Protein Abundance
60000
40000
20000
2h
6h
Time (h)
SA564
SA564−Δ*hptRS*
SA564−Δ*ccpA*
SA564−Δ*hptRS/ccpA*

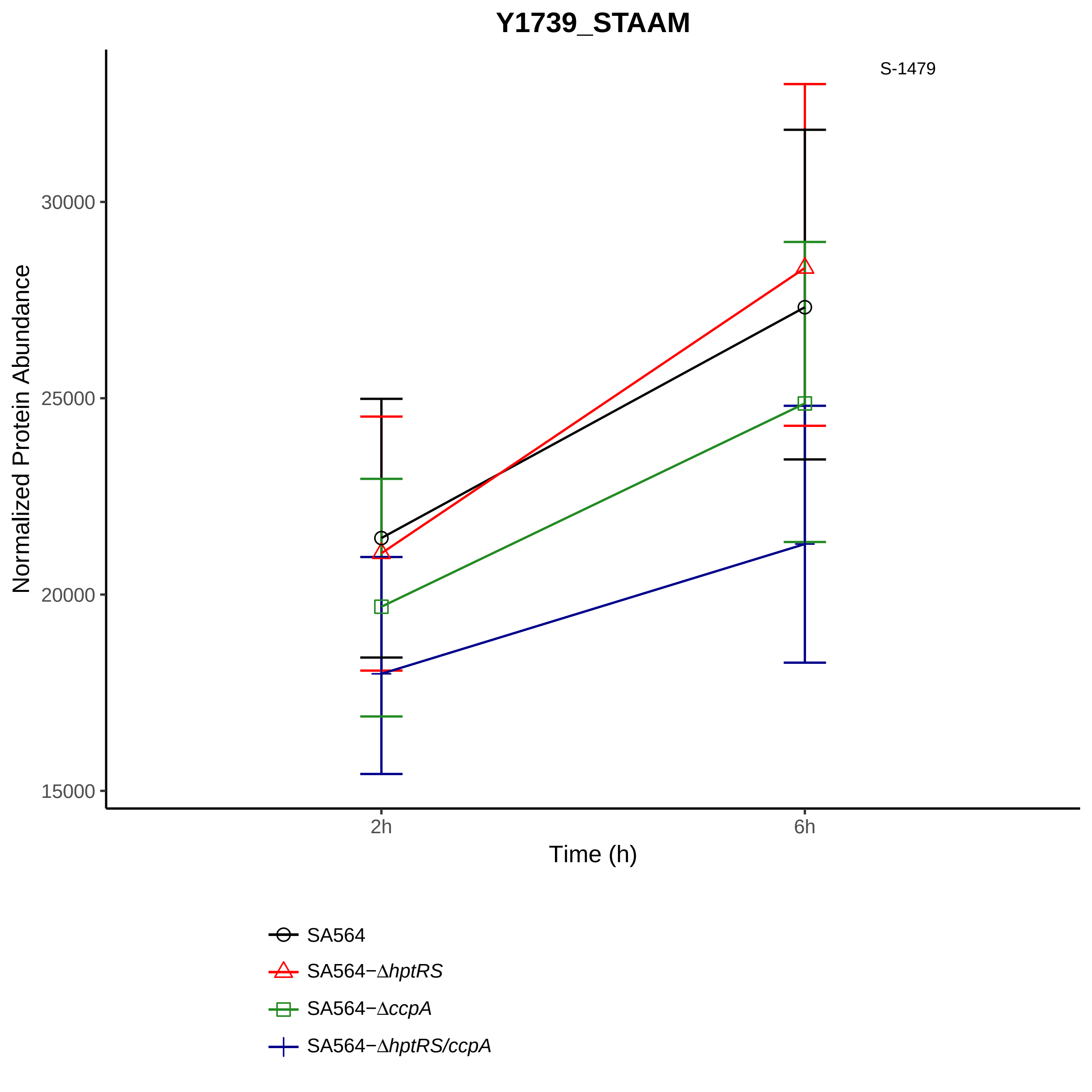

Y1739_STAAM
S-1479
Normalized Protein Abundance
30000
25000
20000
15000
2h
6h
Time (h)
SA564
SA564−ΔhptRS
SA564−ΔccpA
SA564−ΔhptRS/ccpA

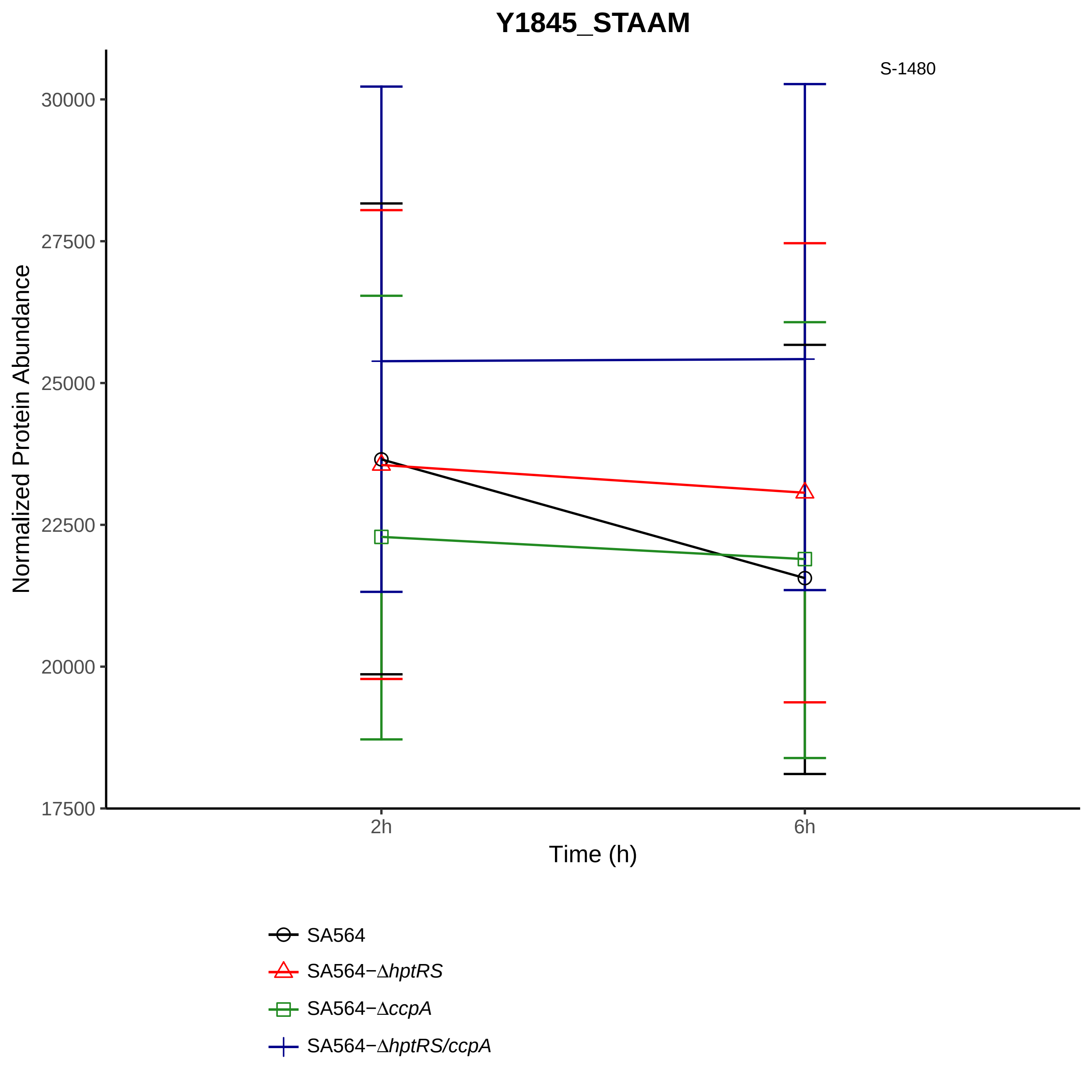

Y1845_STAAM
S-1480
Normalized Protein Abundance
30000
27500
25000
22500
20000
17500
2h
6h
Time (h)
SA564
SA564−Δ*hptRS*
SA564−Δ*ccpA*
SA564−Δ*hptRS/ccpA*

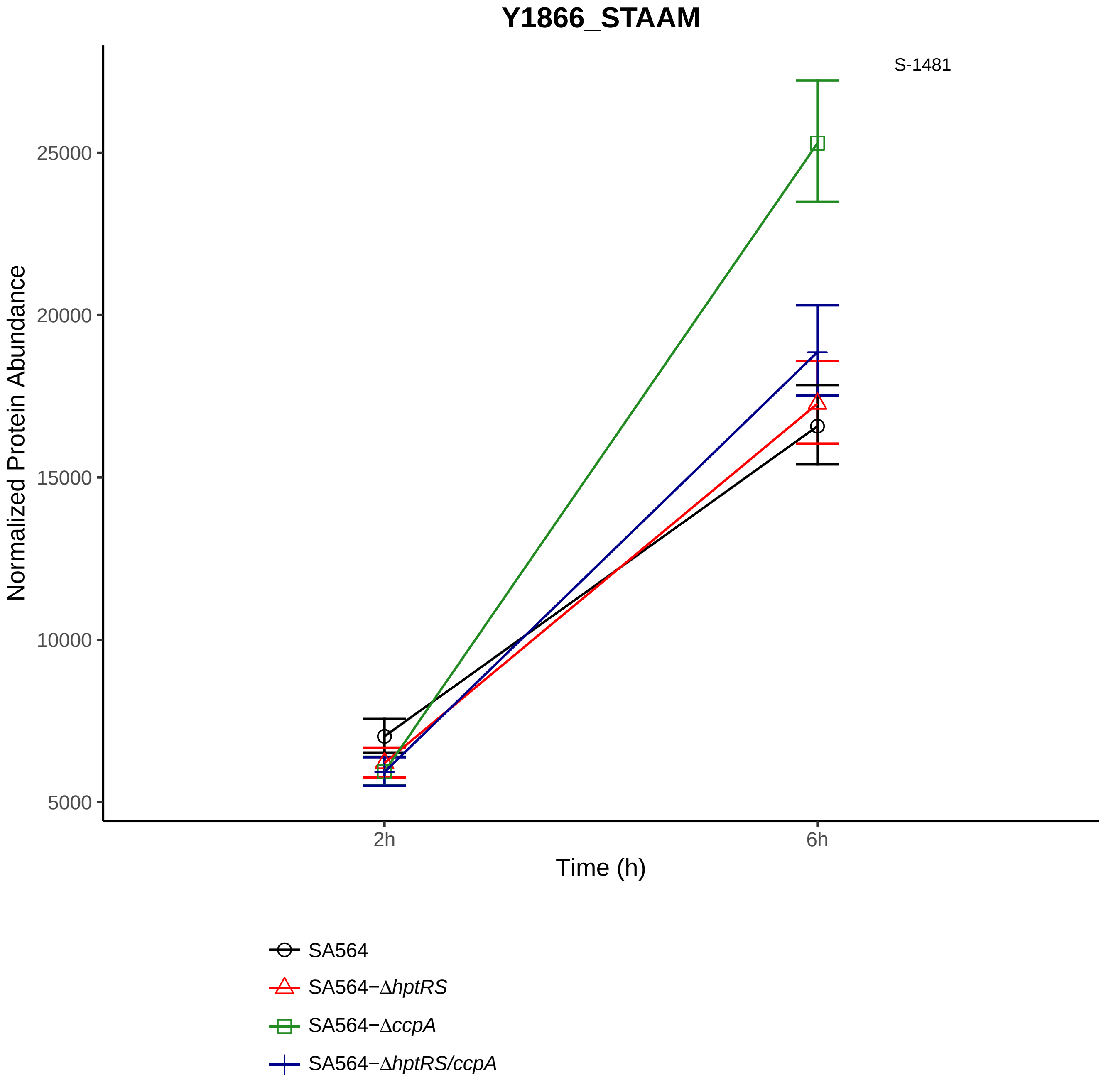

Y1866_STAAM
S-1481
Normalized Protein Abundance
25000
20000
15000
10000
5000
2h
6h
Time (h)
SA564
SA564−Δ*hptRS*
SA564−Δ*ccpA*
SA564−Δ*hptRS/ccpA*

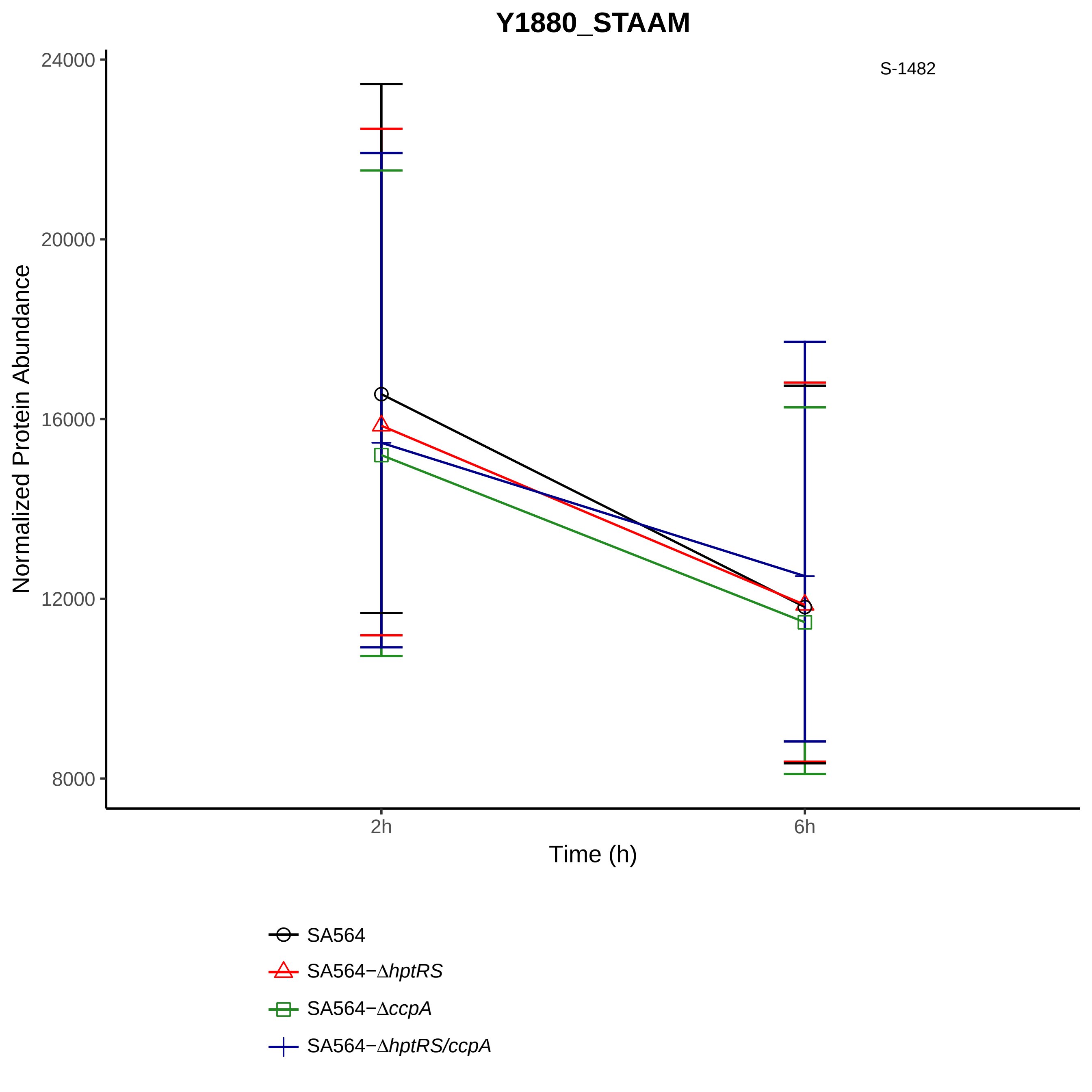

Y1880_STAAM
S-1482
Normalized Protein Abundance
24000
20000
16000
12000
8000
2h
6h
Time (h)
SA564
SA564−ΔhptRS
SA564−ΔccpA
SA564−ΔhptRS/ccpA

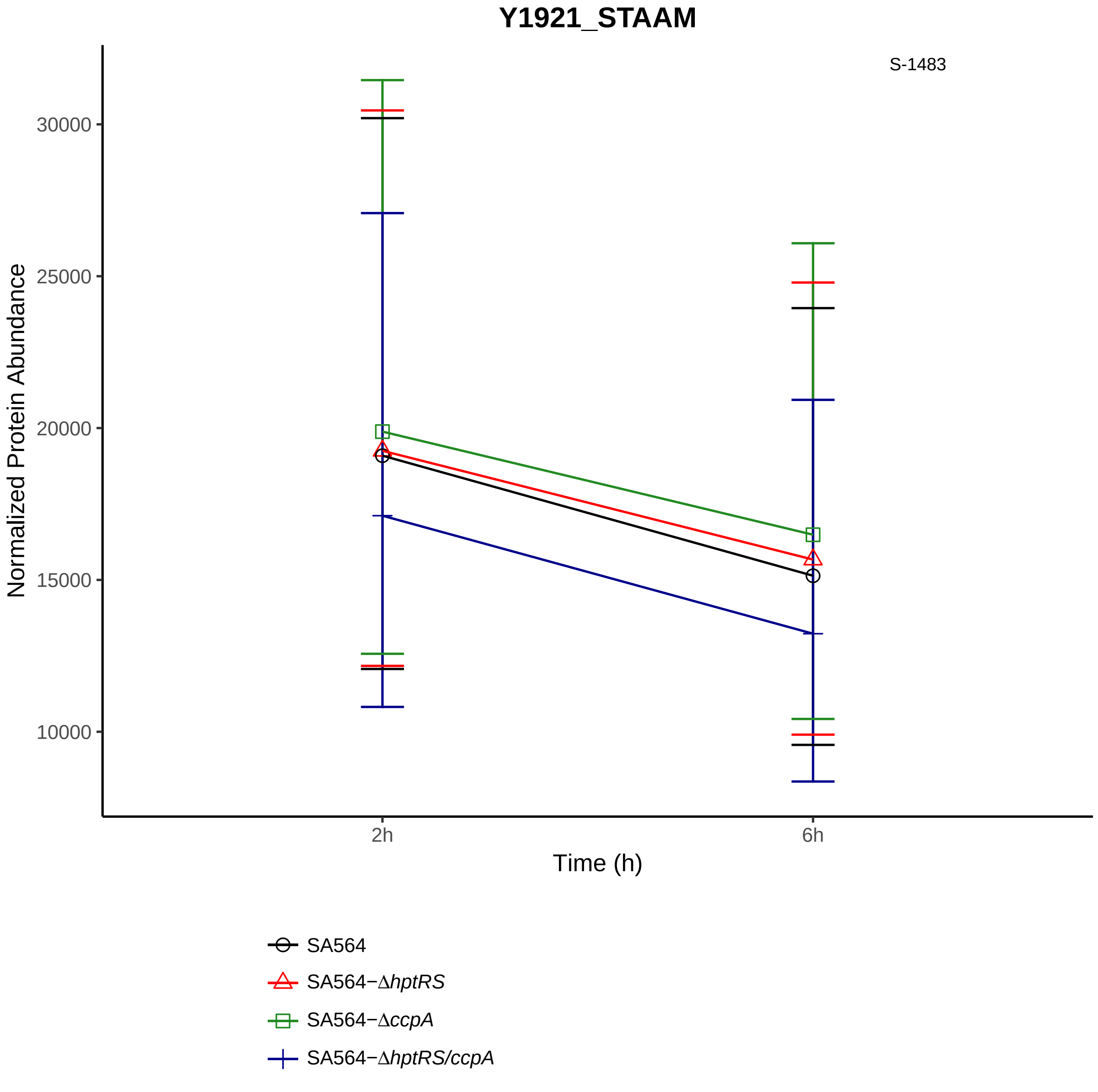

Y1921_STAAM
S-1483
Normalized Protein Abundance
30000
25000
20000
15000
10000
2h
6h
Time (h)
SA564
SA564−ΔhptRS
SA564−ΔccpA
SA564−ΔhptRS/ccpA

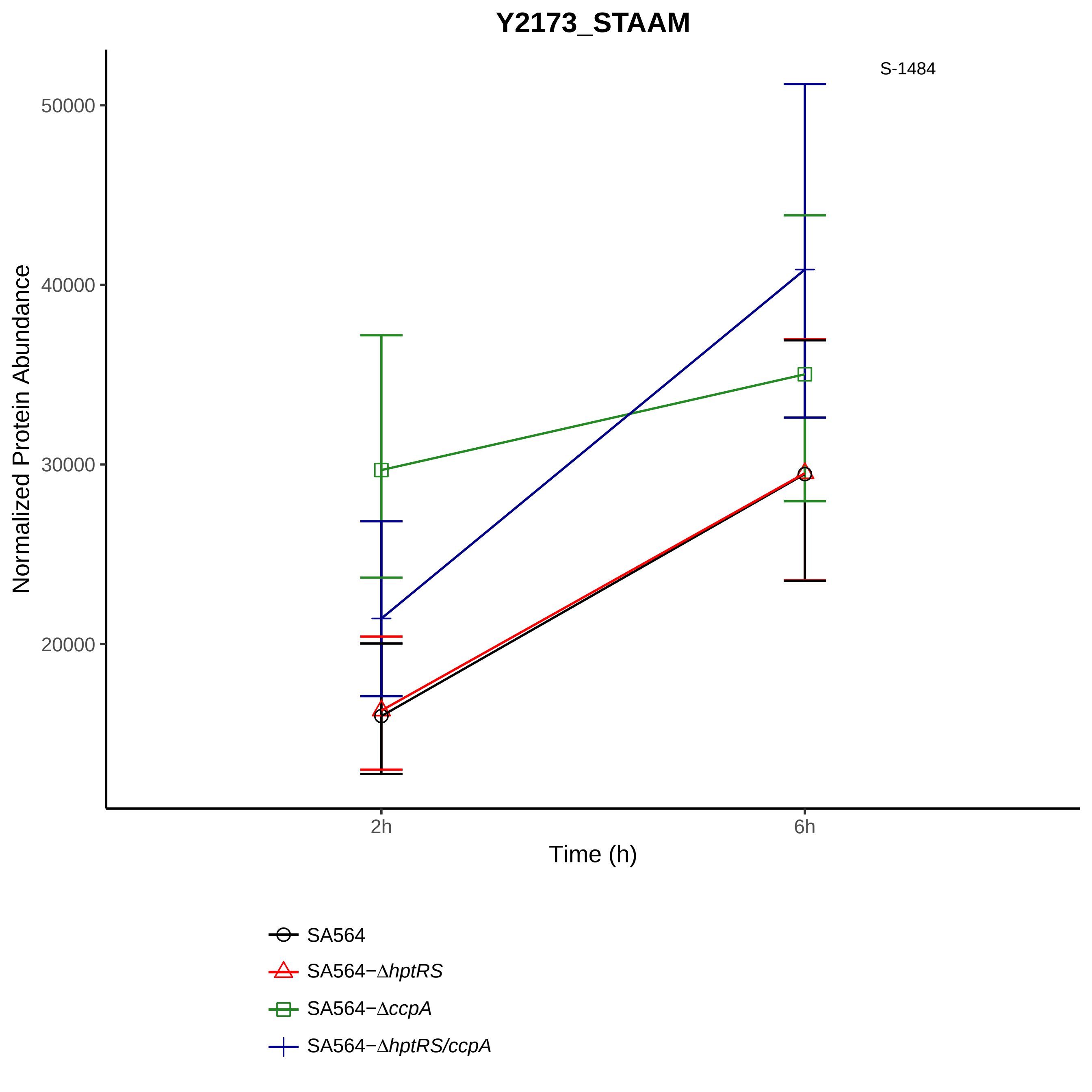

Y2173_STAAM
S-1484
Normalized Protein Abundance
50000
40000
30000
20000
2h
6h
Time (h)
SA564
SA564−ΔhptRS
SA564−ΔccpA
SA564−ΔhptRS/ccpA

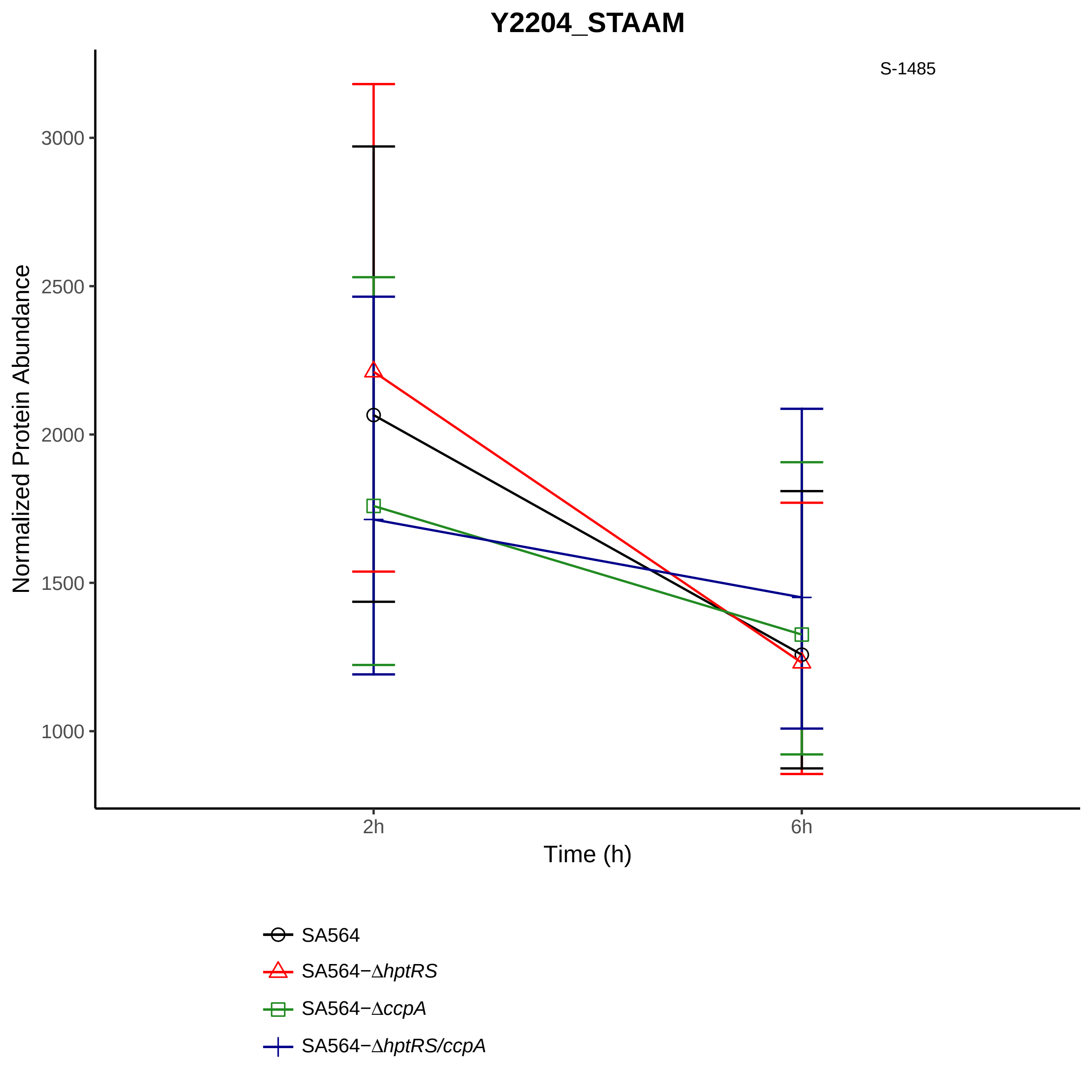

Y2204_STAAM
S-1485
Normalized Protein Abundance
3000
2500
2000
1500
1000
2h
6h
Time (h)
SA564
SA564−Δ*hptRS*
SA564−Δ*ccpA*
SA564−Δ*hptRS/ccpA*

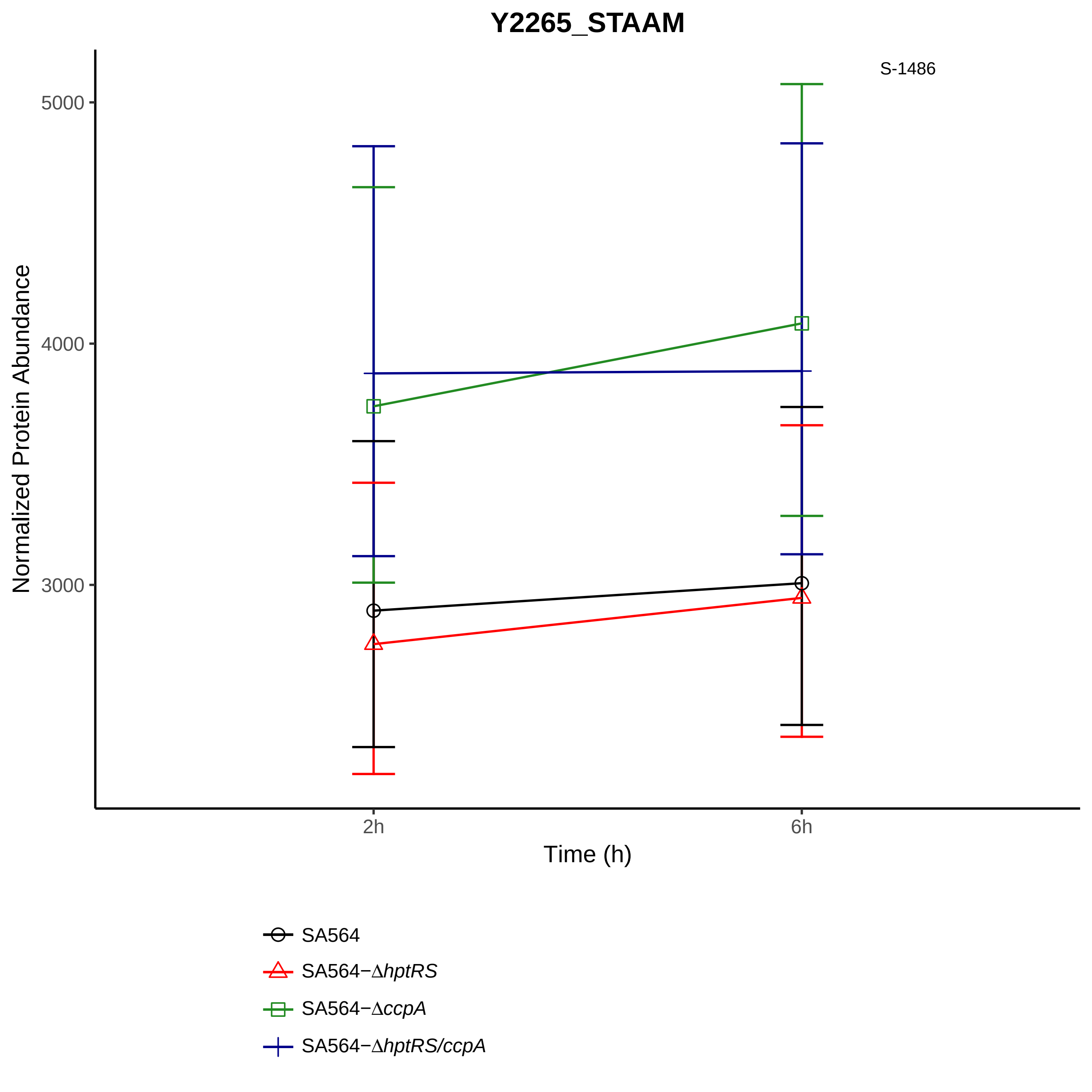

Y2265_STAAM
S-1486
Normalized Protein Abundance
5000
4000
3000
2h
6h
Time (h)
SA564
SA564−Δ*hptRS*
SA564−Δ*ccpA*
SA564−Δ*hptRS/ccpA*

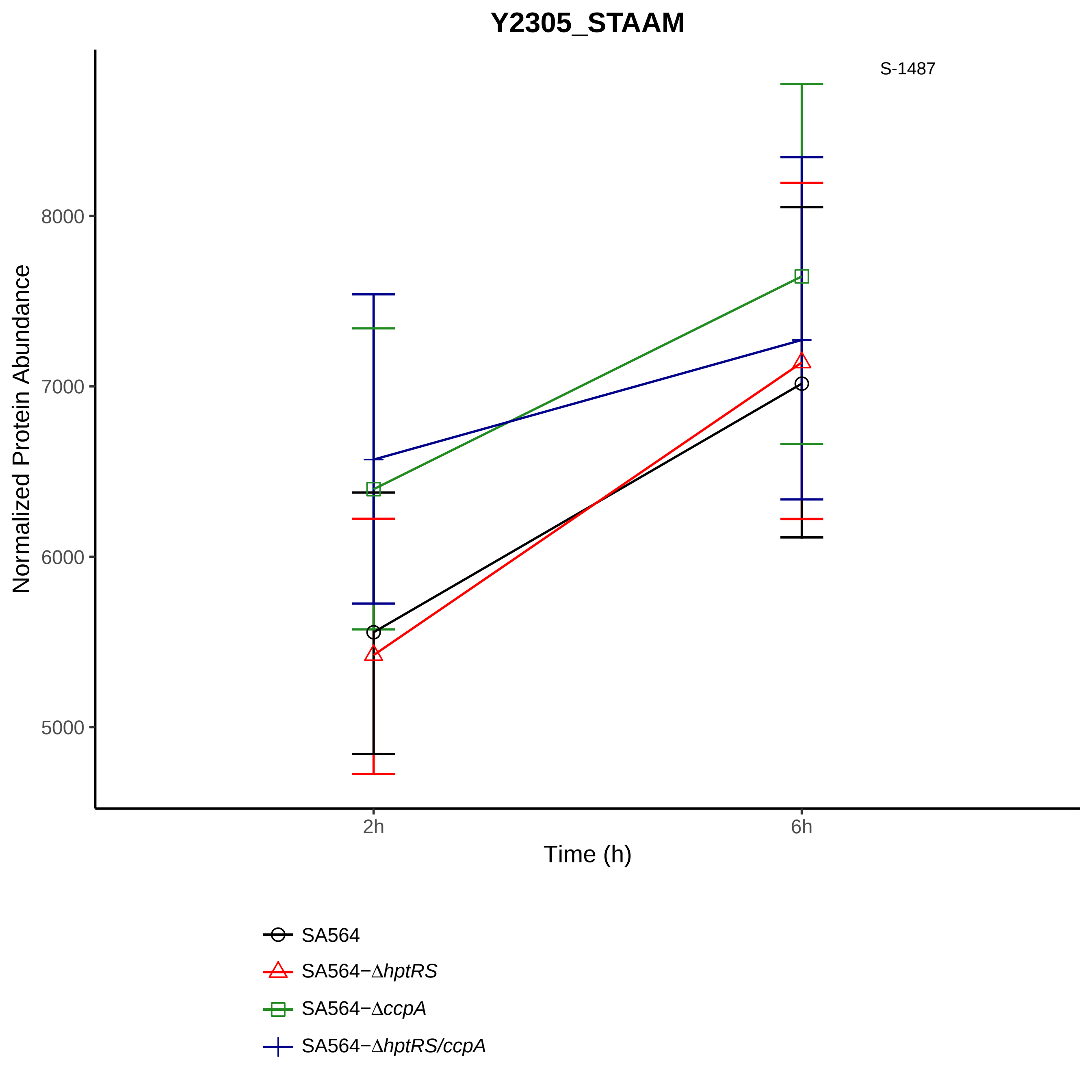

Y2305_STAAM
S-1487
Normalized Protein Abundance
8000
7000
6000
5000
2h
6h
Time (h)
SA564
SA564−Δ*hptRS*
SA564−Δ*ccpA*
SA564−Δ*hptRS/ccpA*

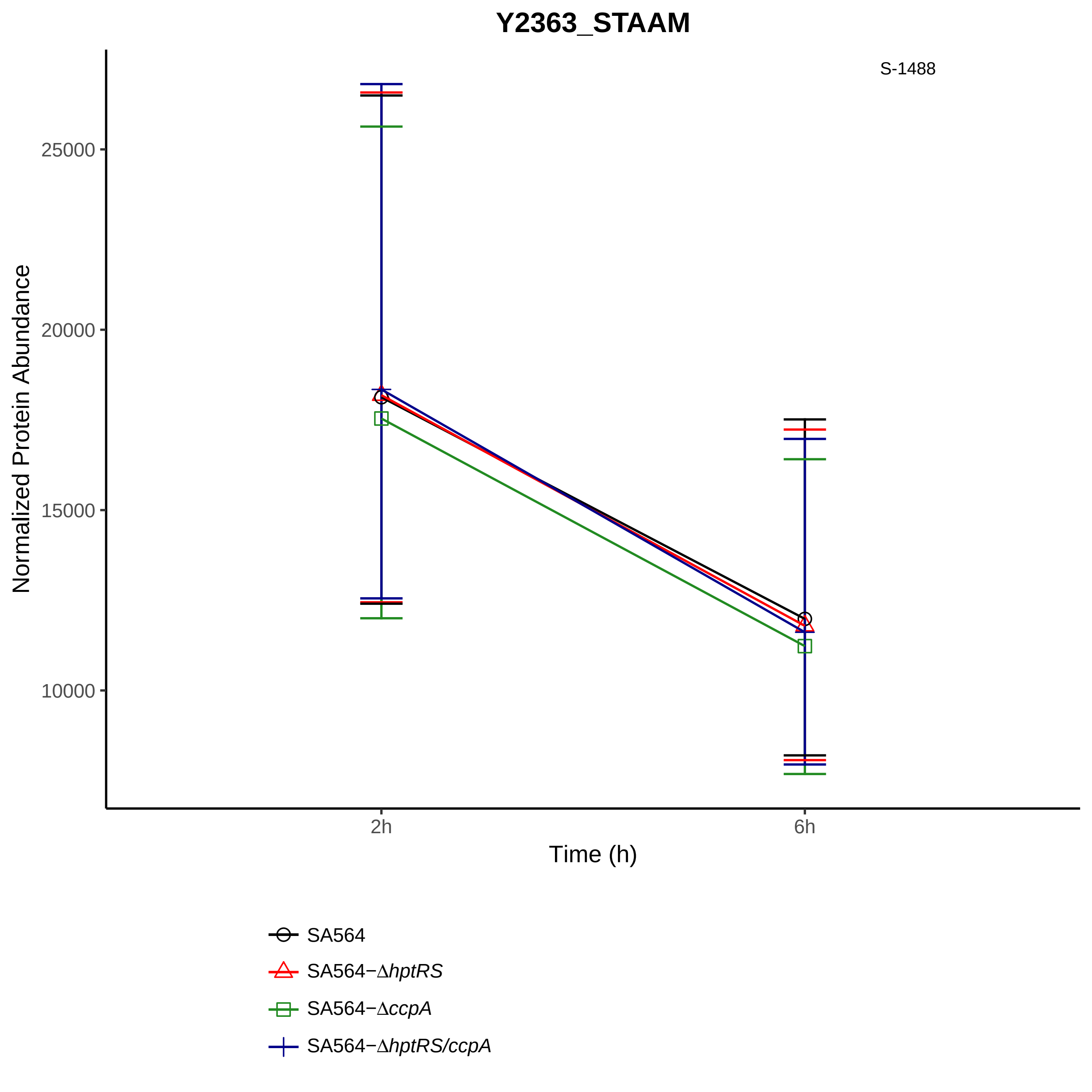

Y2363_STAAM
S-1488
Normalized Protein Abundance
25000
20000
15000
10000
2h
6h
Time (h)
SA564
SA564−Δ*hptRS*
SA564−Δ*ccpA*
SA564−Δ*hptRS/ccpA*

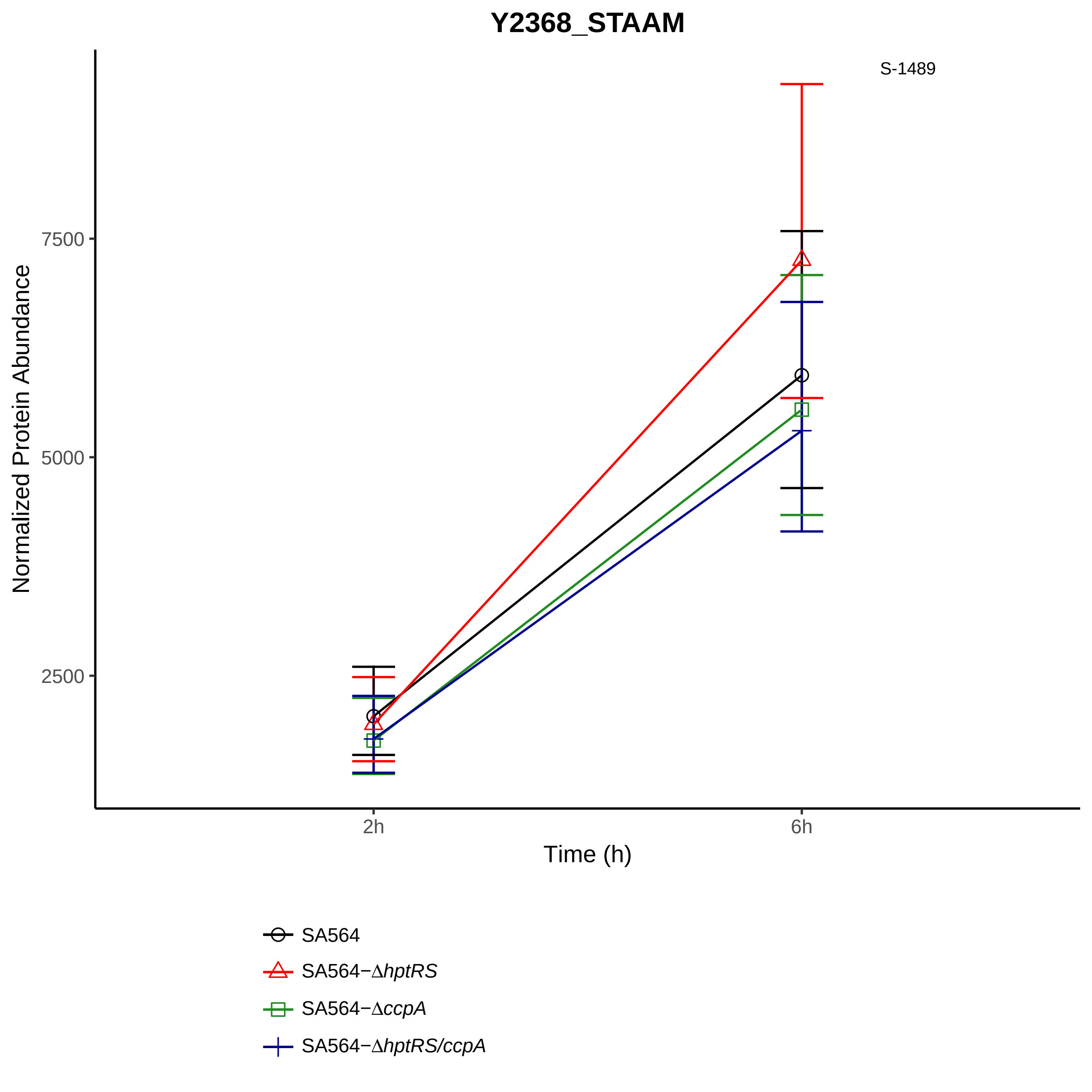

Y2368_STAAM
S-1489
Normalized Protein Abundance
7500
5000
2500
2h
6h
Time (h)
SA564
SA564−Δ*hptRS*
SA564−Δ*ccpA*
SA564−Δ*hptRS/ccpA*

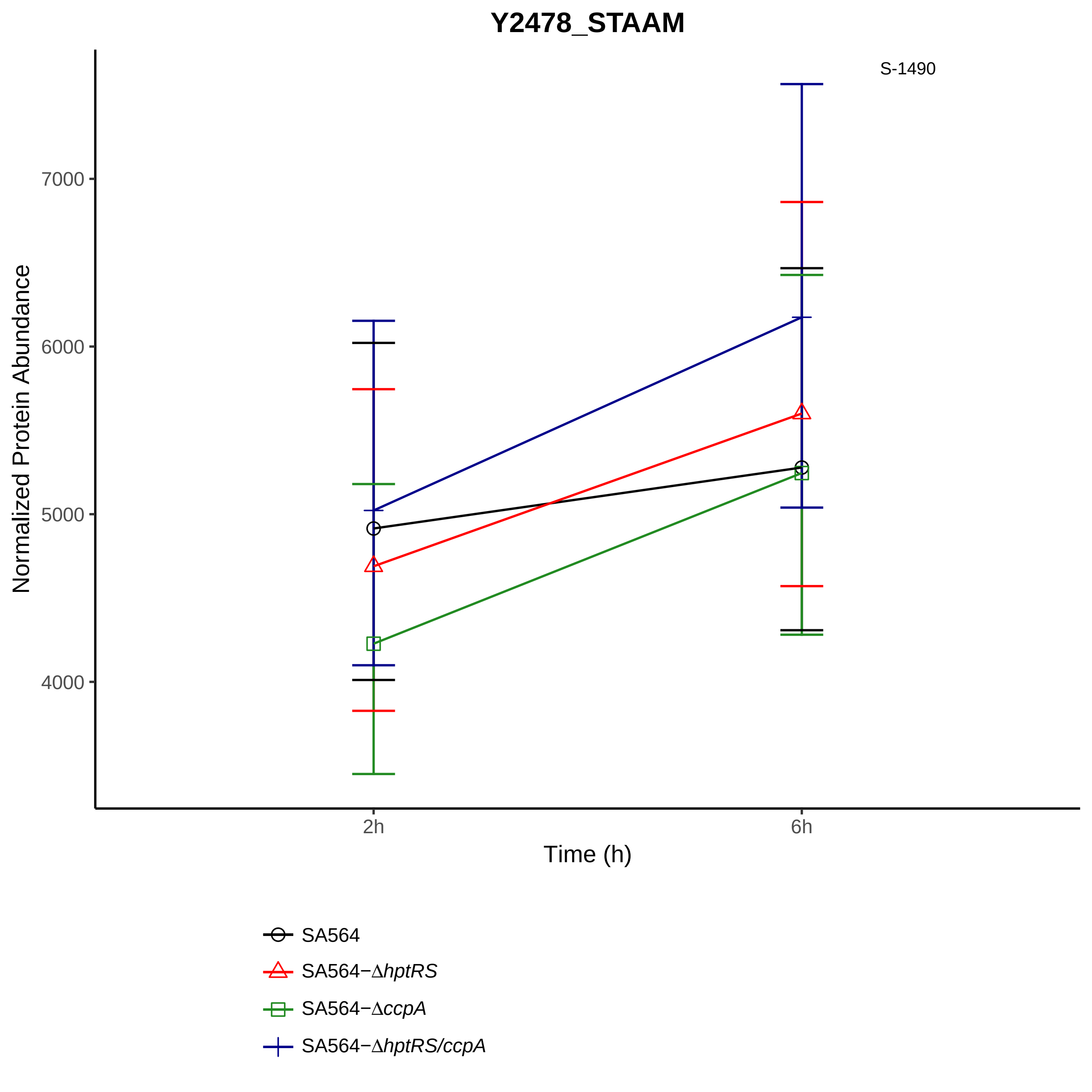

Y2478_STAAM
S-1490
Normalized Protein Abundance
7000
6000
5000
4000
2h
6h
Time (h)
SA564
SA564−Δ*hptRS*
SA564−Δ*ccpA*
SA564−Δ*hptRS/ccpA*

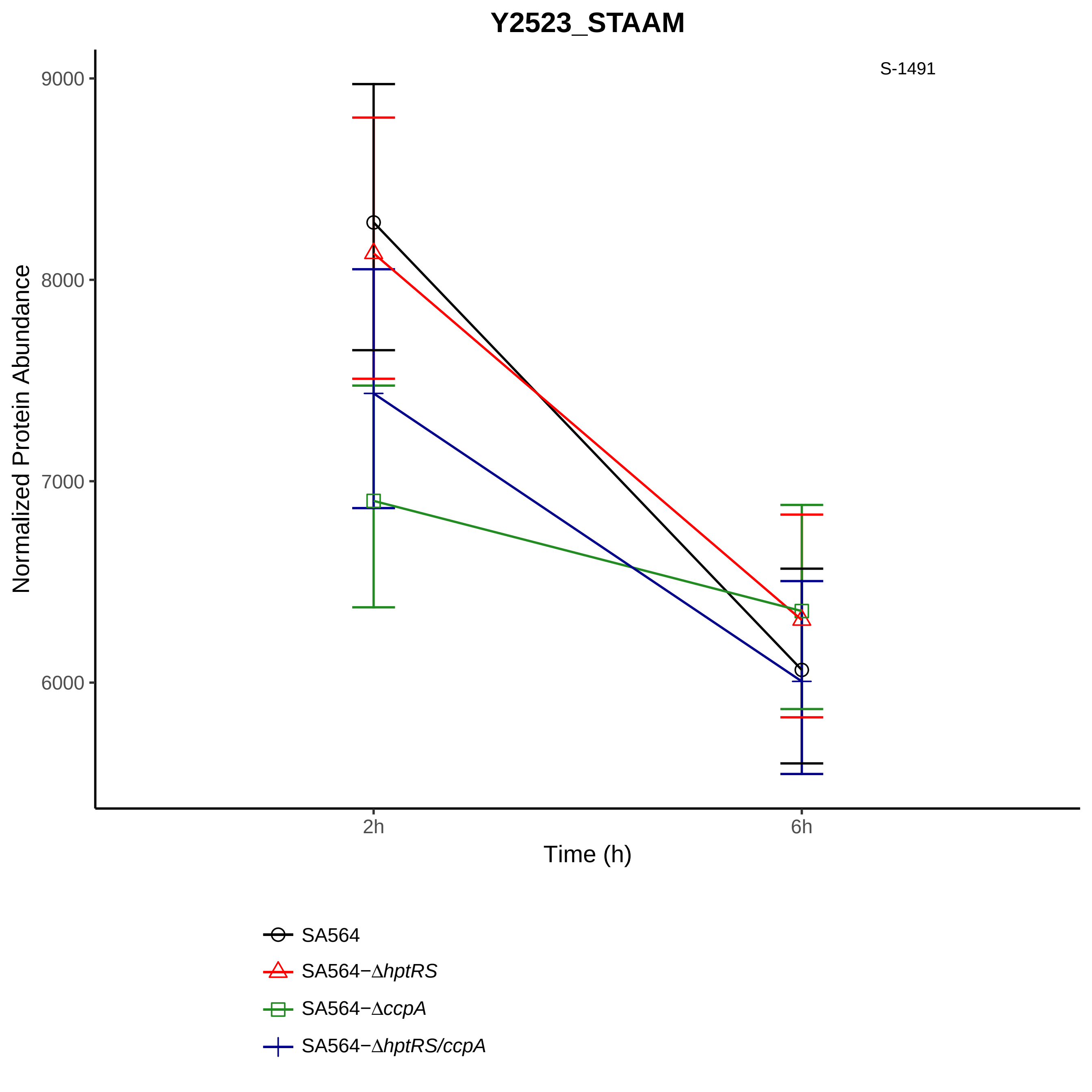

Y2523_STAAM
S-1491
Normalized Protein Abundance
9000
8000
7000
6000
2h
6h
Time (h)
SA564
SA564−ΔhptRS
SA564−ΔccpA
SA564−ΔhptRS/ccpA

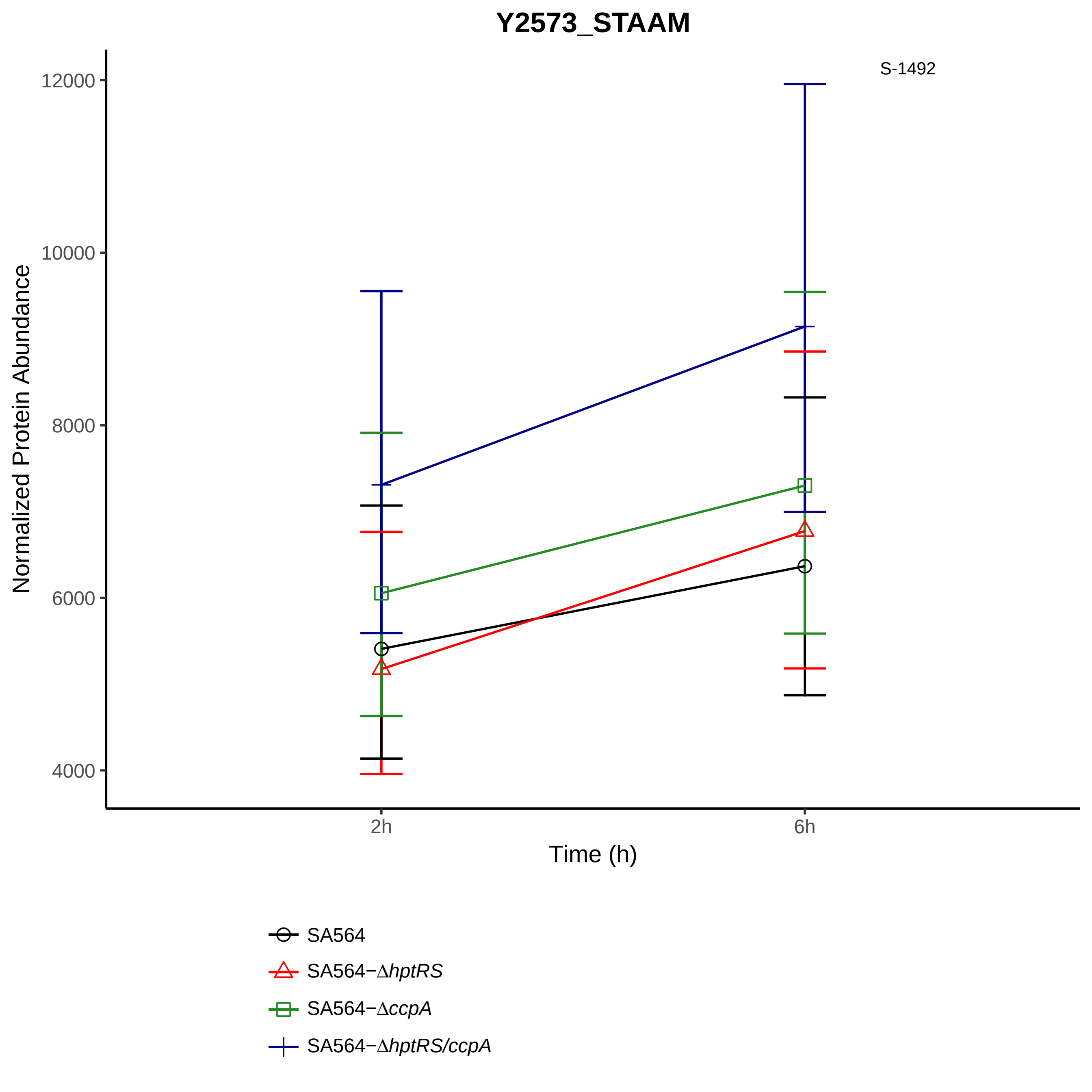

Y2573_STAAM
S-1492
Normalized Protein Abundance
12000
10000
8000
6000
4000
2h
6h
Time (h)
SA564
SA564−Δ*hptRS*
SA564−Δ*ccpA*
SA564−Δ*hptRS/ccpA*

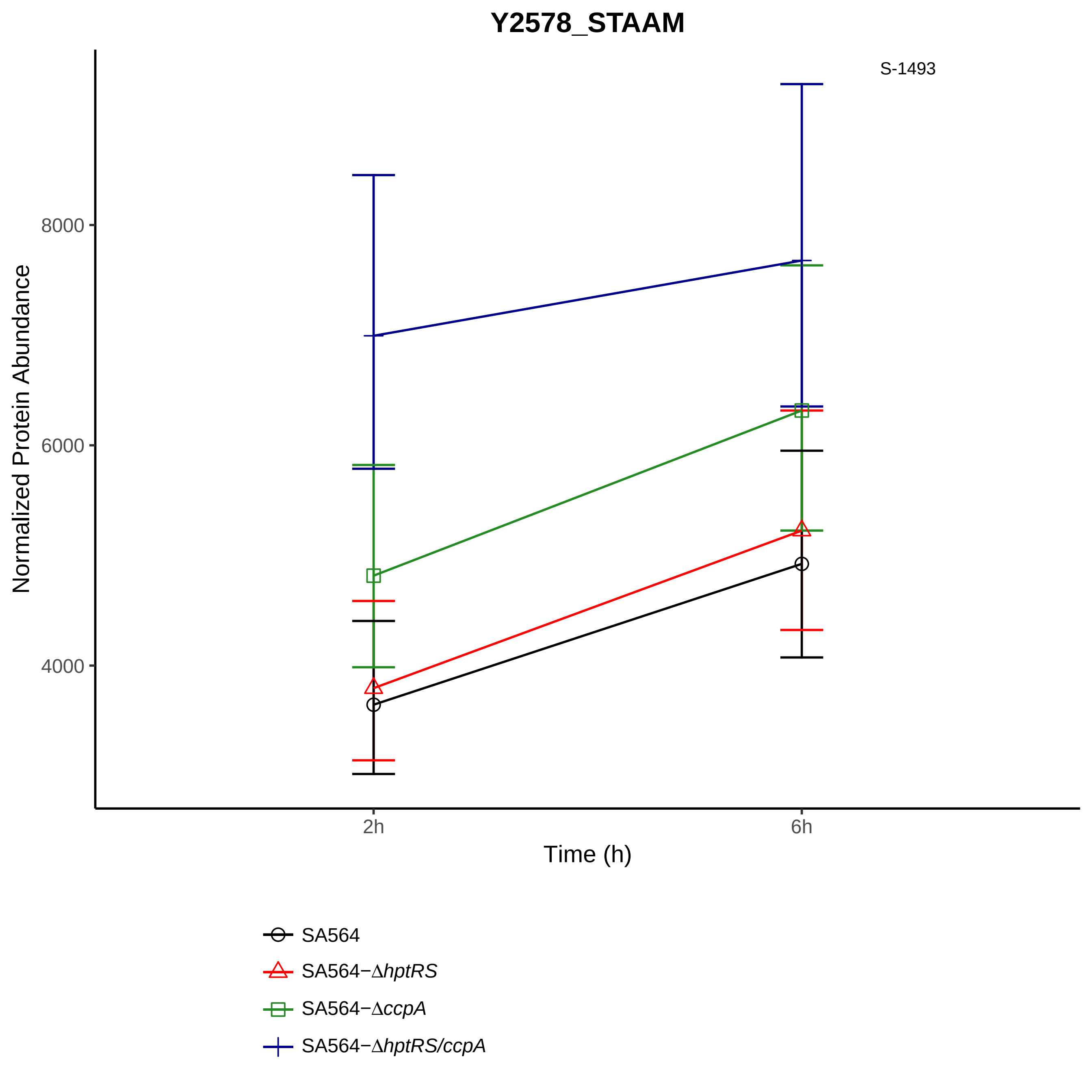

Y2578_STAAM
S-1493
Normalized Protein Abundance
8000
6000
4000
2h
6h
Time (h)
SA564
SA564−Δ*hptRS*
SA564−Δ*ccpA*
SA564−Δ*hptRS/ccpA*

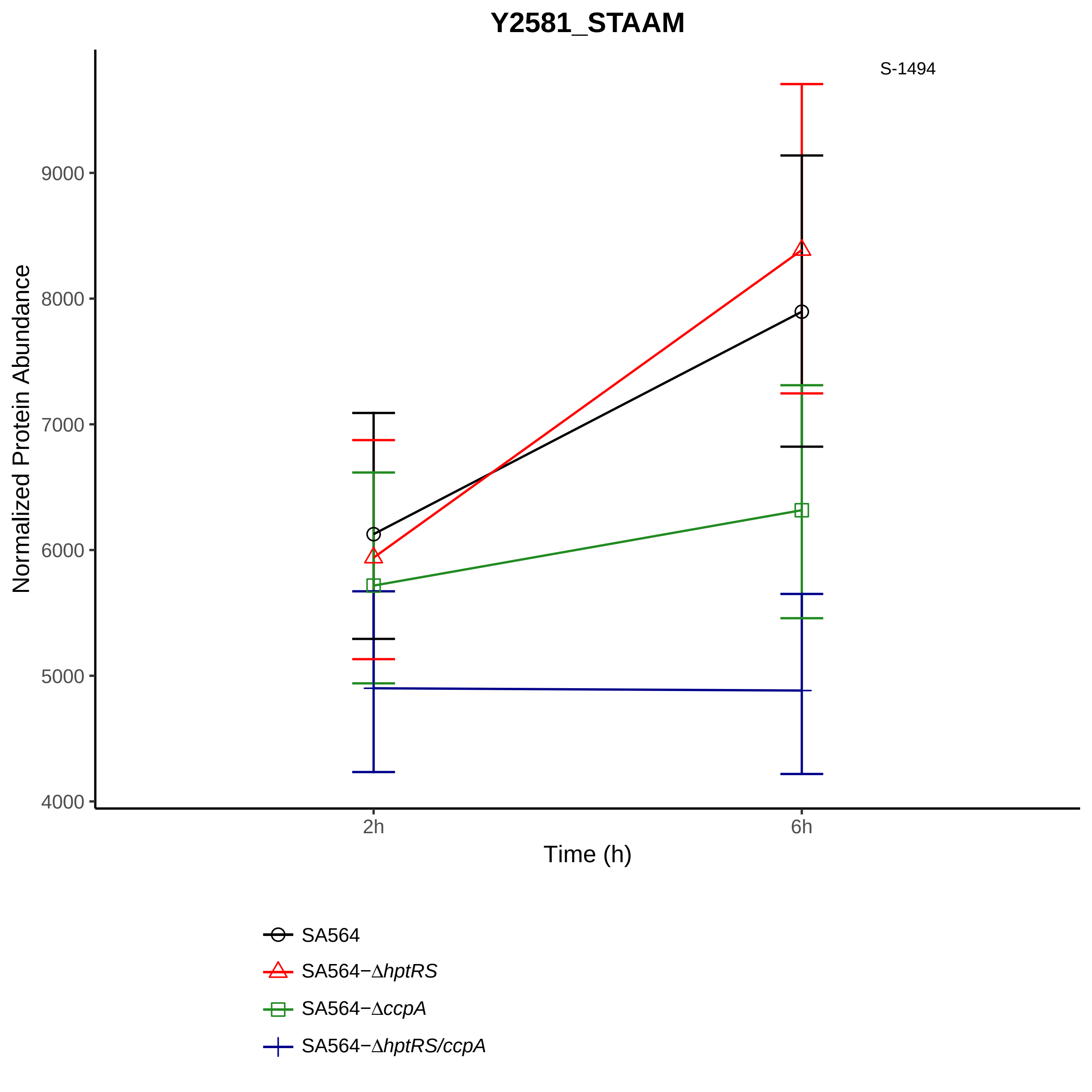

Y2581_STAAM
S-1494
Normalized Protein Abundance
9000
8000
7000
6000
5000
4000
2h
6h
Time (h)
SA564
SA564−Δ*hptRS*
SA564−Δ*ccpA*
SA564−Δ*hptRS/ccpA*

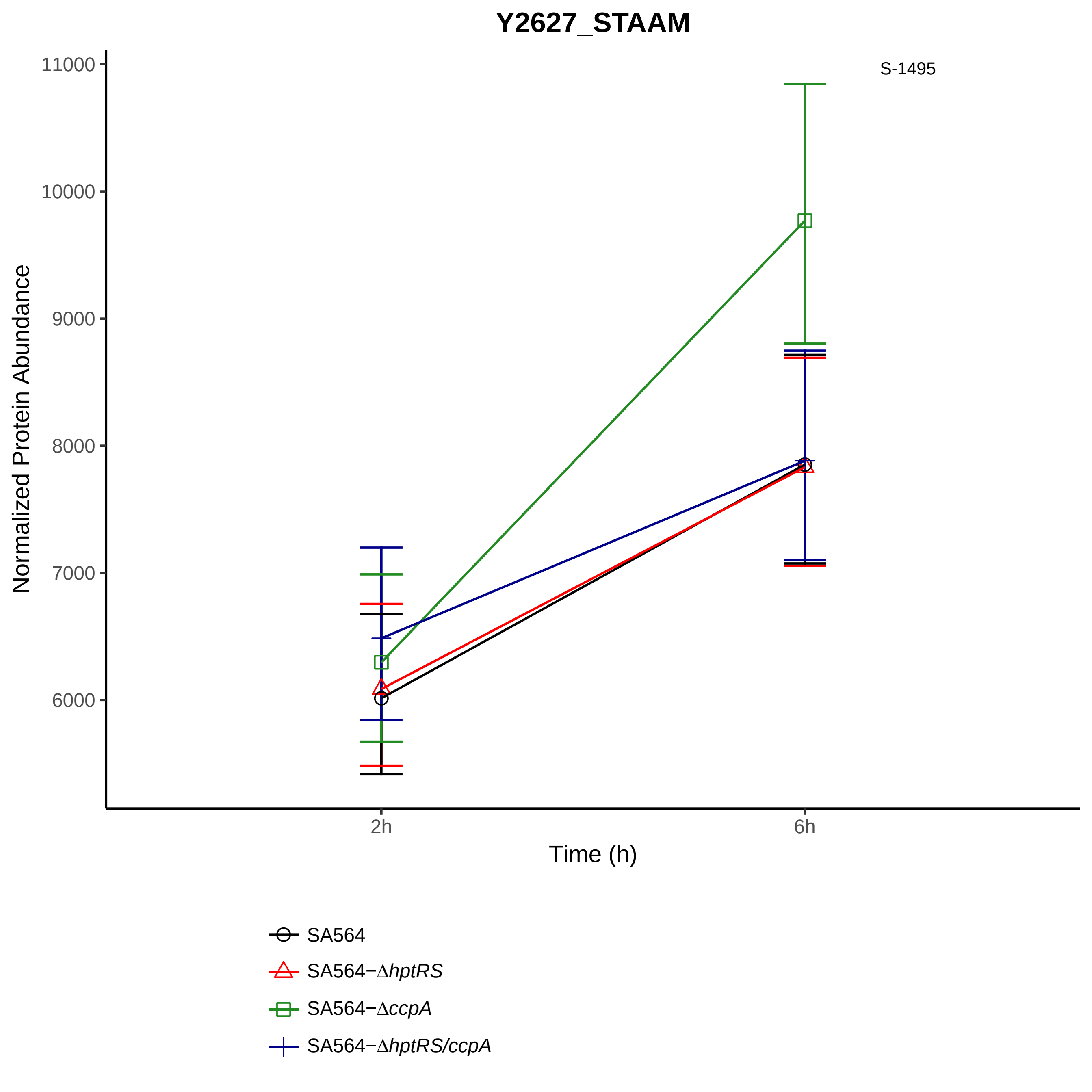

Y2627_STAAM
S-1495
11000
10000
9000
8000
7000
6000
Normalized Protein Abundance
2h
6h
Time (h)
SA564
SA564−Δ*hptRS*
SA564−Δ*ccpA*
SA564−Δ*hptRS/ccpA*

Y2687_STAAM

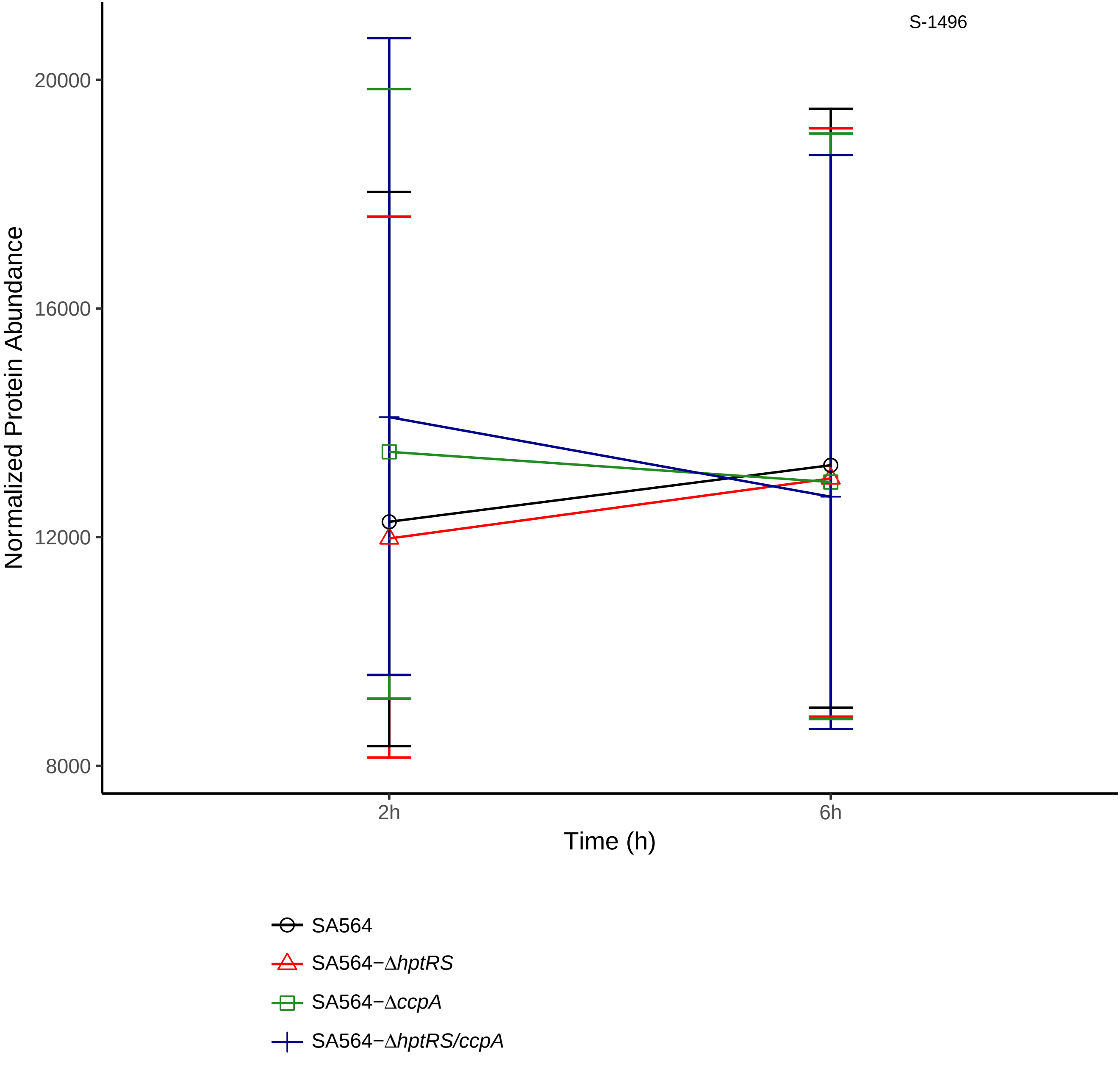

S-1496
Normalized Protein Abundance
20000
16000
12000
8000
2h
6h
Time (h)
SA564
SA564−Δ*hptRS*
SA564−Δ*ccpA*
SA564−Δ*hptRS/ccpA*

Y2689_STAAM

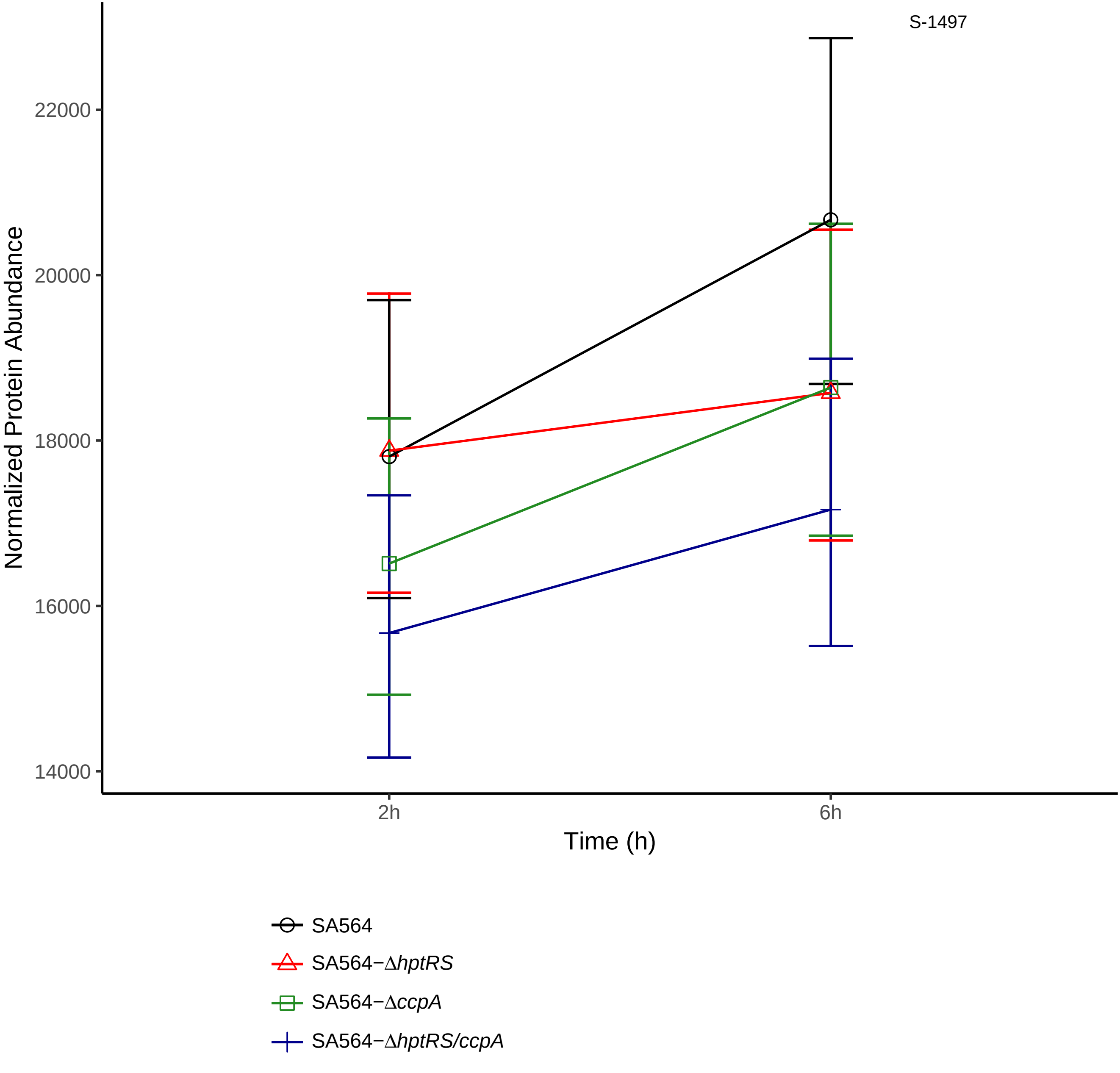

S-1497
Normalized Protein Abundance
22000
20000
18000
16000
14000
2h
6h
Time (h)
SA564
SA564−Δ*hptRS*
SA564−Δ*ccpA*
SA564−Δ*hptRS/ccpA*

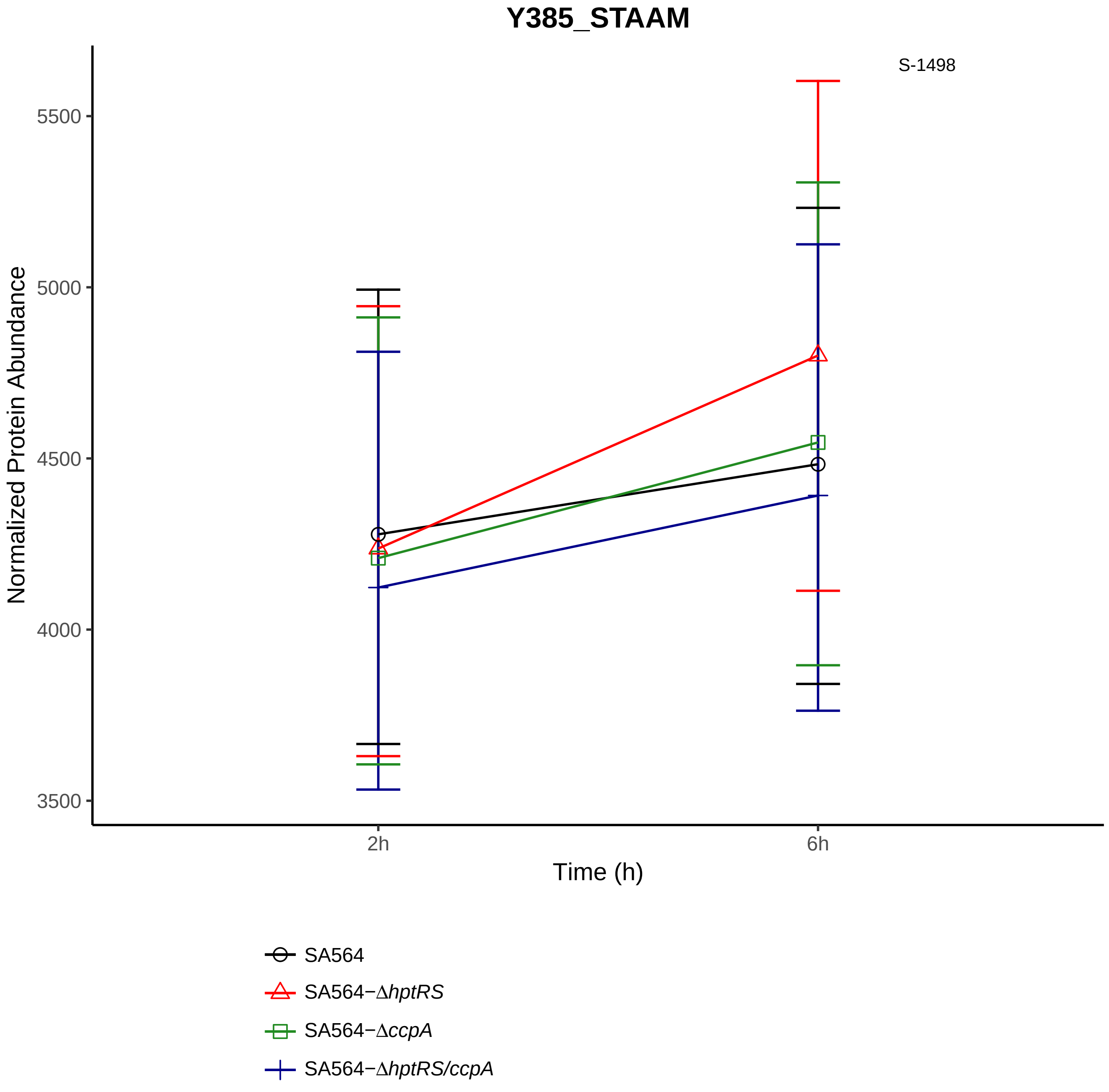

Y385_STAAM
S-1498
Normalized Protein Abundance
5500
5000
4500
4000
3500
2h
6h
Time (h)
SA564
SA564−Δ*hptRS*
SA564−Δ*ccpA*
SA564−Δ*hptRS/ccpA*

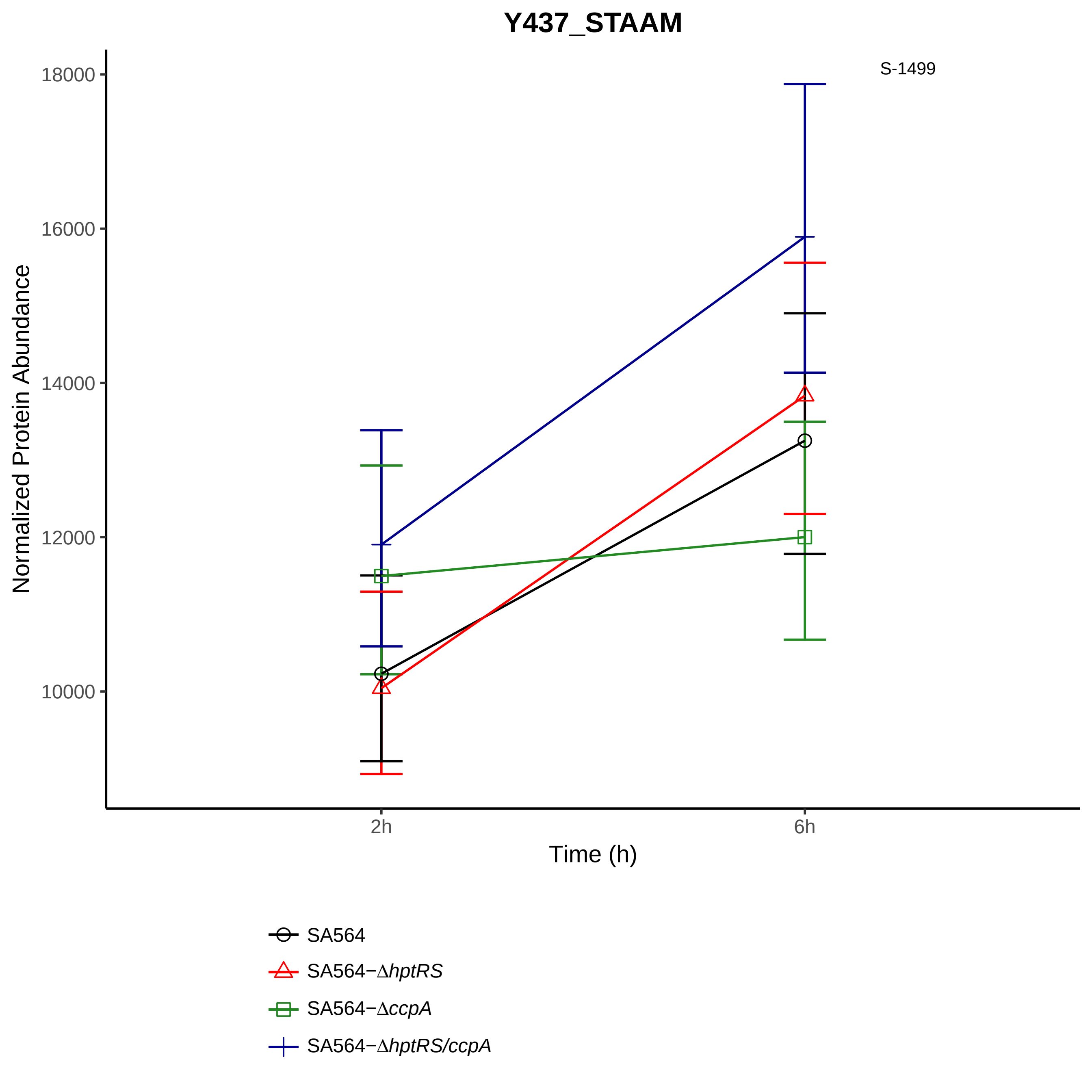

Y437_STAAM
S-1499
18000
16000
14000
12000
10000
Normalized Protein Abundance
2h
6h
Time (h)
SA564
SA564−Δ*hptRS*
SA564−Δ*ccpA*
SA564−Δ*hptRS/ccpA*

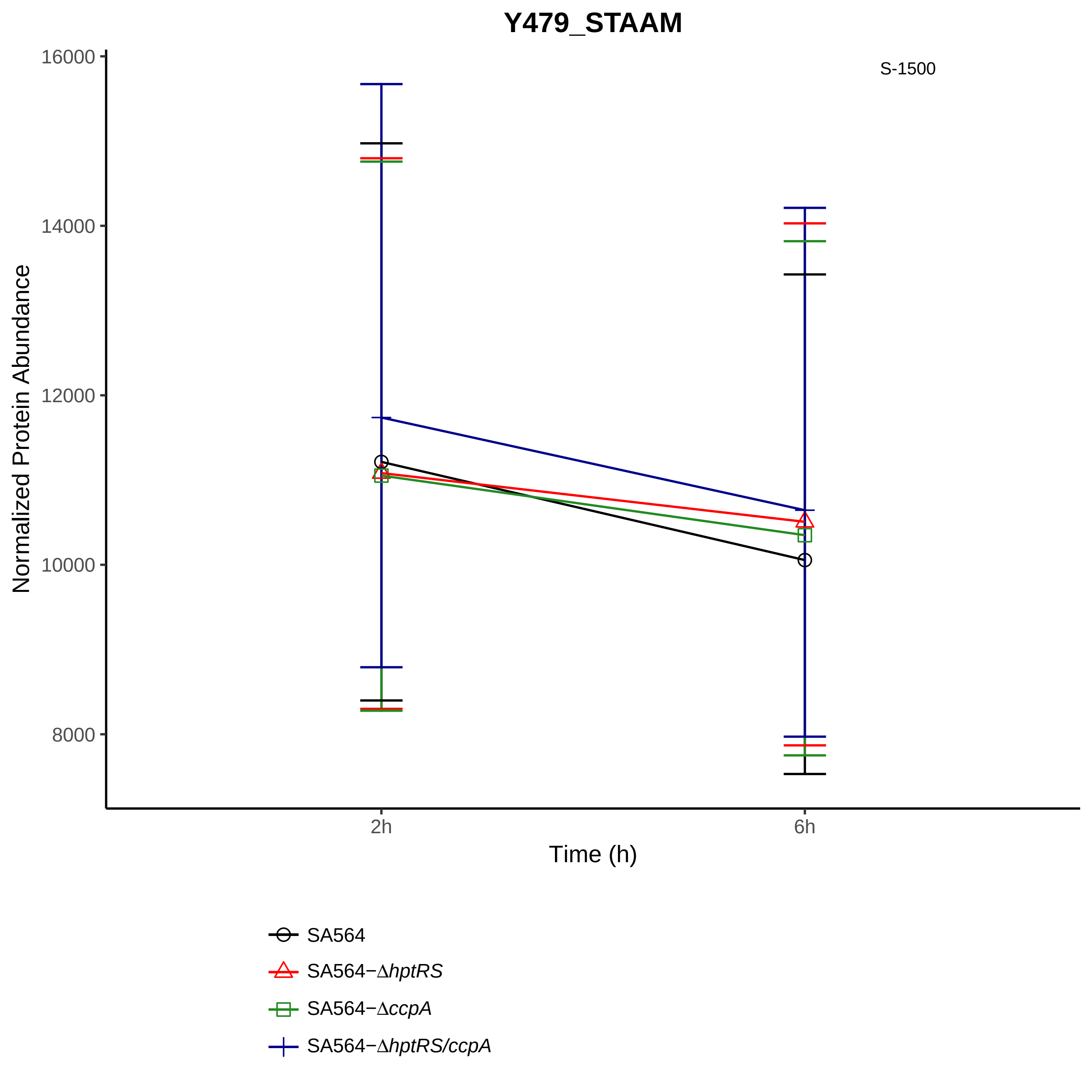
Y479_STAAM
S-1500
16000
14000
12000
10000
8000
Normalized Protein Abundance
2h
6h
Time (h)
SA564
SA564−Δ*hptRS*
SA564−Δ*ccpA*
SA564−Δ*hptRS/ccpA*

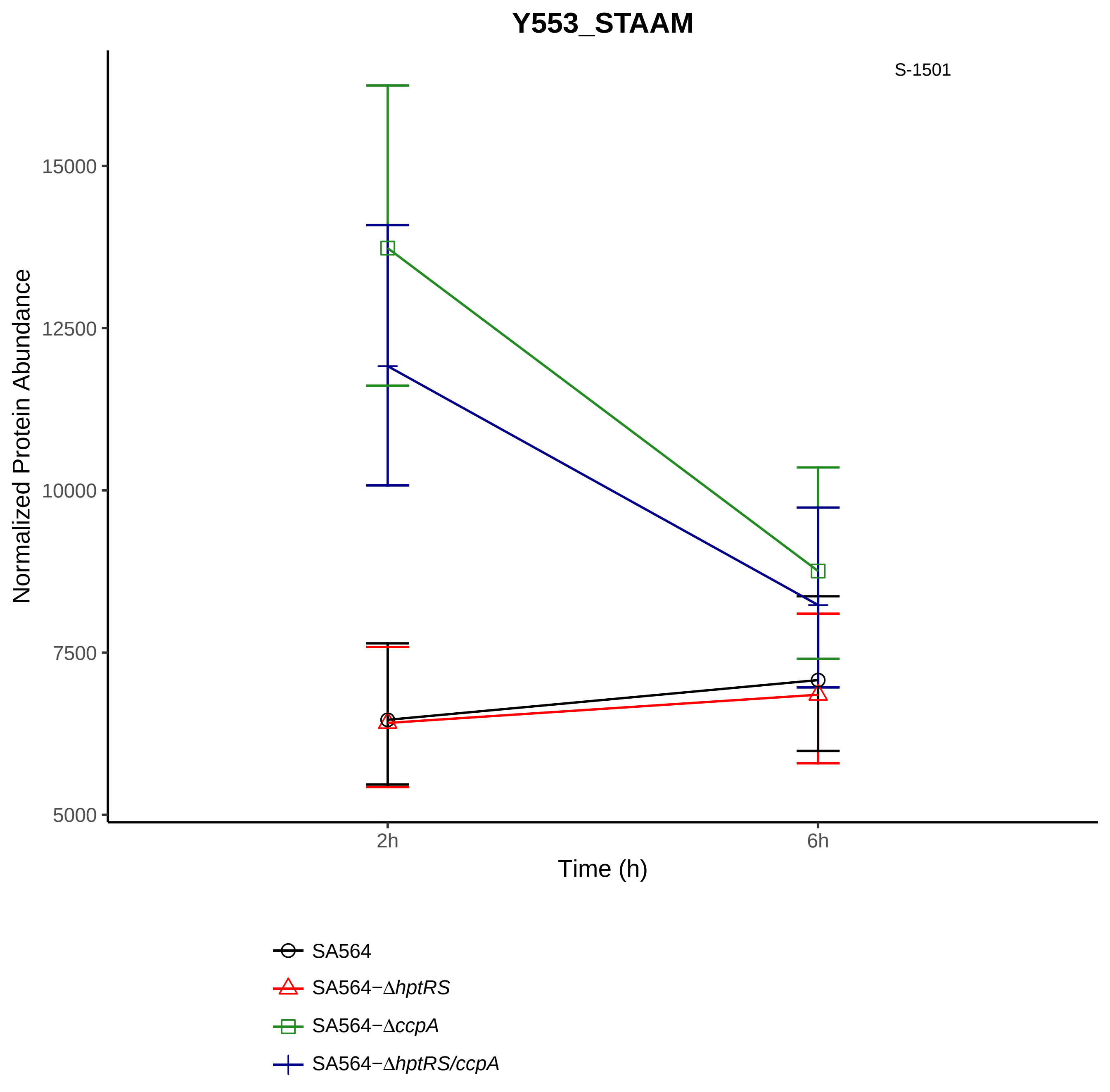

Y553_STAAM
S-1501
Normalized Protein Abundance
15000
12500
10000
7500
5000
2h
6h
Time (h)
SA564
SA564−Δ*hptRS*
SA564−Δ*ccpA*
SA564−Δ*hptRS/ccpA*

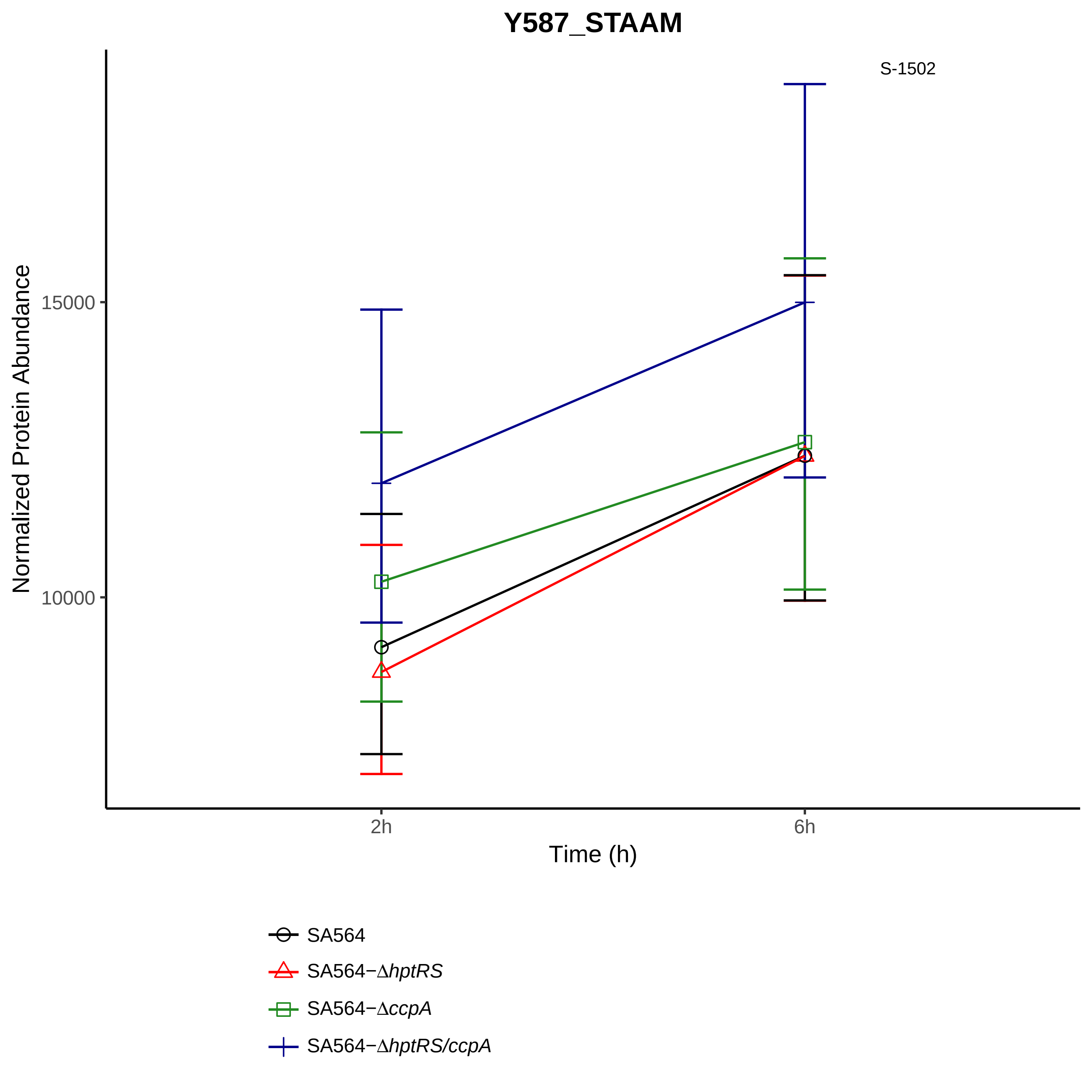
Y587_STAAM
S-1502
Normalized Protein Abundance
15000
10000
2h
6h
Time (h)
SA564
SA564−ΔhptRS
SA564−ΔccpA
SA564−ΔhptRS/ccpA

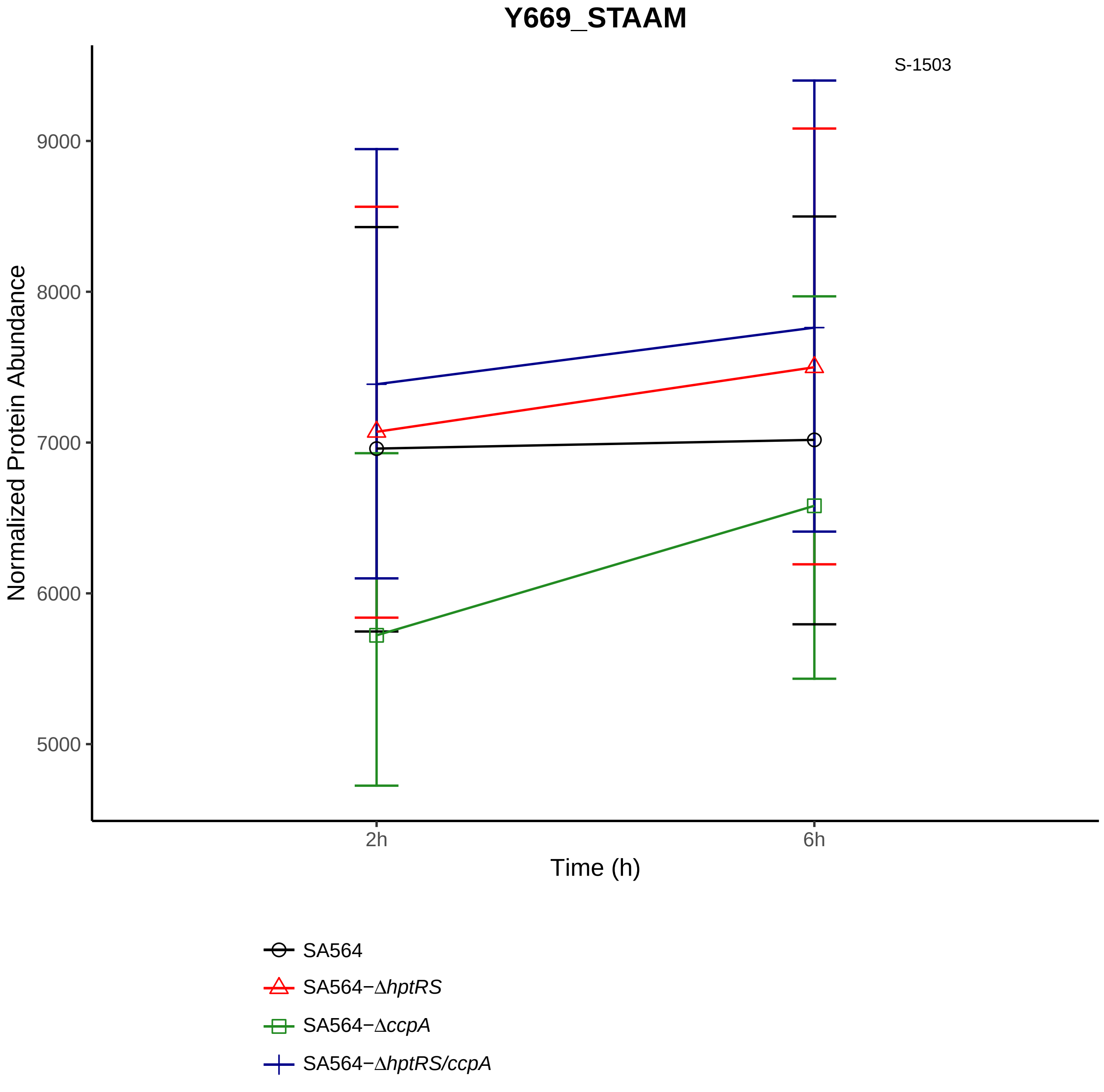

Y669_STAAM
S-1503
Normalized Protein Abundance
9000
8000
7000
6000
5000
2h
6h
Time (h)
SA564
SA564−Δ*hptRS*
SA564−Δ*ccpA*
SA564−Δ*hptRS/ccpA*

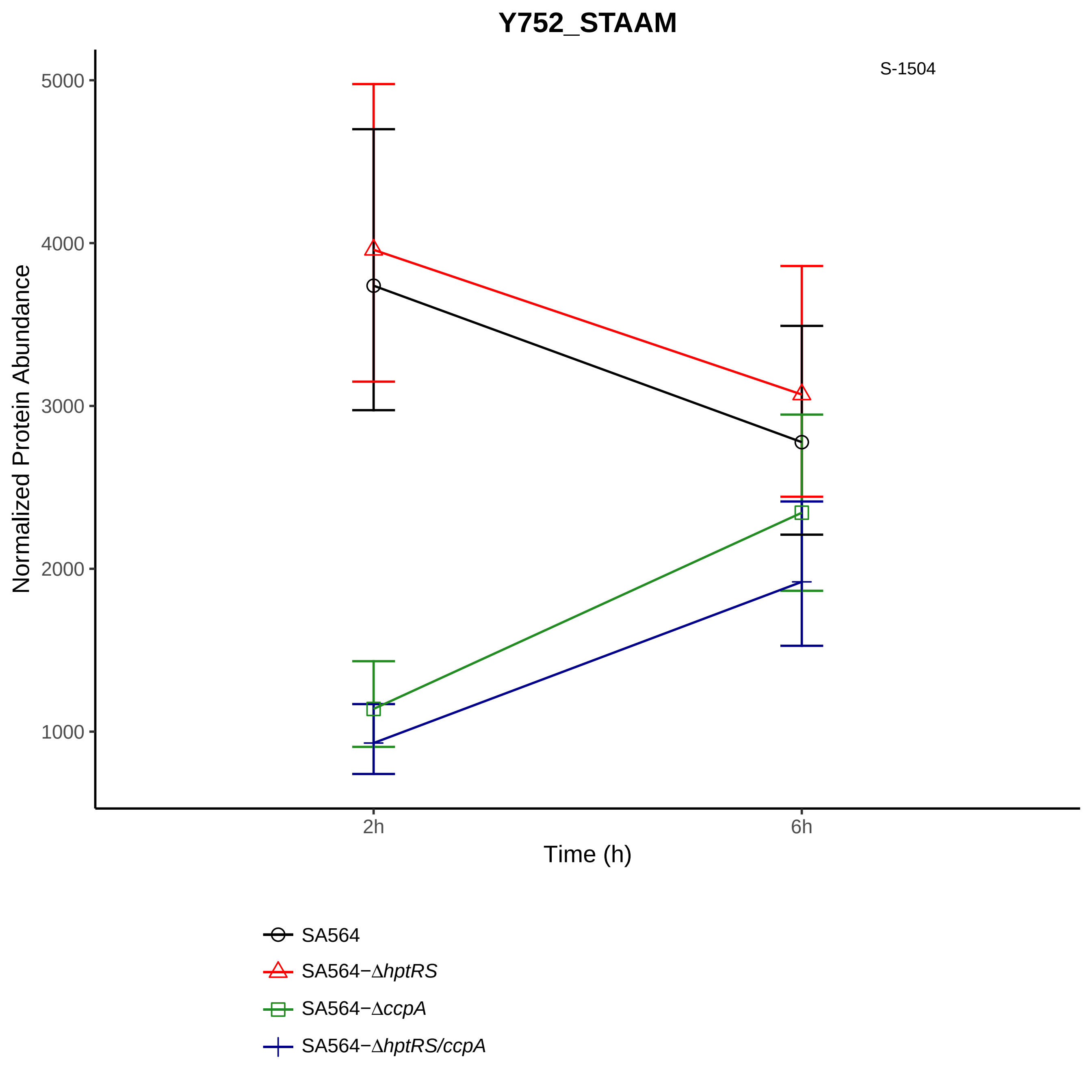
Y752_STAAM
S-1504
Normalized Protein Abundance
5000
4000
3000
2000
1000
2h
6h
Time (h)
SA564
SA564−Δ*hptRS*
SA564−Δ*ccpA*
SA564−Δ*hptRS/ccpA*

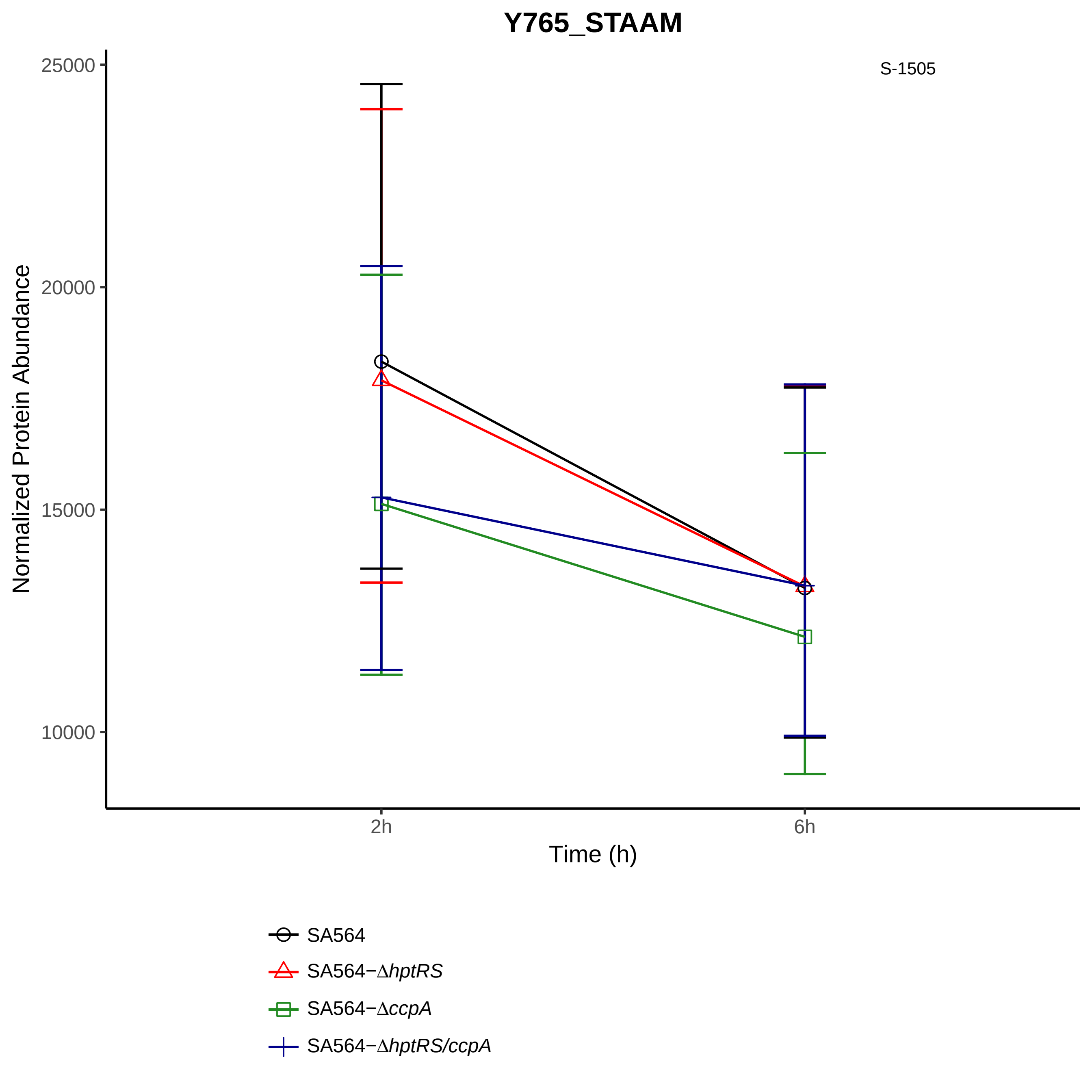

Y765_STAAM
S-1505
25000
20000
15000
10000
Normalized Protein Abundance
2h
6h
Time (h)
SA564
SA564−ΔhptRS
SA564−ΔccpA
SA564−ΔhptRS/ccpA

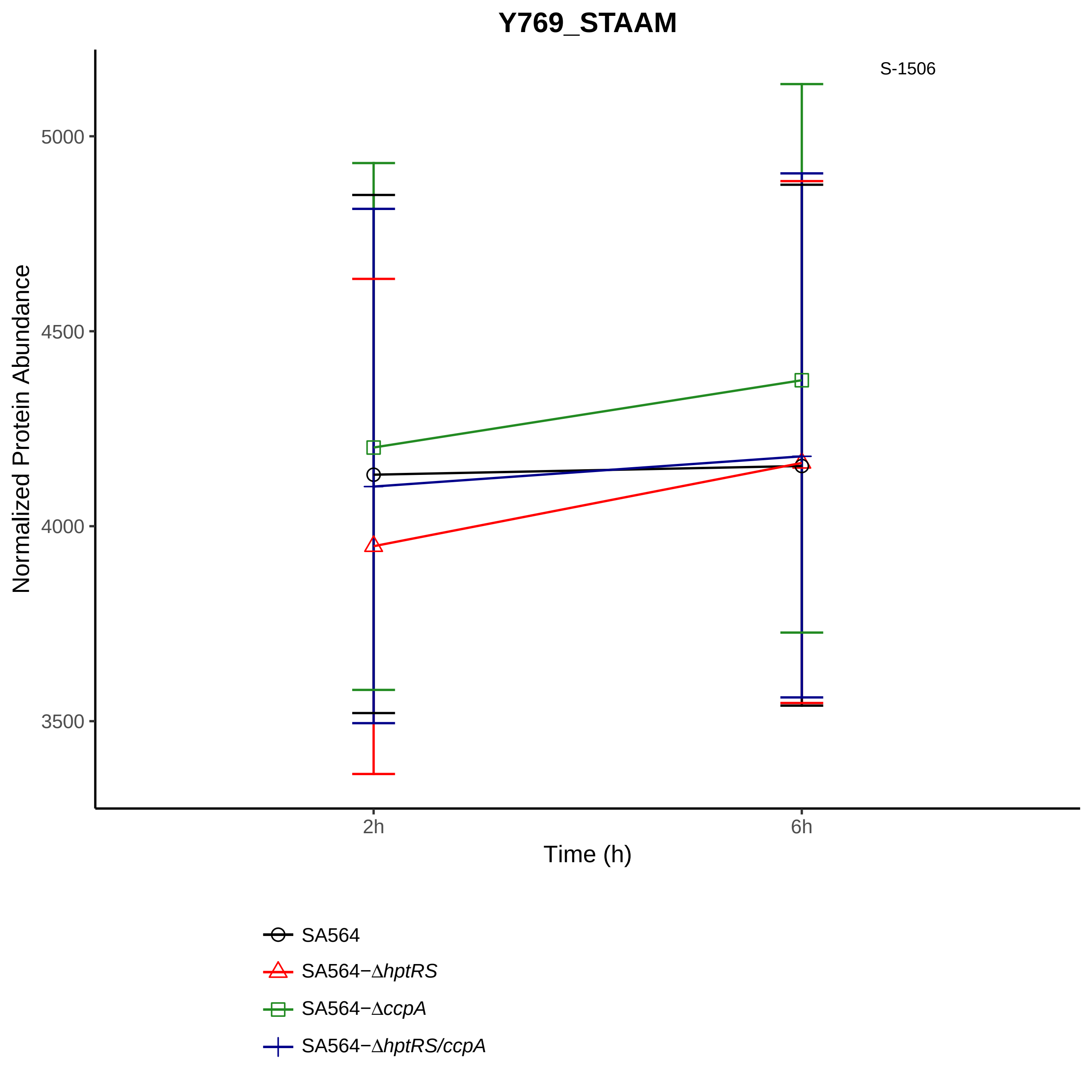

Y769_STAAM
S-1506
Normalized Protein Abundance
5000
4500
4000
3500
2h
6h
Time (h)
SA564
SA564−Δ*hptRS*
SA564−Δ*ccpA*
SA564−Δ*hptRS/ccpA*

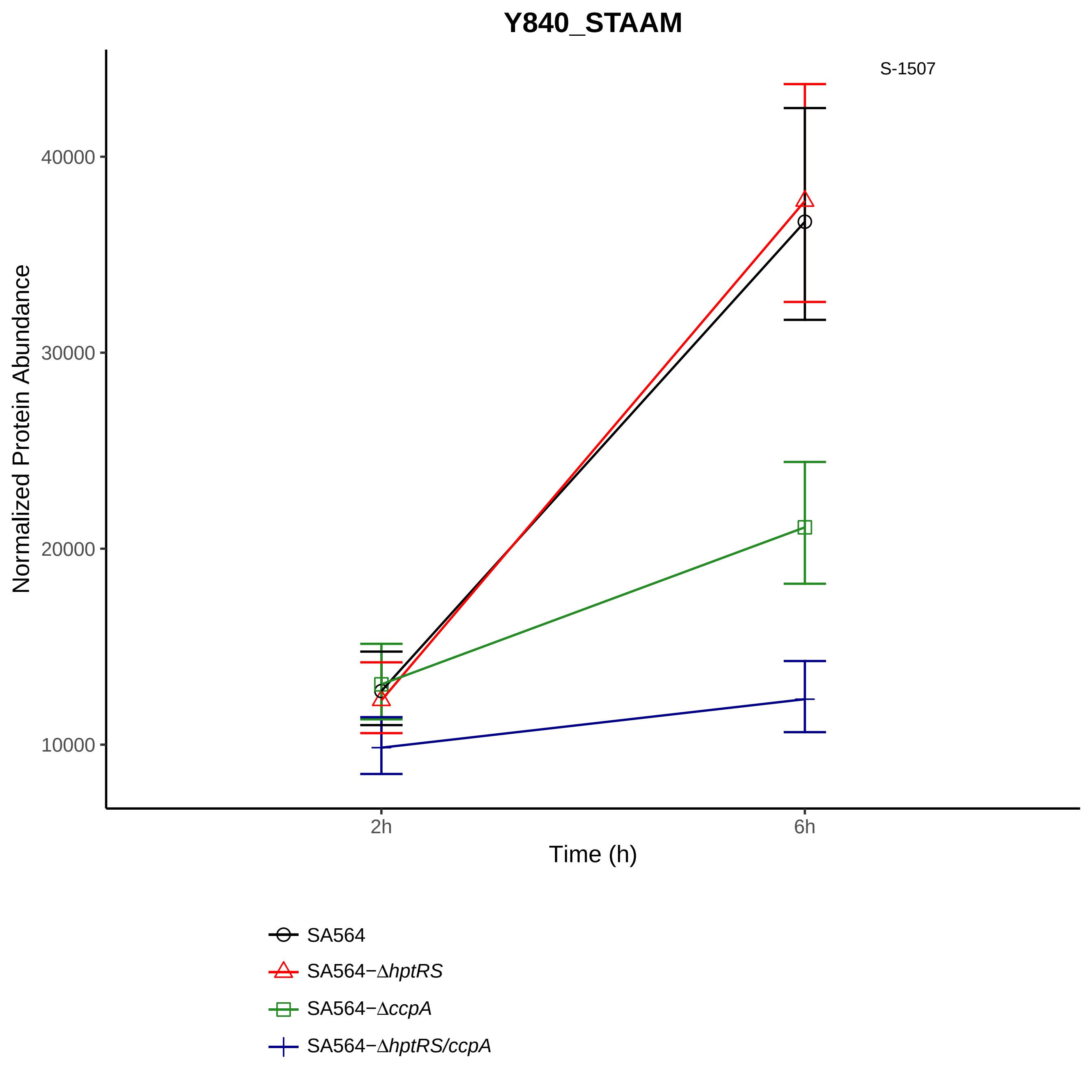

Y840_STAAM
S-1507
Normalized Protein Abundance
40000
30000
20000
10000
2h
6h
Time (h)
SA564
SA564−Δ*hptRS*
SA564−Δ*ccpA*
SA564−Δ*hptRS/ccpA*

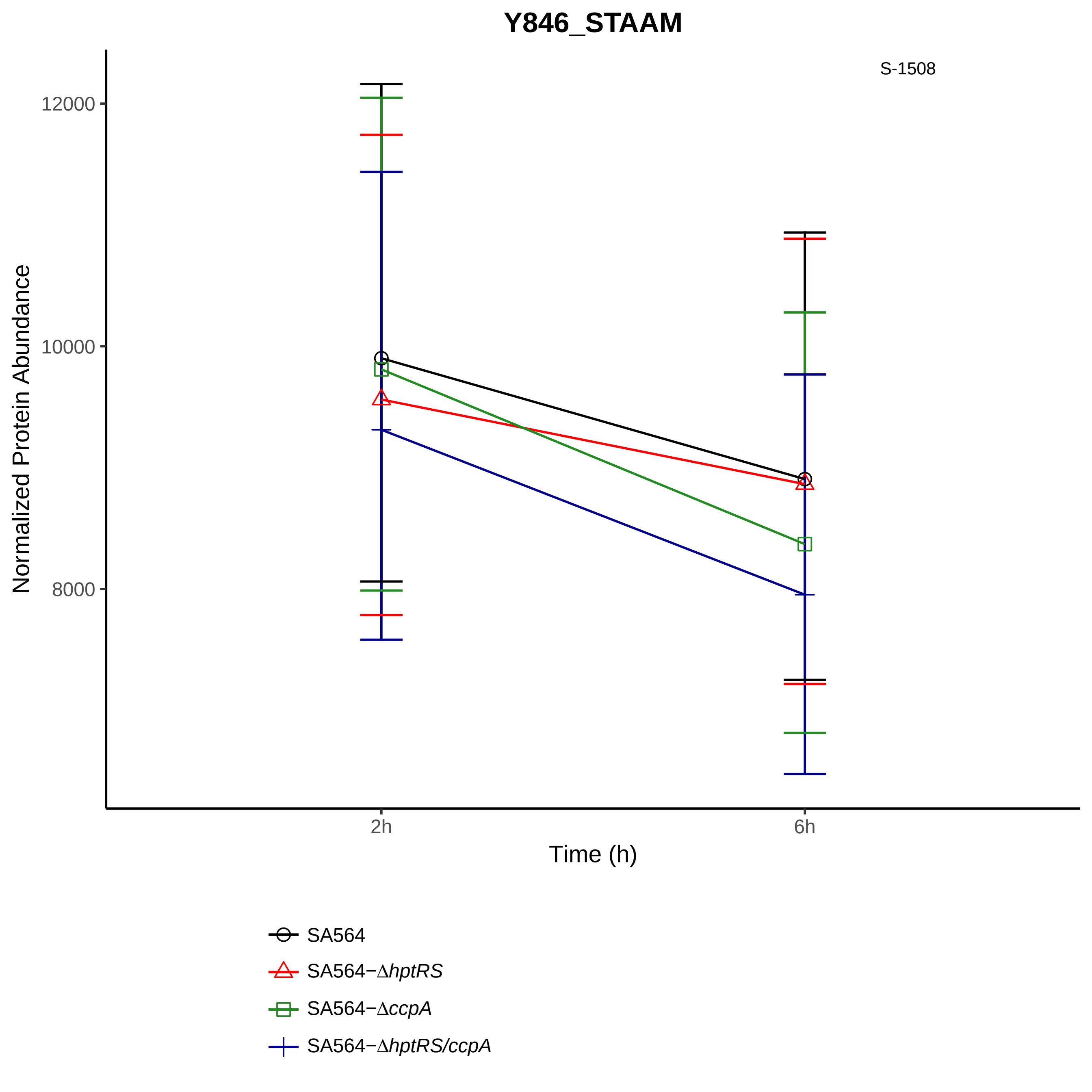

Y846_STAAM
S-1508
Normalized Protein Abundance
12000
10000
8000
2h
6h
Time (h)
SA564
SA564−Δ*hptRS*
SA564−Δ*ccpA*
SA564−Δ*hptRS/ccpA*

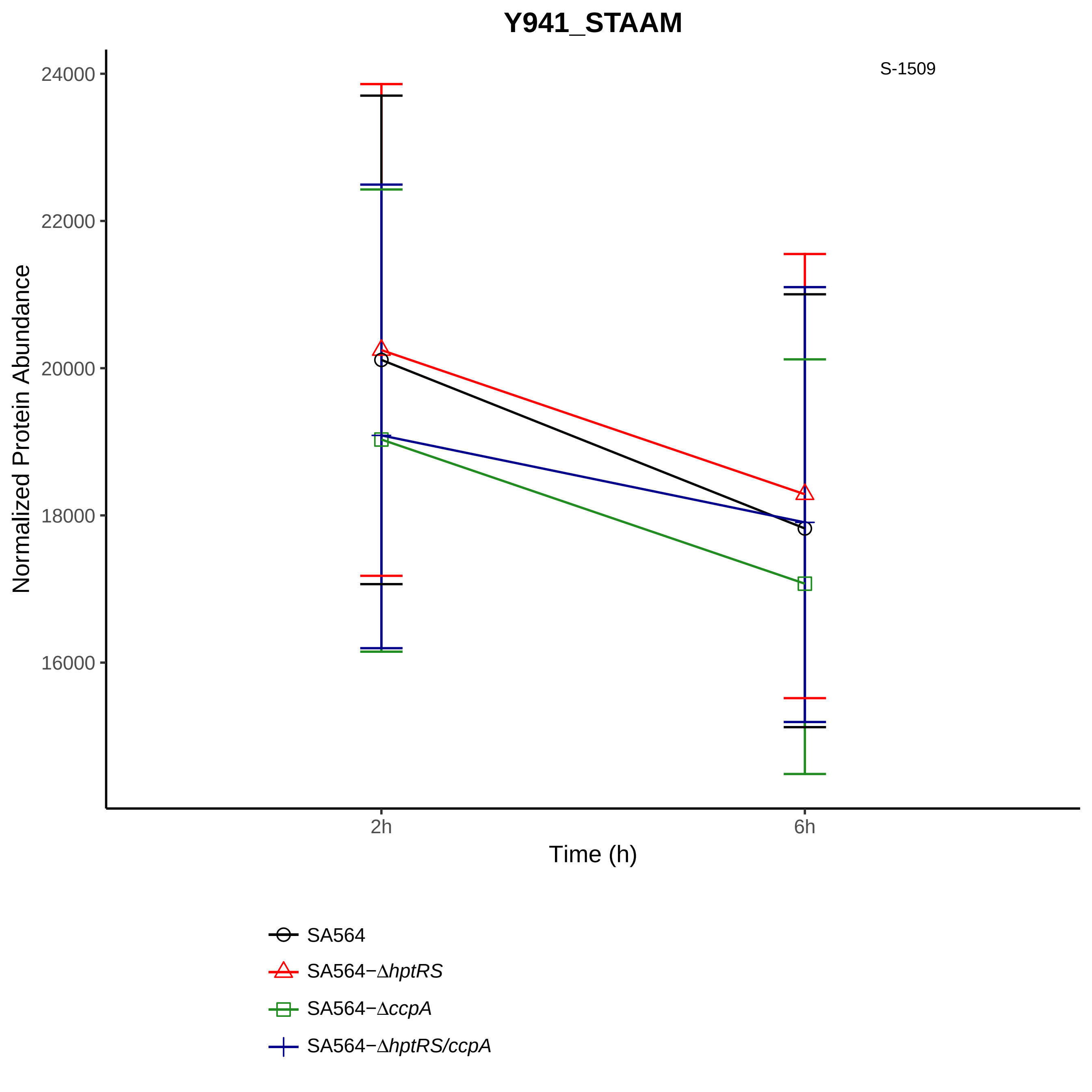

Y941_STAAM
S-1509
Normalized Protein Abundance
24000
22000
20000
18000
16000
2h
6h
Time (h)
SA564
SA564−Δ*hptRS*
SA564−Δ*ccpA*
SA564−Δ*hptRS/ccpA*

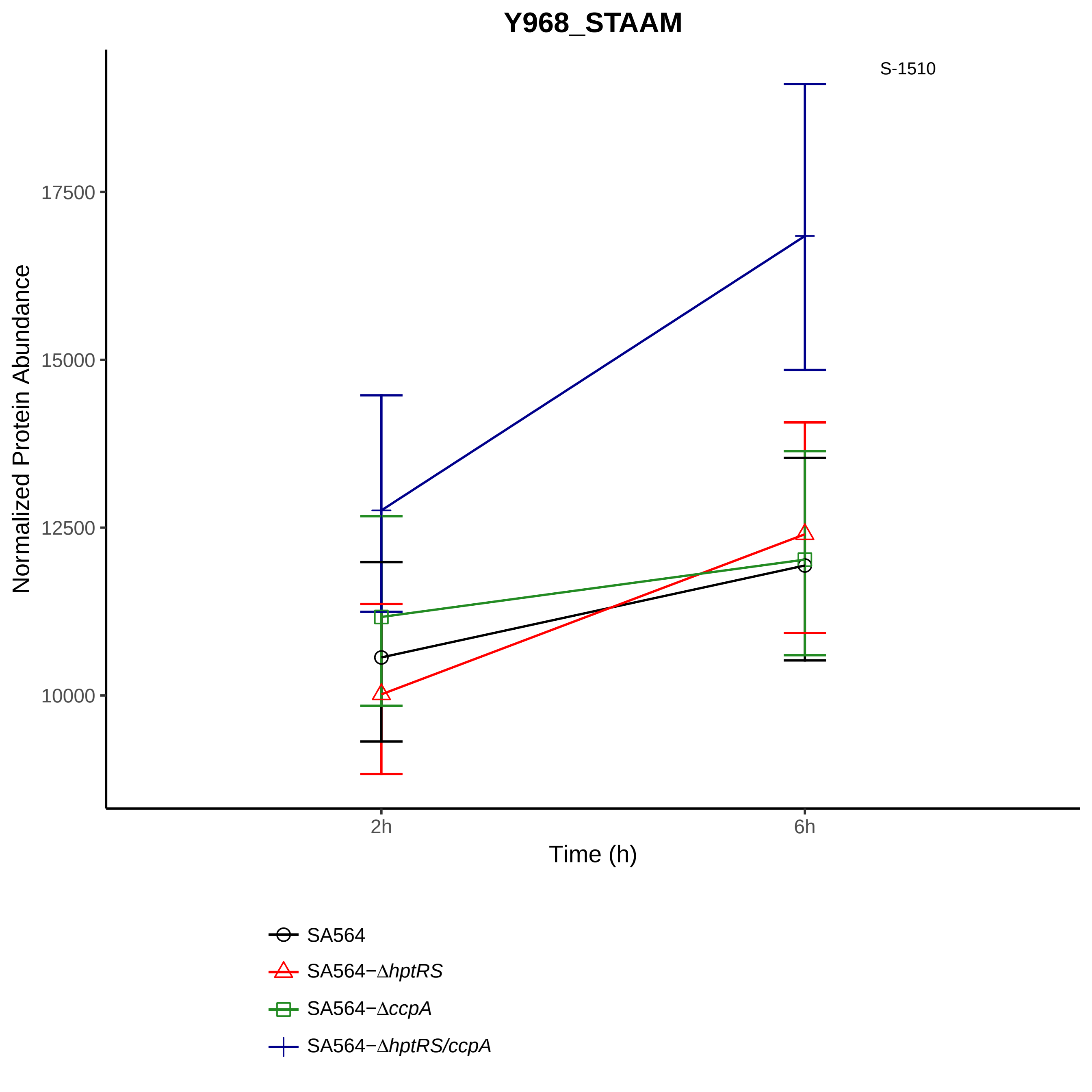
Y968_STAAM
S-1510
Normalized Protein Abundance
17500
15000
12500
10000
2h
6h
Time (h)
SA564
SA564−Δ*hptRS*
SA564−Δ*ccpA*
SA564−Δ*hptRS/ccpA*

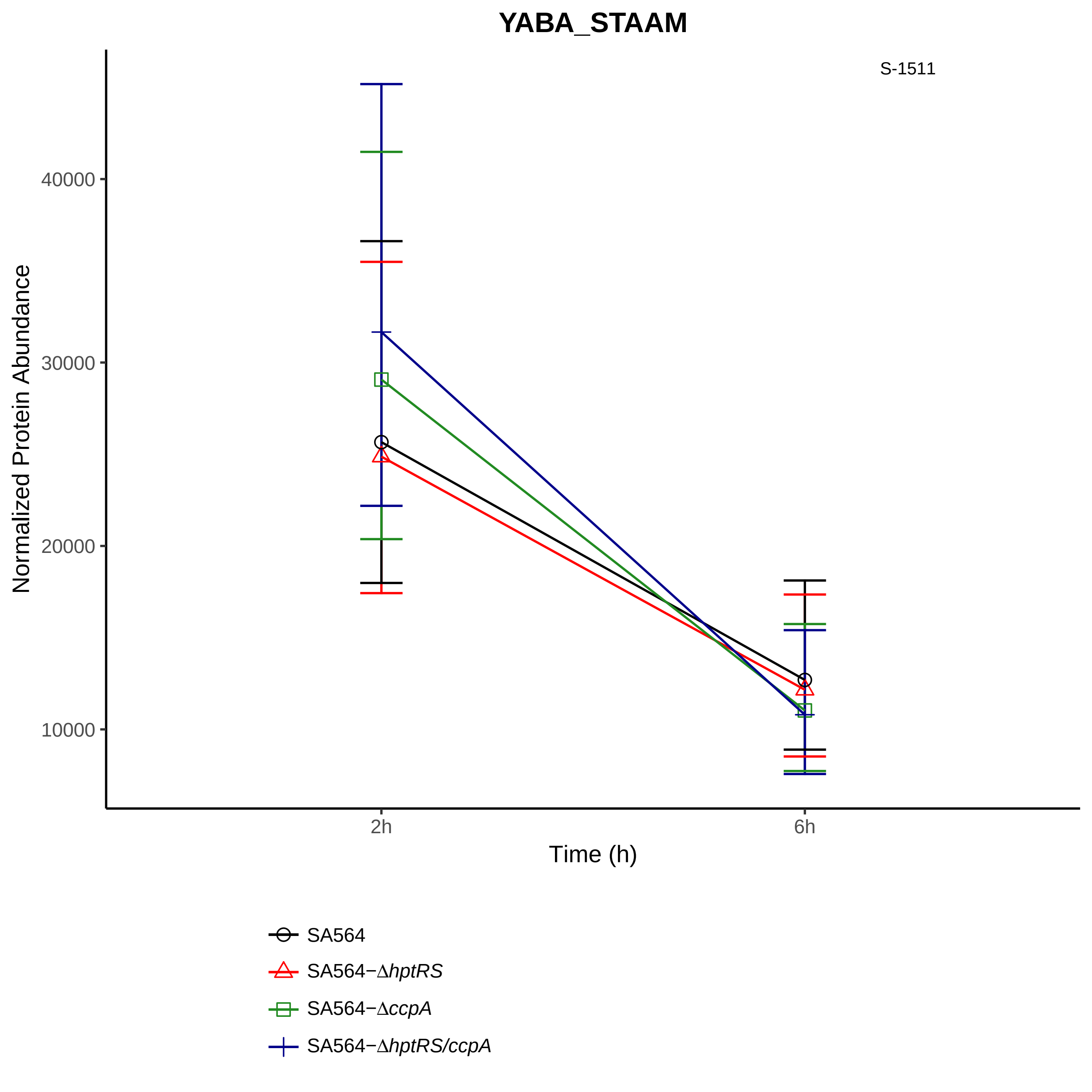

YABA_STAAM
S-1511
Normalized Protein Abundance
40000
30000
20000
10000
2h
6h
Time (h)
SA564
SA564−Δ*hptRS*
SA564−Δ*ccpA*
SA564−Δ*hptRS/ccpA*

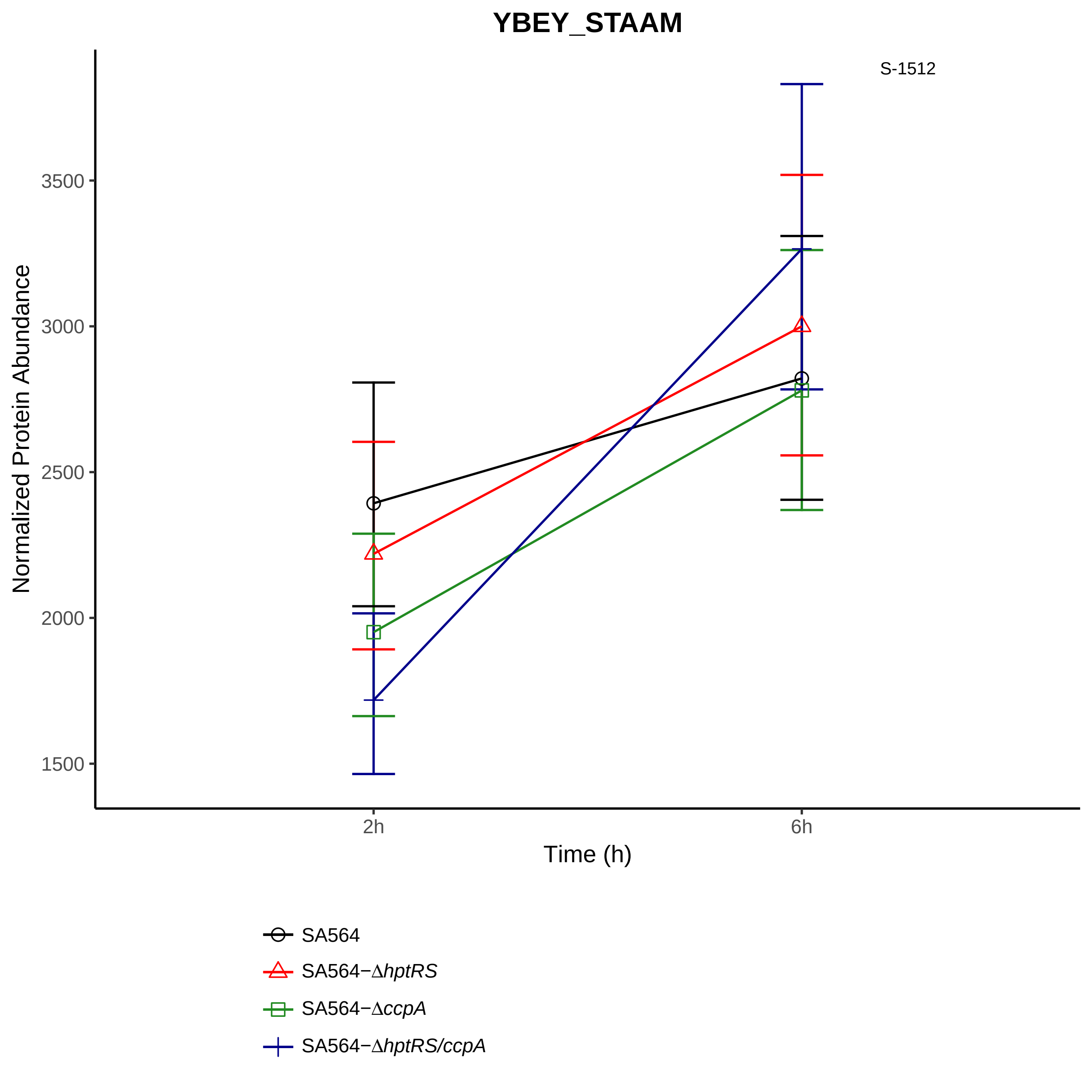

YBEY_STAAM
S-1512
Normalized Protein Abundance
3500
3000
2500
2000
1500
2h
6h
Time (h)
SA564
SA564−Δ*hptRS*
SA564−Δ*ccpA*
SA564−Δ*hptRS/ccpA*

Figure I.

2-way analysis: m x h | s

Pages: S1513-S2014

The normalized protein abundance, lower 95% confidence interval, and upper 95% confidence interval were plotted for each protein. These plots were analyzed if a 3-way interaction was rejected and 2-way interaction was identified between media and hour.

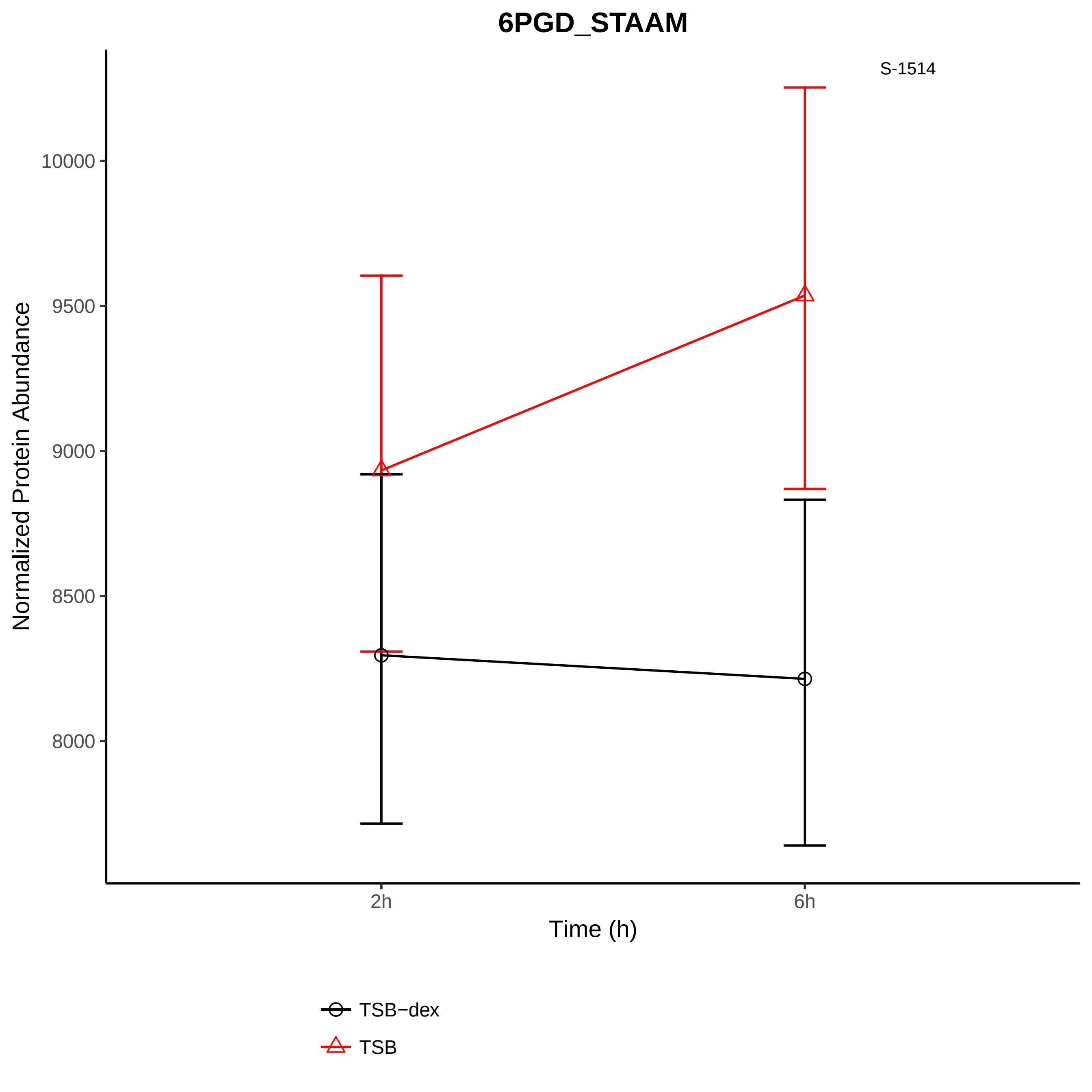

6PGD_STAAM
S-1514
10000
9500
9000
8500
8000
Normalized Protein Abundance
2h
6h
Time (h)
TSB−dex
TSB

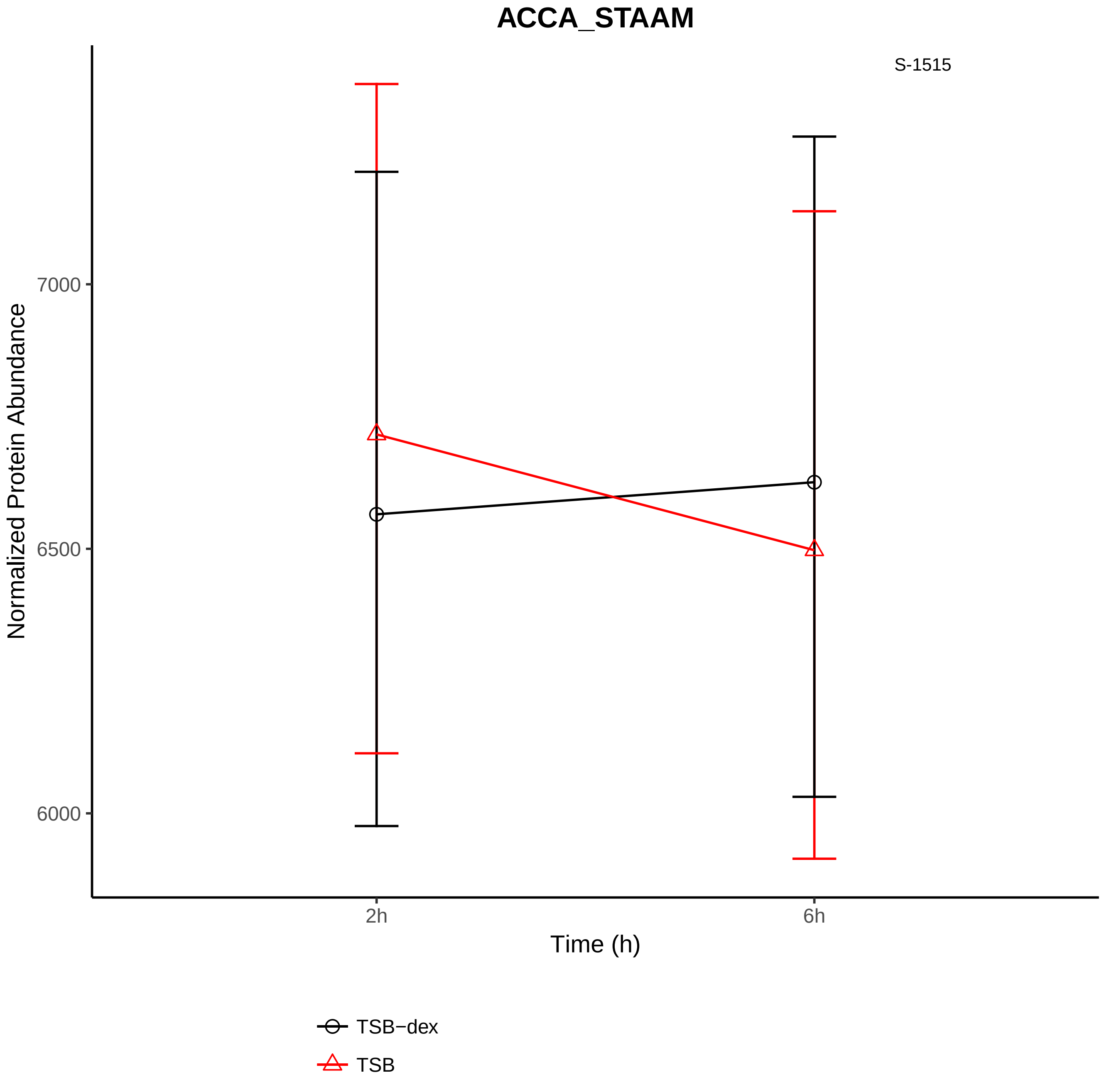
ACCA_STAAM
S-1515
Normalized Protein Abundance
7000
6500
6000
2h
6h
Time (h)
TSB−dex
TSB

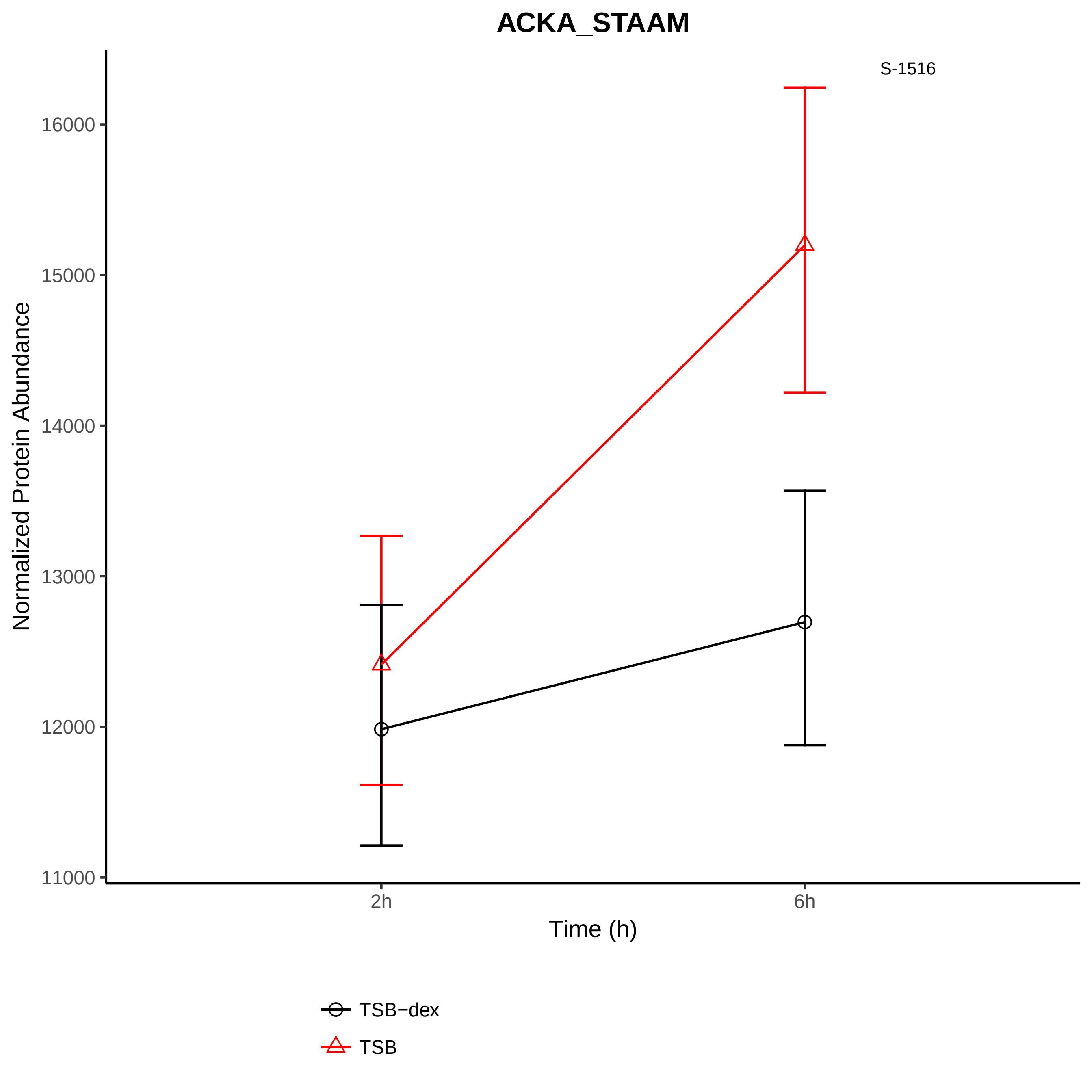

ACKA_STAAM
S-1516
16000
15000
14000
13000
12000
11000
Normalized Protein Abundance
2h
6h
Time (h)
TSB−dex
TSB

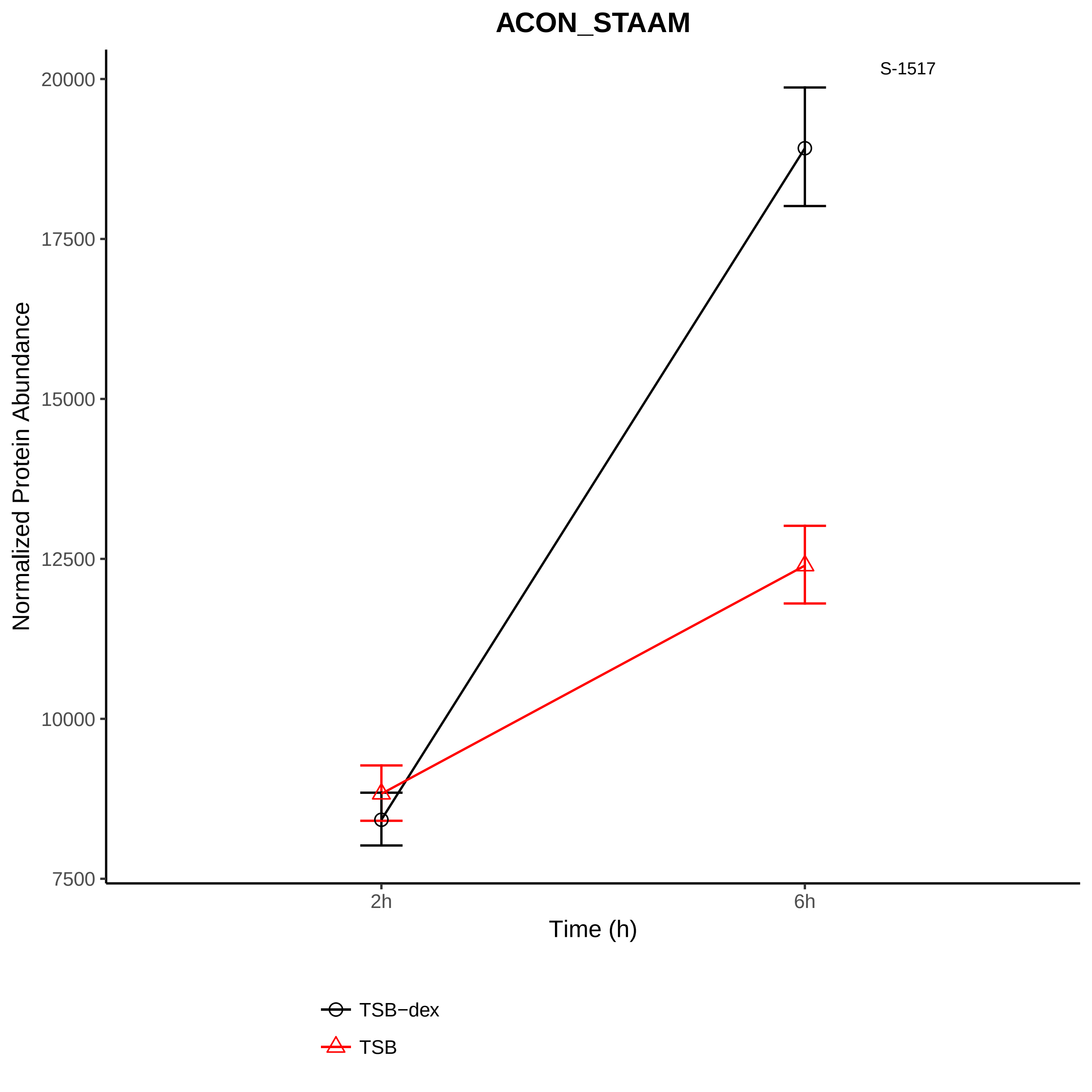

ACON_STAAM
S-1517
20000
17500
15000
12500
10000
7500
Normalized Protein Abundance
2h
6h
Time (h)
TSB−dex
TSB

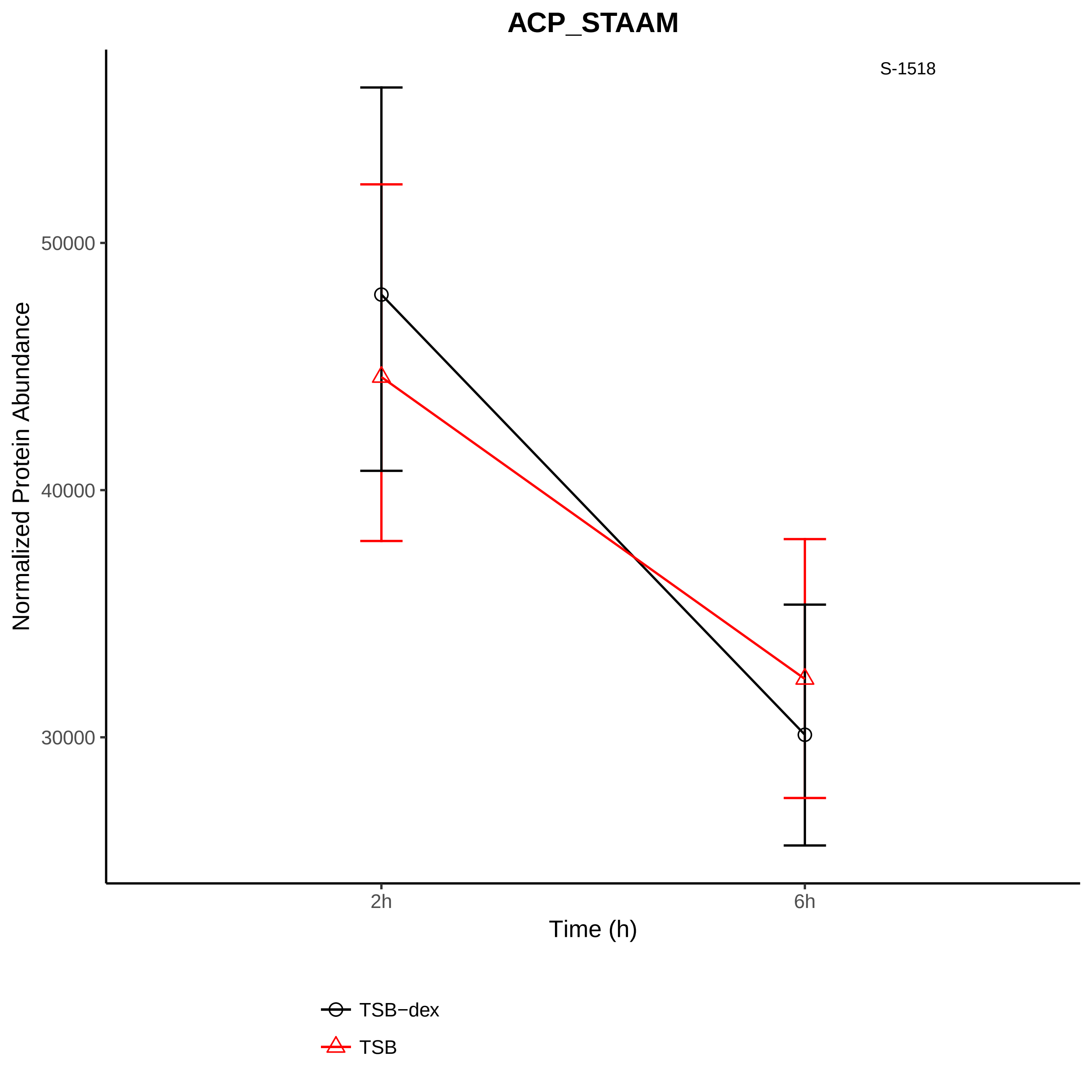

ACP_STAAM
S-1518
50000
40000
30000
Normalized Protein Abundance
2h
6h
Time (h)
TSB−dex
TSB

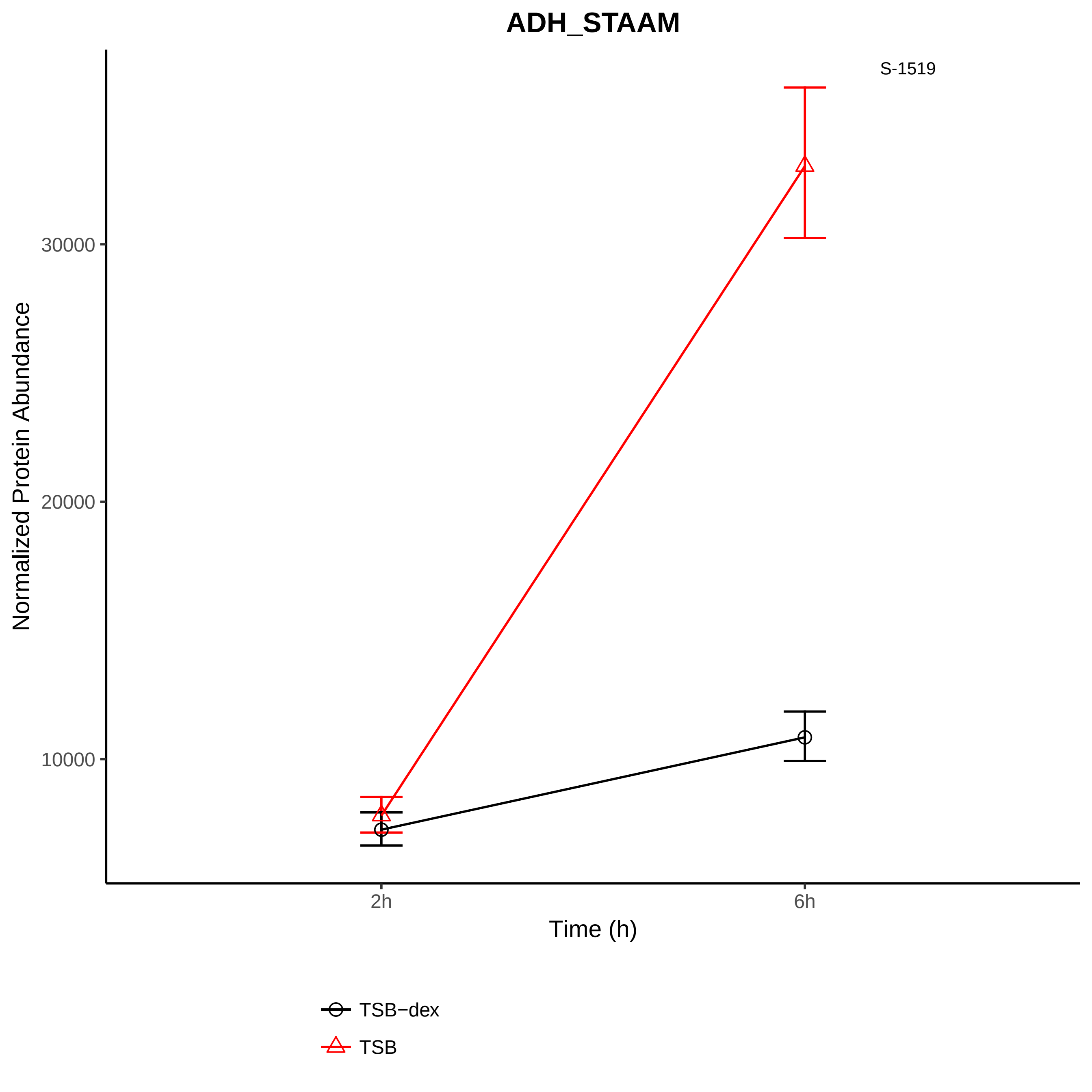
ADH_STAAM
S-1519
Normalized Protein Abundance
30000
20000
10000
2h
6h
Time (h)
TSB−dex
TSB

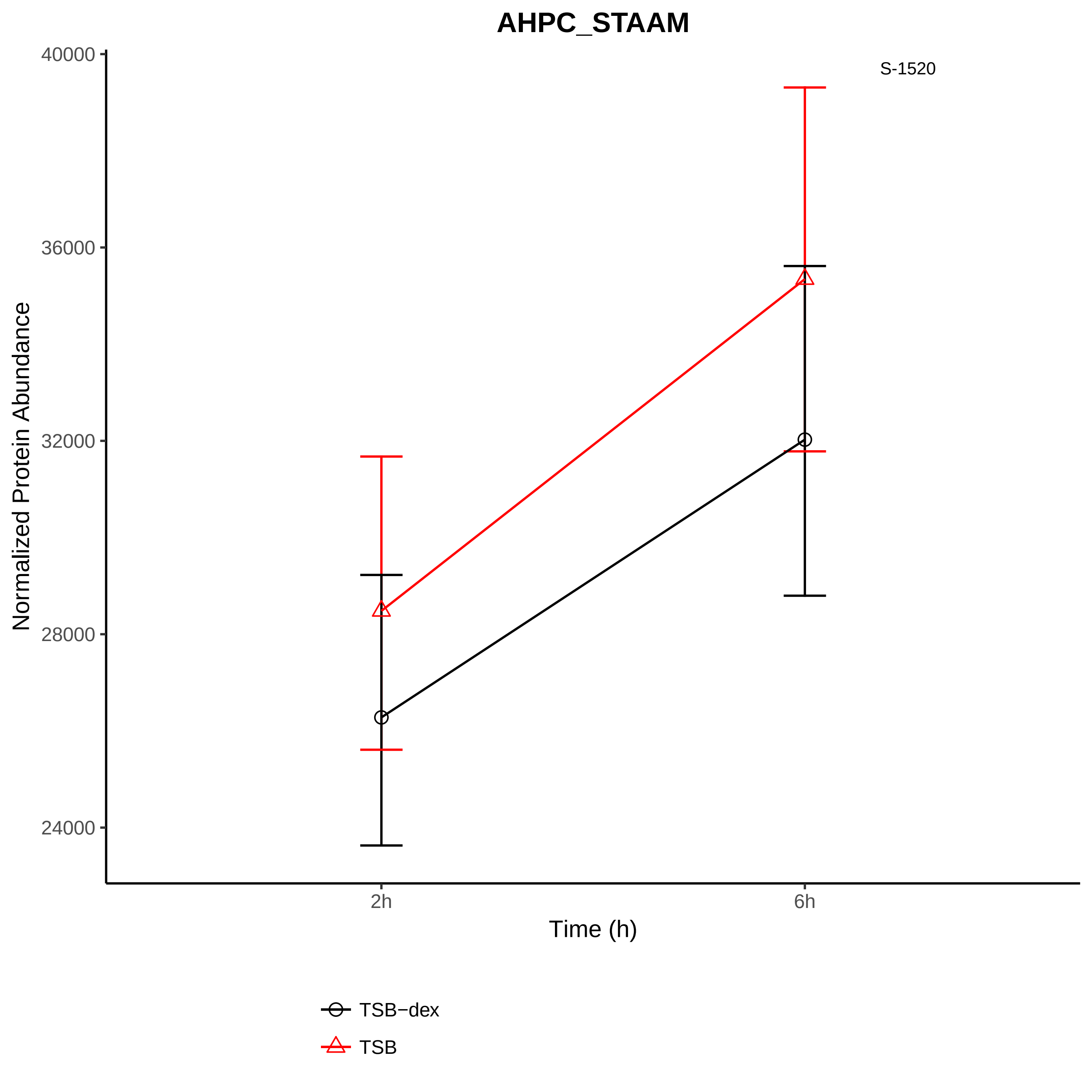
AHPC_STAAM
S-1520
Normalized Protein Abundance
40000
36000
32000
28000
24000
2h
6h
Time (h)
TSB−dex
TSB

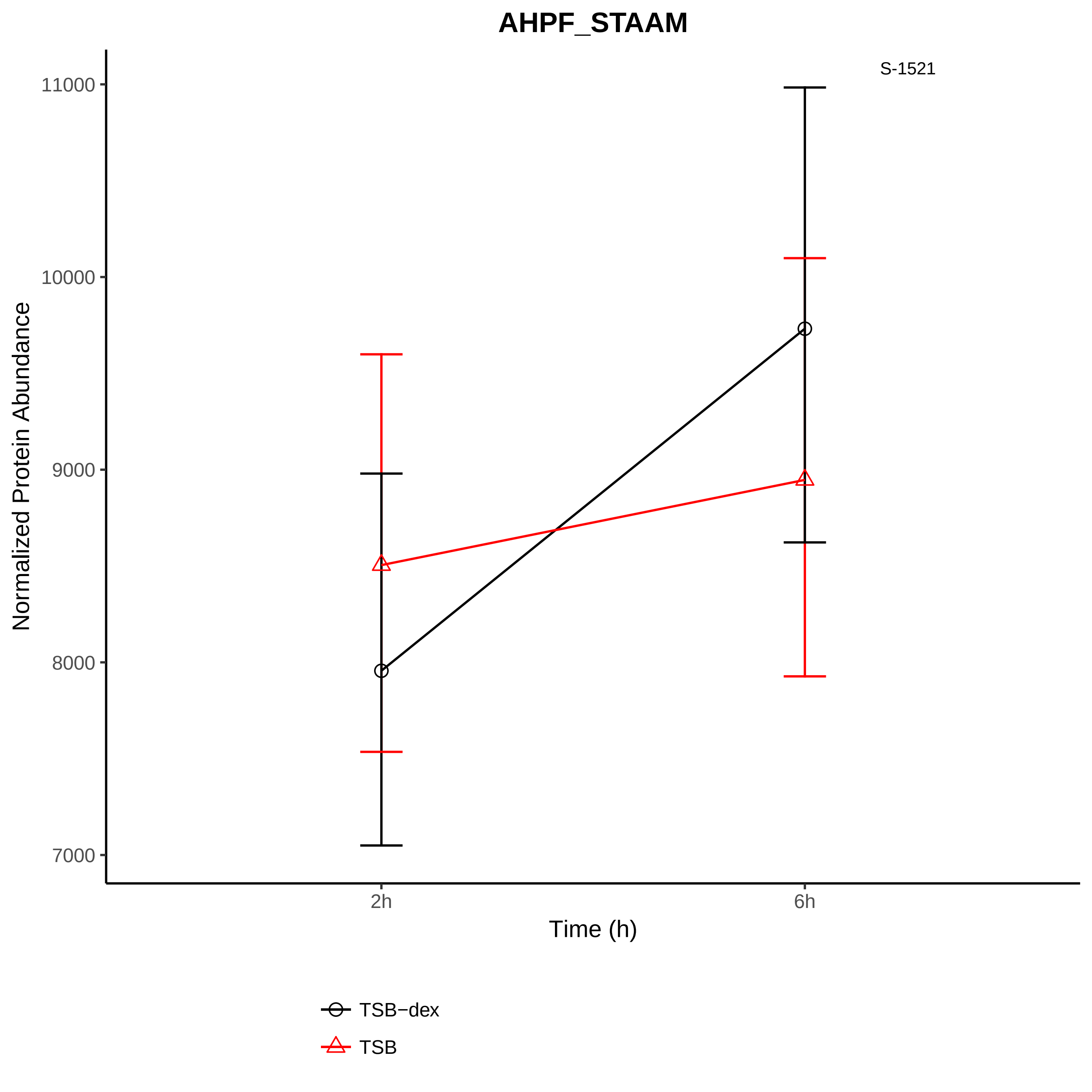

AHPF_STAAM
S-1521
Normalized Protein Abundance
11000
10000
9000
8000
7000
2h
6h
Time (h)
TSB−dex
TSB

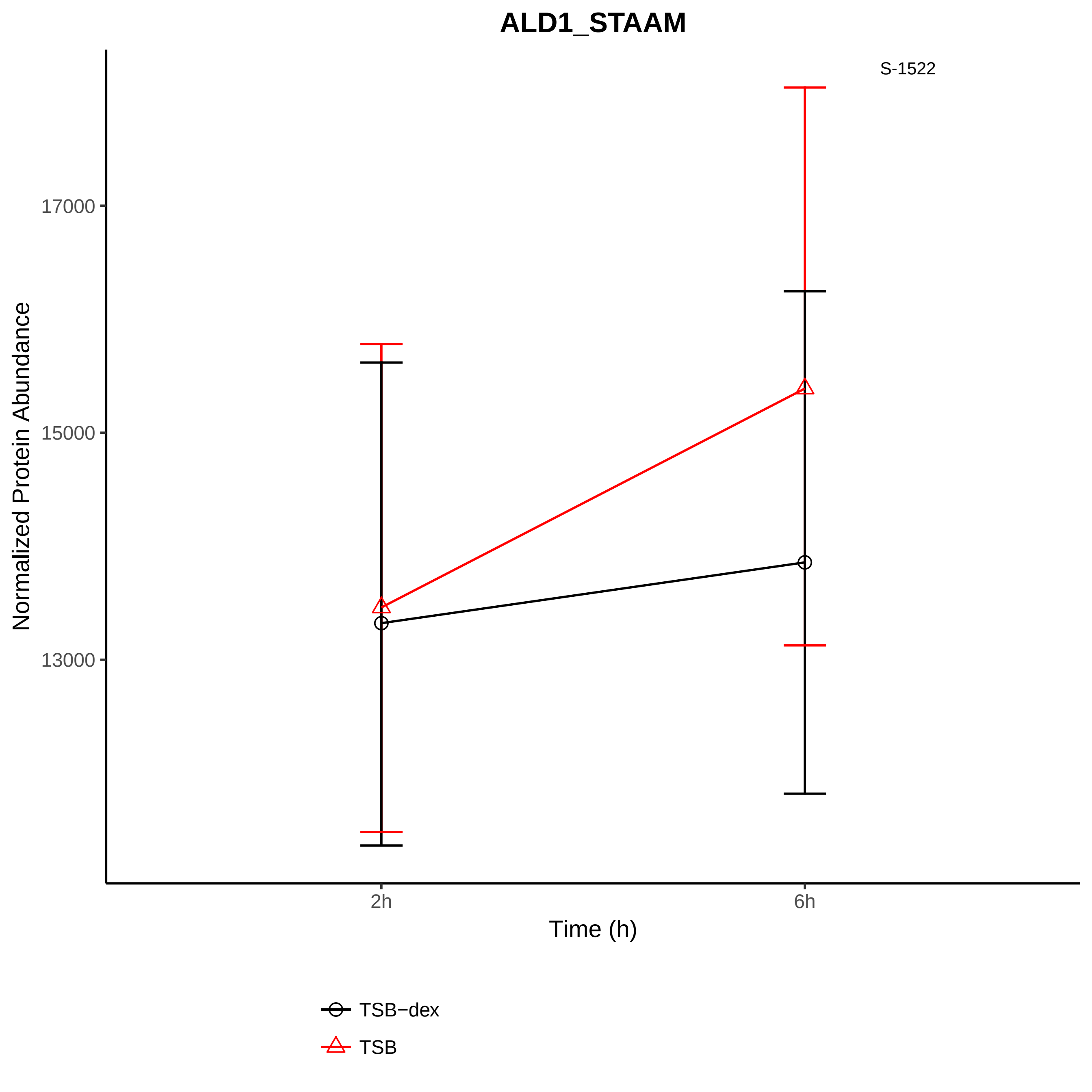

ALD1_STAAM
S-1522
Normalized Protein Abundance
17000
15000
13000
2h
6h
Time (h)
TSB−dex
TSB

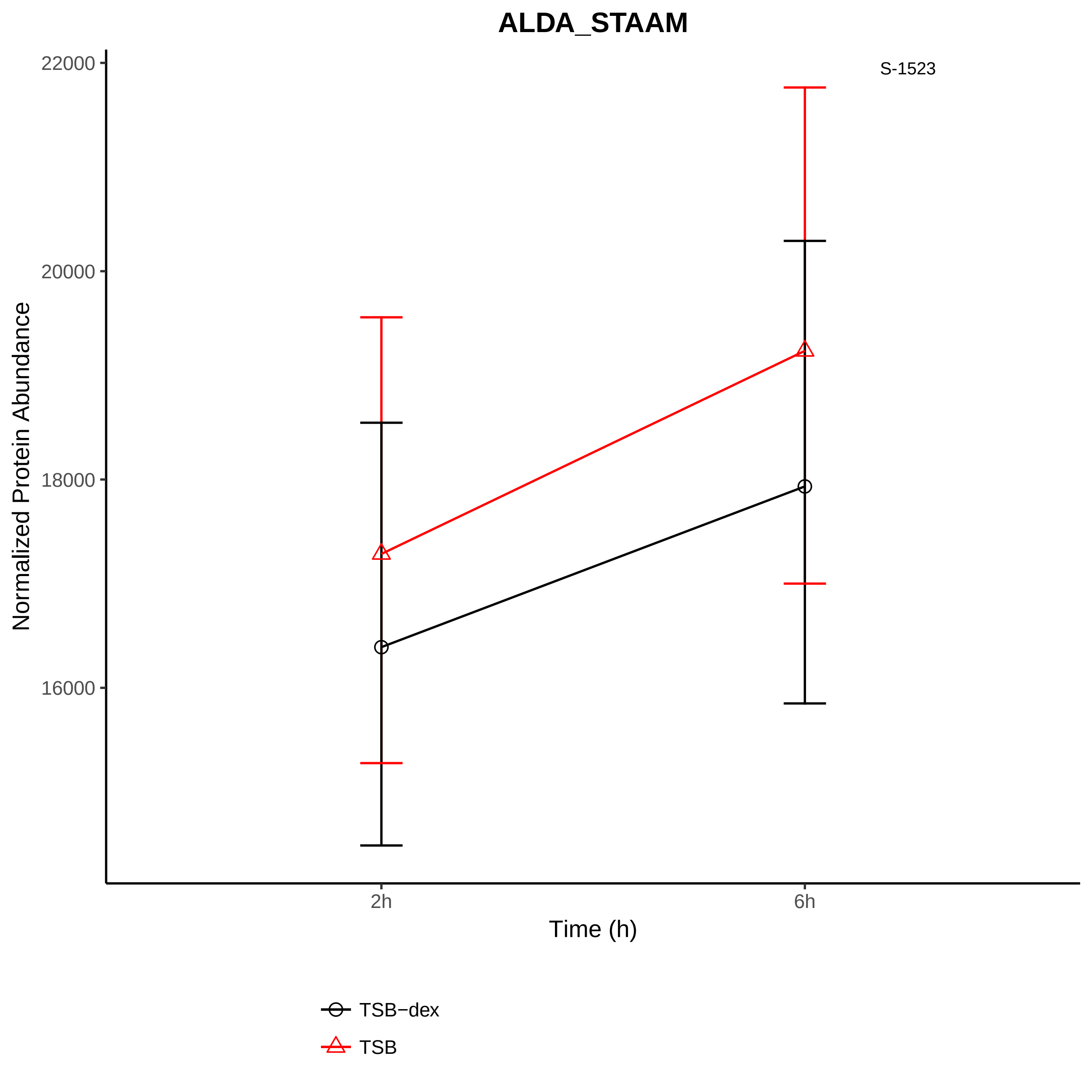

ALDA_STAAM
S-1523
Normalized Protein Abundance
22000
20000
18000
16000
2h
6h
Time (h)
TSB−dex
TSB

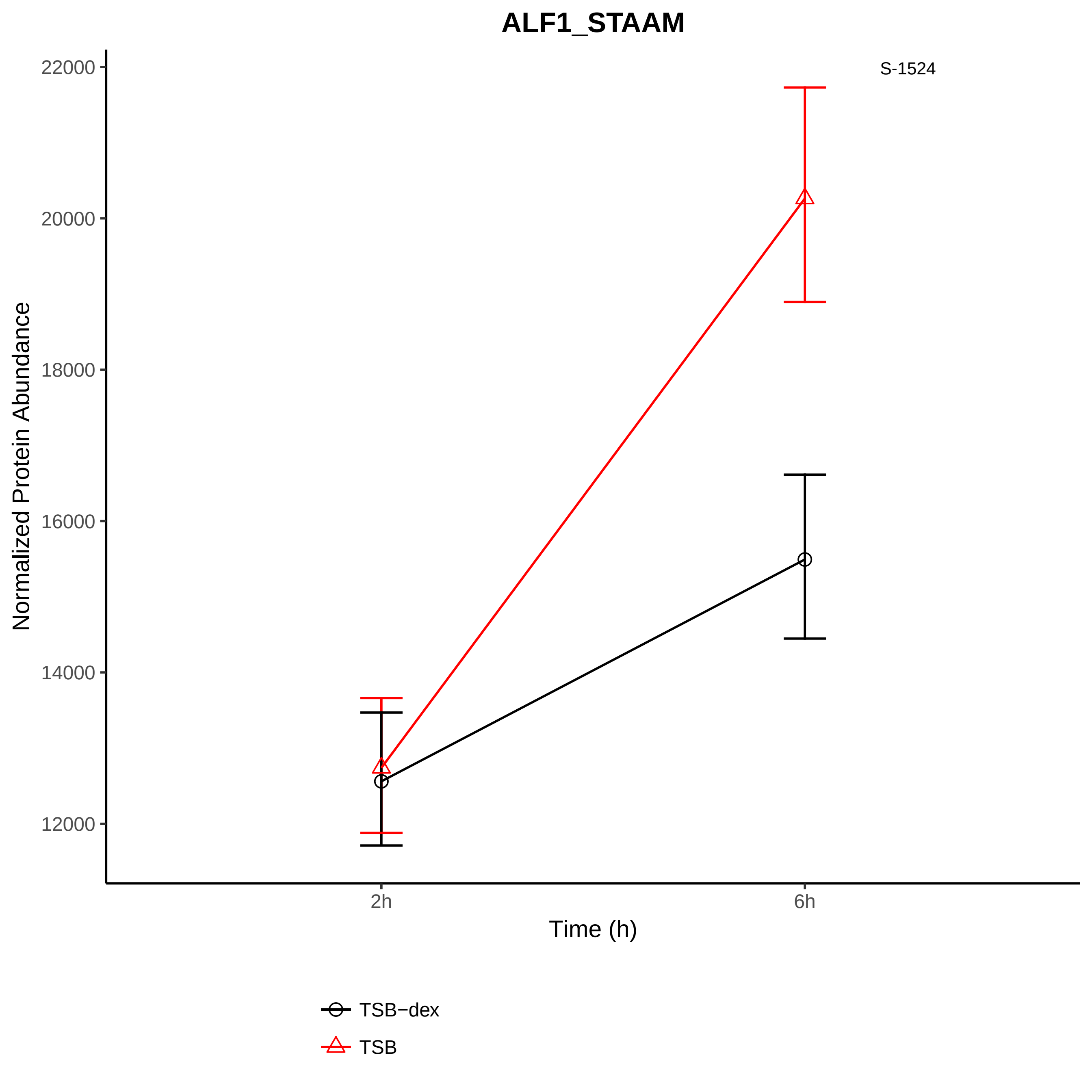
ALF1_STAAM
S-1524
Normalized Protein Abundance
22000
20000
18000
16000
14000
12000
2h
6h
Time (h)
TSB−dex
TSB

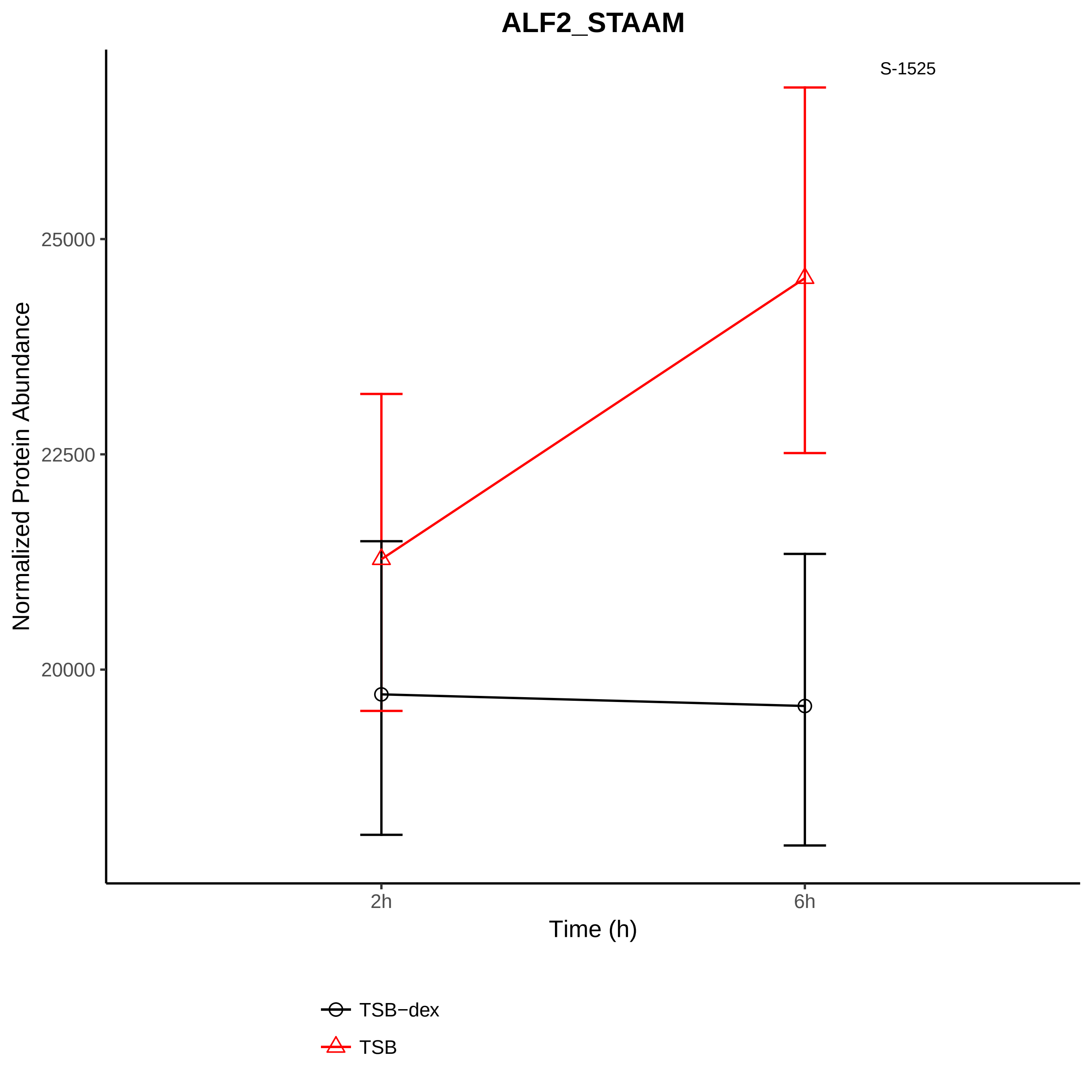
ALF2_STAAM
S-1525
25000
22500
20000
Normalized Protein Abundance
2h
6h
Time (h)
TSB−dex
TSB

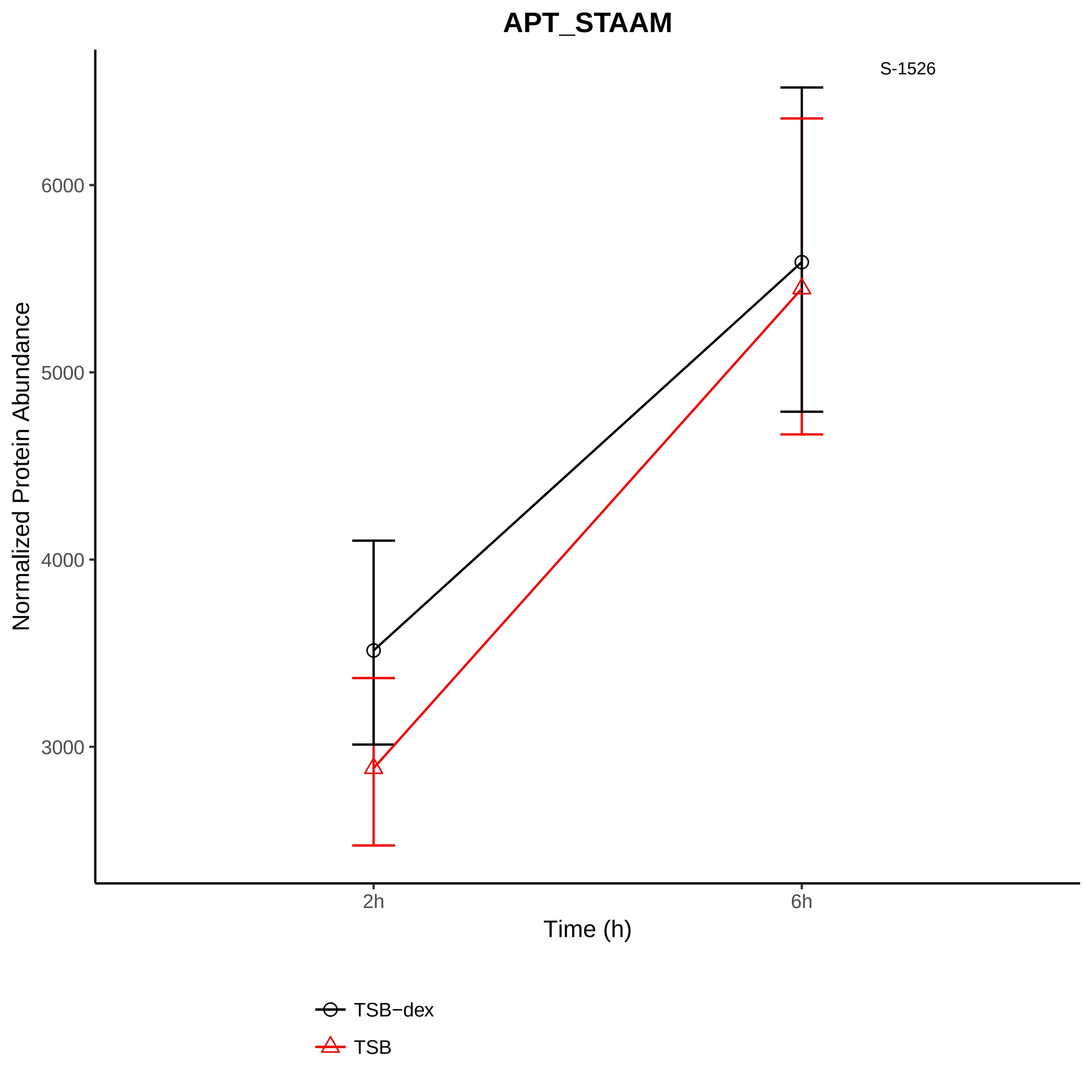

APT_STAAM
S-1526
6000
5000
4000
3000
Normalized Protein Abundance
2h
6h
Time (h)
TSB−dex
TSB

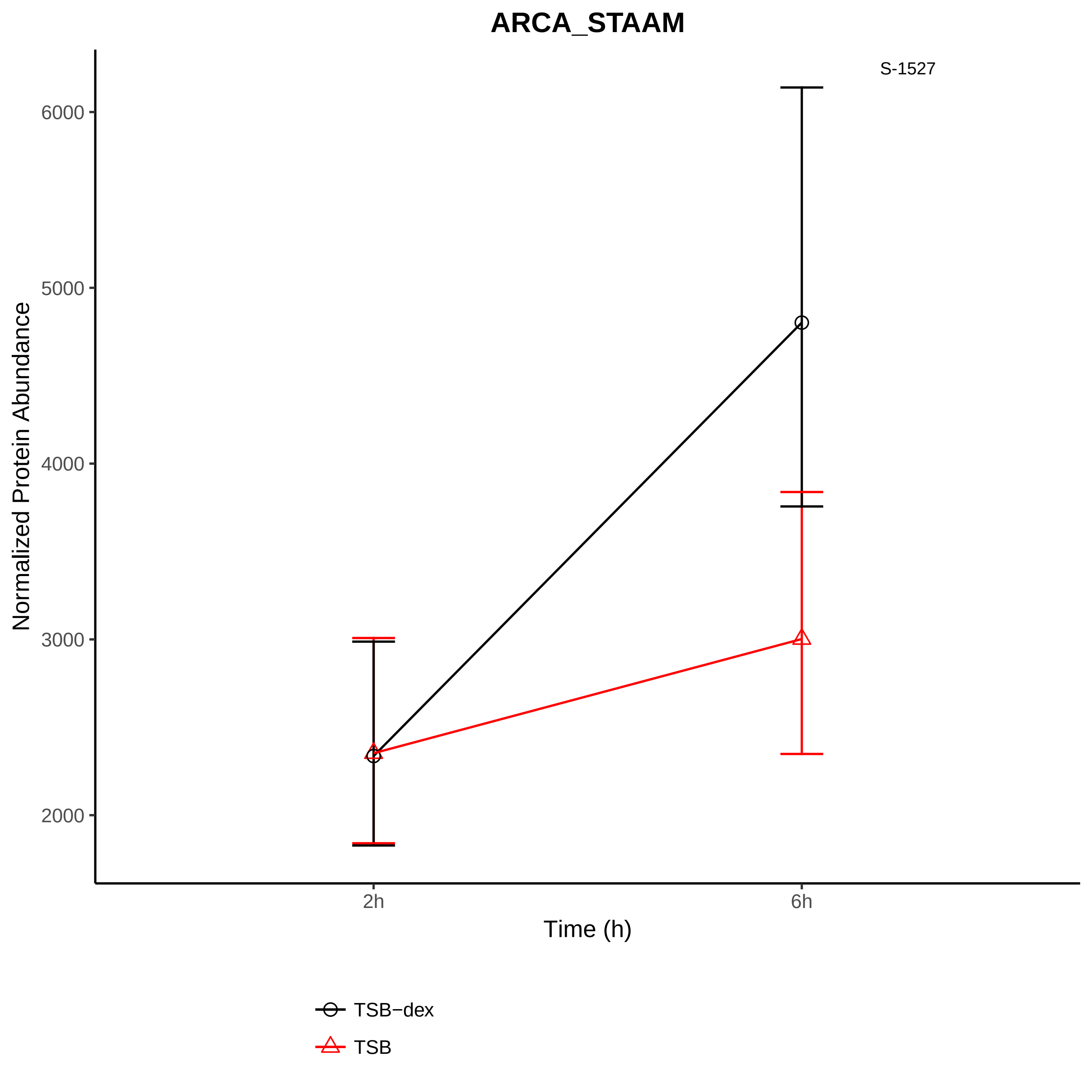

ARCA_STAAM
S-1527
6000
5000
4000
3000
2000
Normalized Protein Abundance
2h
6h
Time (h)
TSB−dex
TSB

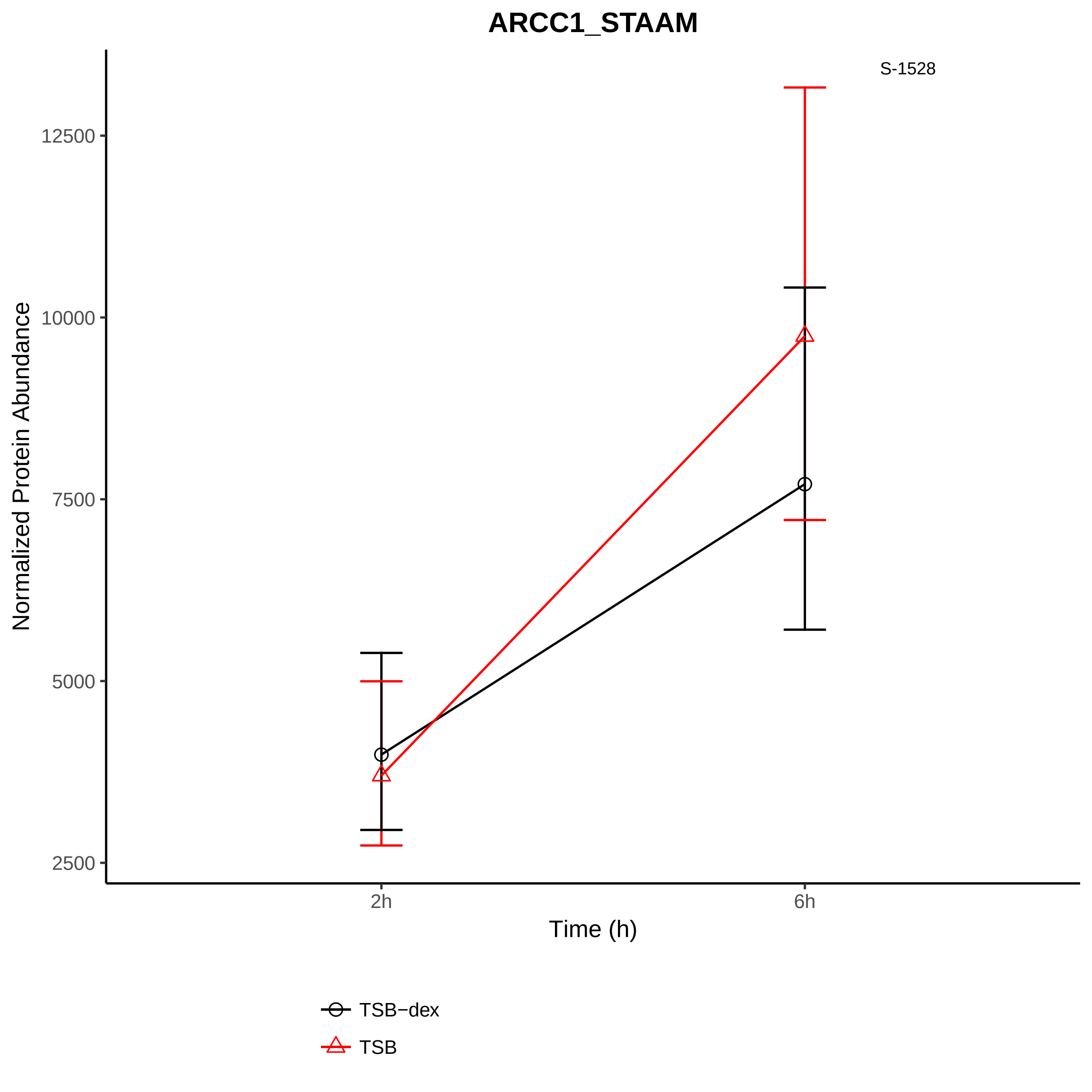
ARCC1_STAAM
S-1528
Normalized Protein Abundance
12500
10000
7500
5000
2500
2h
6h
Time (h)
TSB−dex
TSB

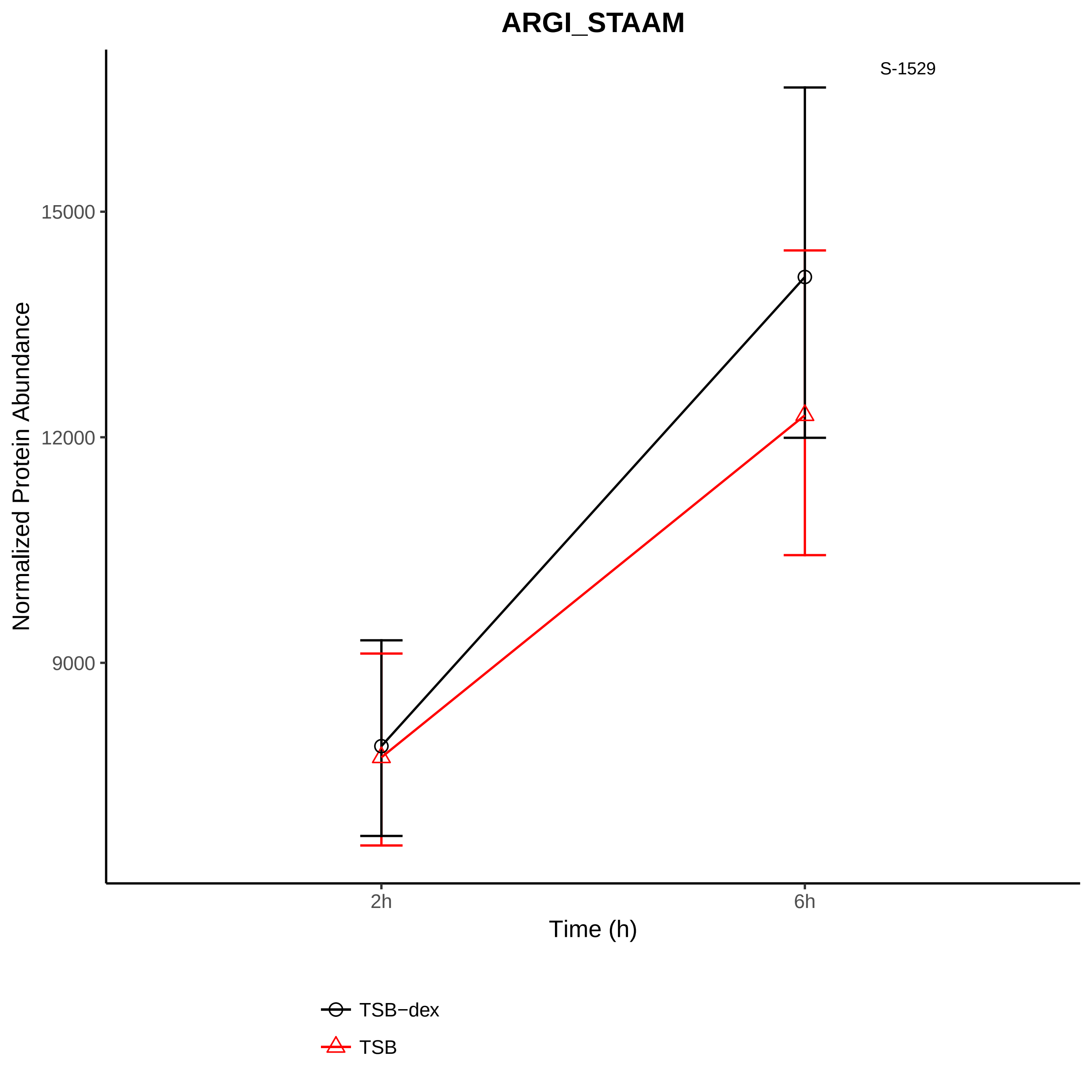
ARGI_STAAM
S-1529
Normalized Protein Abundance
15000
12000
9000
2h
6h
Time (h)
TSB−dex
TSB

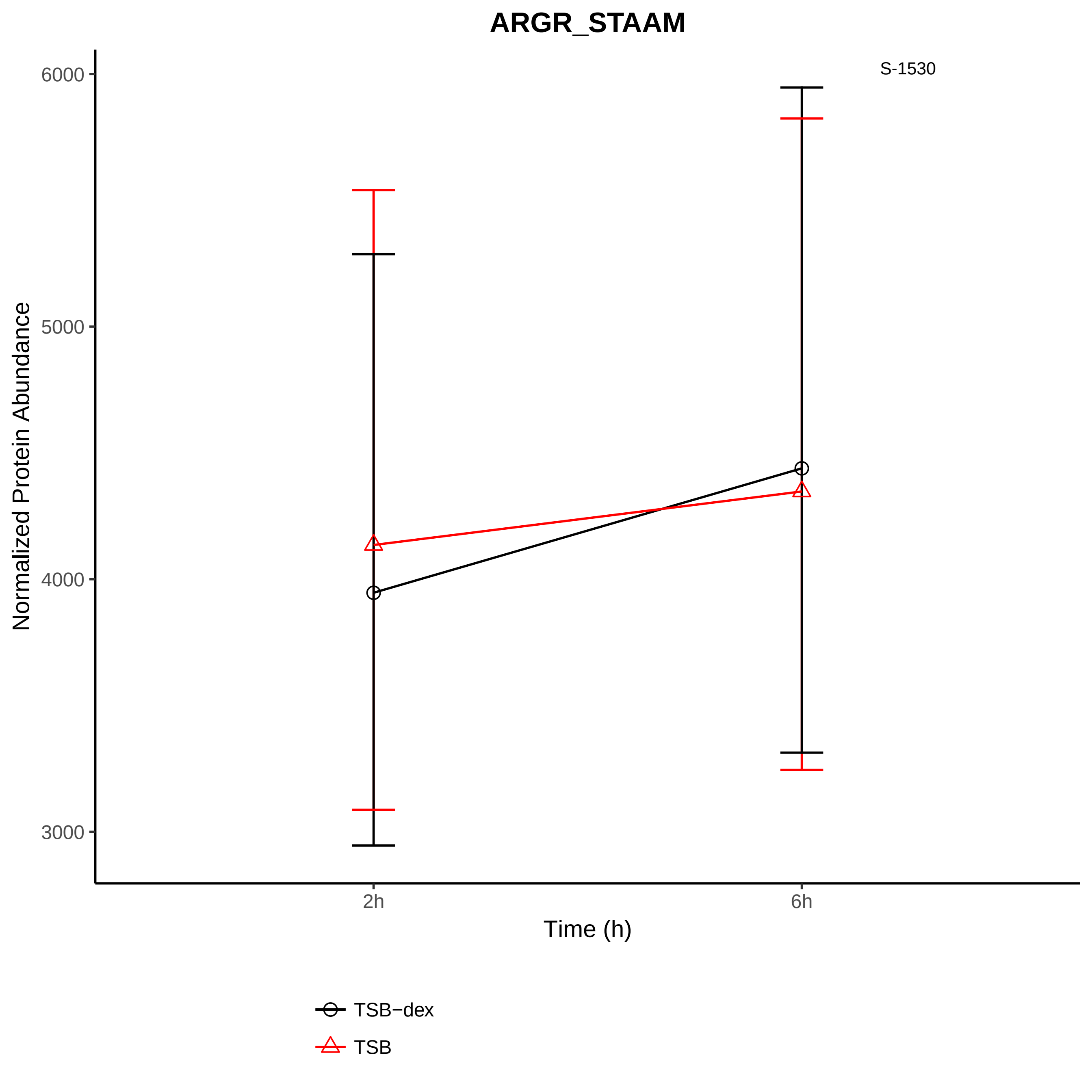

ARGR_STAAM
S-1530
6000
5000
4000
3000
Normalized Protein Abundance
2h
6h
Time (h)
TSB−dex
TSB

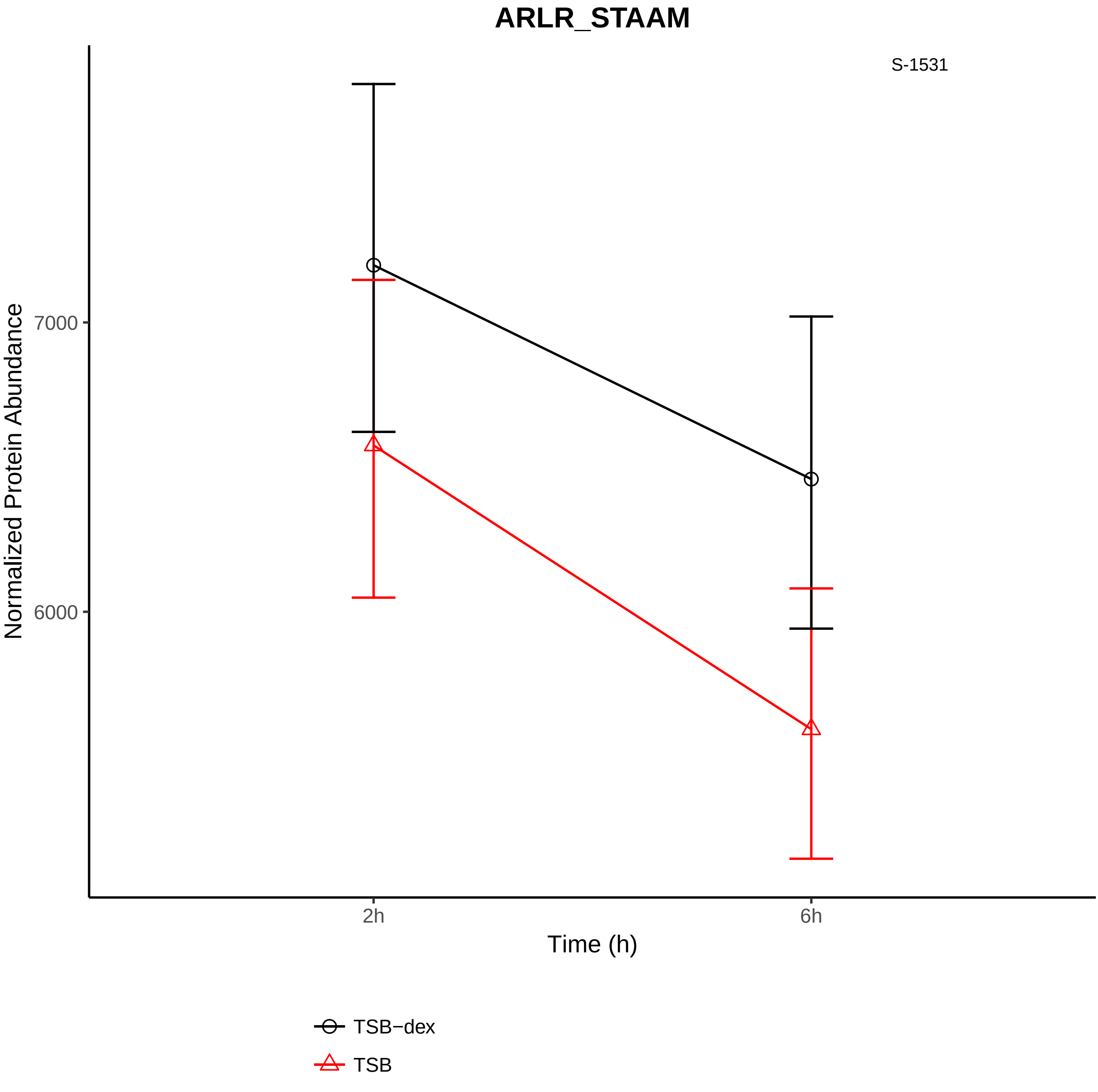

ARLR_STAAM
S-1531
Normalized Protein Abundance
7000
6000
2h
6h
Time (h)
TSB−dex
TSB

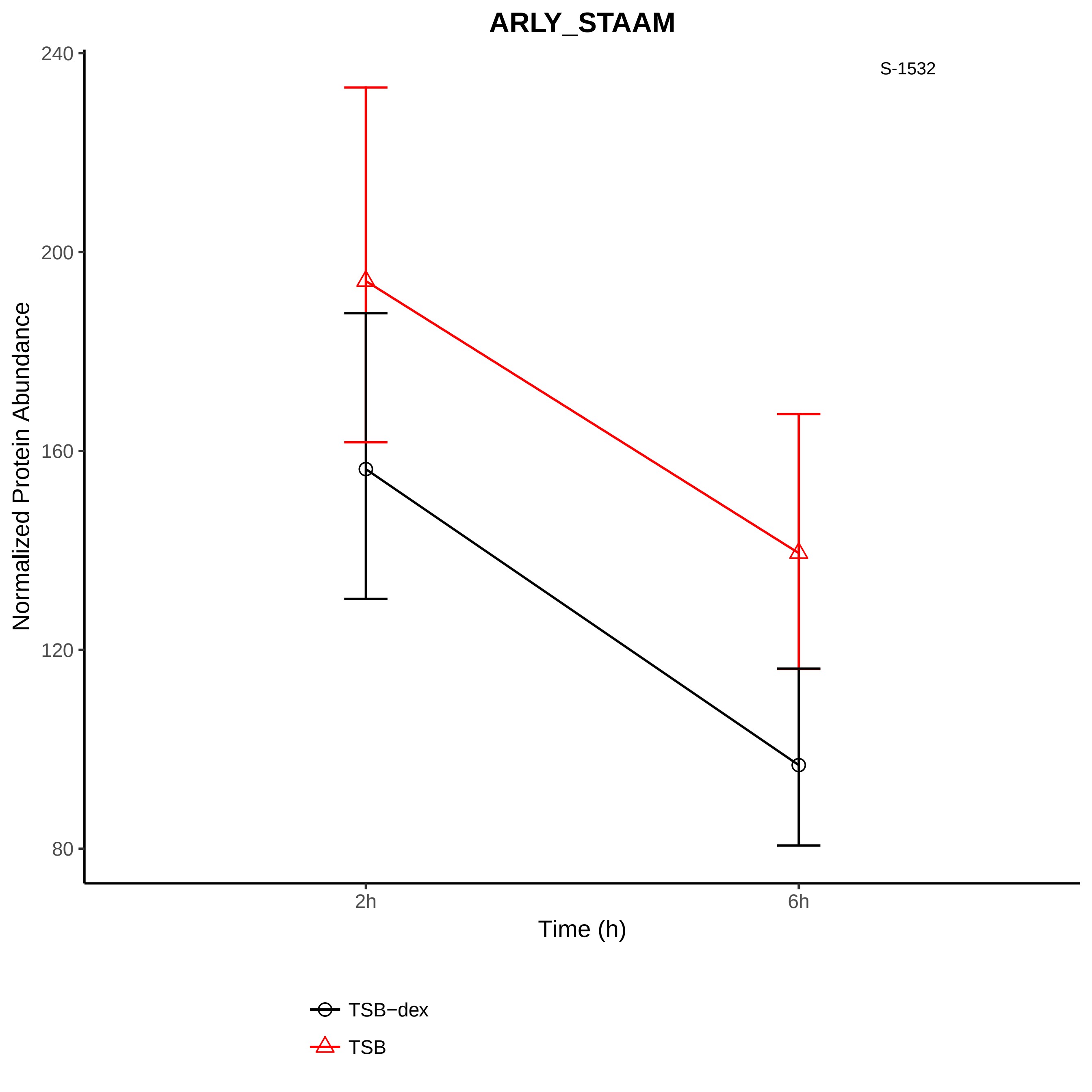

ARLY_STAAM
S-1532
240
200
160
120
80
Normalized Protein Abundance
2h
6h
Time (h)
TSB−dex
TSB

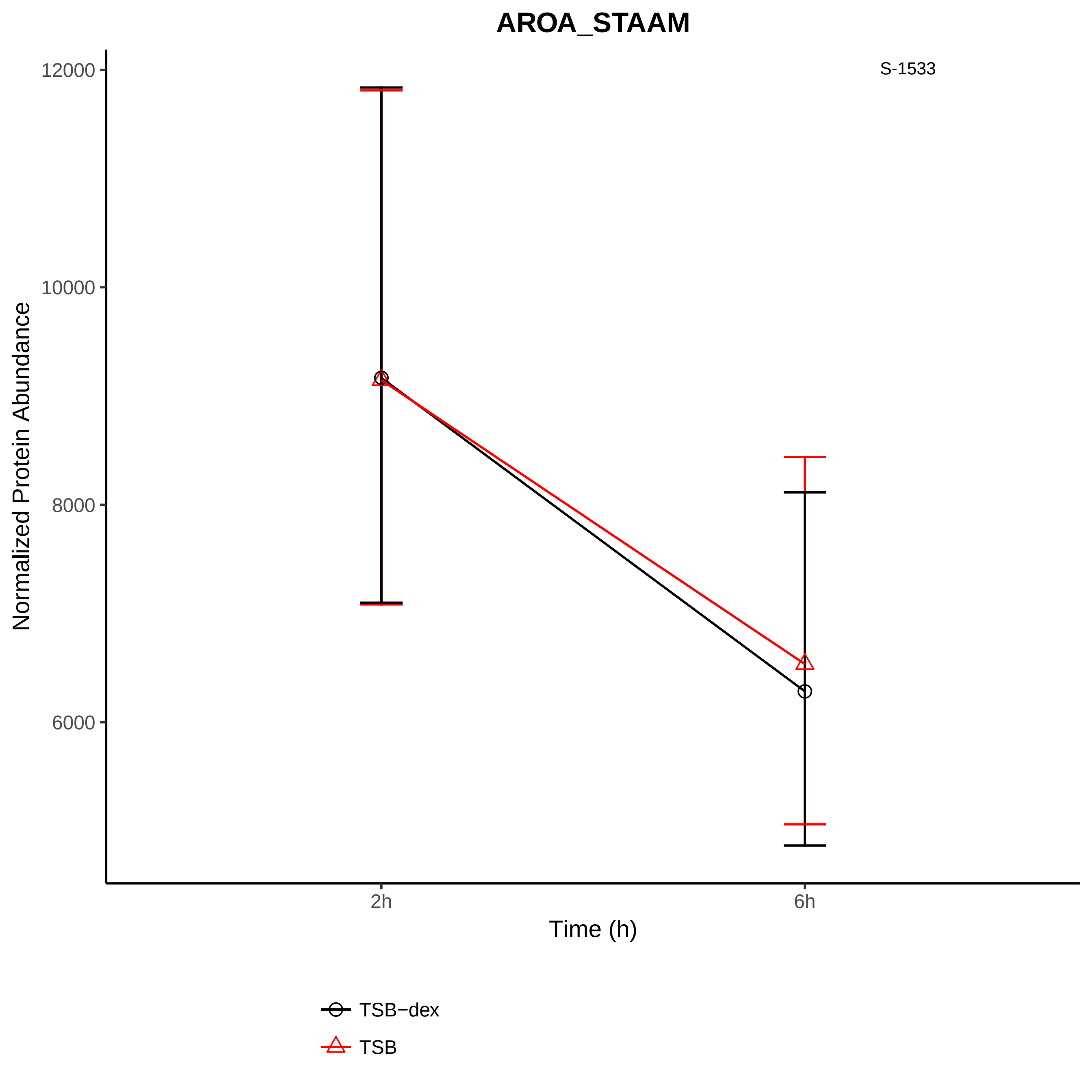

AROA_STAAM
S-1533
12000
10000
8000
6000
Normalized Protein Abundance
2h
6h
Time (h)
TSB−dex
TSB

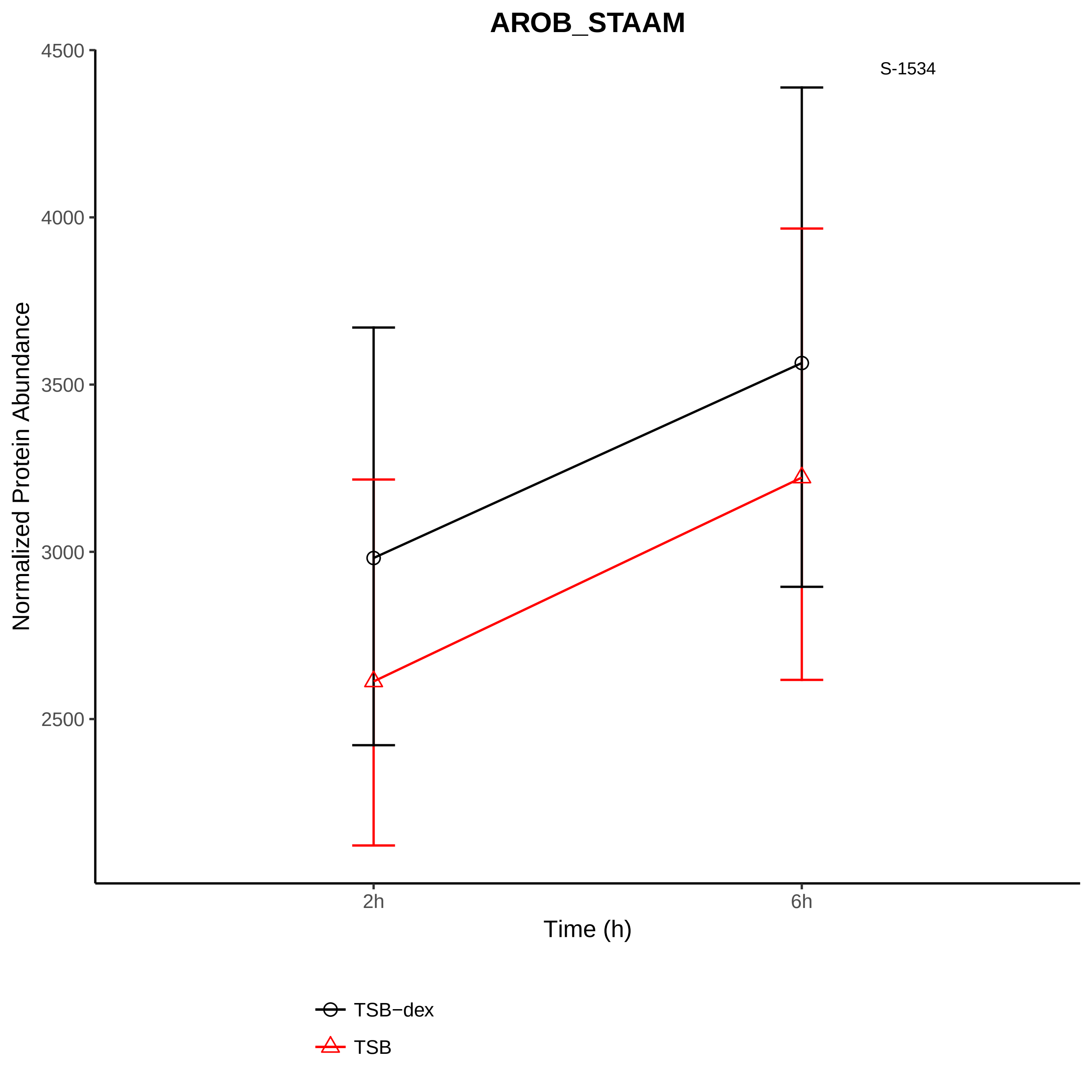

AROB_STAAM
S-1534
4500
4000
3500
3000
2500
Normalized Protein Abundance
2h
6h
Time (h)
TSB−dex
TSB

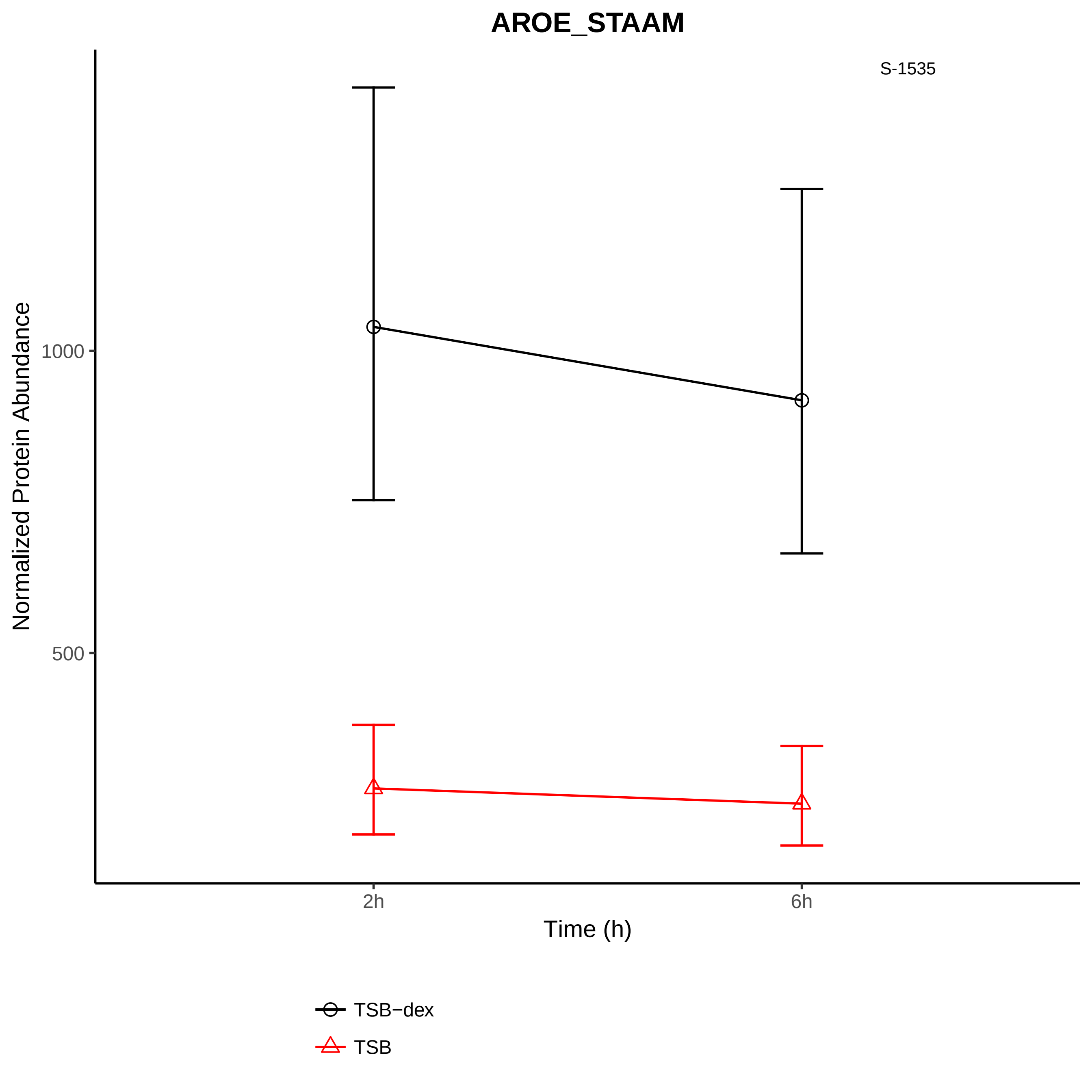
AROE_STAAM
S-1535
Normalized Protein Abundance
1000
500
2h
6h
Time (h)
TSB−dex
TSB

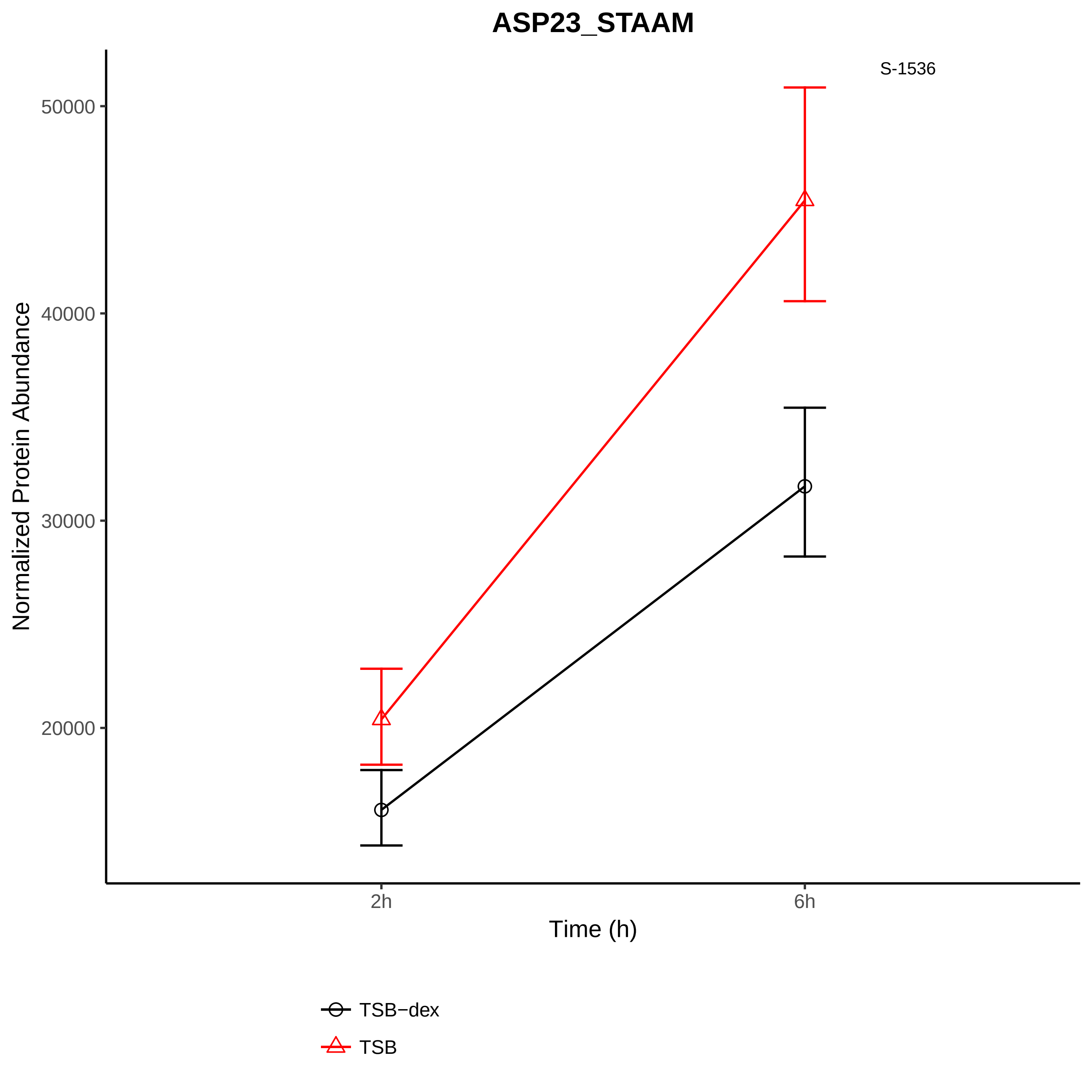
ASP23_STAAM
S-1536
50000
40000
30000
20000
Normalized Protein Abundance
2h
6h
Time (h)
TSB−dex
TSB

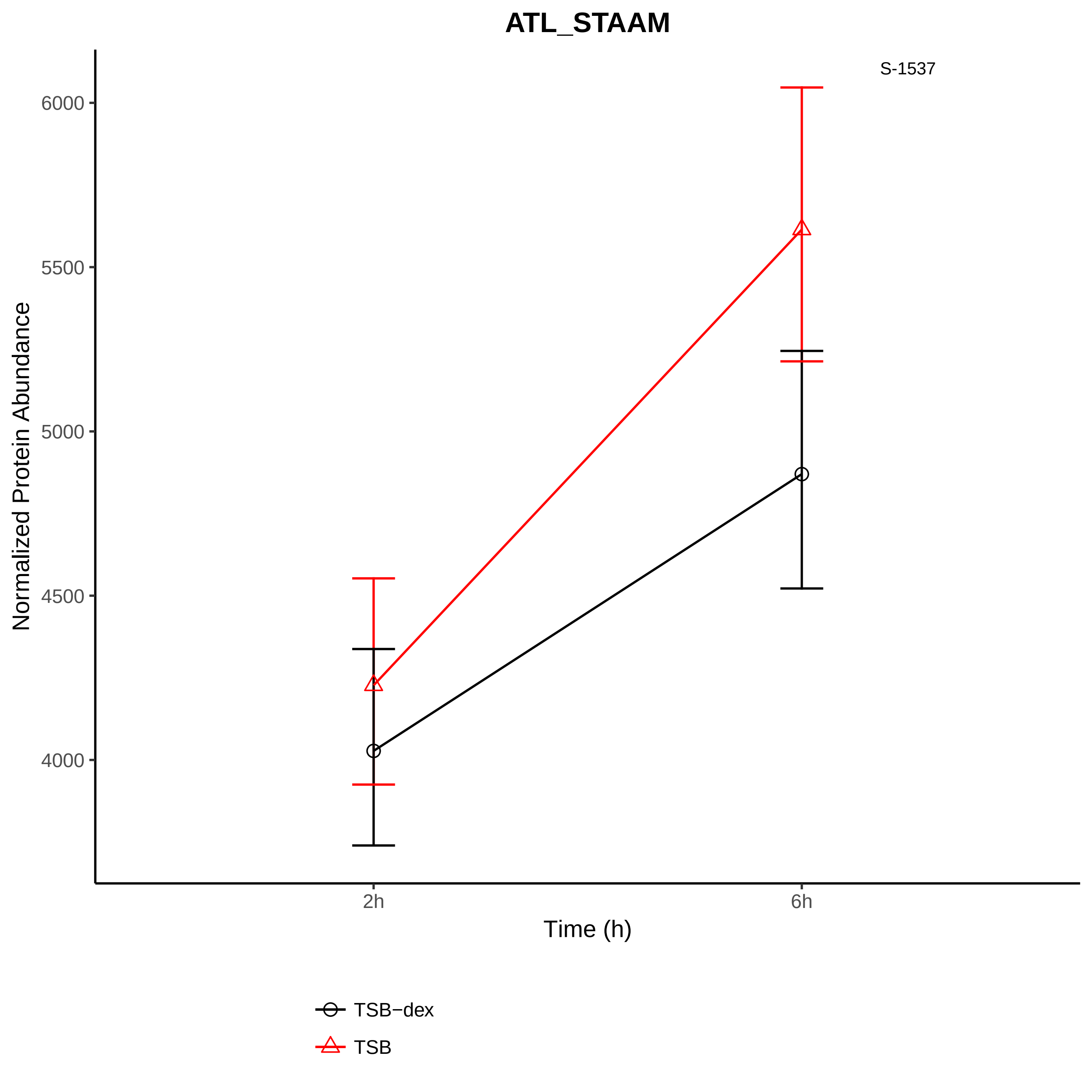
ATL_STAAM
S-1537
6000
5500
5000
4500
4000
Normalized Protein Abundance
2h
6h
Time (h)
TSB−dex
TSB

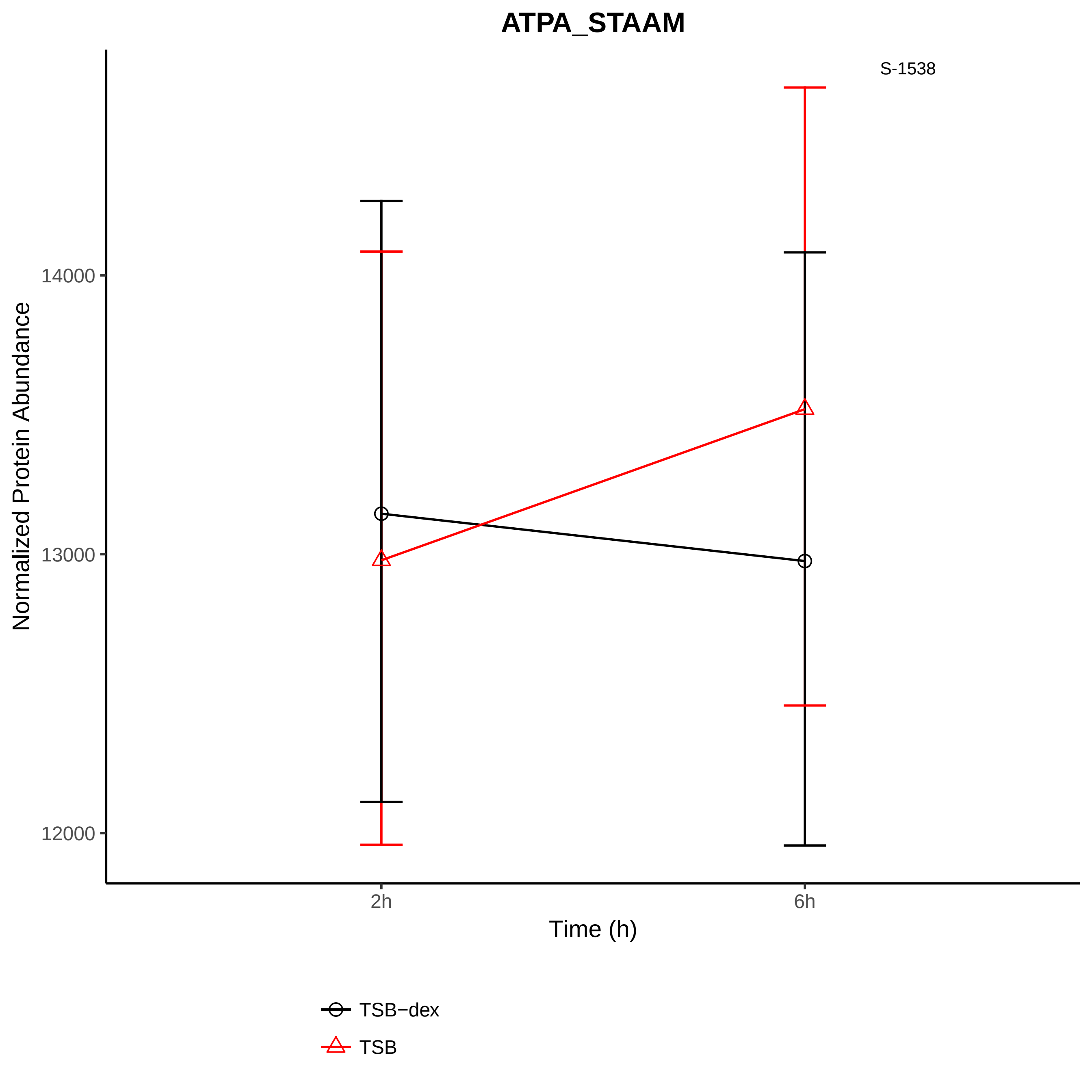

ATPA_STAAM
S-1538
Normalized Protein Abundance
14000
13000
12000
2h
6h
Time (h)
TSB−dex
TSB

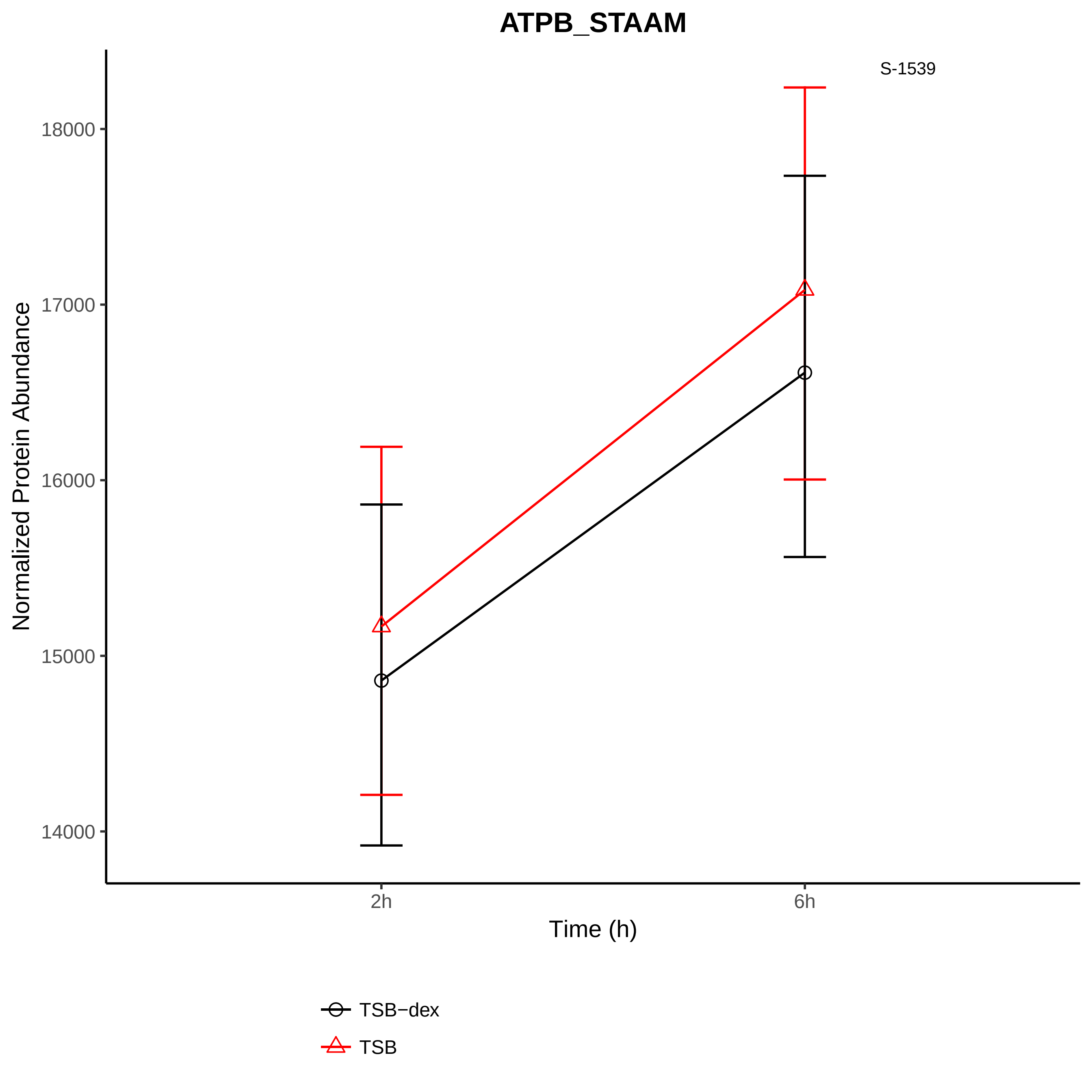
ATPB_STAAM
S-1539
Normalized Protein Abundance
18000
17000
16000
15000
14000
2h
6h
Time (h)
TSB−dex
TSB

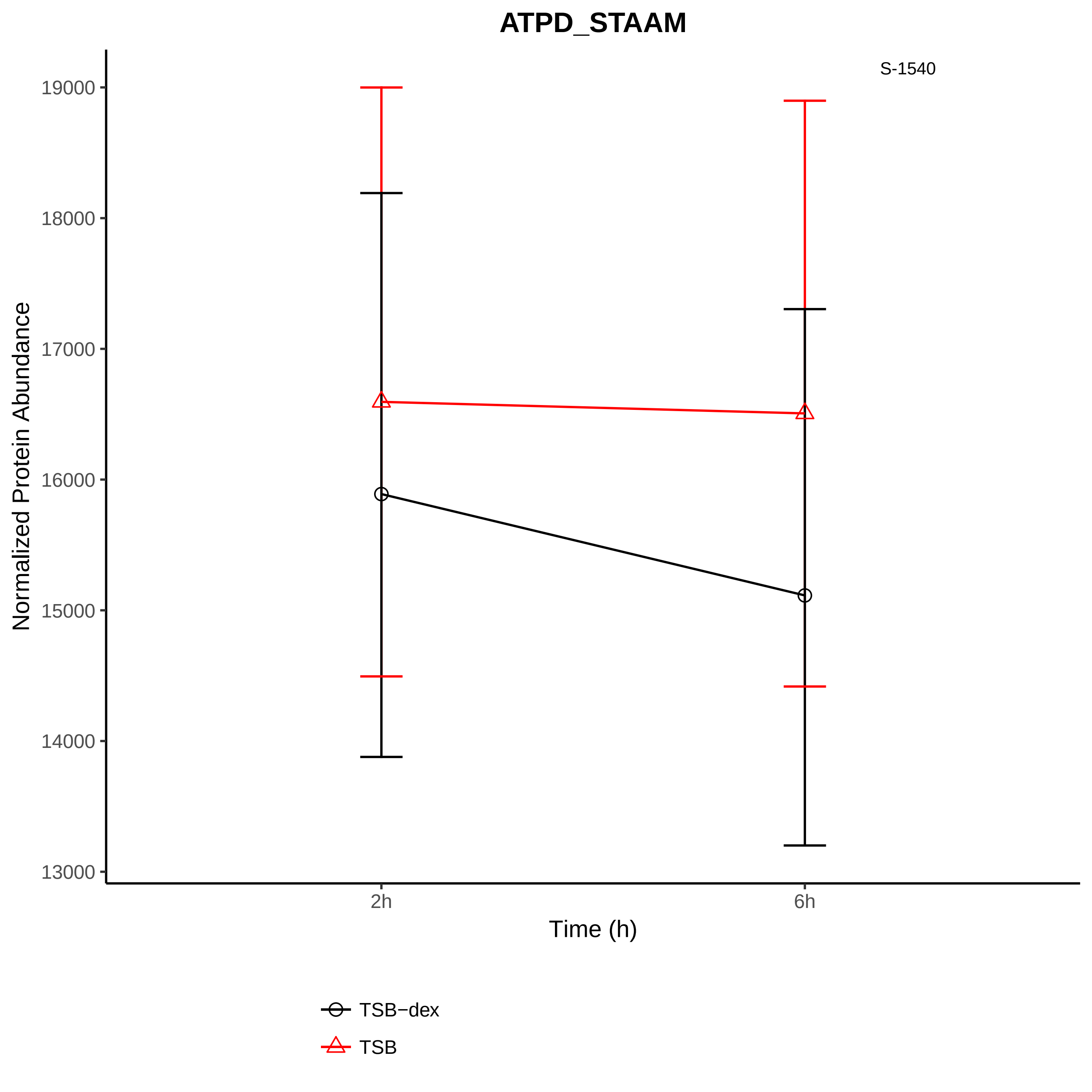

ATPD_STAAM
S-1540
Normalized Protein Abundance
19000
18000
17000
16000
15000
14000
13000
2h
6h
Time (h)
TSB−dex
TSB

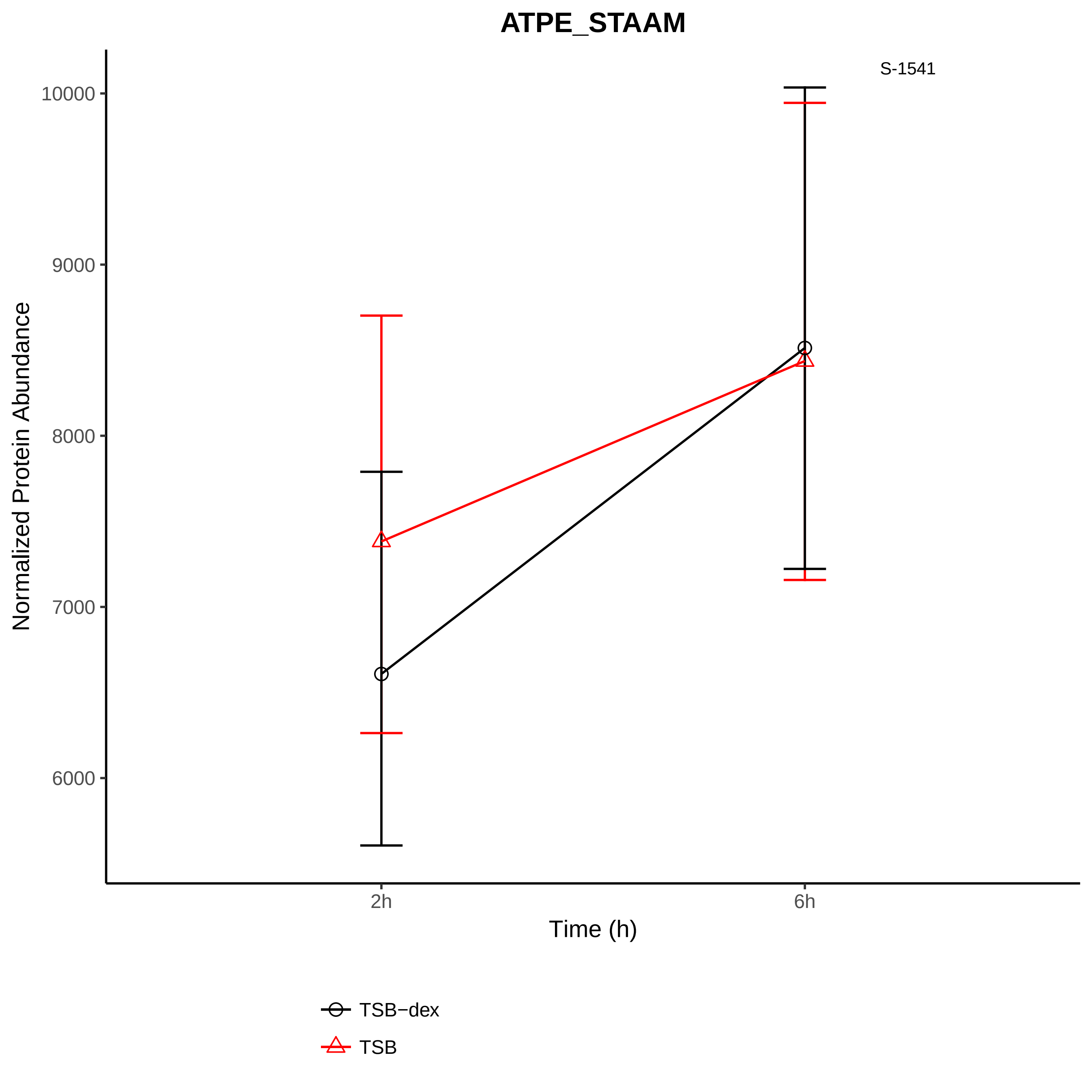

ATPE_STAAM
S-1541
Normalized Protein Abundance
10000
9000
8000
7000
6000
2h
6h
Time (h)
TSB−dex
TSB

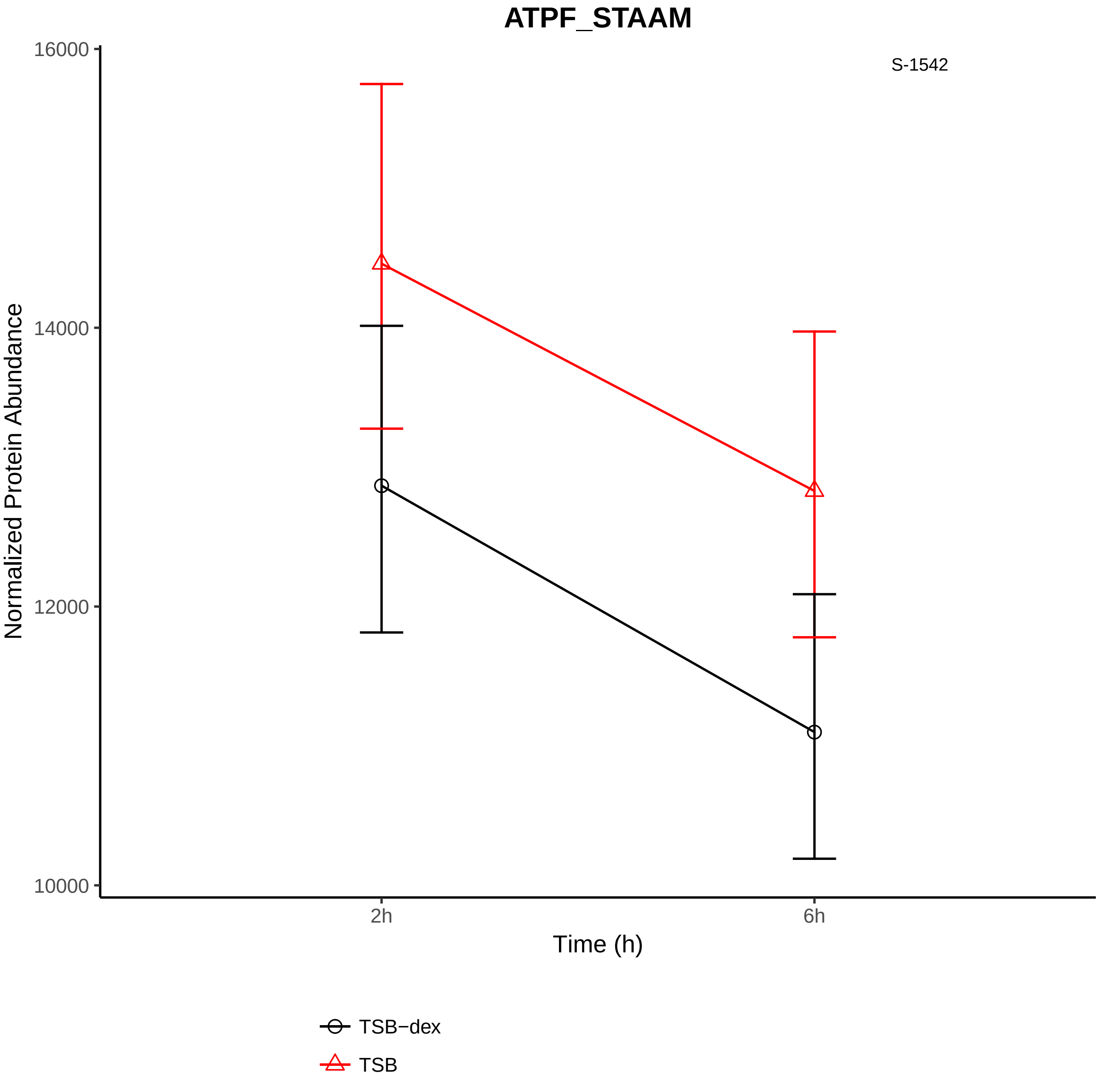
ATPF_STAAM
S-1542
16000
14000
12000
10000
Normalized Protein Abundance
2h
6h
Time (h)
TSB−dex
TSB

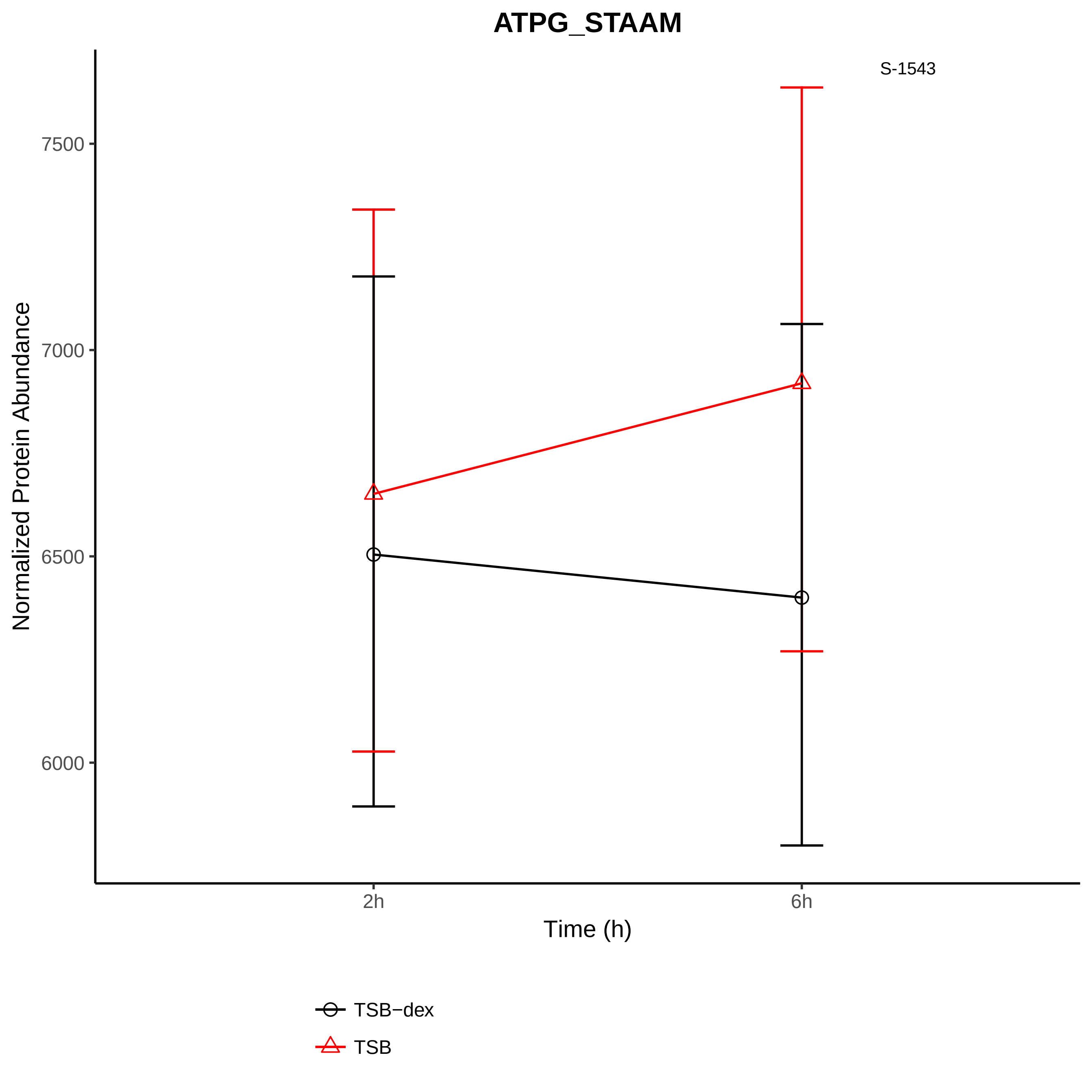

ATPG_STAAM
S-1543
7500
7000
6500
6000
Normalized Protein Abundance
2h
6h
Time (h)
TSB−dex
TSB

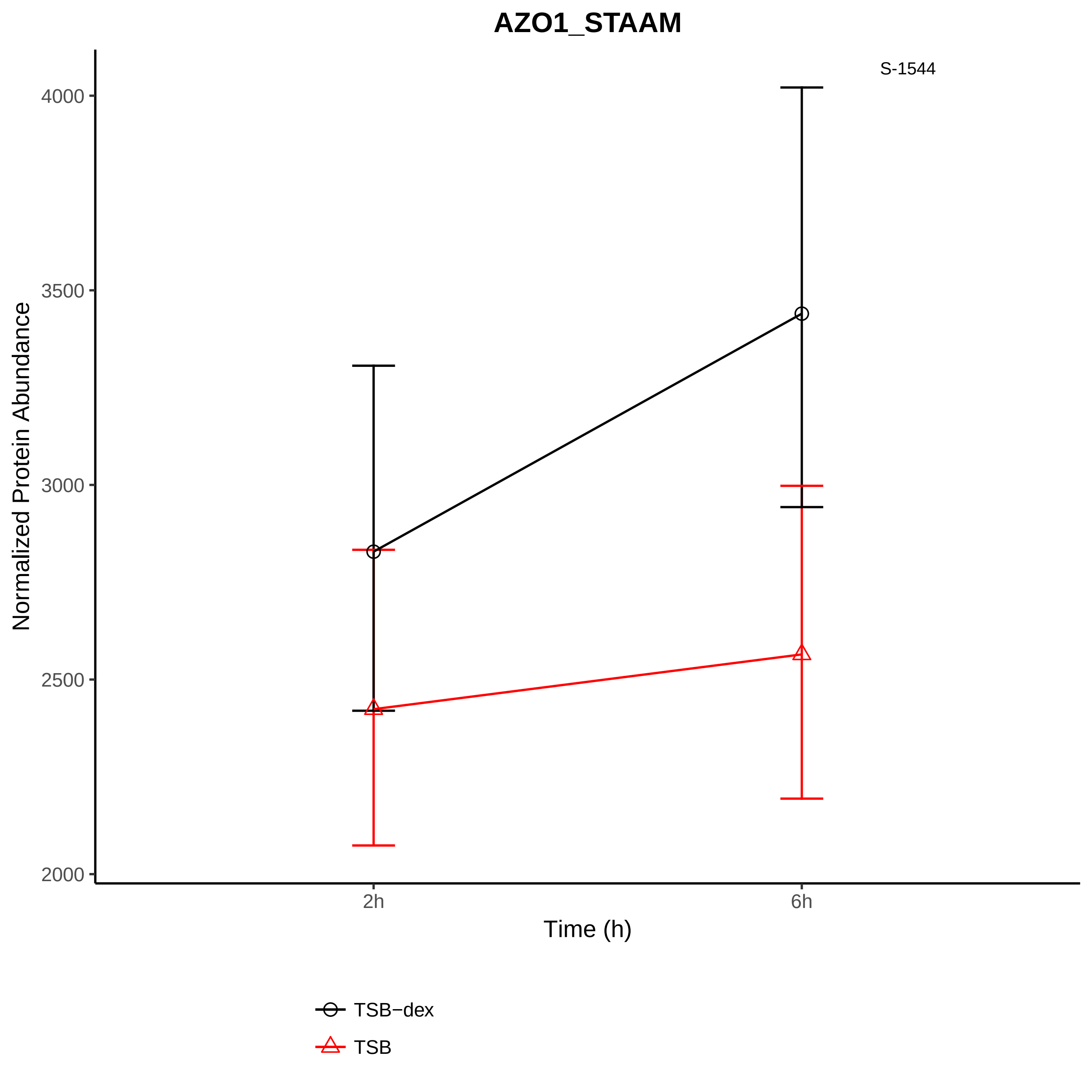
AZO1_STAAM
S-1544
Normalized Protein Abundance
4000
3500
3000
2500
2000
2h
6h
Time (h)
TSB−dex
TSB

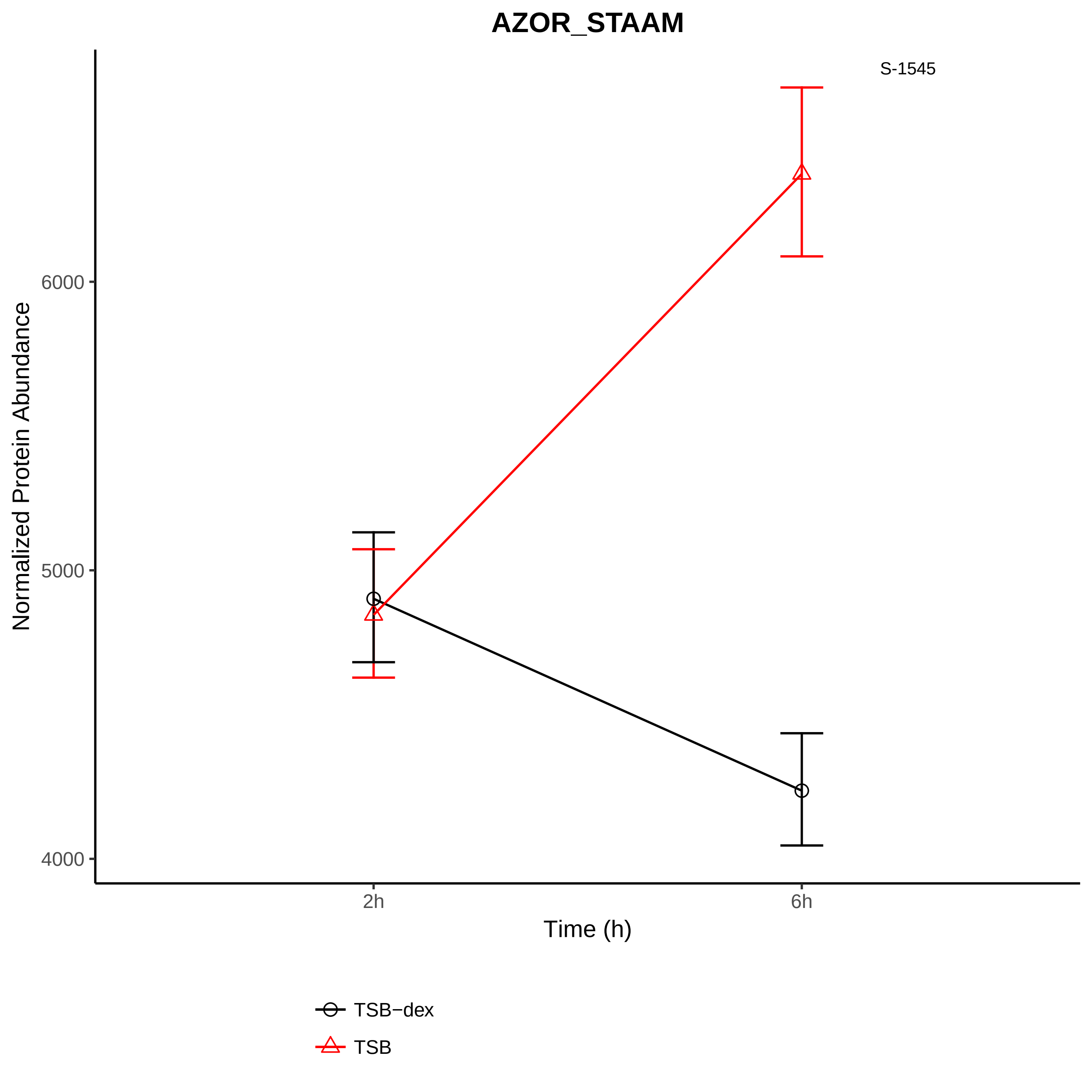
AZOR_STAAM
S-1545
Normalized Protein Abundance
6000
5000
4000
2h
6h
Time (h)
TSB−dex
TSB

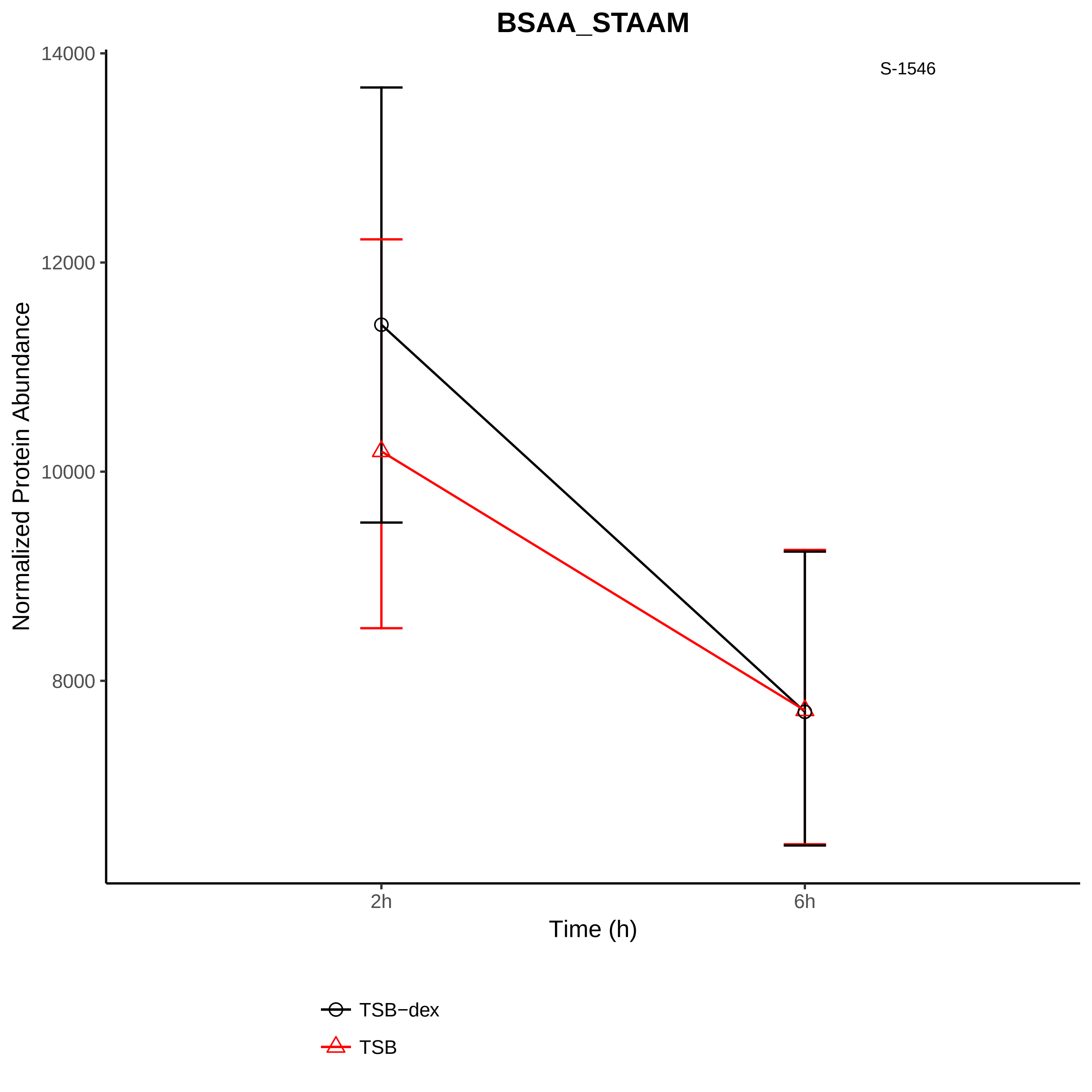

BSAA_STAAM
S-1546
Normalized Protein Abundance
14000
12000
10000
8000
2h
6h
Time (h)
TSB−dex
TSB

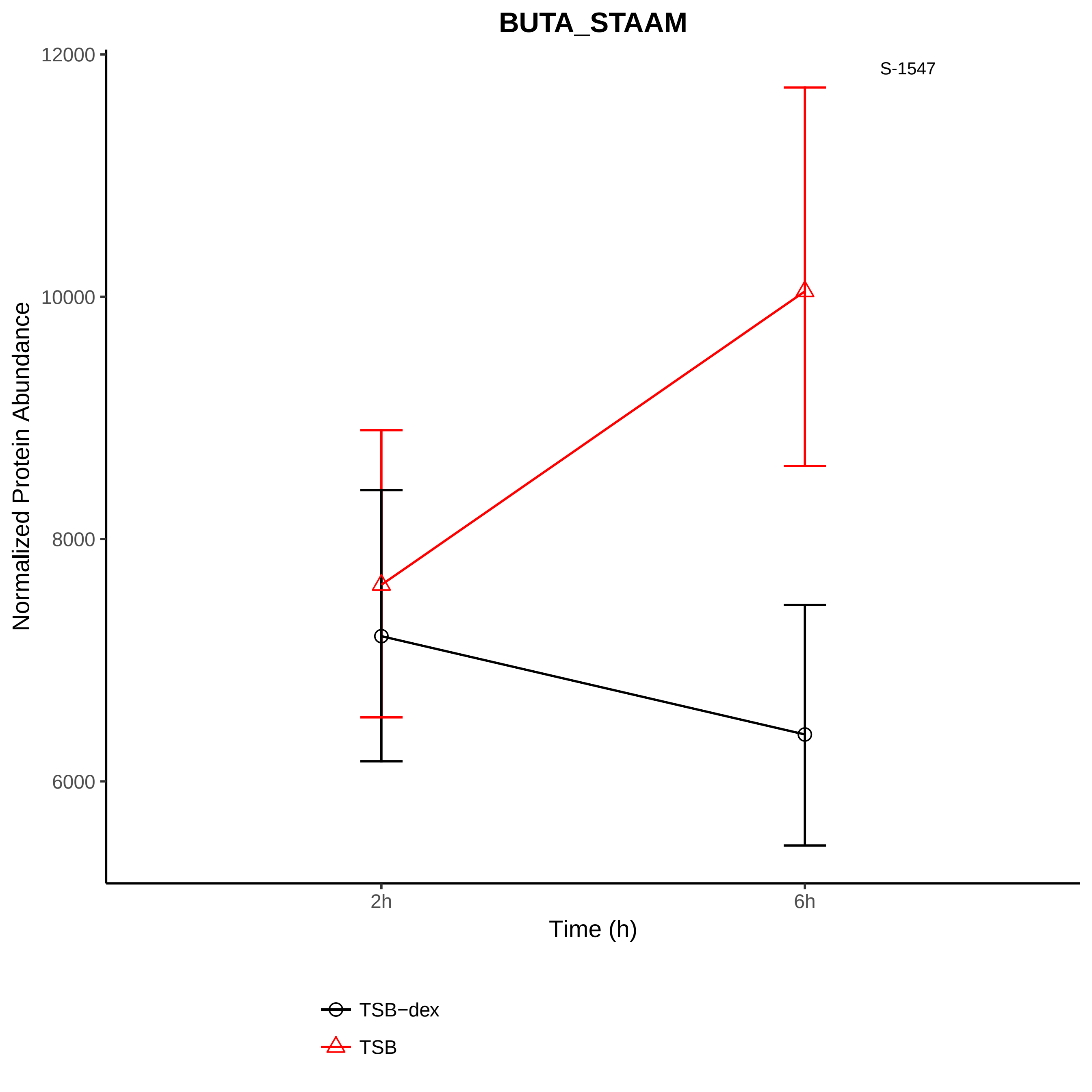
BUTA_STAAM
S-1547
Normalized Protein Abundance
12000
10000
8000
6000
2h
6h
Time (h)
TSB−dex
TSB

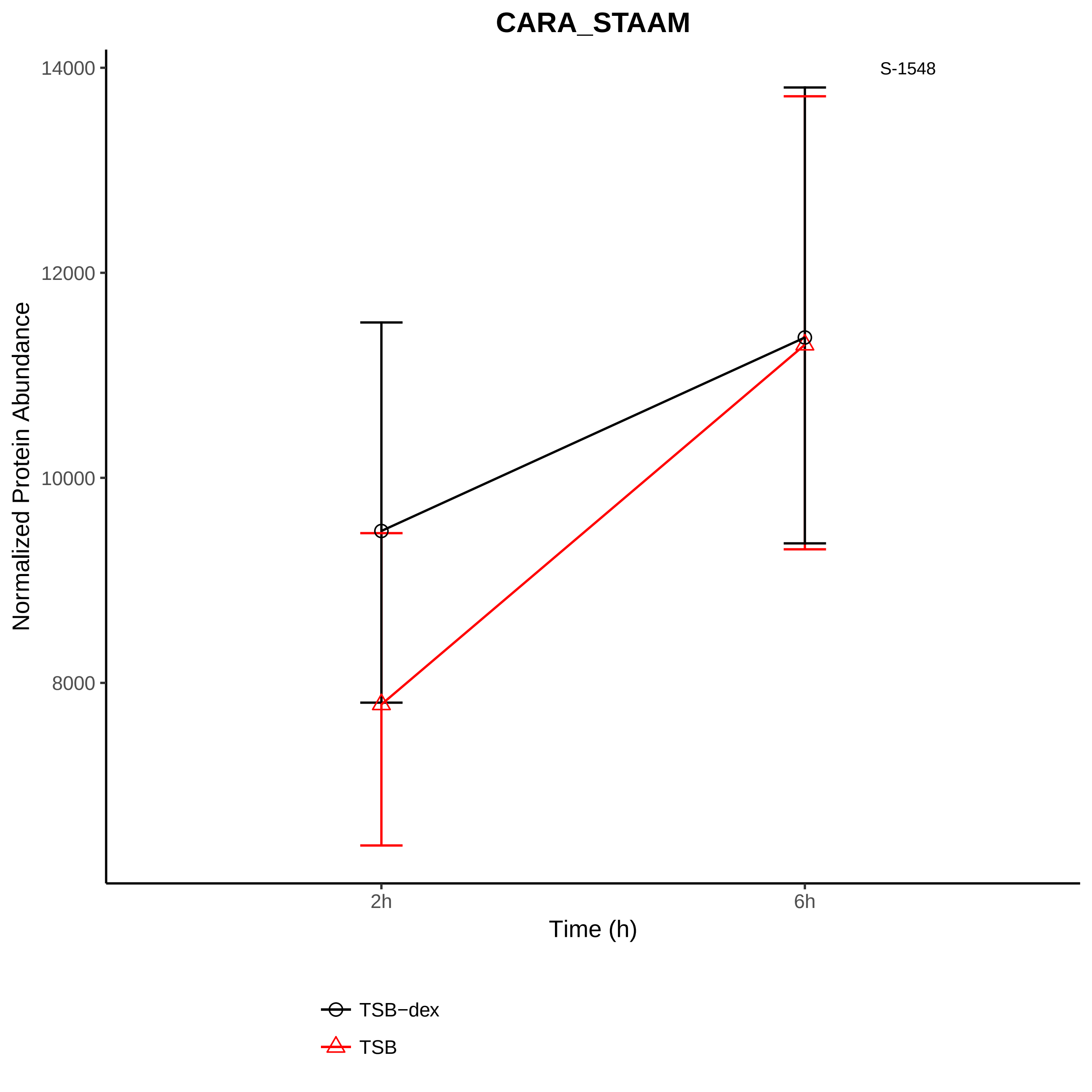

CARA_STAAM
S-1548
14000
12000
10000
8000
Normalized Protein Abundance
2h
6h
Time (h)
TSB−dex
TSB

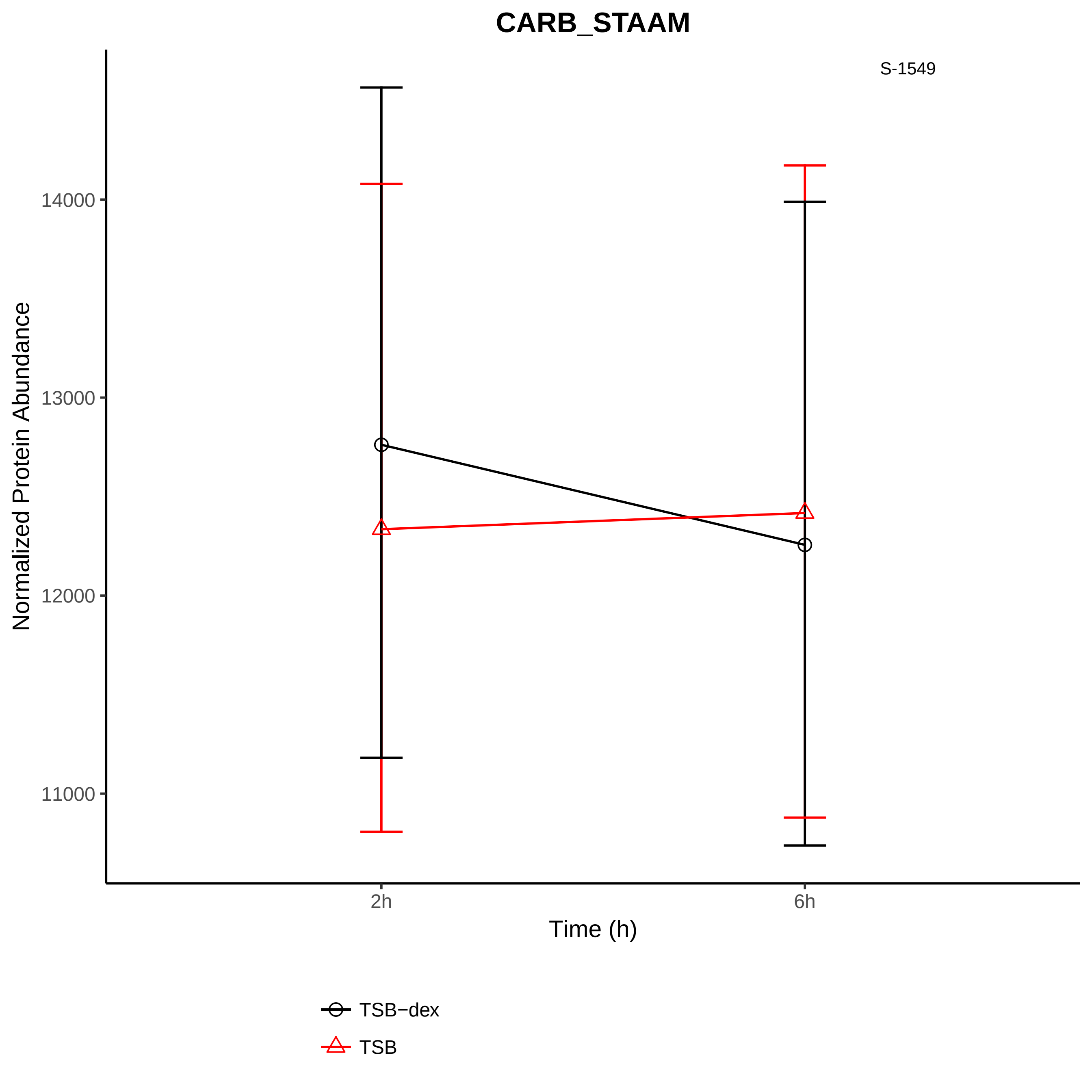
CARB_STAAM
S-1549
Normalized Protein Abundance
14000
13000
12000
11000
2h
6h
Time (h)
TSB−dex
TSB

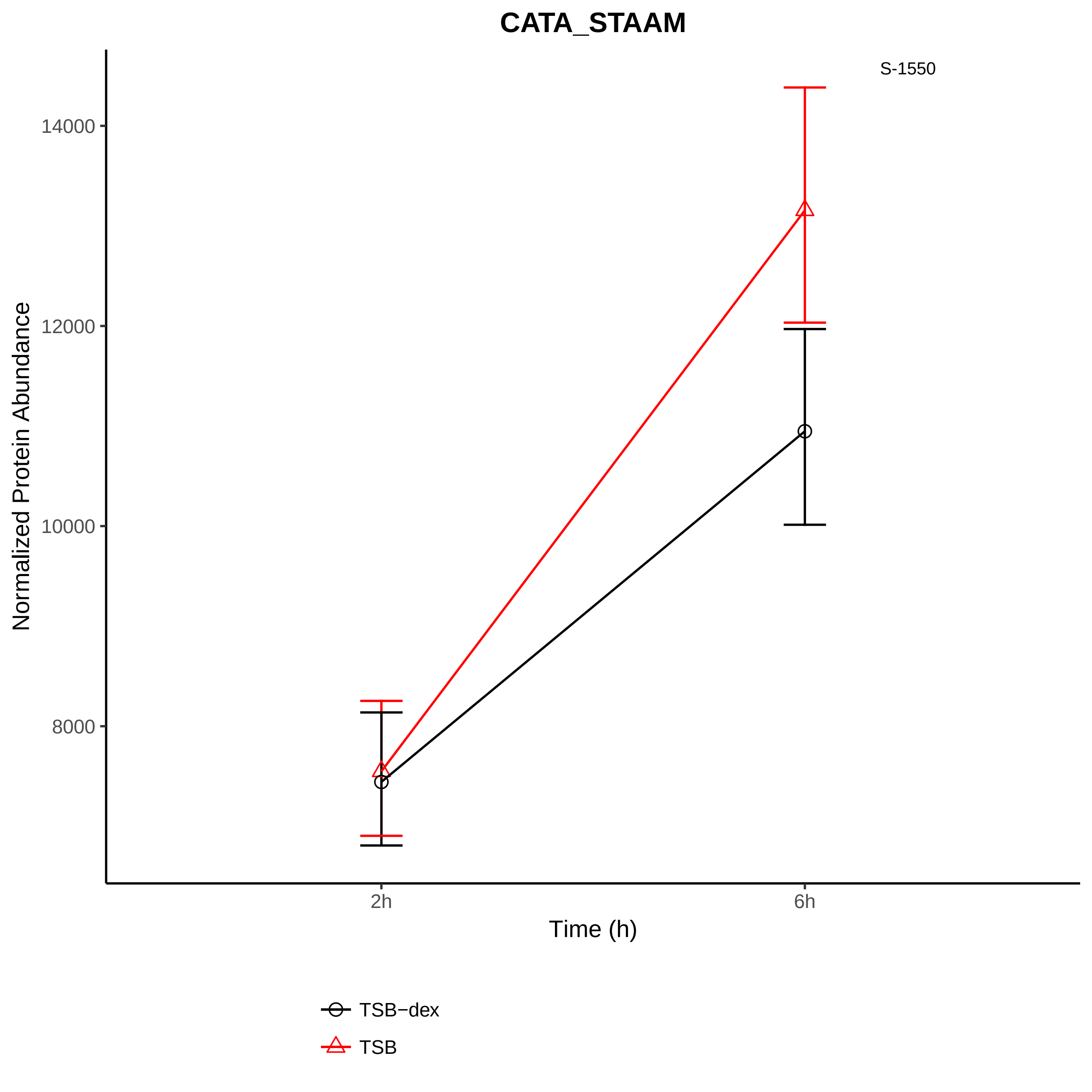

CATA_STAAM
S-1550
14000
12000
10000
8000
Normalized Protein Abundance
2h
6h
Time (h)
TSB−dex
TSB

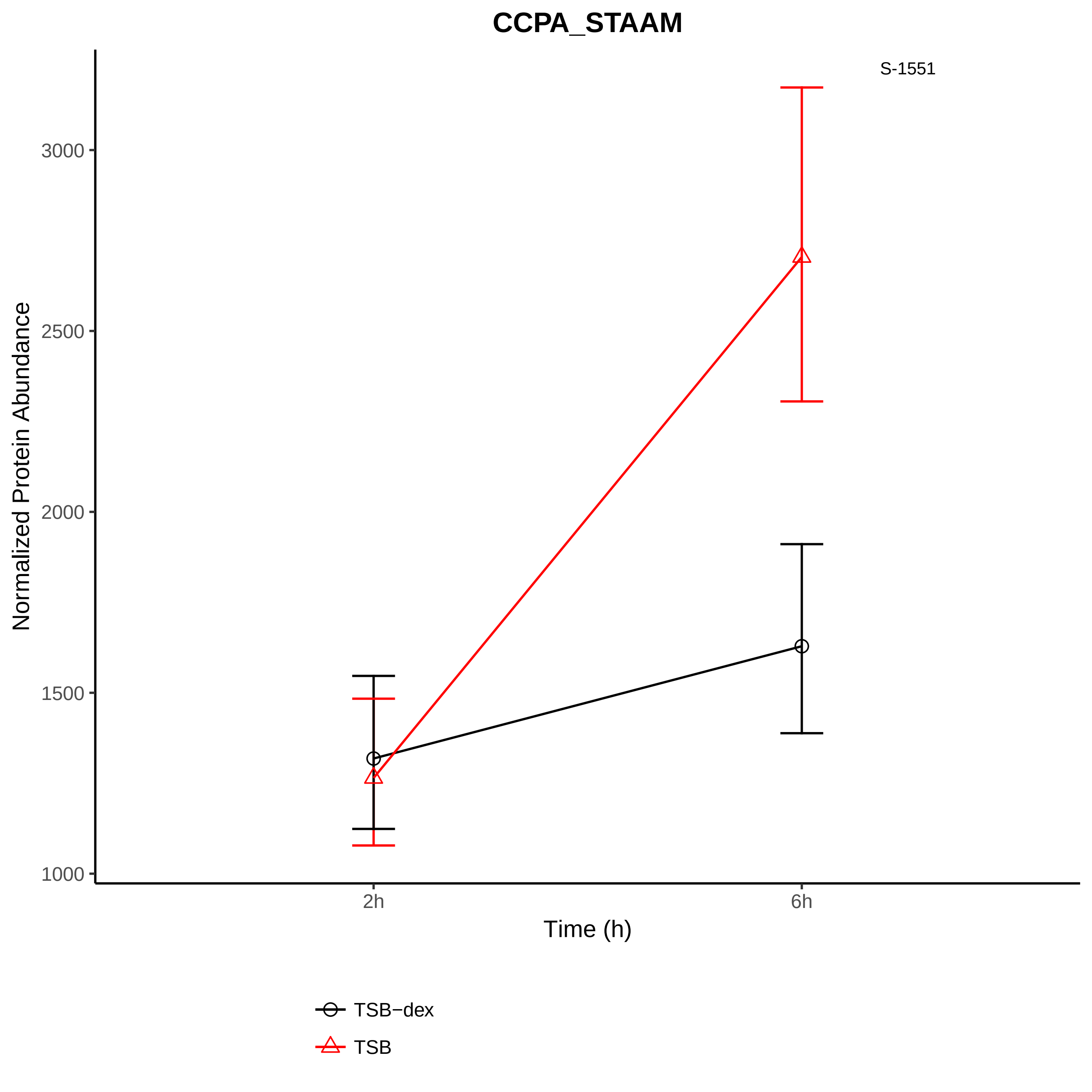

CCPA_STAAM
S-1551
Normalized Protein Abundance
3000
2500
2000
1500
1000
2h
6h
Time (h)
TSB−dex
TSB

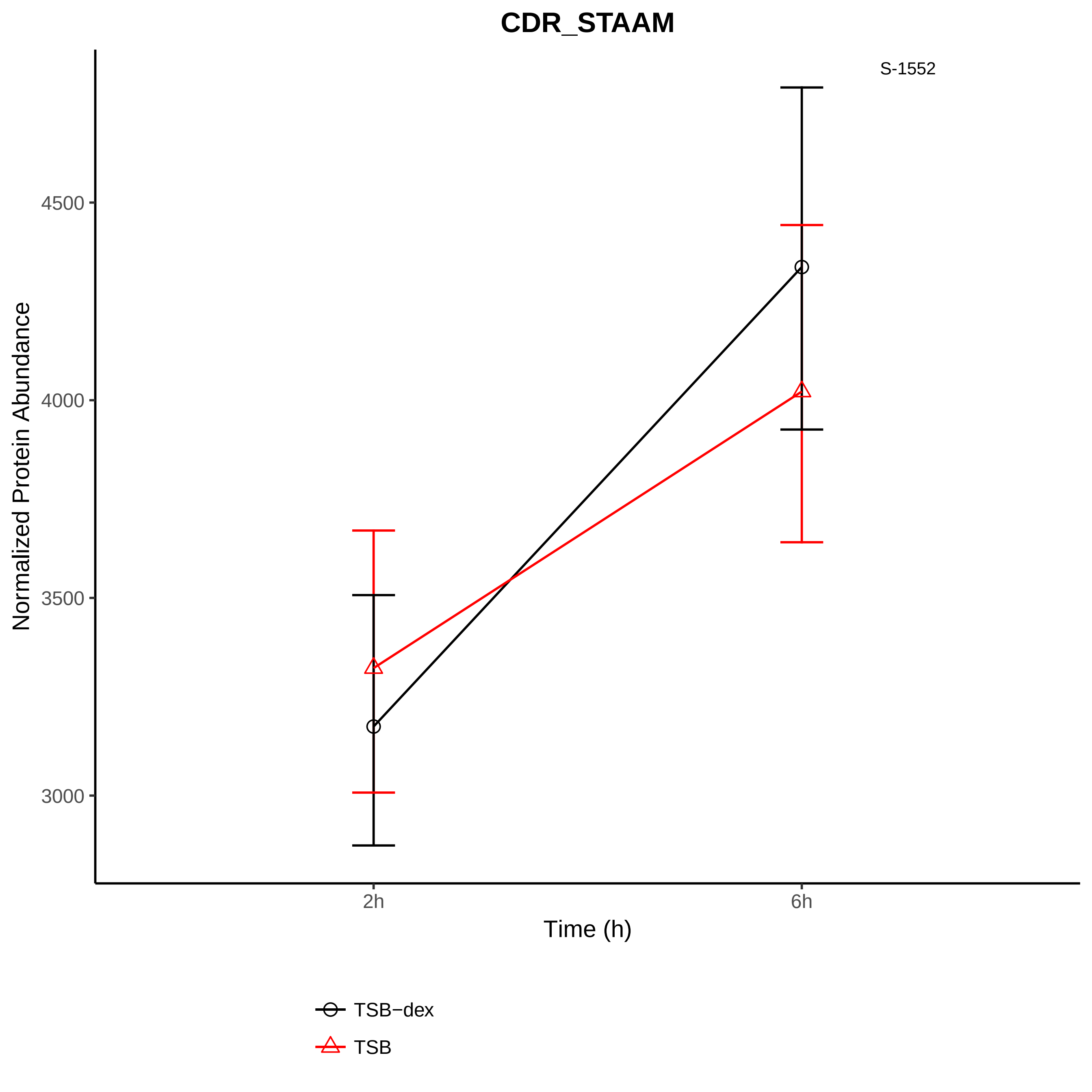

CDR_STAAM
S-1552
Normalized Protein Abundance
4500
4000
3500
3000
2h
6h
Time (h)
TSB−dex
TSB

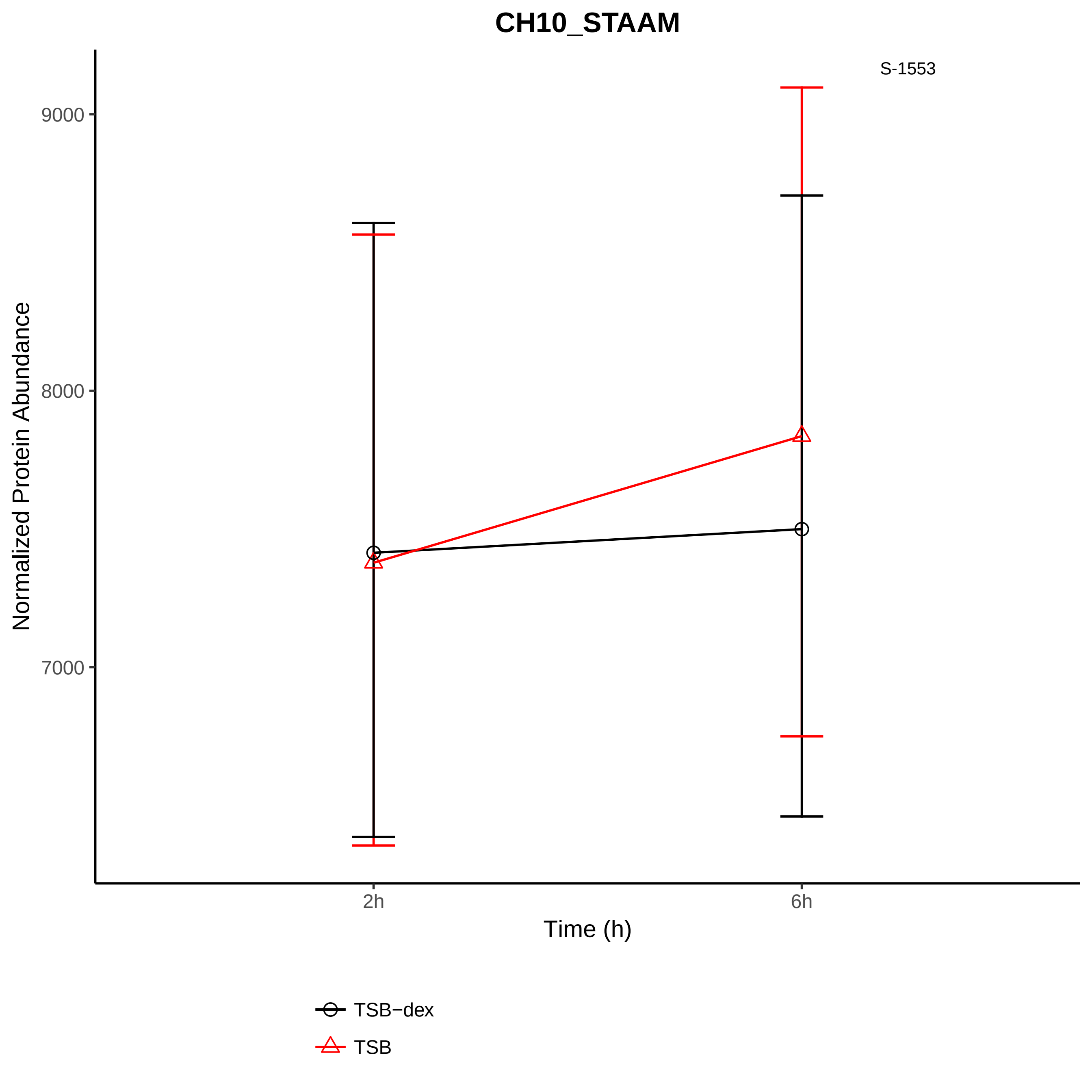

CH10_STAAM
S-1553
9000
8000
7000
Normalized Protein Abundance
2h
6h
Time (h)
TSB−dex
TSB

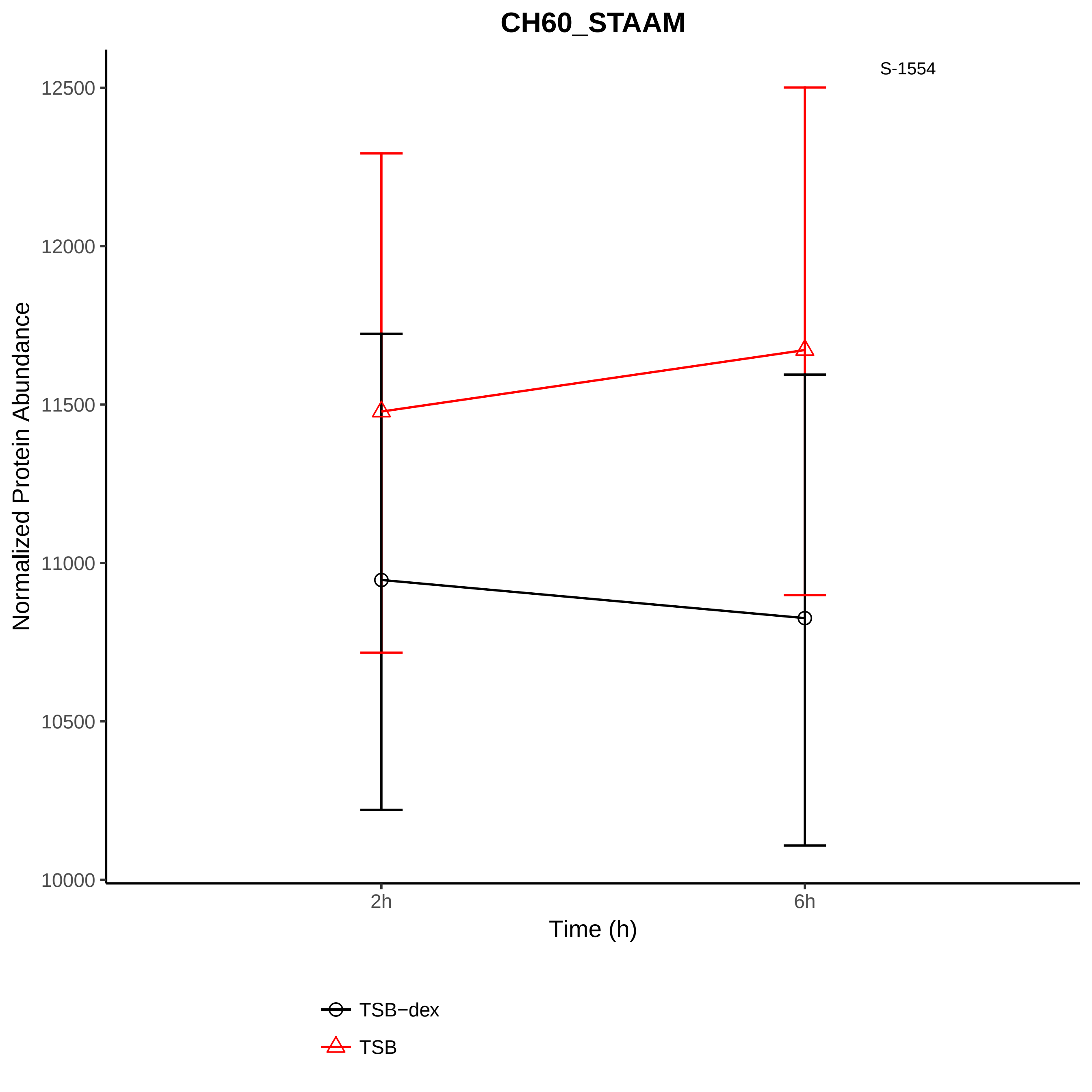
CH60_STAAM
S-1554
12500
12000
11500
11000
10500
10000
Normalized Protein Abundance
2h
6h
Time (h)
TSB−dex
TSB

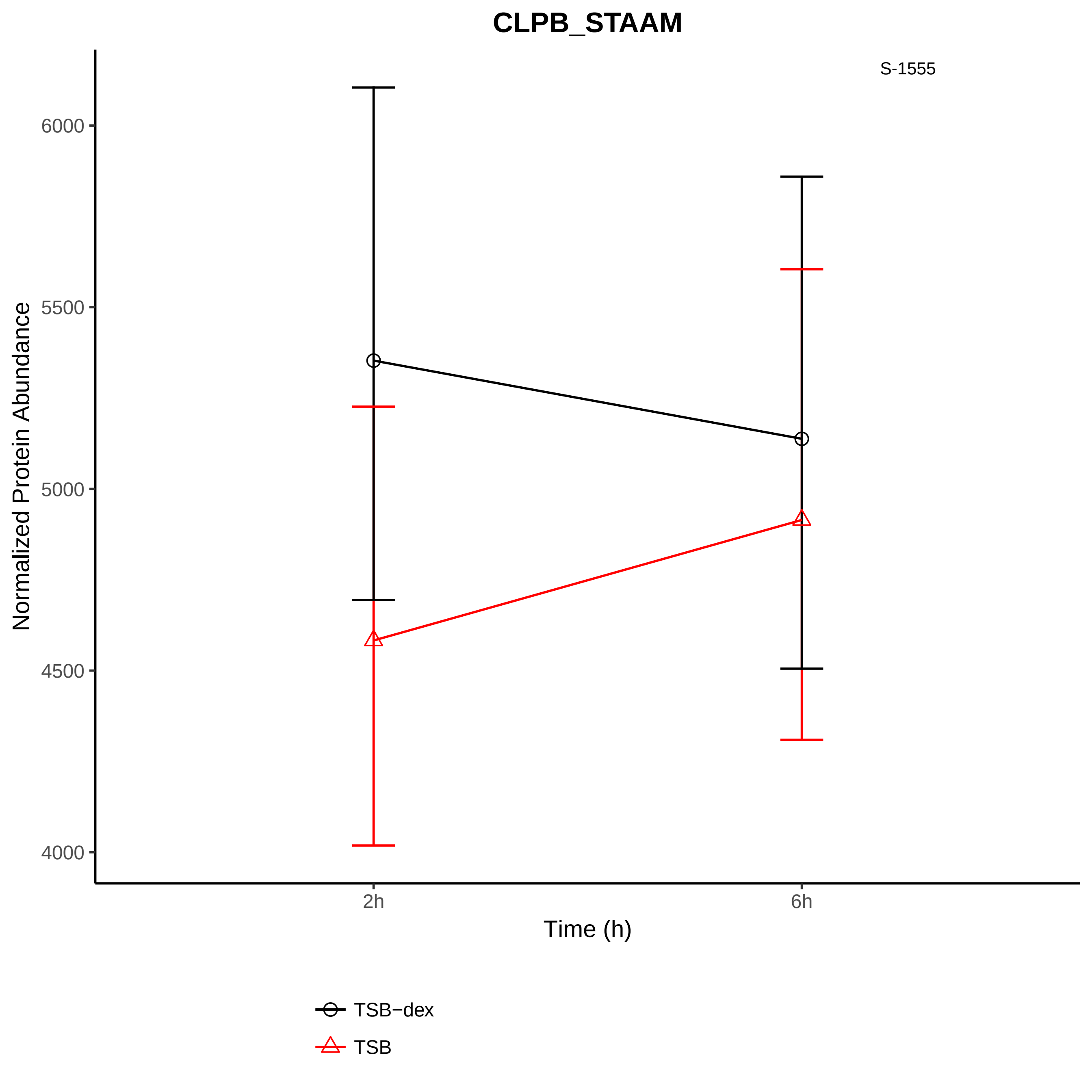

CLPB_STAAM
S-1555
6000
5500
5000
4500
4000
Normalized Protein Abundance
2h
6h
Time (h)
TSB−dex
TSB

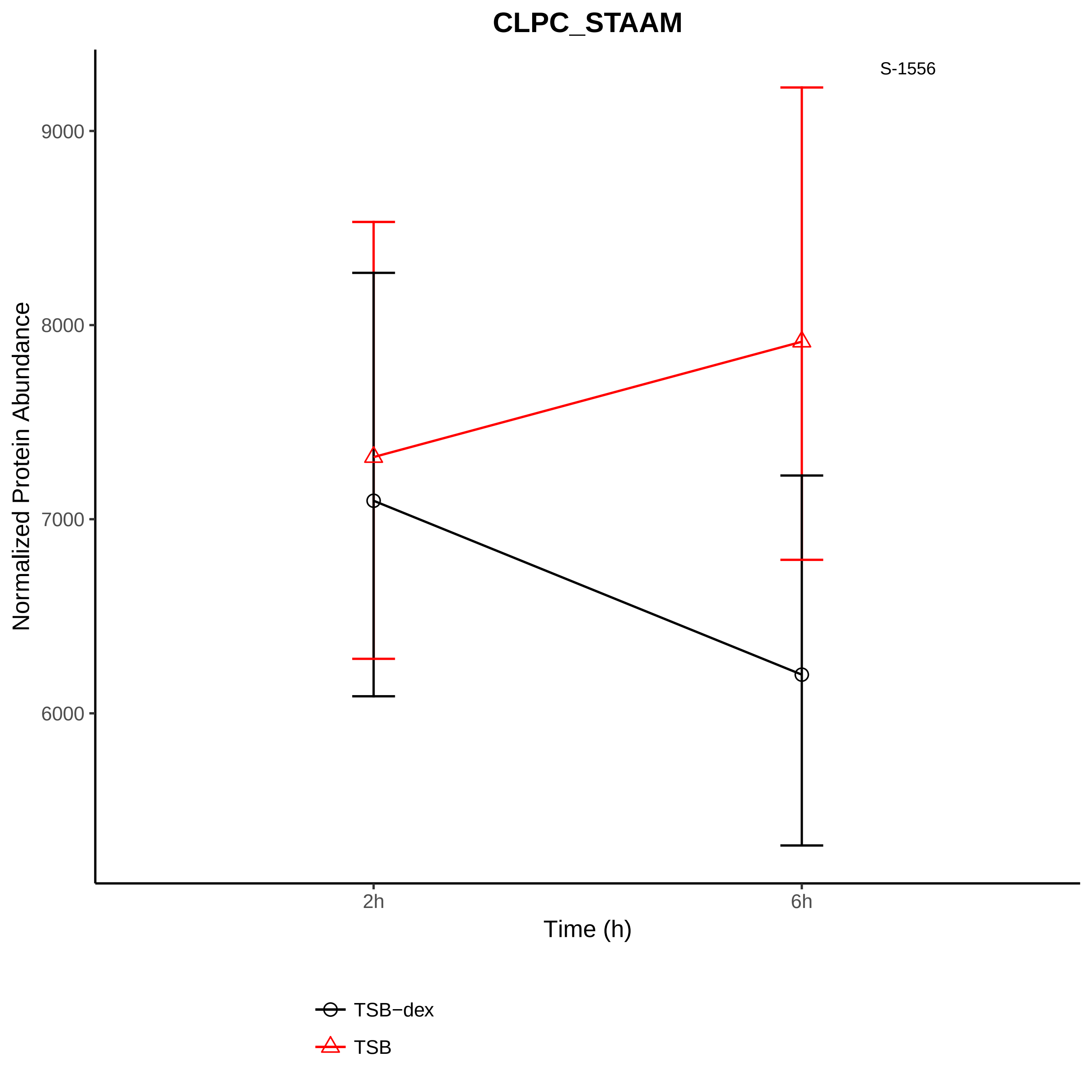
CLPC_STAAM
S-1556
9000
8000
7000
6000
Normalized Protein Abundance
2h
6h
Time (h)
TSB−dex
TSB

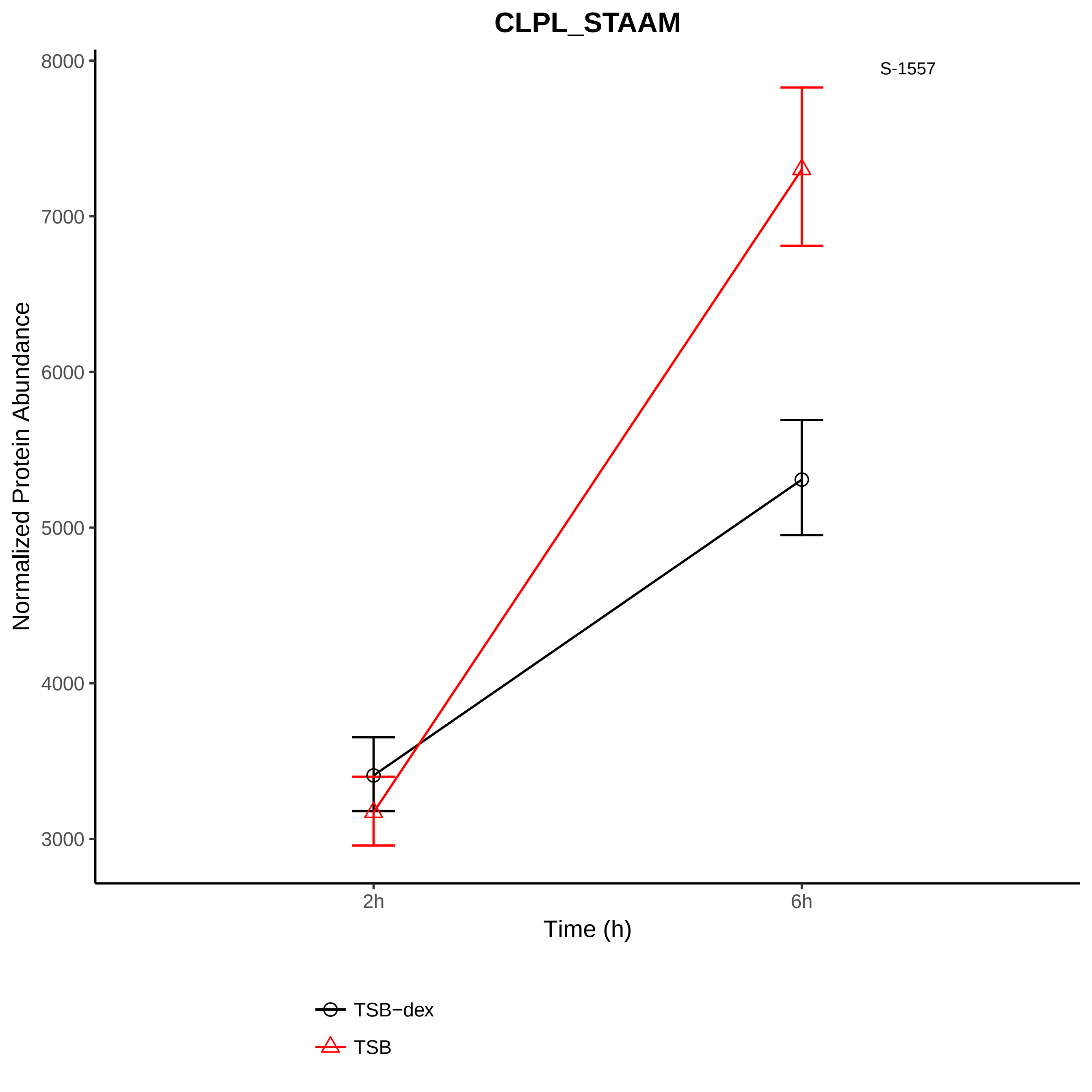

CLPL_STAAM
S-1557
8000
7000
6000
5000
4000
3000
Normalized Protein Abundance
2h
6h
Time (h)
TSB−dex
TSB

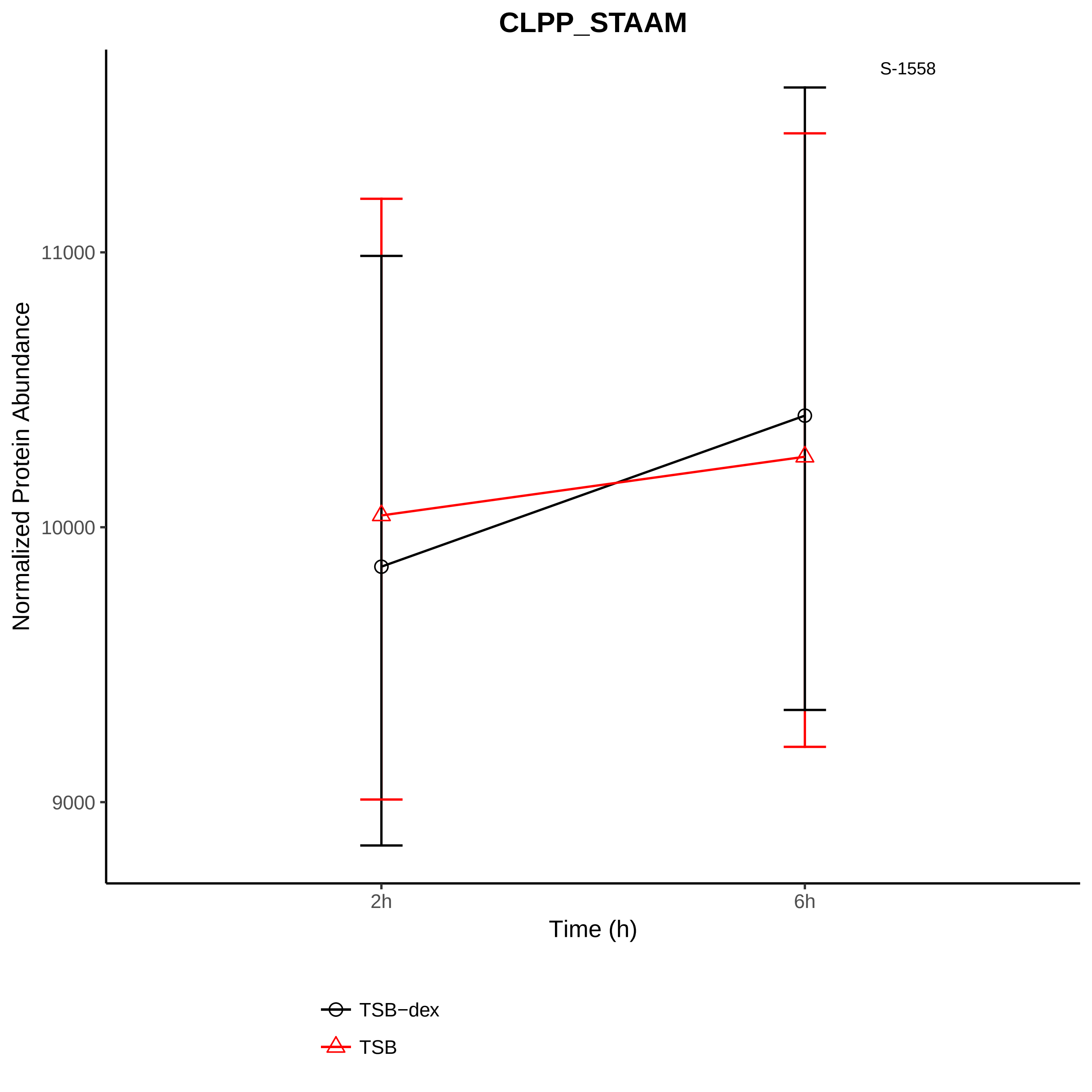

CLPP_STAAM
S-1558
Normalized Protein Abundance
11000
10000
9000
2h
6h
Time (h)
TSB−dex
TSB

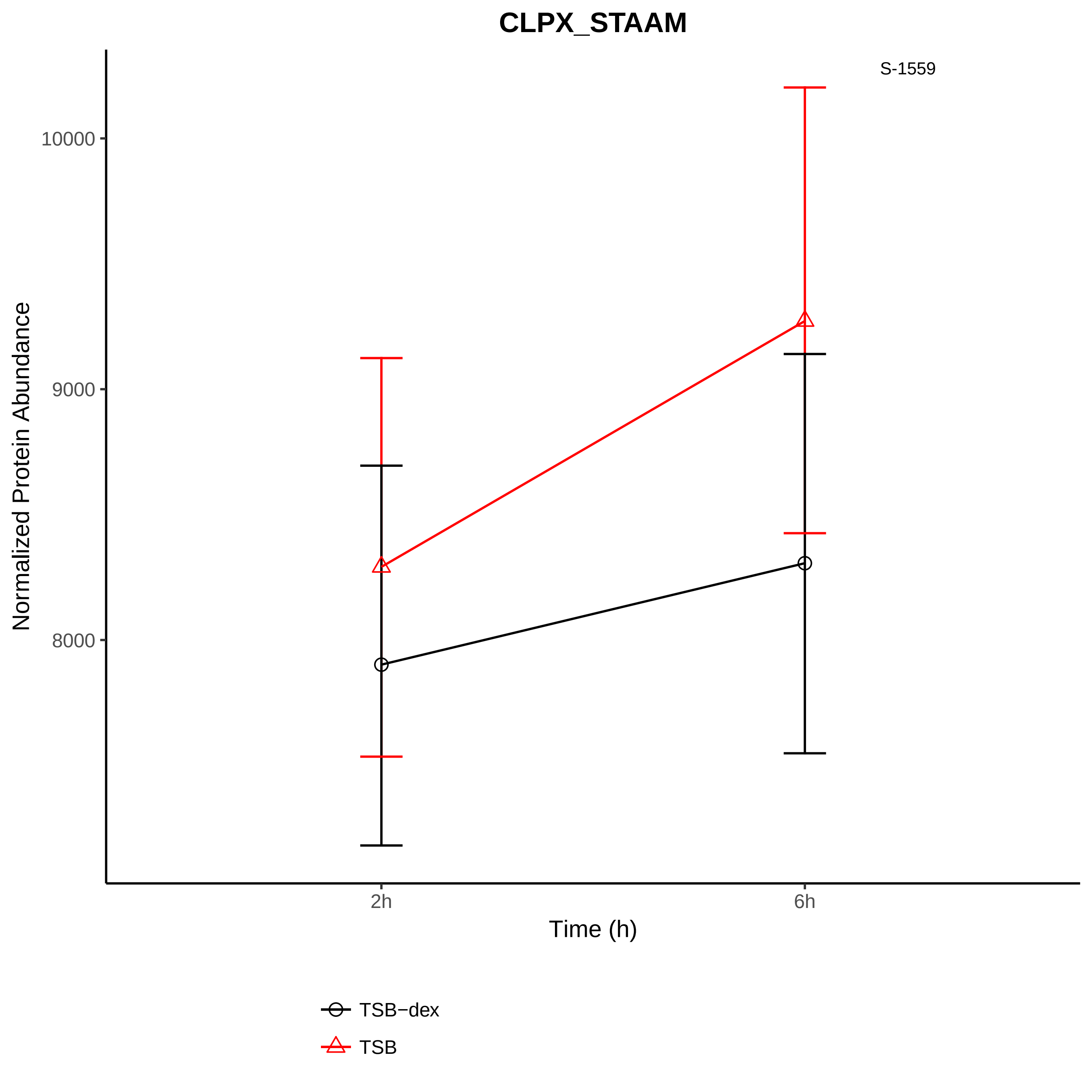

CLPX_STAAM
S-1559
10000
9000
8000
Normalized Protein Abundance
2h
6h
Time (h)
TSB−dex
TSB

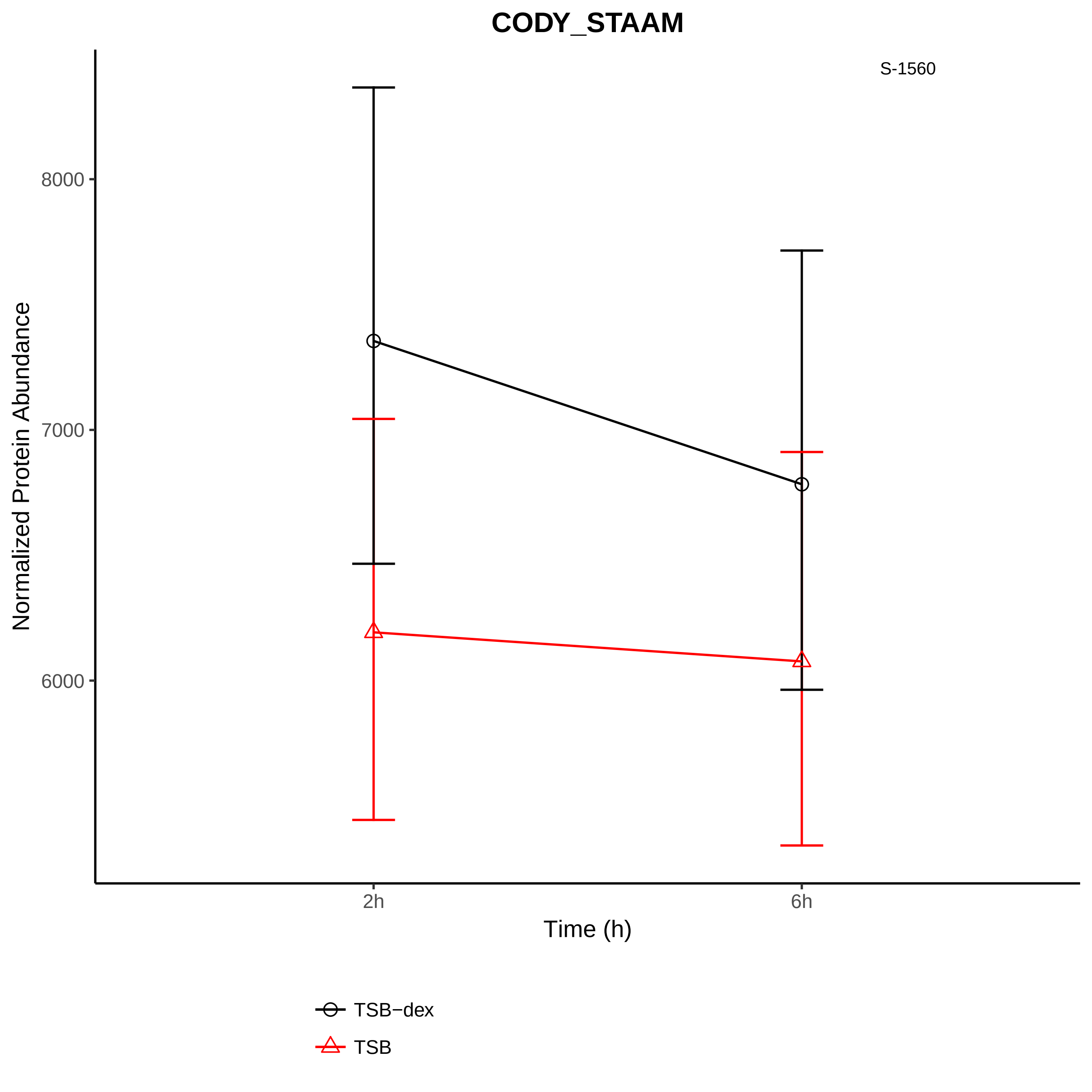

CODY_STAAM
S-1560
8000
7000
6000
Normalized Protein Abundance
2h
6h
Time (h)
TSB−dex
TSB

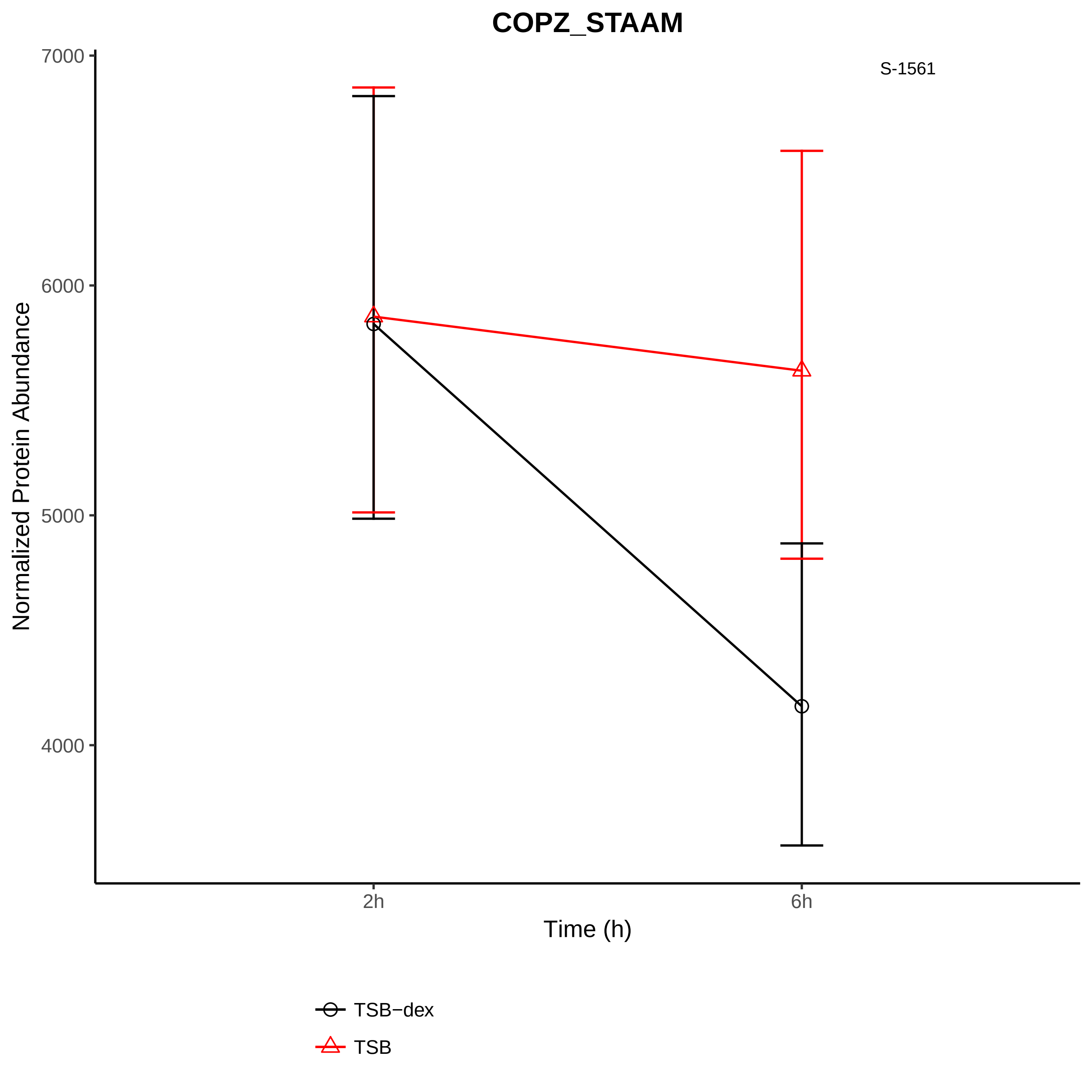

COPZ_STAAM
S-1561
7000
6000
5000
4000
Normalized Protein Abundance
2h
6h
Time (h)
TSB−dex
TSB

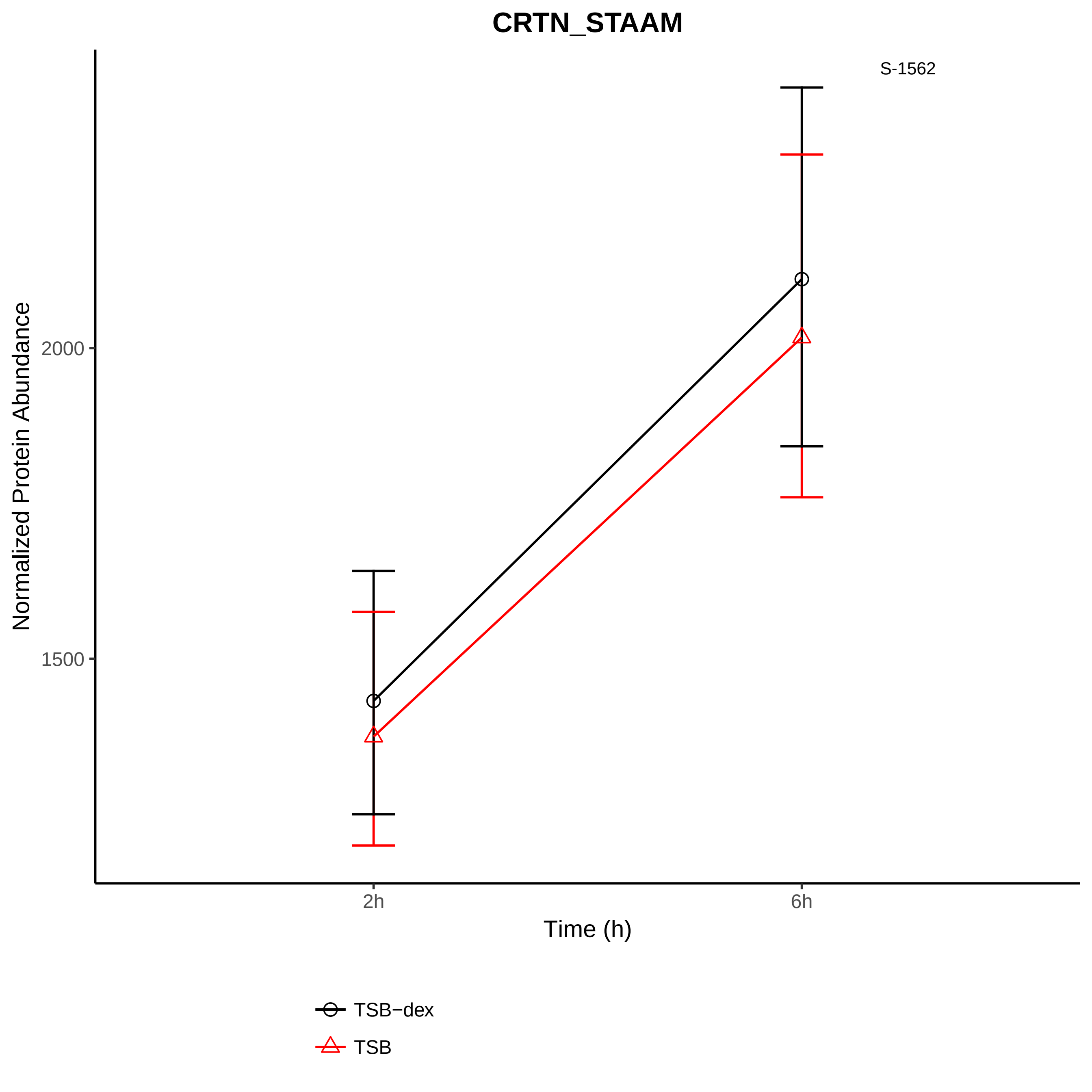

CRTN_STAAM
S-1562
Normalized Protein Abundance
2000
1500
2h
6h
Time (h)
TSB−dex
TSB

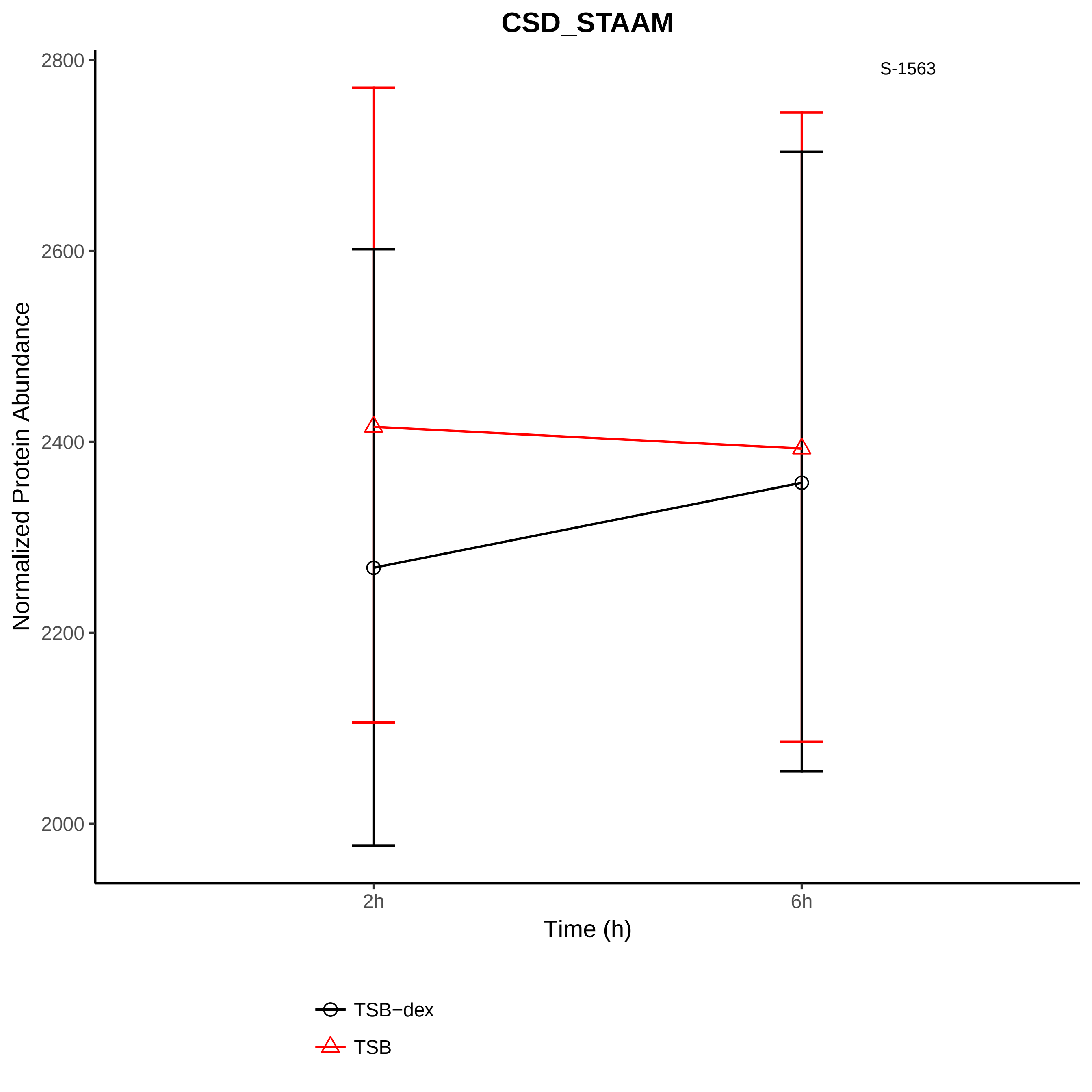

CSD_STAAM
S-1563
Normalized Protein Abundance
2800
2600
2400
2200
2000
2h
6h
Time (h)
TSB−dex
TSB

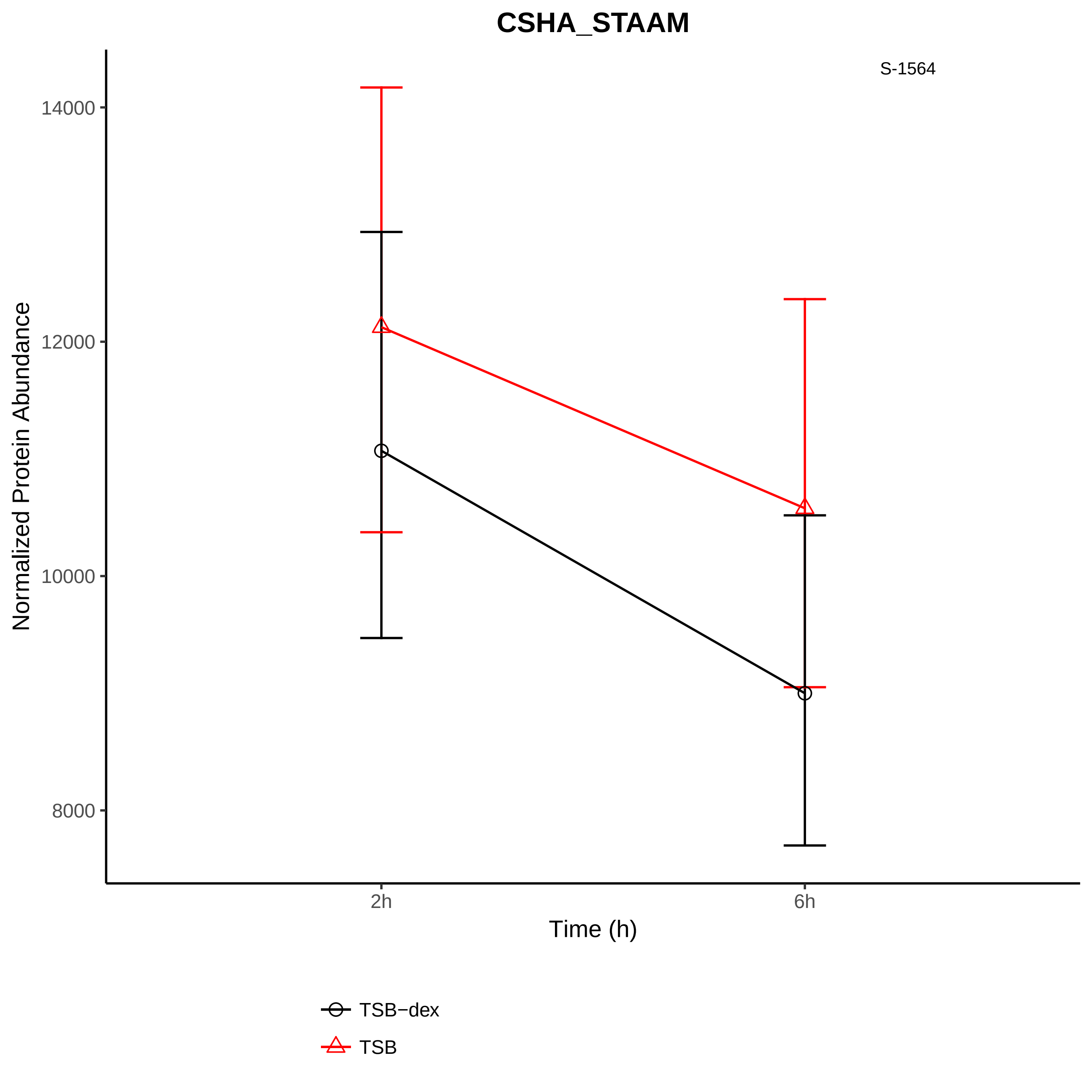
CSHA_STAAM
S-1564
Normalized Protein Abundance
14000
12000
10000
8000
2h
6h
Time (h)
TSB−dex
TSB

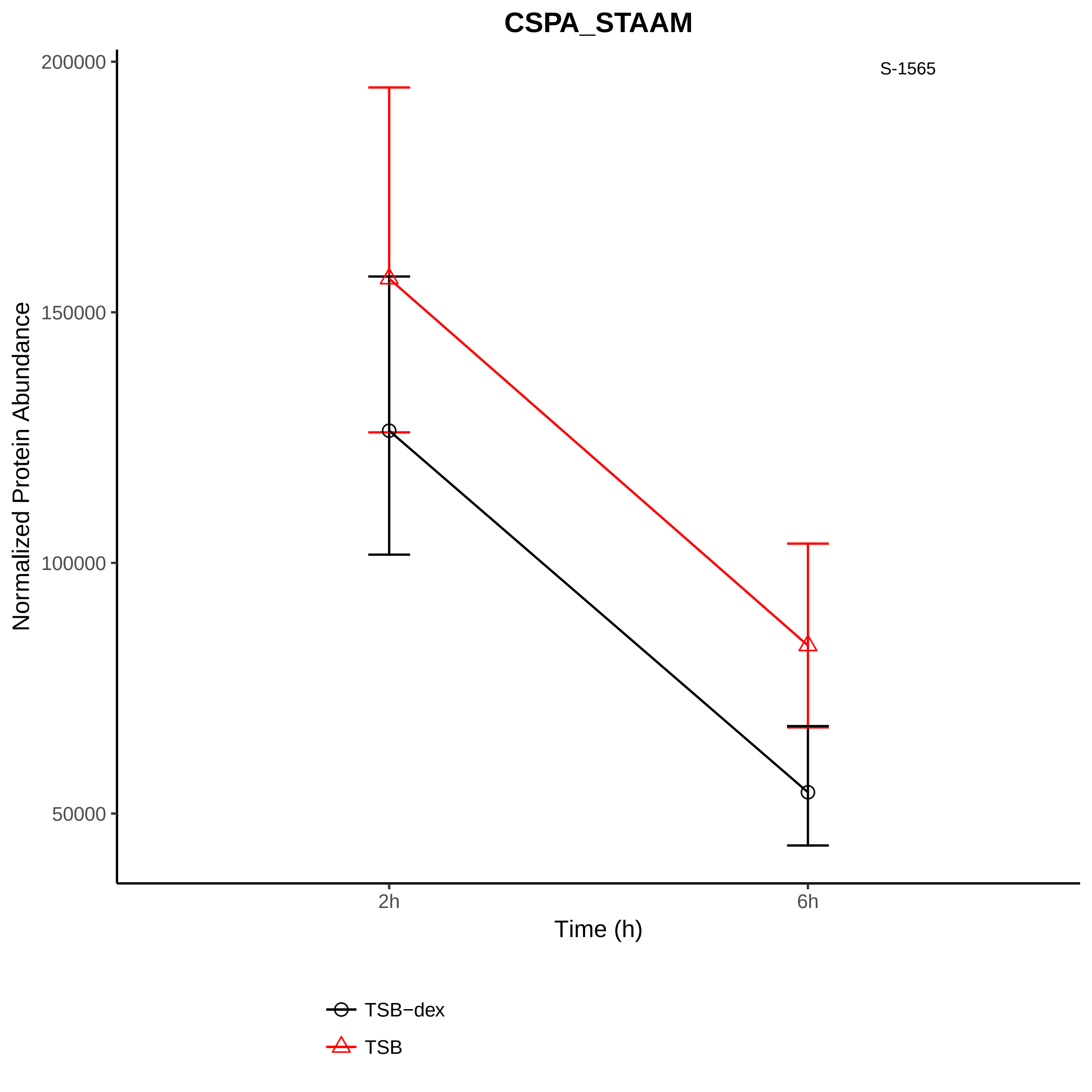
CSPA_STAAM
S-1565
200000
150000
100000
50000
Normalized Protein Abundance
2h
6h
Time (h)
TSB−dex
TSB

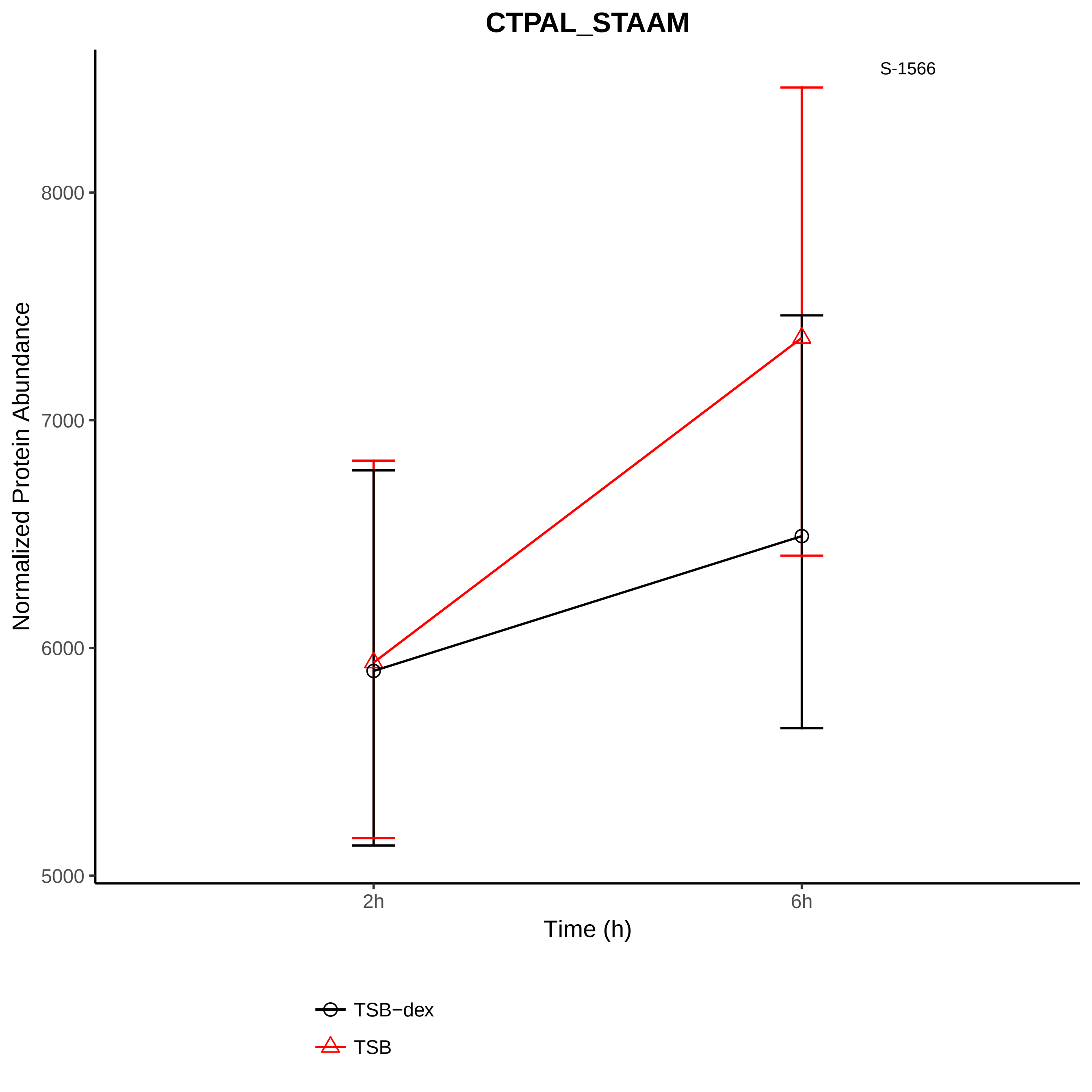

CTPAL_STAAM
S-1566
Normalized Protein Abundance
8000
7000
6000
5000
2h
6h
Time (h)
TSB−dex
TSB

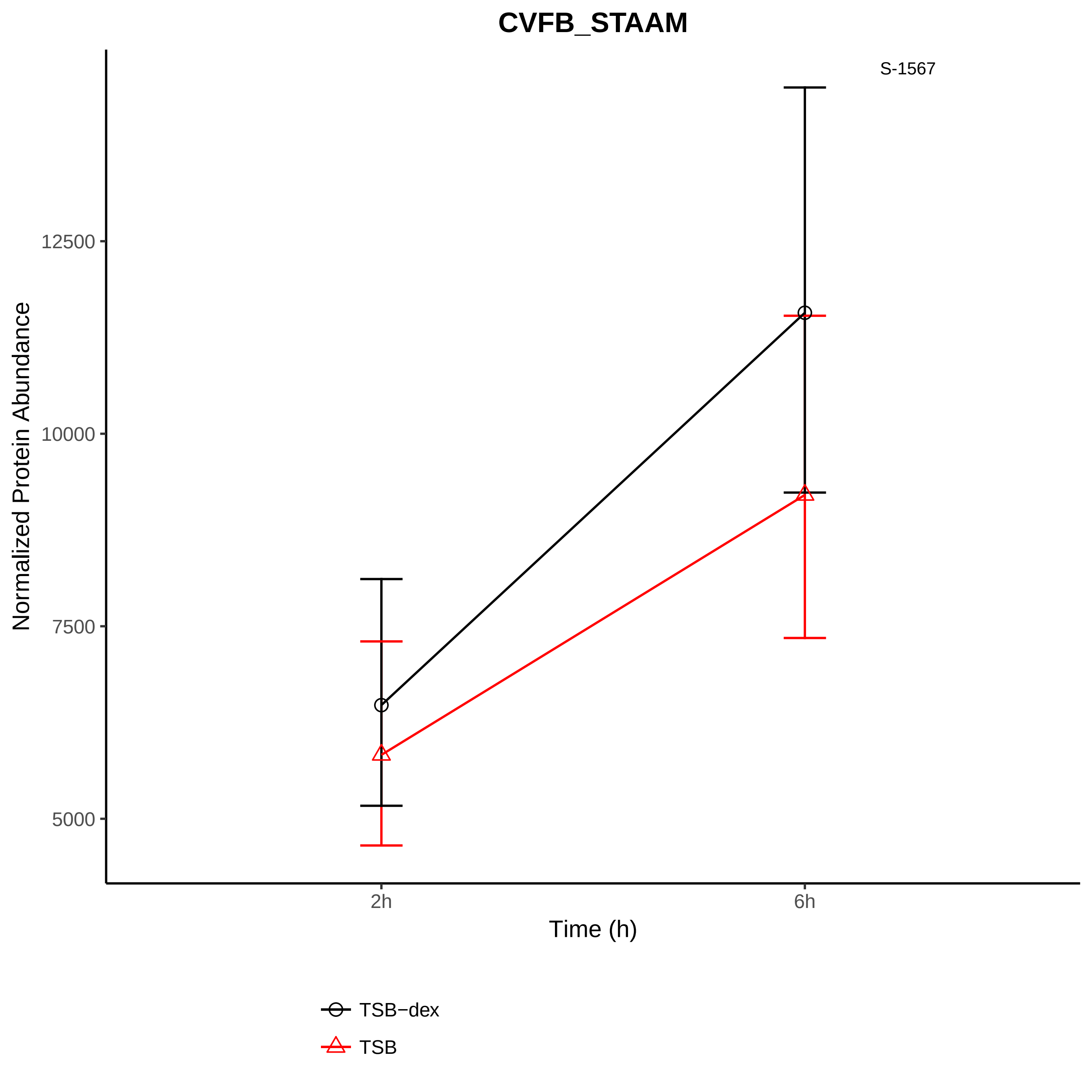

CVFB_STAAM
S-1567
Normalized Protein Abundance
12500
10000
7500
5000
2h
6h
Time (h)
TSB−dex
TSB

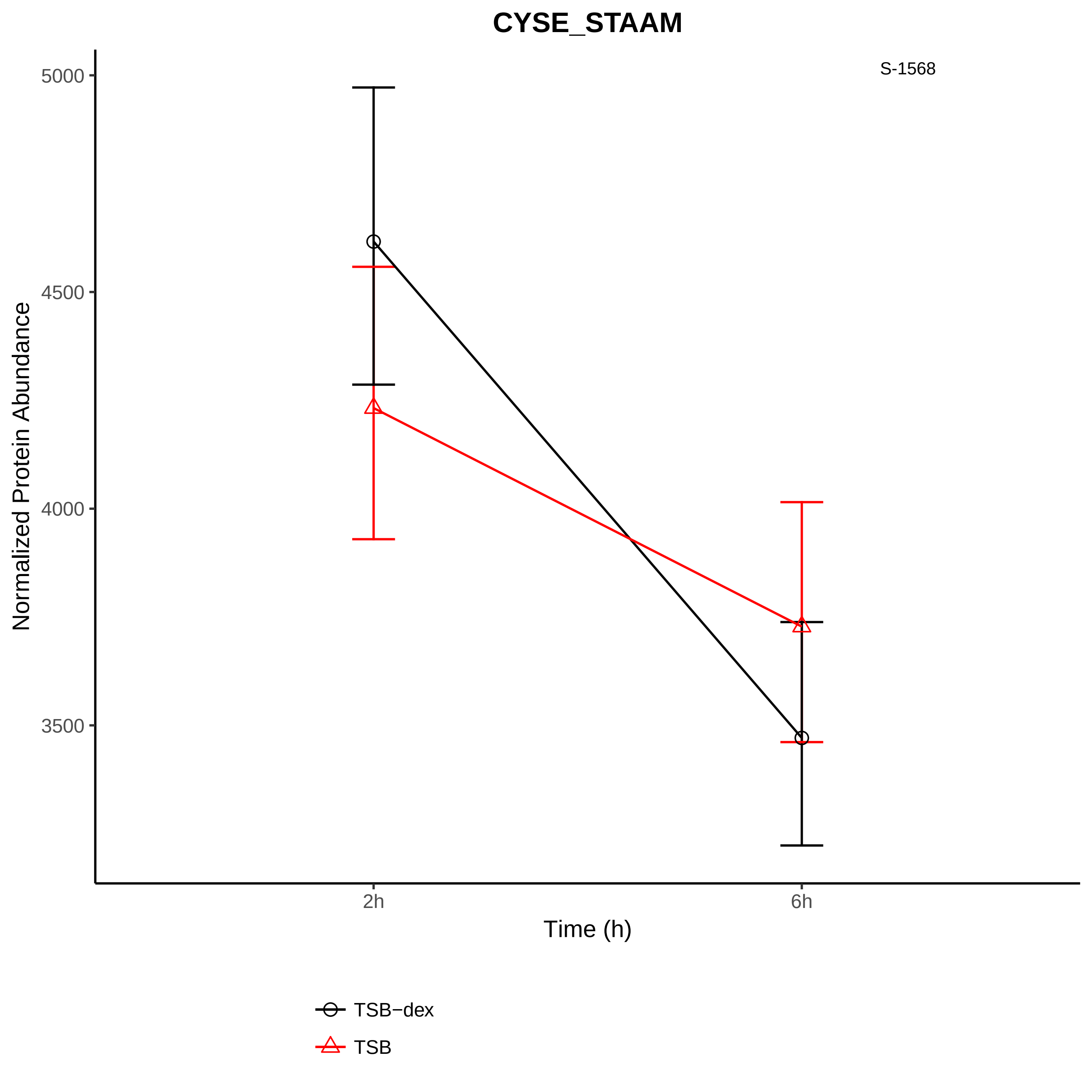

CYSE_STAAM
S-1568
5000
4500
4000
3500
Normalized Protein Abundance
2h
6h
Time (h)
TSB−dex
TSB

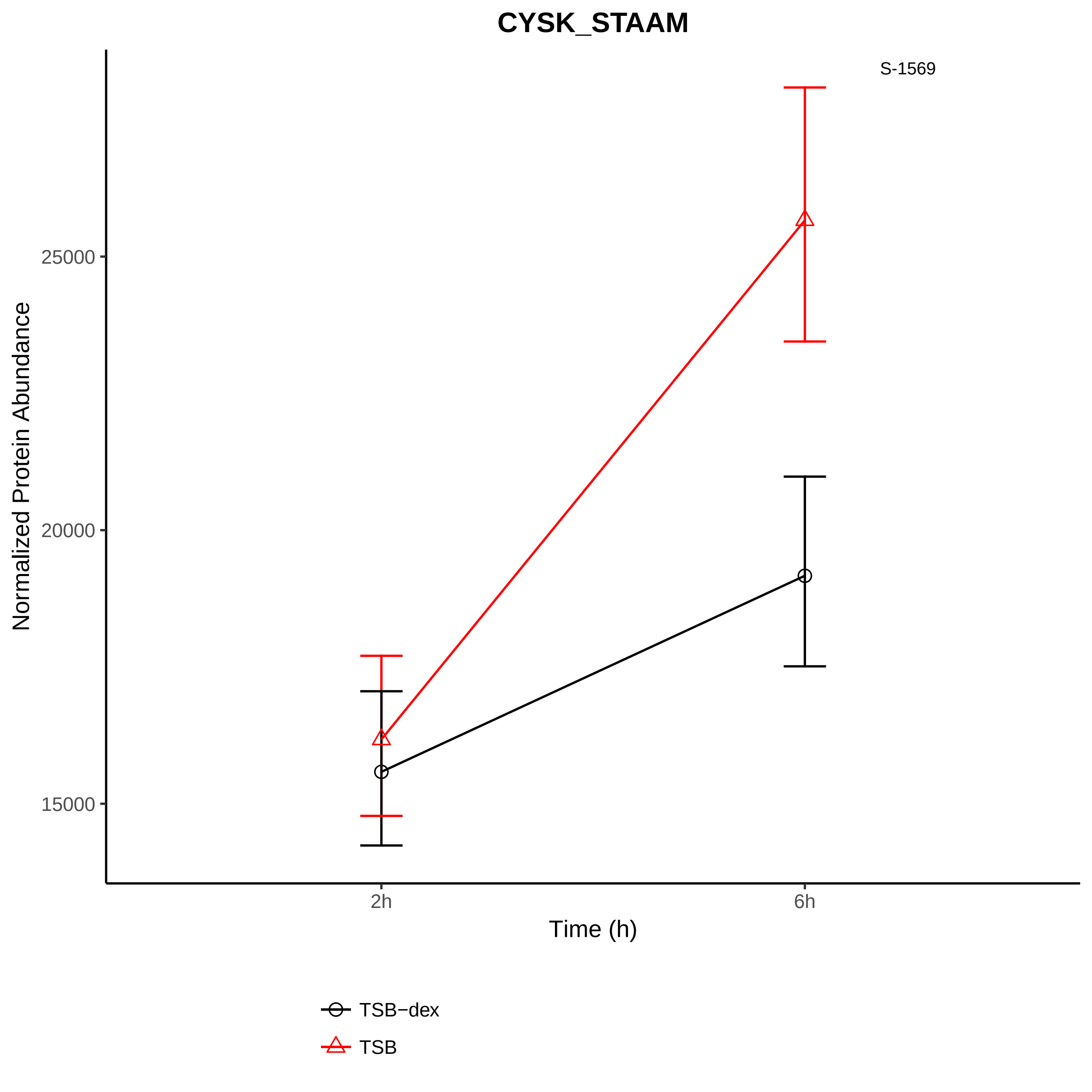

CYSK_STAAM
S-1569
Normalized Protein Abundance
25000
20000
15000
2h
6h
Time (h)
TSB−dex
TSB

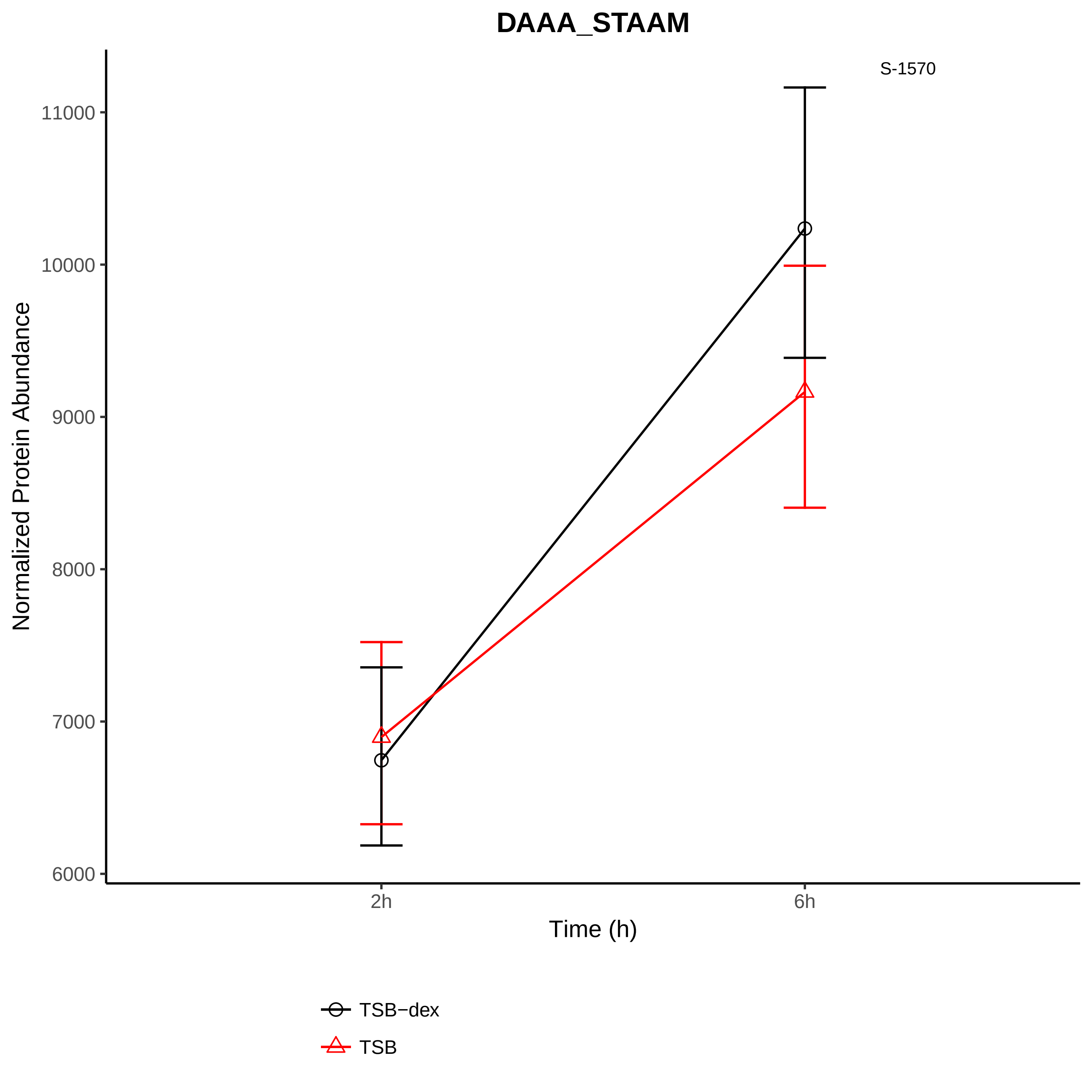

DAAA_STAAM
S-1570
Normalized Protein Abundance
11000
10000
9000
8000
7000
6000
2h
6h
Time (h)
TSB−dex
TSB

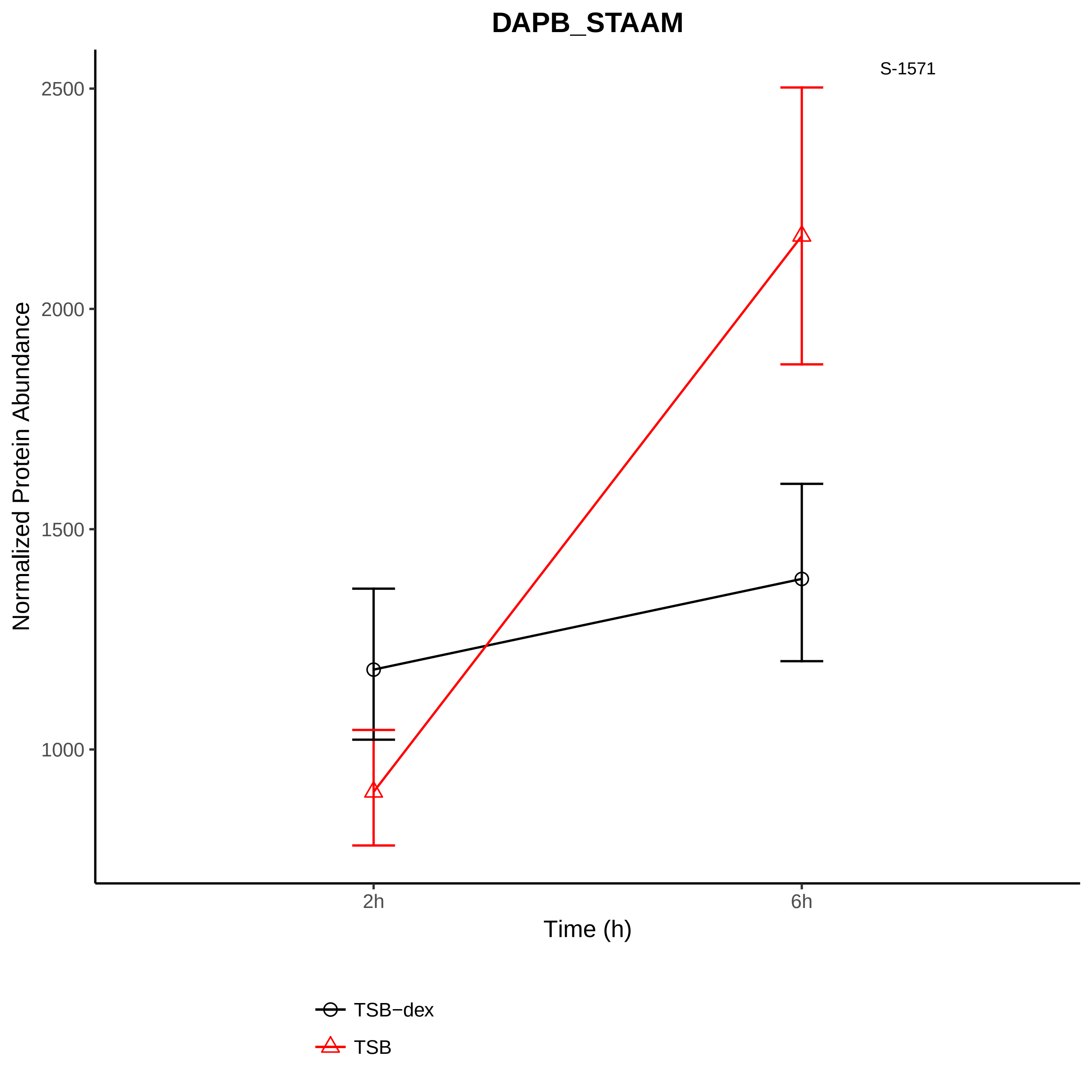

DAPB_STAAM
S-1571
2500
2000
1500
1000
Normalized Protein Abundance
2h
6h
Time (h)
TSB−dex
TSB

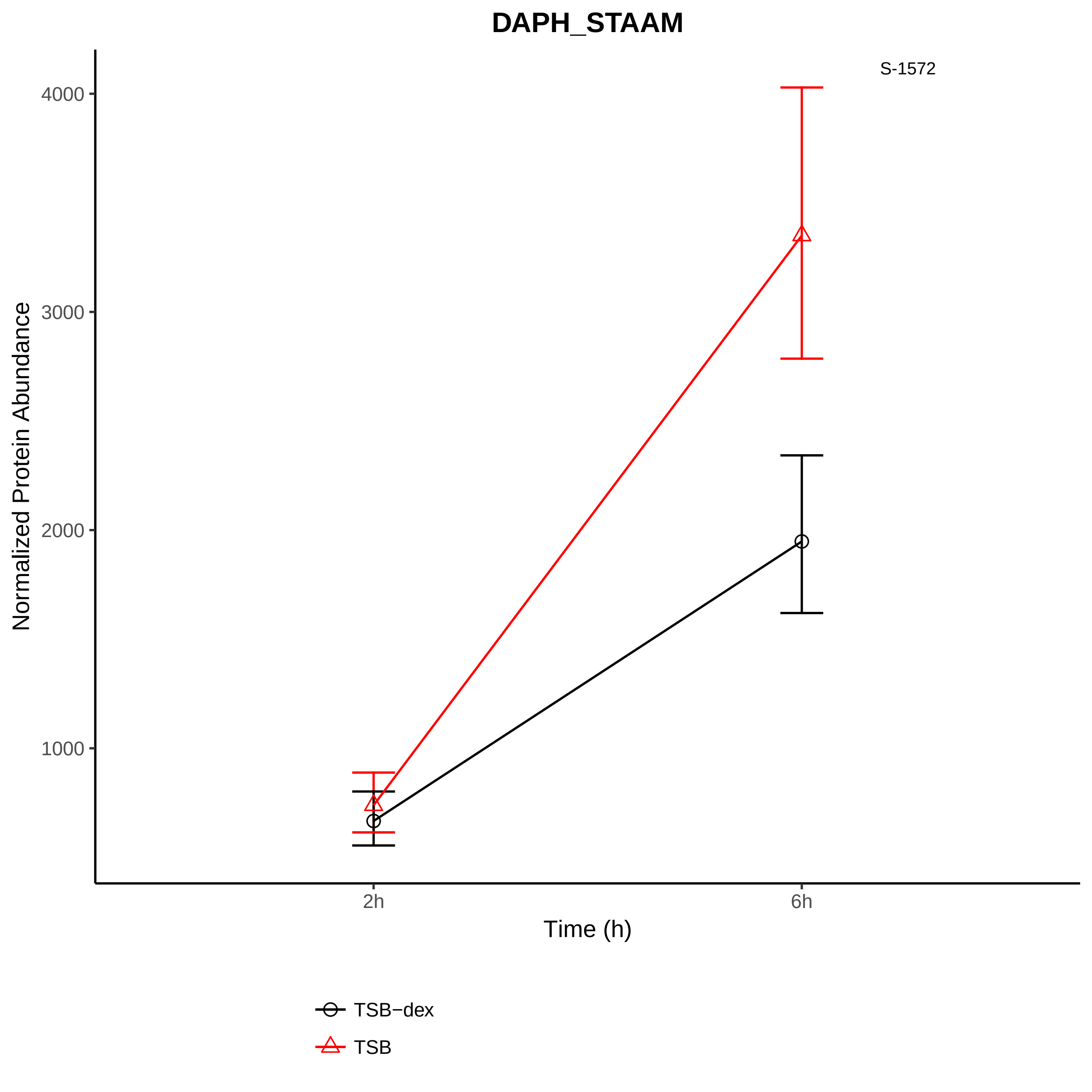
DAPH_STAAM
S-1572
4000
3000
2000
1000
Normalized Protein Abundance
2h
6h
Time (h)
TSB−dex
TSB

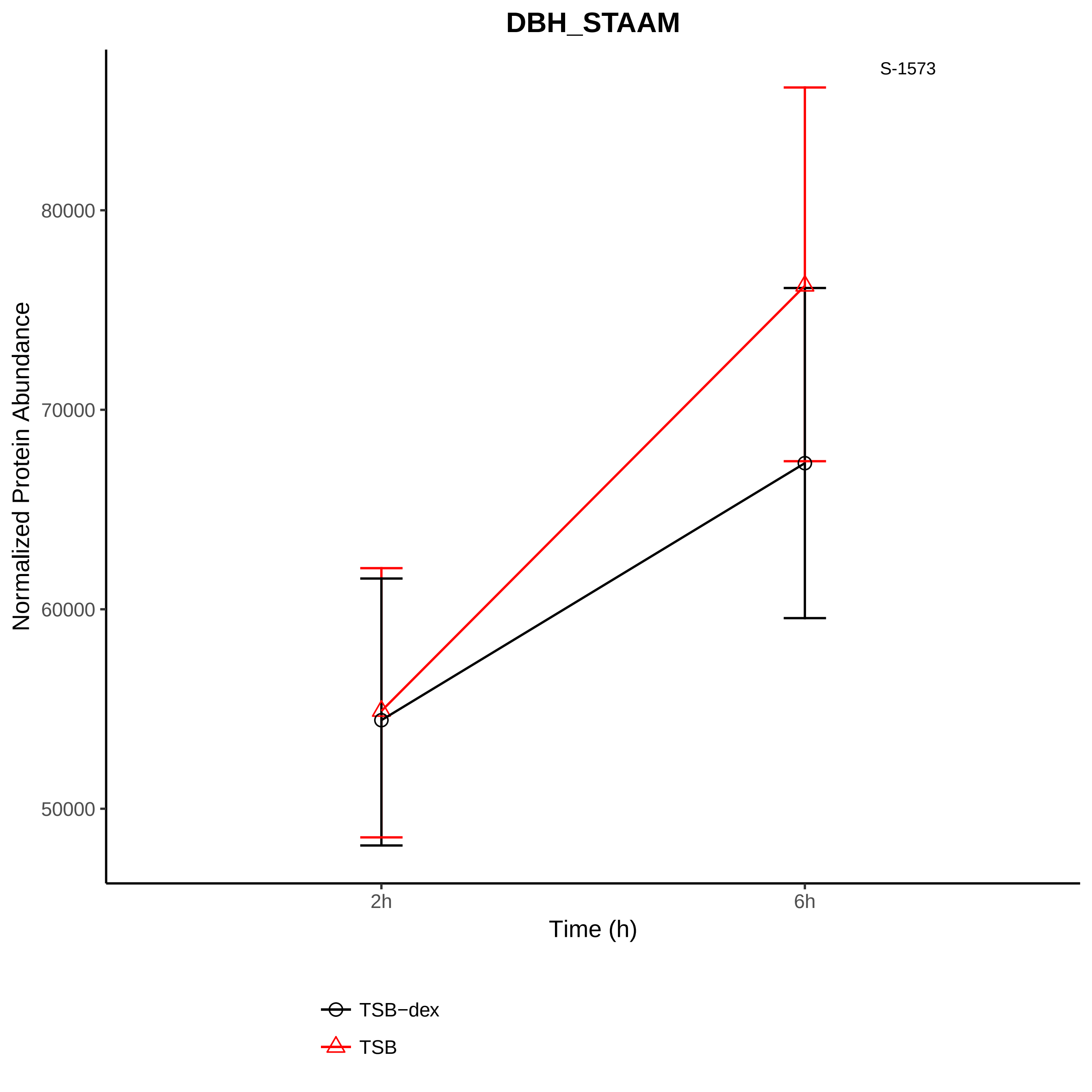
DBH_STAAM
S-1573
Normalized Protein Abundance
80000
70000
60000
50000
2h
6h
Time (h)
TSB−dex
TSB

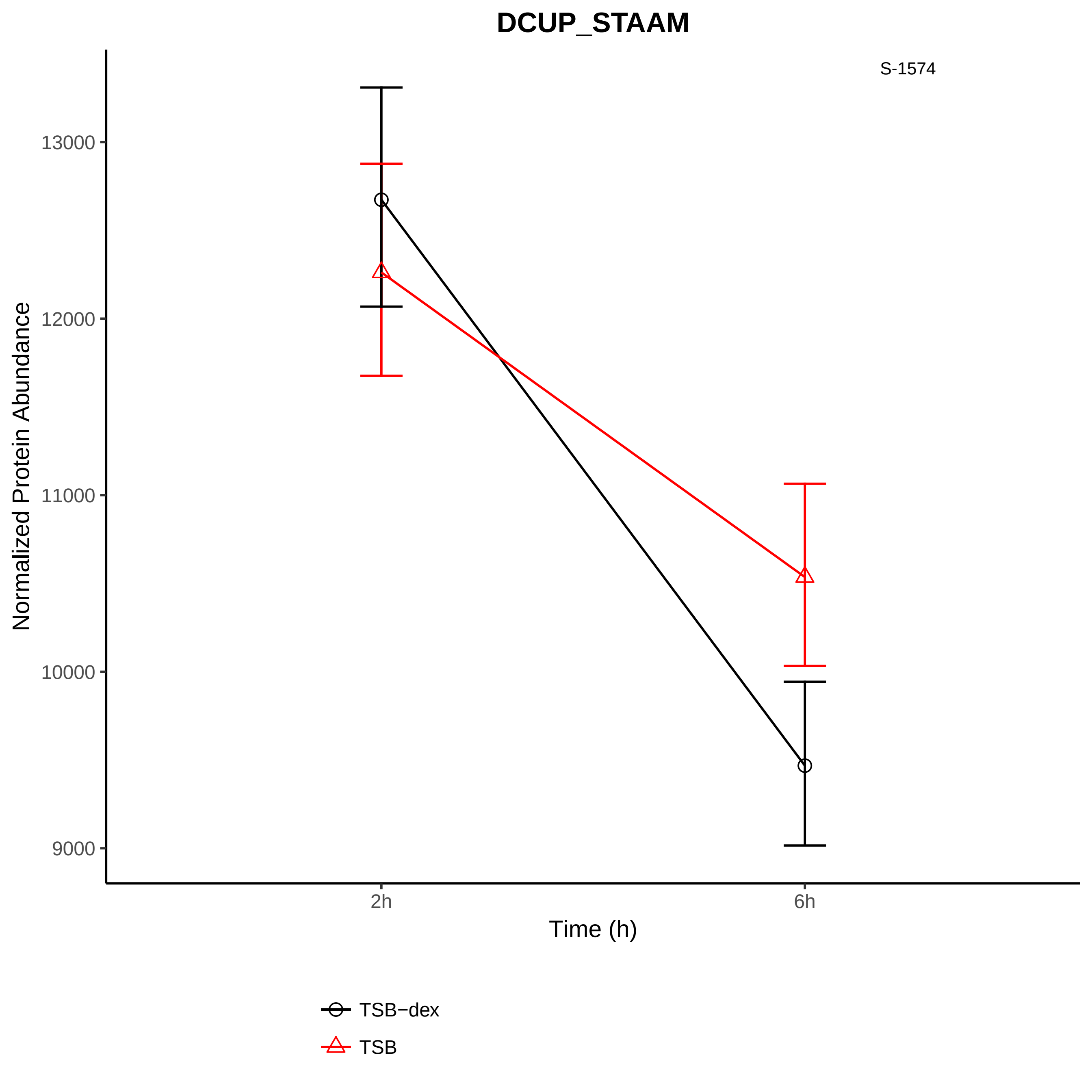

DCUP_STAAM
S-1574
Normalized Protein Abundance
13000
12000
11000
10000
9000
2h
6h
Time (h)
TSB−dex
TSB

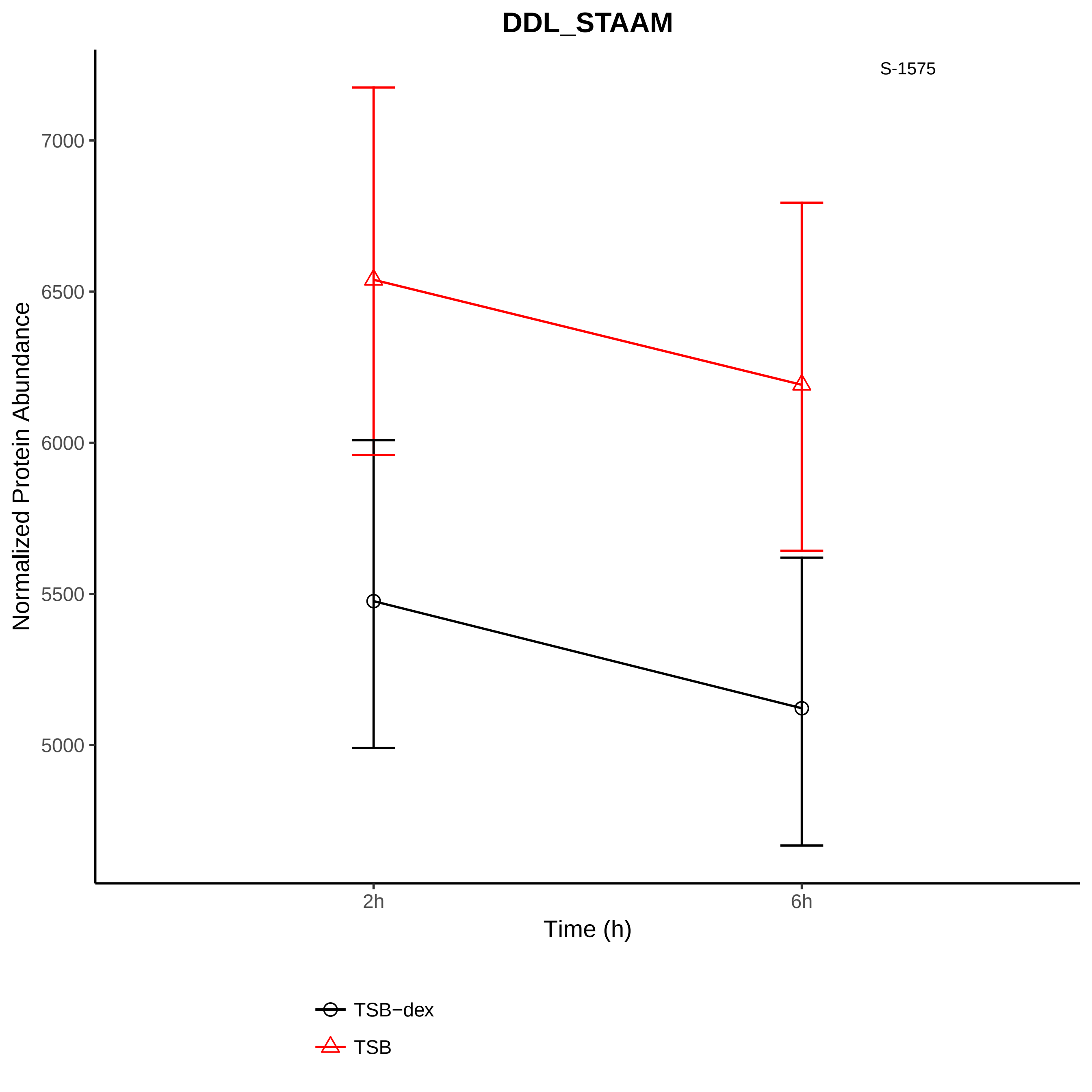
DDL_STAAM
S-1575
7000
6500
6000
5500
5000
Normalized Protein Abundance
2h
6h
Time (h)
TSB−dex
TSB

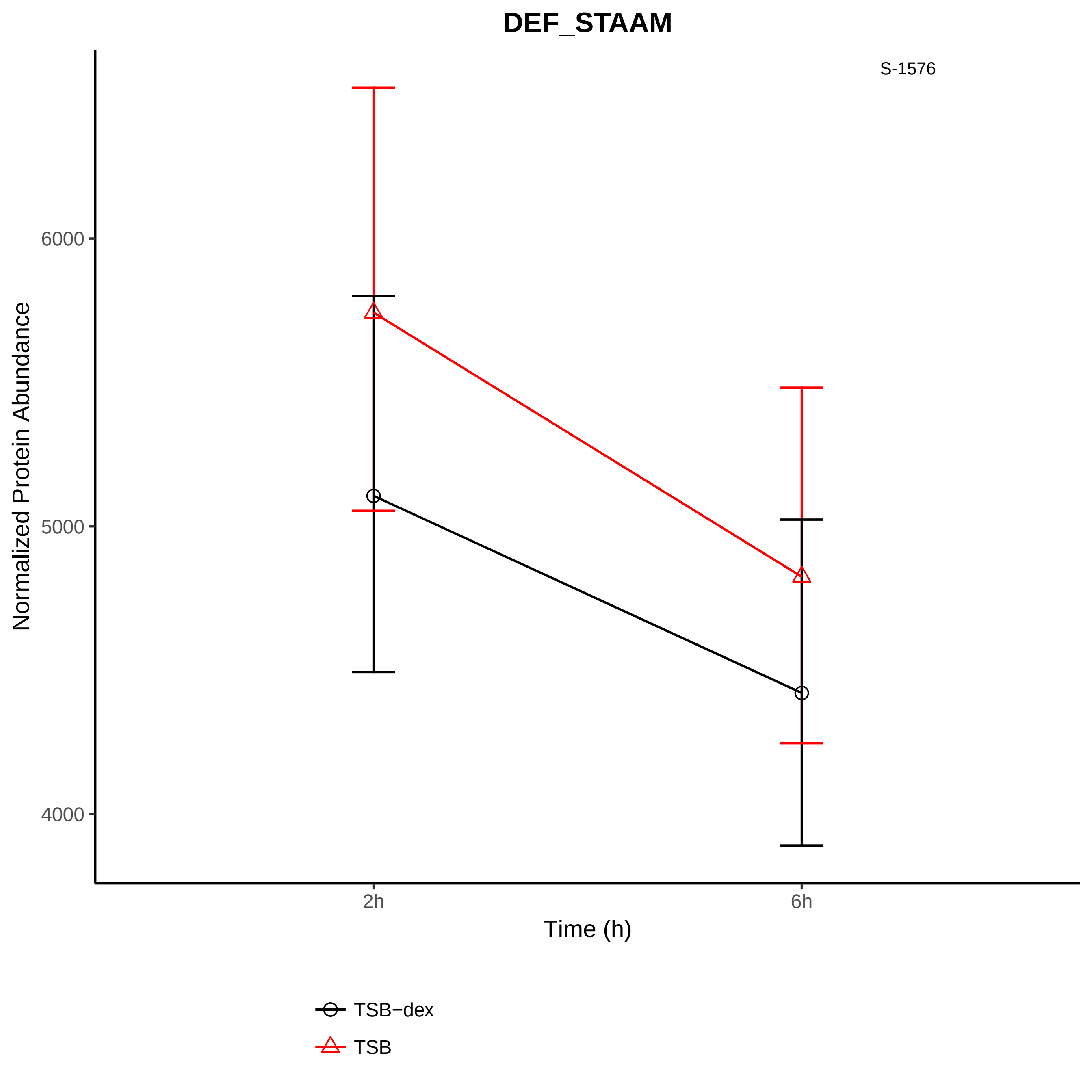
DEF_STAAM
S-1576
Normalized Protein Abundance
6000
5000
4000
2h
6h
Time (h)
TSB−dex
TSB

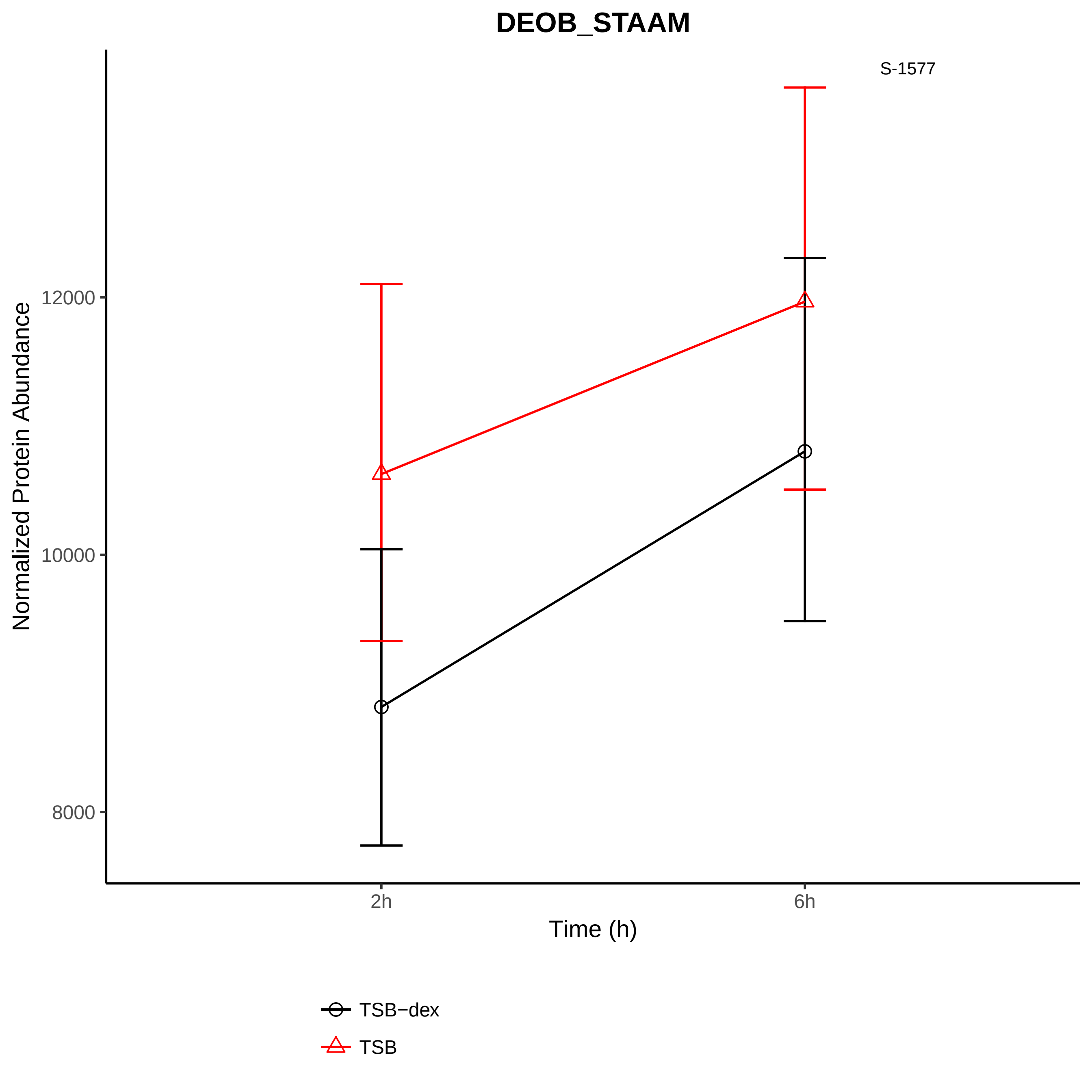
DEOB_STAAM
S-1577
Normalized Protein Abundance
12000
10000
8000
2h
6h
Time (h)
TSB−dex
TSB

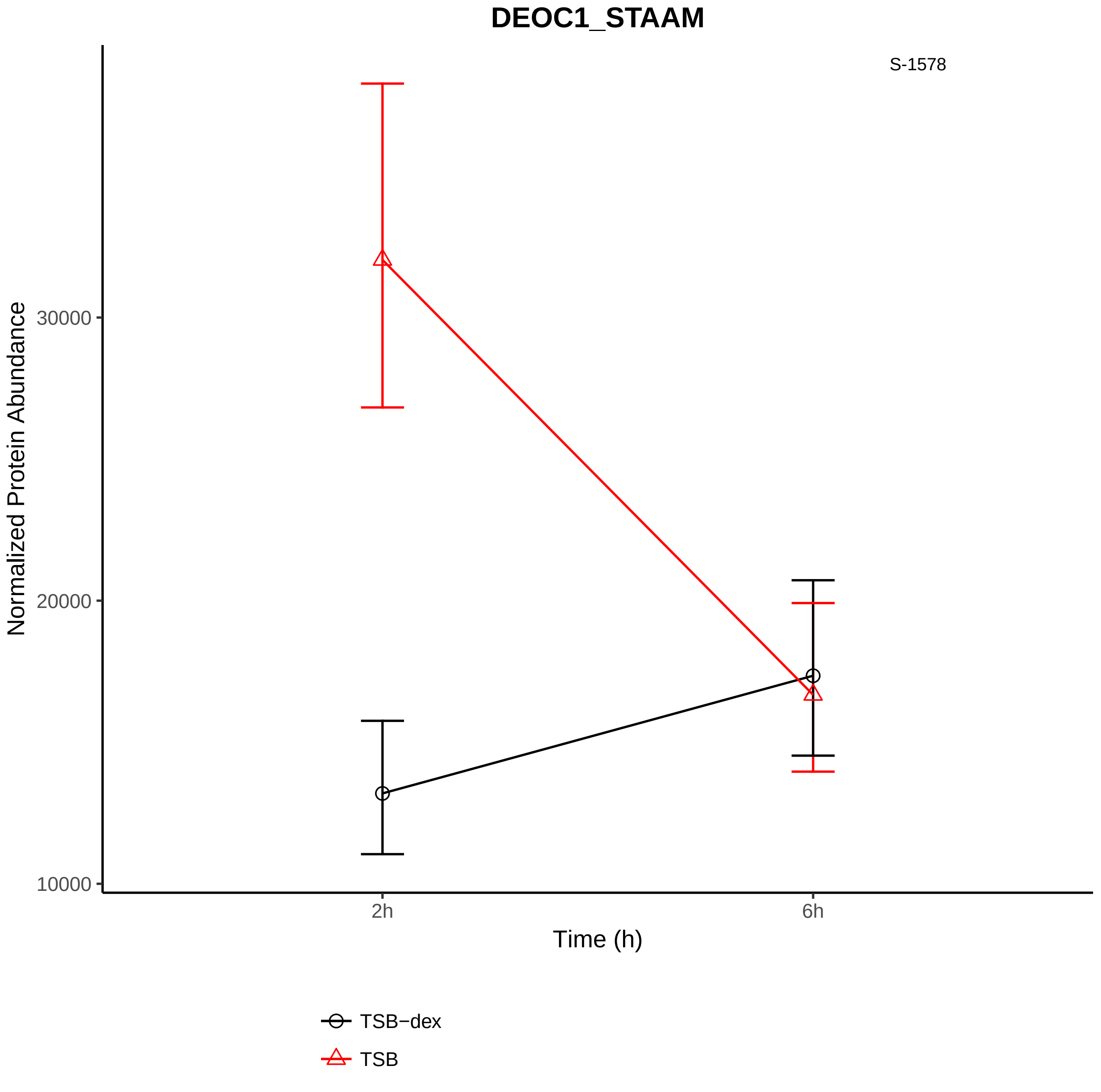

DEOC1_STAAM
S-1578
Normalized Protein Abundance
30000
20000
10000
2h
6h
Time (h)
TSB−dex
TSB

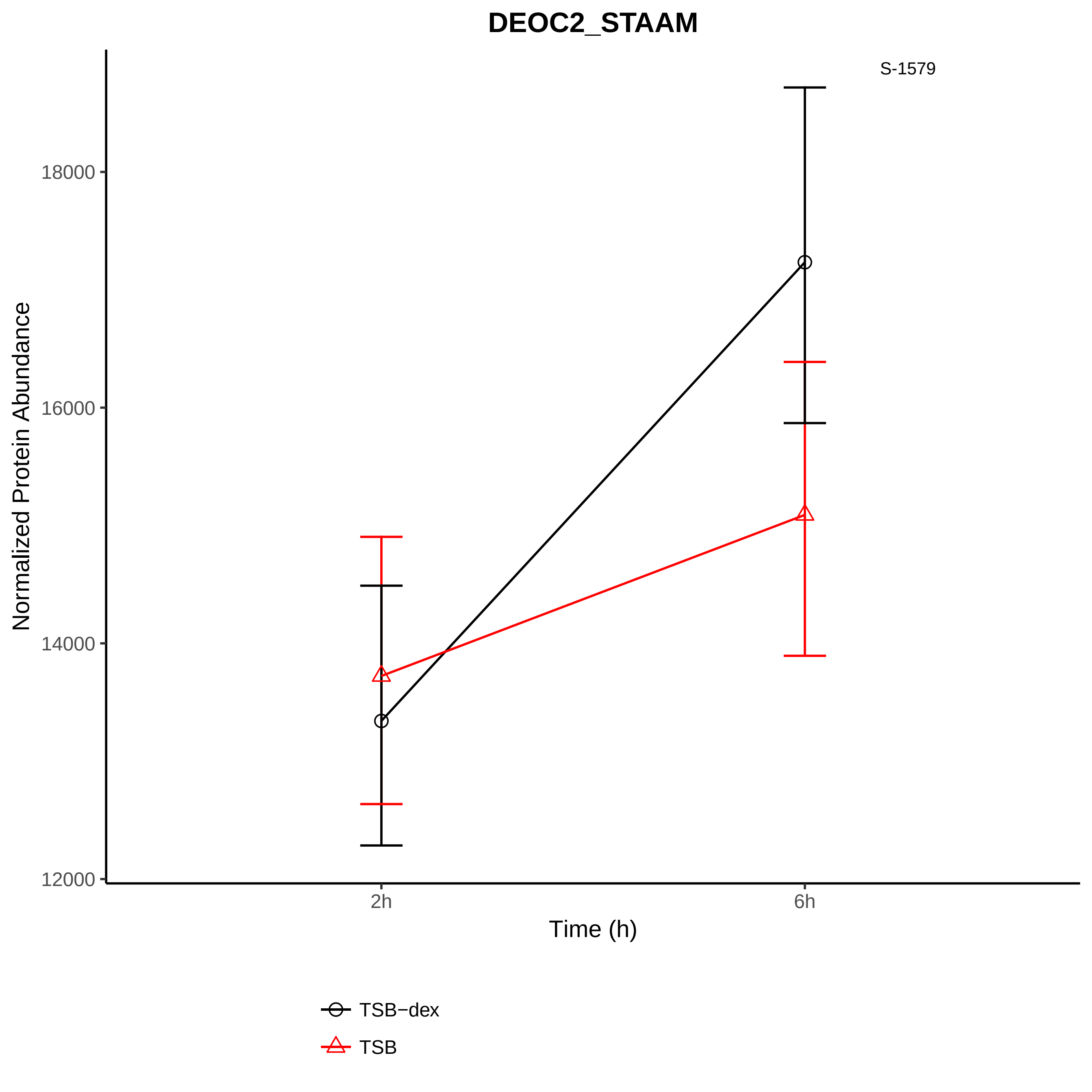

DEOC2_STAAM
S-1579
Normalized Protein Abundance
18000
16000
14000
12000
2h
6h
Time (h)
TSB−dex
TSB

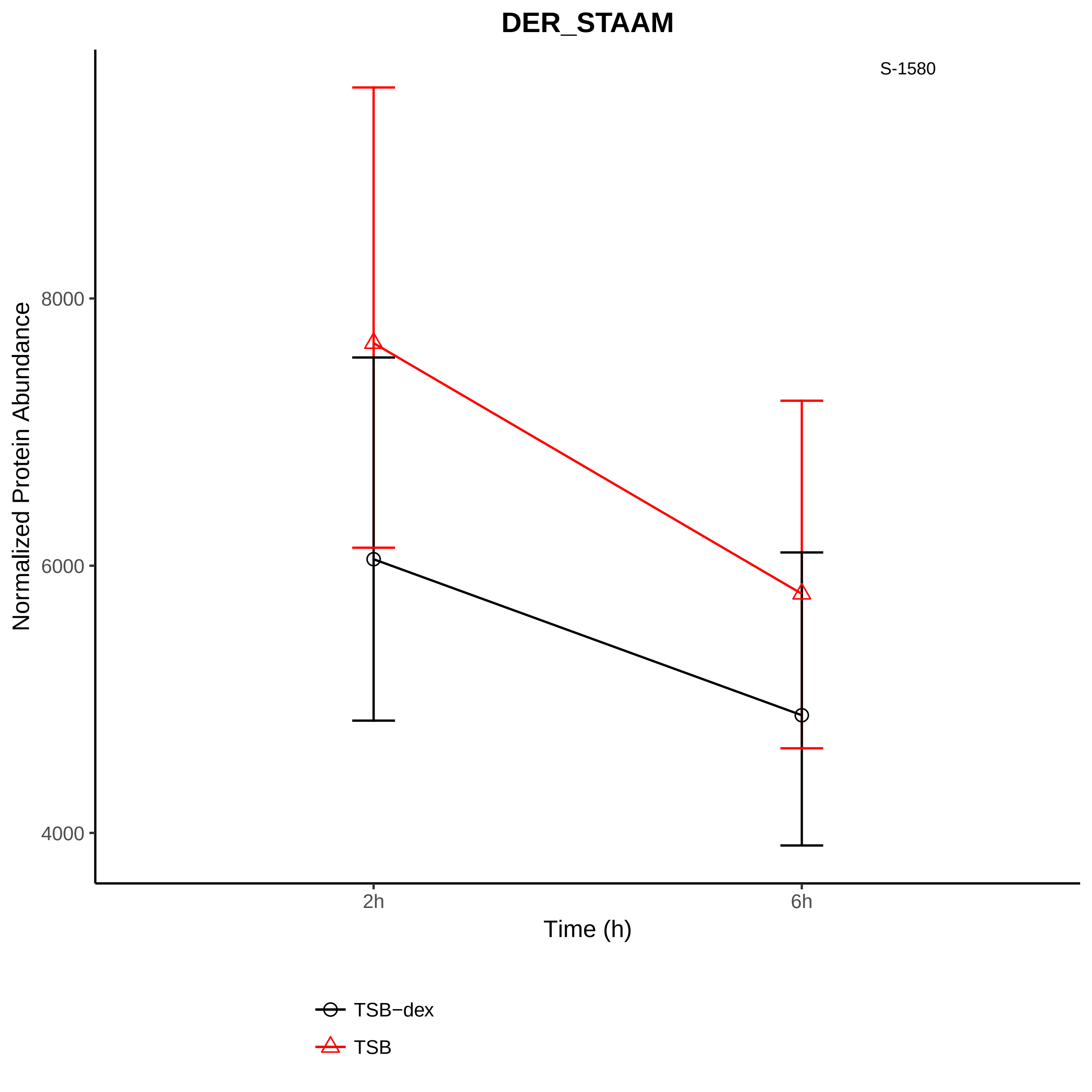

DER_STAAM
S-1580
Normalized Protein Abundance
8000
6000
4000
2h
6h
Time (h)
TSB−dex
TSB

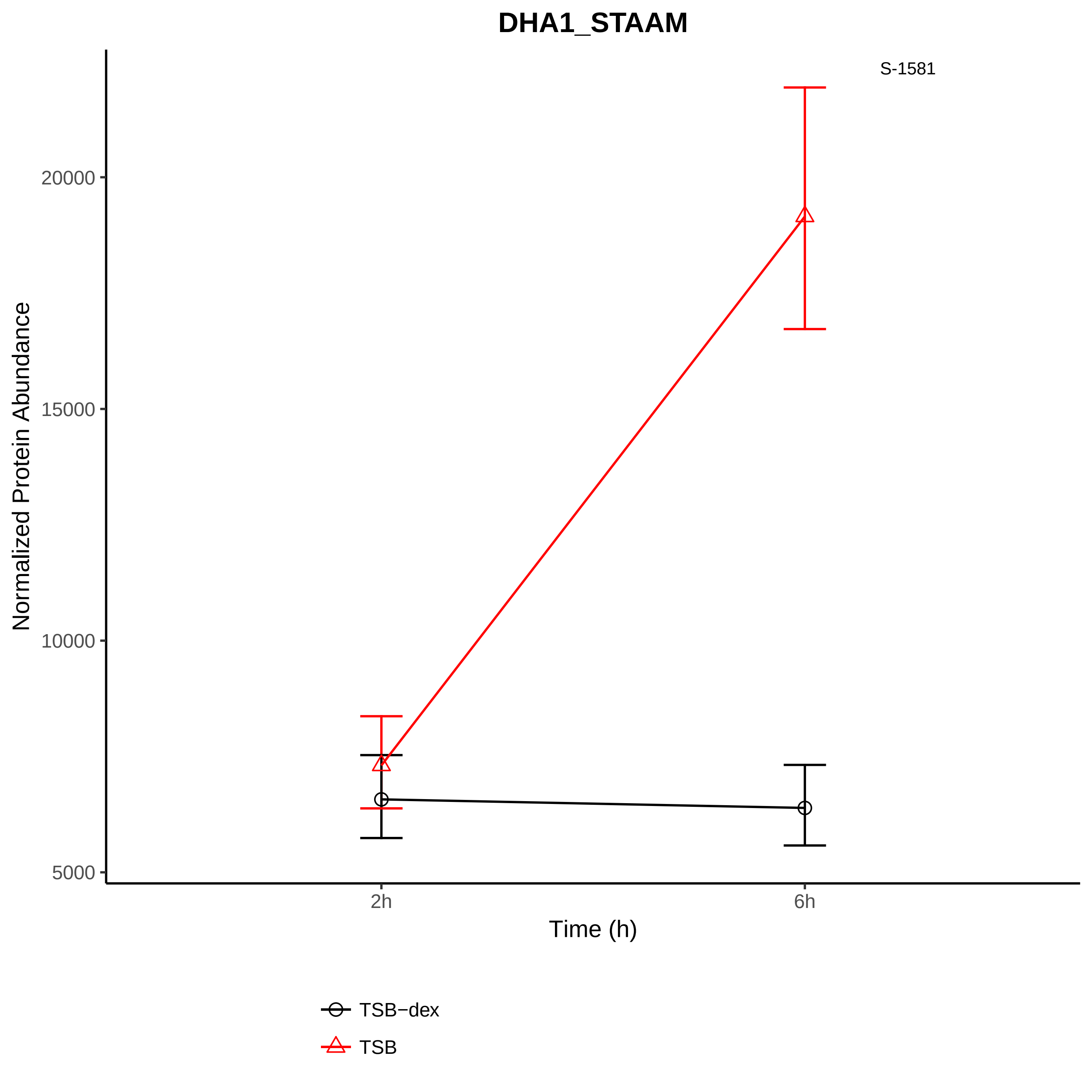

DHA1_STAAM
S-1581
Normalized Protein Abundance
20000
15000
10000
5000
2h
6h
Time (h)
TSB−dex
TSB

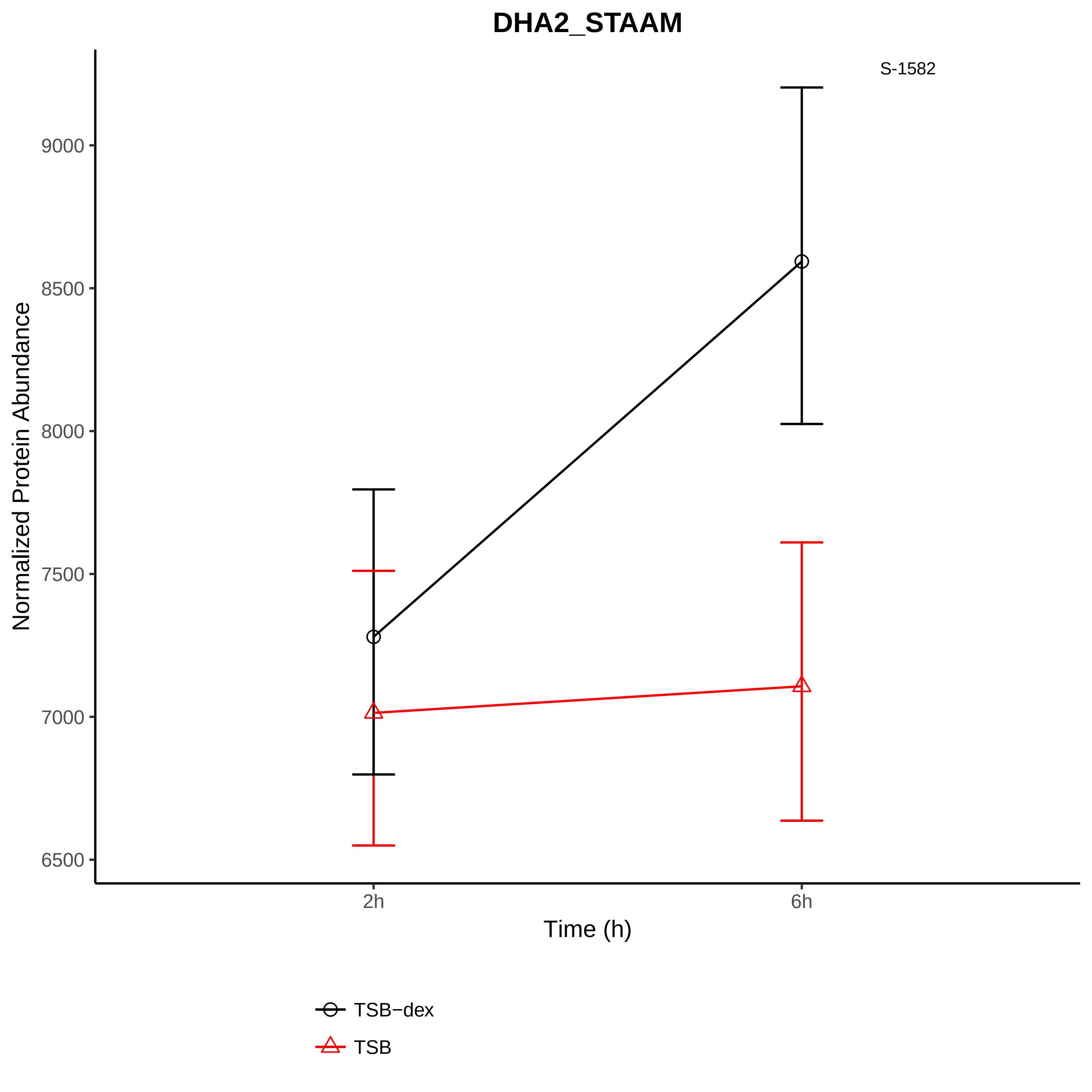

DHA2_STAAM
S-1582
Normalized Protein Abundance
9000
8500
8000
7500
7000
6500
2h
6h
Time (h)
TSB−dex
TSB

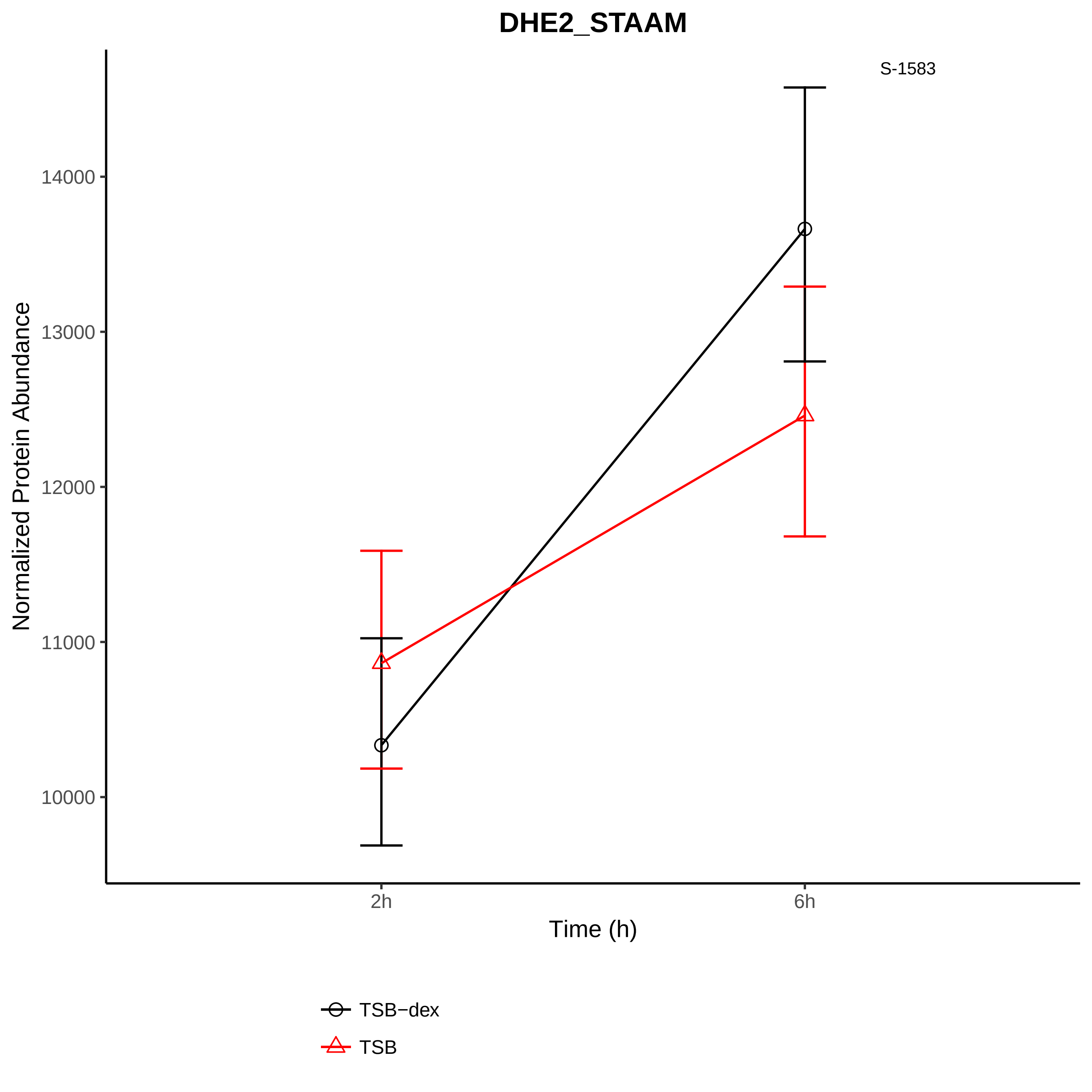

DHE2_STAAM
S-1583
Normalized Protein Abundance
14000
13000
12000
11000
10000
2h
6h
Time (h)
TSB−dex
TSB

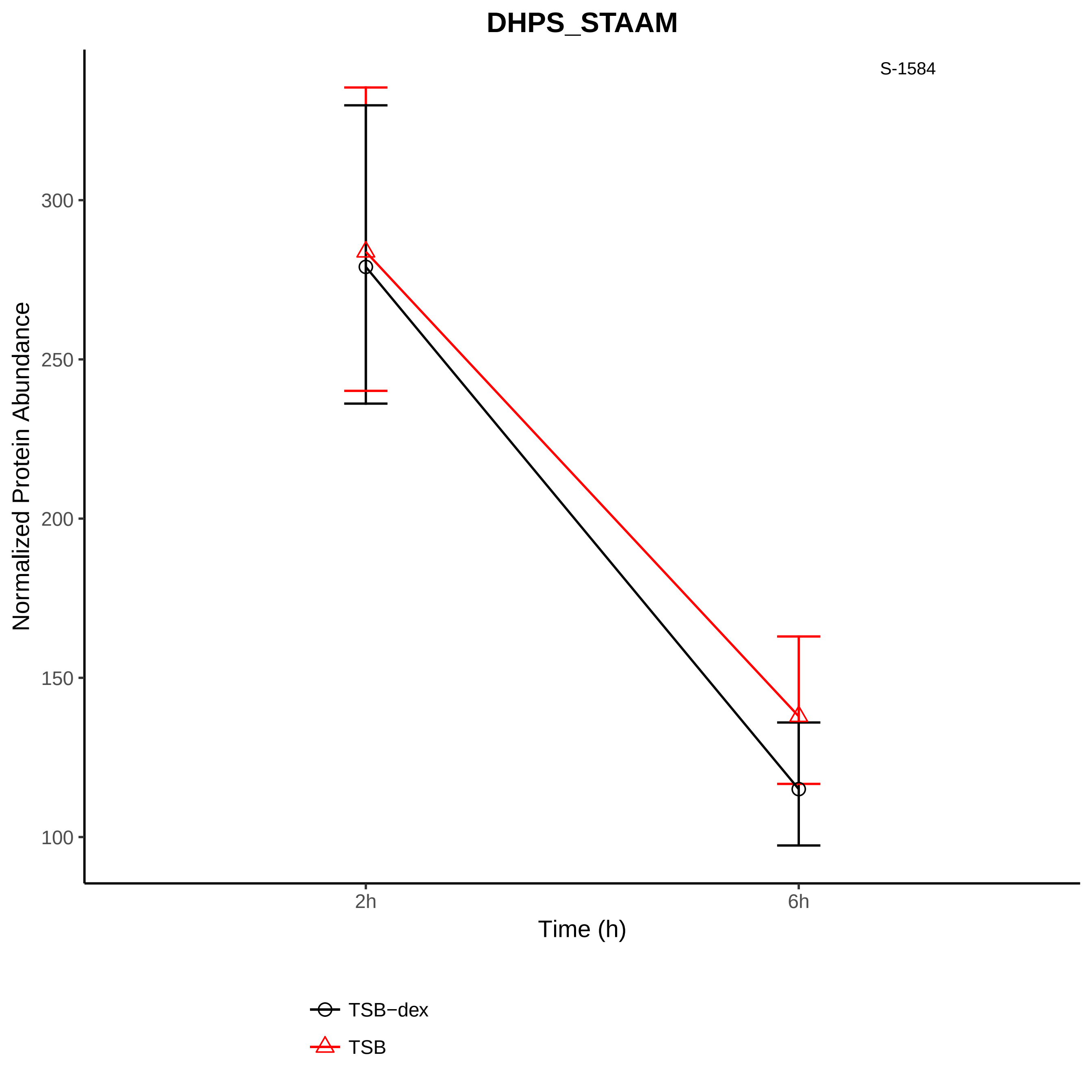

DHPS_STAAM
S-1584
Normalized Protein Abundance
300
250
200
150
100
2h
6h
Time (h)
TSB−dex
TSB

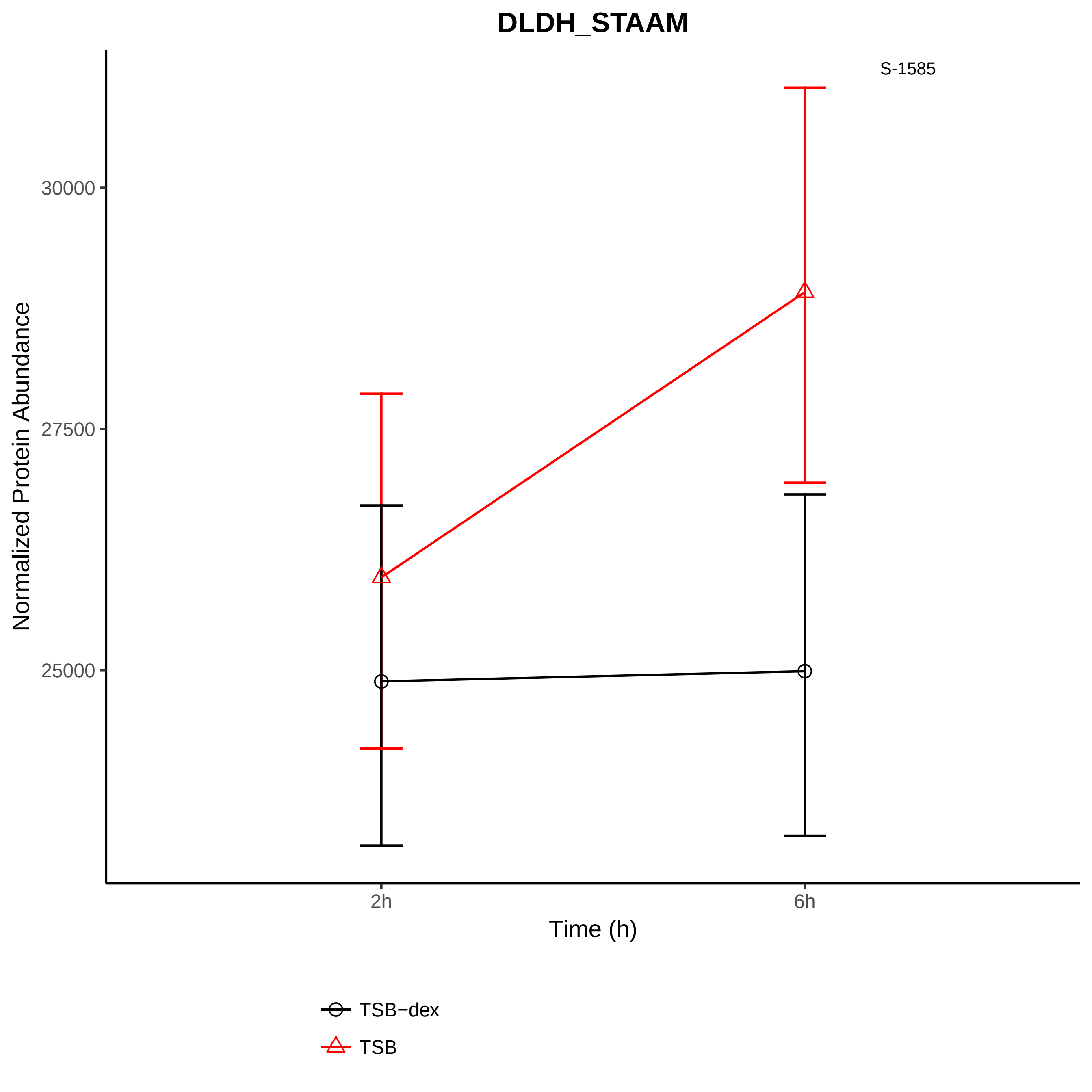
DLDH_STAAM
S-1585
30000
27500
25000
Normalized Protein Abundance
2h
6h
Time (h)
TSB−dex
TSB

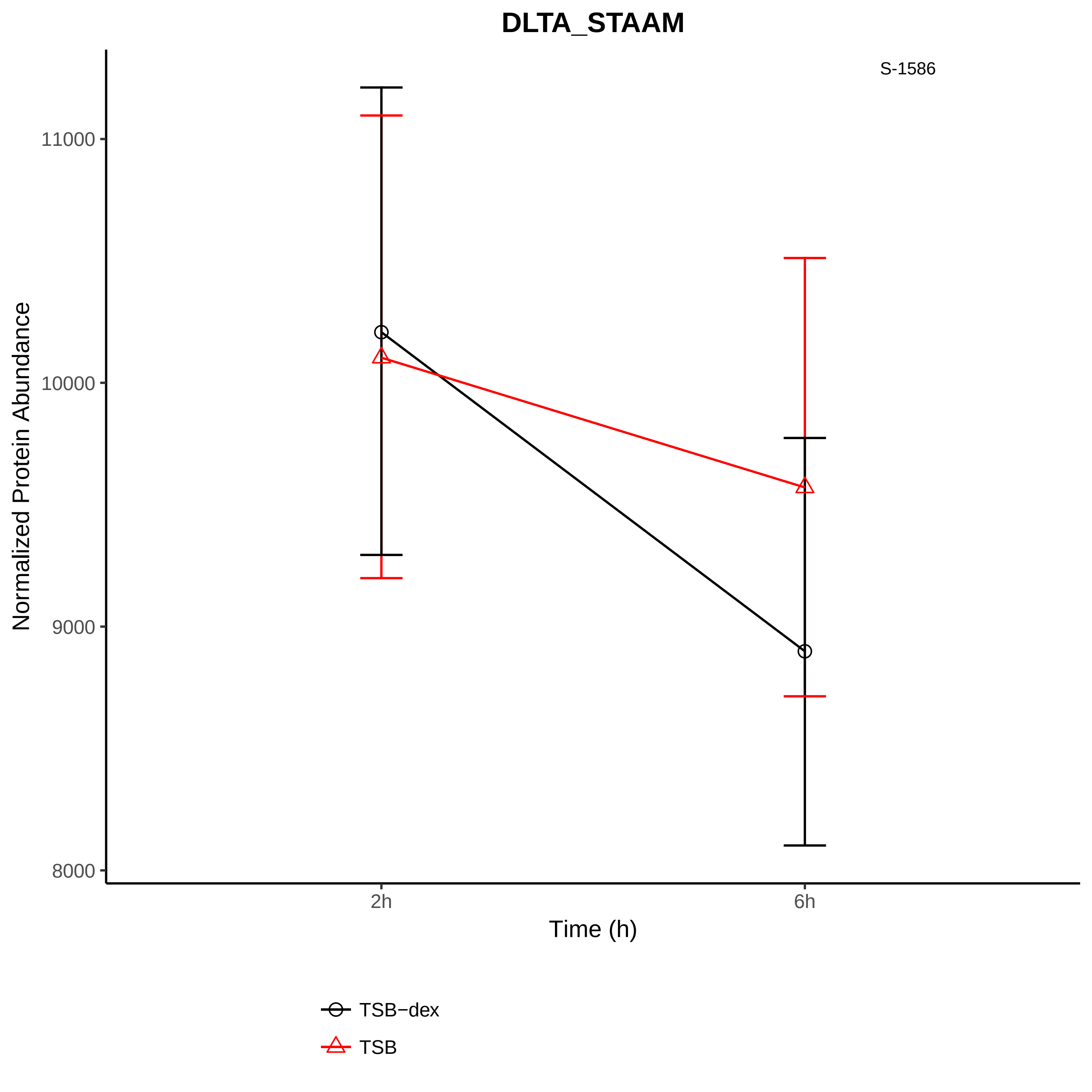

DLTA_STAAM
S-1586
Normalized Protein Abundance
11000
10000
9000
8000
2h
6h
Time (h)
TSB−dex
TSB

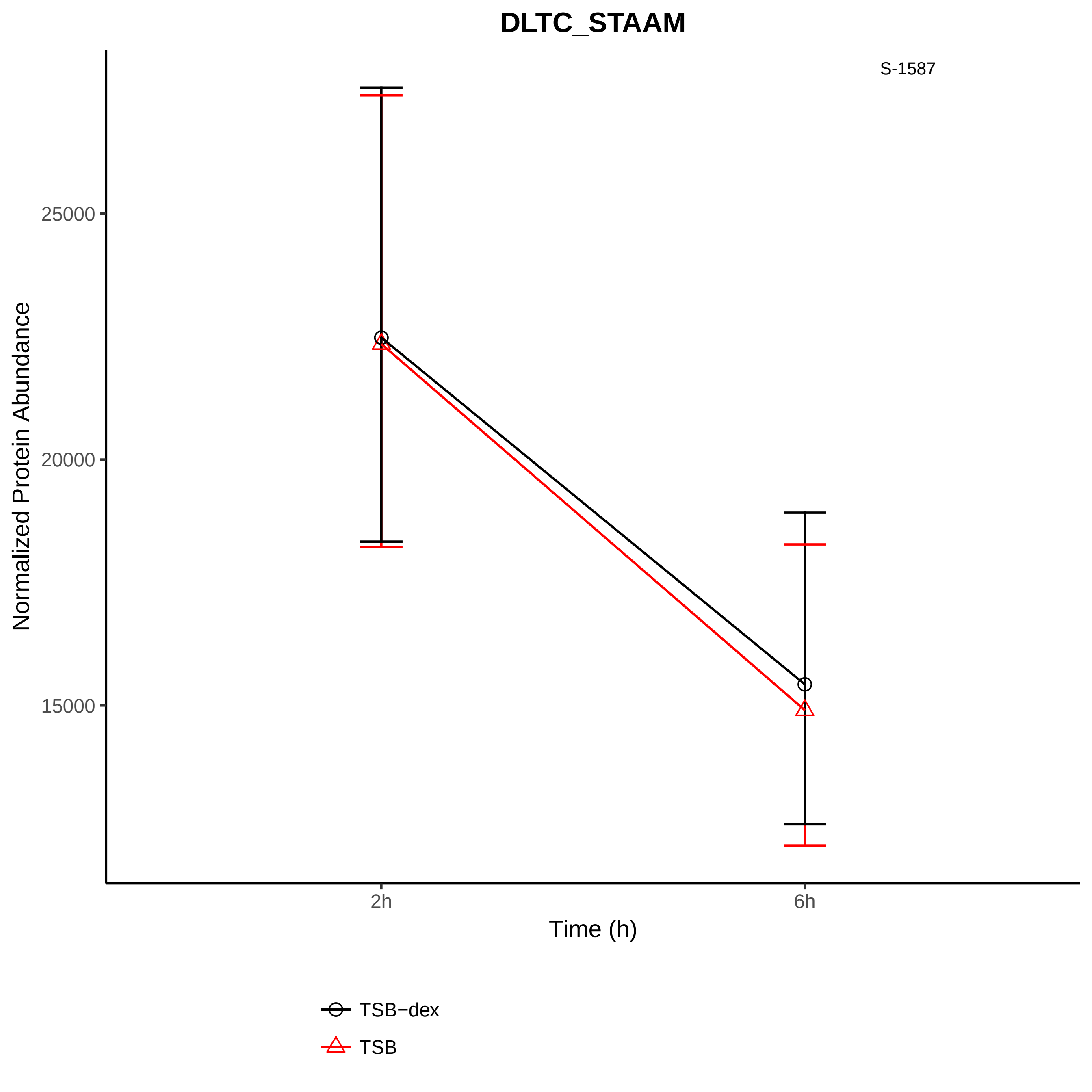

DLTC_STAAM
S-1587
25000
20000
15000
Normalized Protein Abundance
2h
6h
Time (h)
TSB−dex
TSB

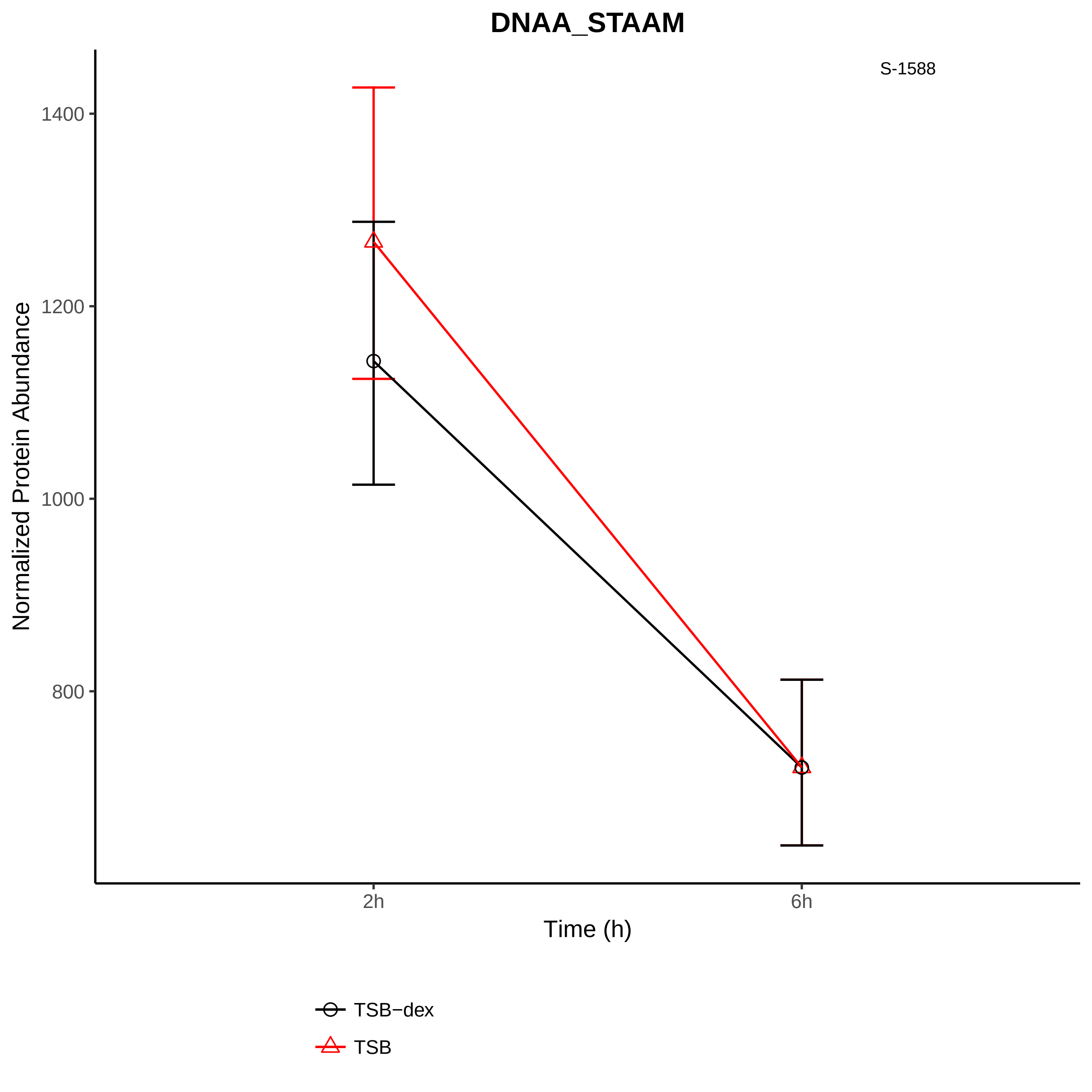
DNAA_STAAM
S-1588
Normalized Protein Abundance
1400
1200
1000
800
2h
6h
Time (h)
TSB−dex
TSB

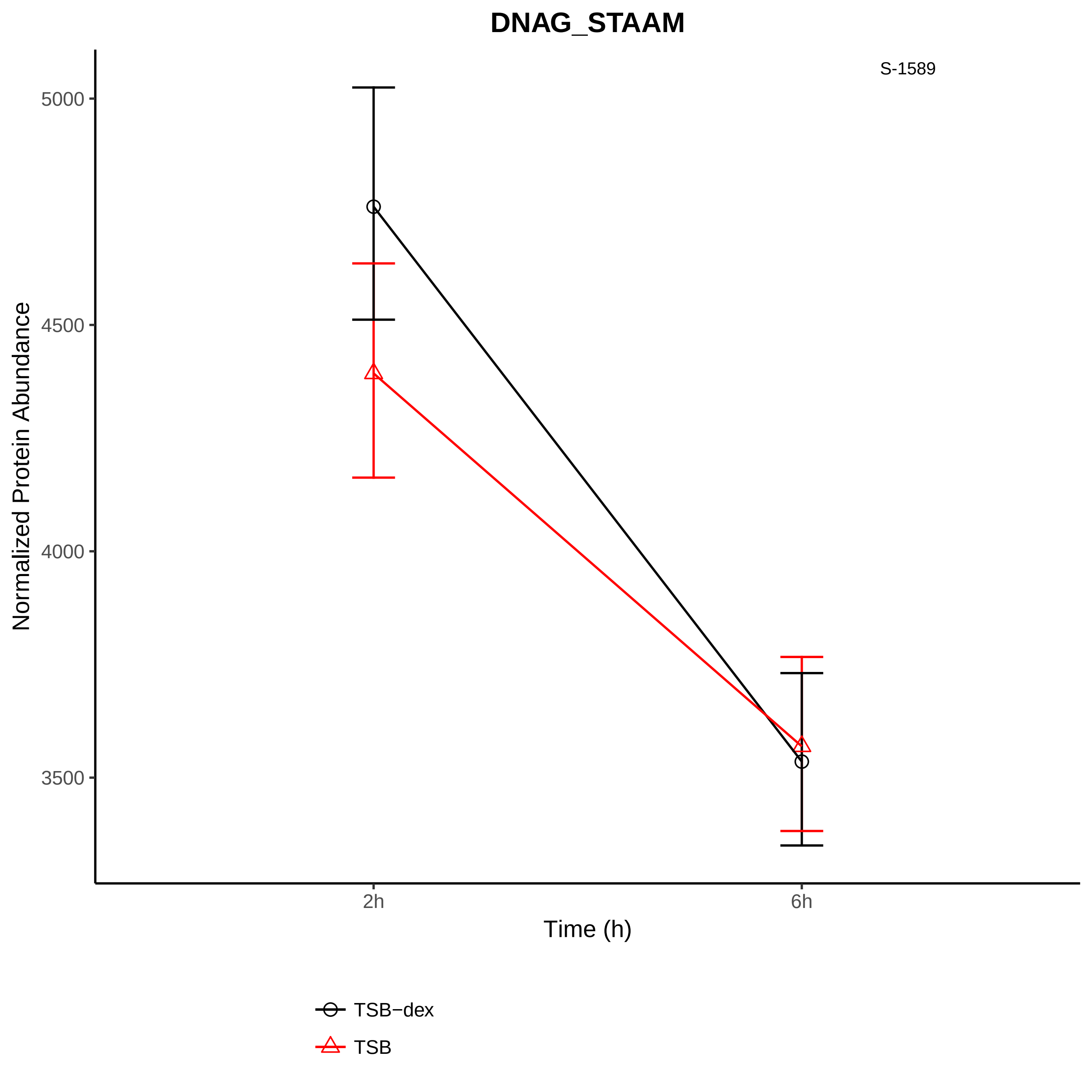
DNAG_STAAM
S-1589
5000
4500
4000
3500
Normalized Protein Abundance
2h
6h
Time (h)
TSB−dex
TSB

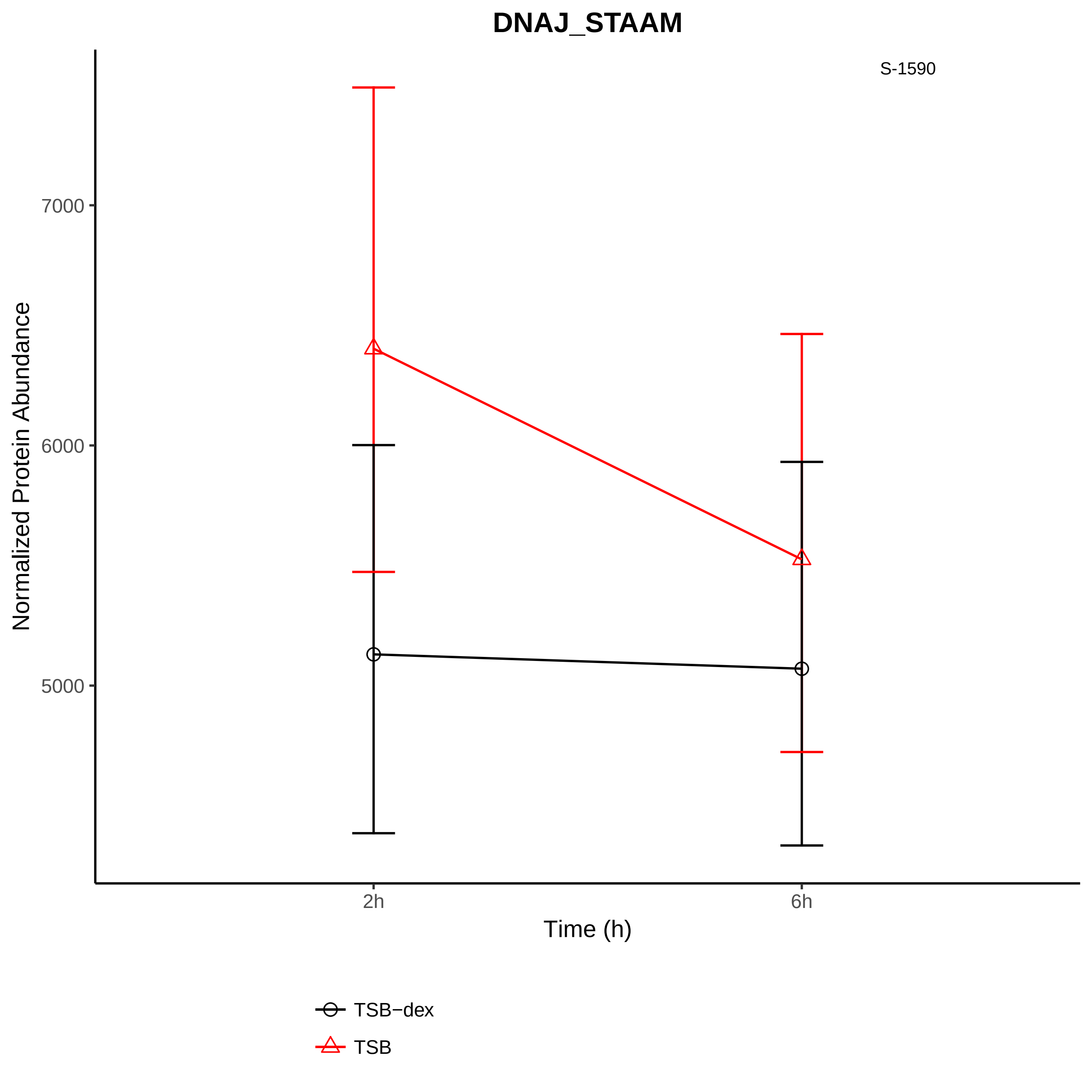

DNAJ_STAAM
S-1590
Normalized Protein Abundance
7000
6000
5000
2h
6h
Time (h)
TSB−dex
TSB

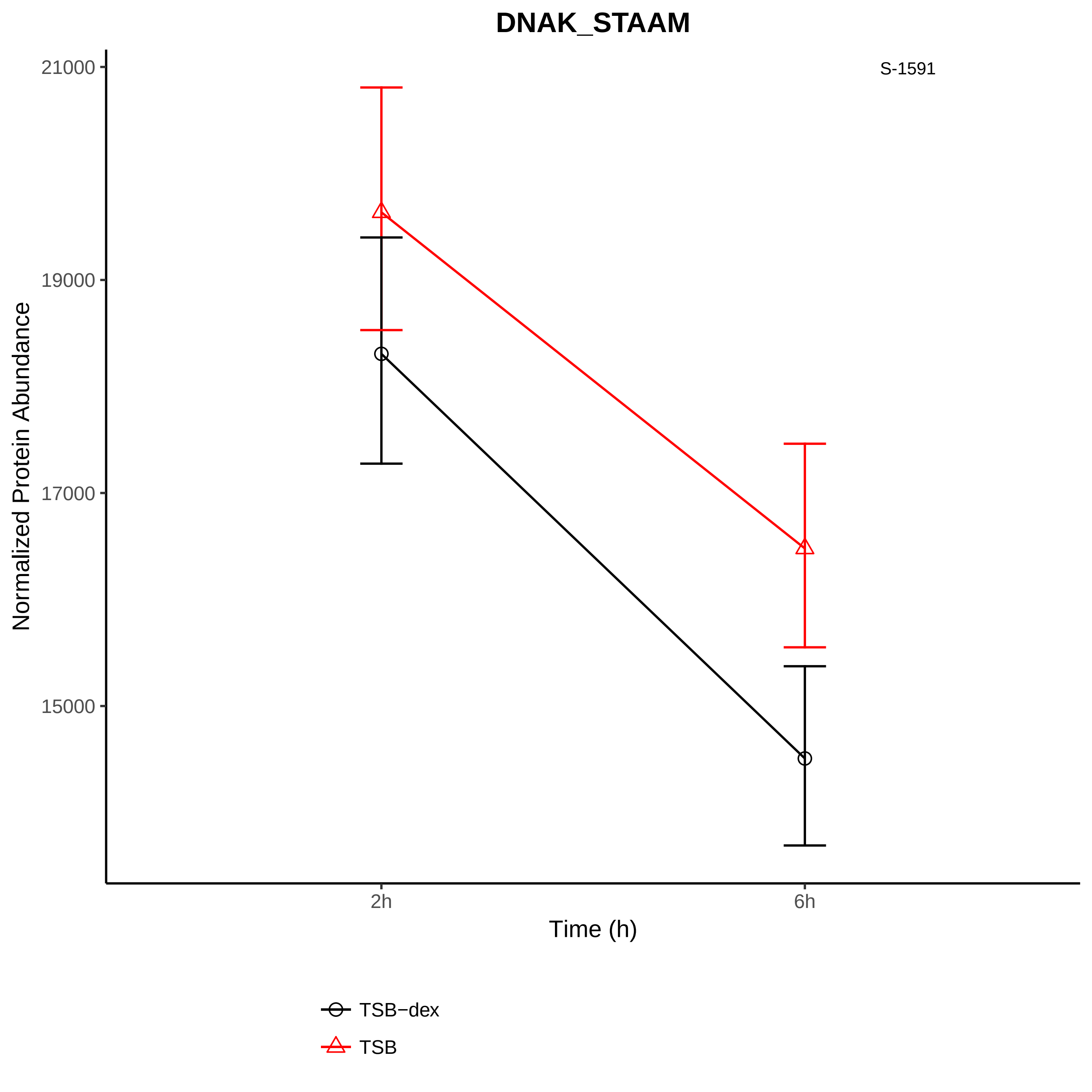
DNAK_STAAM
S-1591
21000
19000
17000
15000
Normalized Protein Abundance
2h
6h
Time (h)
TSB−dex
TSB

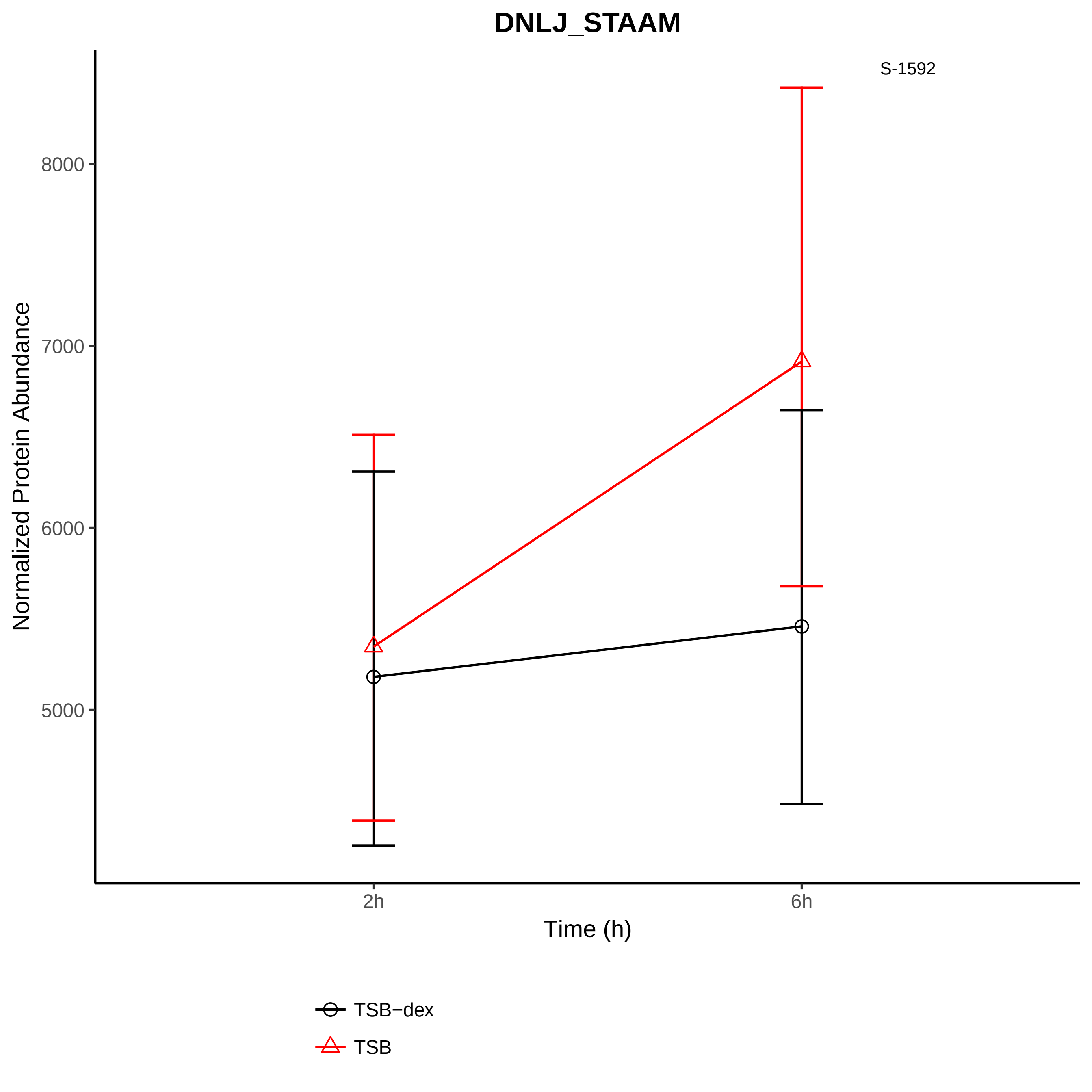
DNLJ_STAAM
S-1592
Normalized Protein Abundance
8000
7000
6000
5000
2h
6h
Time (h)
TSB−dex
TSB

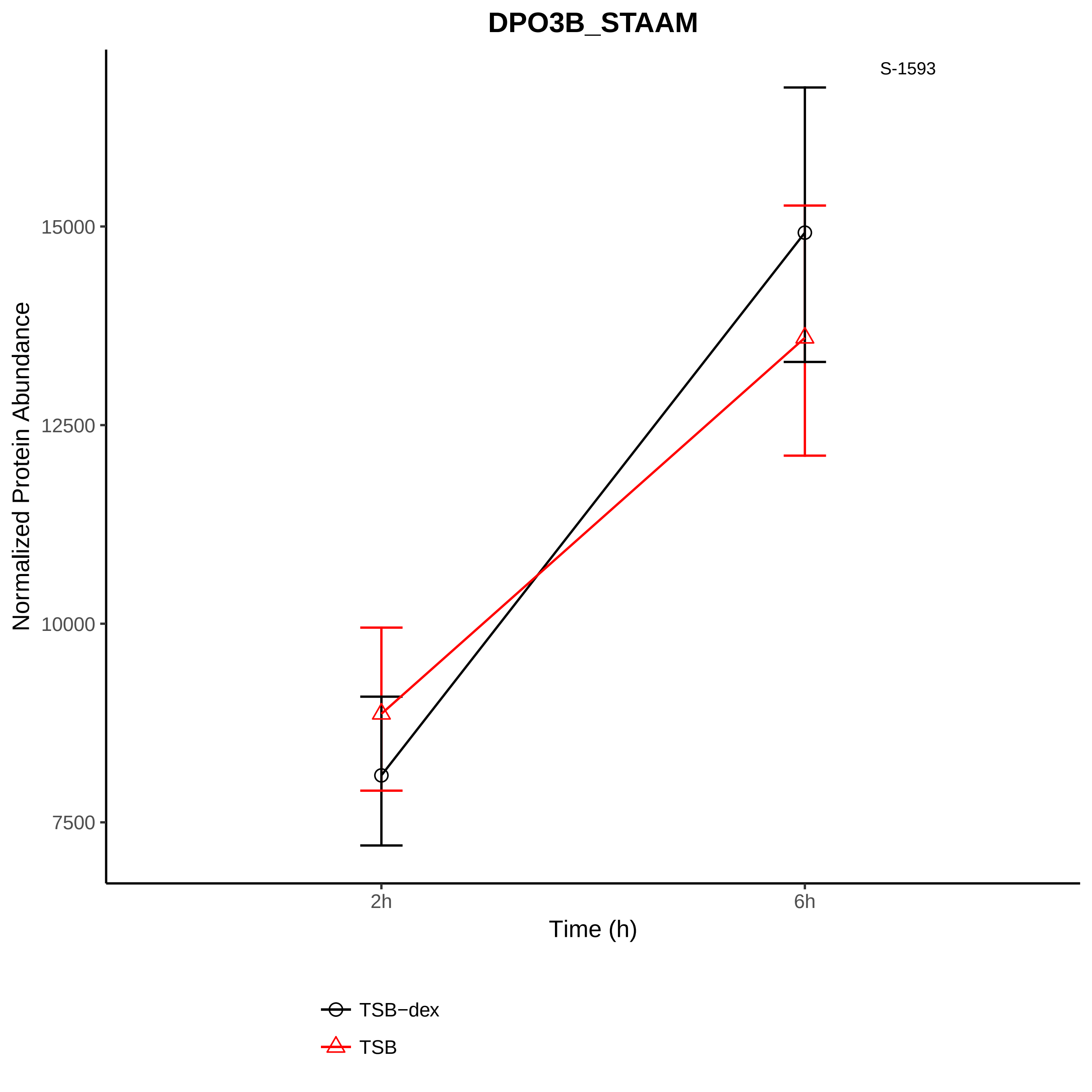

DPO3B_STAAM
S-1593
Normalized Protein Abundance
15000
12500
10000
7500
2h
6h
Time (h)
TSB−dex
TSB

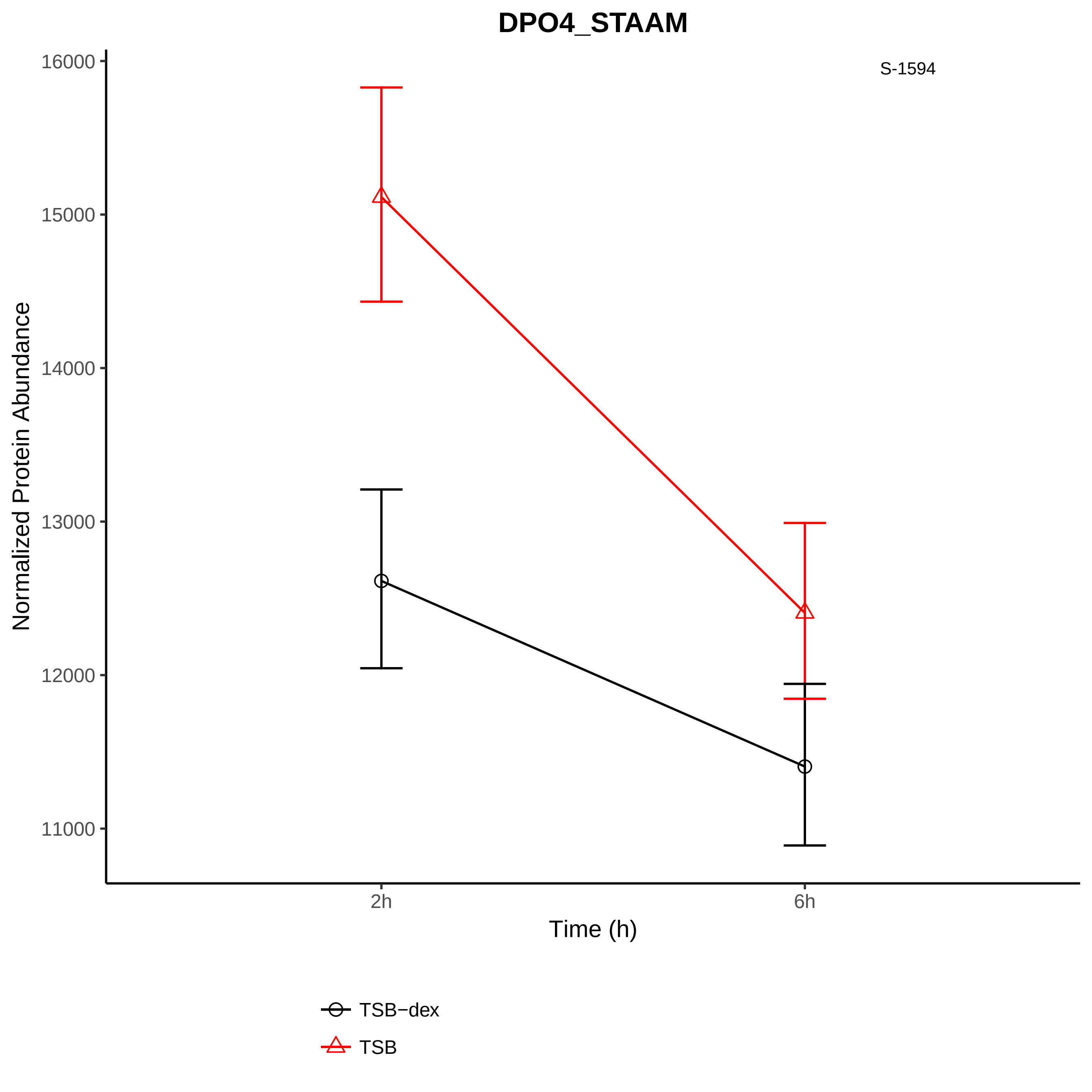

DPO4_STAAM
S-1594
Normalized Protein Abundance
16000
15000
14000
13000
12000
11000
2h
6h
Time (h)
TSB−dex
TSB

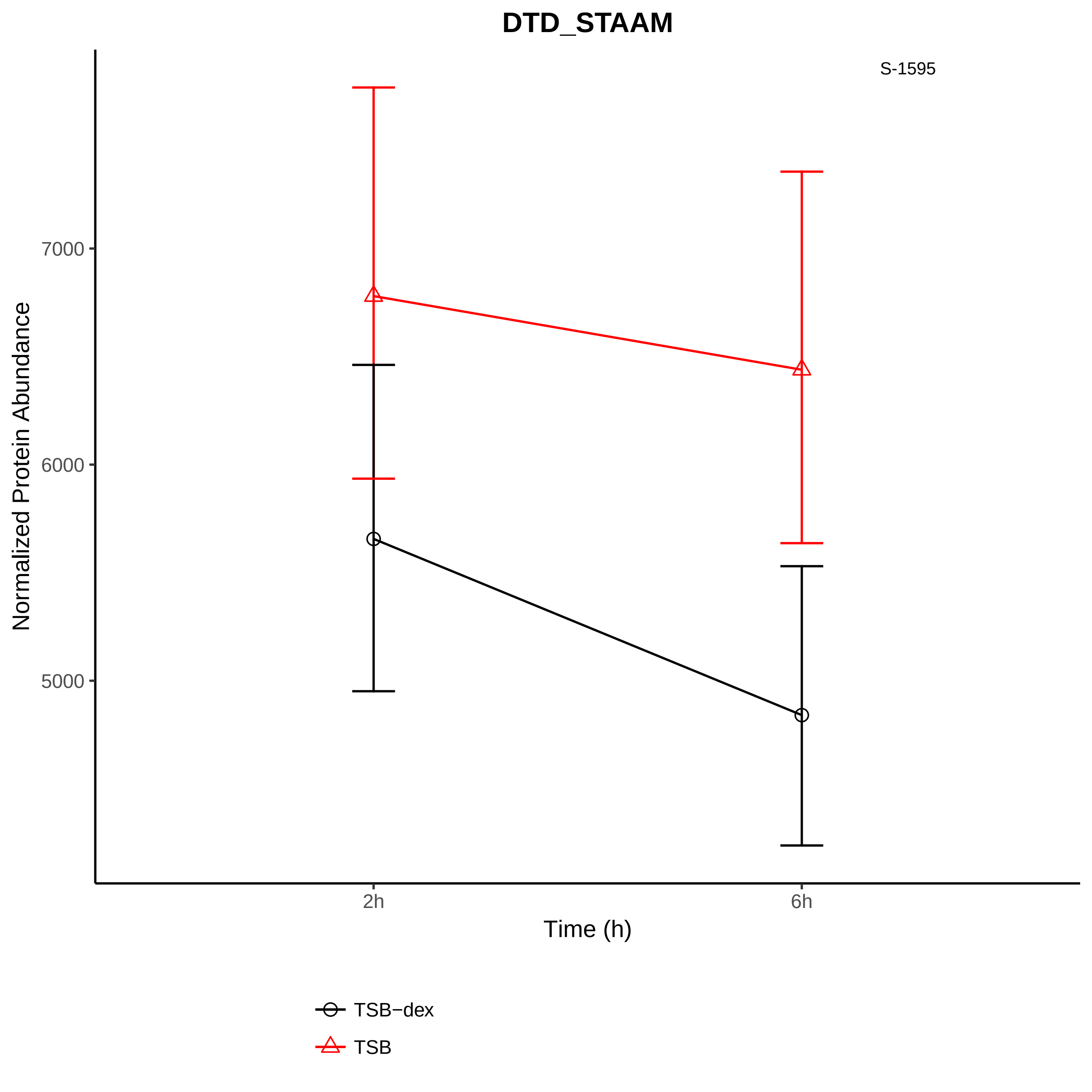
DTD_STAAM
S-1595
Normalized Protein Abundance
7000
6000
5000
2h
6h
Time (h)
TSB−dex
TSB

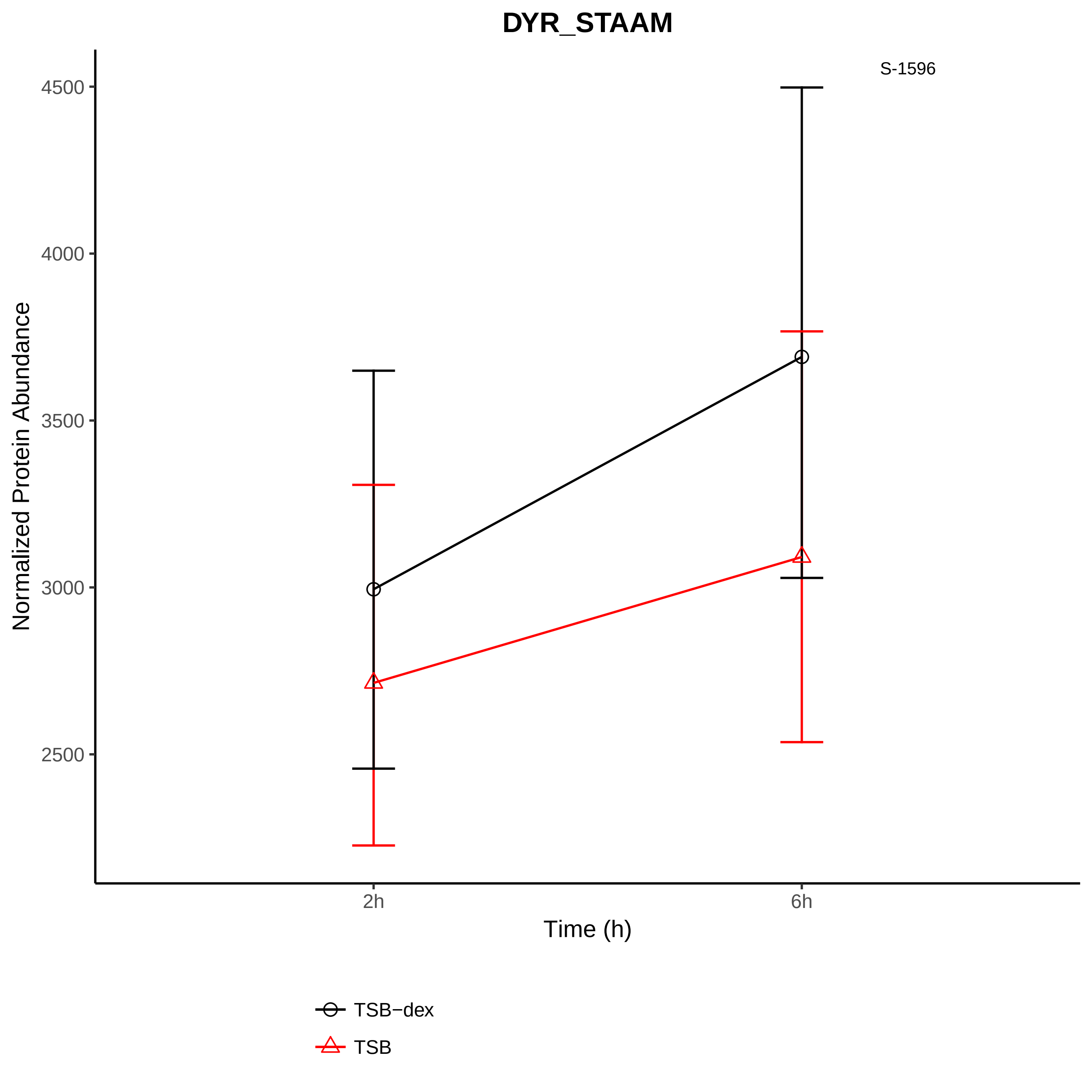
DYR_STAAM
S-1596
Normalized Protein Abundance
4500
4000
3500
3000
2500
2h
6h
Time (h)
TSB−dex
TSB

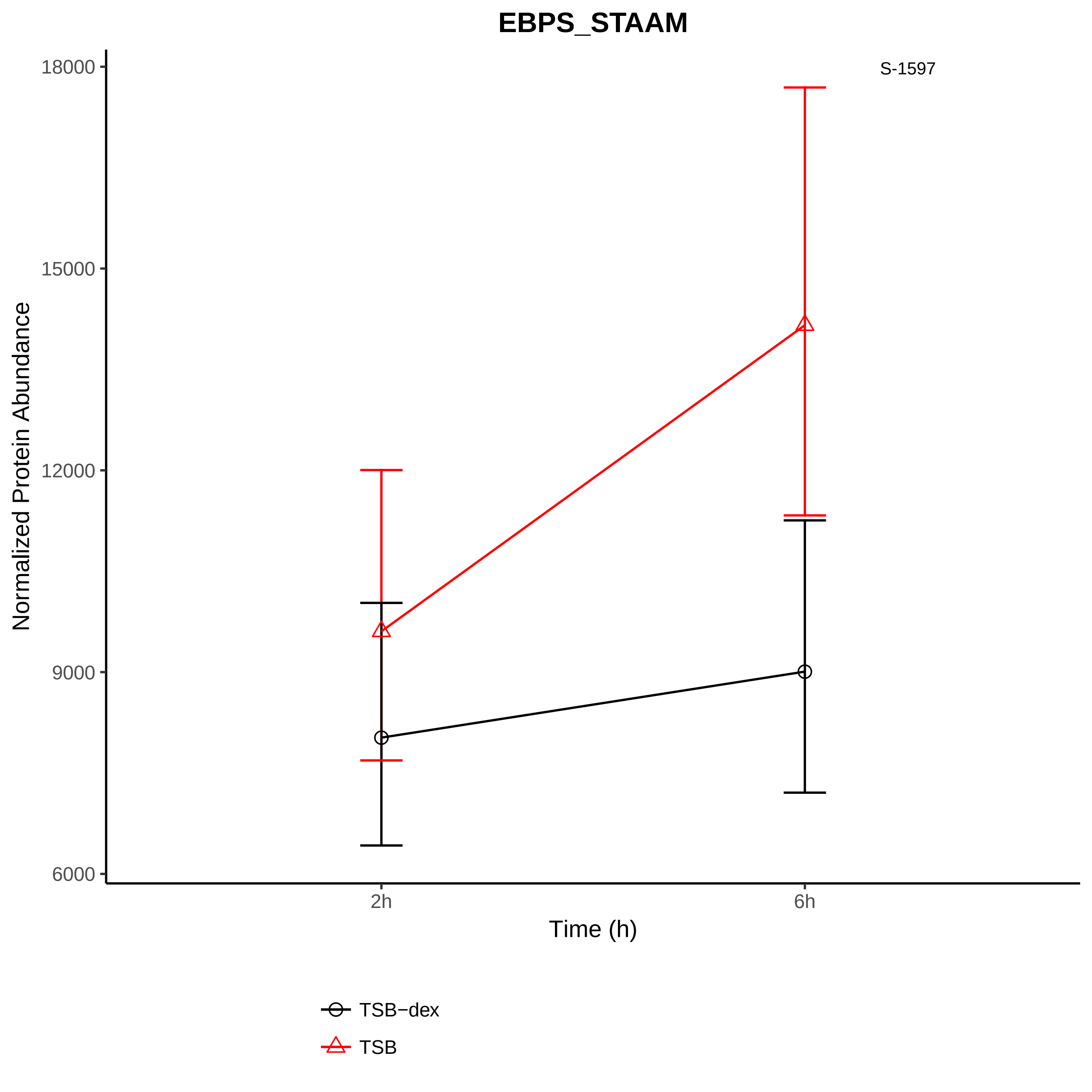
EBPS_STAAM
S-1597
18000
15000
12000
9000
6000
Normalized Protein Abundance
2h
6h
Time (h)
TSB−dex
TSB

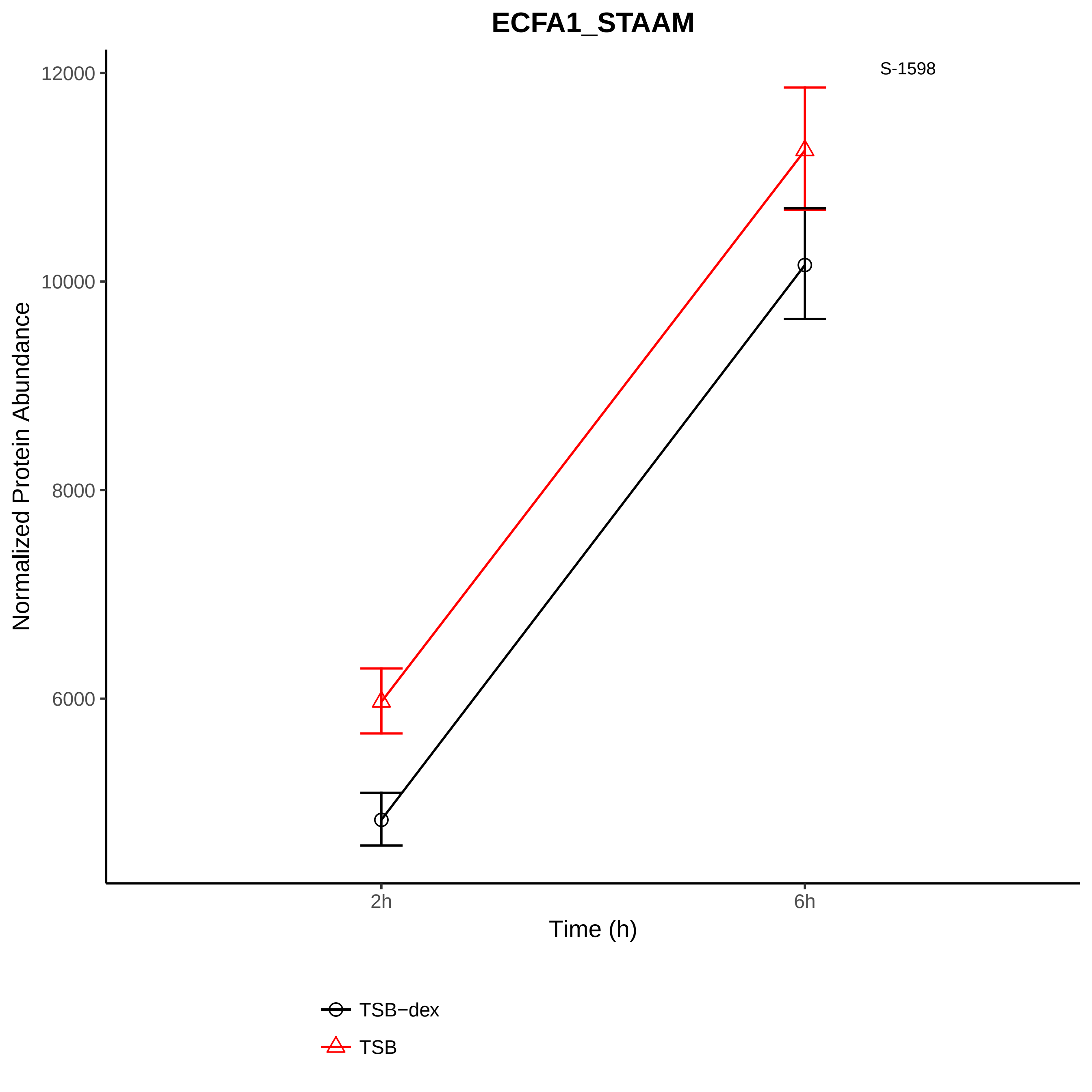

ECFA1_STAAM
S-1598
12000
10000
8000
6000
Normalized Protein Abundance
2h
6h
Time (h)
TSB−dex
TSB

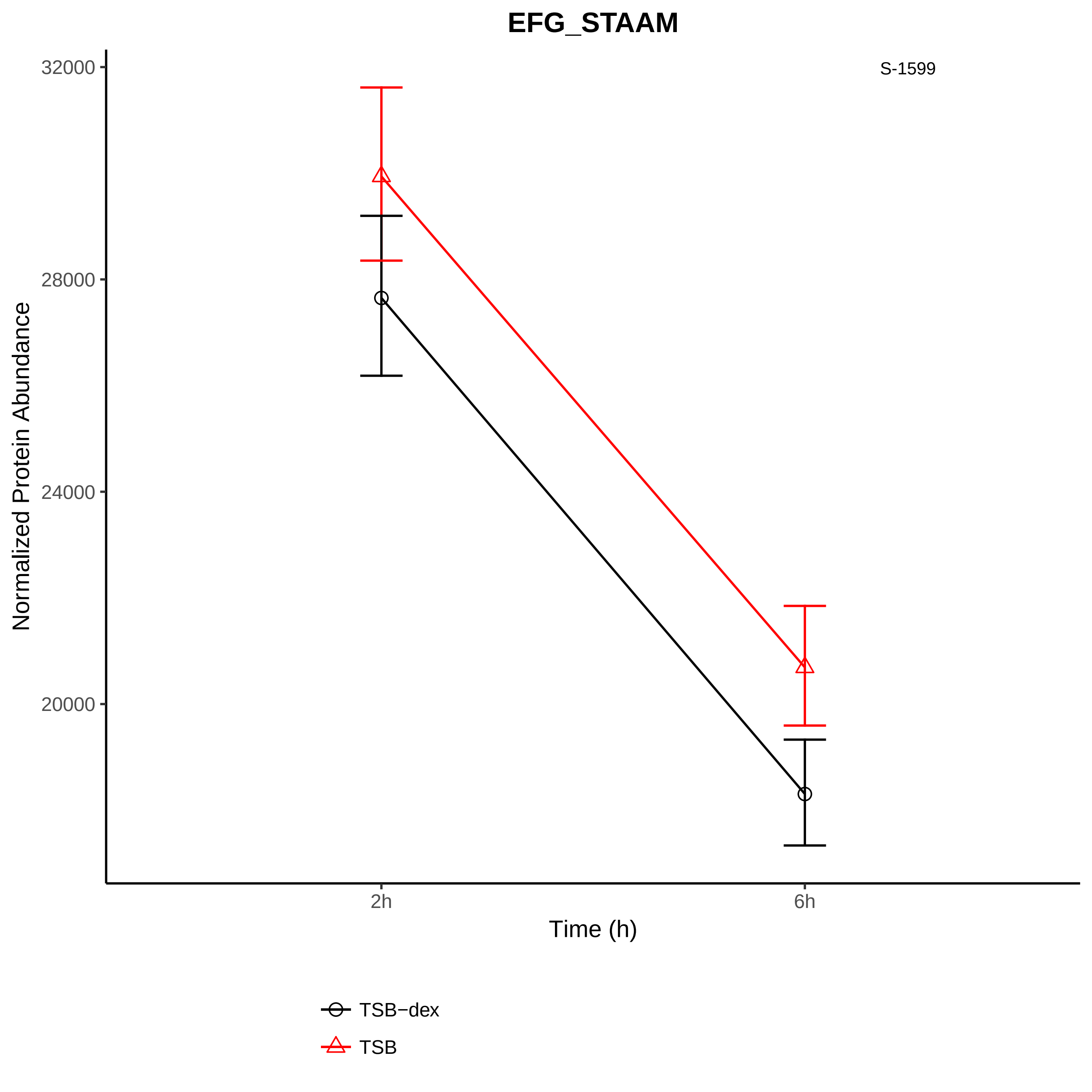

EFG_STAAM
S-1599
32000
28000
24000
20000
Normalized Protein Abundance
2h
6h
Time (h)
TSB−dex
TSB

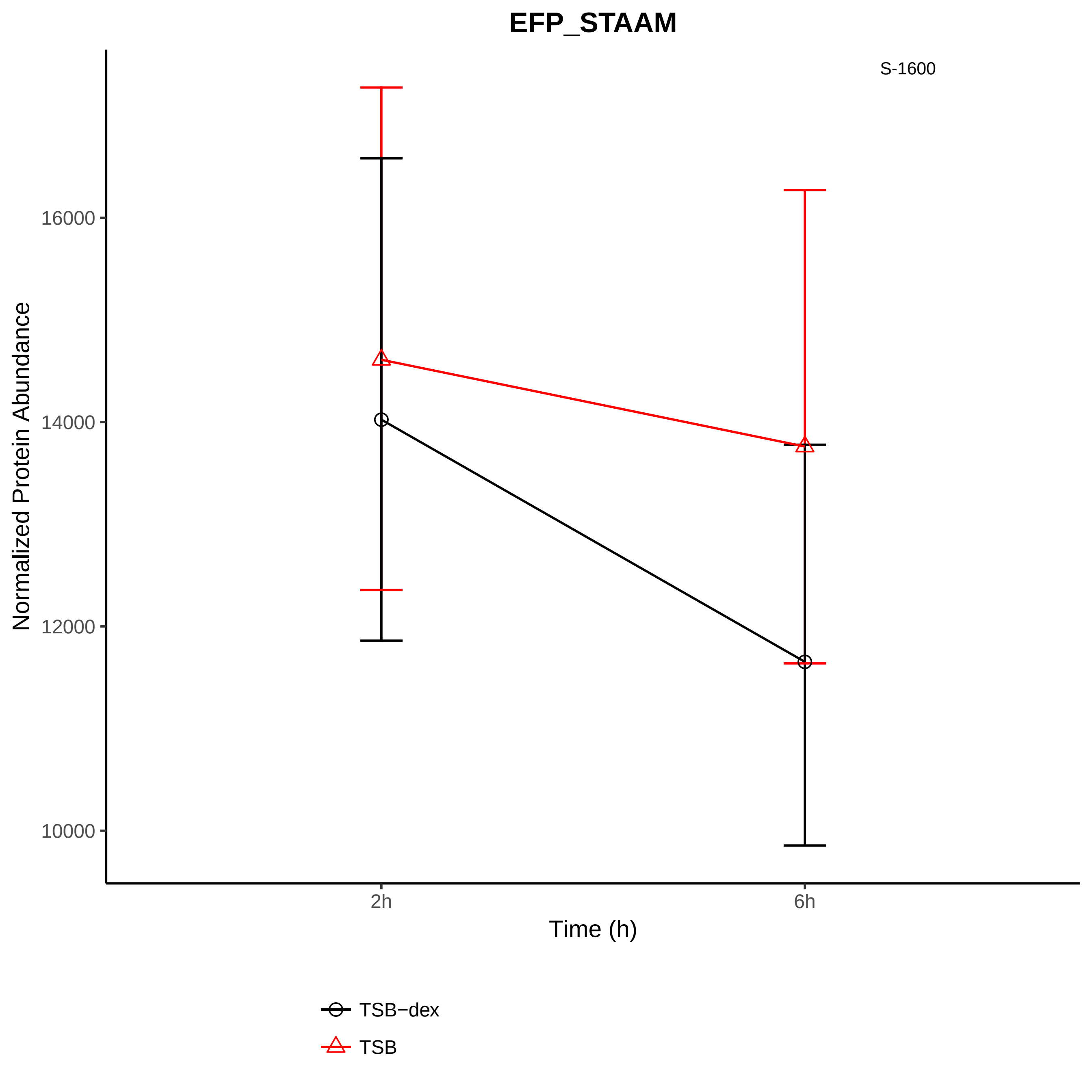

EFP_STAAM
S-1600
Normalized Protein Abundance
16000
14000
12000
10000
2h
6h
Time (h)
TSB−dex
TSB

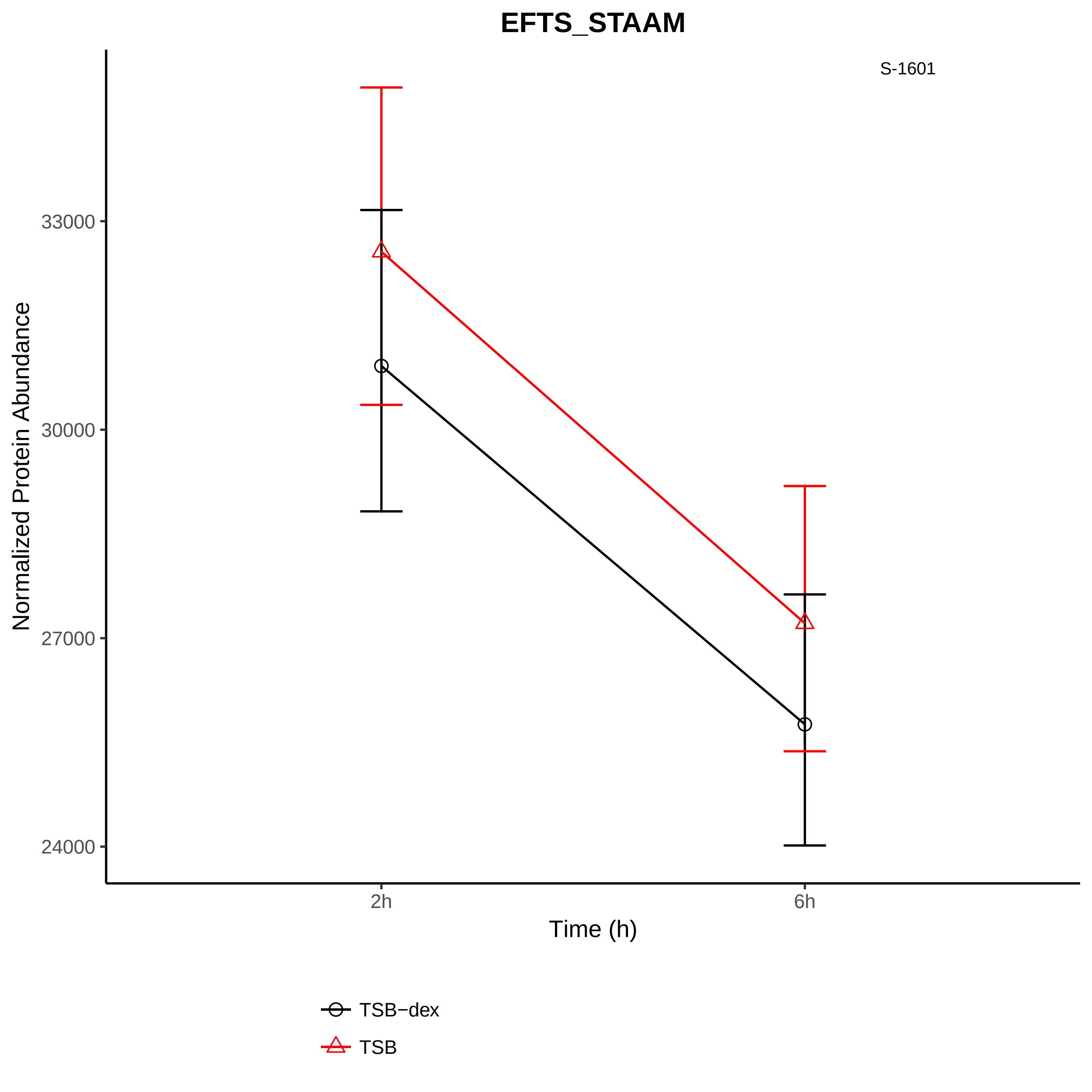

EFTS_STAAM
S-1601
Normalized Protein Abundance
33000
30000
27000
24000
2h
6h
Time (h)
TSB−dex
TSB

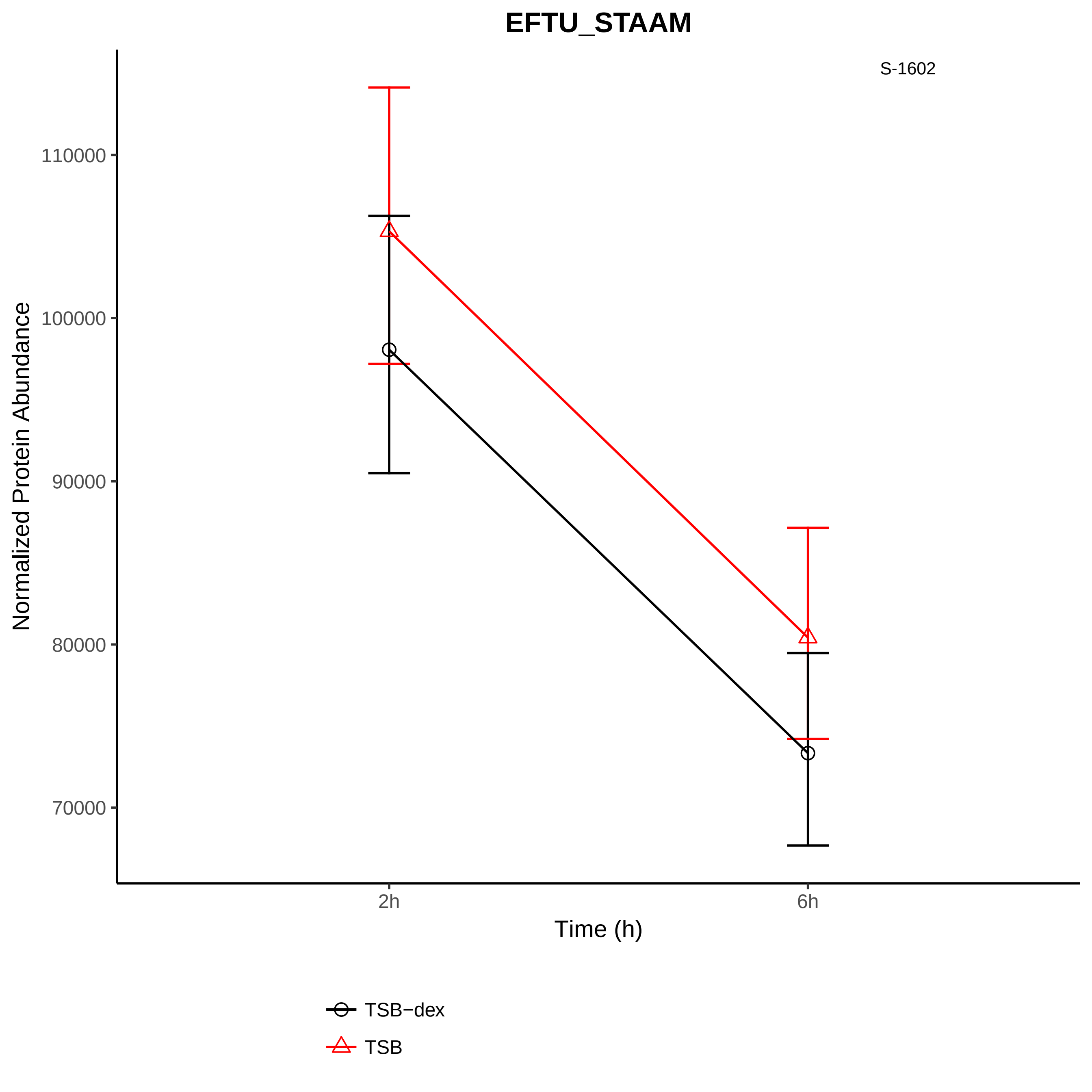

EFTU_STAAM
S-1602
Normalized Protein Abundance
110000
100000
90000
80000
70000
2h
6h
Time (h)
TSB−dex
TSB

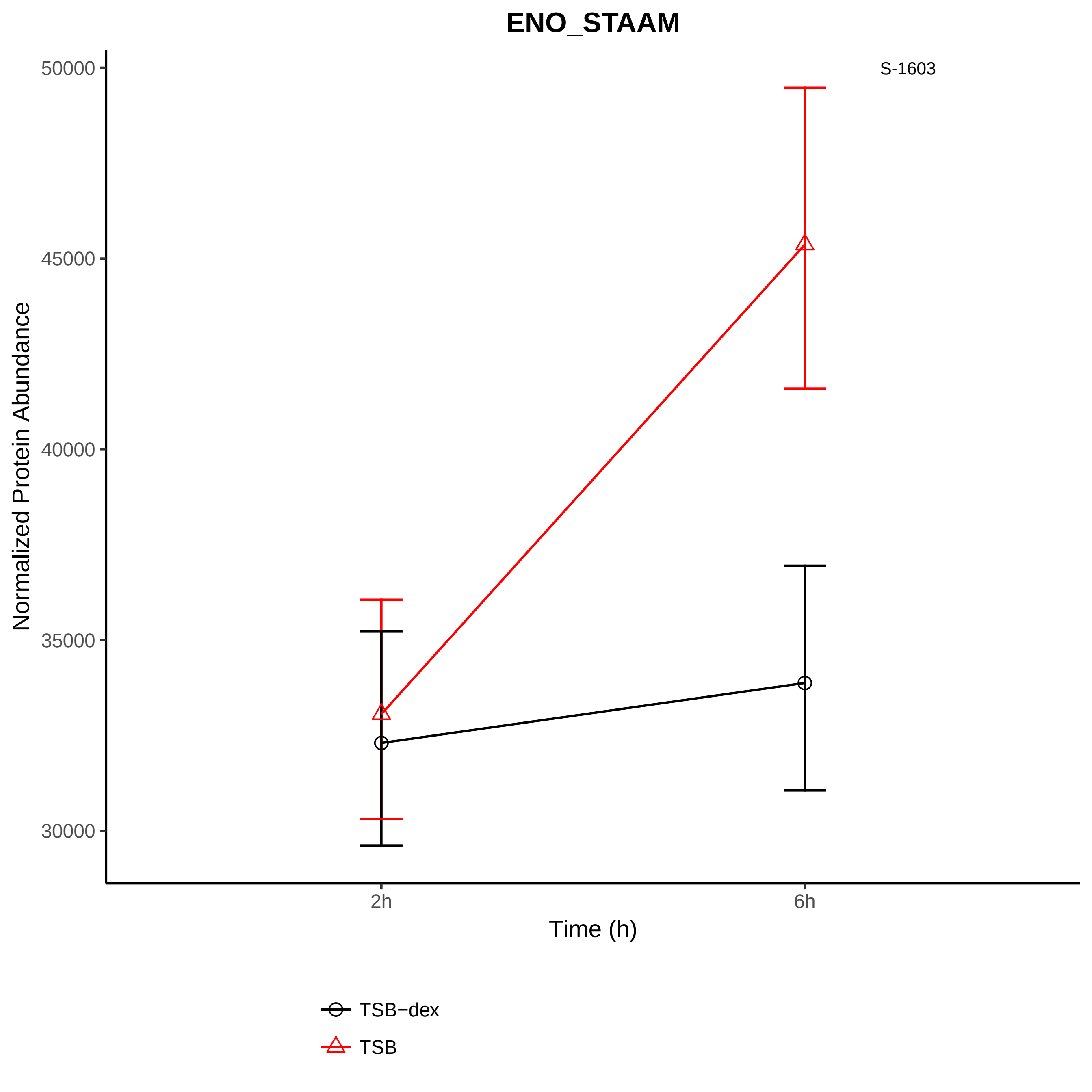

ENO_STAAM
S-1603
50000
45000
40000
35000
30000
Normalized Protein Abundance
2h
6h
Time (h)
TSB−dex
TSB

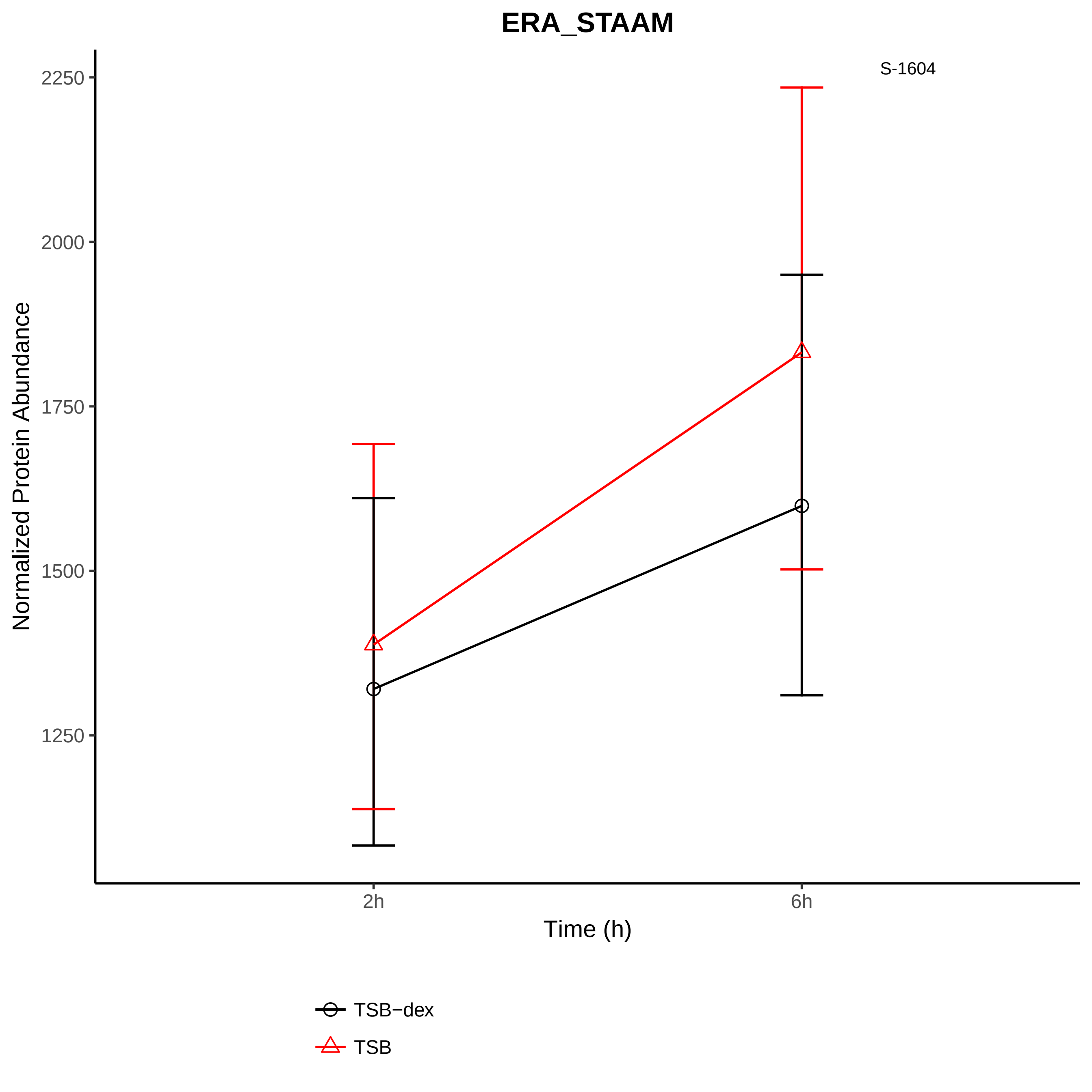
ERA_STAAM
S-1604
Normalized Protein Abundance
2250
2000
1750
1500
1250
2h
6h
Time (h)
TSB−dex
TSB

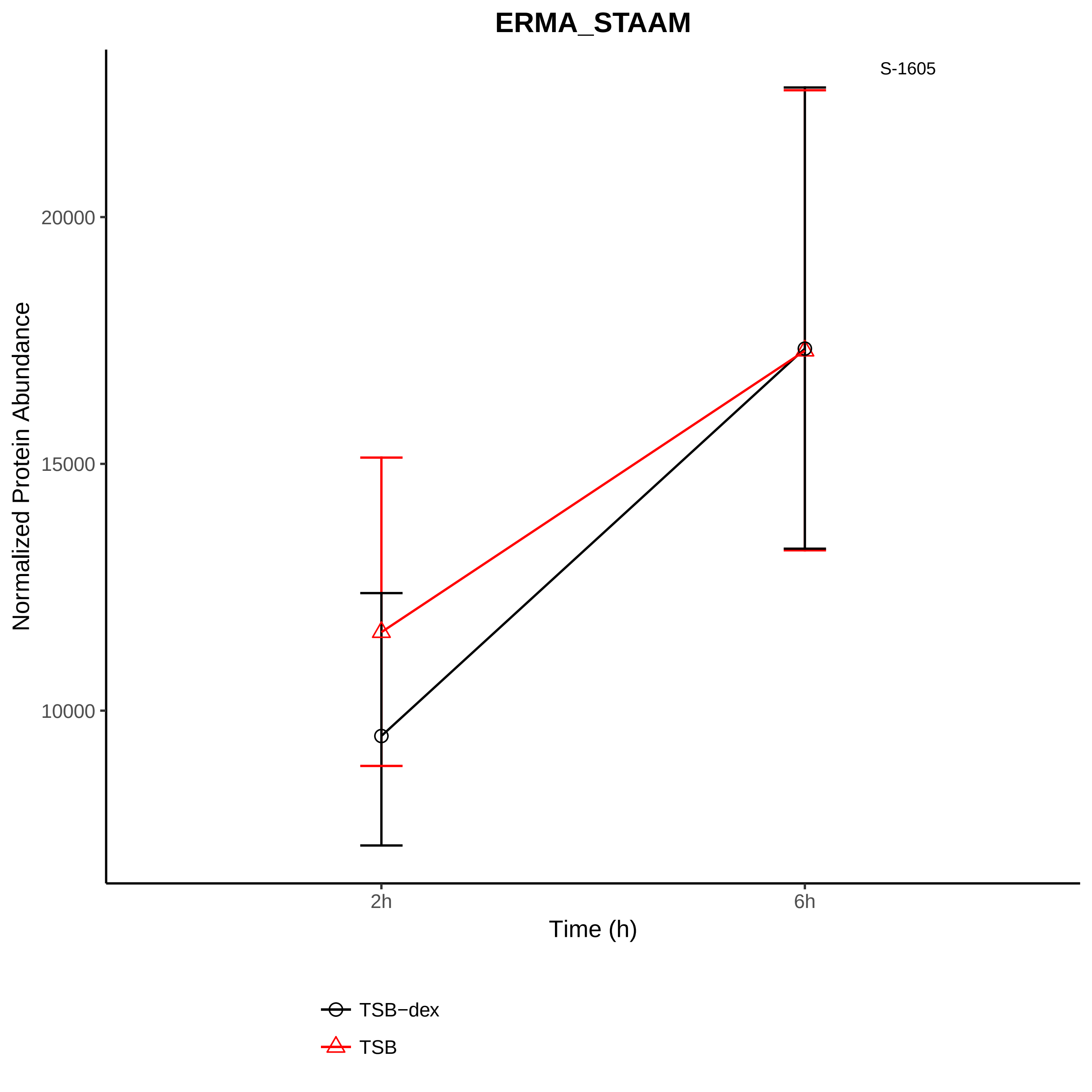
ERMA_STAAM
S-1605
Normalized Protein Abundance
20000
15000
10000
2h
6h
Time (h)
TSB−dex
TSB

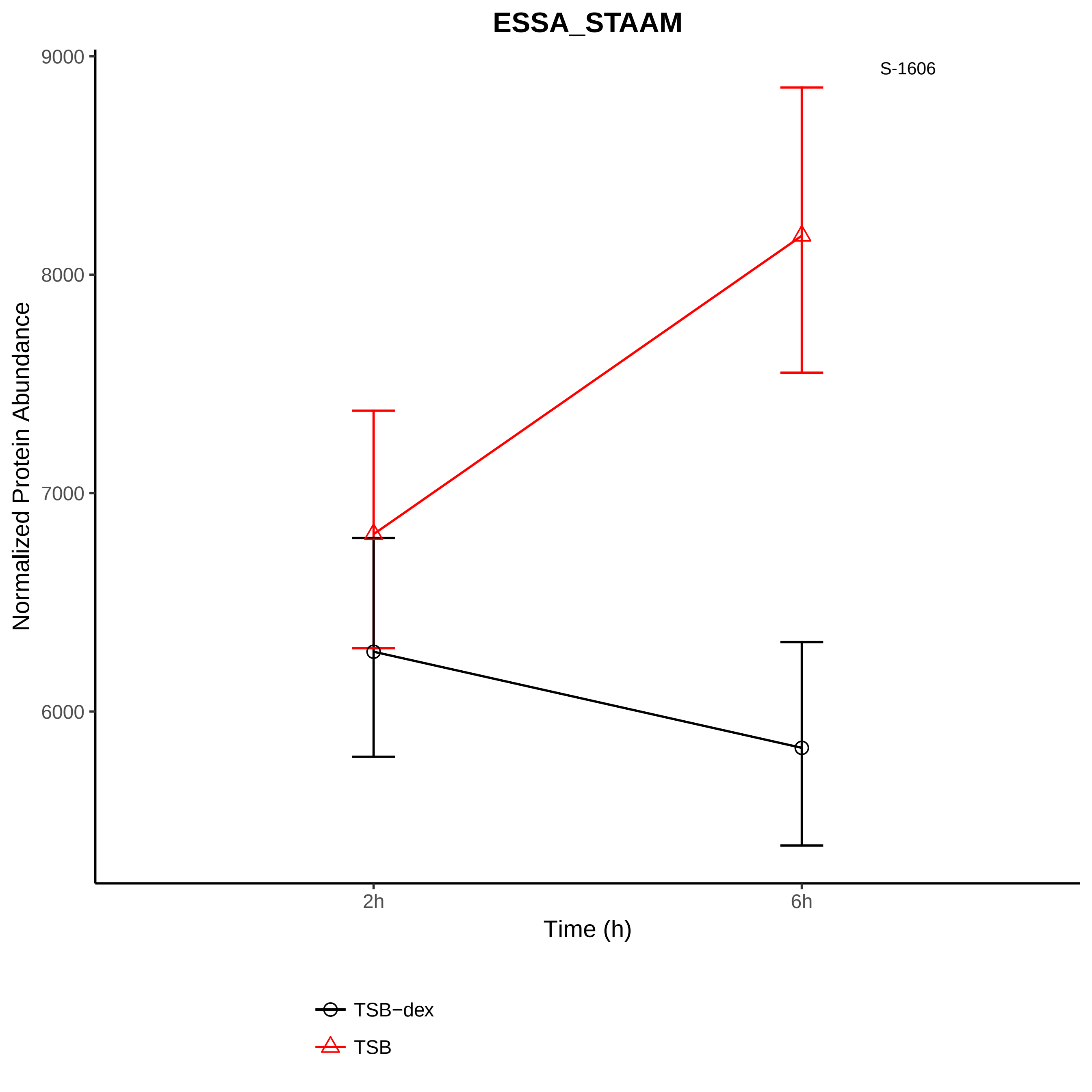
ESSA_STAAM
S-1606
9000
8000
7000
6000
Normalized Protein Abundance
2h
6h
Time (h)
TSB−dex
TSB

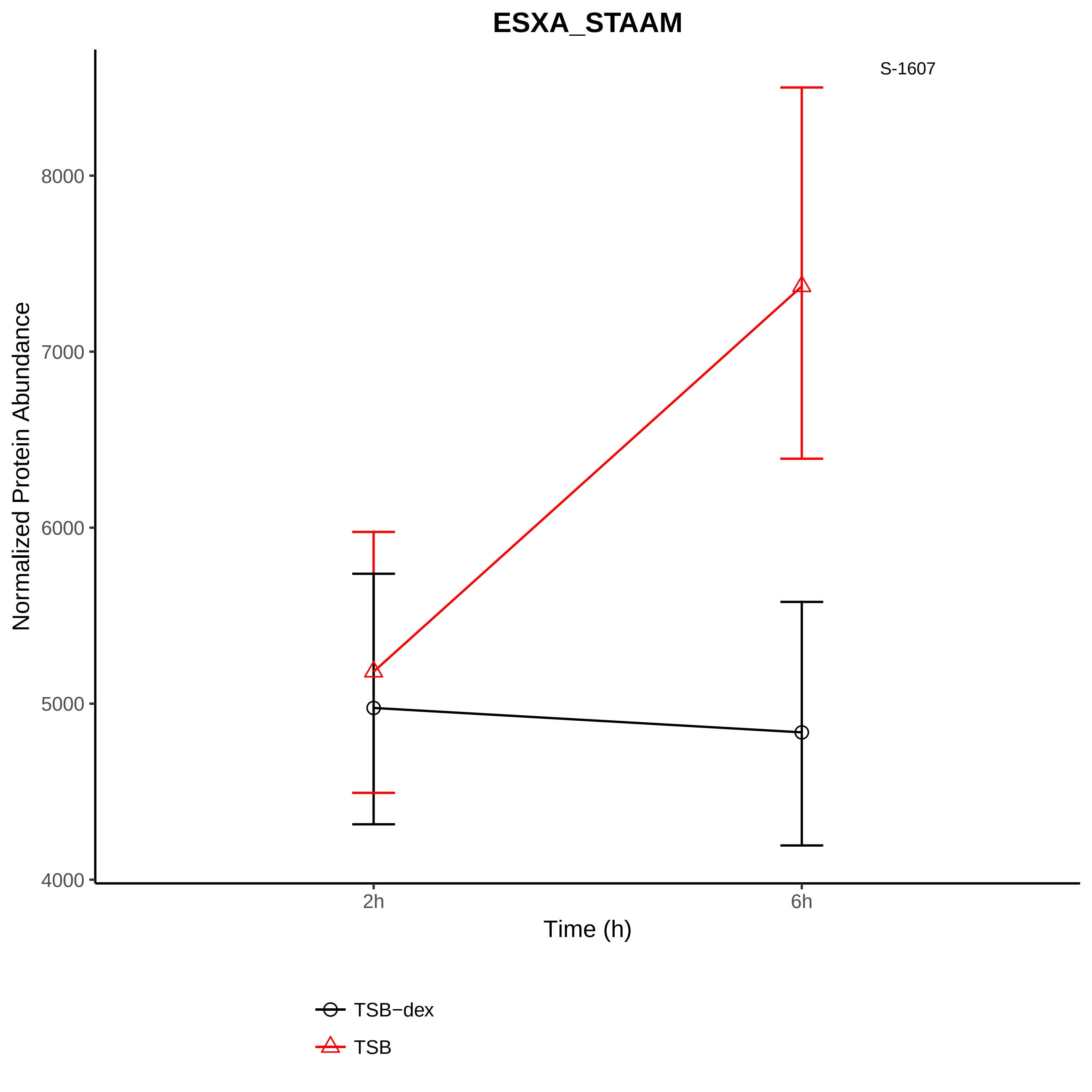
ESXA_STAAM
S-1607
Normalized Protein Abundance
8000
7000
6000
5000
4000
2h
6h
Time (h)
TSB−dex
TSB

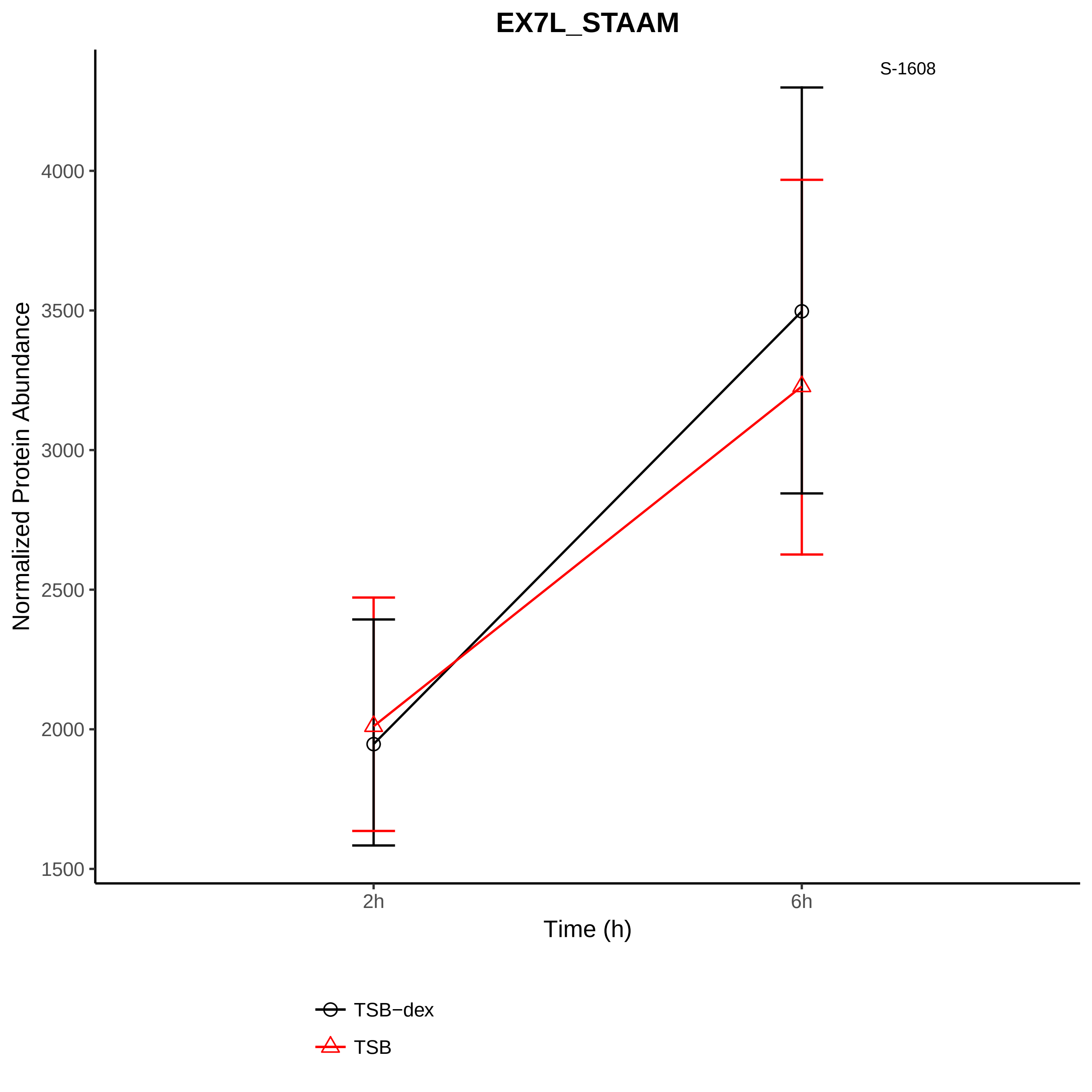

EX7L_STAAM
S-1608
Normalized Protein Abundance
4000
3500
3000
2500
2000
1500
2h
6h
Time (h)
TSB−dex
TSB

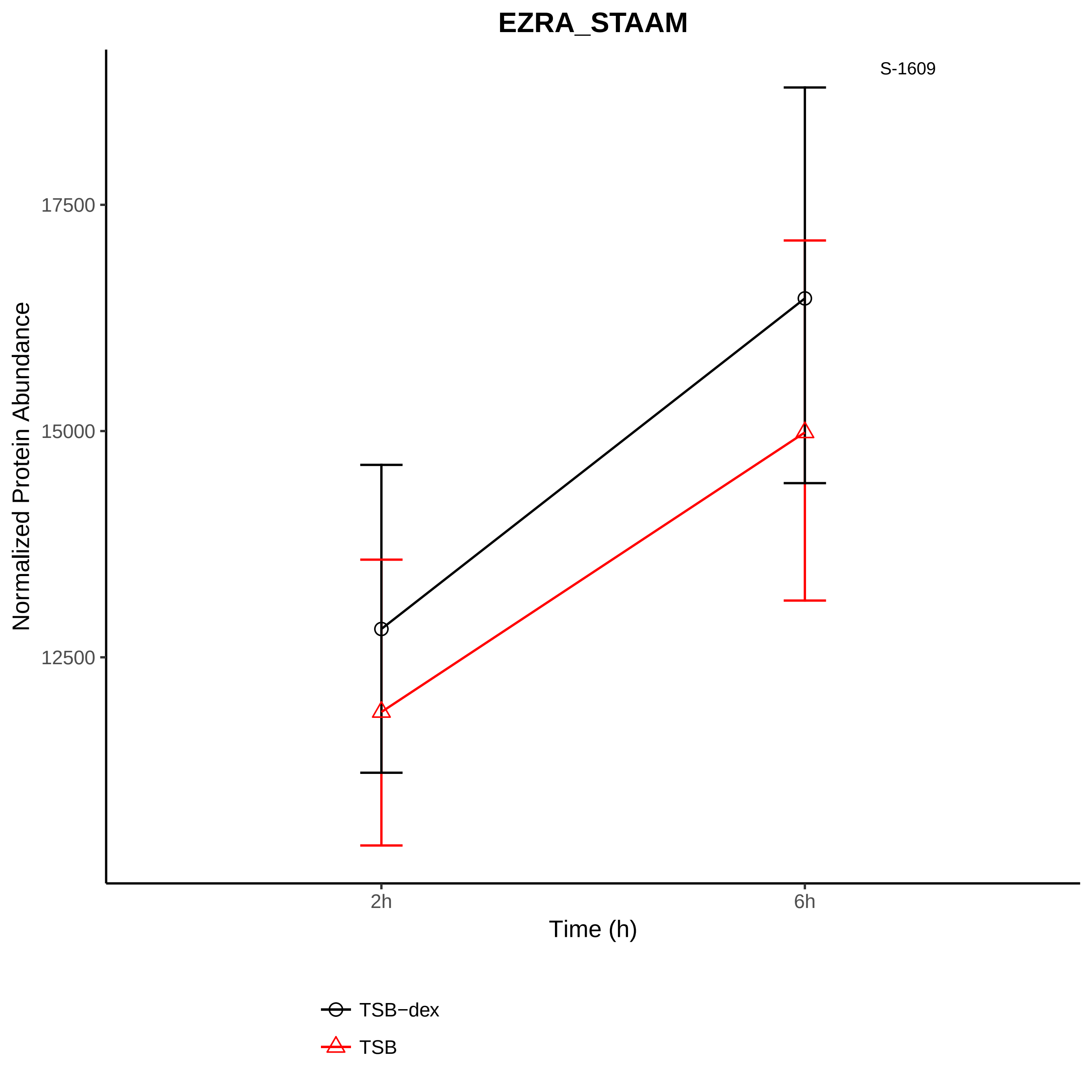
EZRA_STAAM
S-1609
17500
15000
12500
Normalized Protein Abundance
2h
6h
Time (h)
TSB−dex
TSB

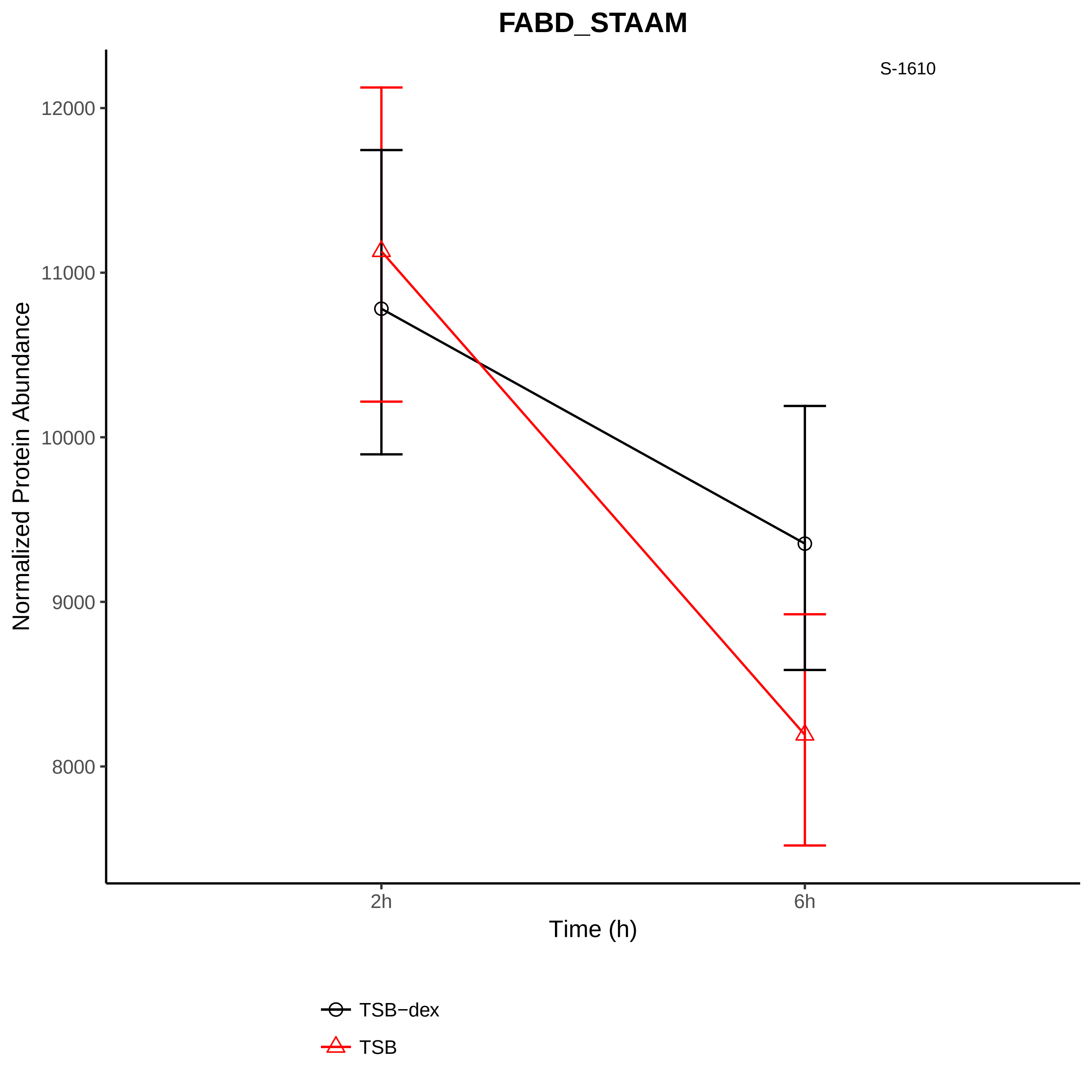
FABD_STAAM
S-1610
Normalized Protein Abundance
12000
11000
10000
9000
8000
2h
6h
Time (h)
TSB−dex
TSB

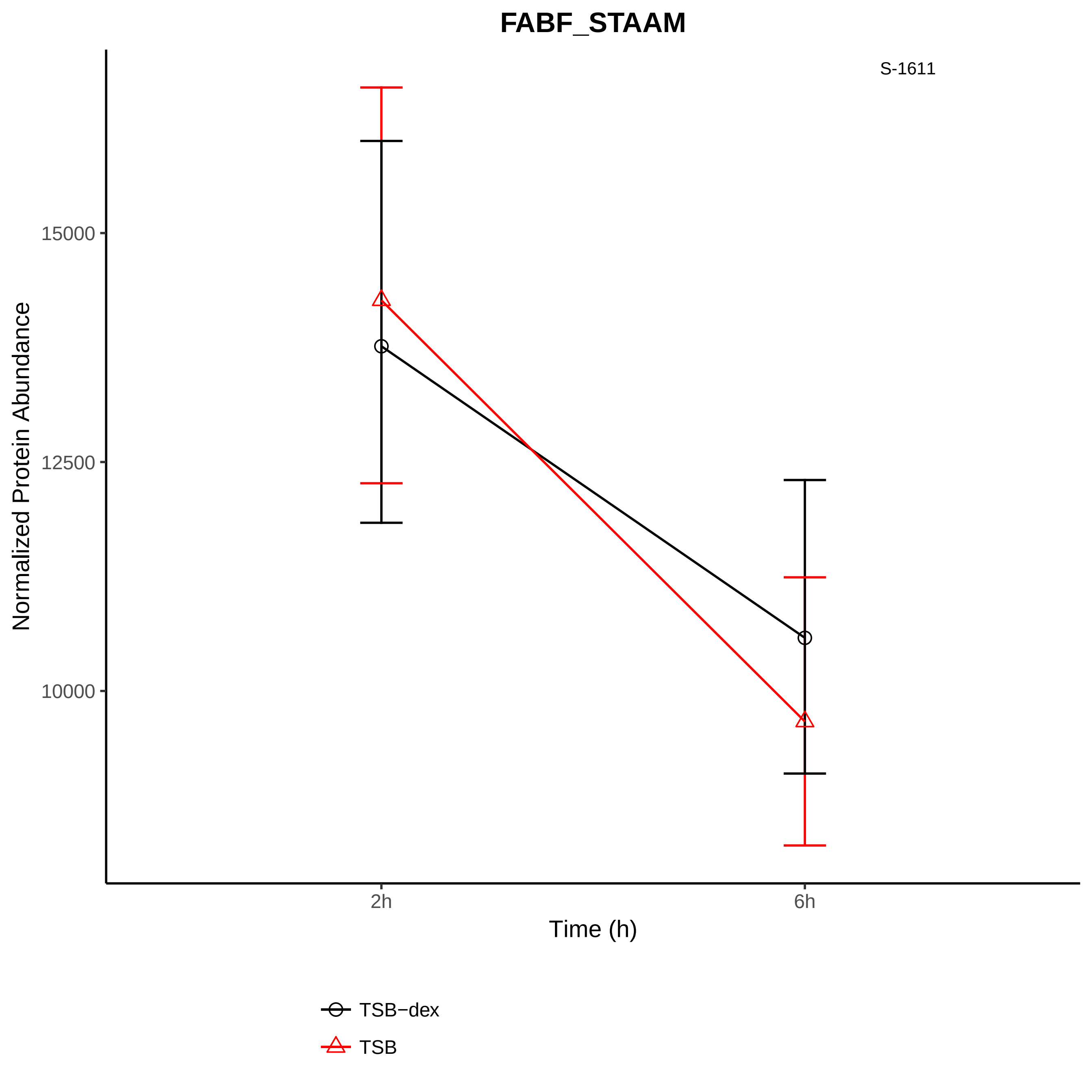
FABF_STAAM
S-1611
Normalized Protein Abundance
15000
12500
10000
2h
6h
Time (h)
TSB−dex
TSB

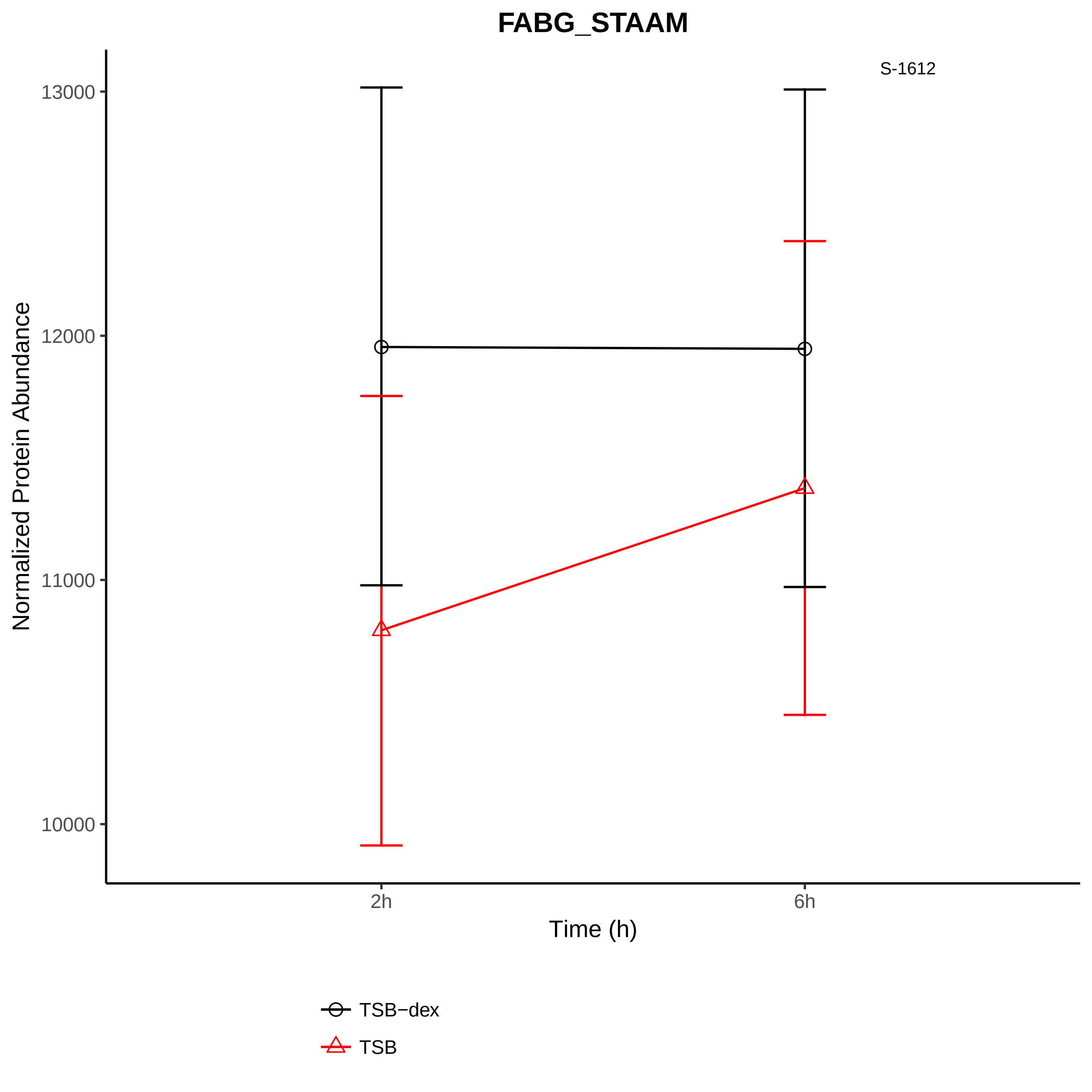

FABG_STAAM
S-1612
13000
12000
11000
10000
Normalized Protein Abundance
2h
6h
Time (h)
TSB−dex
TSB

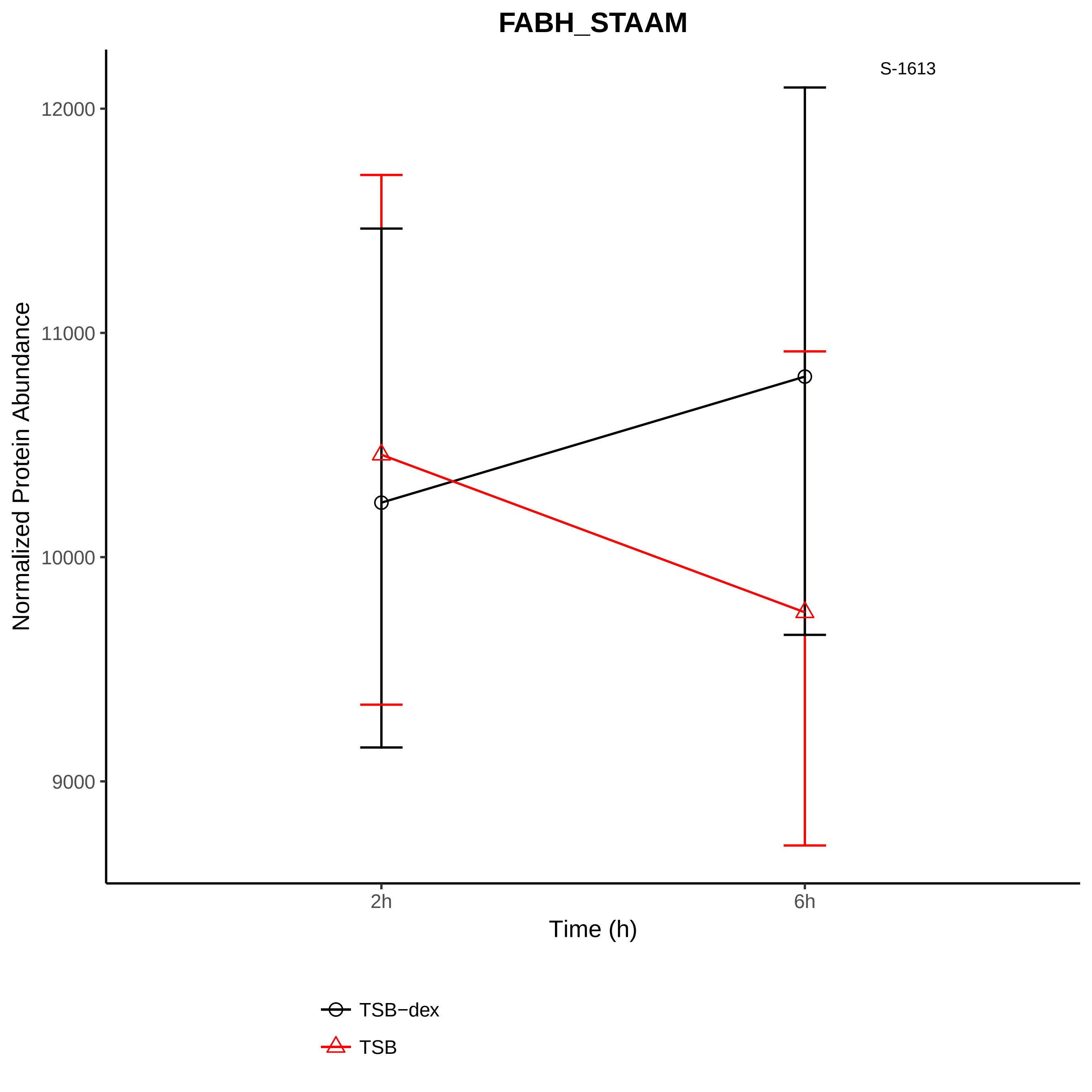

FABH_STAAM
S-1613
12000
11000
10000
9000
Normalized Protein Abundance
2h
6h
Time (h)
TSB−dex
TSB

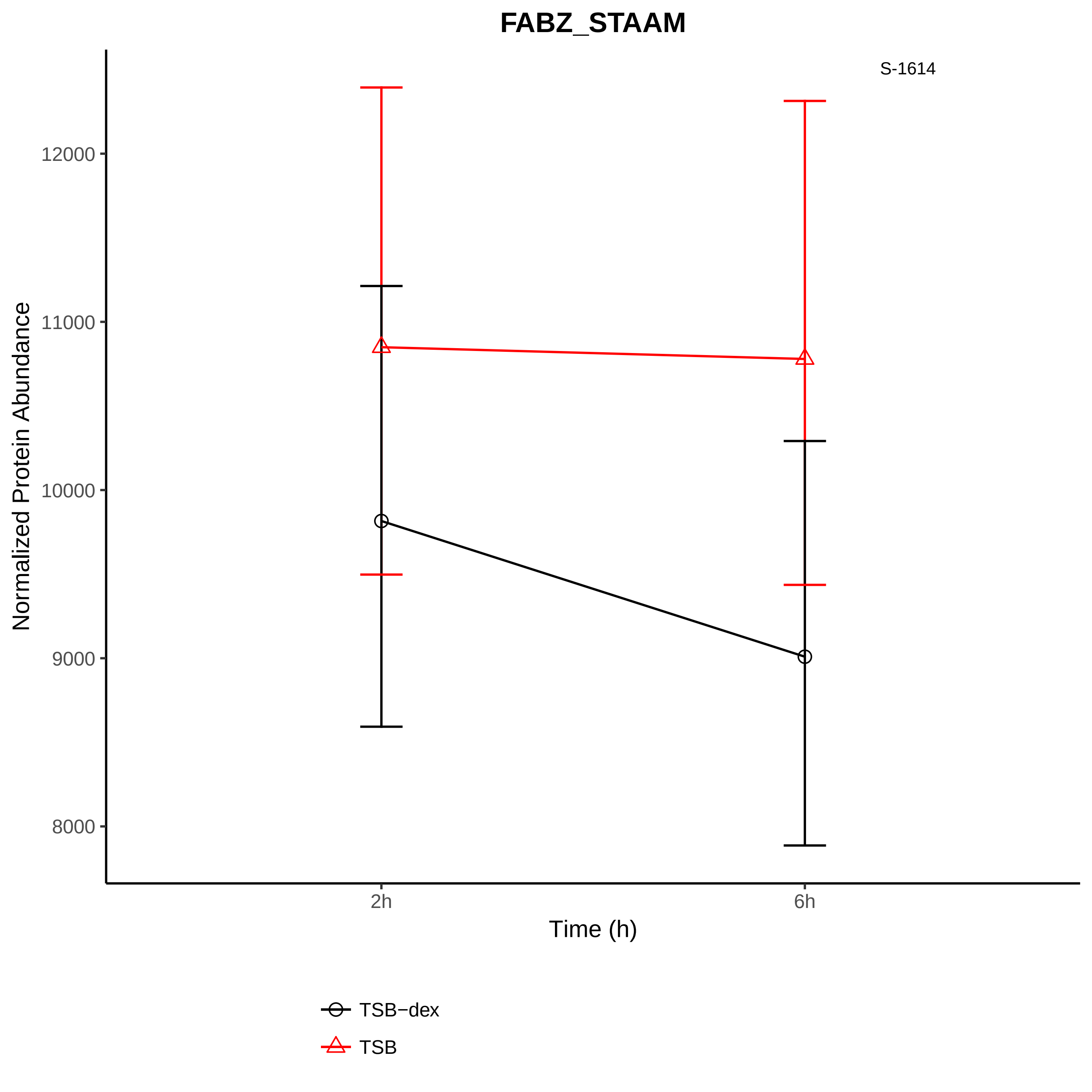

FABZ_STAAM
S-1614
12000
11000
10000
9000
8000
Normalized Protein Abundance
2h
6h
Time (h)
TSB−dex
TSB

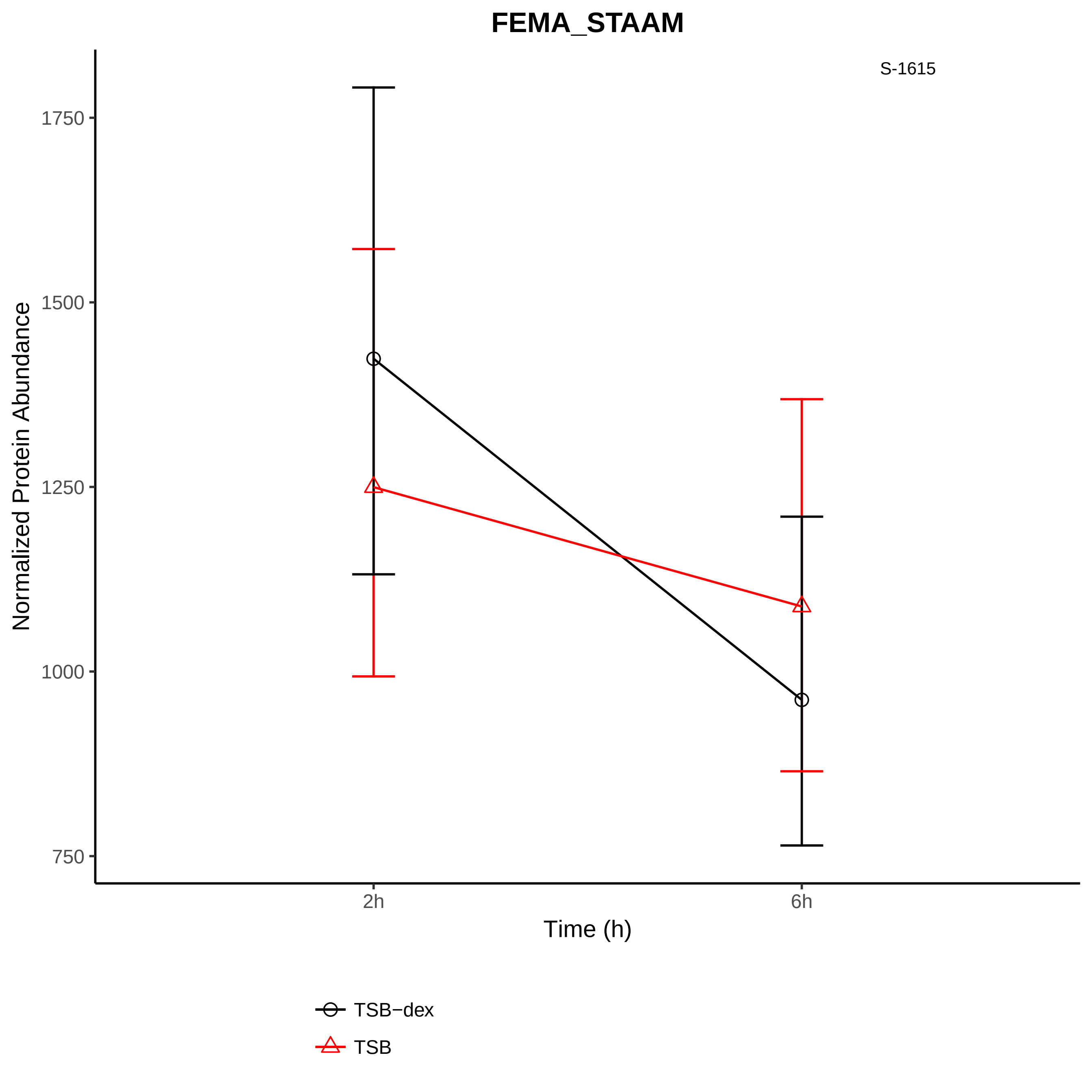

FEMA_STAAM
S-1615
Normalized Protein Abundance
1750
1500
1250
1000
750
2h
6h
Time (h)
TSB−dex
TSB

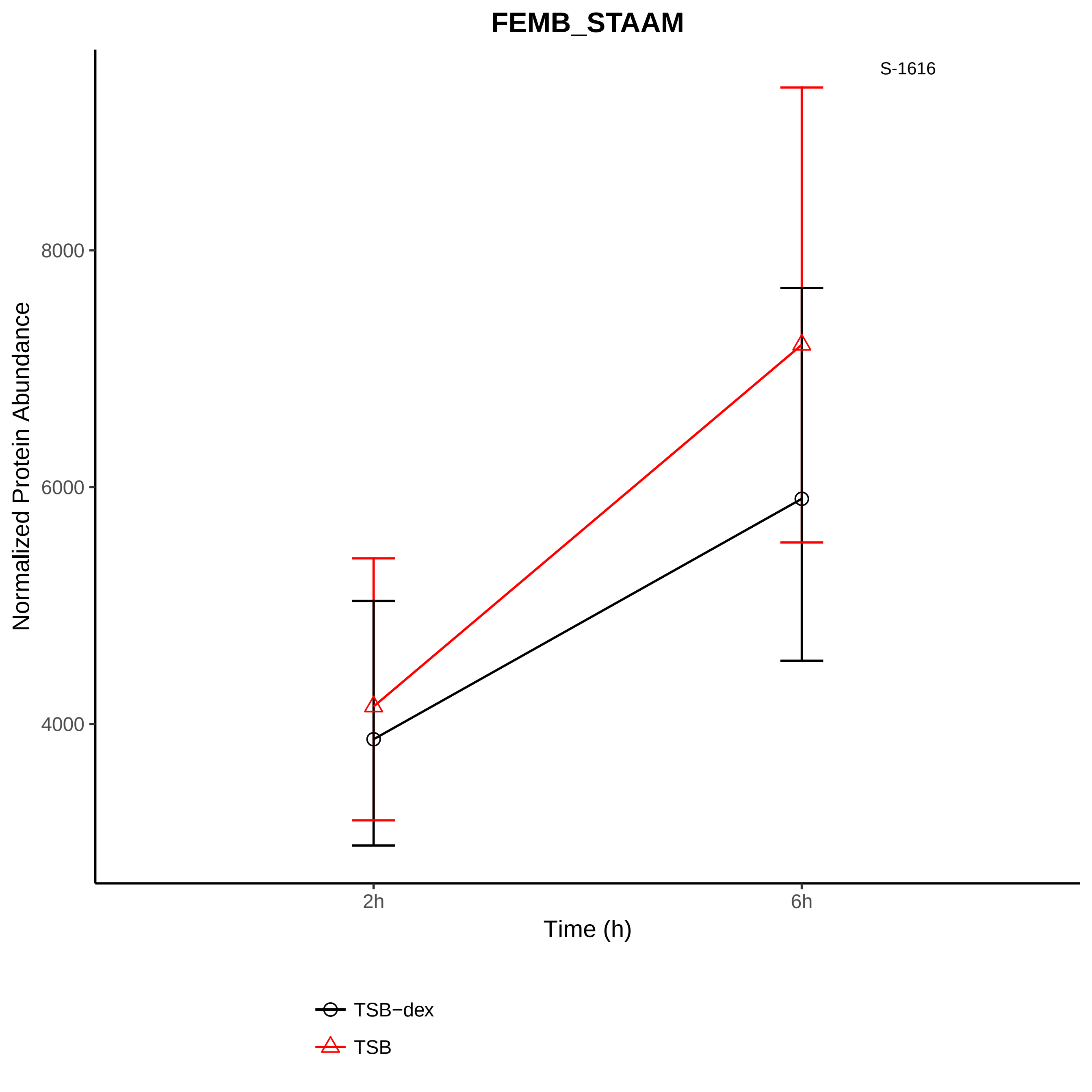
FEMB_STAAM
S-1616
Normalized Protein Abundance
8000
6000
4000
2h
6h
Time (h)
TSB−dex
TSB

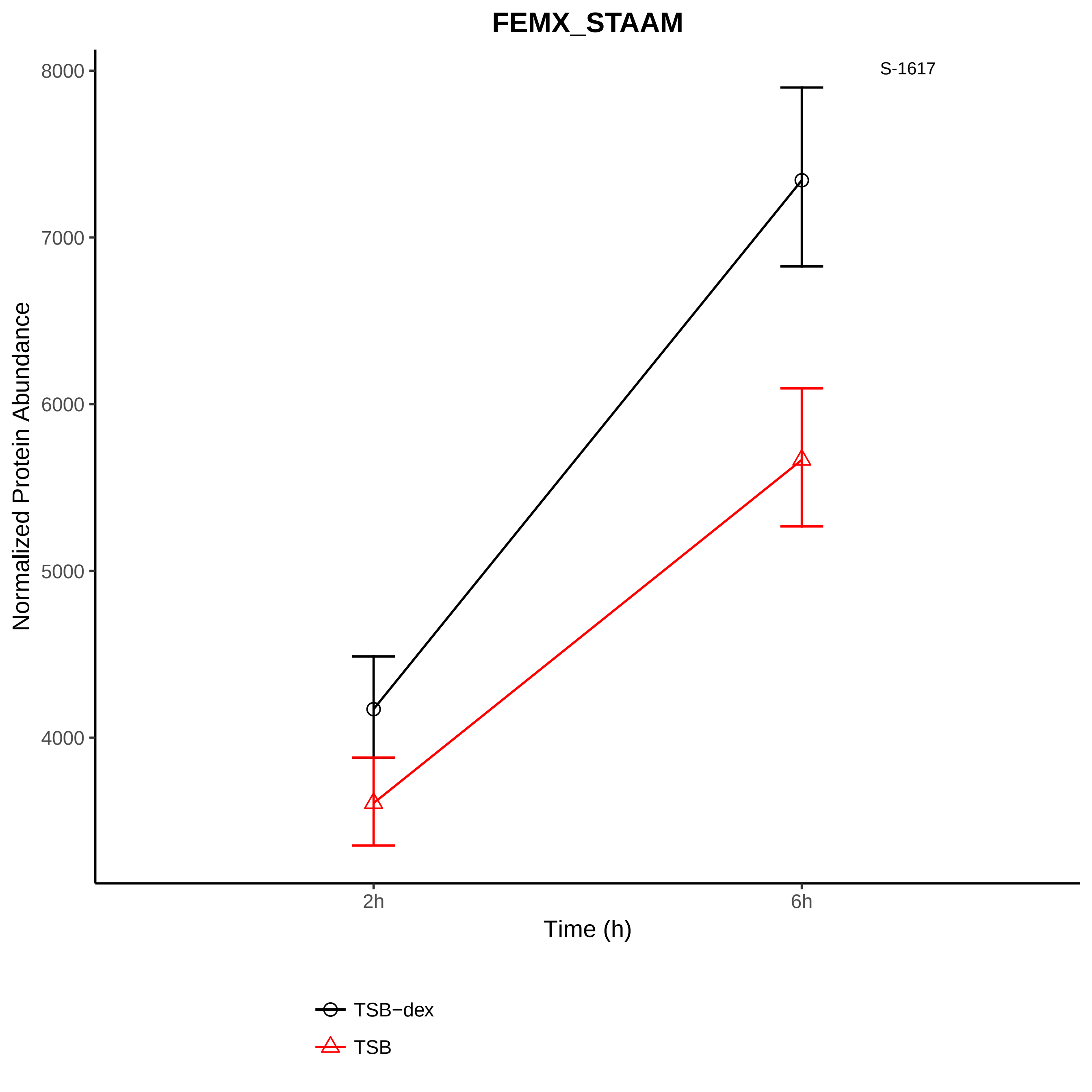

FEMX_STAAM
S-1617
Normalized Protein Abundance
8000
7000
6000
5000
4000
2h
6h
Time (h)
TSB−dex
TSB

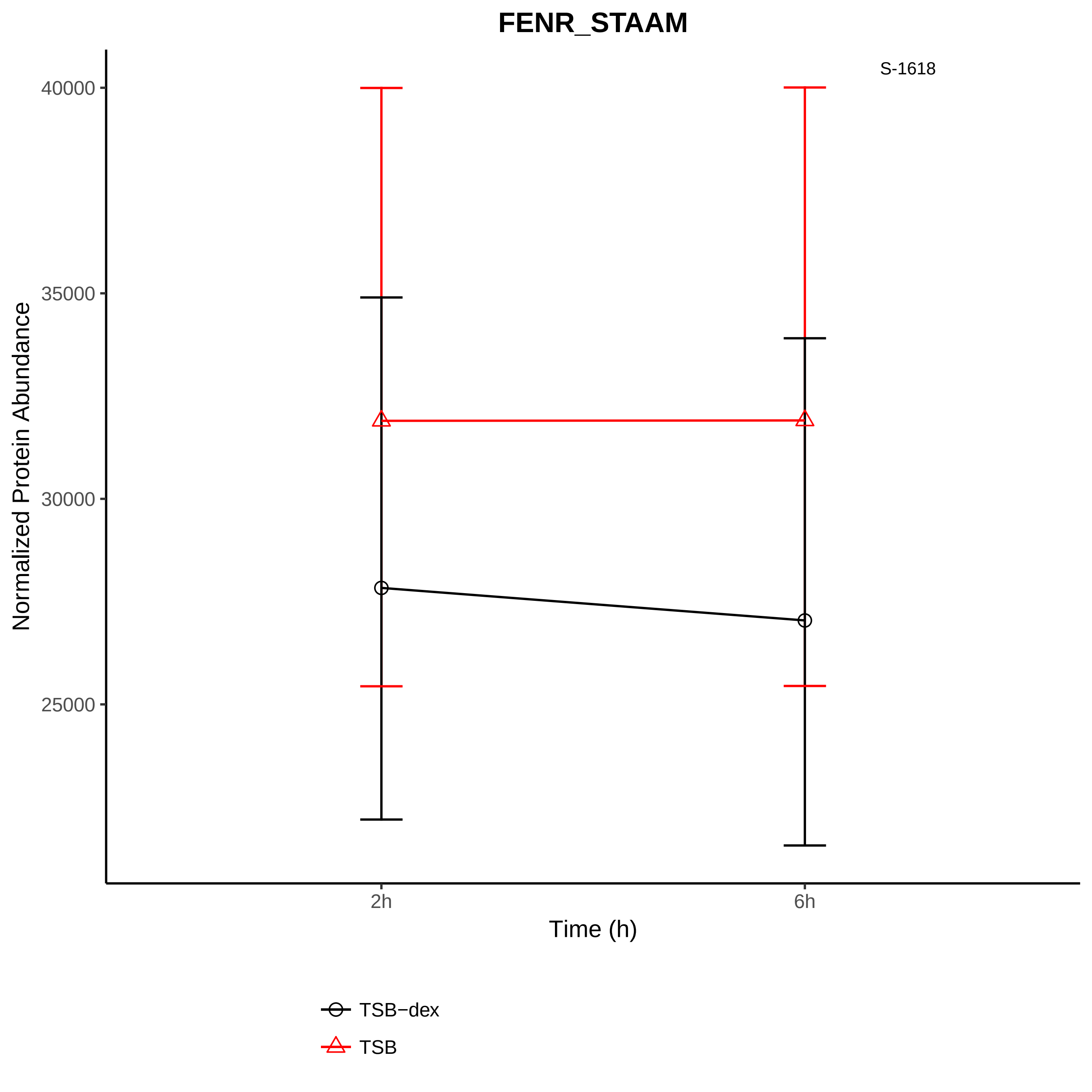
FENR_STAAM
S-1618
40000
35000
30000
25000
Normalized Protein Abundance
2h
6h
Time (h)
TSB−dex
TSB

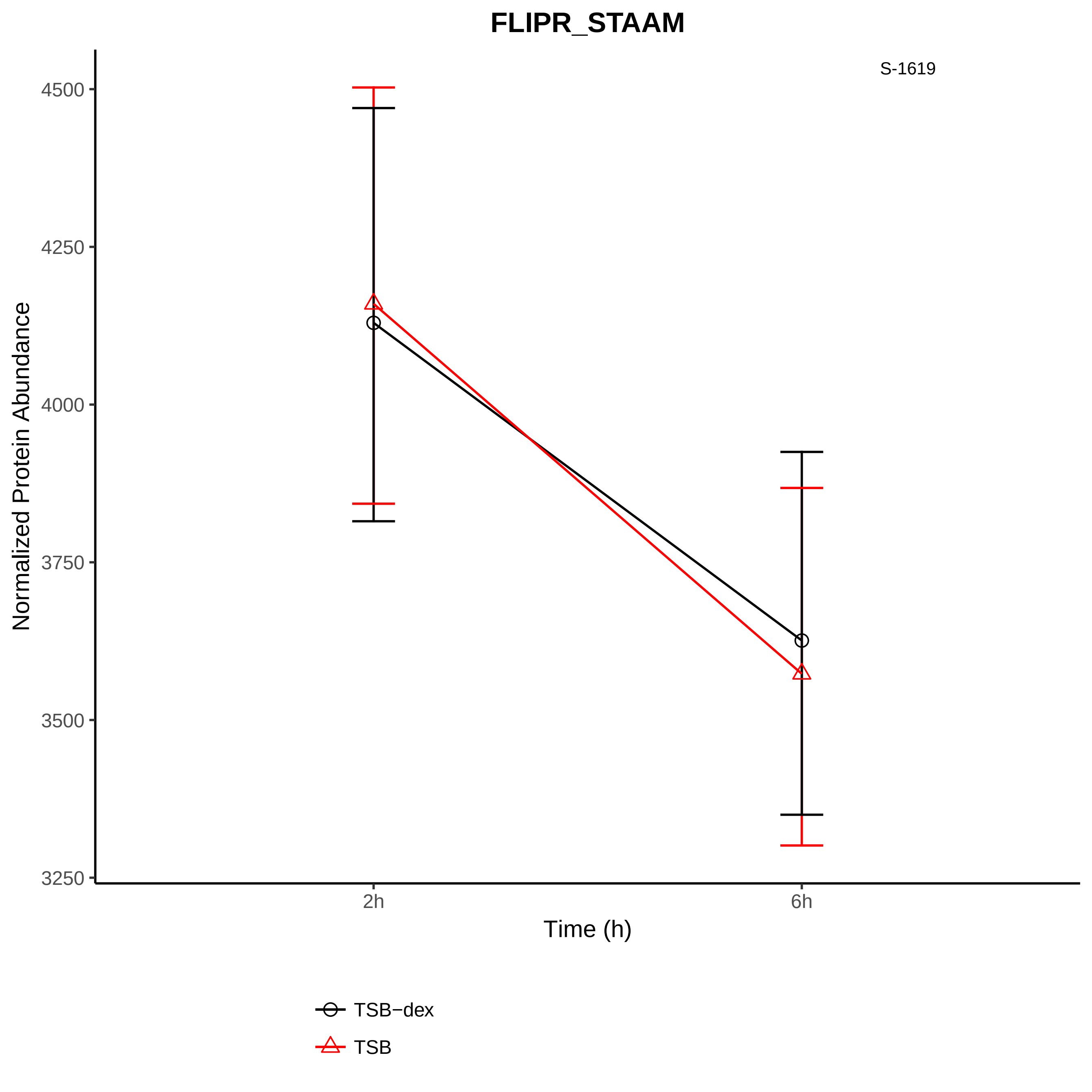

FLIPR_STAAM
S-1619
Normalized Protein Abundance
4500
4250
4000
3750
3500
3250
2h
6h
Time (h)
TSB−dex
TSB

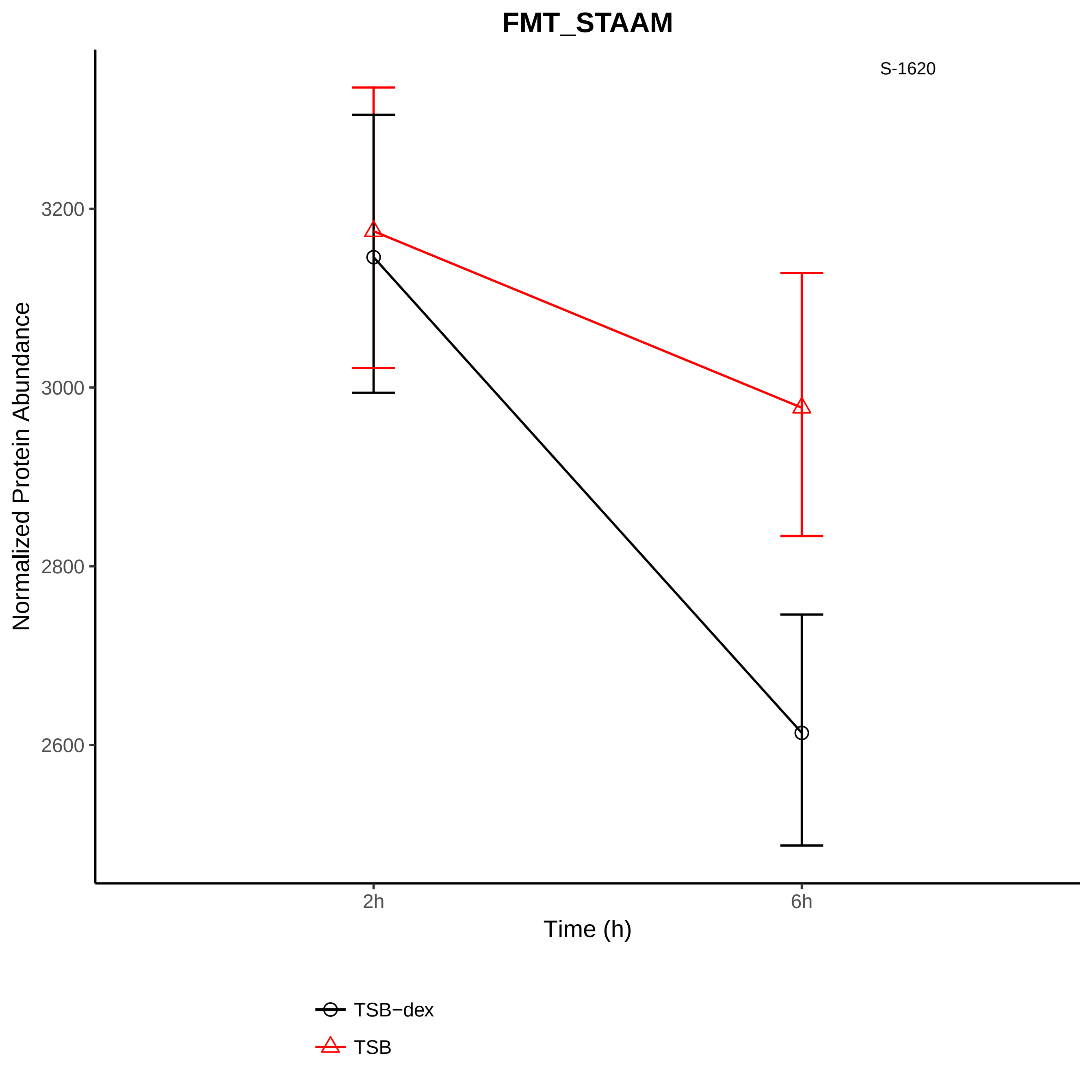

FMT_STAAM
S-1620
Normalized Protein Abundance
3200
3000
2800
2600
2h
6h
Time (h)
TSB−dex
TSB

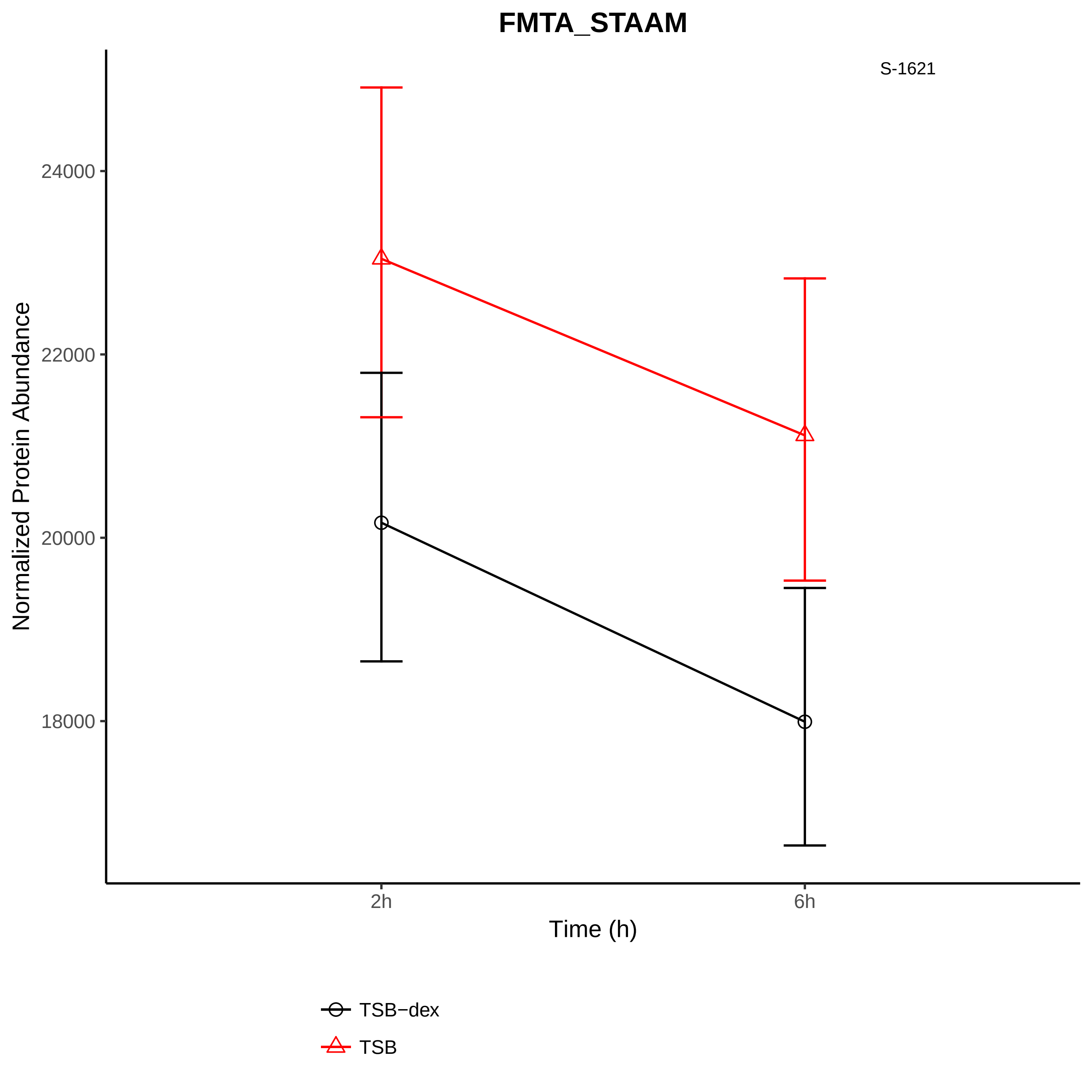

FMTA_STAAM
S-1621
Normalized Protein Abundance
24000
22000
20000
18000
2h
6h
Time (h)
TSB−dex
TSB

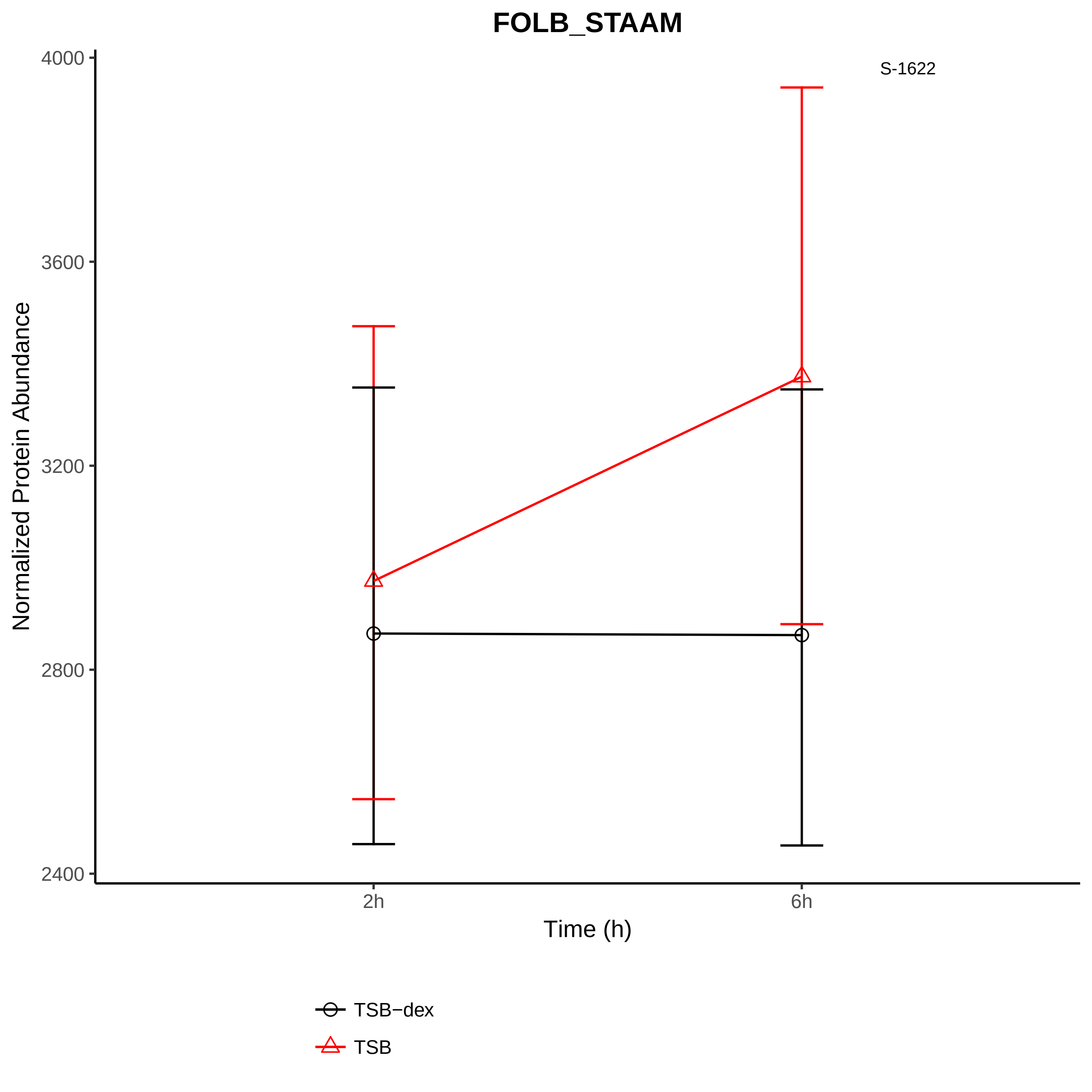

FOLB_STAAM
S-1622
Normalized Protein Abundance
4000
3600
3200
2800
2400
2h
6h
Time (h)
TSB−dex
TSB

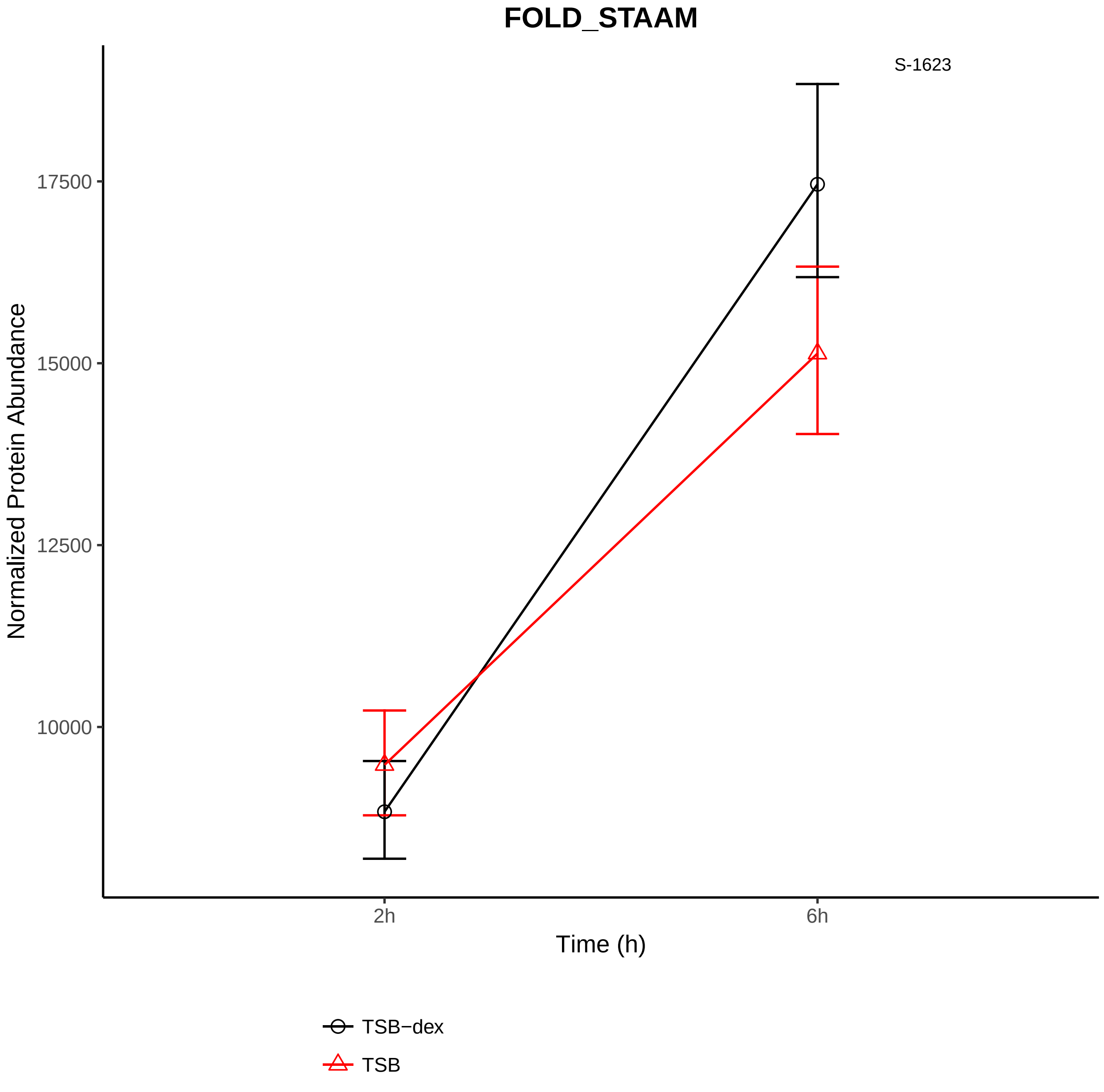
FOLD_STAAM
S-1623
17500
15000
12500
10000
Normalized Protein Abundance
2h
6h
Time (h)
TSB−dex
TSB

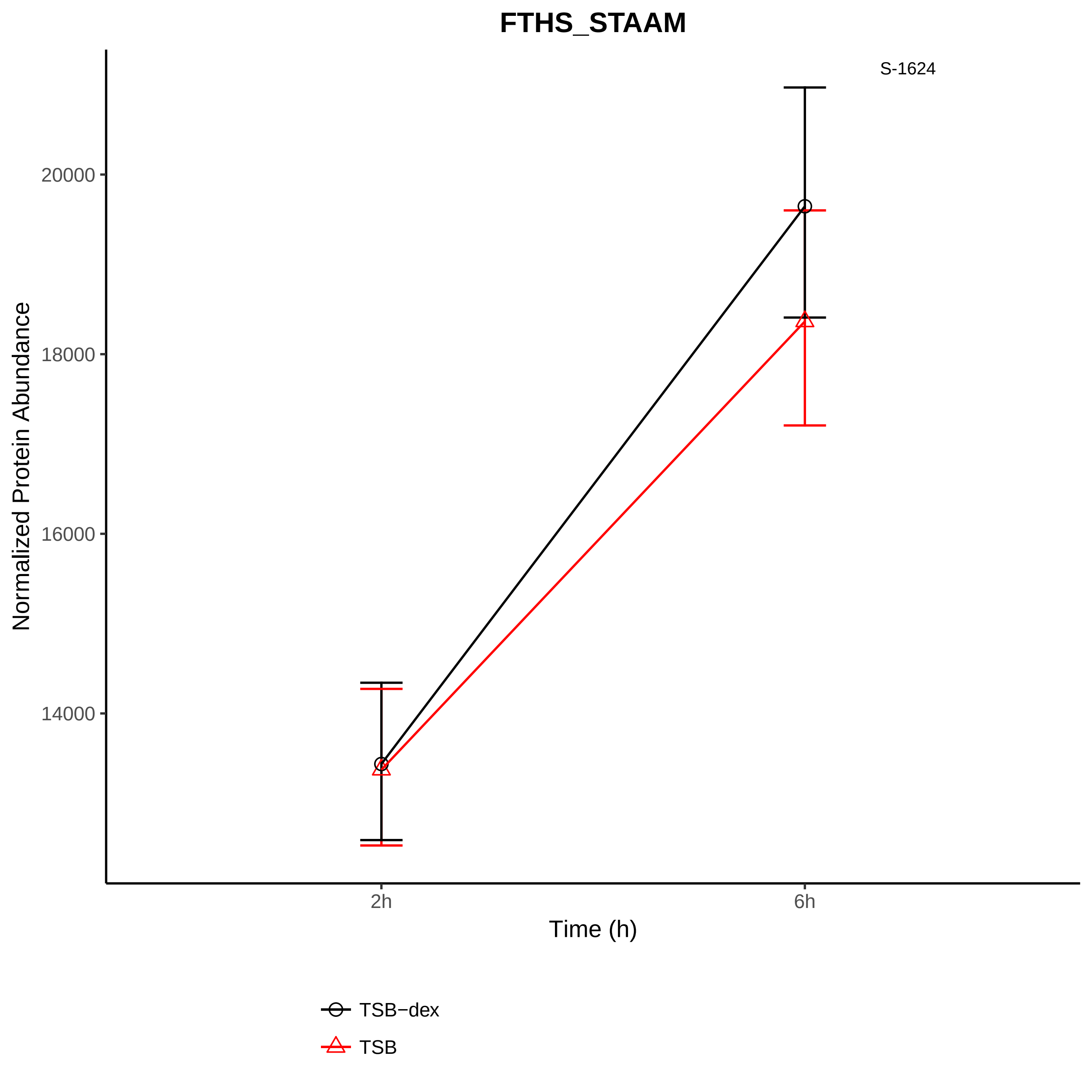

FTHS_STAAM
S-1624
Normalized Protein Abundance
20000
18000
16000
14000
2h
6h
Time (h)
TSB−dex
TSB

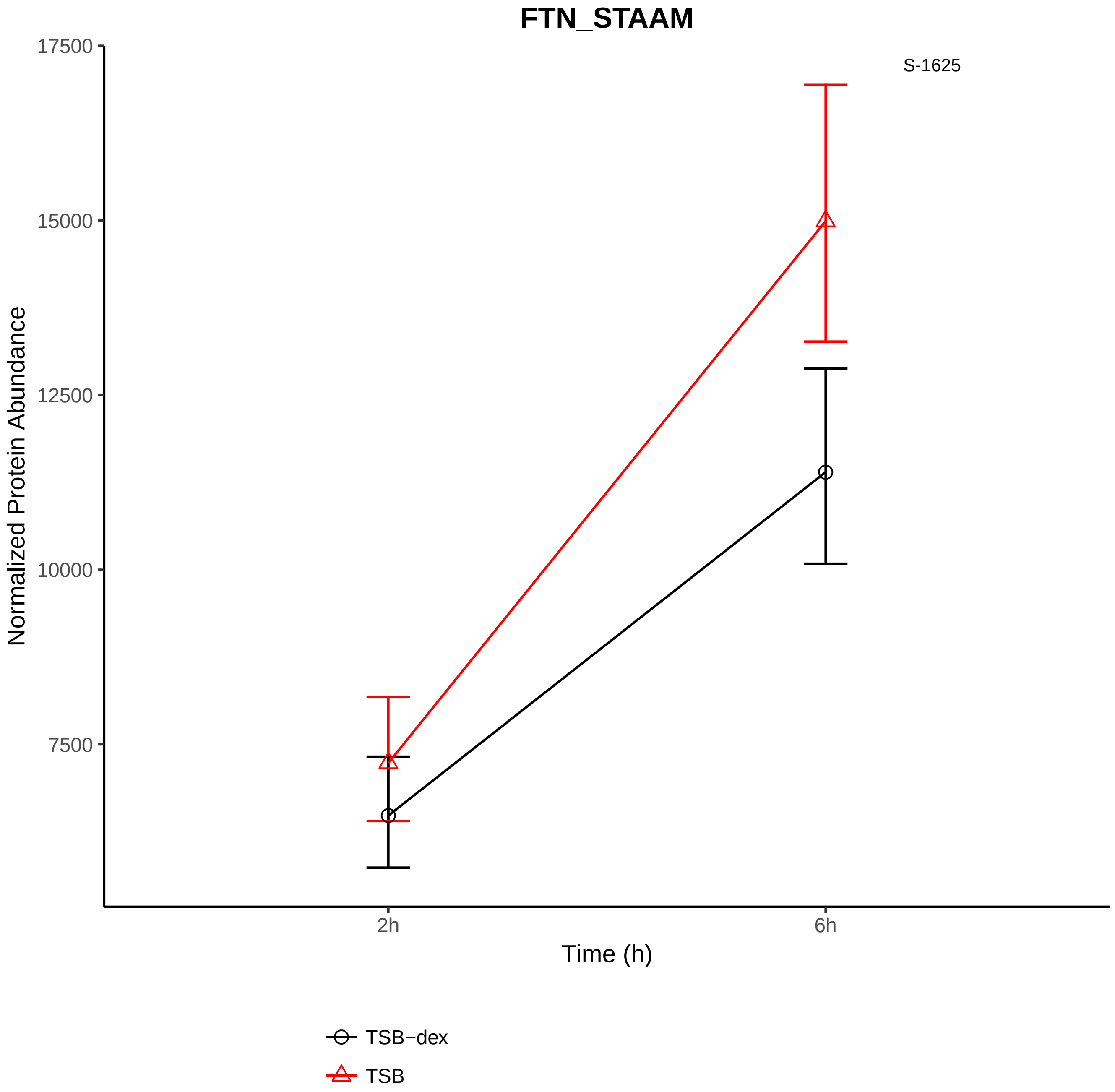

FTN_STAAM
S-1625
17500
15000
12500
10000
7500
Normalized Protein Abundance
2h
6h
Time (h)
TSB−dex
TSB

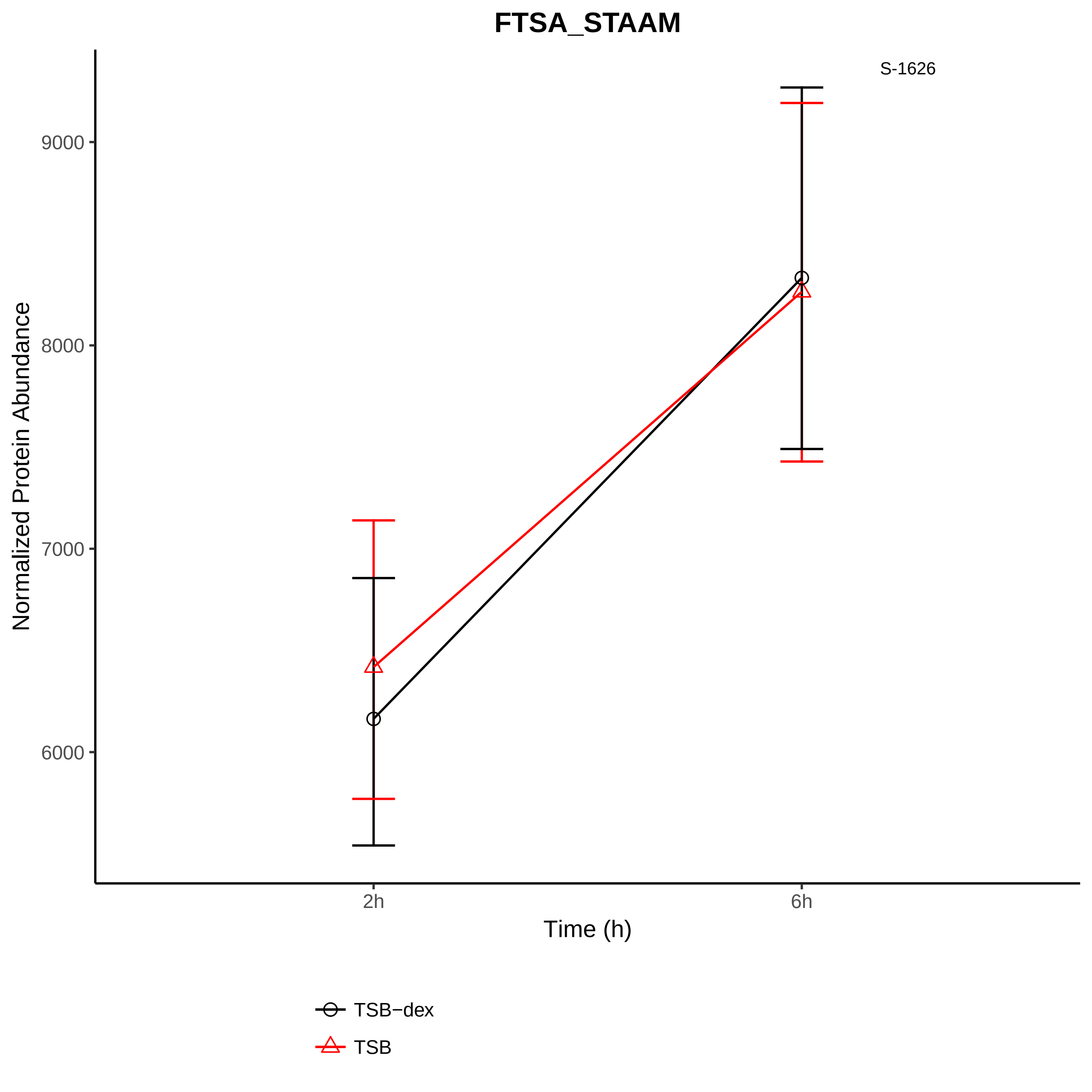
FTSA_STAAM
S-1626
Normalized Protein Abundance
9000
8000
7000
6000
2h
6h
Time (h)
TSB−dex
TSB

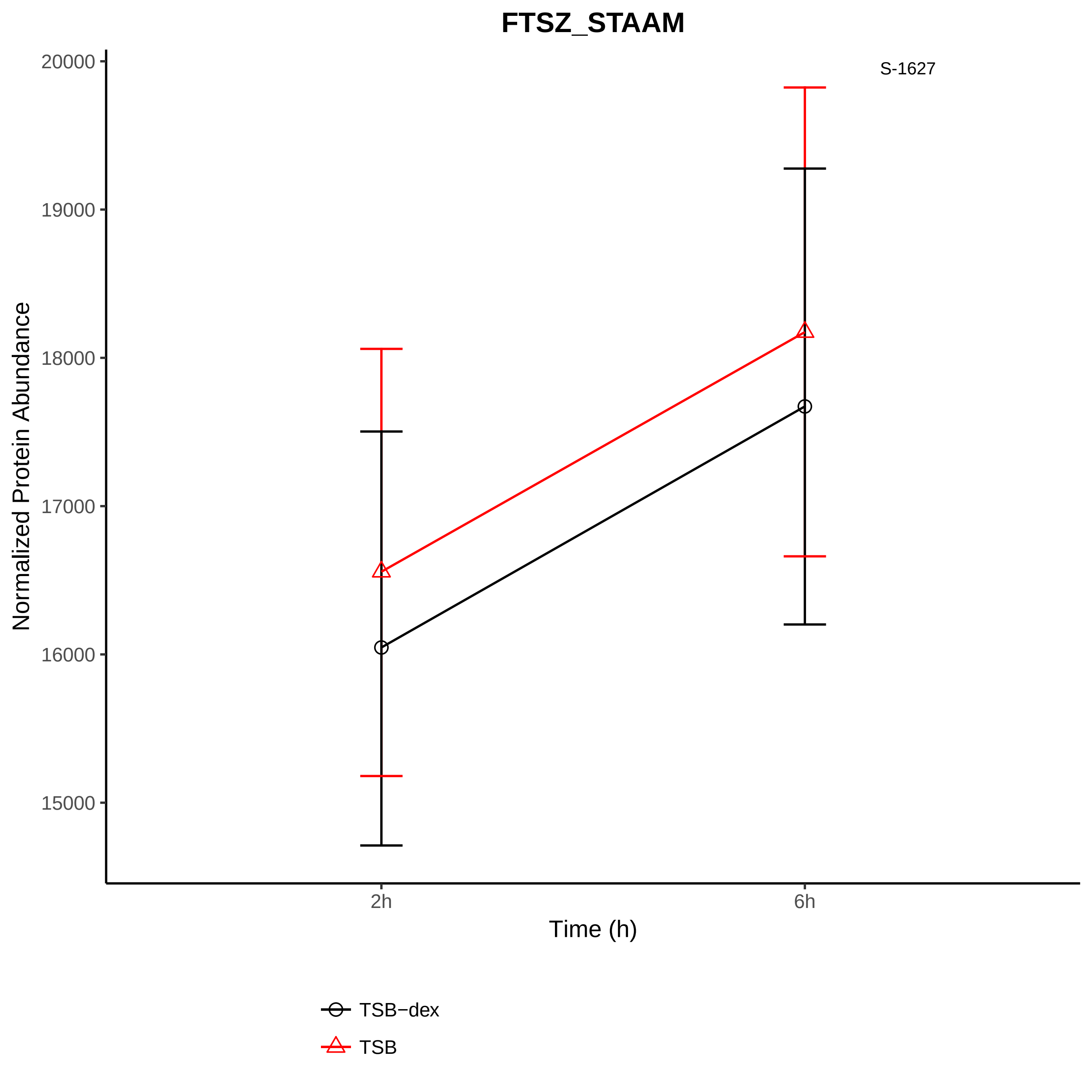

FTSZ_STAAM
S-1627
Normalized Protein Abundance
20000
19000
18000
17000
16000
15000
2h
6h
Time (h)
TSB−dex
TSB

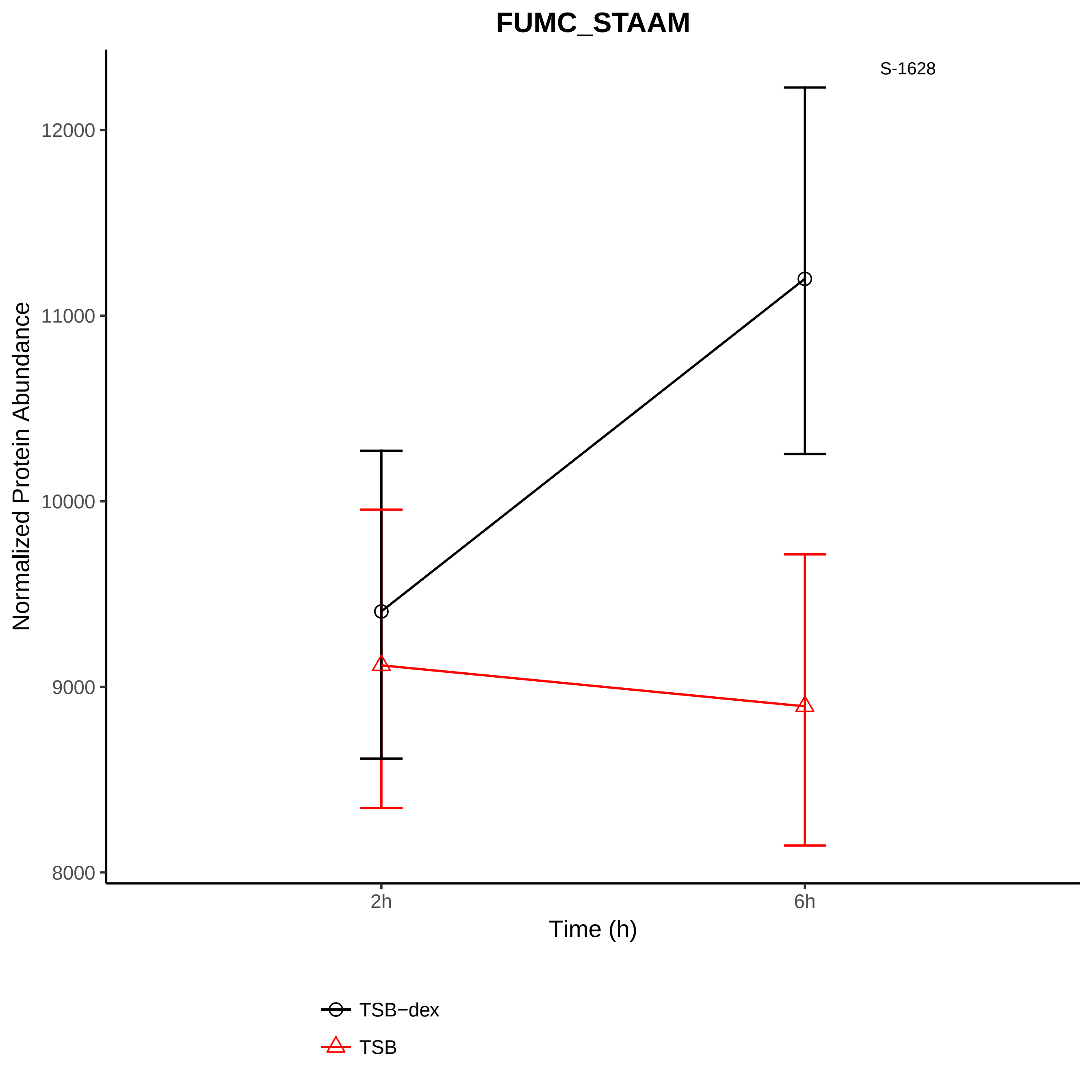

FUMC_STAAM
S-1628
12000
11000
10000
9000
8000
Normalized Protein Abundance
2h
6h
Time (h)
TSB−dex
TSB

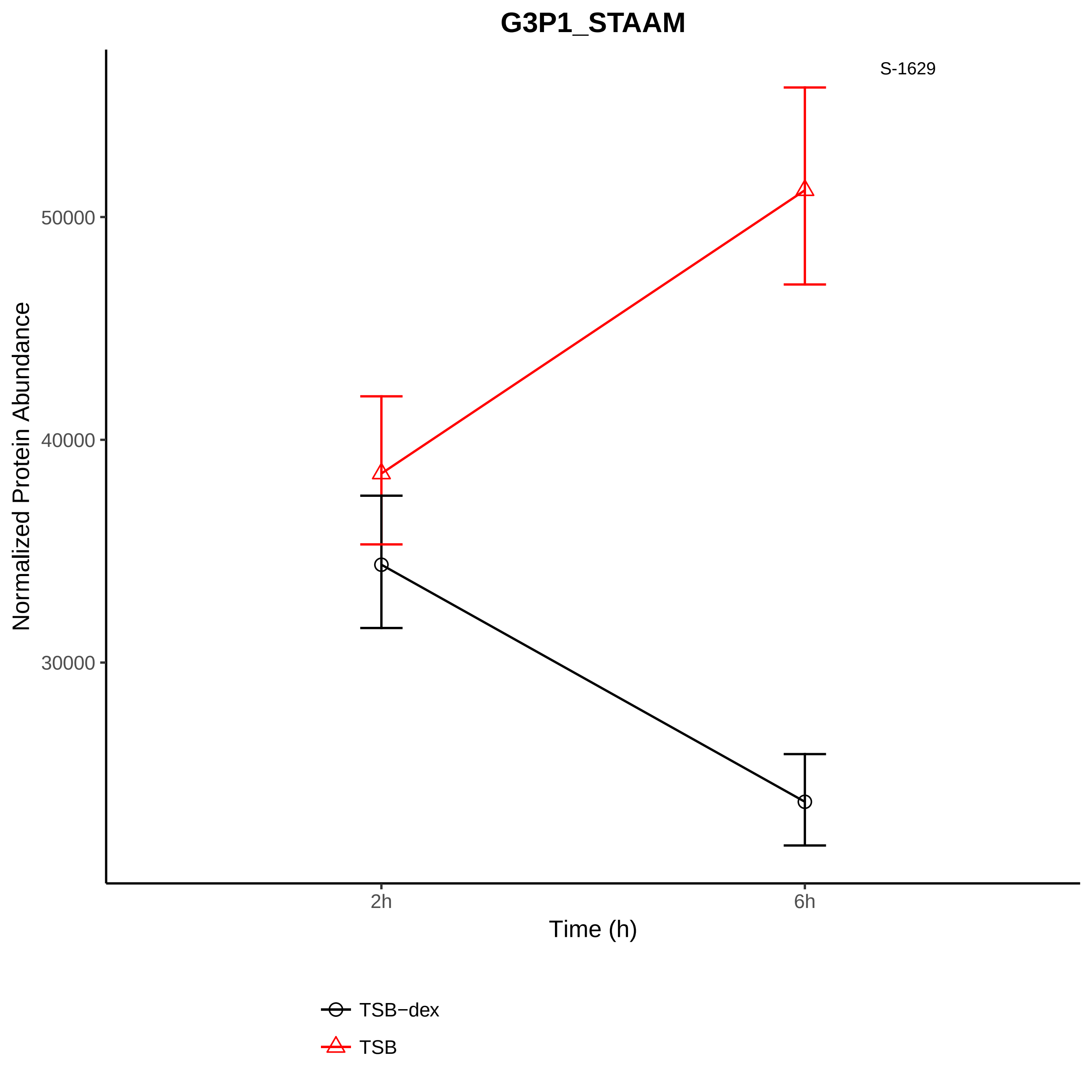

G3P1_STAAM
S-1629
Normalized Protein Abundance
50000
40000
30000
2h
6h
Time (h)
TSB−dex
TSB

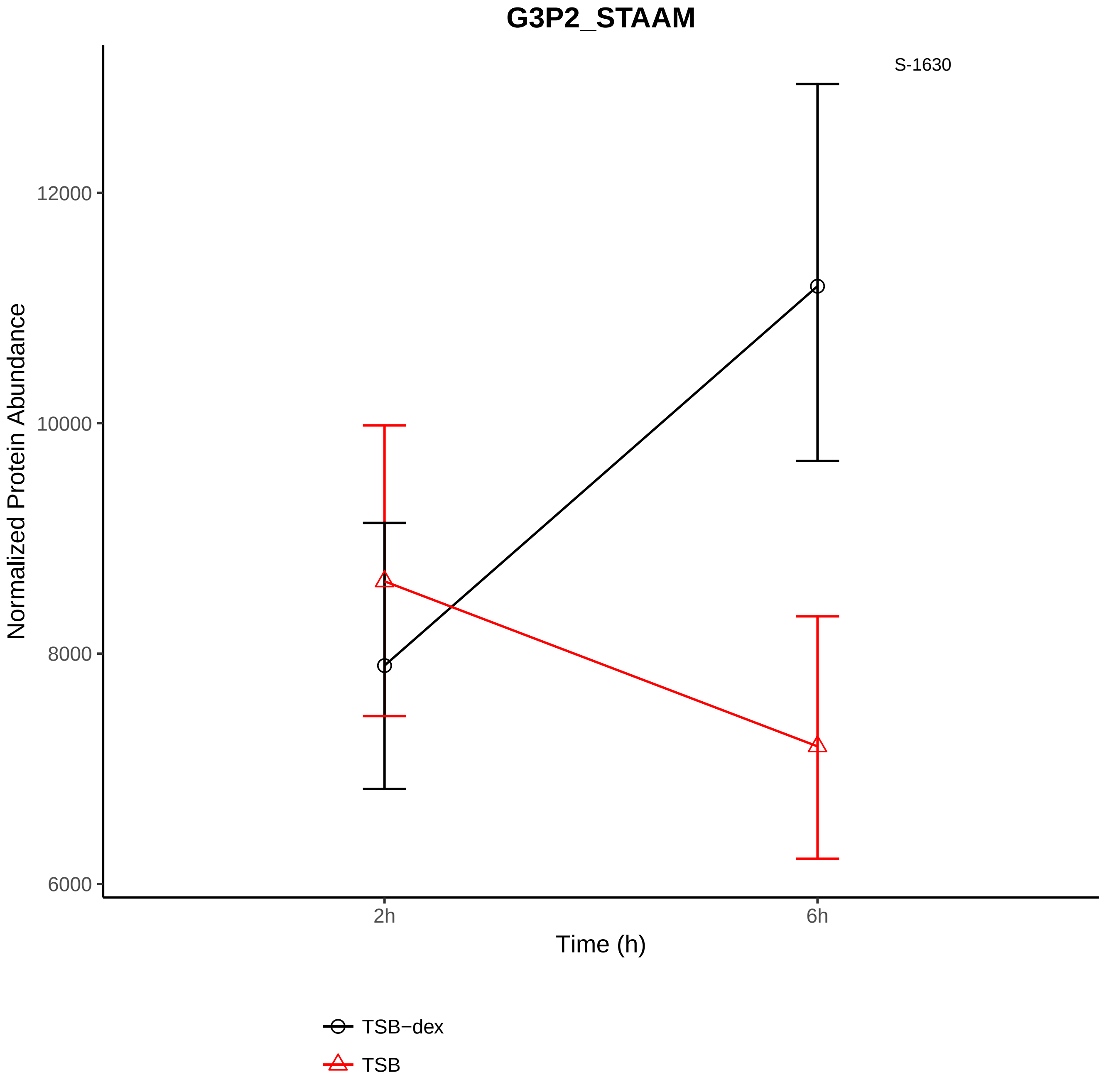

G3P2_STAAM
S-1630
12000
10000
8000
6000
Normalized Protein Abundance
2h
6h
Time (h)
TSB−dex
TSB

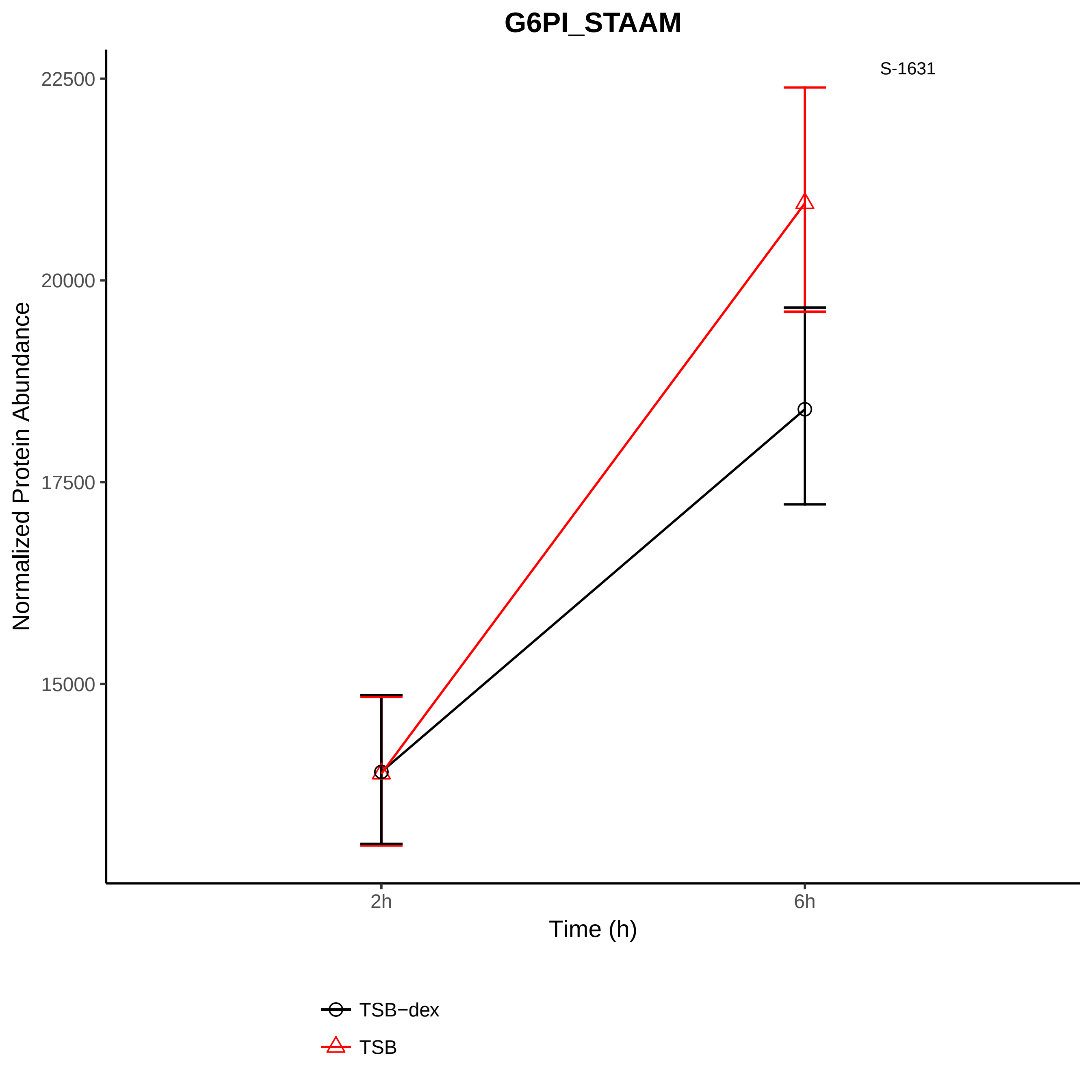

G6PI_STAAM
S-1631
22500
20000
17500
15000
Normalized Protein Abundance
2h
6h
Time (h)
TSB−dex
TSB

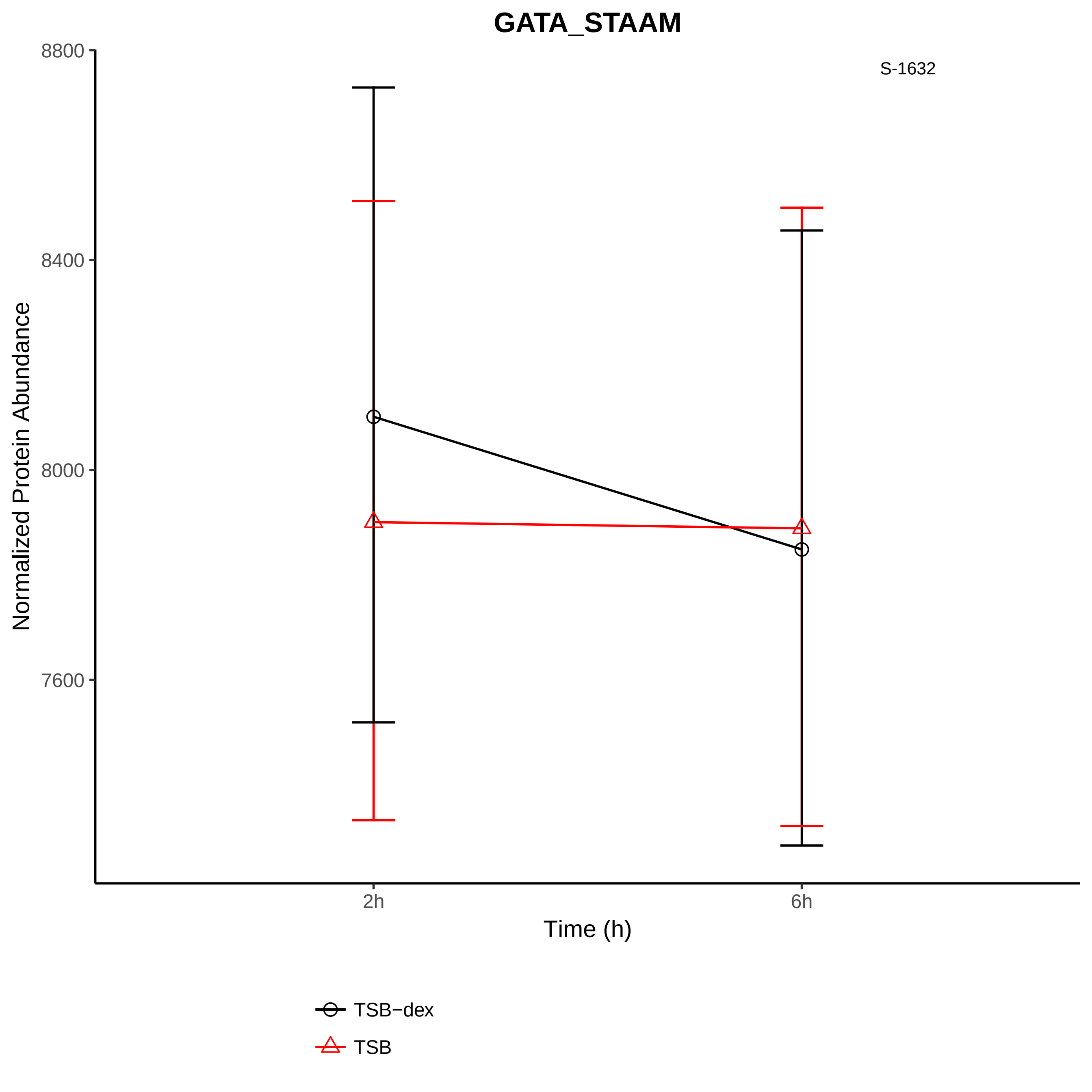
GATA_STAAM
S-1632
Normalized Protein Abundance
8800
8400
8000
7600
2h
6h
Time (h)
TSB−dex
TSB

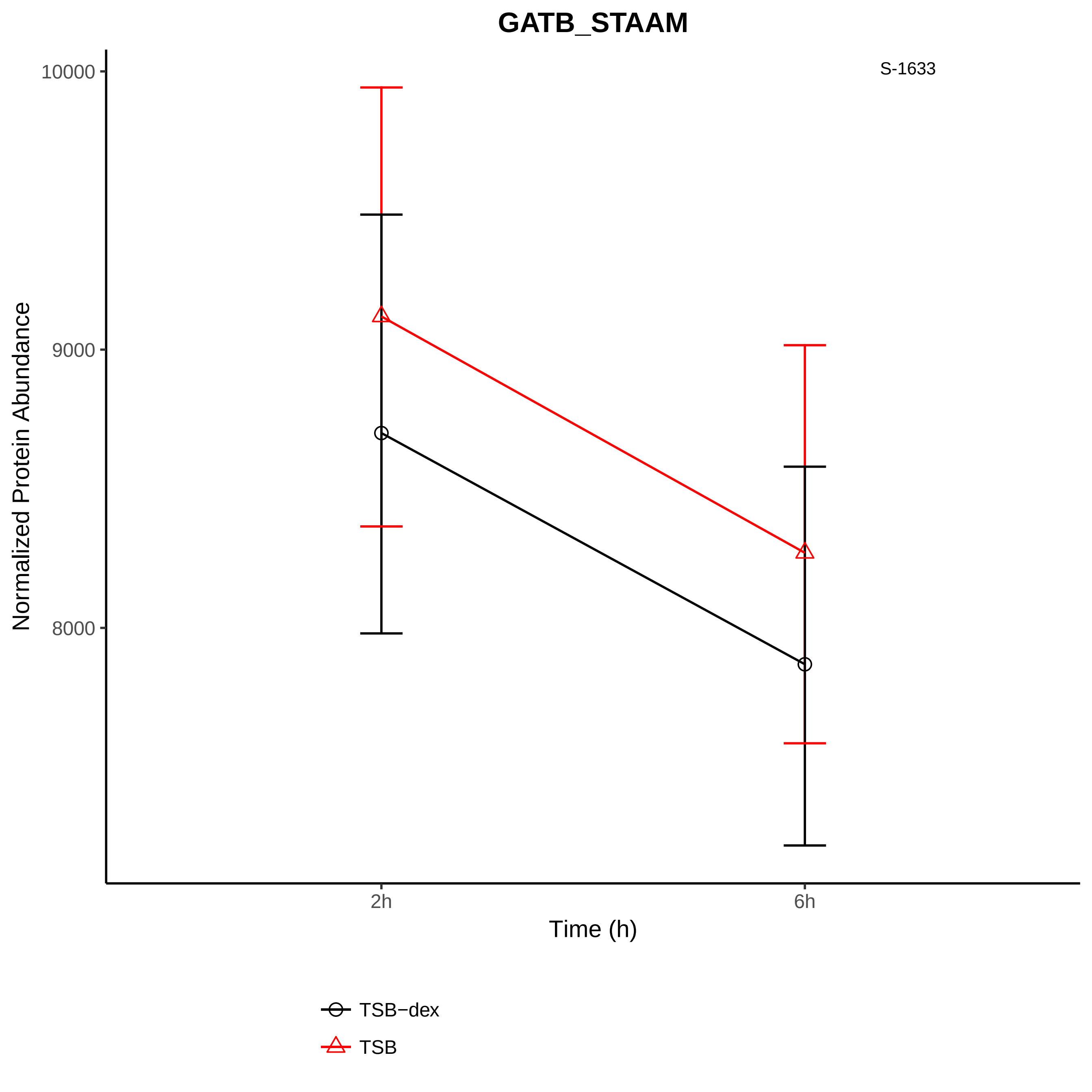

GATB_STAAM
S-1633
10000
9000
8000
Normalized Protein Abundance
2h
6h
Time (h)
TSB−dex
TSB

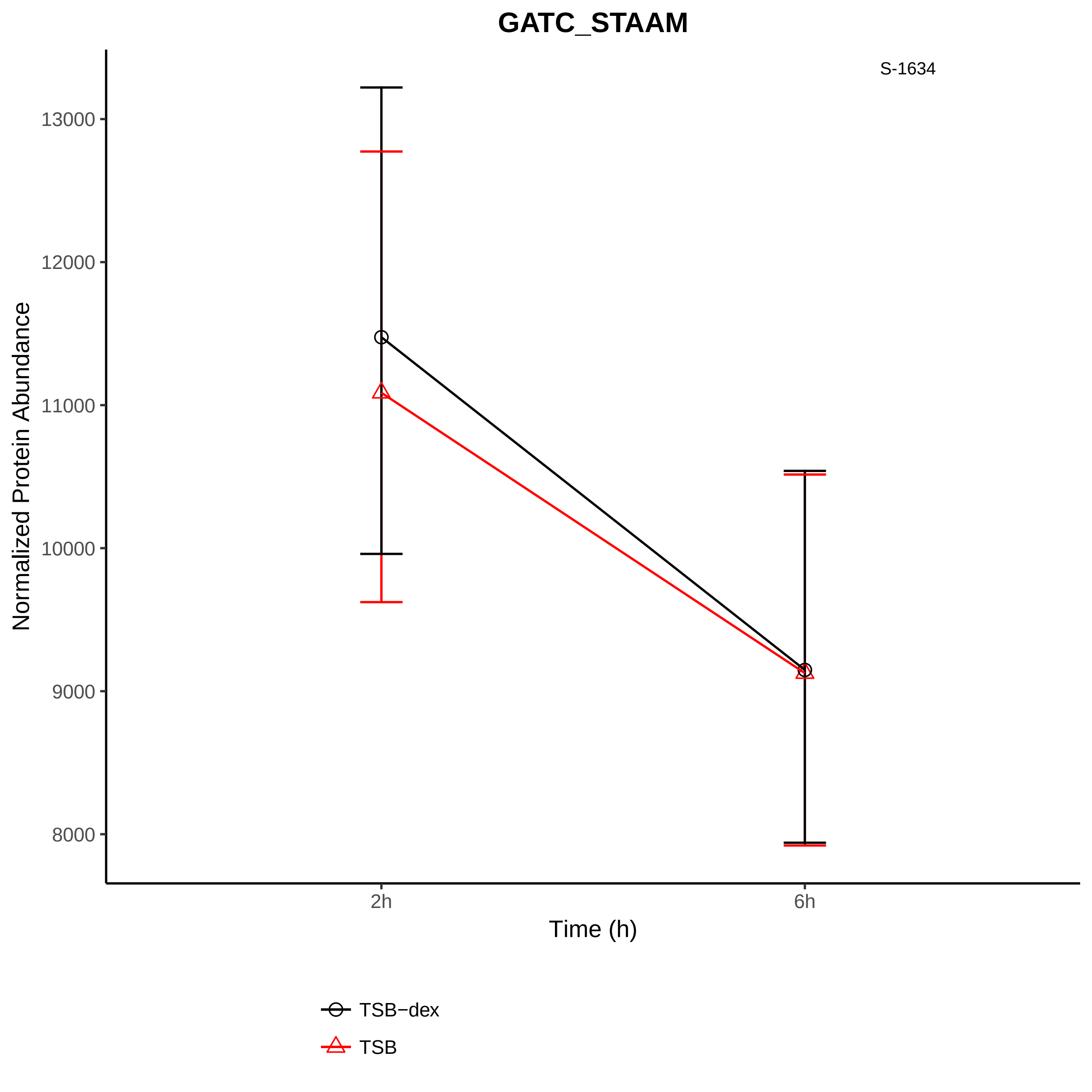

GATC_STAAM
S-1634
Normalized Protein Abundance
13000
12000
11000
10000
9000
8000
2h
6h
Time (h)
TSB−dex
TSB

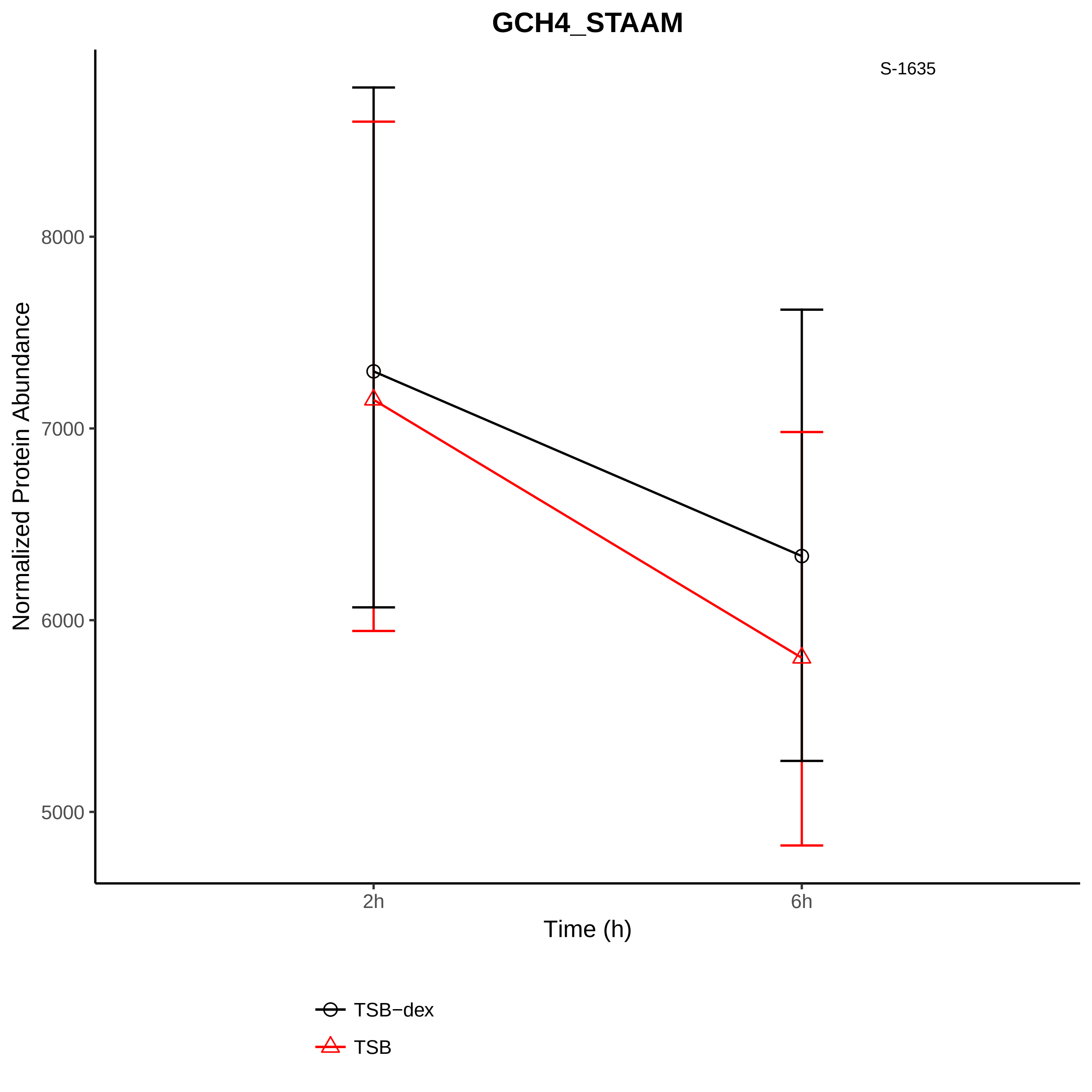

GCH4_STAAM
S-1635
8000
7000
6000
5000
Normalized Protein Abundance
2h
6h
Time (h)
TSB−dex
TSB

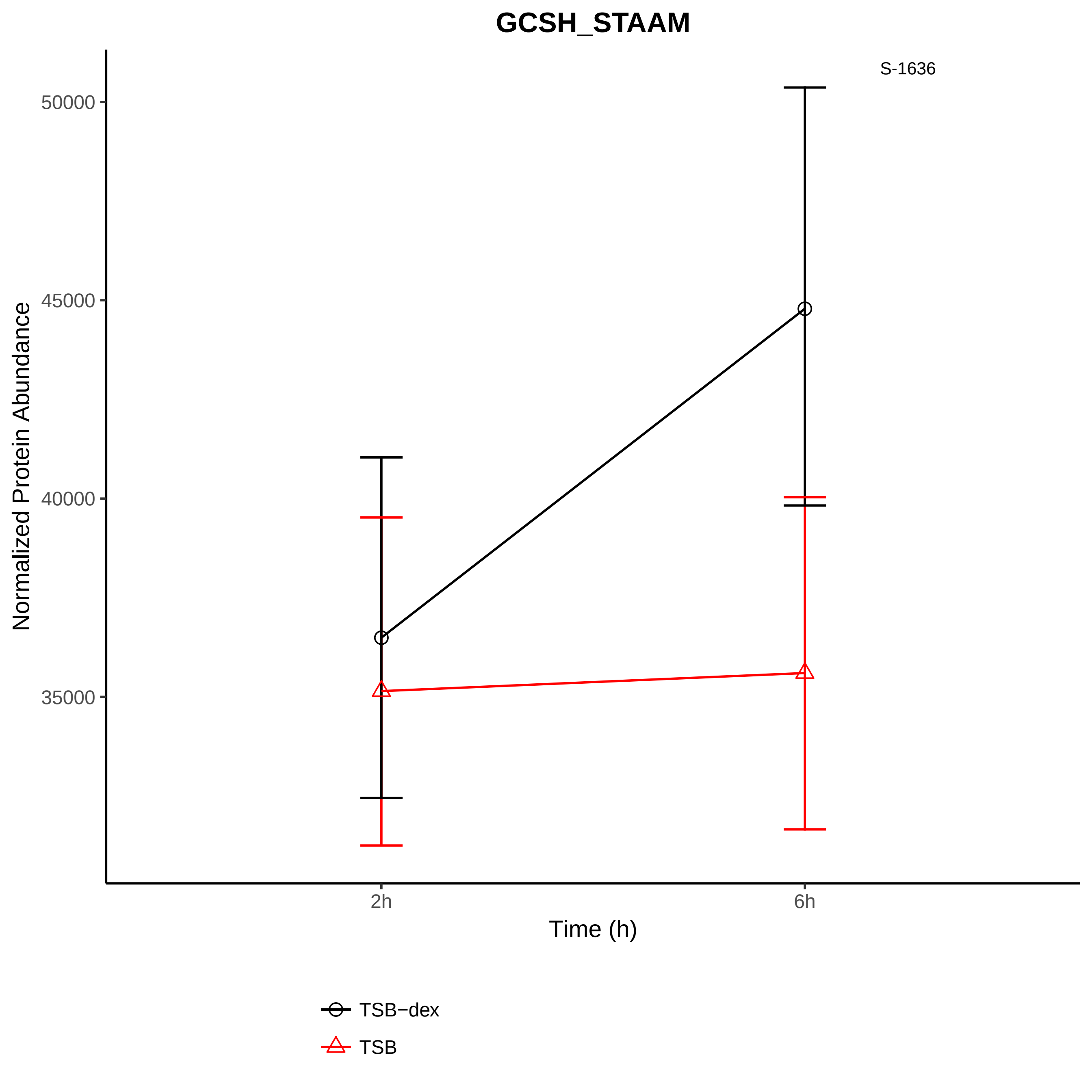

GCSH_STAAM
S-1636
Normalized Protein Abundance
50000
45000
40000
35000
2h
6h
Time (h)
TSB−dex
TSB

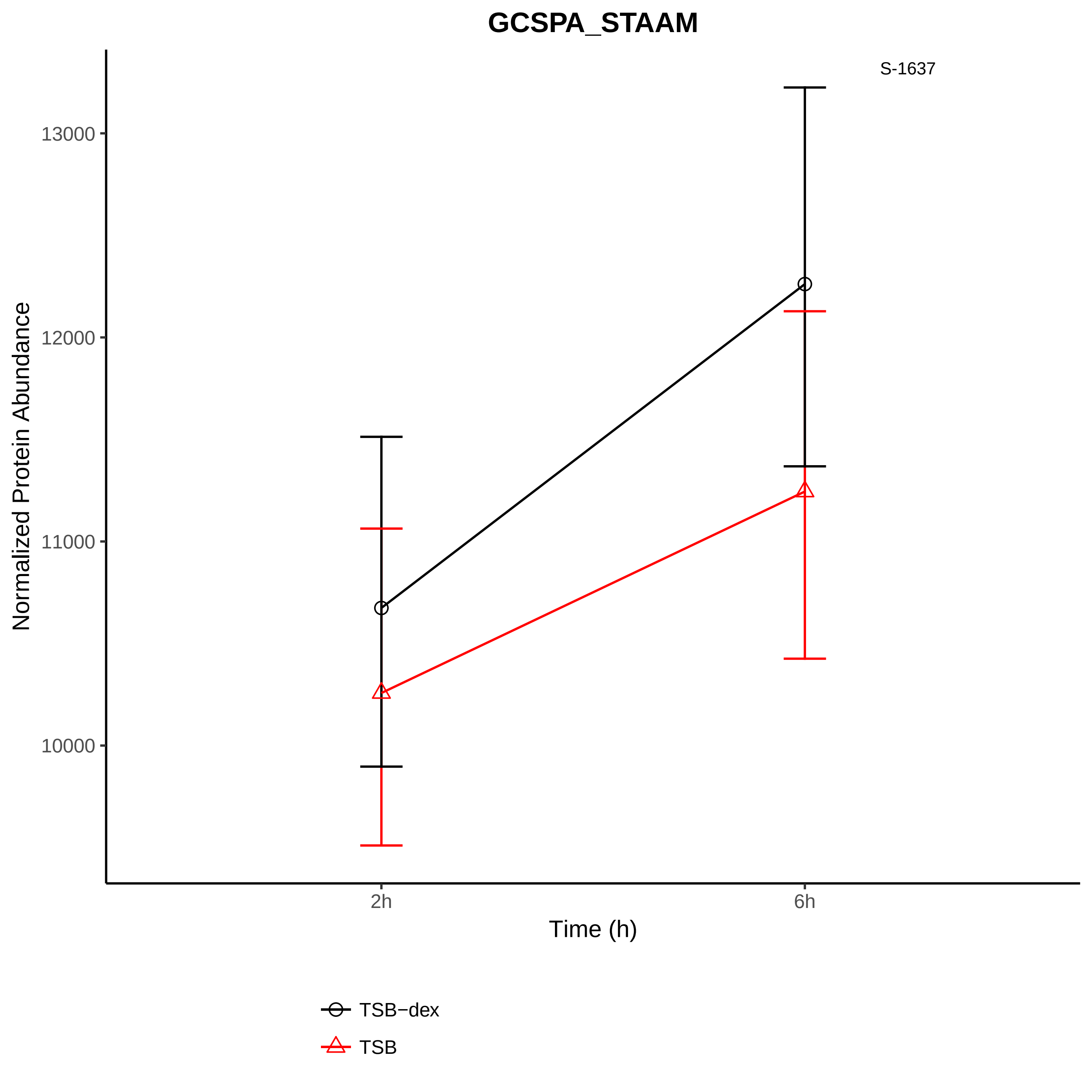
GCSPA_STAAM
S-1637
13000
12000
11000
10000
Normalized Protein Abundance
2h
6h
Time (h)
TSB−dex
TSB

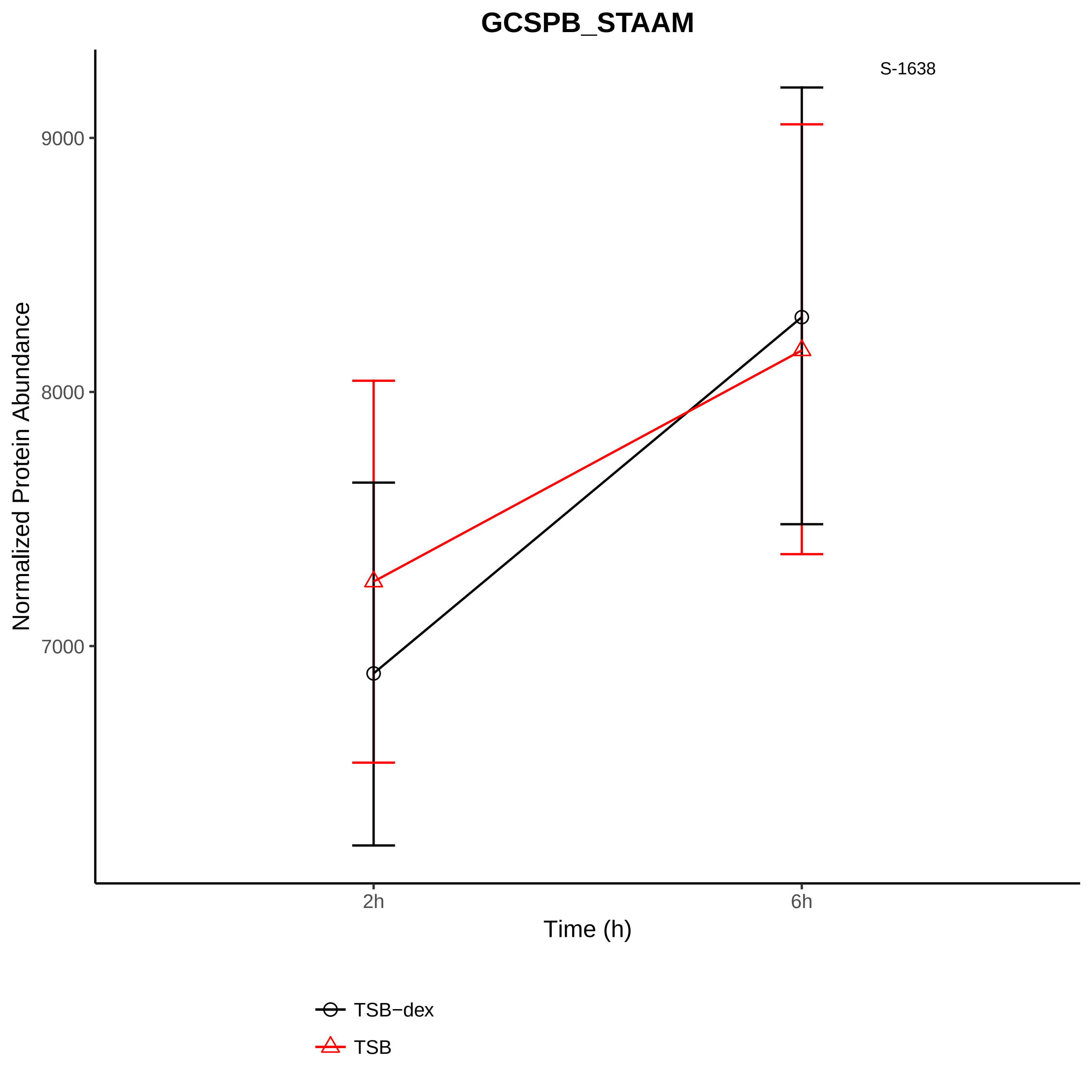
GCSPB_STAAM
S-1638
9000
8000
7000
Normalized Protein Abundance
2h
6h
Time (h)
TSB−dex
TSB

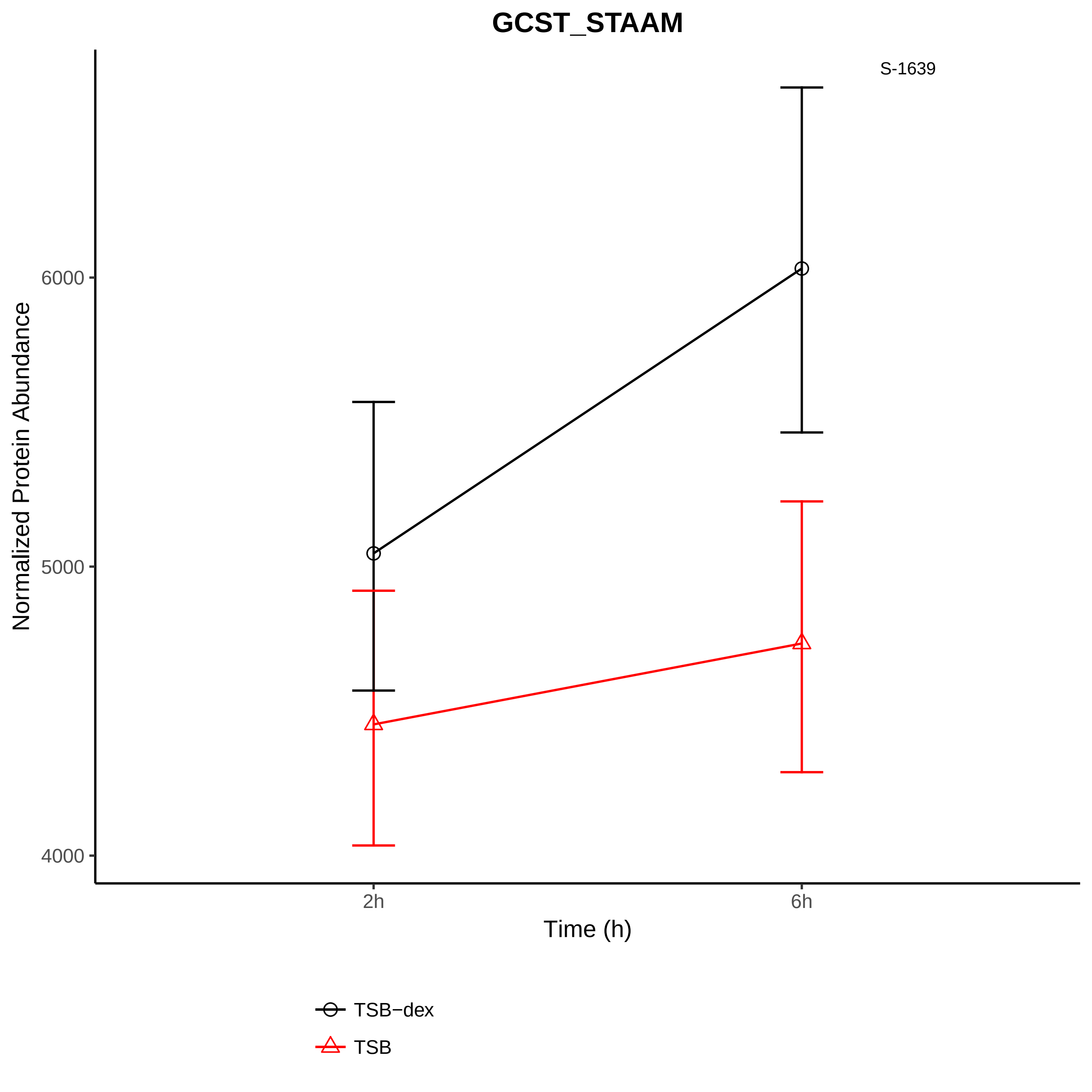
GCST_STAAM
S-1639
Normalized Protein Abundance
6000
5000
4000
2h
6h
Time (h)
TSB−dex
TSB

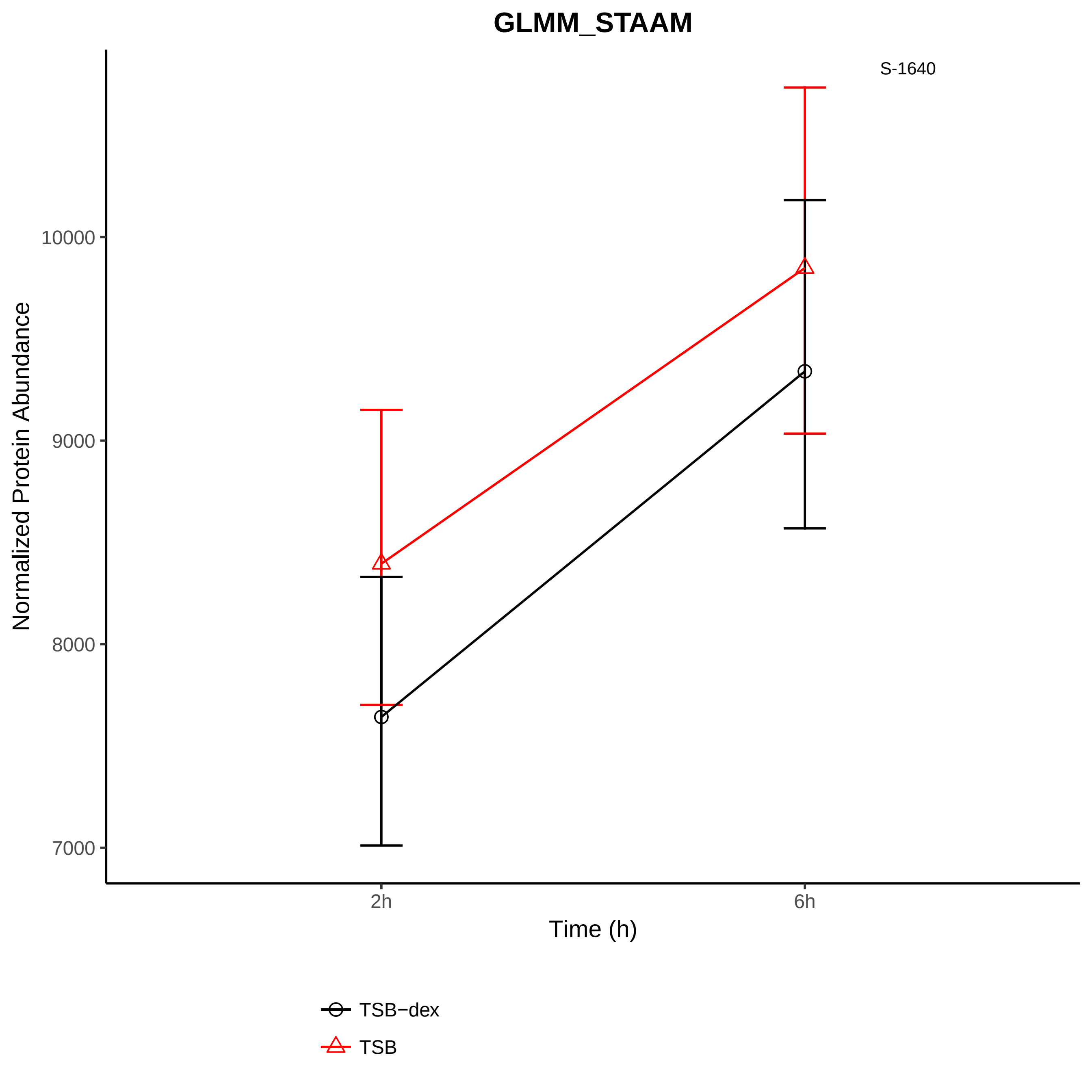

GLMM_STAAM
S-1640
10000
9000
8000
7000
Normalized Protein Abundance
2h
6h
Time (h)
TSB−dex
TSB

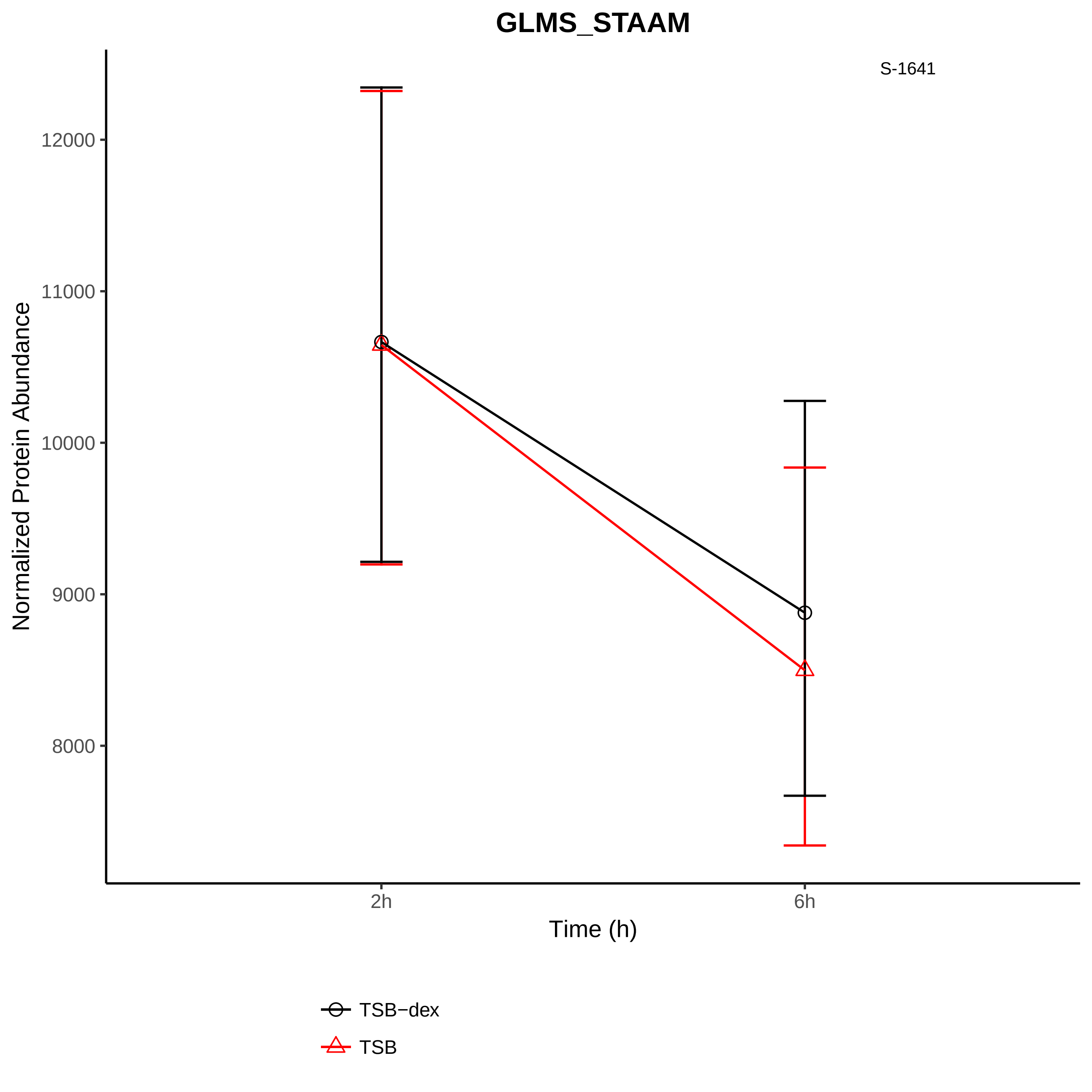
GLMS_STAAM
S-1641
Normalized Protein Abundance
12000
11000
10000
9000
8000
2h
6h
Time (h)
TSB−dex
TSB

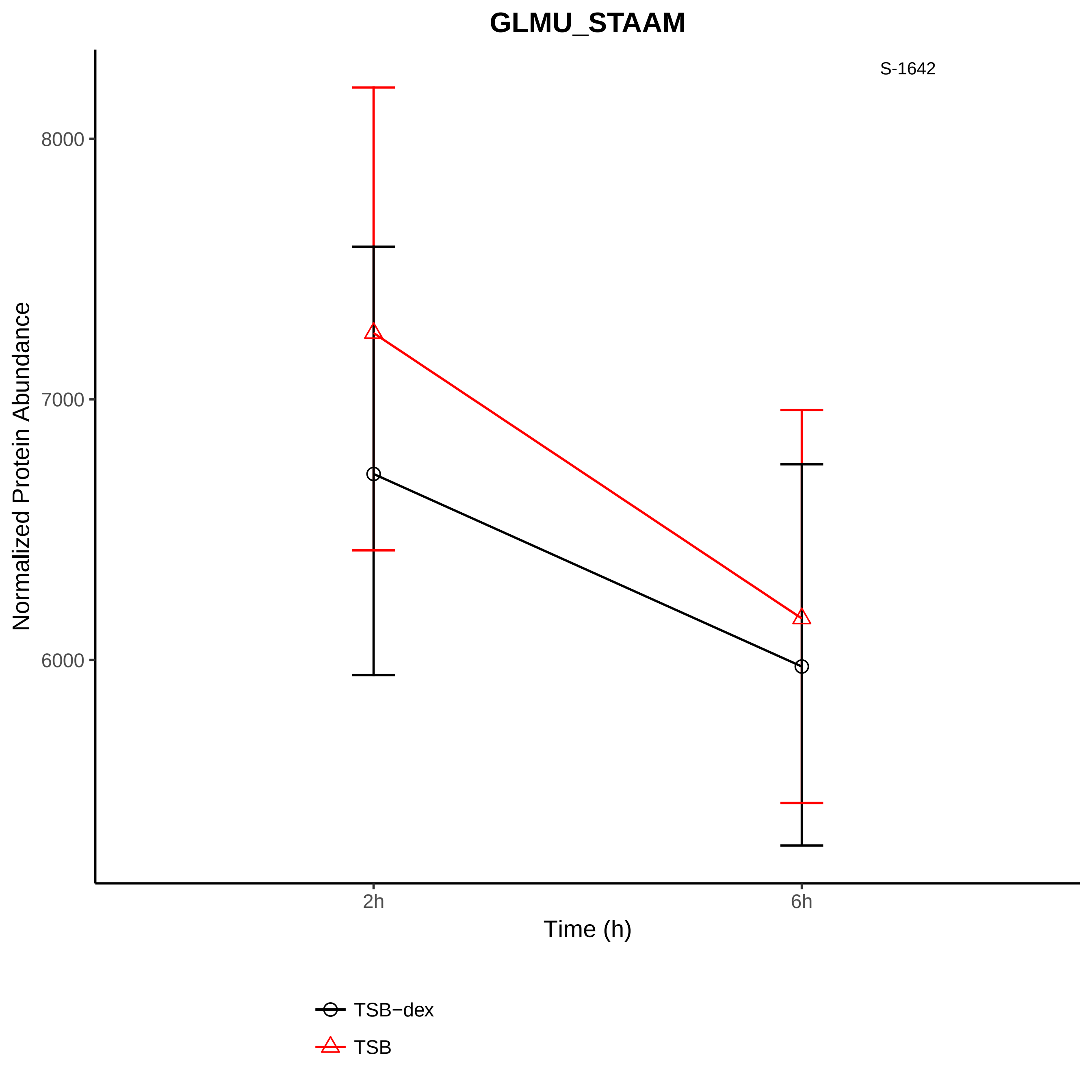

GLMU_STAAM
S-1642
8000
7000
6000
Normalized Protein Abundance
2h
6h
Time (h)
TSB−dex
TSB

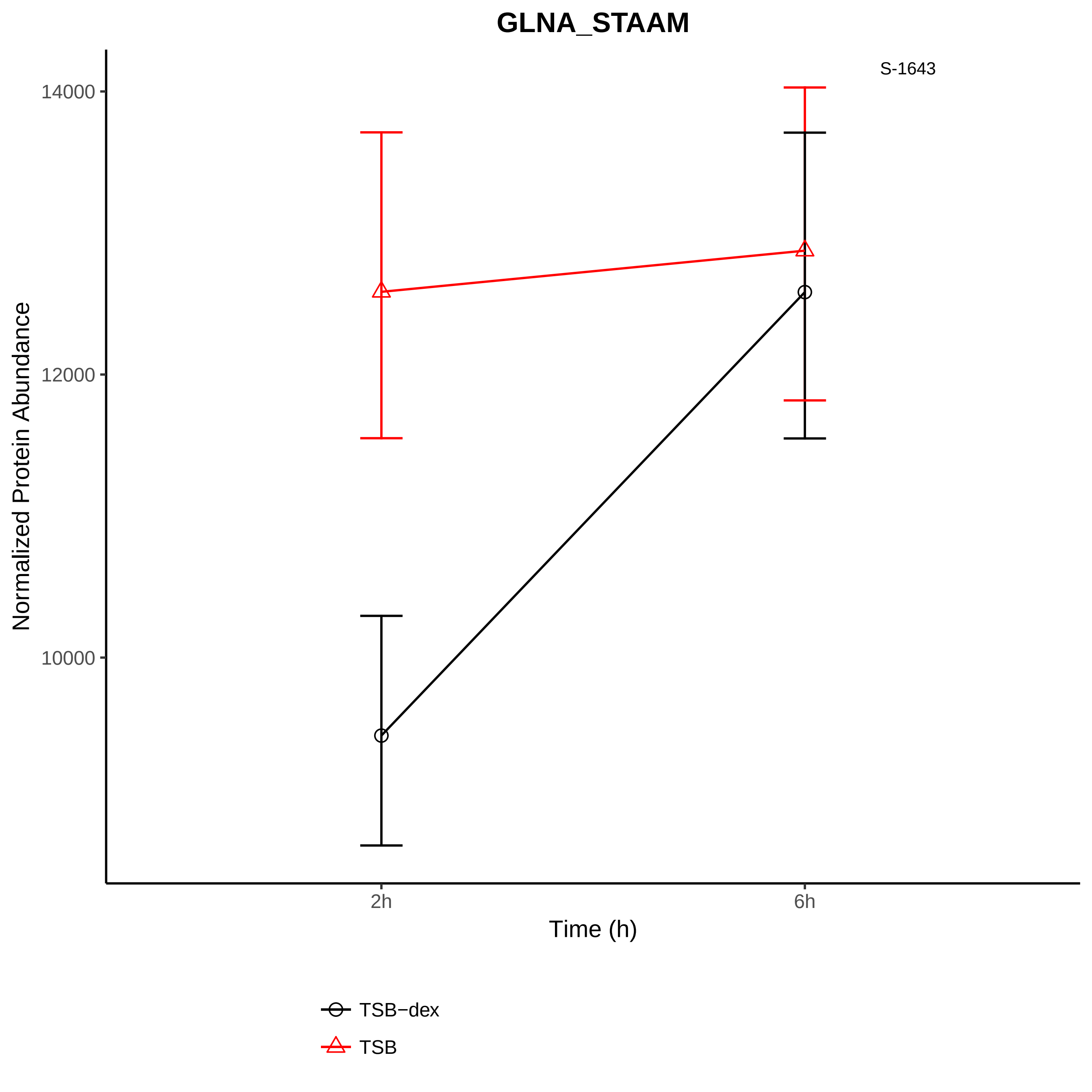

GLNA_STAAM
S-1643
14000
12000
10000
Normalized Protein Abundance
2h
6h
Time (h)
TSB−dex
TSB

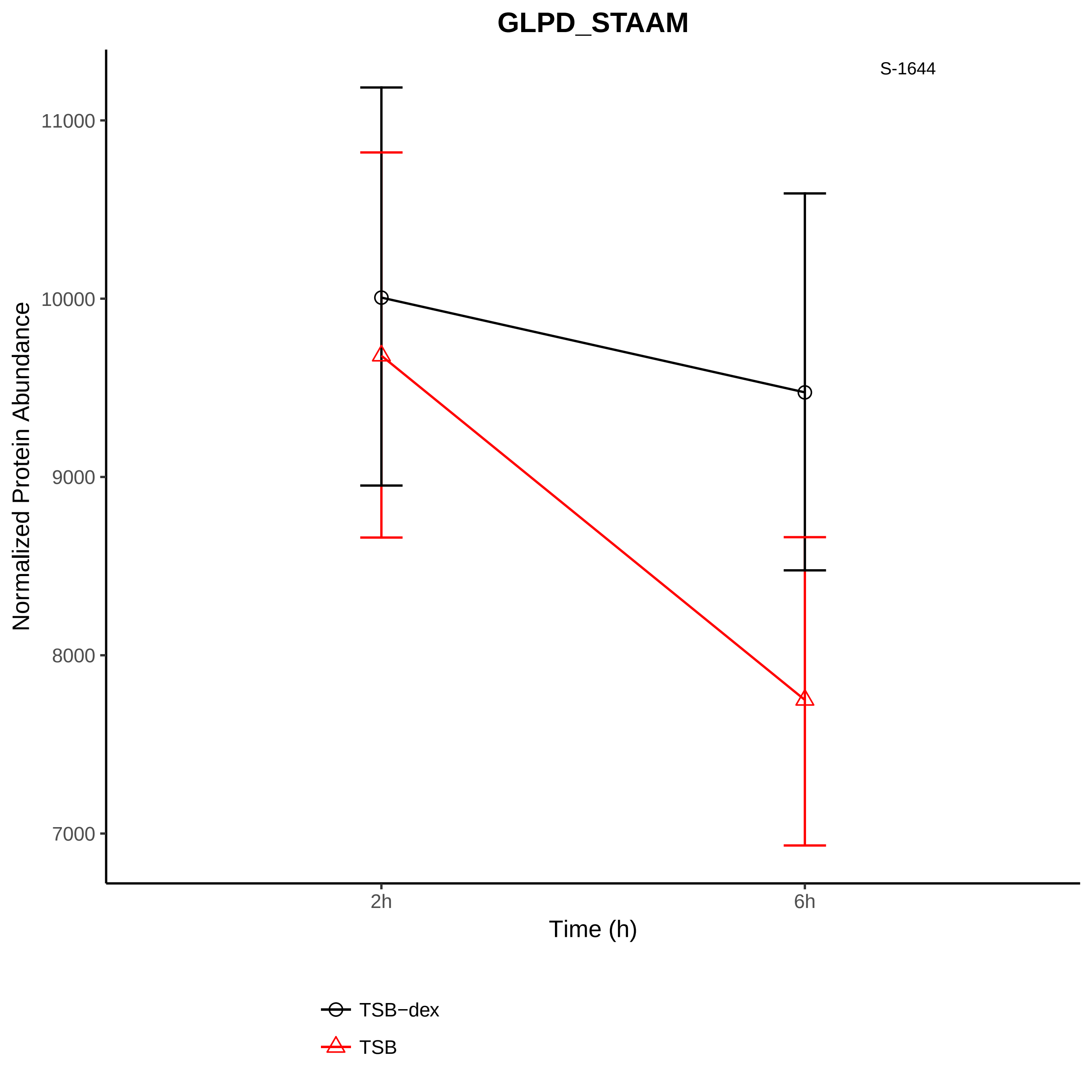

GLPD_STAAM
S-1644
11000
10000
9000
8000
7000
Normalized Protein Abundance
2h
6h
Time (h)
TSB−dex
TSB

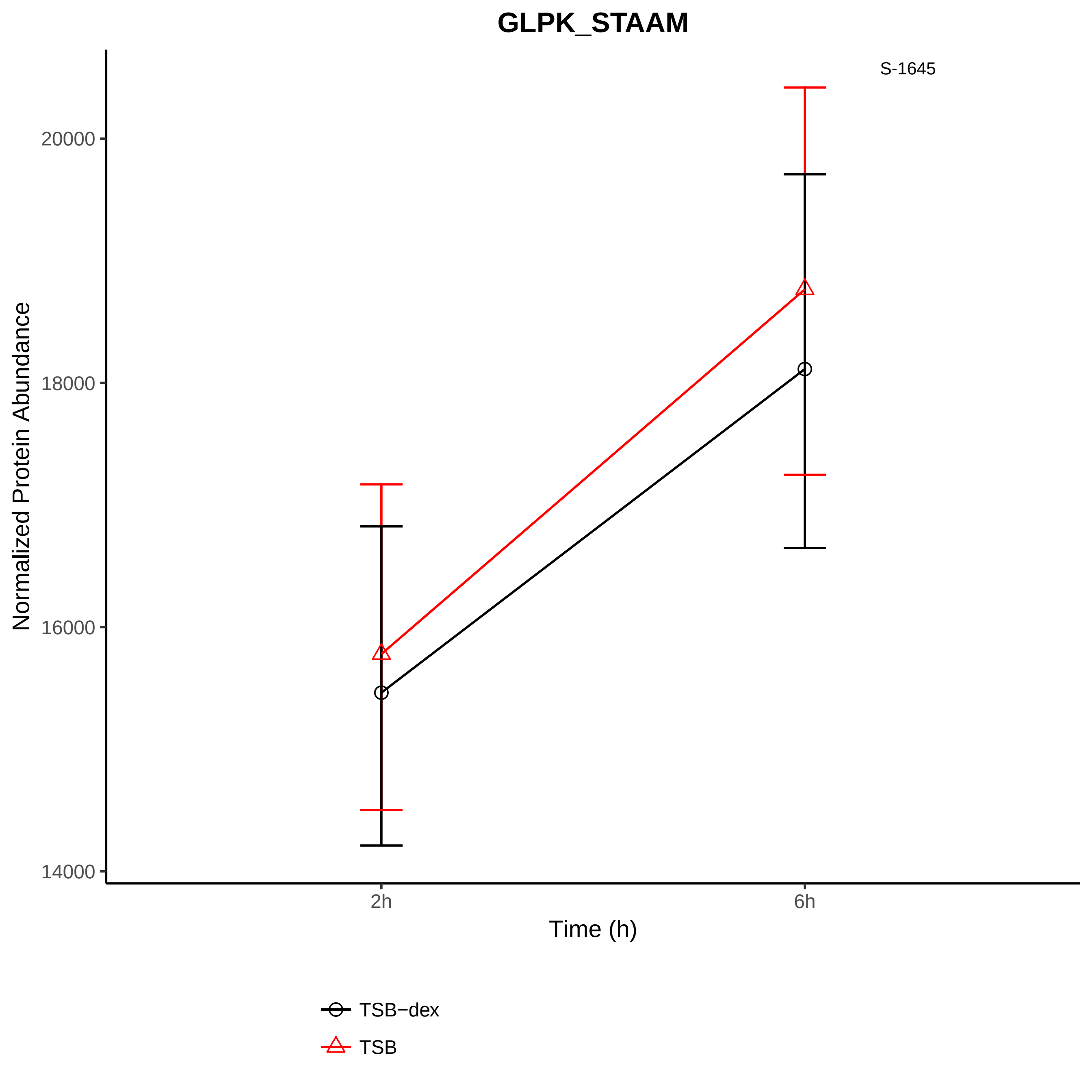
GLPK_STAAM
S-1645
Normalized Protein Abundance
20000
18000
16000
14000
2h
6h
Time (h)
TSB−dex
TSB

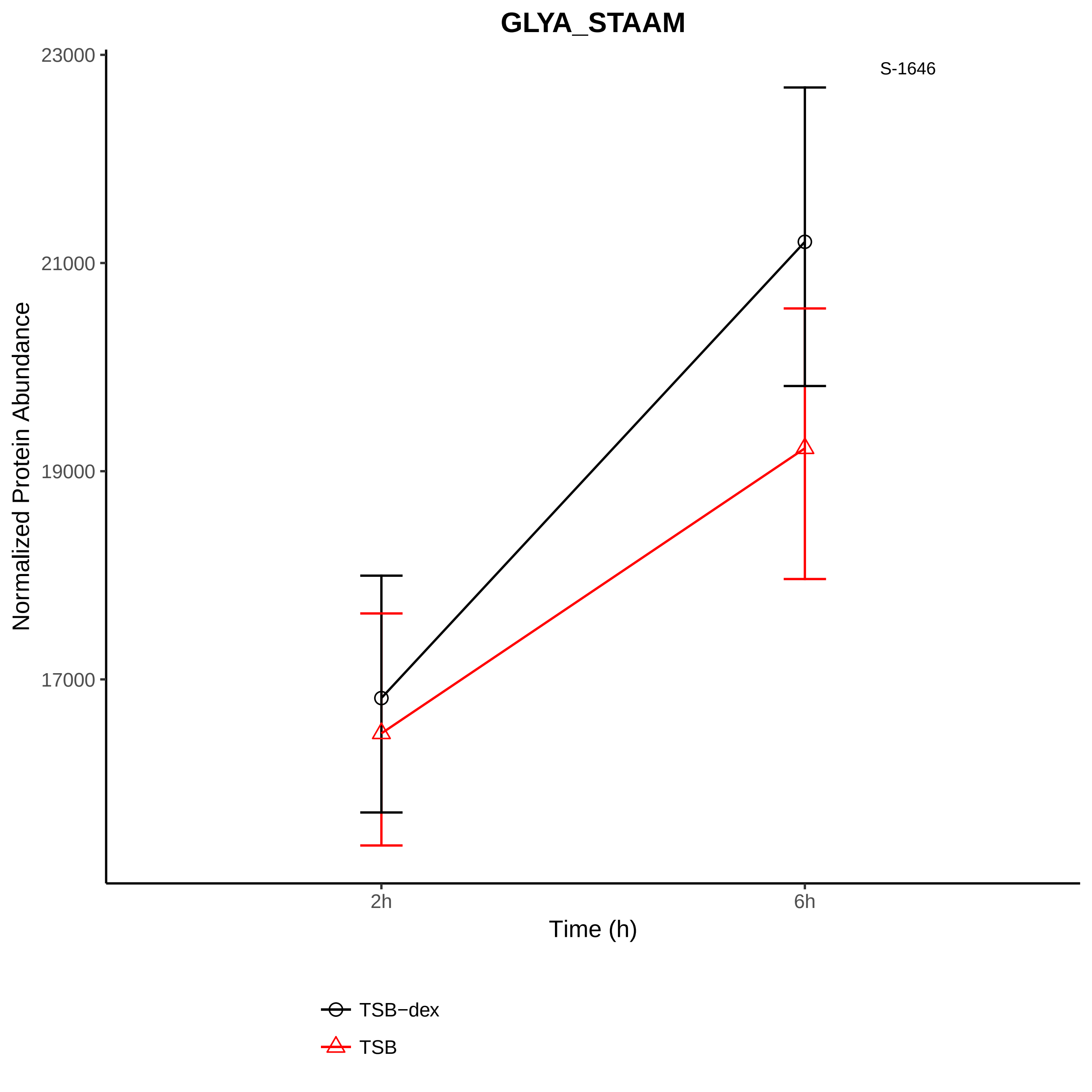

GLYA_STAAM
S-1646
23000
21000
19000
17000
Normalized Protein Abundance
2h
6h
Time (h)
TSB−dex
TSB

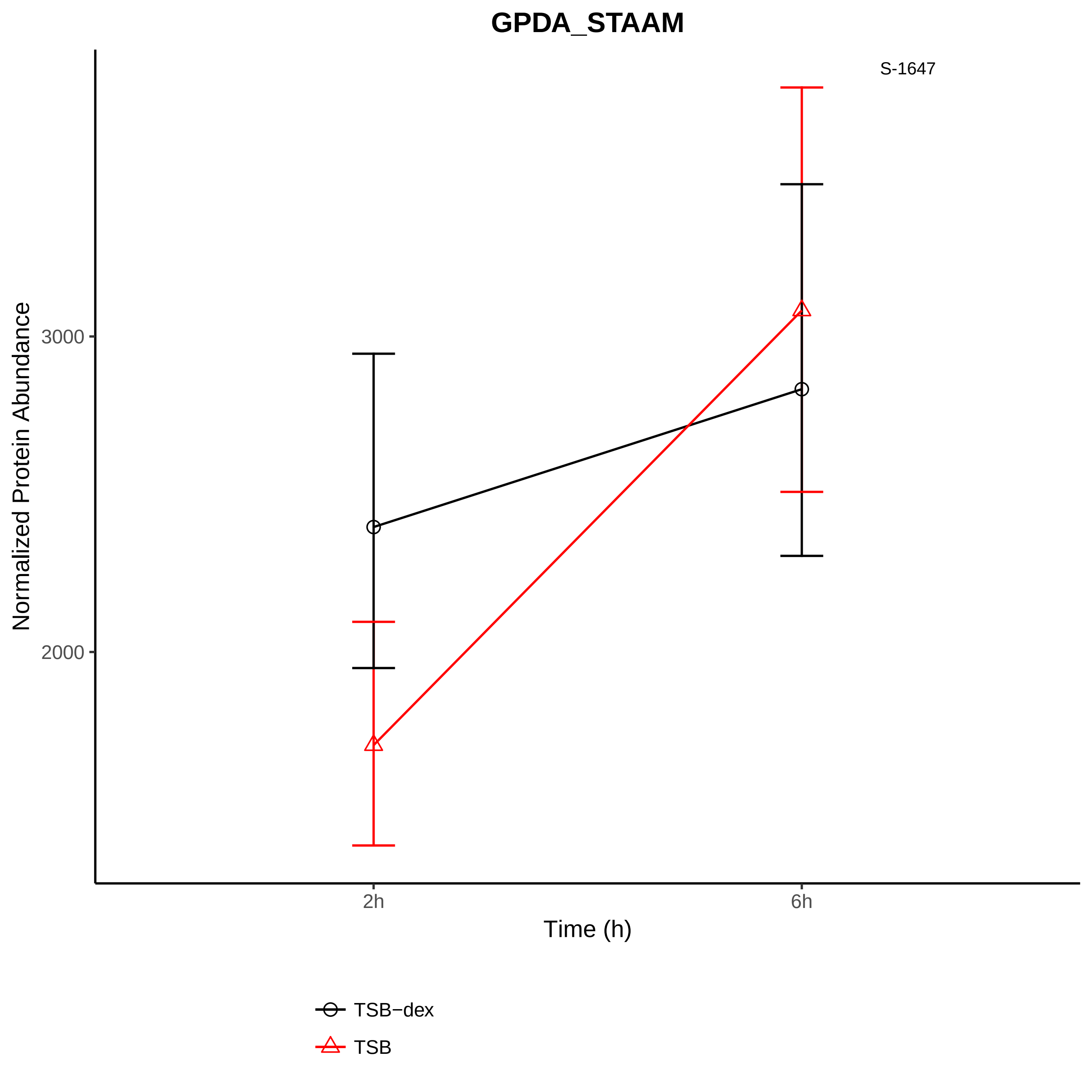

GPDA_STAAM
S-1647
3000
2000
Normalized Protein Abundance
2h
6h
Time (h)
TSB−dex
TSB

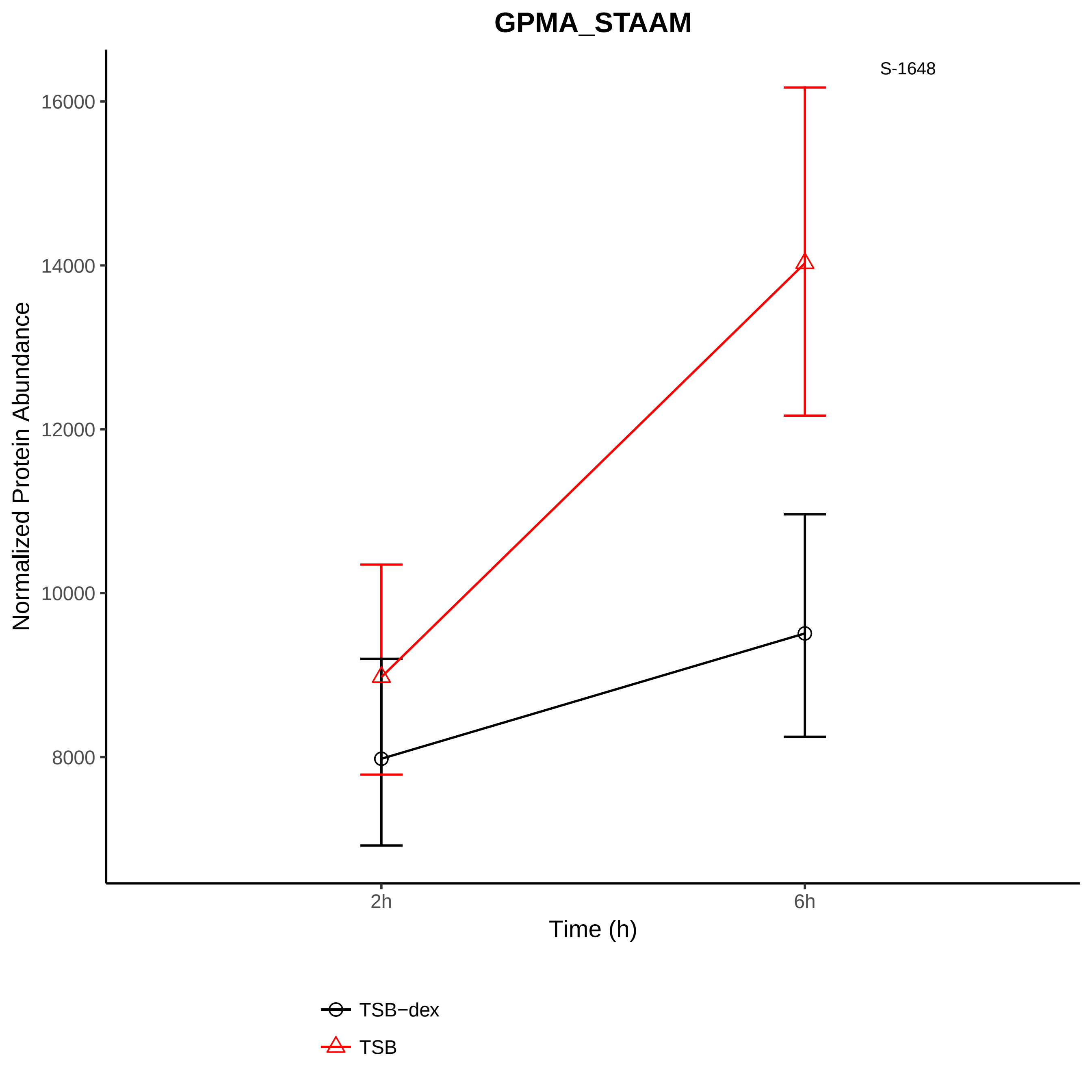

GPMA_STAAM
S-1648
16000
14000
12000
10000
8000
Normalized Protein Abundance
2h
6h
Time (h)
TSB−dex
TSB

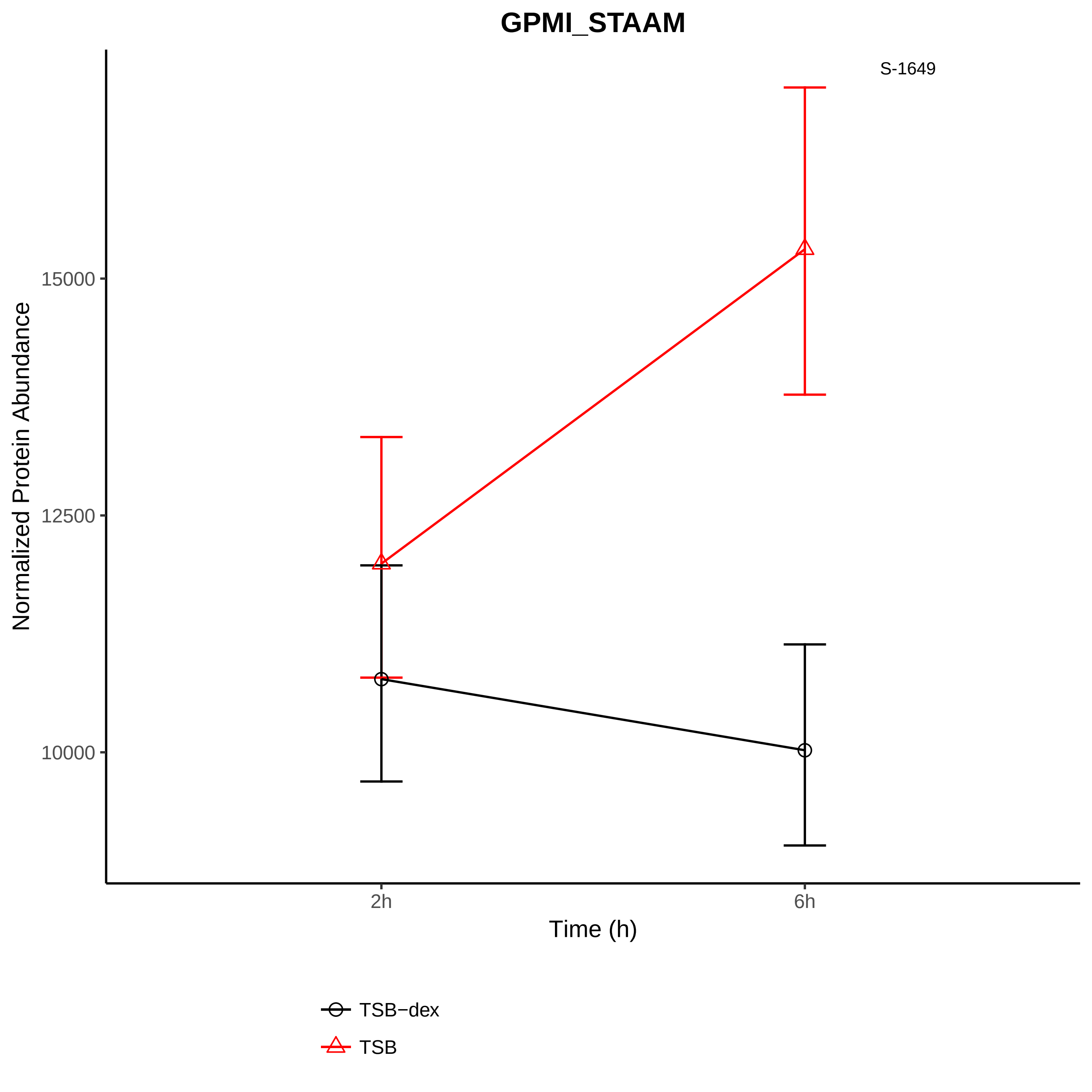
GPMI_STAAM
S-1649
Normalized Protein Abundance
15000
12500
10000
2h
6h
Time (h)
TSB−dex
TSB

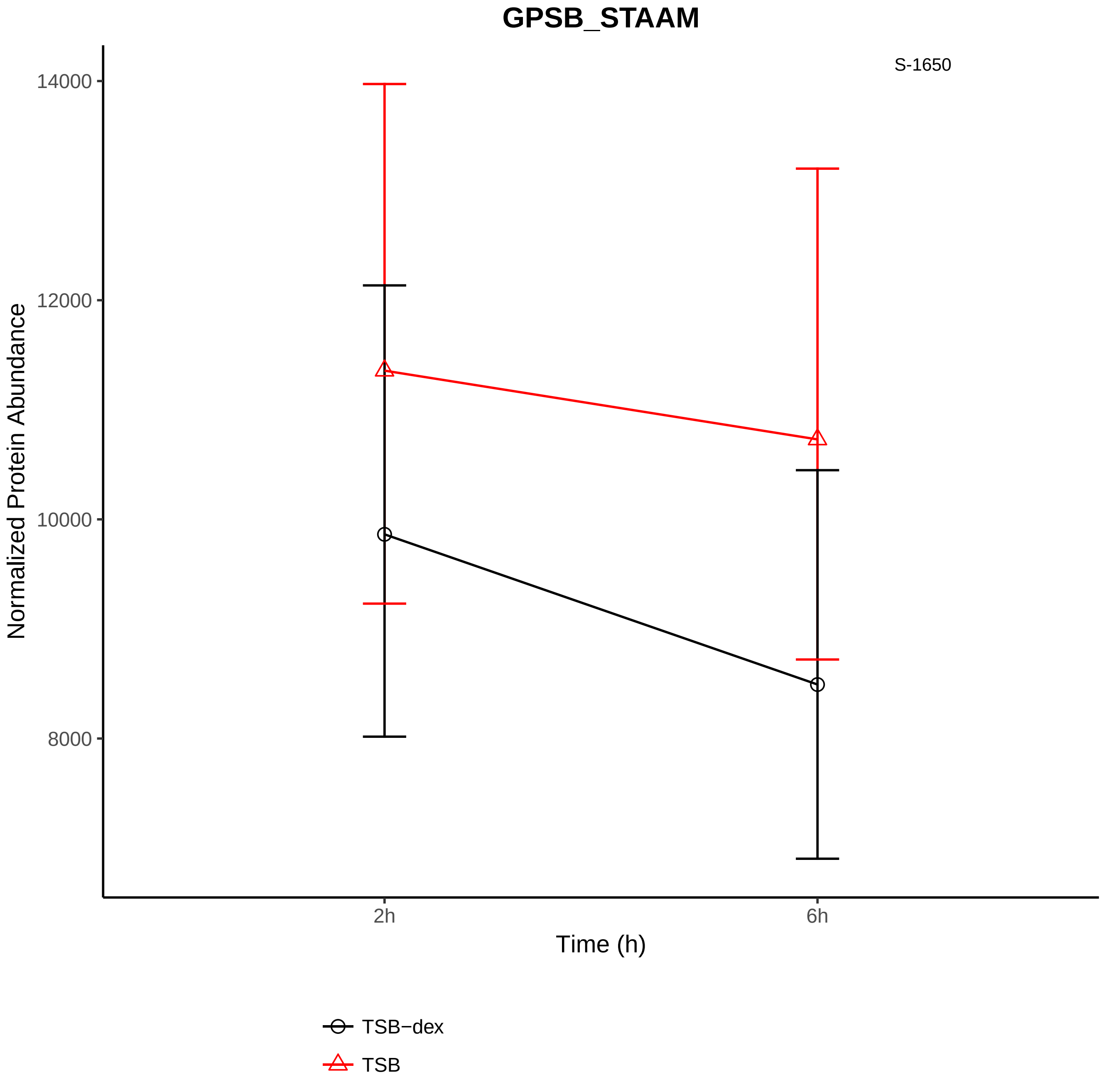
GPSB_STAAM
S-1650
Normalized Protein Abundance
14000
12000
10000
8000
2h
6h
Time (h)
TSB−dex
TSB

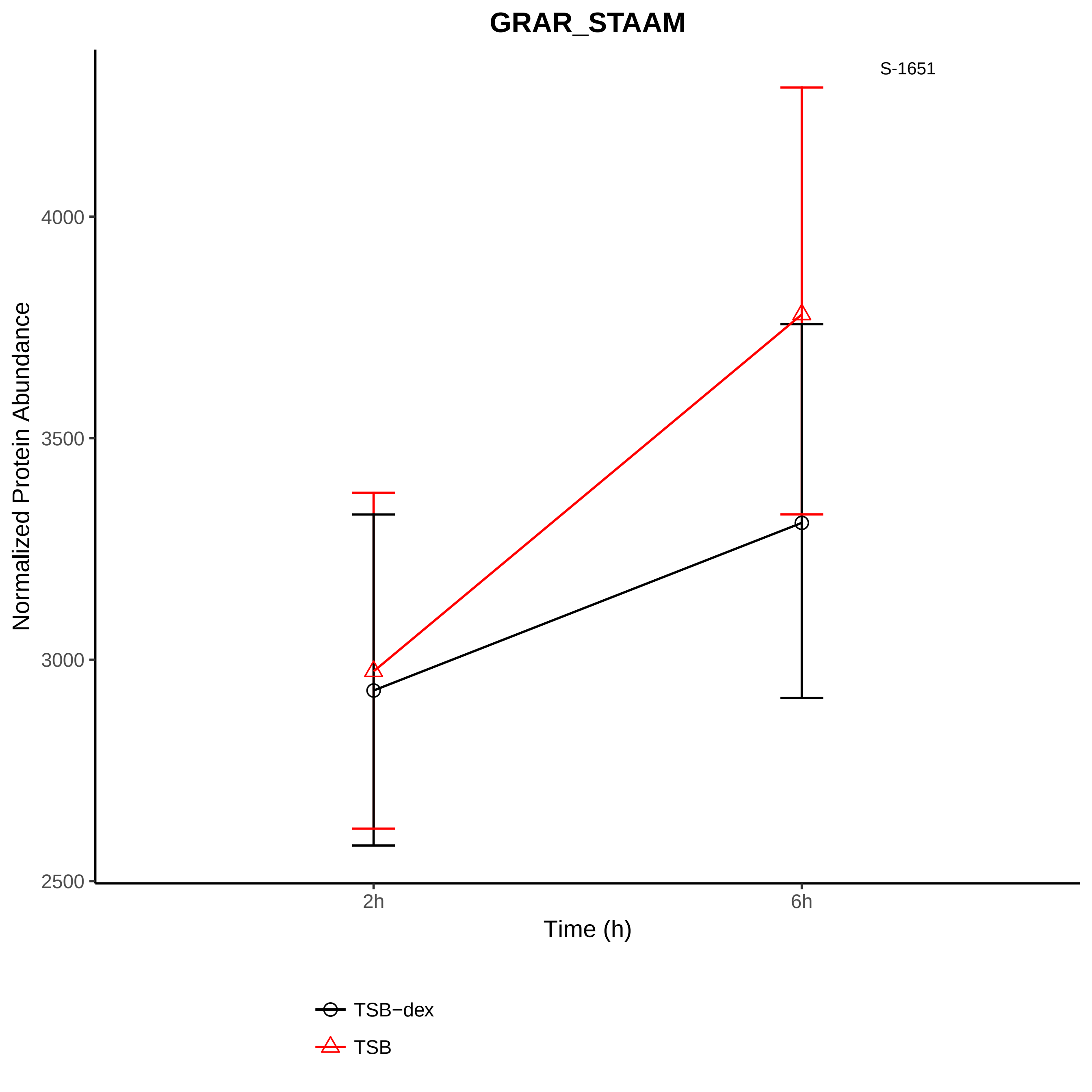

GRAR_STAAM
S-1651
Normalized Protein Abundance
4000
3500
3000
2500
2h
6h
Time (h)
TSB−dex
TSB

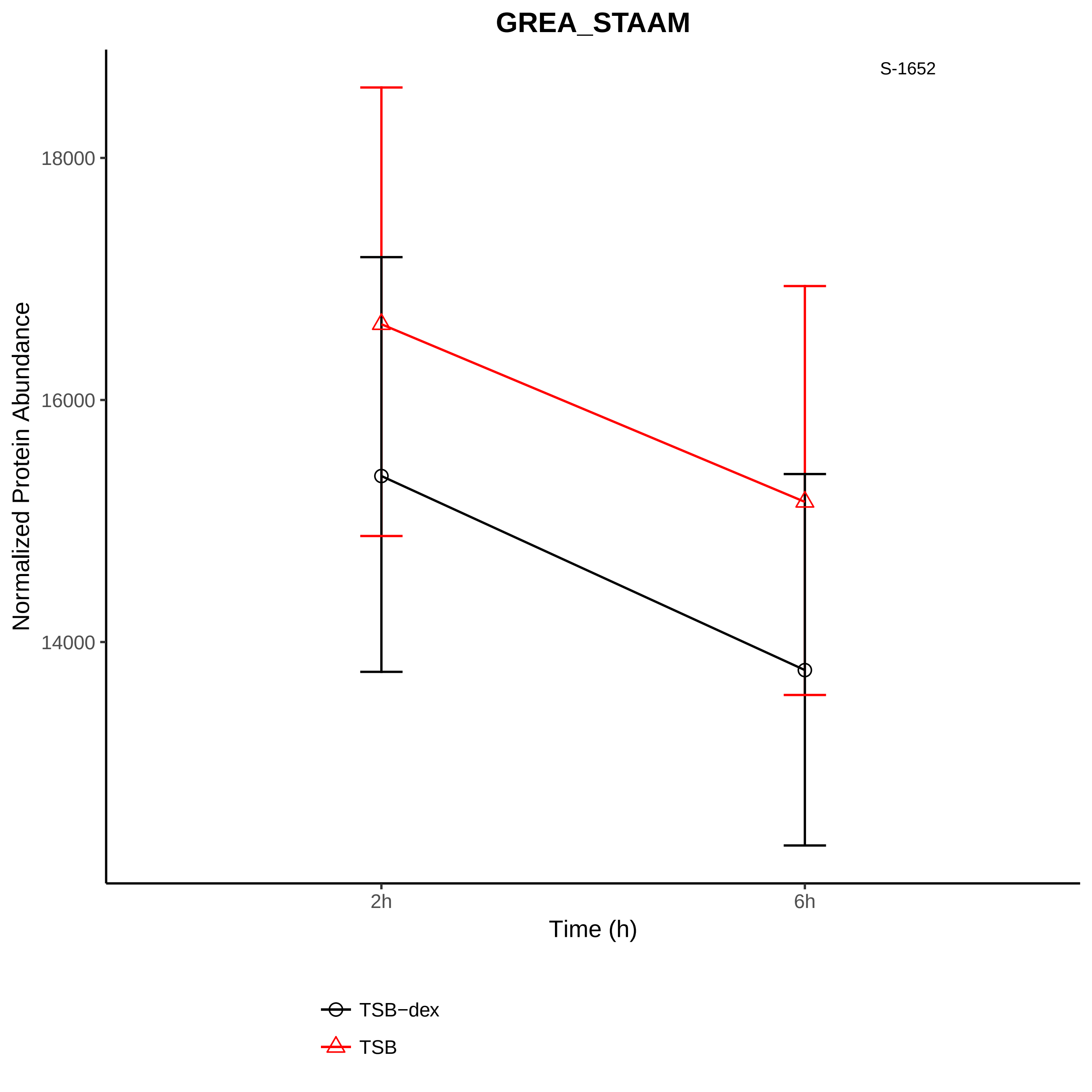

GREA_STAAM
S-1652
Normalized Protein Abundance
18000
16000
14000
2h
6h
Time (h)
TSB−dex
TSB

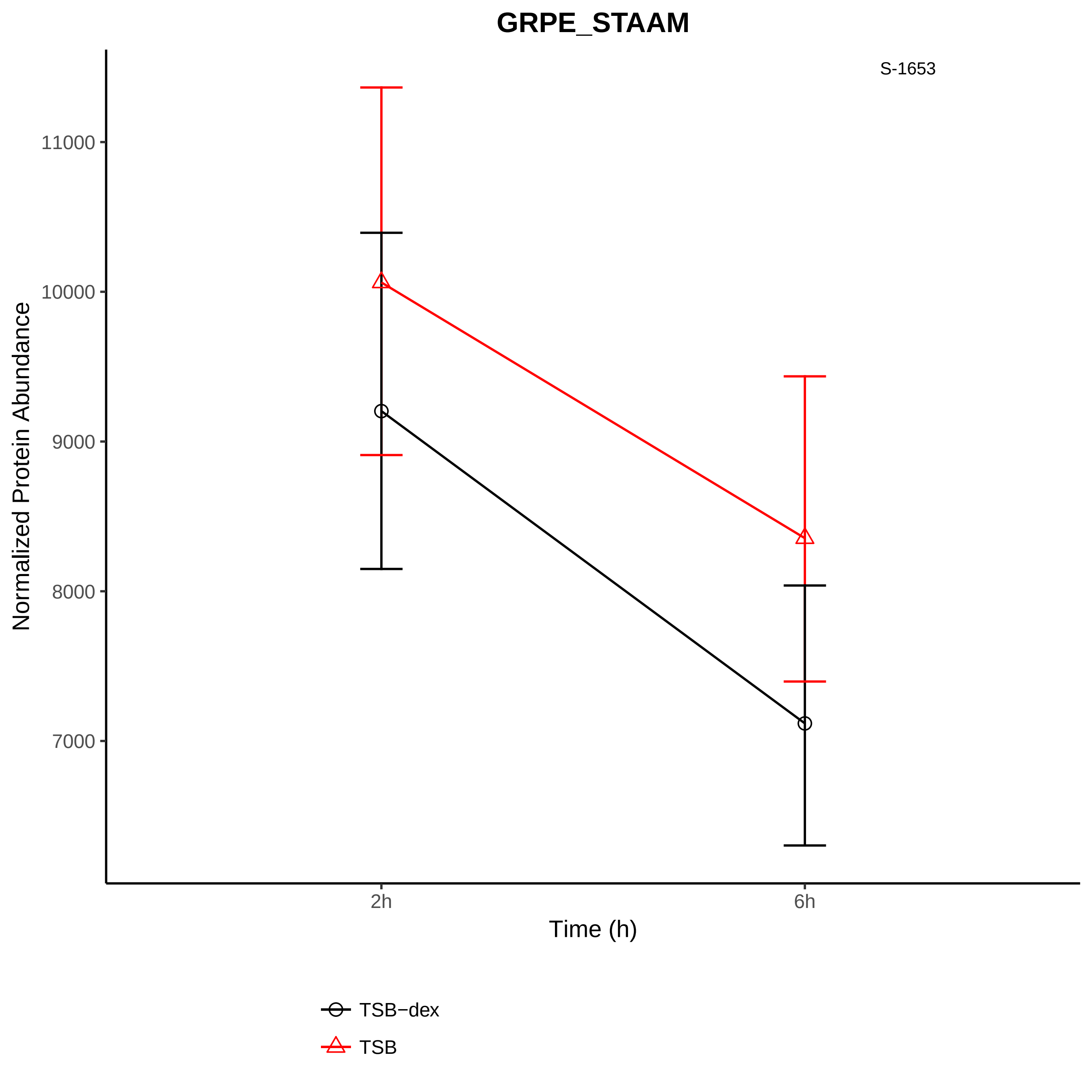

GRPE_STAAM
S-1653
Normalized Protein Abundance
11000
10000
9000
8000
7000
2h
6h
Time (h)
TSB−dex
TSB

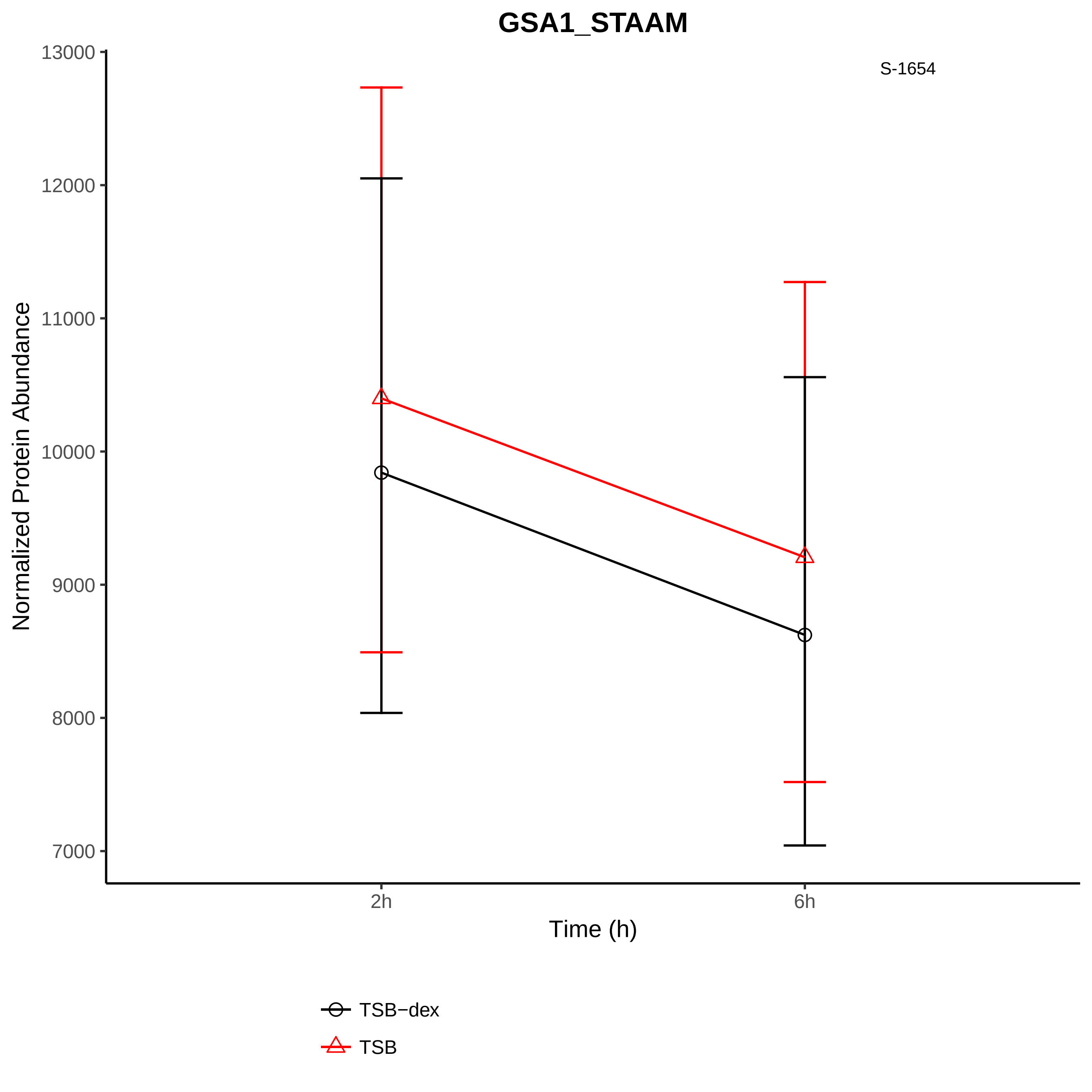

GSA1_STAAM
S-1654
Normalized Protein Abundance
13000
12000
11000
10000
9000
8000
7000
2h
6h
Time (h)
TSB−dex
TSB

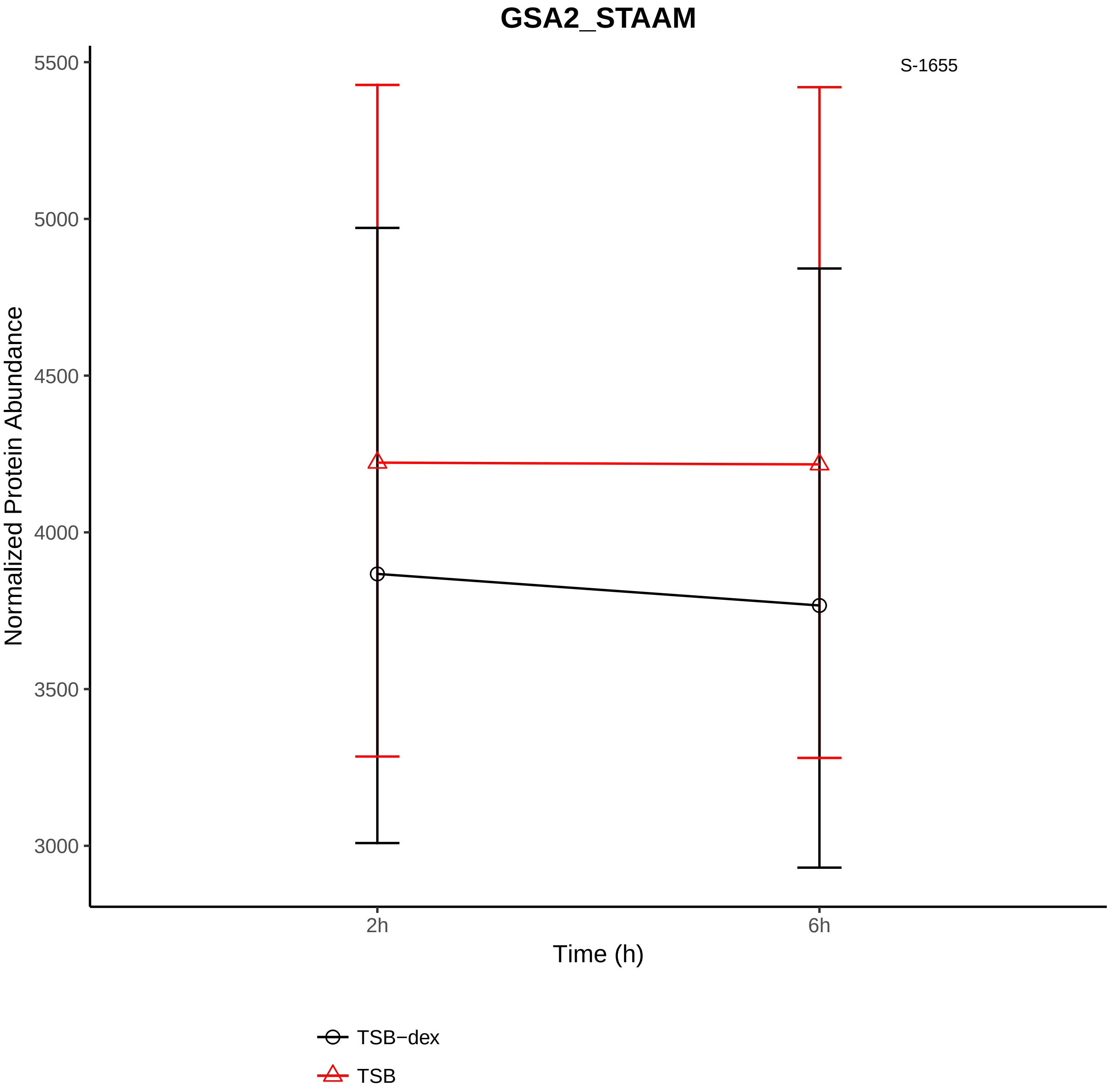

GSA2_STAAM
S-1655
5500
5000
4500
4000
3500
3000
Normalized Protein Abundance
2h
6h
Time (h)
TSB−dex
TSB

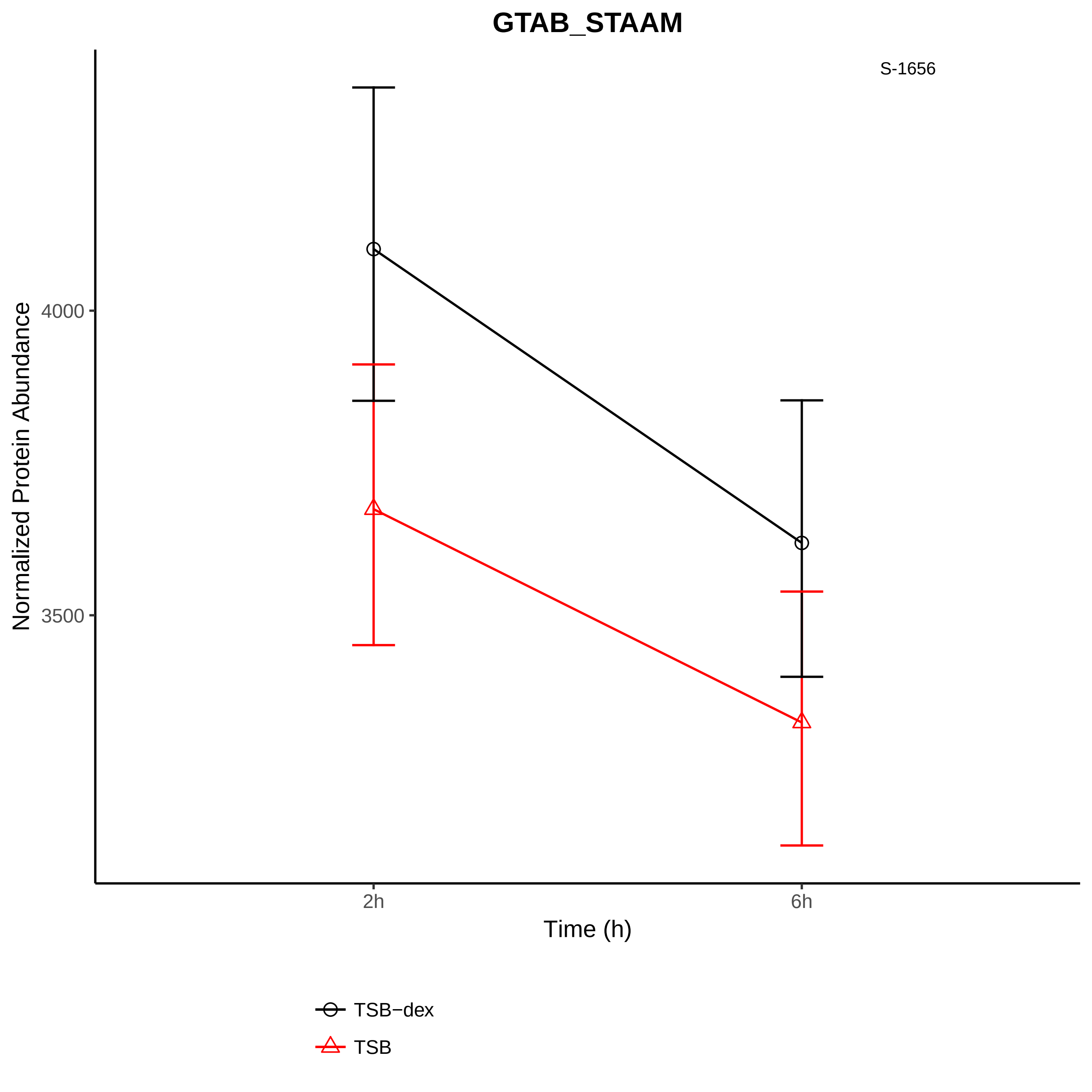
GTAB_STAAM
S-1656
Normalized Protein Abundance
4000
3500
2h
6h
Time (h)
TSB−dex
TSB

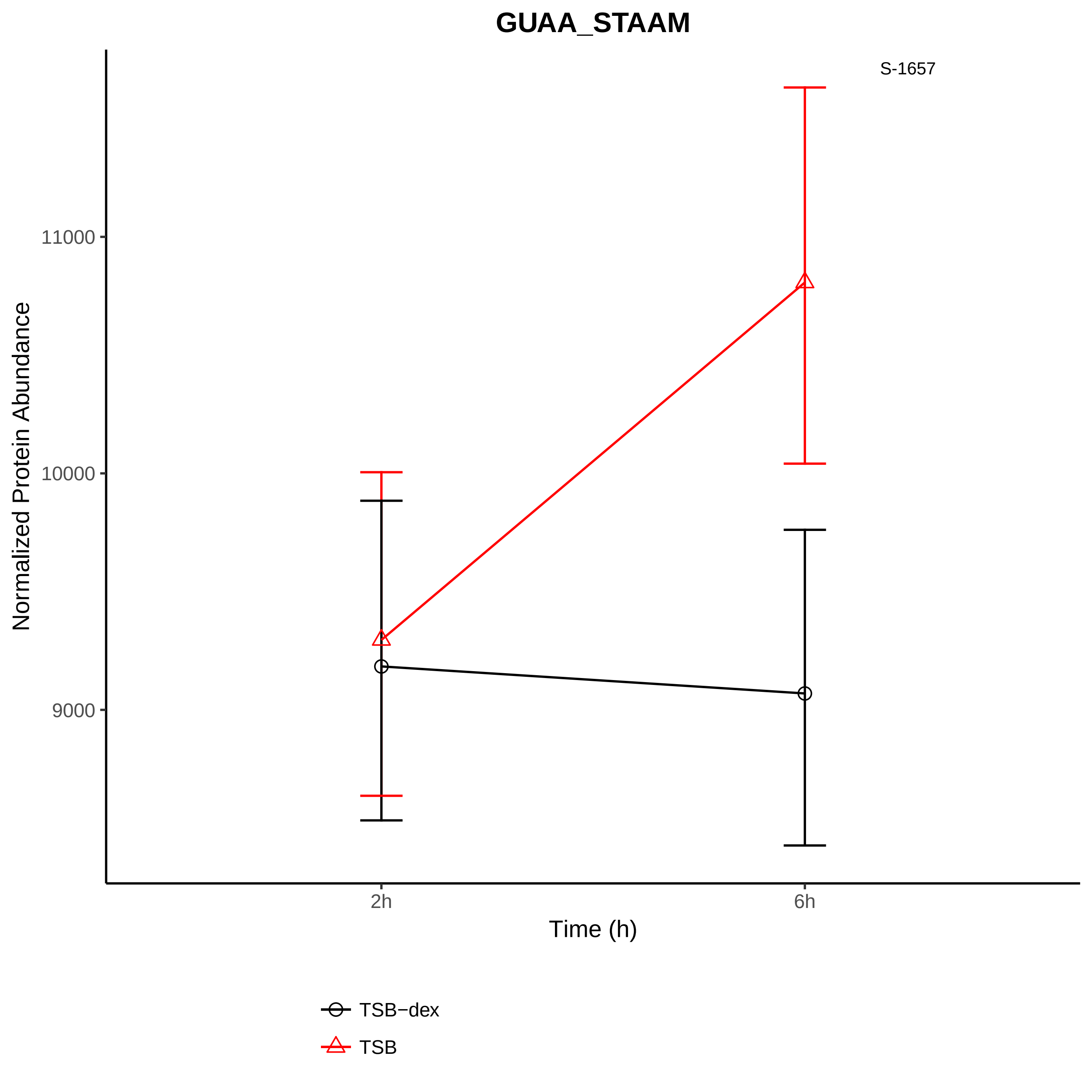
GUAA_STAAM
S-1657
Normalized Protein Abundance
11000
10000
9000
2h
6h
Time (h)
TSB−dex
TSB

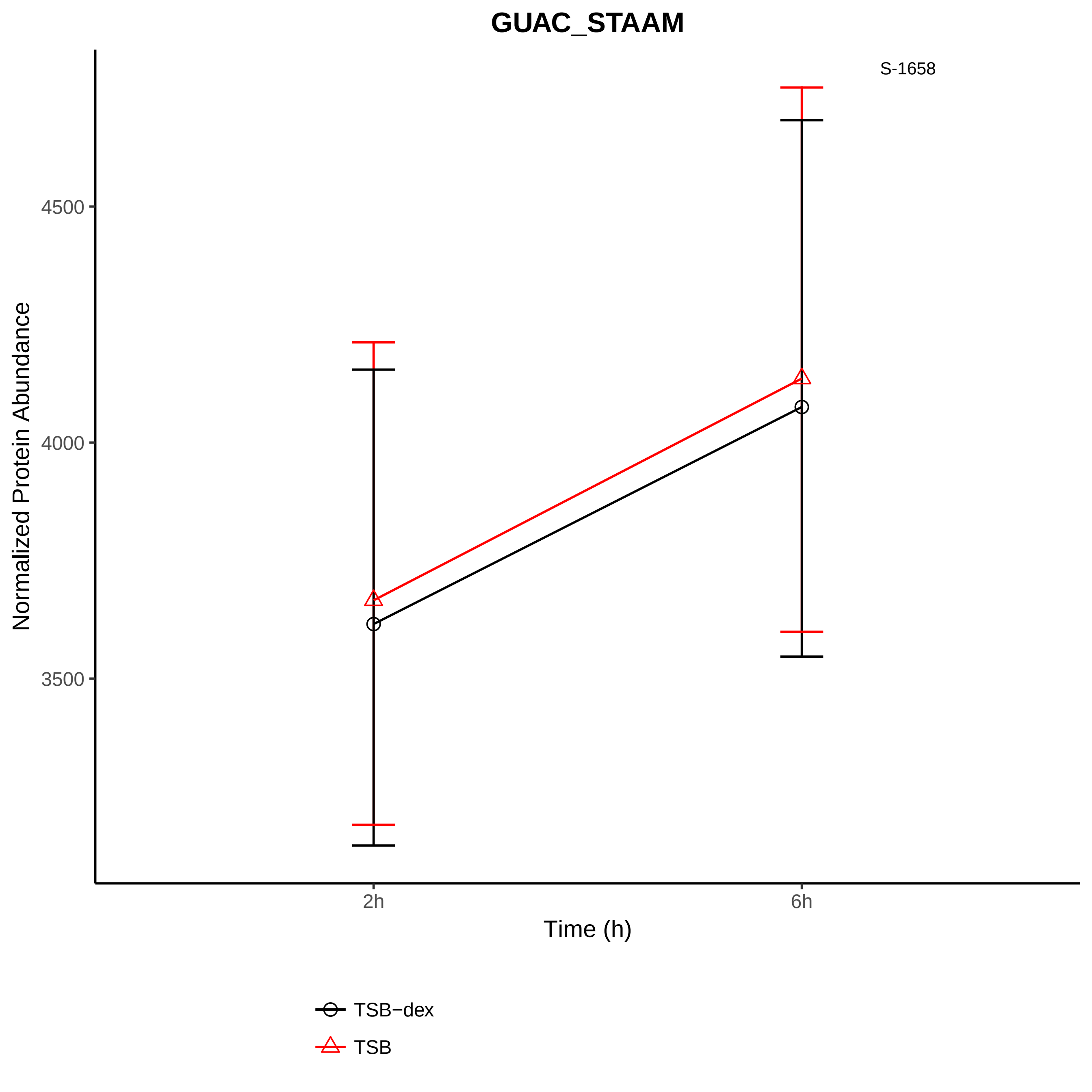

GUAC_STAAM
S-1658
Normalized Protein Abundance
4500
4000
3500
2h
6h
Time (h)
TSB−dex
TSB

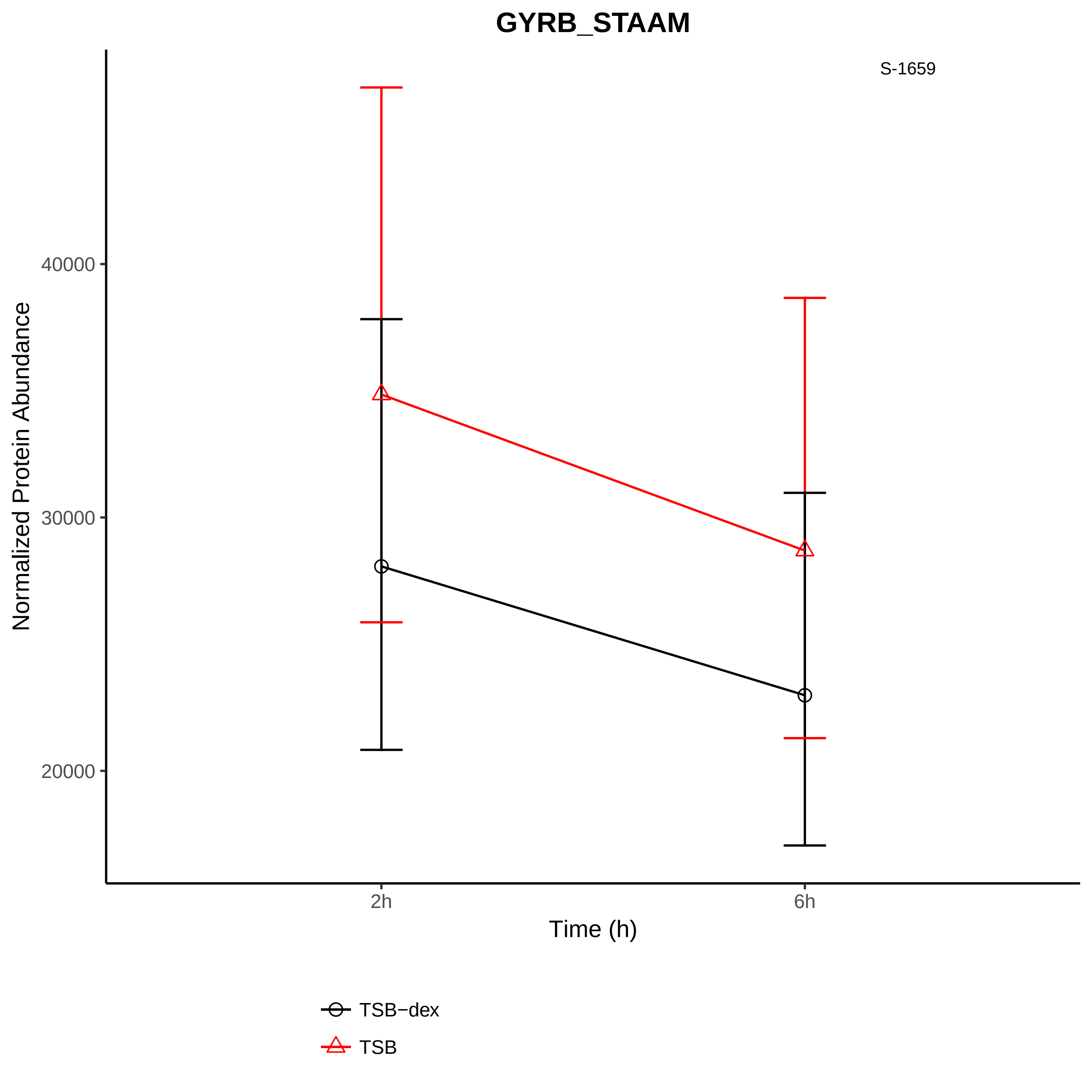

GYRB_STAAM
S-1659
Normalized Protein Abundance
40000
30000
20000
2h
6h
Time (h)
TSB−dex
TSB

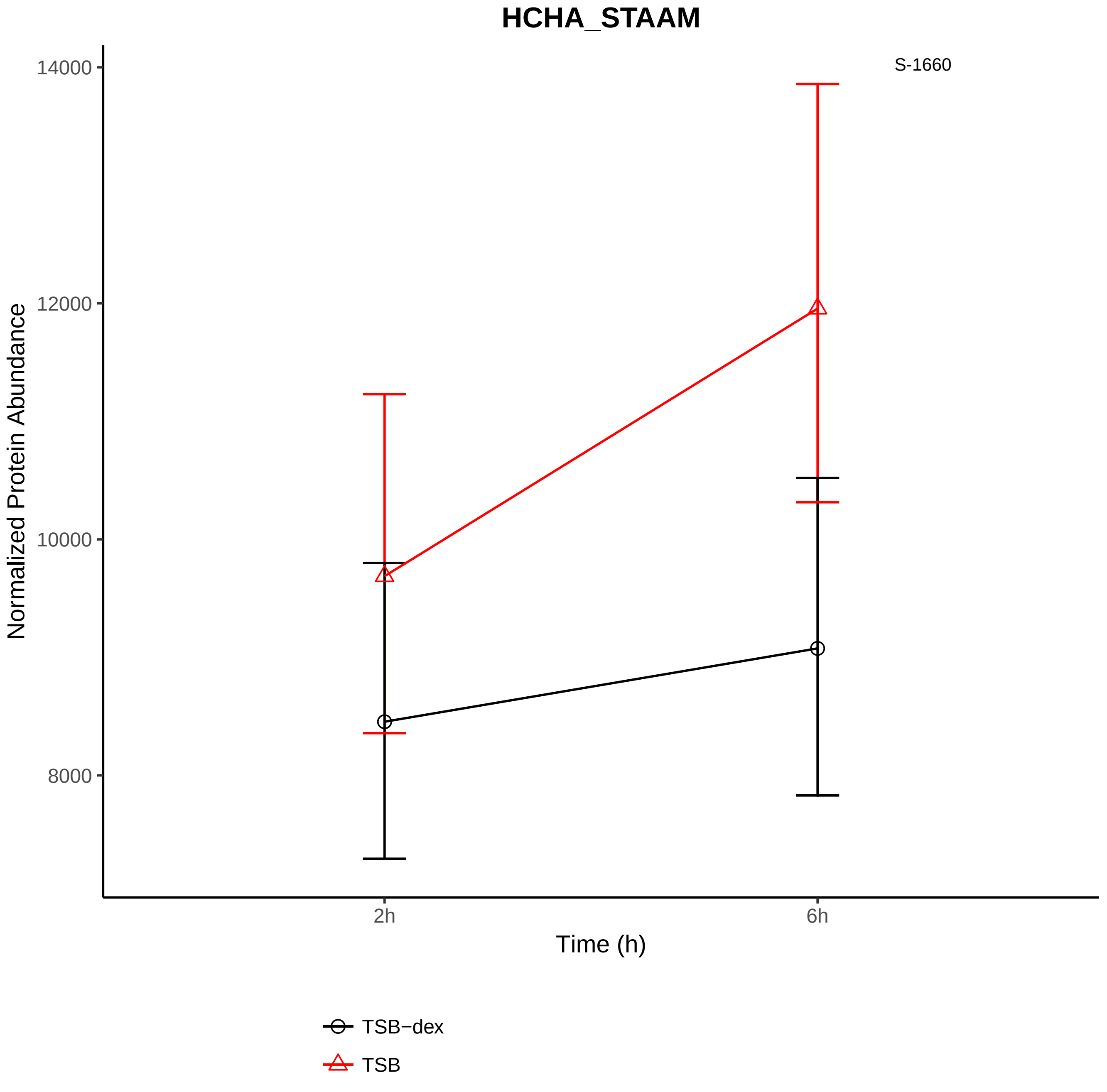
HCHA_STAAM
S-1660
Normalized Protein Abundance
14000
12000
10000
8000
2h
6h
Time (h)
TSB−dex
TSB

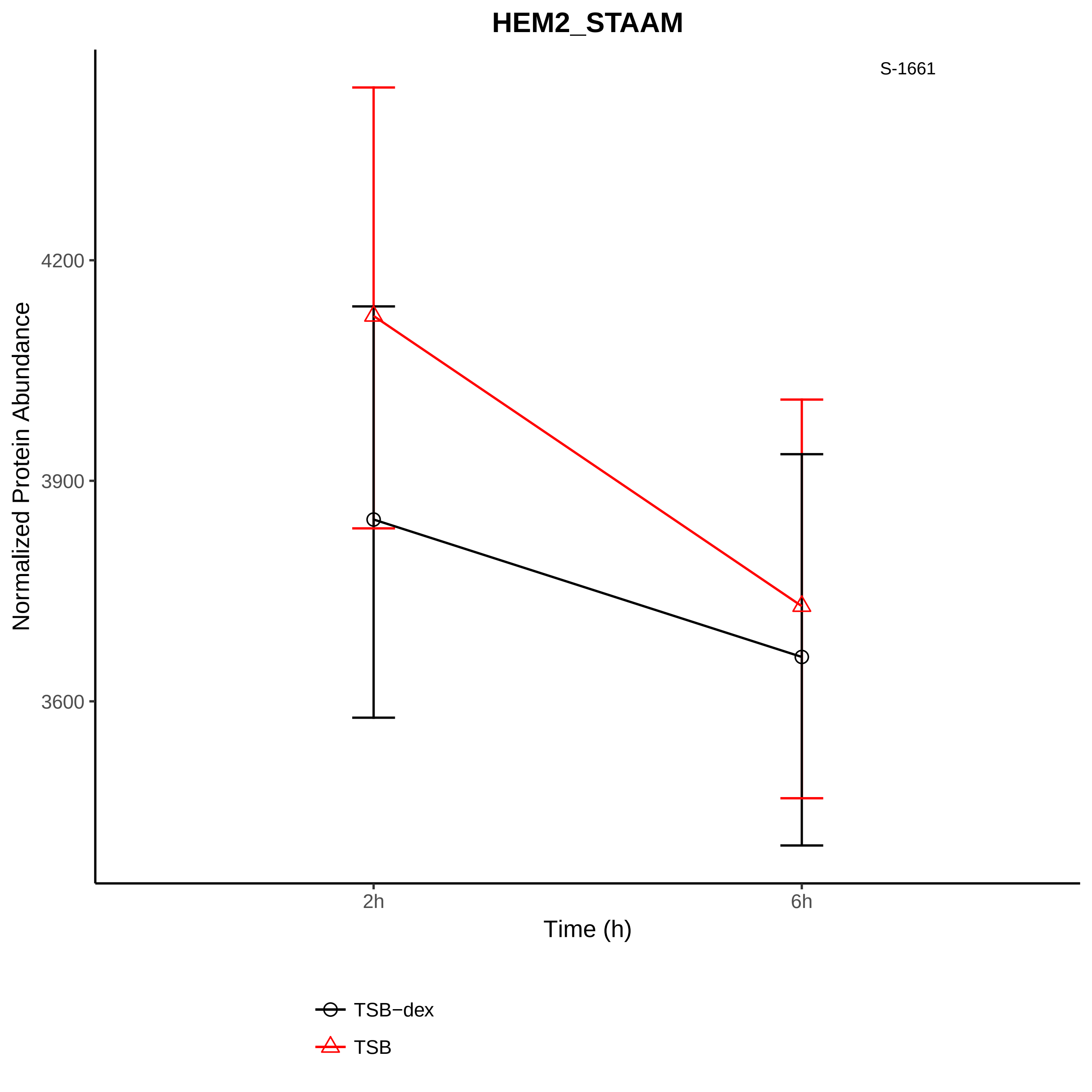
HEM2_STAAM
S-1661
Normalized Protein Abundance
4200
3900
3600
2h
6h
Time (h)
TSB−dex
TSB

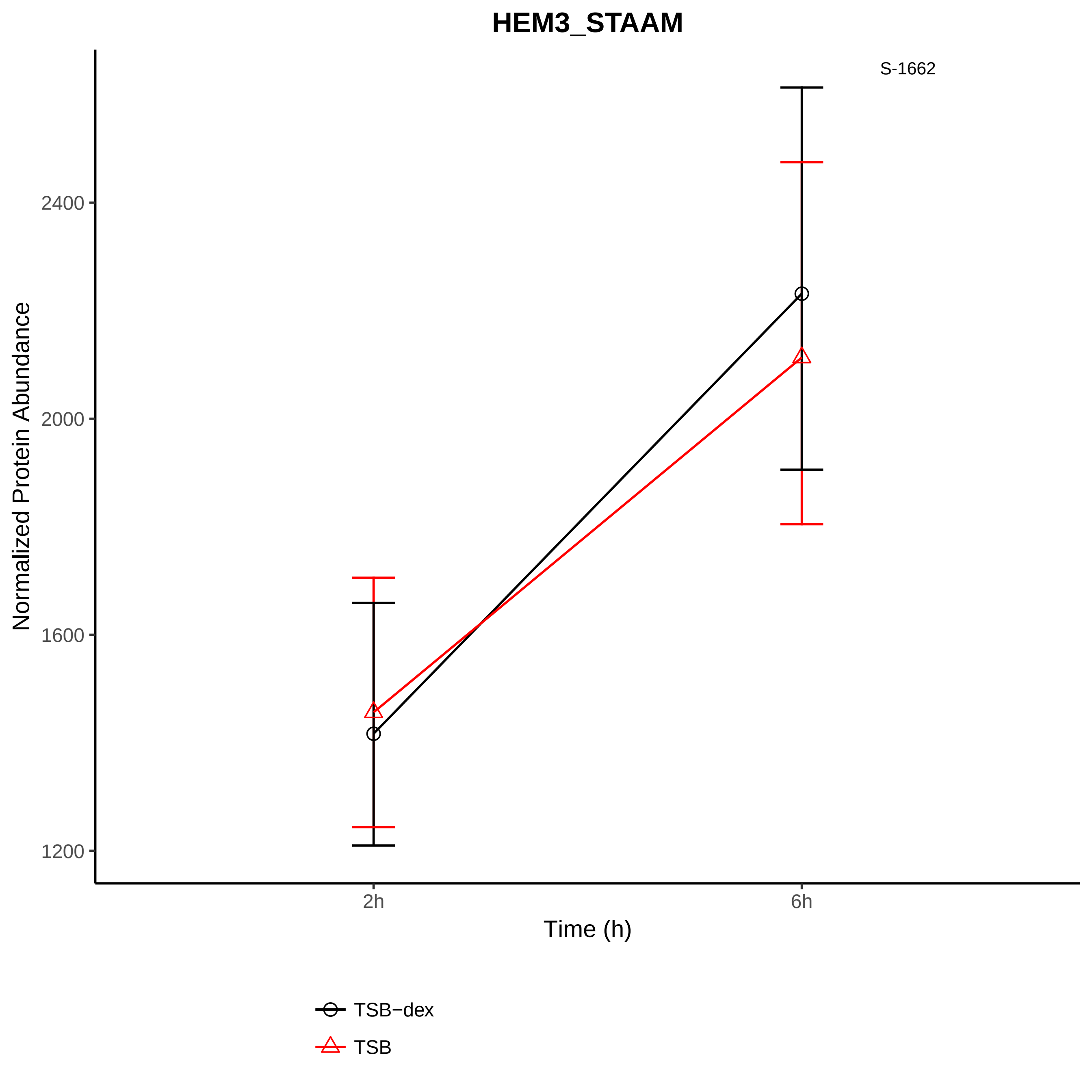
HEM3_STAAM
S-1662
Normalized Protein Abundance
2400
2000
1600
1200
2h
6h
Time (h)
TSB−dex
TSB

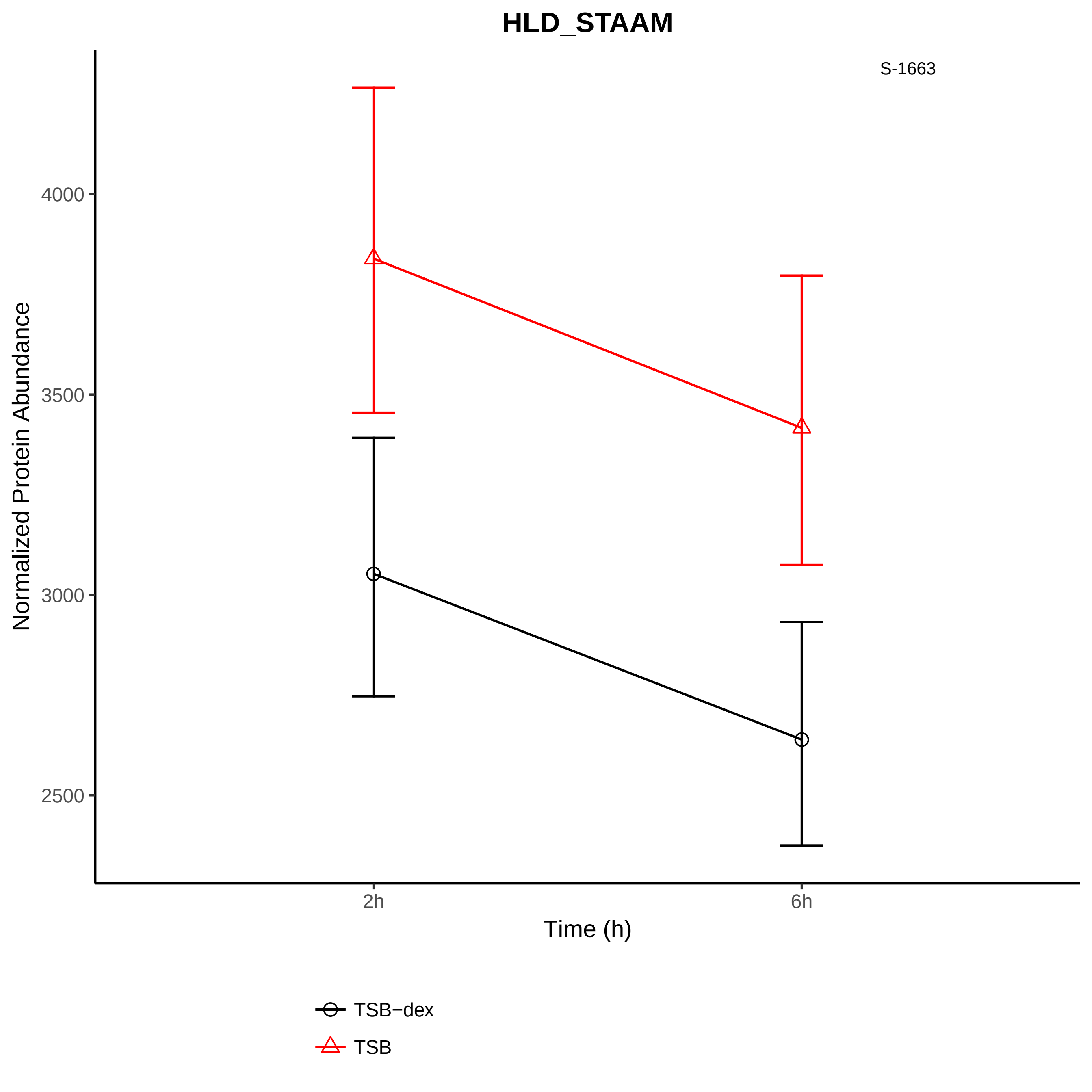
HLD_STAAM
S-1663
Normalized Protein Abundance
4000
3500
3000
2500
2h
6h
Time (h)
TSB−dex
TSB

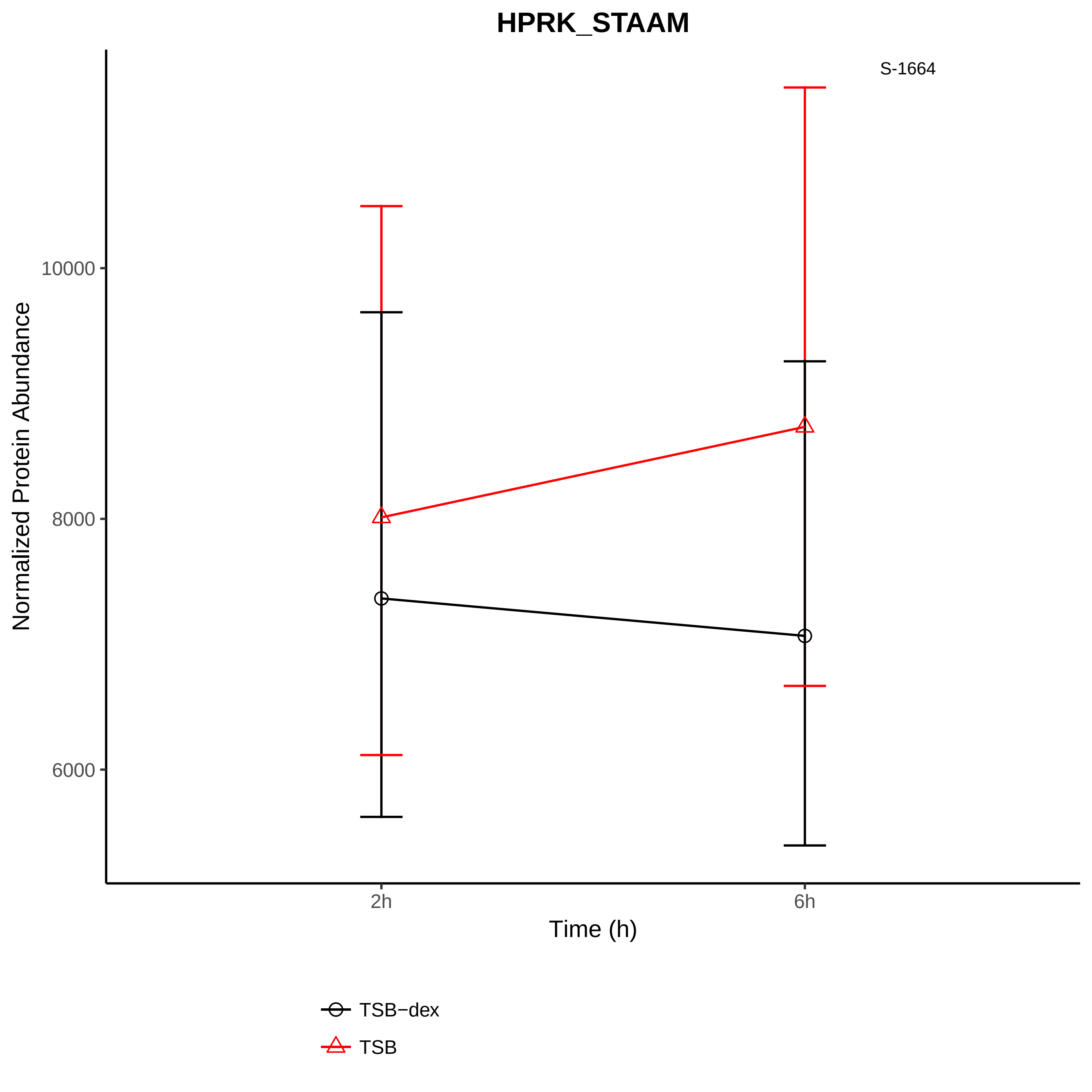

HPRK_STAAM
S-1664
Normalized Protein Abundance
10000
8000
6000
2h
6h
Time (h)
TSB−dex
TSB

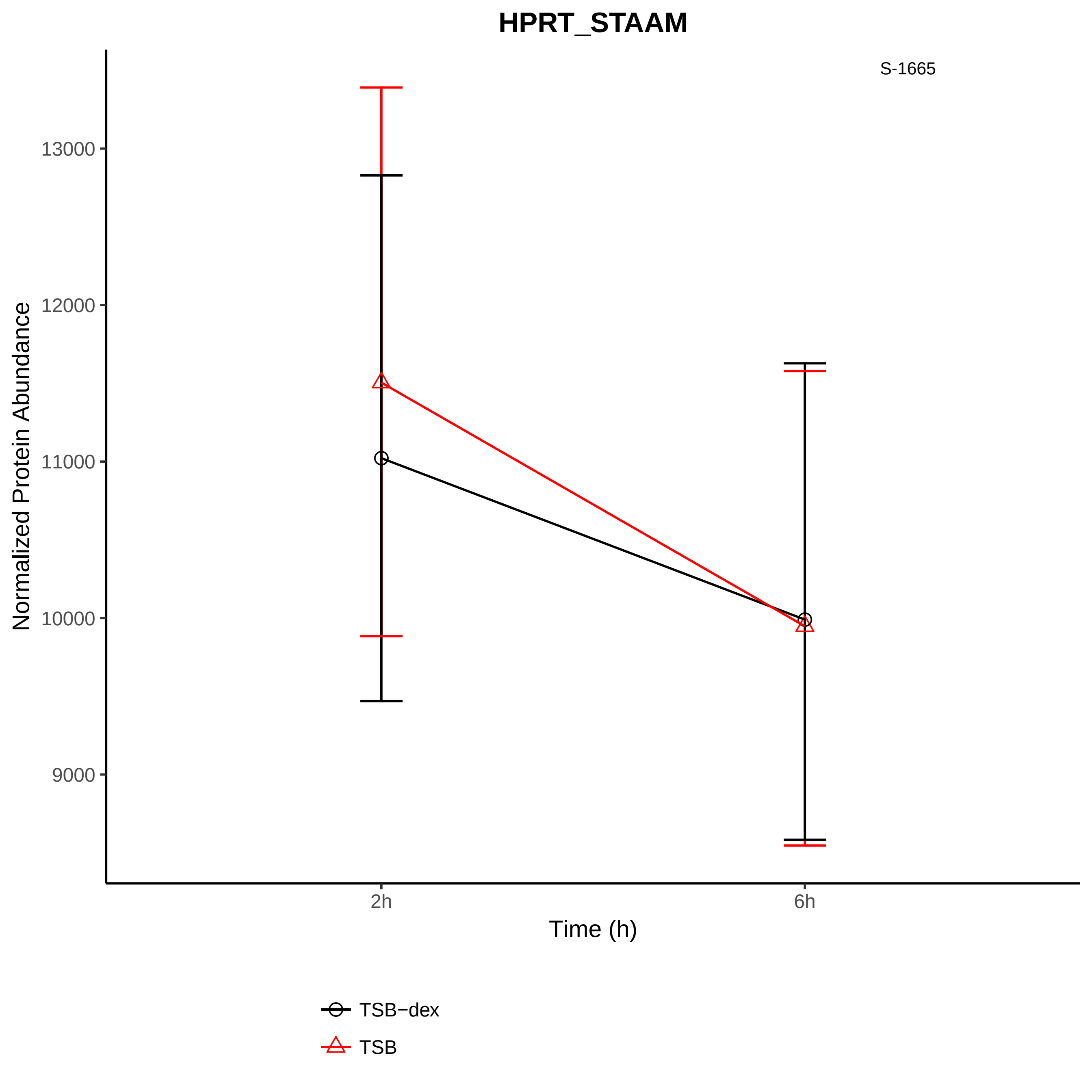
HPRT_STAAM
S-1665
Normalized Protein Abundance
13000
12000
11000
10000
9000
2h
6h
Time (h)
TSB−dex
TSB

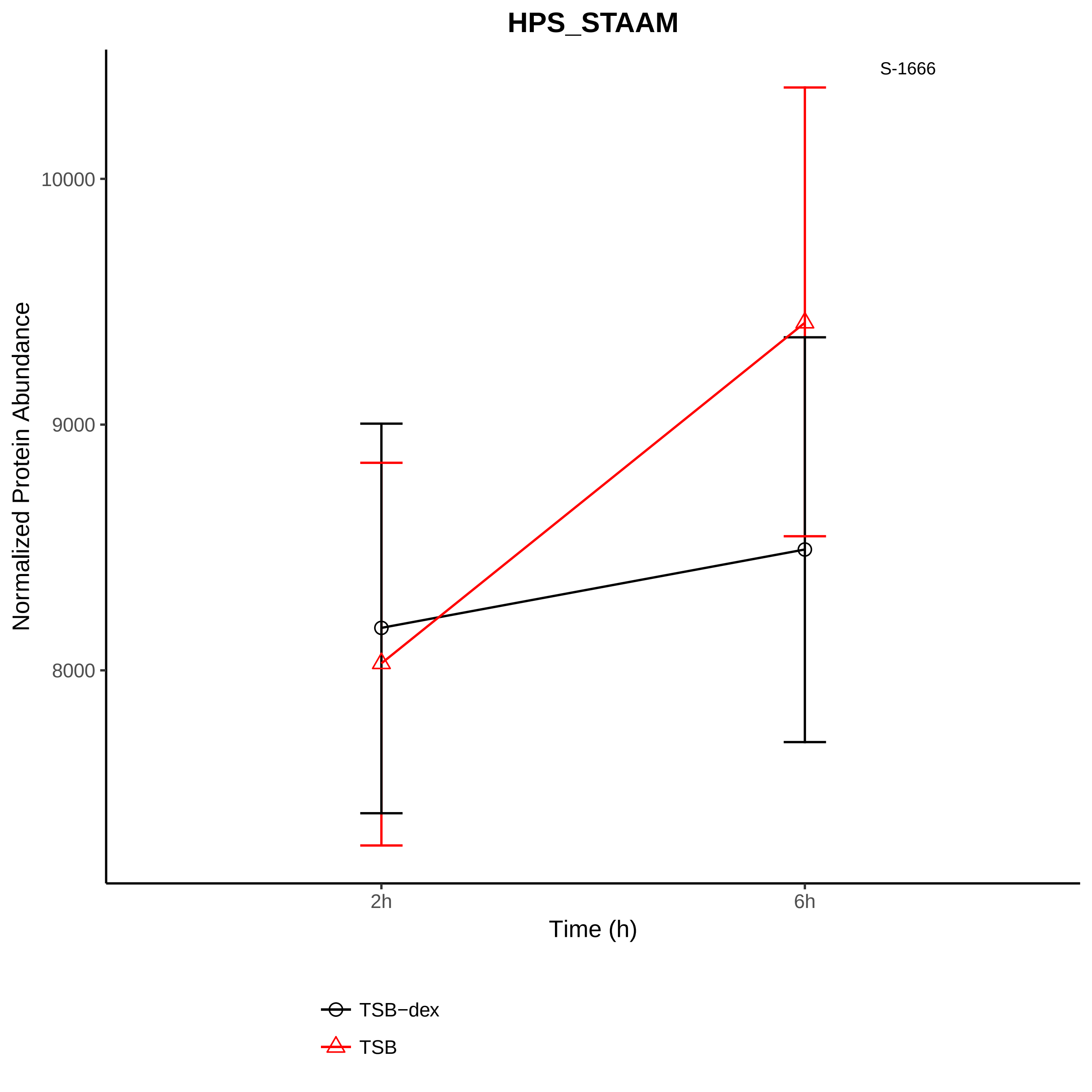

HPS_STAAM
S-1666
10000
9000
8000
Normalized Protein Abundance
2h
6h
Time (h)
TSB−dex
TSB

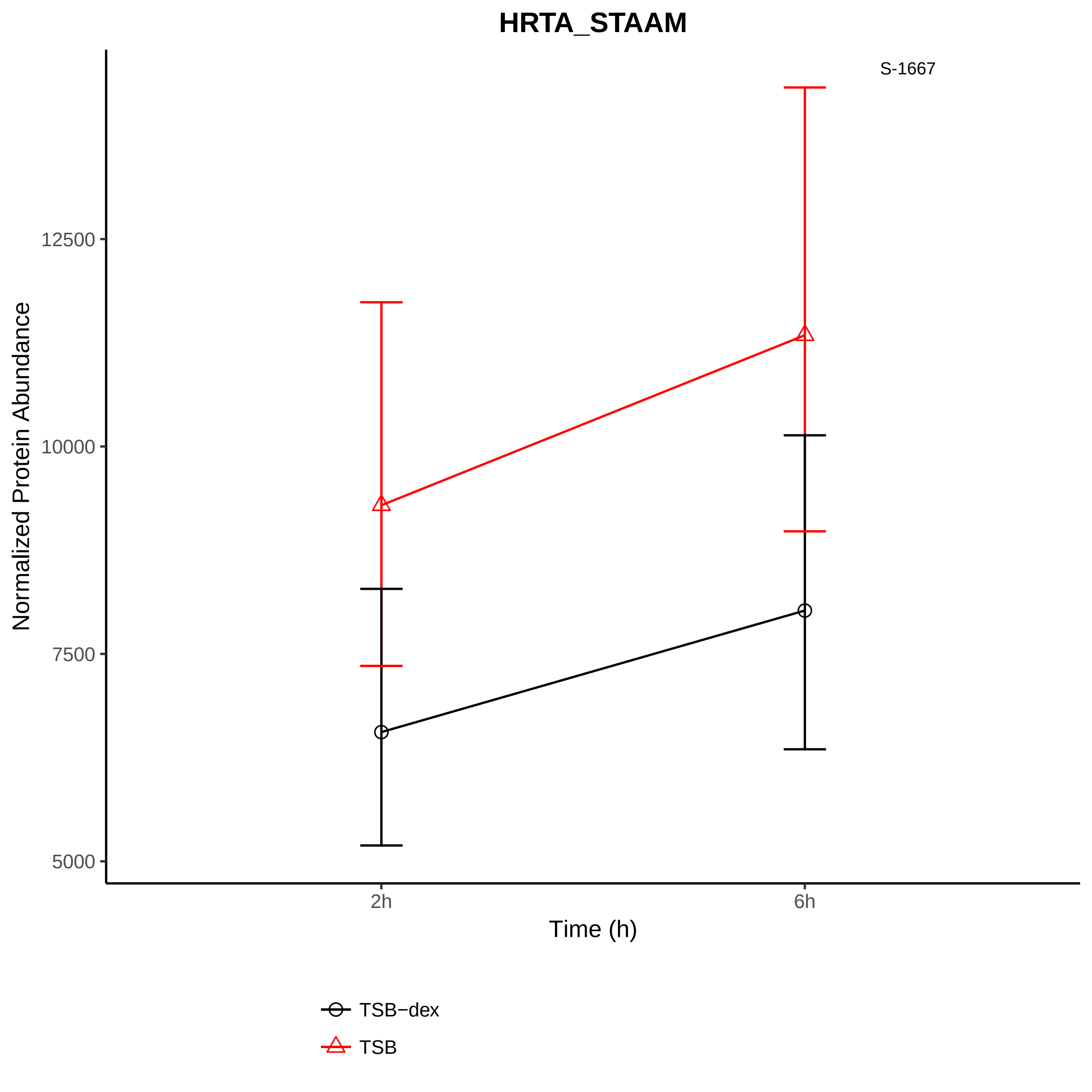

HRTA_STAAM
S-1667
Normalized Protein Abundance
12500
10000
7500
5000
2h
6h
Time (h)
TSB−dex
TSB

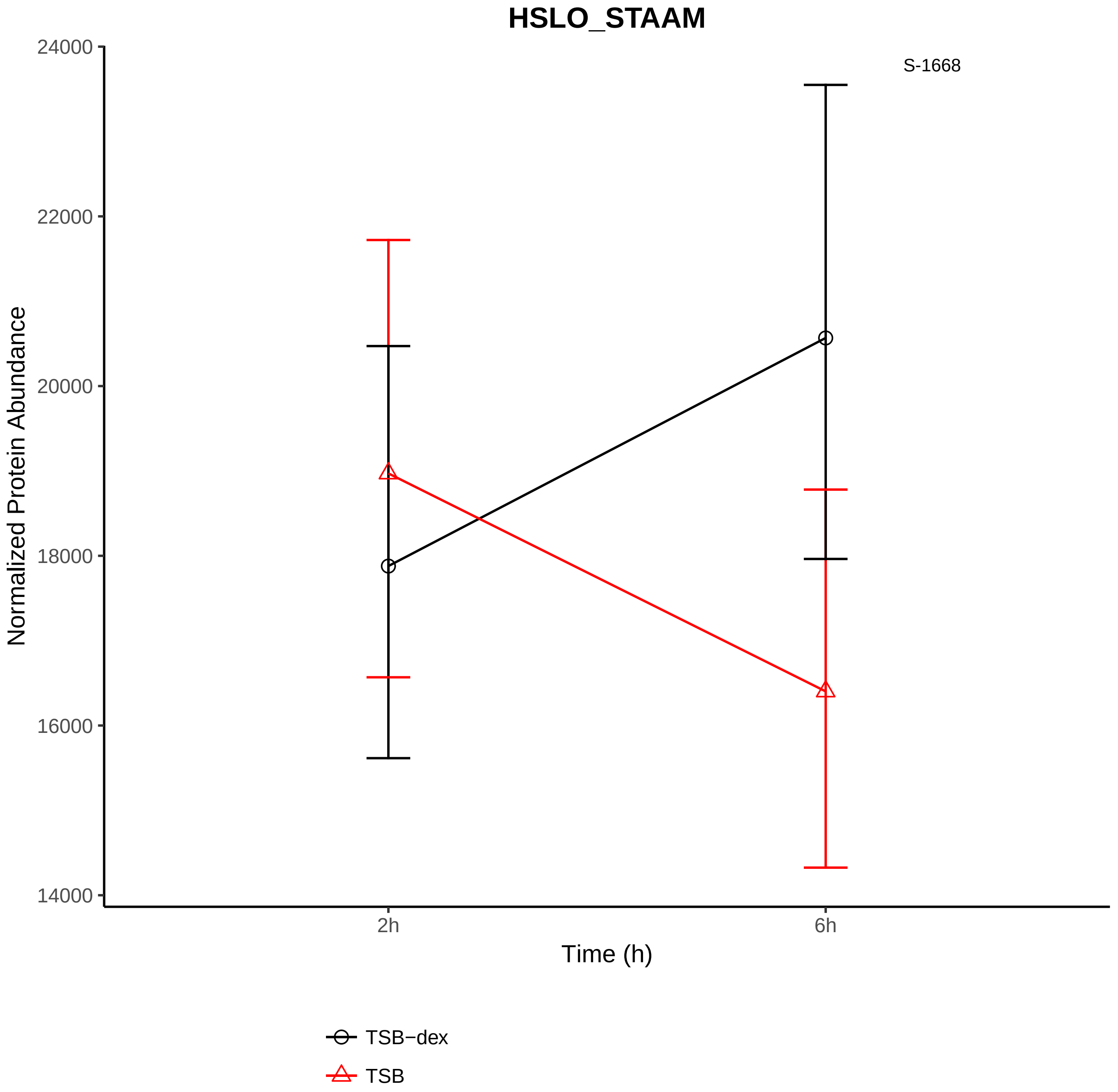
HSLO_STAAM
S-1668
Normalized Protein Abundance
24000
22000
20000
18000
16000
14000
2h
6h
Time (h)
TSB−dex
TSB

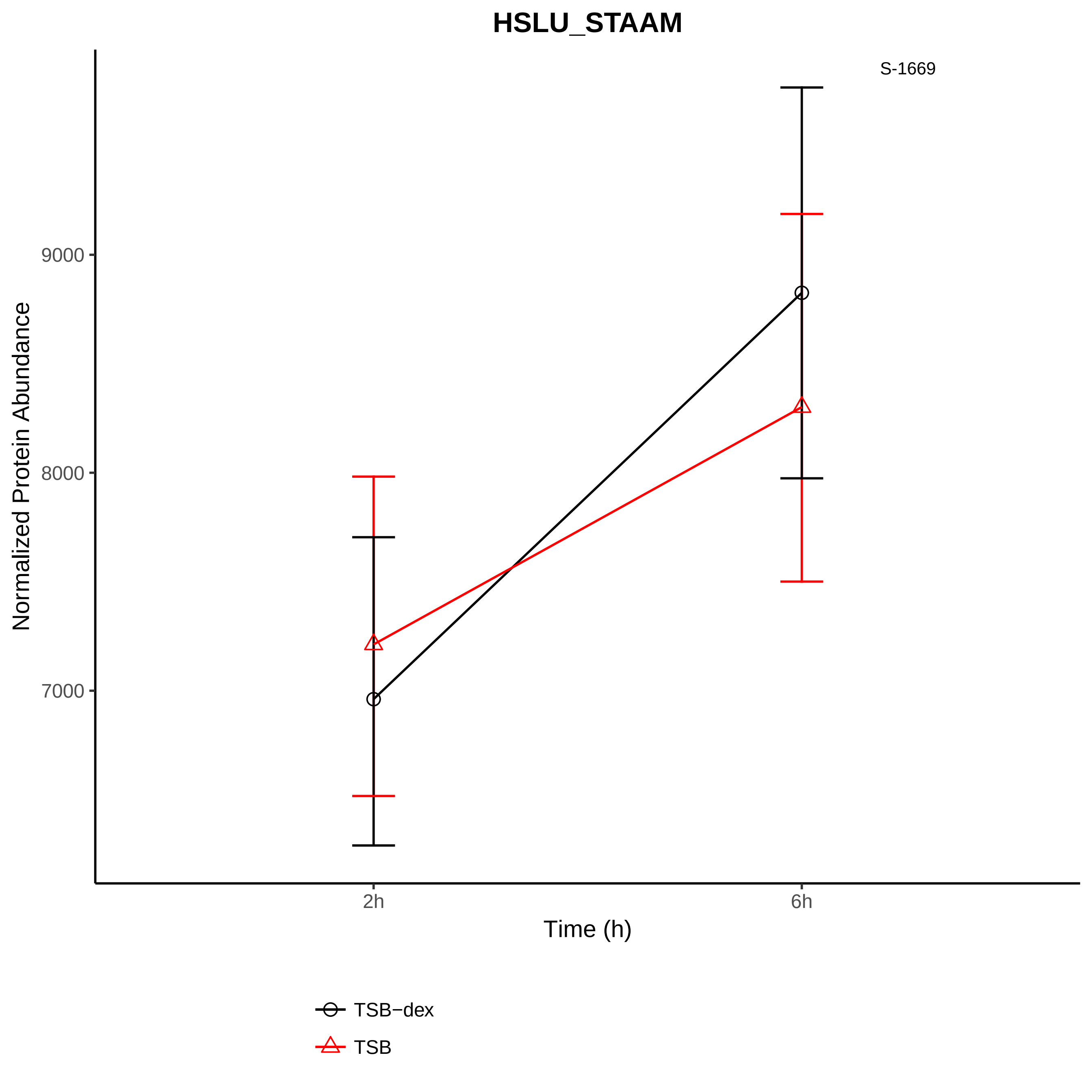

HSLU_STAAM
S-1669
9000
8000
7000
Normalized Protein Abundance
2h
6h
Time (h)
TSB−dex
TSB

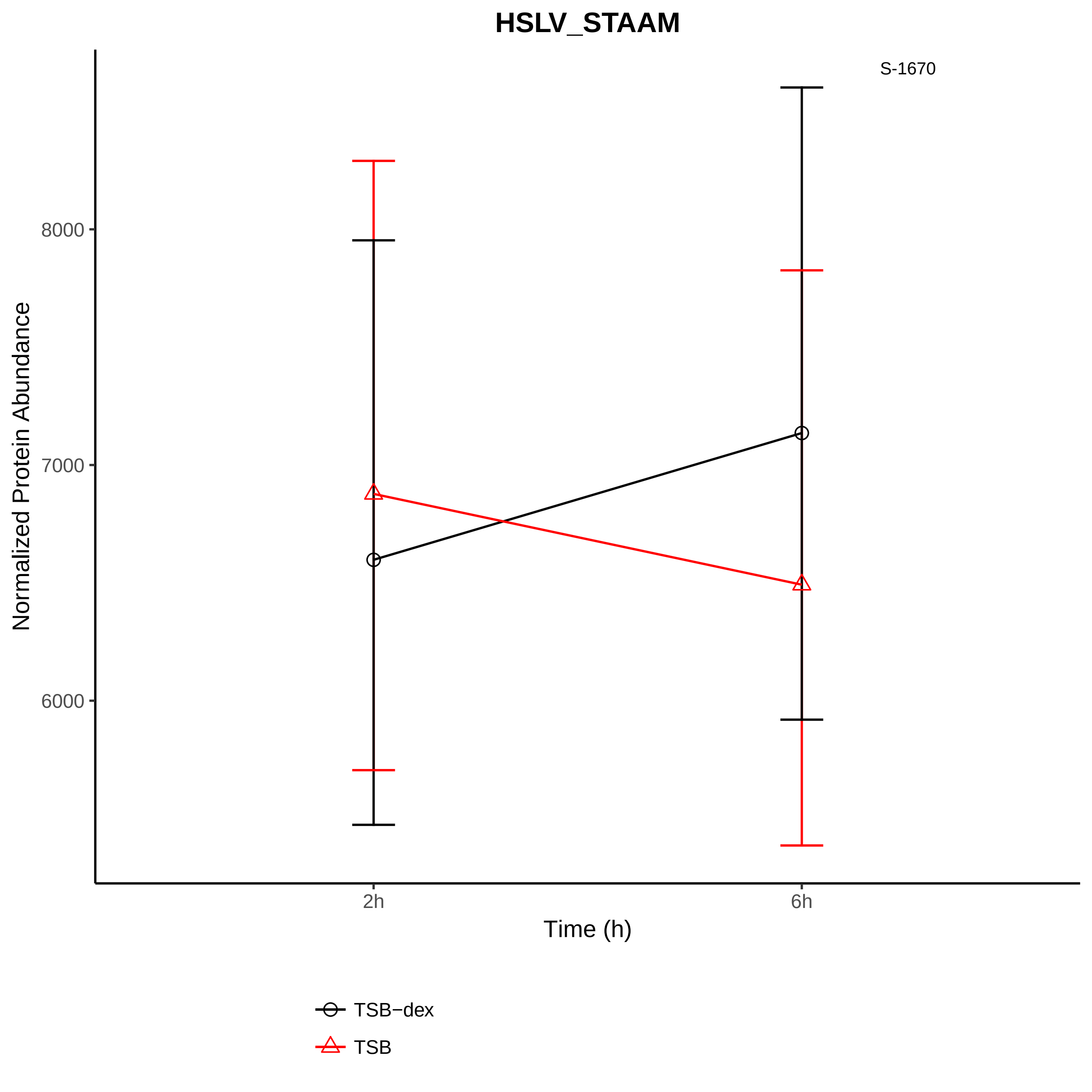
HSLV_STAAM
S-1670
8000
7000
6000
Normalized Protein Abundance
2h
6h
Time (h)
TSB−dex
TSB

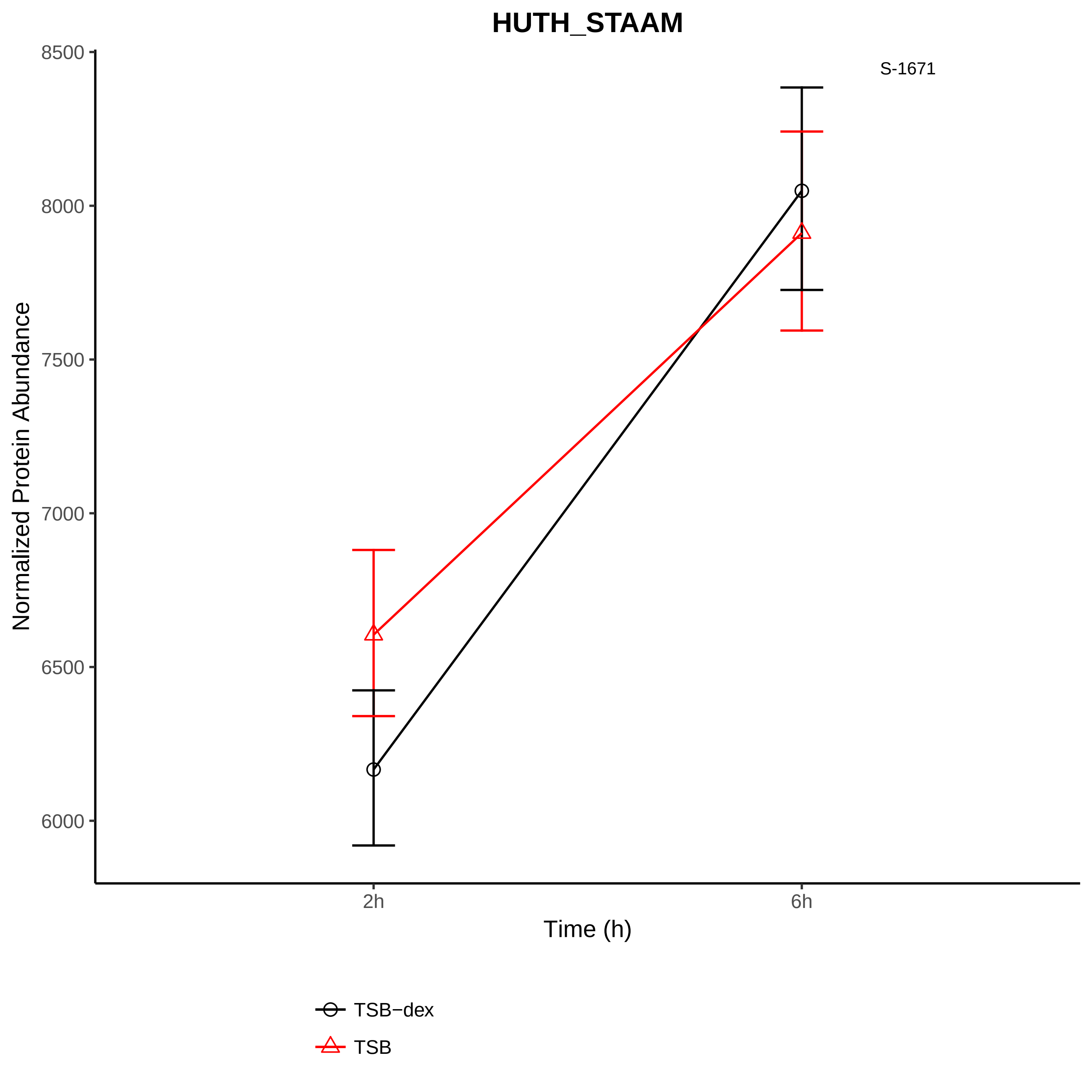

HUTH_STAAM
S-1671
8500
8000
7500
7000
6500
6000
Normalized Protein Abundance
2h
6h
Time (h)
TSB−dex
TSB

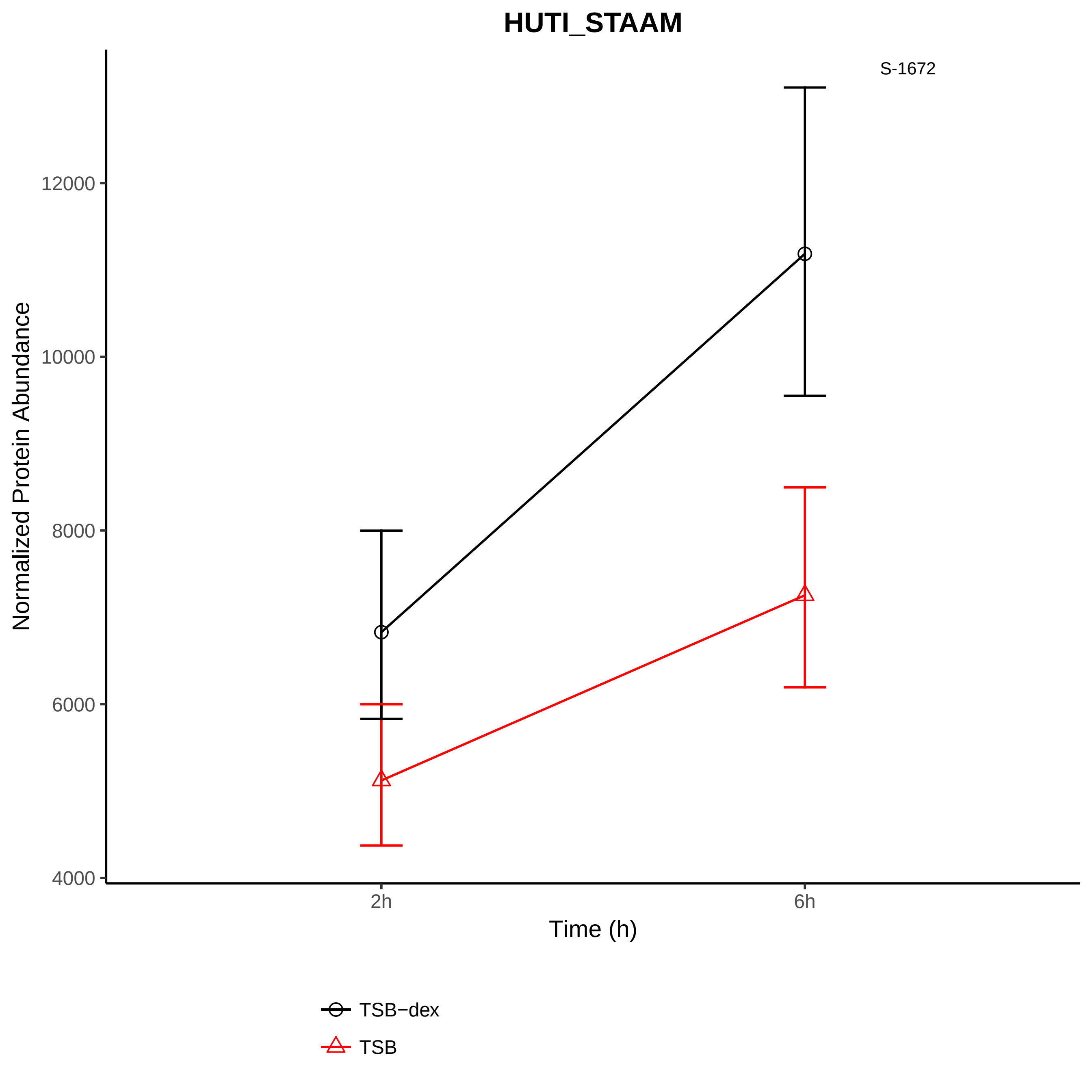

HUTI_STAAM
S-1672
Normalized Protein Abundance
12000
10000
8000
6000
4000
2h
6h
Time (h)
TSB−dex
TSB

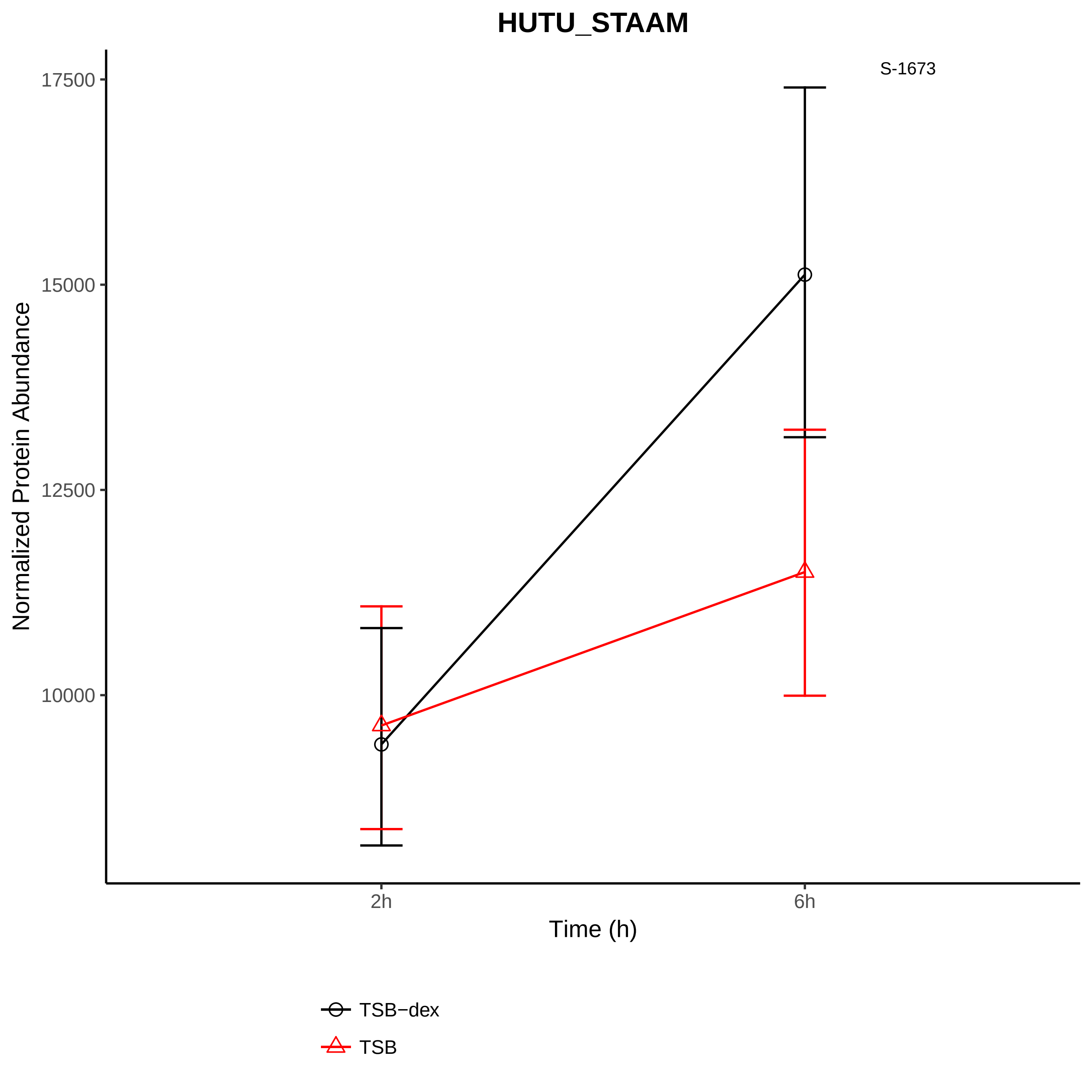

HUTU_STAAM
S-1673
Normalized Protein Abundance
17500
15000
12500
10000
2h
6h
Time (h)
TSB−dex
TSB

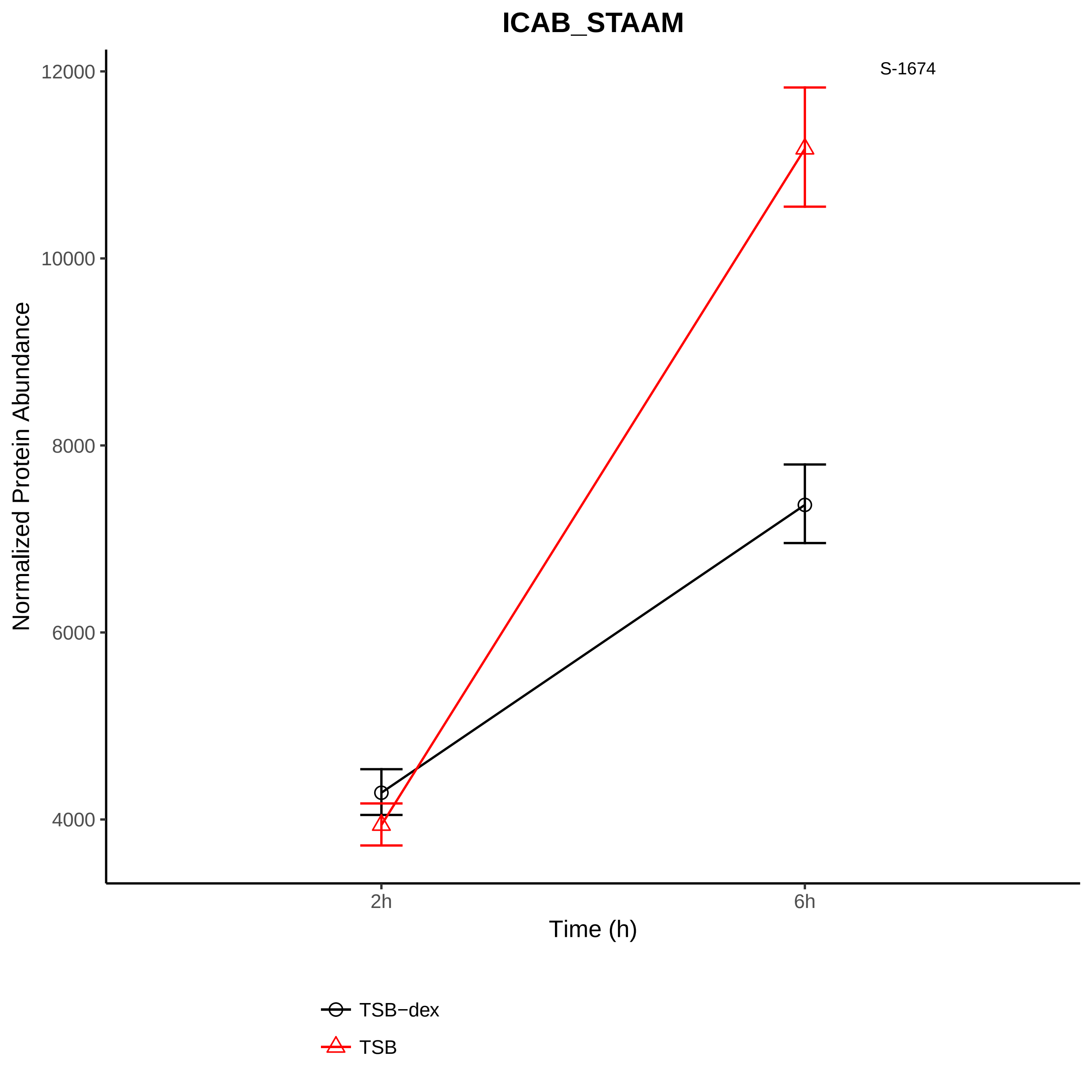
ICAB_STAAM
S-1674
Normalized Protein Abundance
12000
10000
8000
6000
4000
2h
6h
Time (h)
TSB−dex
TSB

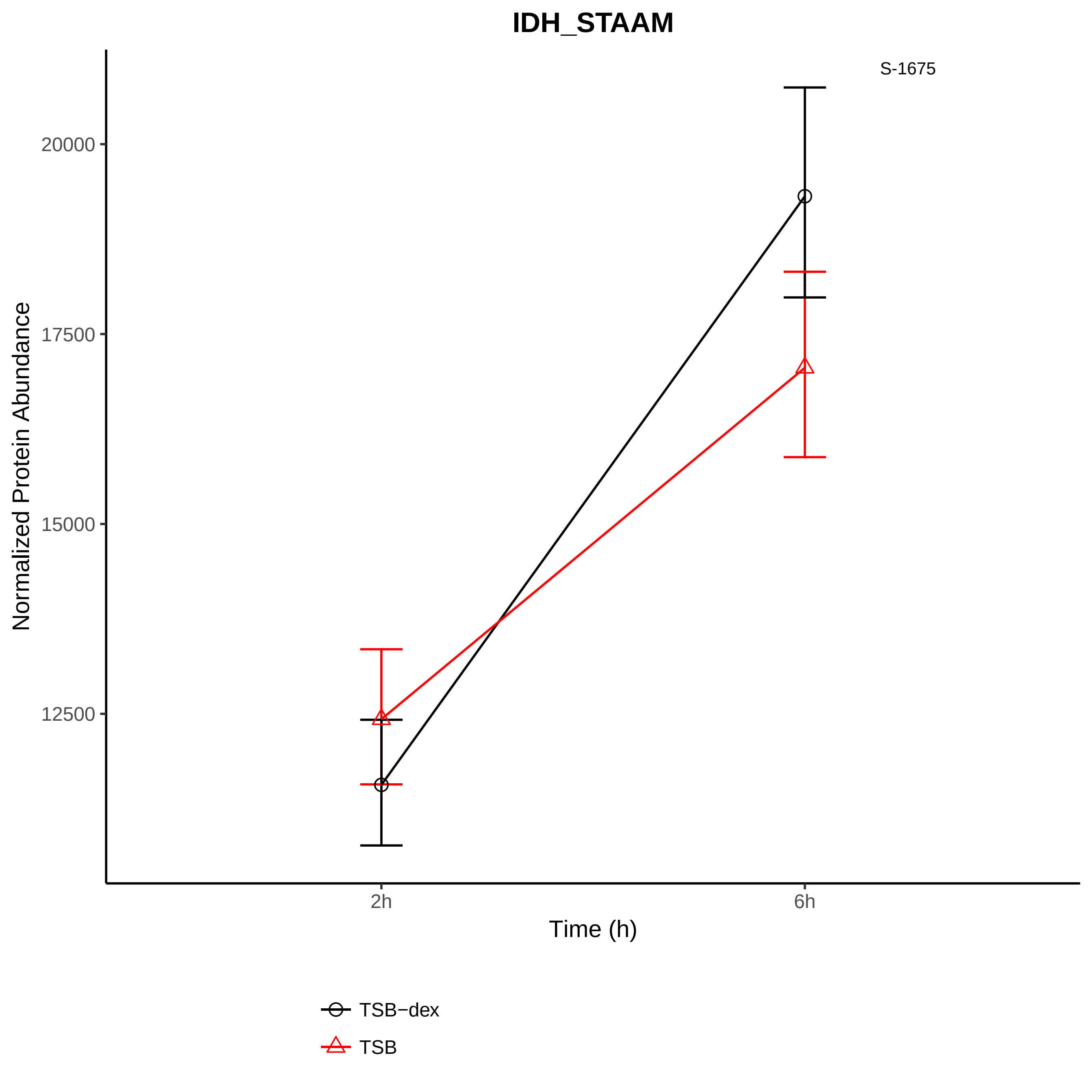
IDH_STAAM
S-1675
Normalized Protein Abundance
20000
17500
15000
12500
2h
6h
Time (h)
TSB−dex
TSB

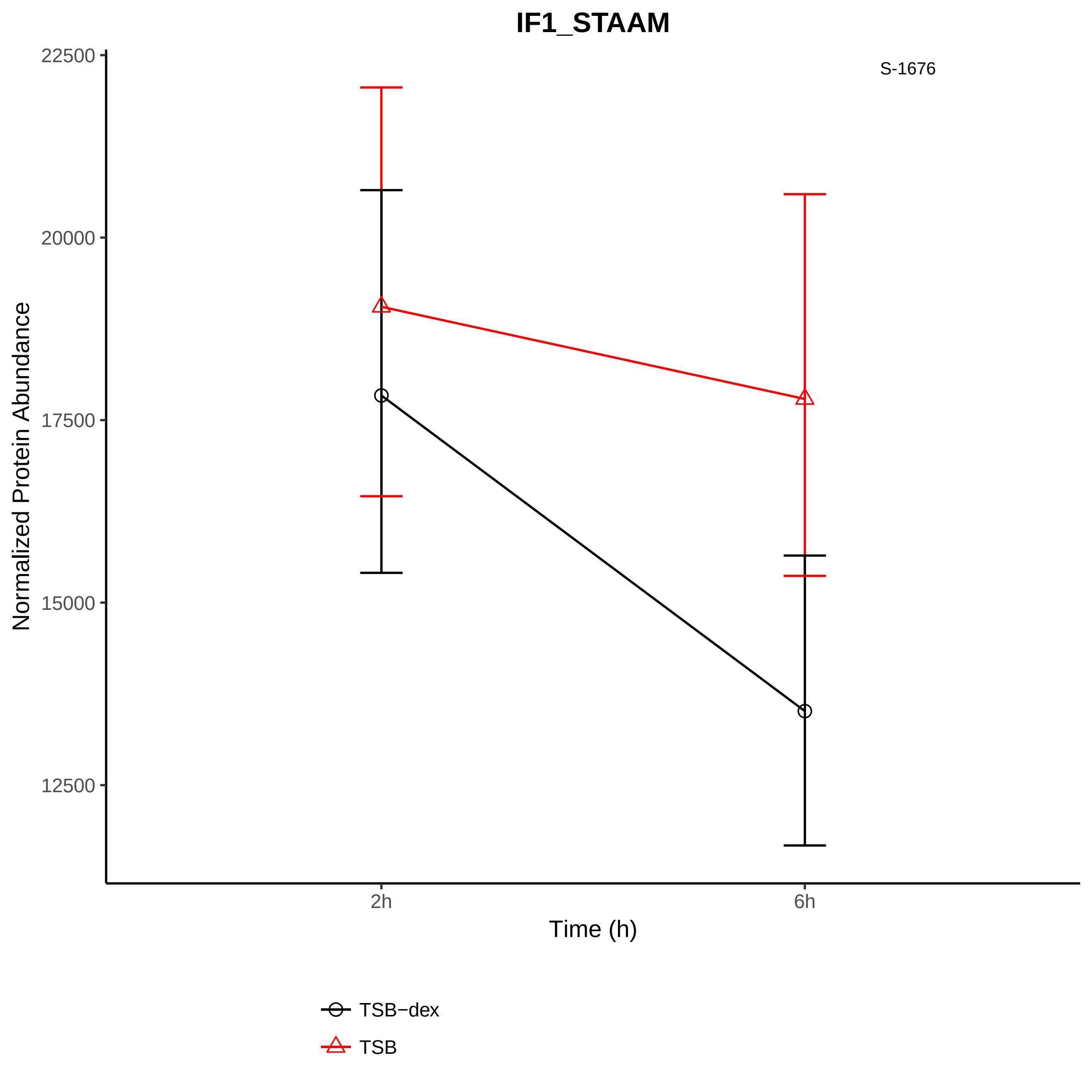
IF1_STAAM
S-1676
Normalized Protein Abundance
22500
20000
17500
15000
12500
2h
6h
Time (h)
TSB−dex
TSB

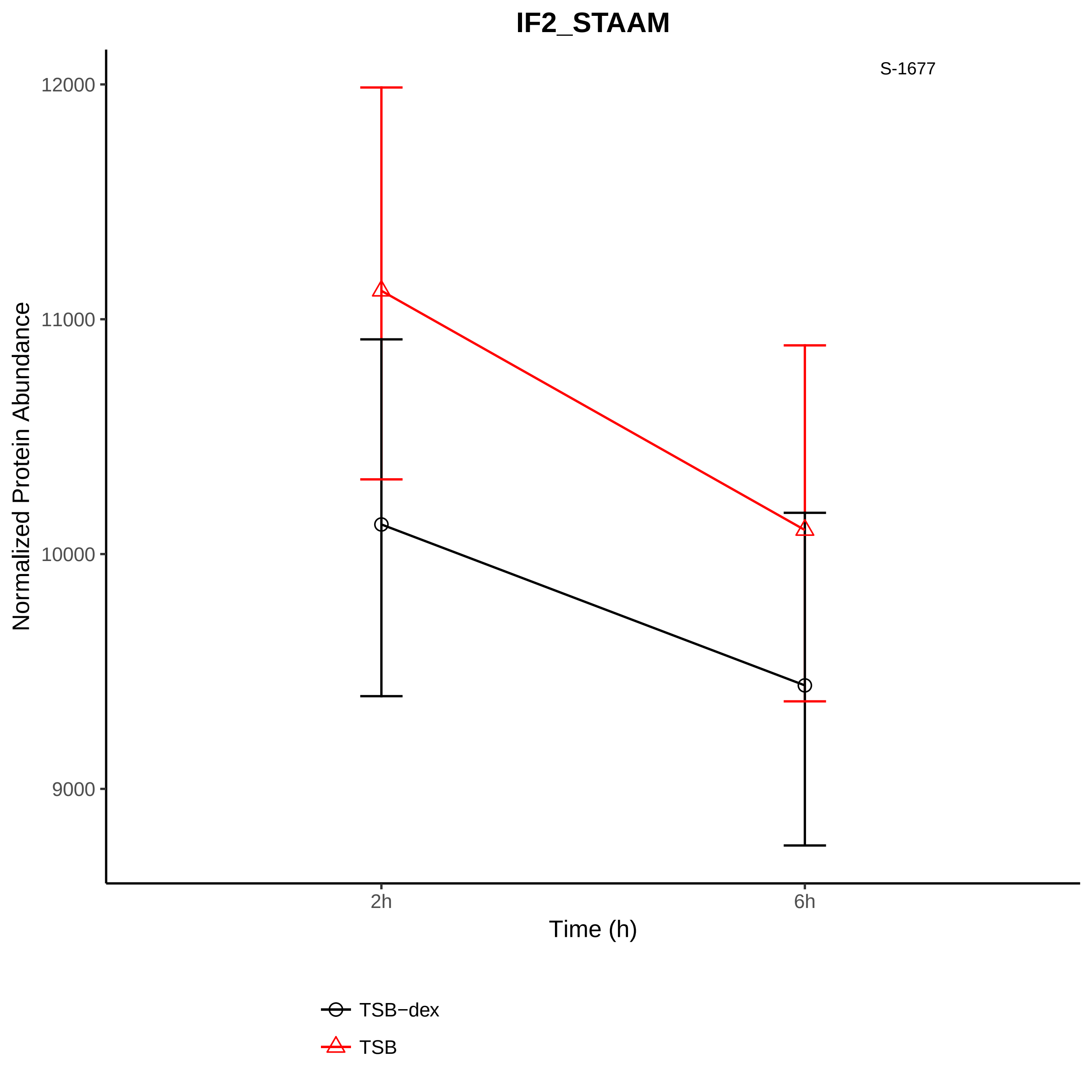

IF2_STAAM
S-1677
12000
11000
10000
9000
Normalized Protein Abundance
2h
6h
Time (h)
TSB−dex
TSB

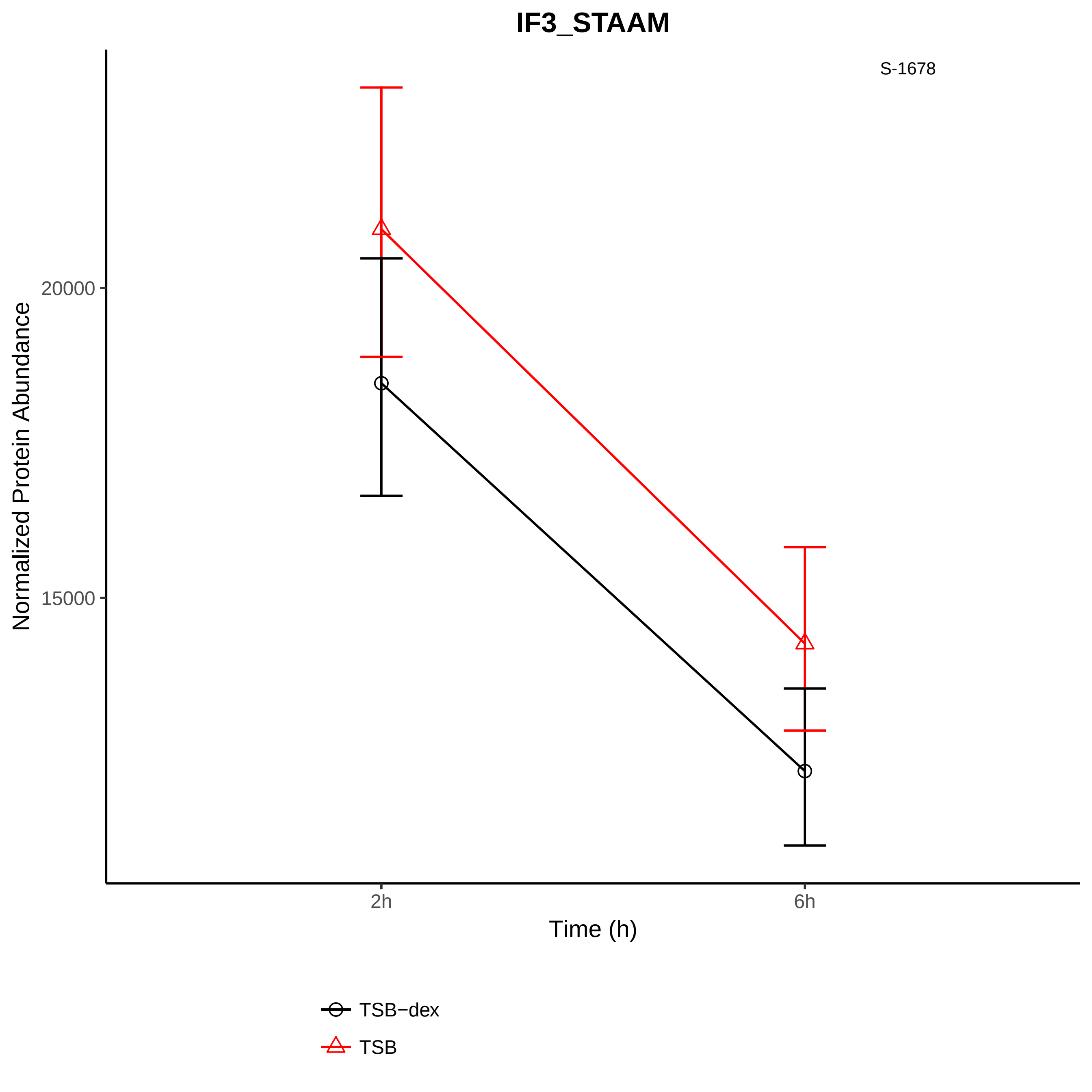

IF3_STAAM
S-1678
Normalized Protein Abundance
20000
15000
2h
6h
Time (h)
TSB−dex
TSB

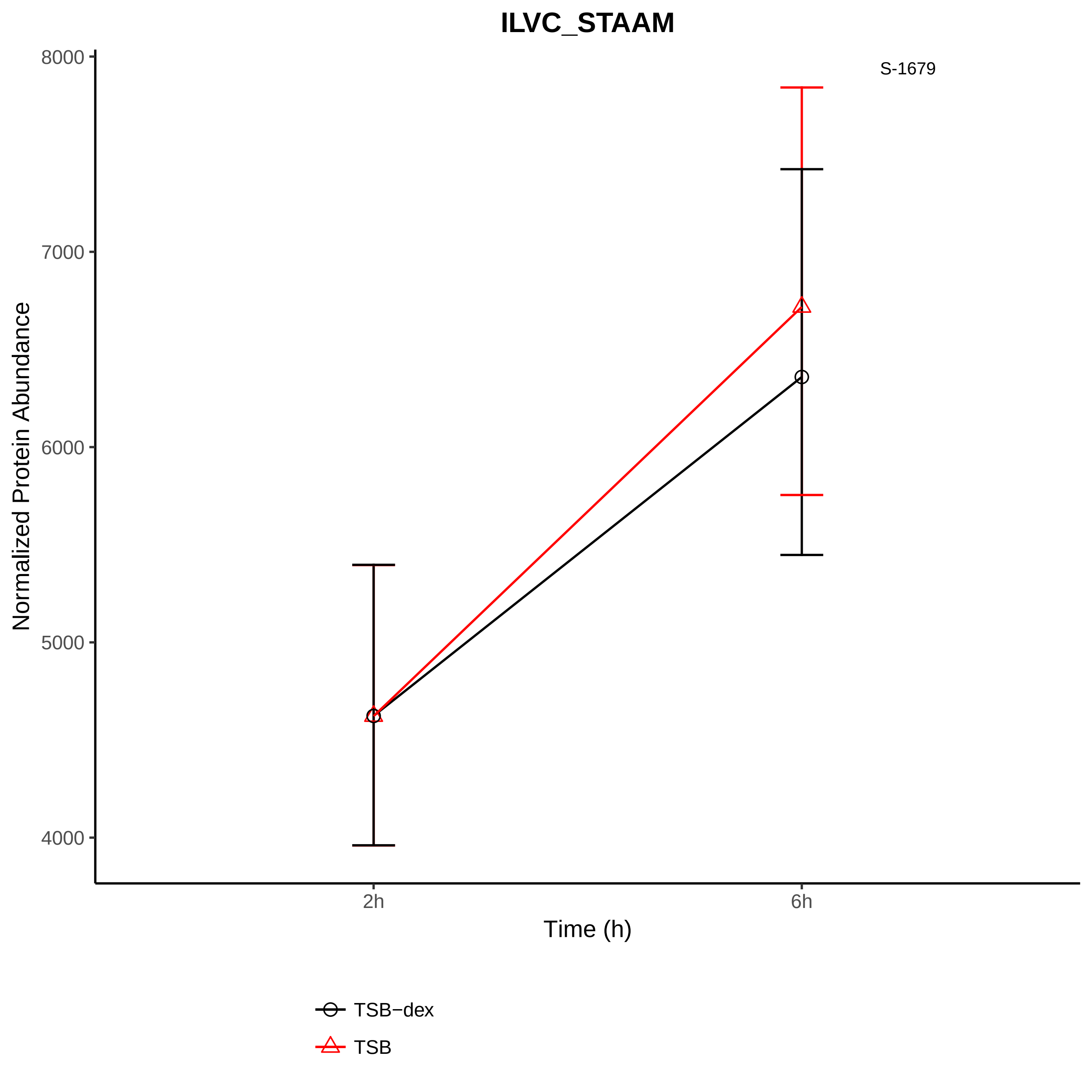

ILVC_STAAM
S-1679
8000
7000
6000
5000
4000
Normalized Protein Abundance
2h
6h
Time (h)
TSB−dex
TSB

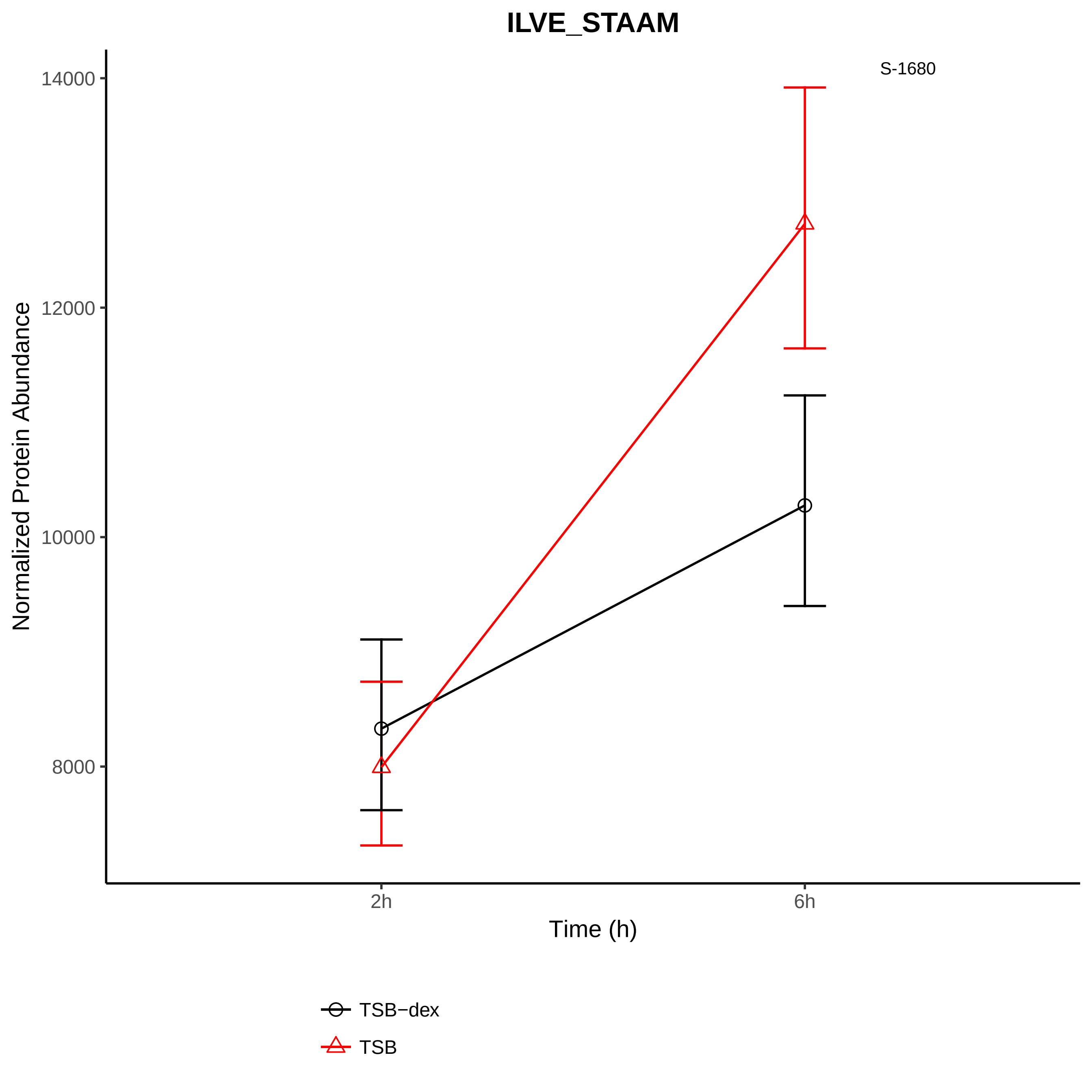

ILVE_STAAM
S-1680
Normalized Protein Abundance
14000
12000
10000
8000
2h
6h
Time (h)
TSB−dex
TSB

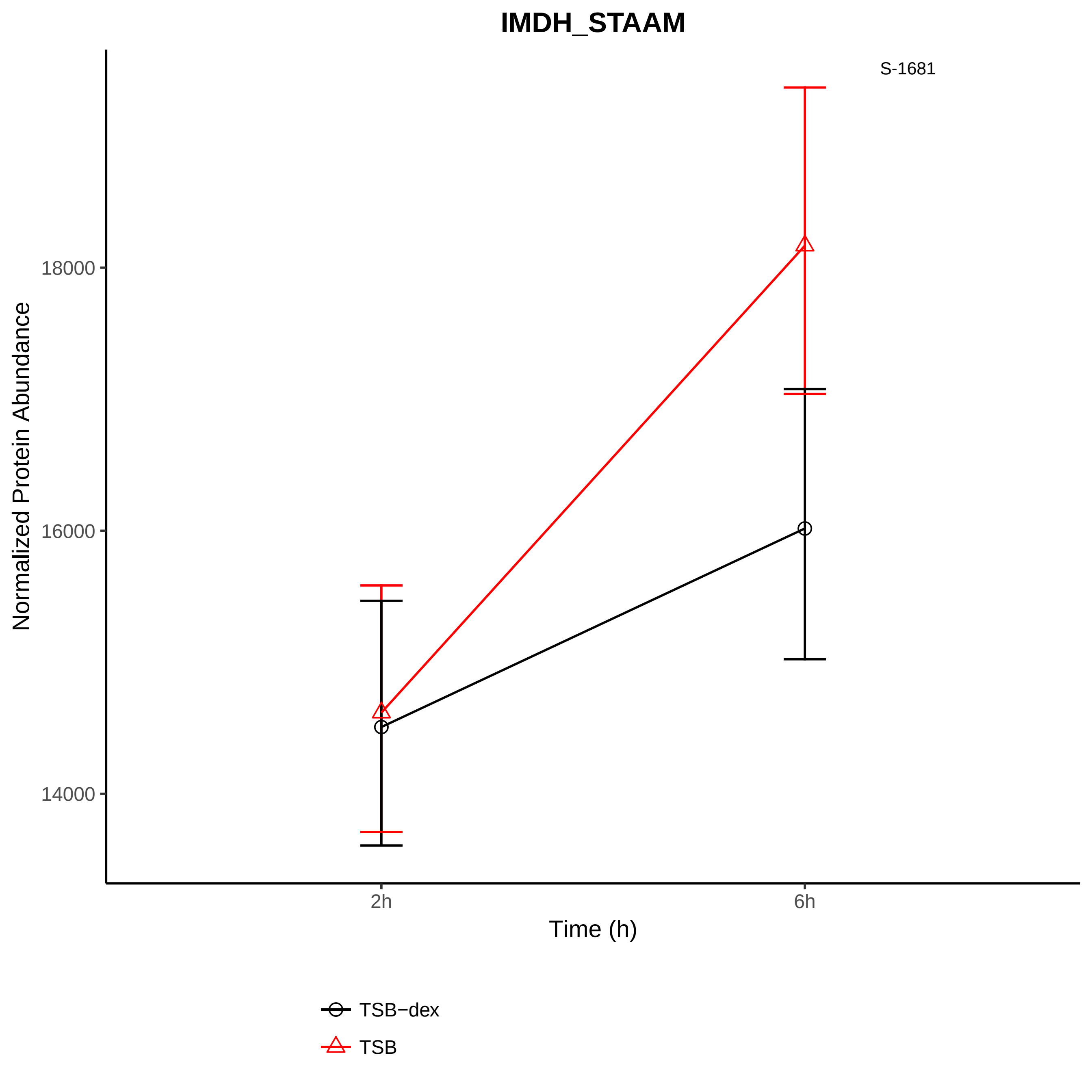

IMDH_STAAM
S-1681
Normalized Protein Abundance
18000
16000
14000
2h
6h
Time (h)
TSB−dex
TSB

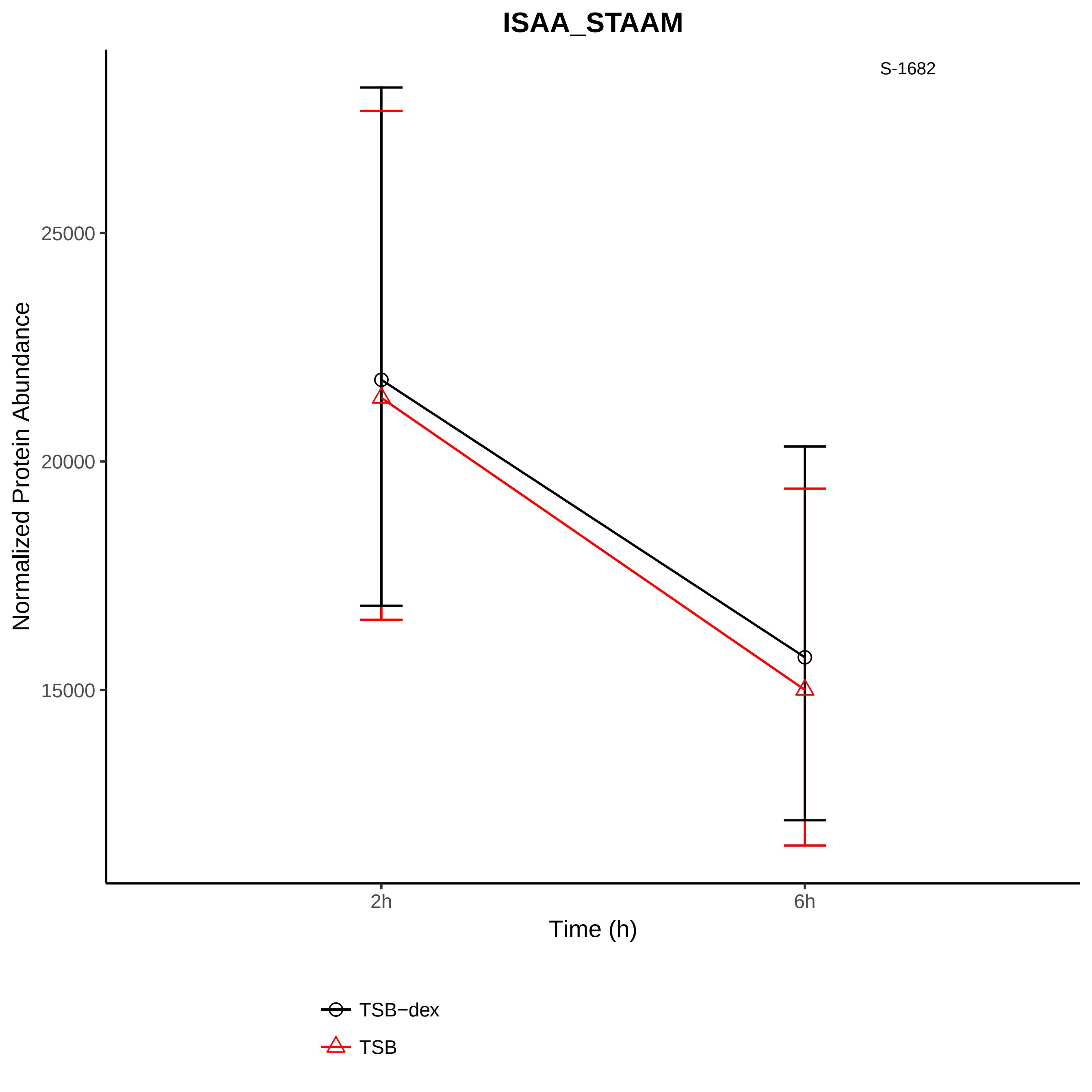

ISAA_STAAM
S-1682
Normalized Protein Abundance
25000
20000
15000
2h
6h
Time (h)
TSB−dex
TSB

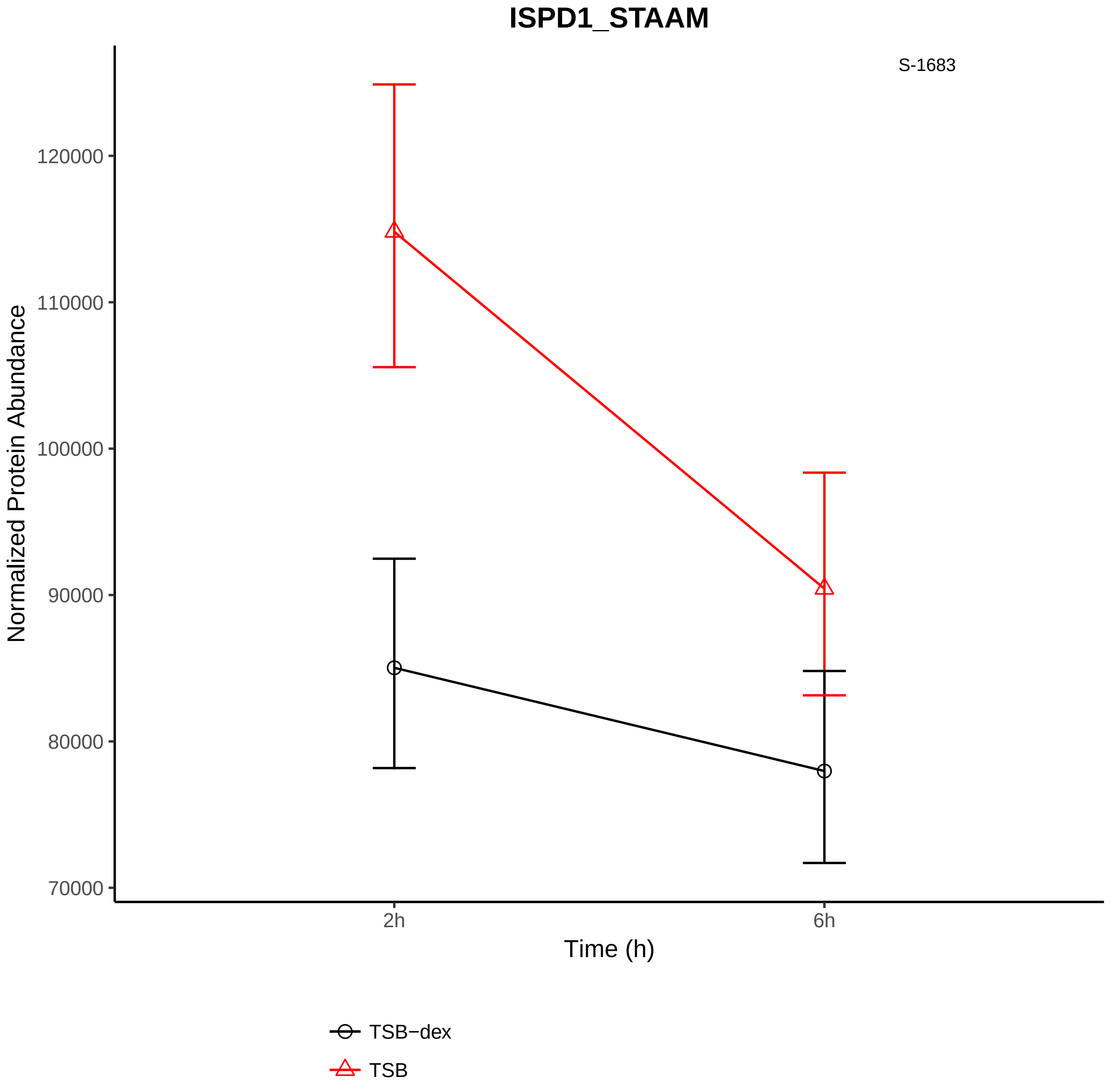

ISPD1_STAAM
S-1683
Normalized Protein Abundance
120000
110000
100000
90000
80000
70000
2h
6h
Time (h)
TSB−dex
TSB

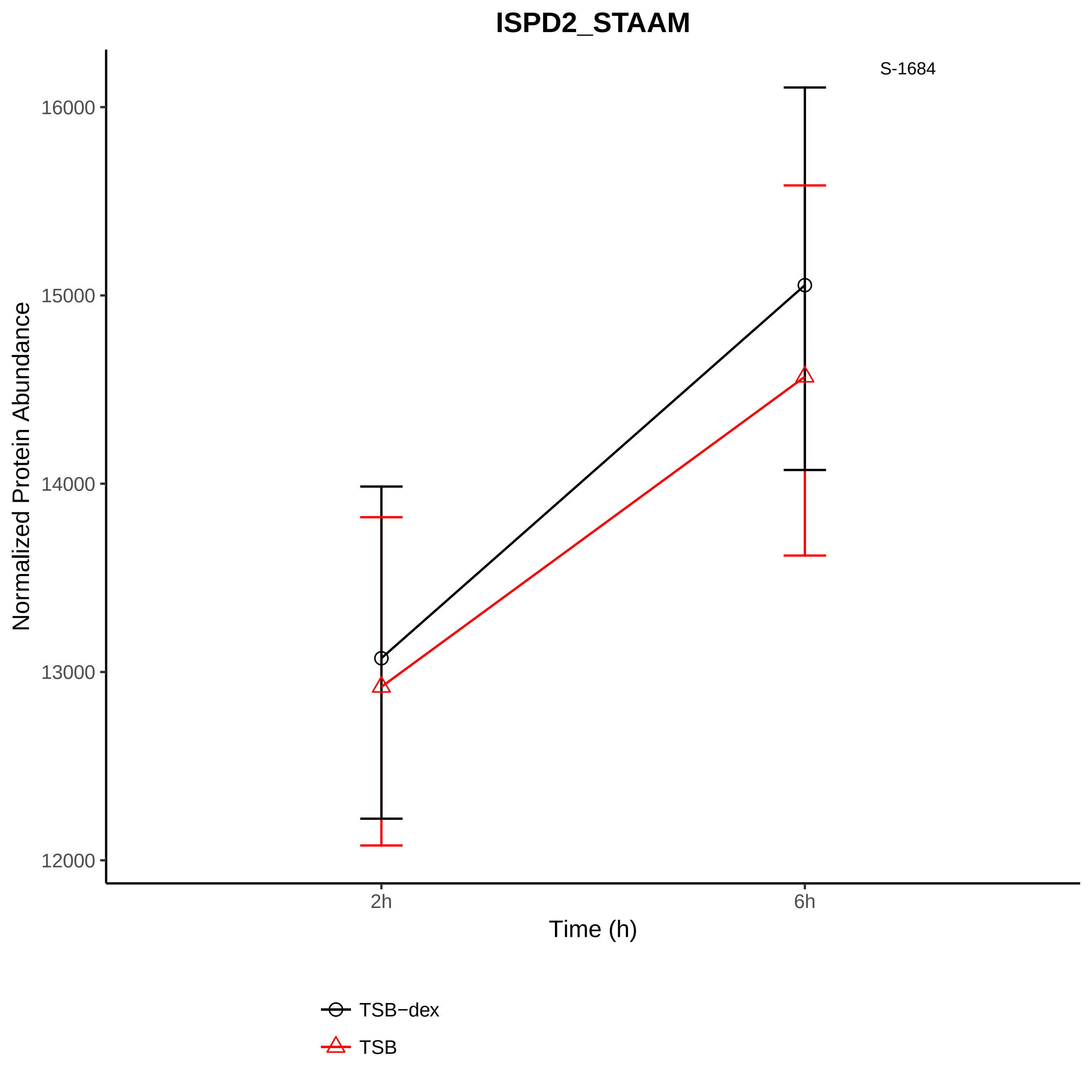
ISPD2_STAAM
S-1684
16000
15000
14000
13000
12000
Normalized Protein Abundance
2h
6h
Time (h)
TSB−dex
TSB

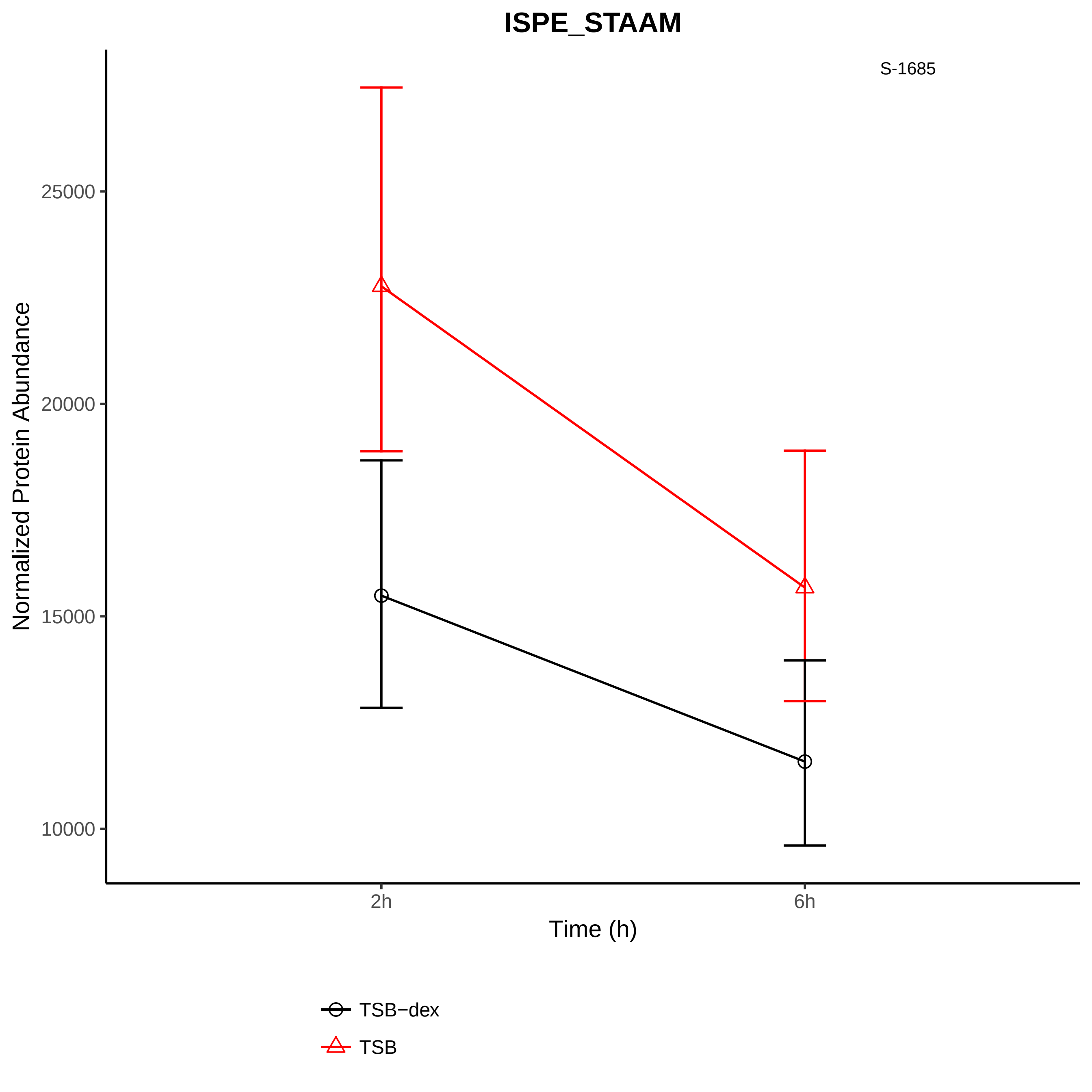

ISPE_STAAM
S-1685
Normalized Protein Abundance
25000
20000
15000
10000
2h
6h
Time (h)
TSB−dex
TSB

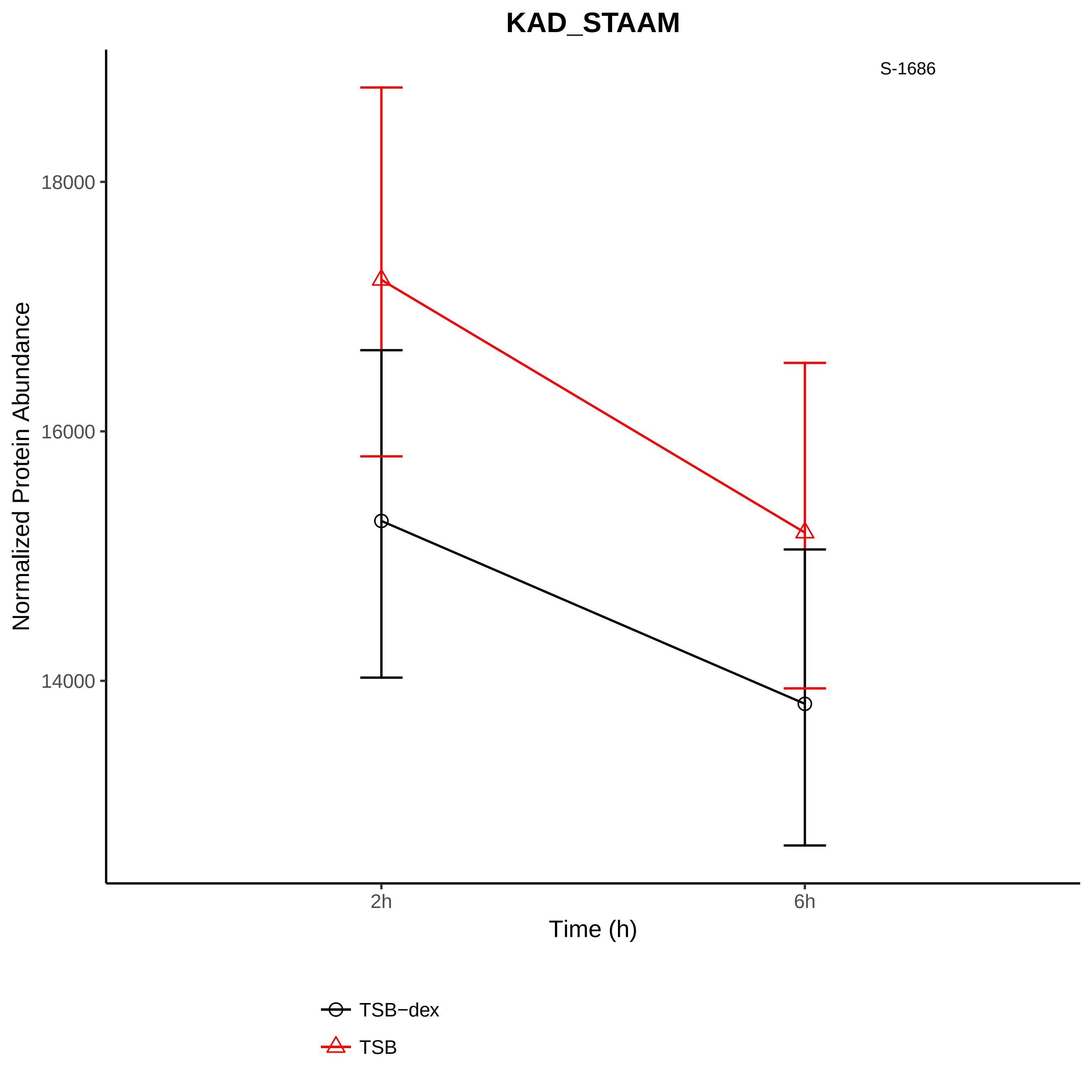

KAD_STAAM
S-1686
Normalized Protein Abundance
18000
16000
14000
2h
6h
Time (h)
TSB−dex
TSB

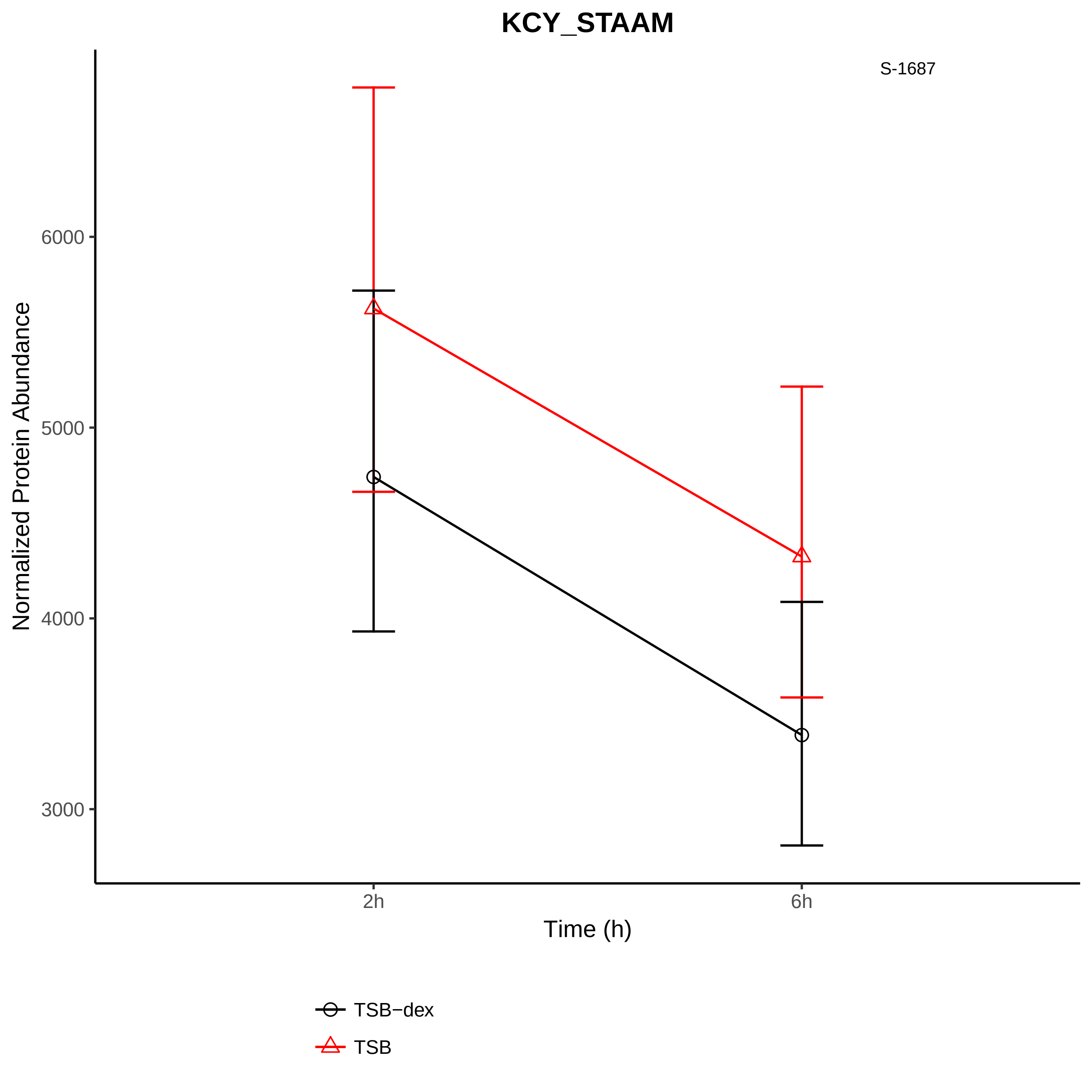
KCY_STAAM
S-1687
Normalized Protein Abundance
6000
5000
4000
3000
2h
6h
Time (h)
TSB−dex
TSB

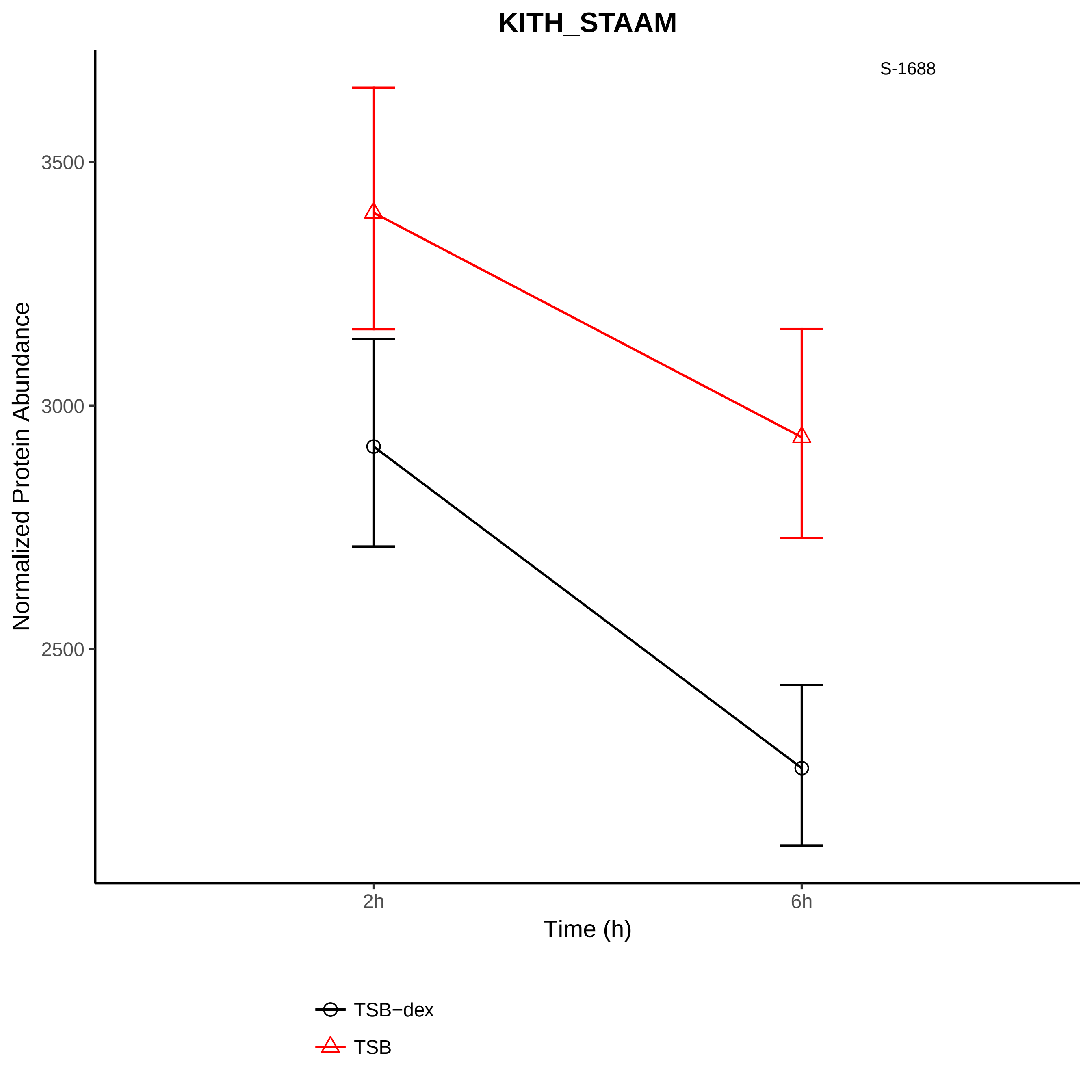

KITH_STAAM
S-1688
3500
3000
2500
Normalized Protein Abundance
2h
6h
Time (h)
TSB−dex
TSB

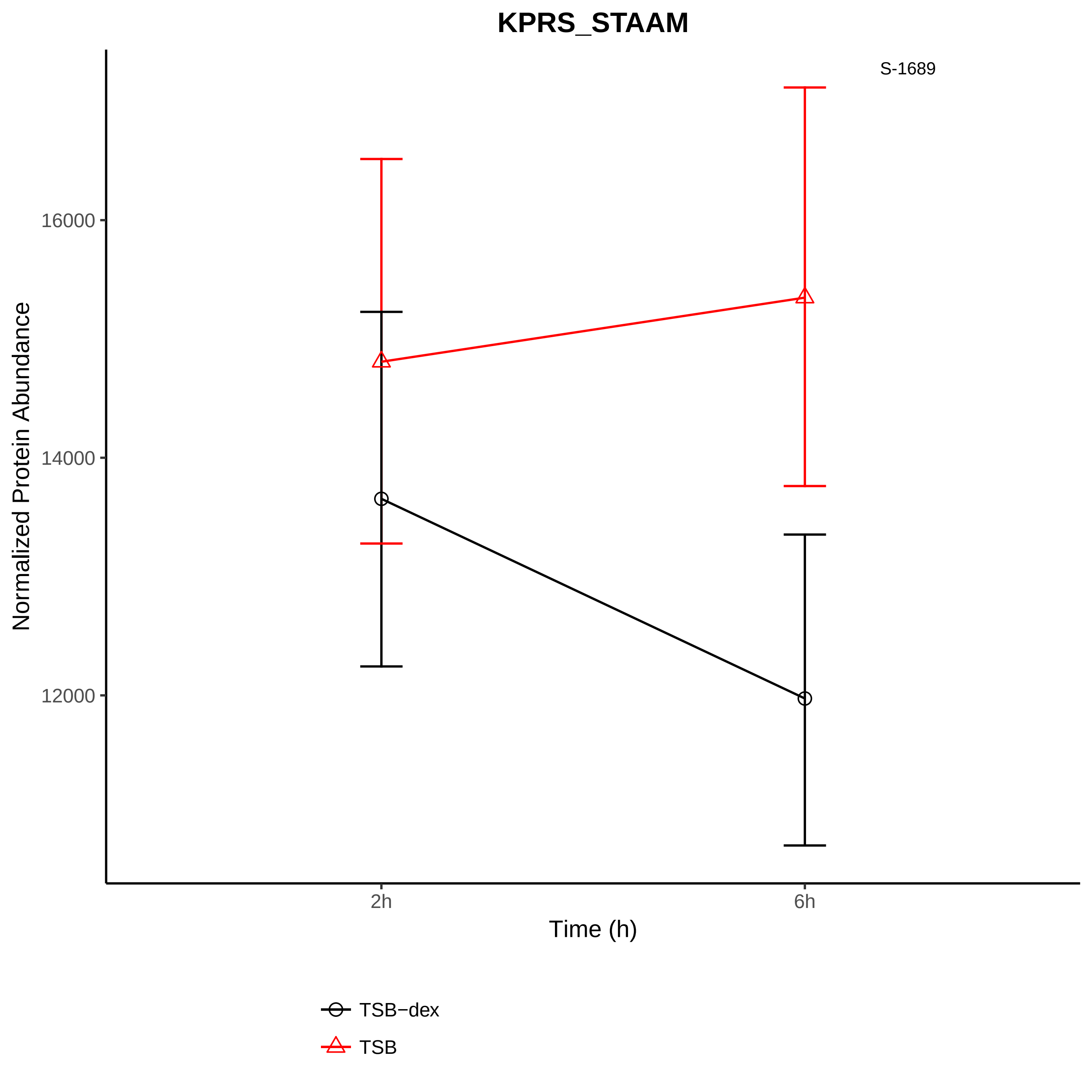
KPRS_STAAM
S-1689
16000
14000
12000
Normalized Protein Abundance
2h
6h
Time (h)
TSB−dex
TSB

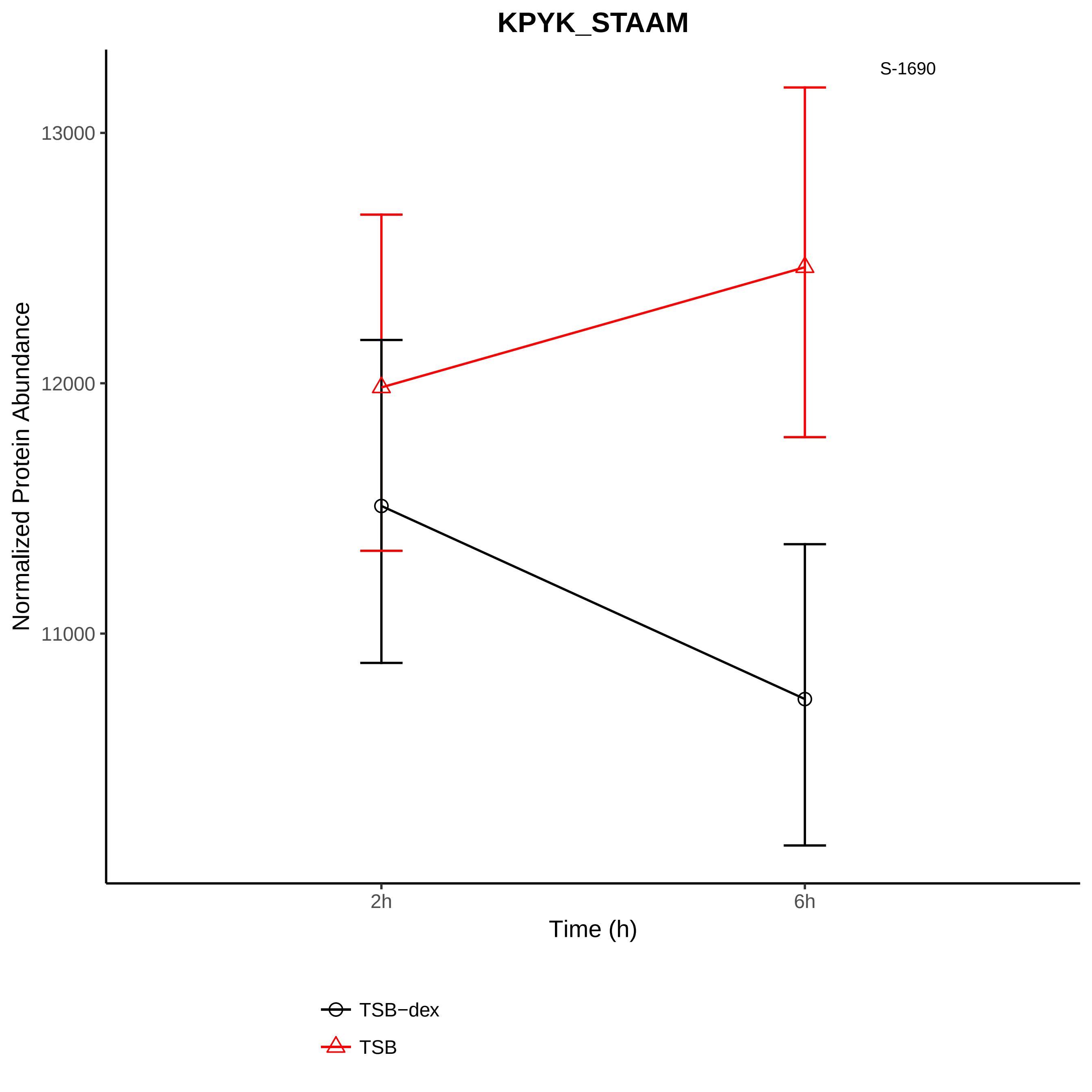

KPYK_STAAM
S-1690
13000
12000
11000
Normalized Protein Abundance
2h
6h
Time (h)
TSB−dex
TSB

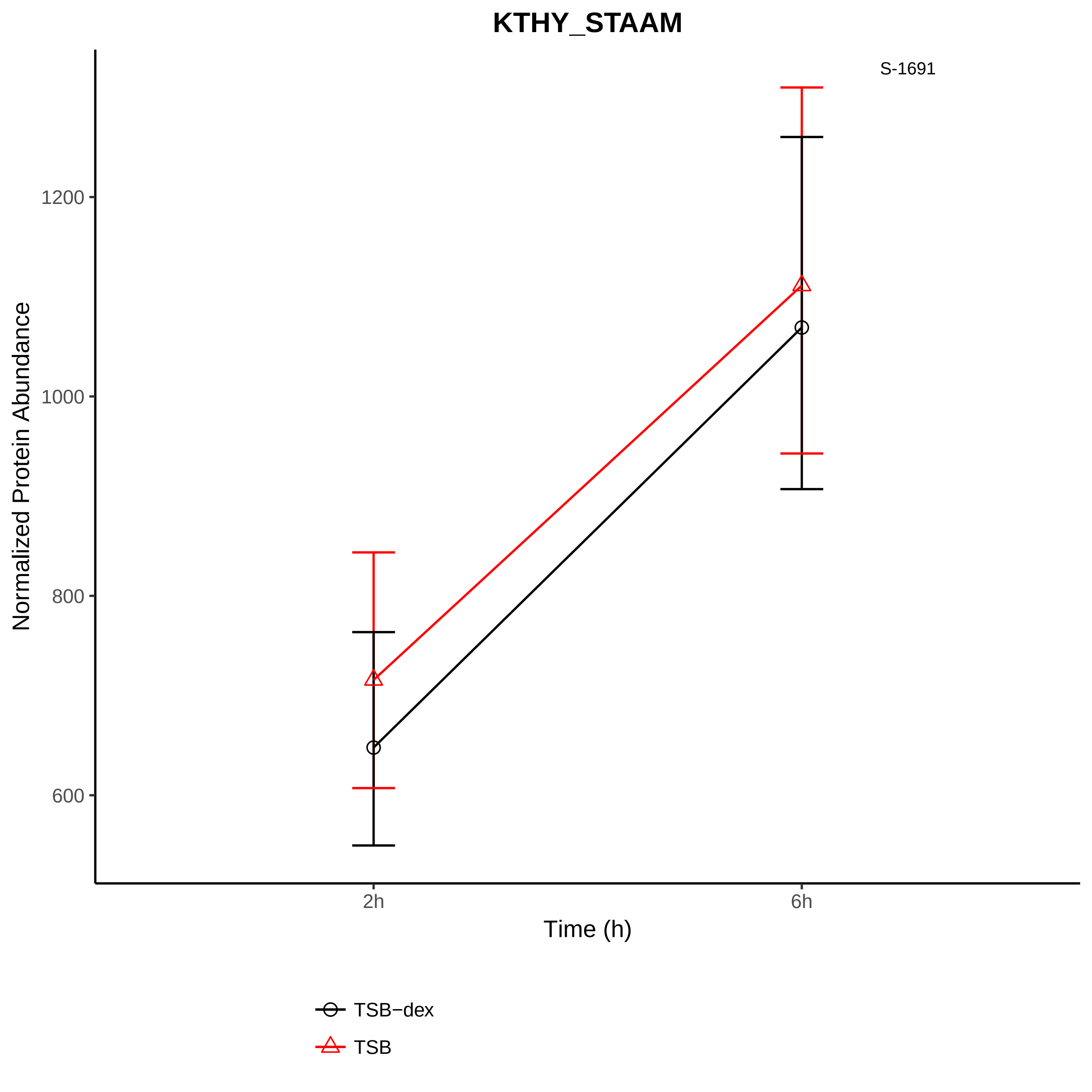

KTHY_STAAM
S-1691
1200
1000
800
600
Normalized Protein Abundance
2h
6h
Time (h)
TSB−dex
TSB

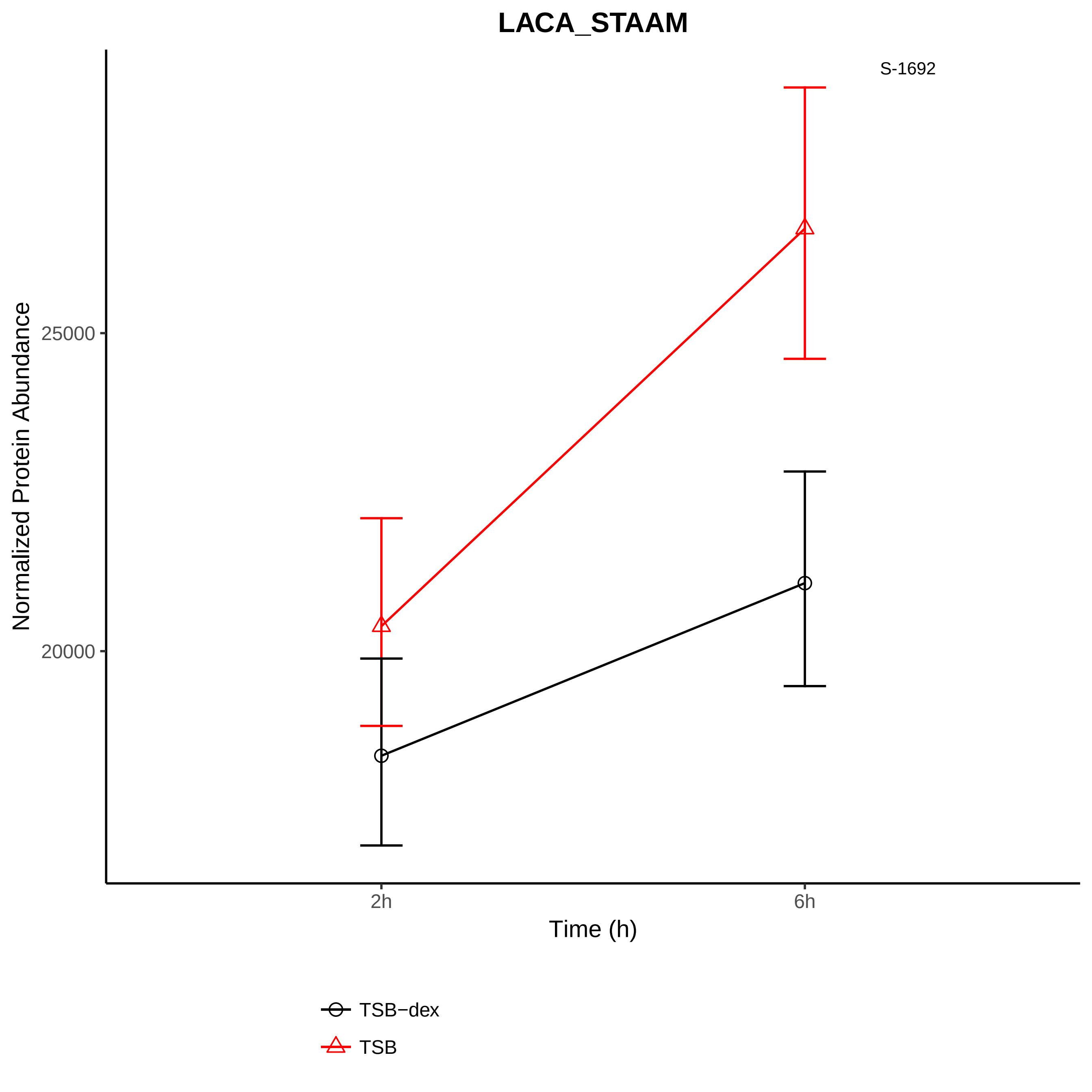
LACA_STAAM
S-1692
Normalized Protein Abundance
25000
20000
2h
6h
Time (h)
TSB−dex
TSB

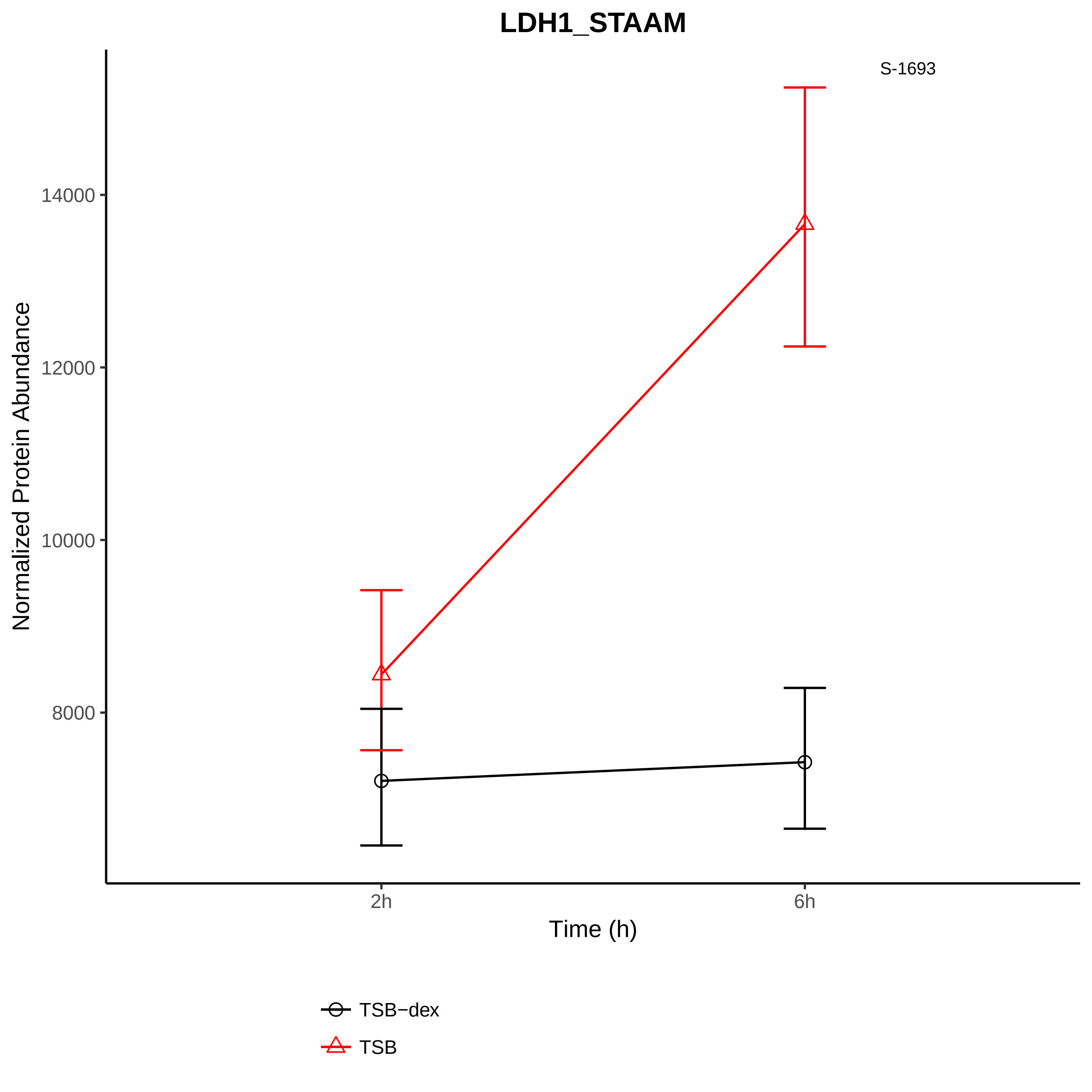

LDH1_STAAM
S-1693
Normalized Protein Abundance
14000
12000
10000
8000
2h
6h
Time (h)
TSB−dex
TSB

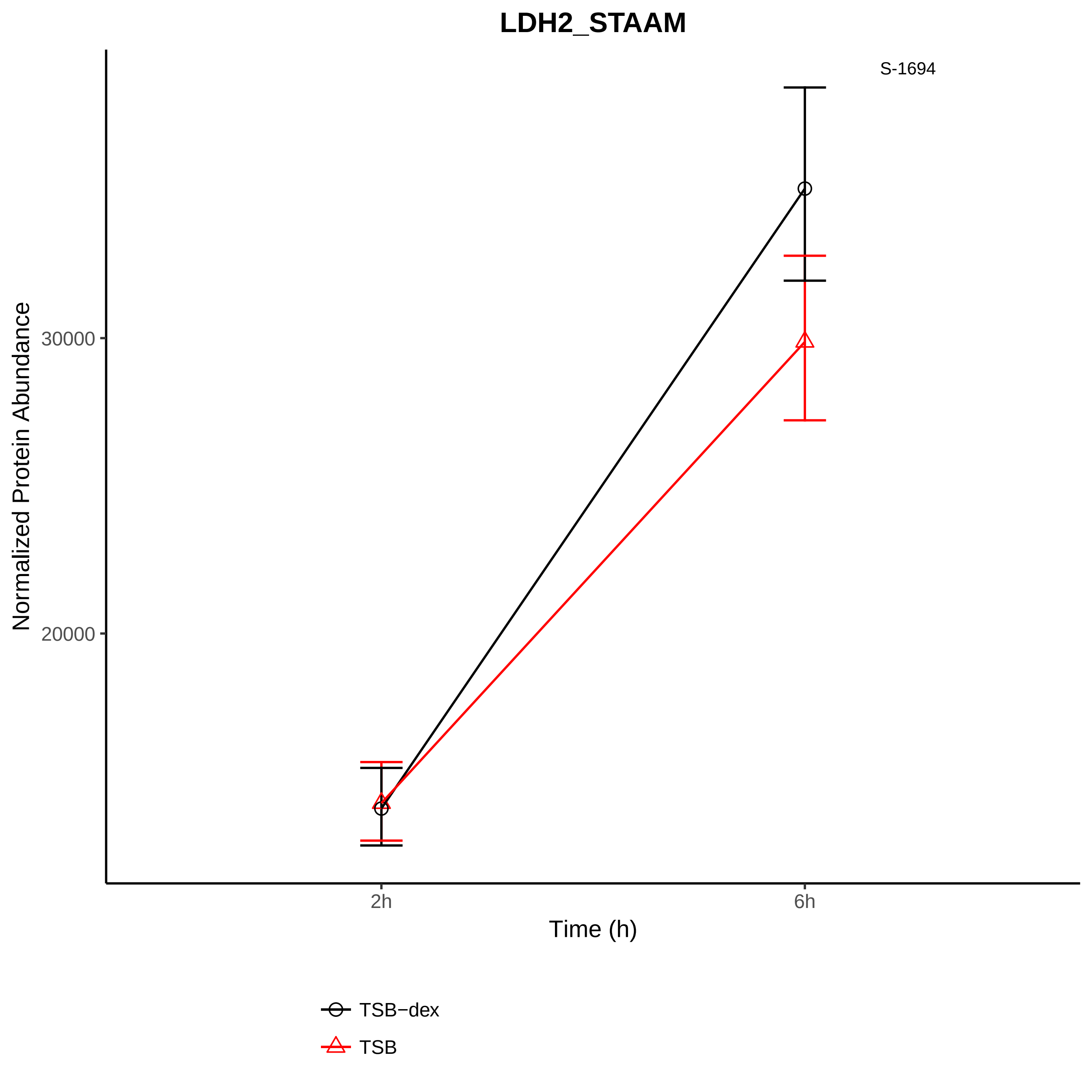

LDH2_STAAM
S-1694
Normalized Protein Abundance
30000
20000
2h
6h
Time (h)
TSB−dex
TSB

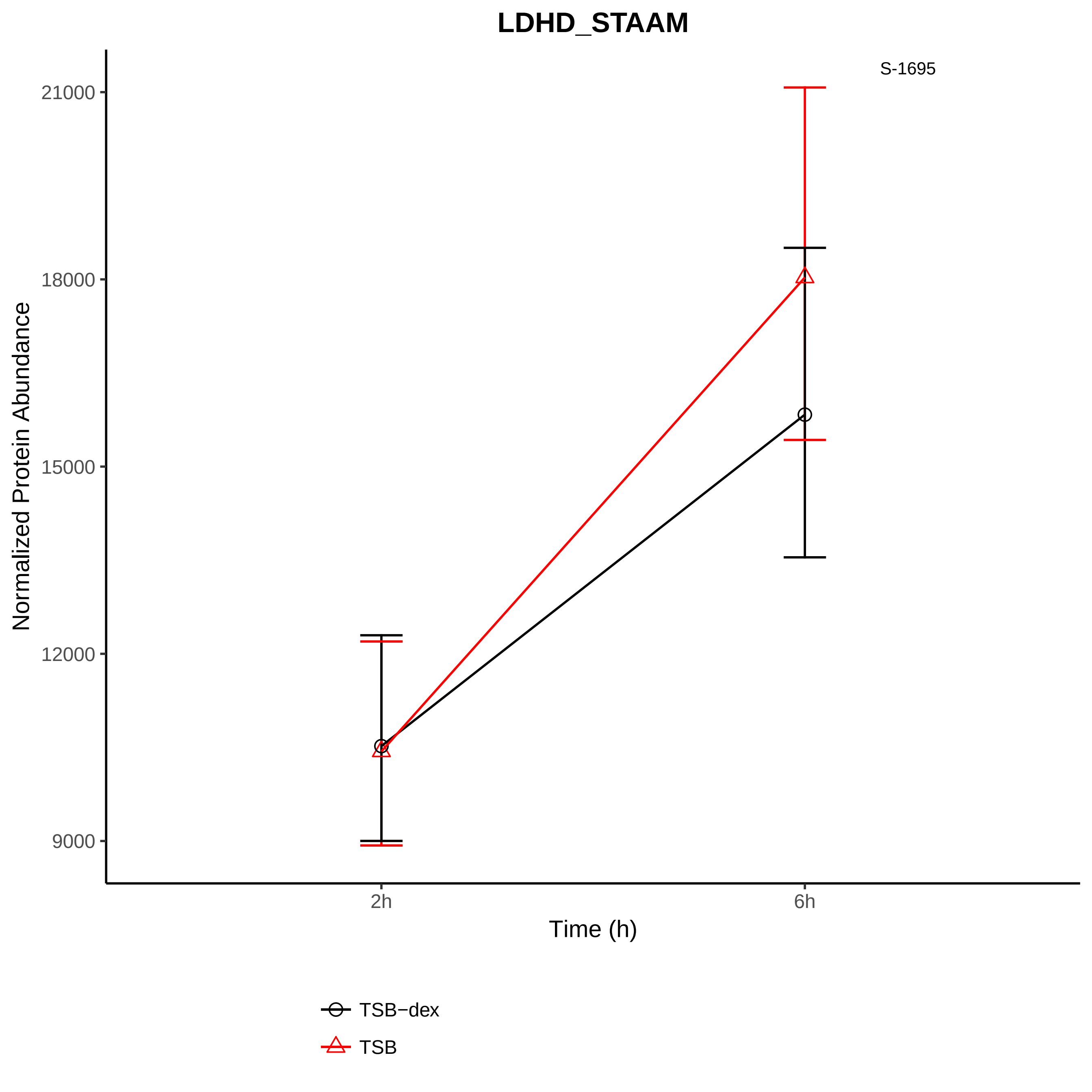

LDHD_STAAM
S-1695
21000
18000
15000
12000
9000
Normalized Protein Abundance
2h
6h
Time (h)
TSB−dex
TSB

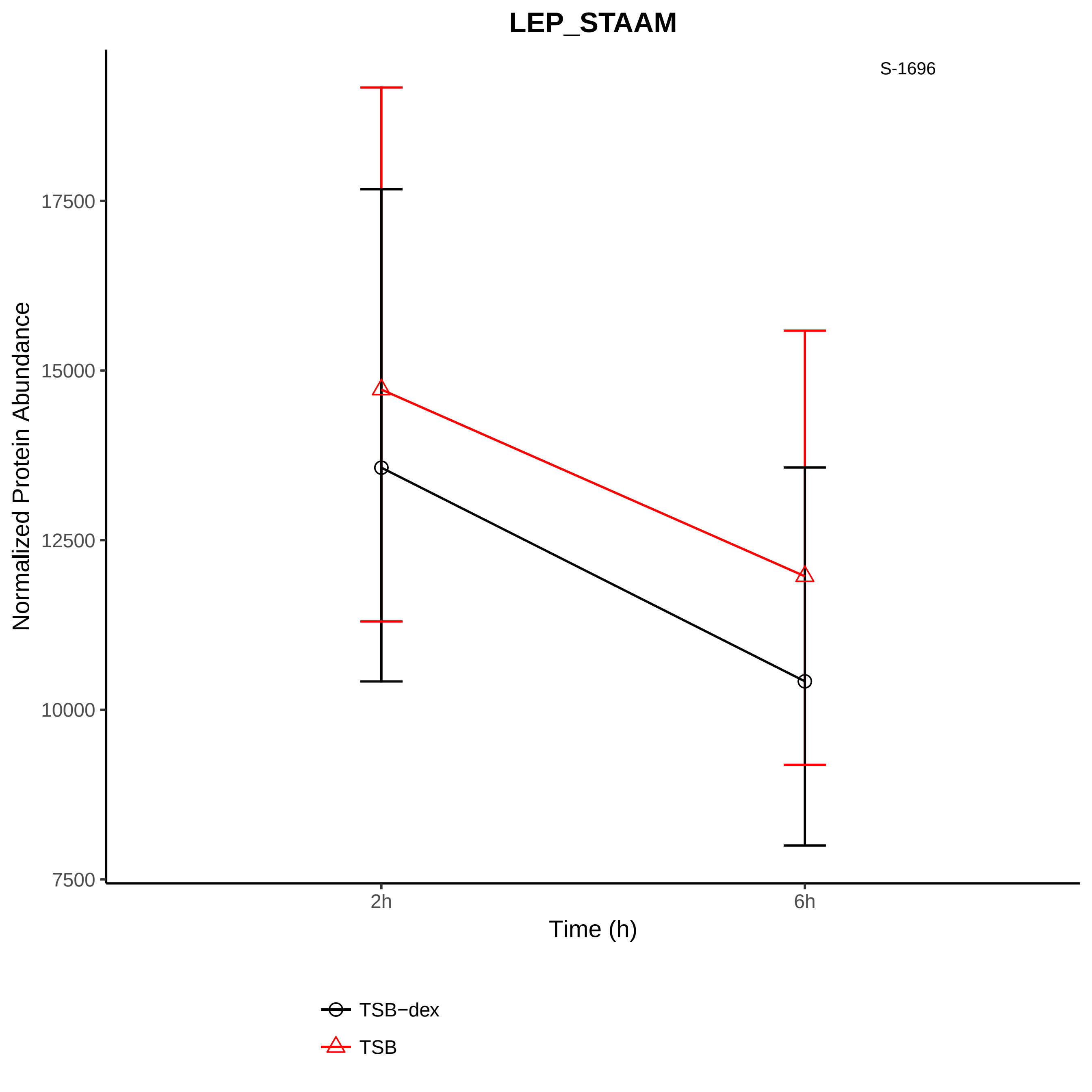

LEP_STAAM
S-1696
Normalized Protein Abundance
17500
15000
12500
10000
7500
2h
6h
Time (h)
TSB−dex
TSB

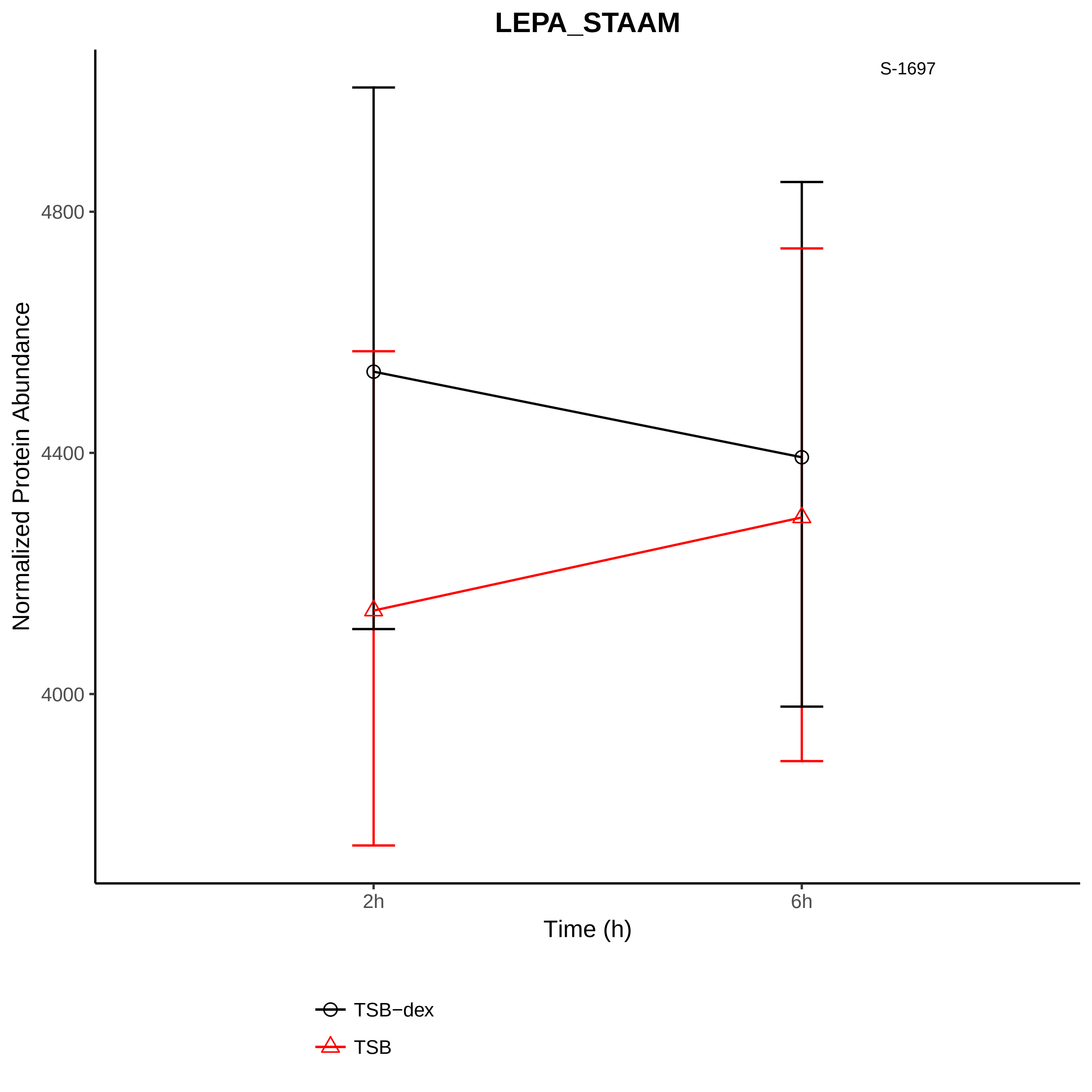
LEPA_STAAM
S-1697
Normalized Protein Abundance
4800
4400
4000
2h
6h
Time (h)
TSB−dex
TSB

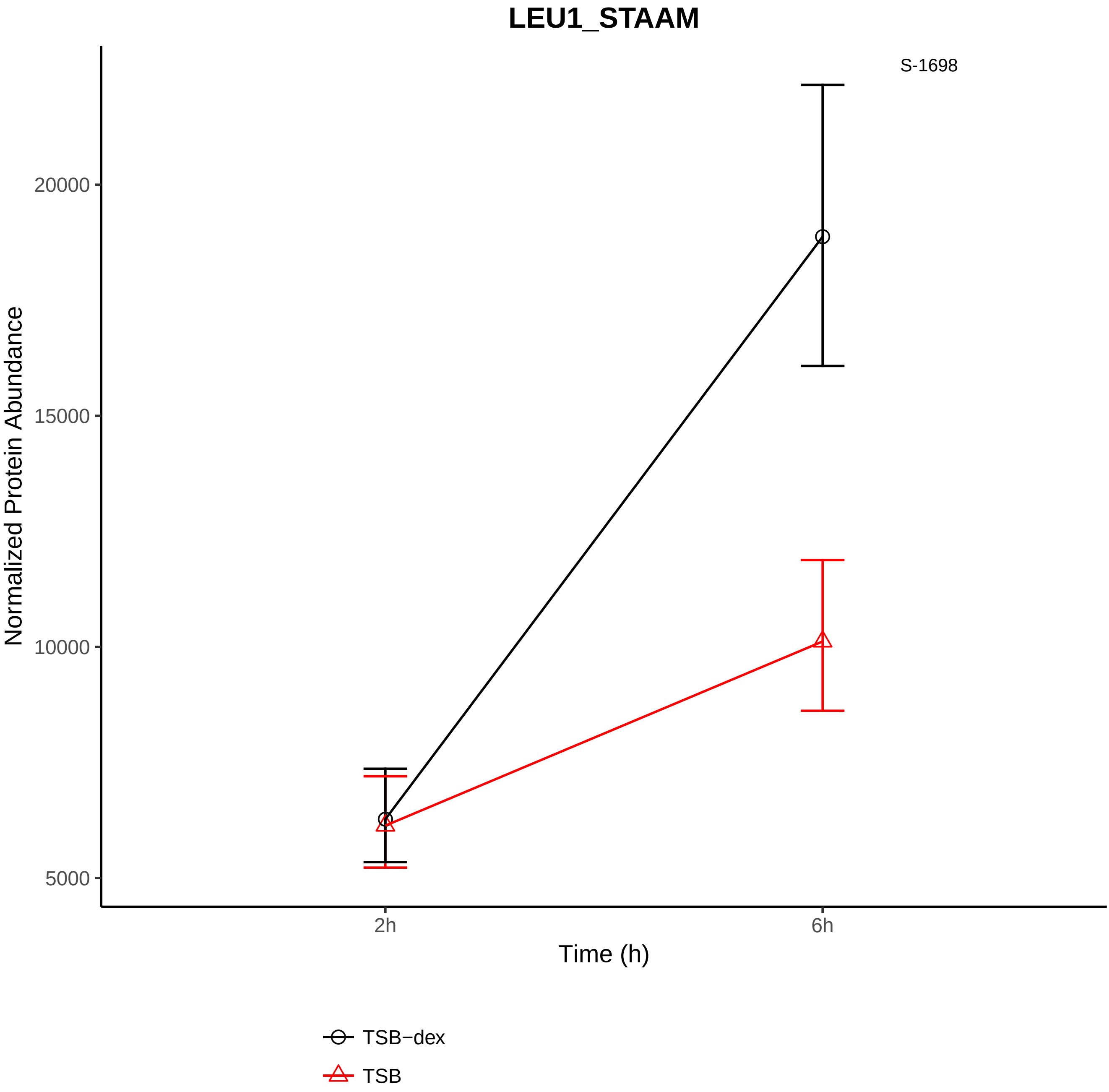
LEU1_STAAM
S-1698
Normalized Protein Abundance
20000
15000
10000
5000
2h
6h
Time (h)
TSB−dex
TSB

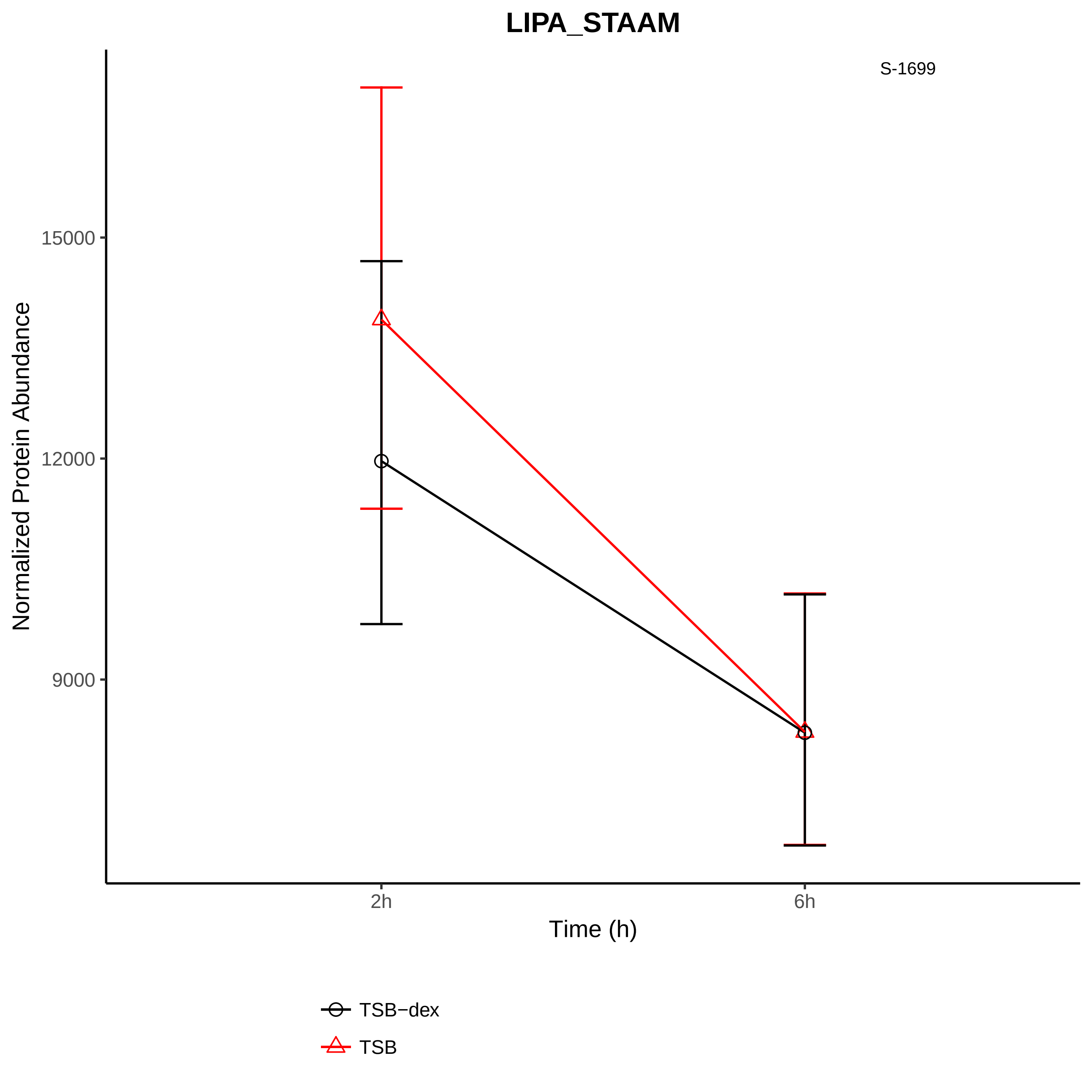

LIPA_STAAM
S-1699
Normalized Protein Abundance
15000
12000
9000
2h
6h
Time (h)
TSB−dex
TSB

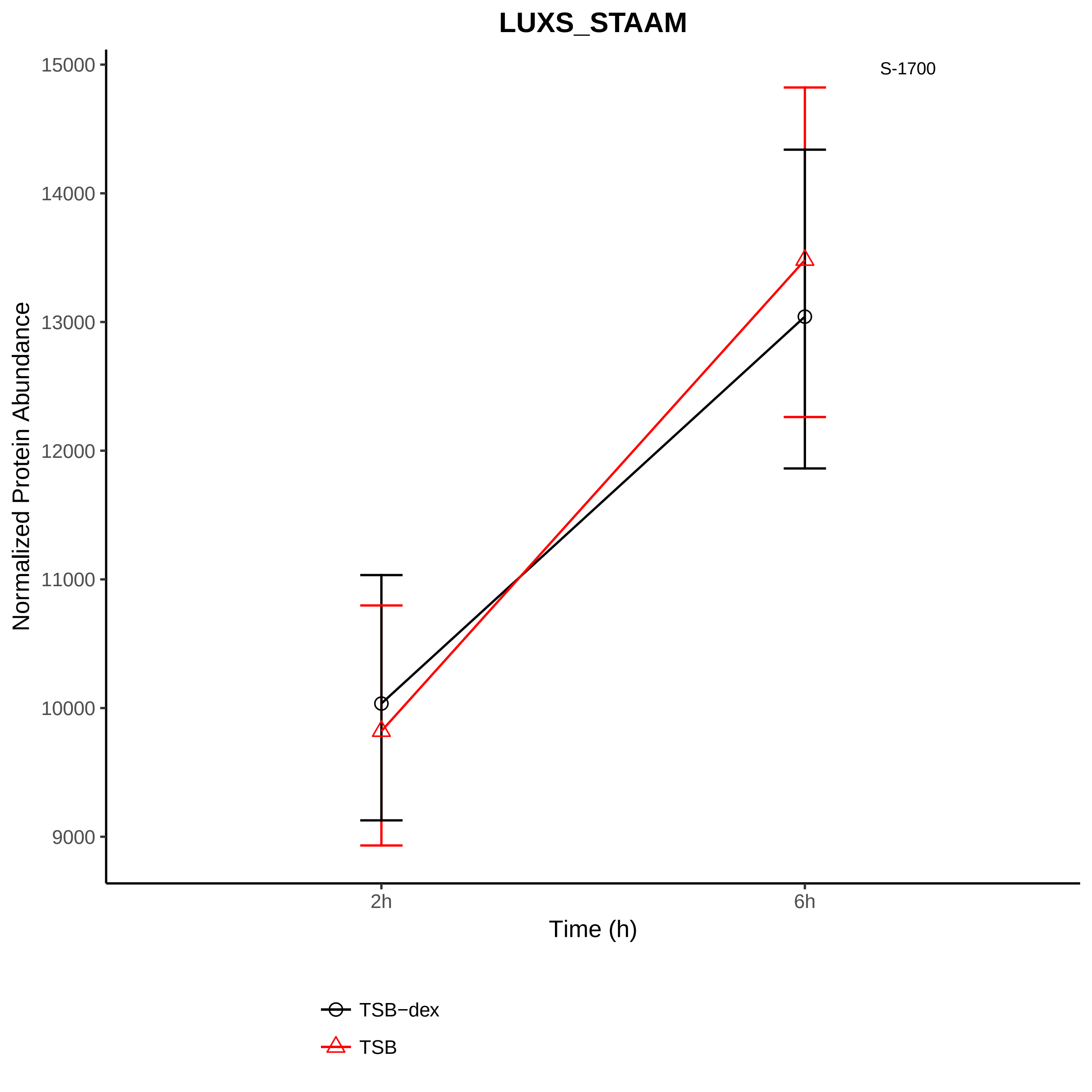

LUXS_STAAM
S-1700
Normalized Protein Abundance
15000
14000
13000
12000
11000
10000
9000
2h
6h
Time (h)
TSB−dex
TSB

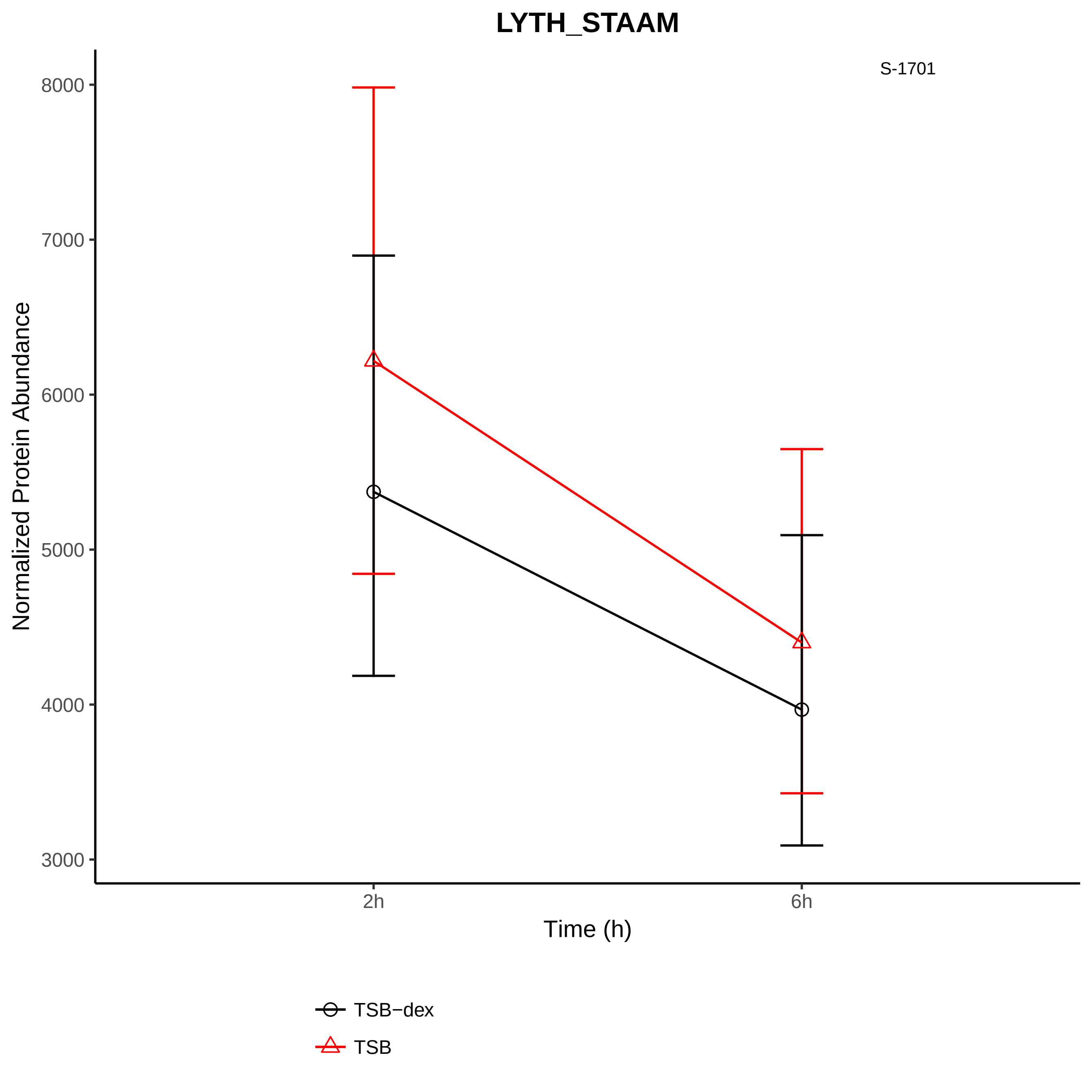

LYTH_STAAM
S-1701
8000
7000
6000
5000
4000
3000
Normalized Protein Abundance
2h
6h
Time (h)
TSB−dex
TSB

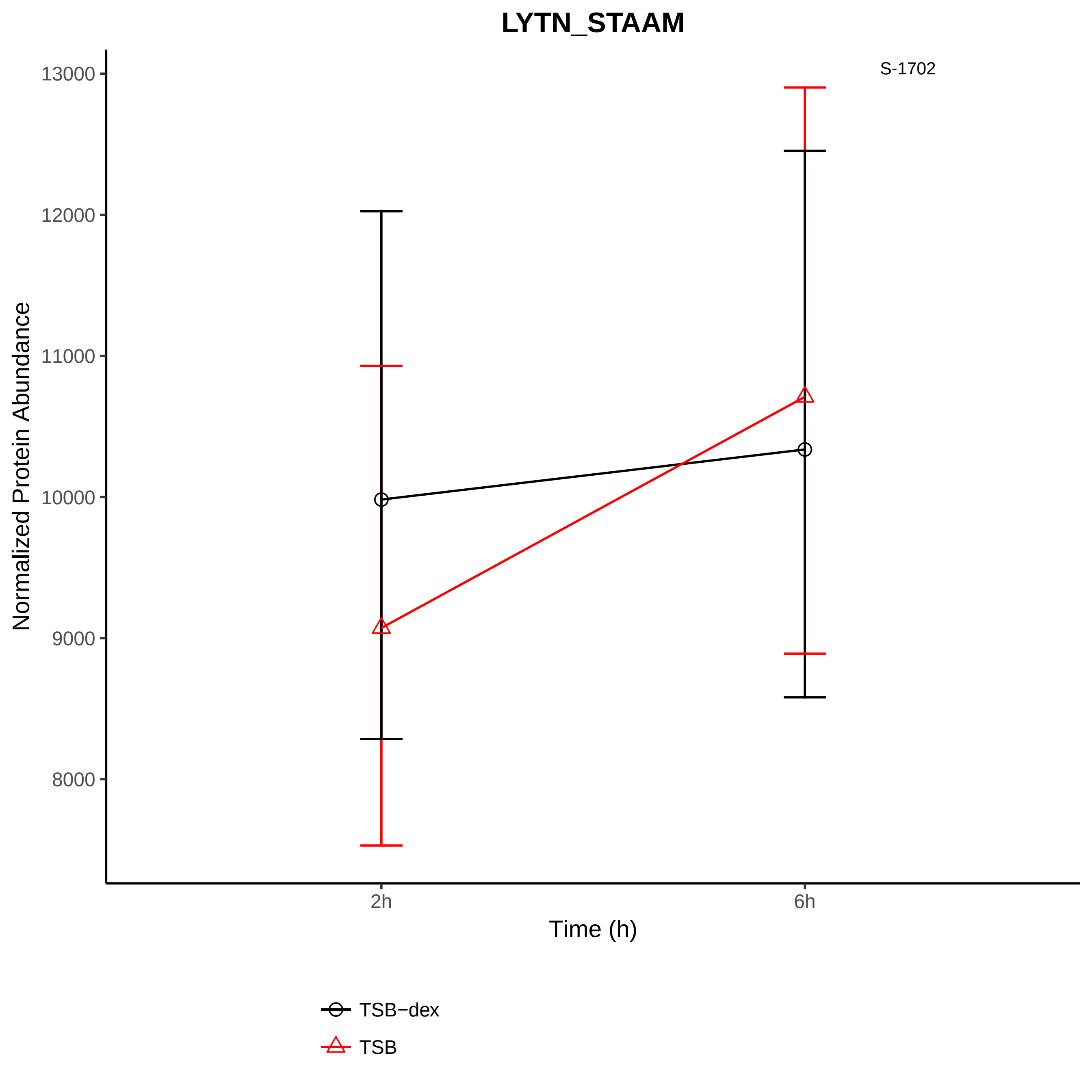

LYTN_STAAM
S-1702
Normalized Protein Abundance
13000
12000
11000
10000
9000
8000
2h
6h
Time (h)
TSB−dex
TSB

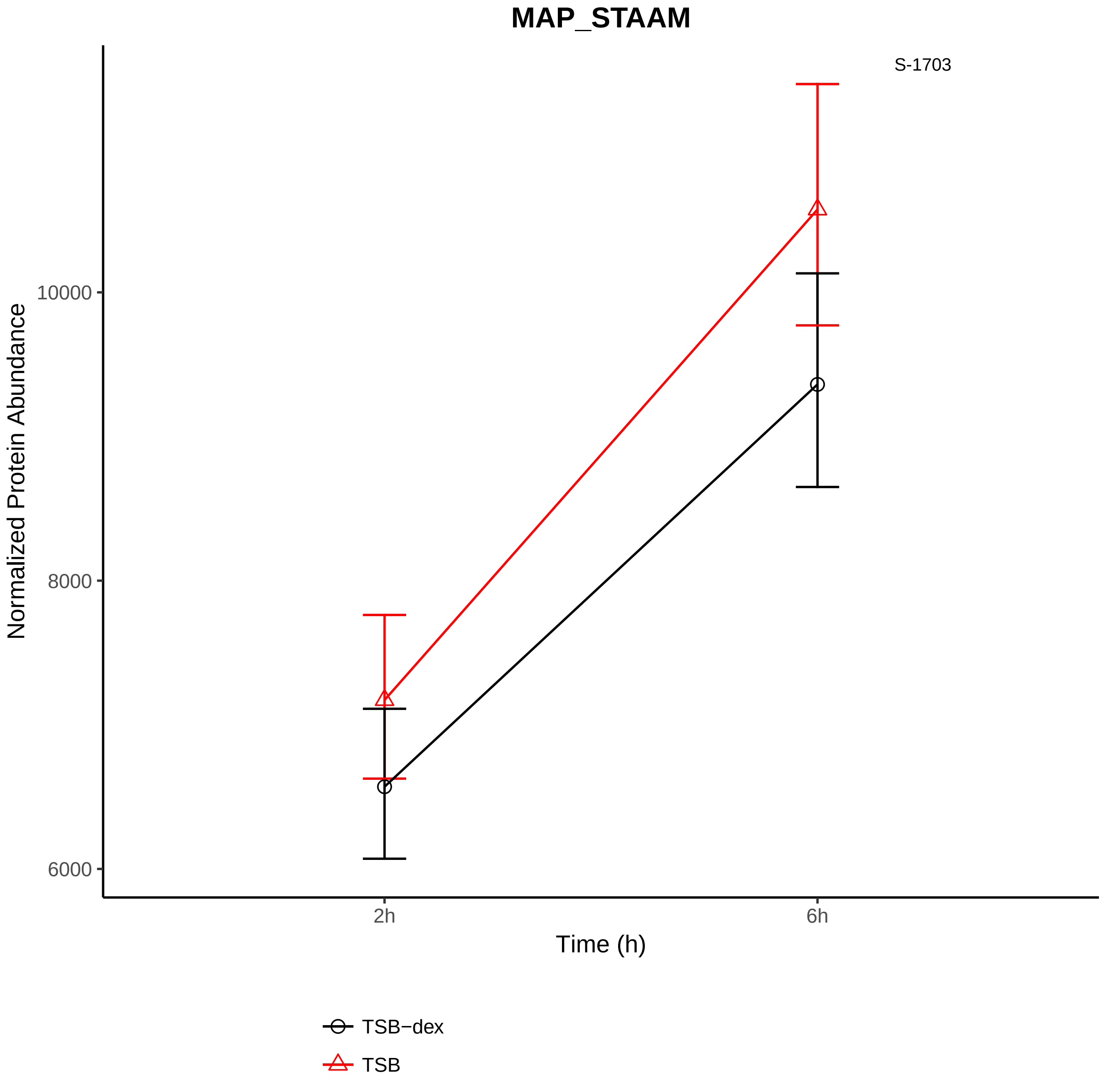

MAP_STAAM
S-1703
Normalized Protein Abundance
10000
8000
6000
2h
6h
Time (h)
TSB−dex
TSB

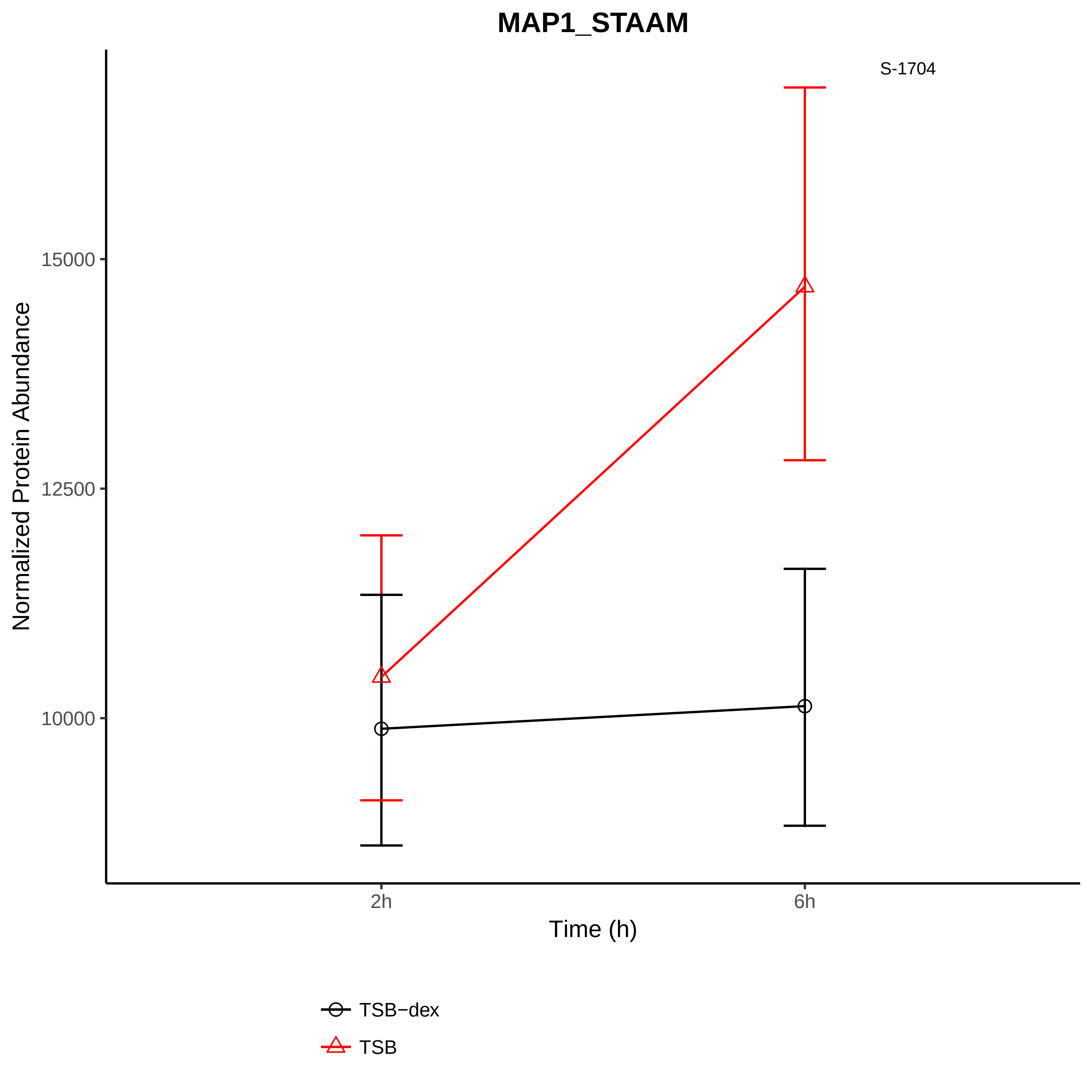

MAP1_STAAM
S-1704
15000
12500
10000
Normalized Protein Abundance
2h
6h
Time (h)
TSB−dex
TSB

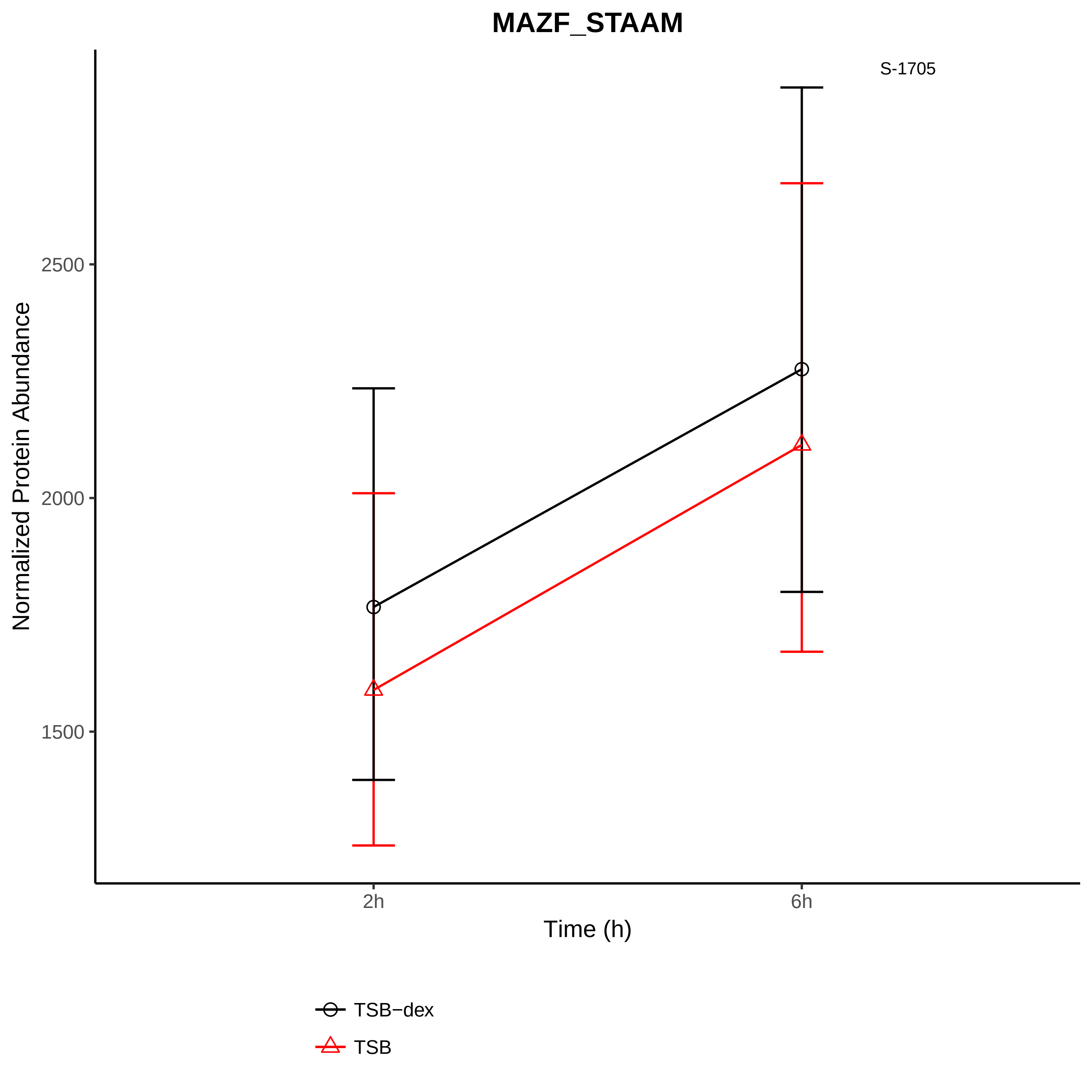

MAZF_STAAM
S-1705
Normalized Protein Abundance
2500
2000
1500
2h
6h
Time (h)
TSB−dex
TSB

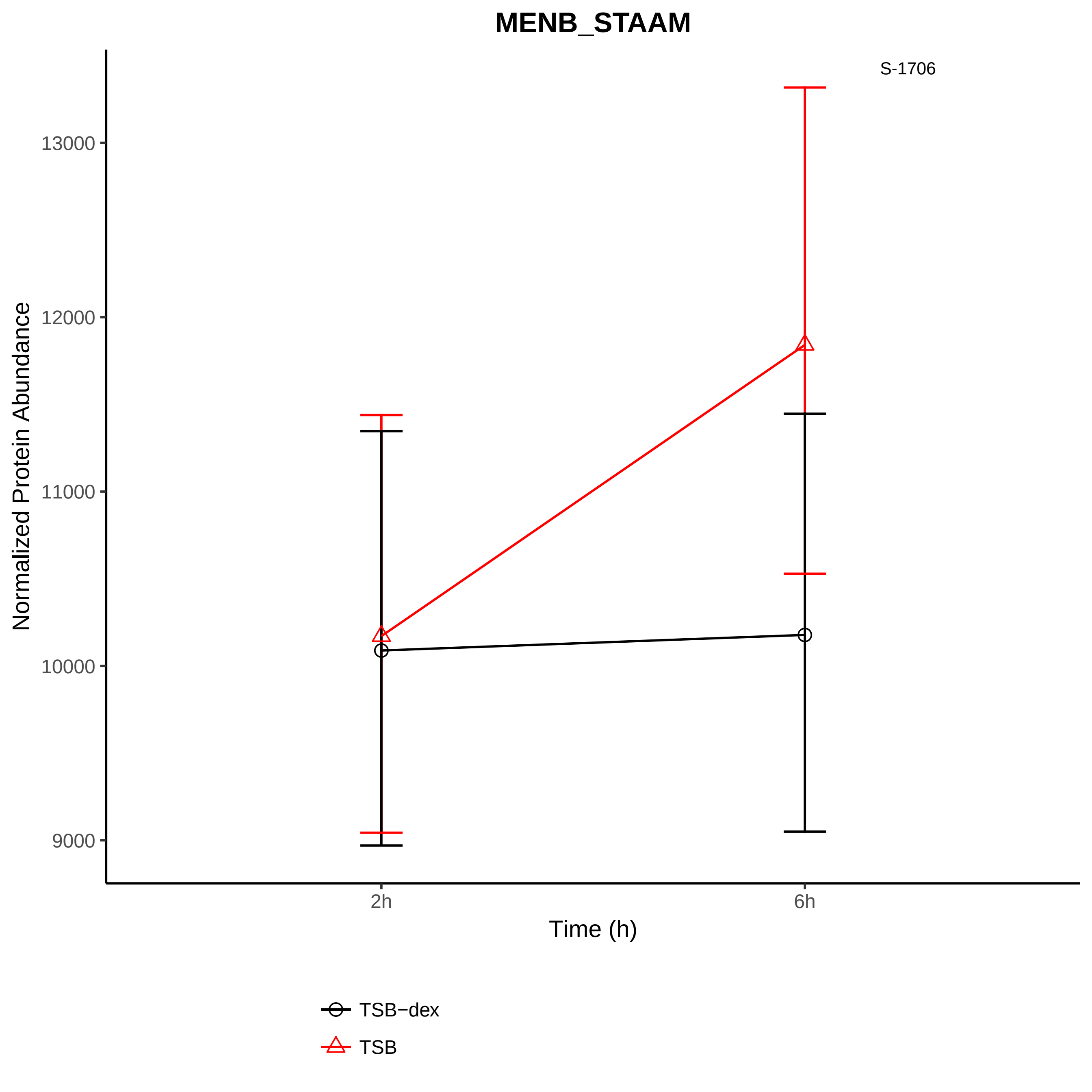

MENB_STAAM
S-1706
Normalized Protein Abundance
13000
12000
11000
10000
9000
2h
6h
Time (h)
TSB−dex
TSB

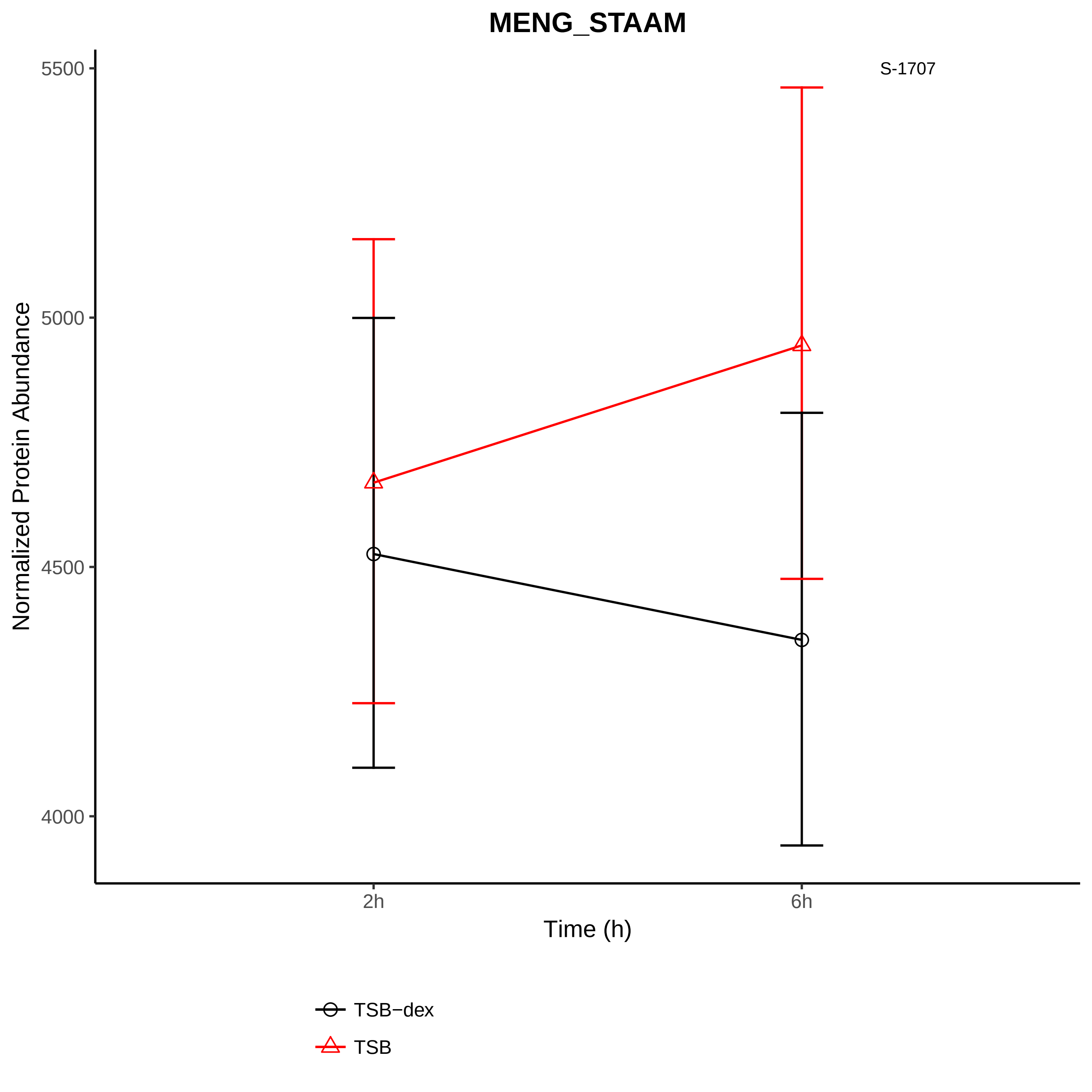

MENG_STAAM
S-1707
5500
5000
4500
4000
Normalized Protein Abundance
2h
6h
Time (h)
TSB−dex
TSB

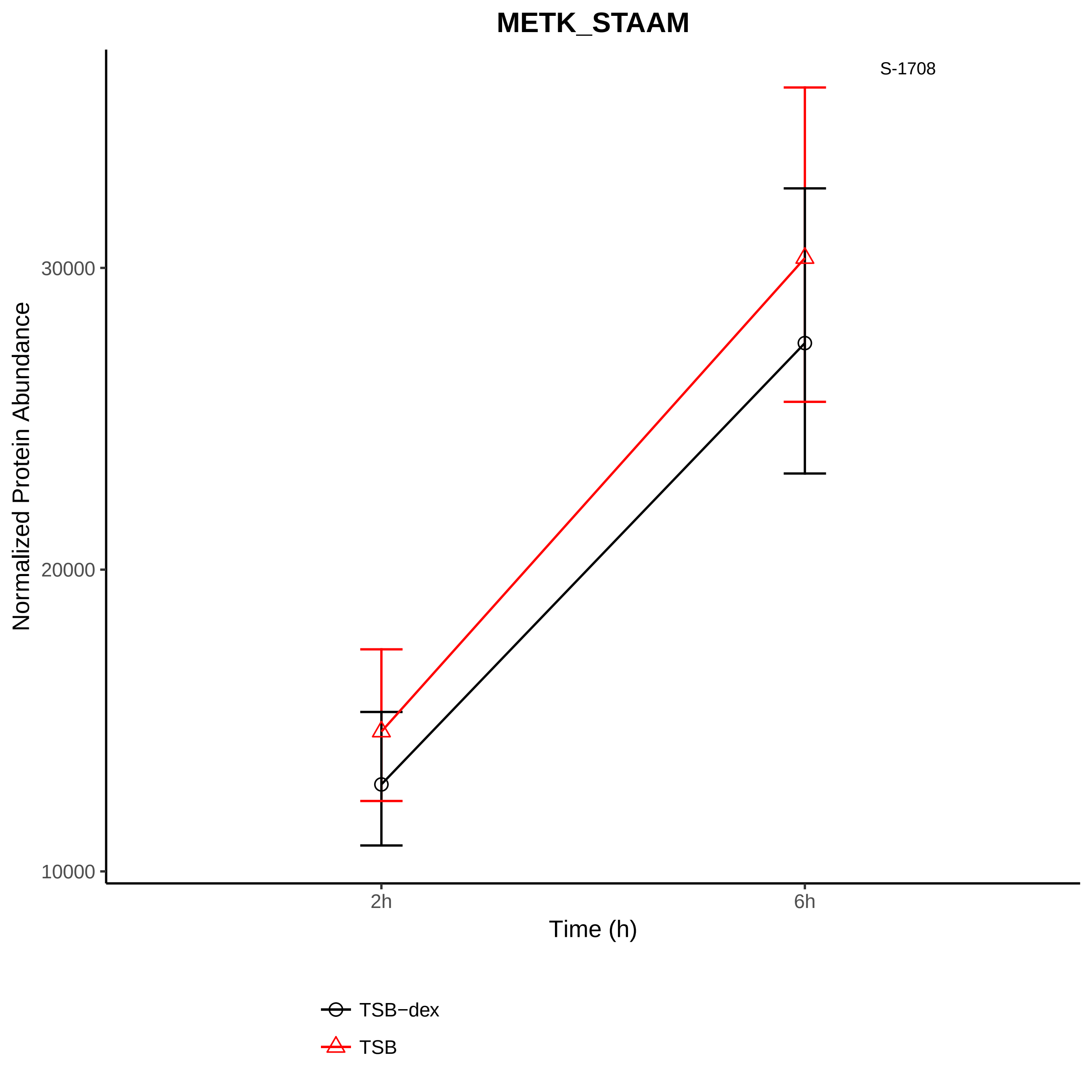

METK_STAAM
S-1708
Normalized Protein Abundance
30000
20000
10000
2h
6h
Time (h)
TSB−dex
TSB

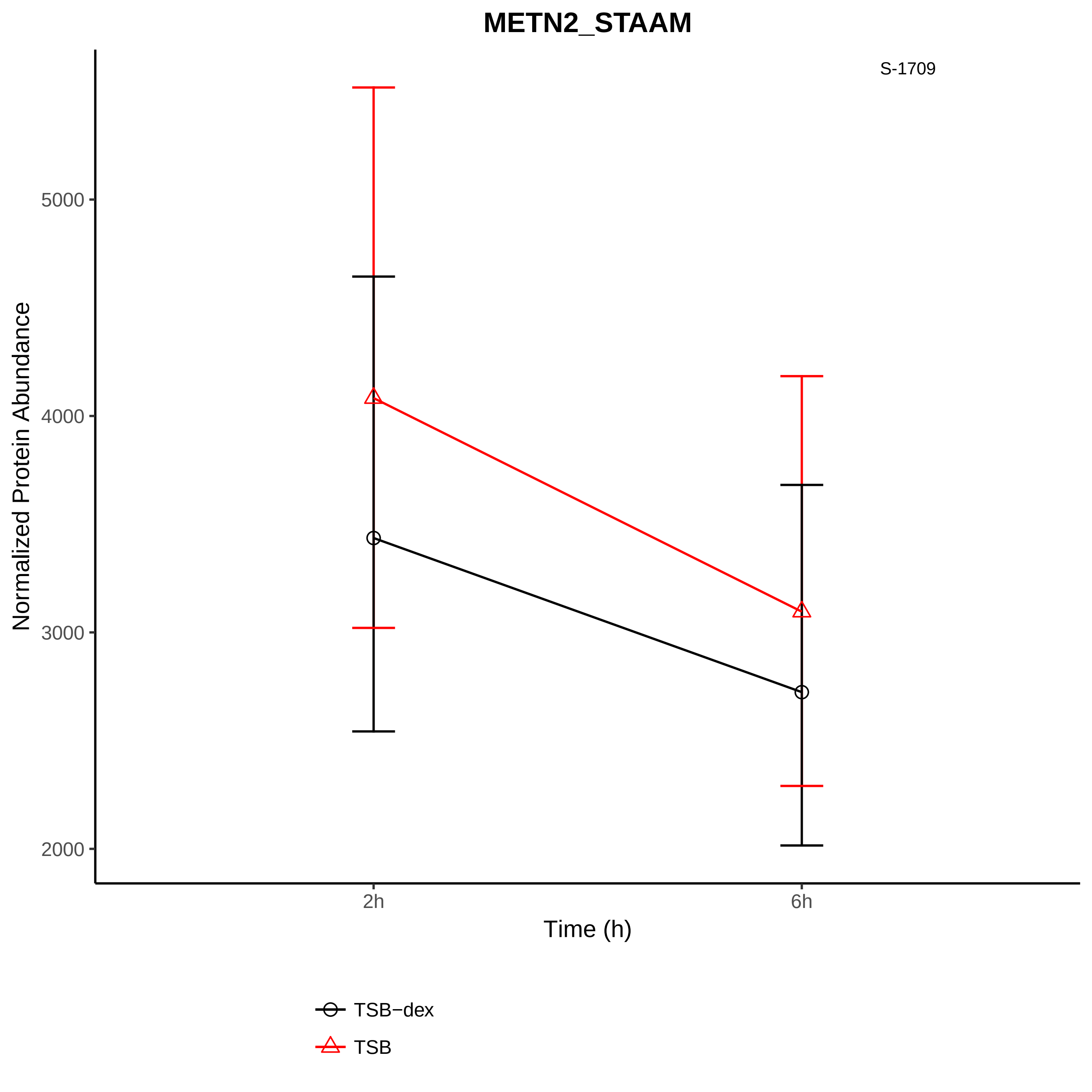

METN2_STAAM
S-1709
Normalized Protein Abundance
5000
4000
3000
2000
2h
6h
Time (h)
TSB−dex
TSB

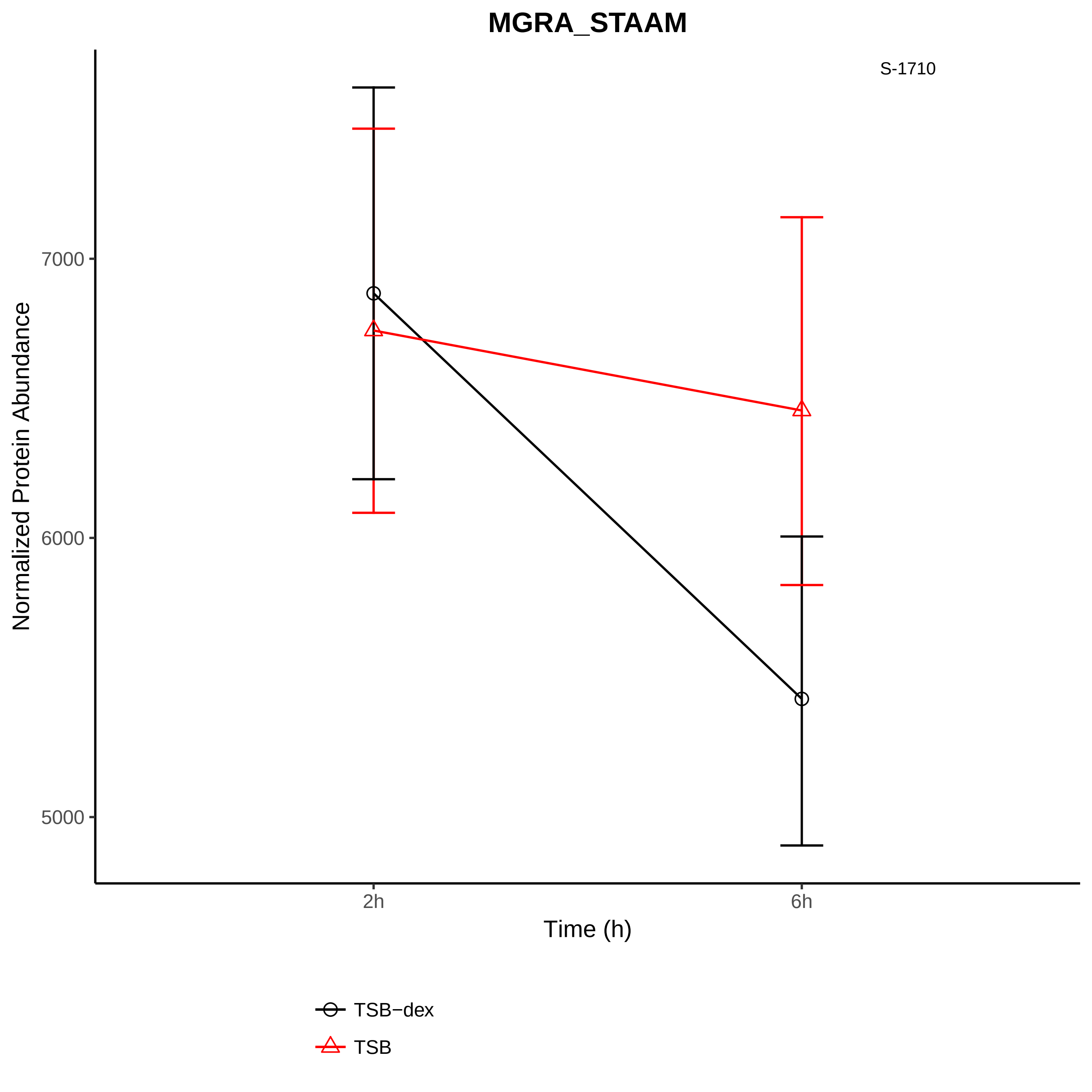

MGRA_STAAM
S-1710
Normalized Protein Abundance
7000
6000
5000
2h
6h
Time (h)
TSB−dex
TSB

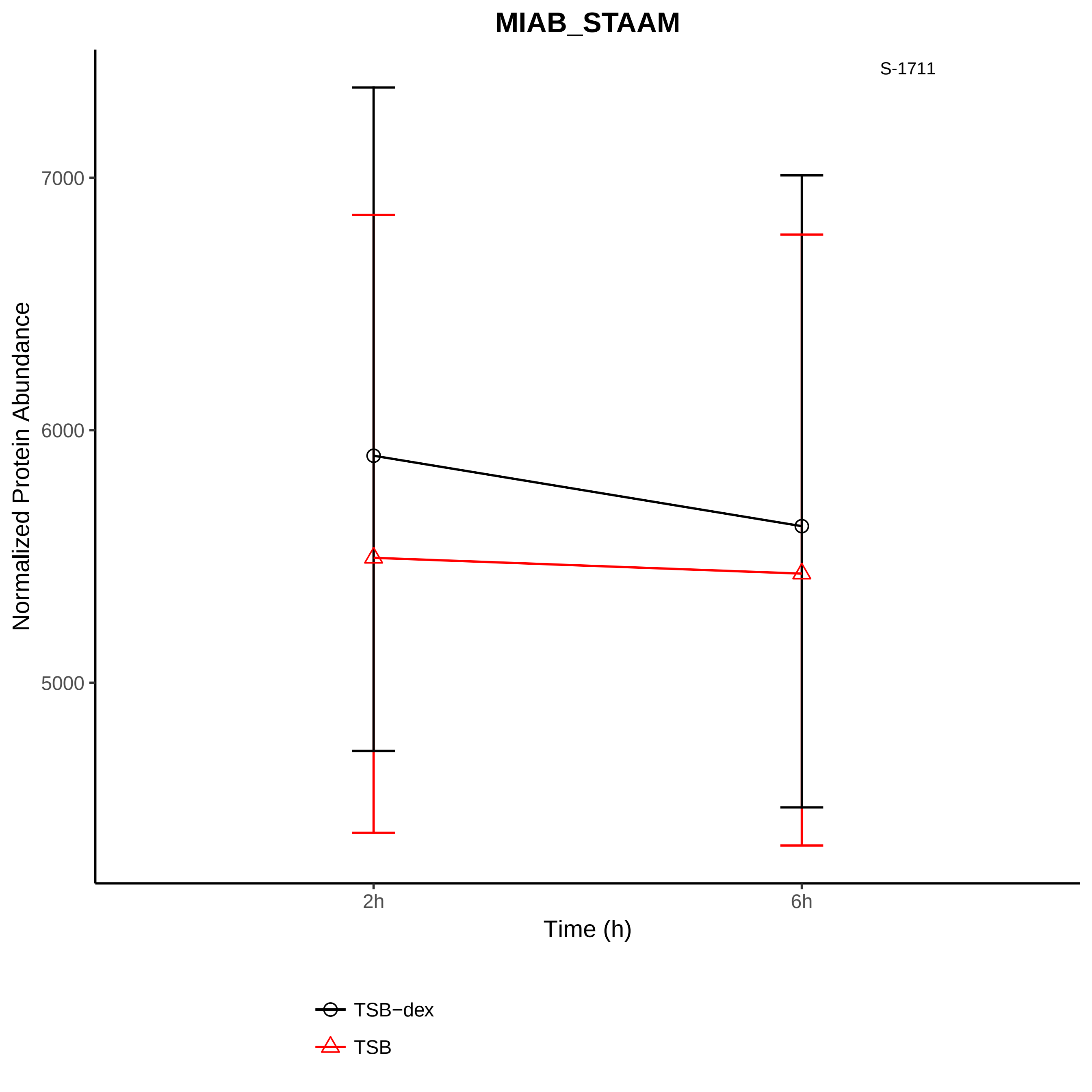

MIAB_STAAM
S-1711
7000
6000
5000
Normalized Protein Abundance
2h
6h
Time (h)
TSB−dex
TSB

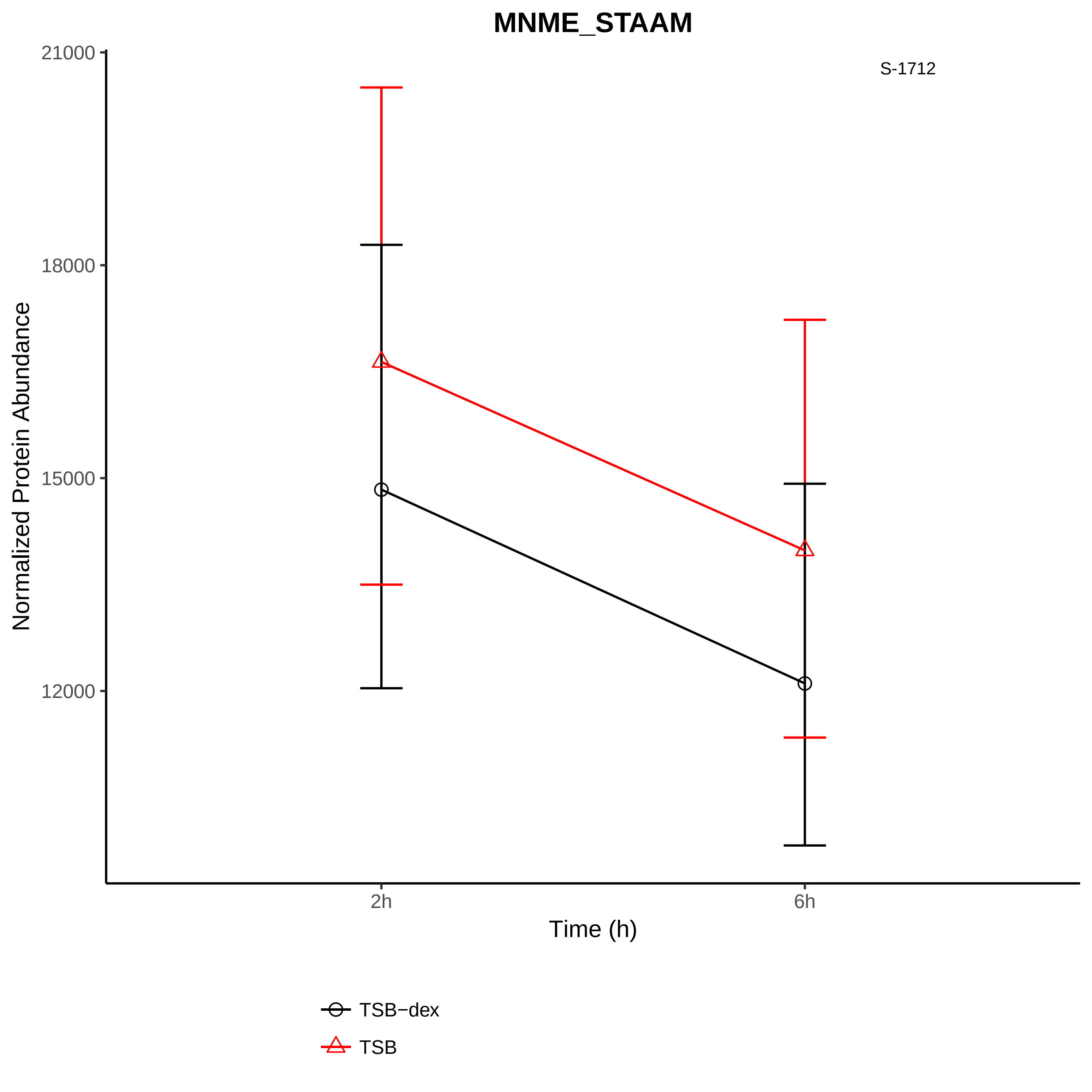

MNME_STAAM
S-1712
21000
18000
15000
12000
Normalized Protein Abundance
2h
6h
Time (h)
TSB−dex
TSB

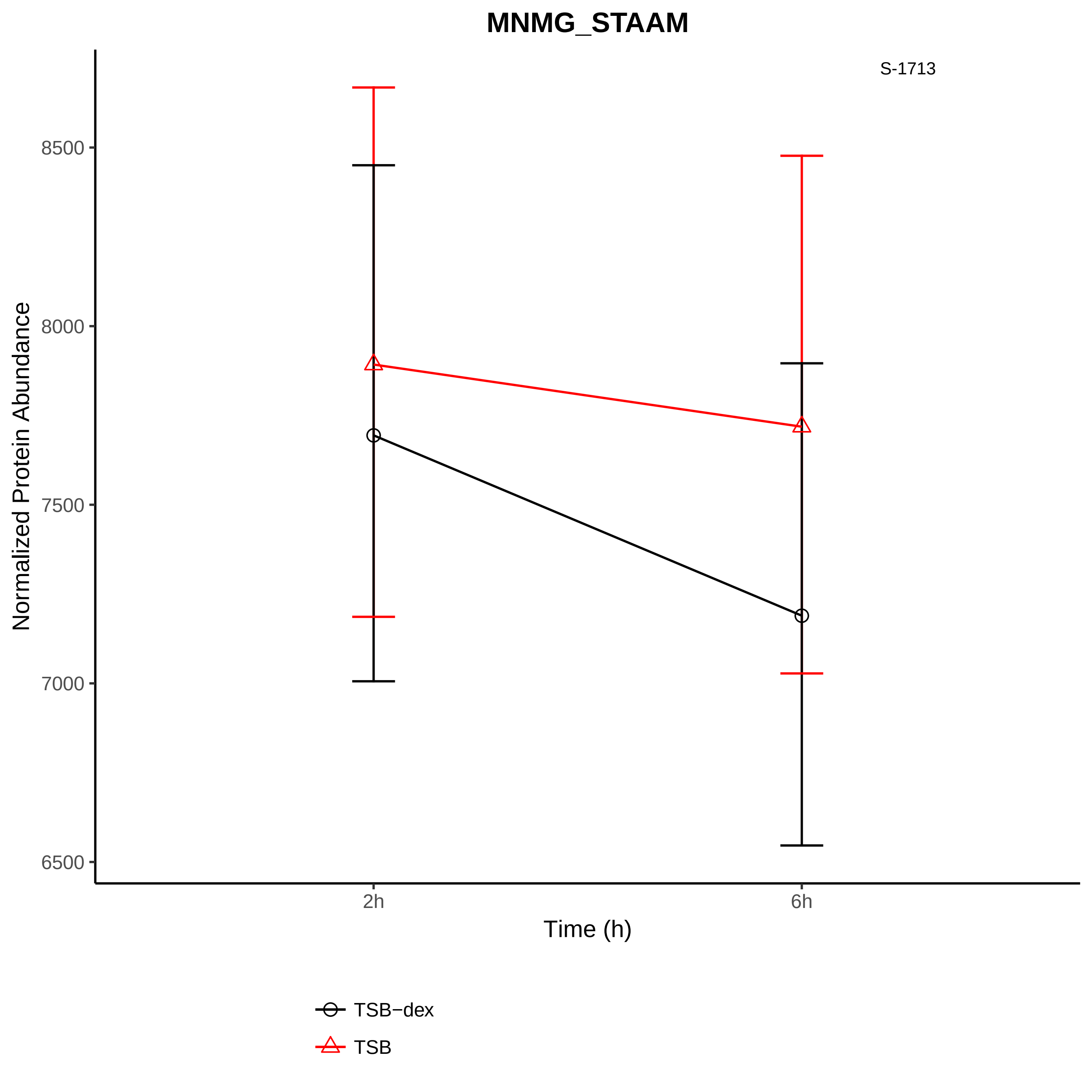

MNMG_STAAM
S-1713
Normalized Protein Abundance
8500
8000
7500
7000
6500
2h
6h
Time (h)
TSB−dex
TSB

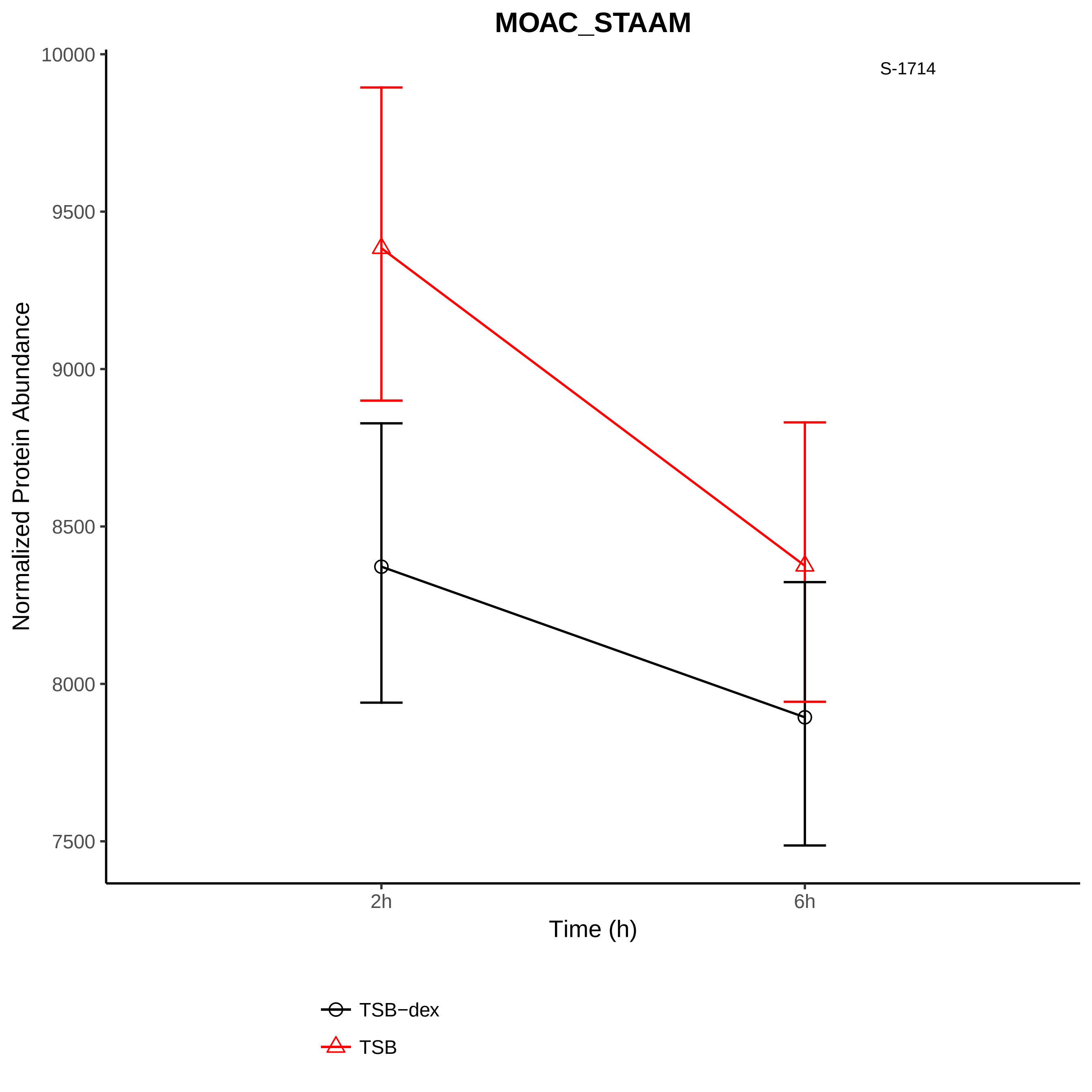
MOAC_STAAM
S-1714
Normalized Protein Abundance
10000
9500
9000
8500
8000
7500
2h
6h
Time (h)
TSB−dex
TSB

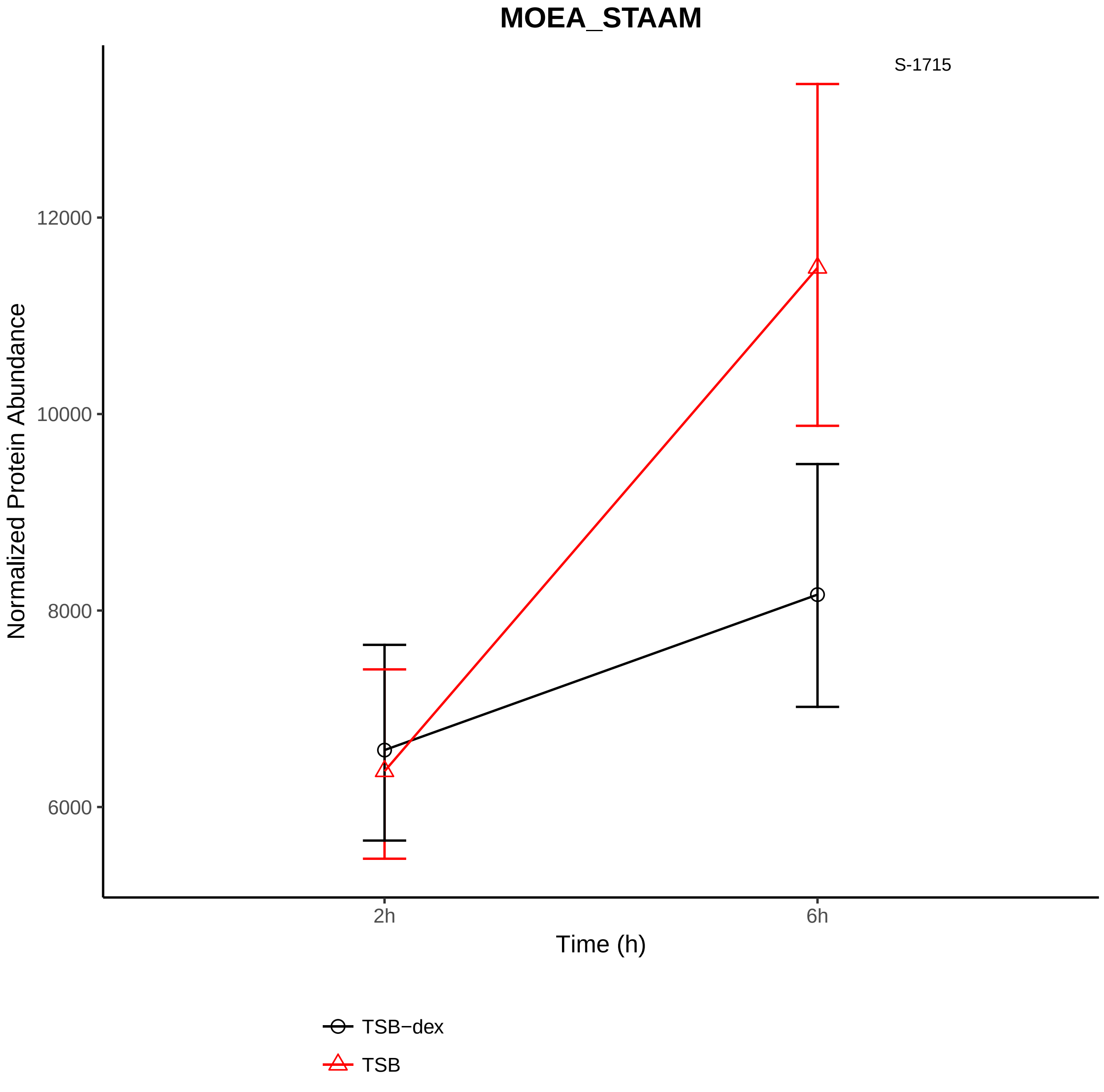

MOEA_STAAM
S-1715
Normalized Protein Abundance
12000
10000
8000
6000
2h
6h
Time (h)
TSB−dex
TSB

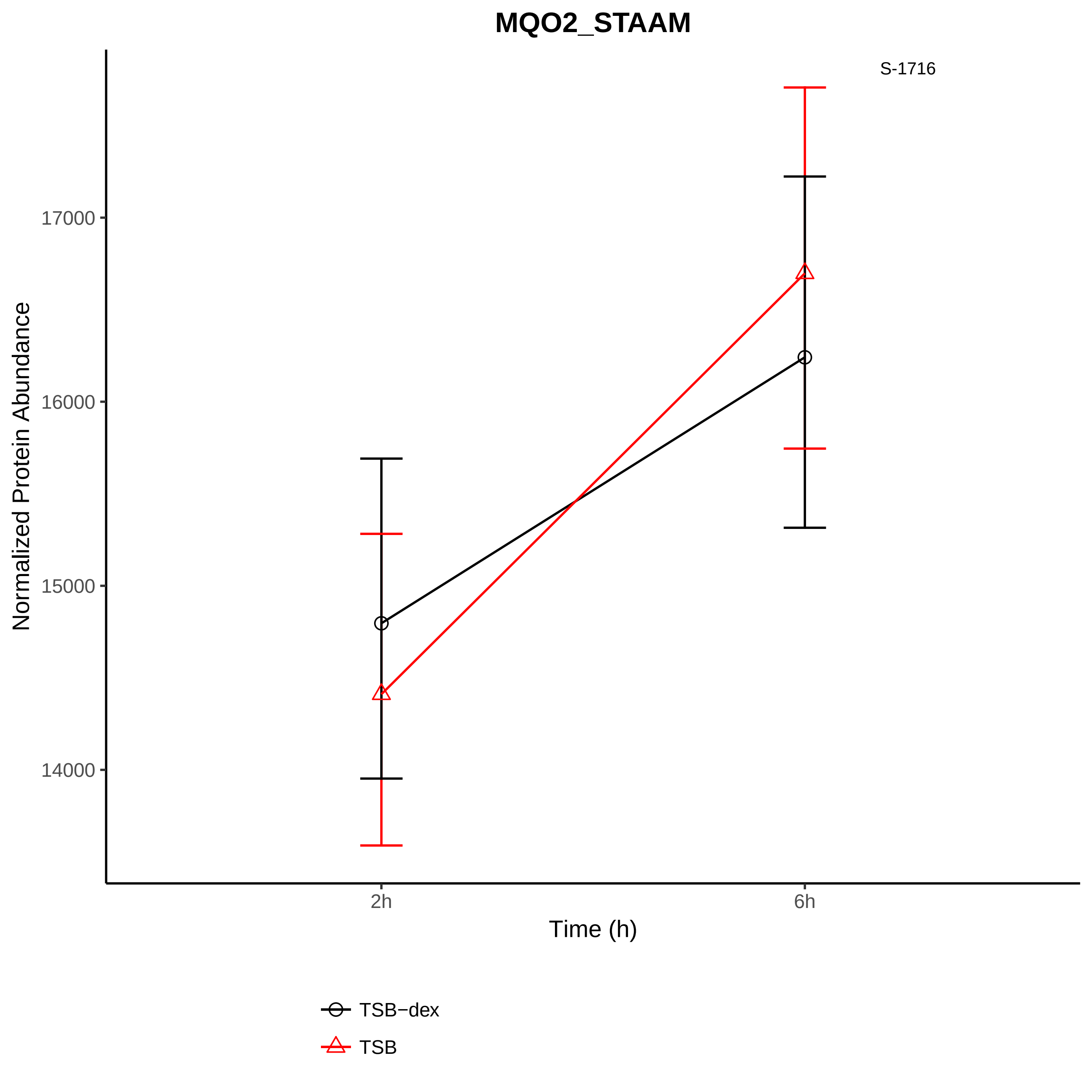

MQO2_STAAM
S-1716
Normalized Protein Abundance
17000
16000
15000
14000
2h
6h
Time (h)
TSB−dex
TSB

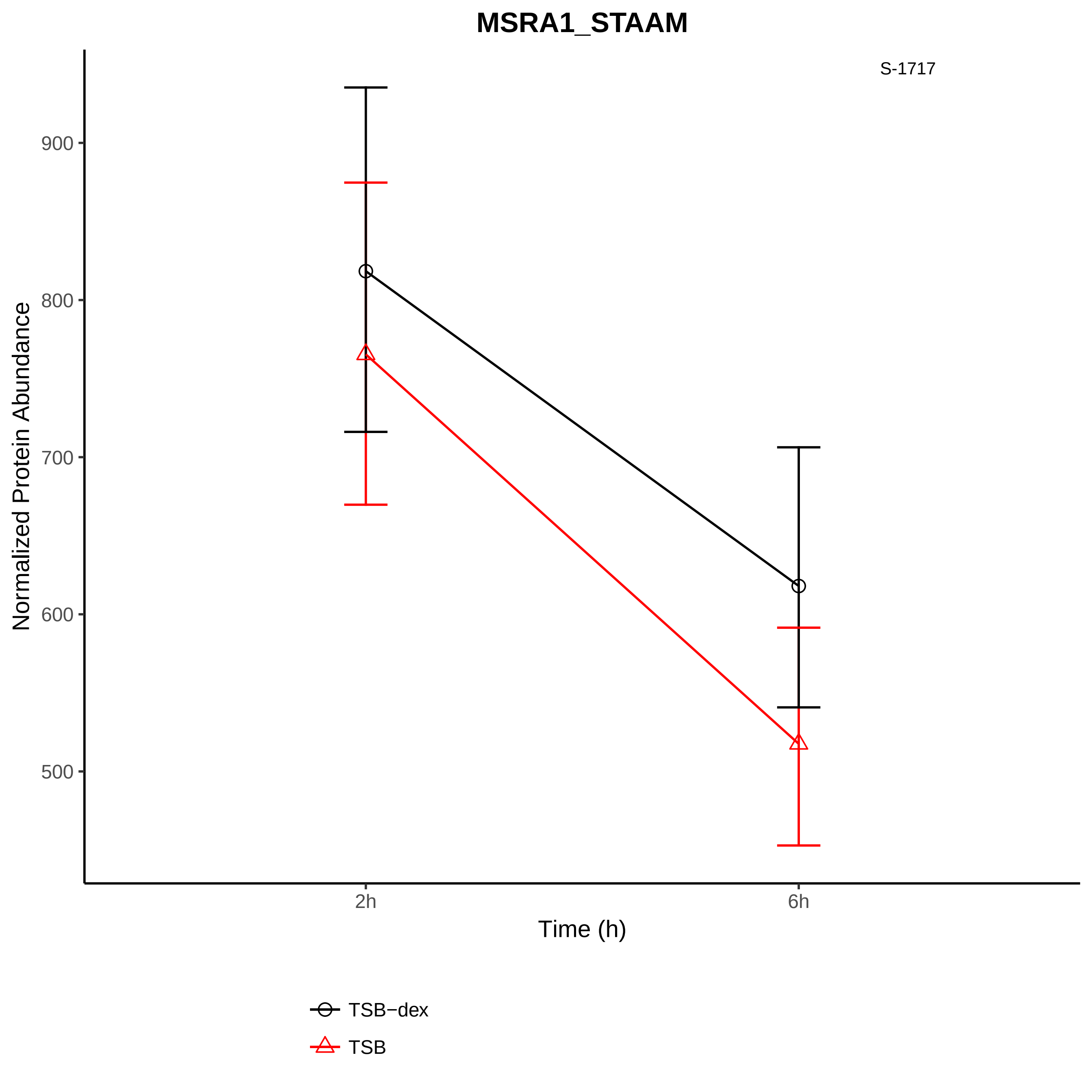

MSRA1_STAAM
S-1717
Normalized Protein Abundance
900
800
700
600
500
2h
6h
Time (h)
TSB−dex
TSB

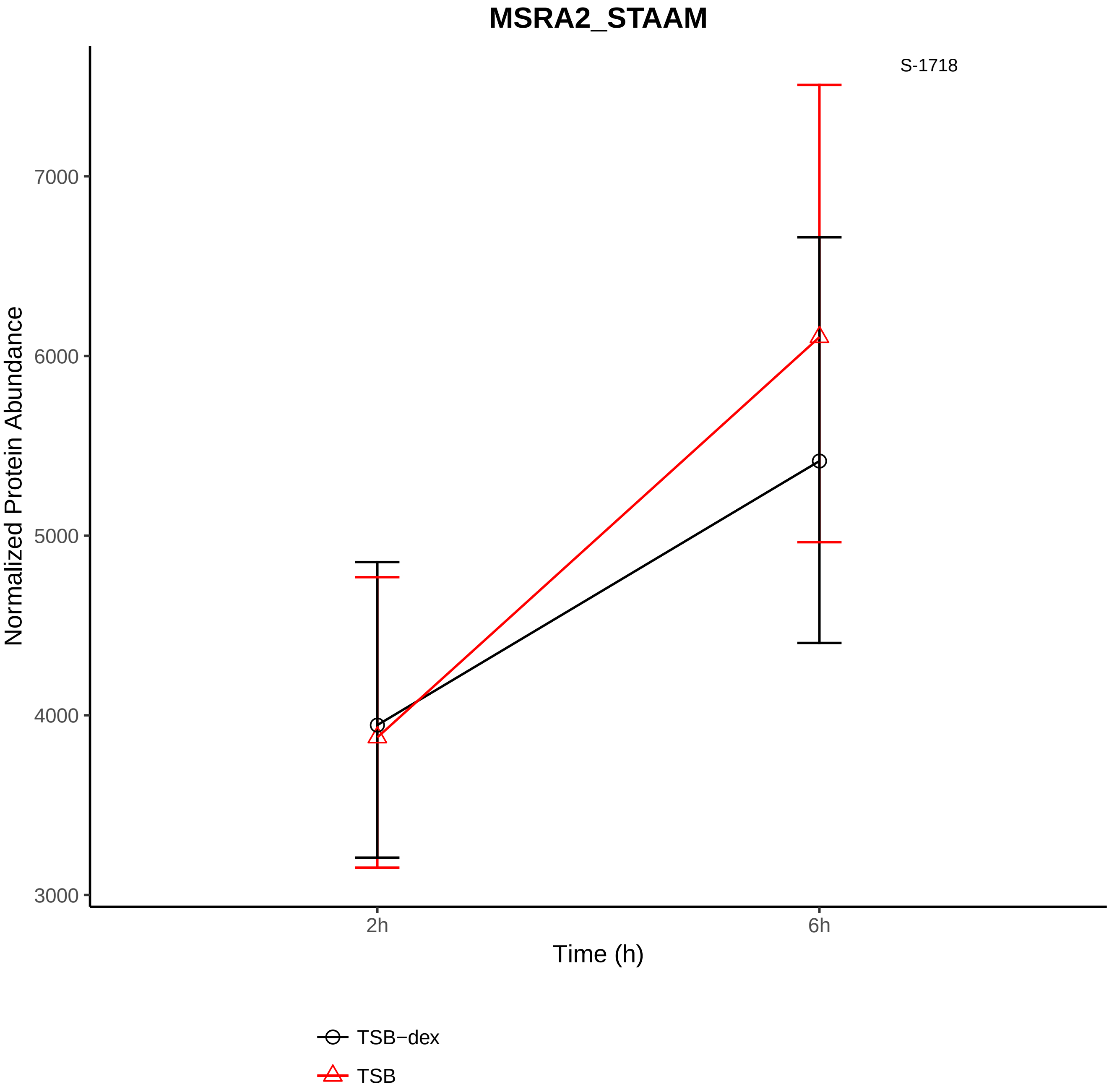

MSRA2_STAAM
S-1718
7000
6000
5000
4000
3000
Normalized Protein Abundance
2h
6h
Time (h)
TSB−dex
TSB

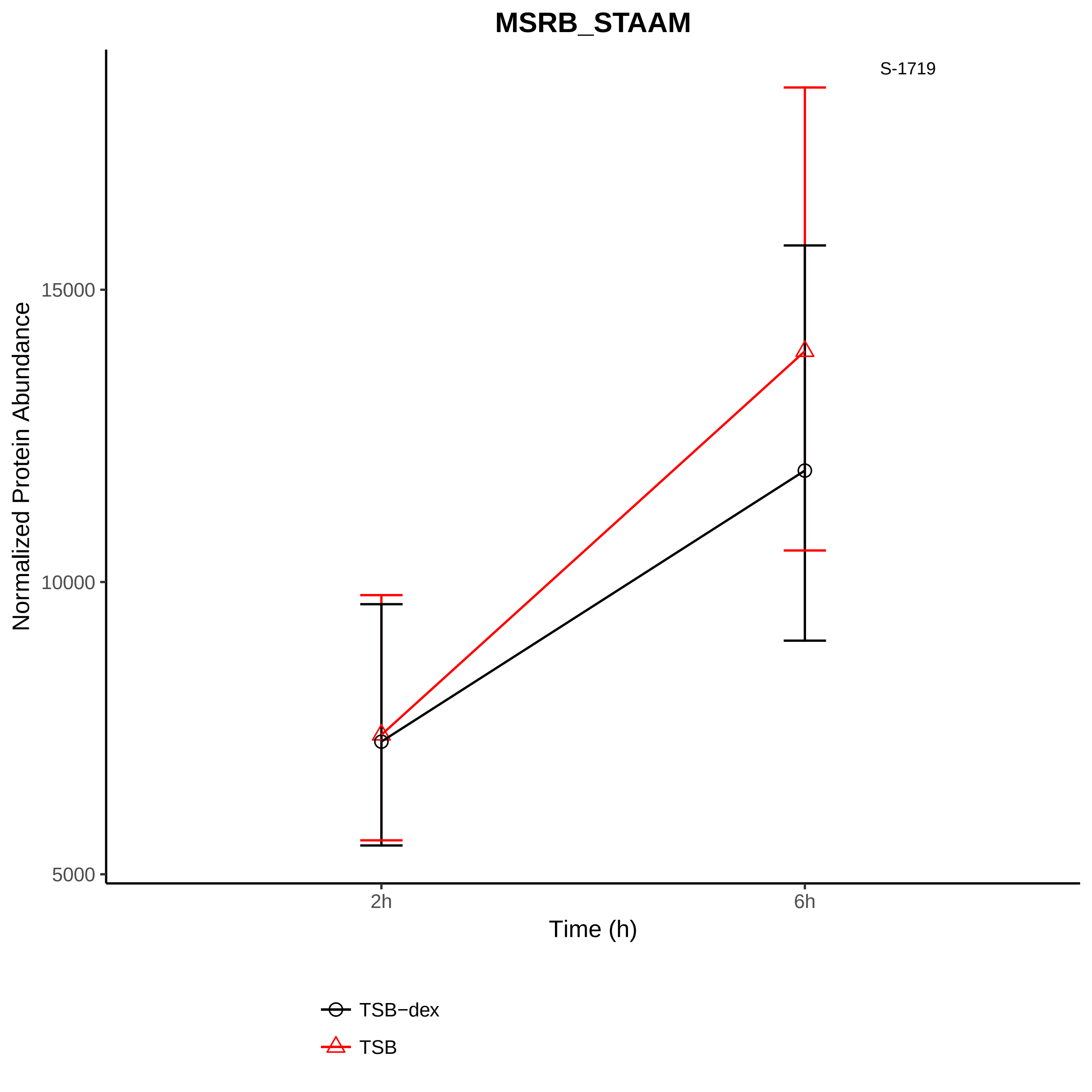
MSRB_STAAM
S-1719
Normalized Protein Abundance
15000
10000
5000
2h
6h
Time (h)
TSB−dex
TSB

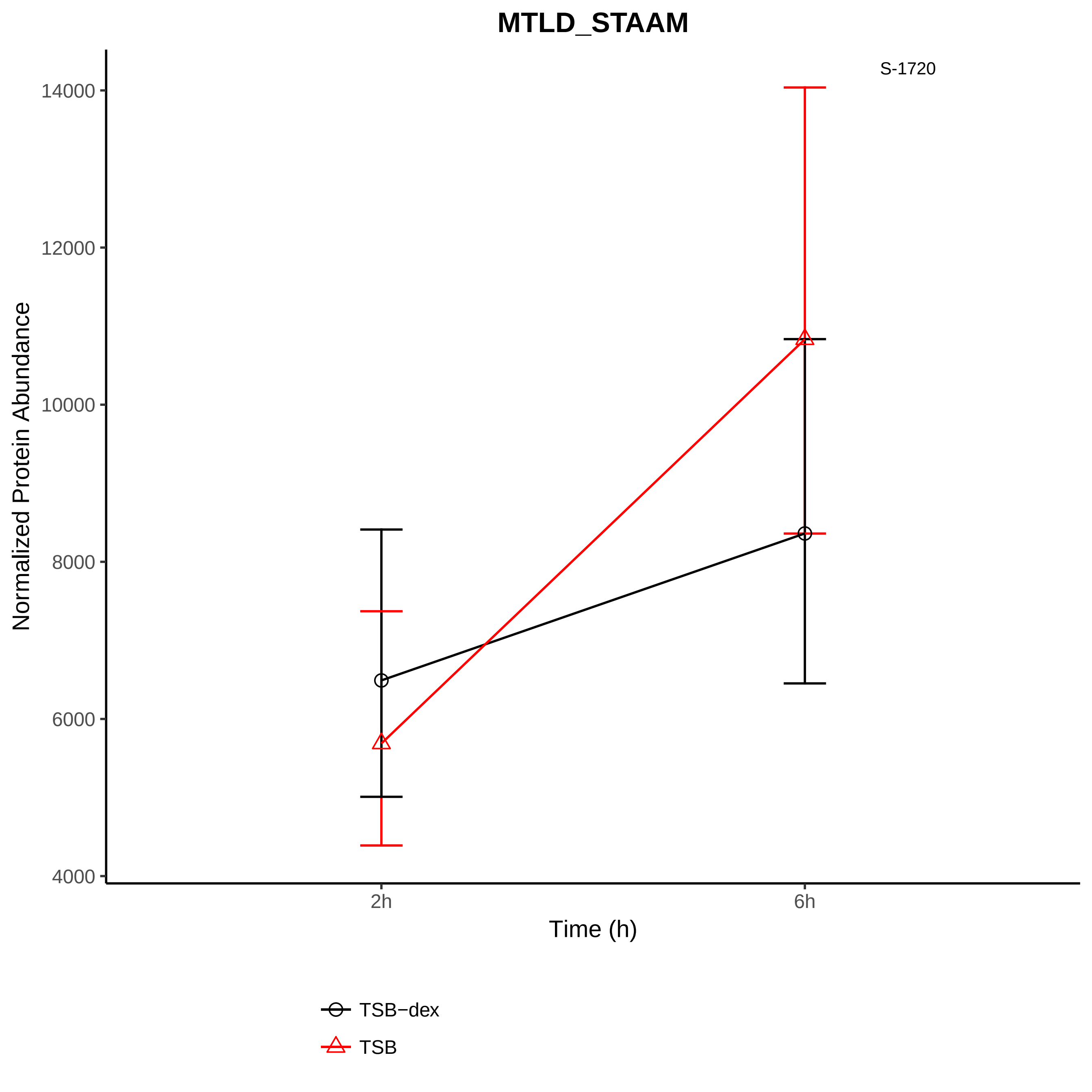

MTLD_STAAM
S-1720
Normalized Protein Abundance
14000
12000
10000
8000
6000
4000
2h
6h
Time (h)
TSB−dex
TSB

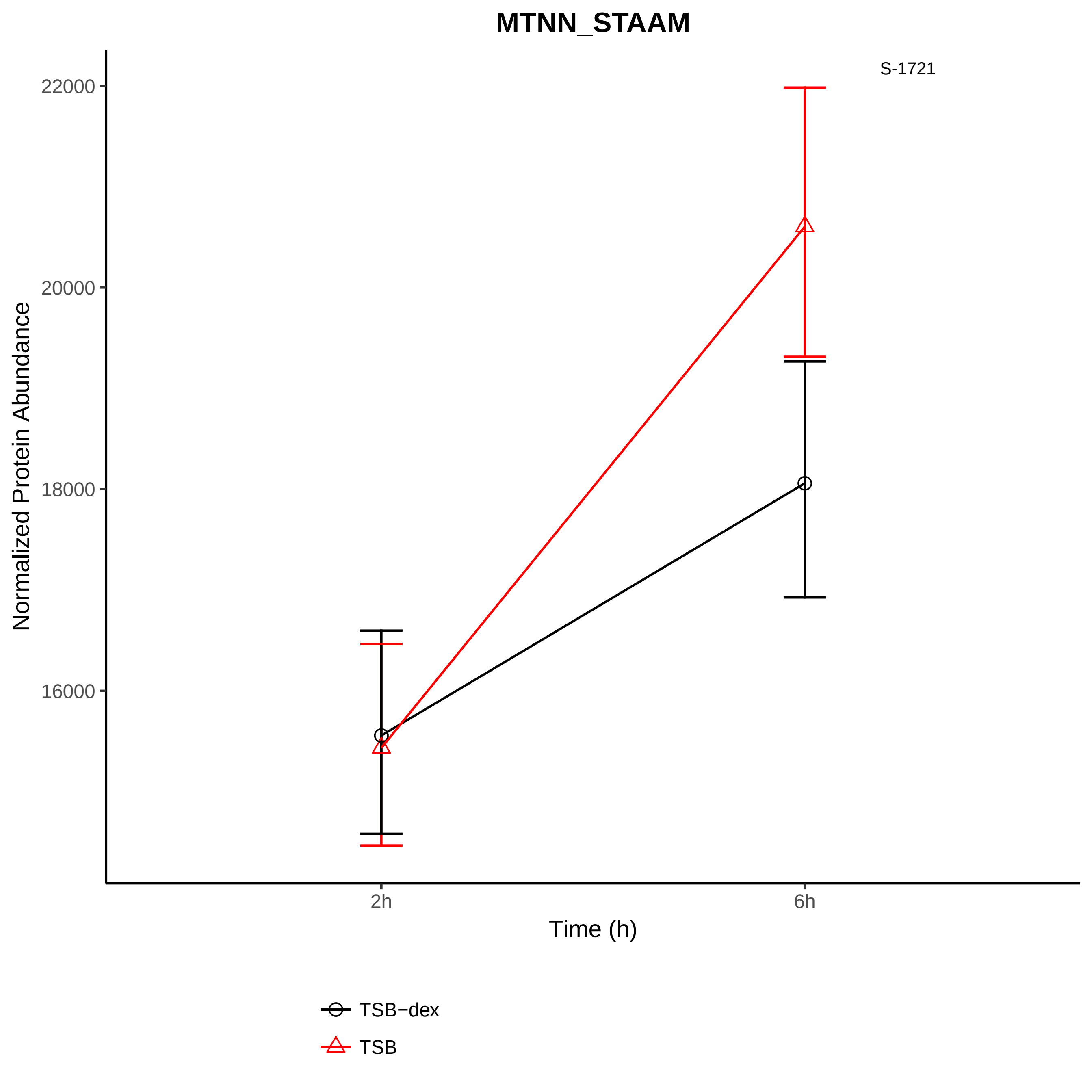
MTNN_STAAM
S-1721
Normalized Protein Abundance
22000
20000
18000
16000
2h
6h
Time (h)
TSB−dex
TSB

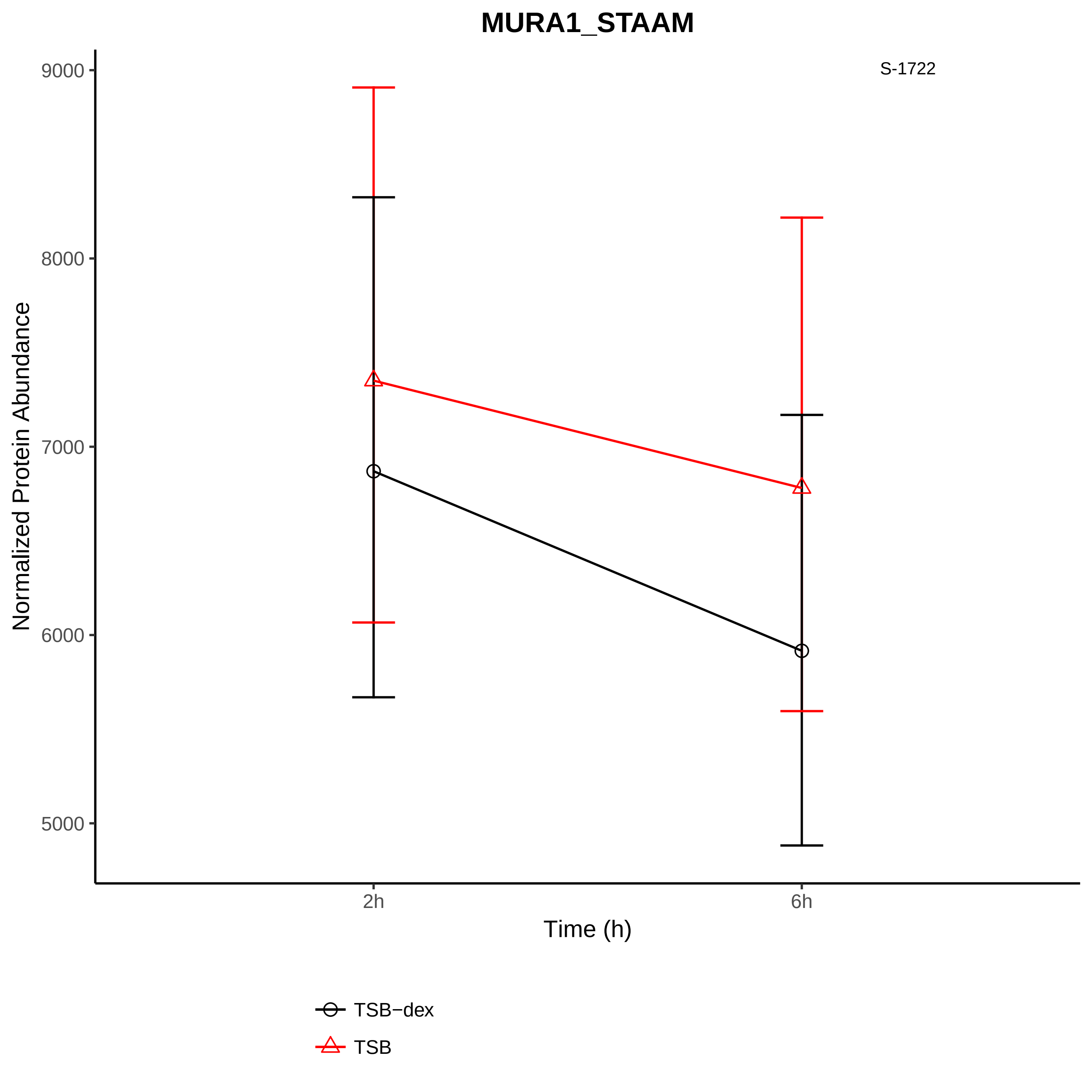

MURA1_STAAM
S-1722
9000
8000
7000
6000
5000
Normalized Protein Abundance
2h
6h
Time (h)
TSB−dex
TSB

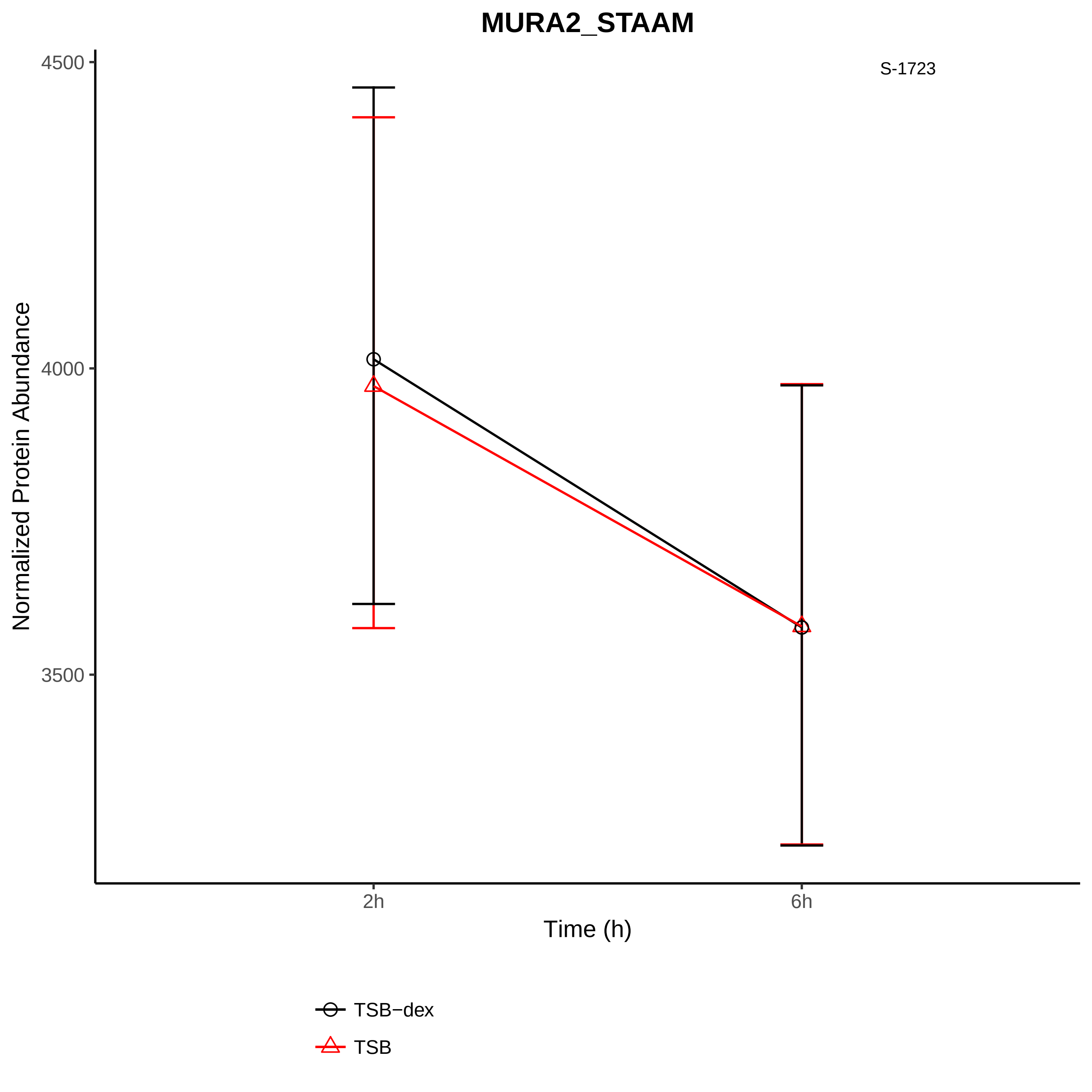
MURA2_STAAM
S-1723
4500
4000
3500
Normalized Protein Abundance
2h
6h
Time (h)
TSB−dex
TSB

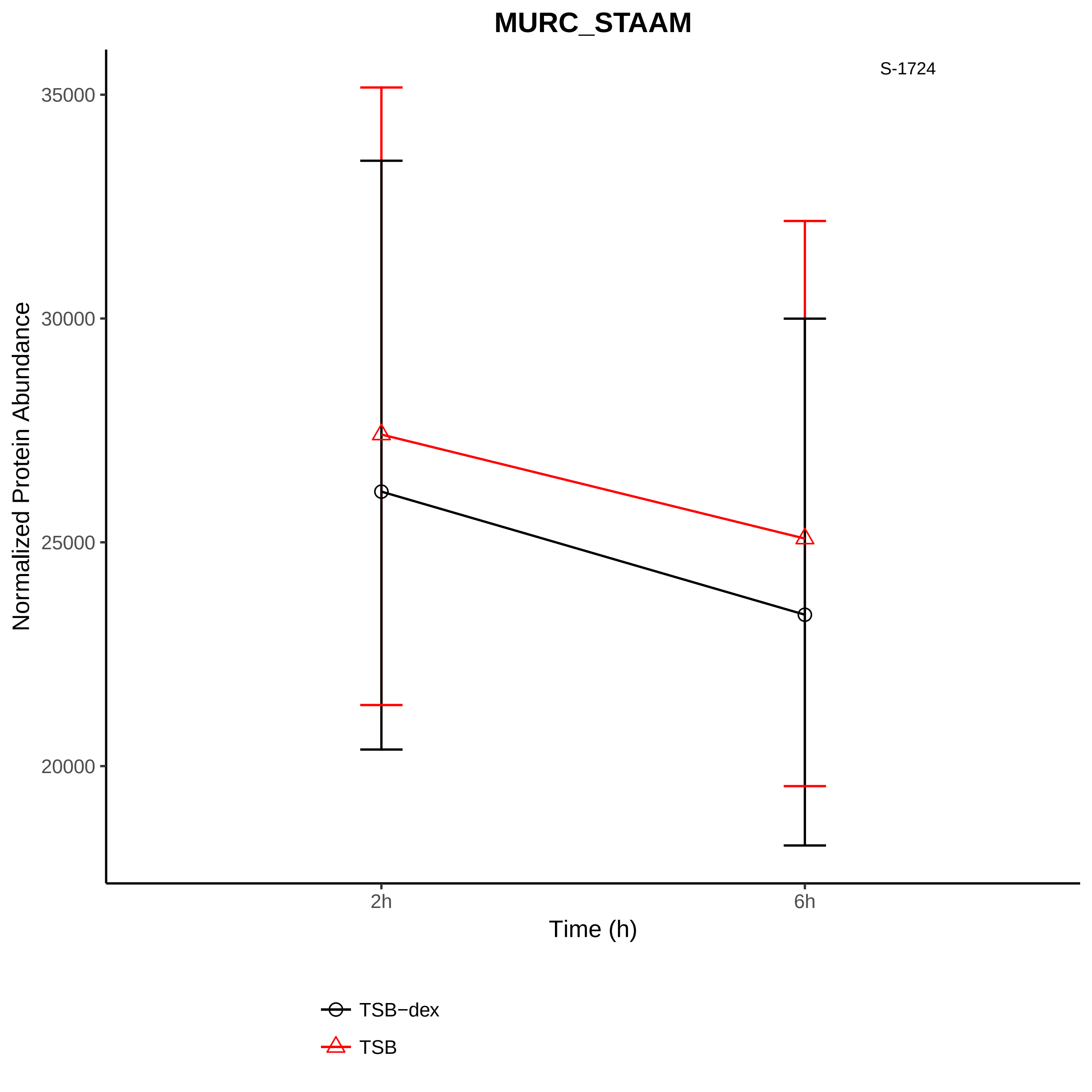

MURC_STAAM
S-1724
Normalized Protein Abundance
35000
30000
25000
20000
2h
6h
Time (h)
TSB−dex
TSB

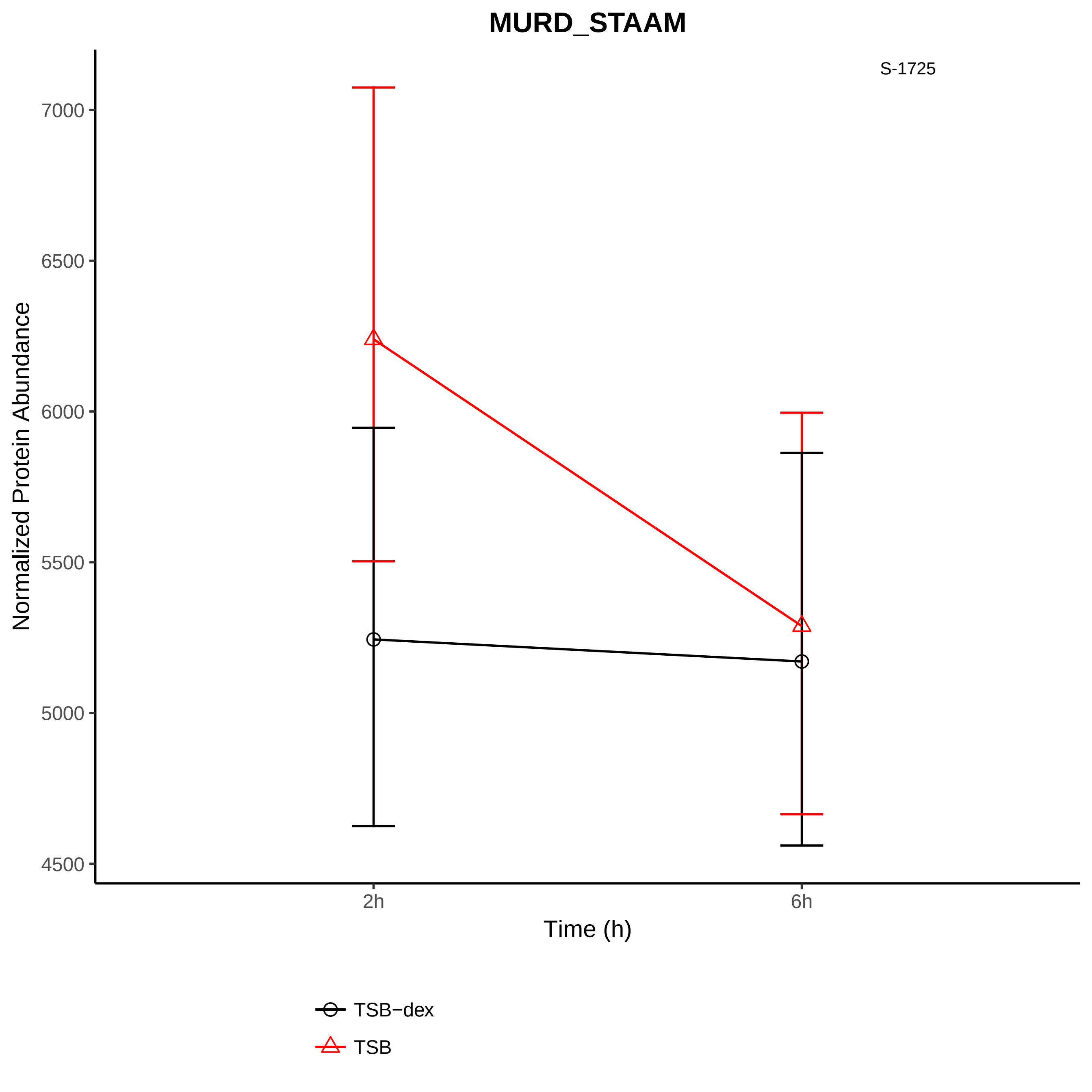

MURD_STAAM
S-1725
Normalized Protein Abundance
7000
6500
6000
5500
5000
4500
2h
6h
Time (h)
TSB−dex
TSB

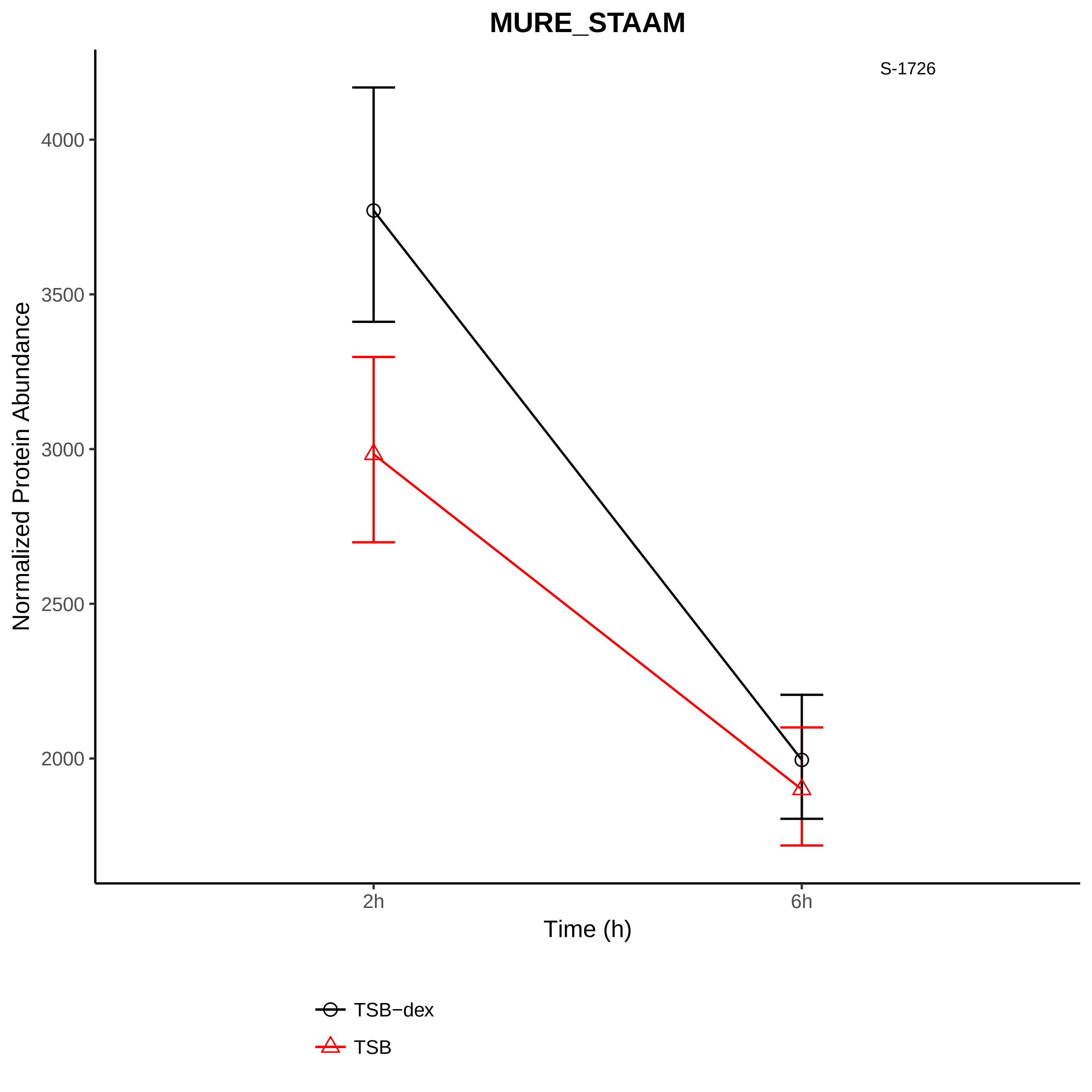

MURE_STAAM
S-1726
Normalized Protein Abundance
4000
3500
3000
2500
2000
2h
6h
Time (h)
TSB−dex
TSB

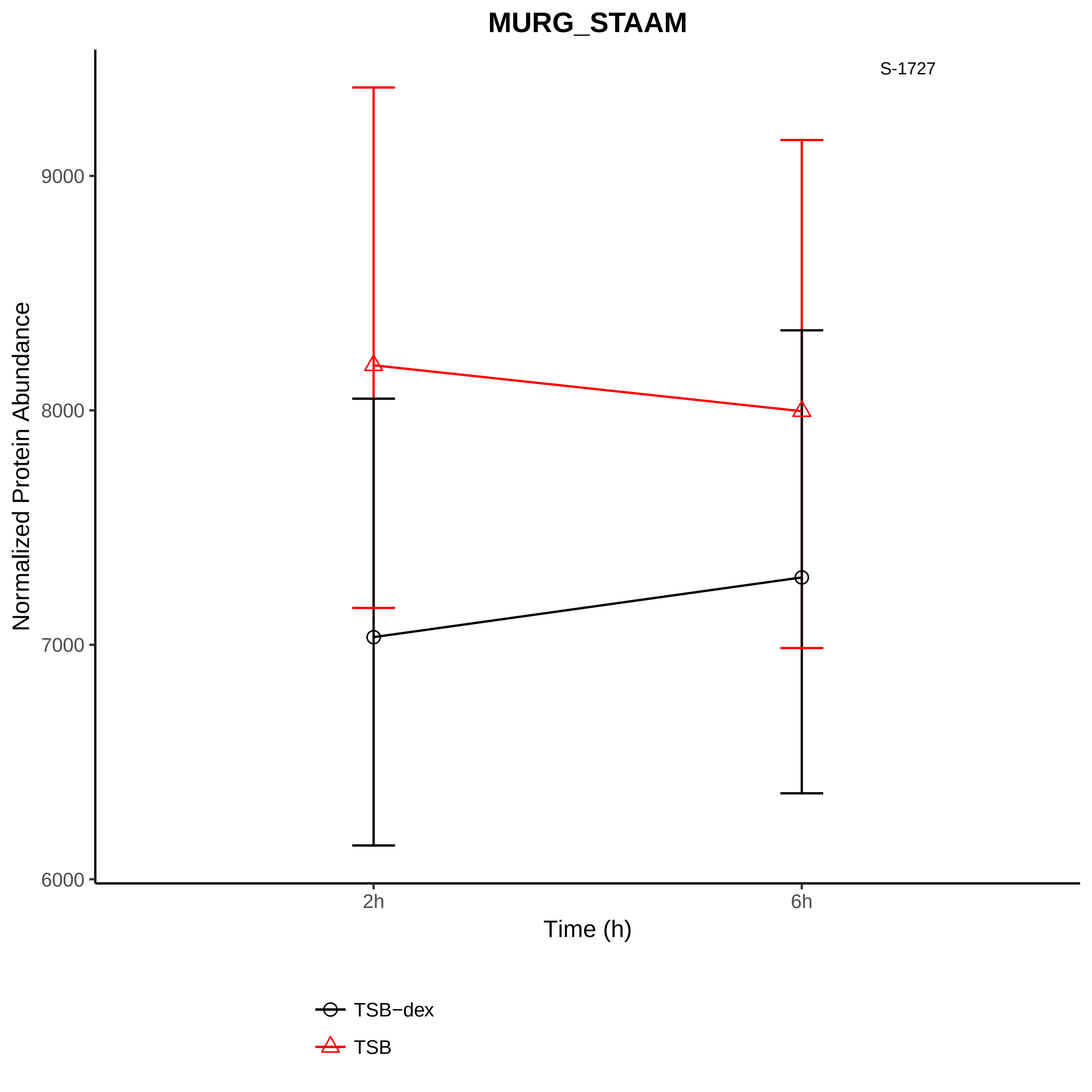
MURG_STAAM
S-1727
Normalized Protein Abundance
9000
8000
7000
6000
2h
6h
Time (h)
TSB−dex
TSB

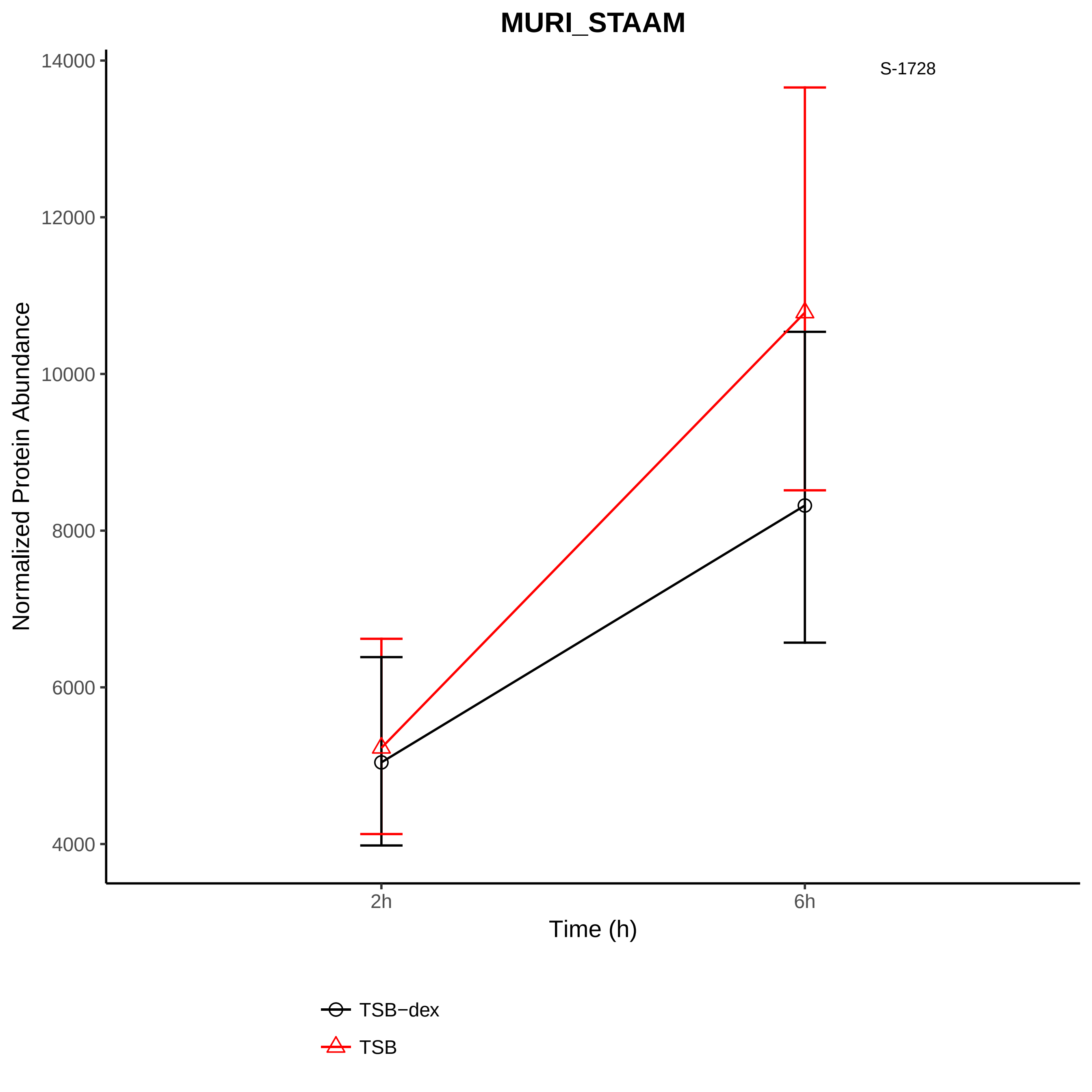
MURI_STAAM
S-1728
Normalized Protein Abundance
14000
12000
10000
8000
6000
4000
2h
6h
Time (h)
TSB−dex
TSB

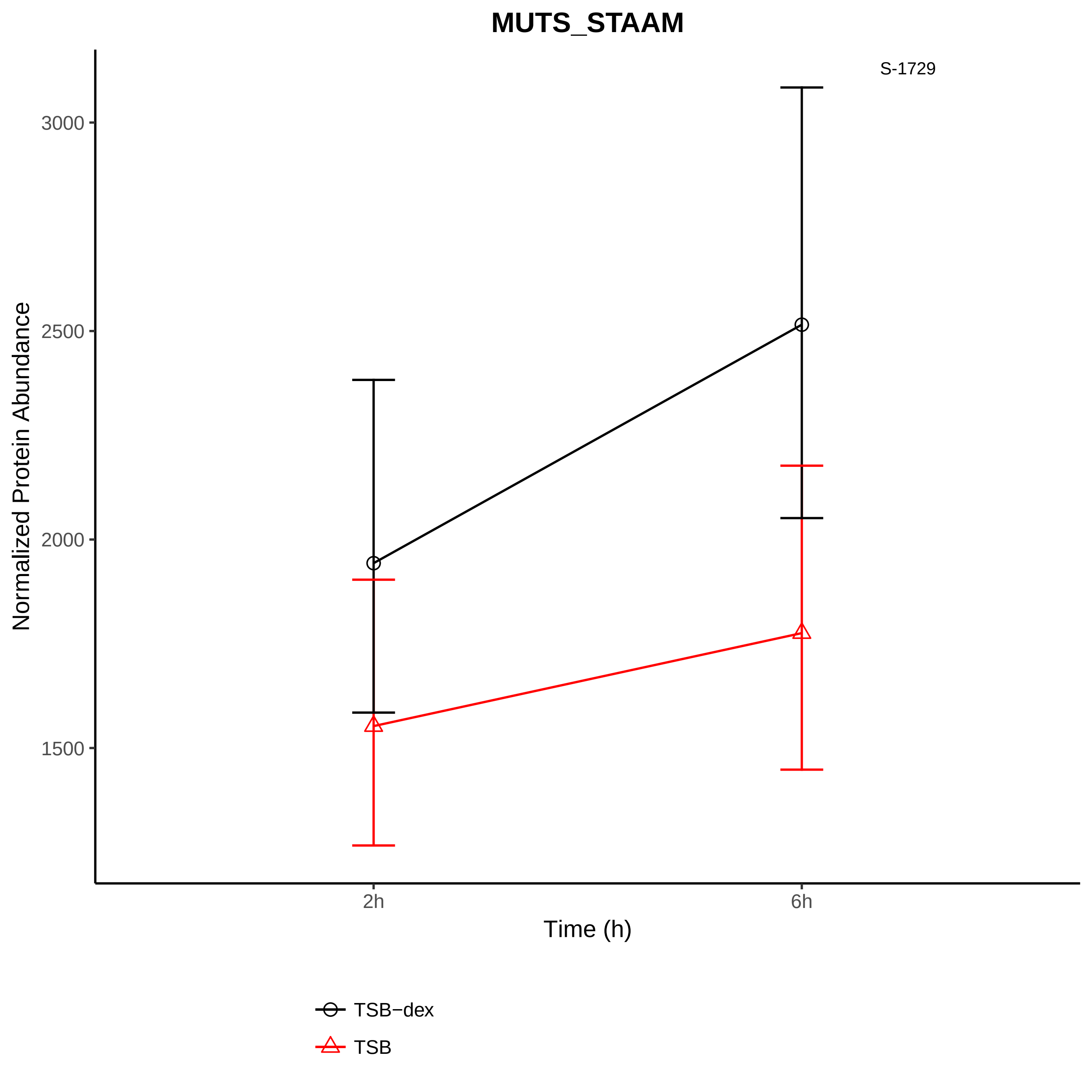

MUTS_STAAM
S-1729
3000
2500
2000
1500
Normalized Protein Abundance
2h
6h
Time (h)
TSB−dex
TSB

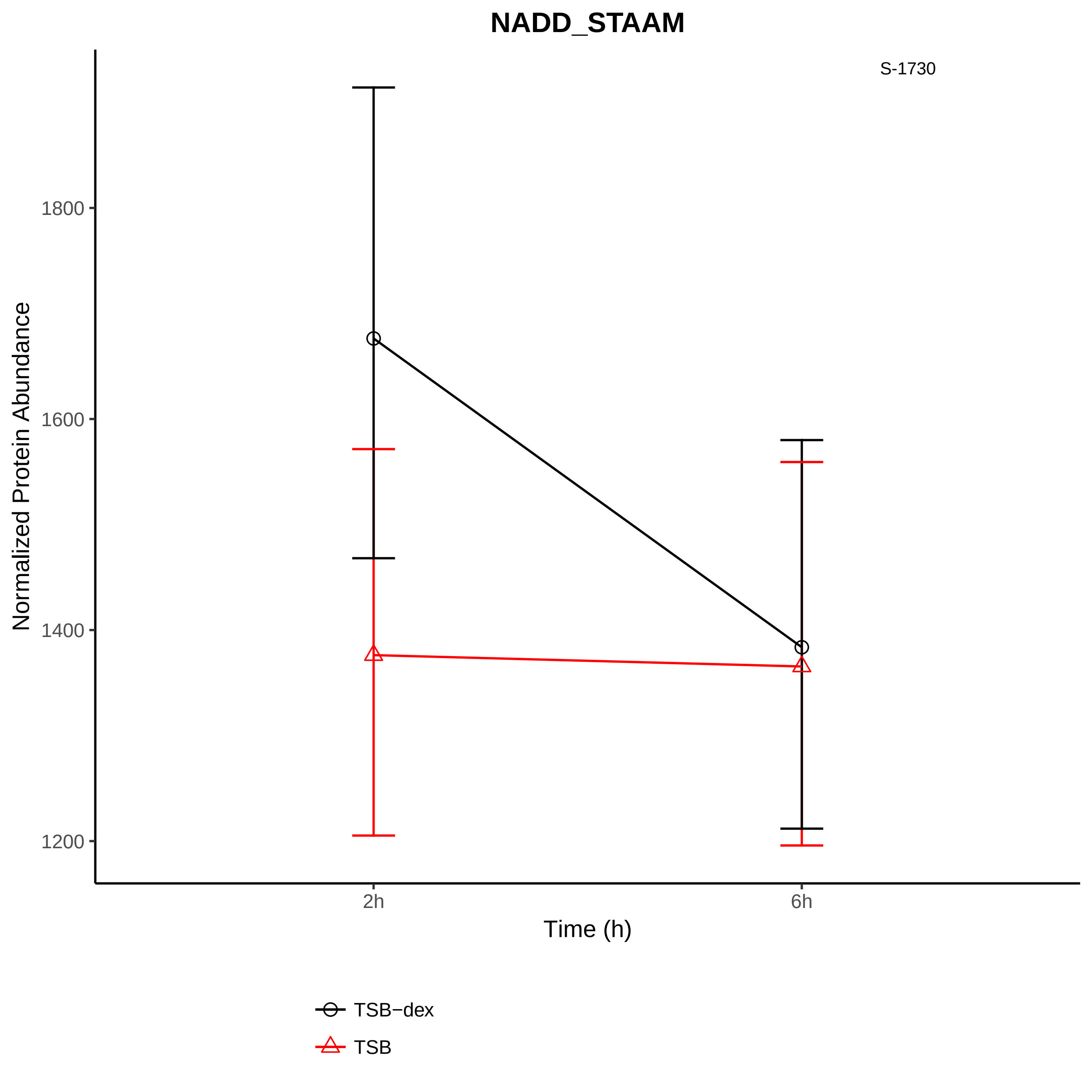
NADD_STAAM
S-1730
Normalized Protein Abundance
1800
1600
1400
1200
2h
6h
Time (h)
TSB−dex
TSB

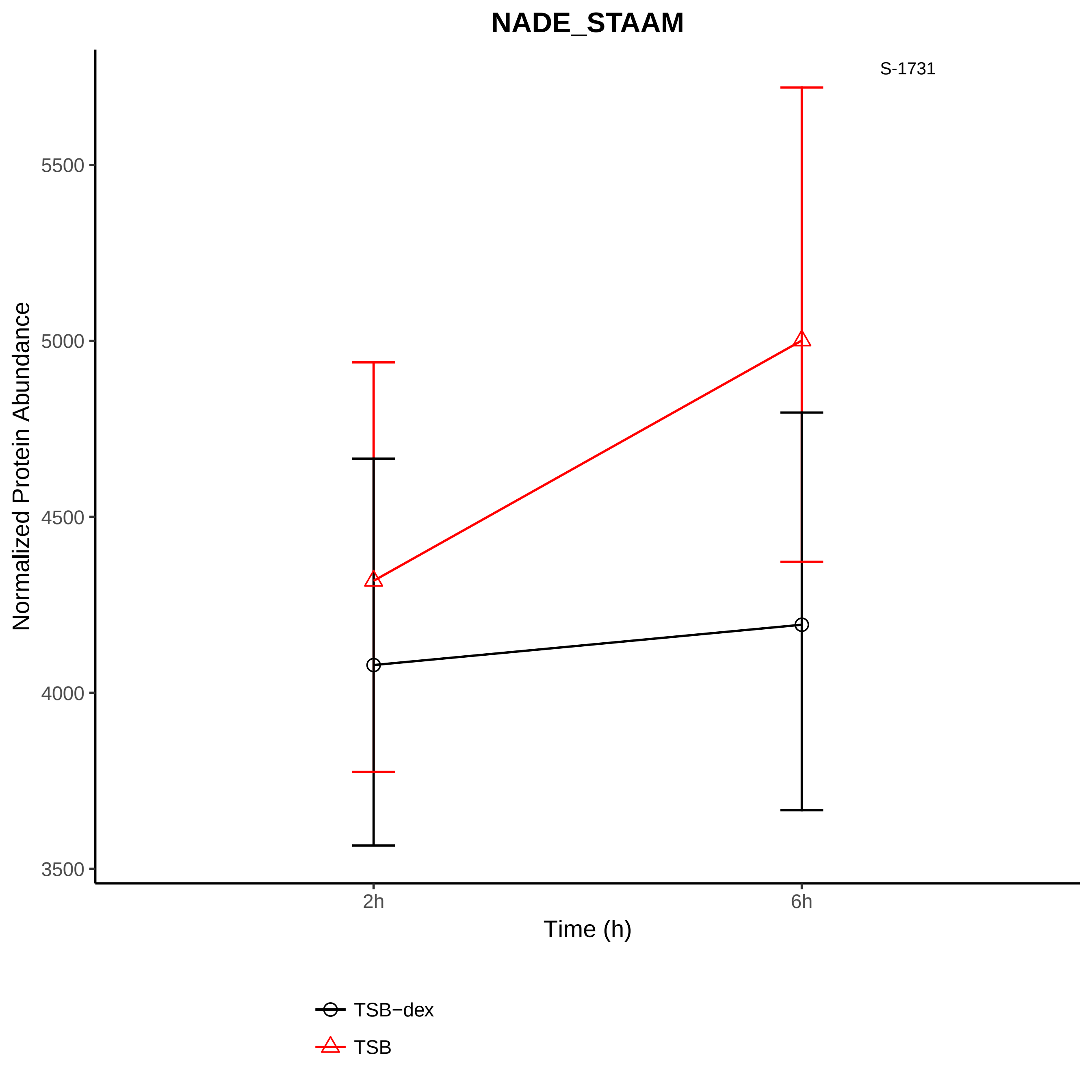

NADE_STAAM
S-1731
Normalized Protein Abundance
5500
5000
4500
4000
3500
2h
6h
Time (h)
TSB−dex
TSB

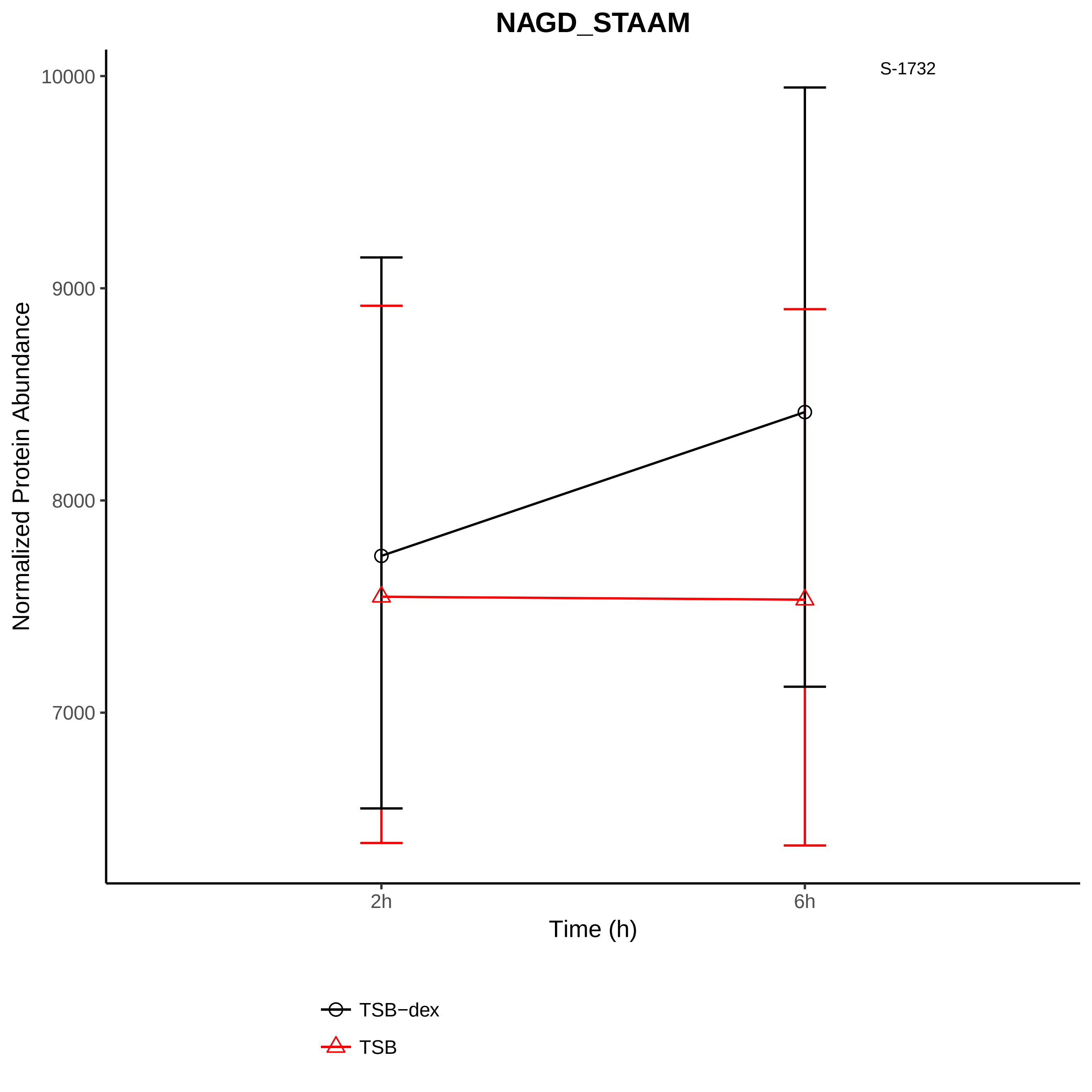
NAGD_STAAM
S-1732
Normalized Protein Abundance
10000
9000
8000
7000
2h
6h
Time (h)
TSB−dex
TSB

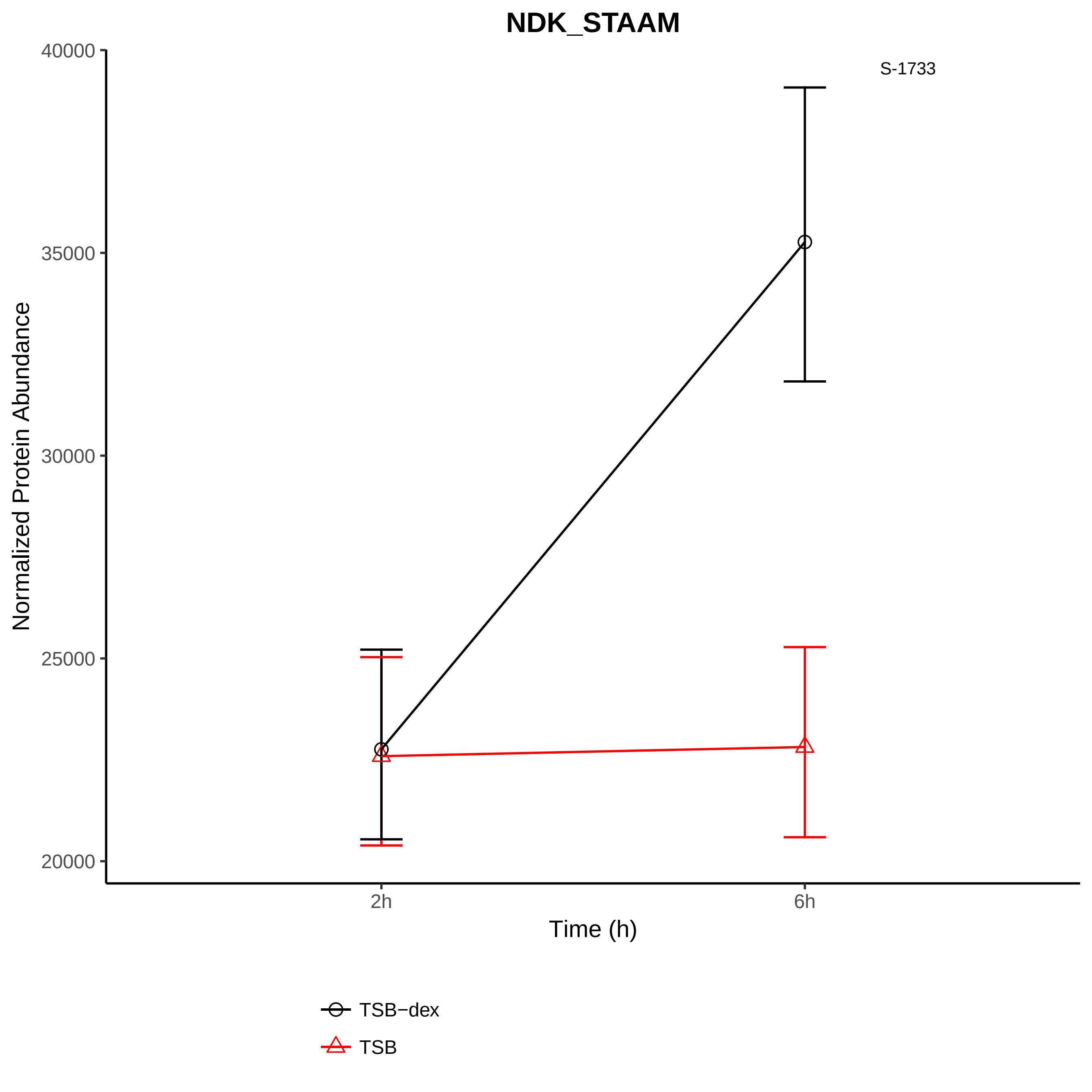

NDK_STAAM
S-1733
Normalized Protein Abundance
40000
35000
30000
25000
20000
2h
6h
Time (h)
TSB−dex
TSB

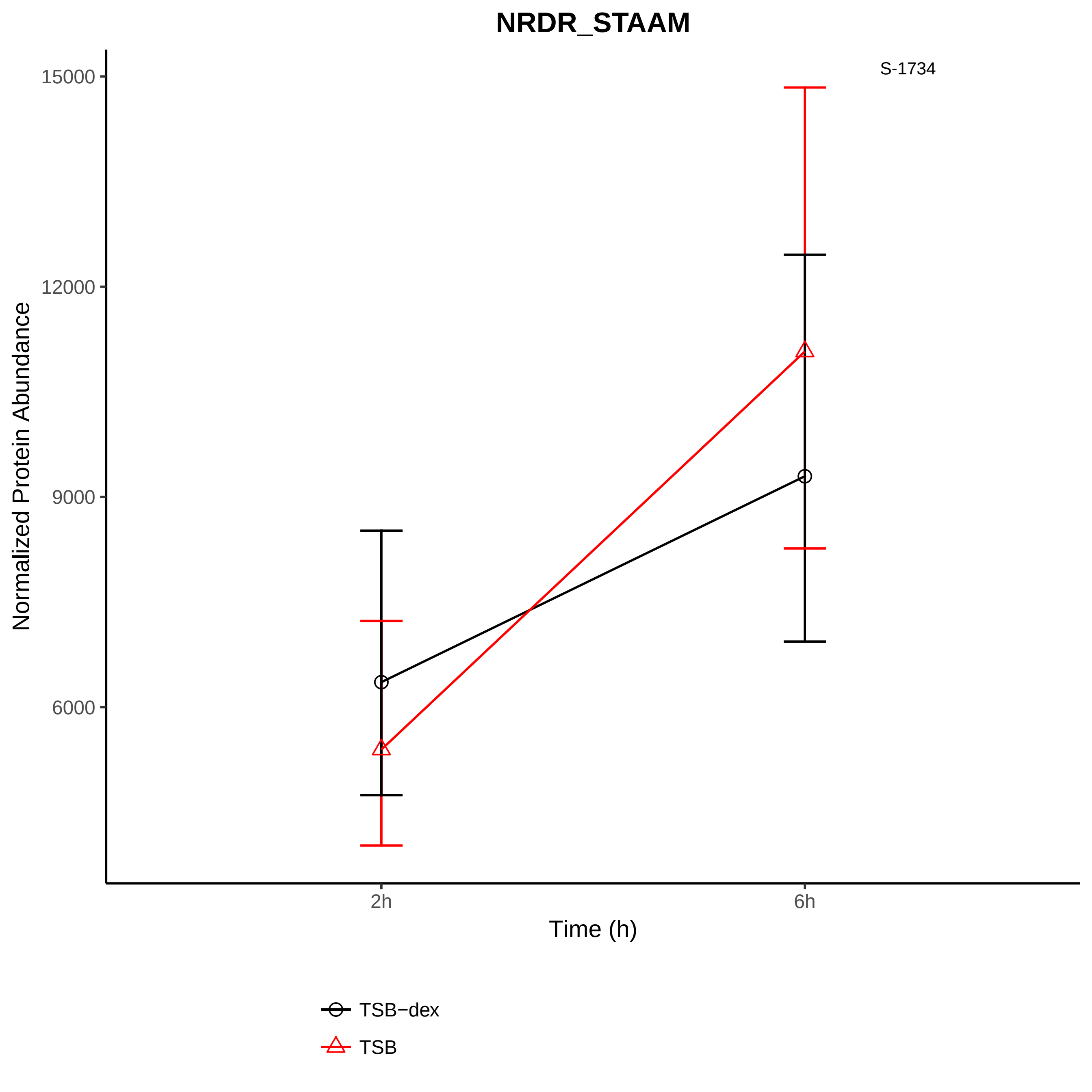

NRDR_STAAM
S-1734
15000
12000
9000
6000
Normalized Protein Abundance
2h
6h
Time (h)
TSB−dex
TSB

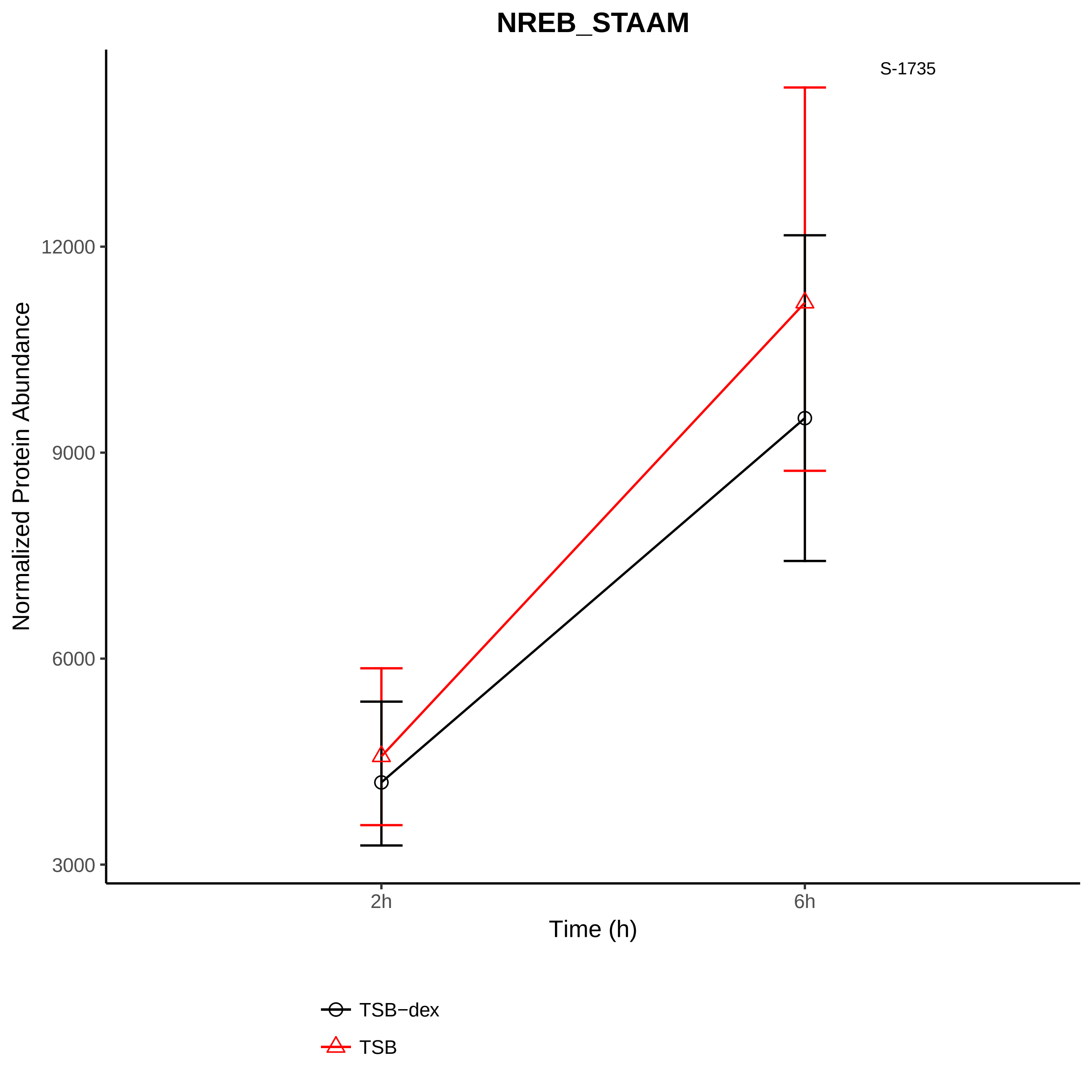
NREB_STAAM
S-1735
Normalized Protein Abundance
12000
9000
6000
3000
2h
6h
Time (h)
TSB−dex
TSB

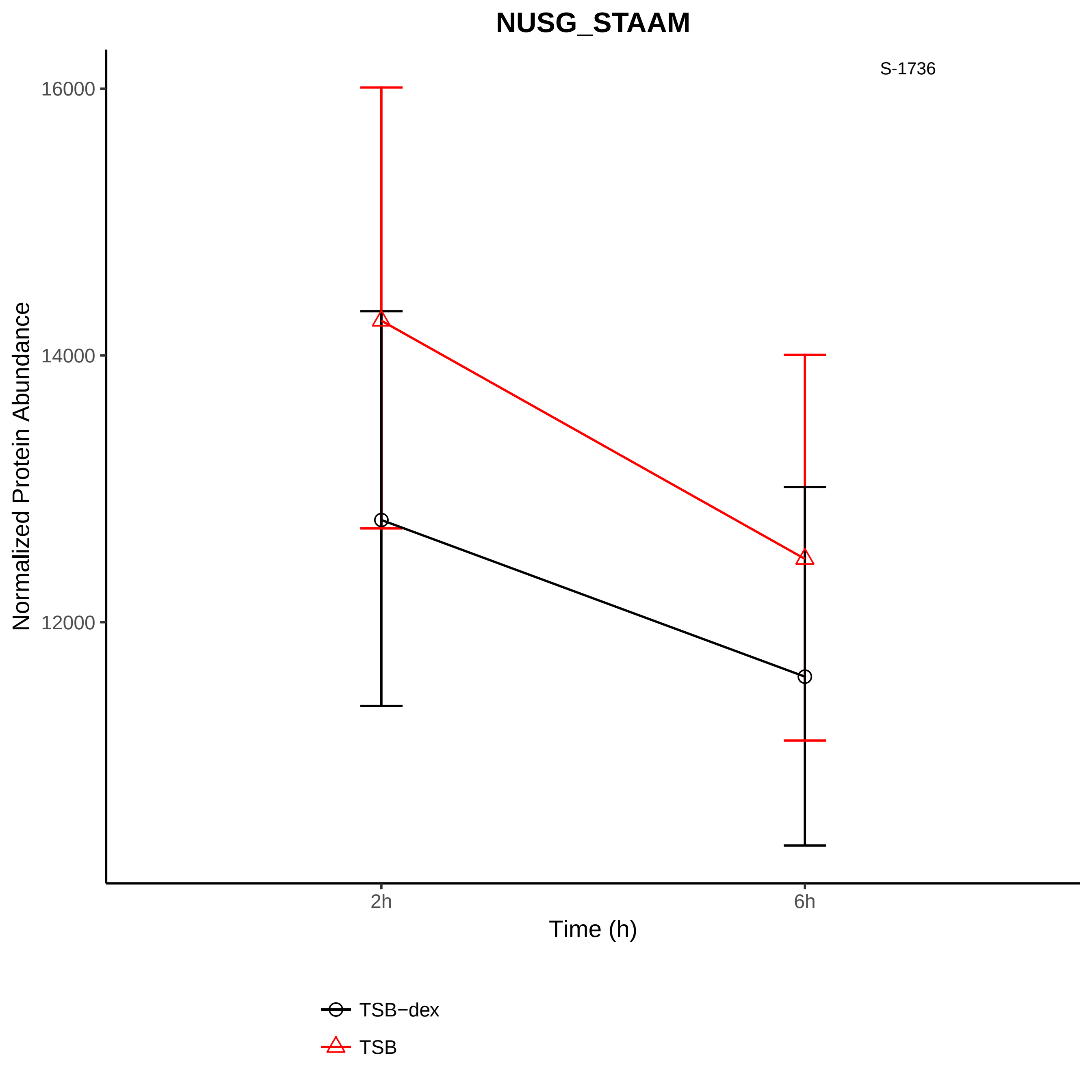

NUSG_STAAM
S-1736
16000
14000
12000
Normalized Protein Abundance
2h
6h
Time (h)
TSB−dex
TSB

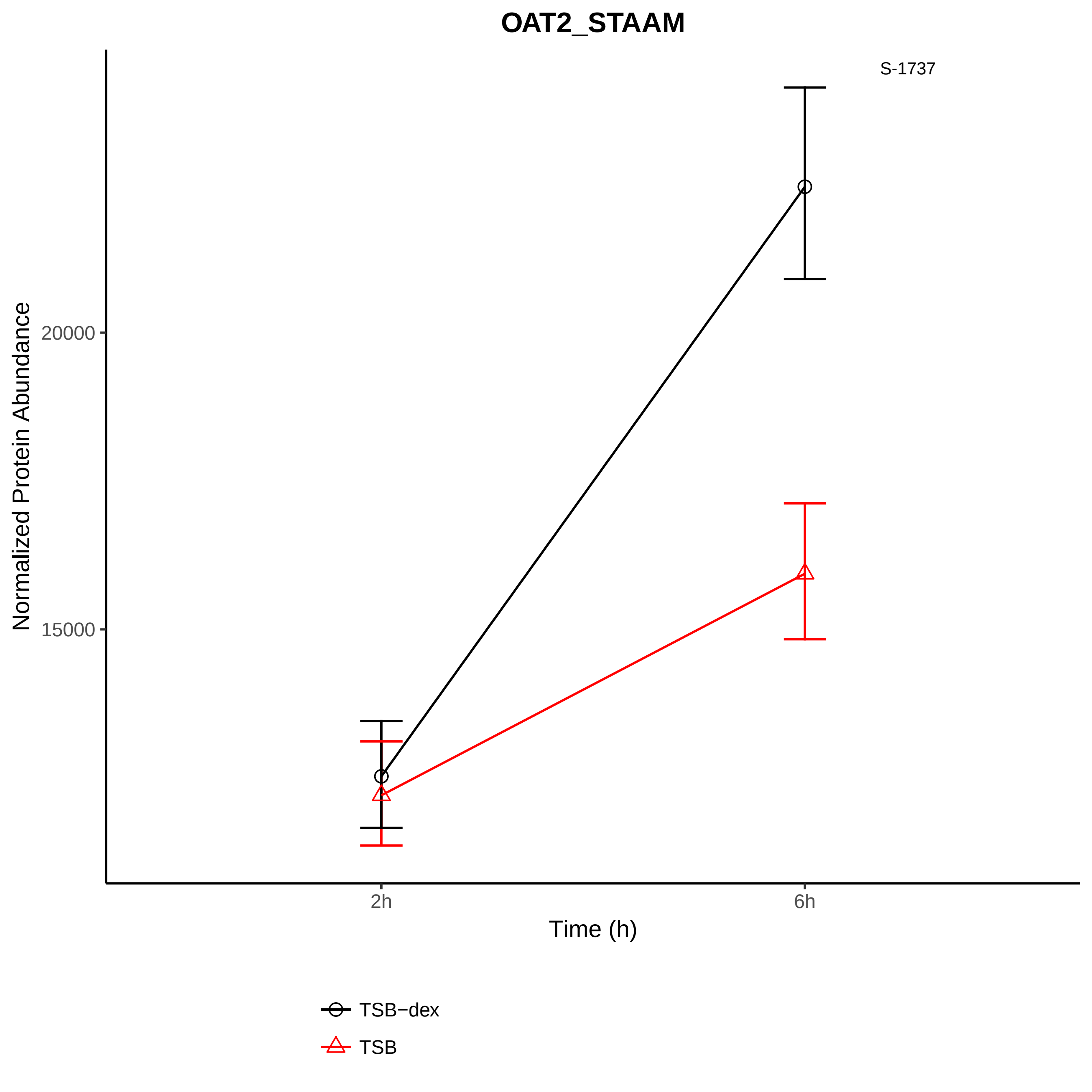
OAT2_STAAM
S-1737
Normalized Protein Abundance
20000
15000
2h
6h
Time (h)
TSB−dex
TSB

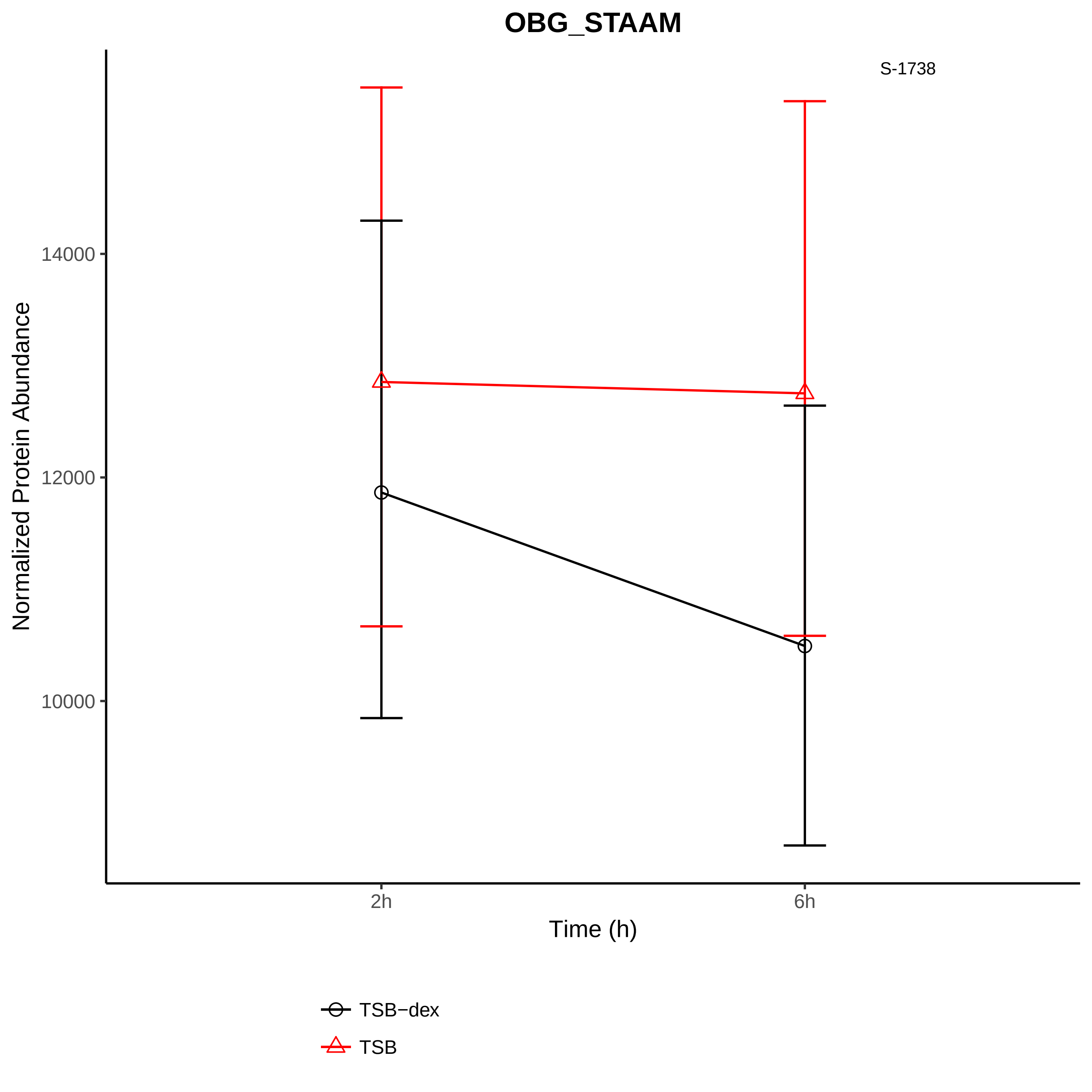

OBG_STAAM
S-1738
Normalized Protein Abundance
14000
12000
10000
2h
6h
Time (h)
TSB−dex
TSB

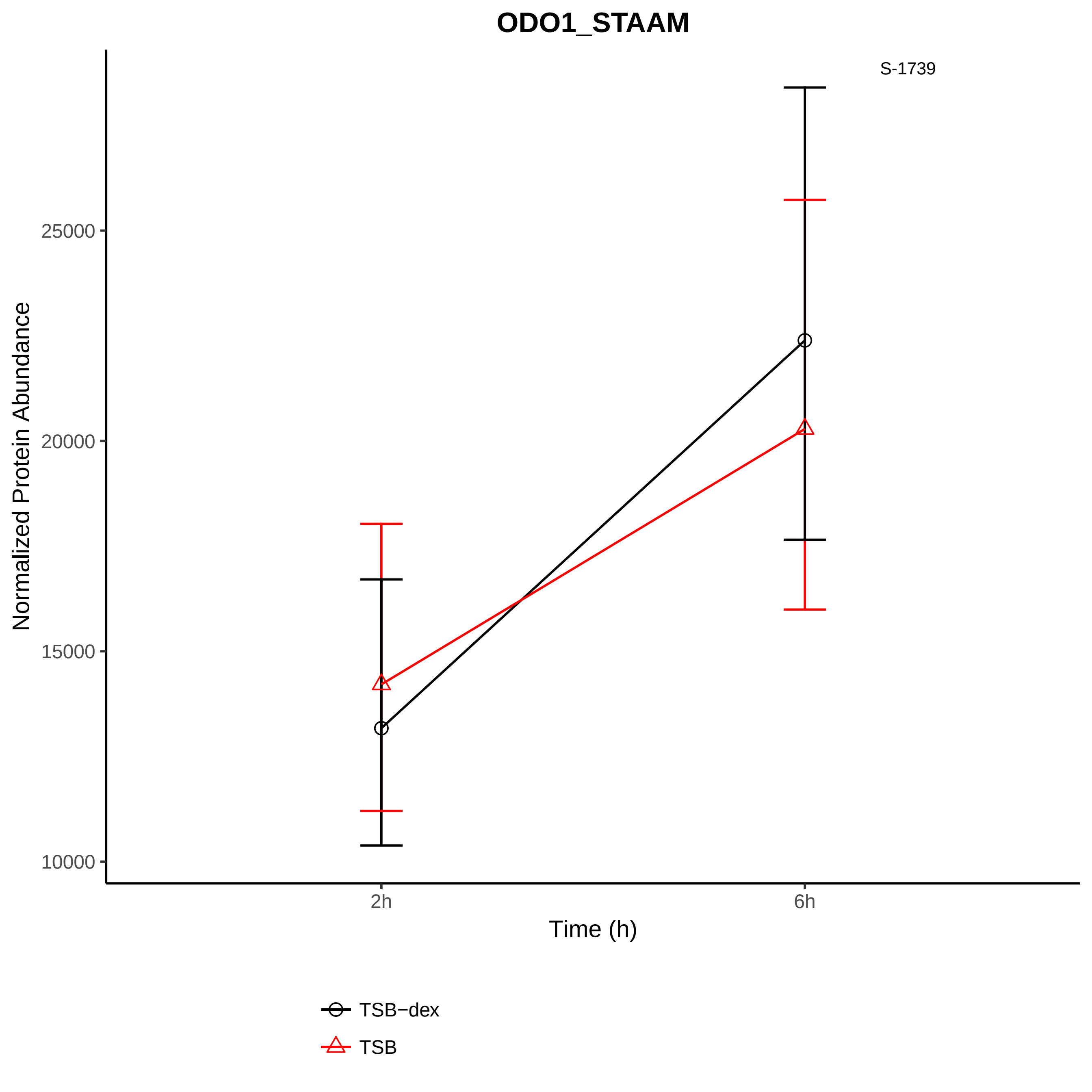
ODO1_STAAM
S-1739
25000
20000
15000
10000
Normalized Protein Abundance
2h
6h
Time (h)
TSB−dex
TSB

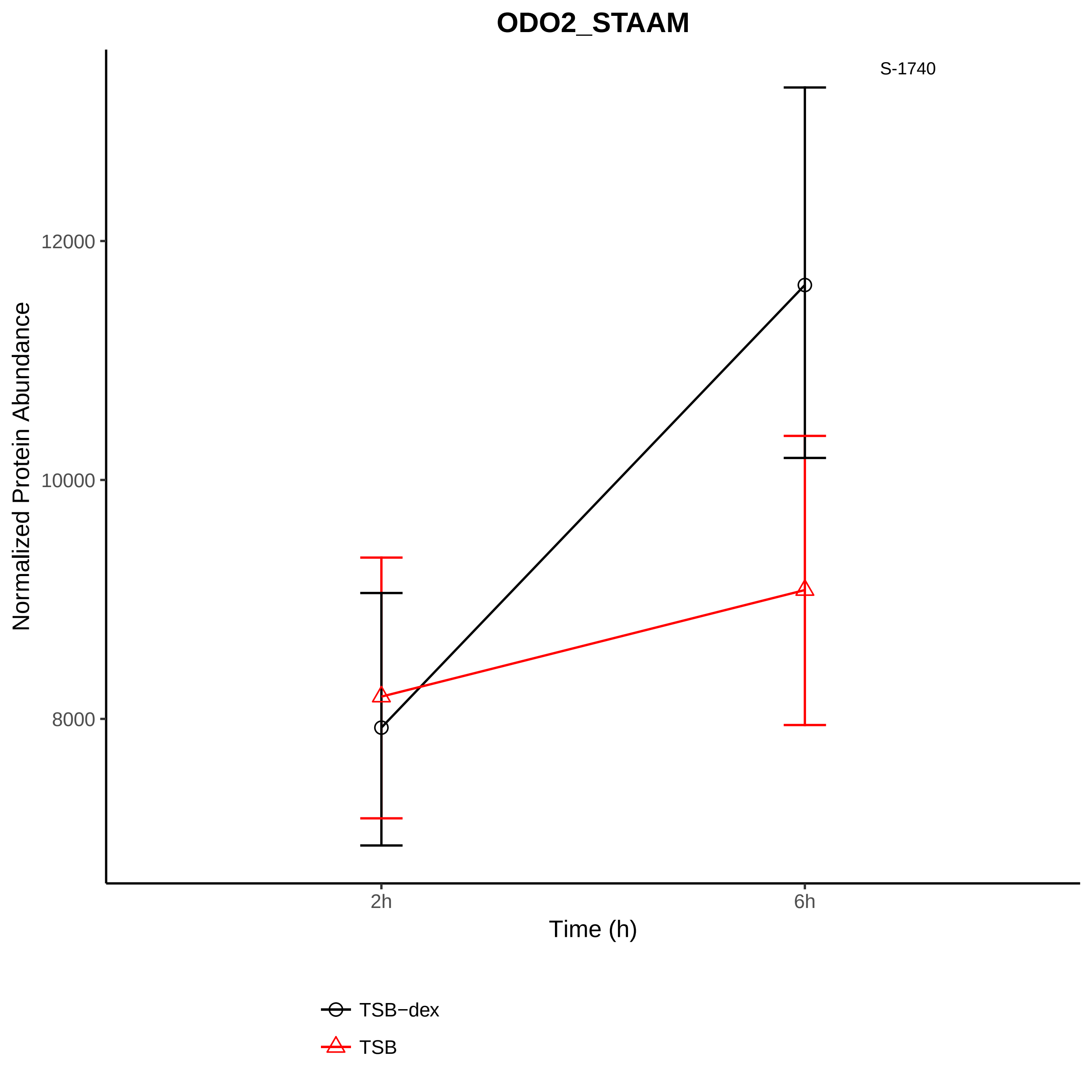

ODO2_STAAM
S-1740
12000
10000
8000
Normalized Protein Abundance
2h
6h
Time (h)
TSB−dex
TSB

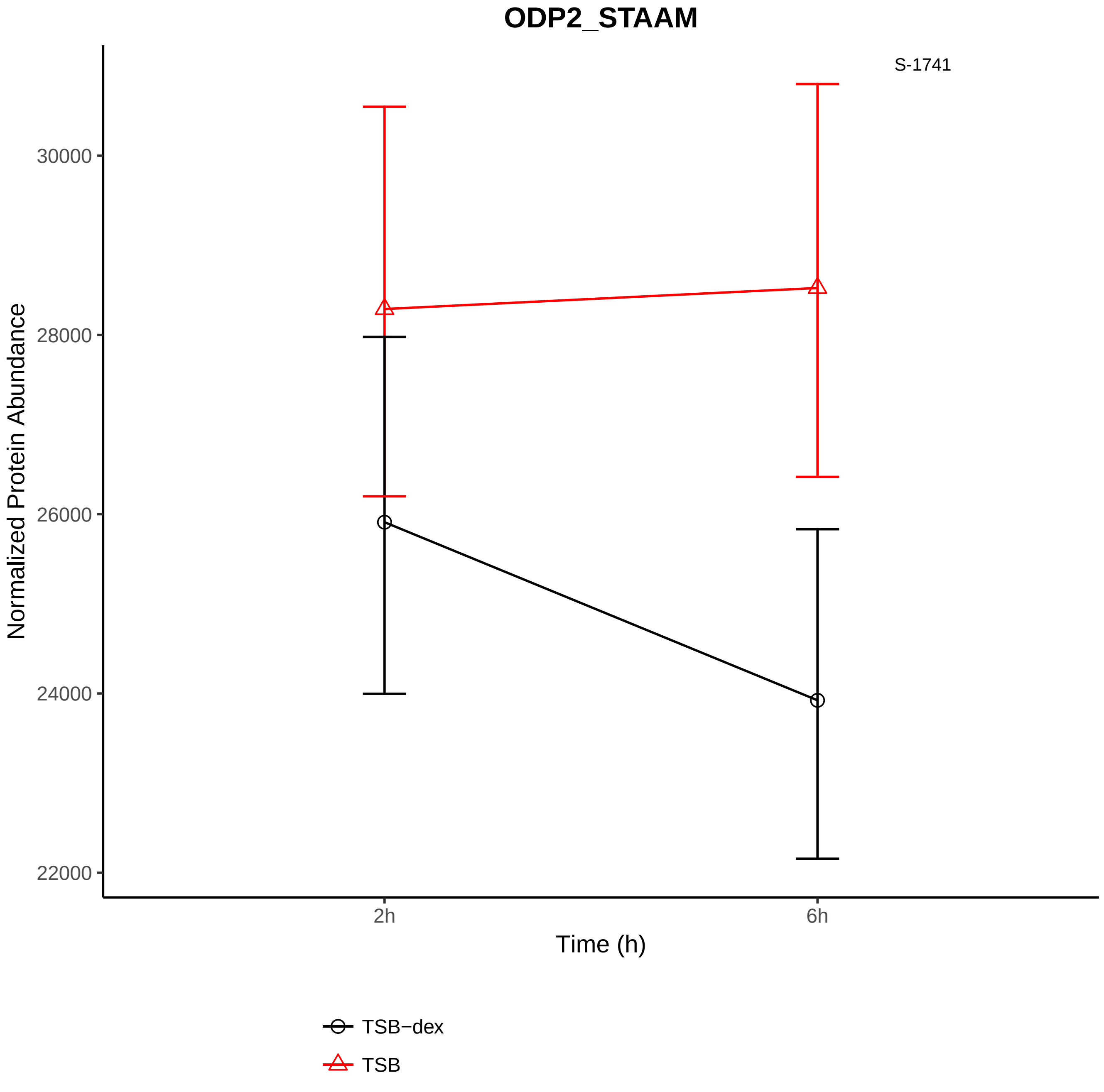
ODP2_STAAM
S-1741
Normalized Protein Abundance
30000
28000
26000
24000
22000
2h
6h
Time (h)
TSB−dex
TSB

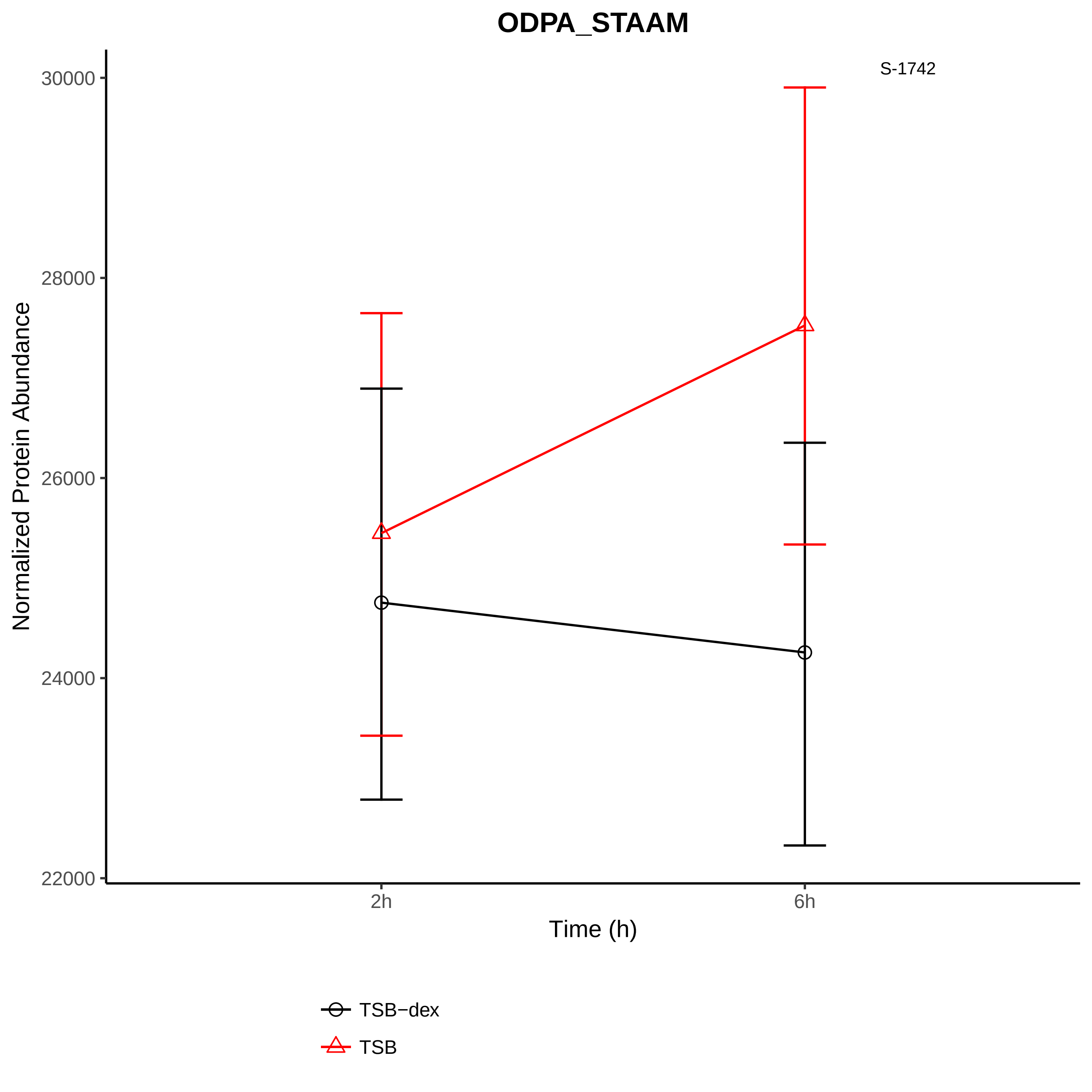

ODPA_STAAM
S-1742
30000
28000
26000
24000
22000
Normalized Protein Abundance
2h
6h
Time (h)
TSB−dex
TSB

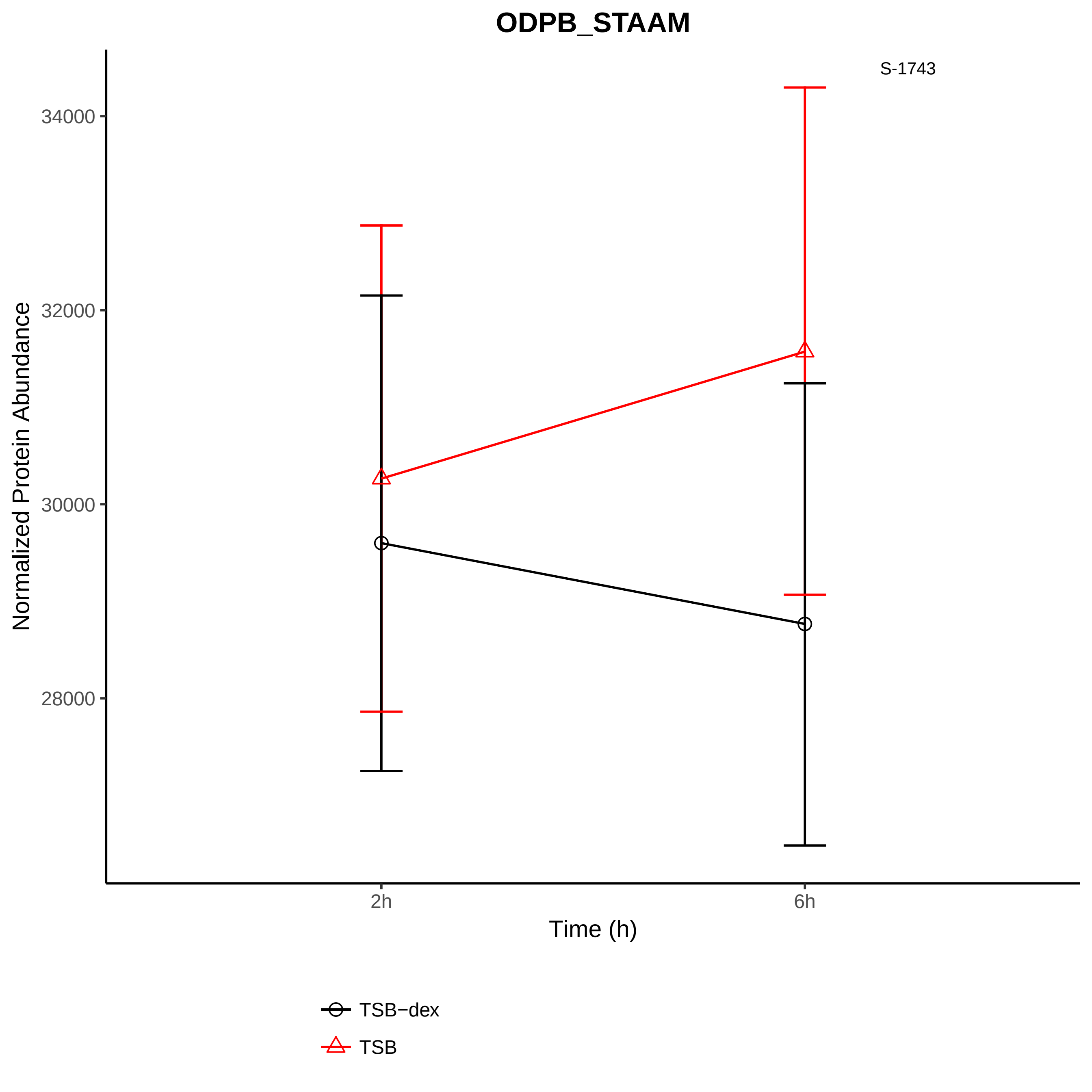

ODPB_STAAM
S-1743
34000
32000
30000
28000
Normalized Protein Abundance
2h
6h
Time (h)
TSB−dex
TSB

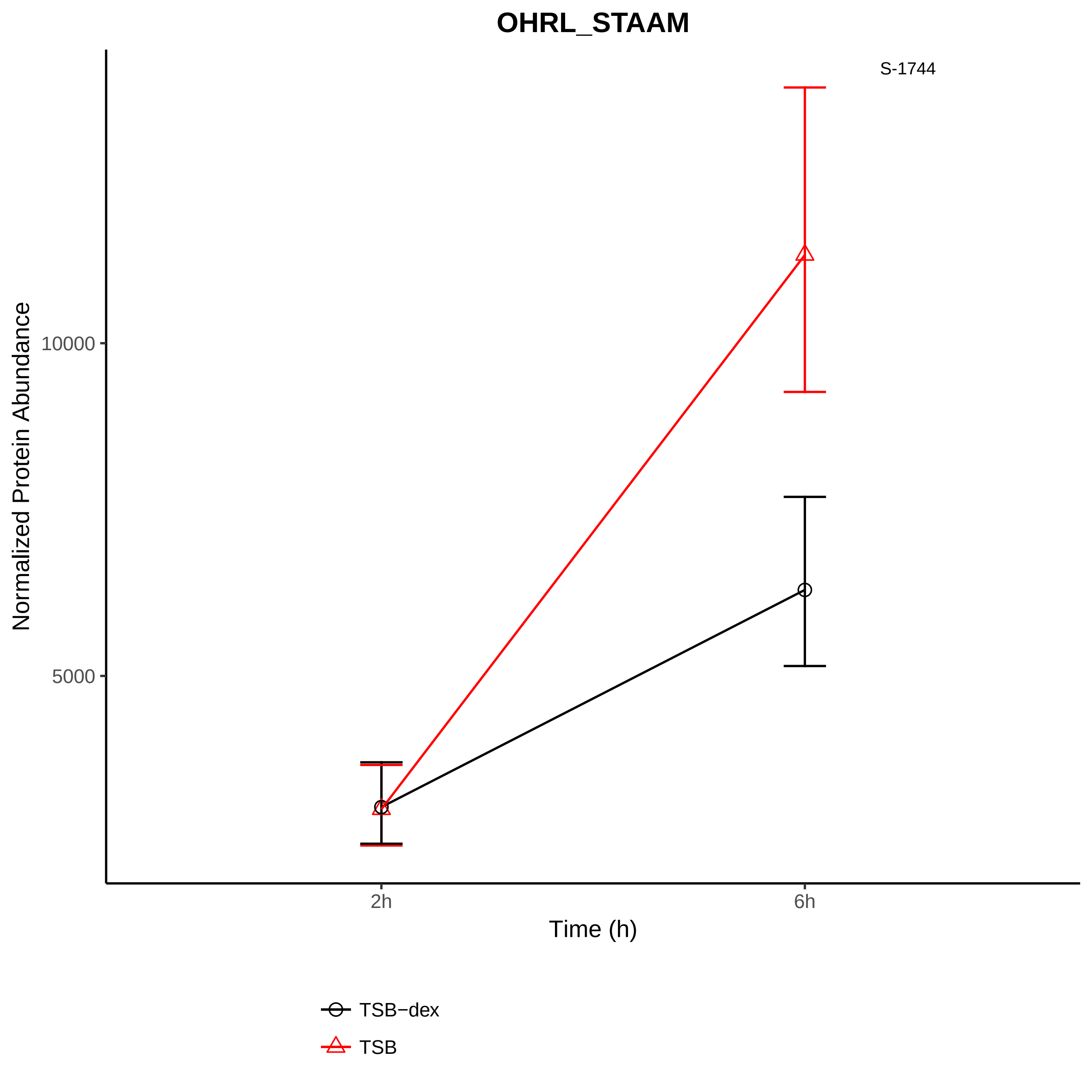

OHRL_STAAM
S-1744
Normalized Protein Abundance
10000
5000
2h
6h
Time (h)
TSB−dex
TSB

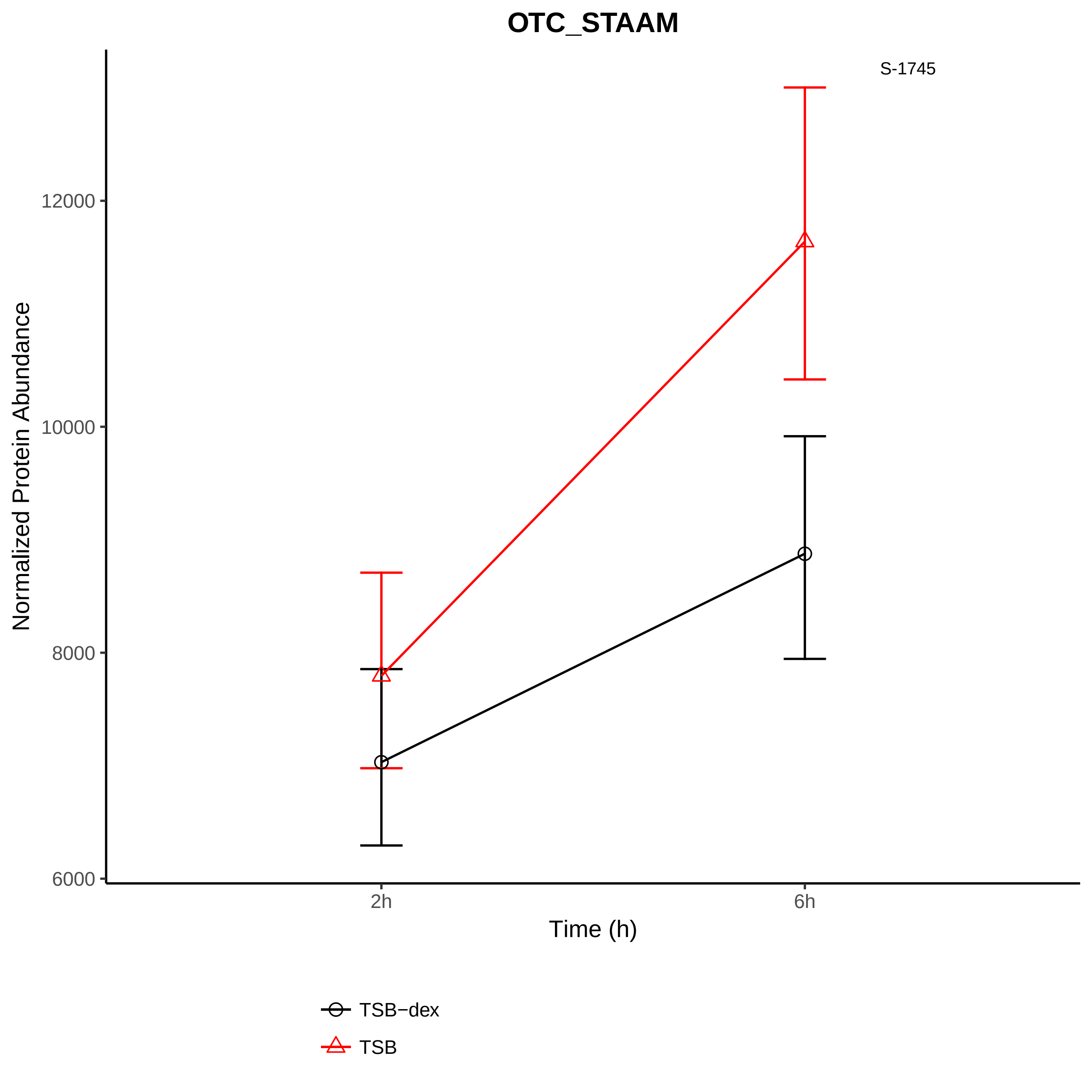

OTC_STAAM
S-1745
Normalized Protein Abundance
12000
10000
8000
6000
2h
6h
Time (h)
TSB−dex
TSB

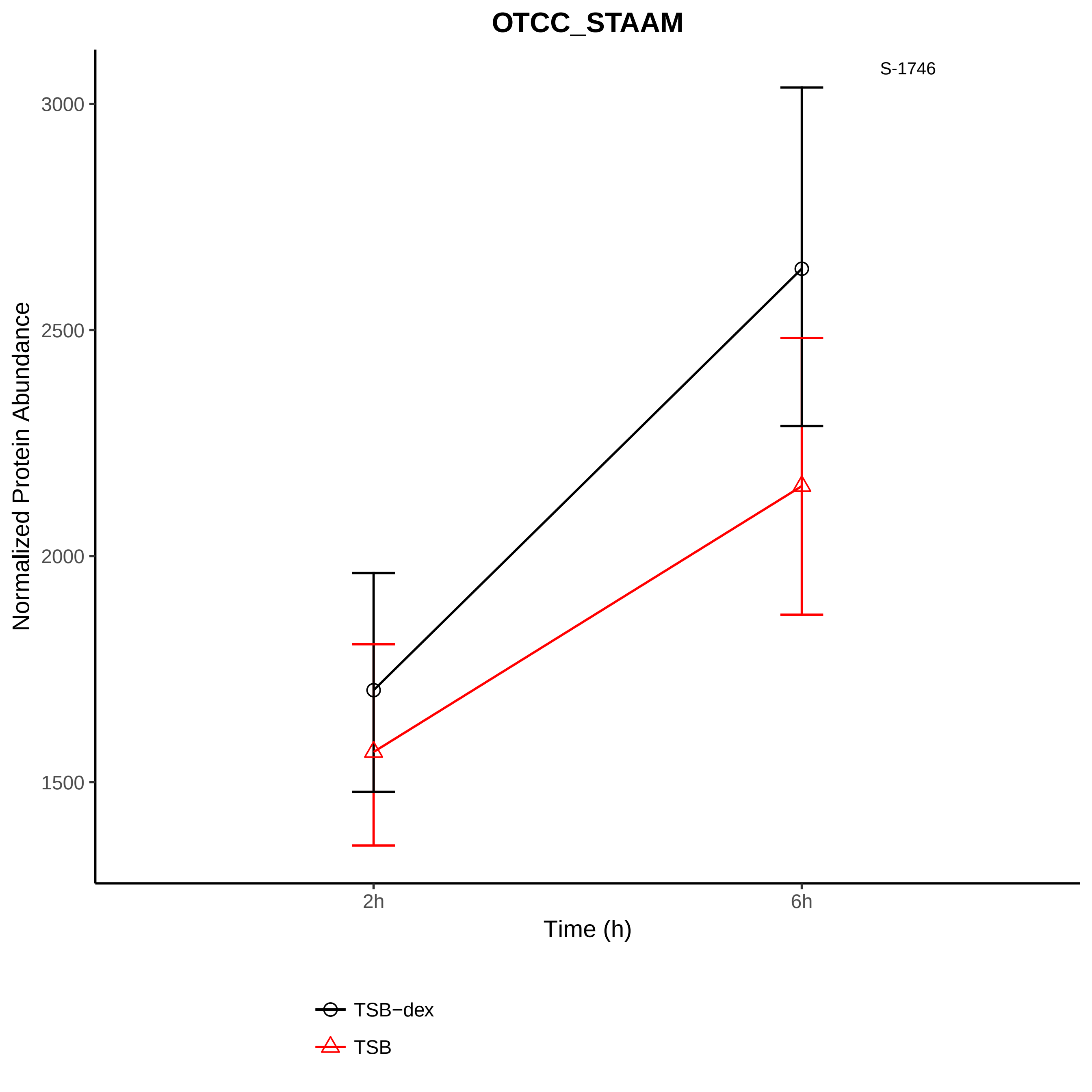

OTCC_STAAM
S-1746
3000
2500
2000
1500
Normalized Protein Abundance
2h
6h
Time (h)
TSB−dex
TSB

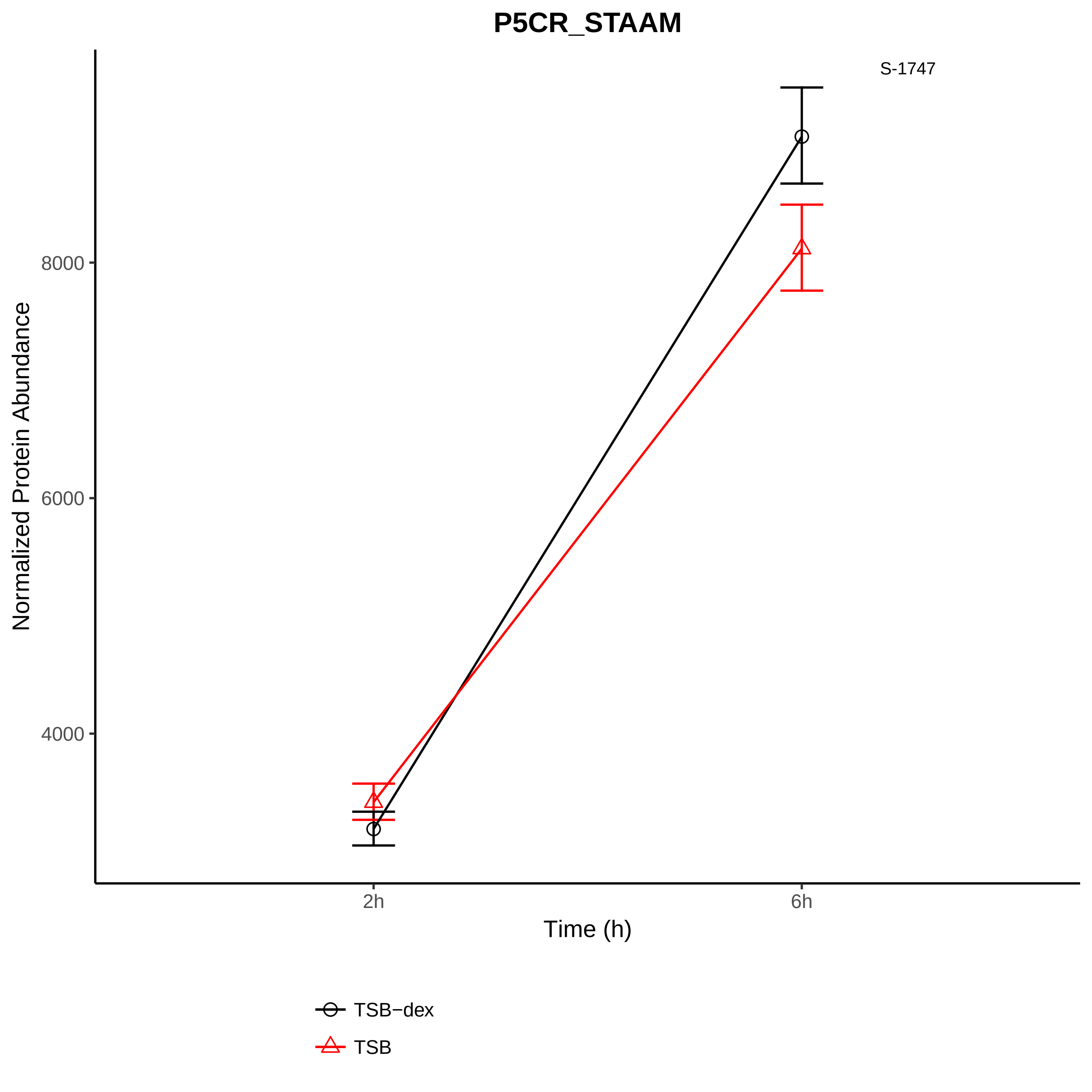

P5CR_STAAM
S-1747
8000
6000
4000
Normalized Protein Abundance
2h
6h
Time (h)
TSB−dex
TSB

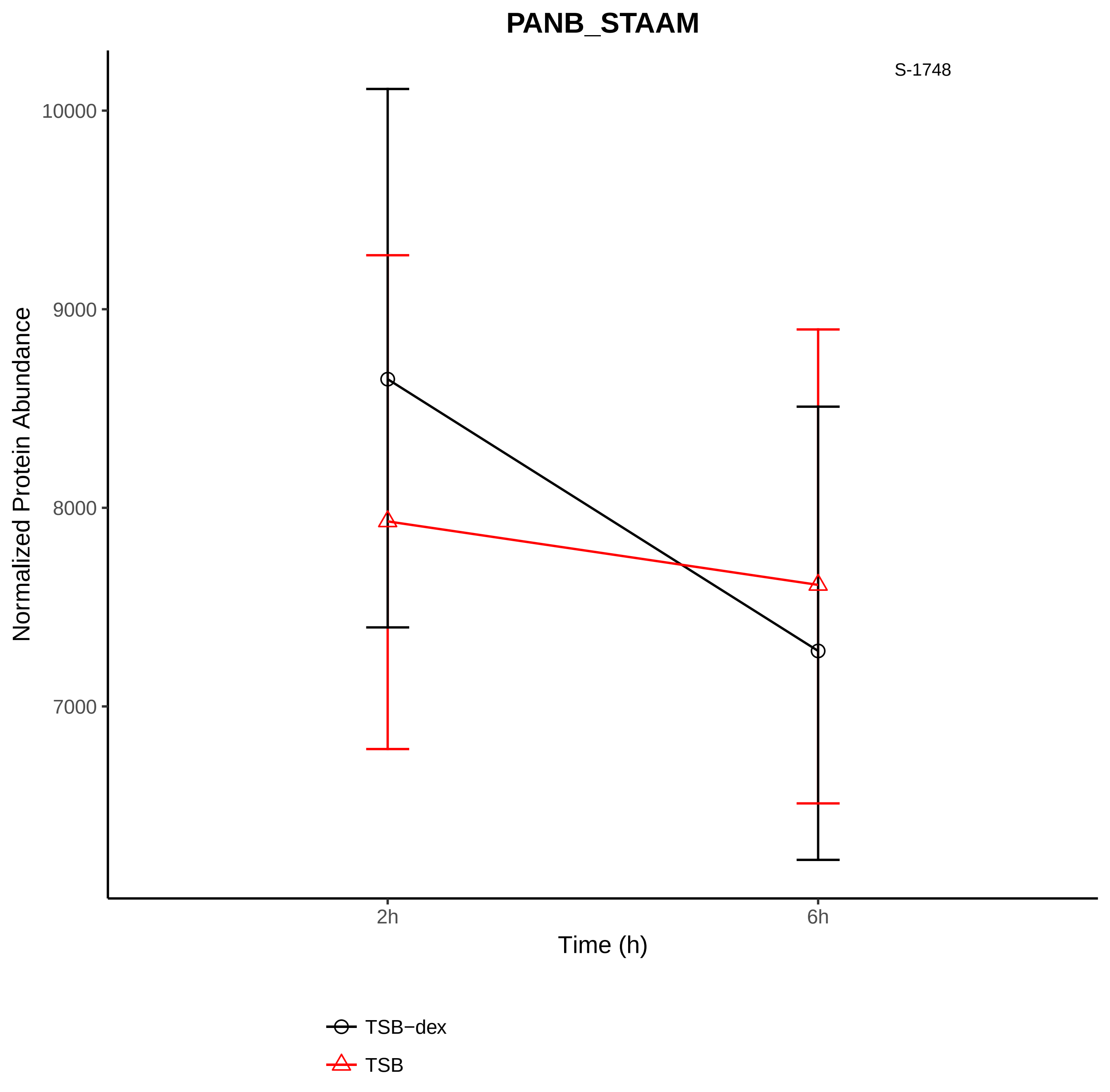

PANB_STAAM
S-1748
Normalized Protein Abundance
10000
9000
8000
7000
2h
6h
Time (h)
TSB−dex
TSB

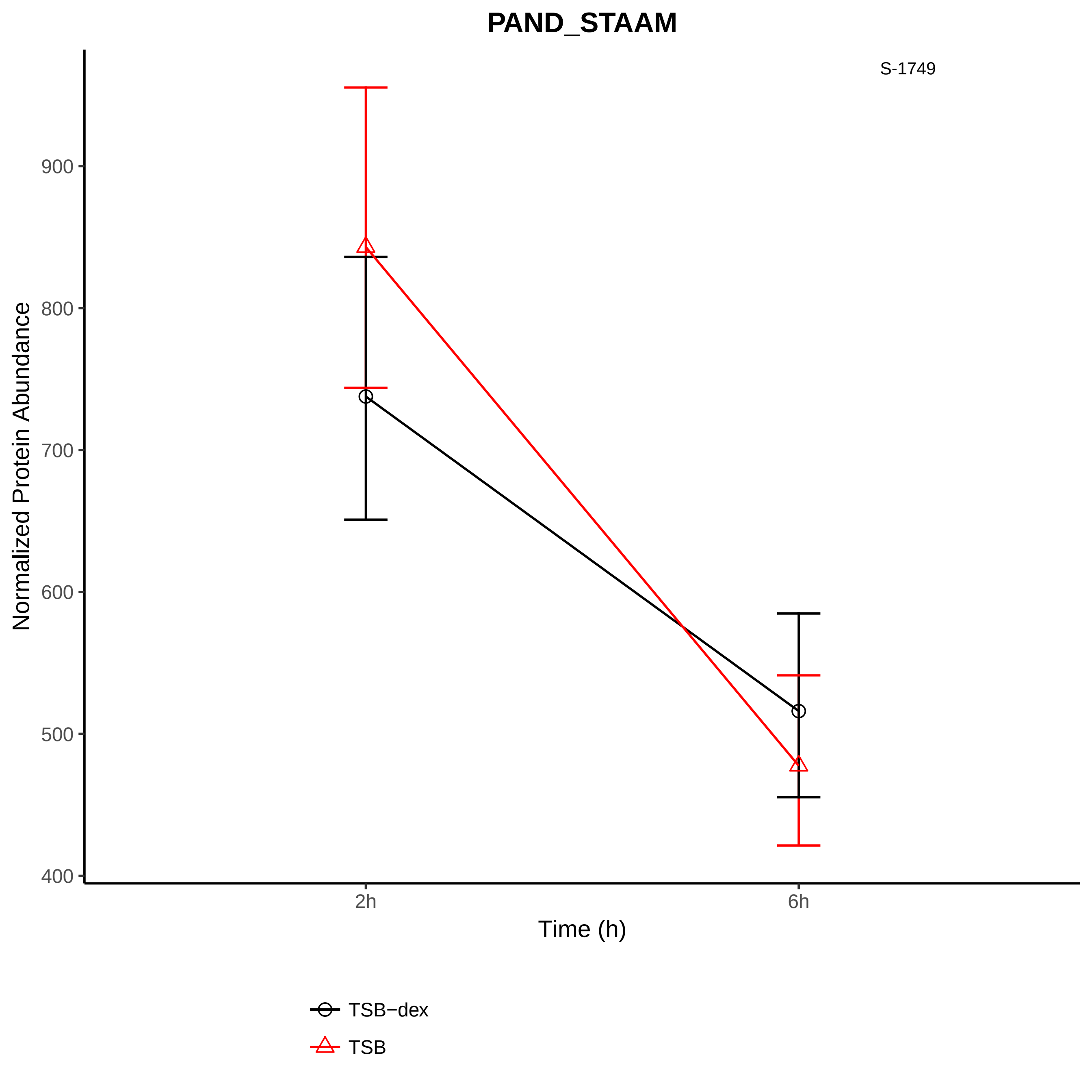

PAND_STAAM
S-1749
Normalized Protein Abundance
900
800
700
600
500
400
2h
6h
Time (h)
TSB−dex
TSB

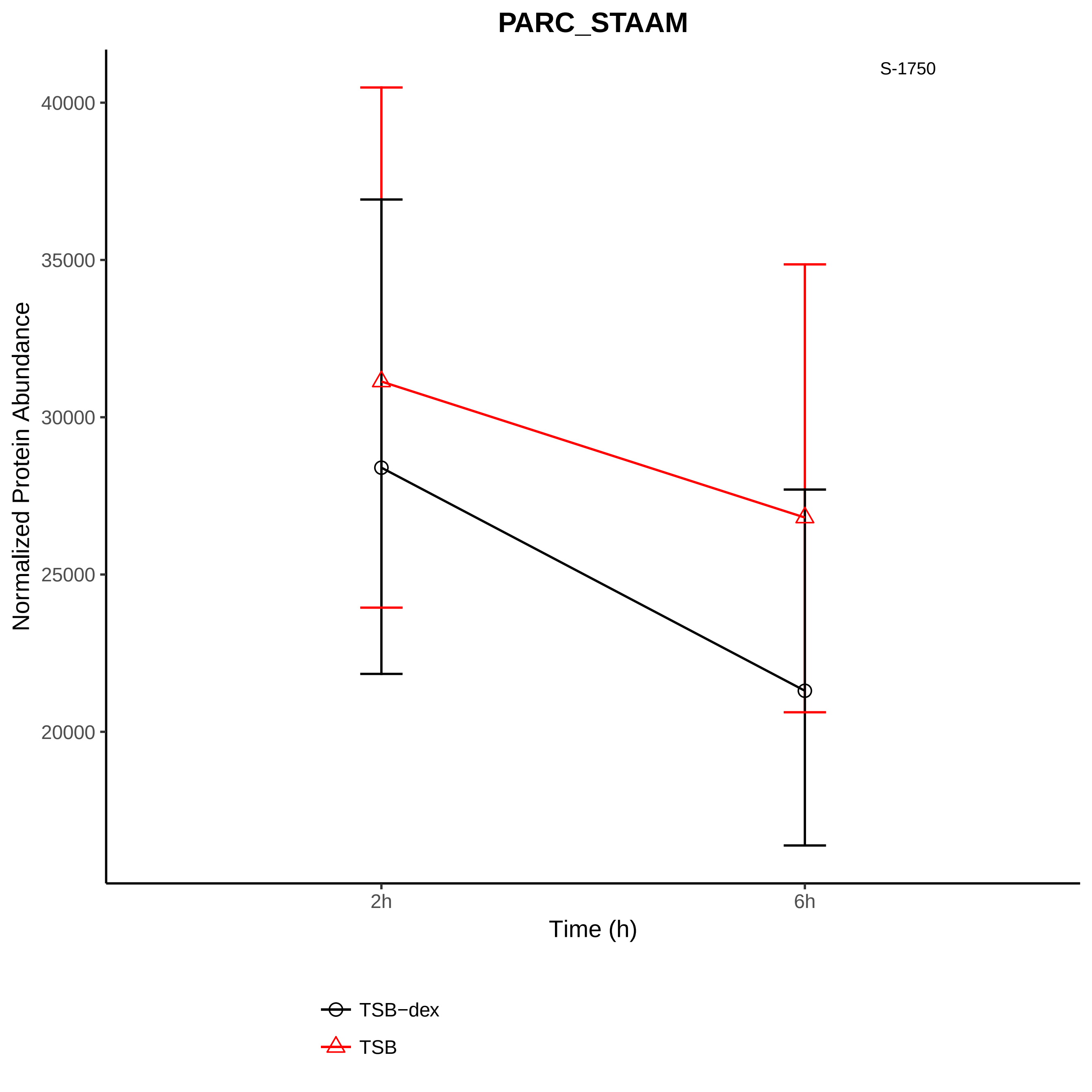
PARC_STAAM
S-1750
Normalized Protein Abundance
40000
35000
30000
25000
20000
2h
6h
Time (h)
TSB−dex
TSB

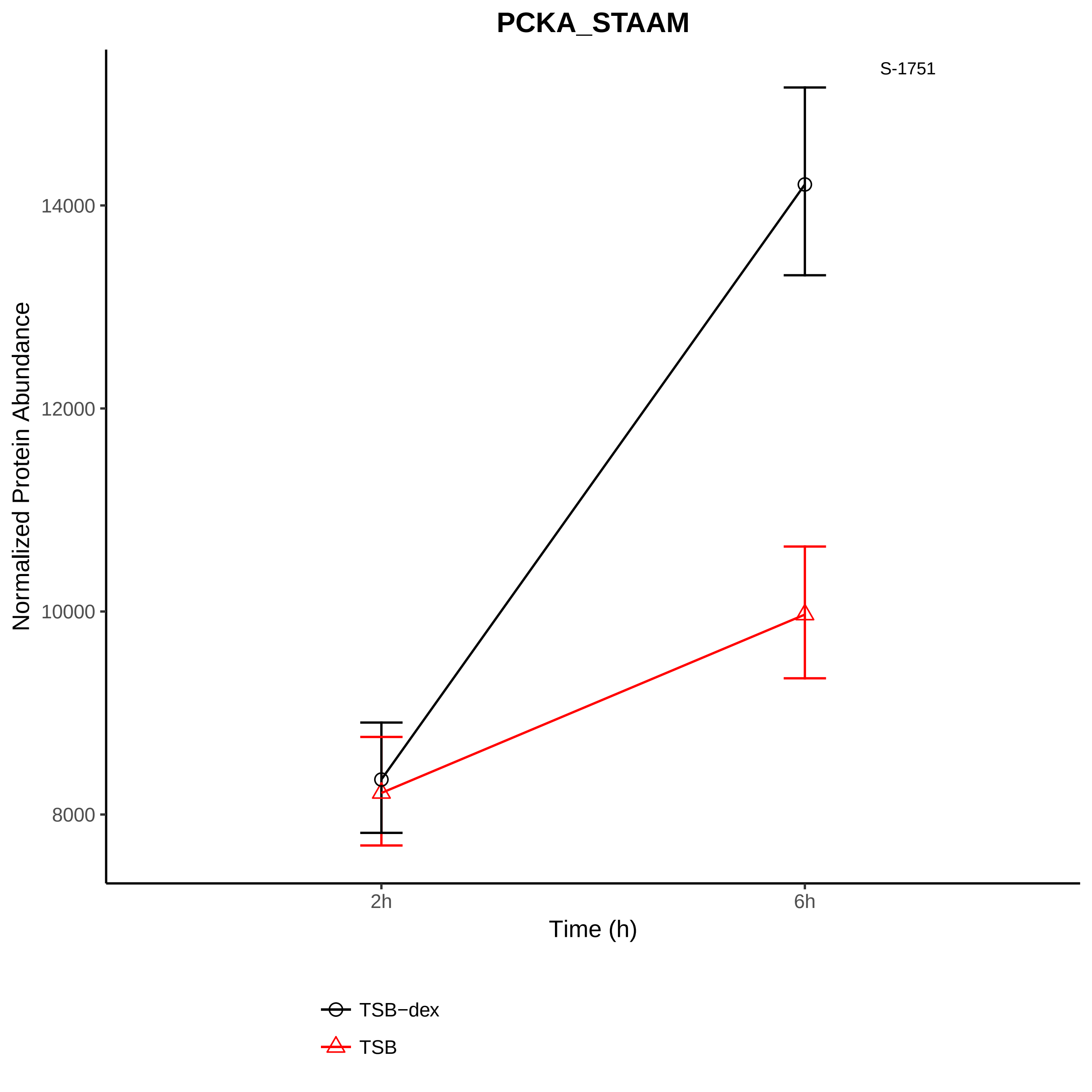

PCKA_STAAM
S-1751
Normalized Protein Abundance
14000
12000
10000
8000
2h
6h
Time (h)
TSB−dex
TSB

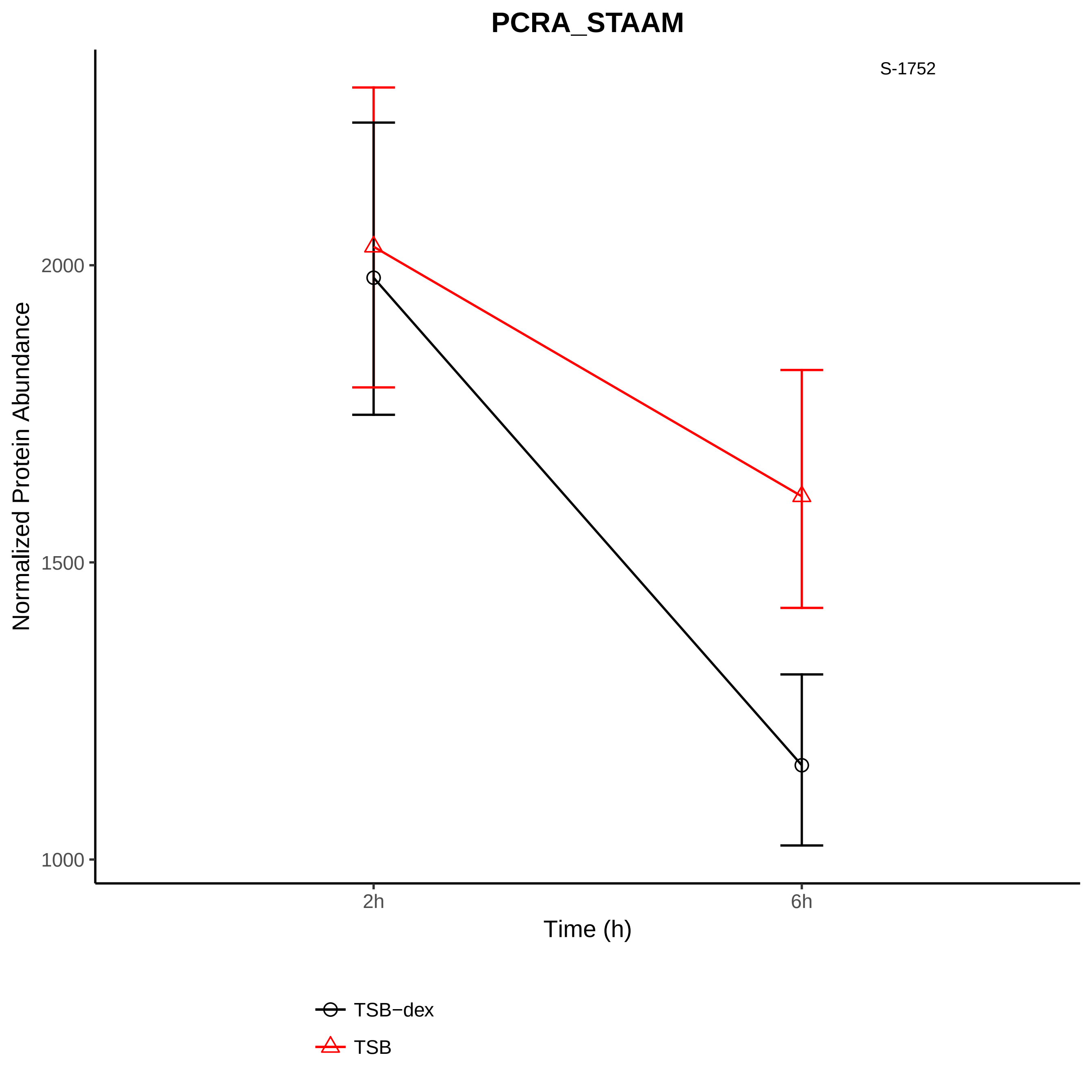

PCRA_STAAM
S-1752
Normalized Protein Abundance
2000
1500
1000
2h
6h
Time (h)
TSB−dex
TSB

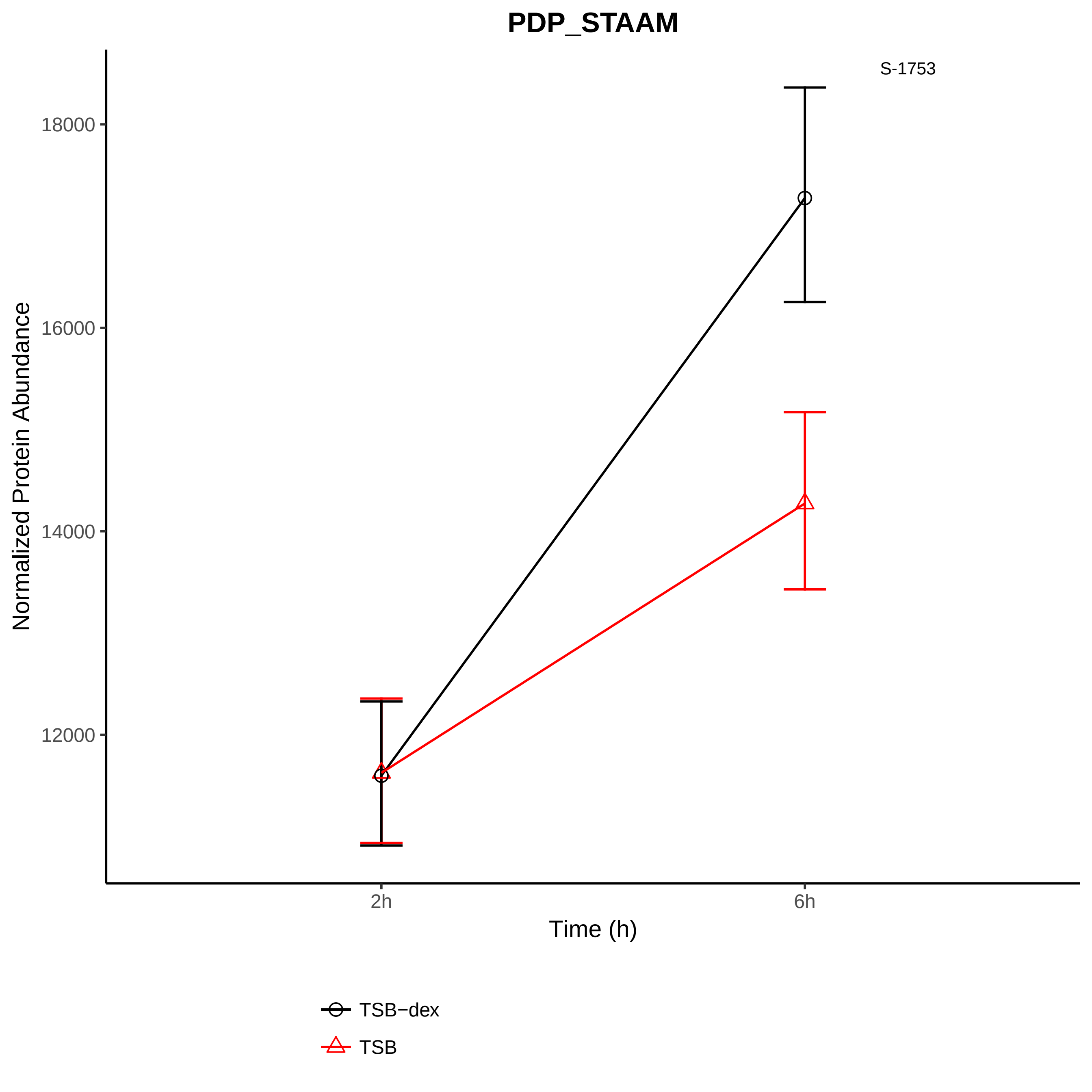
PDP_STAAM
S-1753
Normalized Protein Abundance
18000
16000
14000
12000
2h
6h
Time (h)
TSB−dex
TSB

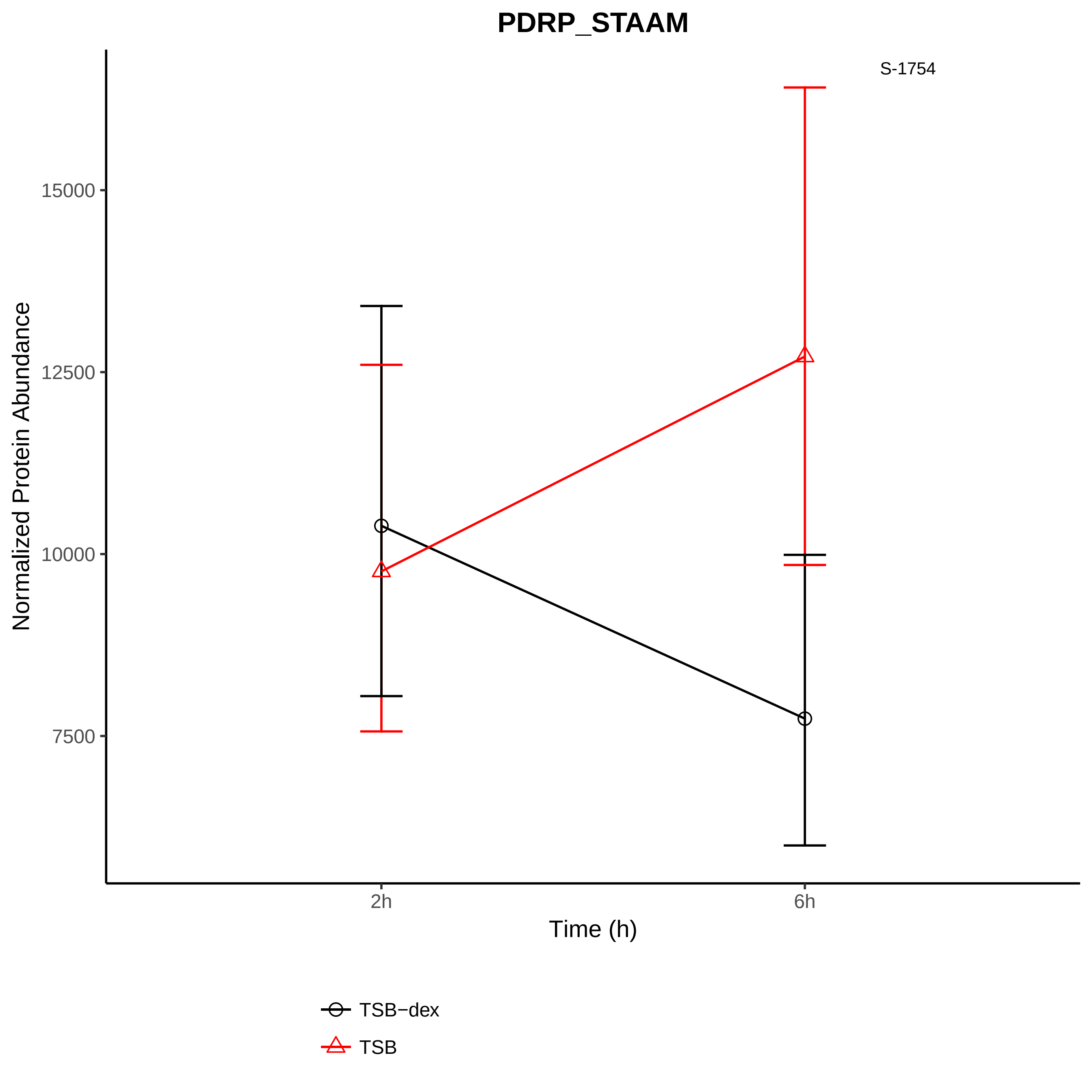

PDRP_STAAM
S-1754
Normalized Protein Abundance
15000
12500
10000
7500
2h
6h
Time (h)
TSB−dex
TSB

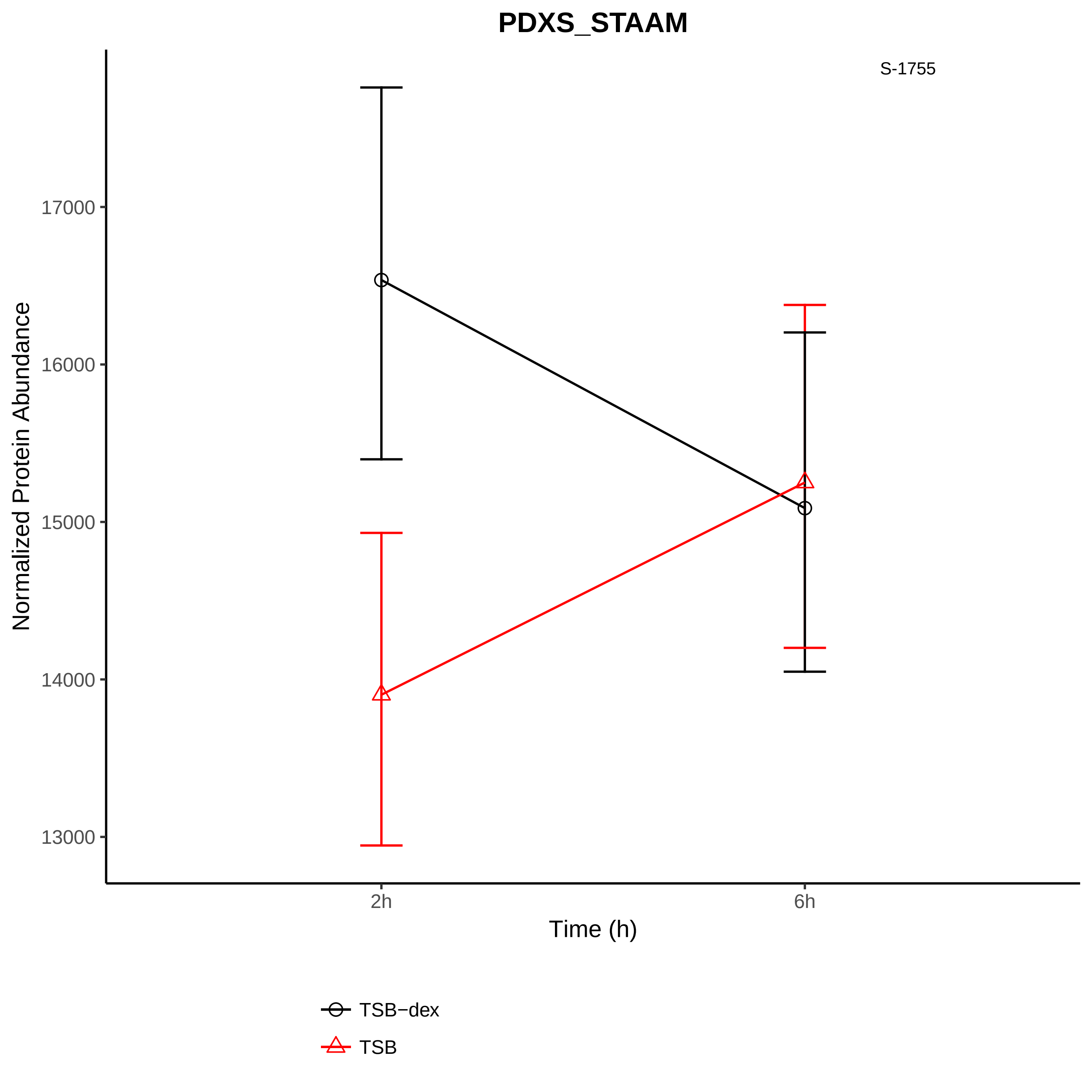

PDXS_STAAM
S-1755
Normalized Protein Abundance
17000
16000
15000
14000
13000
2h
6h
Time (h)
TSB−dex
TSB

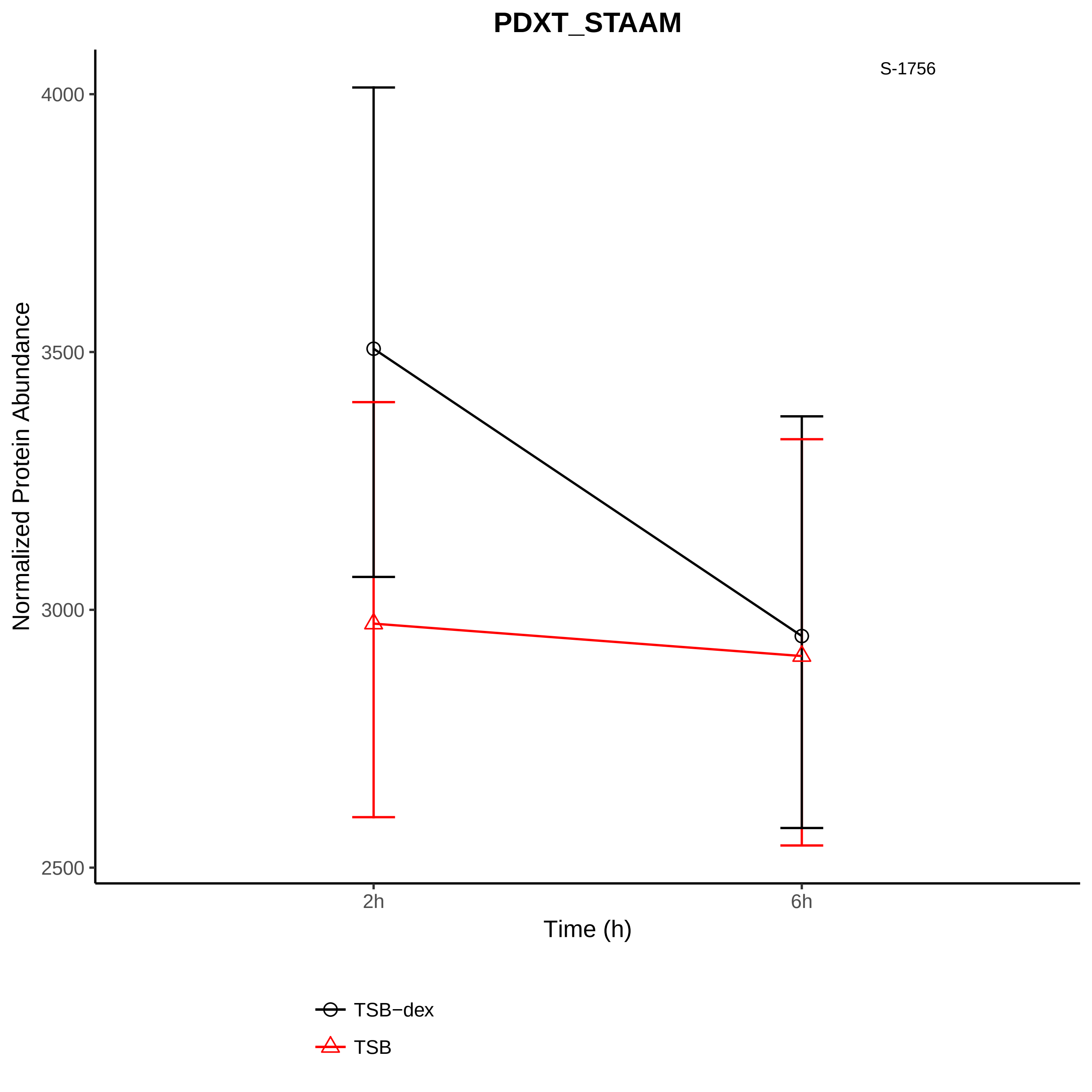

PDXT_STAAM
S-1756
Normalized Protein Abundance
4000
3500
3000
2500
2h
6h
Time (h)
TSB−dex
TSB

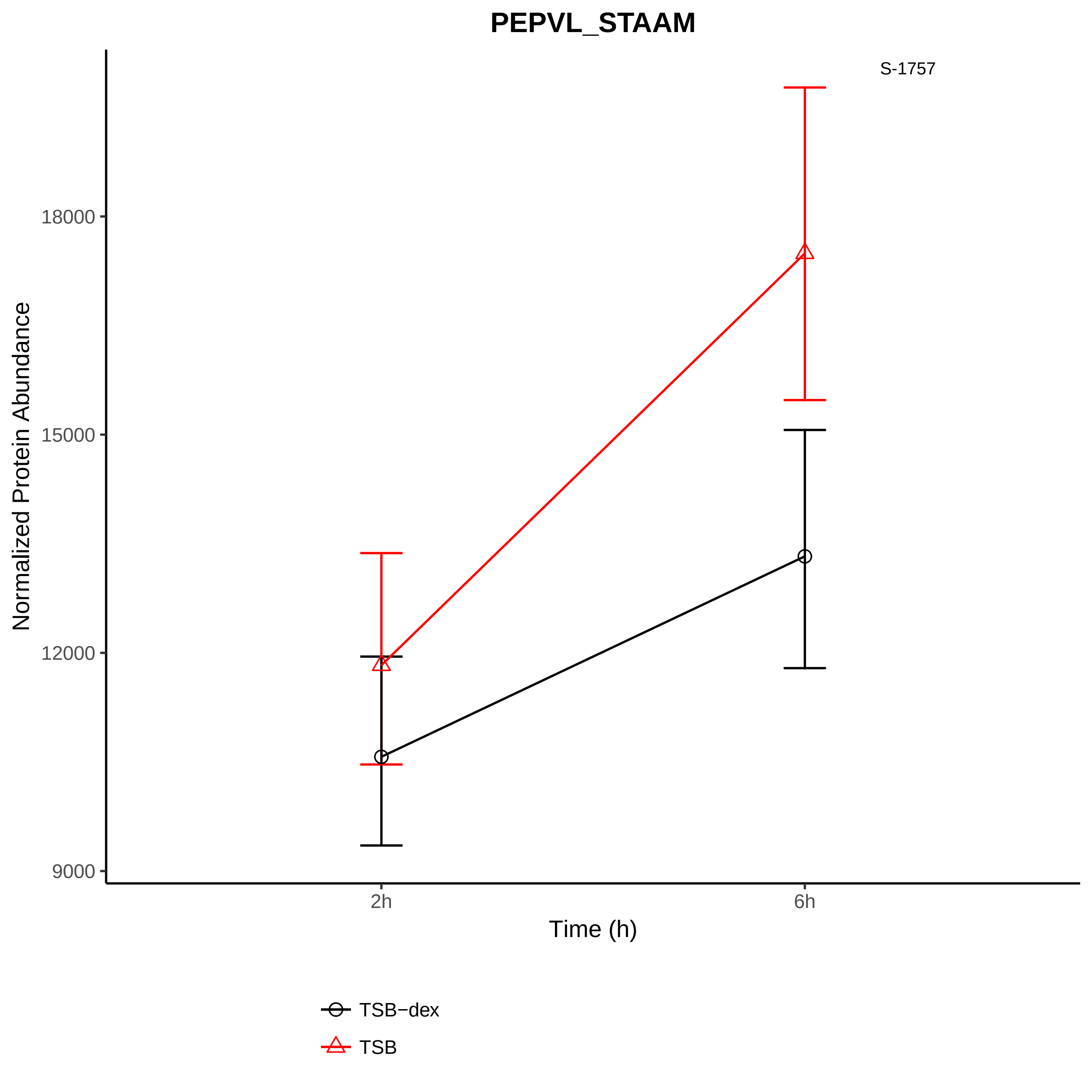

PEPVL_STAAM
S-1757
Normalized Protein Abundance
18000
15000
12000
9000
2h
6h
Time (h)
TSB−dex
TSB

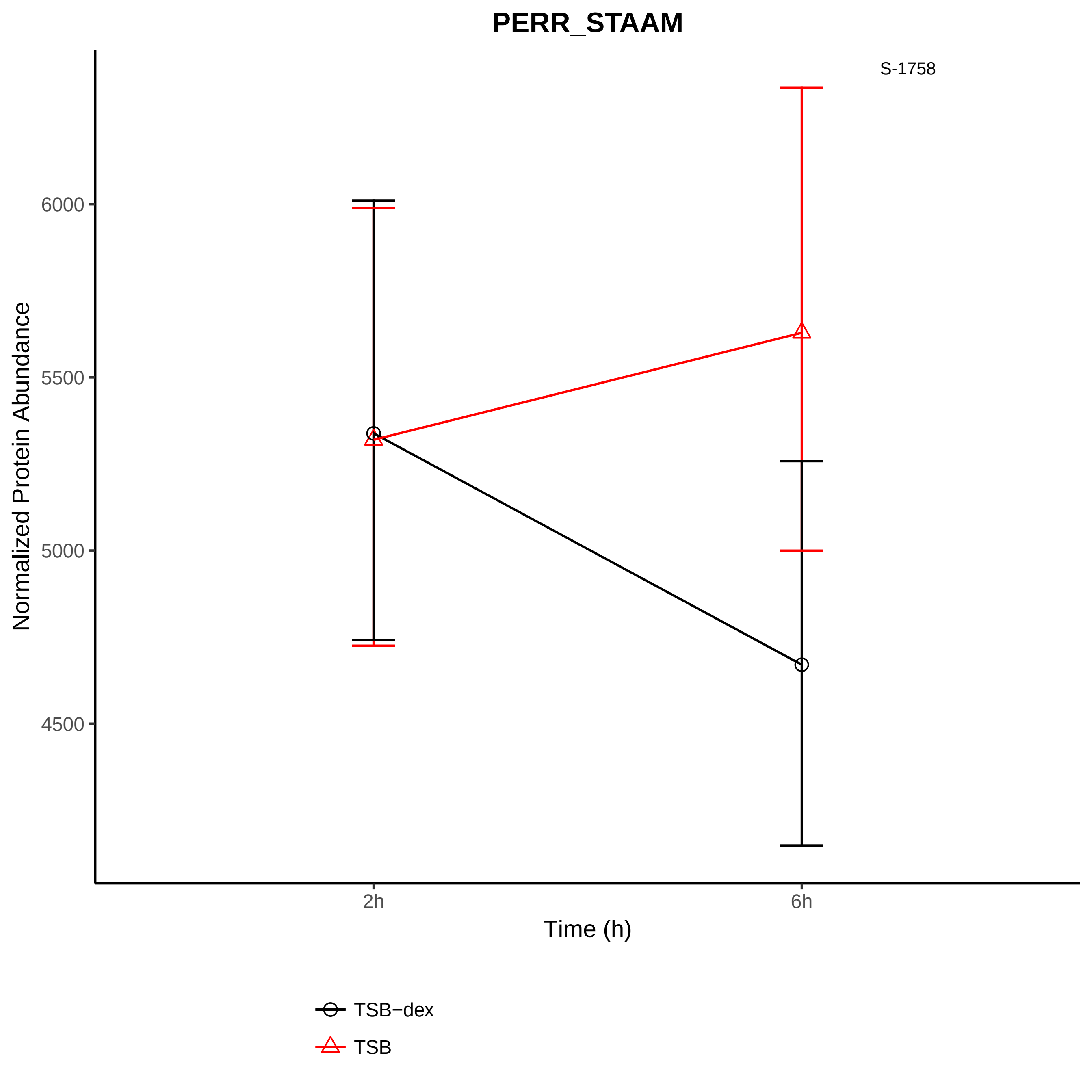

PERR_STAAM
S-1758
6000
5500
5000
4500
Normalized Protein Abundance
2h
6h
Time (h)
TSB−dex
TSB

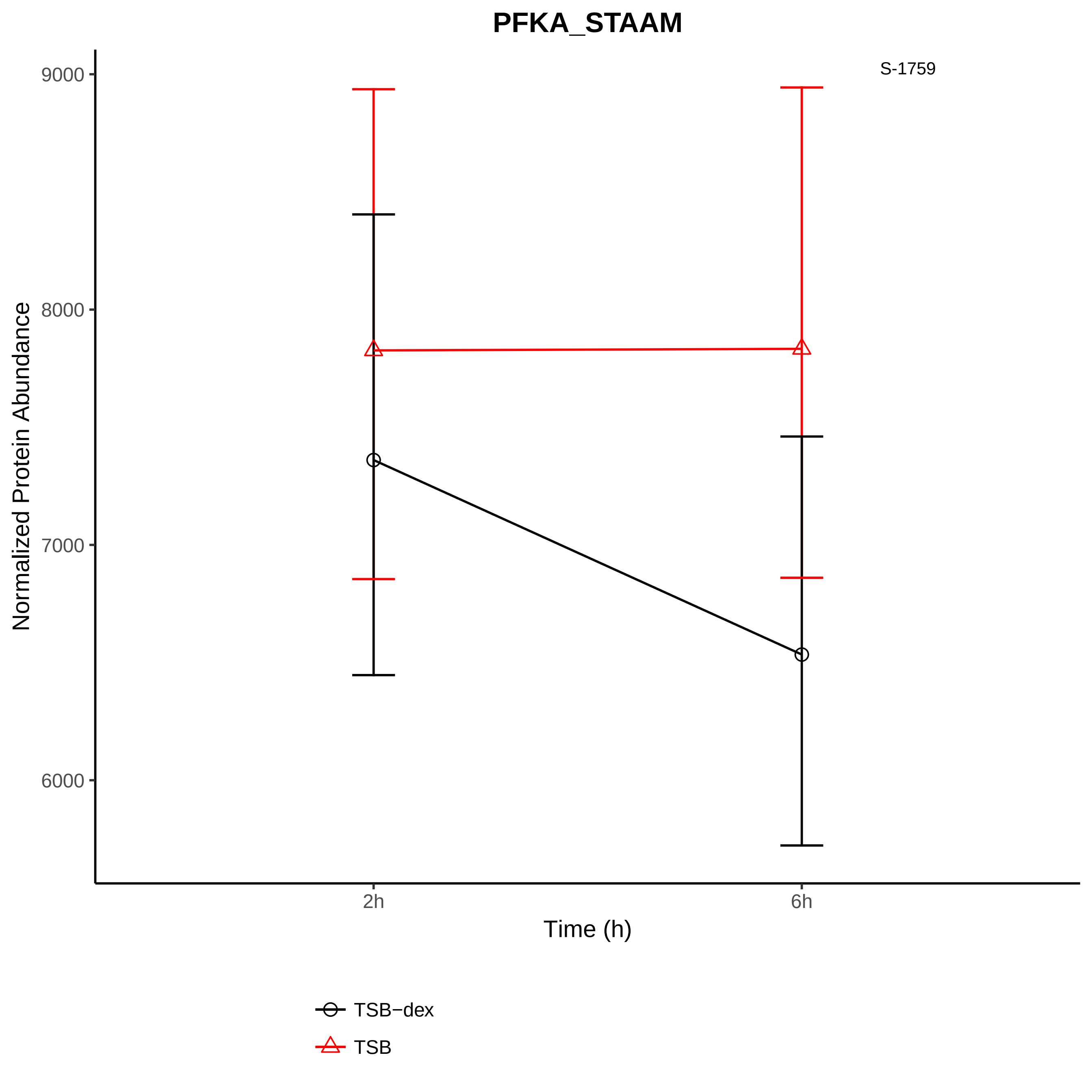
PFKA_STAAM
S-1759
9000
8000
7000
6000
Normalized Protein Abundance
2h
6h
Time (h)
TSB−dex
TSB

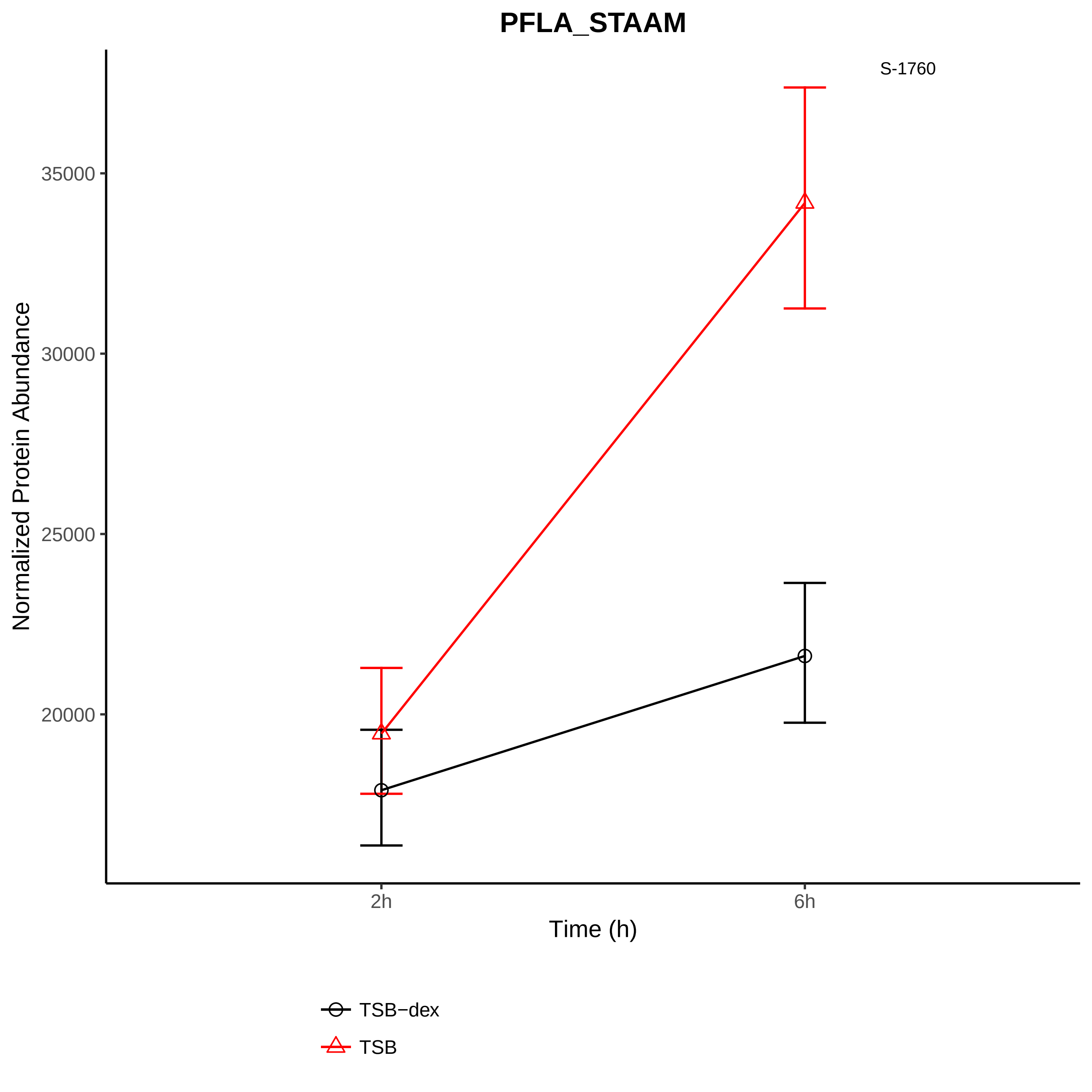

PFLA_STAAM
S-1760
Normalized Protein Abundance
35000
30000
25000
20000
2h
6h
Time (h)
TSB−dex
TSB

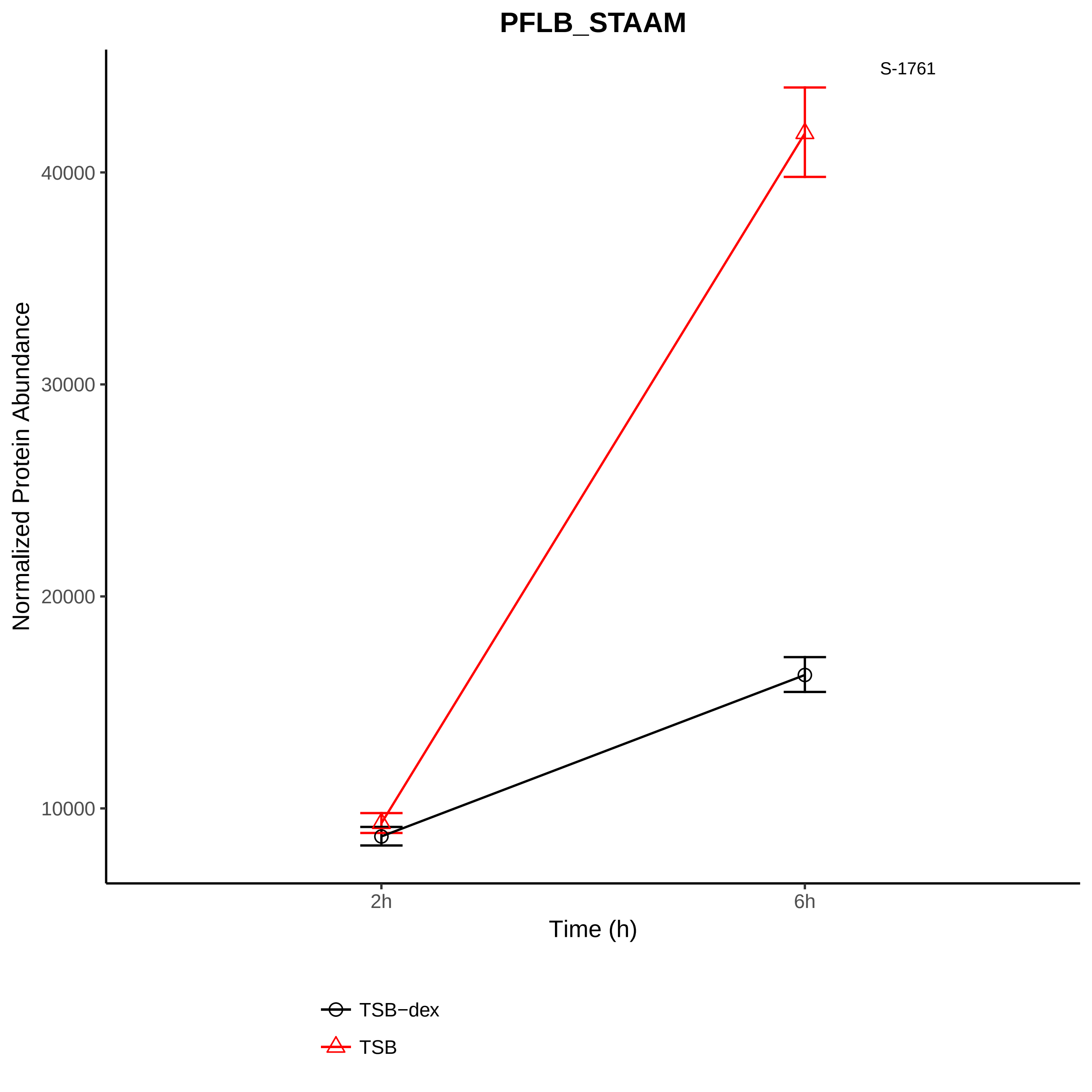

PFLB_STAAM
S-1761
40000
30000
20000
10000
Normalized Protein Abundance
2h
6h
Time (h)
TSB−dex
TSB

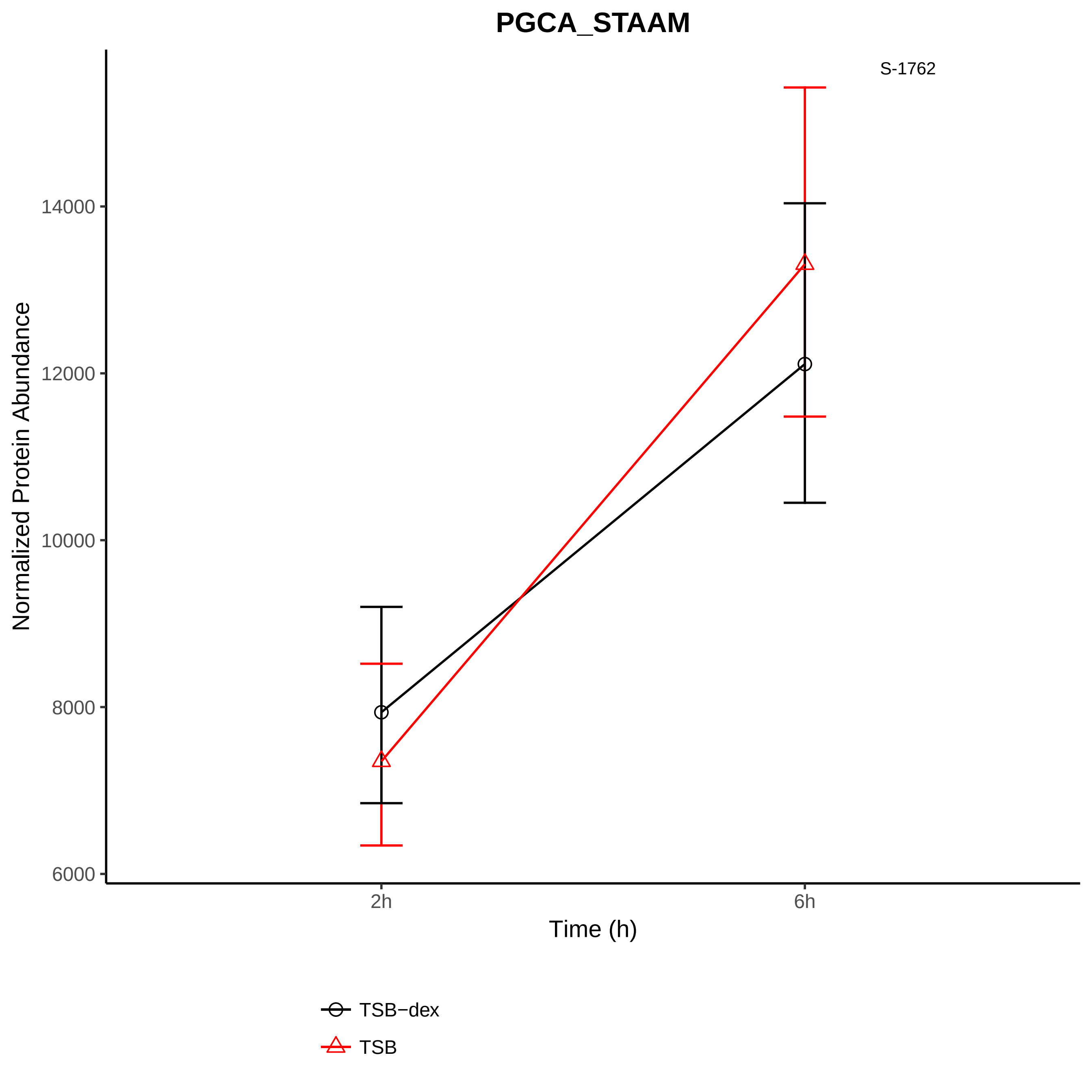
PGCA_STAAM
S-1762
Normalized Protein Abundance
14000
12000
10000
8000
6000
2h
6h
Time (h)
TSB−dex
TSB

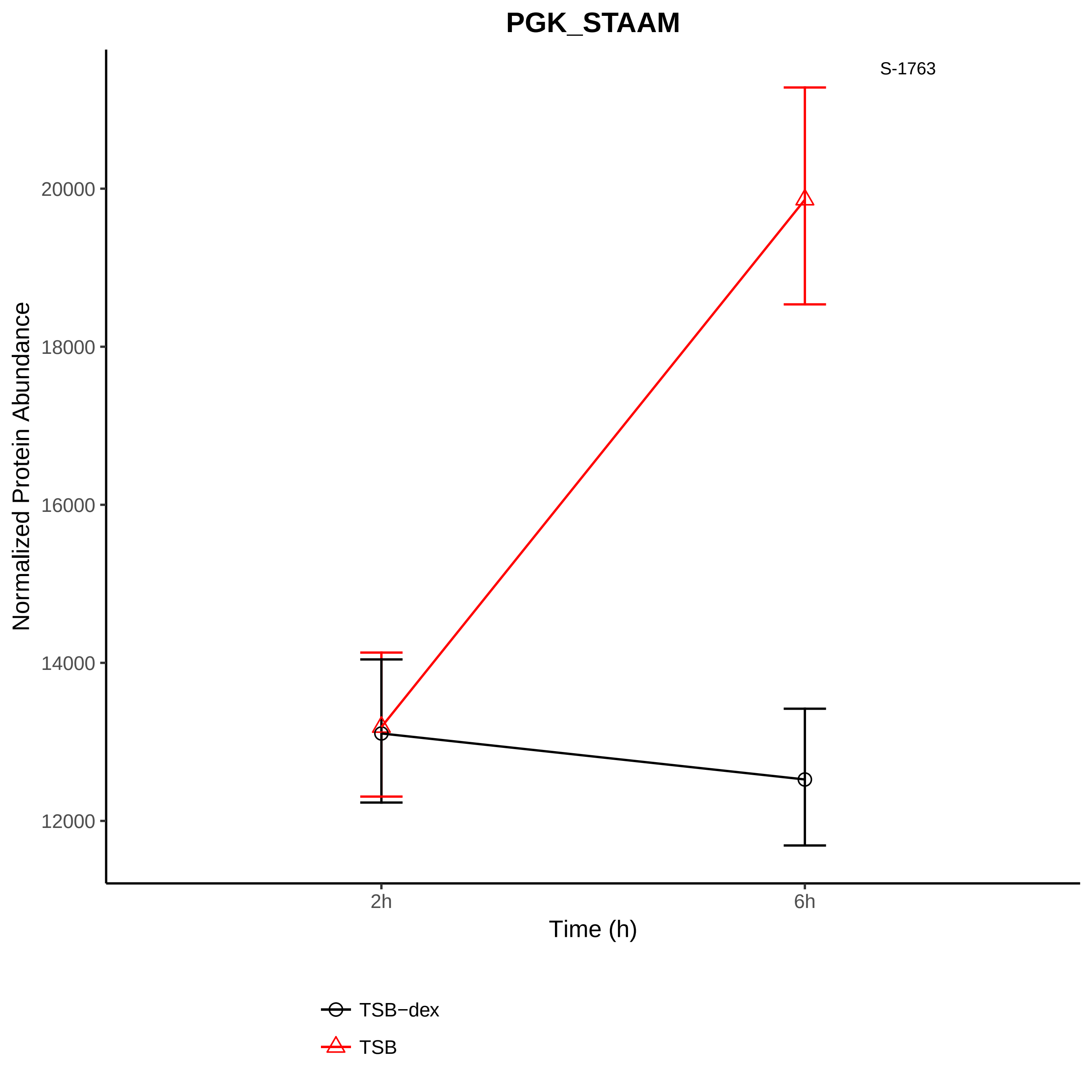

PGK_STAAM
S-1763
Normalized Protein Abundance
20000
18000
16000
14000
12000
2h
6h
Time (h)
TSB−dex
TSB

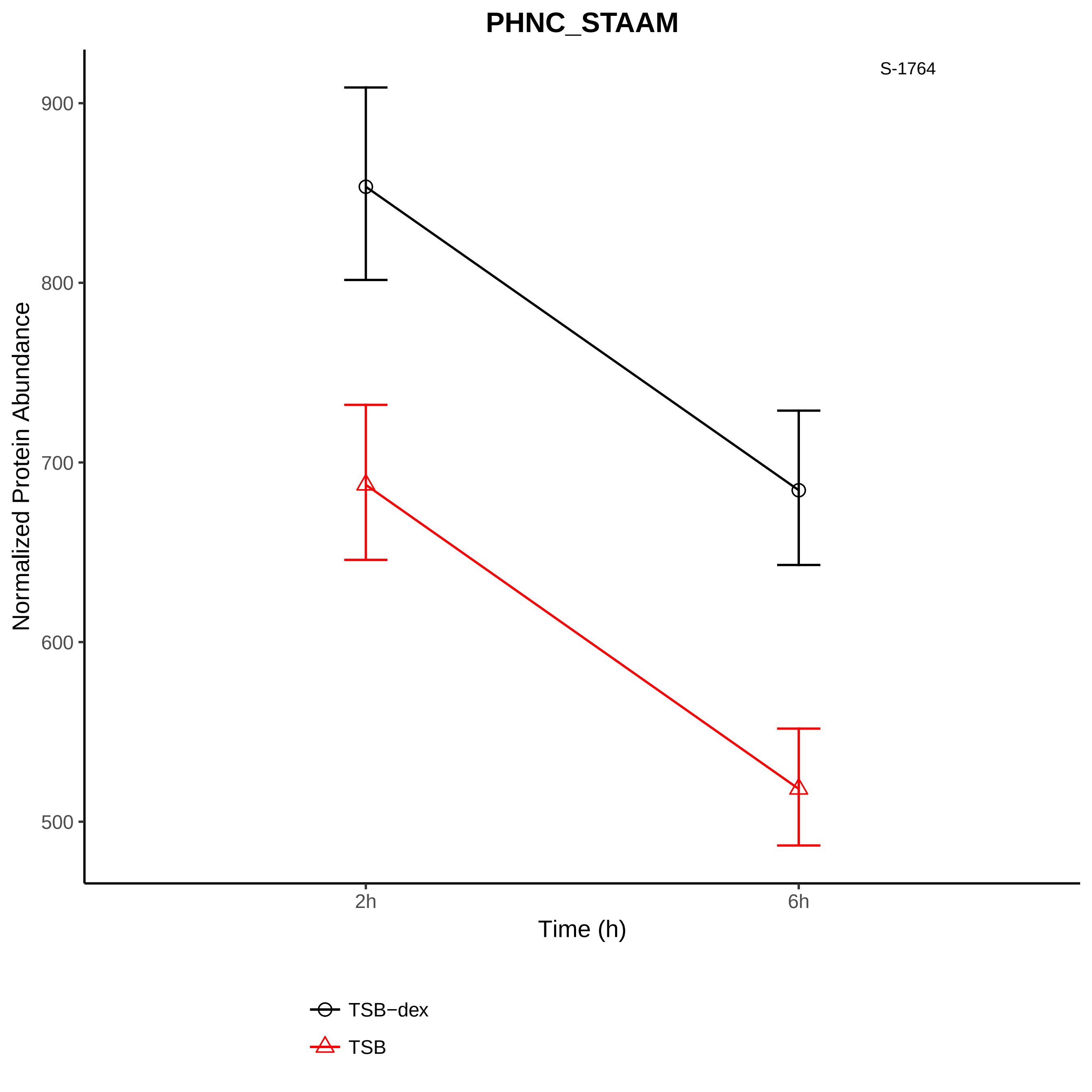
PHNC_STAAM
S-1764
Normalized Protein Abundance
900
800
700
600
500
2h
6h
Time (h)
TSB−dex
TSB

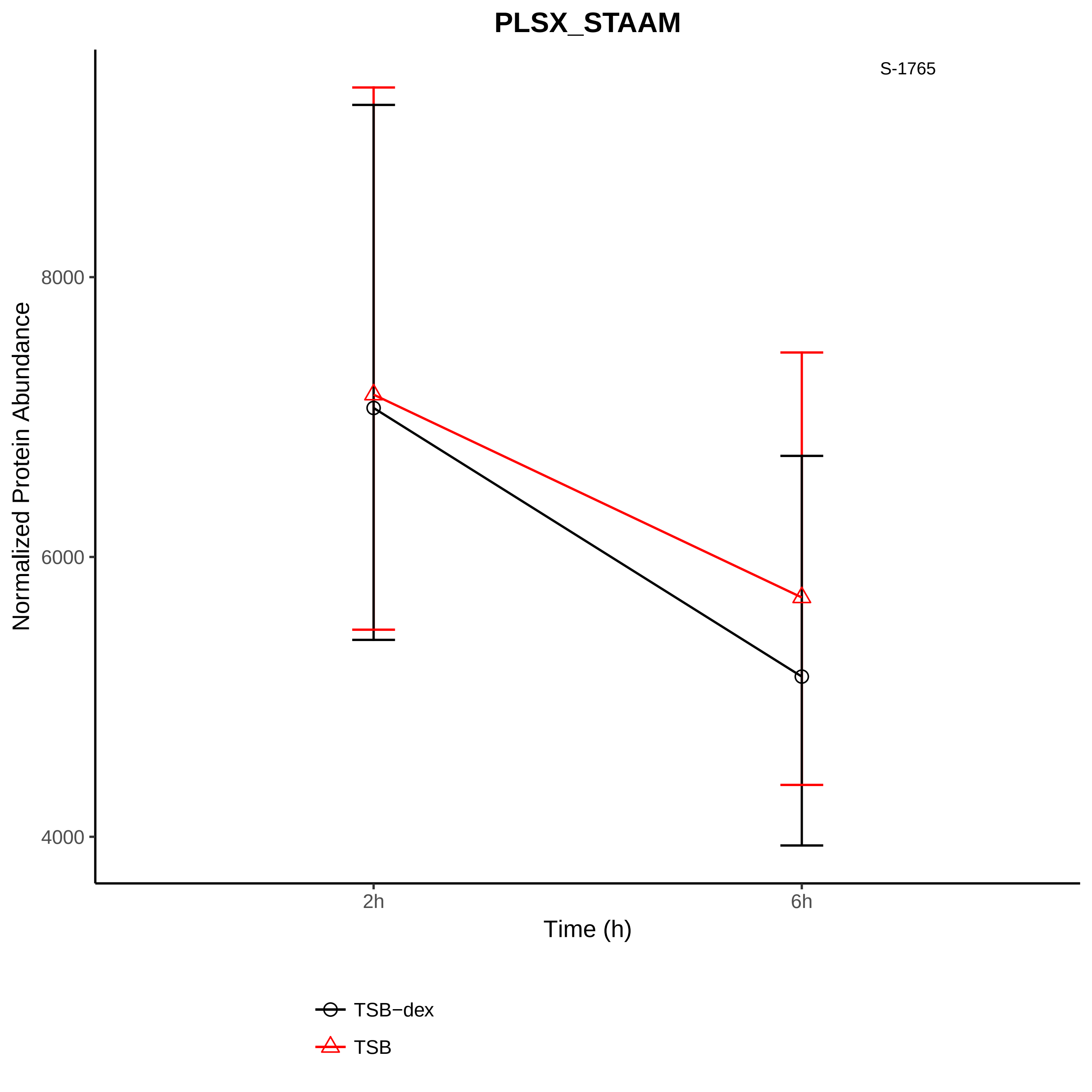
PLSX_STAAM
S-1765
Normalized Protein Abundance
8000
6000
4000
2h
6h
Time (h)
TSB−dex
TSB

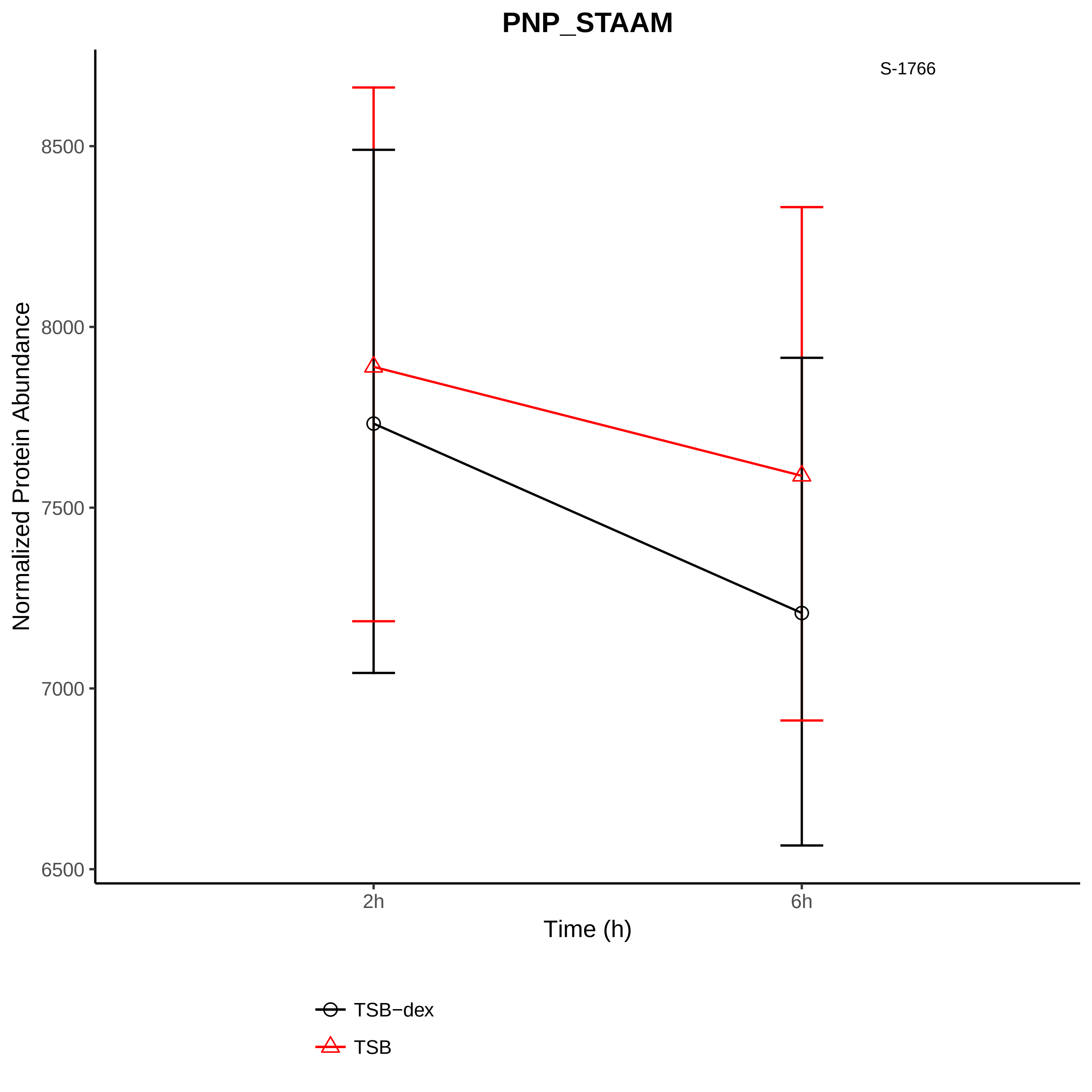

PNP_STAAM
S-1766
Normalized Protein Abundance
8500
8000
7500
7000
6500
2h
6h
Time (h)
TSB−dex
TSB

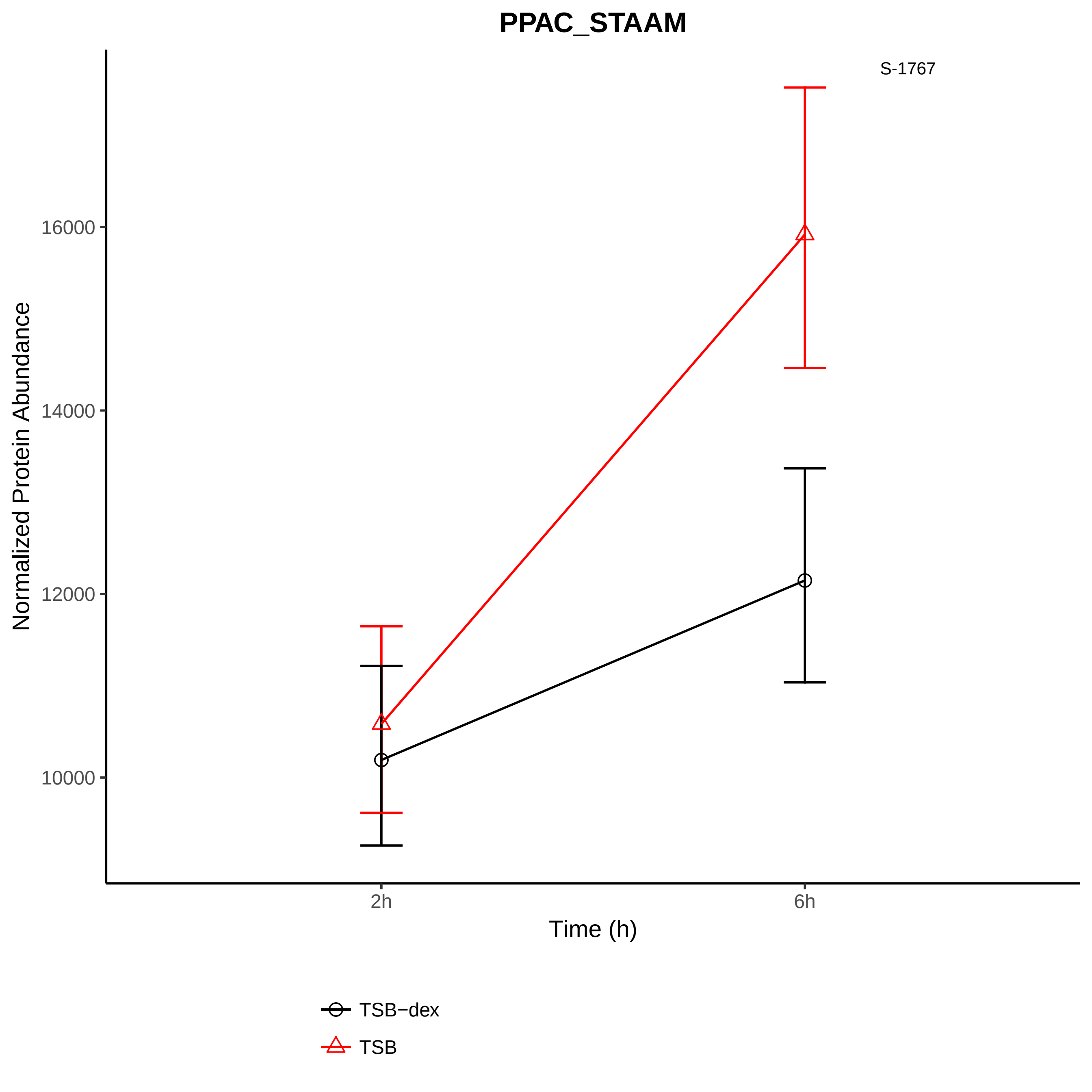
PPAC_STAAM
S-1767
Normalized Protein Abundance
16000
14000
12000
10000
2h
6h
Time (h)
TSB−dex
TSB

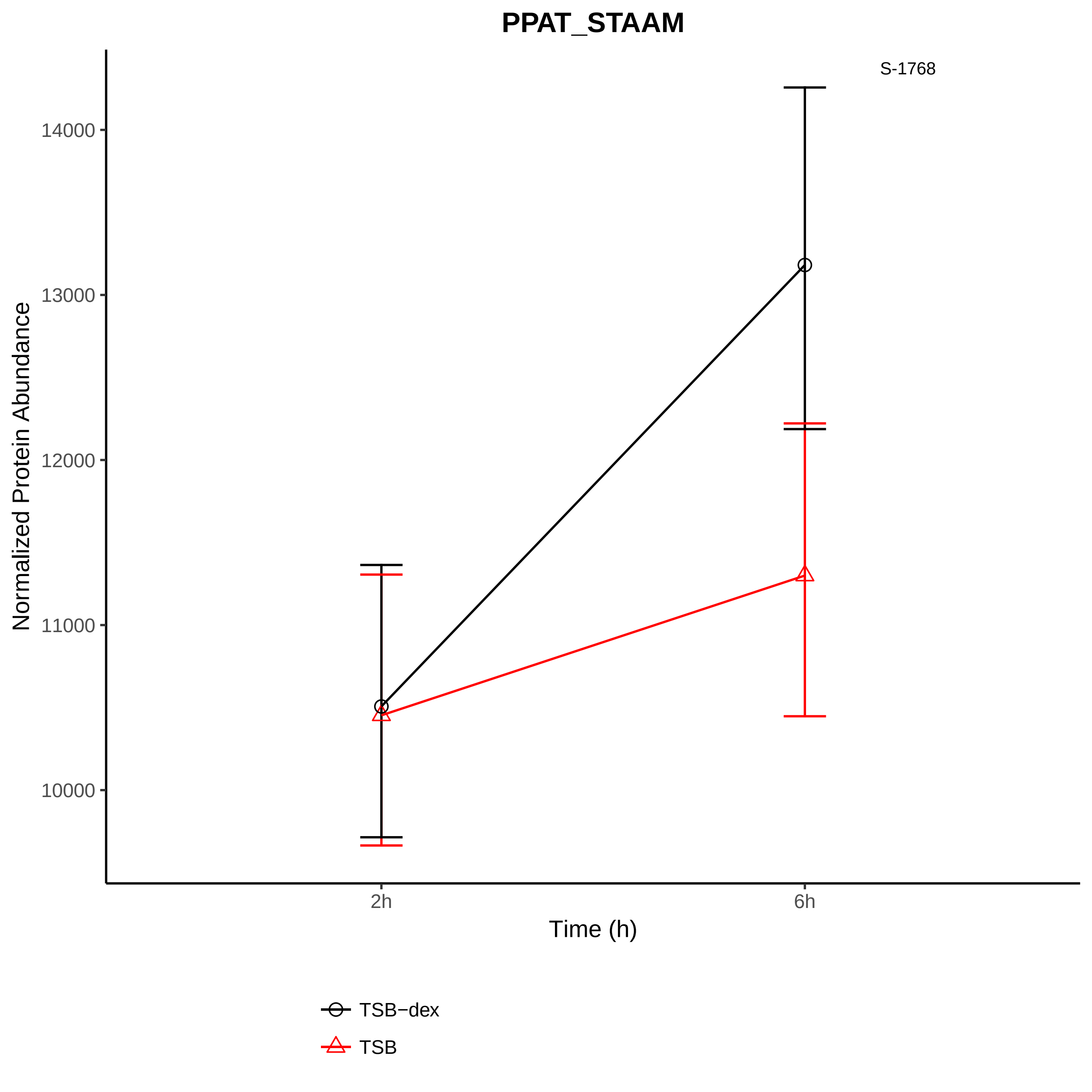

PPAT_STAAM
S-1768
14000
13000
12000
11000
10000
Normalized Protein Abundance
2h
6h
Time (h)
TSB−dex
TSB

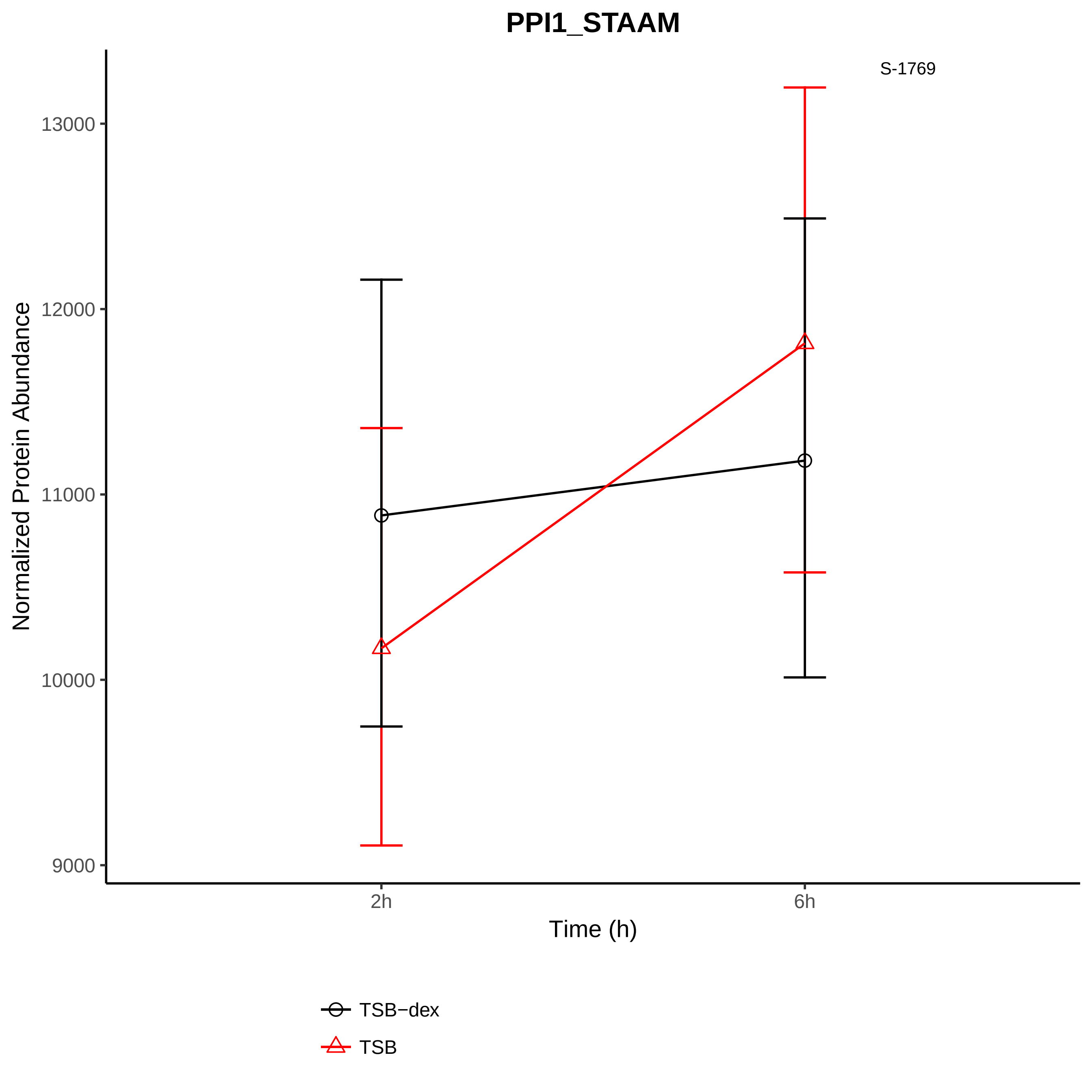

PPI1_STAAM
S-1769
Normalized Protein Abundance
13000
12000
11000
10000
9000
2h
6h
Time (h)
TSB−dex
TSB

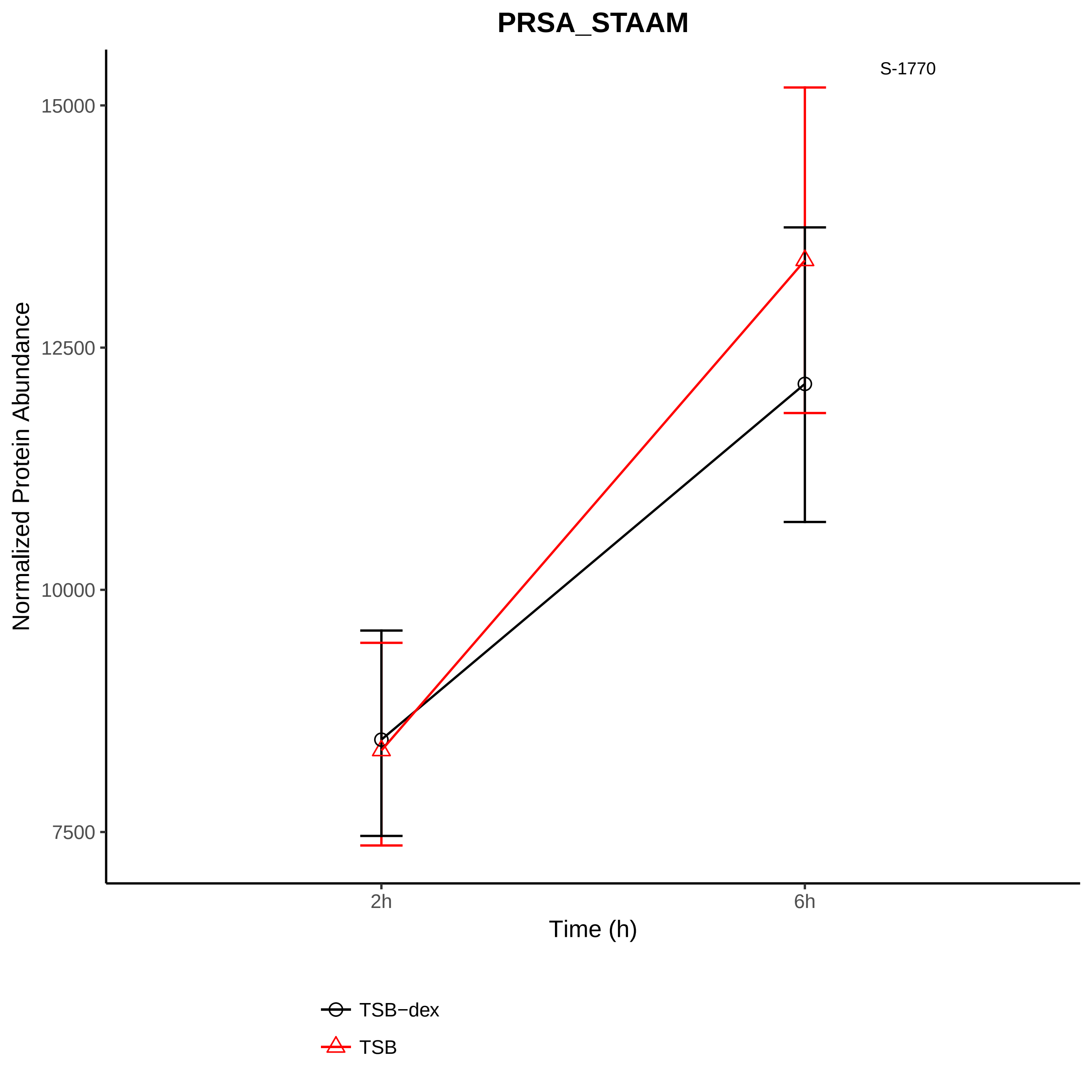
PRSA_STAAM
S-1770
Normalized Protein Abundance
15000
12500
10000
7500
2h
6h
Time (h)
TSB−dex
TSB

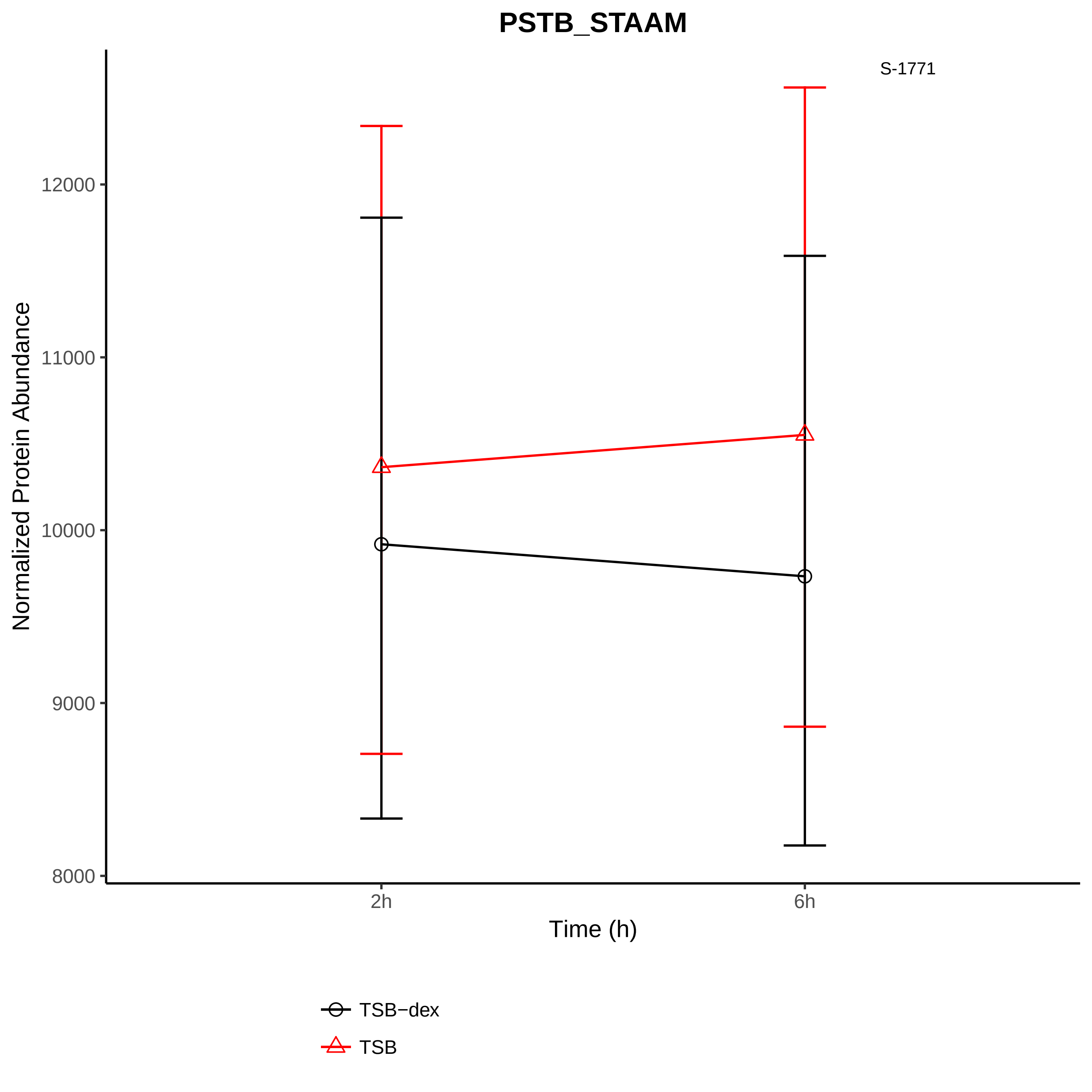

PSTB_STAAM
S-1771
Normalized Protein Abundance
12000
11000
10000
9000
8000
2h
6h
Time (h)
TSB−dex
TSB

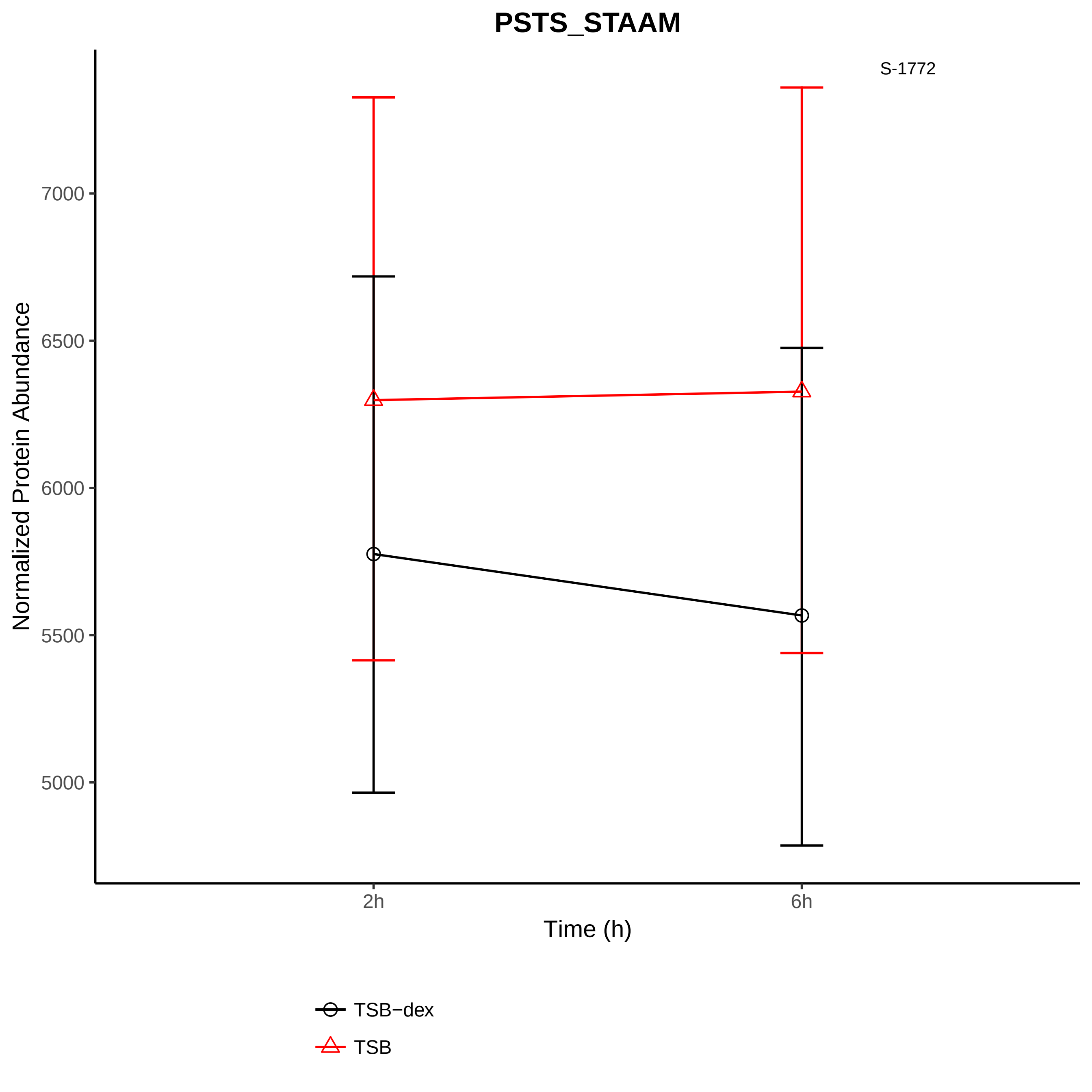

PSTS_STAAM
S-1772
Normalized Protein Abundance
7000
6500
6000
5500
5000
2h
6h
Time (h)
TSB−dex
TSB

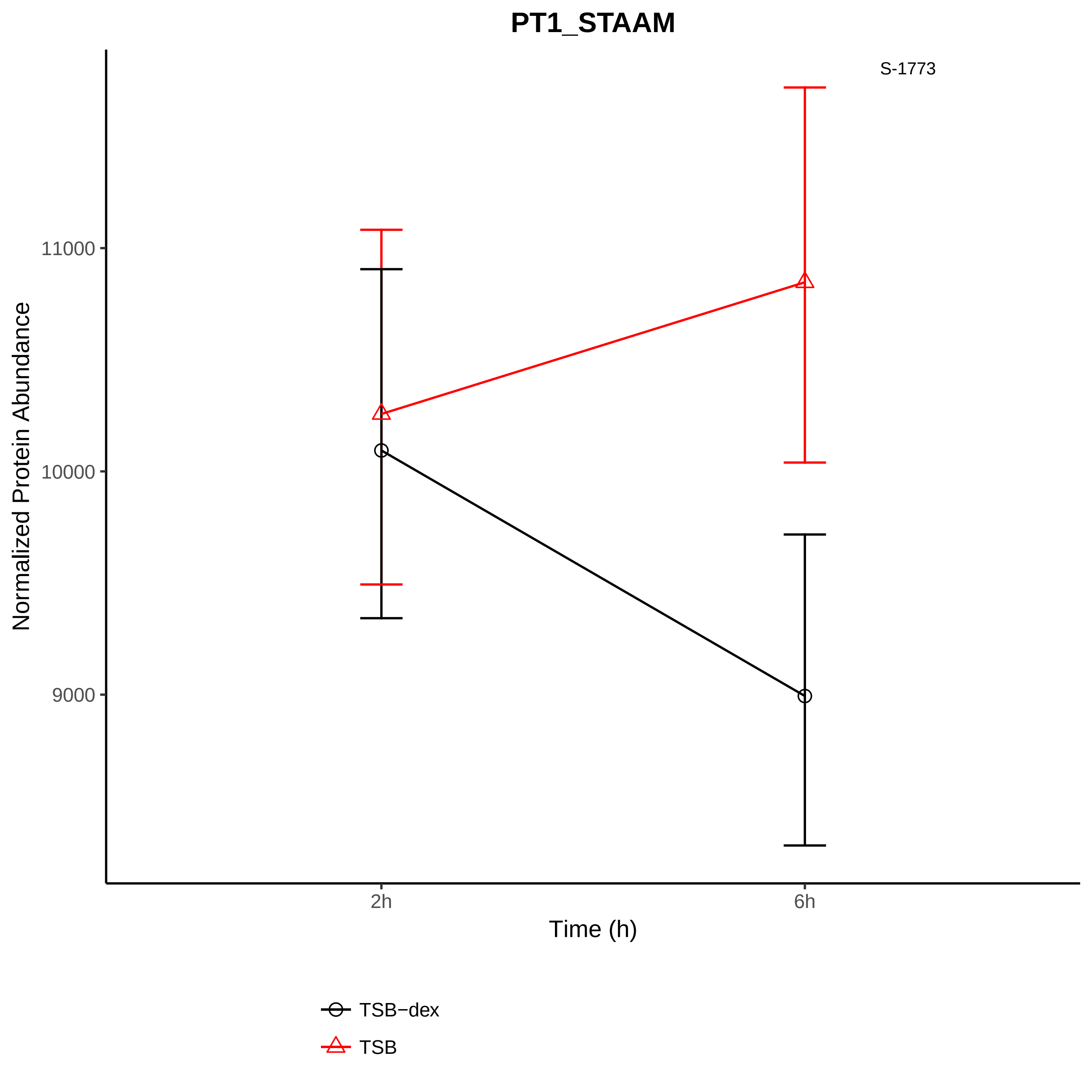
PT1_STAAM
S-1773
Normalized Protein Abundance
11000
10000
9000
2h
6h
Time (h)
TSB−dex
TSB

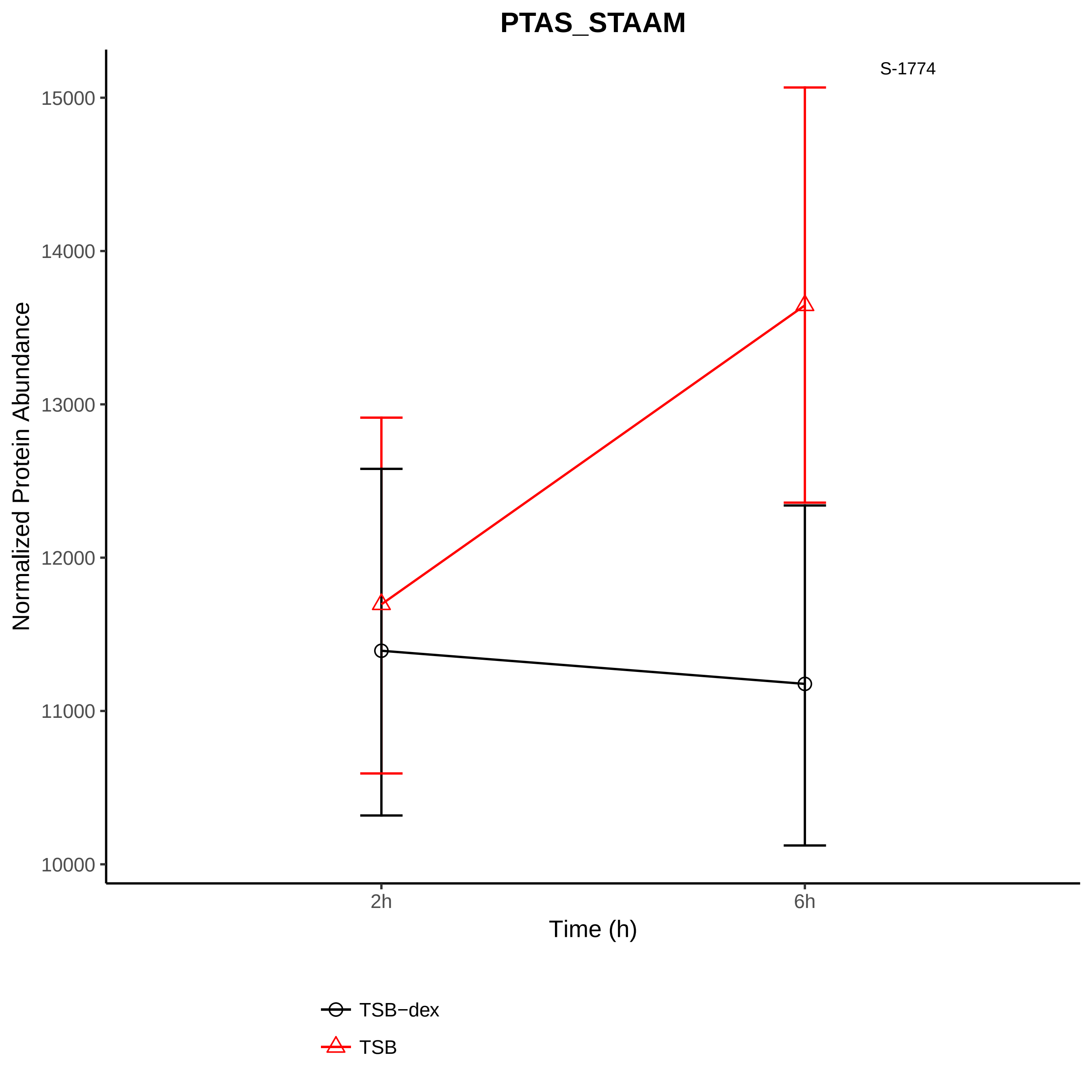

PTAS_STAAM
S-1774
Normalized Protein Abundance
15000
14000
13000
12000
11000
10000
2h
6h
Time (h)
TSB−dex
TSB

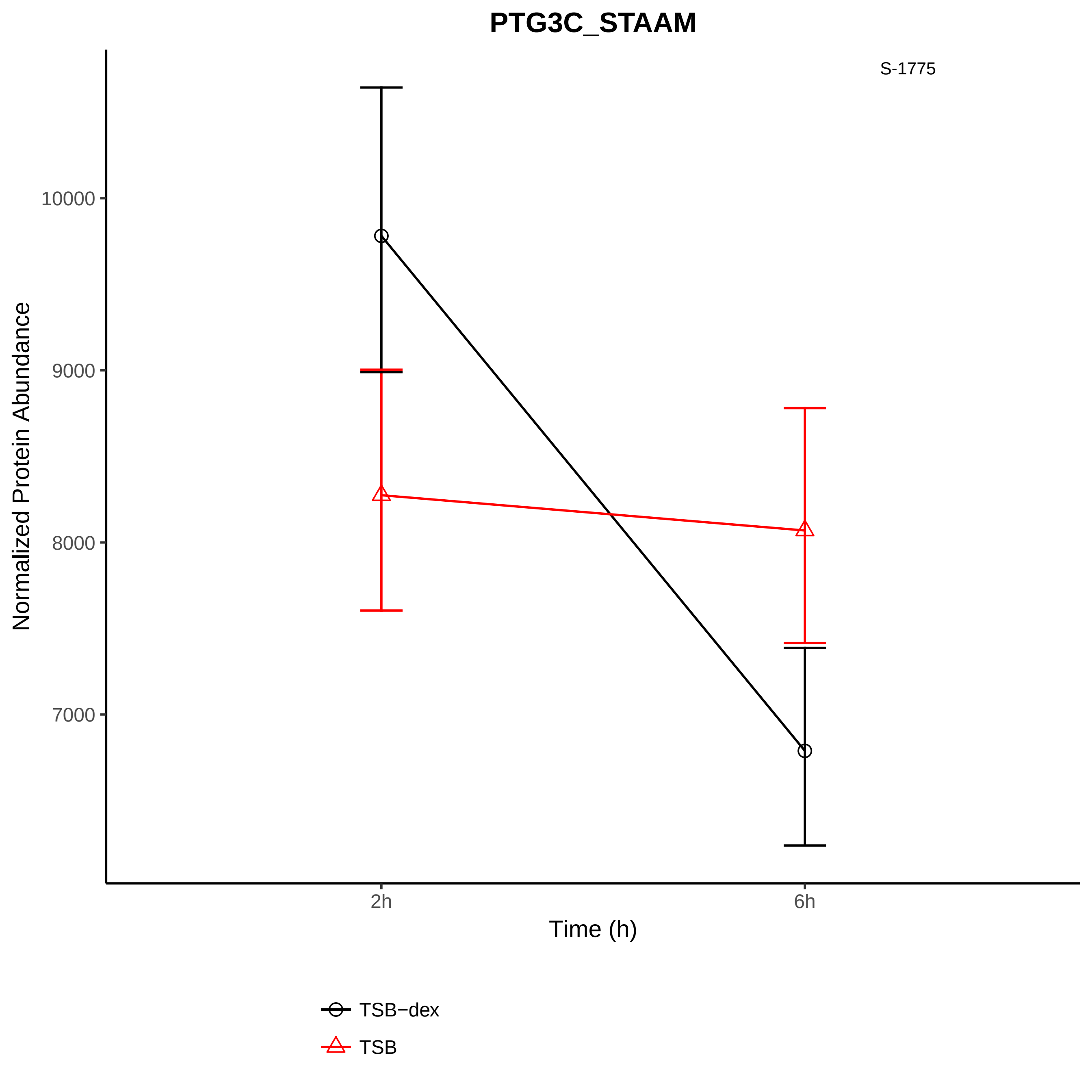

PTG3C_STAAM
S-1775
Normalized Protein Abundance
10000
9000
8000
7000
2h
6h
Time (h)
TSB−dex
TSB

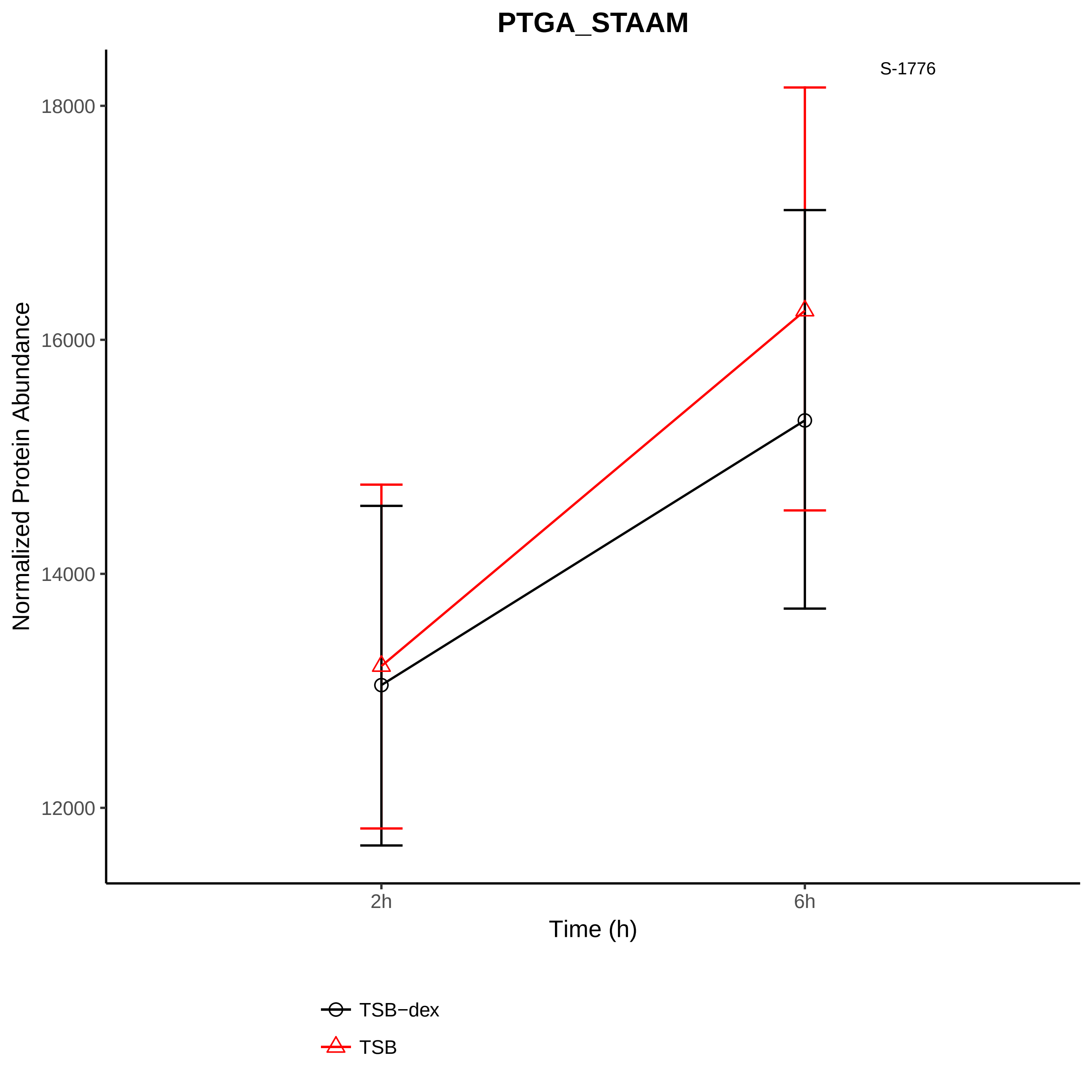
PTGA_STAAM
S-1776
18000
16000
14000
12000
Normalized Protein Abundance
2h
6h
Time (h)
TSB−dex
TSB

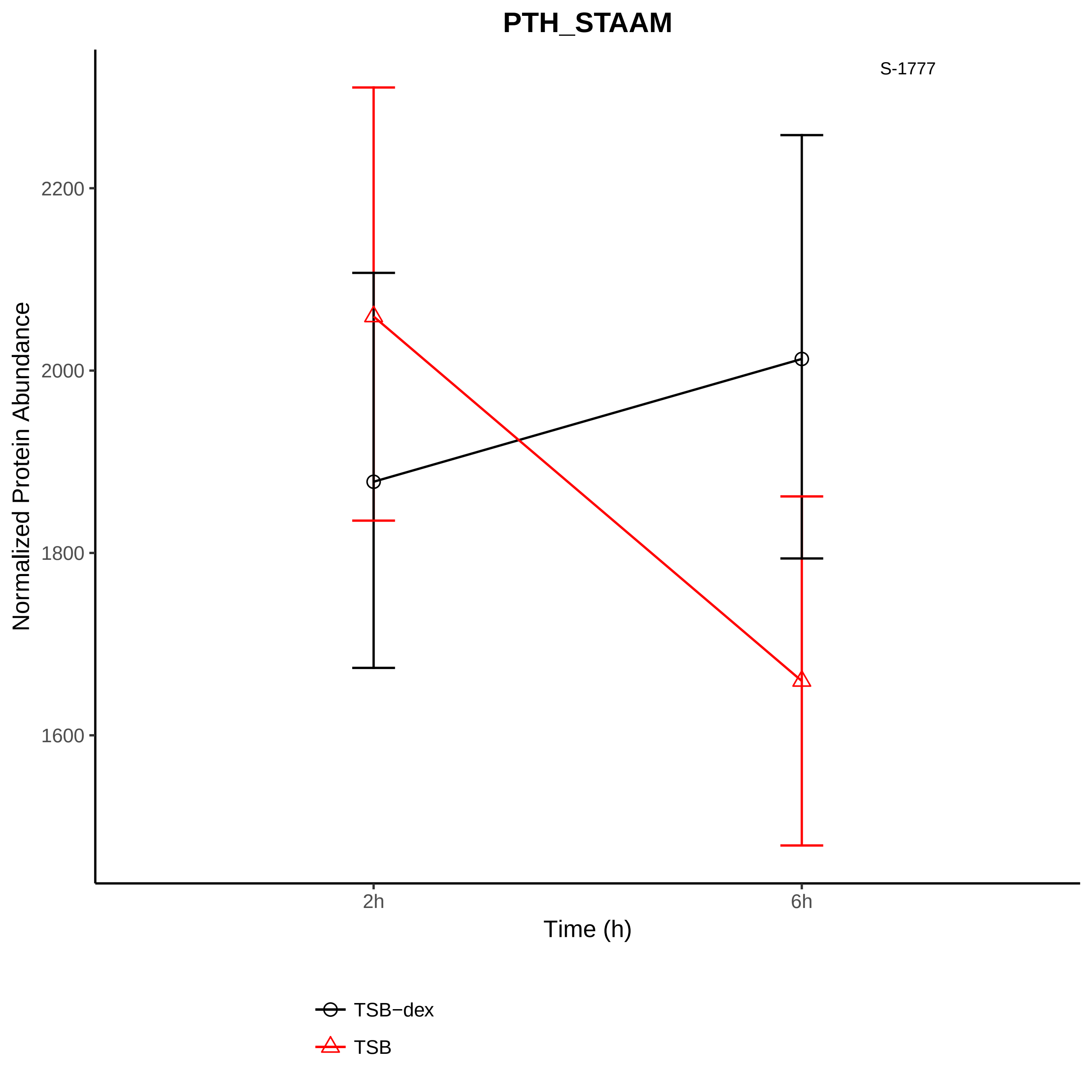
PTH_STAAM
S-1777
Normalized Protein Abundance
2200
2000
1800
1600
2h
6h
Time (h)
TSB−dex
TSB

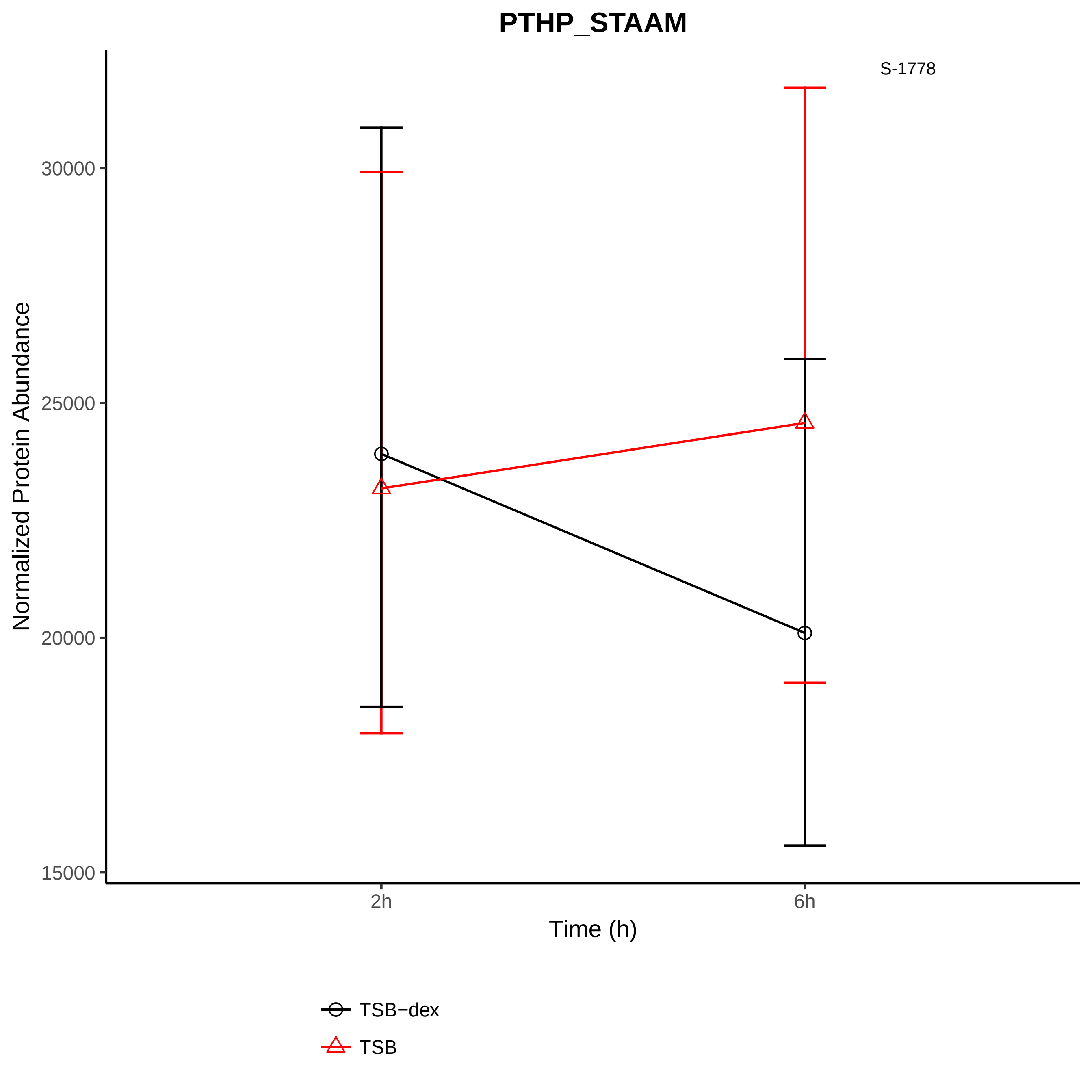

PTHP_STAAM
S-1778
Normalized Protein Abundance
30000
25000
20000
15000
2h
6h
Time (h)
TSB−dex
TSB

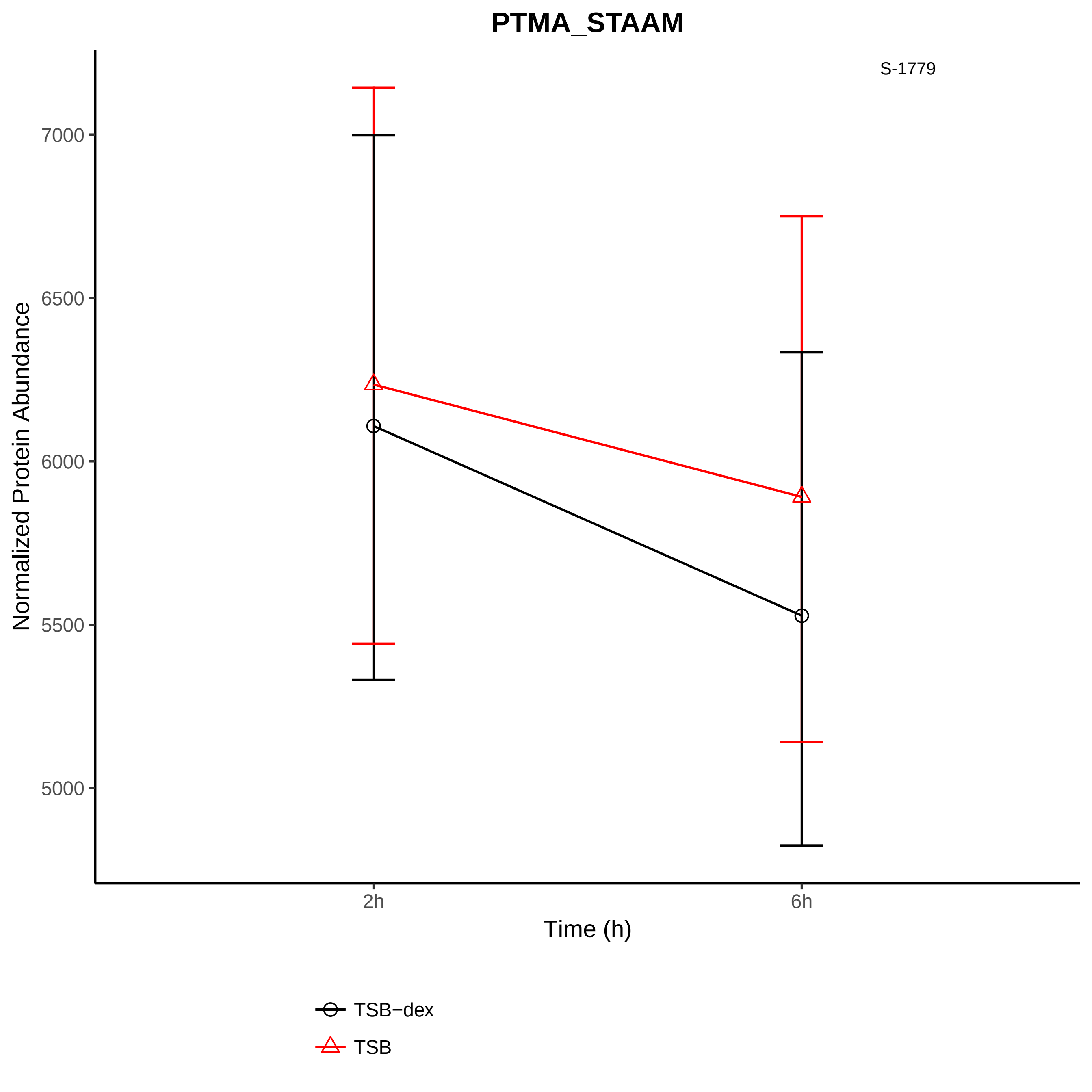

PTMA_STAAM
S-1779
7000
6500
6000
5500
5000
Normalized Protein Abundance
2h
6h
Time (h)
TSB−dex
TSB

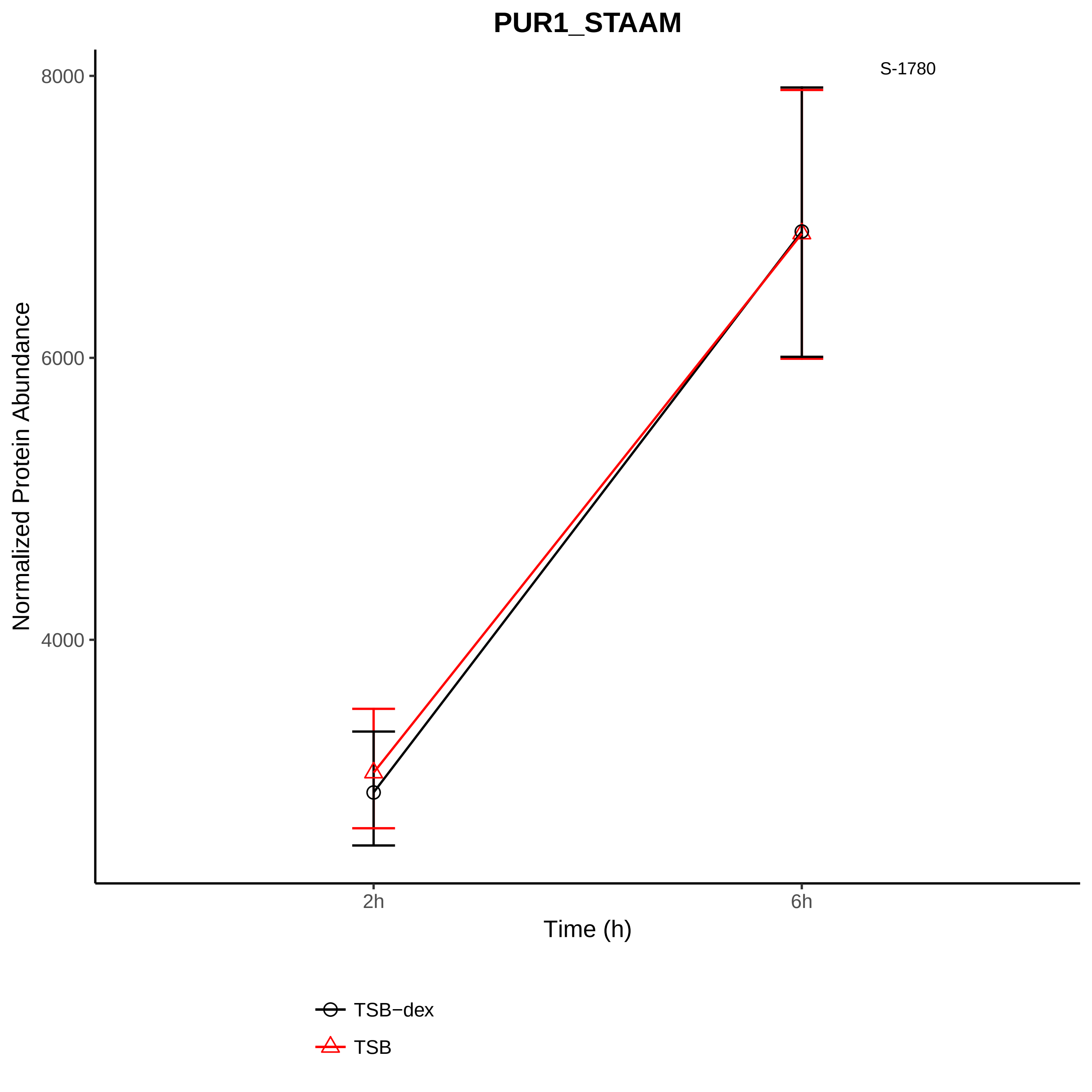
PUR1_STAAM
S-1780
Normalized Protein Abundance
8000
6000
4000
2h
6h
Time (h)
TSB−dex
TSB

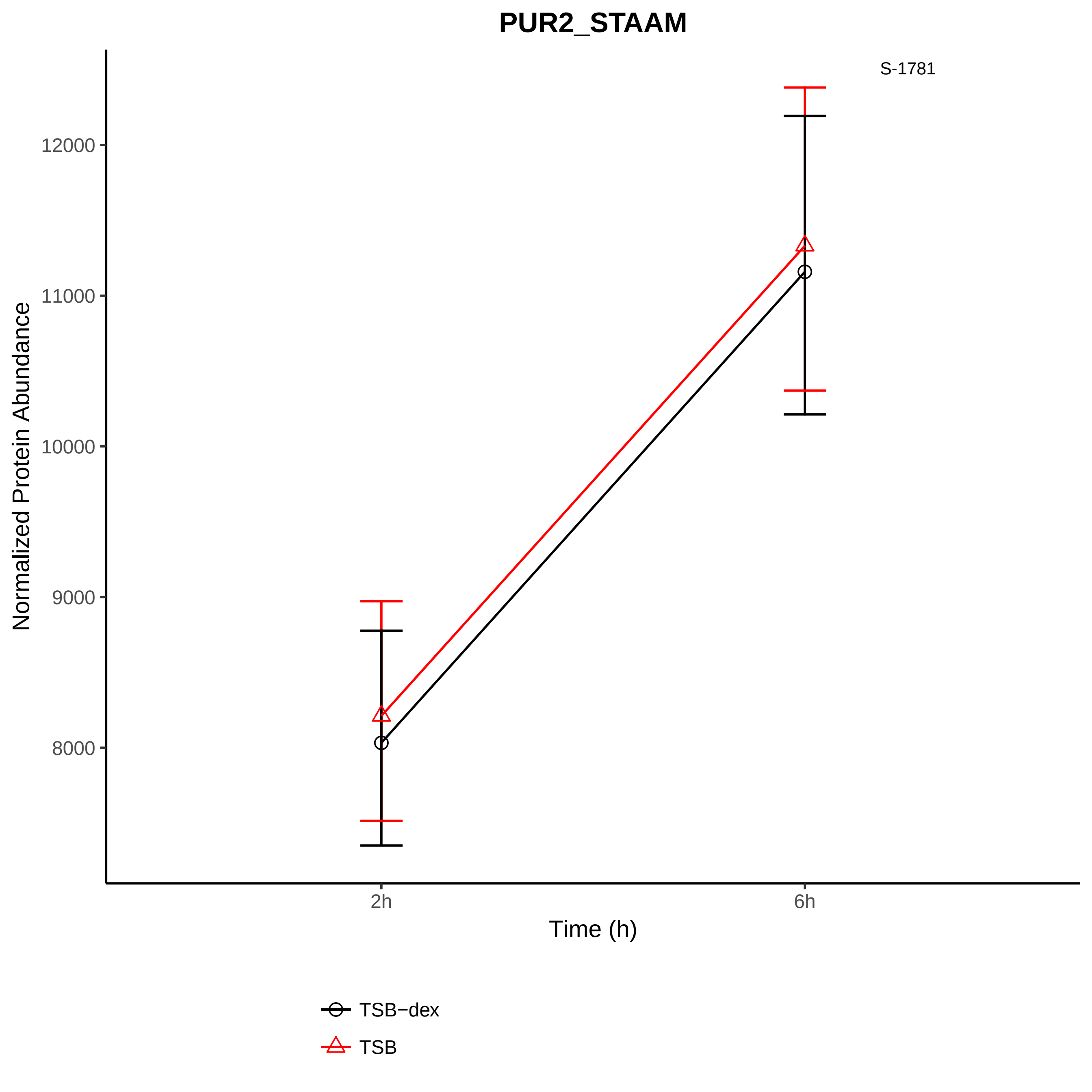

PUR2_STAAM
S-1781
Normalized Protein Abundance
12000
11000
10000
9000
8000
2h
6h
Time (h)
TSB−dex
TSB

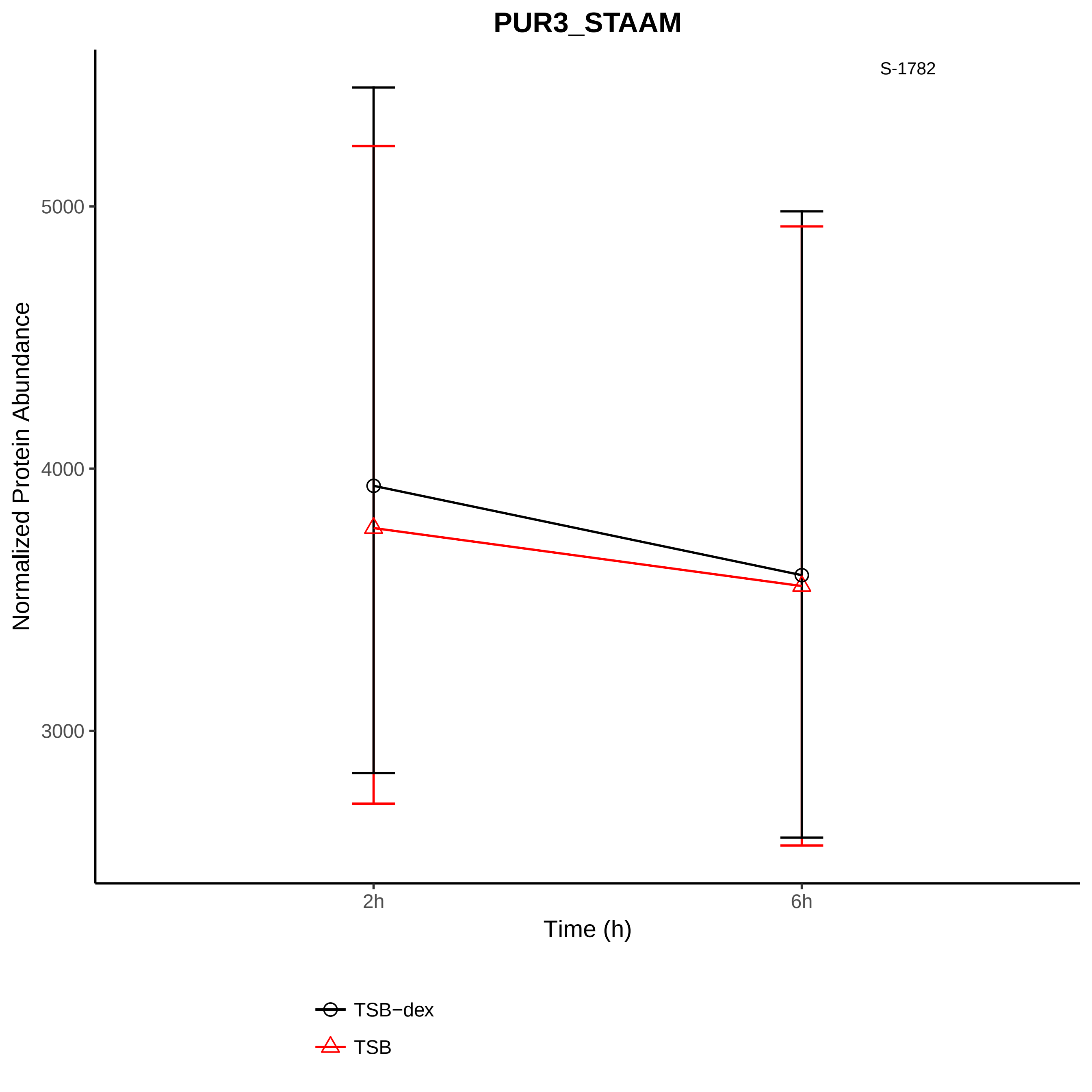

PUR3_STAAM
S-1782
Normalized Protein Abundance
5000
4000
3000
2h
6h
Time (h)
TSB−dex
TSB

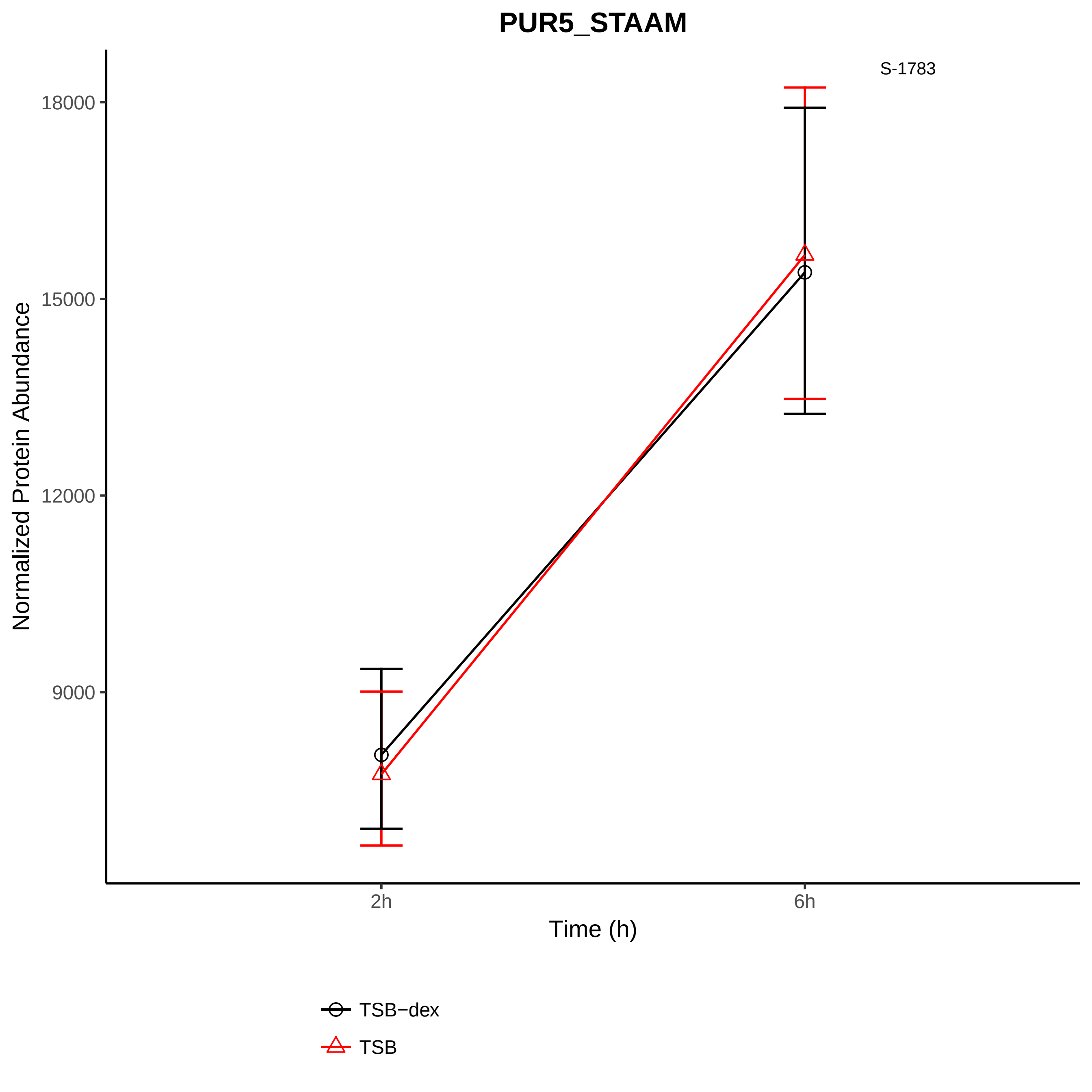

PUR5_STAAM
S-1783
Normalized Protein Abundance
18000
15000
12000
9000
2h
6h
Time (h)
TSB−dex
TSB

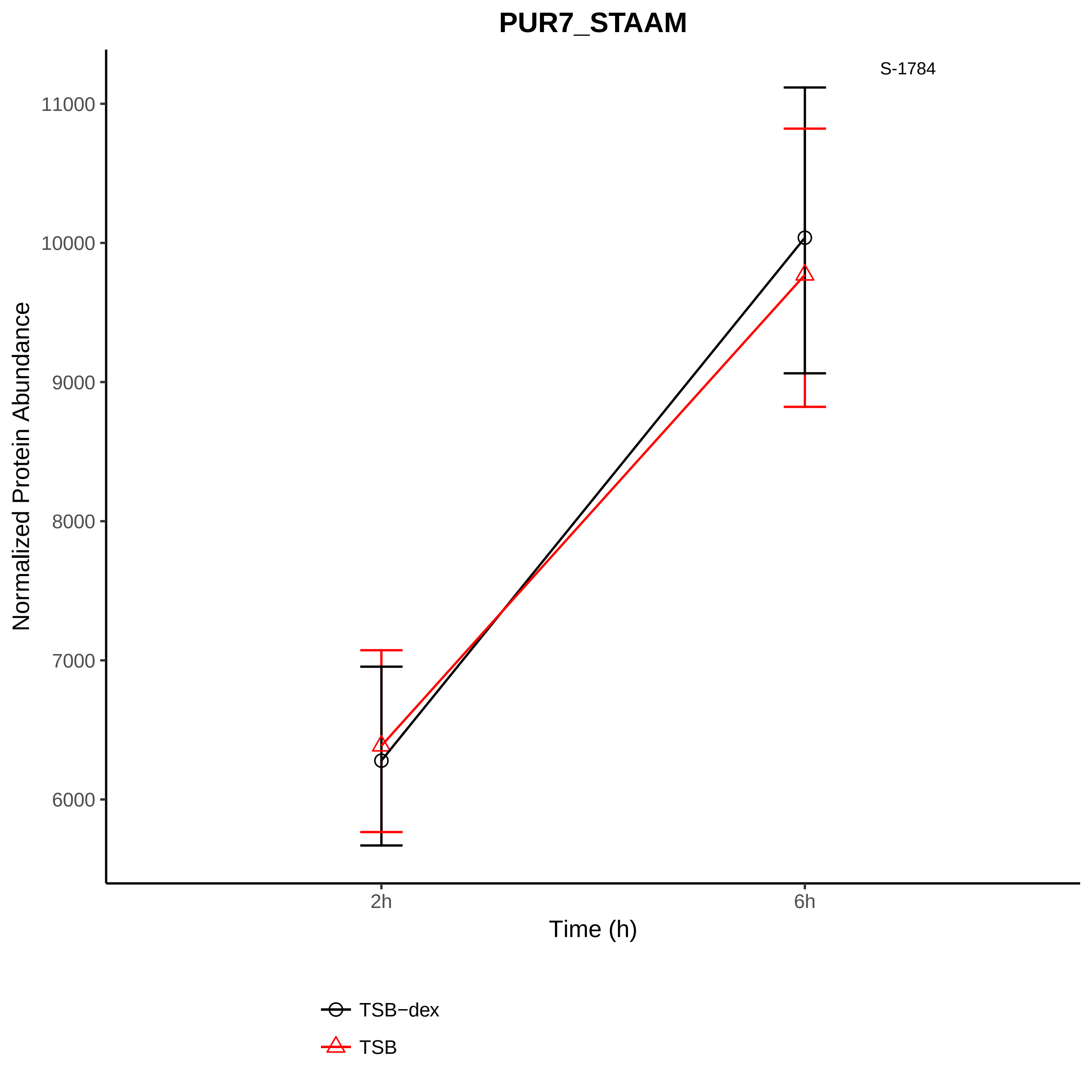

PUR7_STAAM
S-1784
Normalized Protein Abundance
11000
10000
9000
8000
7000
6000
2h
6h
Time (h)
TSB−dex
TSB

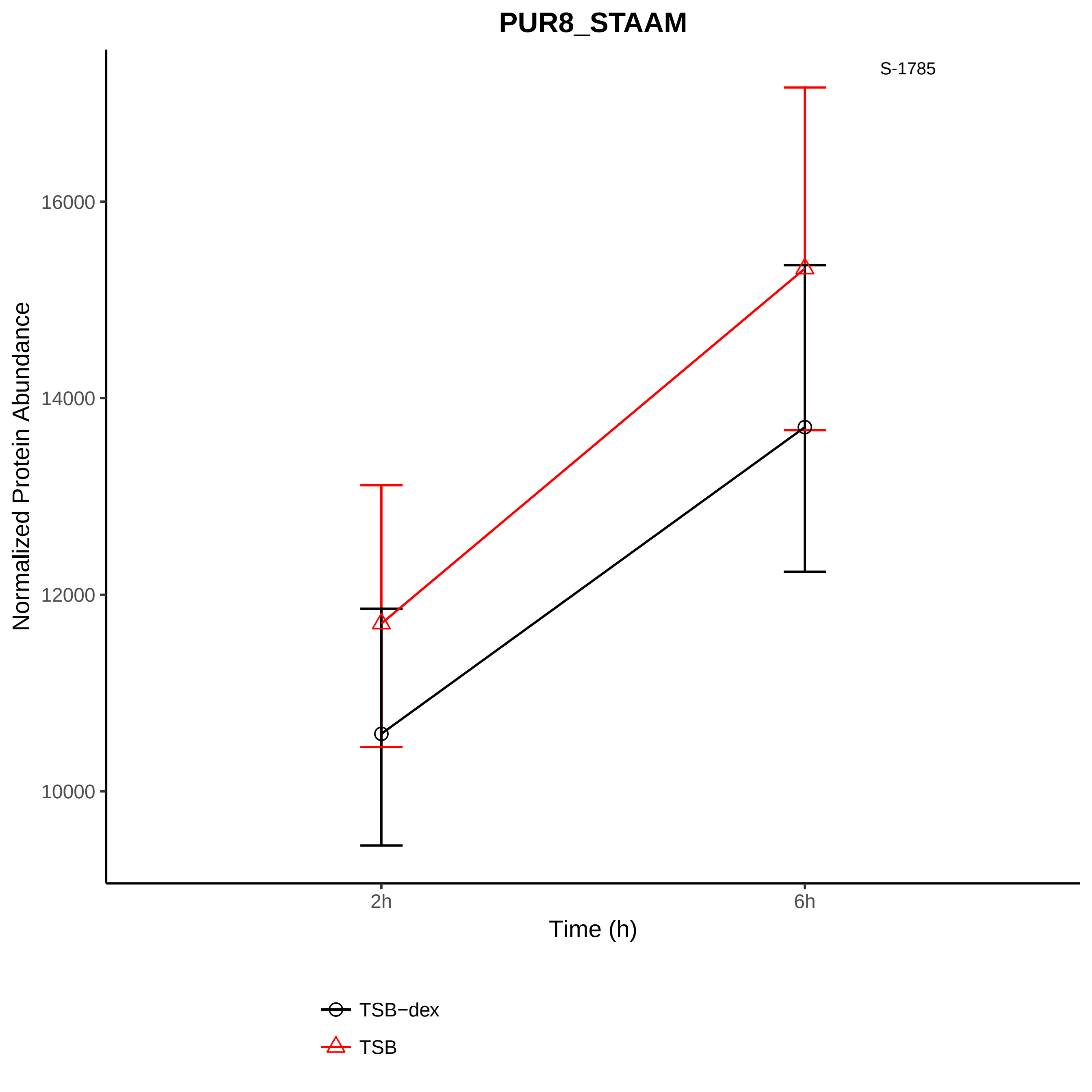
PUR8_STAAM
S-1785
Normalized Protein Abundance
16000
14000
12000
10000
2h
6h
Time (h)
TSB−dex
TSB

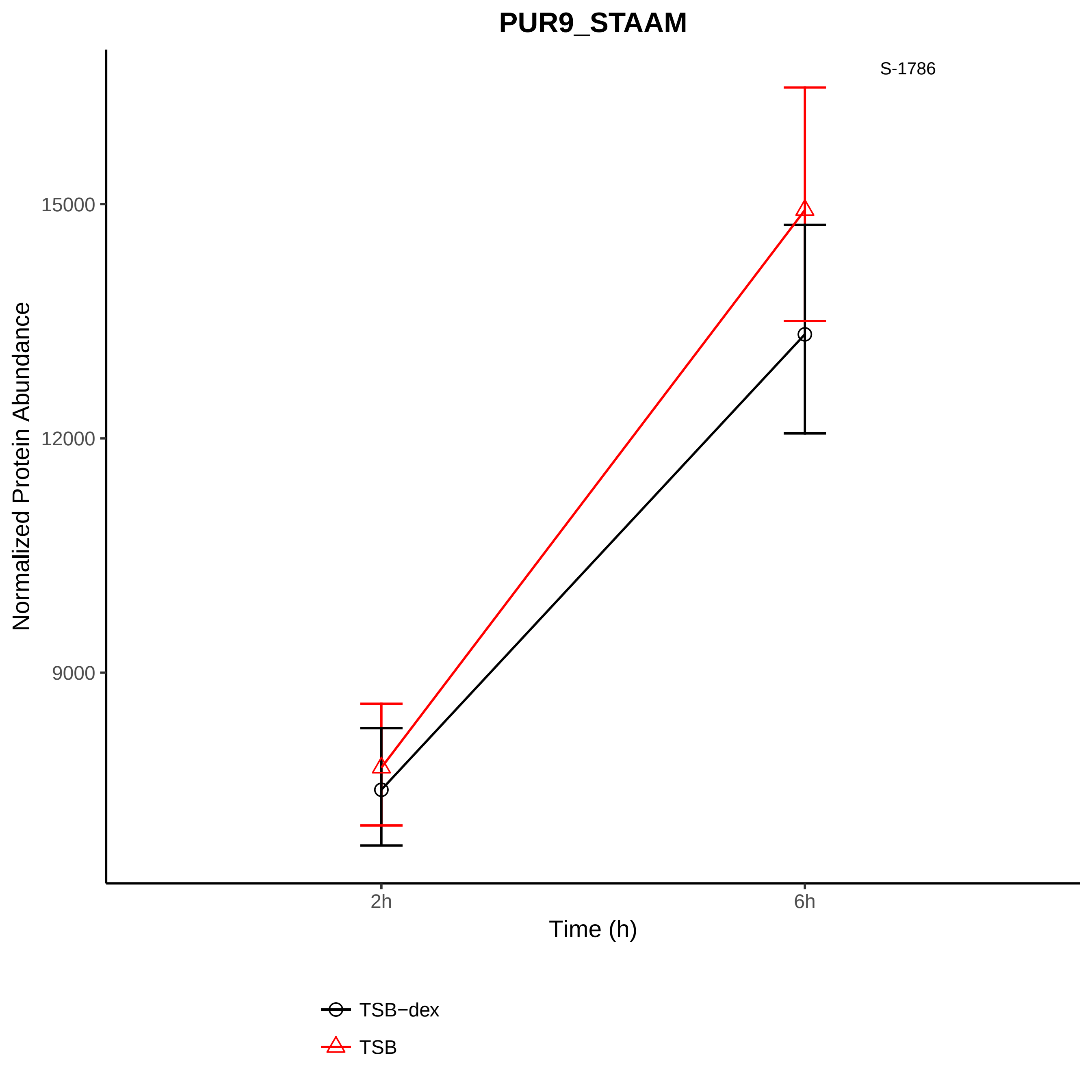
PUR9_STAAM
S-1786
Normalized Protein Abundance
15000
12000
9000
2h
6h
Time (h)
TSB−dex
TSB

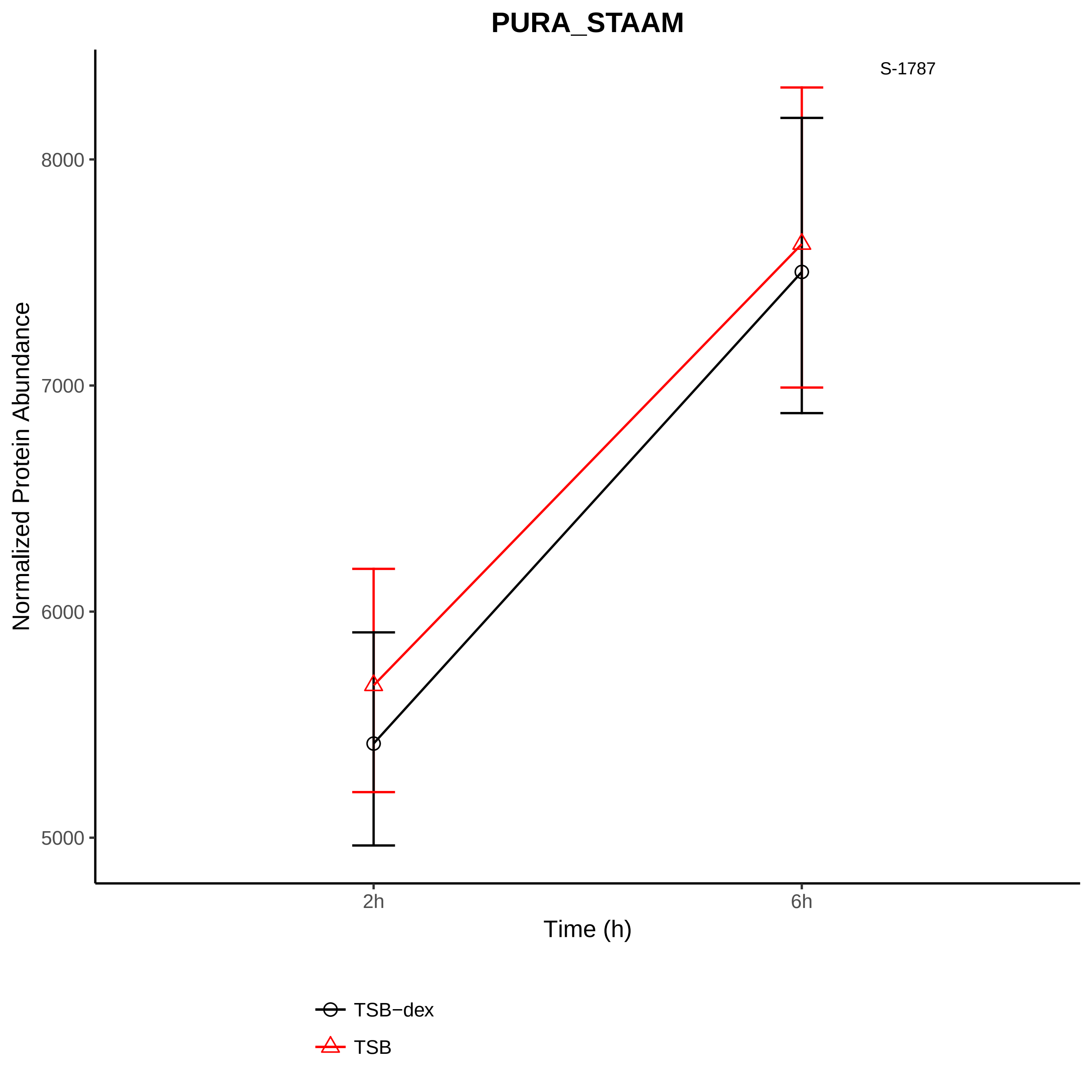
PURA_STAAM
S-1787
Normalized Protein Abundance
8000
7000
6000
5000
2h
6h
Time (h)
TSB−dex
TSB

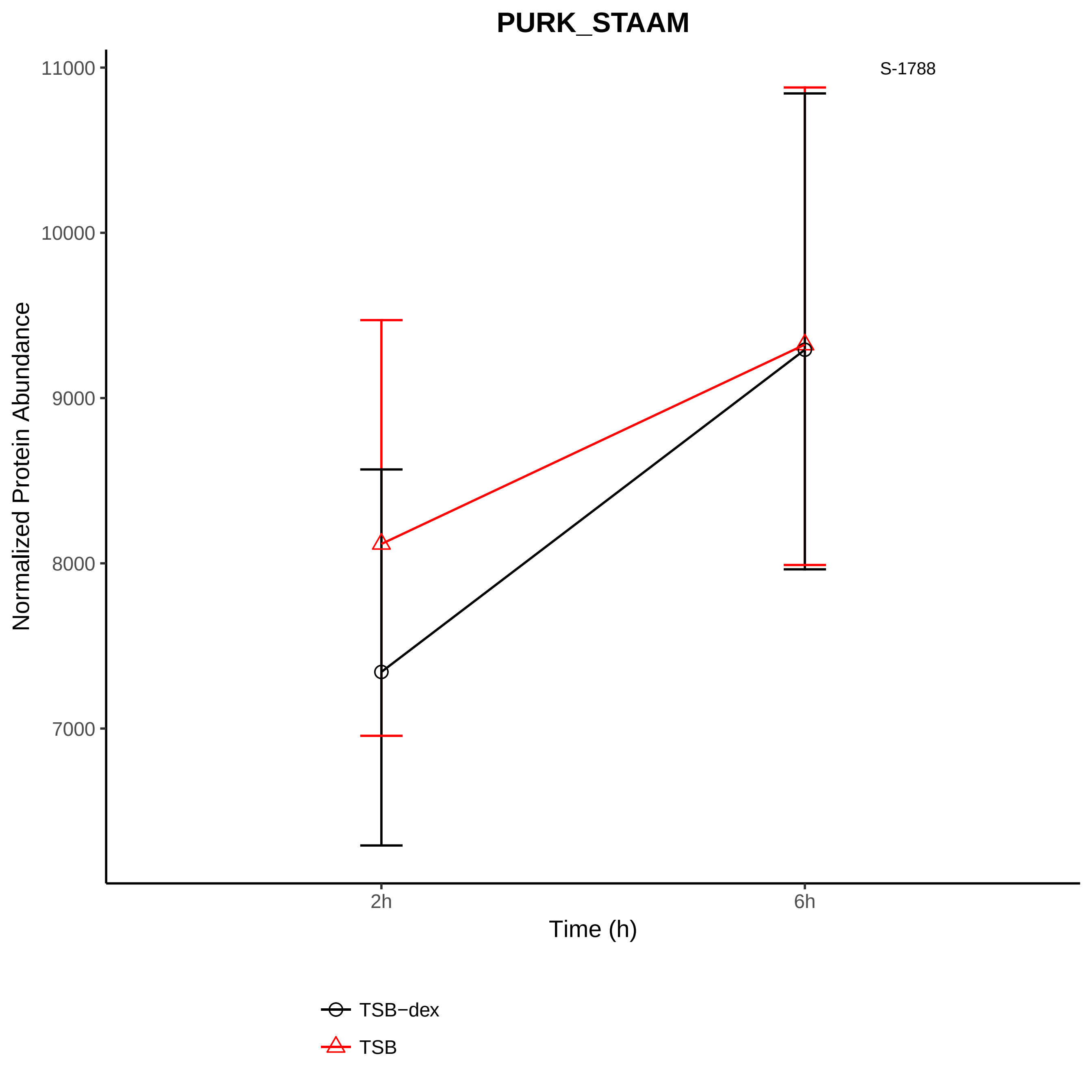

PURK_STAAM
S-1788
Normalized Protein Abundance
11000
10000
9000
8000
7000
2h
6h
Time (h)
TSB−dex
TSB

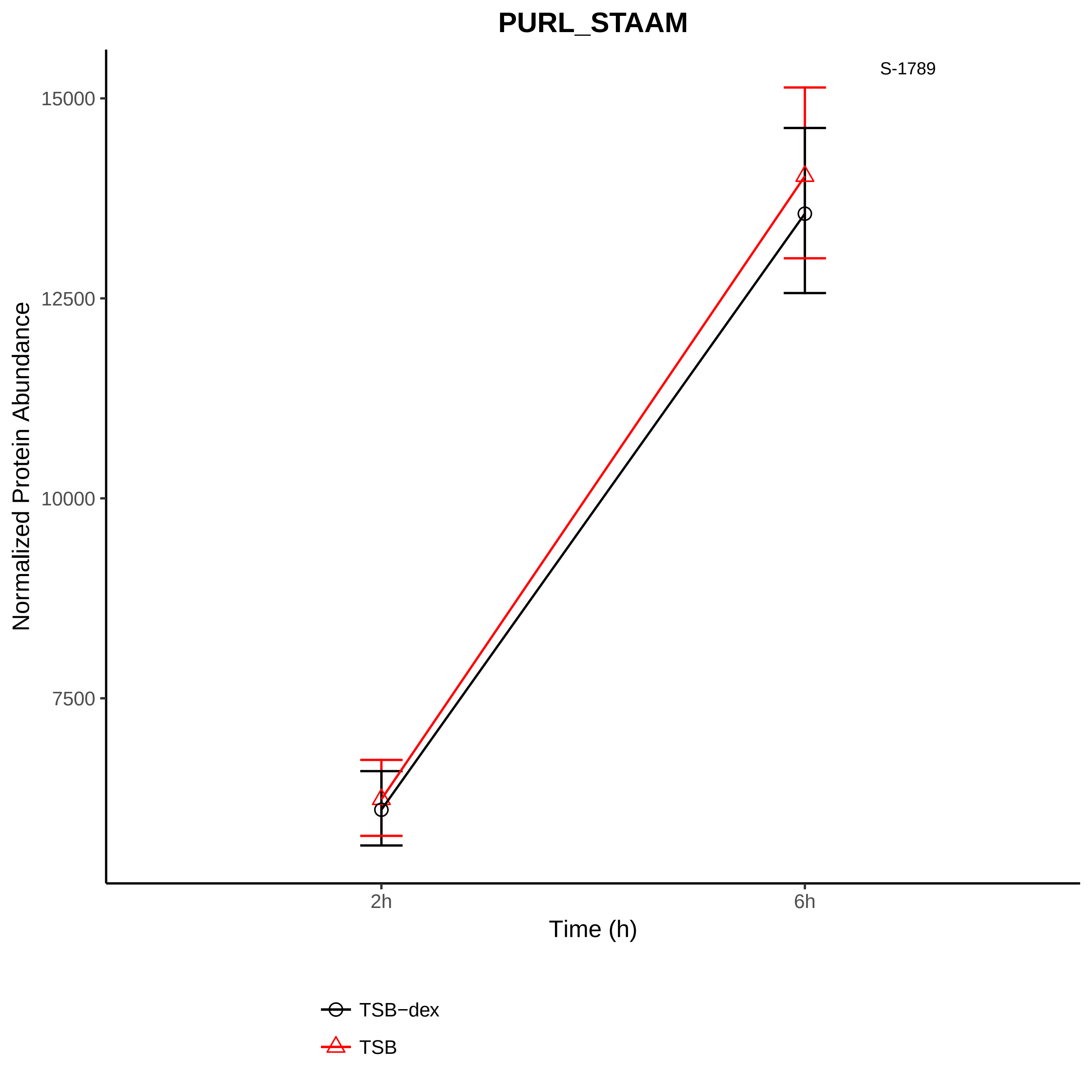

PURL_STAAM
S-1789
15000
12500
10000
7500
Normalized Protein Abundance
2h
6h
Time (h)
TSB−dex
TSB

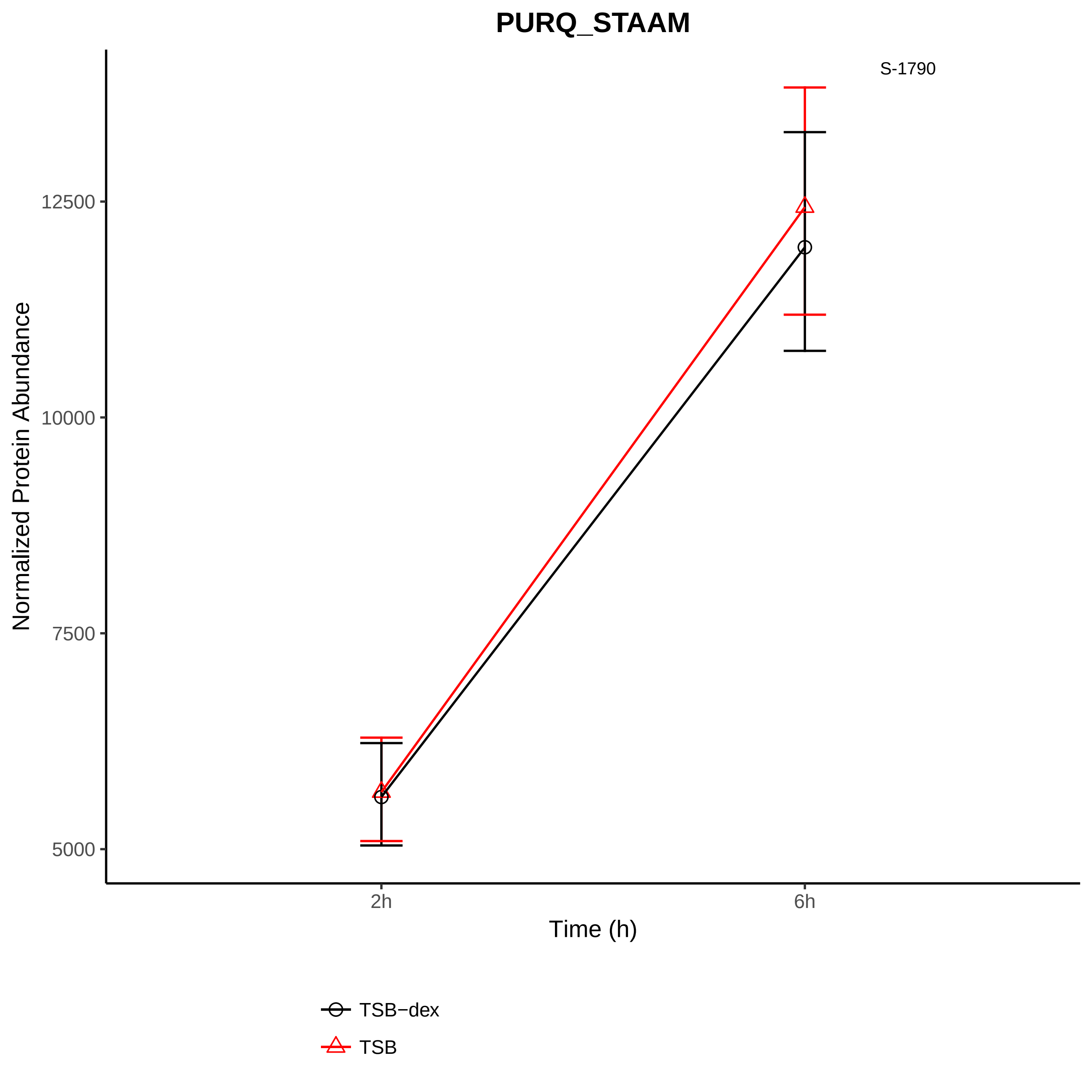

PURQ_STAAM
S-1790
Normalized Protein Abundance
12500
10000
7500
5000
2h
6h
Time (h)
TSB−dex
TSB

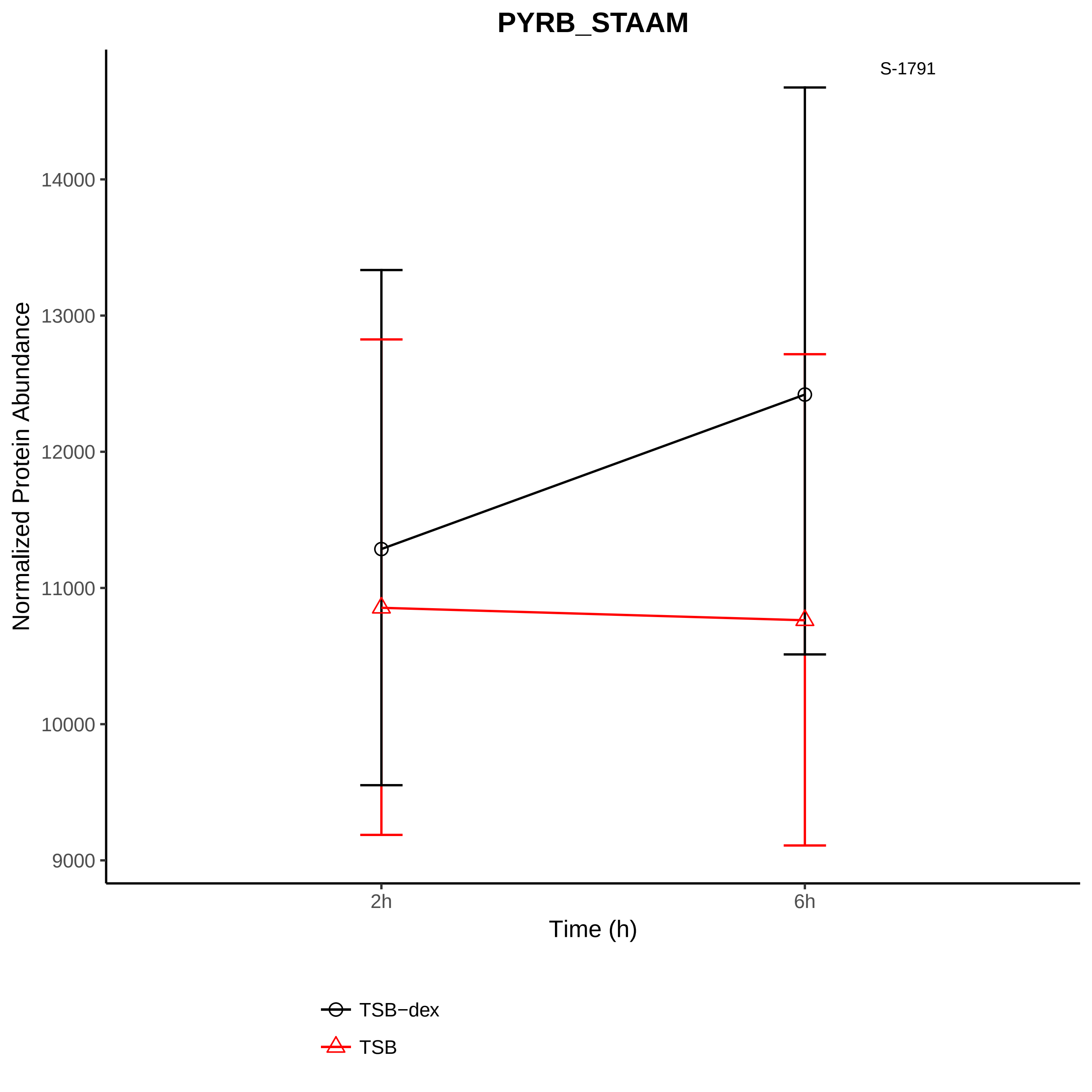
PYRB_STAAM
S-1791
Normalized Protein Abundance
14000
13000
12000
11000
10000
9000
2h
6h
Time (h)
TSB−dex
TSB

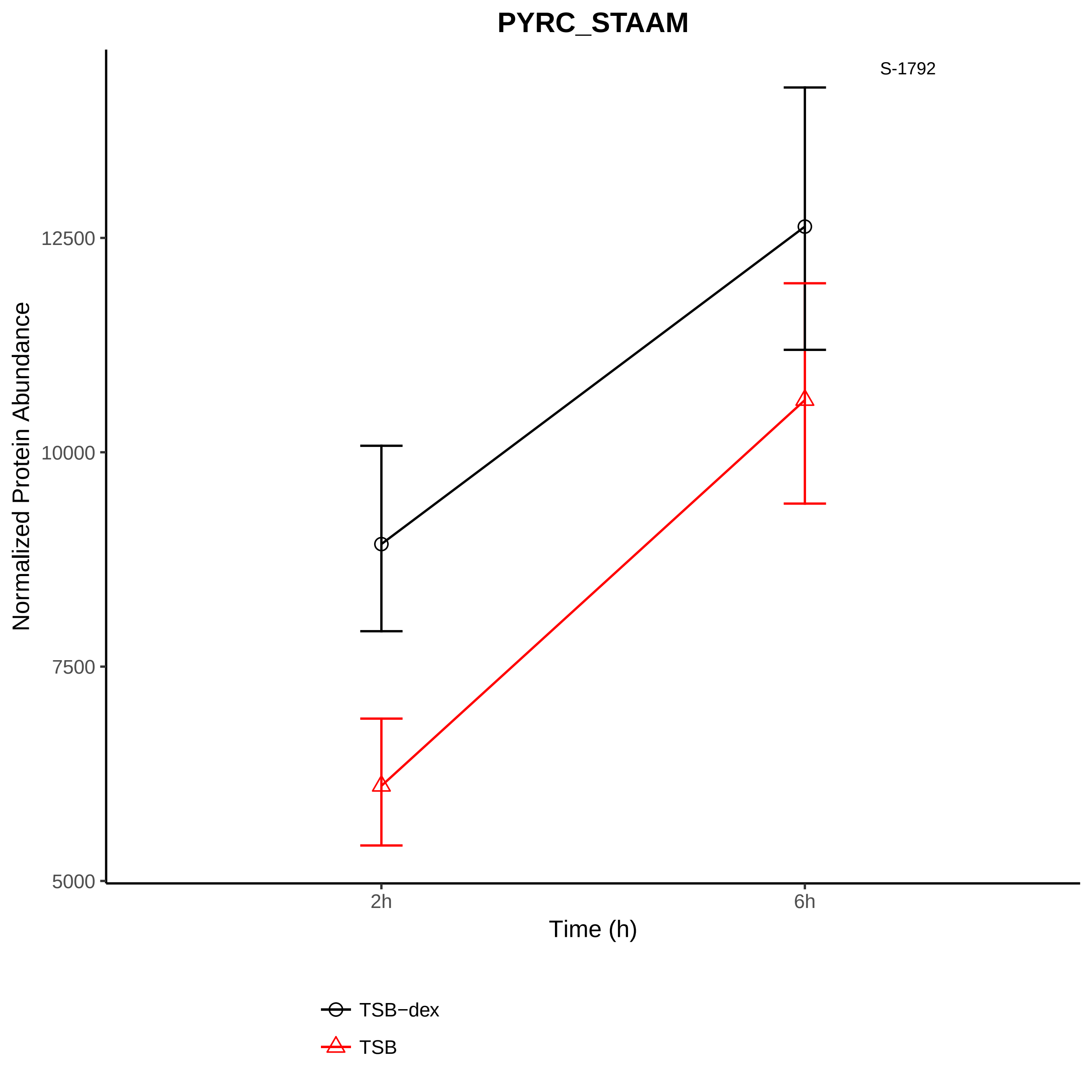

PYRC_STAAM
S-1792
Normalized Protein Abundance
12500
10000
7500
5000
2h
6h
Time (h)
TSB−dex
TSB

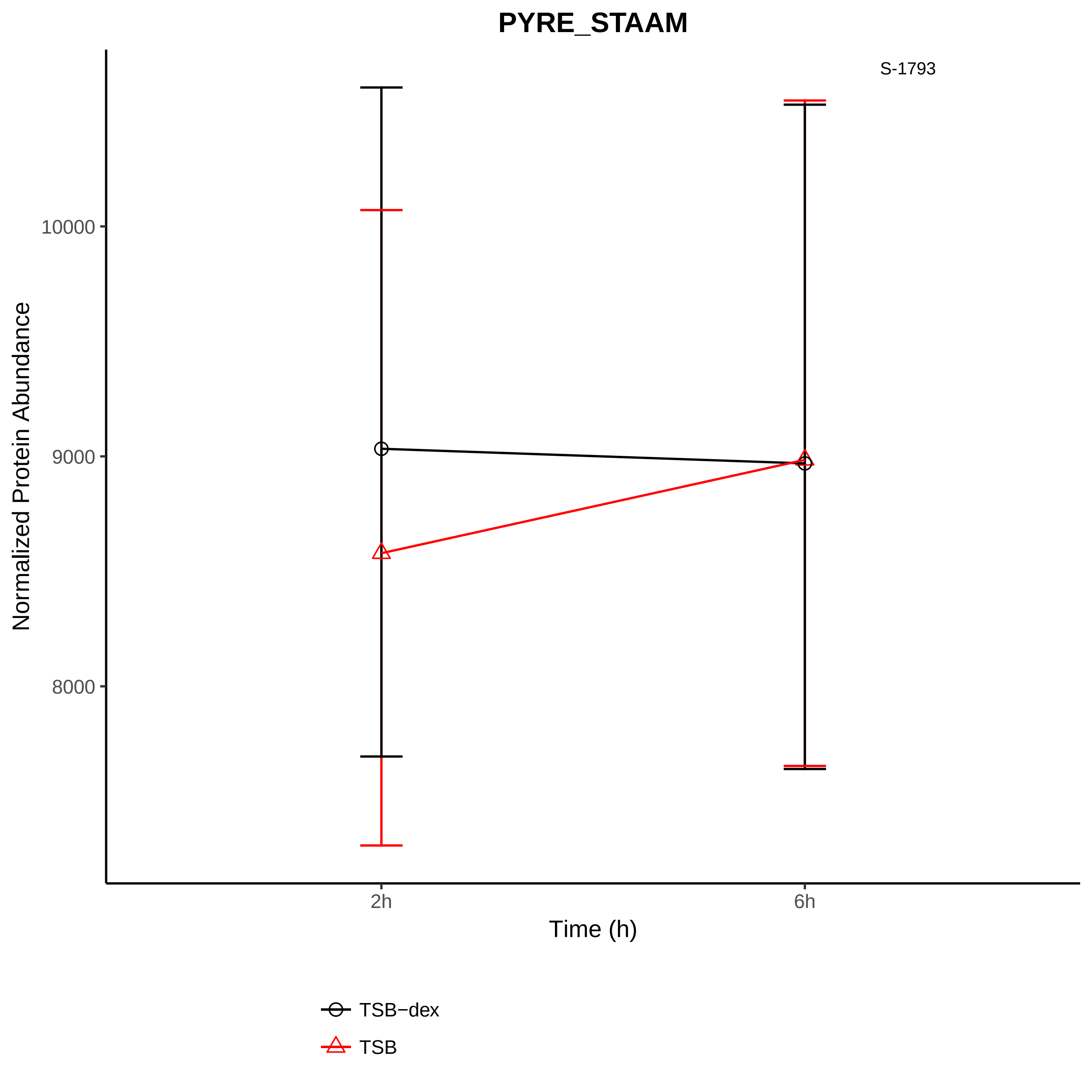

PYRE_STAAM
S-1793
10000
9000
8000
Normalized Protein Abundance
2h
6h
Time (h)
TSB−dex
TSB

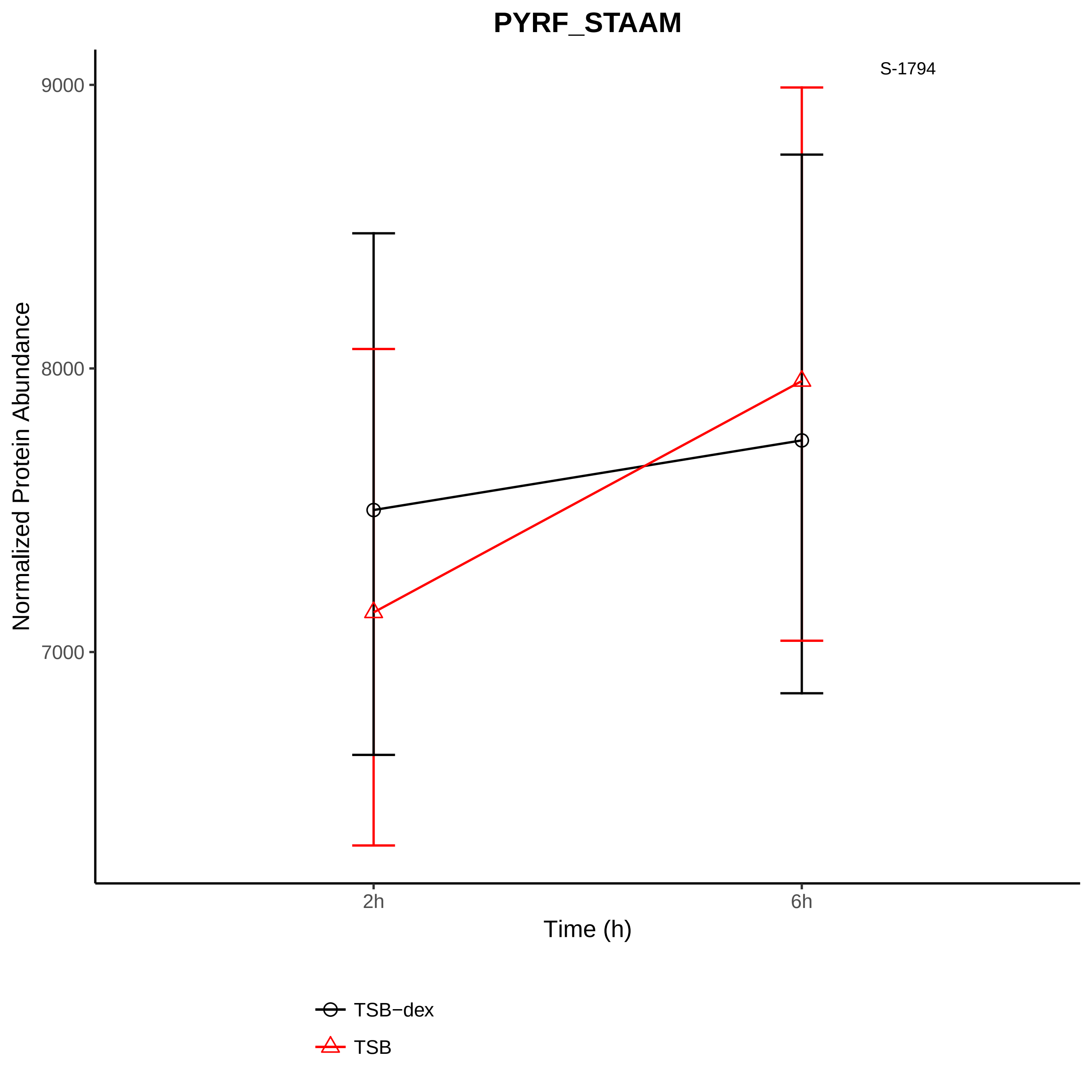

PYRF_STAAM
S-1794
9000
8000
7000
Normalized Protein Abundance
2h
6h
Time (h)
TSB−dex
TSB

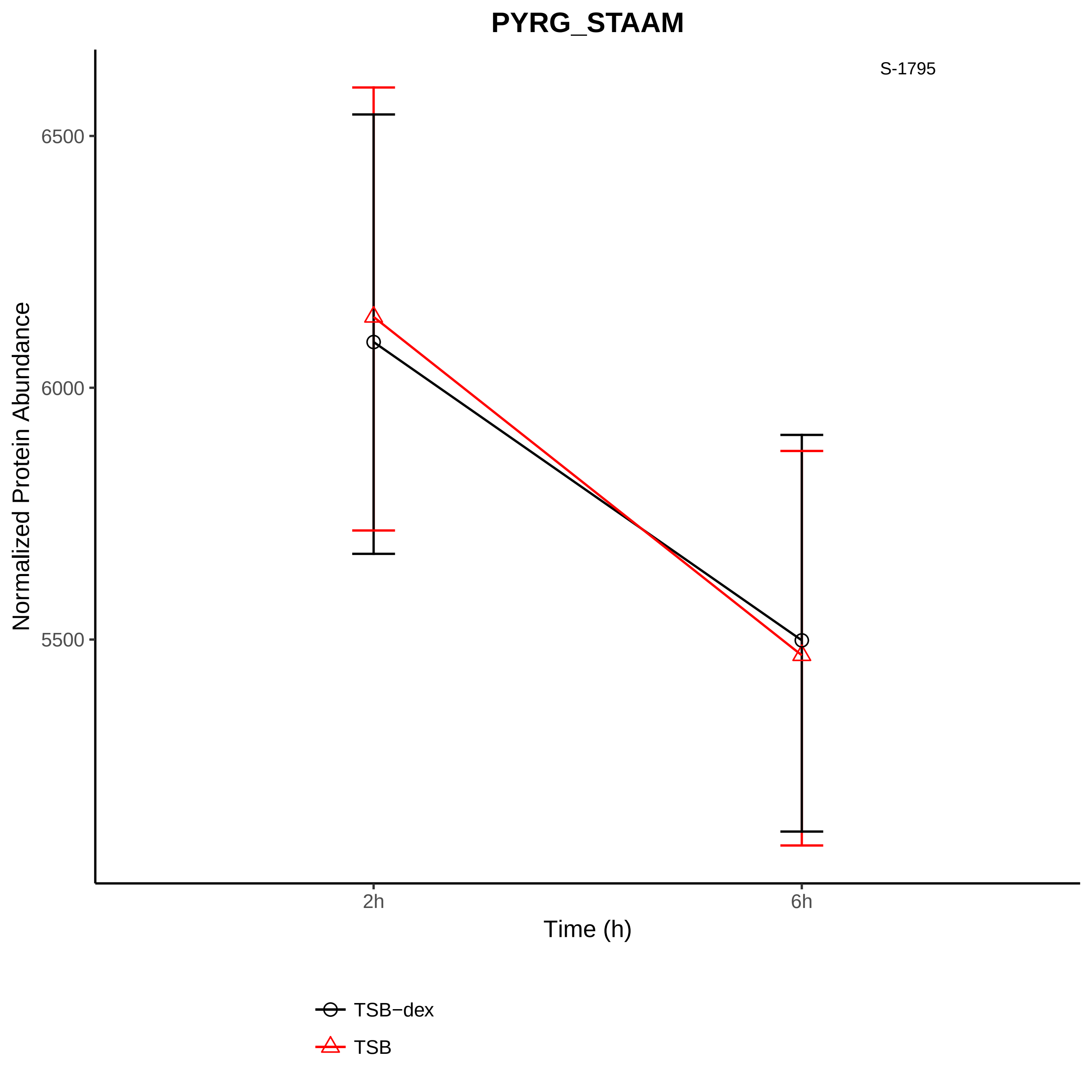
PYRG_STAAM
S-1795
6500
6000
5500
Normalized Protein Abundance
2h
6h
Time (h)
TSB−dex
TSB

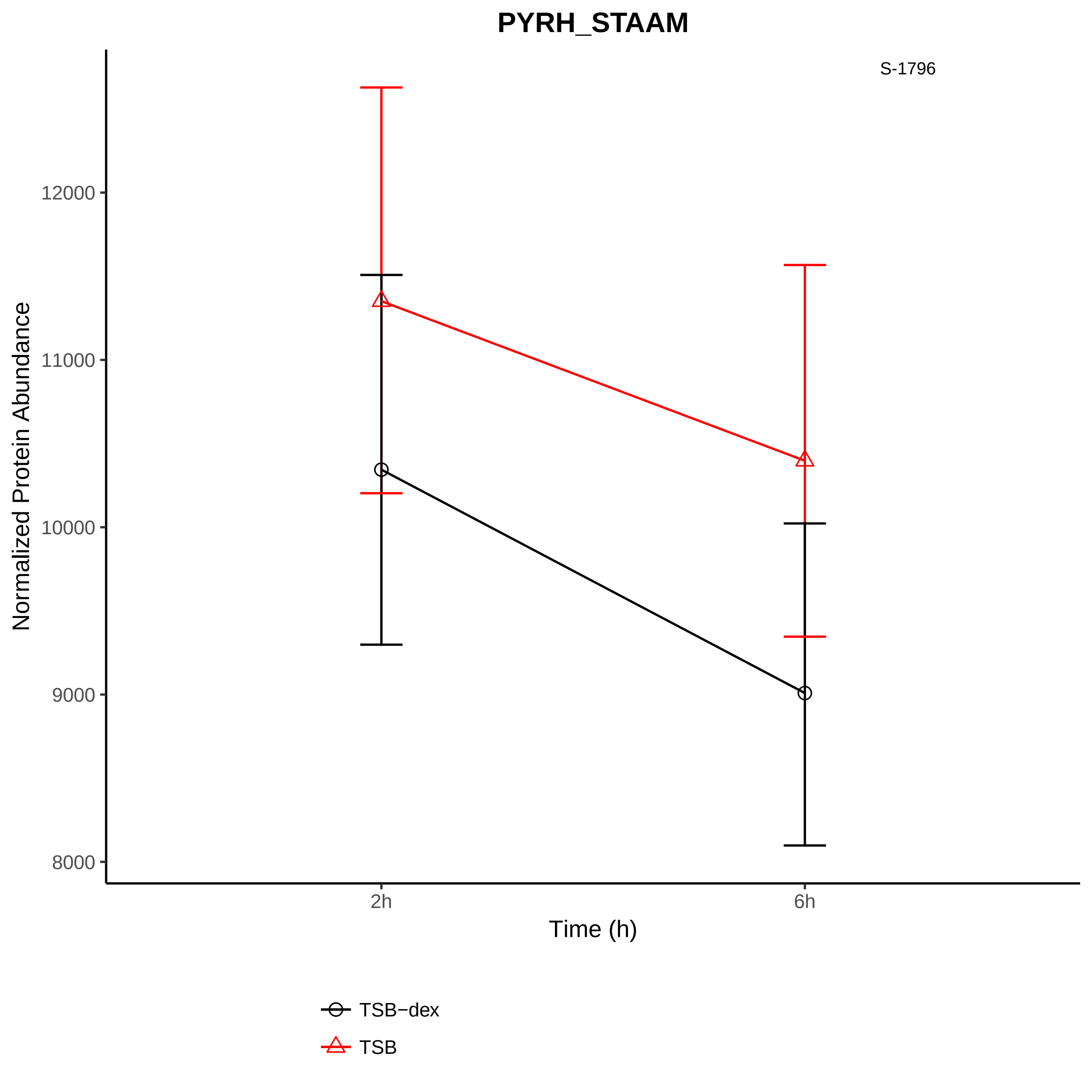
PYRH_STAAM
S-1796
Normalized Protein Abundance
12000
11000
10000
9000
8000
2h
6h
Time (h)
TSB−dex
TSB

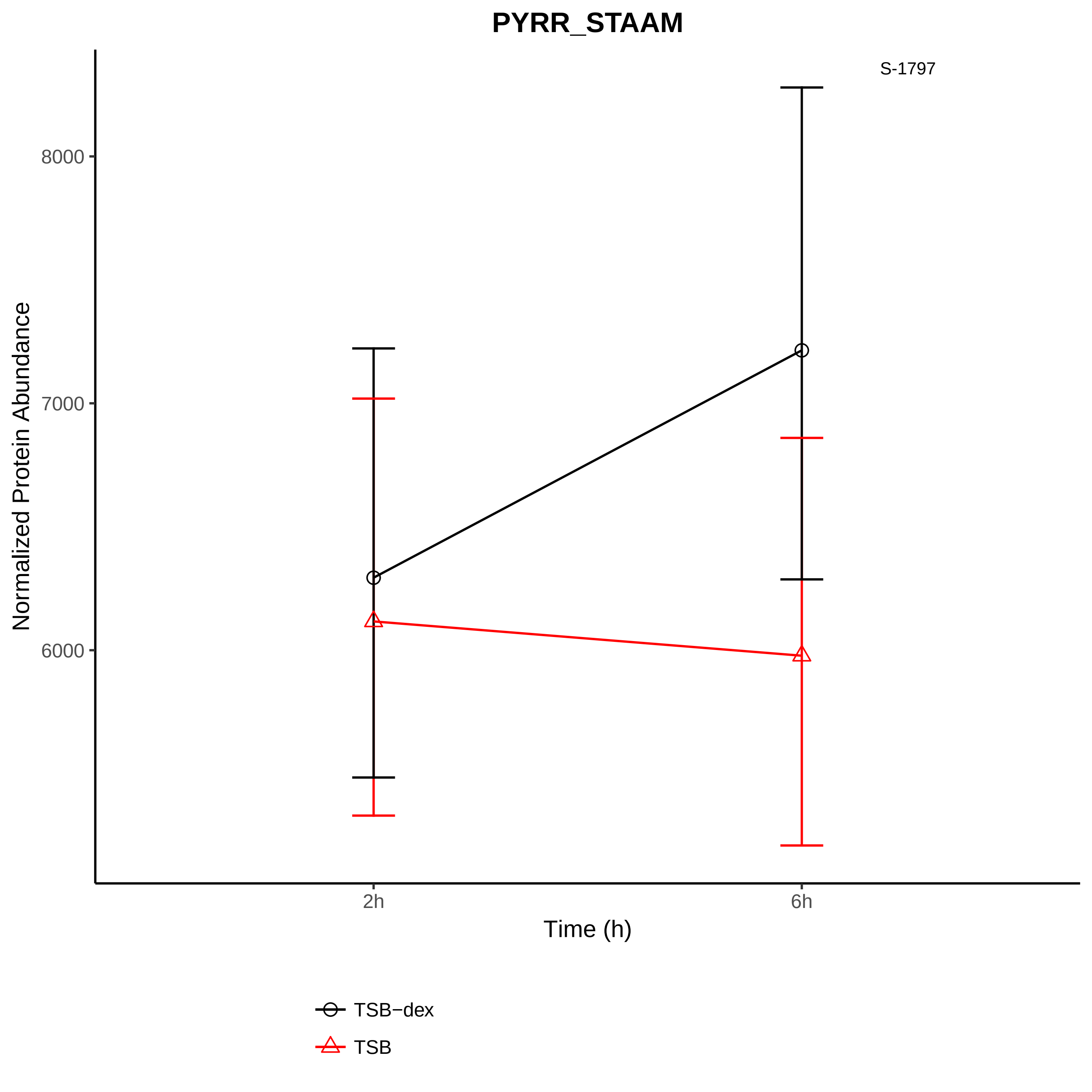

PYRR_STAAM
S-1797
8000
7000
6000
Normalized Protein Abundance
2h
6h
Time (h)
TSB−dex
TSB

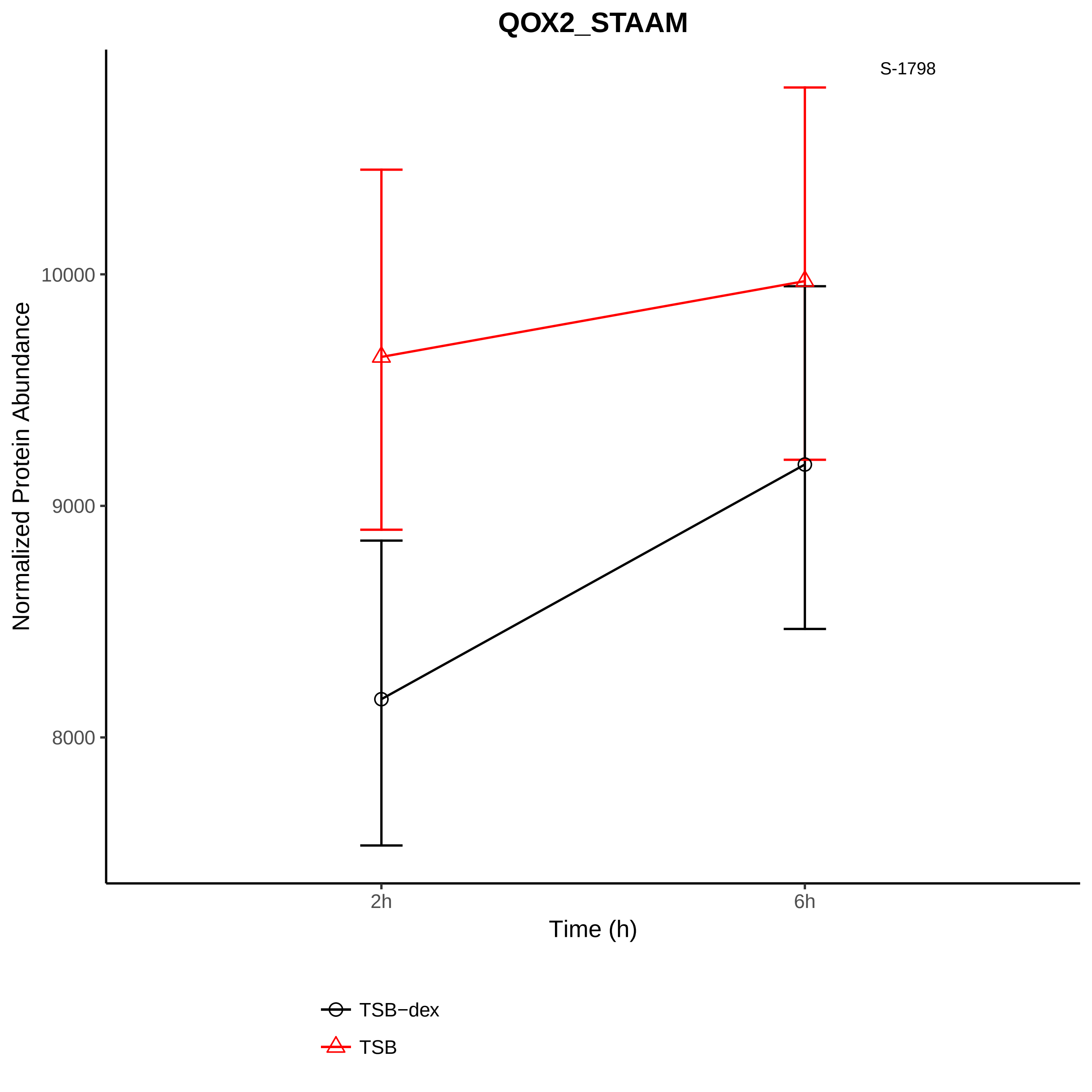
QOX2_STAAM
S-1798
Normalized Protein Abundance
10000
9000
8000
2h
6h
Time (h)
TSB−dex
TSB

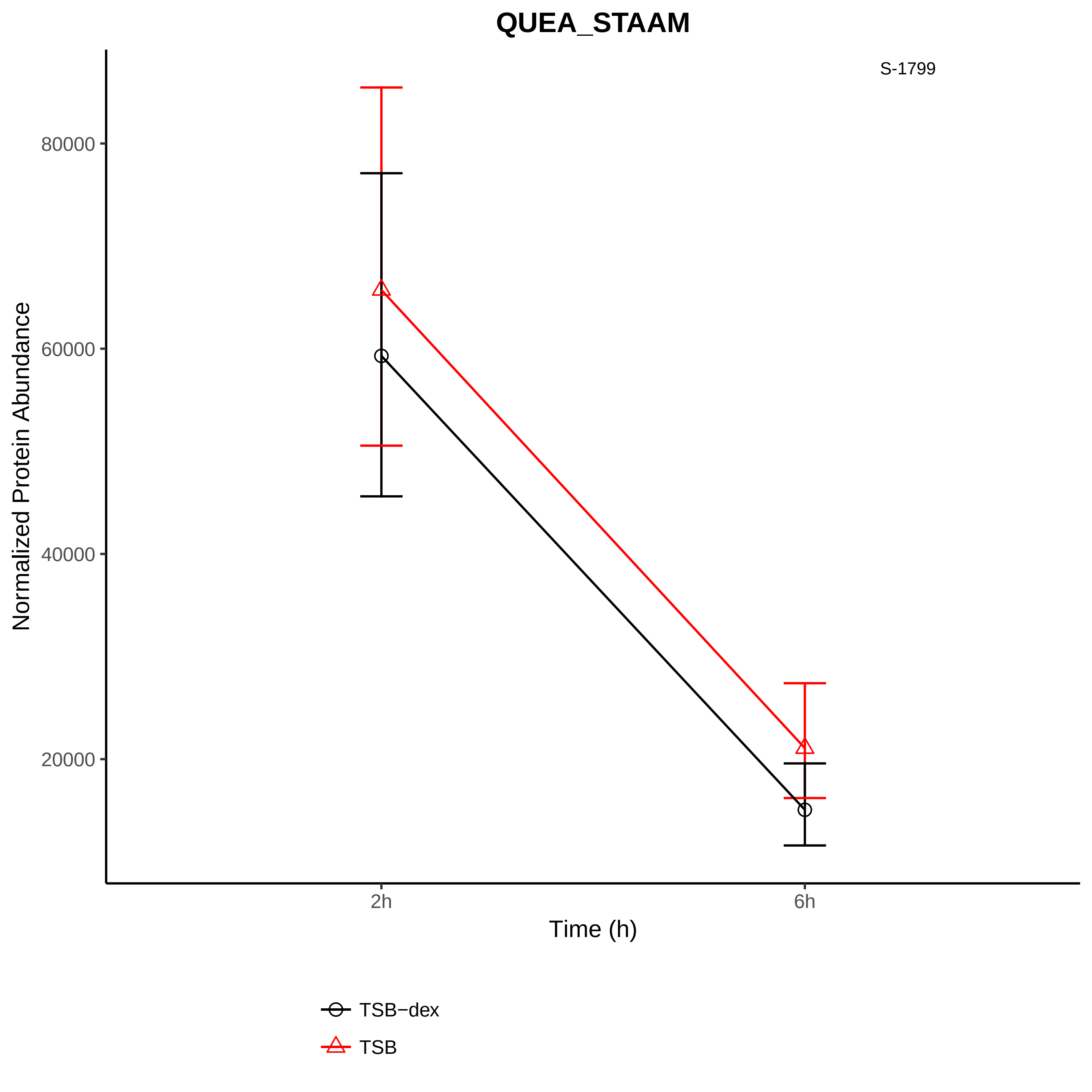
QUEA_STAAM
S-1799
80000
60000
40000
20000
Normalized Protein Abundance
2h
6h
Time (h)
TSB−dex
TSB

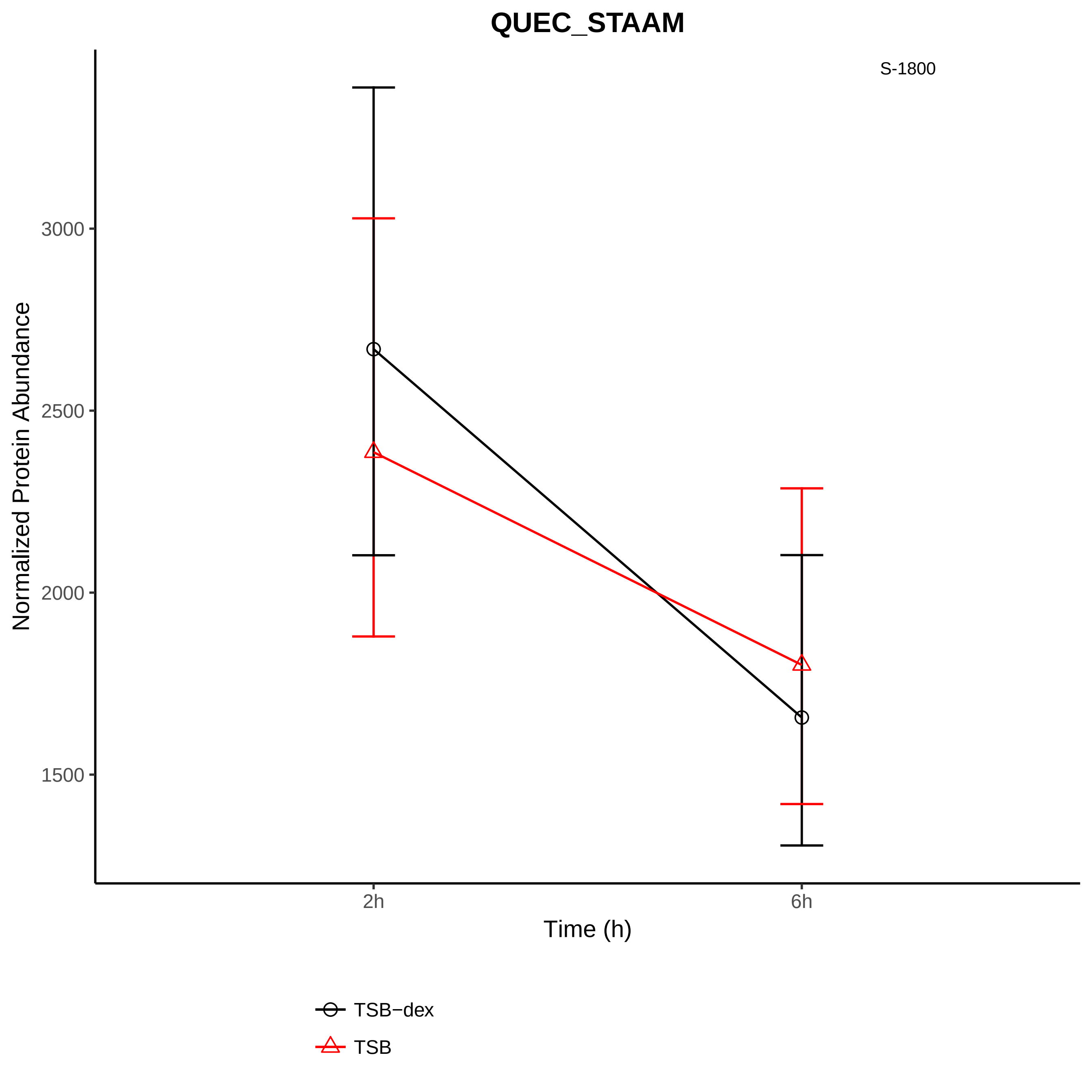
QUEC_STAAM
S-1800
Normalized Protein Abundance
3000
2500
2000
1500
2h
6h
Time (h)
TSB−dex
TSB

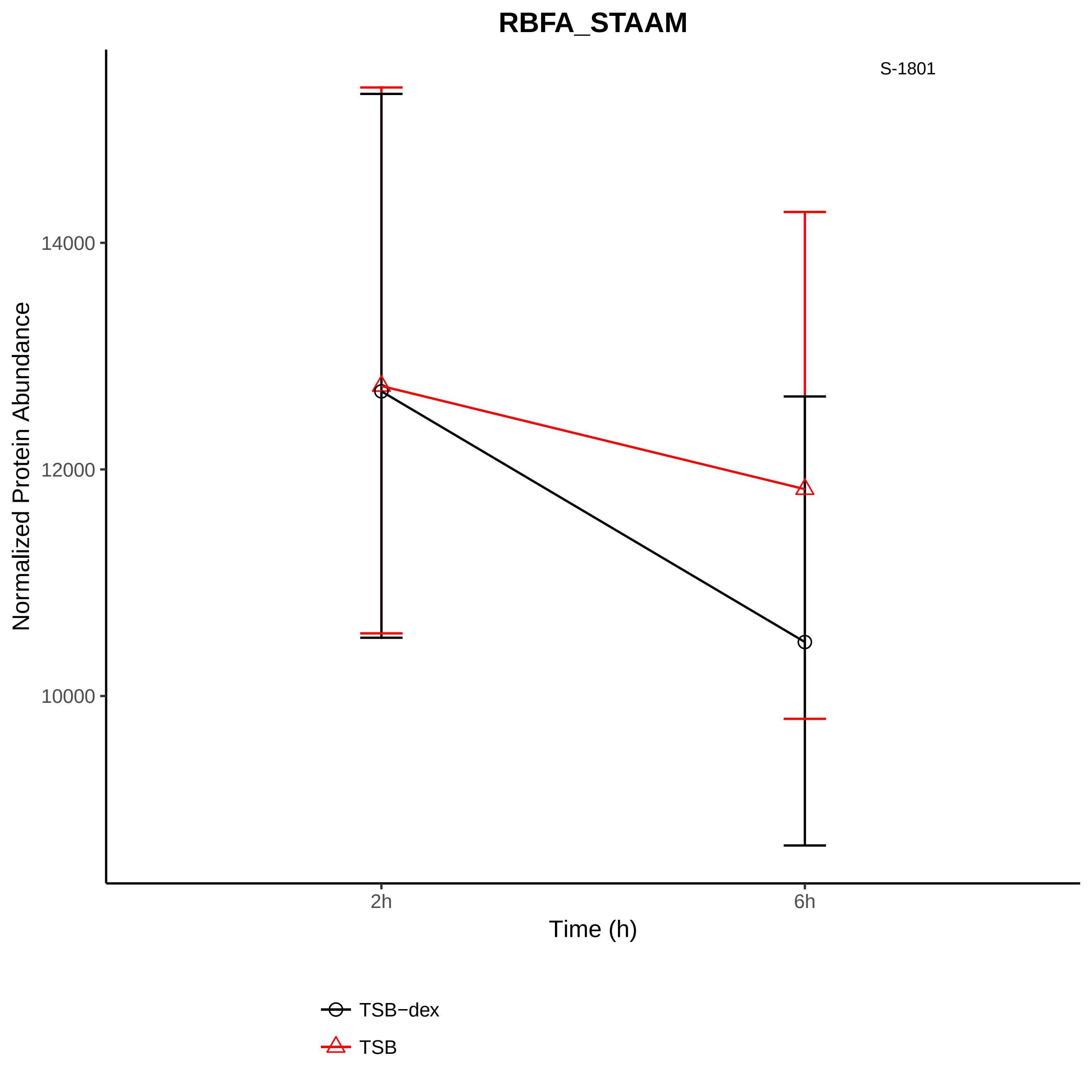
RBFA_STAAM
S-1801
Normalized Protein Abundance
14000
12000
10000
2h
6h
Time (h)
TSB−dex
TSB

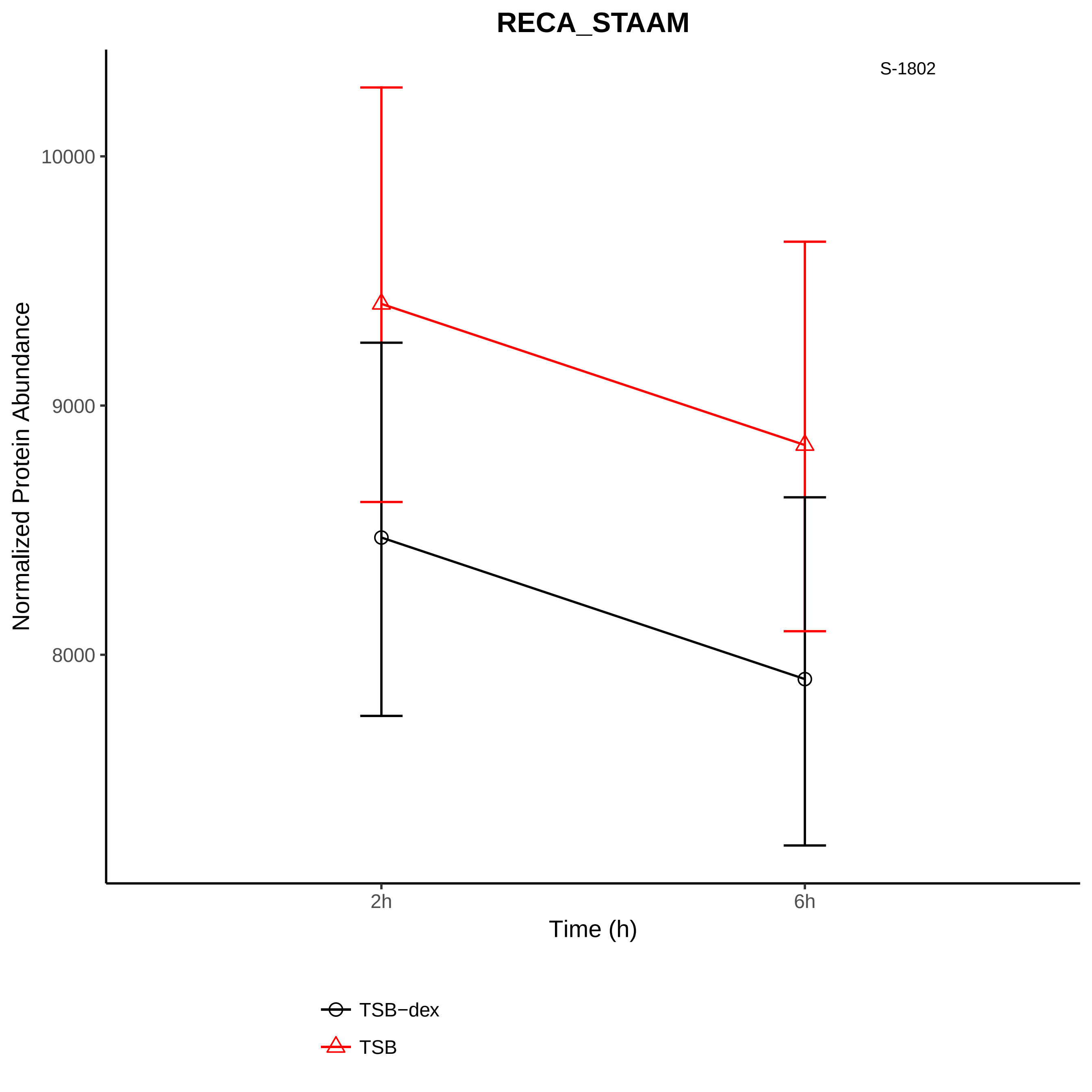

RECA_STAAM
S-1802
Normalized Protein Abundance
10000
9000
8000
2h
6h
Time (h)
TSB−dex
TSB

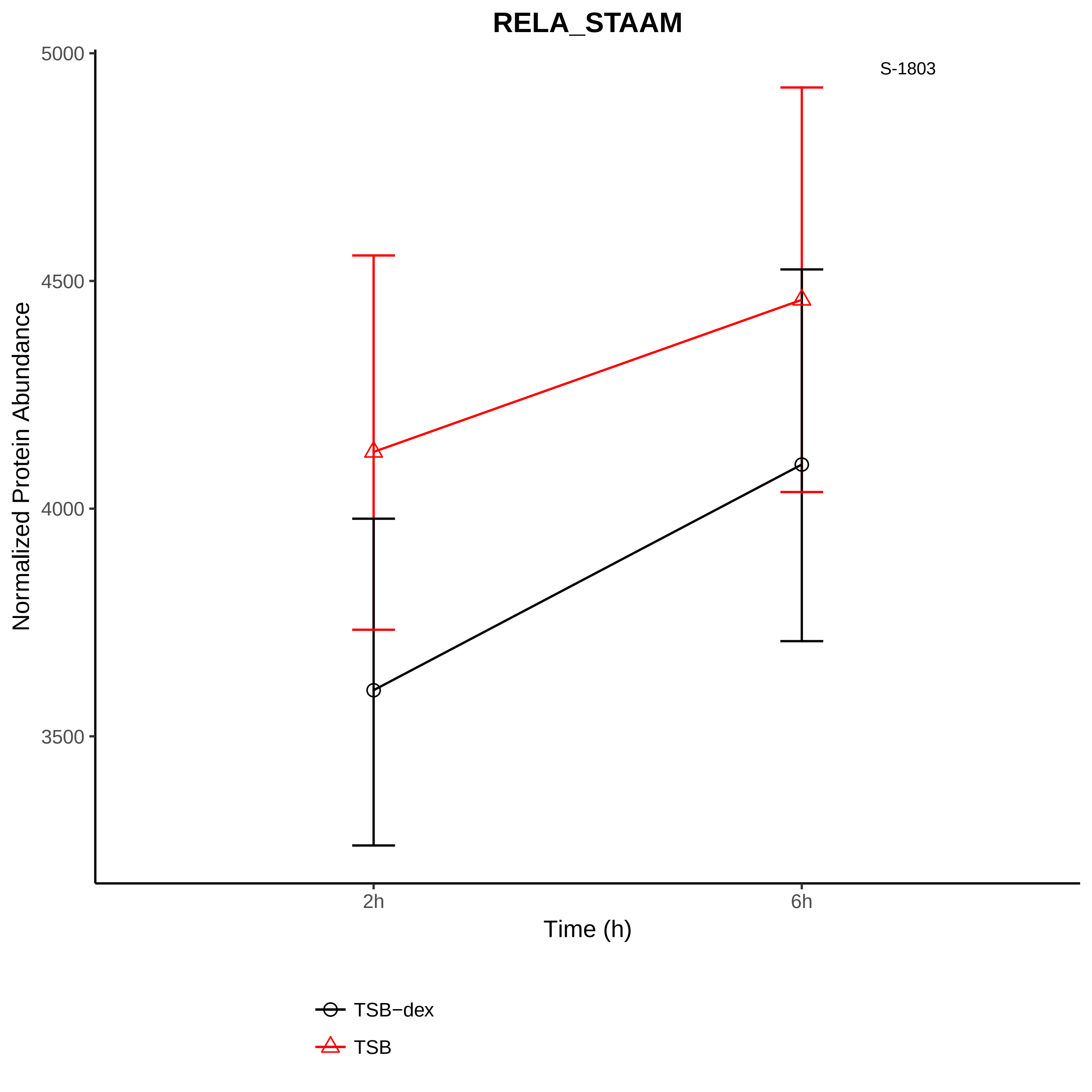

RELA_STAAM
S-1803
5000
4500
4000
3500
Normalized Protein Abundance
2h
6h
Time (h)
TSB−dex
TSB

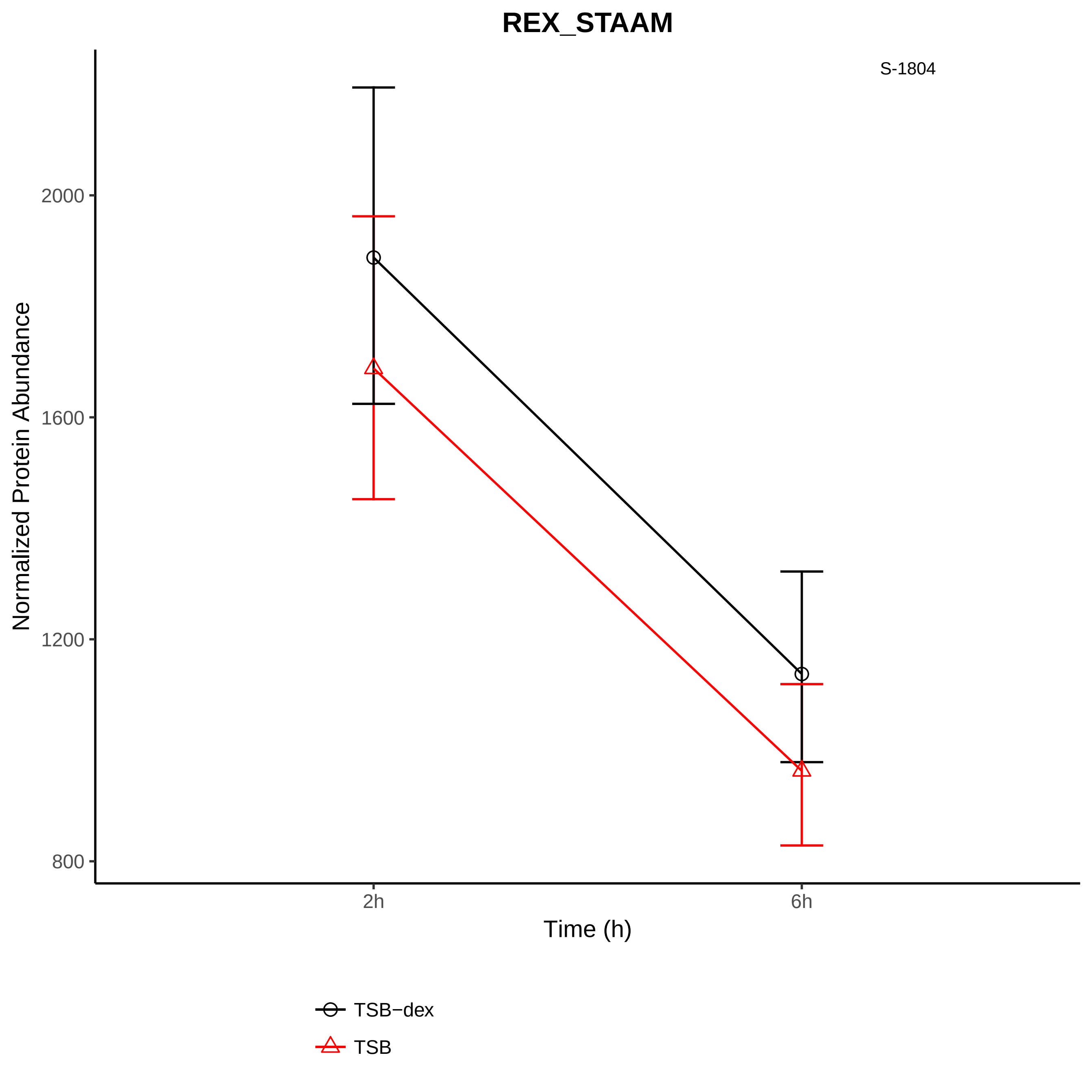
REX_STAAM
S-1804
Normalized Protein Abundance
2000
1600
1200
800
2h
6h
Time (h)
TSB−dex
TSB

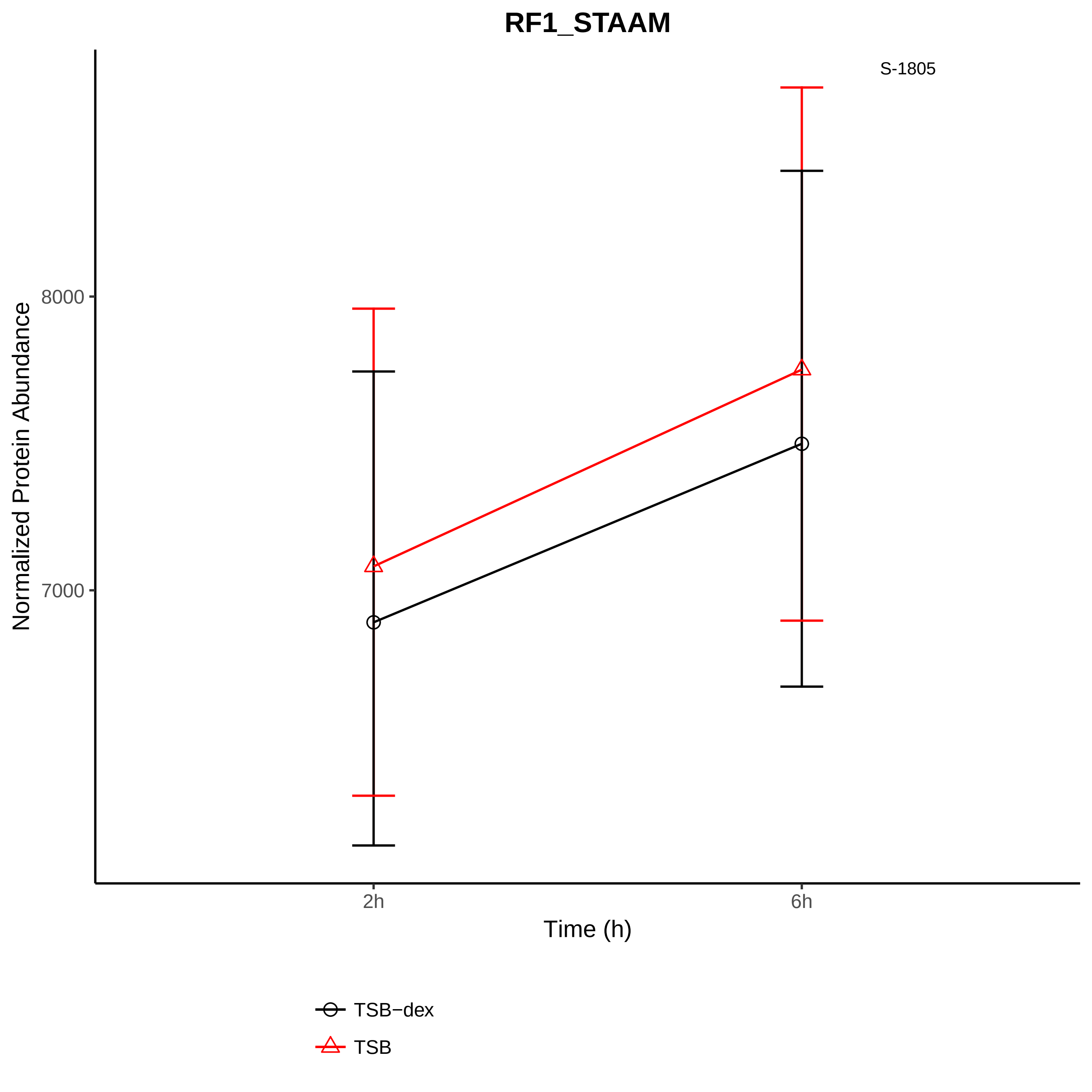

RF1_STAAM
S-1805
Normalized Protein Abundance
8000
7000
2h
6h
Time (h)
TSB−dex
TSB

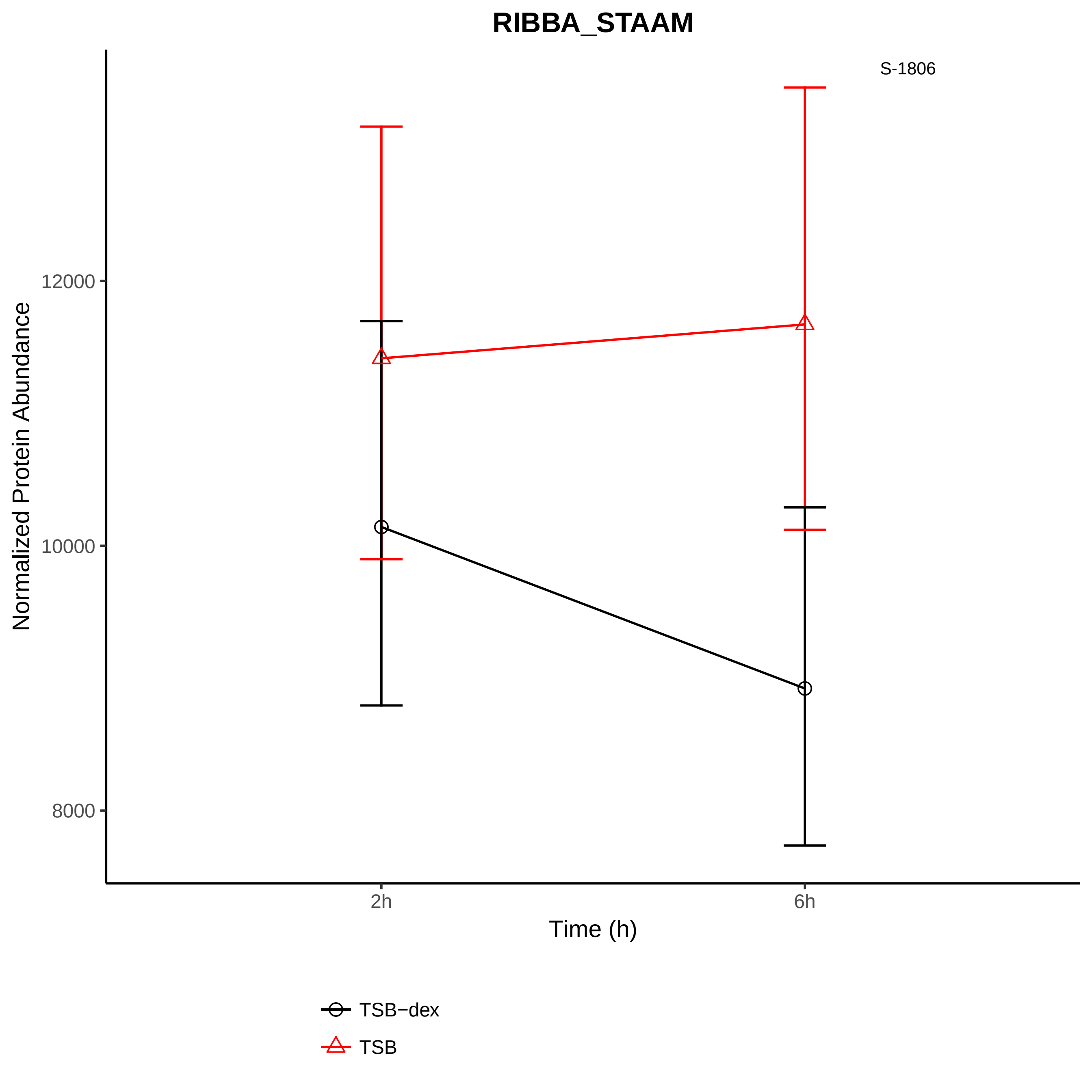

RIBBA_STAAM
S-1806
Normalized Protein Abundance
12000
10000
8000
2h
6h
Time (h)
TSB−dex
TSB

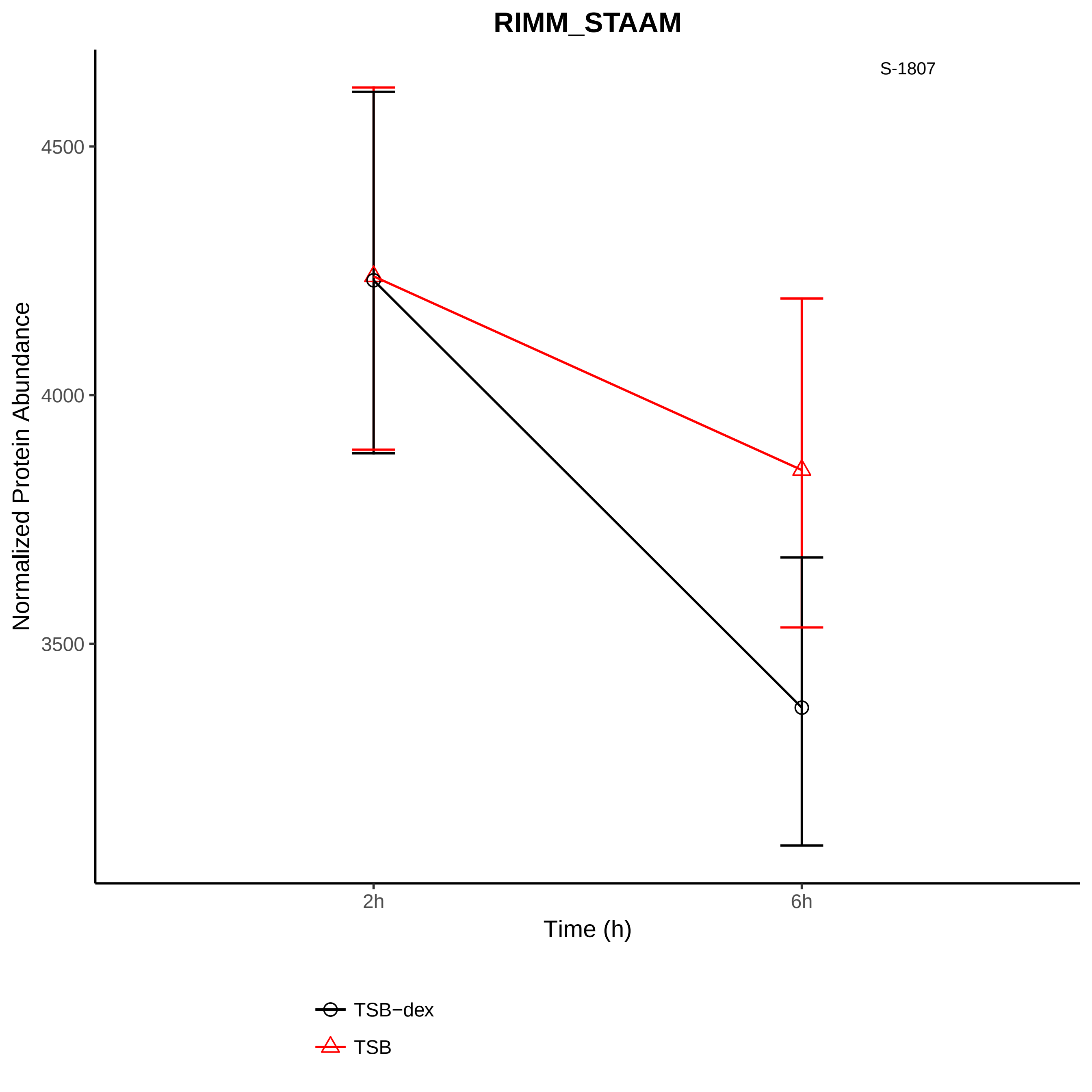

RIMM_STAAM
S-1807
4500
4000
3500
Normalized Protein Abundance
2h
6h
Time (h)
TSB−dex
TSB

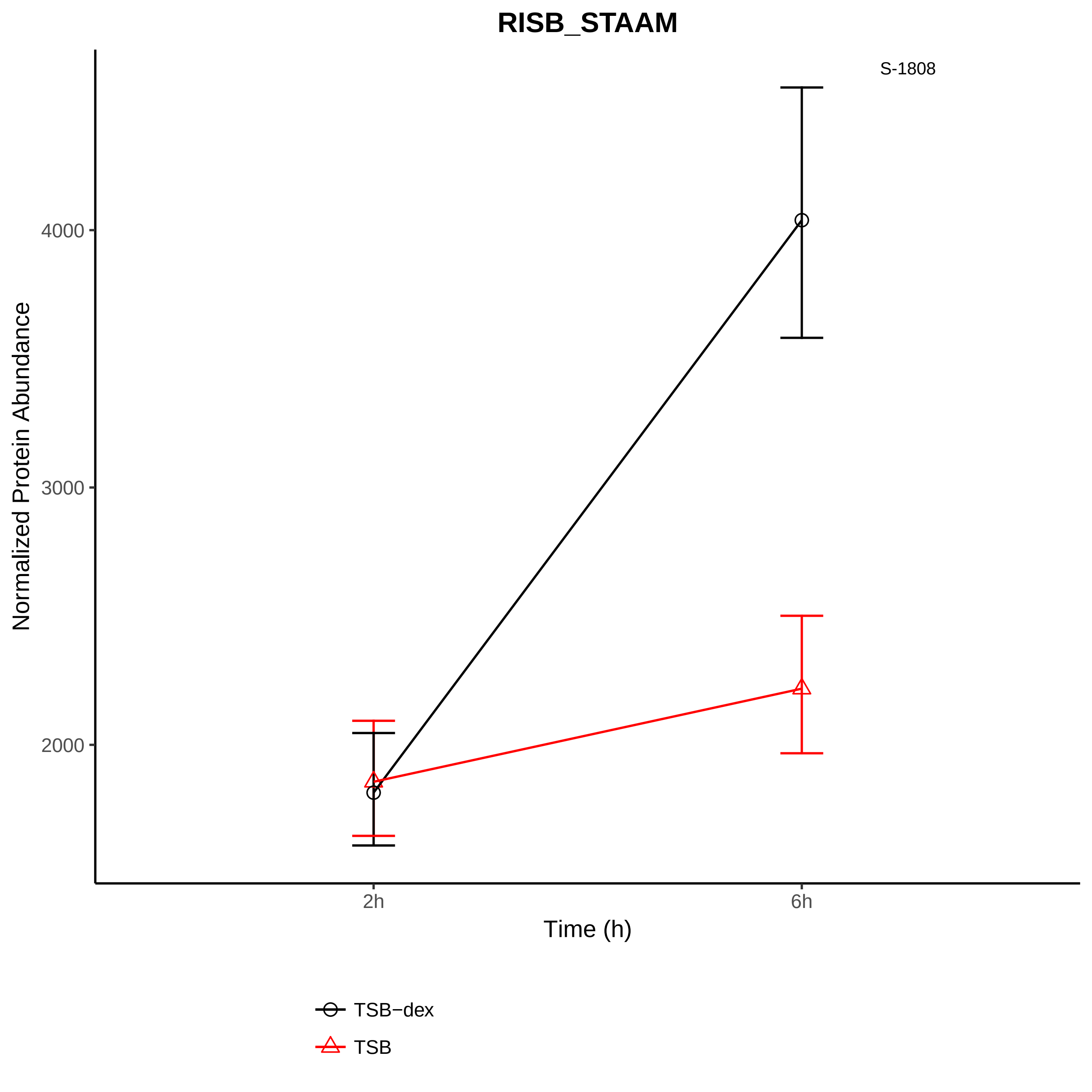

RISB_STAAM
S-1808
4000
3000
2000
Normalized Protein Abundance
2h
6h
Time (h)
TSB−dex
TSB

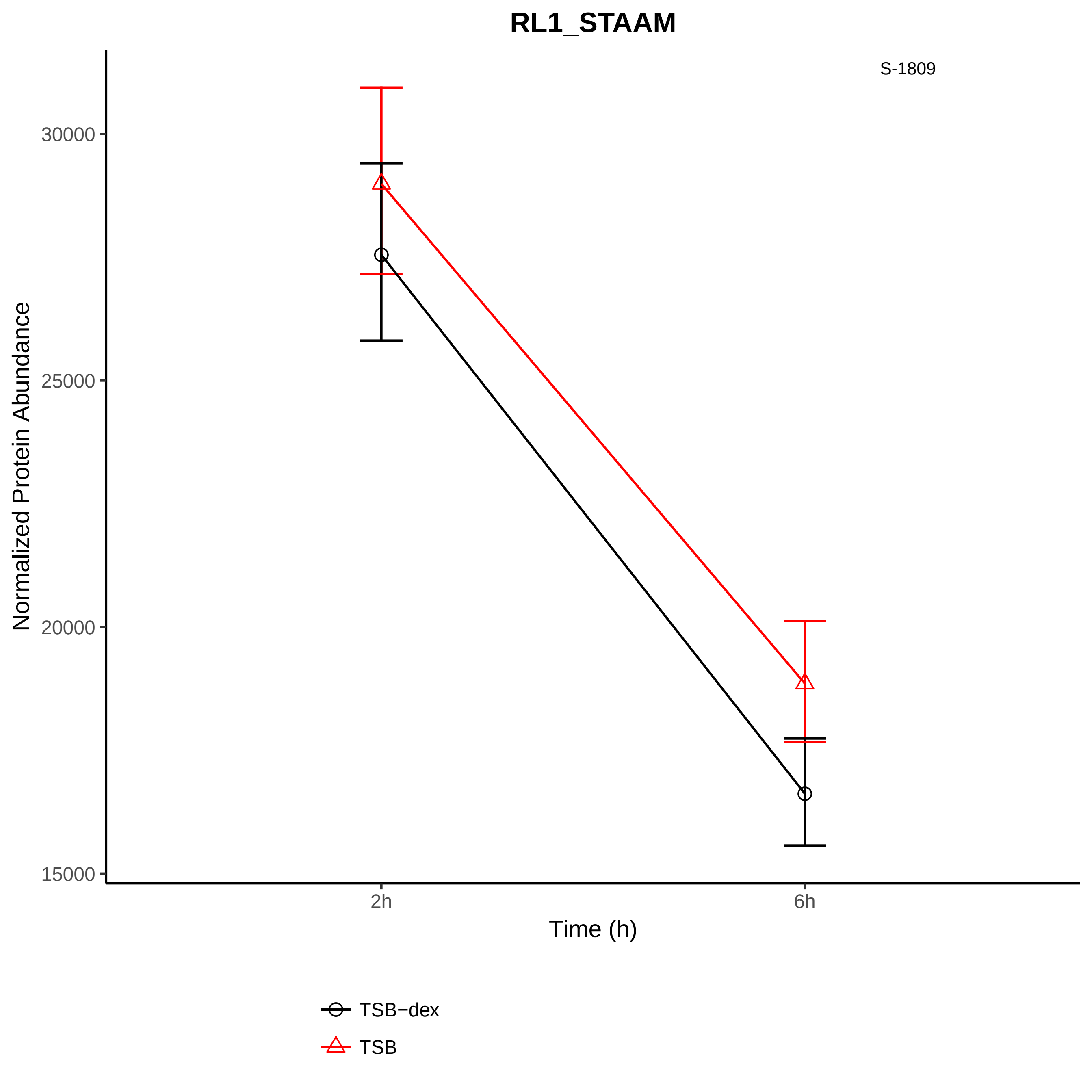

RL1_STAAM
S-1809
Normalized Protein Abundance
30000
25000
20000
15000
2h
6h
Time (h)
TSB−dex
TSB

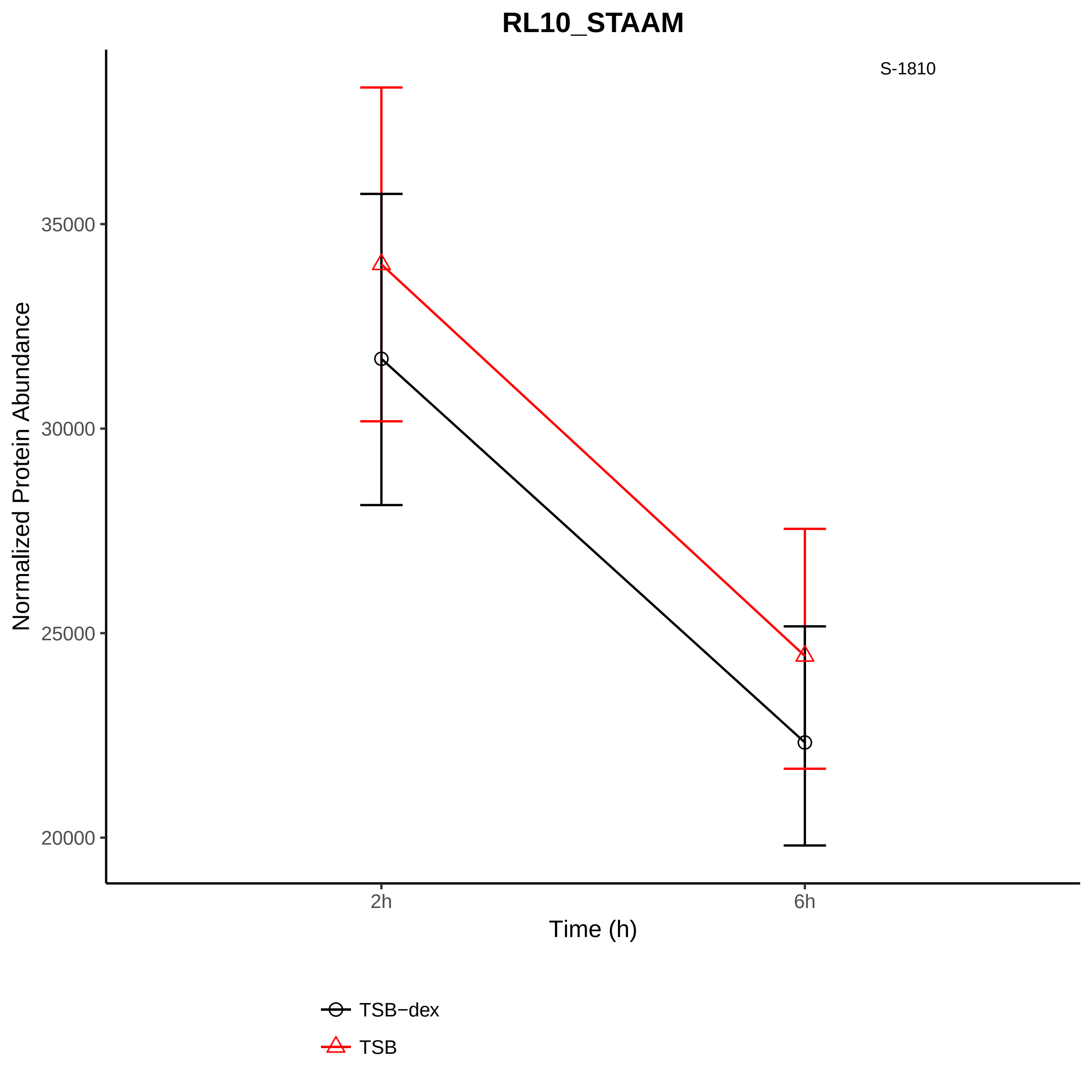

RL10_STAAM
S-1810
Normalized Protein Abundance
35000
30000
25000
20000
2h
6h
Time (h)
TSB−dex
TSB

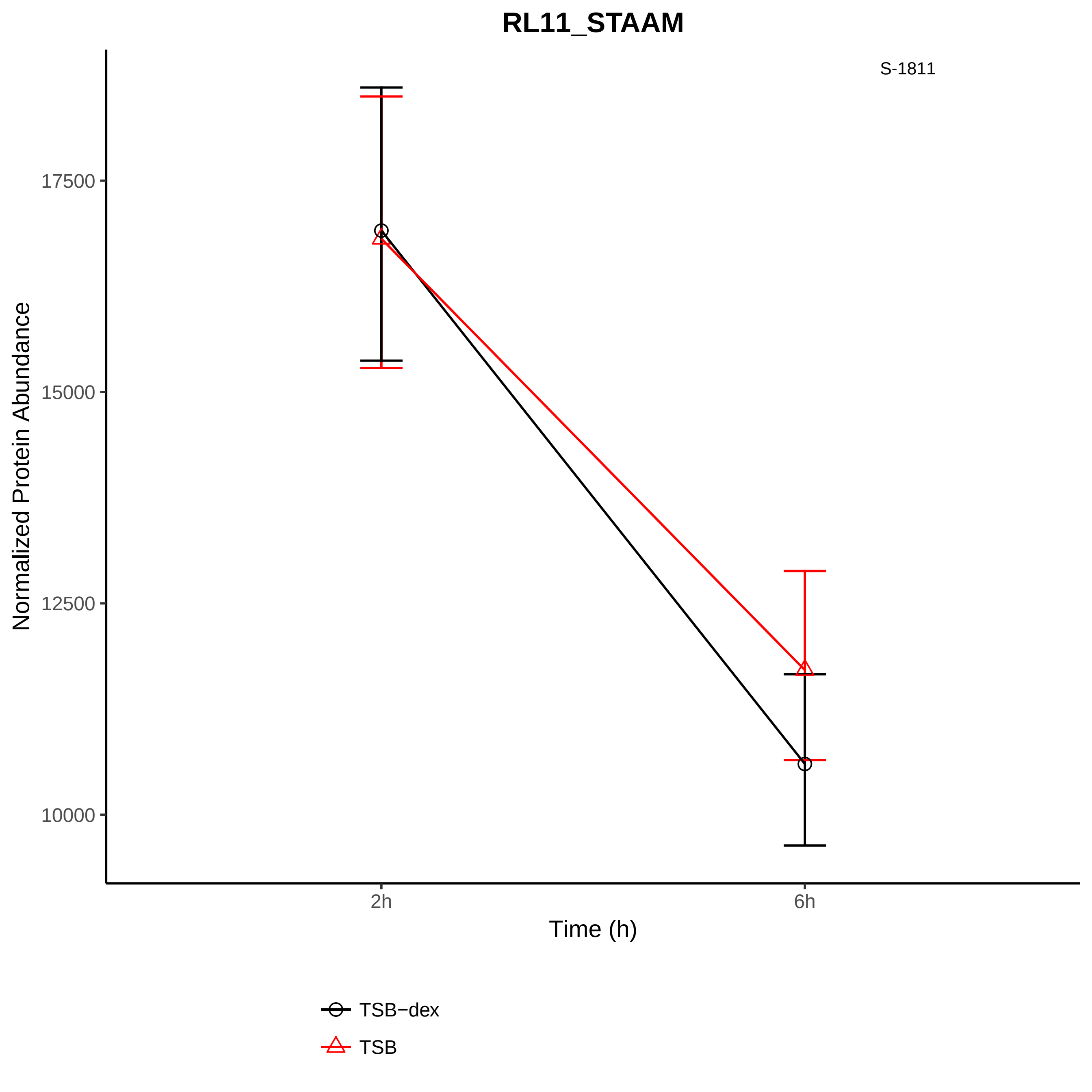

RL11_STAAM
S-1811
17500
15000
12500
10000
Normalized Protein Abundance
2h
6h
Time (h)
TSB−dex
TSB

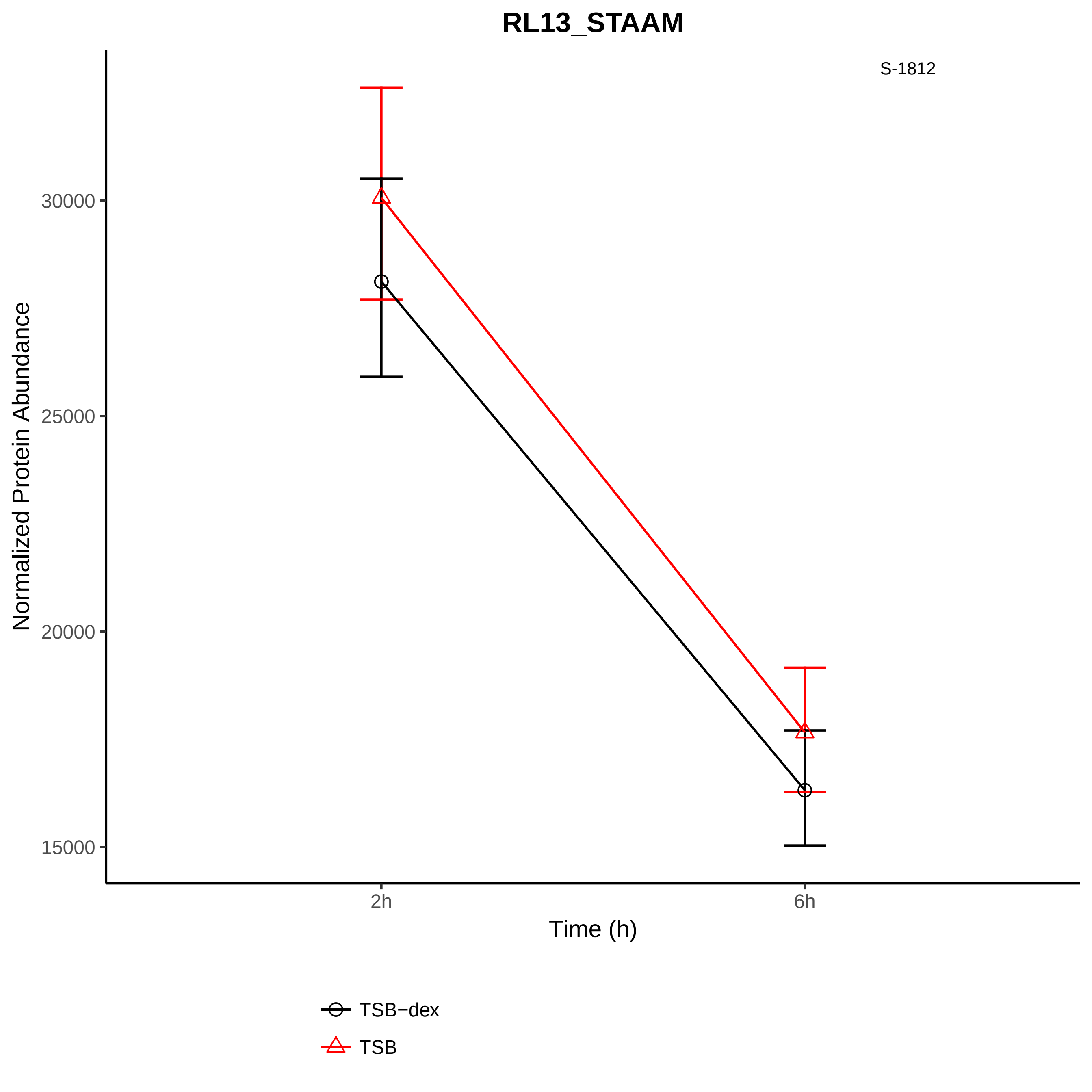
RL13_STAAM
S-1812
Normalized Protein Abundance
30000
25000
20000
15000
2h
6h
Time (h)
TSB−dex
TSB

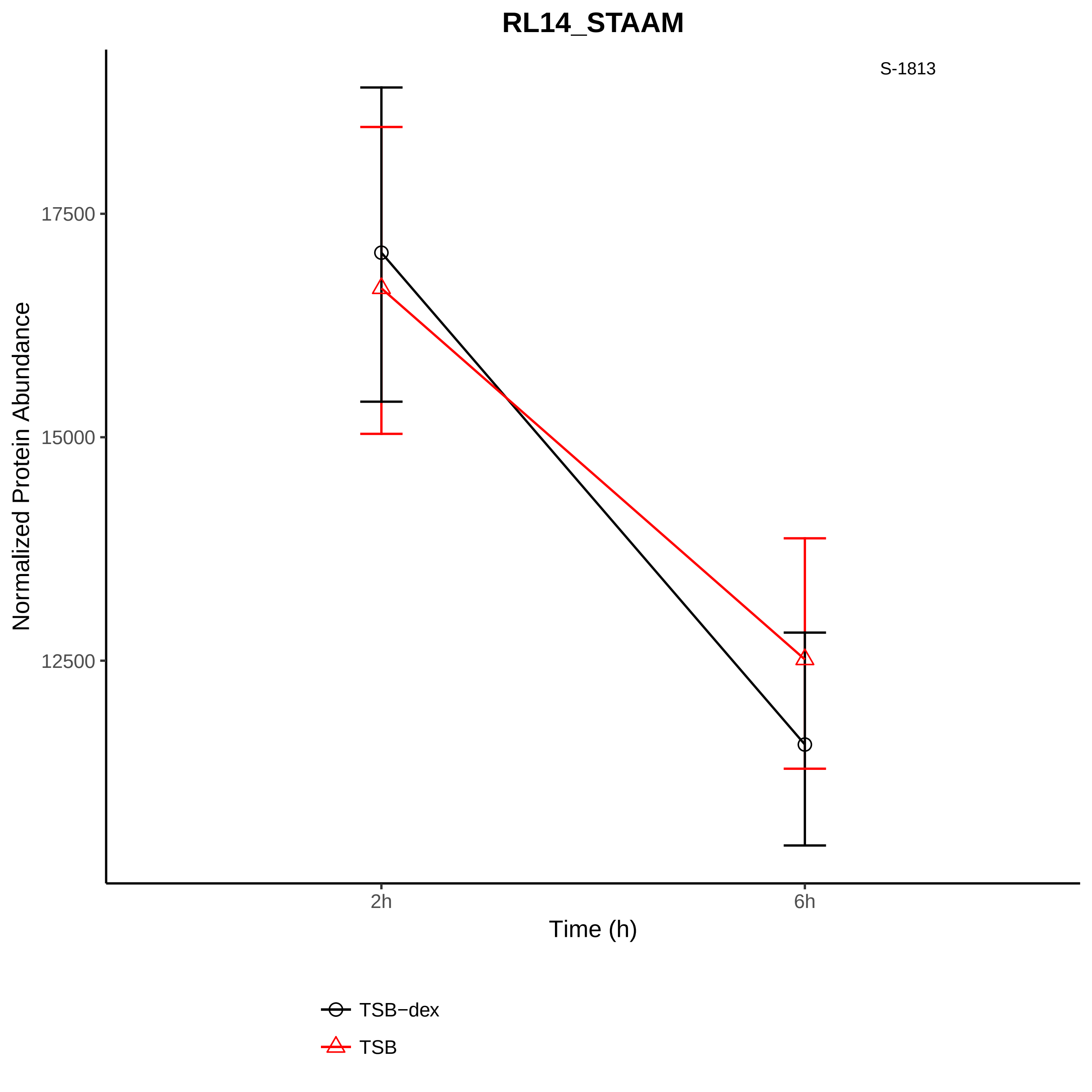

RL14_STAAM
S-1813
17500
15000
12500
Normalized Protein Abundance
2h
6h
Time (h)
TSB−dex
TSB

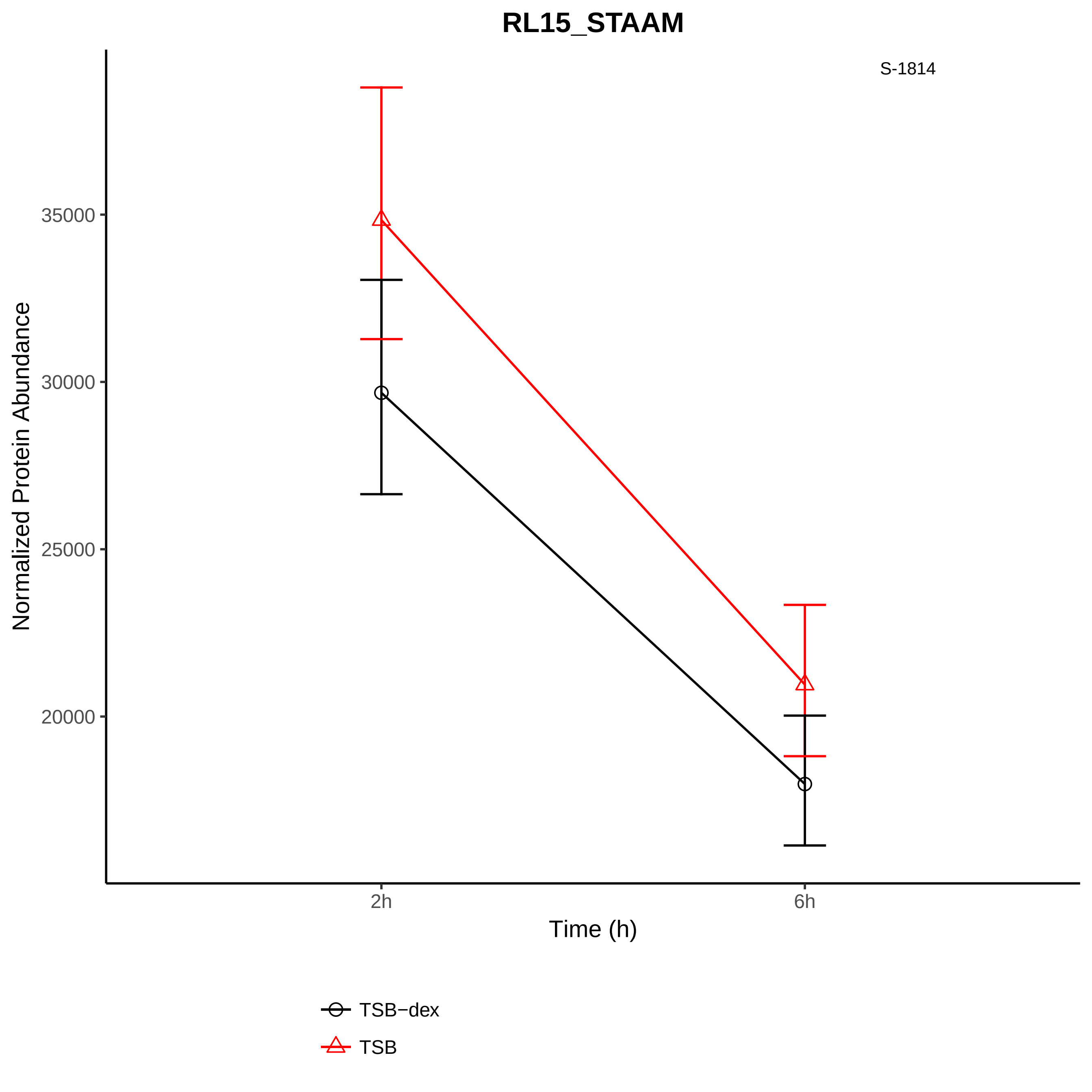
RL15_STAAM
S-1814
Normalized Protein Abundance
35000
30000
25000
20000
2h
6h
Time (h)
TSB−dex
TSB

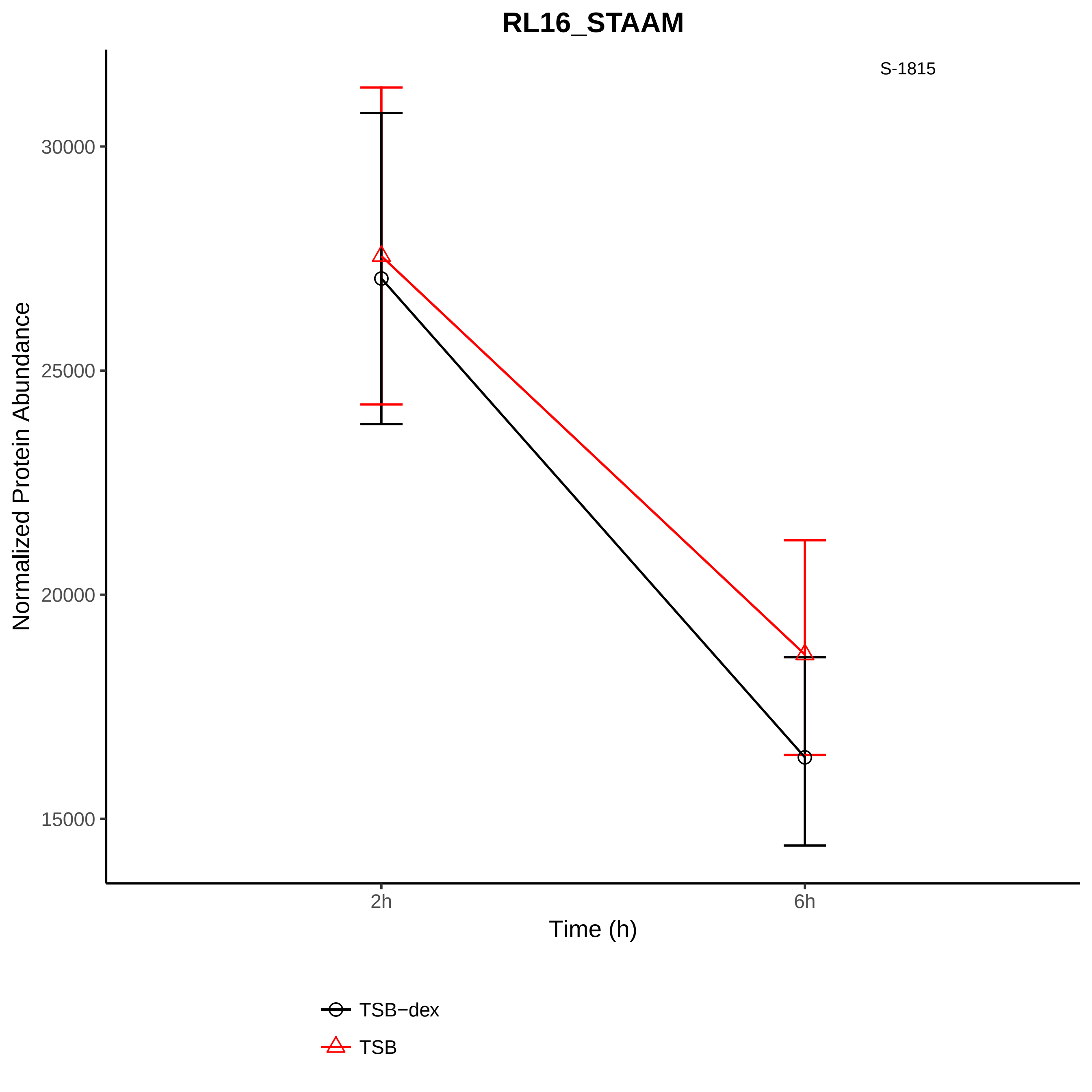

RL16_STAAM
S-1815
30000
25000
20000
15000
Normalized Protein Abundance
2h
6h
Time (h)
TSB−dex
TSB

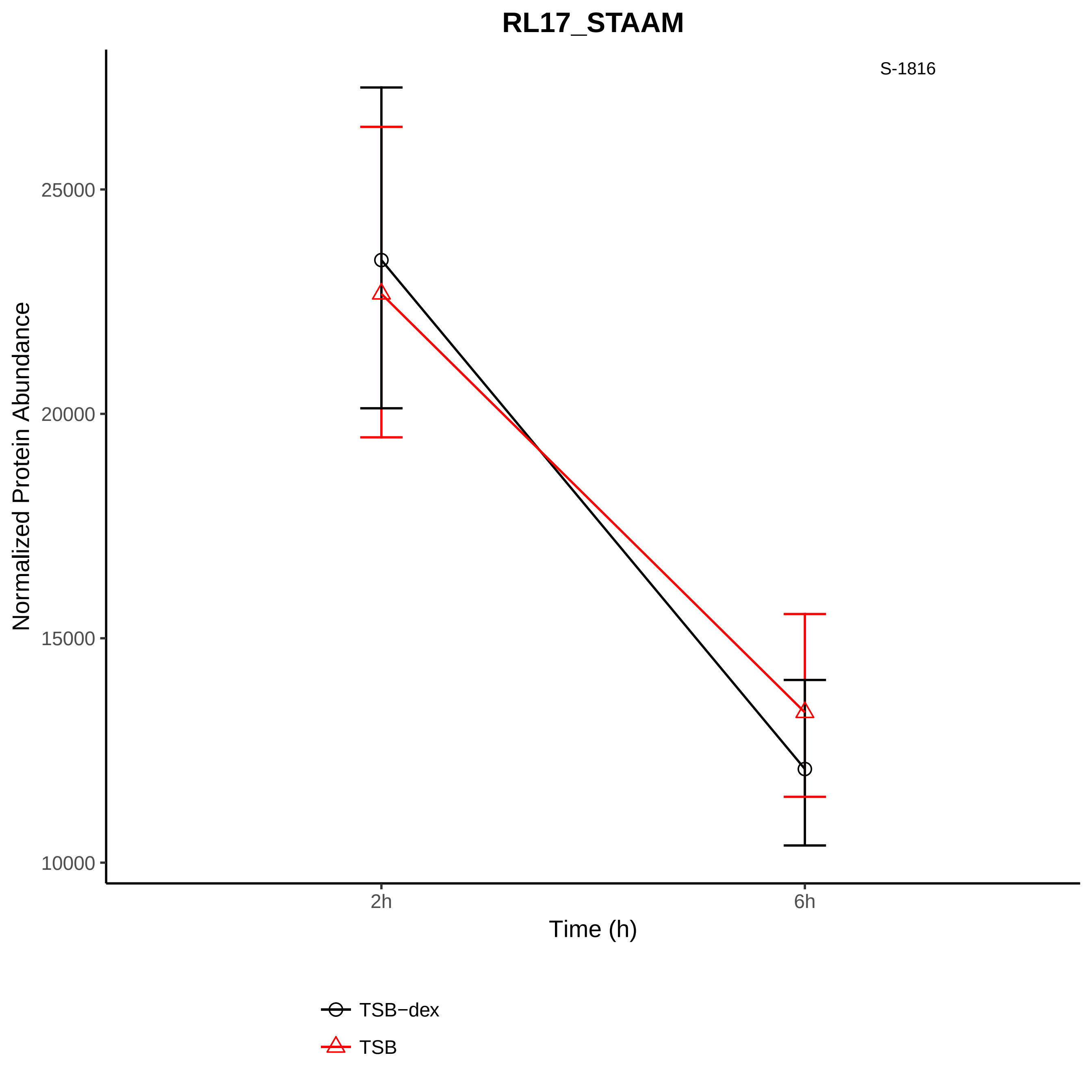
RL17_STAAM
S-1816
Normalized Protein Abundance
25000
20000
15000
10000
2h
6h
Time (h)
TSB−dex
TSB

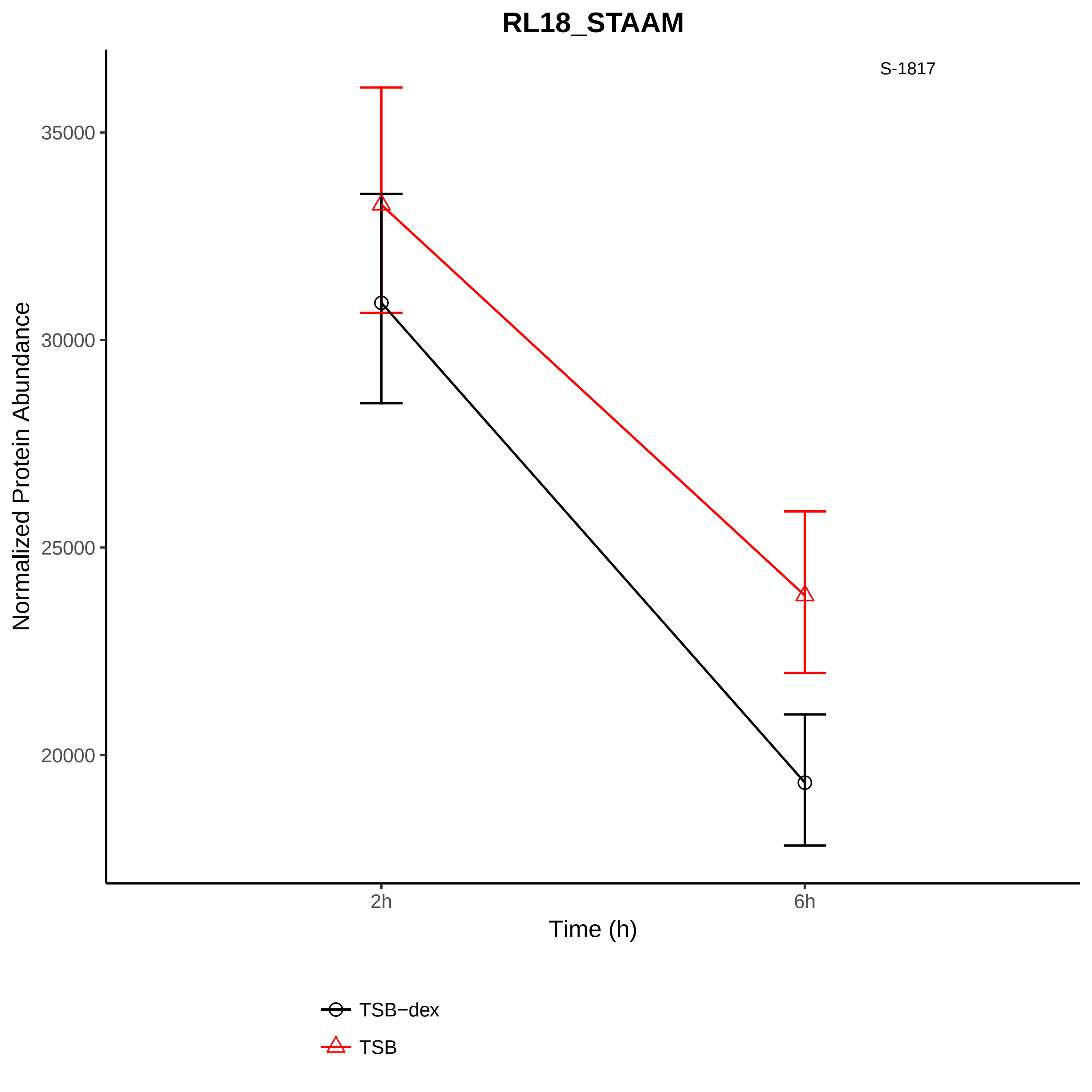

RL18_STAAM
S-1817
Normalized Protein Abundance
35000
30000
25000
20000
2h
6h
Time (h)
TSB−dex
TSB

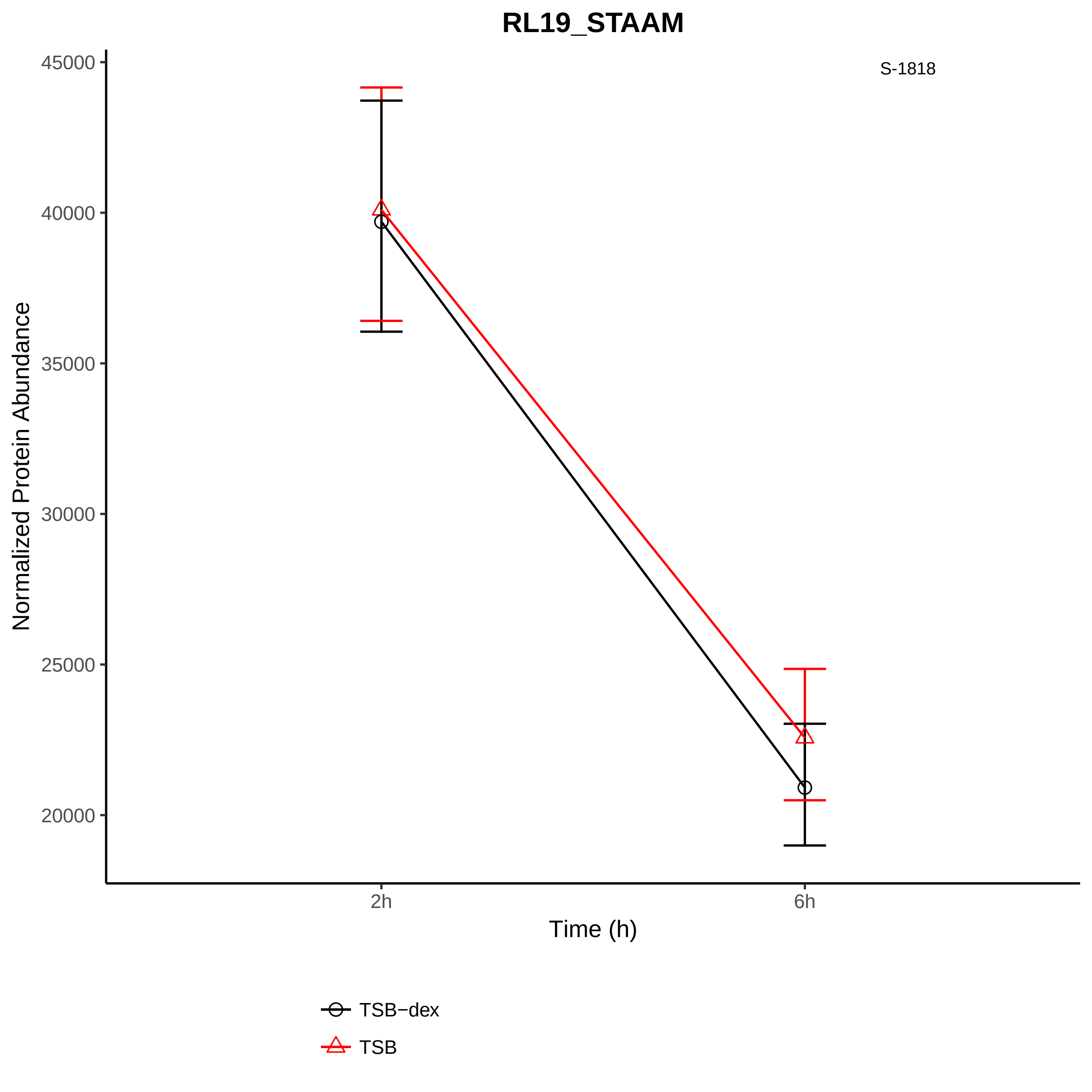

RL19_STAAM
S-1818
Normalized Protein Abundance
45000
40000
35000
30000
25000
20000
2h
6h
Time (h)
TSB−dex
TSB

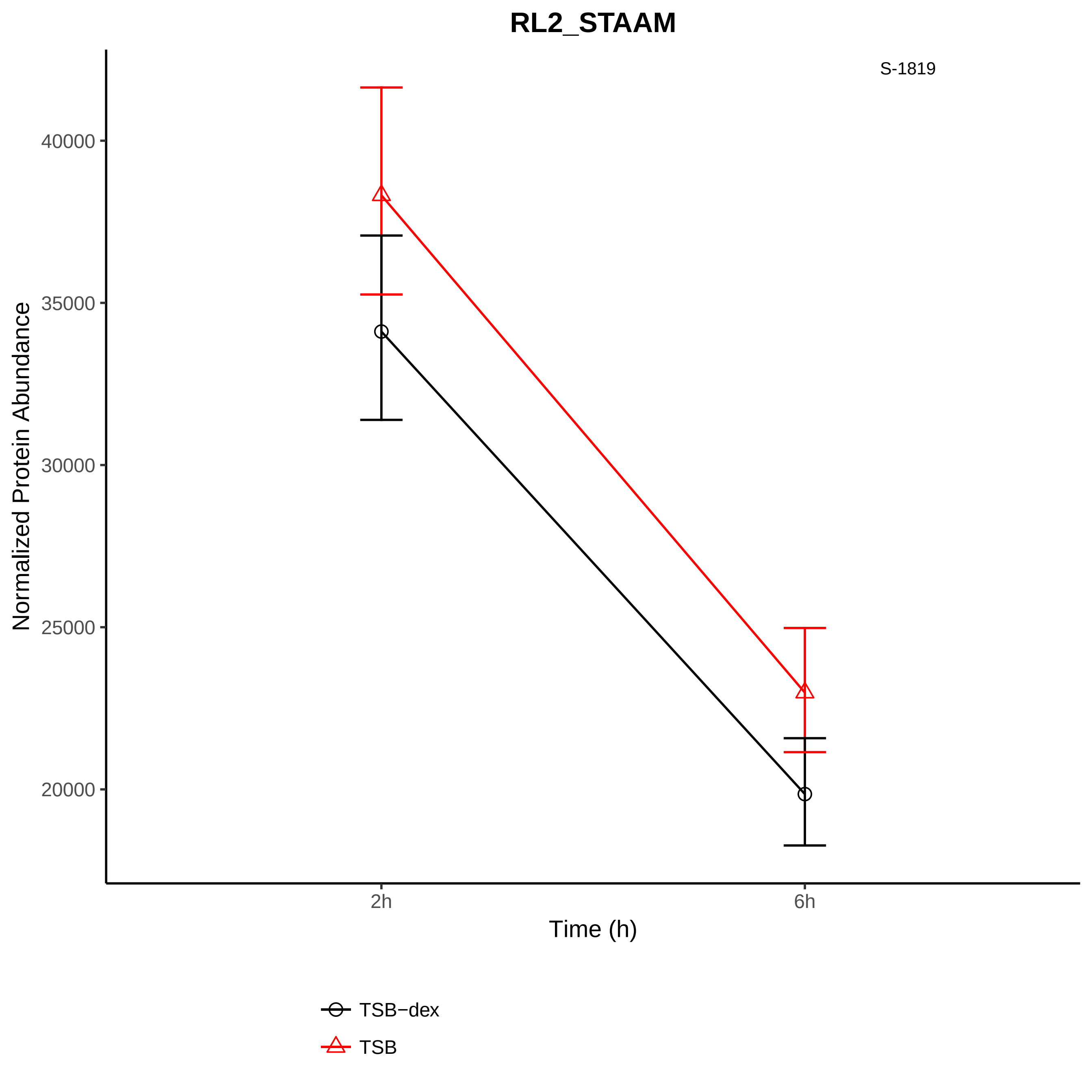

RL2_STAAM
S-1819
Normalized Protein Abundance
40000
35000
30000
25000
20000
2h
6h
Time (h)
TSB−dex
TSB

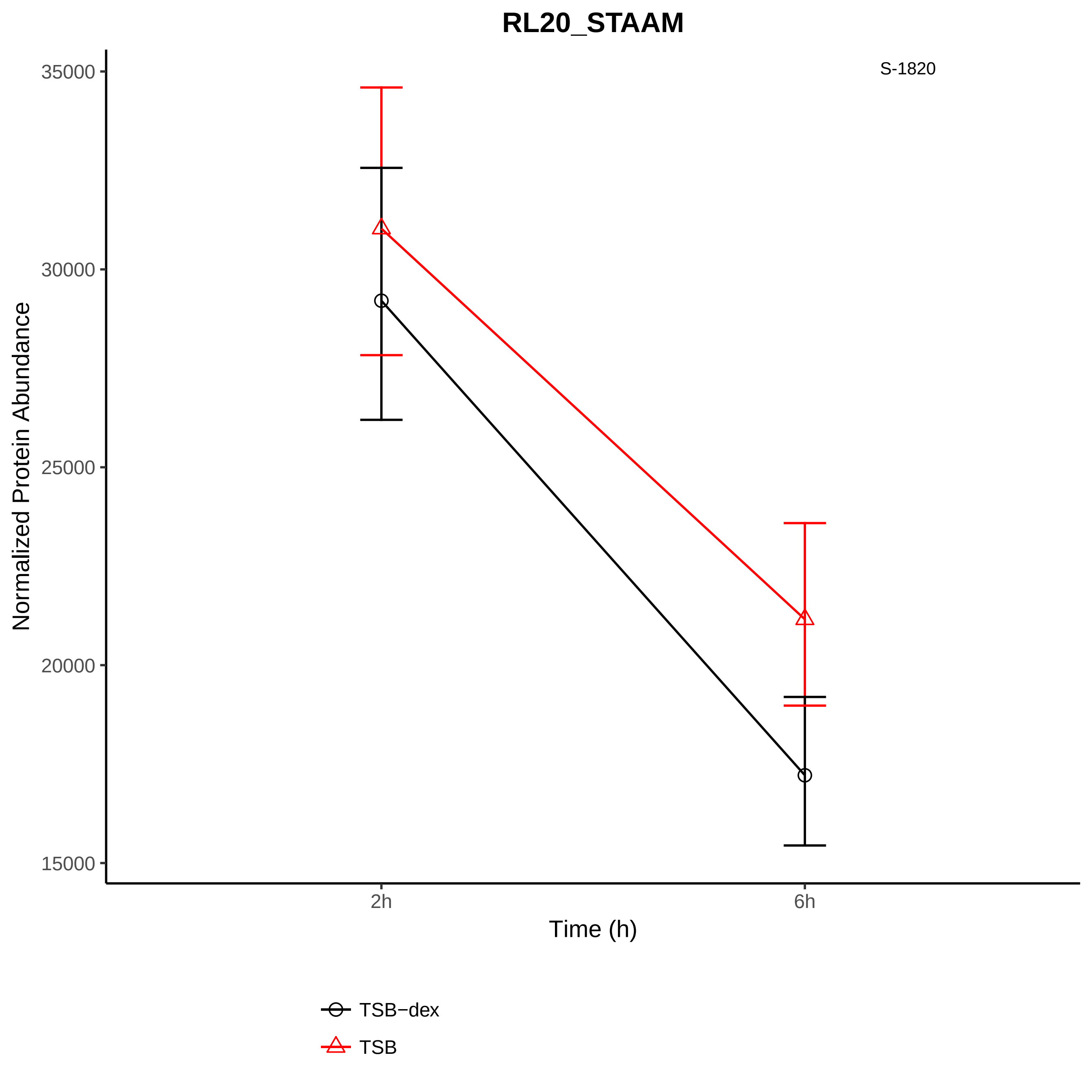
RL20_STAAM
S-1820
35000
30000
25000
20000
15000
Normalized Protein Abundance
2h
6h
Time (h)
TSB−dex
TSB

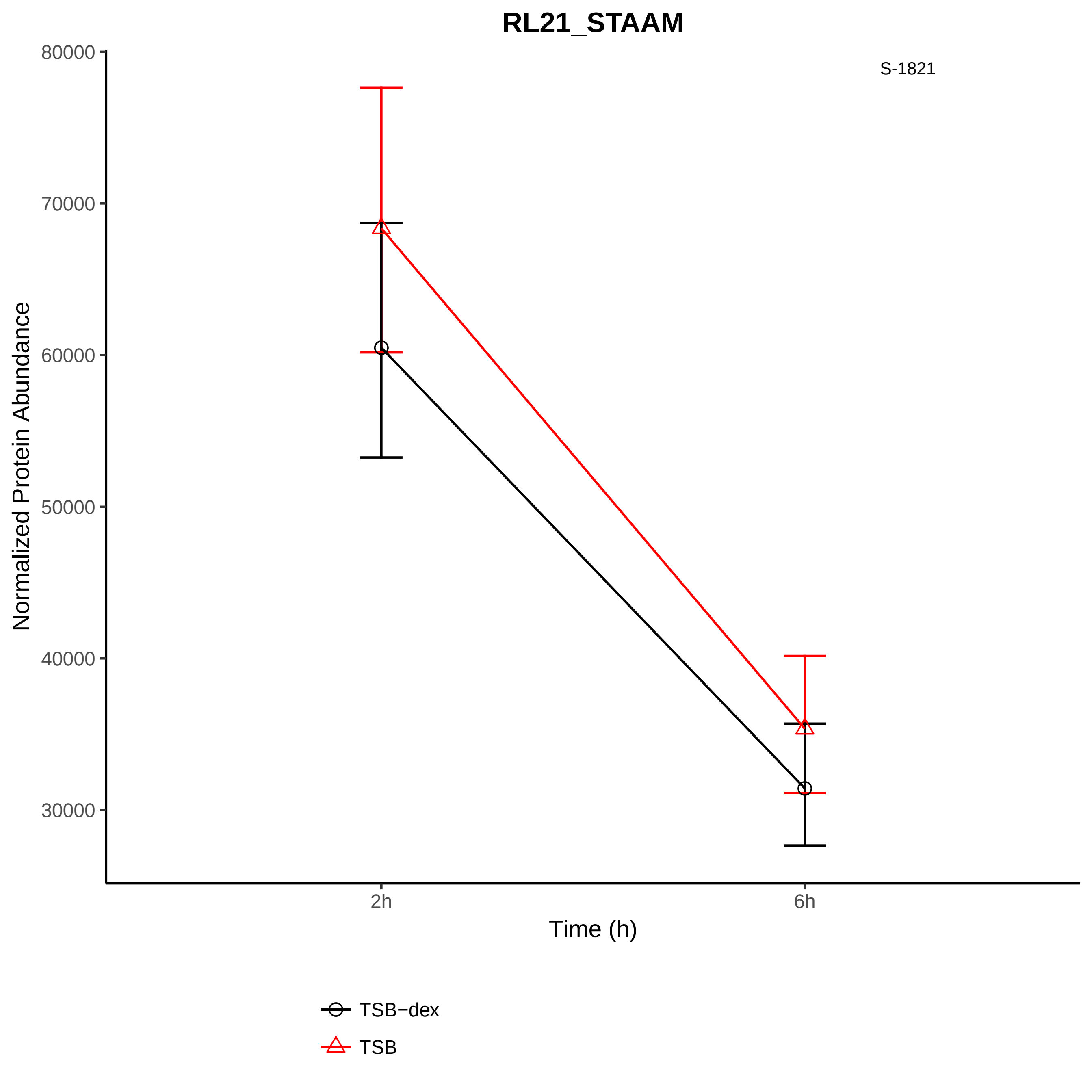

RL21_STAAM
S-1821
80000
70000
60000
50000
40000
30000
Normalized Protein Abundance
2h
6h
Time (h)
TSB−dex
TSB

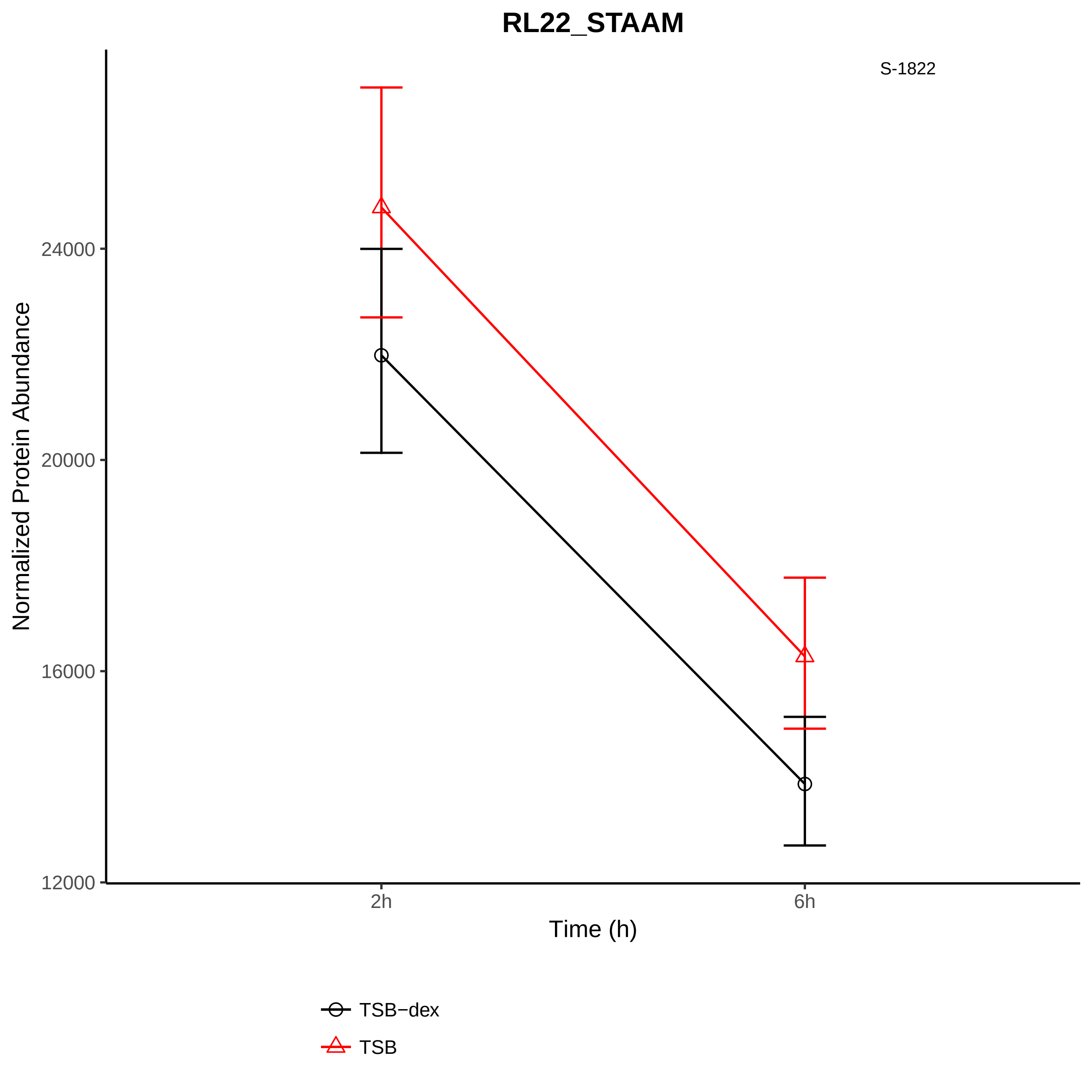
RL22_STAAM
S-1822
Normalized Protein Abundance
24000
20000
16000
12000
2h
6h
Time (h)
TSB−dex
TSB

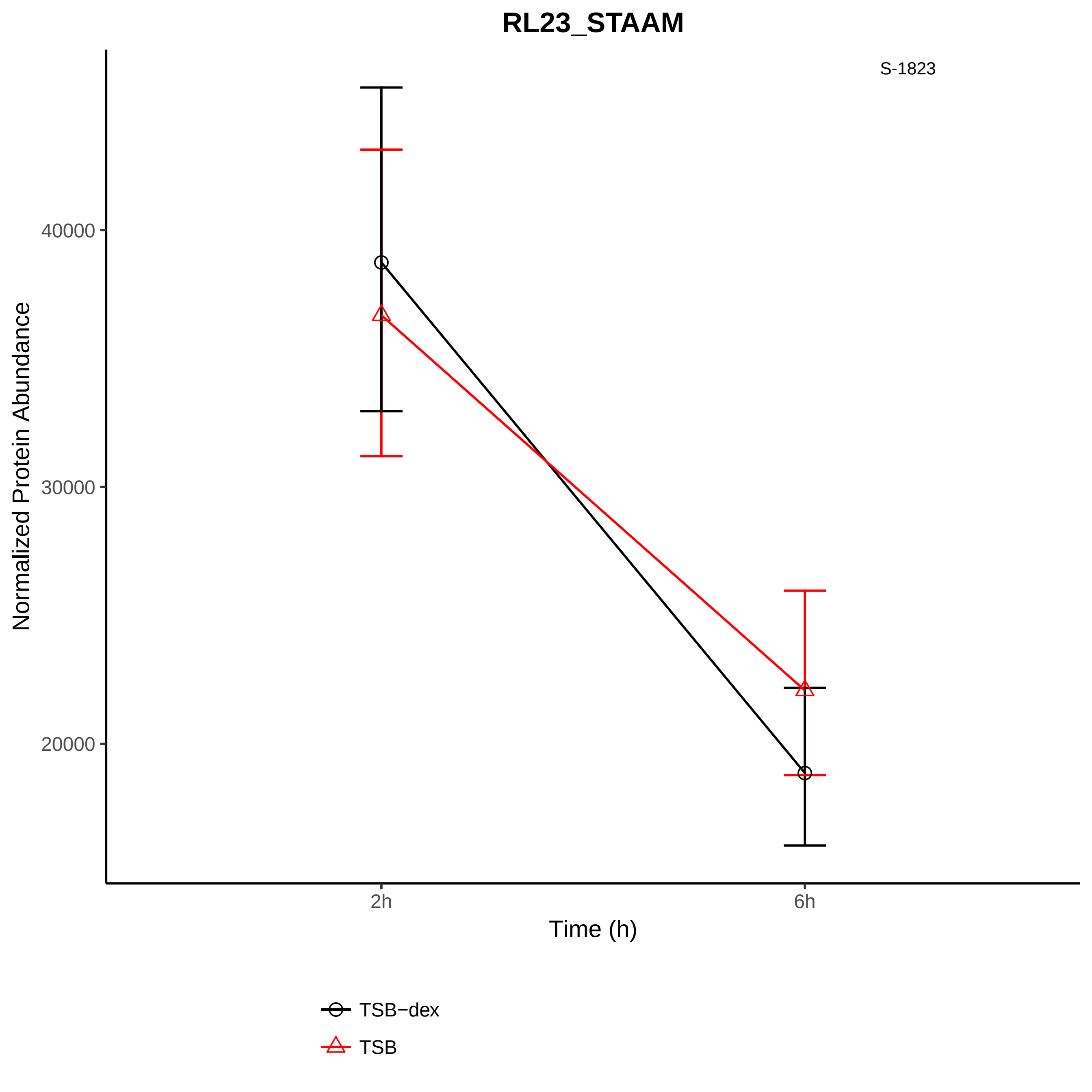

RL23_STAAM
S-1823
Normalized Protein Abundance
40000
30000
20000
2h
6h
Time (h)
TSB−dex
TSB

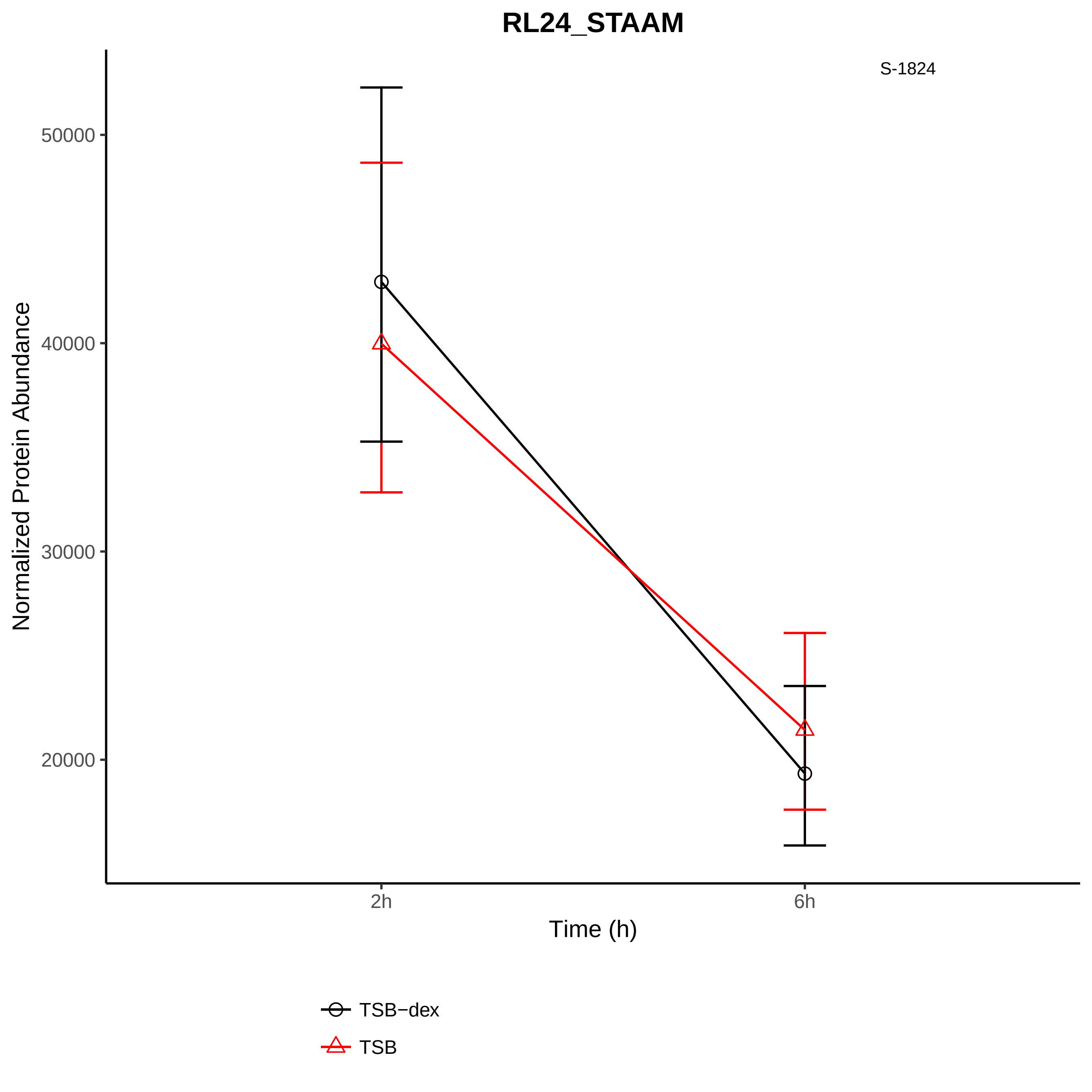

RL24_STAAM
S-1824
Normalized Protein Abundance
50000
40000
30000
20000
2h
6h
Time (h)
TSB−dex
TSB

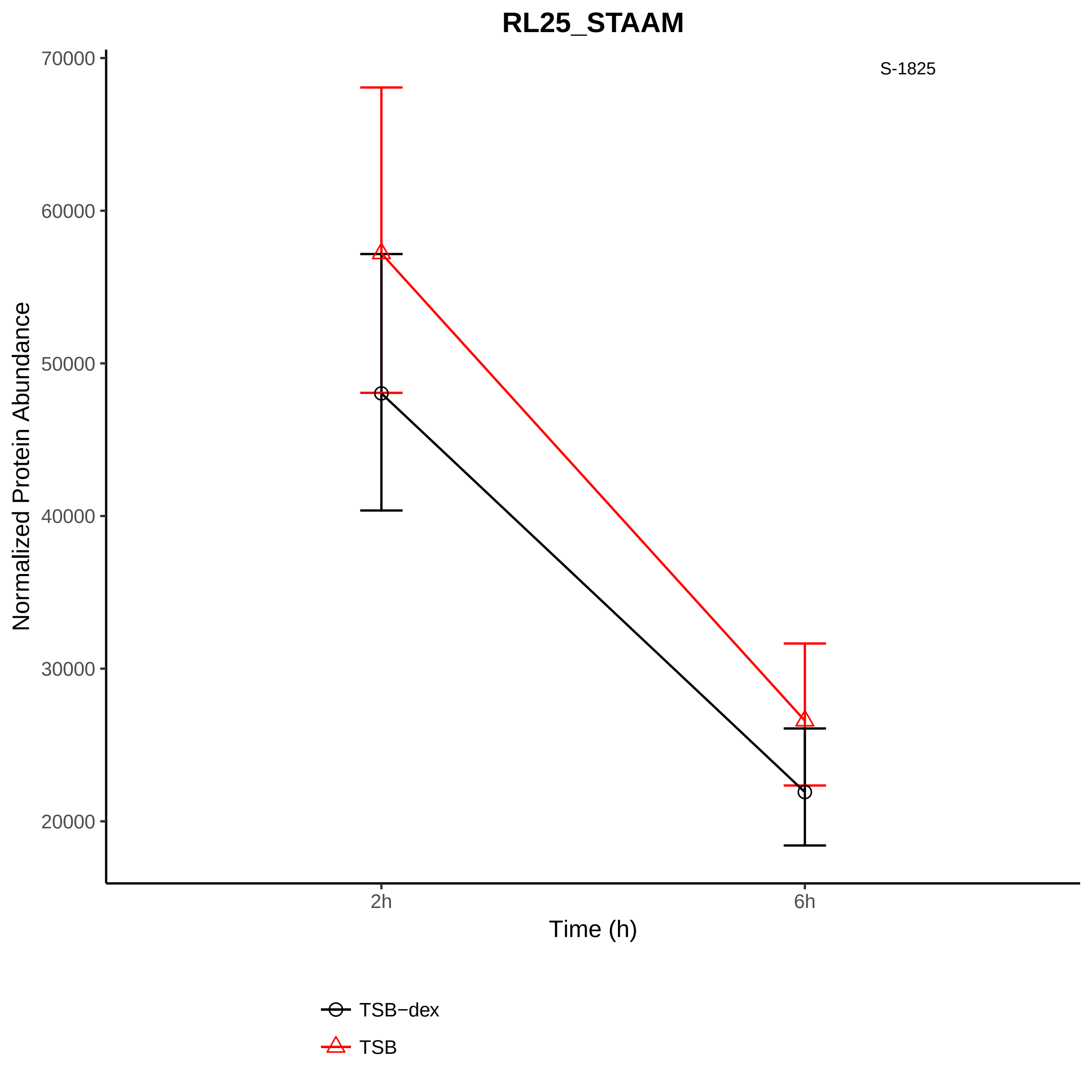
RL25_STAAM
S-1825
70000
60000
50000
40000
30000
20000
Normalized Protein Abundance
2h
6h
Time (h)
TSB−dex
TSB

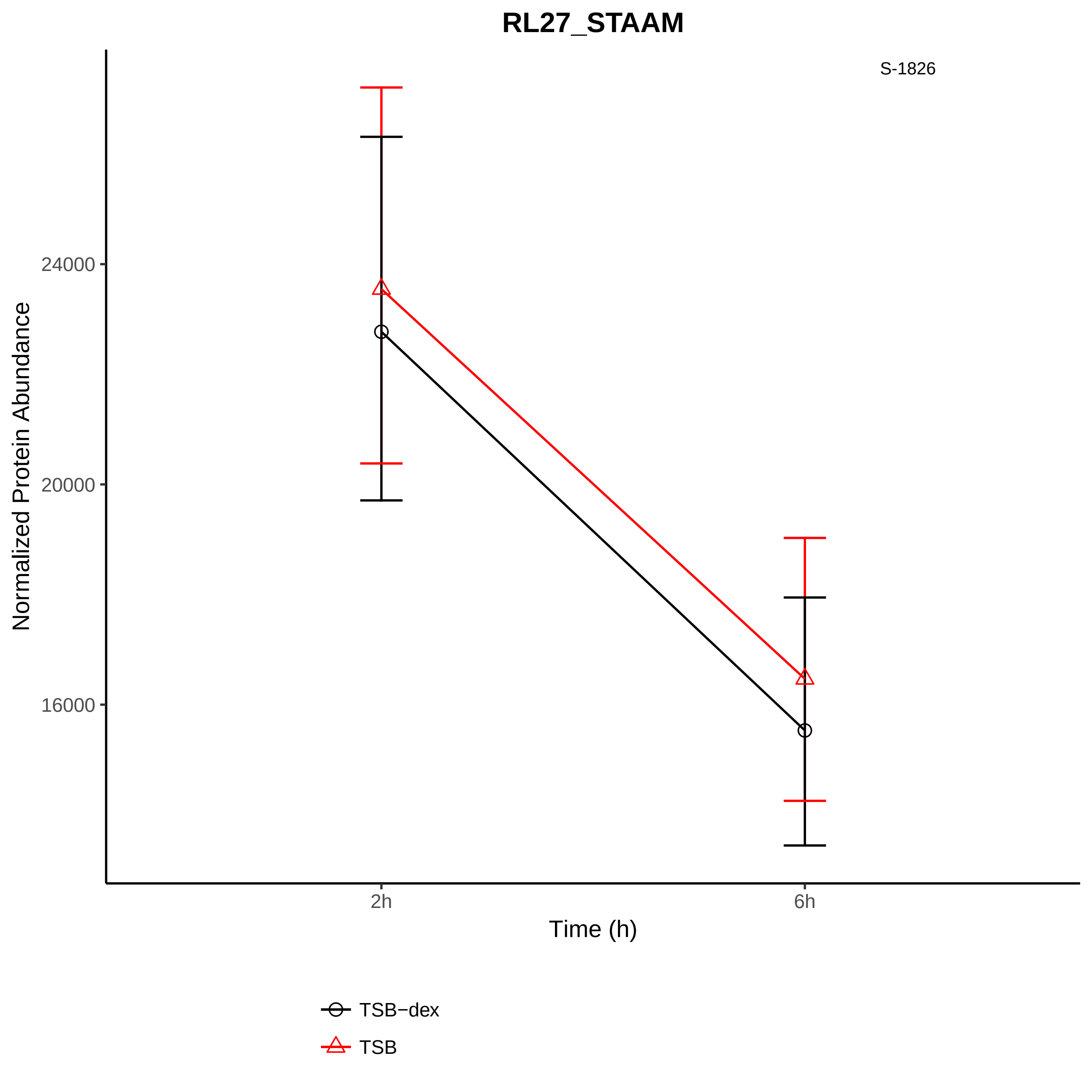

RL27_STAAM
S-1826
Normalized Protein Abundance
24000
20000
16000
2h
6h
Time (h)
TSB−dex
TSB

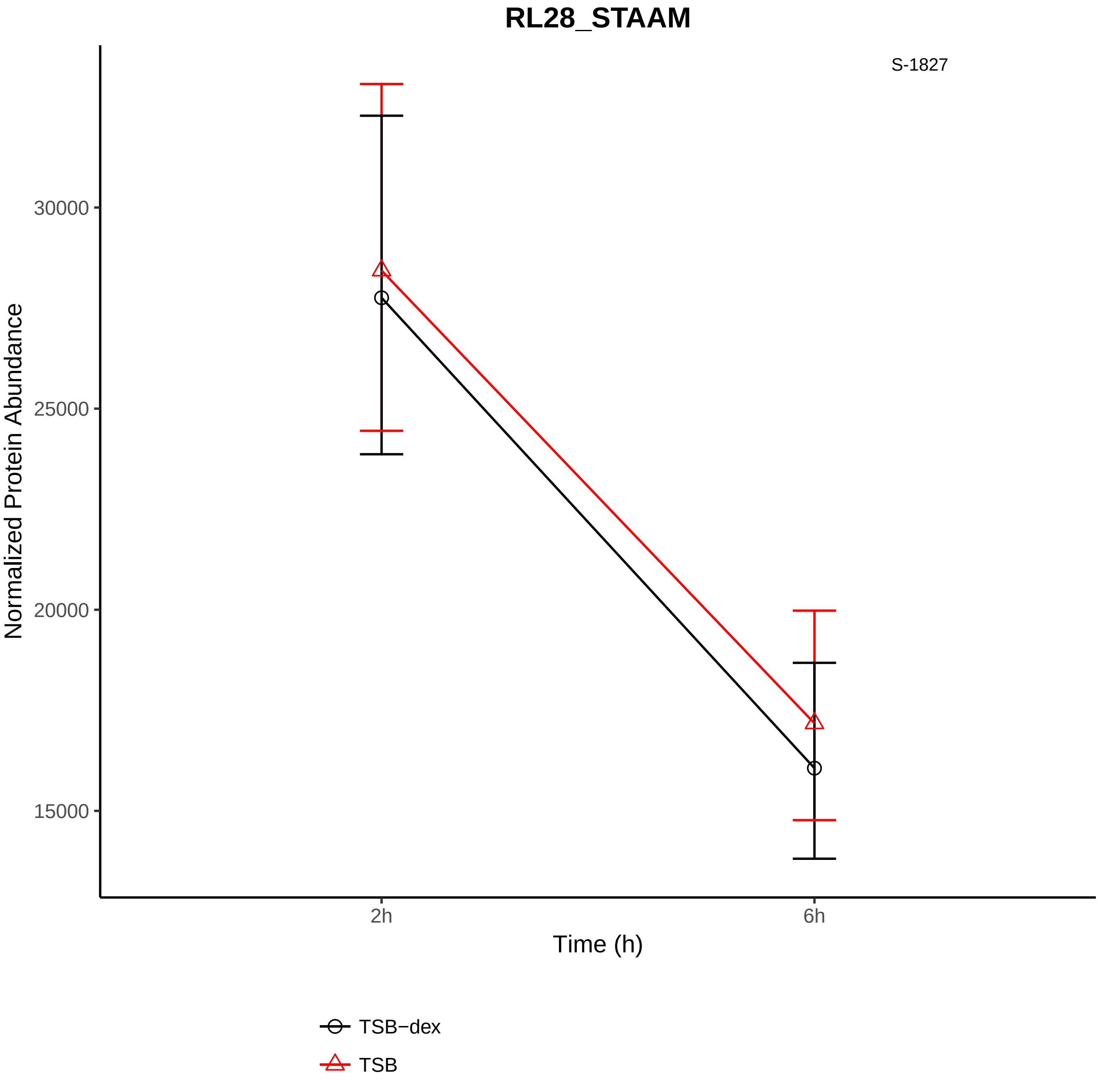

RL28_STAAM
S-1827
30000
25000
20000
15000
Normalized Protein Abundance
2h
6h
Time (h)
TSB−dex
TSB

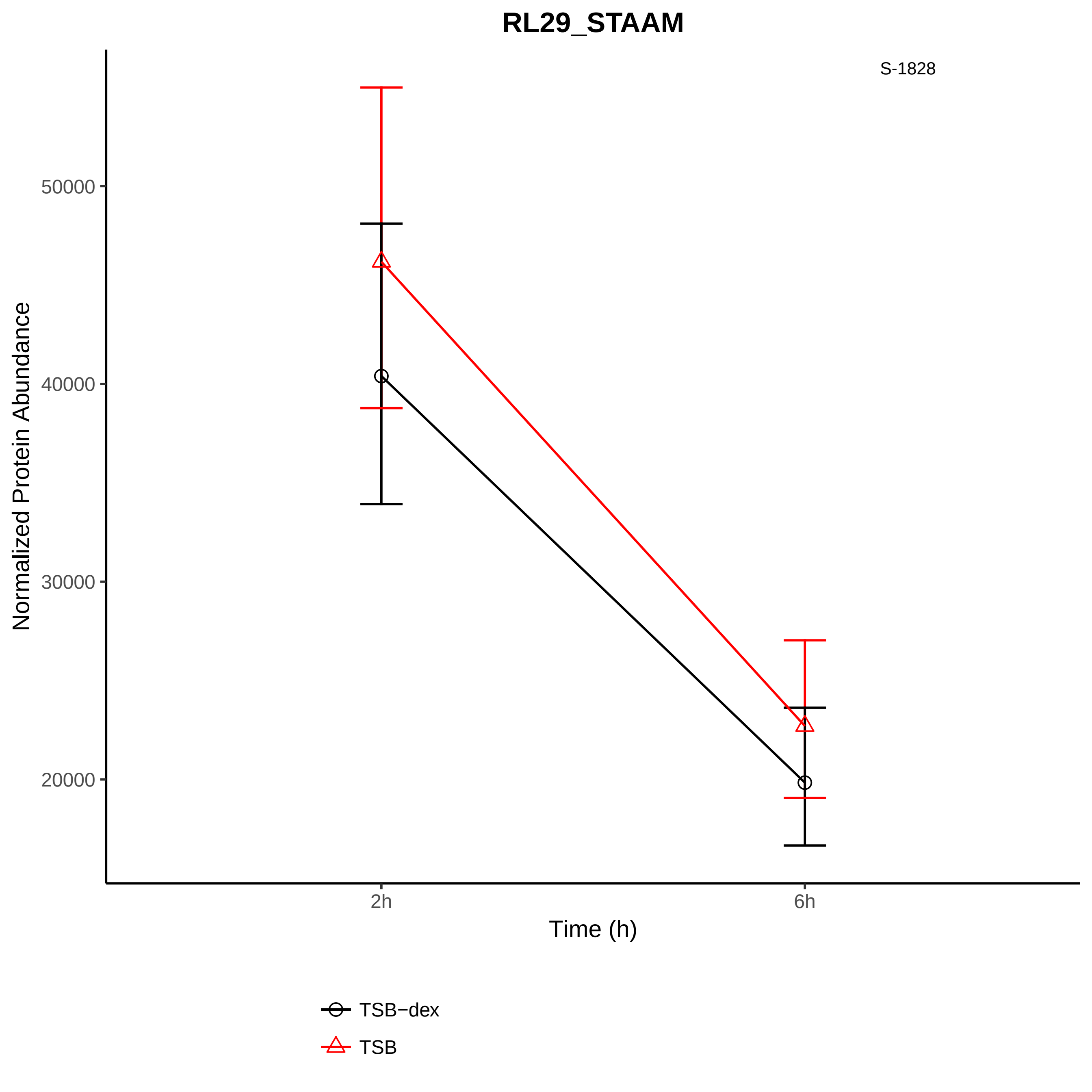
RL29_STAAM
S-1828
Normalized Protein Abundance
50000
40000
30000
20000
2h
6h
Time (h)
TSB−dex
TSB

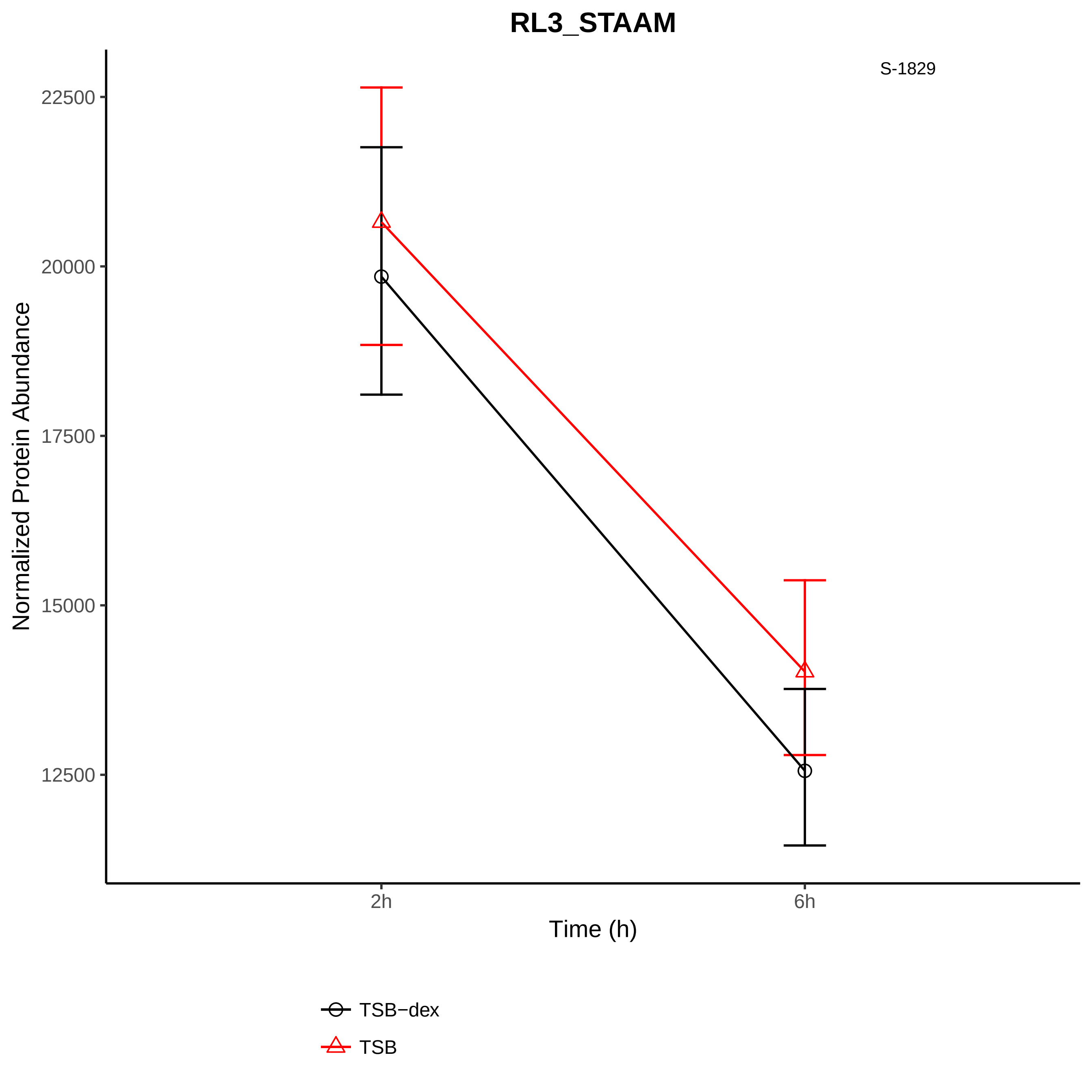

RL3_STAAM
S-1829
Normalized Protein Abundance
22500
20000
17500
15000
12500
2h
6h
Time (h)
TSB−dex
TSB

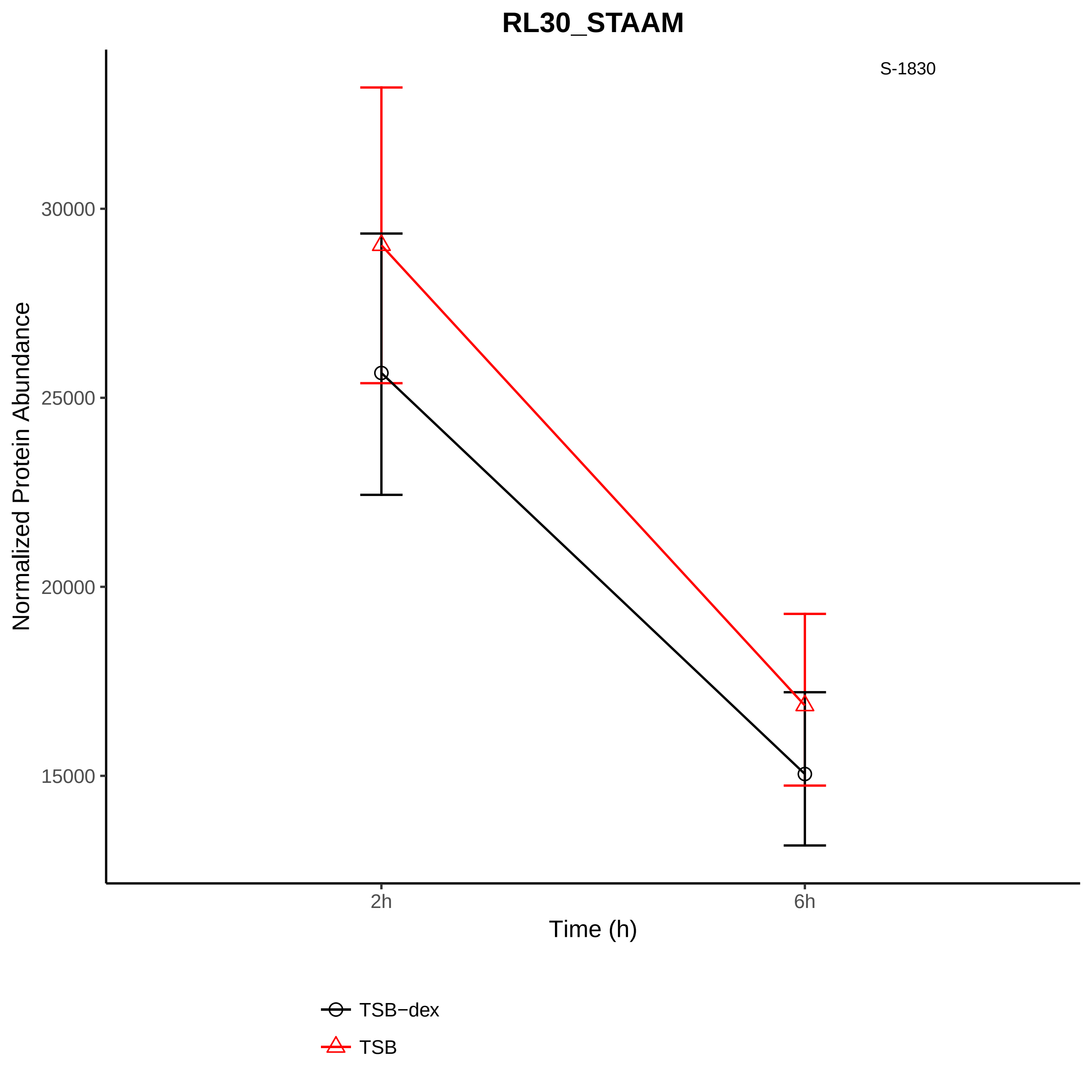
RL30_STAAM
S-1830
Normalized Protein Abundance
30000
25000
20000
15000
2h
6h
Time (h)
TSB−dex
TSB

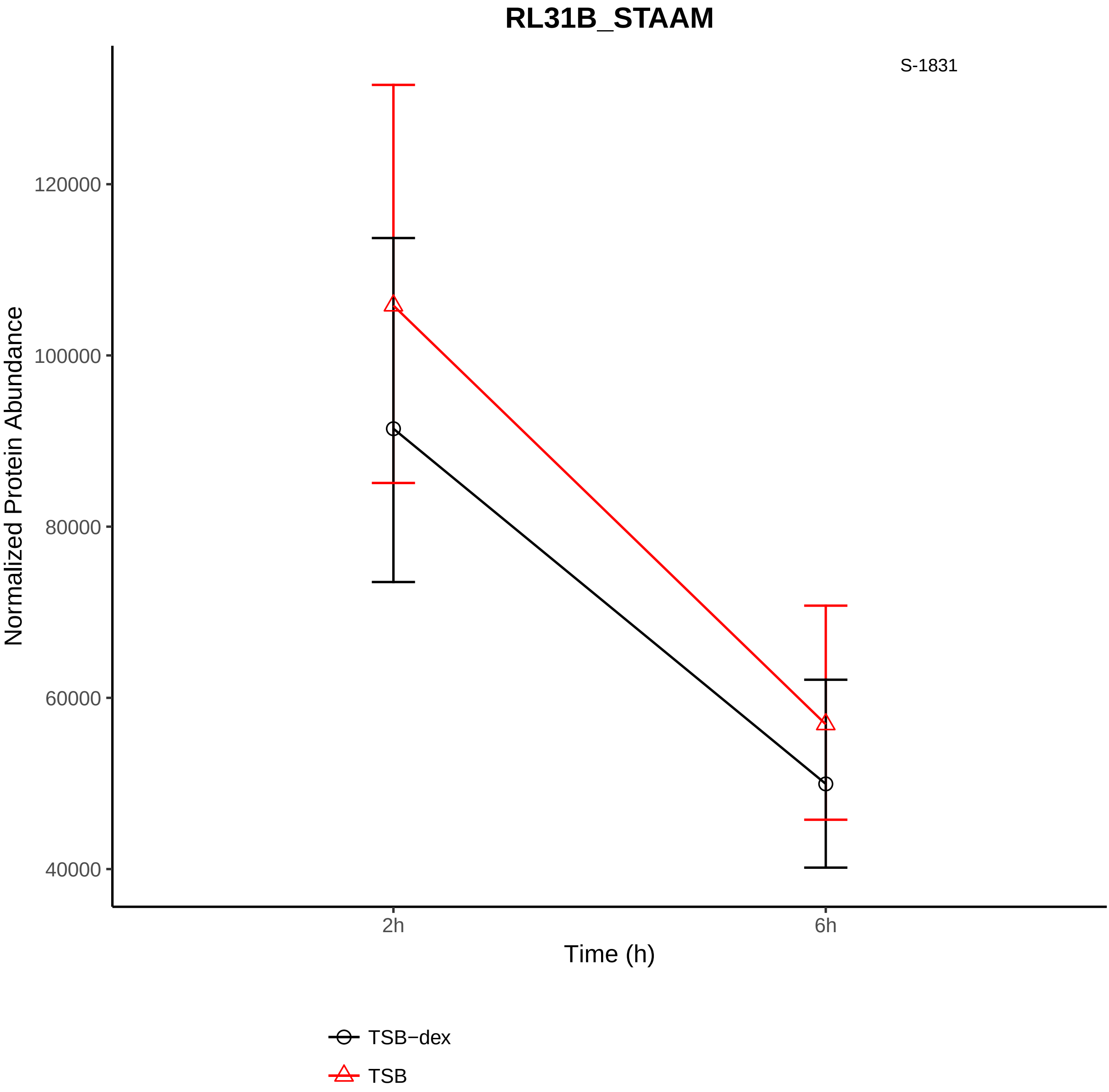
RL31B_STAAM
S-1831
120000
100000
80000
60000
40000
Normalized Protein Abundance
2h
6h
Time (h)
TSB−dex
TSB

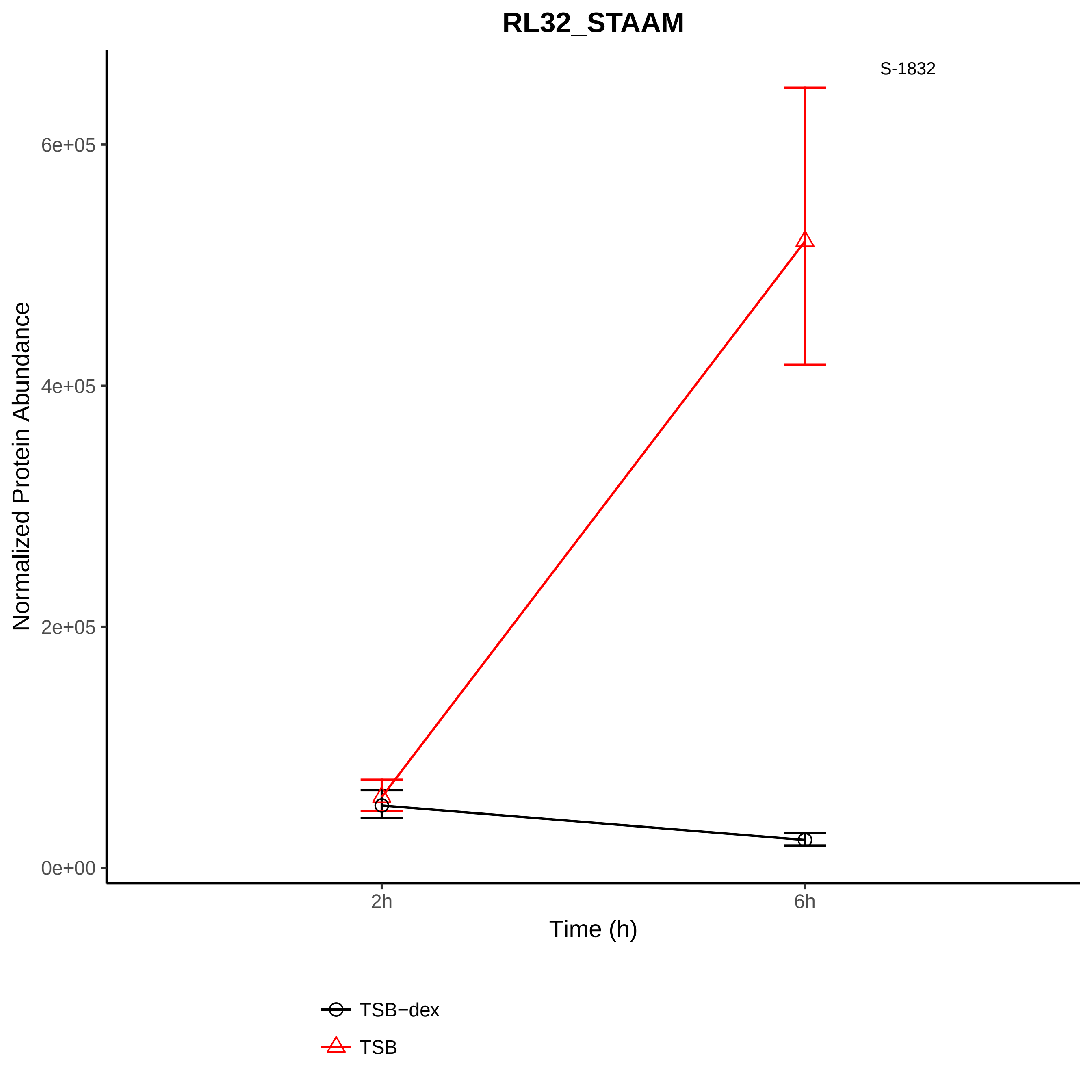

RL32_STAAM
S-1832
Normalized Protein Abundance
6e+05
4e+05
2e+05
0e+00
2h
6h
Time (h)
TSB−dex
TSB

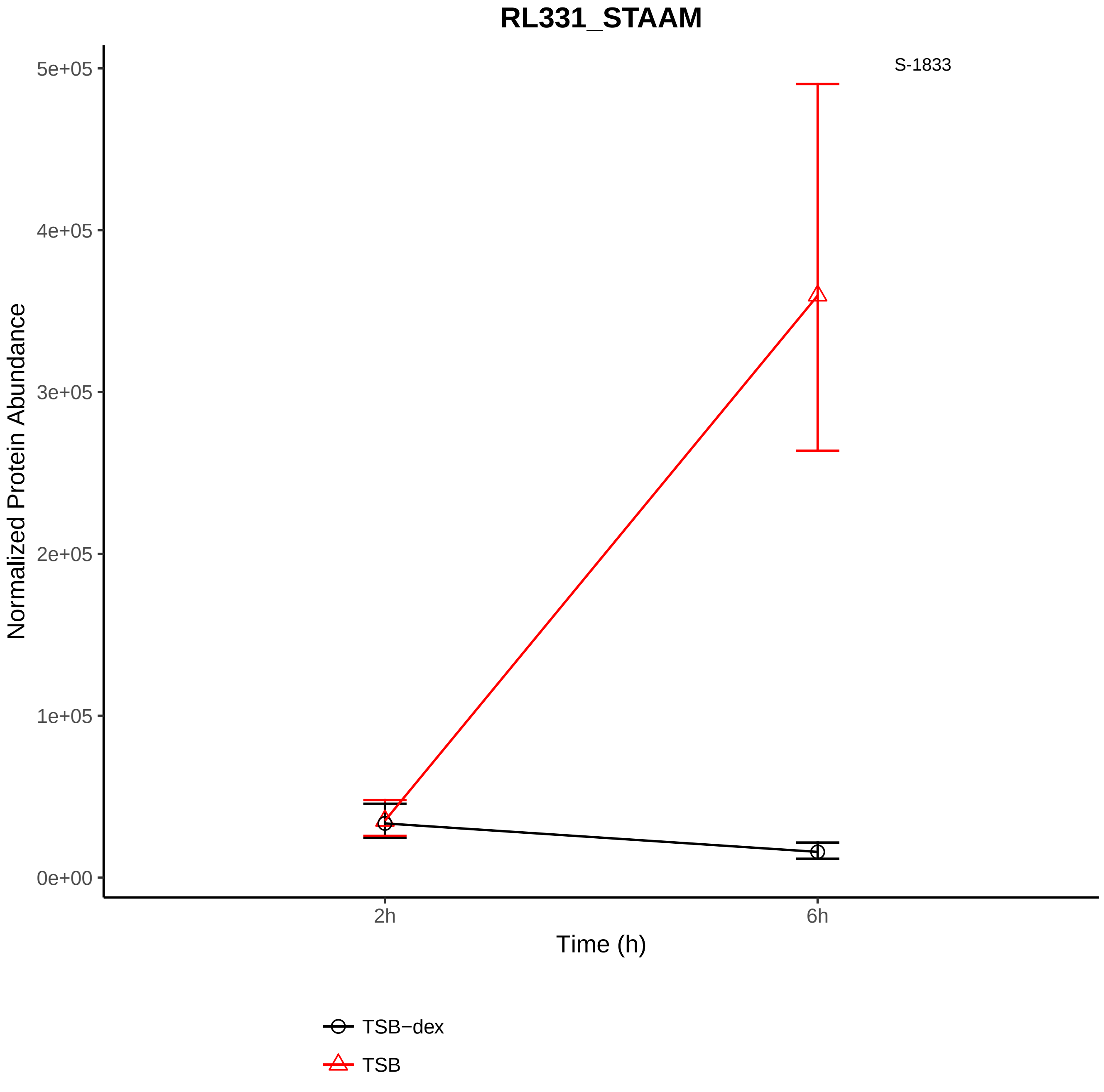

RL331_STAAM
S-1833
Normalized Protein Abundance
5e+05
4e+05
3e+05
2e+05
1e+05
0e+00
2h
6h
Time (h)
TSB−dex
TSB

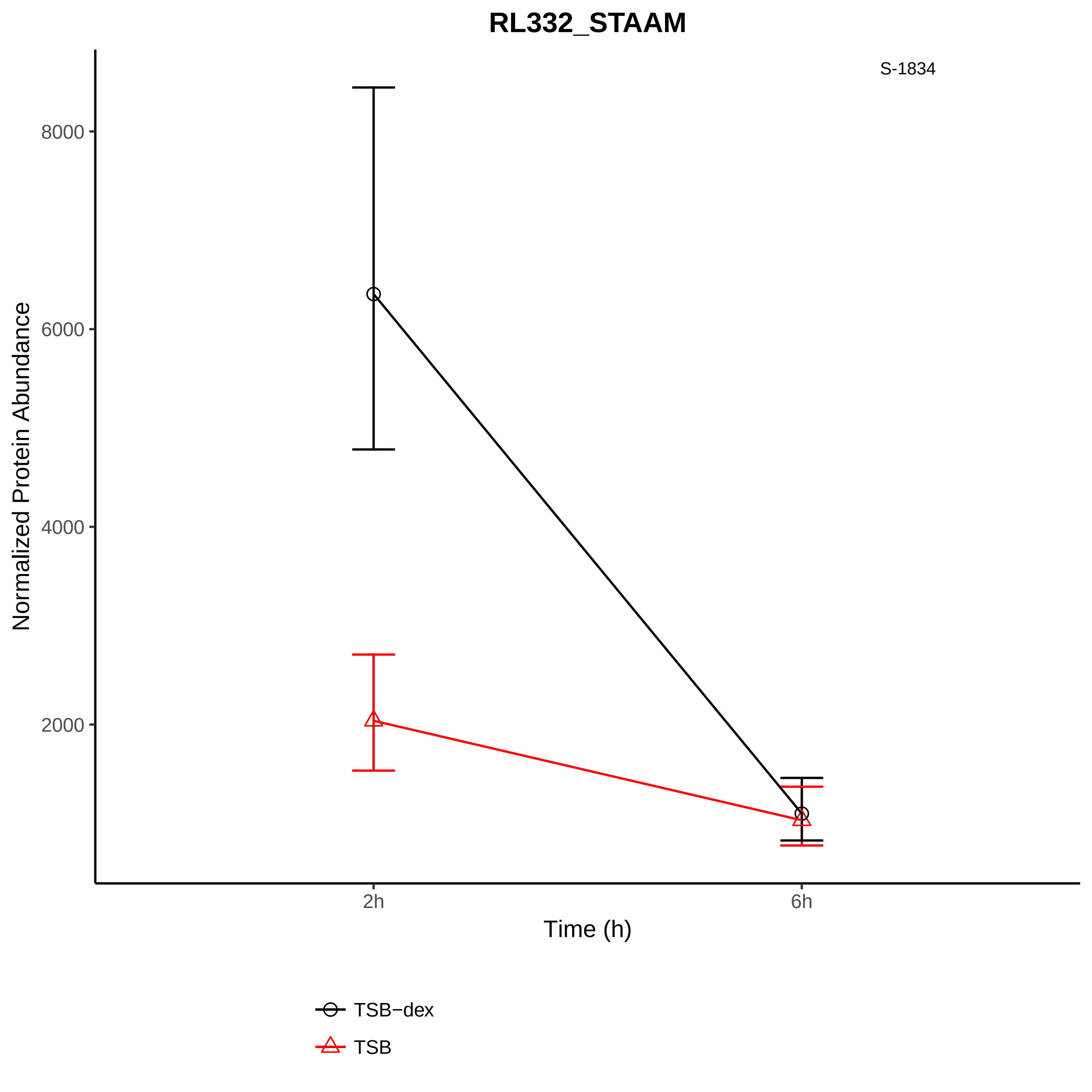

RL332_STAAM
S-1834
8000
6000
4000
2000
Normalized Protein Abundance
2h
6h
Time (h)
TSB−dex
TSB

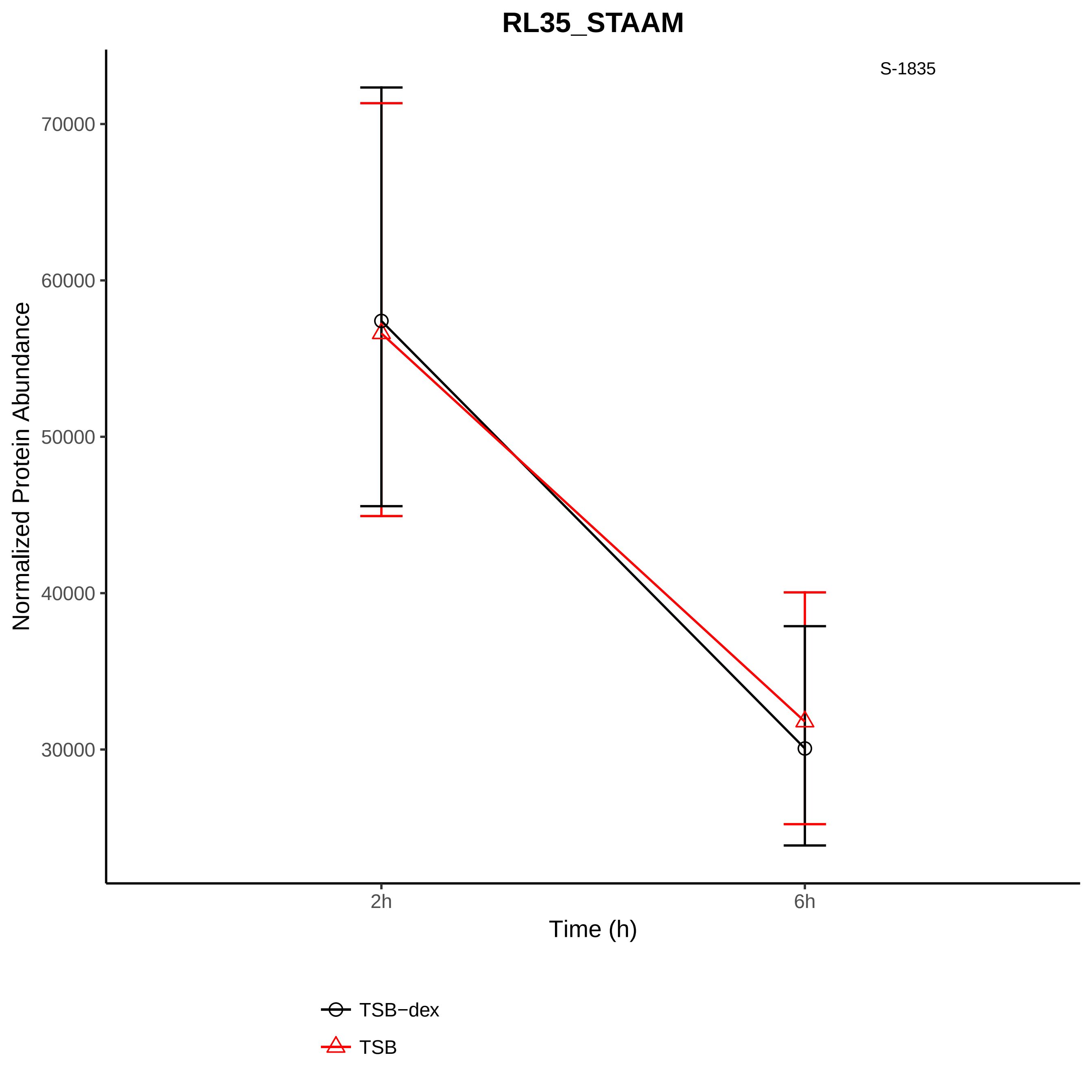

RL35_STAAM
S-1835
Normalized Protein Abundance
70000
60000
50000
40000
30000
2h
6h
Time (h)
TSB−dex
TSB

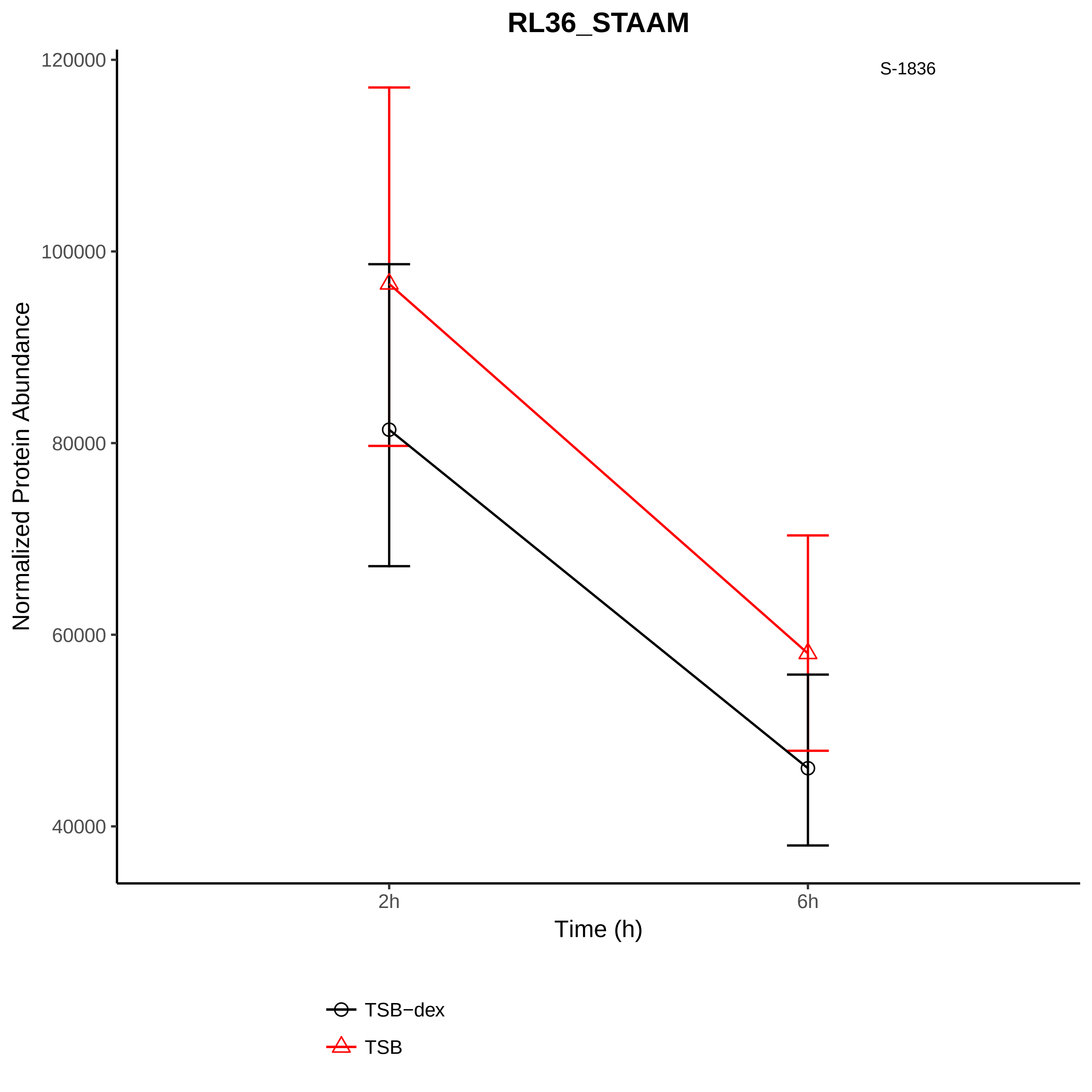

RL36_STAAM
S-1836
120000
100000
80000
60000
40000
Normalized Protein Abundance
2h
6h
Time (h)
TSB−dex
TSB

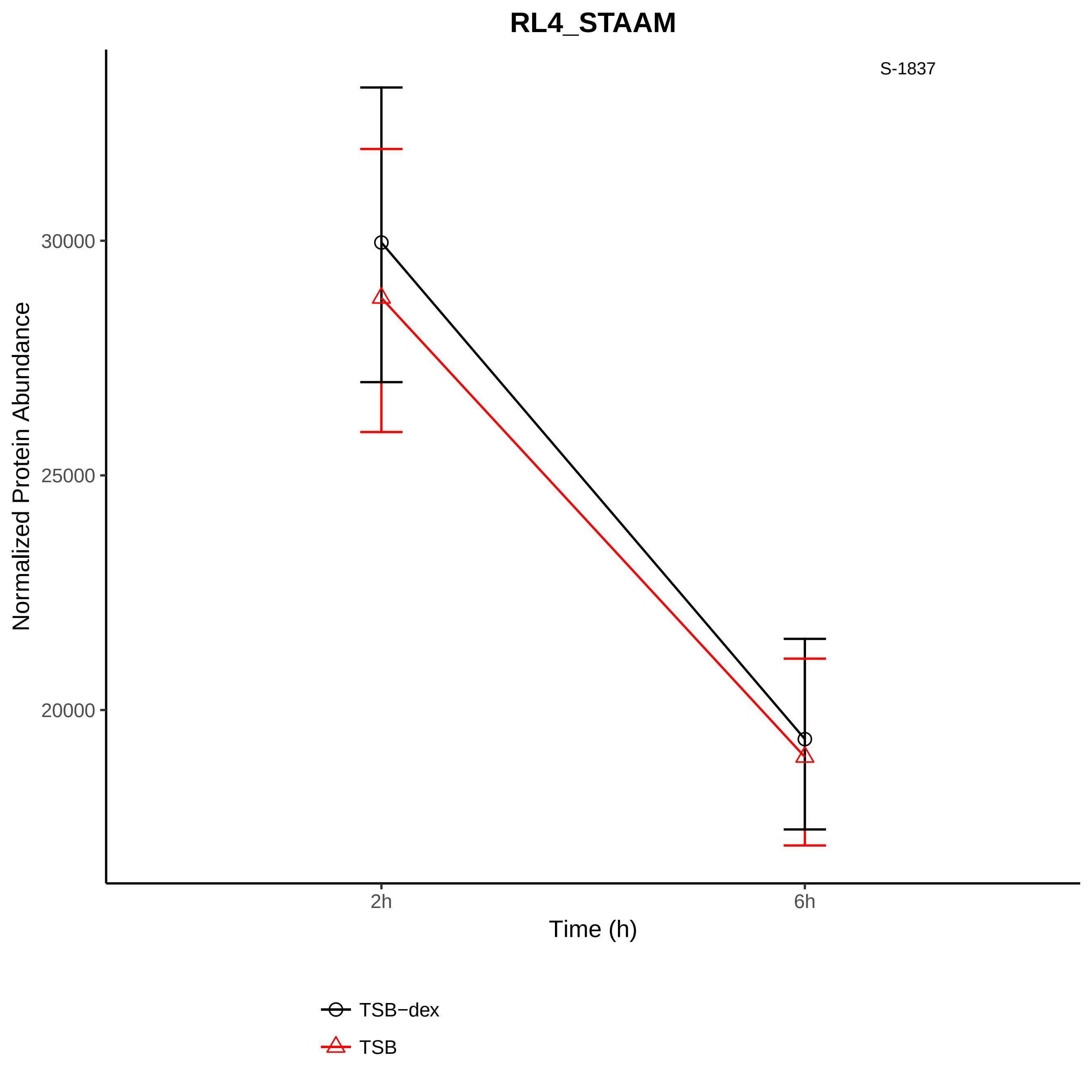
RL4_STAAM
S-1837
Normalized Protein Abundance
30000
25000
20000
2h
6h
Time (h)
TSB−dex
TSB

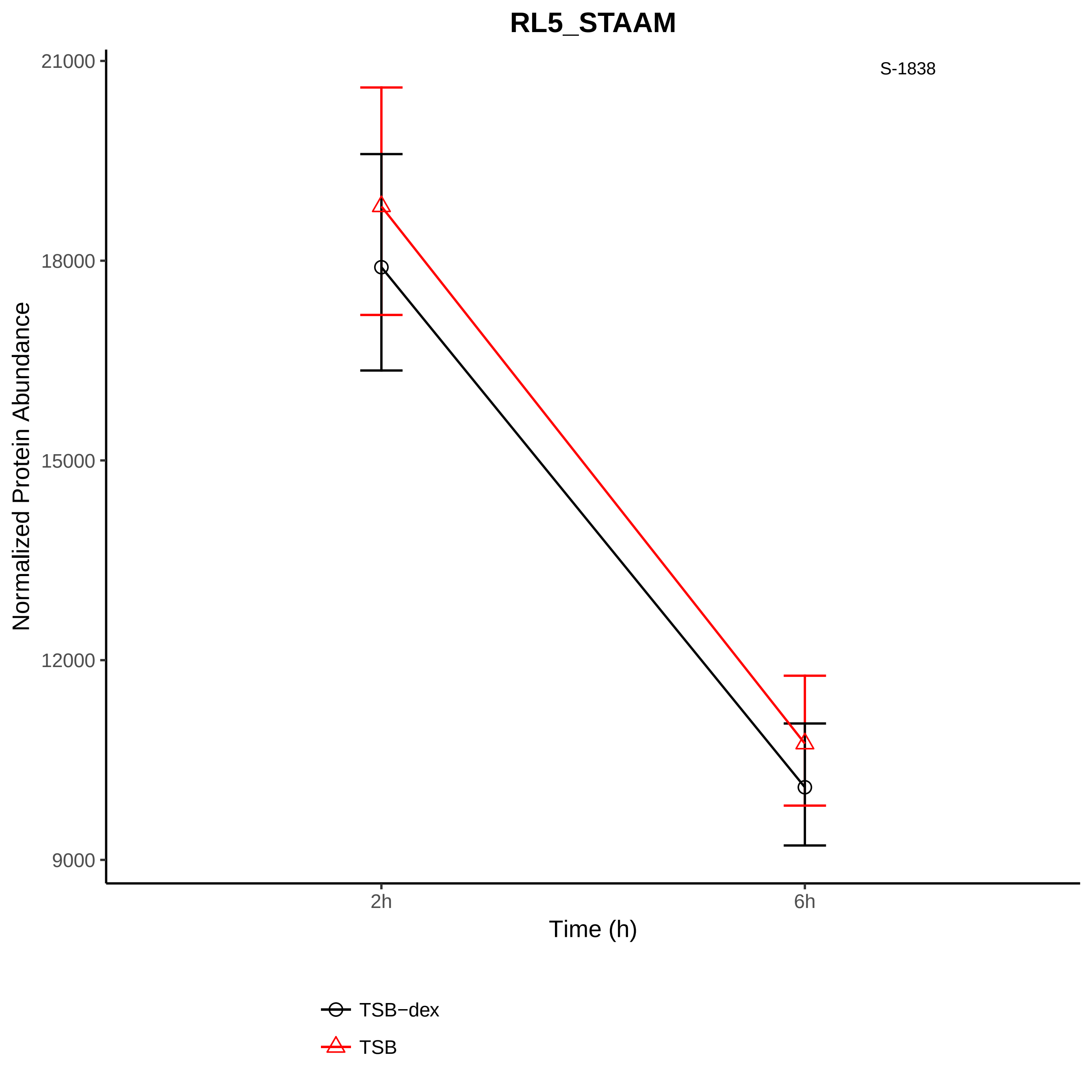

RL5_STAAM
S-1838
21000
18000
15000
12000
9000
Normalized Protein Abundance
2h
6h
Time (h)
TSB−dex
TSB

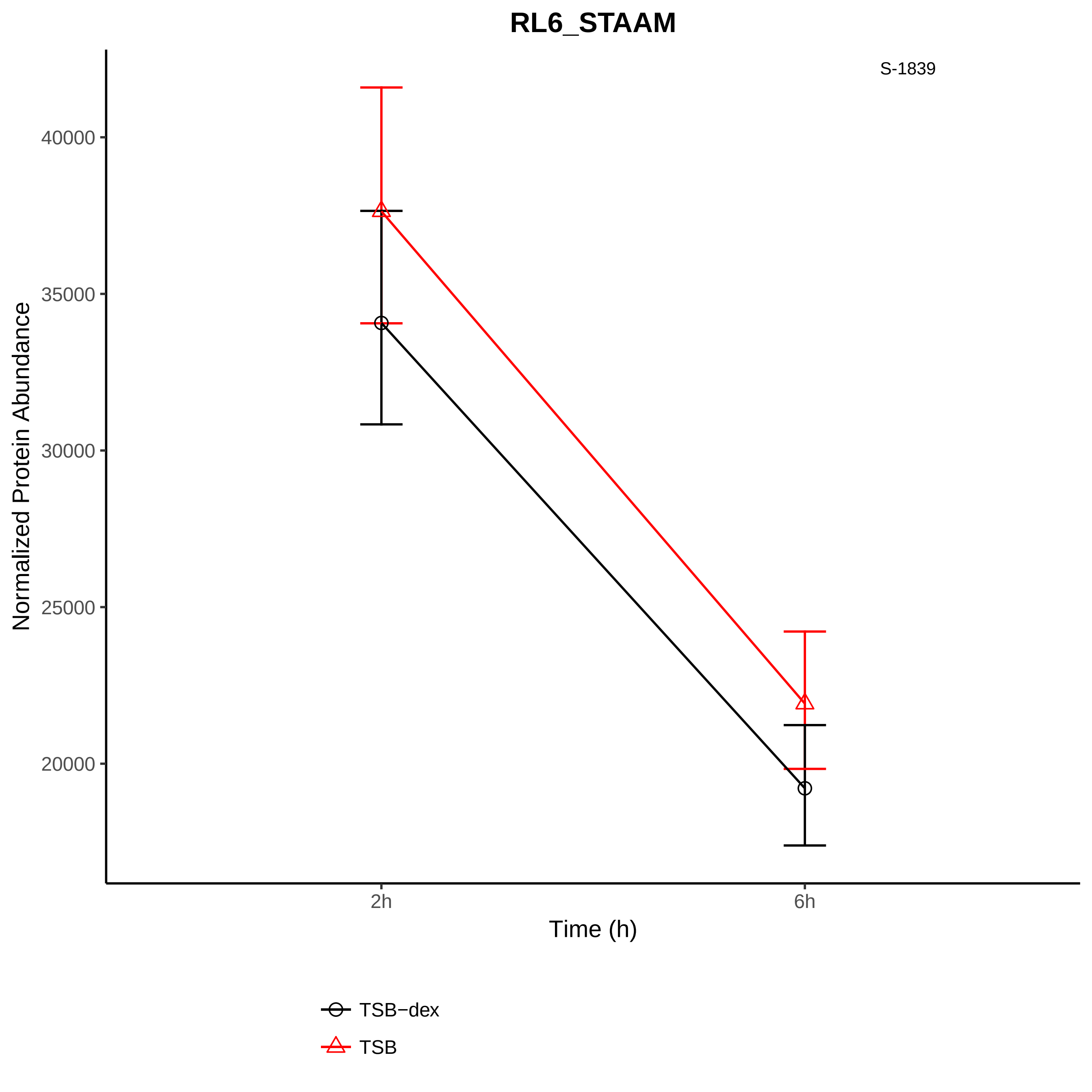

RL6_STAAM
S-1839
40000
35000
30000
25000
20000
Normalized Protein Abundance
2h
6h
Time (h)
TSB−dex
TSB

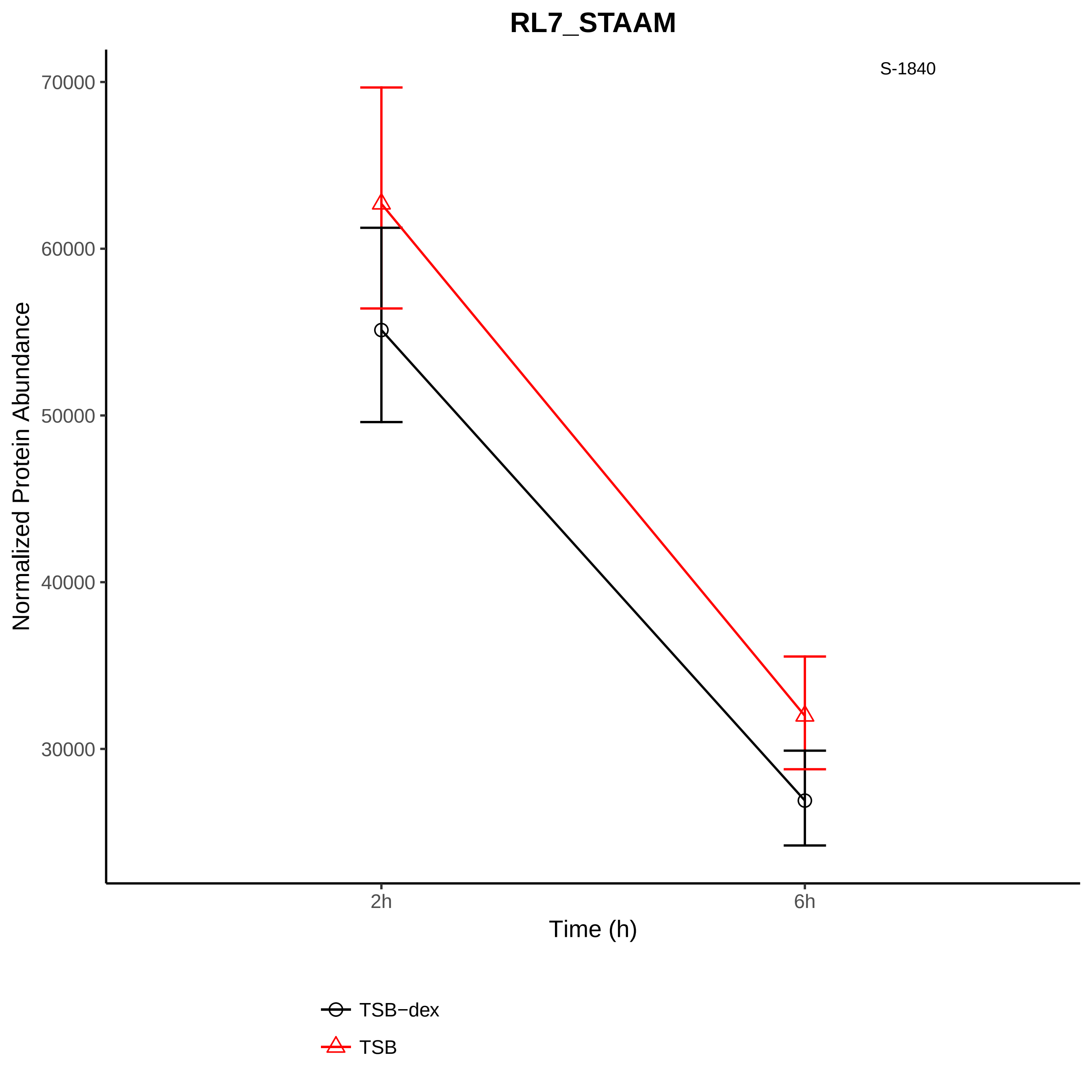
RL7_STAAM
S-1840
Normalized Protein Abundance
70000
60000
50000
40000
30000
2h
6h
Time (h)
TSB−dex
TSB

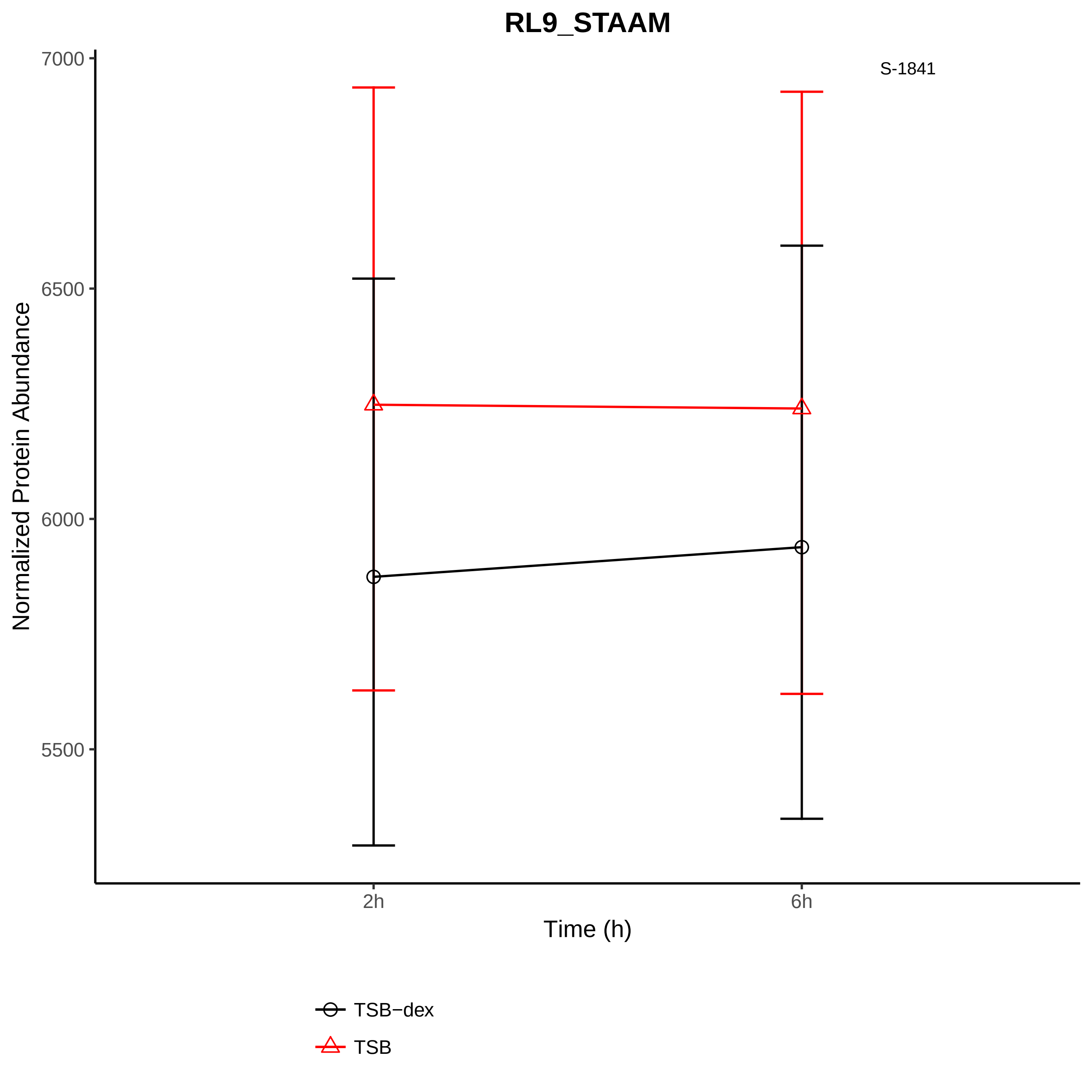

RL9_STAAM
S-1841
7000
6500
6000
5500
Normalized Protein Abundance
2h
6h
Time (h)
TSB−dex
TSB

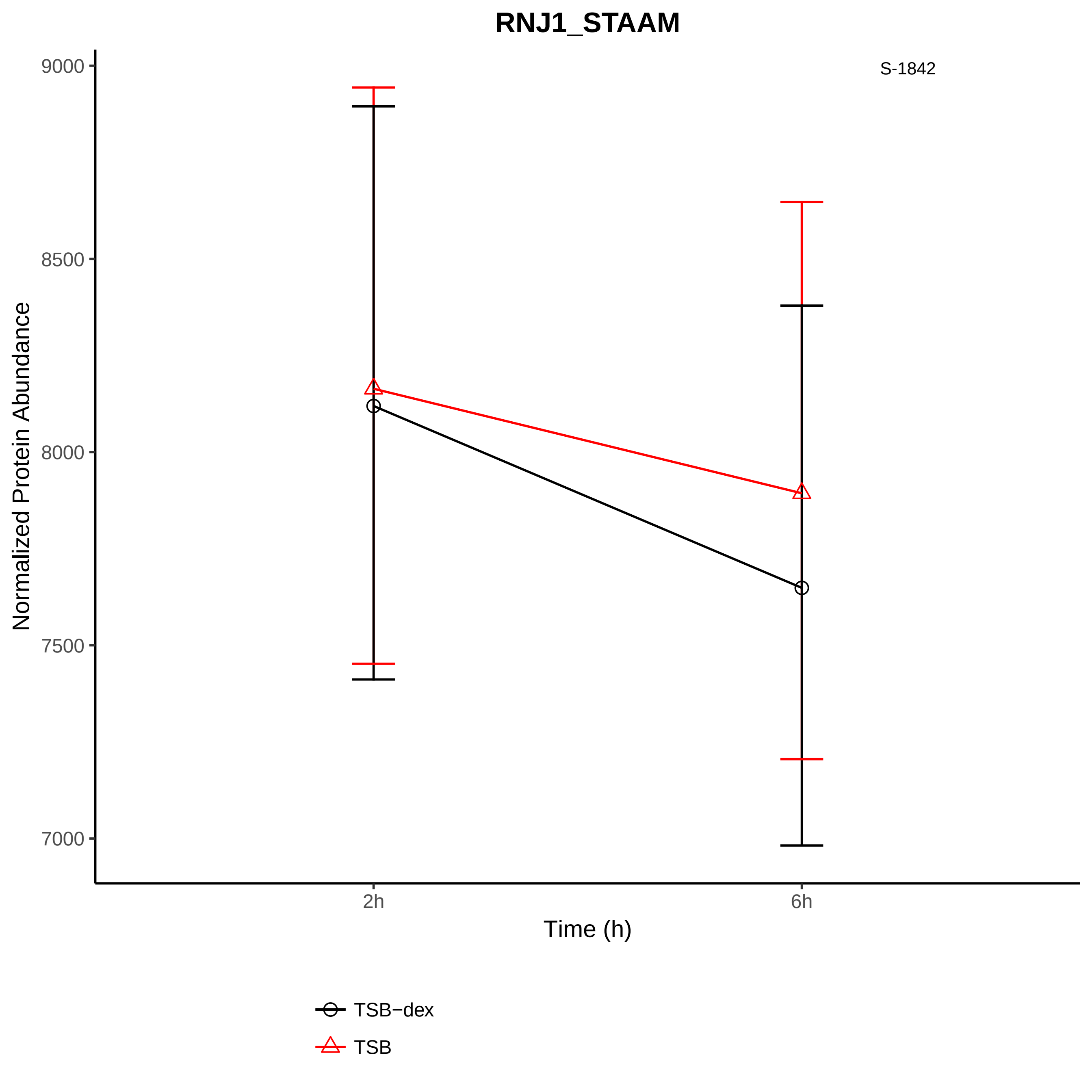

RNJ1_STAAM
S-1842
9000
8500
8000
7500
7000
Normalized Protein Abundance
2h
6h
Time (h)
TSB−dex
TSB

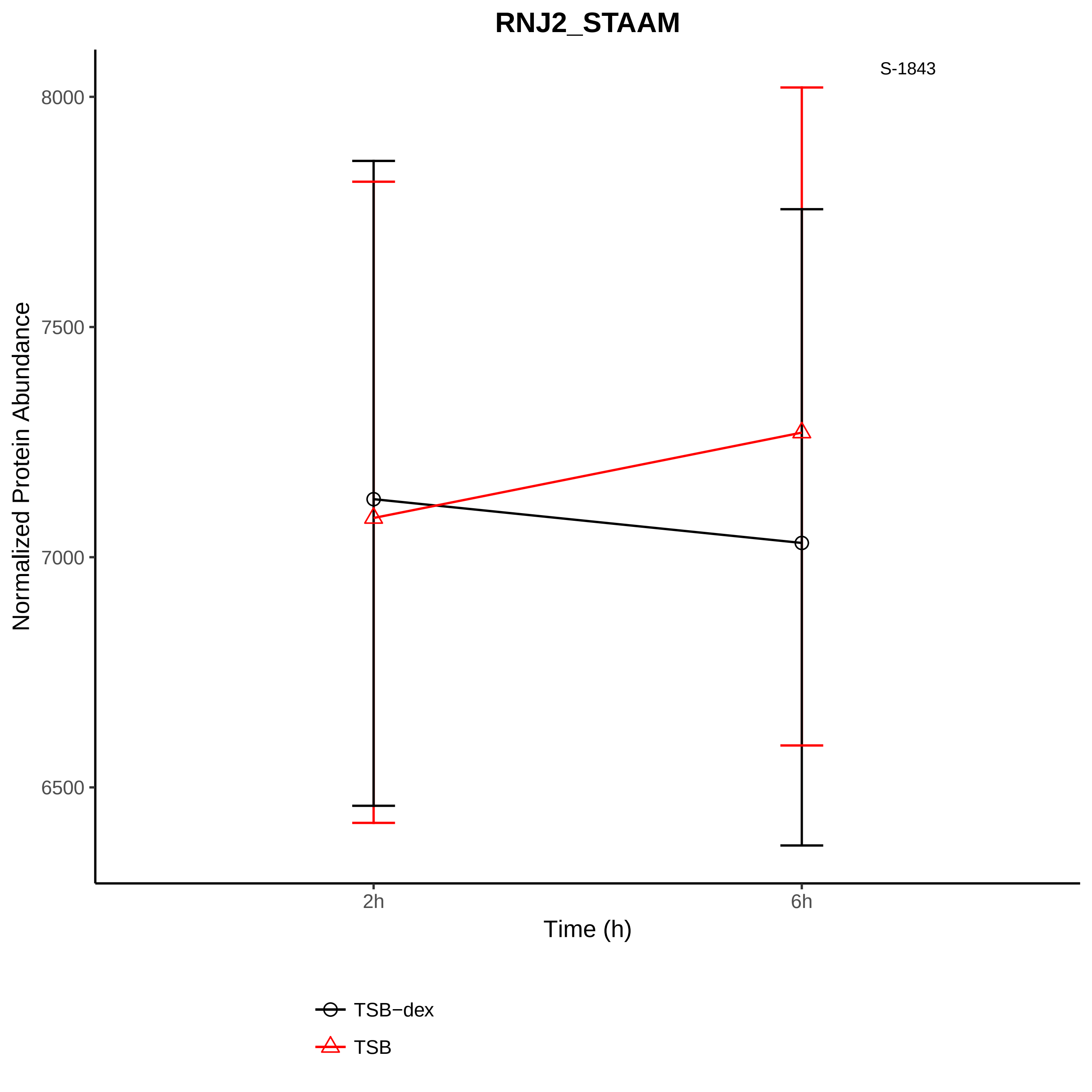
RNJ2_STAAM
S-1843
8000
7500
7000
6500
Normalized Protein Abundance
2h
6h
Time (h)
TSB−dex
TSB

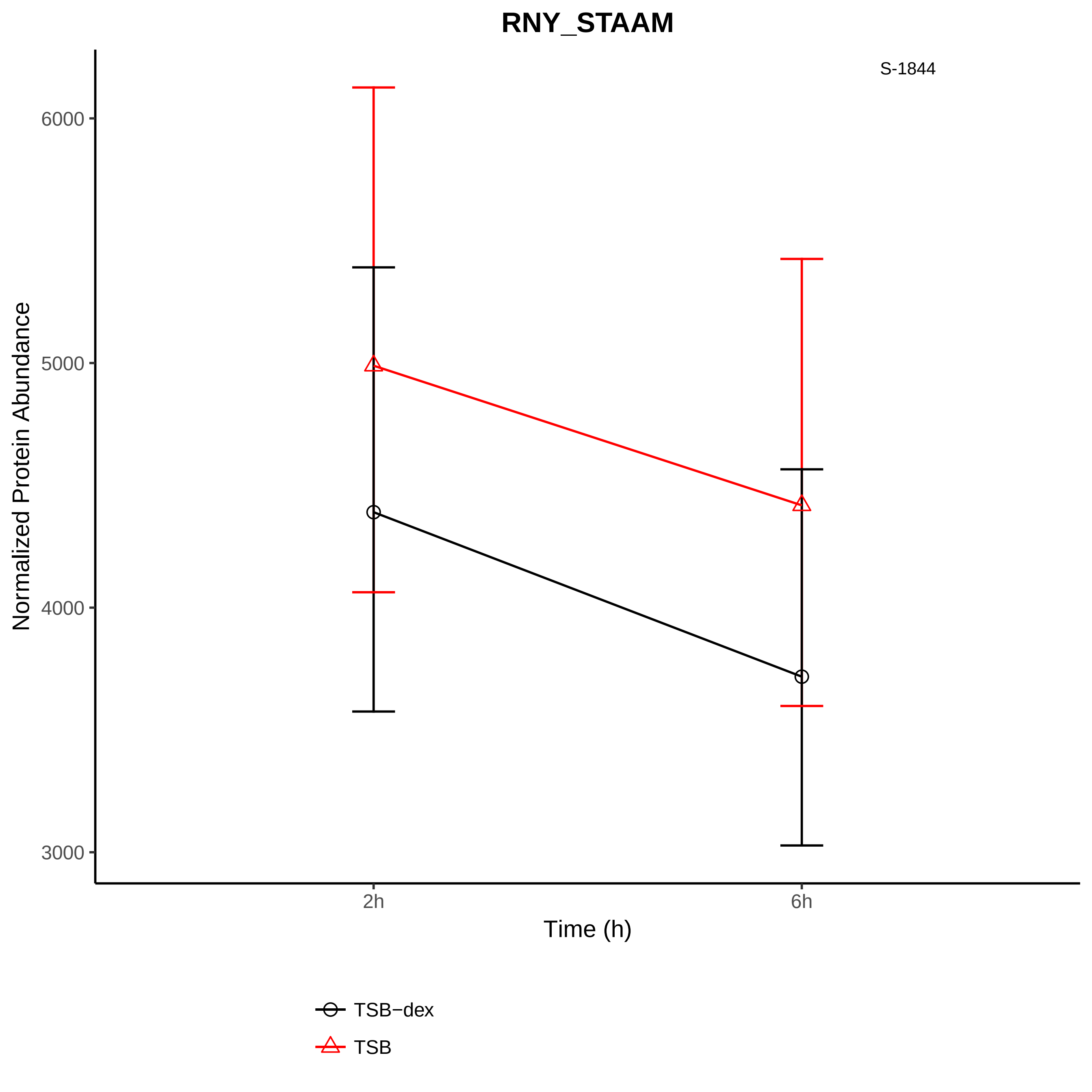

RNY_STAAM
S-1844
6000
5000
4000
3000
Normalized Protein Abundance
2h
6h
Time (h)
TSB−dex
TSB

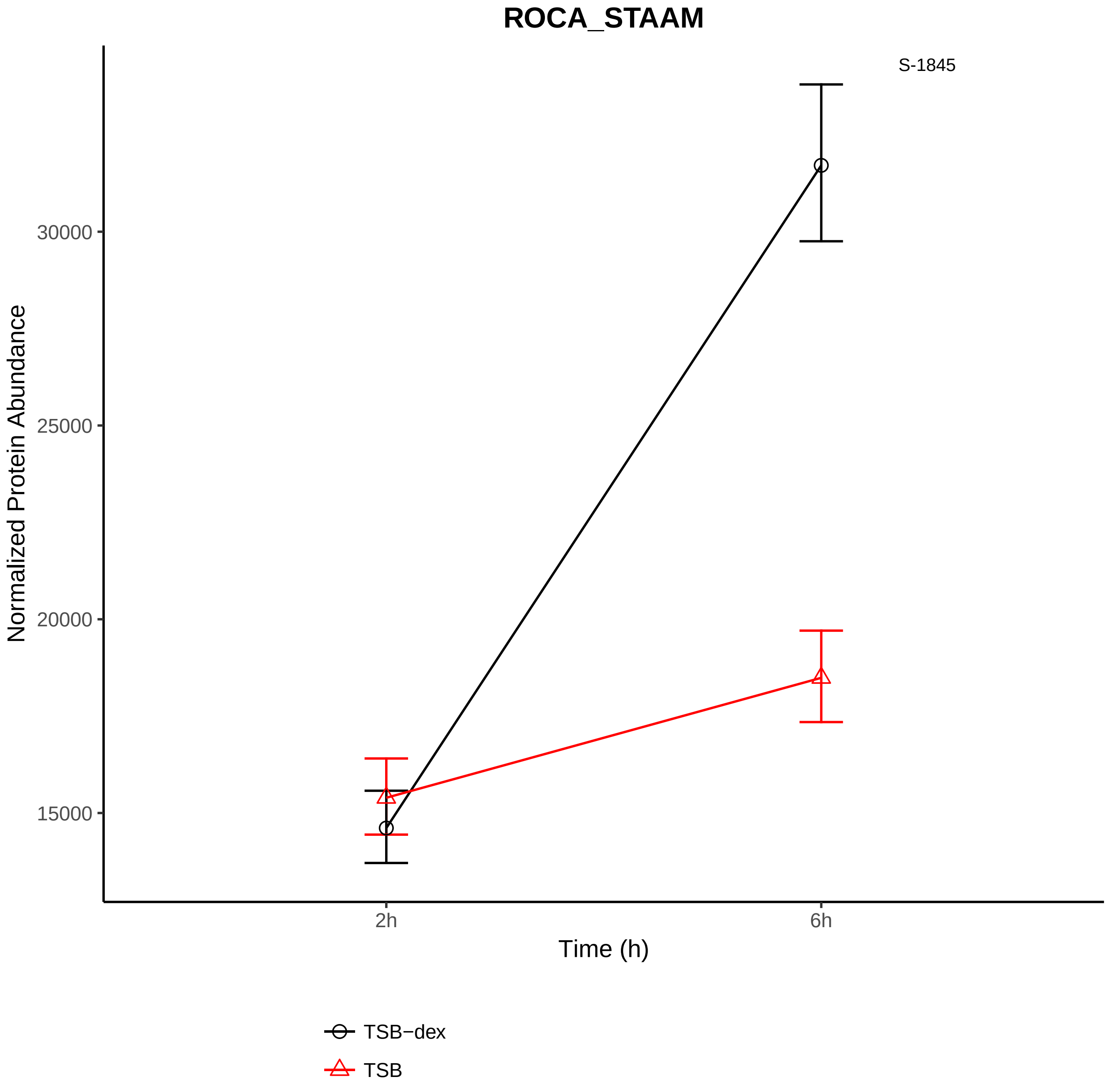

ROCA_STAAM
S-1845
30000
25000
20000
15000
Normalized Protein Abundance
2h
6h
Time (h)
TSB−dex
TSB

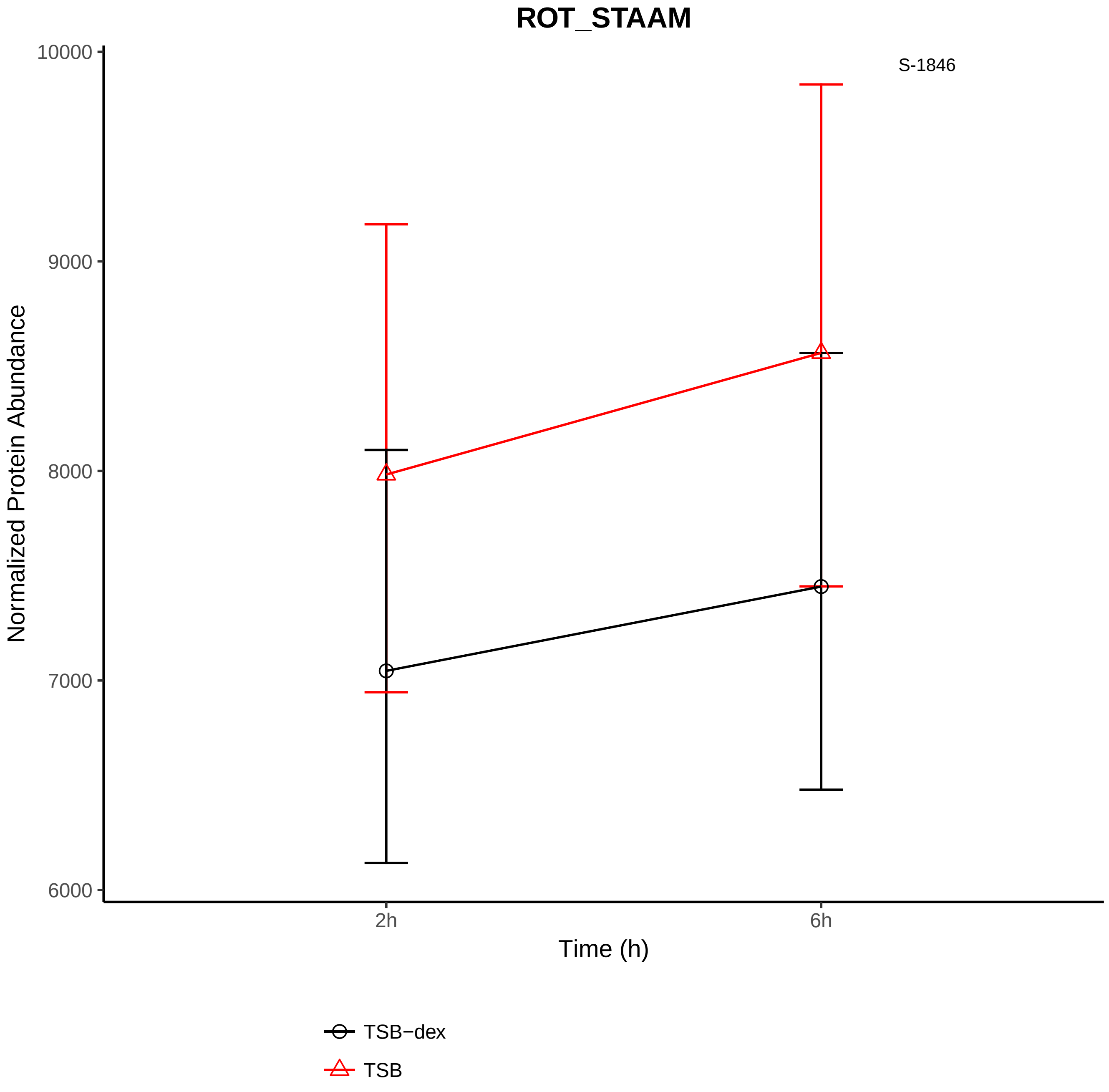
ROT_STAAM
S-1846
Normalized Protein Abundance
10000
9000
8000
7000
6000
2h
6h
Time (h)
TSB−dex
TSB

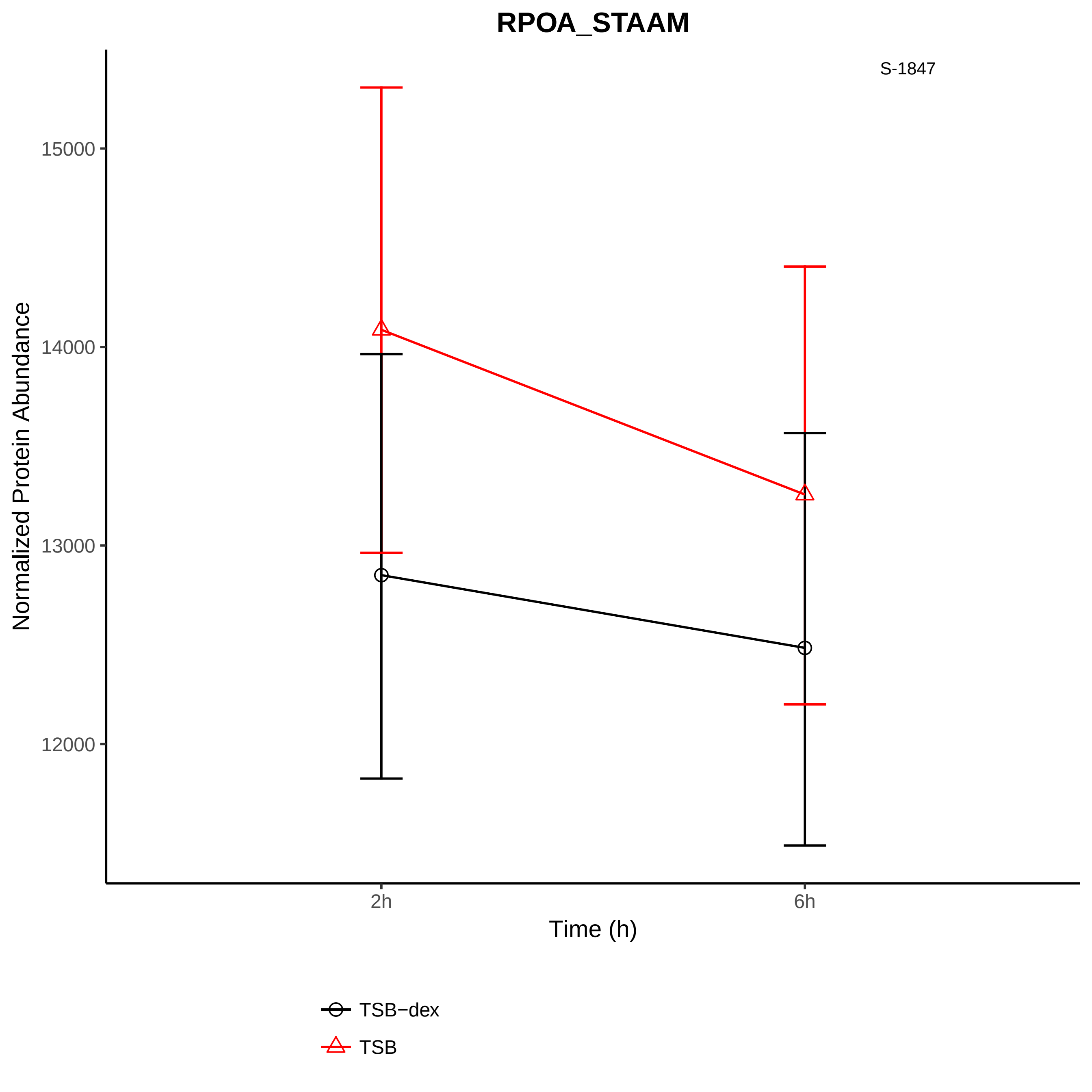

RPOA_STAAM
S-1847
15000
14000
13000
12000
Normalized Protein Abundance
2h
6h
Time (h)
TSB−dex
TSB

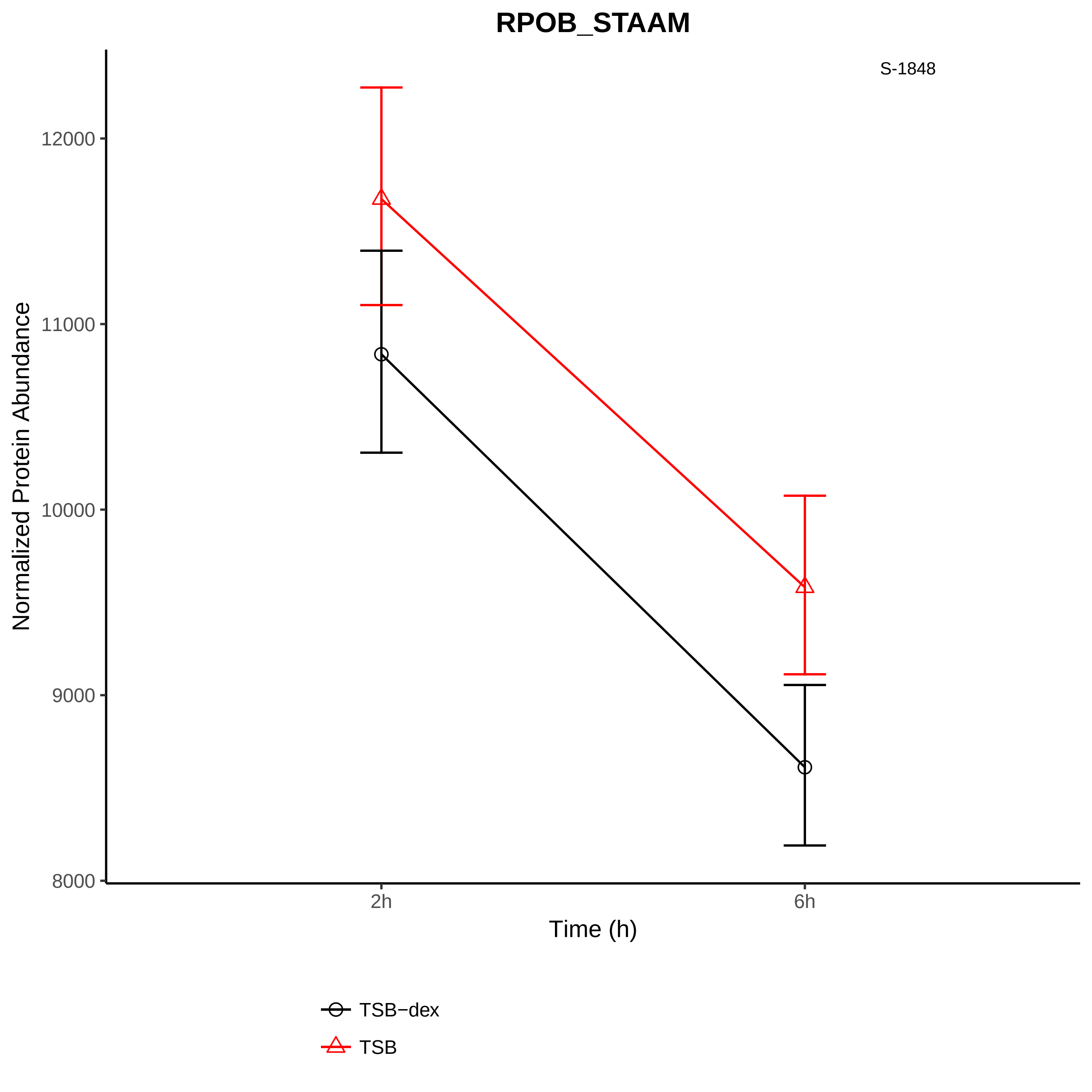

RPOB_STAAM
S-1848
Normalized Protein Abundance
12000
11000
10000
9000
8000
2h
6h
Time (h)
TSB−dex
TSB

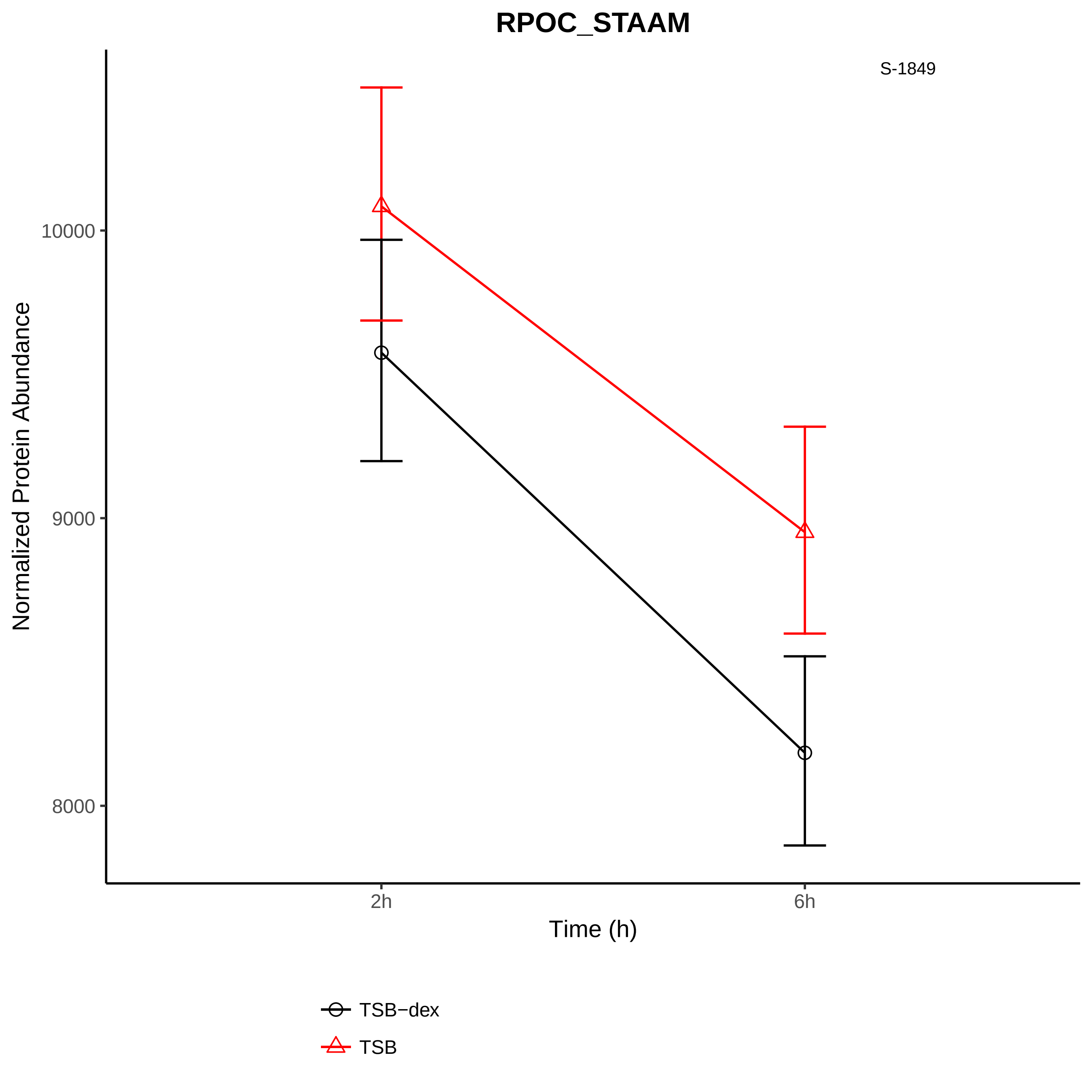
RPOC_STAAM
S-1849
Normalized Protein Abundance
10000
9000
8000
2h
6h
Time (h)
TSB−dex
TSB

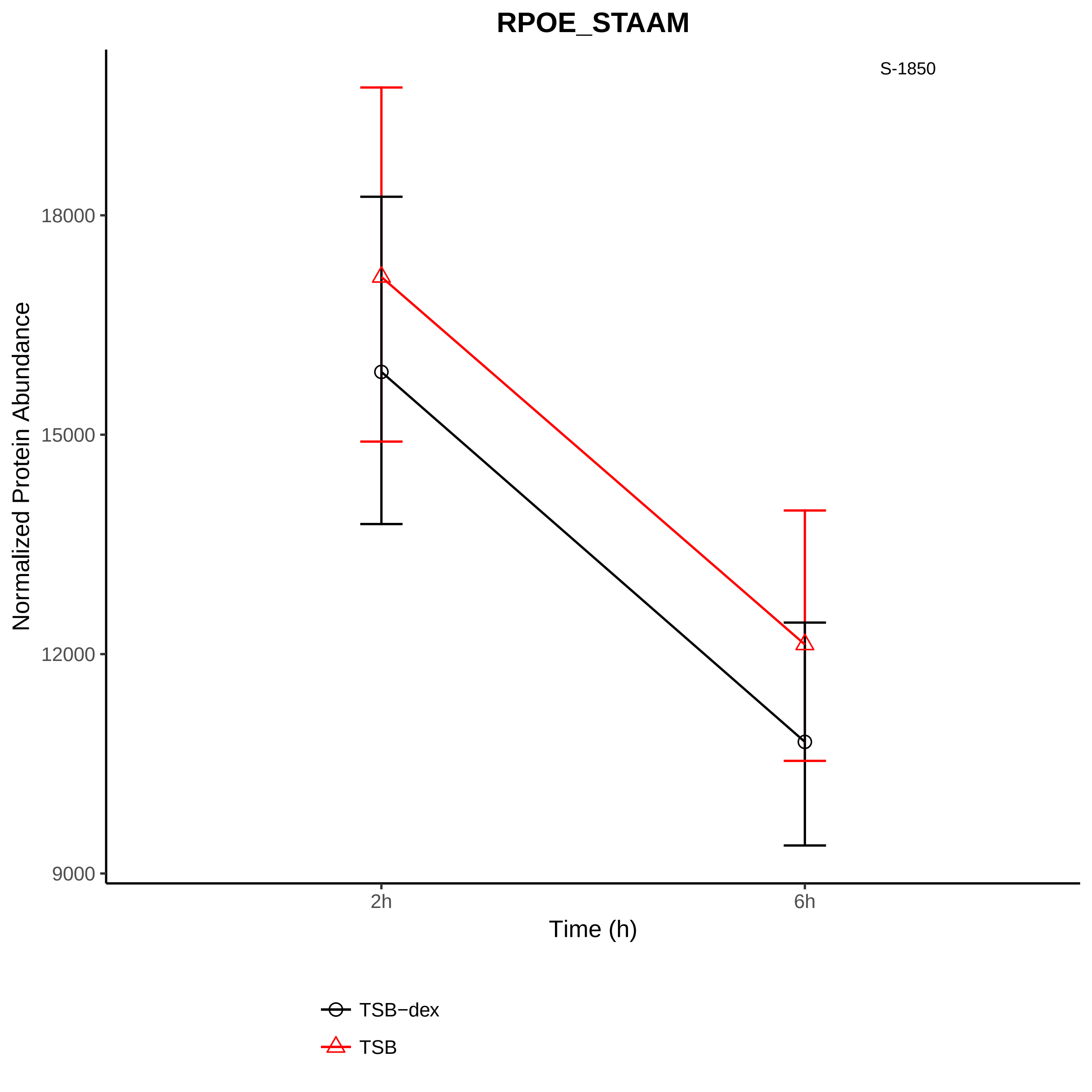

RPOE_STAAM
S-1850
Normalized Protein Abundance
18000
15000
12000
9000
2h
6h
Time (h)
TSB−dex
TSB

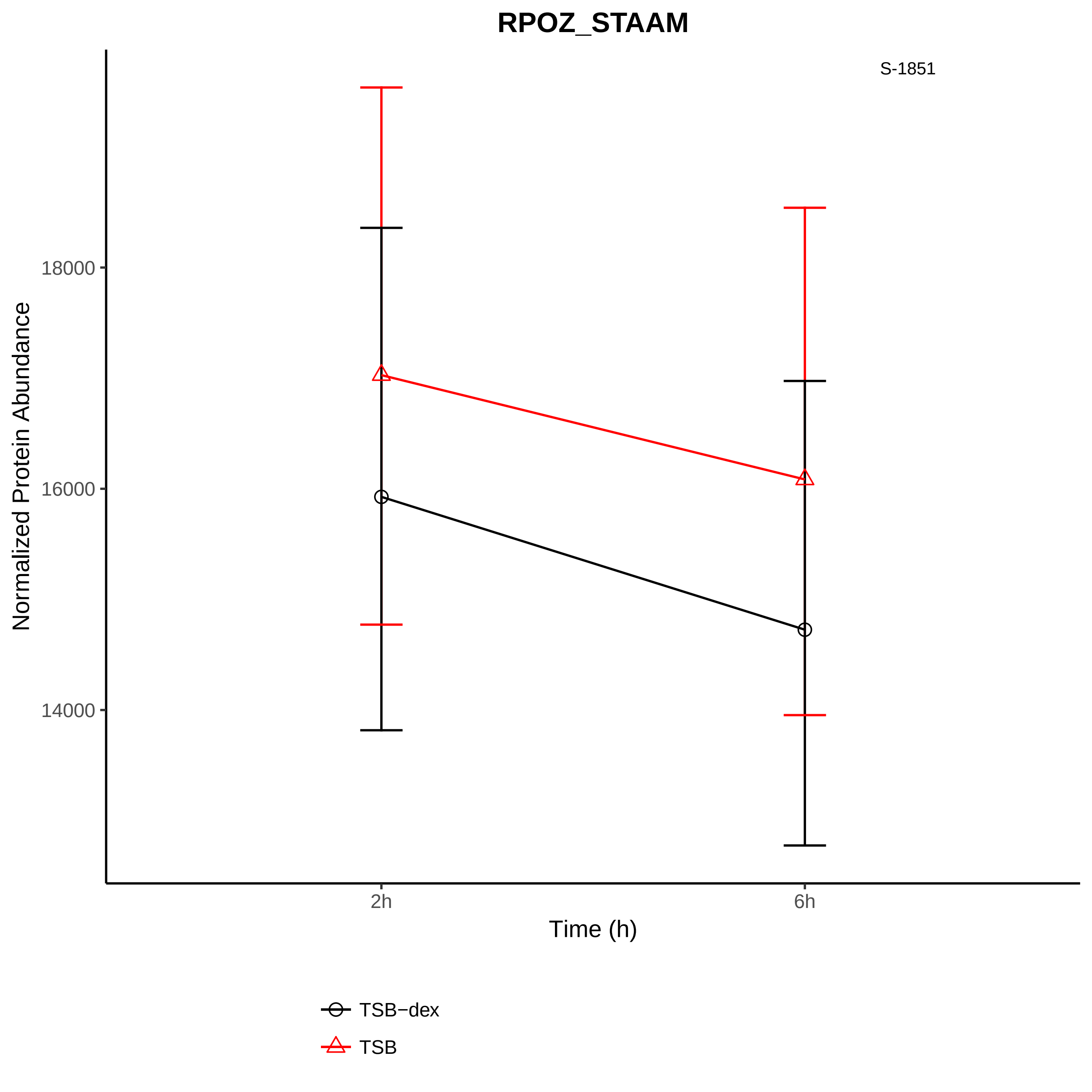
RPOZ_STAAM
S-1851
Normalized Protein Abundance
18000
16000
14000
2h
6h
Time (h)
TSB−dex
TSB

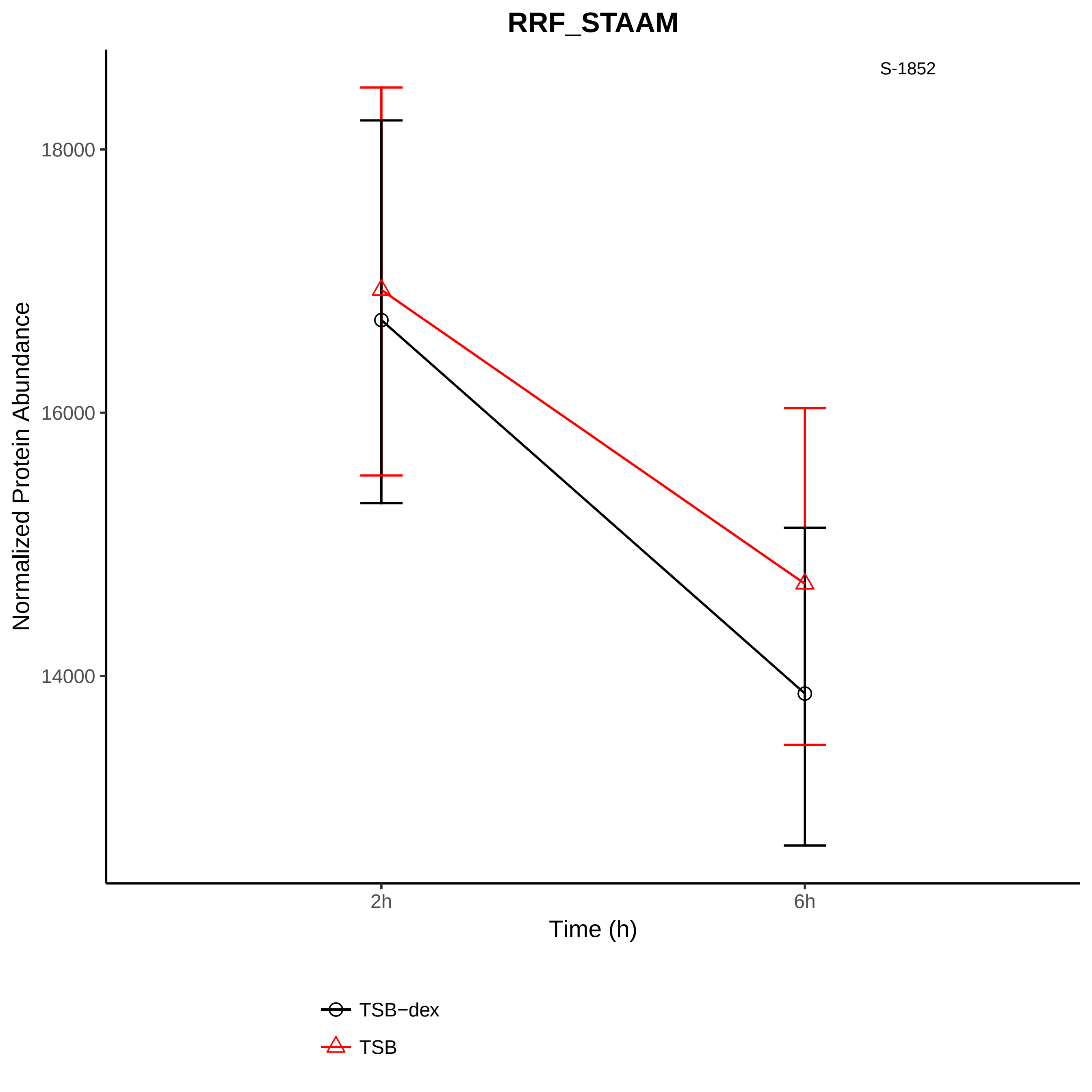

RRF_STAAM
S-1852
18000
16000
14000
Normalized Protein Abundance
2h
6h
Time (h)
TSB−dex
TSB

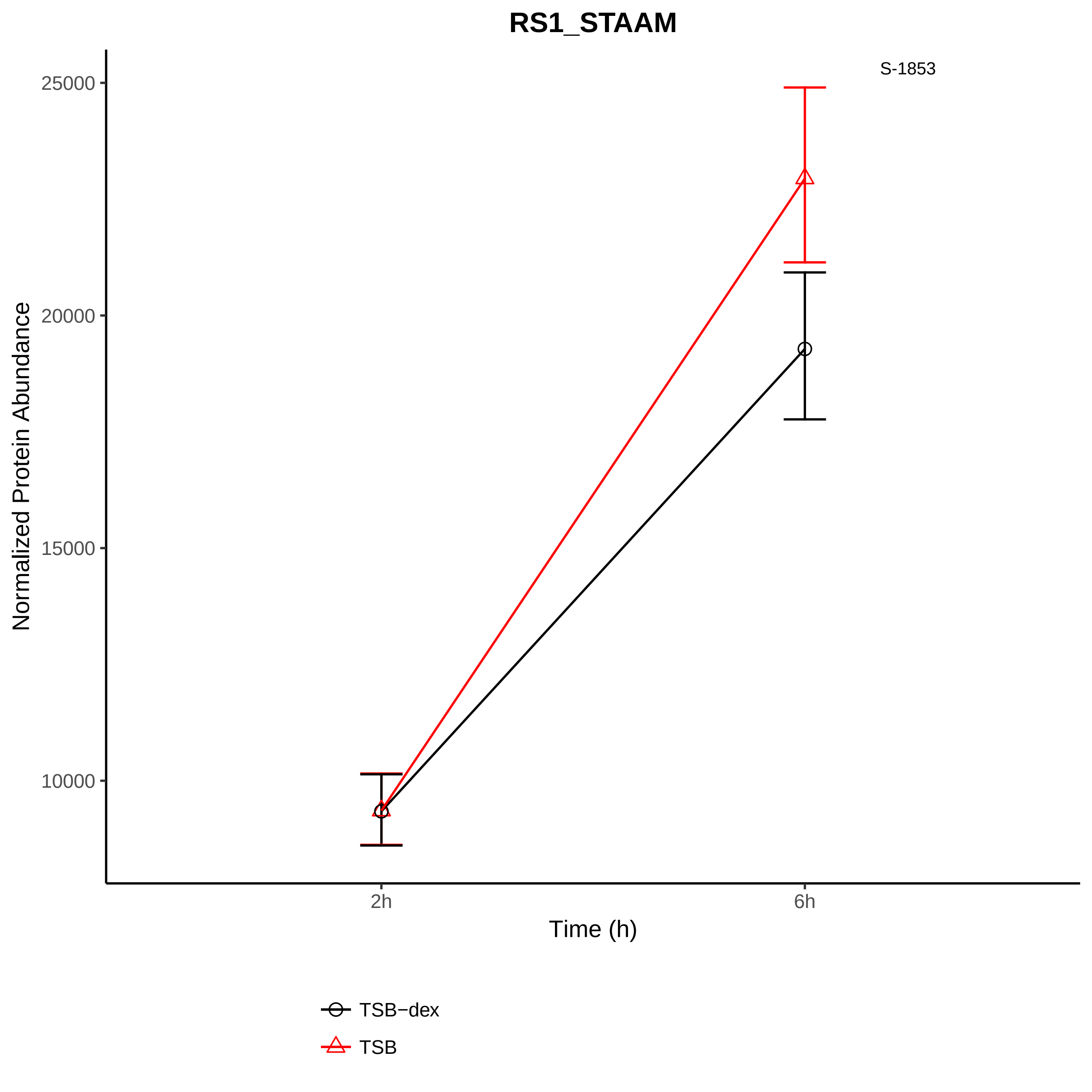

RS1_STAAM
S-1853
Normalized Protein Abundance
25000
20000
15000
10000
2h
6h
Time (h)
TSB−dex
TSB

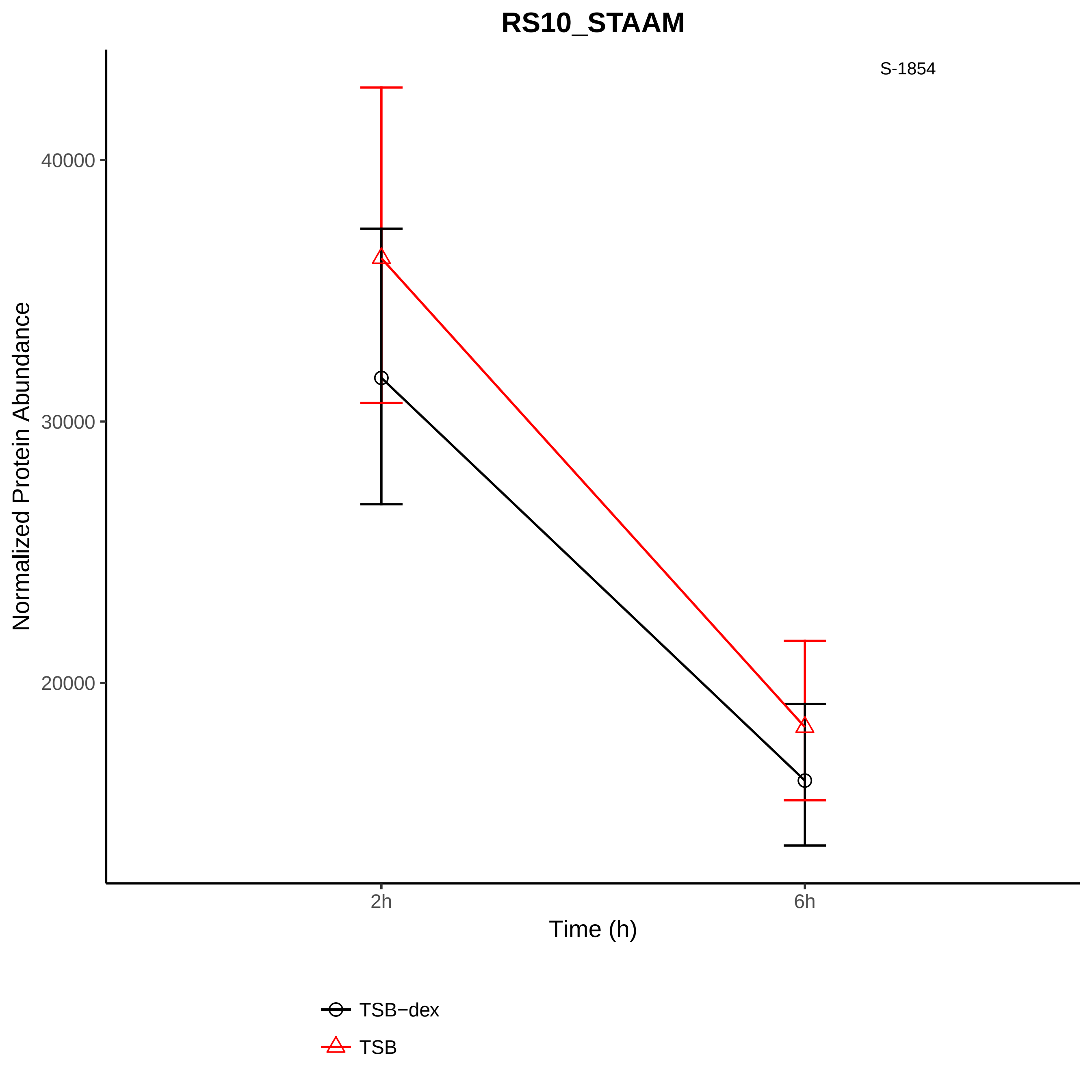

RS10_STAAM
S-1854
40000
30000
20000
Normalized Protein Abundance
2h
6h
Time (h)
TSB−dex
TSB

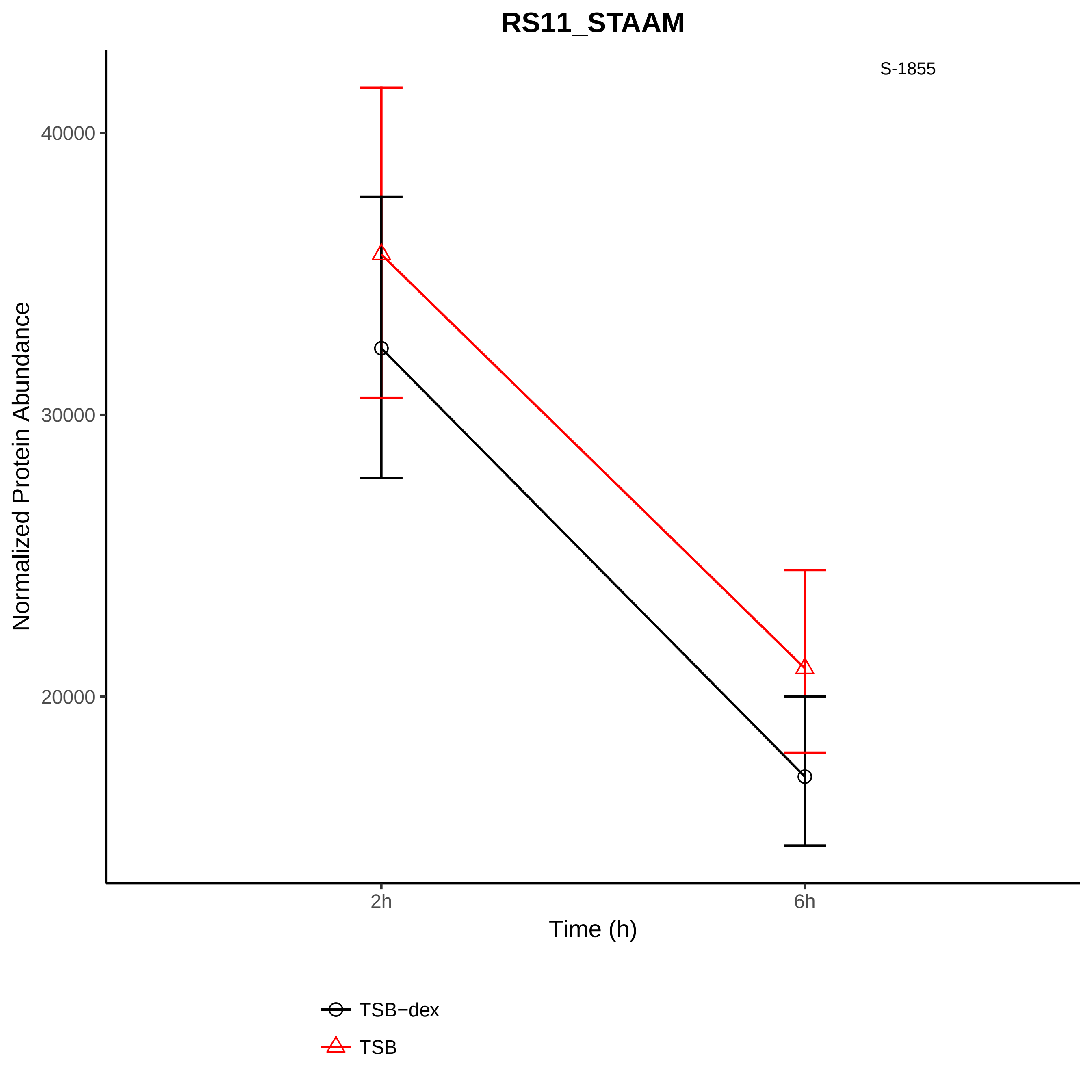

RS11_STAAM
S-1855
40000
30000
20000
Normalized Protein Abundance
2h
6h
Time (h)
TSB−dex
TSB

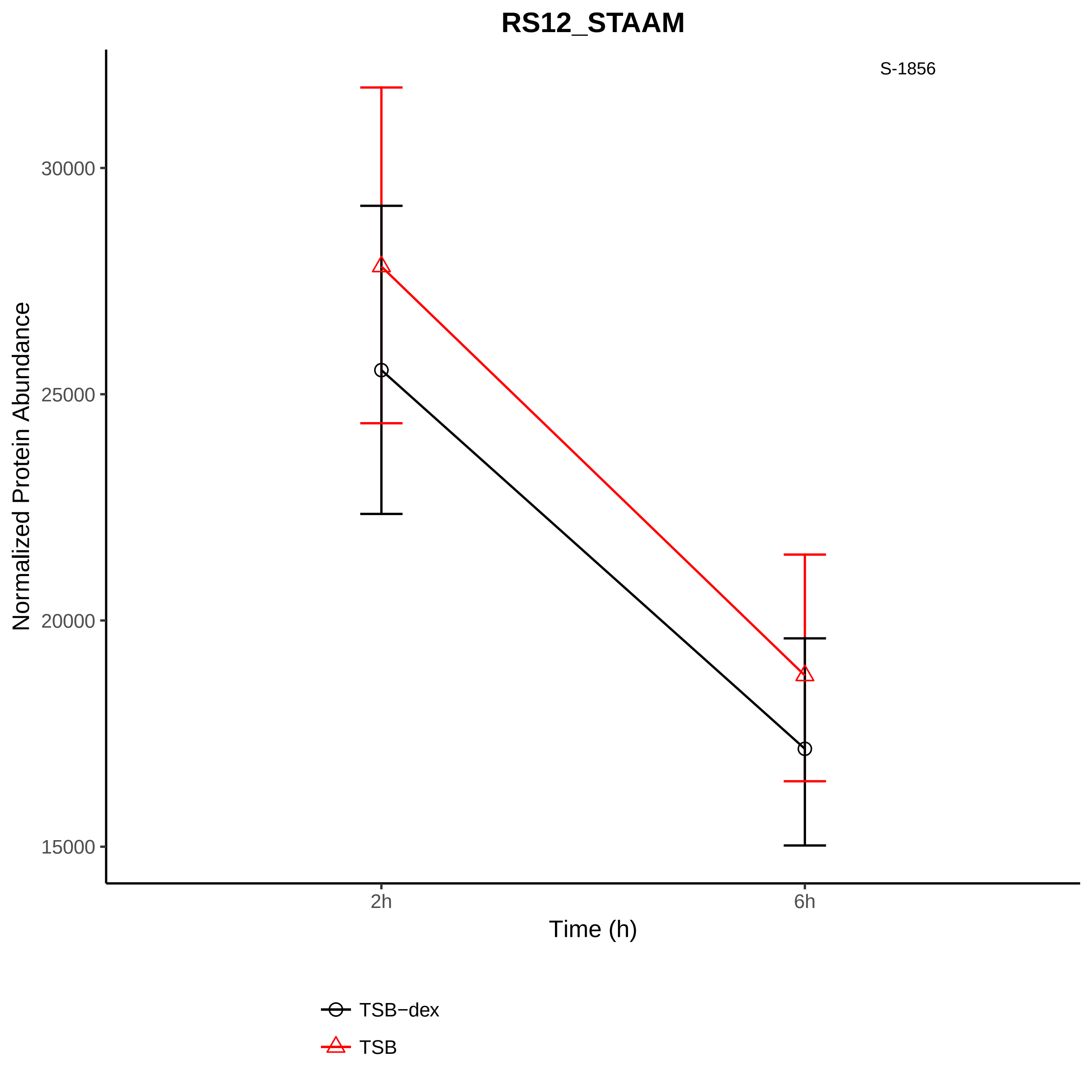
RS12_STAAM
S-1856
Normalized Protein Abundance
30000
25000
20000
15000
2h
6h
Time (h)
TSB−dex
TSB

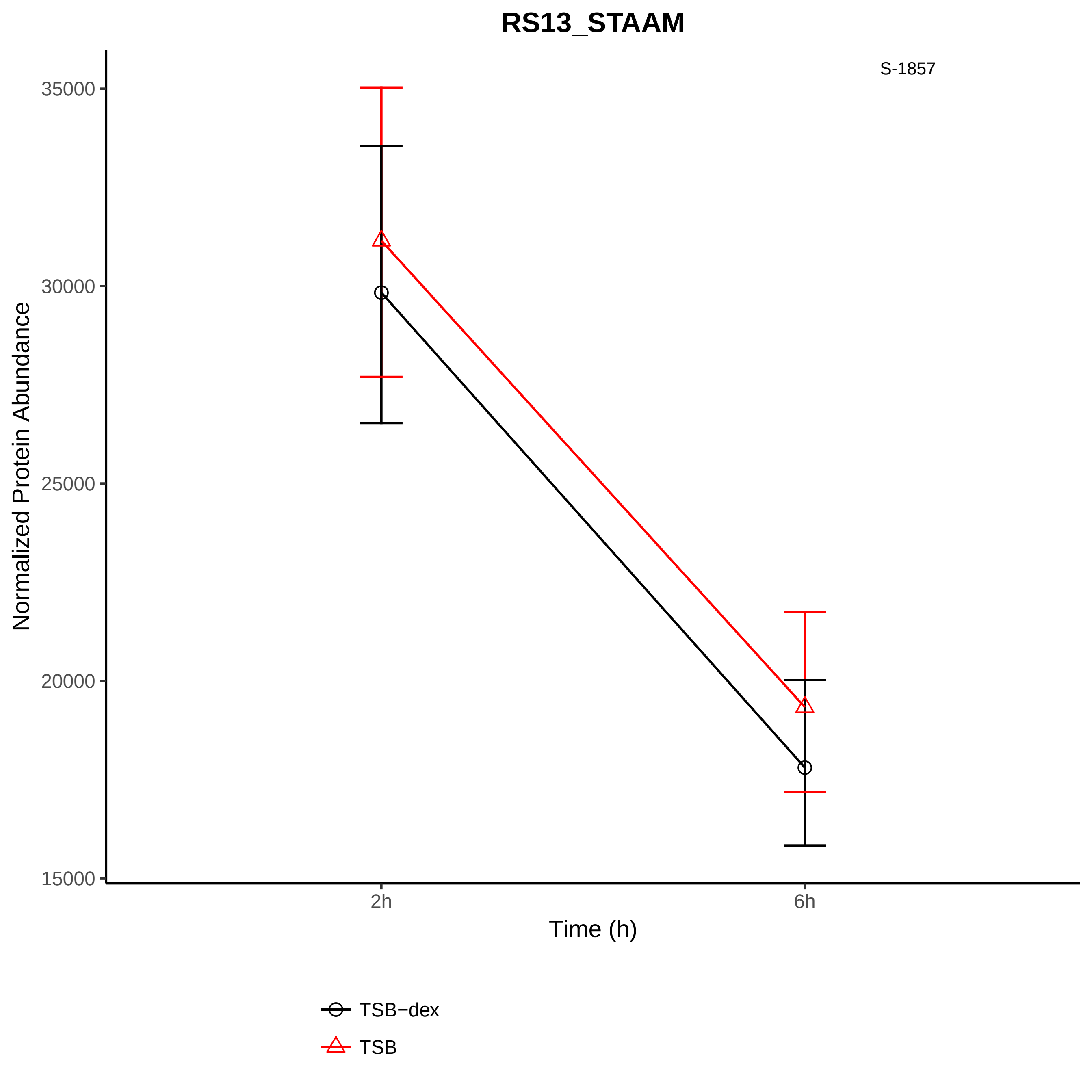
RS13_STAAM
S-1857
Normalized Protein Abundance
35000
30000
25000
20000
15000
2h
6h
Time (h)
TSB−dex
TSB

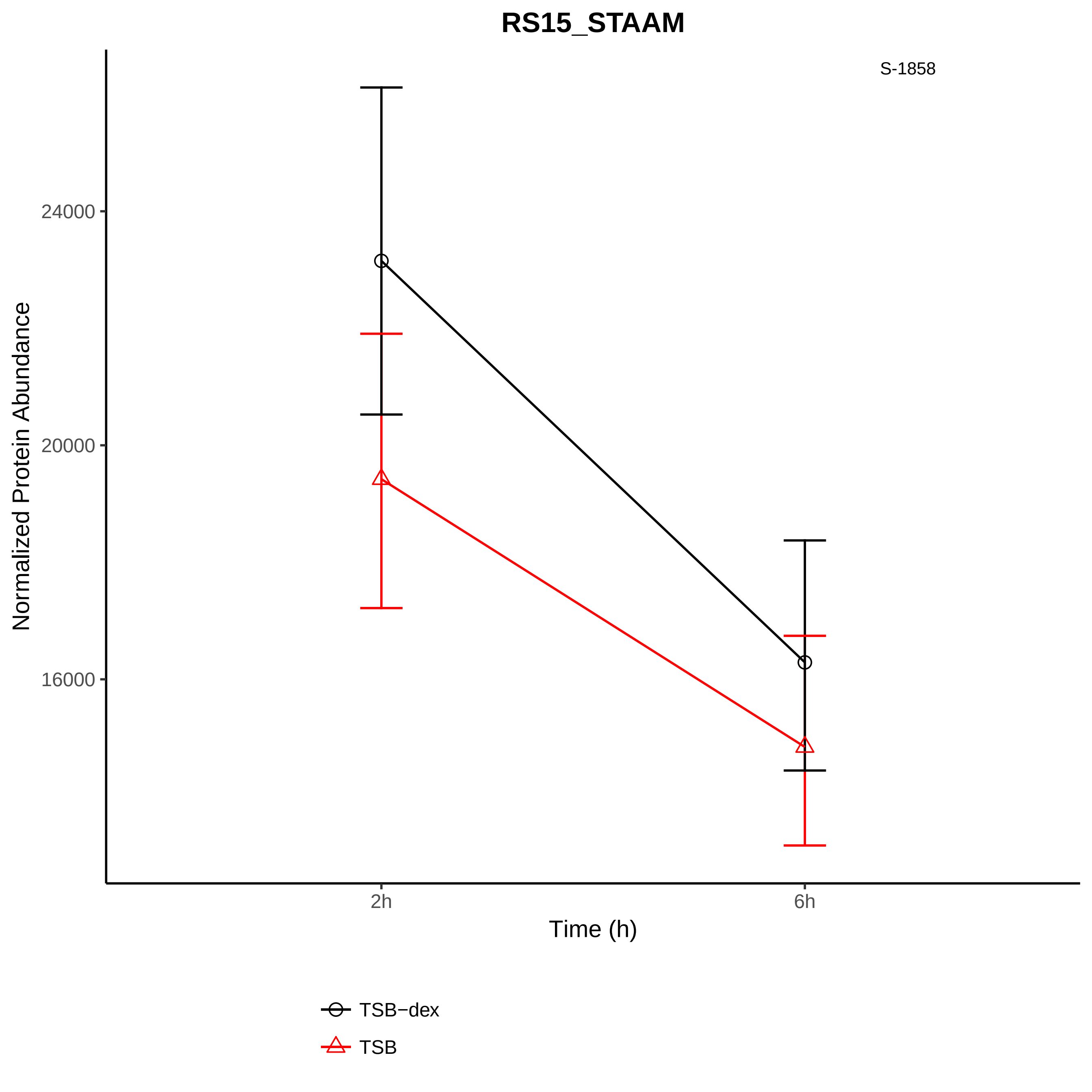

RS15_STAAM
S-1858
24000
20000
16000
Normalized Protein Abundance
2h
6h
Time (h)
TSB−dex
TSB

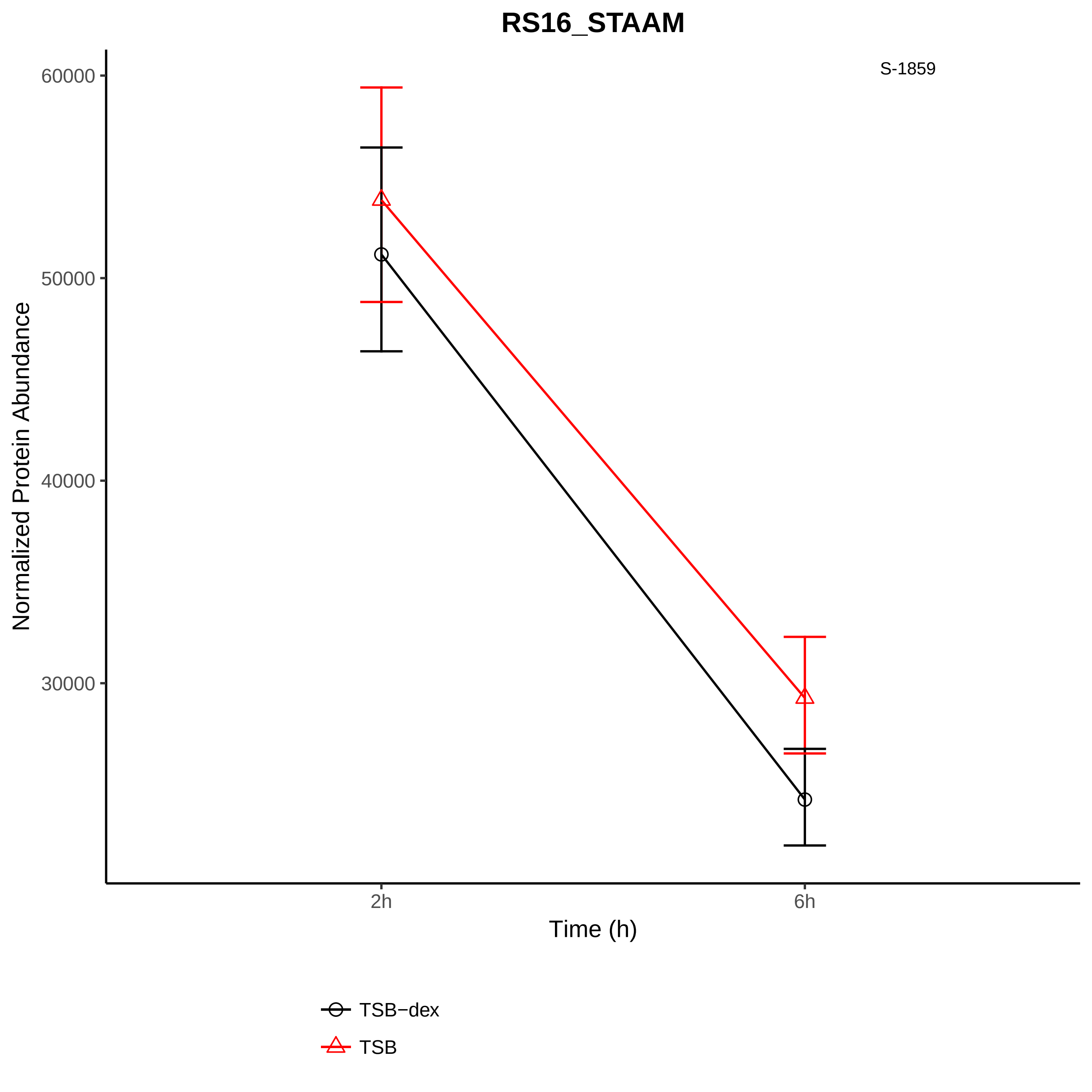

RS16_STAAM
S-1859
60000
50000
40000
30000
Normalized Protein Abundance
2h
6h
Time (h)
TSB−dex
TSB

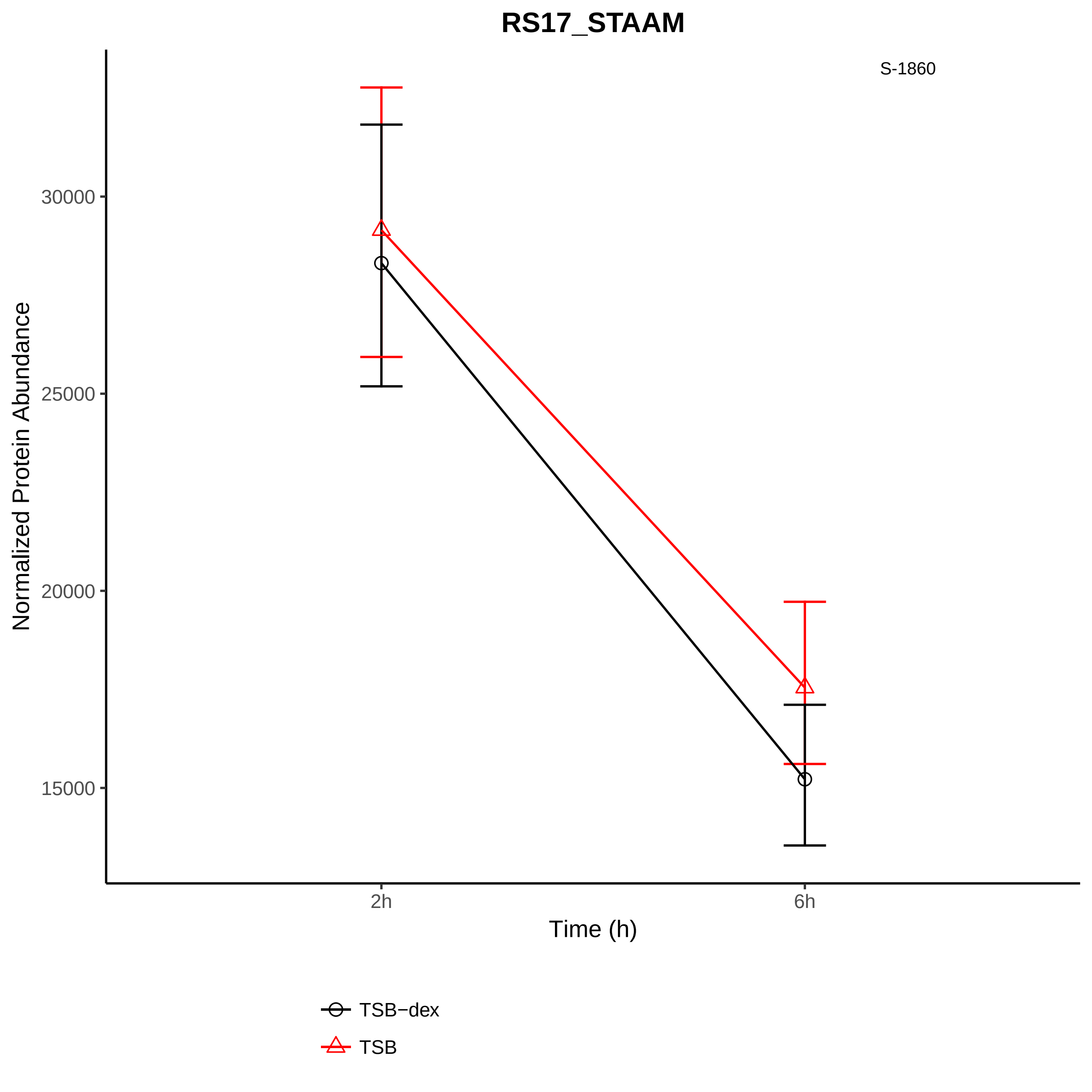

RS17_STAAM
S-1860
Normalized Protein Abundance
30000
25000
20000
15000
2h
6h
Time (h)
TSB−dex
TSB

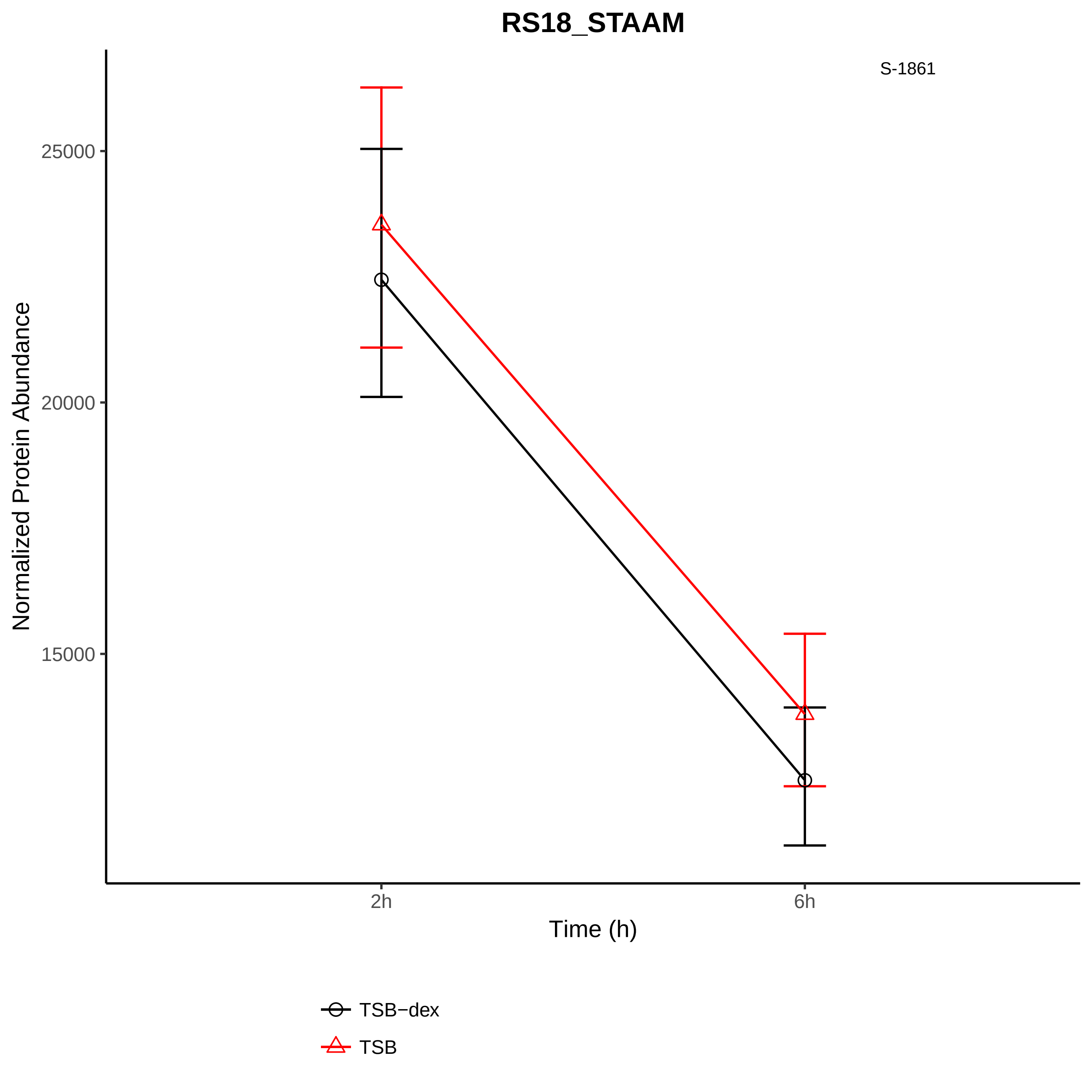
RS18_STAAM
S-1861
25000
20000
15000
Normalized Protein Abundance
2h
6h
Time (h)
TSB−dex
TSB

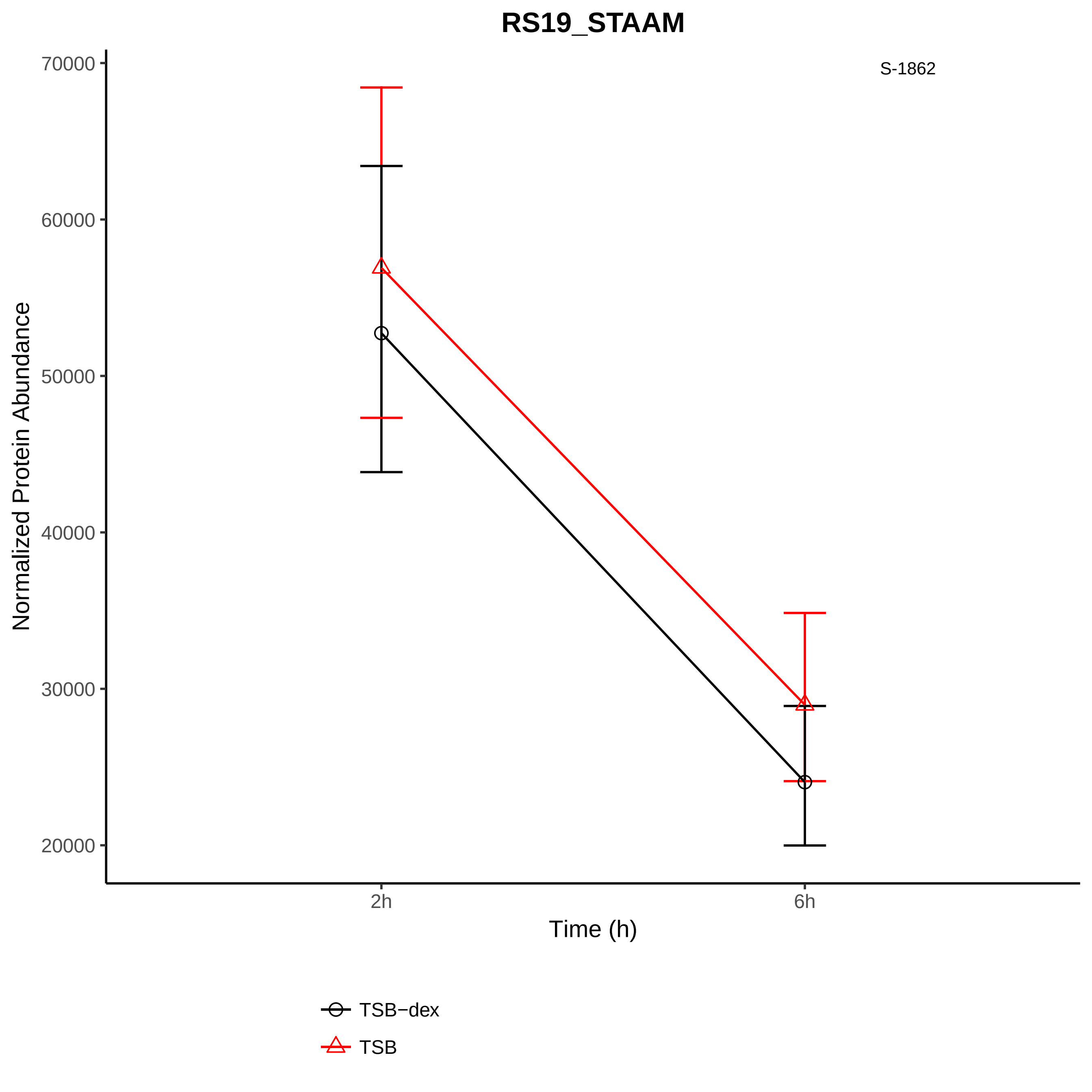
RS19_STAAM
S-1862
Normalized Protein Abundance
70000
60000
50000
40000
30000
20000
2h
6h
Time (h)
TSB−dex
TSB

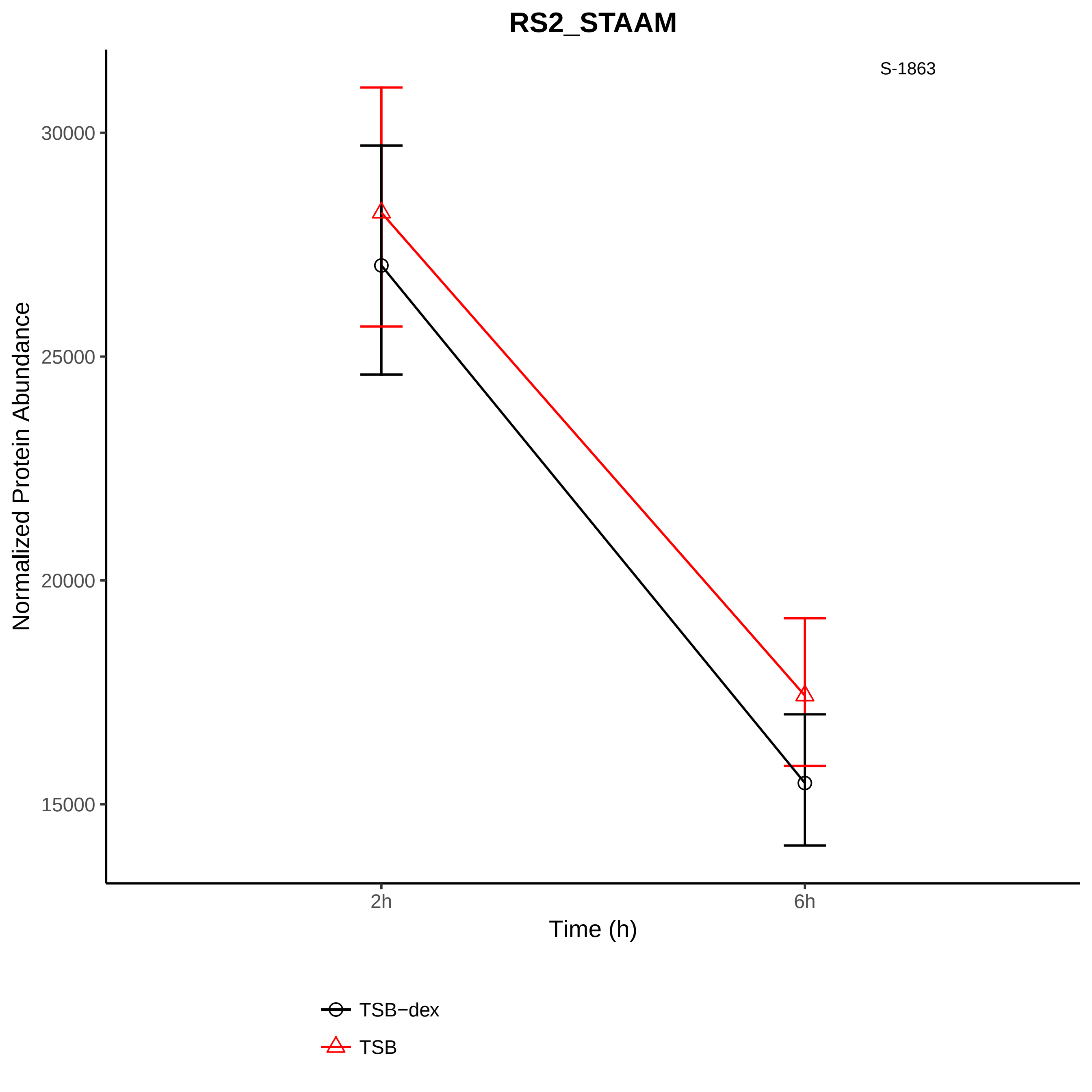

RS2_STAAM
S-1863
Normalized Protein Abundance
30000
25000
20000
15000
2h
6h
Time (h)
TSB−dex
TSB

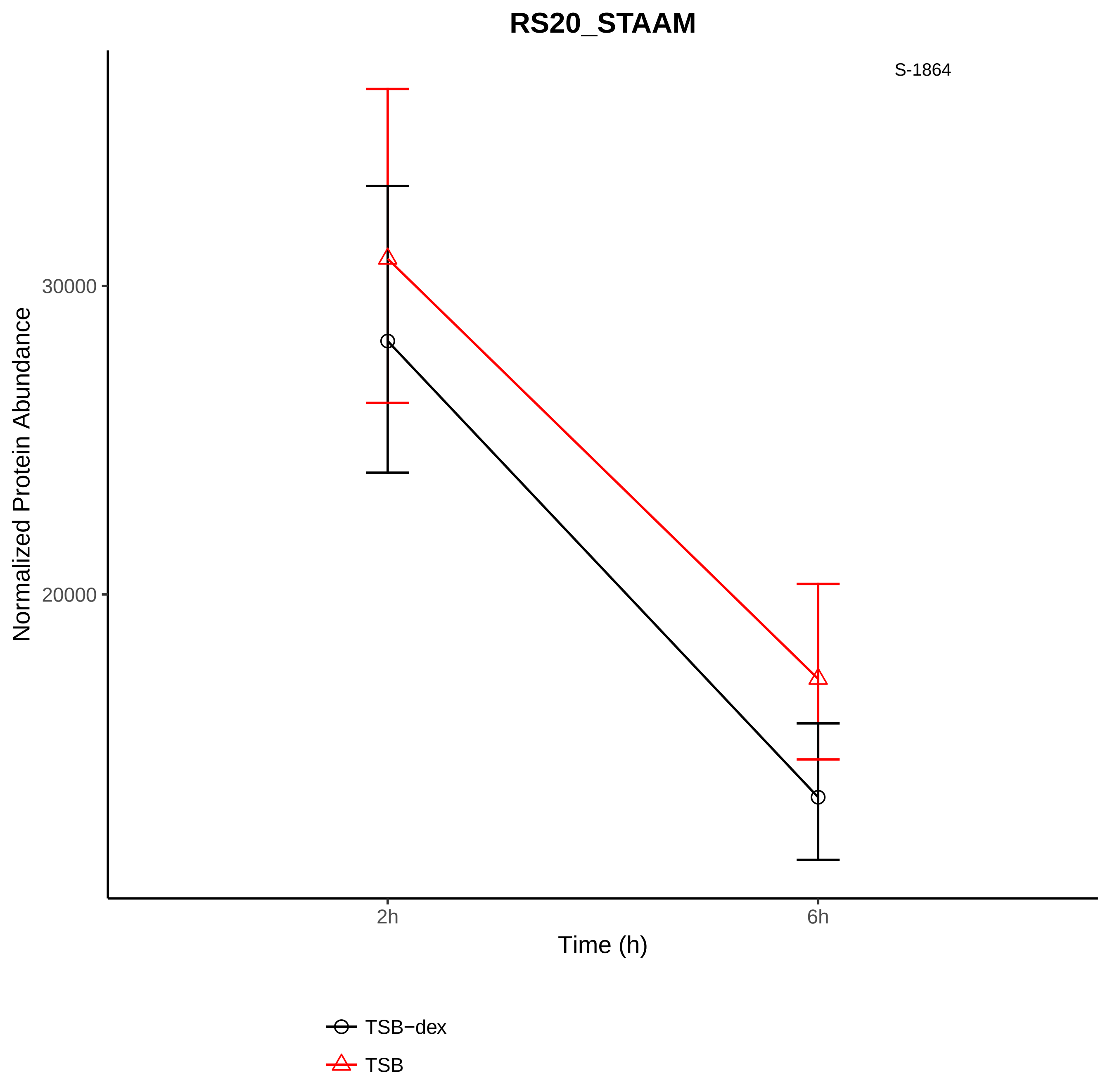
RS20_STAAM
S-1864
Normalized Protein Abundance
30000
20000
2h
6h
Time (h)
TSB−dex
TSB

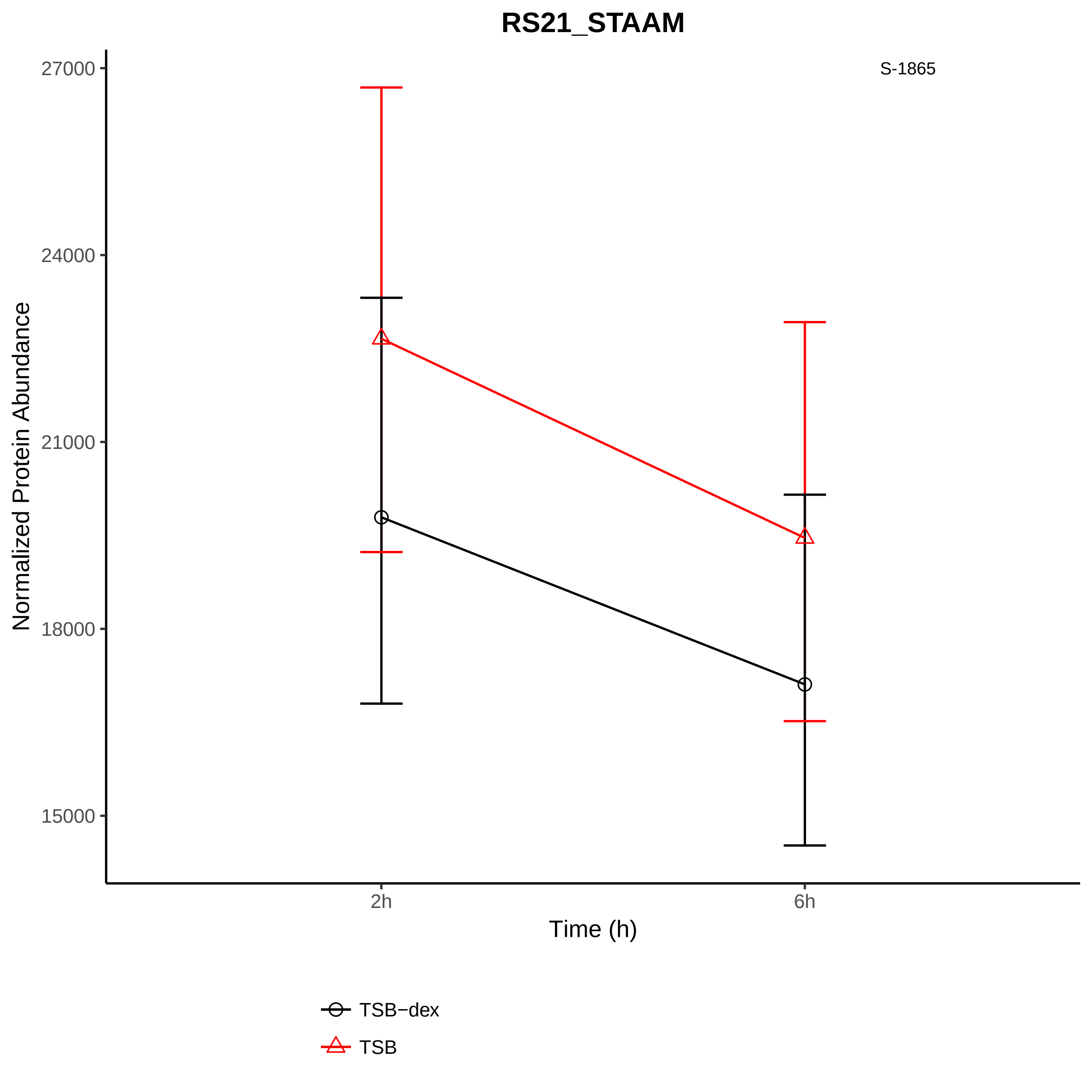

RS21_STAAM
S-1865
27000
24000
21000
18000
15000
Normalized Protein Abundance
2h
6h
Time (h)
TSB−dex
TSB

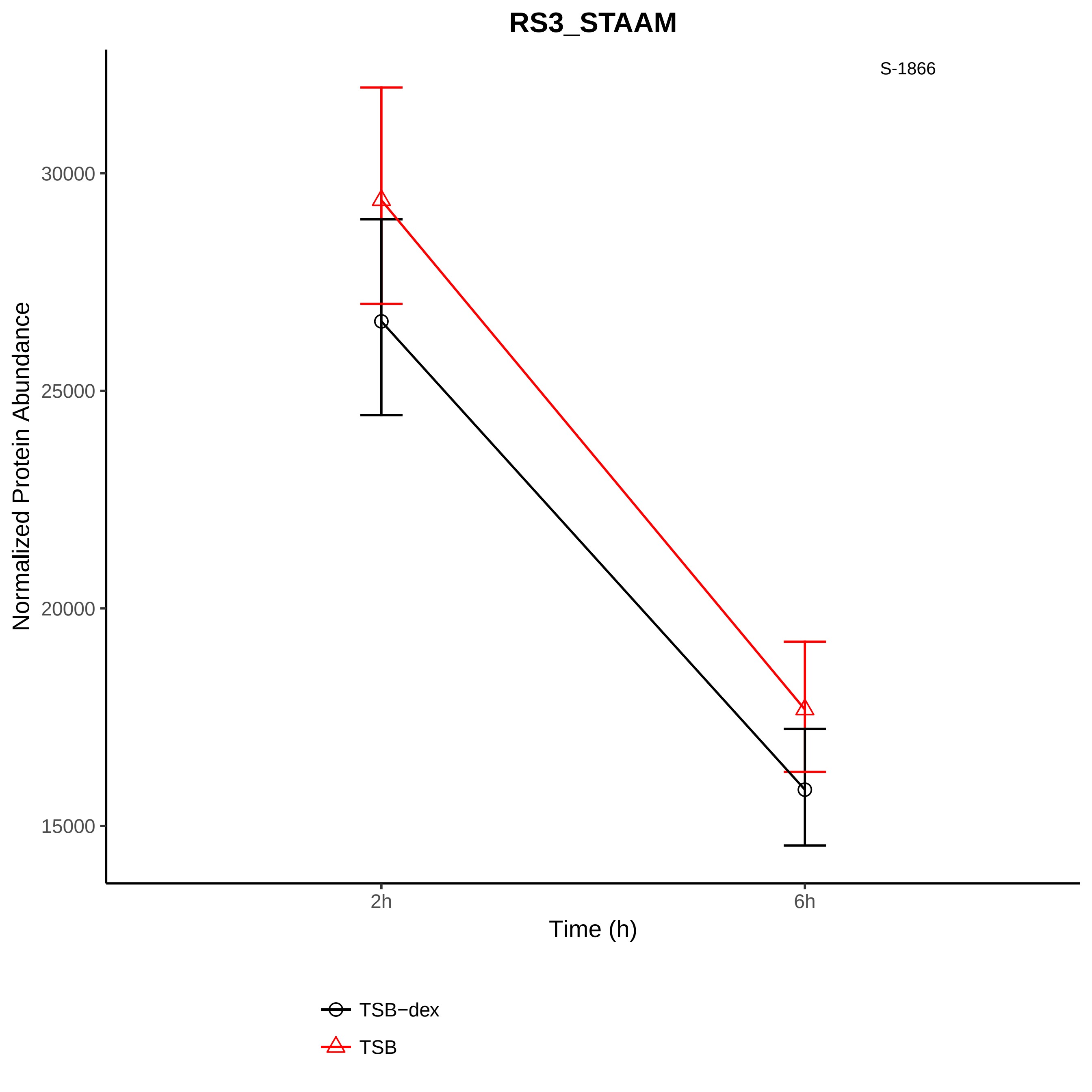

RS3_STAAM
S-1866
Normalized Protein Abundance
30000
25000
20000
15000
2h
6h
Time (h)
TSB−dex
TSB

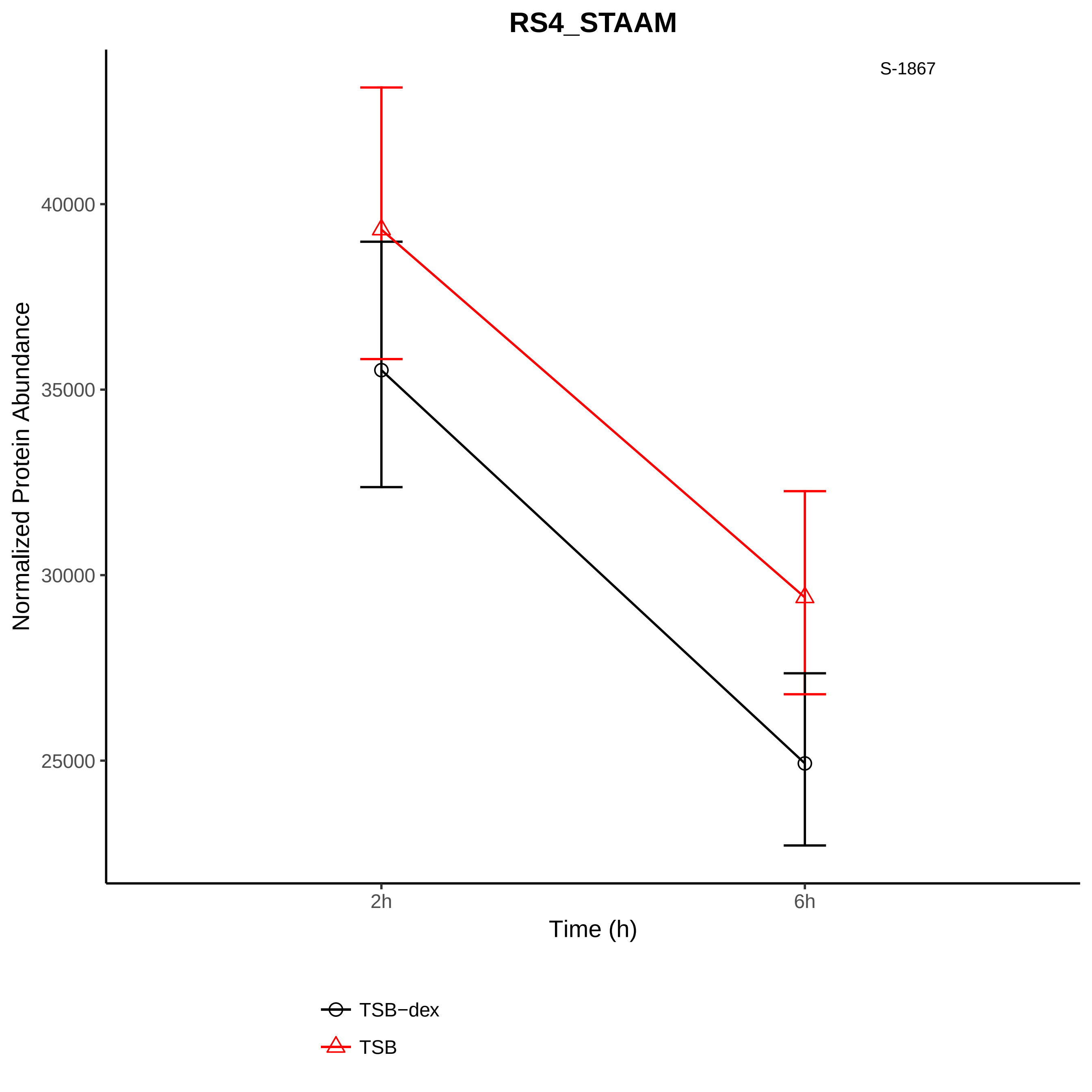
RS4_STAAM
S-1867
Normalized Protein Abundance
40000
35000
30000
25000
2h
6h
Time (h)
TSB−dex
TSB

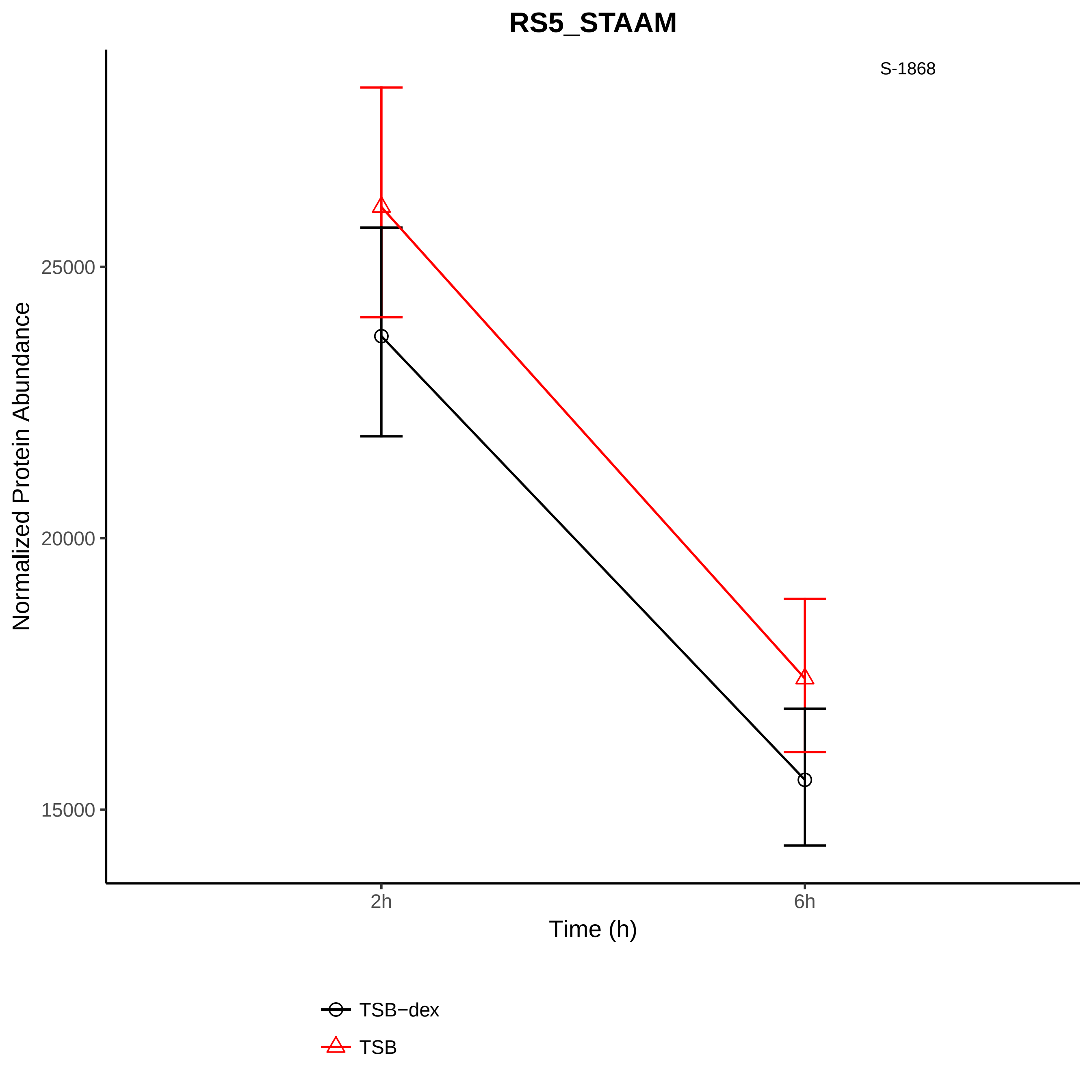
RS5_STAAM
S-1868
Normalized Protein Abundance
25000
20000
15000
2h
6h
Time (h)
TSB−dex
TSB

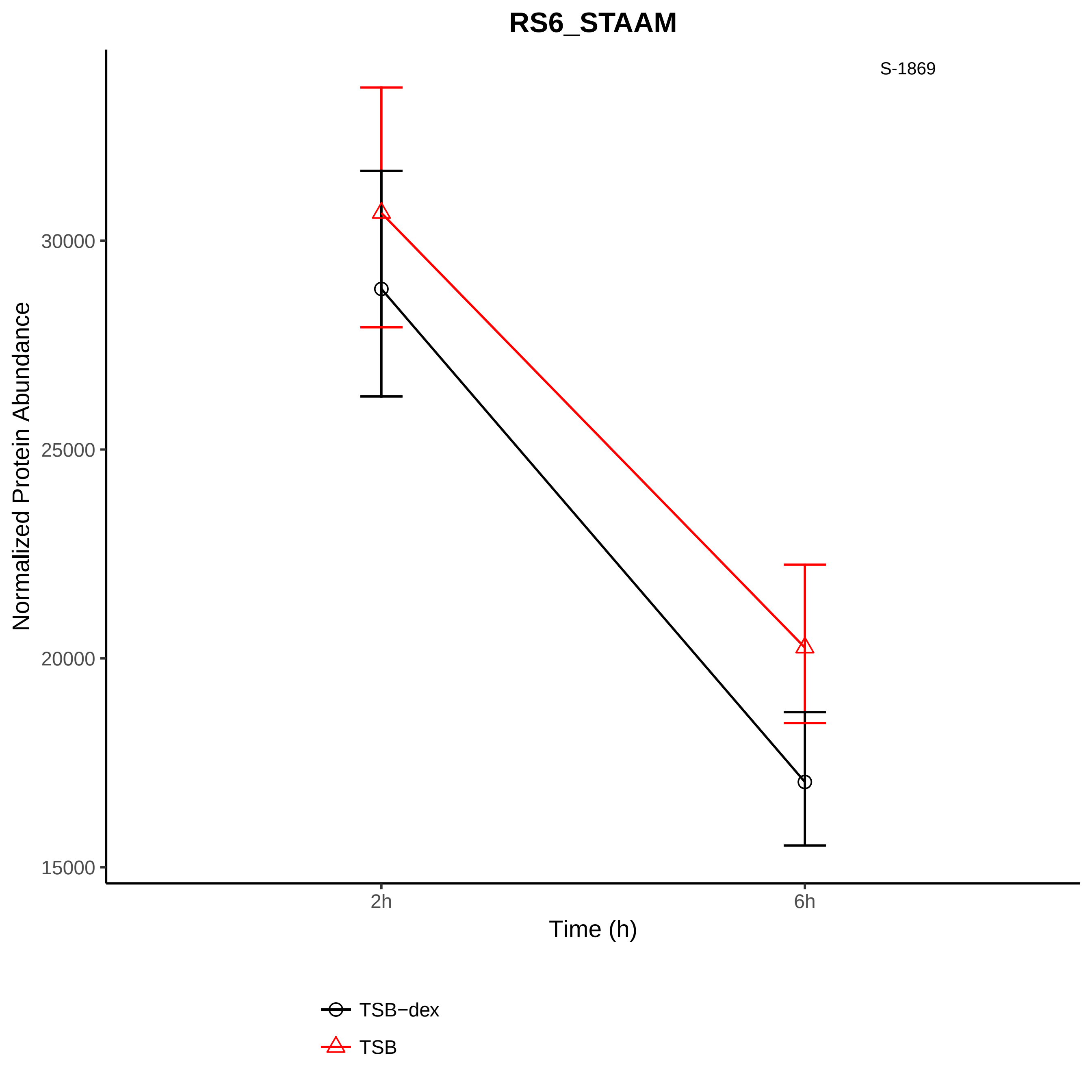

RS6_STAAM
S-1869
Normalized Protein Abundance
30000
25000
20000
15000
2h
6h
Time (h)
TSB−dex
TSB

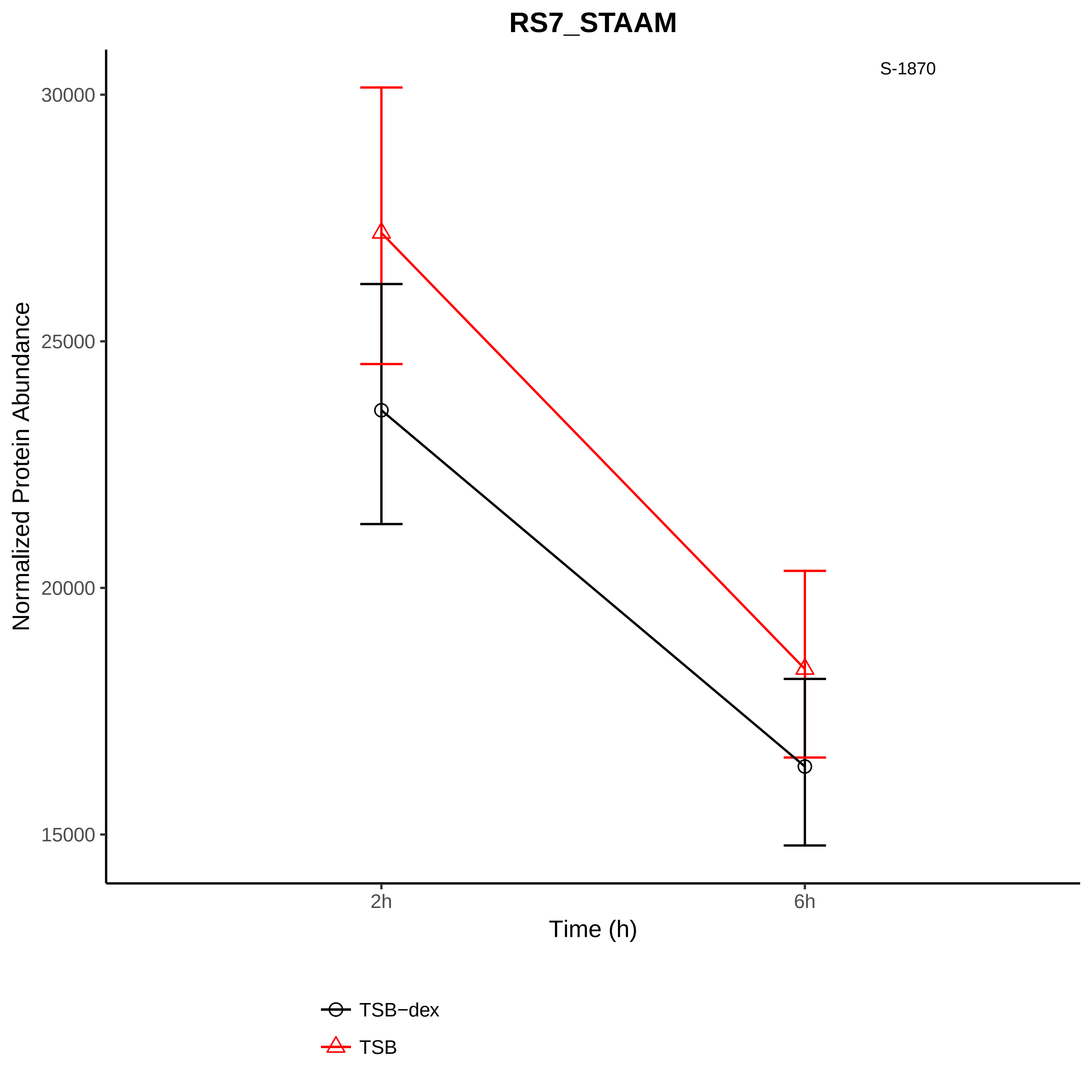
RS7_STAAM
S-1870
30000
25000
20000
15000
Normalized Protein Abundance
2h
6h
Time (h)
TSB−dex
TSB

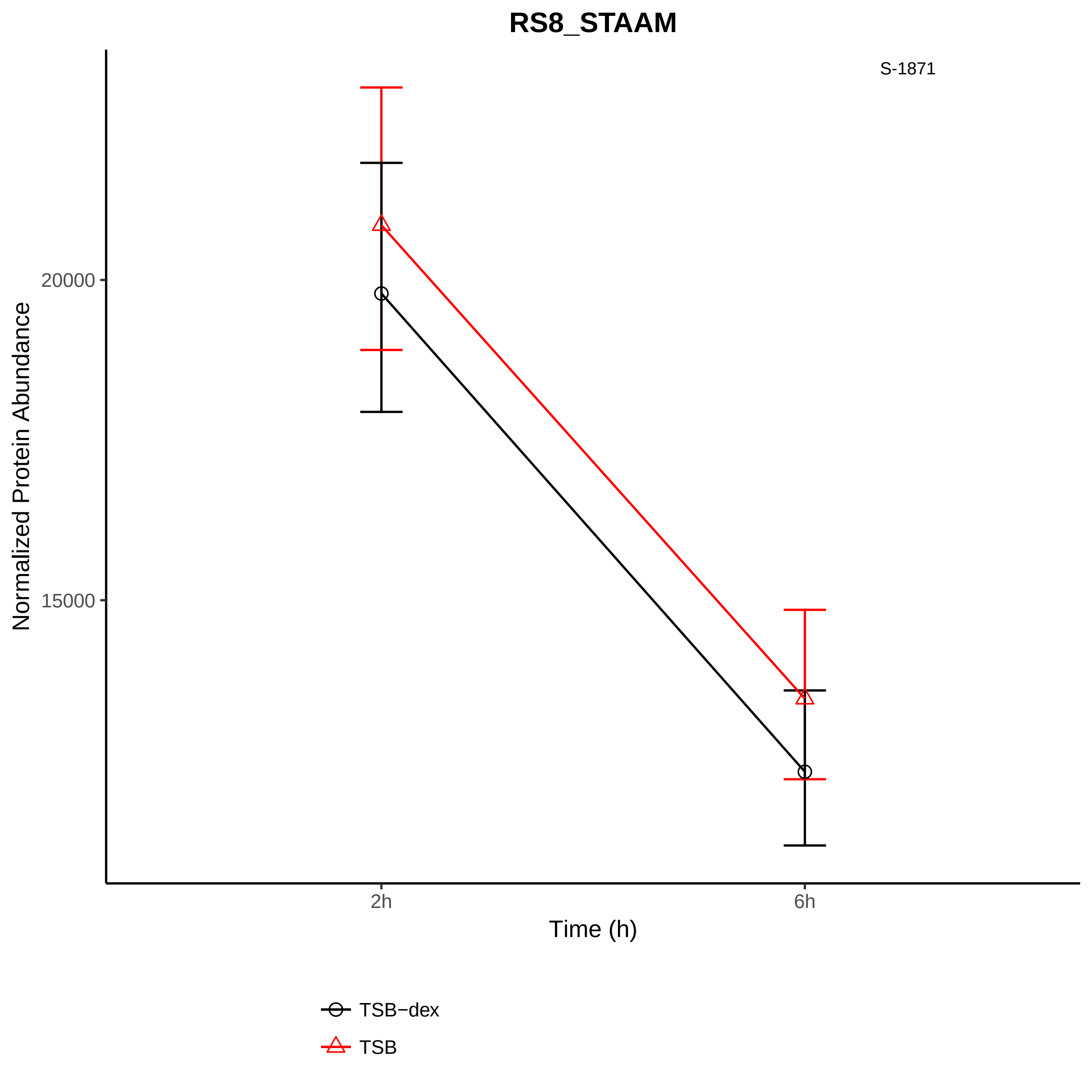
RS8_STAAM
S-1871
20000
15000
Normalized Protein Abundance
2h
6h
Time (h)
TSB−dex
TSB

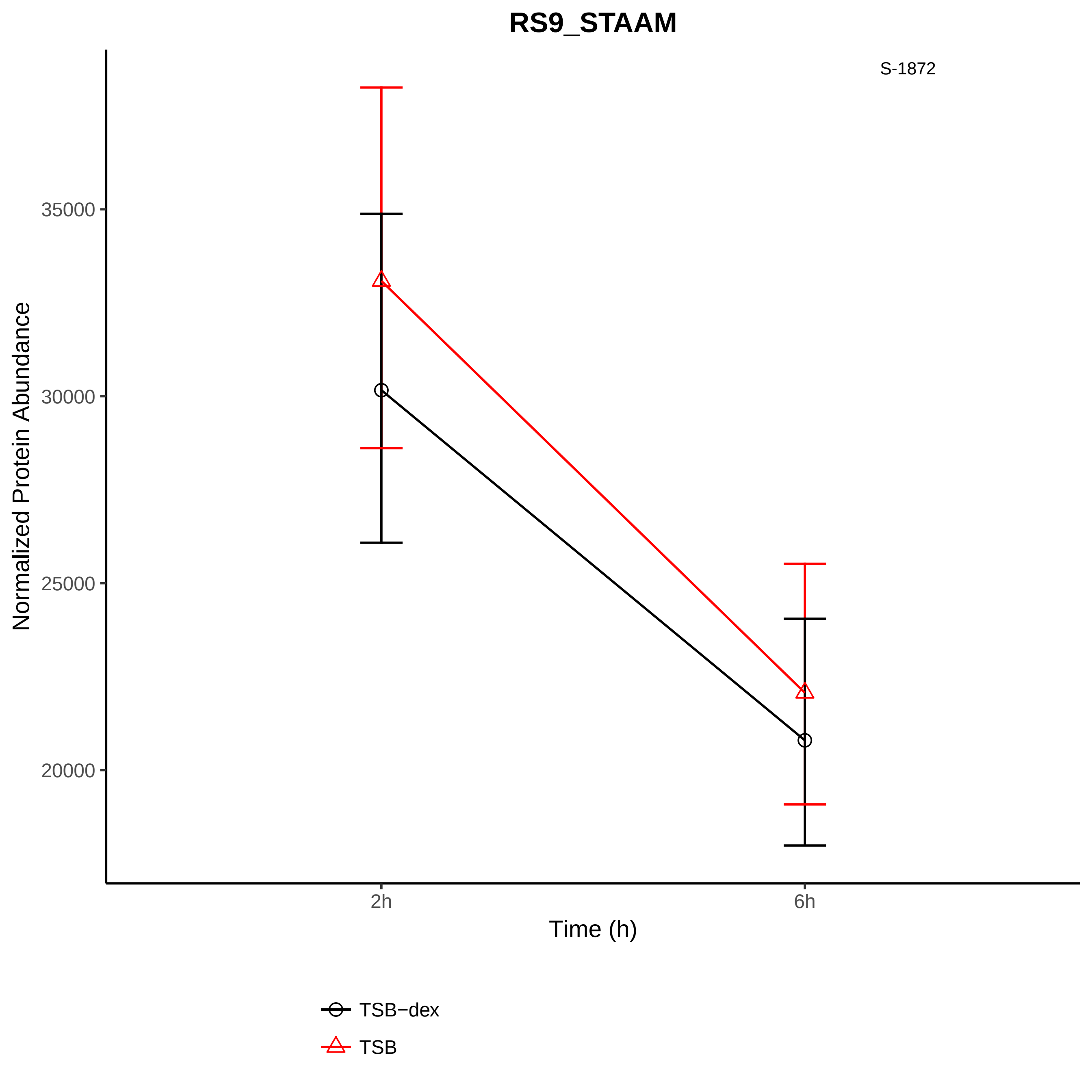

RS9_STAAM
S-1872
35000
30000
25000
20000
Normalized Protein Abundance
2h
6h
Time (h)
TSB−dex
TSB

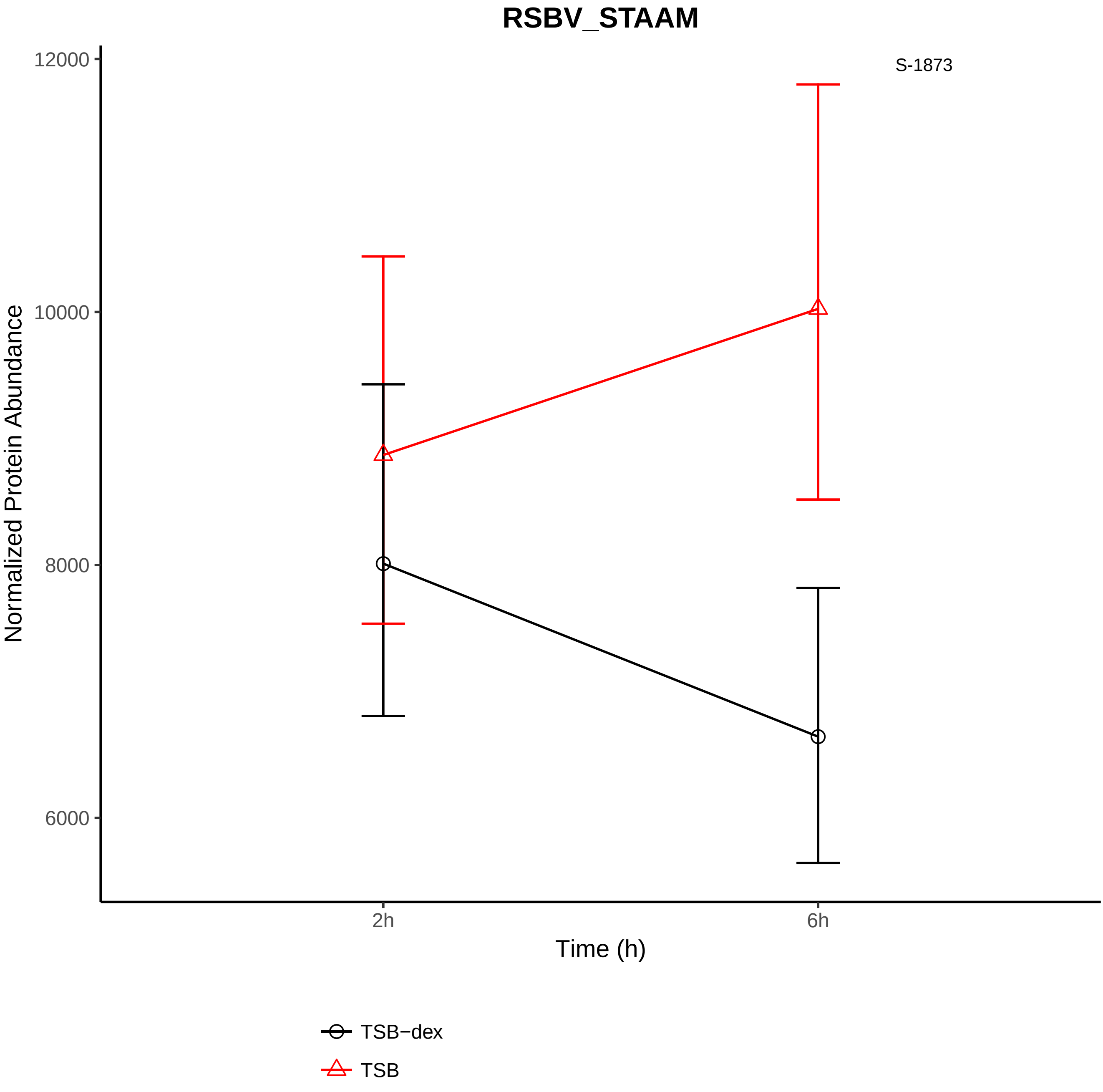

RSBV_STAAM
S-1873
12000
10000
8000
6000
Normalized Protein Abundance
2h
6h
Time (h)
TSB−dex
TSB

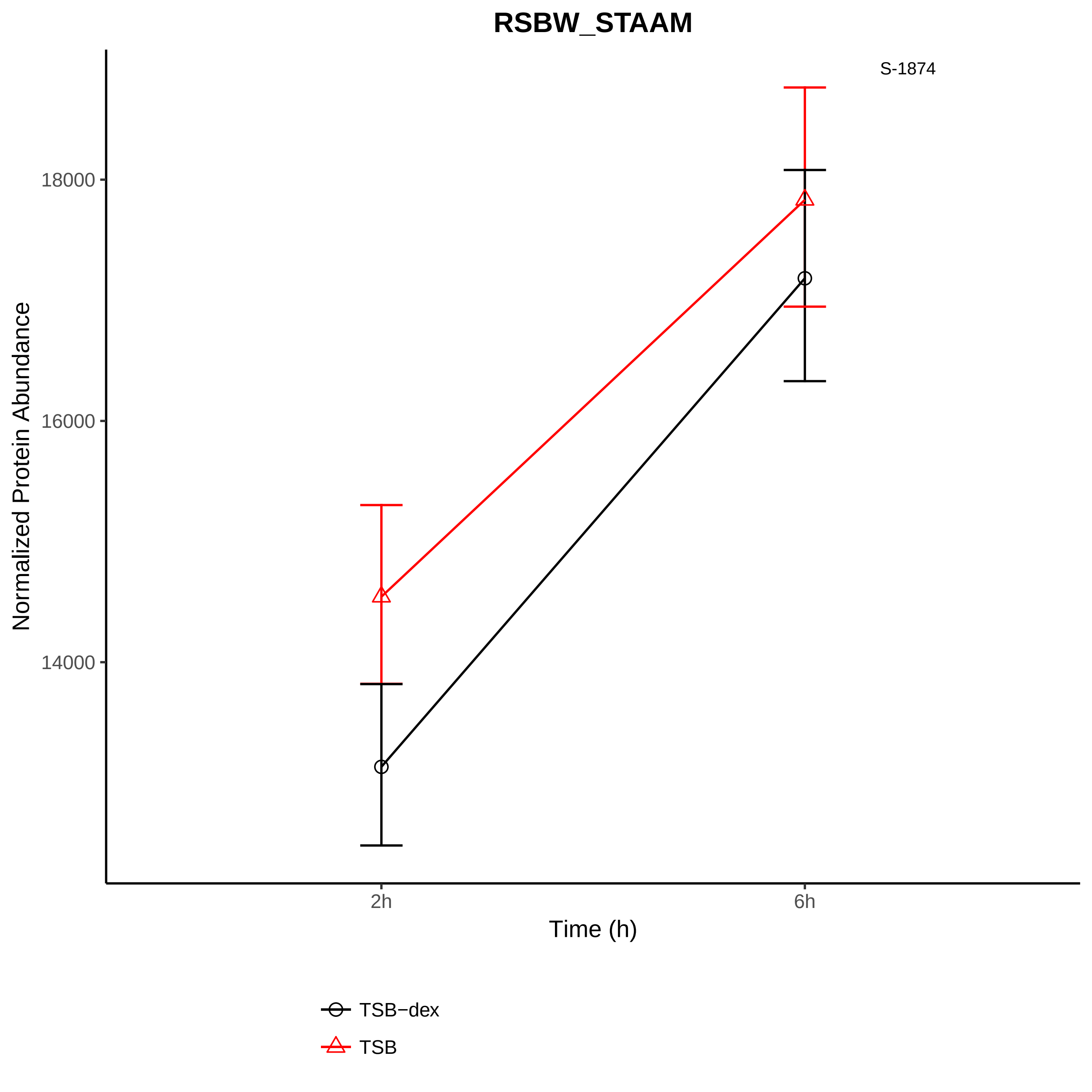

RSBW_STAAM
S-1874
Normalized Protein Abundance
18000
16000
14000
2h
6h
Time (h)
TSB−dex
TSB

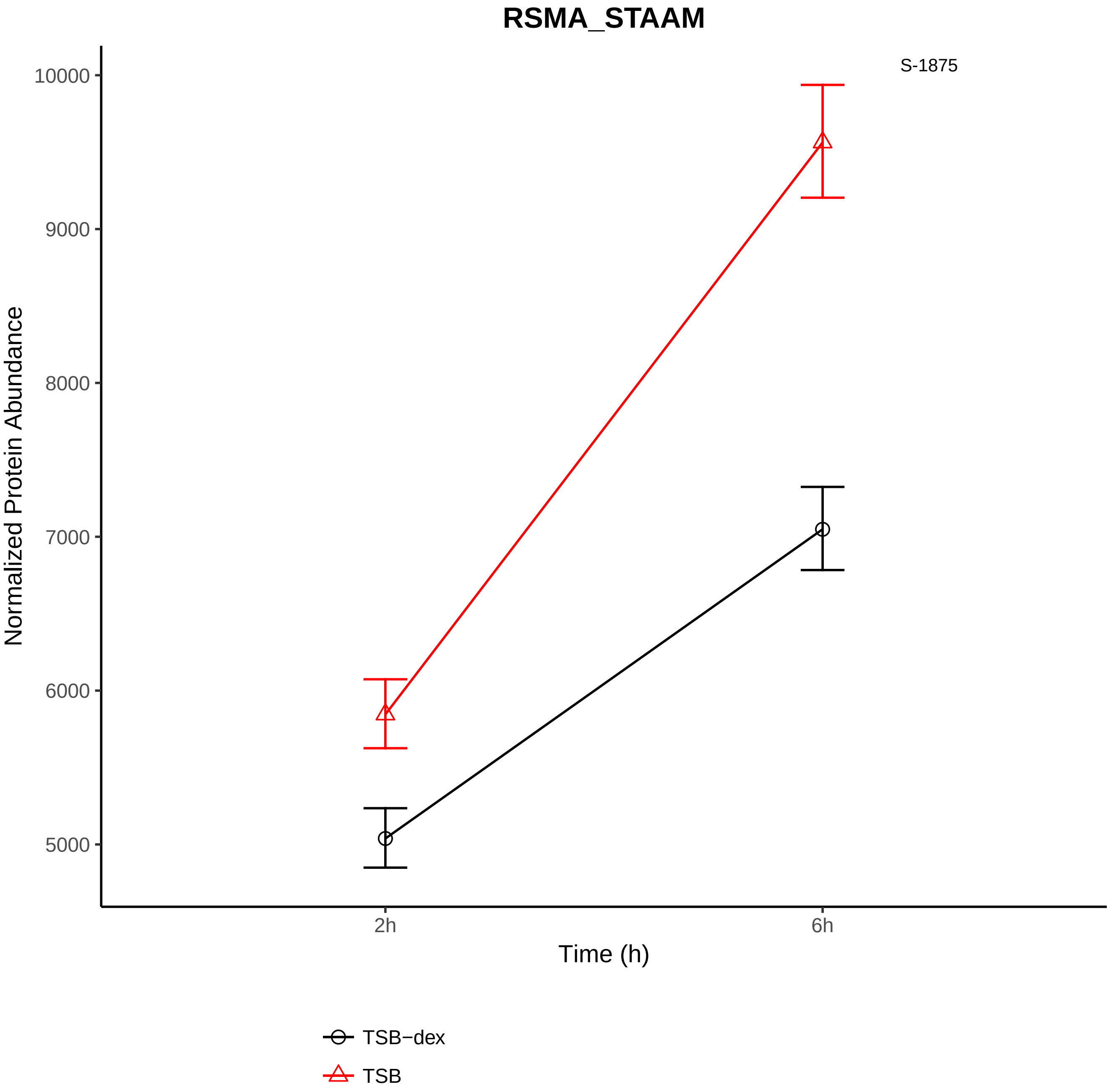
RSMA_STAAM
S-1875
Normalized Protein Abundance
10000
9000
8000
7000
6000
5000
2h
6h
Time (h)
TSB−dex
TSB

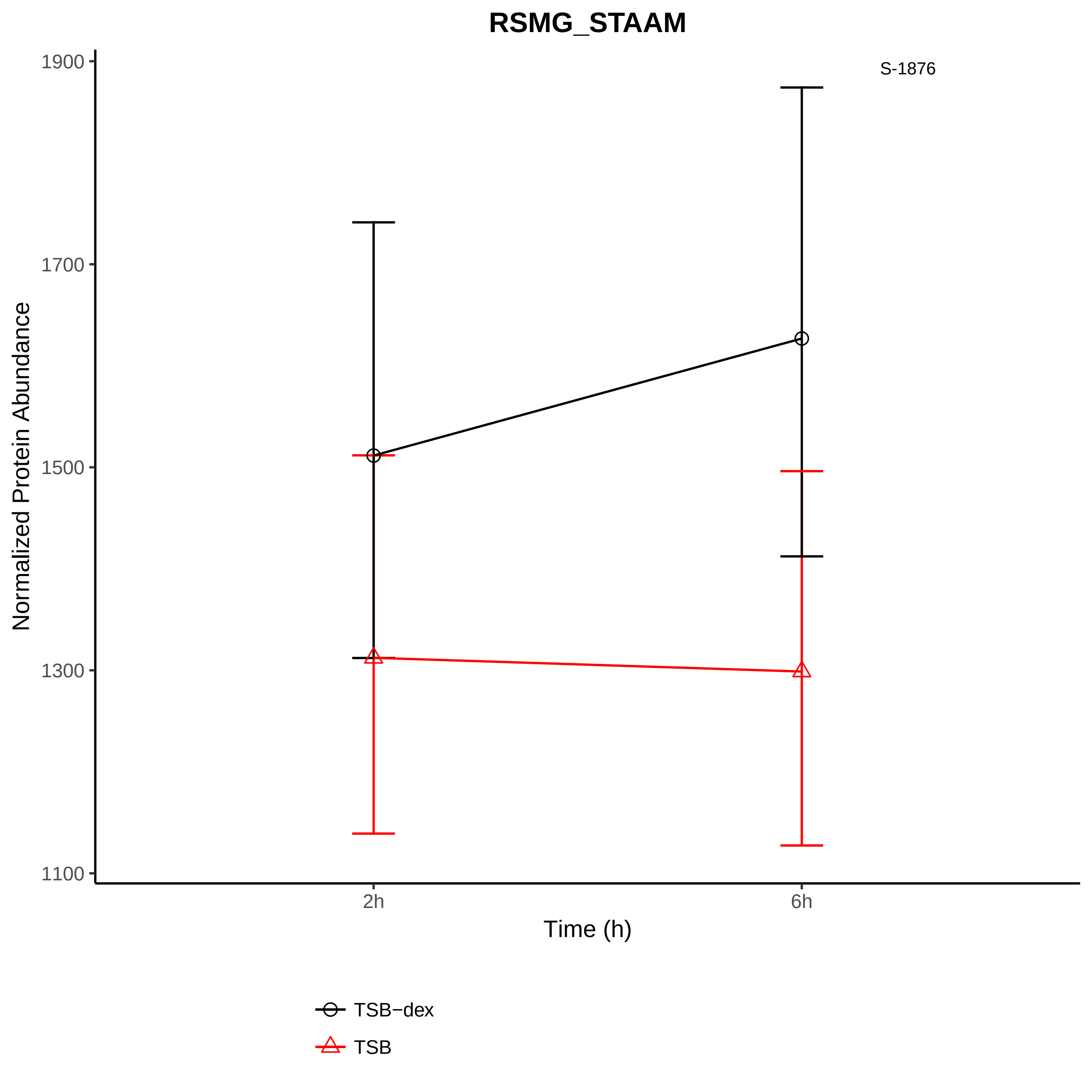

RSMG_STAAM
S-1876
Normalized Protein Abundance
1900
1700
1500
1300
1100
2h
6h
Time (h)
TSB−dex
TSB

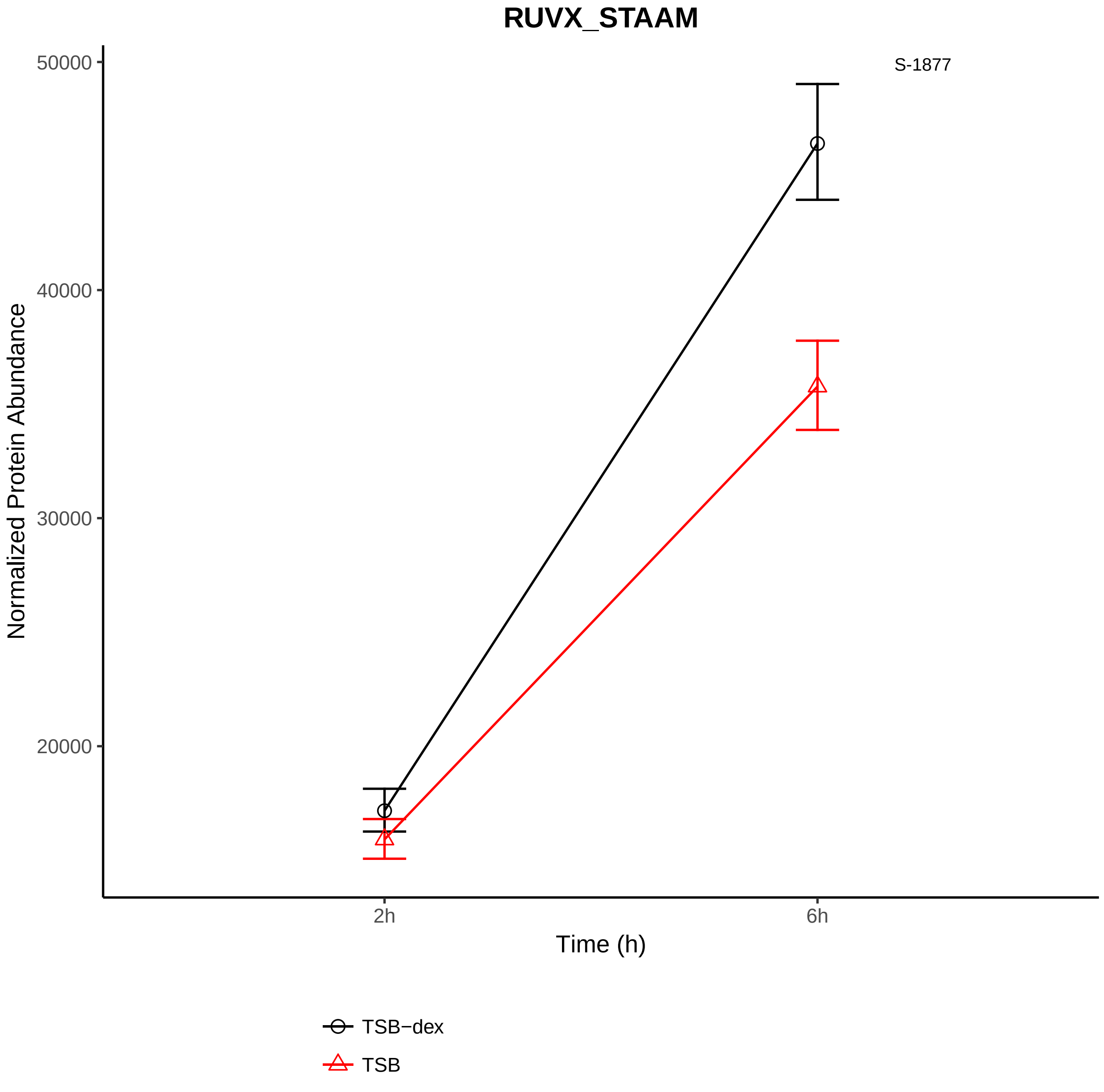
RUVX_STAAM
S-1877
Normalized Protein Abundance
50000
40000
30000
20000
2h
6h
Time (h)
TSB−dex
TSB

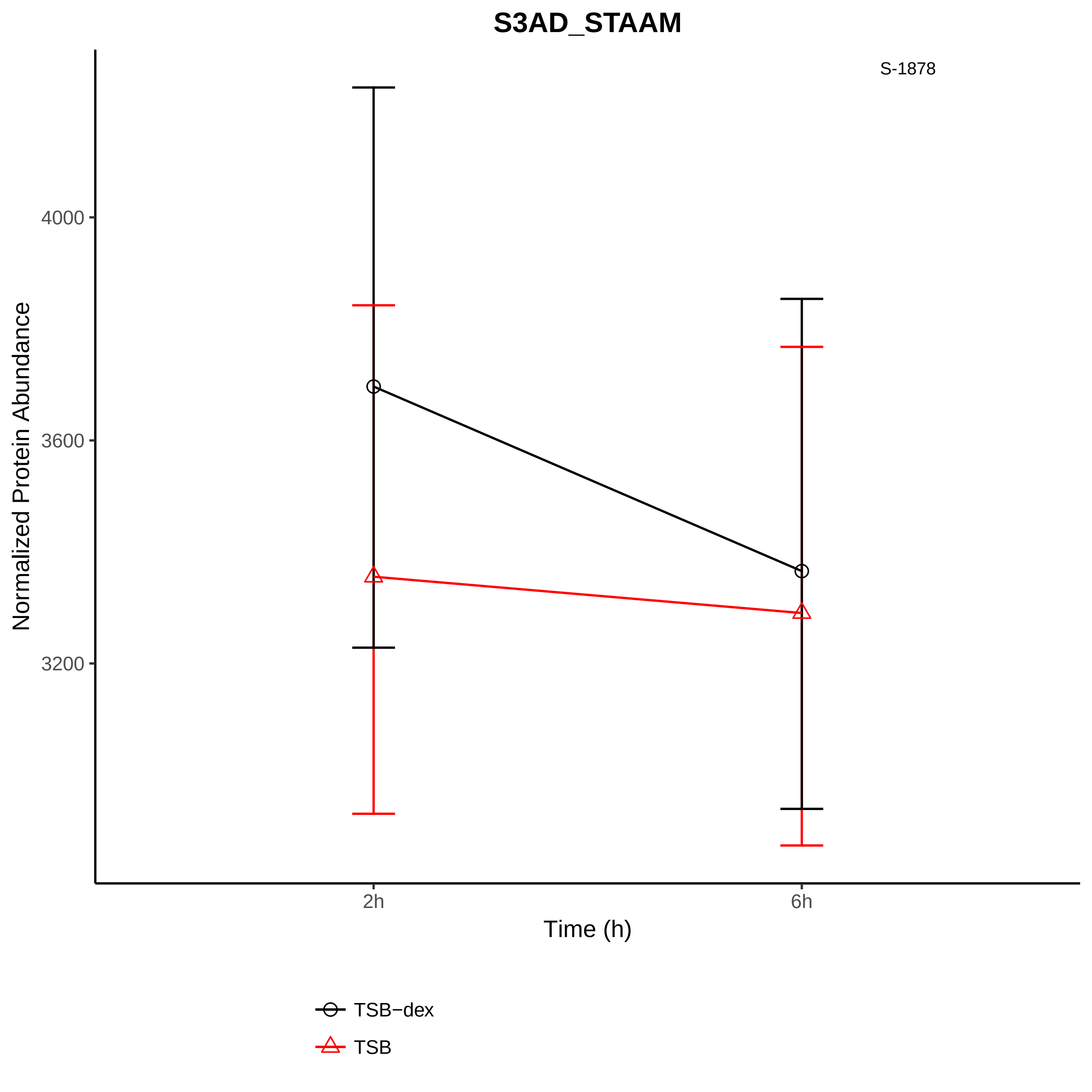

S3AD_STAAM
S-1878
Normalized Protein Abundance
4000
3600
3200
2h
6h
Time (h)
TSB−dex
TSB

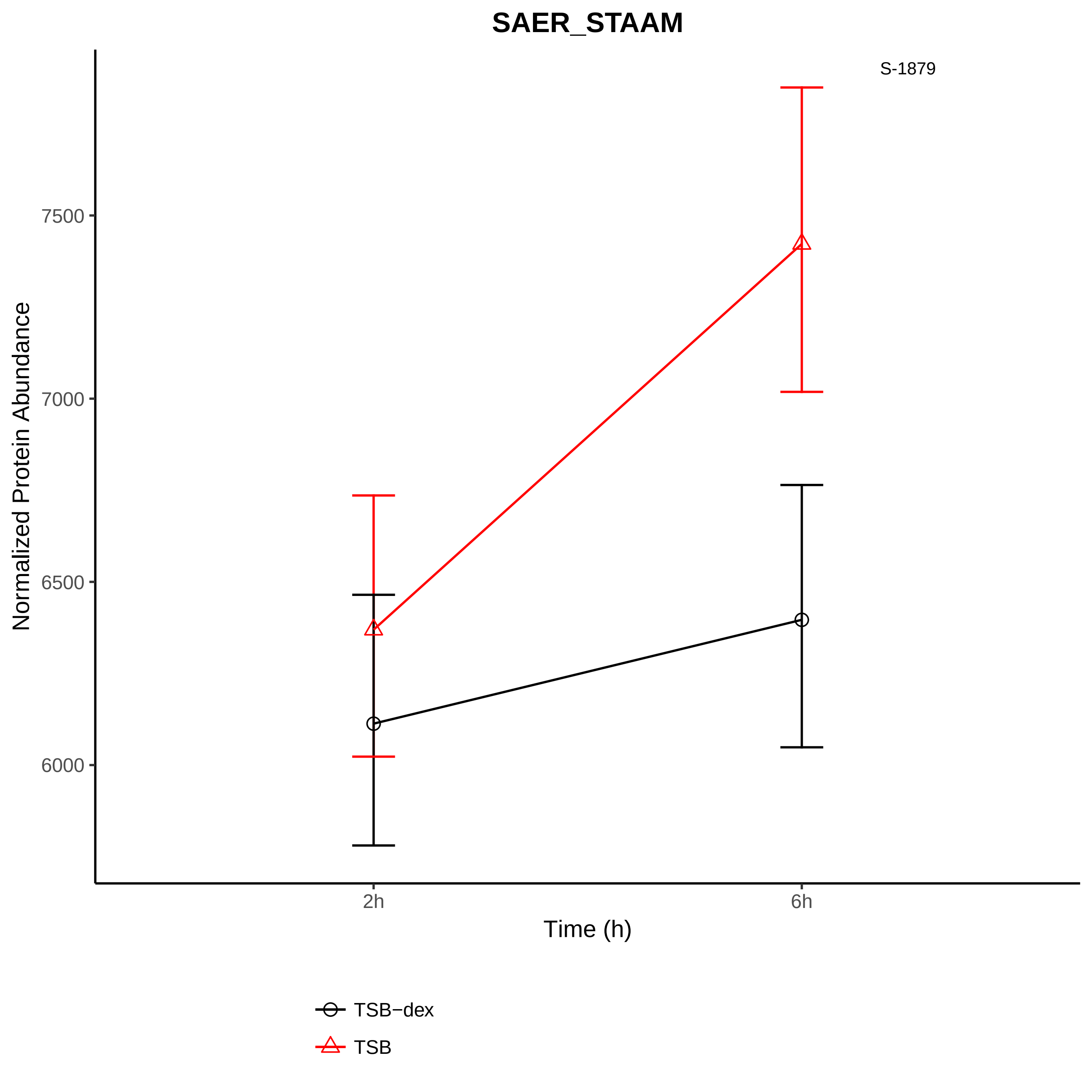

SAER_STAAM
S-1879
Normalized Protein Abundance
7500
7000
6500
6000
2h
6h
Time (h)
TSB−dex
TSB

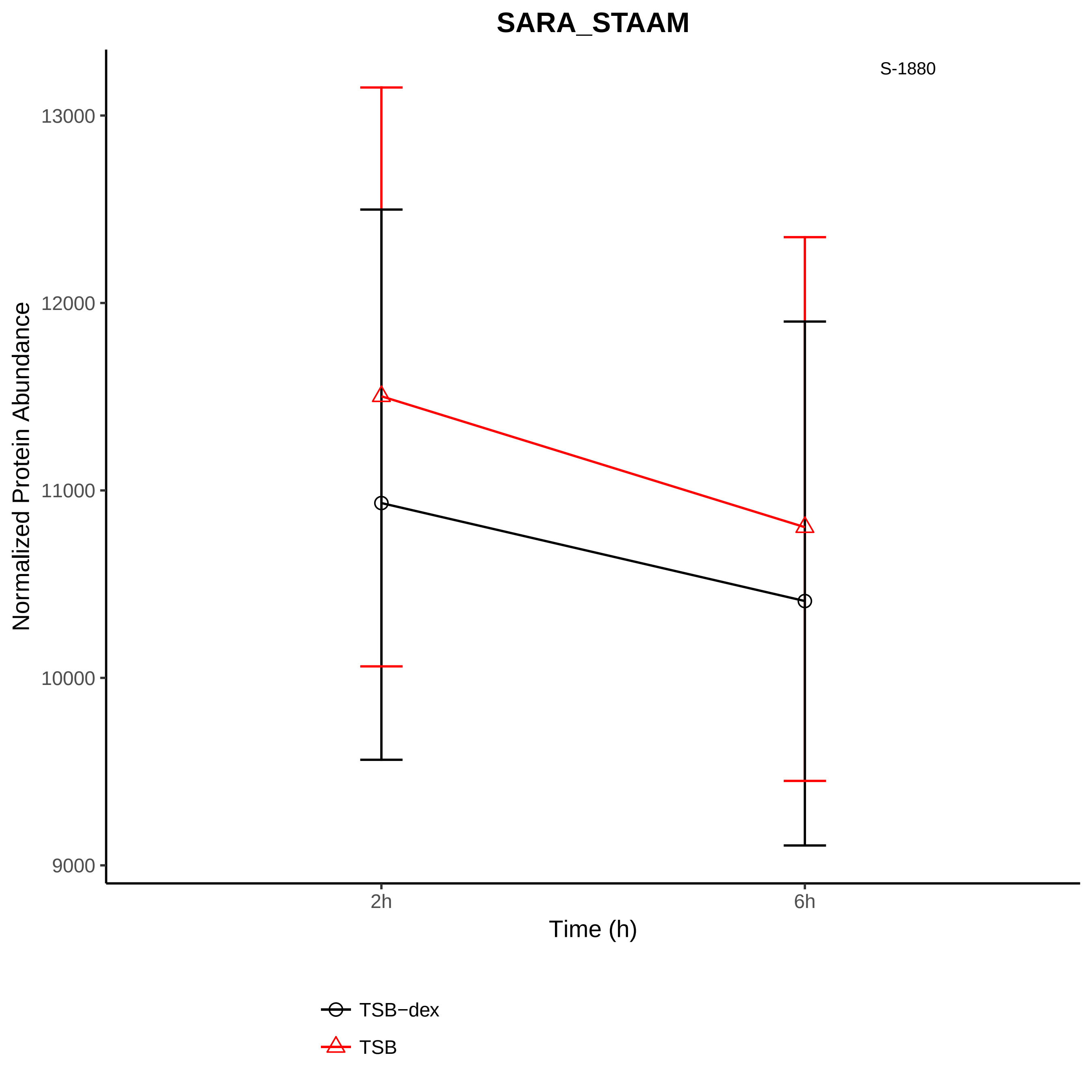

SARA_STAAM
S-1880
Normalized Protein Abundance
13000
12000
11000
10000
9000
2h
6h
Time (h)
TSB−dex
TSB

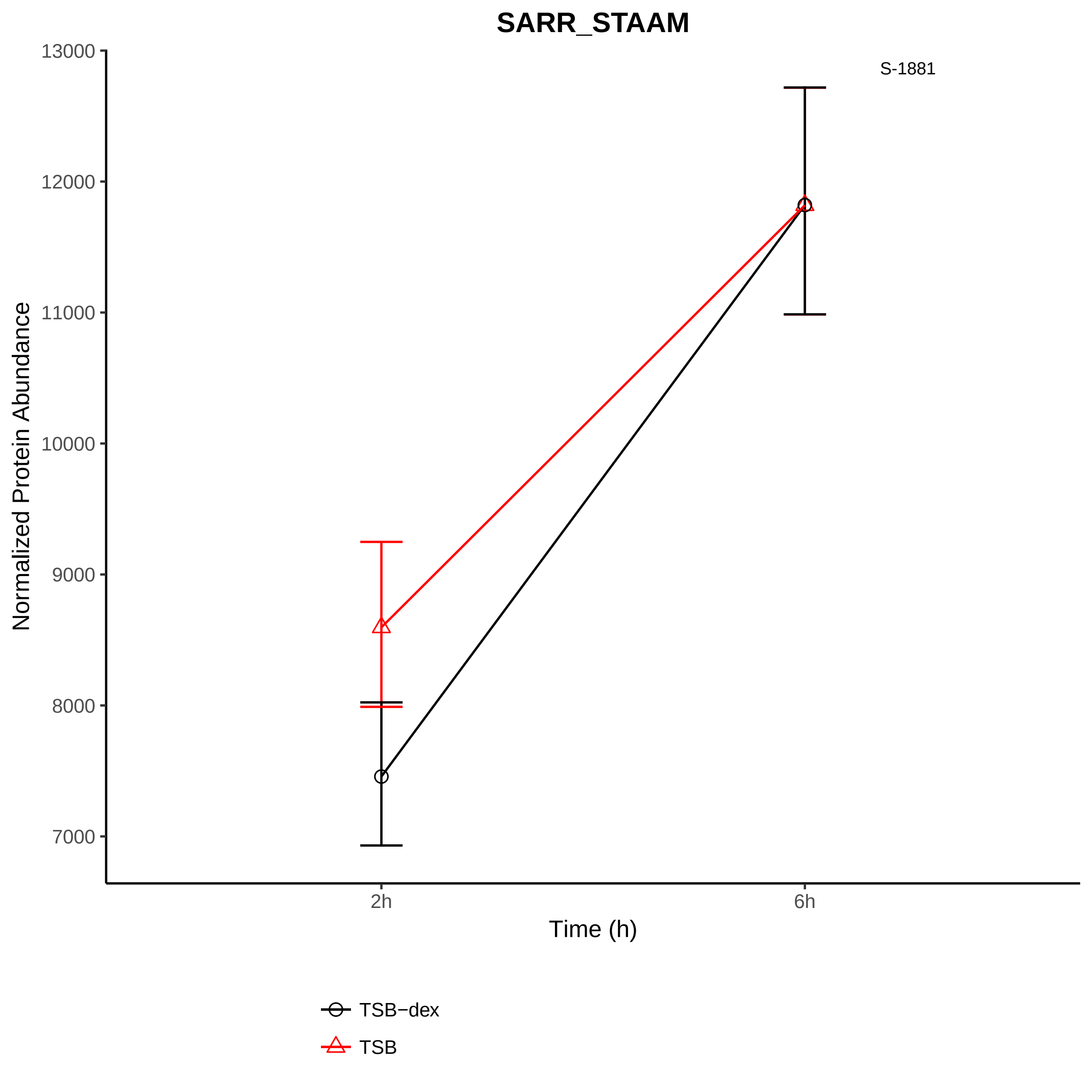

SARR_STAAM
S-1881
13000
12000
11000
10000
9000
8000
7000
Normalized Protein Abundance
2h
6h
Time (h)
TSB−dex
TSB

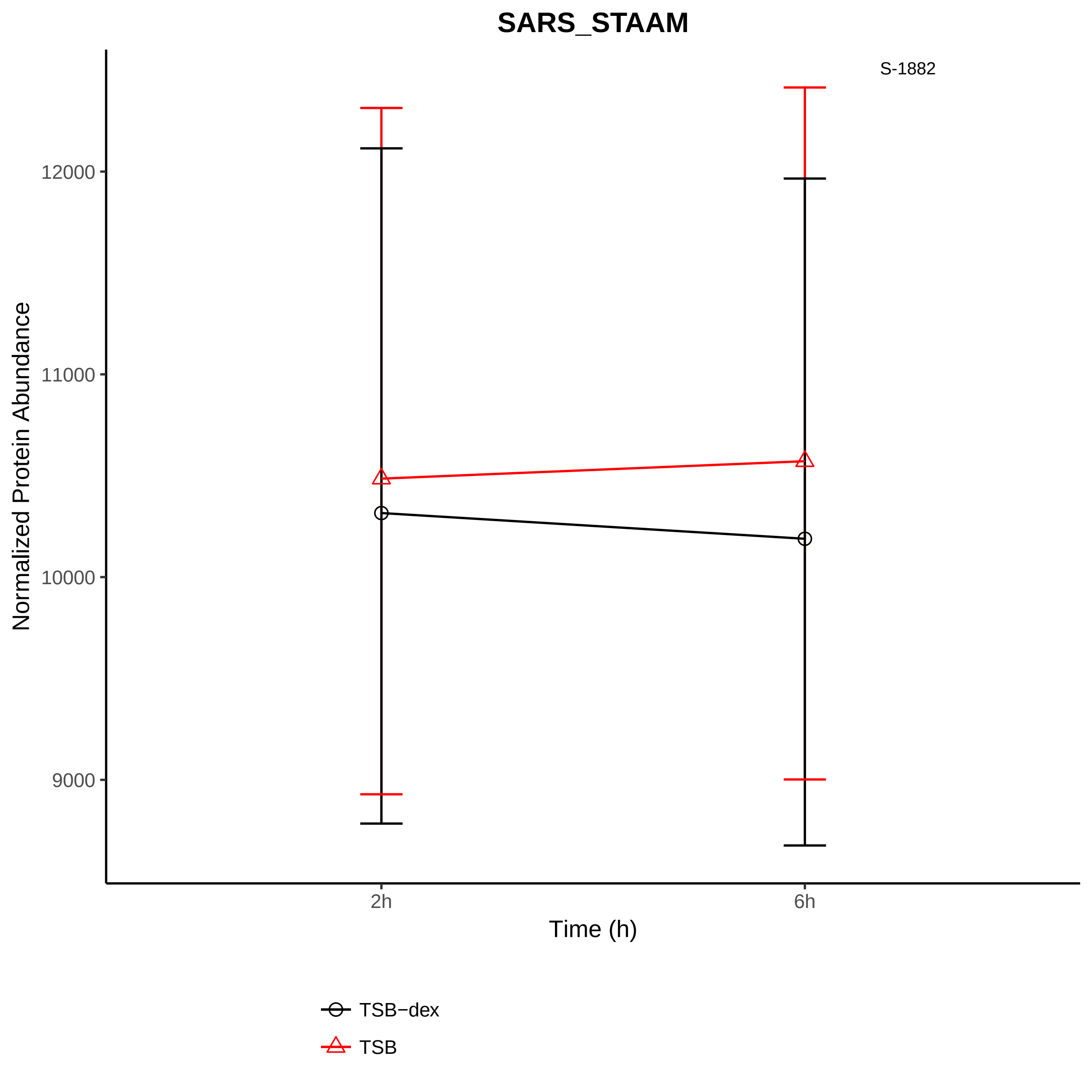

SARS_STAAM
S-1882
12000
11000
10000
9000
Normalized Protein Abundance
2h
6h
Time (h)
TSB−dex
TSB

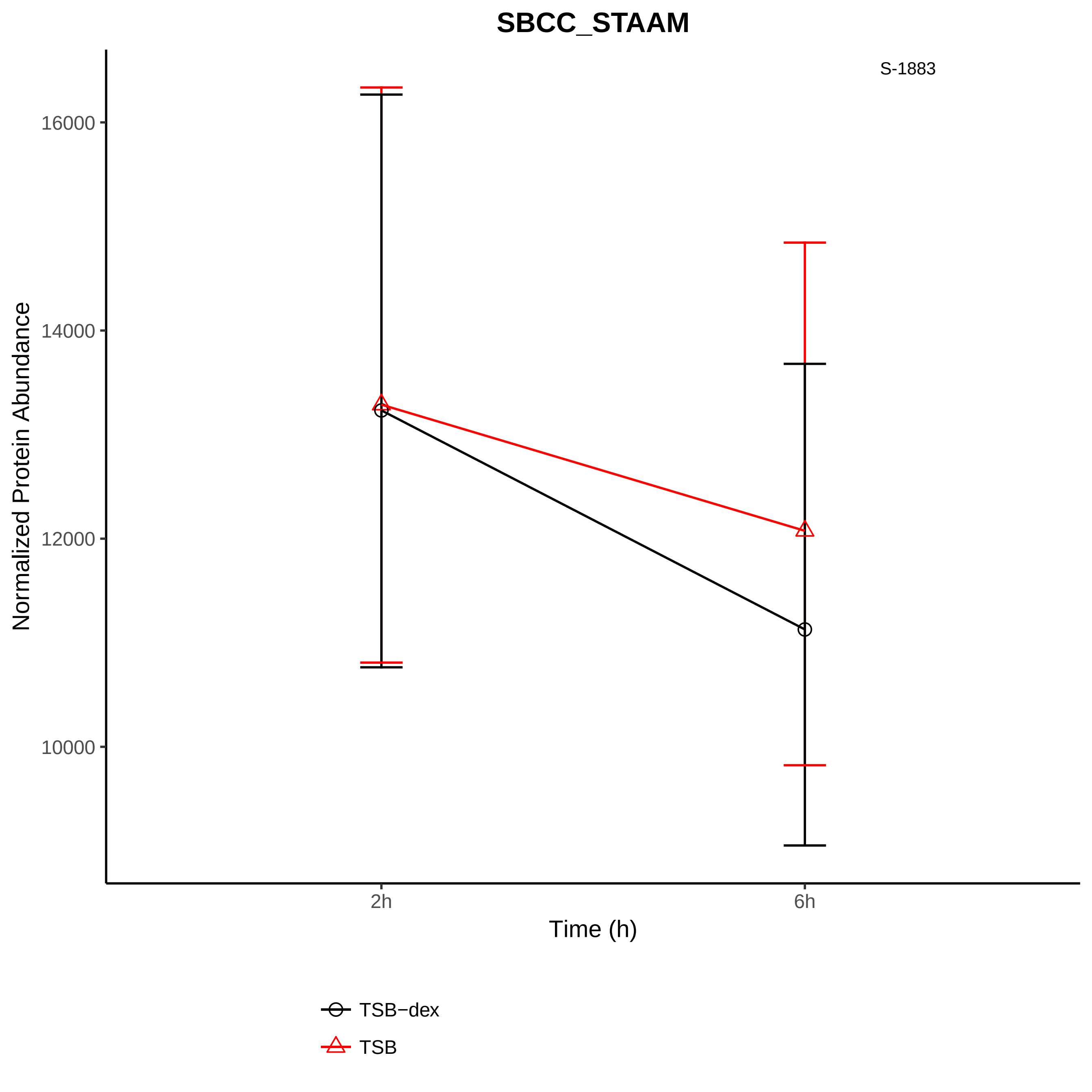

SBCC_STAAM
S-1883
Normalized Protein Abundance
16000
14000
12000
10000
2h
6h
Time (h)
TSB−dex
TSB

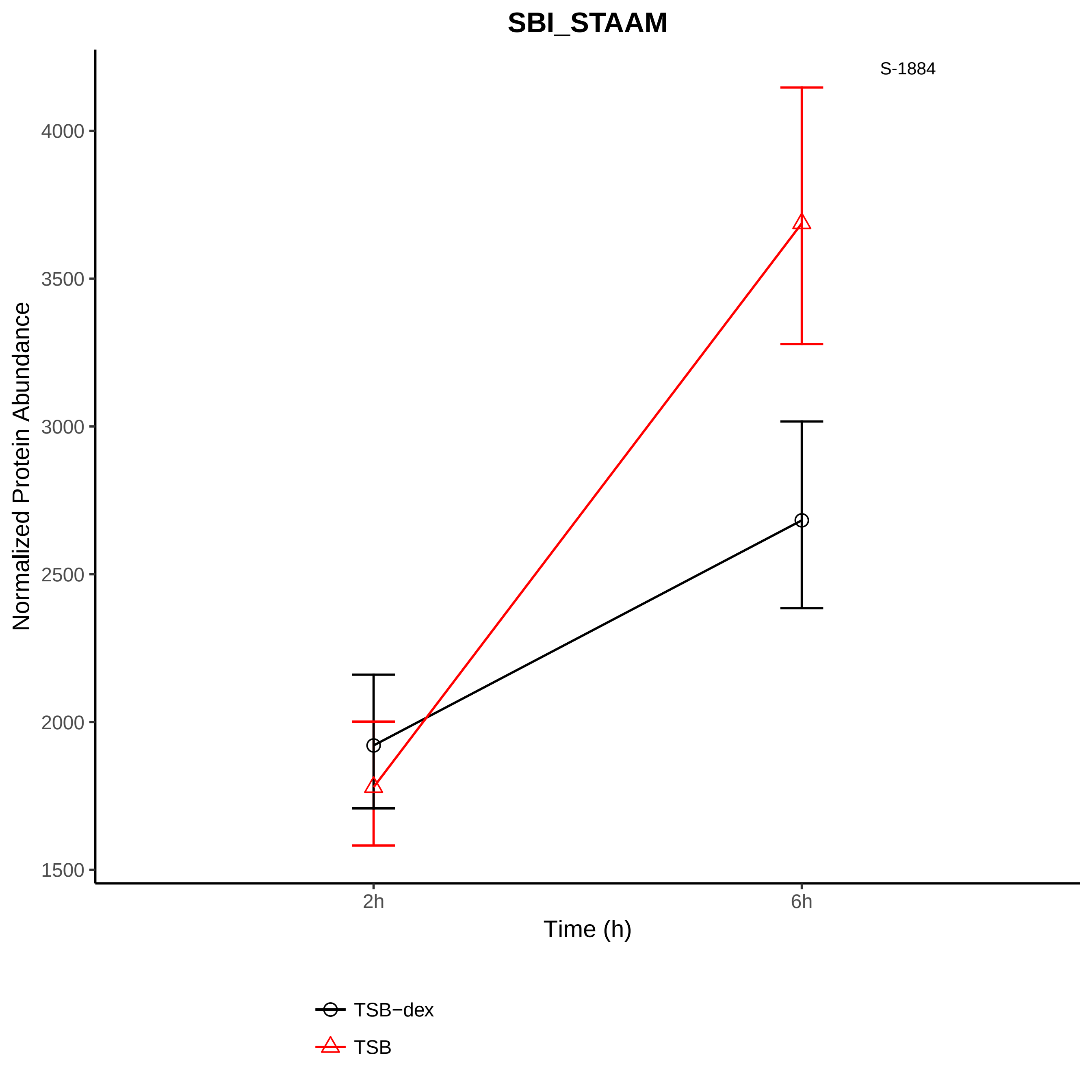

SBI_STAAM
S-1884
4000
3500
3000
2500
2000
1500
Normalized Protein Abundance
2h
6h
Time (h)
TSB−dex
TSB

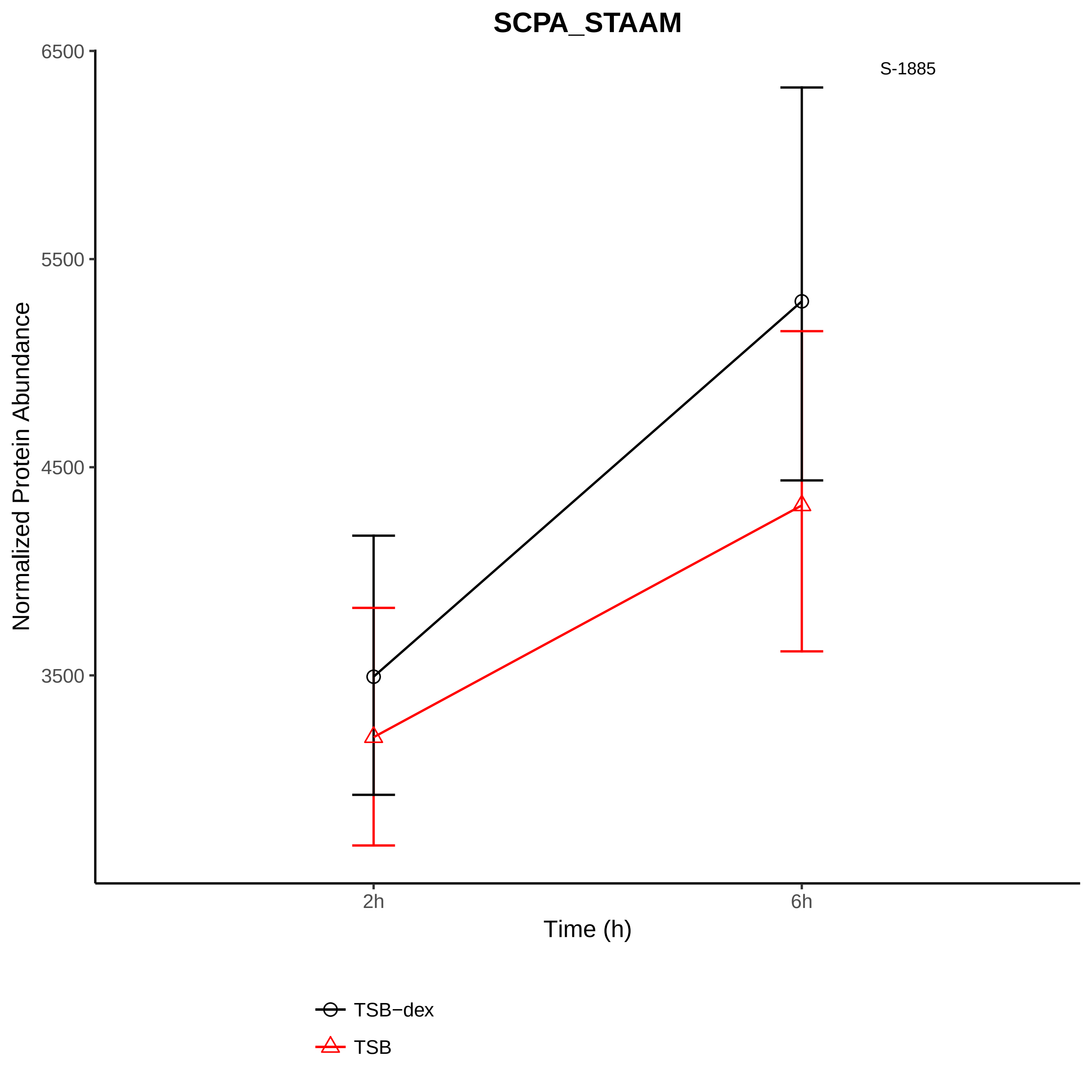

SCPA_STAAM
S-1885
6500
5500
4500
3500
Normalized Protein Abundance
2h
6h
Time (h)
TSB−dex
TSB

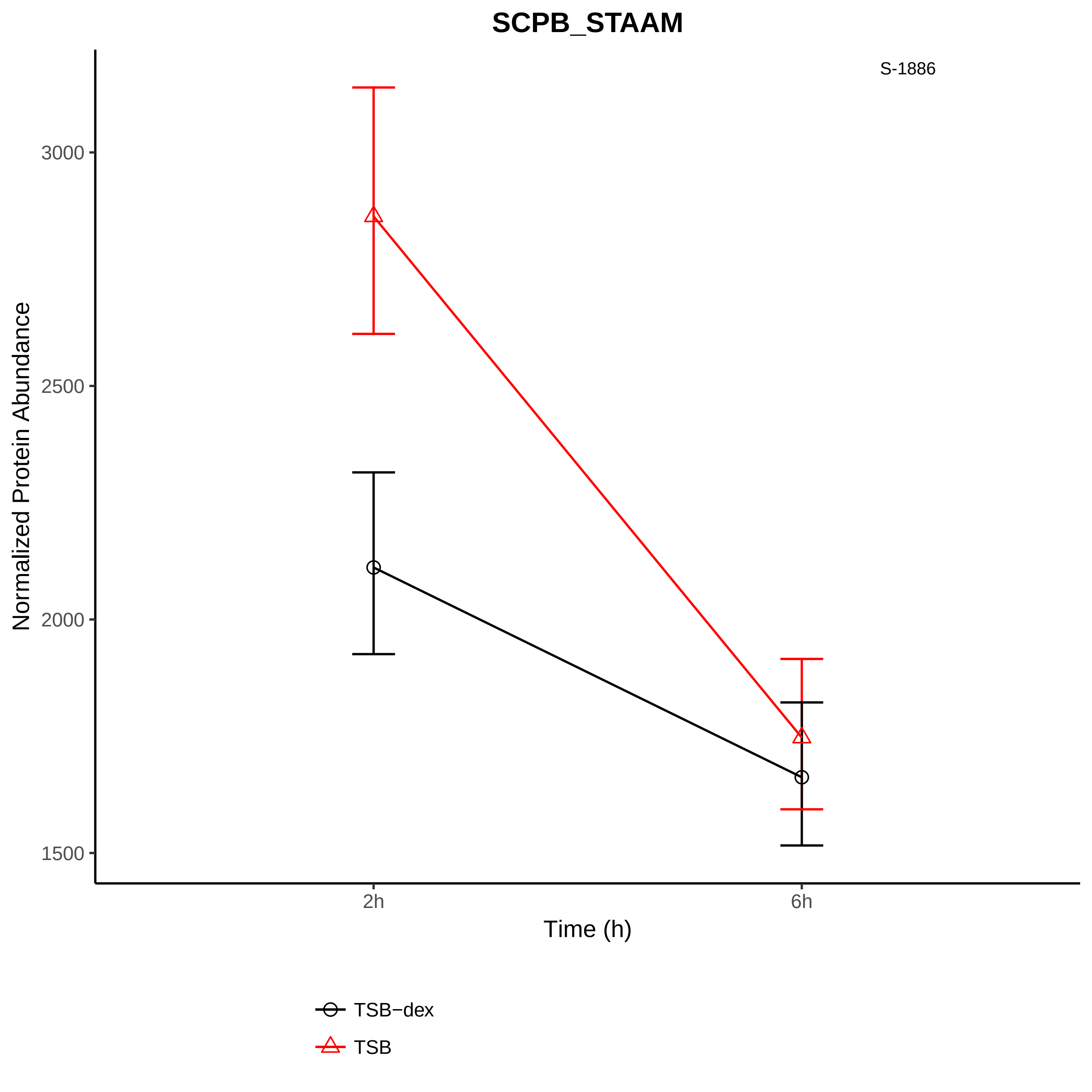

SCPB_STAAM
S-1886
Normalized Protein Abundance
3000
2500
2000
1500
2h
6h
Time (h)
TSB−dex
TSB

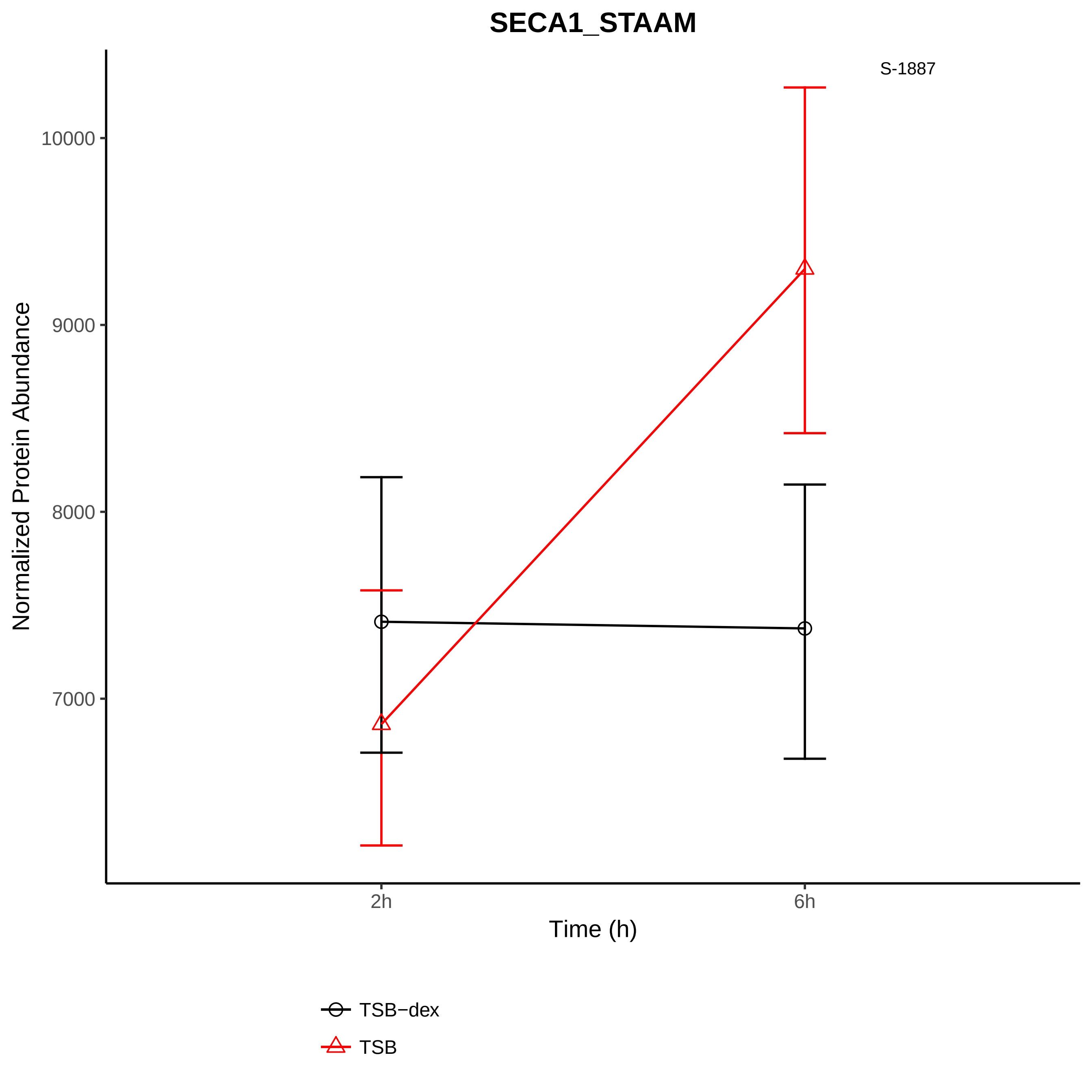

SECA1_STAAM
S-1887
Normalized Protein Abundance
10000
9000
8000
7000
2h
6h
Time (h)
TSB−dex
TSB

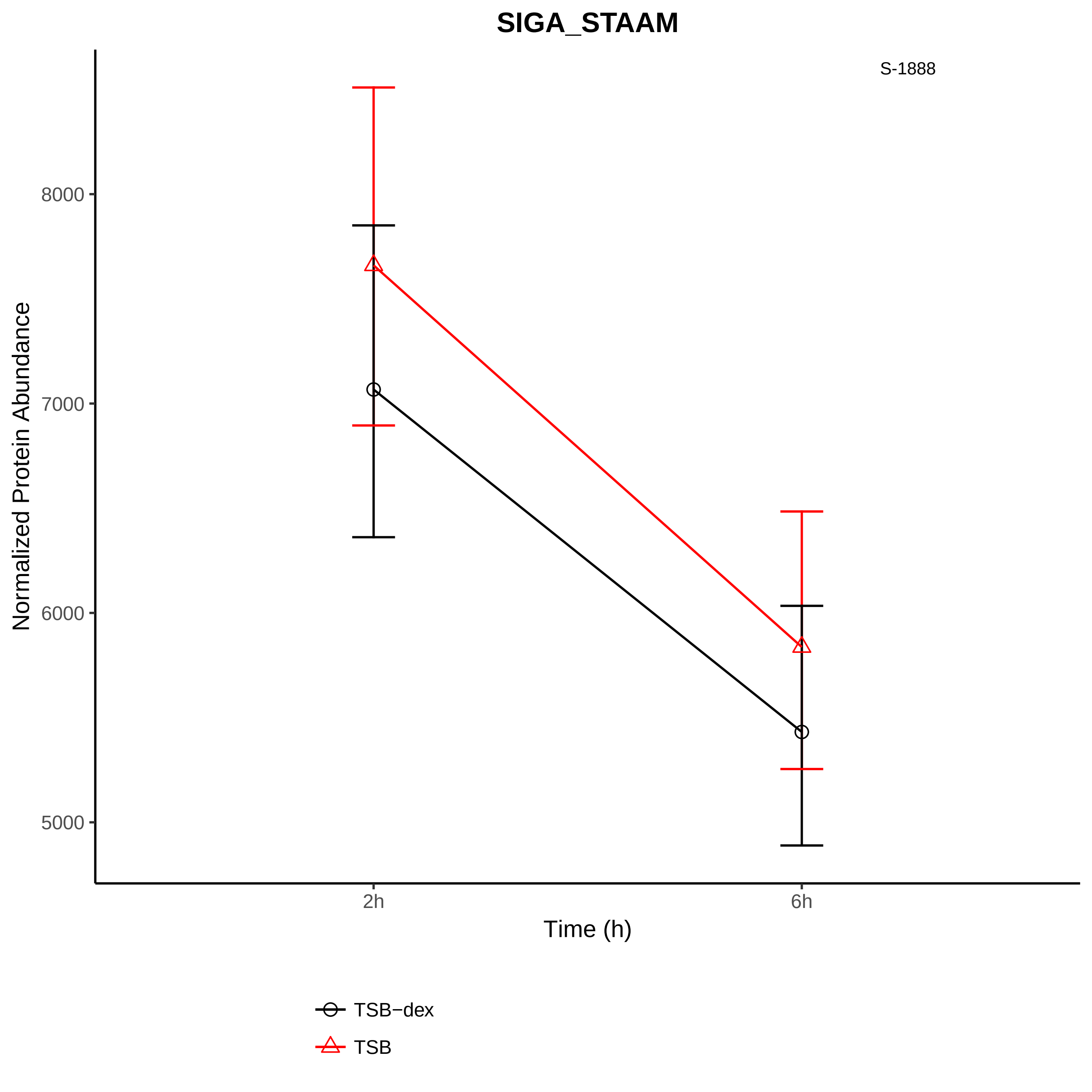

SIGA_STAAM
S-1888
Normalized Protein Abundance
8000
7000
6000
5000
2h
6h
Time (h)
TSB−dex
TSB

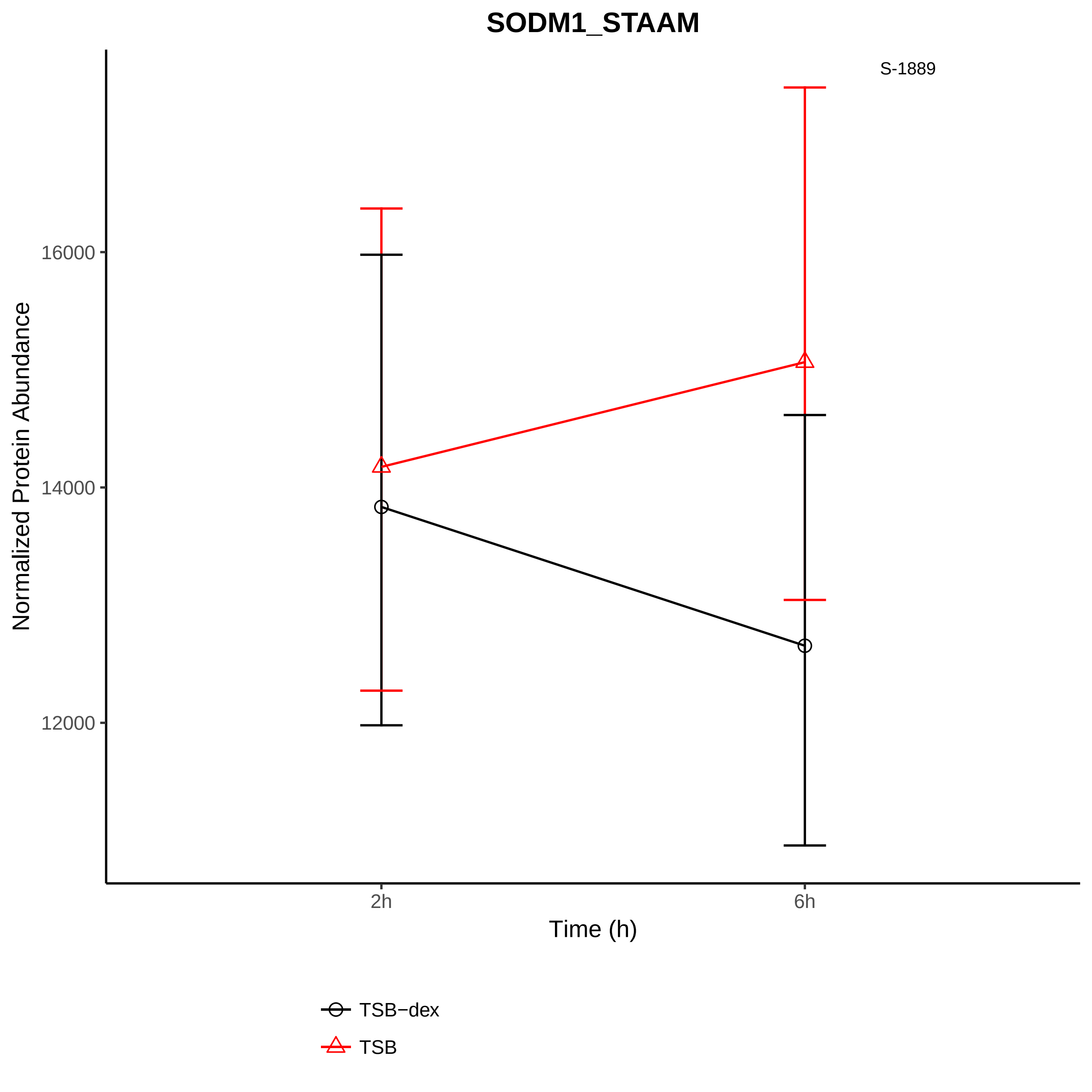

SODM1_STAAM
S-1889
Normalized Protein Abundance
16000
14000
12000
2h
6h
Time (h)
TSB−dex
TSB

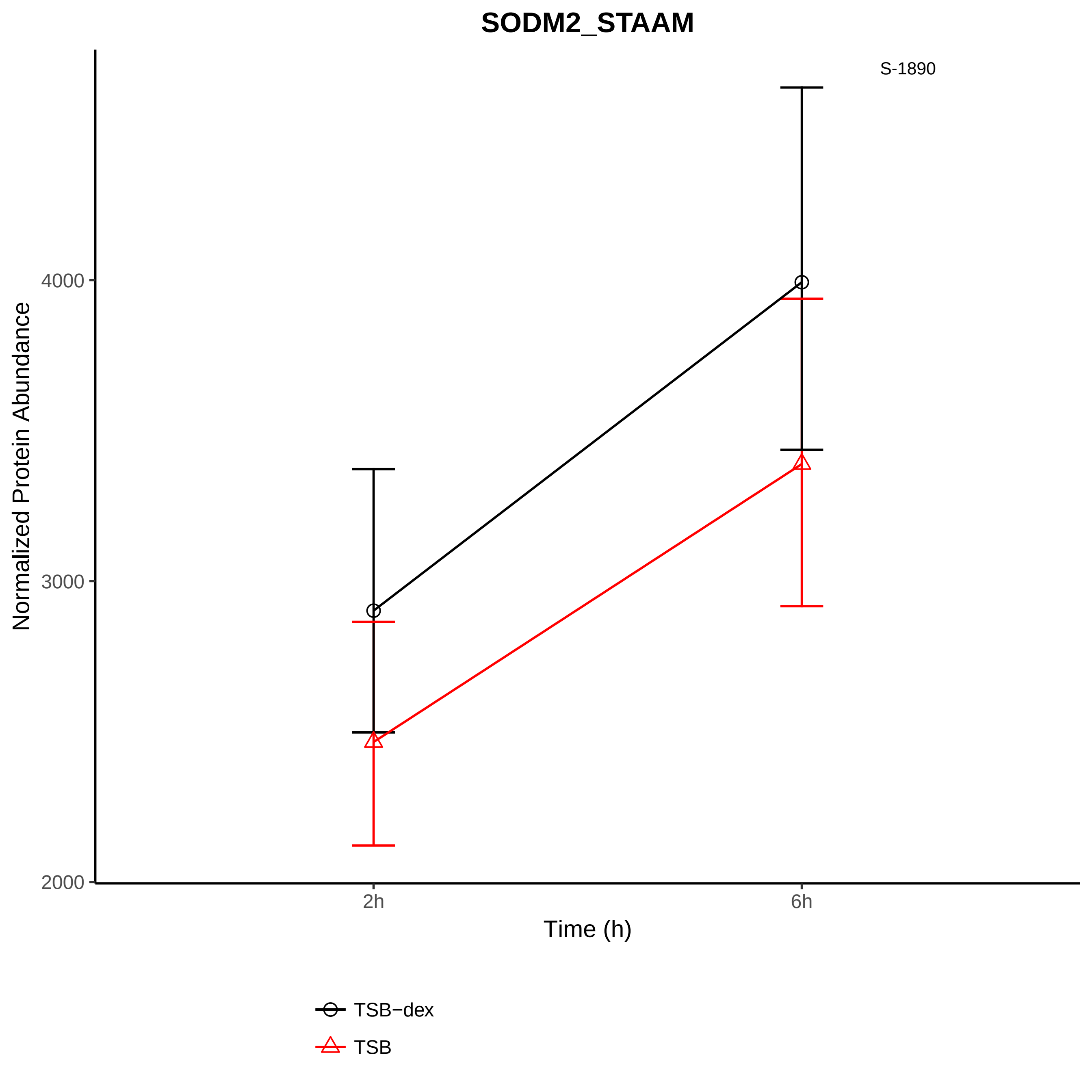

SODM2_STAAM
S-1890
Normalized Protein Abundance
4000
3000
2000
2h
6h
Time (h)
TSB−dex
TSB

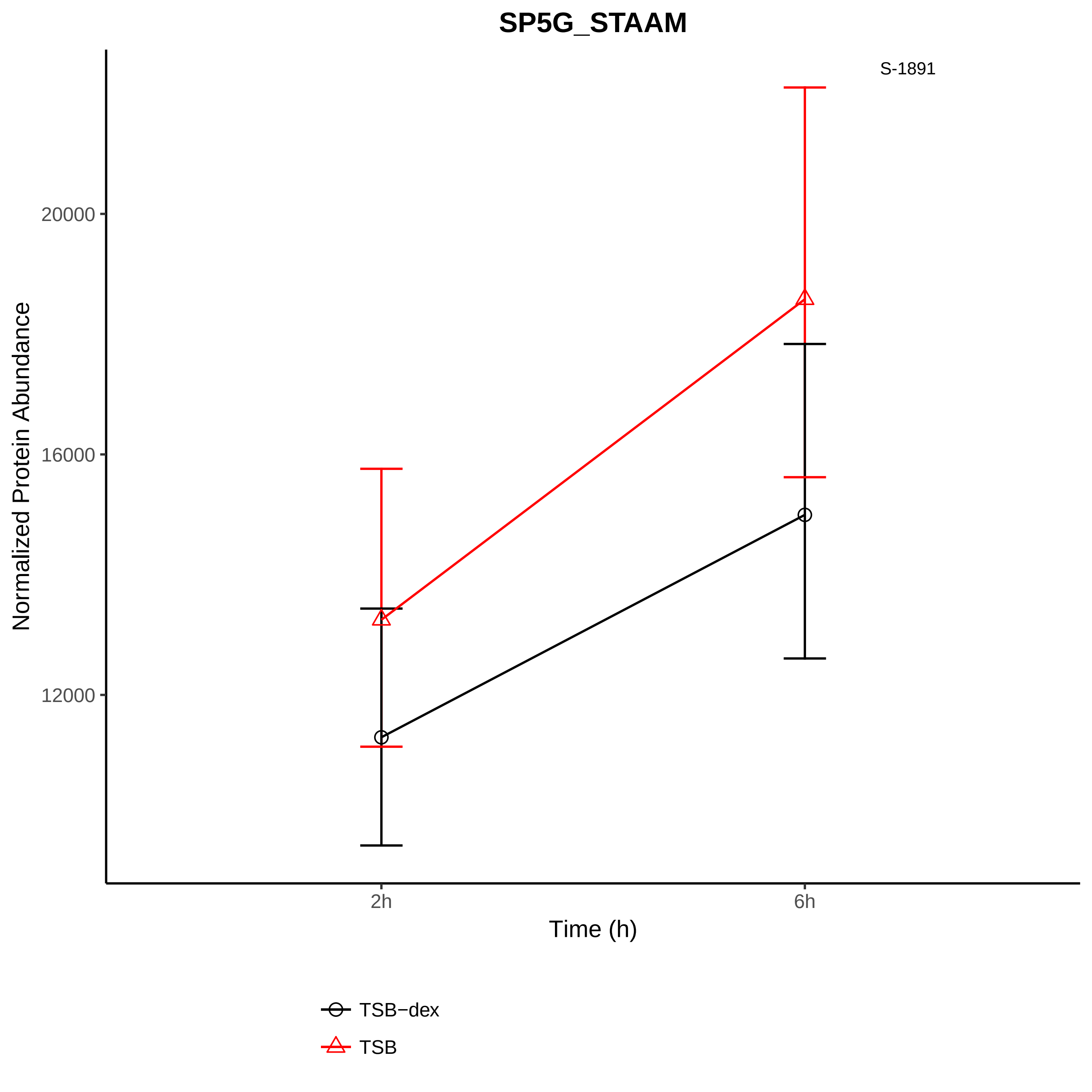

SP5G_STAAM
S-1891
Normalized Protein Abundance
20000
16000
12000
2h
6h
Time (h)
TSB−dex
TSB

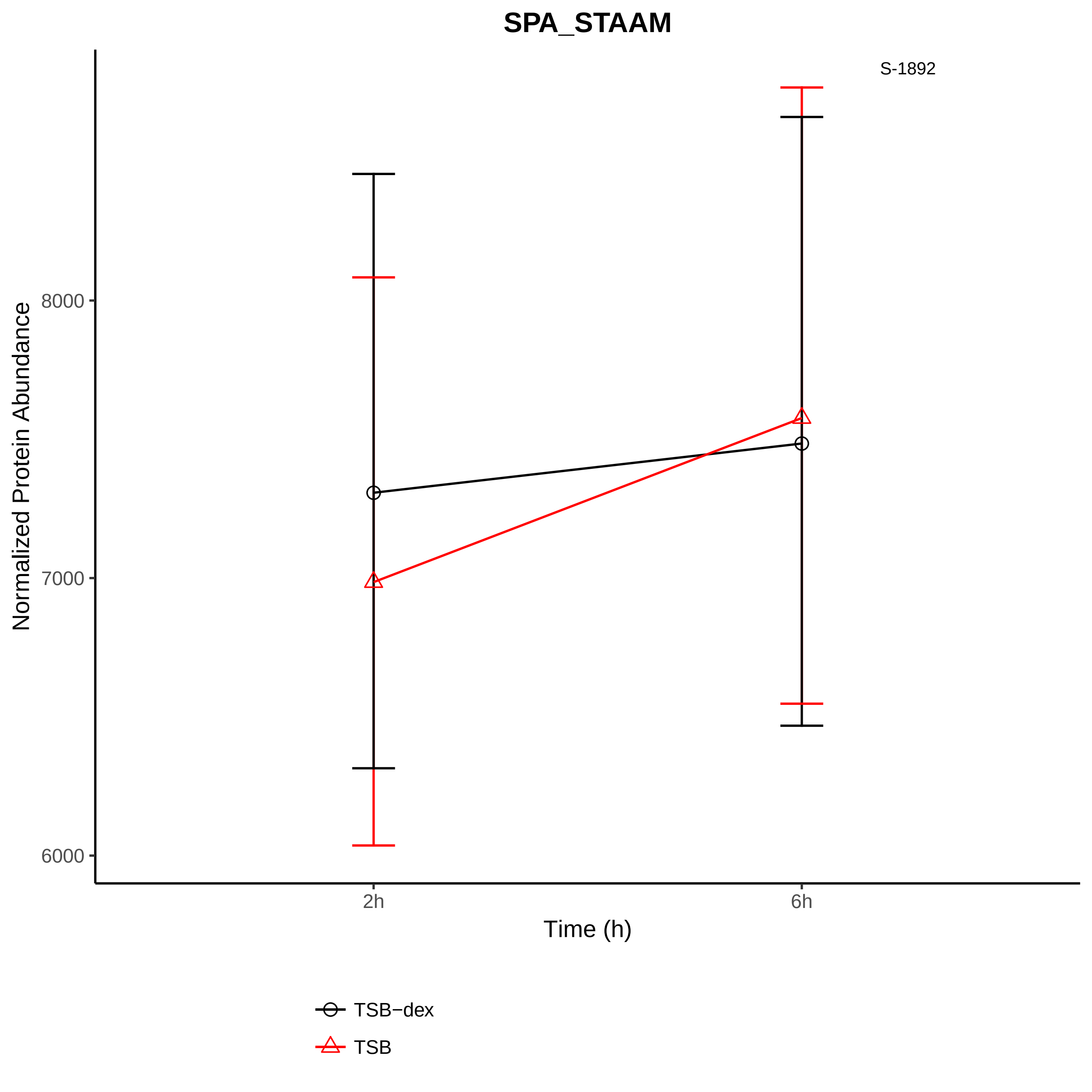

SPA_STAAM
S-1892
Normalized Protein Abundance
8000
7000
6000
2h
6h
Time (h)
TSB−dex
TSB

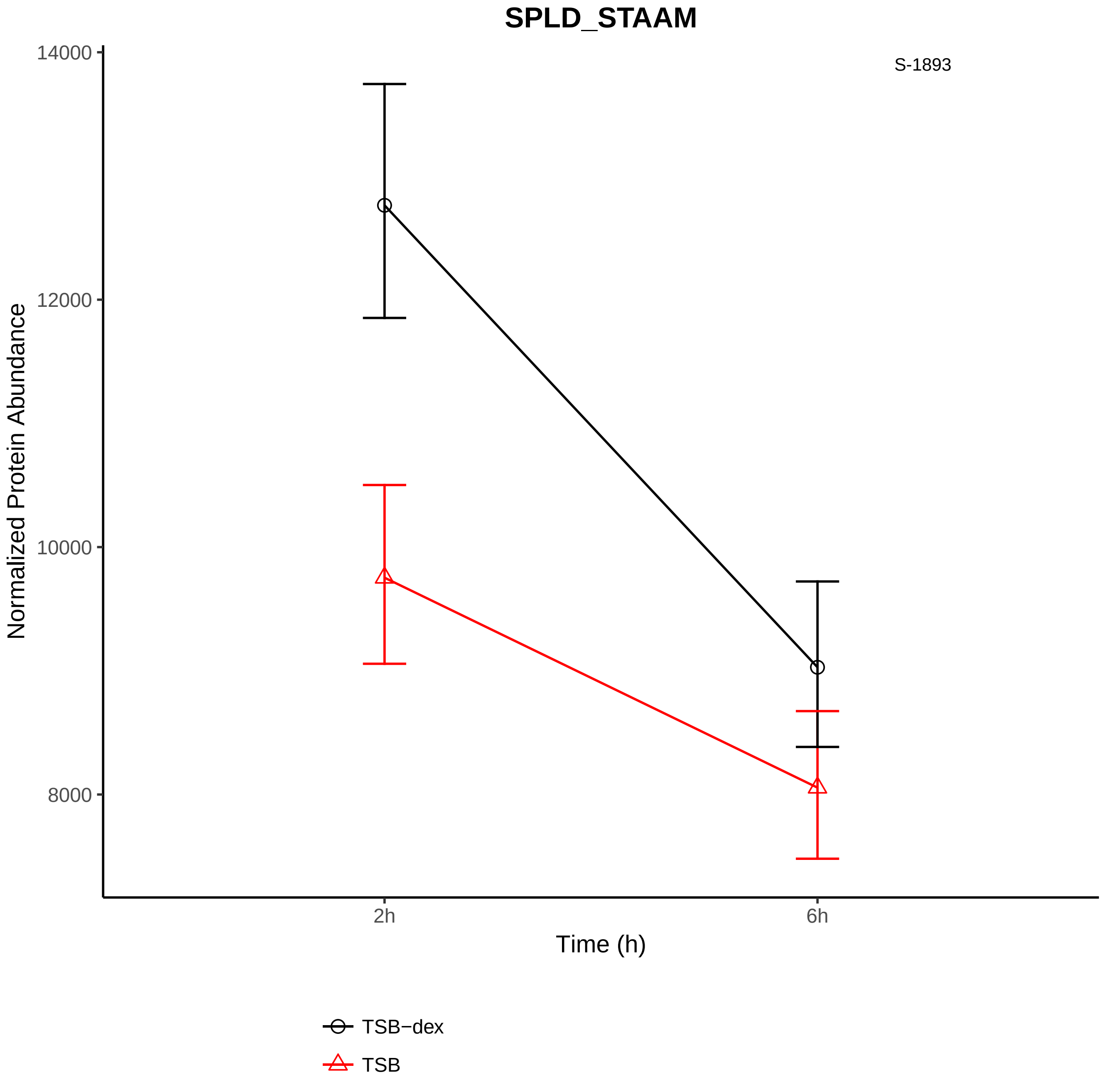

SPLD_STAAM
S-1893
14000
12000
10000
8000
Normalized Protein Abundance
2h
6h
Time (h)
TSB−dex
TSB

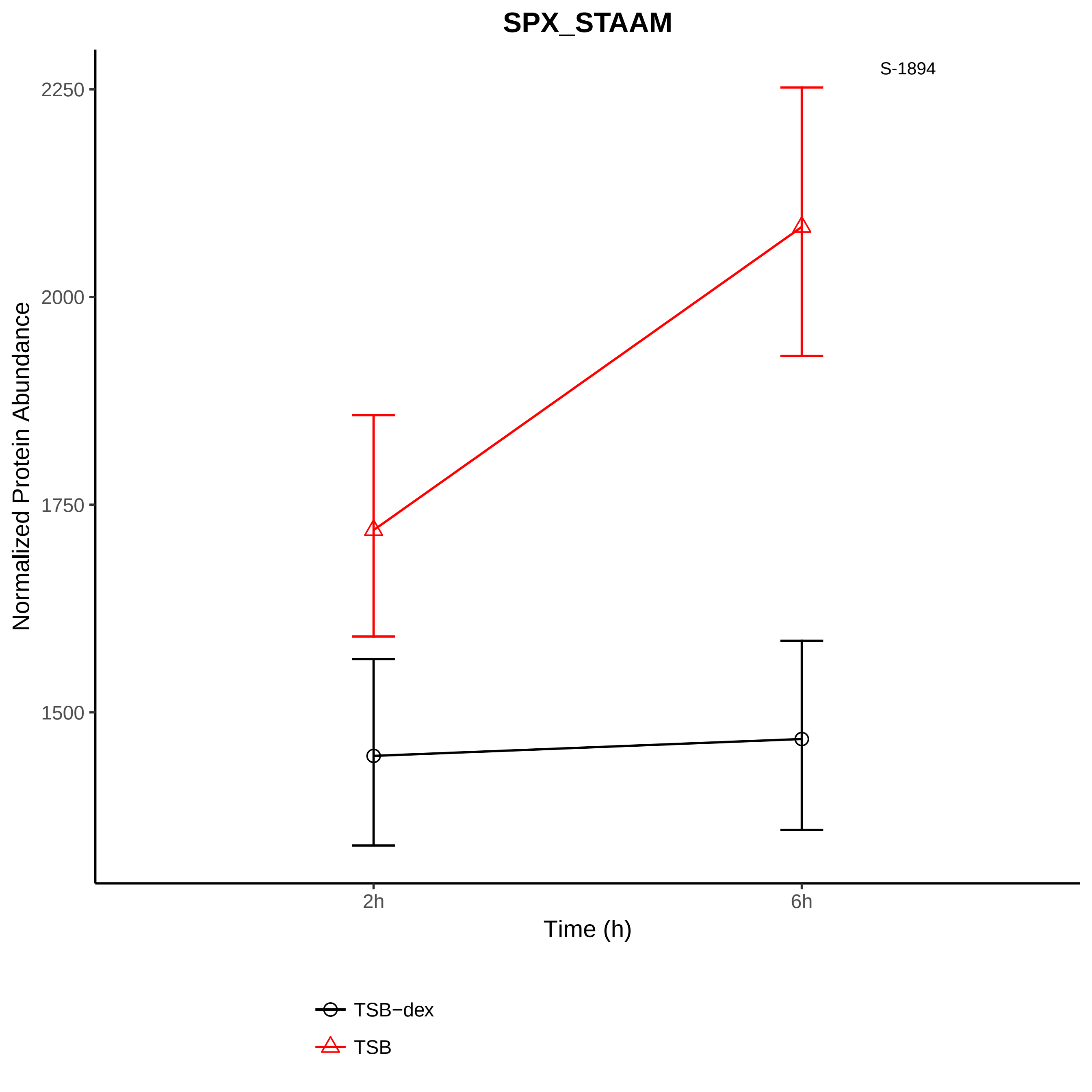

SPX_STAAM
S-1894
Normalized Protein Abundance
2250
2000
1750
1500
2h
6h
Time (h)
TSB−dex
TSB

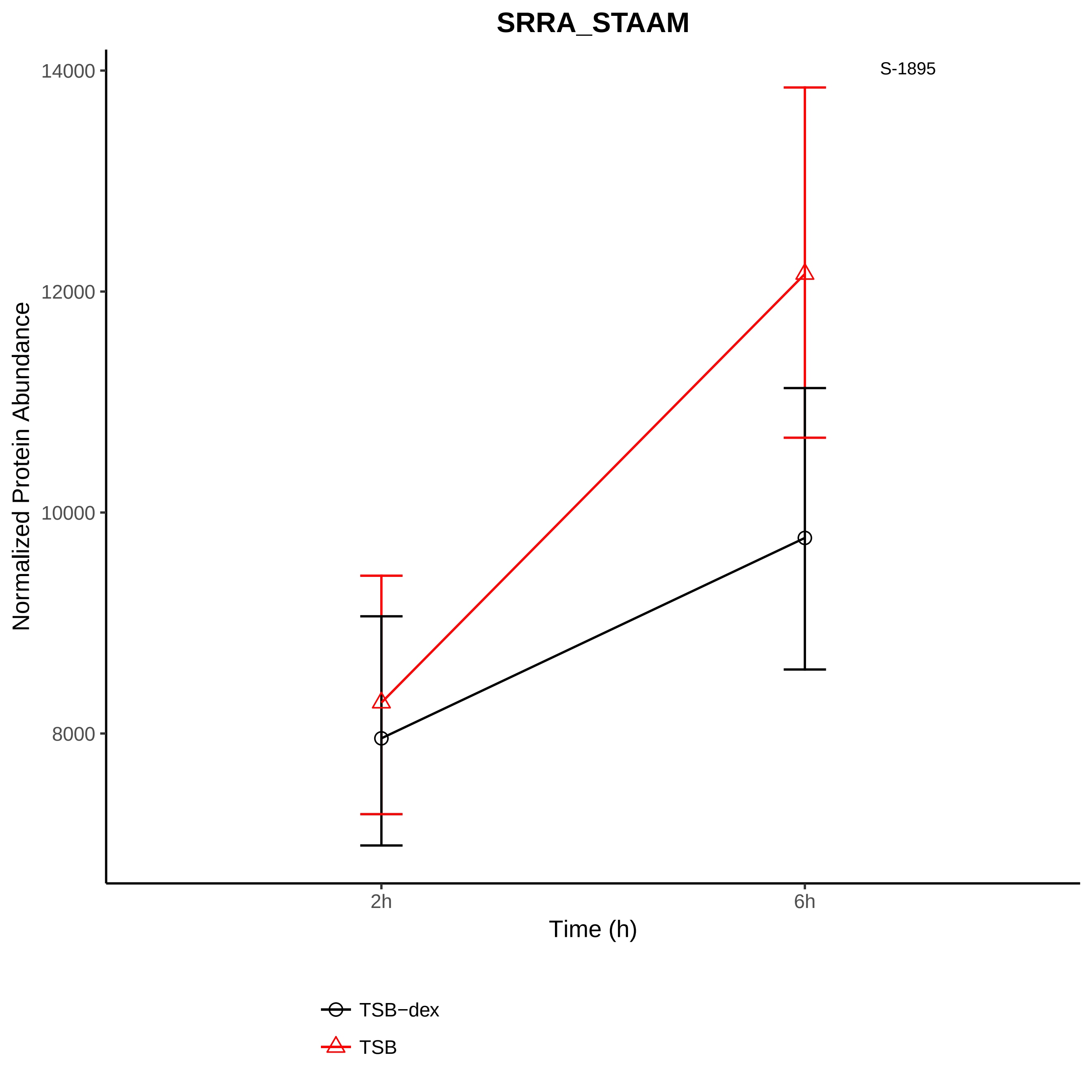
SRRA_STAAM
S-1895
Normalized Protein Abundance
14000
12000
10000
8000
2h
6h
Time (h)
TSB−dex
TSB

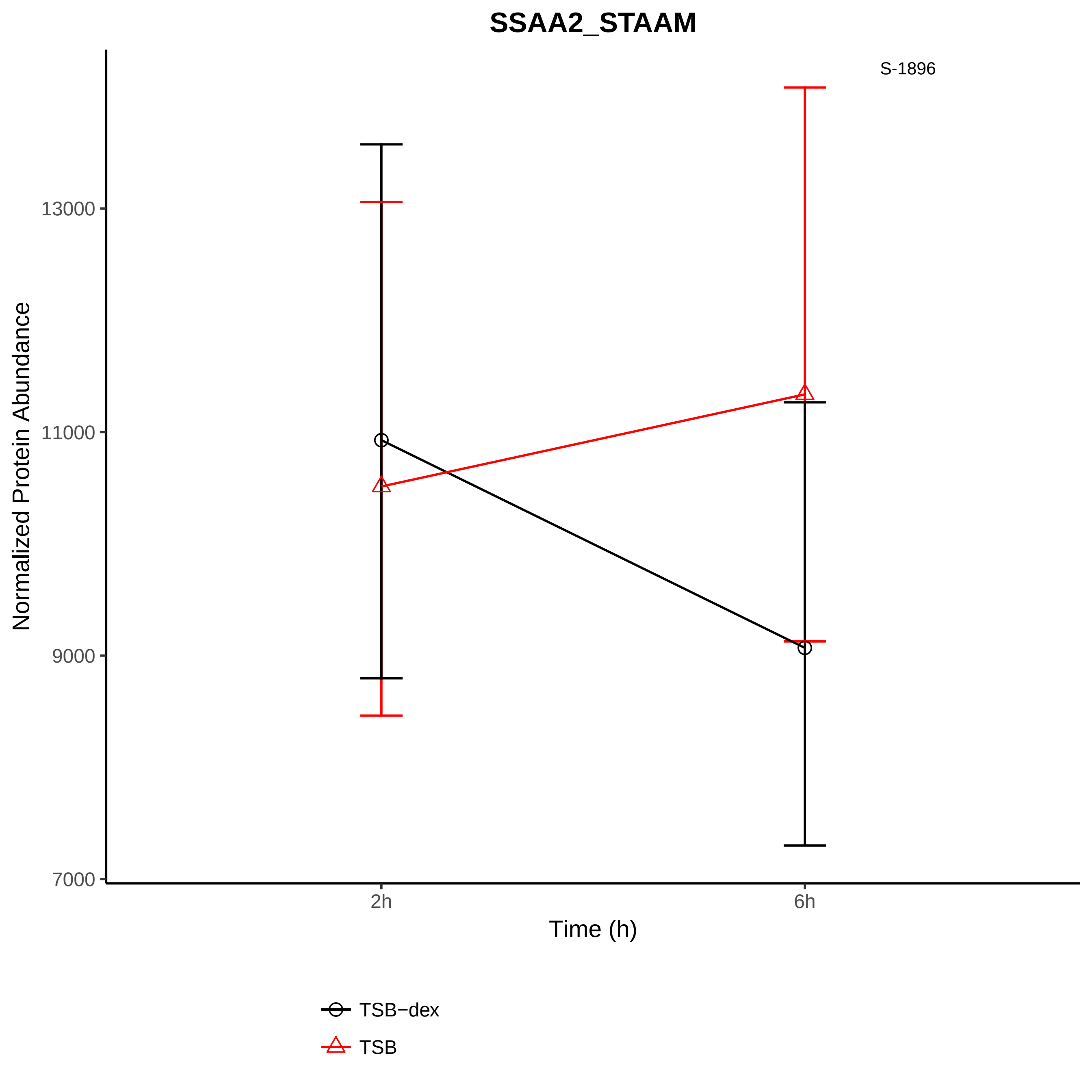

SSAA2_STAAM
S-1896
Normalized Protein Abundance
13000
11000
9000
7000
2h
6h
Time (h)
TSB−dex
TSB

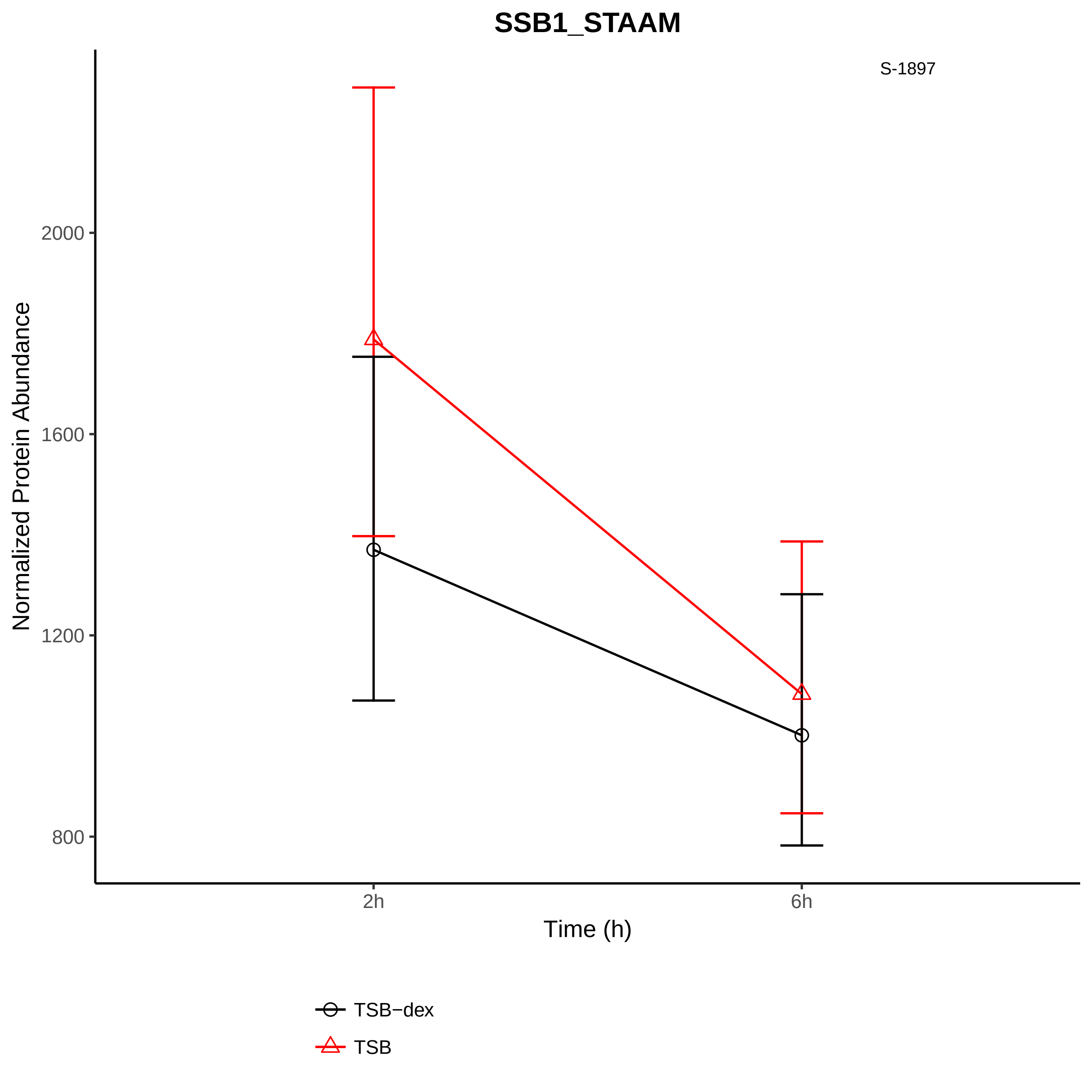

SSB1_STAAM
S-1897
Normalized Protein Abundance
2000
1600
1200
800
2h
6h
Time (h)
TSB−dex
TSB

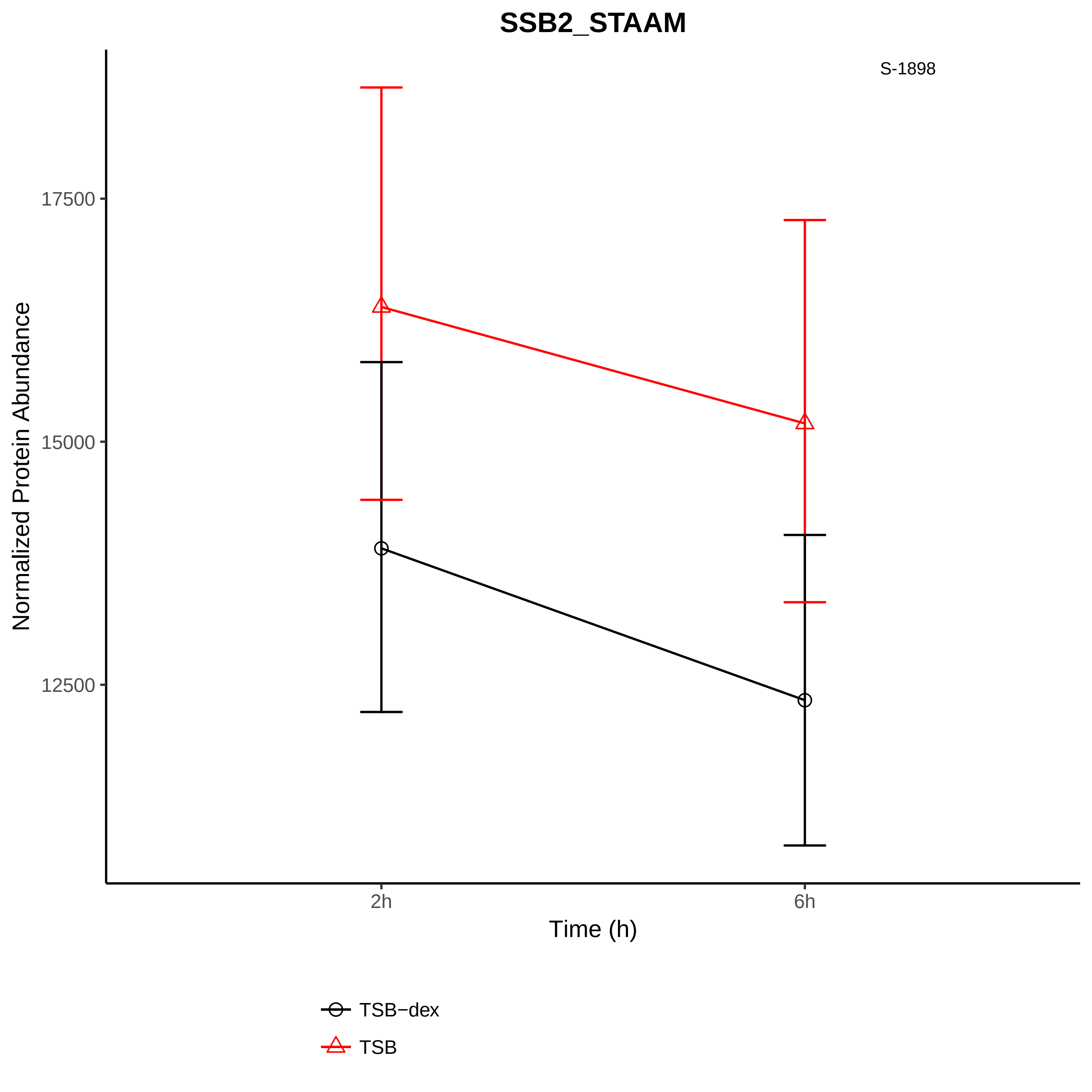

SSB2_STAAM
S-1898
17500
15000
12500
Normalized Protein Abundance
2h
6h
Time (h)
TSB−dex
TSB

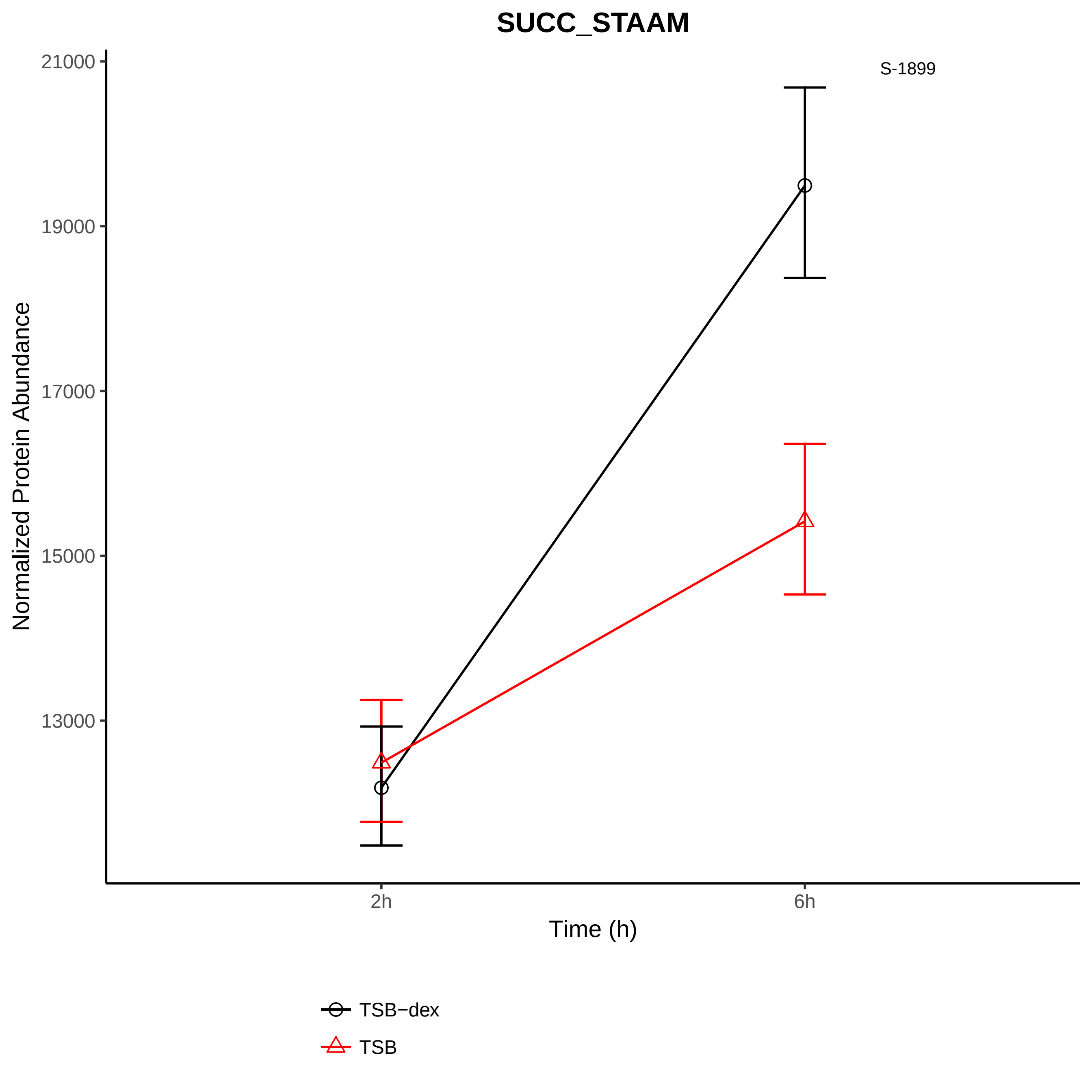
SUCC_STAAM
S-1899
Normalized Protein Abundance
21000
19000
17000
15000
13000
2h
6h
Time (h)
TSB−dex
TSB

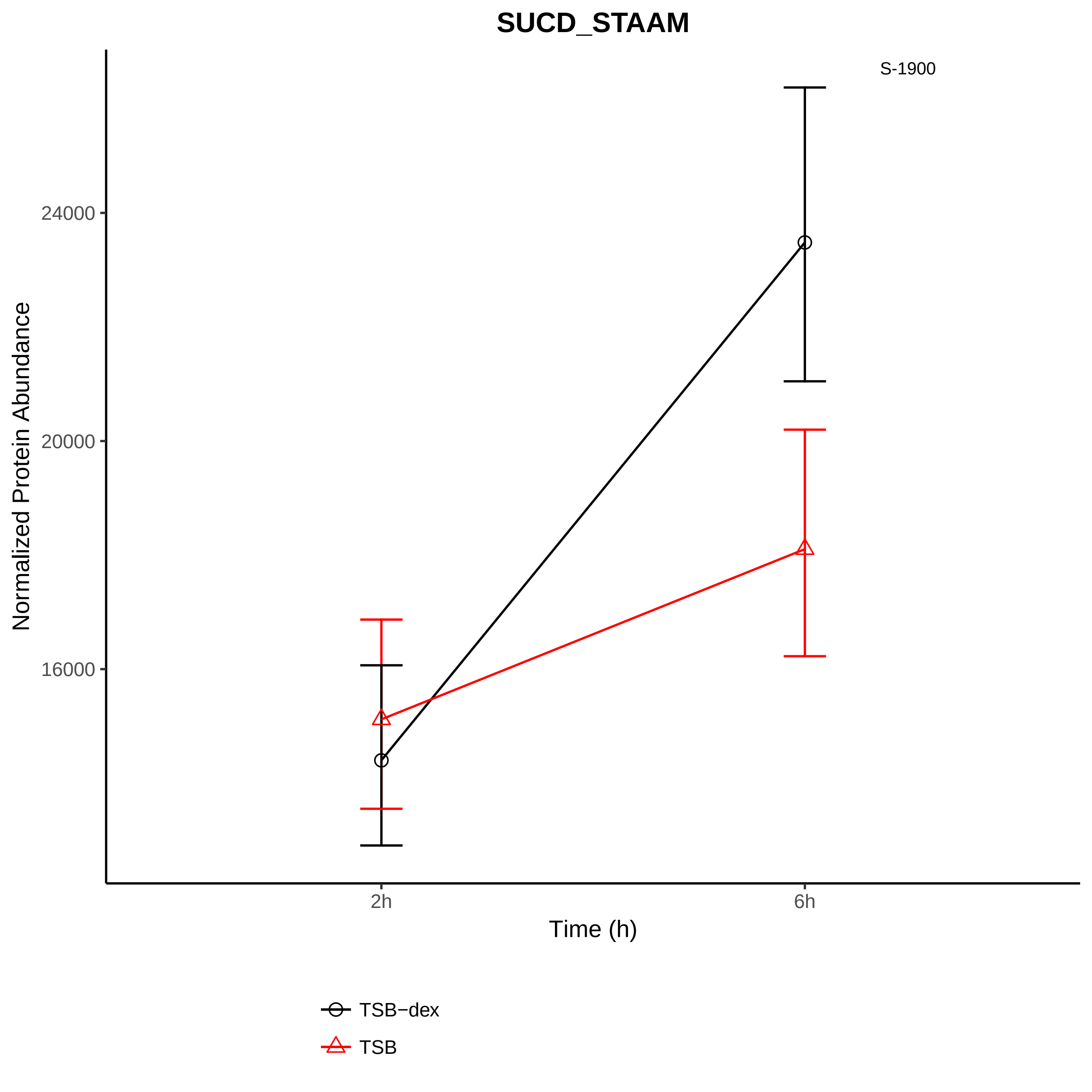
SUCD_STAAM
S-1900
24000
20000
16000
Normalized Protein Abundance
2h
6h
Time (h)
TSB−dex
TSB

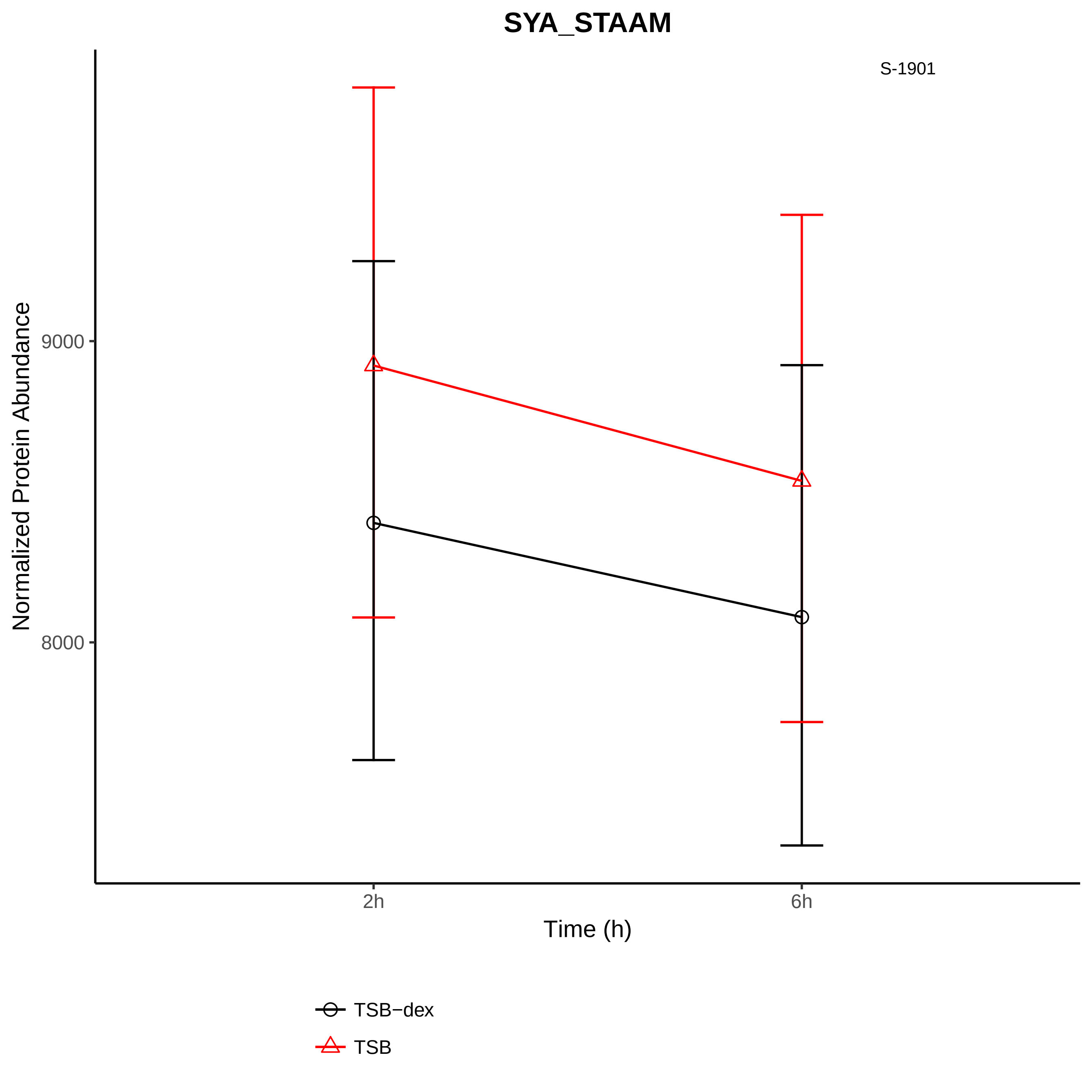
SYA_STAAM
S-1901
Normalized Protein Abundance
9000
8000
2h
6h
Time (h)
TSB−dex
TSB

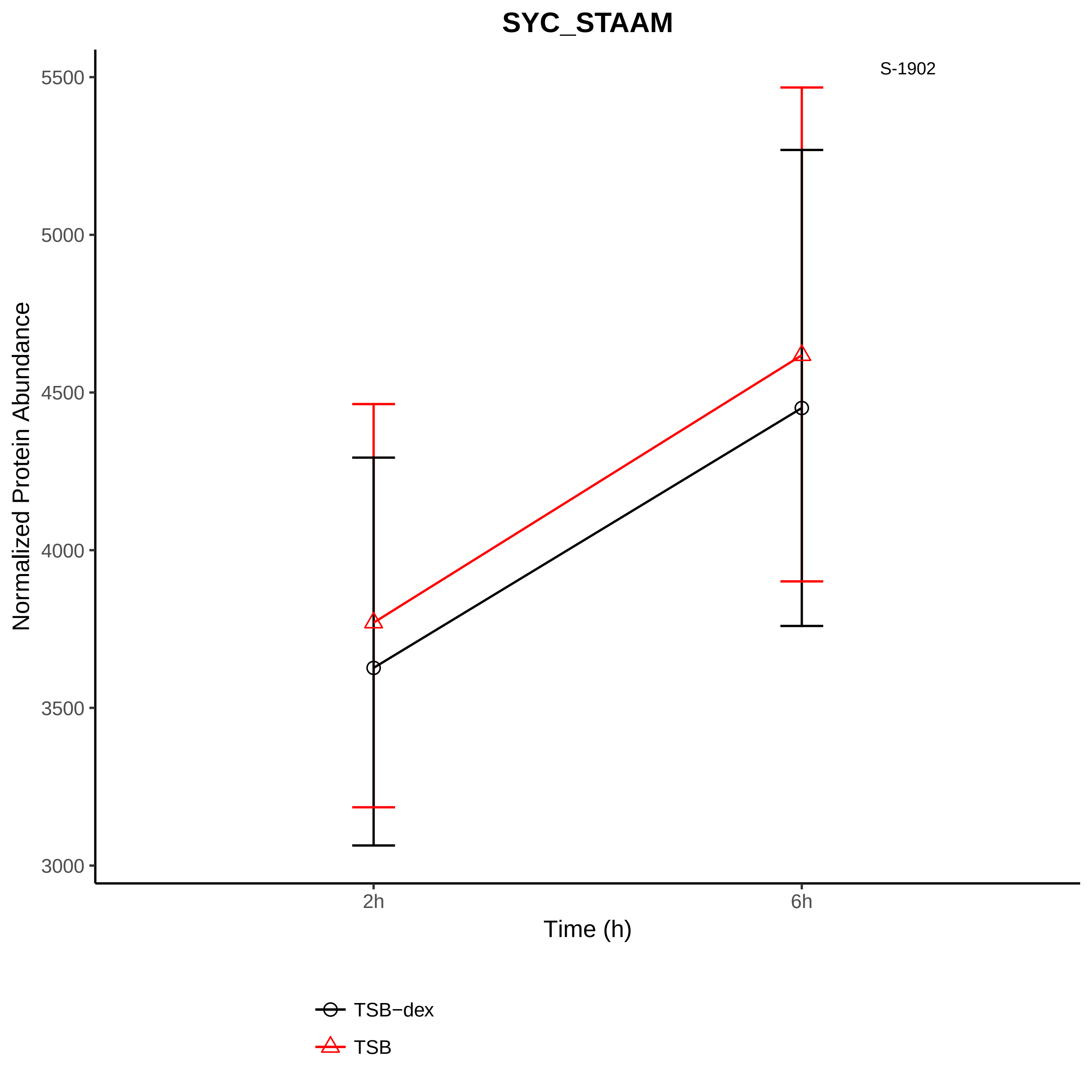
SYC_STAAM
S-1902
Normalized Protein Abundance
5500
5000
4500
4000
3500
3000
2h
6h
Time (h)
TSB−dex
TSB

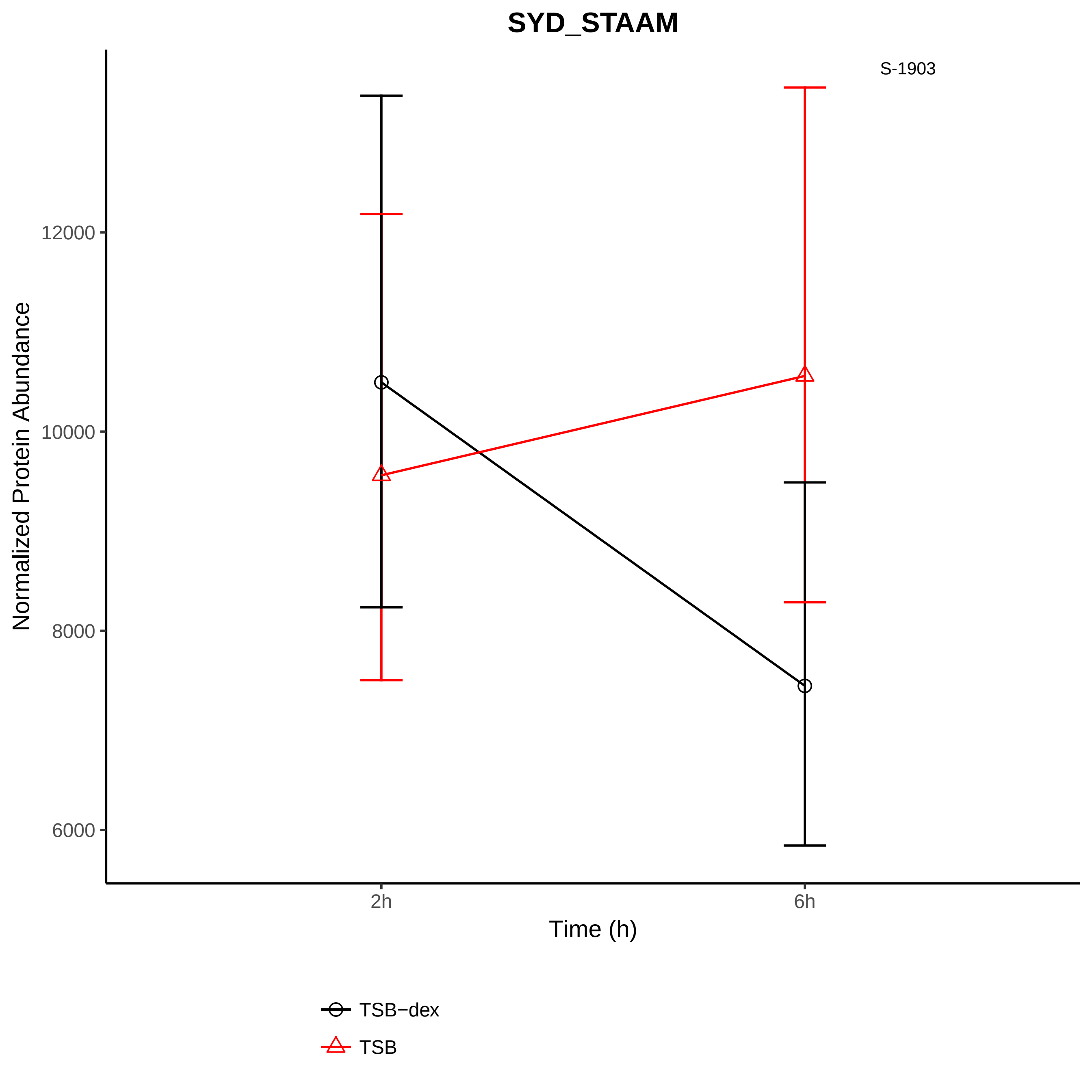

SYD_STAAM
S-1903
Normalized Protein Abundance
12000
10000
8000
6000
2h
6h
Time (h)
TSB−dex
TSB

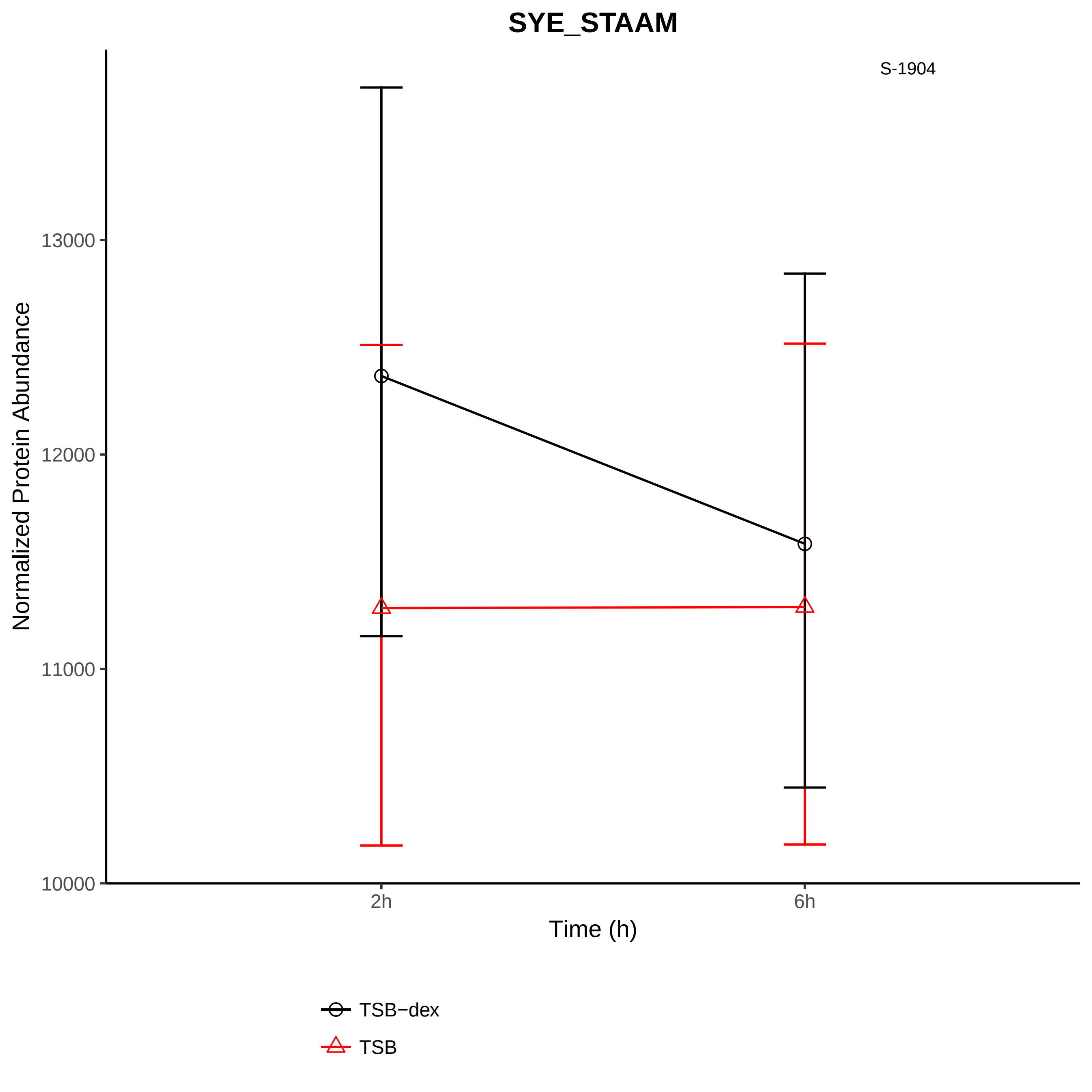

SYE_STAAM
S-1904
Normalized Protein Abundance
13000
12000
11000
10000
2h
6h
Time (h)
TSB−dex
TSB

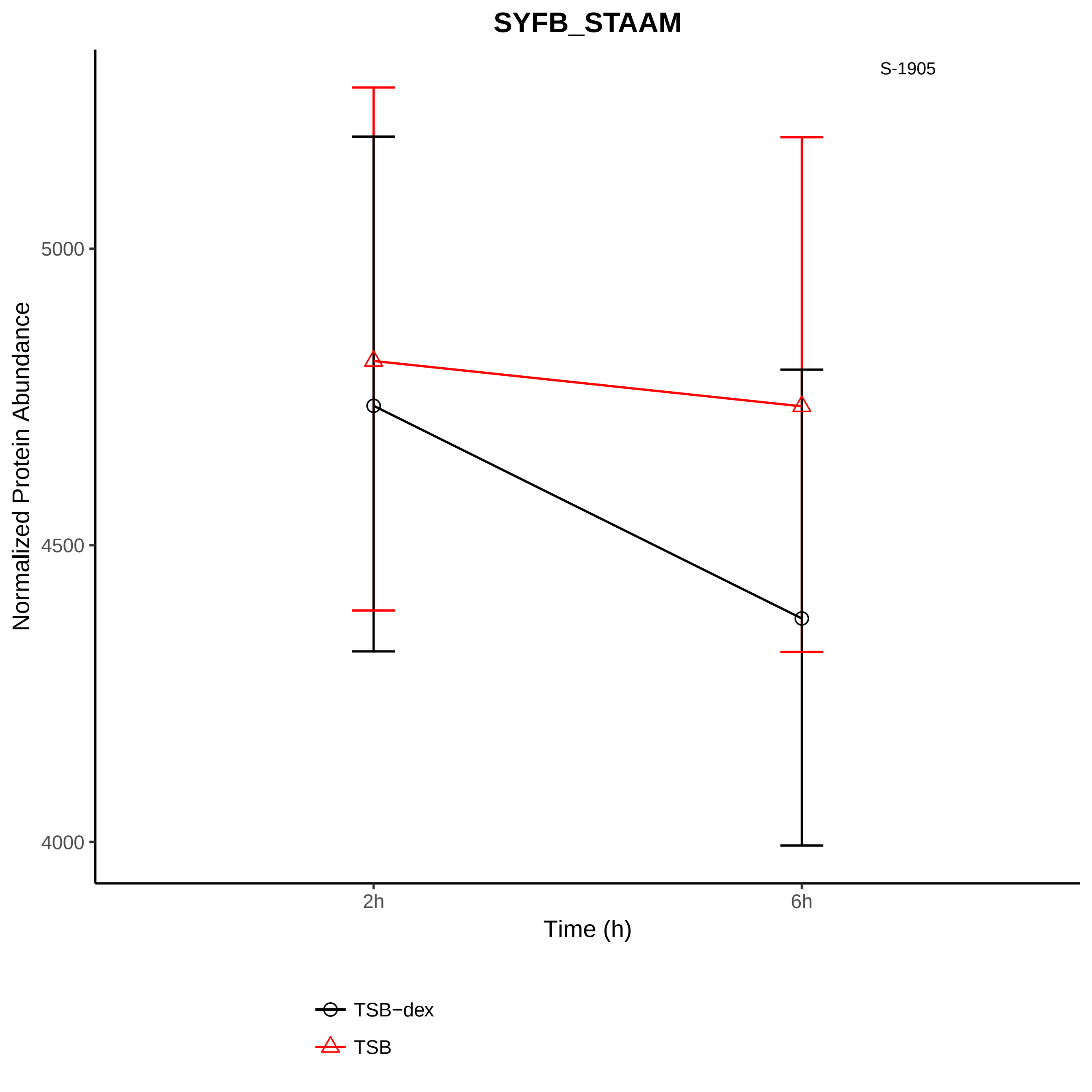
SYFB_STAAM
S-1905
Normalized Protein Abundance
5000
4500
4000
2h
6h
Time (h)
TSB−dex
TSB

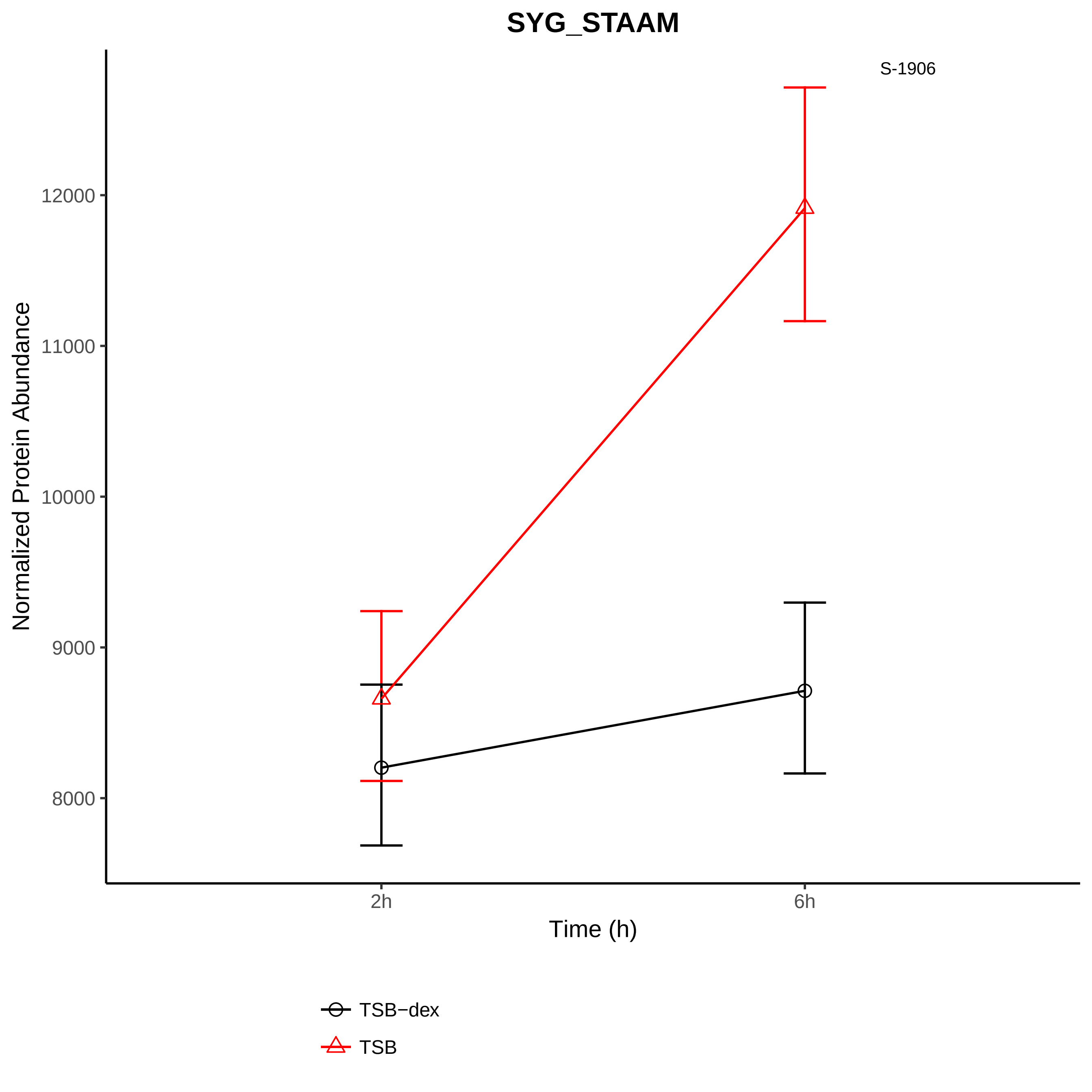

SYG_STAAM
S-1906
Normalized Protein Abundance
12000
11000
10000
9000
8000
2h
6h
Time (h)
TSB−dex
TSB

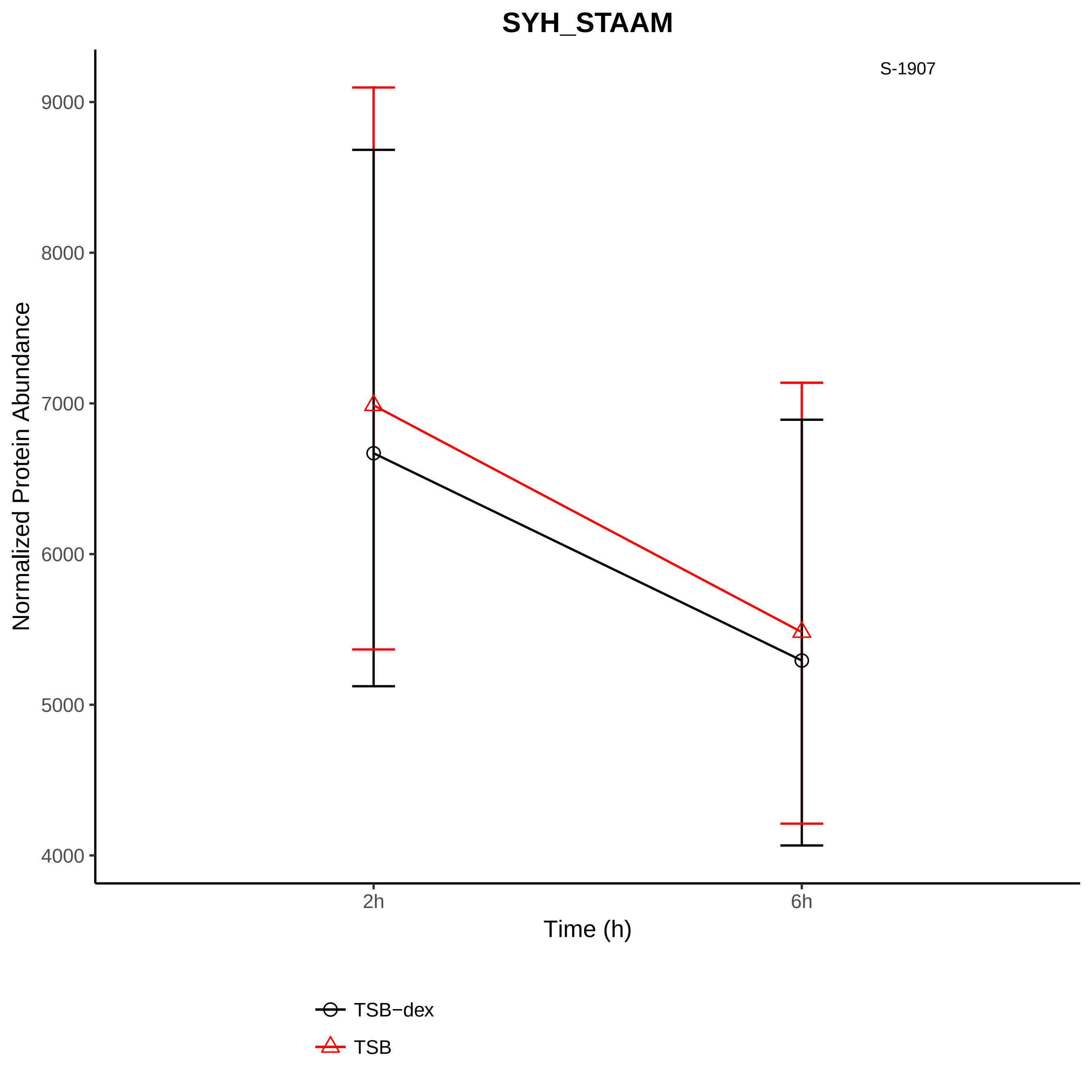

SYH_STAAM
S-1907
Normalized Protein Abundance
9000
8000
7000
6000
5000
4000
2h
6h
Time (h)
TSB−dex
TSB

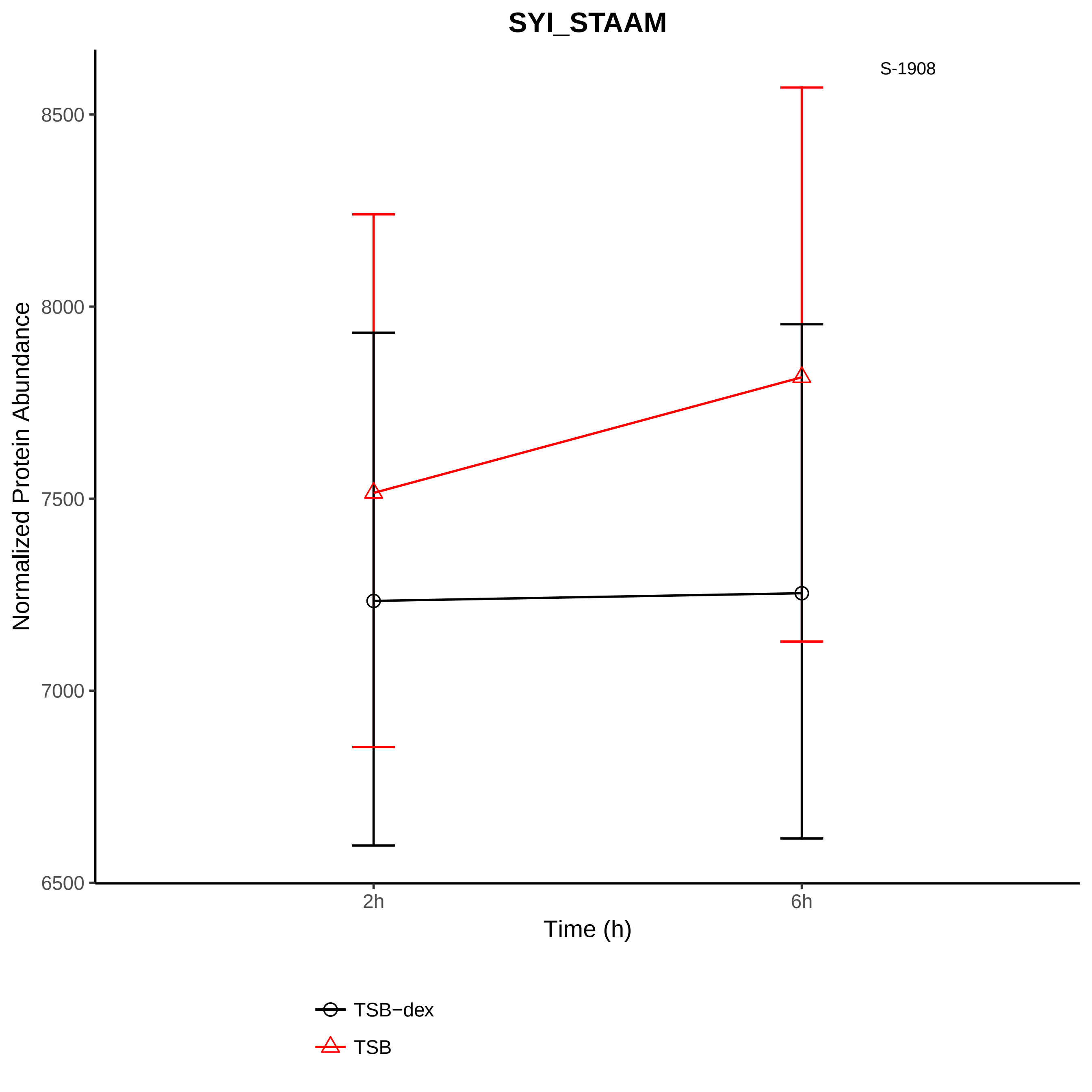
SYI_STAAM
S-1908
Normalized Protein Abundance
8500
8000
7500
7000
6500
2h
6h
Time (h)
TSB−dex
TSB

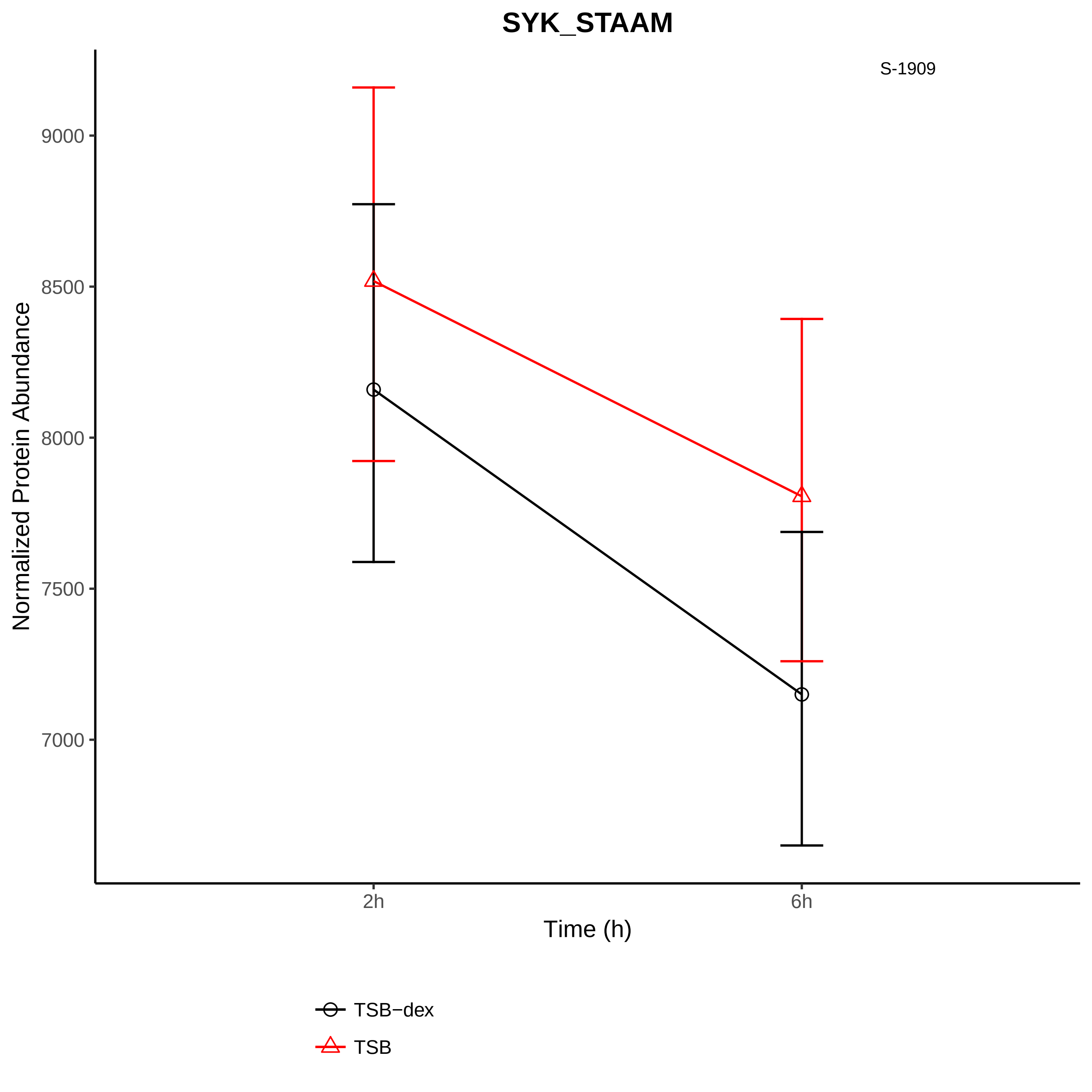
SYK_STAAM
S-1909
Normalized Protein Abundance
9000
8500
8000
7500
7000
2h
6h
Time (h)
TSB−dex
TSB

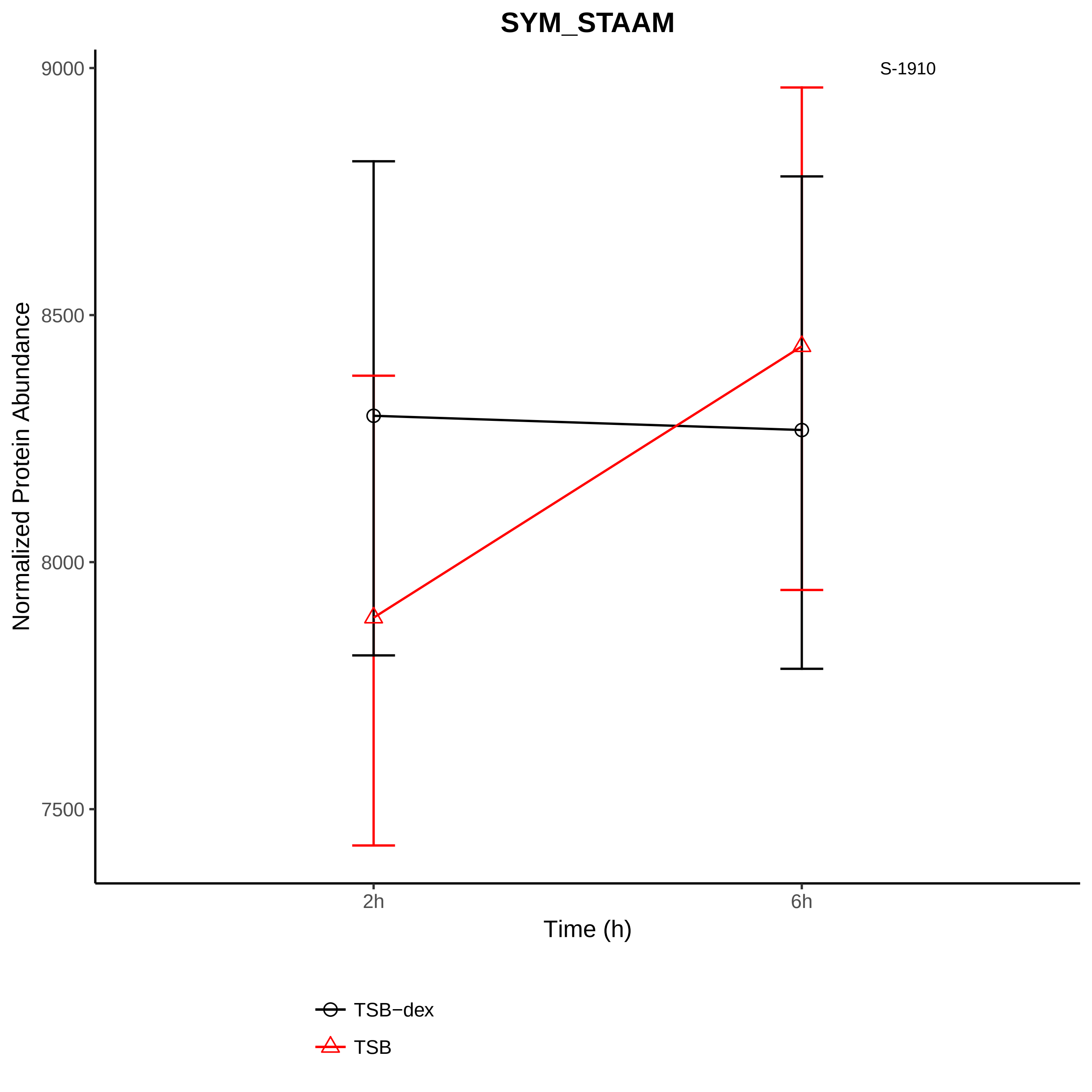
SYM_STAAM
S-1910
Normalized Protein Abundance
9000
8500
8000
7500
2h
6h
Time (h)
TSB−dex
TSB

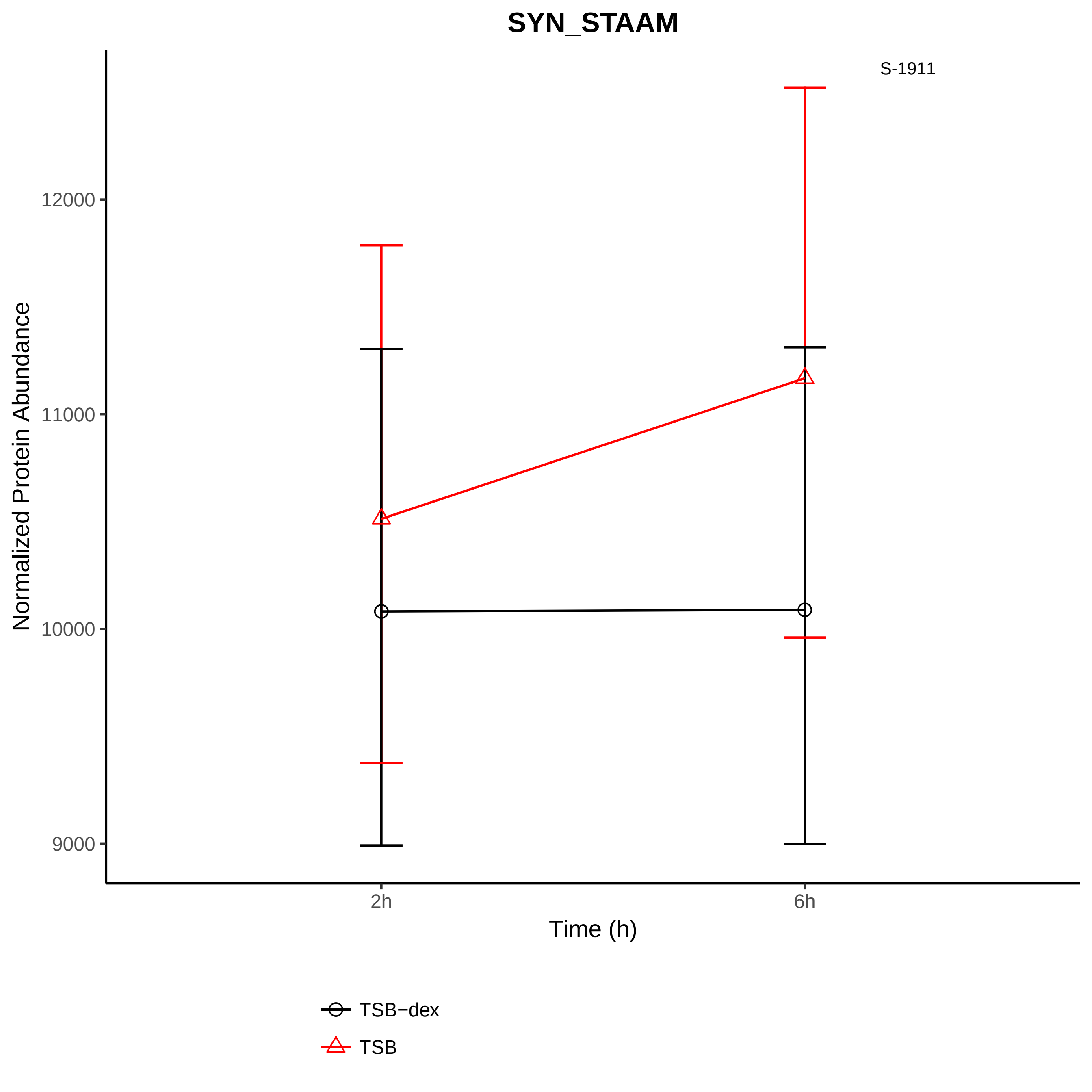
SYN_STAAM
S-1911
Normalized Protein Abundance
12000
11000
10000
9000
2h
6h
Time (h)
TSB−dex
TSB

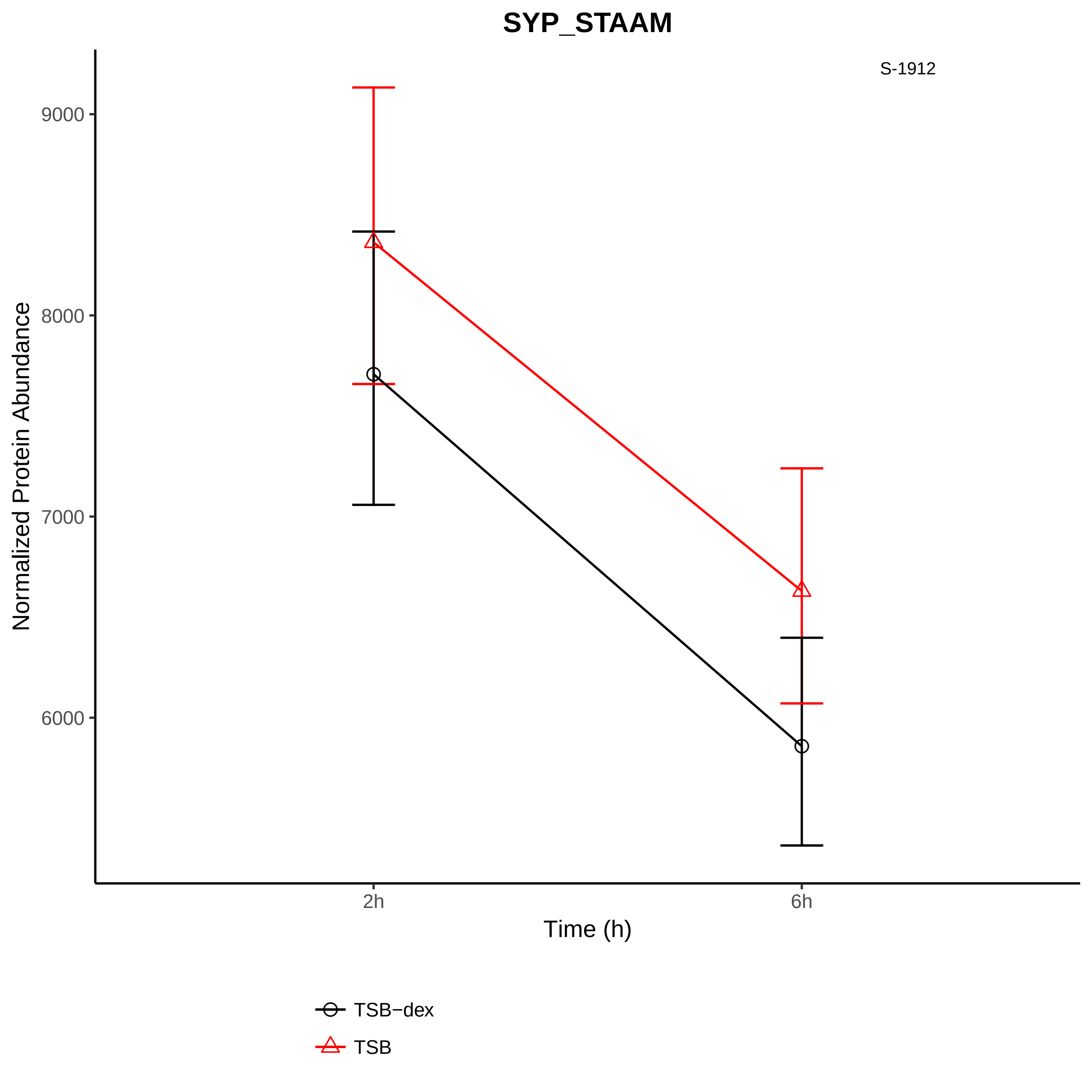

SYP_STAAM
S-1912
9000
8000
7000
6000
Normalized Protein Abundance
2h
6h
Time (h)
TSB−dex
TSB

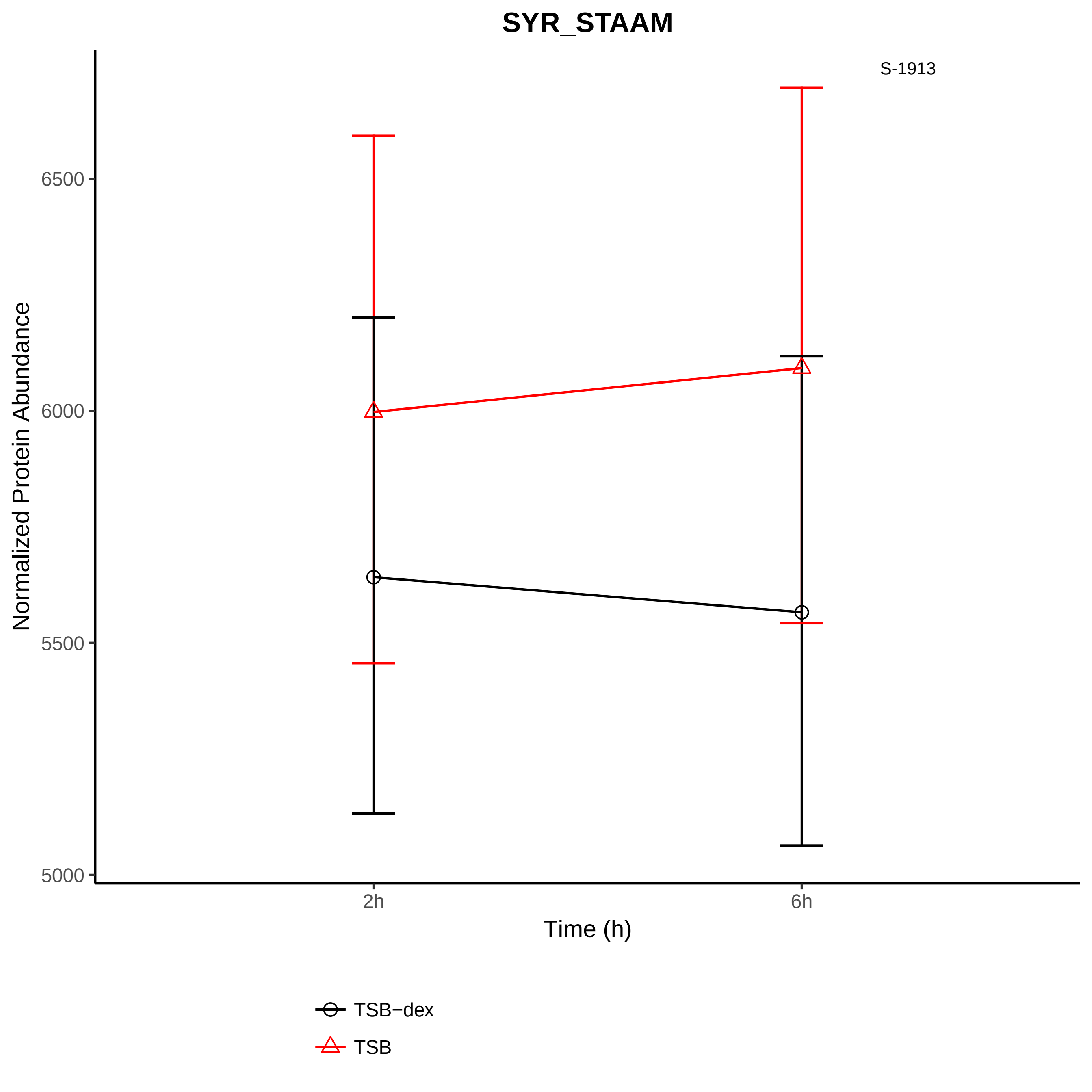

SYR_STAAM
S-1913
6500
6000
5500
5000
Normalized Protein Abundance
2h
6h
Time (h)
TSB−dex
TSB

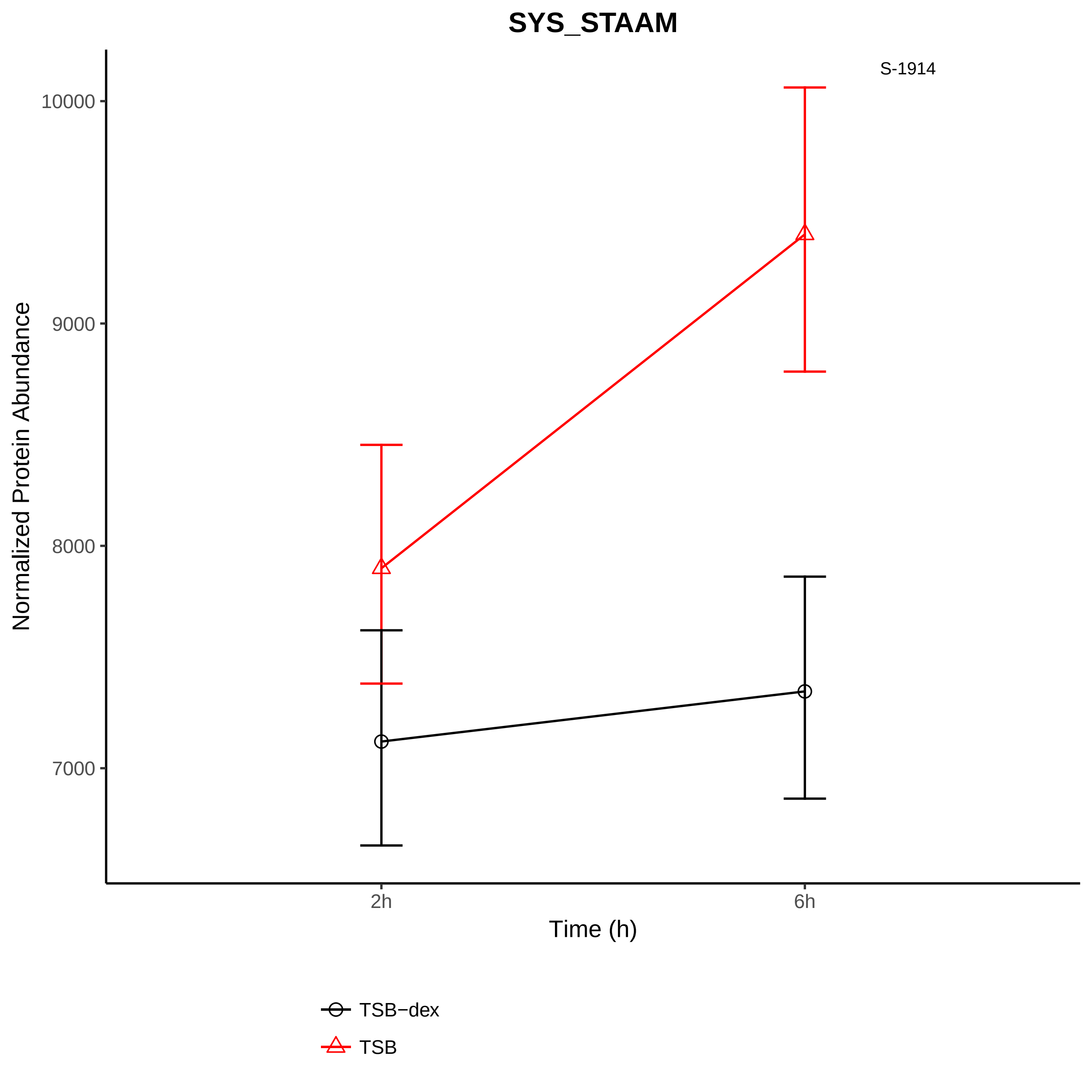
SYS_STAAM
S-1914
10000
9000
8000
7000
Normalized Protein Abundance
2h
6h
Time (h)
TSB−dex
TSB

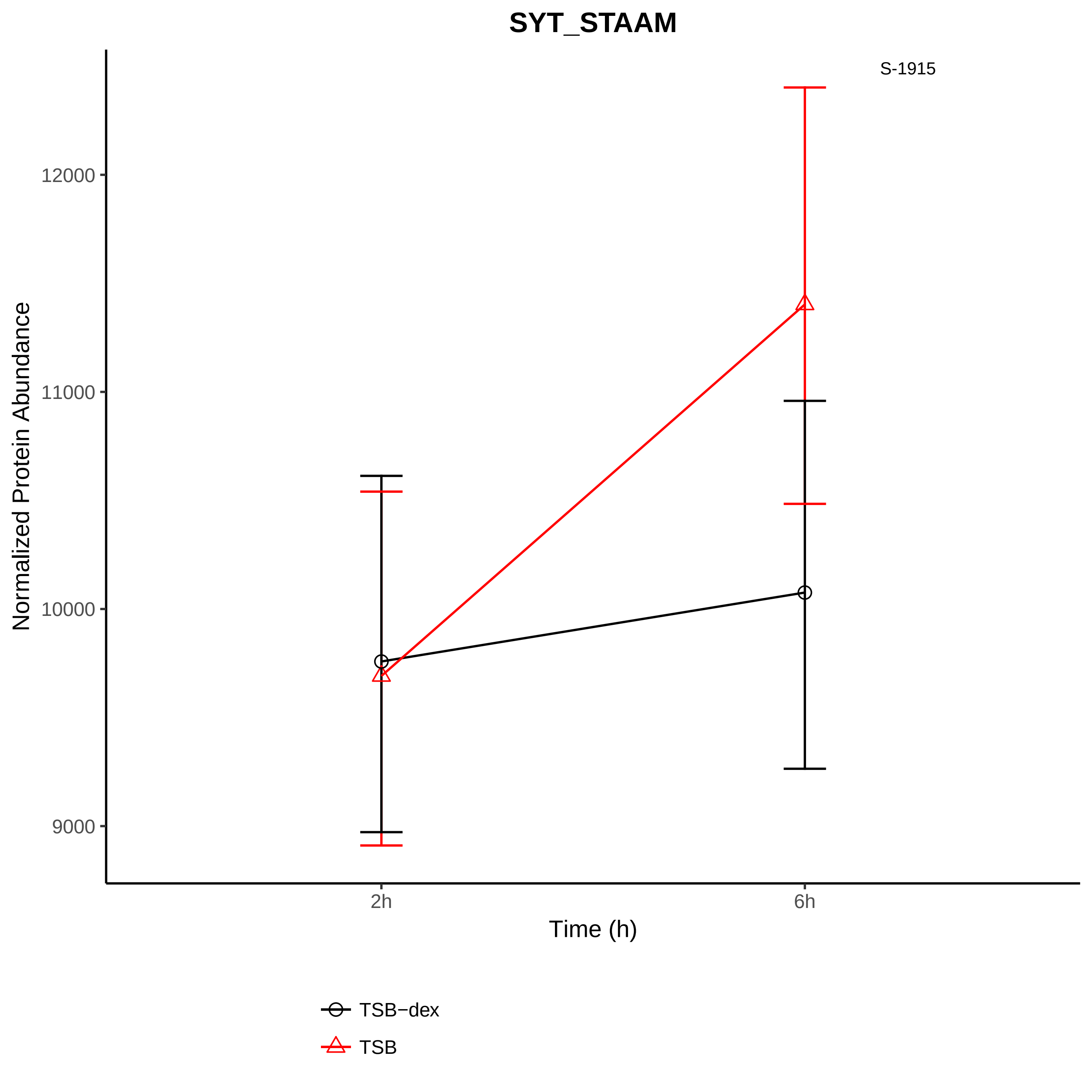

SYT_STAAM
S-1915
12000
11000
10000
9000
Normalized Protein Abundance
2h
6h
Time (h)
TSB−dex
TSB

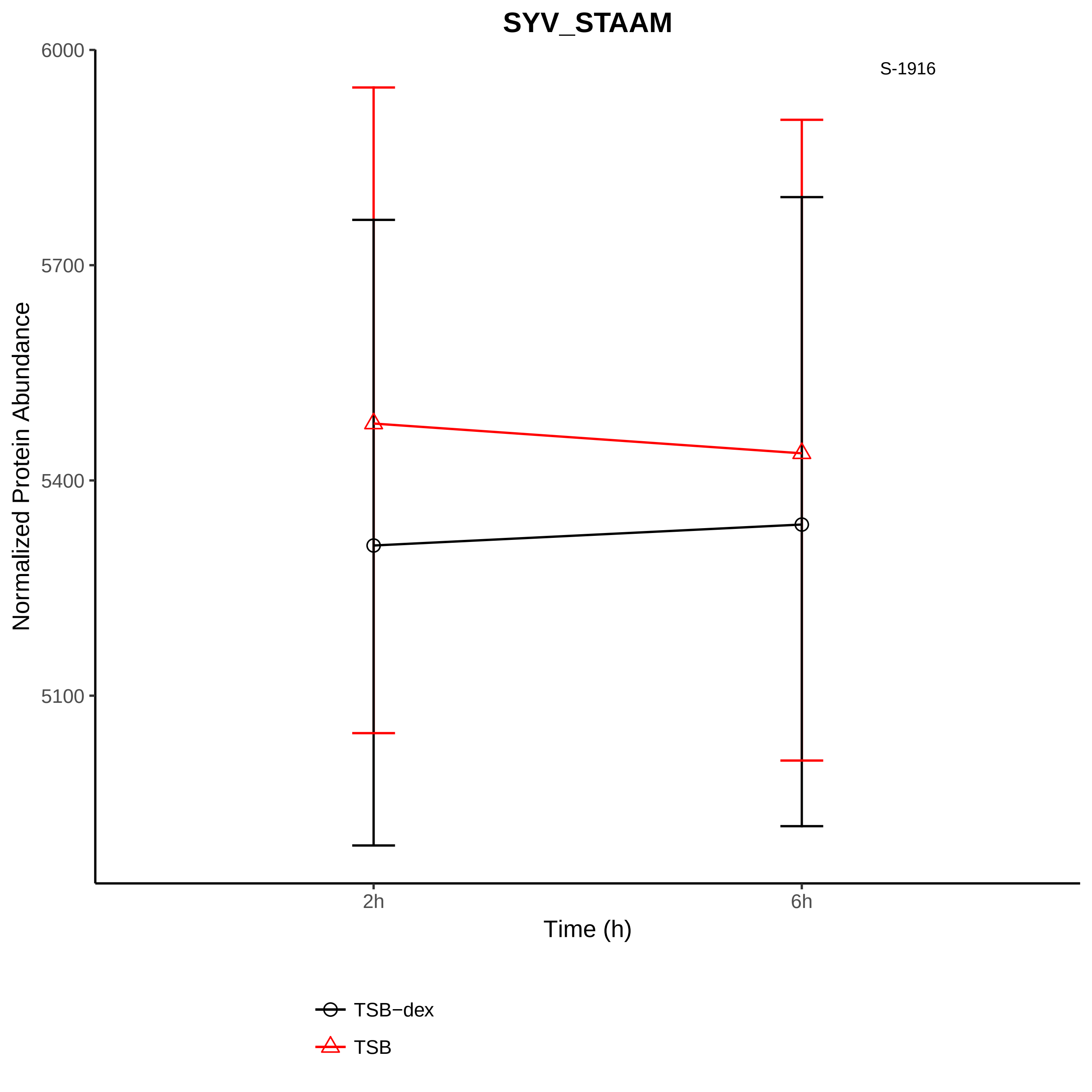

SYV_STAAM
S-1916
6000
5700
5400
5100
Normalized Protein Abundance
2h
6h
Time (h)
TSB−dex
TSB

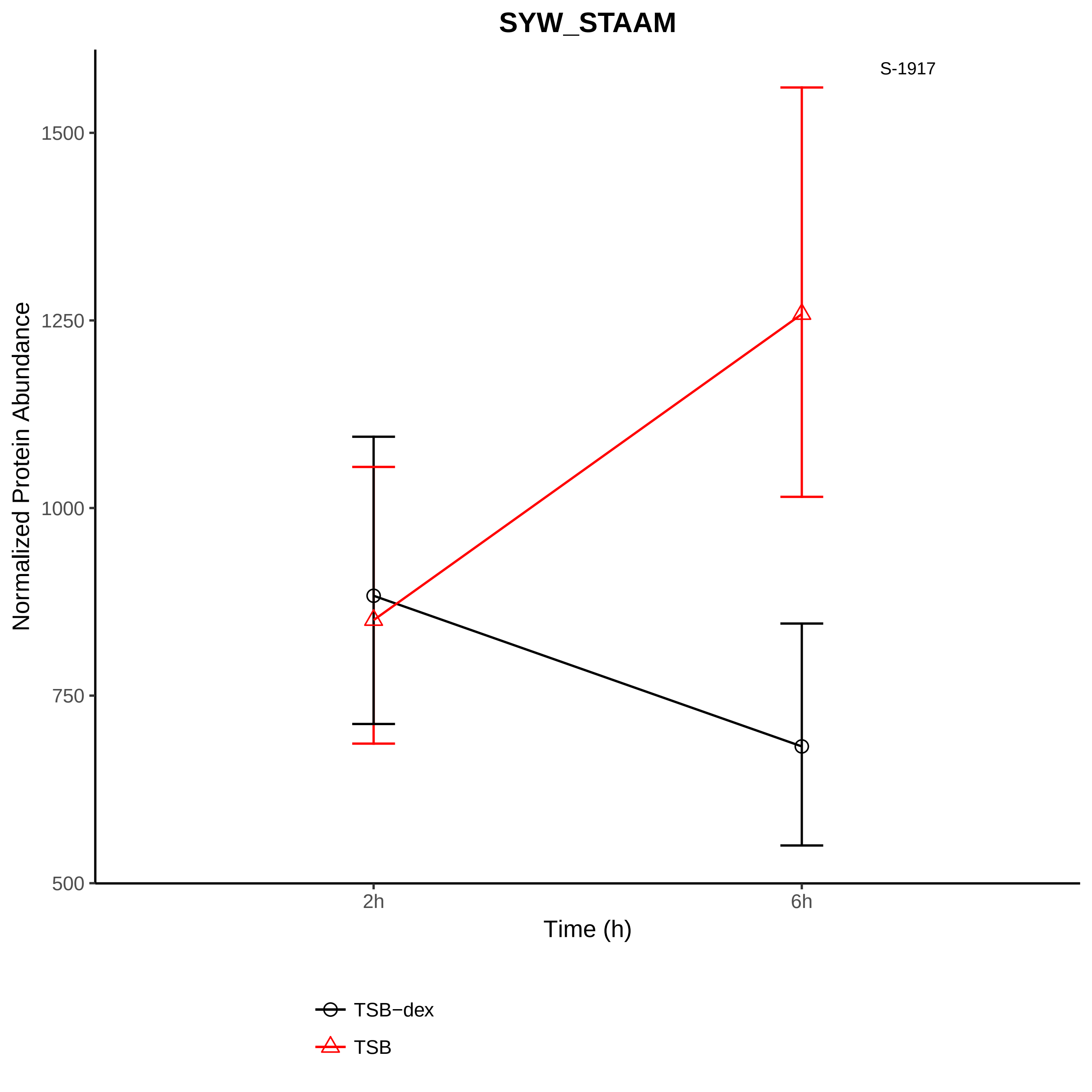
SYW_STAAM
S-1917
Normalized Protein Abundance
1500
1250
1000
750
500
2h
6h
Time (h)
TSB−dex
TSB

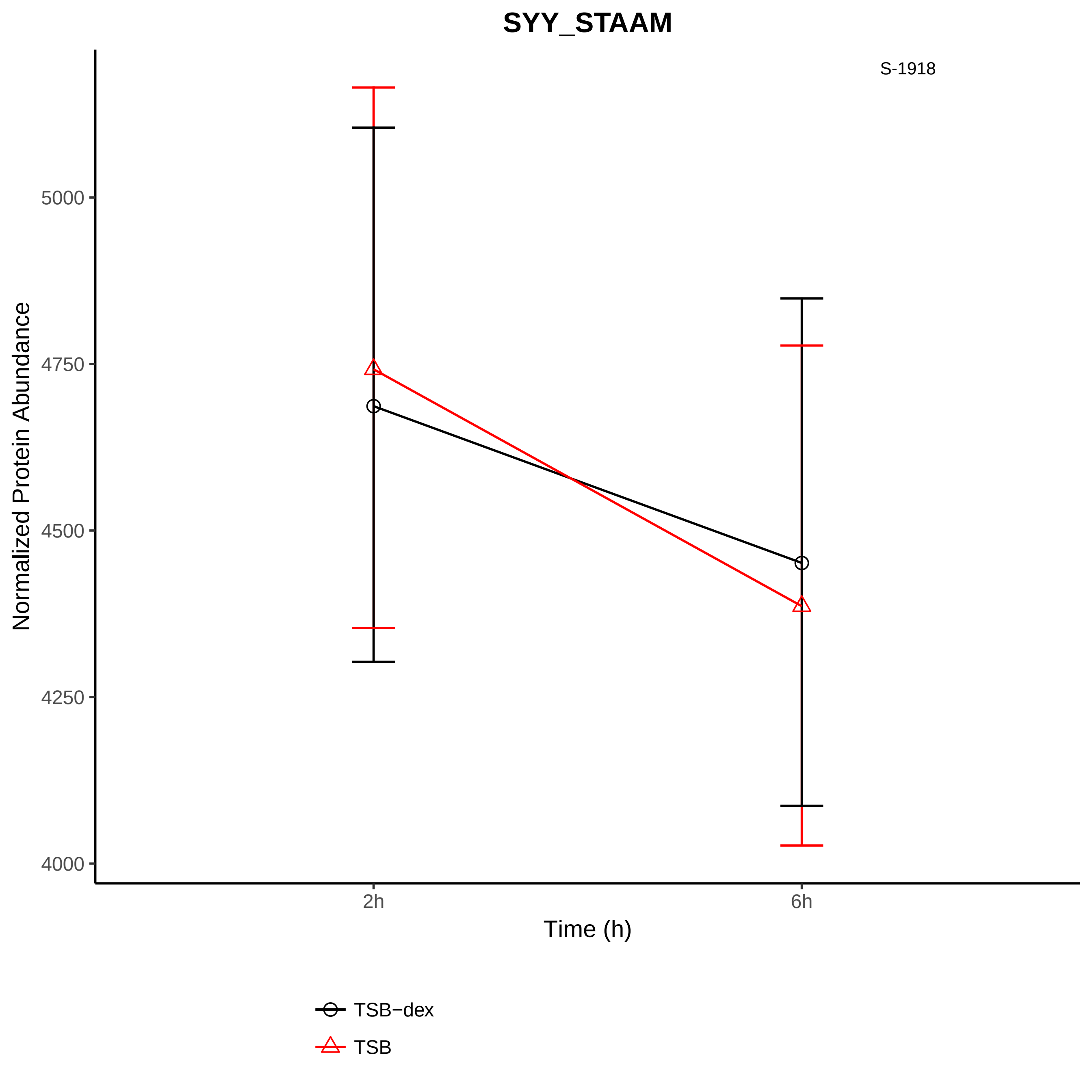

SYY_STAAM
S-1918
Normalized Protein Abundance
5000
4750
4500
4250
4000
2h
6h
Time (h)
TSB−dex
TSB

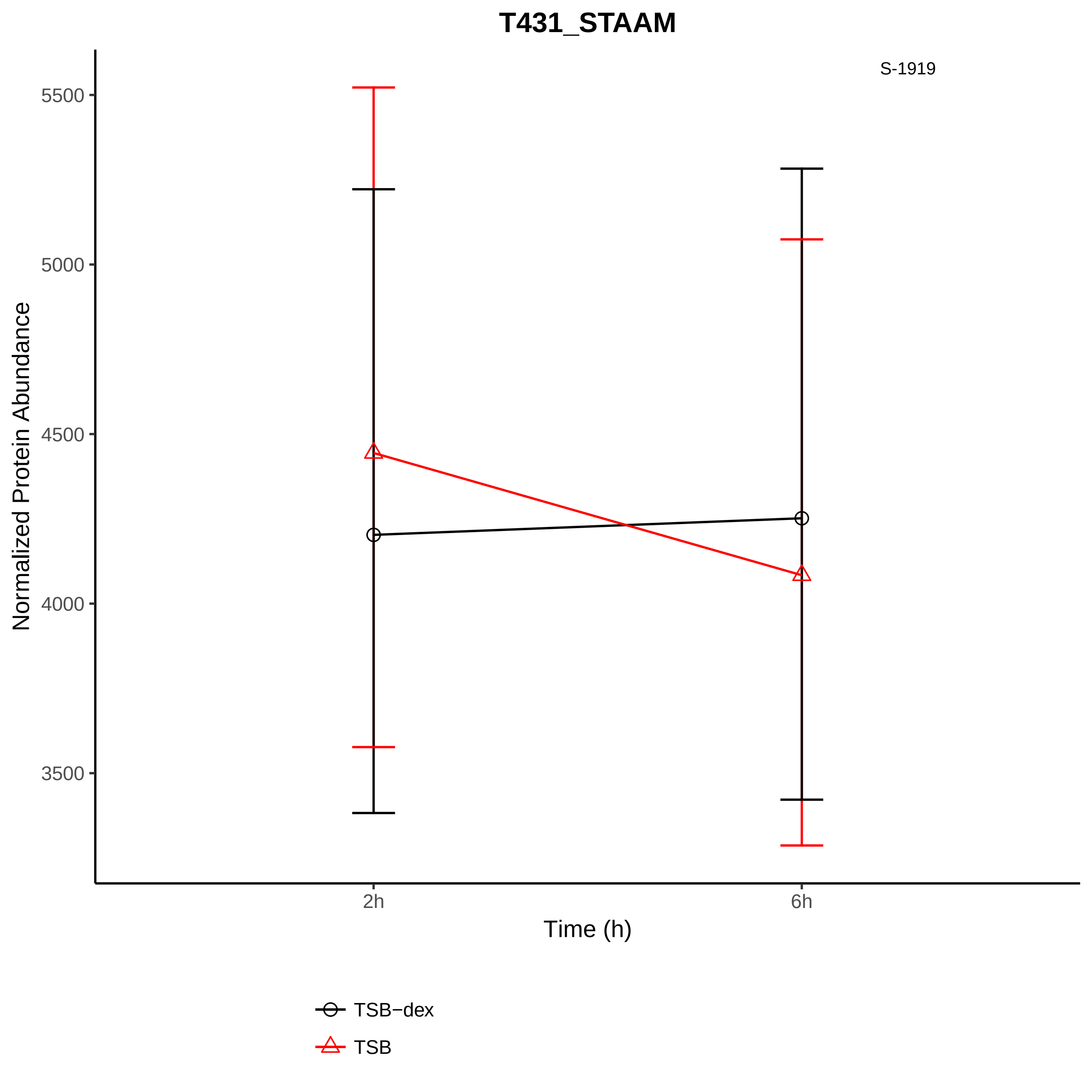
T431_STAAM
S-1919
5500
5000
4500
4000
3500
Normalized Protein Abundance
2h
6h
Time (h)
TSB−dex
TSB

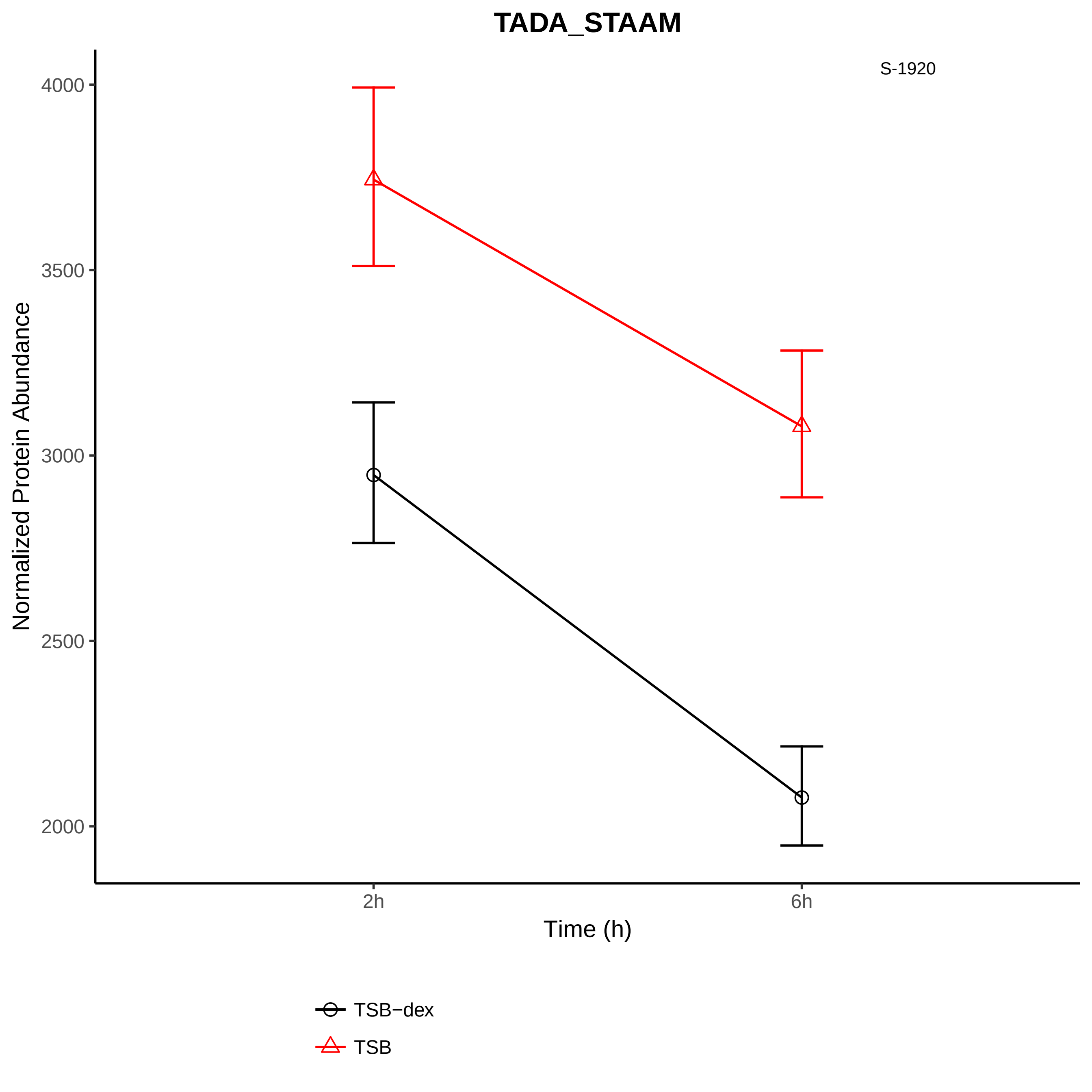

TADA_STAAM
S-1920
4000
3500
3000
2500
2000
Normalized Protein Abundance
2h
6h
Time (h)
TSB−dex
TSB

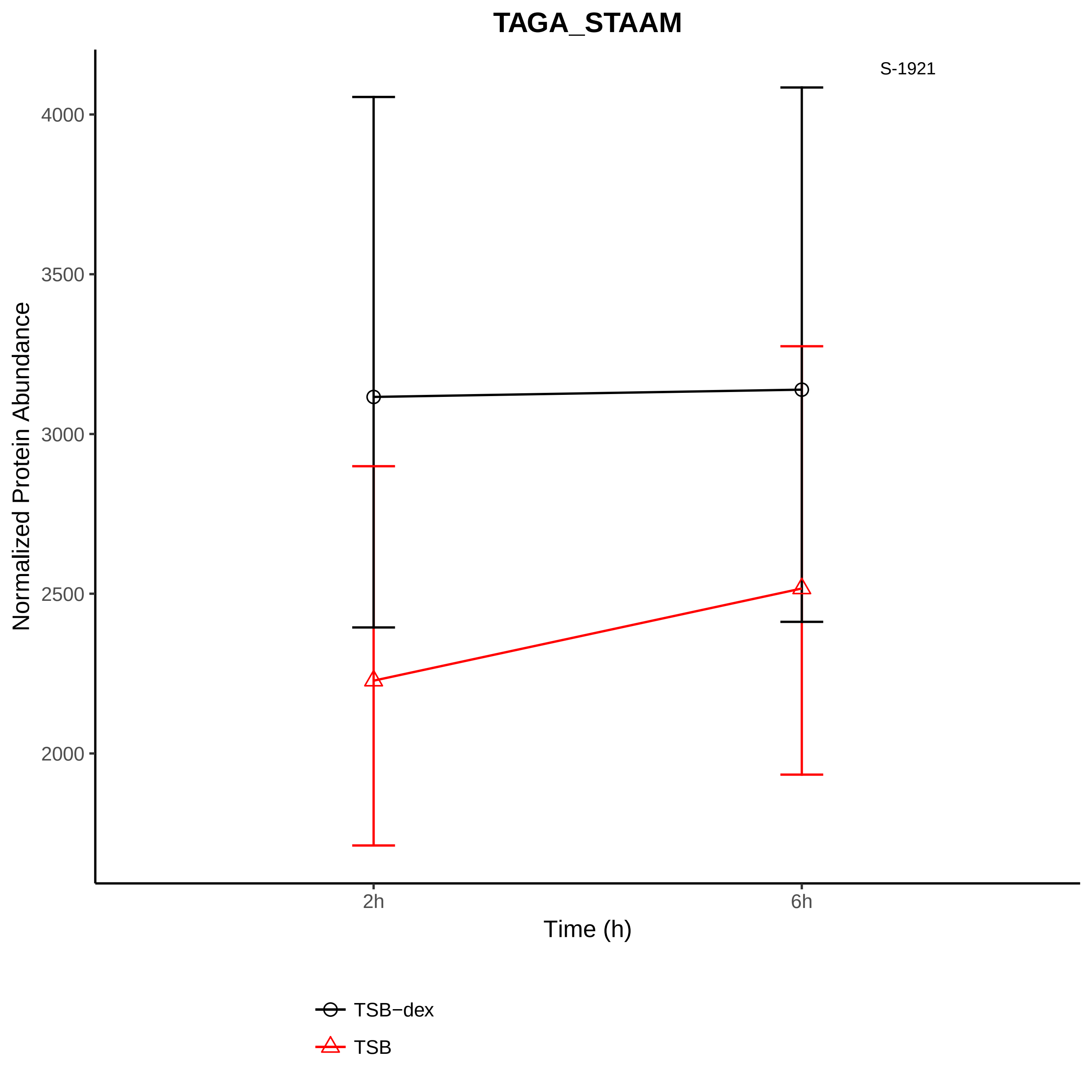
TAGA_STAAM
S-1921
Normalized Protein Abundance
4000
3500
3000
2500
2000
2h
6h
Time (h)
TSB−dex
TSB

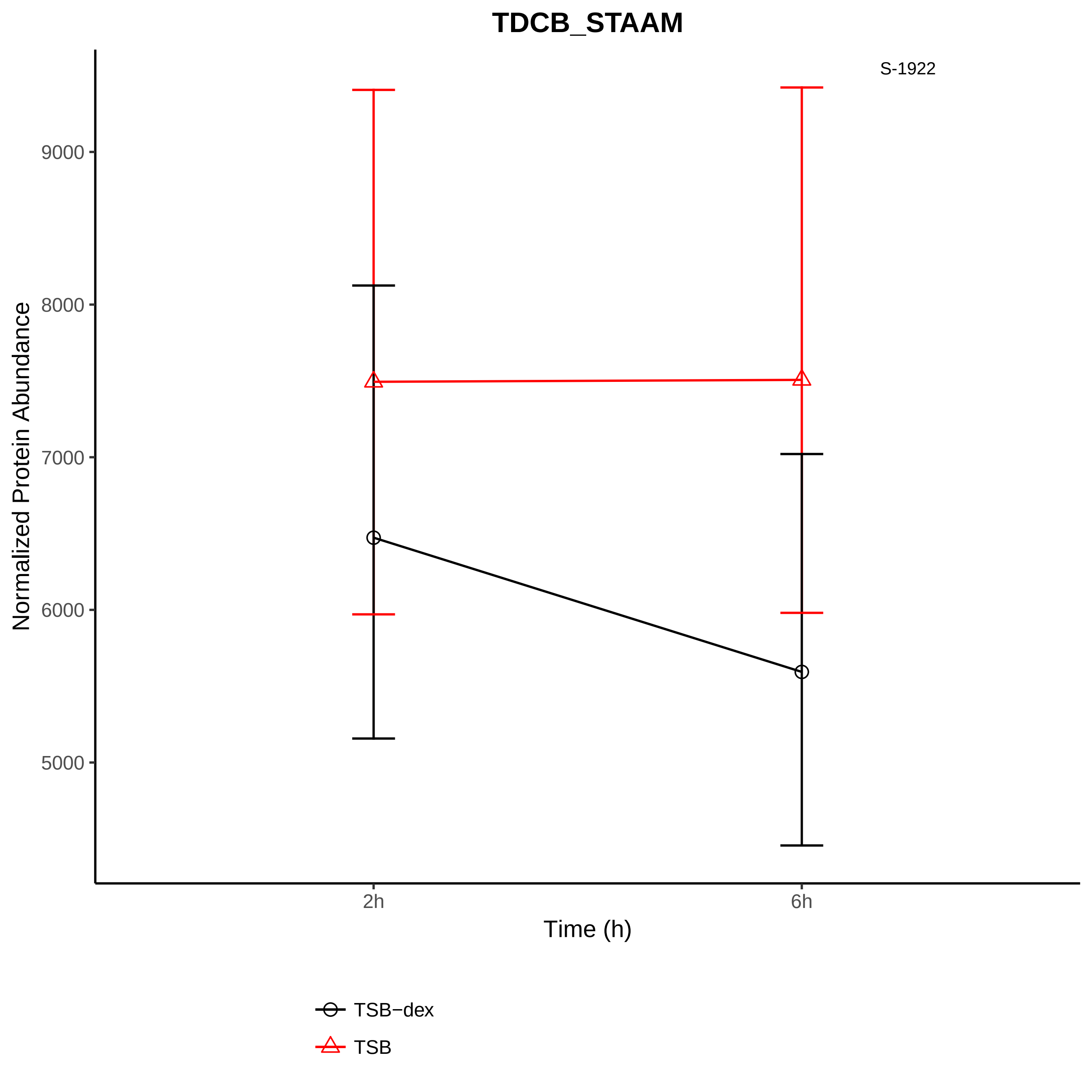
TDCB_STAAM
S-1922
Normalized Protein Abundance
9000
8000
7000
6000
5000
2h
6h
Time (h)
TSB−dex
TSB

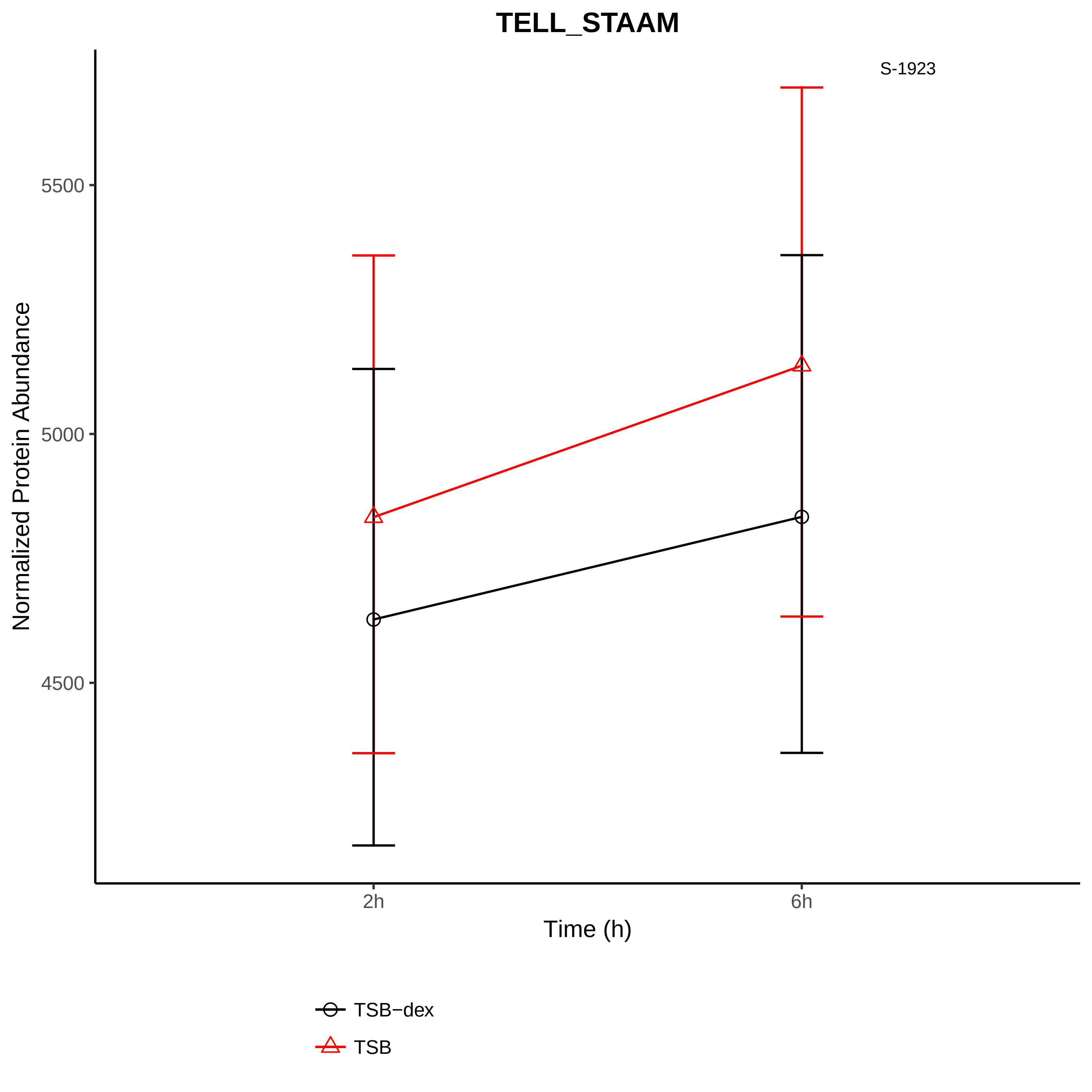

TELL_STAAM
S-1923
Normalized Protein Abundance
5500
5000
4500
2h
6h
Time (h)
TSB−dex
TSB

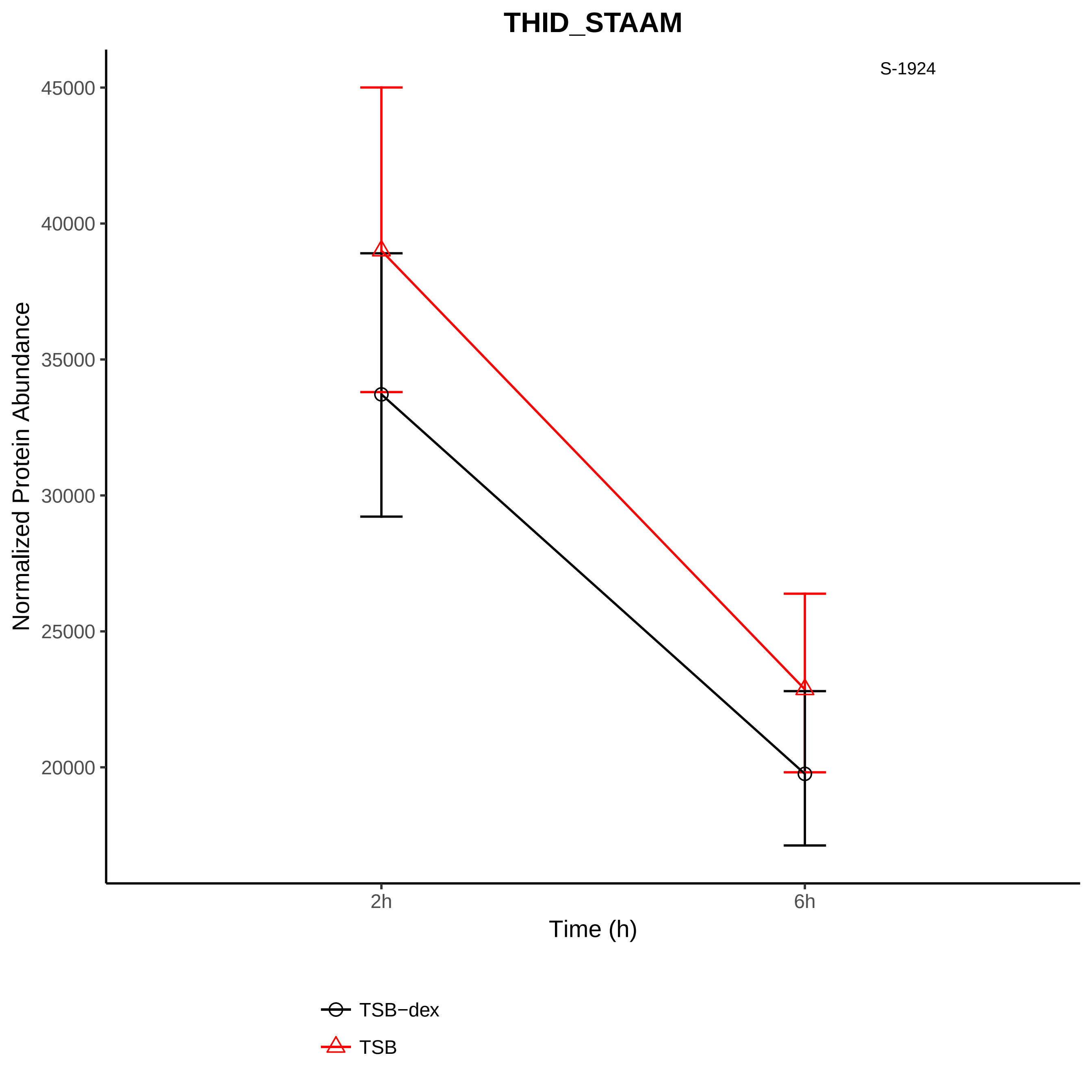
THID_STAAM
S-1924
45000
40000
35000
30000
25000
20000
Normalized Protein Abundance
2h
6h
Time (h)
TSB−dex
TSB

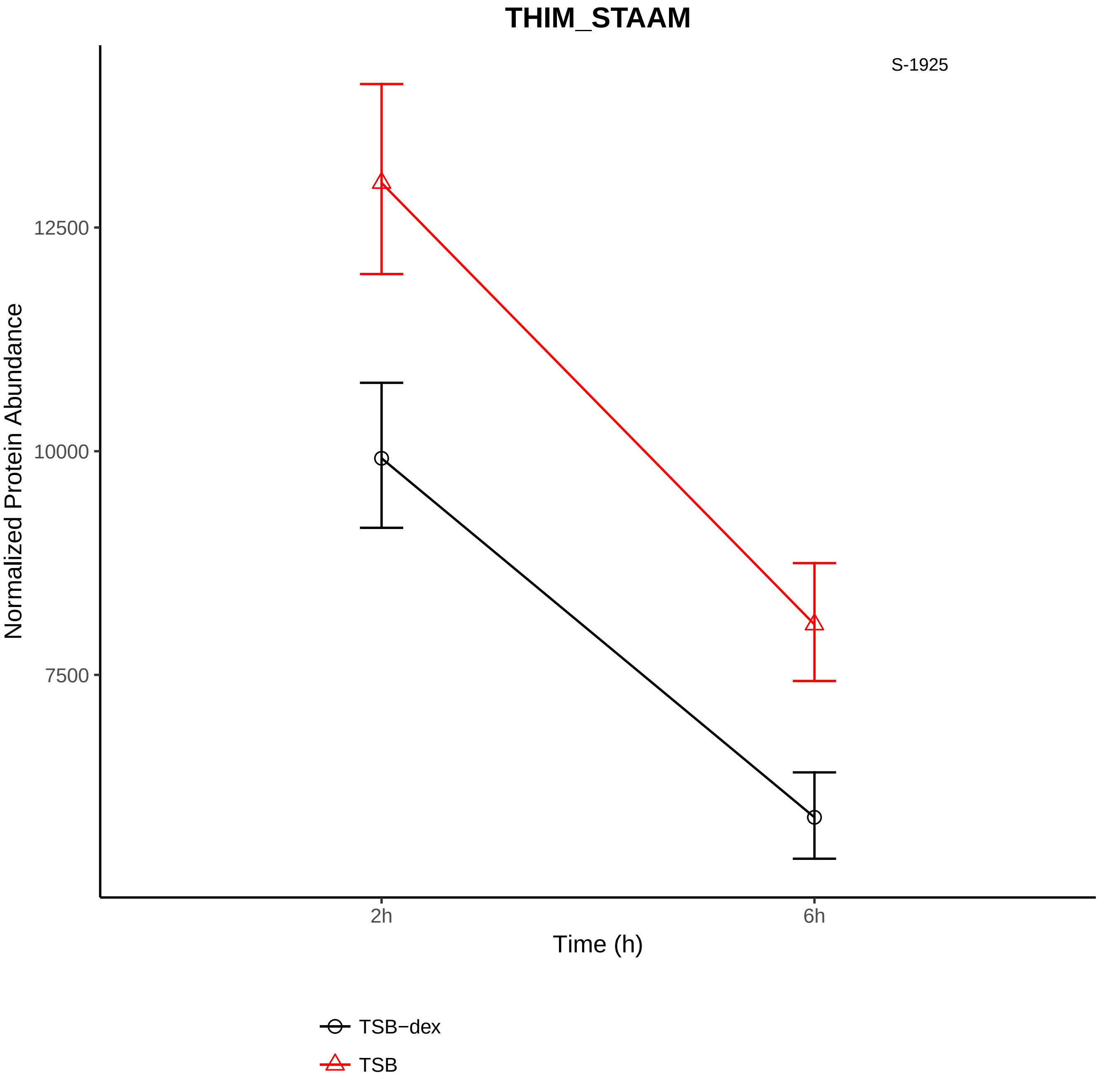
THIM_STAAM
S-1925
Normalized Protein Abundance
12500
10000
7500
2h
6h
Time (h)
TSB−dex
TSB

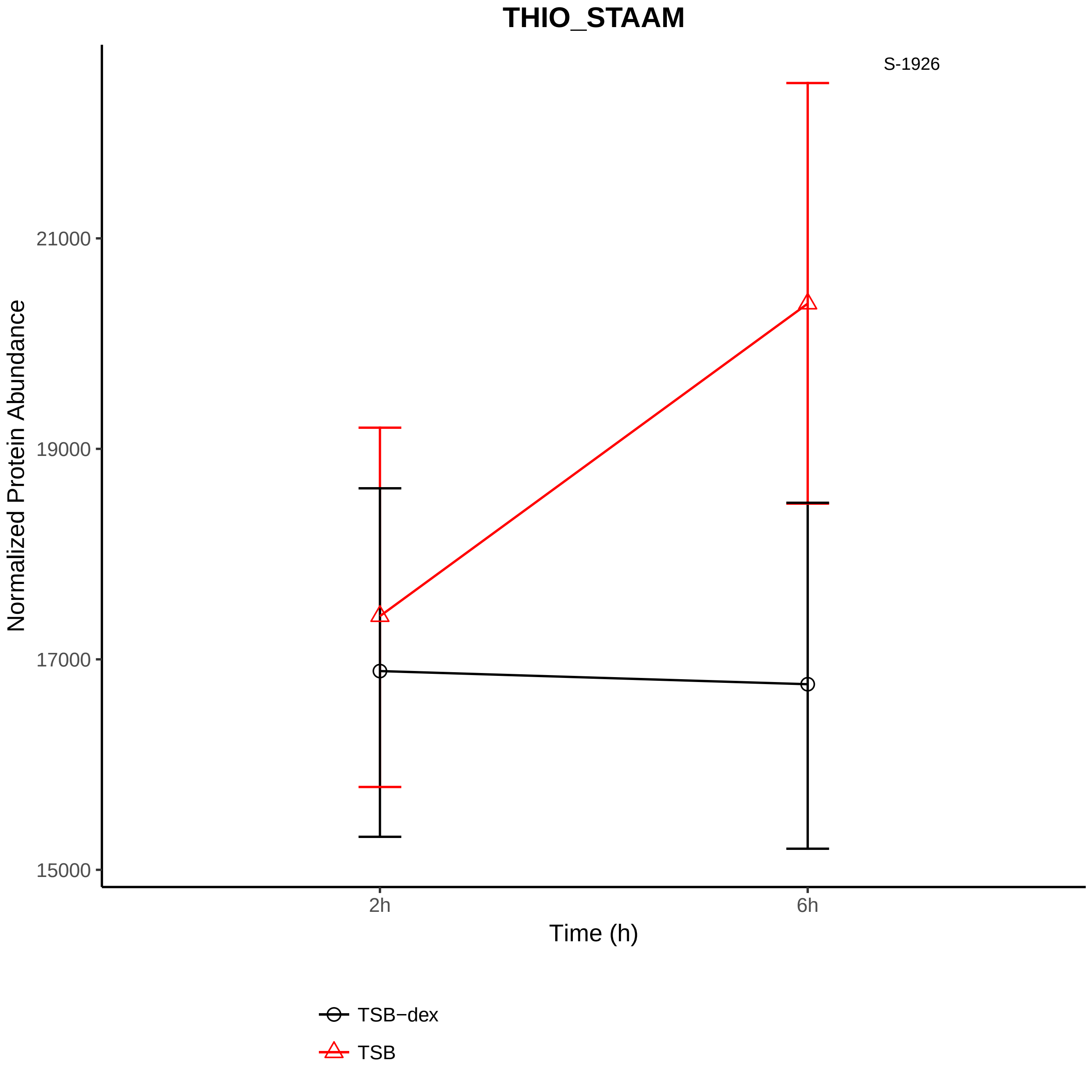

THIO_STAAM
S-1926
Normalized Protein Abundance
21000
19000
17000
15000
2h
6h
Time (h)
TSB−dex
TSB

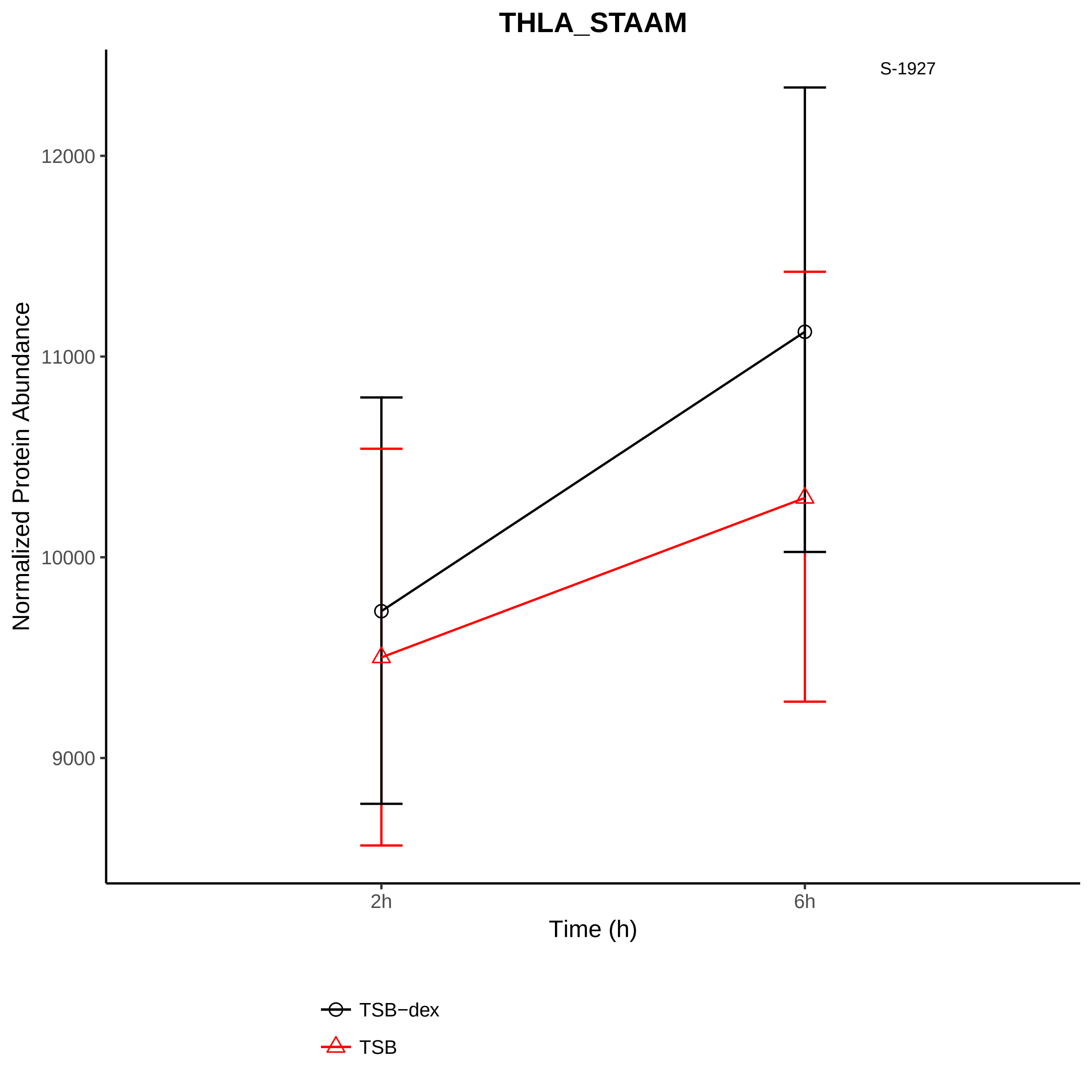

THLA_STAAM
S-1927
Normalized Protein Abundance
12000
11000
10000
9000
2h
6h
Time (h)
TSB−dex
TSB

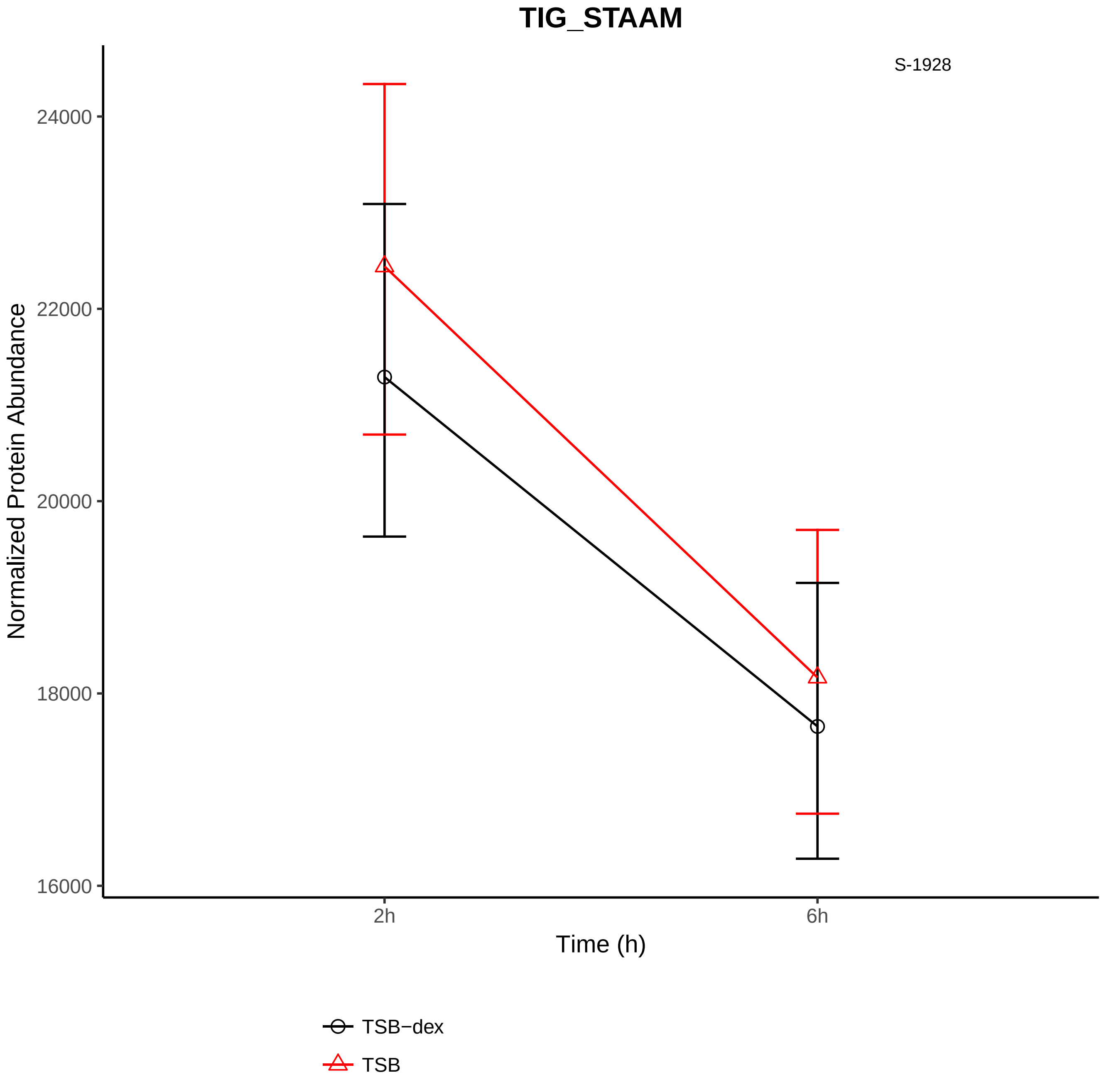

TIG_STAAM
S-1928
Normalized Protein Abundance
24000
22000
20000
18000
16000
2h
6h
Time (h)
TSB−dex
TSB

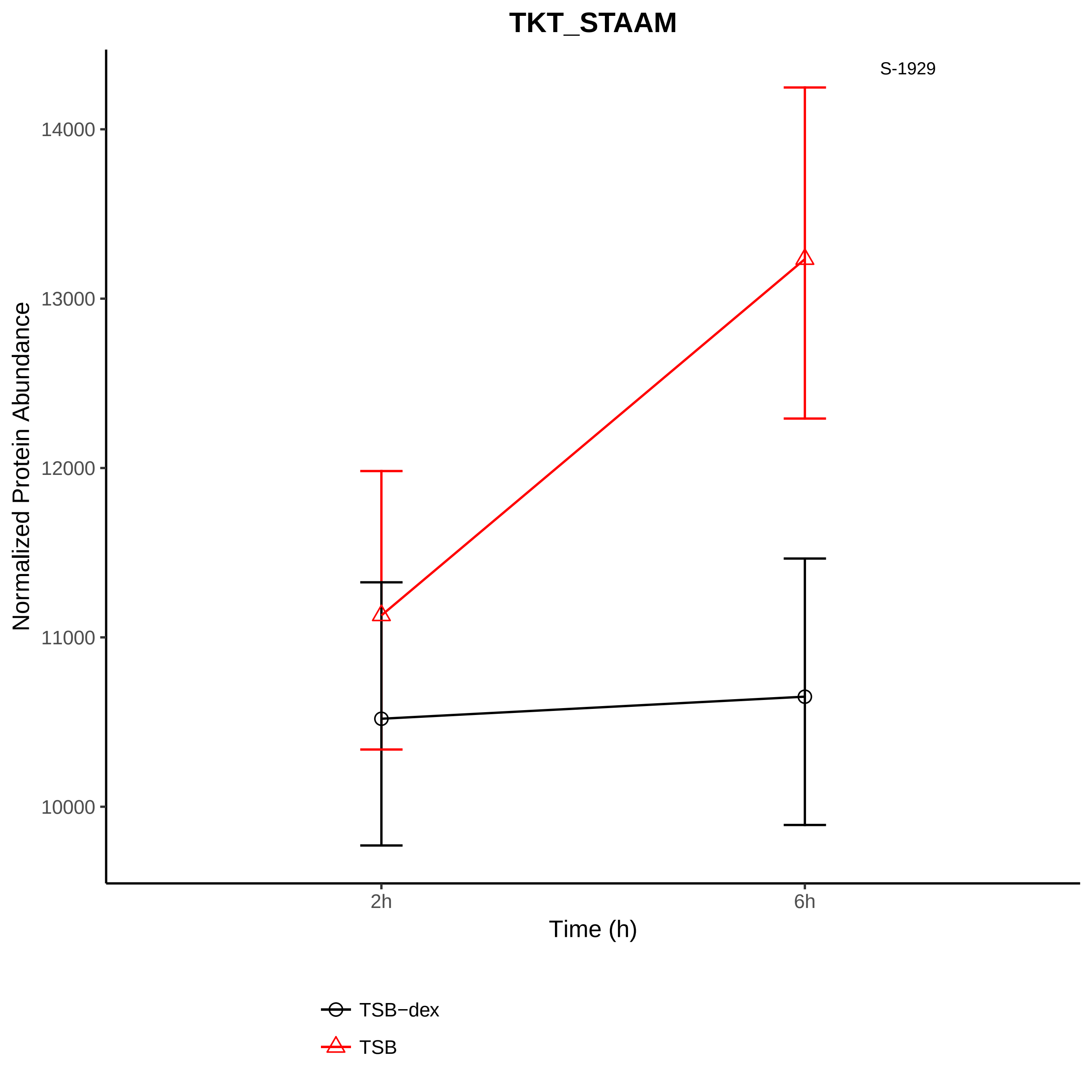

TKT_STAAM
S-1929
Normalized Protein Abundance
14000
13000
12000
11000
10000
2h
6h
Time (h)
TSB−dex
TSB

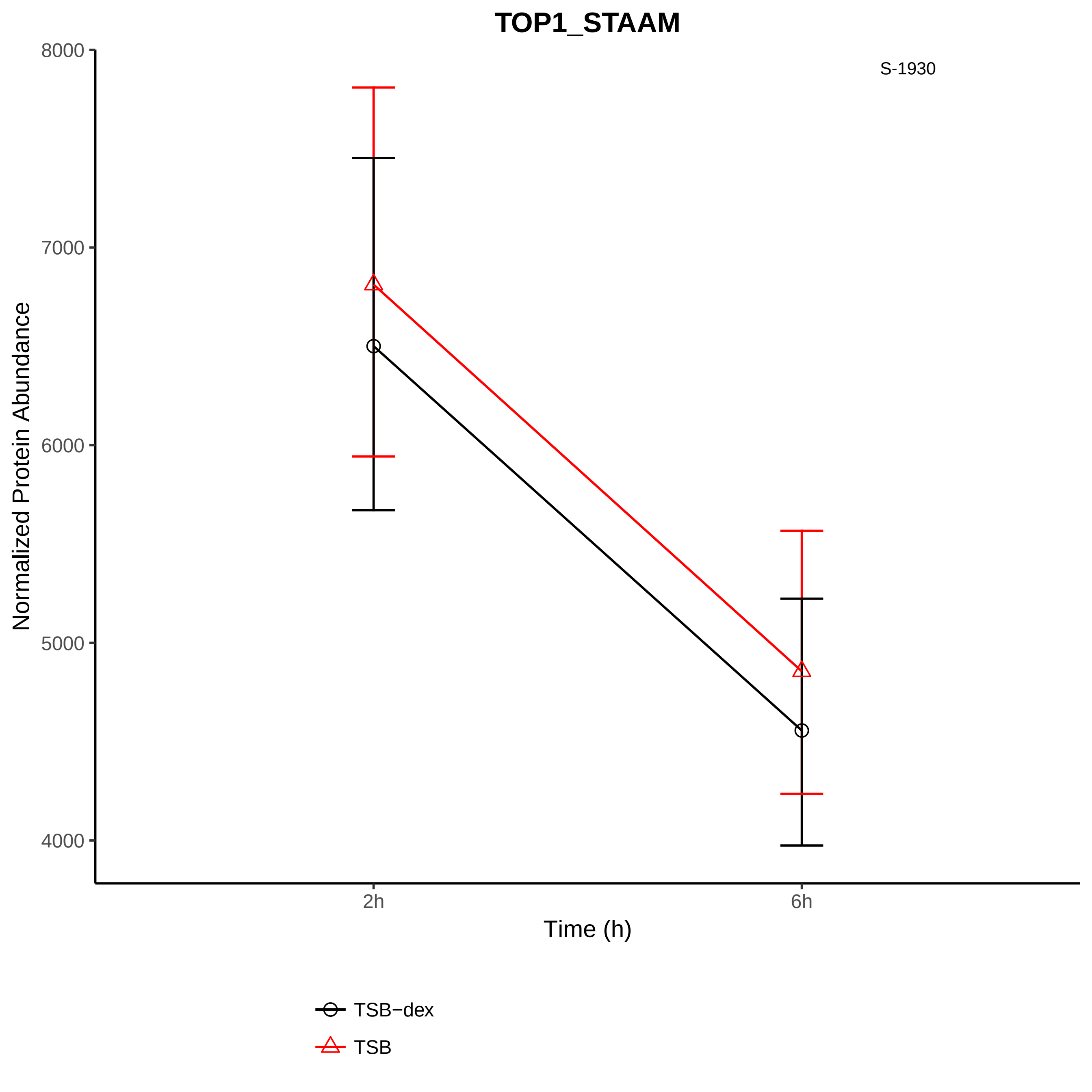

TOP1_STAAM
S-1930
8000
7000
6000
5000
4000
Normalized Protein Abundance
2h
6h
Time (h)
TSB−dex
TSB

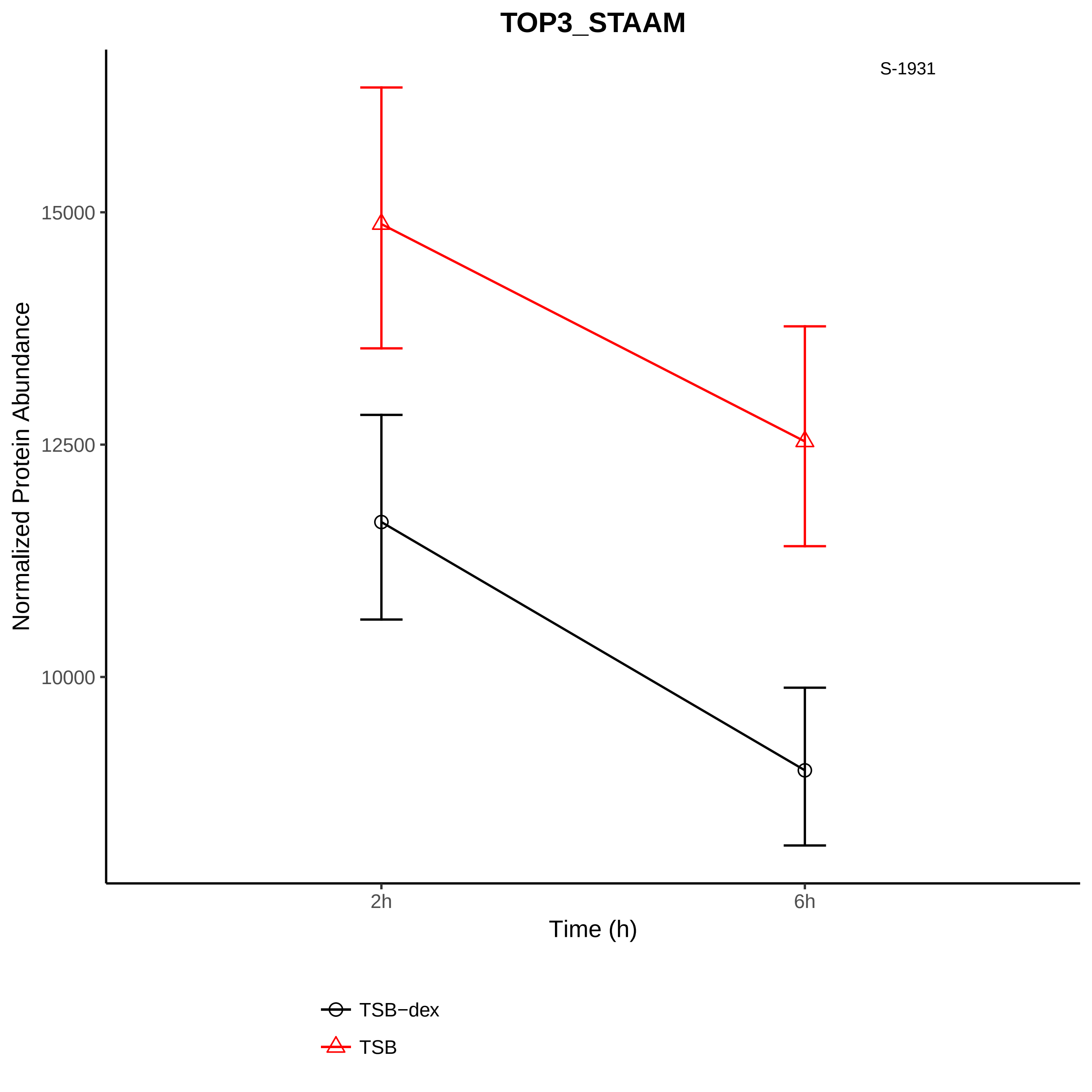

TOP3_STAAM
S-1931
Normalized Protein Abundance
15000
12500
10000
2h
6h
Time (h)
TSB−dex
TSB

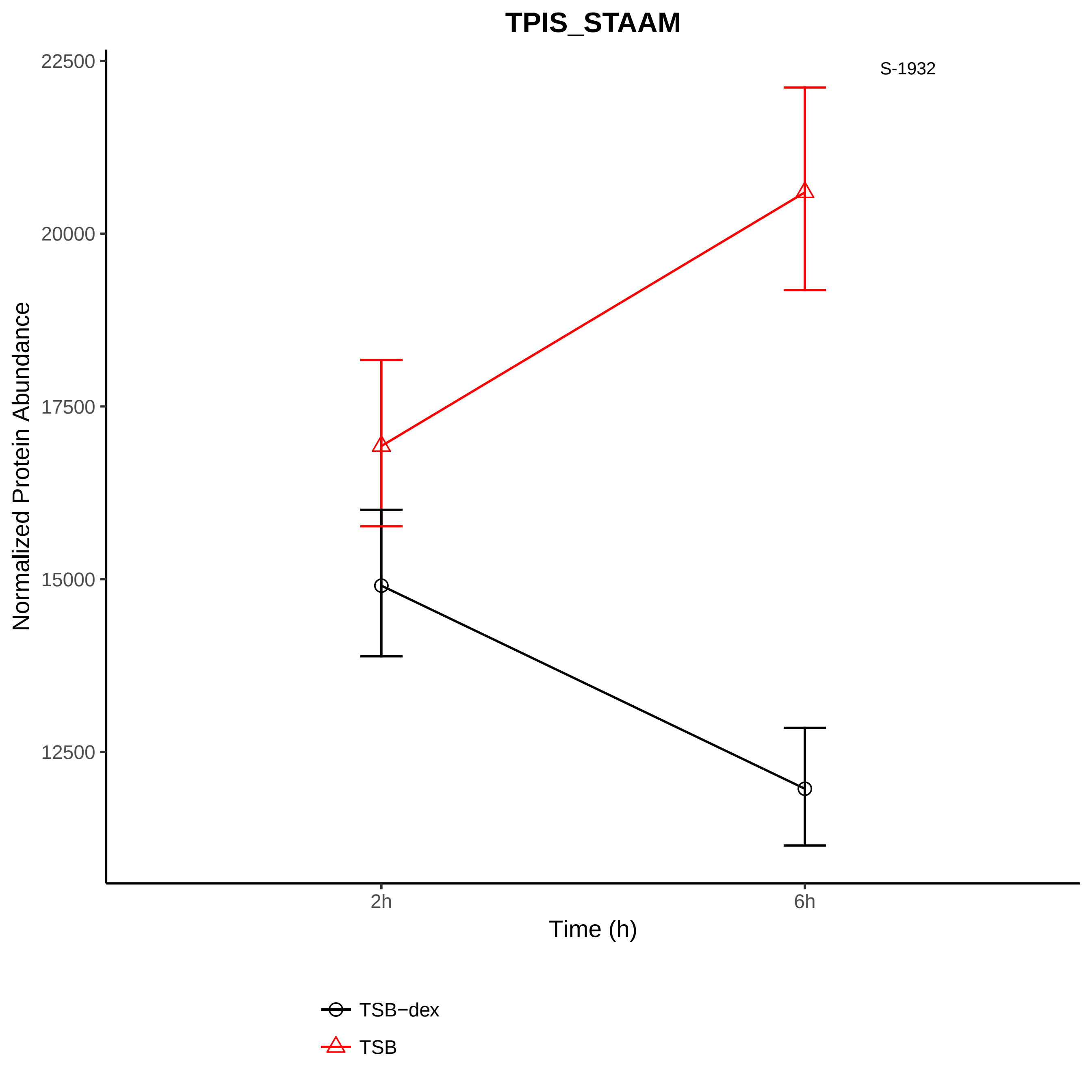
TPIS_STAAM
S-1932
22500
20000
17500
15000
12500
Normalized Protein Abundance
2h
6h
Time (h)
TSB−dex
TSB

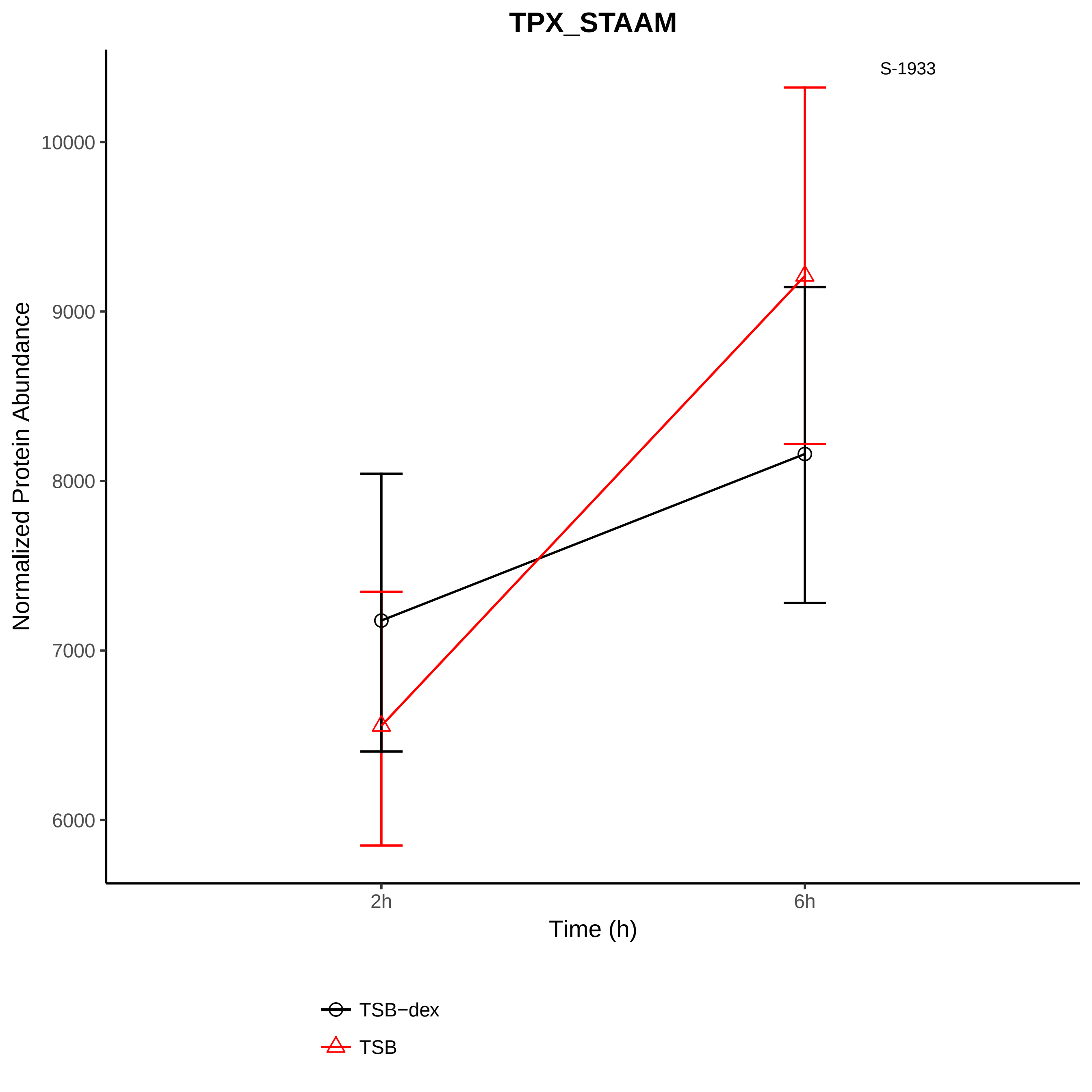

TPX_STAAM
S-1933
Normalized Protein Abundance
10000
9000
8000
7000
6000
2h
6h
Time (h)
TSB−dex
TSB

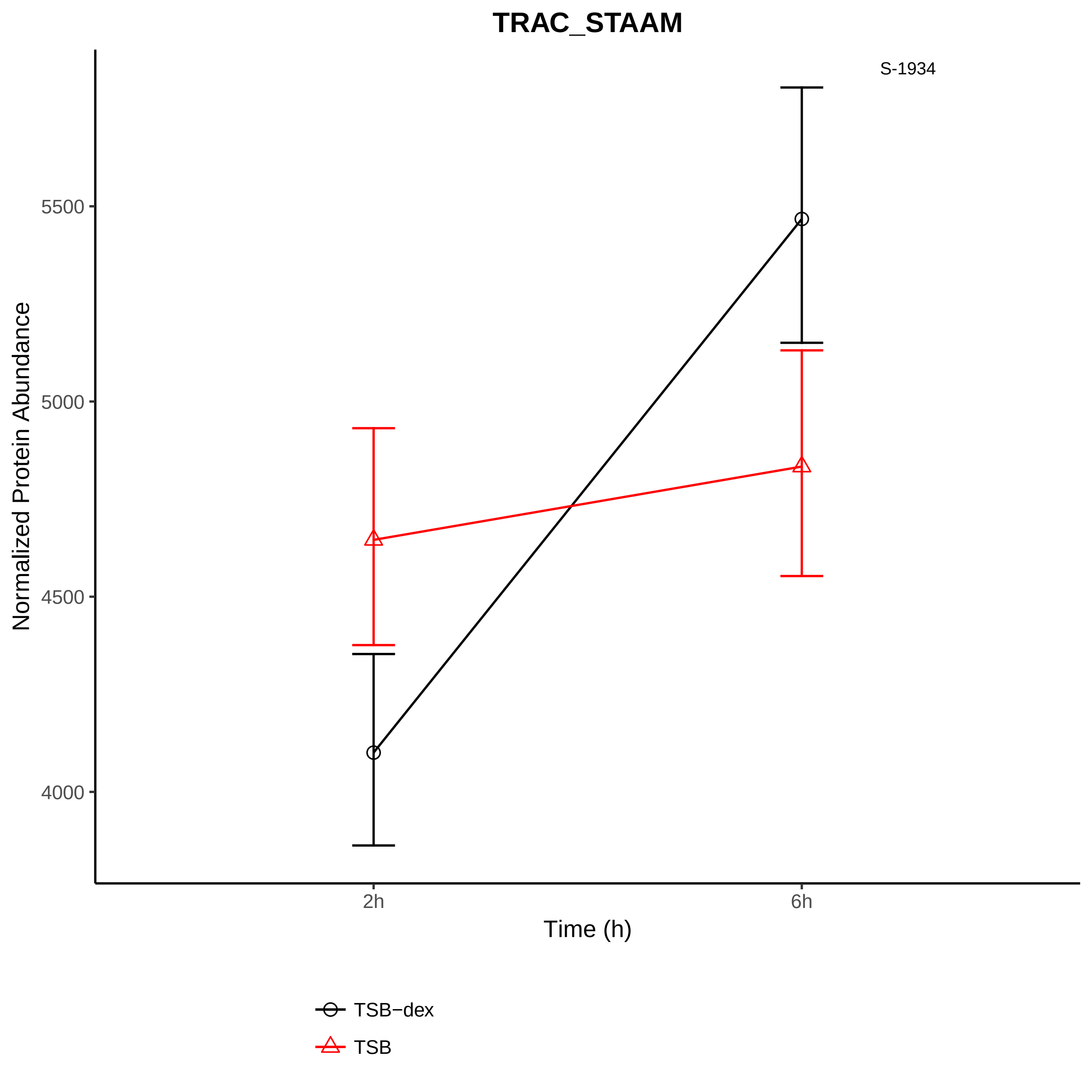

TRAC_STAAM
S-1934
Normalized Protein Abundance
5500
5000
4500
4000
2h
6h
Time (h)
TSB−dex
TSB

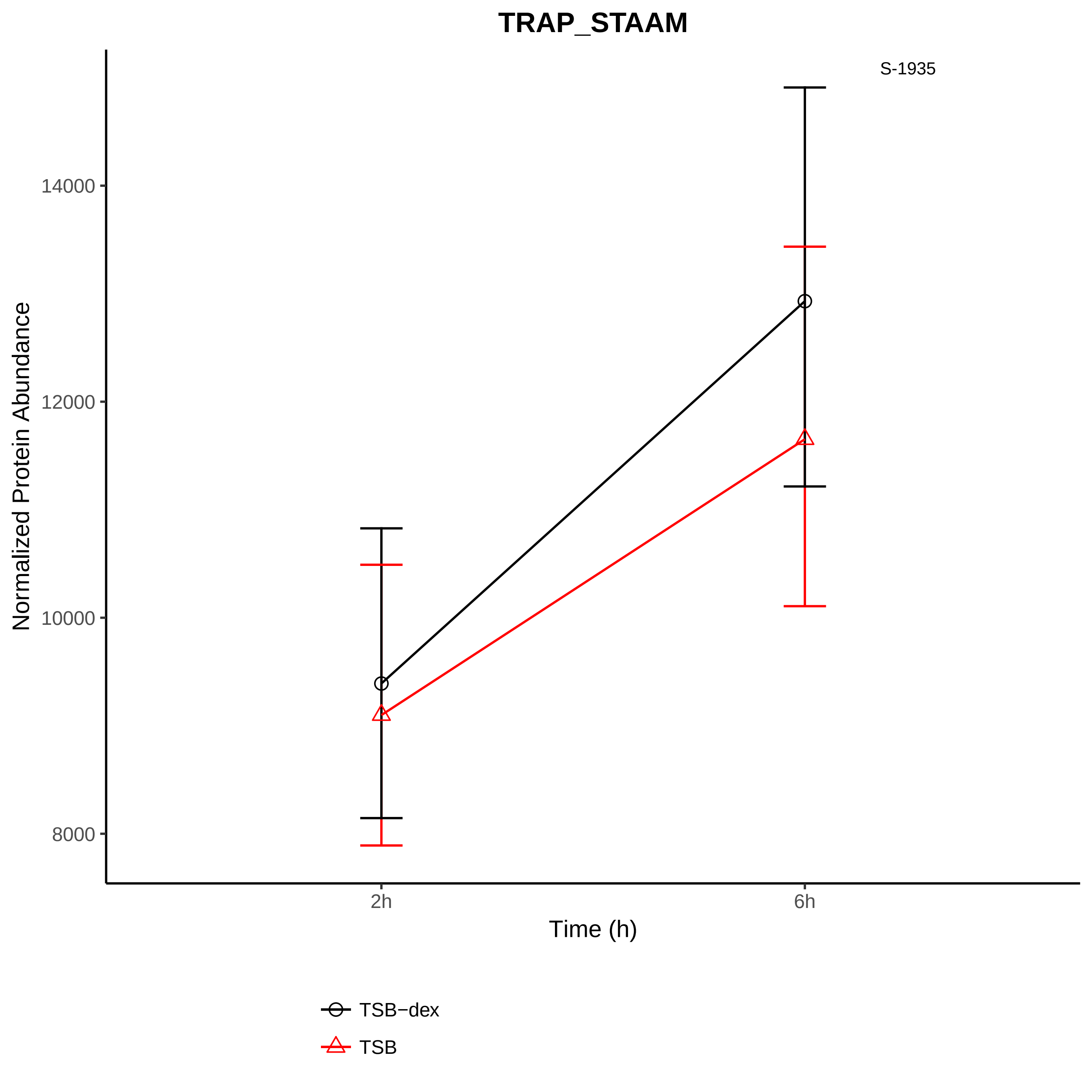
TRAP_STAAM
S-1935
Normalized Protein Abundance
14000
12000
10000
8000
2h
6h
Time (h)
TSB−dex
TSB

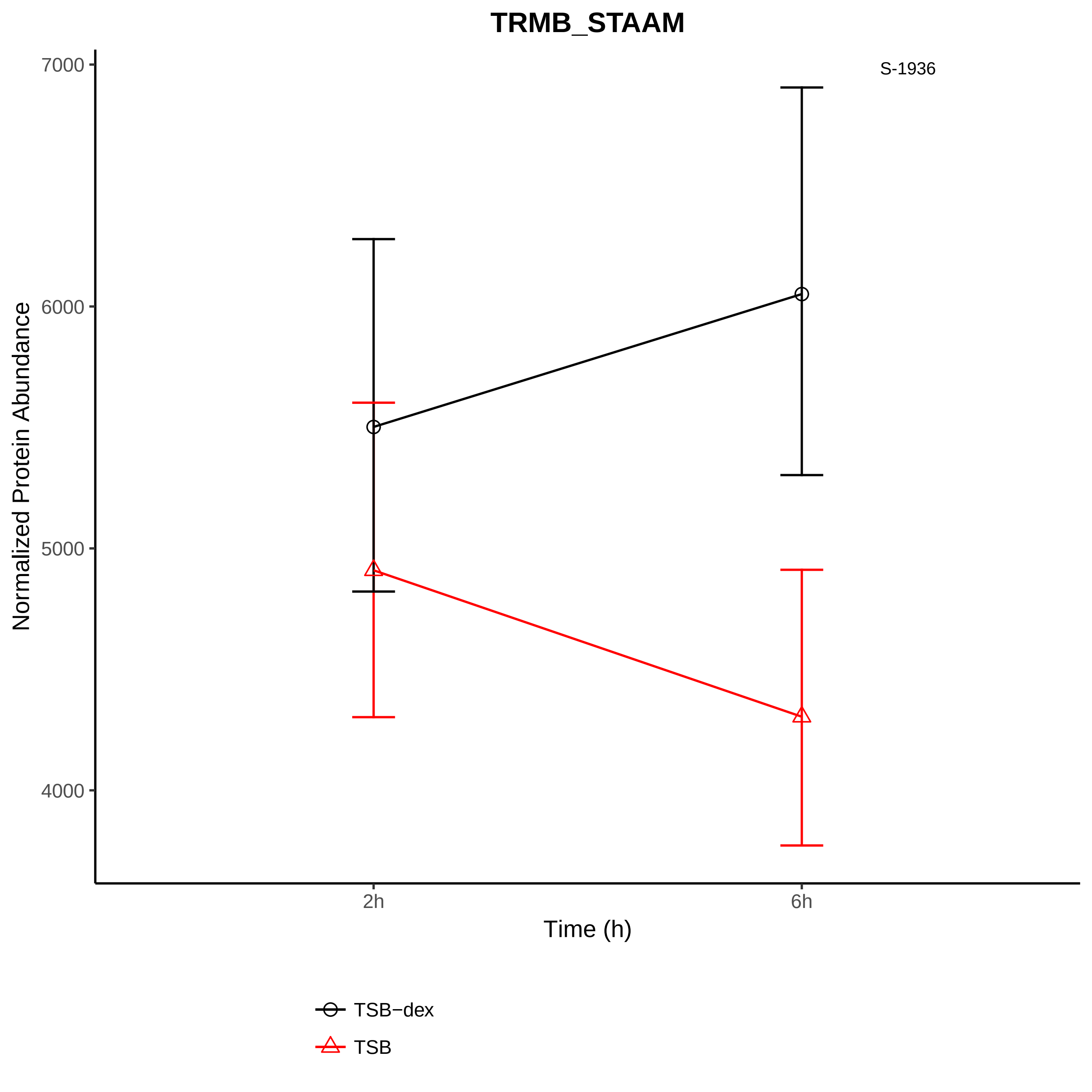

TRMB_STAAM
S-1936
7000
6000
5000
4000
Normalized Protein Abundance
2h
6h
Time (h)
TSB−dex
TSB

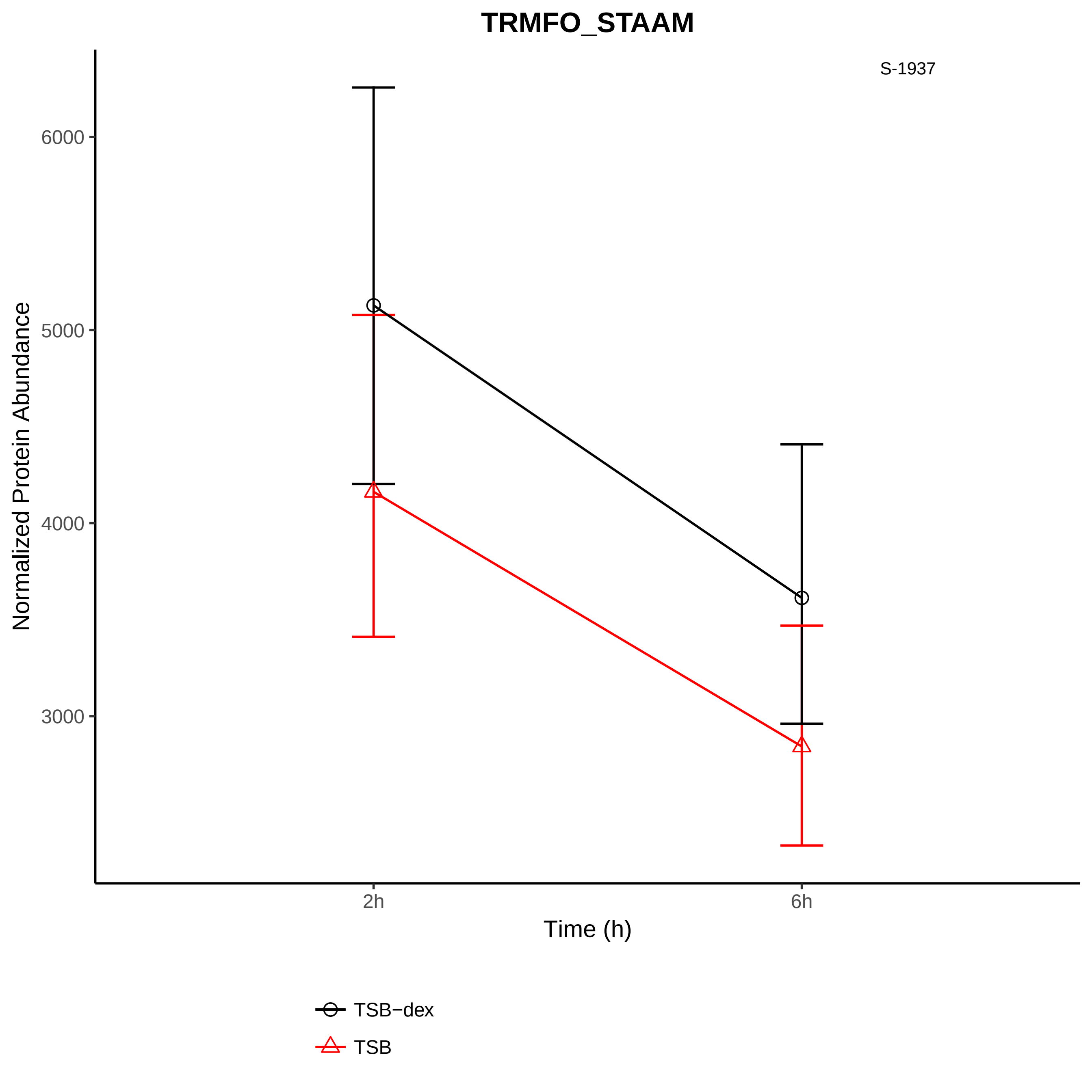

TRMFO_STAAM
S-1937
6000
5000
4000
3000
Normalized Protein Abundance
2h
6h
Time (h)
TSB−dex
TSB

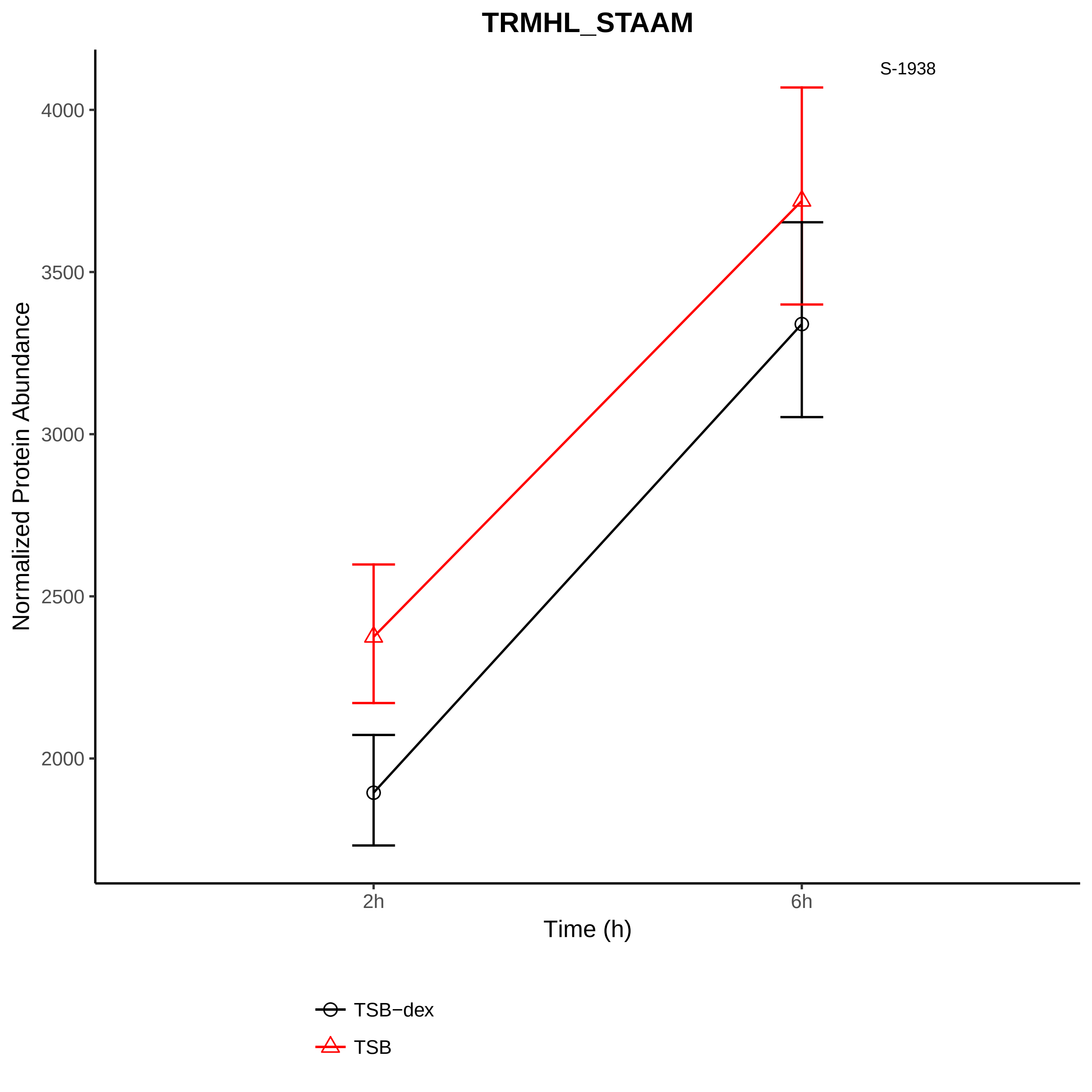

TRMHL_STAAM
S-1938
Normalized Protein Abundance
4000
3500
3000
2500
2000
2h
6h
Time (h)
TSB−dex
TSB

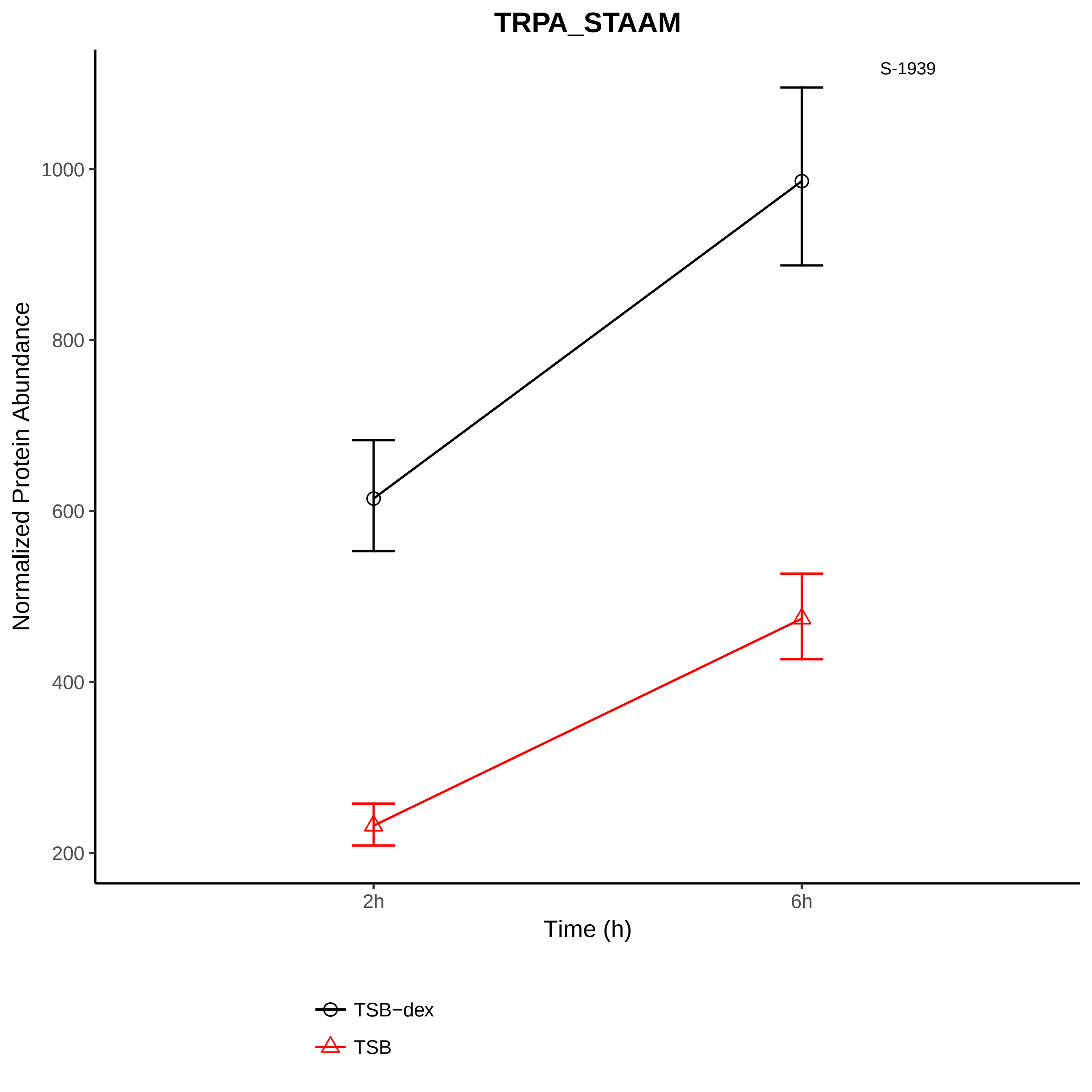

TRPA_STAAM
S-1939
Normalized Protein Abundance
1000
800
600
400
200
2h
6h
Time (h)
TSB−dex
TSB

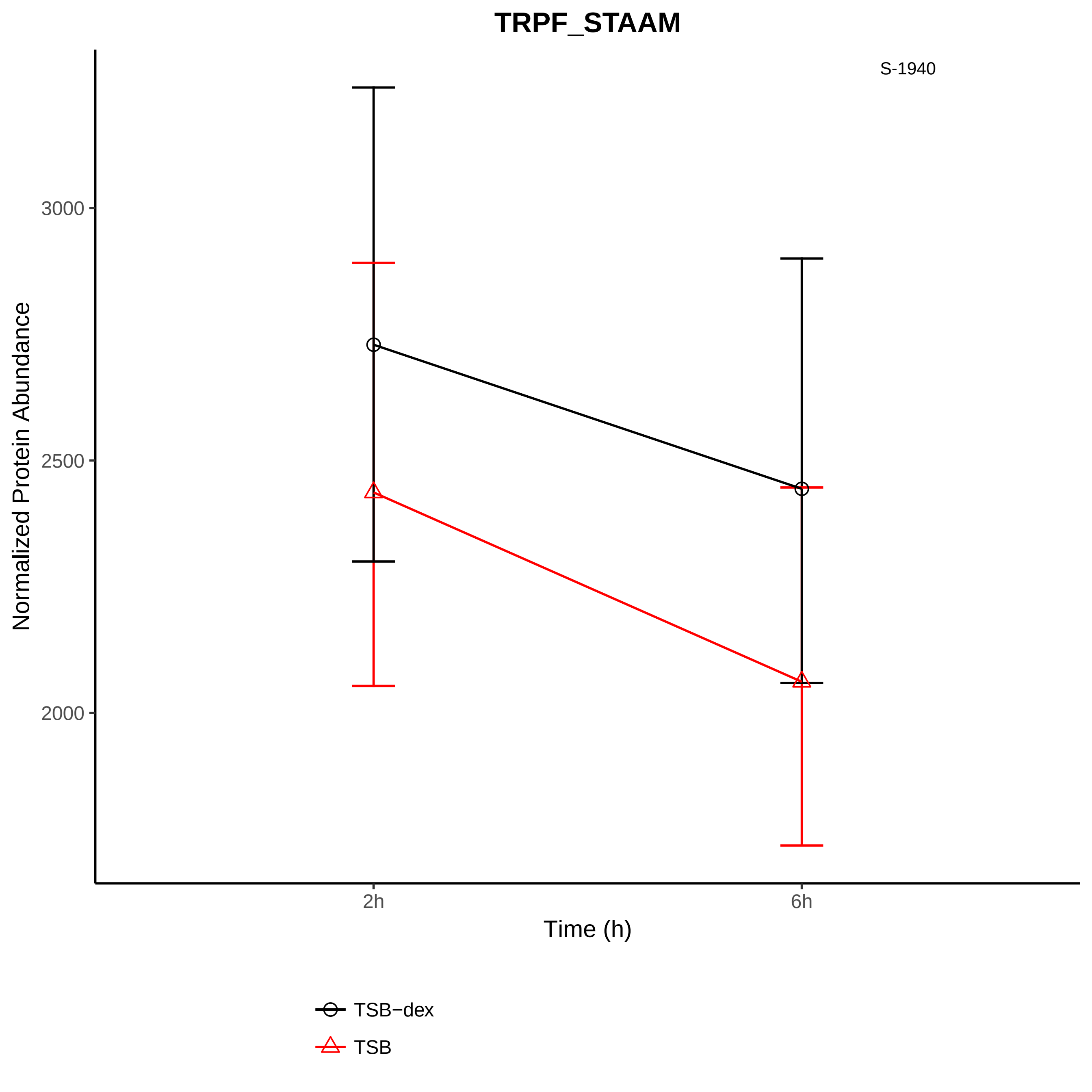

TRPF_STAAM
S-1940
3000
2500
2000
Normalized Protein Abundance
2h
6h
Time (h)
TSB−dex
TSB

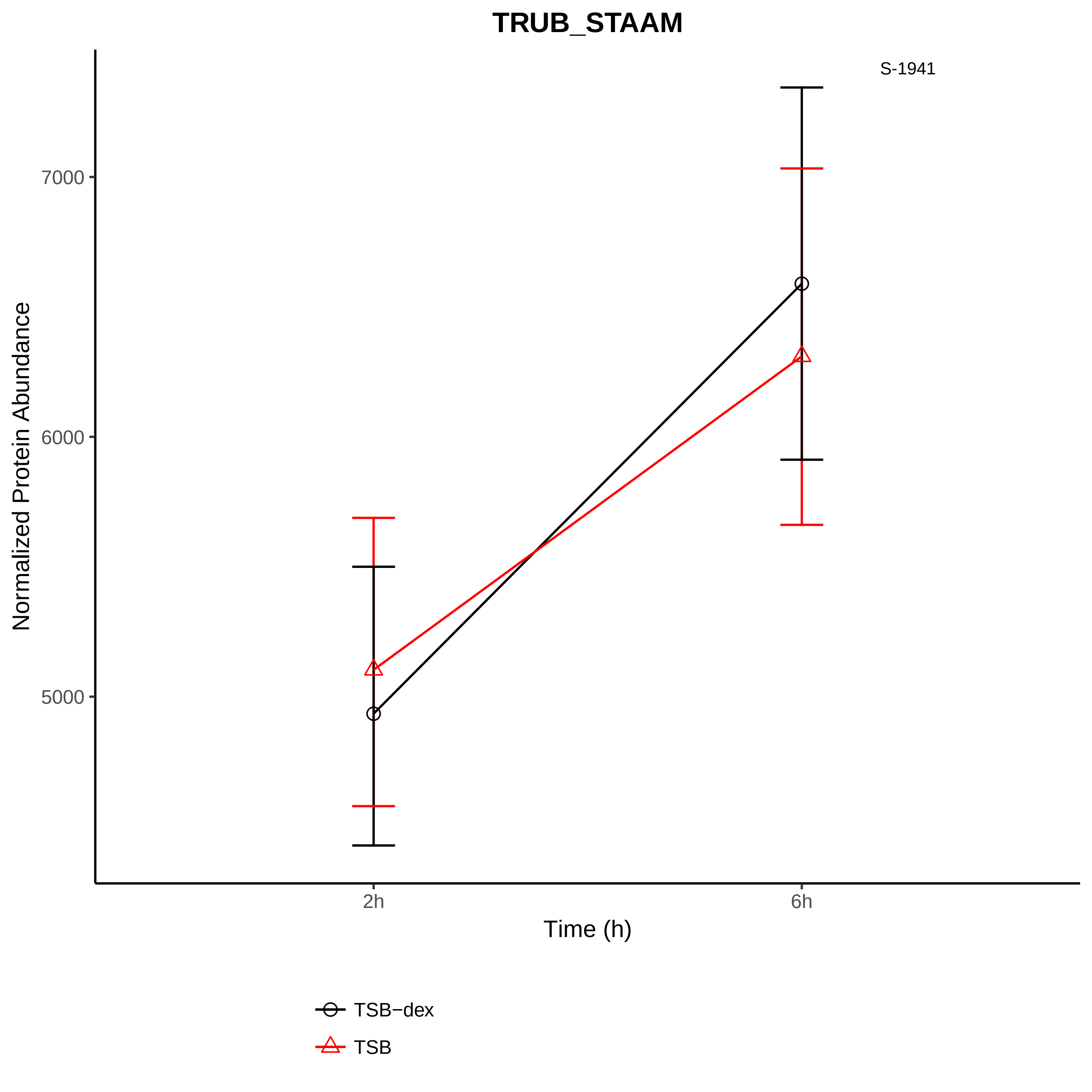

TRUB_STAAM
S-1941
Normalized Protein Abundance
7000
6000
5000
2h
6h
Time (h)
TSB−dex
TSB

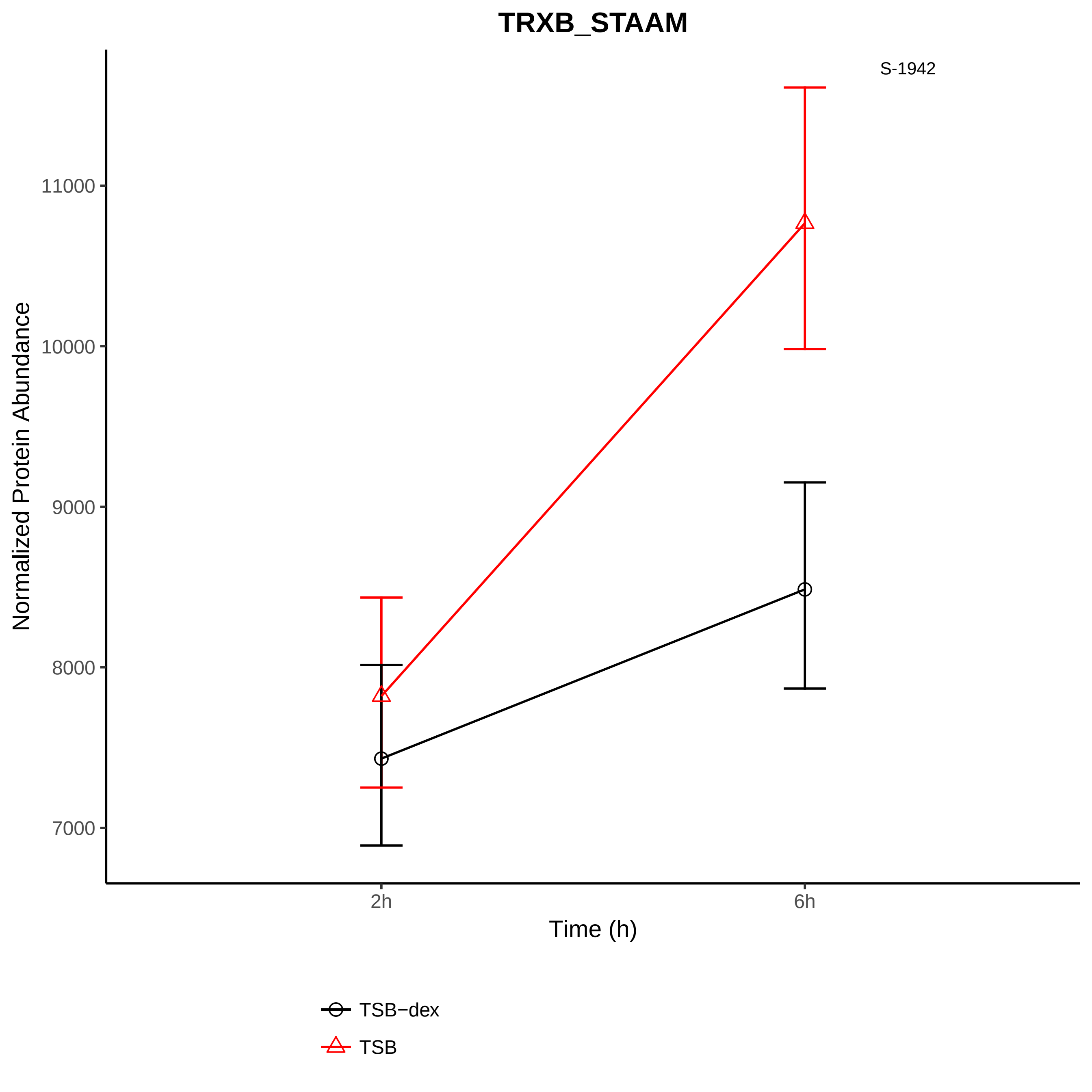

TRXB_STAAM
S-1942
Normalized Protein Abundance
11000
10000
9000
8000
7000
2h
6h
Time (h)
TSB−dex
TSB

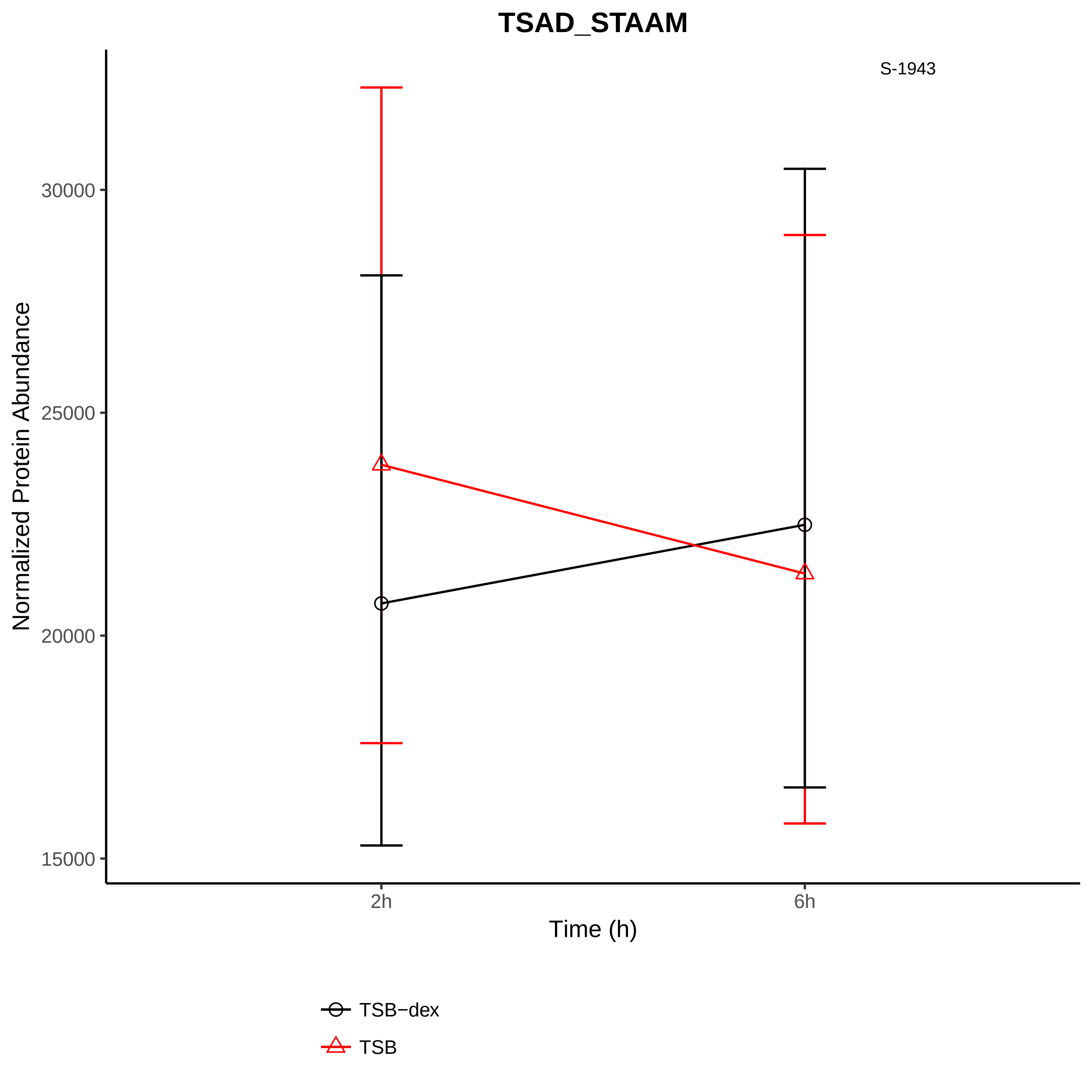

TSAD_STAAM
S-1943
Normalized Protein Abundance
30000
25000
20000
15000
2h
6h
Time (h)
TSB−dex
TSB

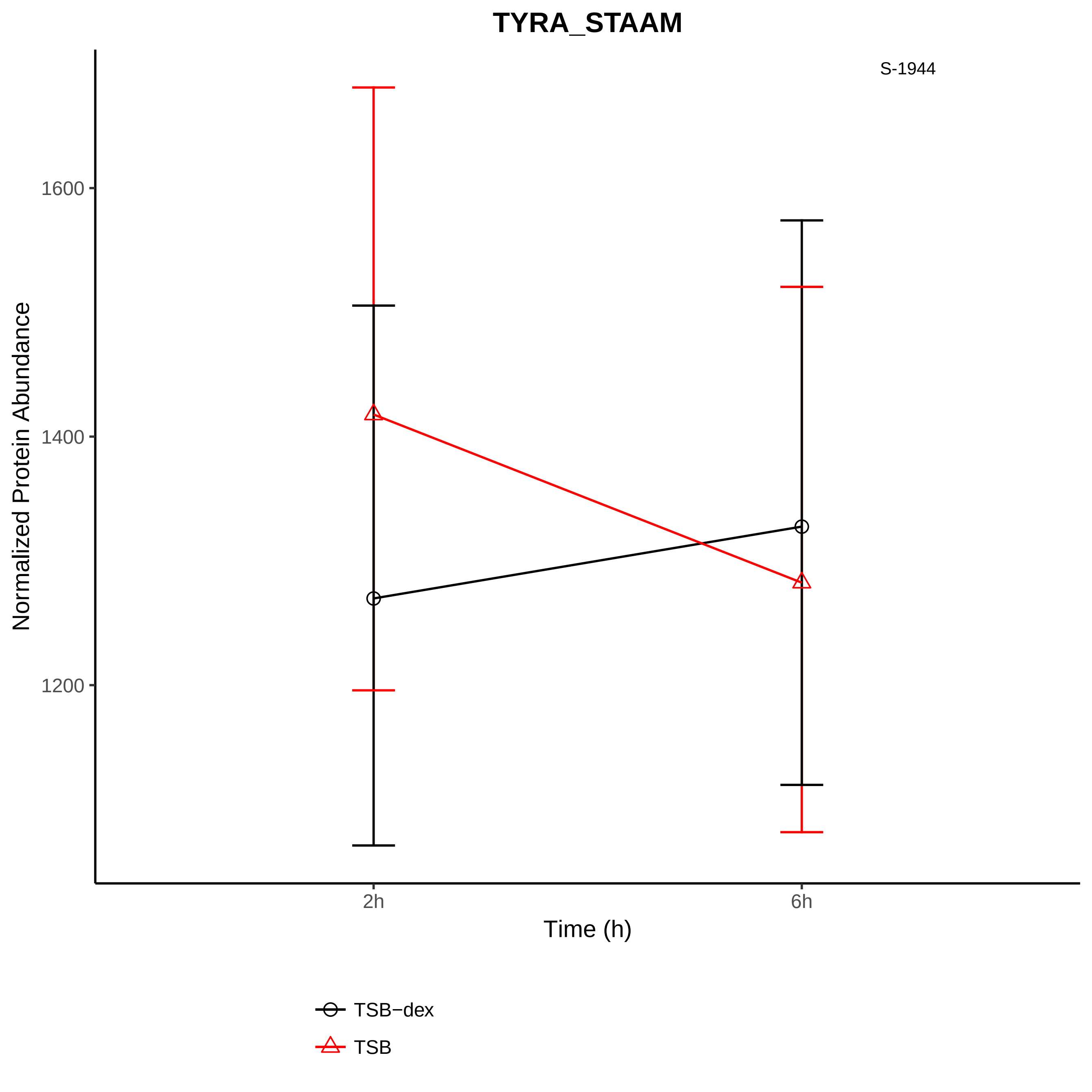
TYRA_STAAM
S-1944
Normalized Protein Abundance
1600
1400
1200
2h
6h
Time (h)
TSB−dex
TSB

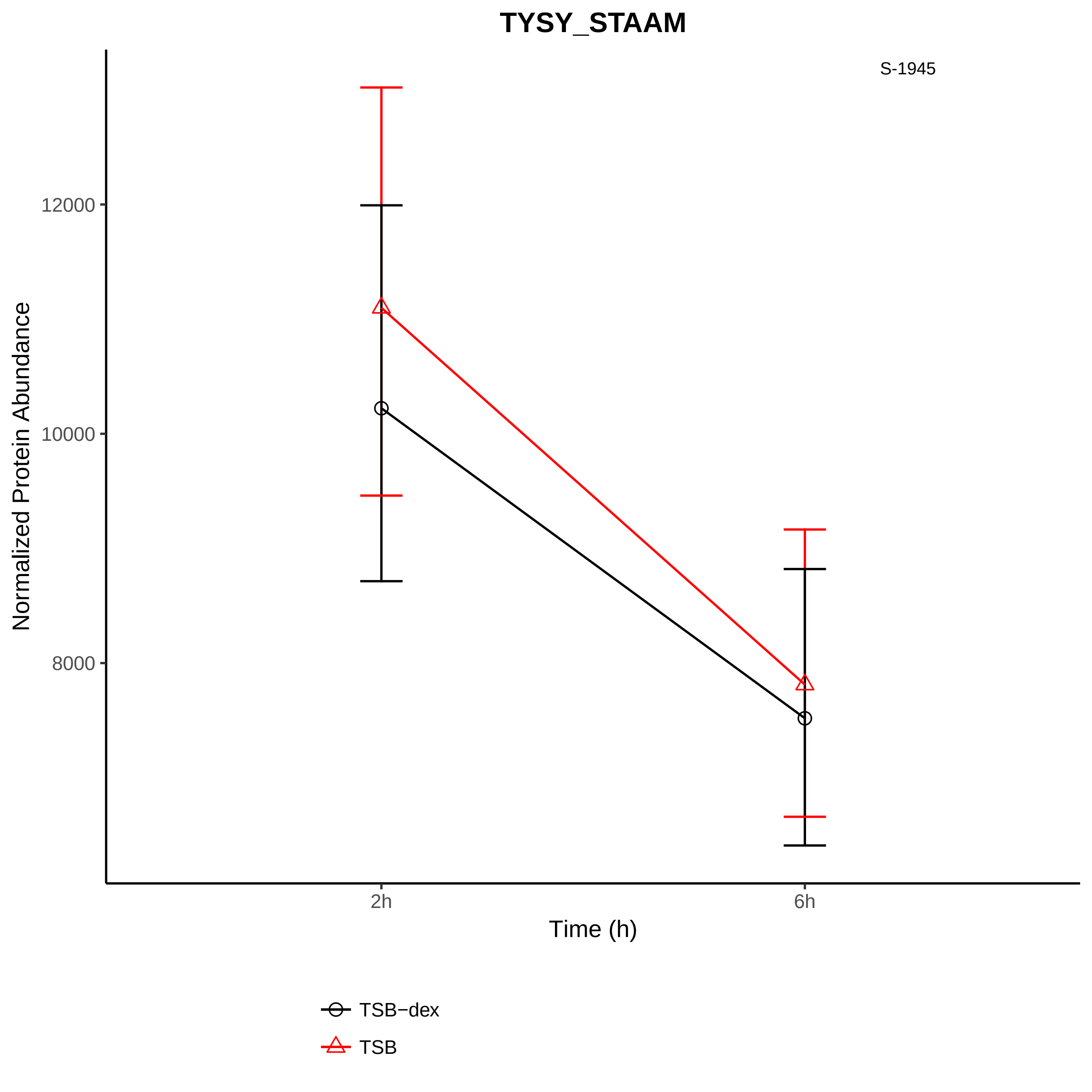

TYSY_STAAM
S-1945
Normalized Protein Abundance
12000
10000
8000
2h
6h
Time (h)
TSB−dex
TSB

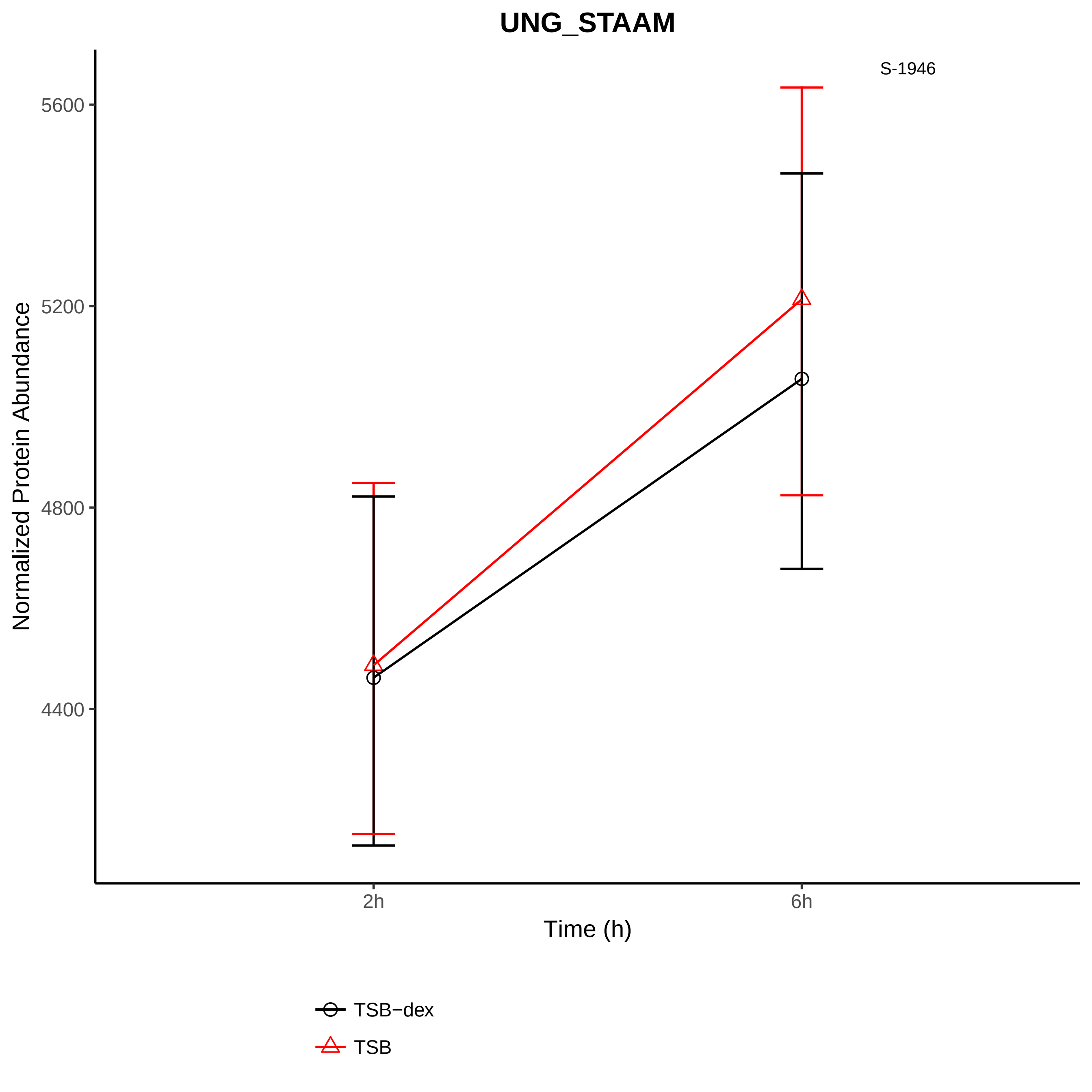
UNG_STAAM
S-1946
Normalized Protein Abundance
5600
5200
4800
4400
2h
6h
Time (h)
TSB−dex
TSB

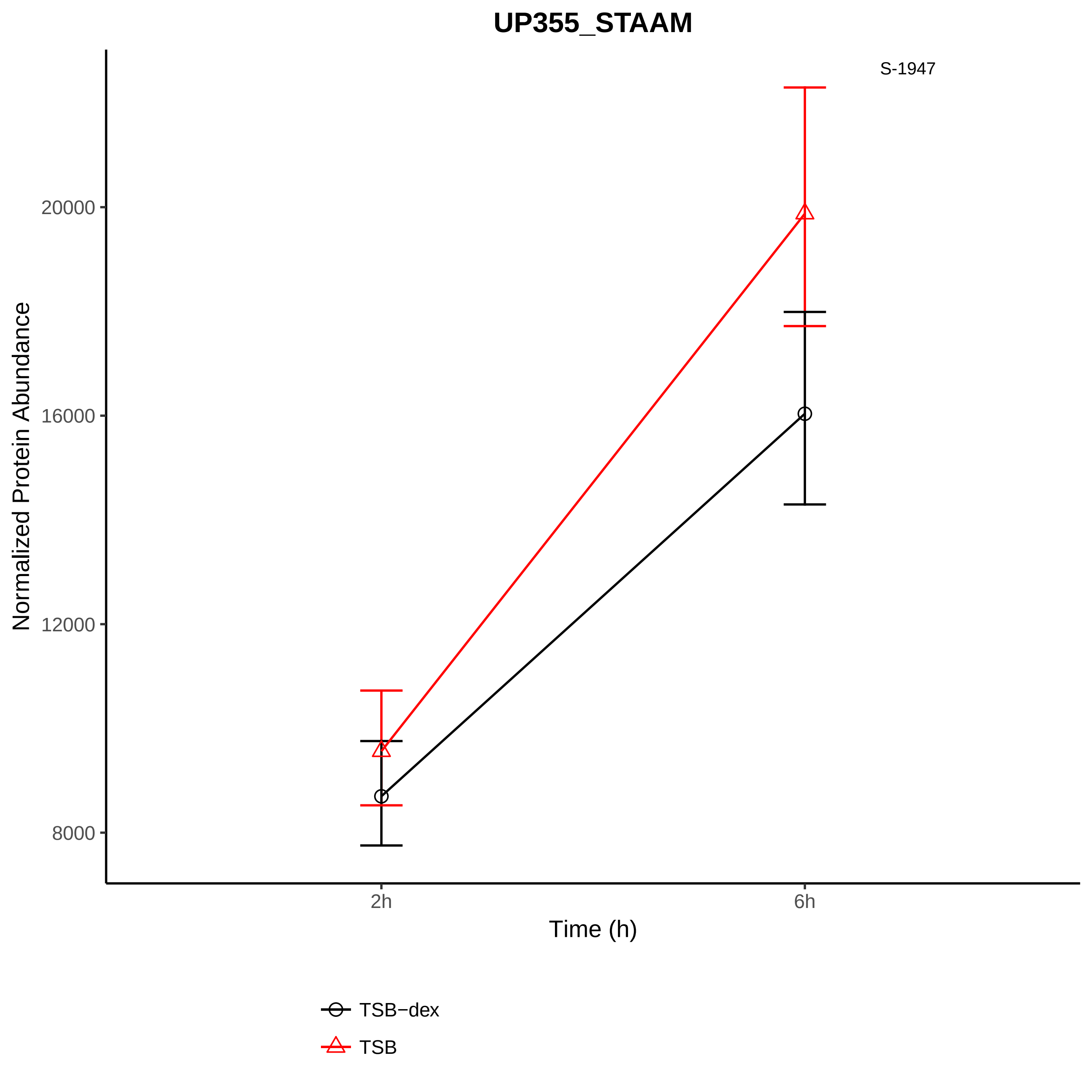
UP355_STAAM
S-1947
Normalized Protein Abundance
20000
16000
12000
8000
2h
6h
Time (h)
TSB−dex
TSB

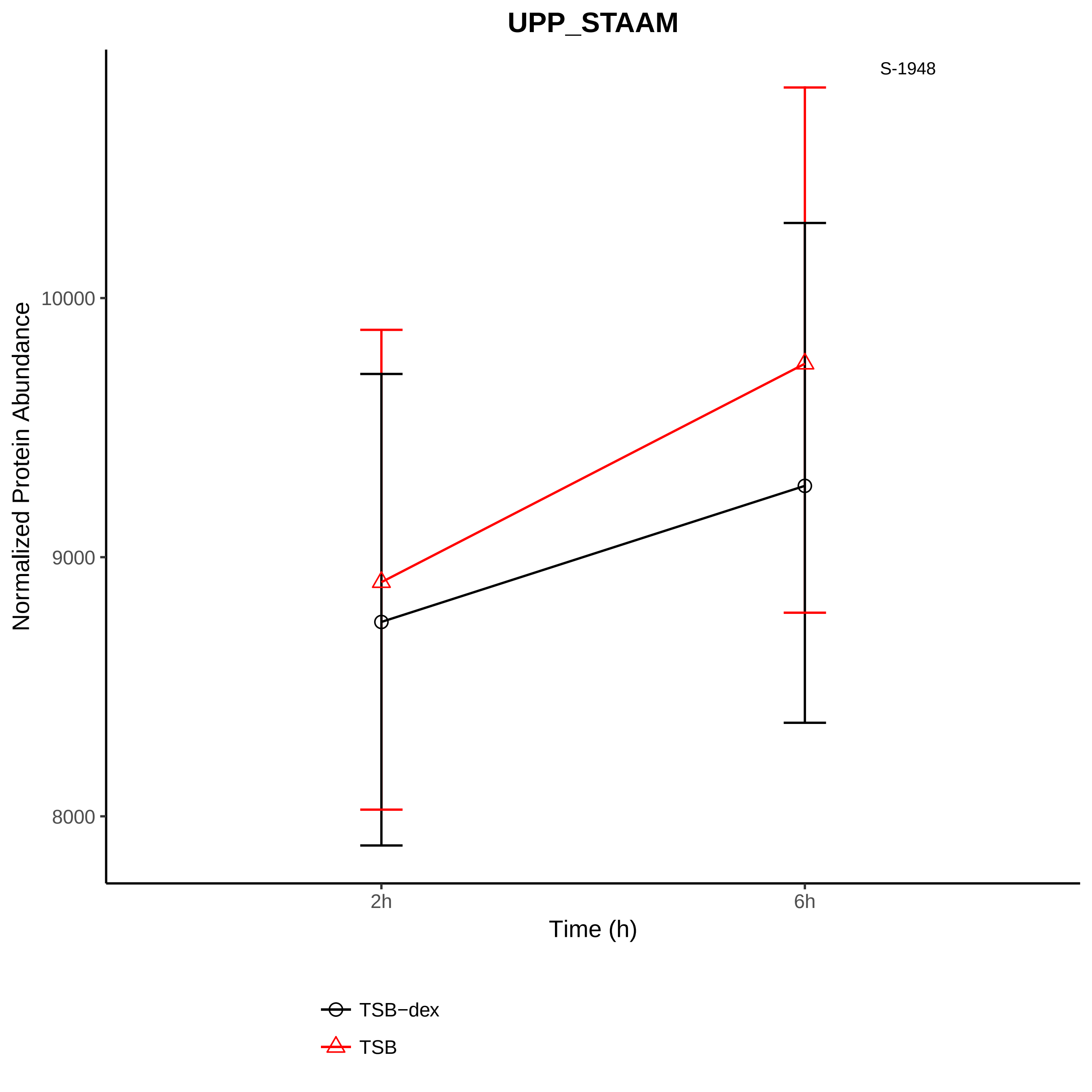
UPP_STAAM
S-1948
Normalized Protein Abundance
10000
9000
8000
2h
6h
Time (h)
TSB−dex
TSB

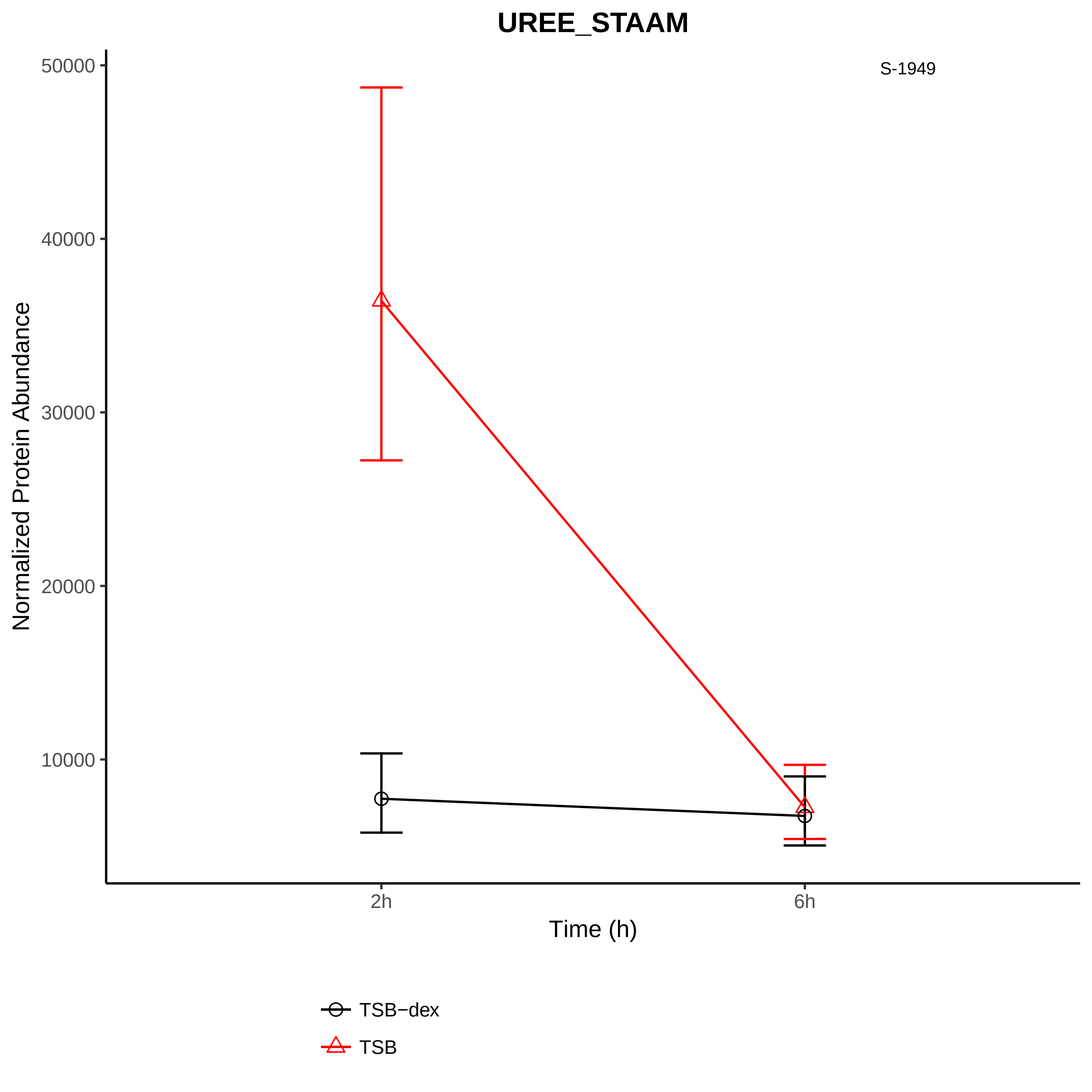

UREE_STAAM
S-1949
50000
40000
30000
20000
10000
Normalized Protein Abundance
2h
6h
Time (h)
TSB−dex
TSB

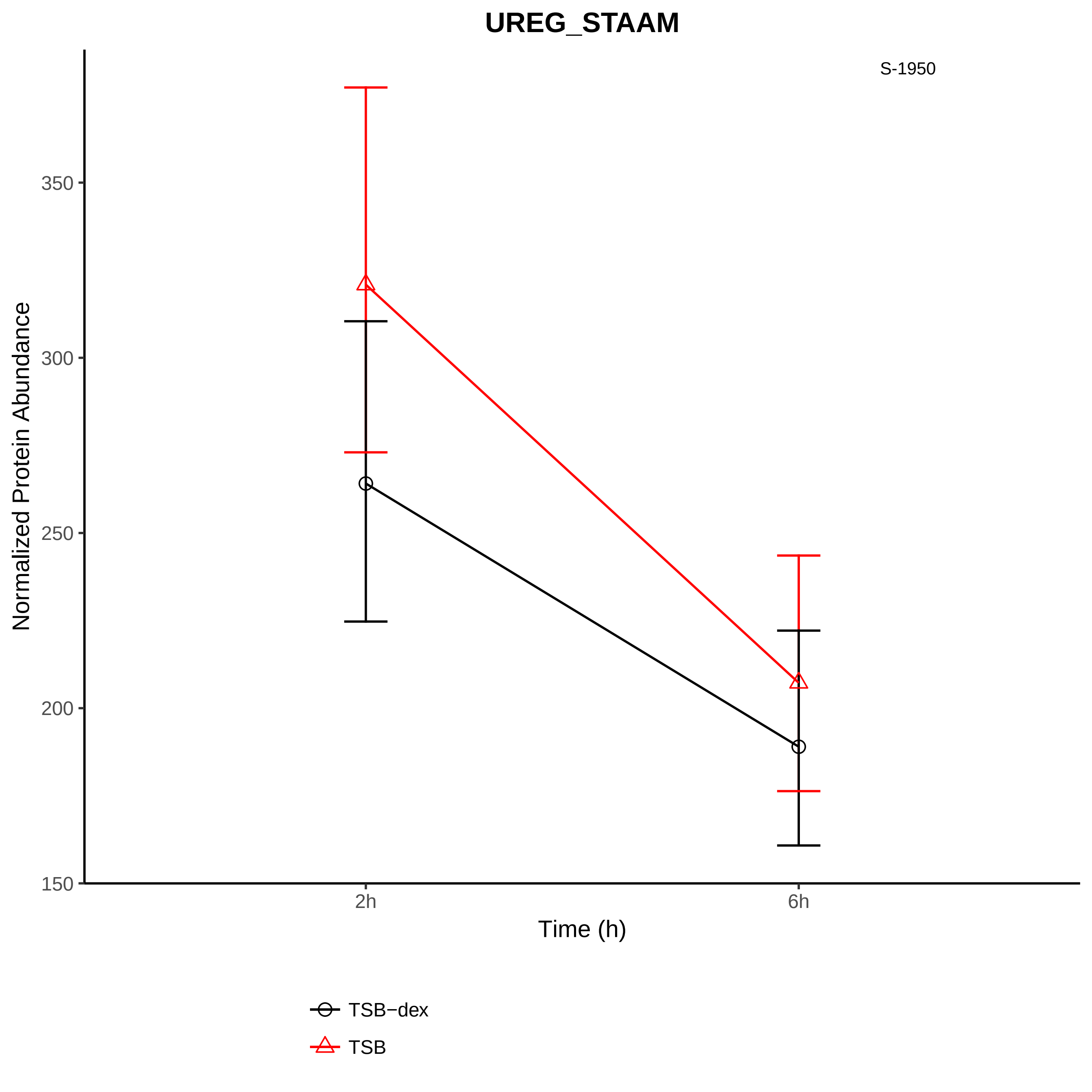
UREG_STAAM
S-1950
Normalized Protein Abundance
350
300
250
200
150
2h
6h
Time (h)
TSB−dex
TSB

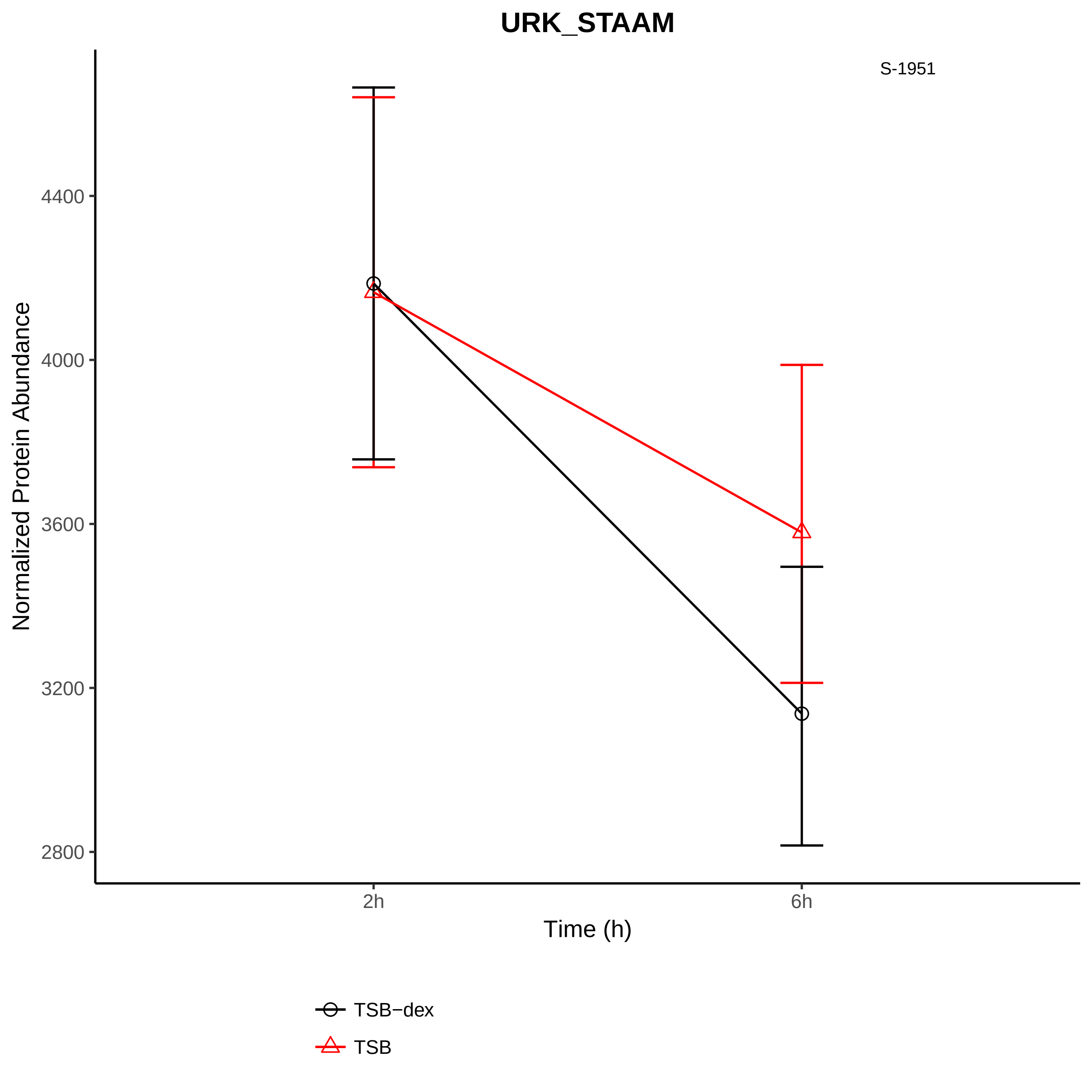
URK_STAAM
S-1951
4400
4000
3600
3200
2800
Normalized Protein Abundance
2h
6h
Time (h)
TSB−dex
TSB

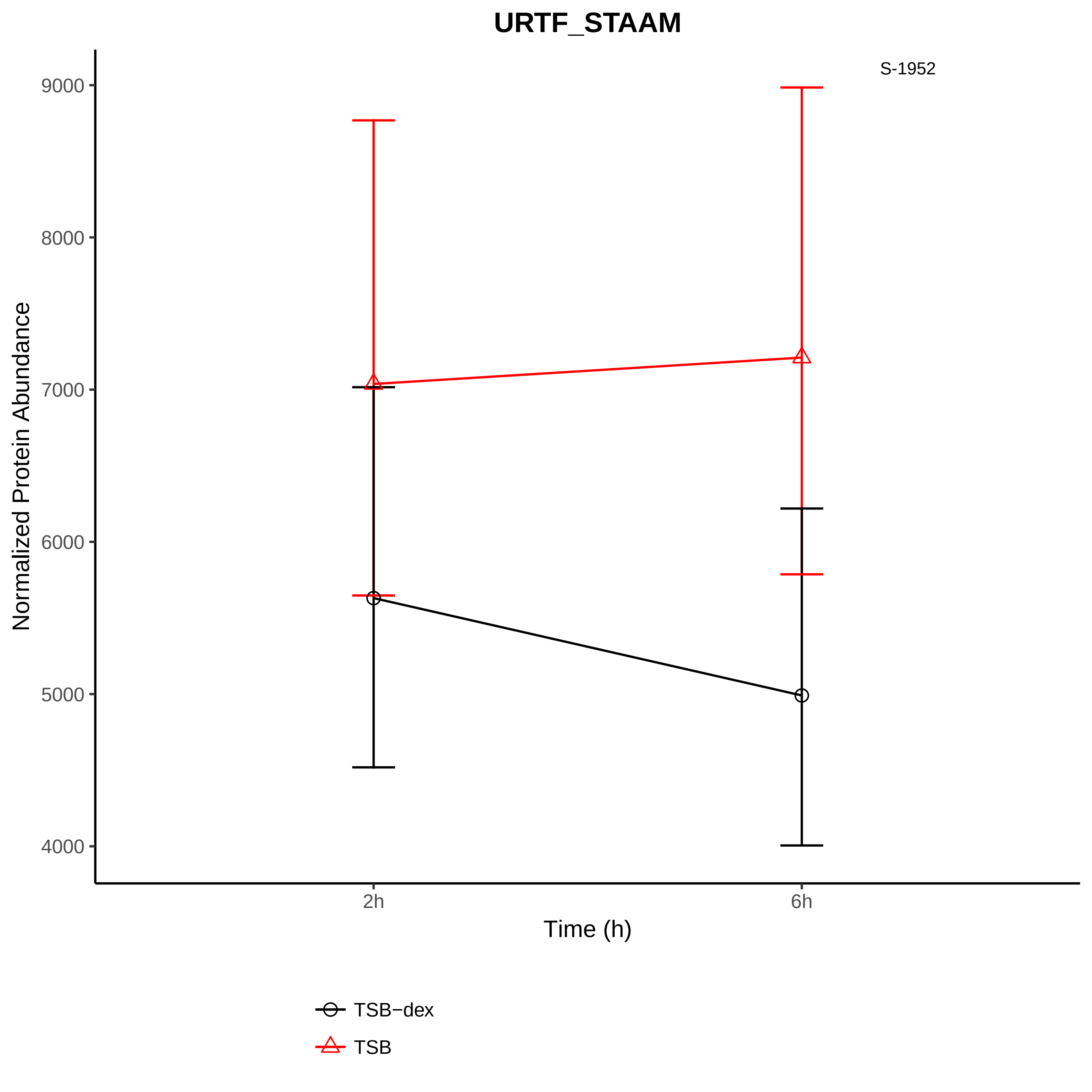
URTF_STAAM
S-1952
Normalized Protein Abundance
9000
8000
7000
6000
5000
4000
2h
6h
Time (h)
TSB−dex
TSB

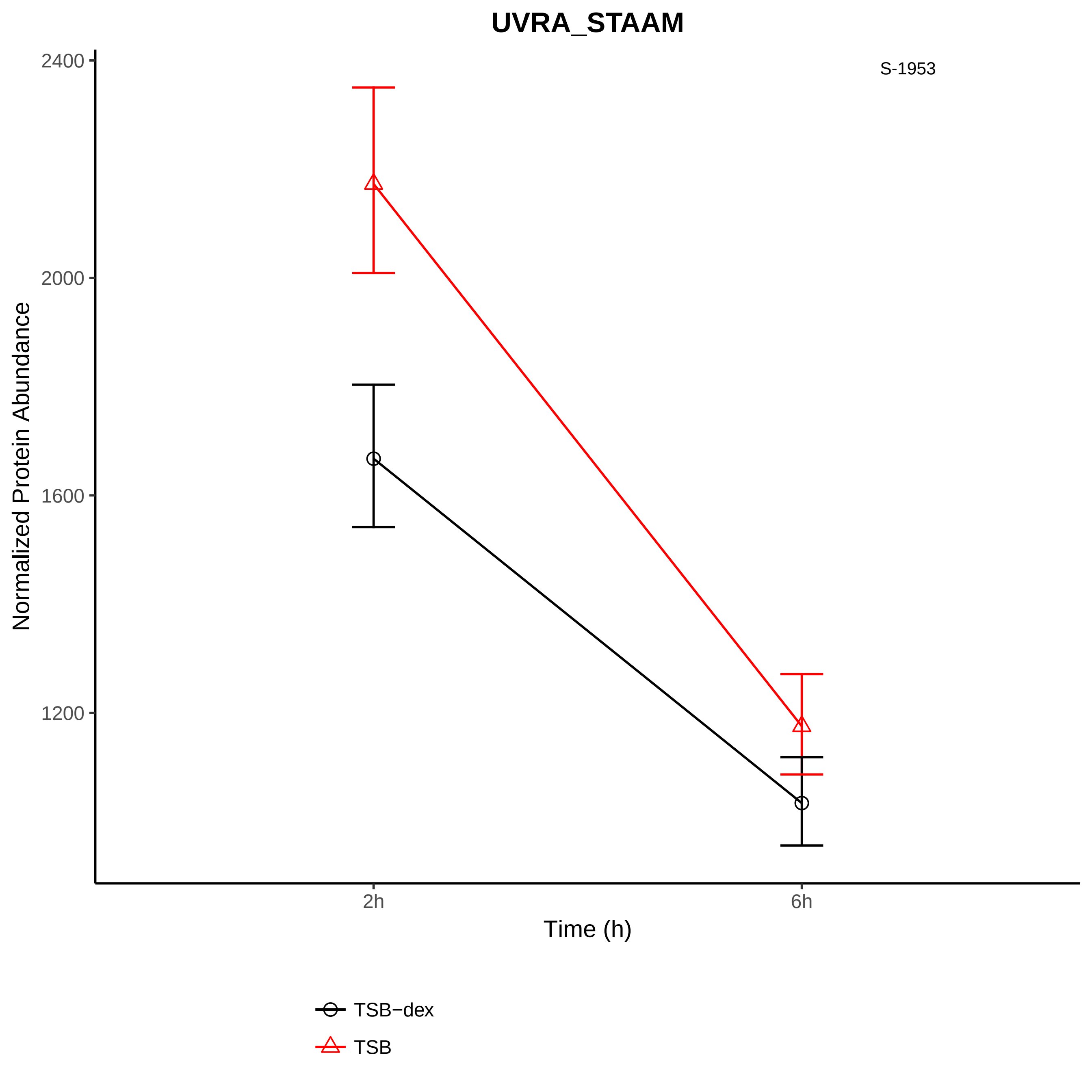

UVRA_STAAM
S-1953
2400
2000
1600
1200
Normalized Protein Abundance
2h
6h
Time (h)
TSB−dex
TSB

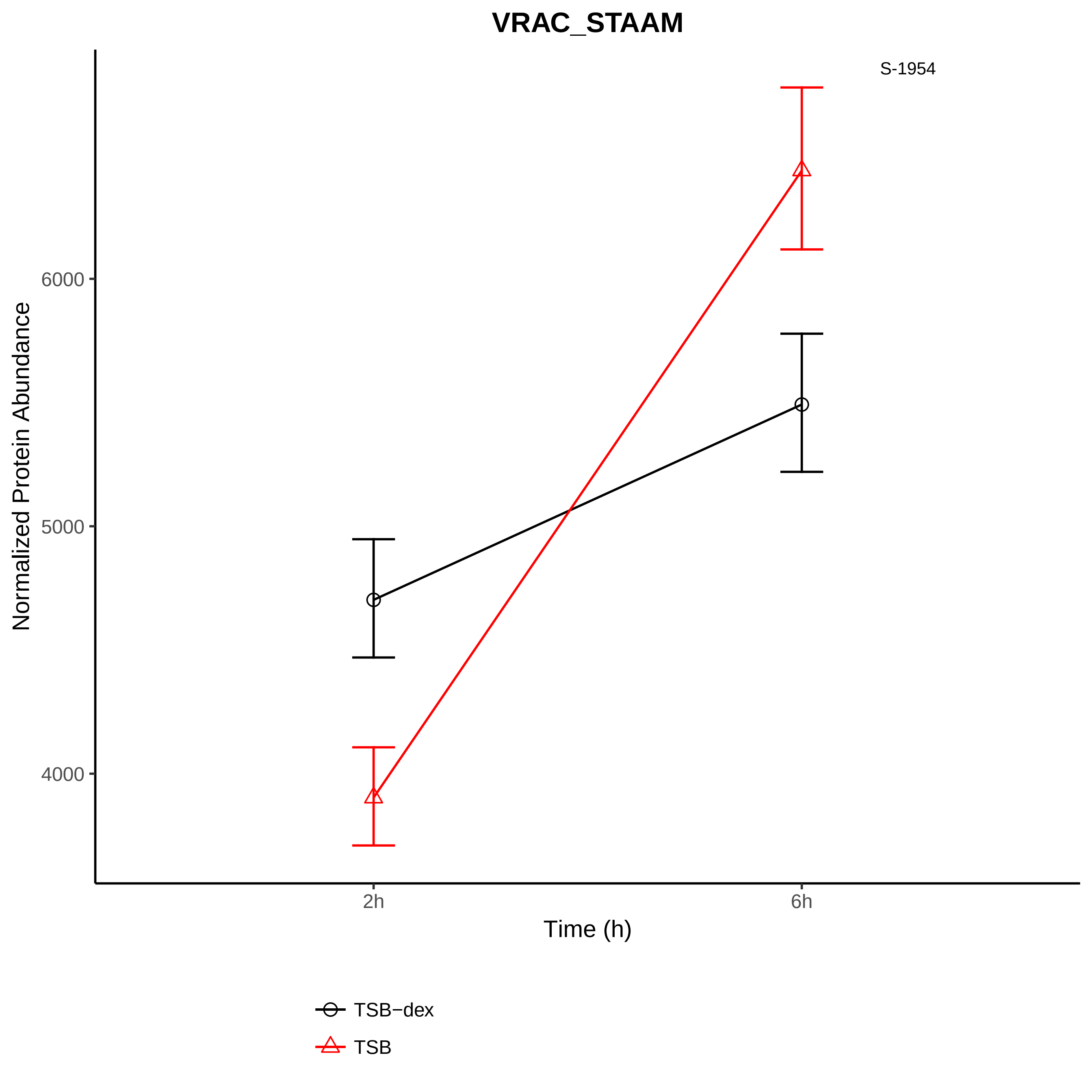

VRAC_STAAM
S-1954
Normalized Protein Abundance
6000
5000
4000
2h
6h
Time (h)
TSB−dex
TSB

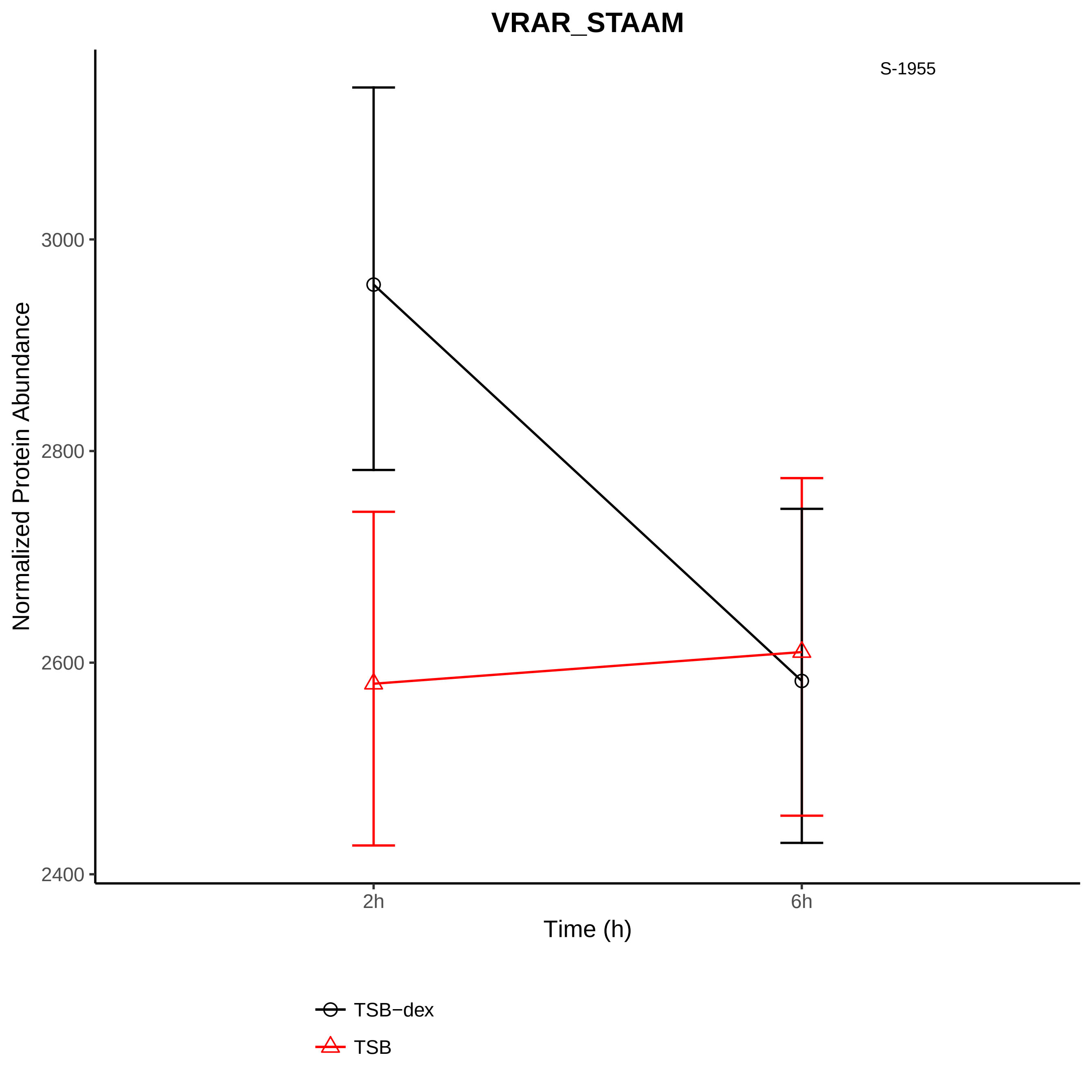

VRAR_STAAM
S-1955
Normalized Protein Abundance
3000
2800
2600
2400
2h
6h
Time (h)
TSB−dex
TSB

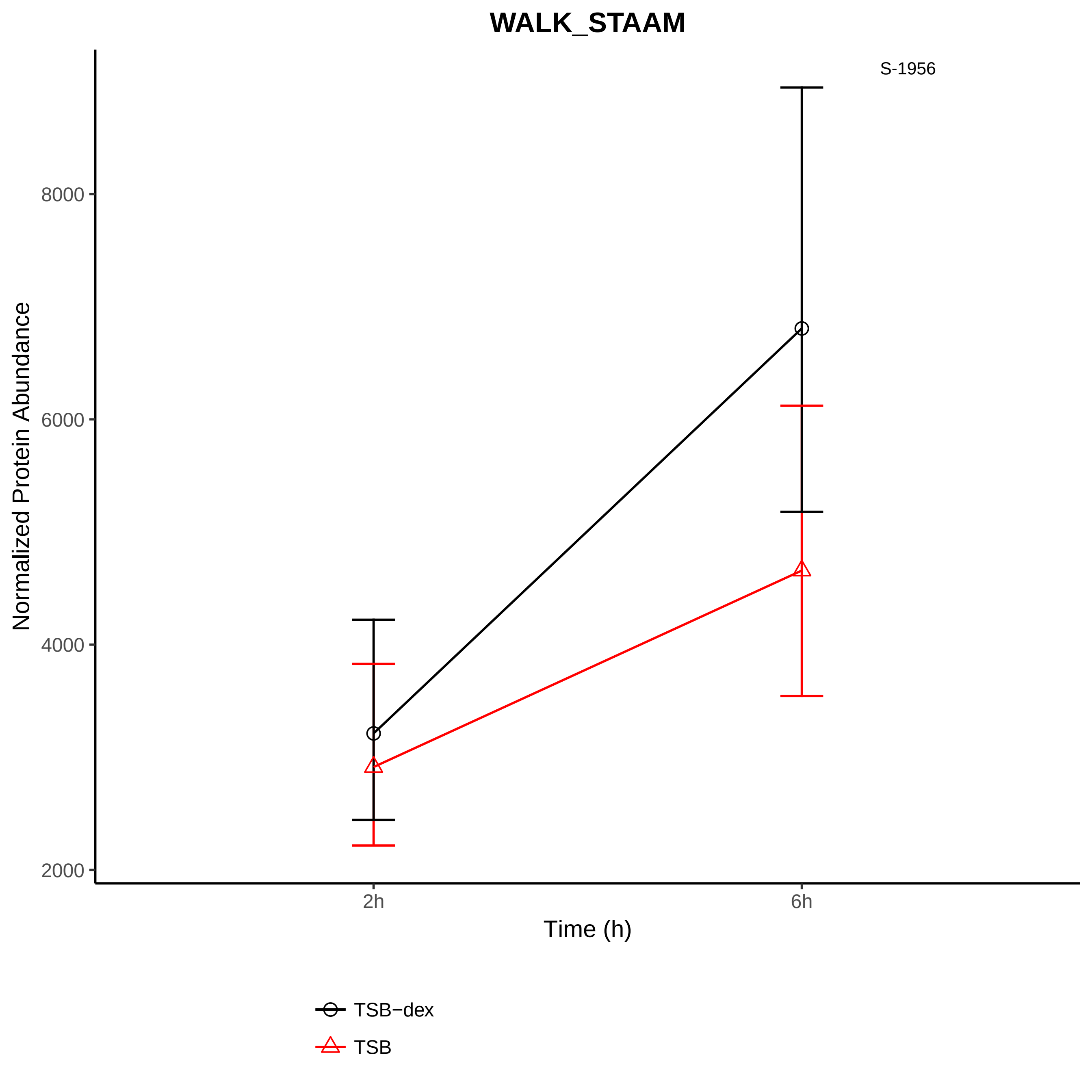
WALK_STAAM
S-1956
Normalized Protein Abundance
8000
6000
4000
2000
2h
6h
Time (h)
TSB−dex
TSB

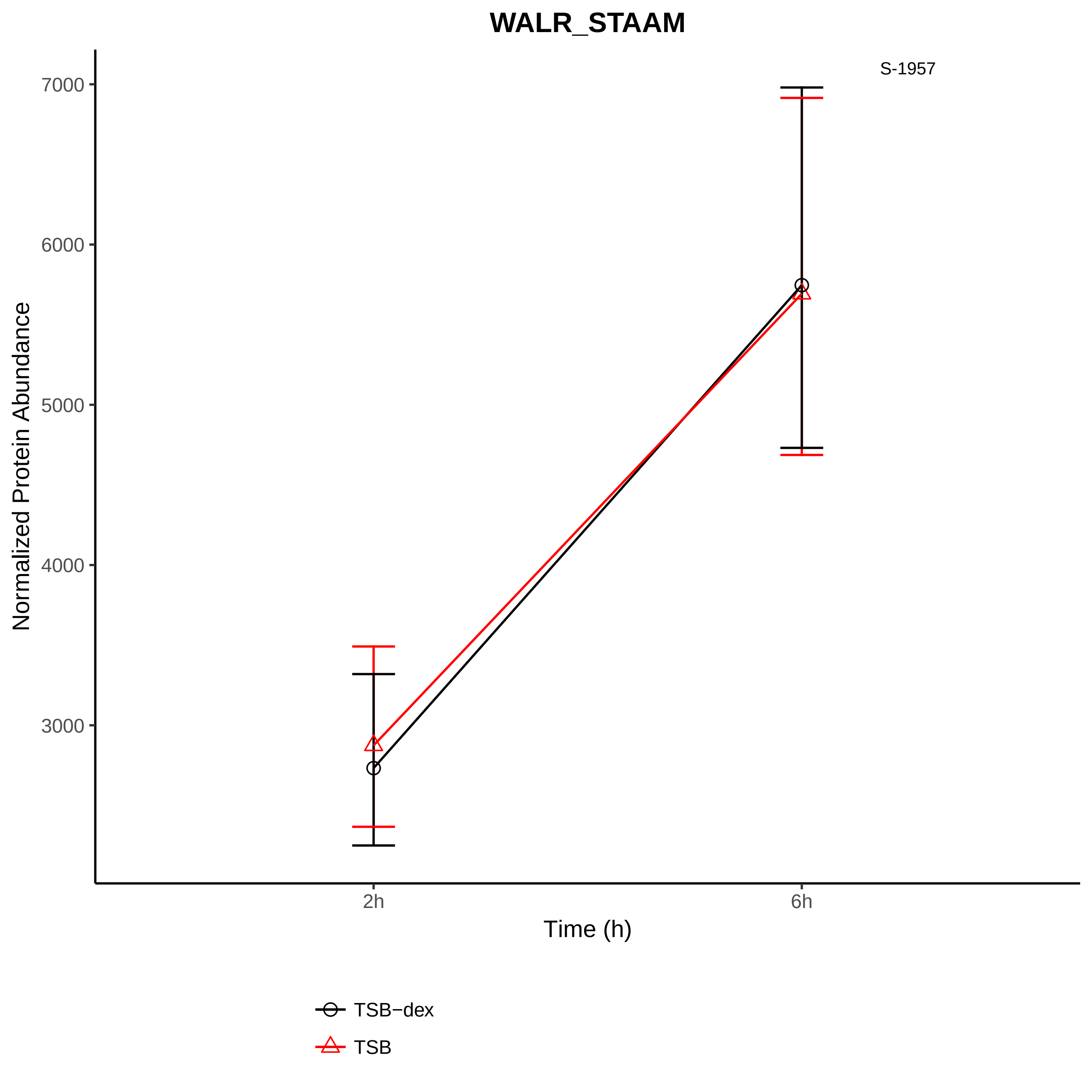

WALR_STAAM
S-1957
Normalized Protein Abundance
7000
6000
5000
4000
3000
2h
6h
Time (h)
TSB−dex
TSB

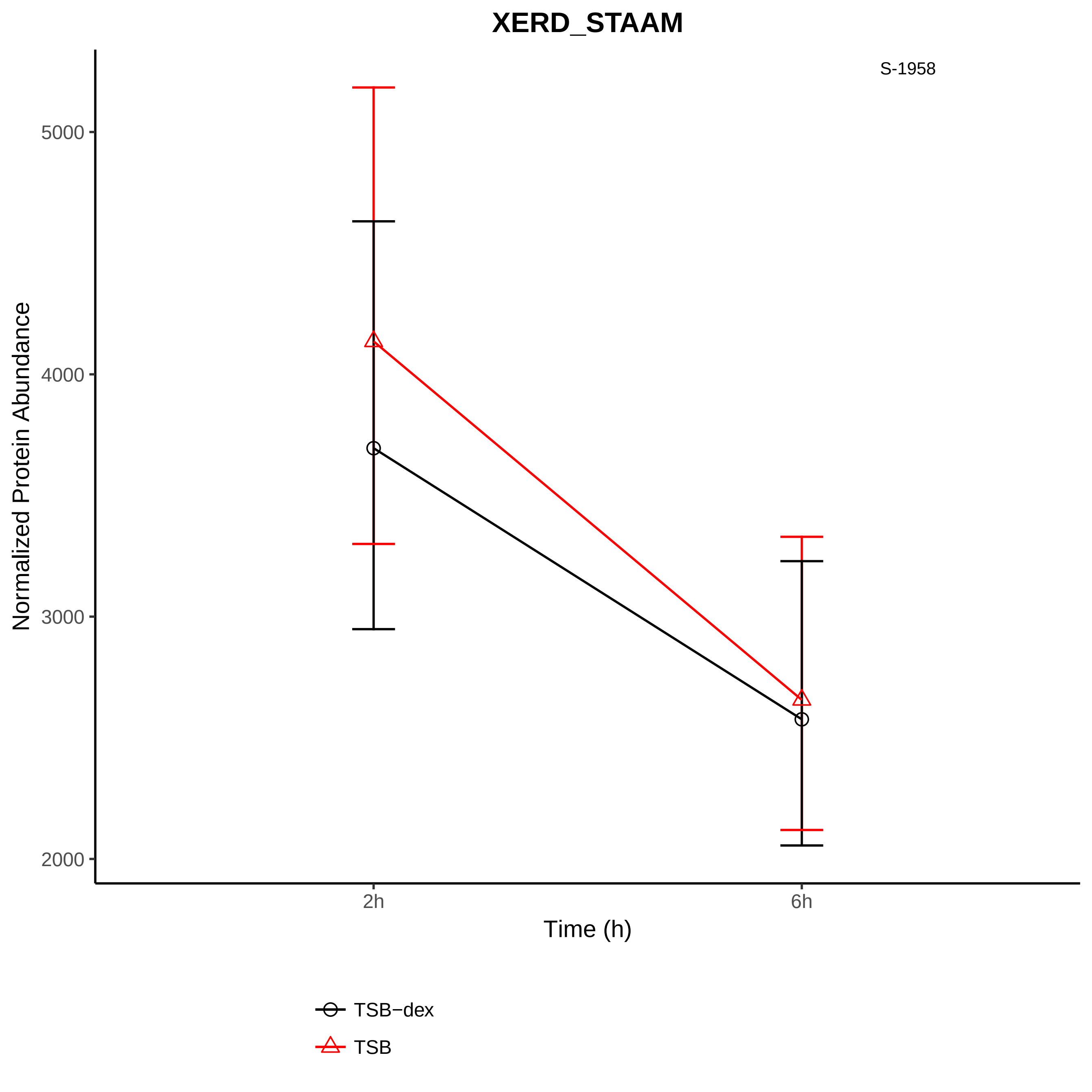

XERD_STAAM
S-1958
Normalized Protein Abundance
5000
4000
3000
2000
2h
6h
Time (h)
TSB−dex
TSB

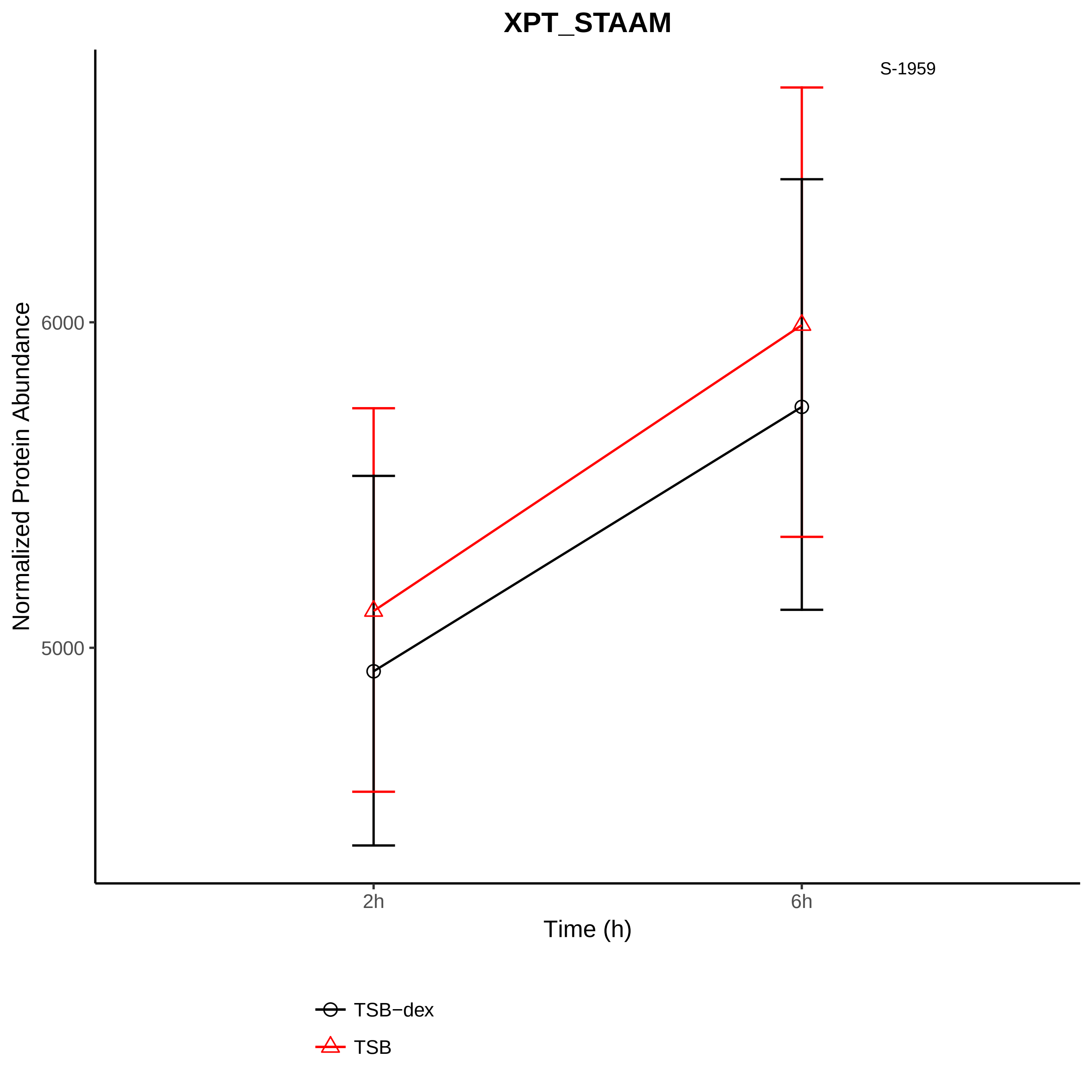

XPT_STAAM
S-1959
Normalized Protein Abundance
6000
5000
2h
6h
Time (h)
TSB−dex
TSB

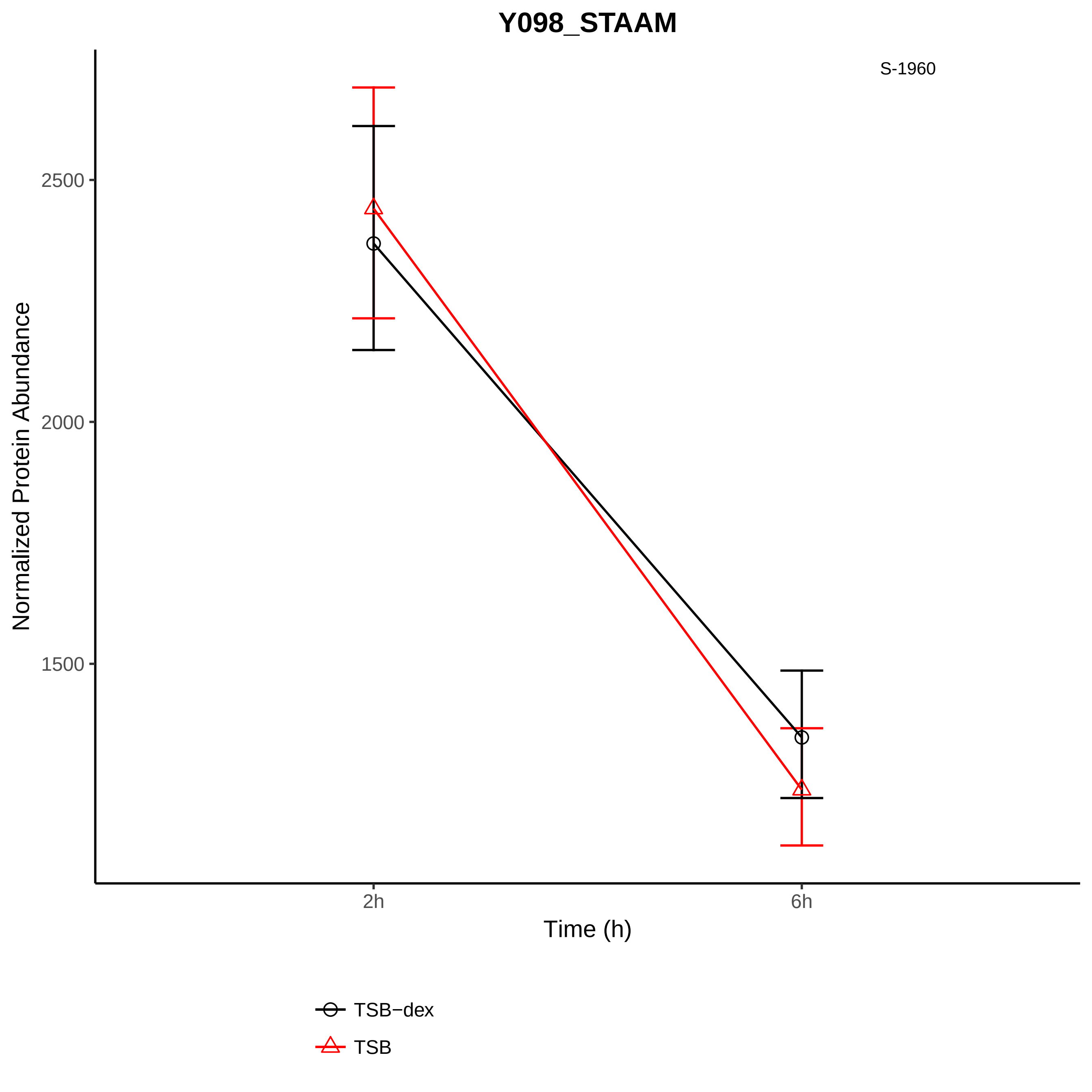

Y098_STAAM
S-1960
2500
2000
1500
Normalized Protein Abundance
2h
6h
Time (h)
TSB−dex
TSB

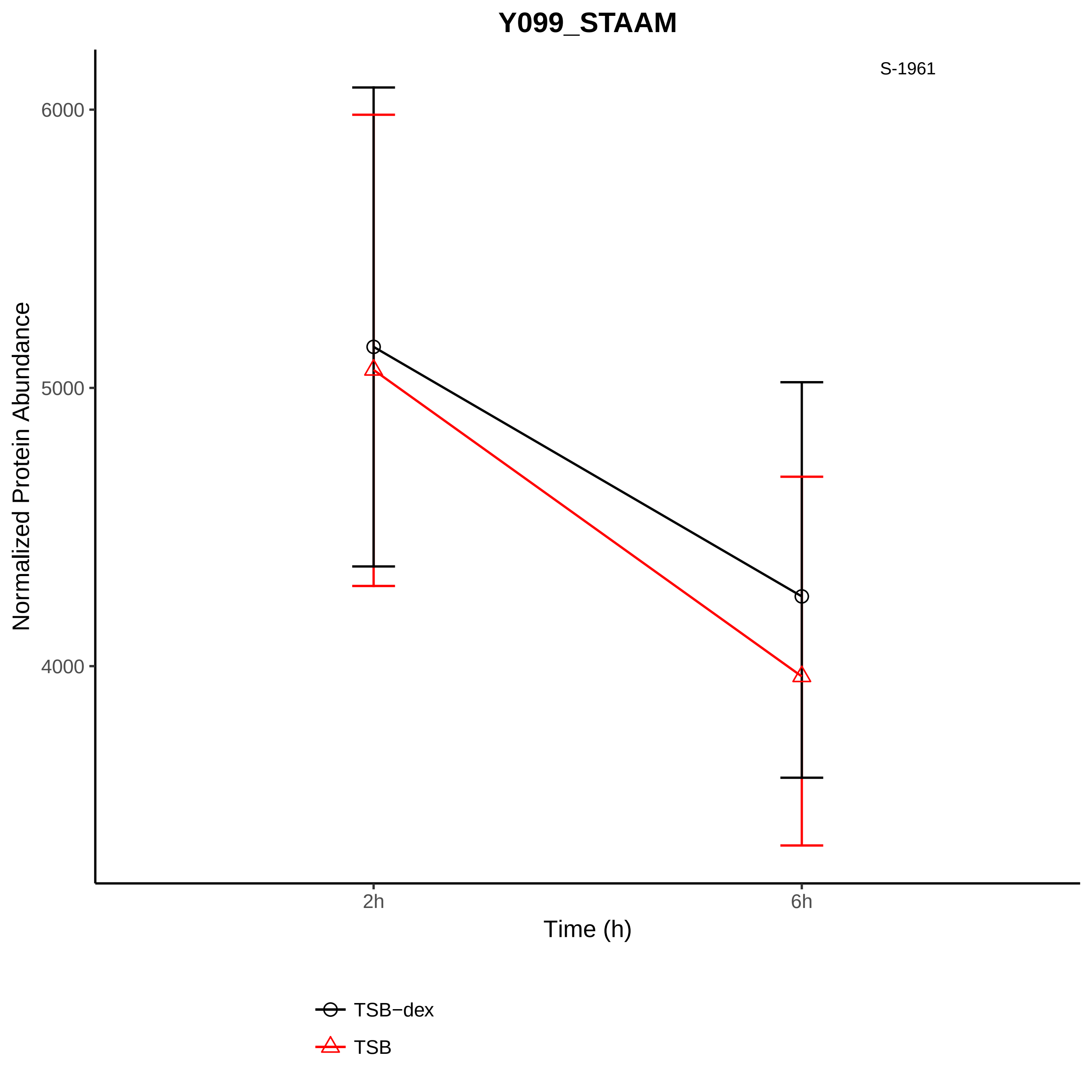
Y099_STAAM
S-1961
6000
5000
4000
Normalized Protein Abundance
2h
6h
Time (h)
TSB−dex
TSB

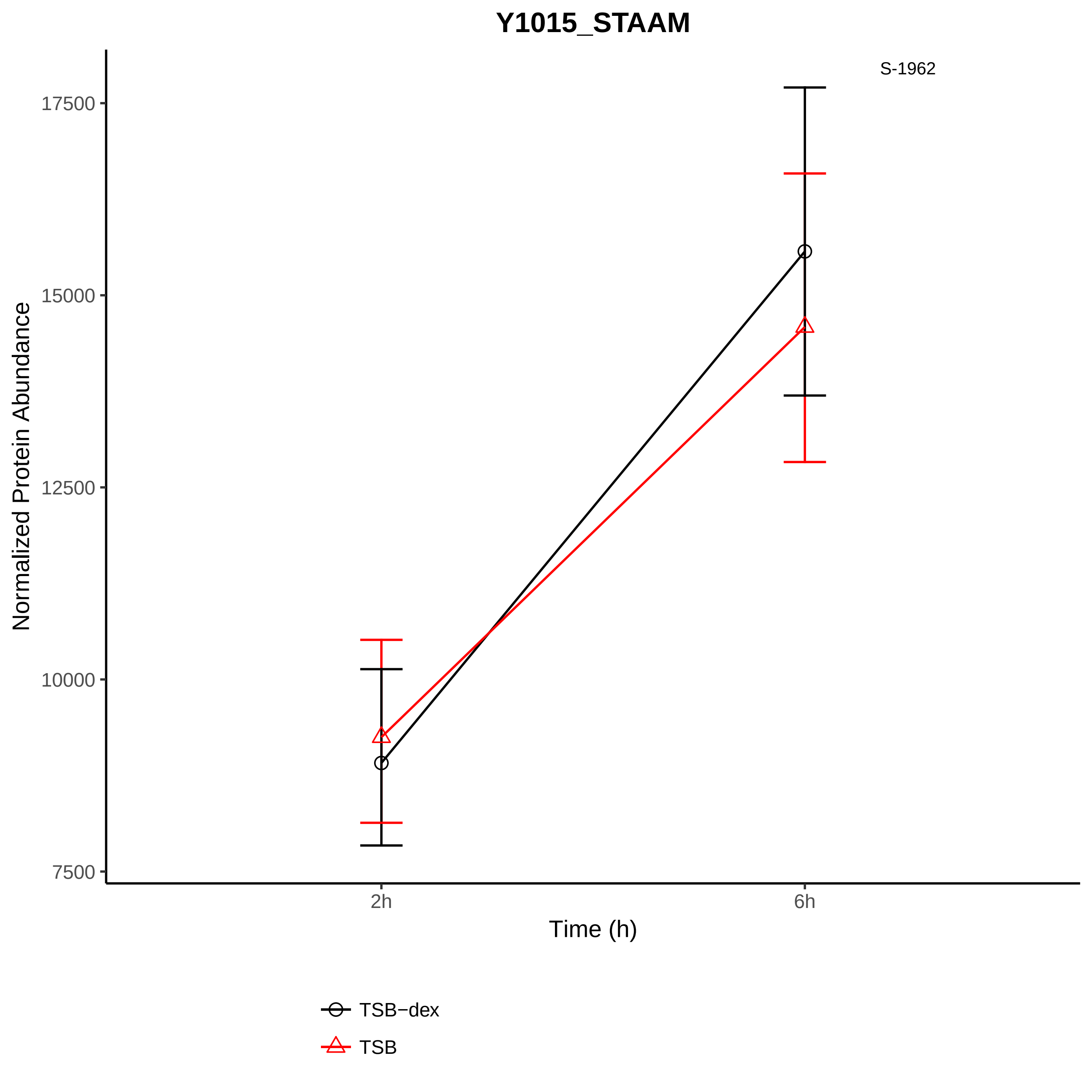

Y1015_STAAM
S-1962
17500
15000
12500
10000
7500
Normalized Protein Abundance
2h
6h
Time (h)
TSB−dex
TSB

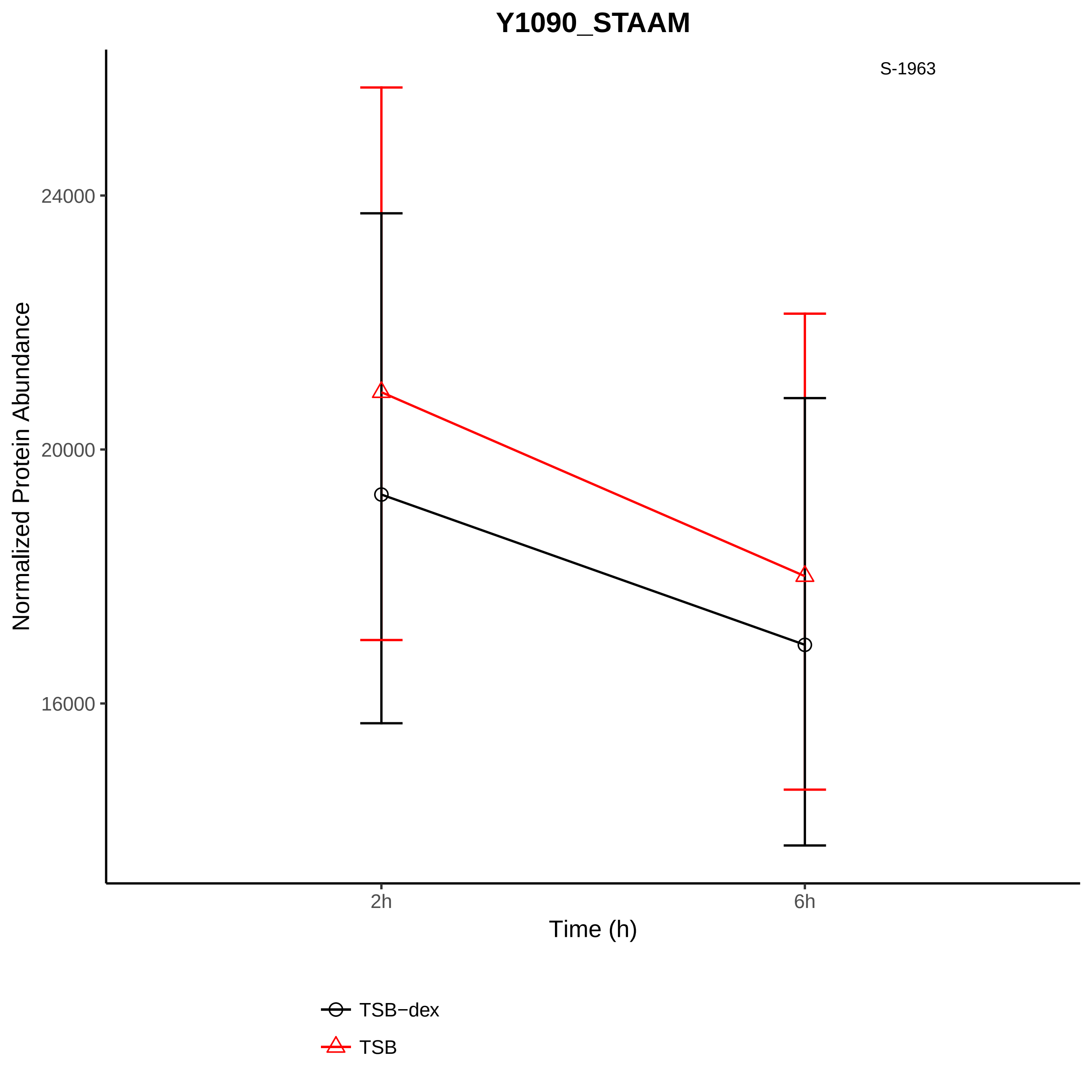

Y1090_STAAM
S-1963
Normalized Protein Abundance
24000
20000
16000
2h
6h
Time (h)
TSB−dex
TSB

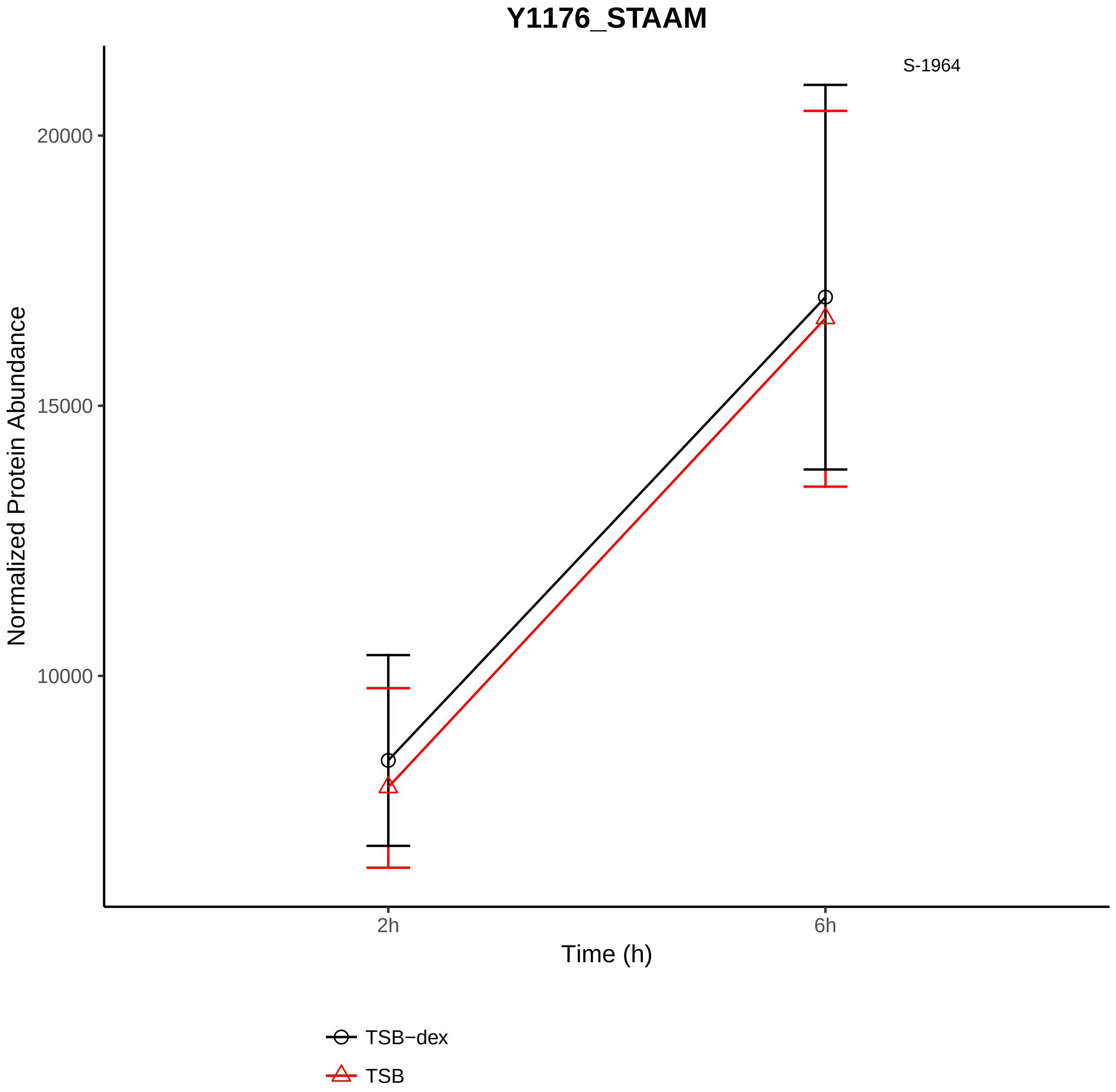

Y1176_STAAM
S-1964
Normalized Protein Abundance
20000
15000
10000
2h
6h
Time (h)
TSB−dex
TSB

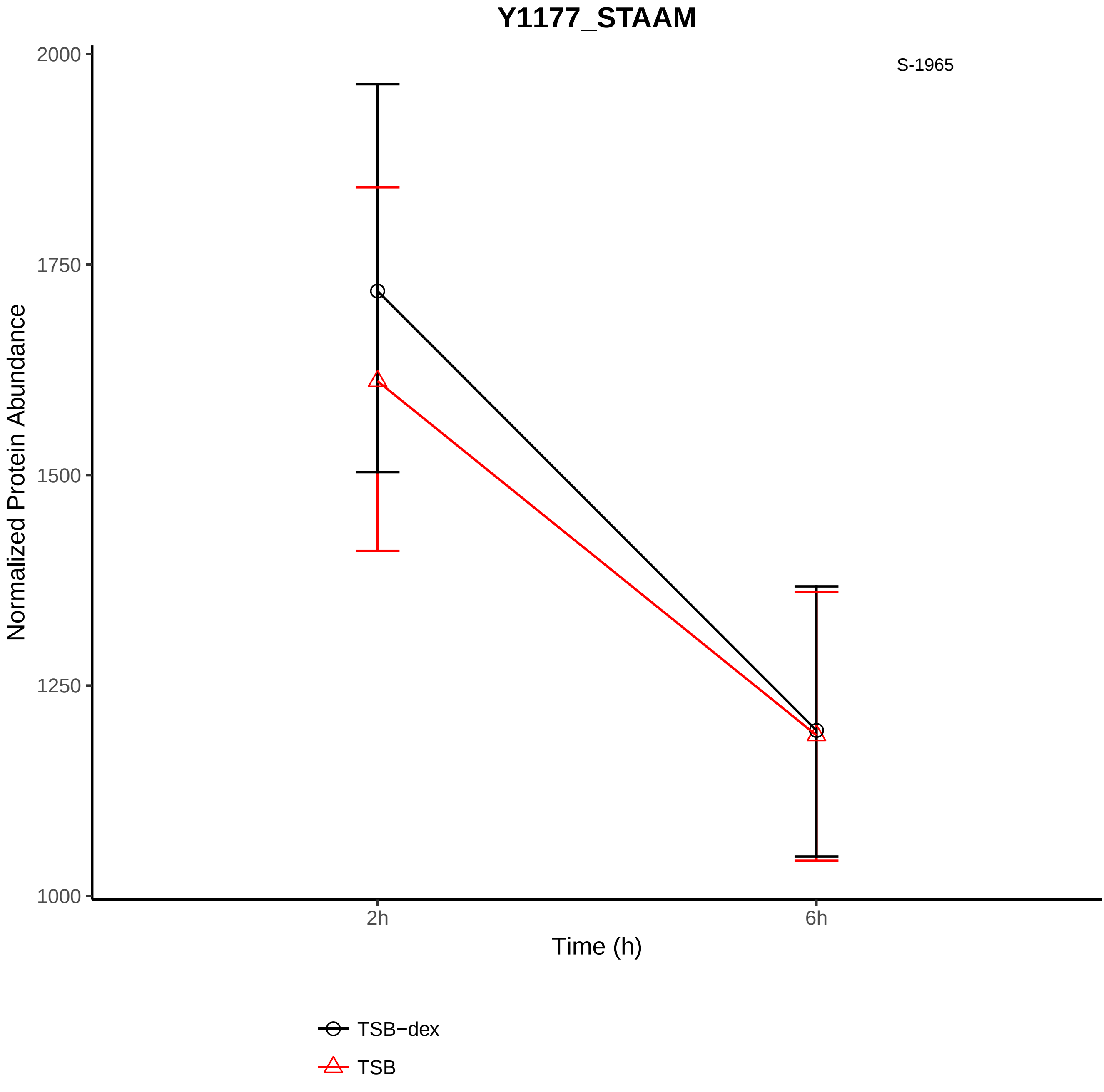
Y1177_STAAM
S-1965
Normalized Protein Abundance
2000
1750
1500
1250
1000
2h
6h
Time (h)
TSB−dex
TSB

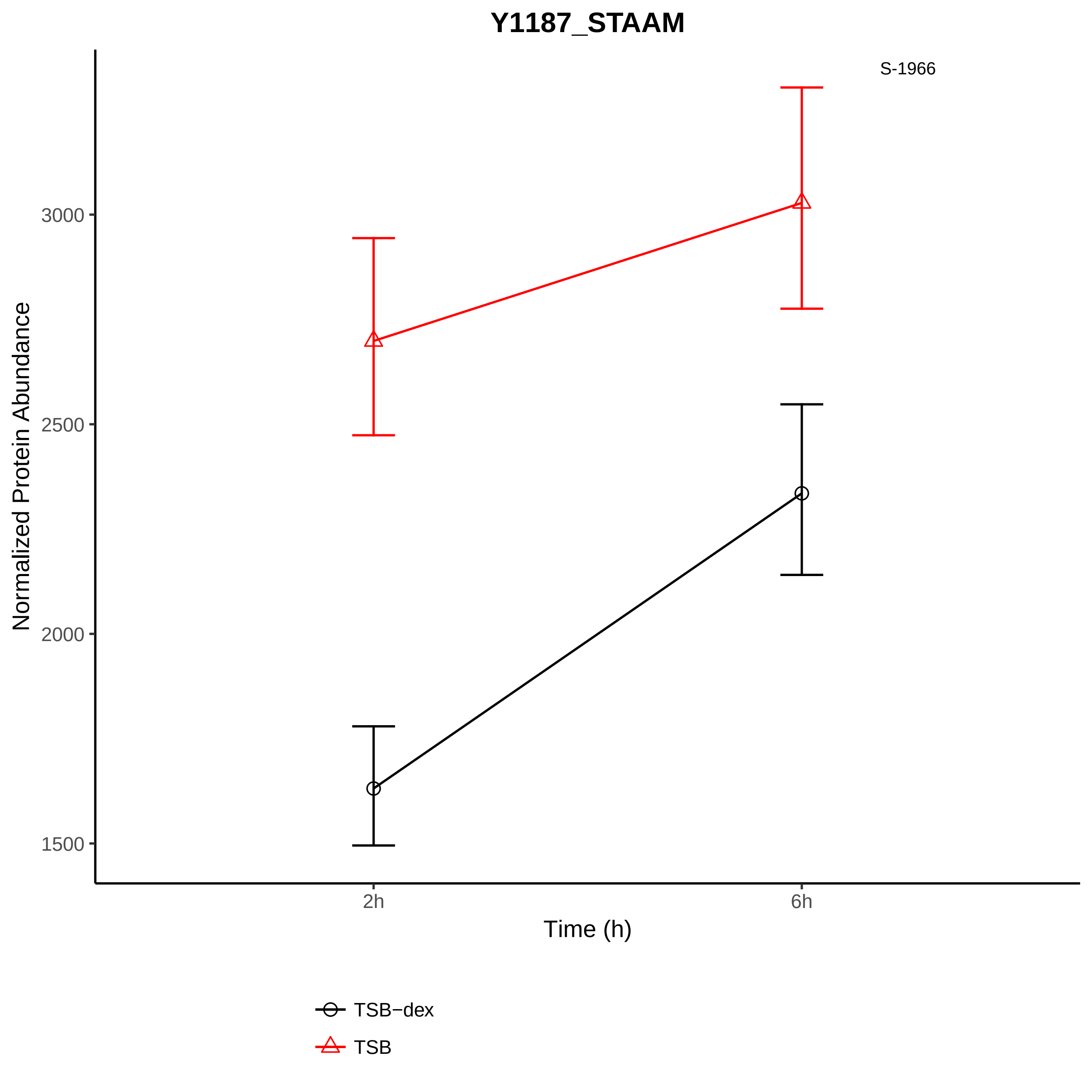

Y1187_STAAM
S-1966
Normalized Protein Abundance
3000
2500
2000
1500
2h
6h
Time (h)
TSB−dex
TSB

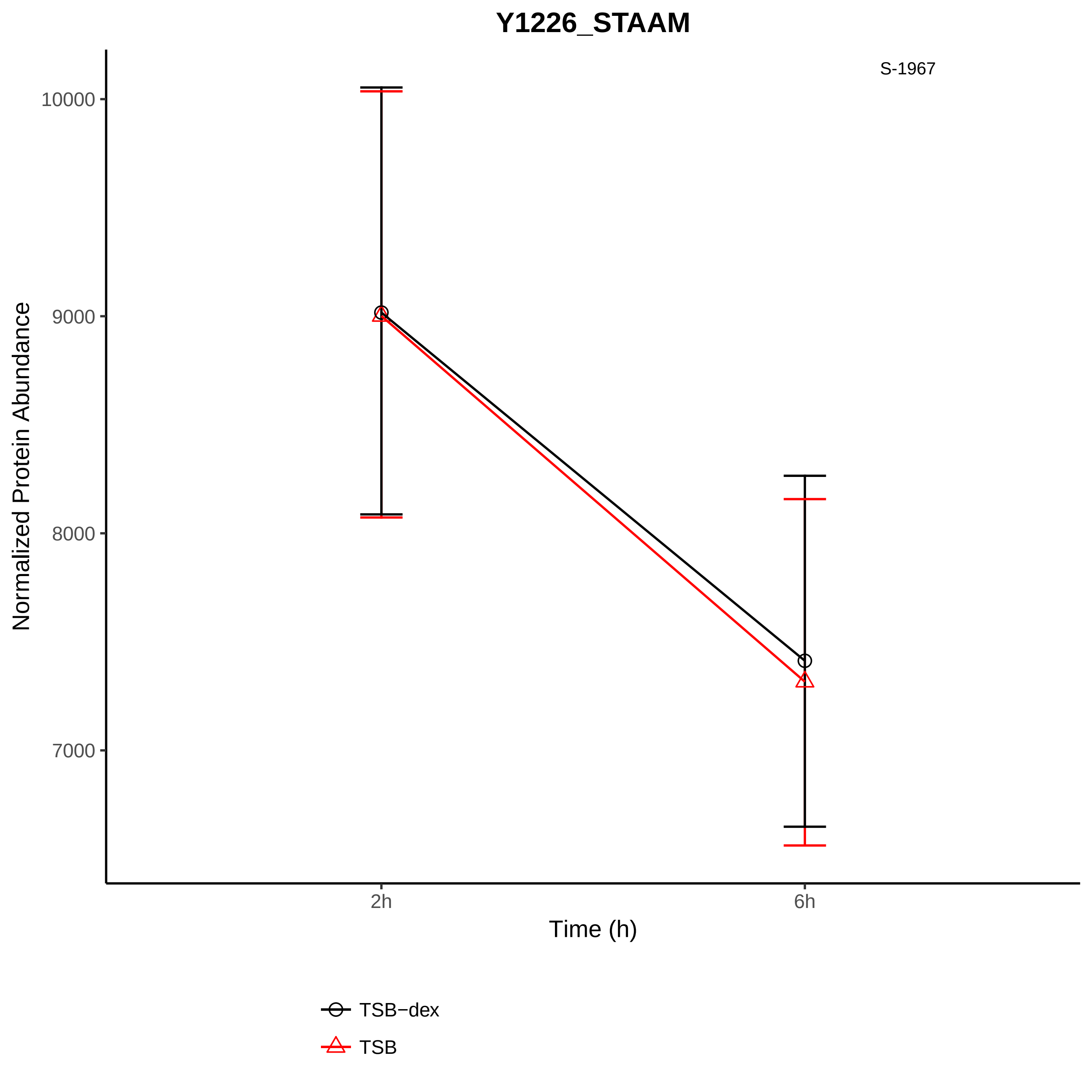

Y1226_STAAM
S-1967
Normalized Protein Abundance
10000
9000
8000
7000
2h
6h
Time (h)
TSB−dex
TSB

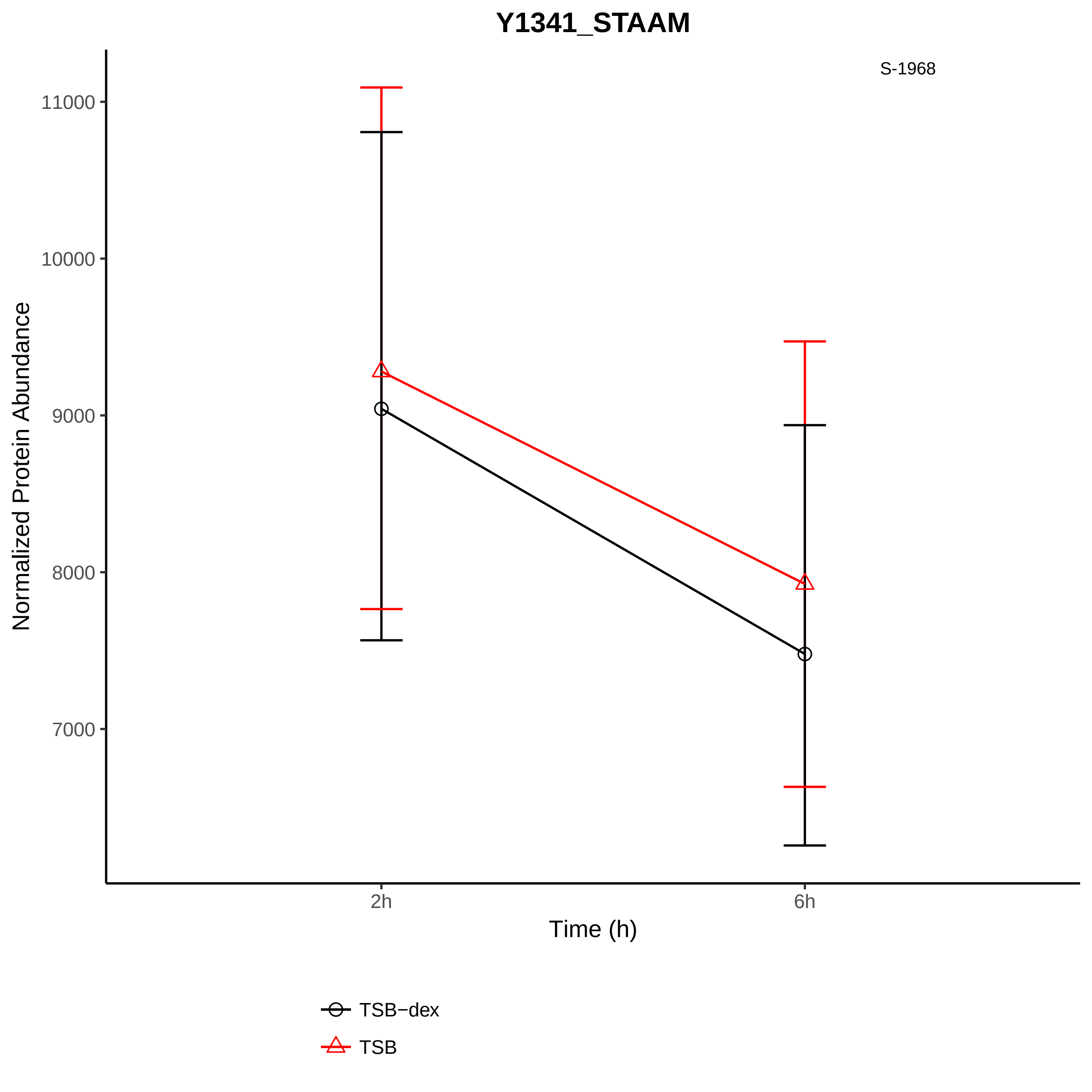

Y1341_STAAM
S-1968
11000
10000
9000
8000
7000
Normalized Protein Abundance
2h
6h
Time (h)
TSB−dex
TSB

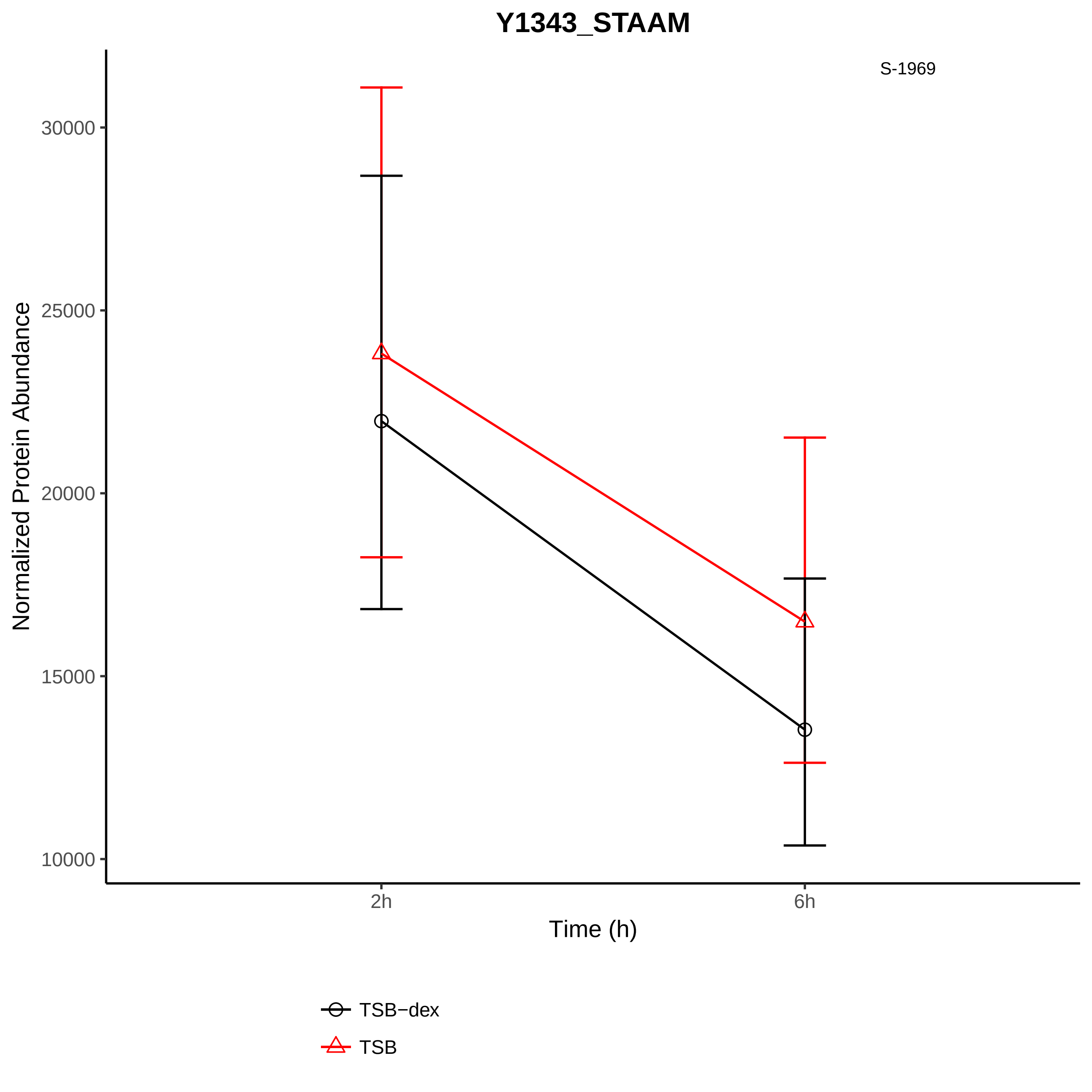

Y1343_STAAM
S-1969
Normalized Protein Abundance
30000
25000
20000
15000
10000
2h
6h
Time (h)
TSB−dex
TSB

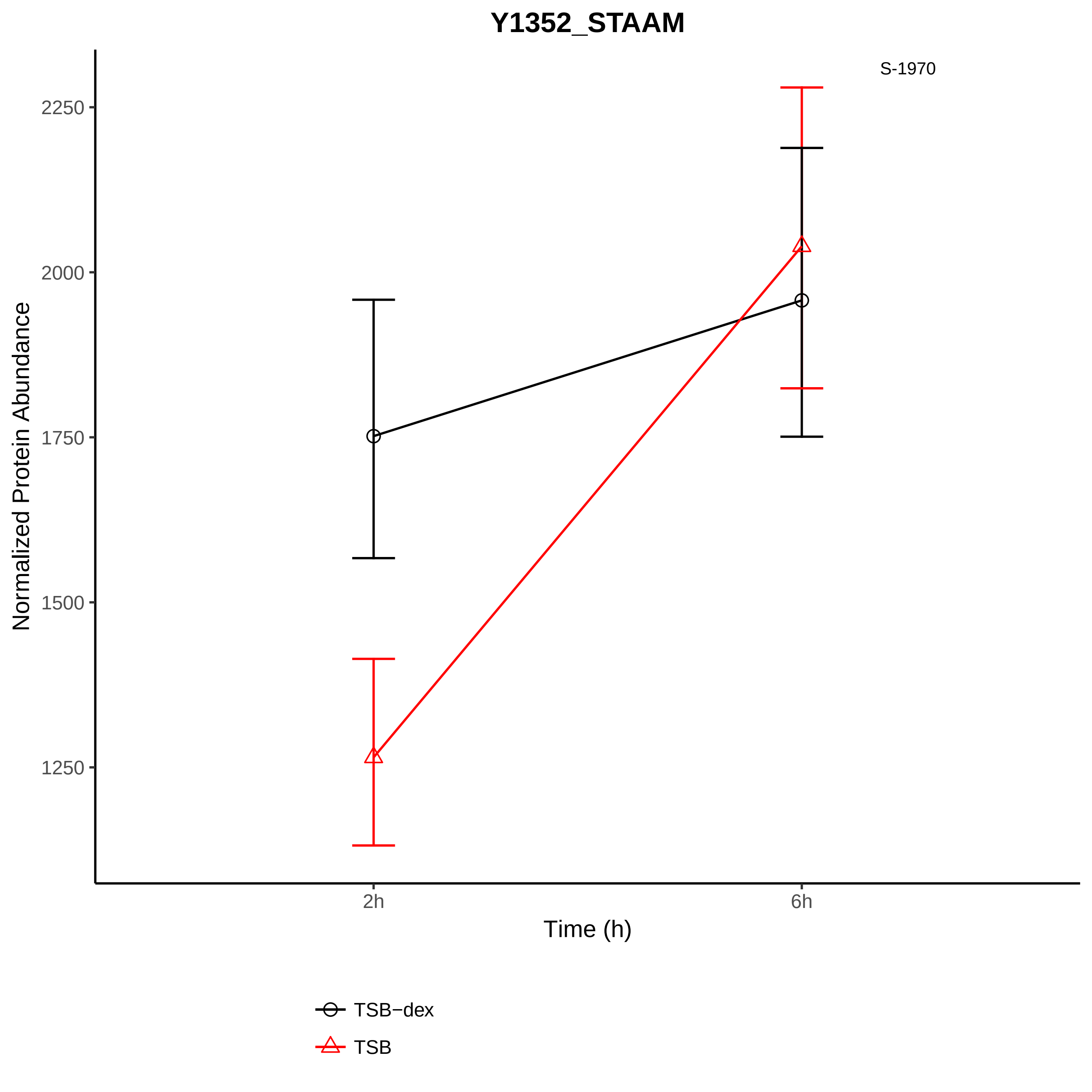

Y1352_STAAM
S-1970
Normalized Protein Abundance
2250
2000
1750
1500
1250
2h
6h
Time (h)
TSB−dex
TSB

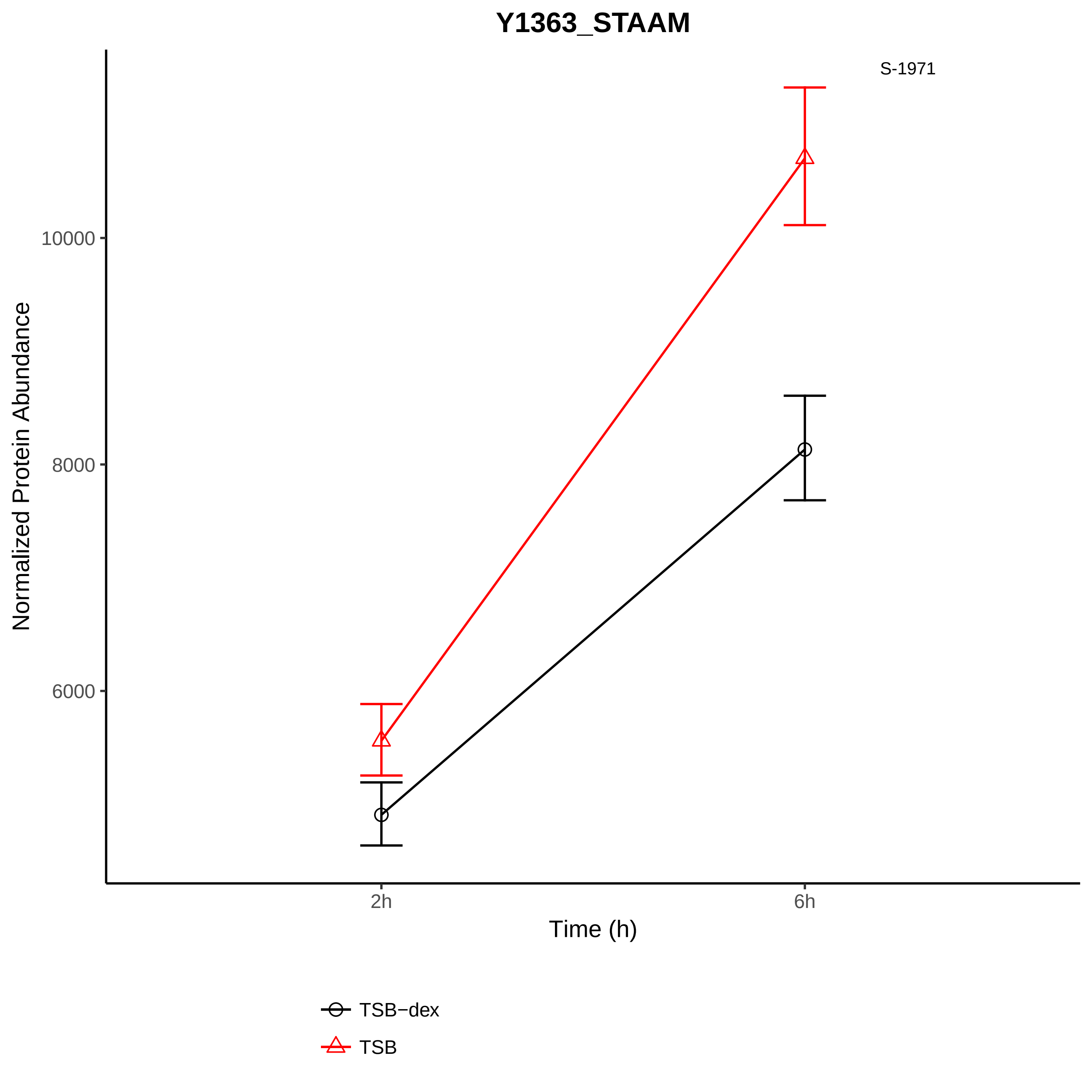

Y1363_STAAM
S-1971
Normalized Protein Abundance
10000
8000
6000
2h
6h
Time (h)
TSB−dex
TSB

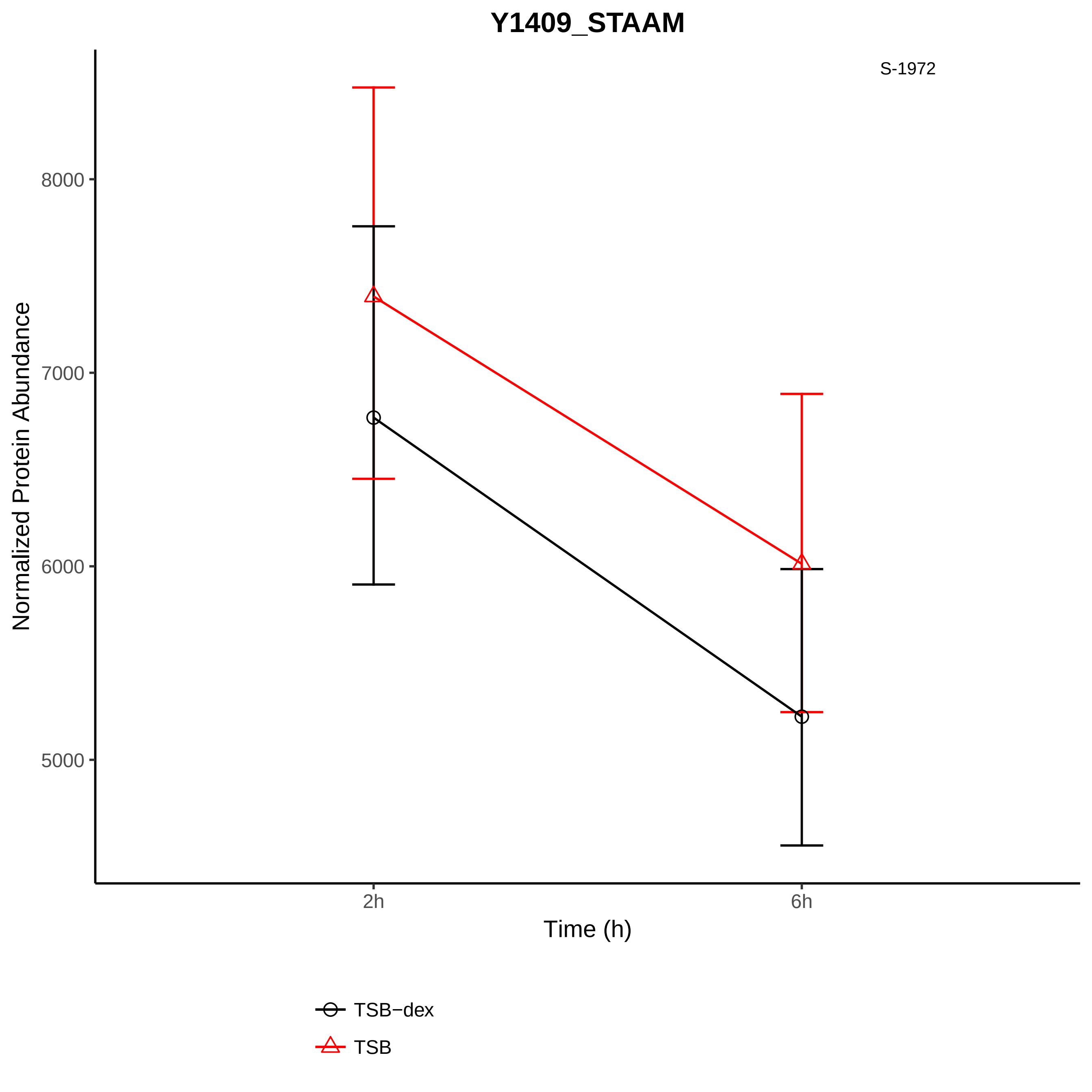

Y1409_STAAM
S-1972
Normalized Protein Abundance
8000
7000
6000
5000
2h
6h
Time (h)
TSB−dex
TSB

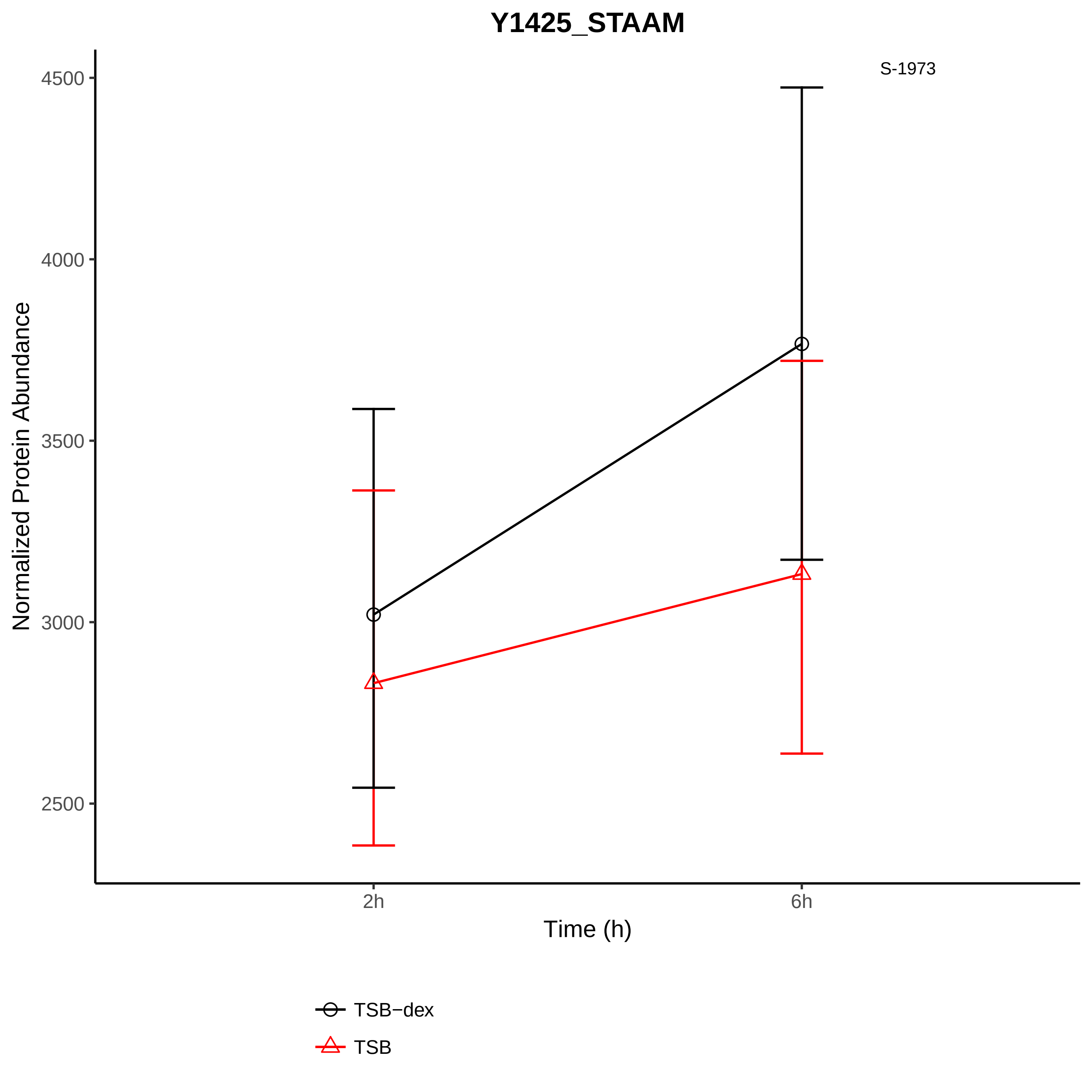
Y1425_STAAM
S-1973
Normalized Protein Abundance
4500
4000
3500
3000
2500
2h
6h
Time (h)
TSB−dex
TSB

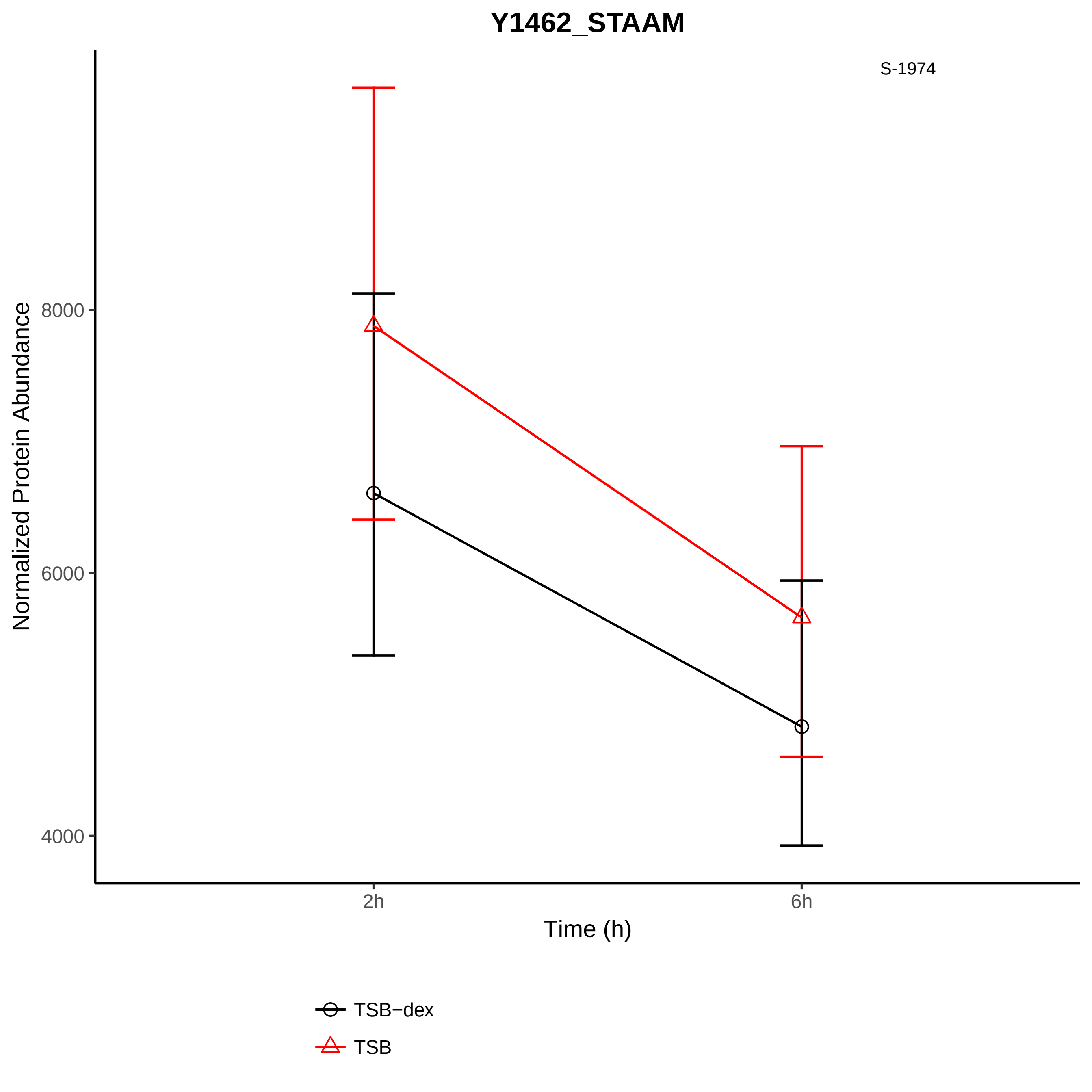

Y1462_STAAM
S-1974
Normalized Protein Abundance
8000
6000
4000
2h
6h
Time (h)
TSB−dex
TSB

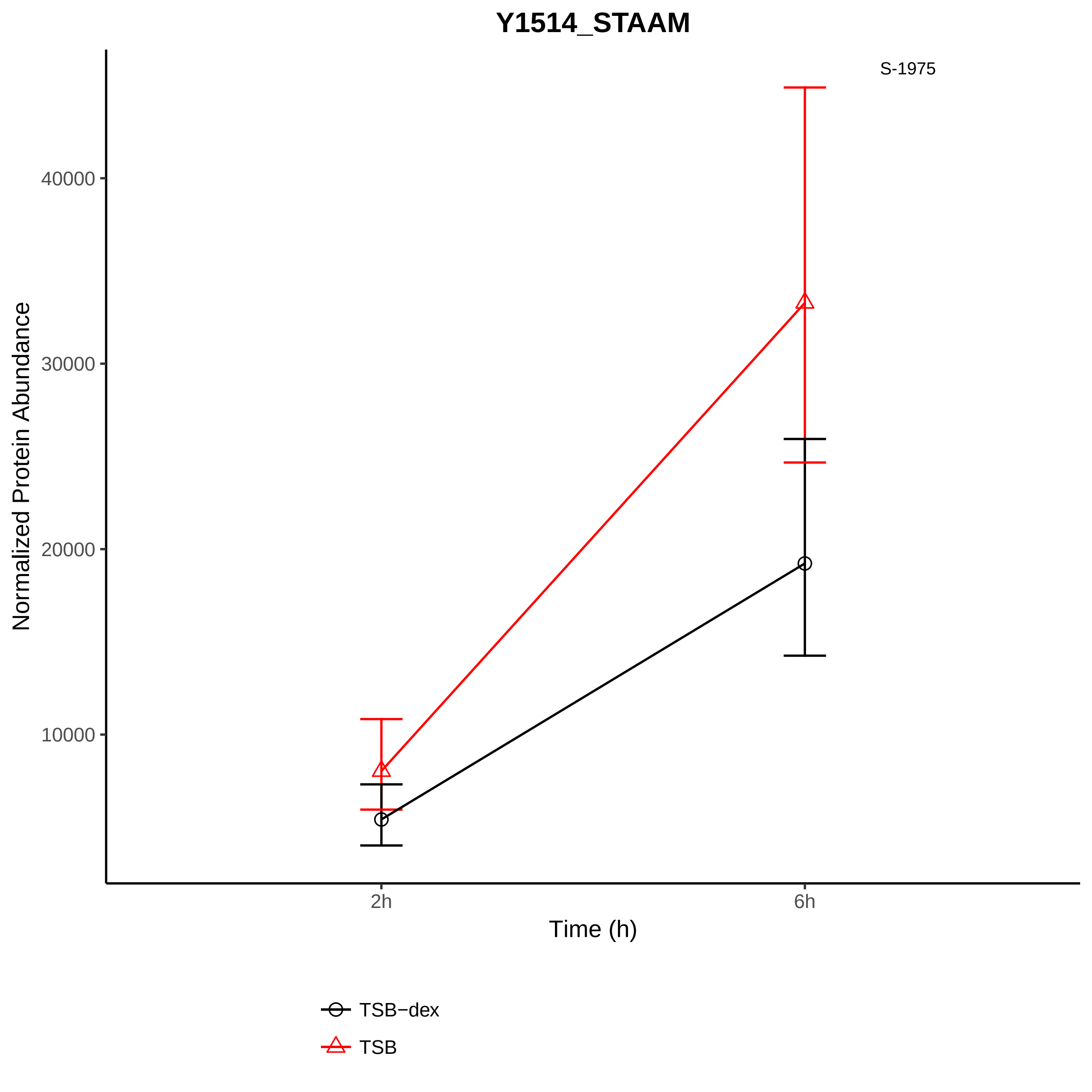

Y1514_STAAM
S-1975
Normalized Protein Abundance
40000
30000
20000
10000
2h
6h
Time (h)
TSB−dex
TSB

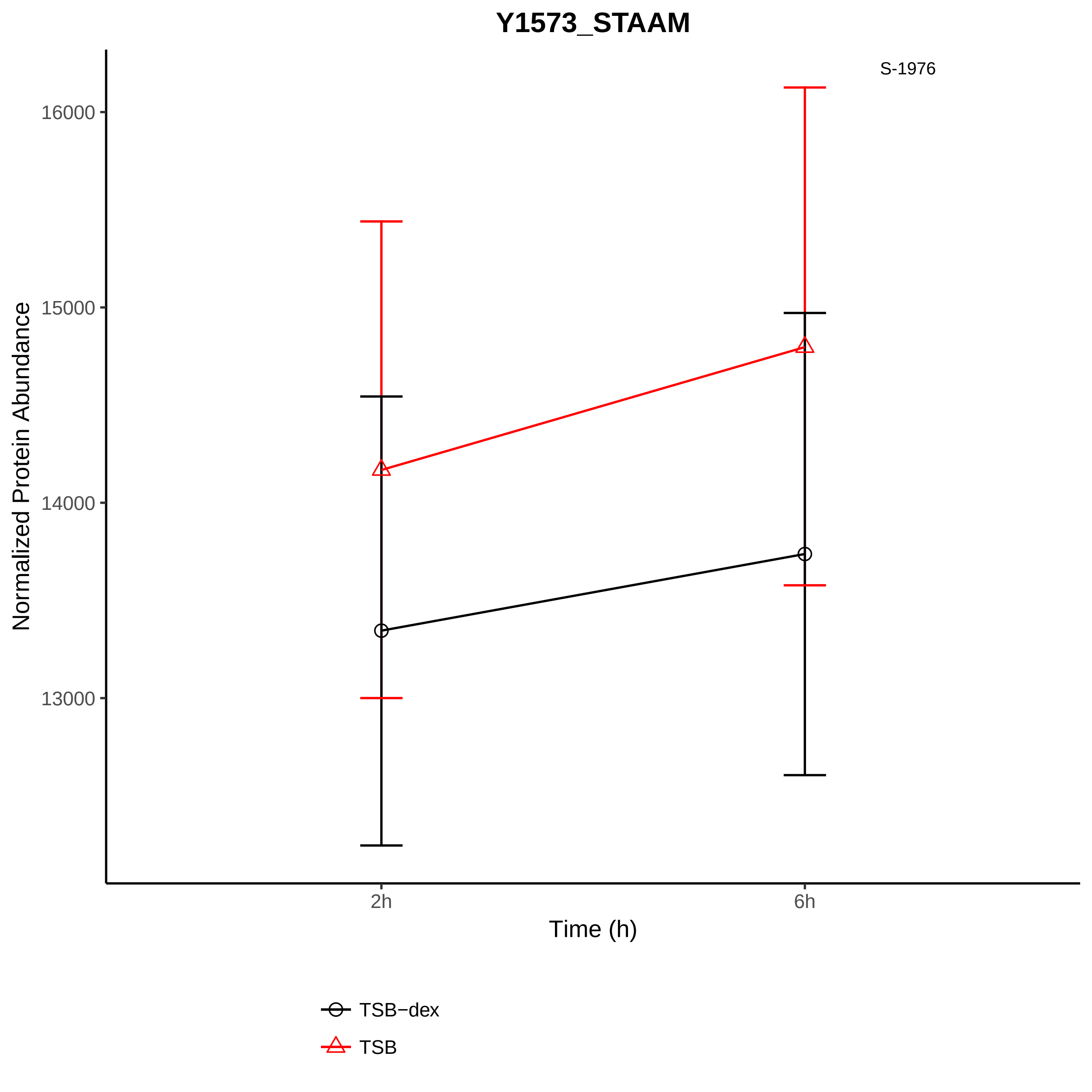
Y1573_STAAM
S-1976
Normalized Protein Abundance
16000
15000
14000
13000
2h
6h
Time (h)
TSB−dex
TSB

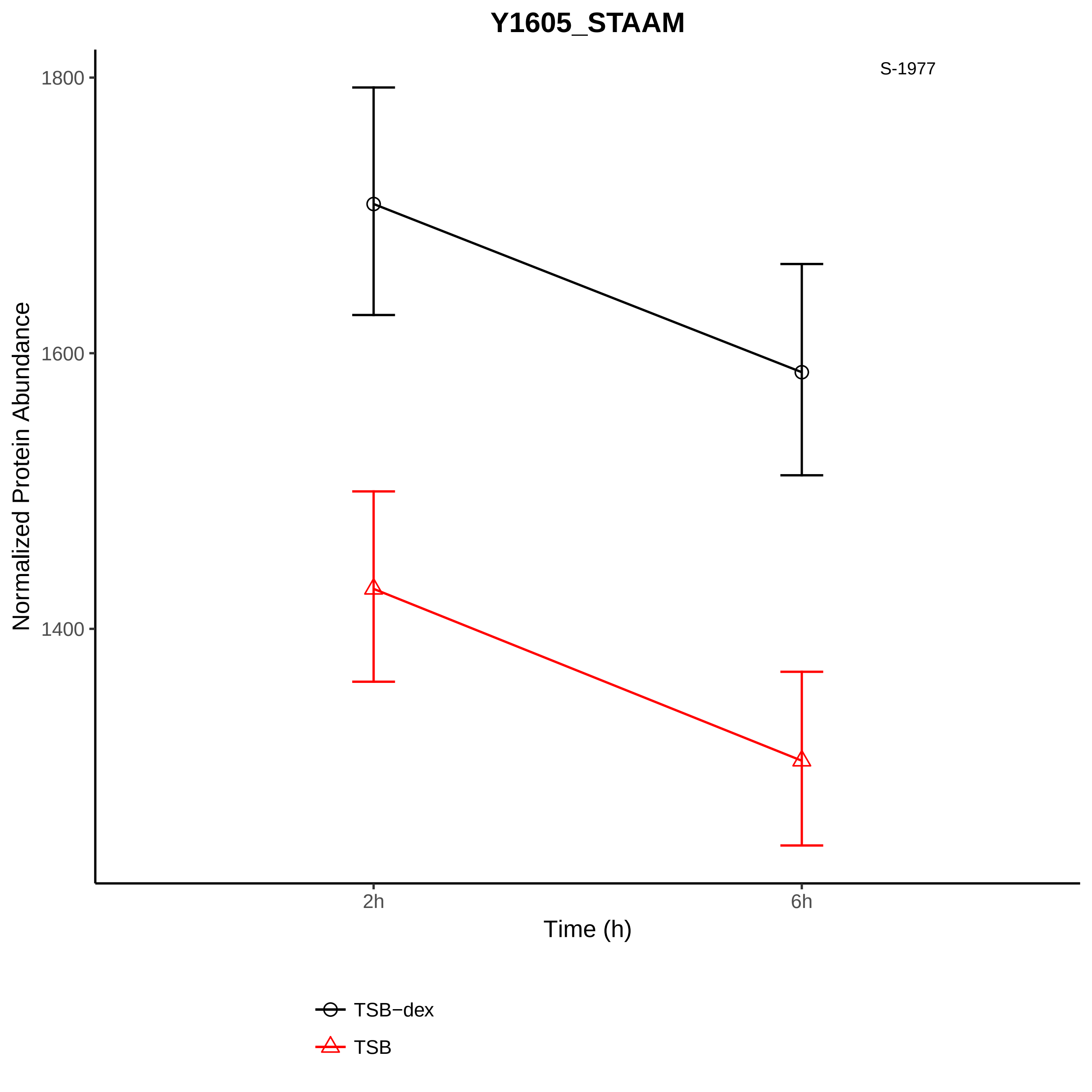
Y1605_STAAM
S-1977
1800
1600
1400
Normalized Protein Abundance
2h
6h
Time (h)
TSB−dex
TSB

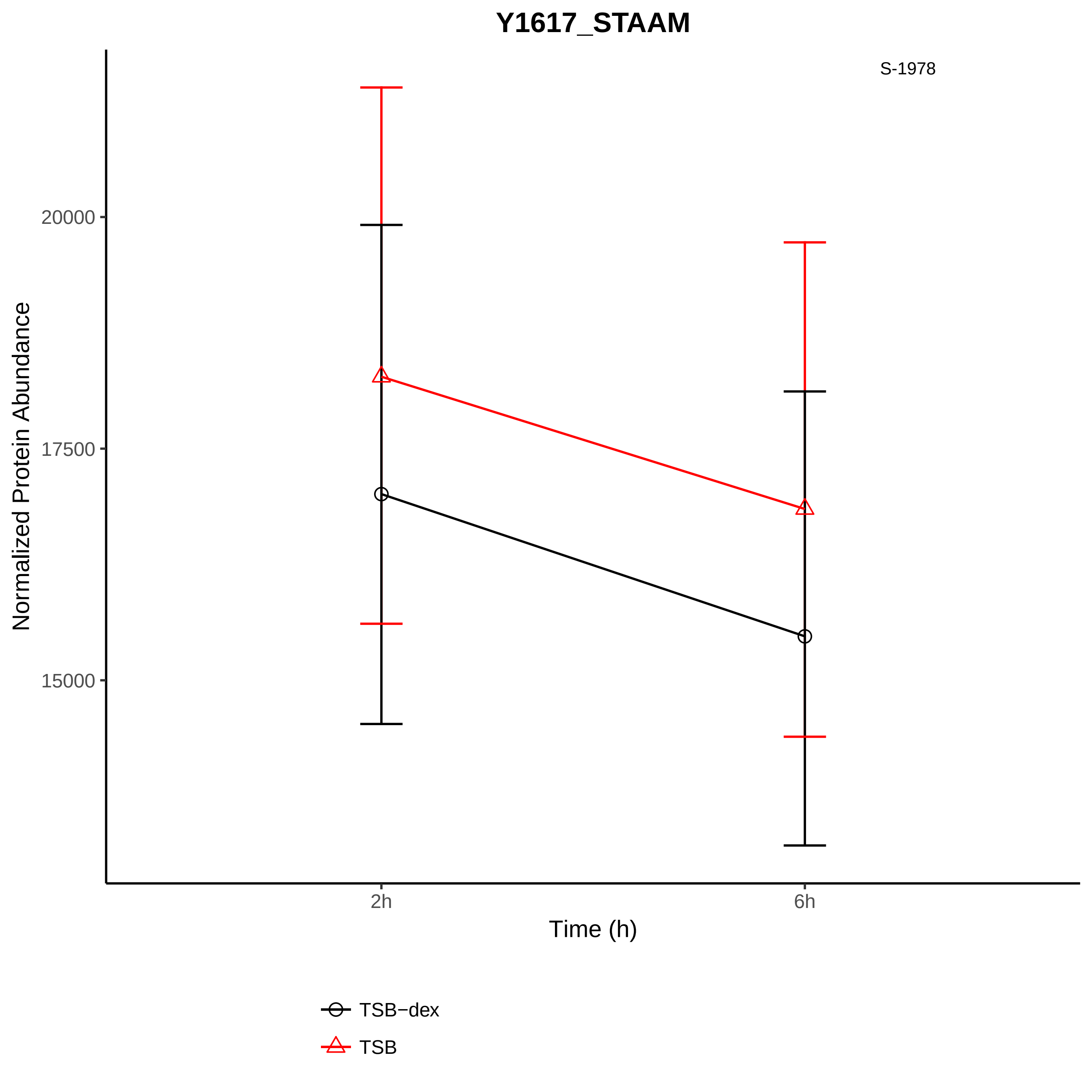

Y1617_STAAM
S-1978
20000
17500
15000
Normalized Protein Abundance
2h
6h
Time (h)
TSB−dex
TSB

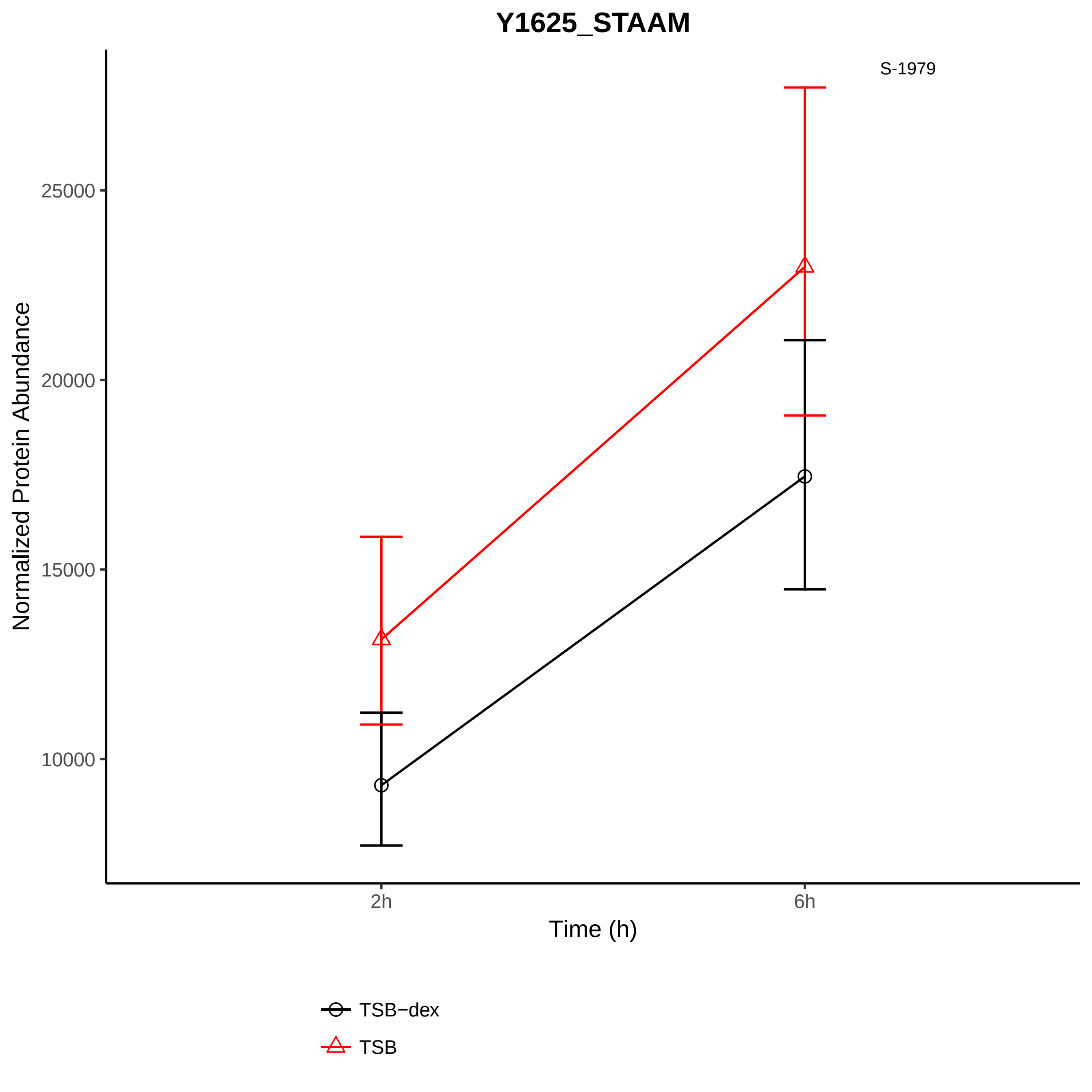

Y1625_STAAM
S-1979
Normalized Protein Abundance
25000
20000
15000
10000
2h
6h
Time (h)
TSB−dex
TSB

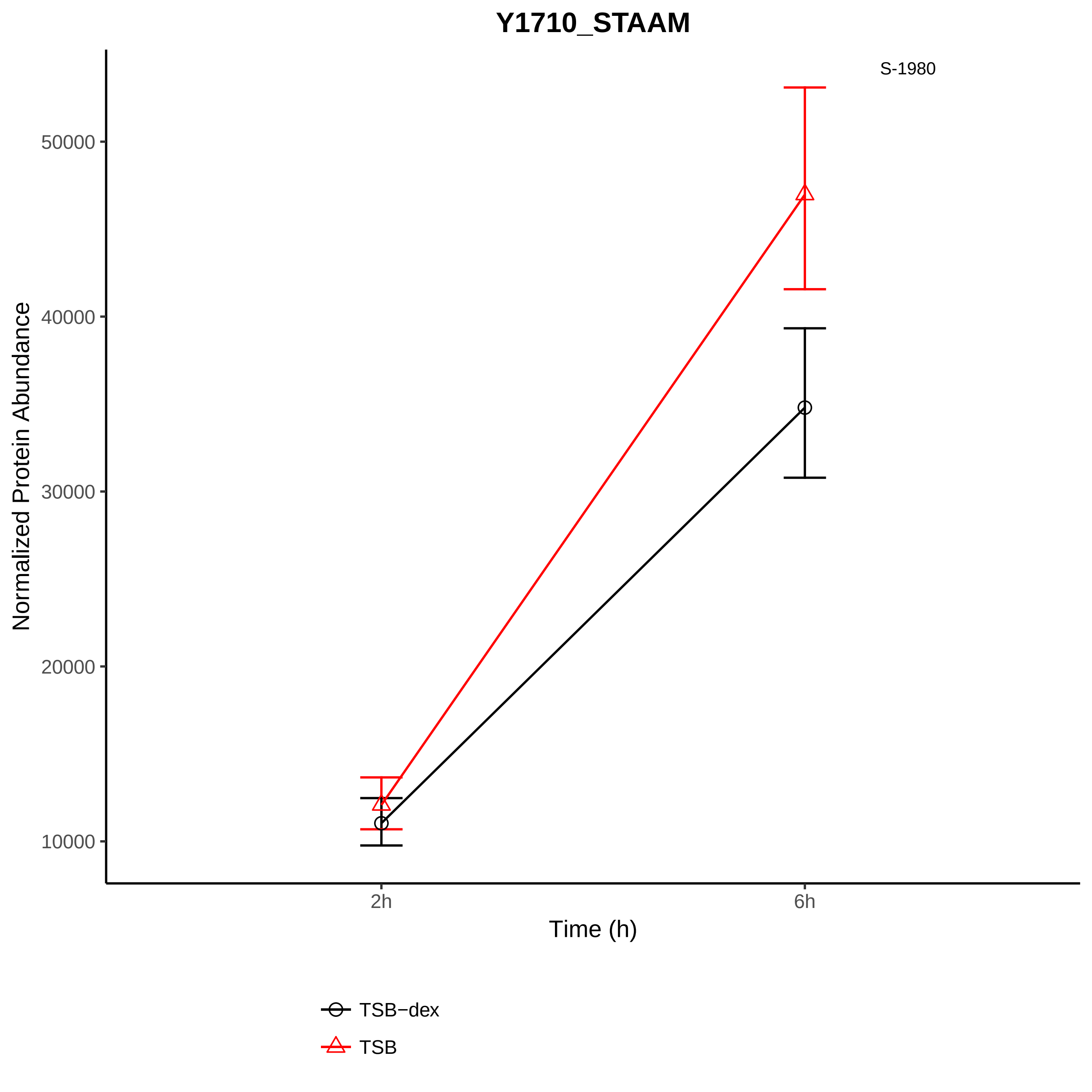

Y1710_STAAM
S-1980
Normalized Protein Abundance
50000
40000
30000
20000
10000
2h
6h
Time (h)
TSB−dex
TSB

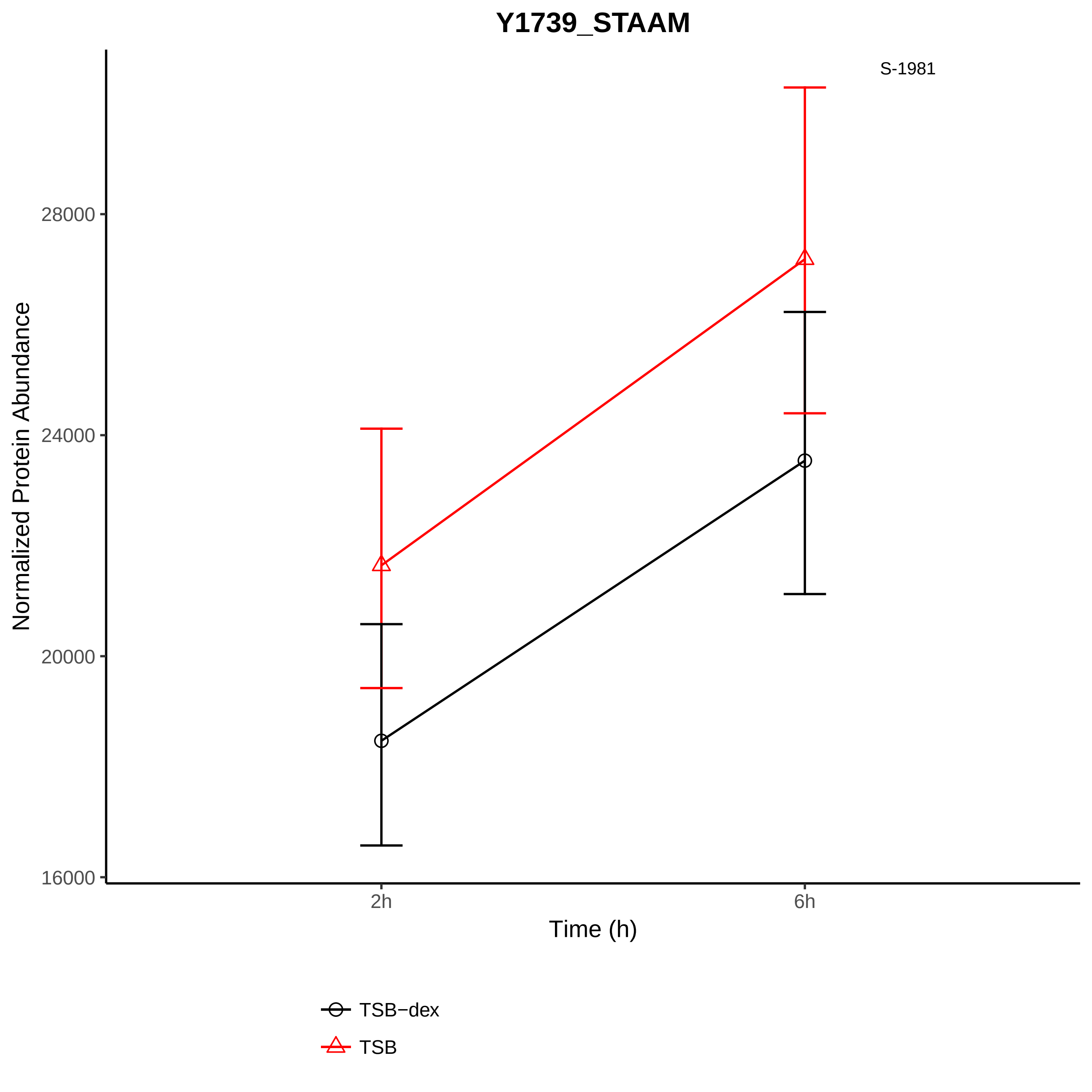

Y1739_STAAM
S-1981
28000
24000
20000
16000
Normalized Protein Abundance
2h
6h
Time (h)
TSB−dex
TSB

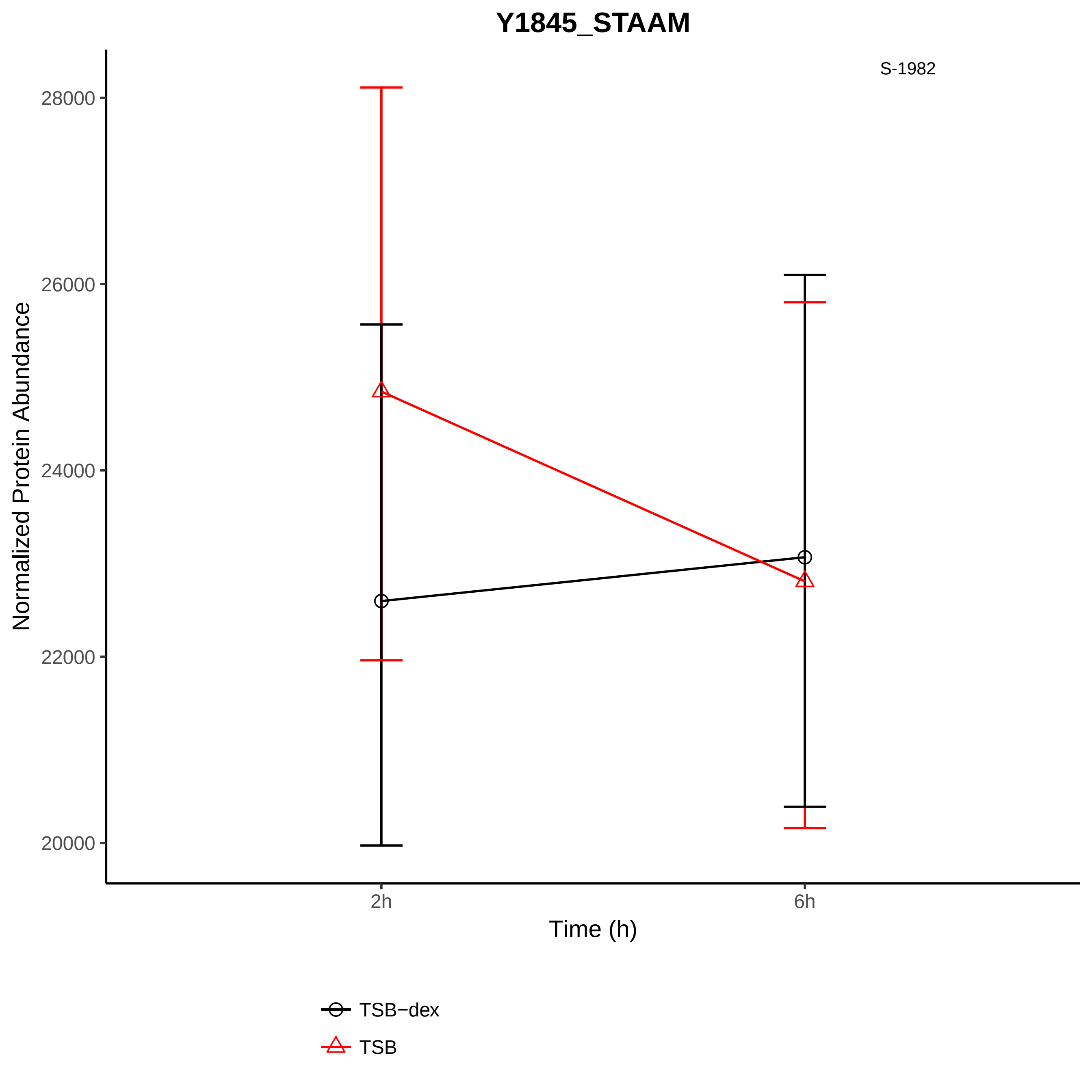

Y1845_STAAM
S-1982
Normalized Protein Abundance
28000
26000
24000
22000
20000
2h
6h
Time (h)
TSB−dex
TSB

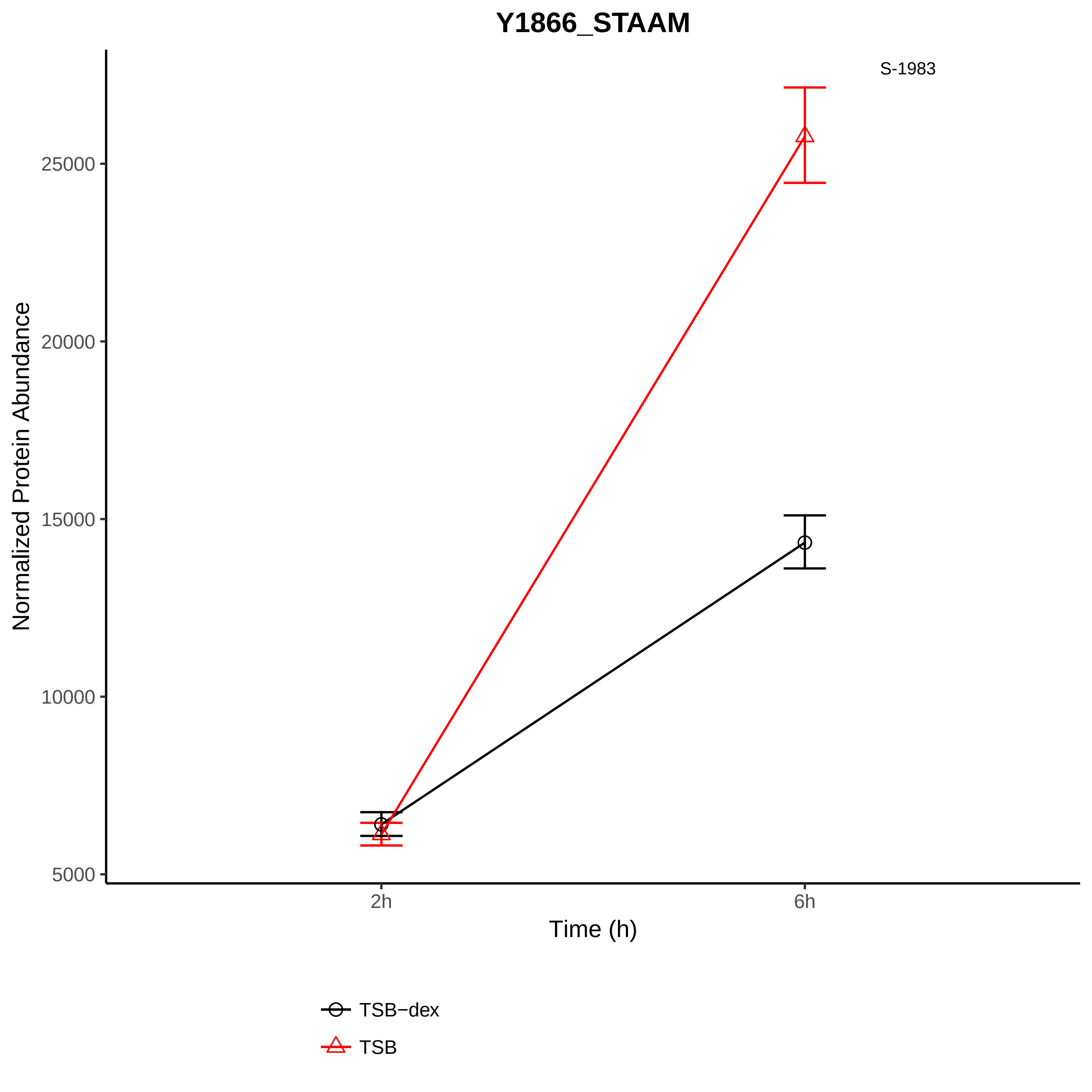
Y1866_STAAM
S-1983
Normalized Protein Abundance
25000
20000
15000
10000
5000
2h
6h
Time (h)
TSB−dex
TSB

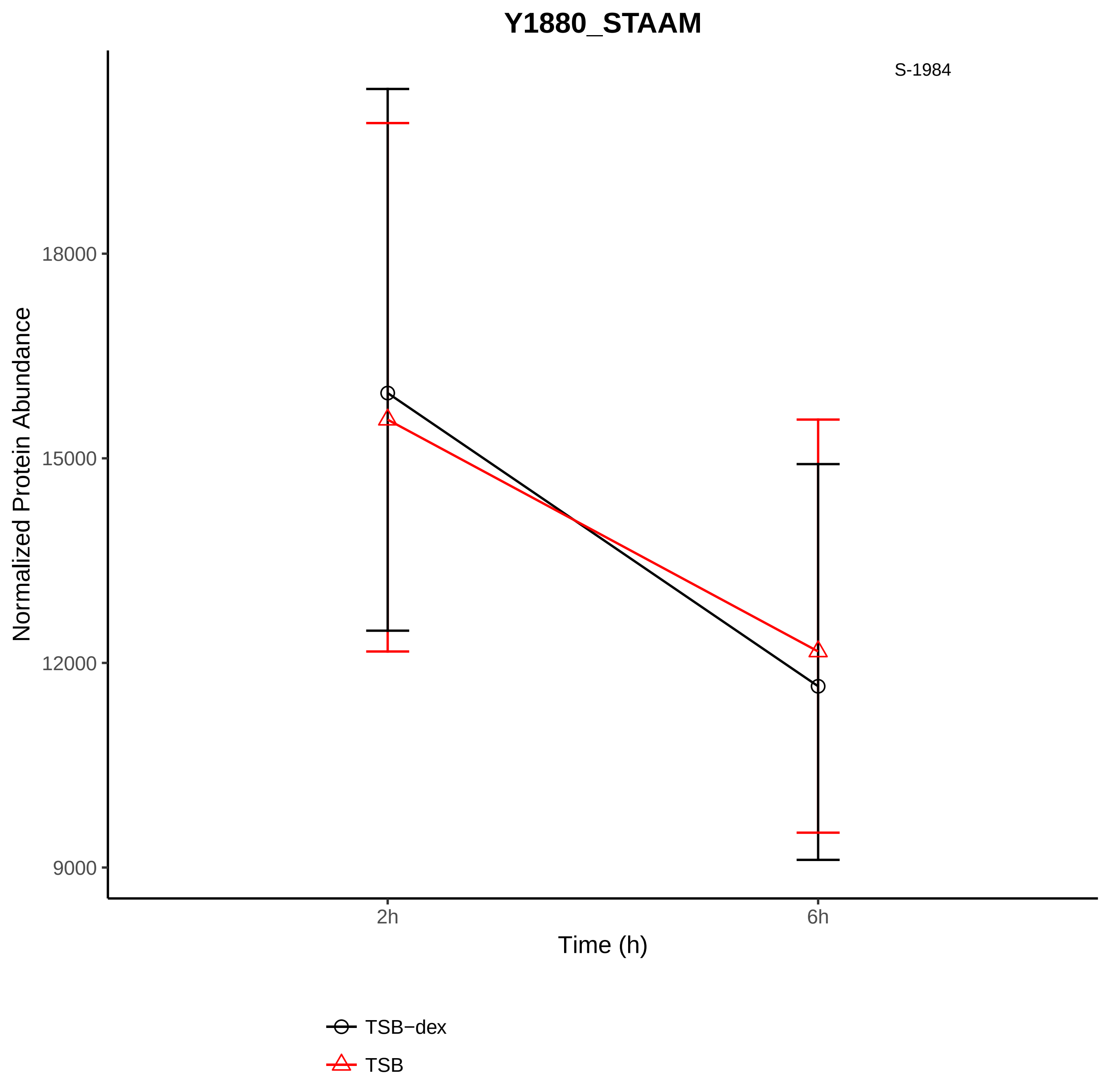

Y1880_STAAM
S-1984
Normalized Protein Abundance
18000
15000
12000
9000
2h
6h
Time (h)
TSB−dex
TSB

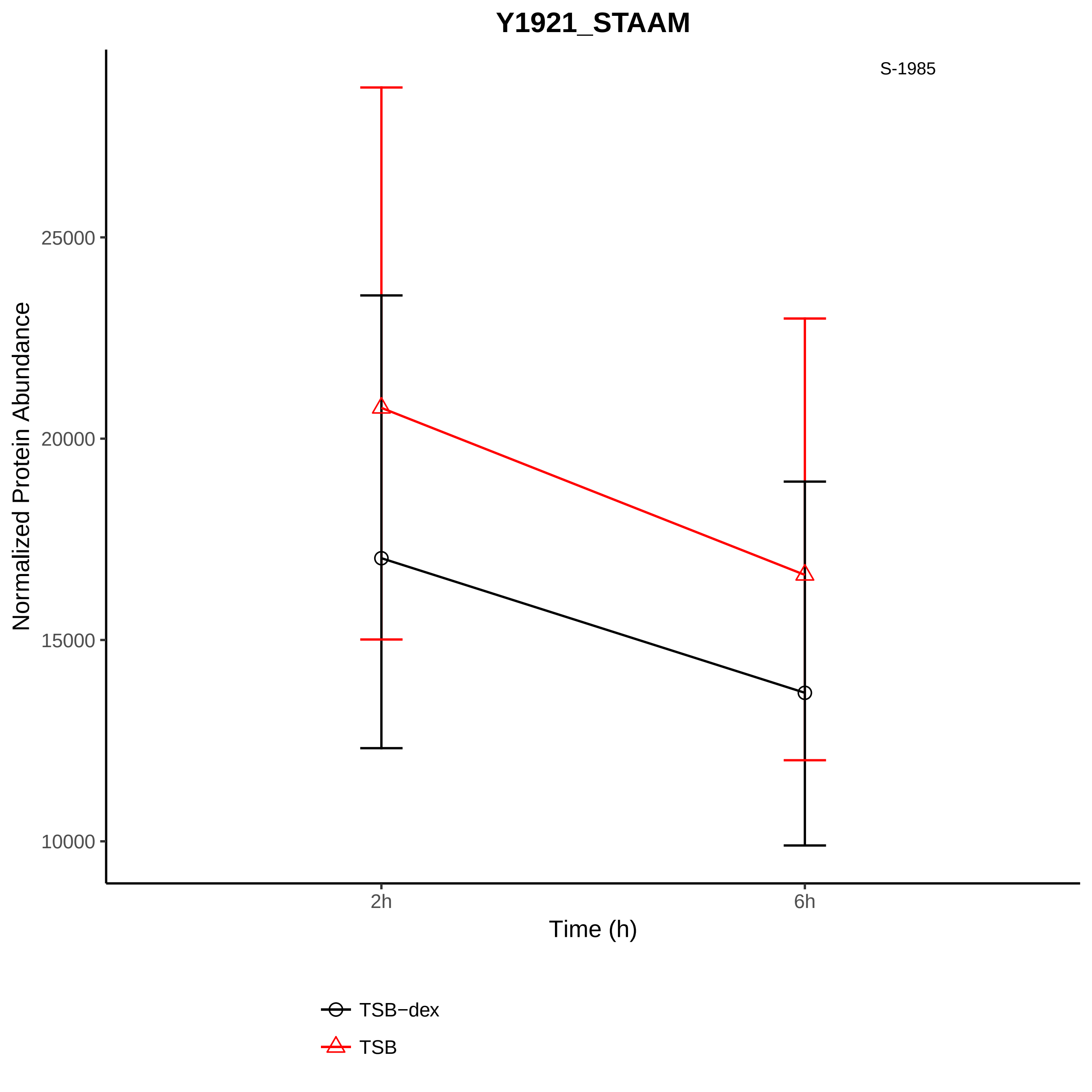

Y1921_STAAM
S-1985
Normalized Protein Abundance
25000
20000
15000
10000
2h
6h
Time (h)
TSB−dex
TSB

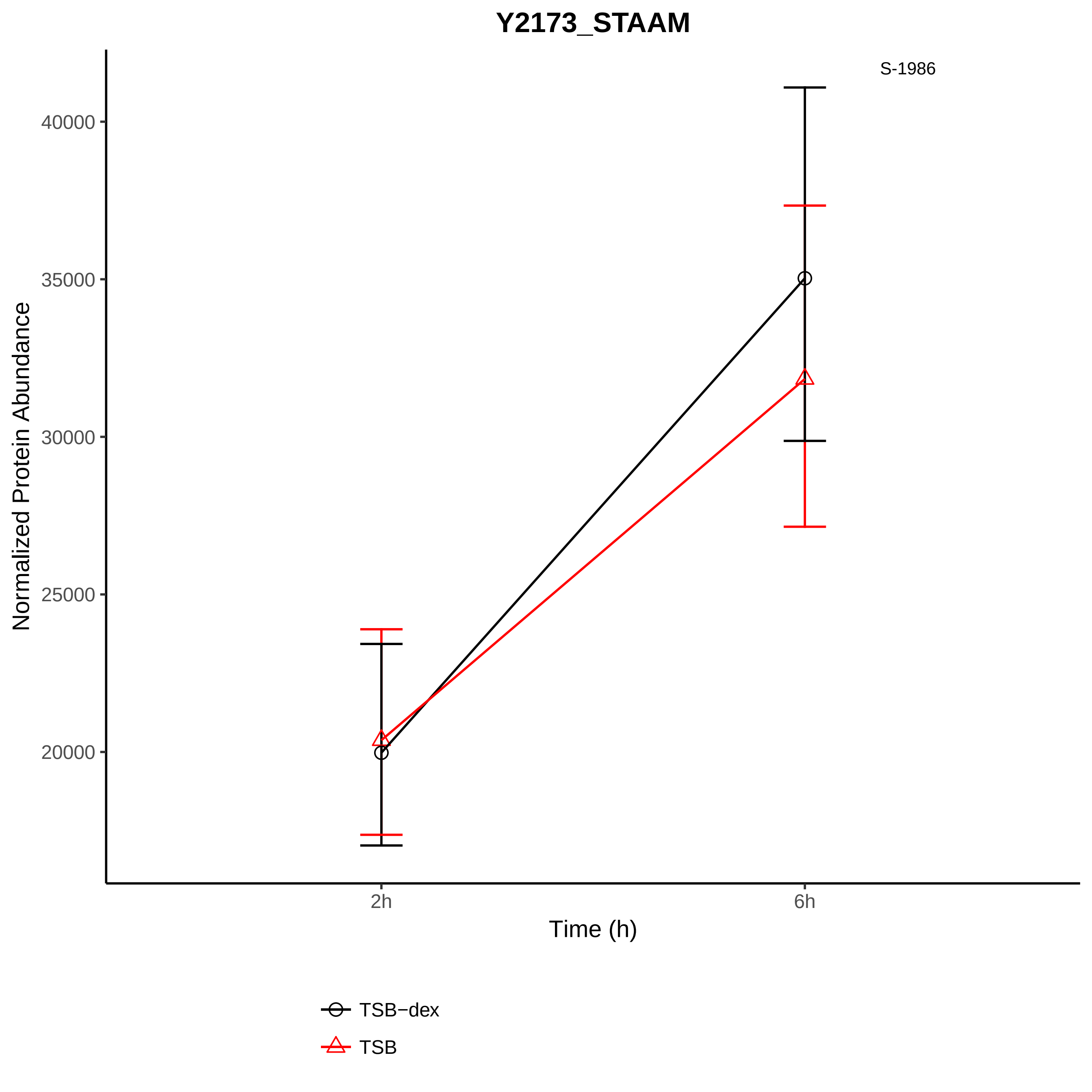

Y2173_STAAM
S-1986
40000
35000
30000
25000
20000
Normalized Protein Abundance
2h
6h
Time (h)
TSB−dex
TSB

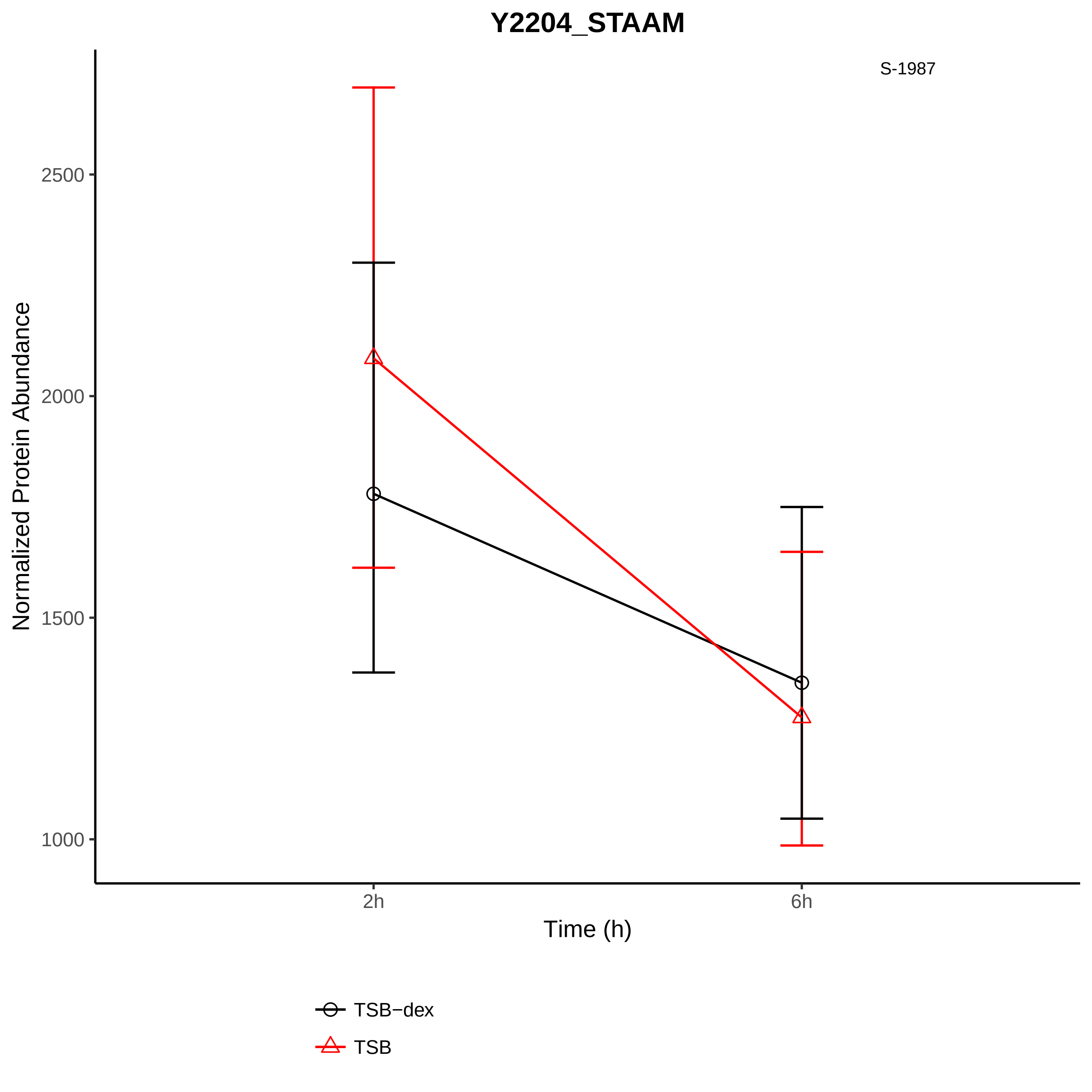
Y2204_STAAM
S-1987
Normalized Protein Abundance
2500
2000
1500
1000
2h
6h
Time (h)
TSB−dex
TSB

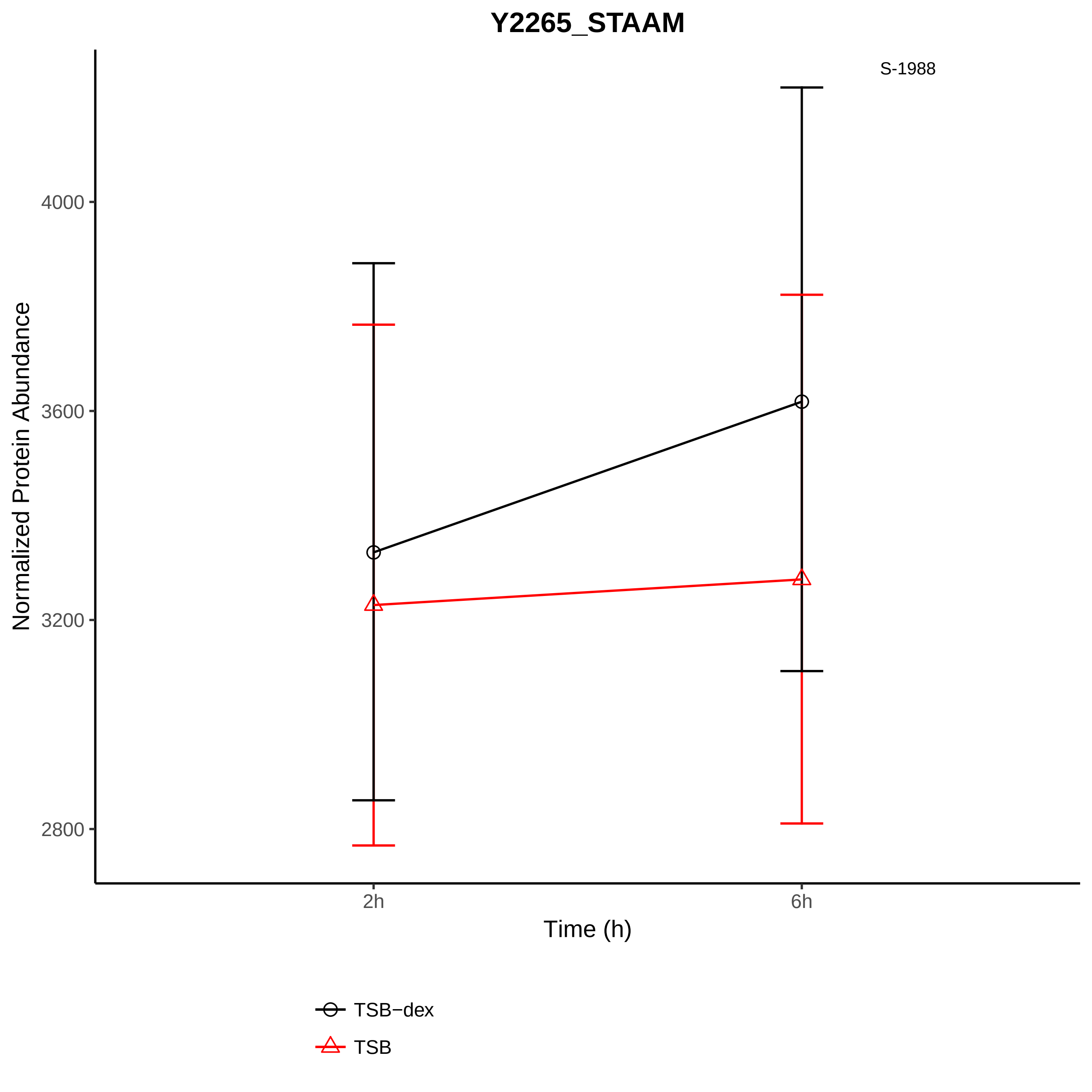

Y2265_STAAM
S-1988
Normalized Protein Abundance
4000
3600
3200
2800
2h
6h
Time (h)
TSB−dex
TSB

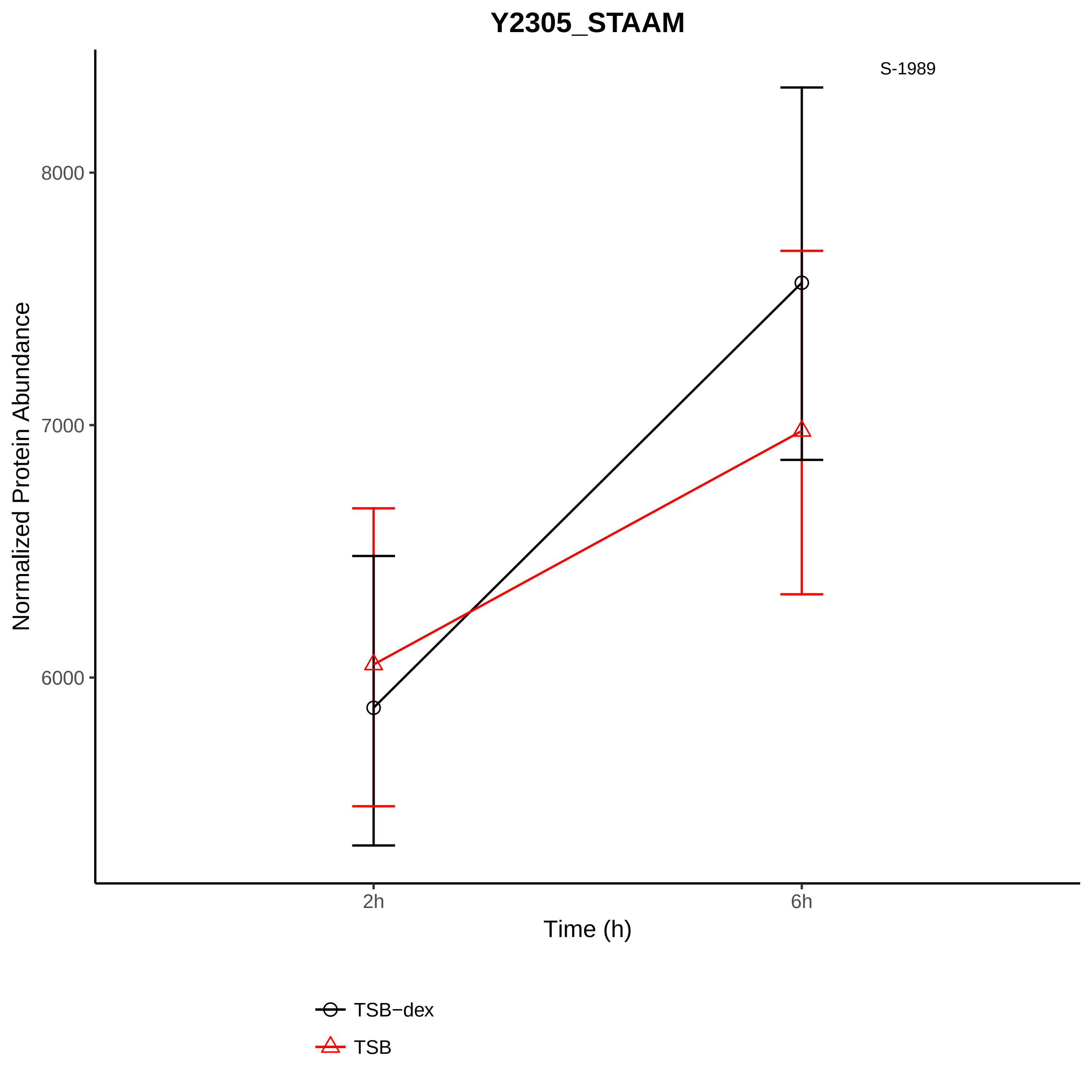

Y2305_STAAM
S-1989
8000
7000
6000
Normalized Protein Abundance
2h
6h
Time (h)
TSB−dex
TSB

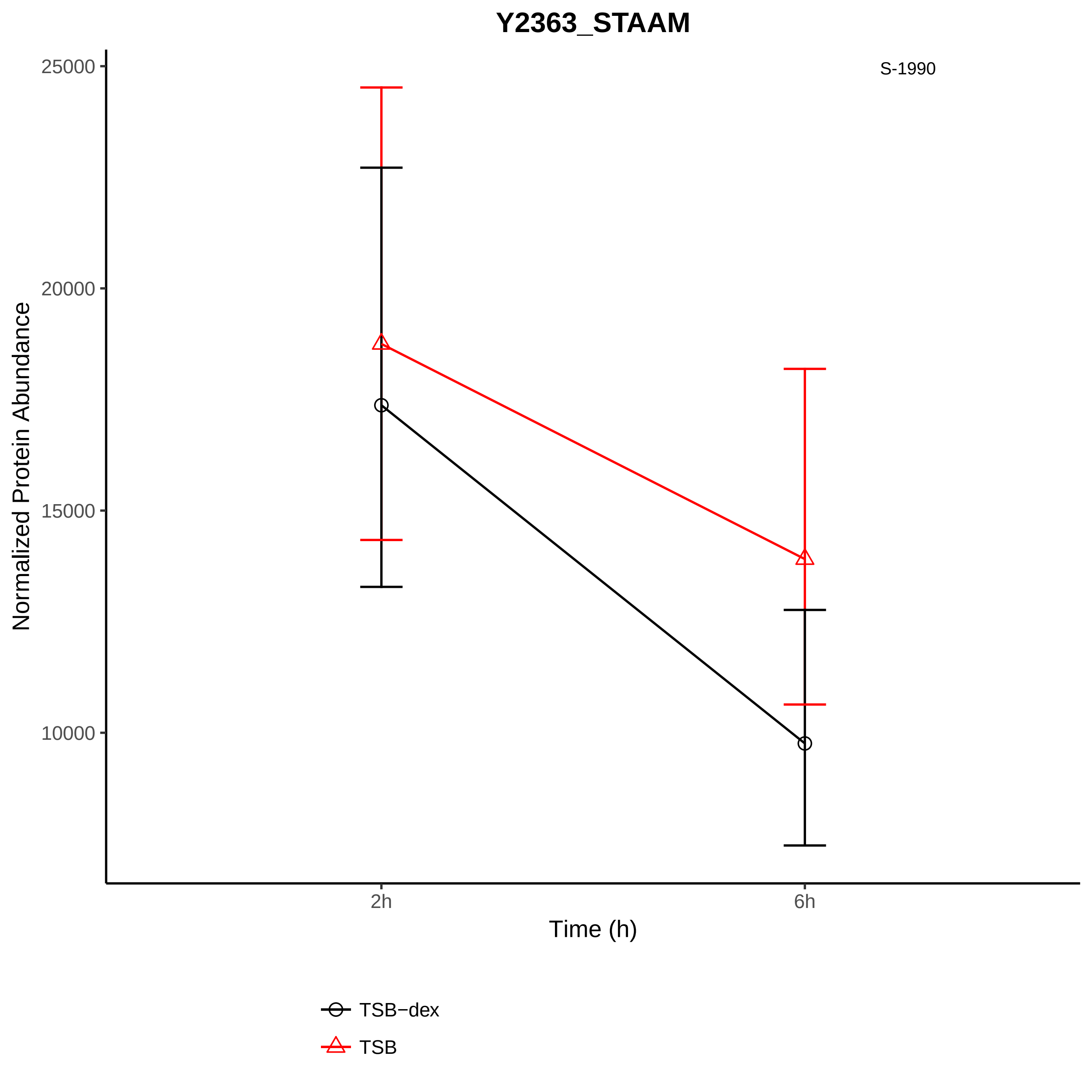

Y2363_STAAM
S-1990
Normalized Protein Abundance
25000
20000
15000
10000
2h
6h
Time (h)
TSB−dex
TSB

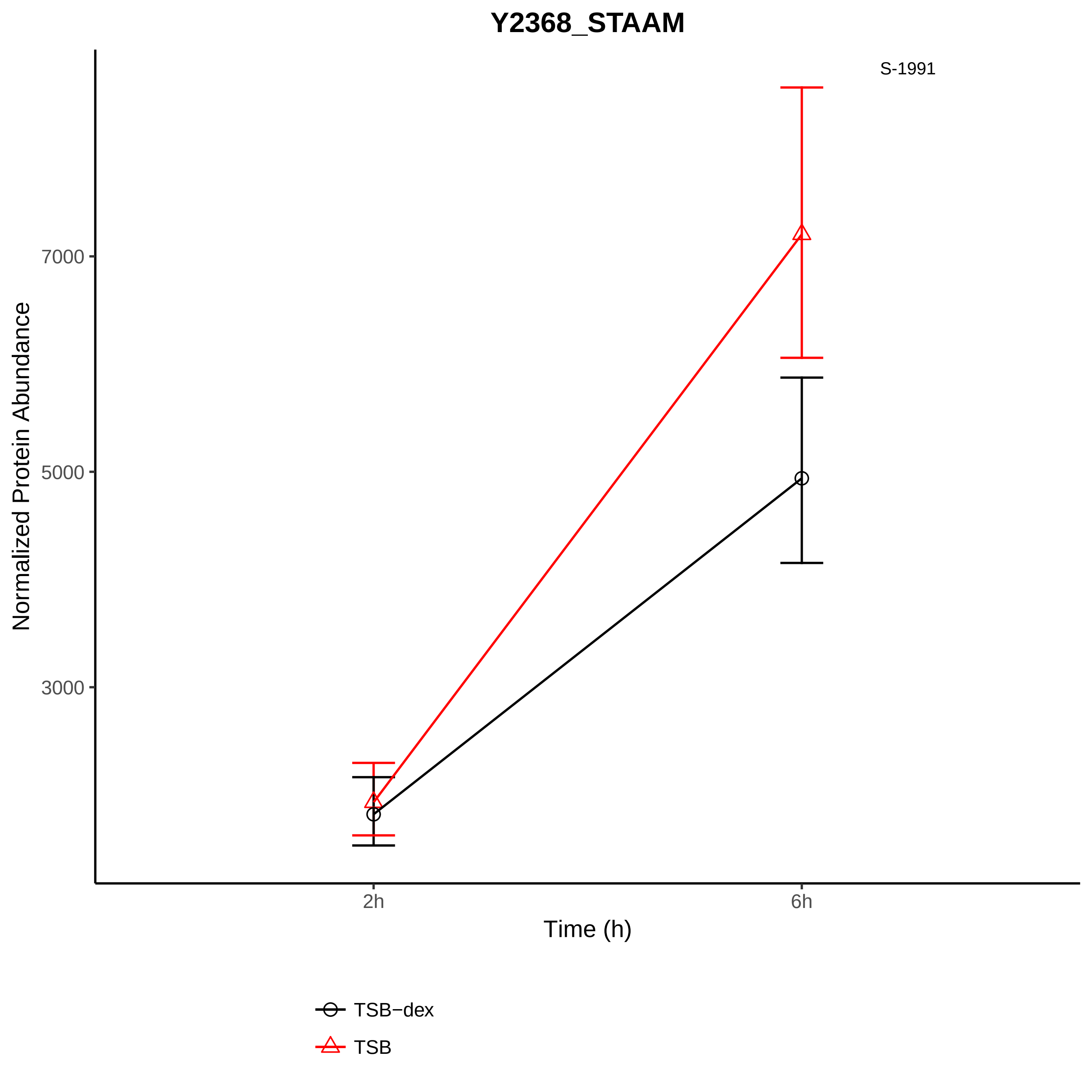

Y2368_STAAM
S-1991
7000
5000
3000
Normalized Protein Abundance
2h
6h
Time (h)
TSB−dex
TSB

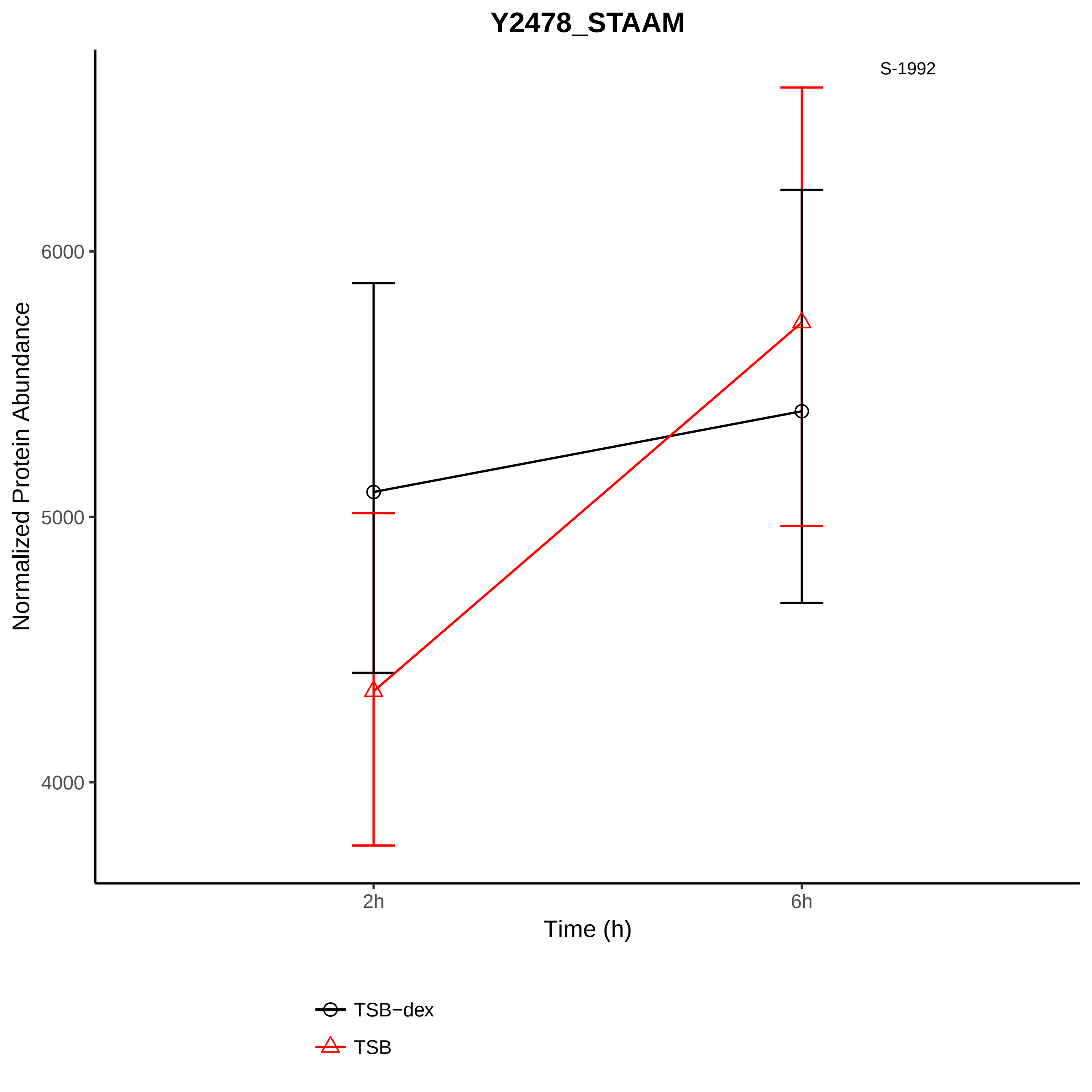
Y2478_STAAM
S-1992
Normalized Protein Abundance
6000
5000
4000
2h
6h
Time (h)
TSB−dex
TSB

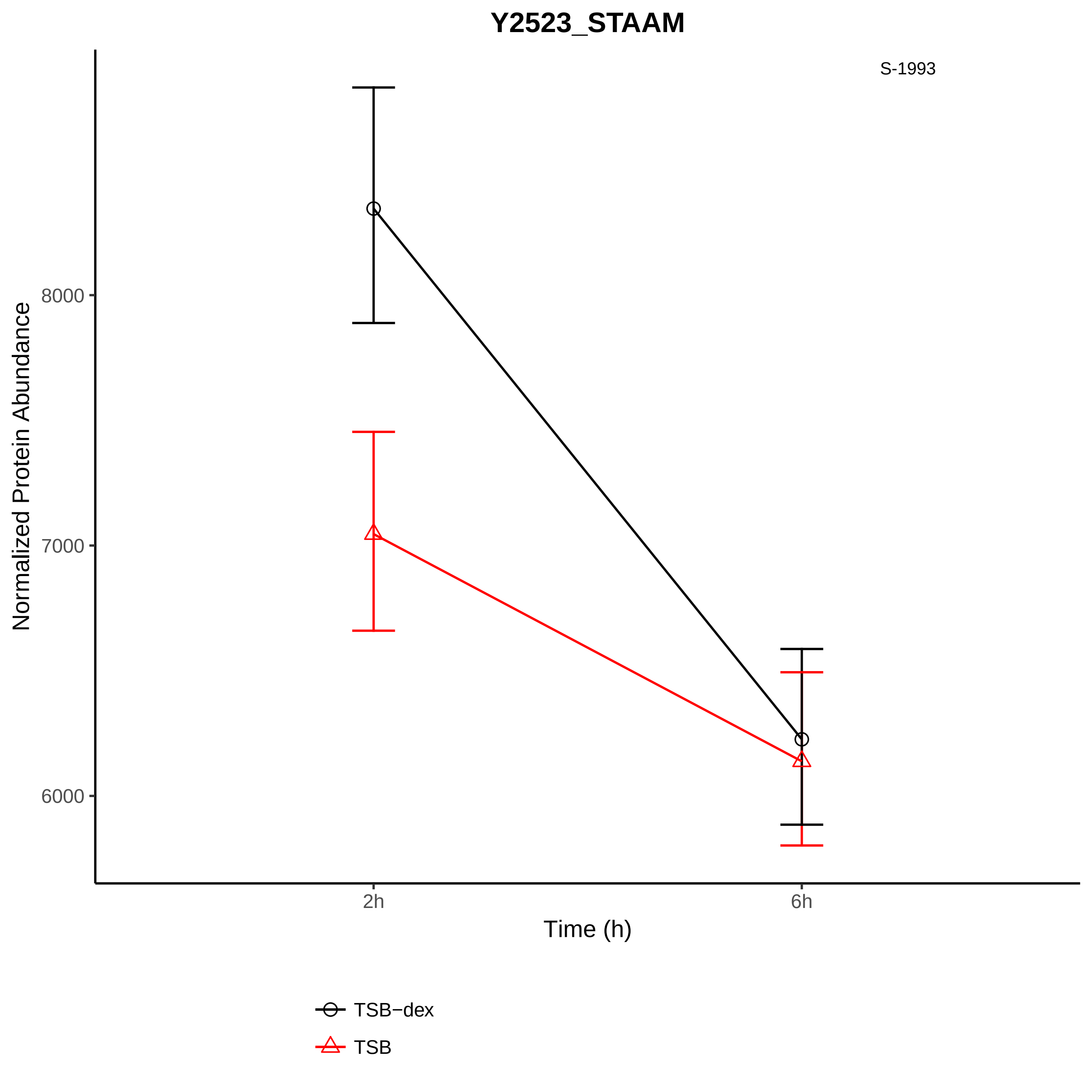

Y2523_STAAM
S-1993
Normalized Protein Abundance
8000
7000
6000
2h
6h
Time (h)
TSB−dex
TSB

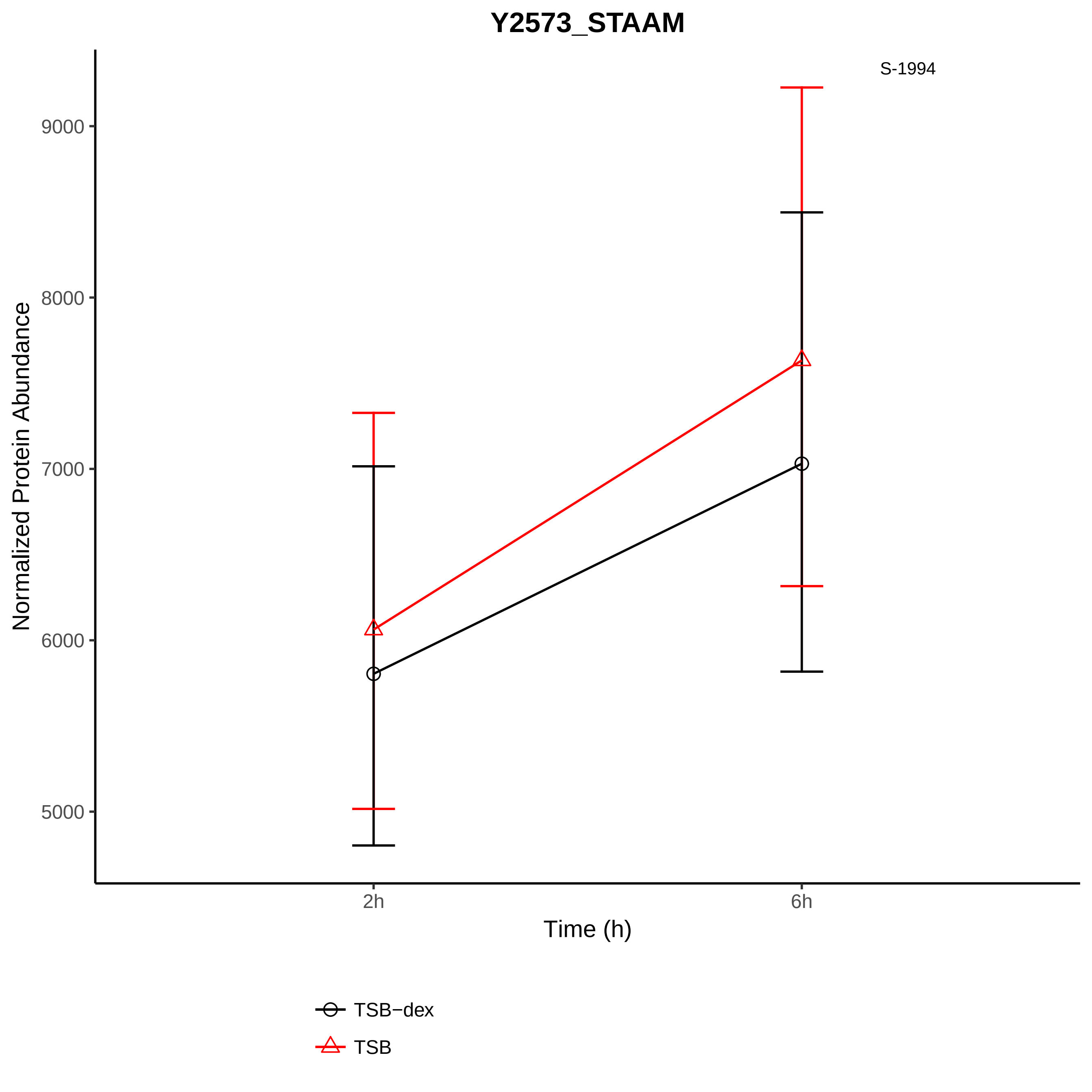

Y2573_STAAM
S-1994
Normalized Protein Abundance
9000
8000
7000
6000
5000
2h
6h
Time (h)
TSB−dex
TSB

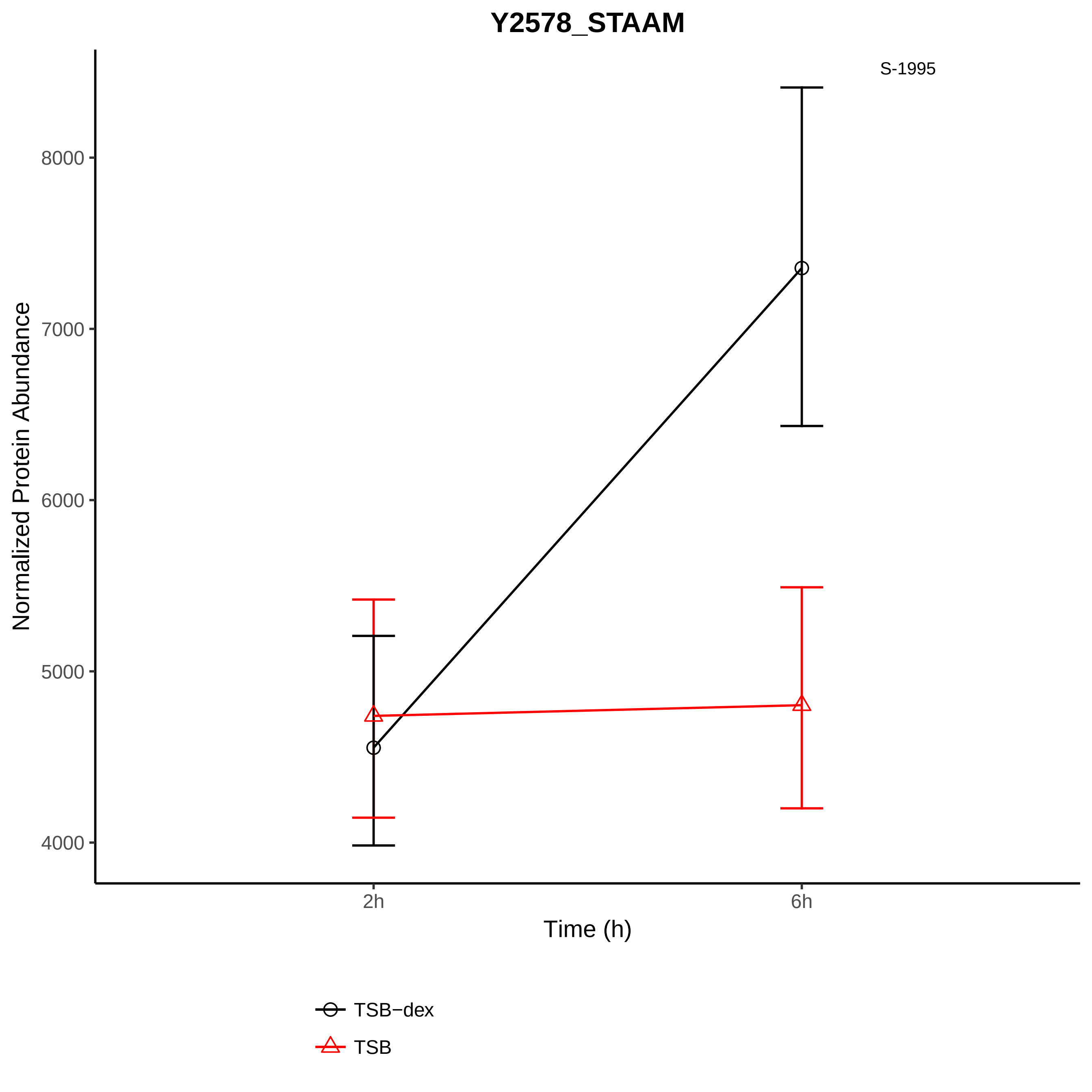

Y2578_STAAM
S-1995
8000
7000
6000
5000
4000
Normalized Protein Abundance
2h
6h
Time (h)
TSB−dex
TSB

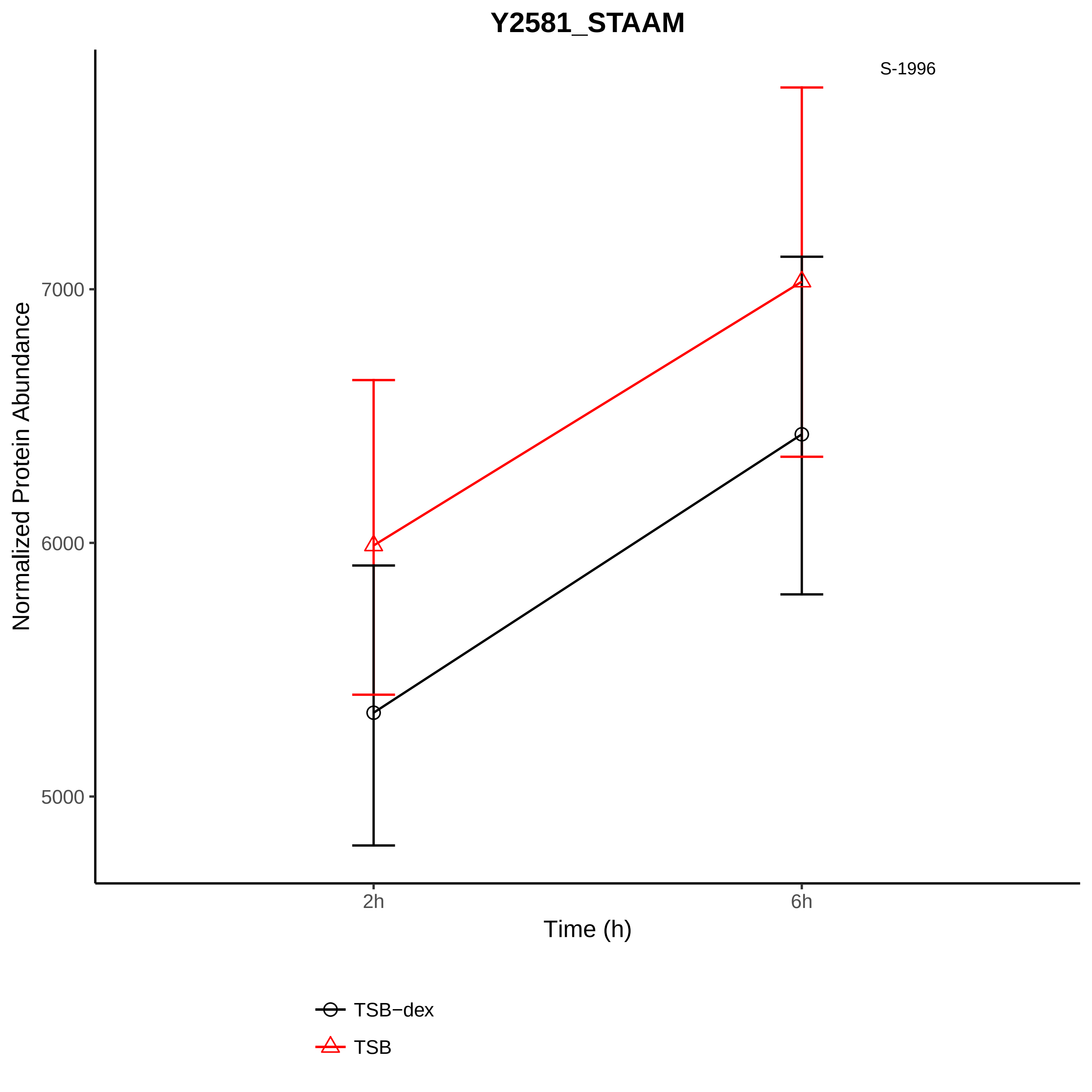

Y2581_STAAM
S-1996
Normalized Protein Abundance
7000
6000
5000
2h
6h
Time (h)
TSB−dex
TSB

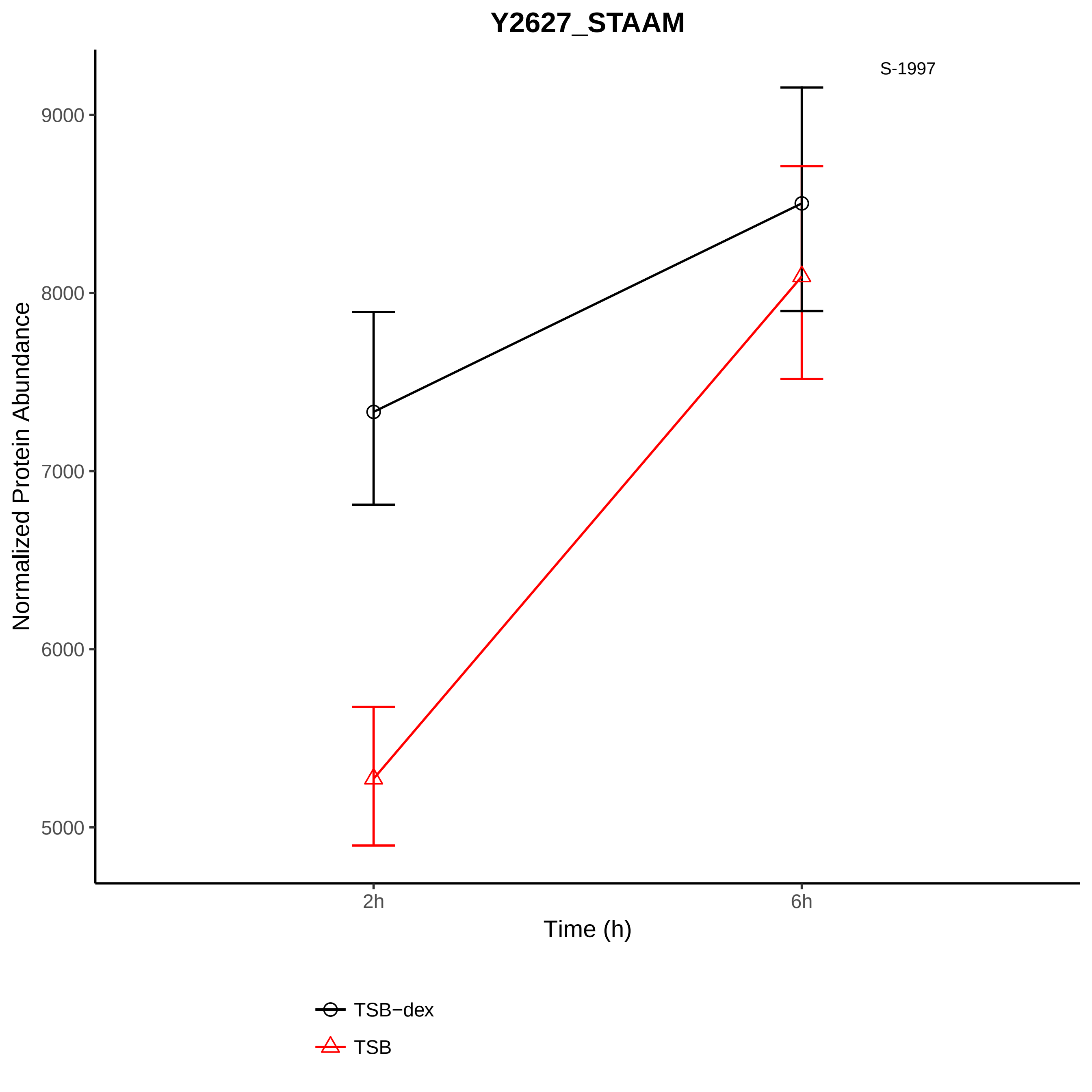
Y2627_STAAM
S-1997
Normalized Protein Abundance
9000
8000
7000
6000
5000
2h
6h
Time (h)
TSB−dex
TSB

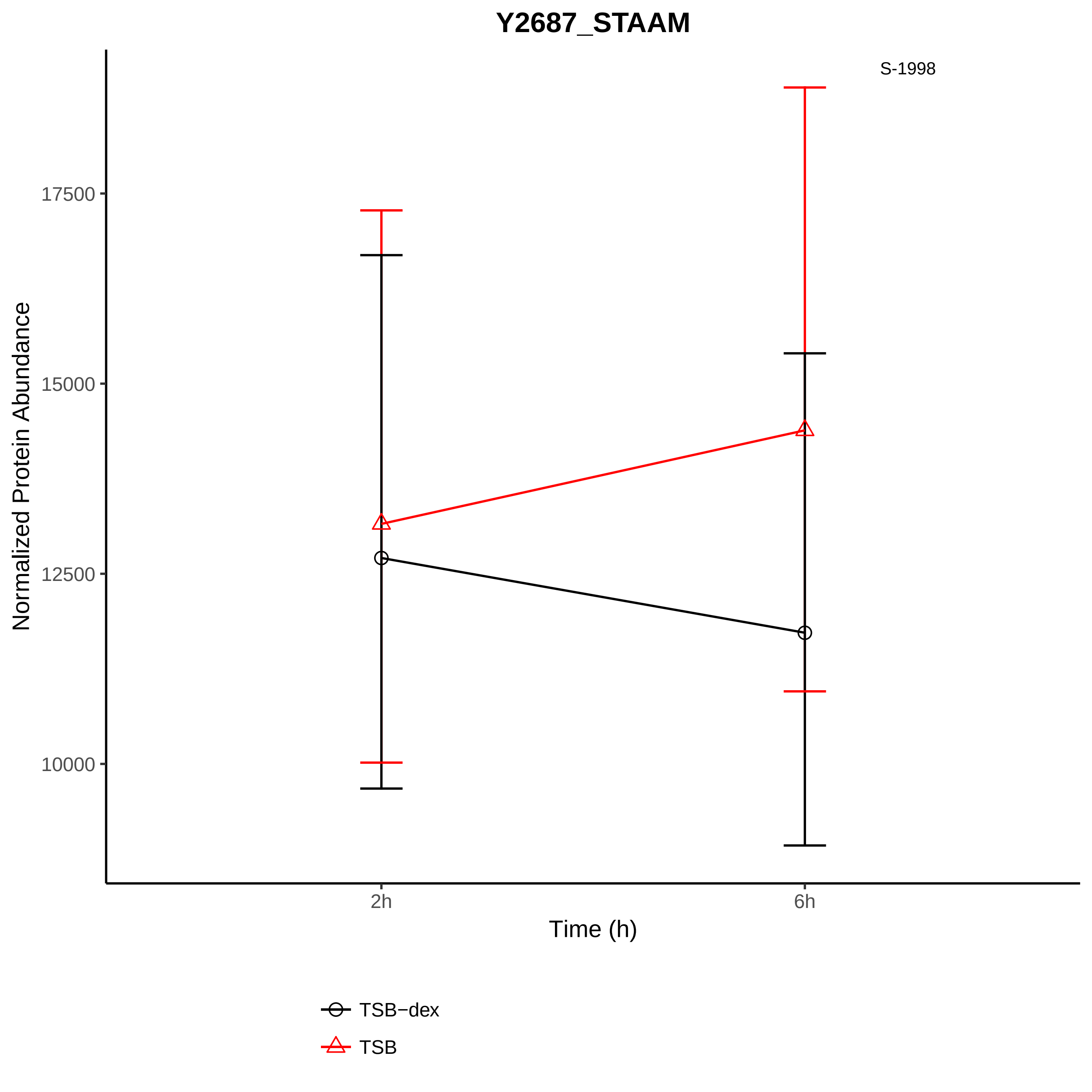

Y2687_STAAM
S-1998
17500
15000
12500
10000
Normalized Protein Abundance
2h
6h
Time (h)
TSB−dex
TSB

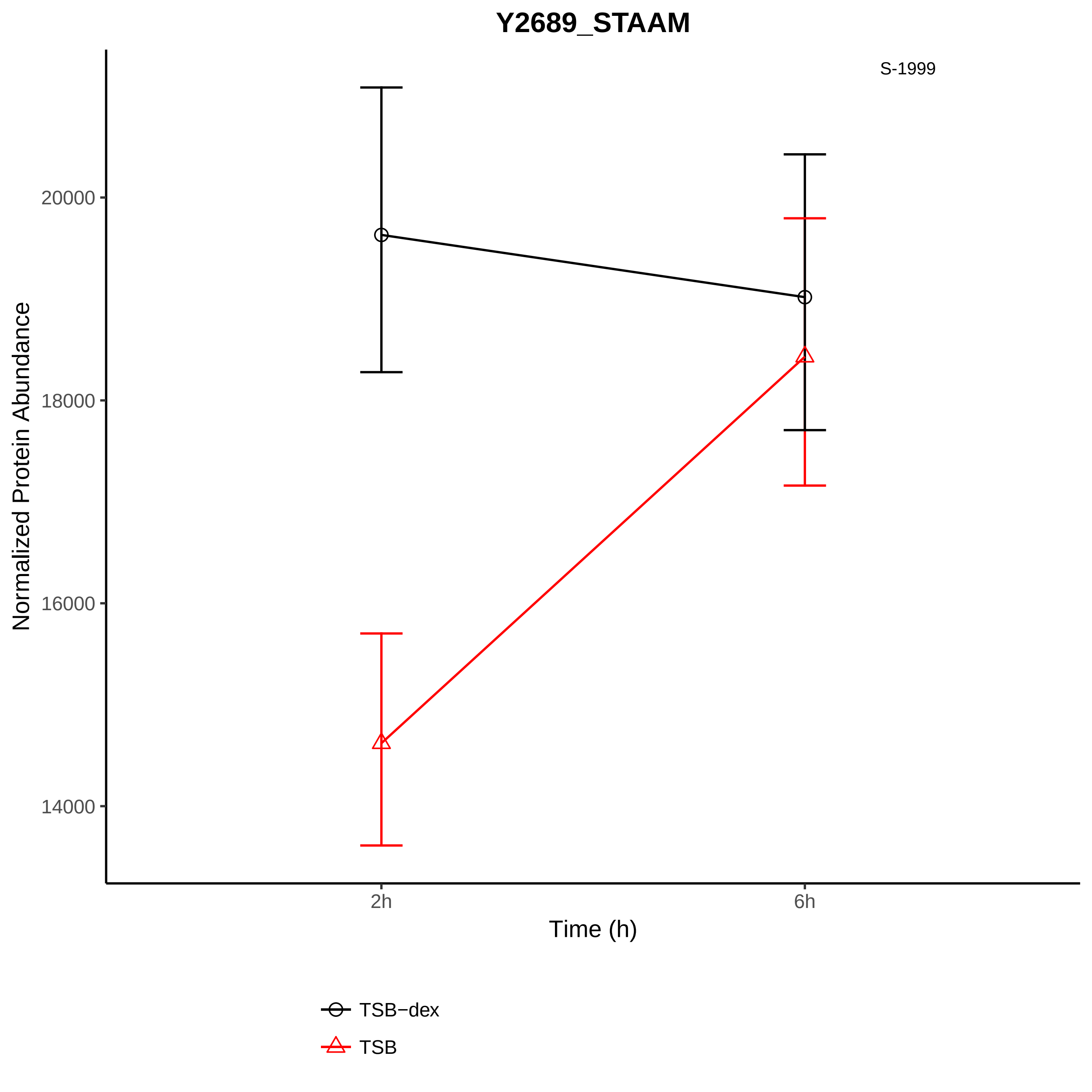

Y2689_STAAM
S-1999
Normalized Protein Abundance
20000
18000
16000
14000
2h
6h
Time (h)
TSB−dex
TSB

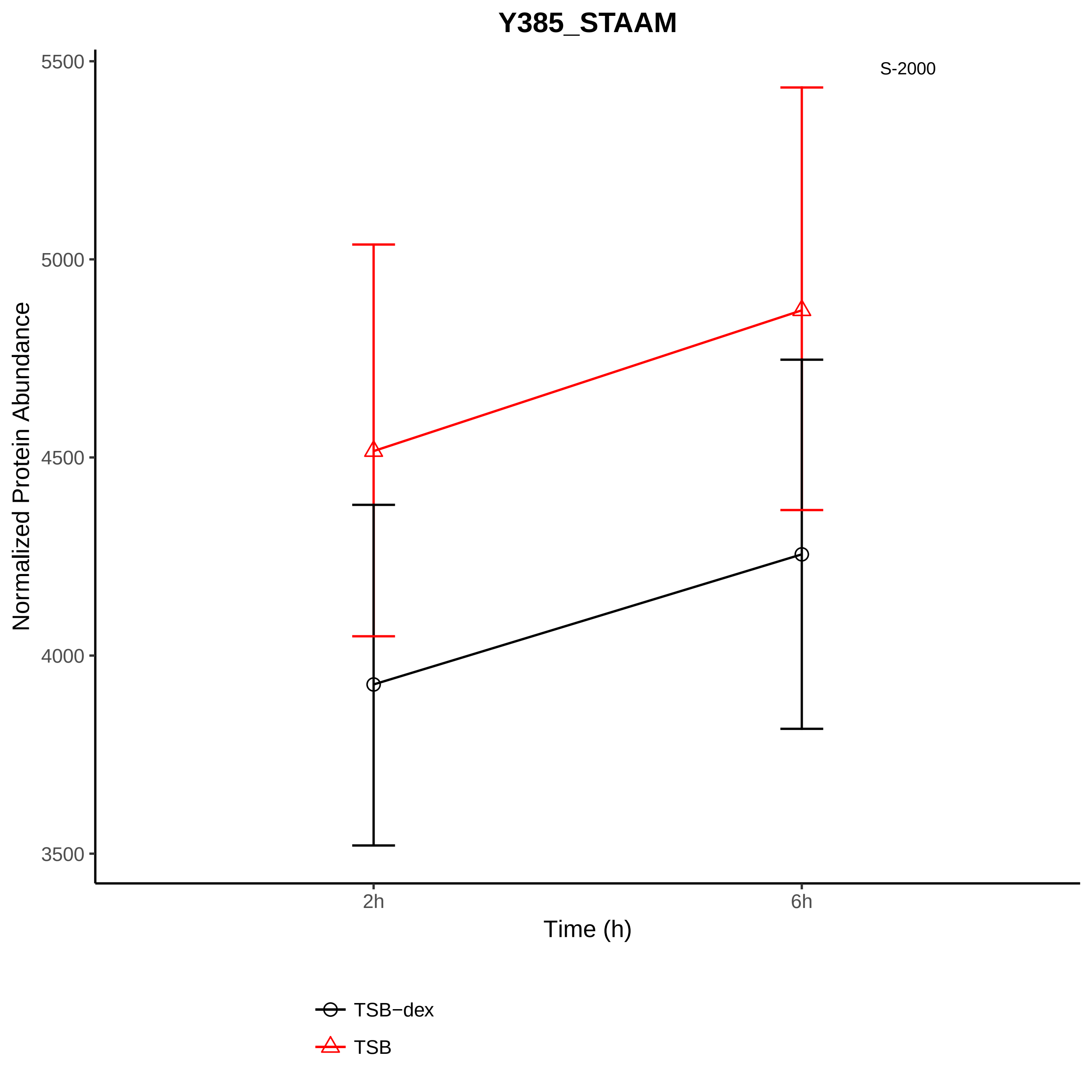

Y385_STAAM
S-2000
5500
5000
4500
4000
3500
Normalized Protein Abundance
2h
6h
Time (h)
TSB−dex
TSB

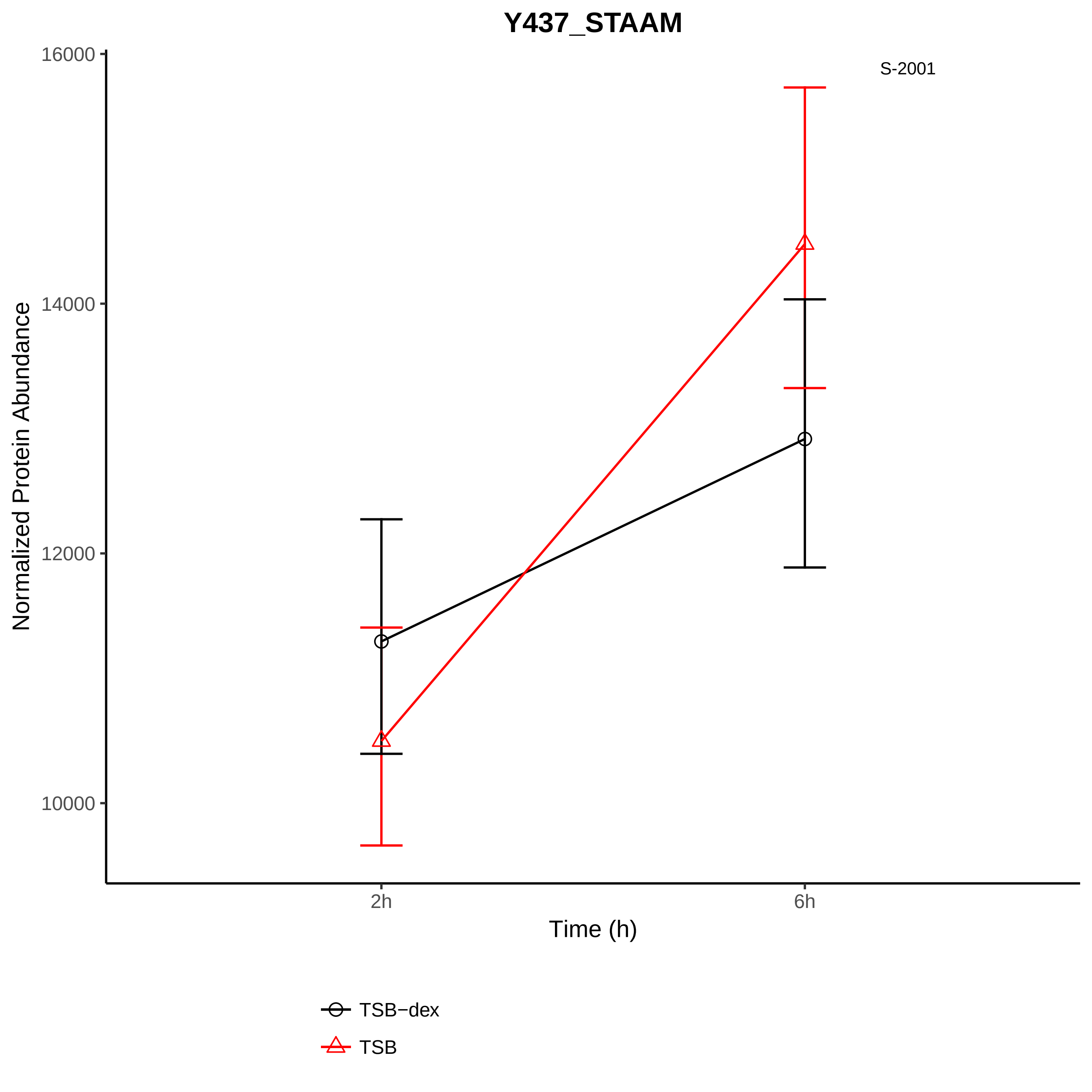
Y437_STAAM
S-2001
16000
14000
12000
10000
Normalized Protein Abundance
2h
6h
Time (h)
TSB−dex
TSB

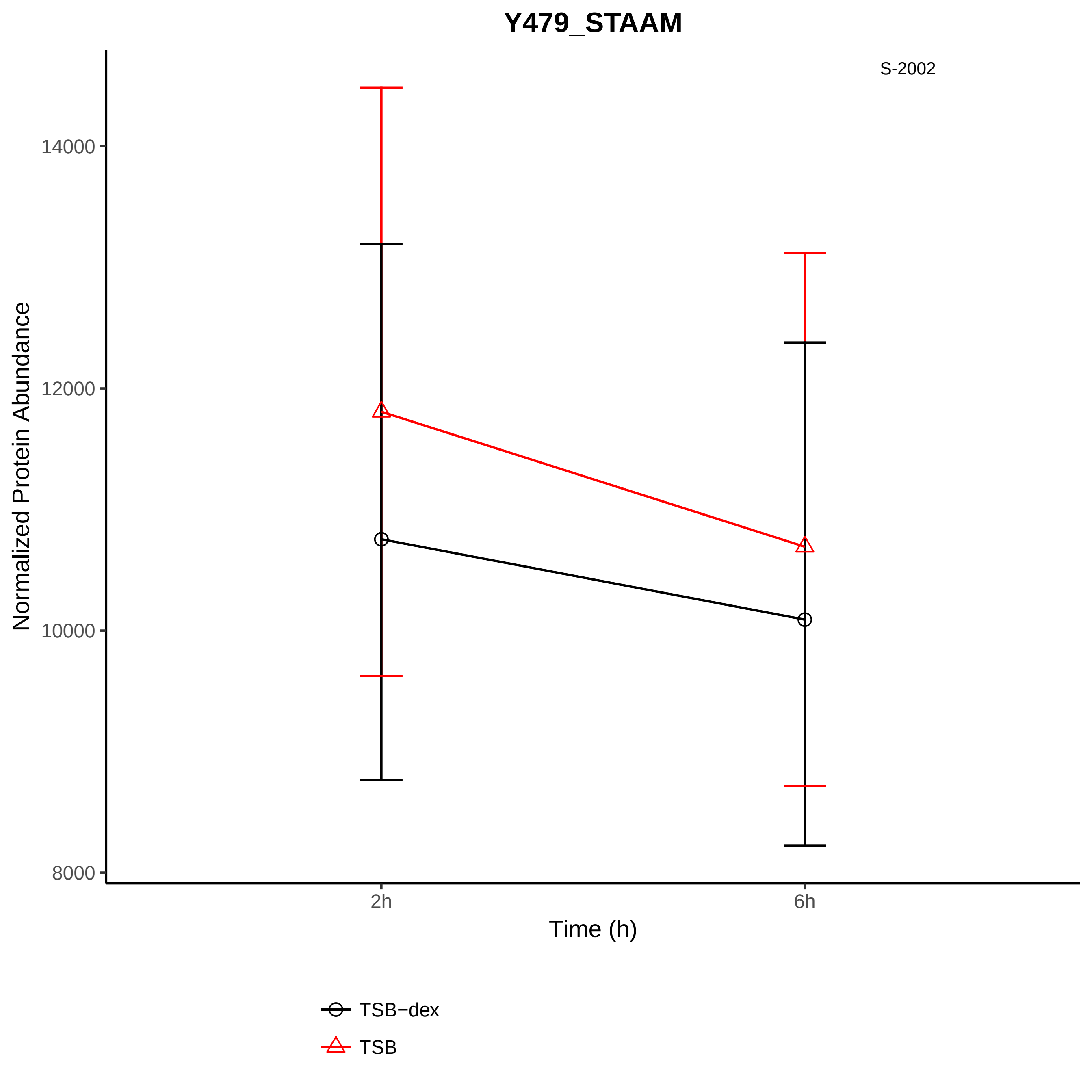

Y479_STAAM
S-2002
14000
12000
10000
8000
Normalized Protein Abundance
2h
6h
Time (h)
TSB−dex
TSB

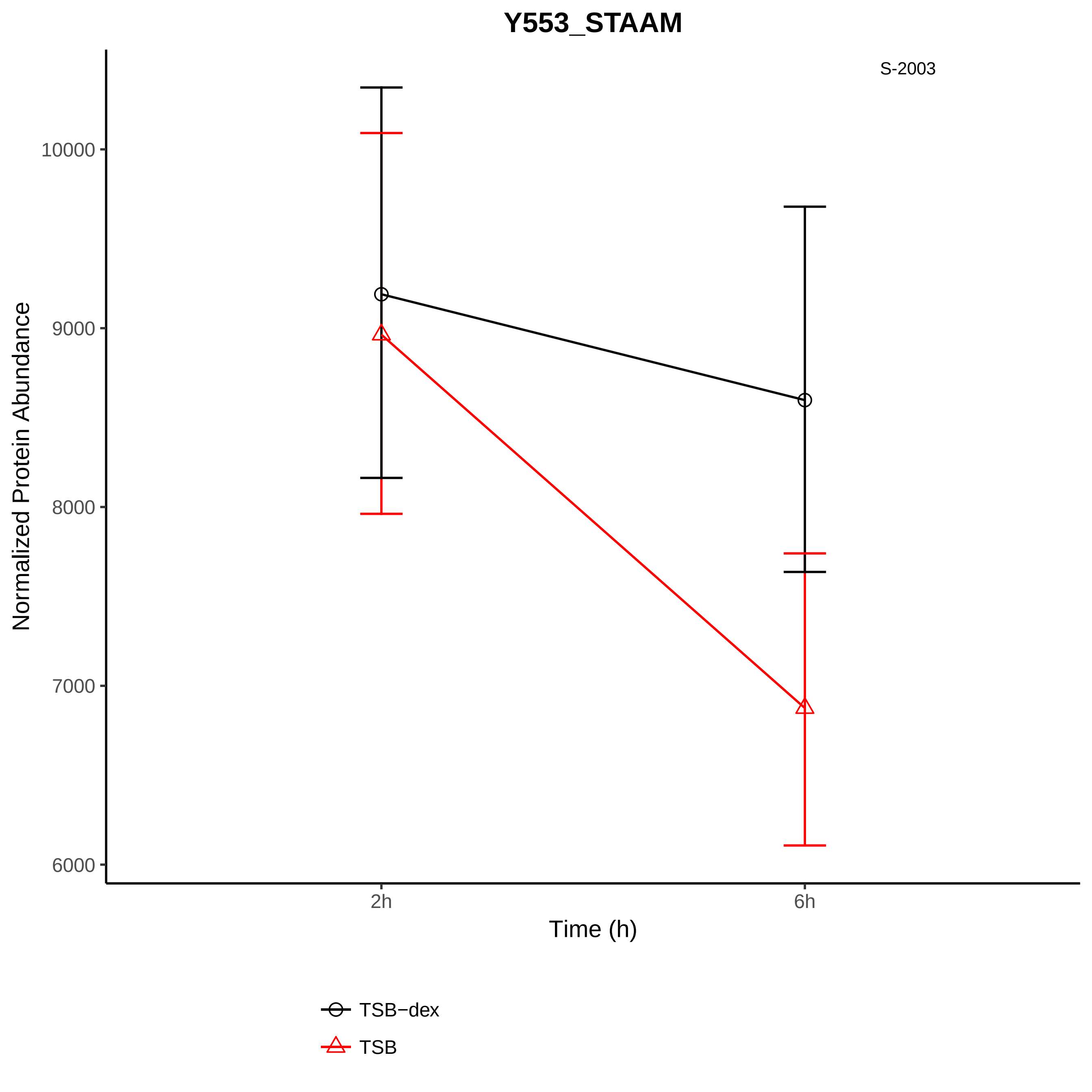

Y553_STAAM
S-2003
Normalized Protein Abundance
10000
9000
8000
7000
6000
2h
6h
Time (h)
TSB−dex
TSB

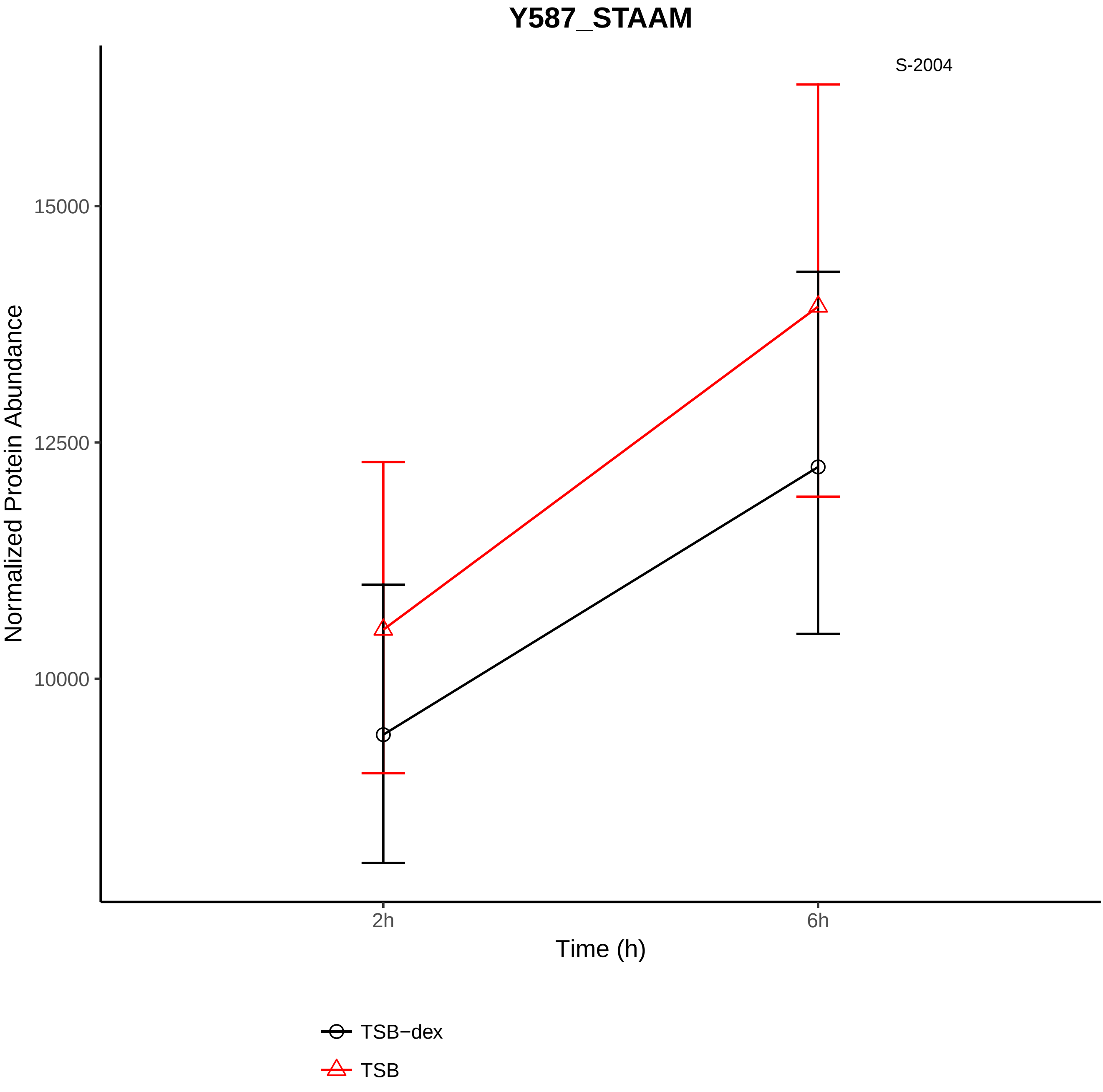

Y587_STAAM
S-2004
15000
12500
10000
Normalized Protein Abundance
2h
6h
Time (h)
TSB−dex
TSB

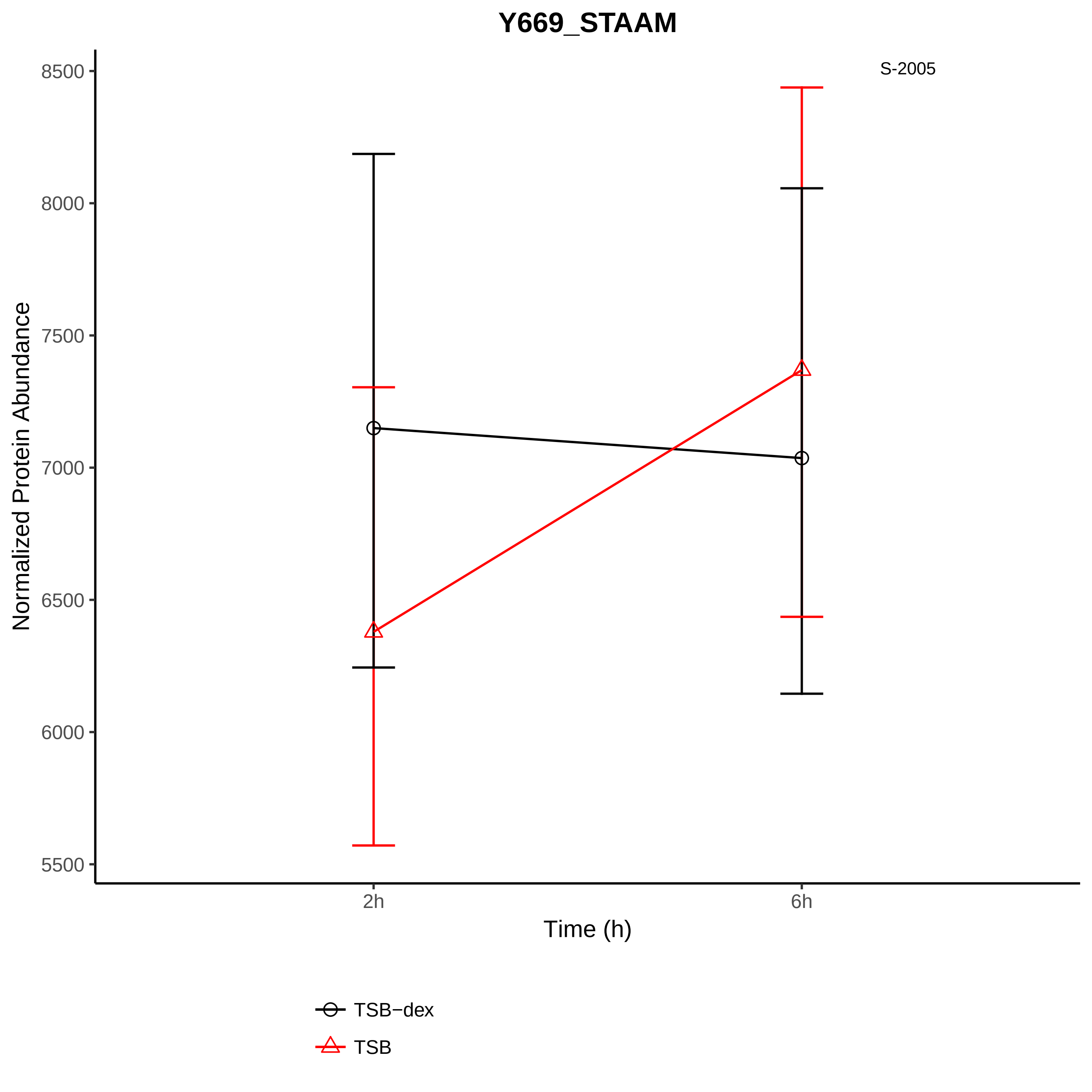

Y669_STAAM
S-2005
Normalized Protein Abundance
8500
8000
7500
7000
6500
6000
5500
2h
6h
Time (h)
TSB−dex
TSB

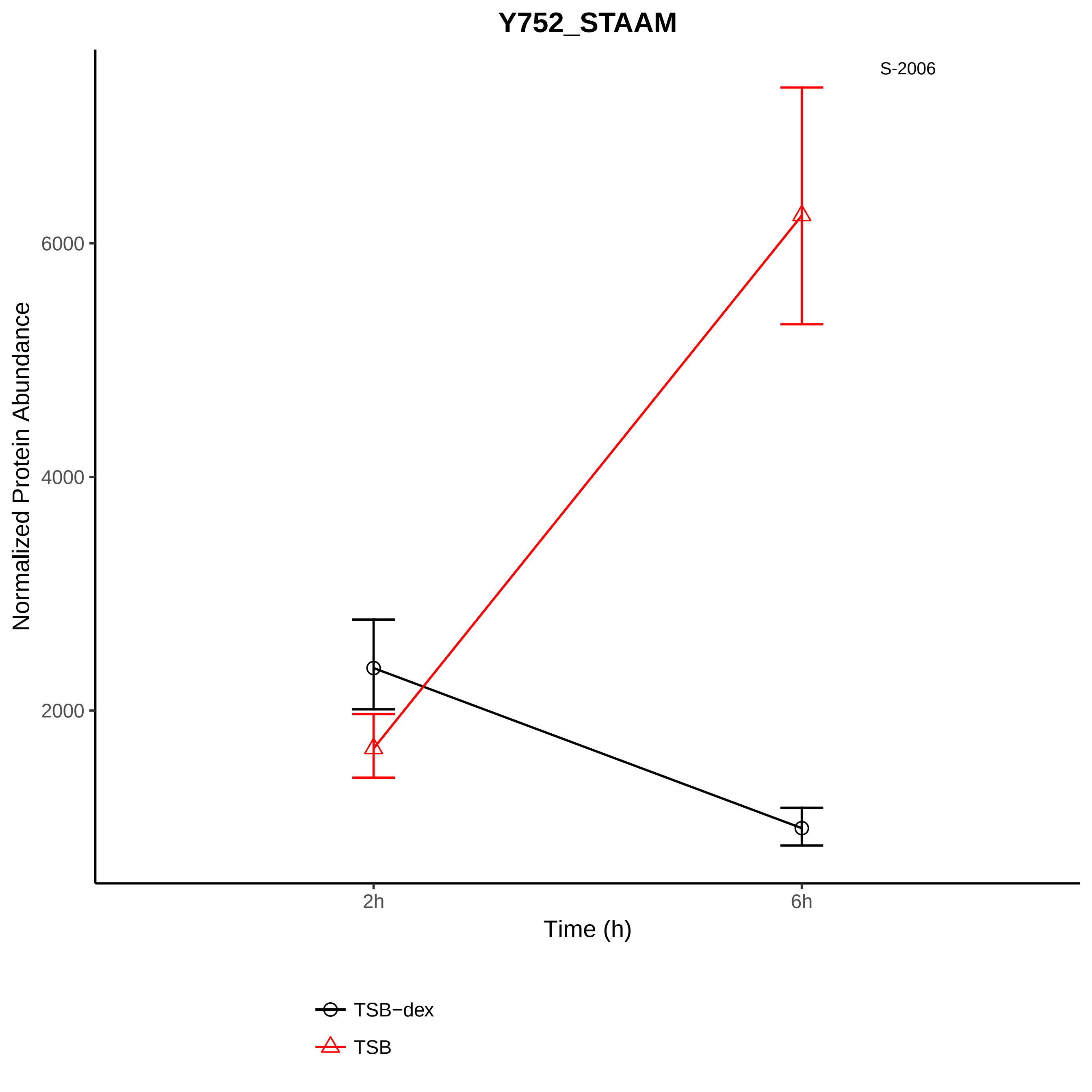
Y752_STAAM
S-2006
Normalized Protein Abundance
6000
4000
2000
2h
6h
Time (h)
TSB−dex
TSB

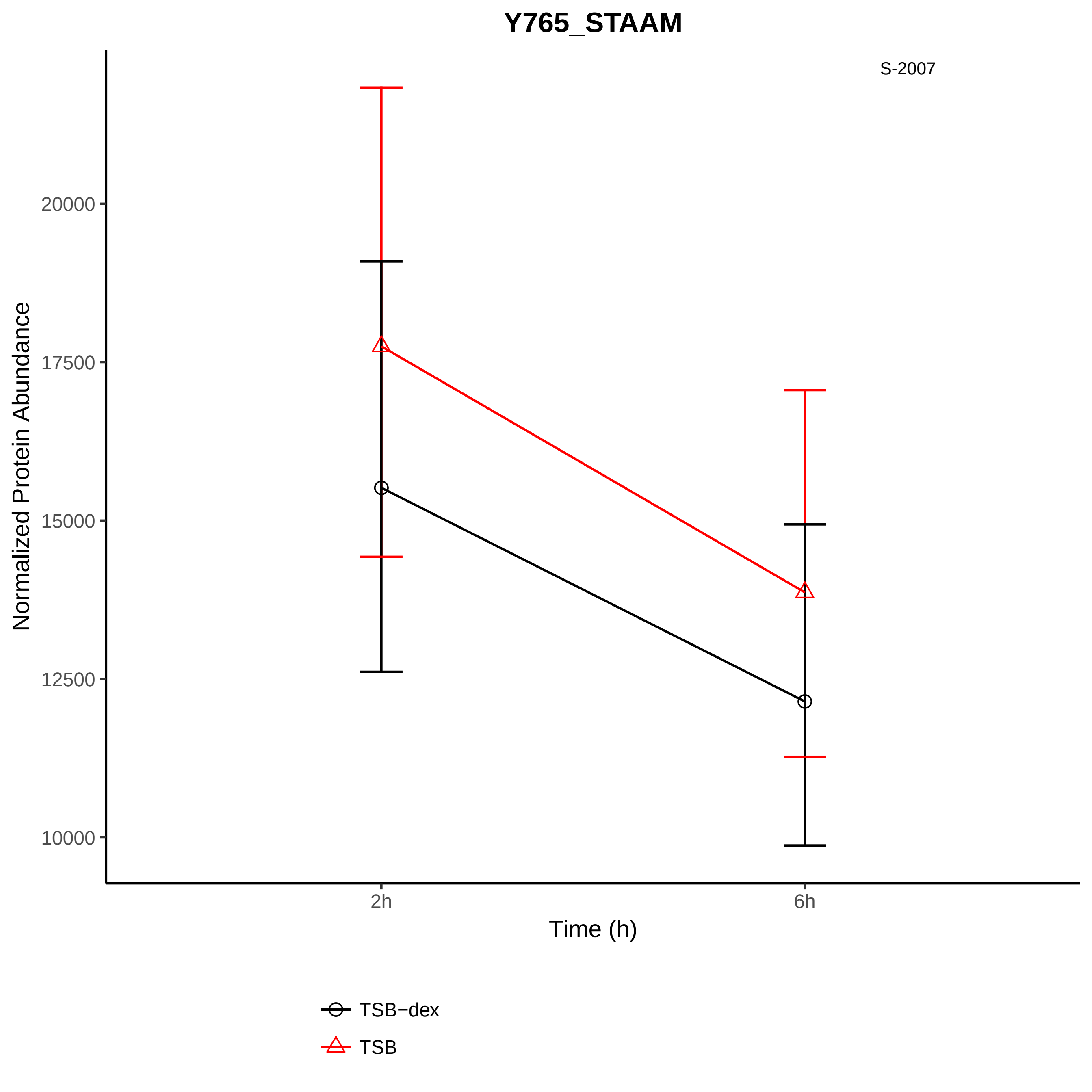

Y765_STAAM
S-2007
Normalized Protein Abundance
20000
17500
15000
12500
10000
2h
6h
Time (h)
TSB−dex
TSB

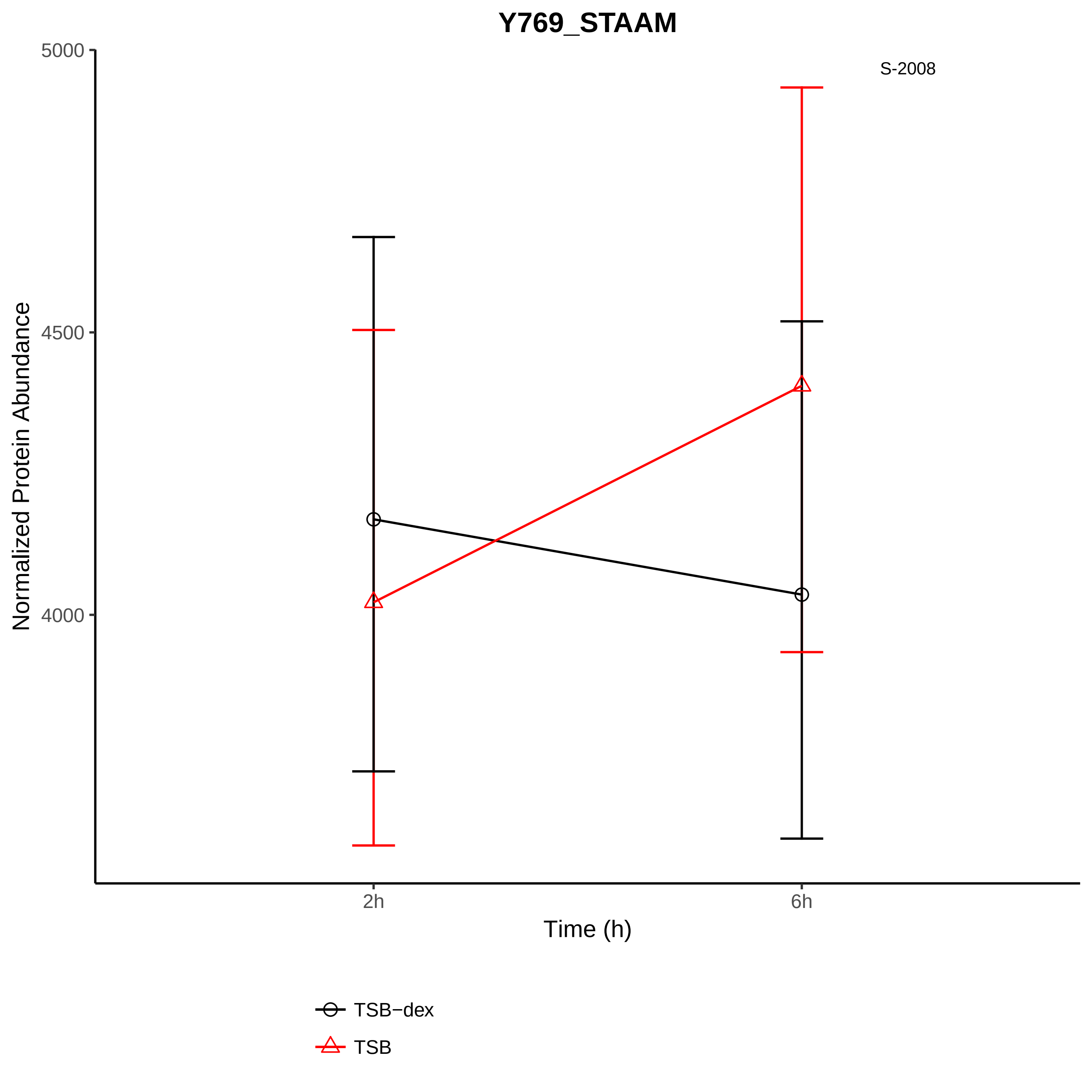

Y769_STAAM
S-2008
5000
4500
4000
Normalized Protein Abundance
2h
6h
Time (h)
TSB−dex
TSB

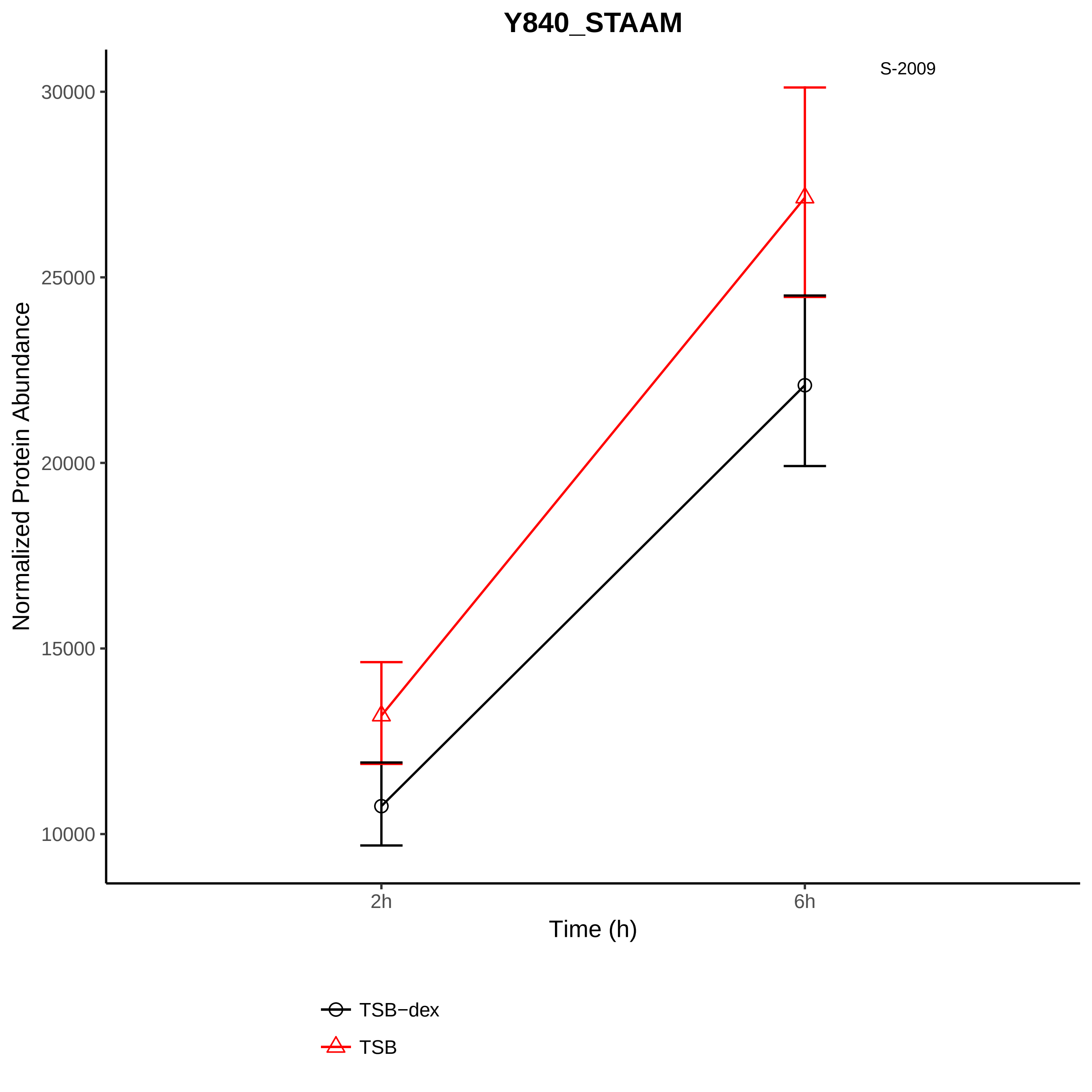
Y840_STAAM
S-2009
Normalized Protein Abundance
30000
25000
20000
15000
10000
2h
6h
Time (h)
TSB−dex
TSB

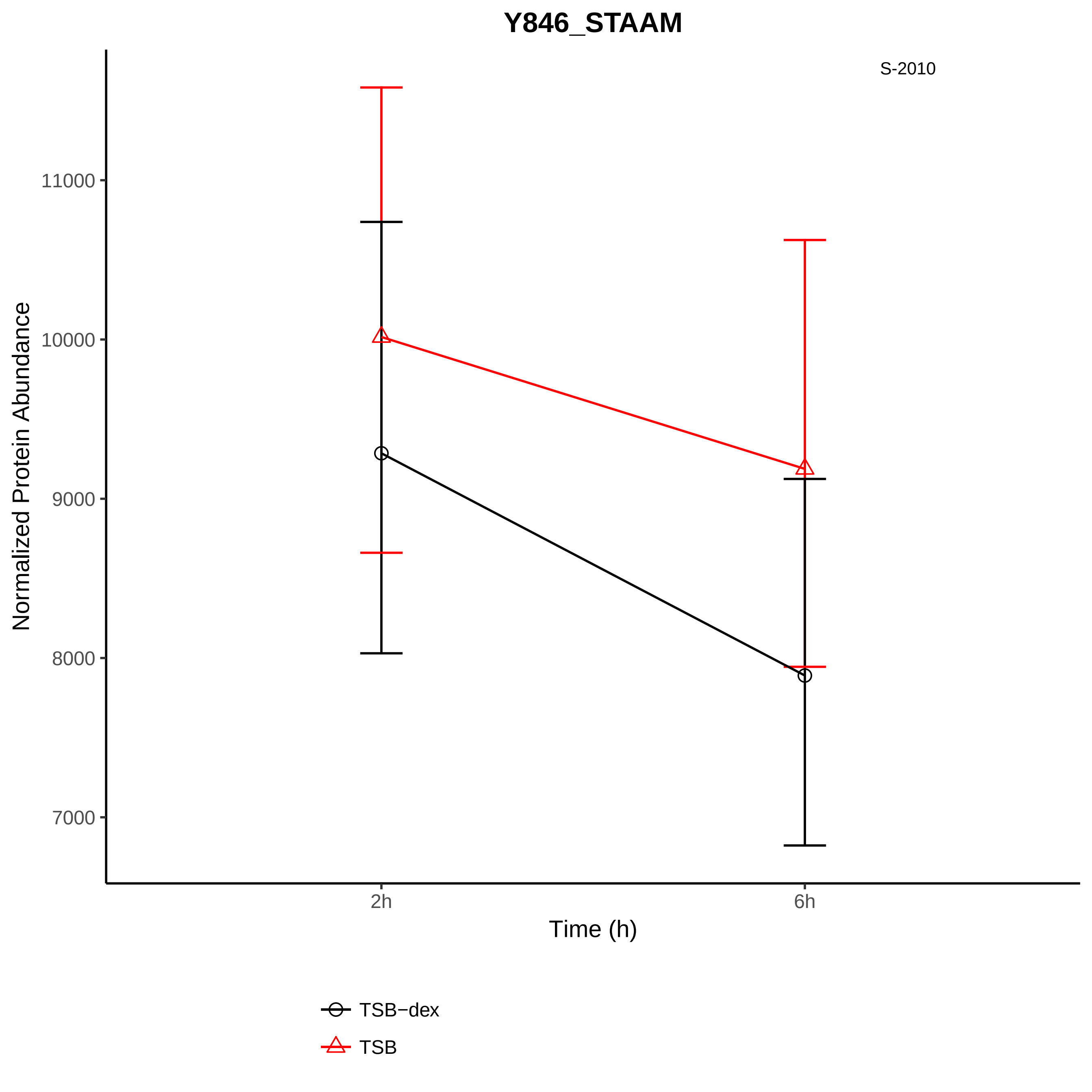

Y846_STAAM
S-2010
Normalized Protein Abundance
11000
10000
9000
8000
7000
2h
6h
Time (h)
TSB−dex
TSB

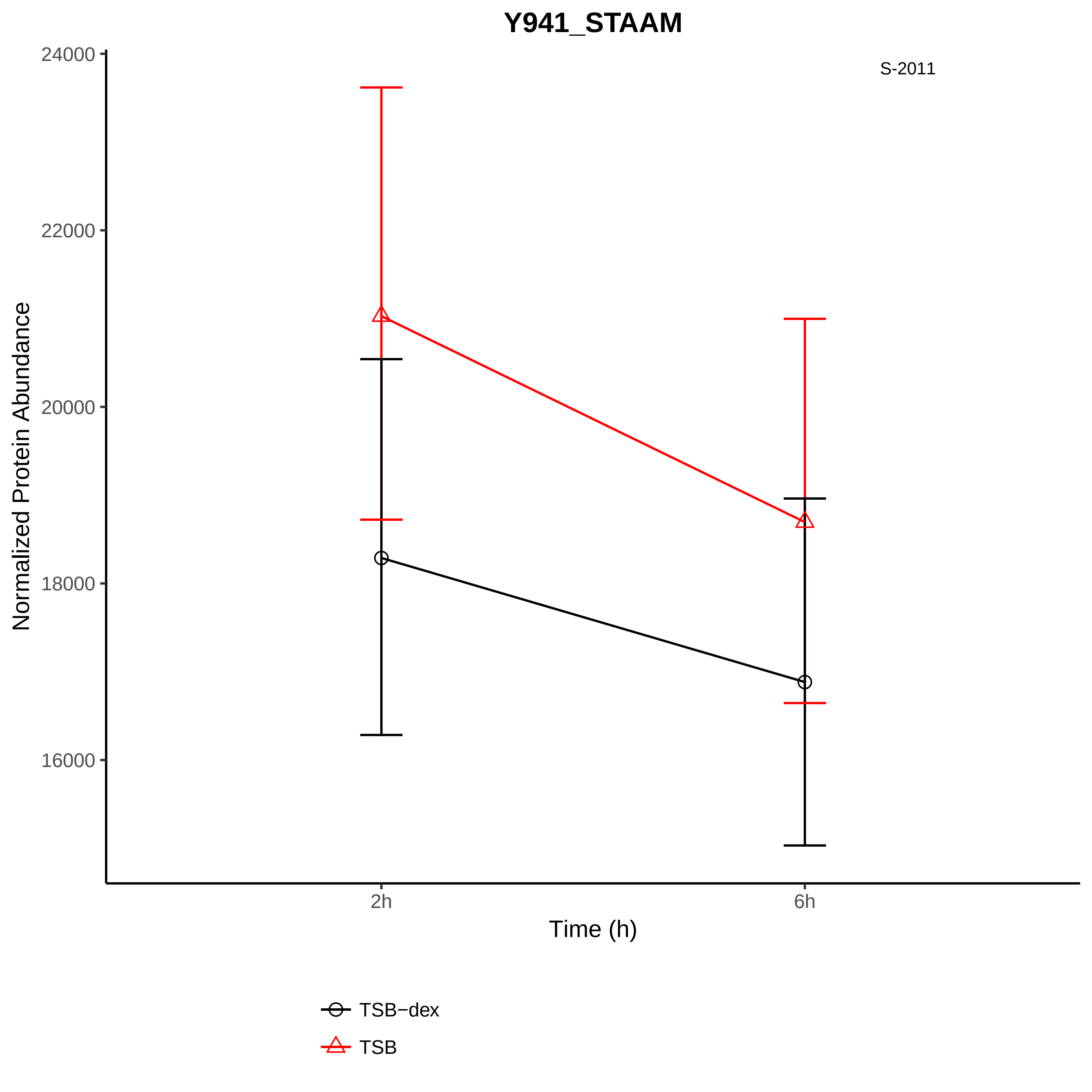

Y941_STAAM
S-2011
24000
22000
20000
18000
16000
Normalized Protein Abundance
2h
6h
Time (h)
TSB−dex
TSB

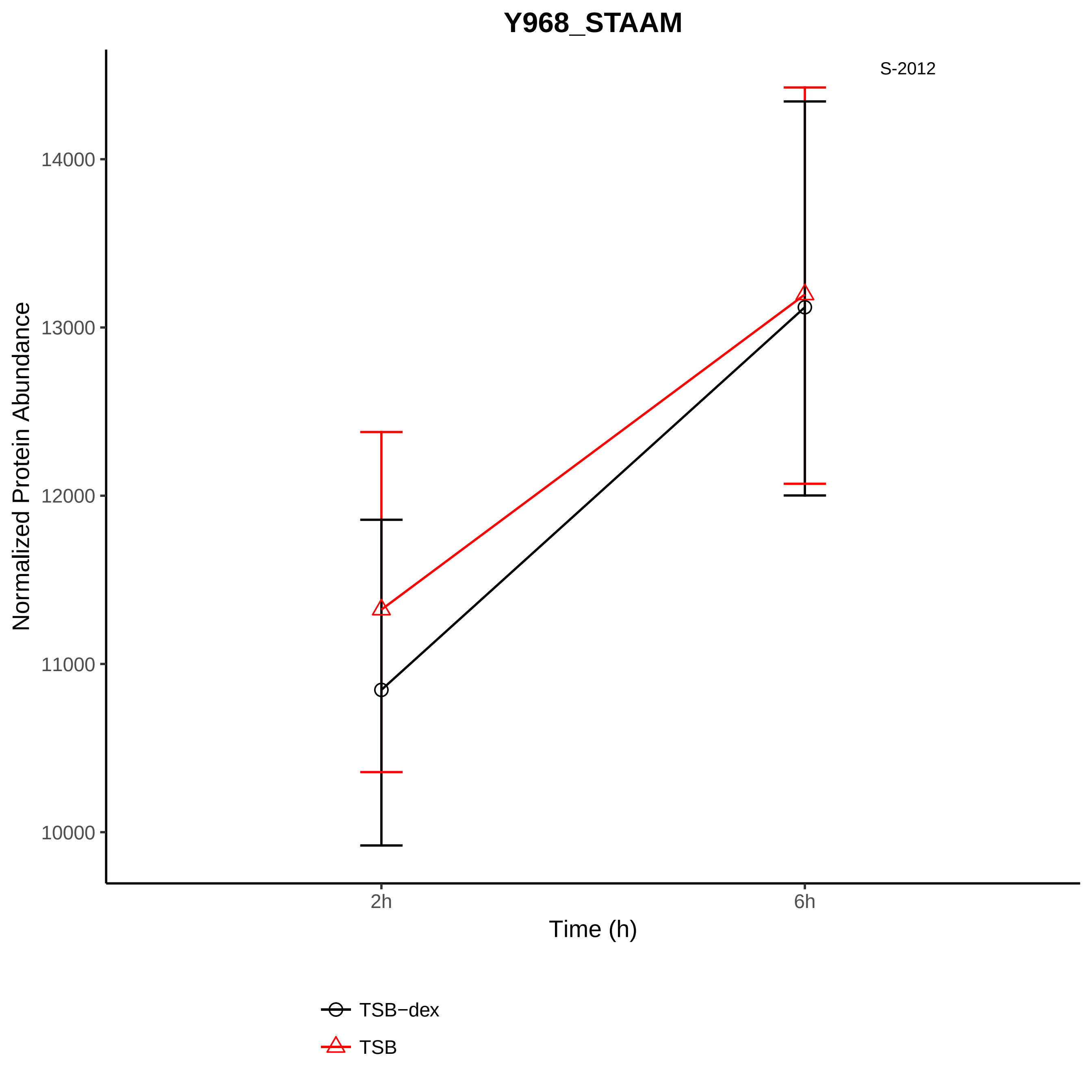
Y968_STAAM
S-2012
Normalized Protein Abundance
14000
13000
12000
11000
10000
2h
6h
Time (h)
TSB−dex
TSB

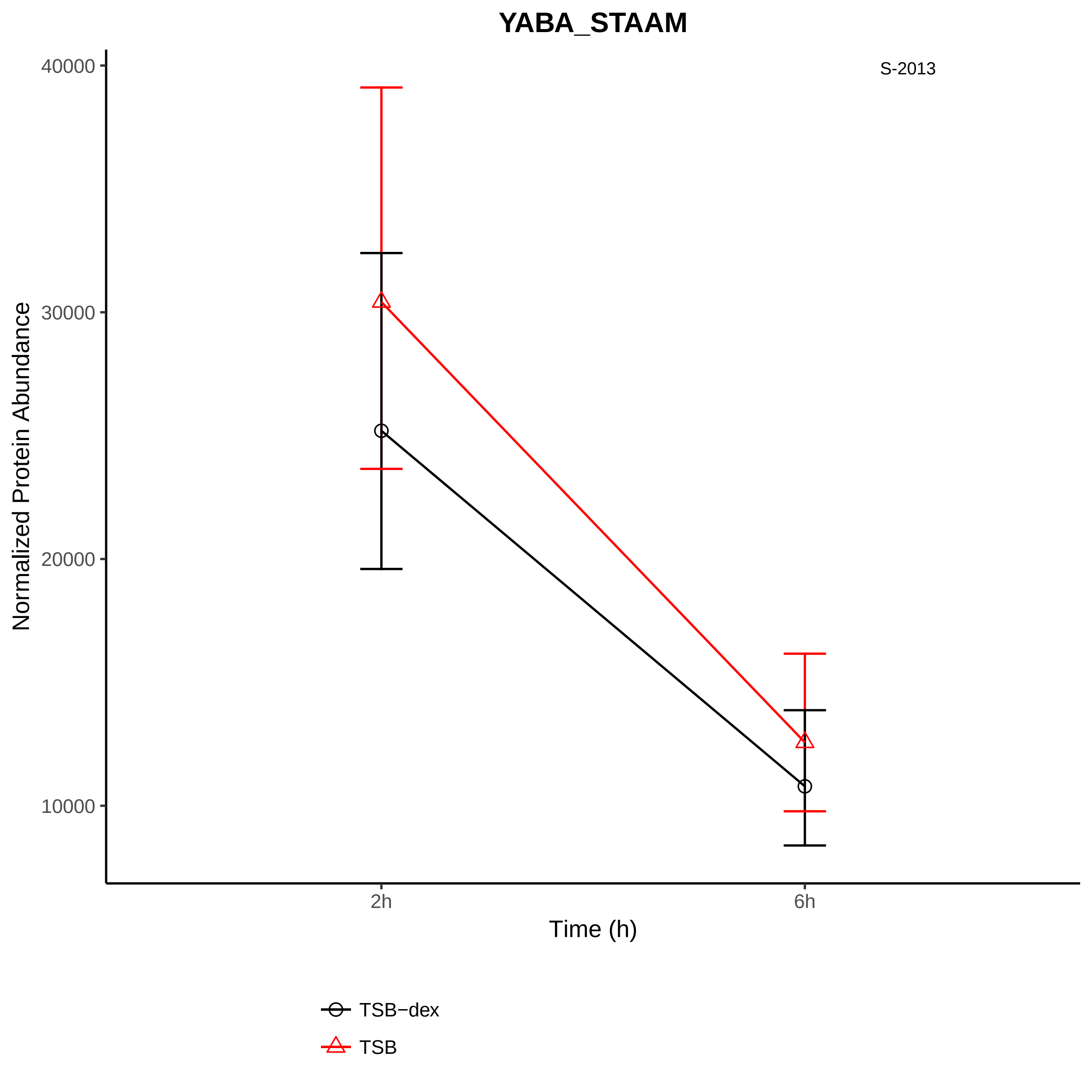
YABA_STAAM
S-2013
Normalized Protein Abundance
40000
30000
20000
10000
2h
6h
Time (h)
TSB−dex
TSB

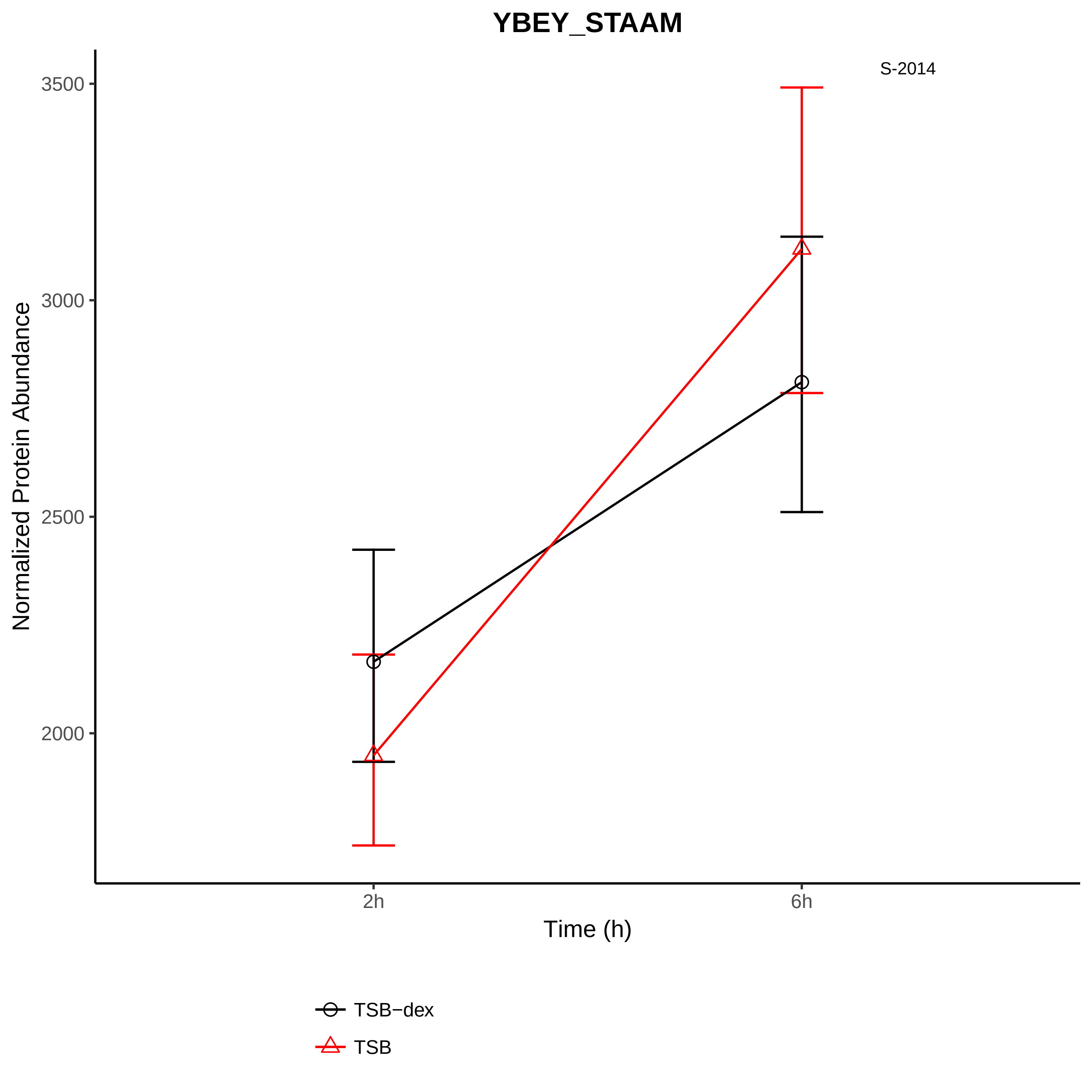

YBEY_STAAM
S-2014
3500
3000
2500
2000
Normalized Protein Abundance
2h
6h
Time (h)
TSB−dex
TSB

Figure J.

1-way analysis: h

Pages: S2015-S2516

The normalized protein abundance, lower 95% confidence interval, and upper 95% confidence interval were plotted for each protein. These plots were analyzed if the 3-way and 2-way interactions were rejected and a significant main effect (1-way) was identified for hour.

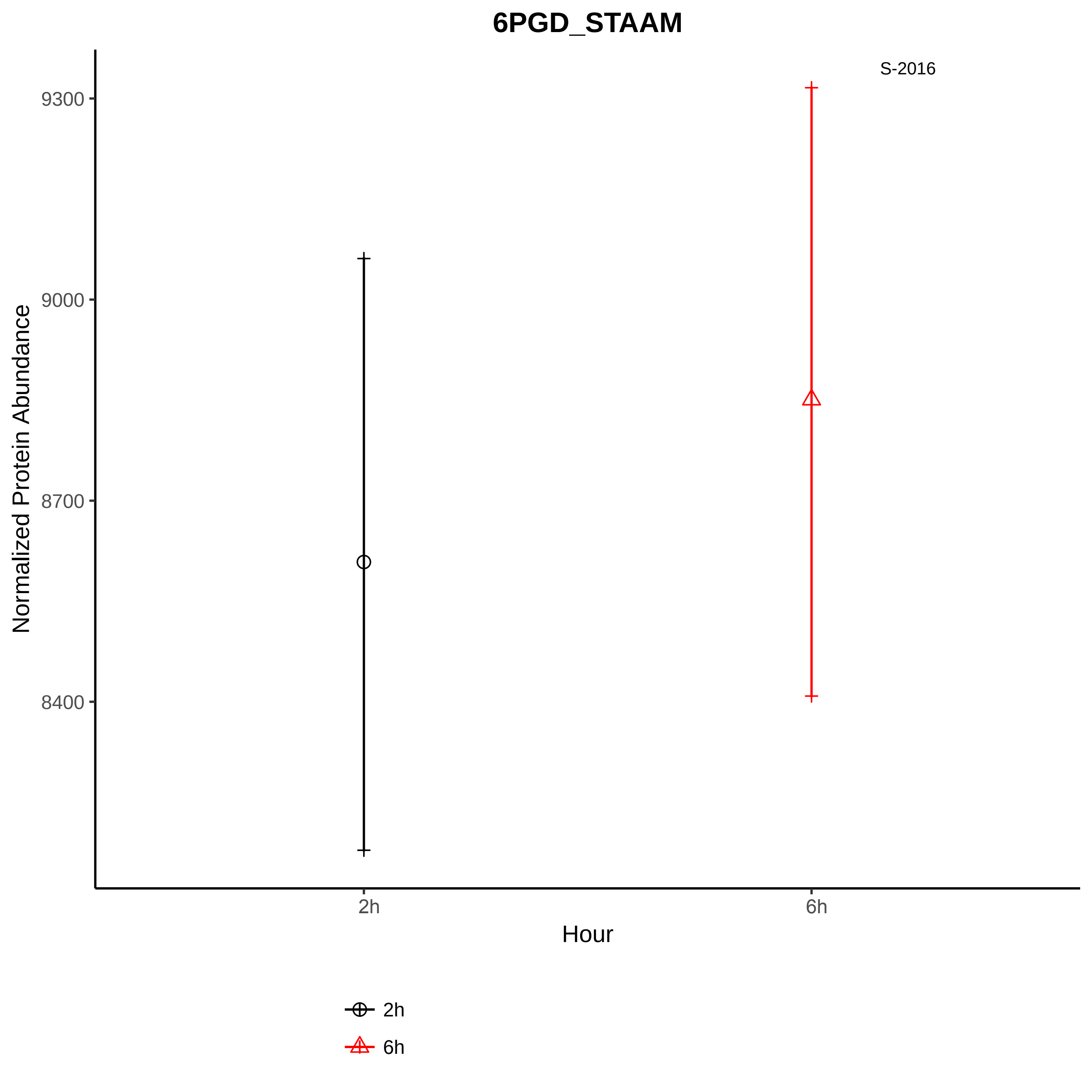

6PGD_STAAM
S-2016
9300
9000
8700
8400
Normalized Protein Abundance
2h
6h
Hour
2h
6h

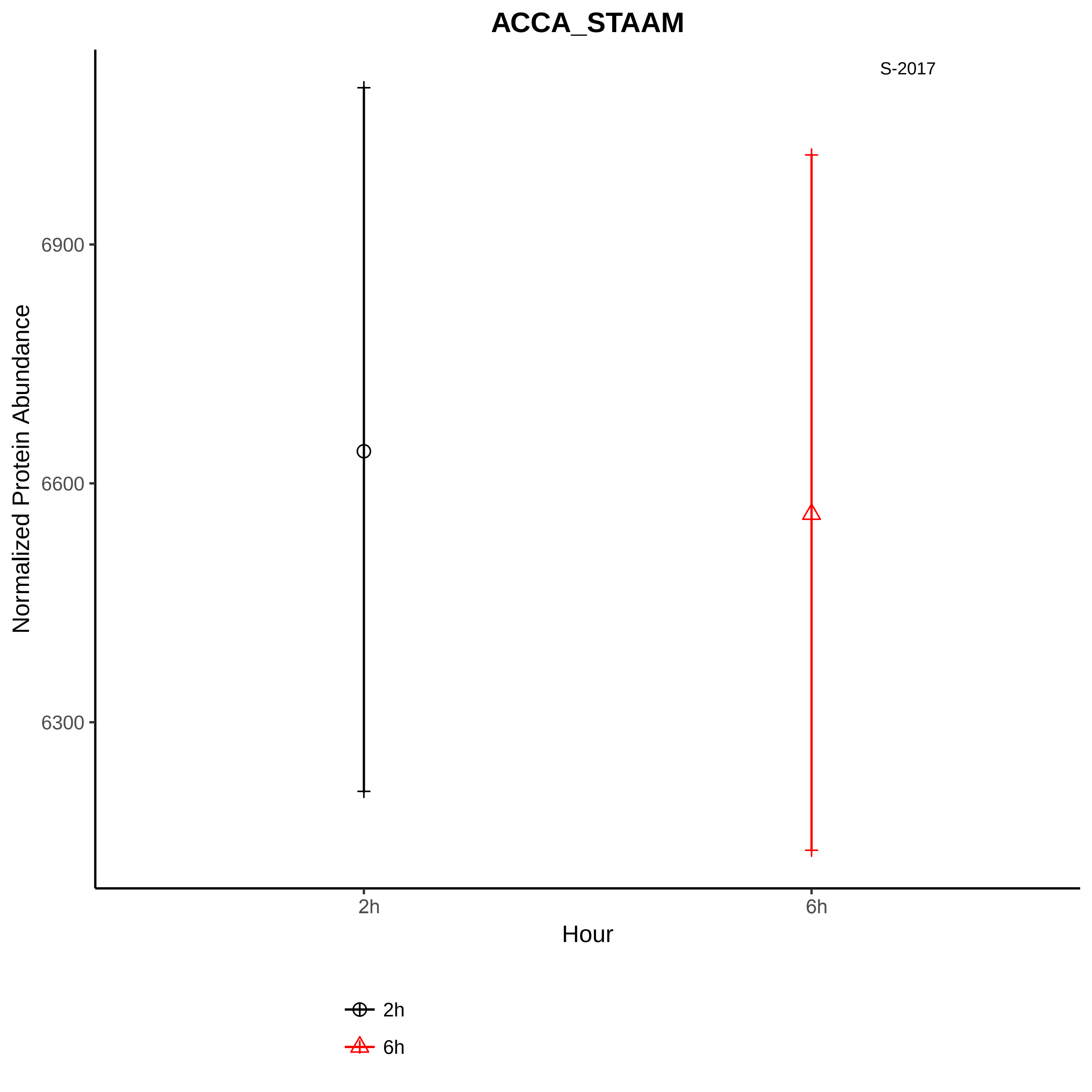

ACCA_STAAM
S-2017
Normalized Protein Abundance
6900
6600
6300
2h
6h
Hour
2h
6h

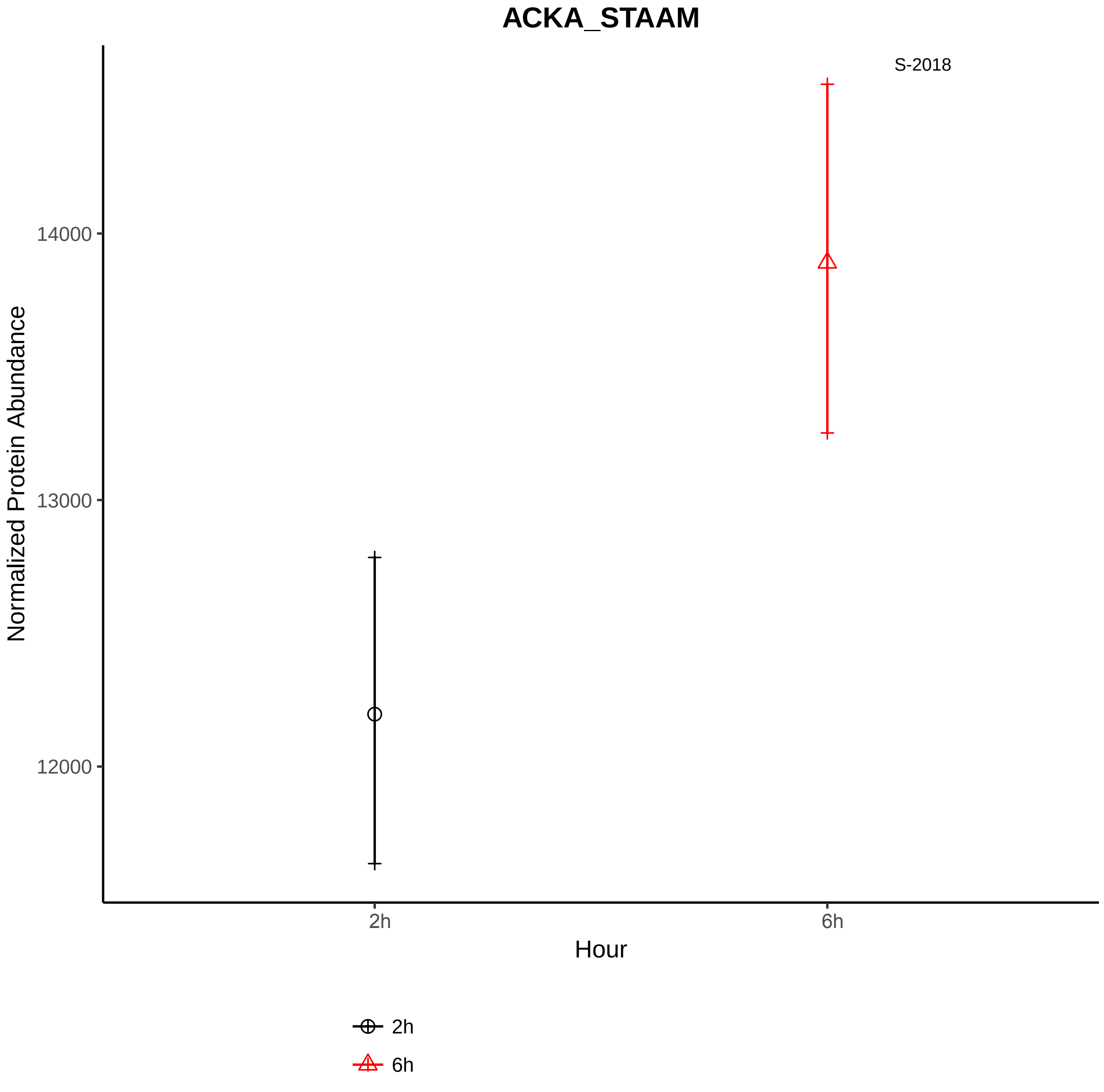
ACKA_STAAM
S-2018
Normalized Protein Abundance
14000
13000
12000
2h
6h
Hour
2h
6h

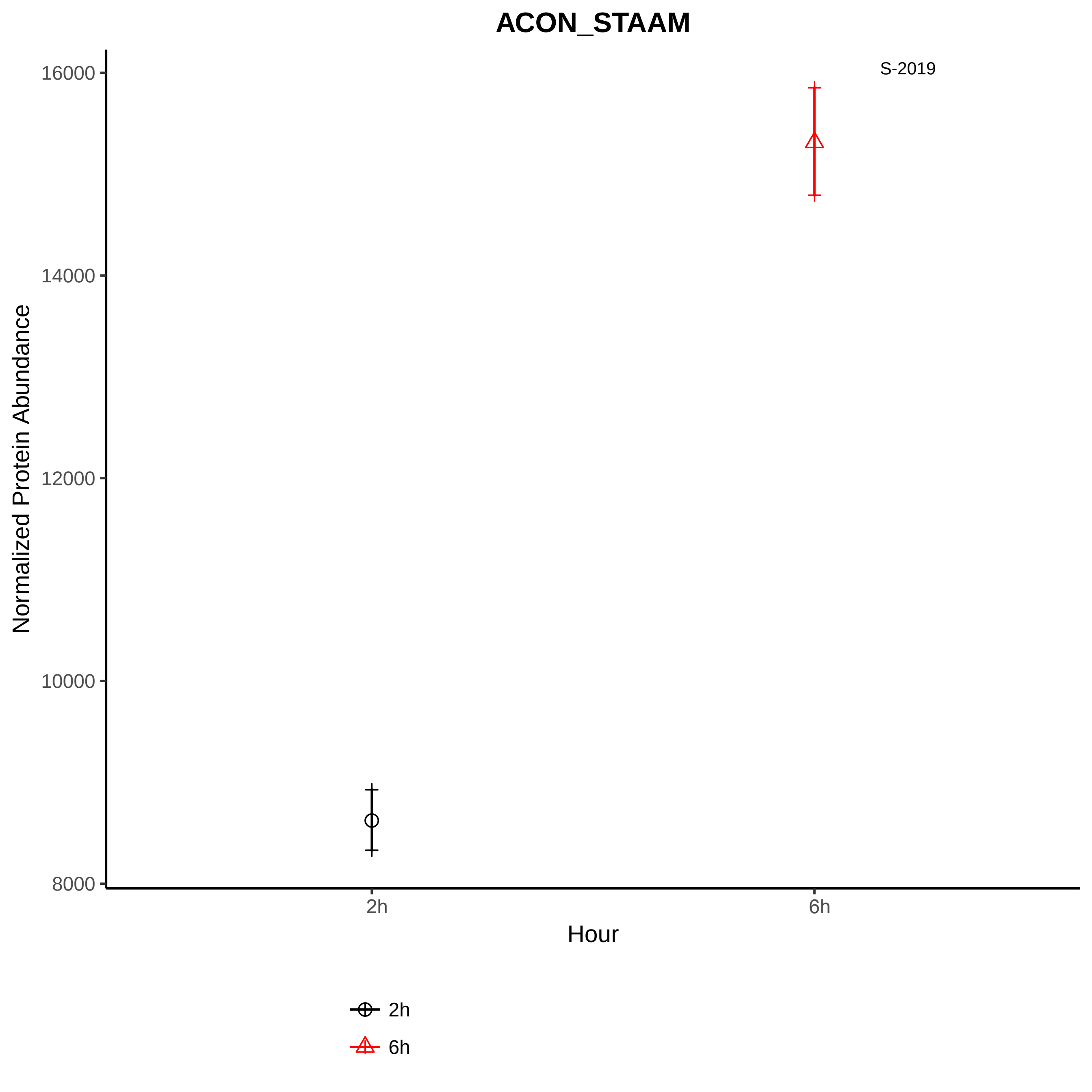
ACON_STAAM
S-2019
16000
14000
12000
10000
8000
Normalized Protein Abundance
2h
6h
Hour
2h
6h

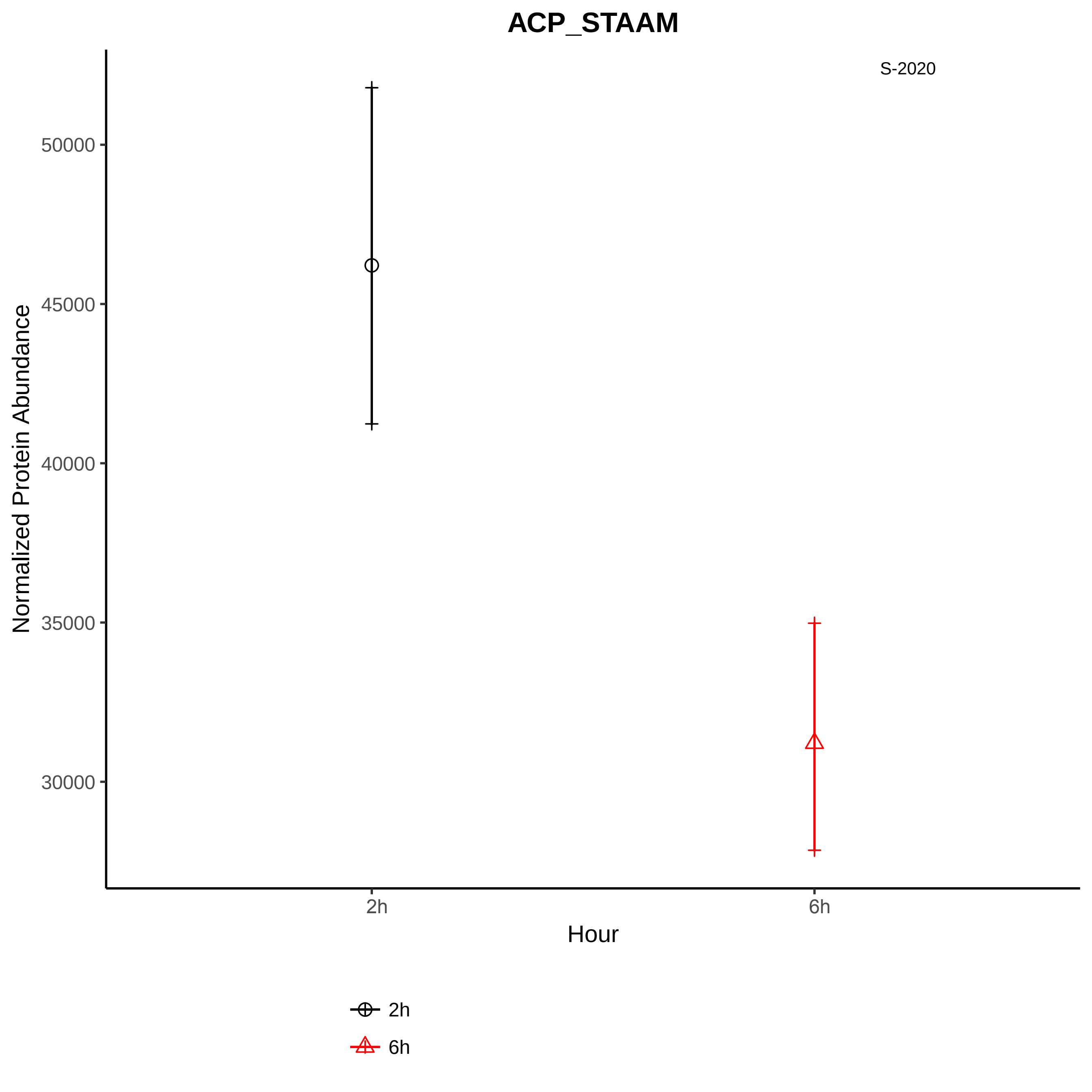

ACP_STAAM
S-2020
Normalized Protein Abundance
50000
45000
40000
35000
30000
2h
6h
Hour
2h
6h

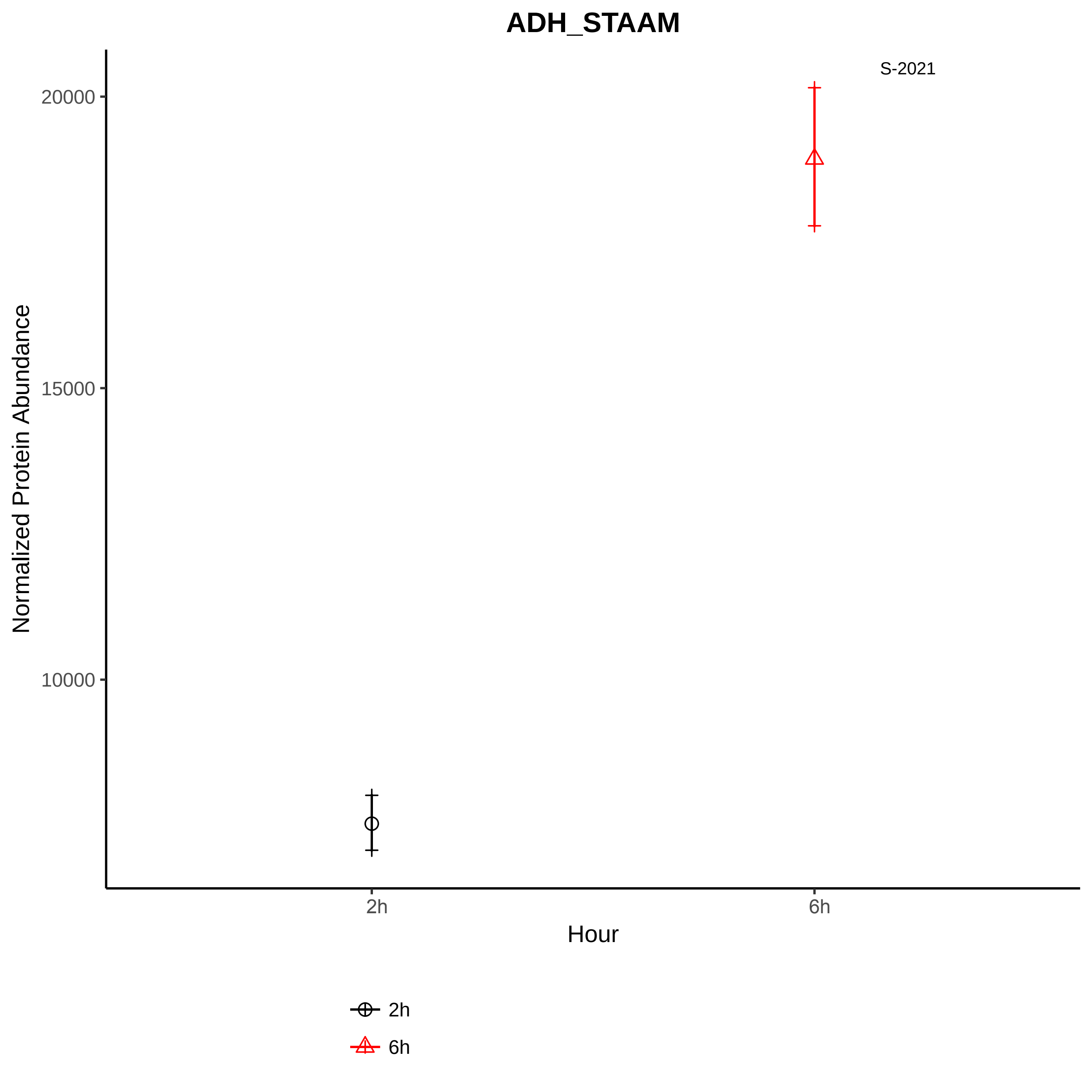
ADH_STAAM
S-2021
Normalized Protein Abundance
20000
15000
10000
2h
6h
Hour
2h
6h

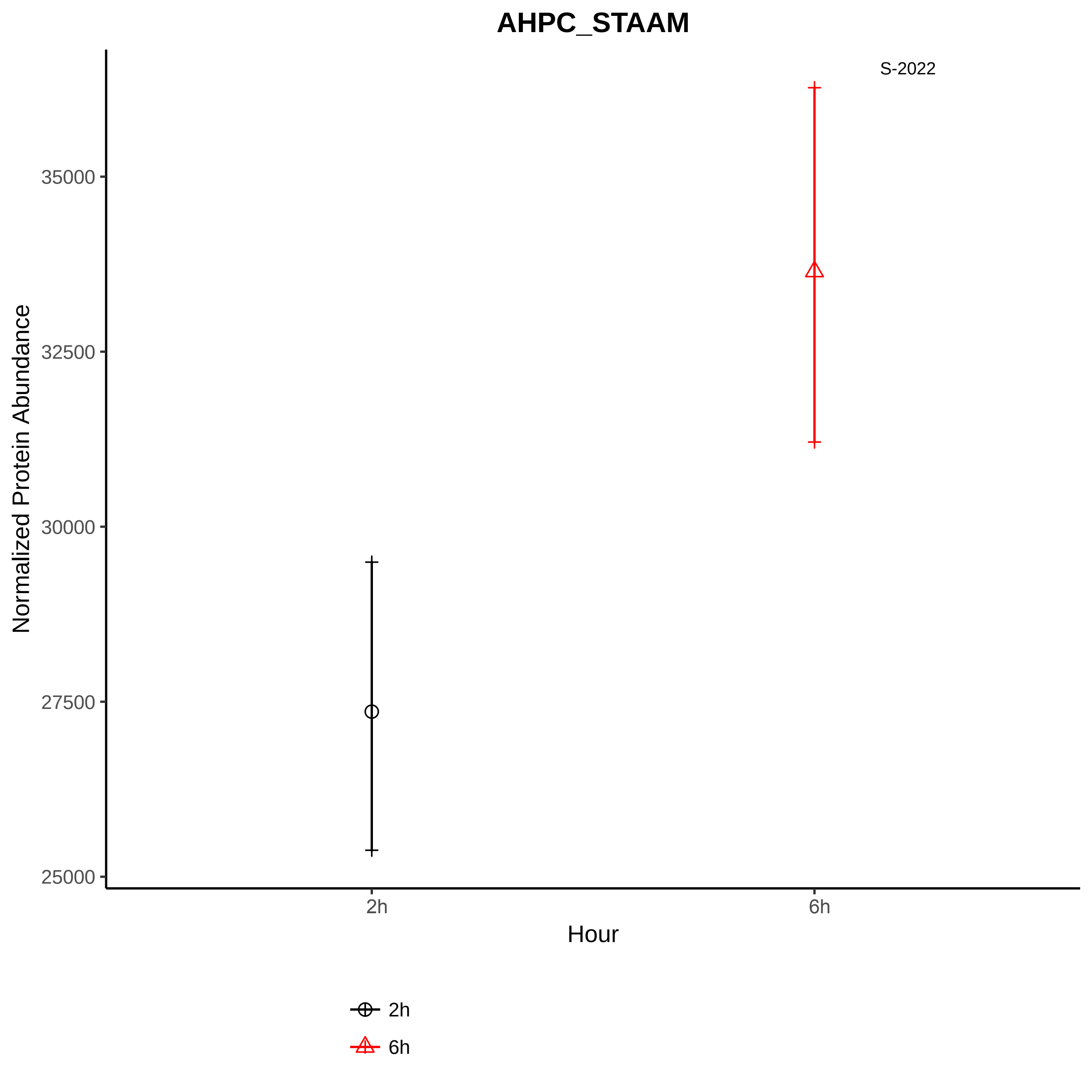
AHPC_STAAM
S-2022
35000
32500
30000
27500
25000
Normalized Protein Abundance
2h
6h
Hour
2h
6h

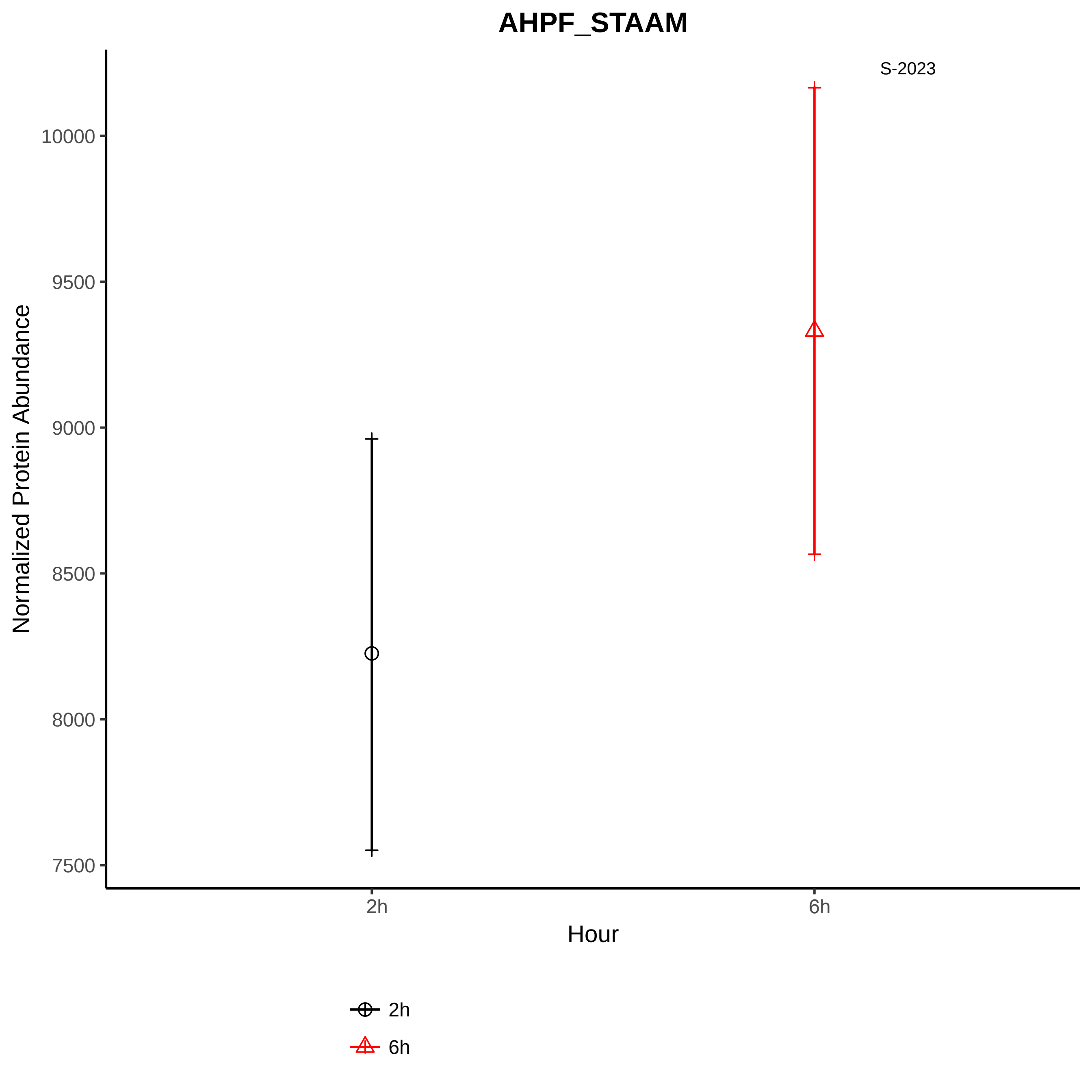
AHPF_STAAM
S-2023
Normalized Protein Abundance
10000
9500
9000
8500
8000
7500
2h
6h
Hour
2h
6h

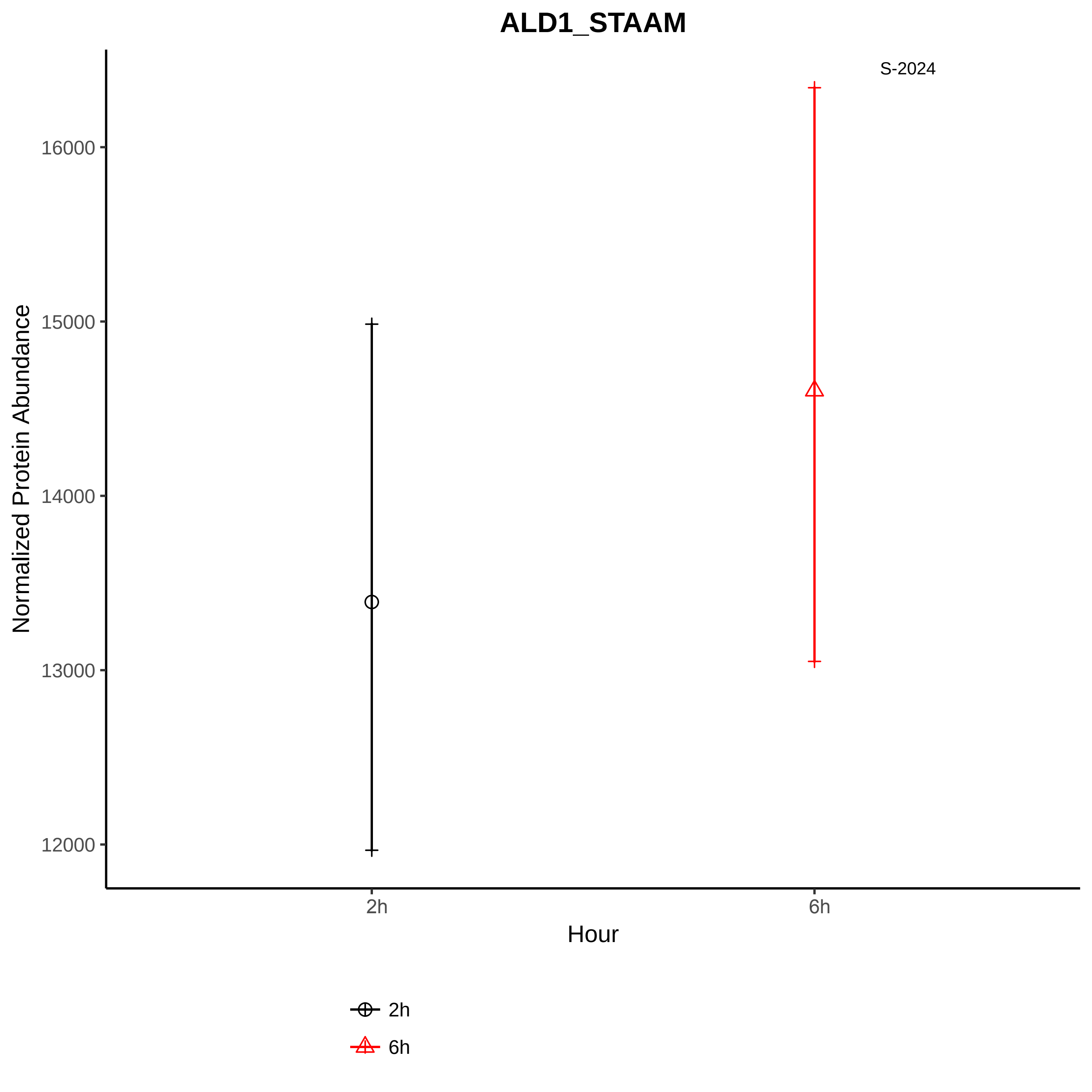

ALD1_STAAM
S-2024
Normalized Protein Abundance
16000
15000
14000
13000
12000
2h
6h
Hour
2h
6h

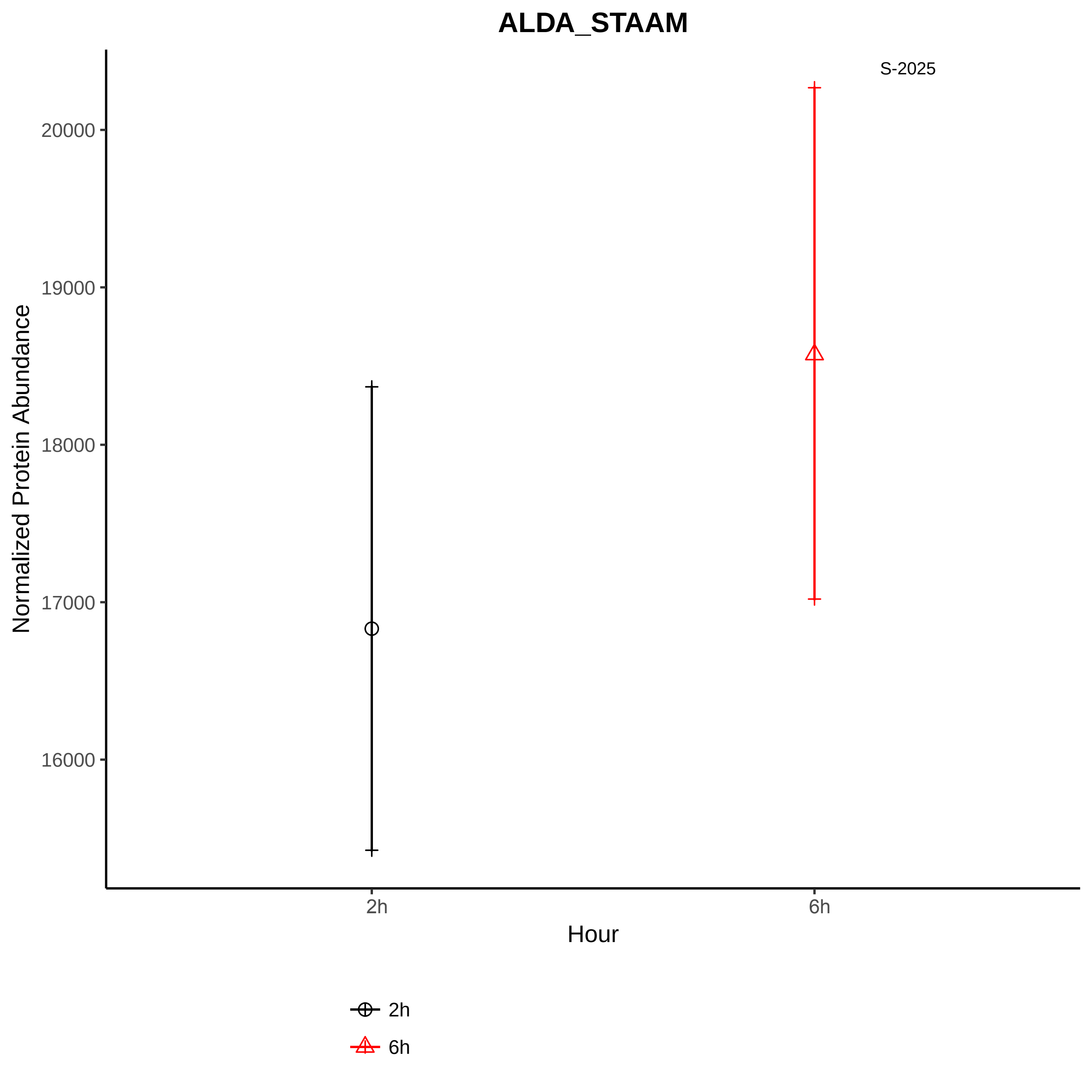

ALDA_STAAM
S-2025
20000
19000
18000
17000
16000
Normalized Protein Abundance
2h
6h
Hour
2h
6h

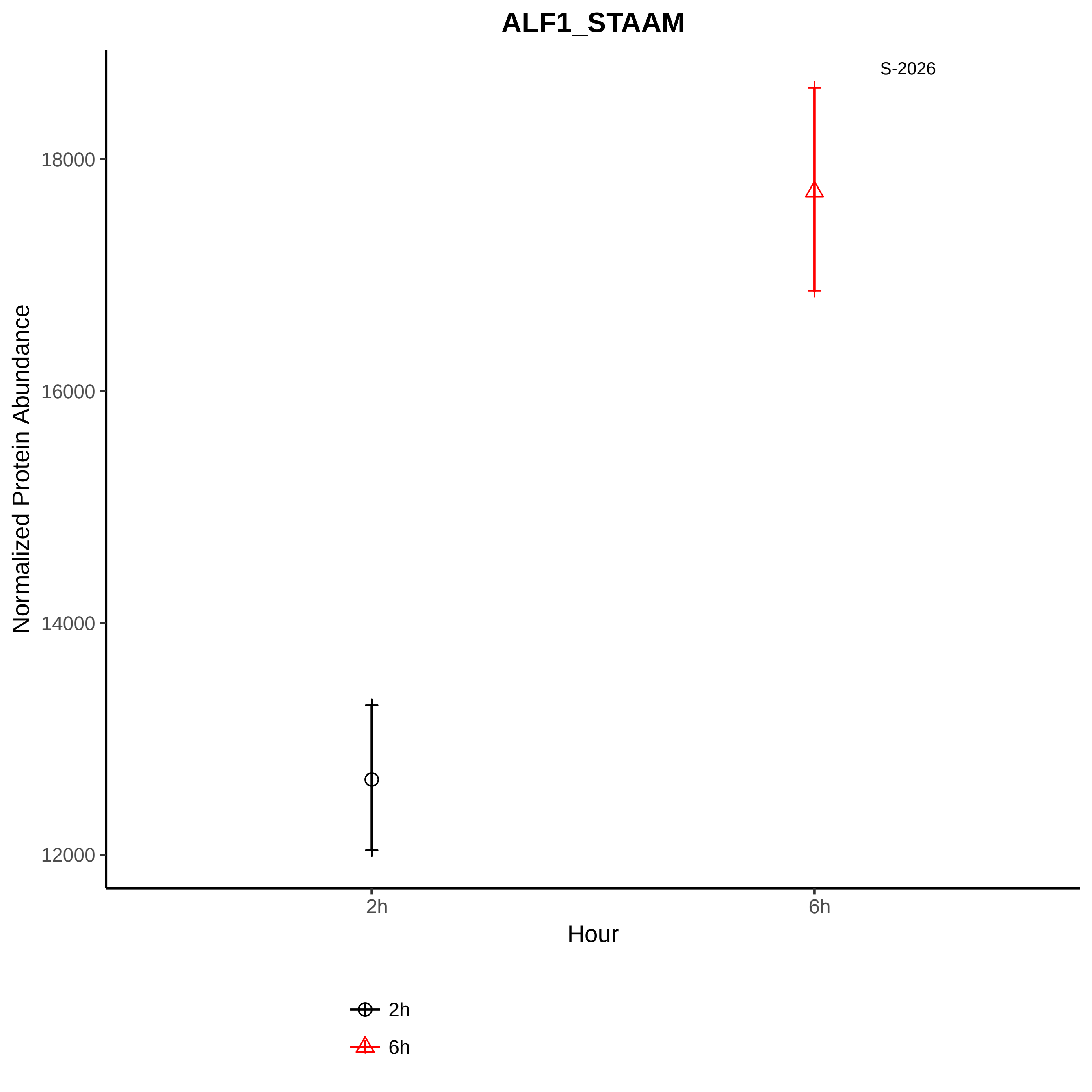

ALF1_STAAM
S-2026
Normalized Protein Abundance
18000
16000
14000
12000
2h
6h
Hour
2h
6h

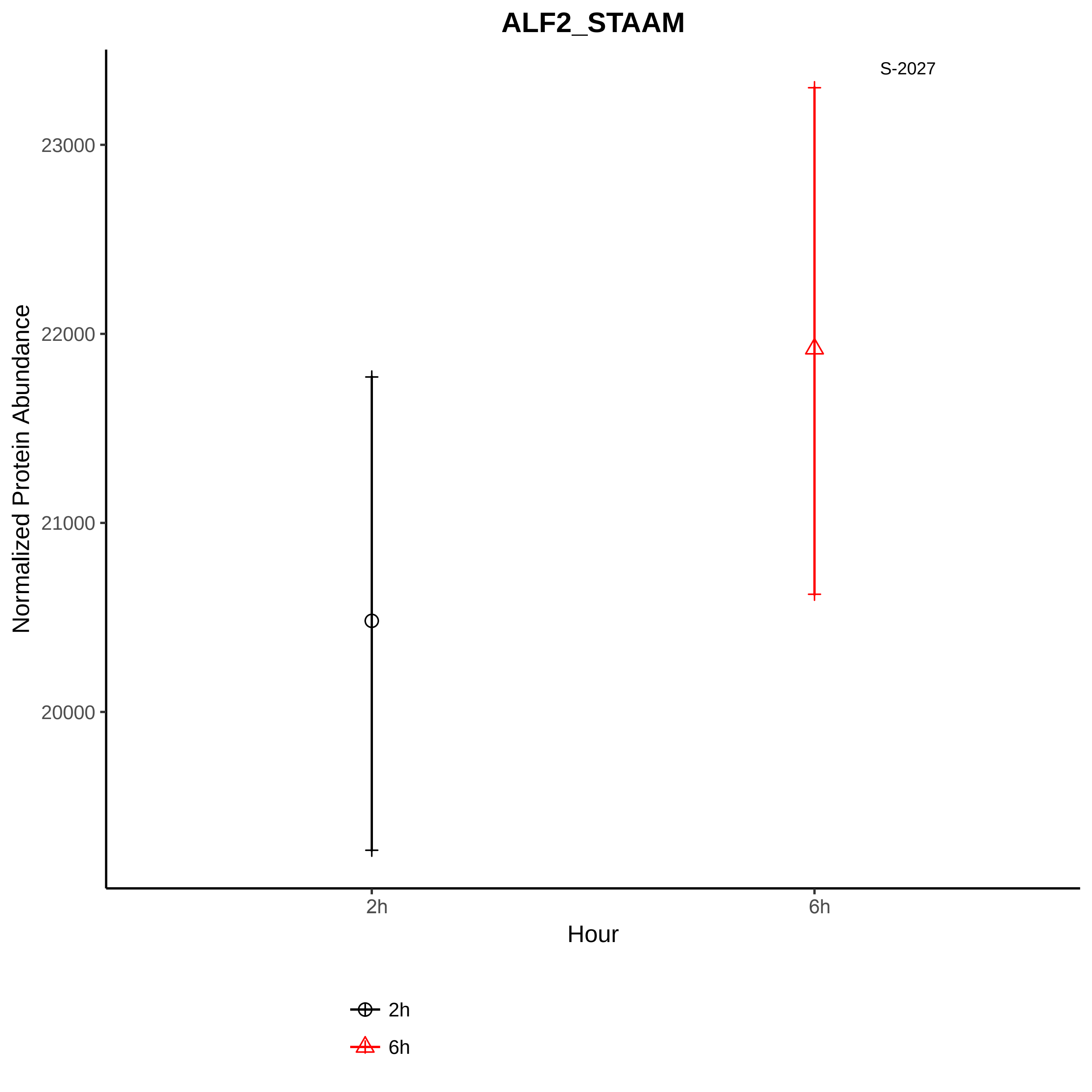

ALF2_STAAM
S-2027
Normalized Protein Abundance
23000
22000
21000
20000
2h
6h
Hour
2h
6h

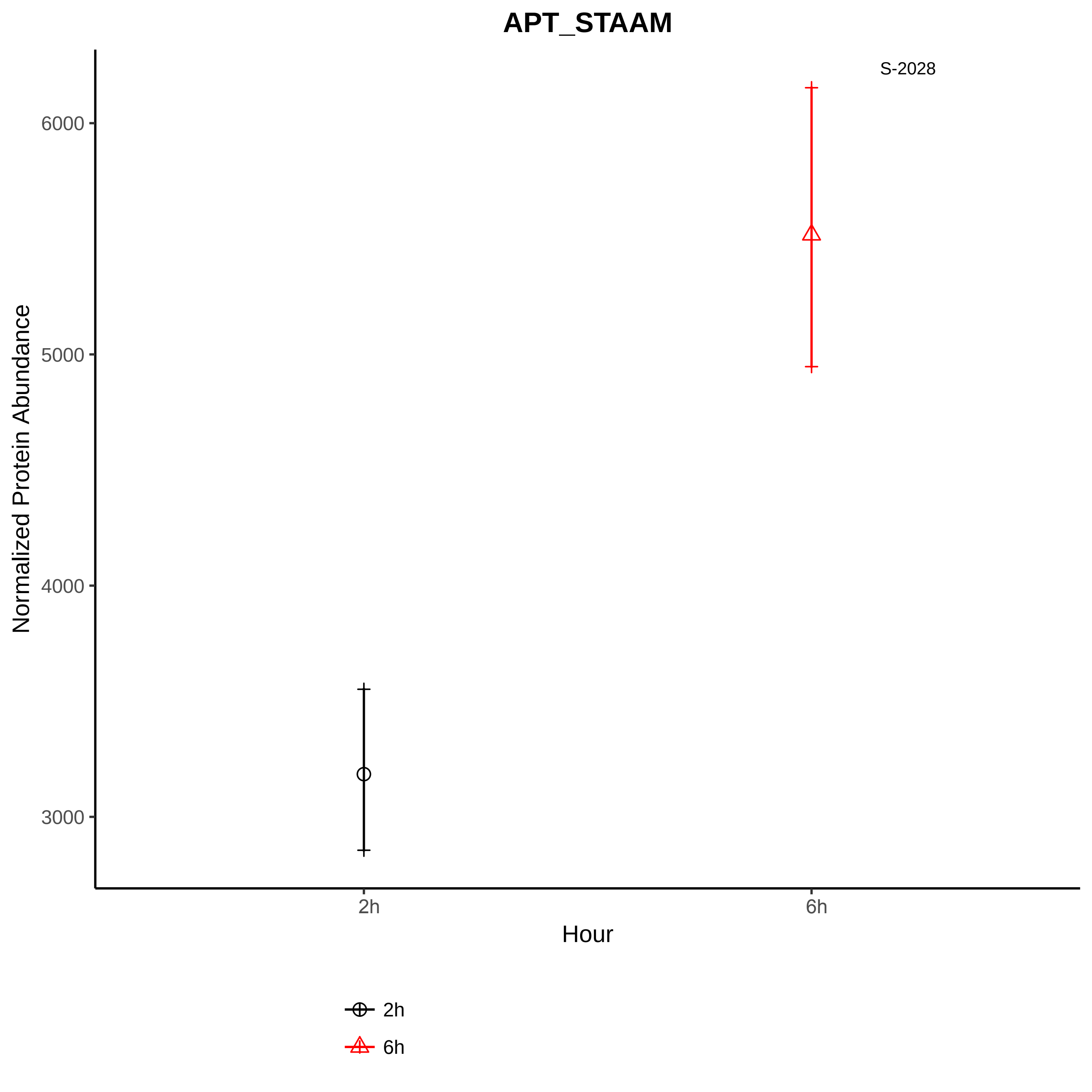
APT_STAAM
S-2028
Normalized Protein Abundance
6000
5000
4000
3000
2h
6h
Hour
2h
6h

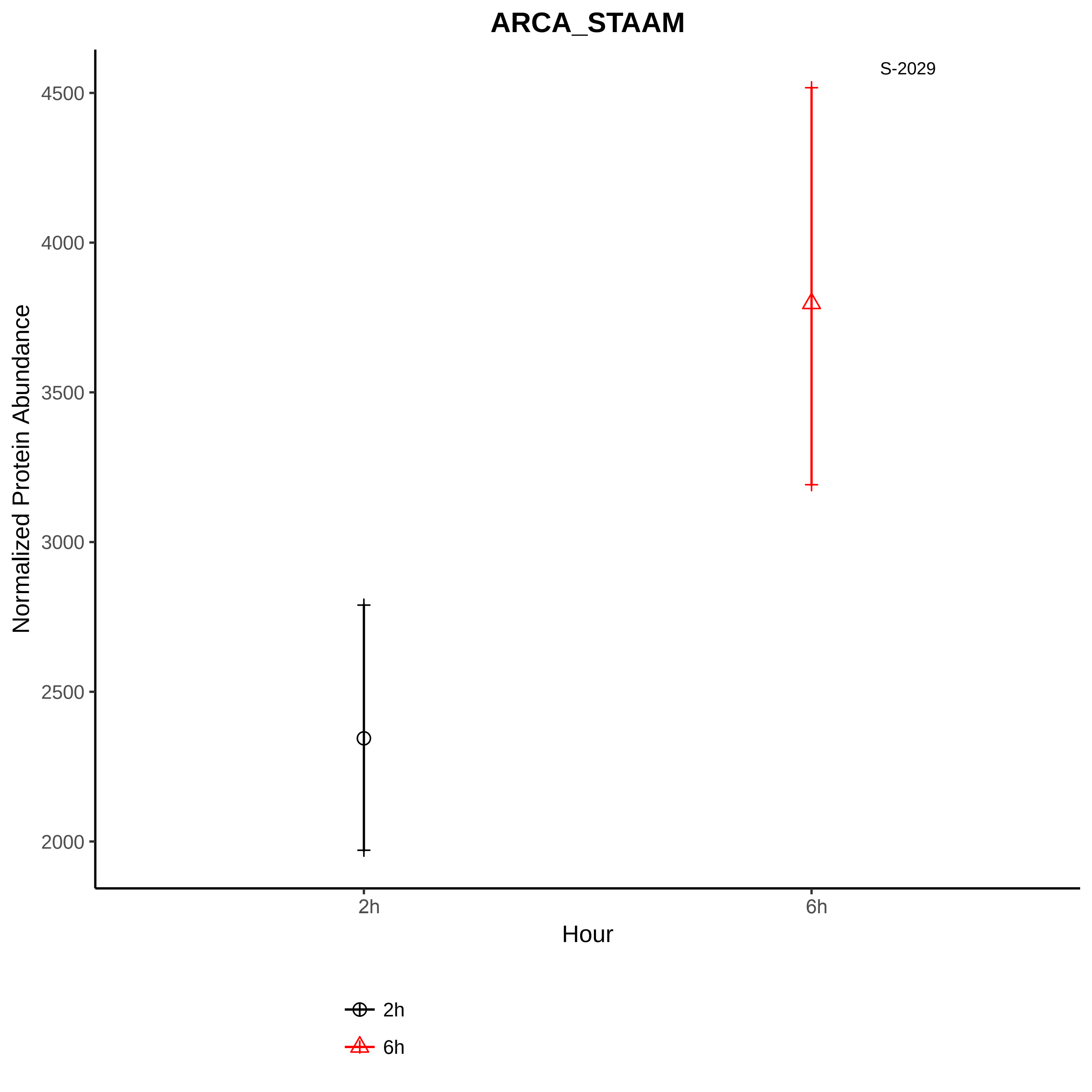
ARCA_STAAM
S-2029
Normalized Protein Abundance
4500
4000
3500
3000
2500
2000
2h
6h
Hour
2h
6h

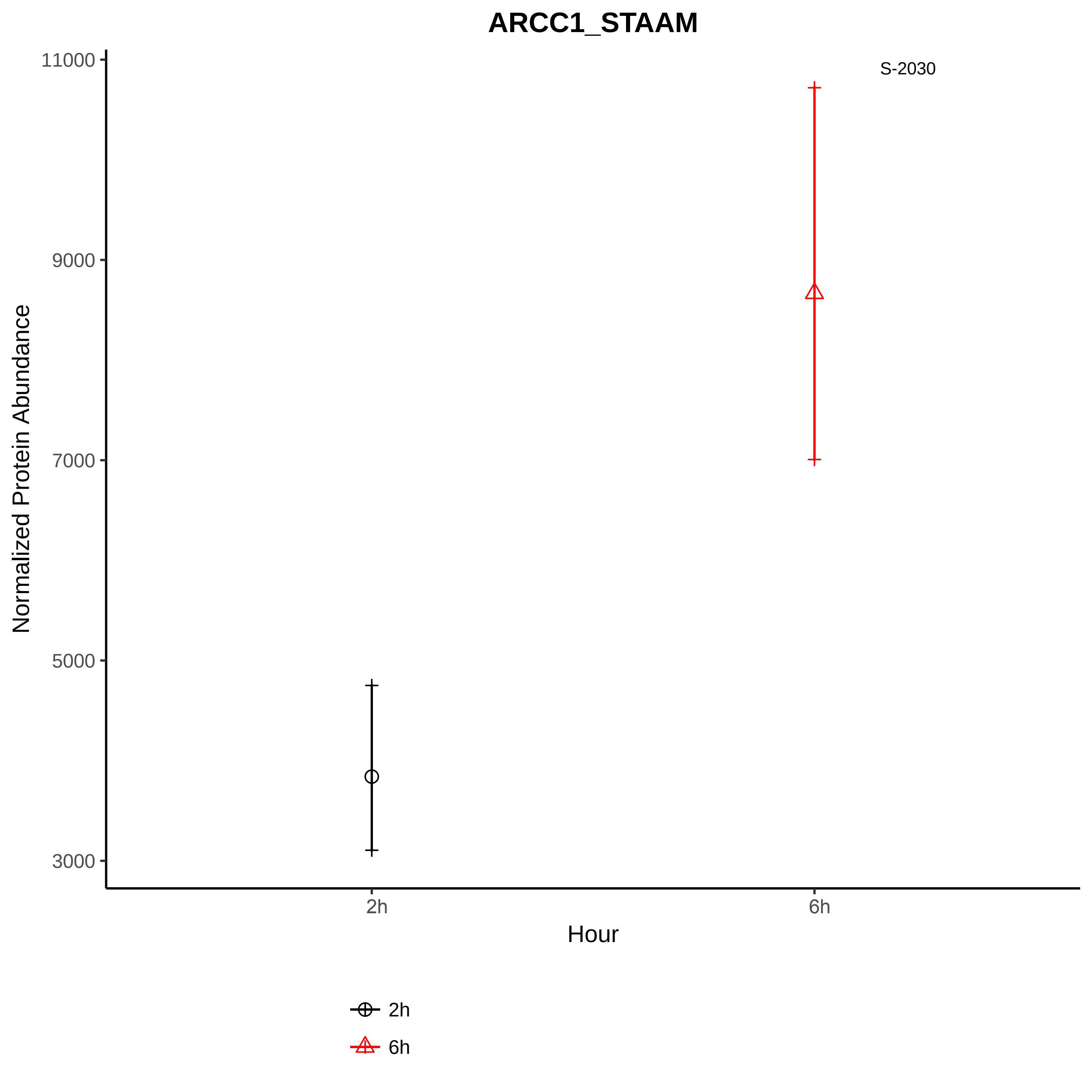
ARCC1_STAAM
S-2030
11000
9000
7000
5000
3000
Normalized Protein Abundance
2h
6h
Hour
2h
6h

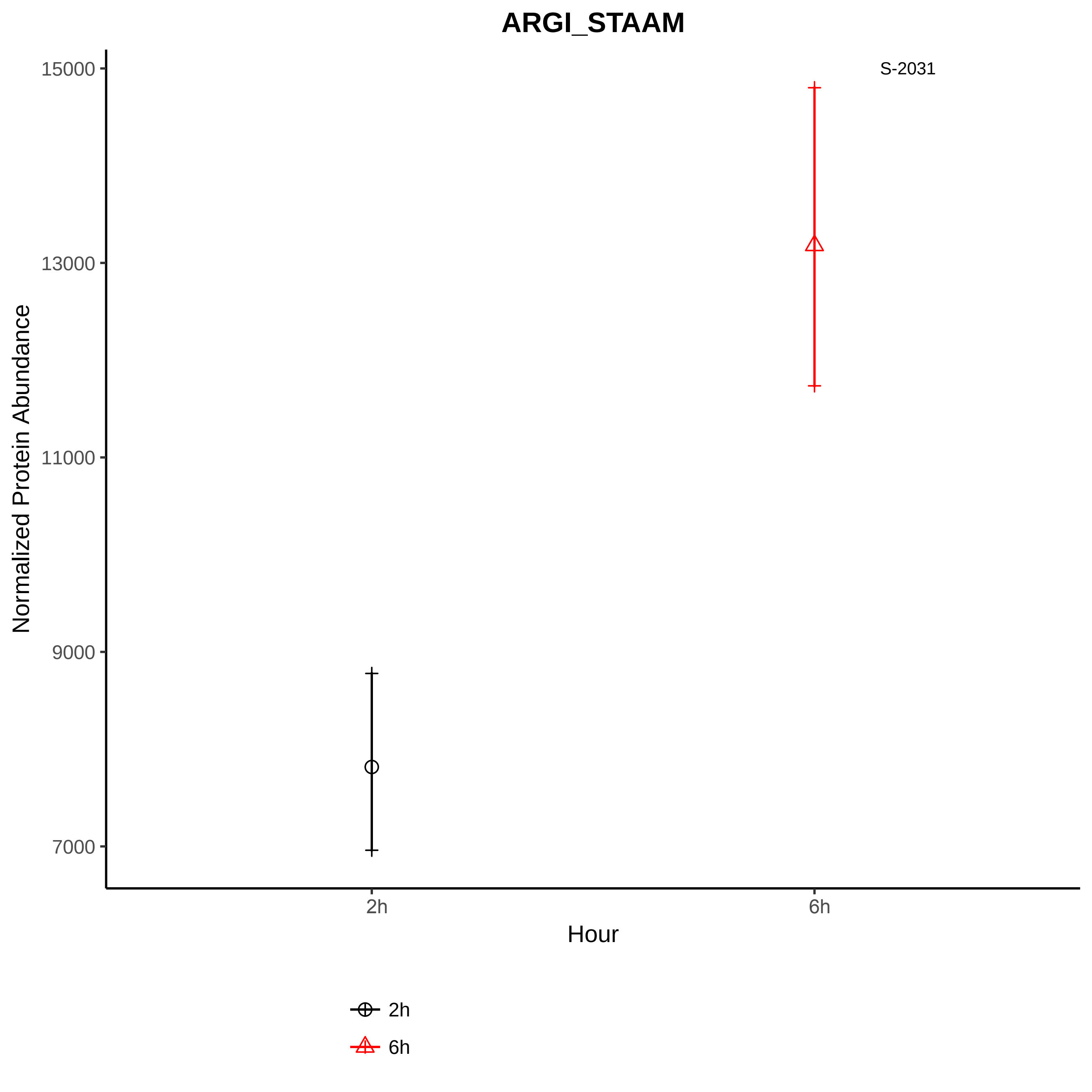

ARGI_STAAM
S-2031
Normalized Protein Abundance
15000
13000
11000
9000
7000
2h
6h
Hour
2h
6h

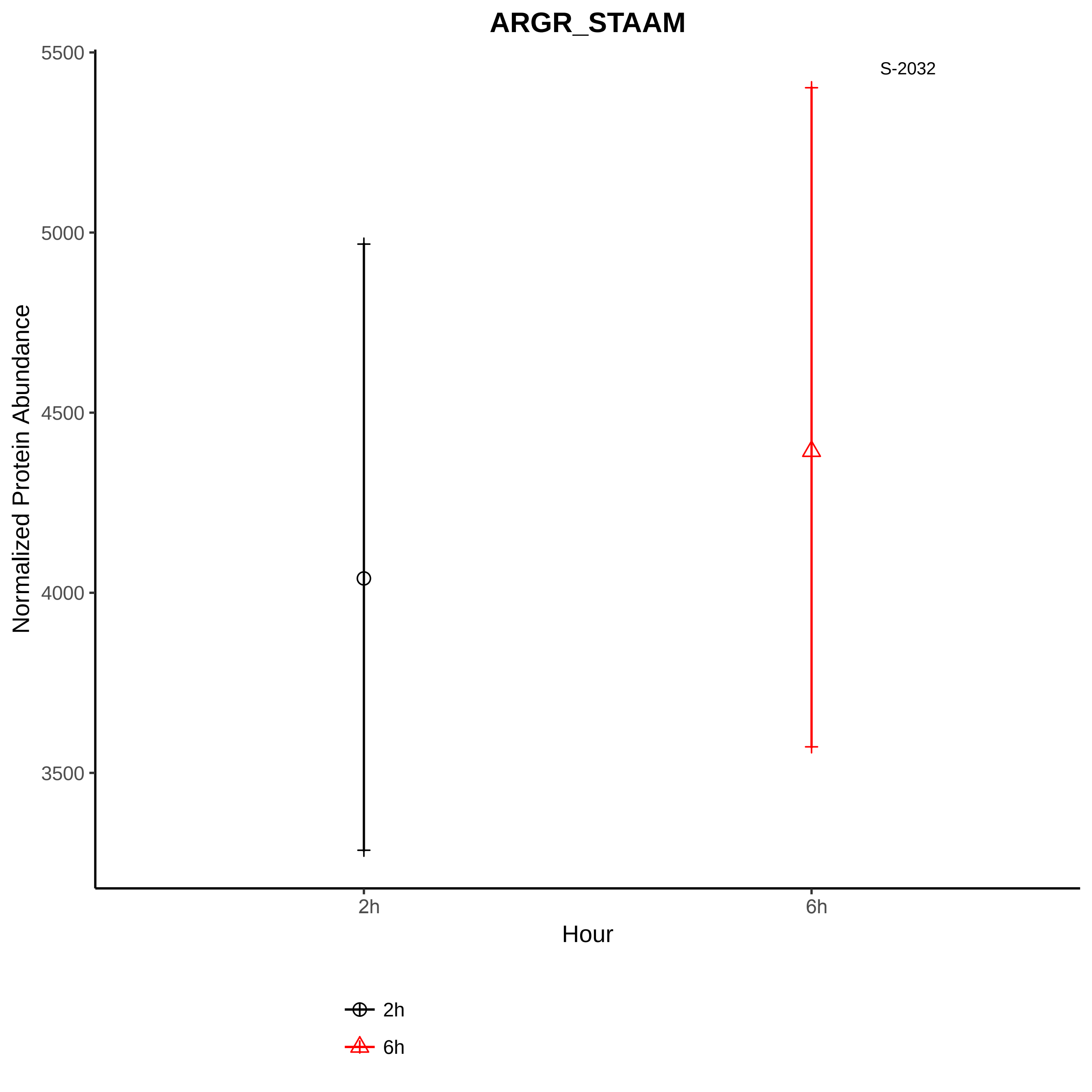
ARGR_STAAM
S-2032
5500
5000
4500
4000
3500
Normalized Protein Abundance
2h
6h
Hour
2h
6h

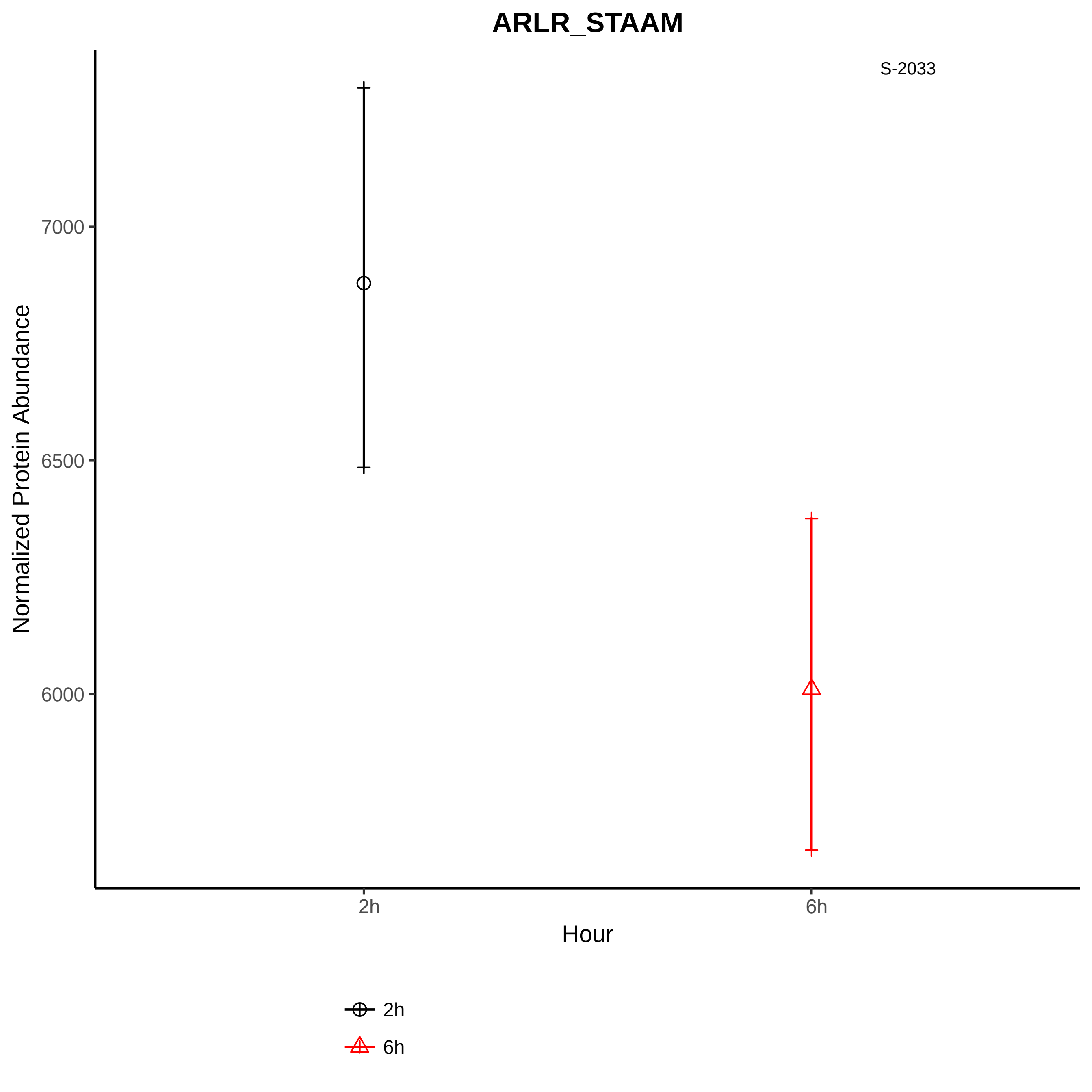

ARLR_STAAM
S-2033
Normalized Protein Abundance
7000
6500
6000
2h
6h
Hour
2h
6h

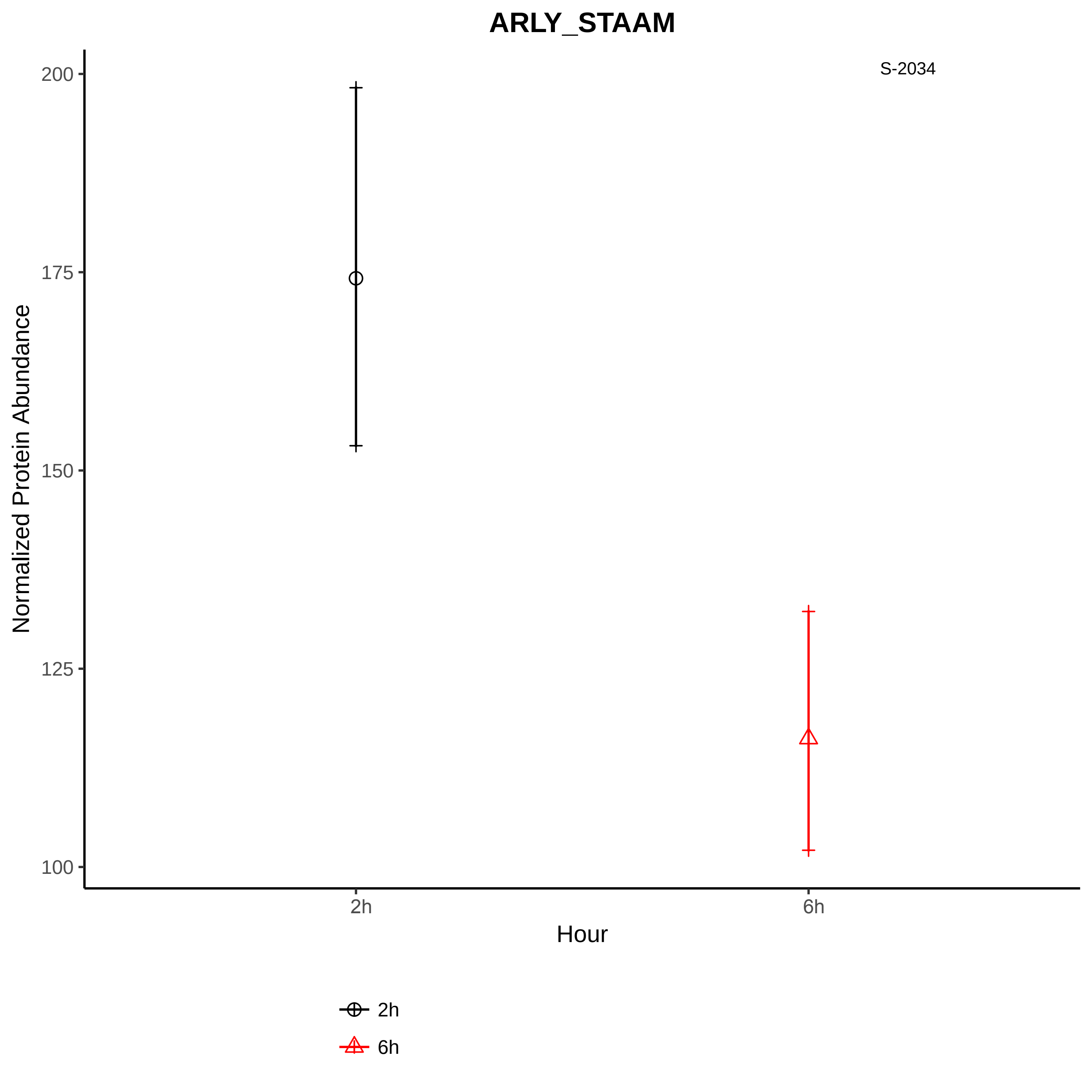
ARLY_STAAM
S-2034
200
175
150
125
100
Normalized Protein Abundance
2h
6h
Hour
2h
6h

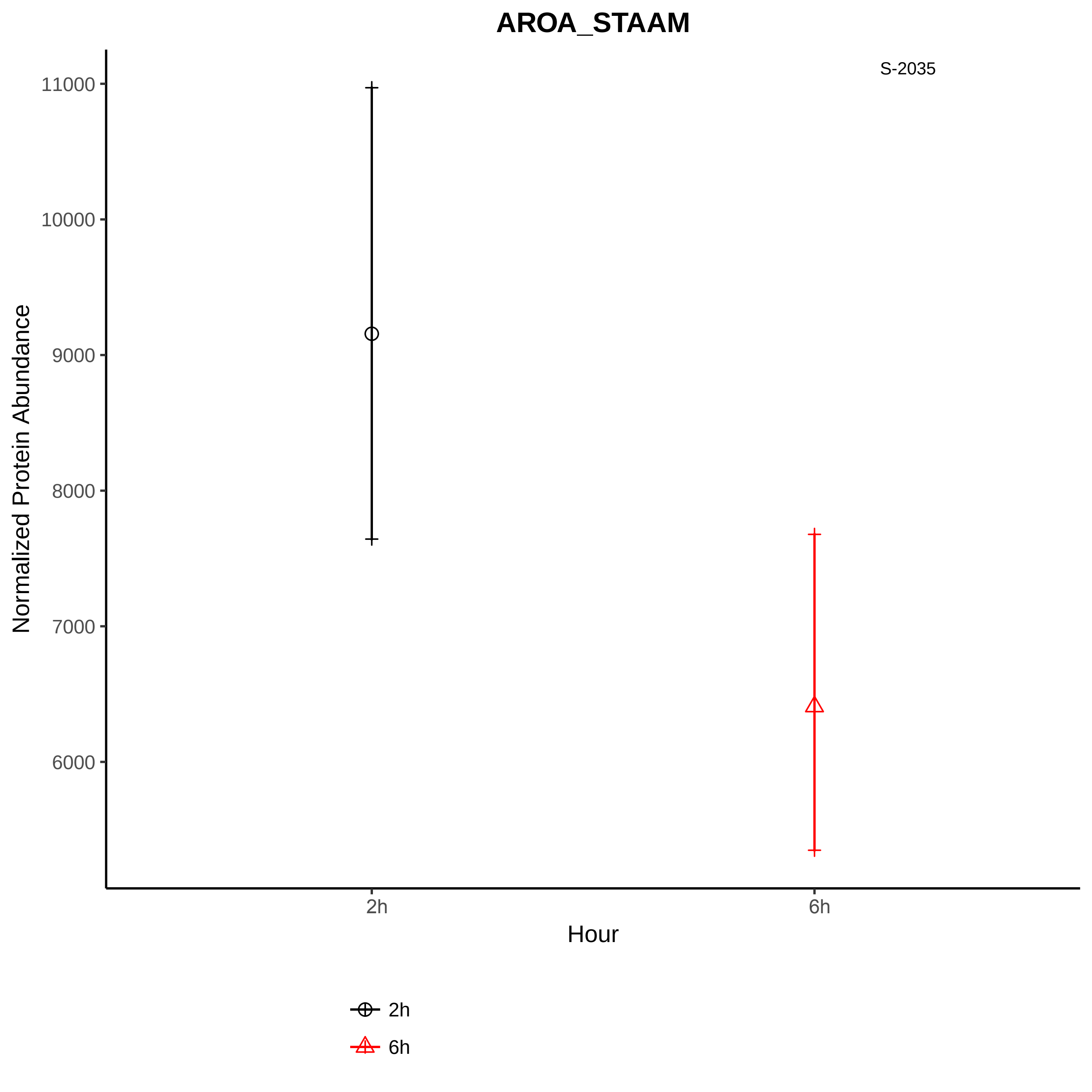
AROA_STAAM
S-2035
Normalized Protein Abundance
11000
10000
9000
8000
7000
6000
2h
6h
Hour
2h
6h

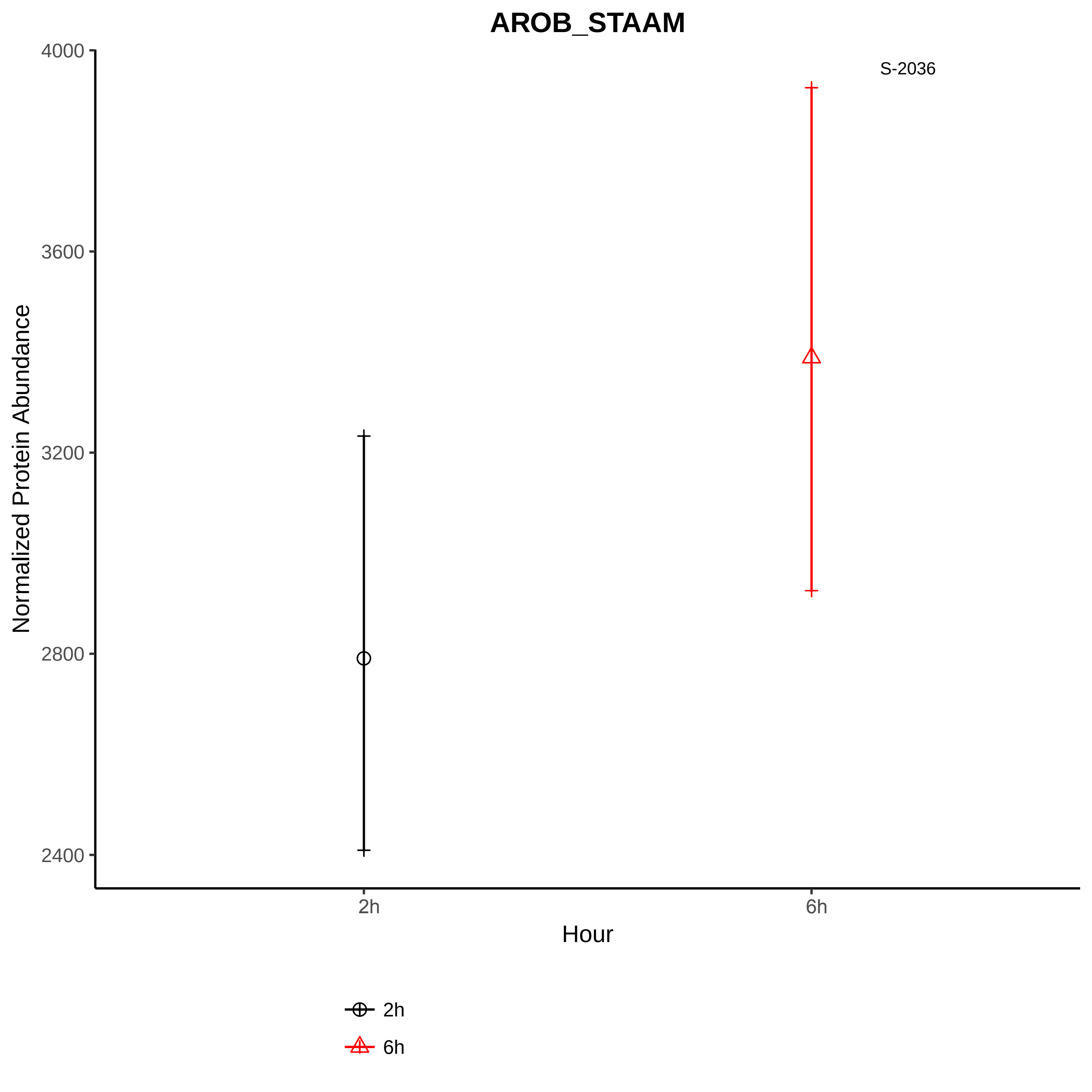
AROB_STAAM
S-2036
Normalized Protein Abundance
4000
3600
3200
2800
2400
2h
6h
Hour
2h
6h

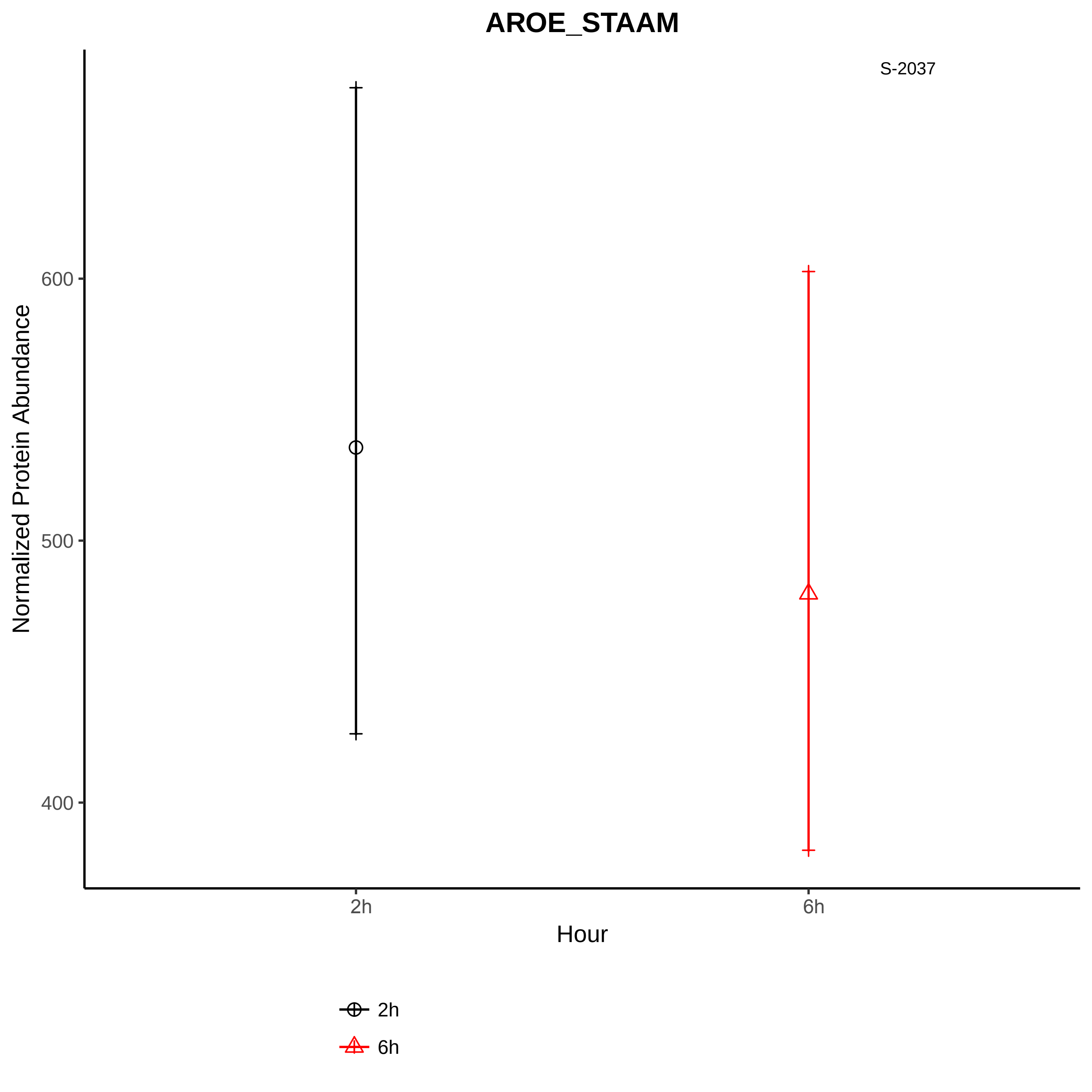

AROE_STAAM
S-2037
Normalized Protein Abundance
600
500
400
2h
6h
Hour
2h
6h

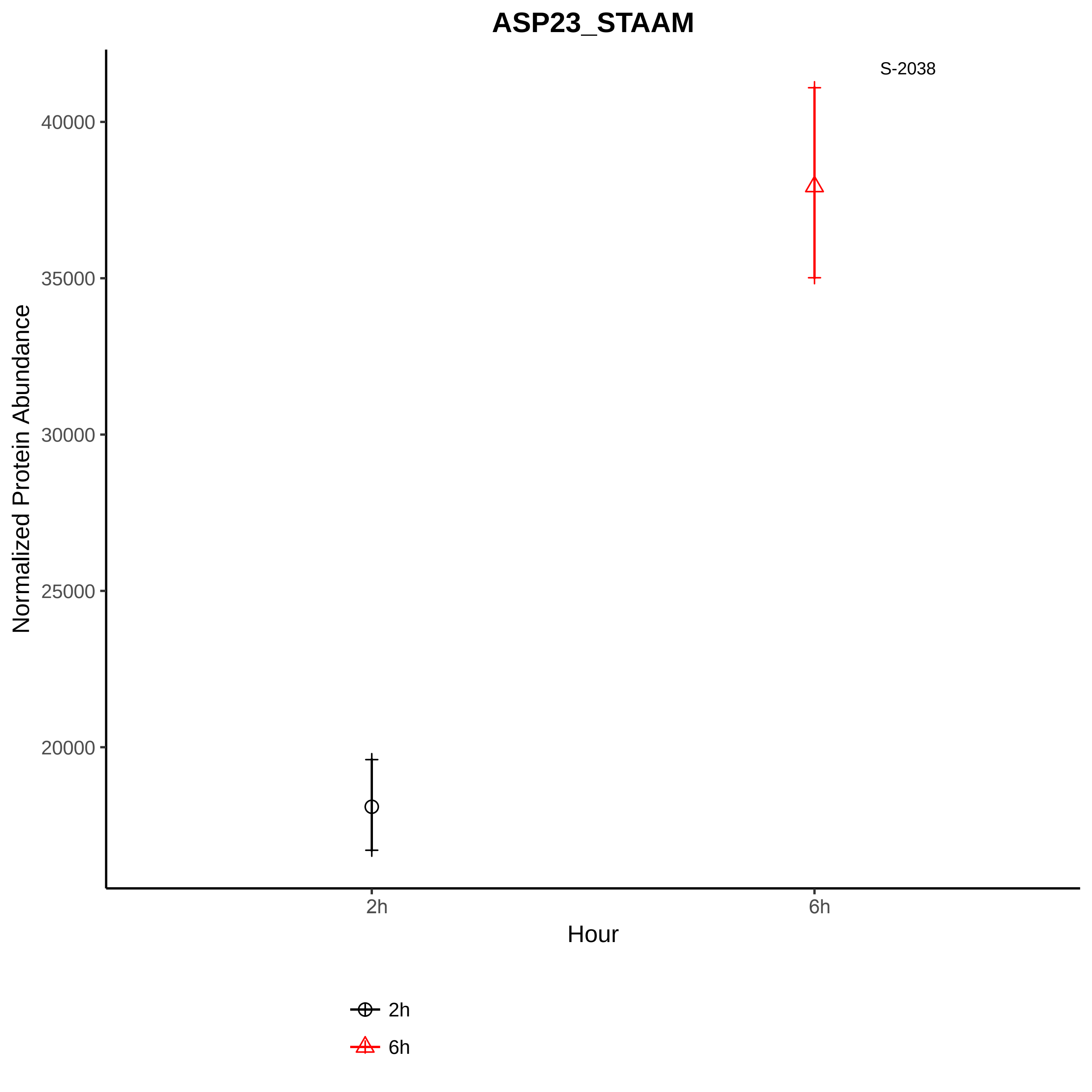
ASP23_STAAM
S-2038
40000
35000
30000
25000
20000
Normalized Protein Abundance
2h
6h
Hour
2h
6h

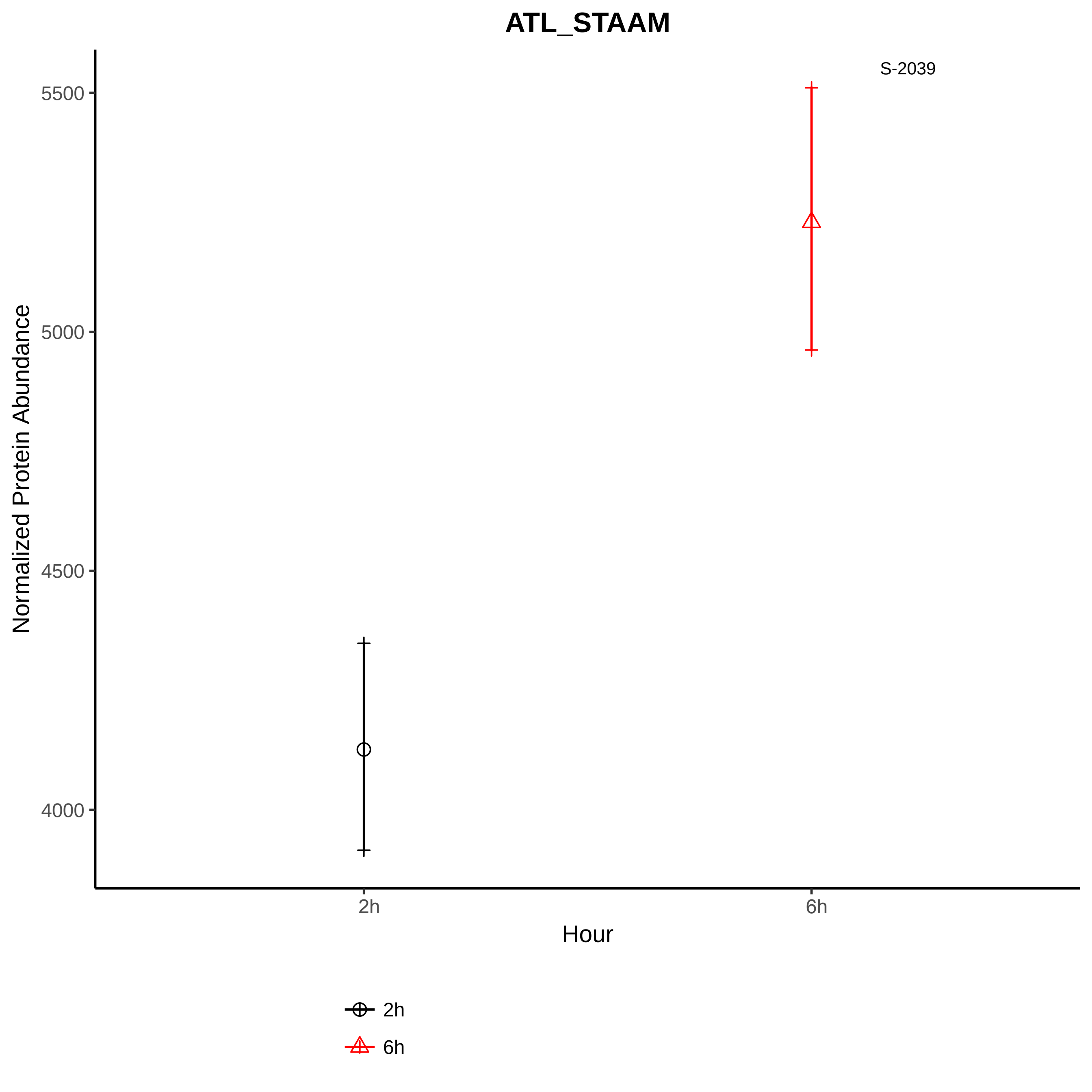

ATL_STAAM
S-2039
Normalized Protein Abundance
5500
5000
4500
4000
2h
6h
Hour
2h
6h

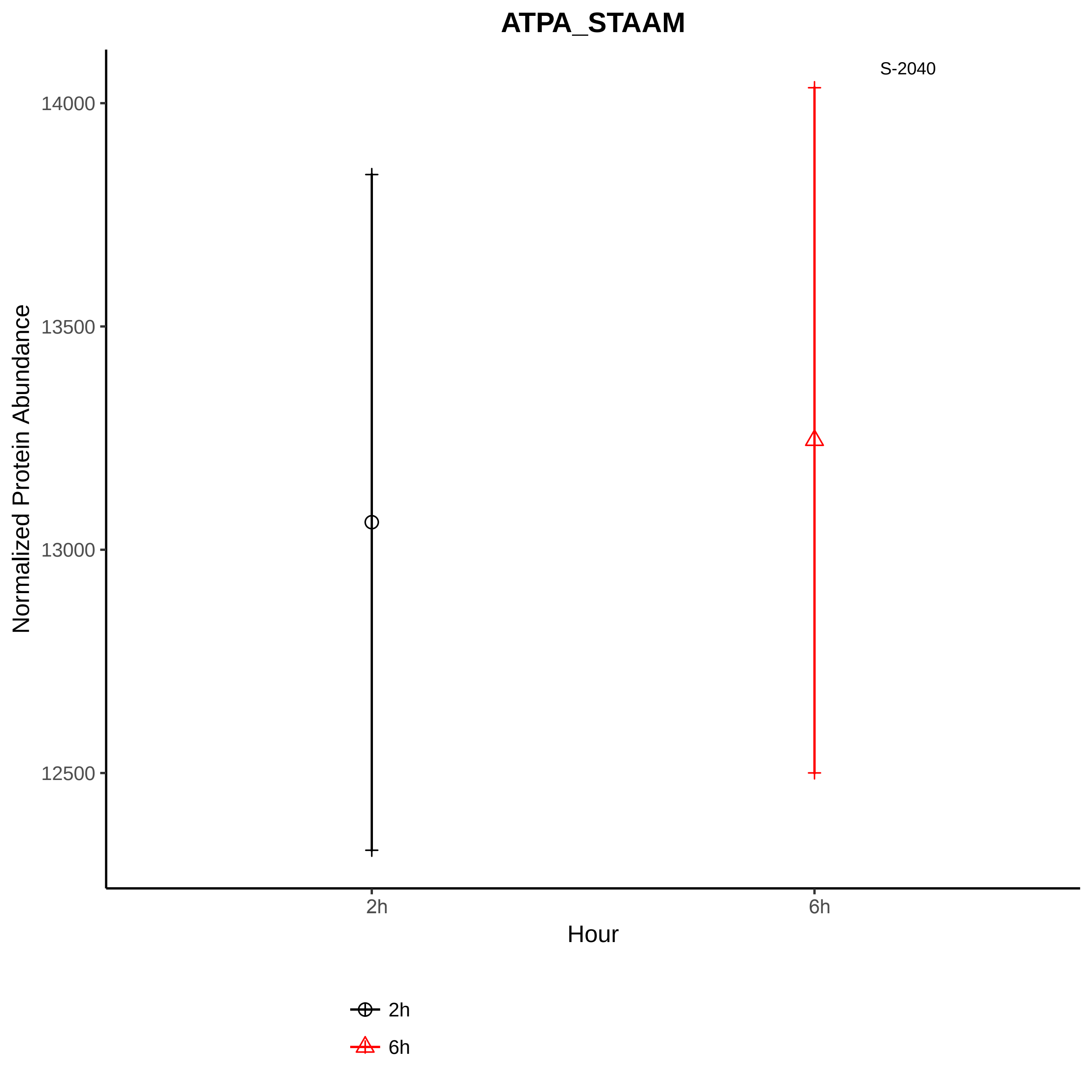

ATPA_STAAM
S-2040
14000
13500
13000
12500
Normalized Protein Abundance
2h
6h
Hour
2h
6h

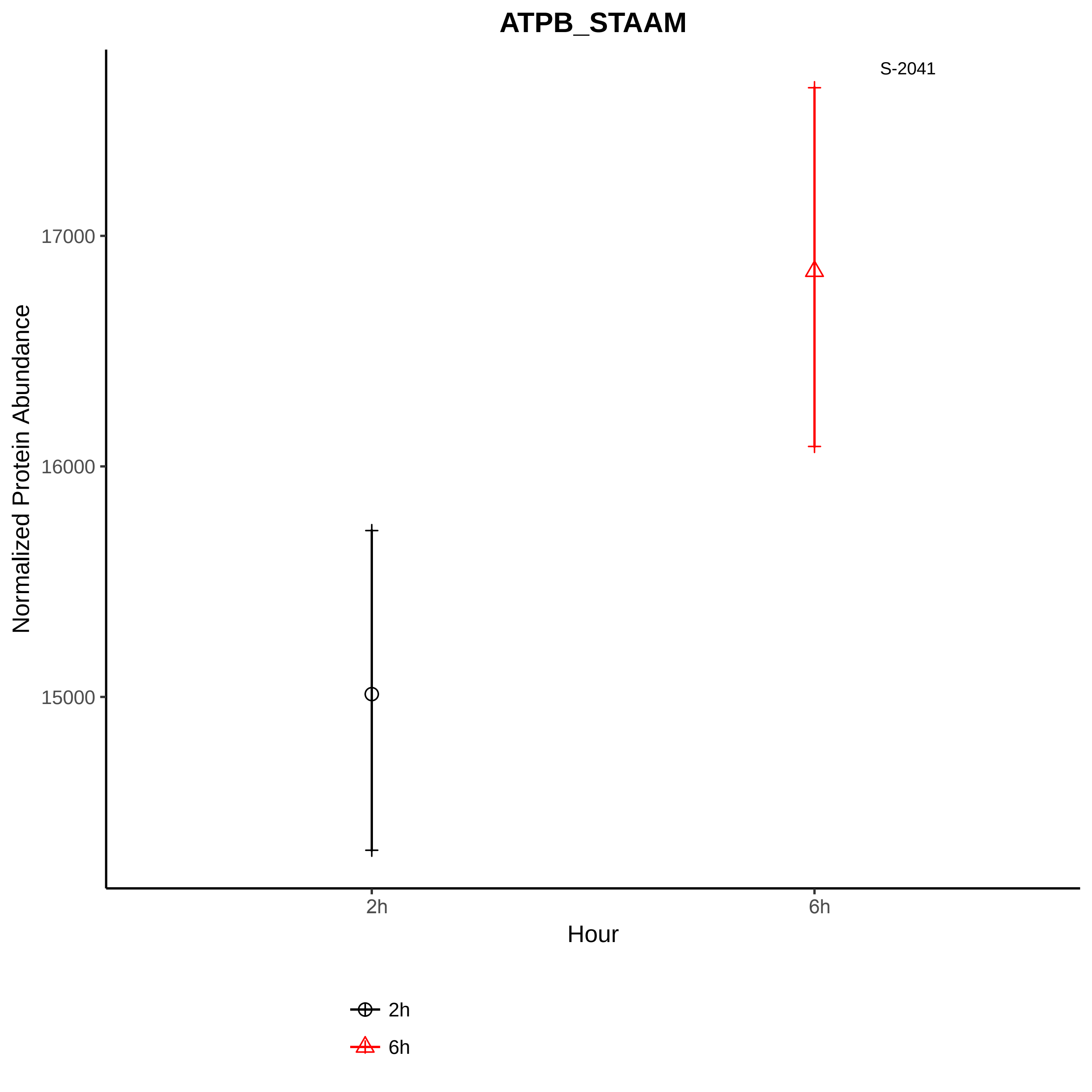
ATPB_STAAM
S-2041
Normalized Protein Abundance
17000
16000
15000
2h
6h
Hour
2h
6h

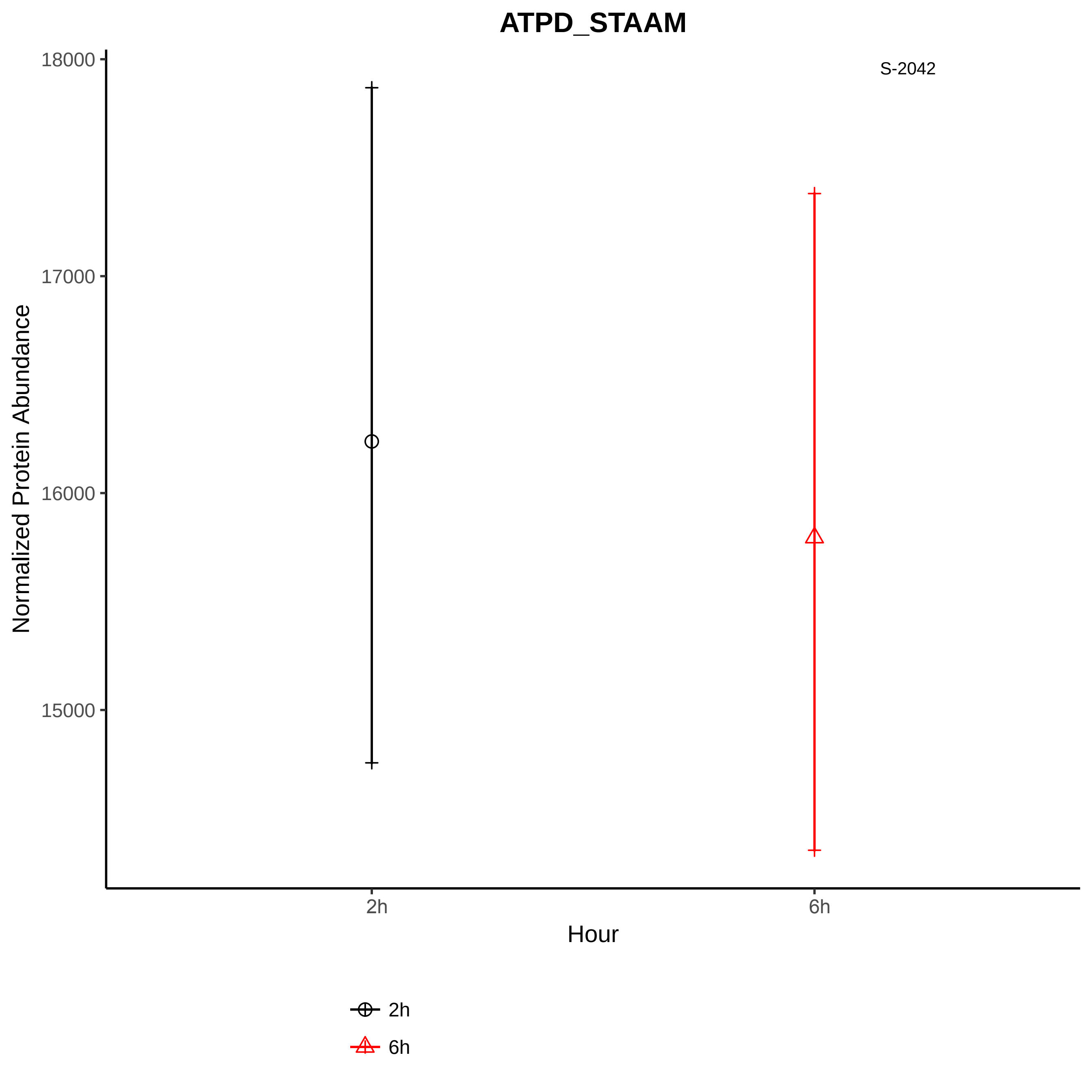

ATPD_STAAM
S-2042
18000
17000
16000
15000
Normalized Protein Abundance
2h
6h
Hour
2h
6h

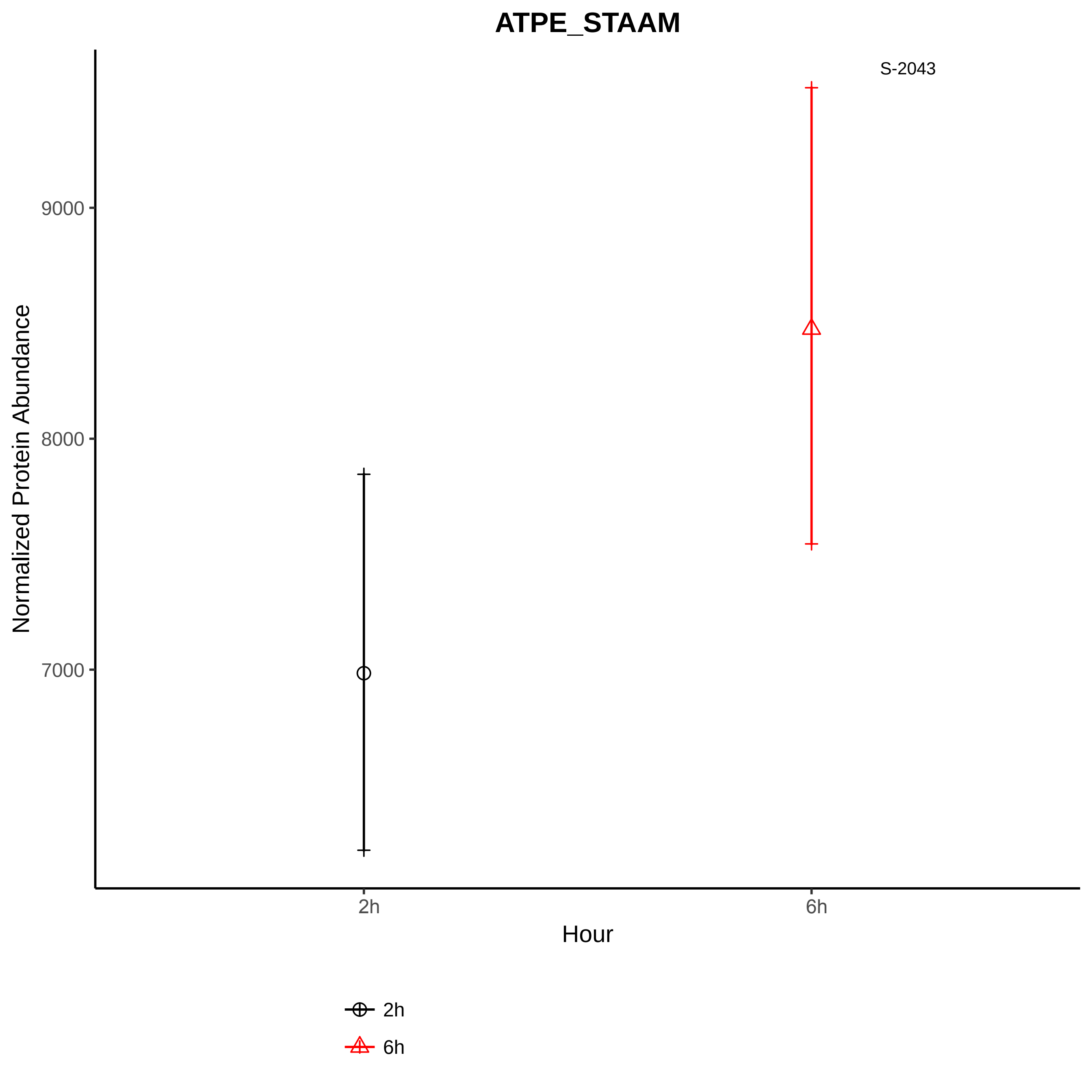

ATPE_STAAM
S-2043
9000
8000
7000
Normalized Protein Abundance
2h
6h
Hour
2h
6h

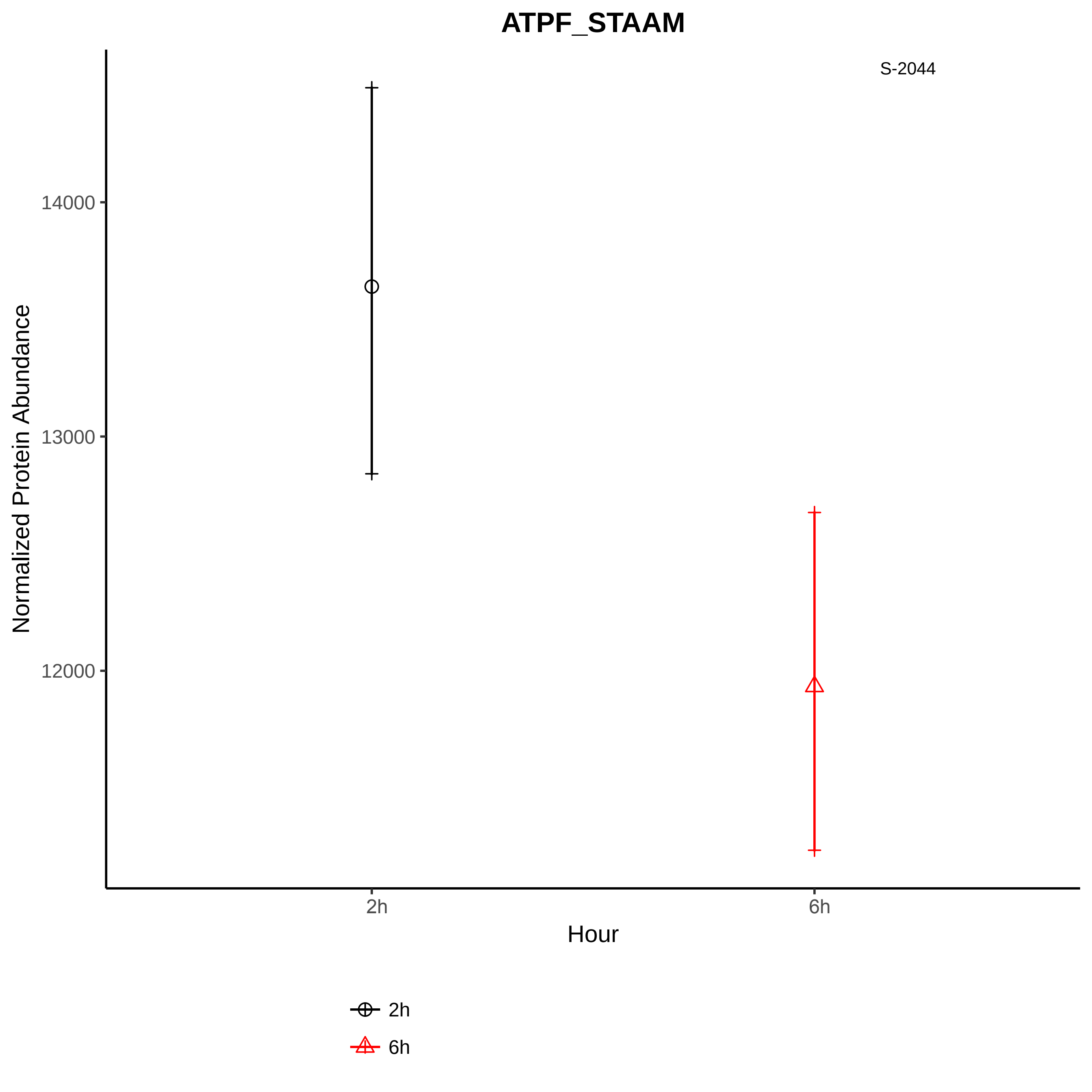

ATPF_STAAM
S-2044
Normalized Protein Abundance
14000
13000
12000
2h
6h
Hour
2h
6h

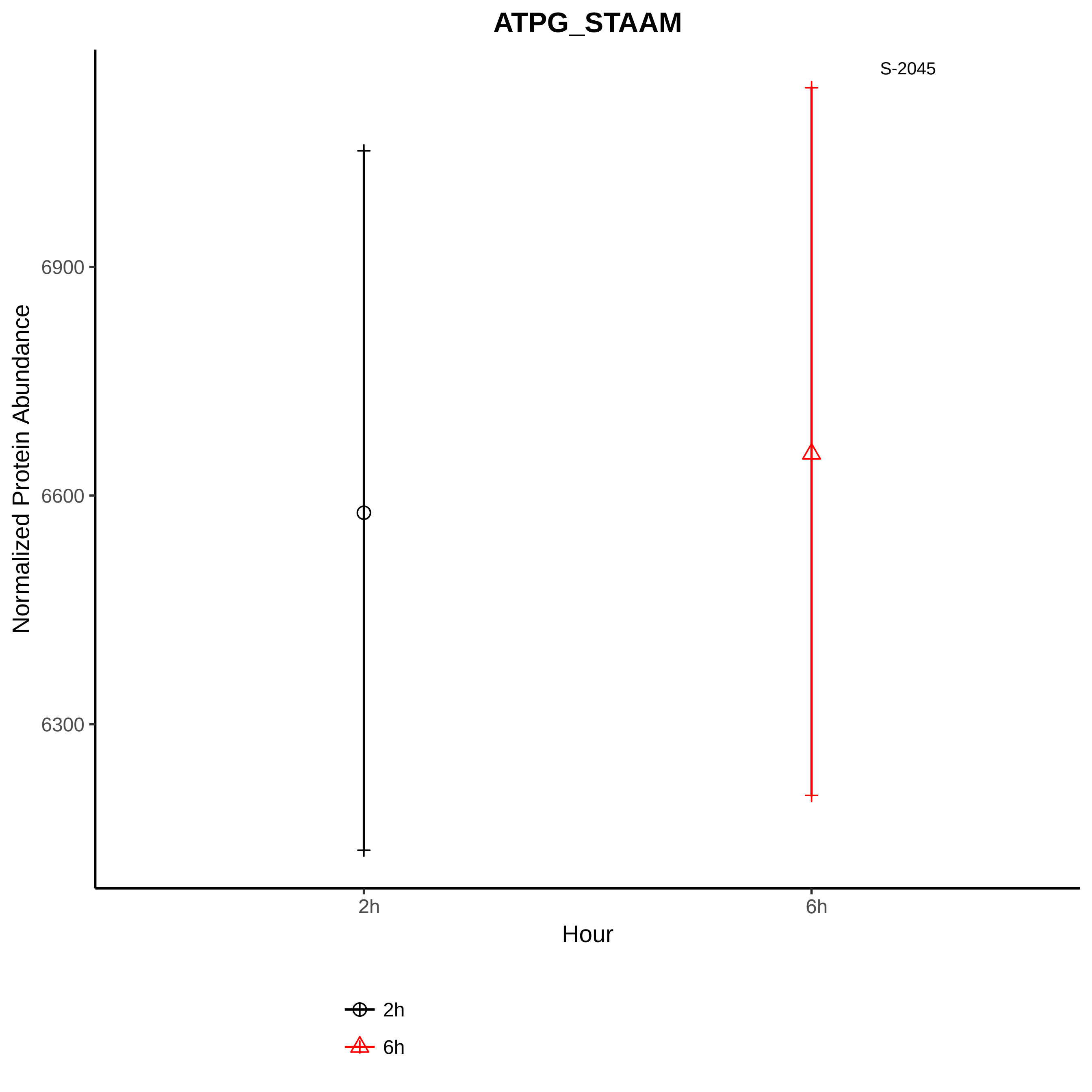

ATPG_STAAM
S-2045
6900
6600
6300
Normalized Protein Abundance
2h
6h
Hour
2h
6h

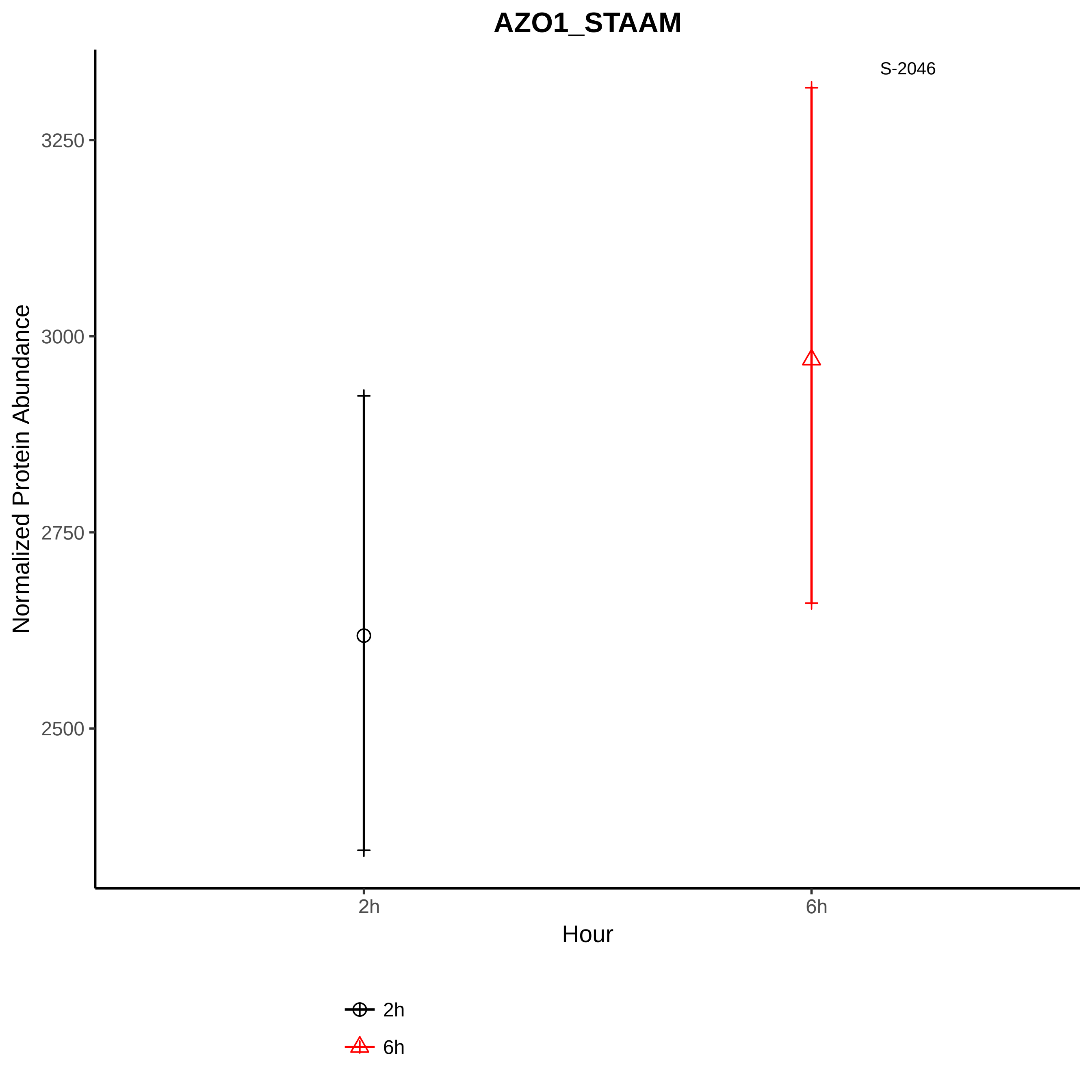
AZO1_STAAM
S-2046
Normalized Protein Abundance
3250
3000
2750
2500
2h
6h
Hour
2h
6h

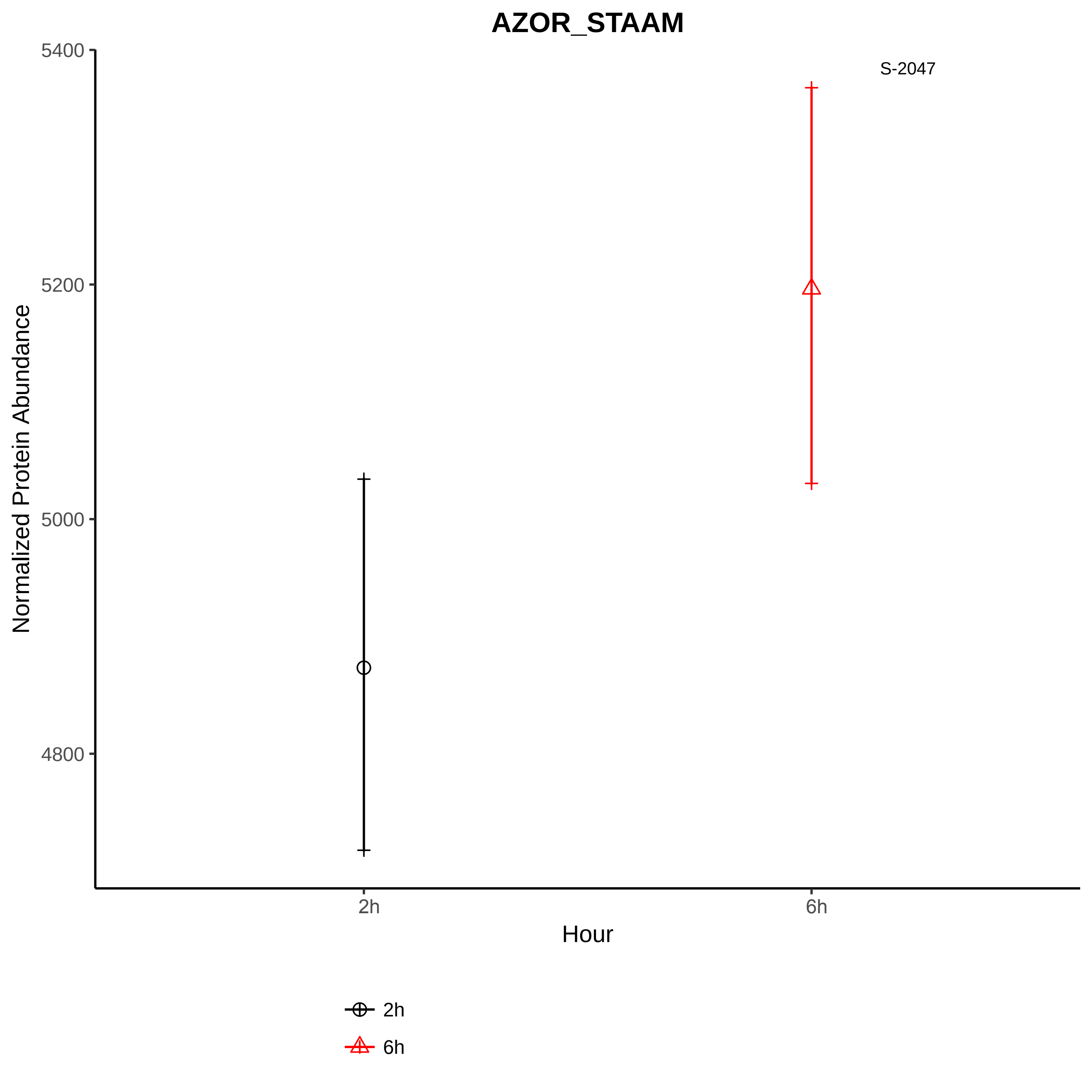

AZOR_STAAM
S-2047
5400
5200
5000
4800
Normalized Protein Abundance
2h
6h
Hour
2h
6h

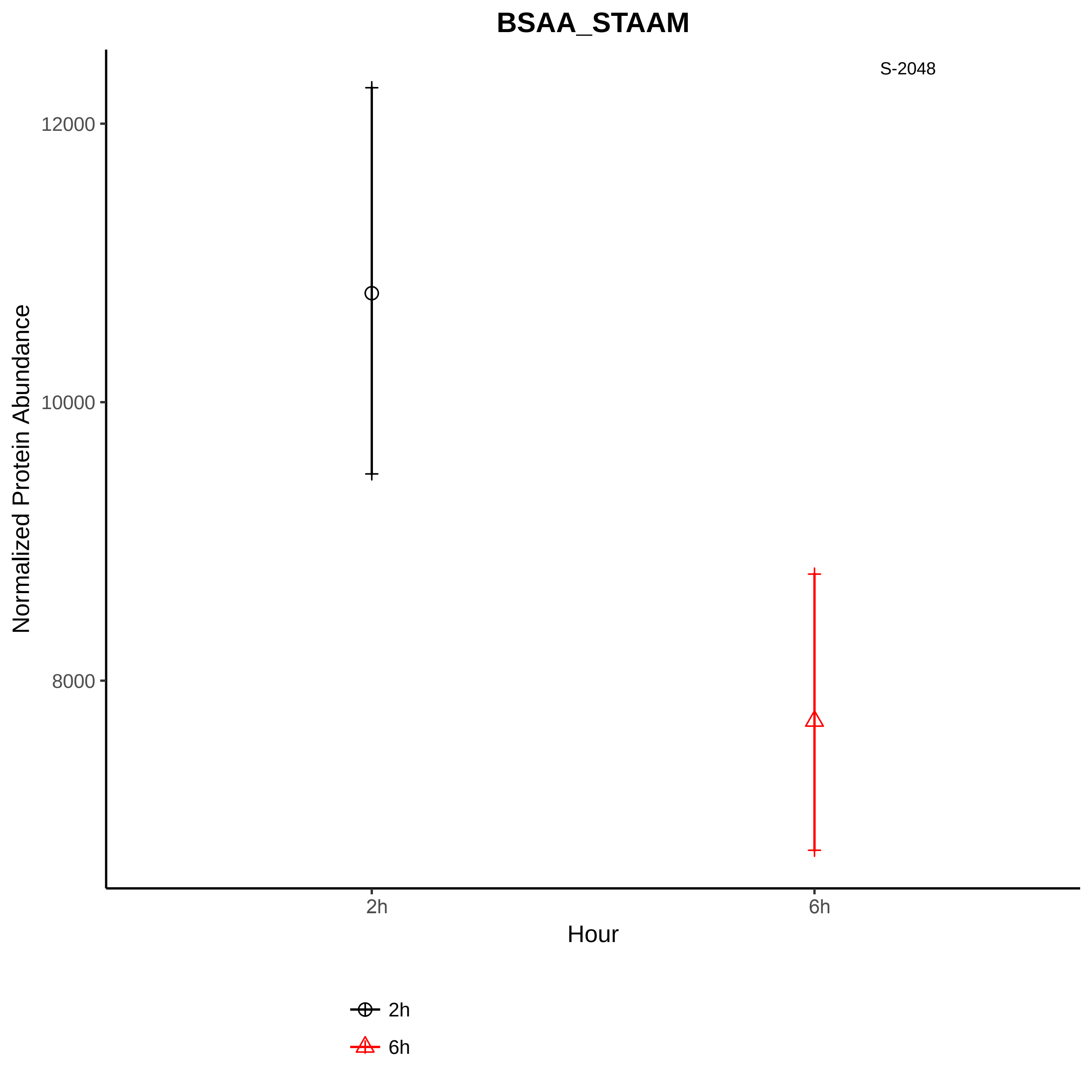
BSAA_STAAM
S-2048
12000
10000
8000
Normalized Protein Abundance
2h
6h
Hour
2h
6h

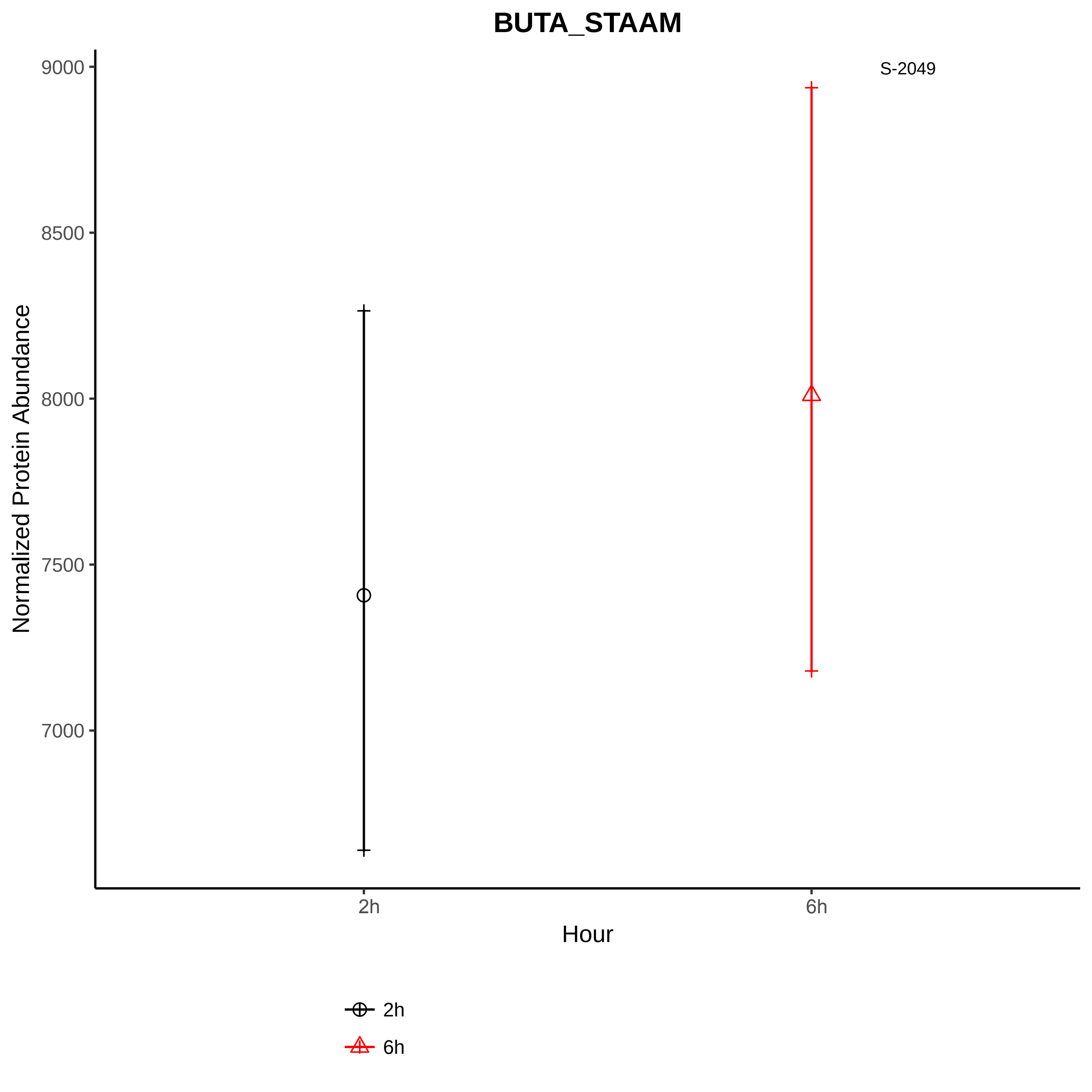

BUTA_STAAM
S-2049
9000
8500
8000
7500
7000
Normalized Protein Abundance
2h
6h
Hour
2h
6h

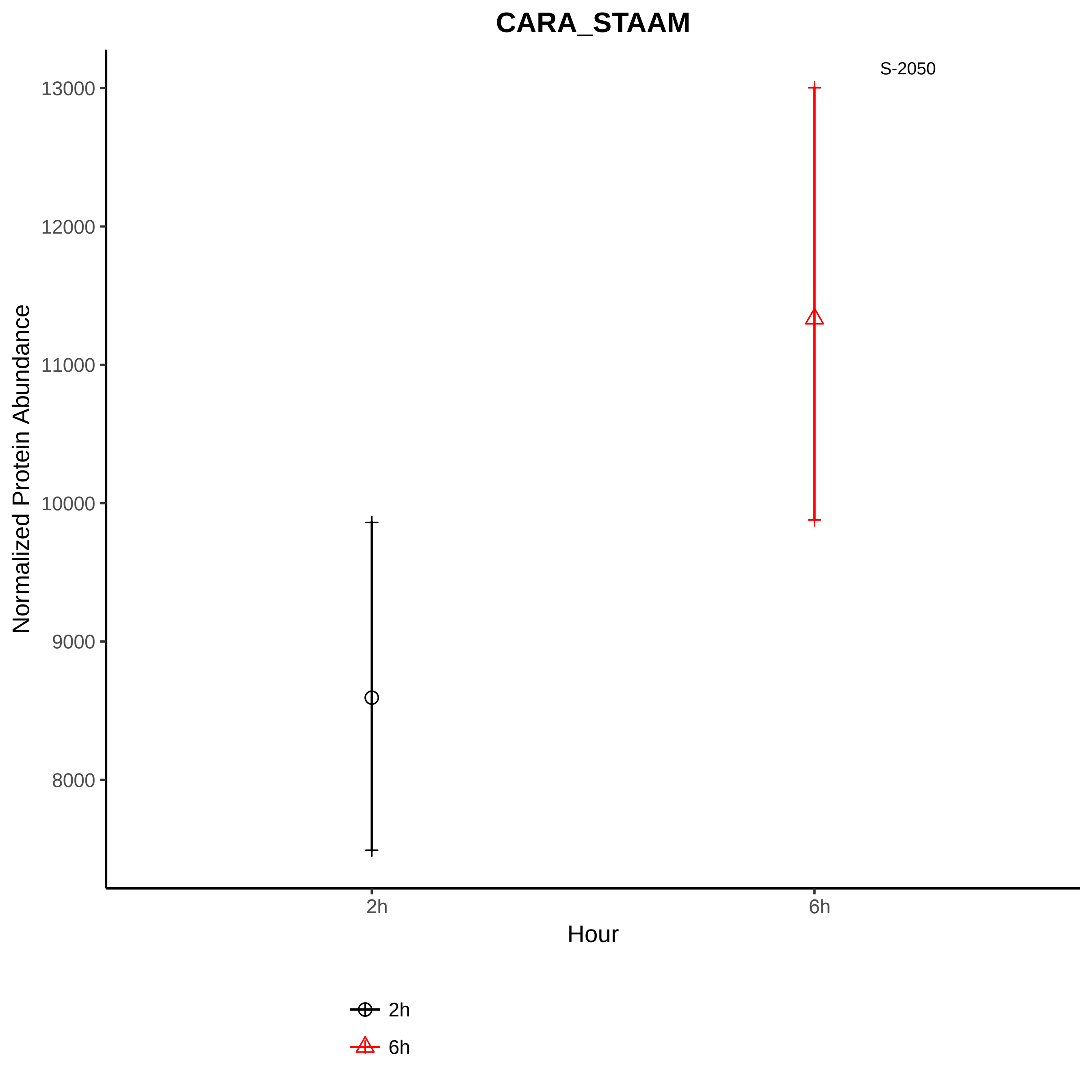

CARA_STAAM
S-2050
Normalized Protein Abundance
13000
12000
11000
10000
9000
8000
2h
6h
Hour
2h
6h

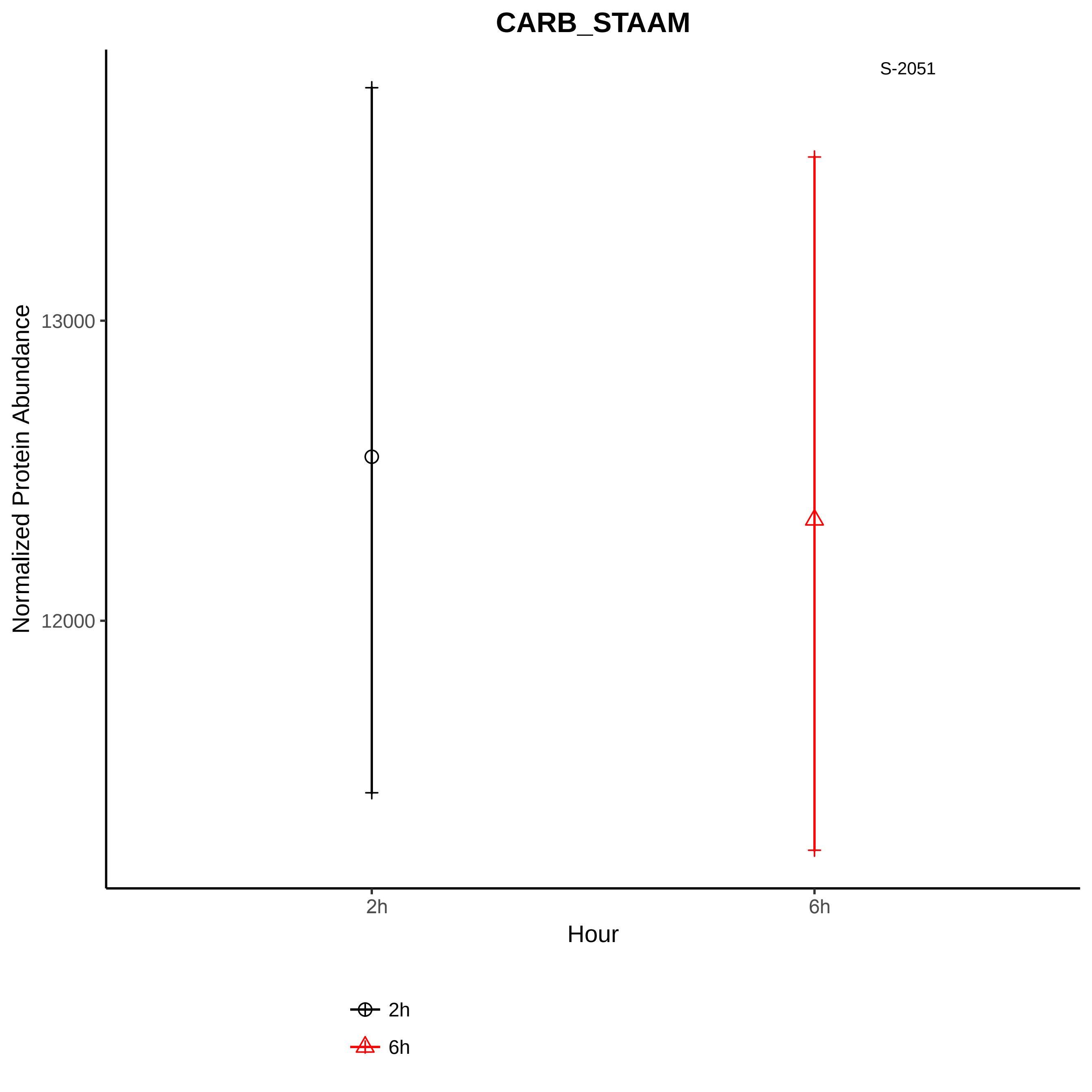
CARB_STAAM
S-2051
Normalized Protein Abundance
13000
12000
2h
6h
Hour
2h
6h

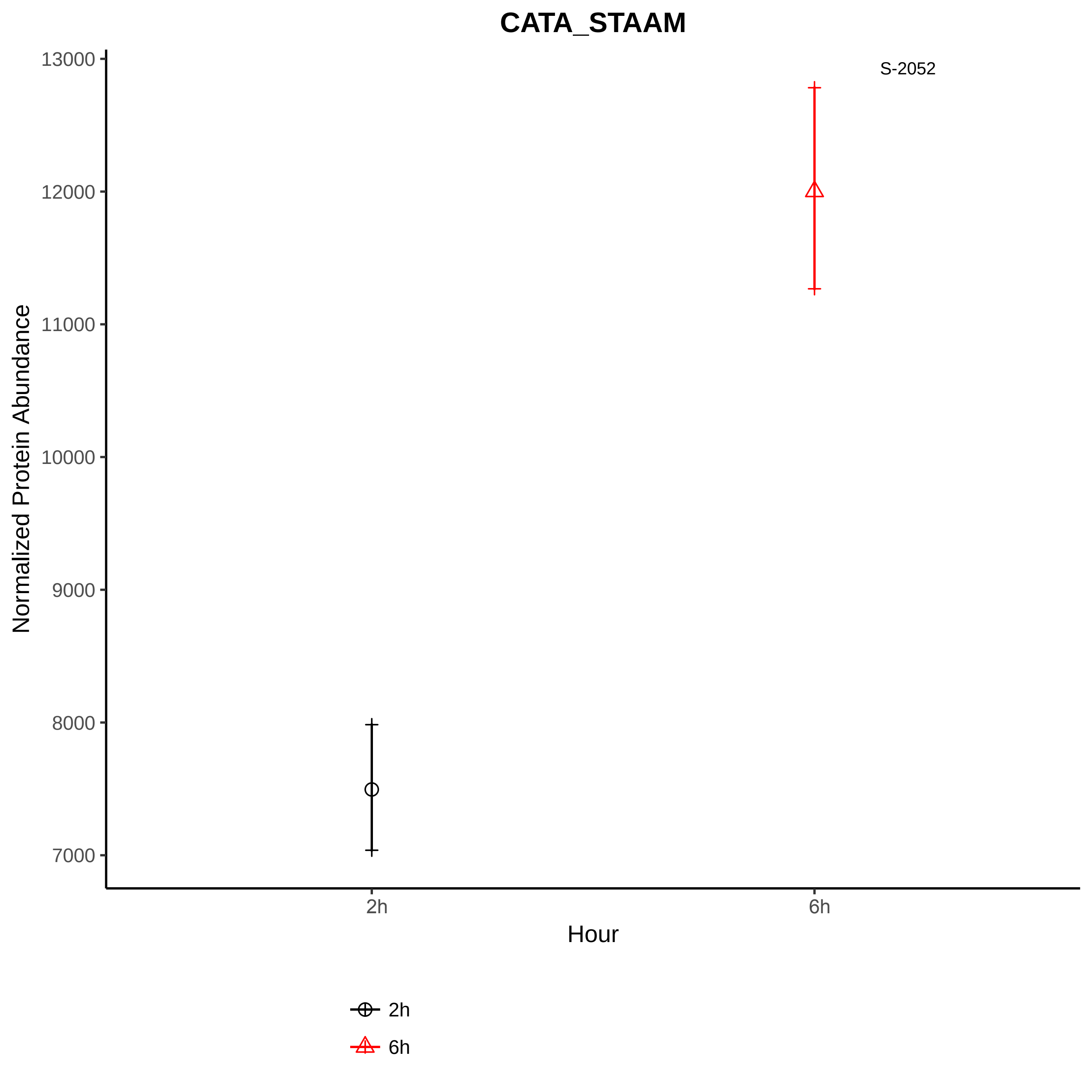

CATA_STAAM
S-2052
Normalized Protein Abundance
13000
12000
11000
10000
9000
8000
7000
2h
6h
Hour
2h
6h

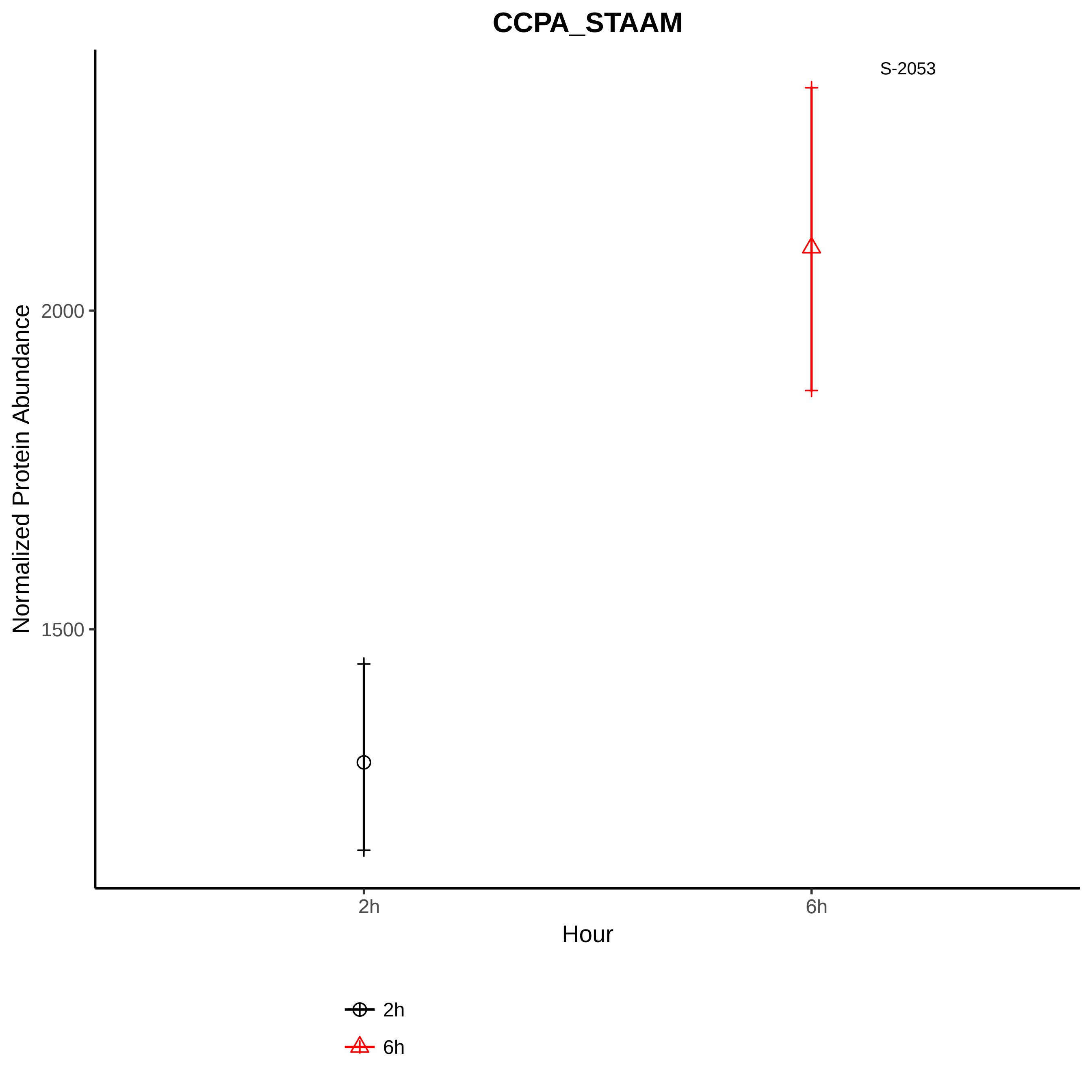
CCPA_STAAM
S-2053
Normalized Protein Abundance
2000
1500
2h
6h
Hour
2h
6h

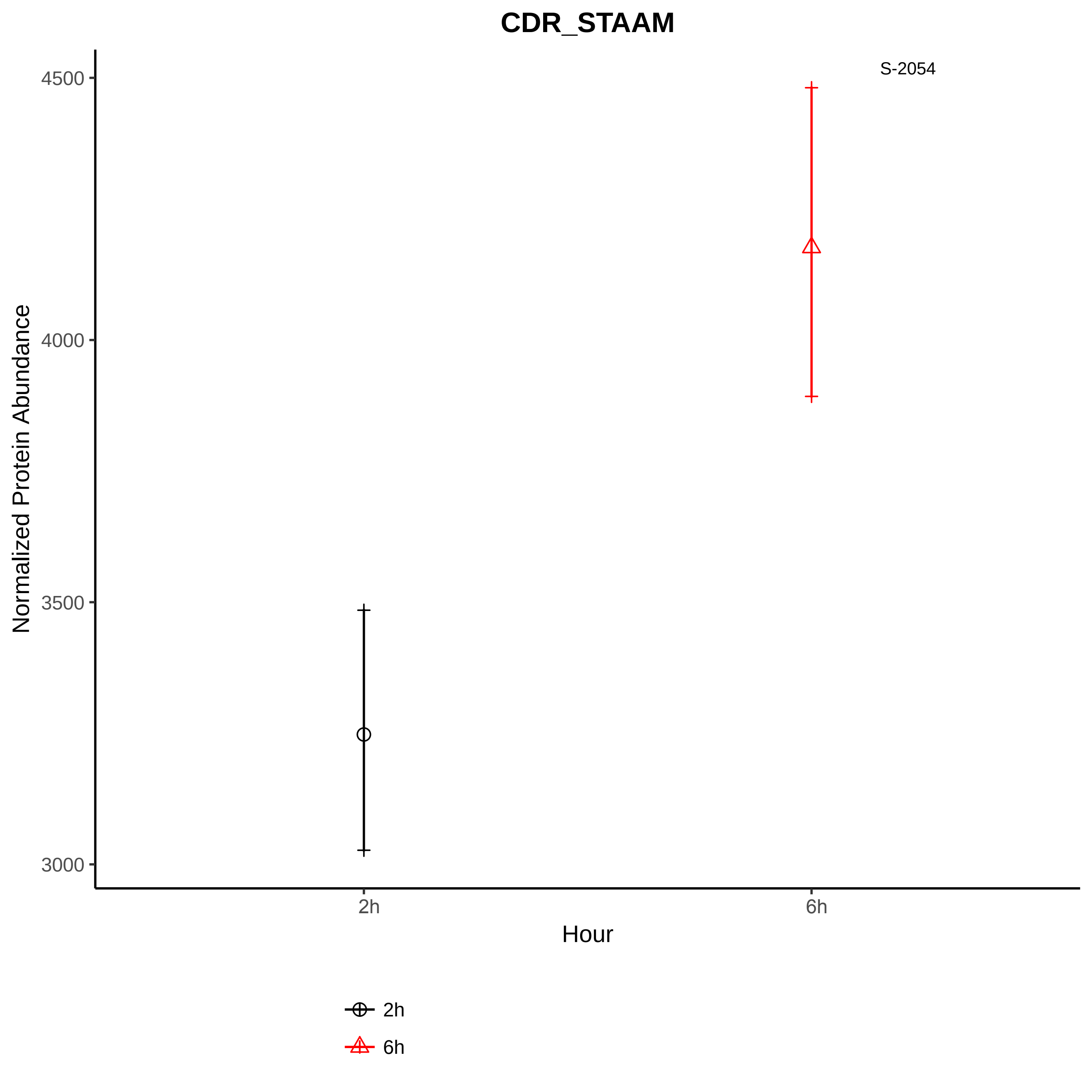
CDR_STAAM
S-2054
Normalized Protein Abundance
4500
4000
3500
3000
2h
6h
Hour
2h
6h

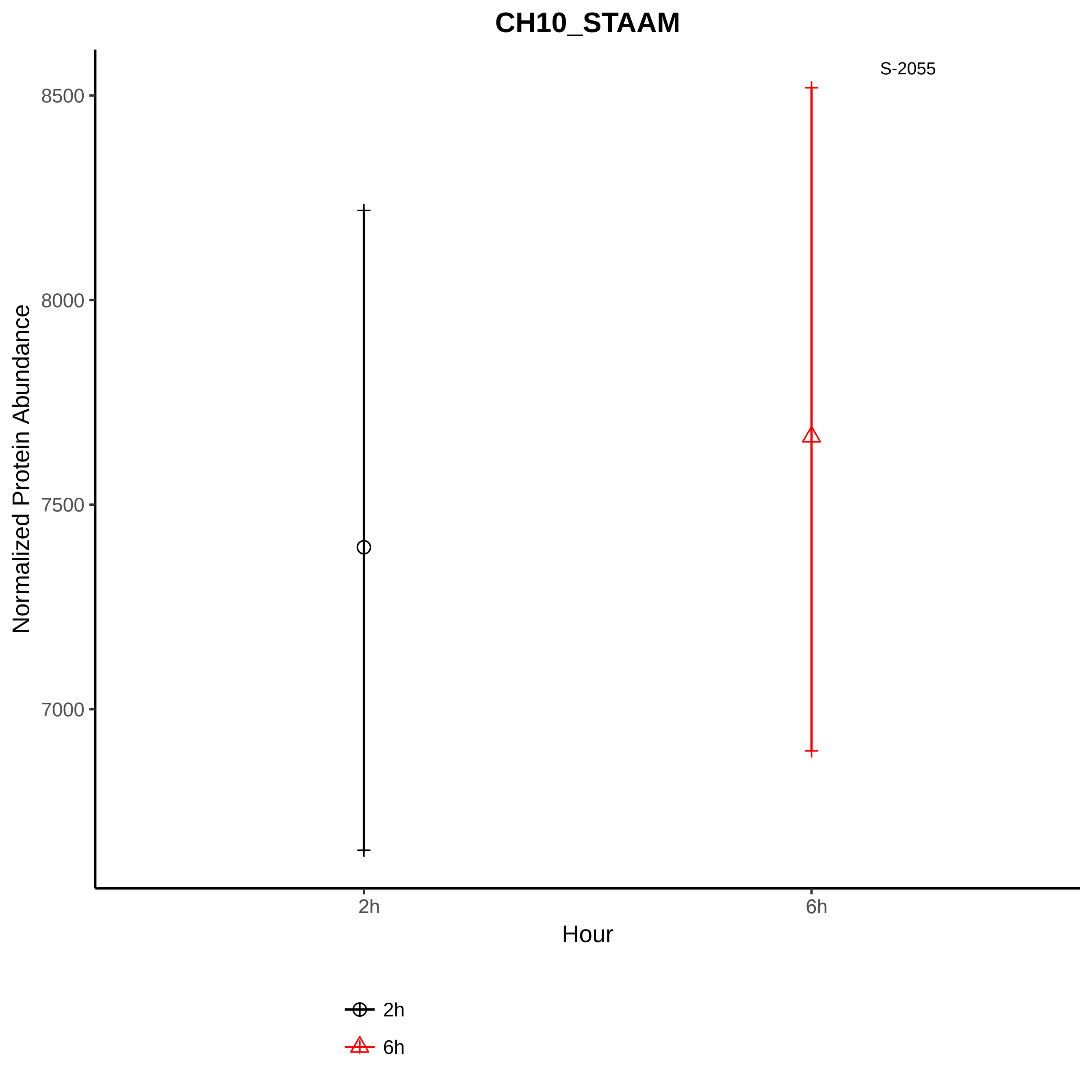
CH10_STAAM
S-2055
Normalized Protein Abundance
8500
8000
7500
7000
2h
6h
Hour
2h
6h

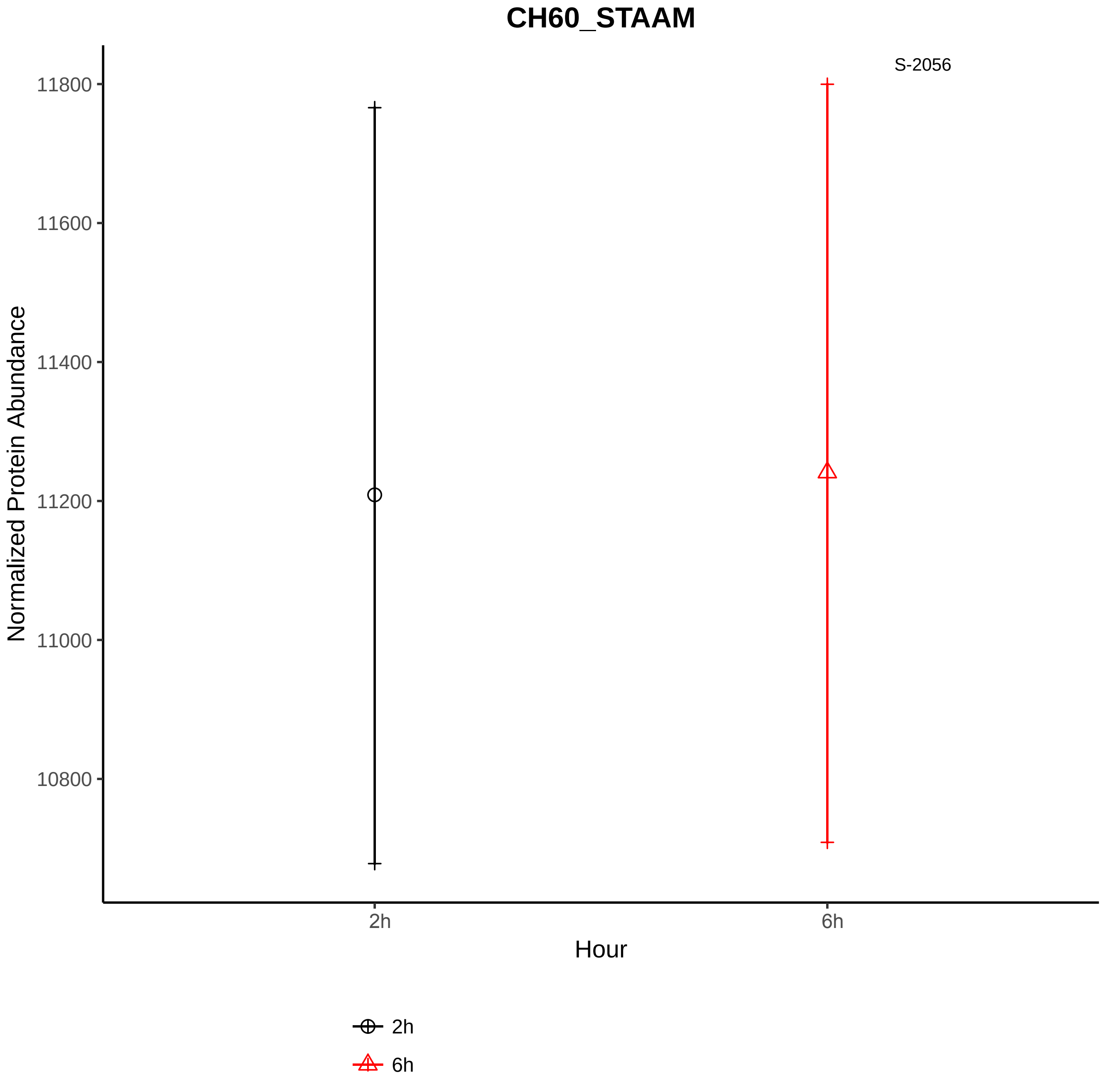

CH60_STAAM
S-2056
Normalized Protein Abundance
11800
11600
11400
11200
11000
10800
2h
6h
Hour
2h
6h

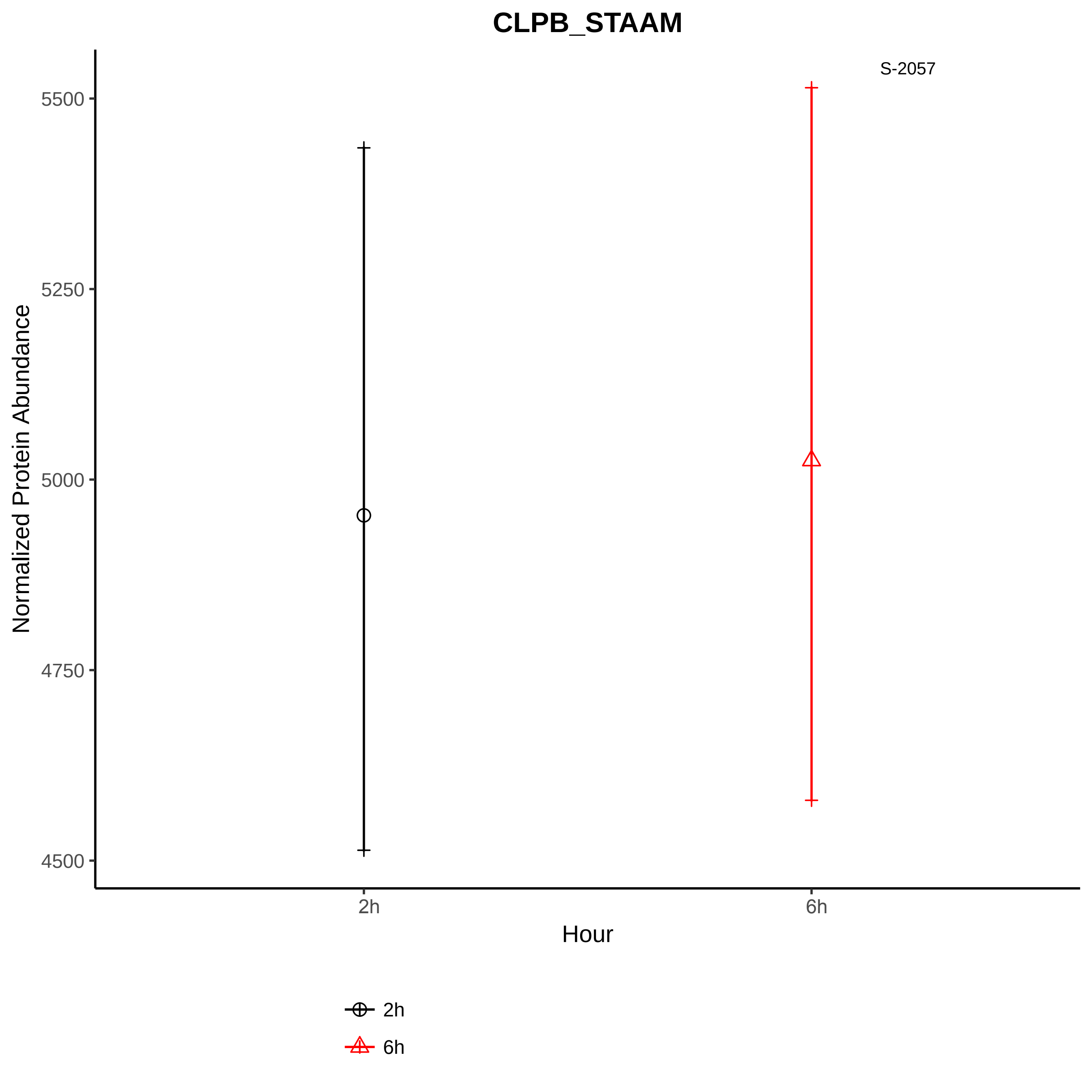

CLPB_STAAM
S-2057
Normalized Protein Abundance
5500
5250
5000
4750
4500
2h
6h
Hour
2h
6h

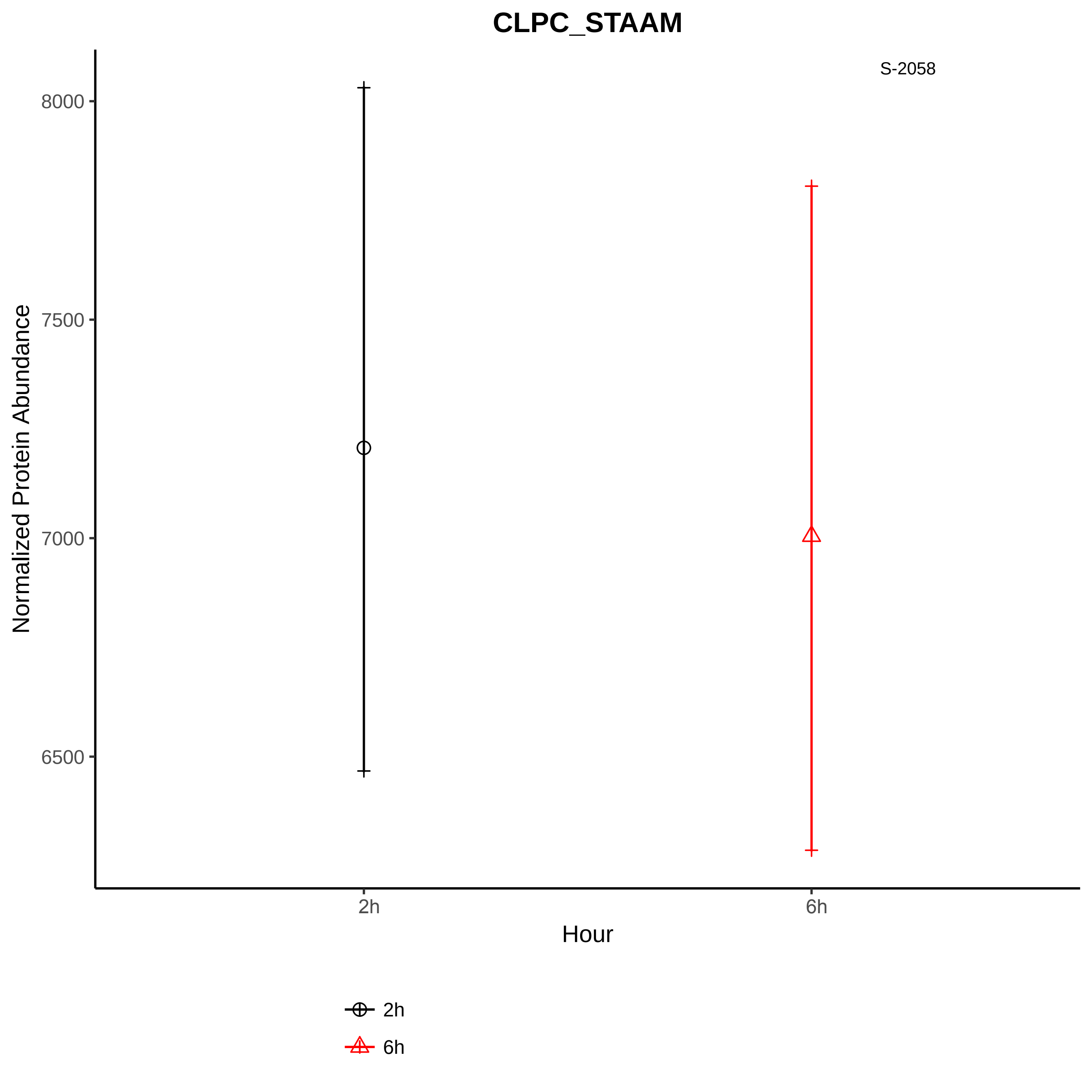

CLPC_STAAM
S-2058
8000
7500
7000
6500
Normalized Protein Abundance
2h
6h
Hour
2h
6h

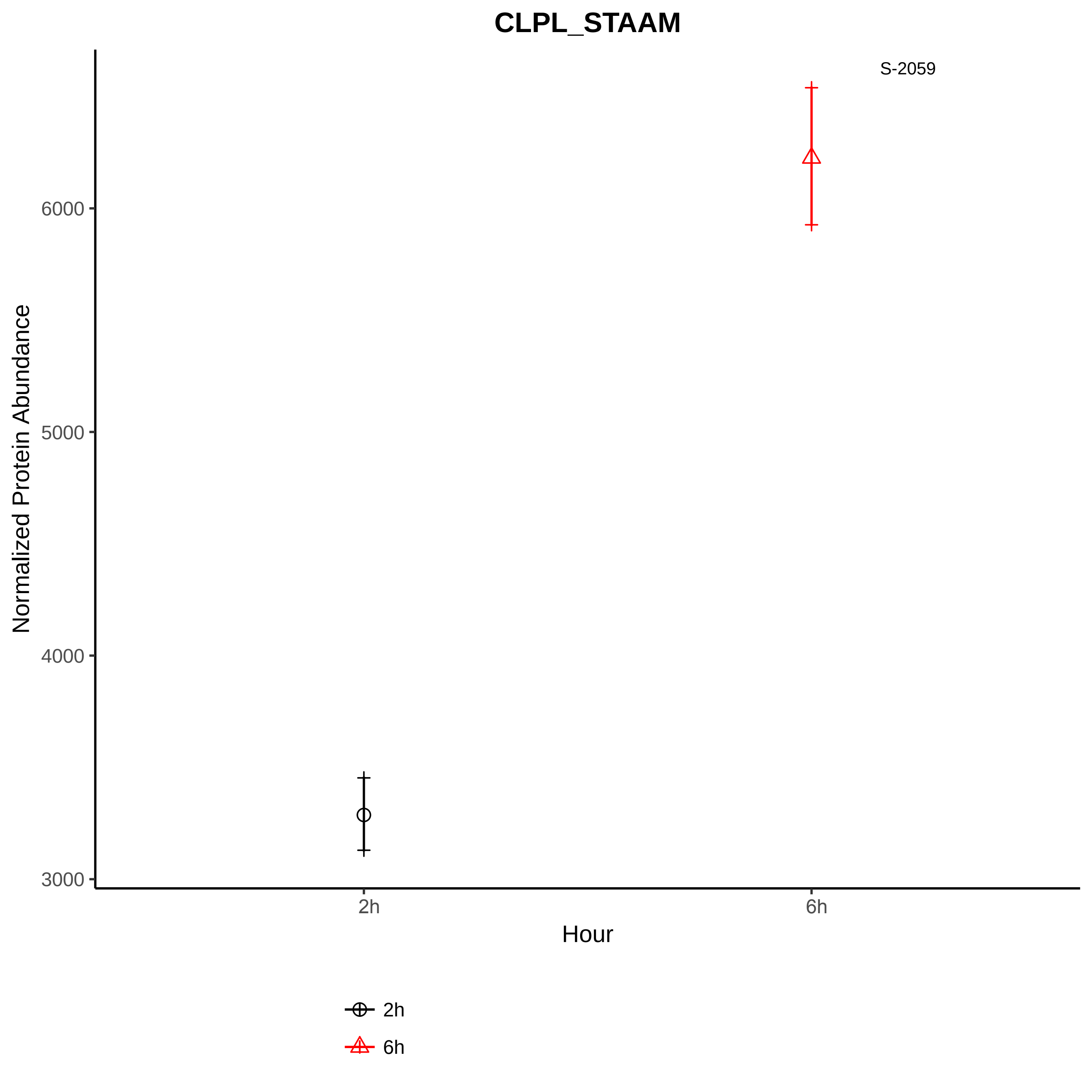

CLPL_STAAM
S-2059
Normalized Protein Abundance
6000
5000
4000
3000
2h
6h
Hour
2h
6h

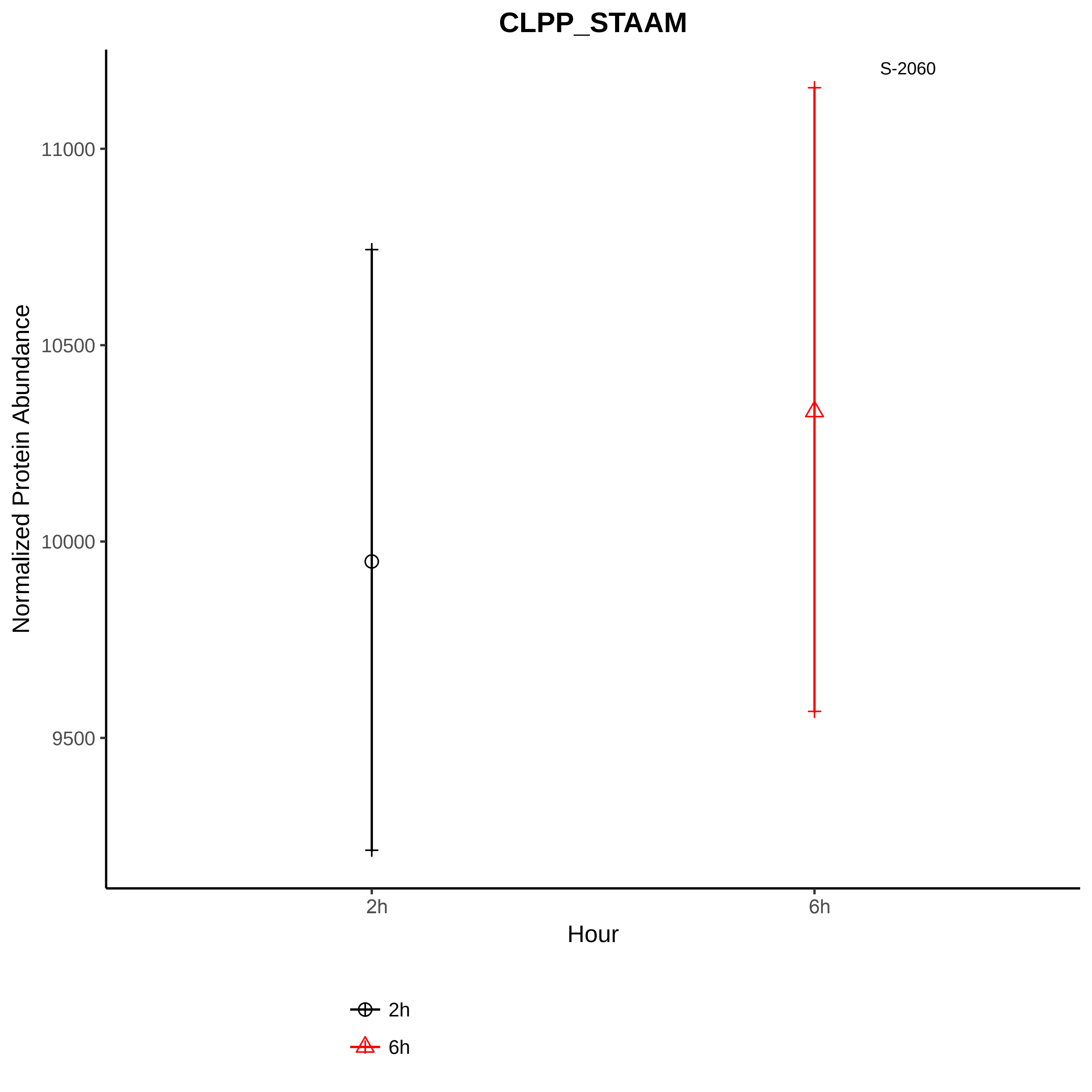
CLPP_STAAM
S-2060
11000
10500
10000
9500
Normalized Protein Abundance
2h
6h
Hour
2h
6h

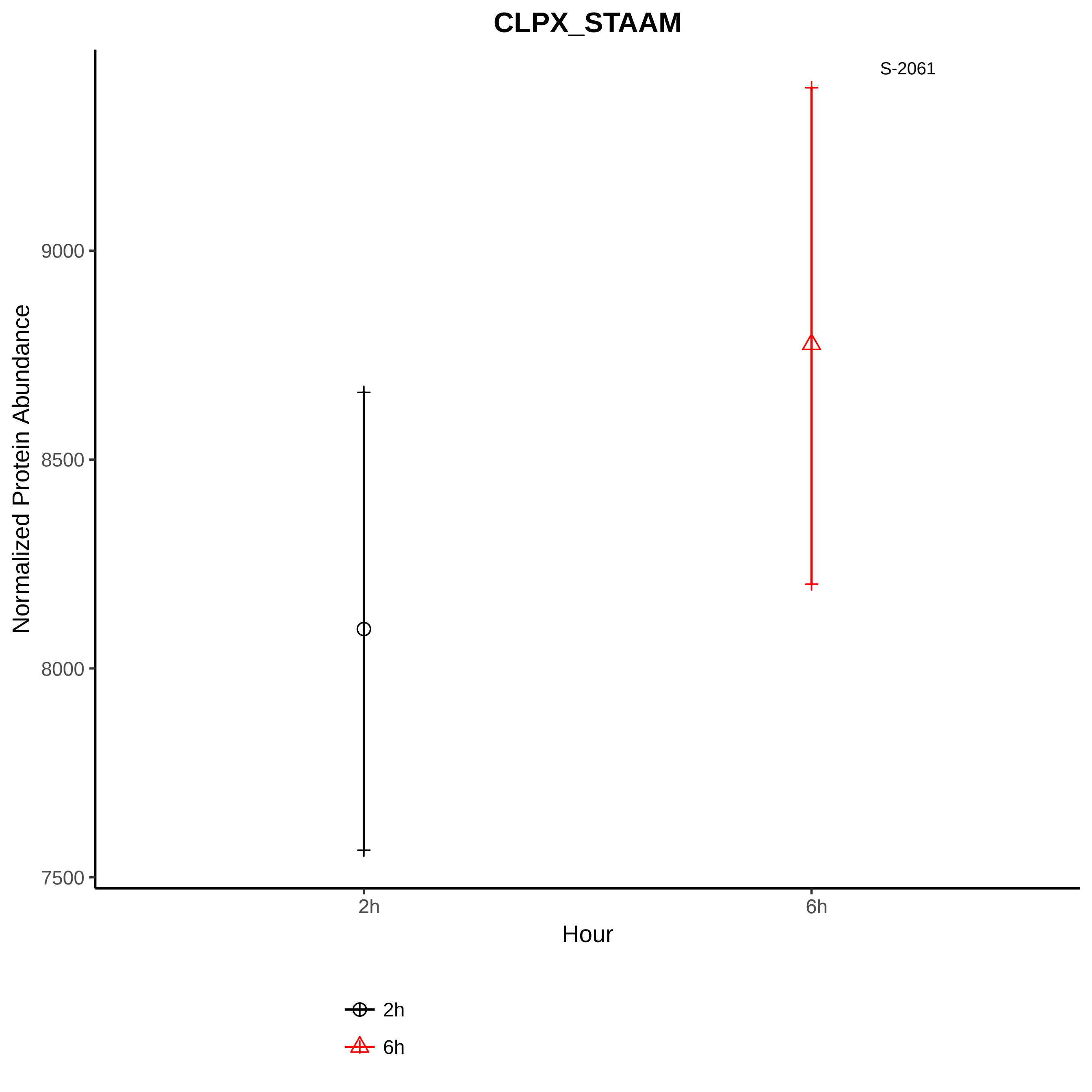
CLPX_STAAM
S-2061
Normalized Protein Abundance
9000
8500
8000
7500
2h
6h
Hour
2h
6h

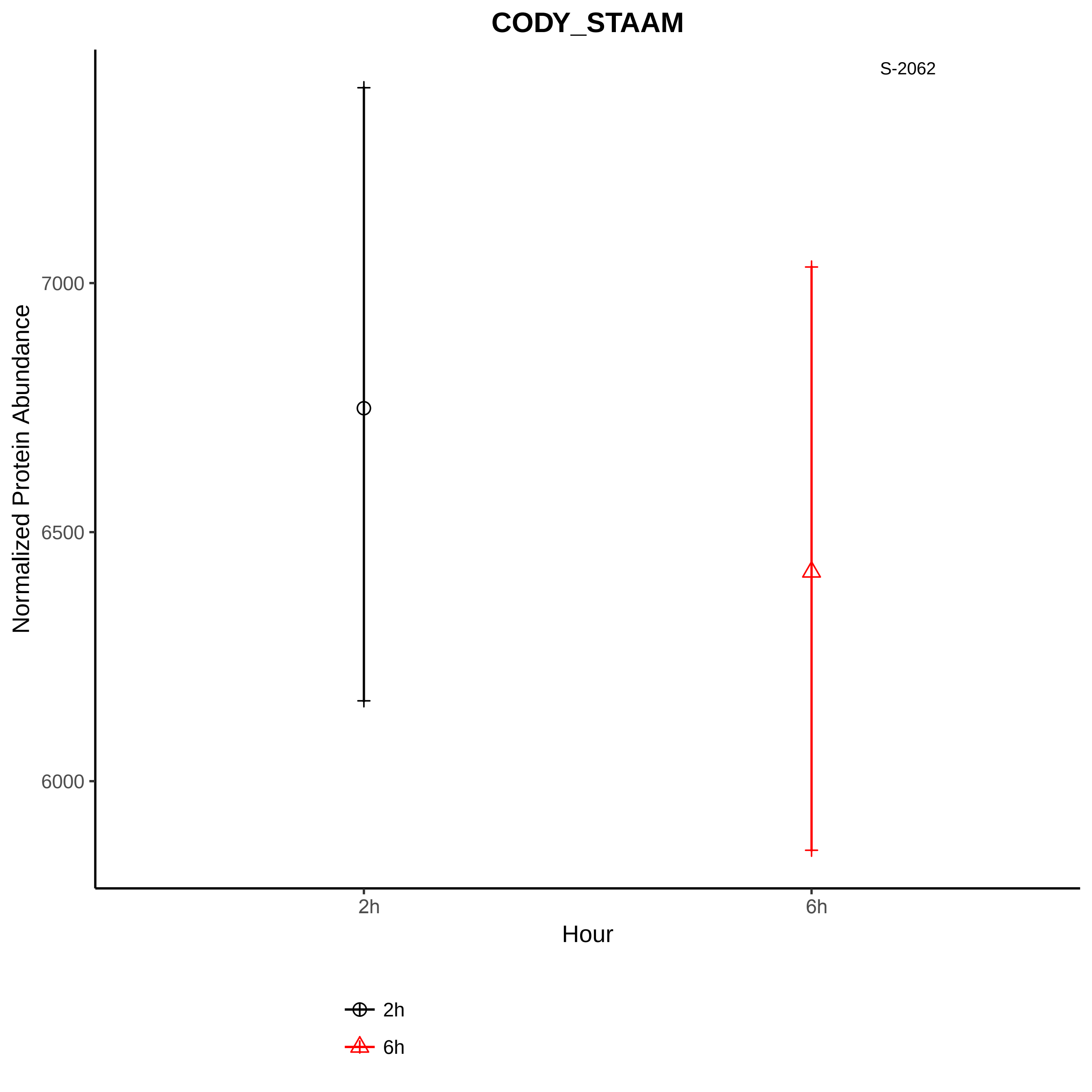

CODY_STAAM
S-2062
7000
6500
6000
Normalized Protein Abundance
2h
6h
Hour
2h
6h

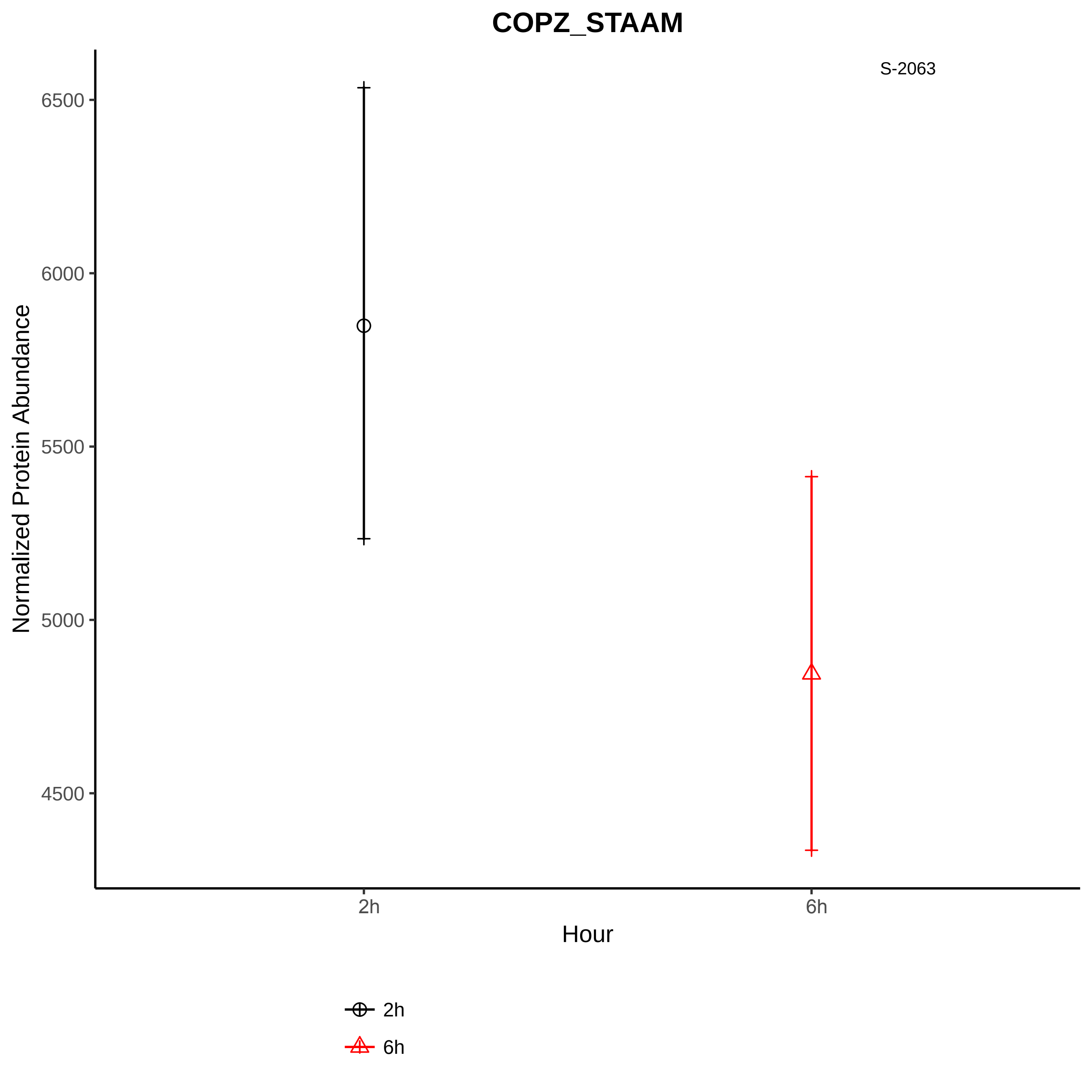

COPZ_STAAM
S-2063
Normalized Protein Abundance
6500
6000
5500
5000
4500
2h
6h
Hour
2h
6h

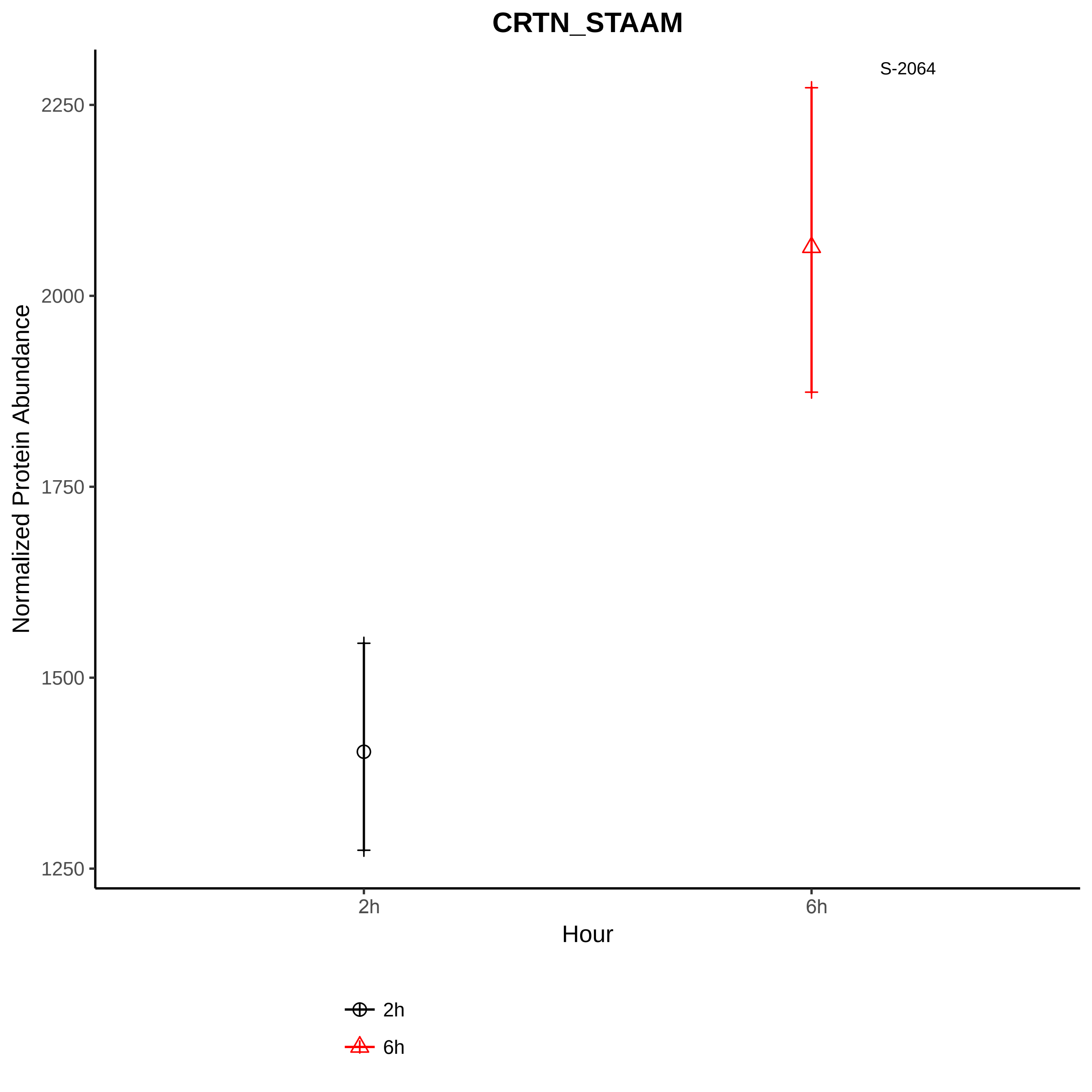

CRTN_STAAM
S-2064
Normalized Protein Abundance
2250
2000
1750
1500
1250
2h
6h
Hour
2h
6h

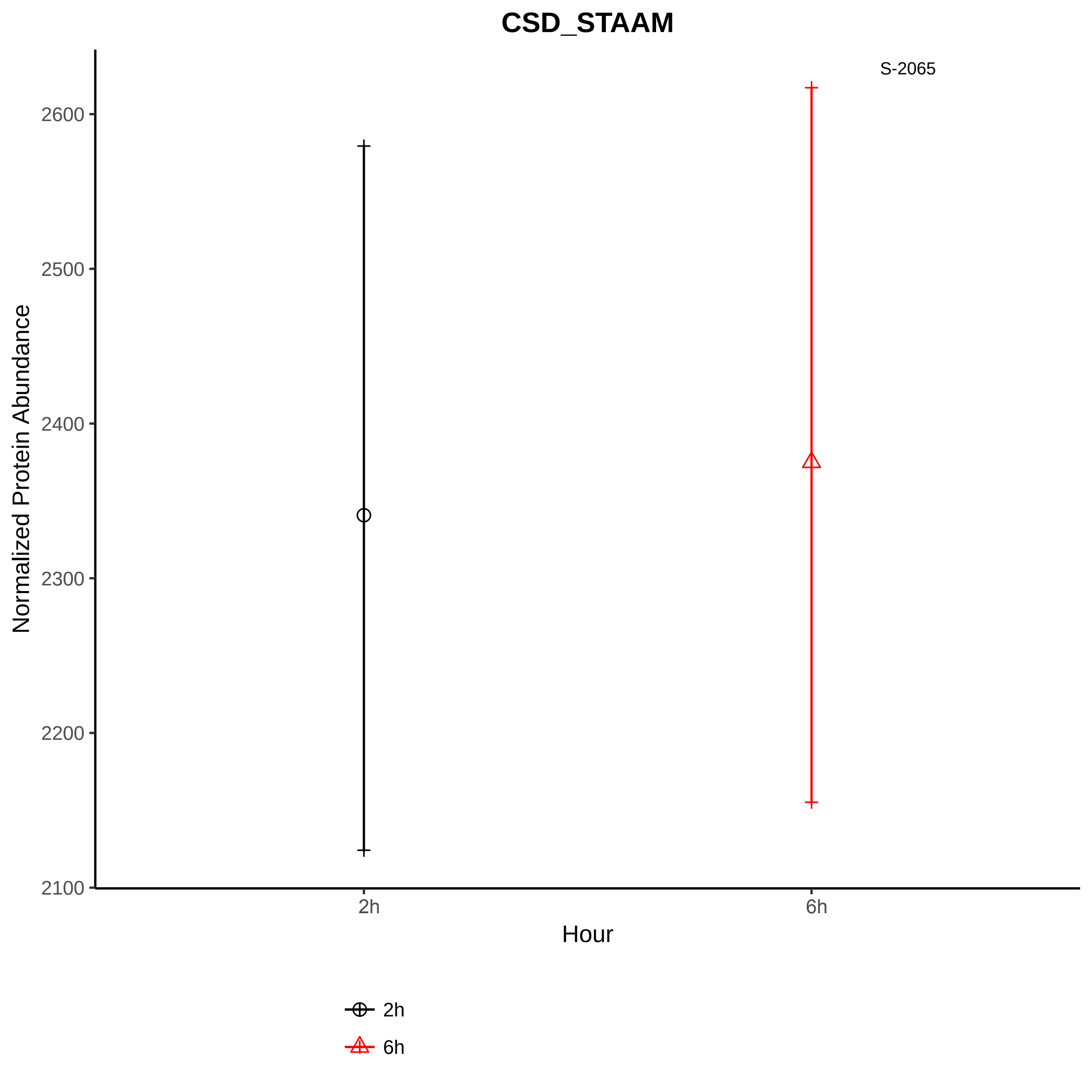

CSD_STAAM
S-2065
Normalized Protein Abundance
2600
2500
2400
2300
2200
2100
2h
6h
Hour
2h
6h

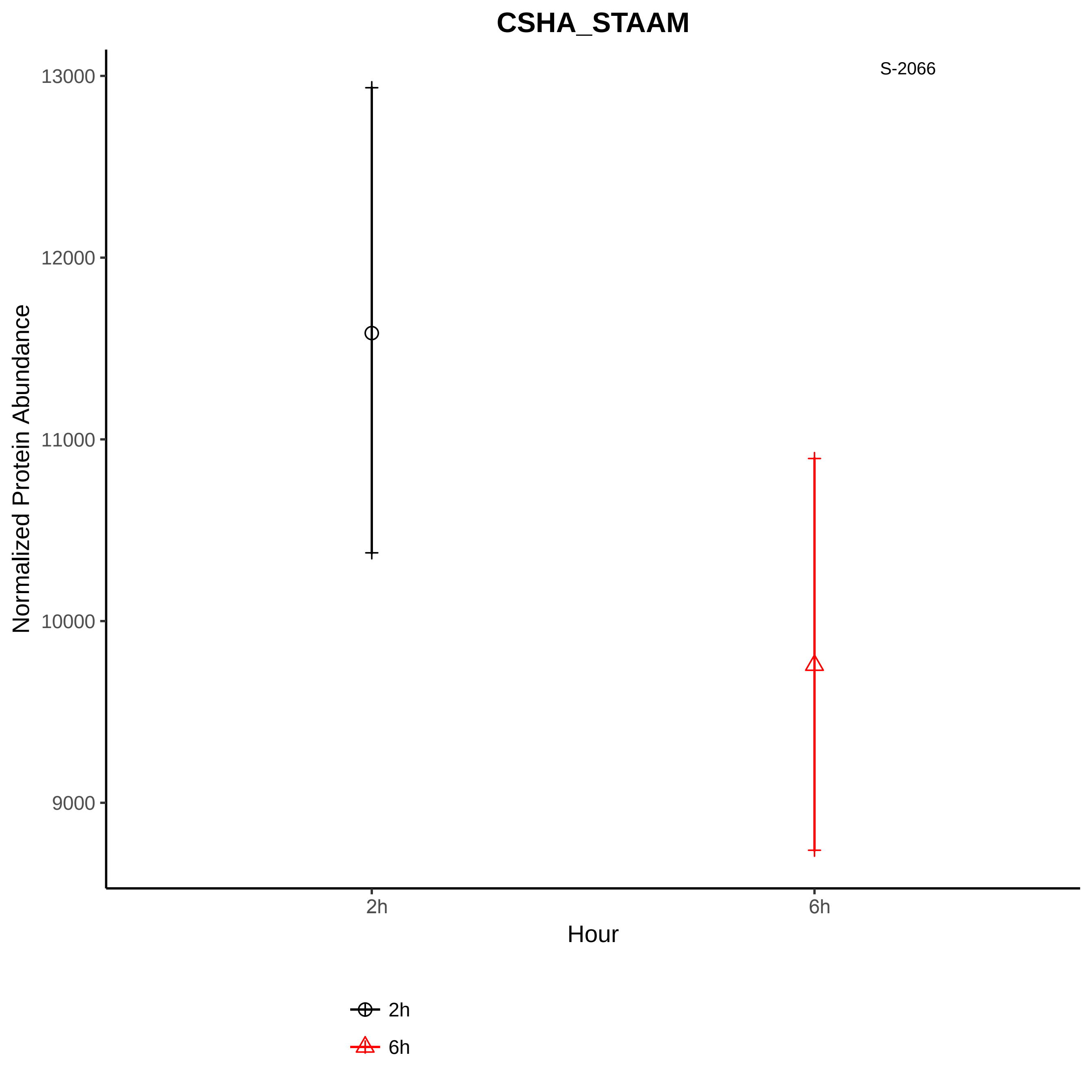

CSHA_STAAM
S-2066
Normalized Protein Abundance
13000
12000
11000
10000
9000
2h
6h
Hour
2h
6h

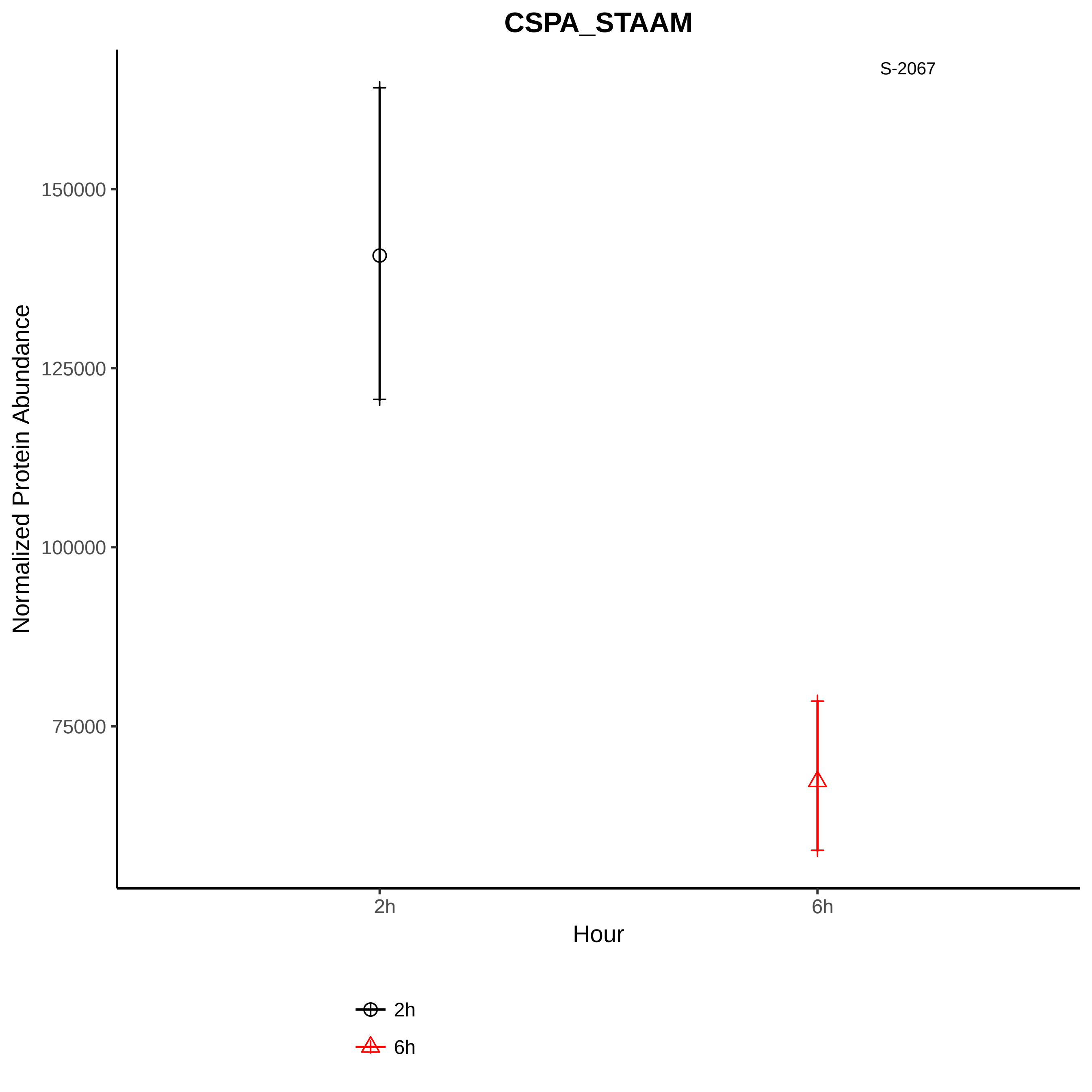
CSPA_STAAM
S-2067
Normalized Protein Abundance
150000
125000
100000
75000
2h
6h
Hour
2h
6h

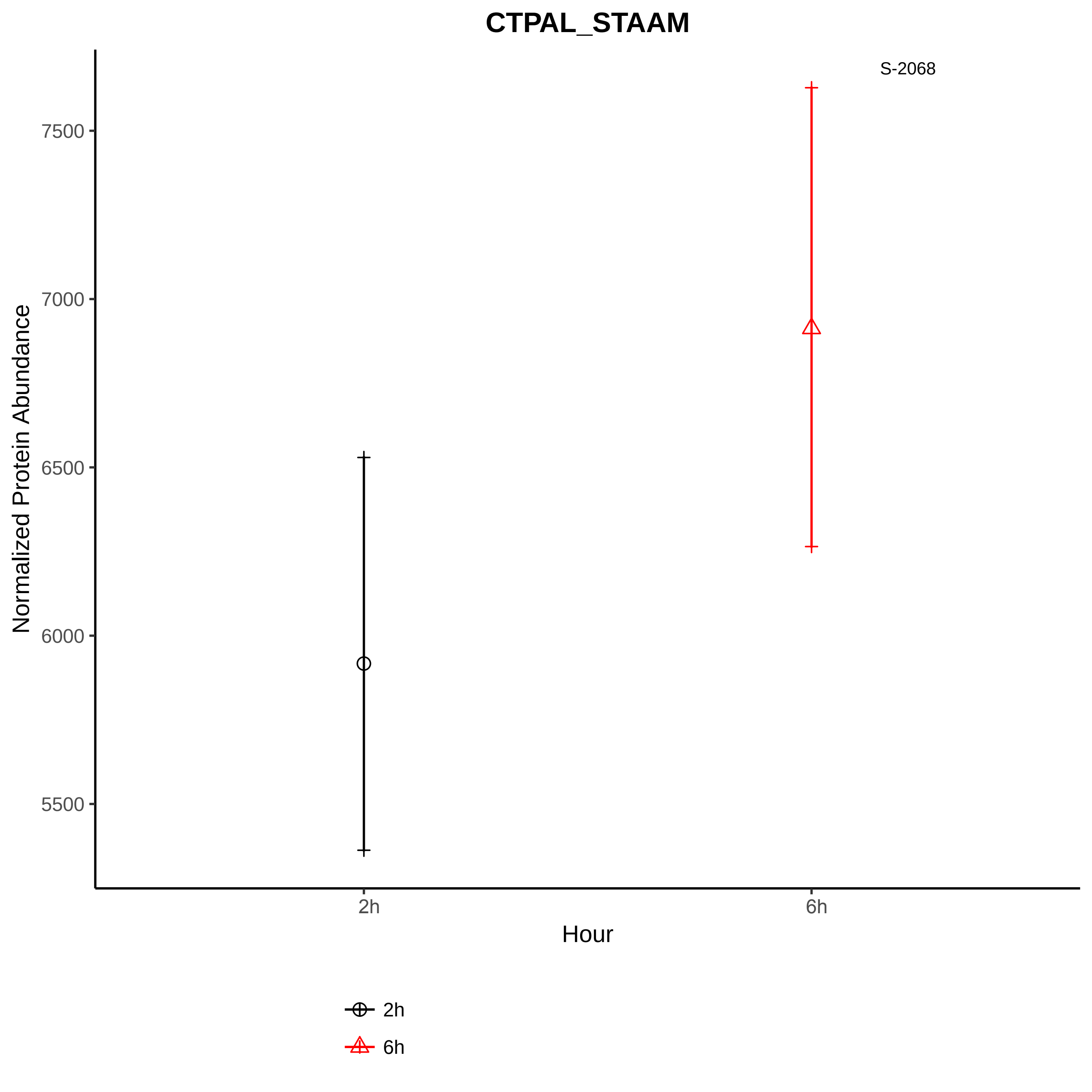

CTPAL_STAAM
S-2068
Normalized Protein Abundance
7500
7000
6500
6000
5500
2h
6h
Hour
2h
6h

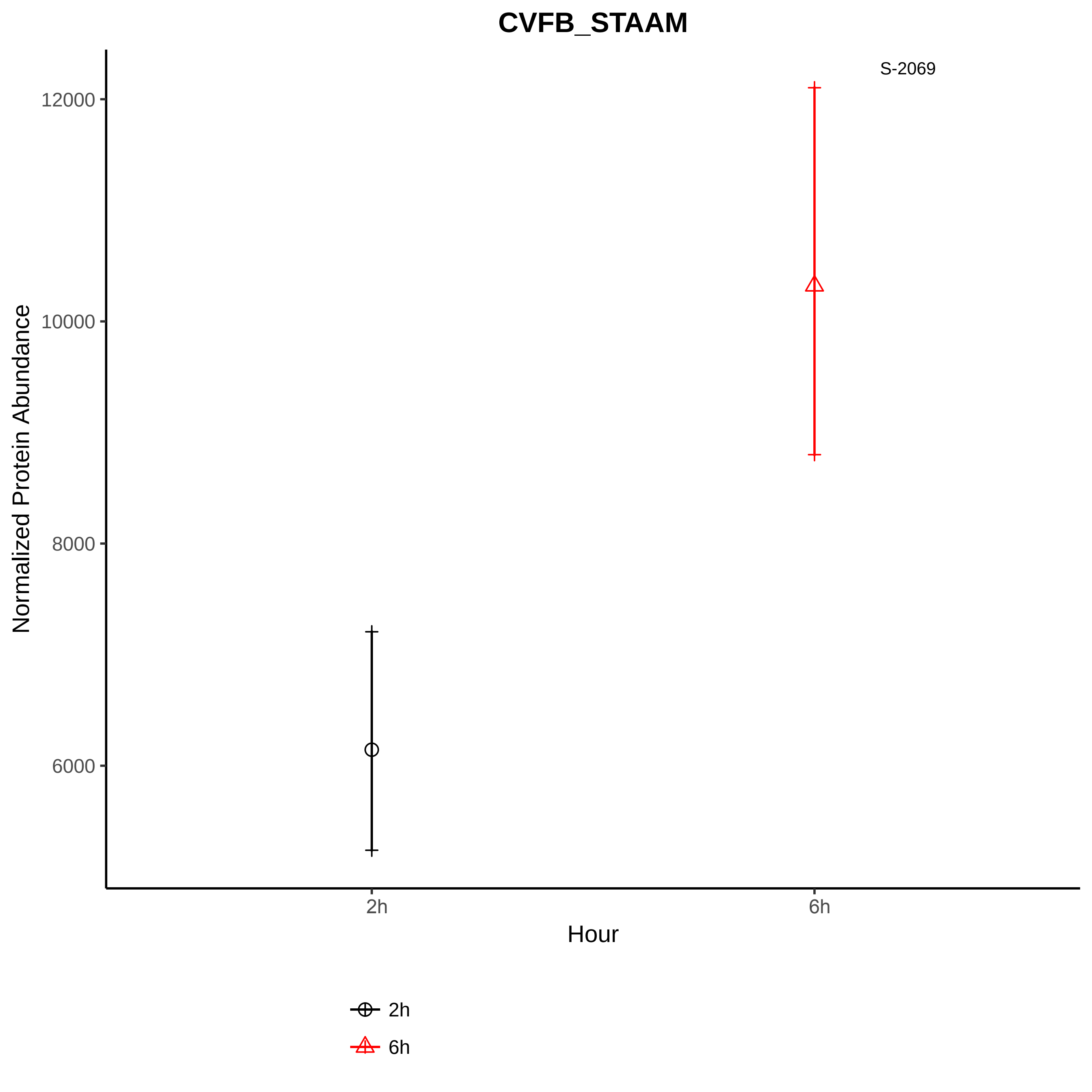
CVFB_STAAM
S-2069
Normalized Protein Abundance
12000
10000
8000
6000
2h
6h
Hour
2h
6h

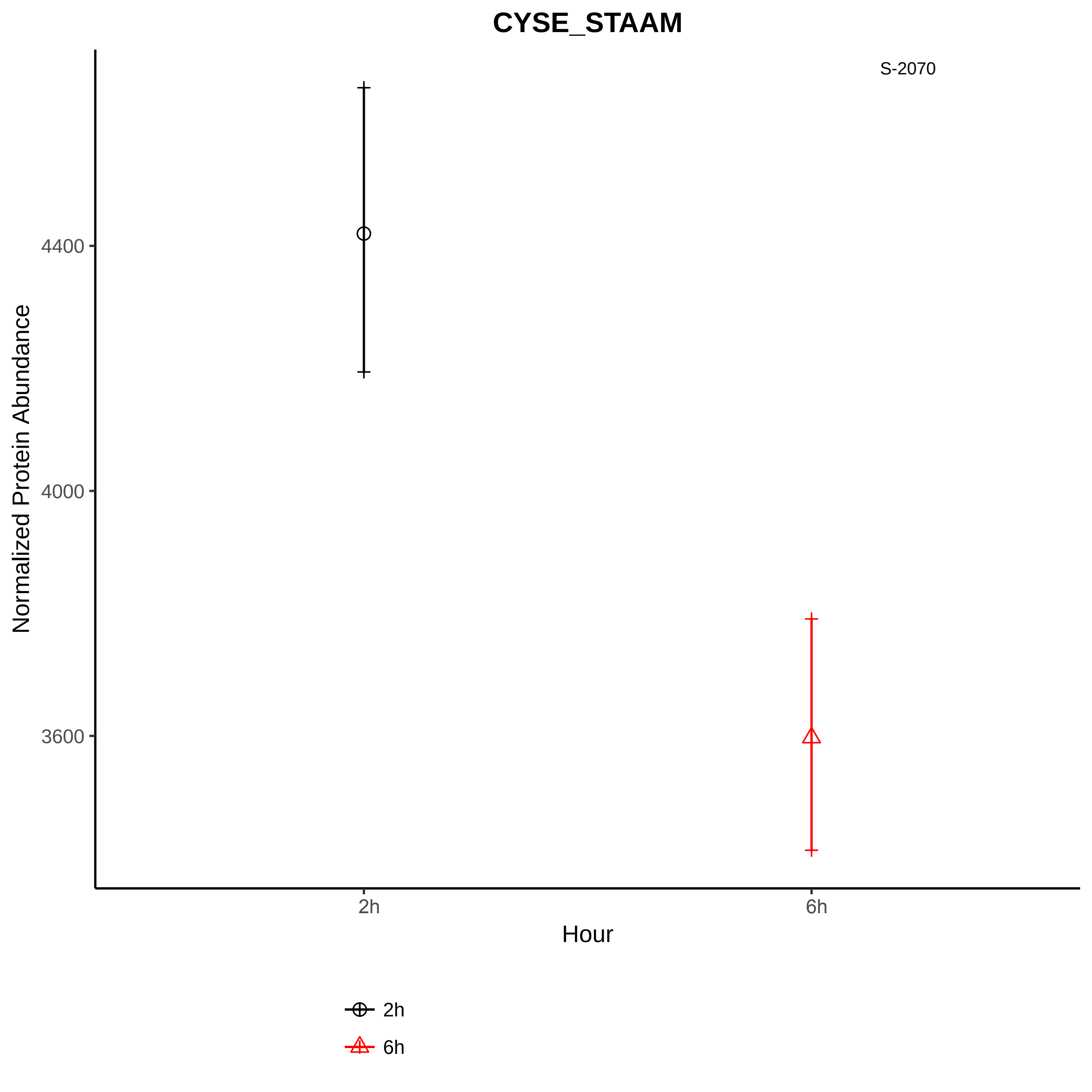
CYSE_STAAM
S-2070
Normalized Protein Abundance
4400
4000
3600
2h
6h
Hour
2h
6h

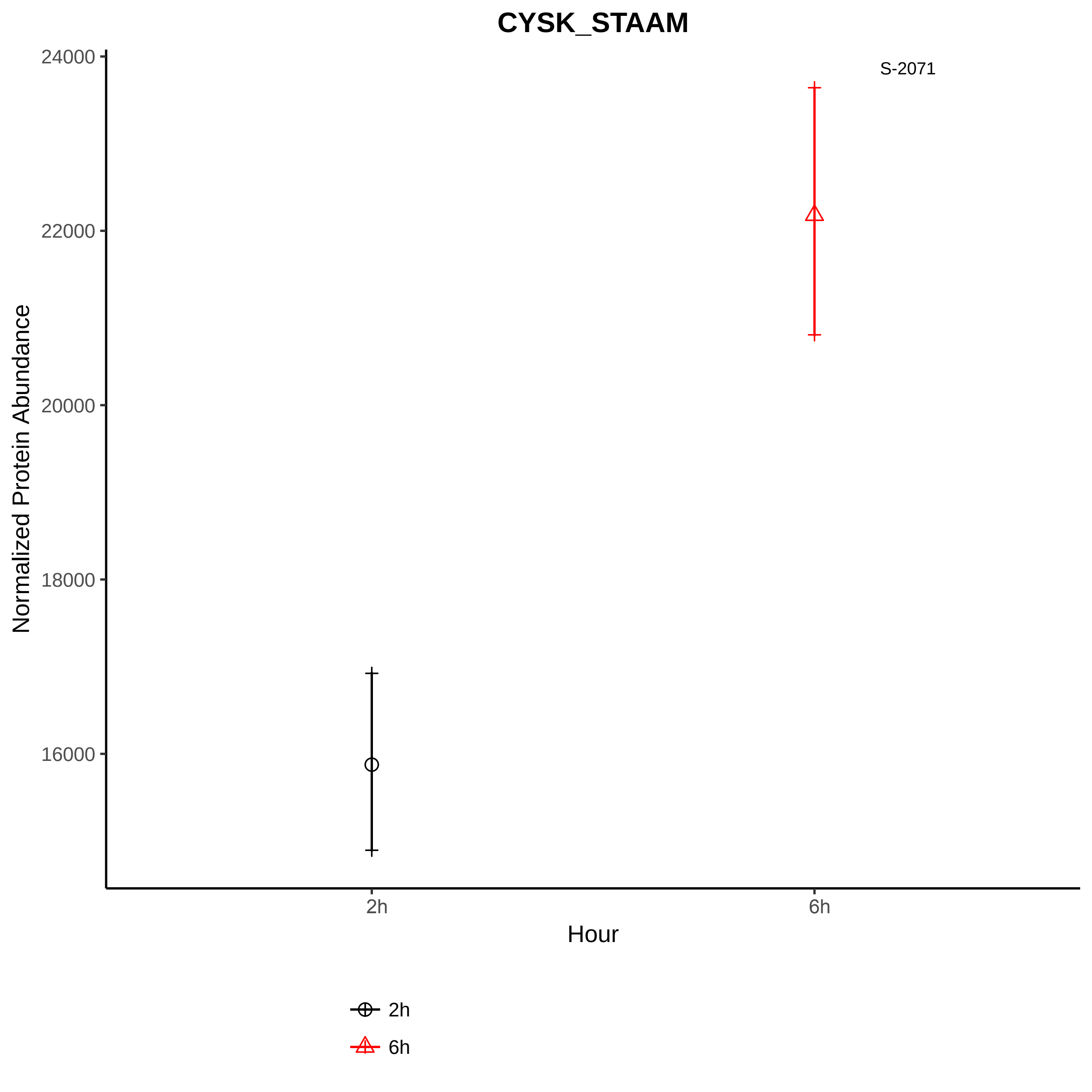

CYSK_STAAM
S-2071
24000
22000
20000
18000
16000
Normalized Protein Abundance
2h
6h
Hour
2h
6h

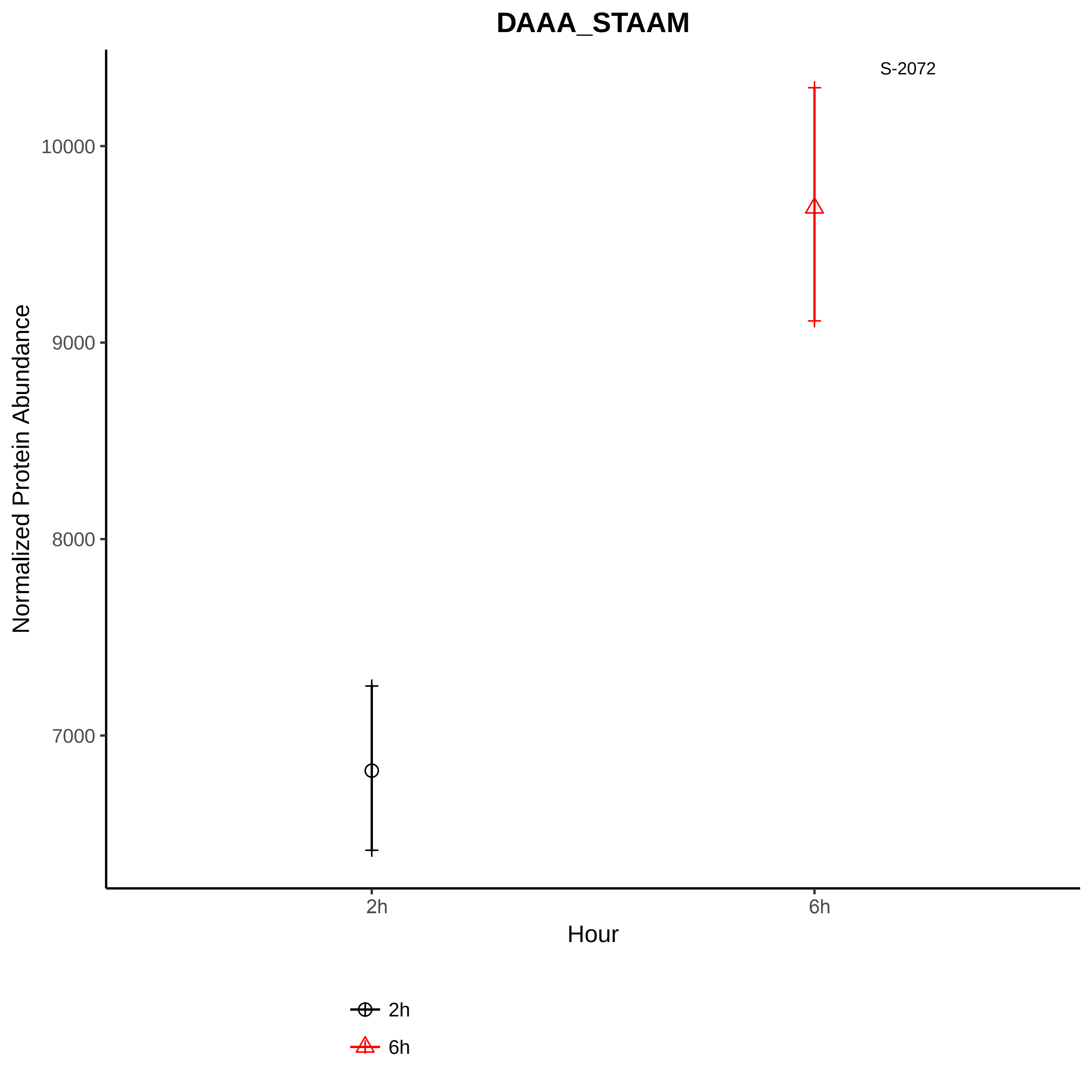
DAAA_STAAM
S-2072
Normalized Protein Abundance
10000
9000
8000
7000
2h
6h
Hour
2h
6h

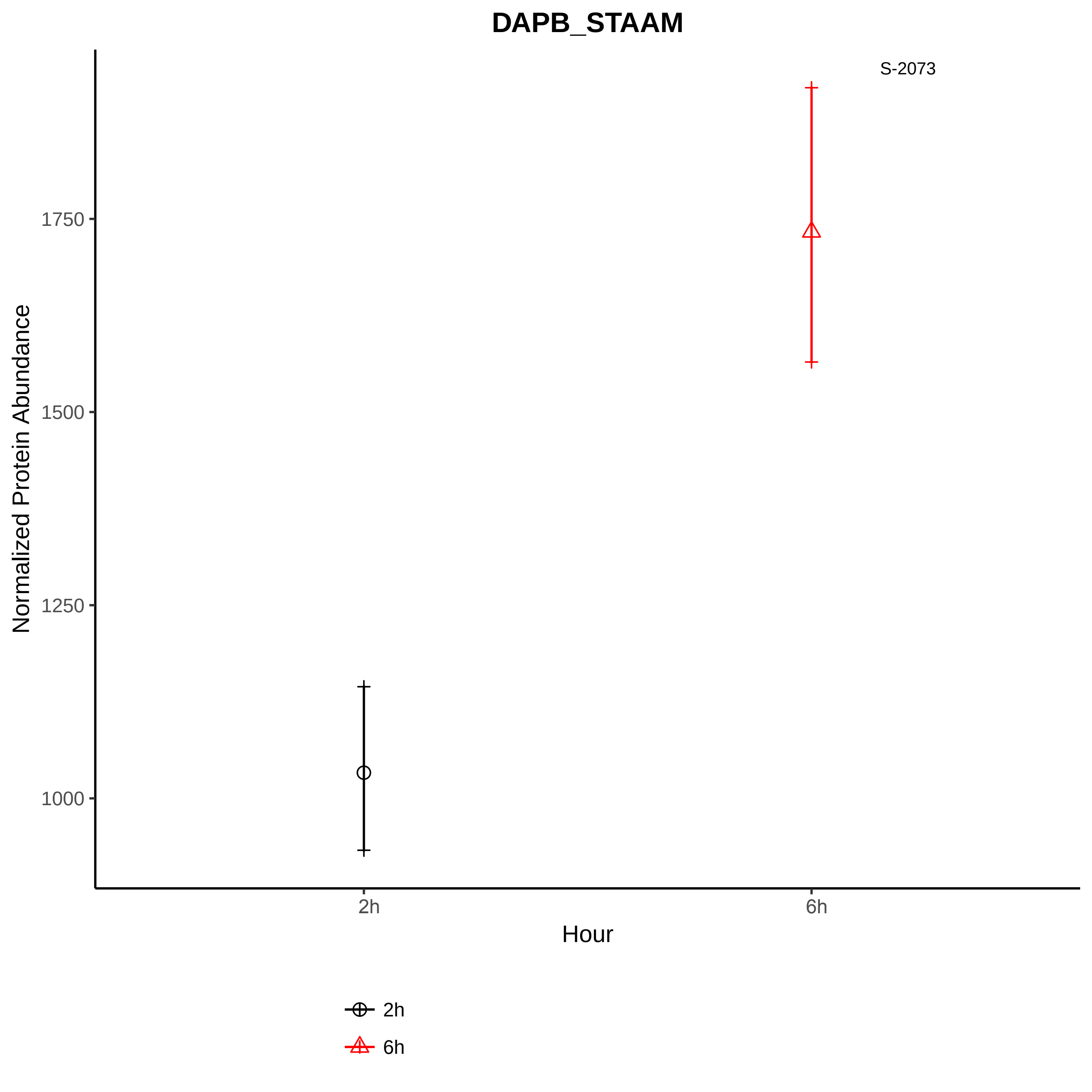

DAPB_STAAM
S-2073
Normalized Protein Abundance
1750
1500
1250
1000
2h
6h
Hour
2h
6h

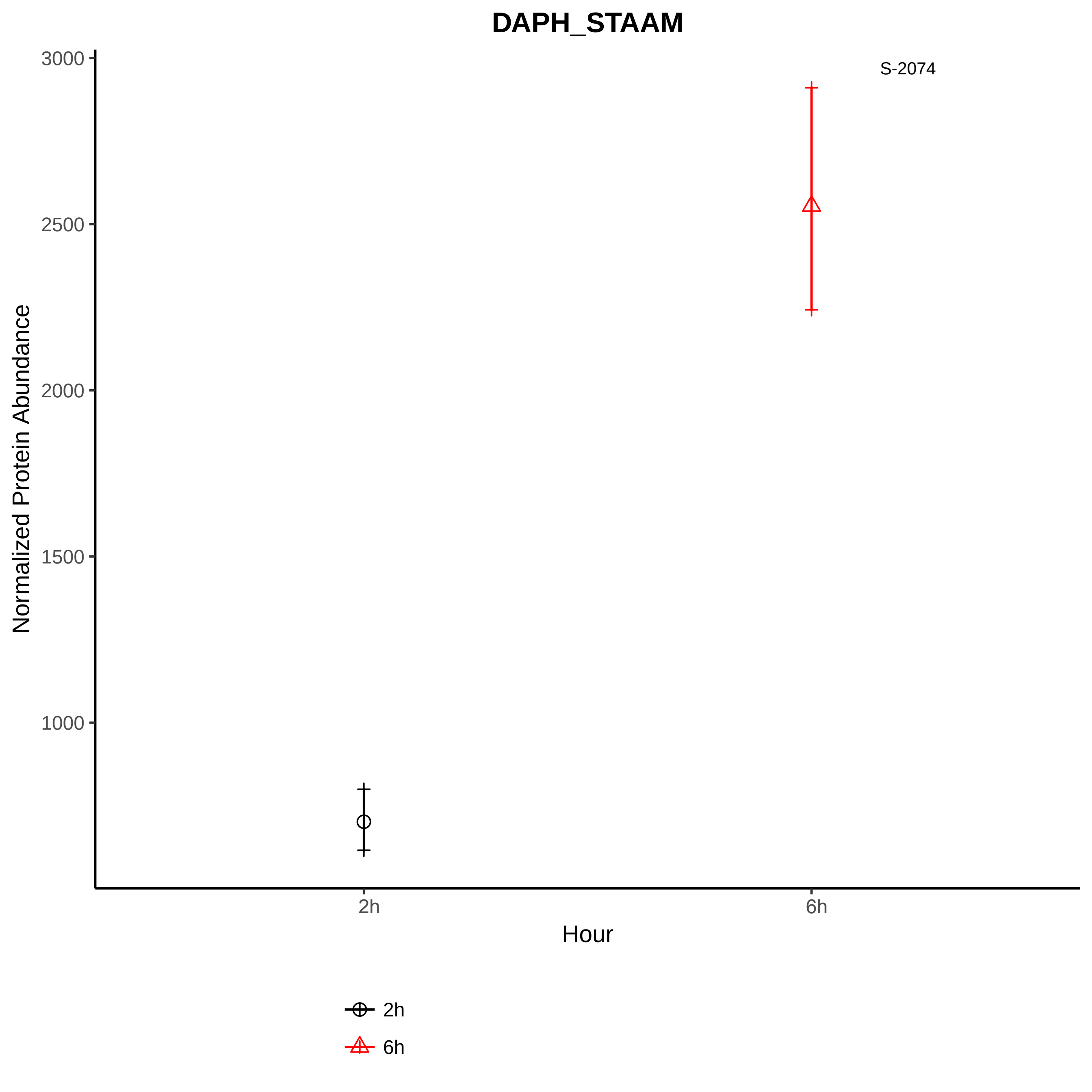
DAPH_STAAM
S-2074
3000
2500
2000
1500
1000
Normalized Protein Abundance
2h
6h
Hour
2h
6h

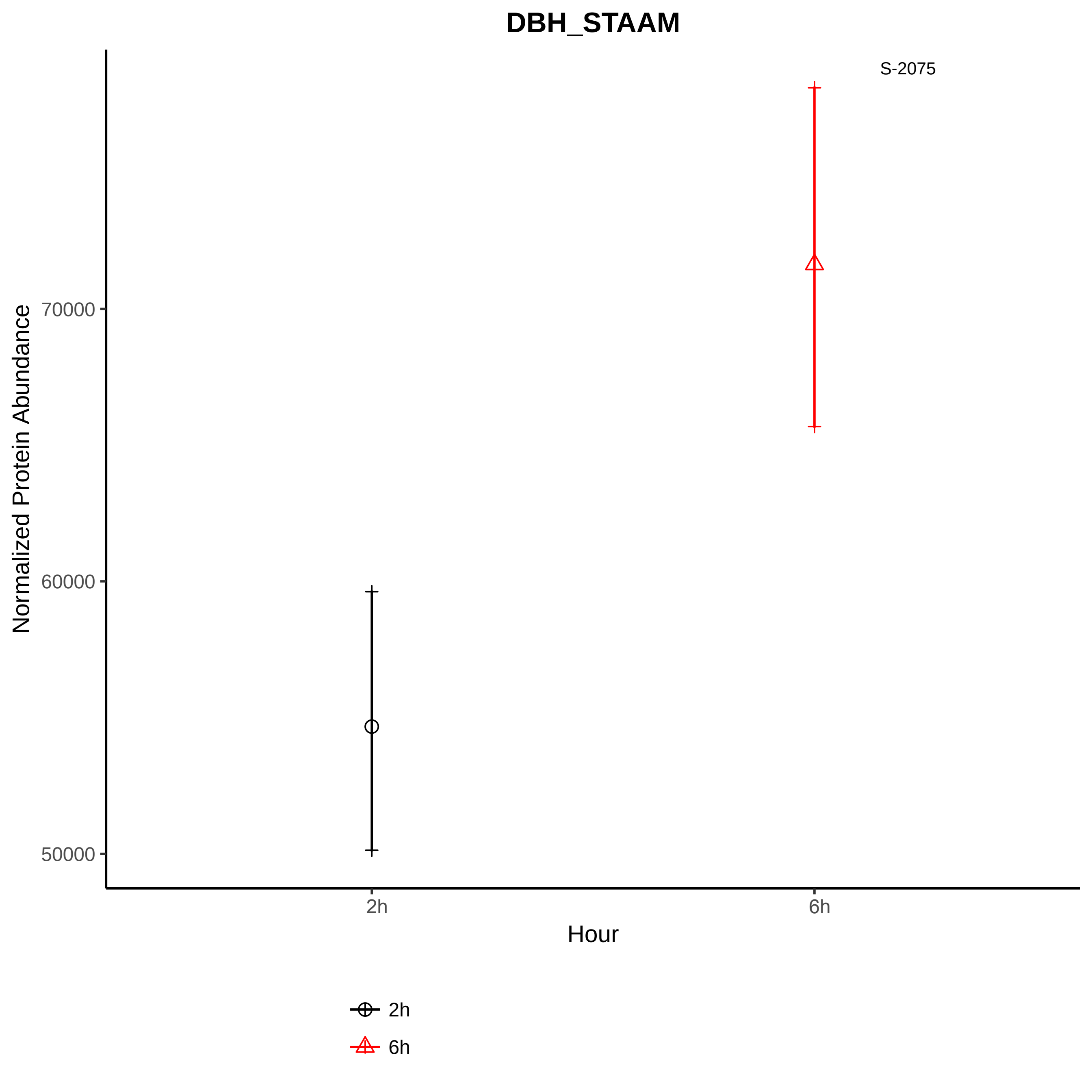
DBH_STAAM
S-2075
Normalized Protein Abundance
70000
60000
50000
2h
6h
Hour
2h
6h

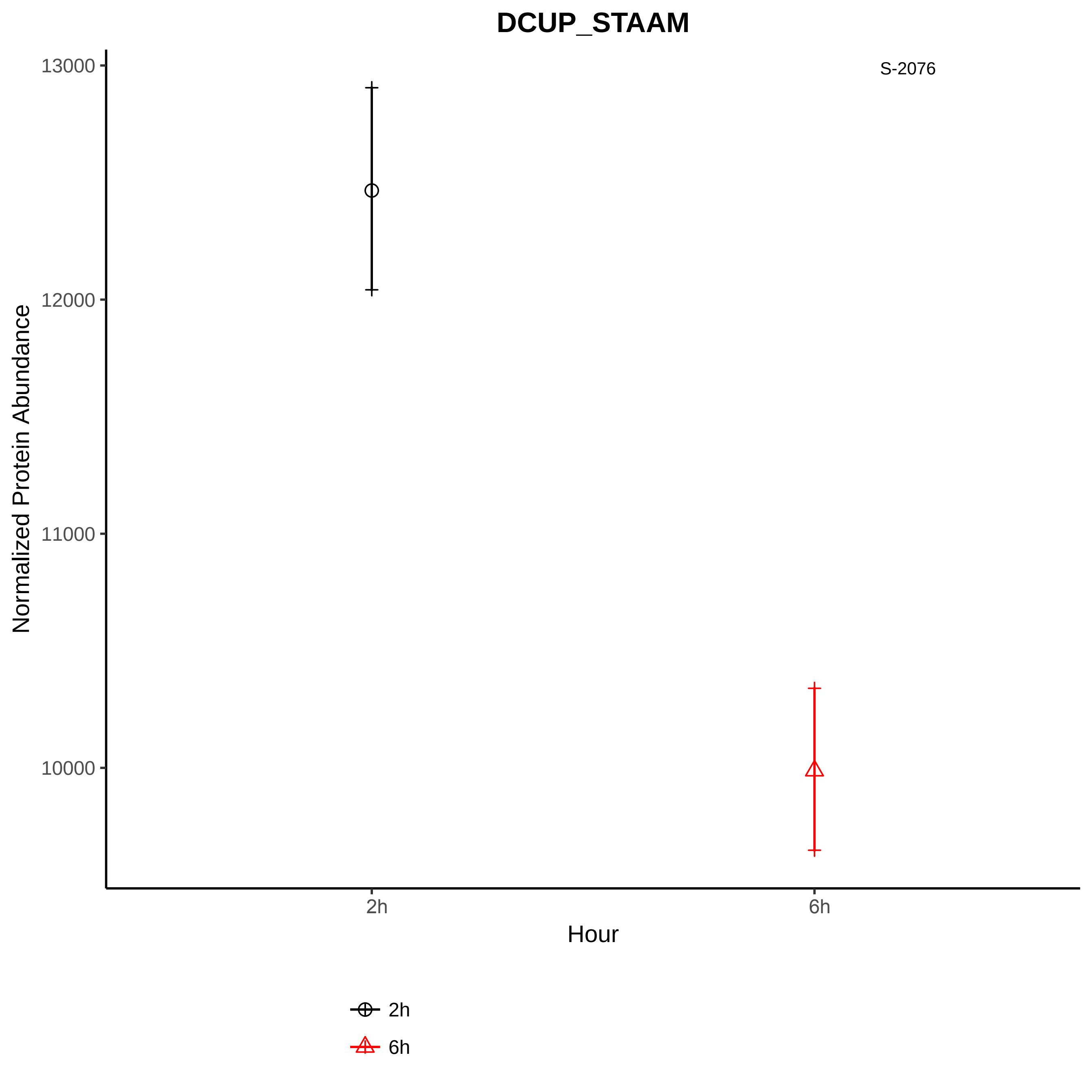

DCUP_STAAM
S-2076
Normalized Protein Abundance
13000
12000
11000
10000
2h
6h
Hour
2h
6h

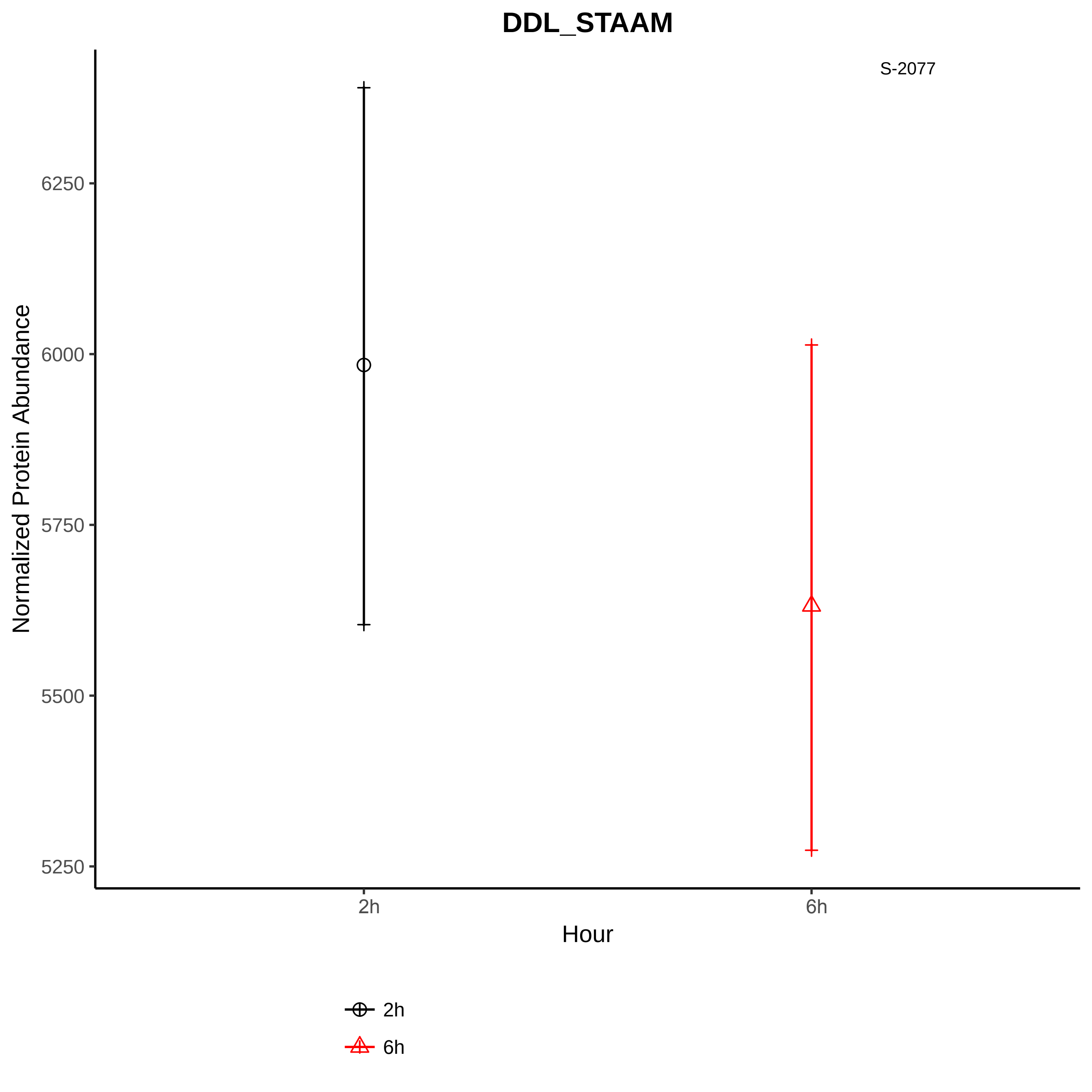
DDL_STAAM
S-2077
Normalized Protein Abundance
6250
6000
5750
5500
5250
2h
6h
Hour
2h
6h

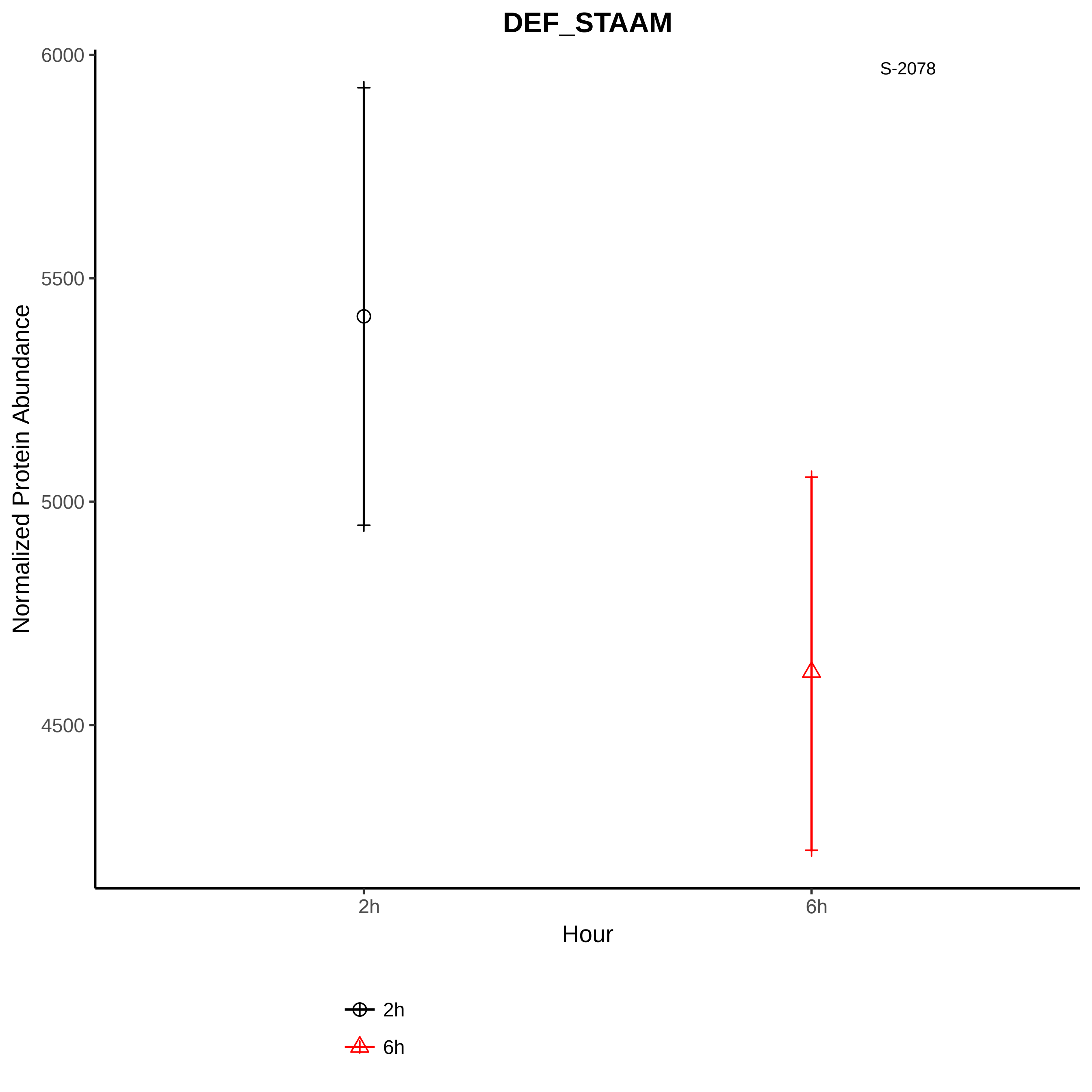
DEF_STAAM
S-2078
Normalized Protein Abundance
6000
5500
5000
4500
2h
6h
Hour
2h
6h

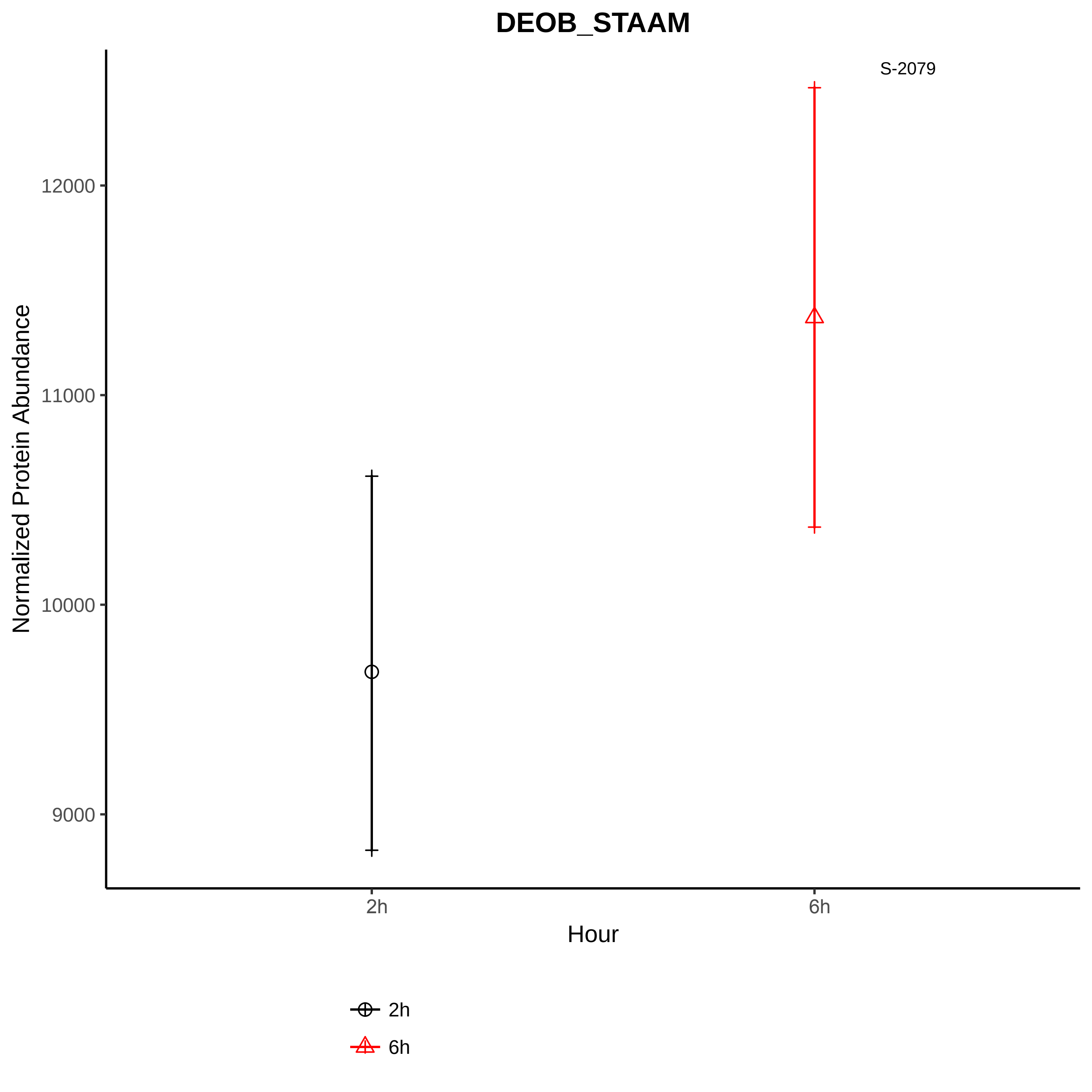

DEOB_STAAM
S-2079
Normalized Protein Abundance
12000
11000
10000
9000
2h
6h
Hour
2h
6h

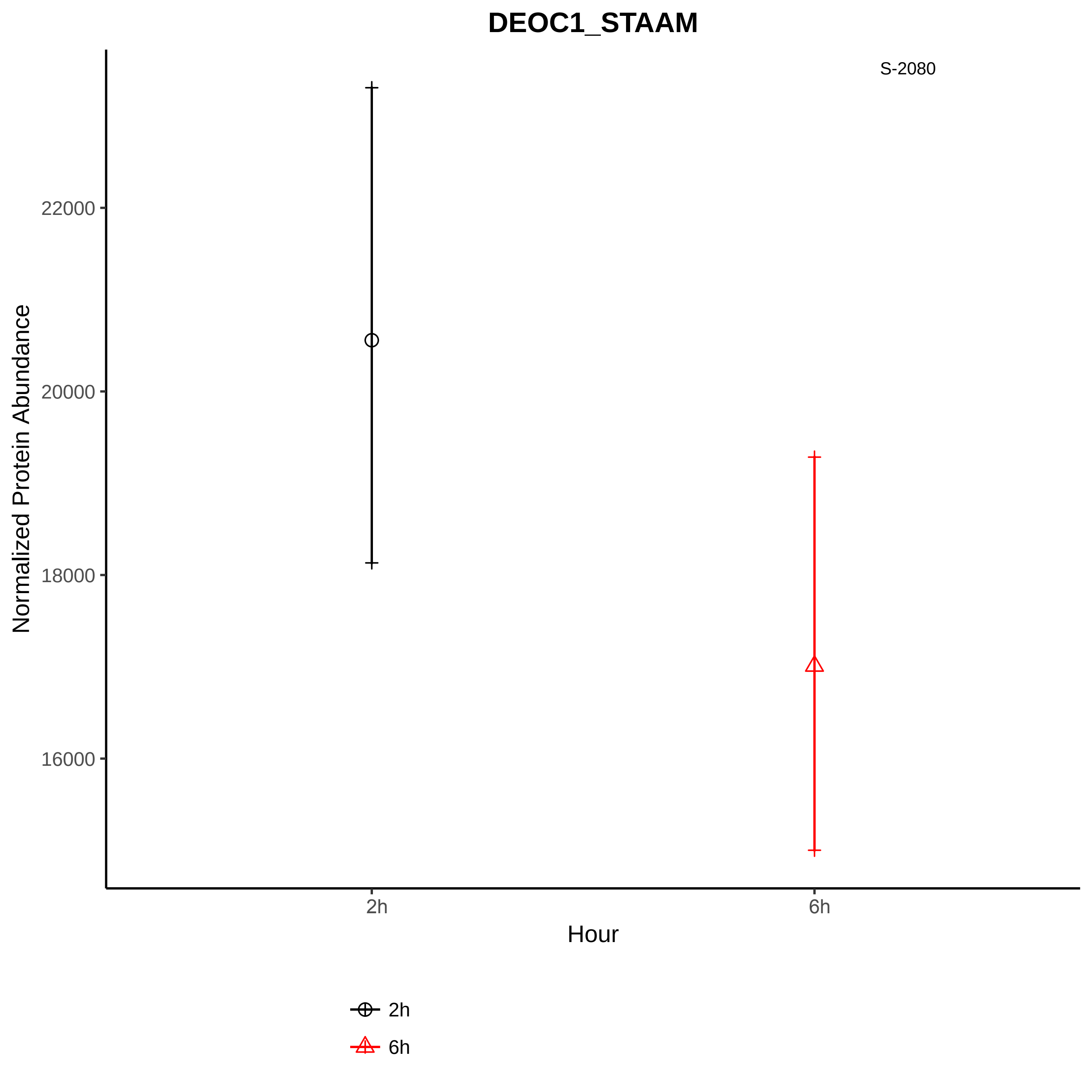
DEOC1_STAAM
S-2080
Normalized Protein Abundance
22000
20000
18000
16000
2h
6h
Hour
2h
6h

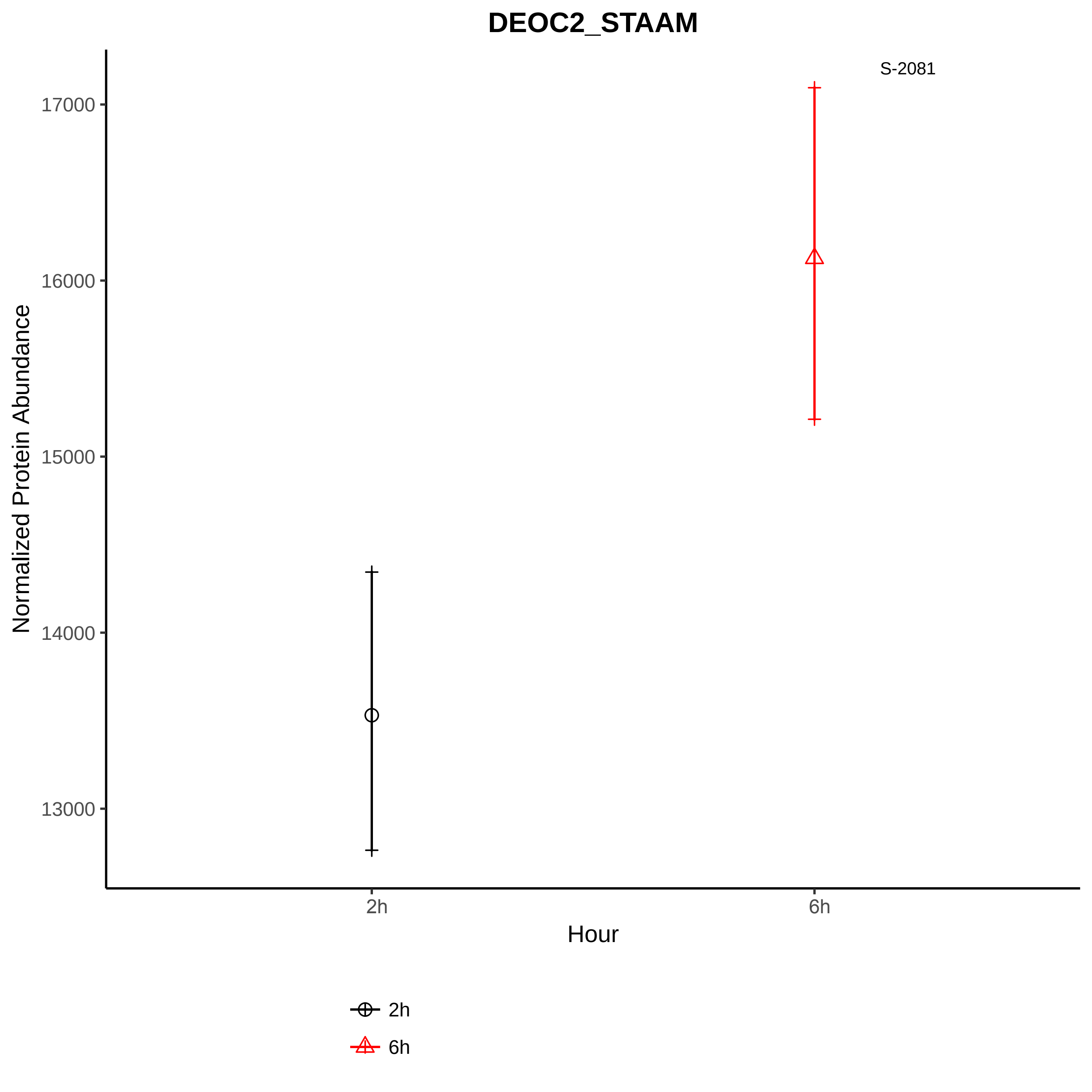

DEOC2_STAAM
S-2081
Normalized Protein Abundance
17000
16000
15000
14000
13000
2h
6h
Hour
2h
6h

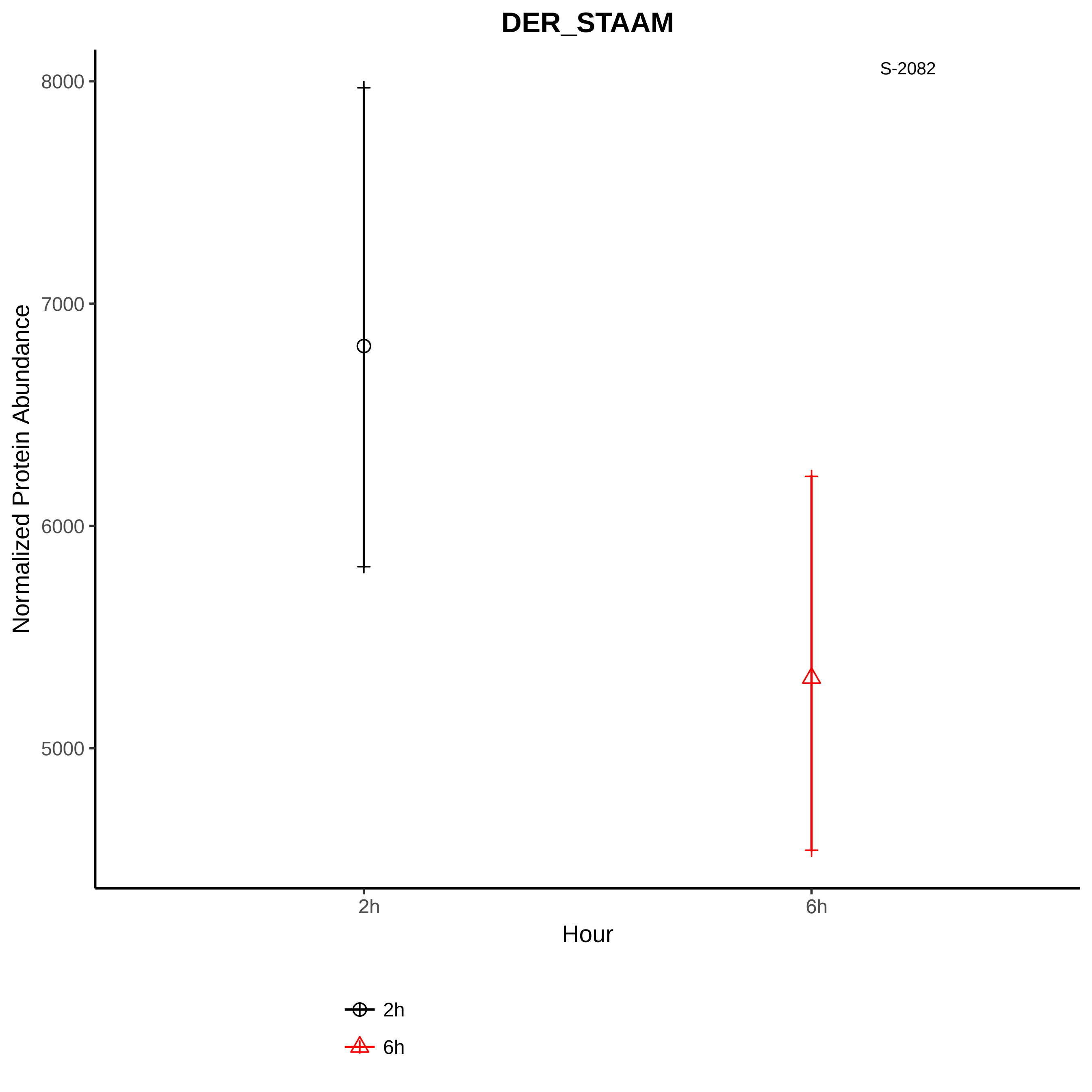

DER_STAAM
S-2082
8000
7000
6000
5000
Normalized Protein Abundance
2h
6h
Hour
2h
6h

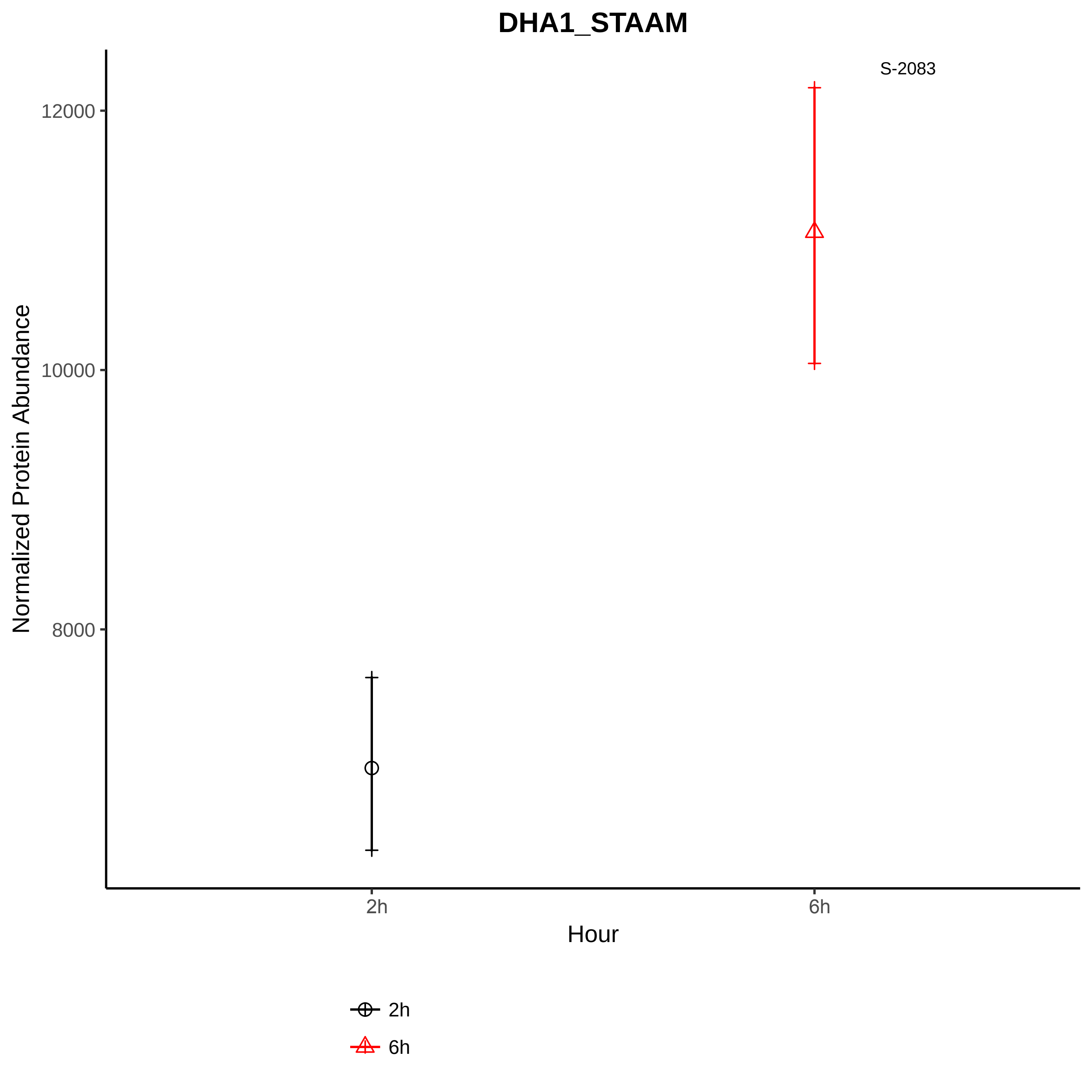
DHA1_STAAM
S-2083
Normalized Protein Abundance
12000
10000
8000
2h
6h
Hour
2h
6h

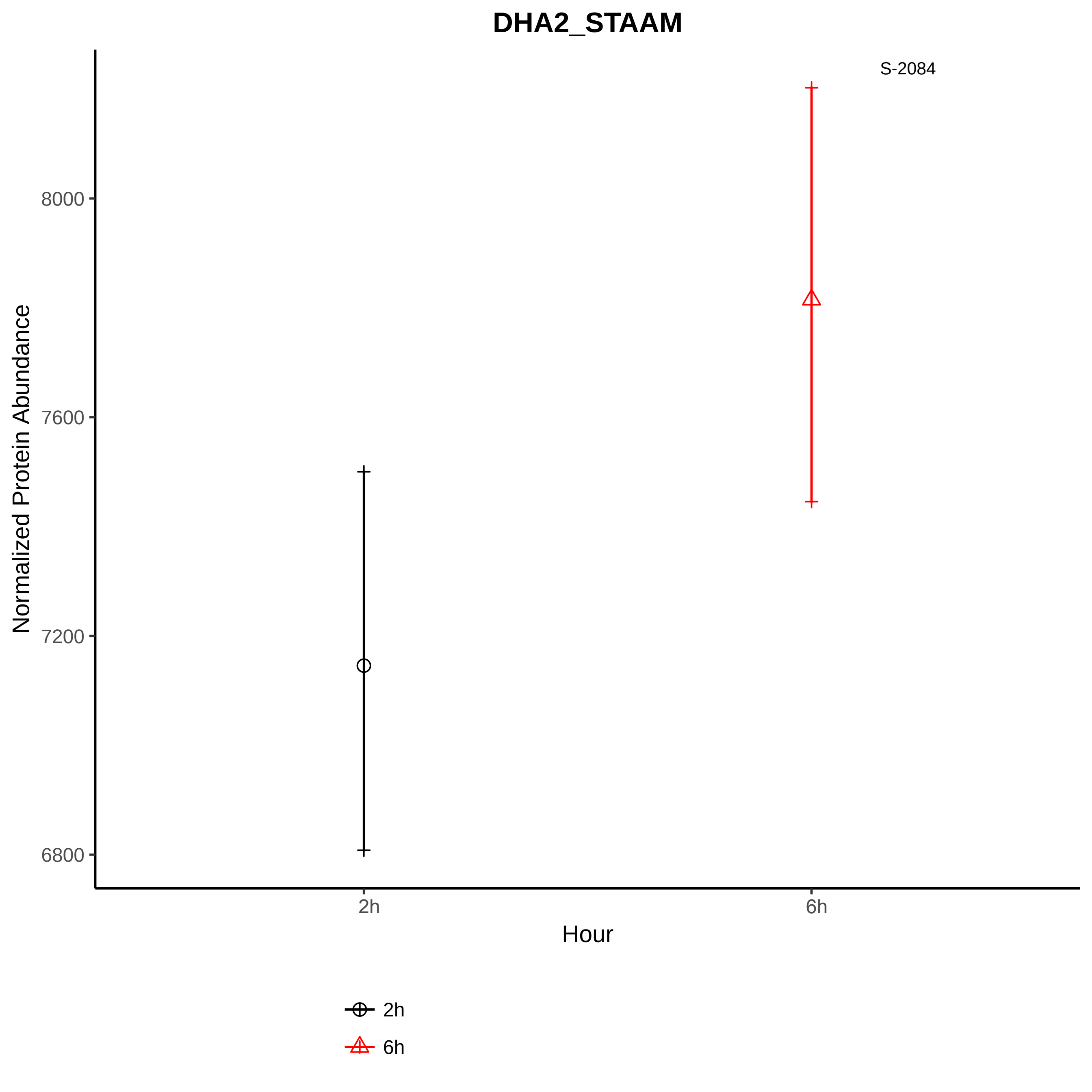

DHA2_STAAM
S-2084
Normalized Protein Abundance
8000
7600
7200
6800
2h
6h
Hour
2h
6h

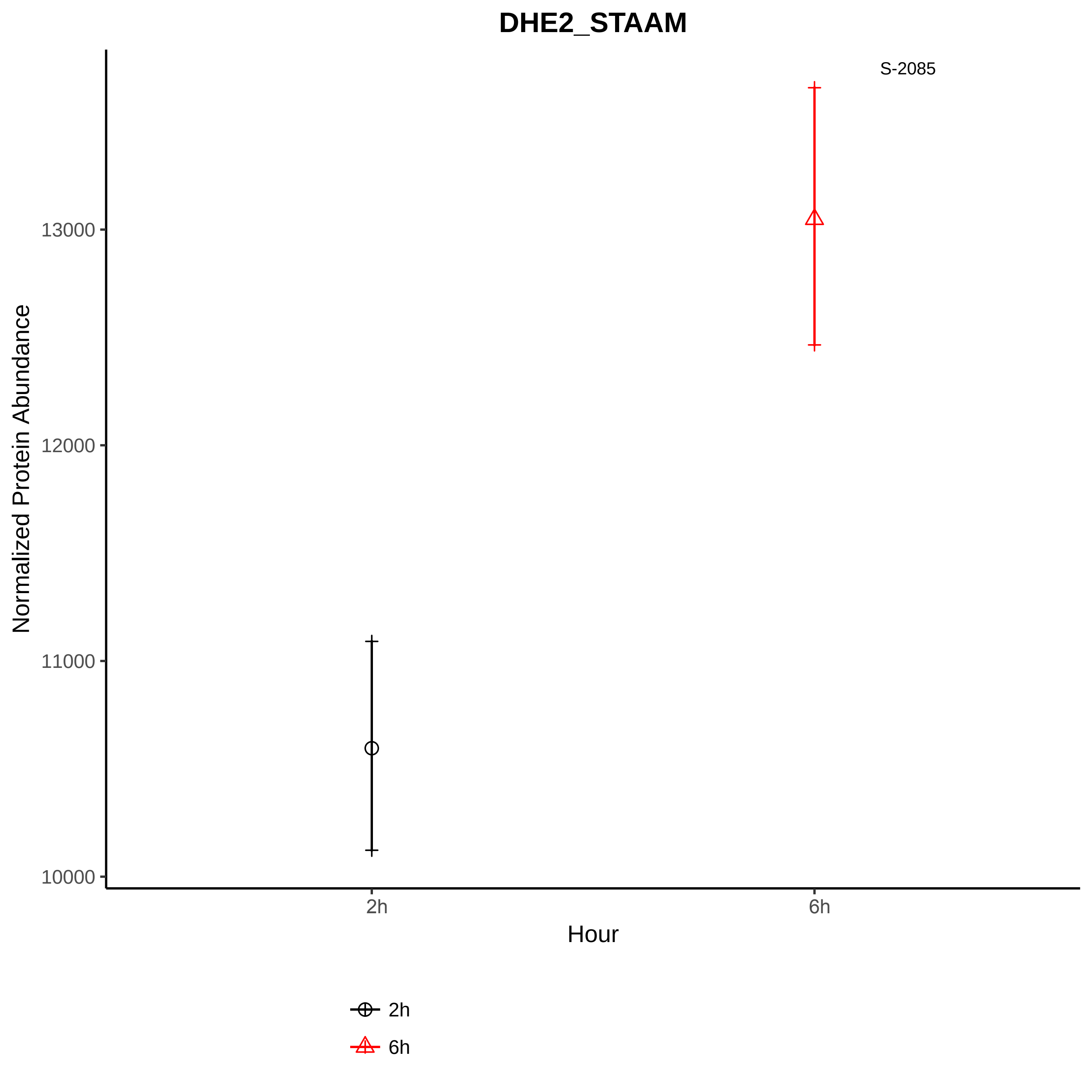
DHE2_STAAM
S-2085
Normalized Protein Abundance
13000
12000
11000
10000
2h
6h
Hour
2h
6h

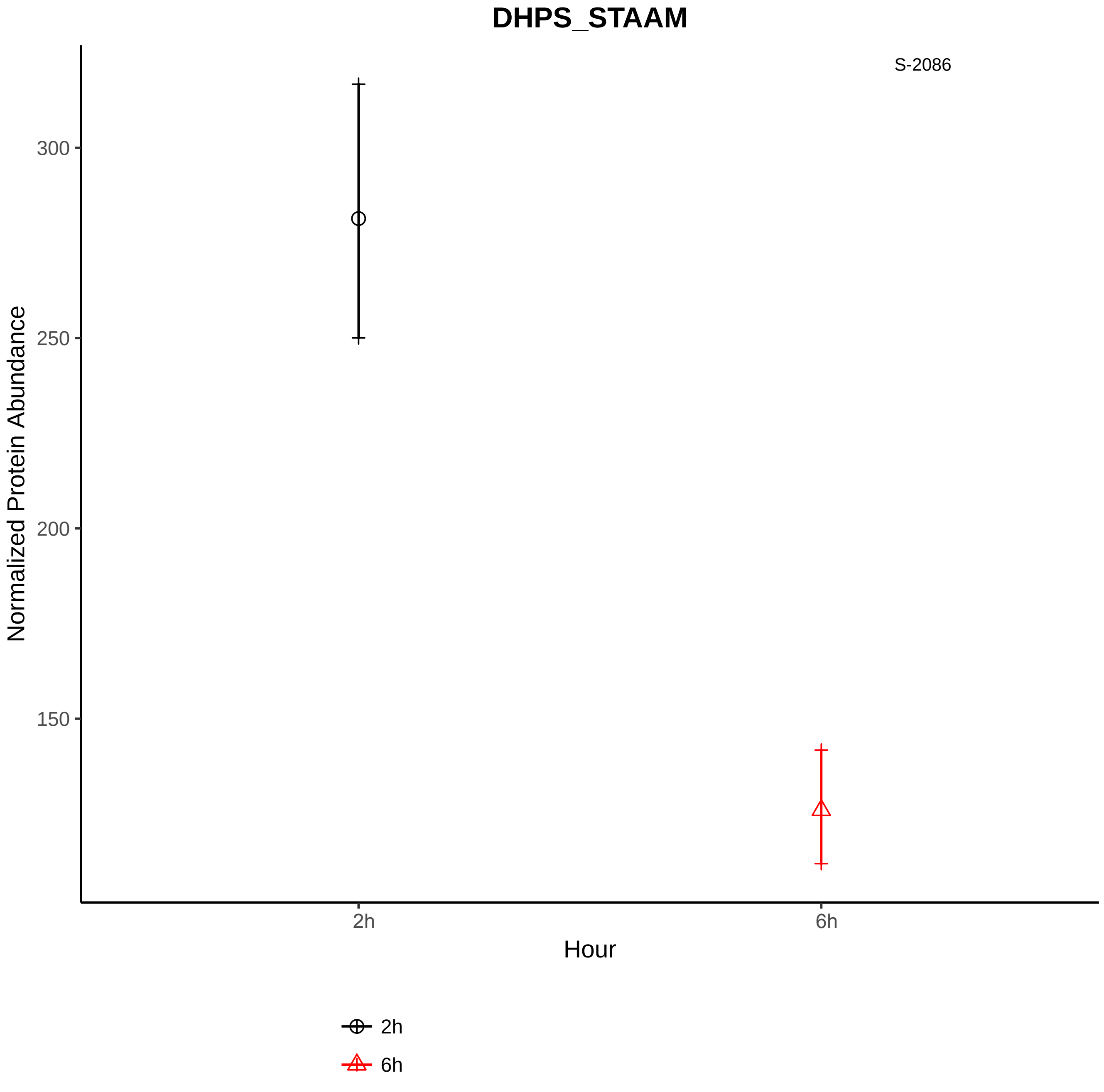
DHPS_STAAM
S-2086
Normalized Protein Abundance
300
250
200
150
2h
6h
Hour
2h
6h

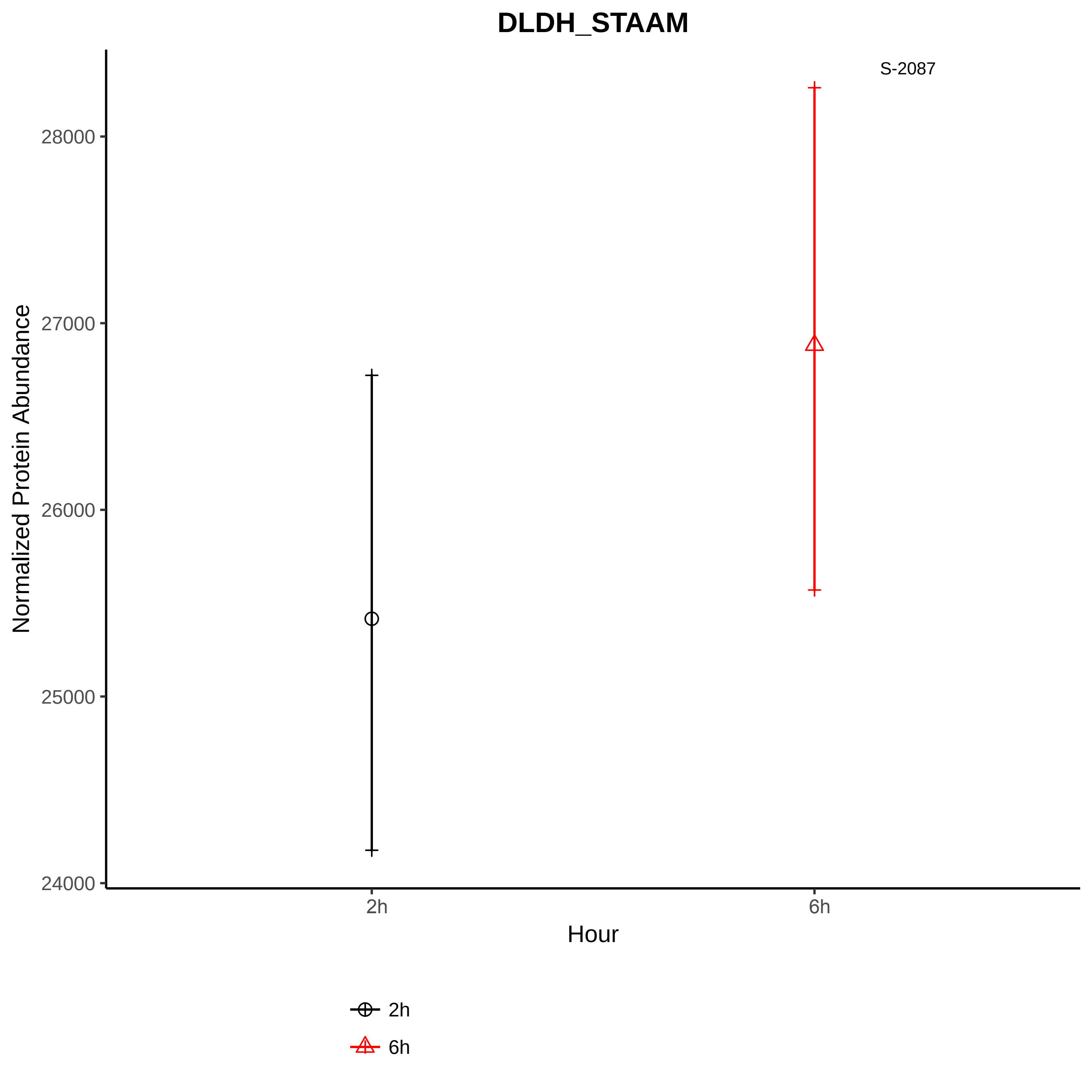

DLDH_STAAM
S-2087
Normalized Protein Abundance
28000
27000
26000
25000
24000
2h
6h
Hour
2h
6h

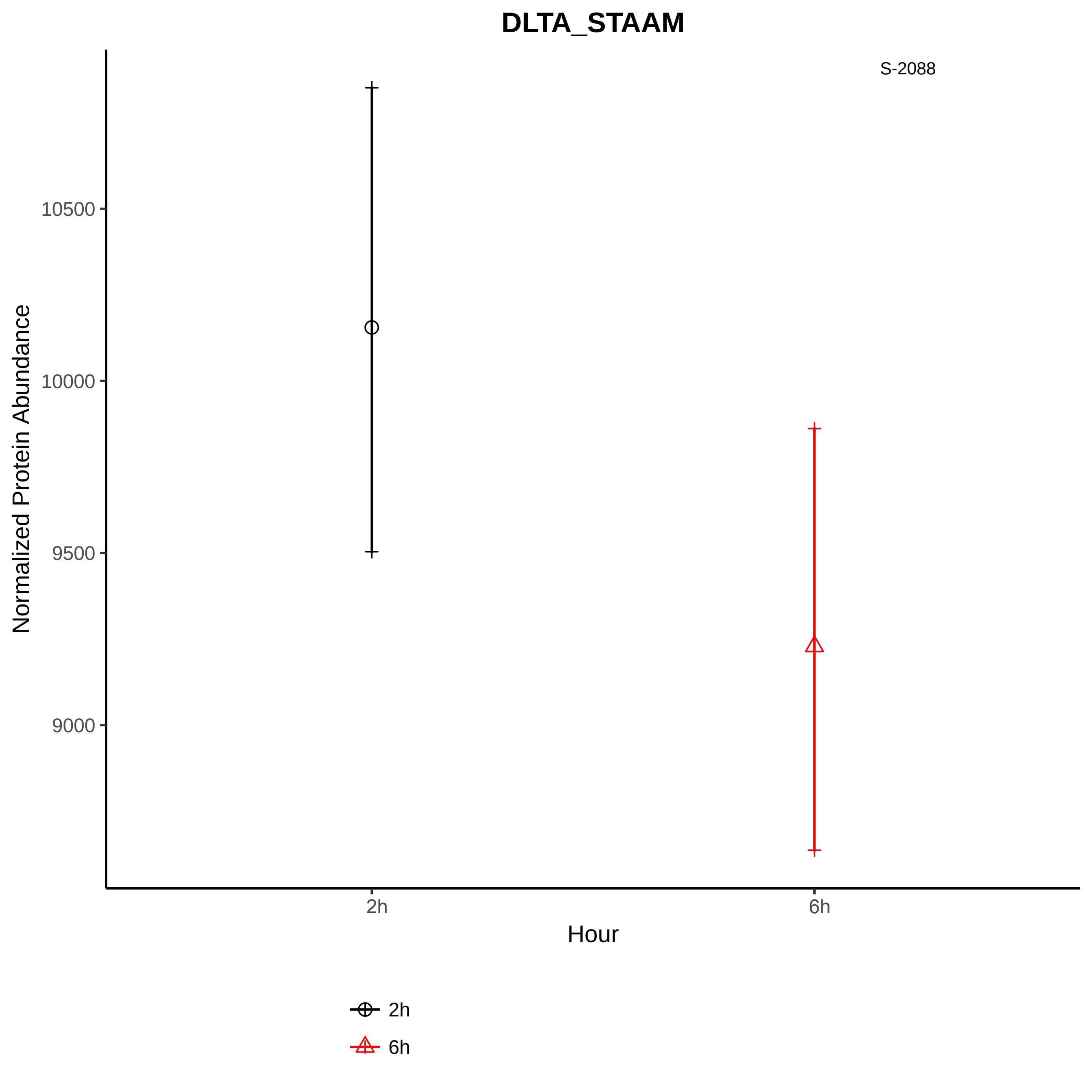
DLTA_STAAM
S-2088
Normalized Protein Abundance
10500
10000
9500
9000
2h
6h
Hour
2h
6h

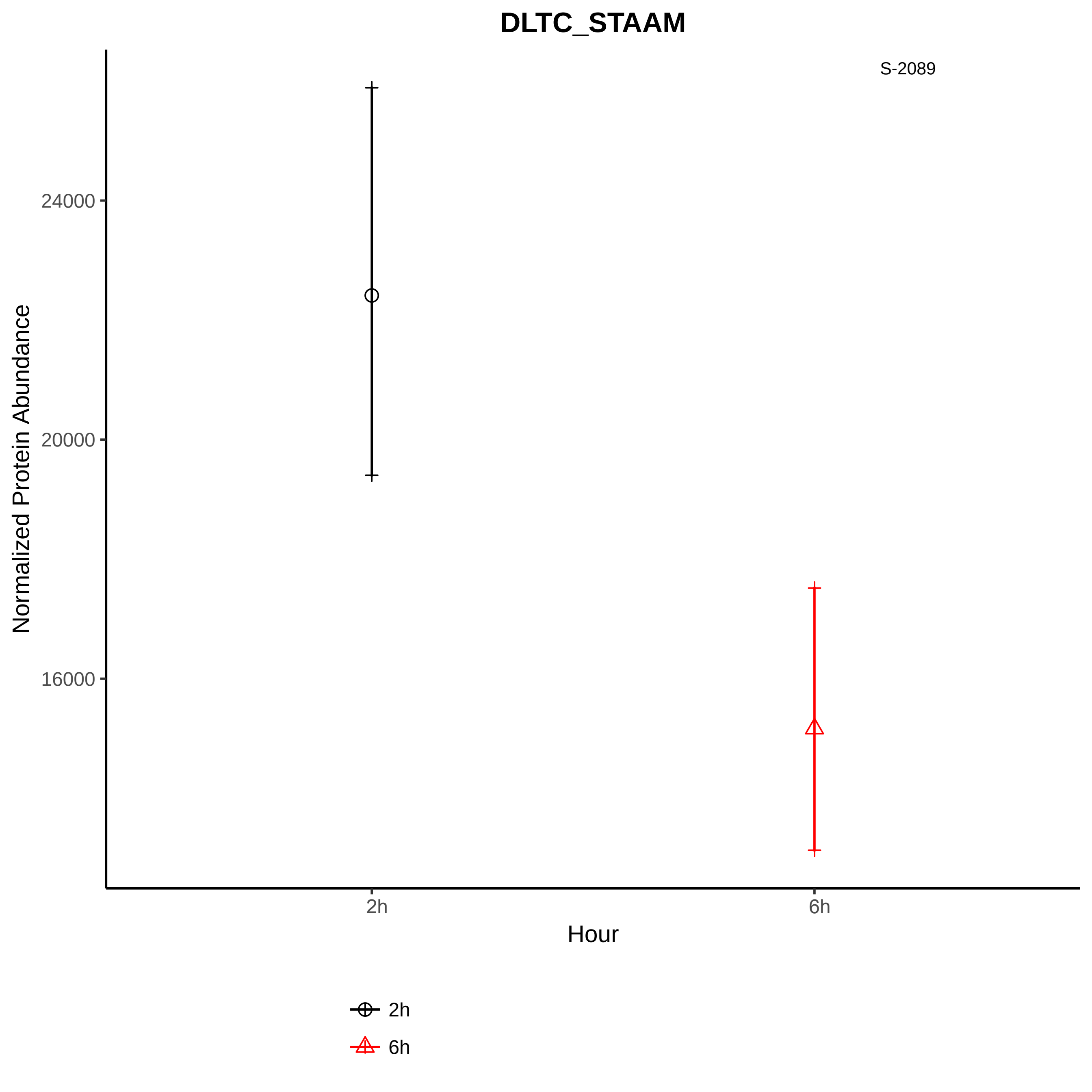

DLTC_STAAM
S-2089
24000
20000
16000
Normalized Protein Abundance
2h
6h
Hour
2h
6h

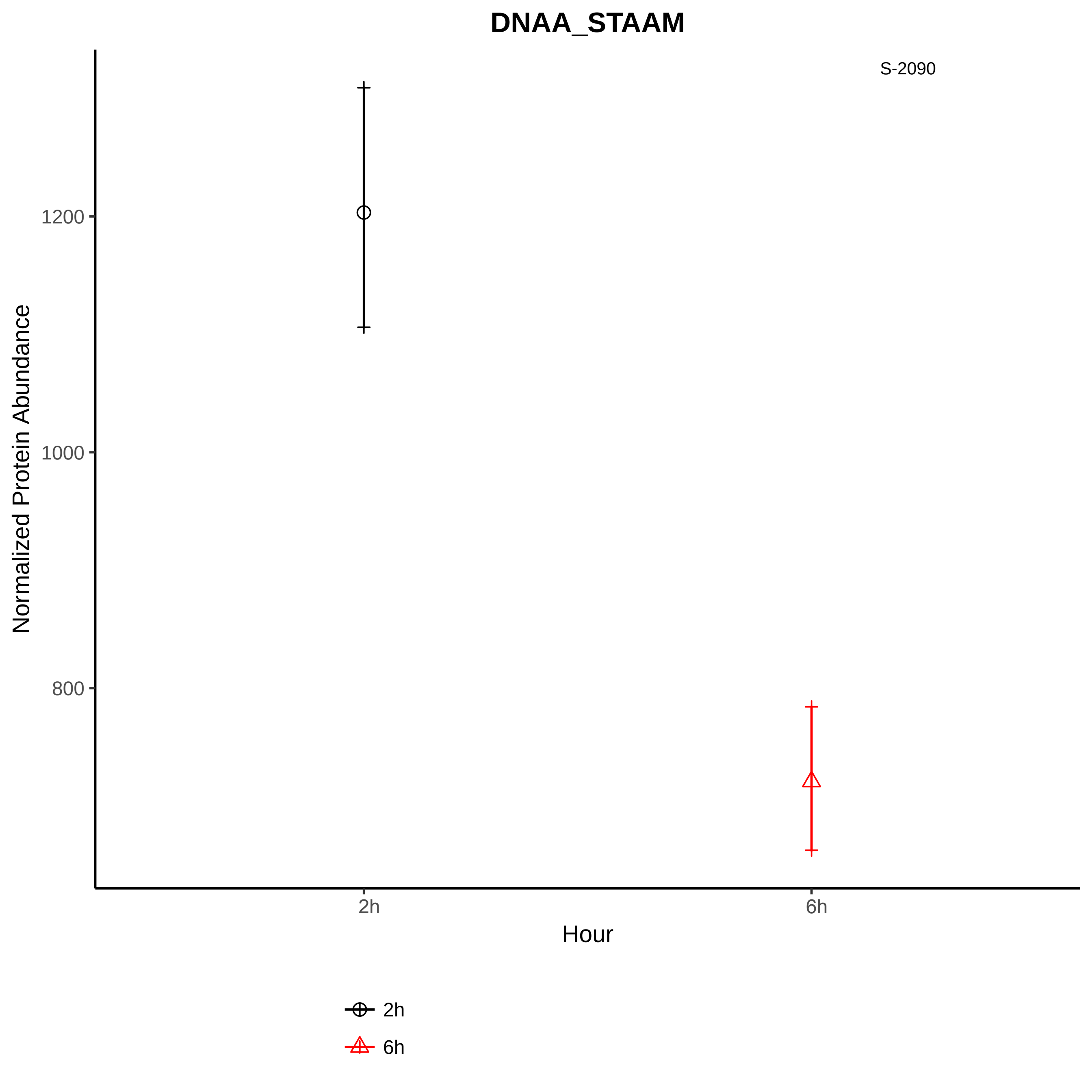
DNAA_STAAM
S-2090
Normalized Protein Abundance
1200
1000
800
2h
6h
Hour
2h
6h

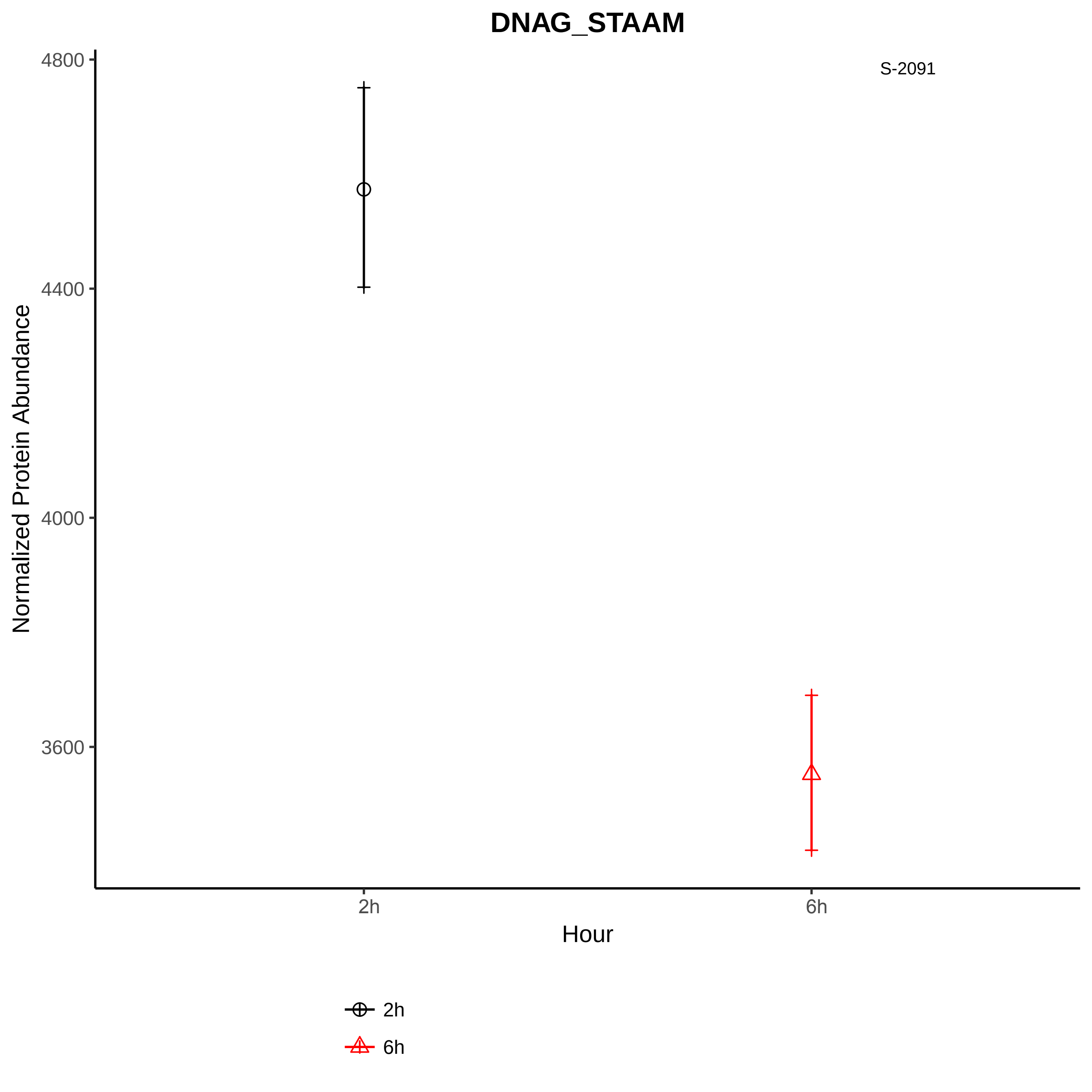

DNAG_STAAM
S-2091
4800
4400
4000
3600
Normalized Protein Abundance
2h
6h
Hour
2h
6h

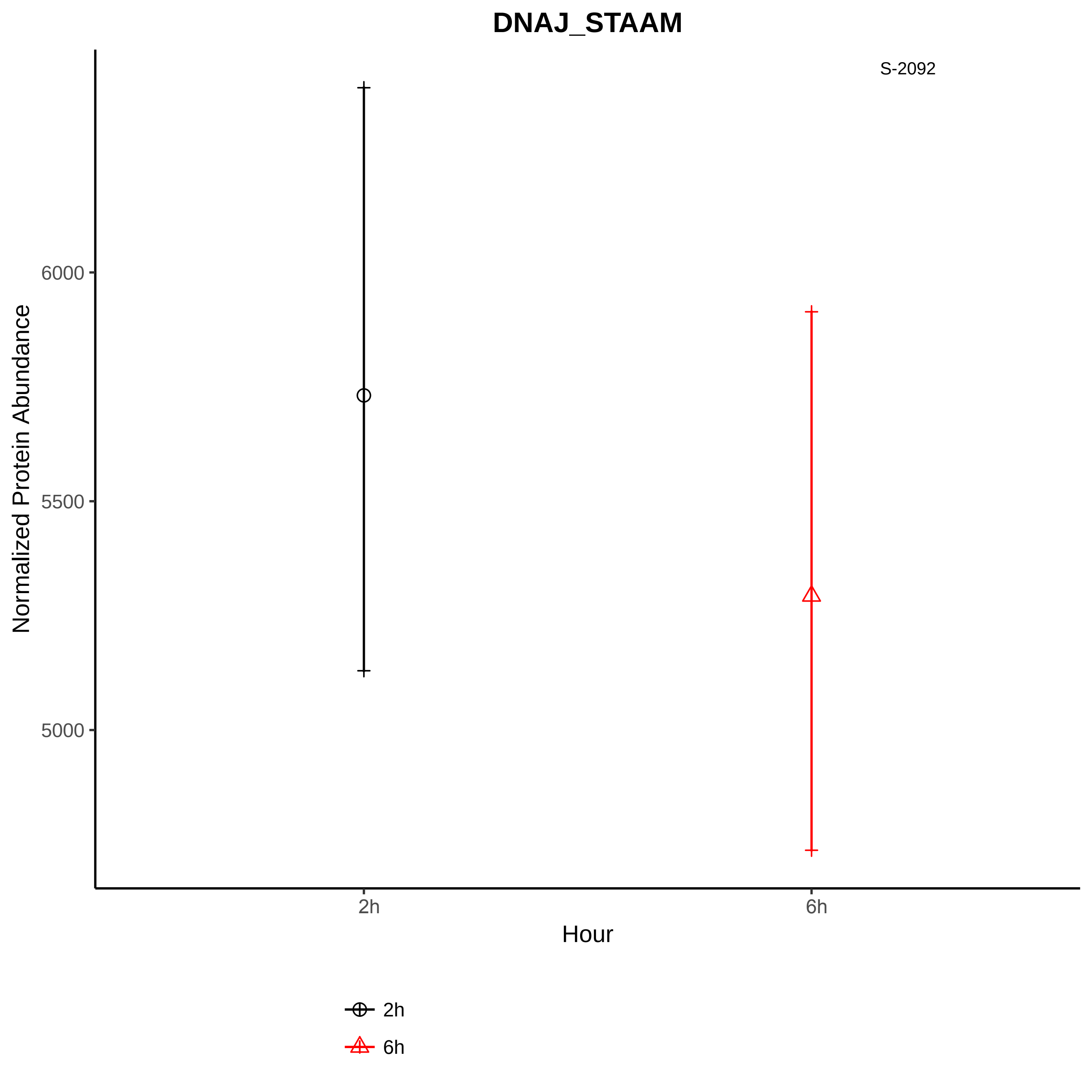

DNAJ_STAAM
S-2092
6000
5500
5000
Normalized Protein Abundance
2h
6h
Hour
2h
6h

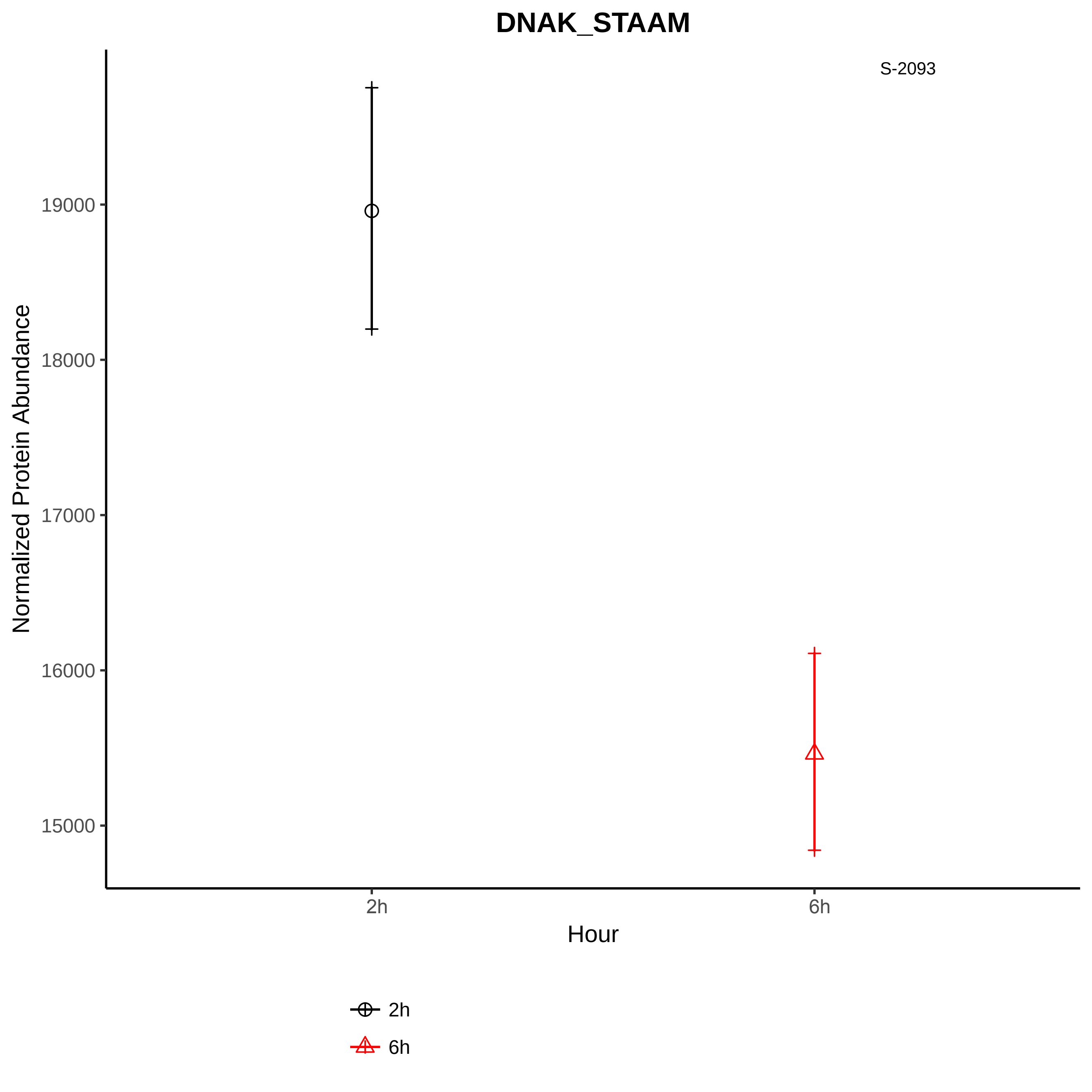

DNAK_STAAM
S-2093
Normalized Protein Abundance
19000
18000
17000
16000
15000
2h
6h
Hour
2h
6h

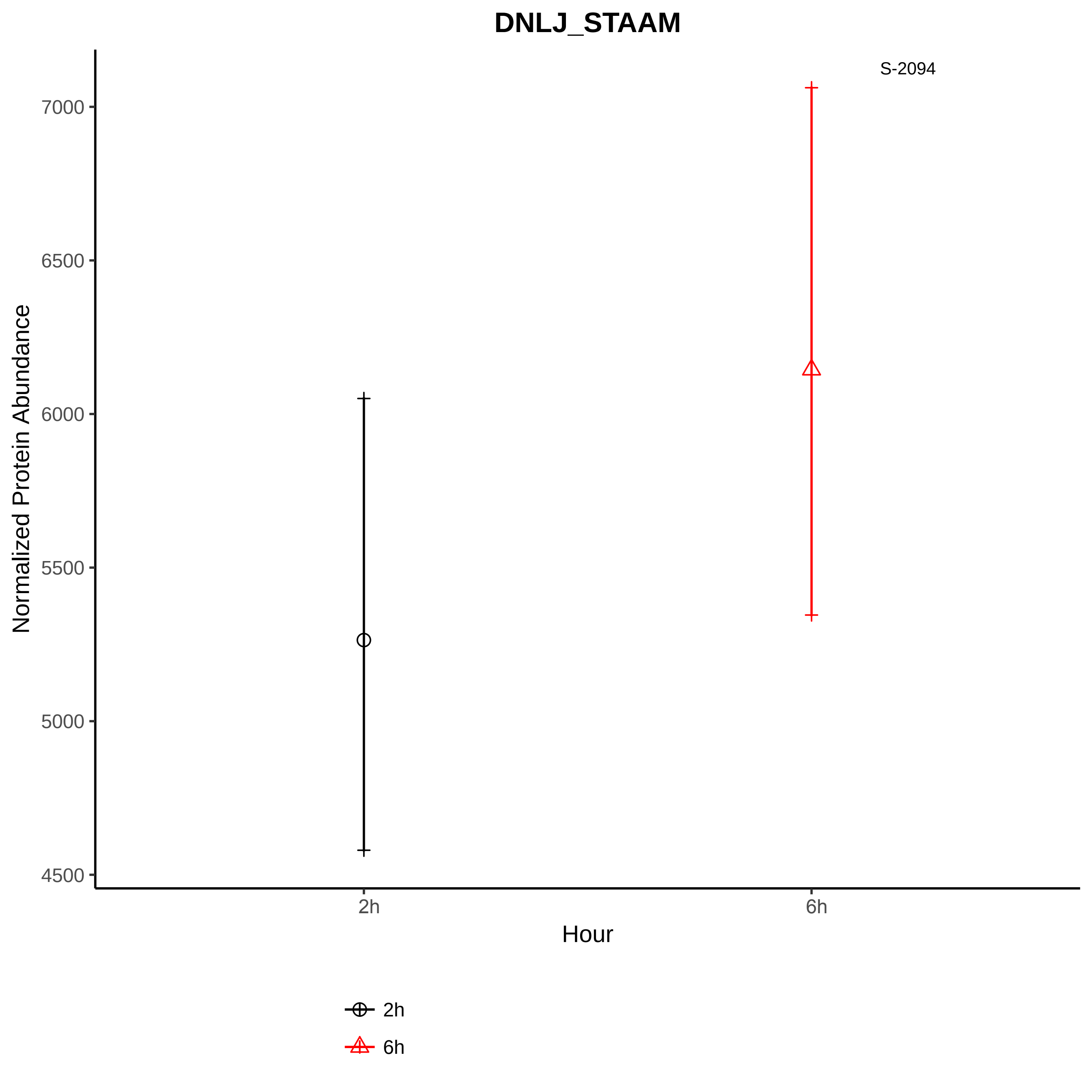

DNLJ_STAAM
S-2094
Normalized Protein Abundance
7000
6500
6000
5500
5000
4500
2h
6h
Hour
2h
6h

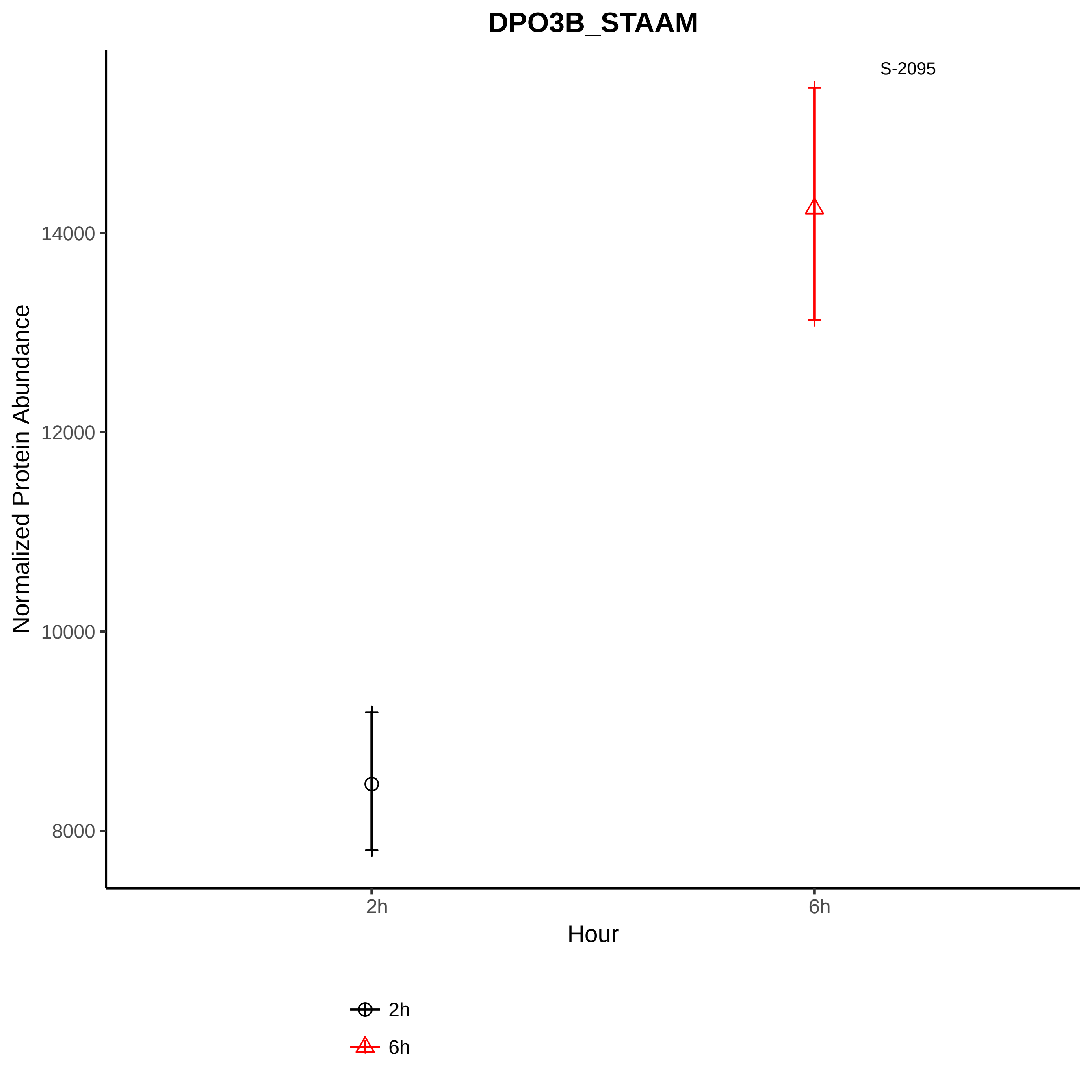

DPO3B_STAAM
S-2095
Normalized Protein Abundance
14000
12000
10000
8000
2h
6h
Hour
2h
6h

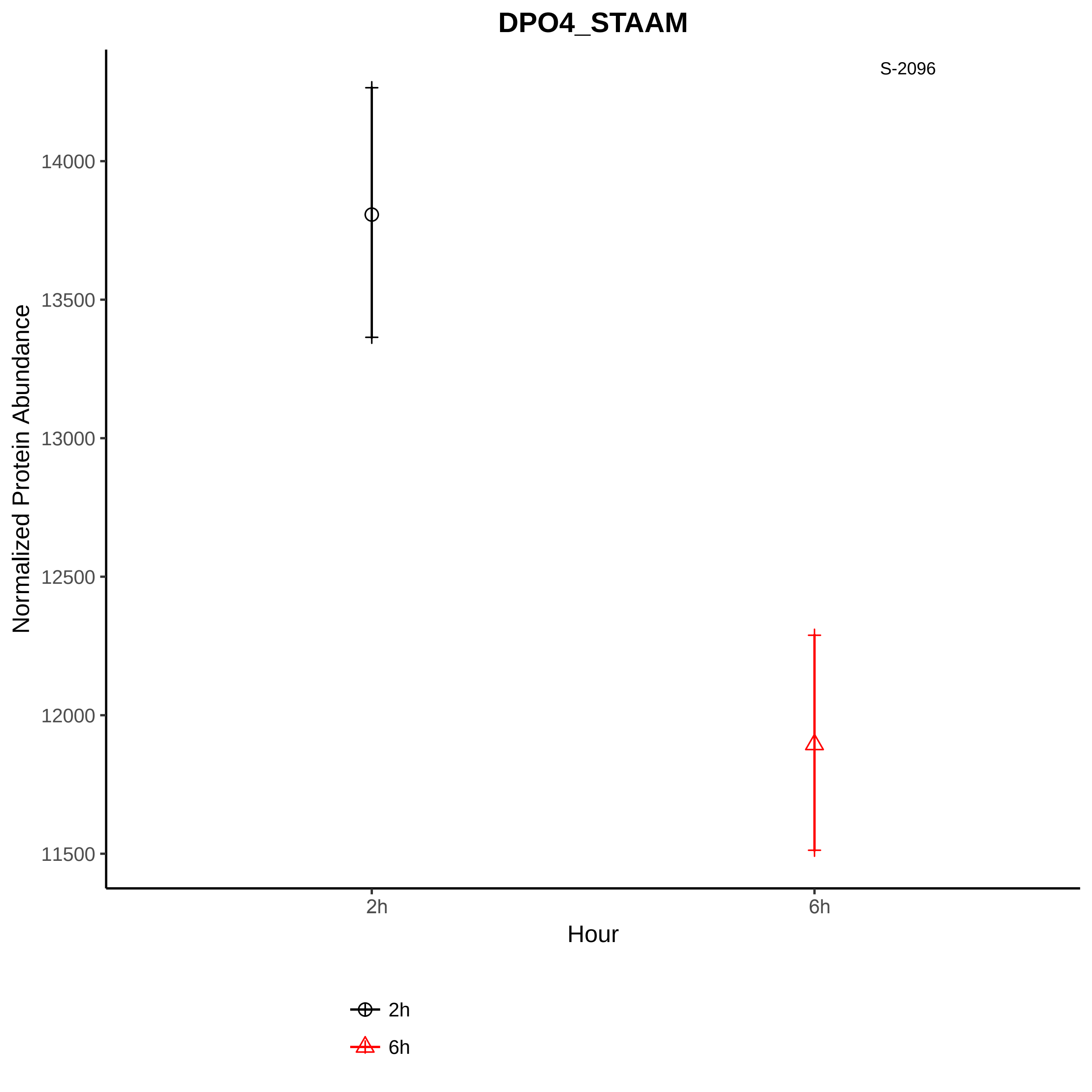
DPO4_STAAM
S-2096
Normalized Protein Abundance
14000
13500
13000
12500
12000
11500
2h
6h
Hour
2h
6h

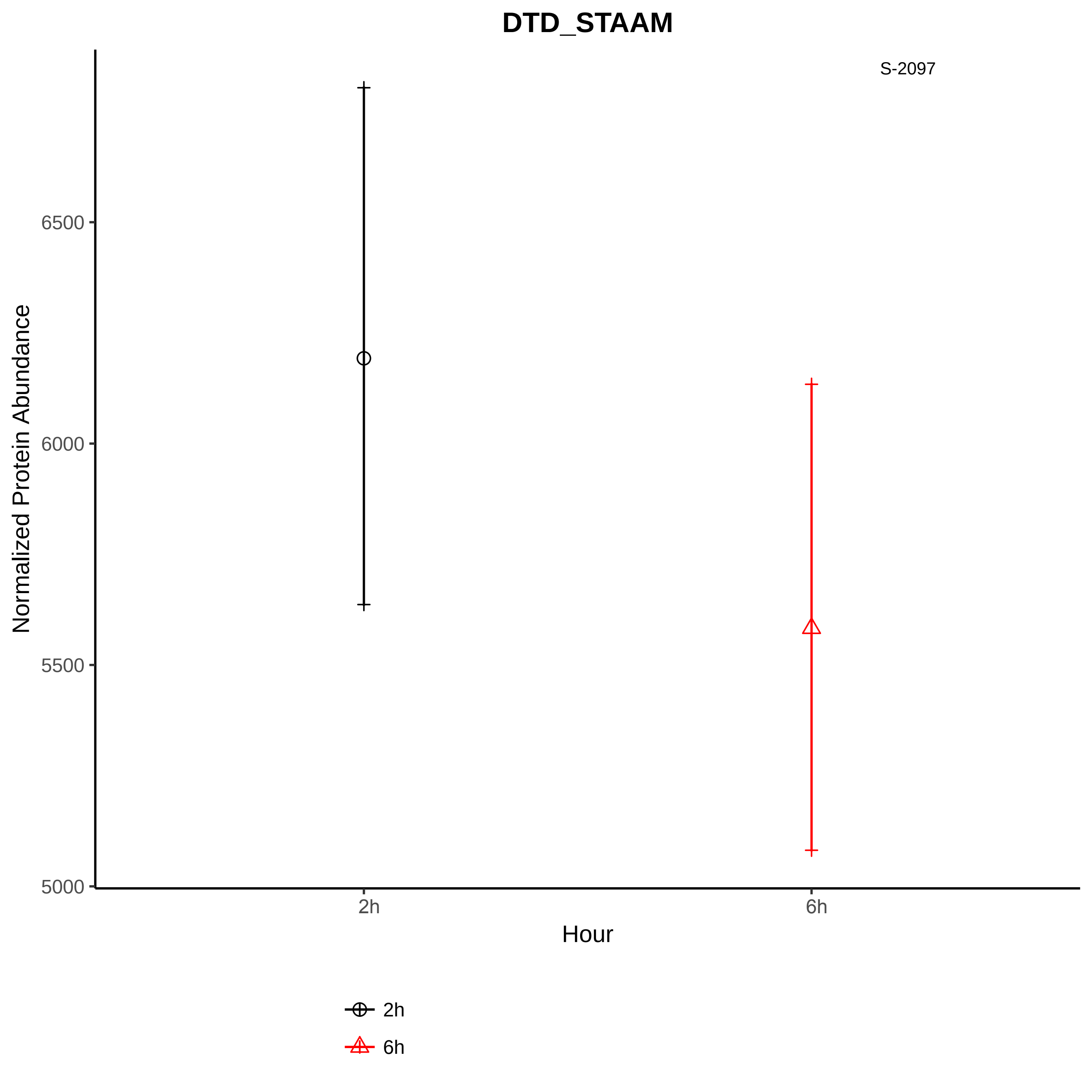

DTD_STAAM
S-2097
Normalized Protein Abundance
6500
6000
5500
5000
2h
6h
Hour
2h
6h

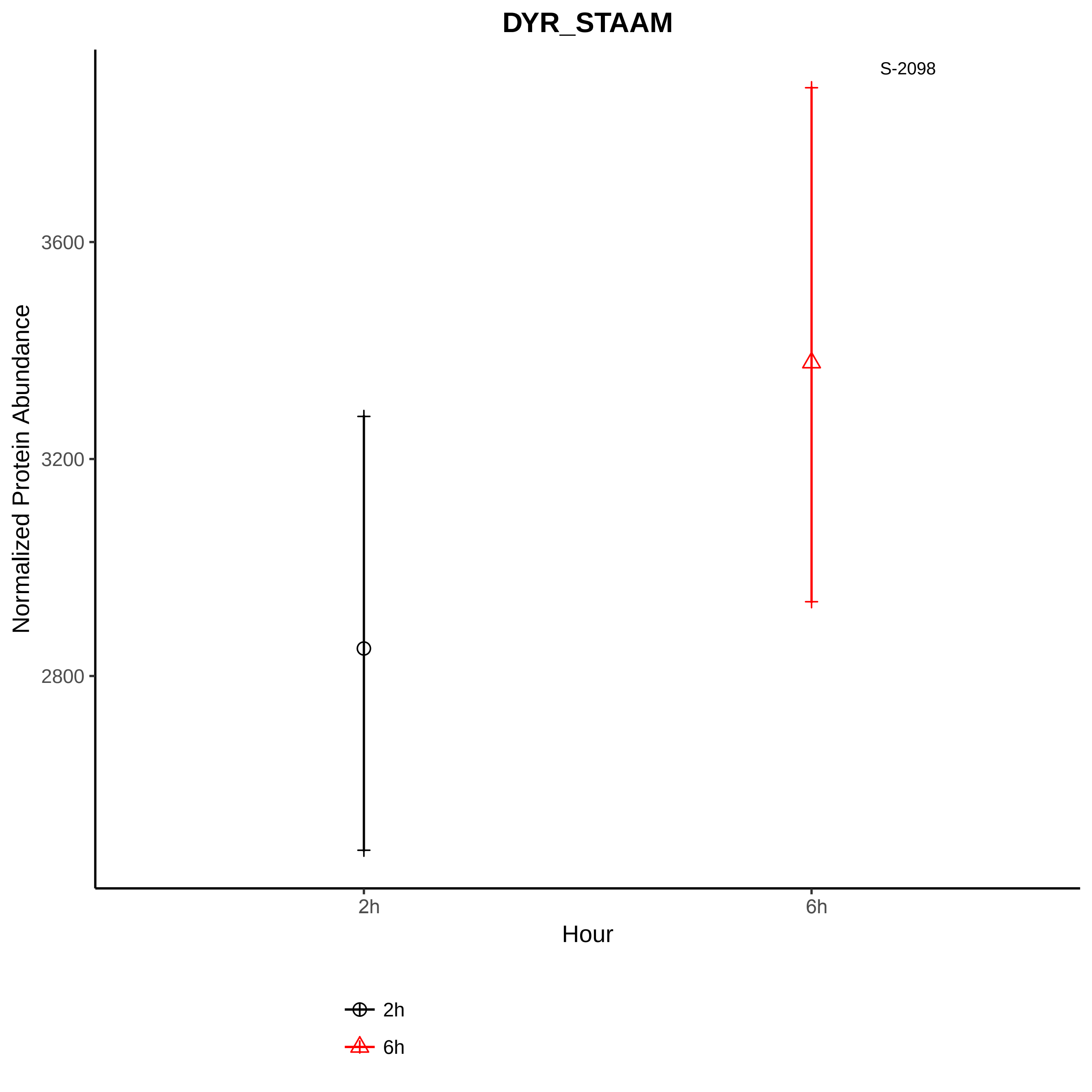

DYR_STAAM
S-2098
Normalized Protein Abundance
3600
3200
2800
2h
6h
Hour
2h
6h

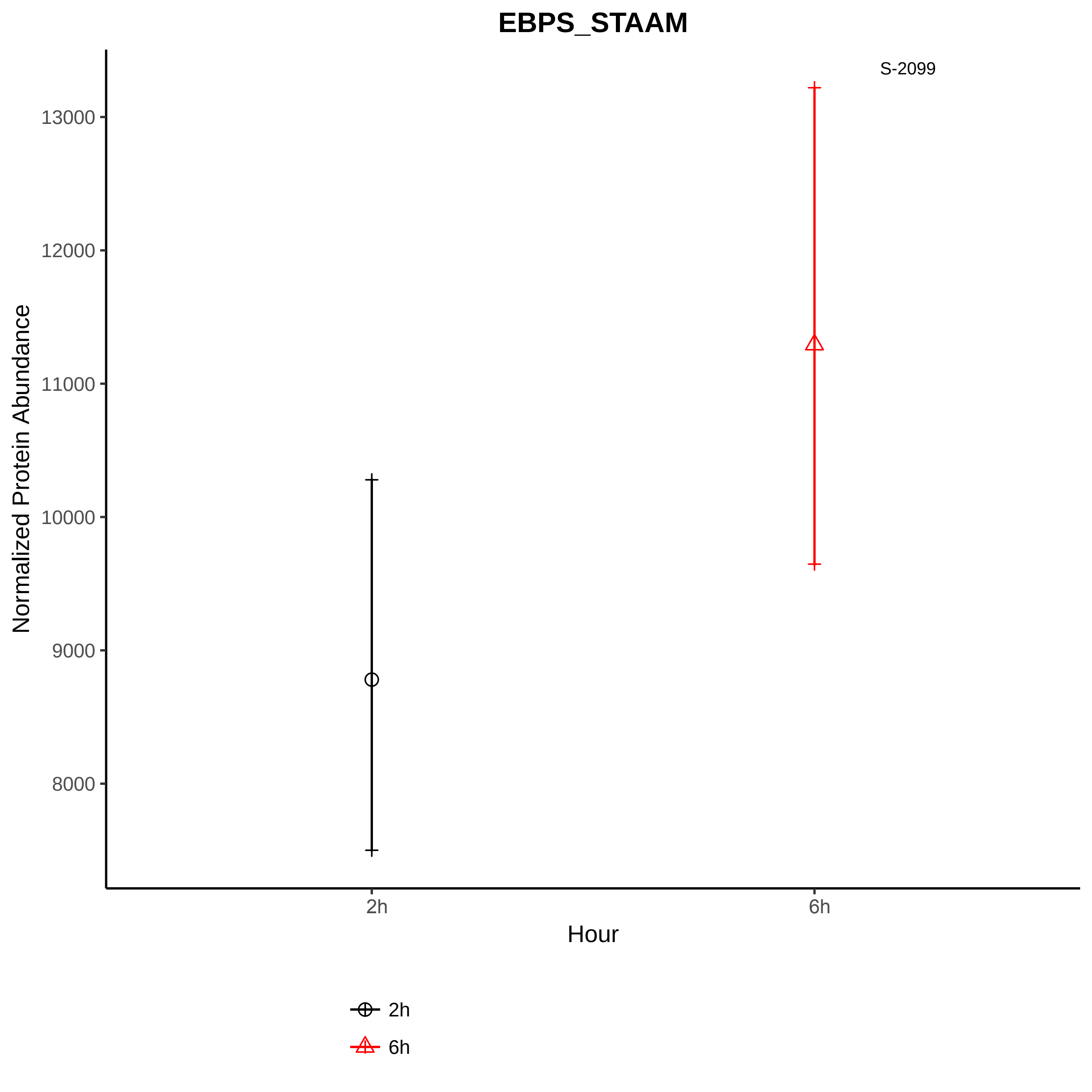

EBPS_STAAM
S-2099
Normalized Protein Abundance
13000
12000
11000
10000
9000
8000
2h
6h
Hour
2h
6h

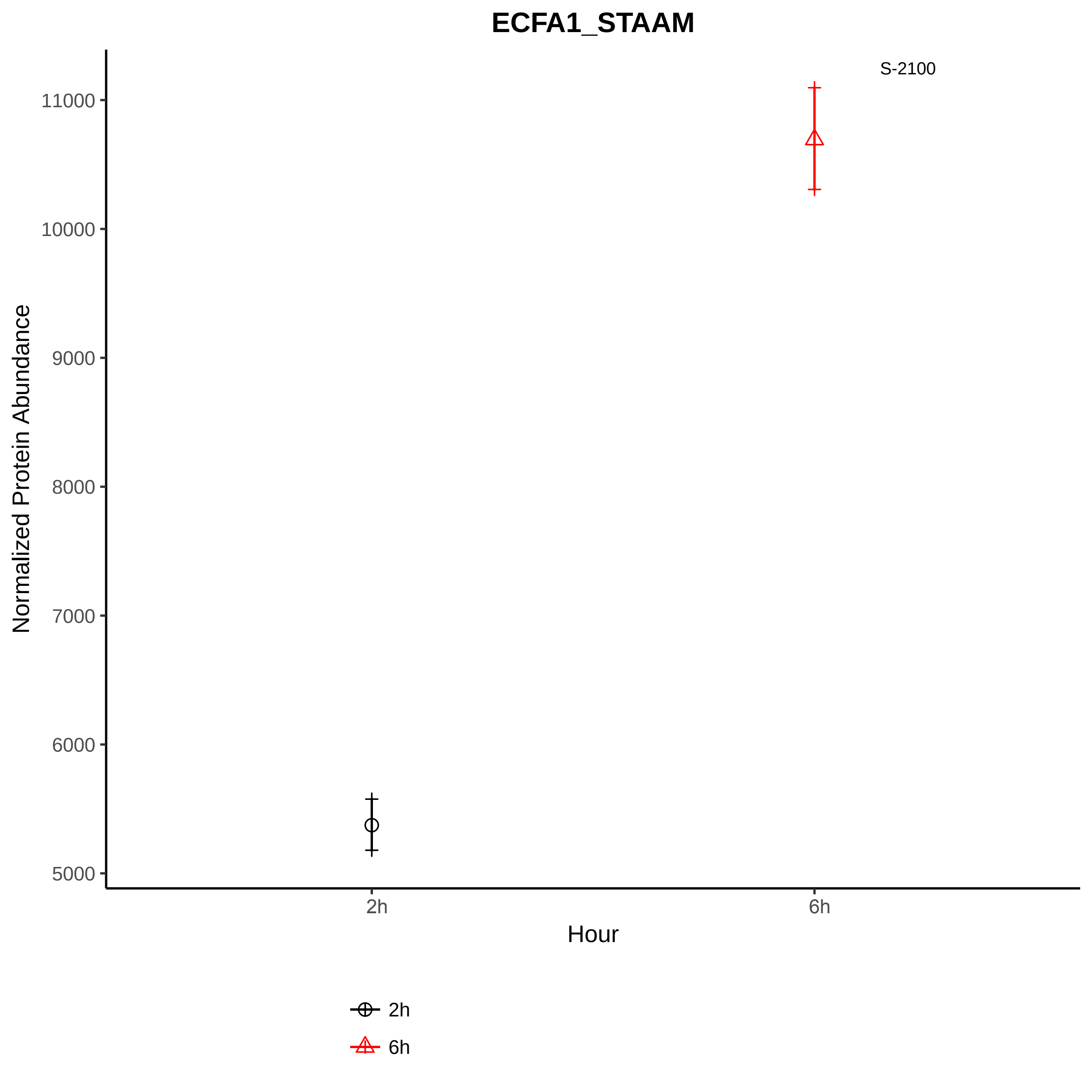

ECFA1_STAAM
S-2100
Normalized Protein Abundance
11000
10000
9000
8000
7000
6000
5000
2h
6h
Hour
2h
6h

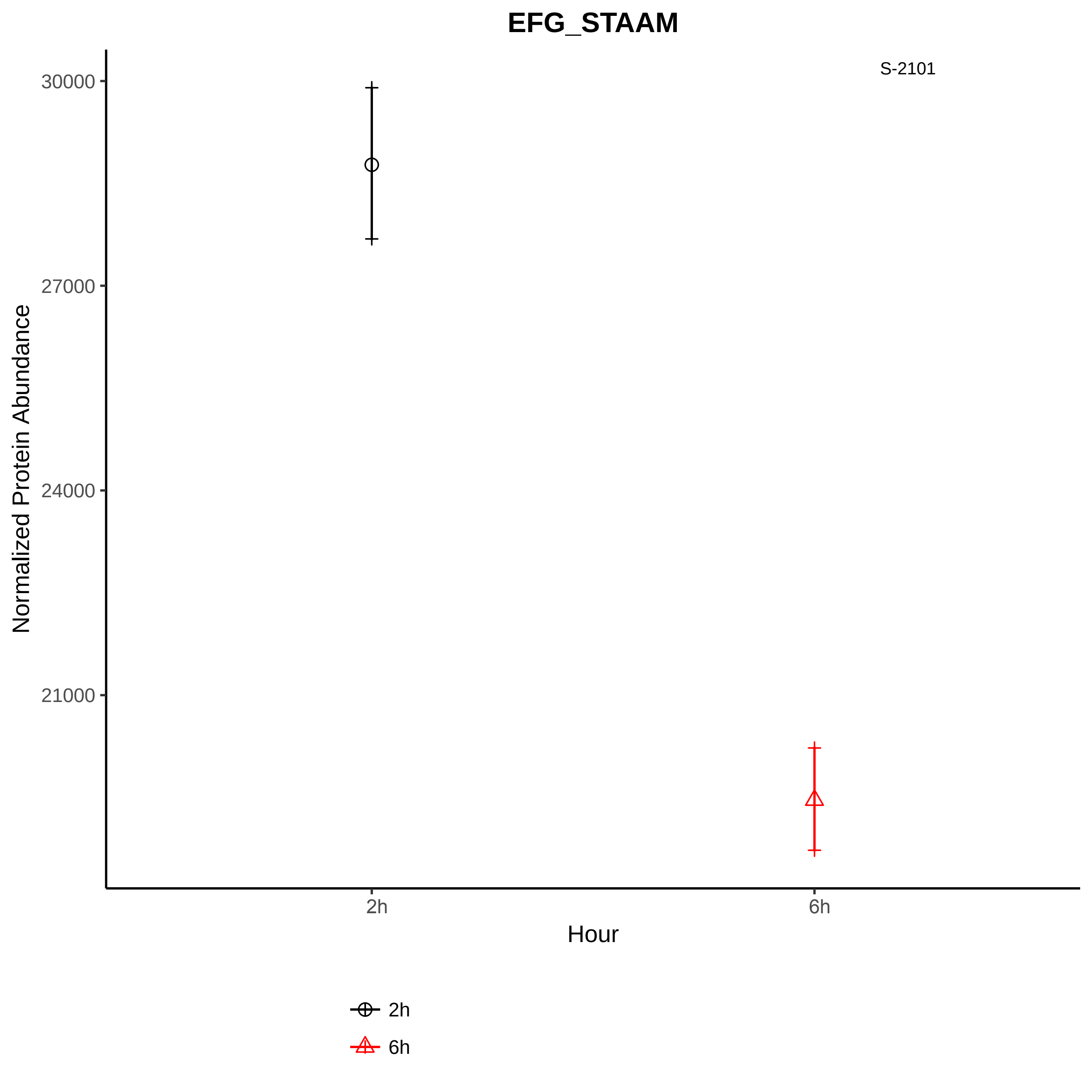
EFG_STAAM
S-2101
30000
27000
24000
21000
Normalized Protein Abundance
2h
6h
Hour
2h
6h

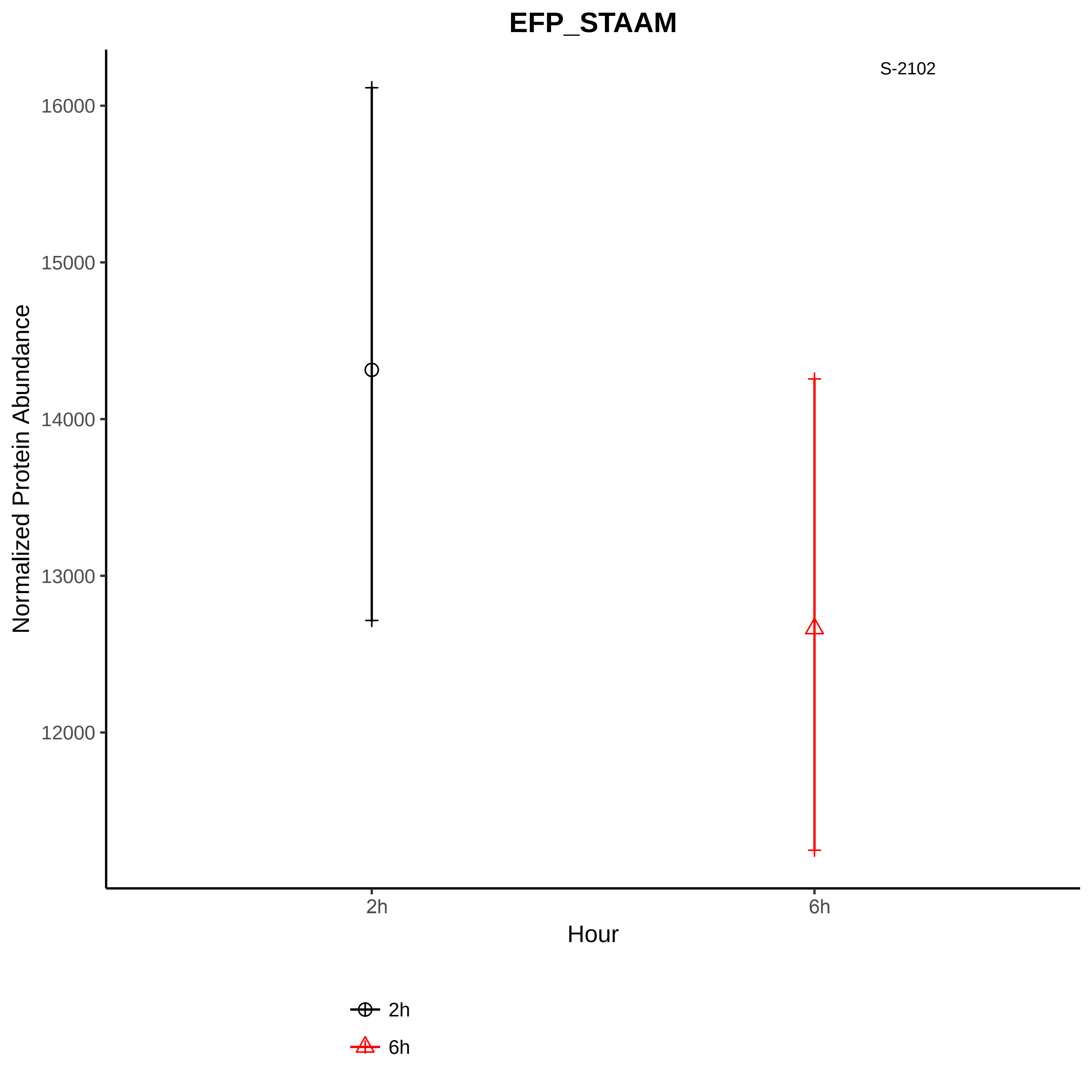

EFP_STAAM
S-2102
Normalized Protein Abundance
16000
15000
14000
13000
12000
2h
6h
Hour
2h
6h

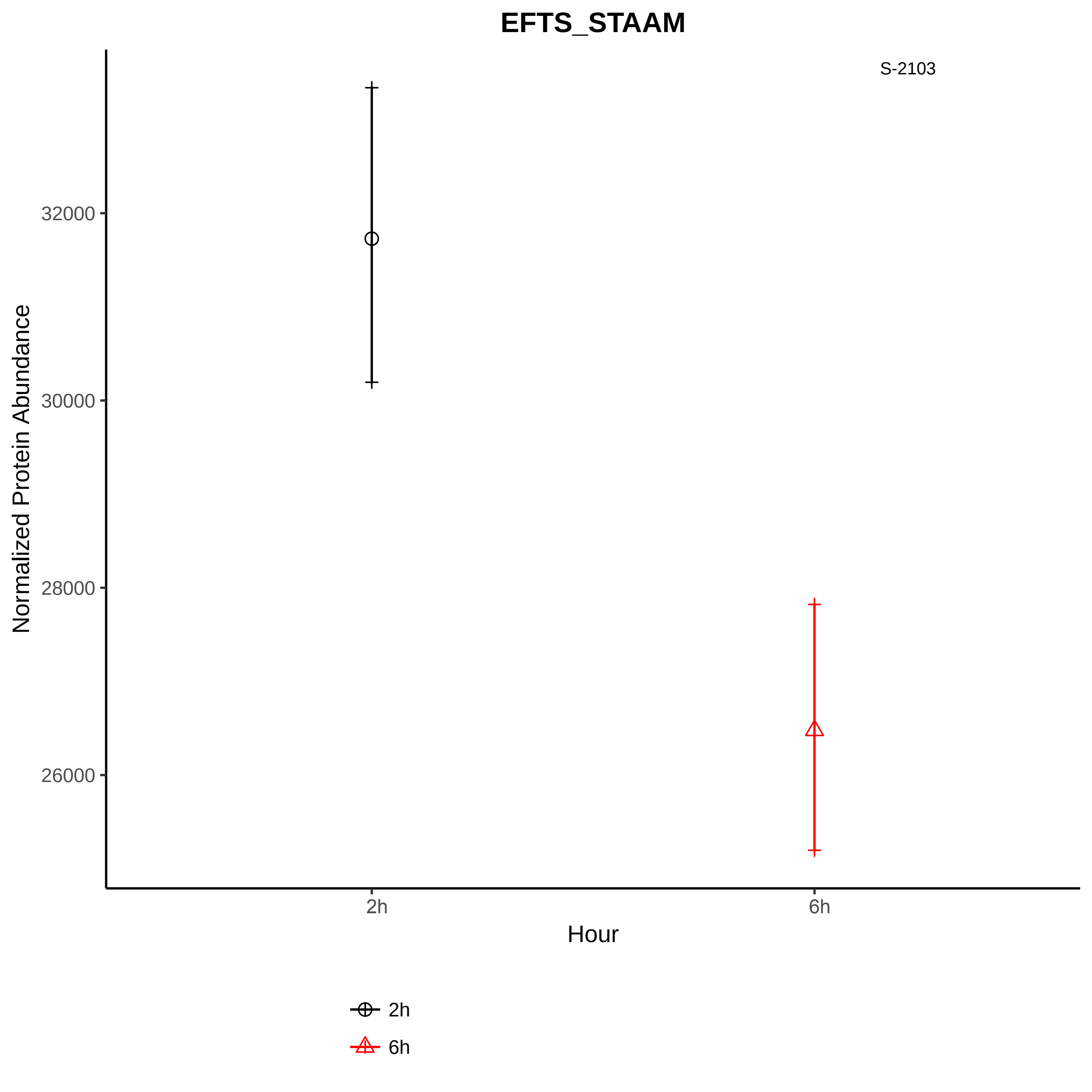
EFTS_STAAM
S-2103
Normalized Protein Abundance
32000
30000
28000
26000
2h
6h
Hour
2h
6h

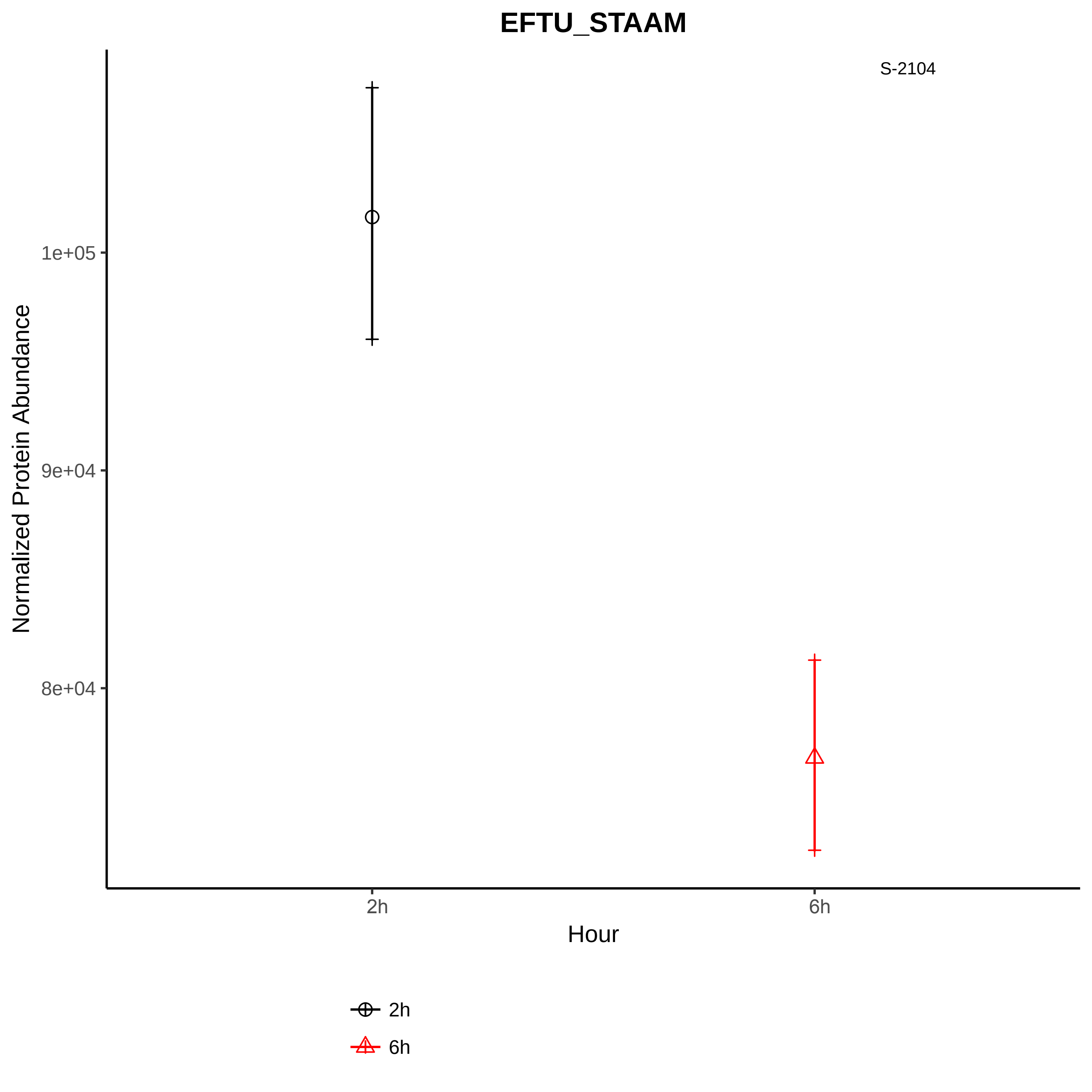

EFTU_STAAM
S-2104
Normalized Protein Abundance
1e+05
9e+04
8e+04
2h
6h
Hour
2h
6h

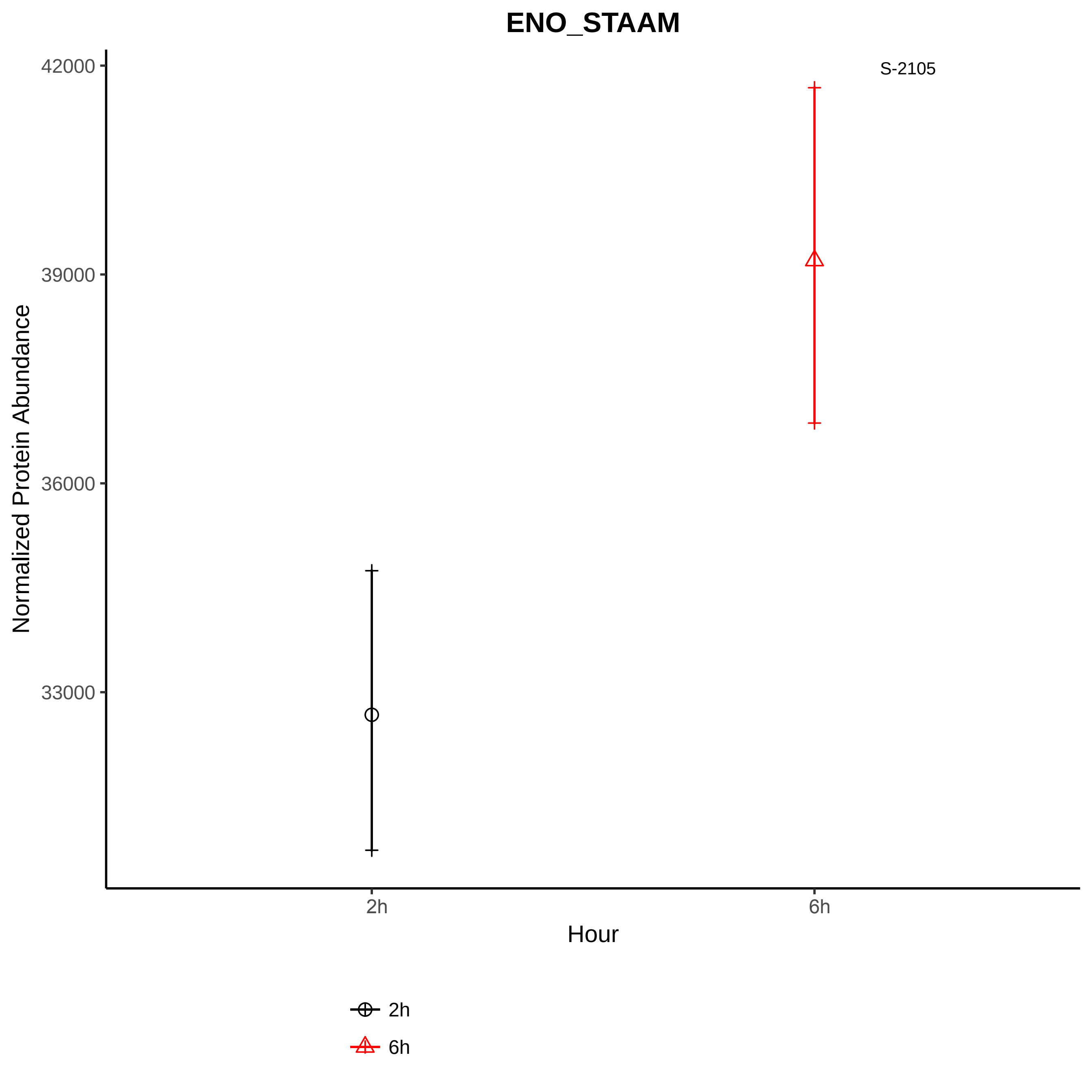
ENO_STAAM
S-2105
42000
39000
36000
33000
Normalized Protein Abundance
2h
6h
Hour
2h
6h

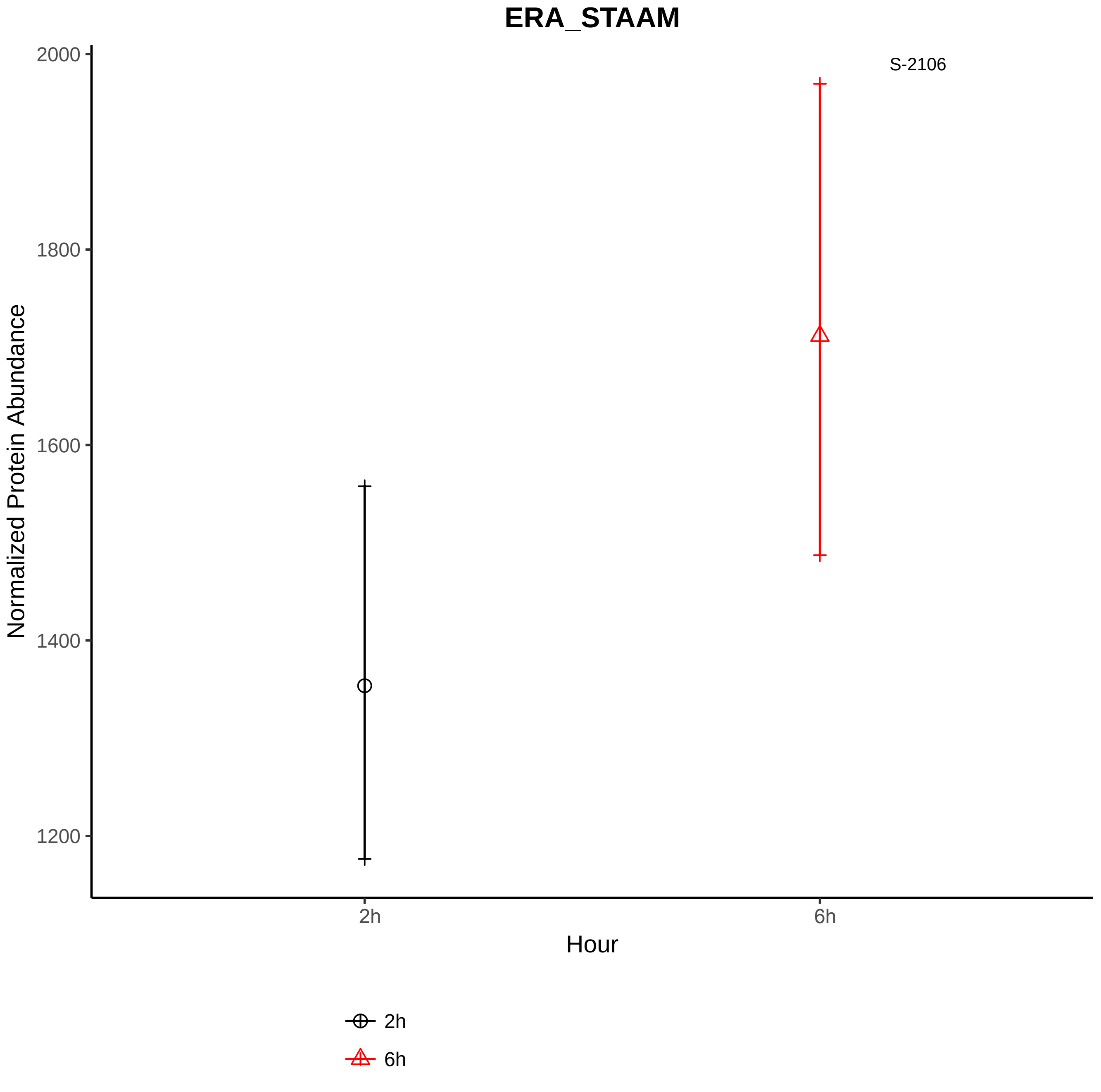
ERA_STAAM
S-2106
Normalized Protein Abundance
2000
1800
1600
1400
1200
2h
6h
Hour
2h
6h

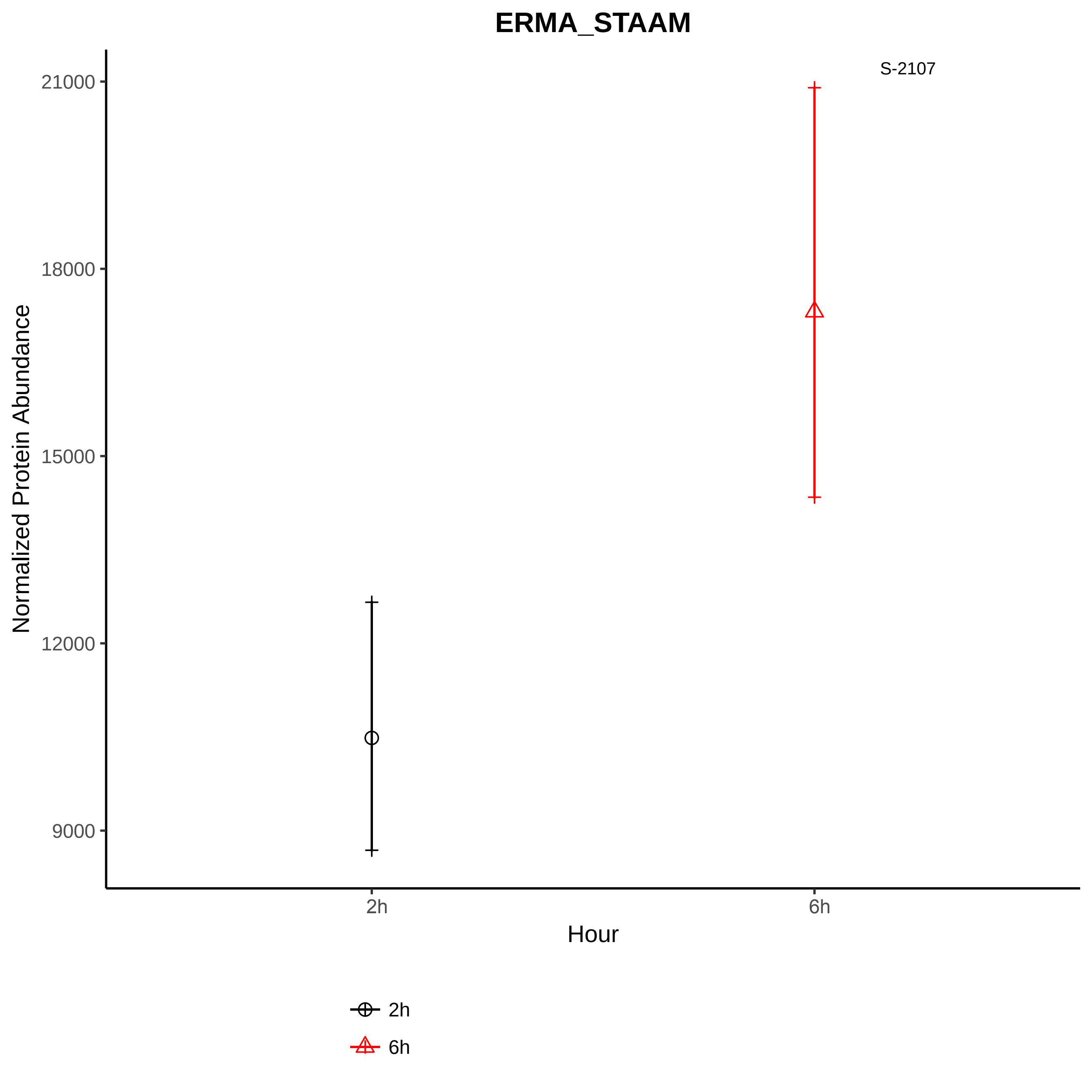

ERMA_STAAM
S-2107
21000
18000
15000
12000
9000
Normalized Protein Abundance
2h
6h
Hour
2h
6h

ESSA_STAAM

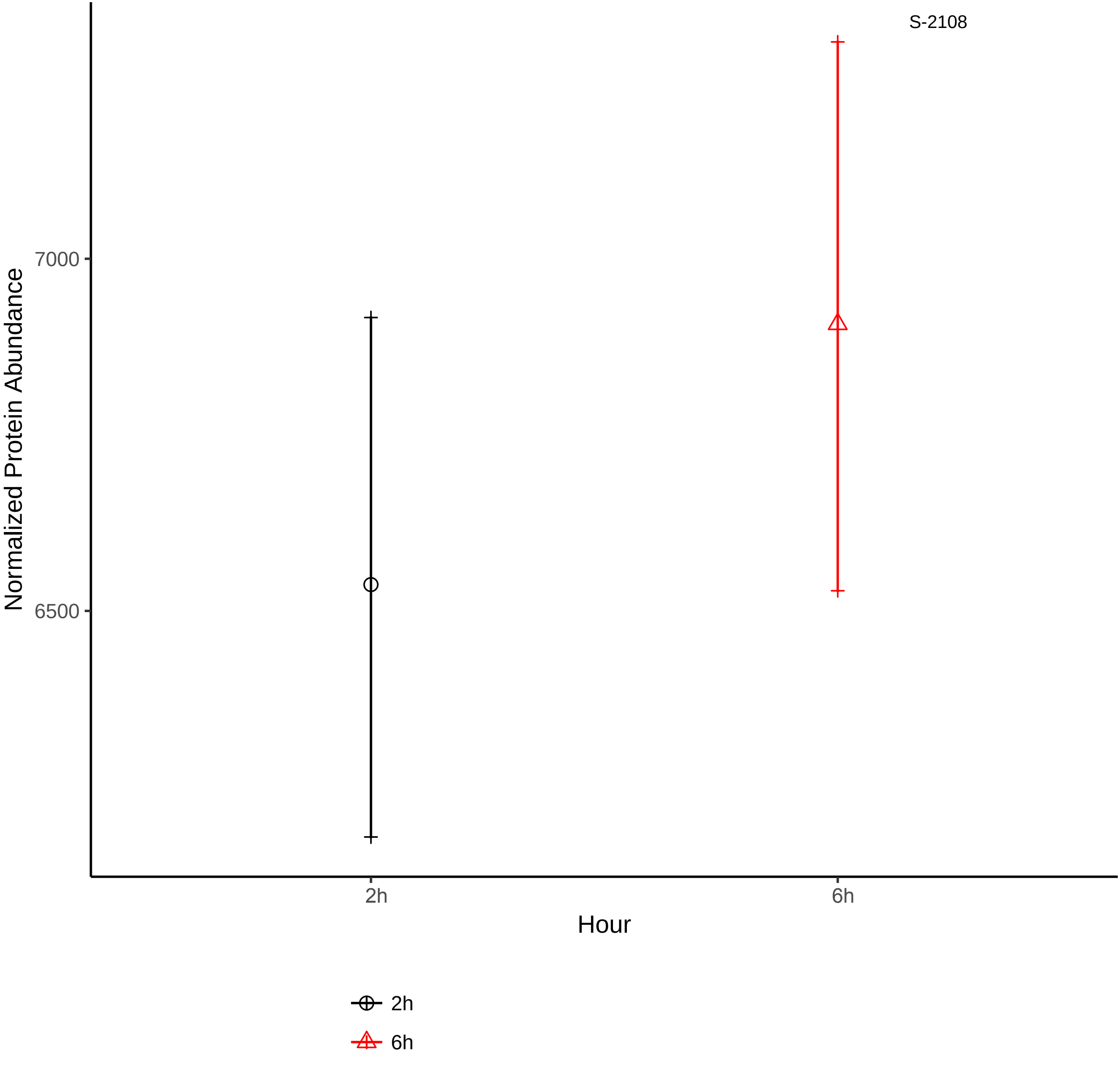

S-2108
7000
6500
Normalized Protein Abundance
2h
6h
Hour
2h
6h

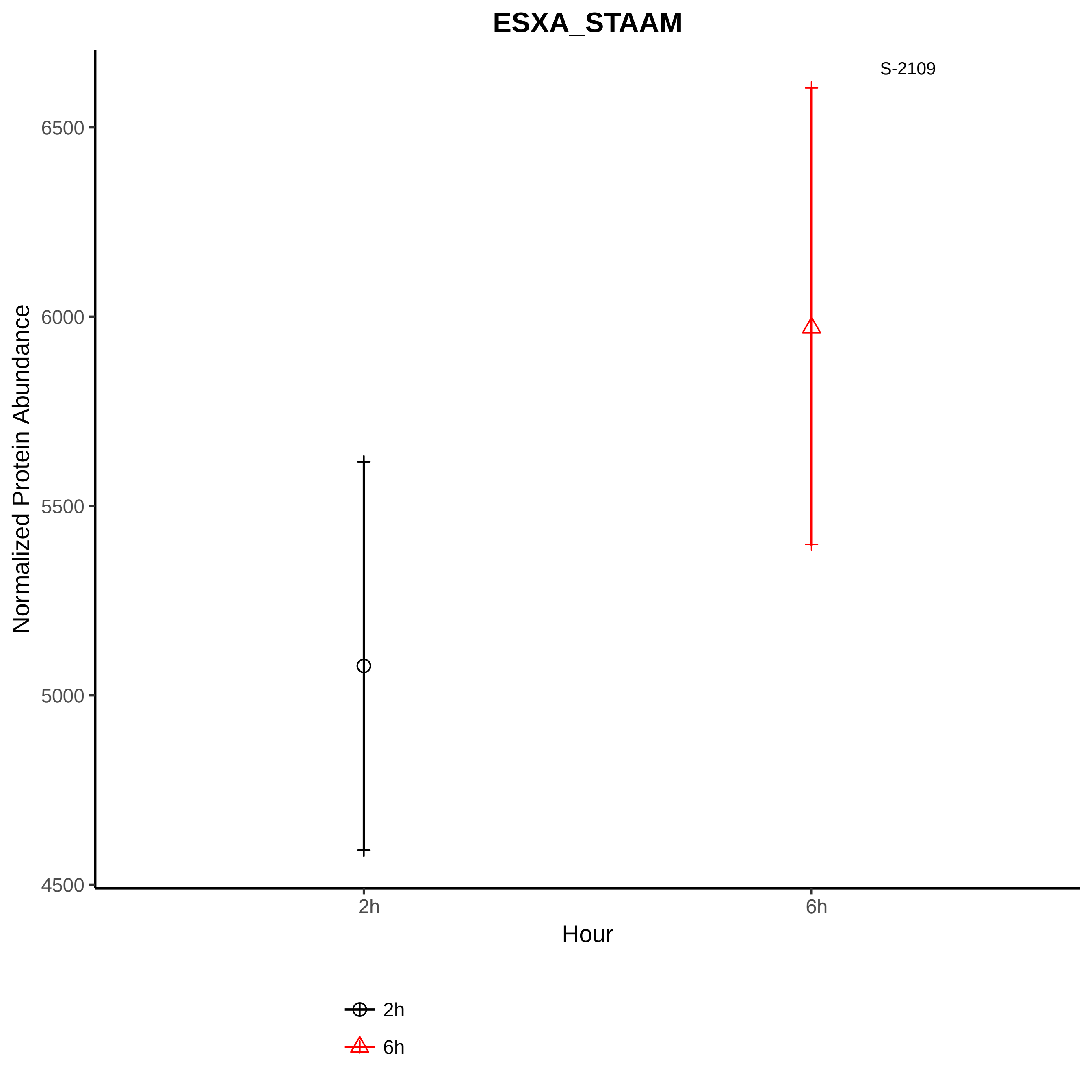

ESXA_STAAM
S-2109
Normalized Protein Abundance
6500
6000
5500
5000
4500
2h
6h
Hour
2h
6h

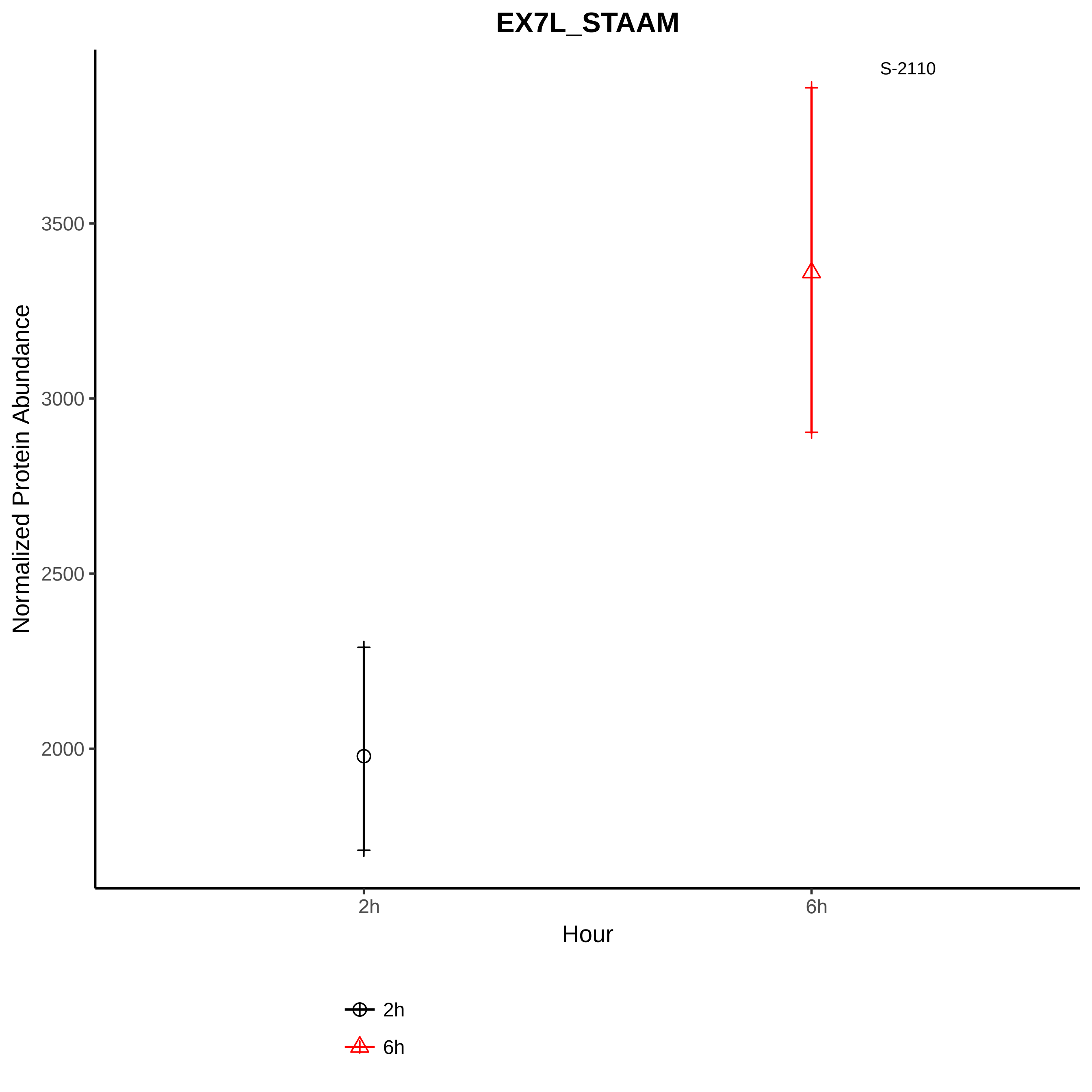
EX7L_STAAM
S-2110
Normalized Protein Abundance
3500
3000
2500
2000
2h
6h
Hour
2h
6h

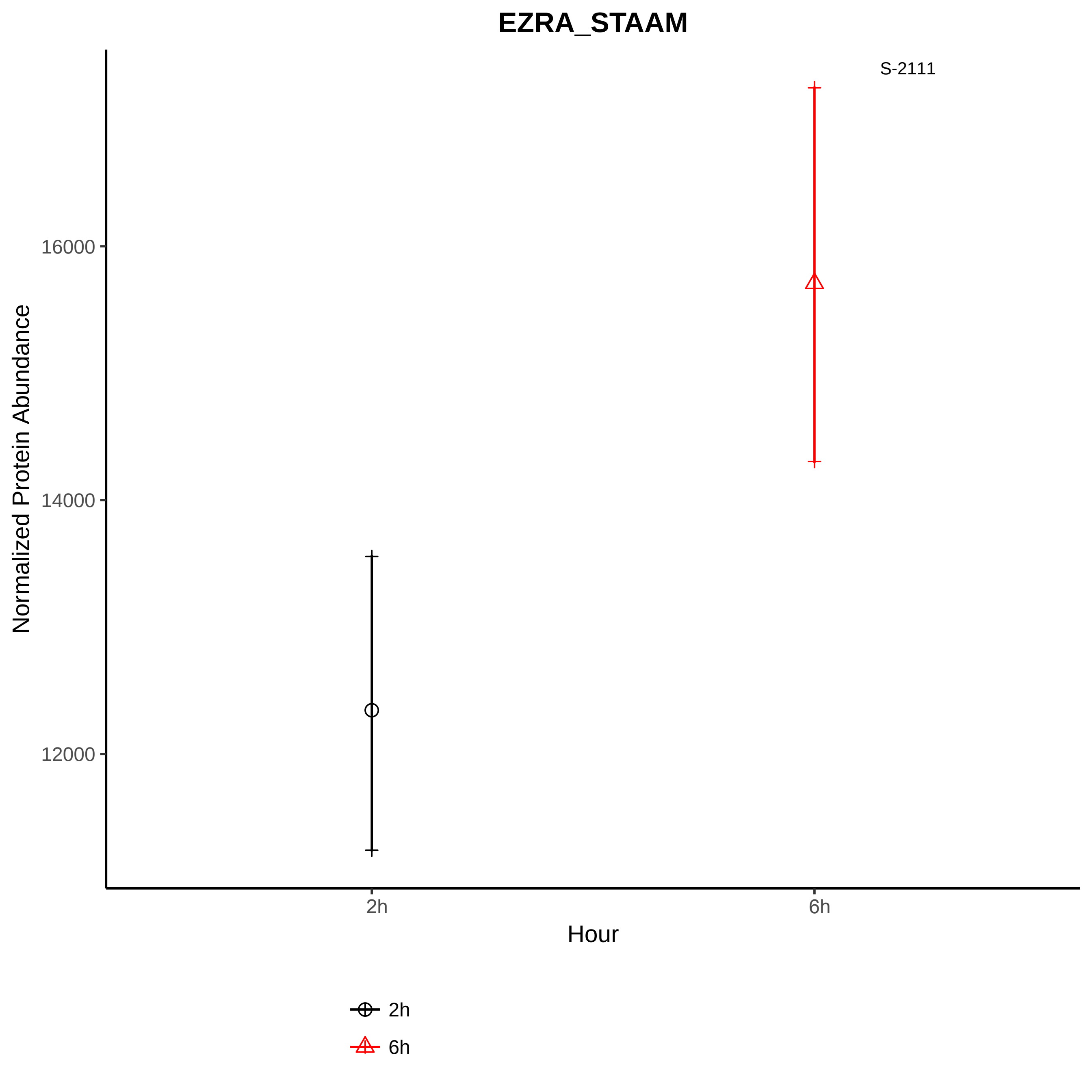
EZRA_STAAM
S-2111
Normalized Protein Abundance
16000
14000
12000
2h
6h
Hour
2h
6h

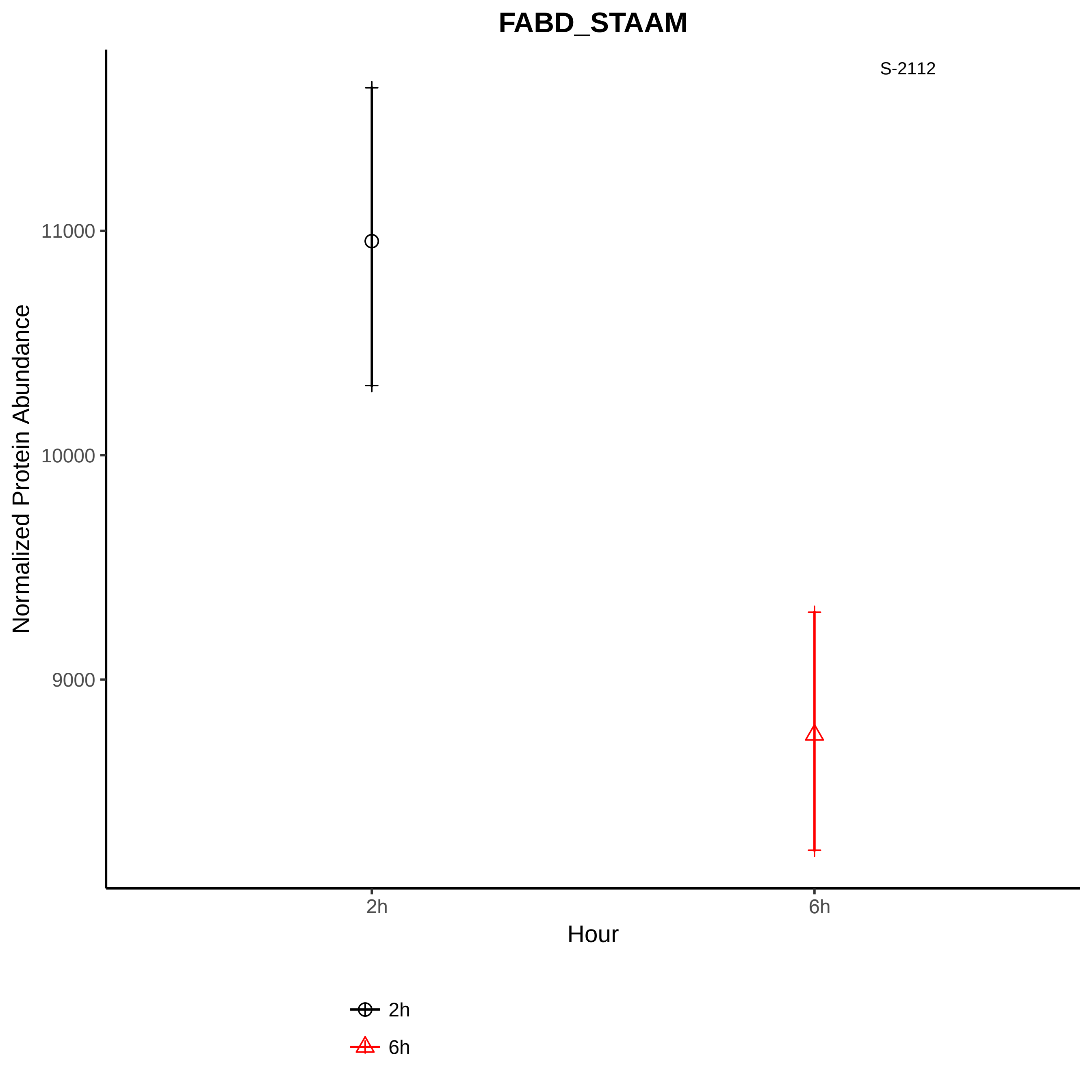
FABD_STAAM
S-2112
Normalized Protein Abundance
11000
10000
9000
2h
6h
Hour
2h
6h

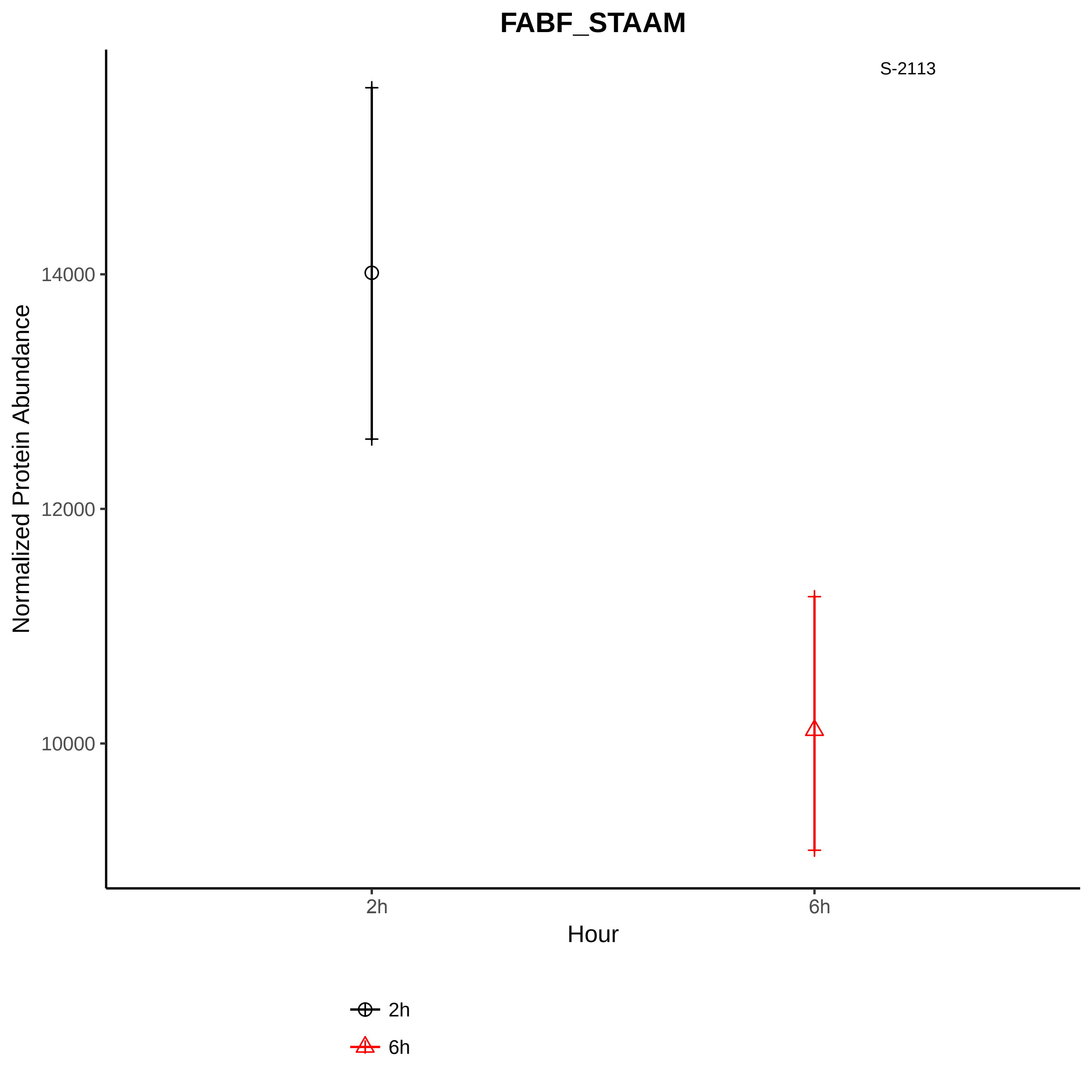

FABF_STAAM
S-2113
Normalized Protein Abundance
14000
12000
10000
2h
6h
Hour
2h
6h

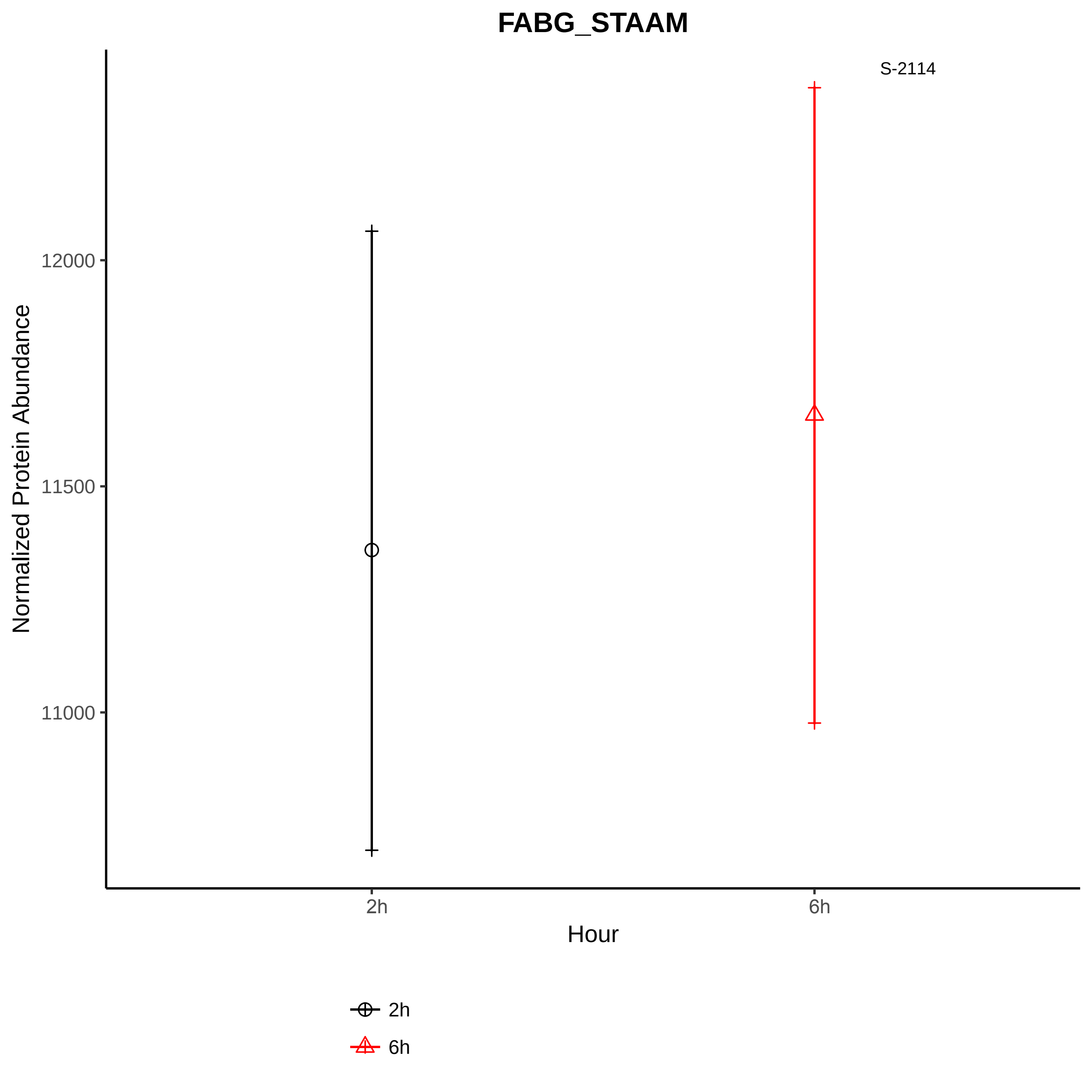
FABG_STAAM
S-2114
Normalized Protein Abundance
12000
11500
11000
2h
6h
Hour
2h
6h

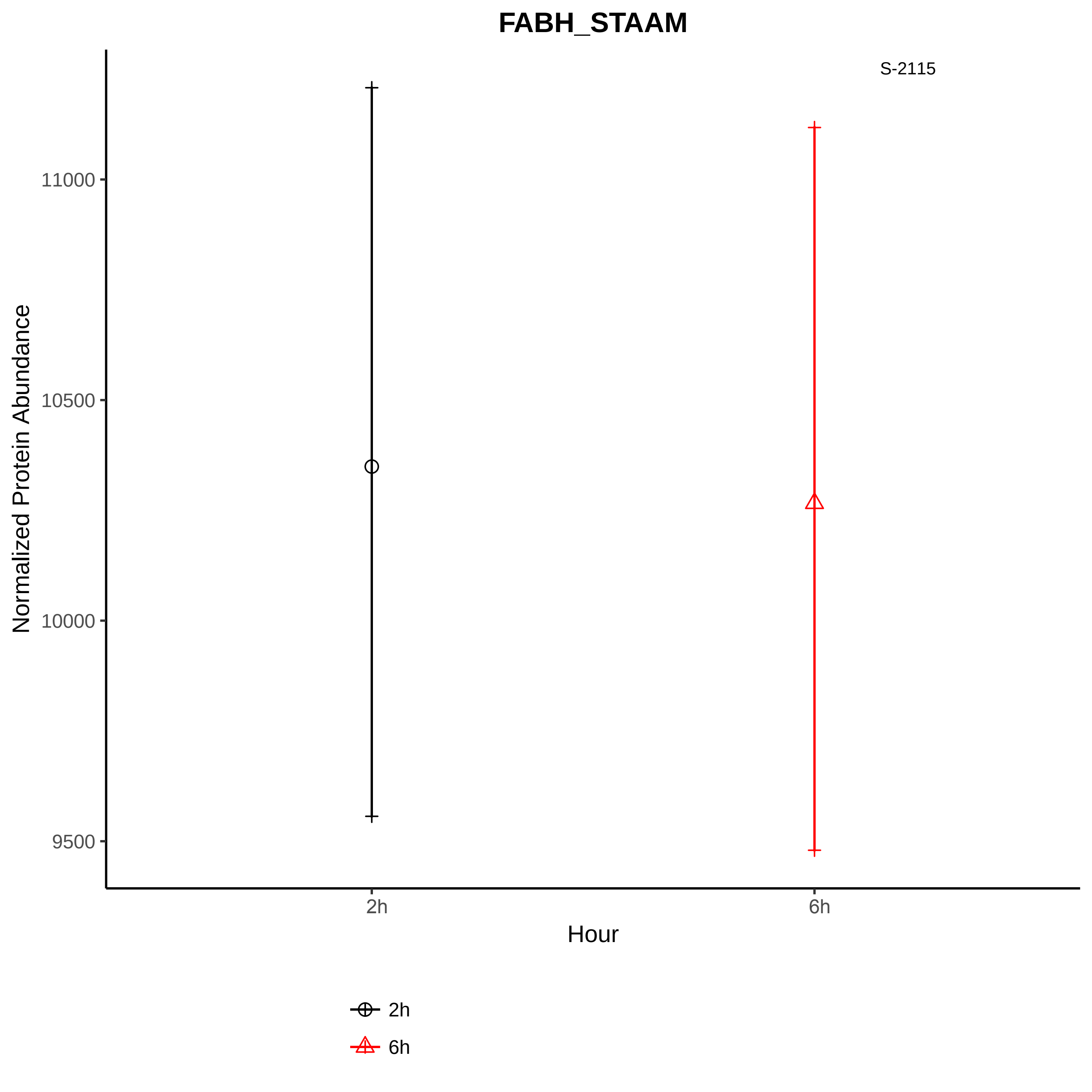

FABH_STAAM
S-2115
Normalized Protein Abundance
11000
10500
10000
9500
2h
6h
Hour
2h
6h

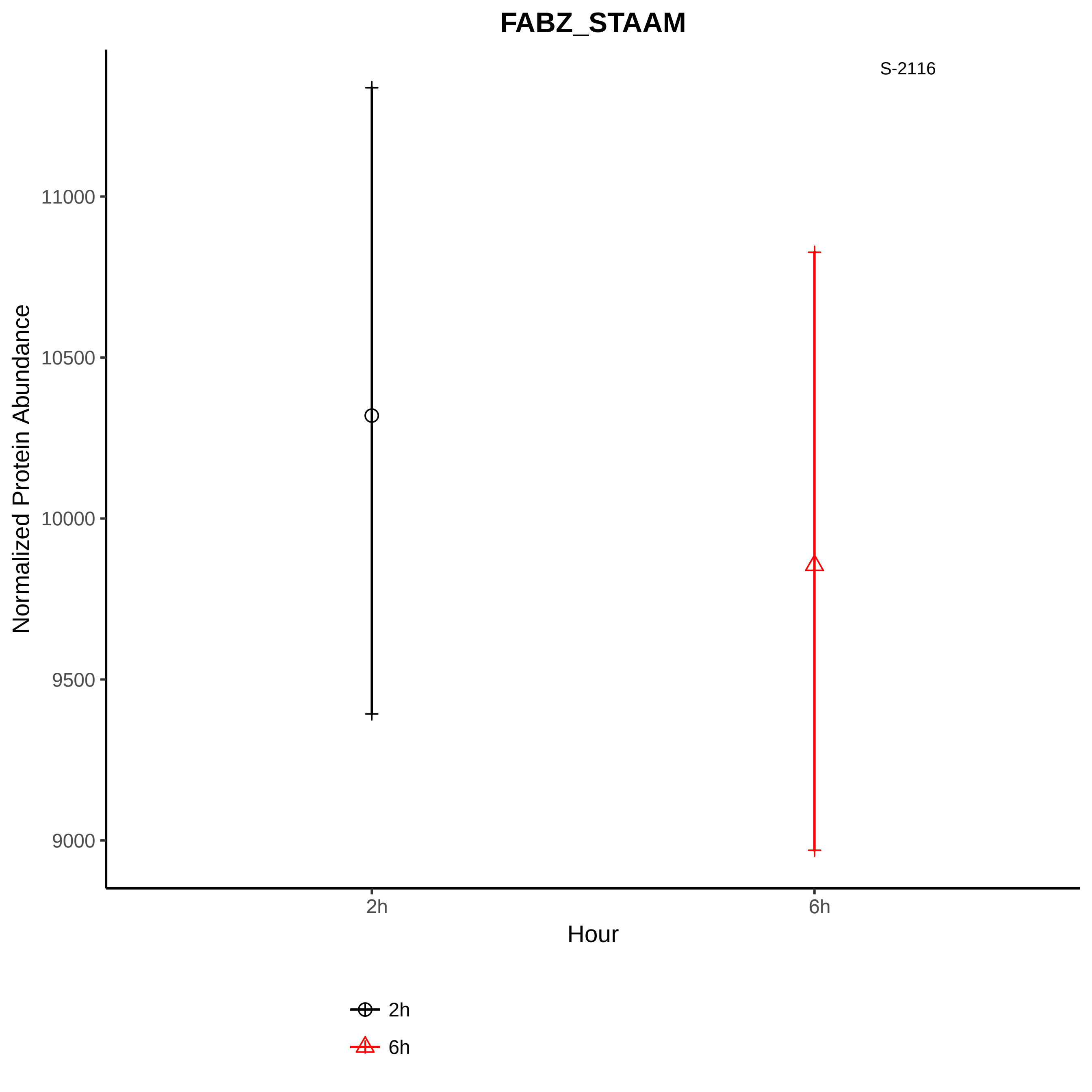

FABZ_STAAM
S-2116
Normalized Protein Abundance
11000
10500
10000
9500
9000
2h
6h
Hour
2h
6h

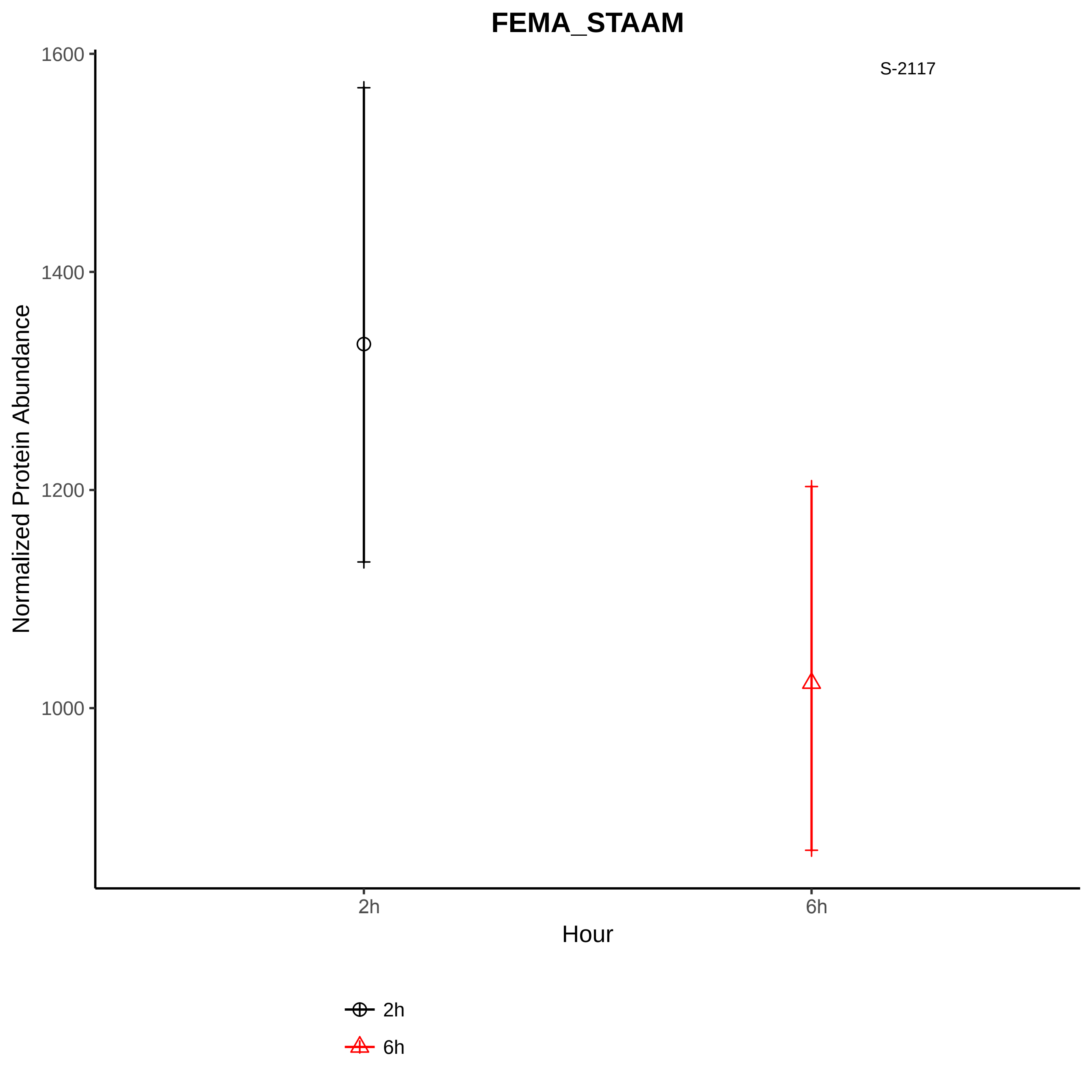

FEMA_STAAM
S-2117
1600
1400
1200
1000
Normalized Protein Abundance
2h
6h
Hour
2h
6h

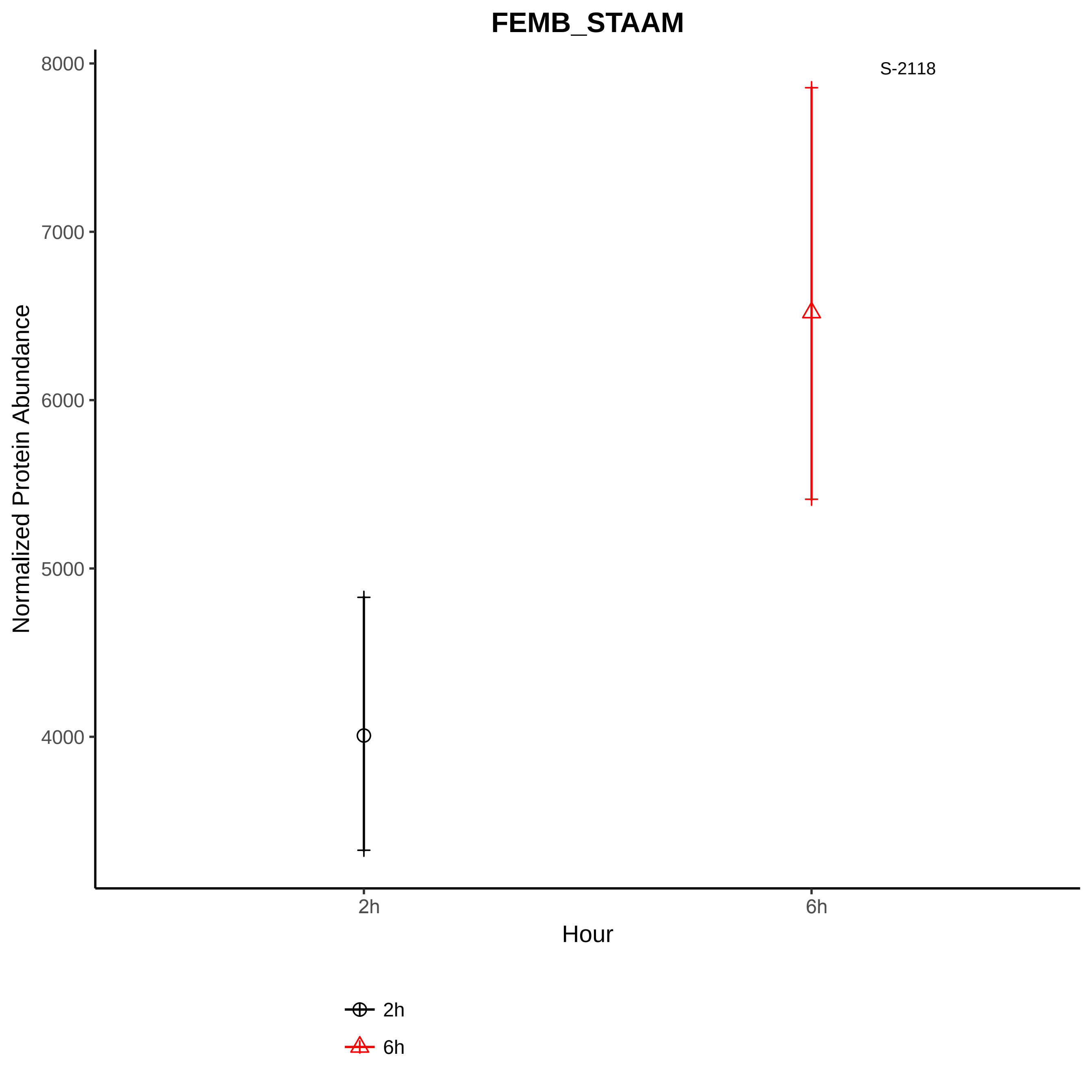
FEMB_STAAM
S-2118
Normalized Protein Abundance
8000
7000
6000
5000
4000
2h
6h
Hour
2h
6h

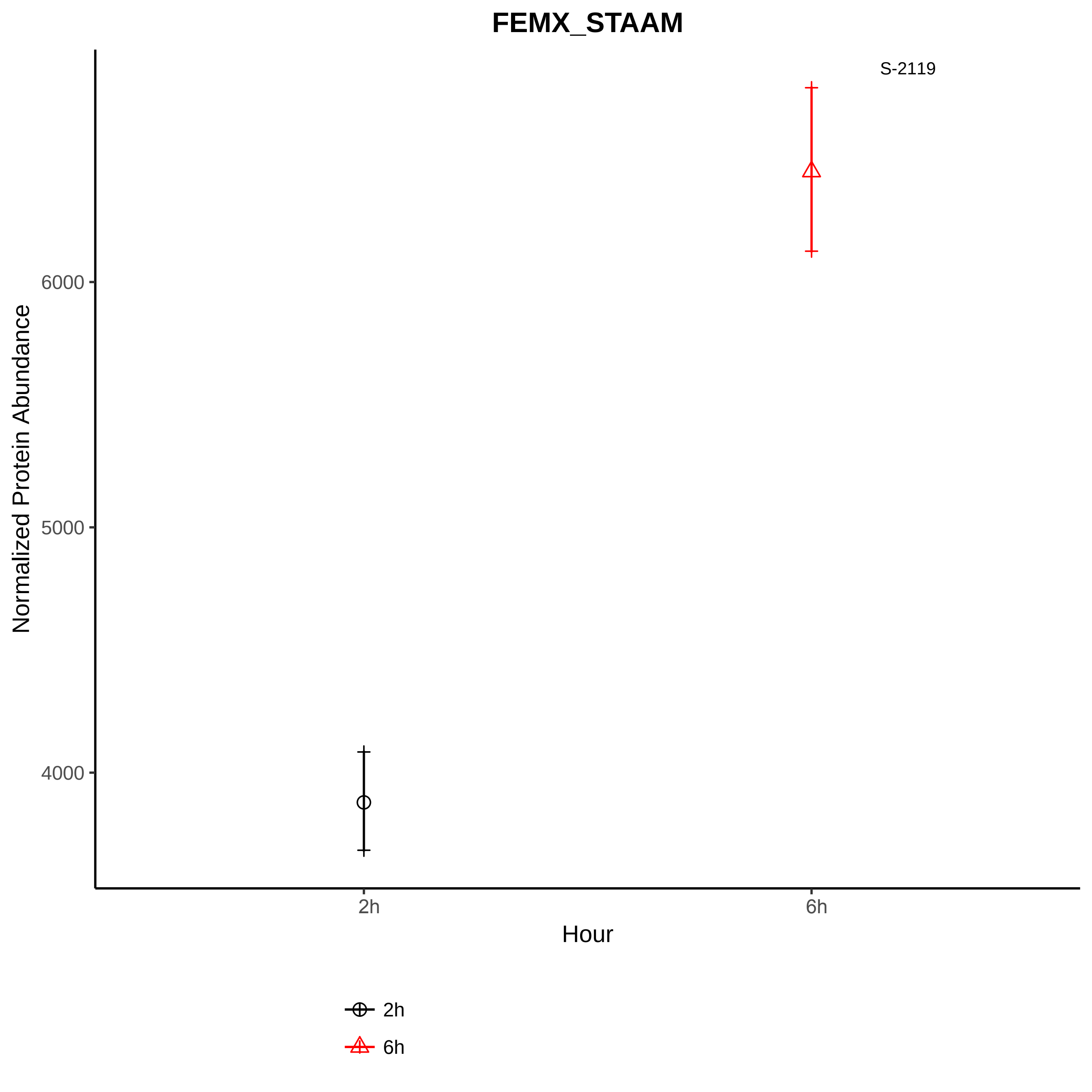

FEMX_STAAM
S-2119
Normalized Protein Abundance
6000
5000
4000
2h
6h
Hour
2h
6h

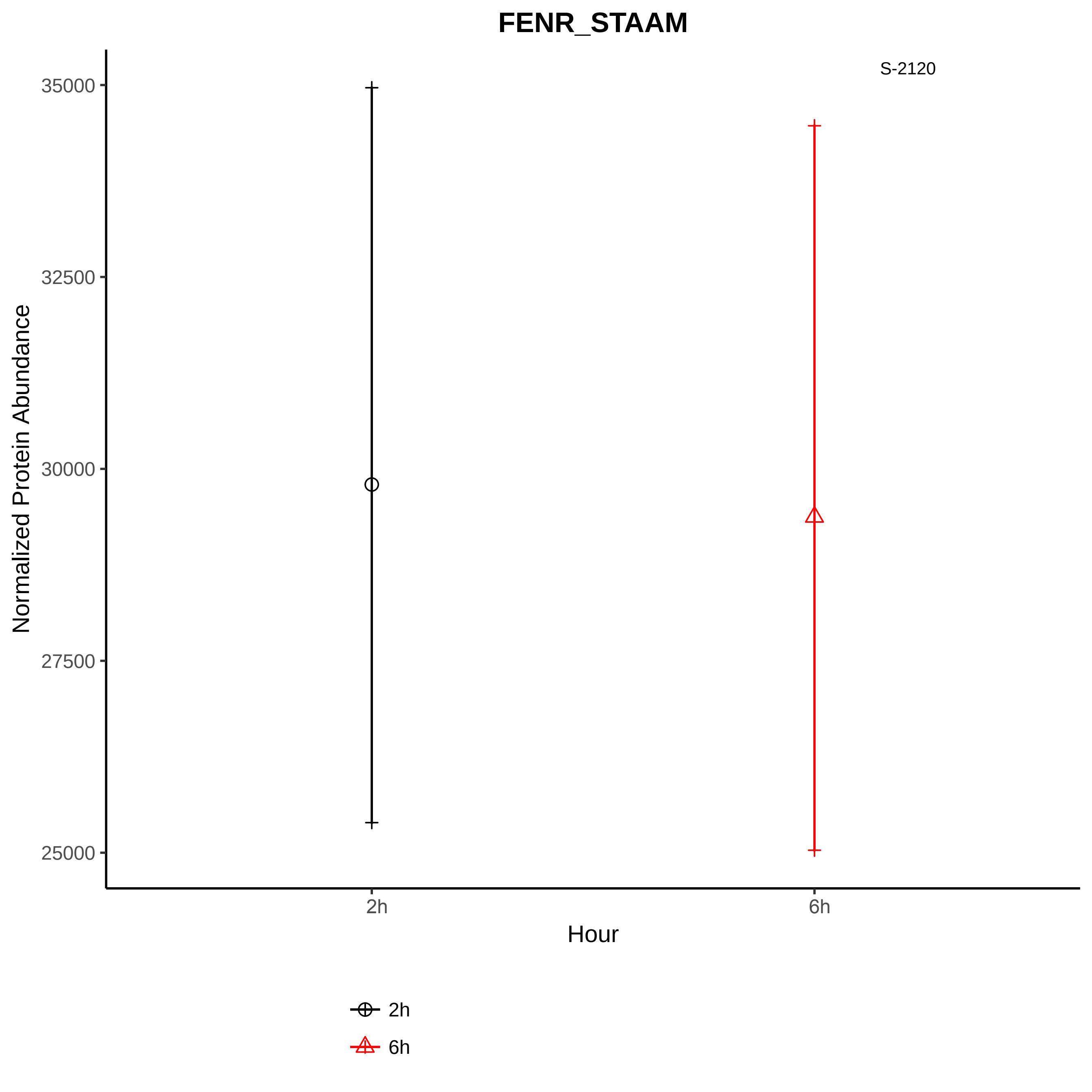

FENR_STAAM
S-2120
Normalized Protein Abundance
35000
32500
30000
27500
25000
2h
6h
Hour
2h
6h

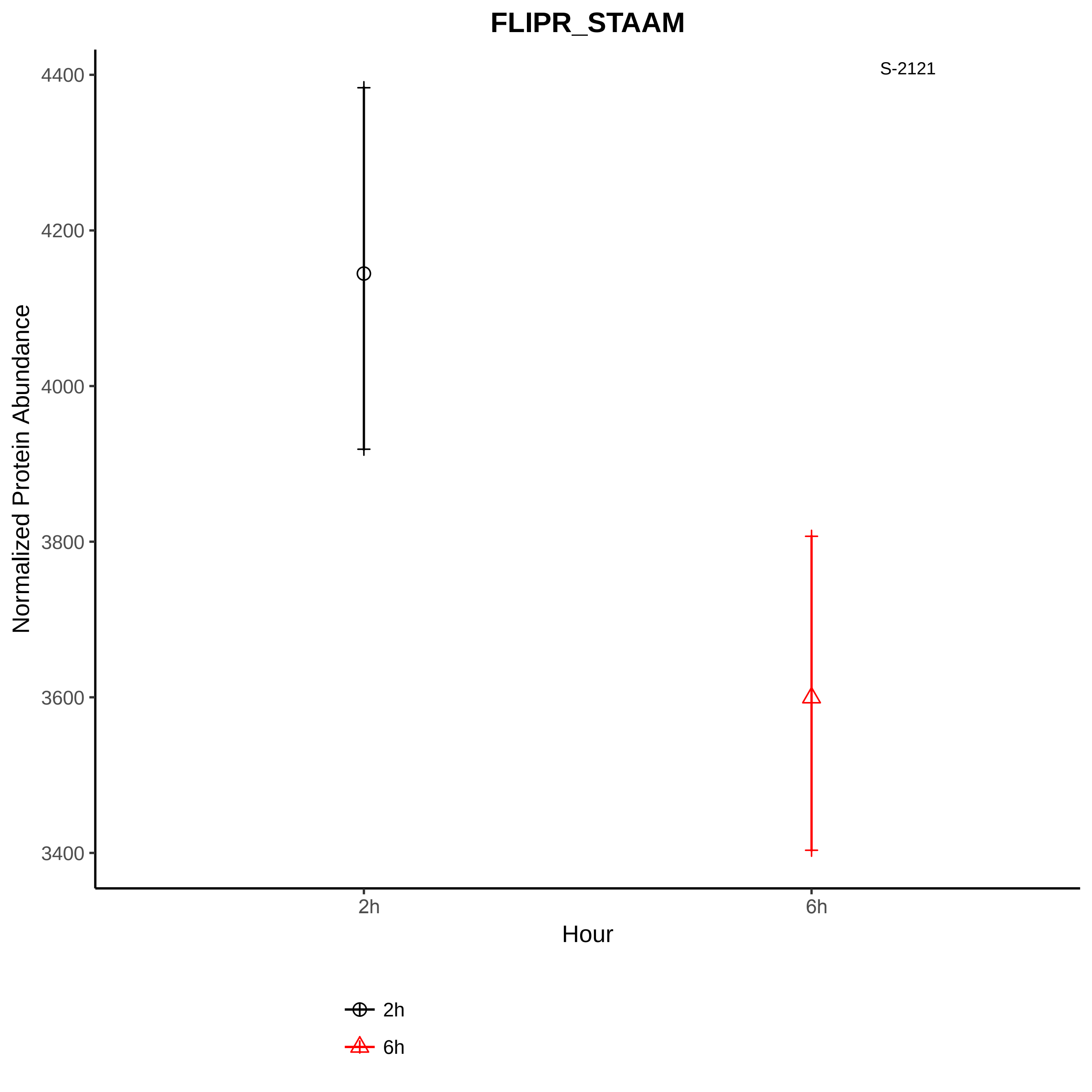
FLIPR_STAAM
S-2121
Normalized Protein Abundance
4400
4200
4000
3800
3600
3400
2h
6h
Hour
2h
6h

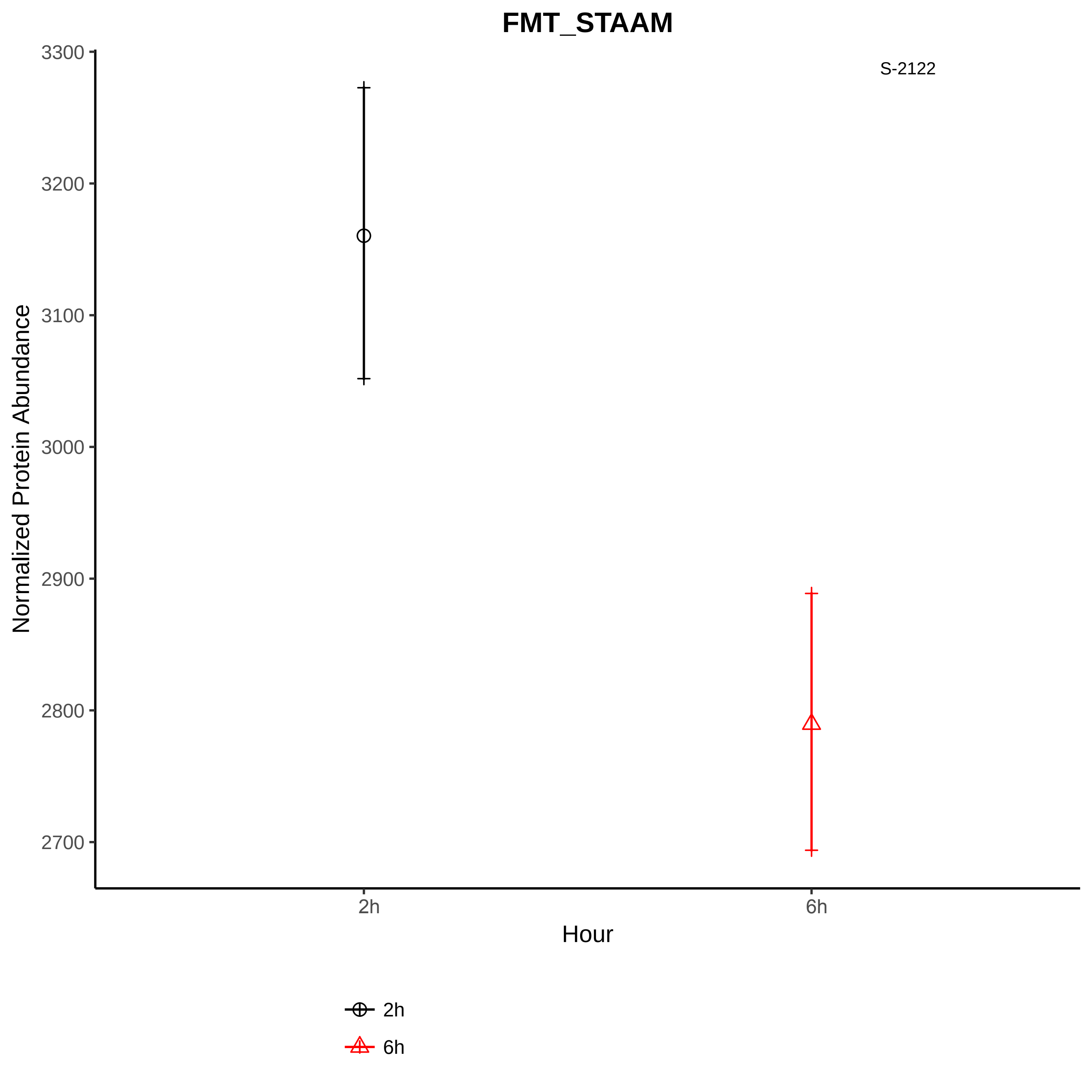
FMT_STAAM
S-2122
Normalized Protein Abundance
3300
3200
3100
3000
2900
2800
2700
2h
6h
Hour
2h
6h

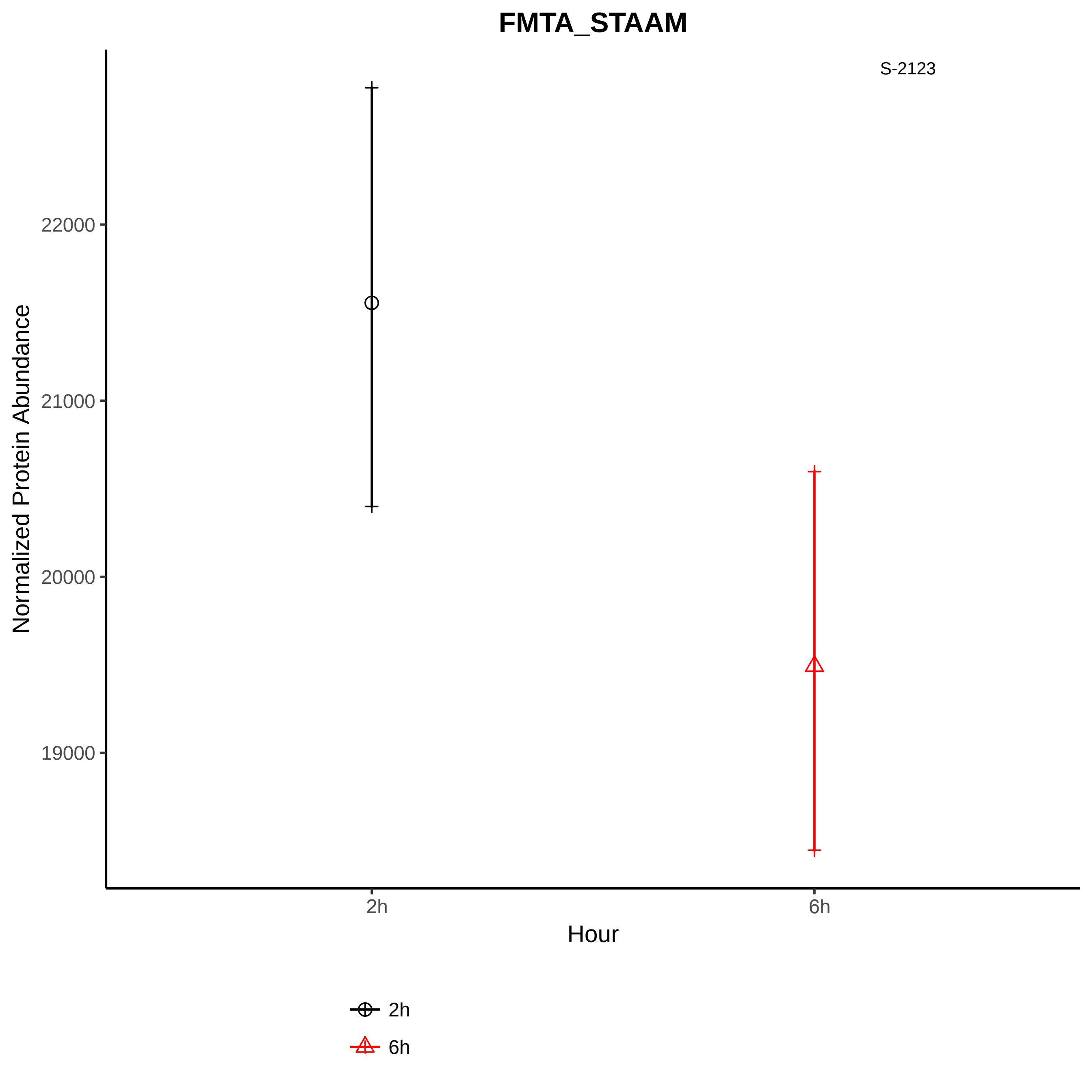

FMTA_STAAM
S-2123
Normalized Protein Abundance
22000
21000
20000
19000
2h
6h
Hour
2h
6h

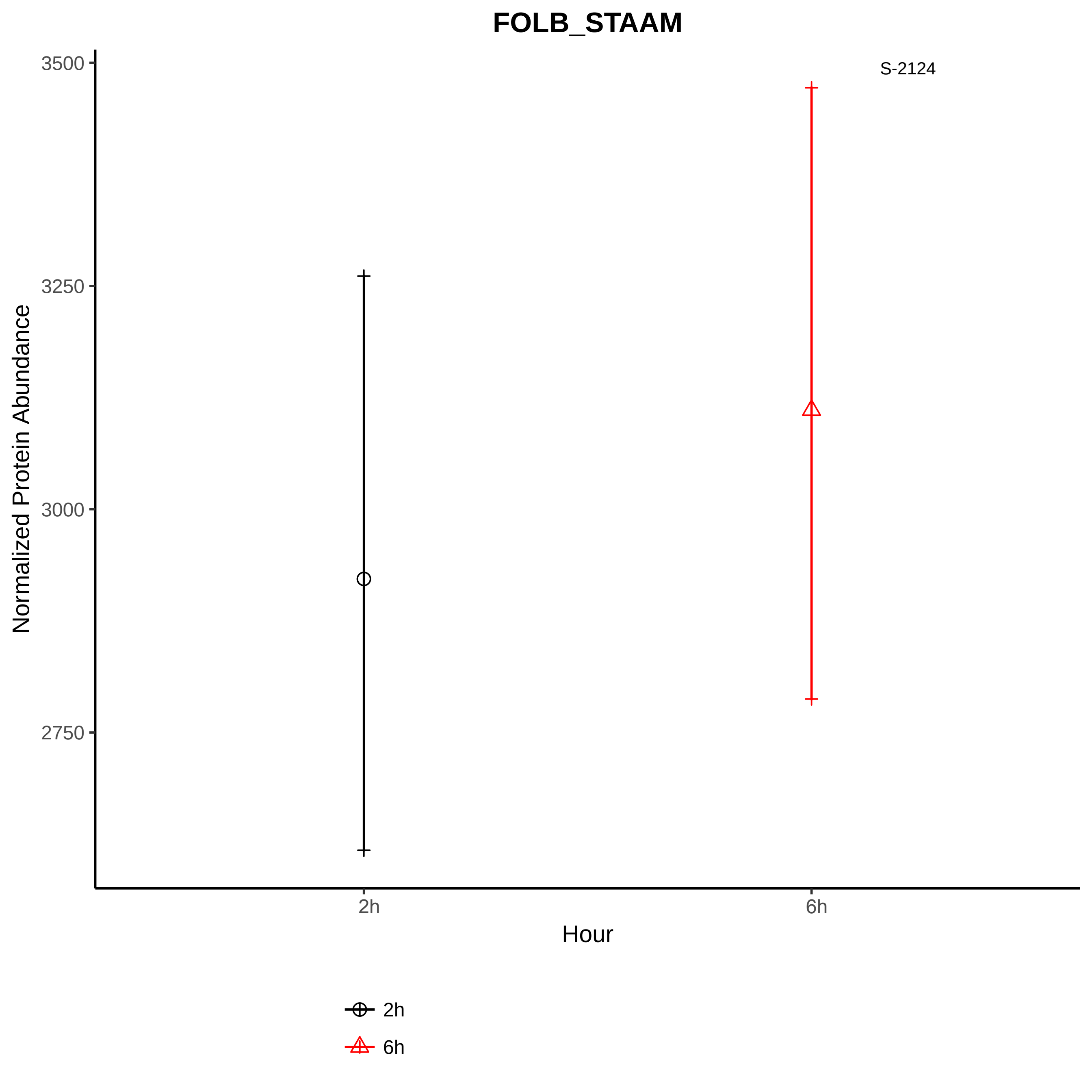

FOLB_STAAM
S-2124
Normalized Protein Abundance
3500
3250
3000
2750
2h
6h
Hour
2h
6h

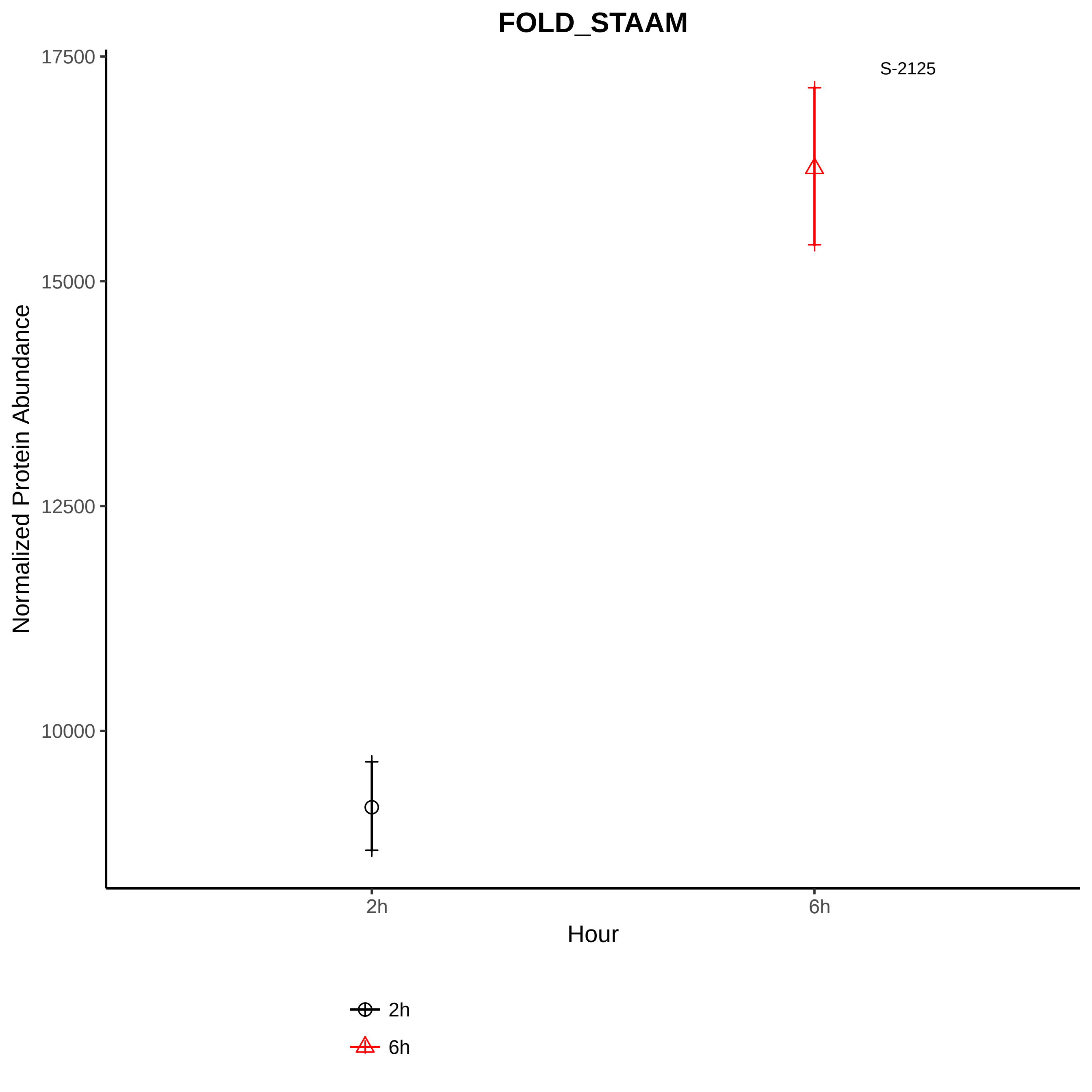
FOLD_STAAM
S-2125
Normalized Protein Abundance
17500
15000
12500
10000
2h
6h
Hour
2h
6h

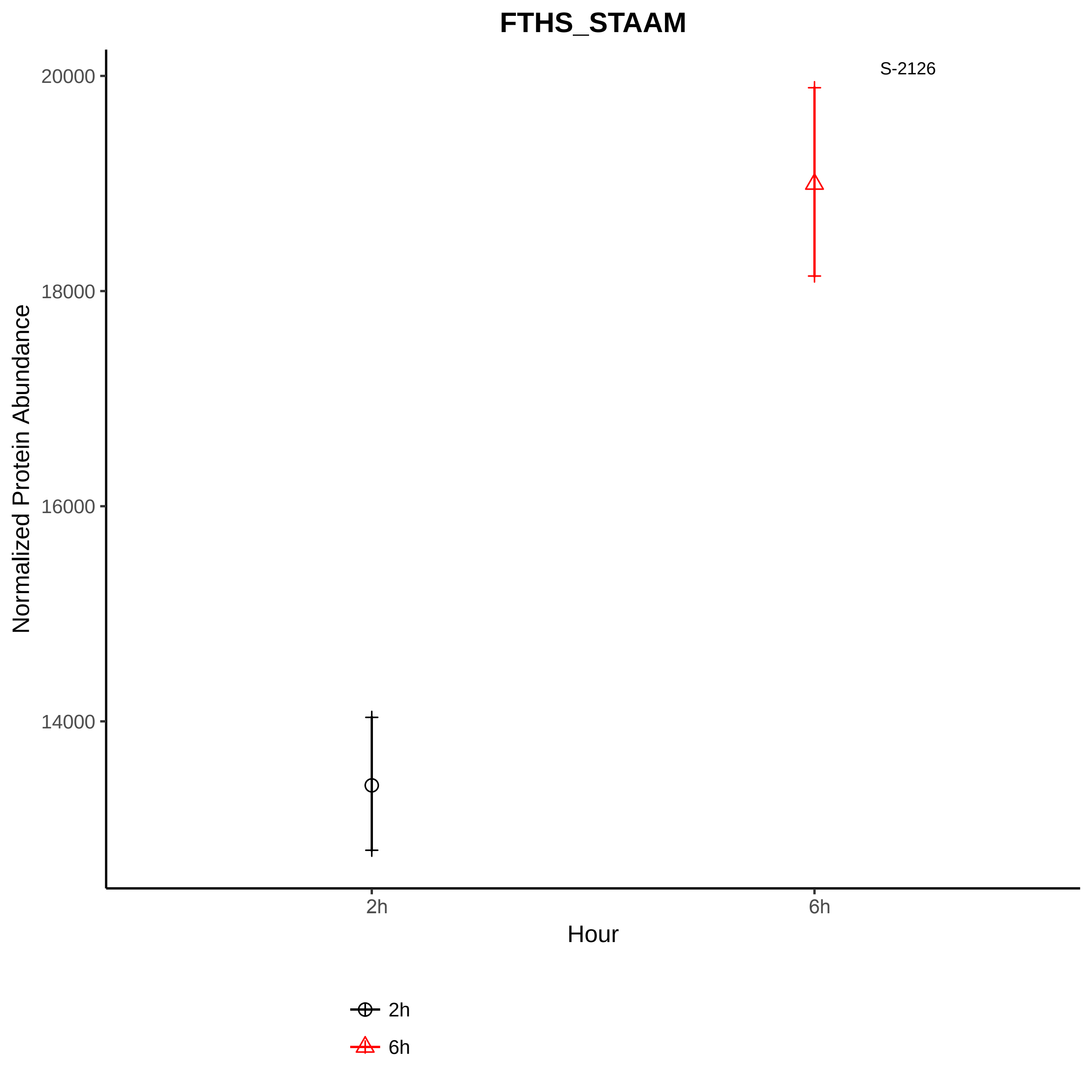

FTHS_STAAM
S-2126
Normalized Protein Abundance
20000
18000
16000
14000
2h
6h
Hour
2h
6h

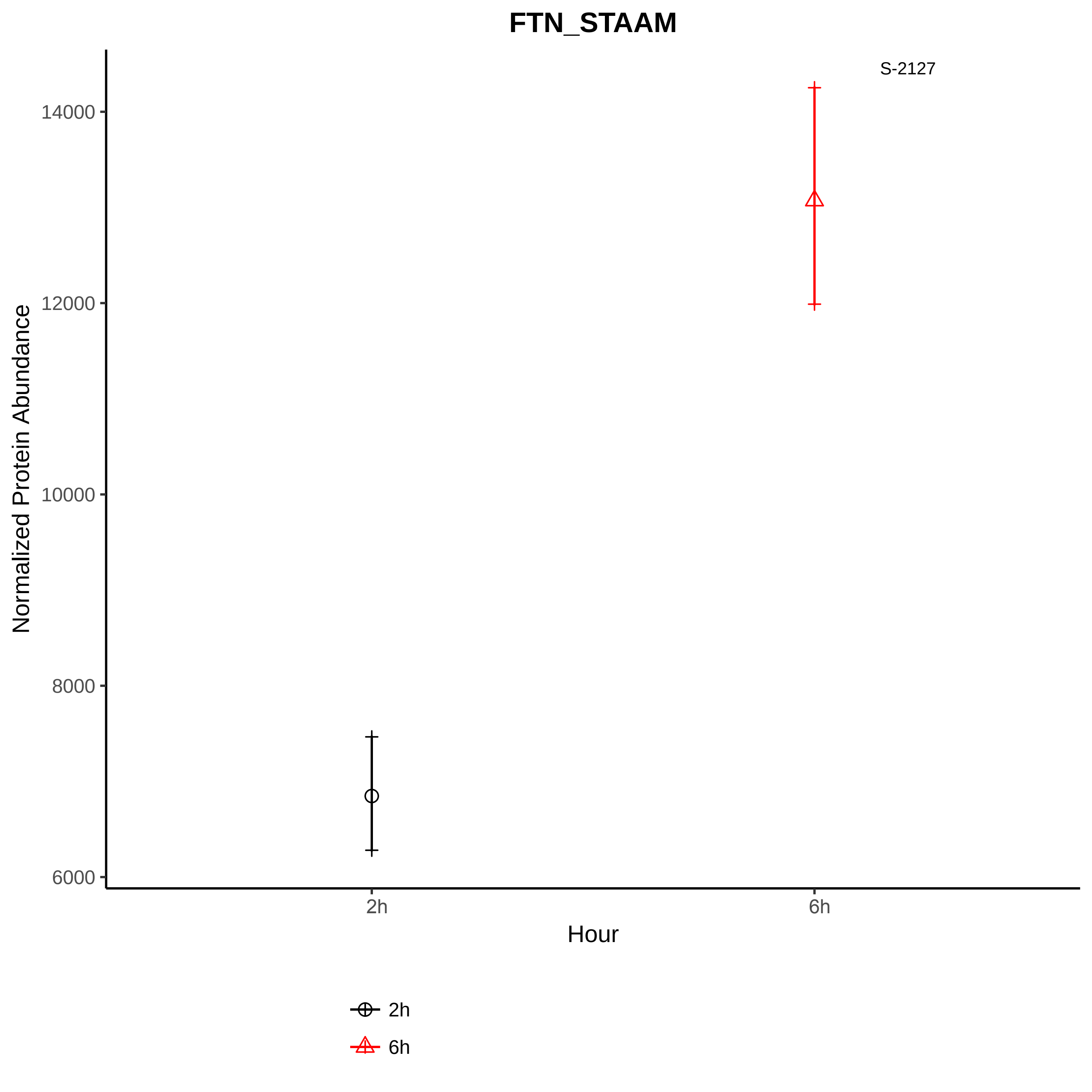

FTN_STAAM
S-2127
14000
12000
10000
8000
6000
Normalized Protein Abundance
2h
6h
Hour
2h
6h

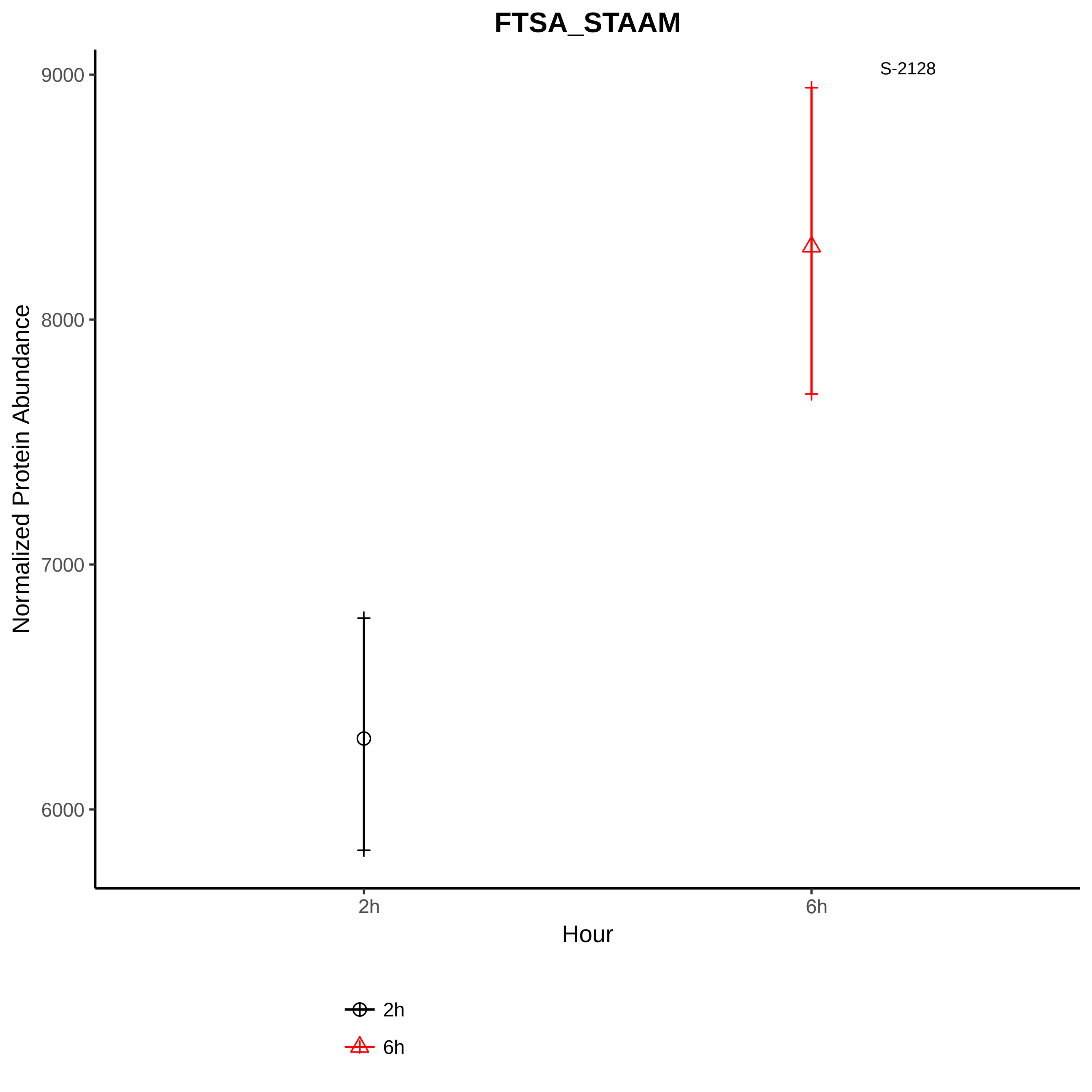

FTSA_STAAM
S-2128
9000
8000
7000
6000
Normalized Protein Abundance
2h
6h
Hour
2h
6h

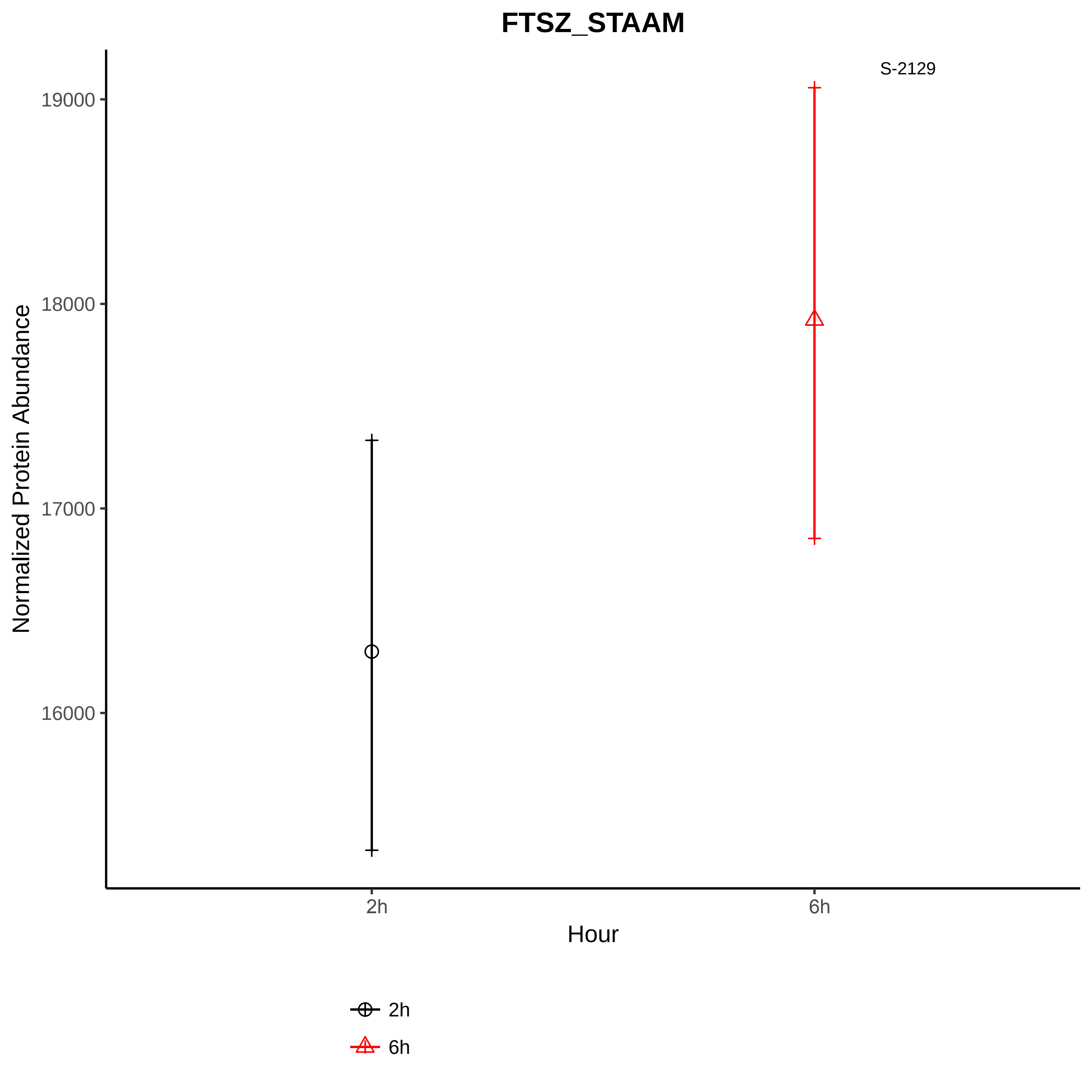

FTSZ_STAAM
S-2129
19000
18000
17000
16000
Normalized Protein Abundance
2h
6h
Hour
2h
6h

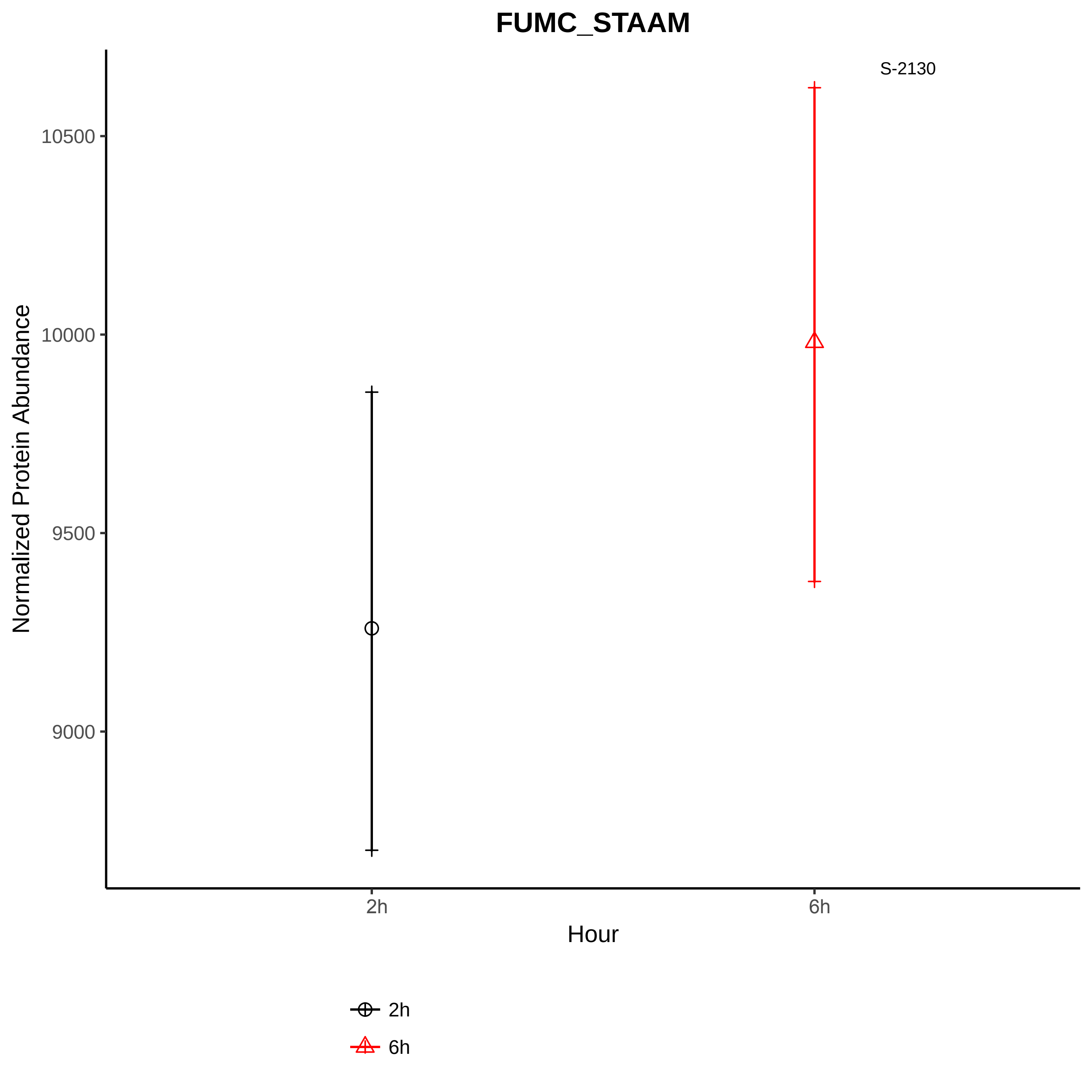

FUMC_STAAM
S-2130
Normalized Protein Abundance
10500
10000
9500
9000
2h
6h
Hour
2h
6h

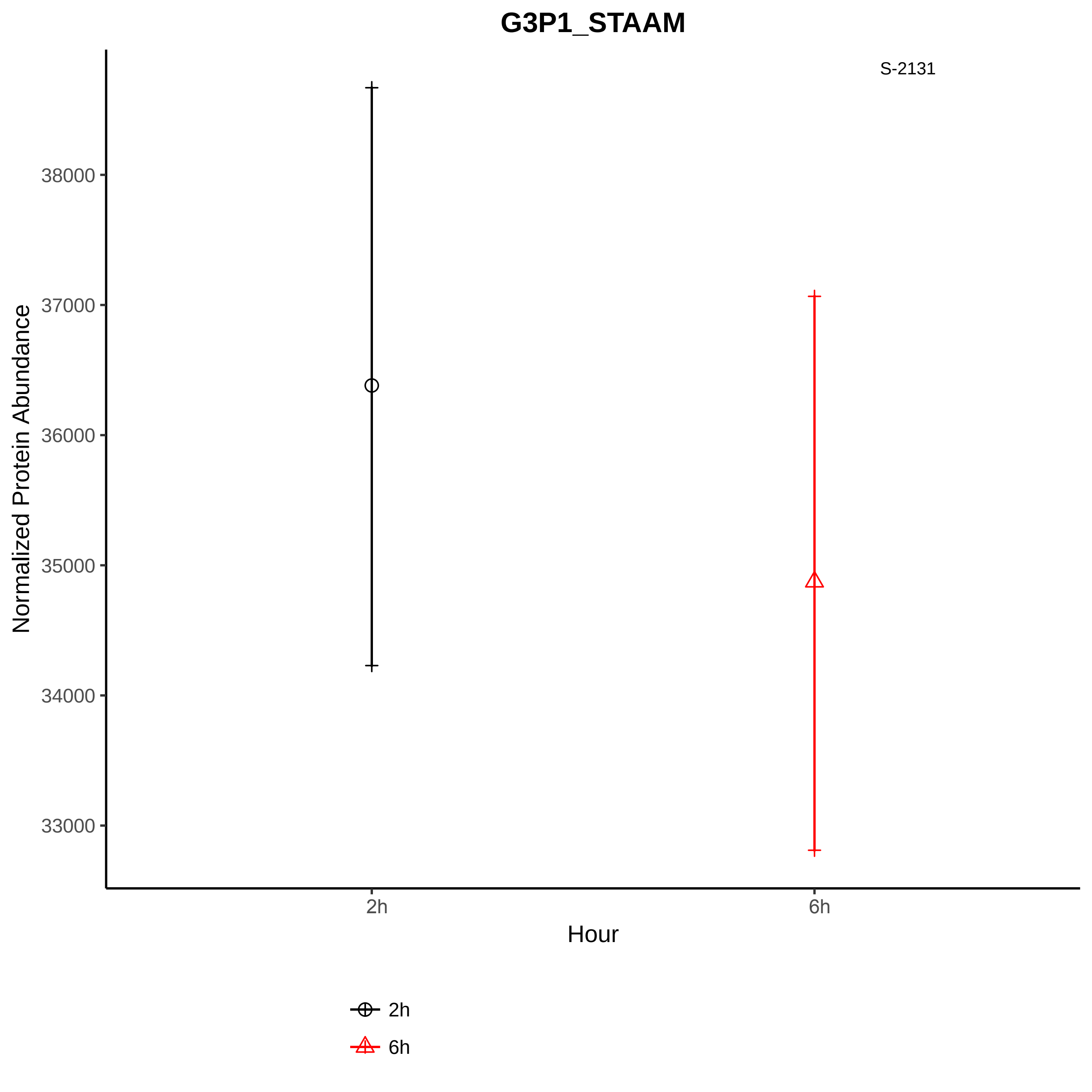

G3P1_STAAM
S-2131
Normalized Protein Abundance
38000
37000
36000
35000
34000
33000
2h
6h
Hour
2h
6h

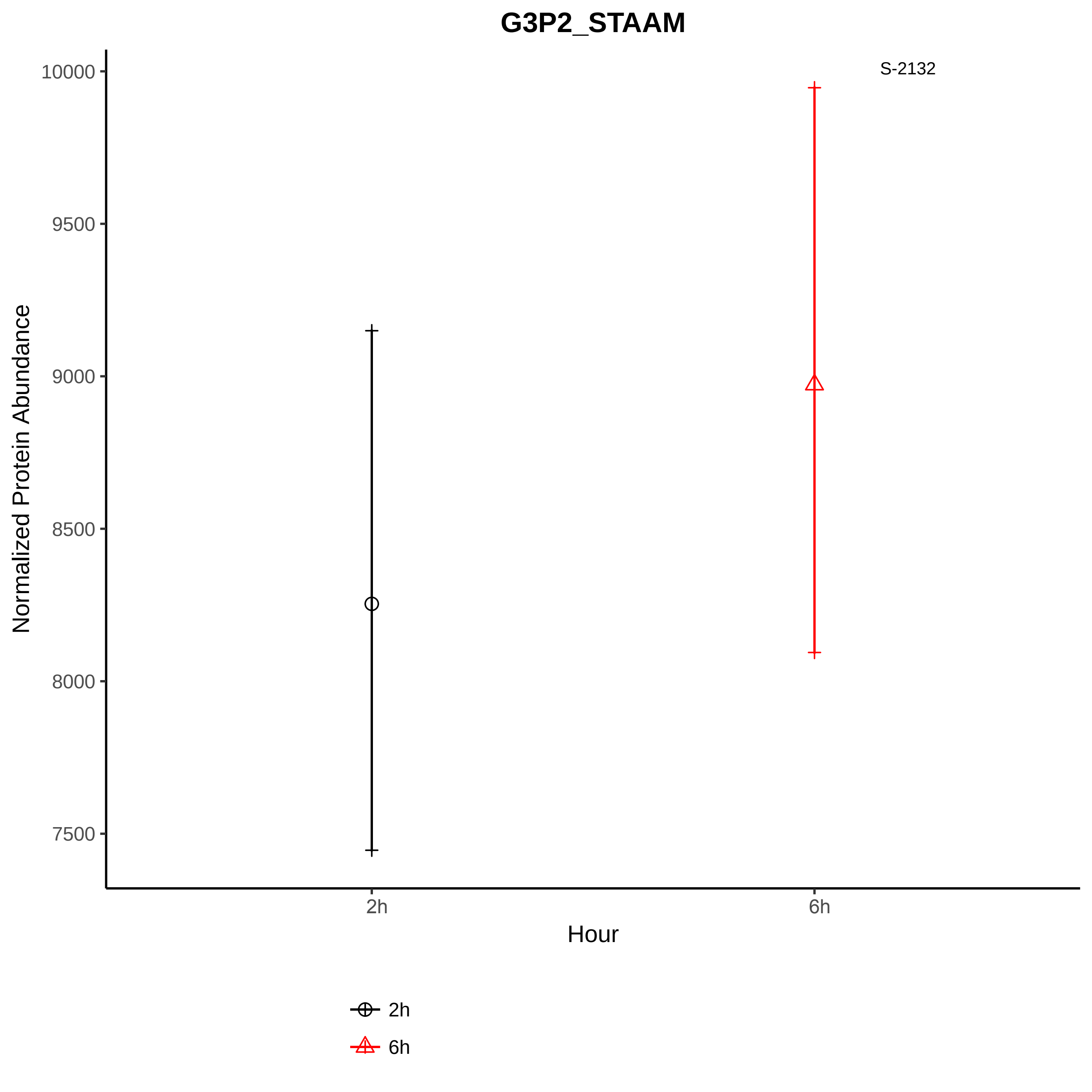
G3P2_STAAM
S-2132
Normalized Protein Abundance
10000
9500
9000
8500
8000
7500
2h
6h
Hour
2h
6h

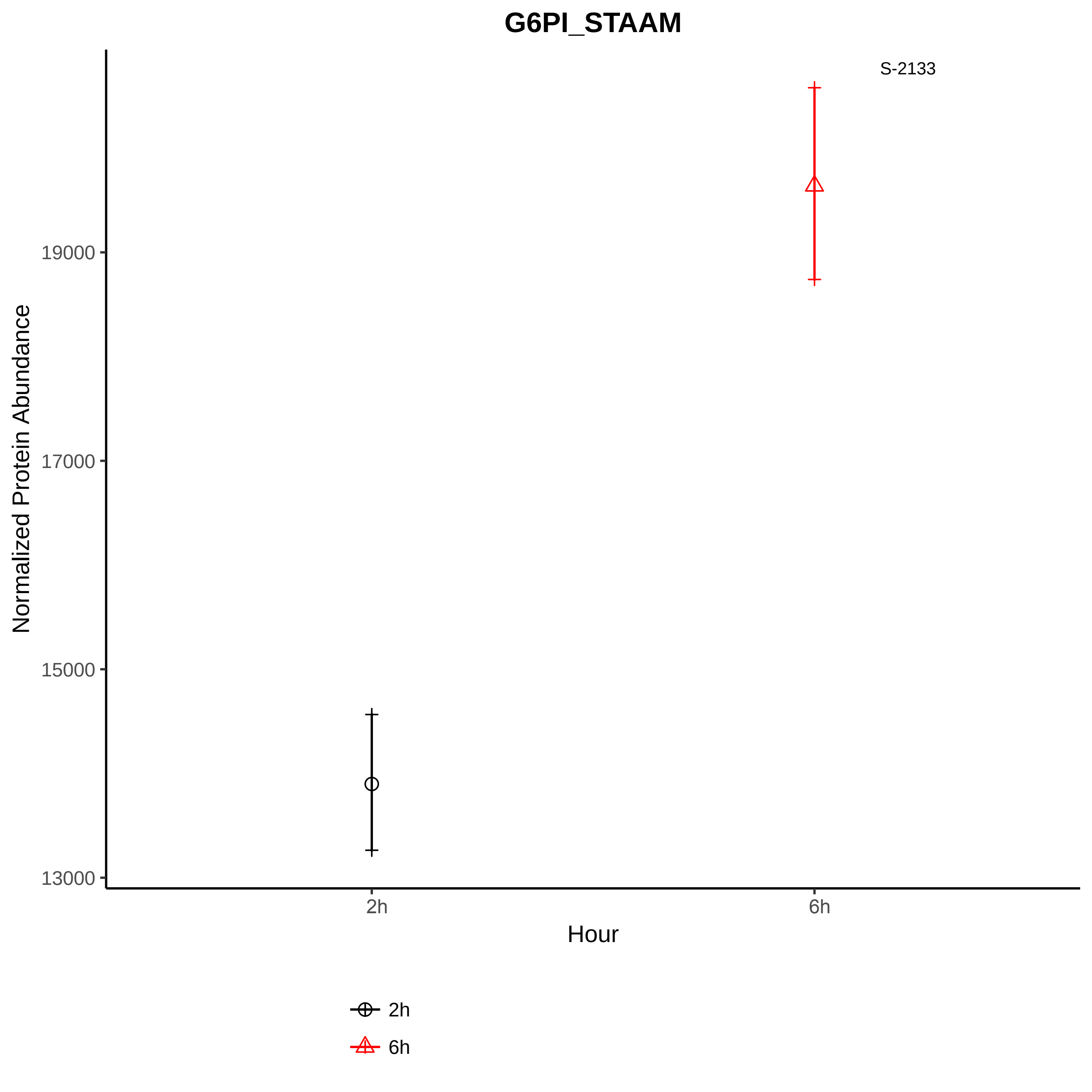
G6PI_STAAM
S-2133
Normalized Protein Abundance
19000
17000
15000
13000
2h
6h
Hour
2h
6h

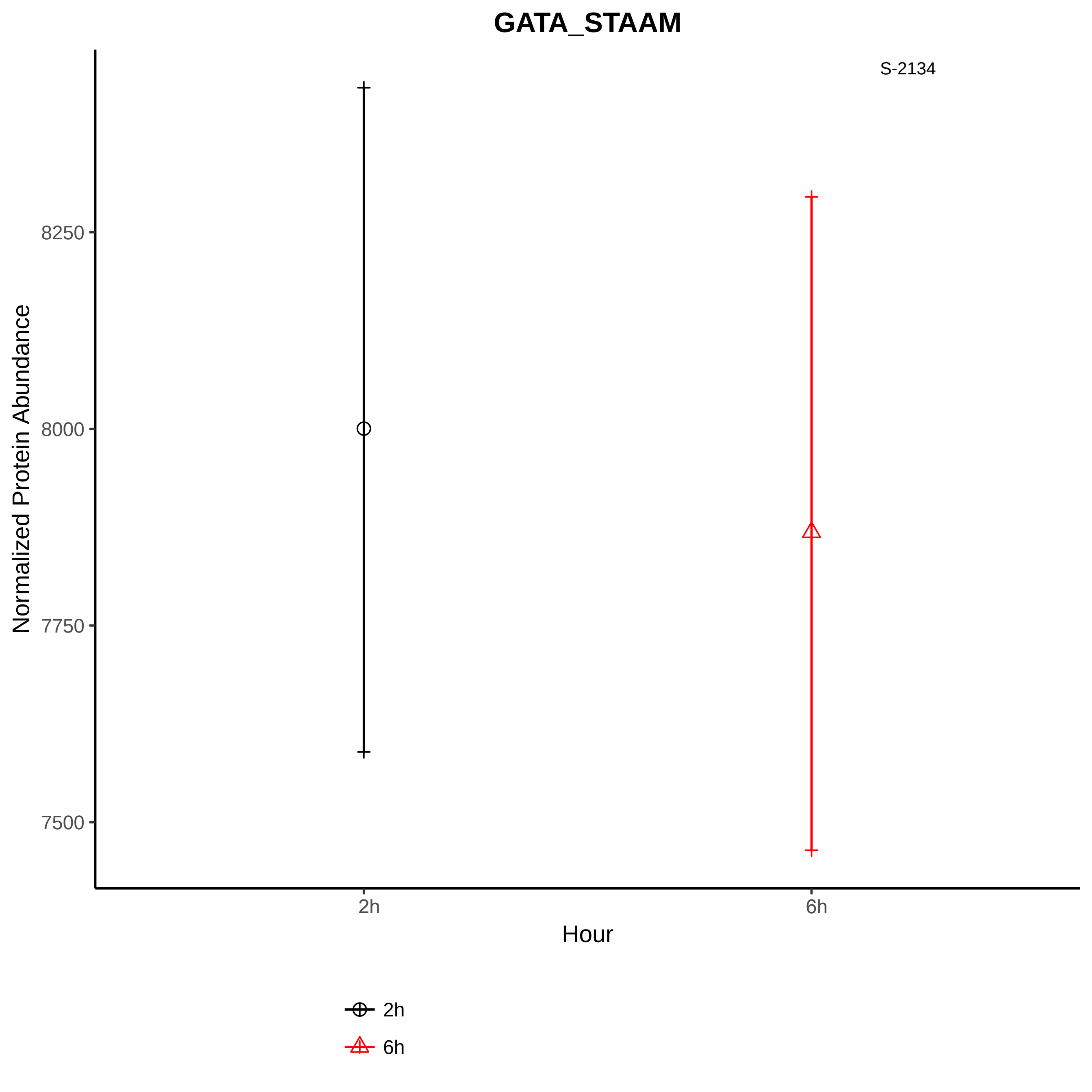

GATA_STAAM
S-2134
Normalized Protein Abundance
8250
8000
7750
7500
2h
6h
Hour
2h
6h

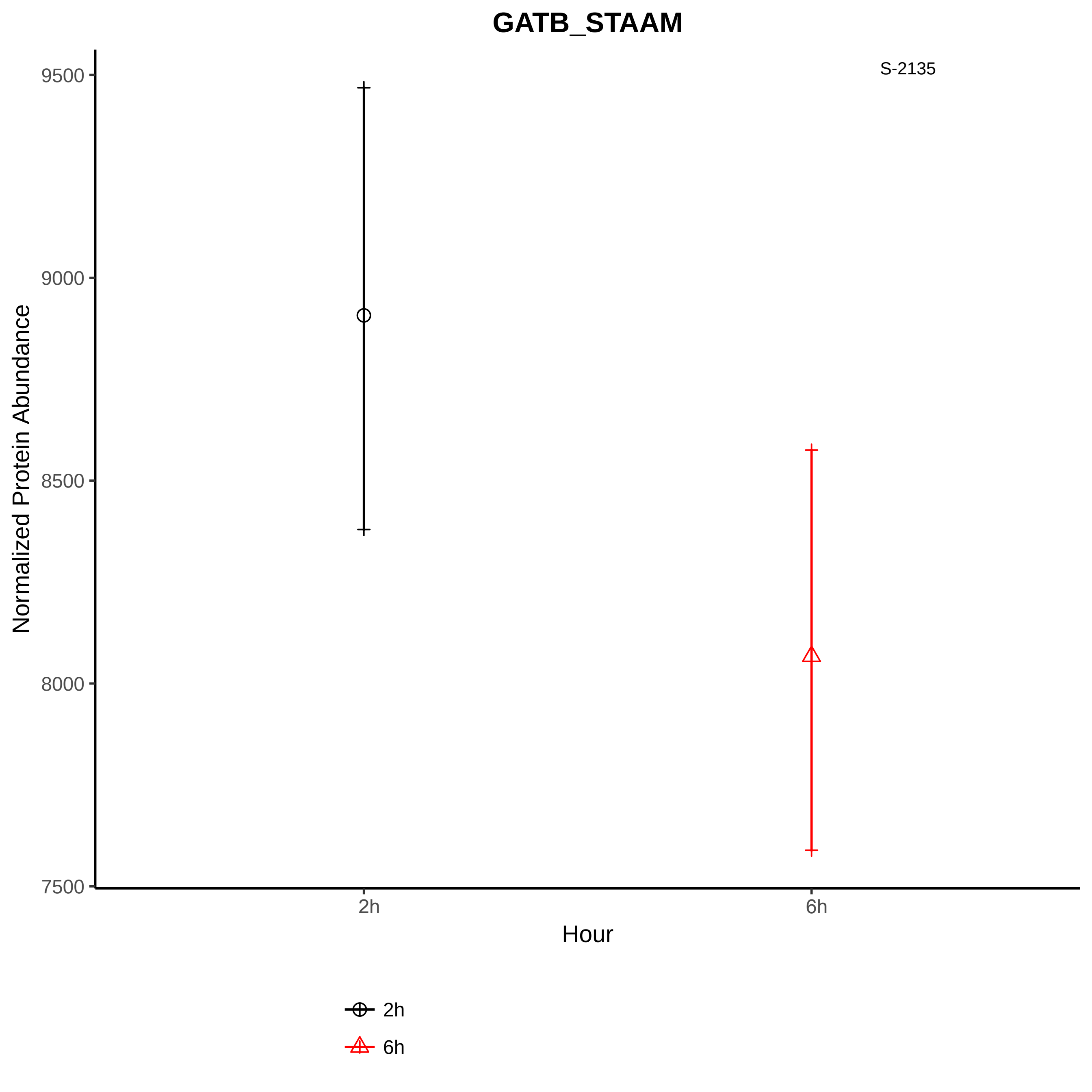
GATB_STAAM
S-2135
Normalized Protein Abundance
9500
9000
8500
8000
7500
2h
6h
Hour
2h
6h

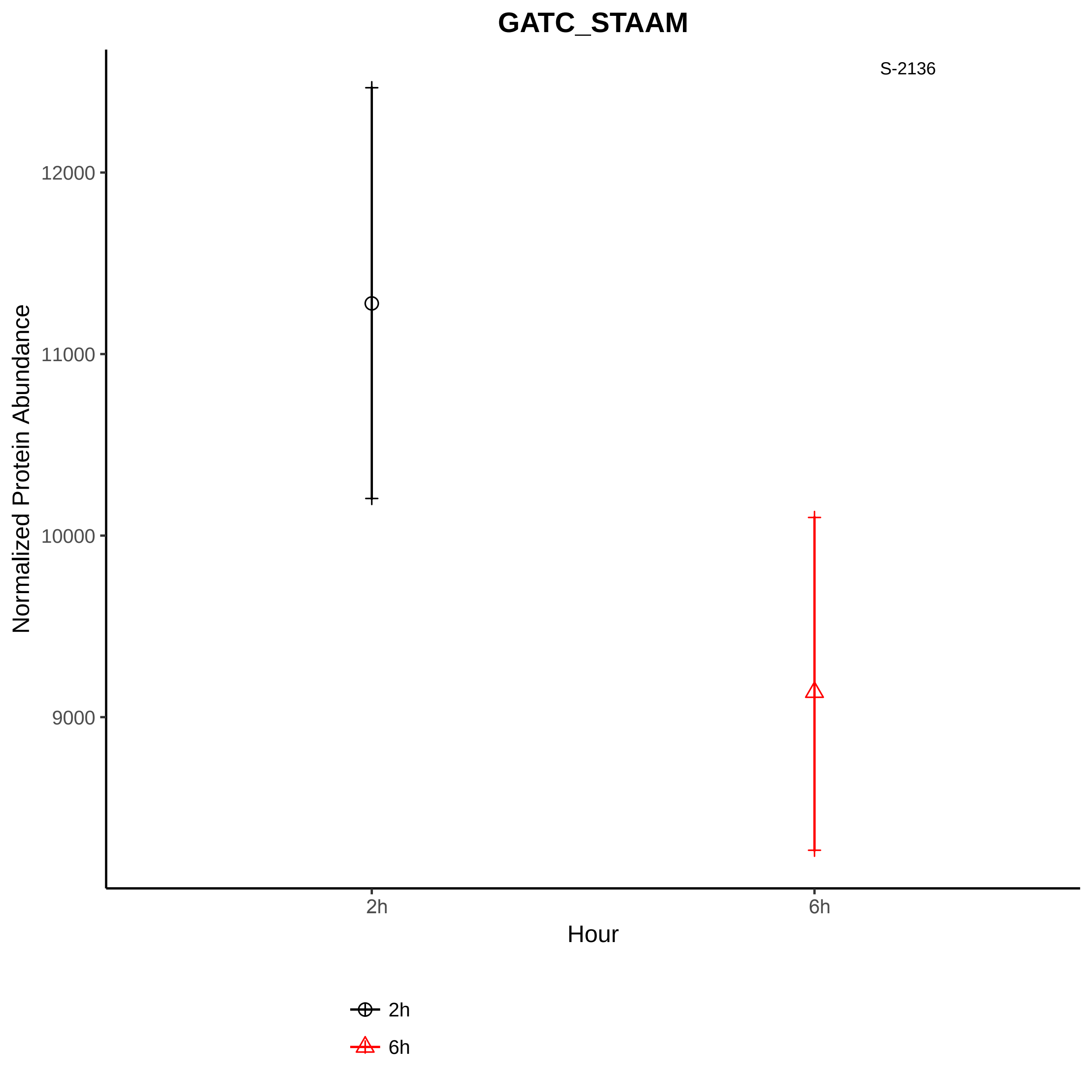

GATC_STAAM
S-2136
Normalized Protein Abundance
12000
11000
10000
9000
2h
6h
Hour
2h
6h

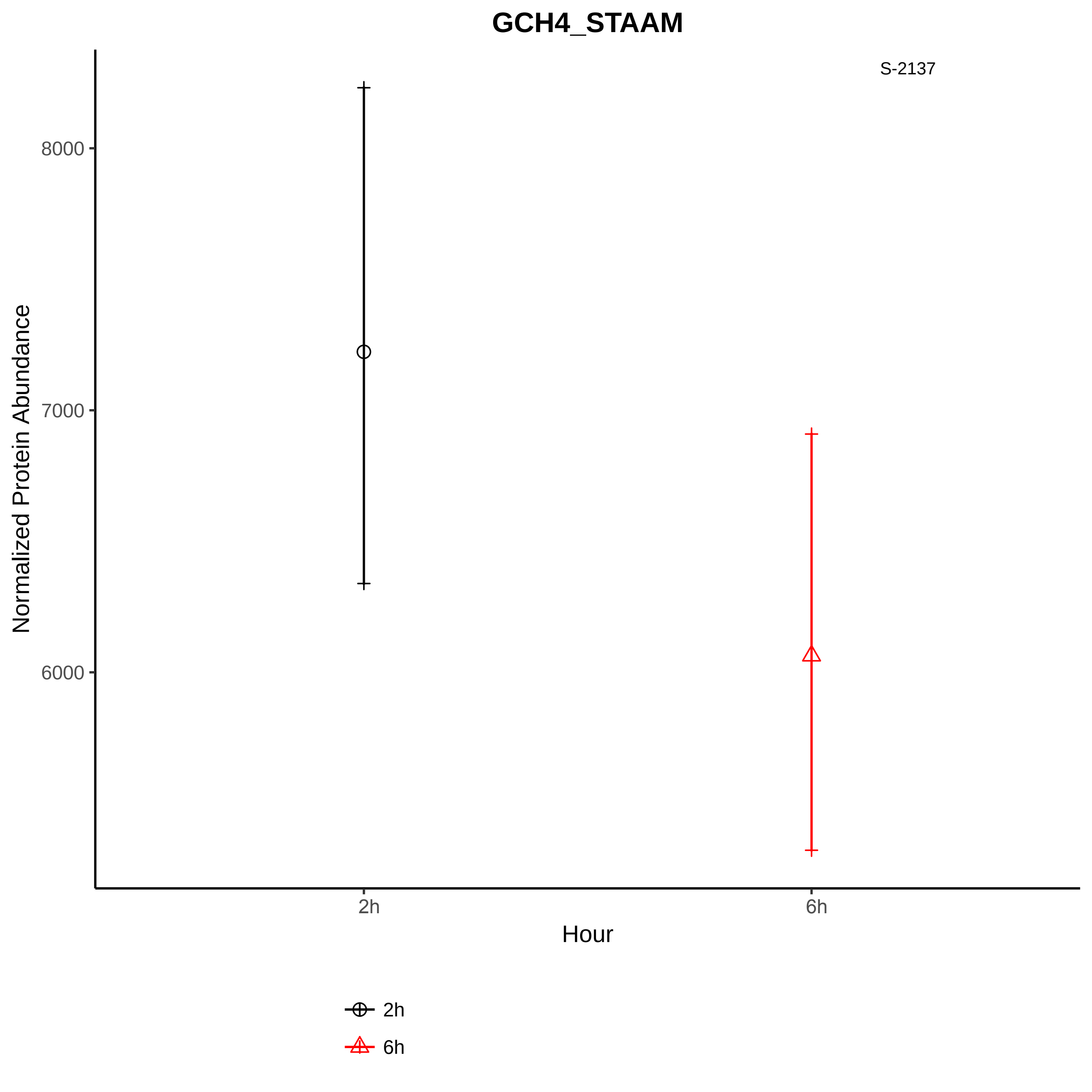

GCH4_STAAM
S-2137
8000
7000
6000
Normalized Protein Abundance
2h
6h
Hour
2h
6h

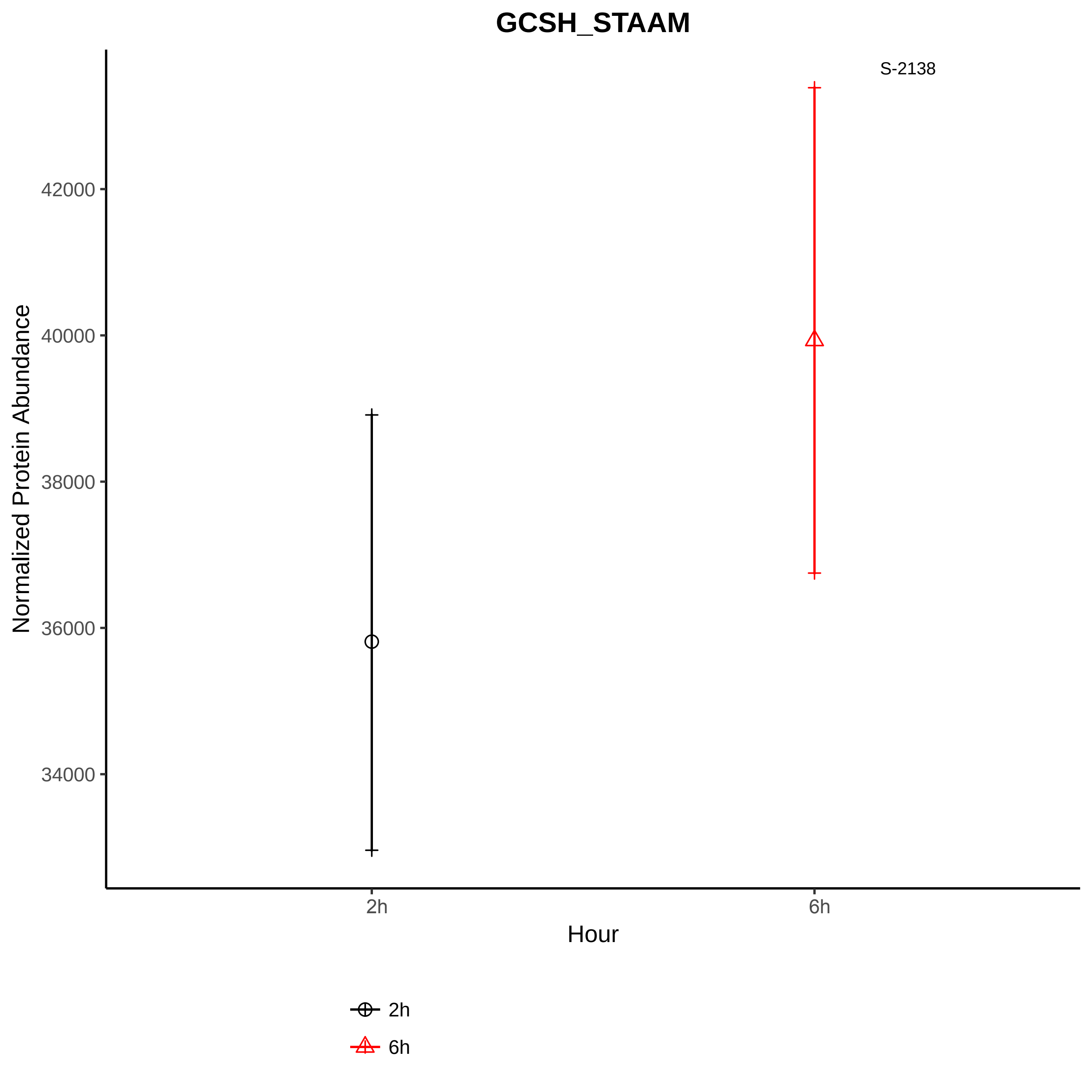
GCSH_STAAM
S-2138
Normalized Protein Abundance
42000
40000
38000
36000
34000
2h
6h
Hour
2h
6h

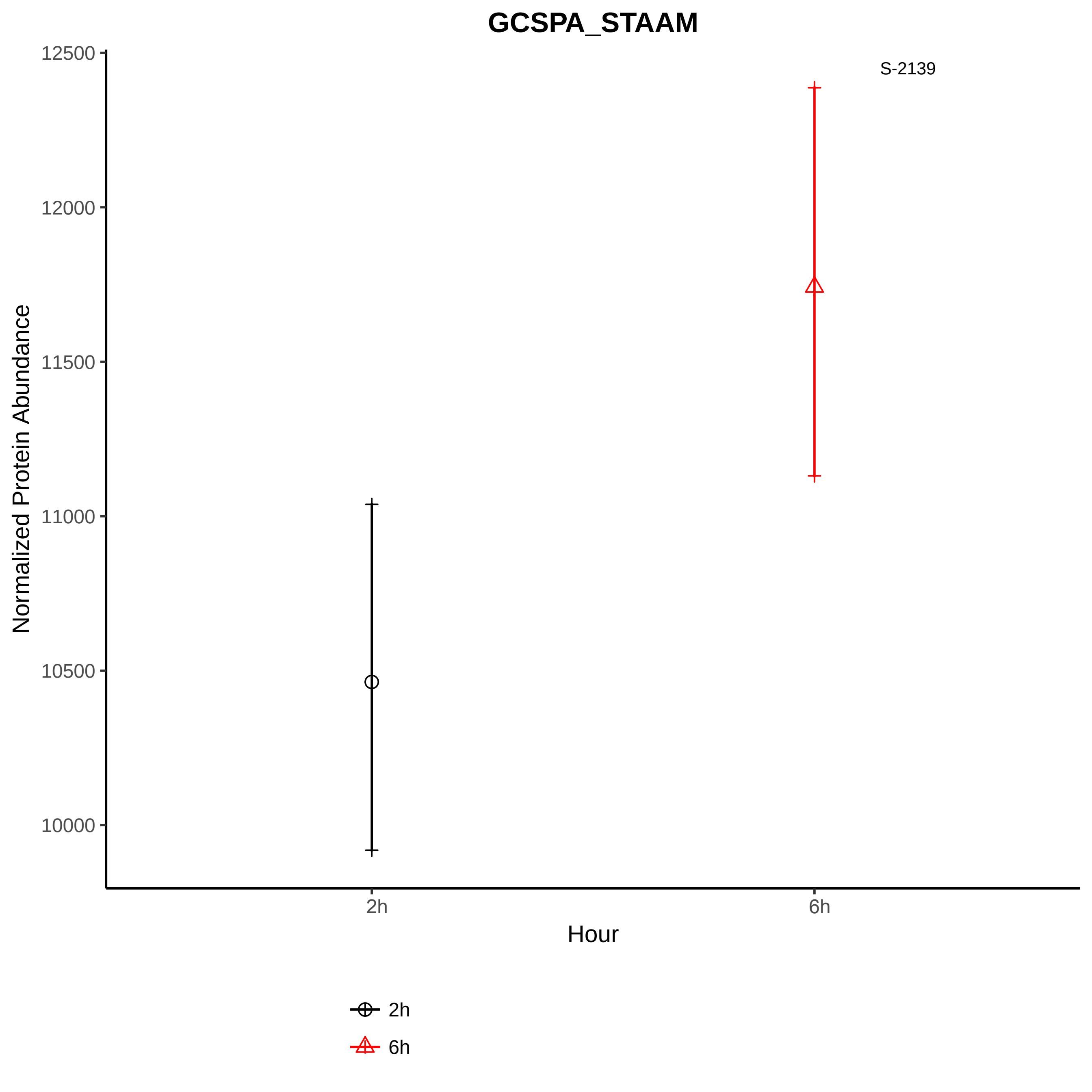

GCSPA_STAAM
S-2139
12500
12000
11500
11000
10500
10000
Normalized Protein Abundance
2h
6h
Hour
2h
6h

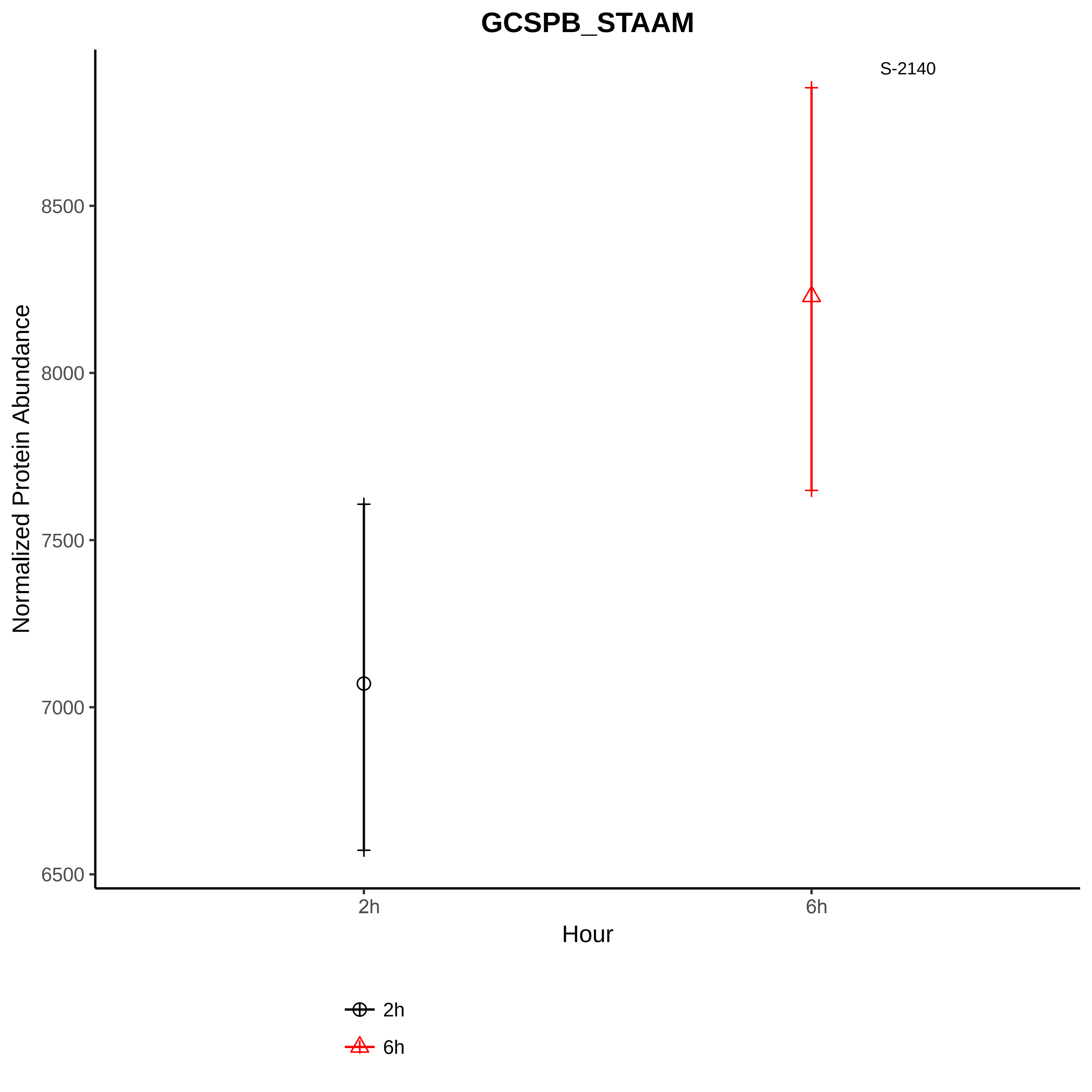

GCSPB_STAAM
S-2140
Normalized Protein Abundance
8500
8000
7500
7000
6500
2h
6h
Hour
2h
6h

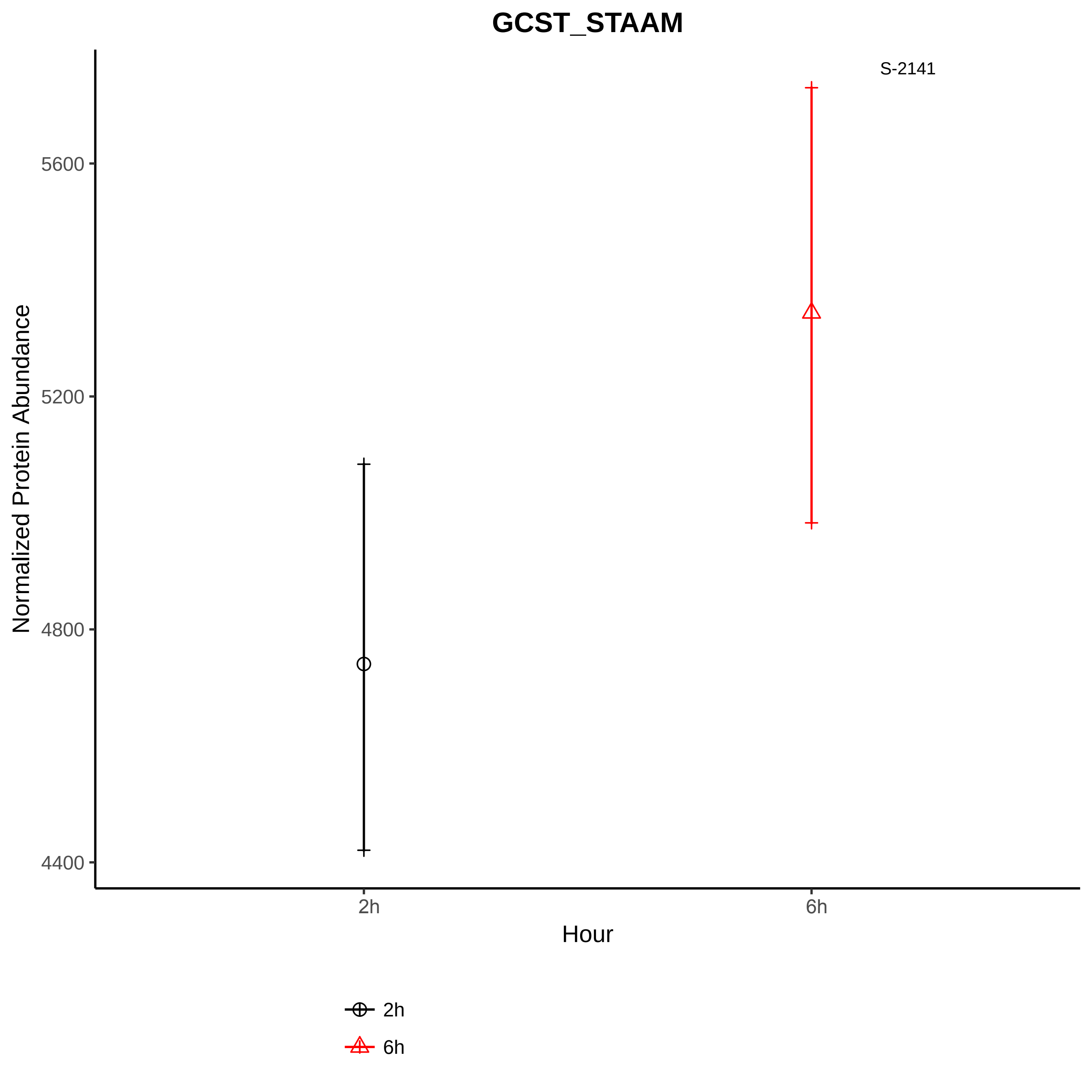
GCST_STAAM
S-2141
Normalized Protein Abundance
5600
5200
4800
4400
2h
6h
Hour
2h
6h

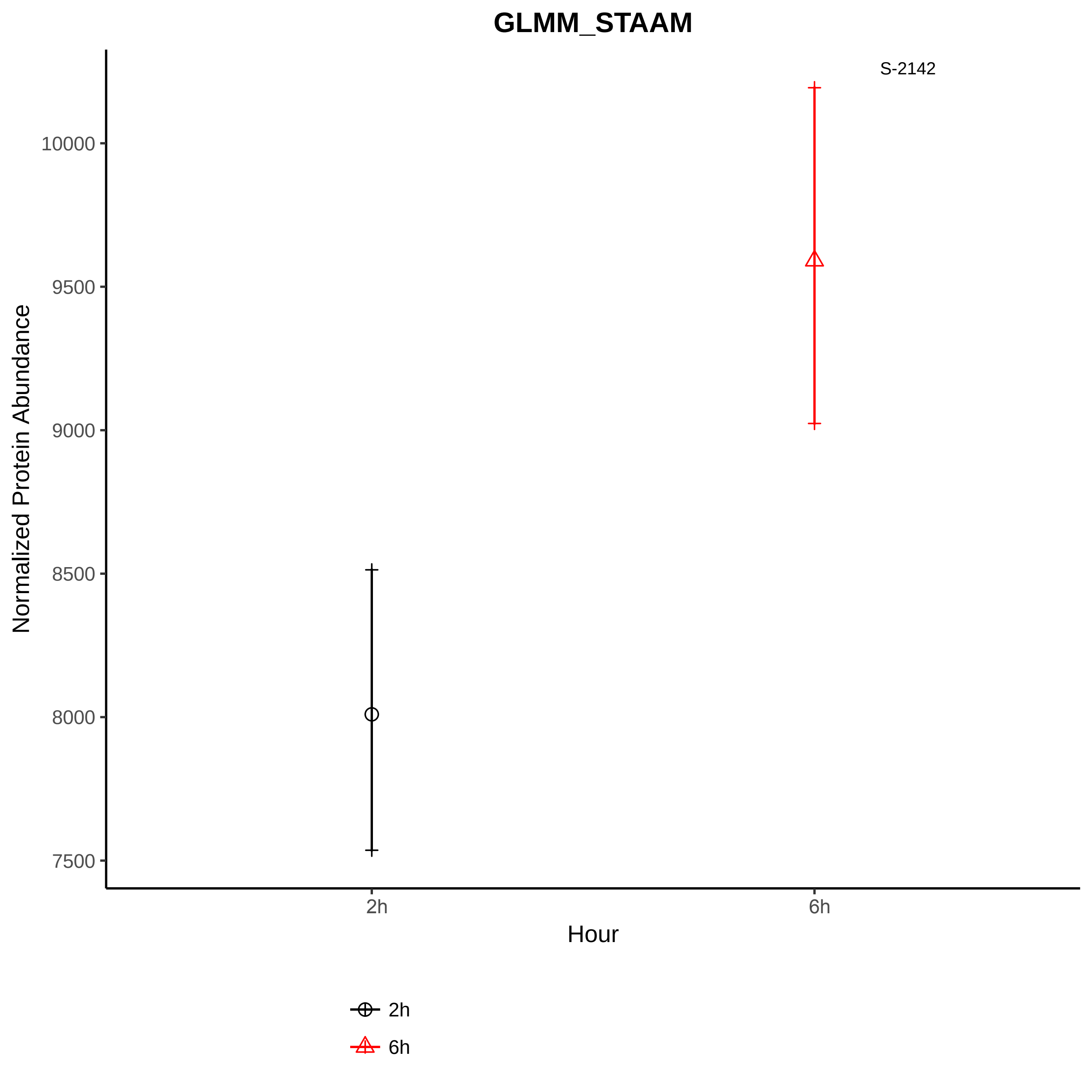

GLMM_STAAM
S-2142
10000
9500
9000
8500
8000
7500
2h
6h
Hour
Normalized Protein Abundance
2h
6h

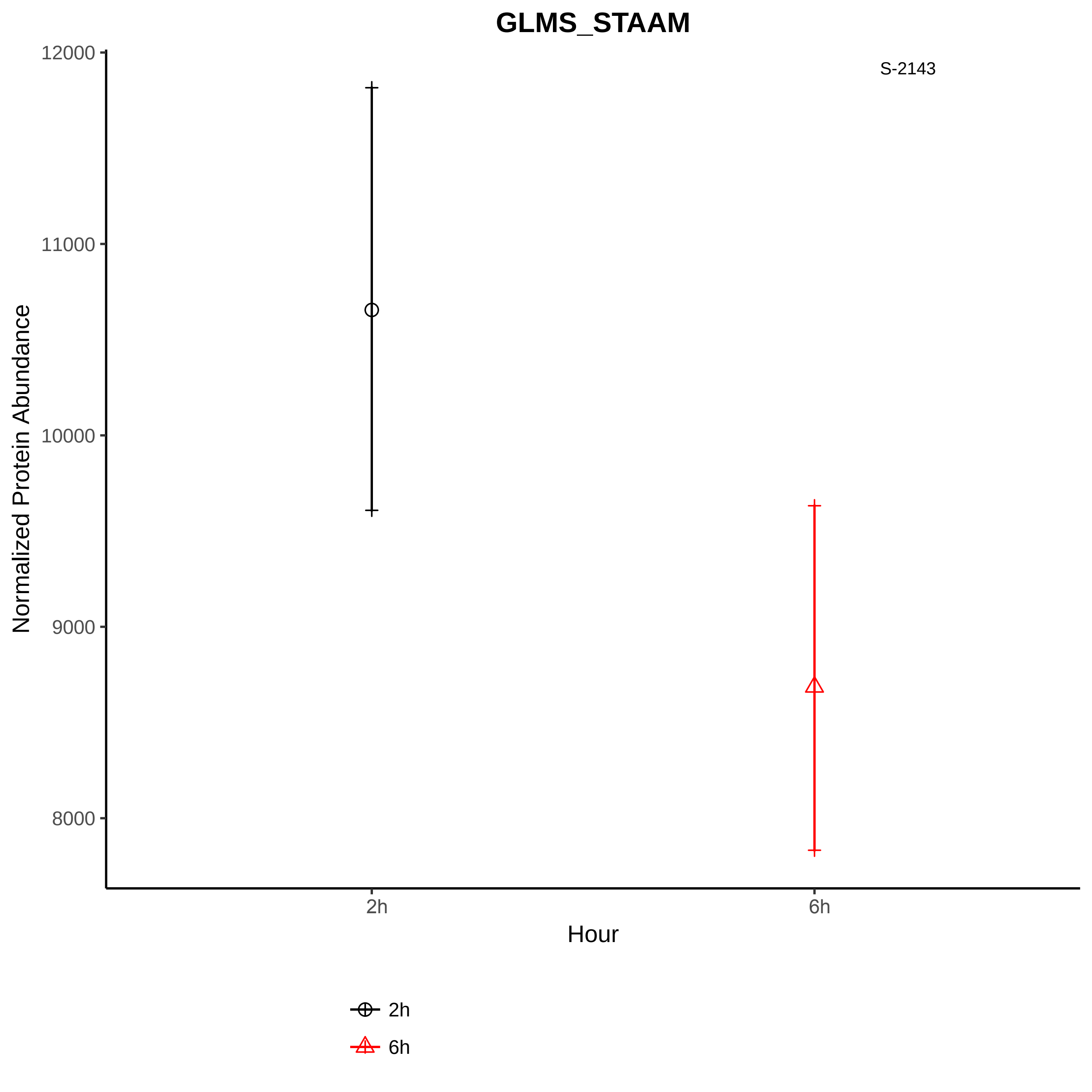

GLMS_STAAM
S-2143
Normalized Protein Abundance
12000
11000
10000
9000
8000
2h
6h
Hour
2h
6h

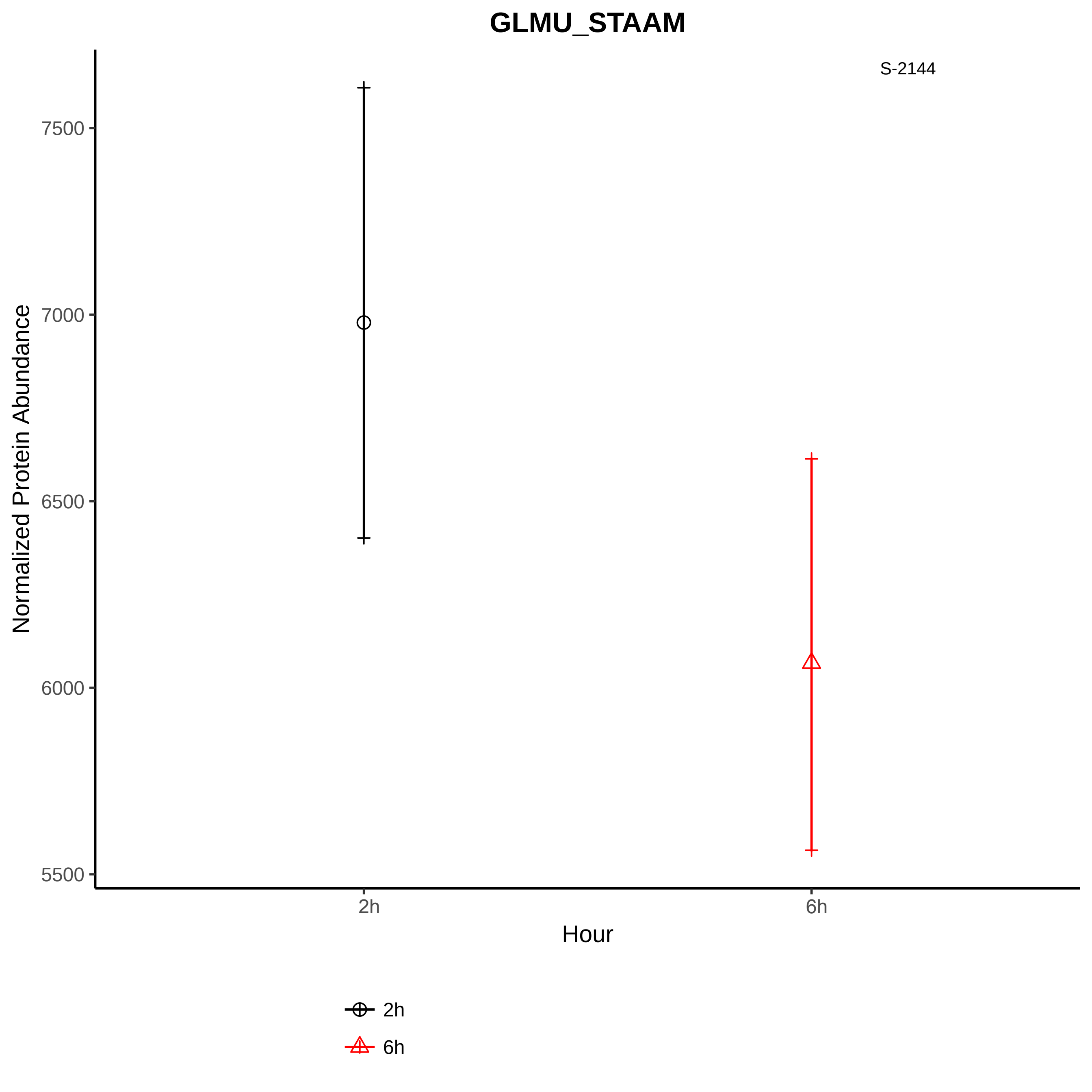
GLMU_STAAM
S-2144
Normalized Protein Abundance
7500
7000
6500
6000
5500
2h
6h
Hour
2h
6h

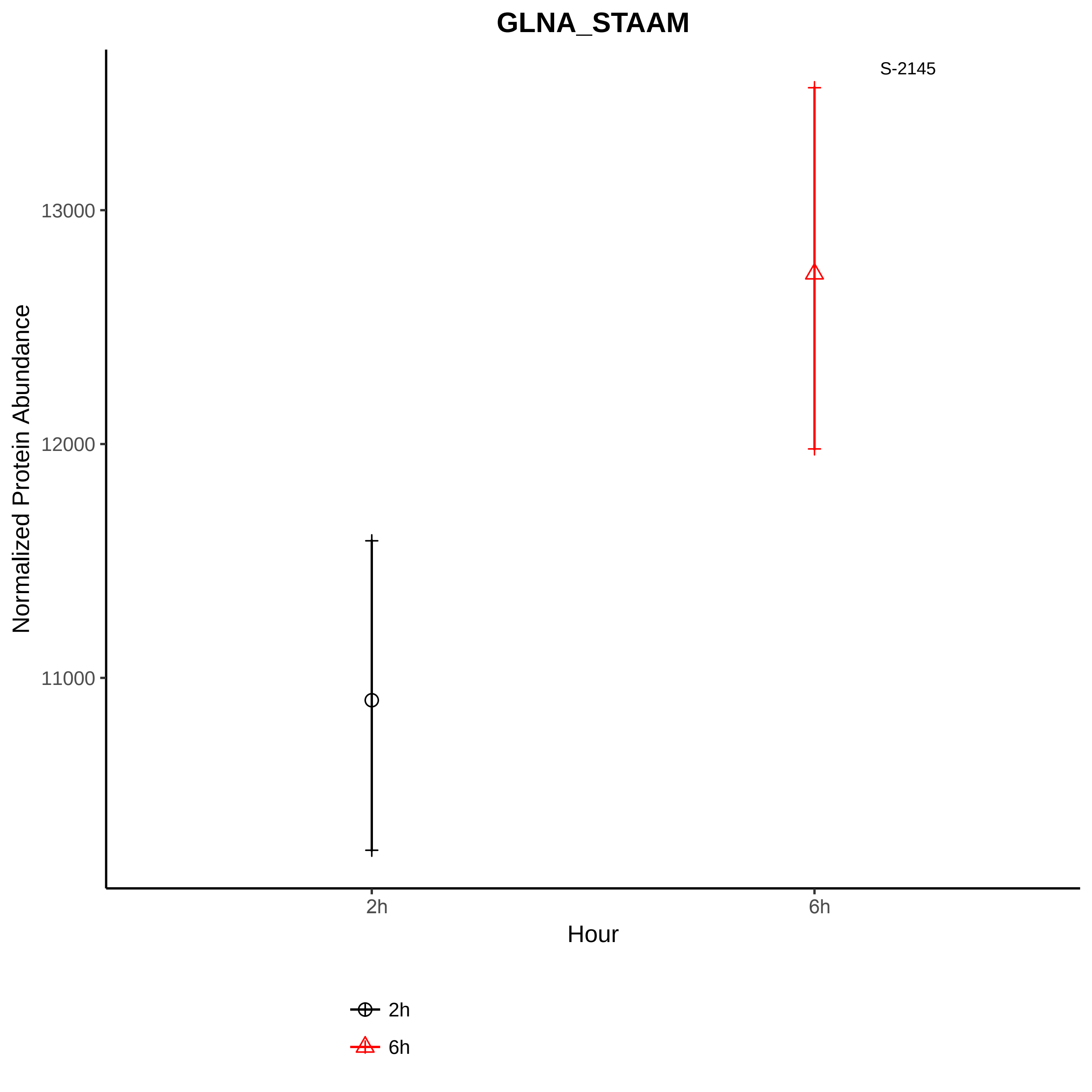
GLNA_STAAM
S-2145
Normalized Protein Abundance
13000
12000
11000
2h
6h
Hour
2h
6h

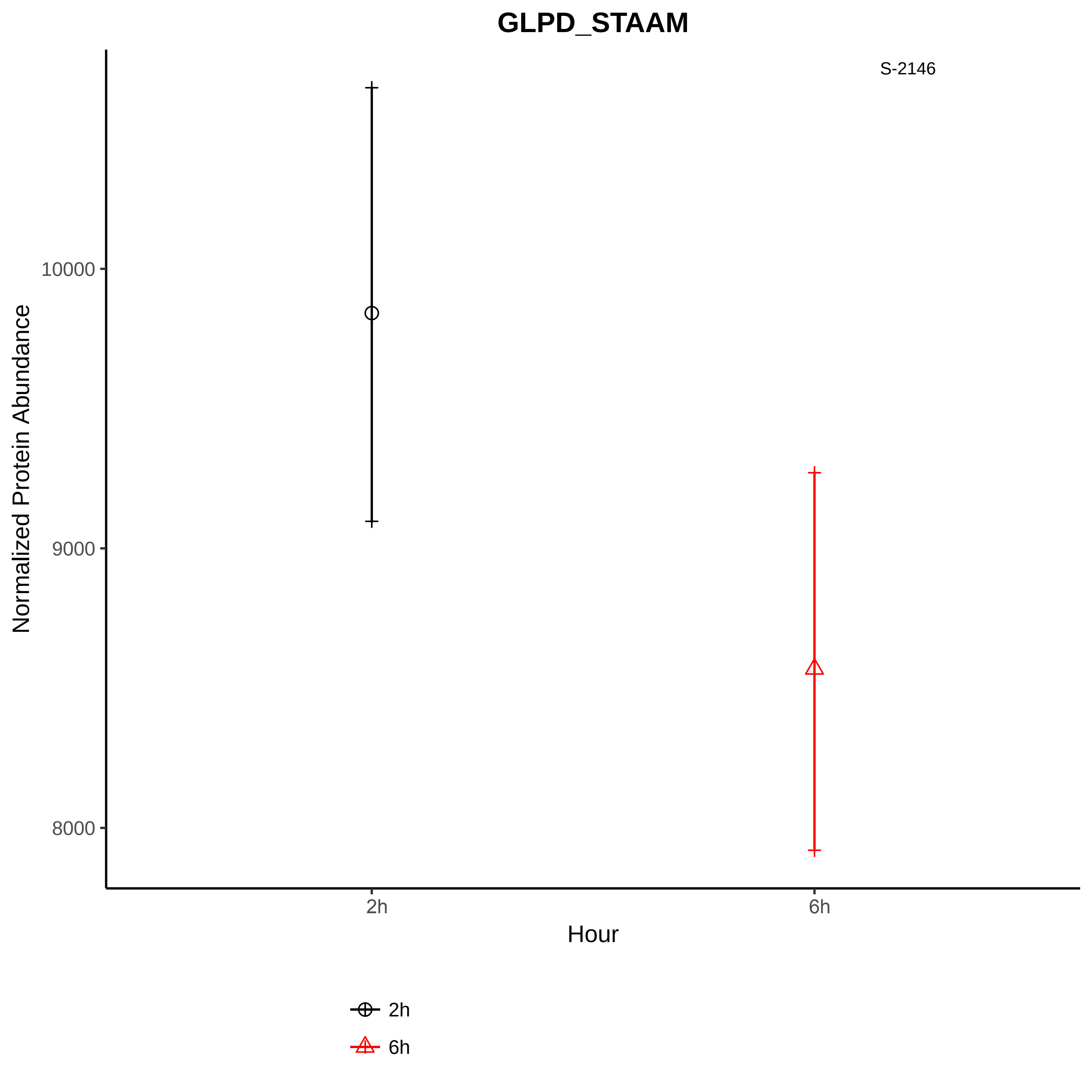
GLPD_STAAM
S-2146
Normalized Protein Abundance
10000
9000
8000
2h
6h
Hour
2h
6h

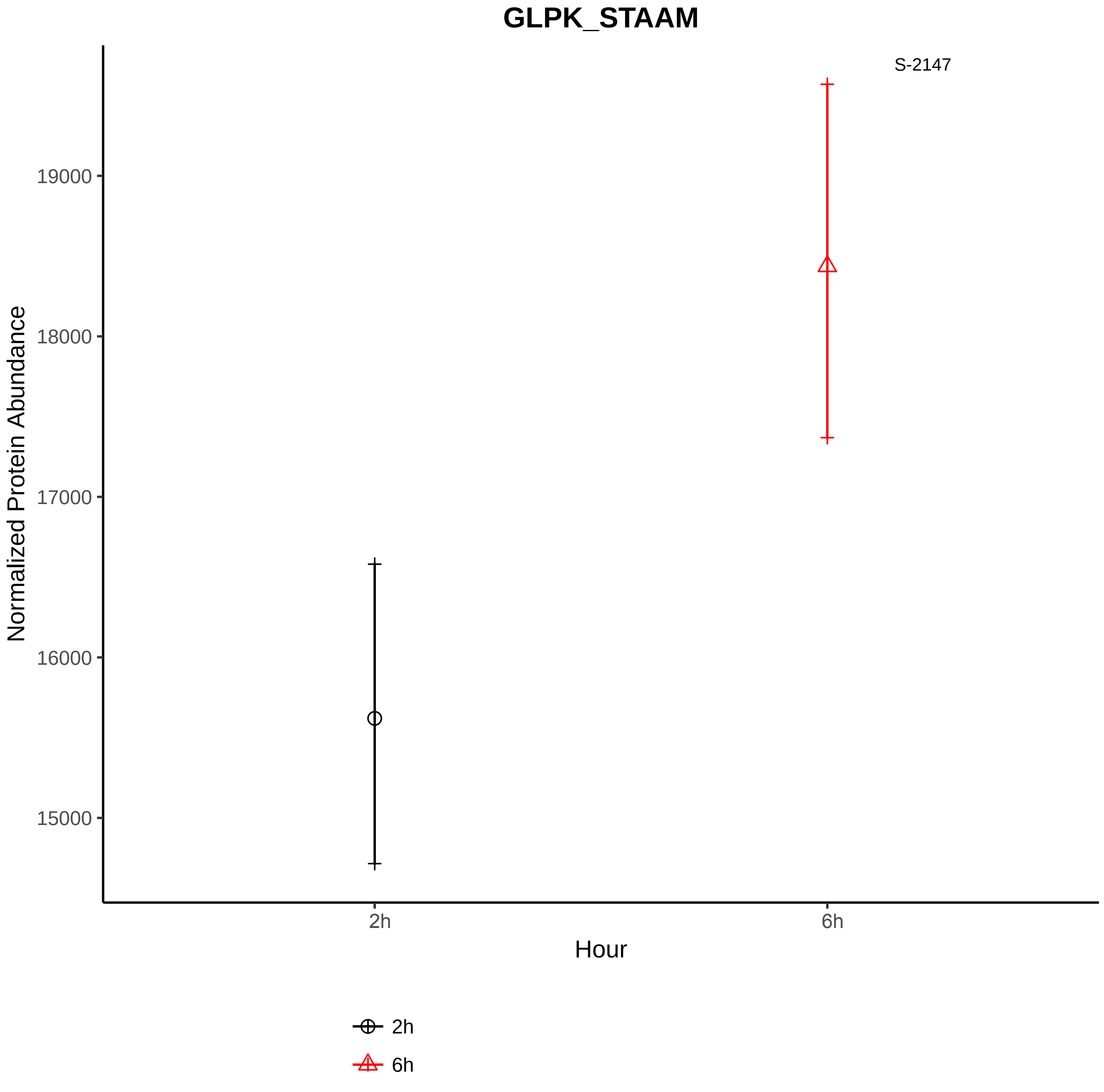

GLPK_STAAM
S-2147
Normalized Protein Abundance
19000
18000
17000
16000
15000
2h
6h
Hour
2h
6h

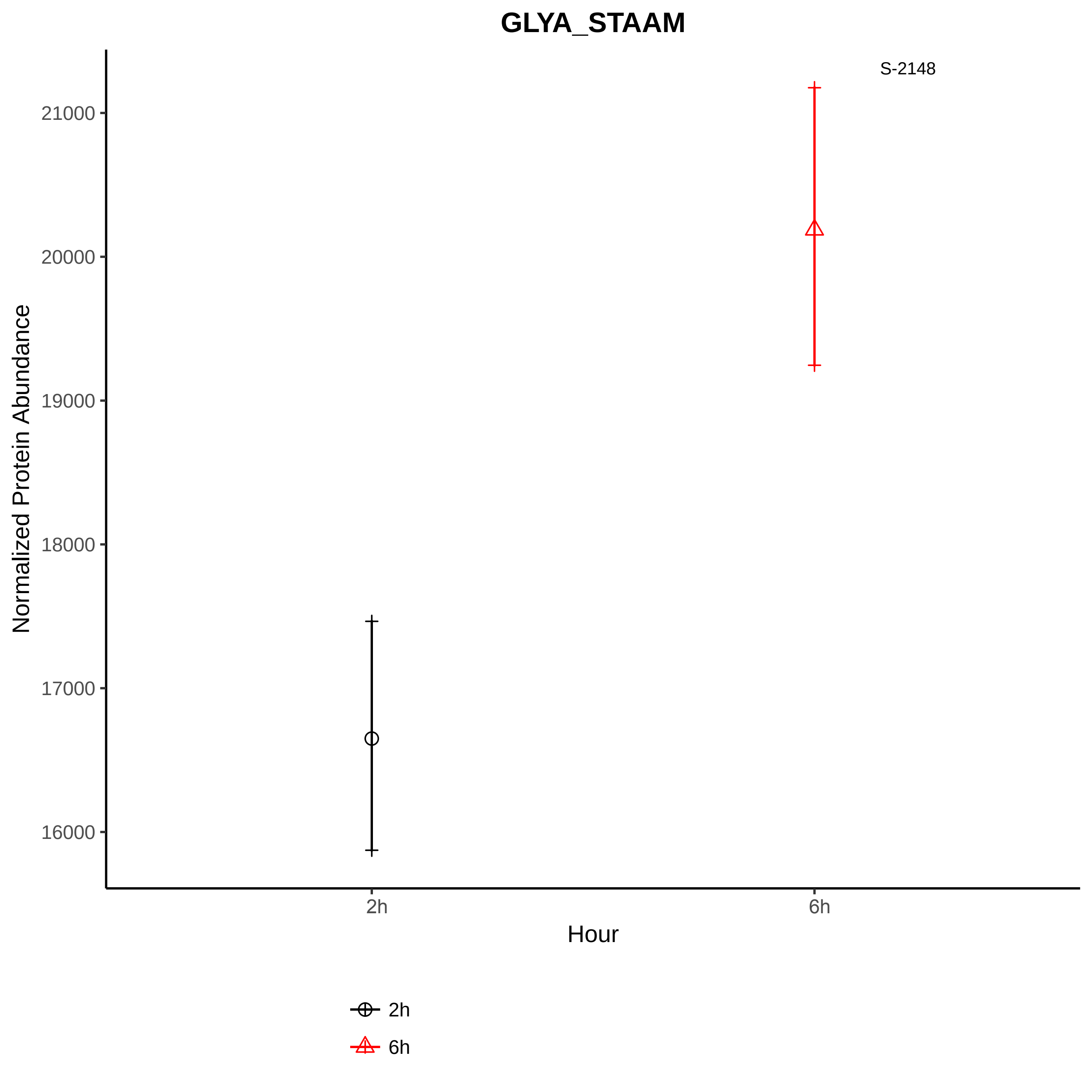
GLYA_STAAM
S-2148
Normalized Protein Abundance
21000
20000
19000
18000
17000
16000
2h
6h
Hour
2h
6h

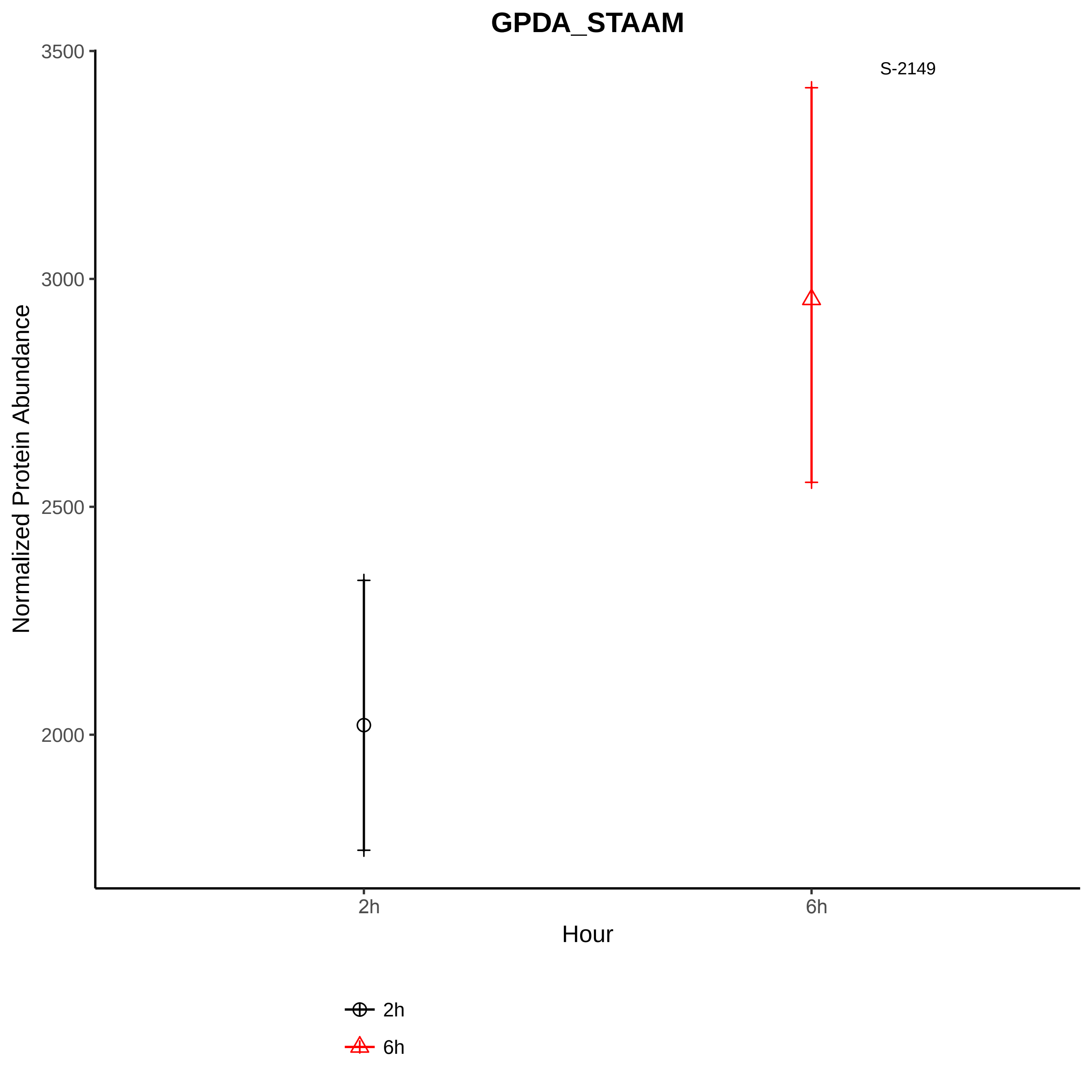

GPDA_STAAM
S-2149
3500
3000
2500
2000
Normalized Protein Abundance
2h
6h
Hour
2h
6h

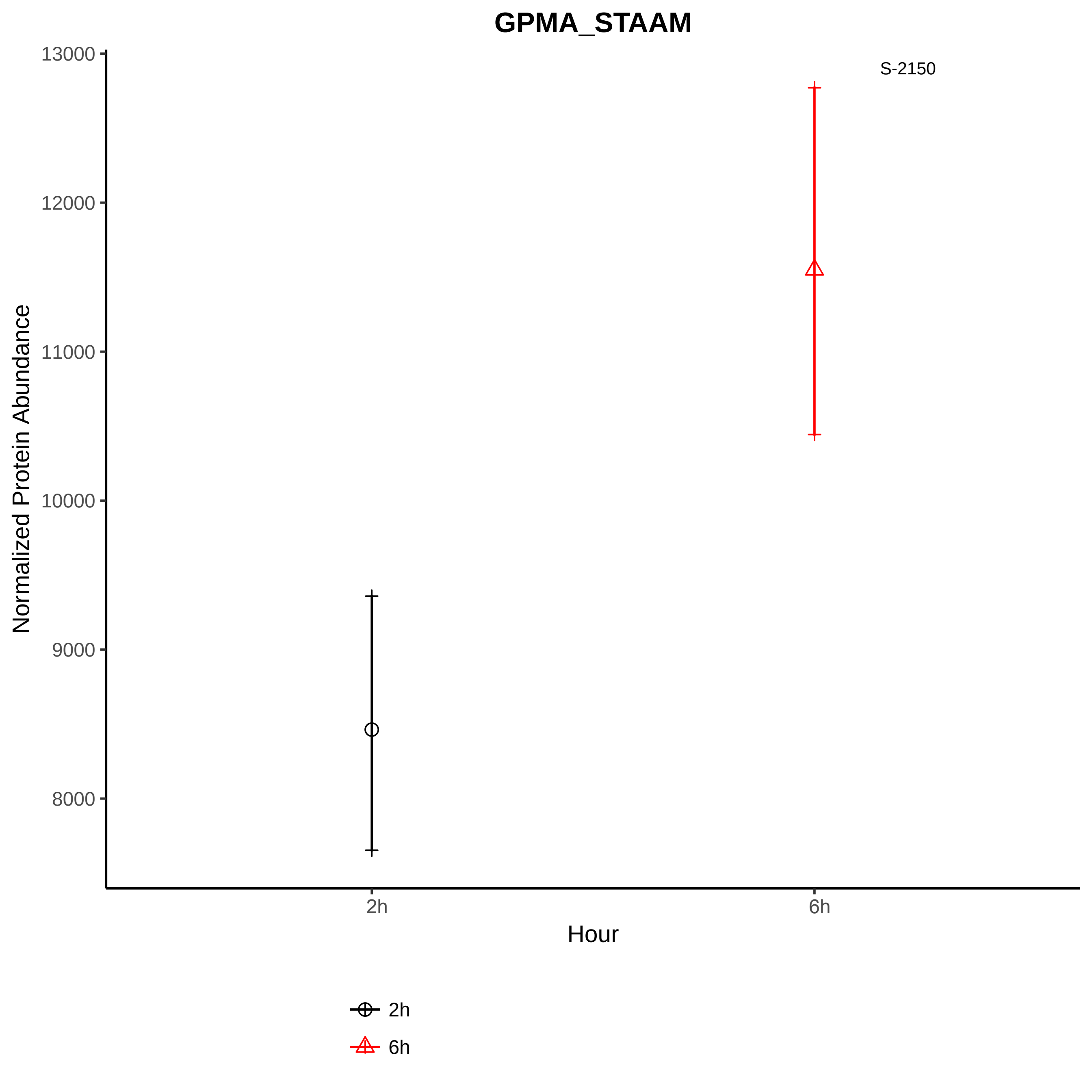
GPMA_STAAM
S-2150
Normalized Protein Abundance
13000
12000
11000
10000
9000
8000
2h
6h
Hour
2h
6h

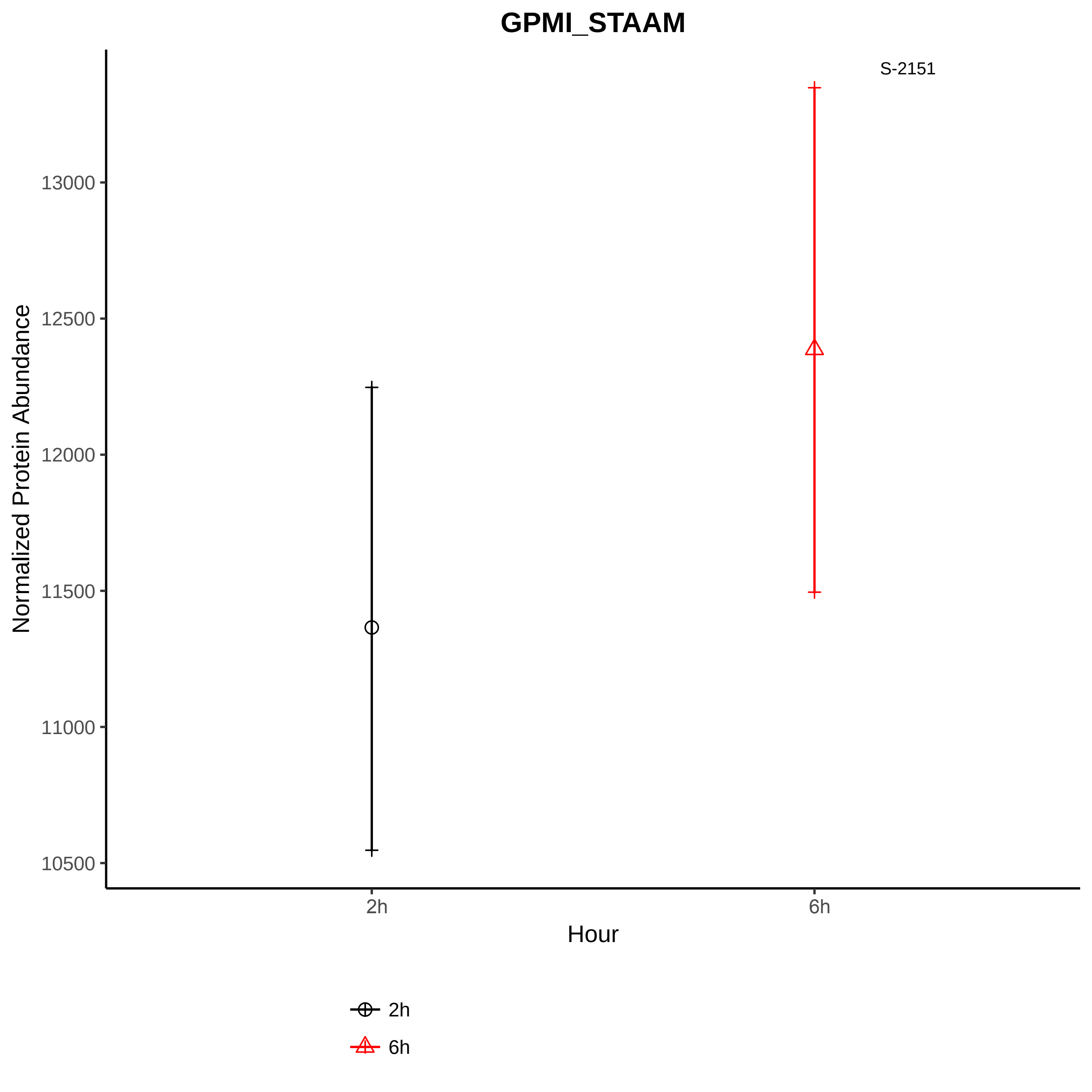

GPMI_STAAM
S-2151
Normalized Protein Abundance
13000
12500
12000
11500
11000
10500
2h
6h
Hour
2h
6h

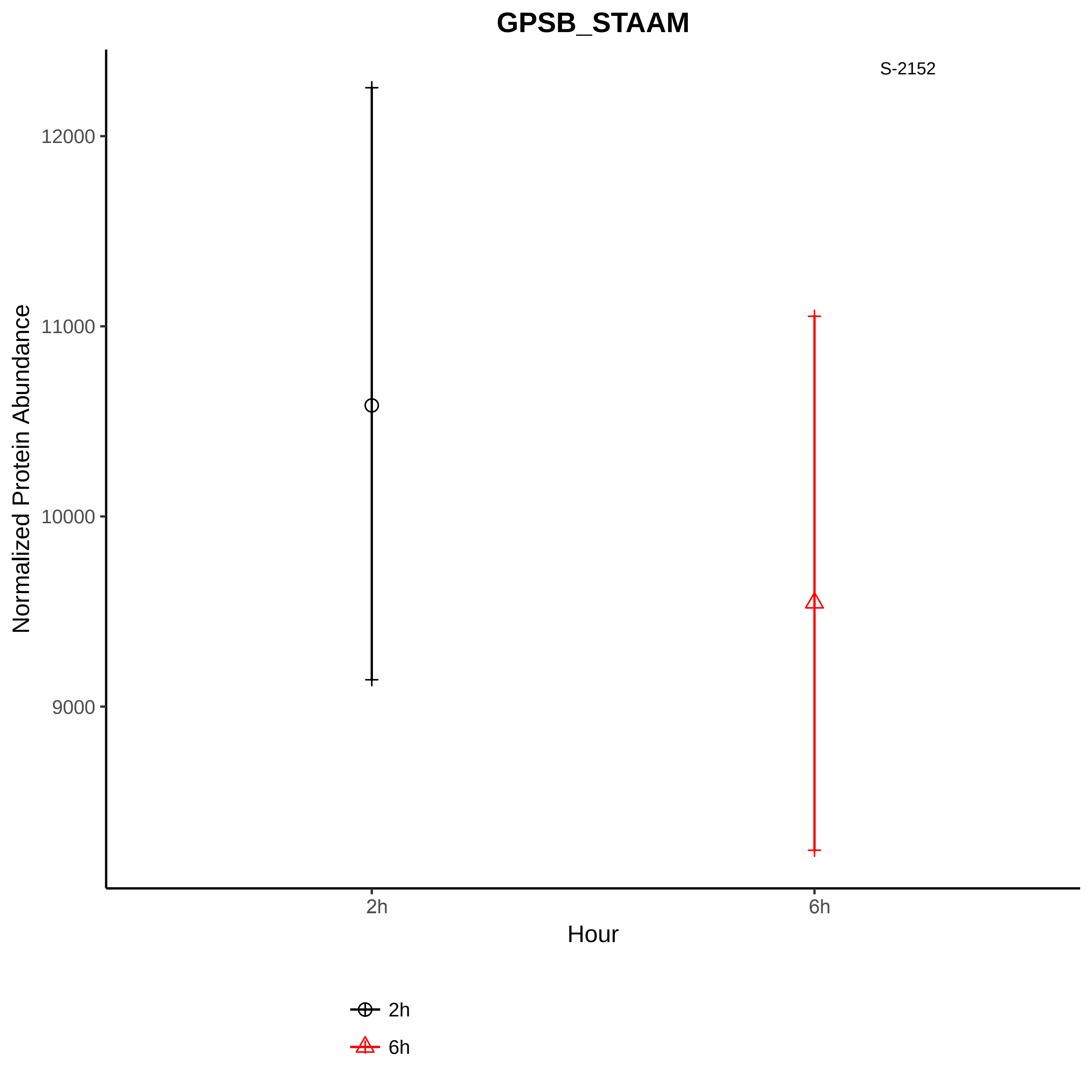

GPSB_STAAM
S-2152
Normalized Protein Abundance
12000
11000
10000
9000
2h
6h
Hour
2h
6h

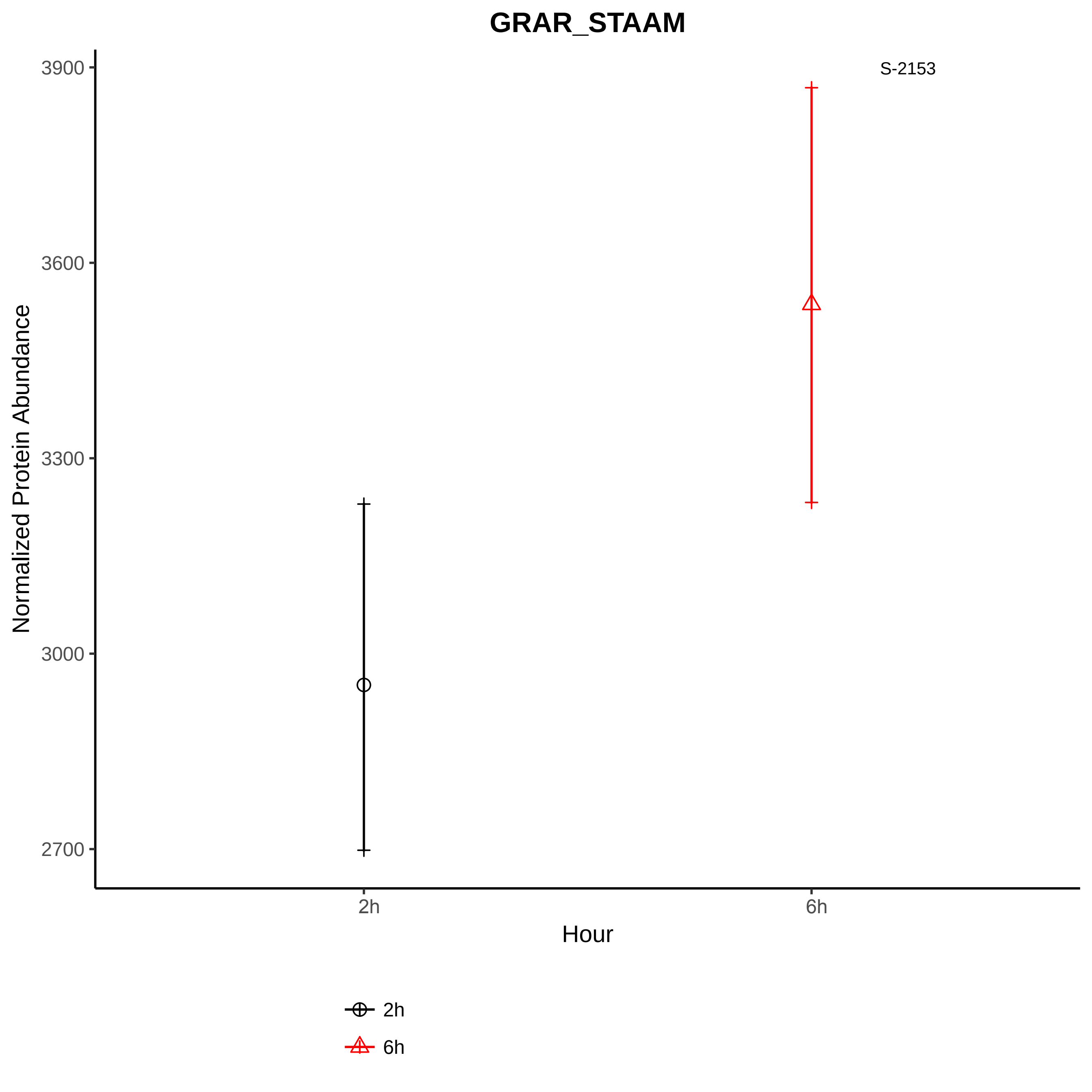
GRAR_STAAM
S-2153
Normalized Protein Abundance
3900
3600
3300
3000
2700
2h
6h
Hour
2h
6h

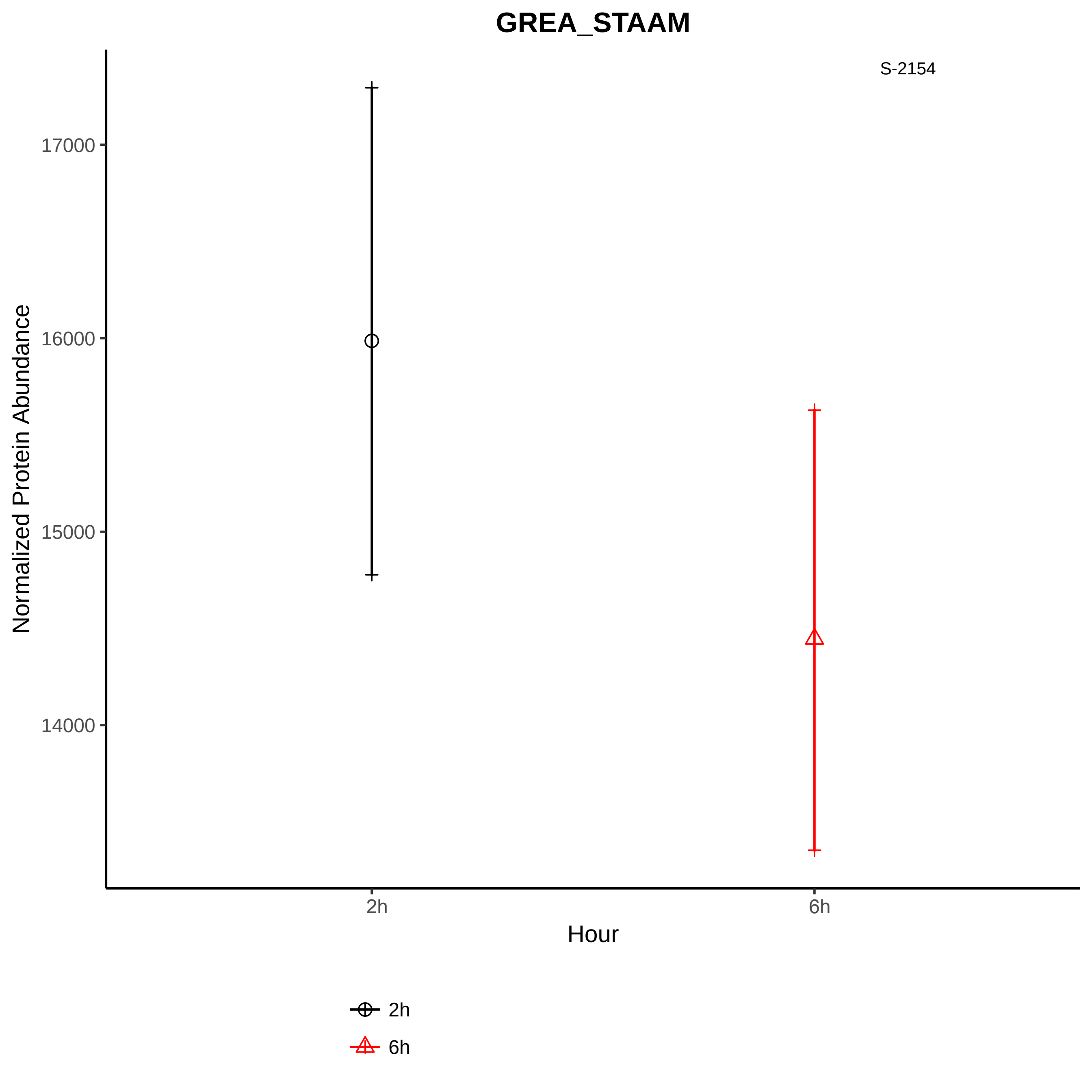
GREA_STAAM
S-2154
17000
16000
15000
14000
Normalized Protein Abundance
2h
6h
Hour
2h
6h

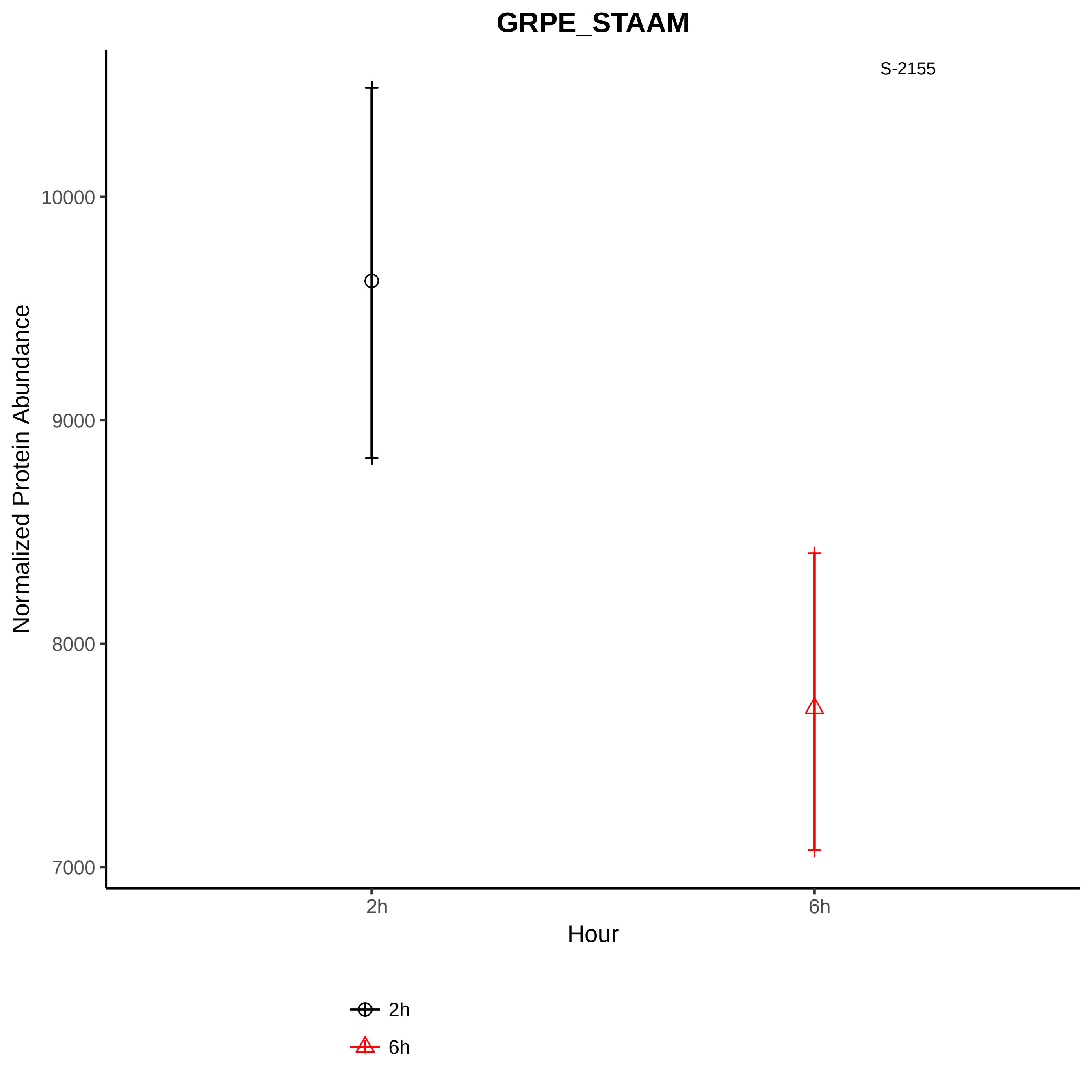
GRPE_STAAM
S-2155
Normalized Protein Abundance
10000
9000
8000
7000
2h
6h
Hour
2h
6h

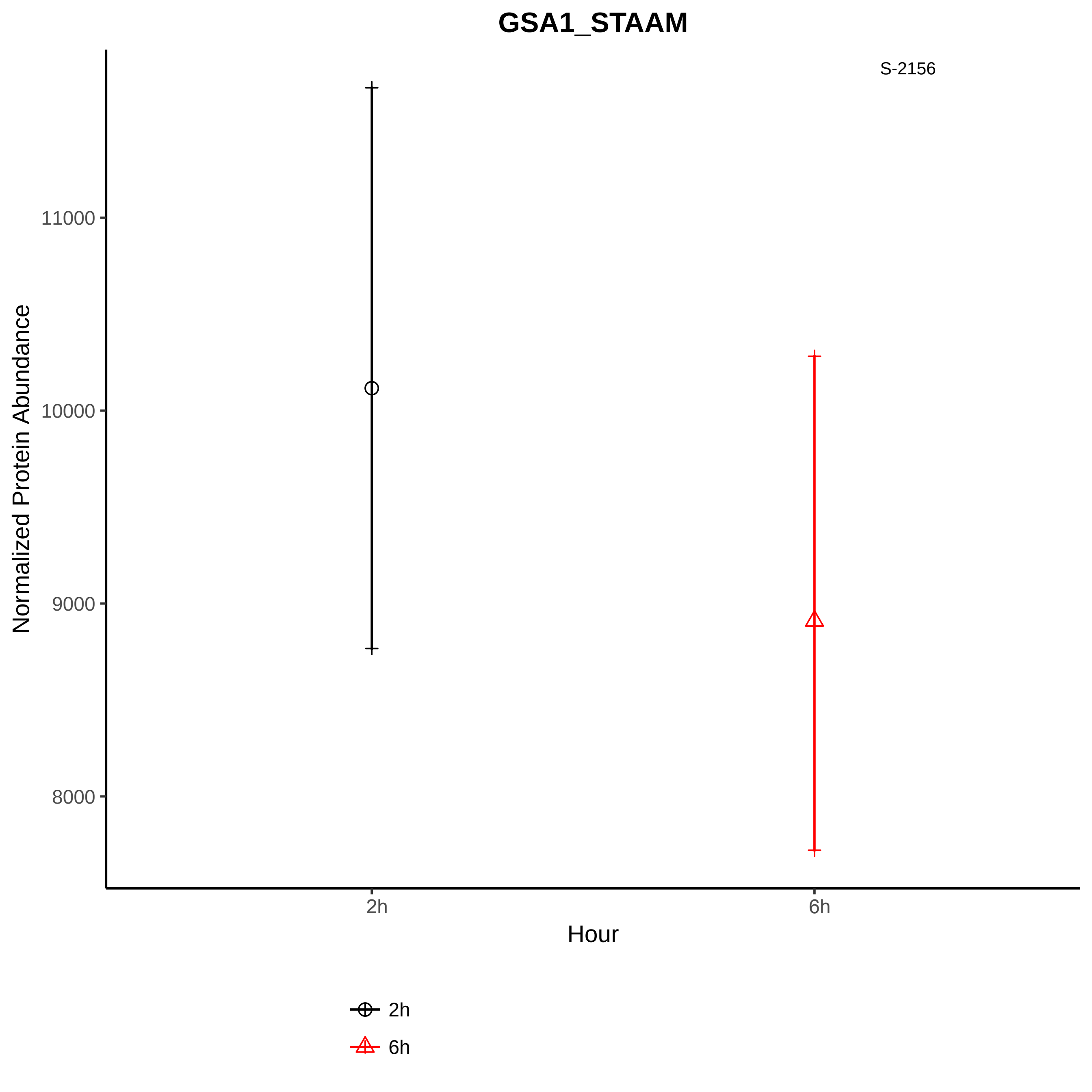
GSA1_STAAM
S-2156
Normalized Protein Abundance
11000
10000
9000
8000
2h
6h
Hour
2h
6h

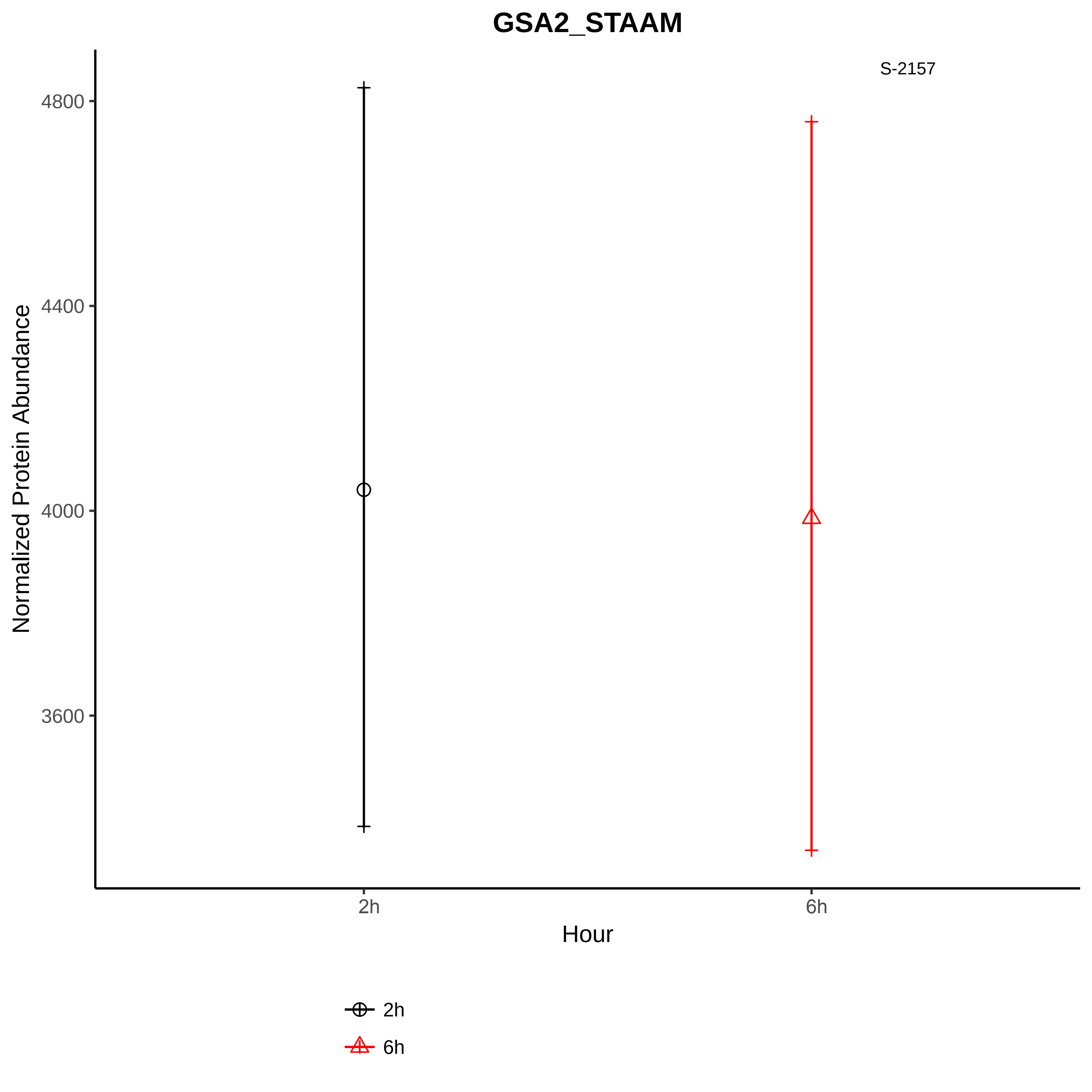

GSA2_STAAM
S-2157
Normalized Protein Abundance
4800
4400
4000
3600
2h
6h
Hour
2h
6h

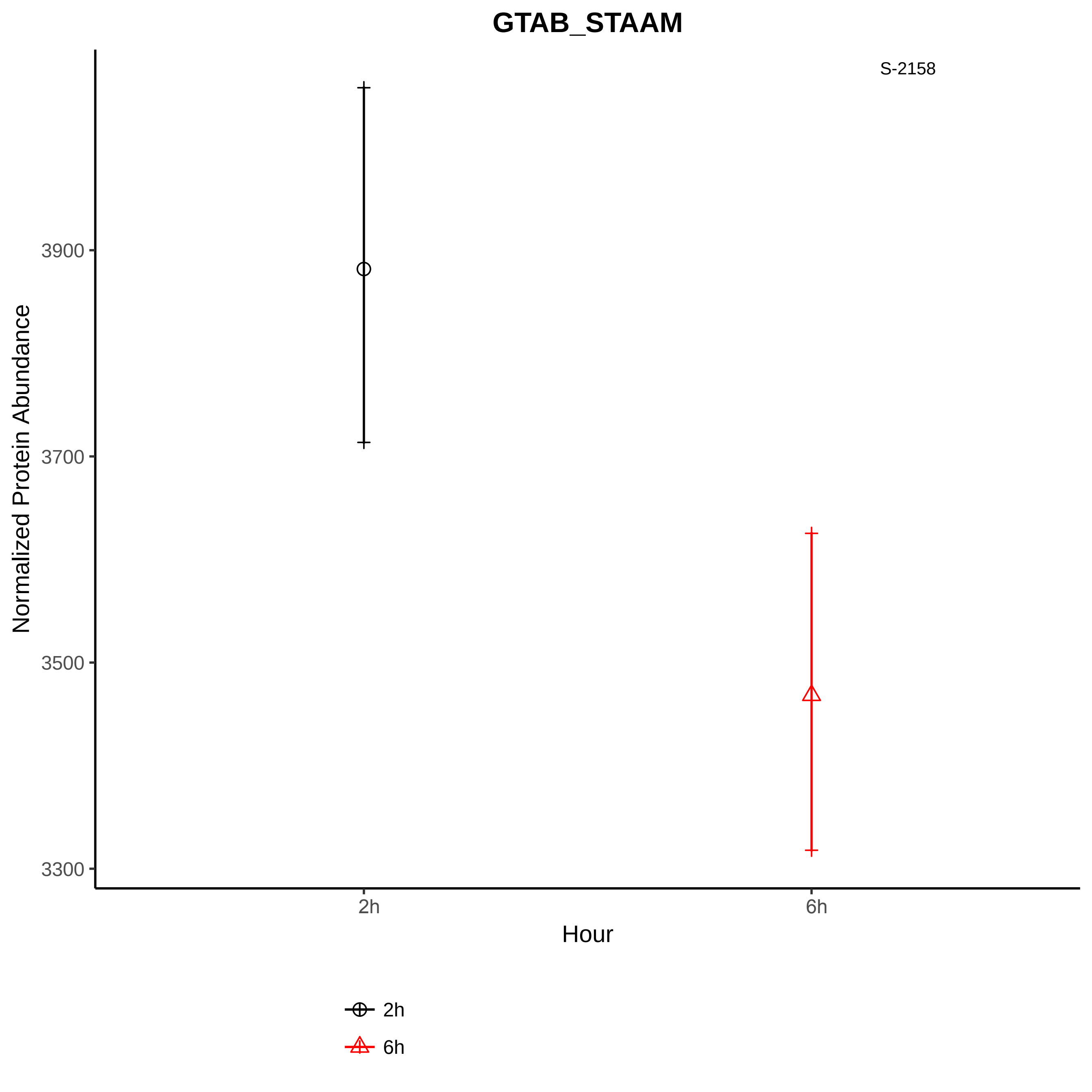
GTAB_STAAM
S-2158
Normalized Protein Abundance
3900
3700
3500
3300
2h
6h
Hour
2h
6h

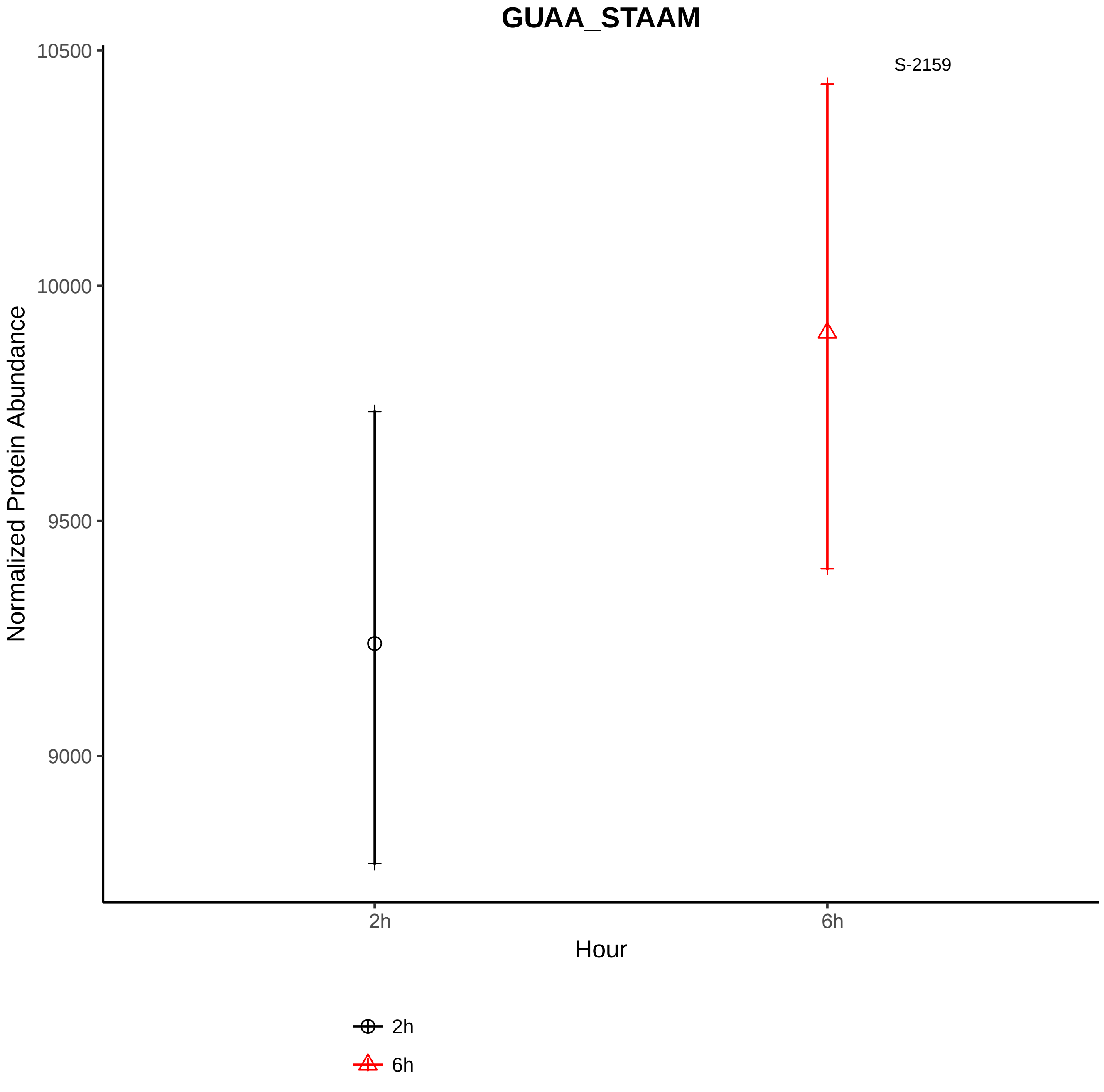

GUAA_STAAM
S-2159
10500
10000
9500
9000
Normalized Protein Abundance
2h
6h
Hour
2h
6h

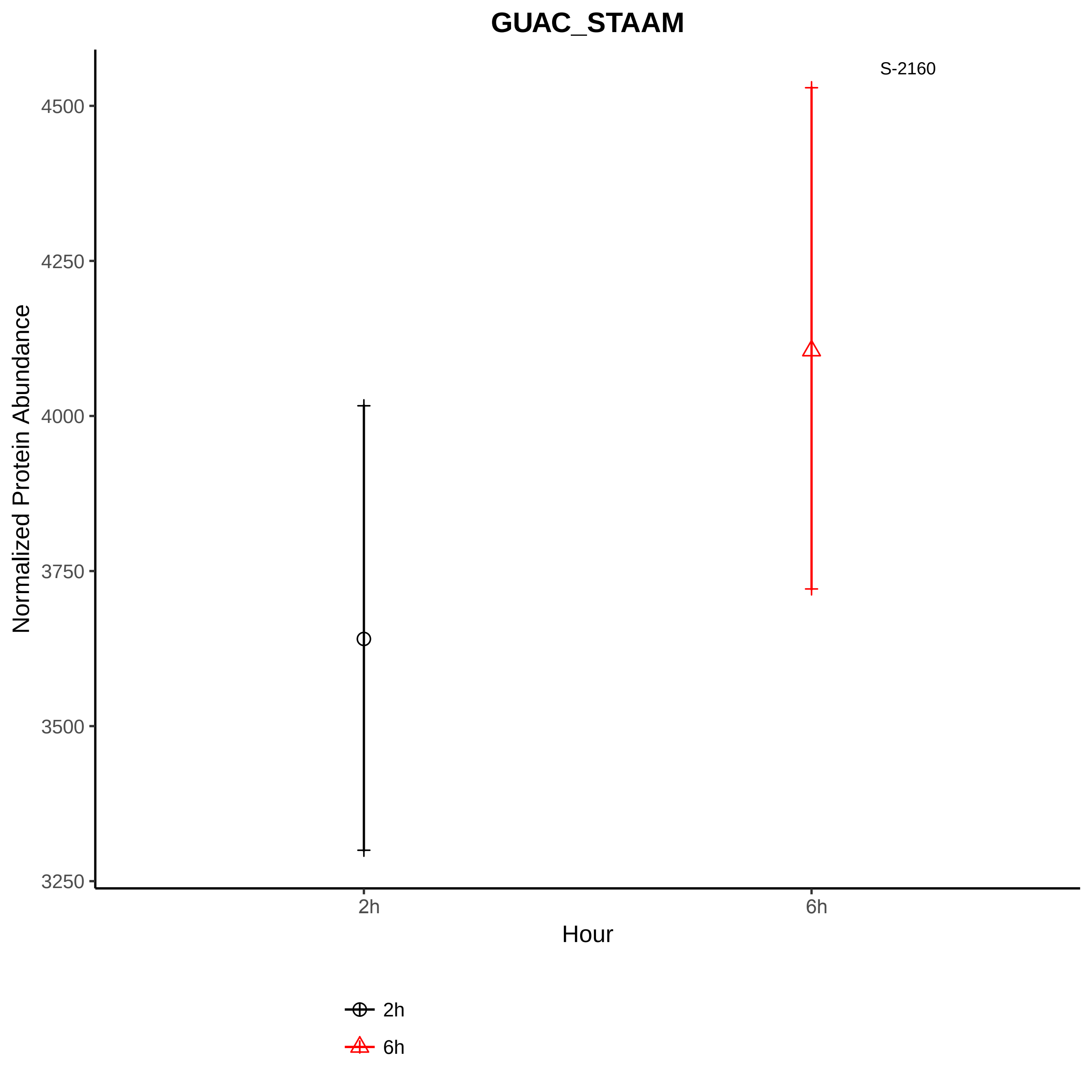

GUAC_STAAM
S-2160
4500
4250
4000
3750
3500
3250
Normalized Protein Abundance
2h
6h
Hour
2h
6h

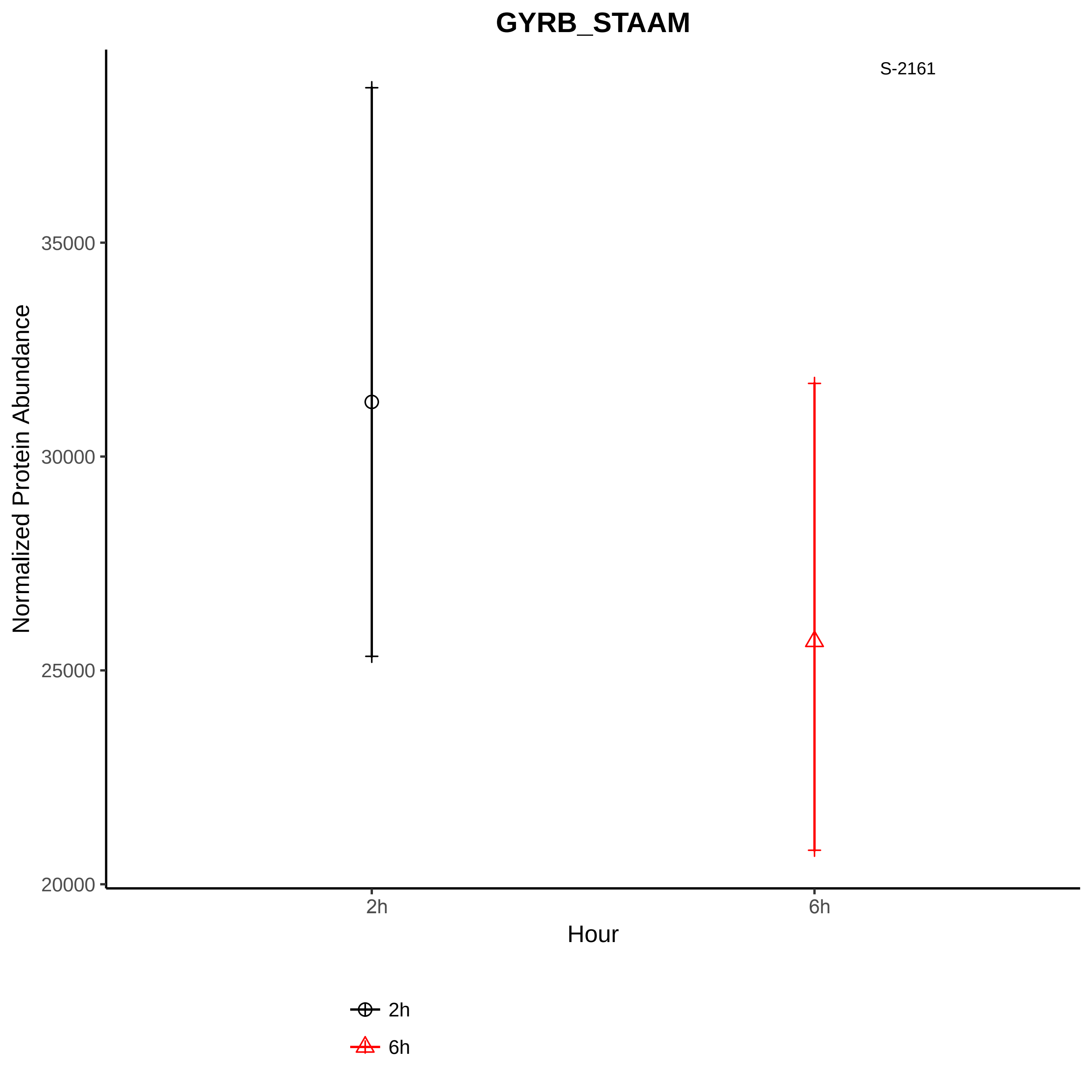
GYRB_STAAM
S-2161
Normalized Protein Abundance
35000
30000
25000
20000
2h
6h
Hour
2h
6h

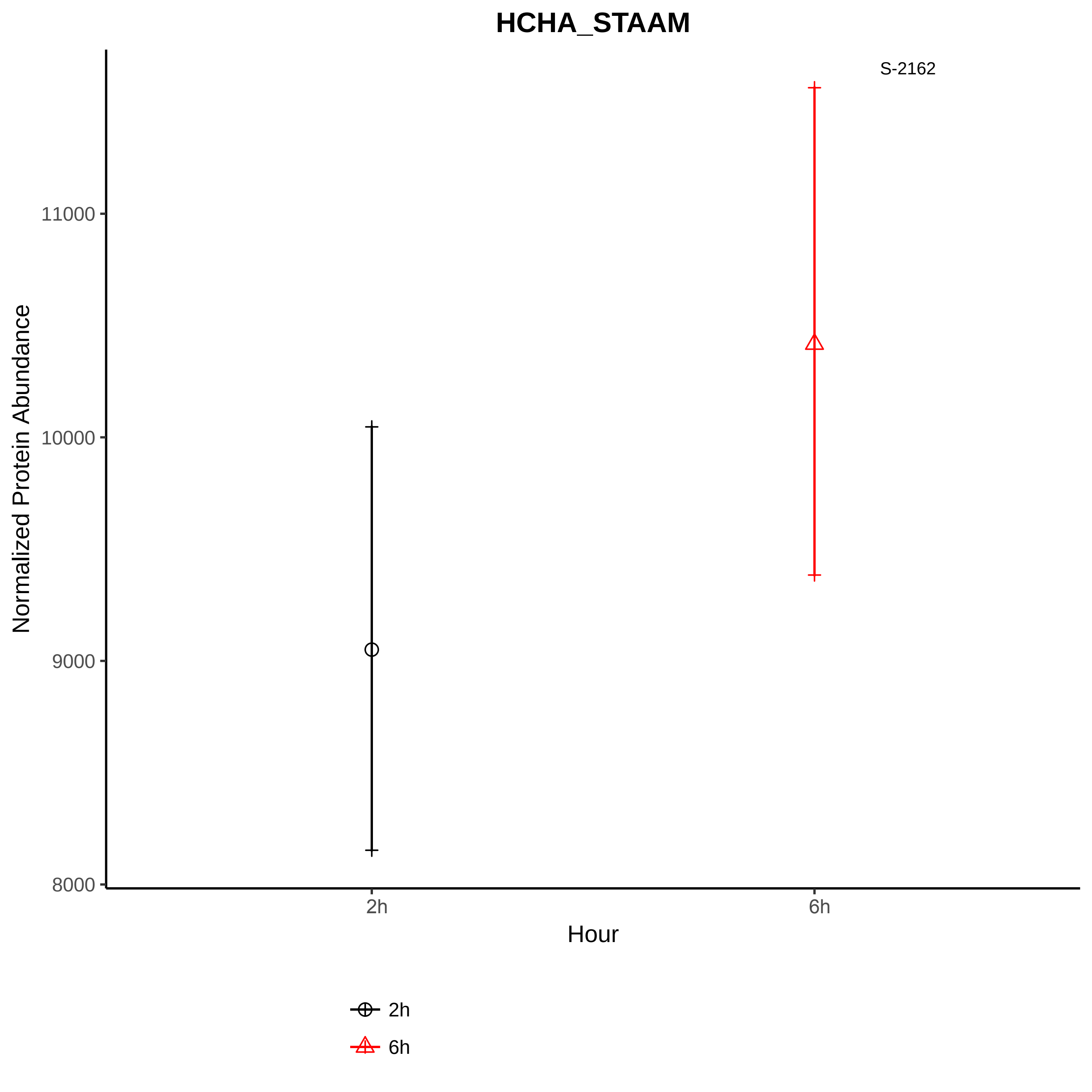

HCHA_STAAM
S-2162
Normalized Protein Abundance
11000
10000
9000
8000
2h
6h
Hour
2h
6h

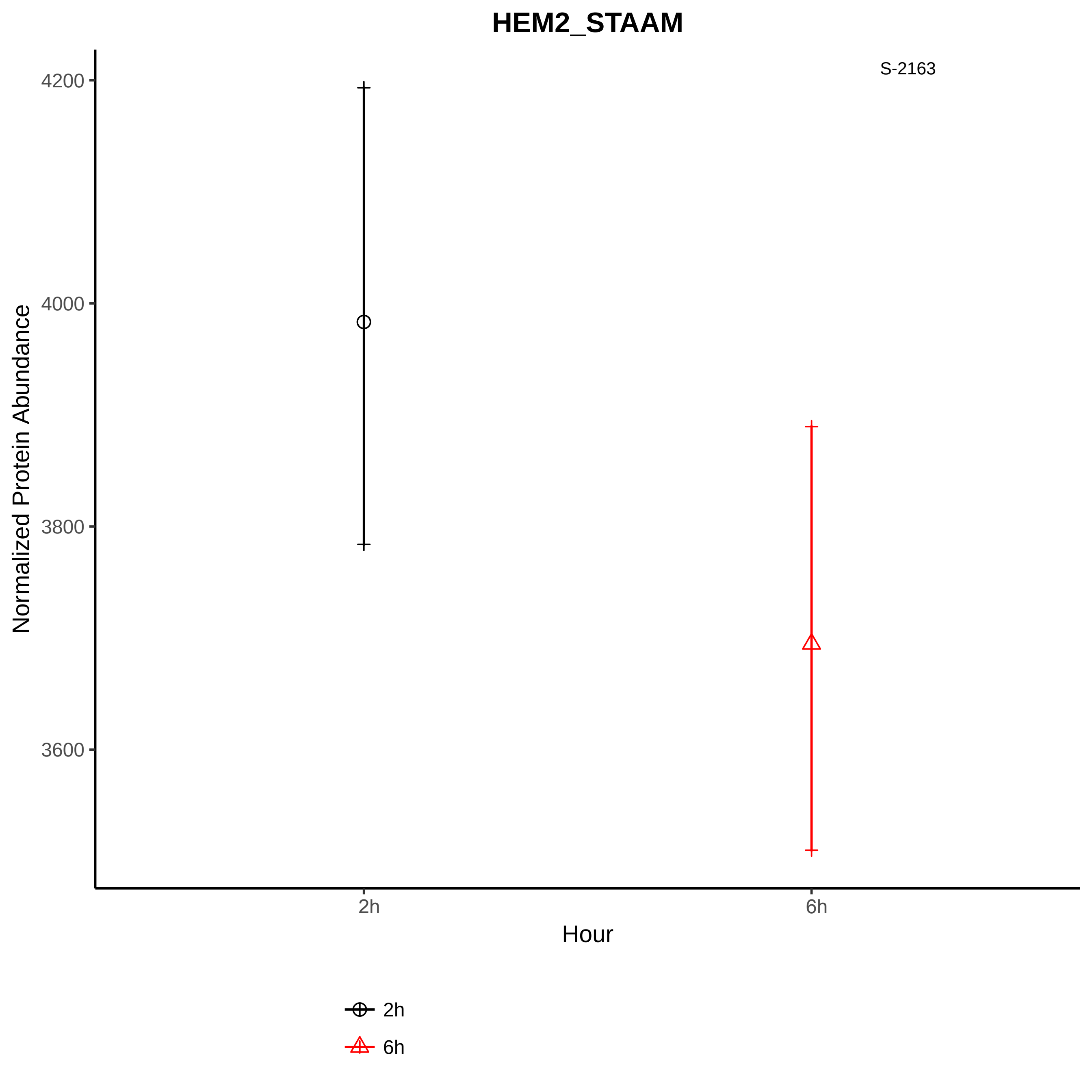
HEM2_STAAM
S-2163
Normalized Protein Abundance
4200
4000
3800
3600
2h
6h
Hour
2h
6h

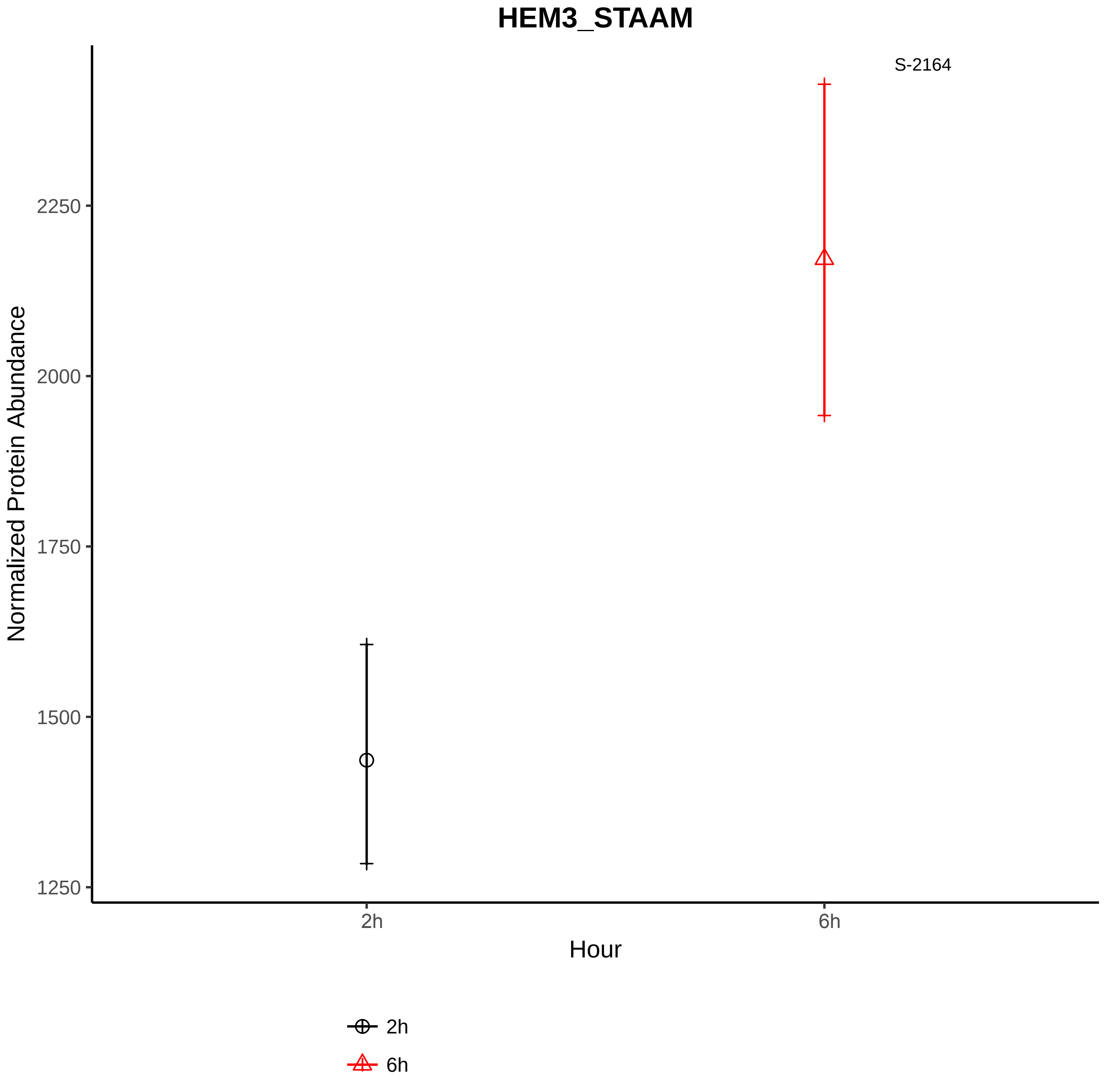
HEM3_STAAM
S-2164
Normalized Protein Abundance
2250
2000
1750
1500
1250
2h
6h
Hour
2h
6h

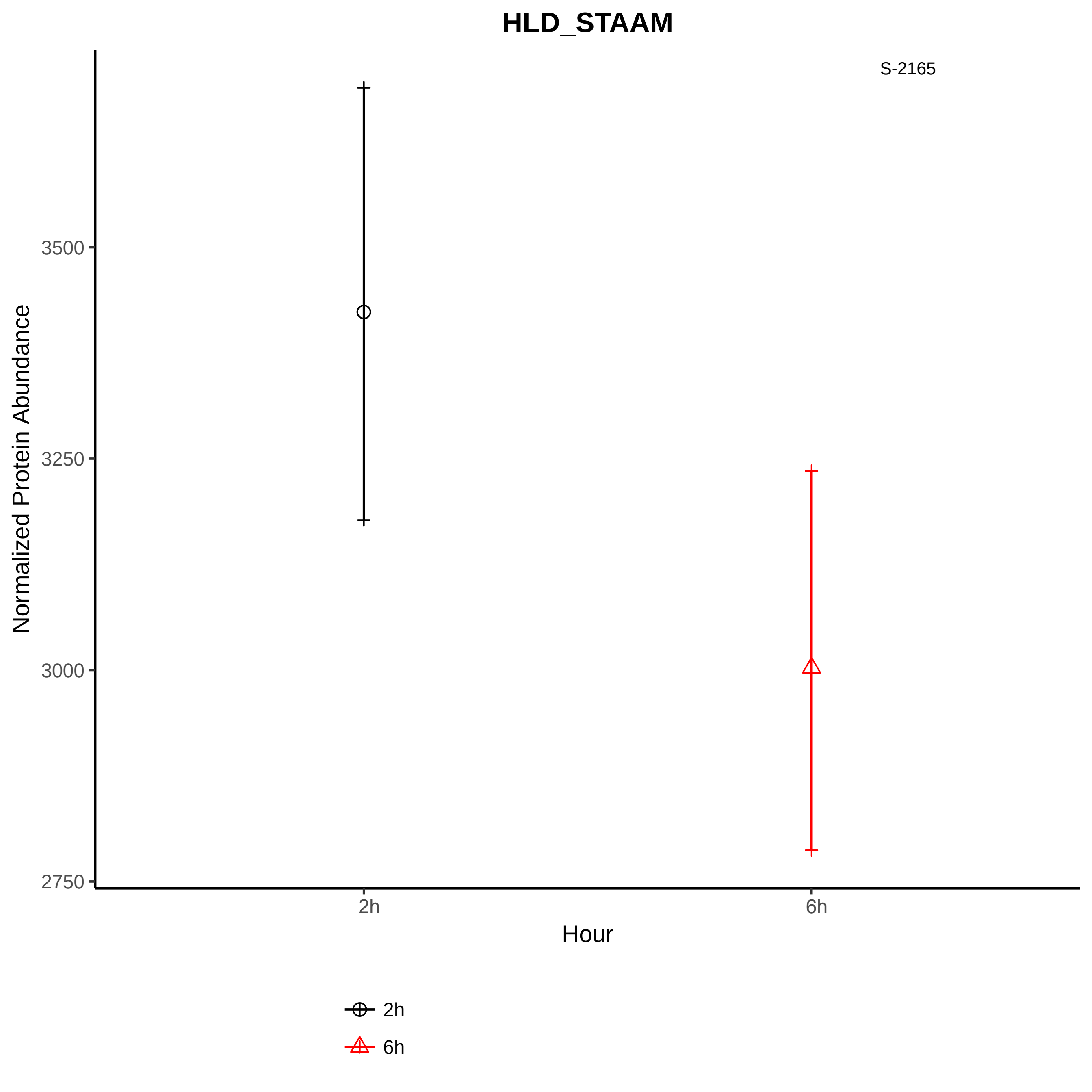

HLD_STAAM
S-2165
Normalized Protein Abundance
3500
3250
3000
2750
2h
6h
Hour
2h
6h

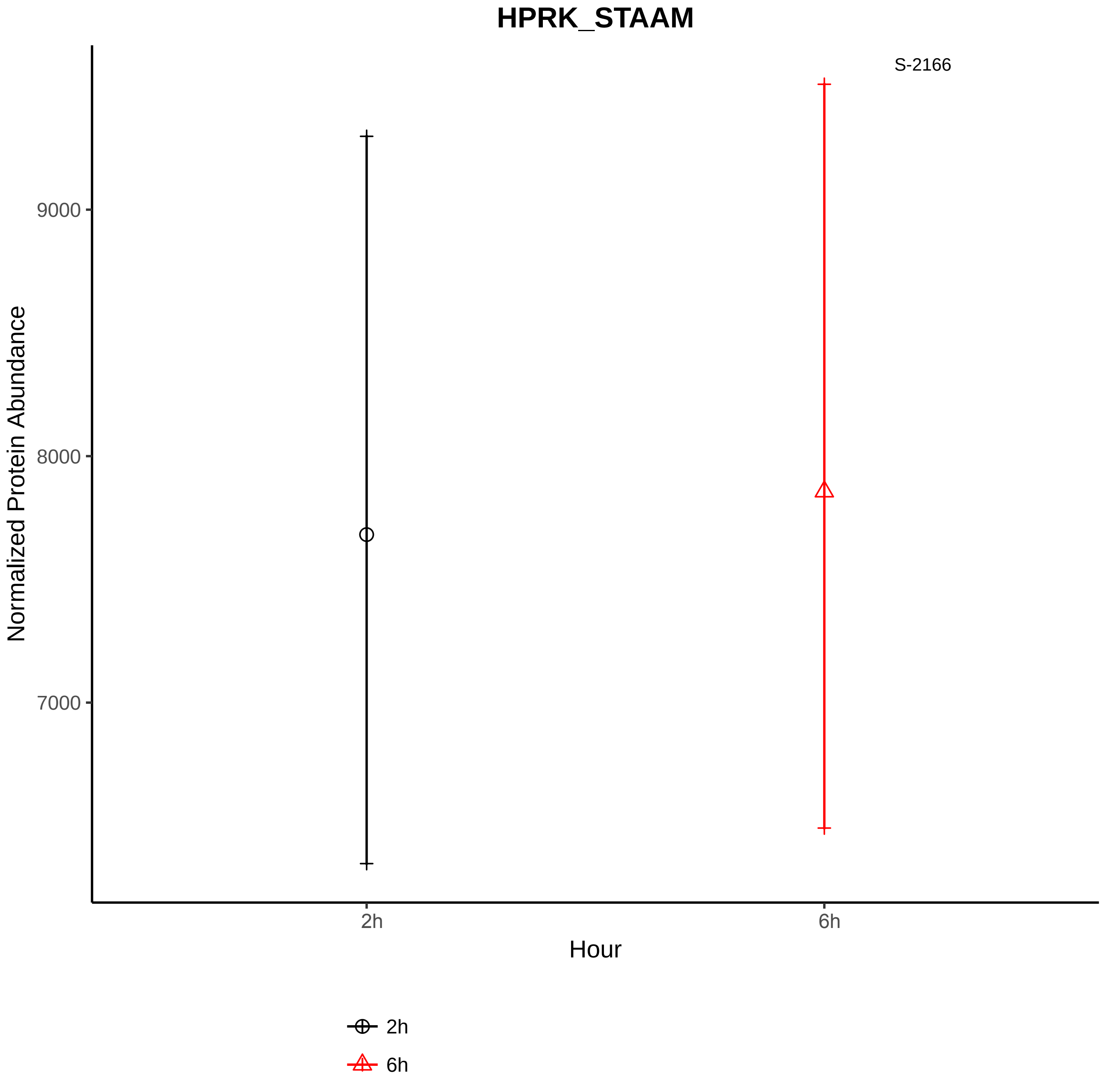

HPRK_STAAM
S-2166
9000
8000
7000
Normalized Protein Abundance
2h
6h
Hour
2h
6h

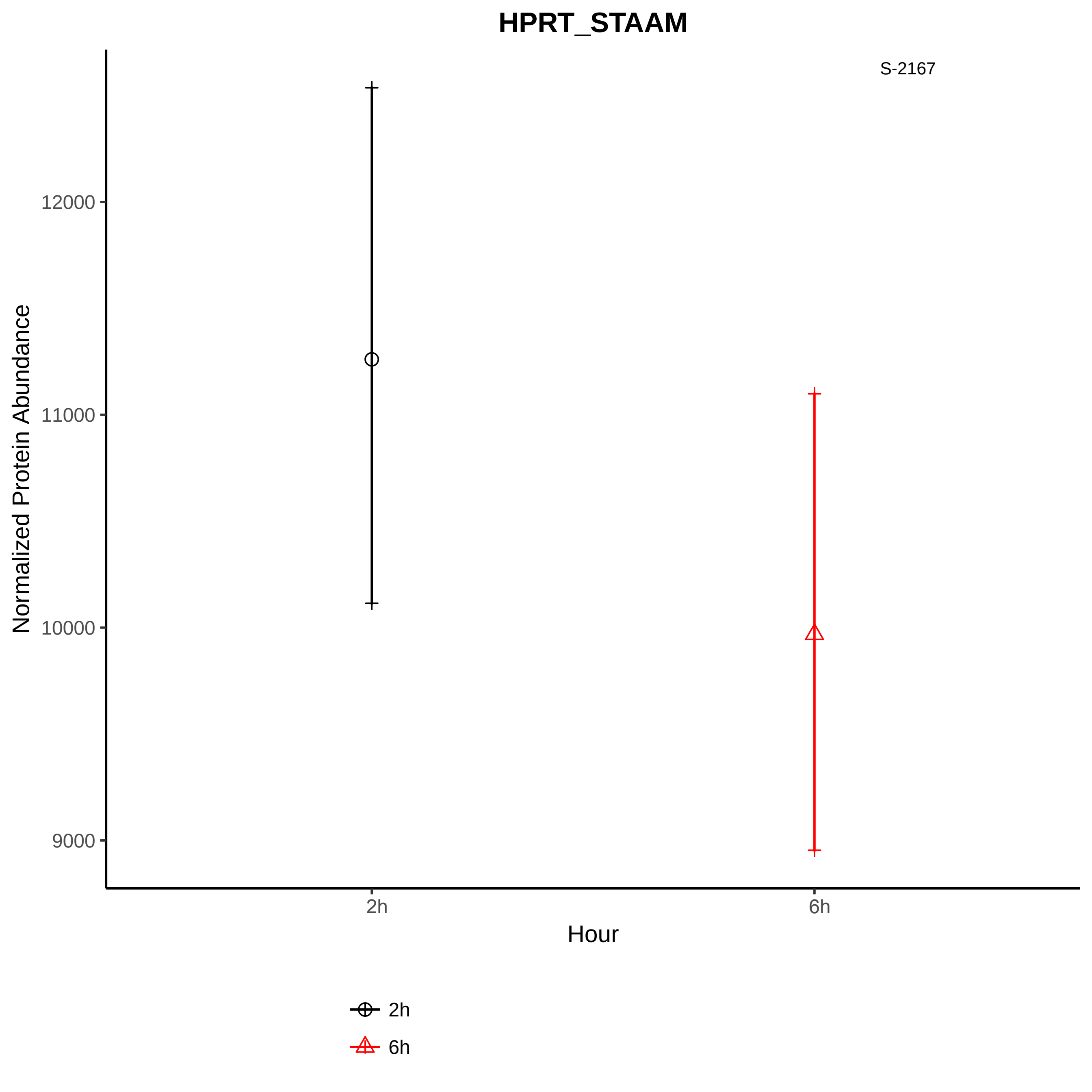

HPRT_STAAM
S-2167
Normalized Protein Abundance
12000
11000
10000
9000
2h
6h
Hour
2h
6h

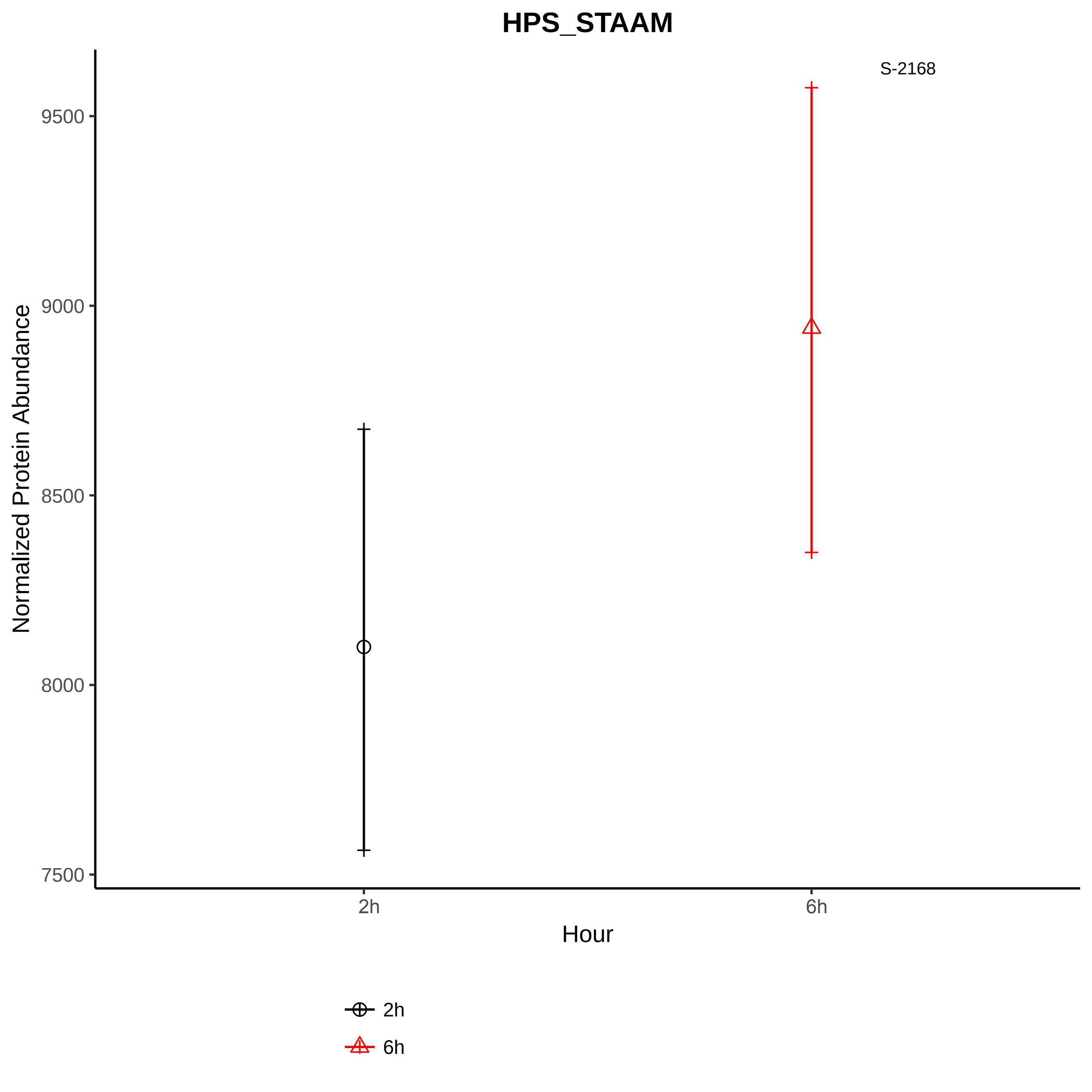
HPS_STAAM
S-2168
Normalized Protein Abundance
9500
9000
8500
8000
7500
2h
6h
Hour
2h
6h

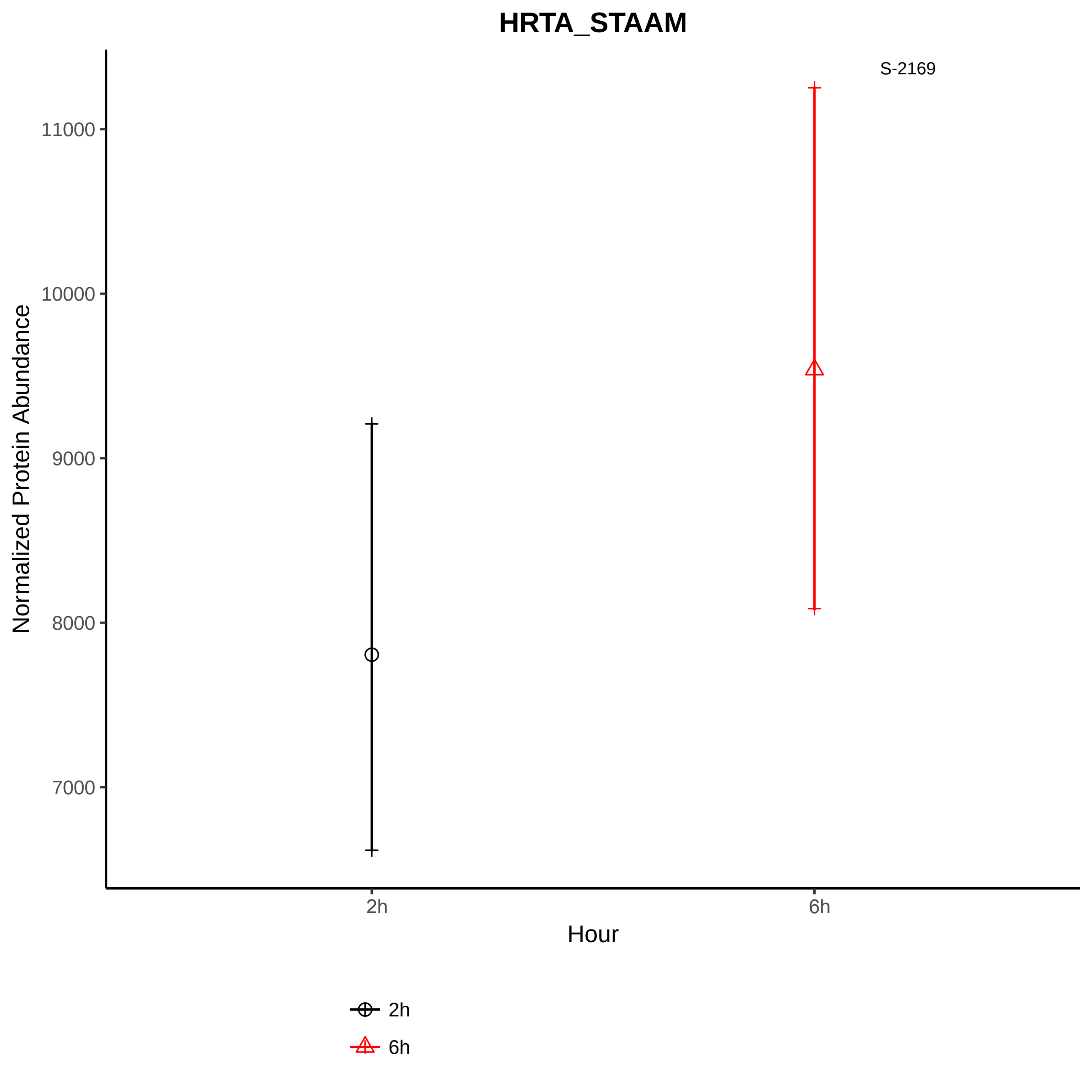
HRTA_STAAM
S-2169
11000
10000
9000
8000
7000
Normalized Protein Abundance
2h
6h
Hour
2h
6h

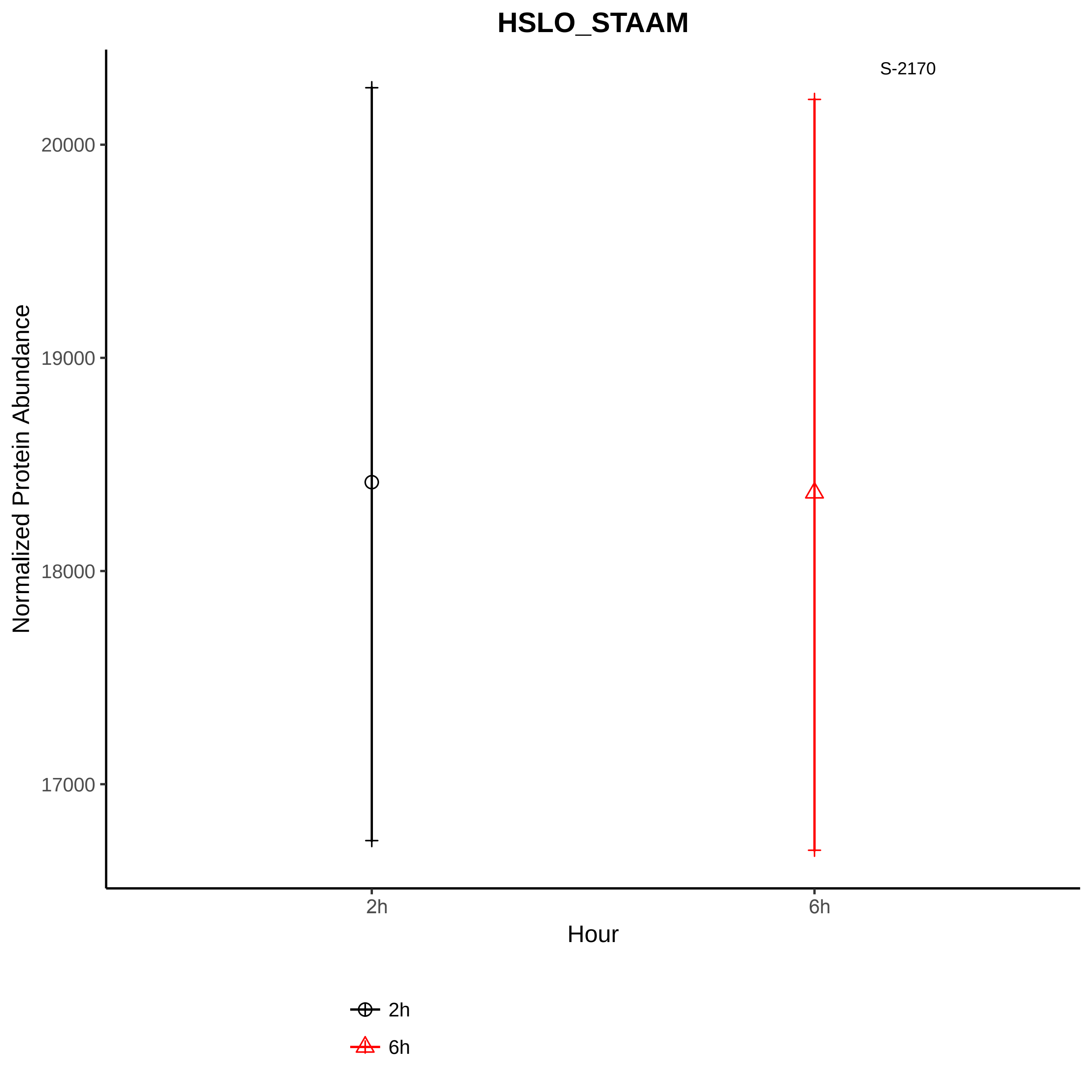

HSLO_STAAM
S-2170
20000
19000
18000
17000
Normalized Protein Abundance
2h
6h
Hour
2h
6h

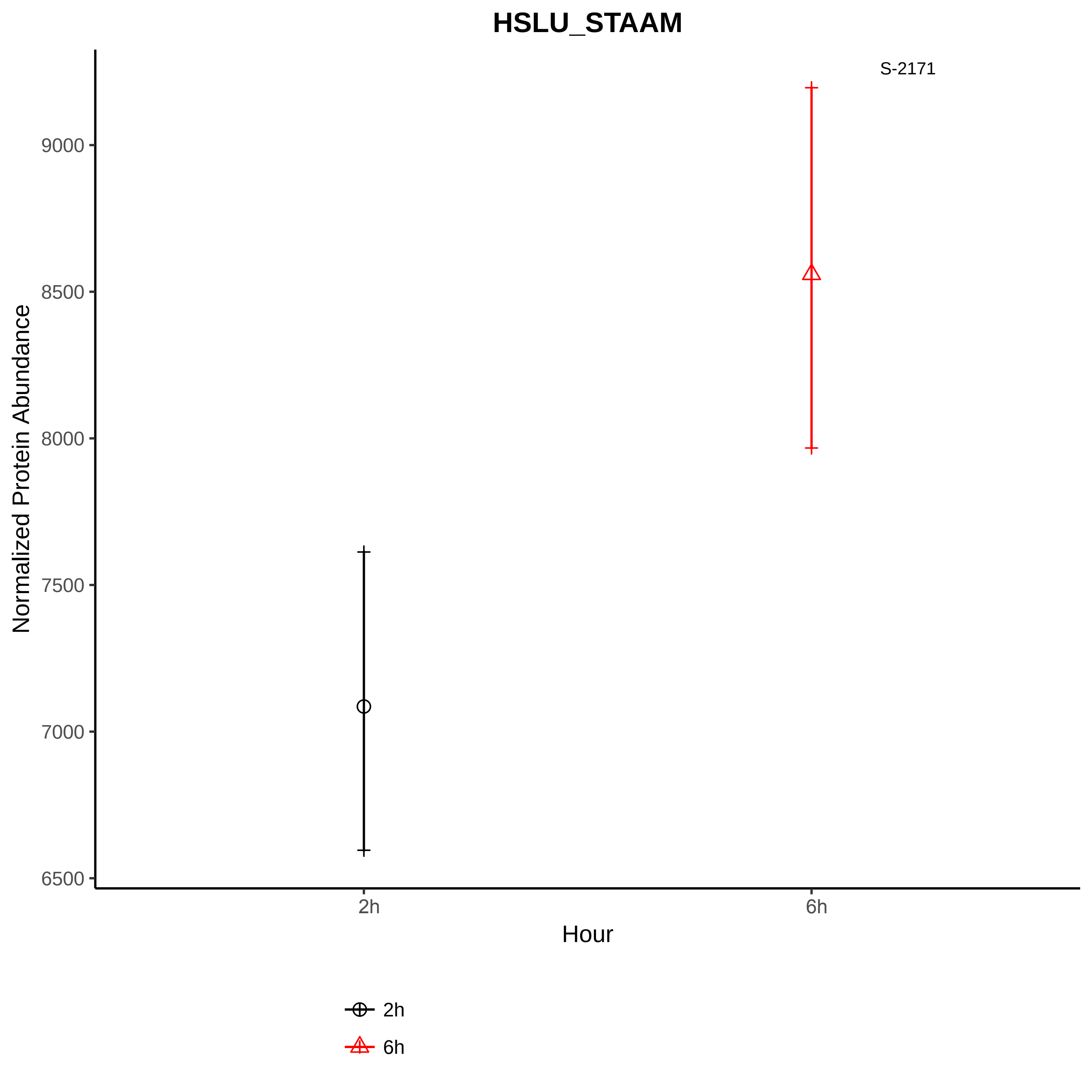
HSLU_STAAM
S-2171
9000
8500
8000
7500
7000
6500
Normalized Protein Abundance
2h
6h
Hour
2h
6h

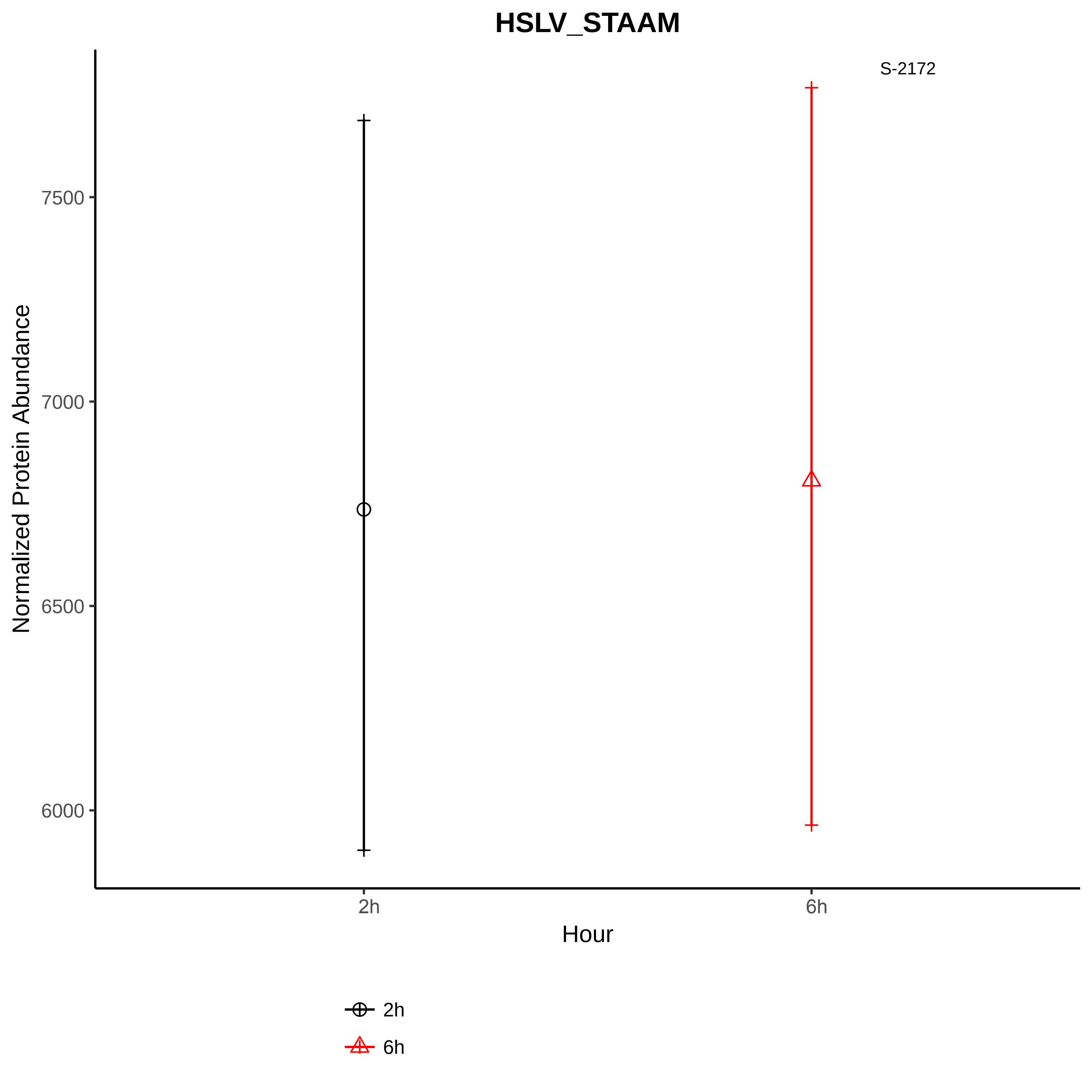

HSLV_STAAM
S-2172
Normalized Protein Abundance
7500
7000
6500
6000
2h
6h
Hour
2h
6h

HUTH_STAAM

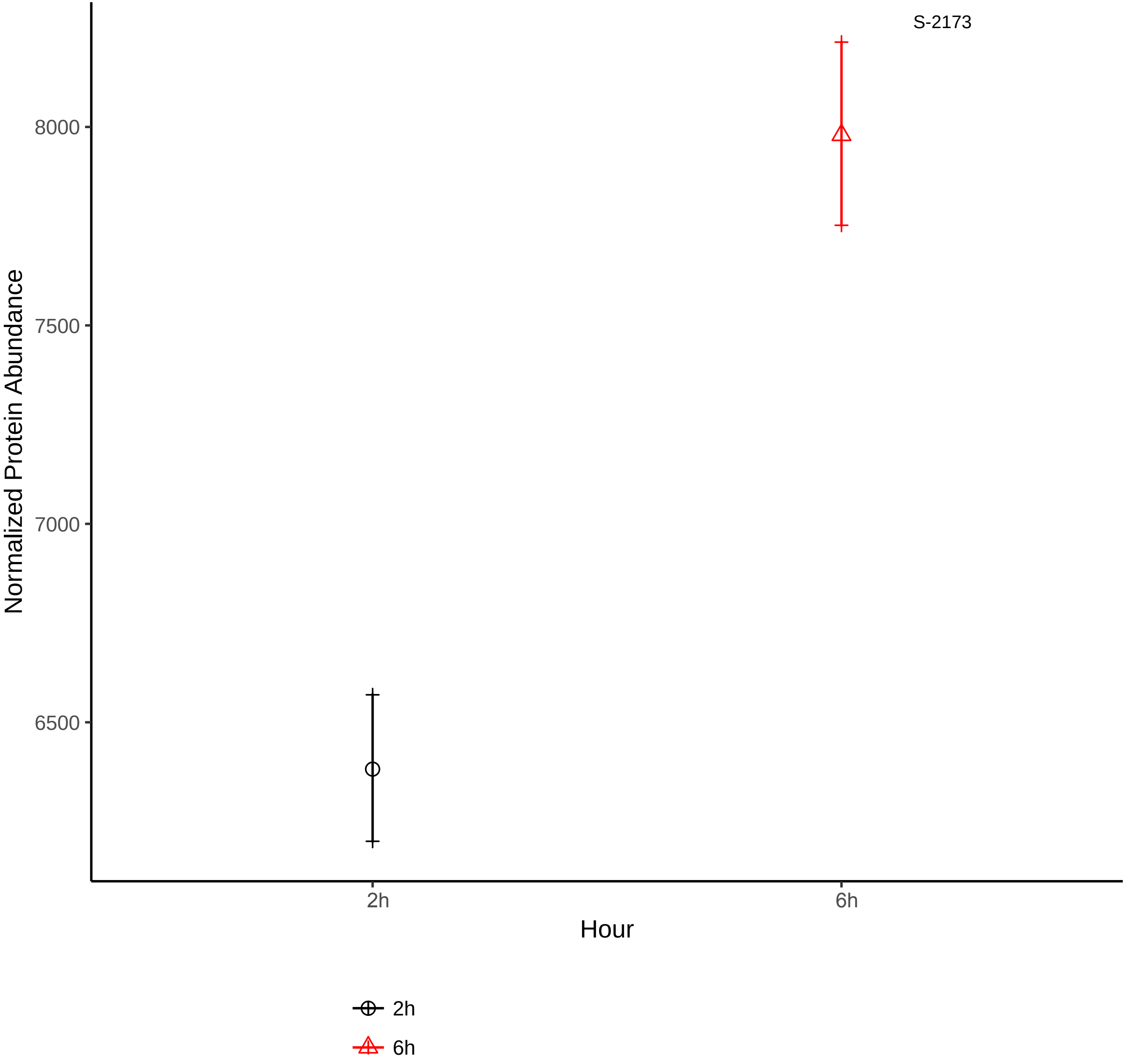
S-2173
8000
7500
7000
6500
Normalized Protein Abundance
2h
6h
Hour
2h
6h

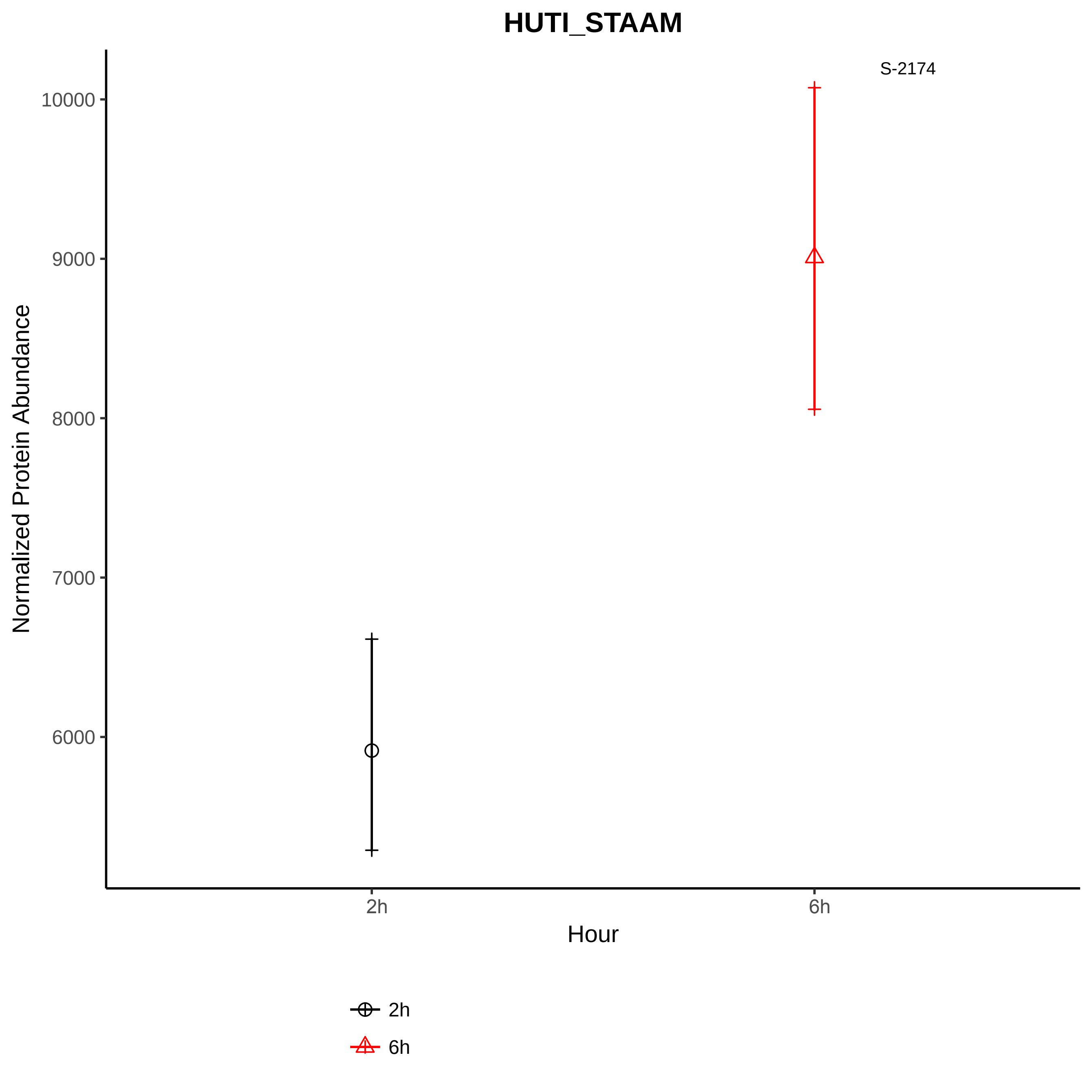

HUTI_STAAM
S-2174
10000
9000
8000
7000
6000
Normalized Protein Abundance
2h
6h
Hour
2h
6h

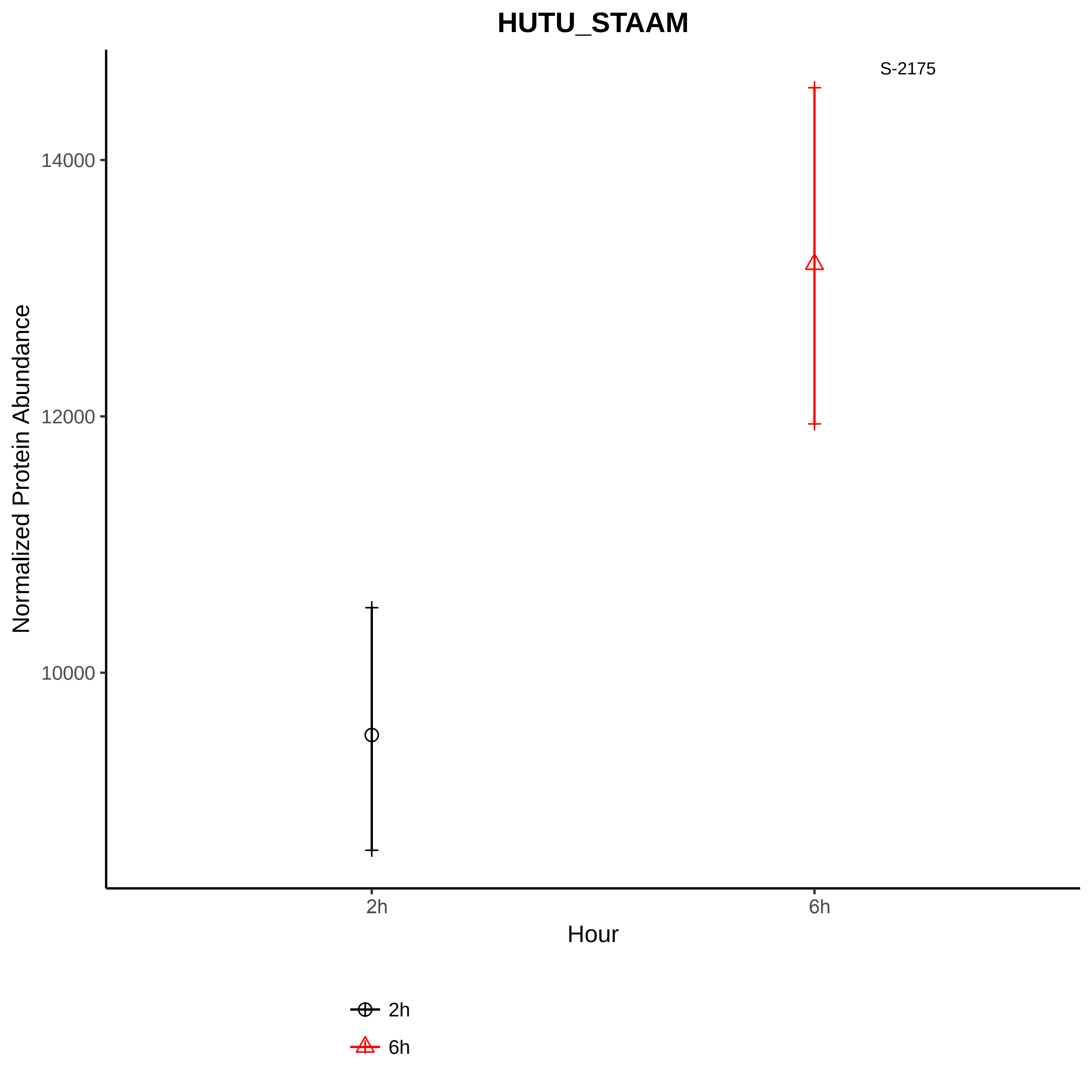

HUTU_STAAM
S-2175
Normalized Protein Abundance
14000
12000
10000
2h
6h
Hour
2h
6h

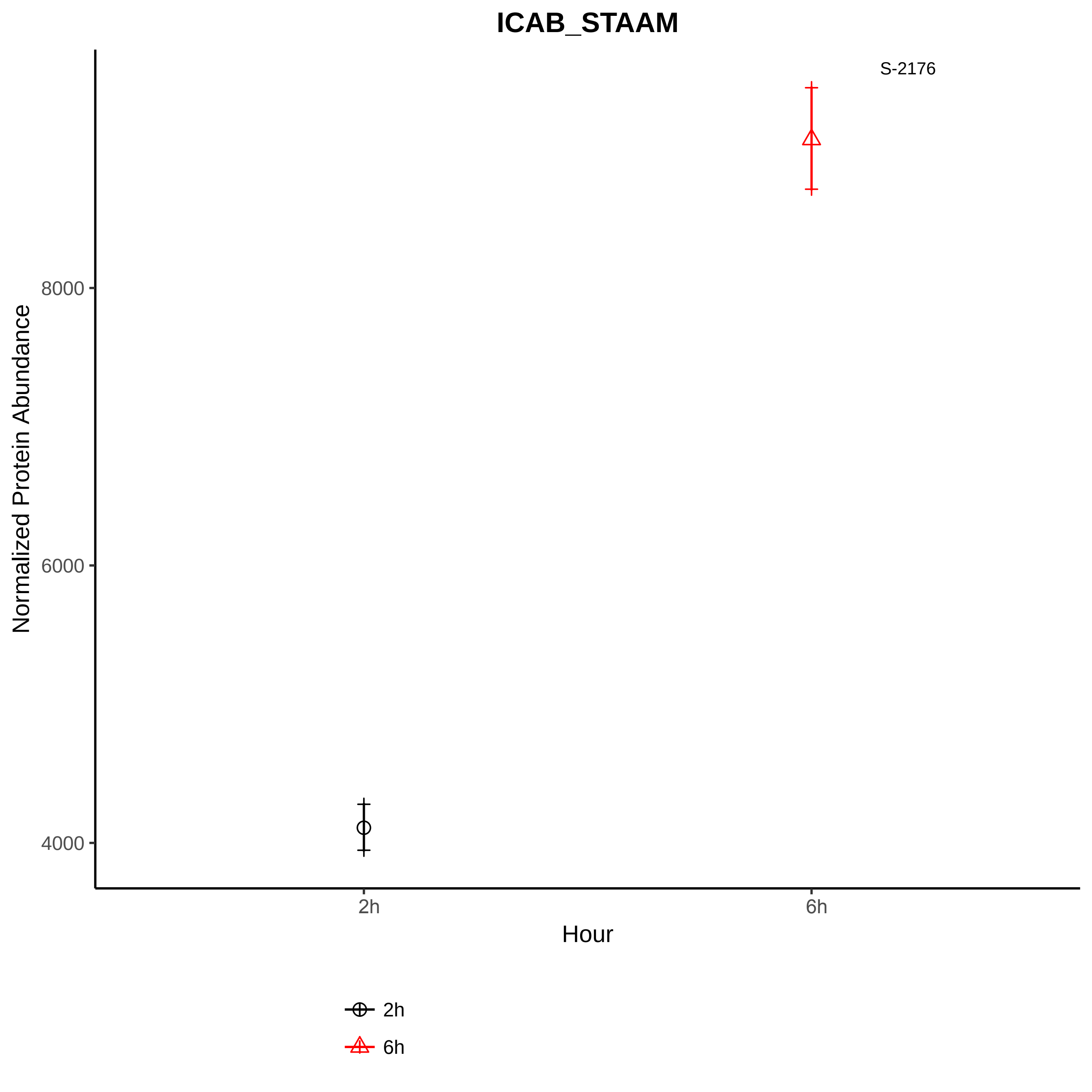

ICAB_STAAM
S-2176
Normalized Protein Abundance
8000
6000
4000
2h
6h
Hour
2h
6h

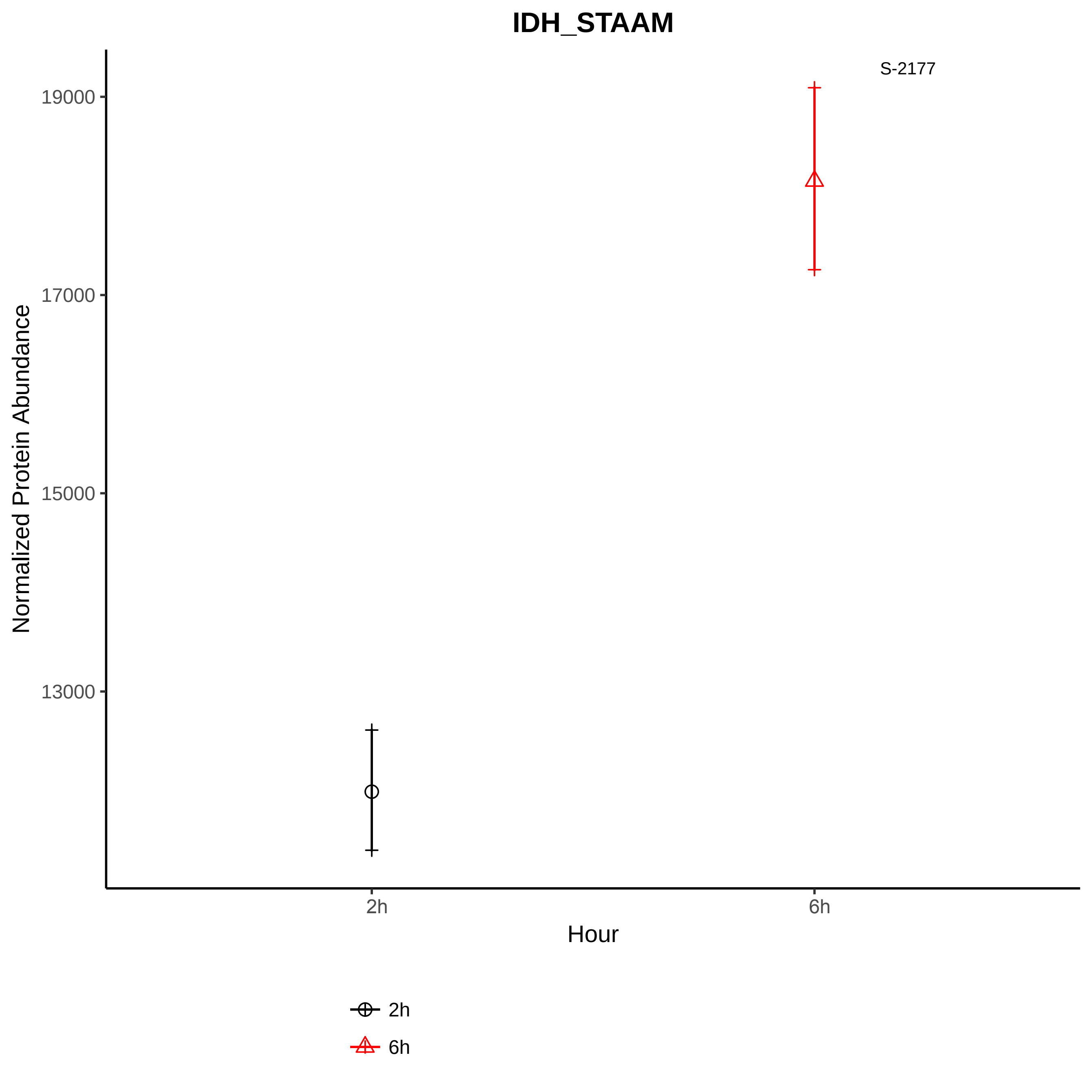
IDH_STAAM
S-2177
19000
17000
15000
13000
Normalized Protein Abundance
2h
6h
Hour
2h
6h

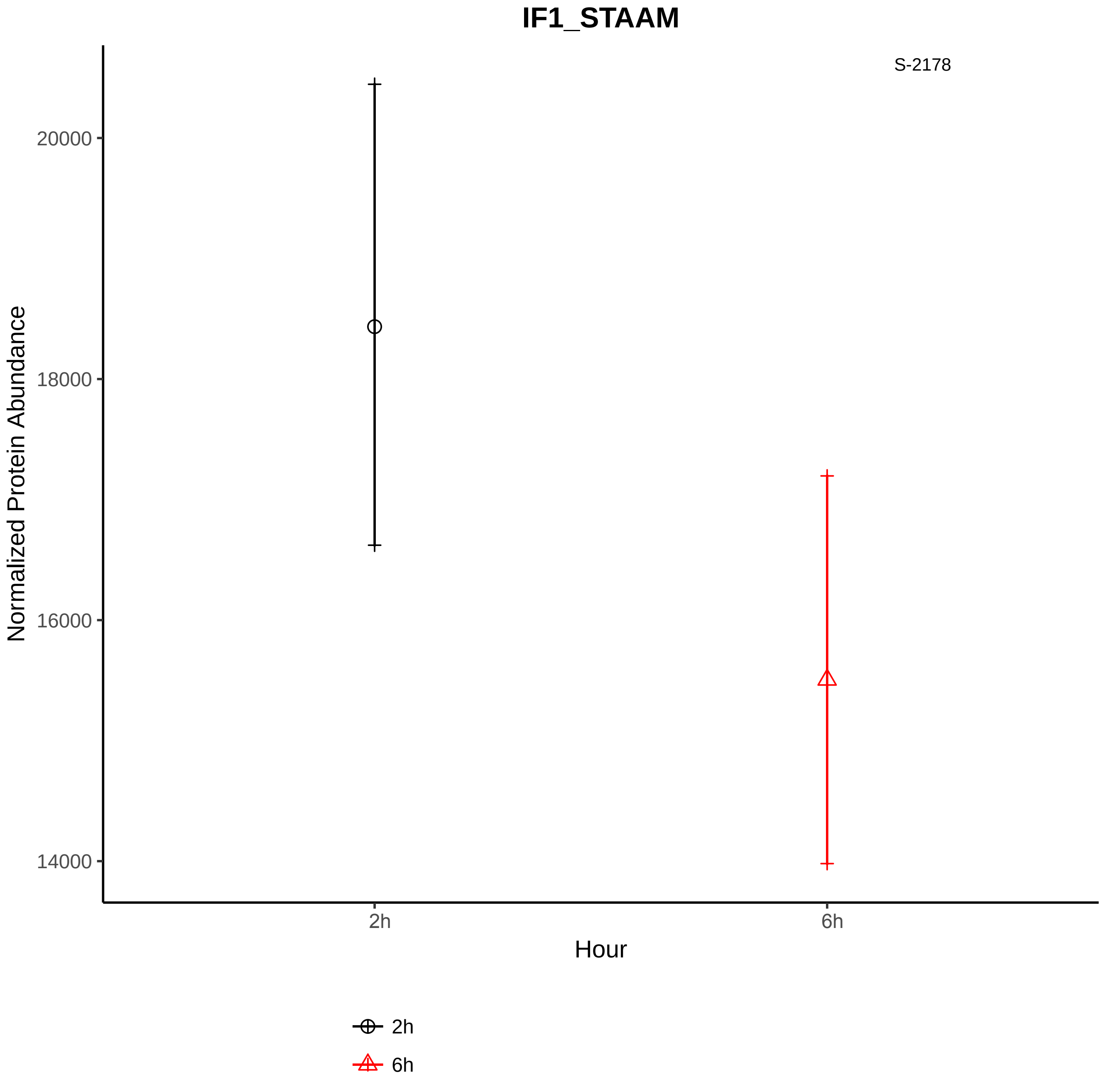

IF1_STAAM
S-2178
Normalized Protein Abundance
20000
18000
16000
14000
2h
6h
Hour
2h
6h

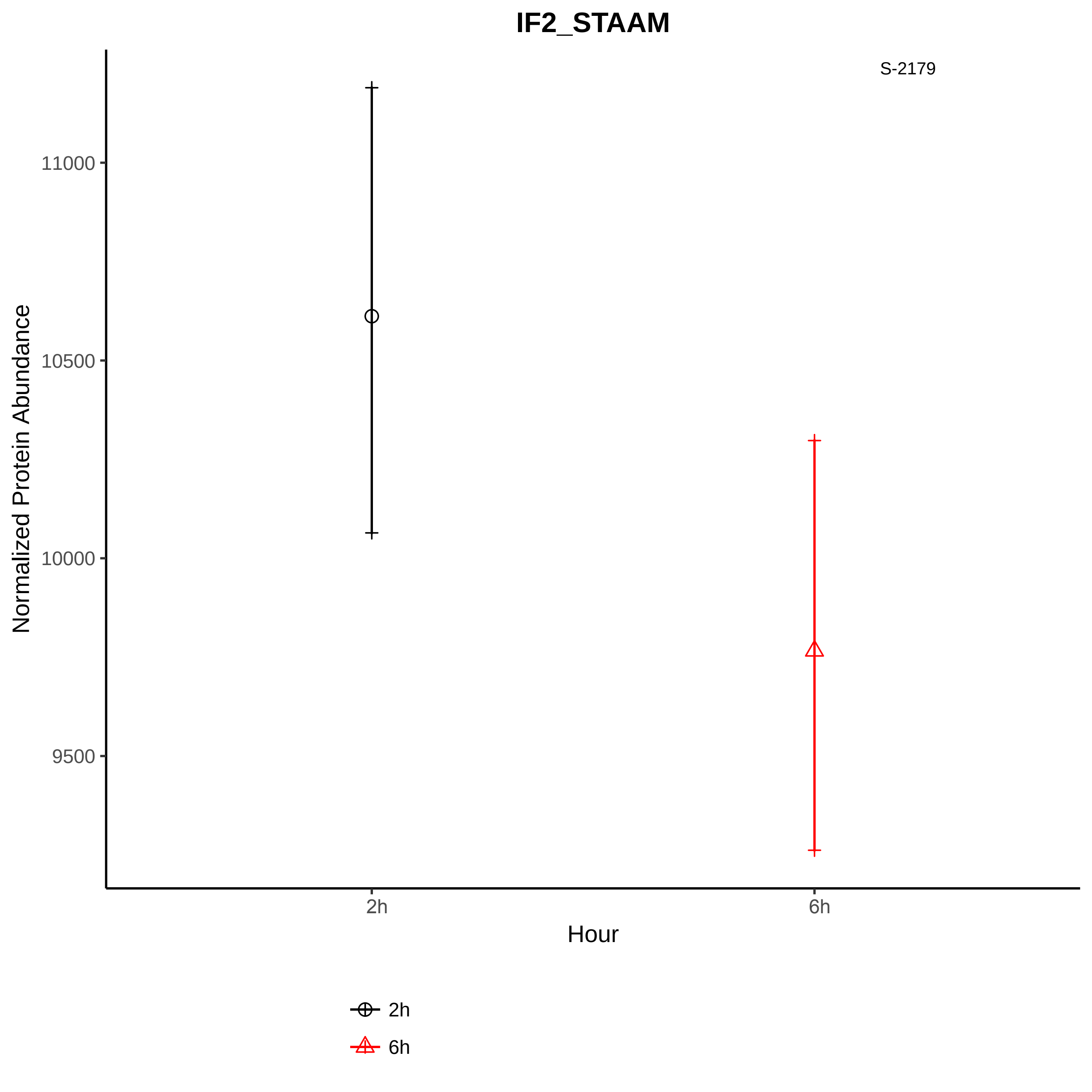
IF2_STAAM
S-2179
Normalized Protein Abundance
11000
10500
10000
9500
2h
6h
Hour
2h
6h

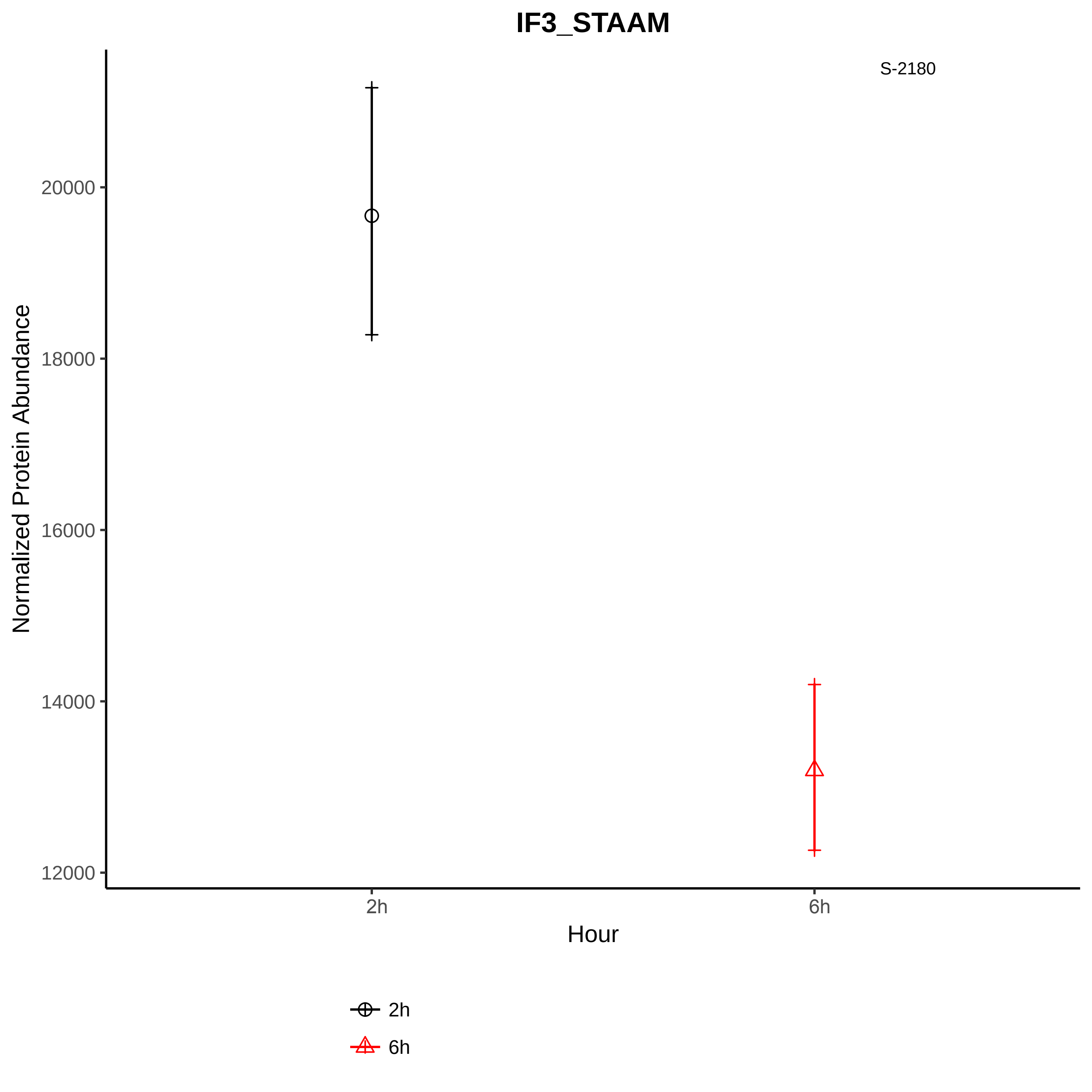

IF3_STAAM
S-2180
Normalized Protein Abundance
20000
18000
16000
14000
12000
2h
6h
Hour
2h
6h

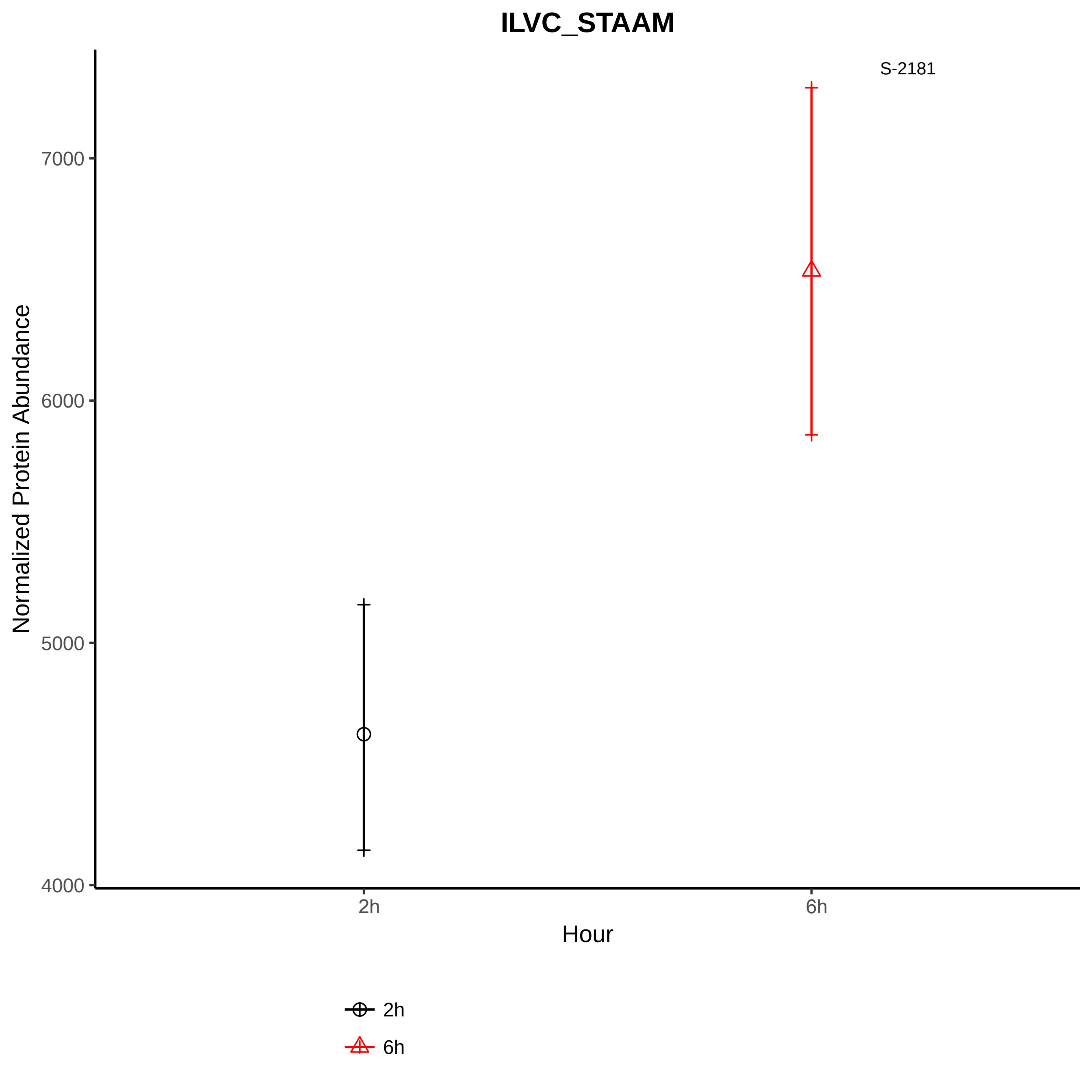
ILVC_STAAM
S-2181
7000
6000
5000
4000
Normalized Protein Abundance
2h
6h
Hour
2h
6h

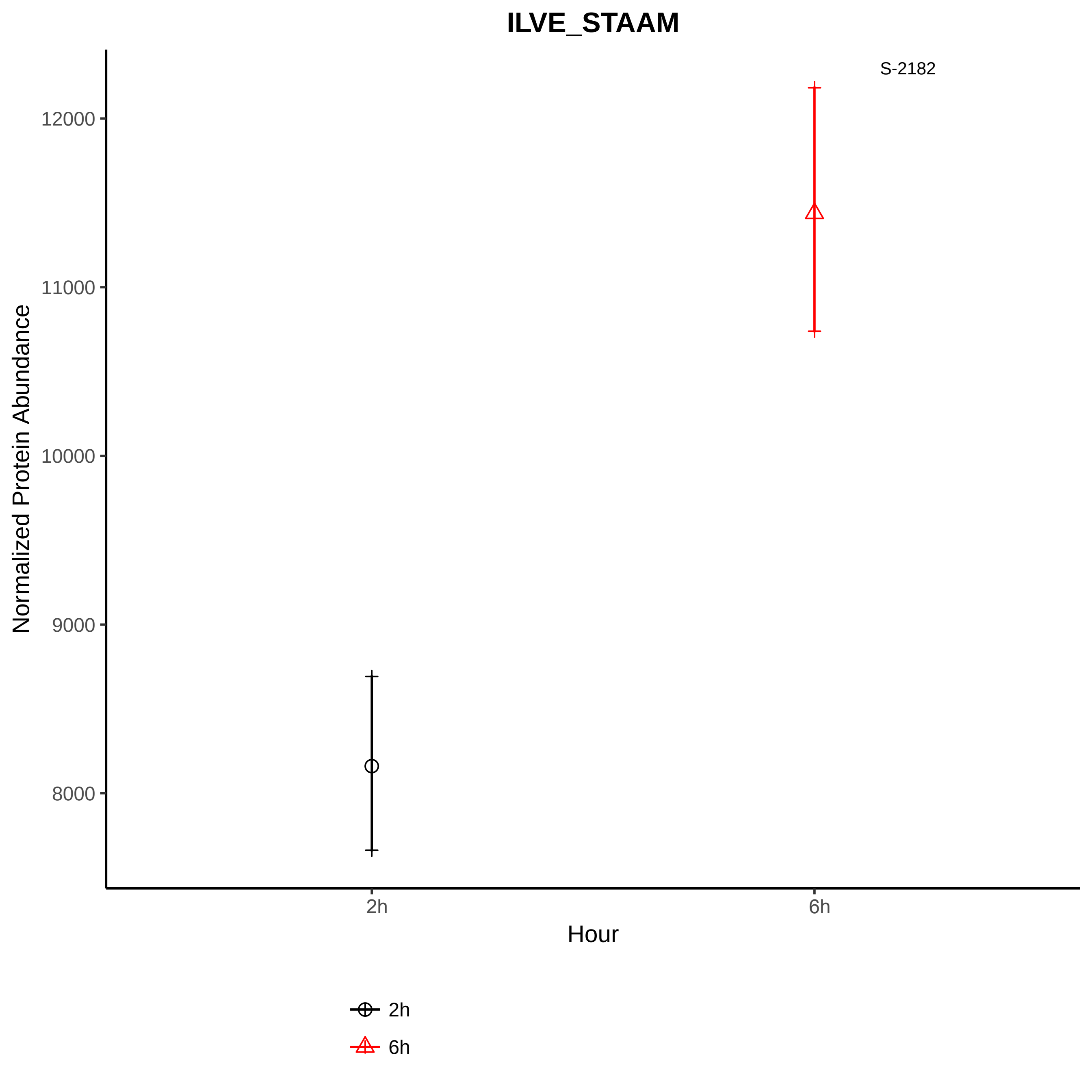

ILVE_STAAM
S-2182
Normalized Protein Abundance
12000
11000
10000
9000
8000
2h
6h
Hour
2h
6h

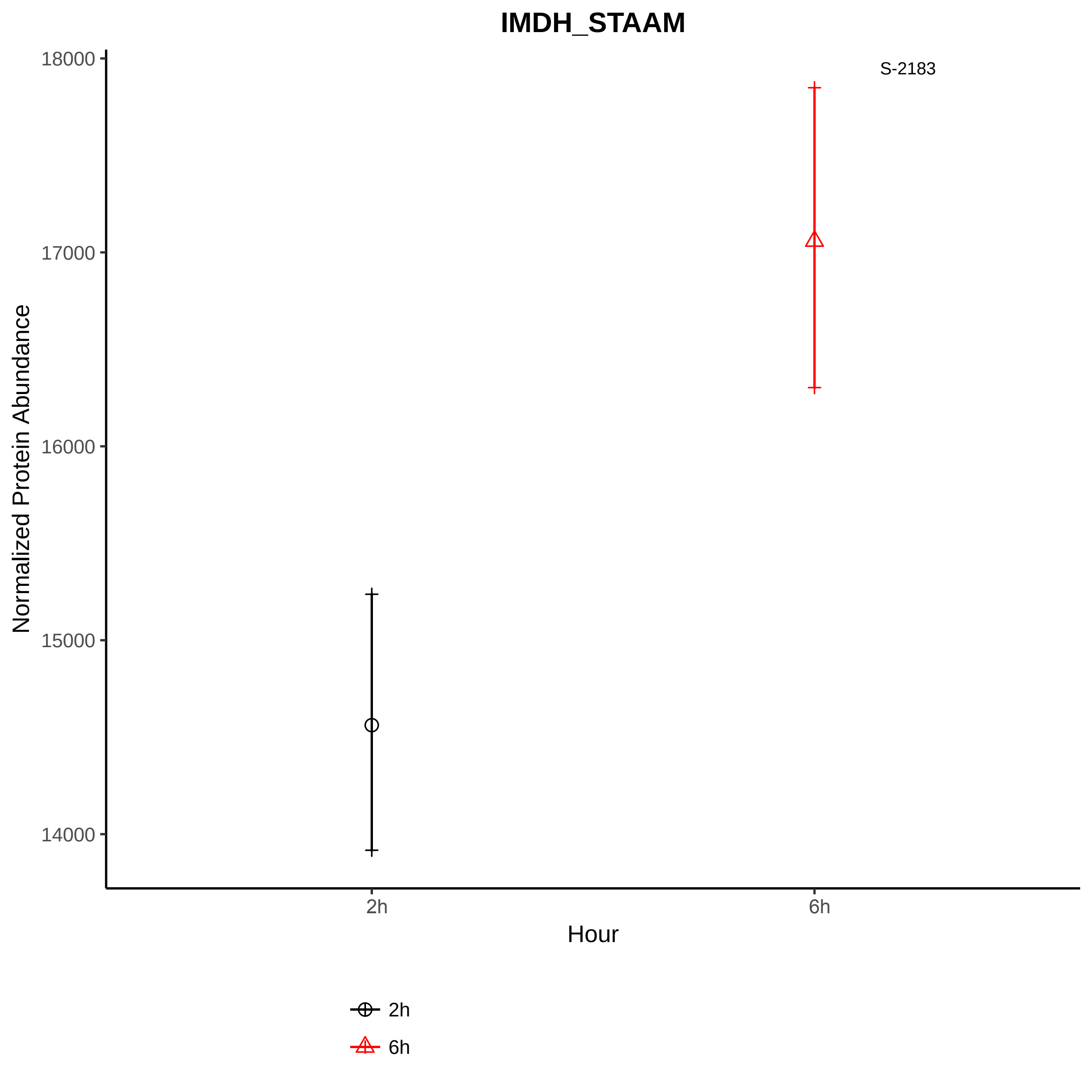
IMDH_STAAM
S-2183
Normalized Protein Abundance
18000
17000
16000
15000
14000
2h
6h
Hour
2h
6h

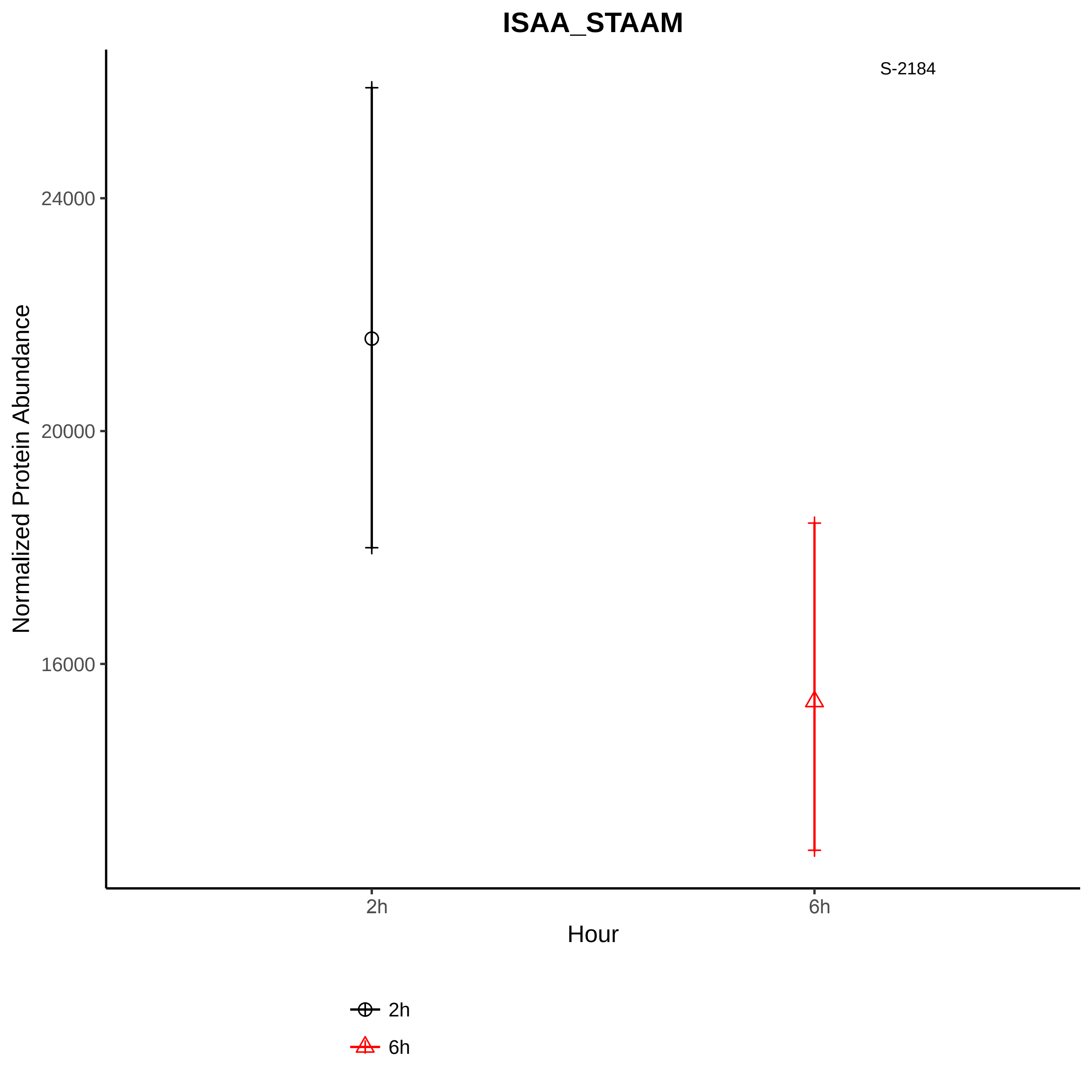

ISAA_STAAM
S-2184
Normalized Protein Abundance
24000
20000
16000
2h
6h
Hour
2h
6h

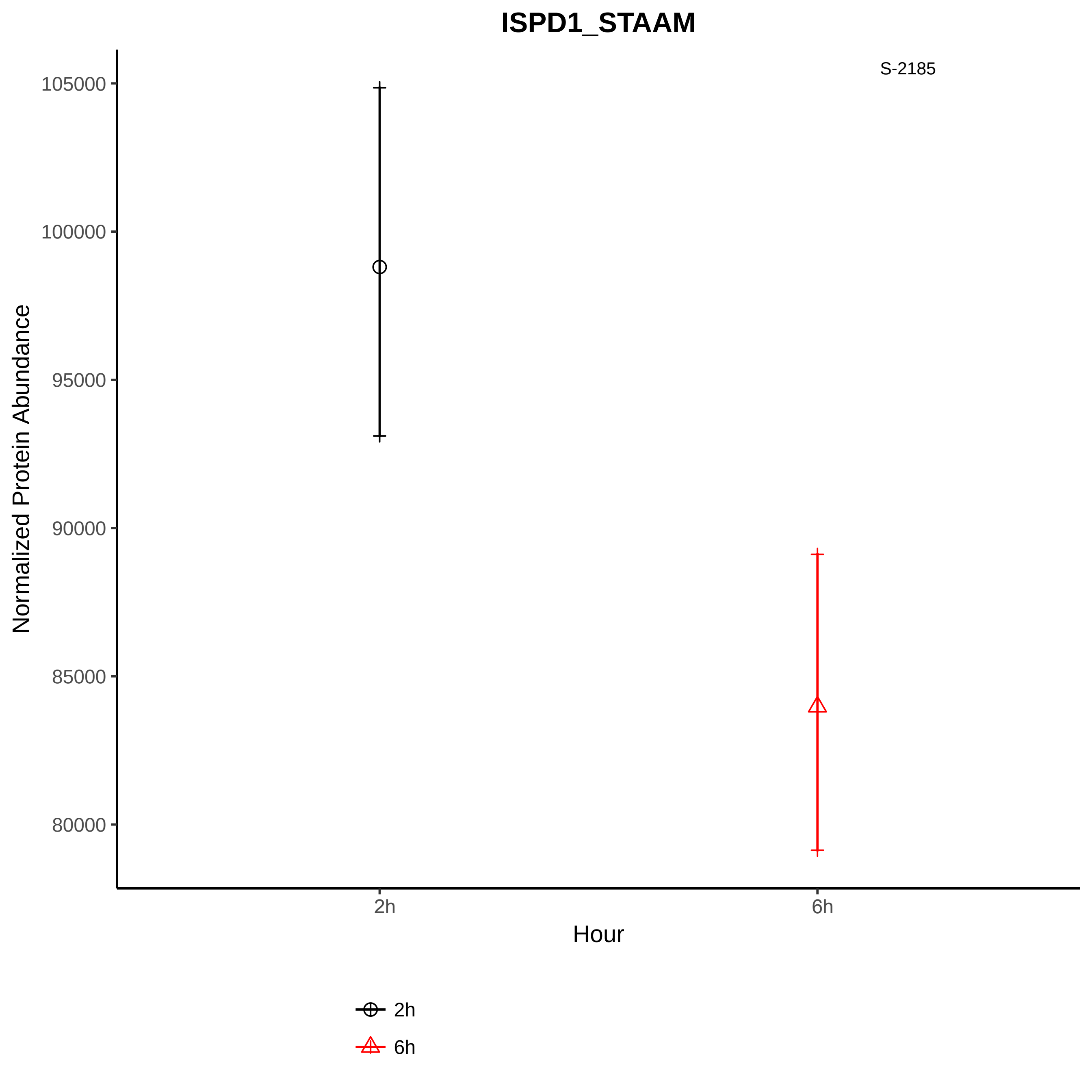

ISPD1_STAAM
S-2185
Normalized Protein Abundance
105000
100000
95000
90000
85000
80000
2h
6h
Hour
2h
6h

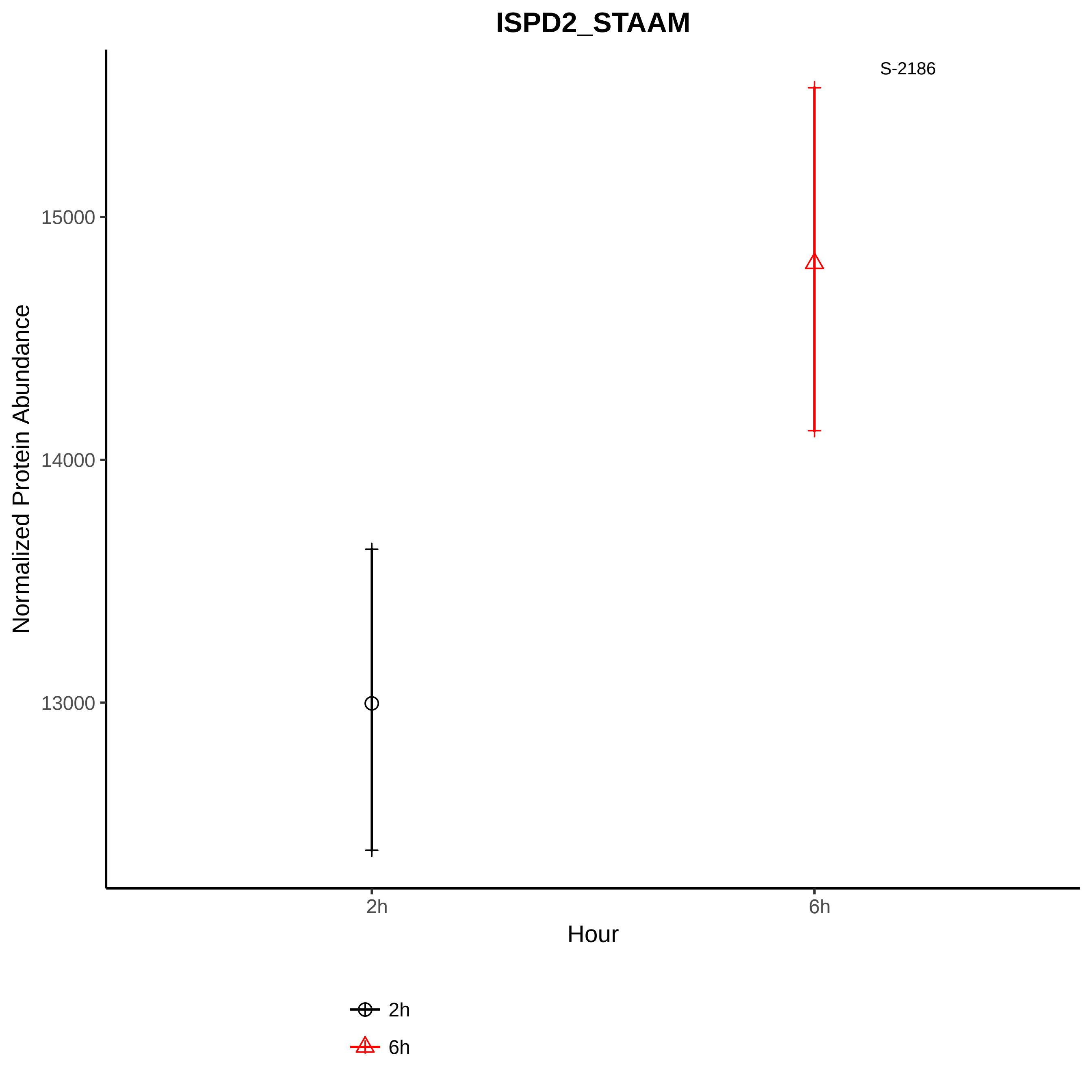

ISPD2_STAAM
S-2186
Normalized Protein Abundance
15000
14000
13000
2h
6h
Hour
2h
6h

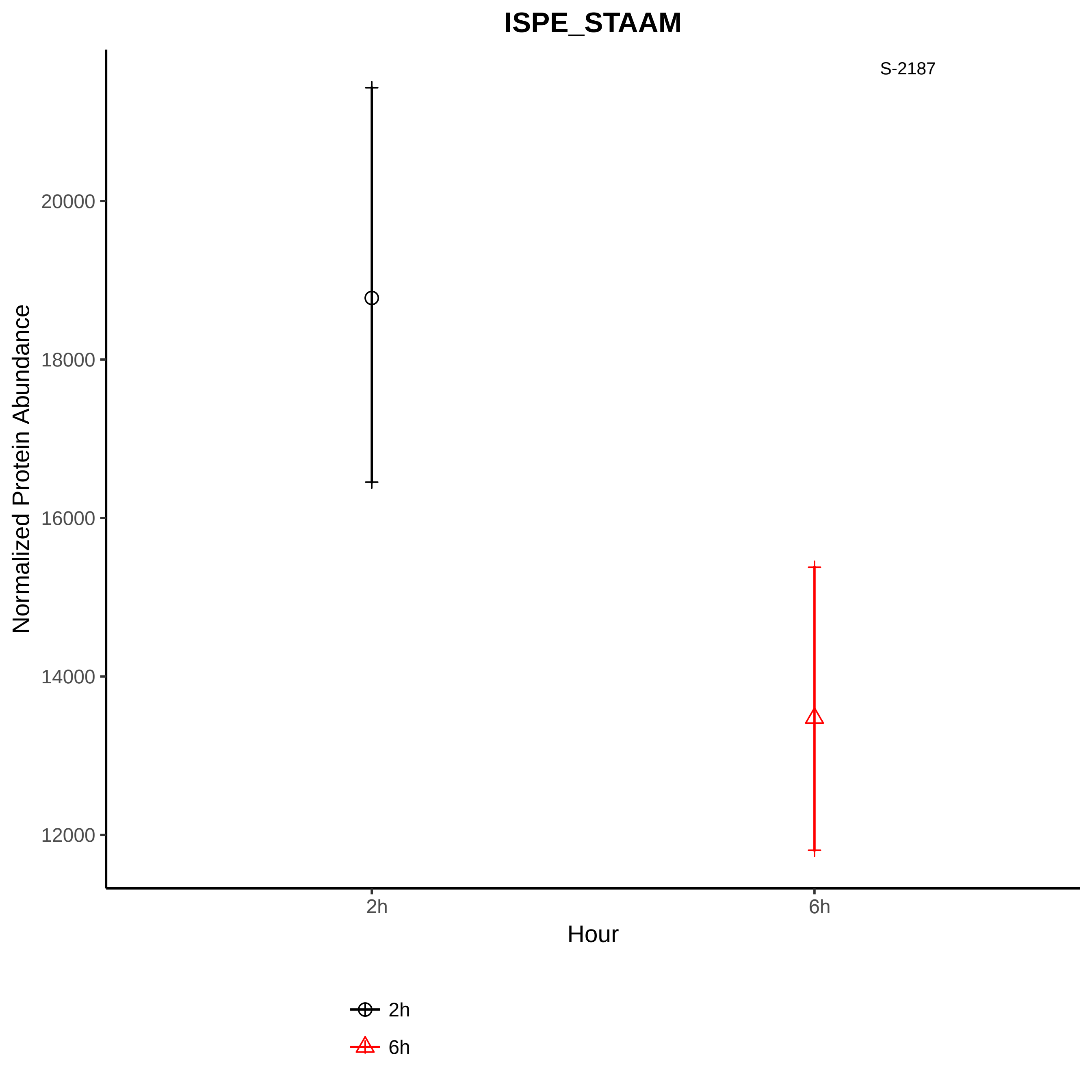

ISPE_STAAM
S-2187
Normalized Protein Abundance
20000
18000
16000
14000
12000
2h
6h
Hour
2h
6h

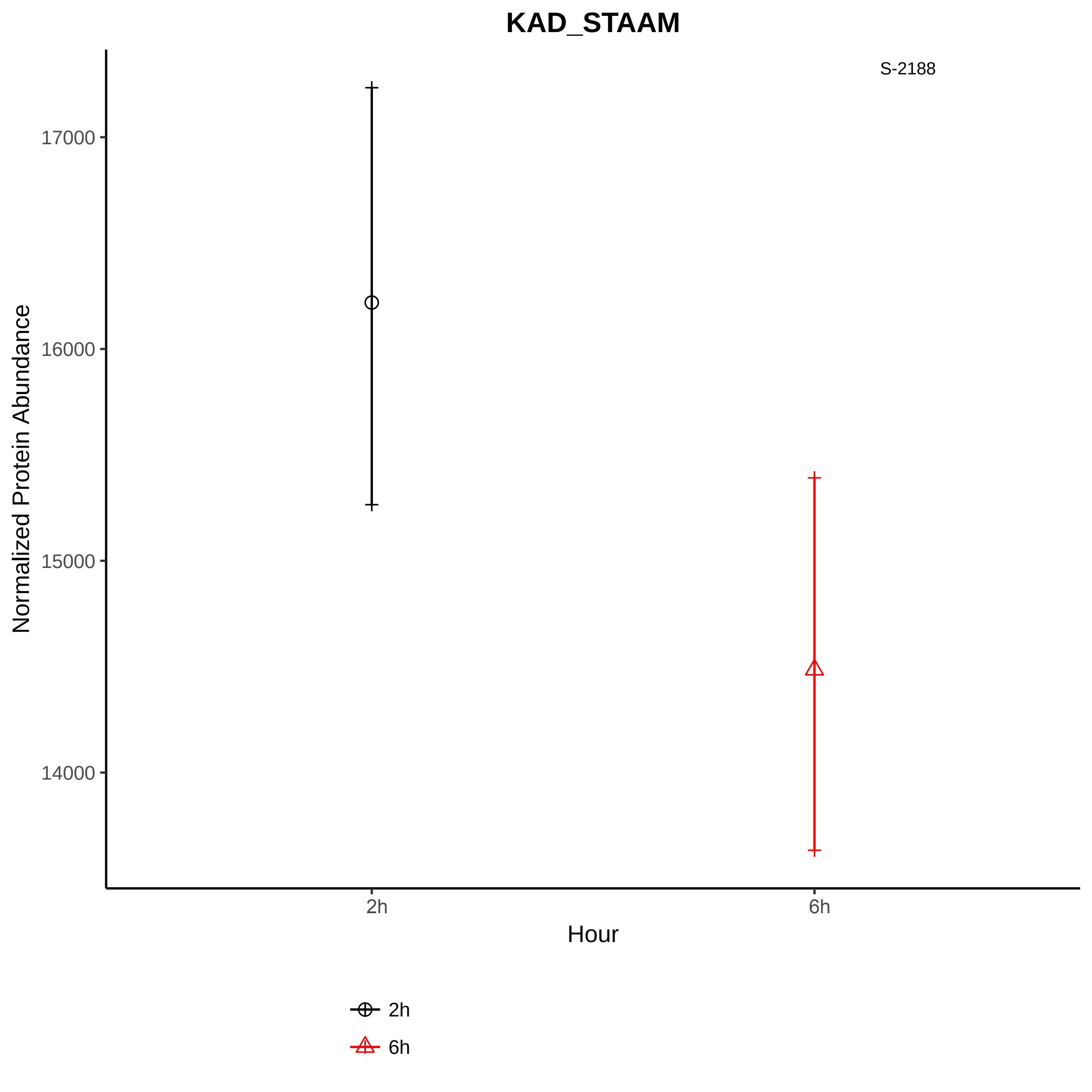

KAD_STAAM
S-2188
Normalized Protein Abundance
17000
16000
15000
14000
2h
6h
Hour
2h
6h

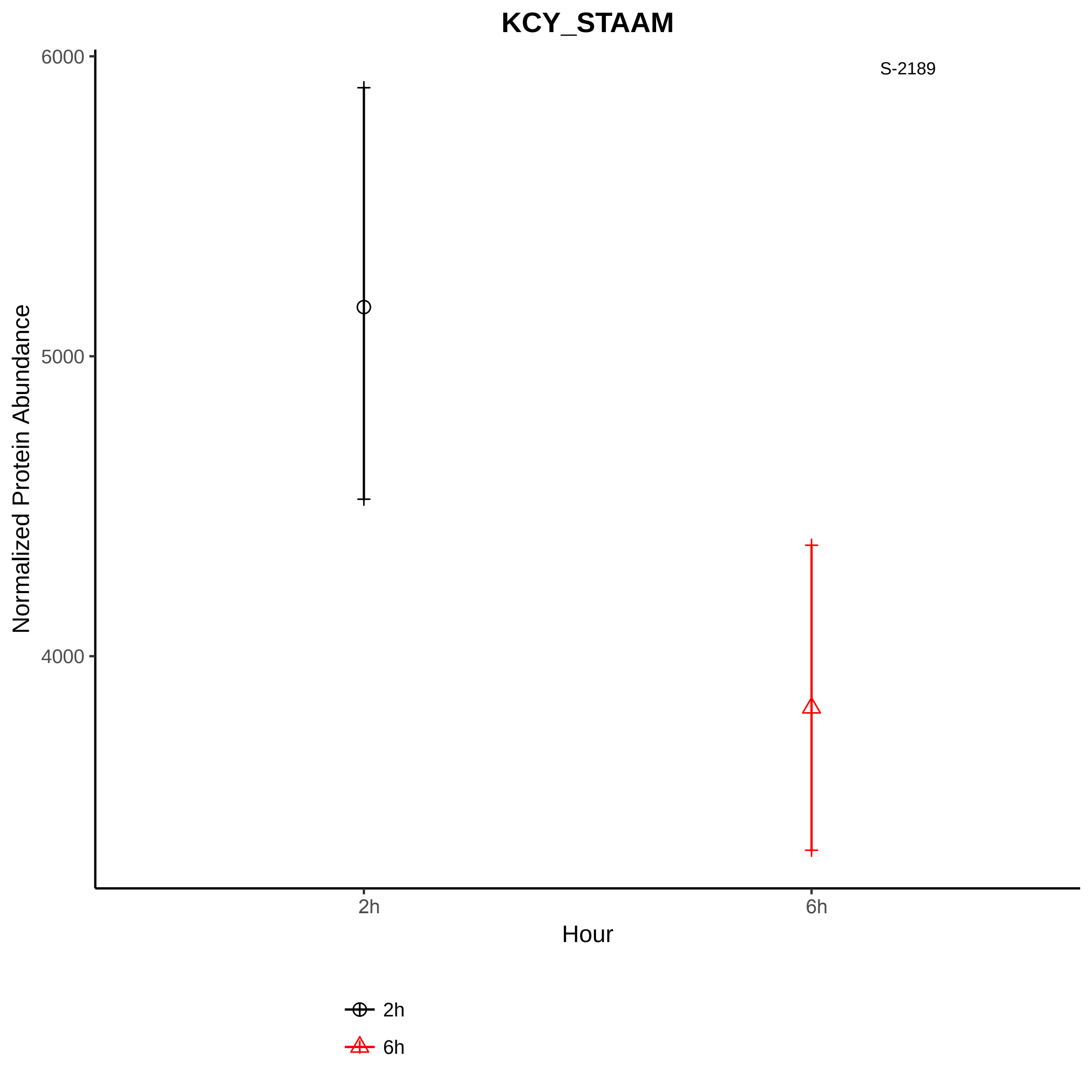
KCY_STAAM
S-2189
Normalized Protein Abundance
6000
5000
4000
2h
6h
Hour
2h
6h

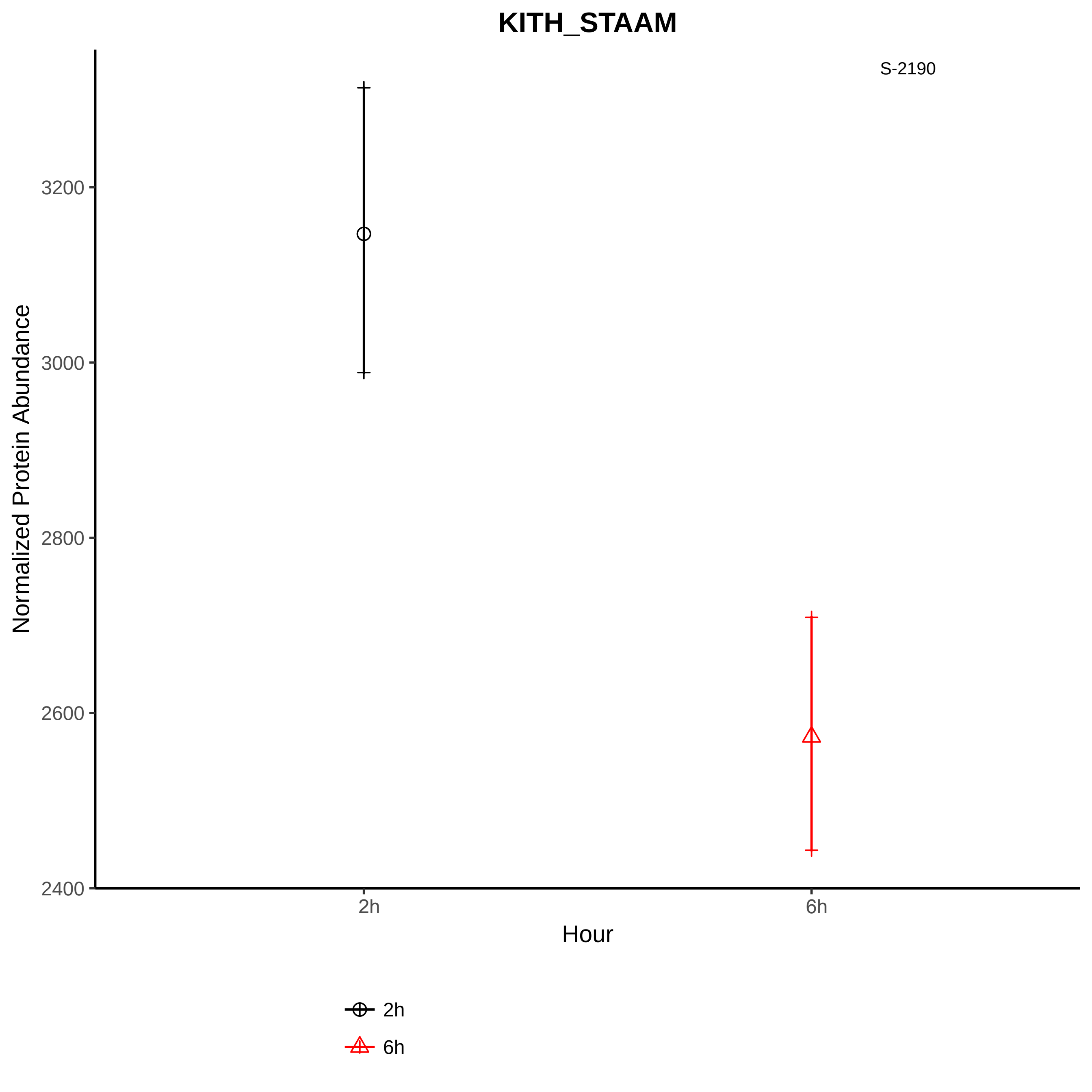

KITH_STAAM
S-2190
Normalized Protein Abundance
3200
3000
2800
2600
2400
2h
6h
Hour
2h
6h

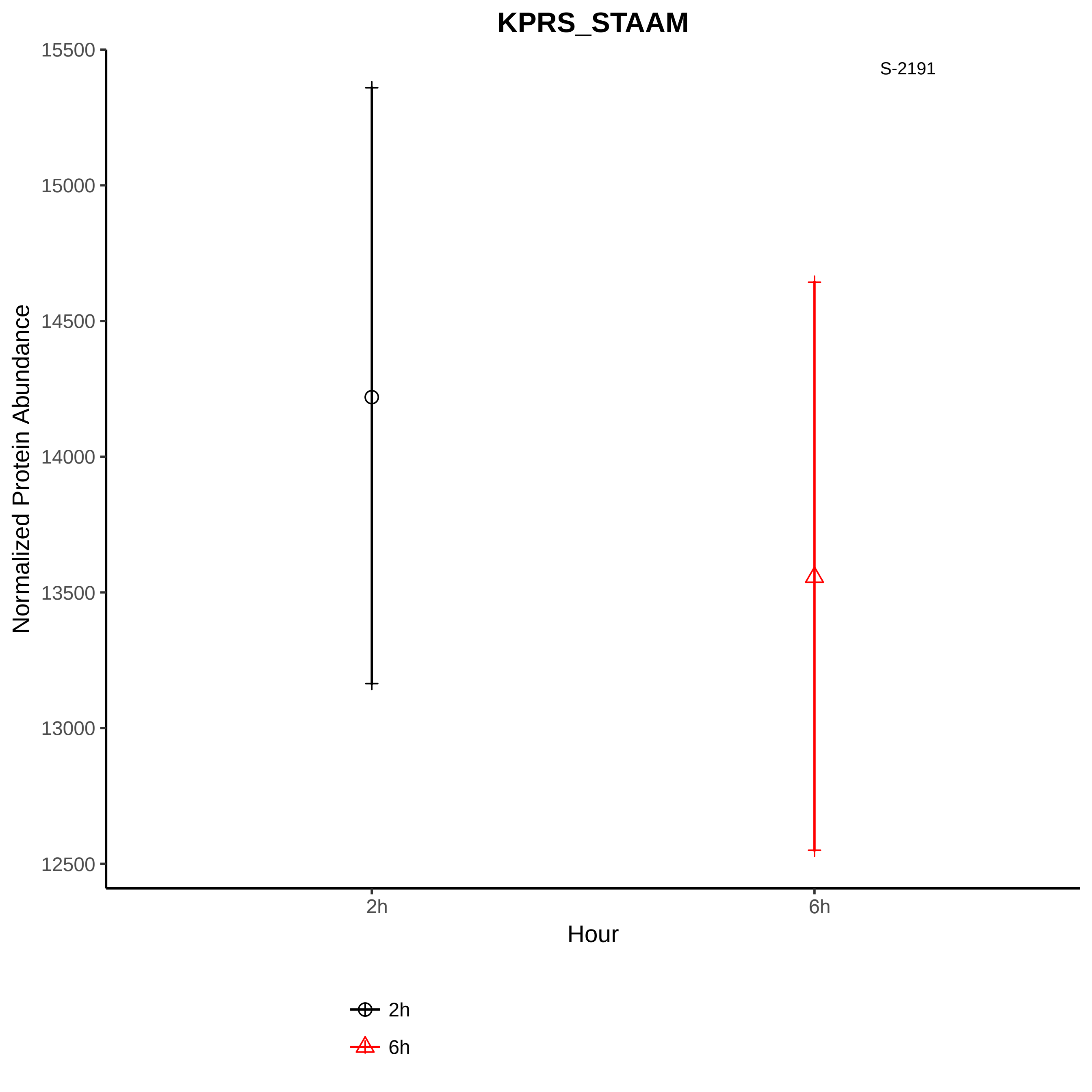
KPRS_STAAM
S-2191
Normalized Protein Abundance
15500
15000
14500
14000
13500
13000
12500
2h
6h
Hour
2h
6h

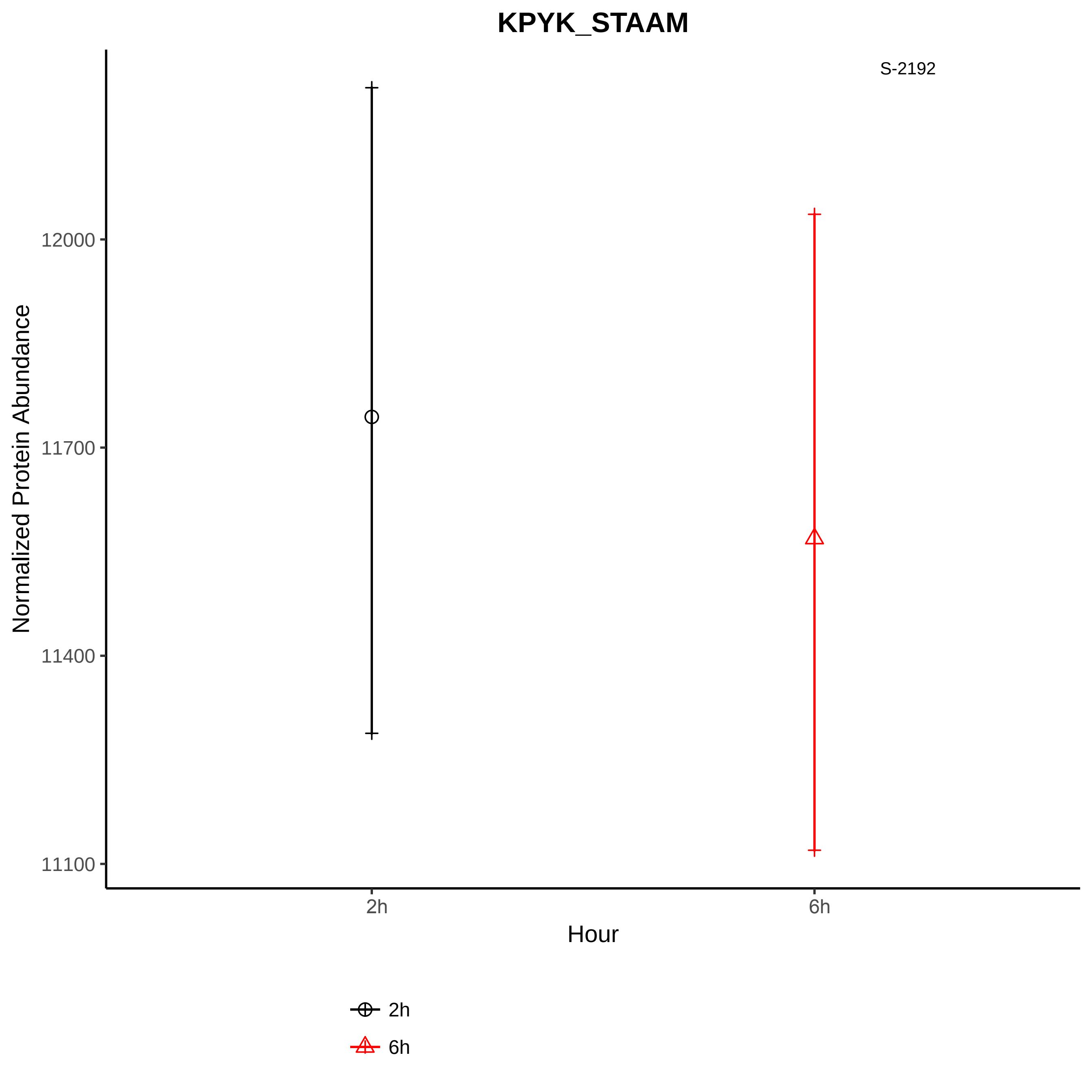

KPYK_STAAM
S-2192
Normalized Protein Abundance
12000
11700
11400
11100
2h
6h
Hour
2h
6h

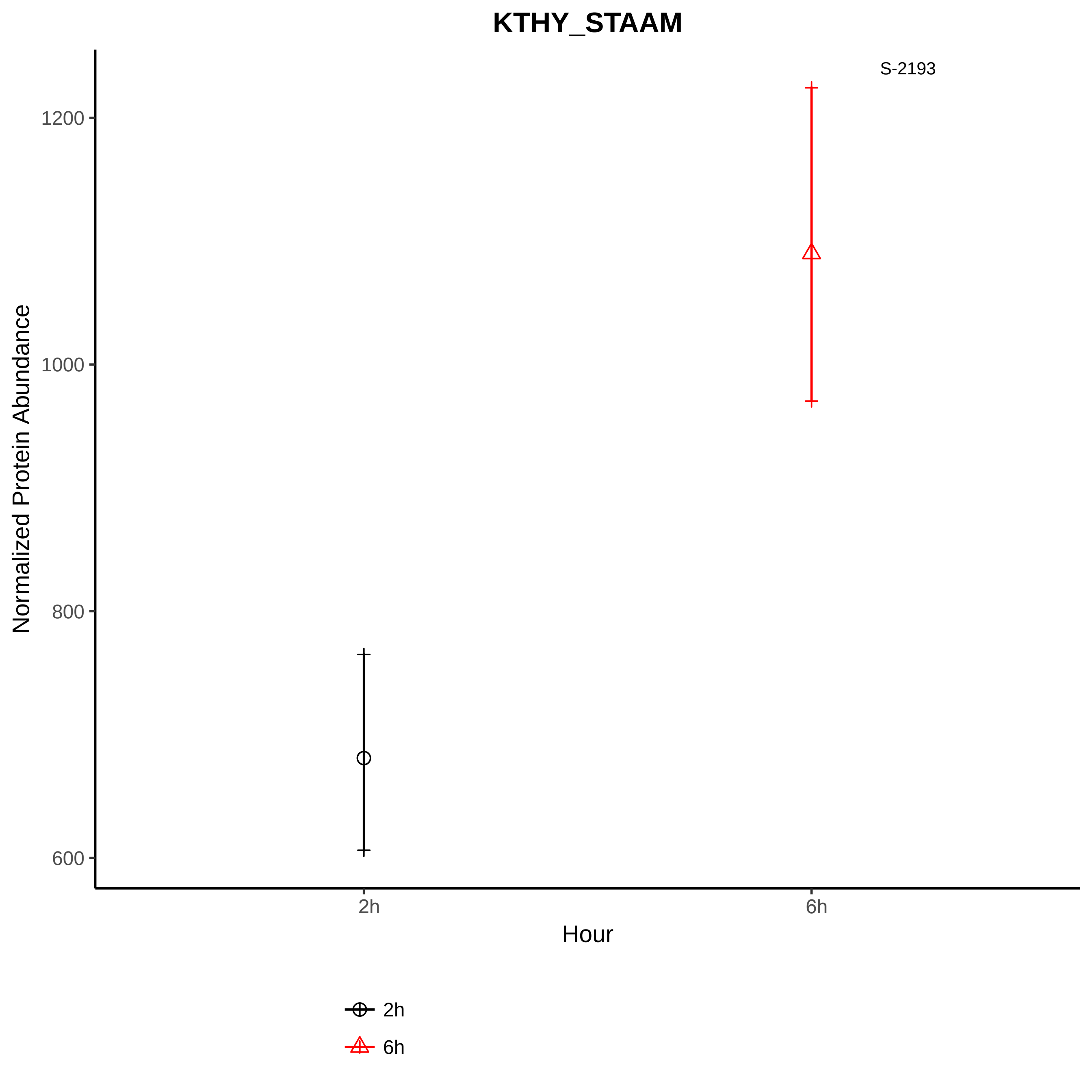

KTHY_STAAM
S-2193
1200
1000
800
600
Normalized Protein Abundance
2h
6h
Hour
2h
6h

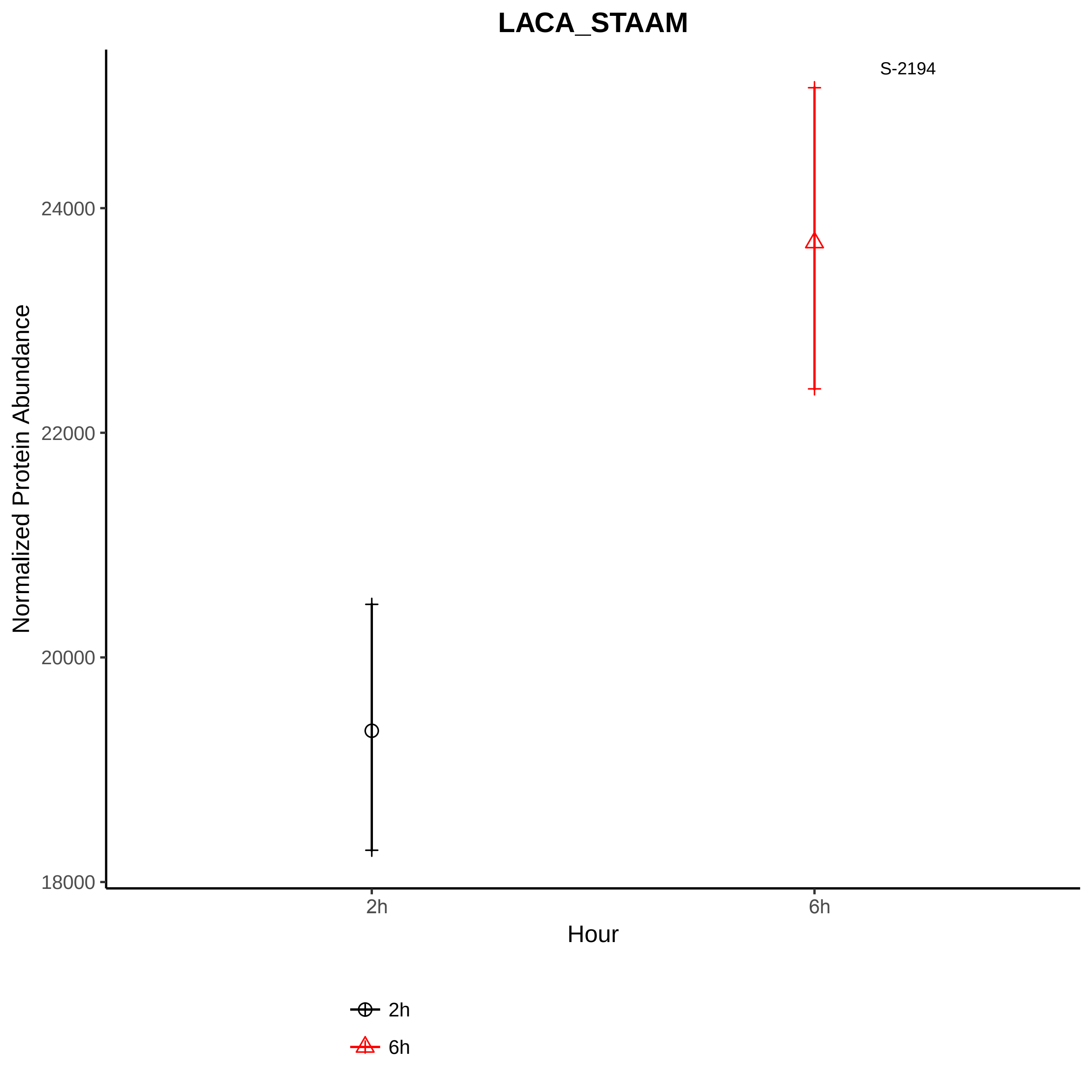

LACA_STAAM
S-2194
Normalized Protein Abundance
24000
22000
20000
18000
2h
6h
Hour
2h
6h

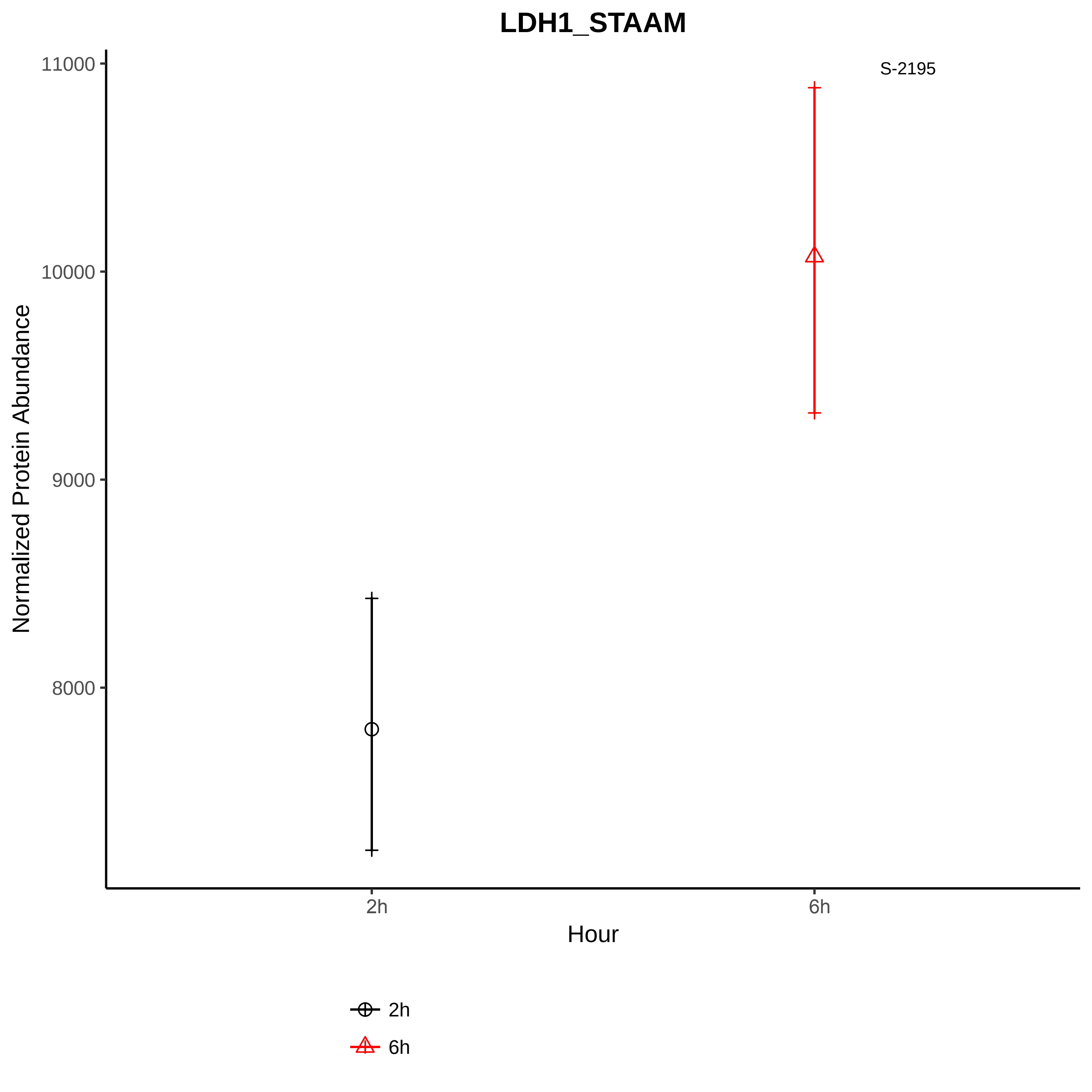

LDH1_STAAM
S-2195
11000
10000
9000
8000
Normalized Protein Abundance
2h
6h
Hour
2h
6h

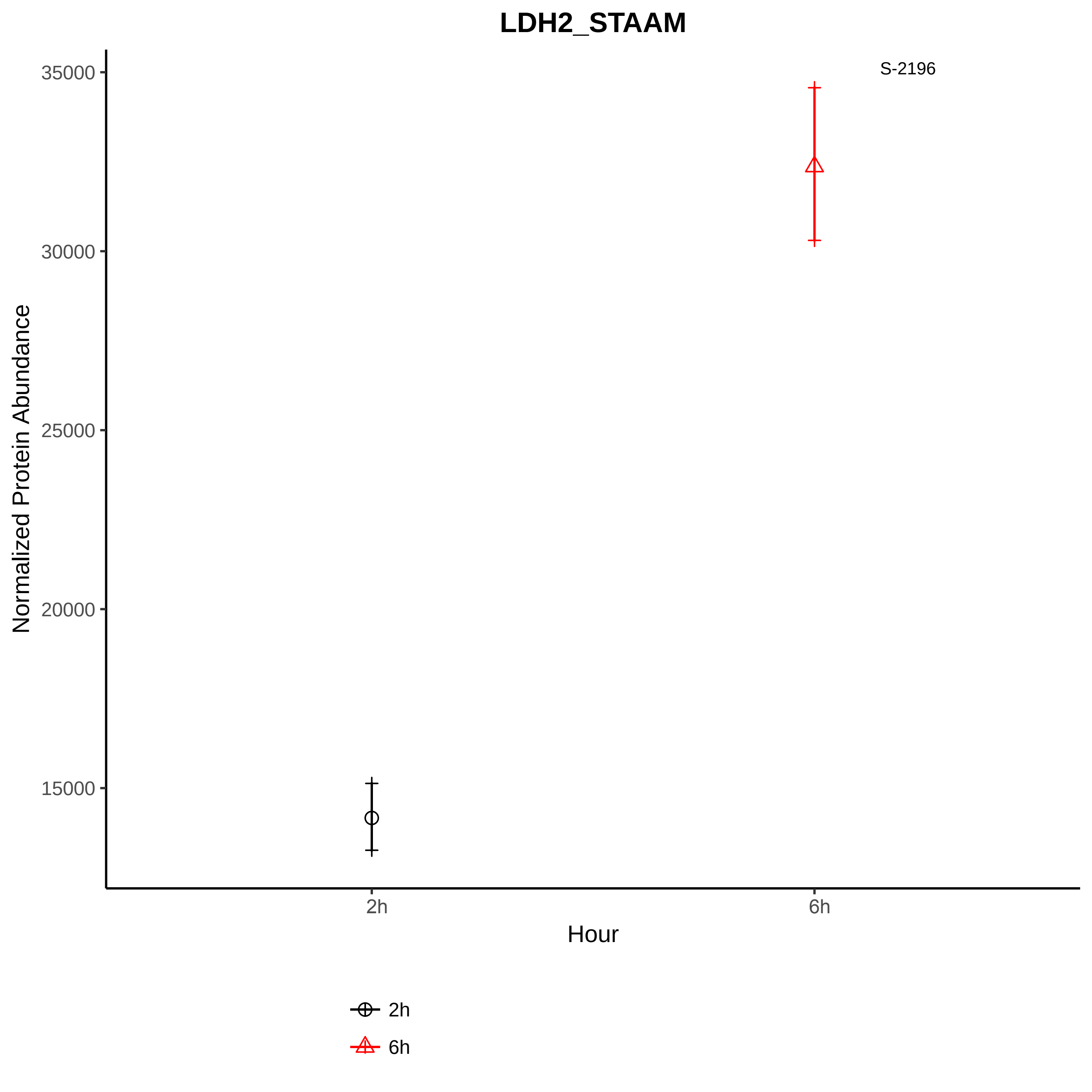
LDH2_STAAM
S-2196
35000
30000
25000
20000
15000
Normalized Protein Abundance
2h
6h
Hour
2h
6h

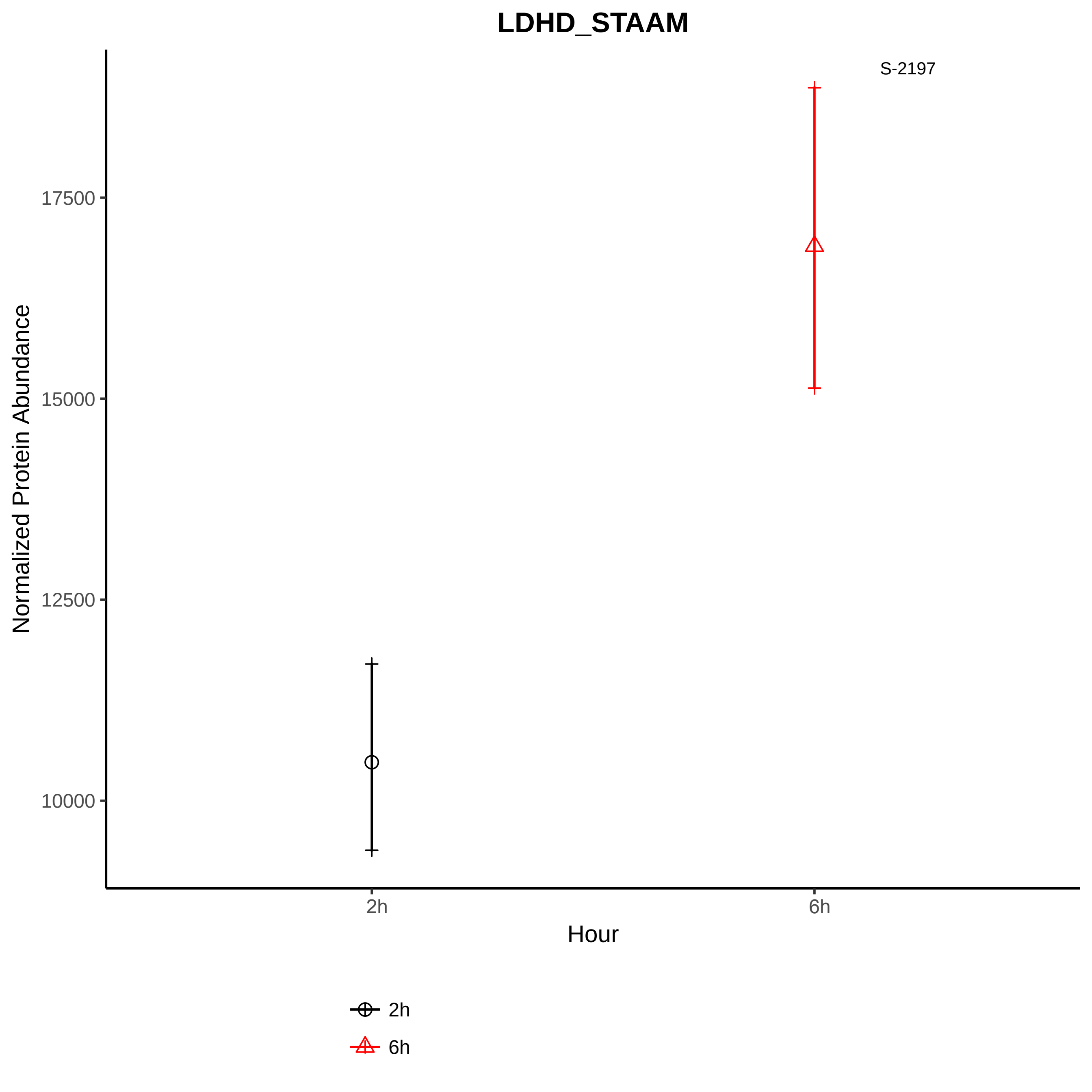

LDHD_STAAM
S-2197
17500
15000
12500
10000
Normalized Protein Abundance
2h
6h
Hour
2h
6h

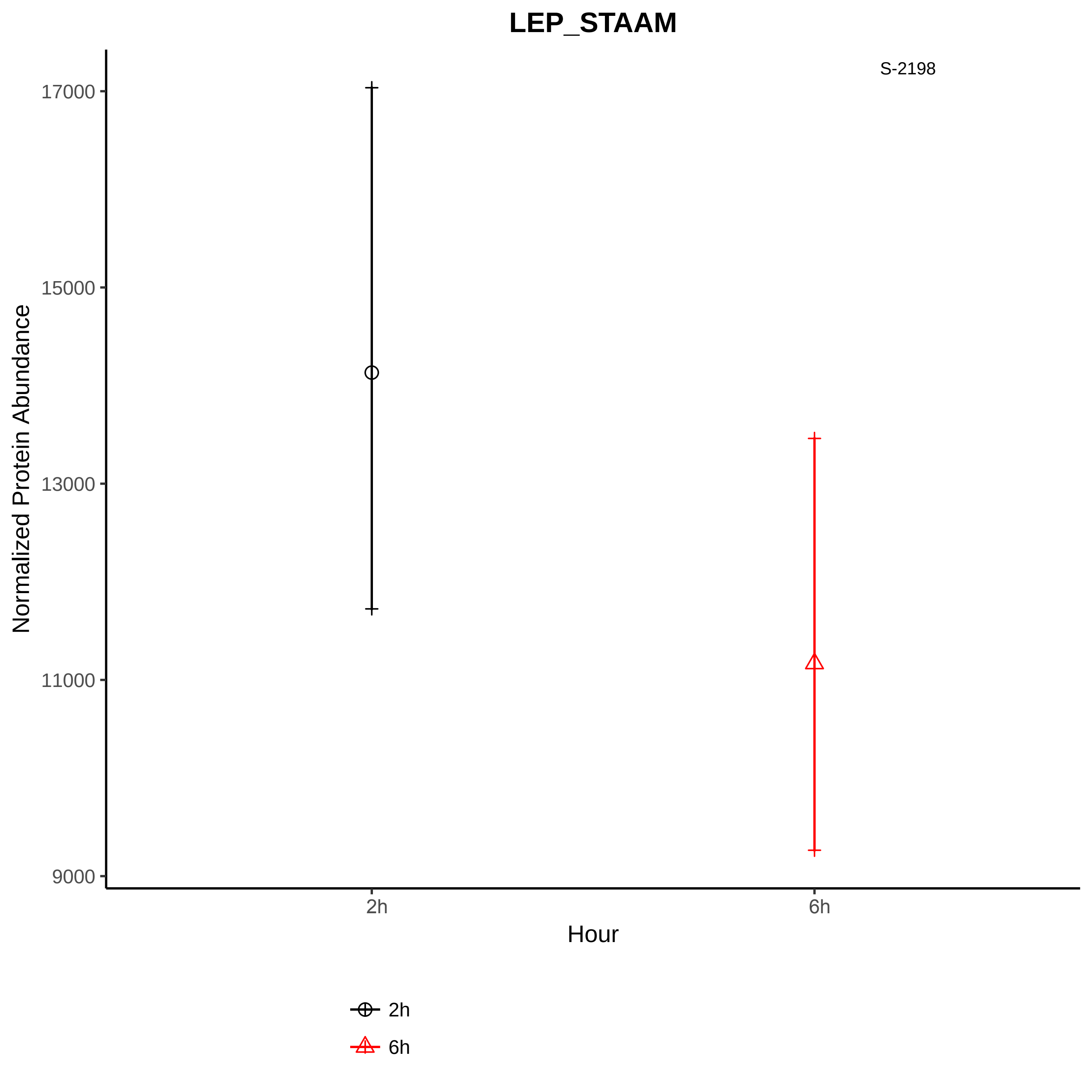

LEP_STAAM
S-2198
Normalized Protein Abundance
17000
15000
13000
11000
9000
2h
6h
Hour
2h
6h

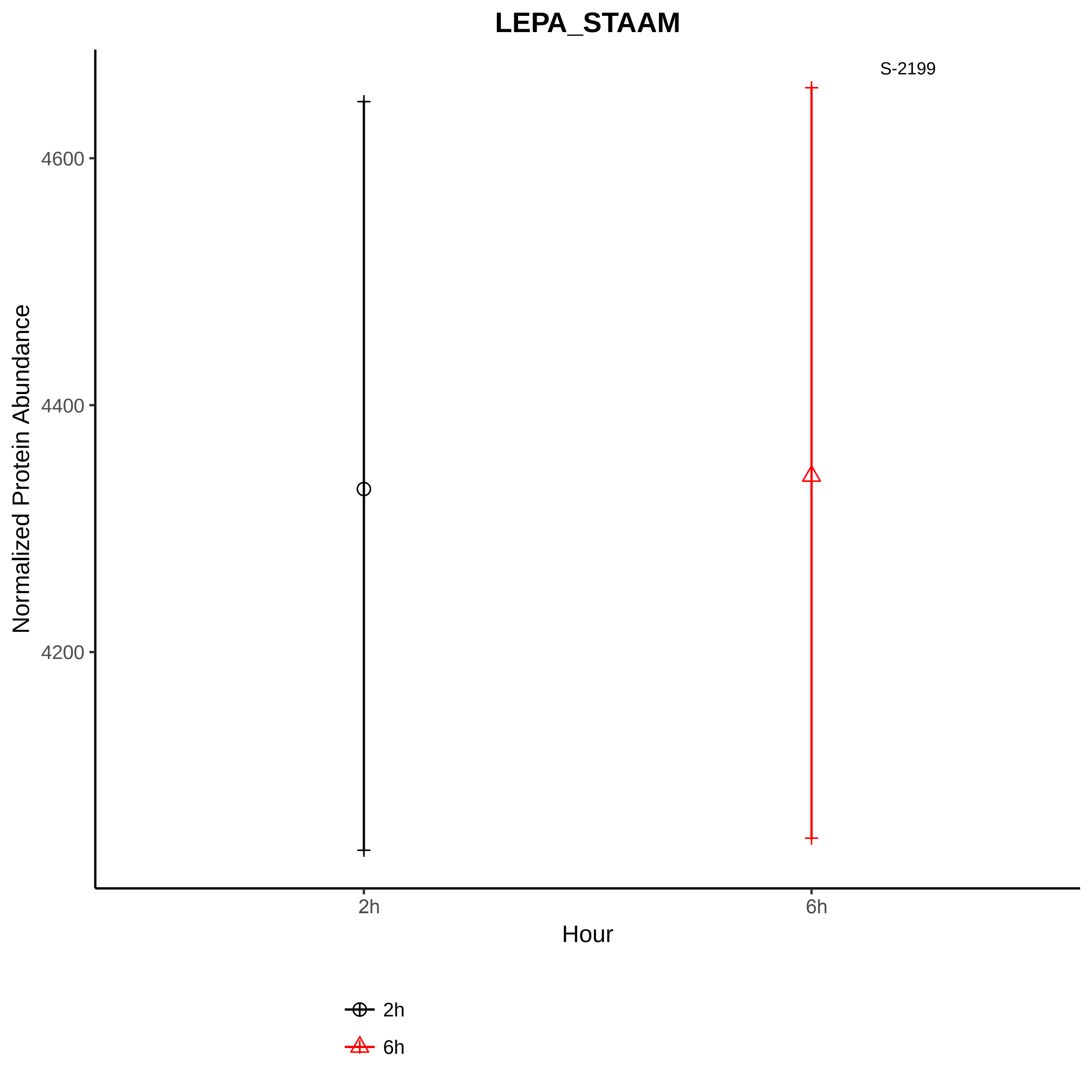

LEPA_STAAM
S-2199
Normalized Protein Abundance
4600
4400
4200
2h
6h
Hour
2h
6h

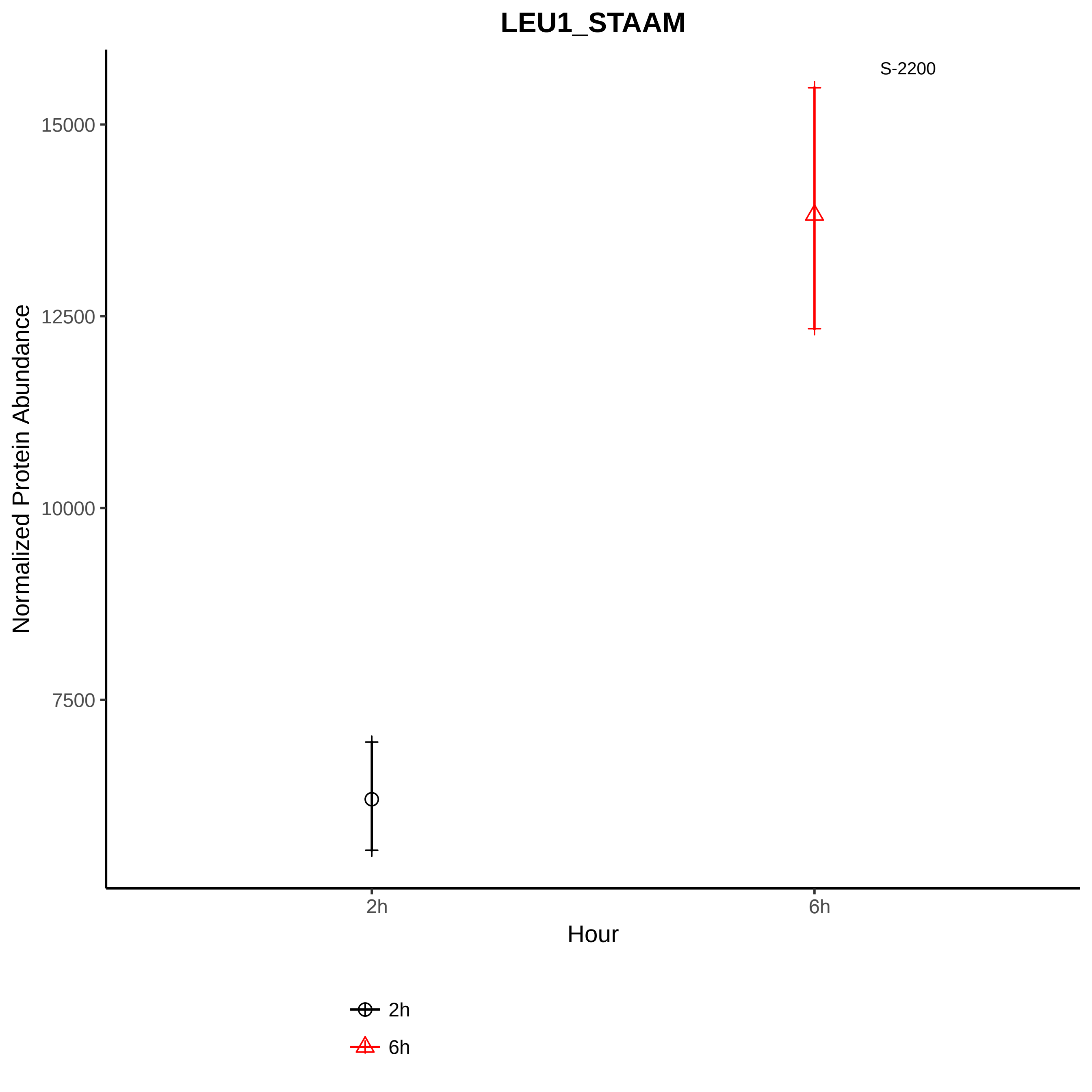

LEU1_STAAM
S-2200
Normalized Protein Abundance
15000
12500
10000
7500
2h
6h
Hour
2h
6h

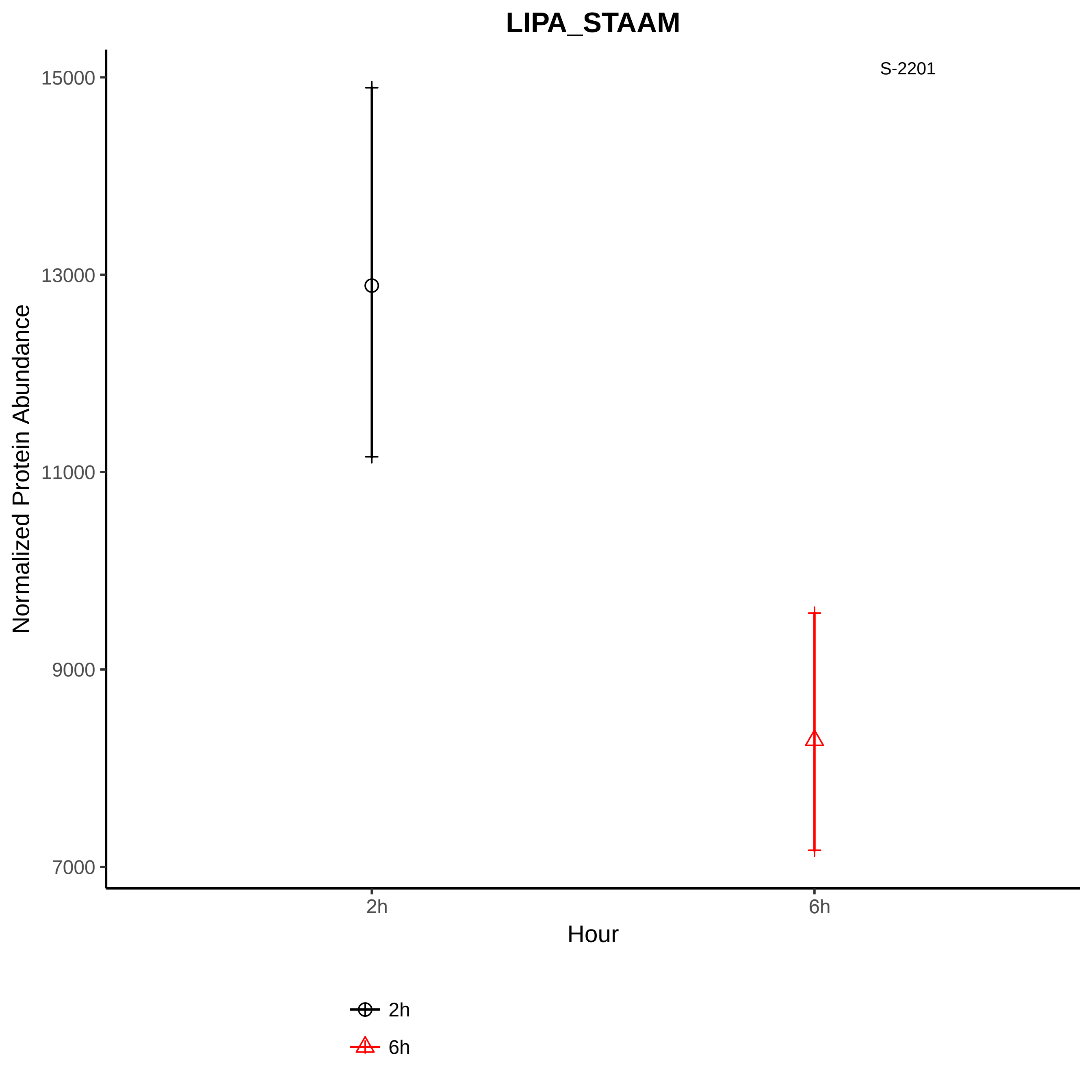

LIPA_STAAM
S-2201
Normalized Protein Abundance
15000
13000
11000
9000
7000
2h
6h
Hour
2h
6h

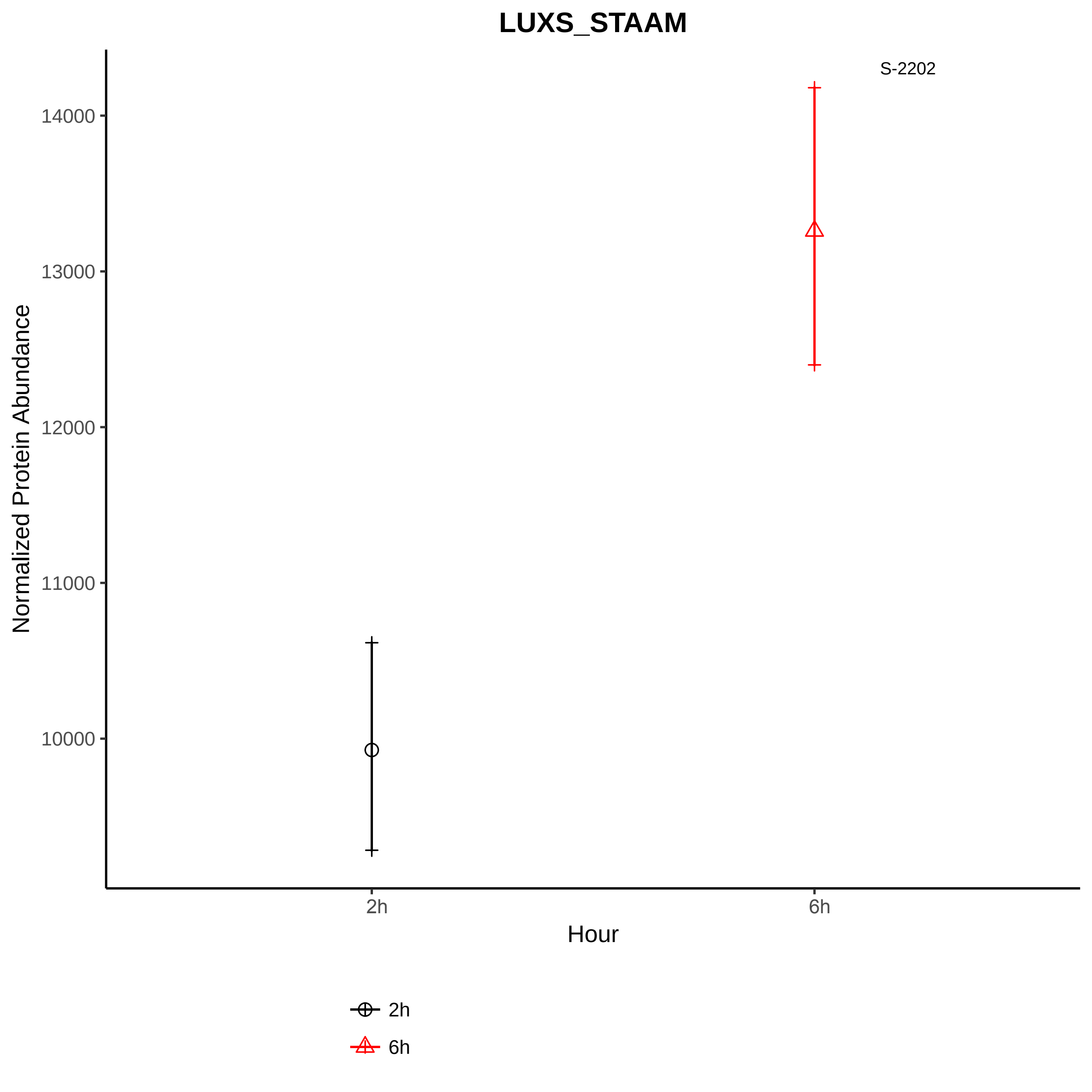

LUXS_STAAM
S-2202
14000
13000
12000
11000
10000
Normalized Protein Abundance
2h
6h
Hour
2h
6h

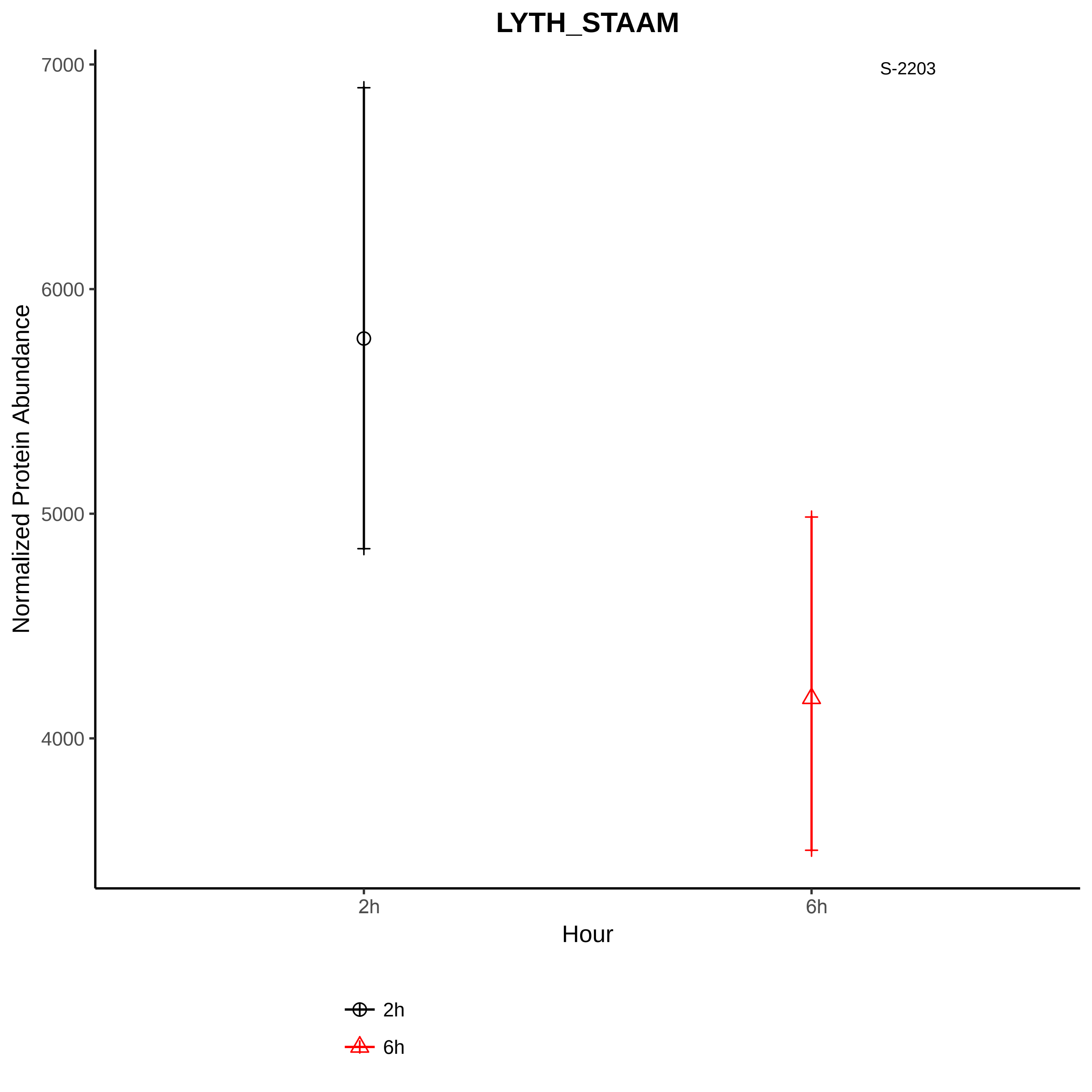

LYTH_STAAM
S-2203
7000
6000
5000
4000
Normalized Protein Abundance
2h
6h
Hour
2h
6h

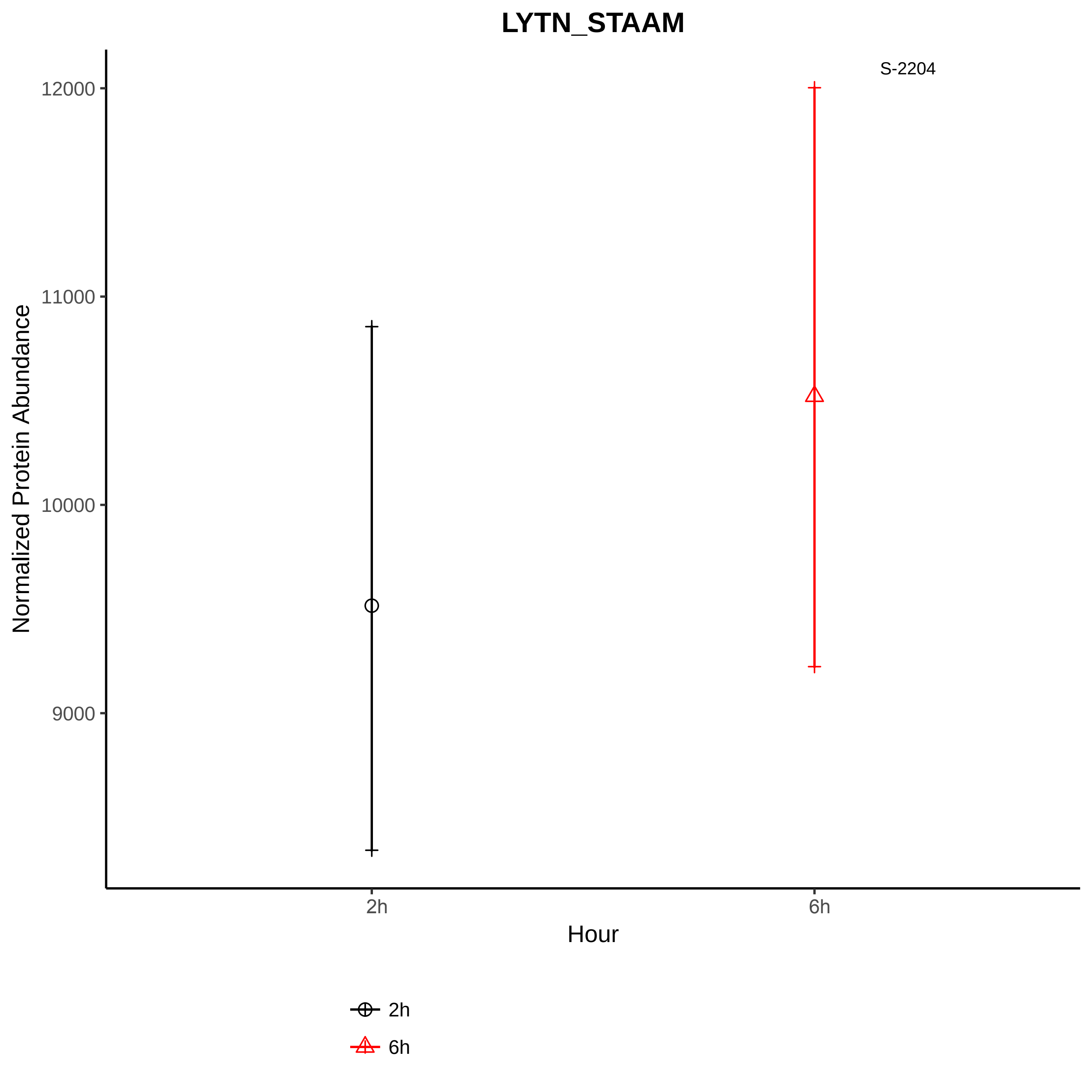
LYTN_STAAM
S-2204
Normalized Protein Abundance
12000
11000
10000
9000
2h
6h
Hour
2h
6h

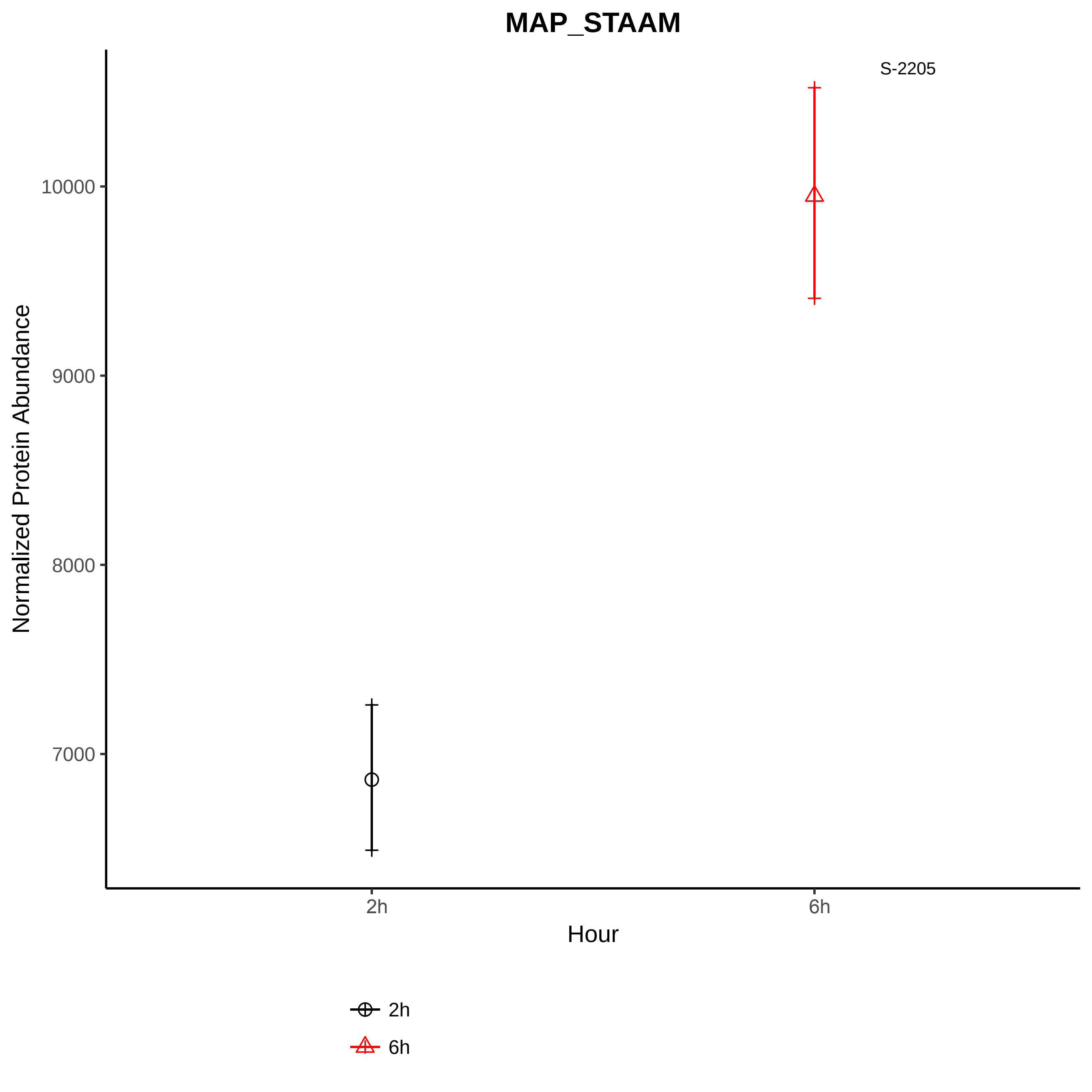
MAP_STAAM
S-2205
Normalized Protein Abundance
10000
9000
8000
7000
2h
6h
Hour
2h
6h

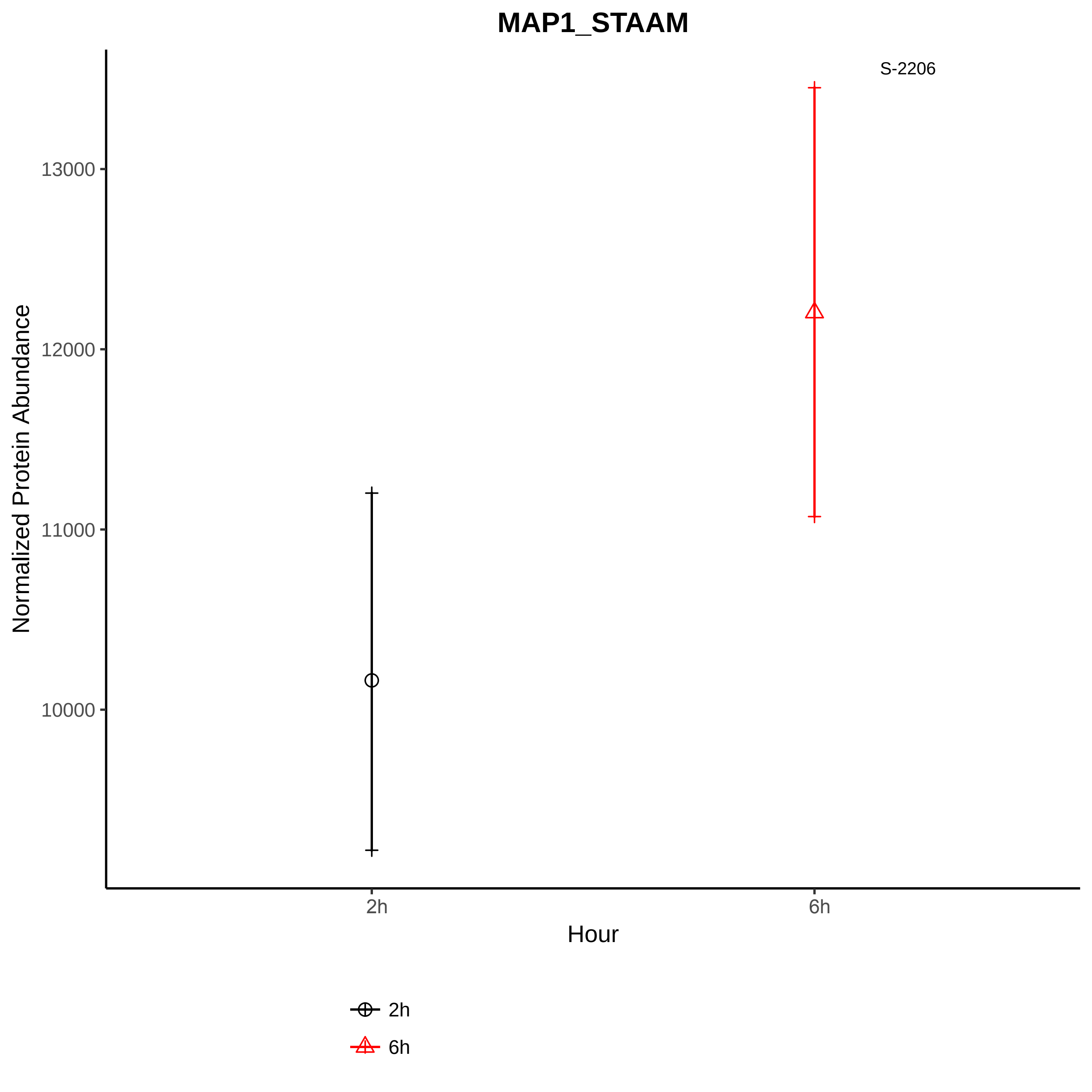

MAP1_STAAM
S-2206
Normalized Protein Abundance
13000
12000
11000
10000
2h
6h
Hour
2h
6h

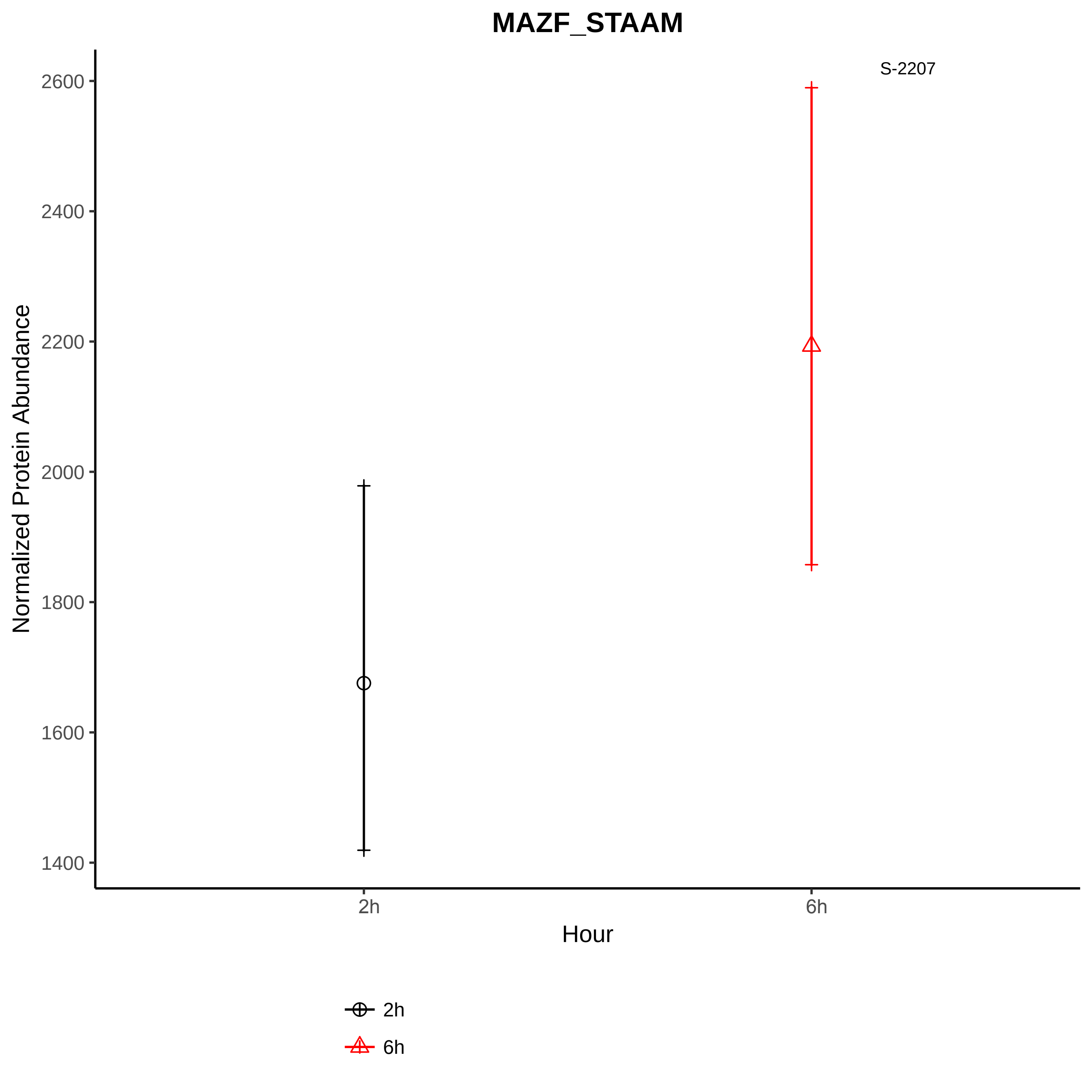

MAZF_STAAM
S-2207
Normalized Protein Abundance
2600
2400
2200
2000
1800
1600
1400
2h
6h
Hour
2h
6h

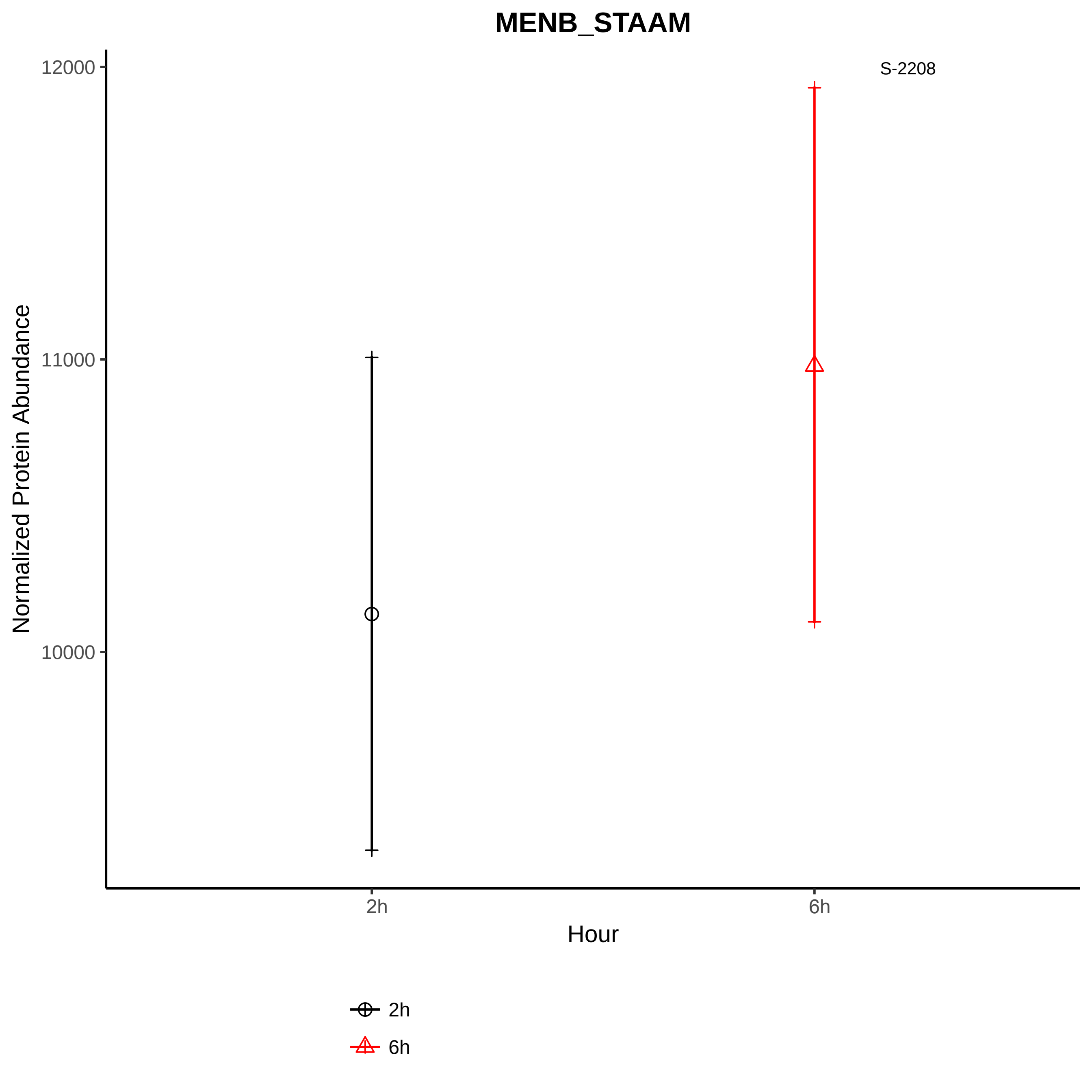
MENB_STAAM
S-2208
12000
11000
10000
Normalized Protein Abundance
2h
6h
Hour
2h
6h

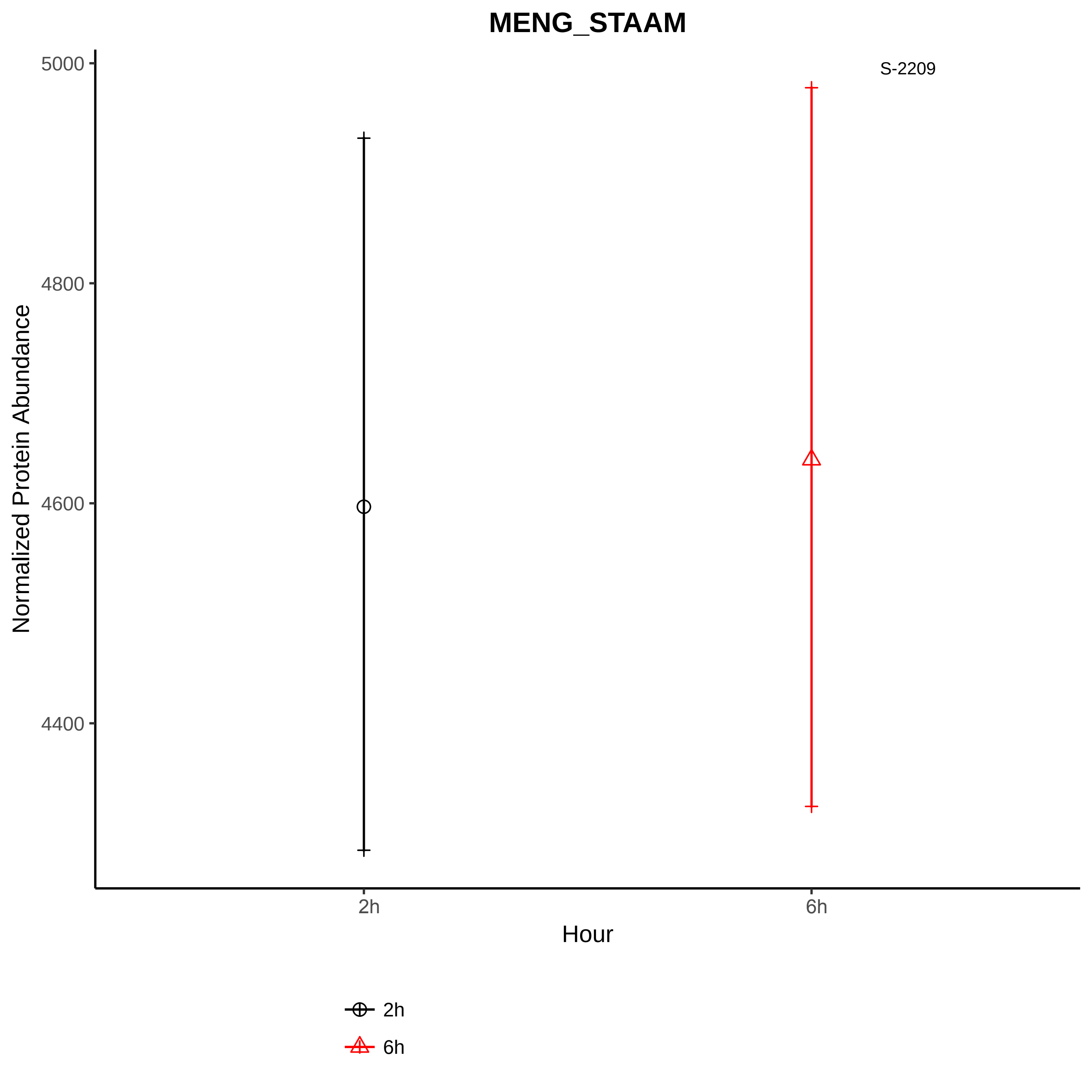

MENG_STAAM
S-2209
5000
4800
4600
4400
Normalized Protein Abundance
2h
6h
Hour
2h
6h

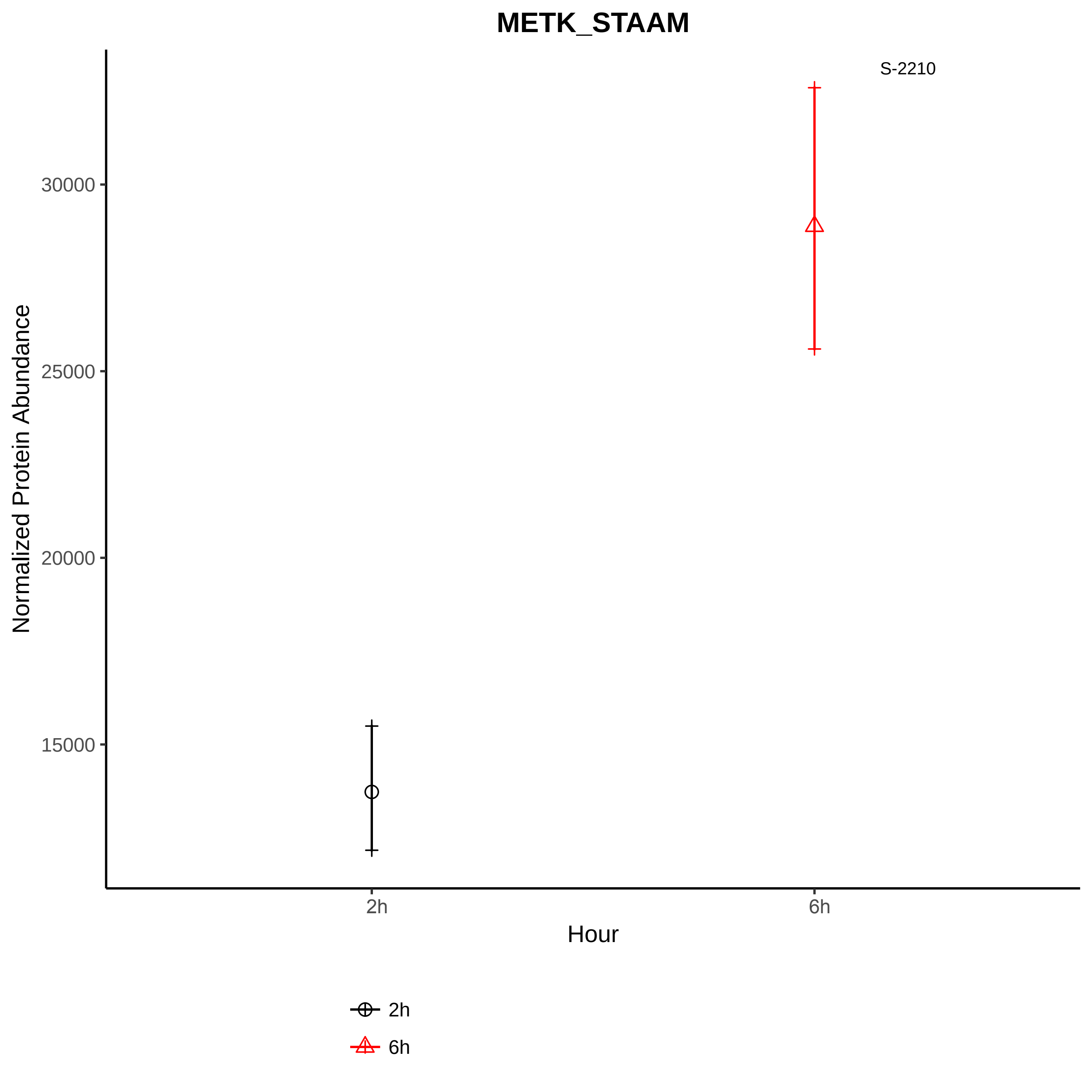
METK_STAAM
S-2210
Normalized Protein Abundance
30000
25000
20000
15000
2h
6h
Hour
2h
6h

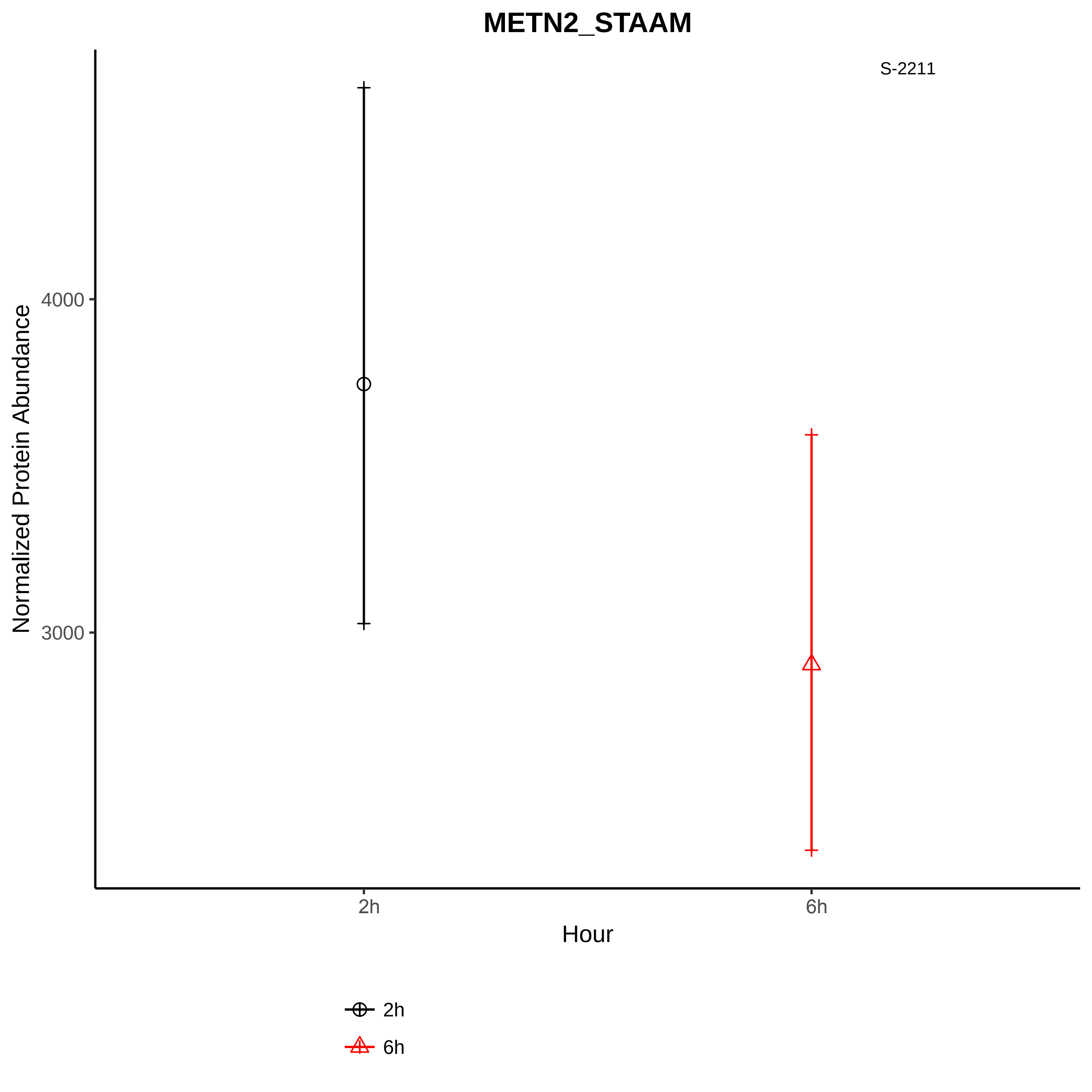

METN2_STAAM
S-2211
Normalized Protein Abundance
4000
3000
2h
6h
Hour
2h
6h

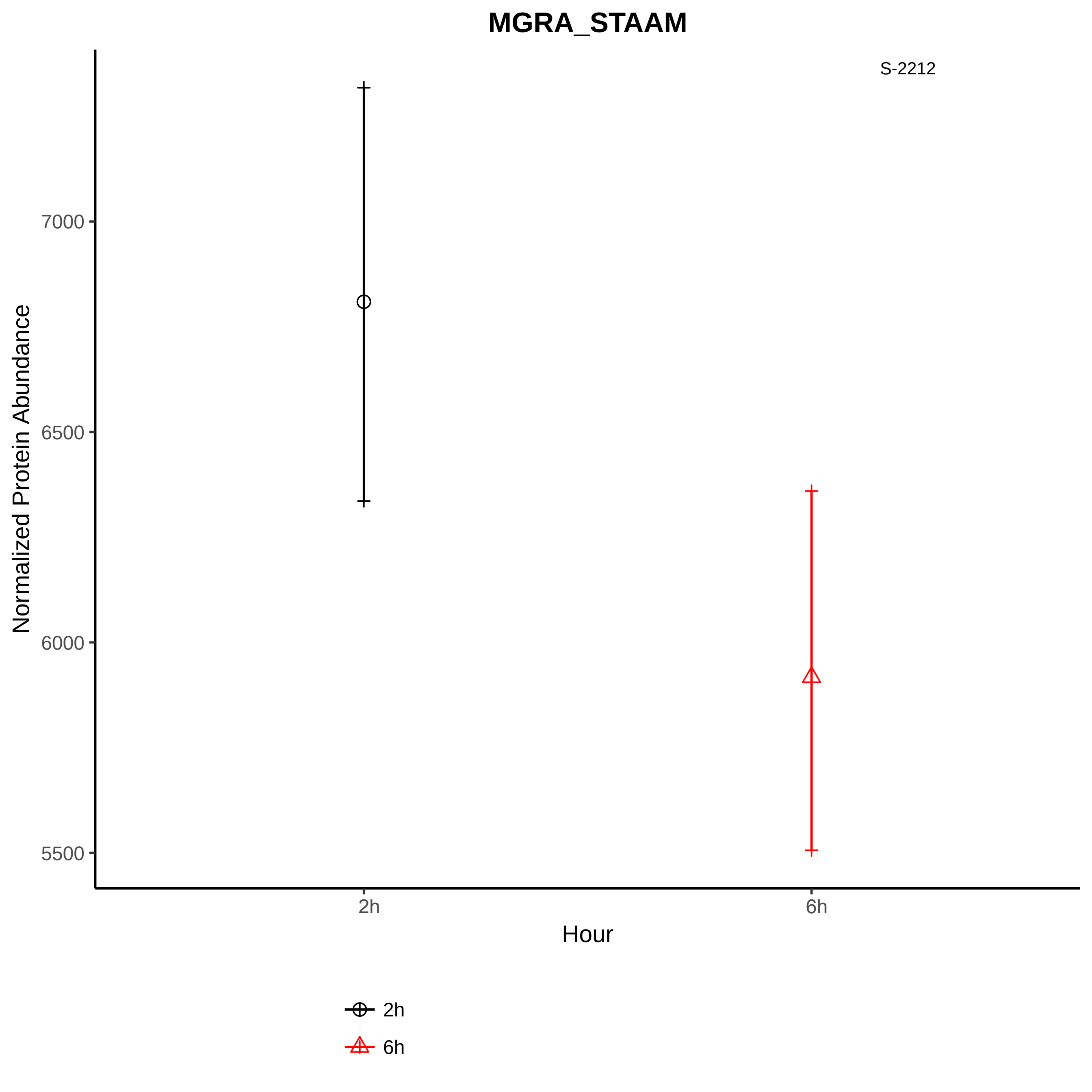
MGRA_STAAM
S-2212
Normalized Protein Abundance
7000
6500
6000
5500
2h
6h
Hour
2h
6h

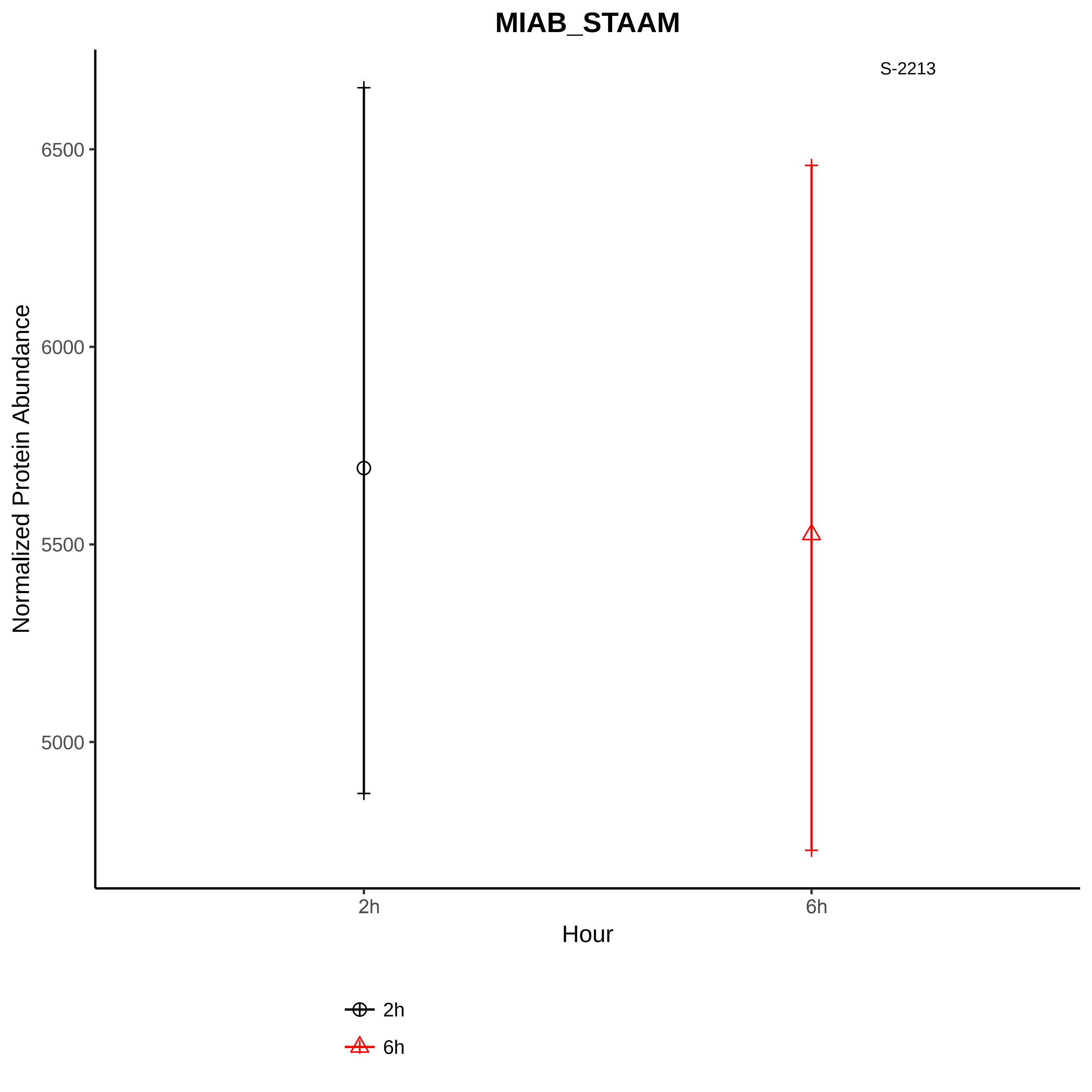
MIAB_STAAM
S-2213
Normalized Protein Abundance
6500
6000
5500
5000
2h
6h
Hour
2h
6h

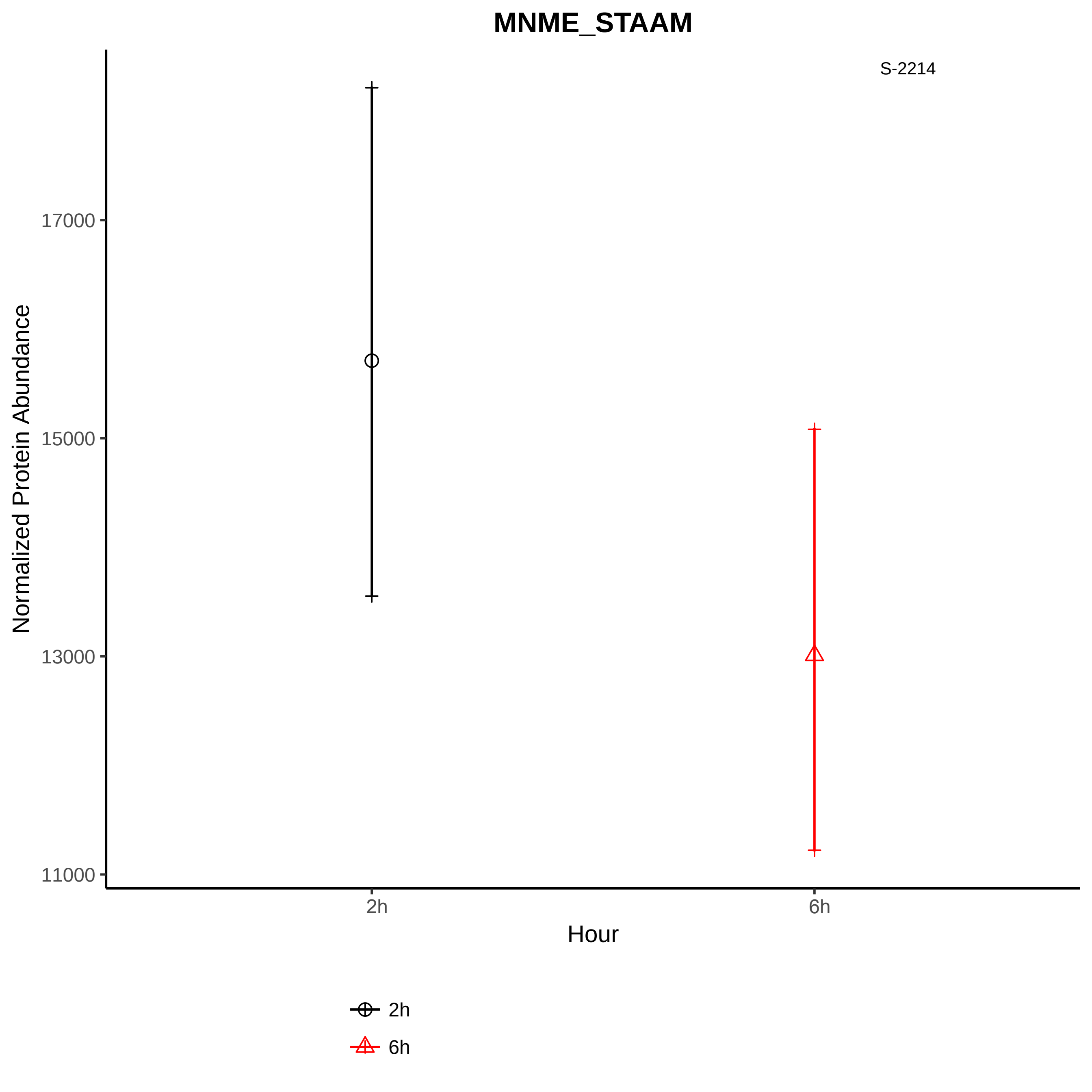

MNME_STAAM
S-2214
Normalized Protein Abundance
17000
15000
13000
11000
2h
6h
Hour
2h
6h

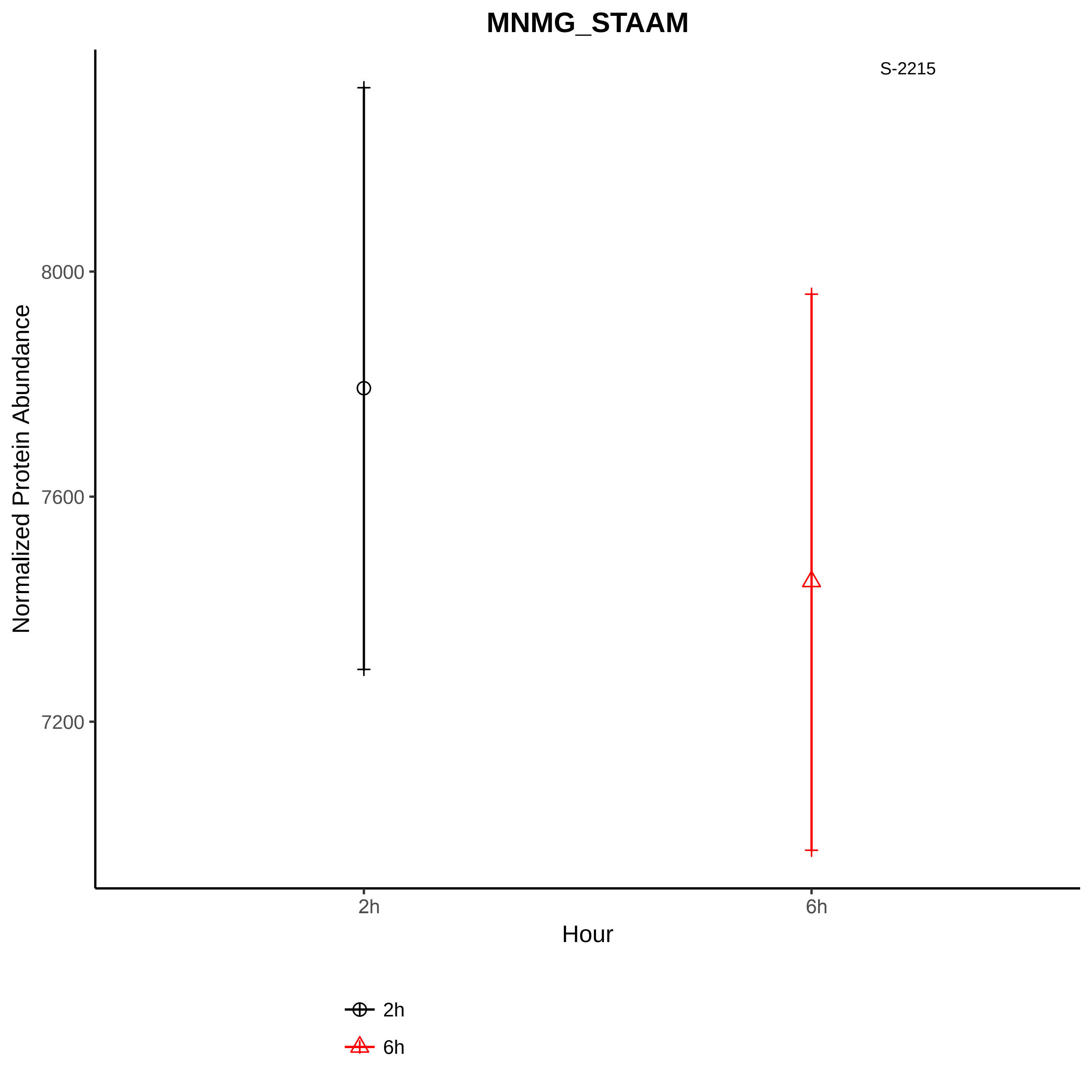

MNMG_STAAM
S-2215
Normalized Protein Abundance
8000
7600
7200
2h
6h
Hour
2h
6h

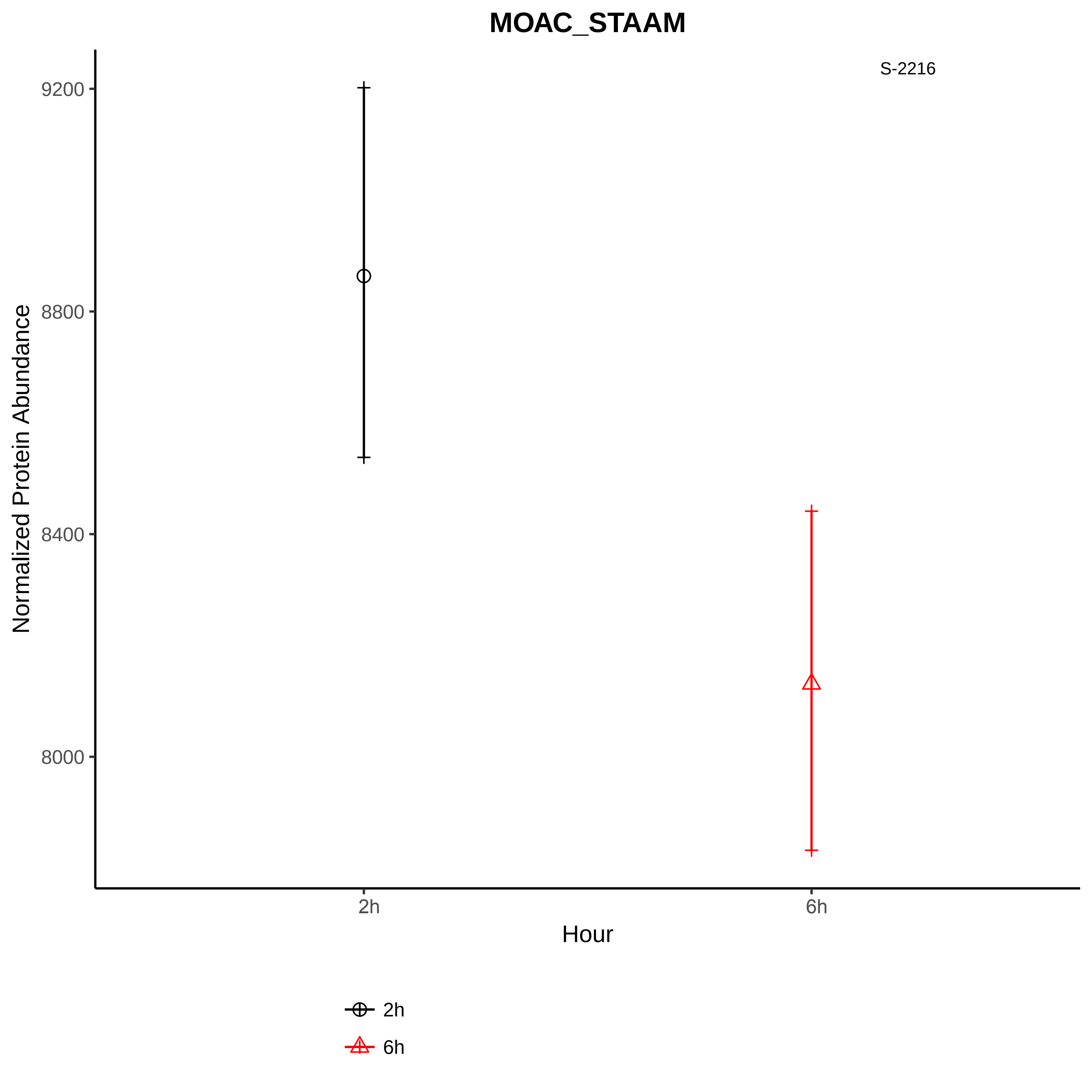

MOAC_STAAM
S-2216
9200
8800
8400
8000
Normalized Protein Abundance
2h
6h
Hour
2h
6h

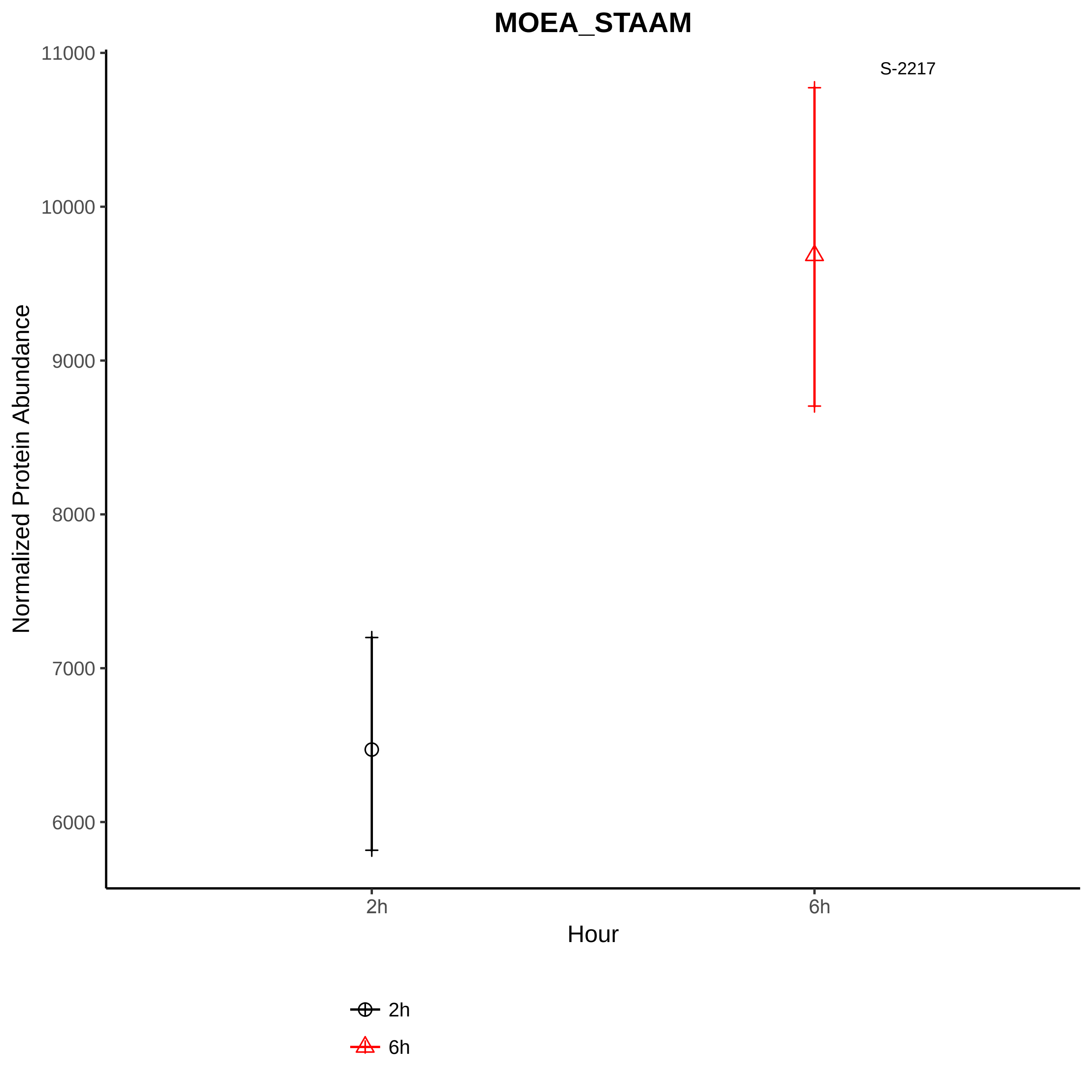
MOEA_STAAM
S-2217
Normalized Protein Abundance
11000
10000
9000
8000
7000
6000
2h
6h
Hour
2h
6h

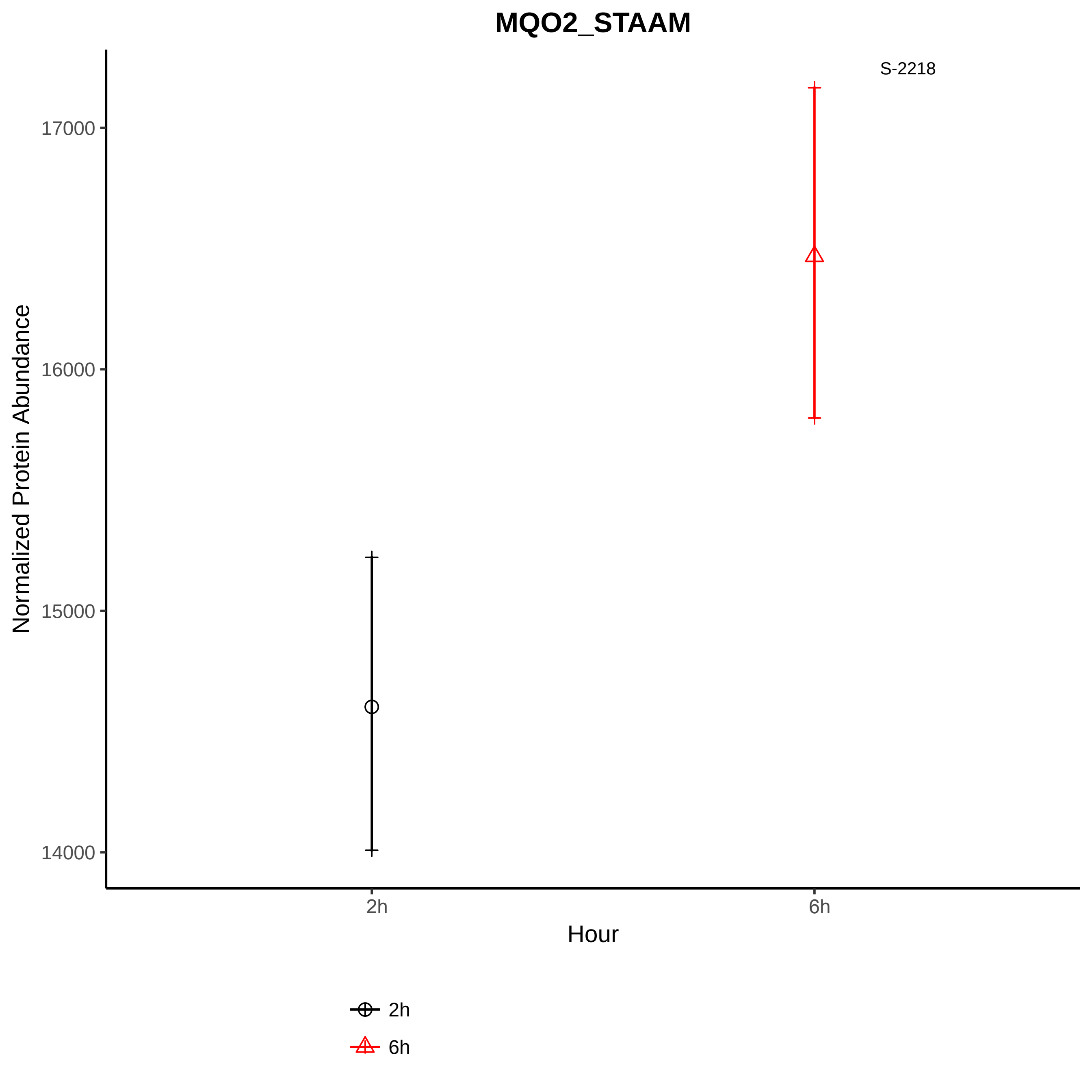
MQO2_STAAM
S-2218
Normalized Protein Abundance
17000
16000
15000
14000
2h
6h
Hour
2h
6h

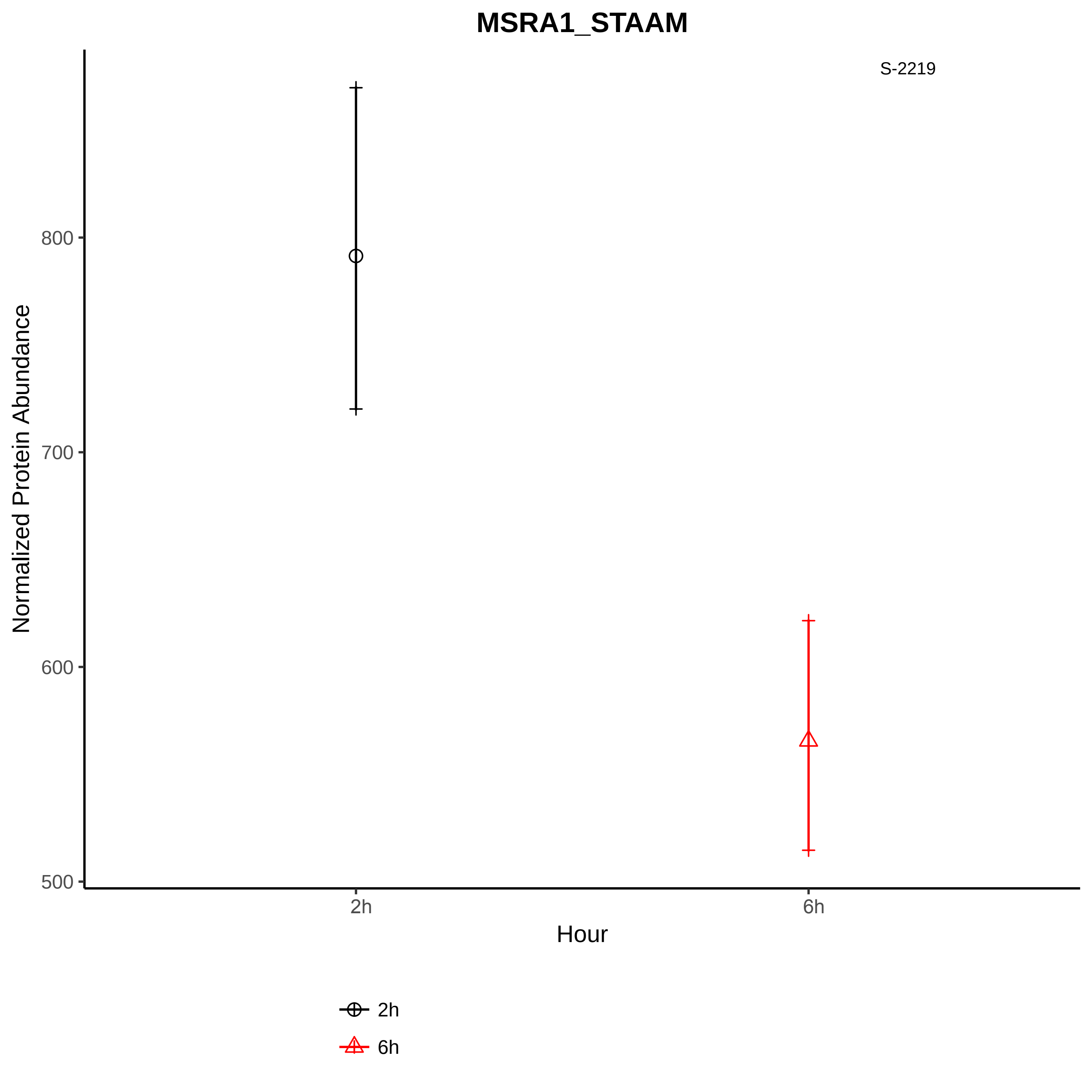

MSRA1_STAAM
S-2219
Normalized Protein Abundance
800
700
600
500
2h
6h
Hour
2h
6h

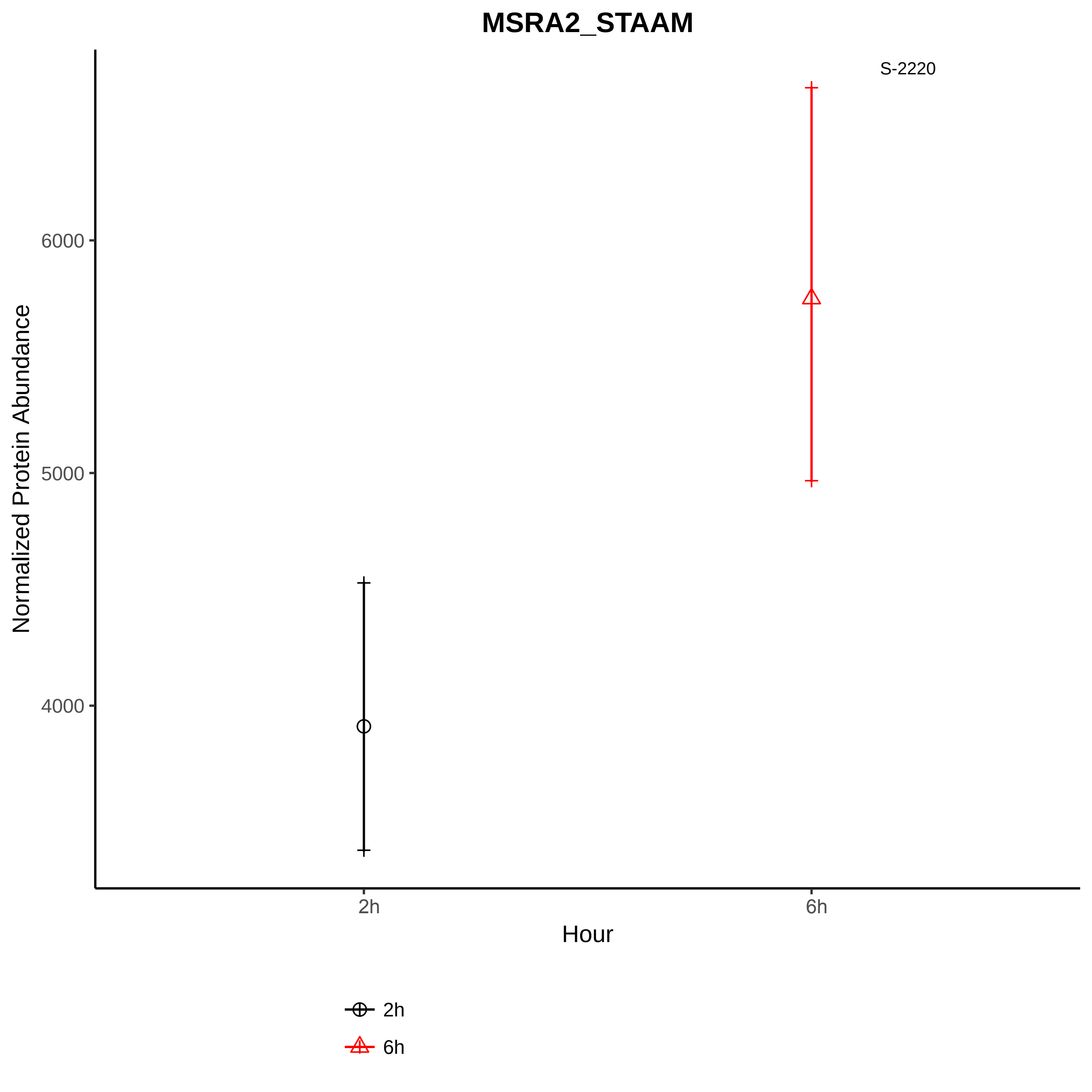
MSRA2_STAAM
S-2220
Normalized Protein Abundance
6000
5000
4000
2h
6h
Hour
2h
6h

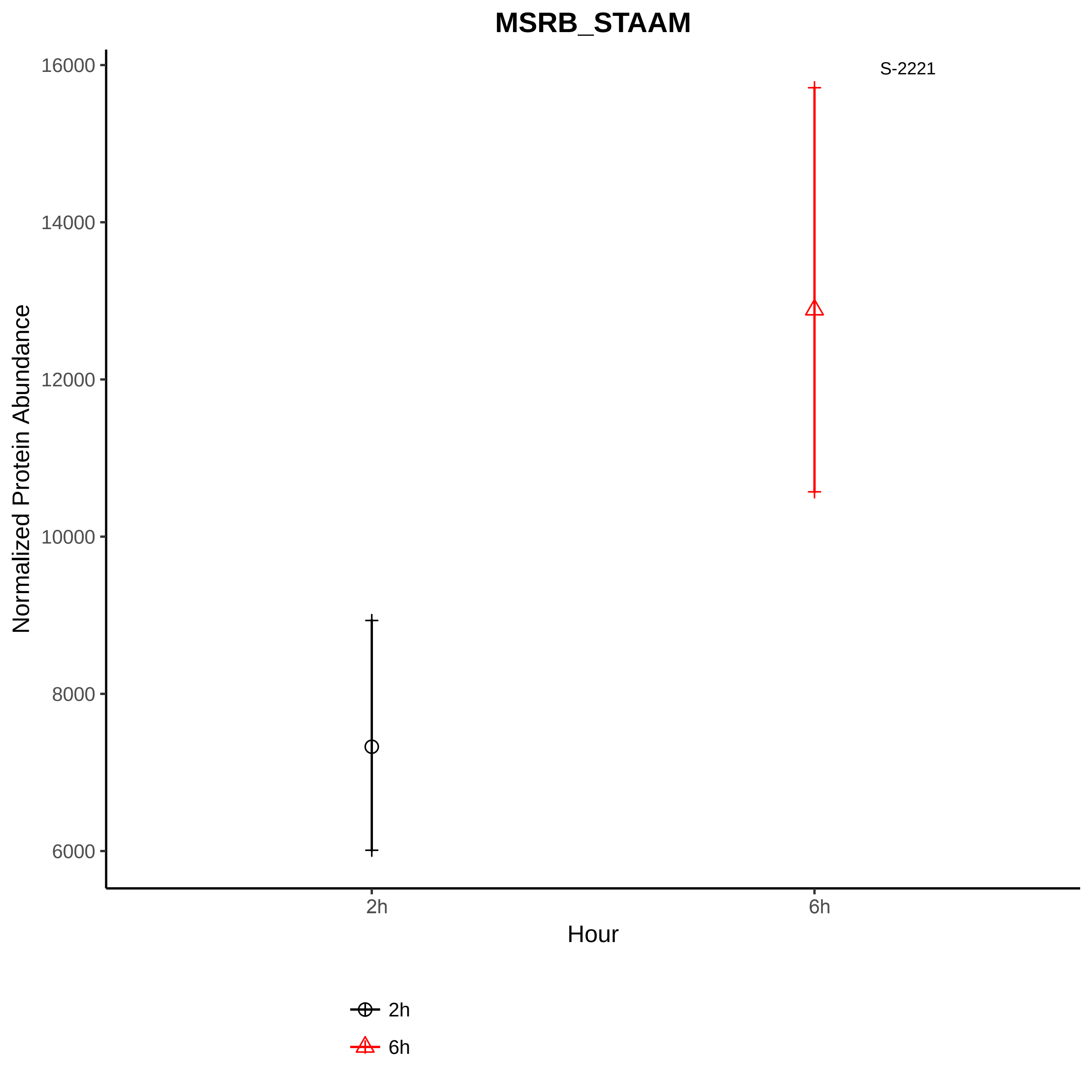
MSRB_STAAM
S-2221
Normalized Protein Abundance
16000
14000
12000
10000
8000
6000
2h
6h
Hour
2h
6h

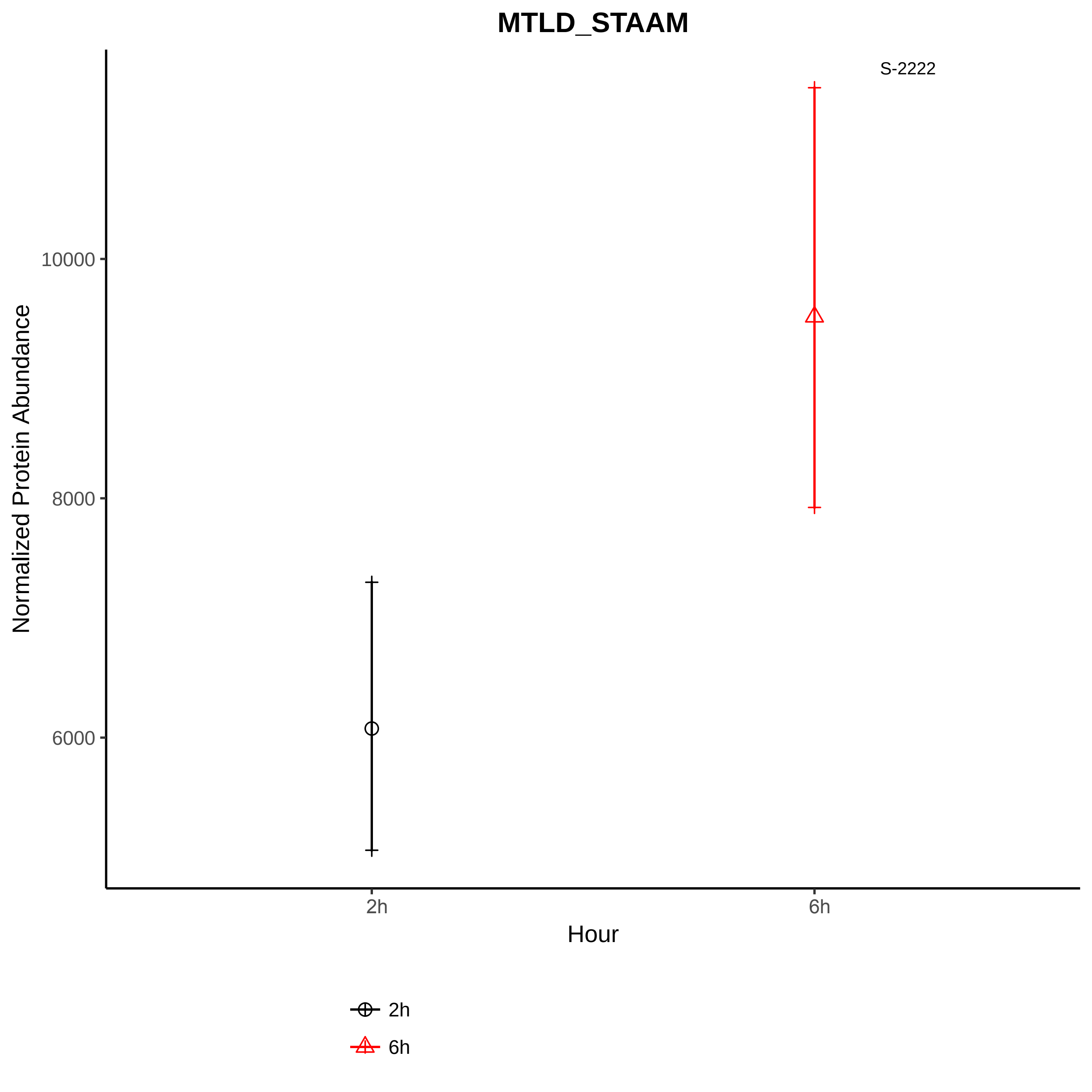
MTLD_STAAM
S-2222
Normalized Protein Abundance
10000
8000
6000
2h
6h
Hour
2h
6h

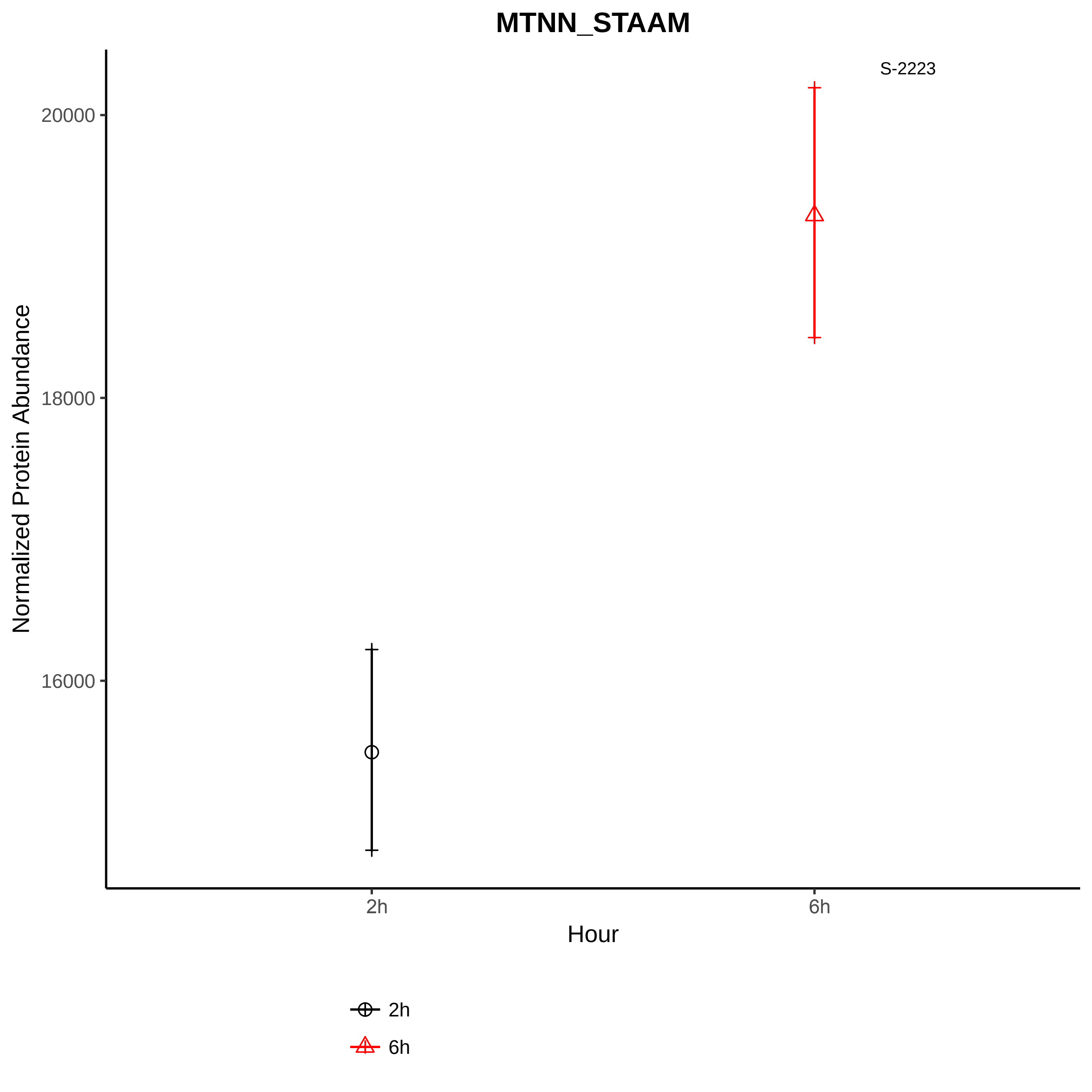
MTNN_STAAM
S-2223
20000
18000
16000
Normalized Protein Abundance
2h
6h
Hour
2h
6h

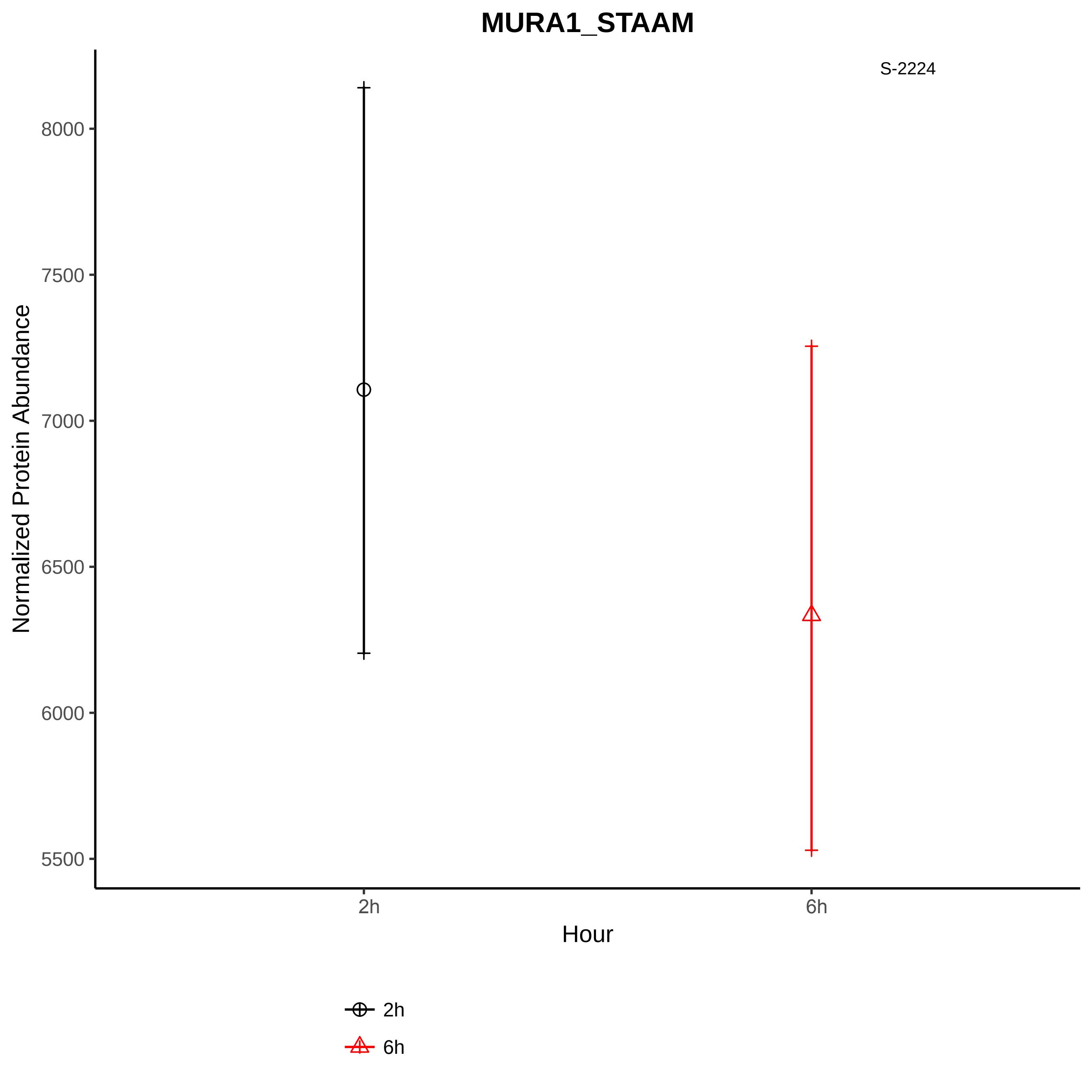

MURA1_STAAM
S-2224
Normalized Protein Abundance
8000
7500
7000
6500
6000
5500
2h
6h
Hour
2h
6h

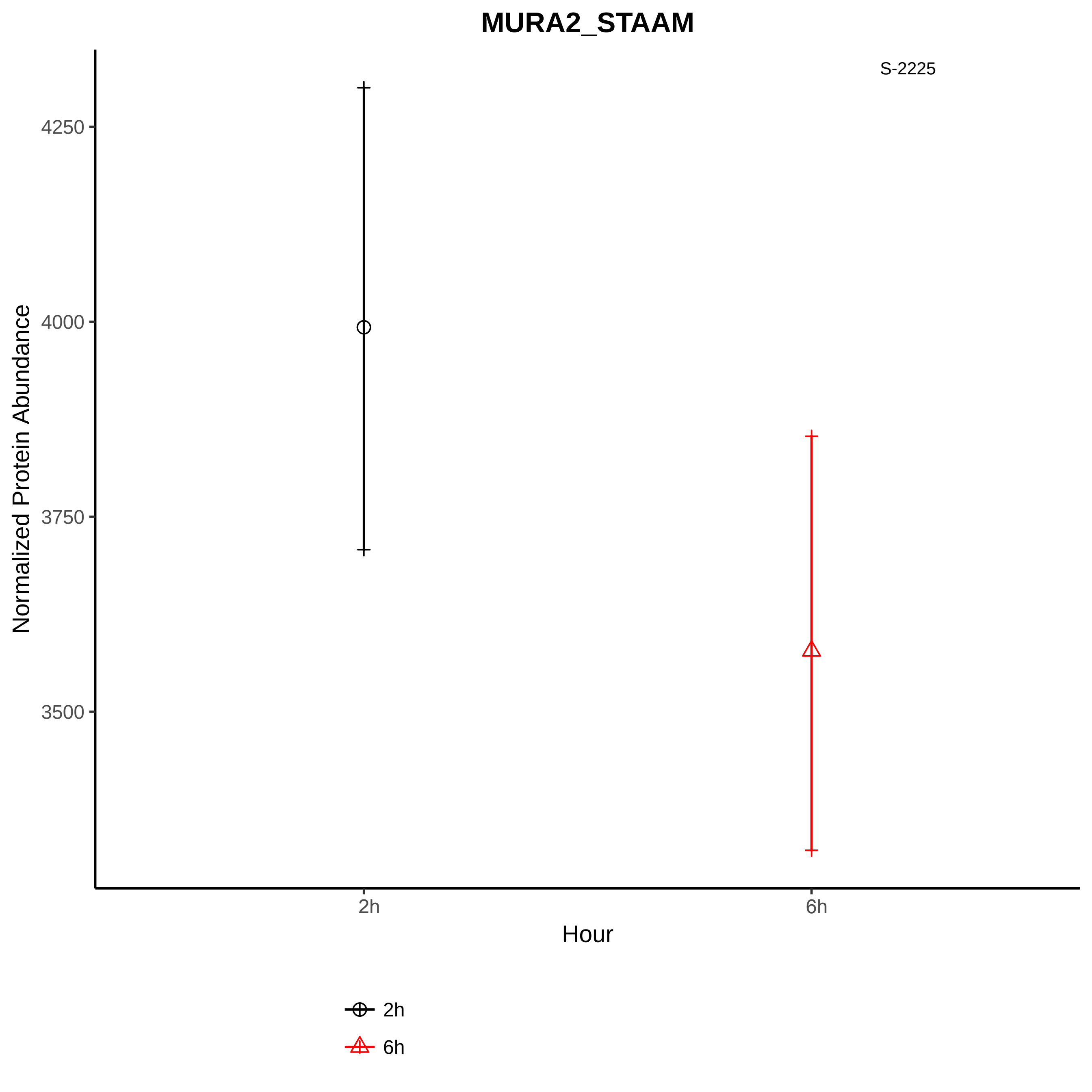
MURA2_STAAM
S-2225
Normalized Protein Abundance
4250
4000
3750
3500
2h
6h
Hour
2h
6h

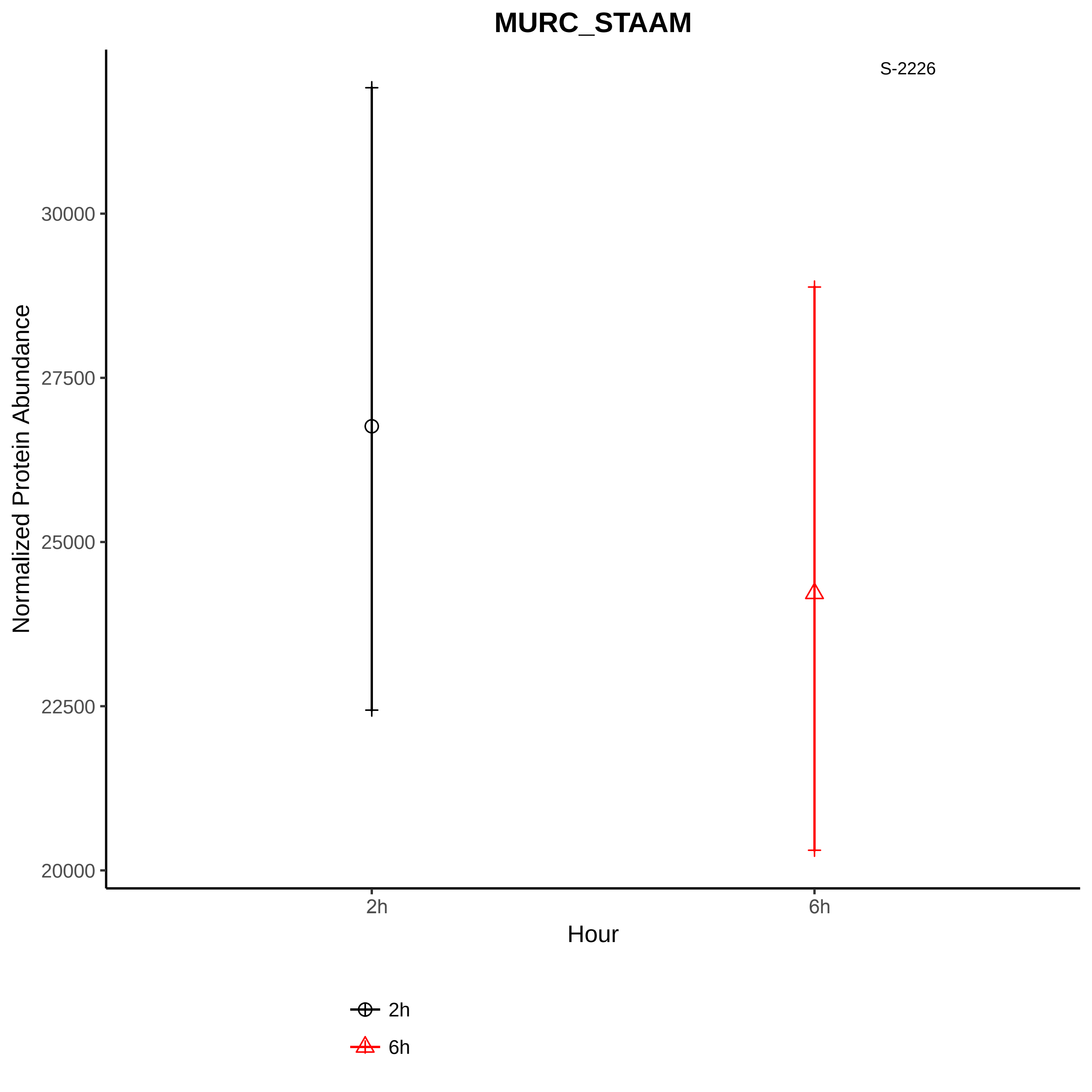

MURC_STAAM
S-2226
Normalized Protein Abundance
30000
27500
25000
22500
20000
2h
6h
Hour
2h
6h

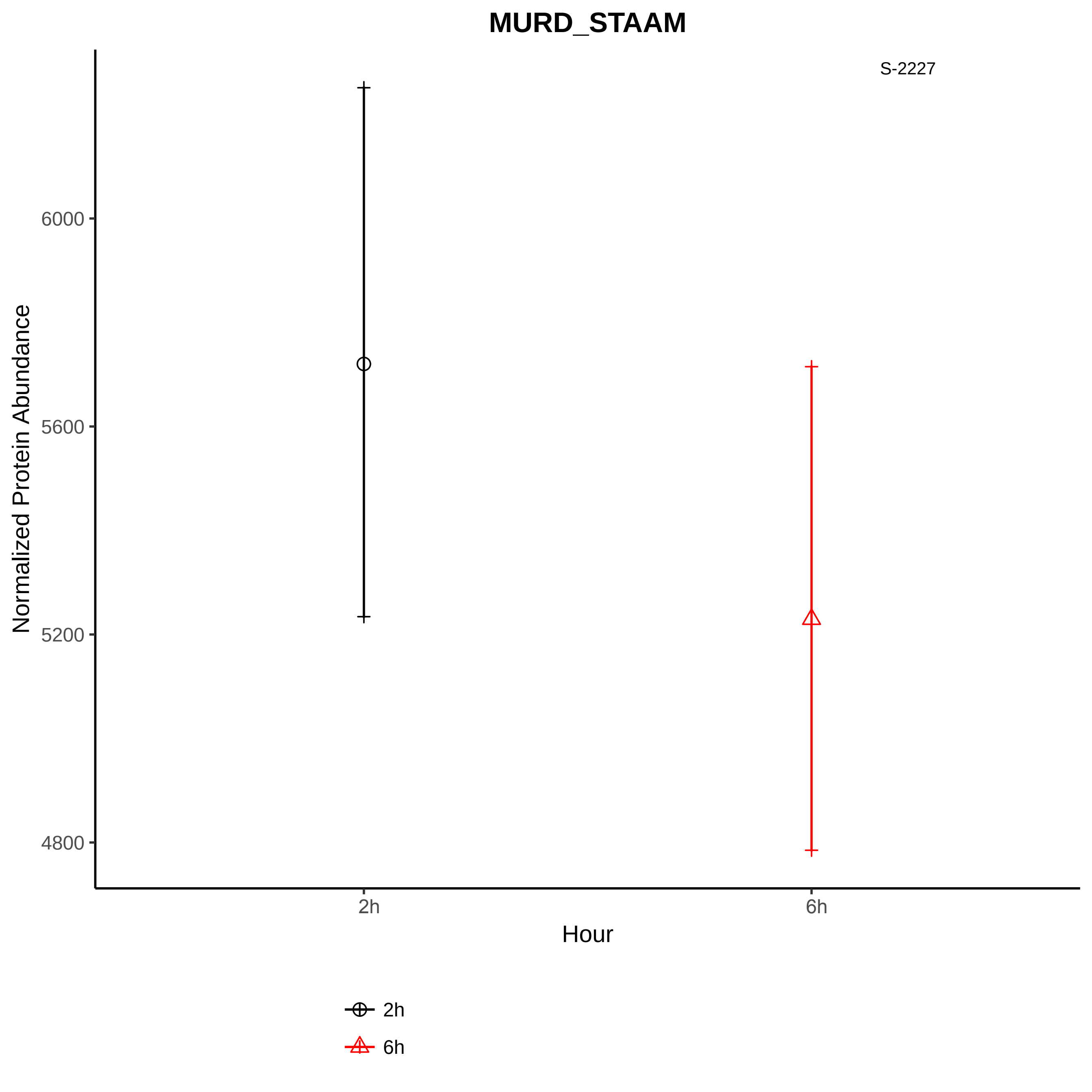

MURD_STAAM
S-2227
Normalized Protein Abundance
6000
5600
5200
4800
2h
6h
Hour
2h
6h

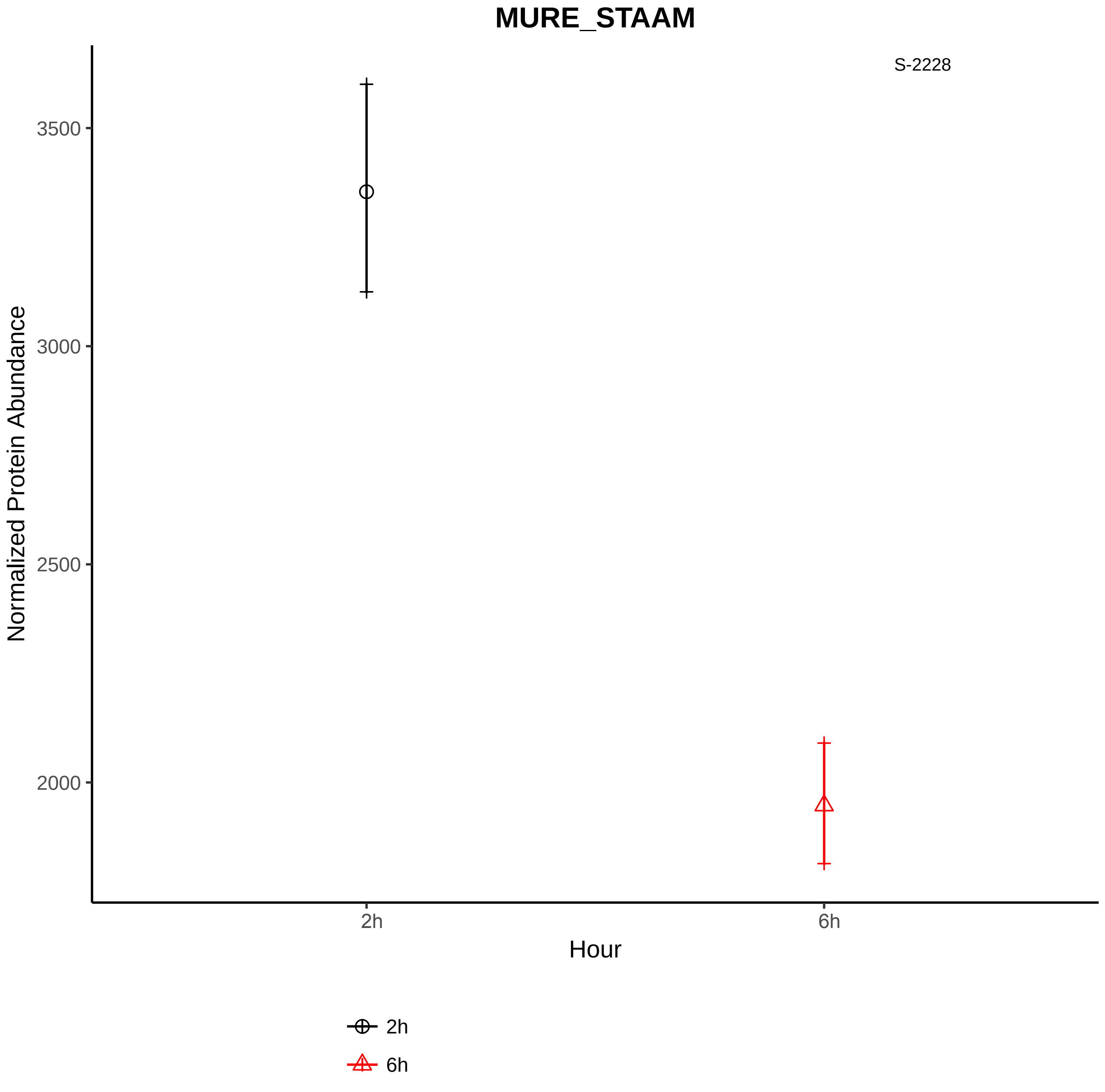
MURE_STAAM
S-2228
3500
3000
2500
2000
Normalized Protein Abundance
2h
6h
Hour
2h
6h

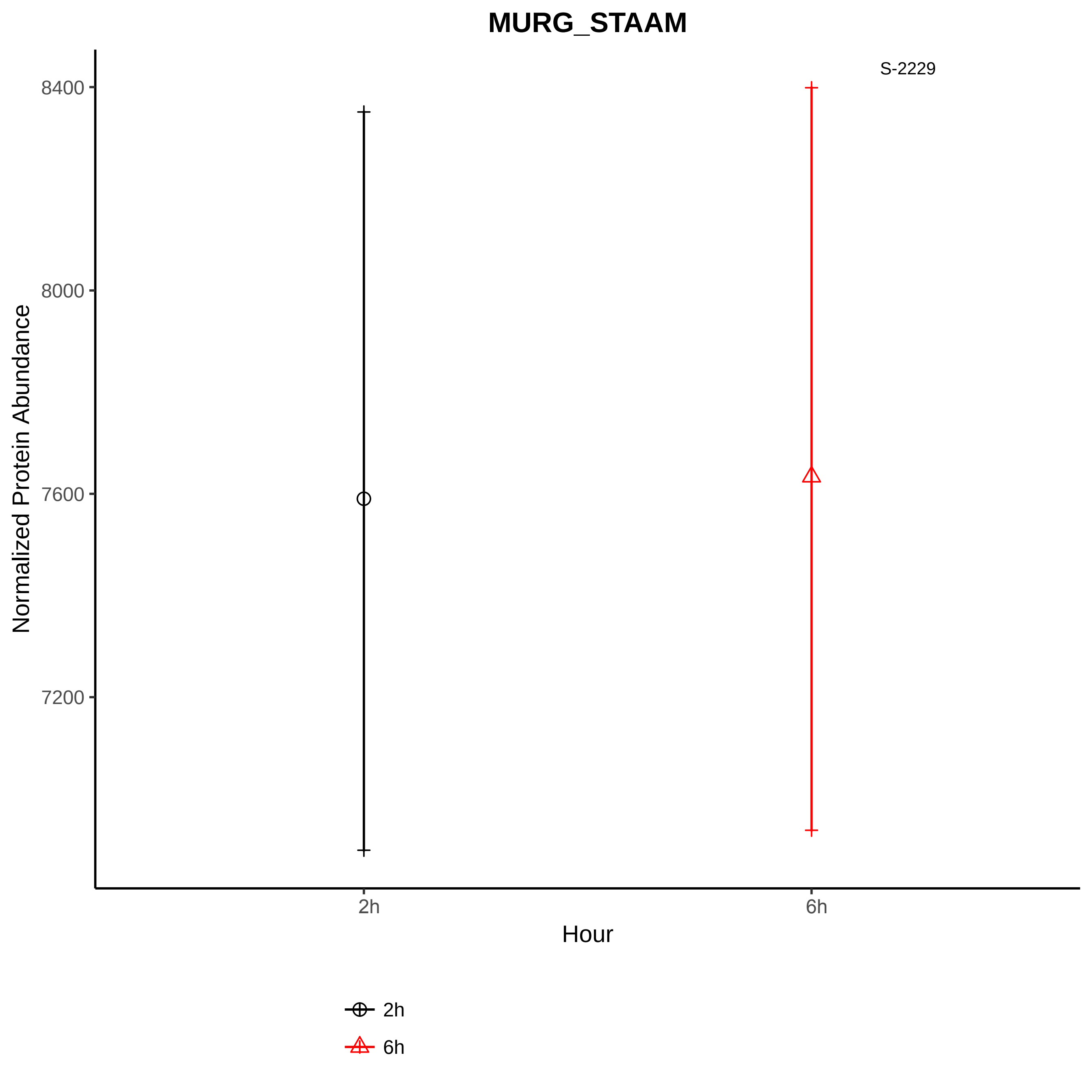

MURG_STAAM
S-2229
8400
8000
7600
7200
Normalized Protein Abundance
2h
6h
Hour
2h
6h

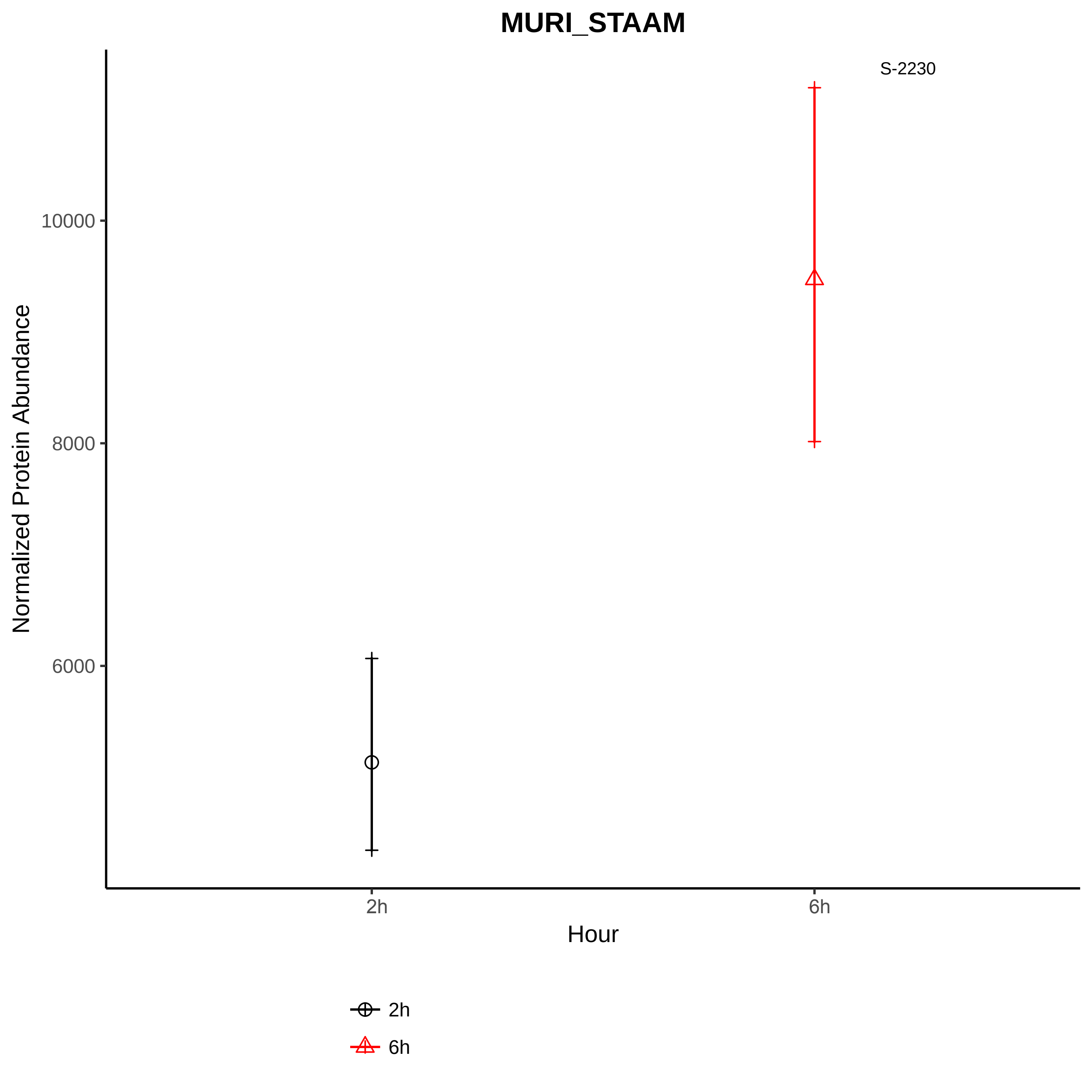
MURI_STAAM
S-2230
10000
8000
6000
Normalized Protein Abundance
2h
6h
Hour
2h
6h

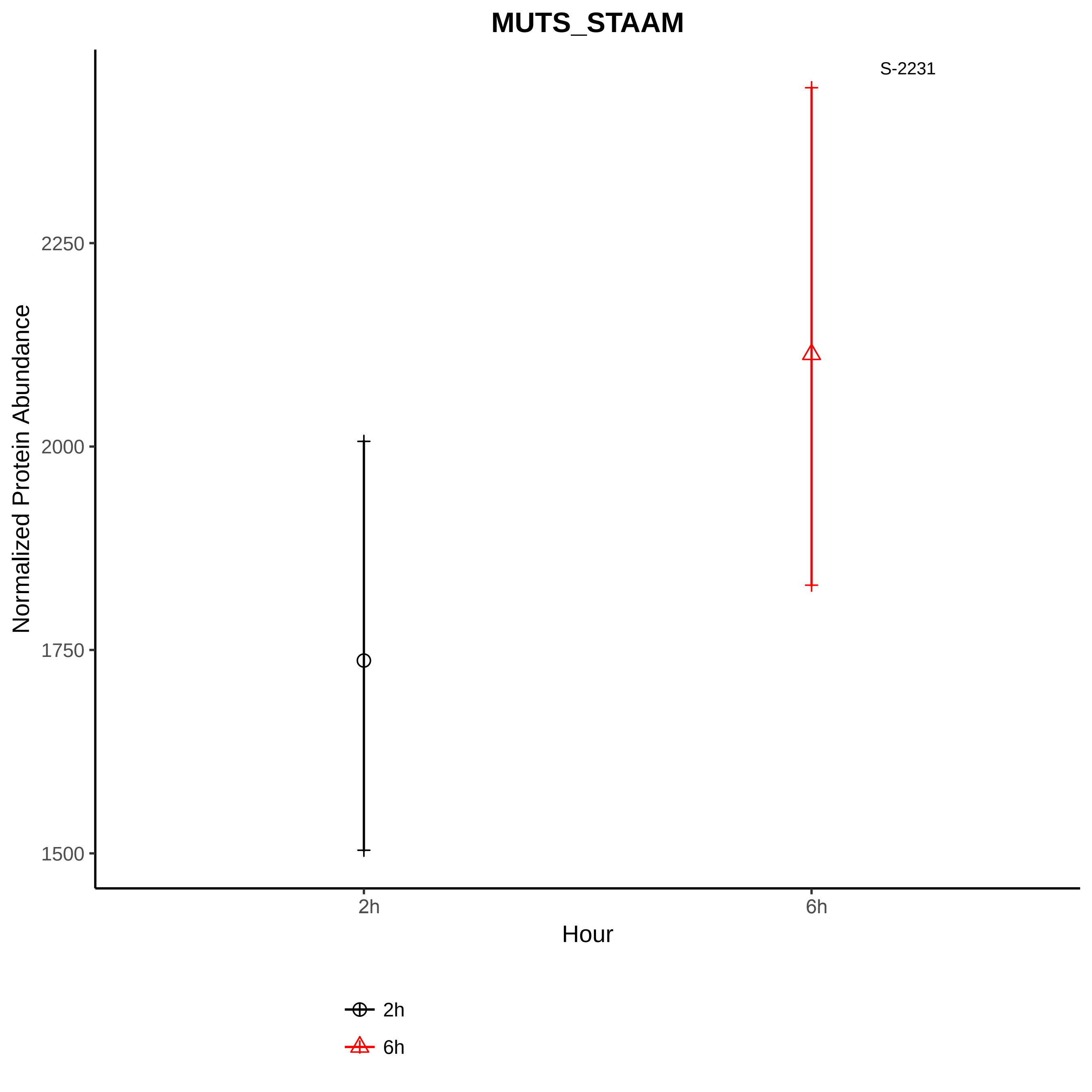

MUTS_STAAM
S-2231
Normalized Protein Abundance
2250
2000
1750
1500
2h
6h
Hour
2h
6h

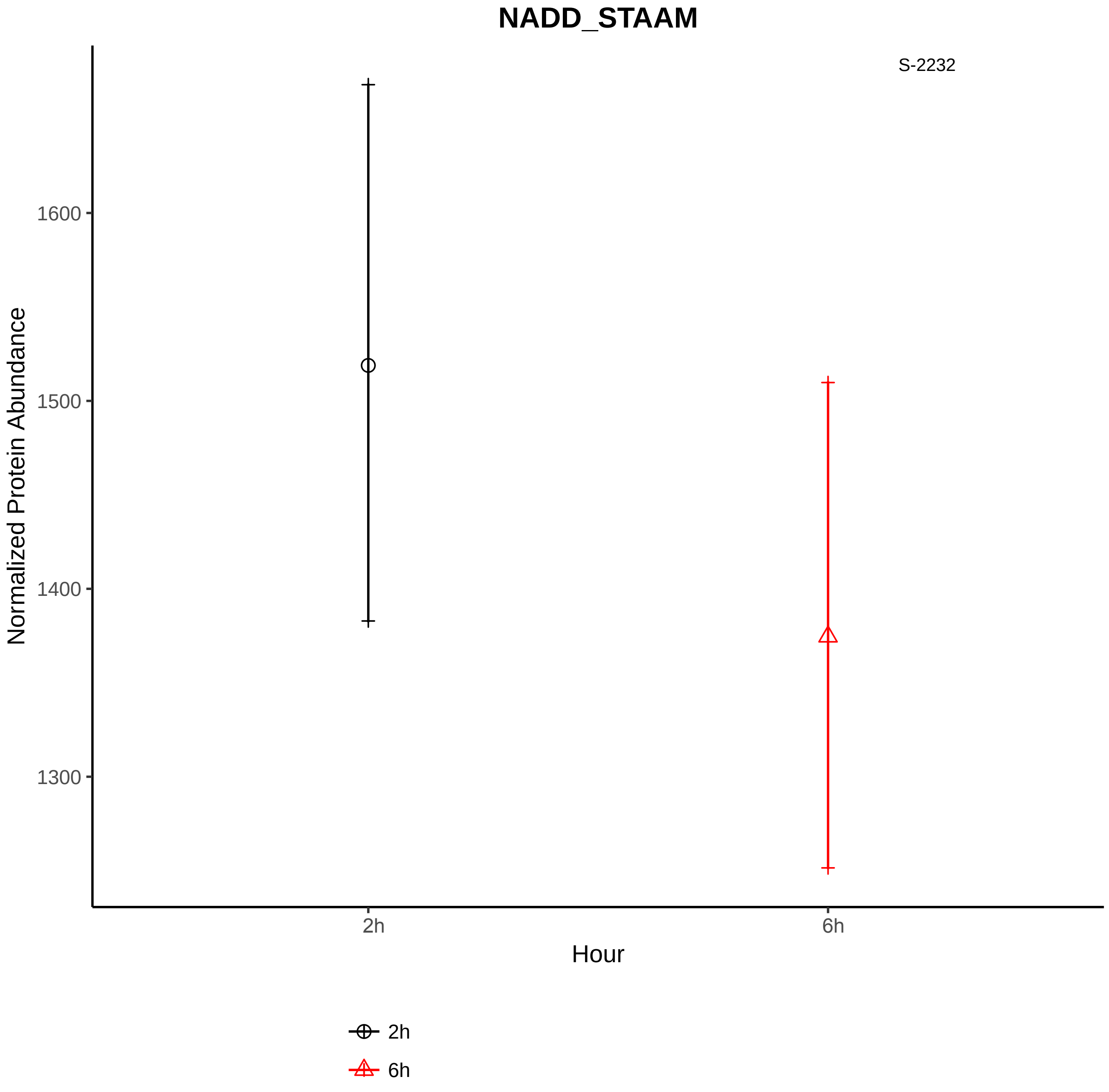

NADD_STAAM
S-2232
1600
1500
1400
1300
Normalized Protein Abundance
2h
6h
Hour
2h
6h

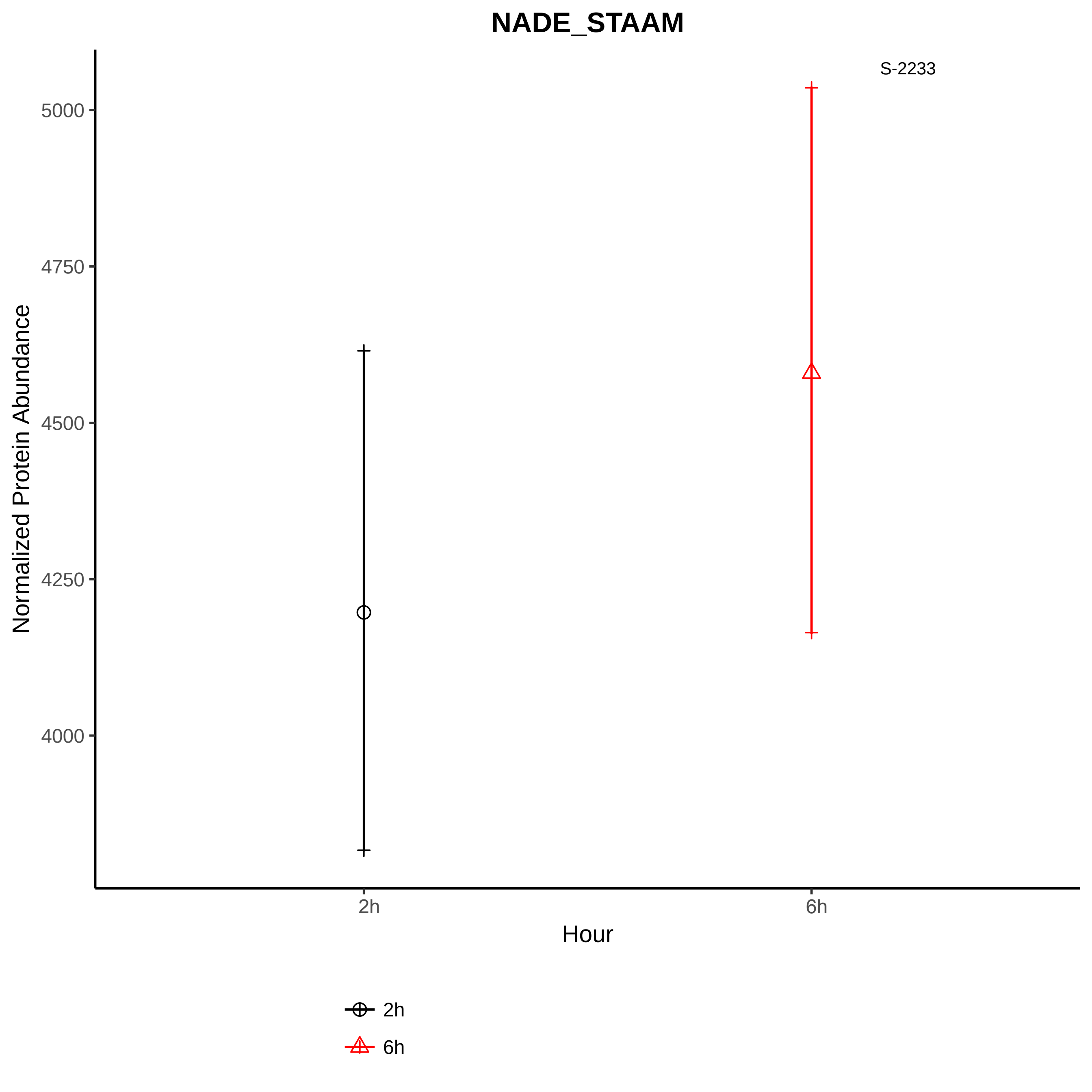

NADE_STAAM
S-2233
Normalized Protein Abundance
5000
4750
4500
4250
4000
2h
6h
Hour
2h
6h

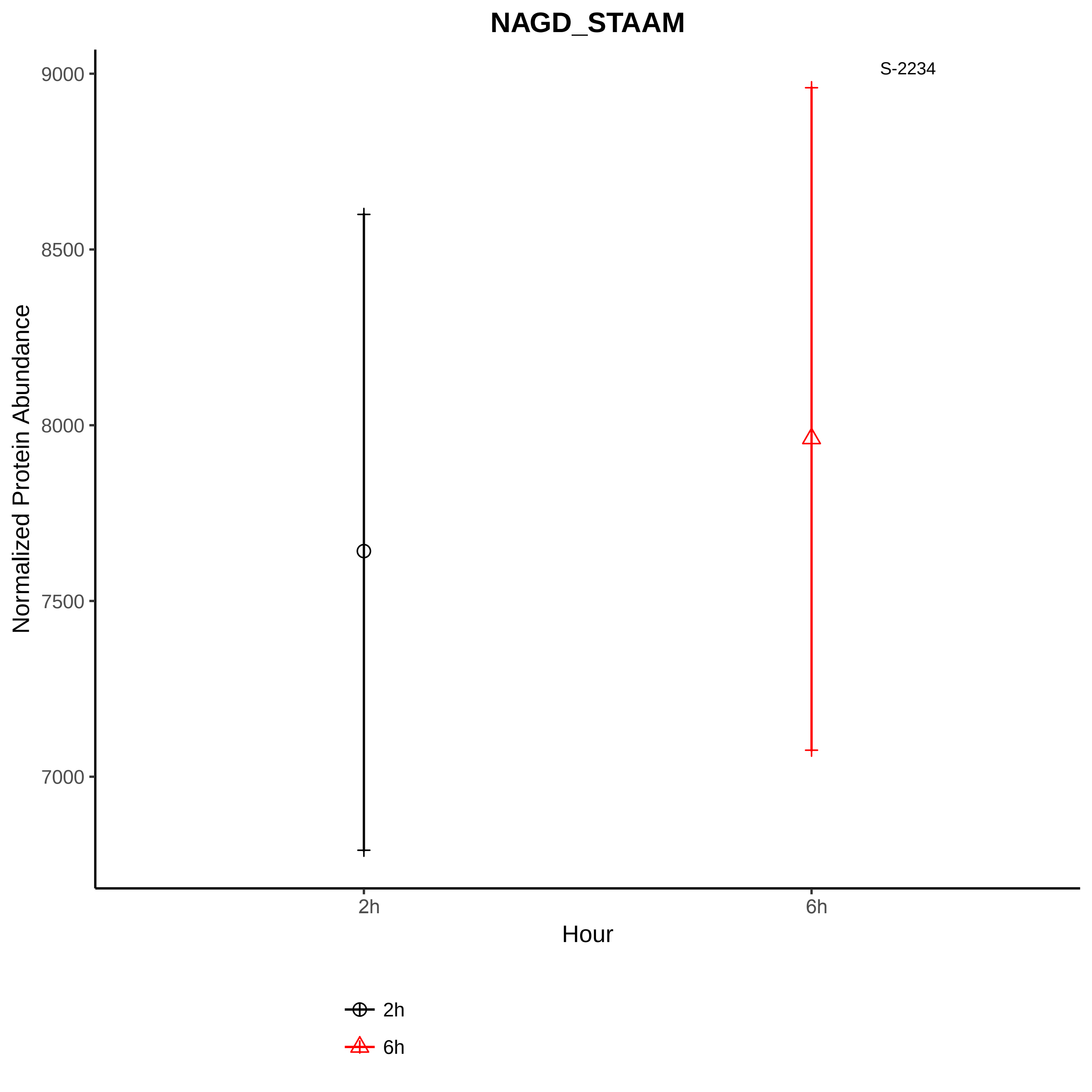

NAGD_STAAM
S-2234
Normalized Protein Abundance
9000
8500
8000
7500
7000
2h
6h
Hour
2h
6h

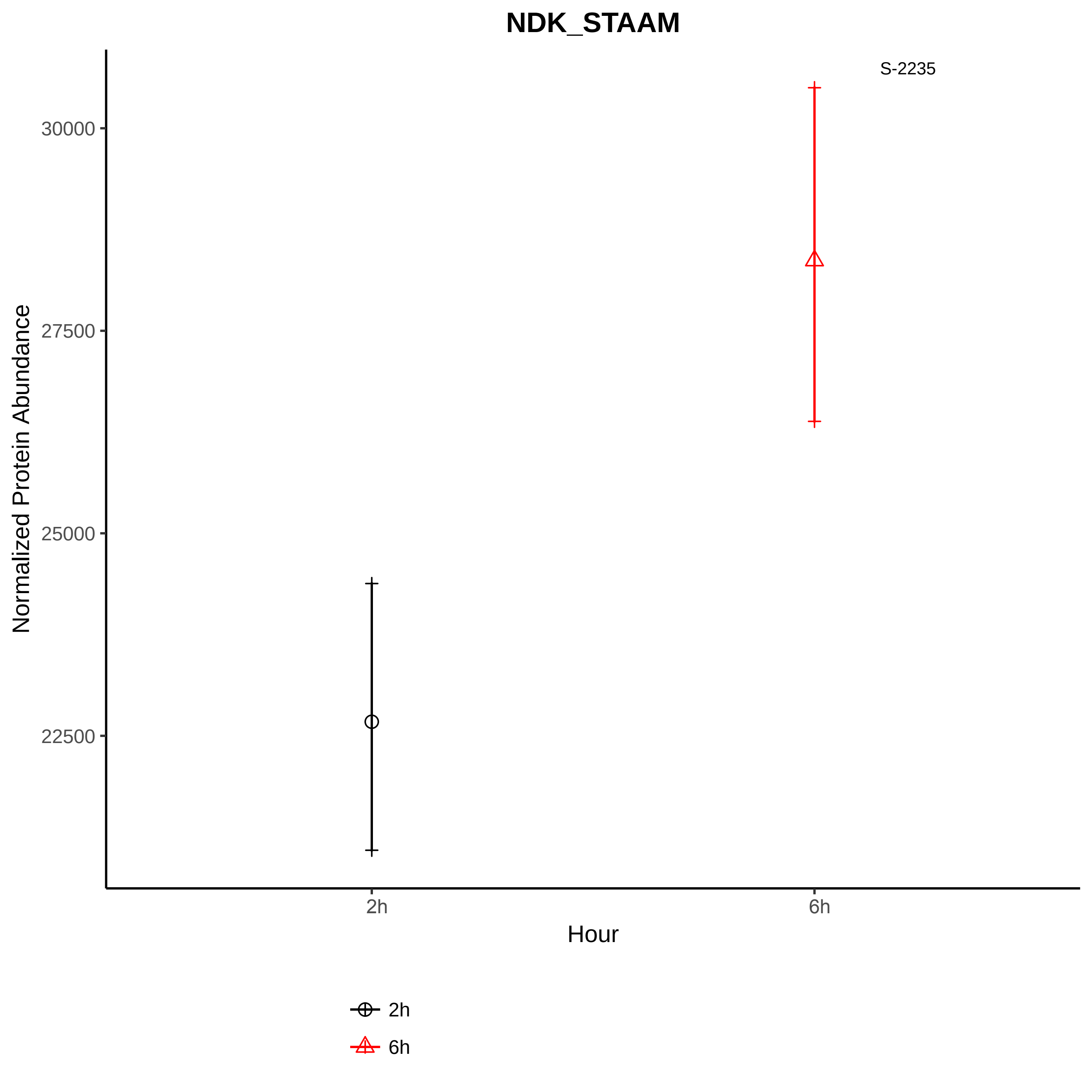
NDK_STAAM
S-2235
Normalized Protein Abundance
30000
27500
25000
22500
2h
6h
Hour
2h
6h

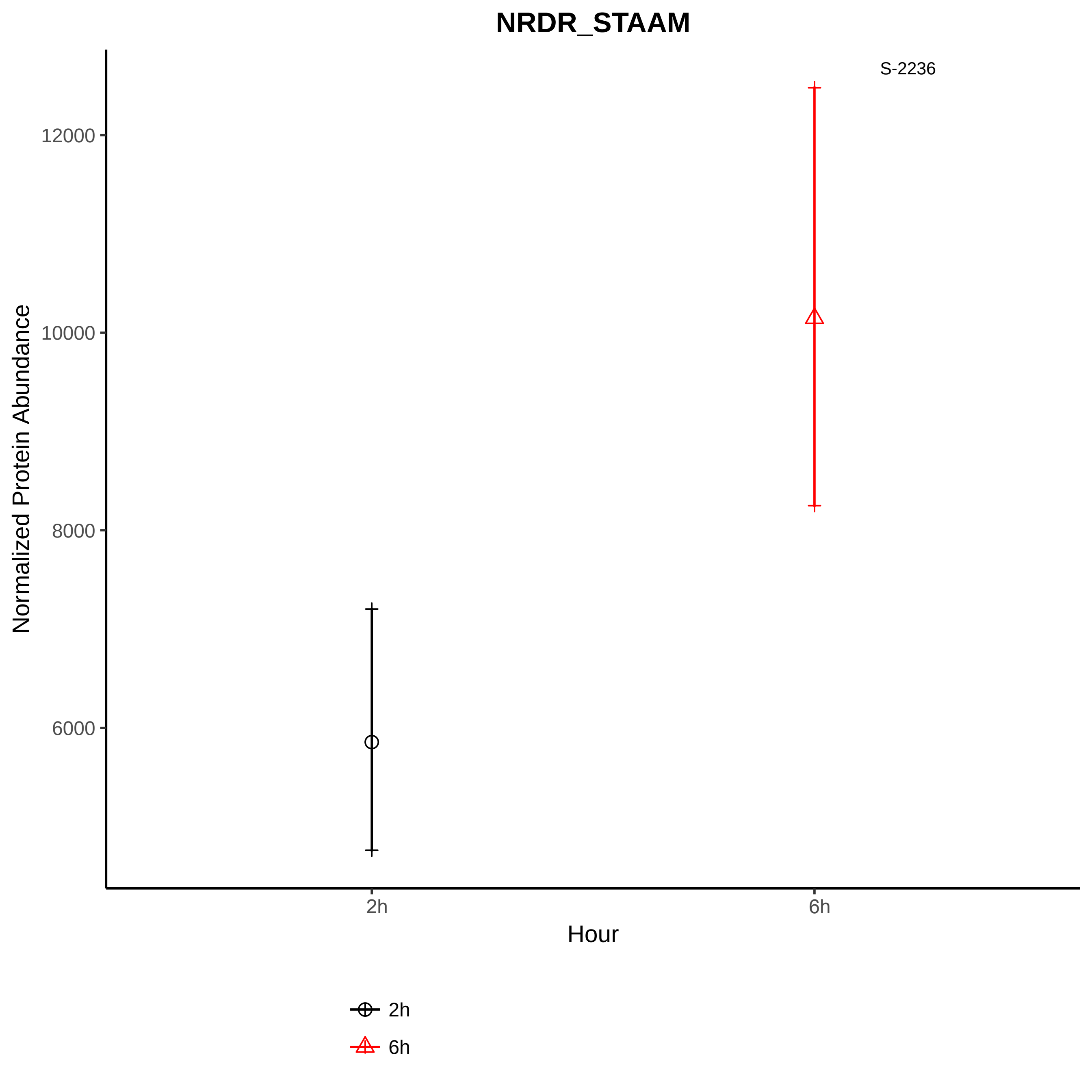
NRDR_STAAM
S-2236
Normalized Protein Abundance
12000
10000
8000
6000
2h
6h
Hour
2h
6h

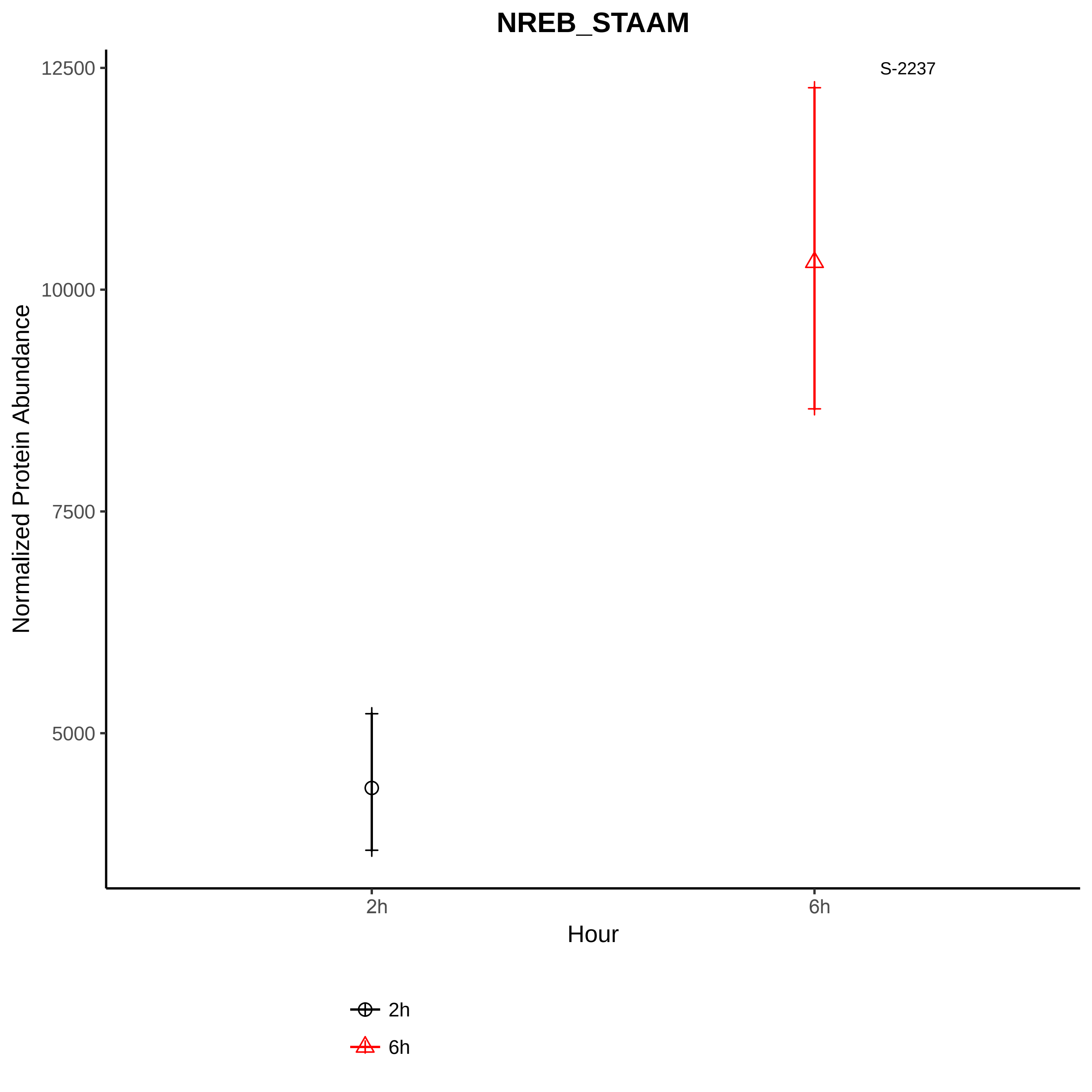

NREB_STAAM
S-2237
12500
10000
7500
5000
Normalized Protein Abundance
2h
6h
Hour
2h
6h

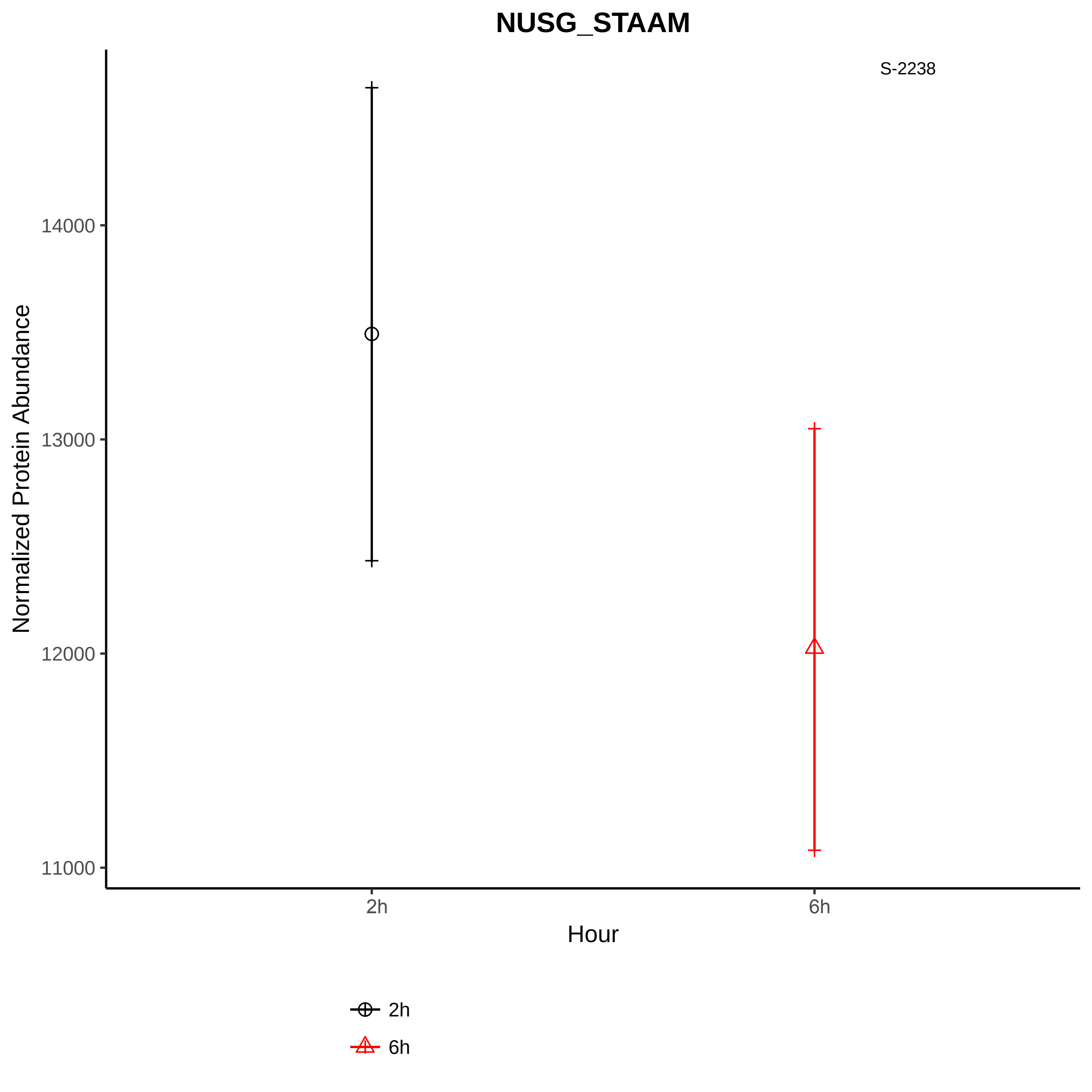
NUSG_STAAM
S-2238
Normalized Protein Abundance
14000
13000
12000
11000
2h
6h
Hour
2h
6h

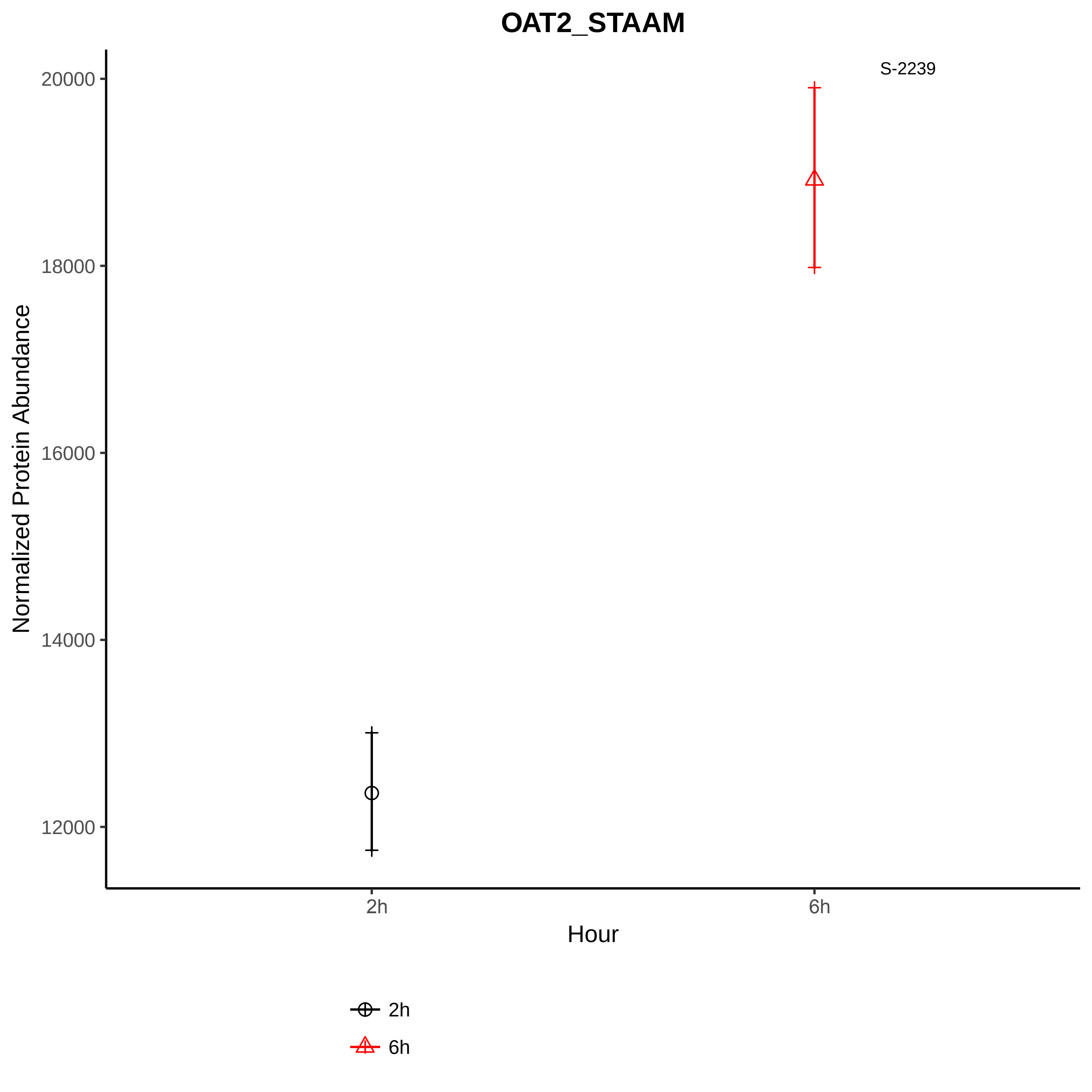

OAT2_STAAM
S-2239
20000
18000
16000
14000
12000
Normalized Protein Abundance
2h
6h
Hour
2h
6h

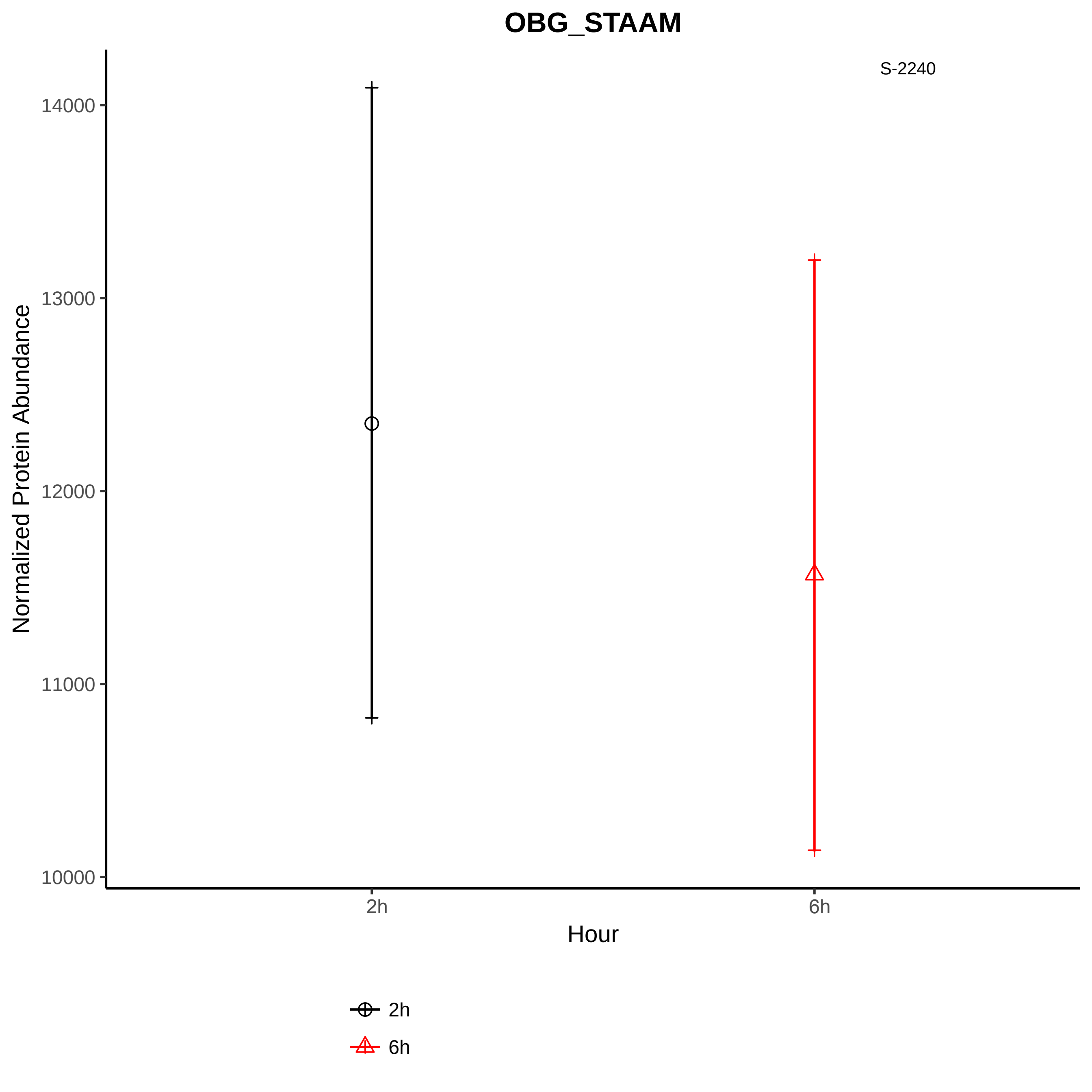
OBG_STAAM
S-2240
Normalized Protein Abundance
14000
13000
12000
11000
10000
2h
6h
Hour
2h
6h

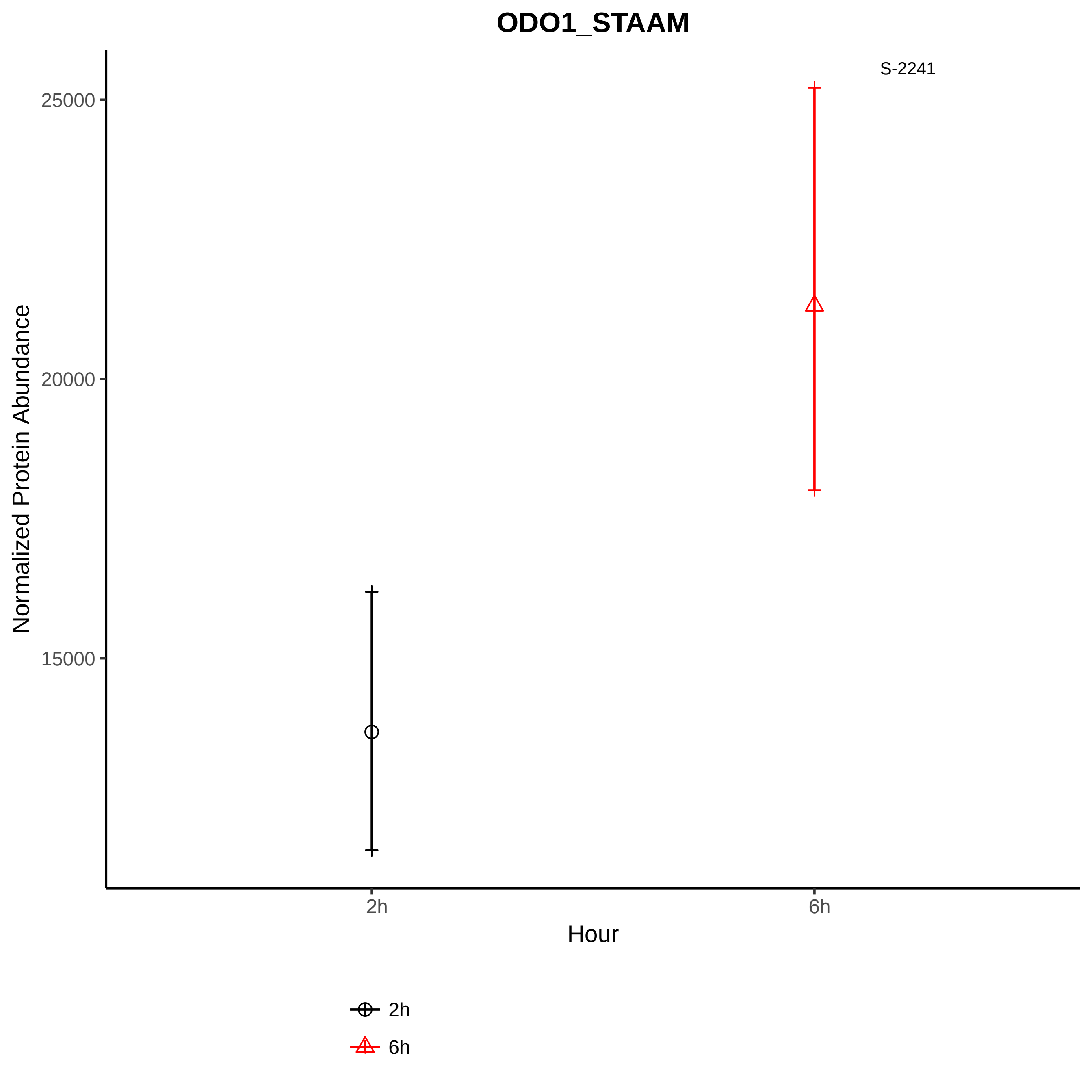
ODO1_STAAM
S-2241
25000
20000
15000
Normalized Protein Abundance
2h
6h
Hour
2h
6h

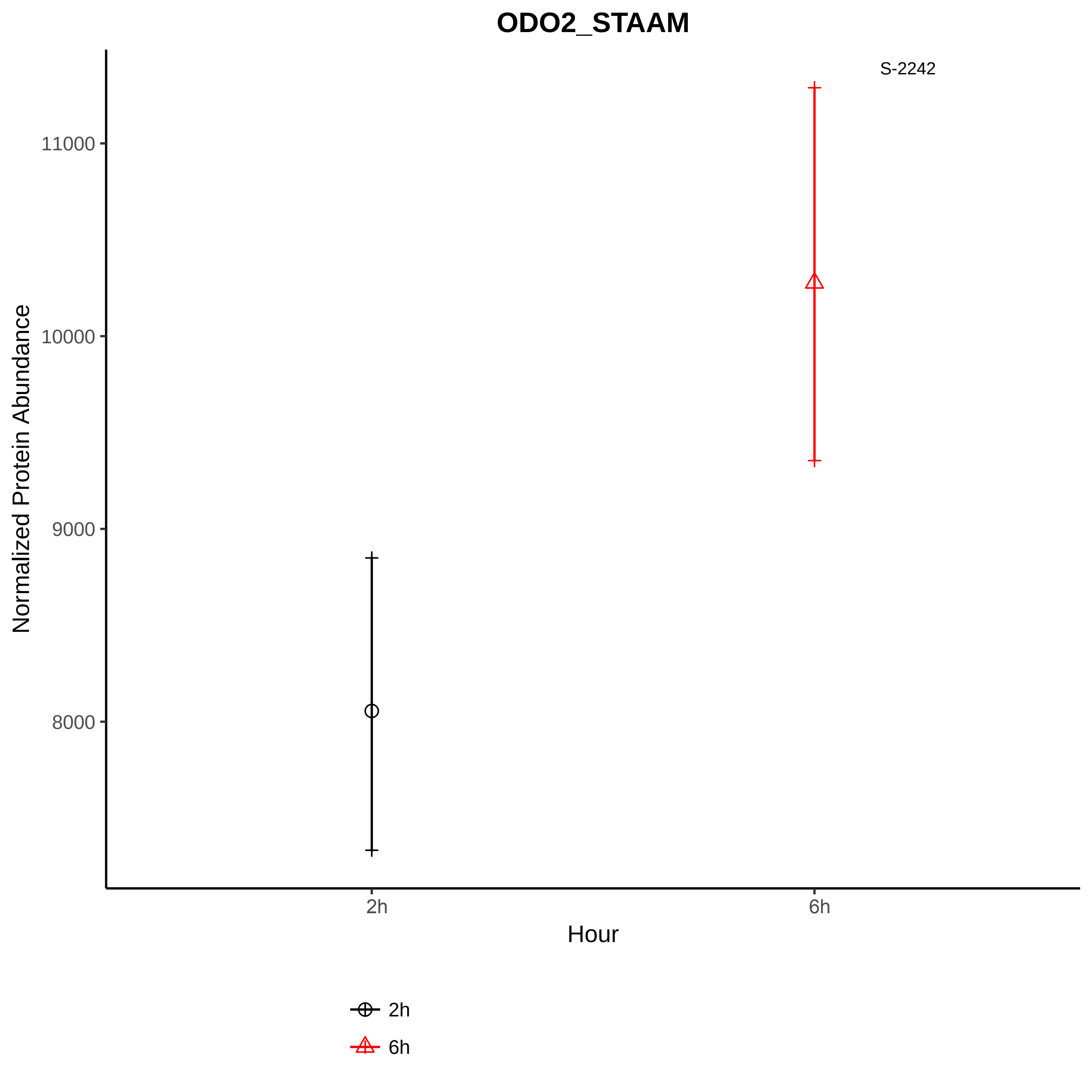

ODO2_STAAM
S-2242
Normalized Protein Abundance
11000
10000
9000
8000
2h
6h
Hour
2h
6h

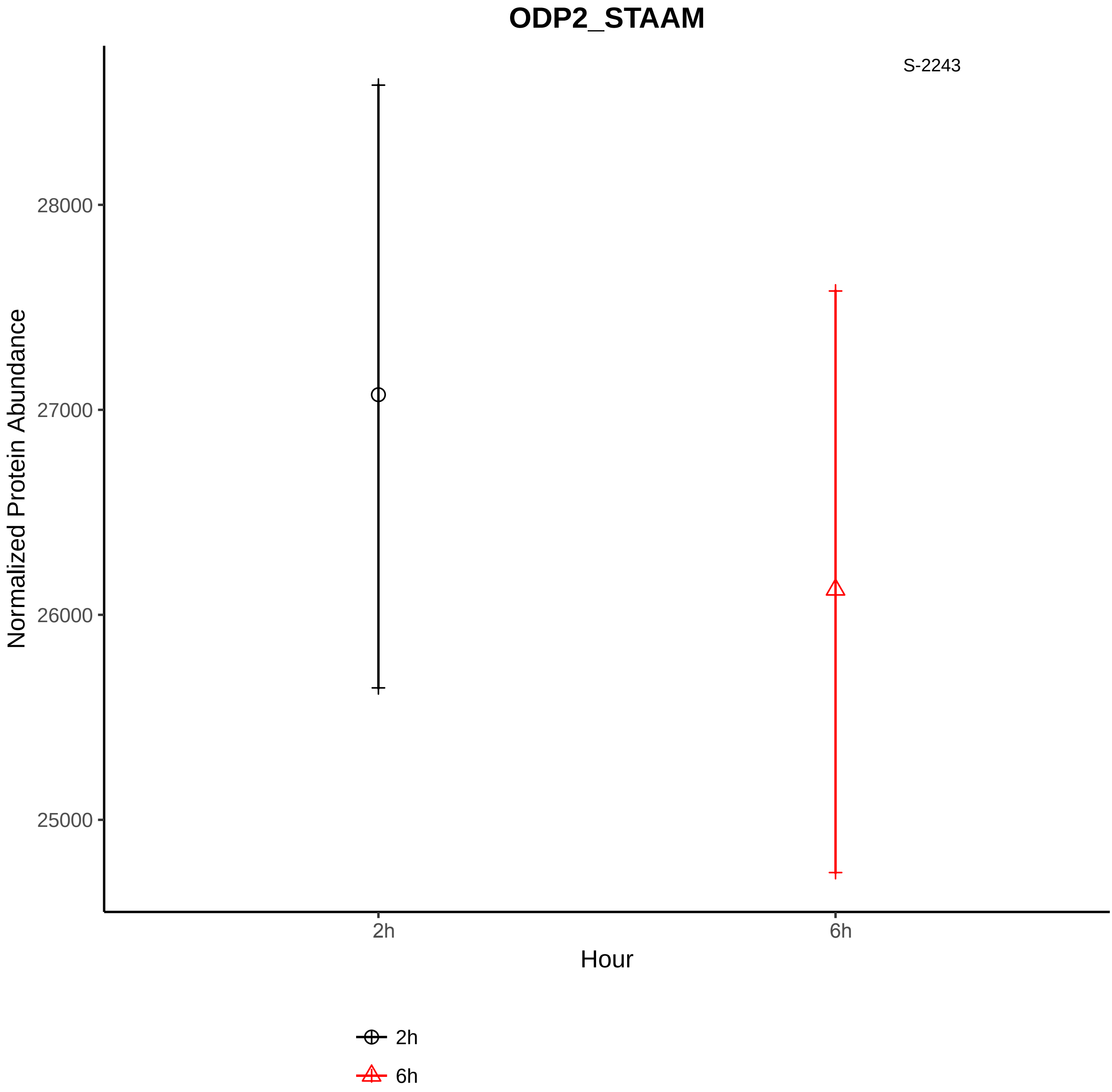

ODP2_STAAM
S-2243
28000
27000
26000
25000
Normalized Protein Abundance
2h
6h
Hour
2h
6h

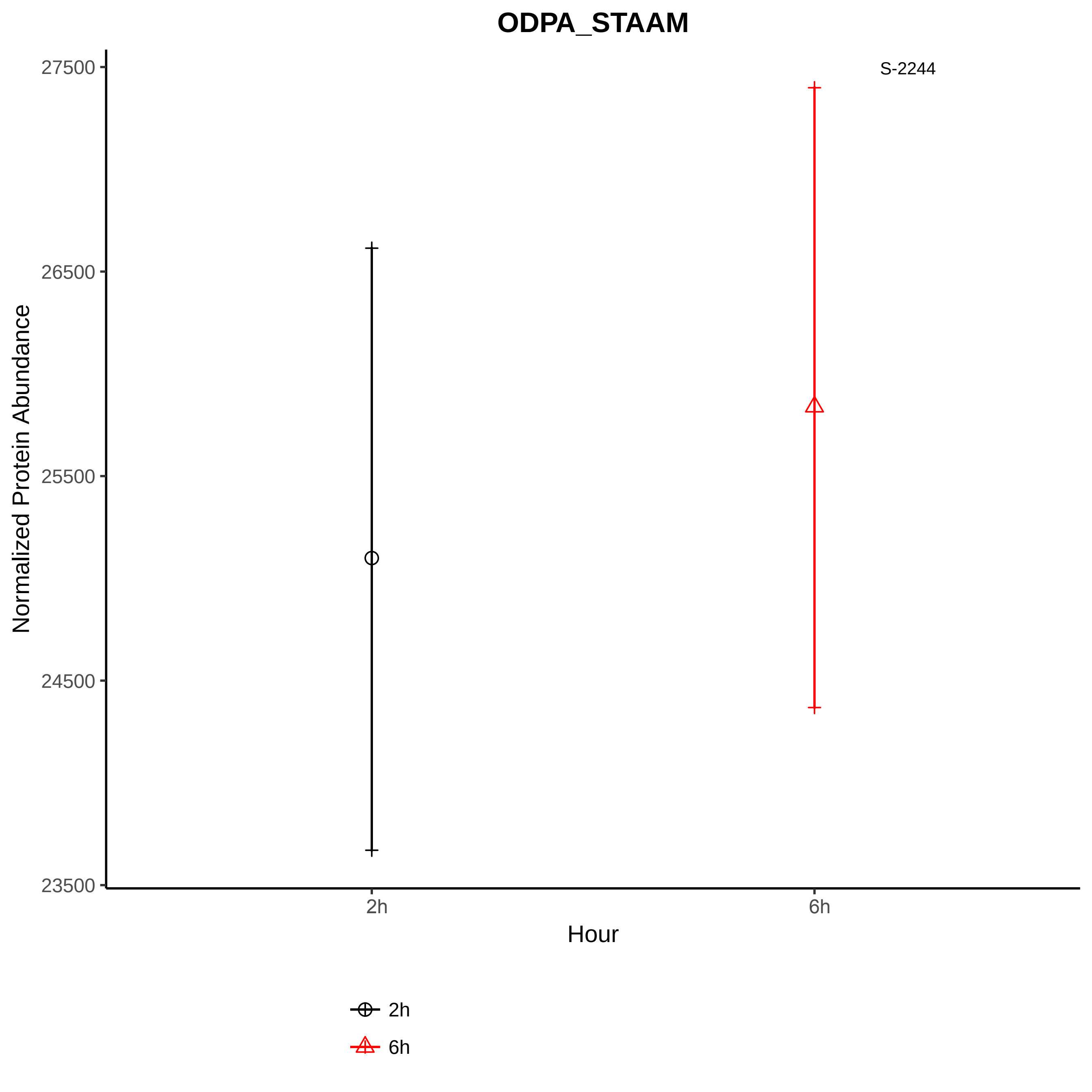

ODPA_STAAM
S-2244
27500
26500
25500
24500
23500
Normalized Protein Abundance
2h
6h
Hour
2h
6h

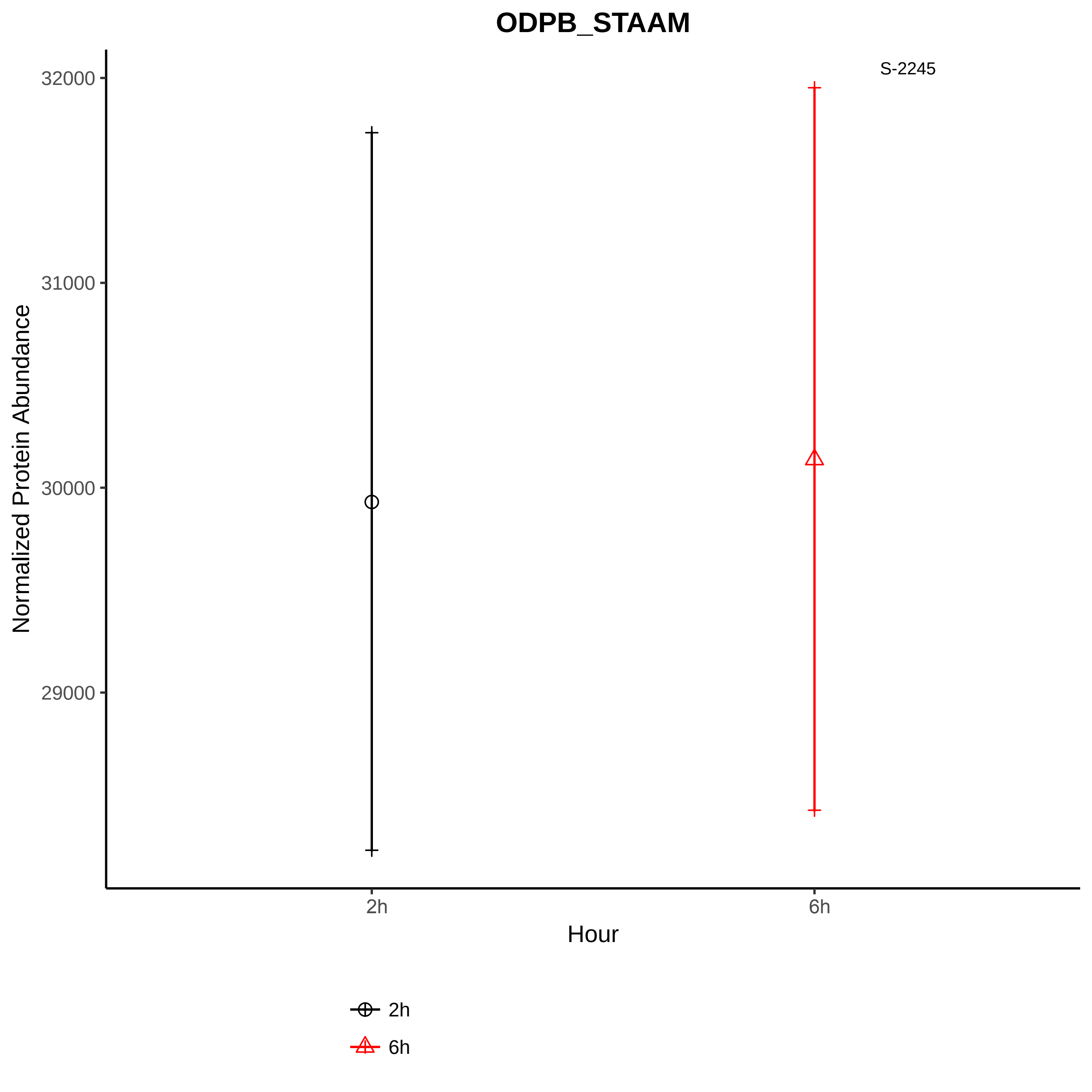

ODPB_STAAM
S-2245
32000
31000
30000
29000
Normalized Protein Abundance
2h
6h
Hour
2h
6h

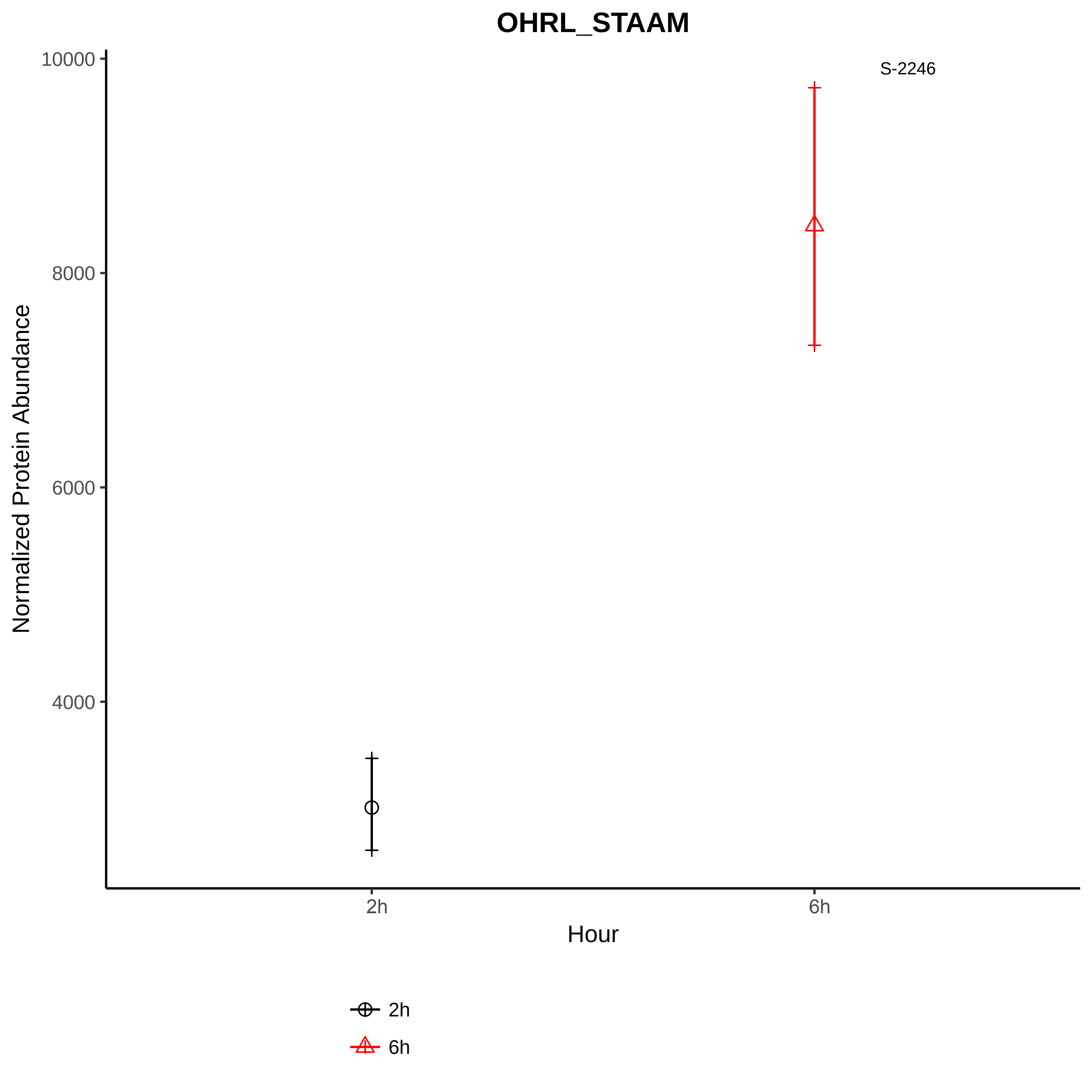

OHRL_STAAM
S-2246
Normalized Protein Abundance
10000
8000
6000
4000
2h
6h
Hour
2h
6h

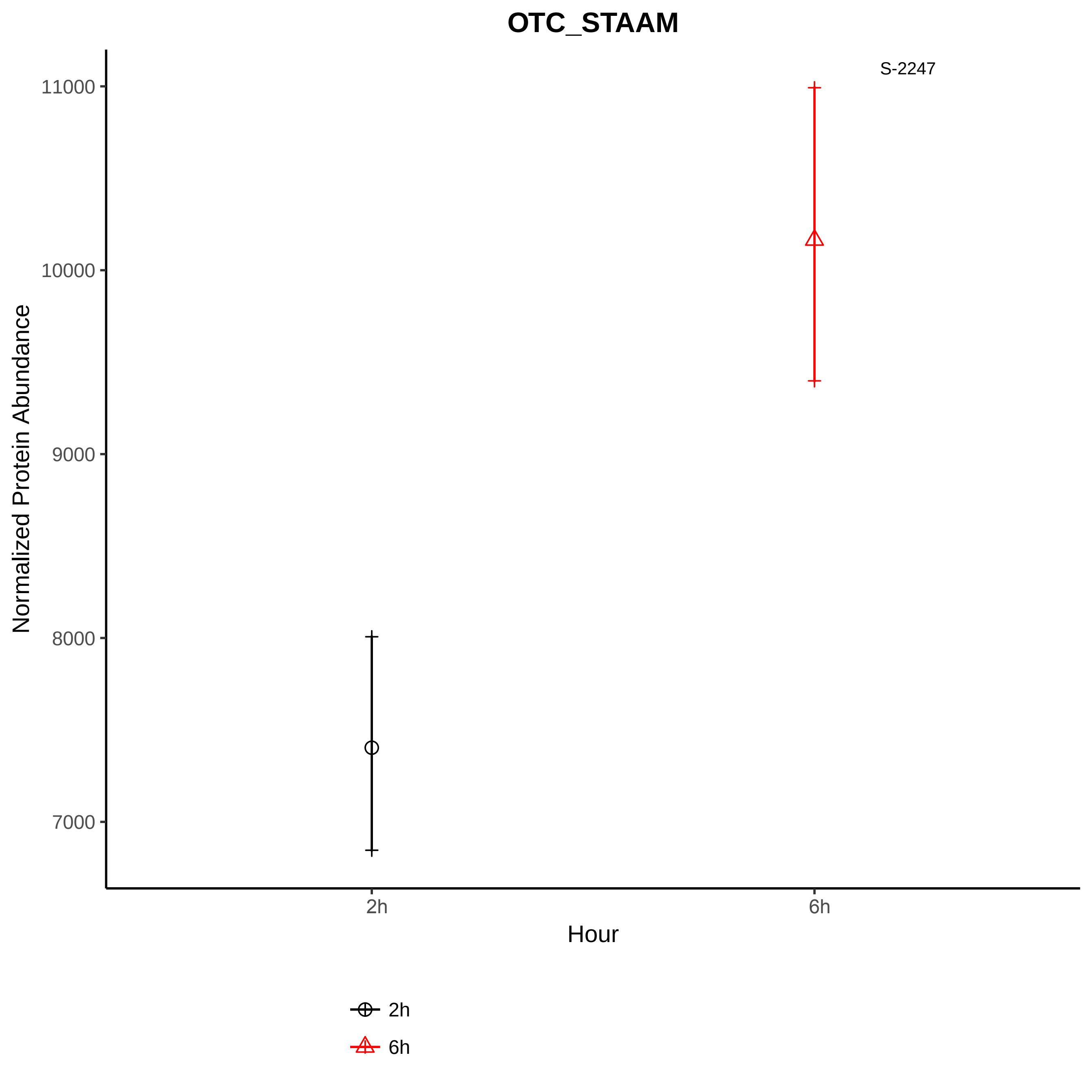
OTC_STAAM
S-2247
Normalized Protein Abundance
11000
10000
9000
8000
7000
2h
6h
Hour
2h
6h

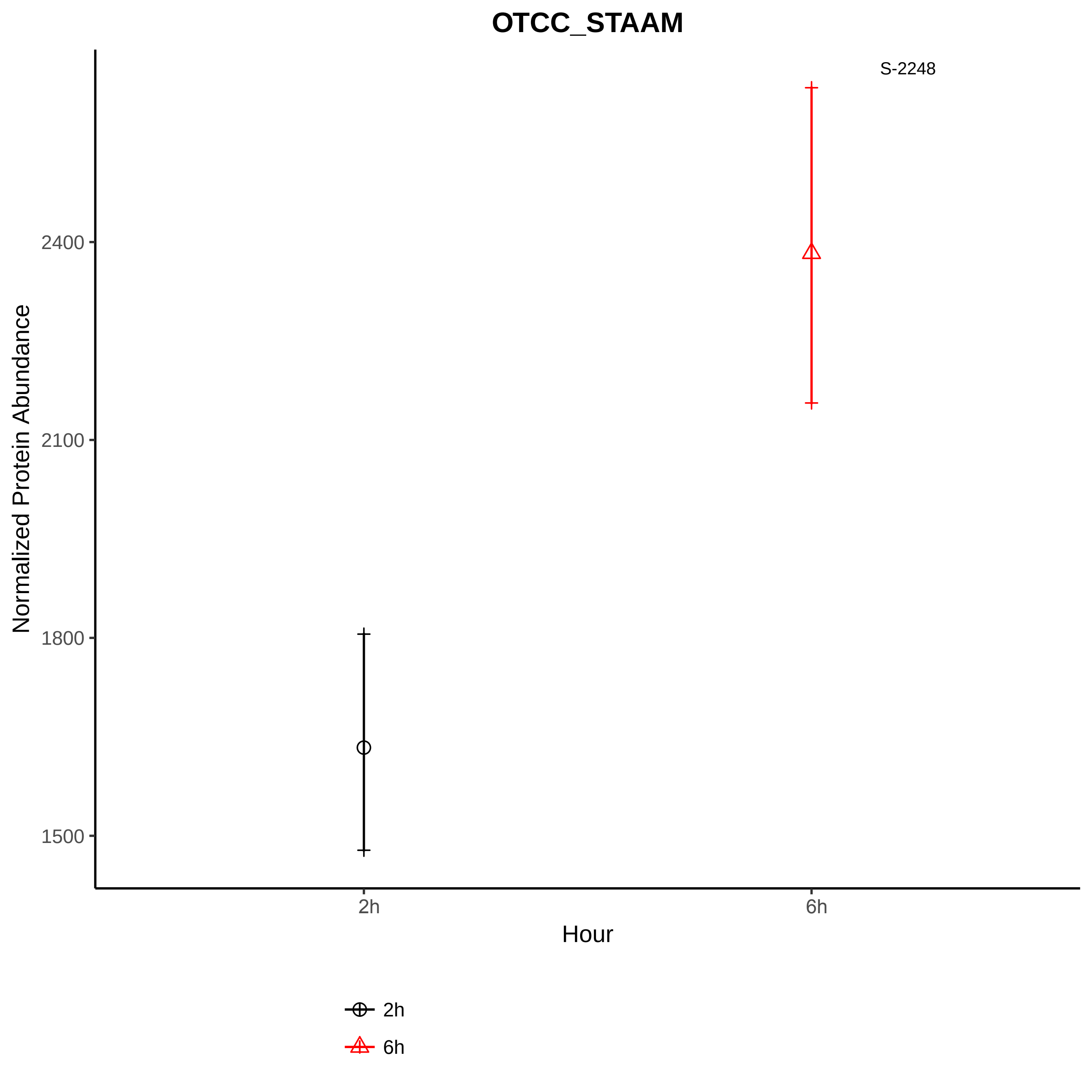

OTCC_STAAM
S-2248
2400
2100
1800
1500
Normalized Protein Abundance
2h
6h
Hour
2h
6h

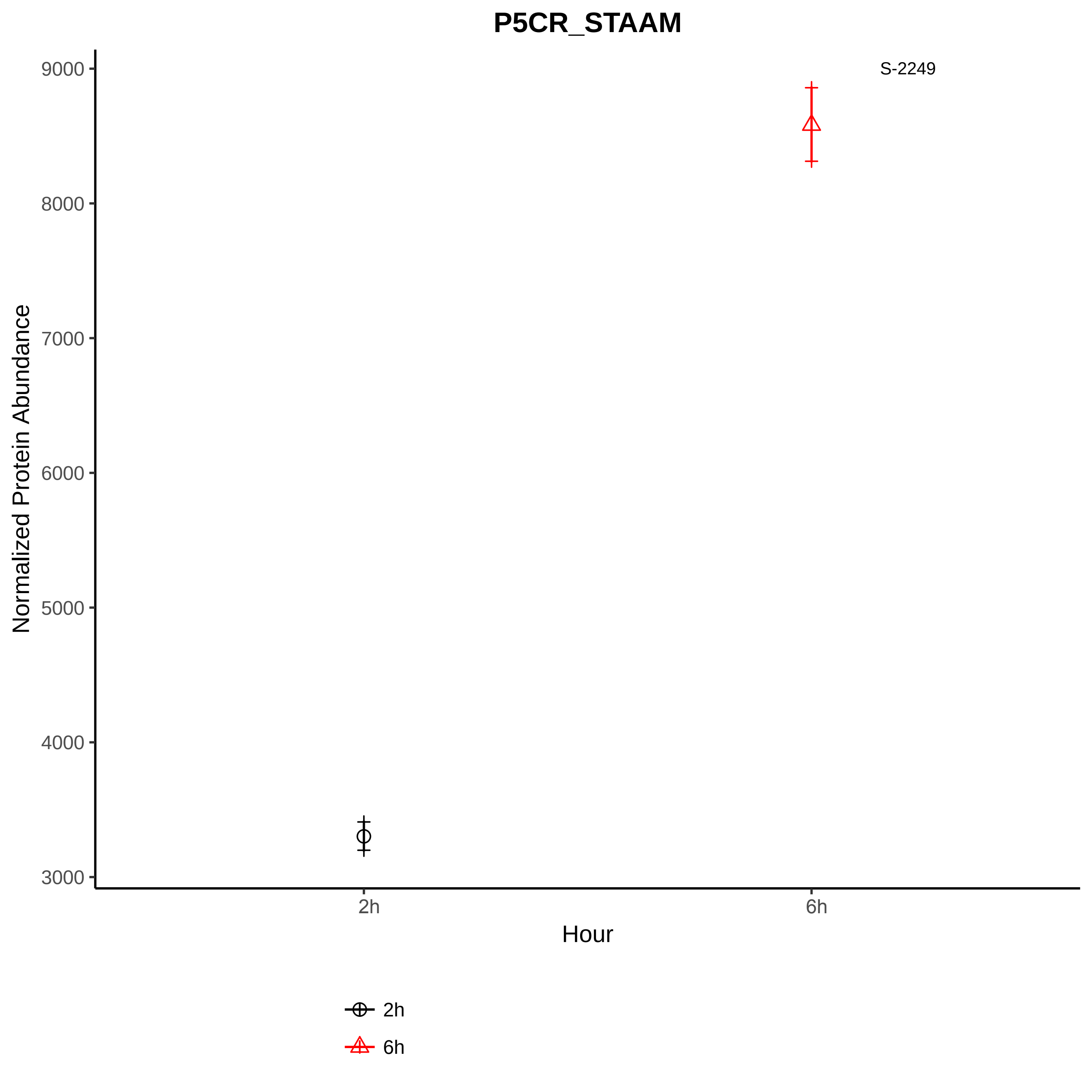

P5CR_STAAM
S-2249
Normalized Protein Abundance
9000
8000
7000
6000
5000
4000
3000
2h
6h
Hour
2h
6h

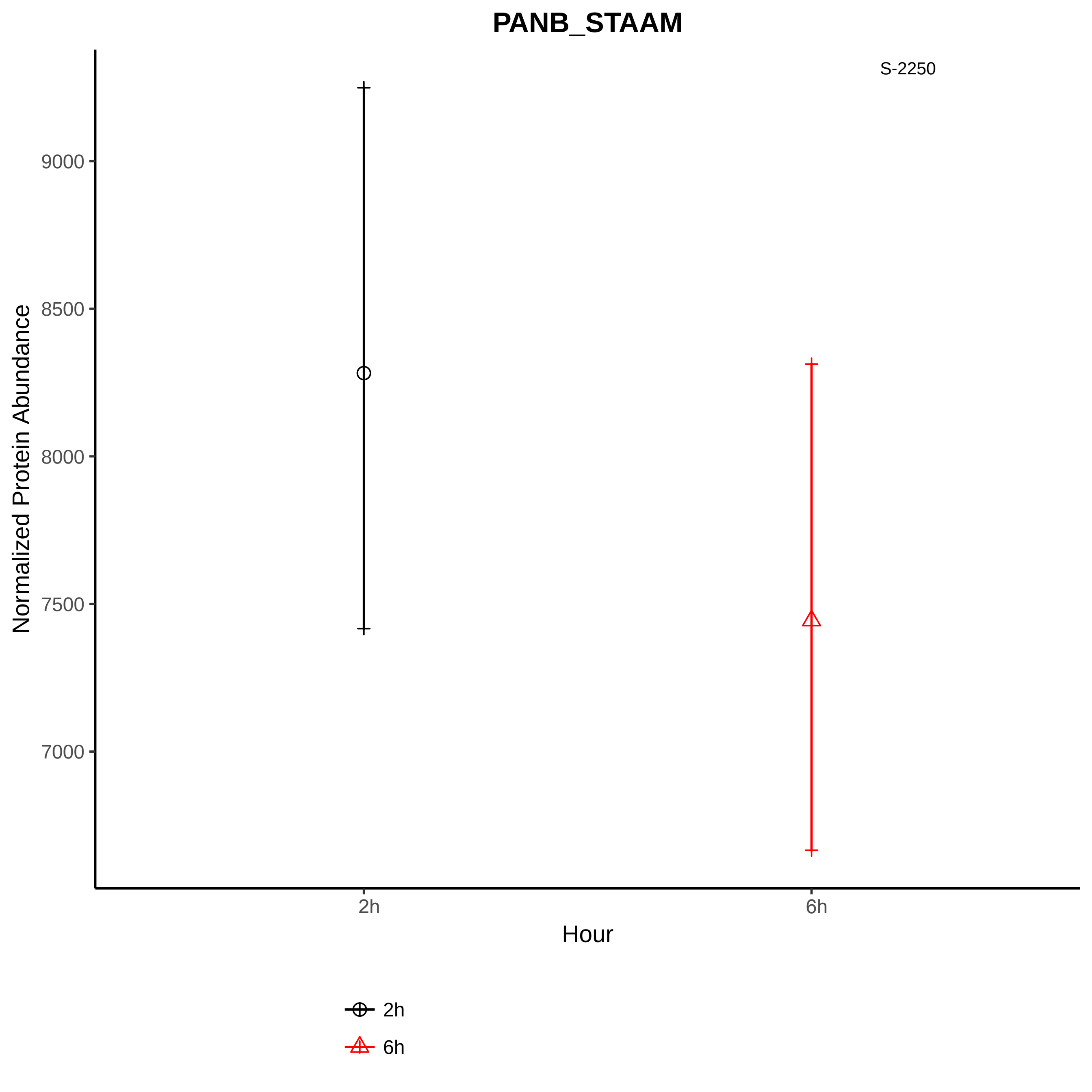

PANB_STAAM
S-2250
Normalized Protein Abundance
9000
8500
8000
7500
7000
2h
6h
Hour
2h
6h

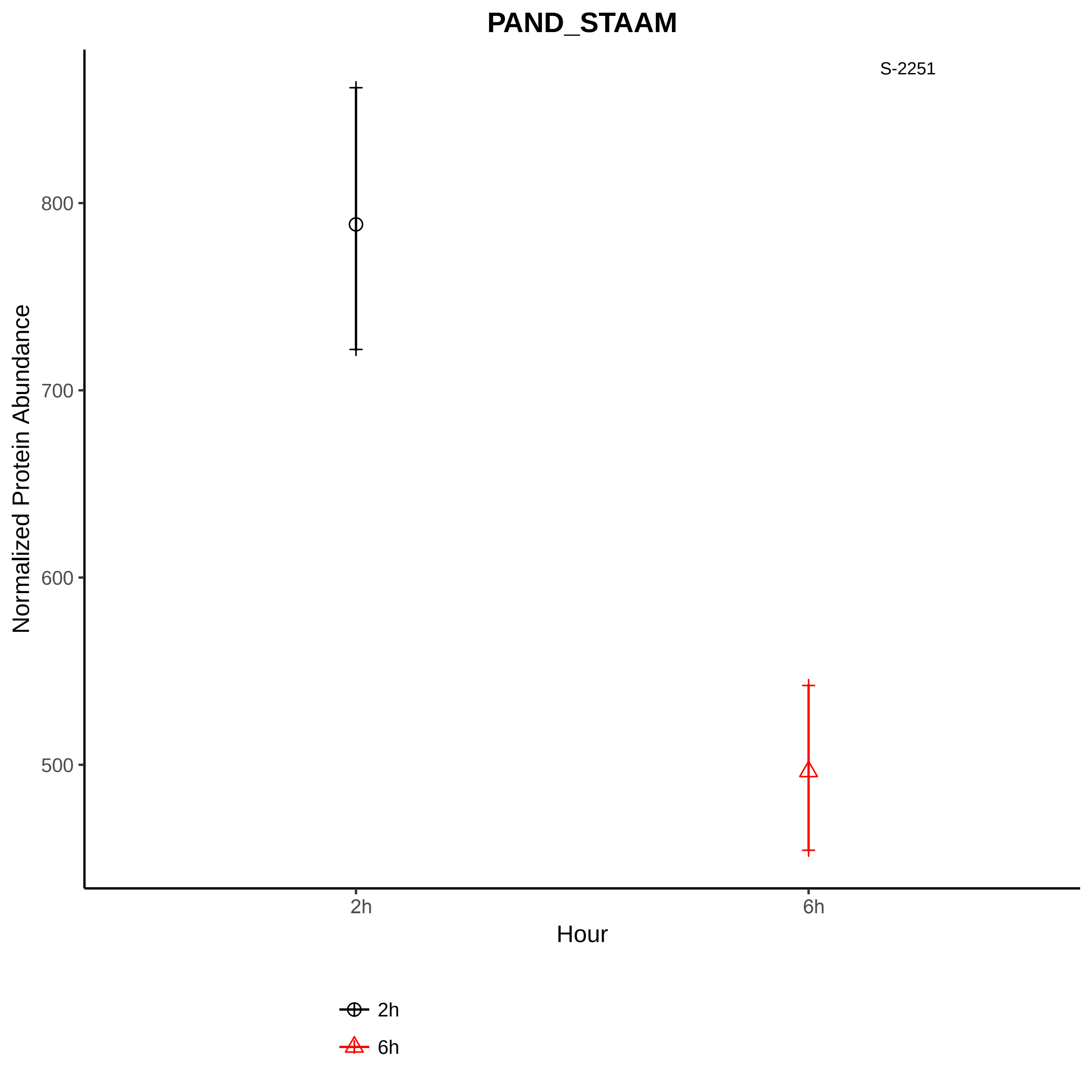
PAND_STAAM
S-2251
Normalized Protein Abundance
800
700
600
500
2h
6h
Hour
2h
6h

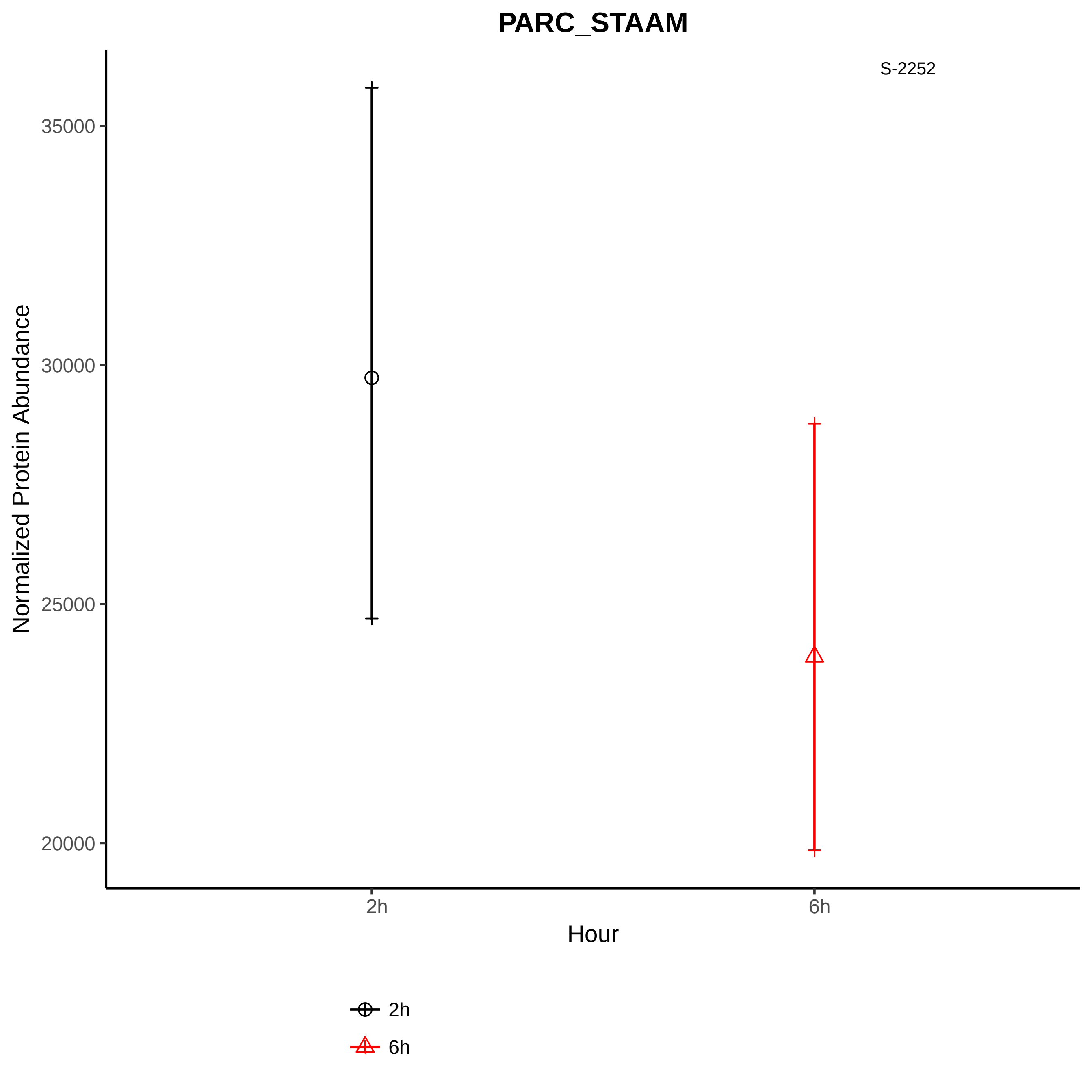

PARC_STAAM
S-2252
35000
30000
25000
20000
Normalized Protein Abundance
2h
6h
Hour
2h
6h

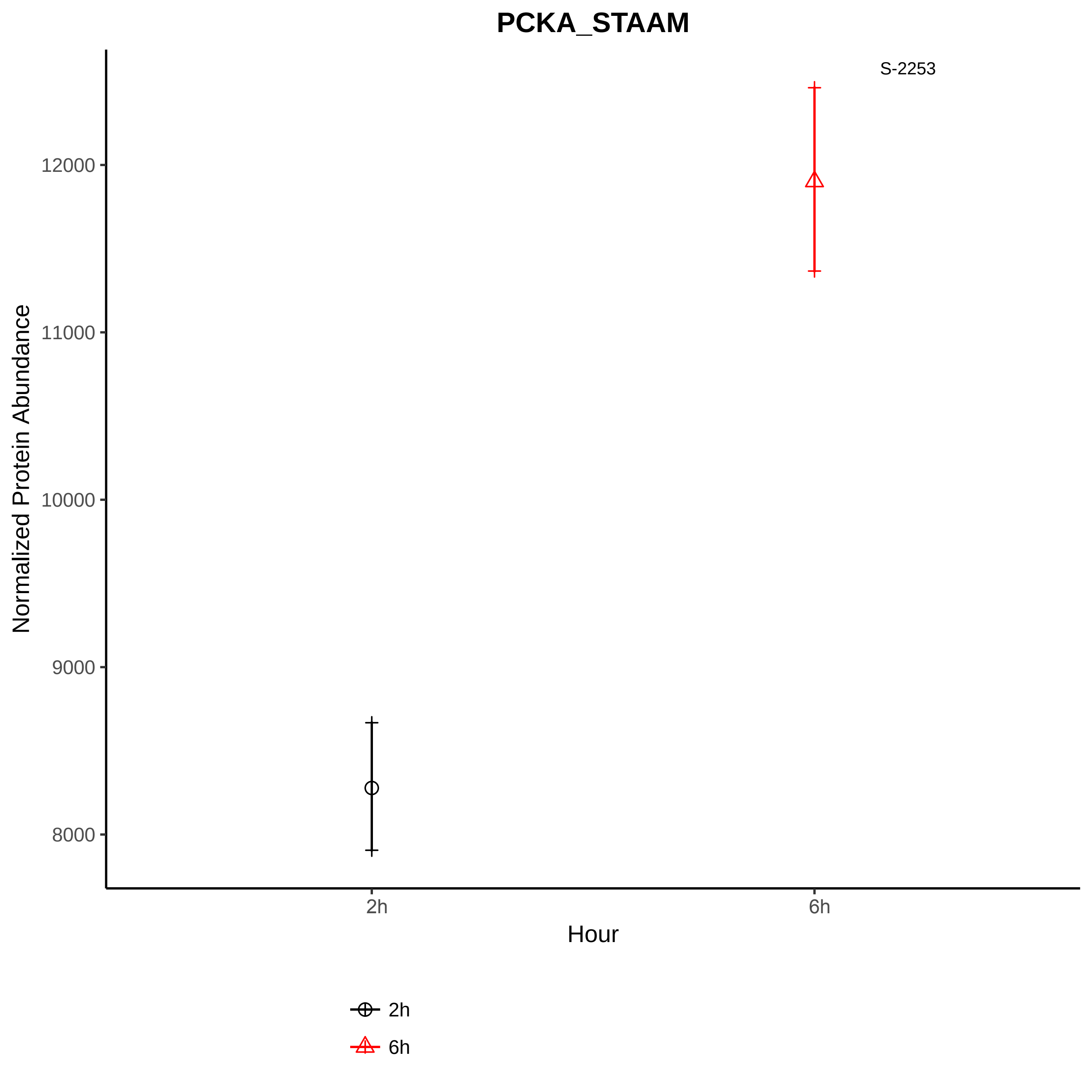
PCKA_STAAM
S-2253
Normalized Protein Abundance
12000
11000
10000
9000
8000
2h
6h
Hour
2h
6h

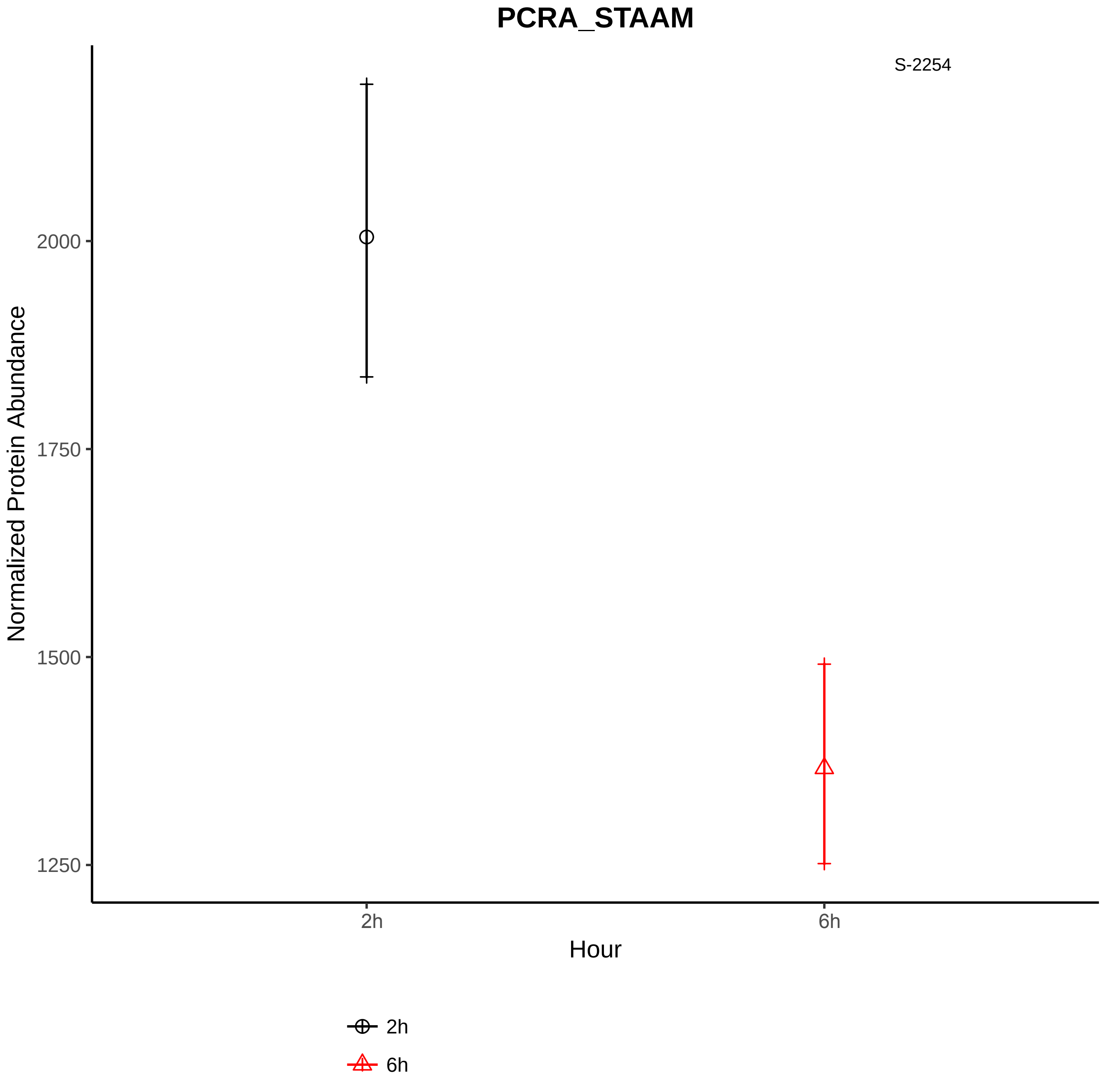
PCRA_STAAM
S-2254
Normalized Protein Abundance
2000
1750
1500
1250
2h
6h
Hour
2h
6h

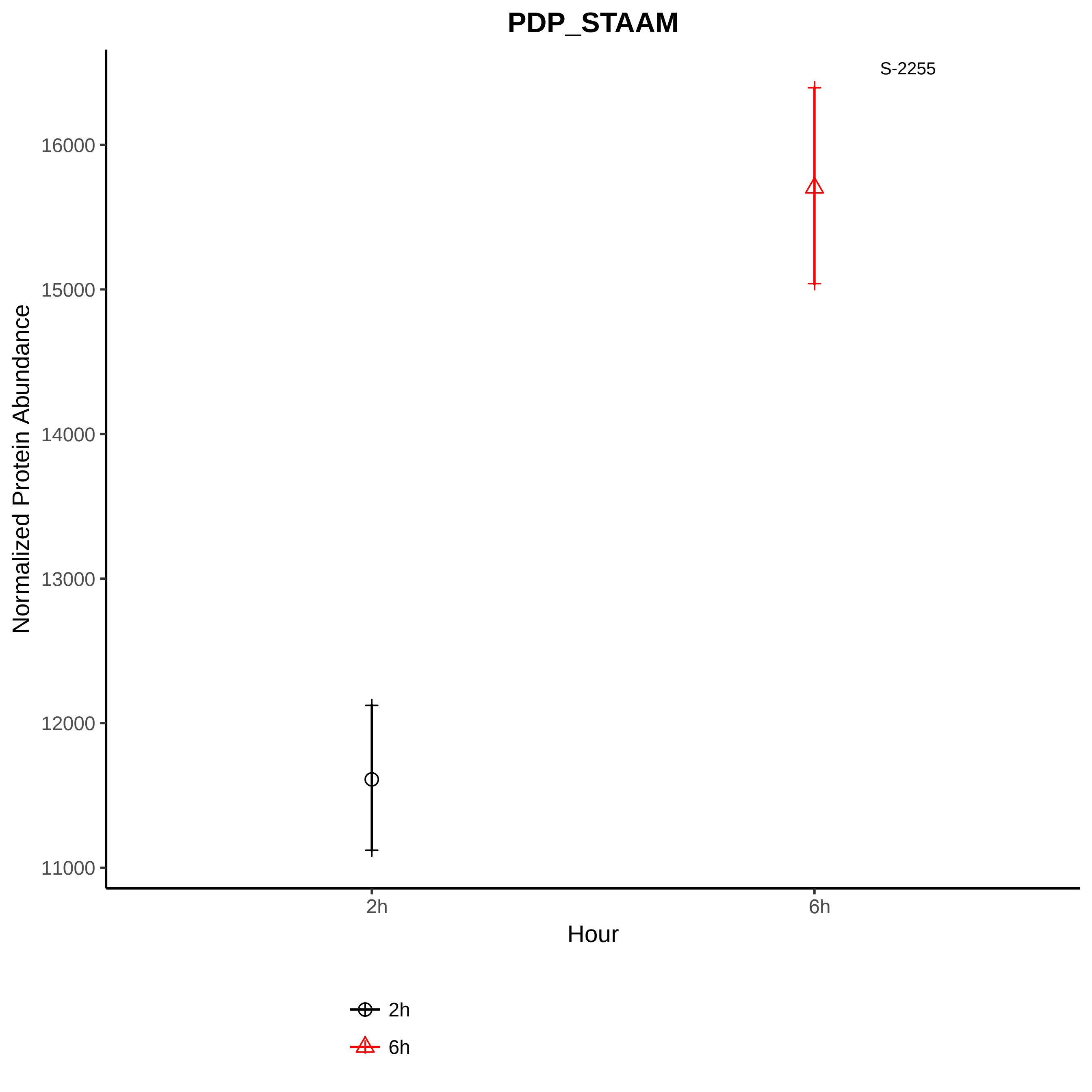
PDP_STAAM
S-2255
Normalized Protein Abundance
16000
15000
14000
13000
12000
11000
2h
6h
Hour
2h
6h

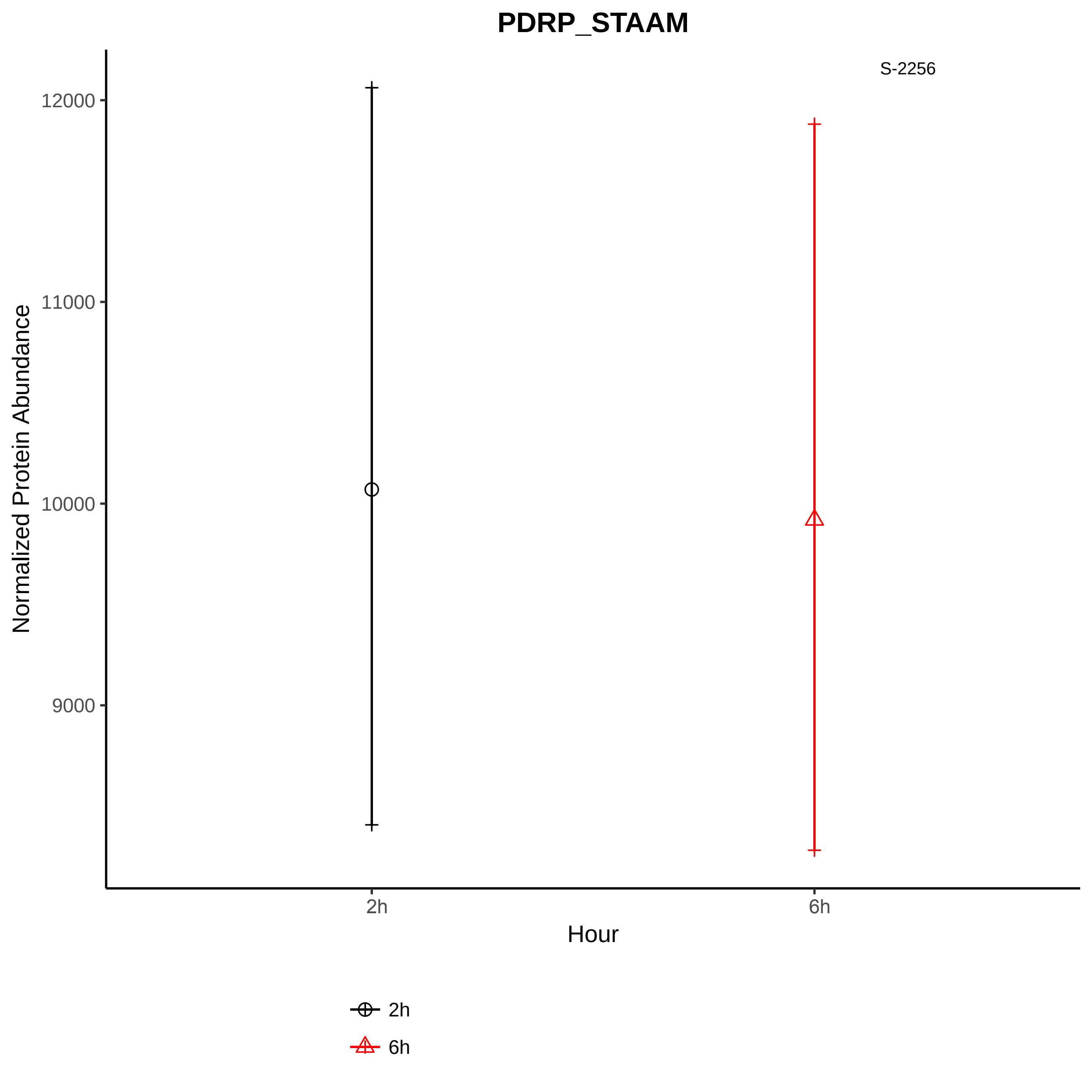
PDRP_STAAM
S-2256
12000
11000
10000
9000
Normalized Protein Abundance
2h
6h
Hour
2h
6h

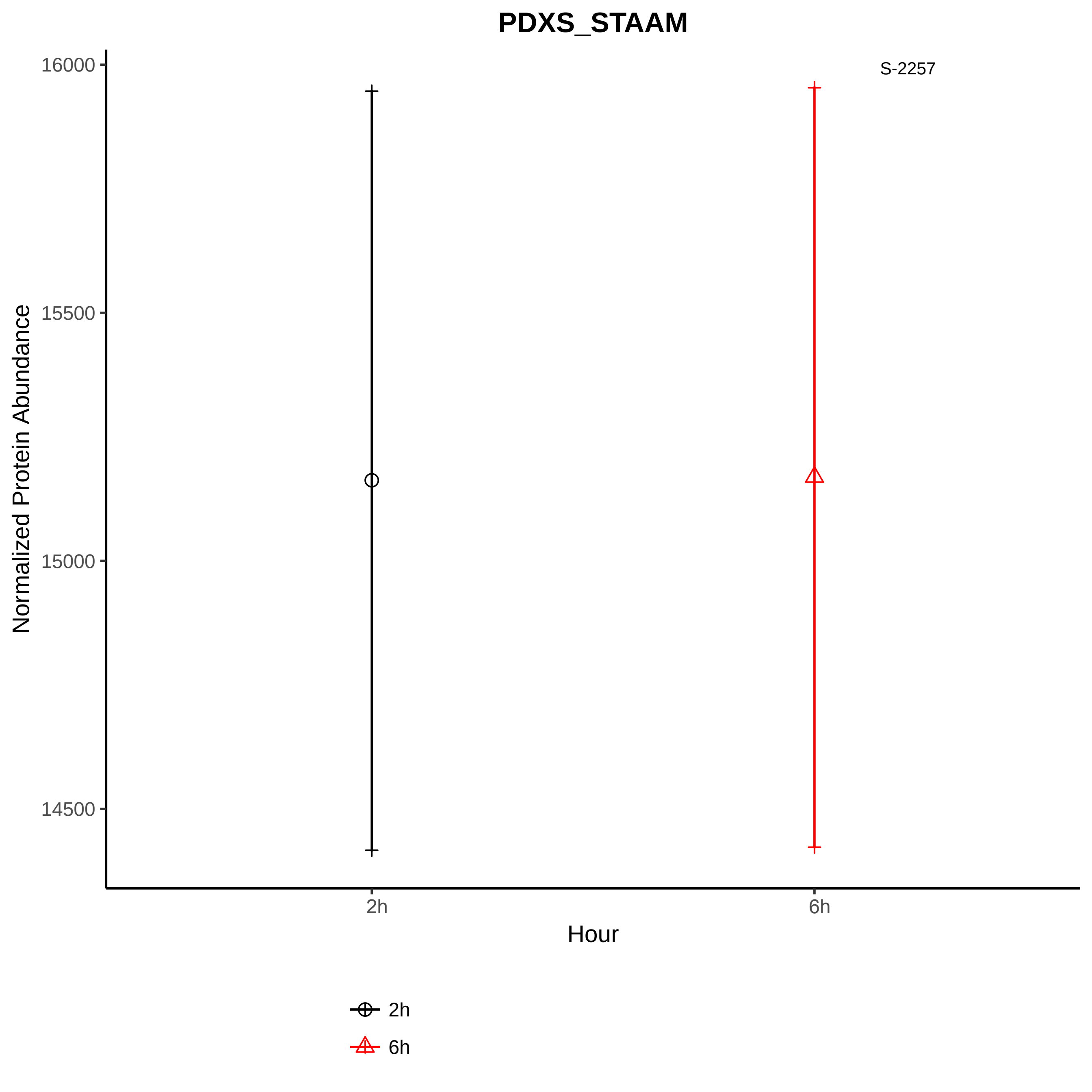

PDXS_STAAM
S-2257
16000
15500
15000
14500
Normalized Protein Abundance
2h
6h
Hour
2h
6h

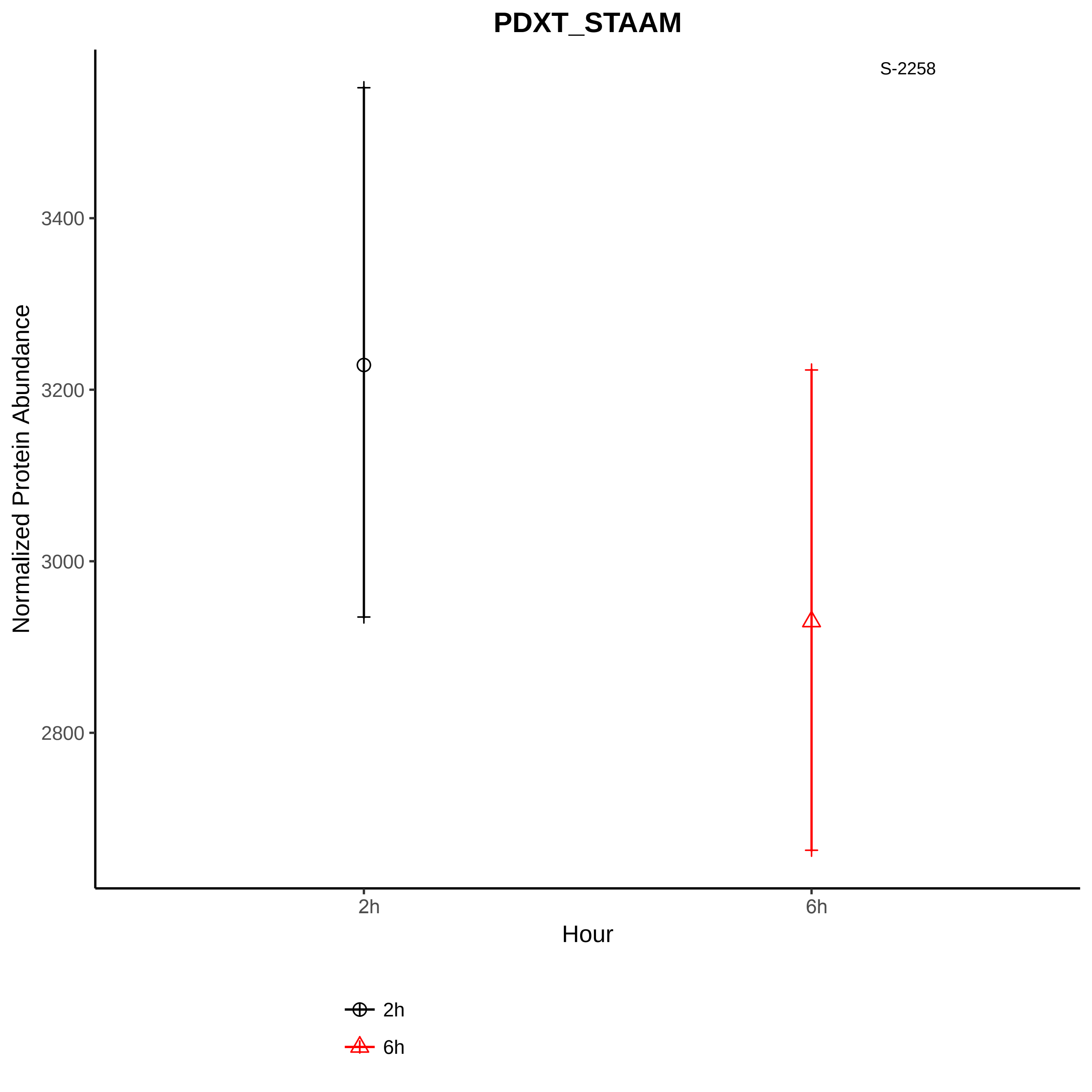

PDXT_STAAM
S-2258
Normalized Protein Abundance
3400
3200
3000
2800
2h
6h
Hour
2h
6h

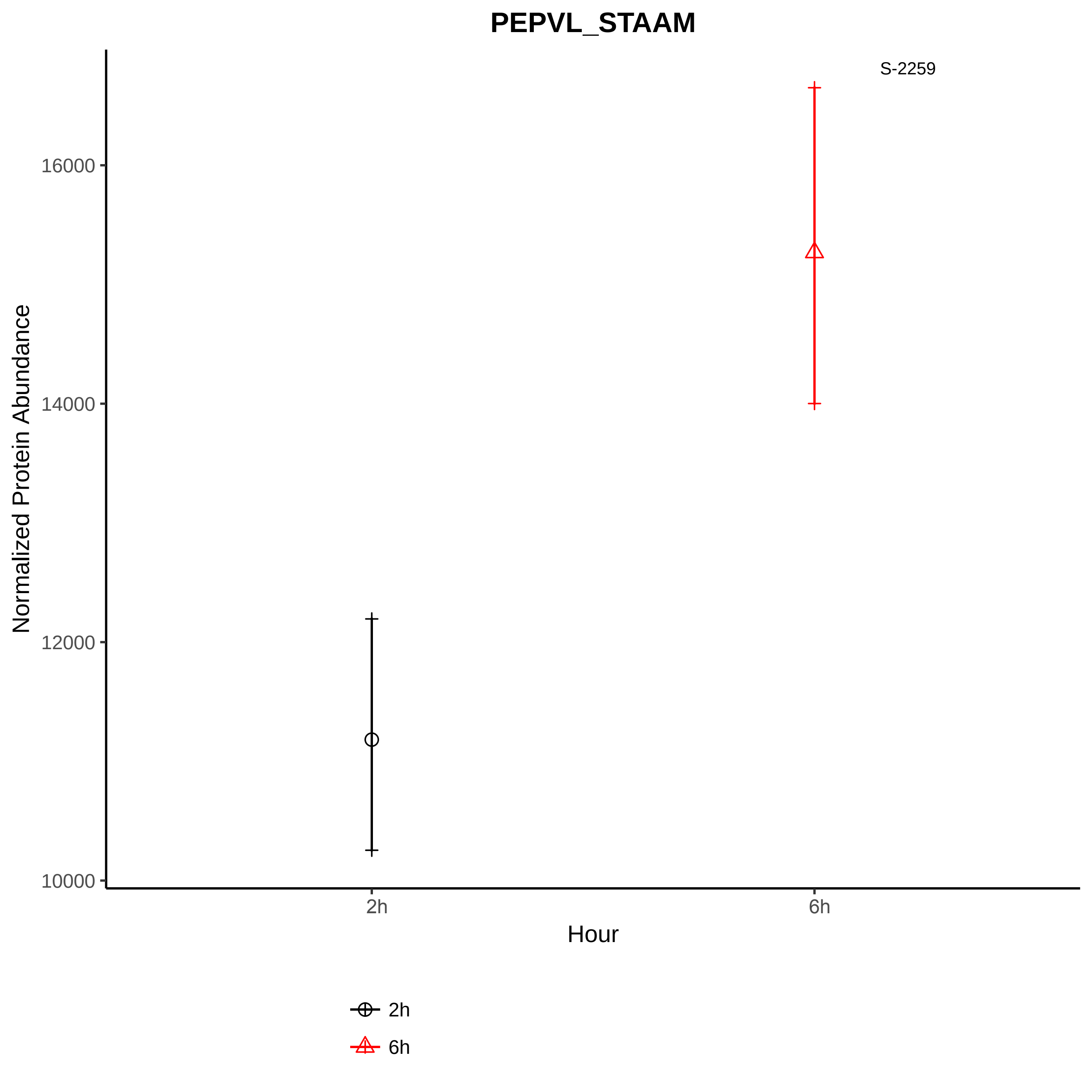
PEPVL_STAAM
S-2259
Normalized Protein Abundance
16000
14000
12000
10000
2h
6h
Hour
2h
6h

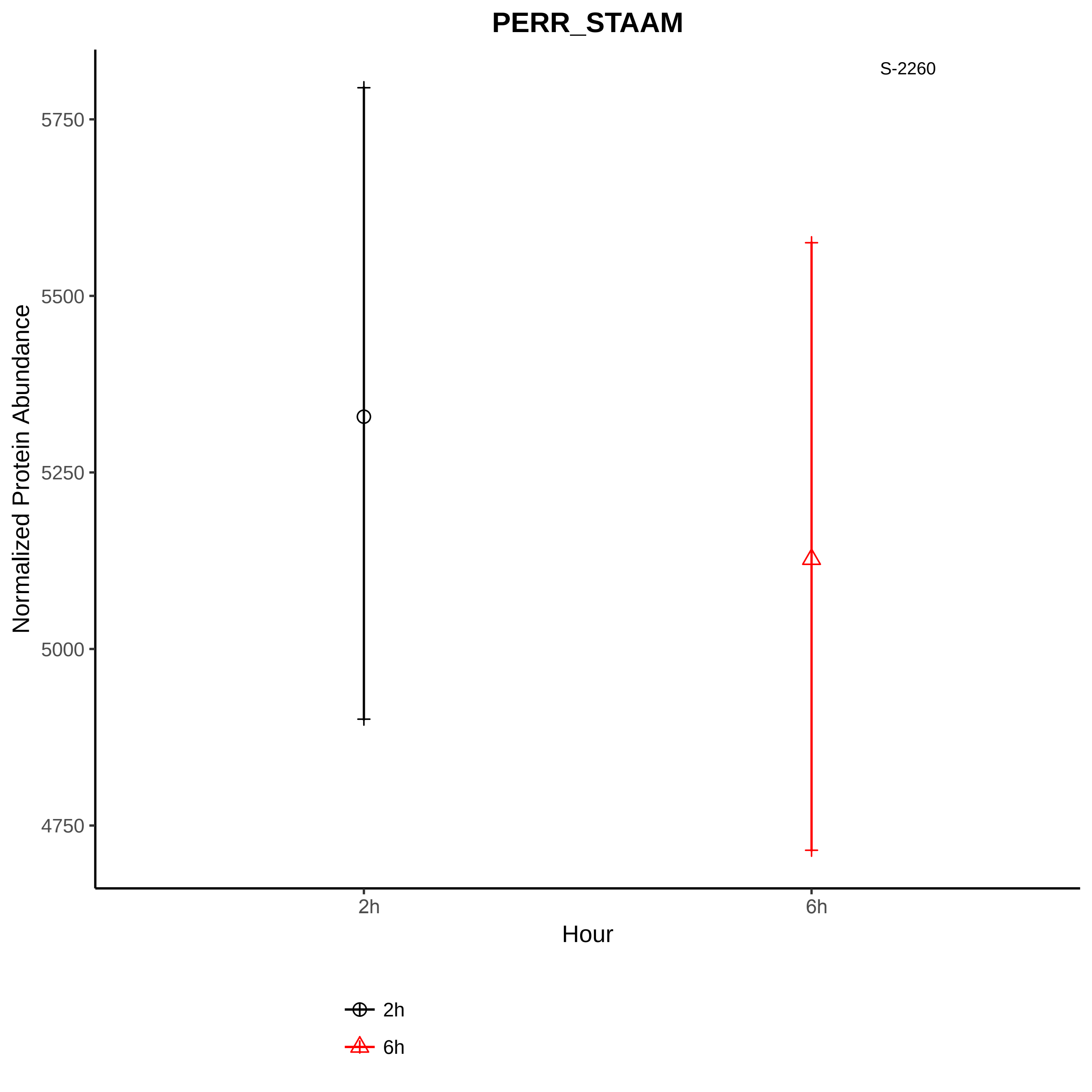
PERR_STAAM
S-2260
Normalized Protein Abundance
5750
5500
5250
5000
4750
2h
6h
Hour
2h
6h

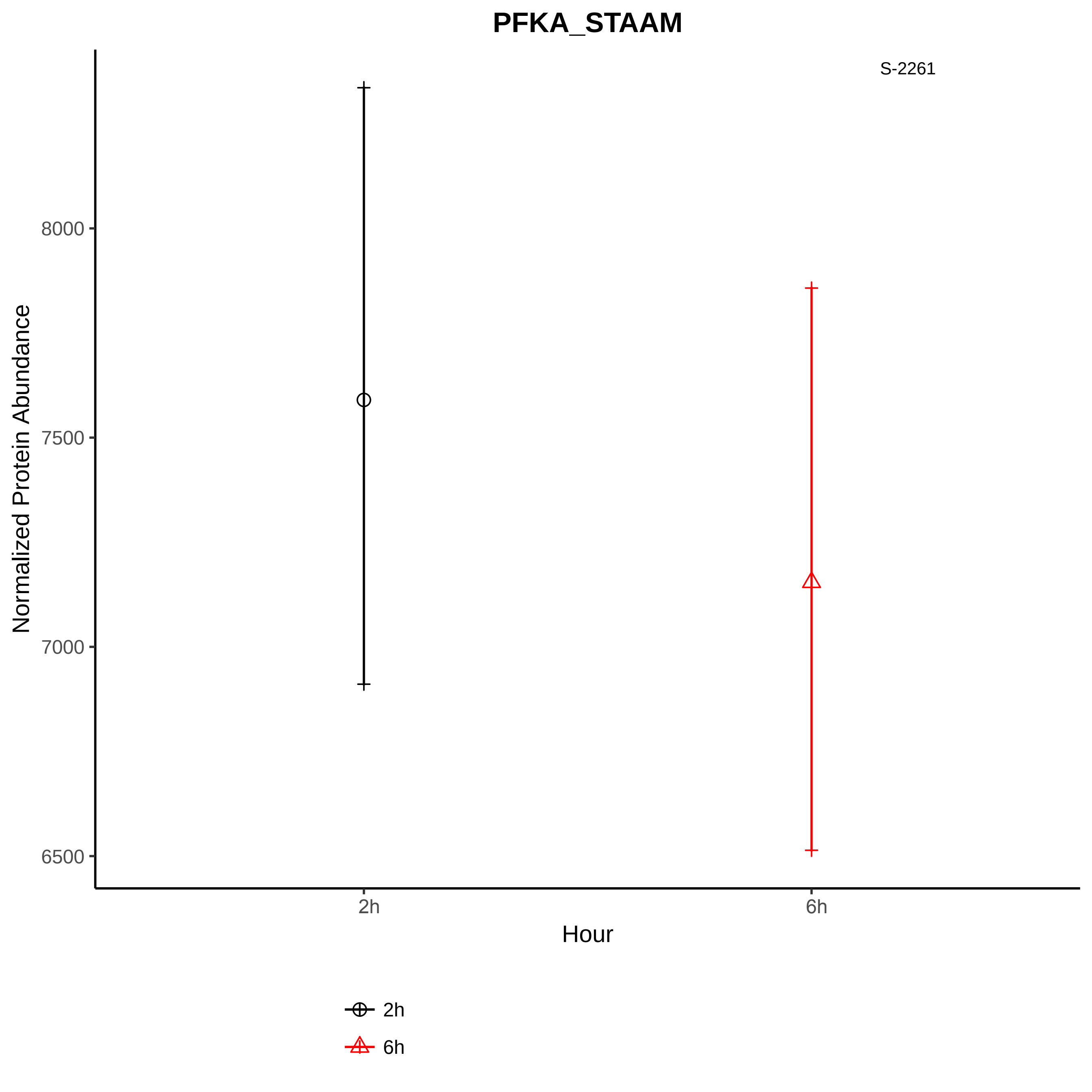

PFKA_STAAM
S-2261
Normalized Protein Abundance
8000
7500
7000
6500
2h
6h
Hour
2h
6h

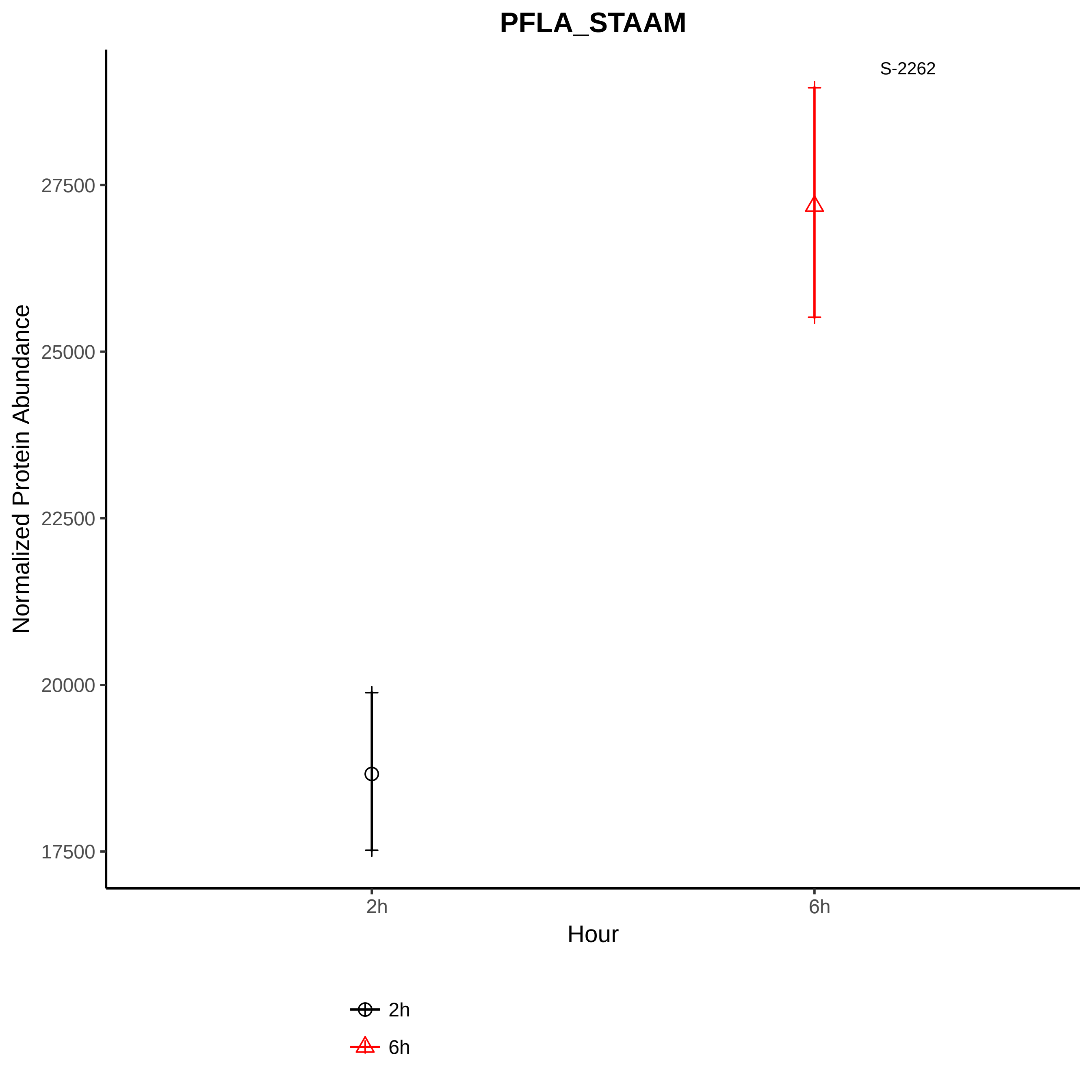

PFLA_STAAM
S-2262
Normalized Protein Abundance
27500
25000
22500
20000
17500
2h
6h
Hour
2h
6h

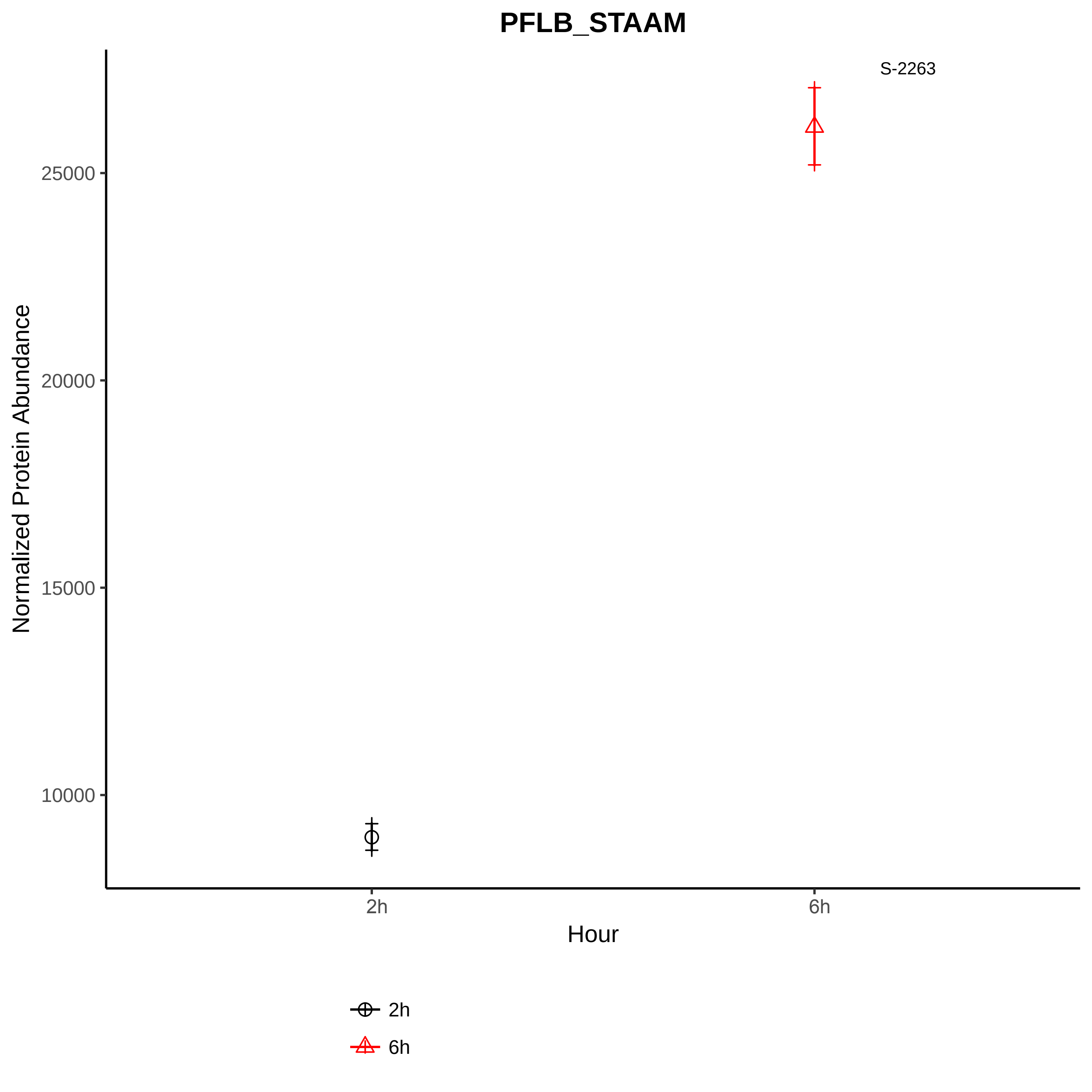
PFLB_STAAM
S-2263
Normalized Protein Abundance
25000
20000
15000
10000
2h
6h
Hour
2h
6h

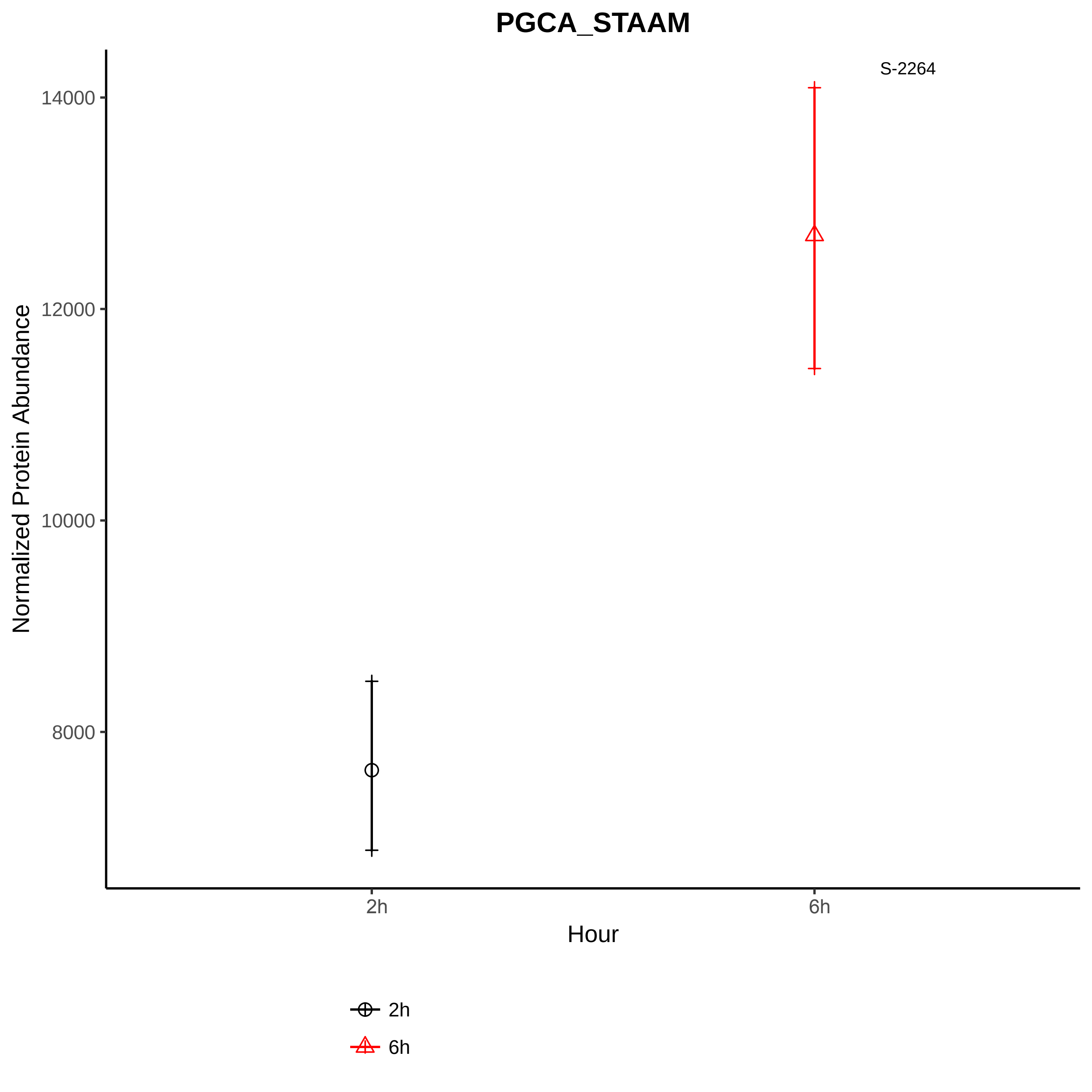

PGCA_STAAM
S-2264
14000
12000
10000
8000
Normalized Protein Abundance
2h
6h
Hour
2h
6h

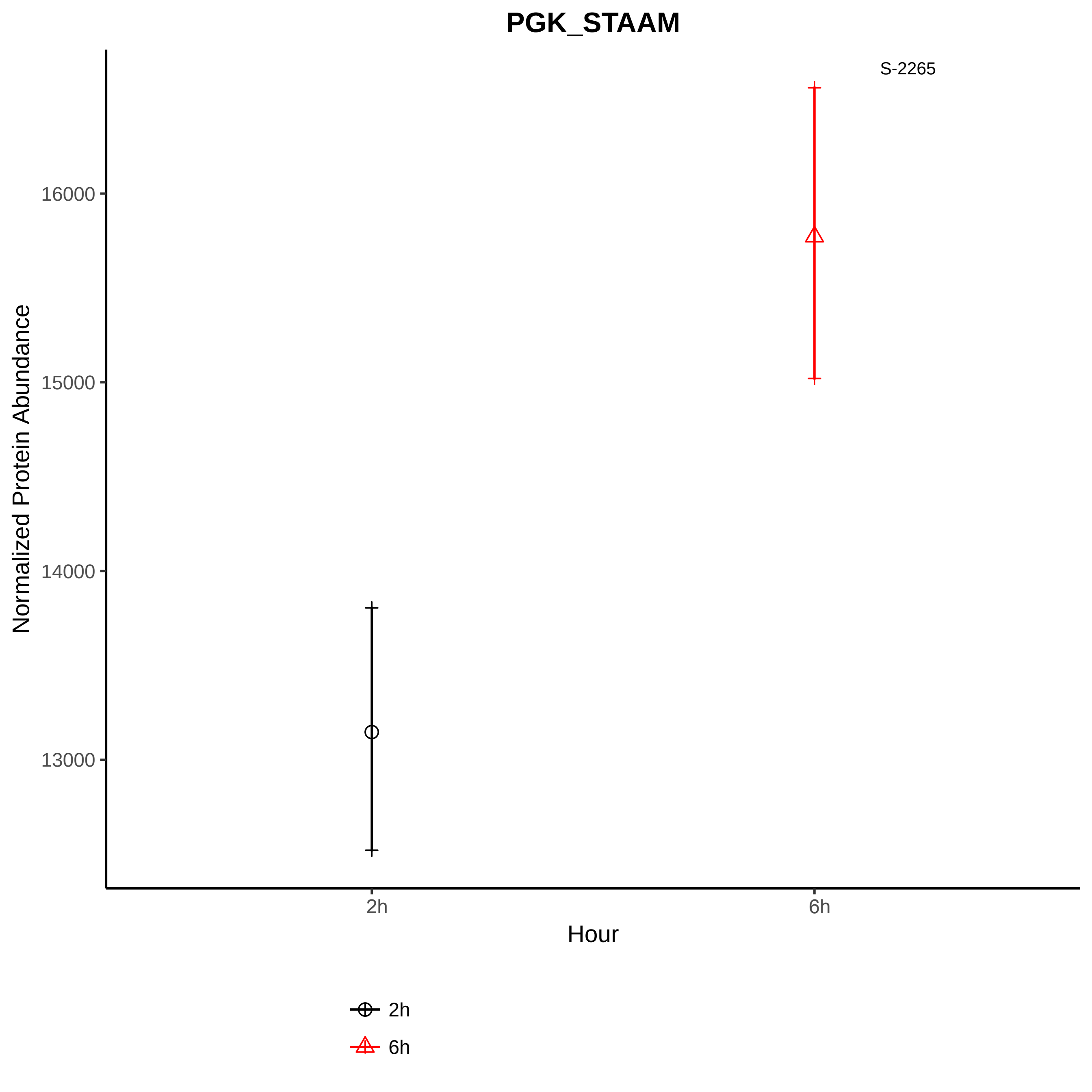

PGK_STAAM
S-2265
Normalized Protein Abundance
16000
15000
14000
13000
2h
6h
Hour
2h
6h

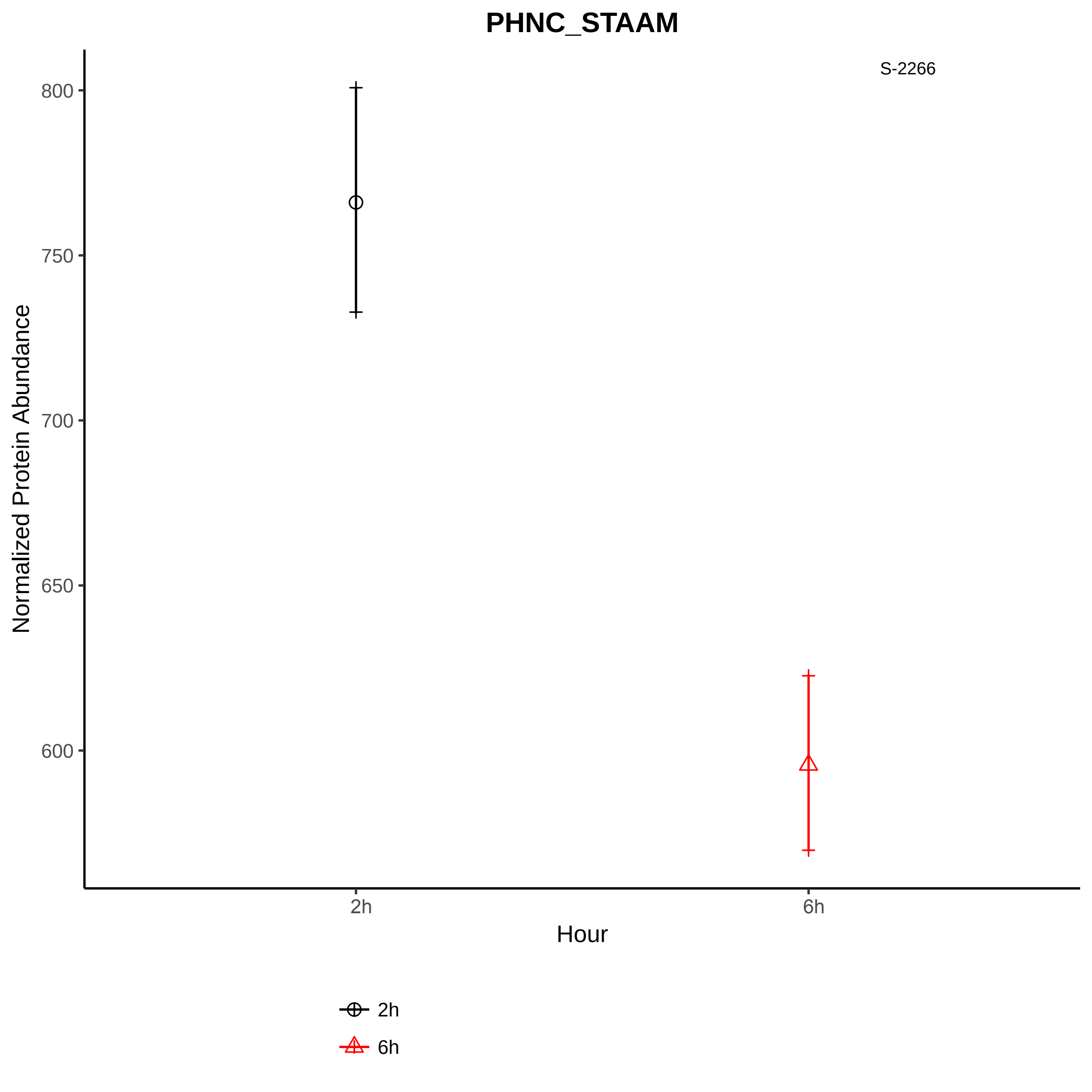

PHNC_STAAM
S-2266
Normalized Protein Abundance
800
750
700
650
600
2h
6h
Hour
2h
6h

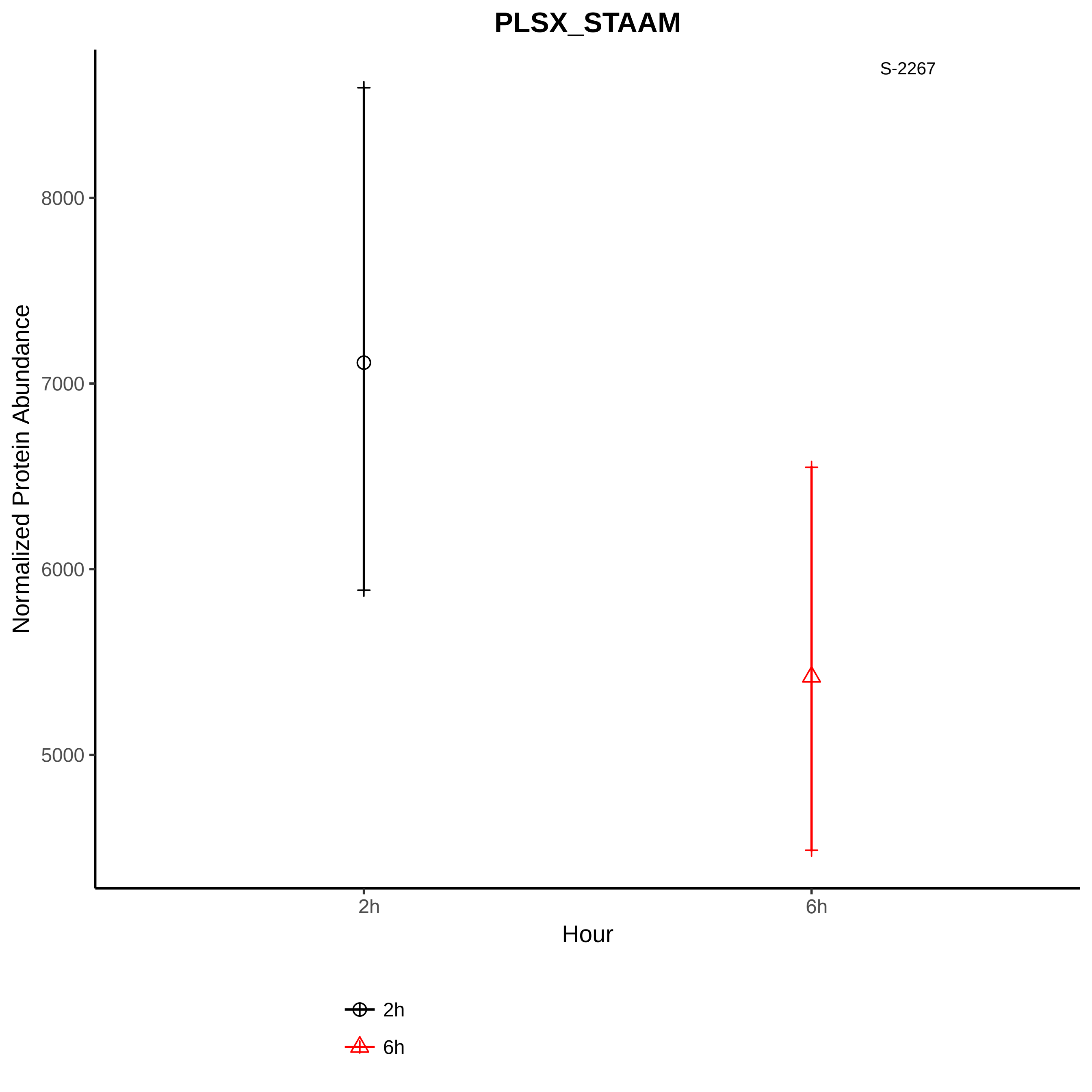
PLSX_STAAM
S-2267
Normalized Protein Abundance
8000
7000
6000
5000
2h
6h
Hour
2h
6h

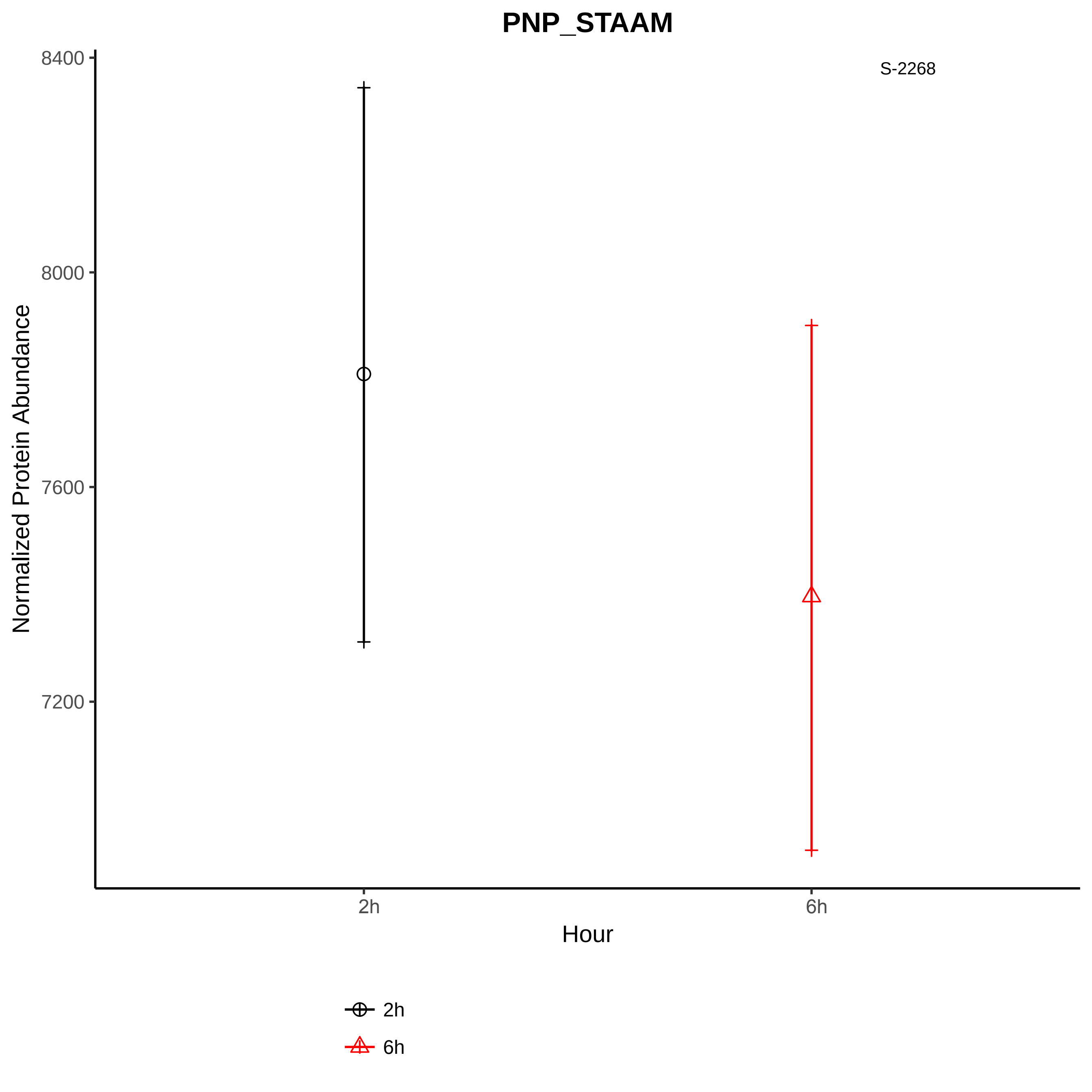
PNP_STAAM
S-2268
8400
8000
7600
7200
Normalized Protein Abundance
2h
6h
Hour
2h
6h

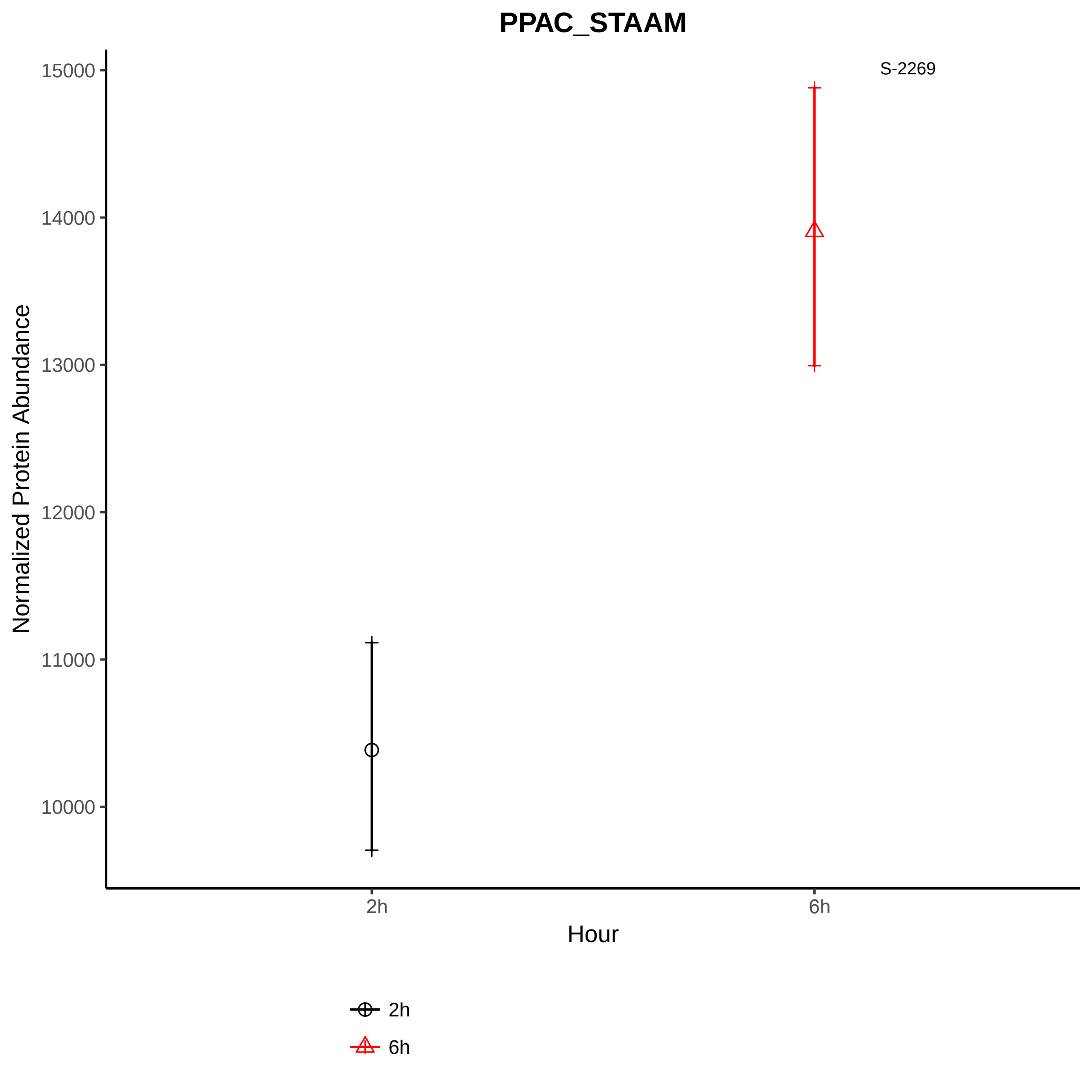

PPAC_STAAM
S-2269
15000
14000
13000
12000
11000
10000
Normalized Protein Abundance
2h
6h
Hour
2h
6h

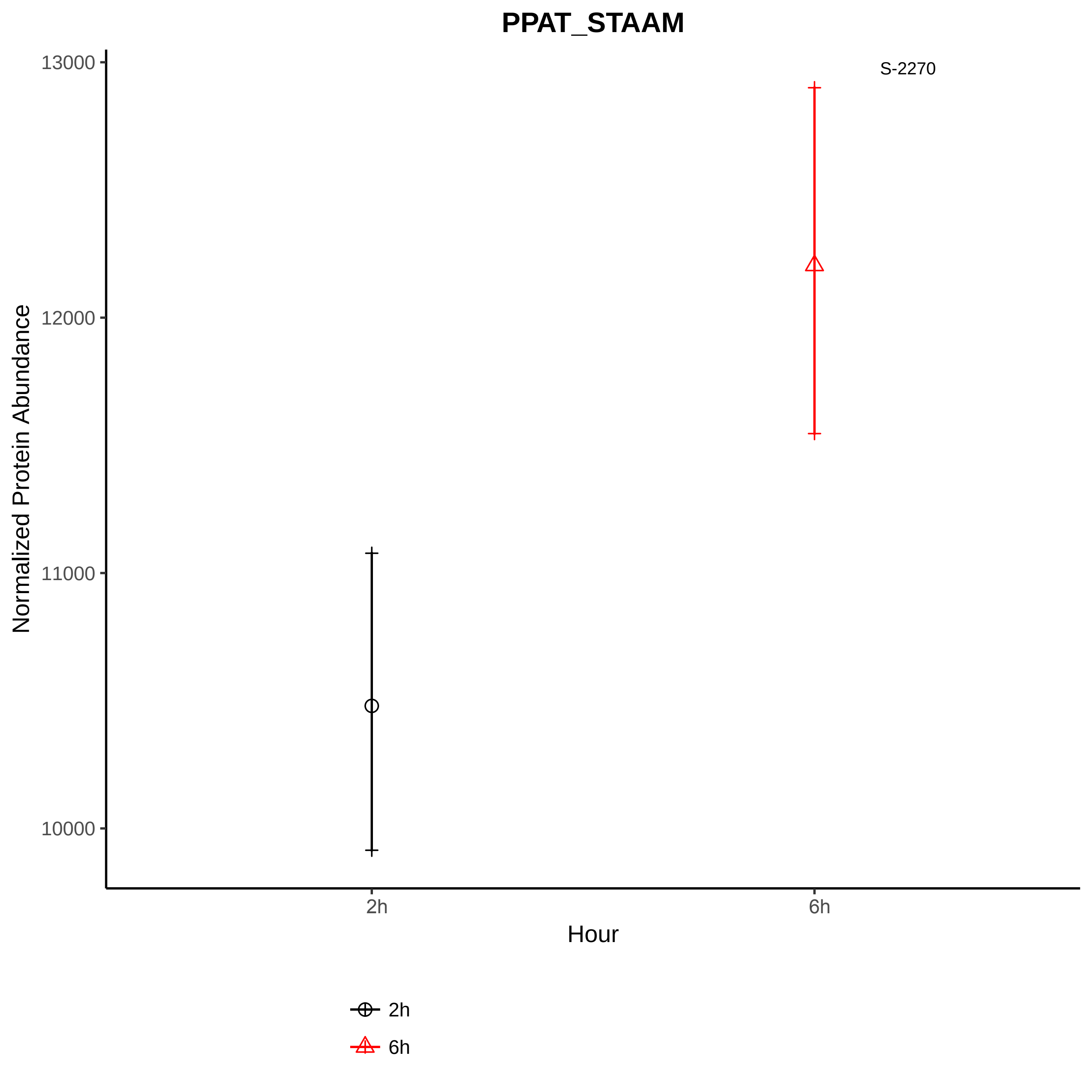

PPAT_STAAM
S-2270
Normalized Protein Abundance
13000
12000
11000
10000
2h
6h
Hour
2h
6h

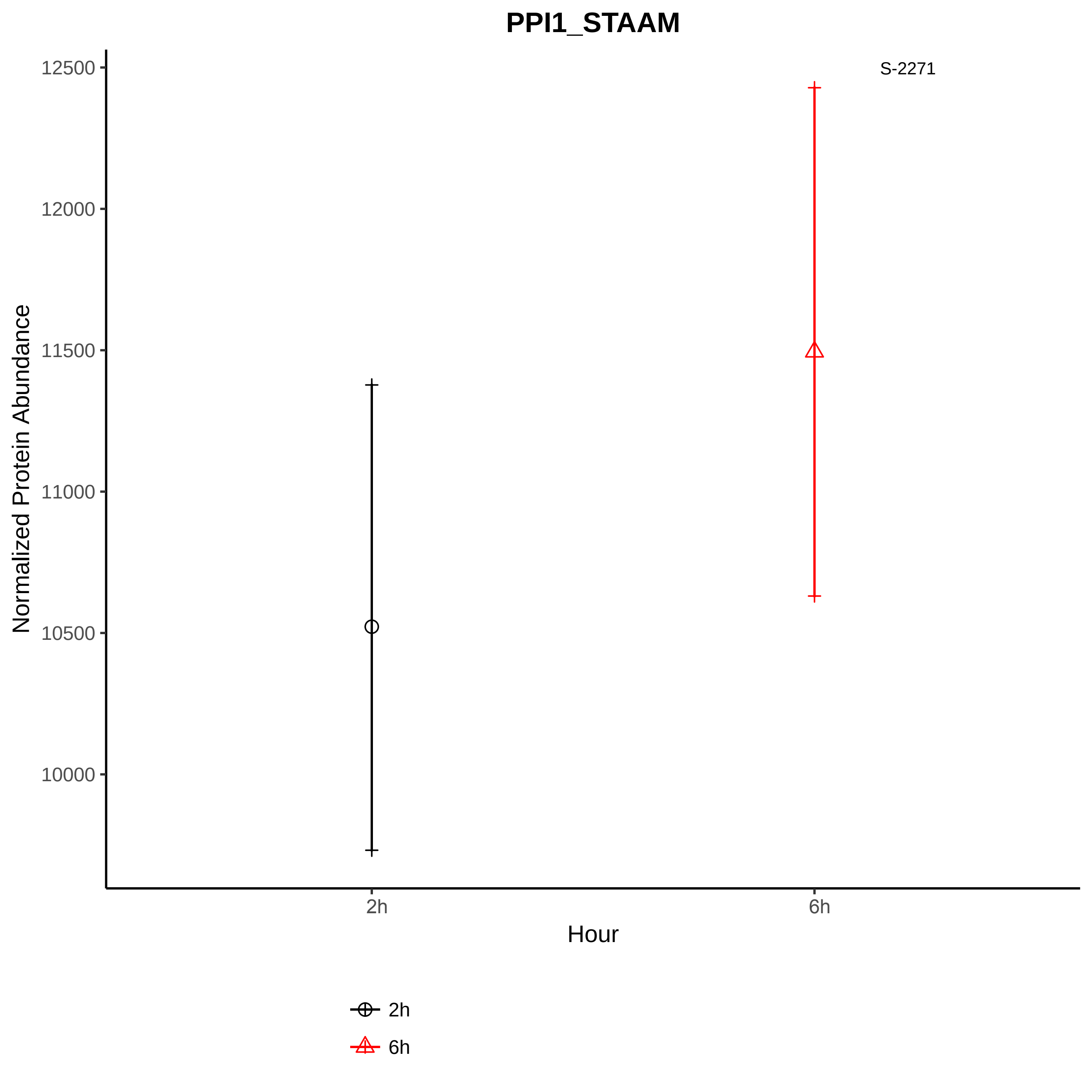

PPI1_STAAM
S-2271
12500
12000
11500
11000
10500
10000
Normalized Protein Abundance
2h
6h
Hour
2h
6h

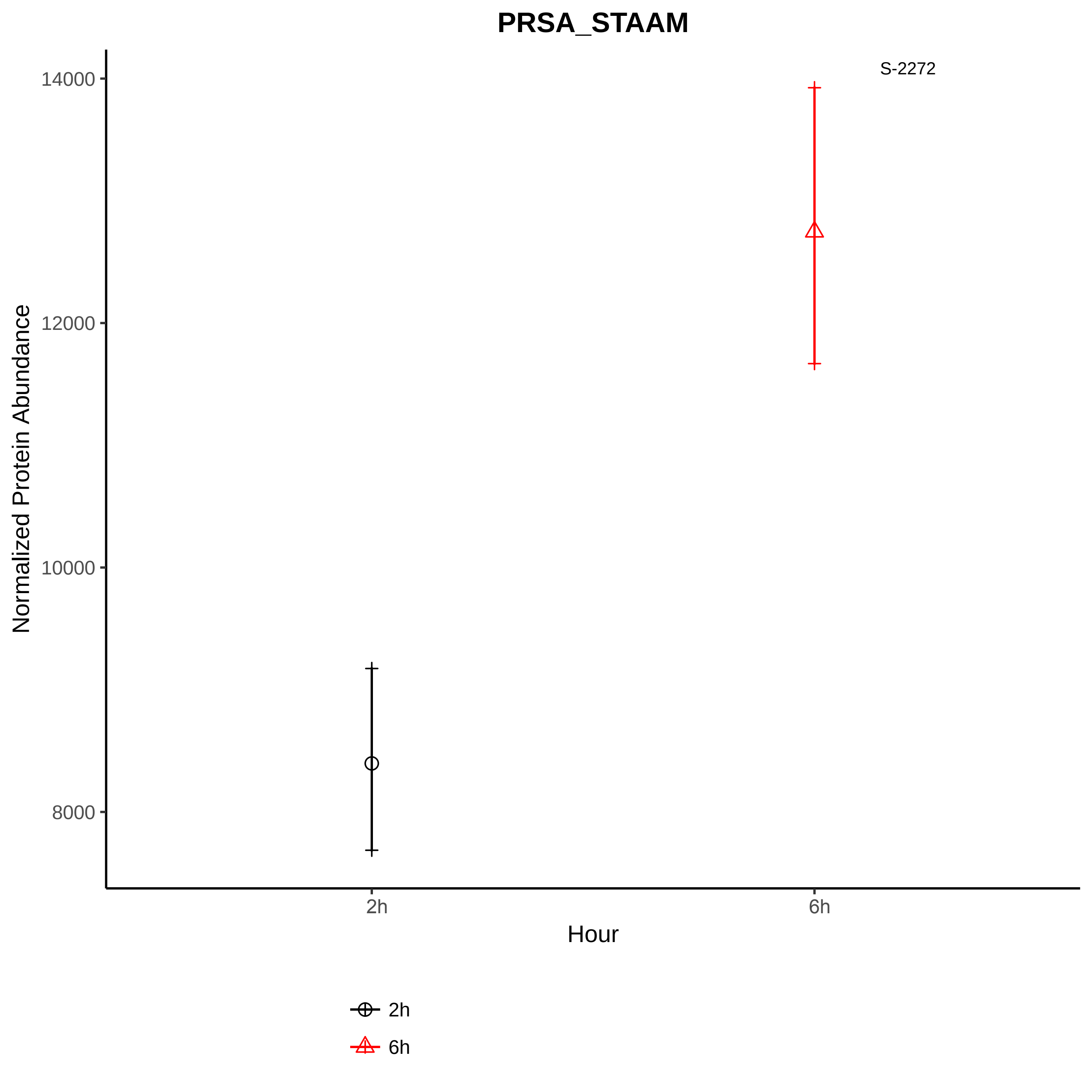

PRSA_STAAM
S-2272
Normalized Protein Abundance
14000
12000
10000
8000
2h
6h
Hour
2h
6h

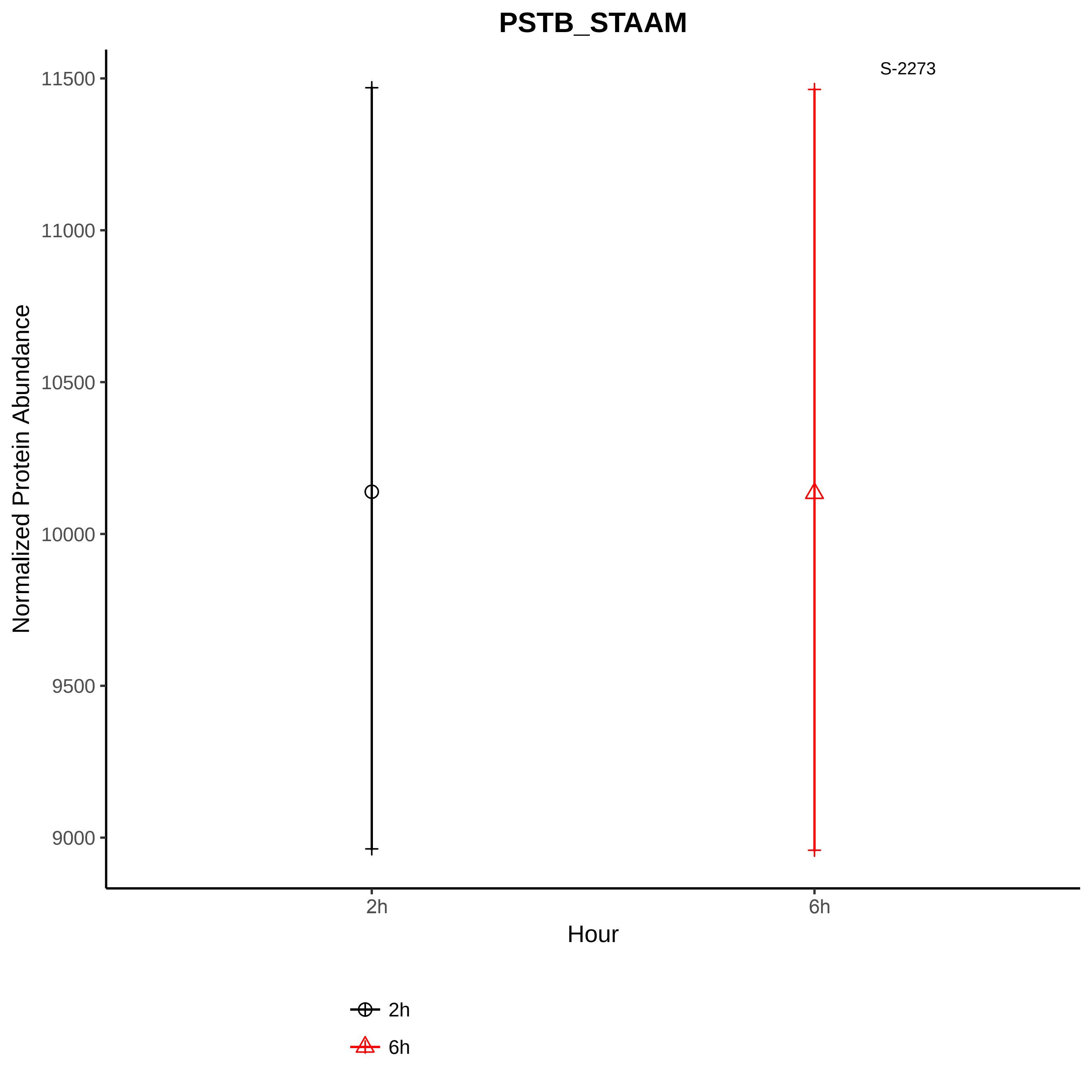

PSTB_STAAM
S-2273
Normalized Protein Abundance
11500
11000
10500
10000
9500
9000
2h
6h
Hour
2h
6h

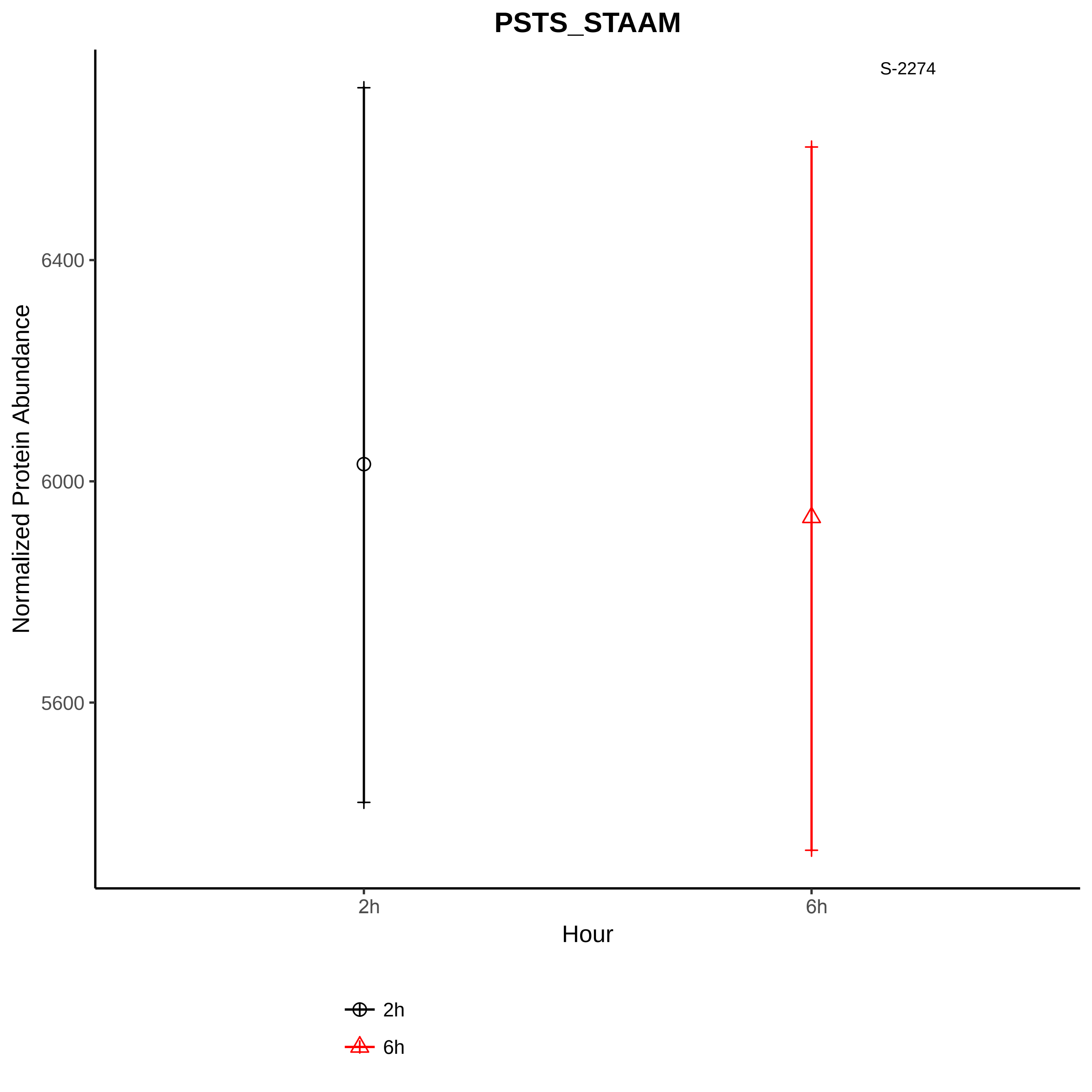
PSTS_STAAM
S-2274
Normalized Protein Abundance
6400
6000
5600
2h
6h
Hour
2h
6h

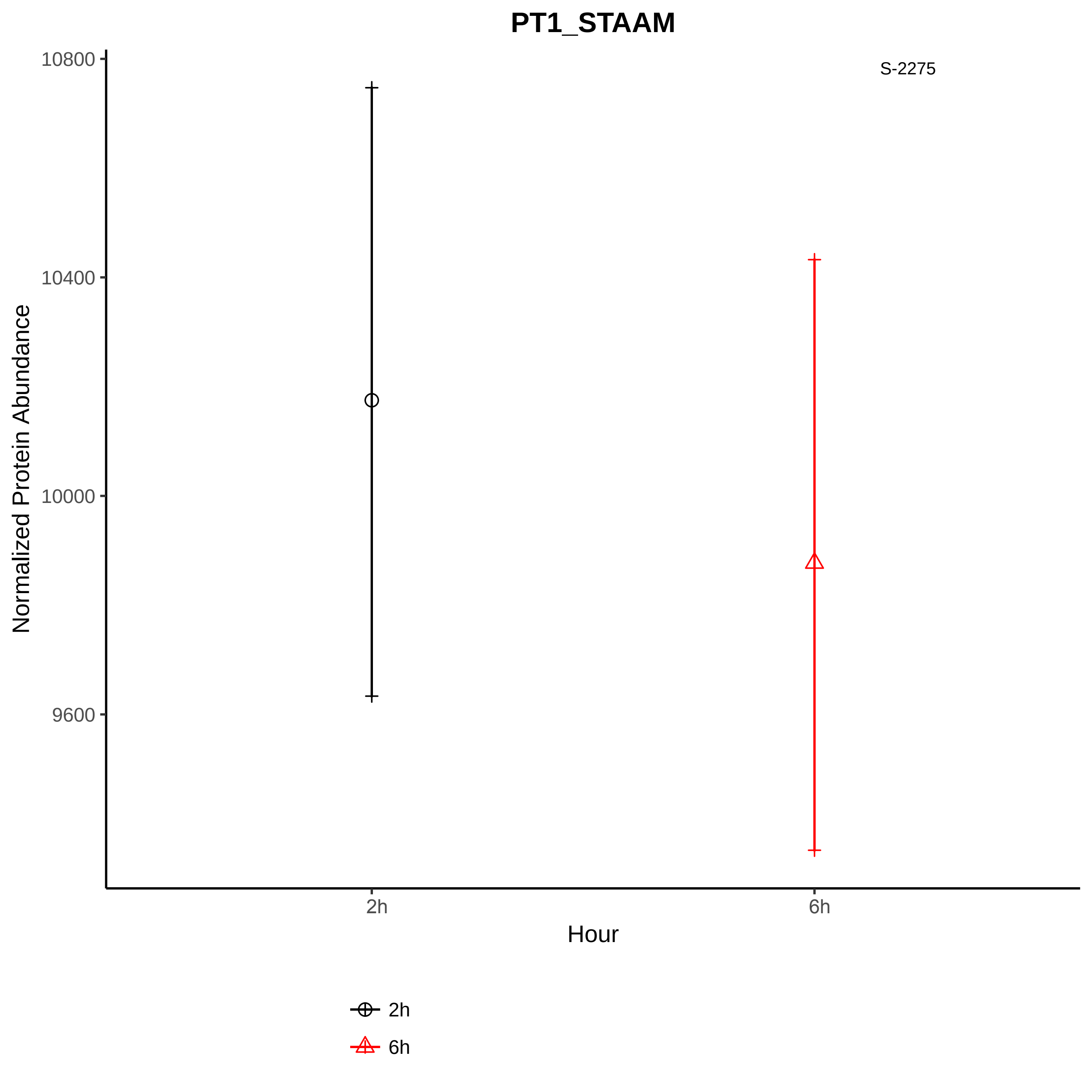
PT1_STAAM
S-2275
Normalized Protein Abundance
10800
10400
10000
9600
2h
6h
Hour
2h
6h

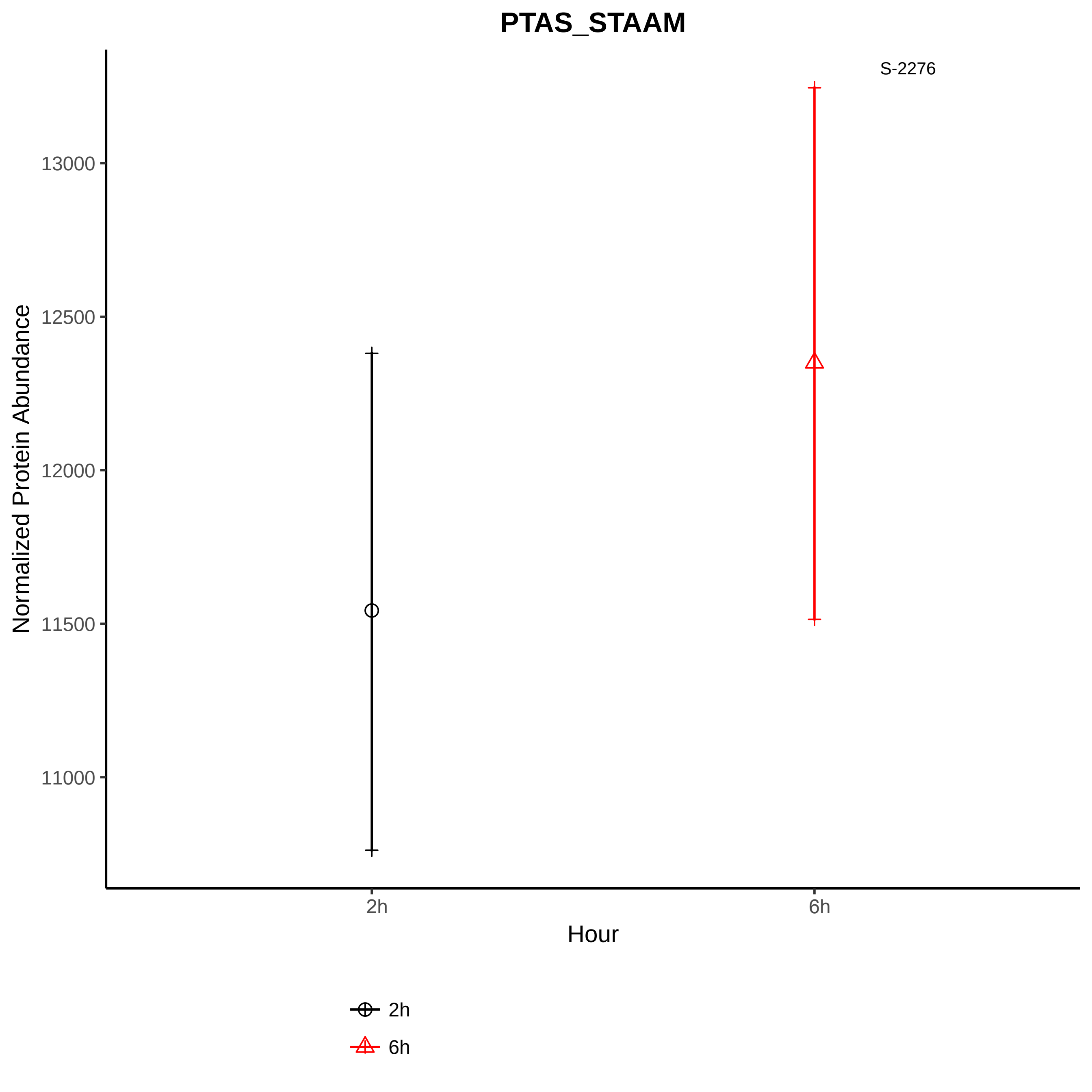

PTAS_STAAM
S-2276
13000
12500
12000
11500
11000
Normalized Protein Abundance
2h
6h
Hour
2h
6h

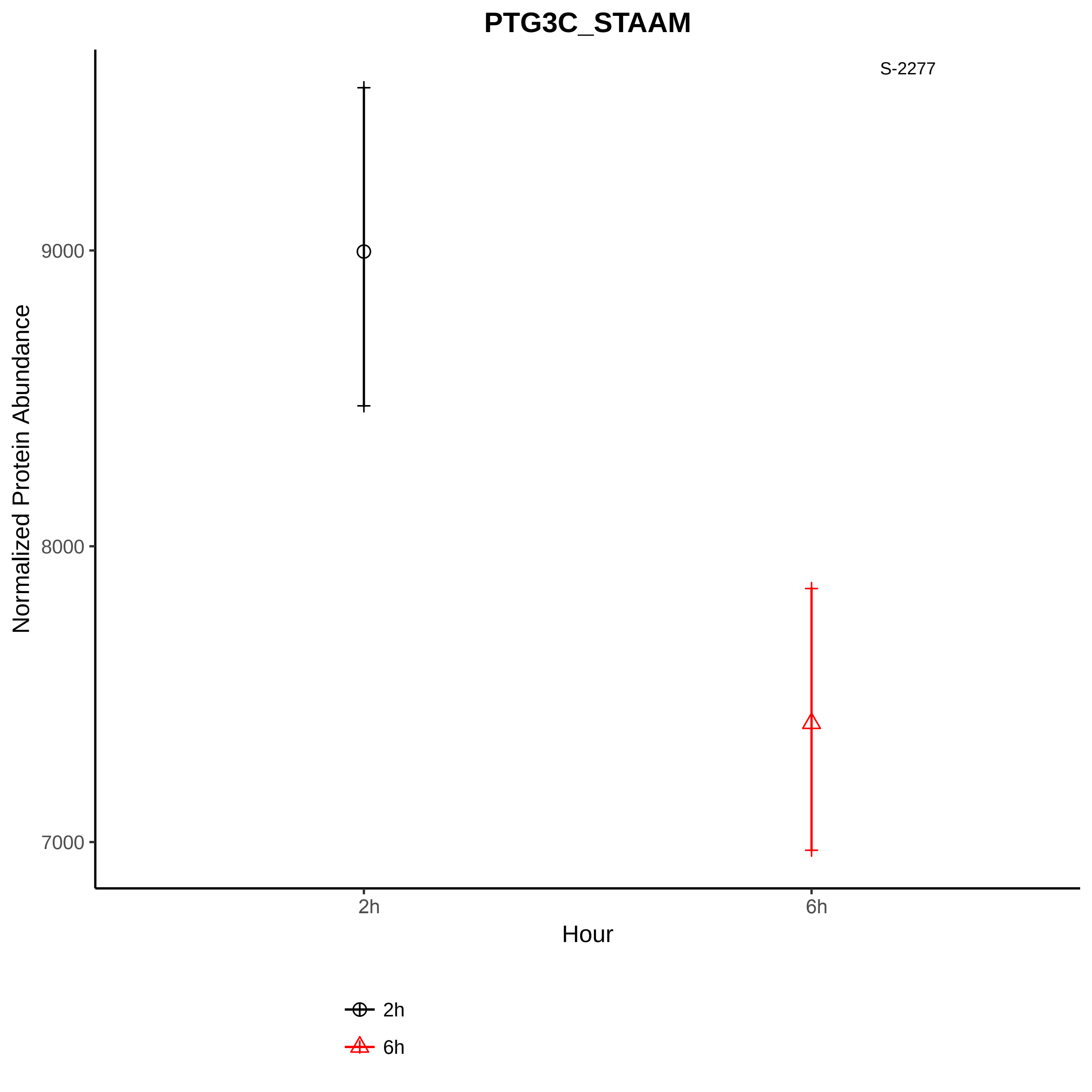
PTG3C_STAAM
S-2277
Normalized Protein Abundance
9000
8000
7000
2h
6h
Hour
2h
6h

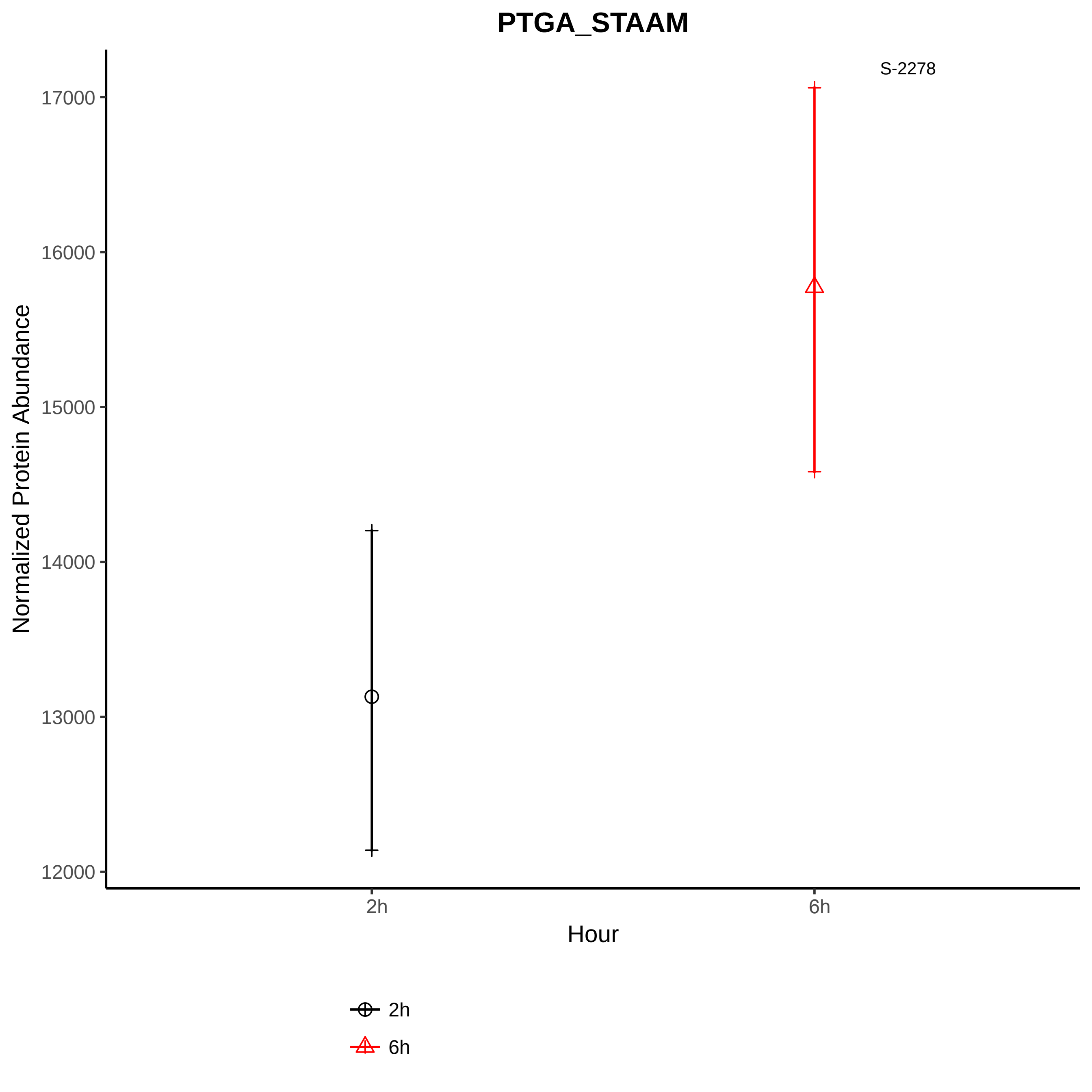
PTGA_STAAM
S-2278
Normalized Protein Abundance
17000
16000
15000
14000
13000
12000
2h
6h
Hour
2h
6h

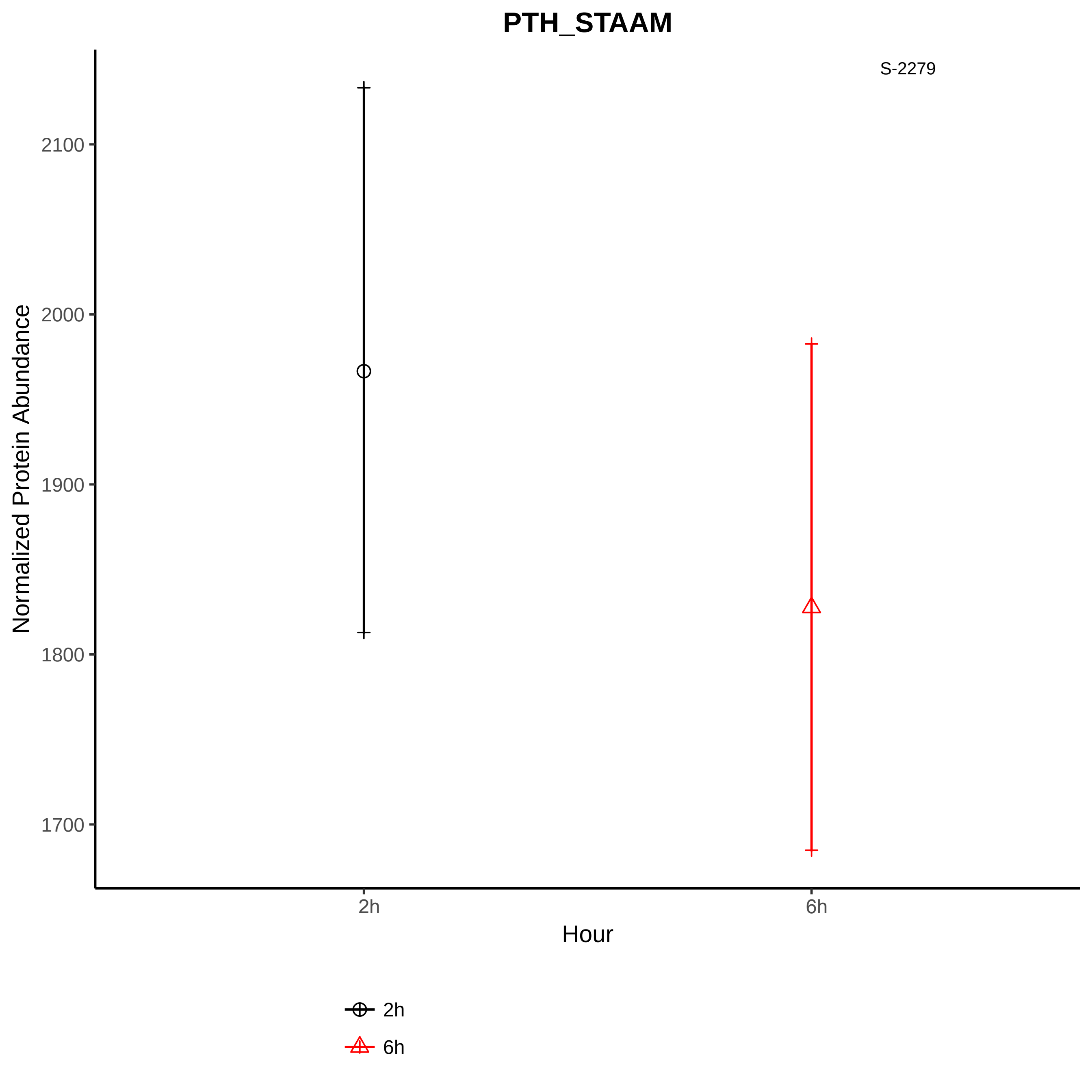

PTH_STAAM
S-2279
2100
2000
1900
1800
1700
Normalized Protein Abundance
2h
6h
Hour
2h
6h

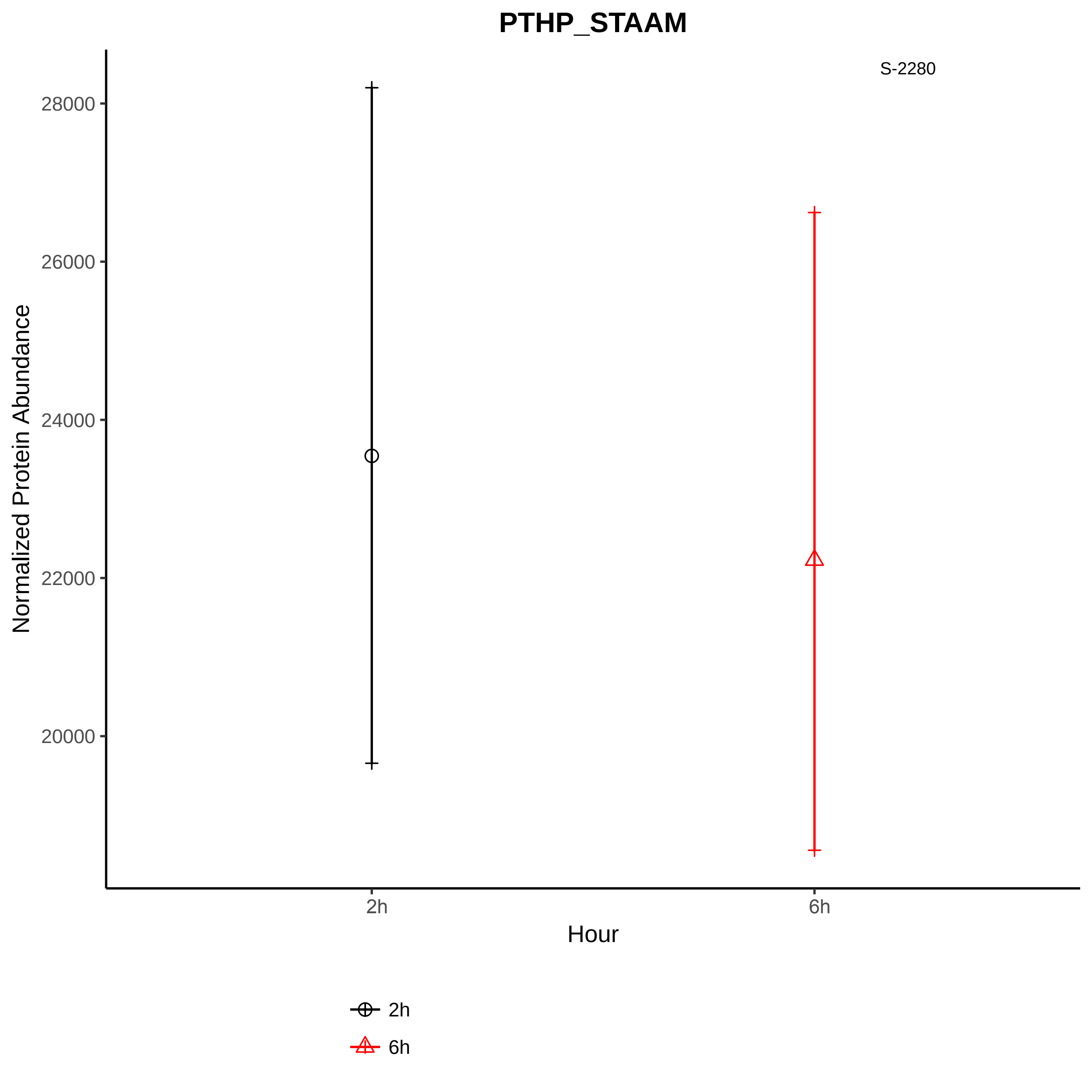

PTHP_STAAM
S-2280
28000
26000
24000
22000
20000
Normalized Protein Abundance
2h
6h
Hour
2h
6h

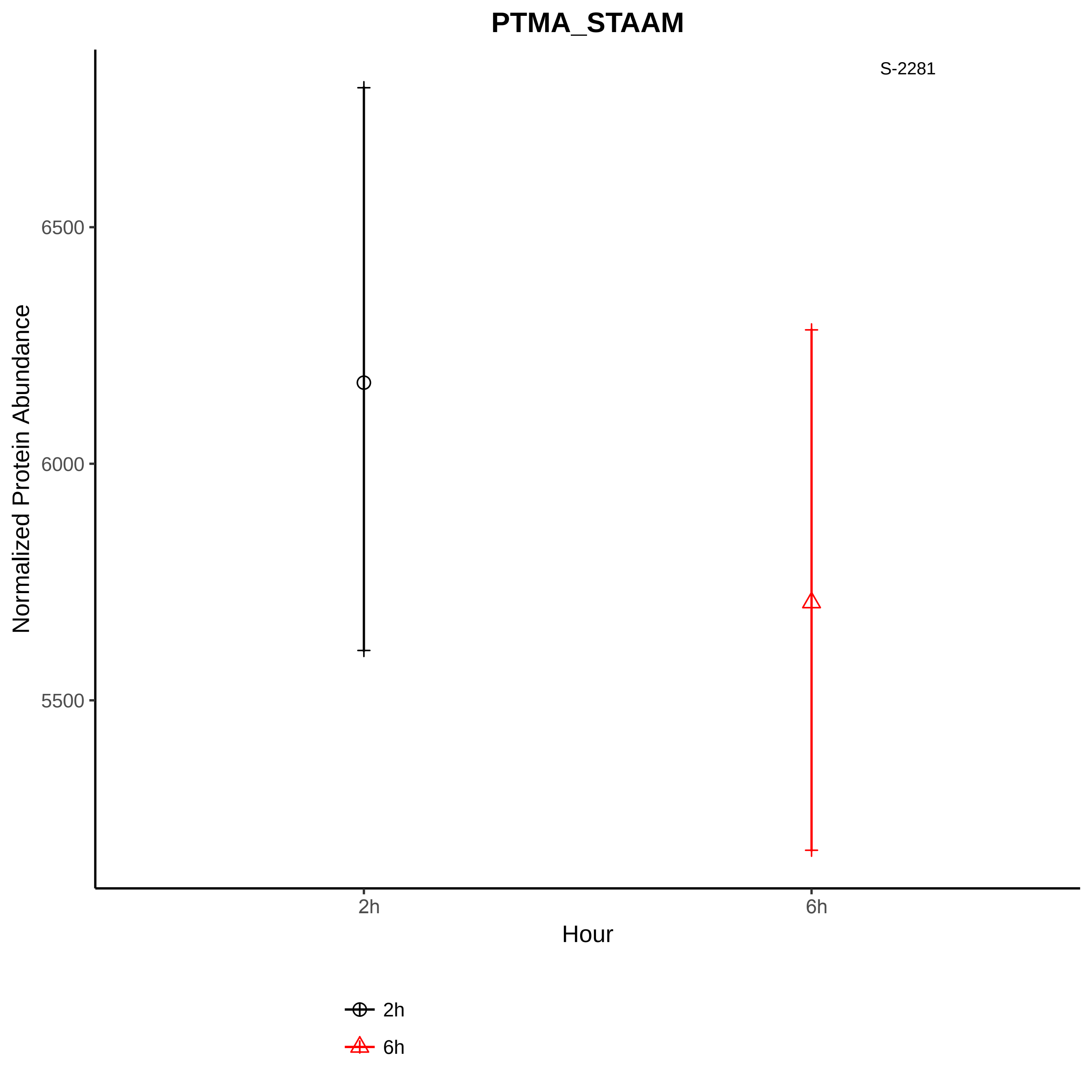

PTMA_STAAM
S-2281
6500
6000
5500
Normalized Protein Abundance
2h
6h
Hour
2h
6h

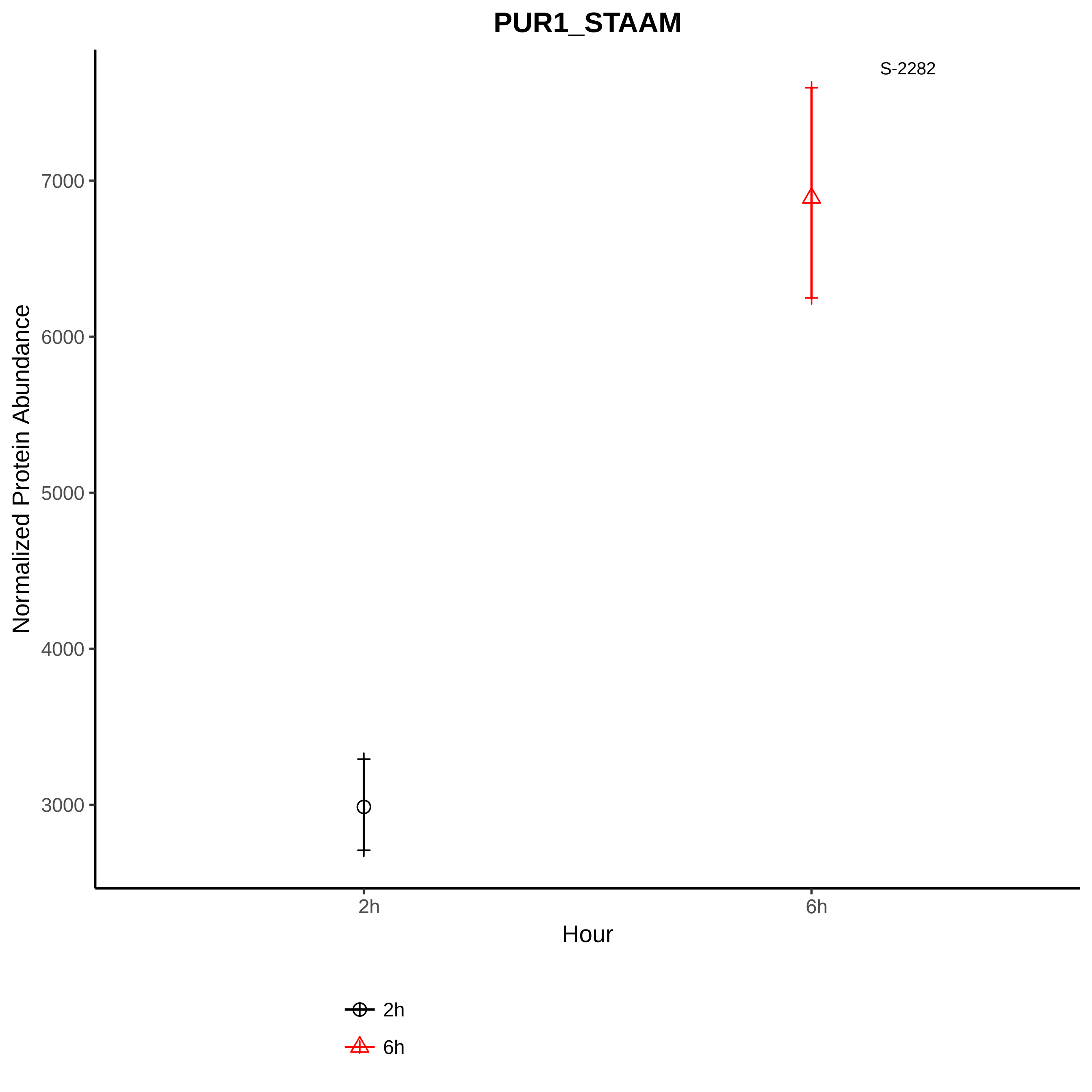
PUR1_STAAM
S-2282
Normalized Protein Abundance
7000
6000
5000
4000
3000
2h
6h
Hour
2h
6h

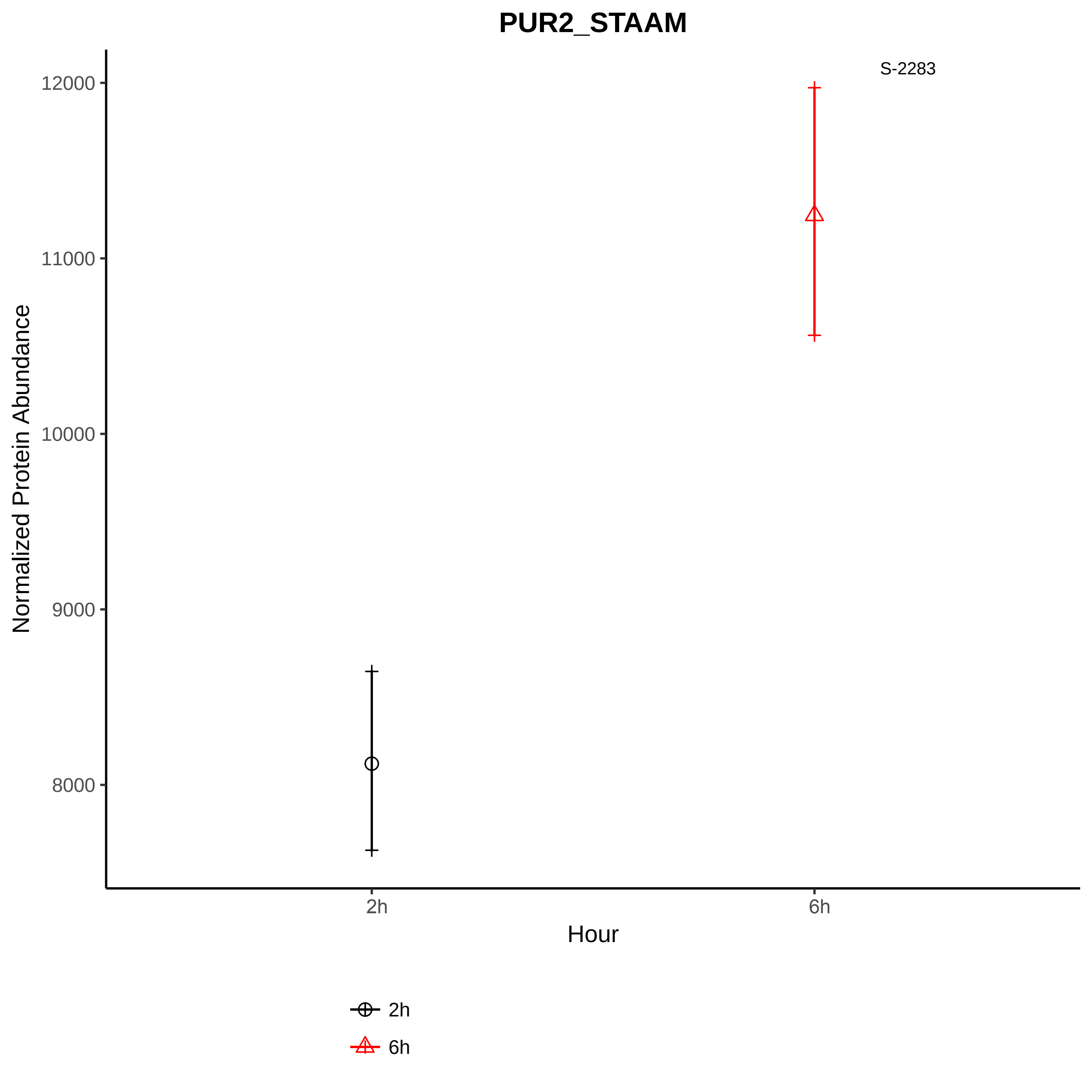
PUR2_STAAM
S-2283
Normalized Protein Abundance
12000
11000
10000
9000
8000
2h
6h
Hour
2h
6h

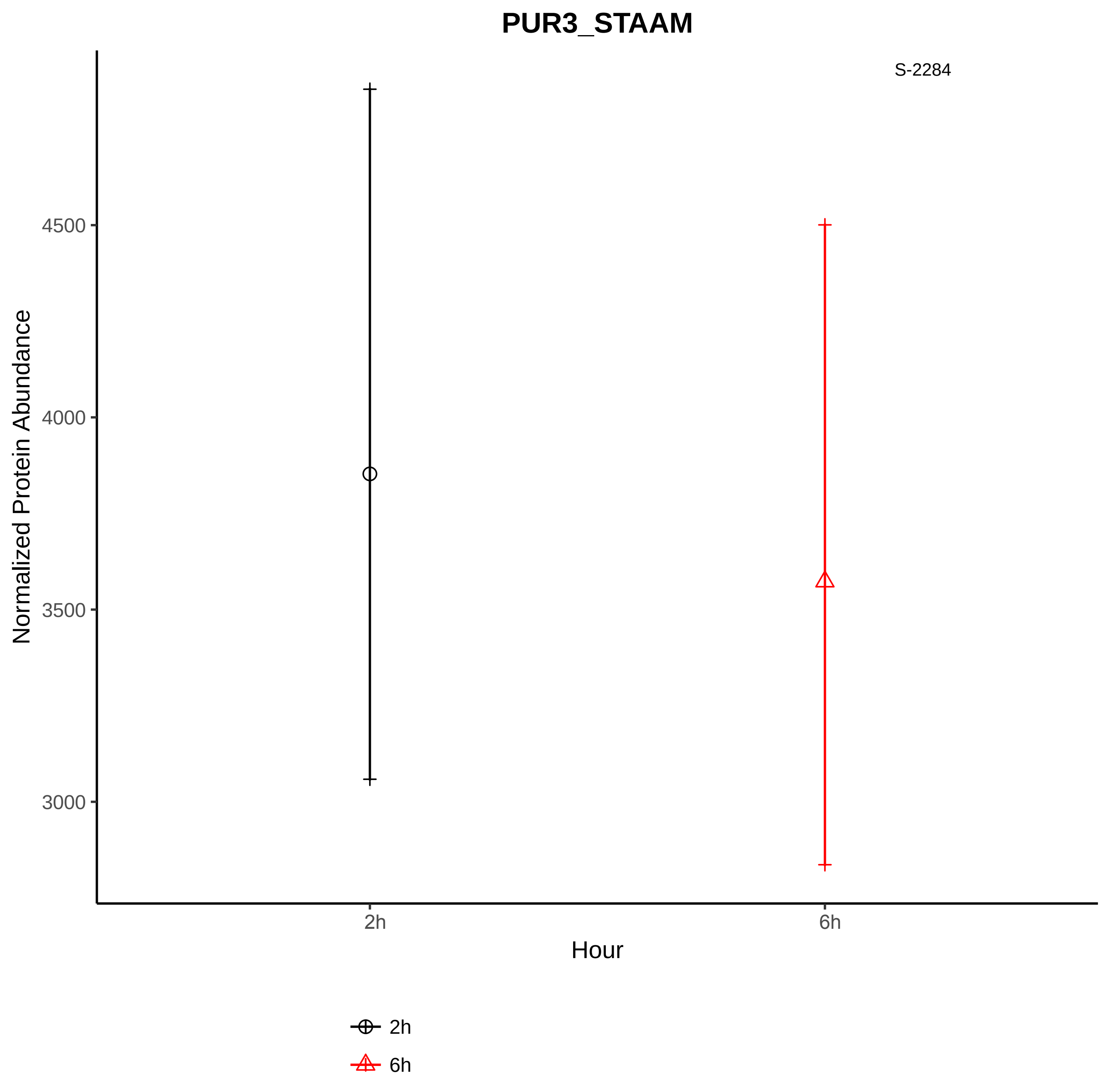
PUR3_STAAM
S-2284
Normalized Protein Abundance
4500
4000
3500
3000
2h
6h
Hour
2h
6h

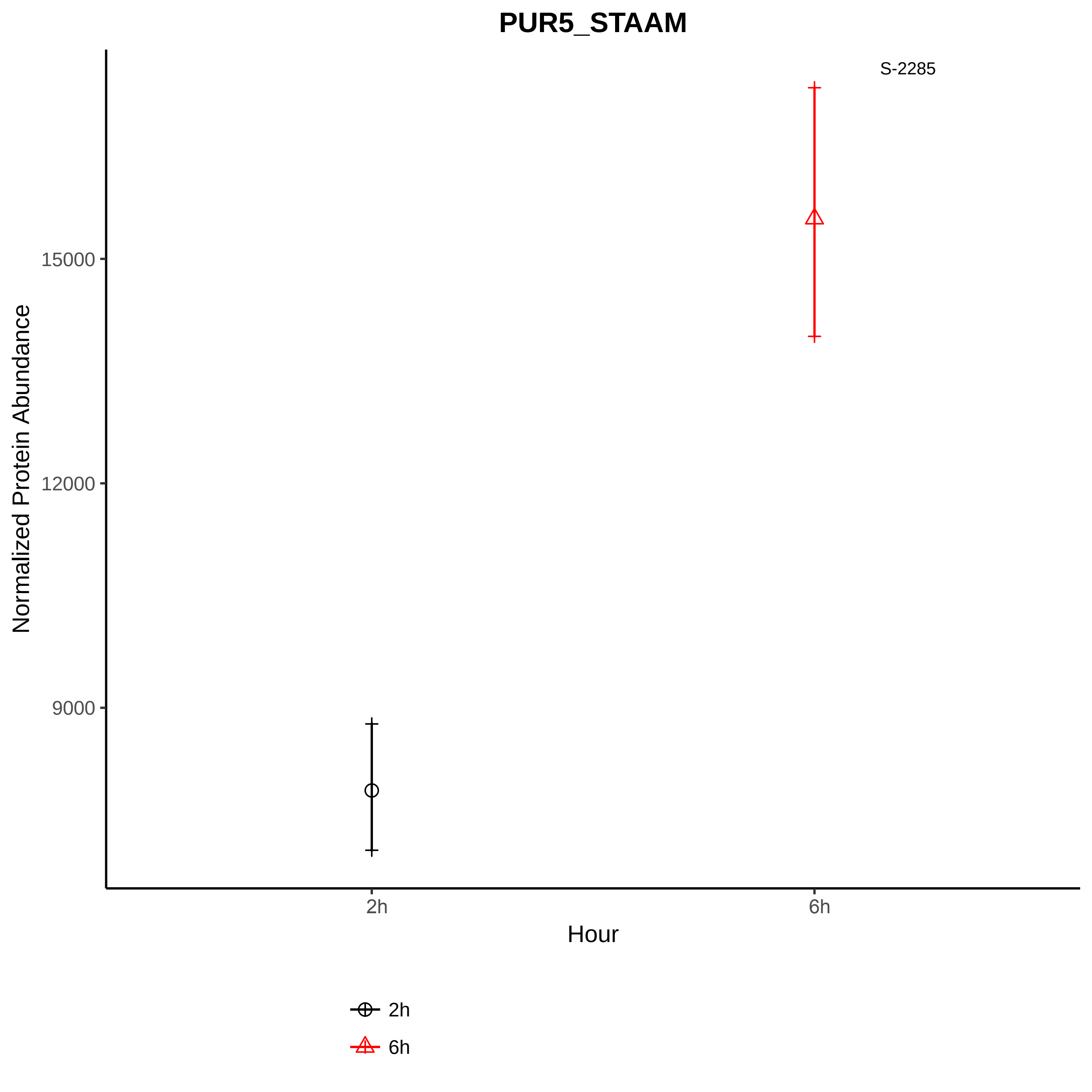

PUR5_STAAM
S-2285
Normalized Protein Abundance
15000
12000
9000
2h
6h
Hour
2h
6h

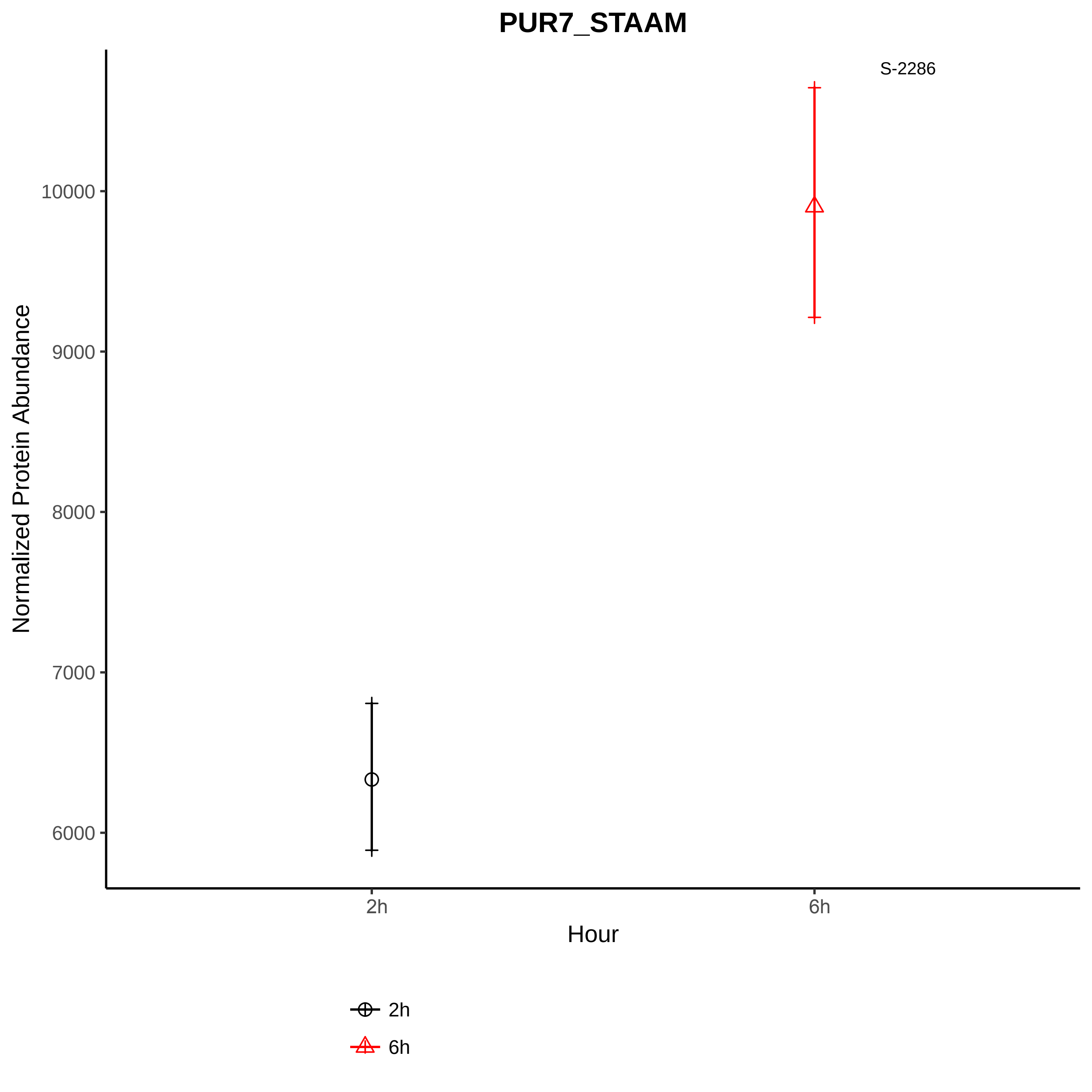

PUR7_STAAM
S-2286
Normalized Protein Abundance
10000
9000
8000
7000
6000
2h
6h
Hour
2h
6h

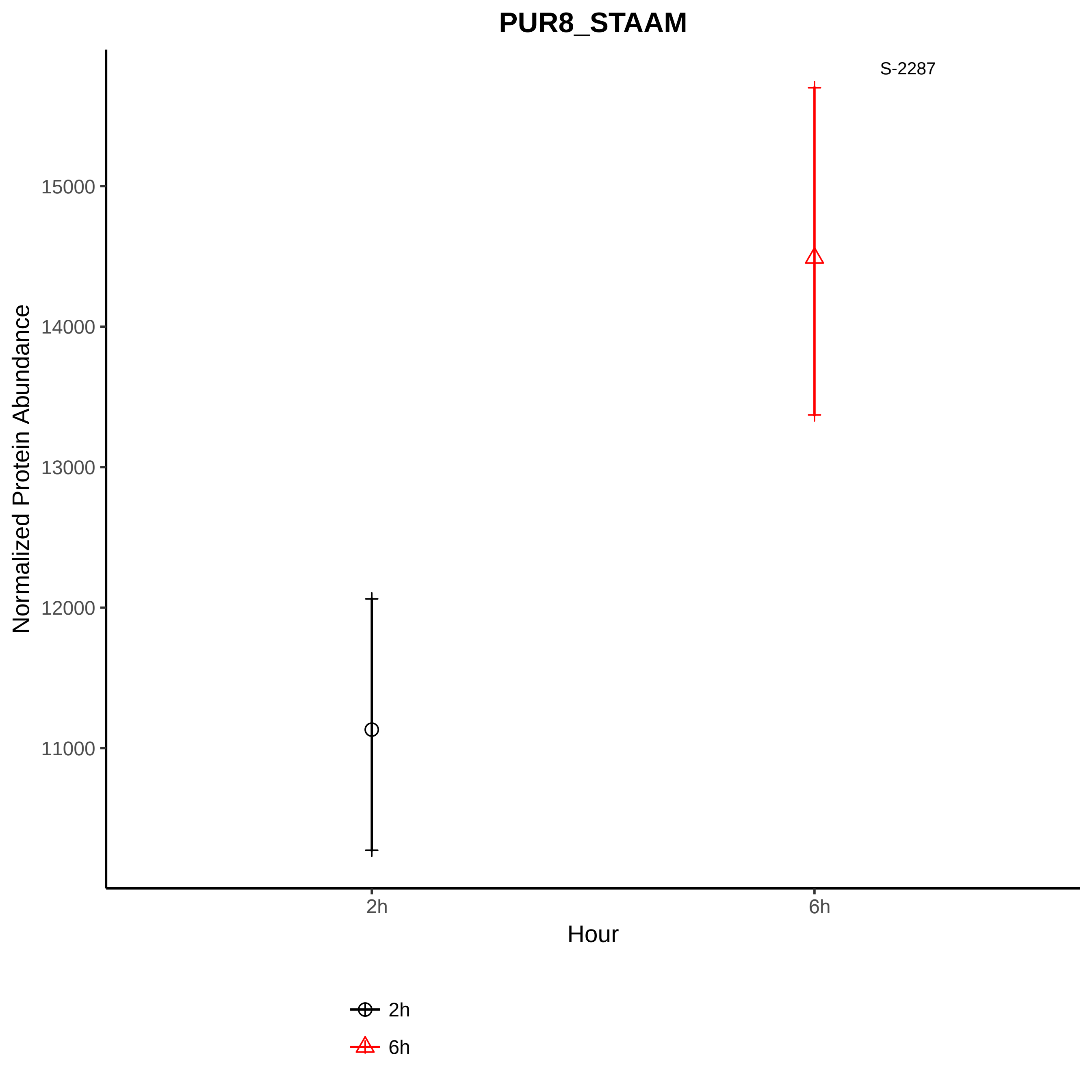

PUR8_STAAM
S-2287
Normalized Protein Abundance
15000
14000
13000
12000
11000
2h
6h
Hour
2h
6h

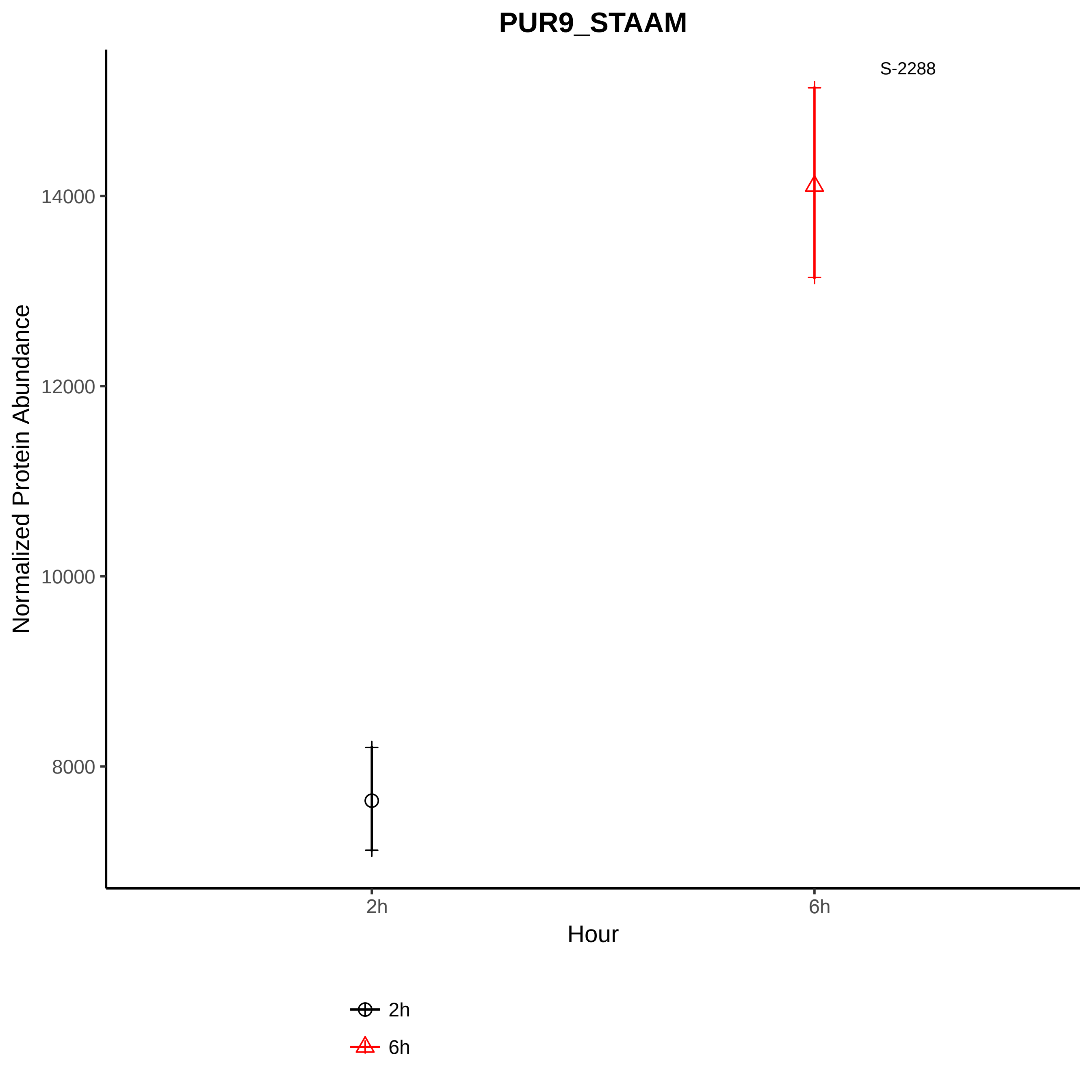

PUR9_STAAM
S-2288
Normalized Protein Abundance
14000
12000
10000
8000
2h
6h
Hour
2h
6h

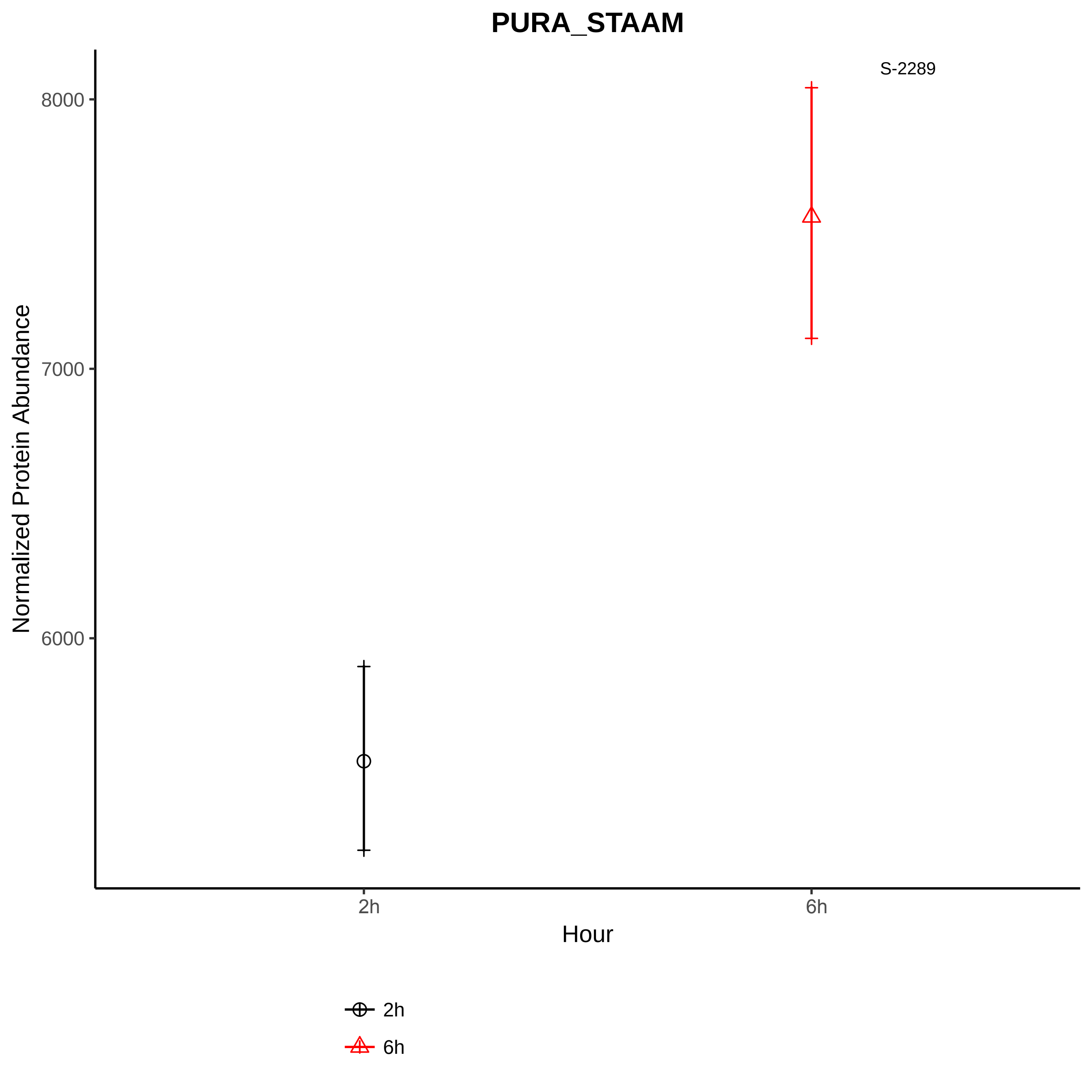

PURA_STAAM
S-2289
8000
7000
6000
Normalized Protein Abundance
2h
6h
Hour
2h
6h

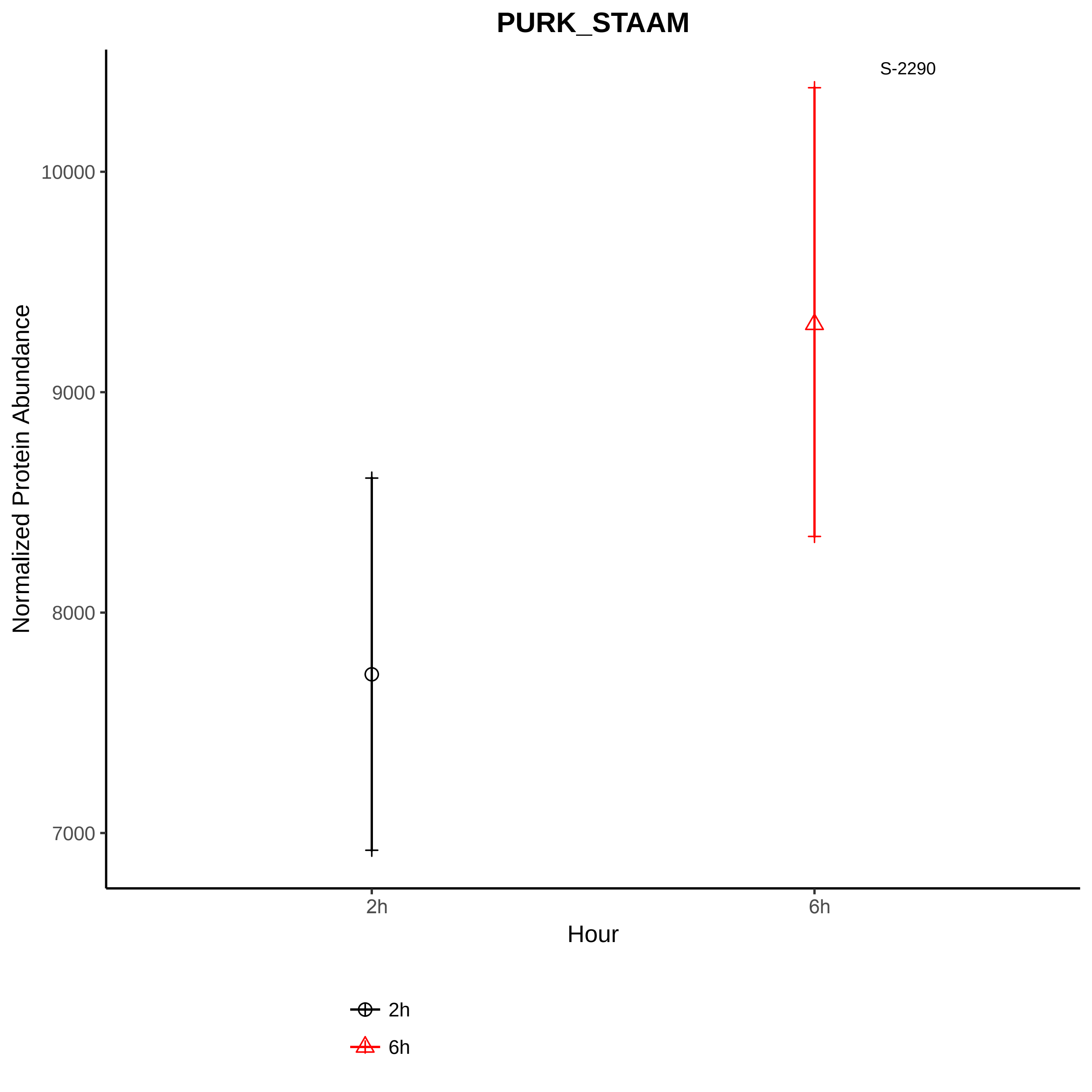

PURK_STAAM
S-2290
Normalized Protein Abundance
10000
9000
8000
7000
2h
6h
Hour
2h
6h

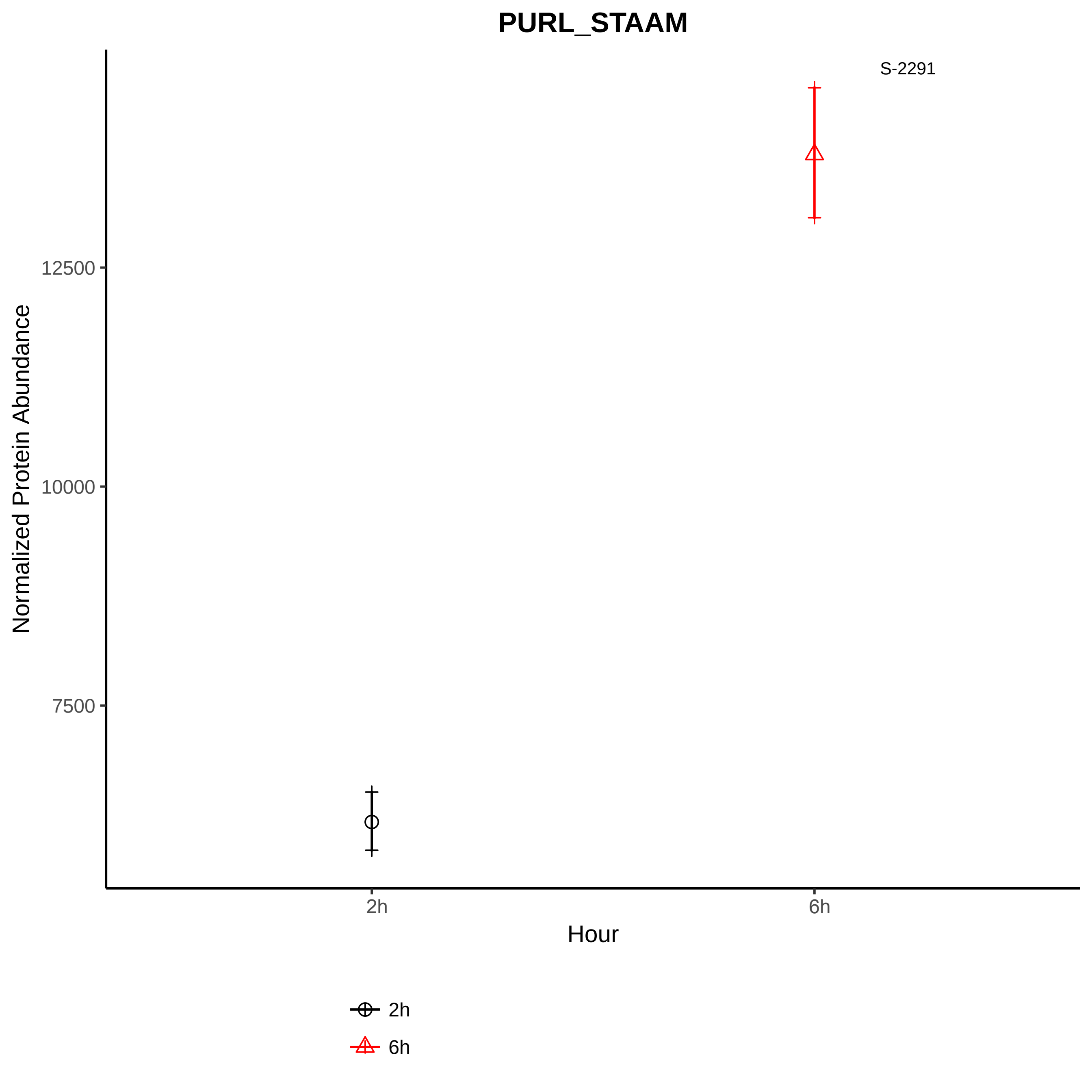
PURL_STAAM
S-2291
Normalized Protein Abundance
12500
10000
7500
2h
6h
Hour
2h
6h

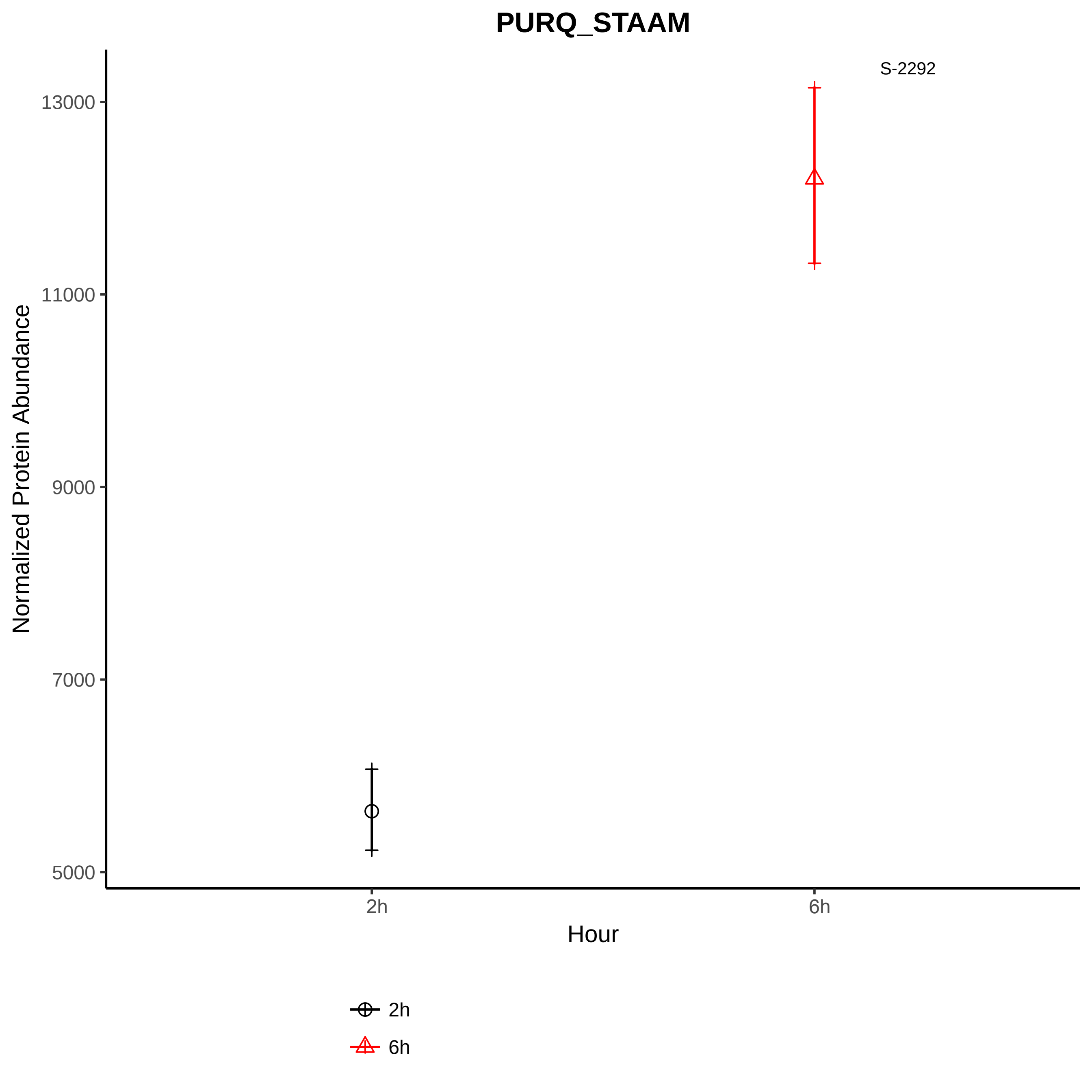
PURQ_STAAM
S-2292
Normalized Protein Abundance
13000
11000
9000
7000
5000
2h
6h
Hour
2h
6h

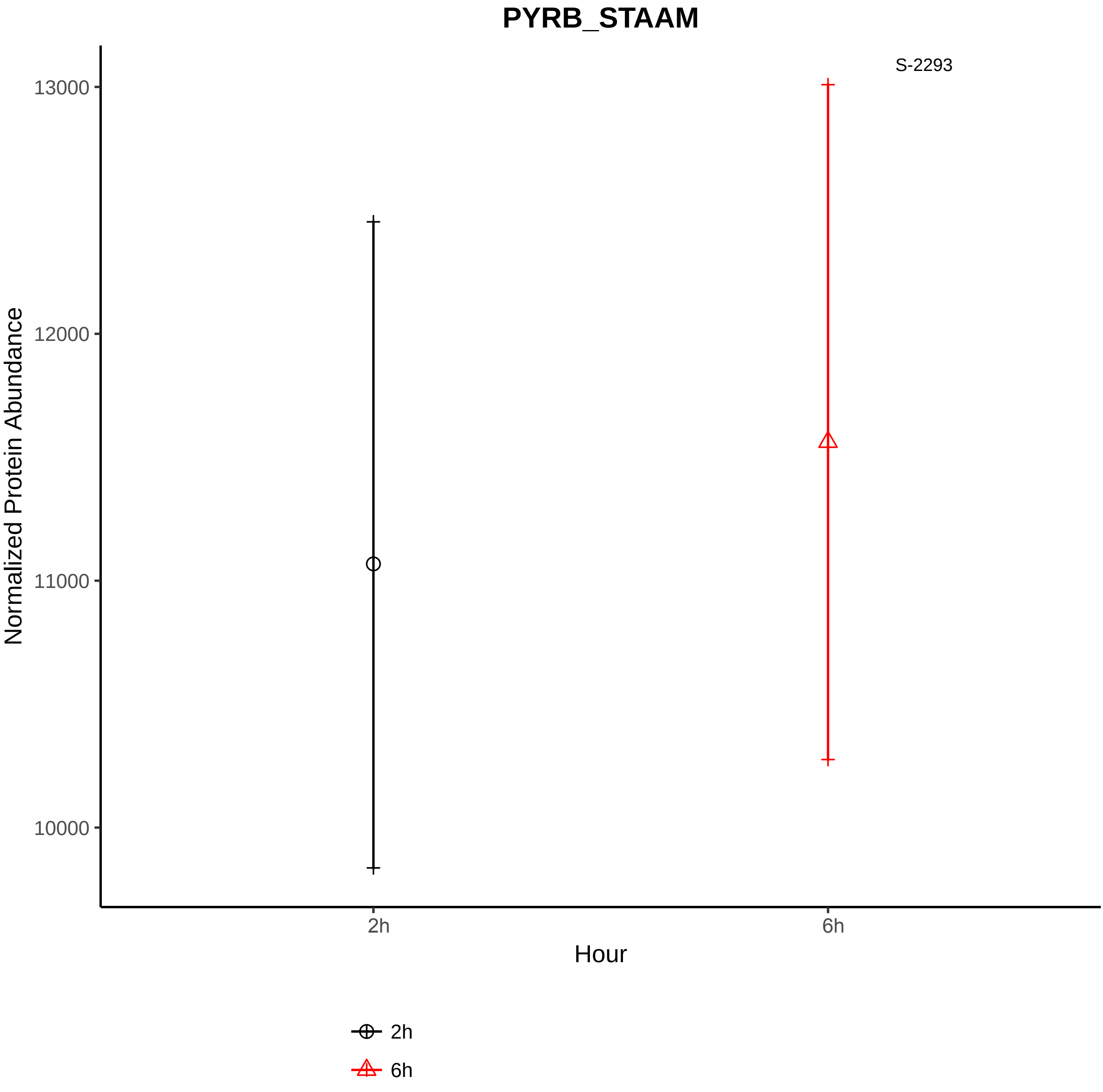

PYRB_STAAM
S-2293
13000
12000
11000
10000
Normalized Protein Abundance
2h
6h
Hour
2h
6h

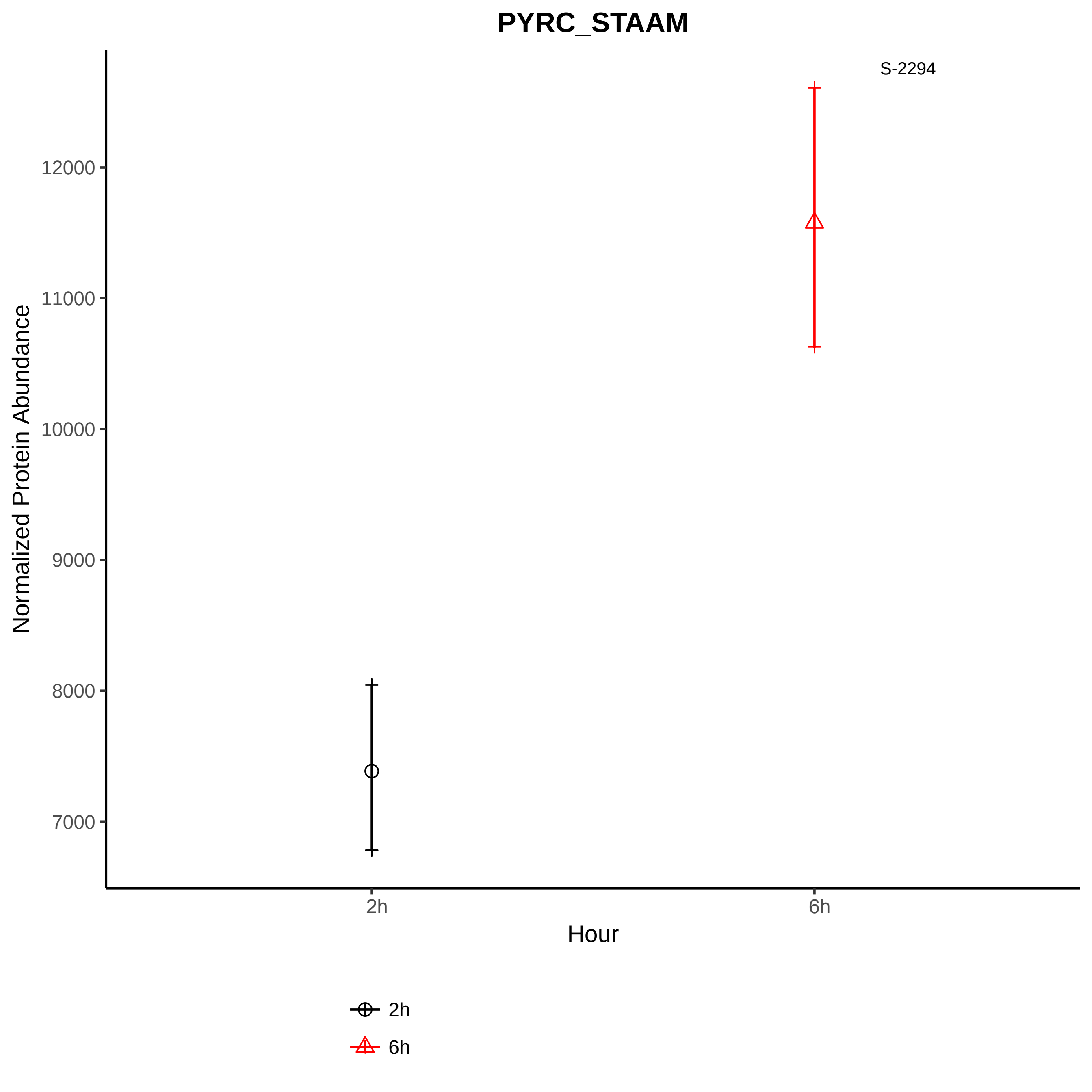
PYRC_STAAM
S-2294
Normalized Protein Abundance
12000
11000
10000
9000
8000
7000
2h
6h
Hour
2h
6h

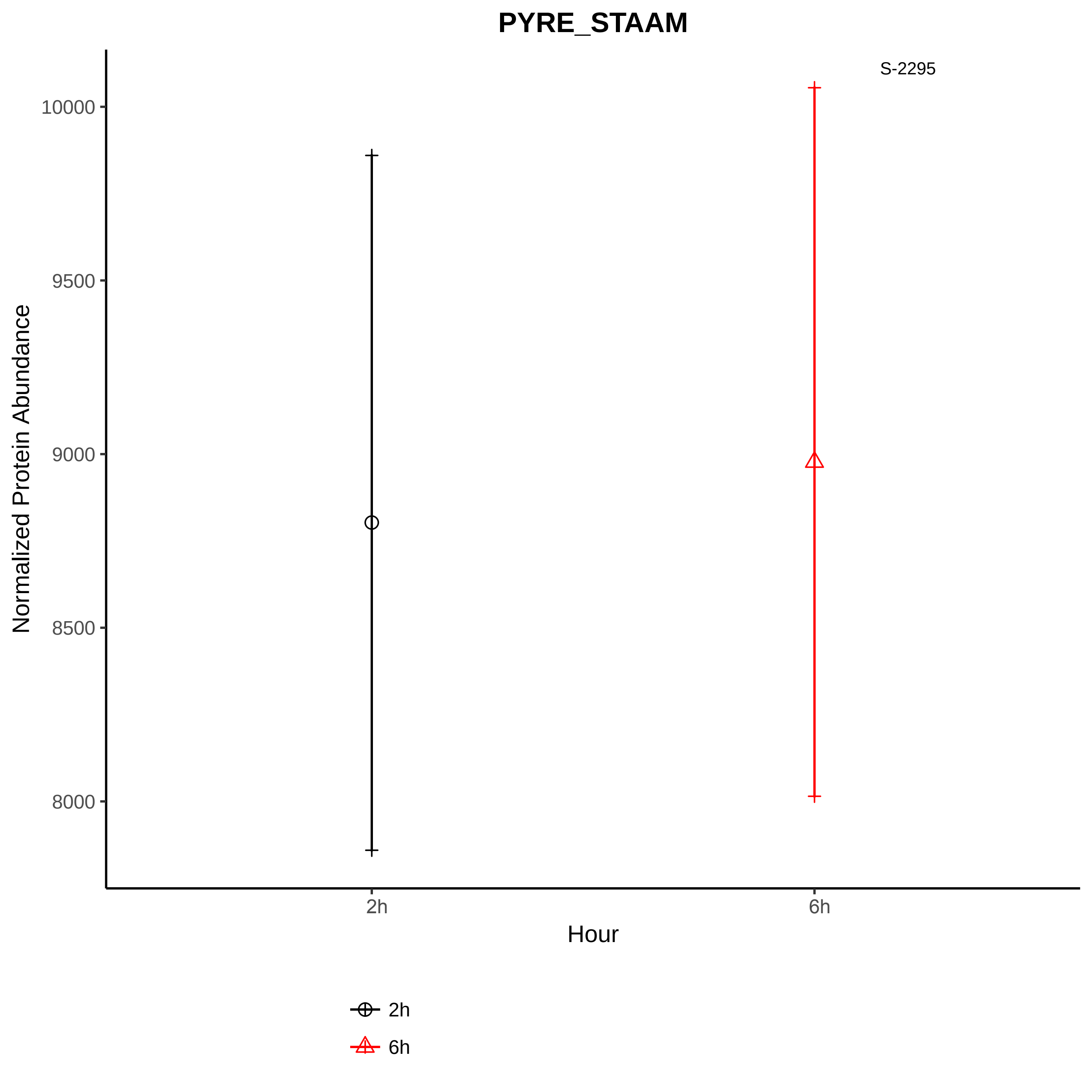

PYRE_STAAM
S-2295
10000
9500
9000
8500
8000
Normalized Protein Abundance
2h
6h
Hour
2h
6h

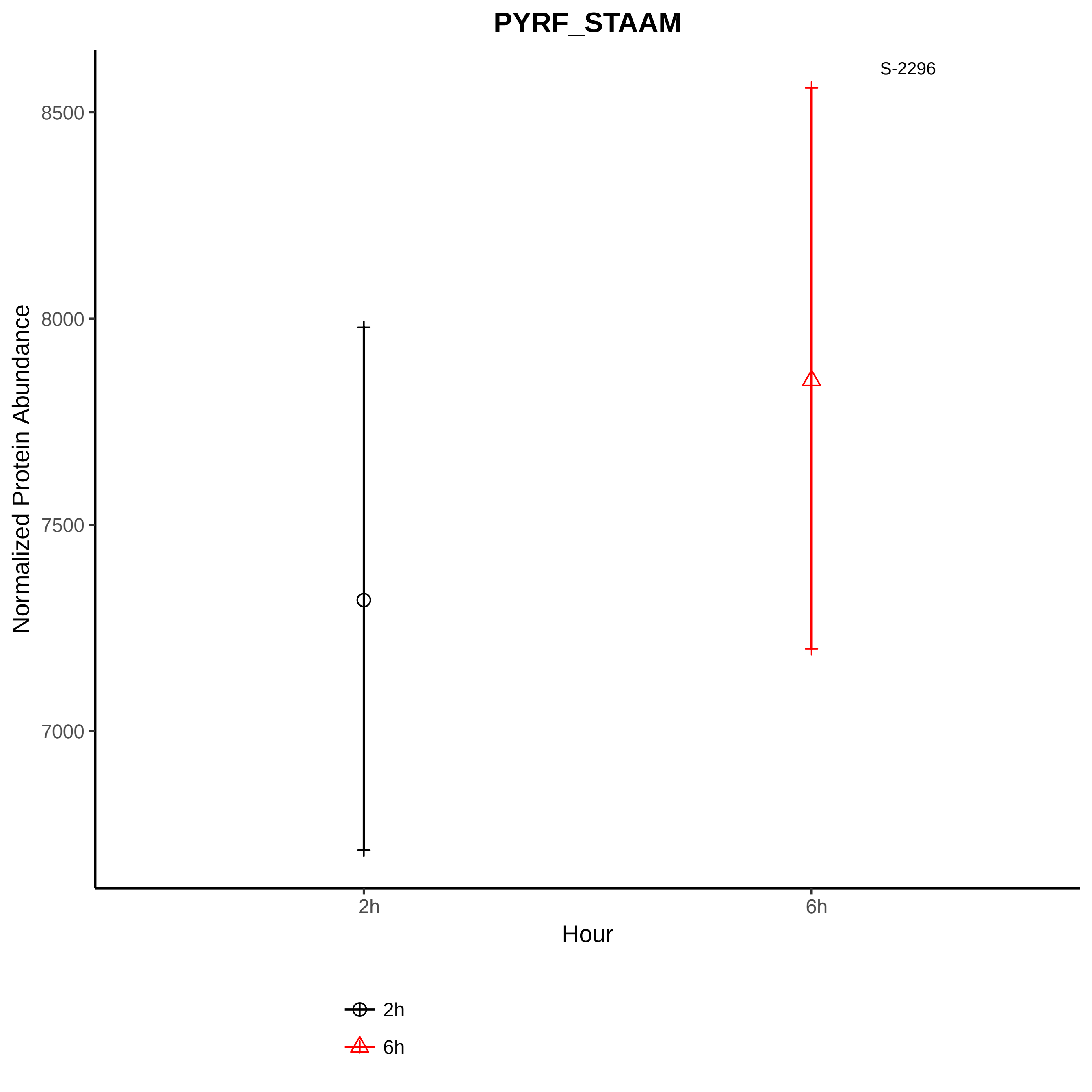

PYRF_STAAM
S-2296
8500
8000
7500
7000
Normalized Protein Abundance
2h
6h
Hour
2h
6h

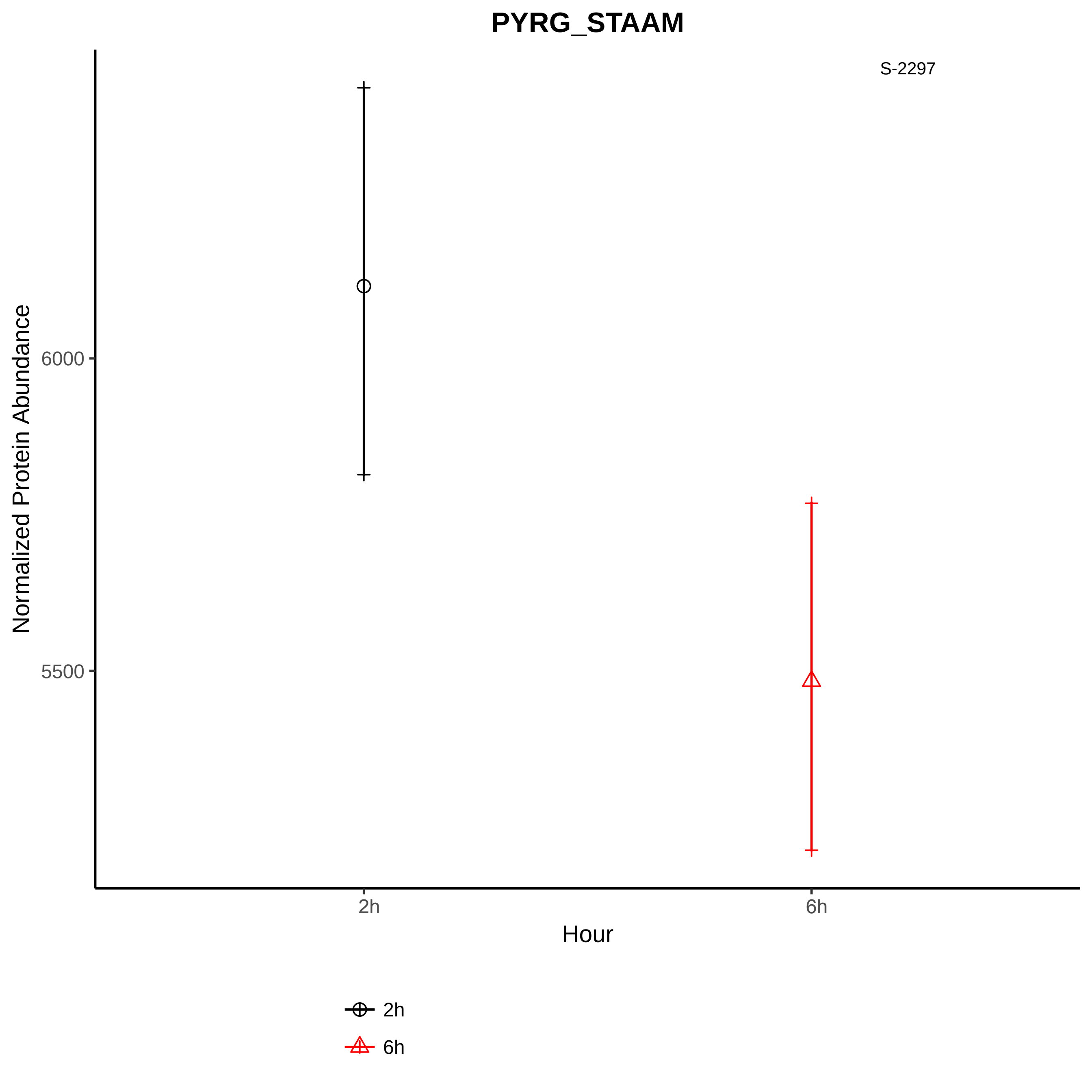

PYRG_STAAM
S-2297
Normalized Protein Abundance
6000
5500
2h
6h
Hour
2h
6h

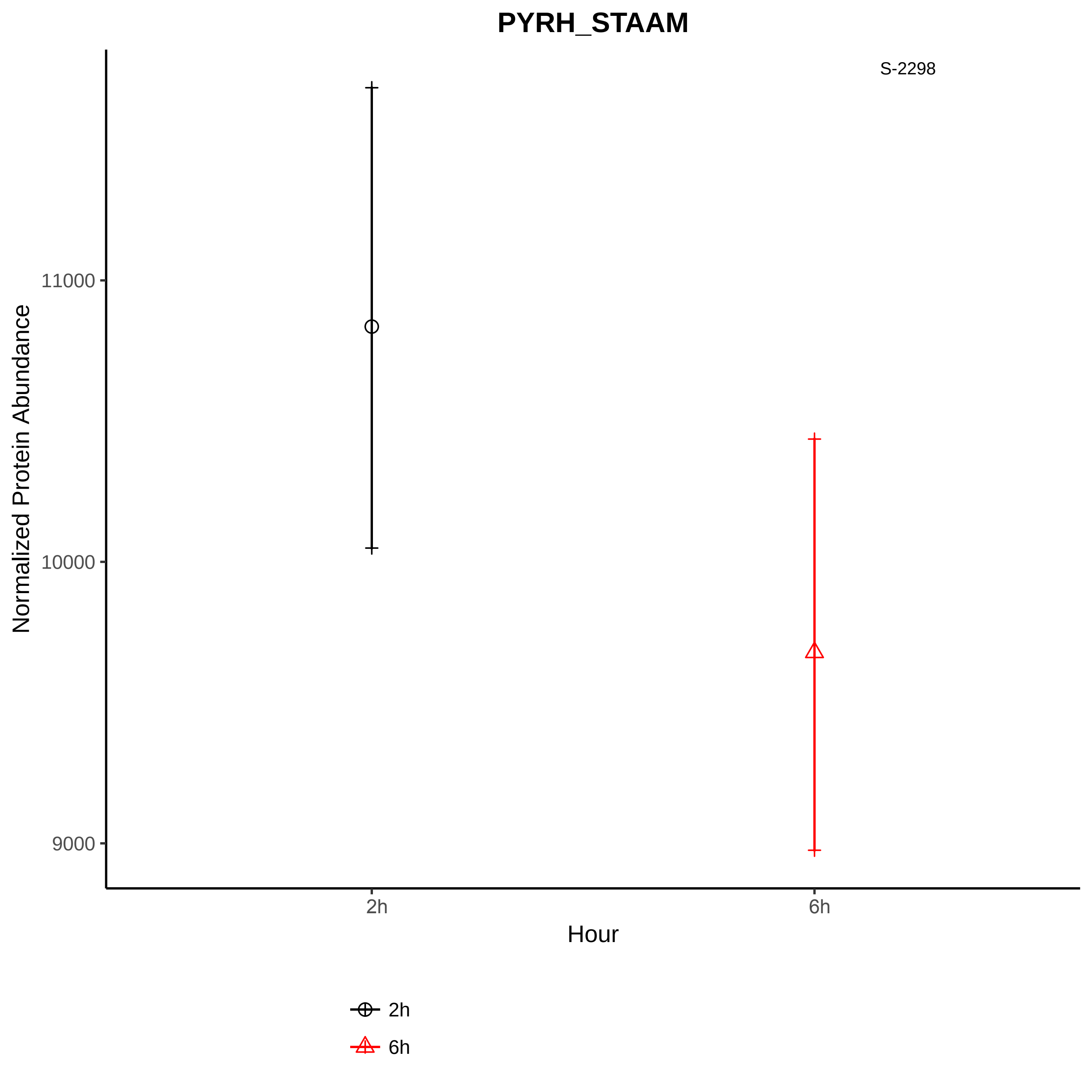
PYRH_STAAM
S-2298
Normalized Protein Abundance
11000
10000
9000
2h
6h
Hour
2h
6h

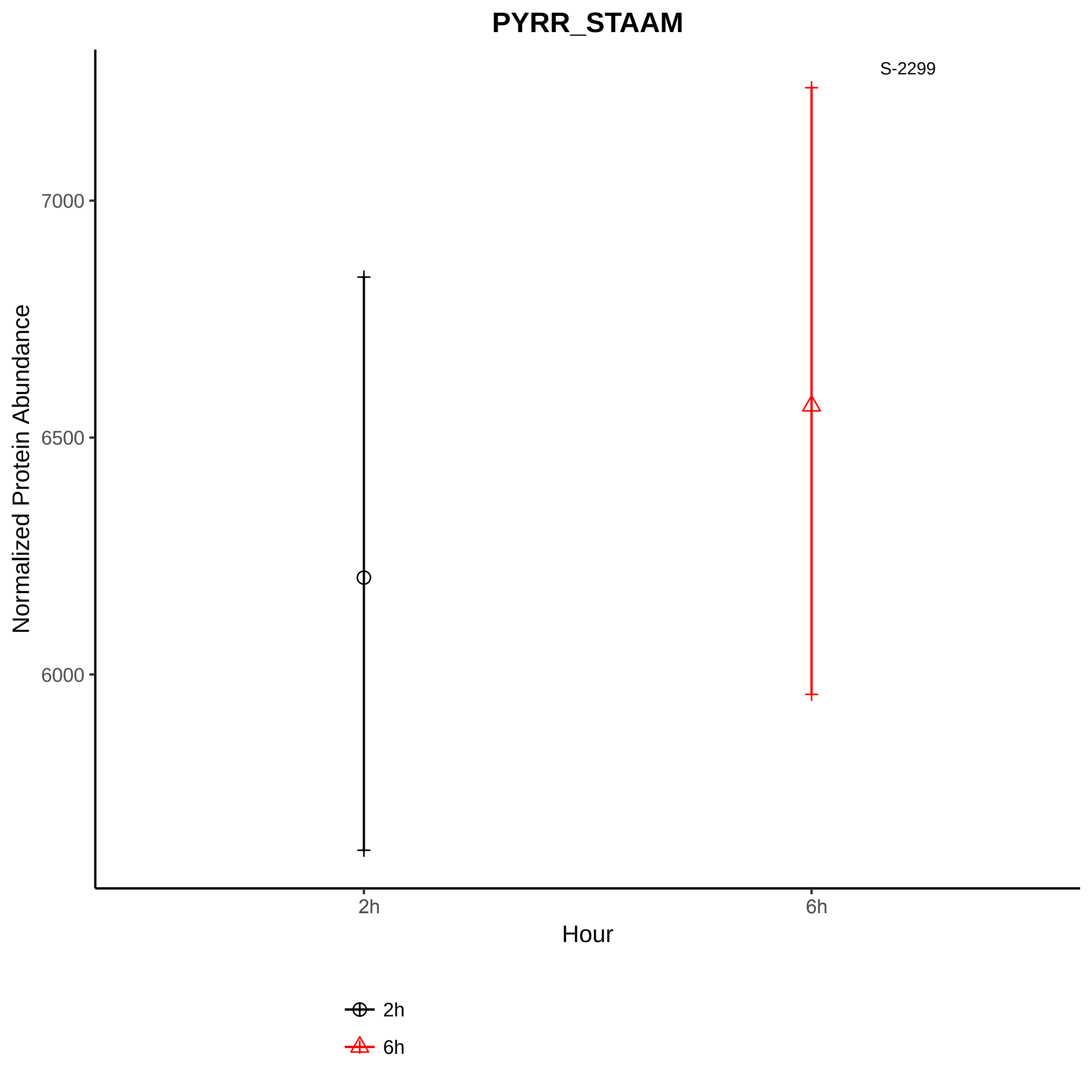

PYRR_STAAM
S-2299
Normalized Protein Abundance
7000
6500
6000
2h
6h
Hour
2h
6h

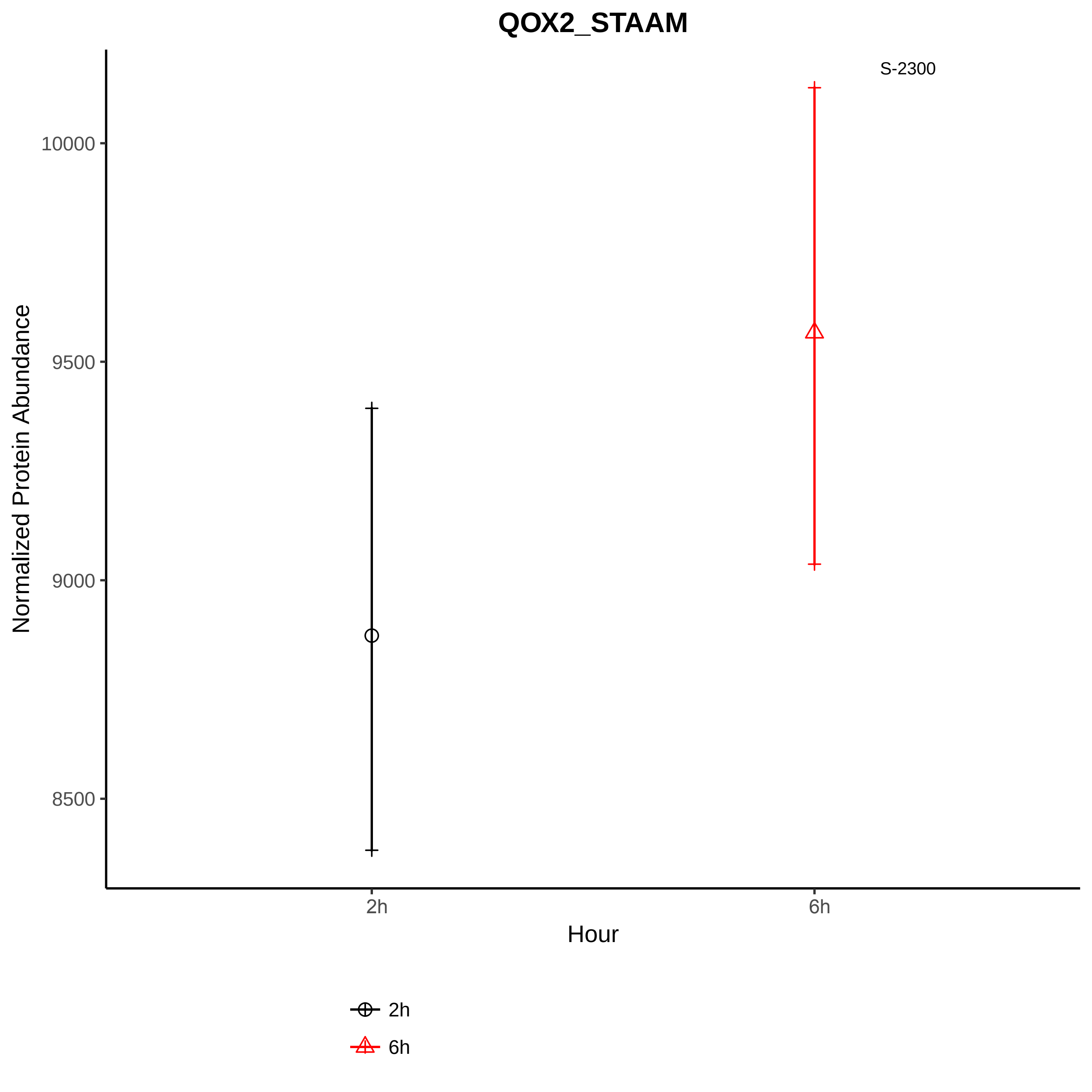
QOX2_STAAM
S-2300
Normalized Protein Abundance
10000
9500
9000
8500
2h
6h
Hour
2h
6h

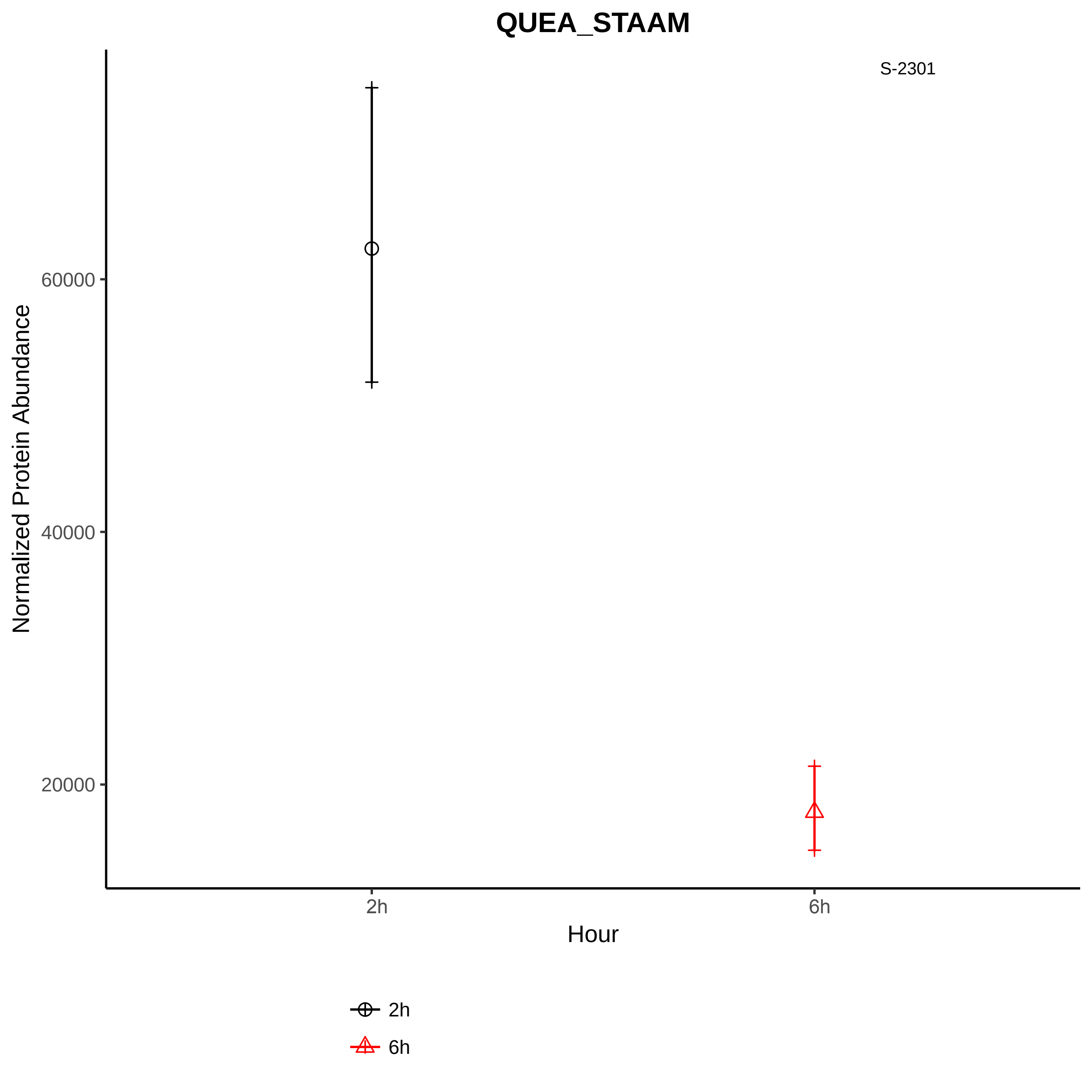

QUEA_STAAM
S-2301
Normalized Protein Abundance
60000
40000
20000
2h
6h
Hour
2h
6h

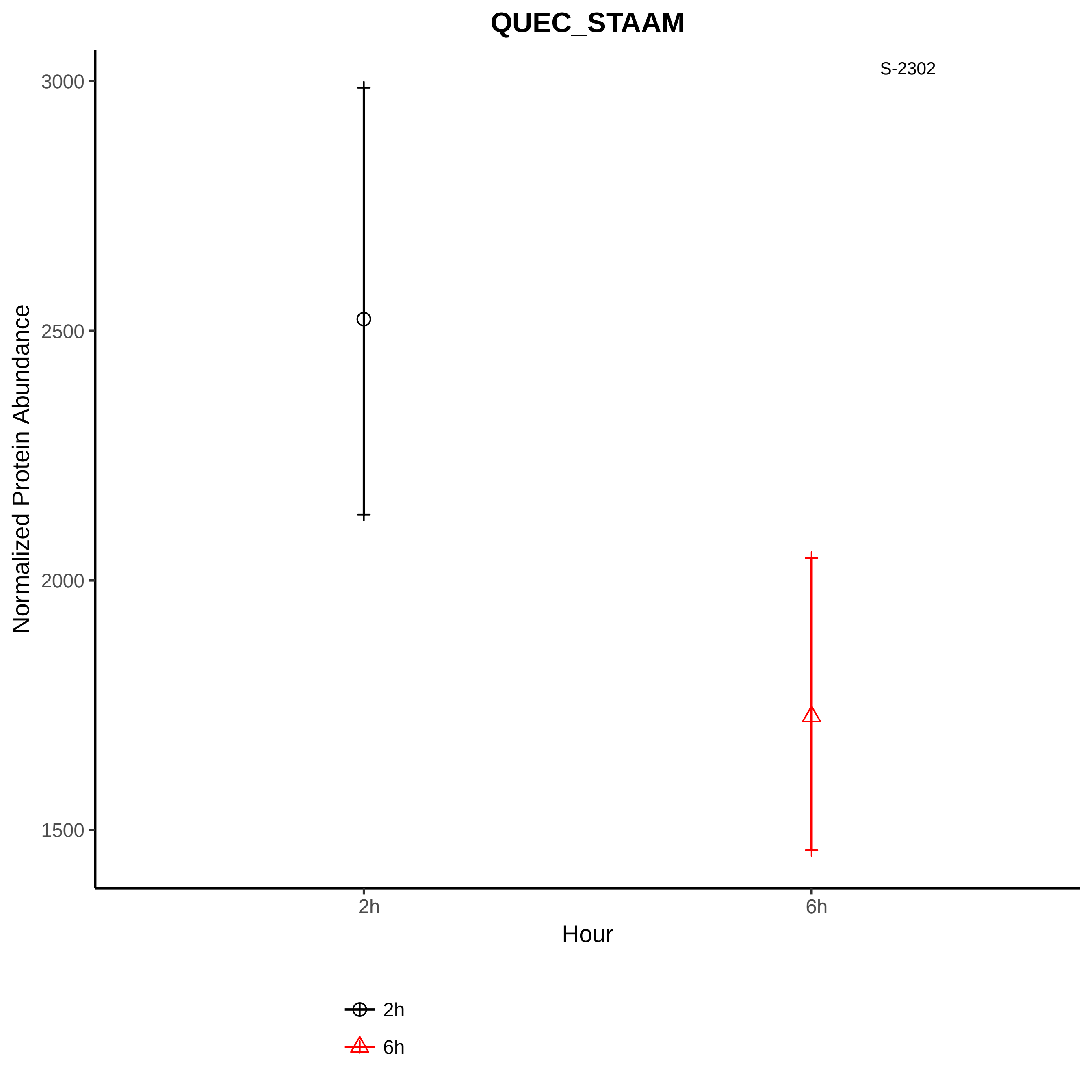

QUEC_STAAM
S-2302
3000
2500
2000
1500
Normalized Protein Abundance
2h
6h
Hour
2h
6h

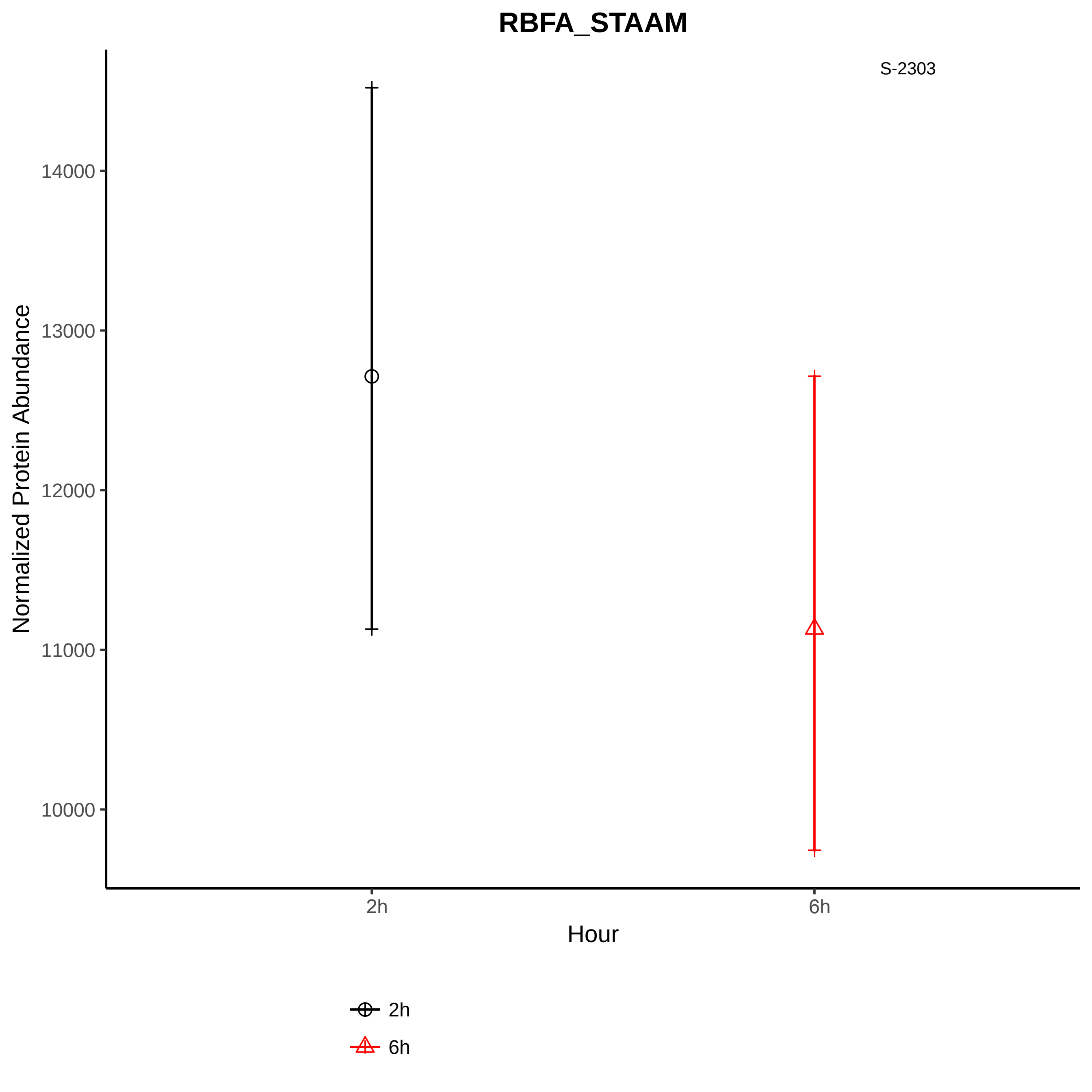

RBFA_STAAM
S-2303
Normalized Protein Abundance
14000
13000
12000
11000
10000
2h
6h
Hour
2h
6h

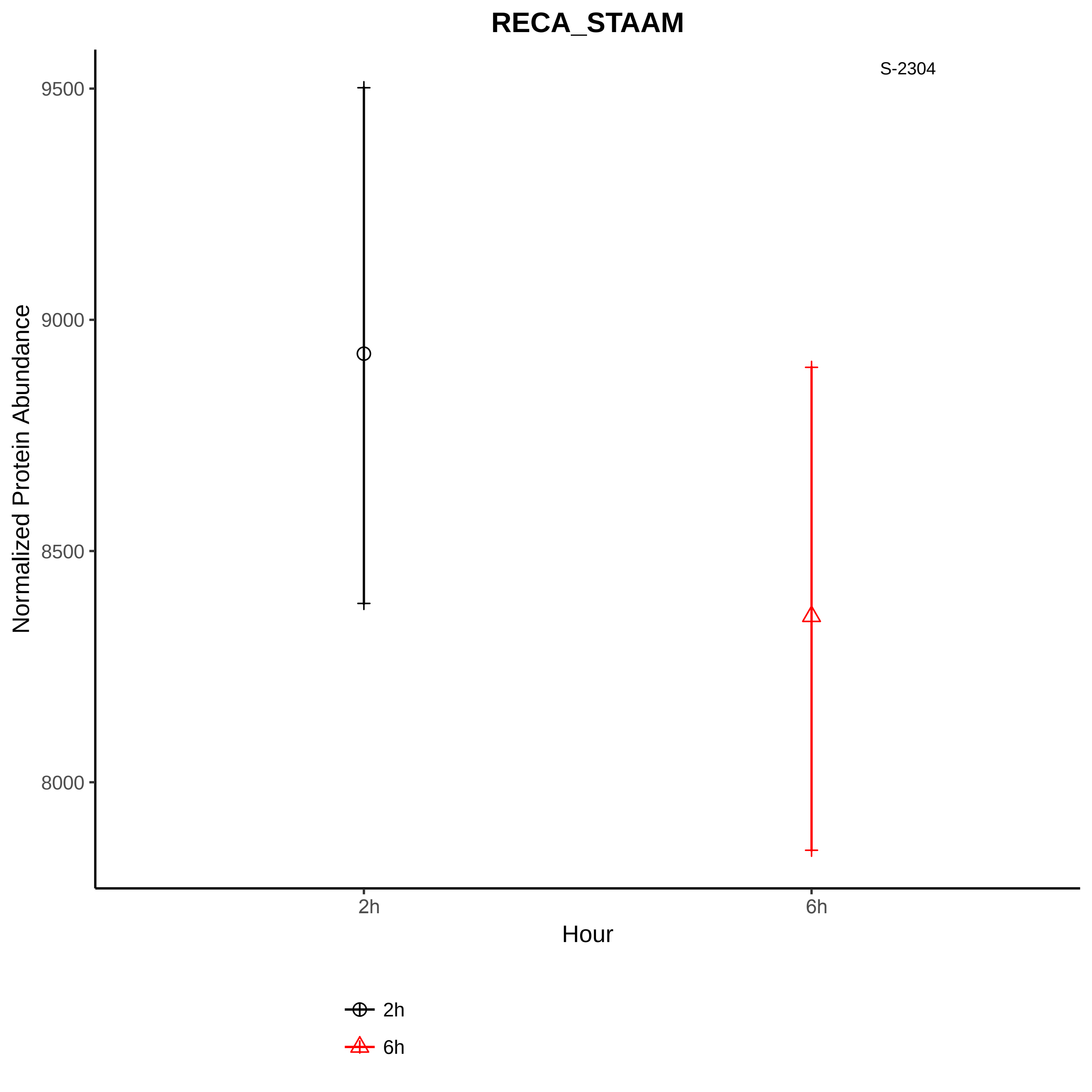

RECA_STAAM
S-2304
Normalized Protein Abundance
9500
9000
8500
8000
2h
6h
Hour
2h
6h

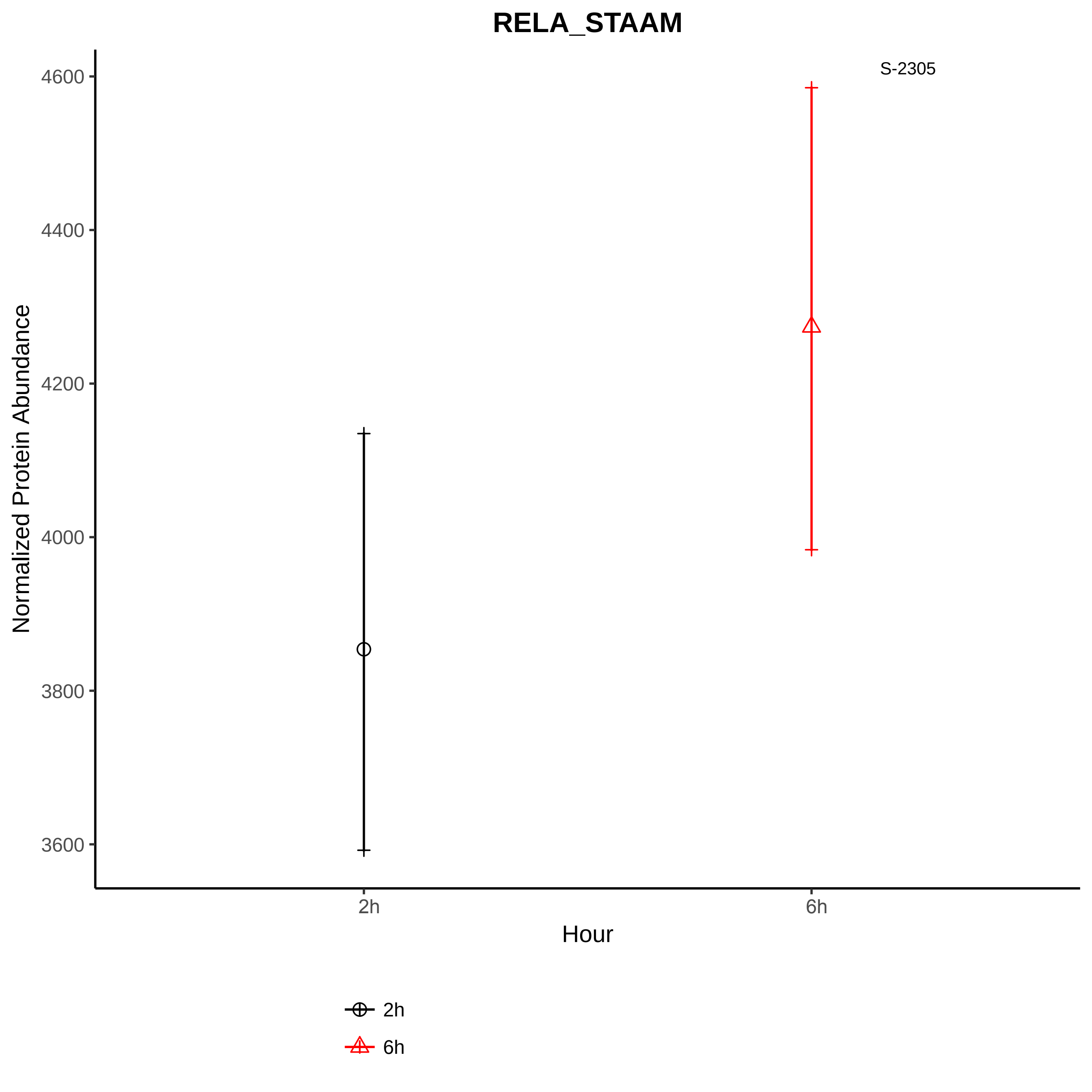
RELA_STAAM
S-2305
Normalized Protein Abundance
4600
4400
4200
4000
3800
3600
2h
6h
Hour
2h
6h

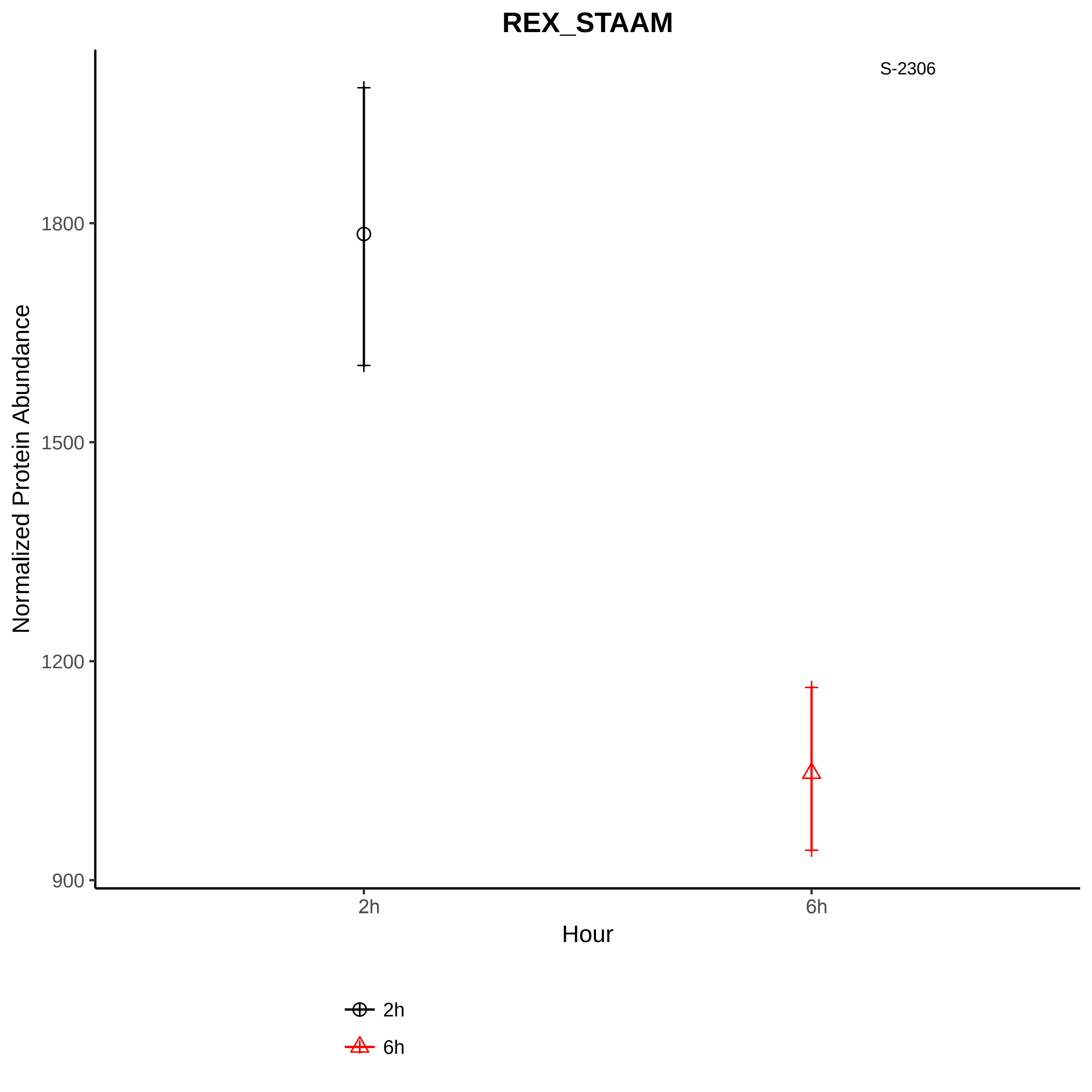

REX_STAAM
S-2306
Normalized Protein Abundance
1800
1500
1200
900
2h
6h
Hour
2h
6h

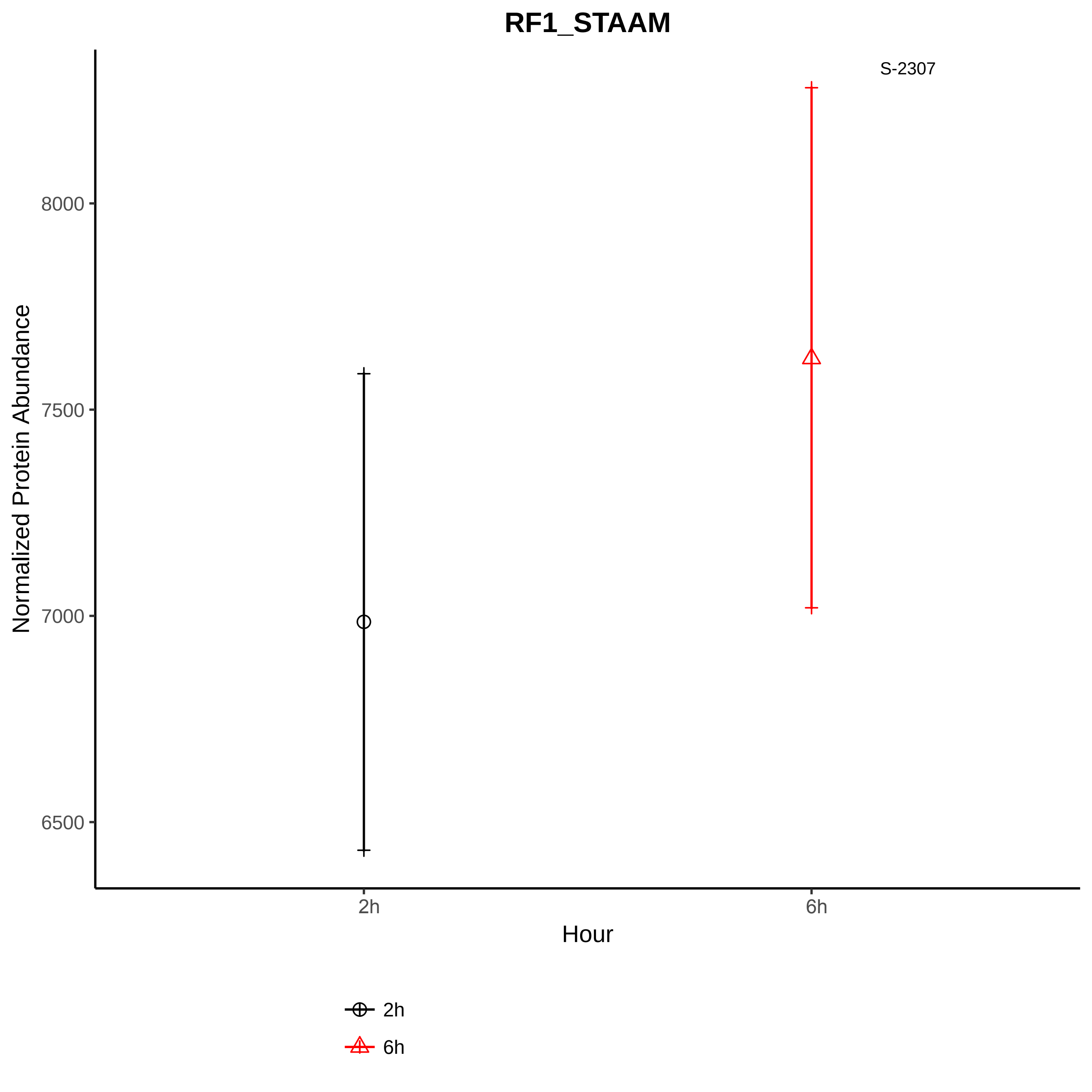
RF1_STAAM
S-2307
Normalized Protein Abundance
8000
7500
7000
6500
2h
6h
Hour
2h
6h

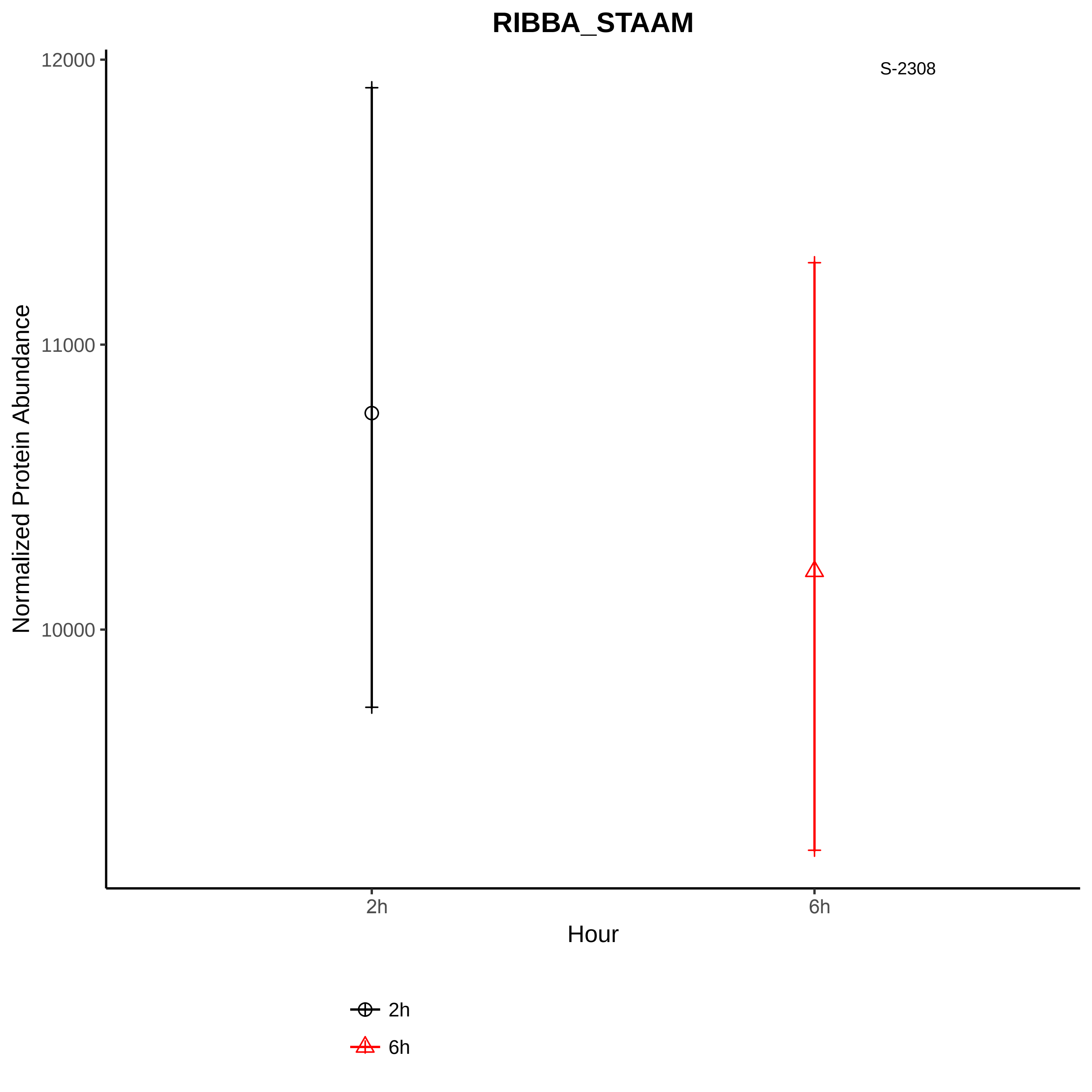

RIBBA_STAAM
S-2308
Normalized Protein Abundance
12000
11000
10000
2h
6h
Hour
2h
6h

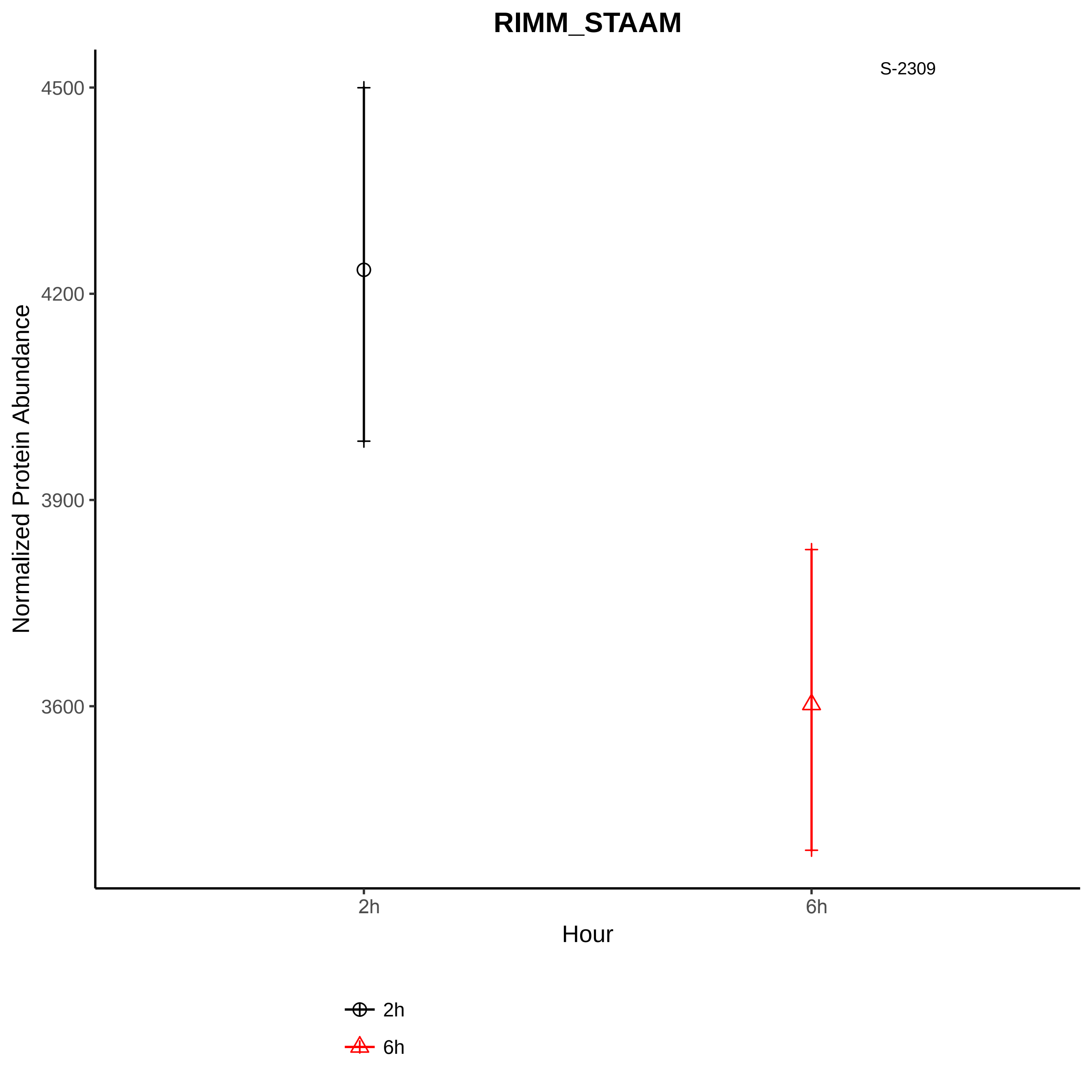

RIMM_STAAM
S-2309
4500
4200
3900
3600
Normalized Protein Abundance
2h
6h
Hour
2h
6h

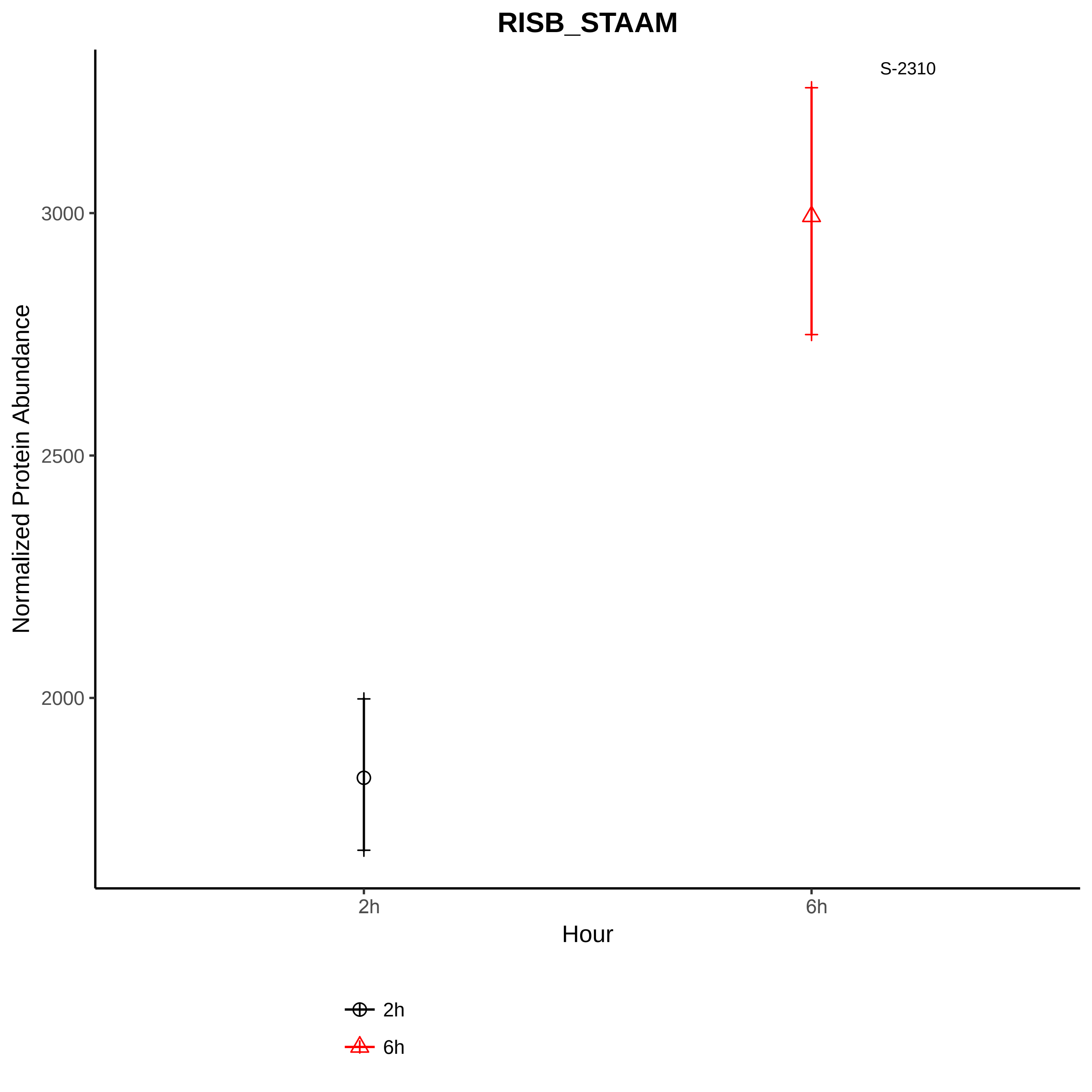

RISB_STAAM
S-2310
Normalized Protein Abundance
3000
2500
2000
2h
6h
Hour
2h
6h

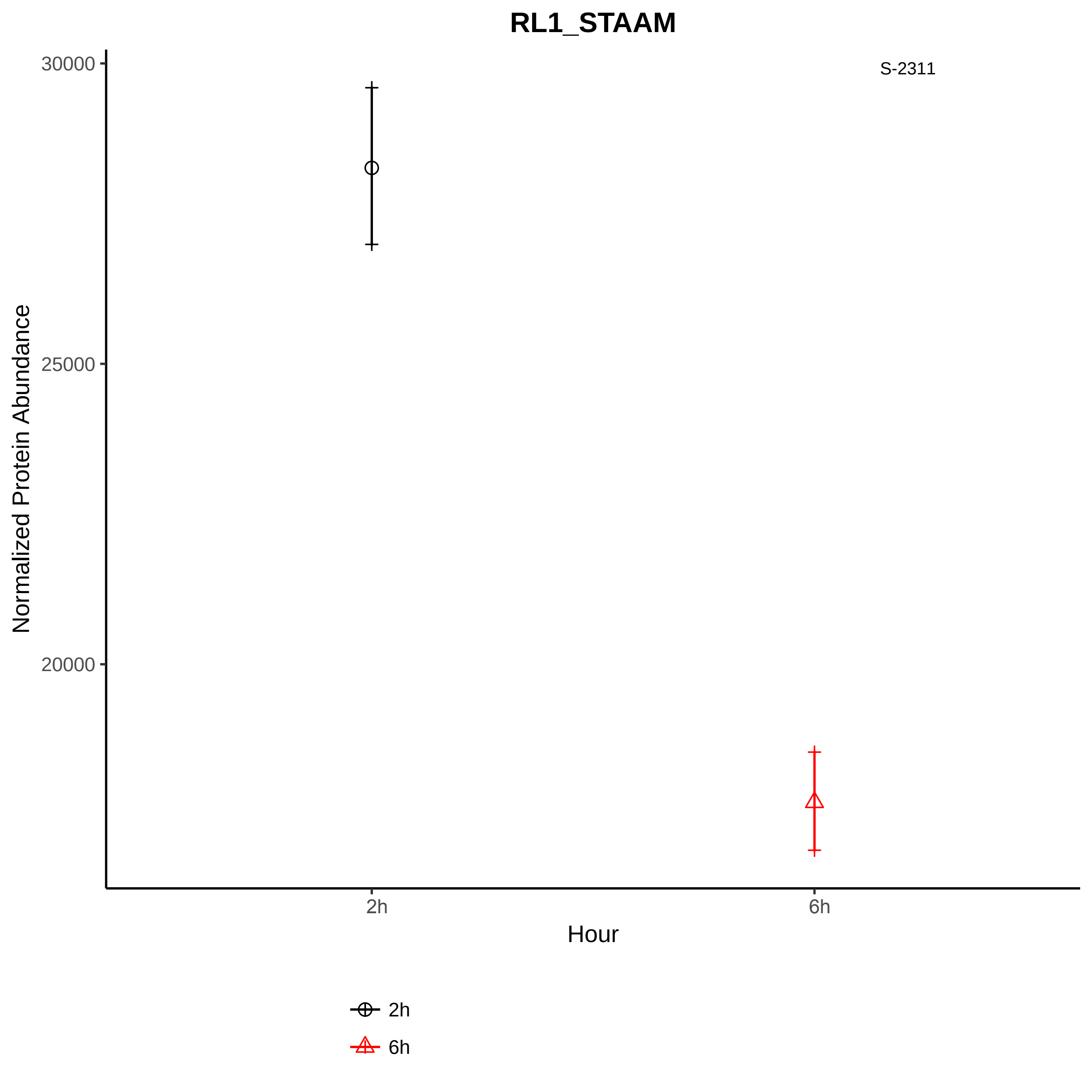

RL1_STAAM
S-2311
30000
25000
20000
Normalized Protein Abundance
2h
6h
Hour
2h
6h

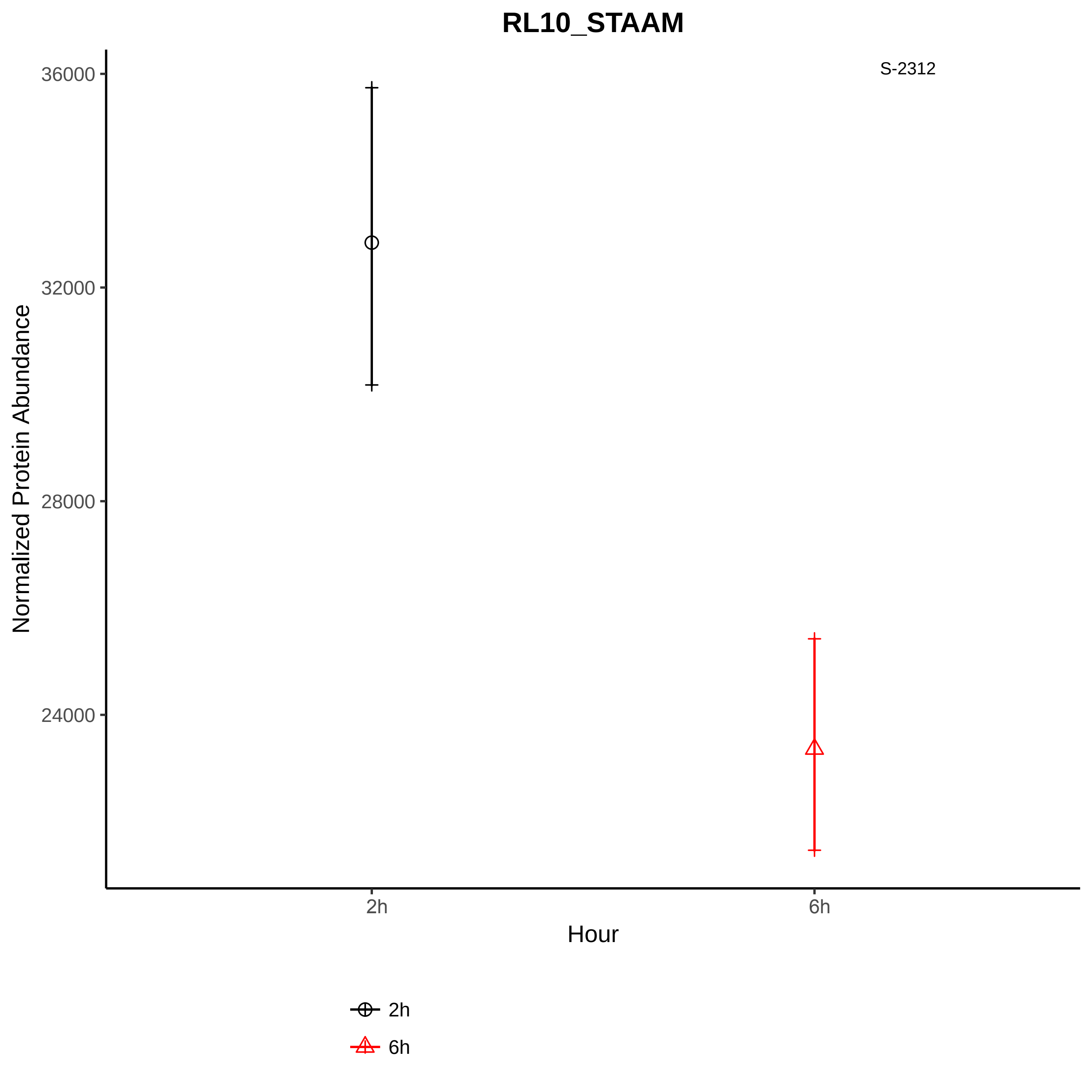

RL10_STAAM
S-2312
36000
32000
28000
24000
Normalized Protein Abundance
2h
6h
Hour
2h
6h

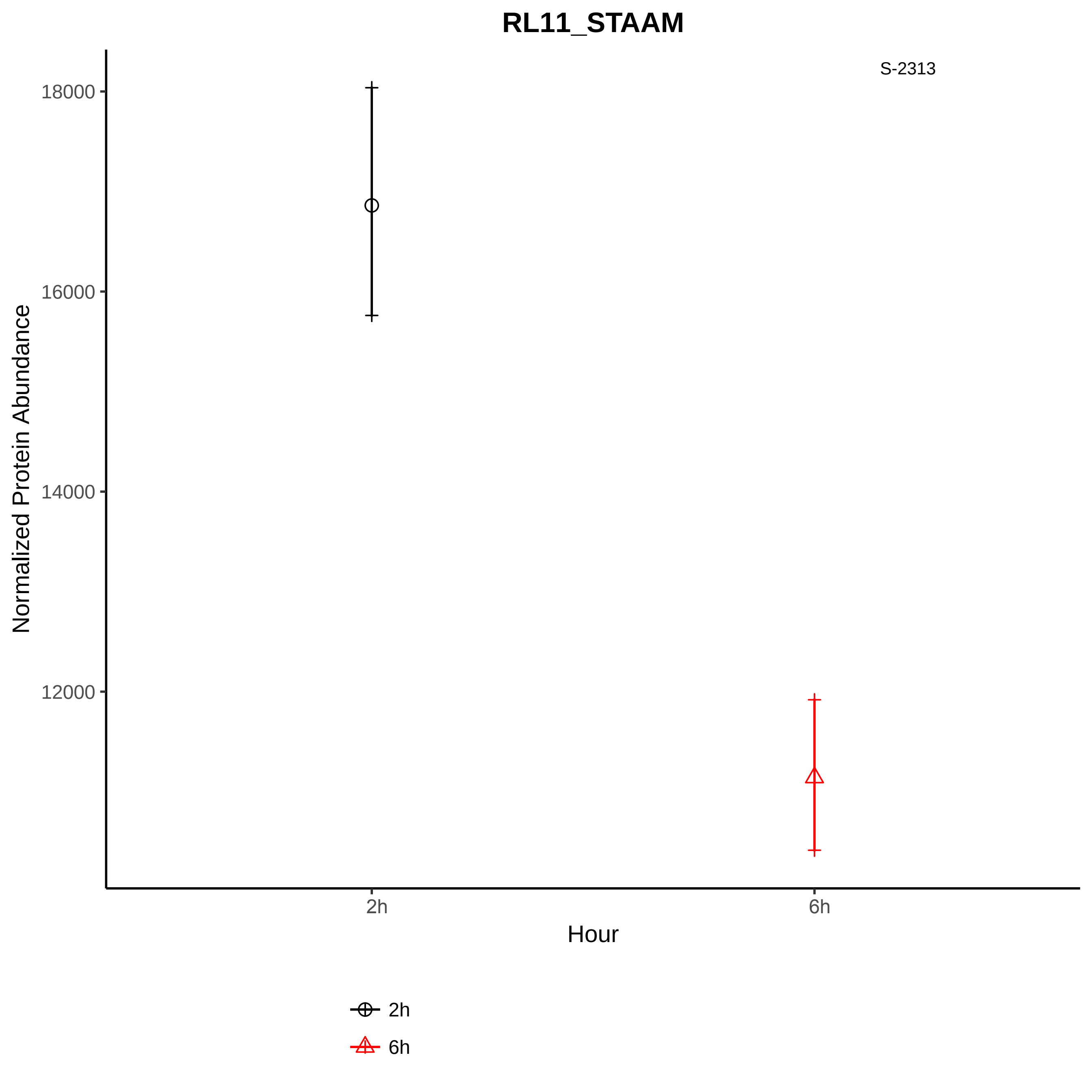

RL11_STAAM
S-2313
Normalized Protein Abundance
18000
16000
14000
12000
2h
6h
Hour
2h
6h

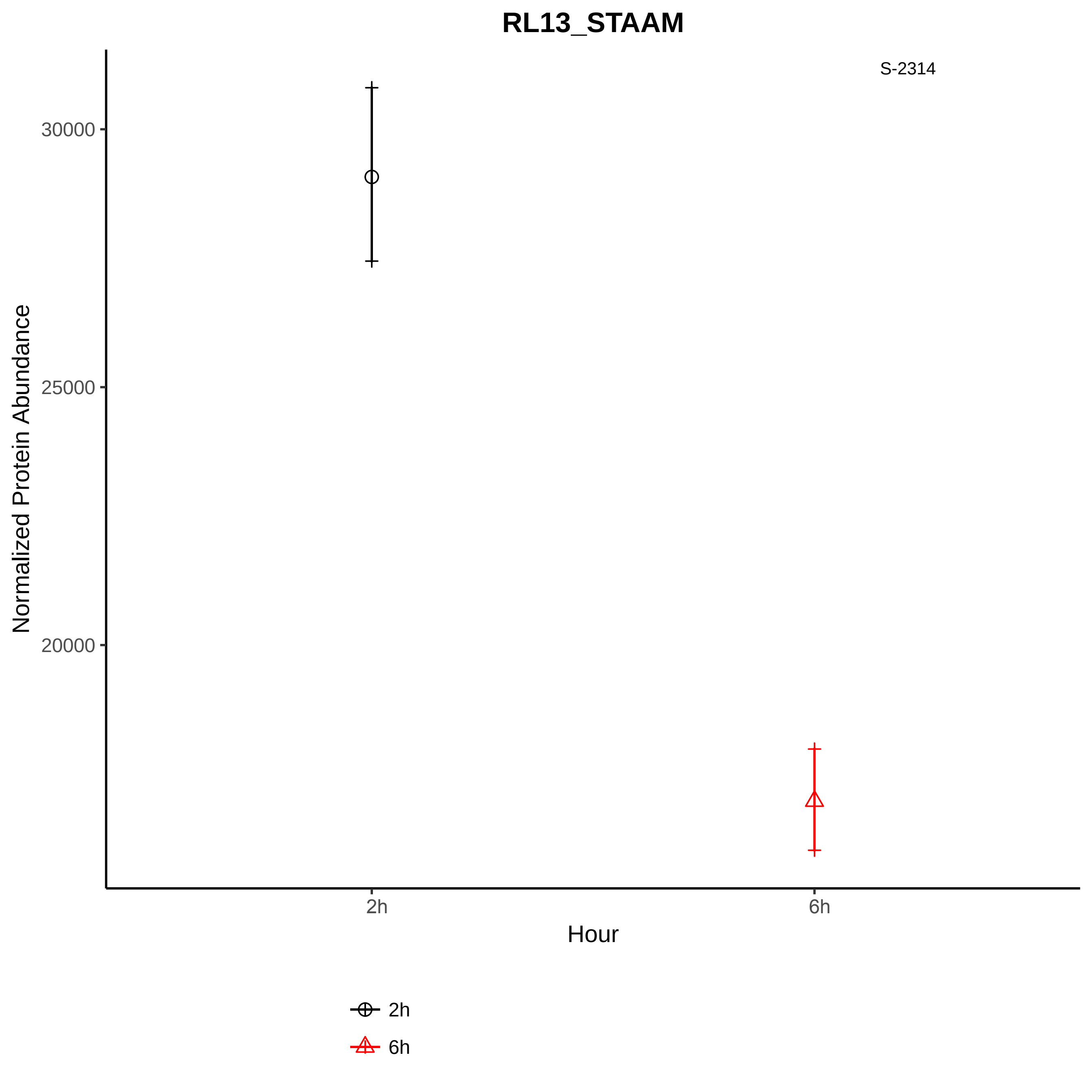

RL13_STAAM
S-2314
30000
25000
20000
Normalized Protein Abundance
2h
6h
Hour
2h
6h

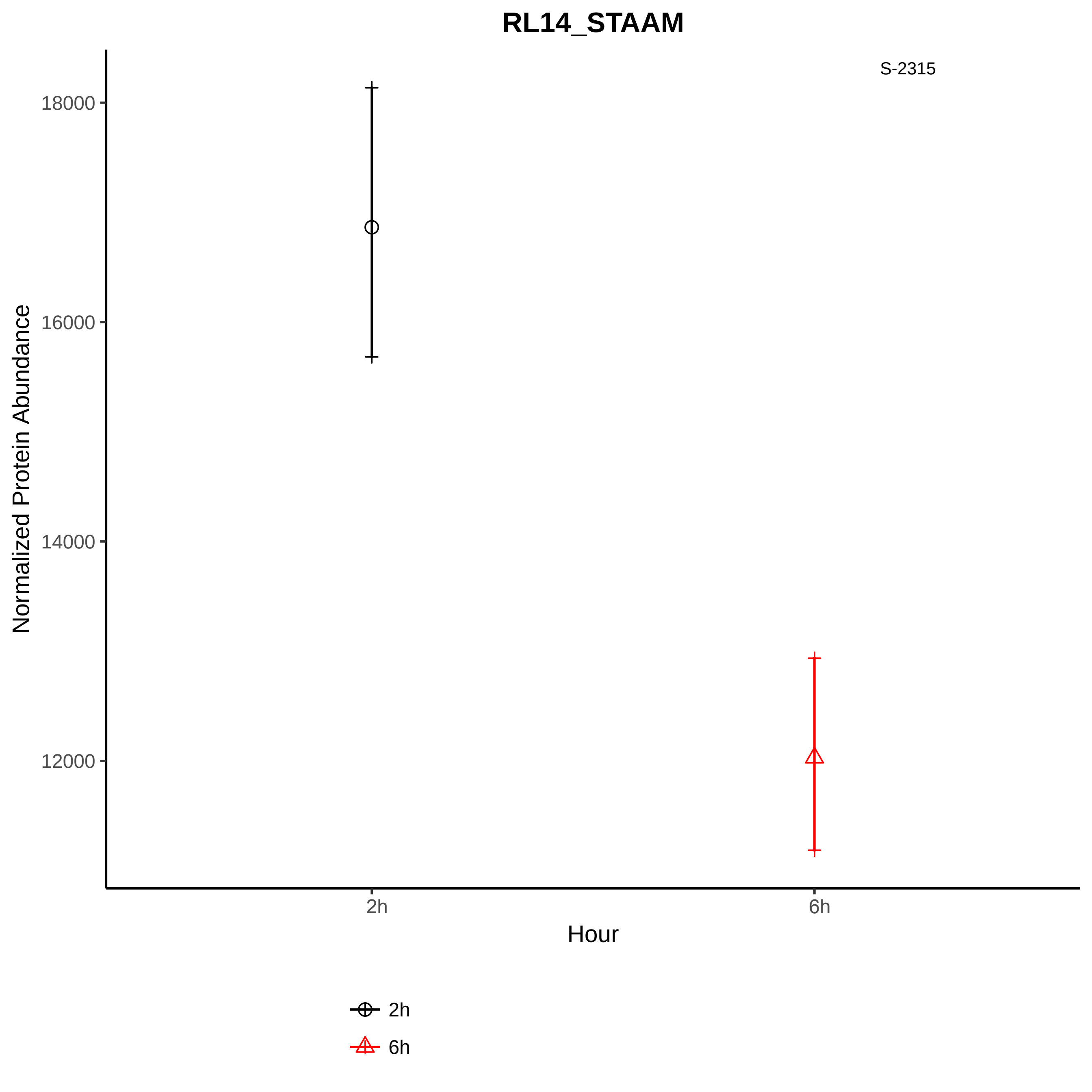

RL14_STAAM
S-2315
18000
16000
14000
12000
Normalized Protein Abundance
2h
6h
Hour
2h
6h

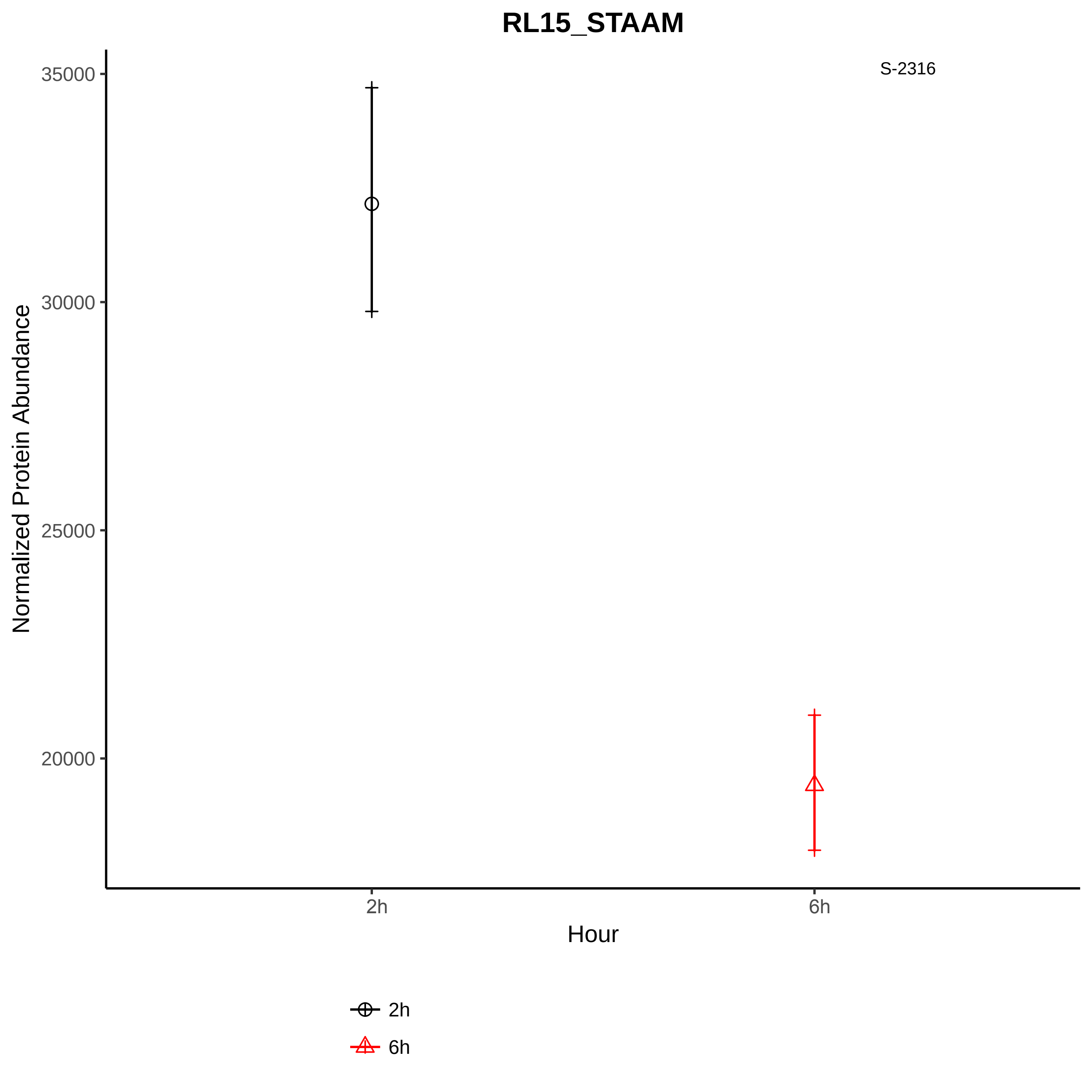
RL15_STAAM
S-2316
Normalized Protein Abundance
35000
30000
25000
20000
2h
6h
Hour
2h
6h

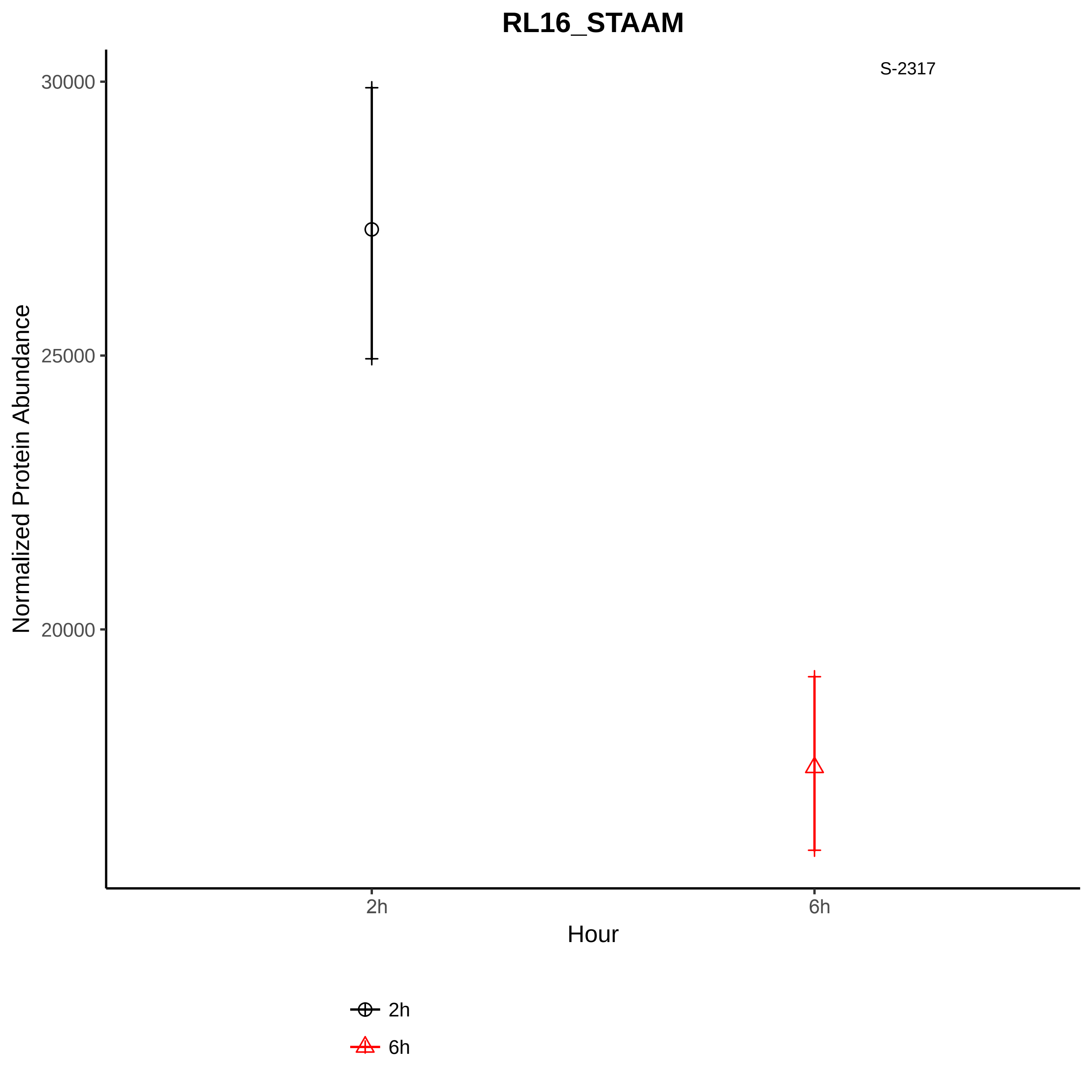
RL16_STAAM
S-2317
30000
25000
20000
Normalized Protein Abundance
2h
6h
Hour
2h
6h

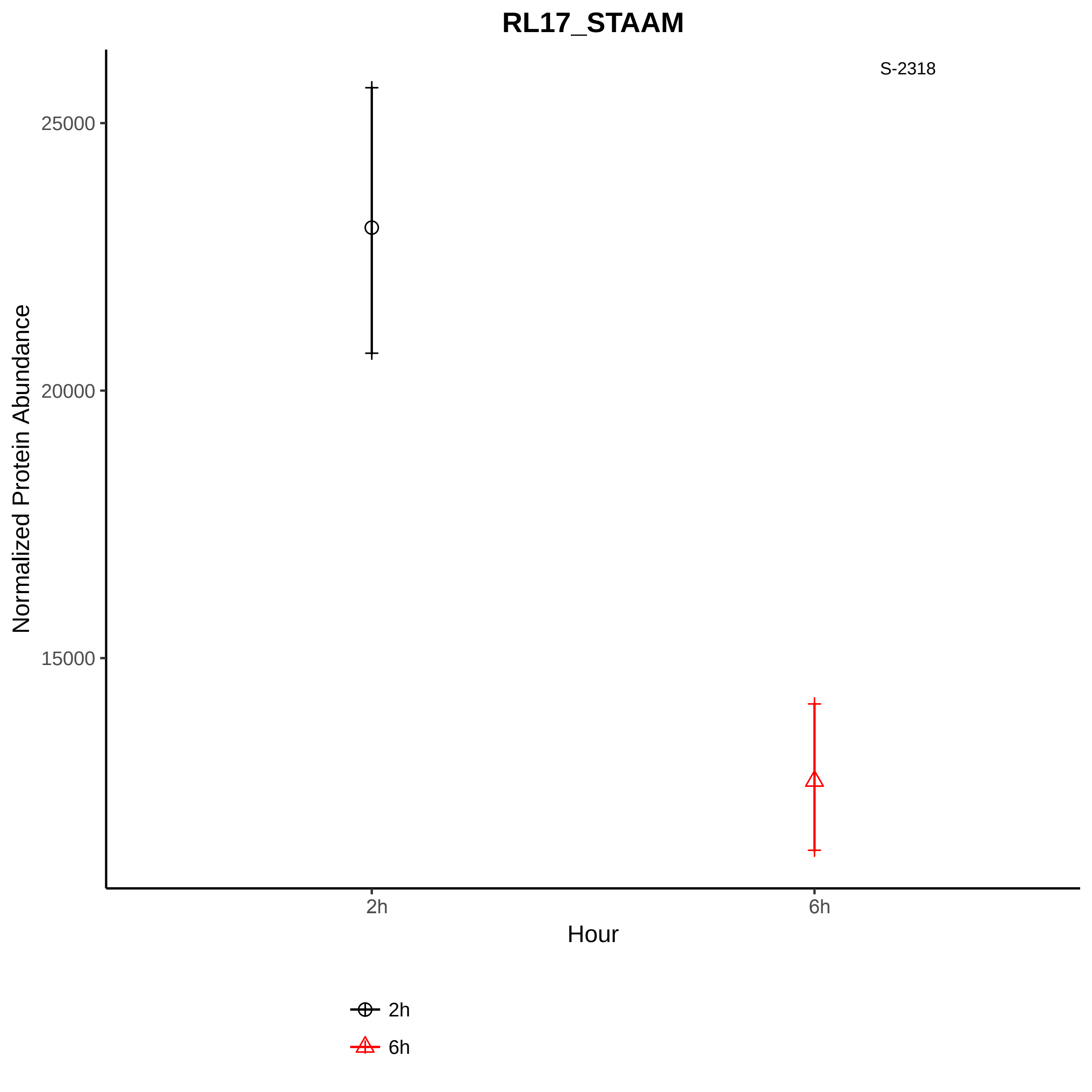
RL17_STAAM
S-2318
25000
20000
15000
Normalized Protein Abundance
2h
6h
Hour
2h
6h

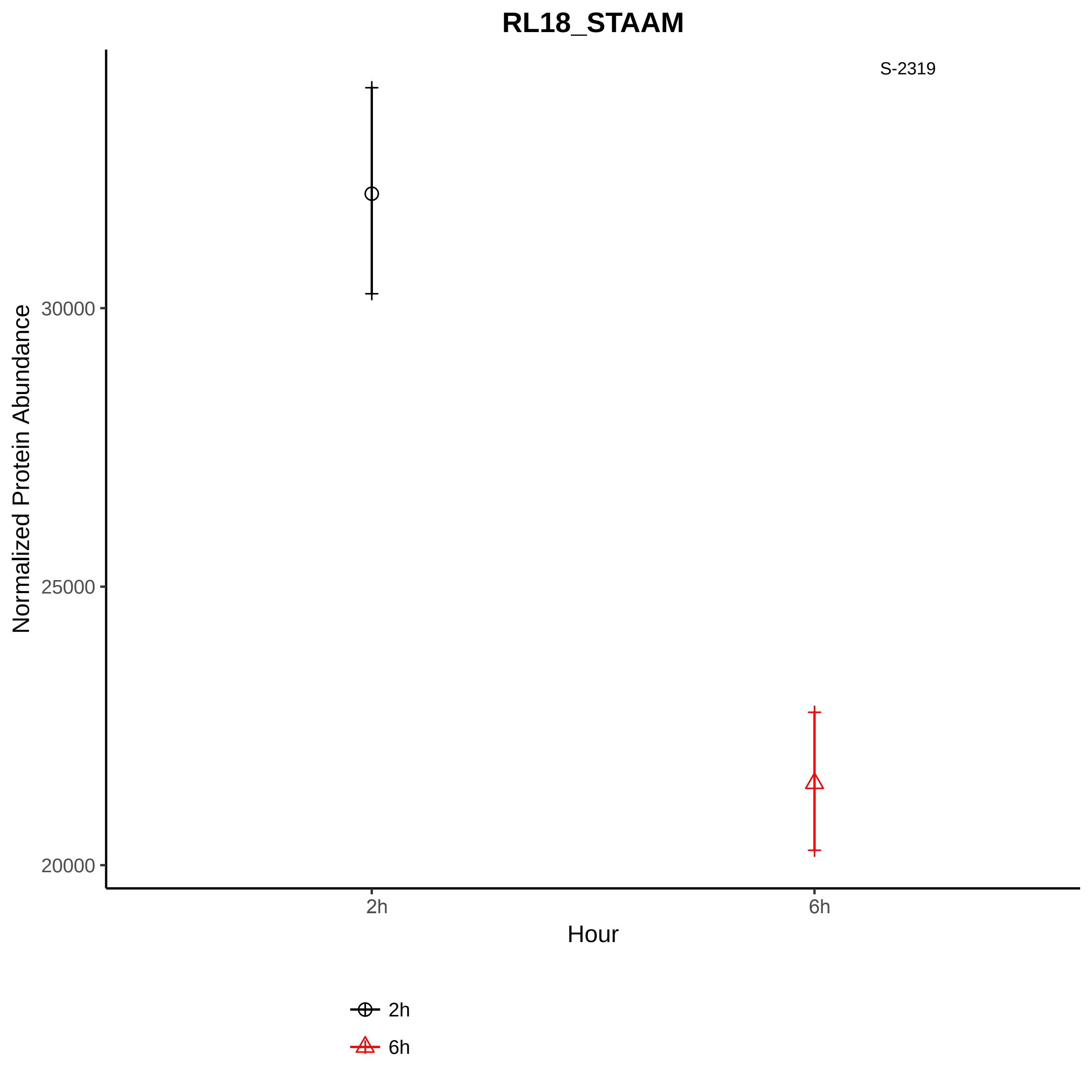
RL18_STAAM
S-2319
Normalized Protein Abundance
30000
25000
20000
2h
6h
Hour
2h
6h

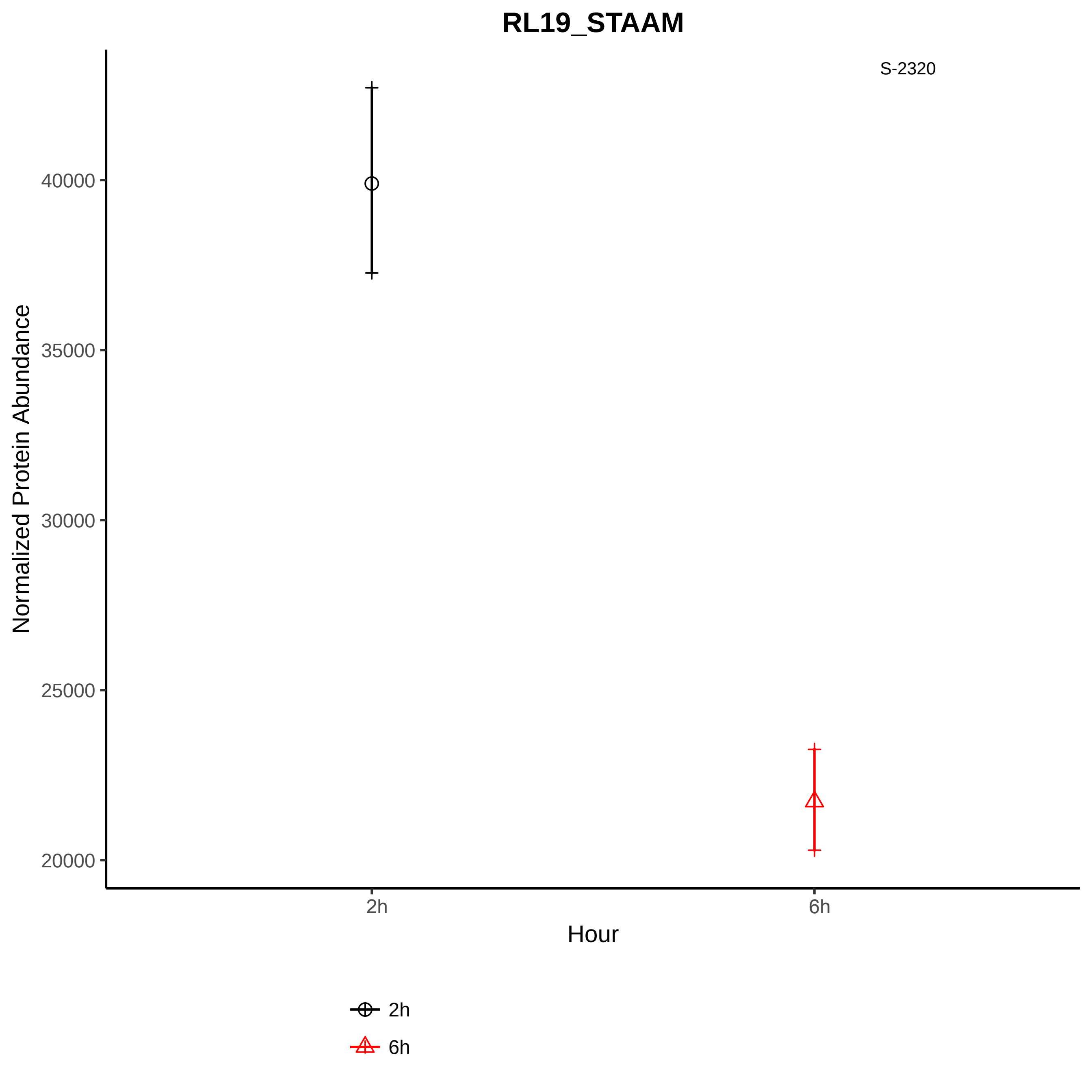

RL19_STAAM
S-2320
Normalized Protein Abundance
40000
35000
30000
25000
20000
2h
6h
Hour
2h
6h

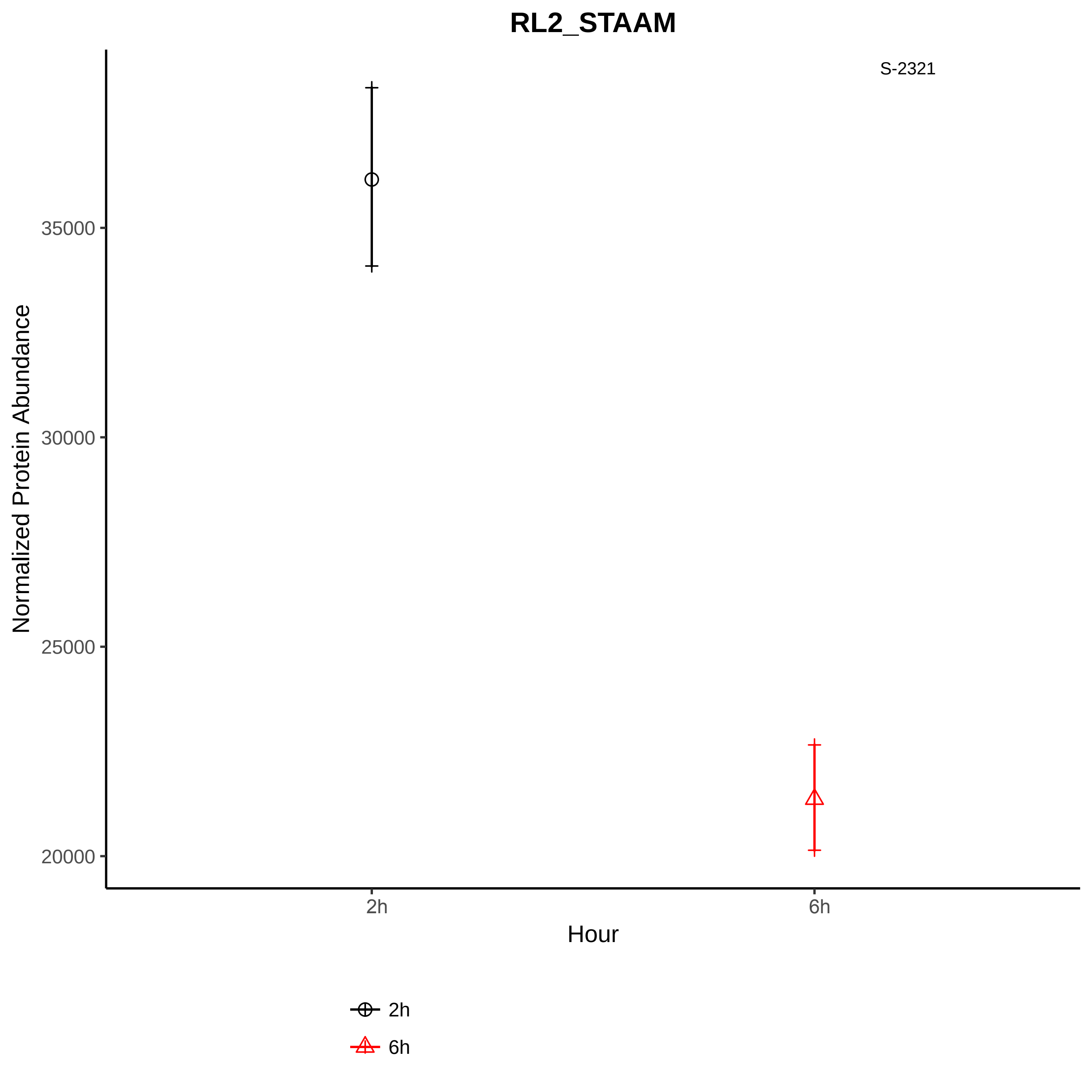

RL2_STAAM
S-2321
Normalized Protein Abundance
35000
30000
25000
20000
2h
6h
Hour
2h
6h

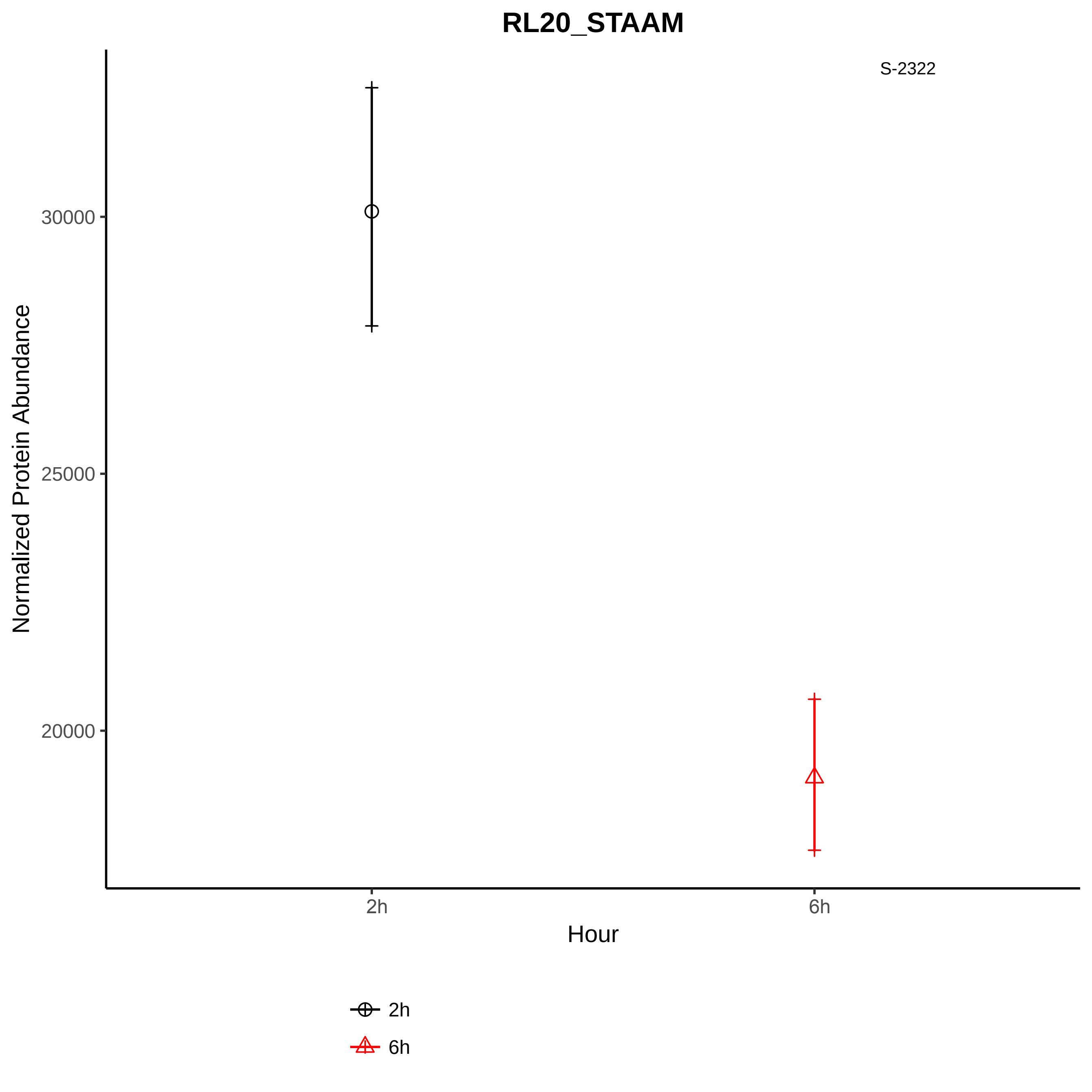

RL20_STAAM
S-2322
Normalized Protein Abundance
30000
25000
20000
2h
6h
Hour
2h
6h

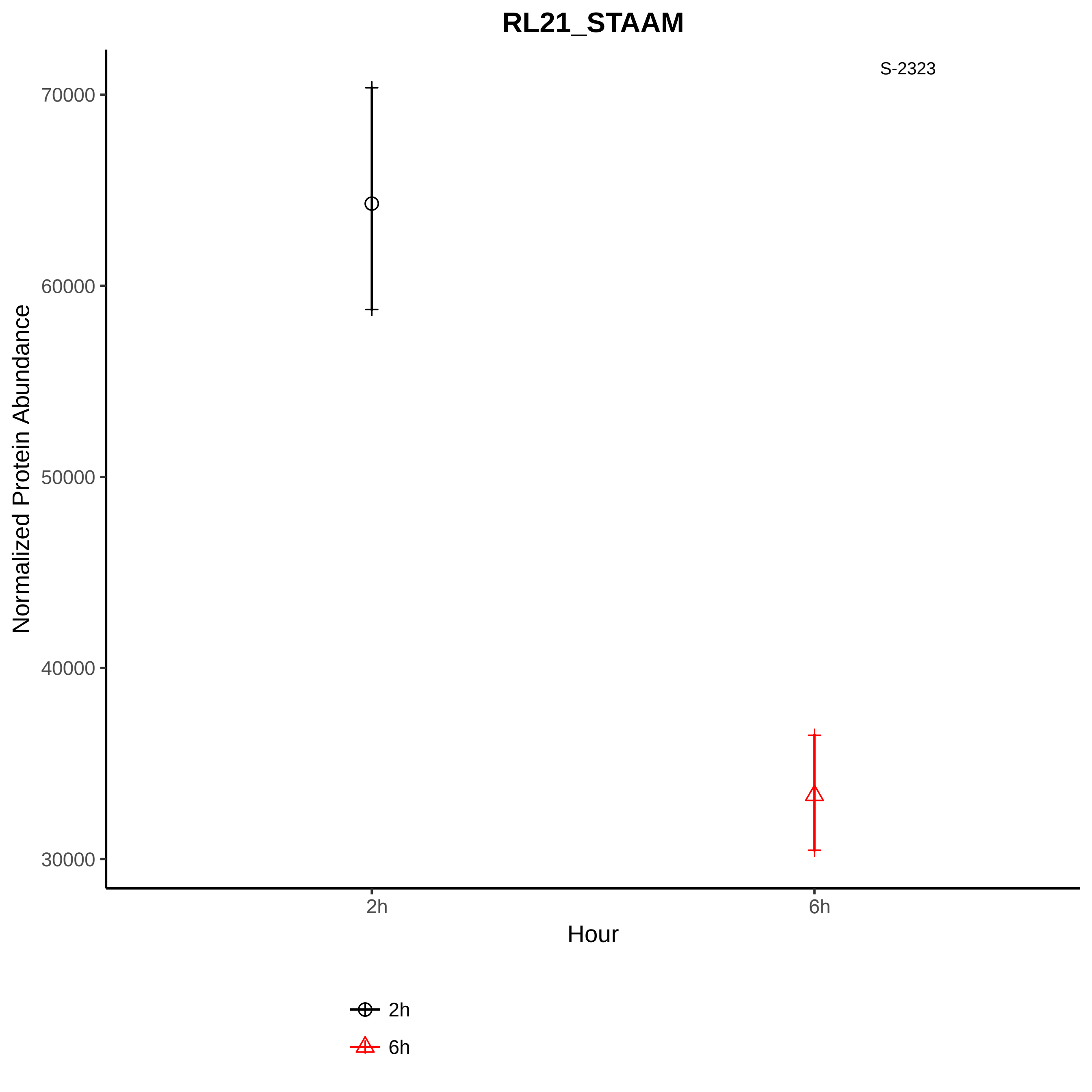
RL21_STAAM
S-2323
Normalized Protein Abundance
70000
60000
50000
40000
30000
2h
6h
Hour
2h
6h

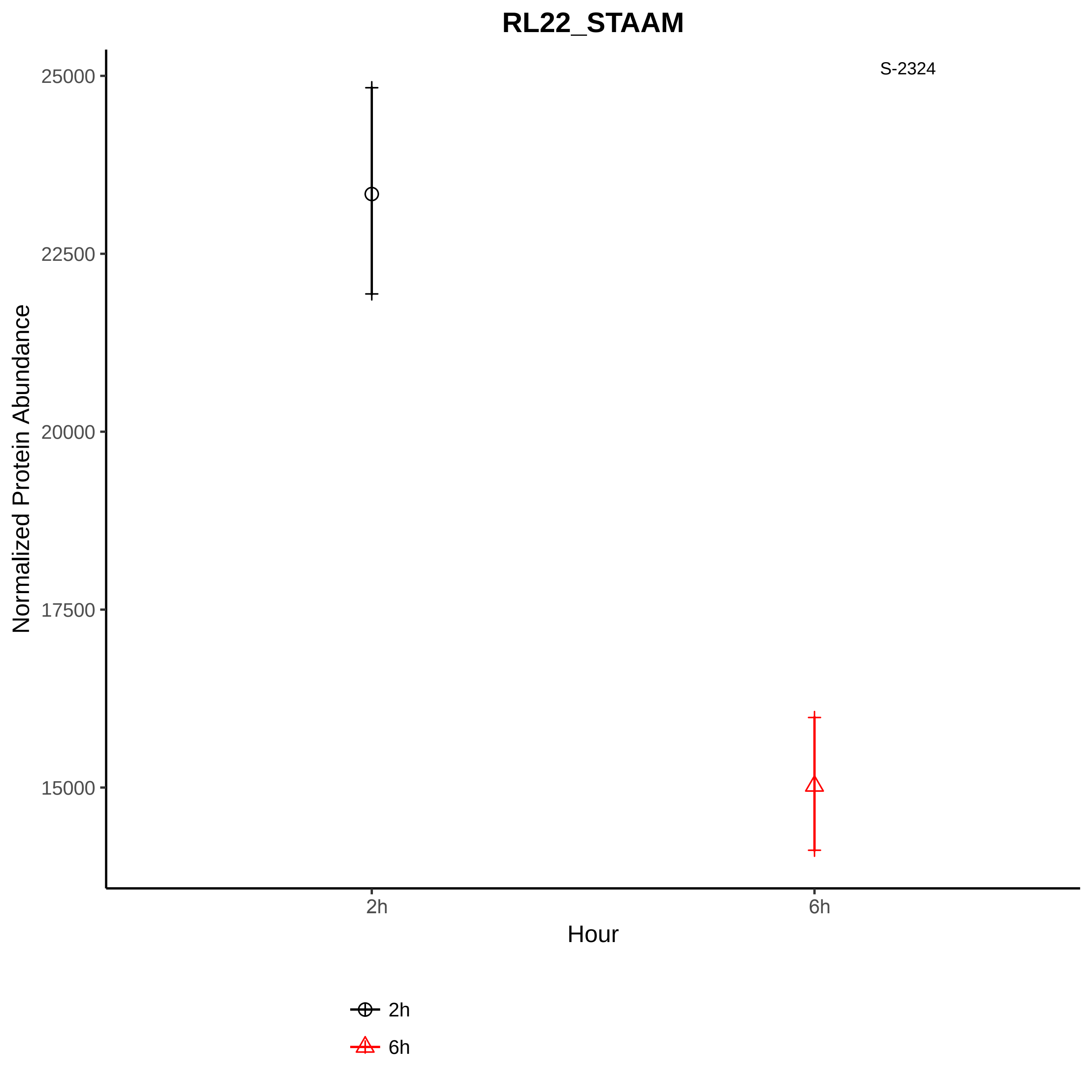
RL22_STAAM
S-2324
Normalized Protein Abundance
25000
22500
20000
17500
15000
2h
6h
Hour
2h
6h

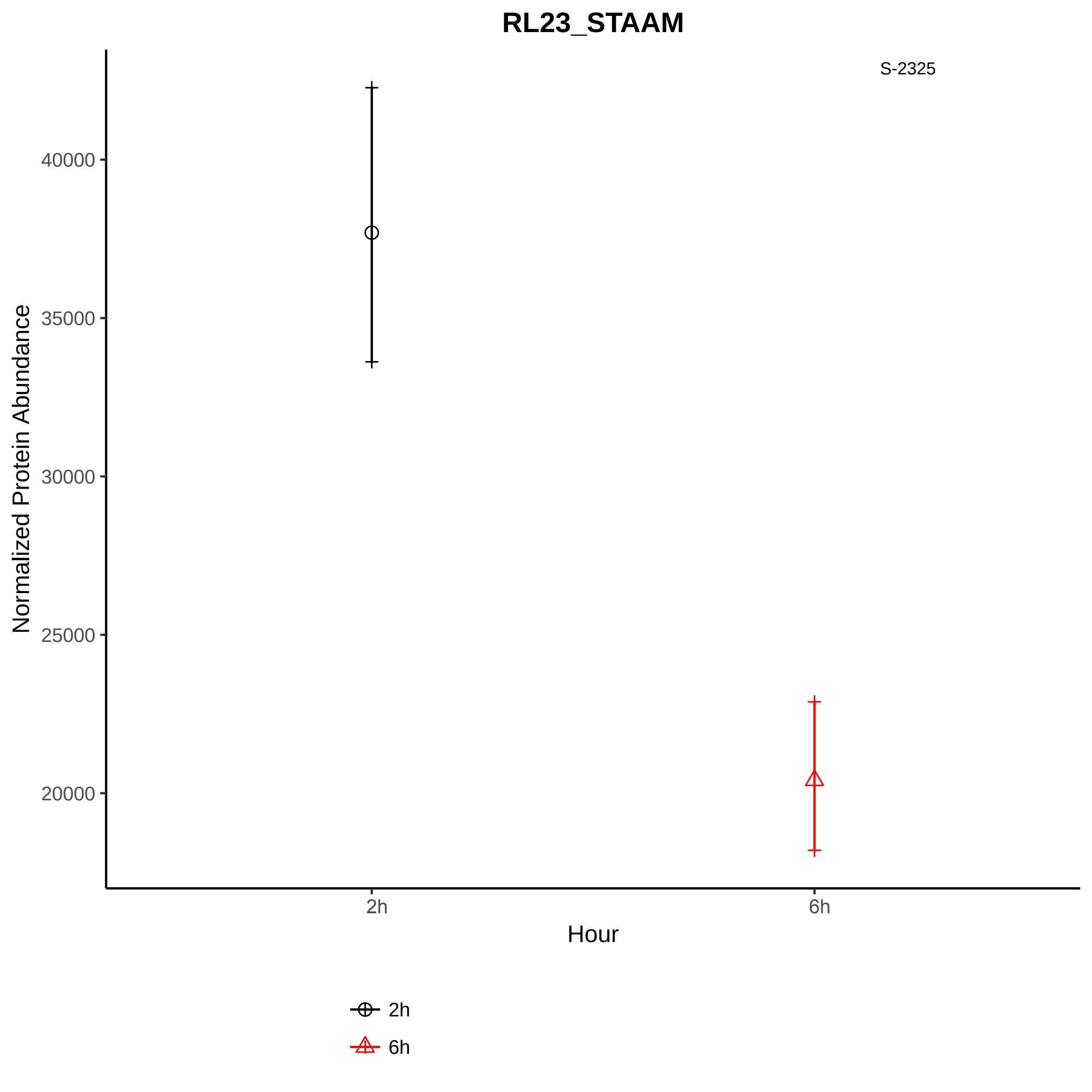
RL23_STAAM
S-2325
40000
35000
30000
25000
20000
Normalized Protein Abundance
2h
6h
Hour
2h
6h

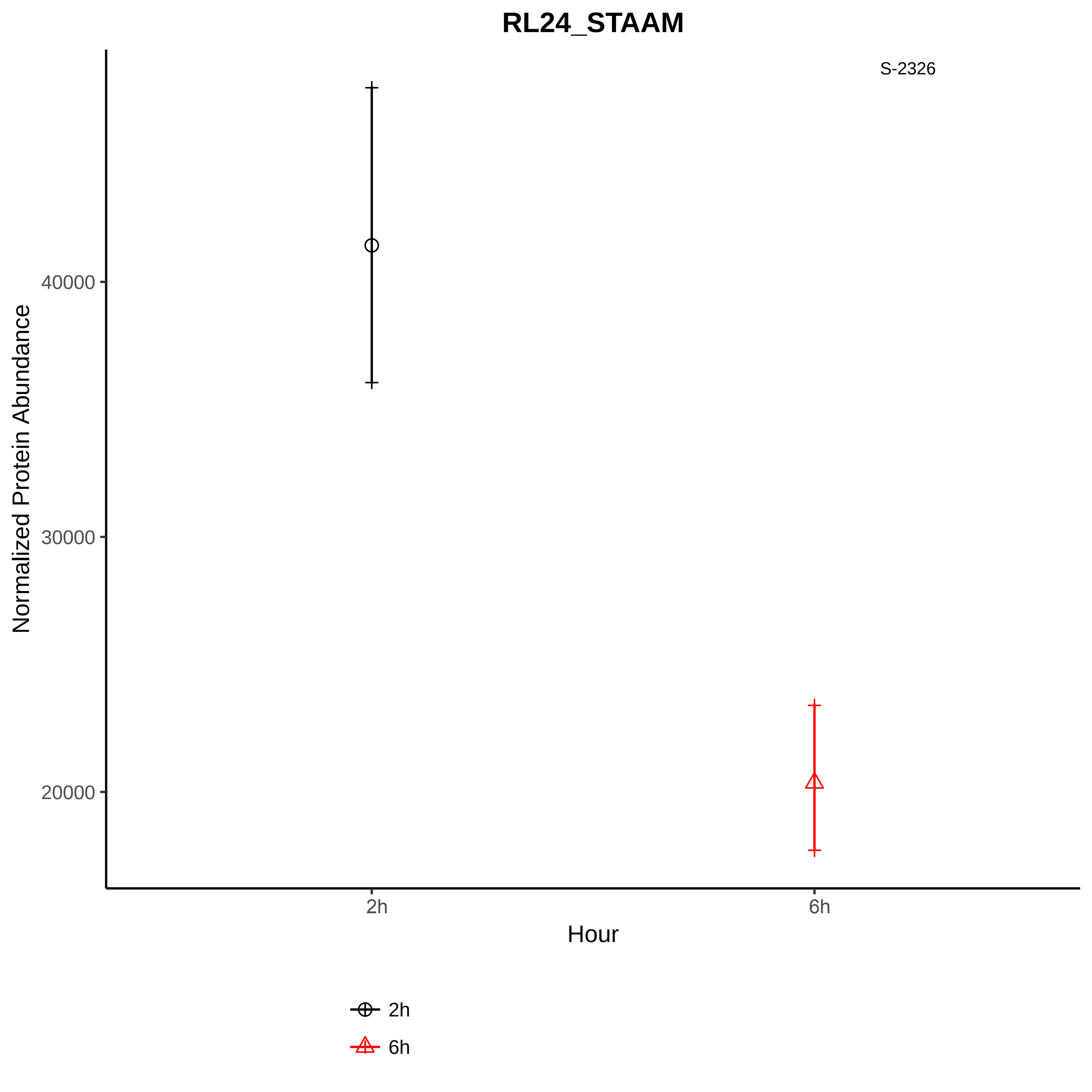

RL24_STAAM
S-2326
Normalized Protein Abundance
40000
30000
20000
2h
6h
Hour
2h
6h

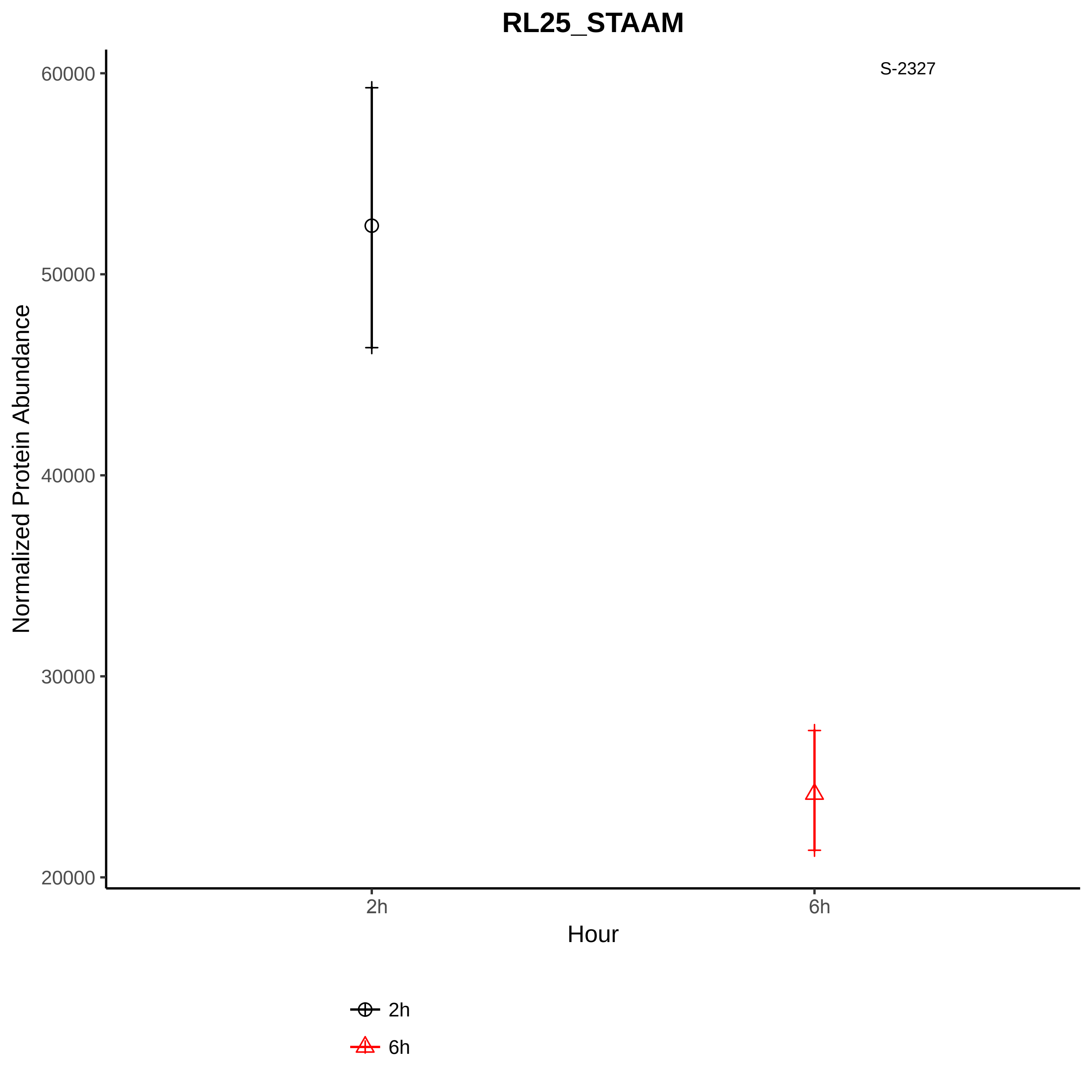

RL25_STAAM
S-2327
Normalized Protein Abundance
60000
50000
40000
30000
20000
2h
6h
Hour
2h
6h

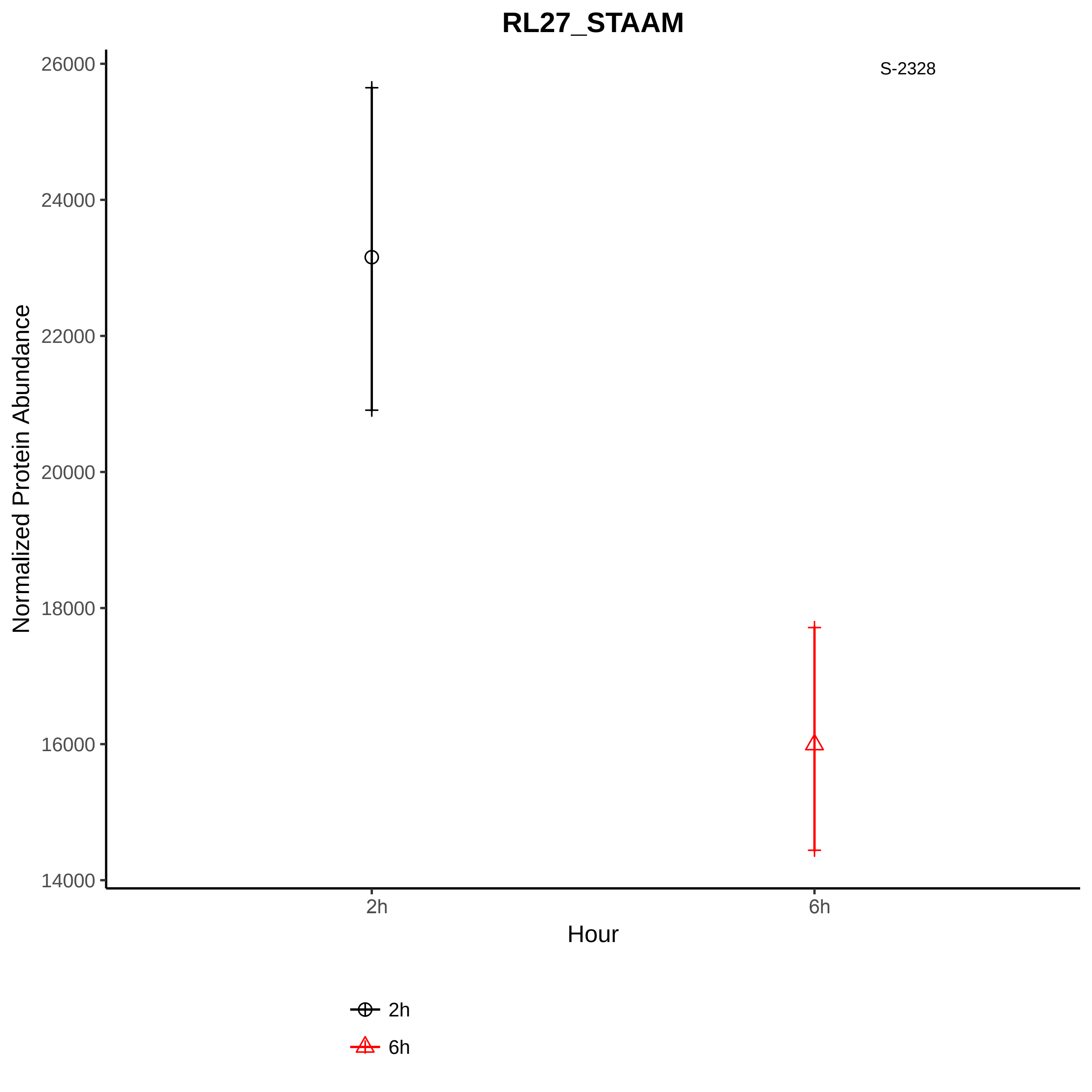

RL27_STAAM
S-2328
Normalized Protein Abundance
26000
24000
22000
20000
18000
16000
14000
2h
6h
Hour
2h
6h

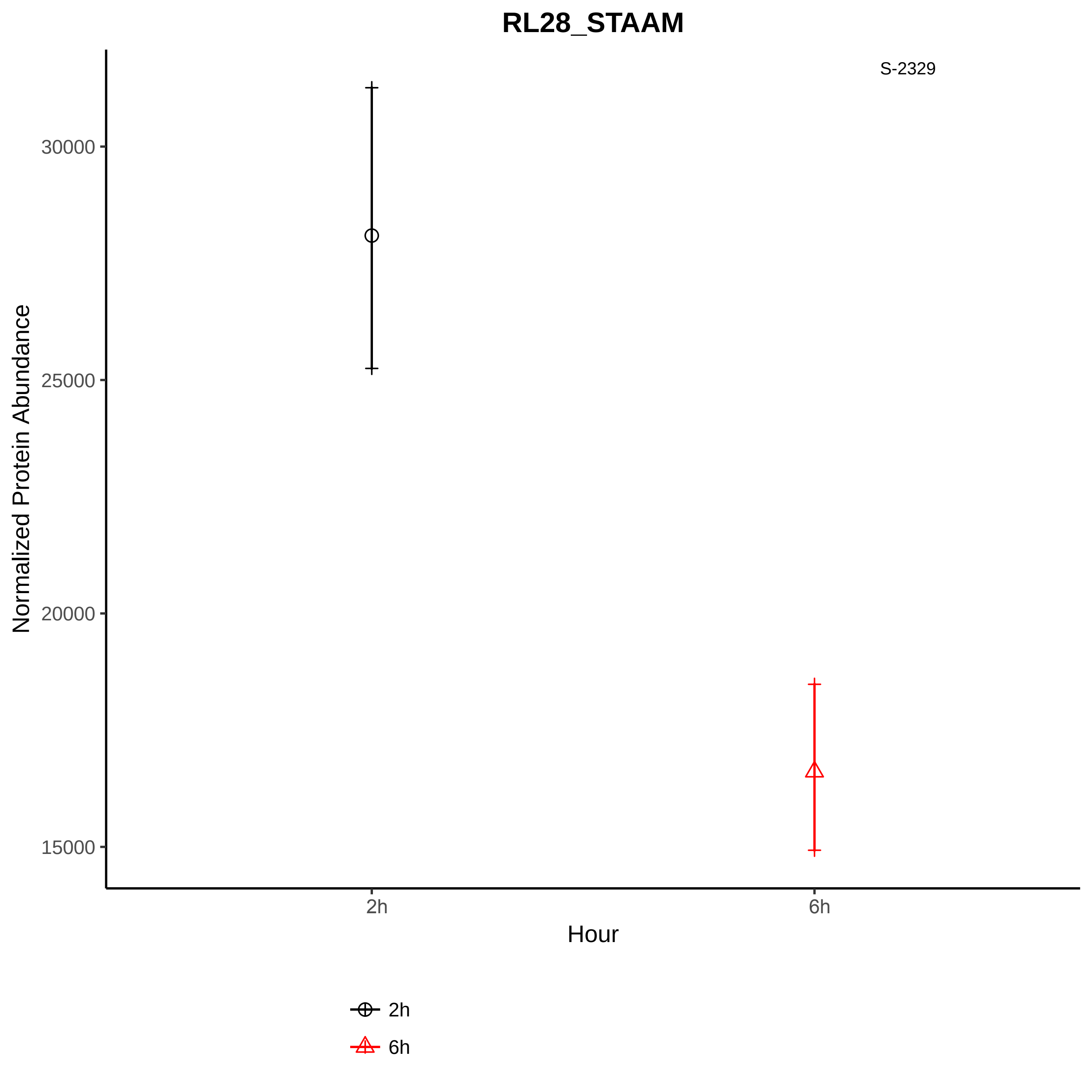
RL28_STAAM
S-2329
Normalized Protein Abundance
30000
25000
20000
15000
2h
6h
Hour
2h
6h

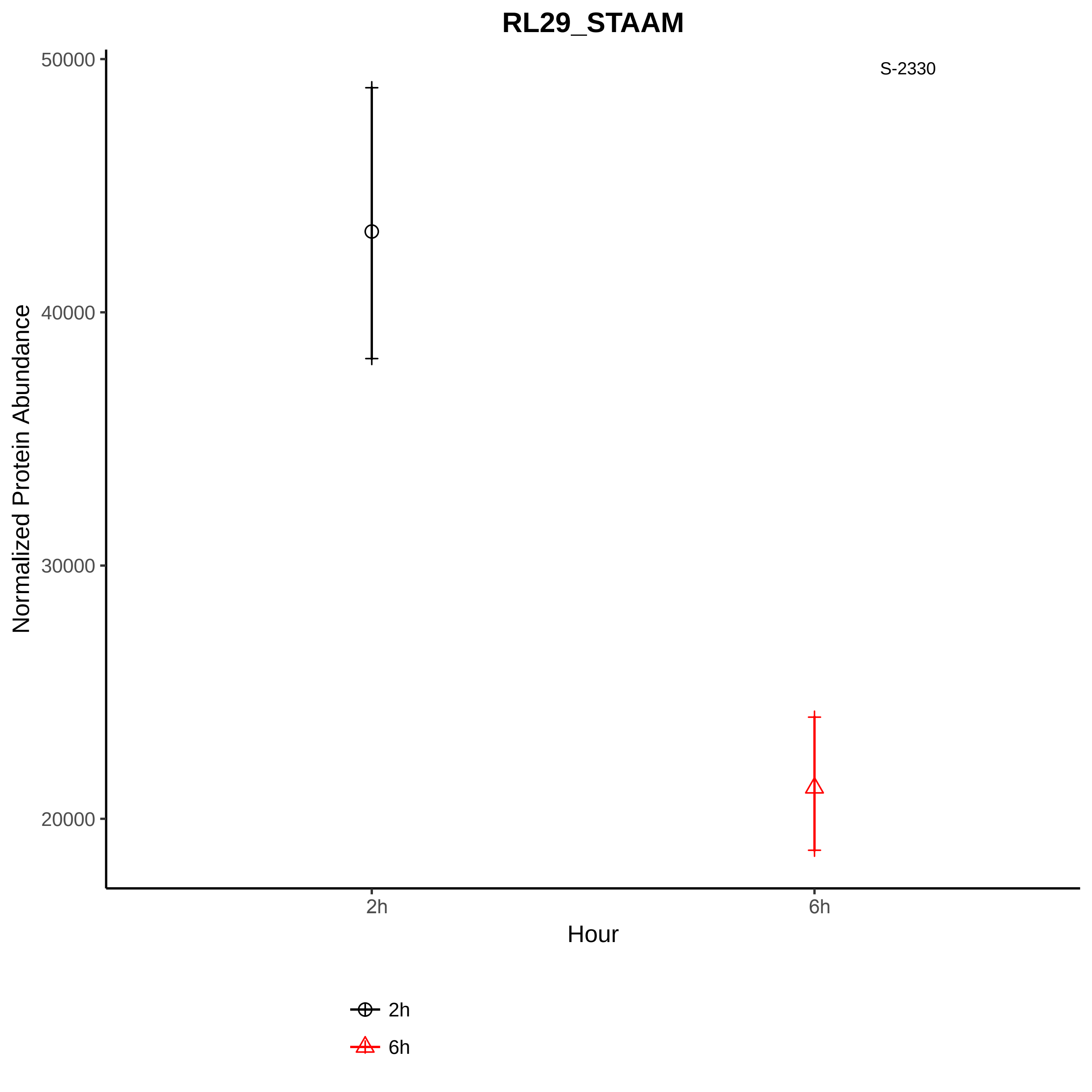

RL29_STAAM
S-2330
50000
40000
30000
20000
Normalized Protein Abundance
2h
6h
Hour
2h
6h

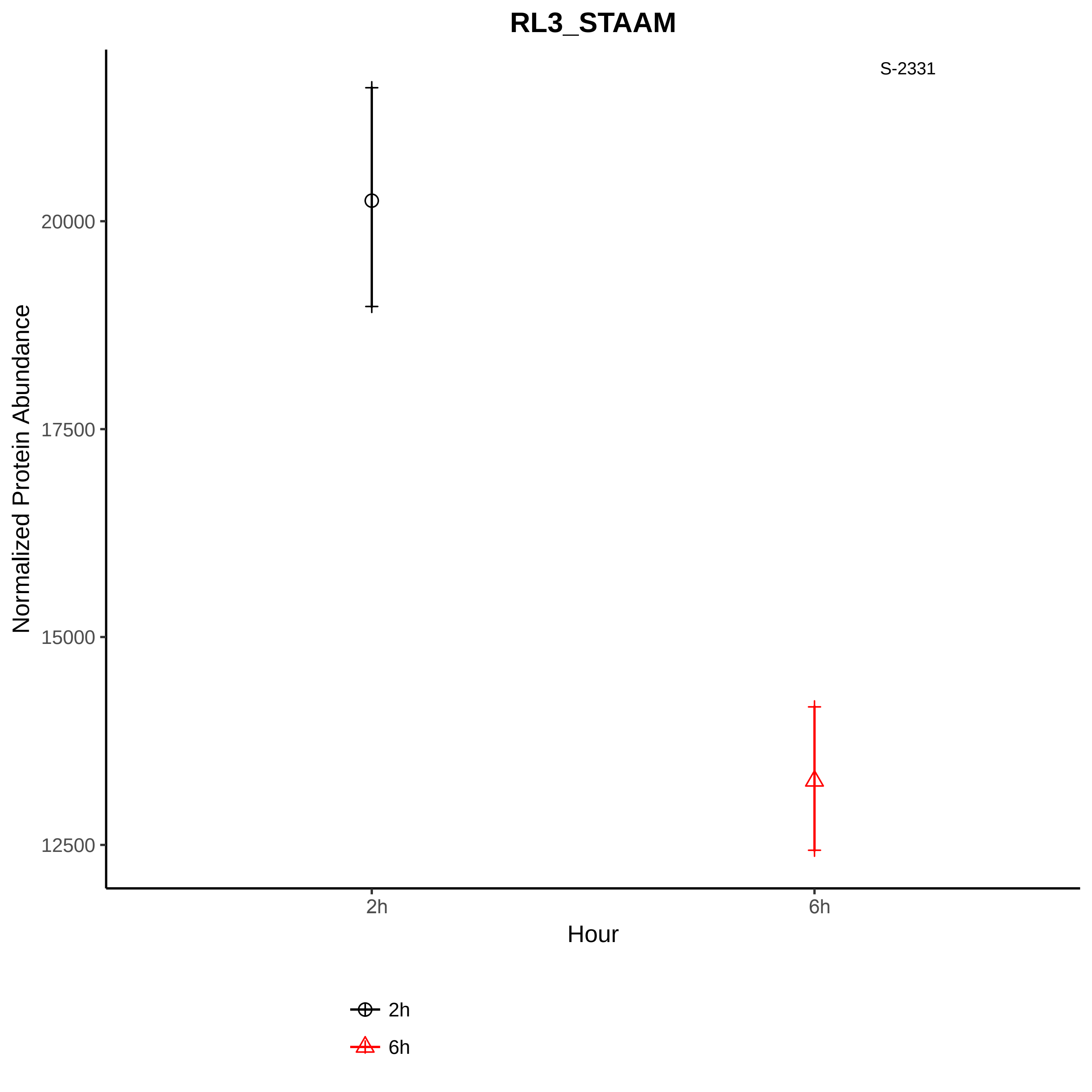

RL3_STAAM
S-2331
Normalized Protein Abundance
20000
17500
15000
12500
2h
6h
Hour
2h
6h

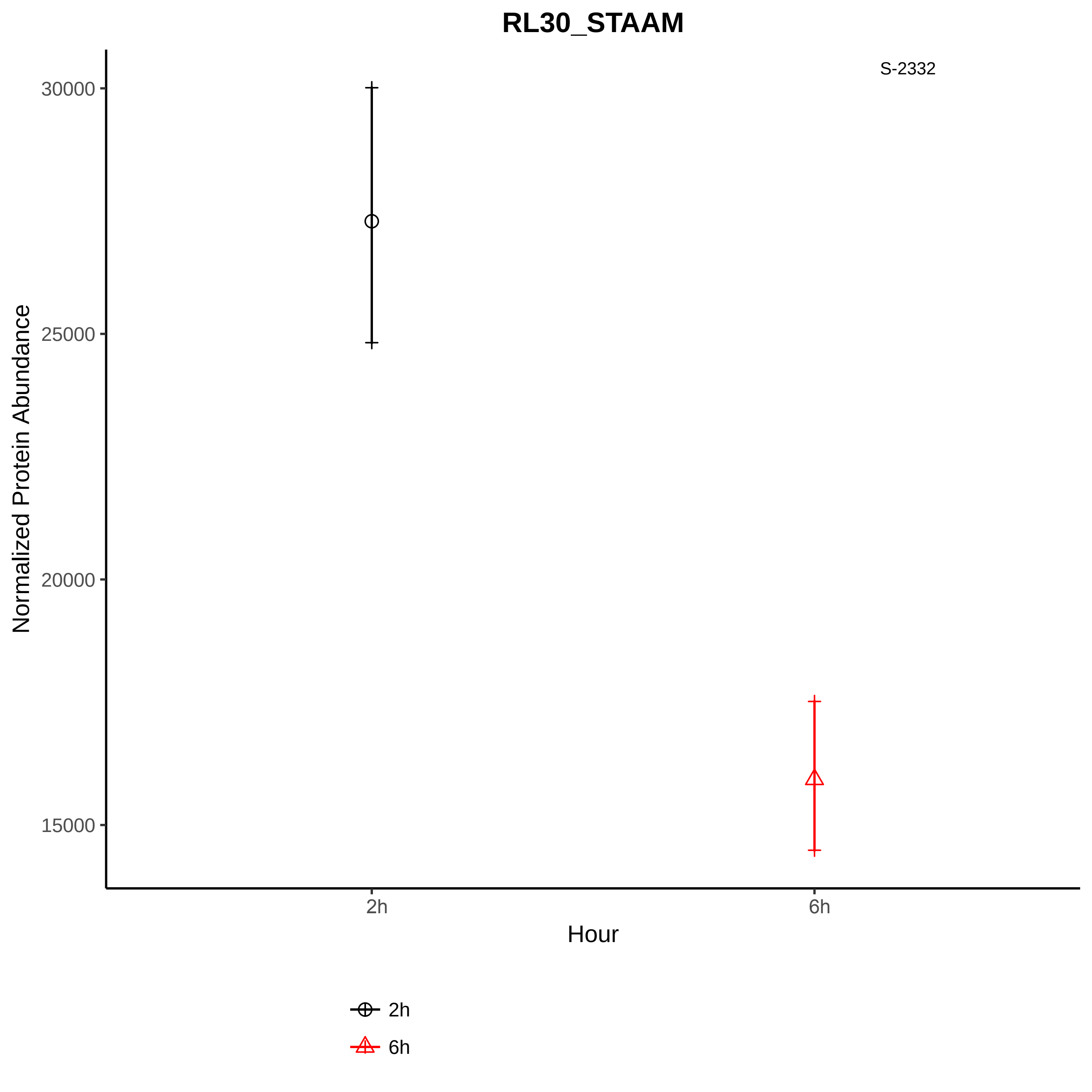
RL30_STAAM
S-2332
30000
25000
20000
15000
Normalized Protein Abundance
2h
6h
Hour
2h
6h

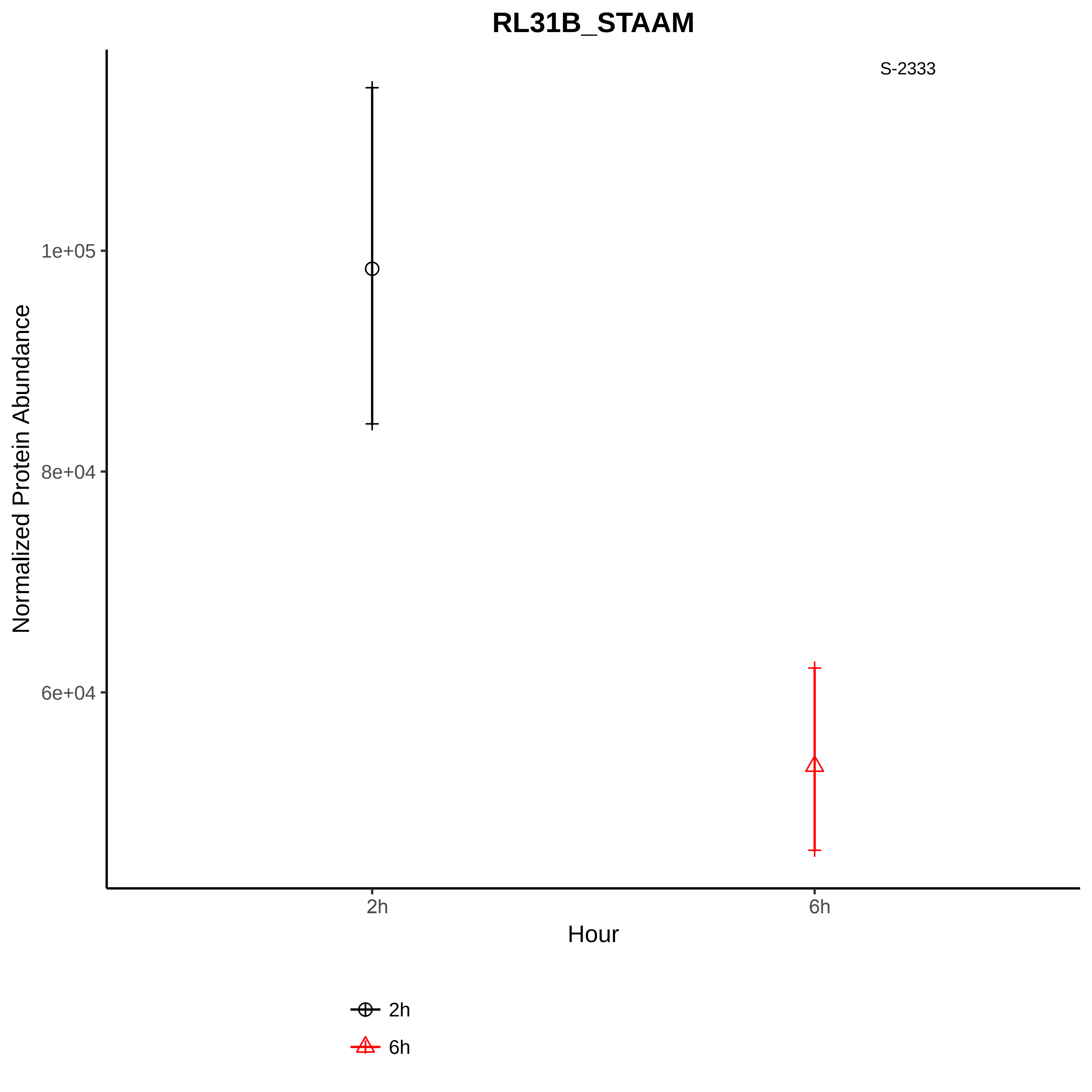

RL31B_STAAM
S-2333
Normalized Protein Abundance
1e+05
8e+04
6e+04
2h
6h
Hour
2h
6h

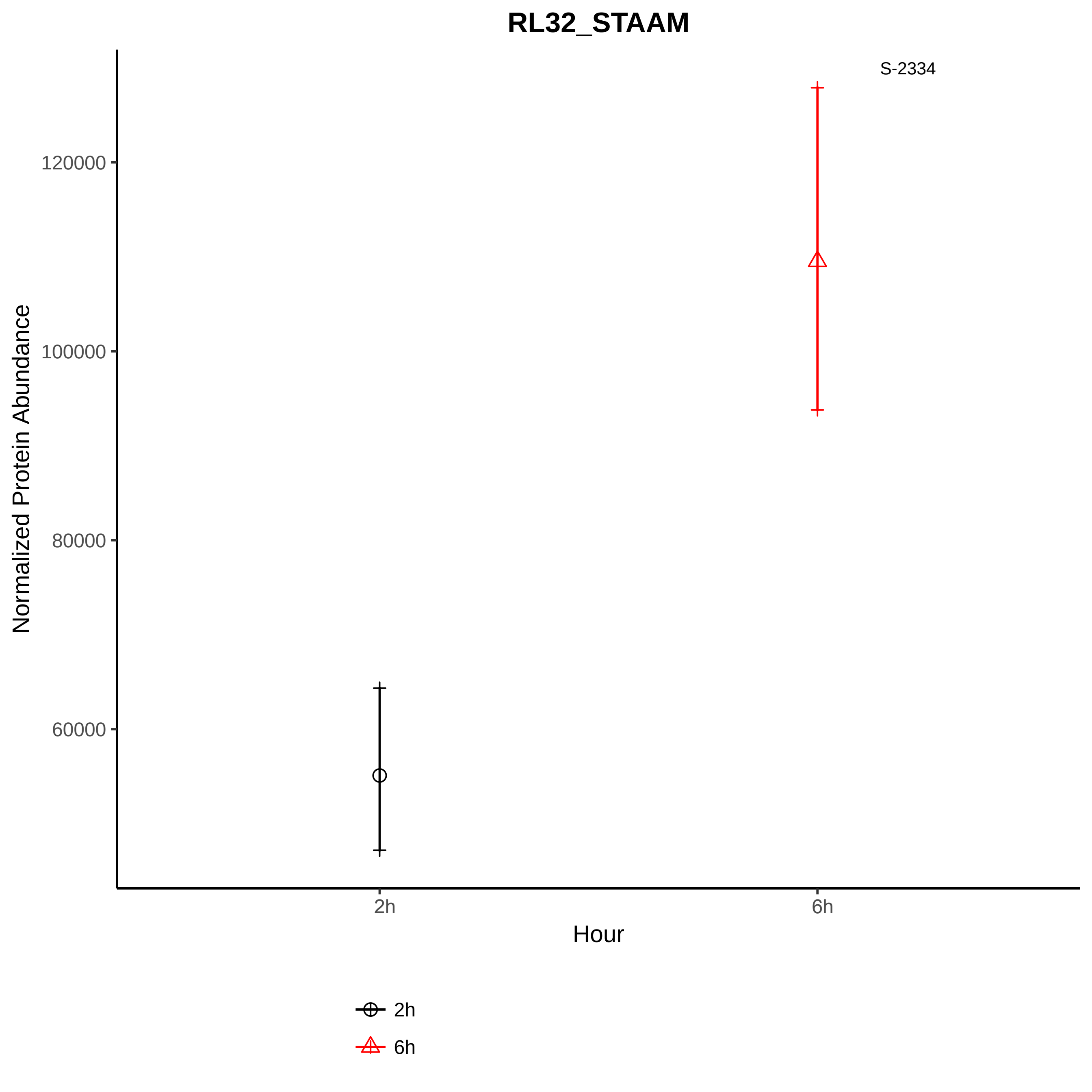

RL32_STAAM
S-2334
Normalized Protein Abundance
120000
100000
80000
60000
2h
6h
Hour
2h
6h

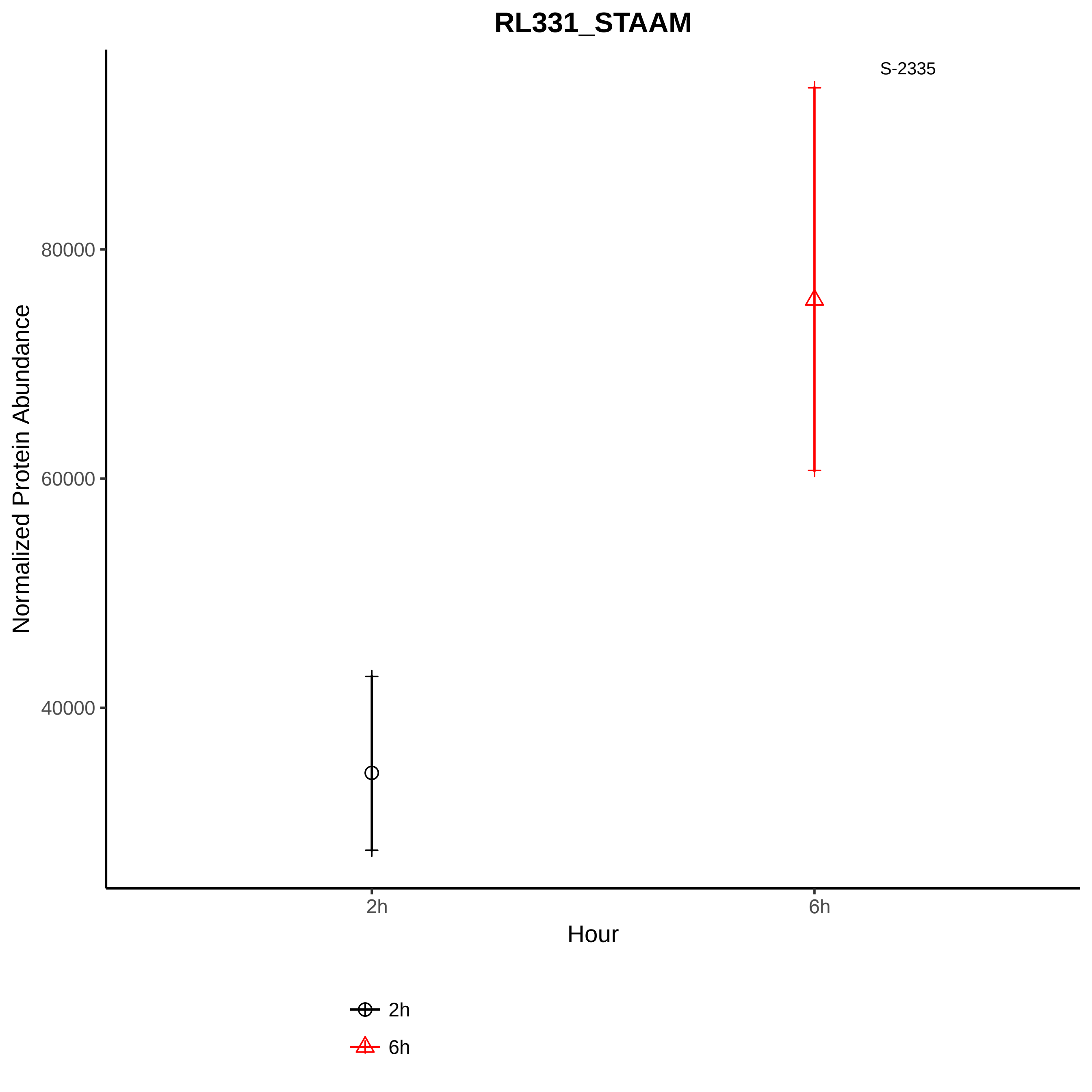

RL331_STAAM
S-2335
Normalized Protein Abundance
80000
60000
40000
2h
6h
Hour
2h
6h

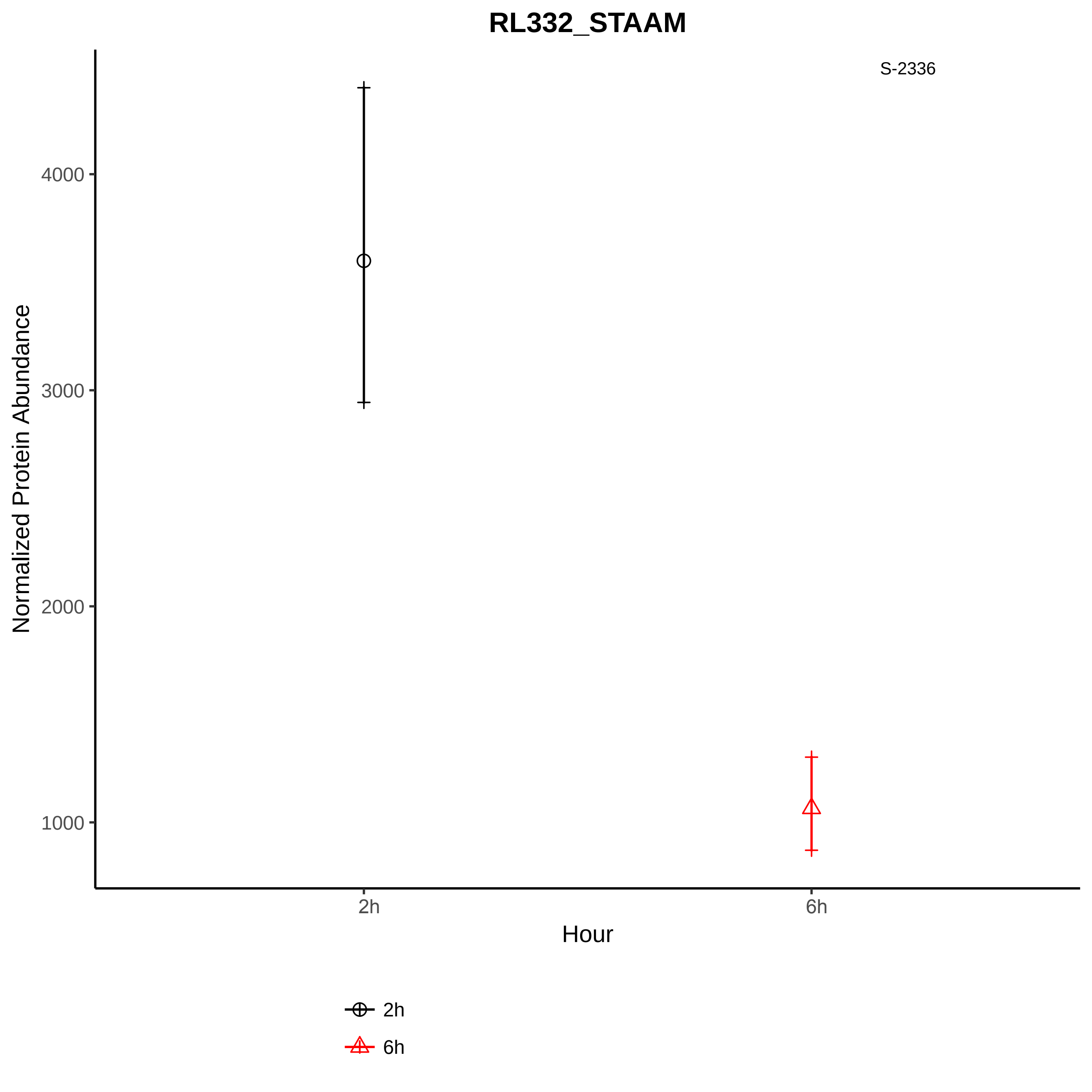
RL332_STAAM
S-2336
Normalized Protein Abundance
4000
3000
2000
1000
2h
6h
Hour
2h
6h

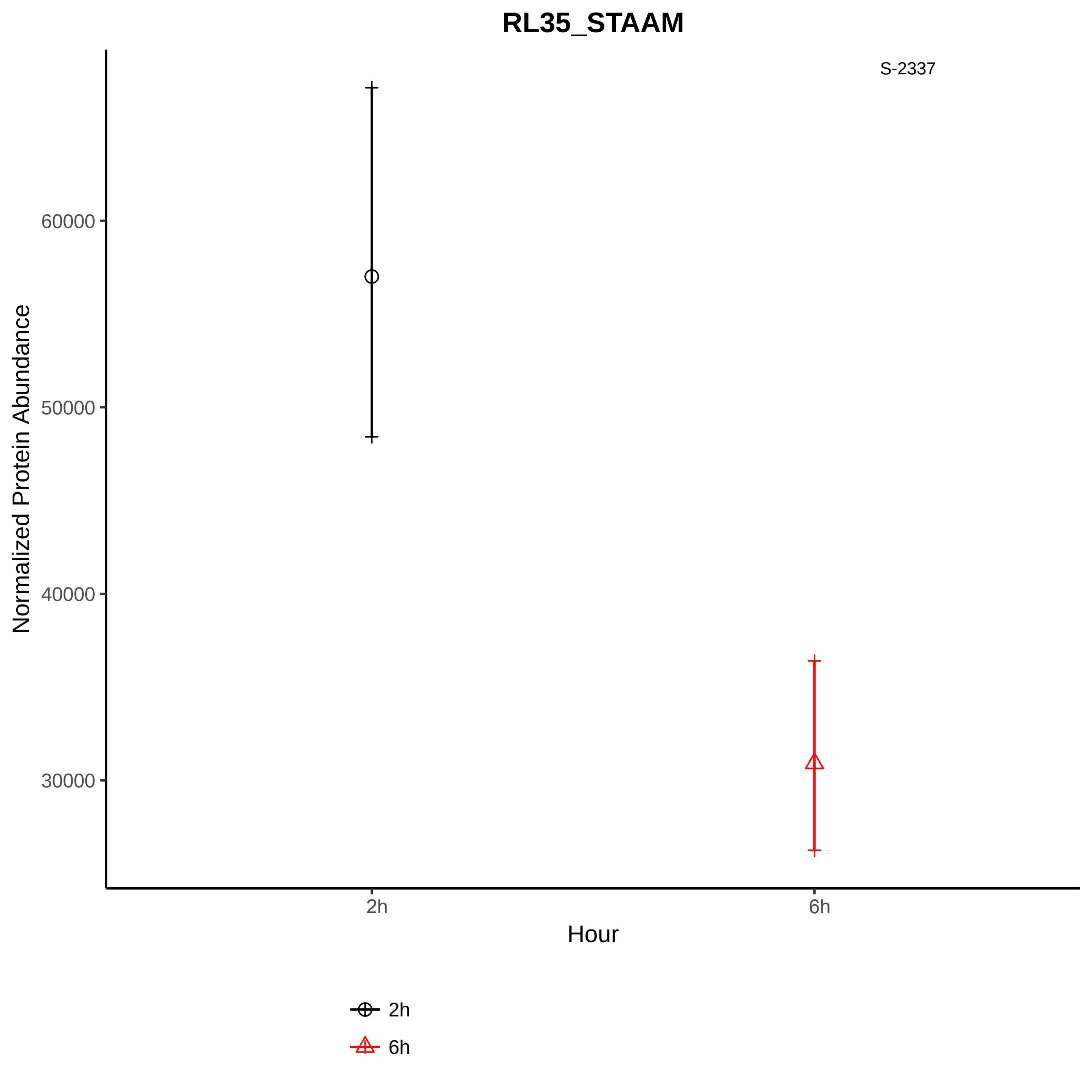

RL35_STAAM
S-2337
Normalized Protein Abundance
60000
50000
40000
30000
2h
6h
Hour
2h
6h

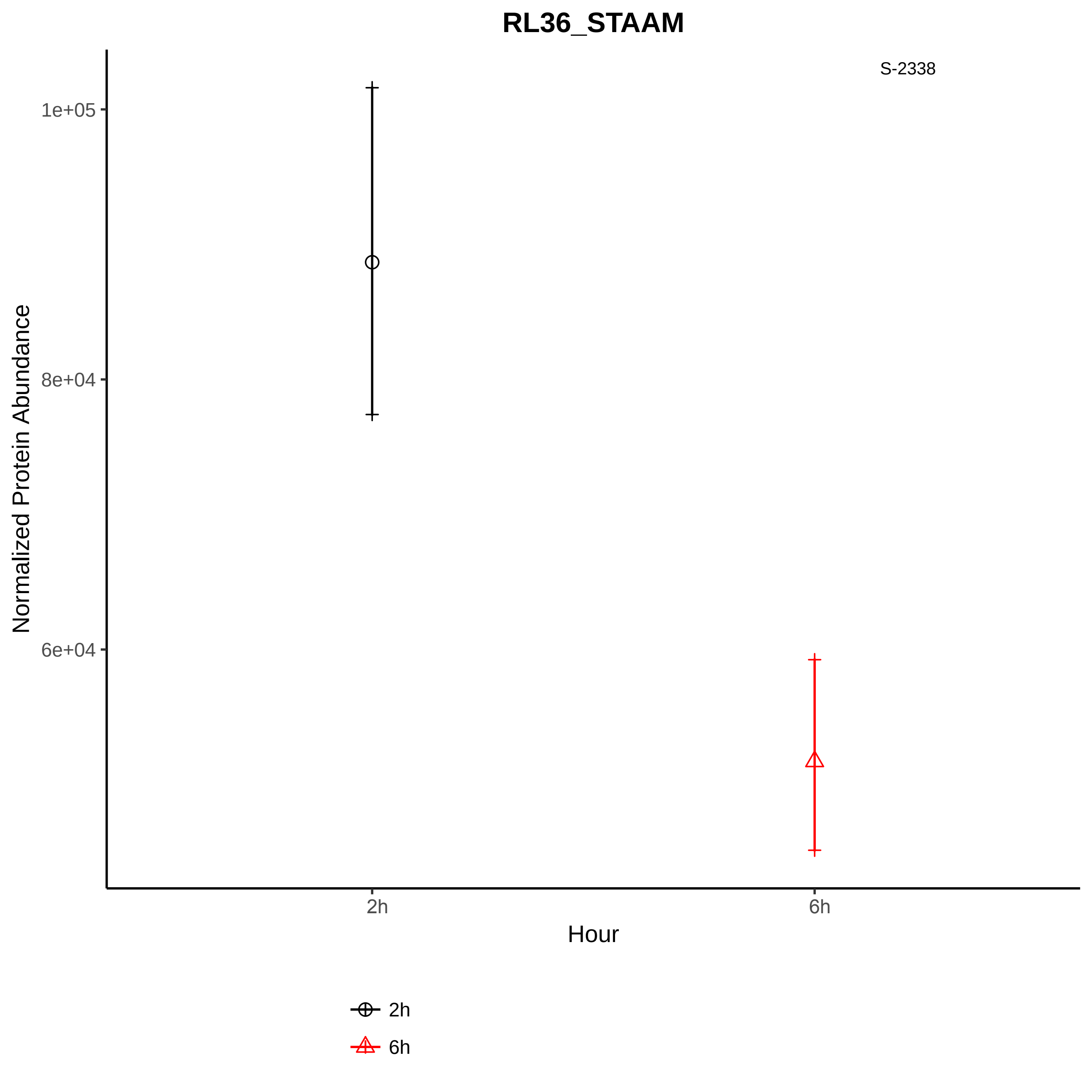

RL36_STAAM
S-2338
1e+05
8e+04
6e+04
Normalized Protein Abundance
2h
6h
Hour
2h
6h

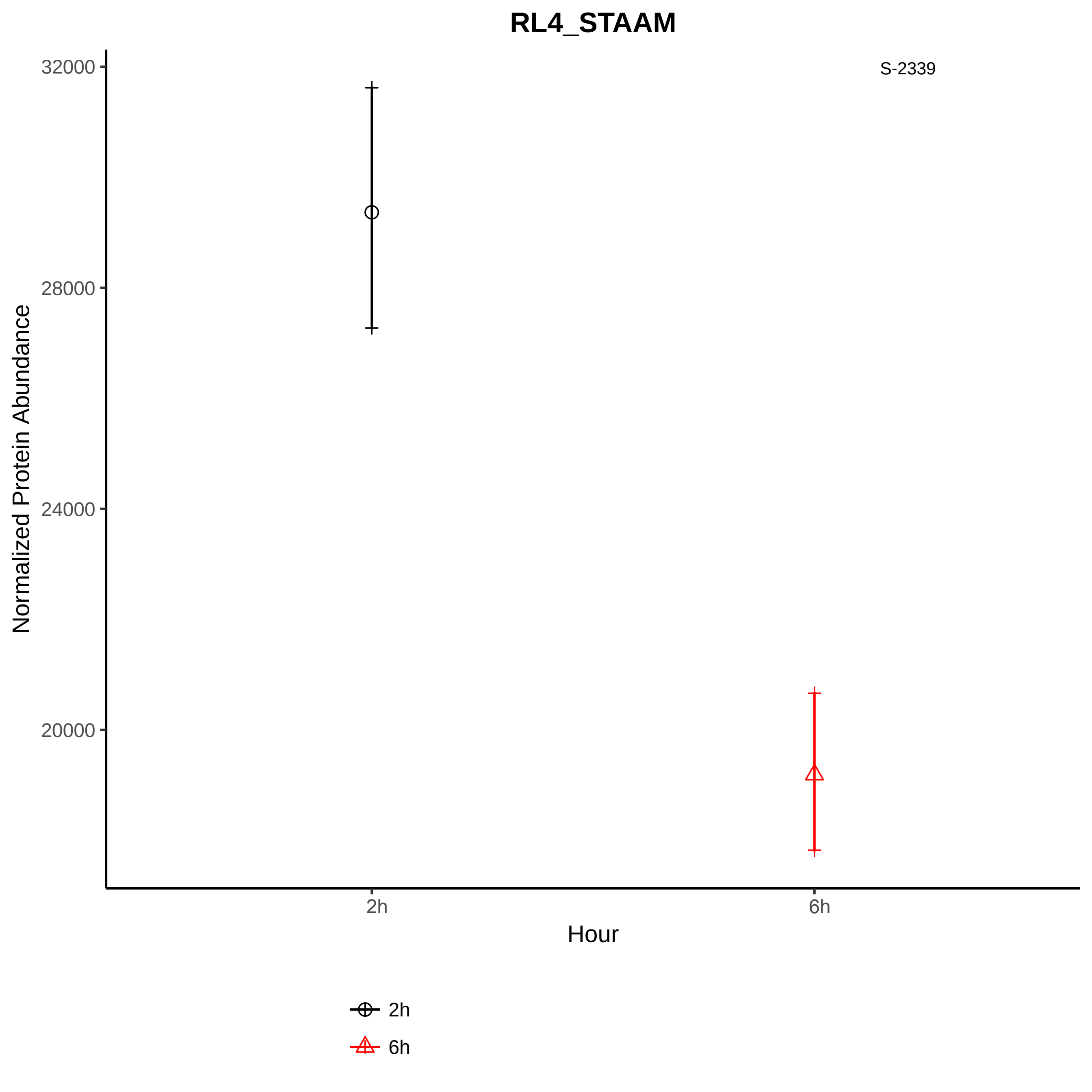

RL4_STAAM
S-2339
Normalized Protein Abundance
32000
28000
24000
20000
2h
6h
Hour
2h
6h

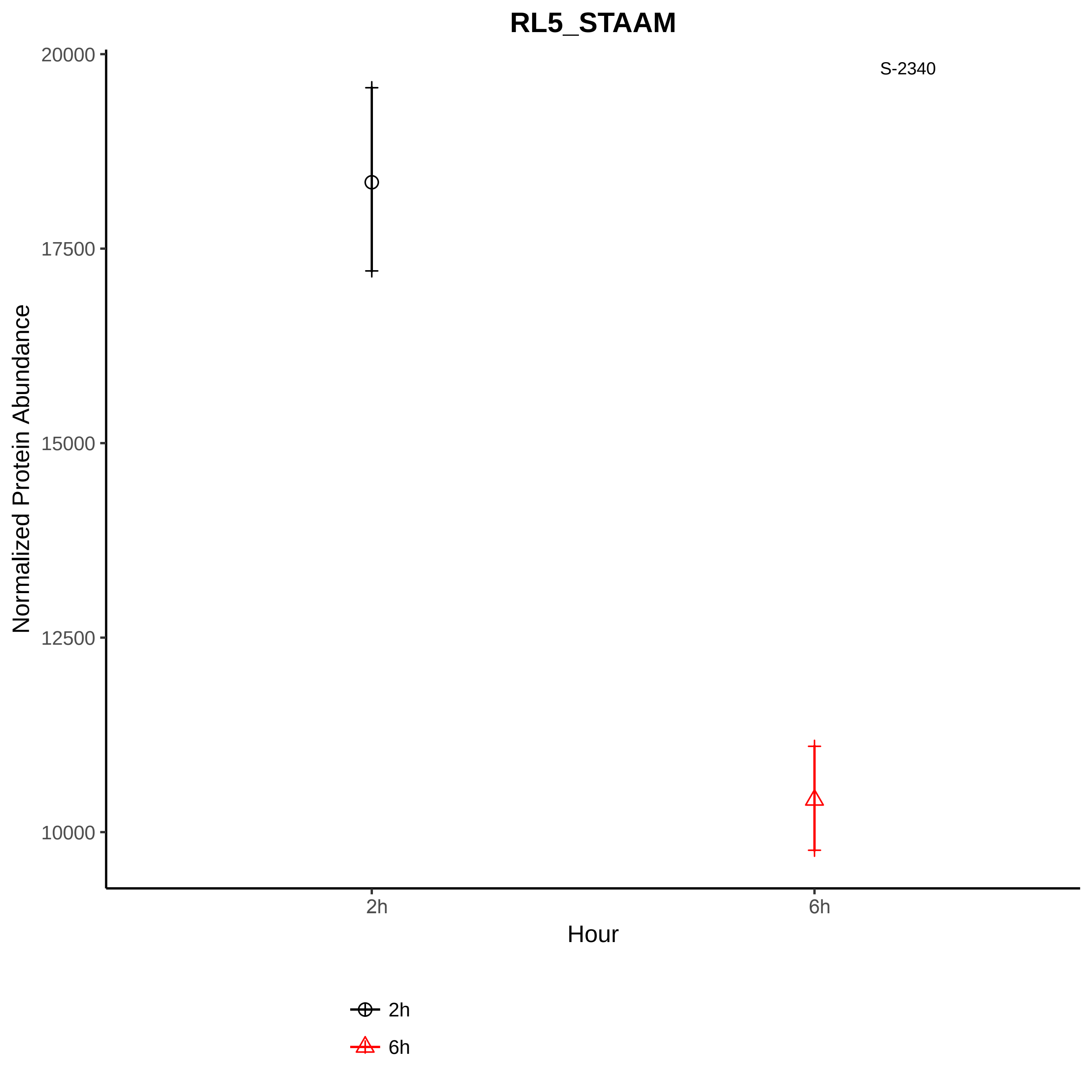
RL5_STAAM
S-2340
20000
17500
15000
12500
10000
Normalized Protein Abundance
2h
6h
Hour
2h
6h

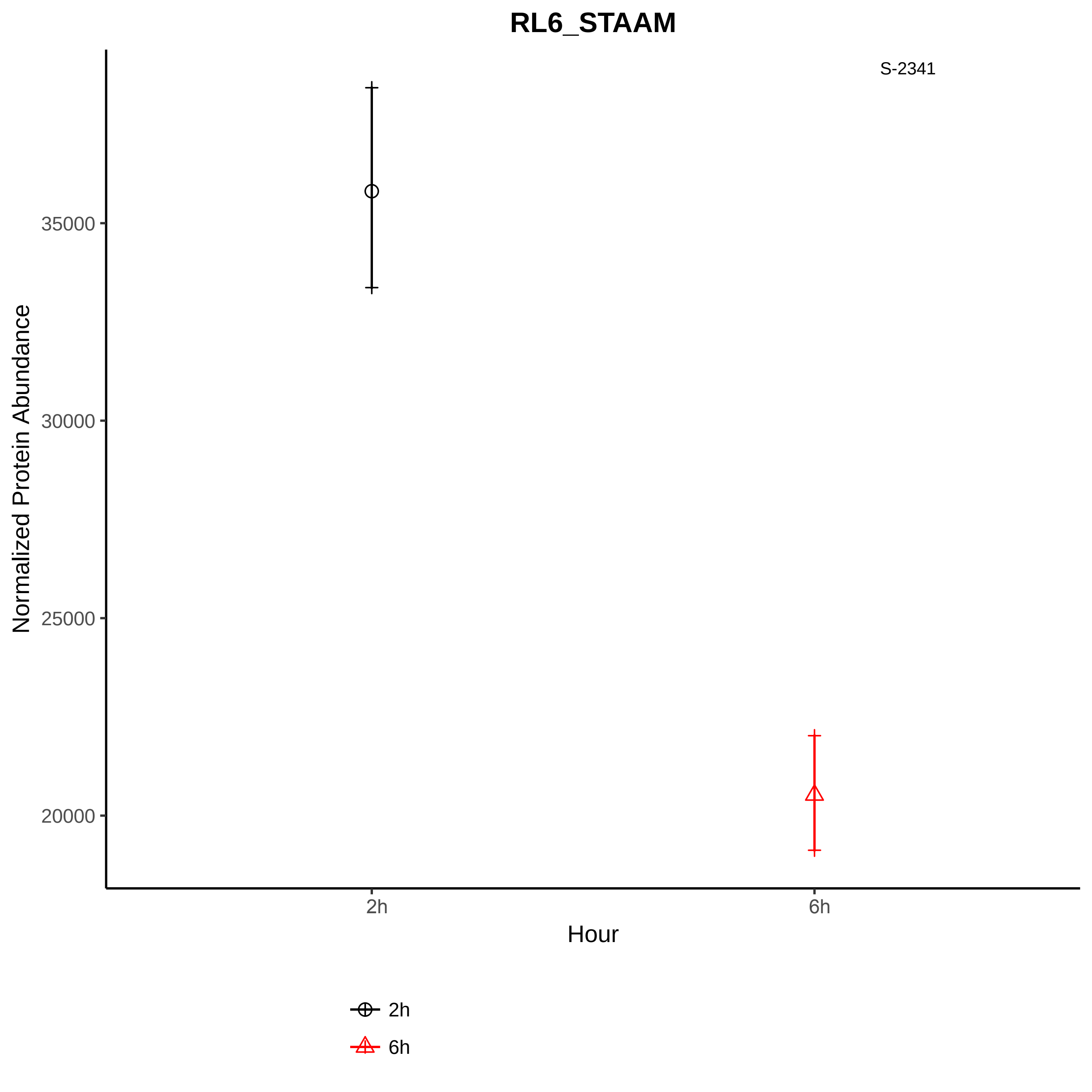

RL6_STAAM
S-2341
Normalized Protein Abundance
35000
30000
25000
20000
2h
6h
Hour
2h
6h

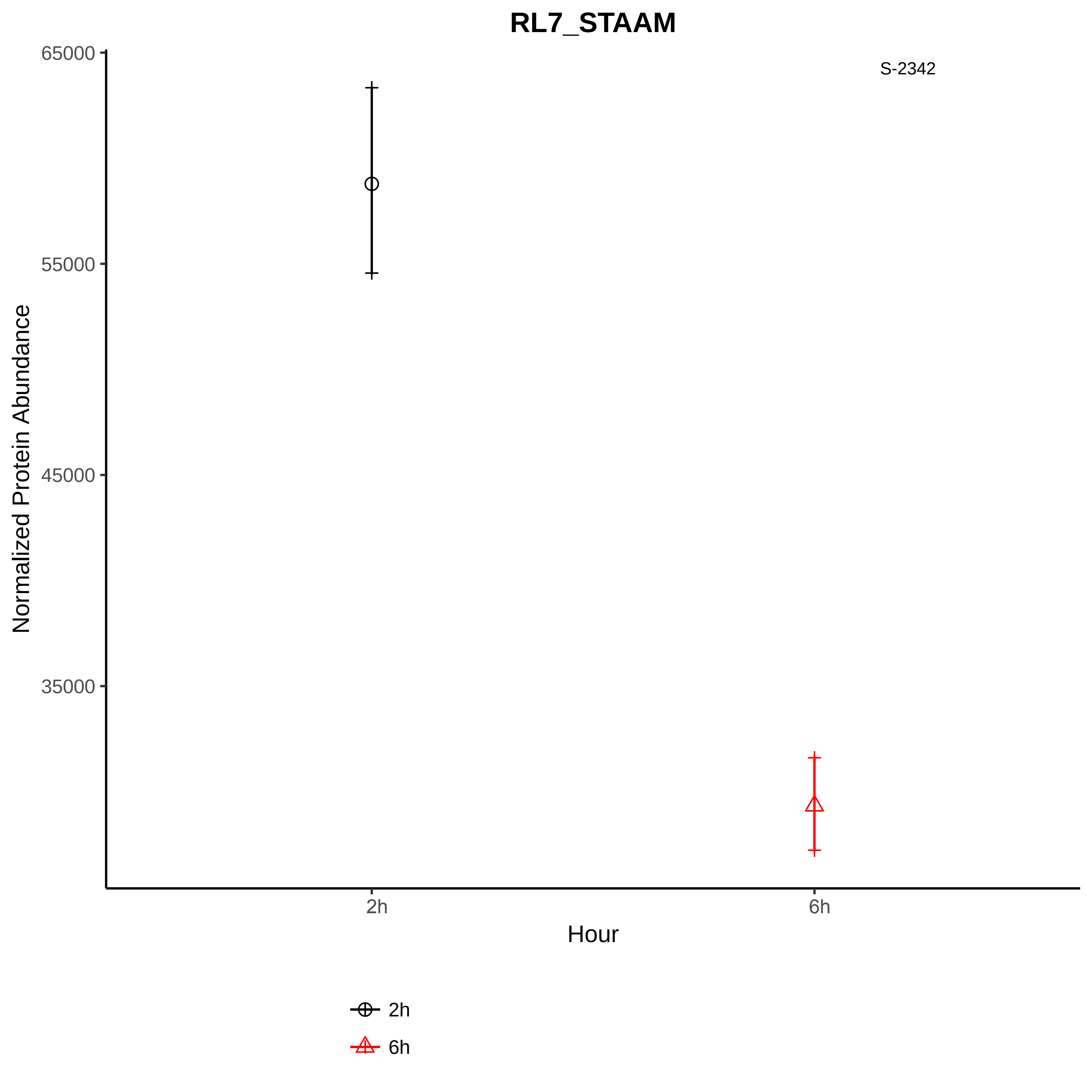

RL7_STAAM
S-2342
65000
55000
45000
35000
Normalized Protein Abundance
2h
6h
Hour
2h
6h

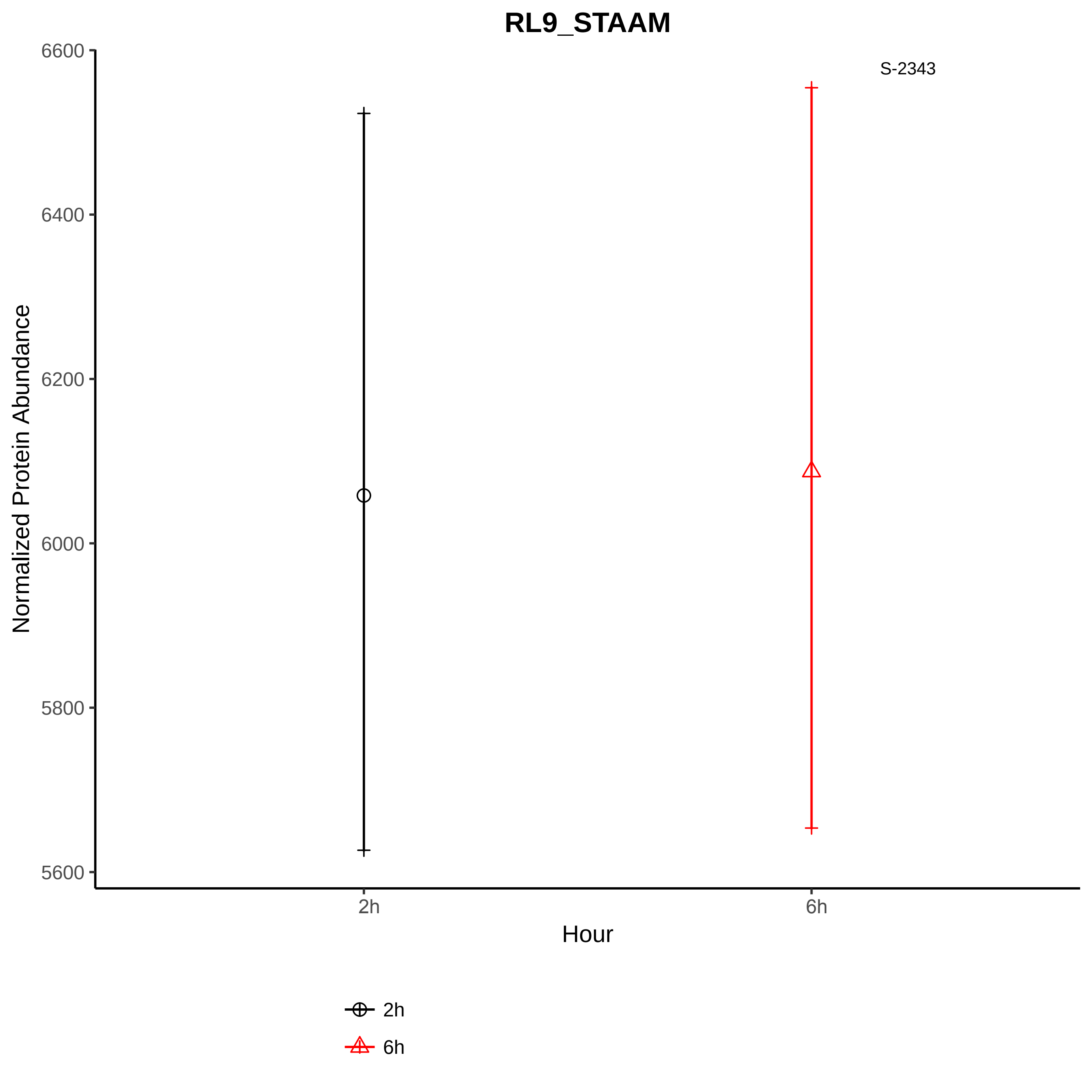
RL9_STAAM
S-2343
Normalized Protein Abundance
6600
6400
6200
6000
5800
5600
2h
6h
Hour
2h
6h

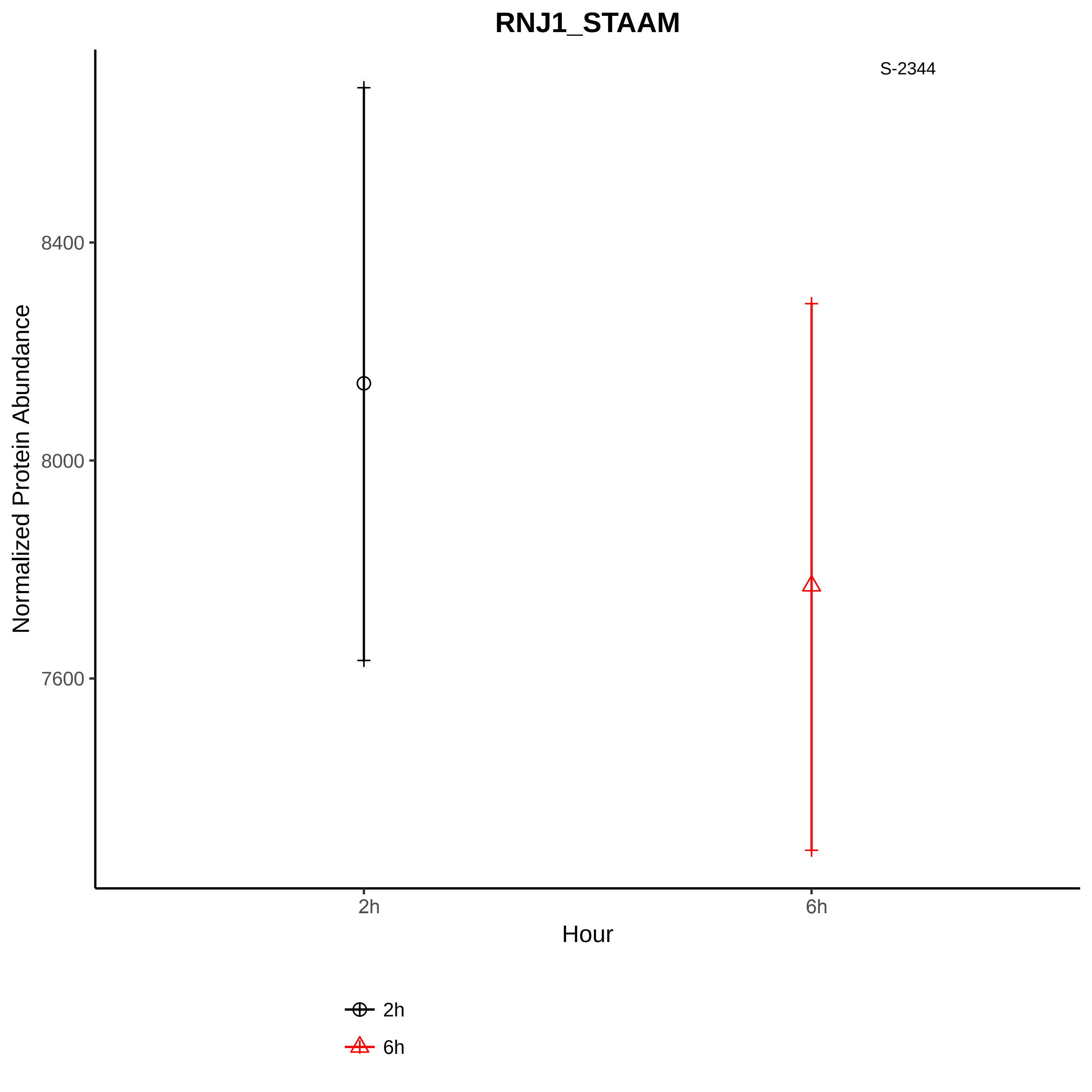

RNJ1_STAAM
S-2344
Normalized Protein Abundance
8400
8000
7600
2h
6h
Hour
2h
6h

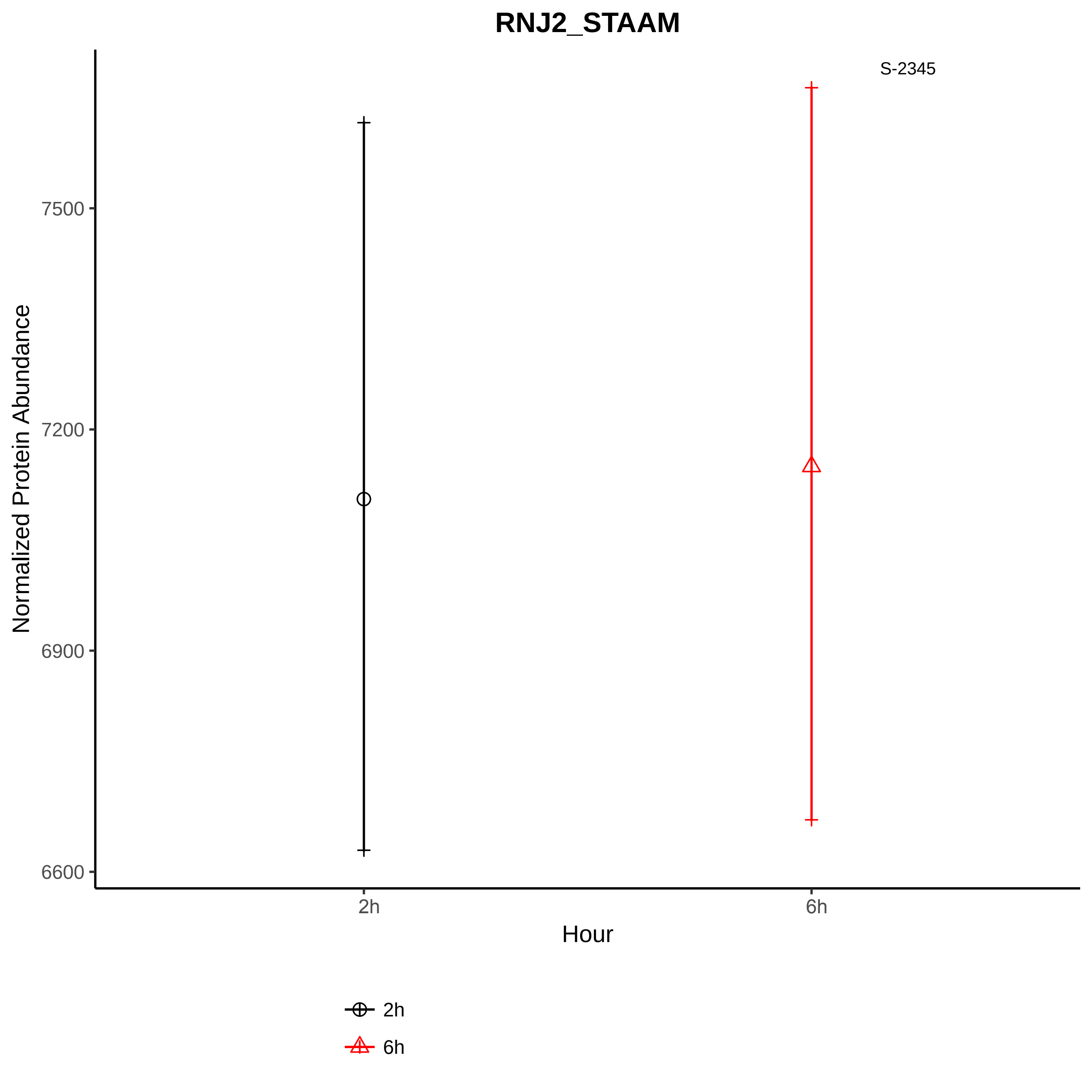
RNJ2_STAAM
S-2345
Normalized Protein Abundance
7500
7200
6900
6600
2h
6h
Hour
2h
6h

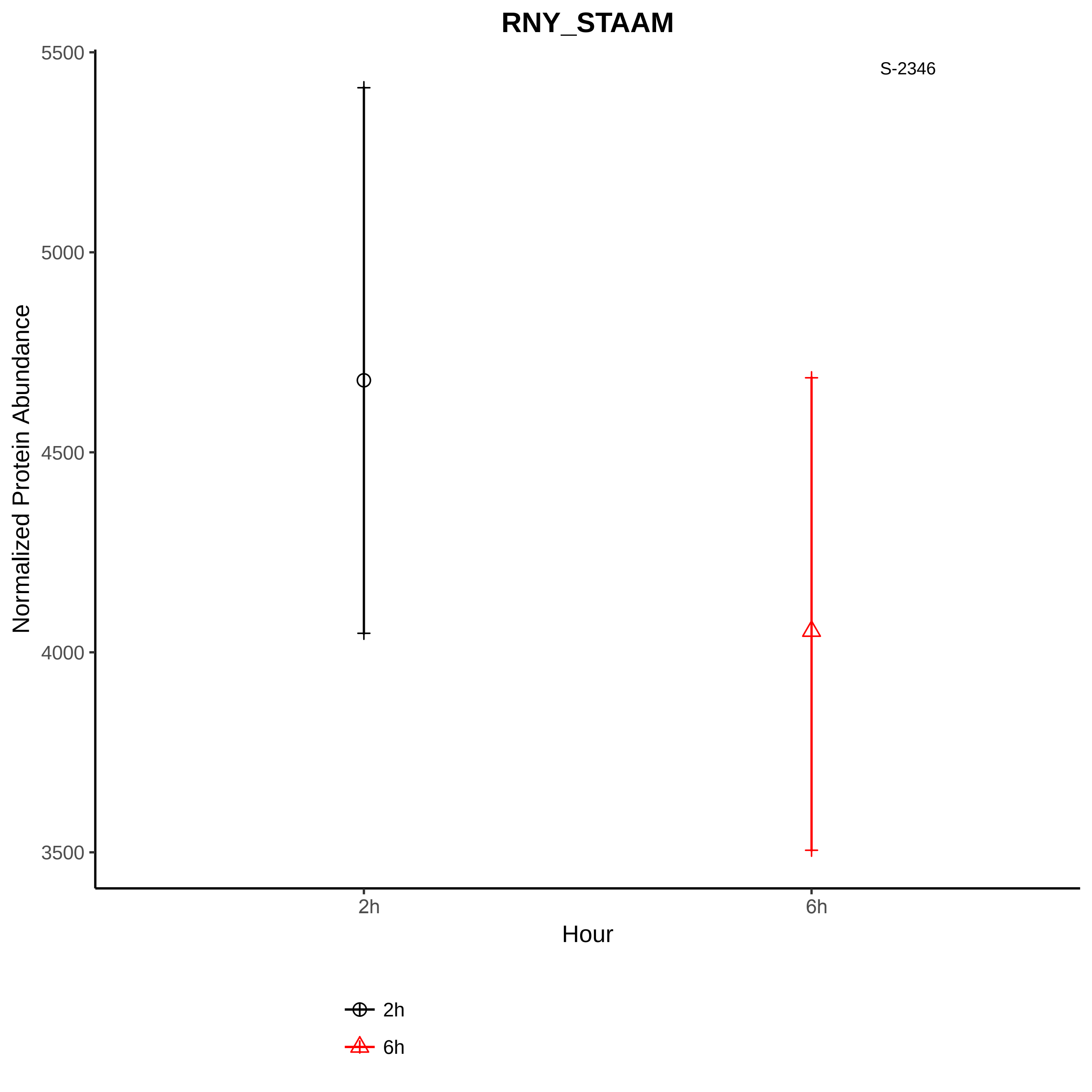

RNY_STAAM
S-2346
Normalized Protein Abundance
5500
5000
4500
4000
3500
2h
6h
Hour
2h
6h

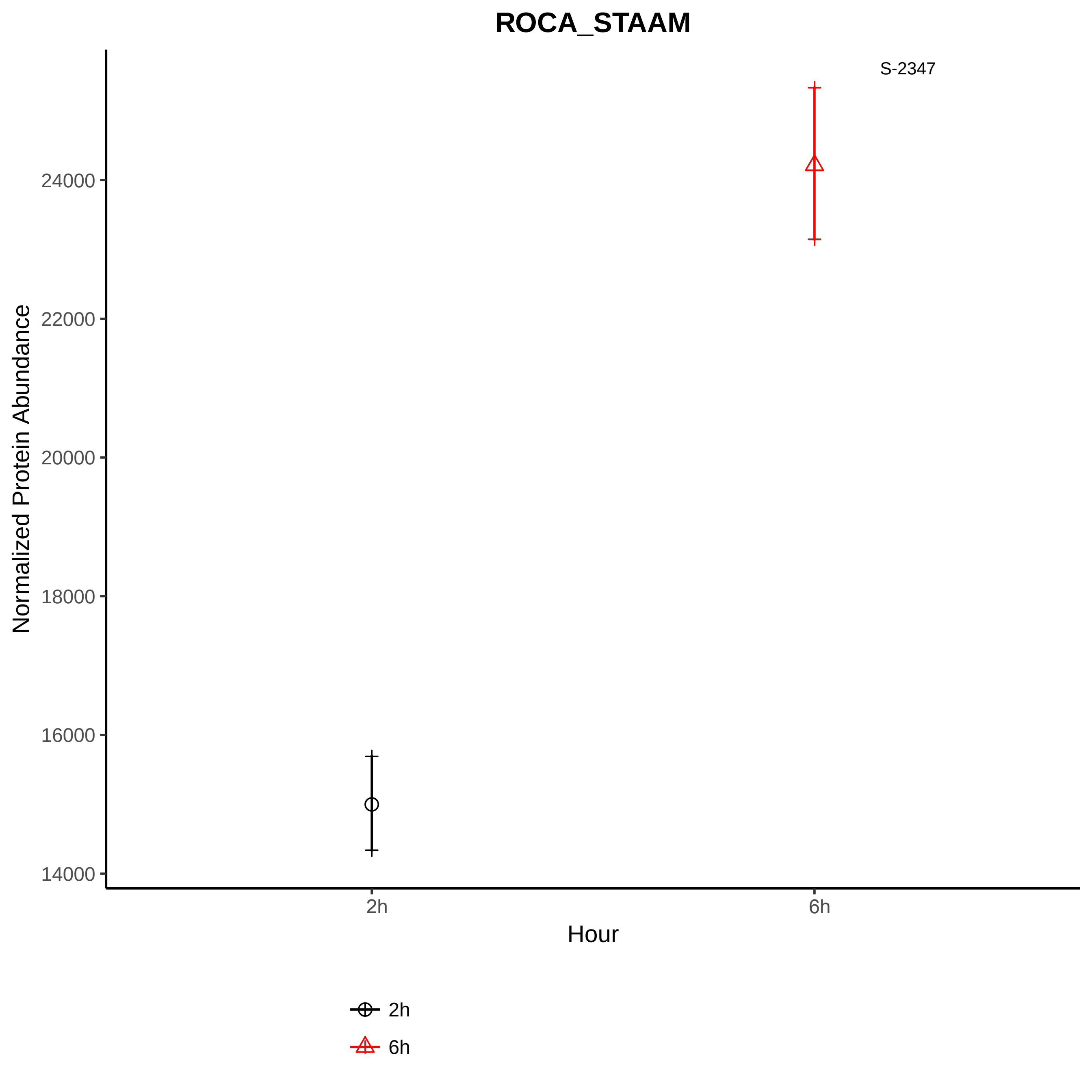

ROCA_STAAM
S-2347
Normalized Protein Abundance
24000
22000
20000
18000
16000
14000
2h
6h
Hour
2h
6h

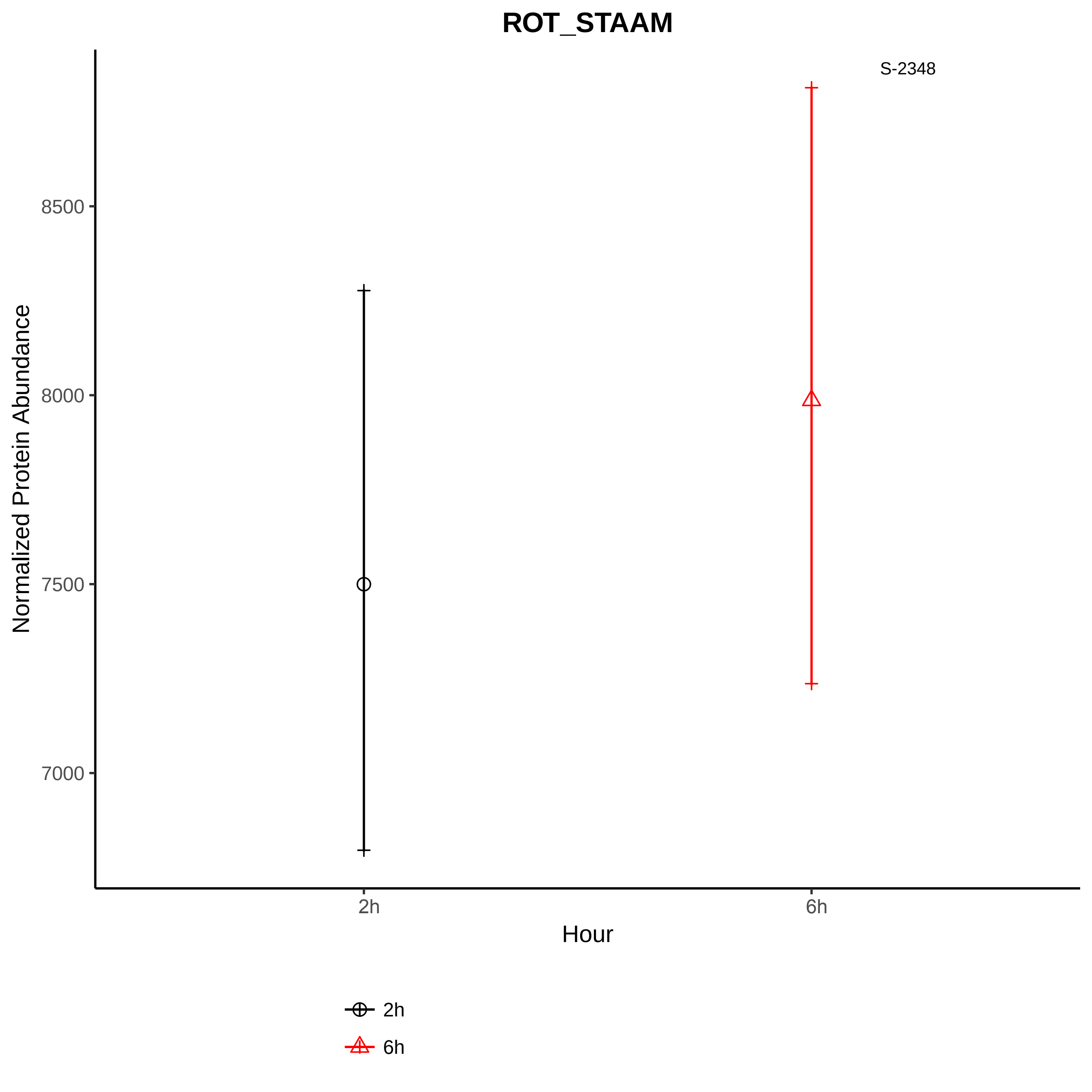

ROT_STAAM
S-2348
Normalized Protein Abundance
8500
8000
7500
7000
2h
6h
Hour
2h
6h

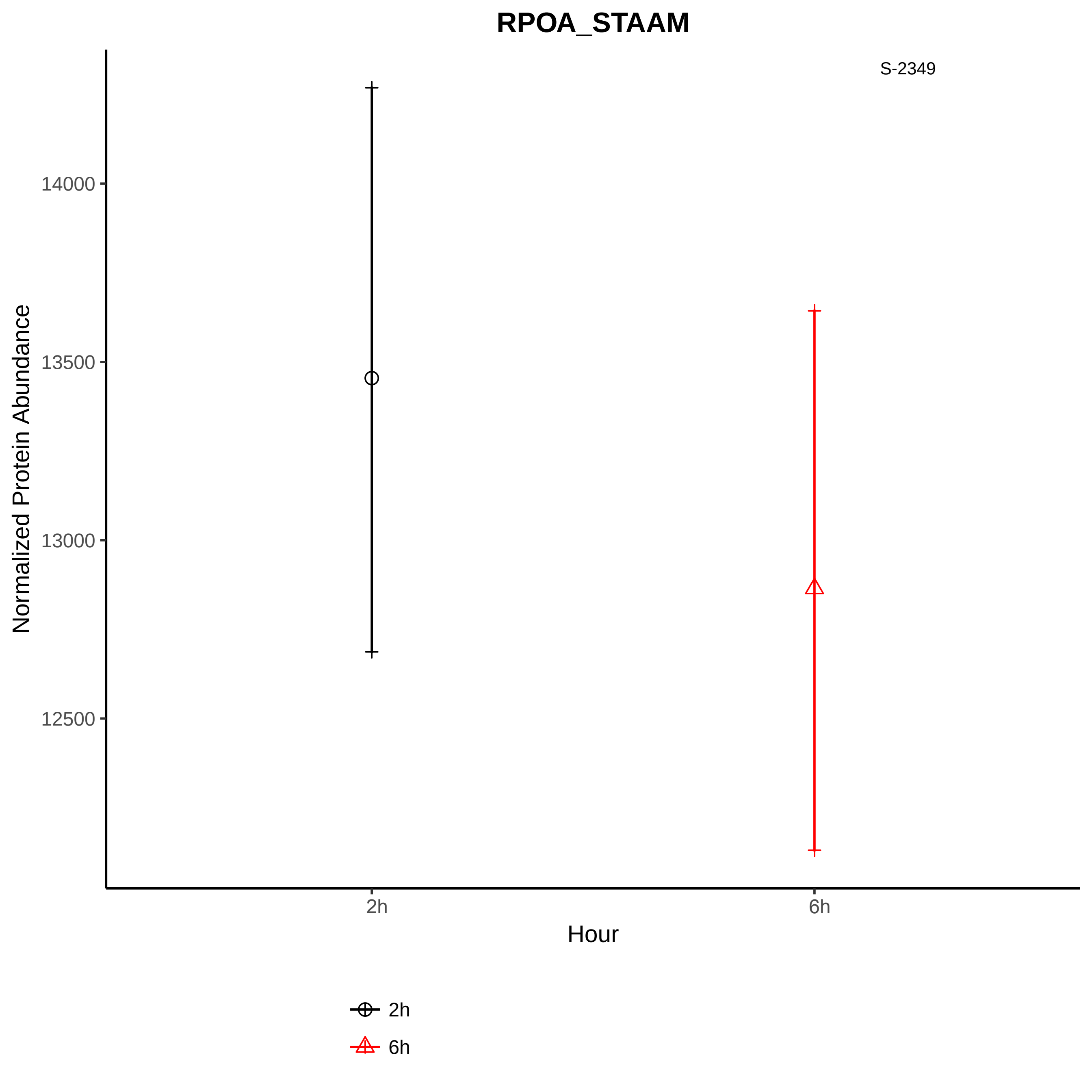
RPOA_STAAM
S-2349
Normalized Protein Abundance
14000
13500
13000
12500
2h
6h
Hour
2h
6h

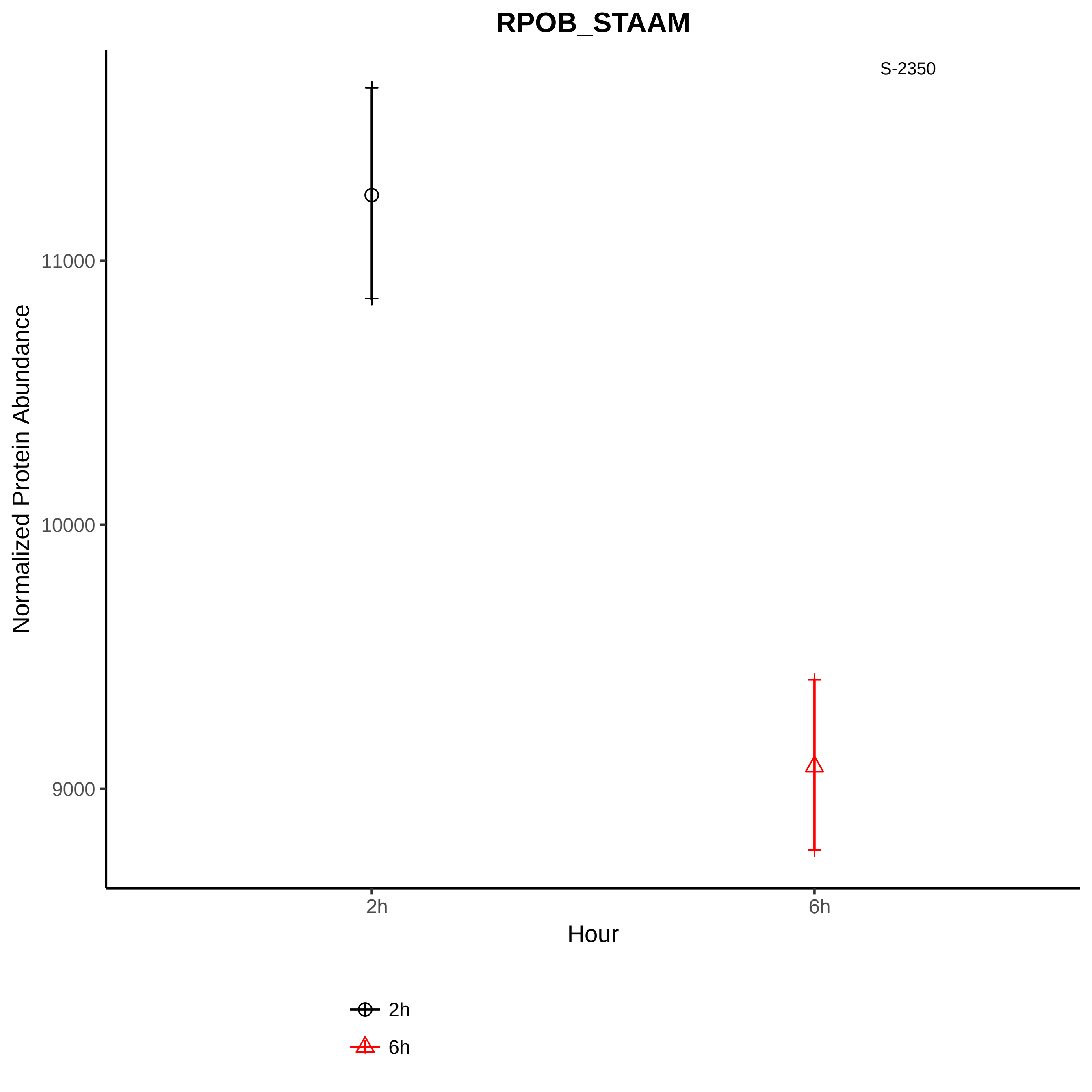
RPOB_STAAM
S-2350
Normalized Protein Abundance
11000
10000
9000
2h
6h
Hour
2h
6h

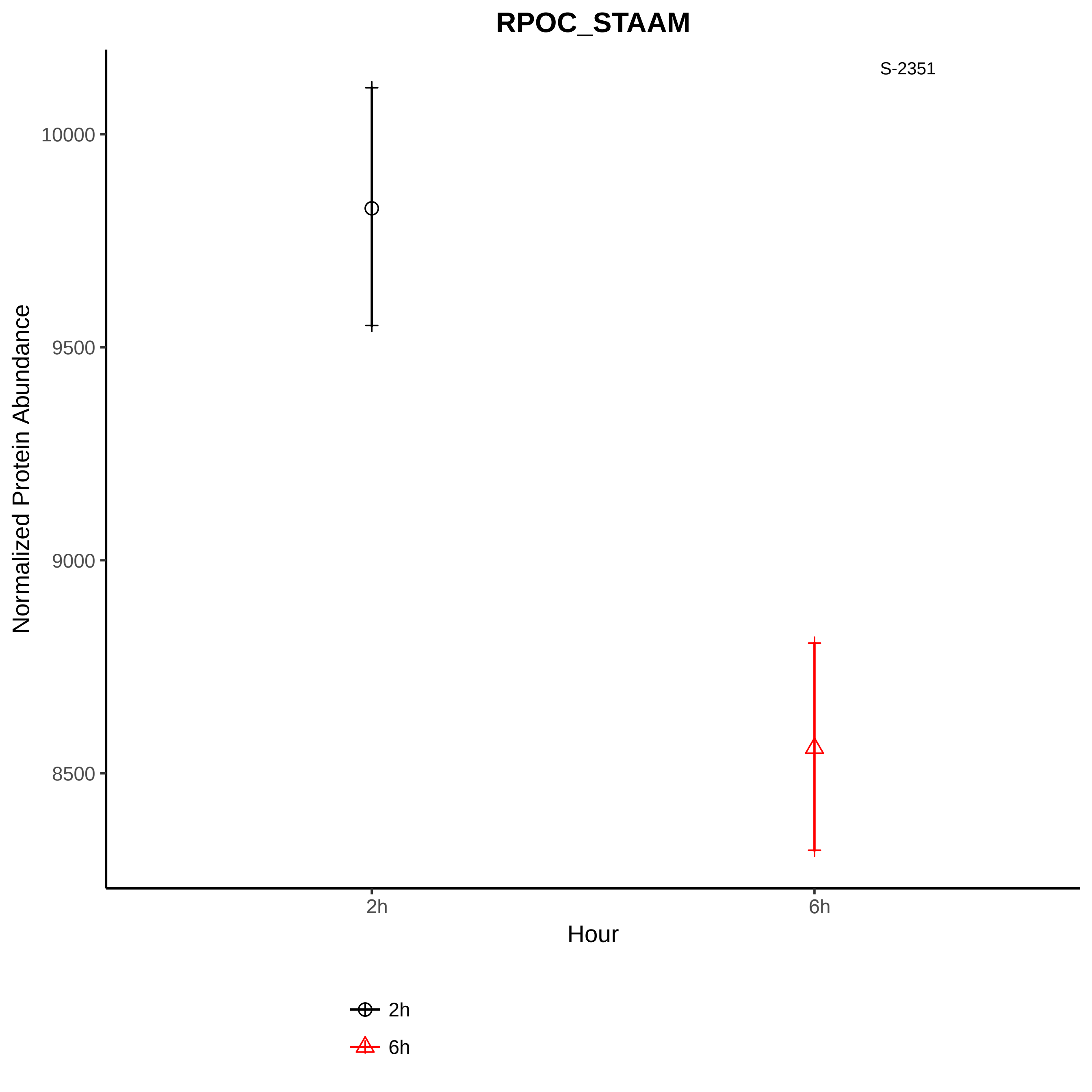

RPOC_STAAM
S-2351
10000
9500
9000
8500
Normalized Protein Abundance
2h
6h
Hour
2h
6h

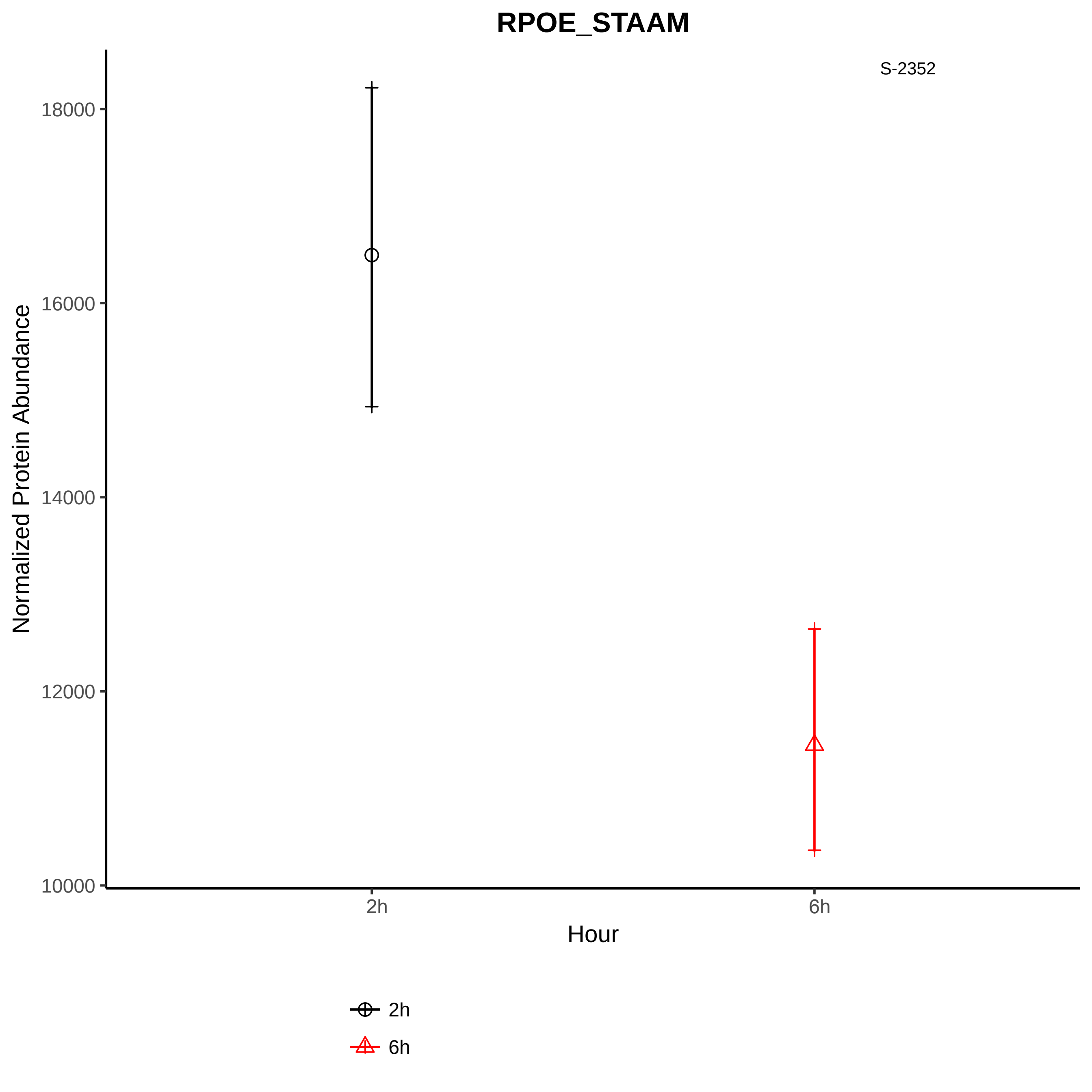

RPOE_STAAM
S-2352
Normalized Protein Abundance
18000
16000
14000
12000
10000
2h
6h
Hour
2h
6h

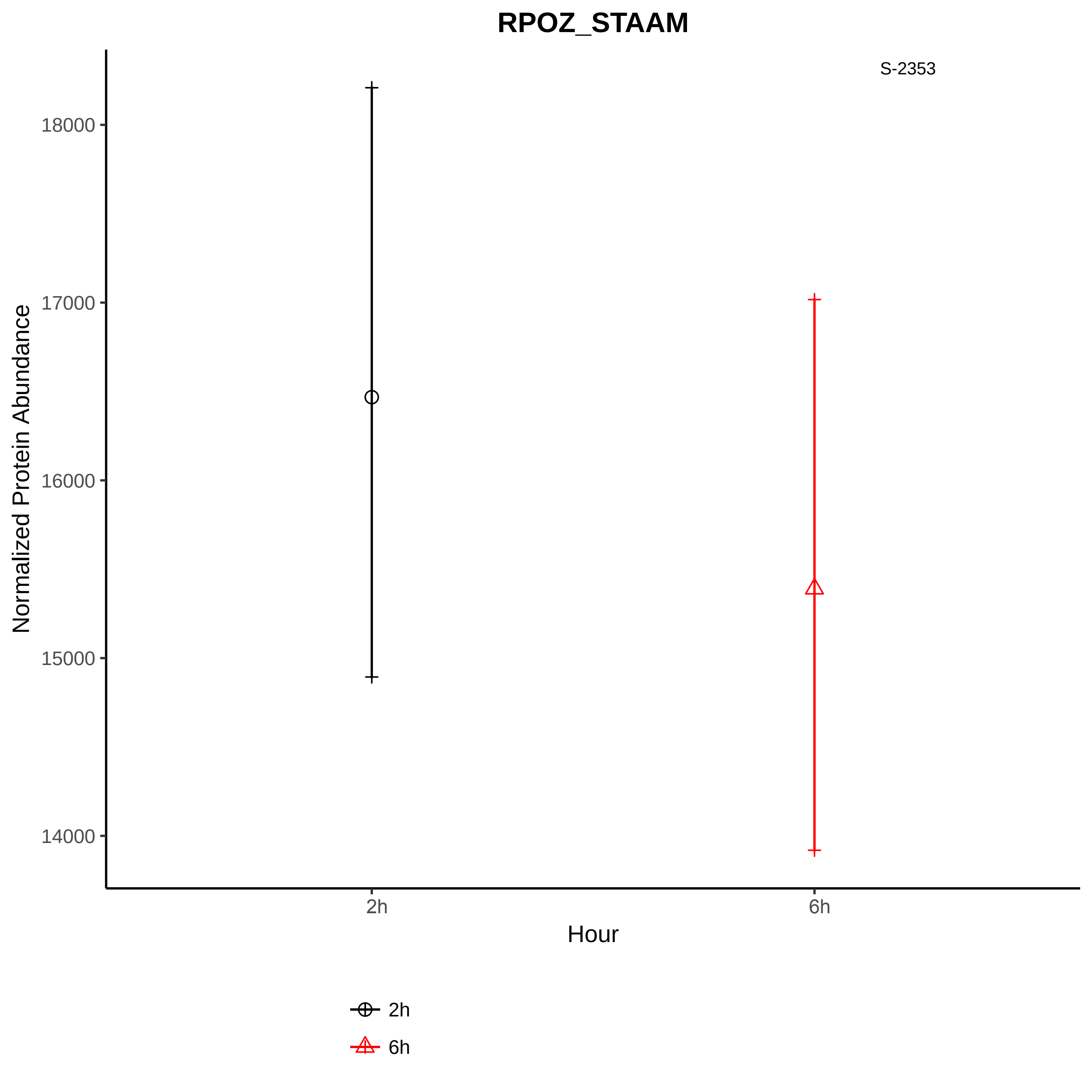
RPOZ_STAAM
S-2353
Normalized Protein Abundance
18000
17000
16000
15000
14000
2h
6h
Hour
2h
6h

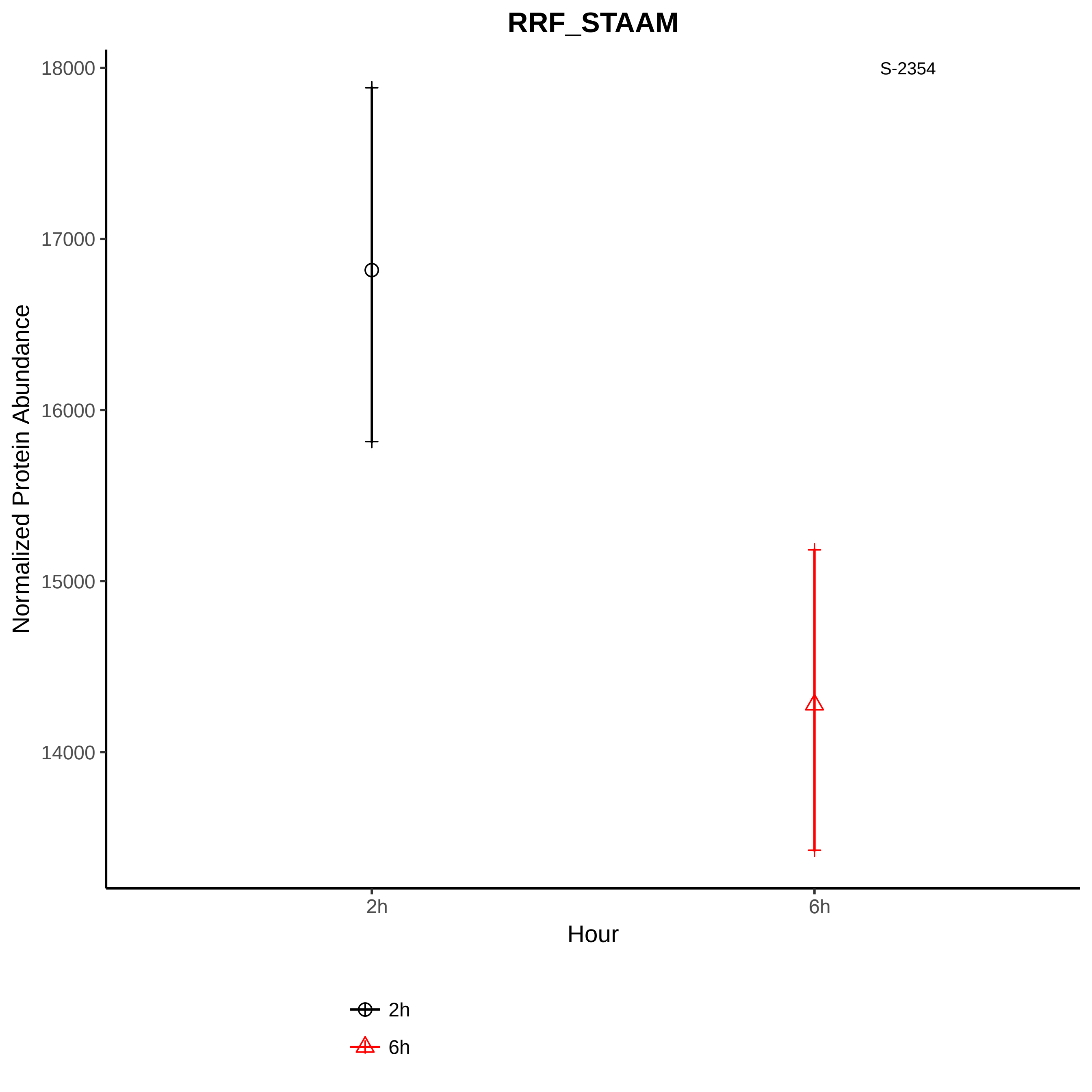

RRF_STAAM
S-2354
18000
17000
16000
15000
14000
Normalized Protein Abundance
2h
6h
Hour
2h
6h

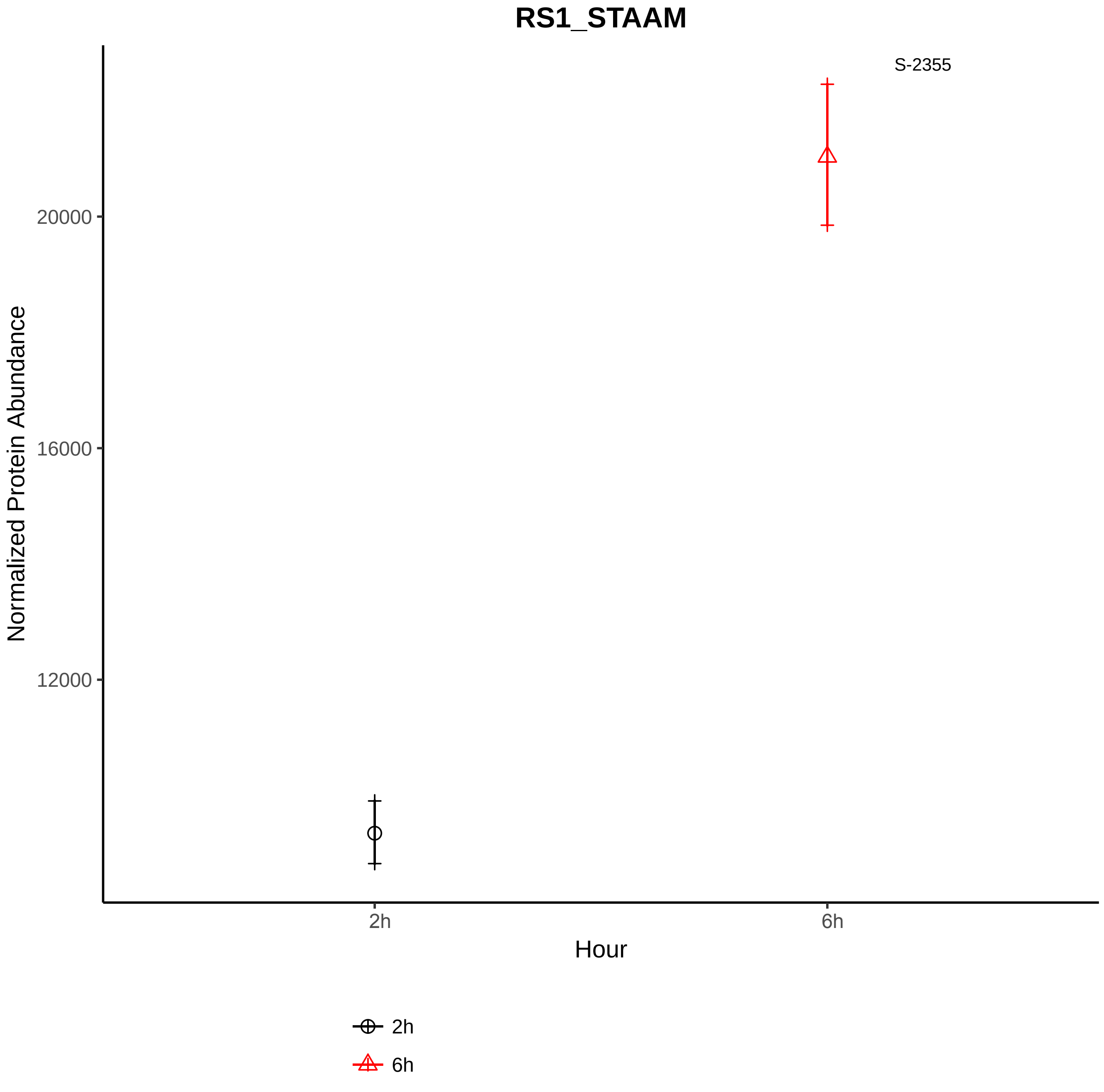
RS1_STAAM
S-2355
Normalized Protein Abundance
20000
16000
12000
2h
6h
Hour
2h
6h

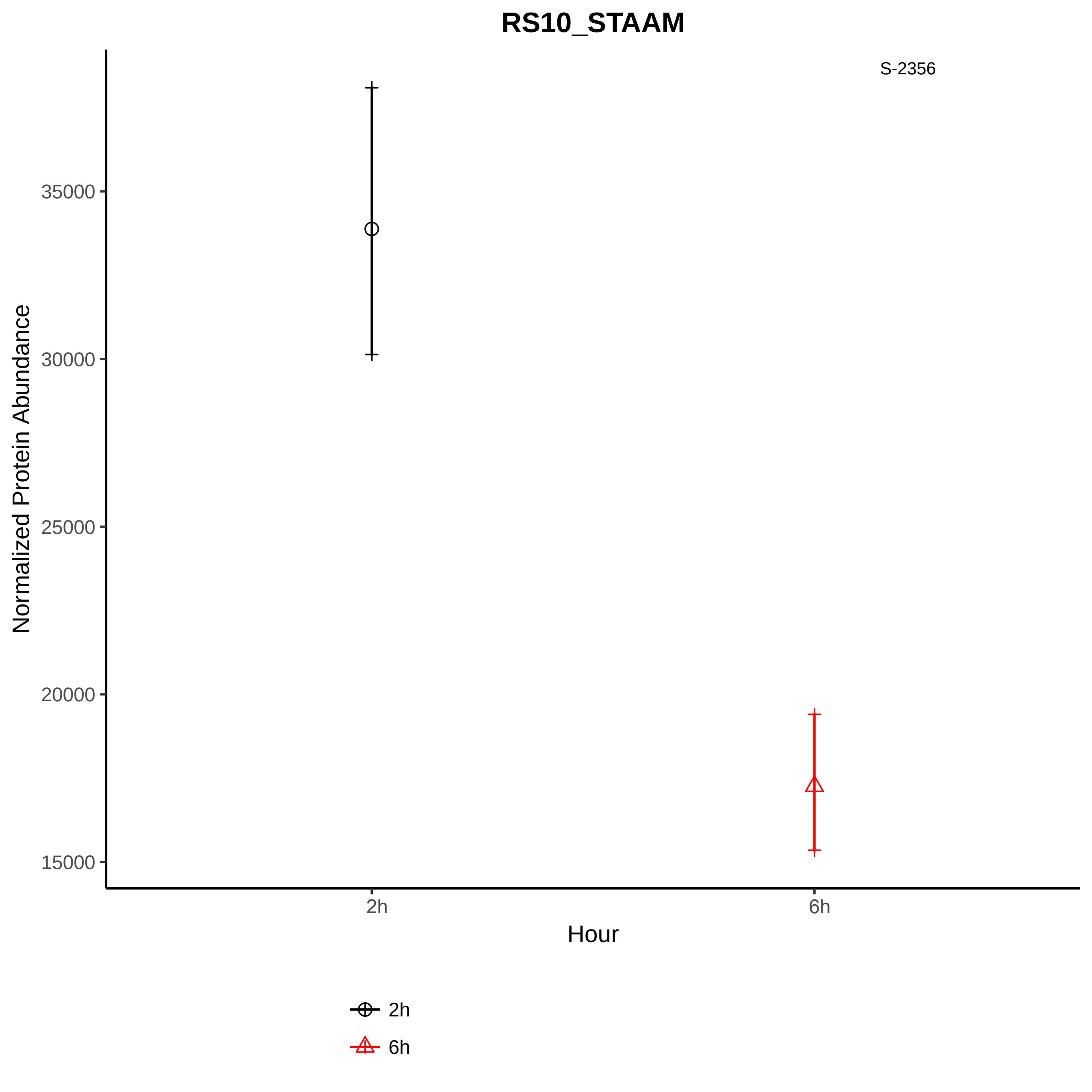

RS10_STAAM
S-2356
35000
30000
25000
20000
15000
Normalized Protein Abundance
2h
6h
Hour
2h
6h

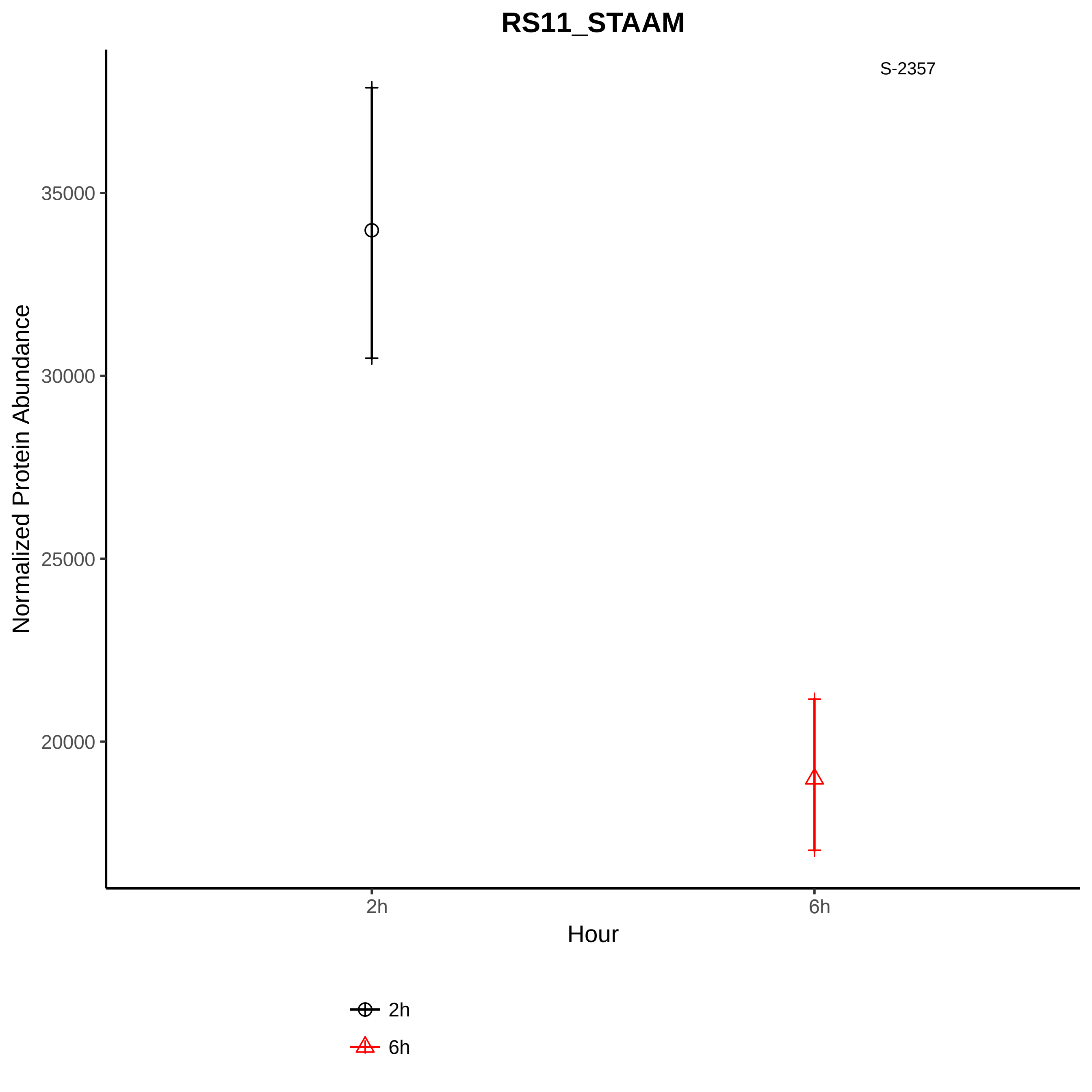

RS11_STAAM
S-2357
Normalized Protein Abundance
35000
30000
25000
20000
2h
6h
Hour
2h
6h

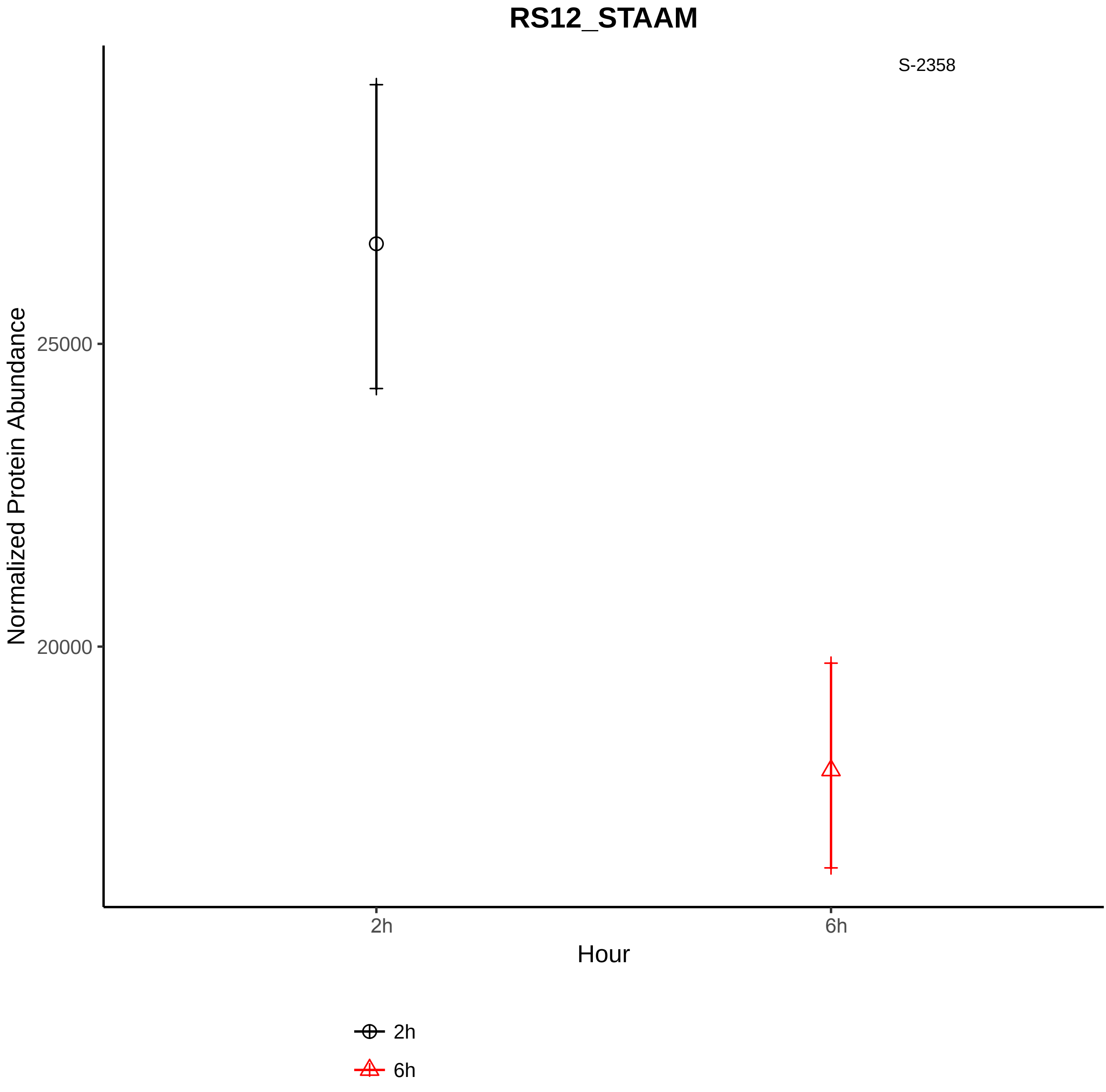

RS12_STAAM
S-2358
Normalized Protein Abundance
25000
20000
2h
6h
Hour
2h
6h

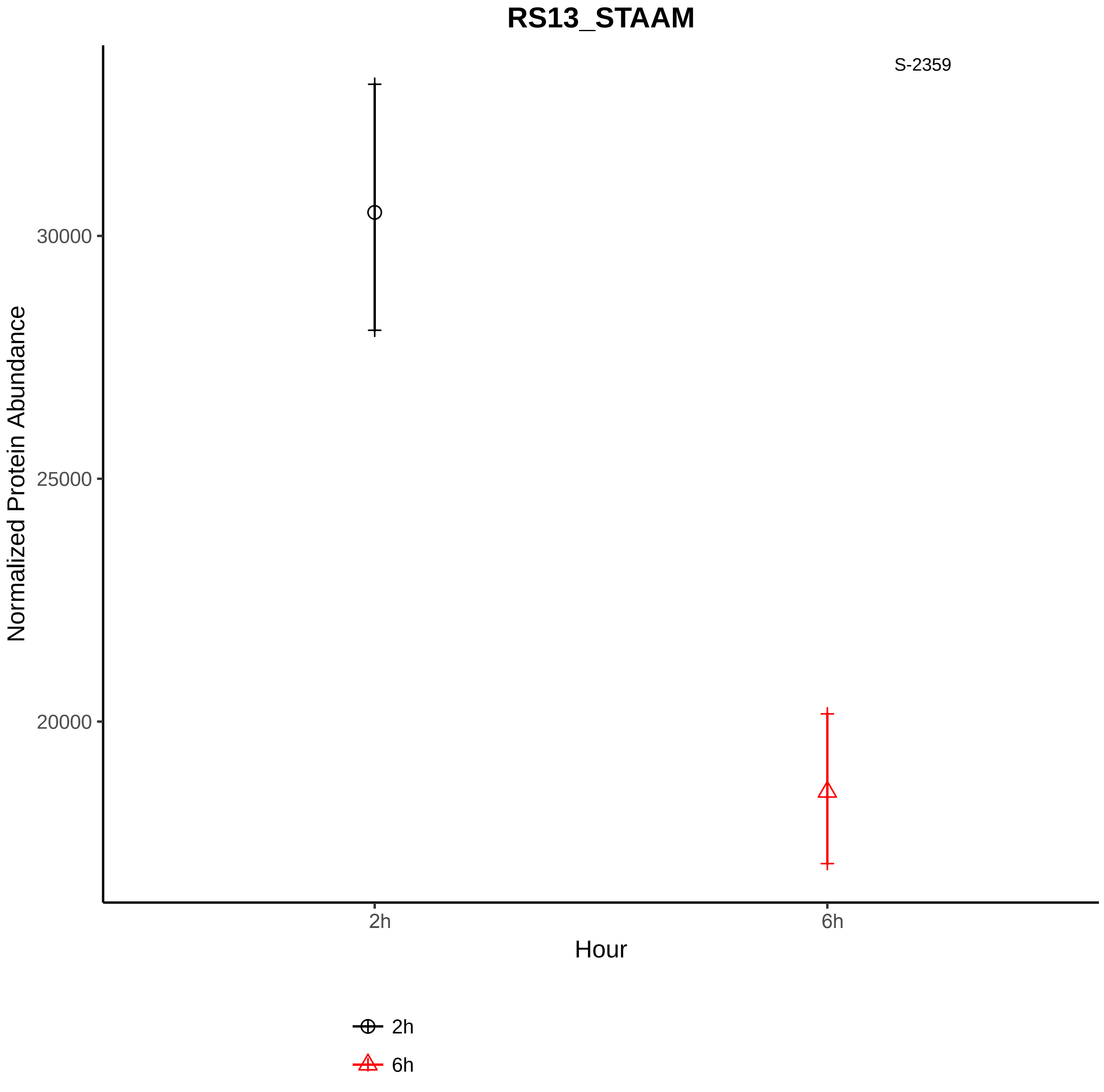

RS13_STAAM
S-2359
Normalized Protein Abundance
30000
25000
20000
2h
6h
Hour
2h
6h

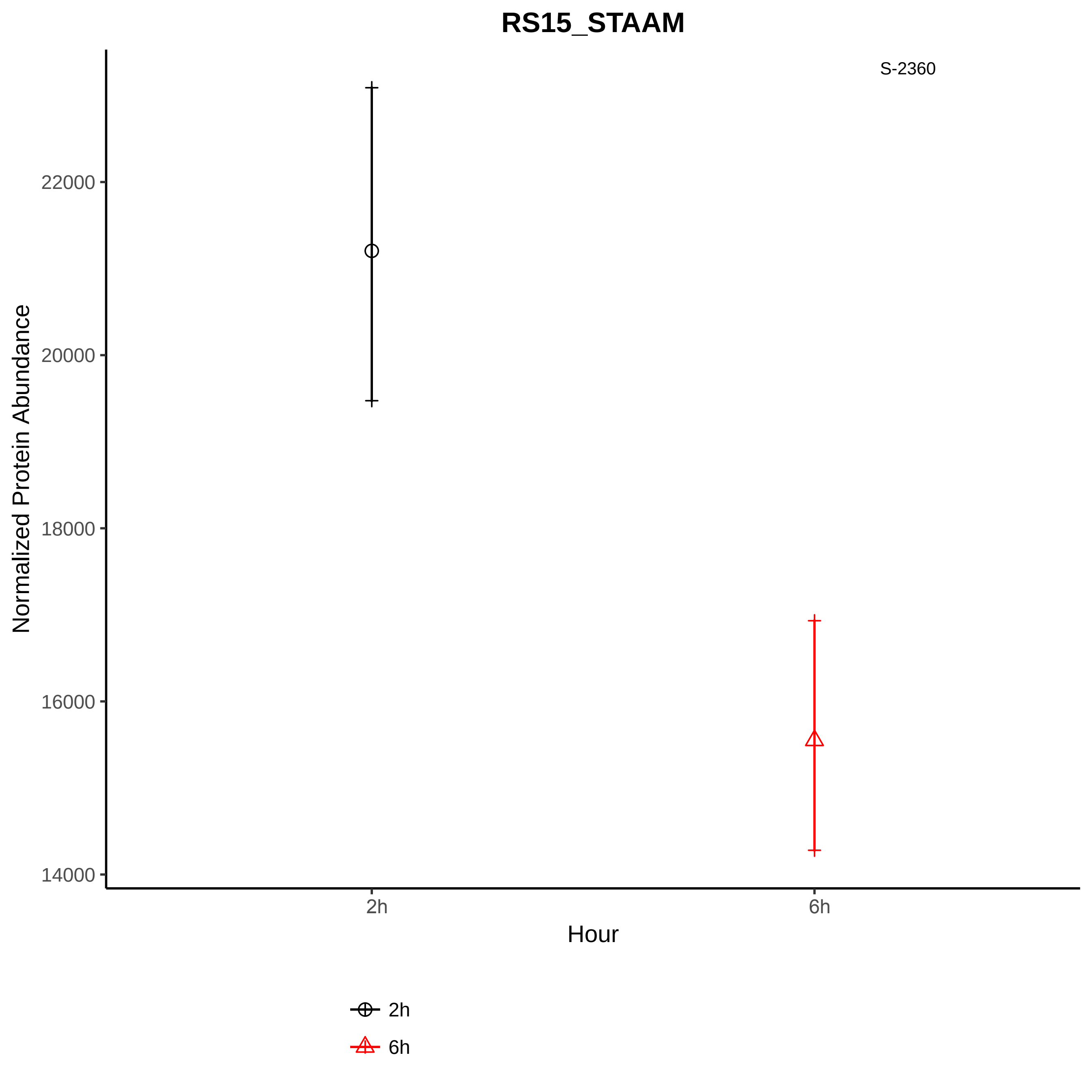

RS15_STAAM
S-2360
Normalized Protein Abundance
22000
20000
18000
16000
14000
2h
6h
Hour
2h
6h

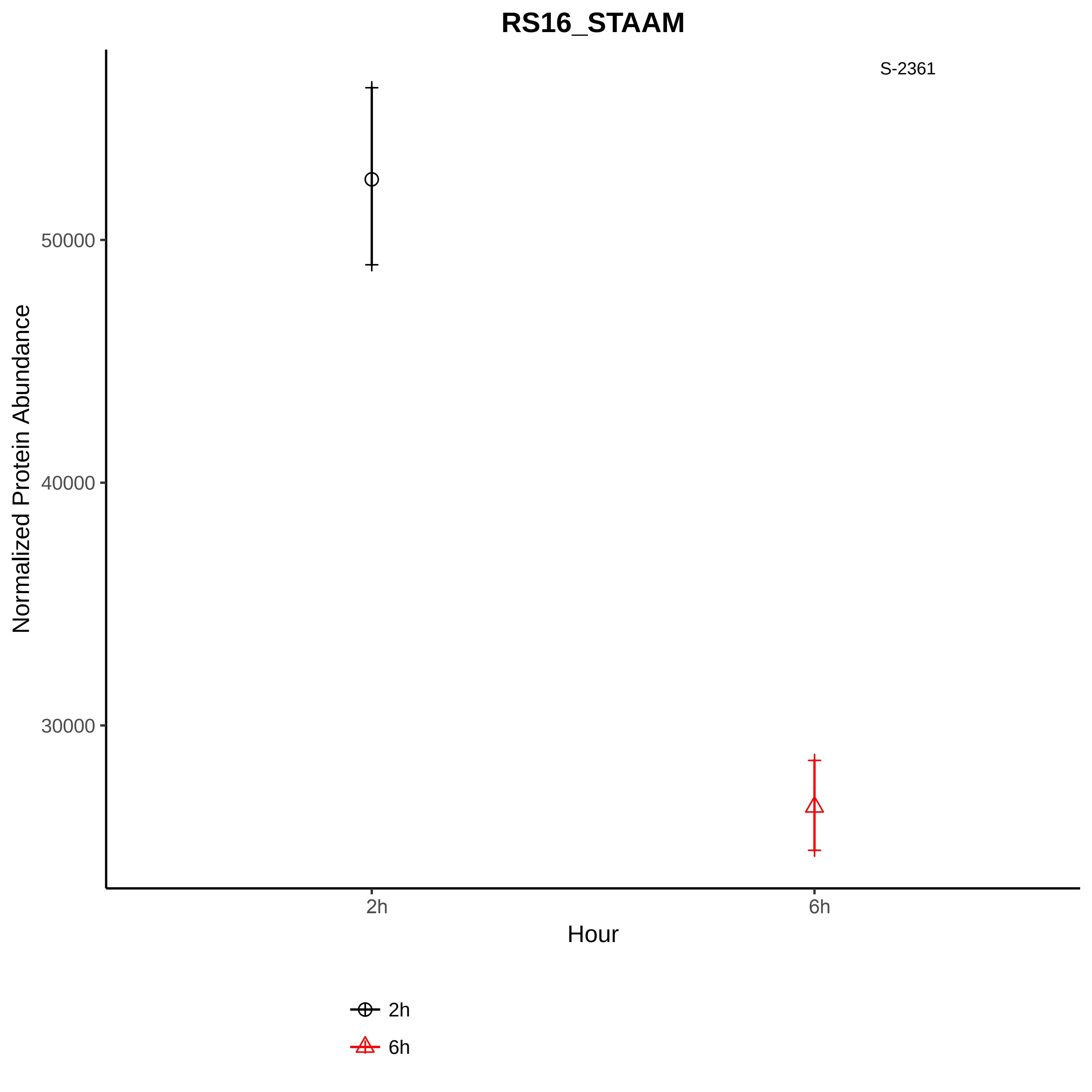

RS16_STAAM
S-2361
Normalized Protein Abundance
50000
40000
30000
2h
6h
Hour
2h
6h

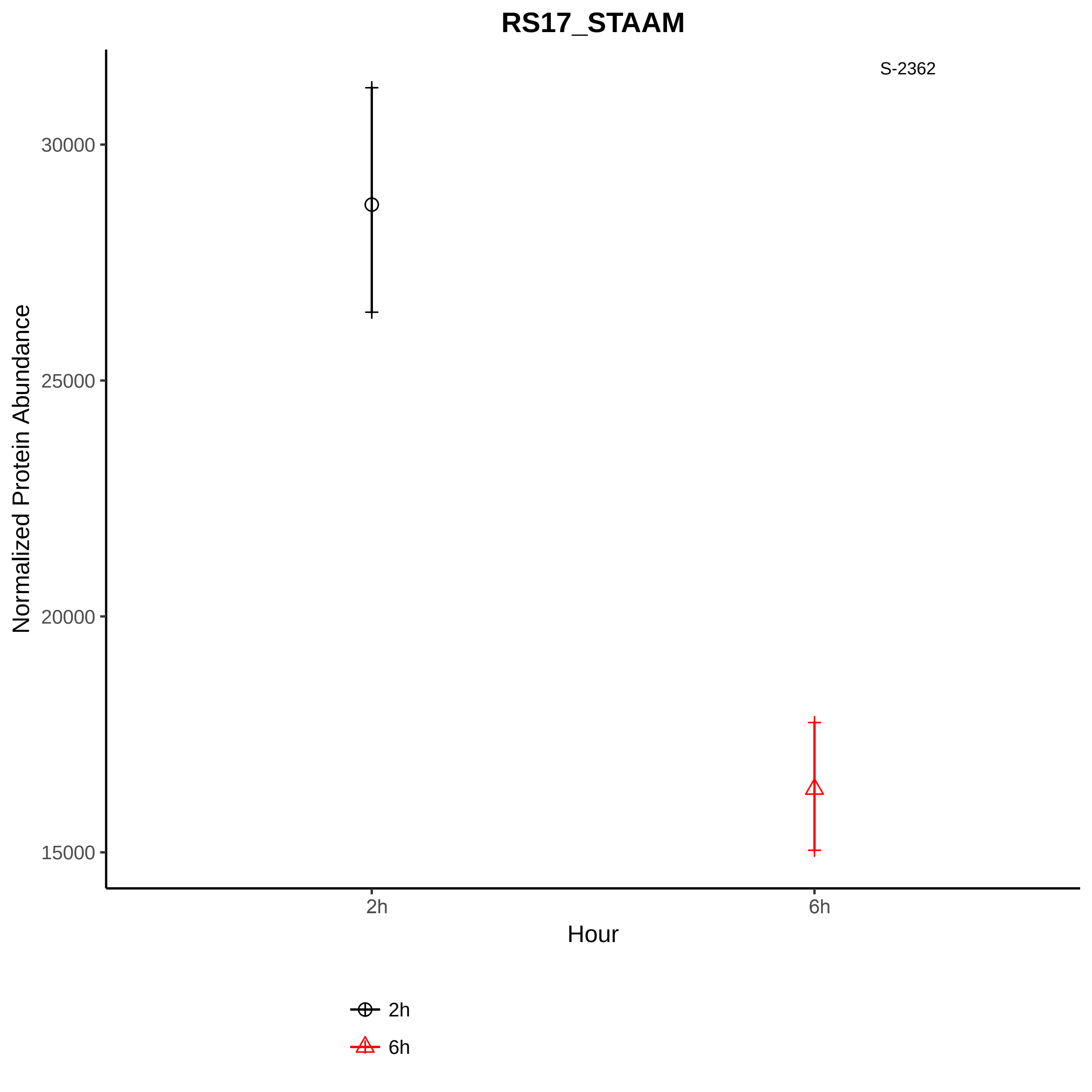

RS17_STAAM
S-2362
Normalized Protein Abundance
30000
25000
20000
15000
2h
6h
Hour
2h
6h

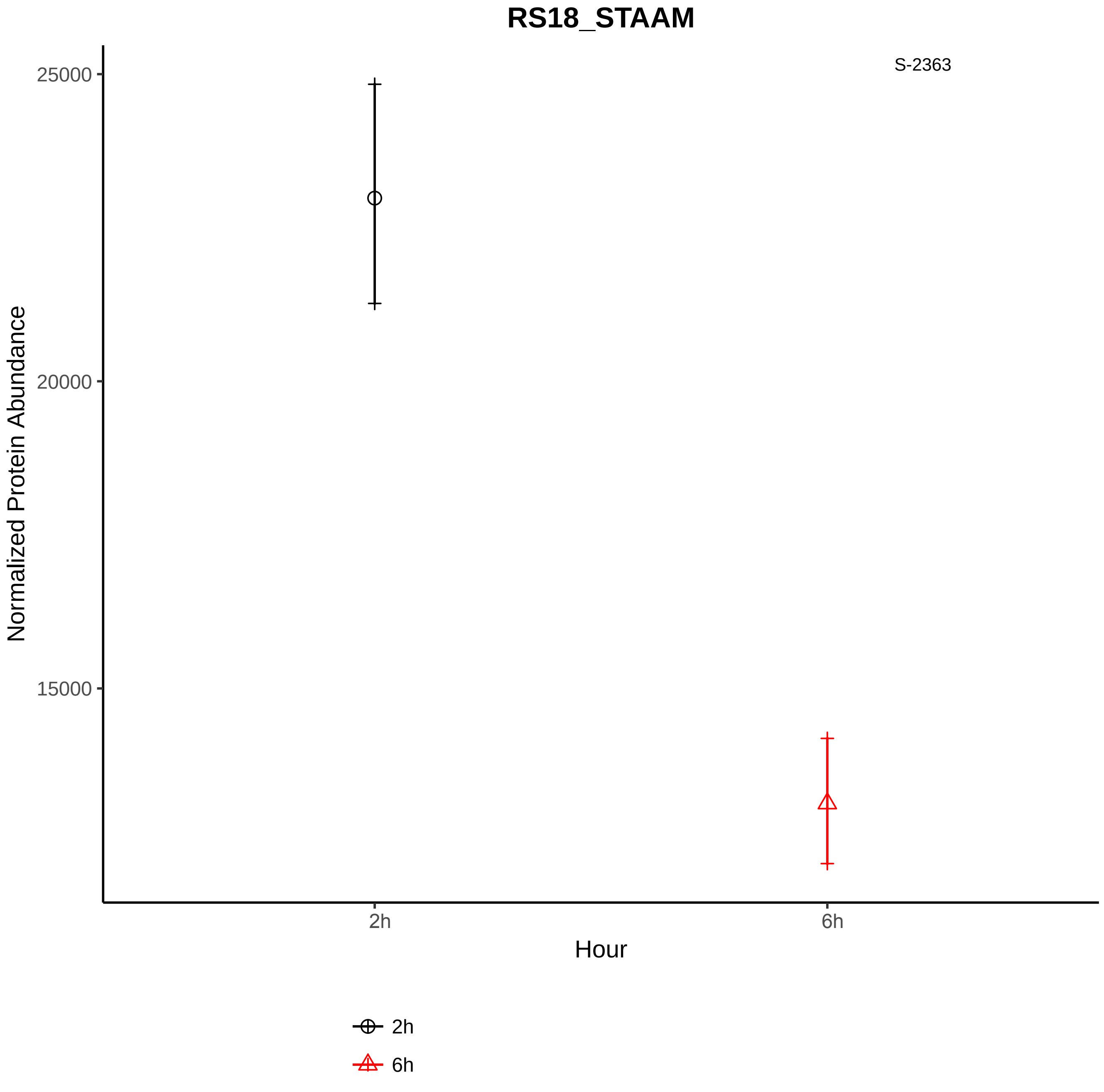
RS18_STAAM
S-2363
25000
20000
15000
Normalized Protein Abundance
2h
6h
Hour
2h
6h

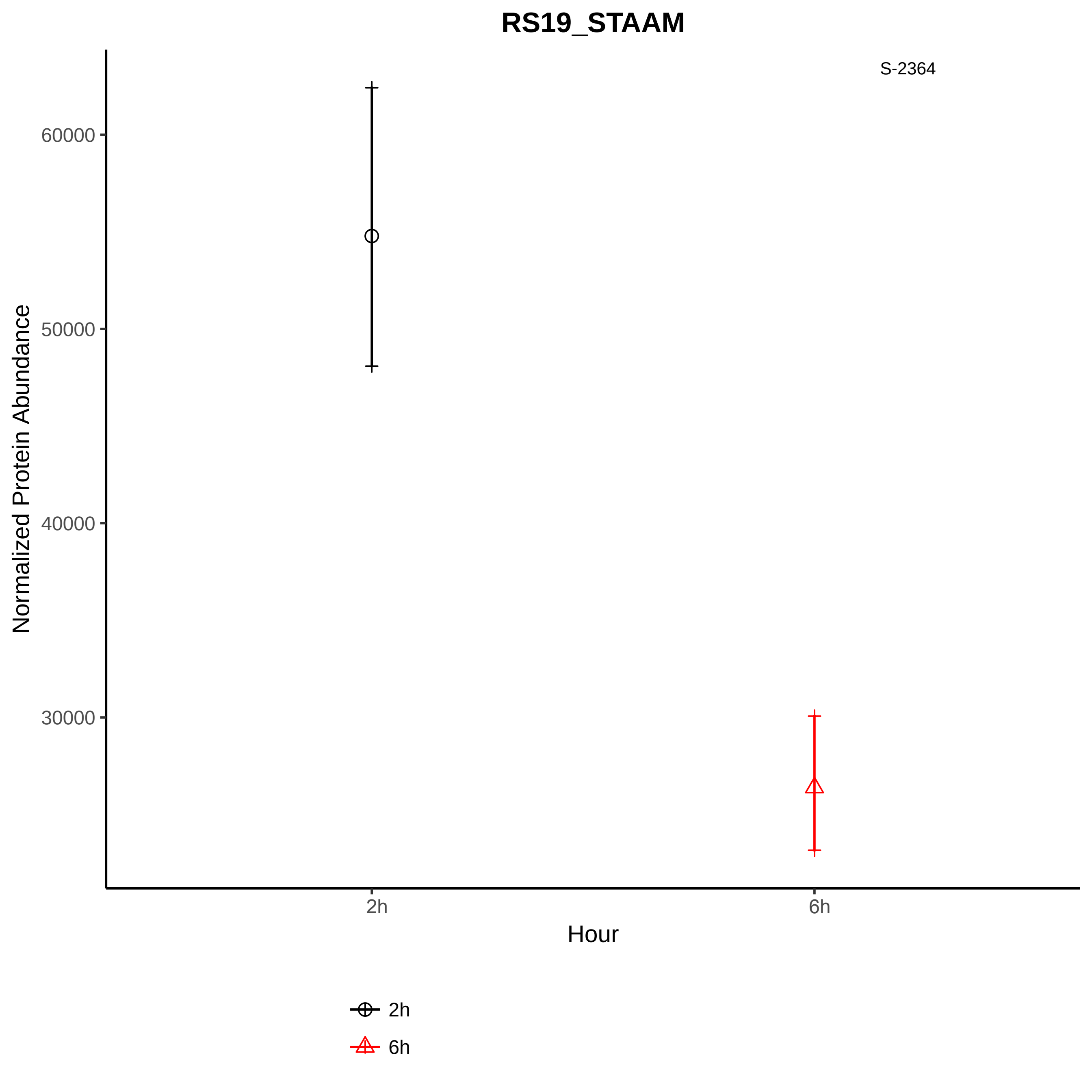
RS19_STAAM
S-2364
Normalized Protein Abundance
60000
50000
40000
30000
2h
6h
Hour
2h
6h

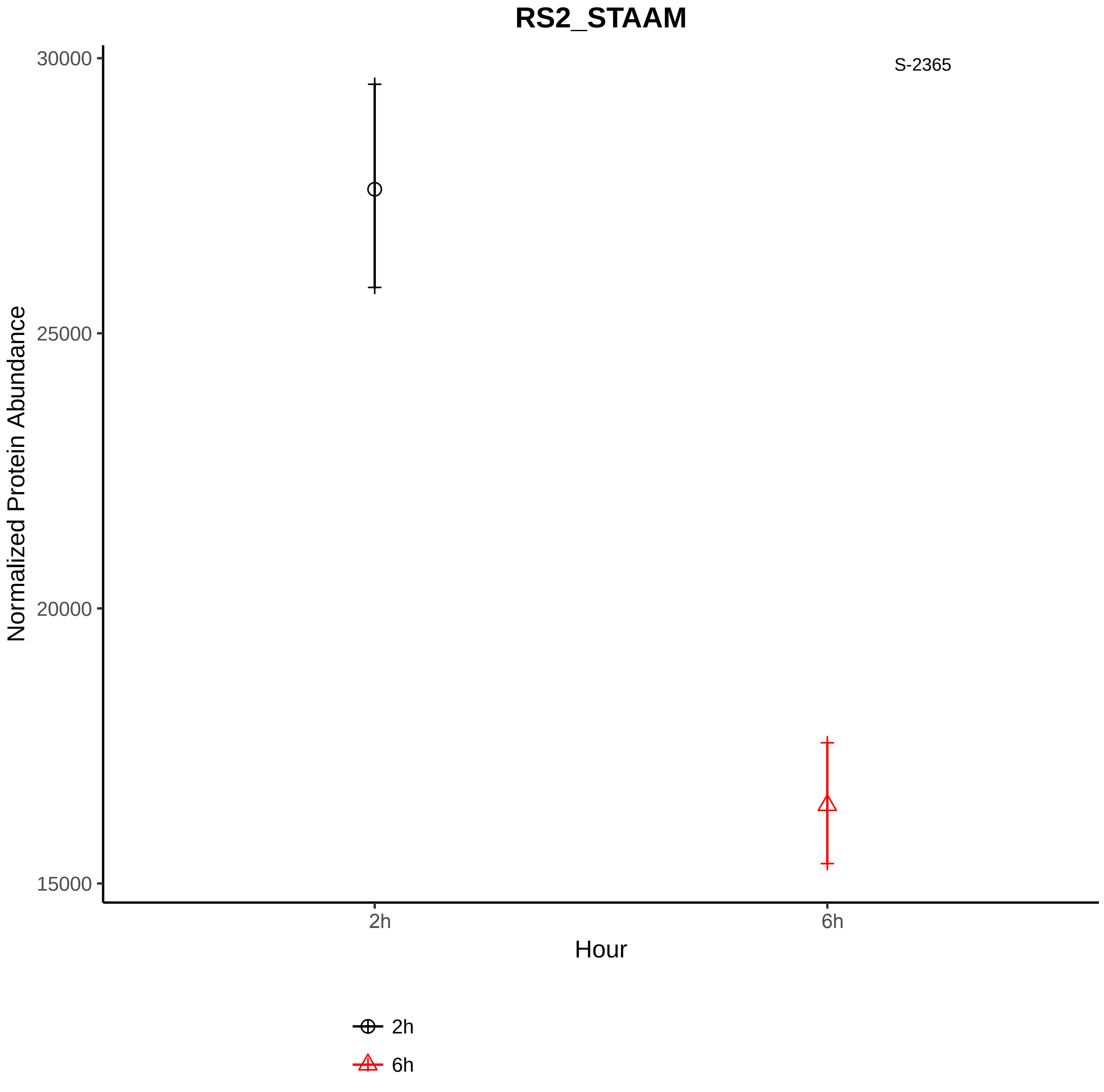

RS2_STAAM
S-2365
Normalized Protein Abundance
30000
25000
20000
15000
2h
6h
Hour
2h
6h

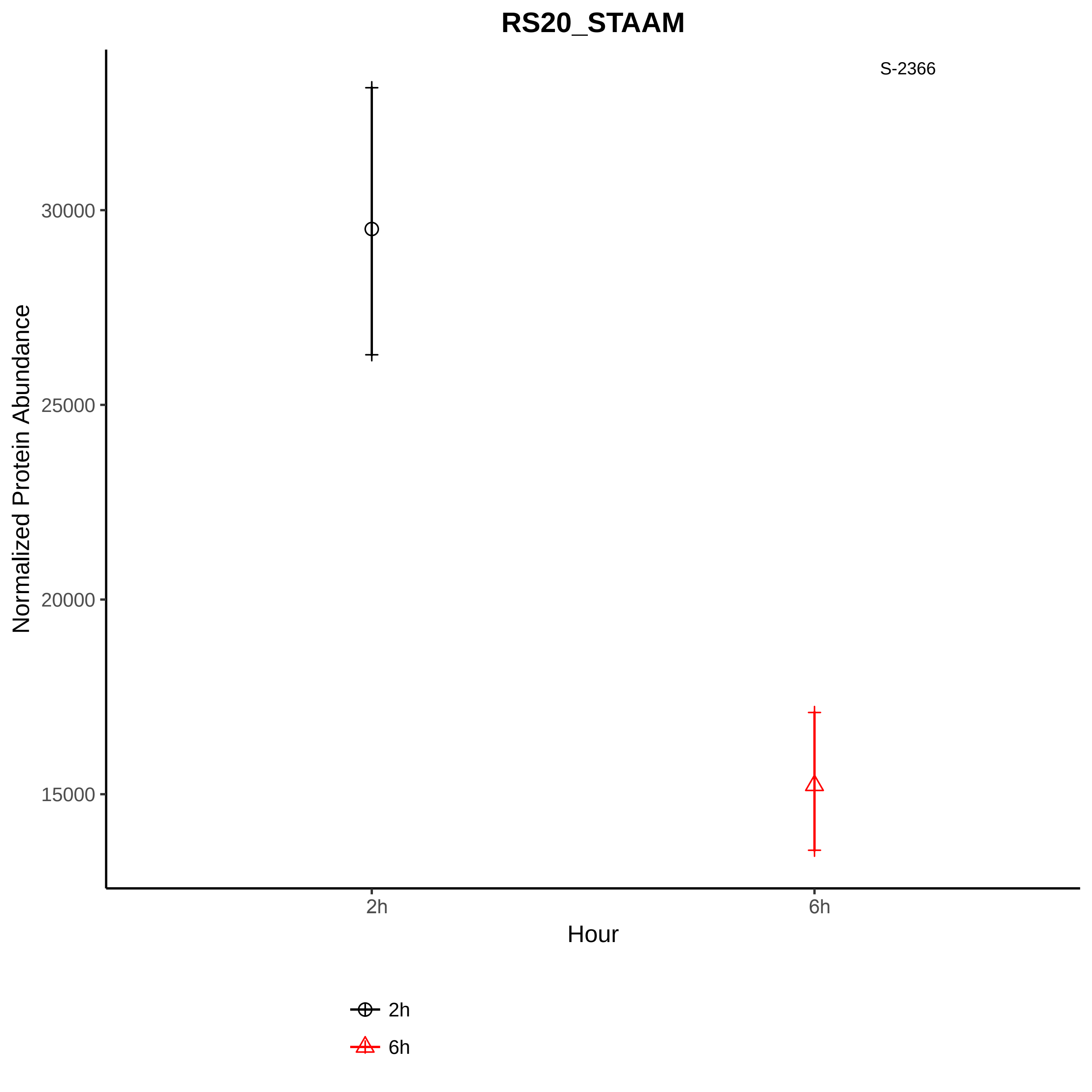

RS20_STAAM
S-2366
30000
25000
20000
15000
Normalized Protein Abundance
2h
6h
Hour
2h
6h

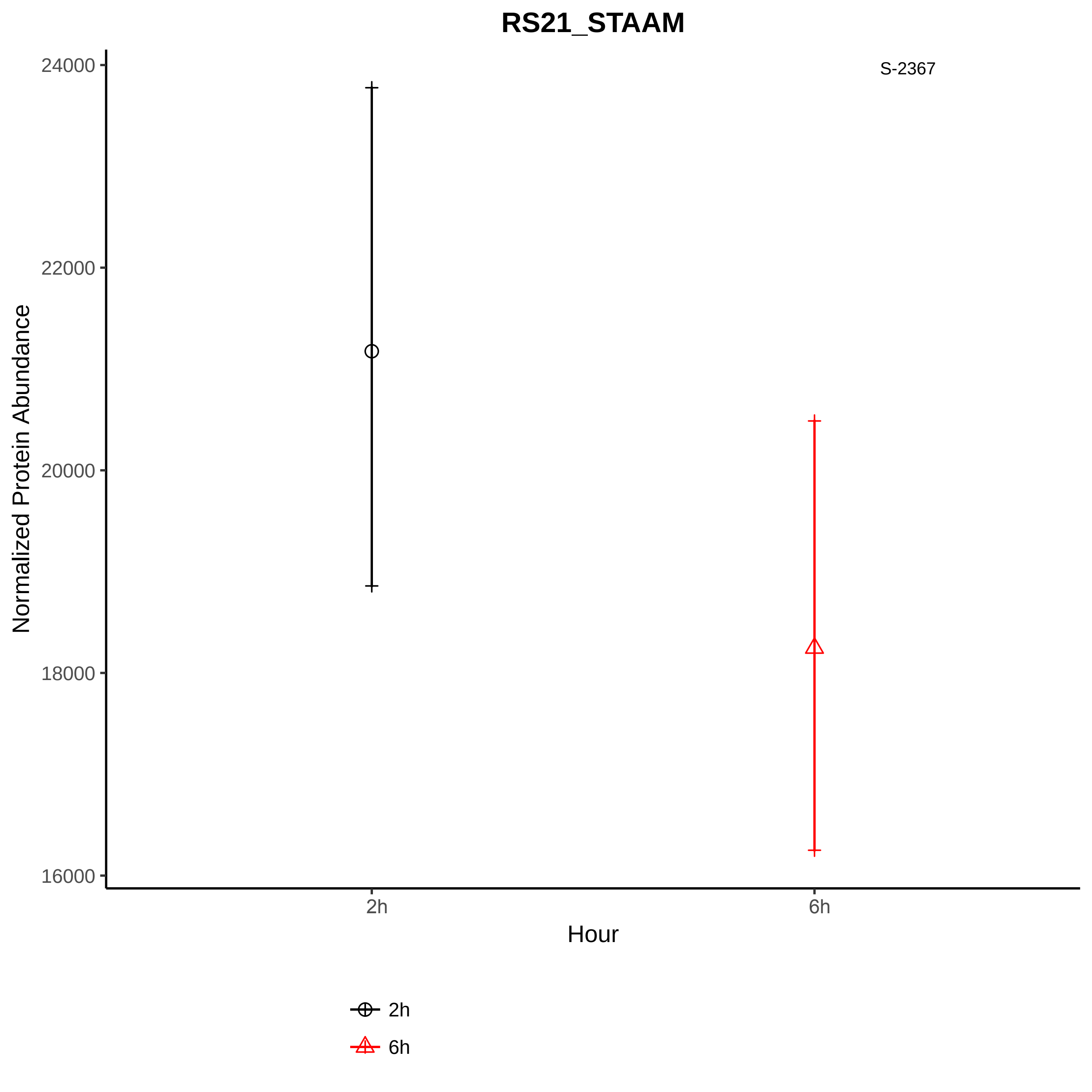
RS21_STAAM
S-2367
Normalized Protein Abundance
24000
22000
20000
18000
16000
2h
6h
Hour
2h
6h

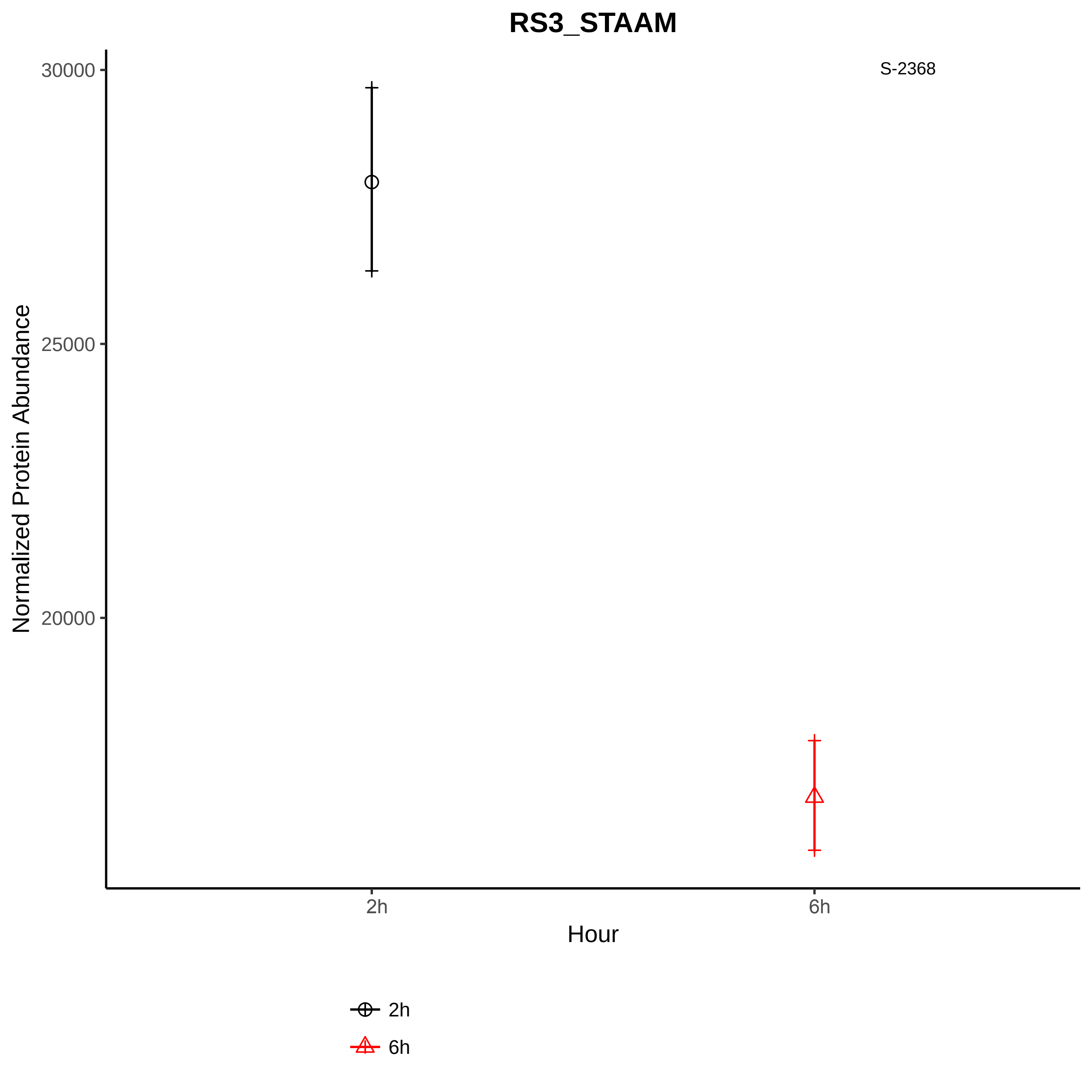
RS3_STAAM
S-2368
Normalized Protein Abundance
30000
25000
20000
2h
6h
Hour
2h
6h

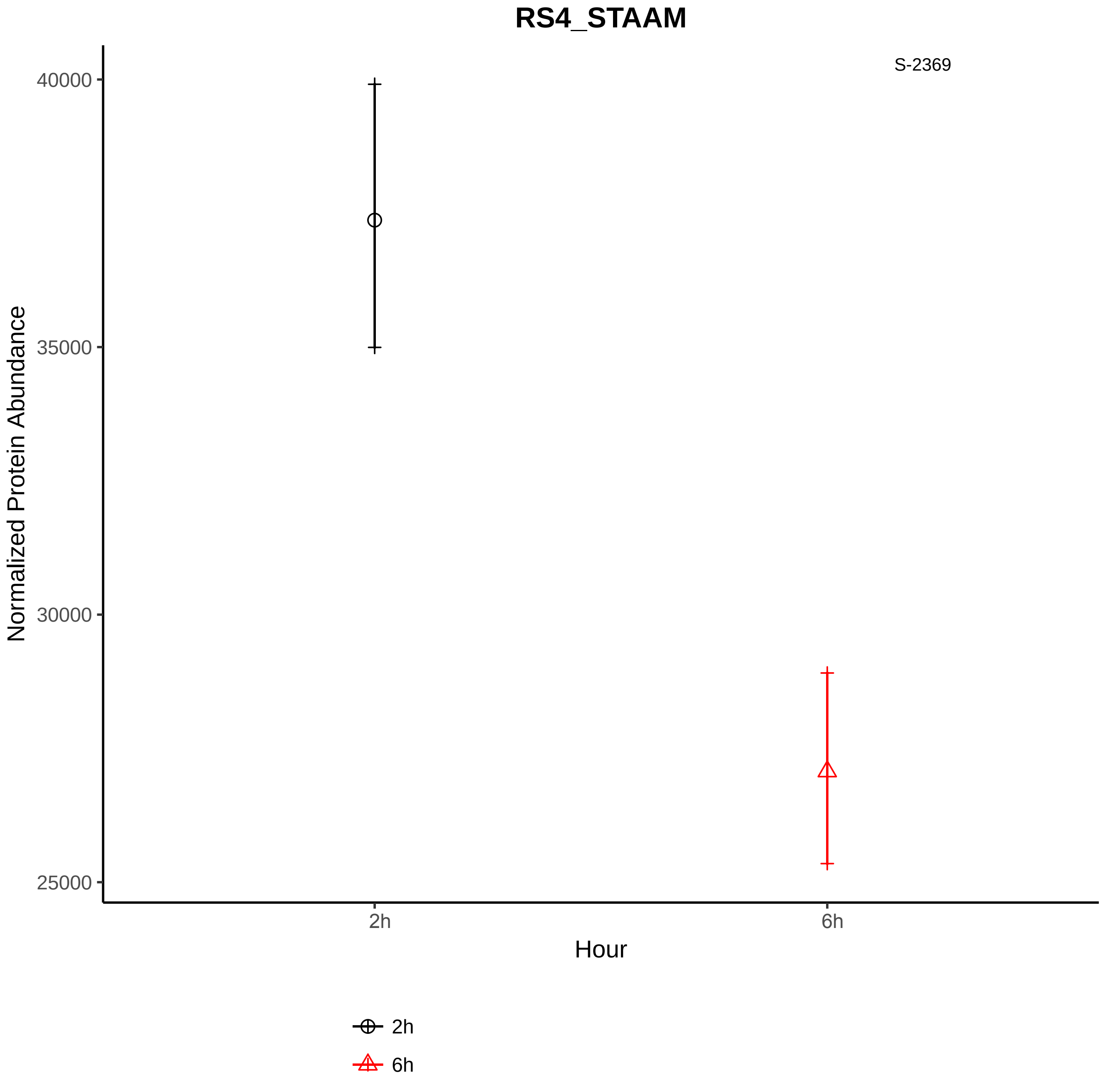

RS4_STAAM
S-2369
Normalized Protein Abundance
40000
35000
30000
25000
2h
6h
Hour
2h
6h

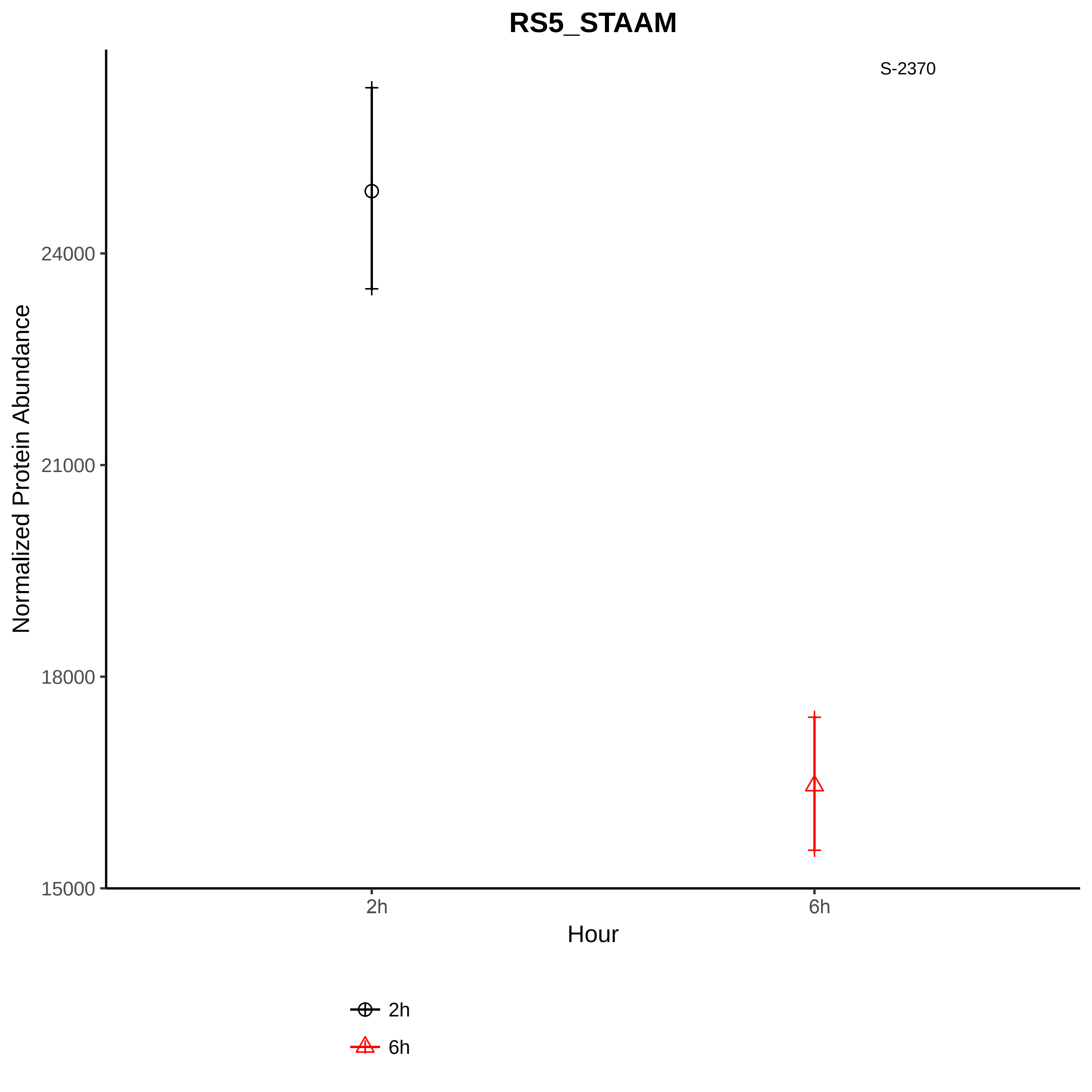

RS5_STAAM
S-2370
Normalized Protein Abundance
24000
21000
18000
15000
2h
6h
Hour
2h
6h

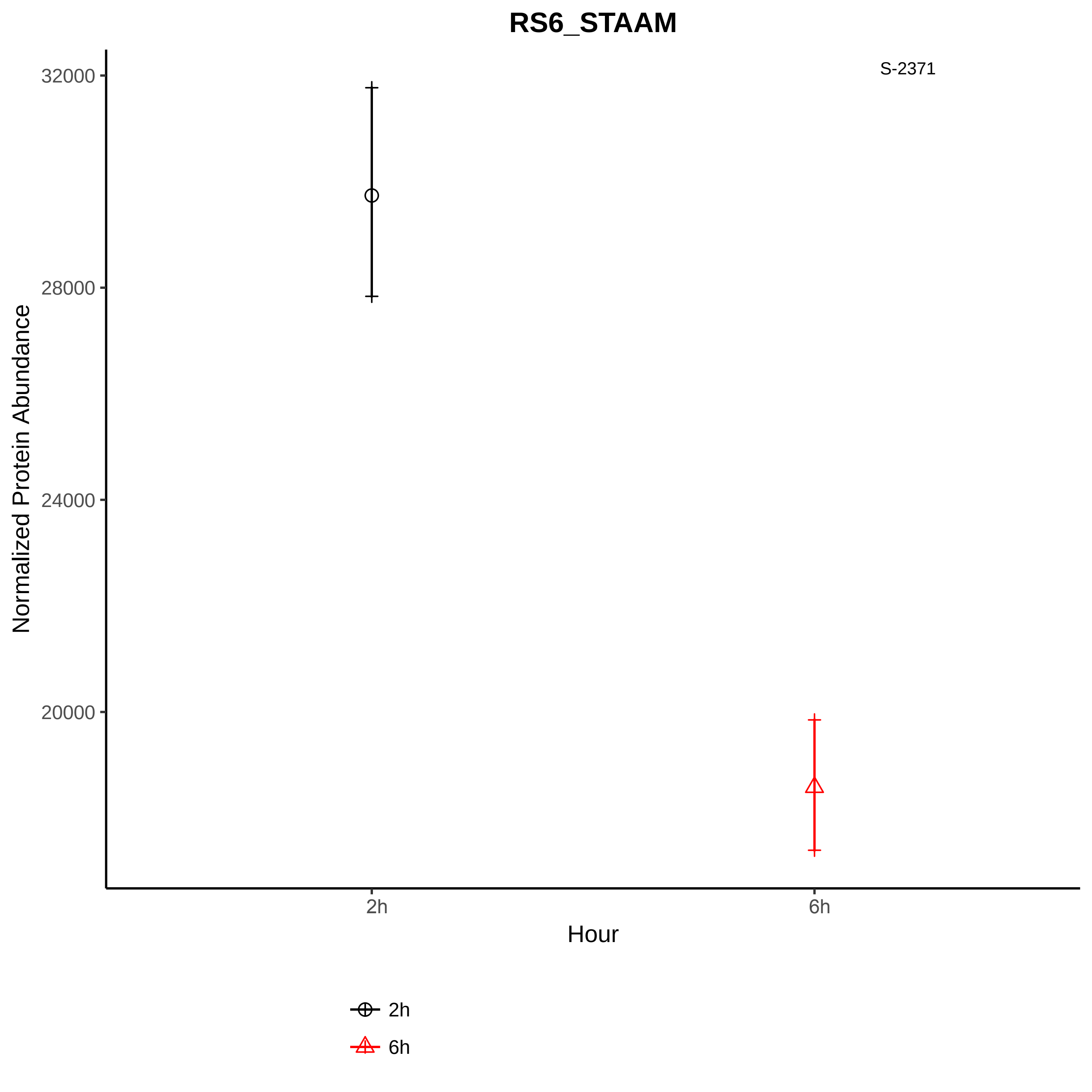

RS6_STAAM
S-2371
32000
28000
24000
20000
Normalized Protein Abundance
2h
6h
Hour
2h
6h

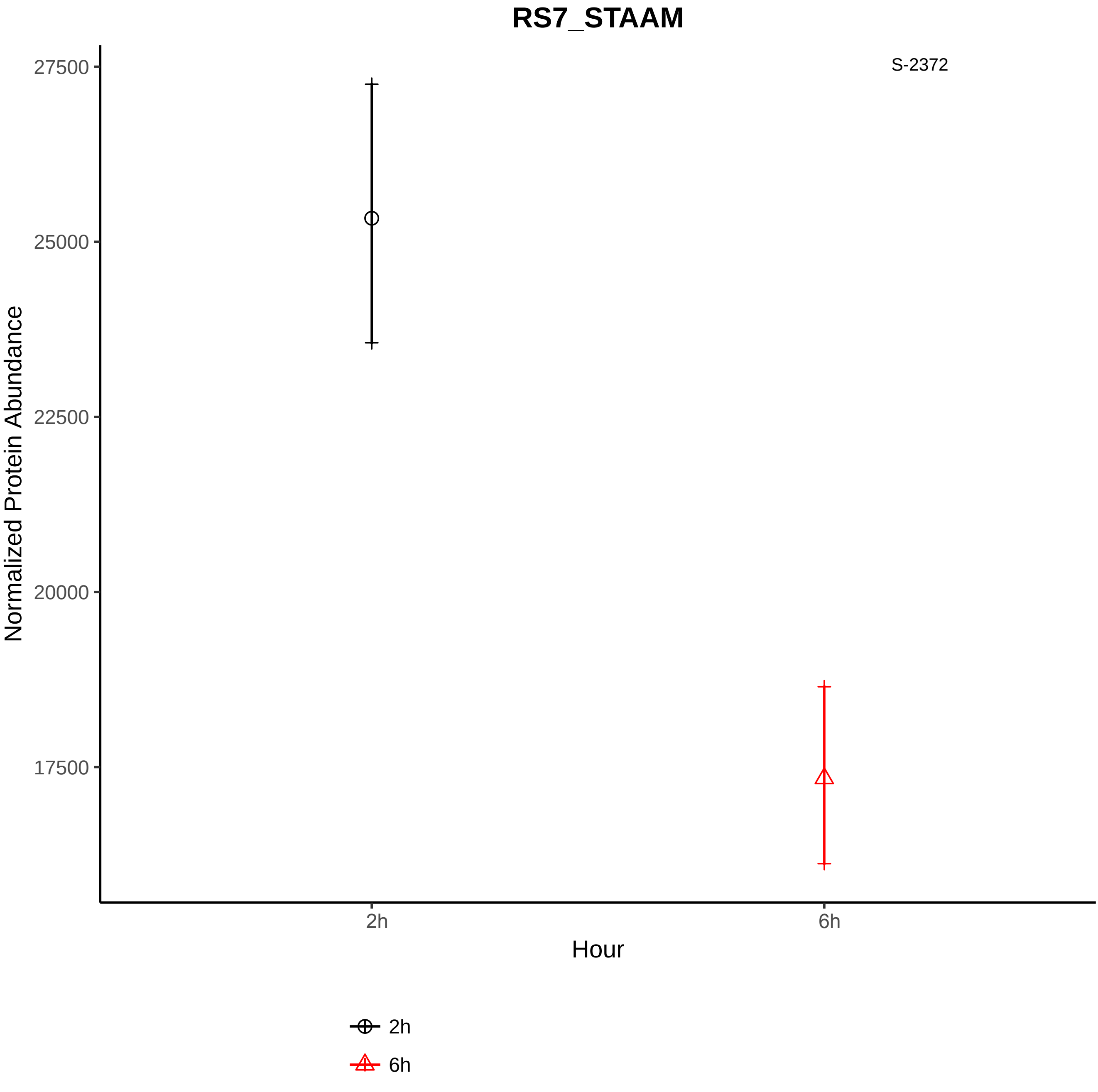

RS7_STAAM
S-2372
Normalized Protein Abundance
27500
25000
22500
20000
17500
2h
6h
Hour
2h
6h

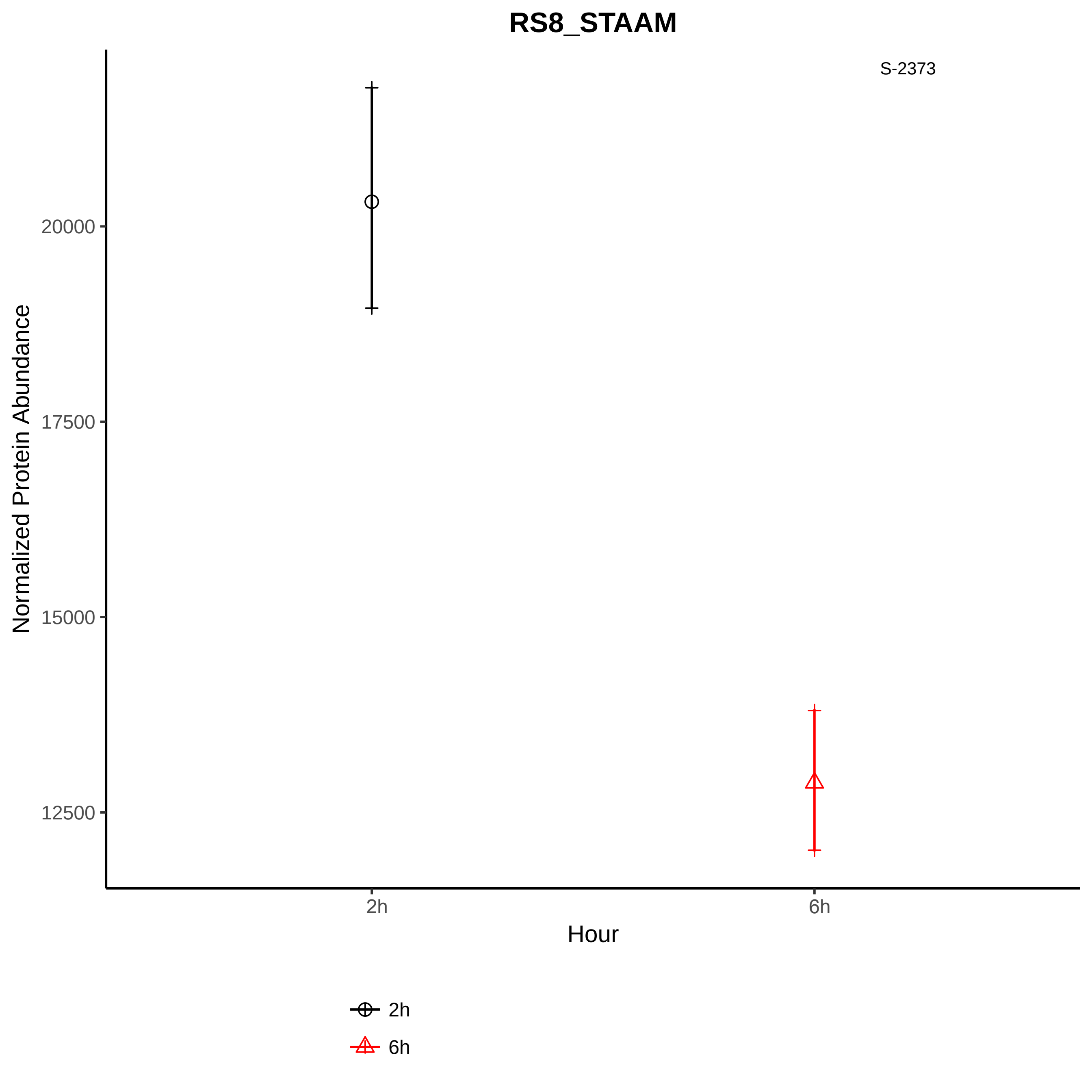

RS8_STAAM
S-2373
Normalized Protein Abundance
20000
17500
15000
12500
2h
6h
Hour
2h
6h

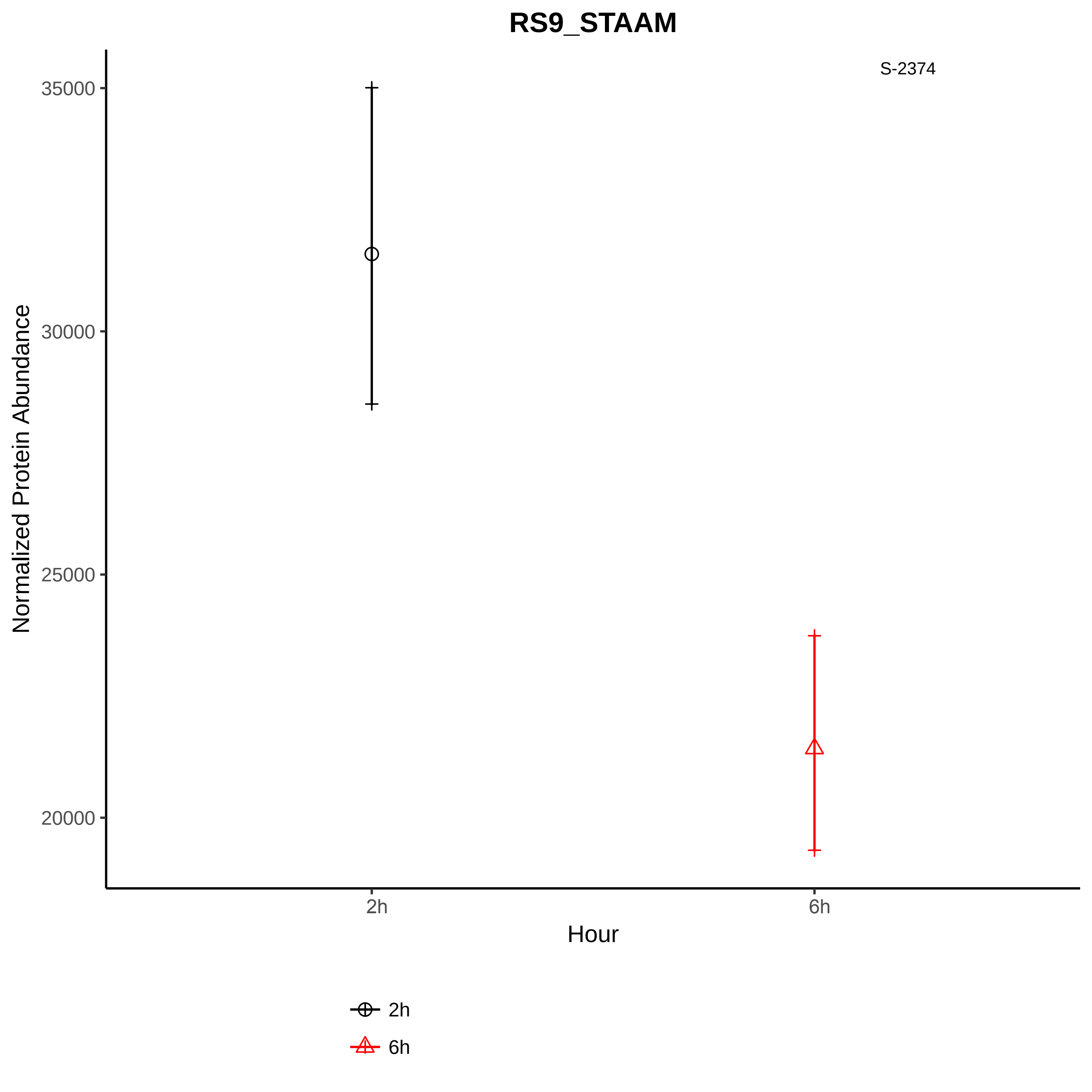
RS9_STAAM
S-2374
35000
30000
25000
20000
Normalized Protein Abundance
2h
6h
Hour
2h
6h

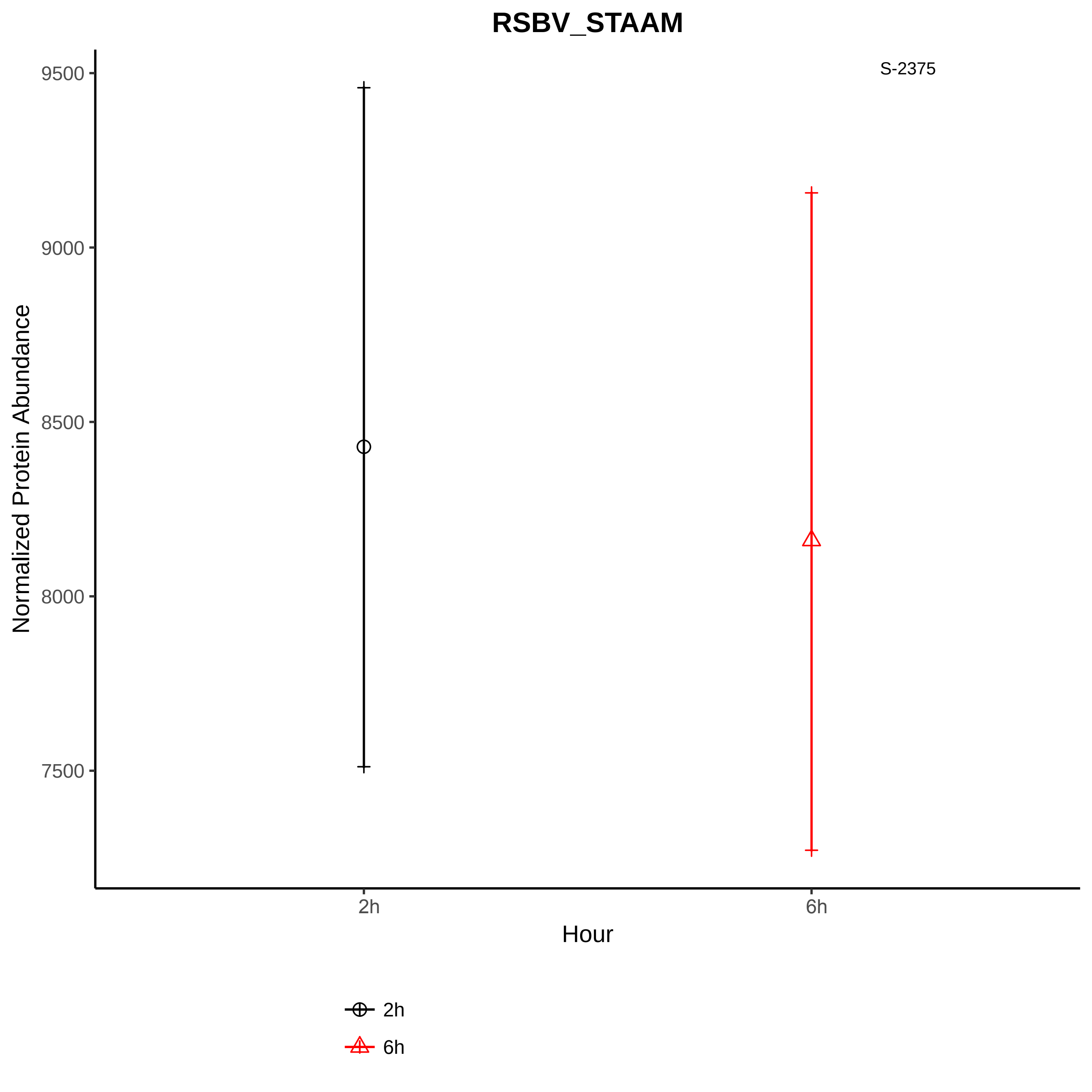

RSBV_STAAM
S-2375
9500
9000
8500
8000
7500
Normalized Protein Abundance
2h
6h
Hour
2h
6h

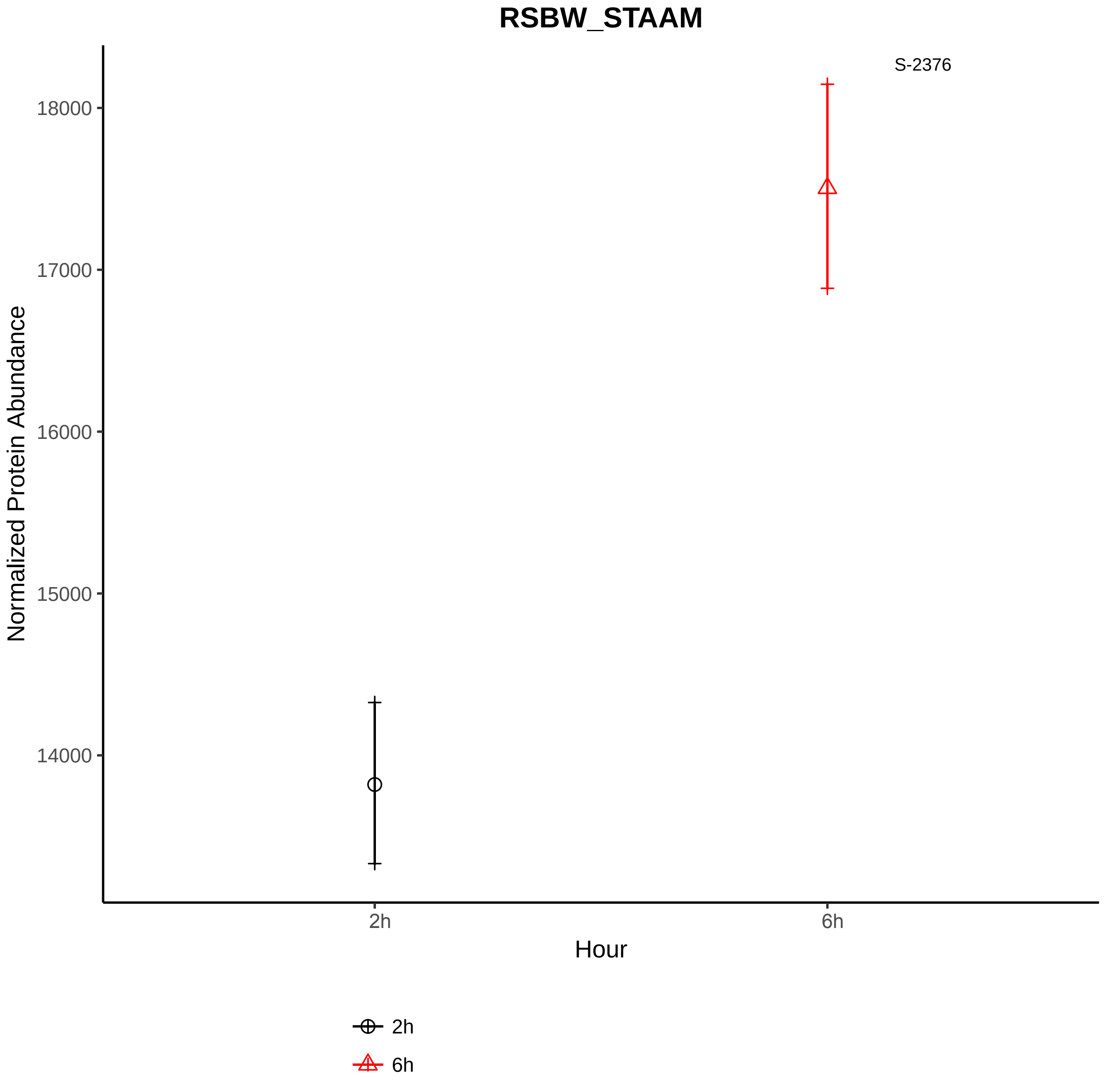

RSBW_STAAM
S-2376
18000
17000
16000
15000
14000
Normalized Protein Abundance
2h
6h
Hour
2h
6h

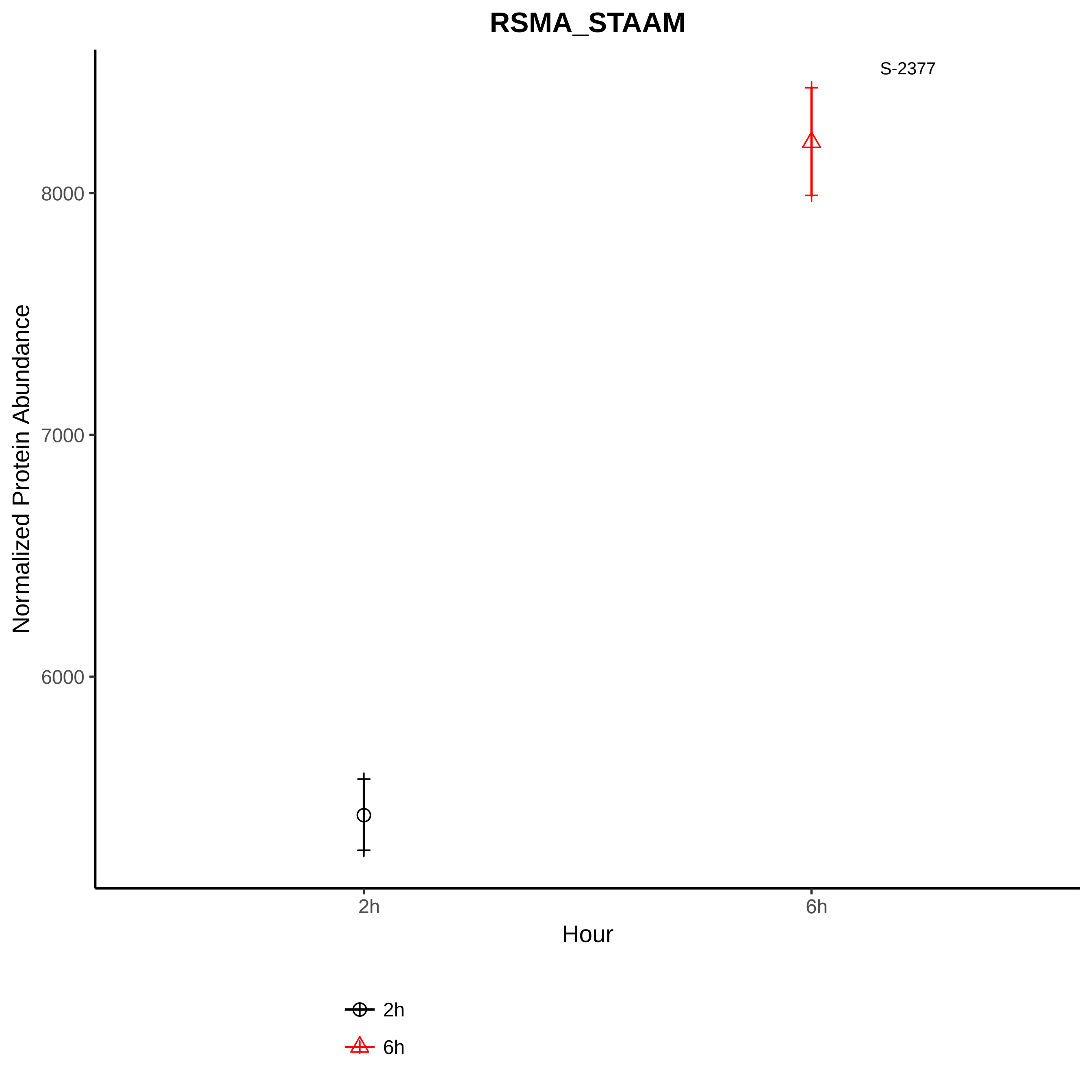
RSMA_STAAM
S-2377
Normalized Protein Abundance
8000
7000
6000
2h
6h
Hour
2h
6h

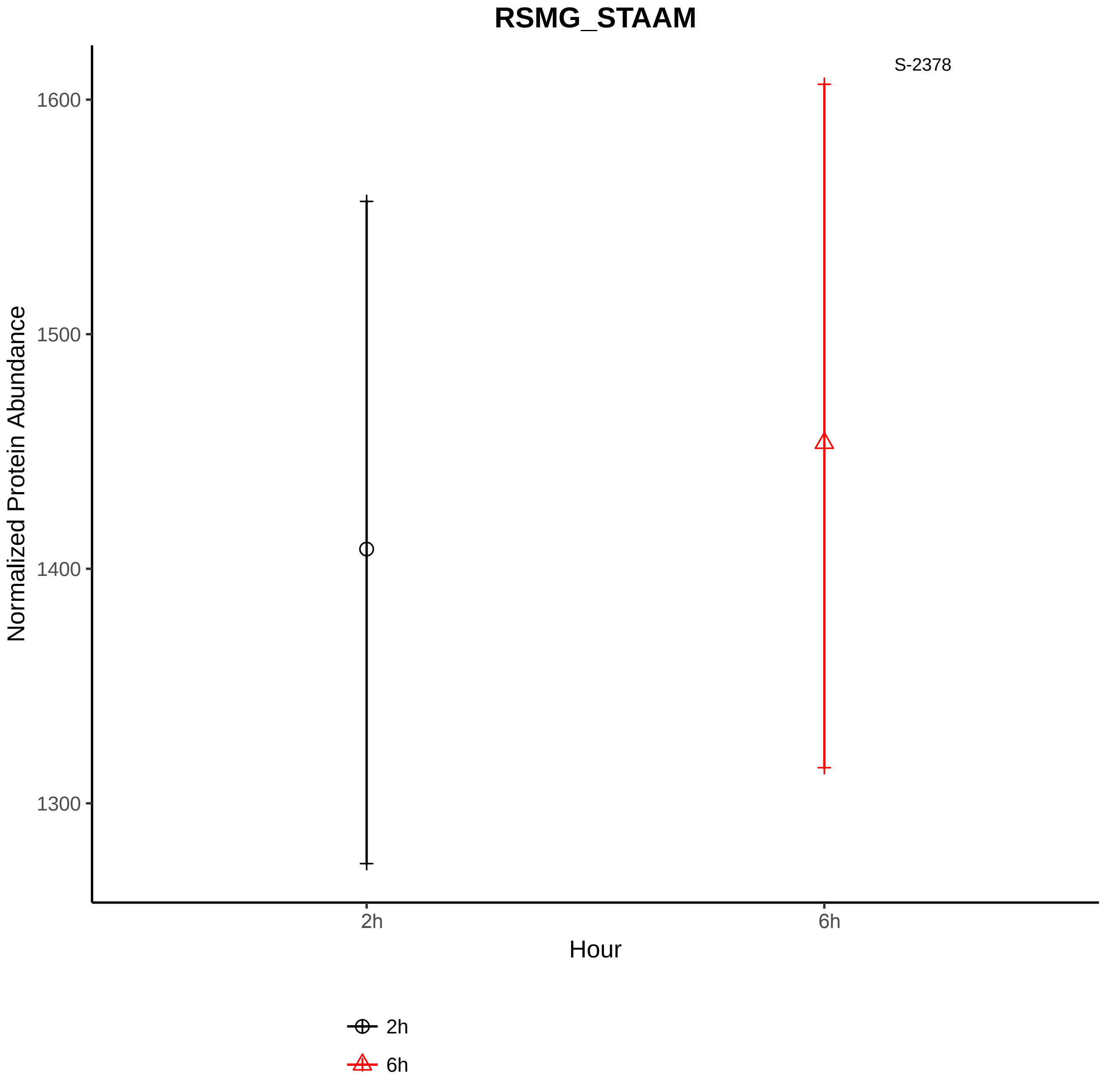

RSMG_STAAM
S-2378
Normalized Protein Abundance
1600
1500
1400
1300
2h
6h
Hour
2h
6h

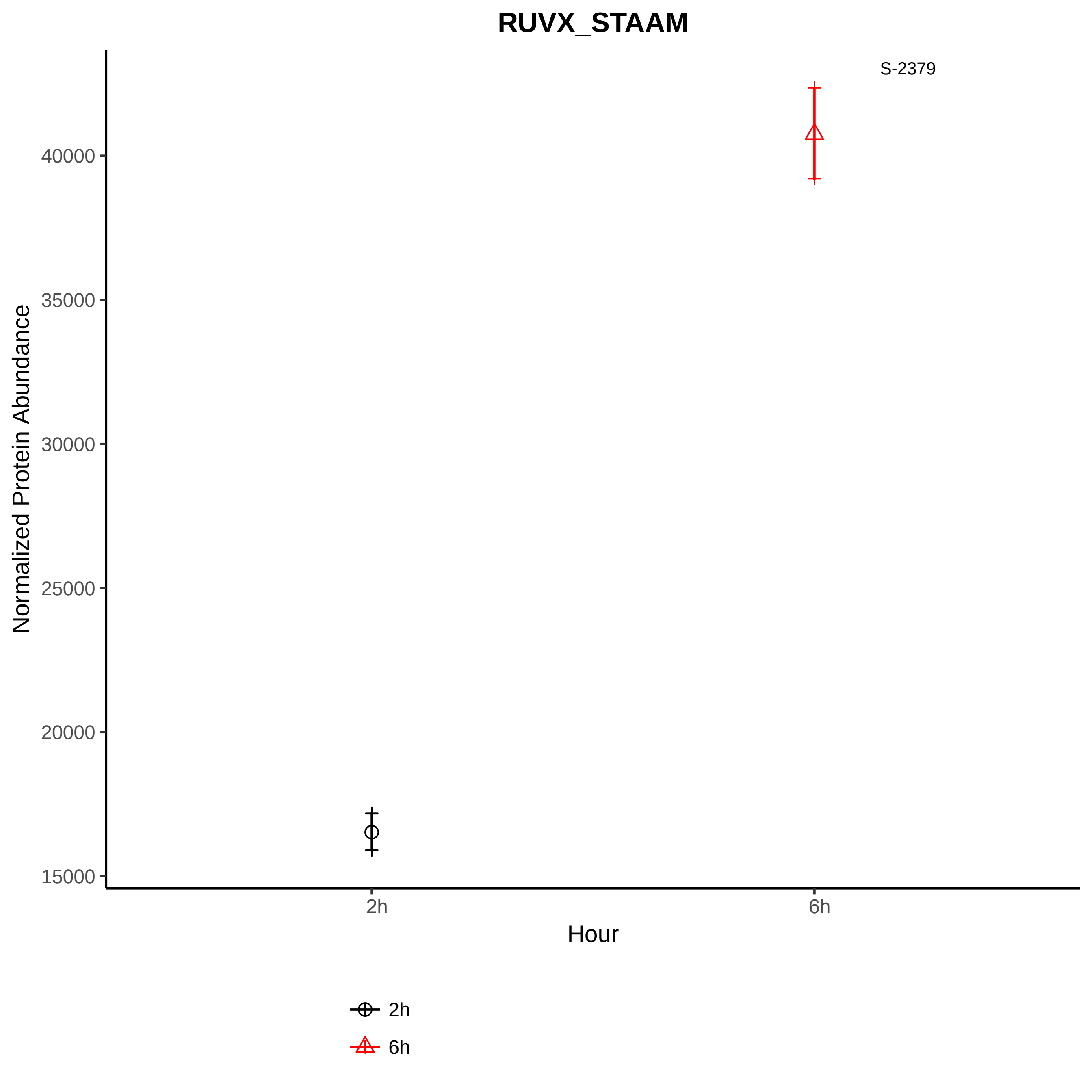
RUVX_STAAM
S-2379
Normalized Protein Abundance
40000
35000
30000
25000
20000
15000
2h
6h
Hour
2h
6h

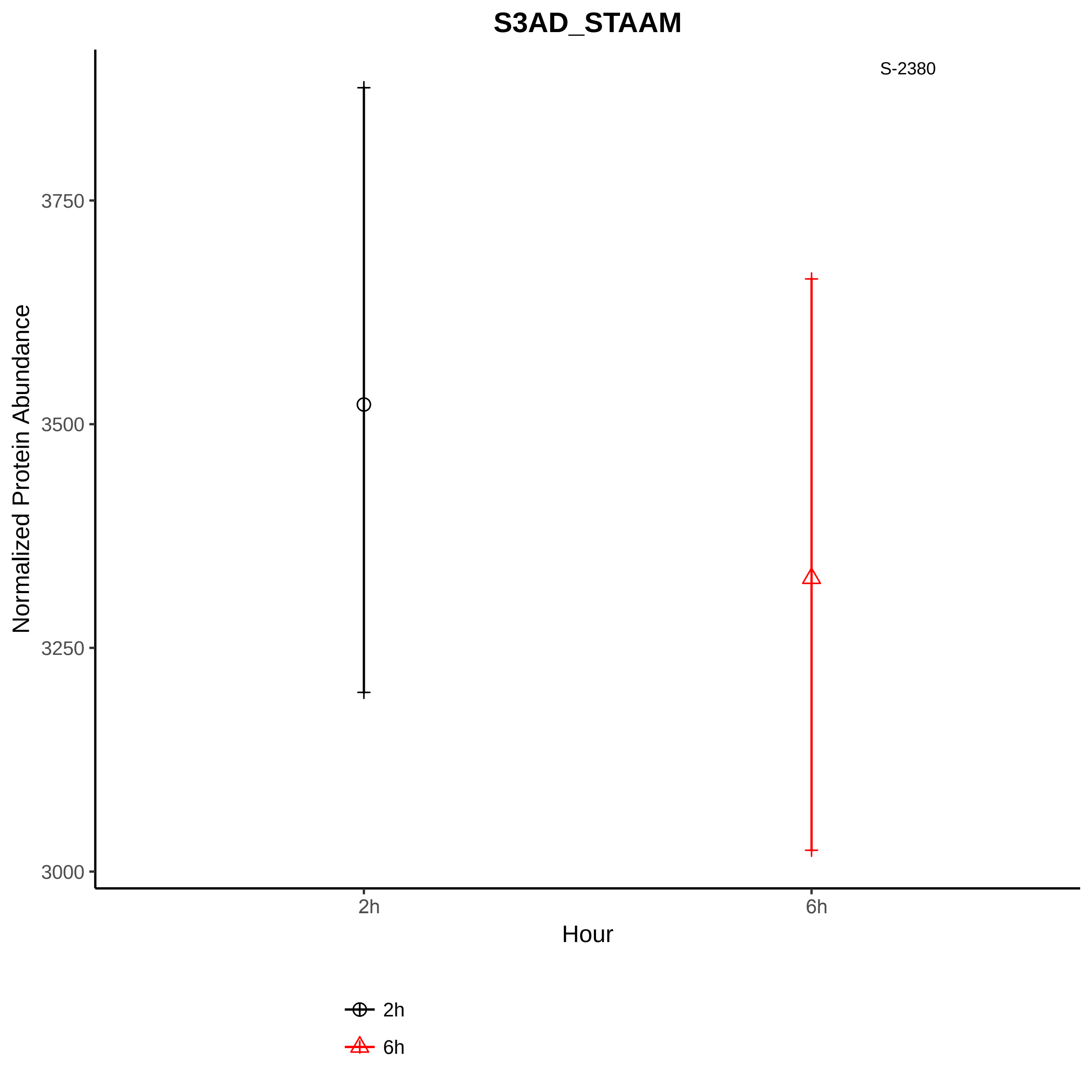

S3AD_STAAM
S-2380
Normalized Protein Abundance
3750
3500
3250
3000
2h
6h
Hour
2h
6h

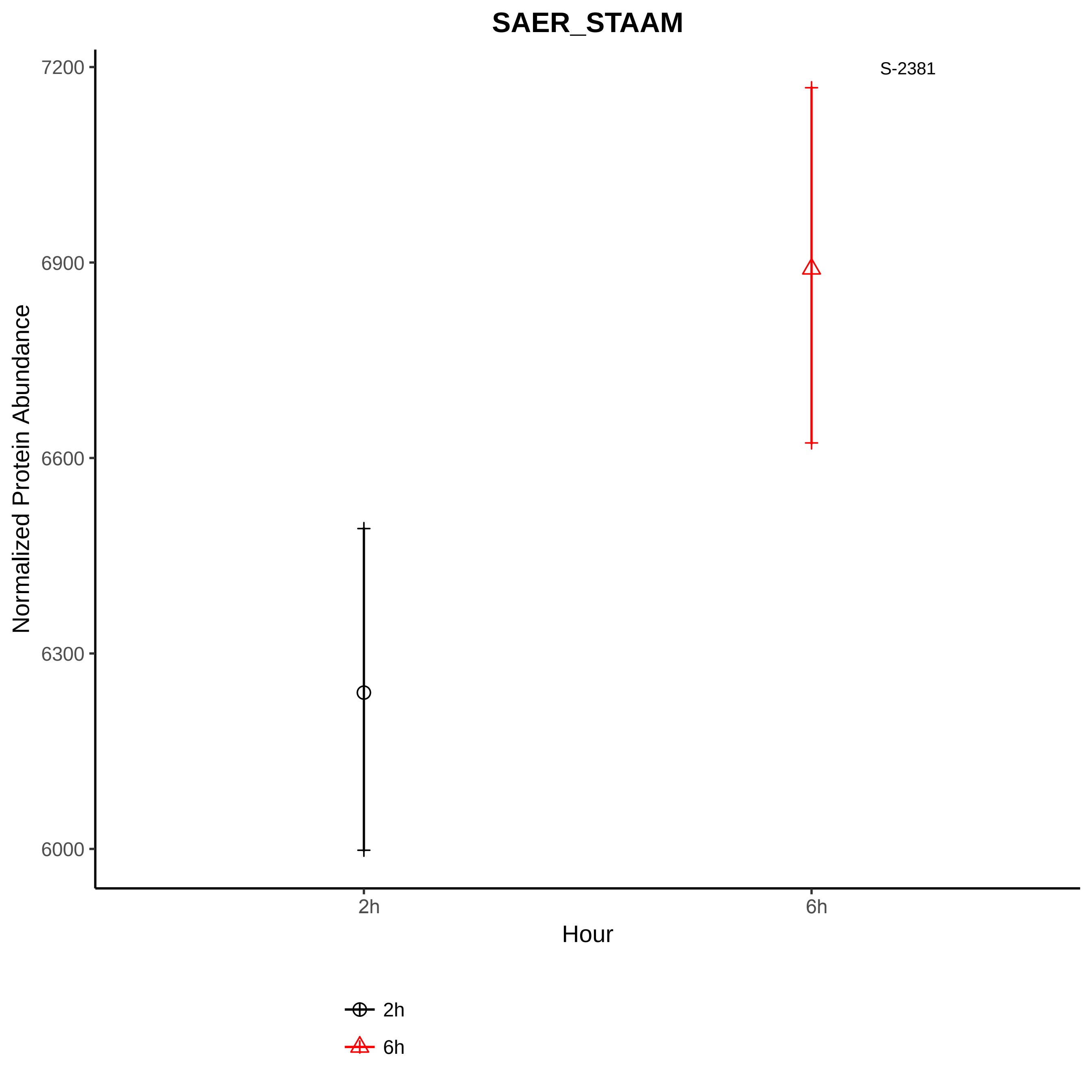

SAER_STAAM
S-2381
7200
6900
6600
6300
6000
Normalized Protein Abundance
2h
6h
Hour
2h
6h

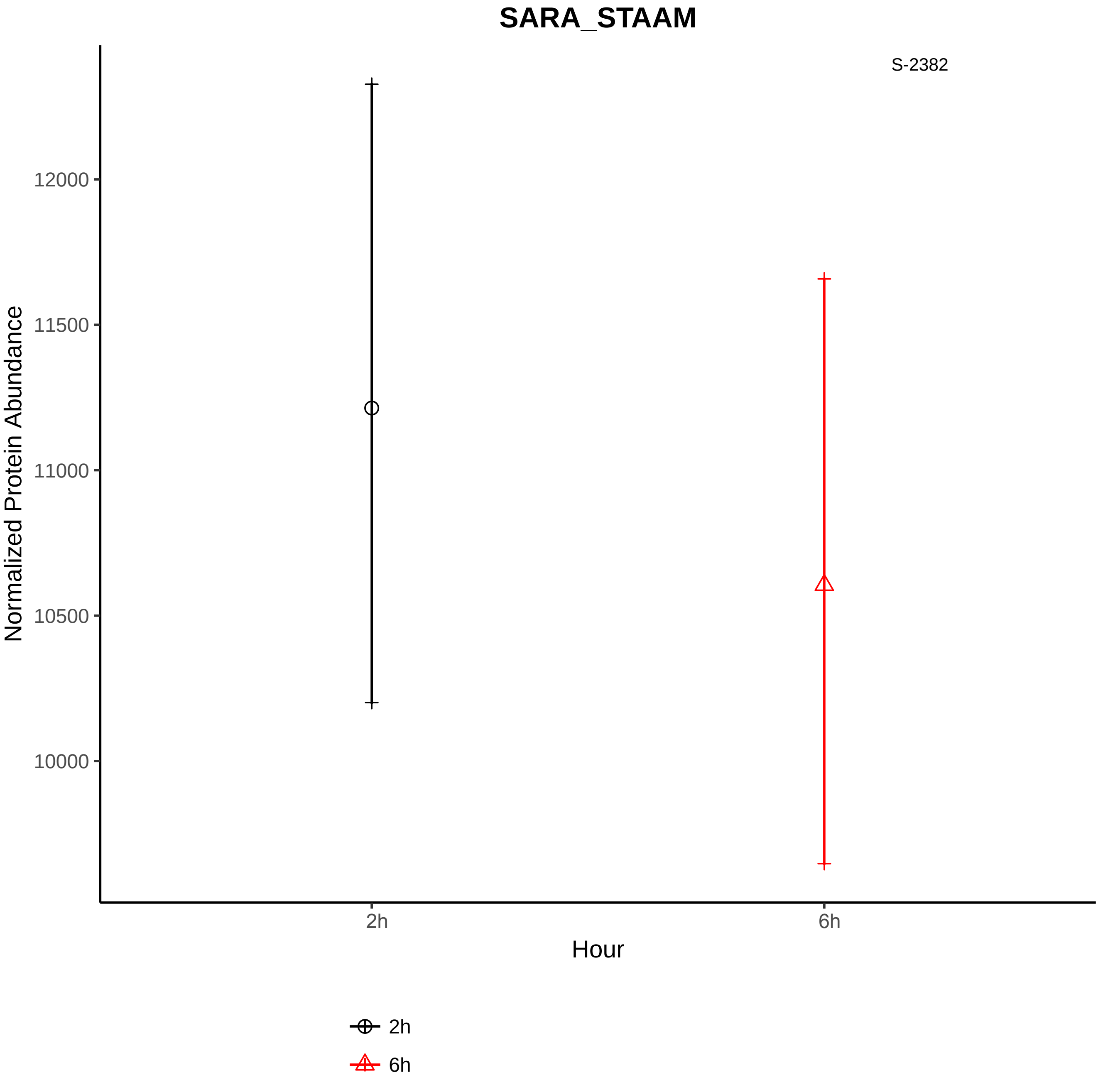

SARA_STAAM
S-2382
Normalized Protein Abundance
12000
11500
11000
10500
10000
2h
6h
Hour
2h
6h

SARR_STAAM

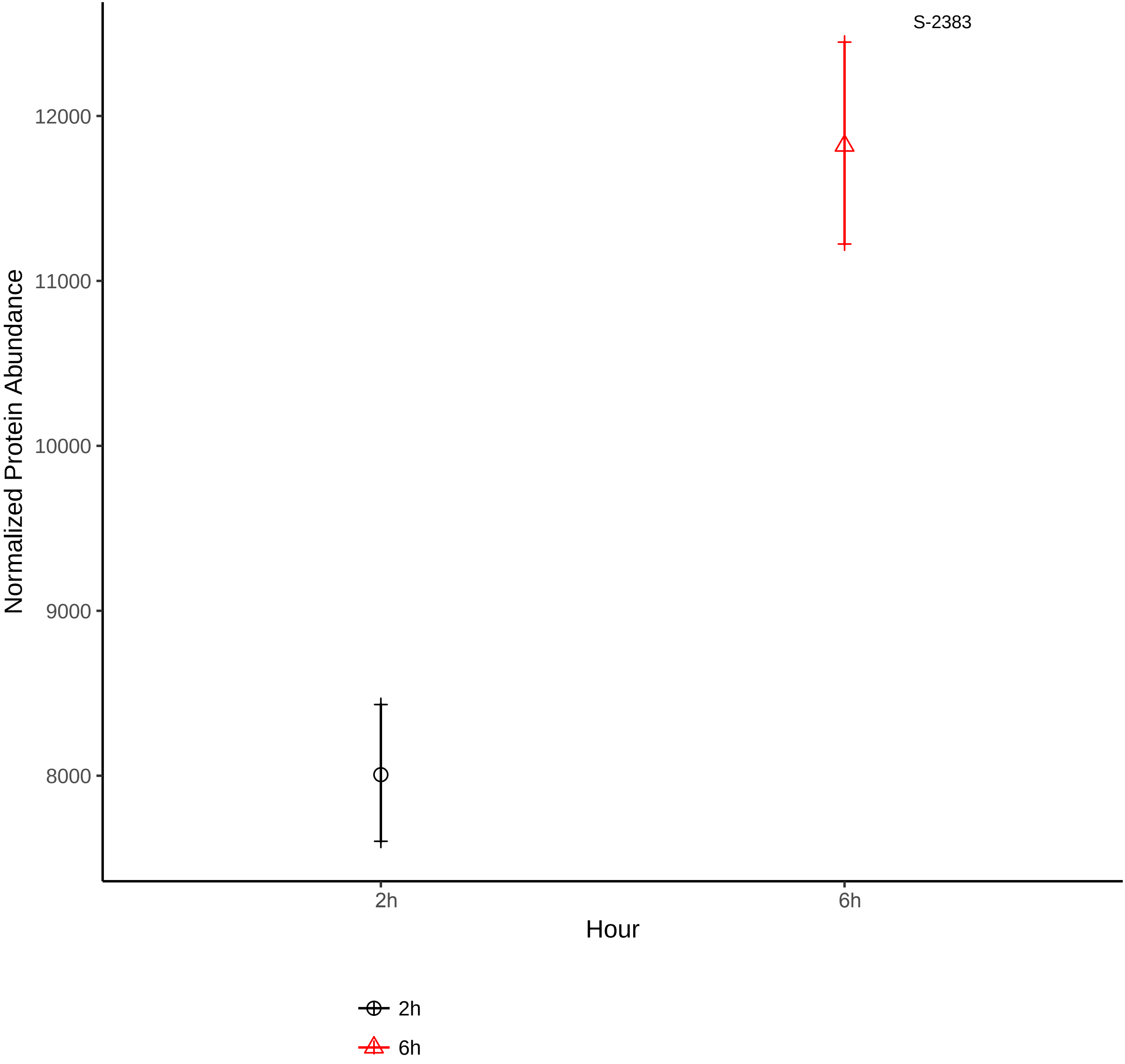

S-2383
Normalized Protein Abundance
12000
11000
10000
9000
8000
2h
6h
Hour
2h
6h

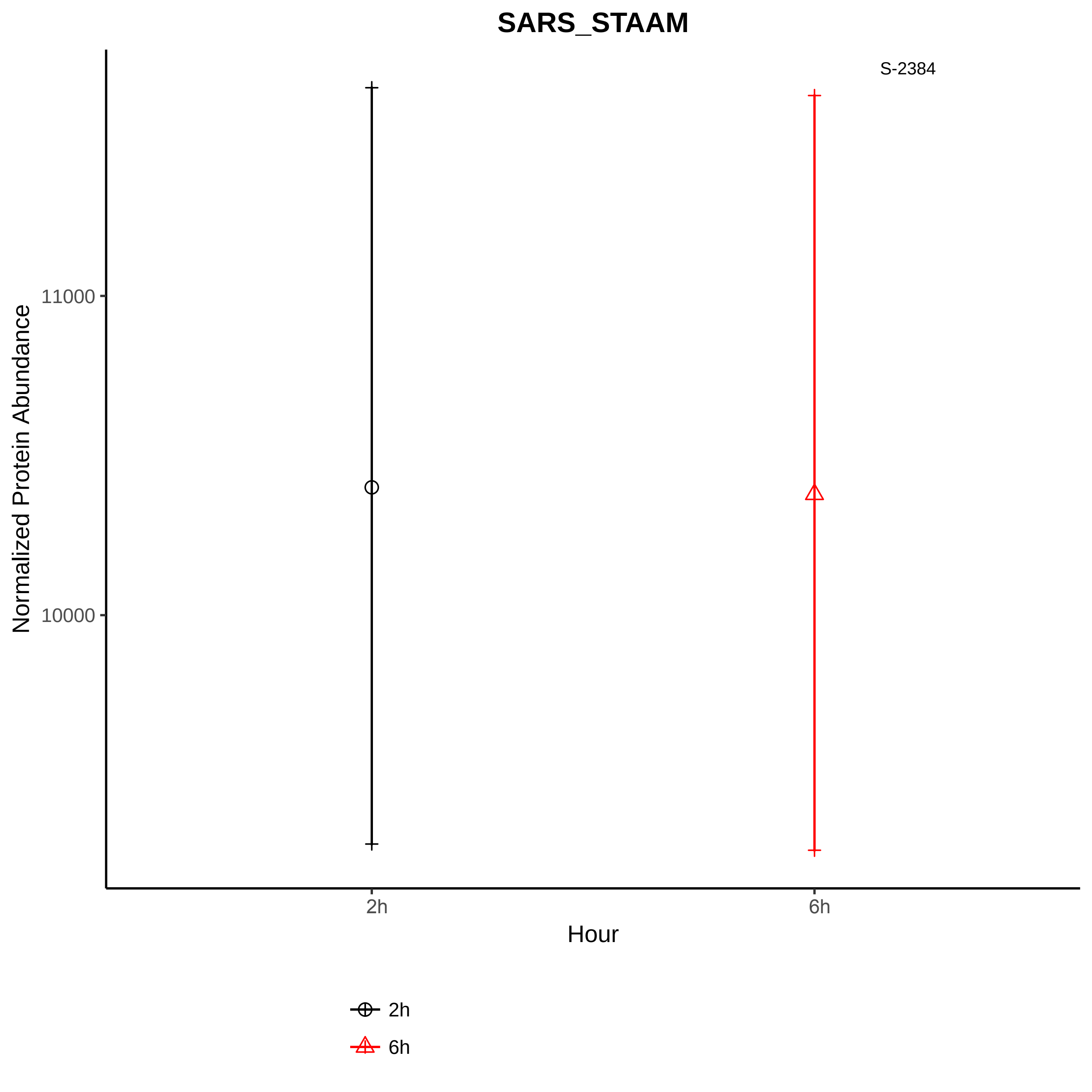
SARS_STAAM
S-2384
Normalized Protein Abundance
11000
10000
2h
6h
Hour
2h
6h

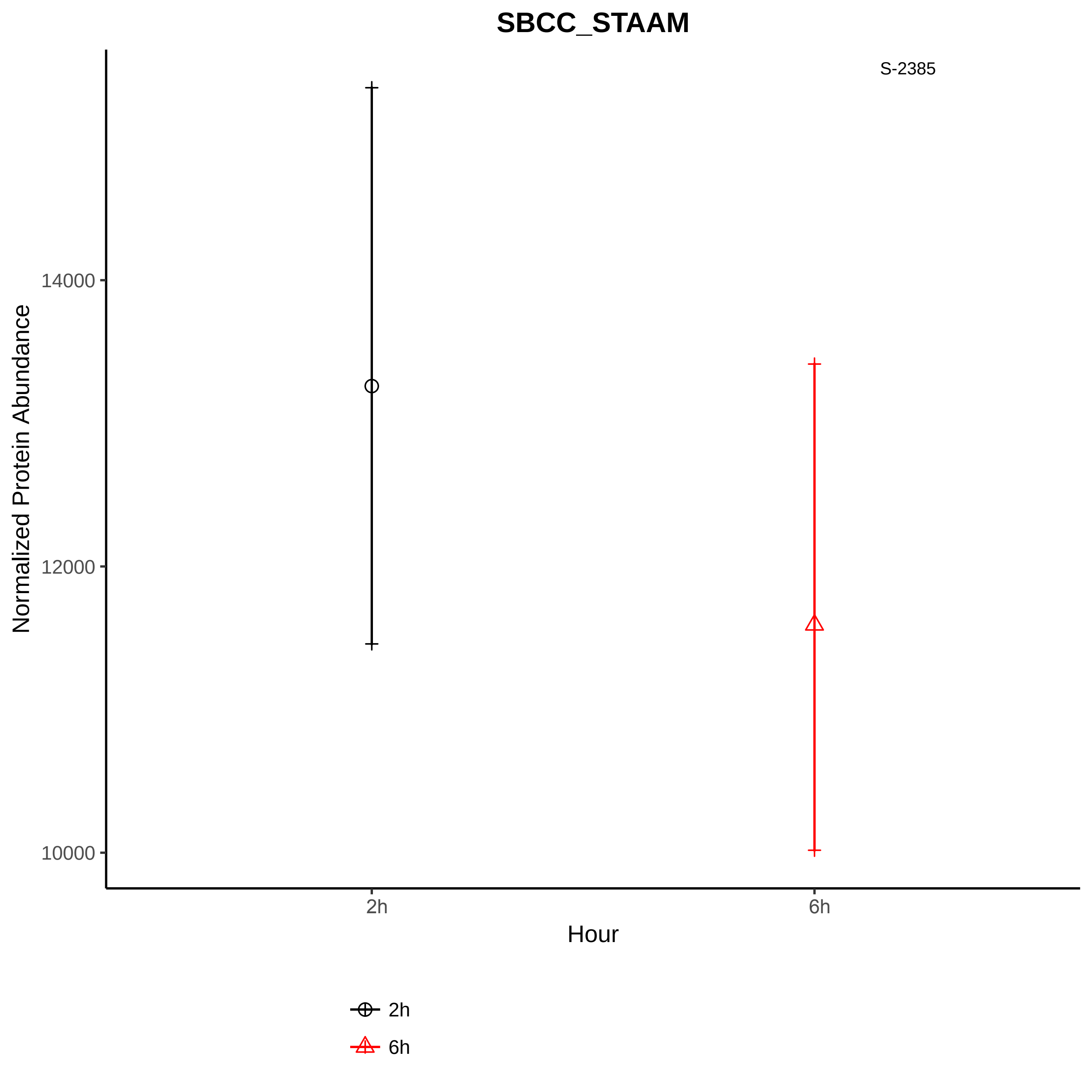

SBCC_STAAM
S-2385
Normalized Protein Abundance
14000
12000
10000
2h
6h
Hour
2h
6h

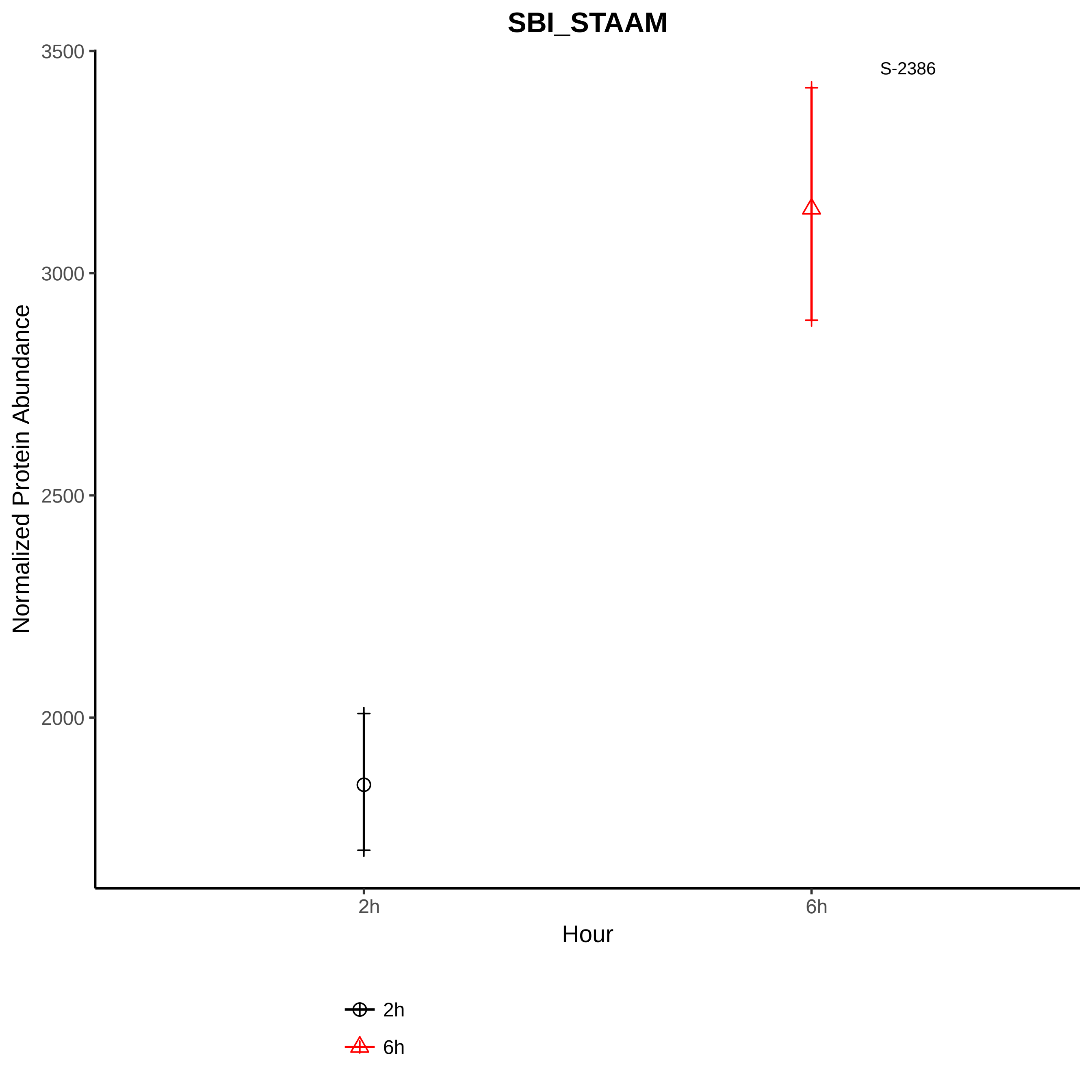

SBI_STAAM
S-2386
3500
3000
2500
2000
Normalized Protein Abundance
2h
6h
Hour
2h
6h

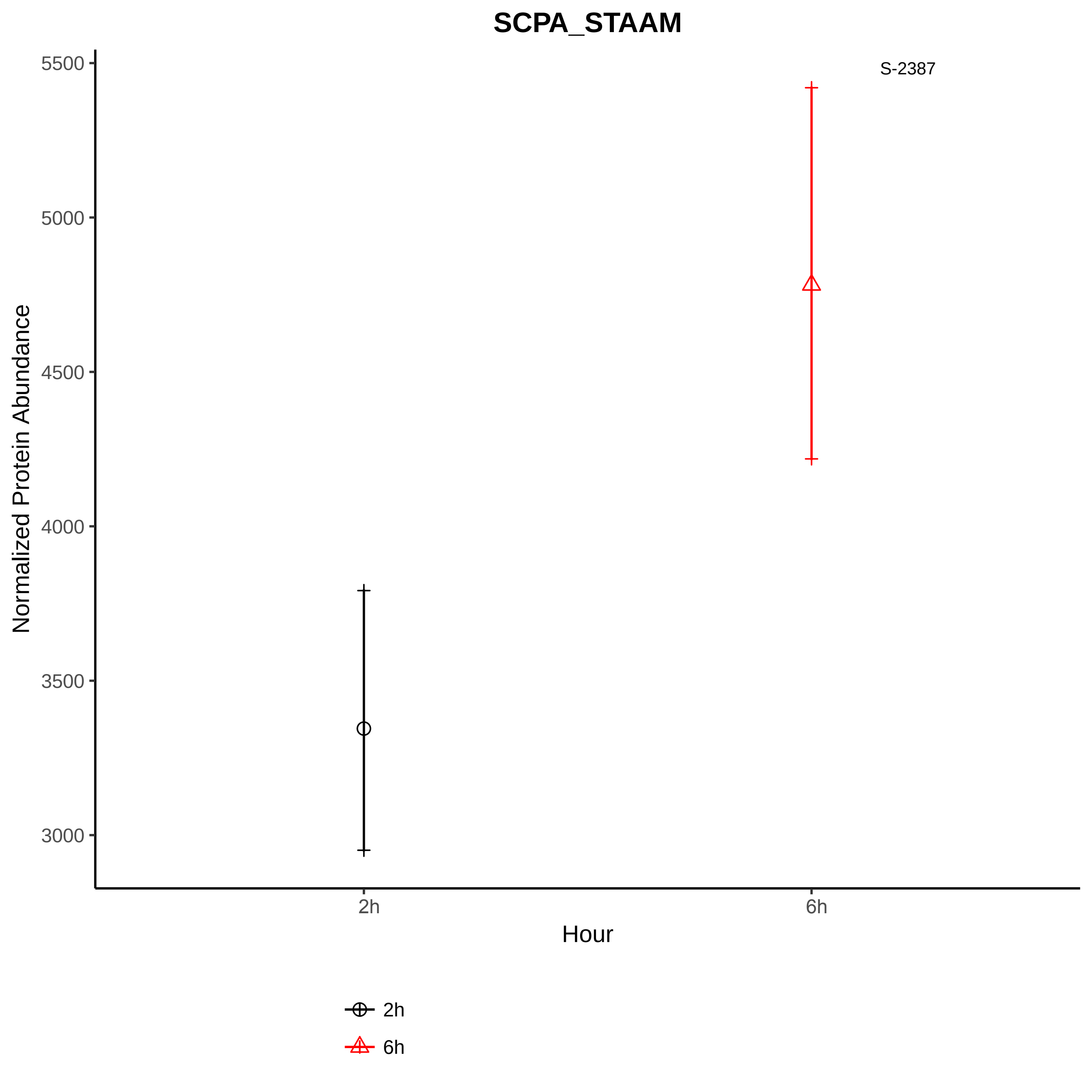

SCPA_STAAM
S-2387
Normalized Protein Abundance
5500
5000
4500
4000
3500
3000
2h
6h
Hour
2h
6h

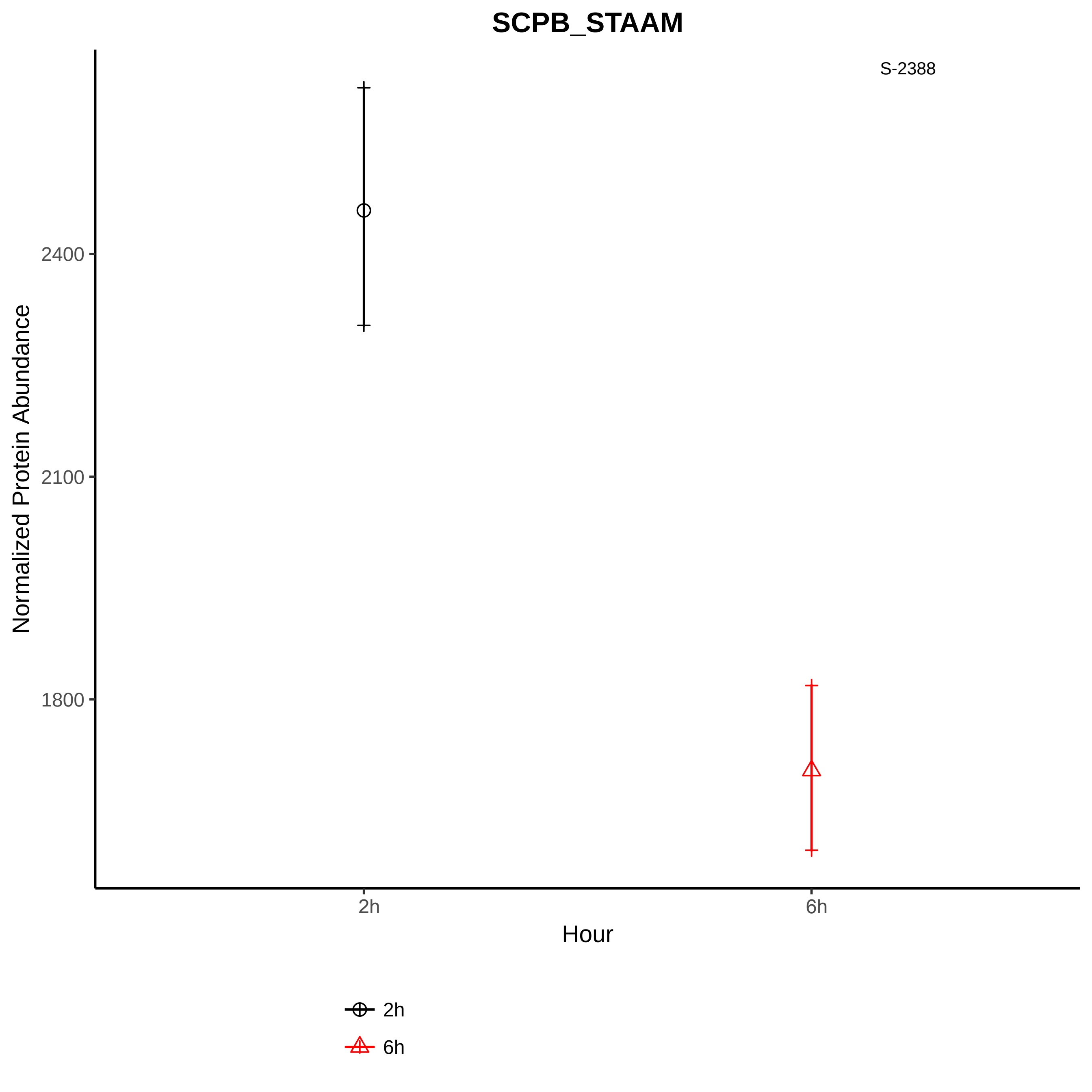

SCPB_STAAM
S-2388
2400
2100
1800
Normalized Protein Abundance
2h
6h
Hour
2h
6h

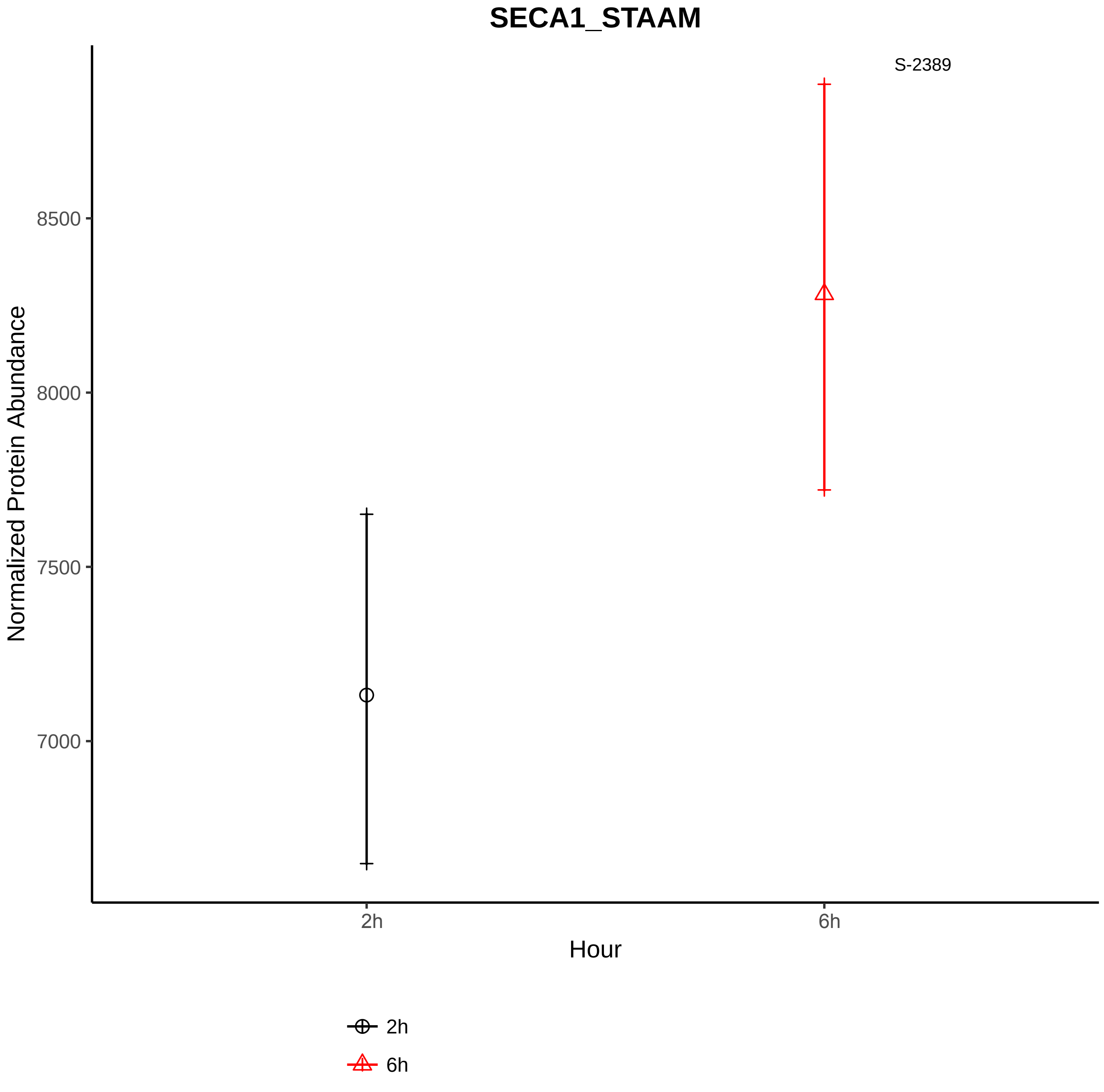

SECA1_STAAM
S-2389
Normalized Protein Abundance
8500
8000
7500
7000
2h
6h
Hour
2h
6h

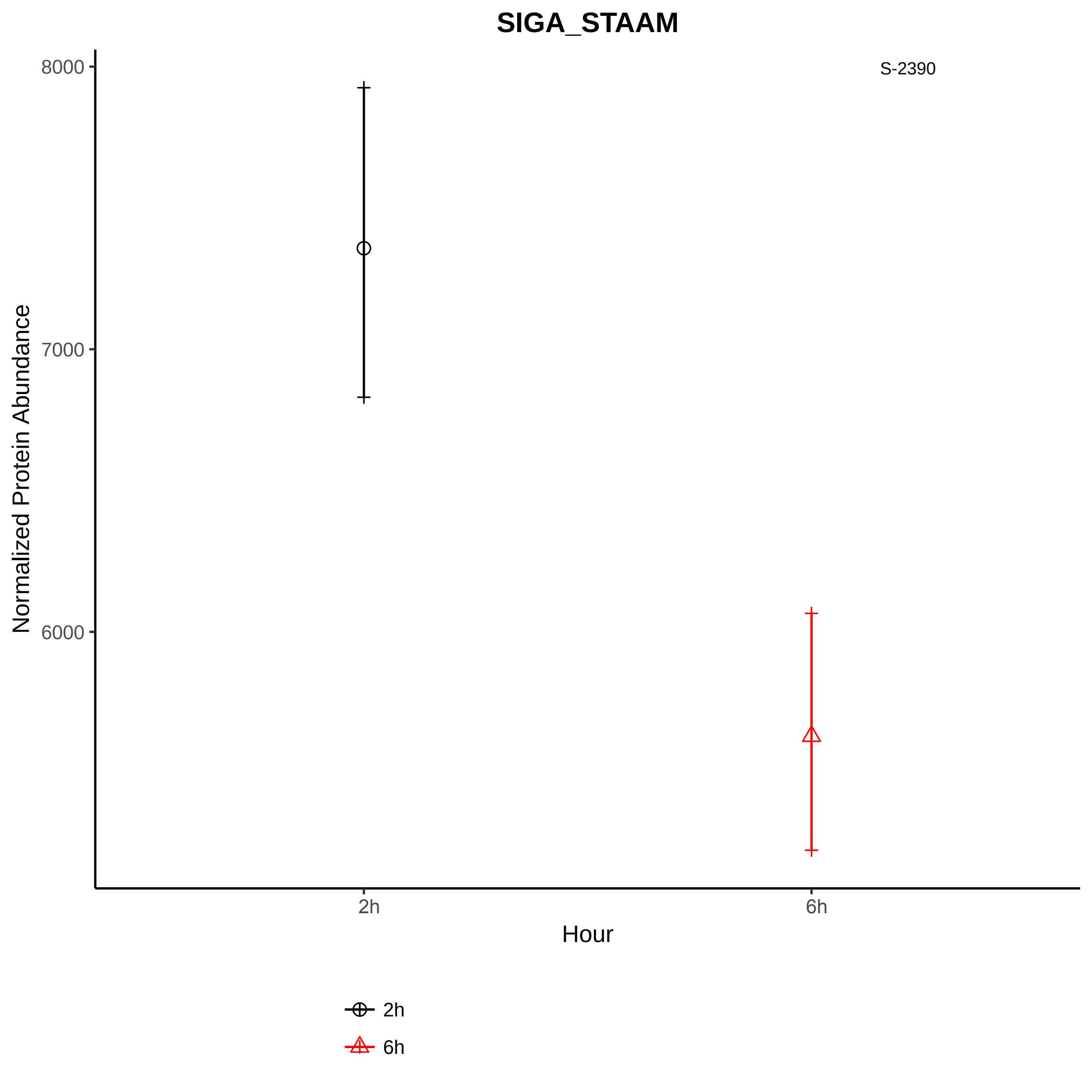

SIGA_STAAM
S-2390
8000
7000
6000
Normalized Protein Abundance
2h
6h
Hour
2h
6h

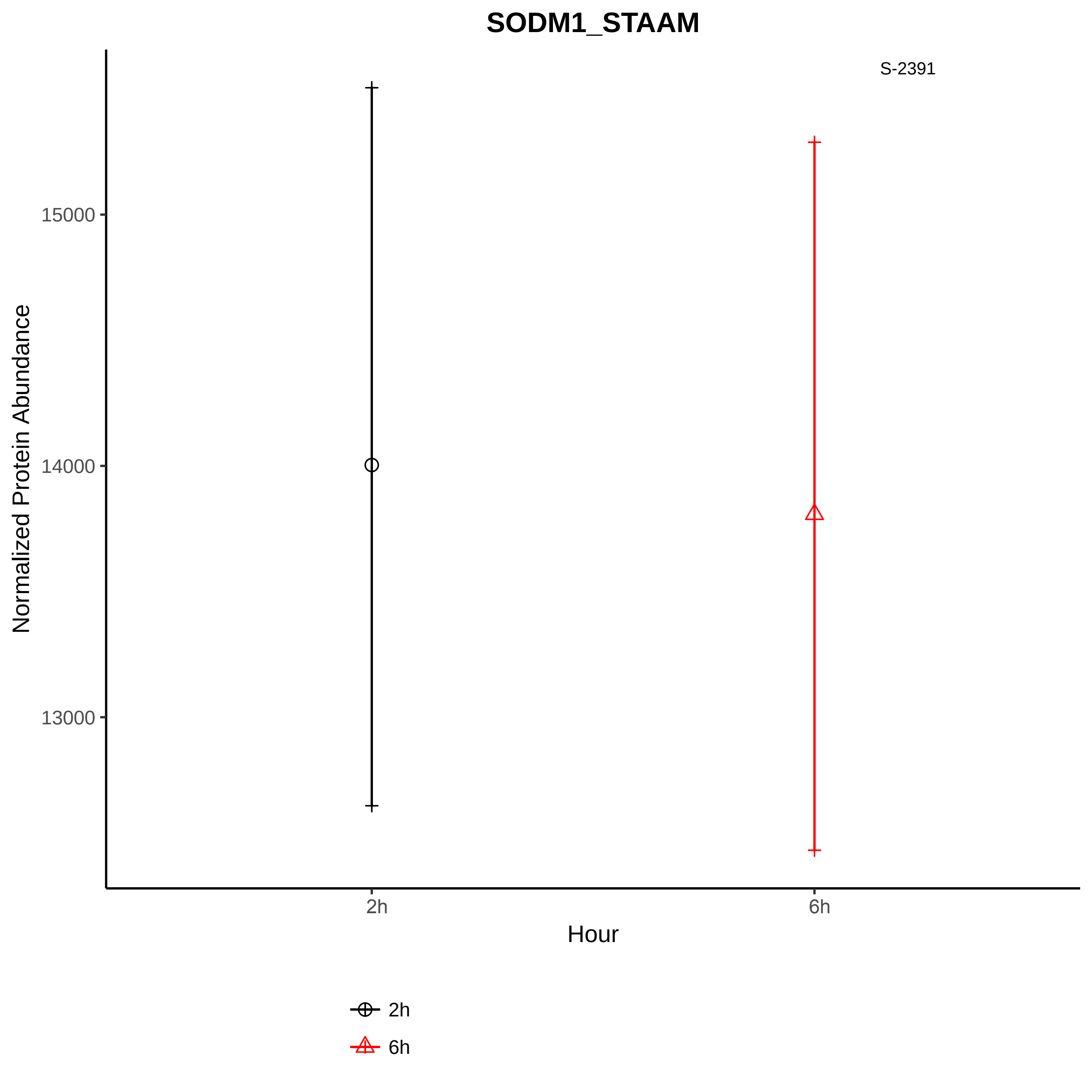

SODM1_STAAM
S-2391
Normalized Protein Abundance
15000
14000
13000
2h
6h
Hour
2h
6h

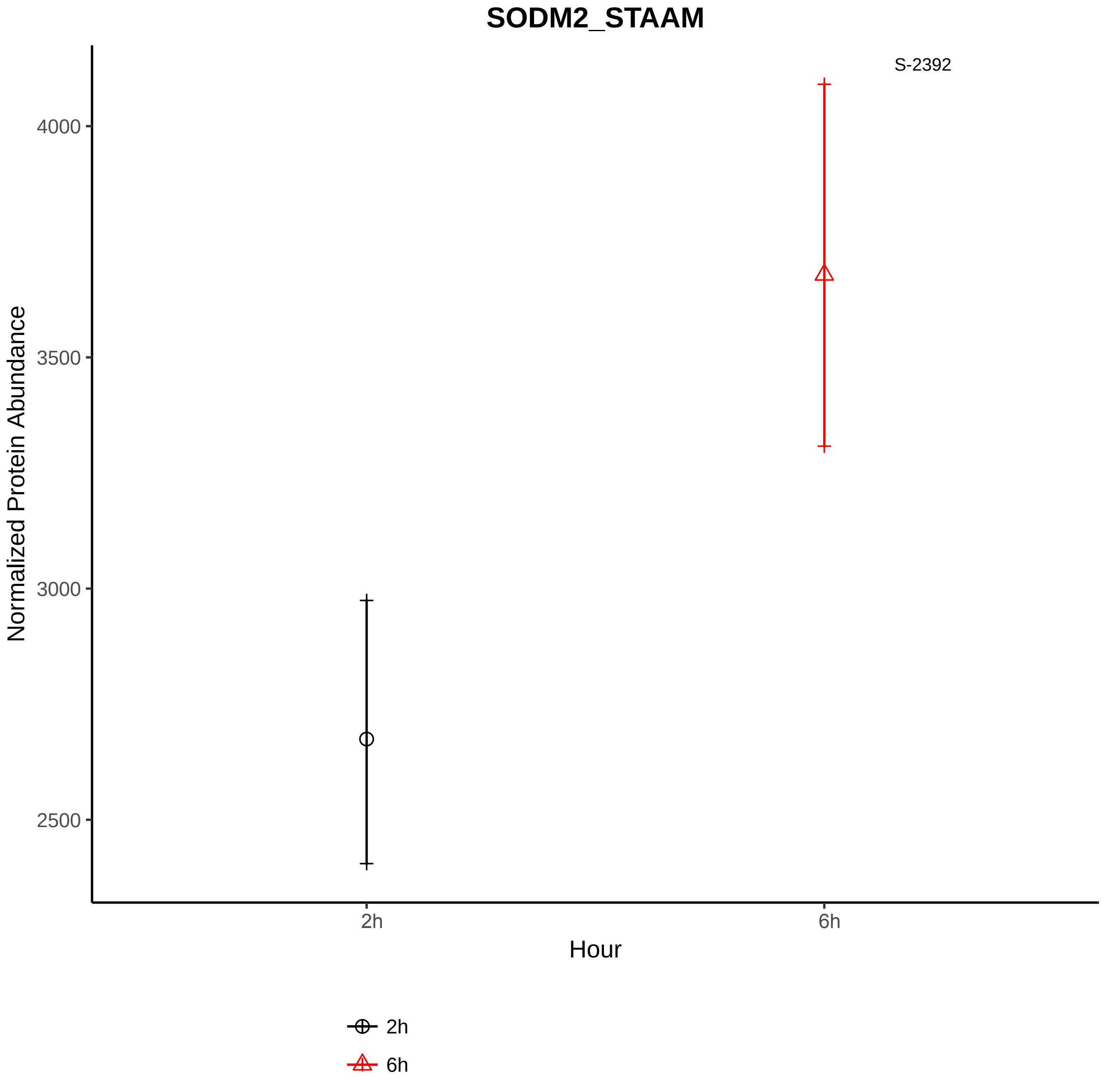

SODM2_STAAM
S-2392
Normalized Protein Abundance
4000
3500
3000
2500
2h
6h
Hour
2h
6h

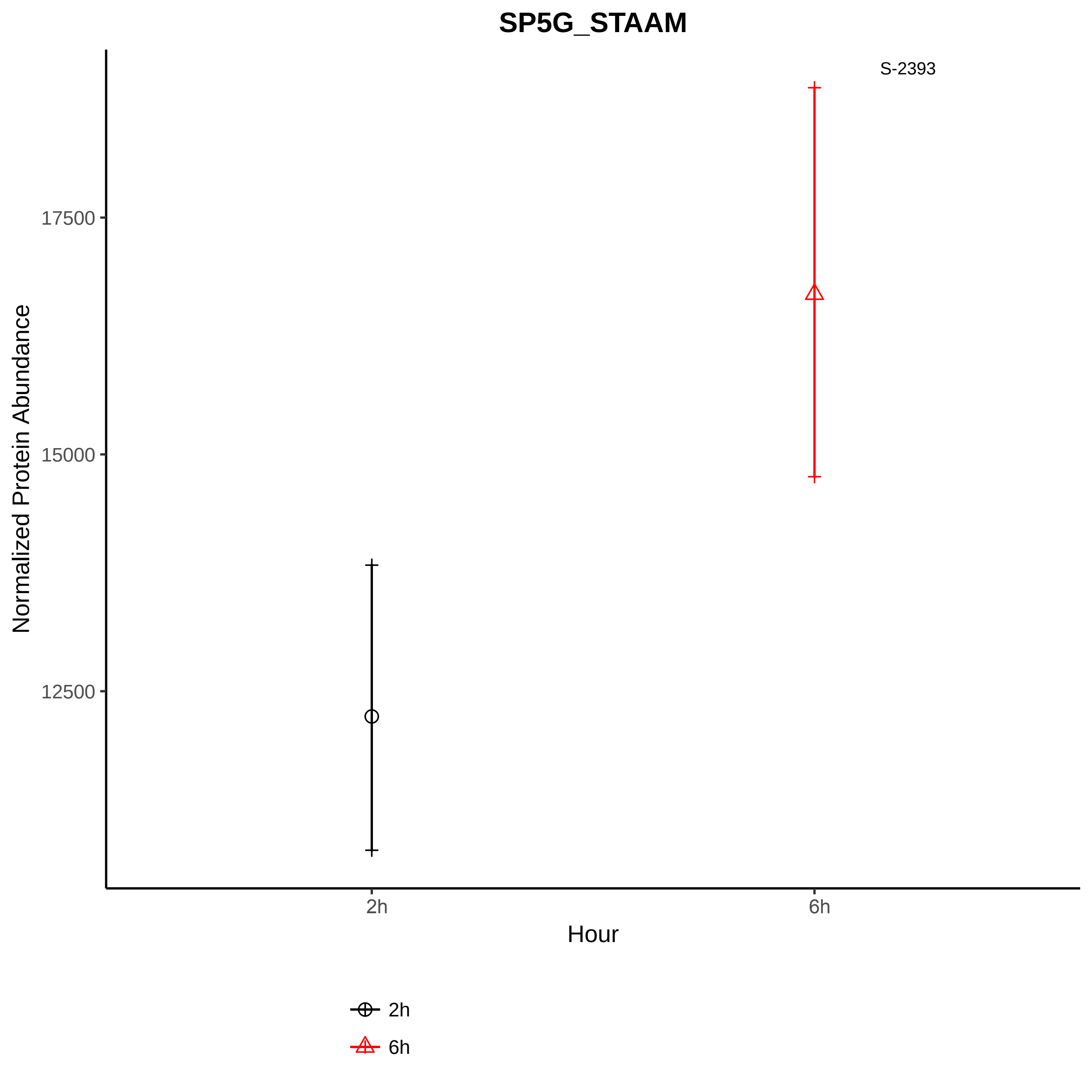

SP5G_STAAM
S-2393
Normalized Protein Abundance
17500
15000
12500
2h
6h
Hour
2h
6h

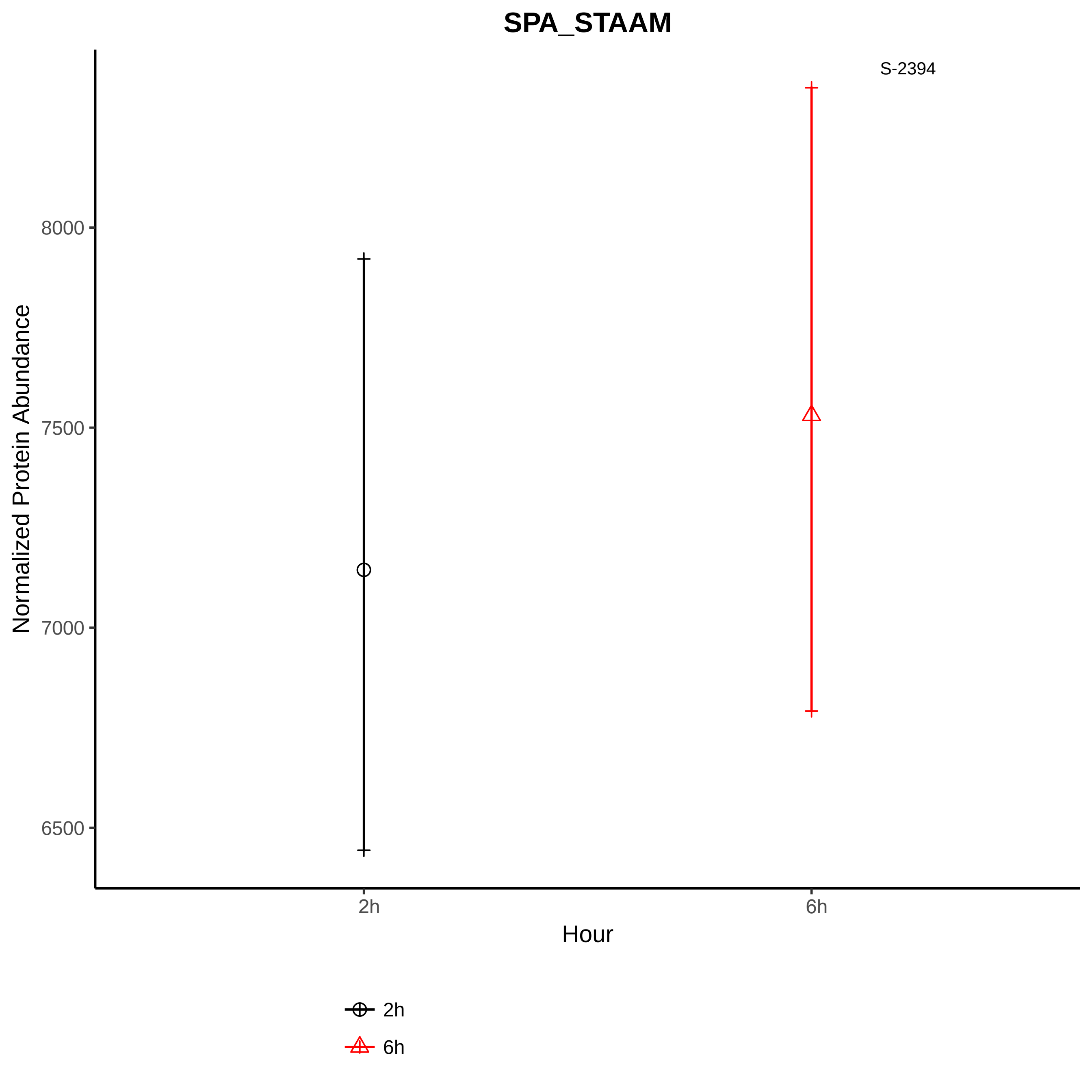
SPA_STAAM
S-2394
Normalized Protein Abundance
8000
7500
7000
6500
2h
6h
Hour
2h
6h

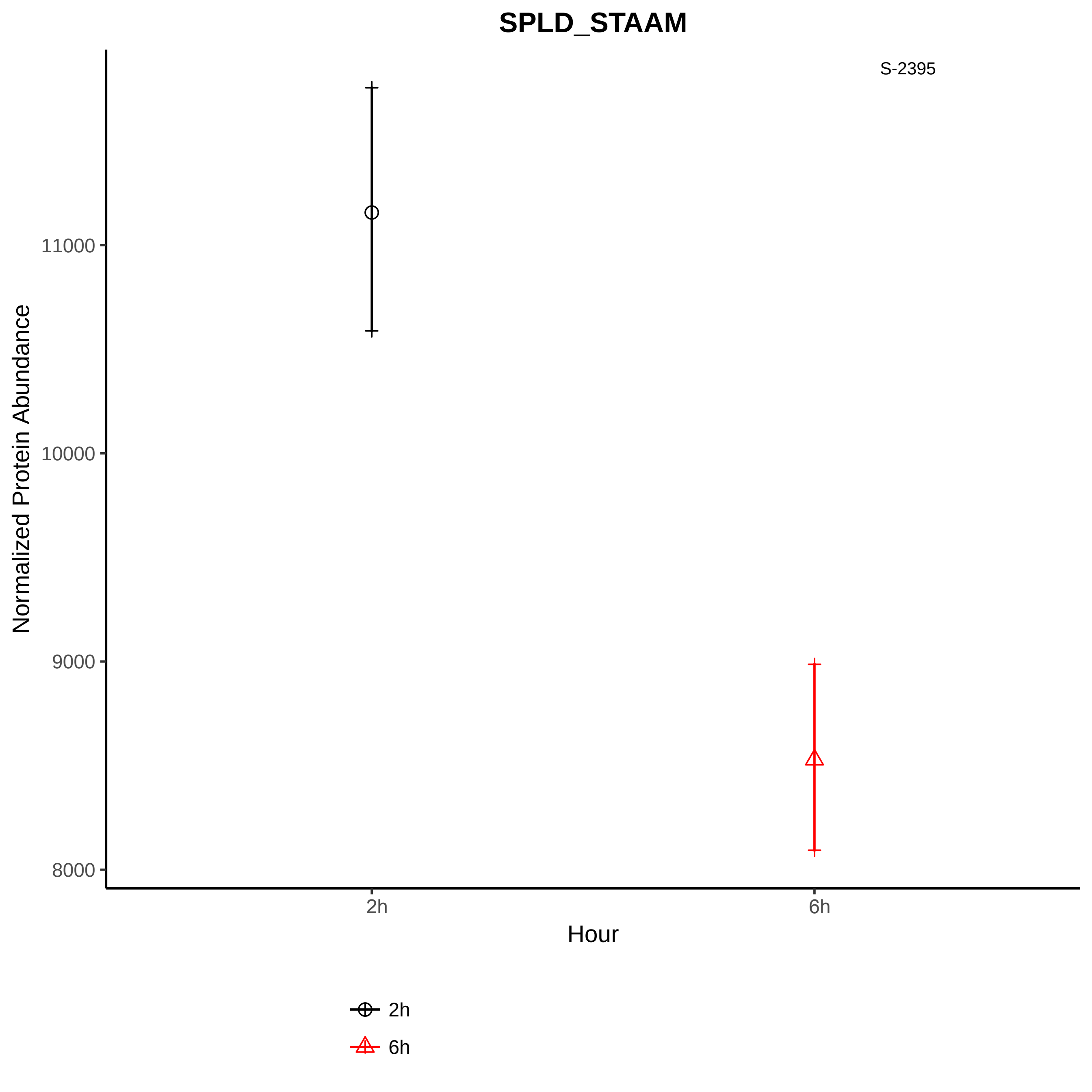

SPLD_STAAM
S-2395
Normalized Protein Abundance
11000
10000
9000
8000
2h
6h
Hour
2h
6h

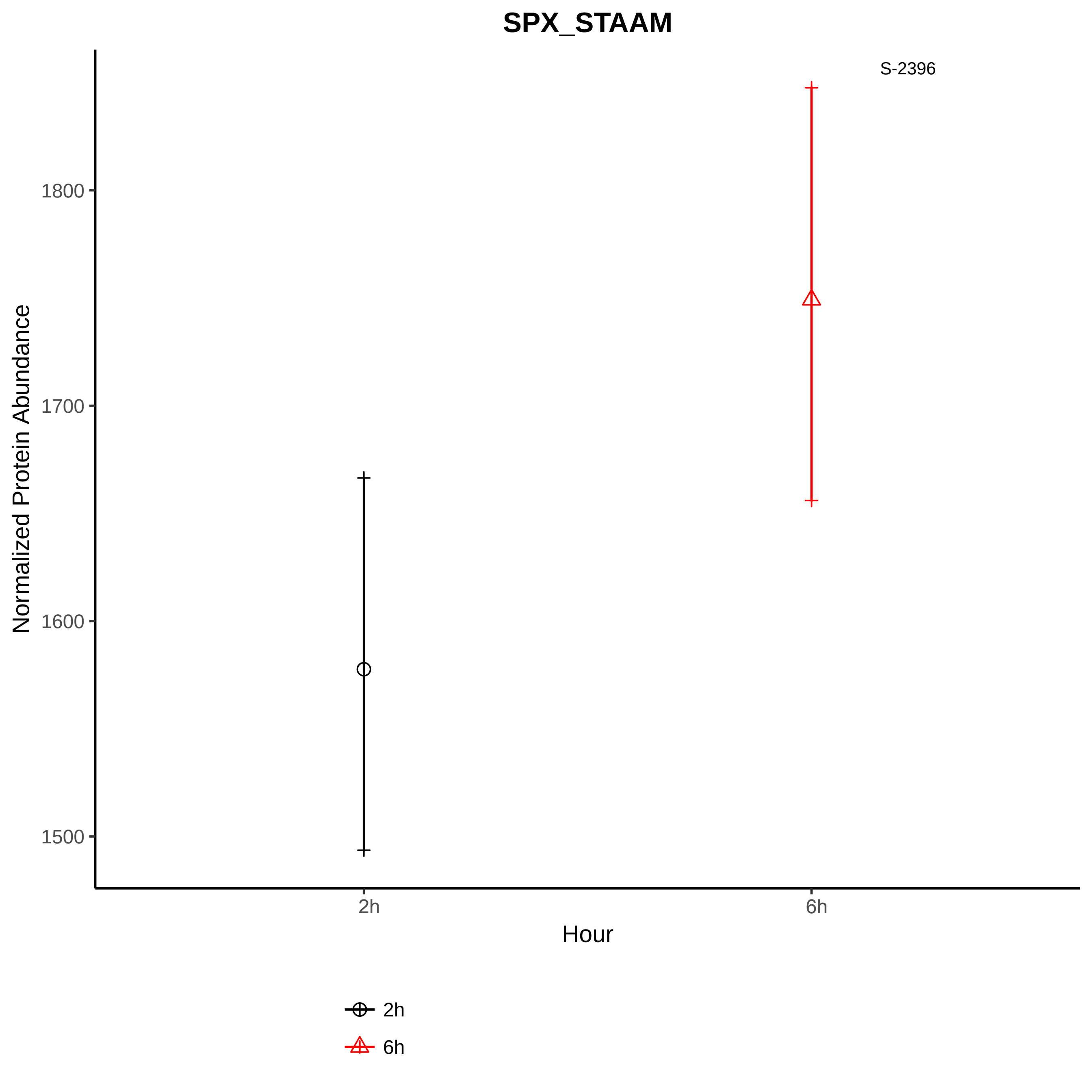

SPX_STAAM
S-2396
Normalized Protein Abundance
1800
1700
1600
1500
2h
6h
Hour
2h
6h

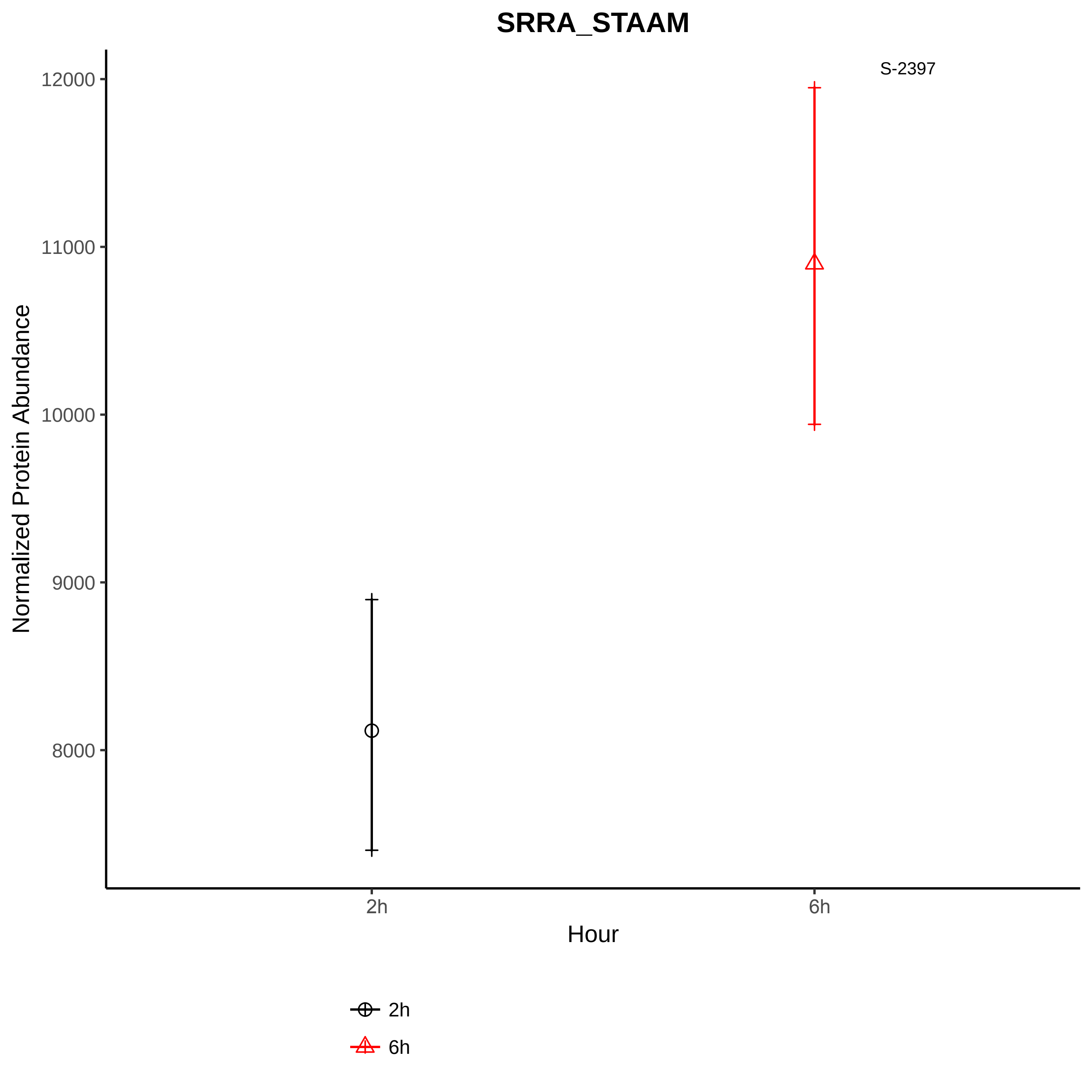
SRRA_STAAM
S-2397
Normalized Protein Abundance
12000
11000
10000
9000
8000
2h
6h
Hour
2h
6h

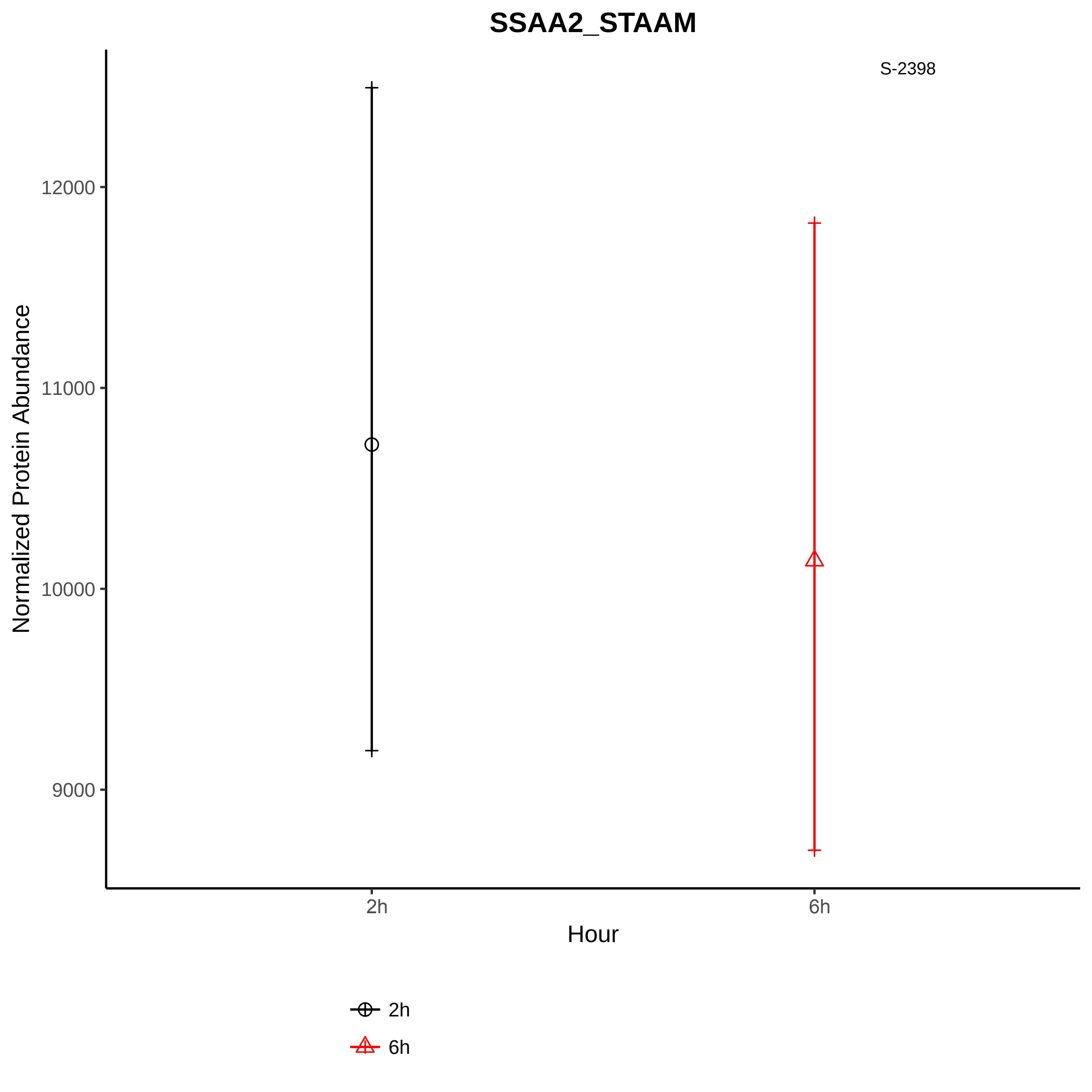

SSAA2_STAAM
S-2398
Normalized Protein Abundance
12000
11000
10000
9000
2h
6h
Hour
2h
6h

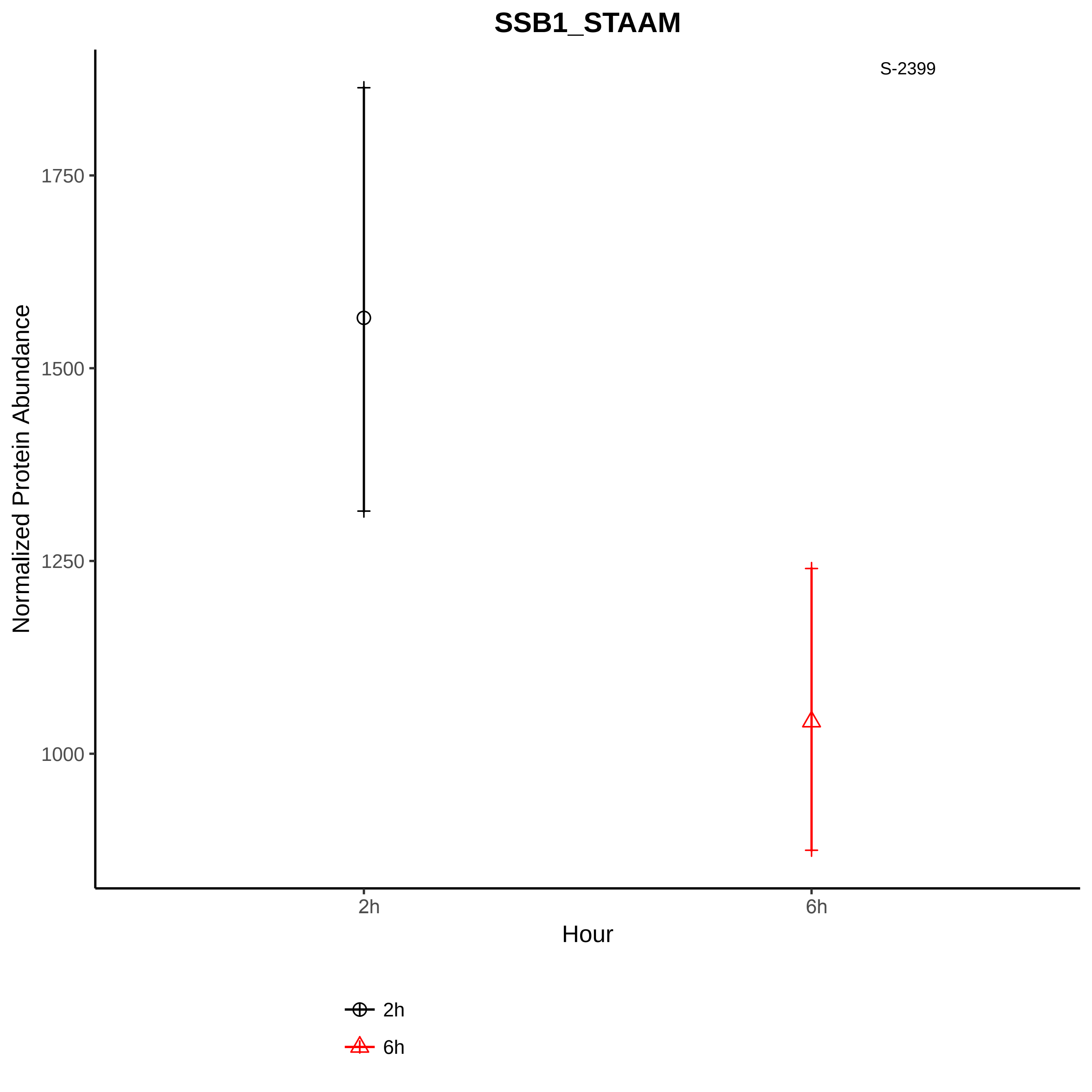

SSB1_STAAM
S-2399
Normalized Protein Abundance
1750
1500
1250
1000
2h
6h
Hour
2h
6h

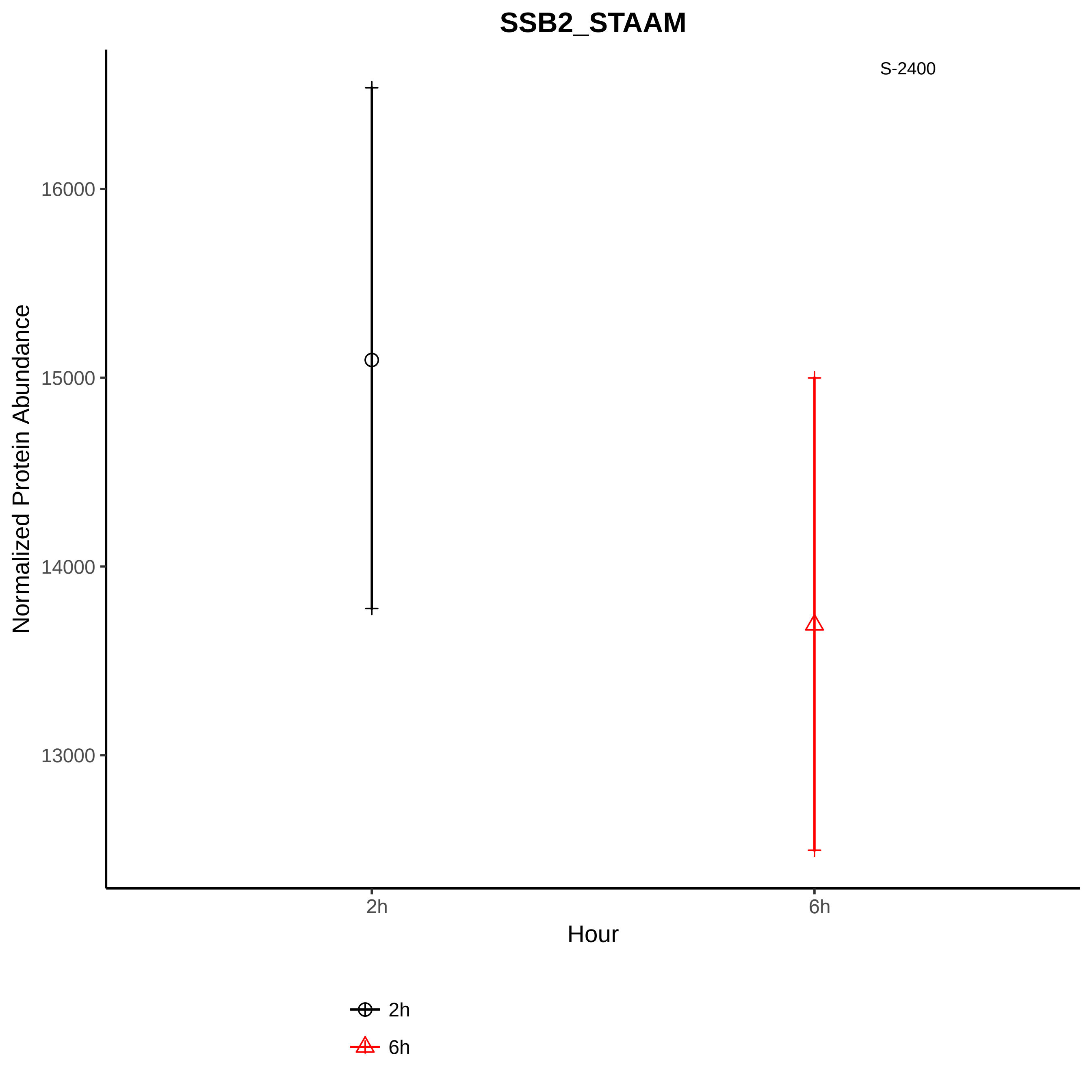

SSB2_STAAM
S-2400
Normalized Protein Abundance
16000
15000
14000
13000
2h
6h
Hour
2h
6h

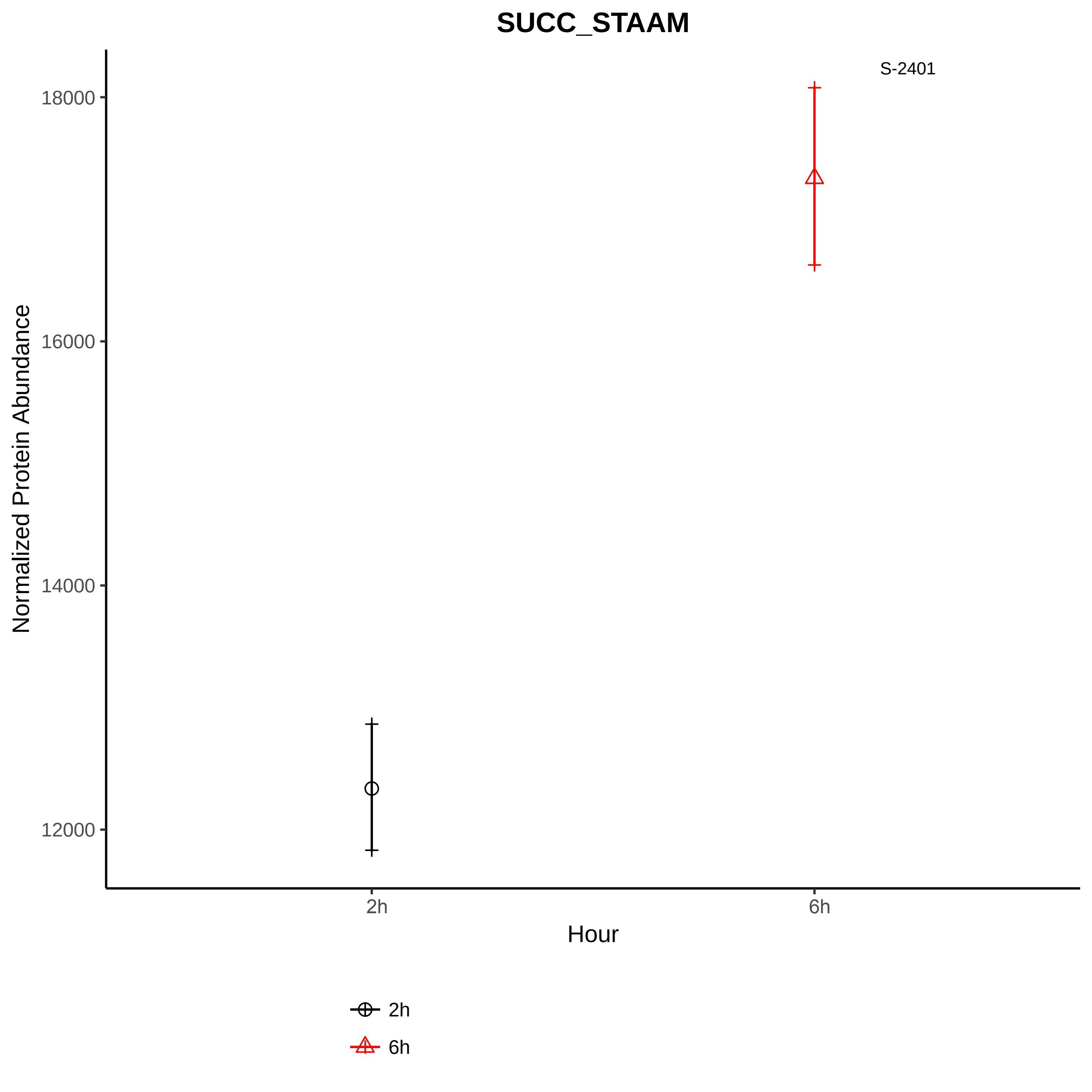
SUCC_STAAM
S-2401
Normalized Protein Abundance
18000
16000
14000
12000
2h
6h
Hour
2h
6h

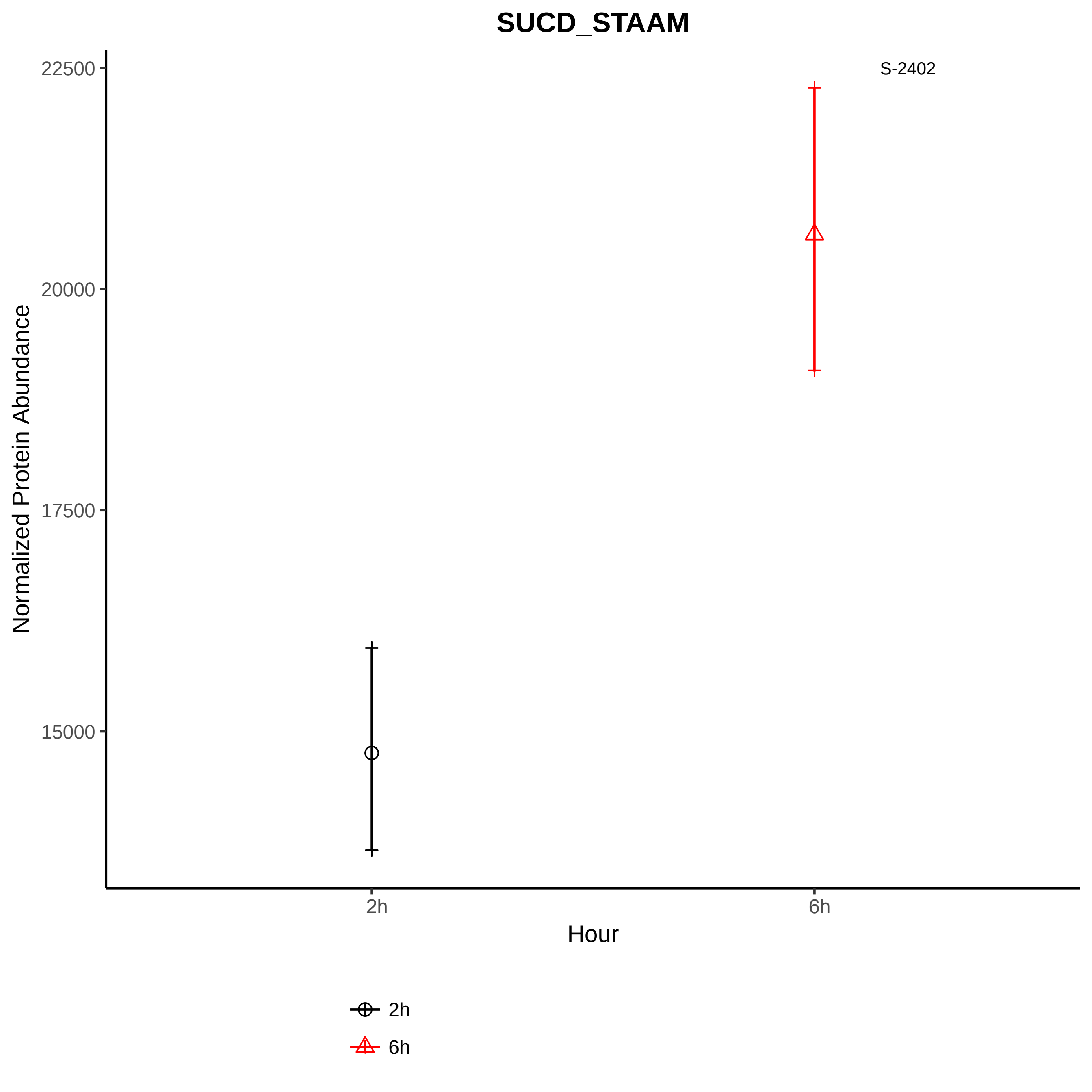
SUCD_STAAM
S-2402
22500
20000
17500
15000
Normalized Protein Abundance
2h
6h
Hour
2h
6h

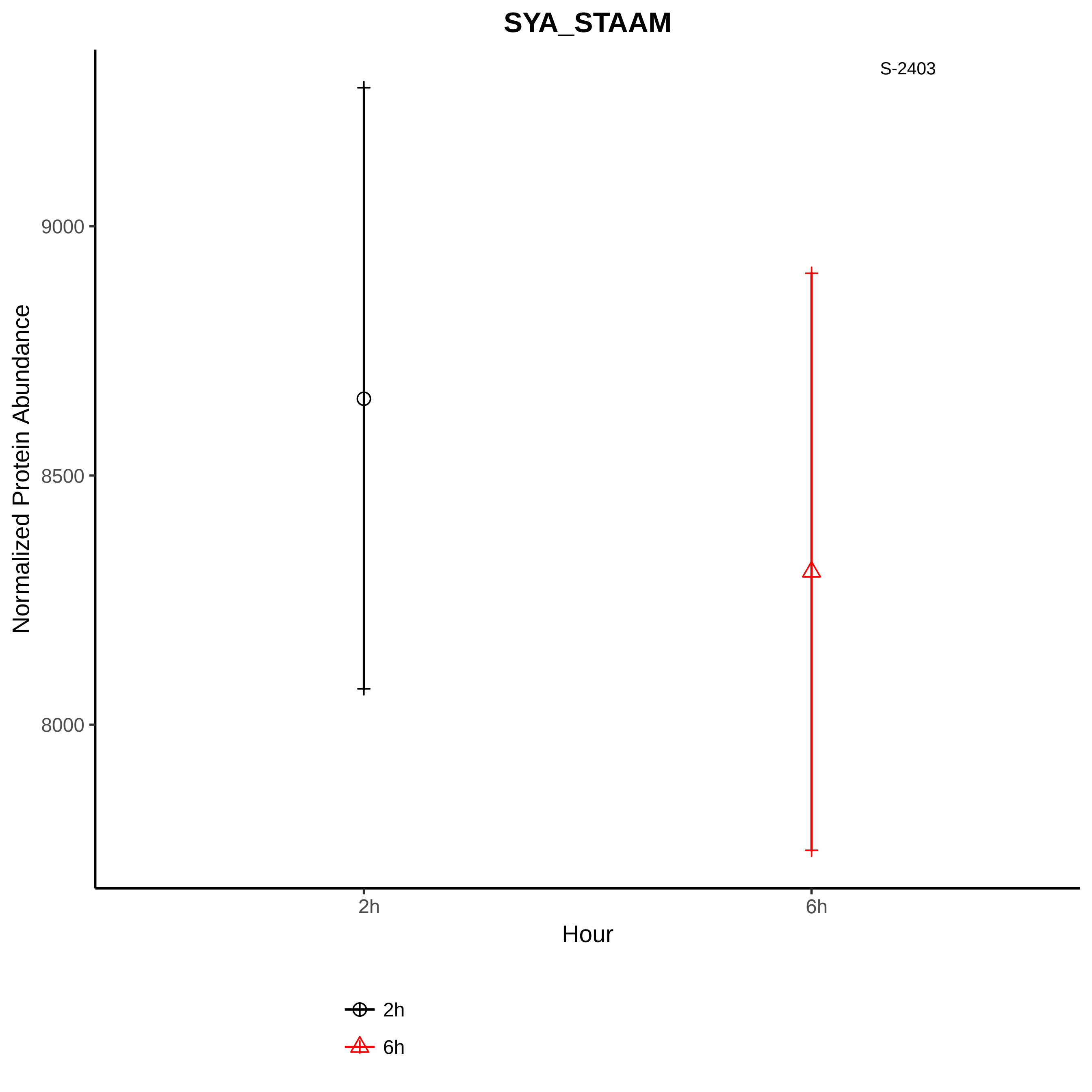

SYA_STAAM
S-2403
9000
8500
8000
Normalized Protein Abundance
2h
6h
Hour
2h
6h

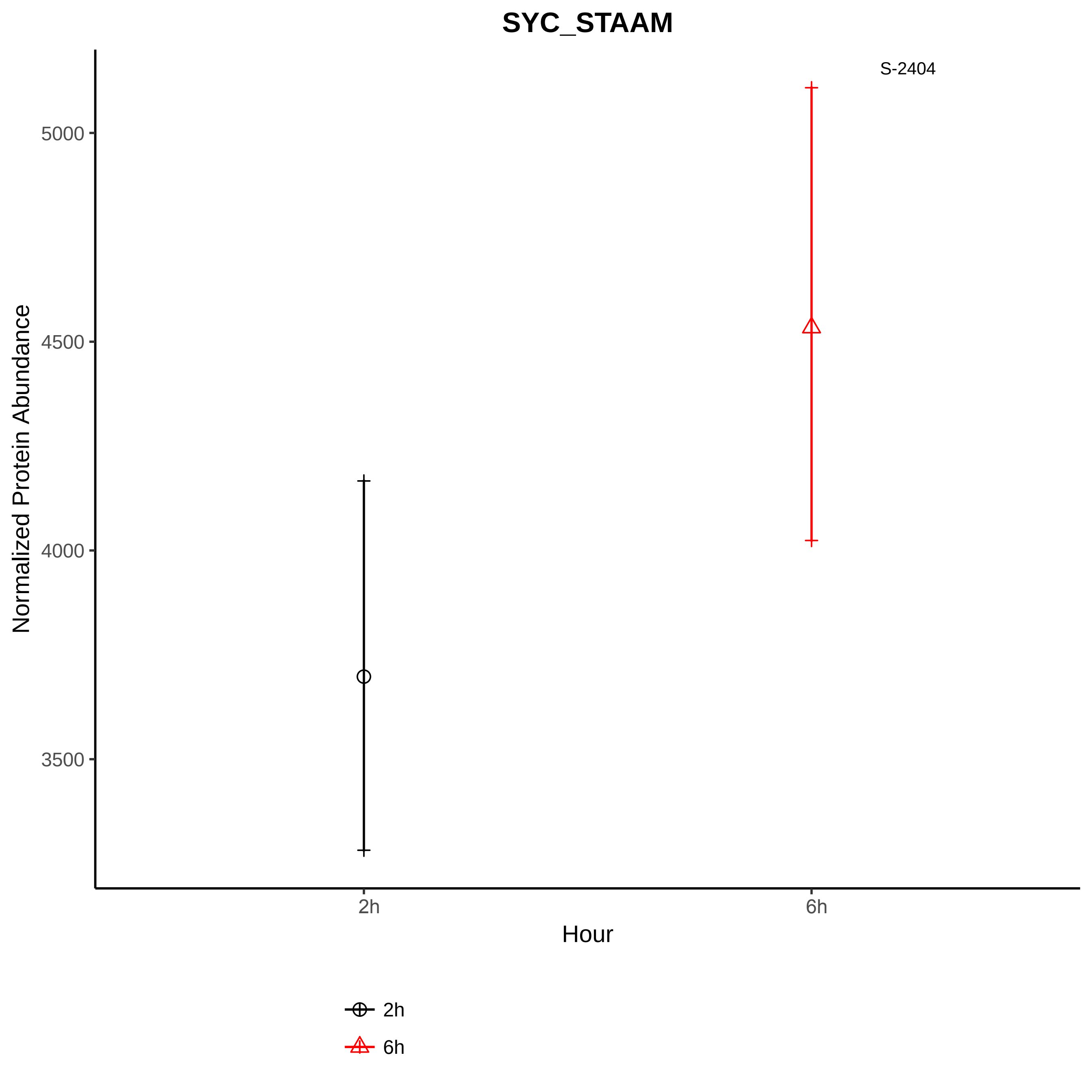
SYC_STAAM
S-2404
Normalized Protein Abundance
5000
4500
4000
3500
2h
6h
Hour
2h
6h

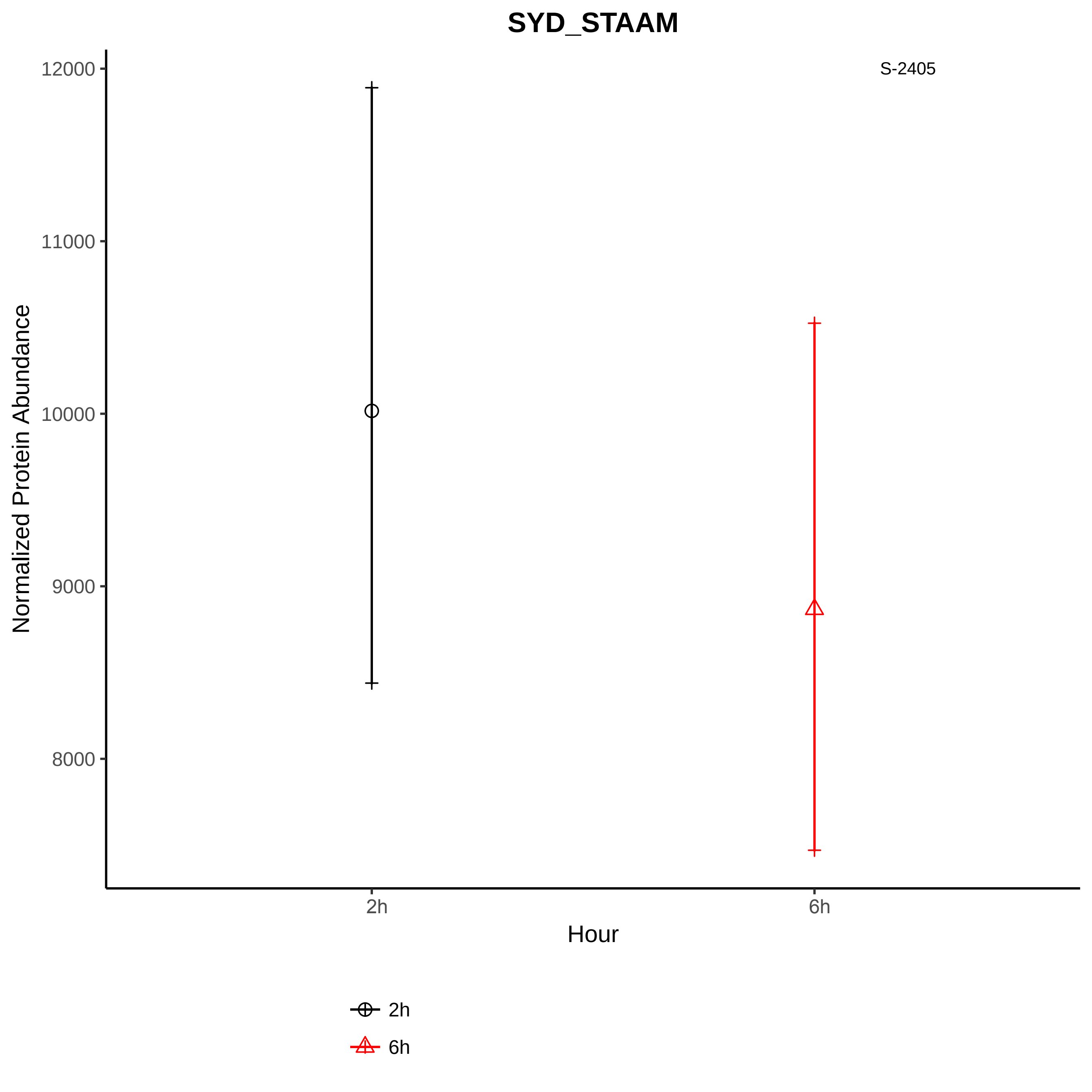

SYD_STAAM
S-2405
12000
11000
10000
9000
8000
Normalized Protein Abundance
2h
6h
Hour
2h
6h

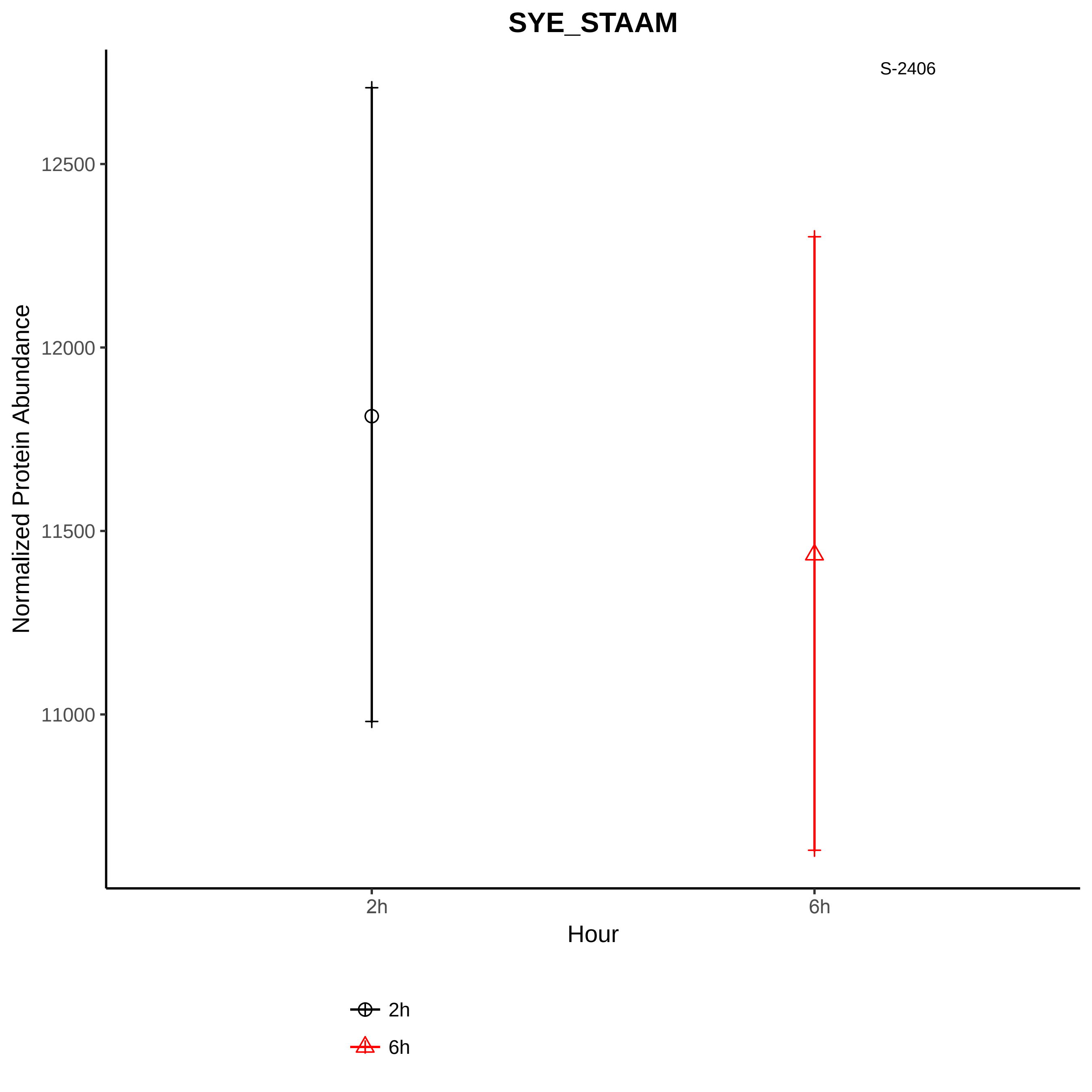

SYE_STAAM
S-2406
Normalized Protein Abundance
12500
12000
11500
11000
2h
6h
Hour
2h
6h

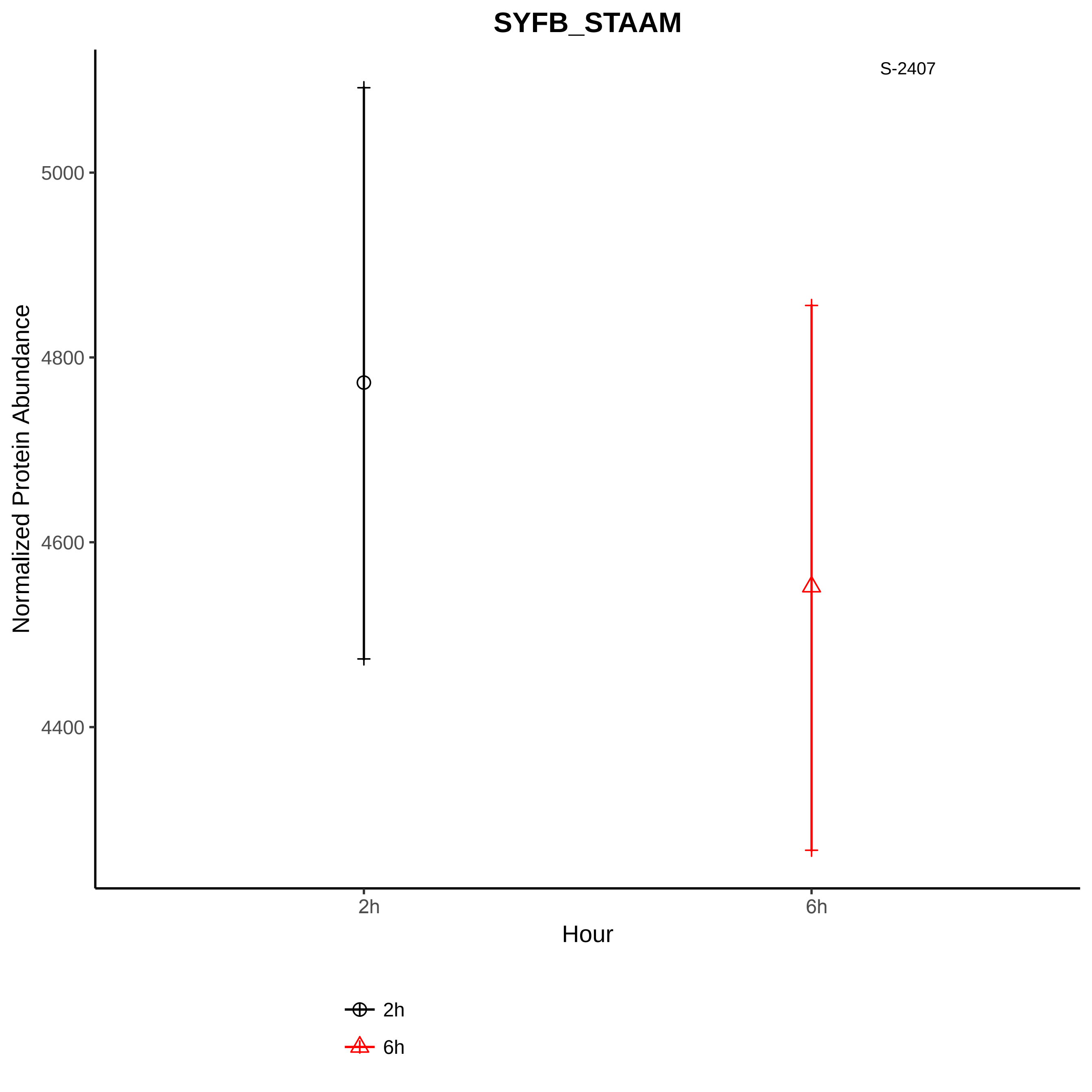
SYFB_STAAM
S-2407
Normalized Protein Abundance
5000
4800
4600
4400
2h
6h
Hour
2h
6h

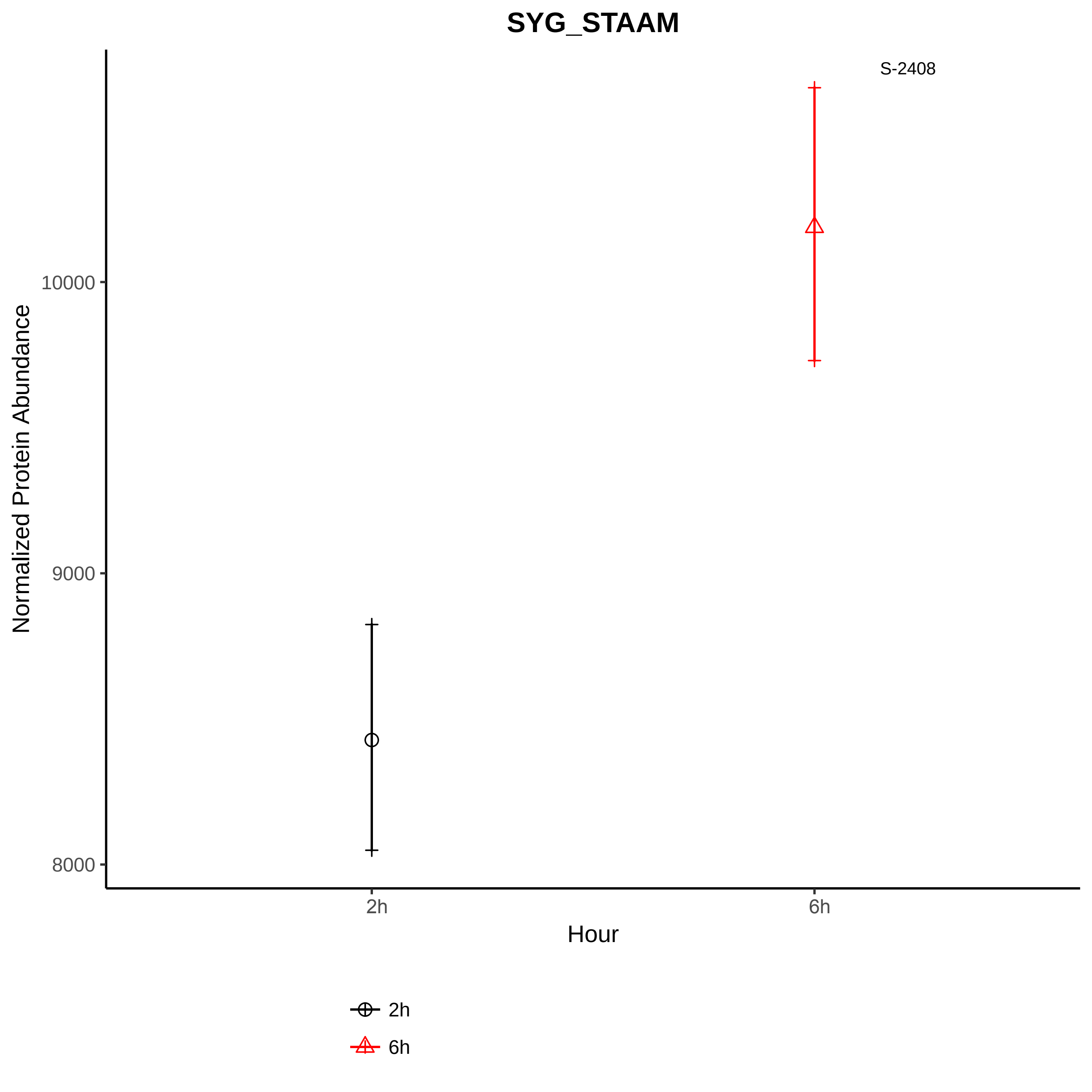

SYG_STAAM
S-2408
Normalized Protein Abundance
10000
9000
8000
2h
6h
Hour
2h
6h

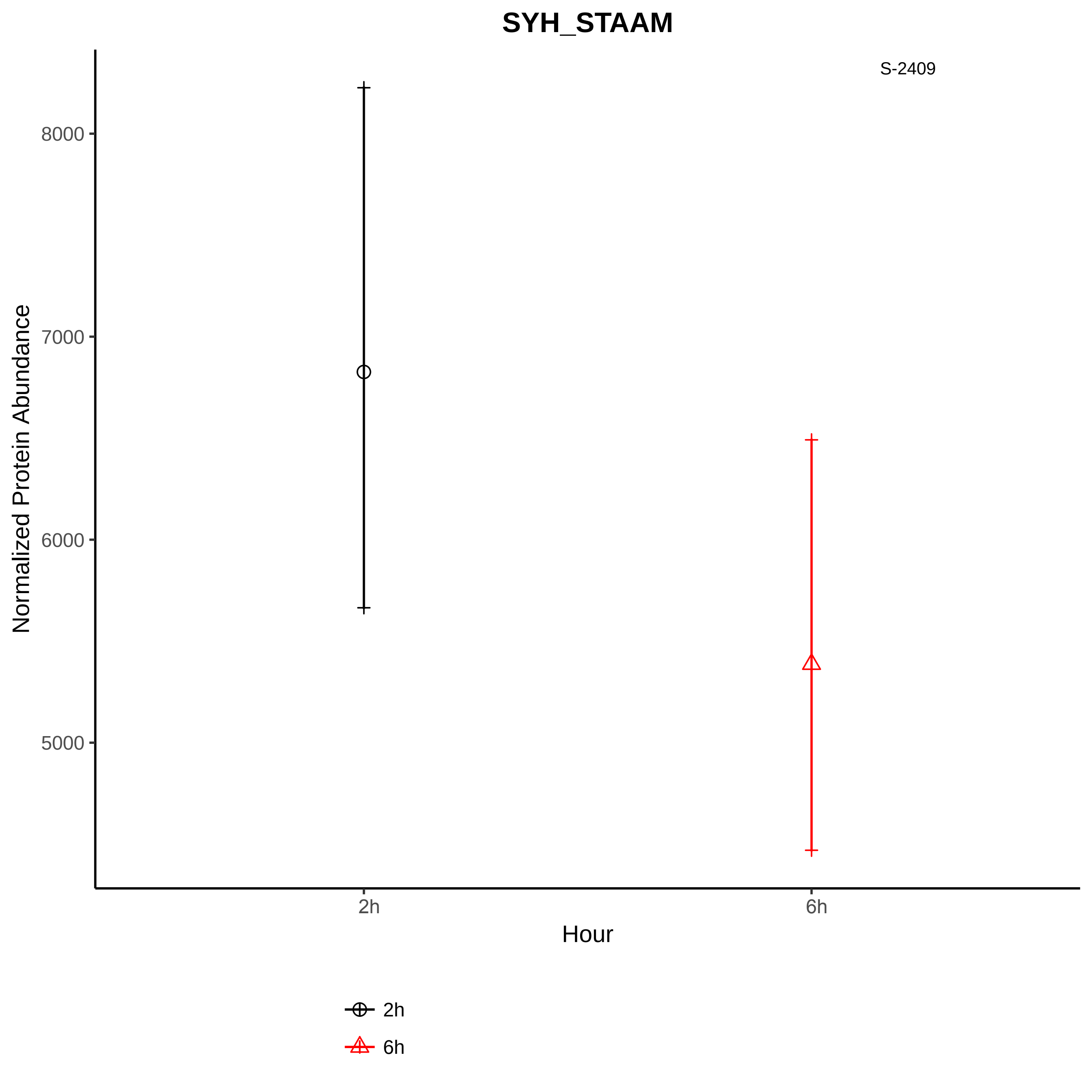

SYH_STAAM
S-2409
Normalized Protein Abundance
8000
7000
6000
5000
2h
6h
Hour
2h
6h

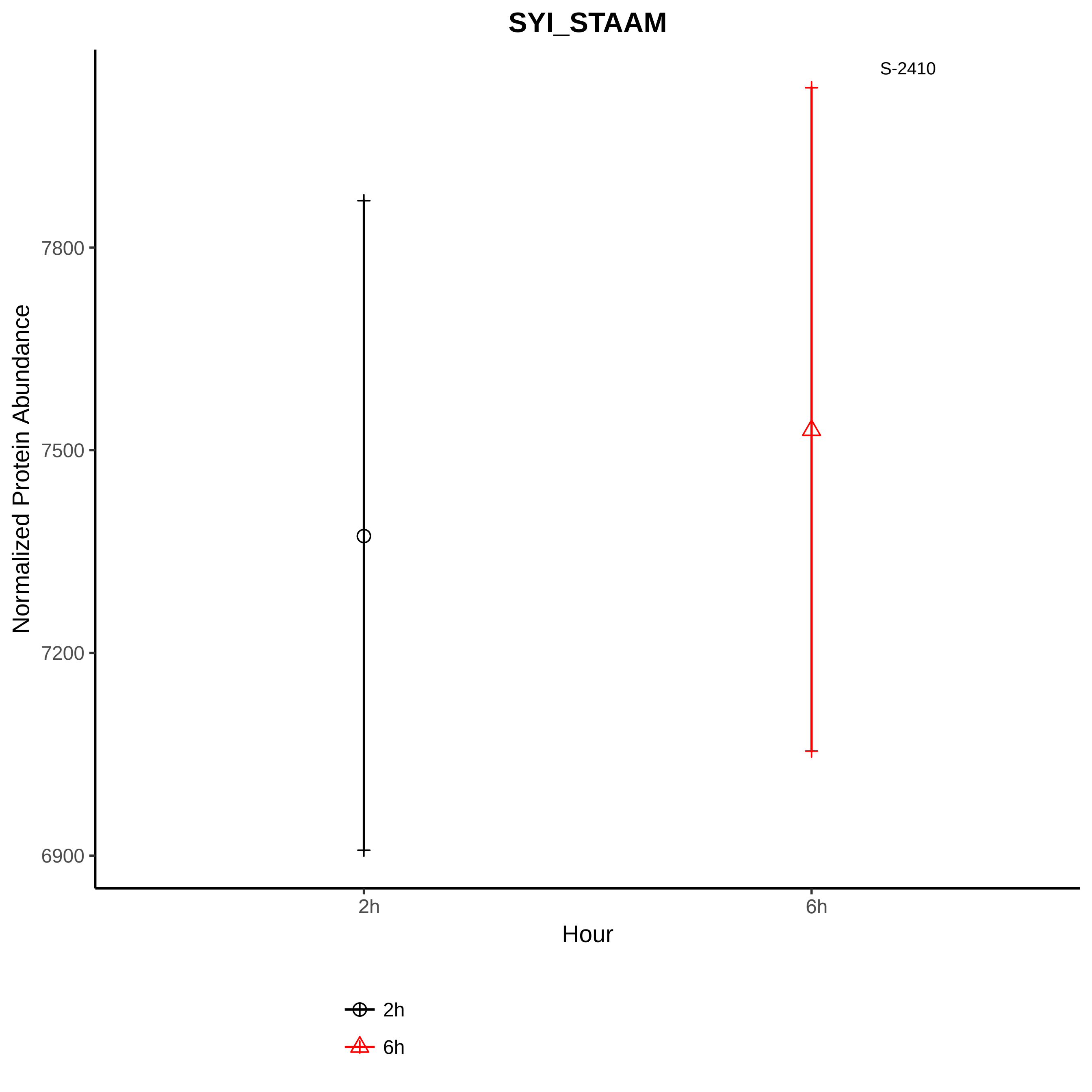

SYI_STAAM
S-2410
Normalized Protein Abundance
7800
7500
7200
6900
2h
6h
Hour
2h
6h

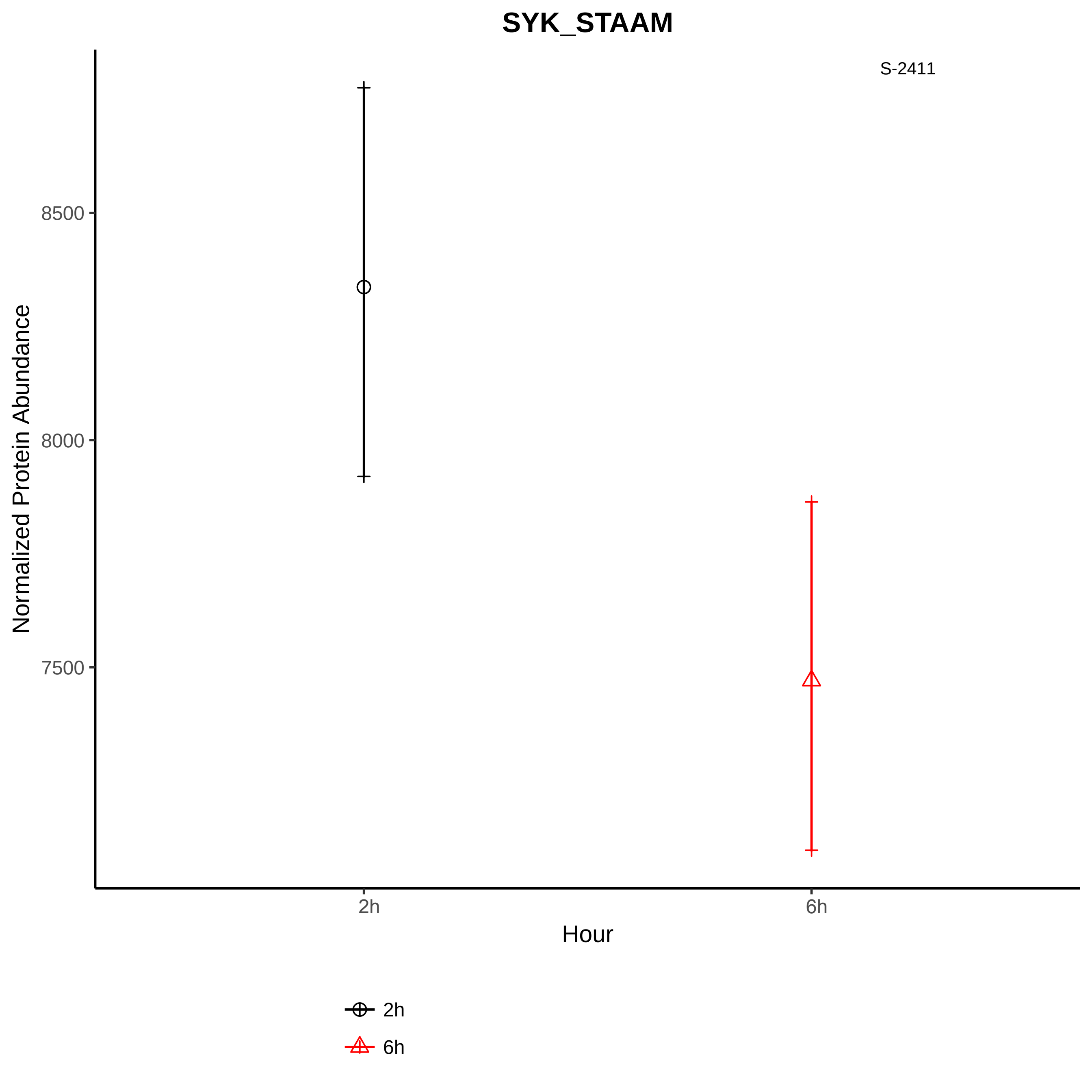
SYK_STAAM
S-2411
8500
8000
7500
Normalized Protein Abundance
2h
6h
Hour
2h
6h

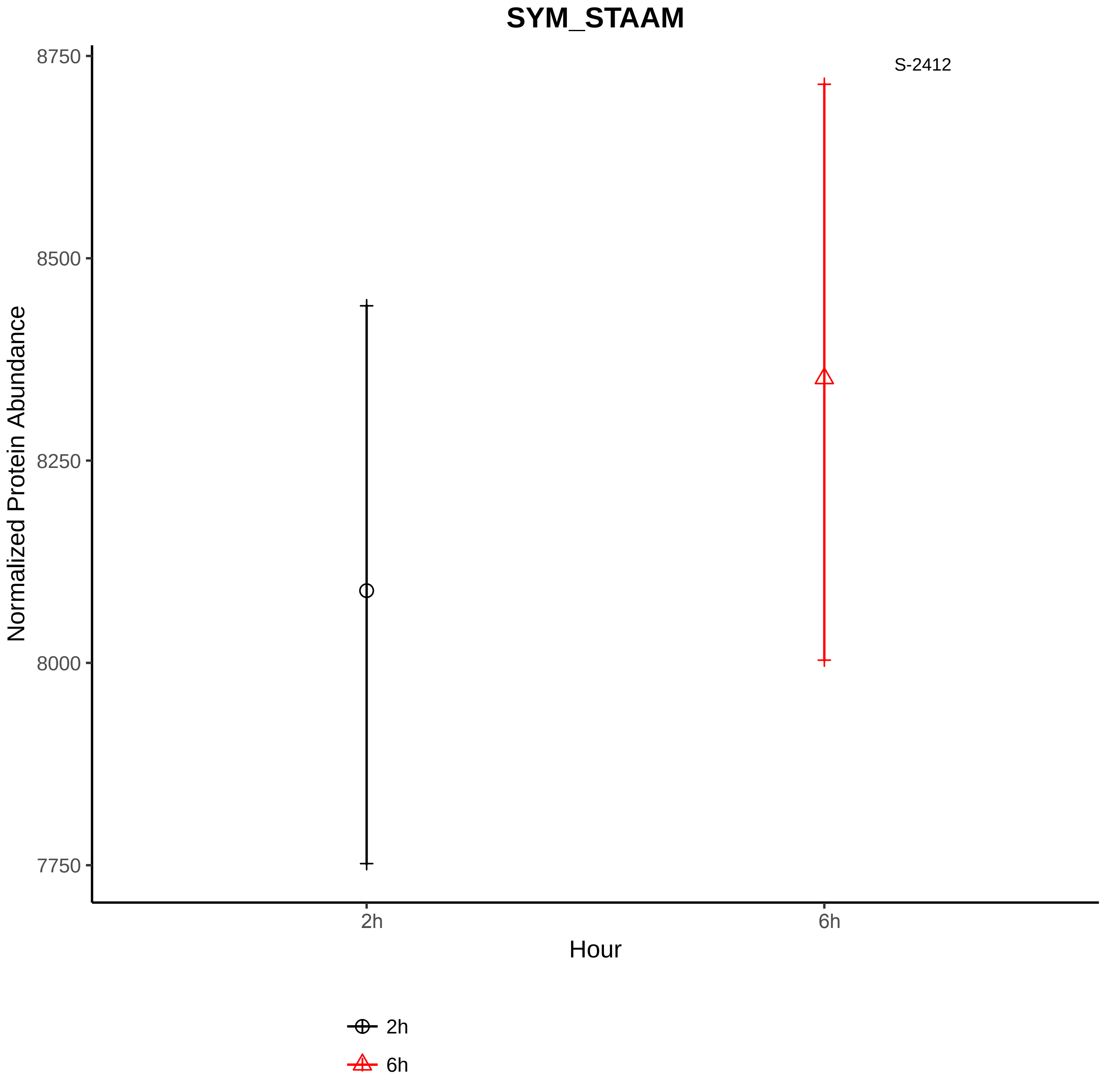

SYM_STAAM
S-2412
Normalized Protein Abundance
8750
8500
8250
8000
7750
2h
6h
Hour
2h
6h

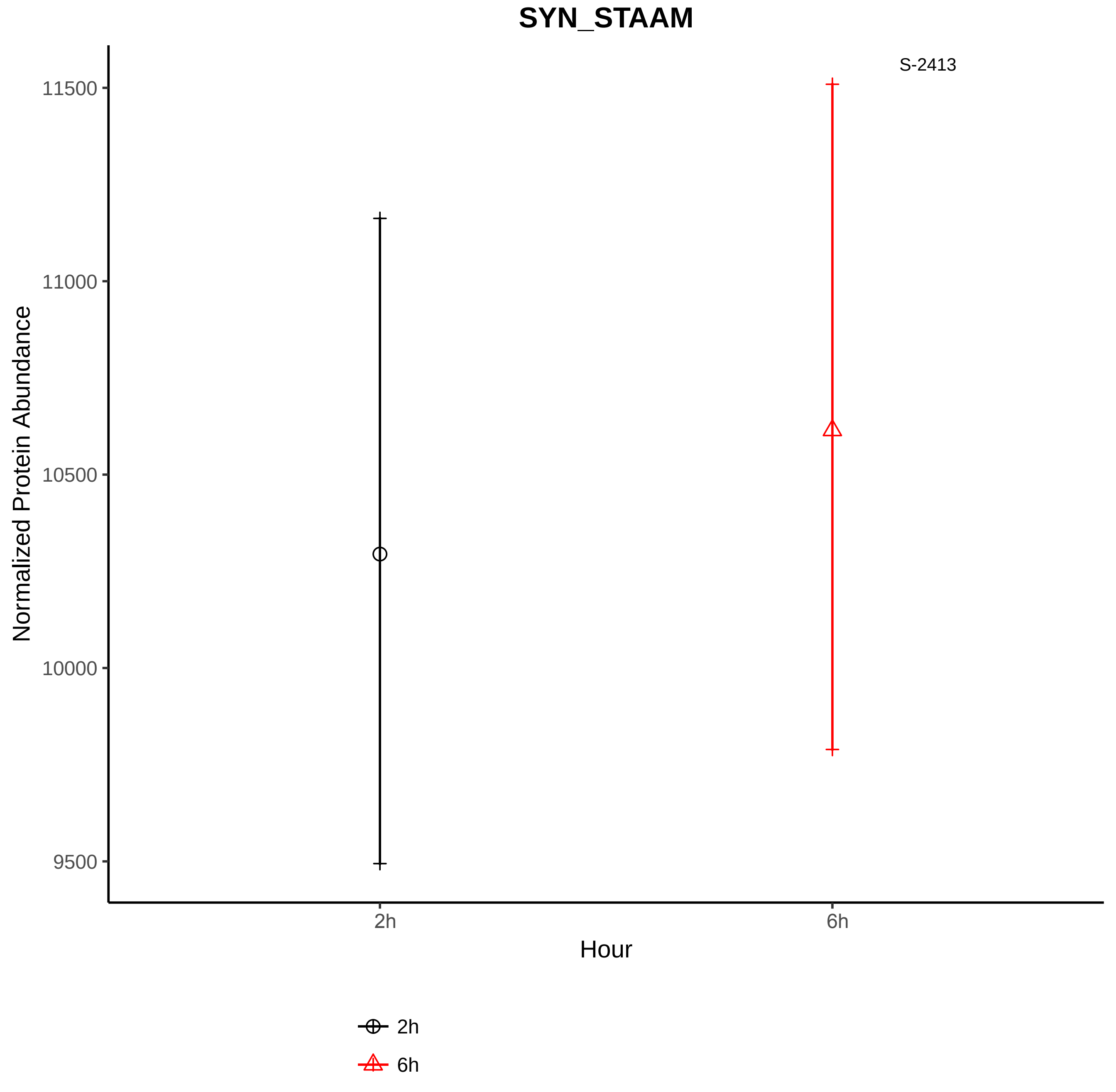
SYN_STAAM
S-2413
Normalized Protein Abundance
11500
11000
10500
10000
9500
2h
6h
Hour
2h
6h

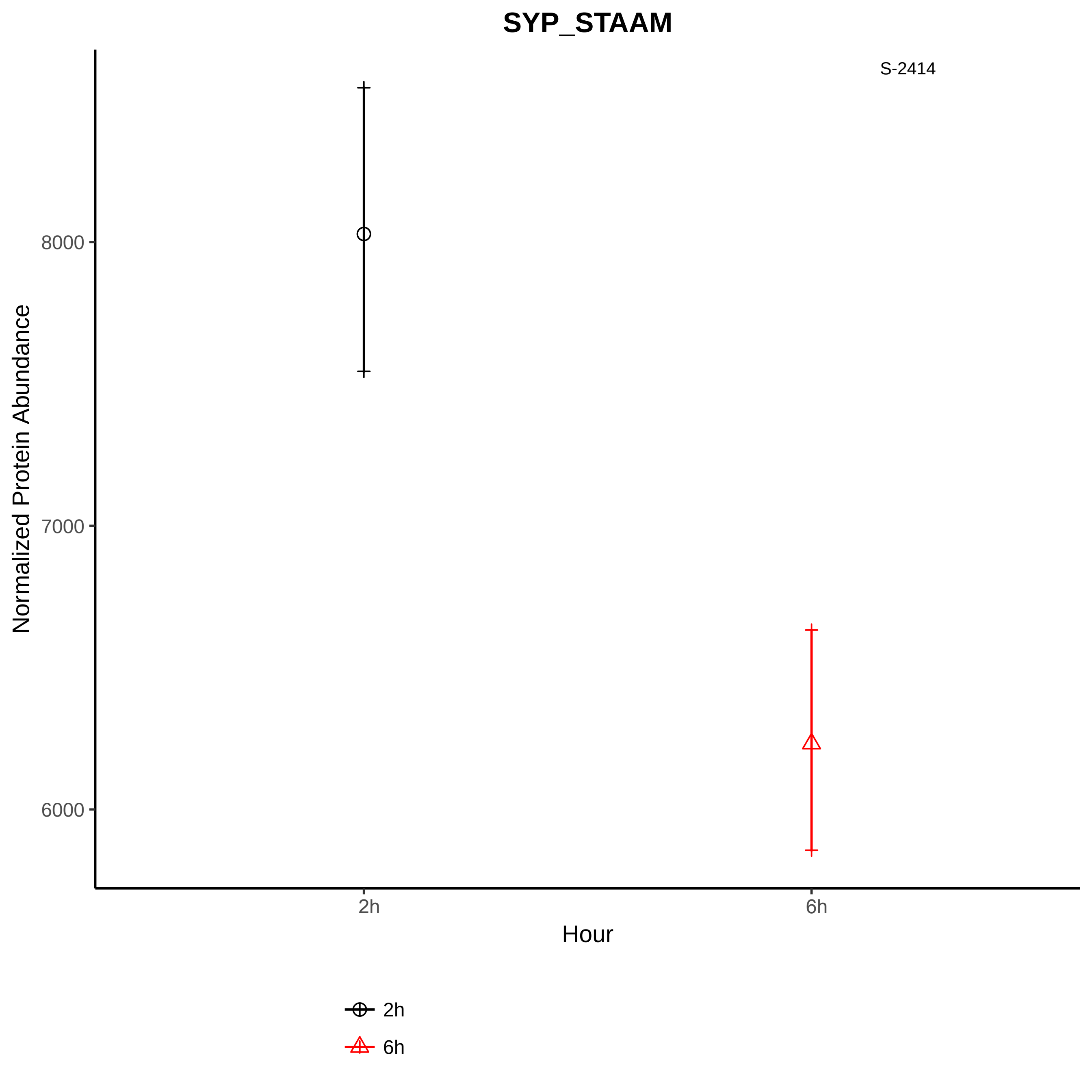

SYP_STAAM
S-2414
Normalized Protein Abundance
8000
7000
6000
2h
6h
Hour
2h
6h

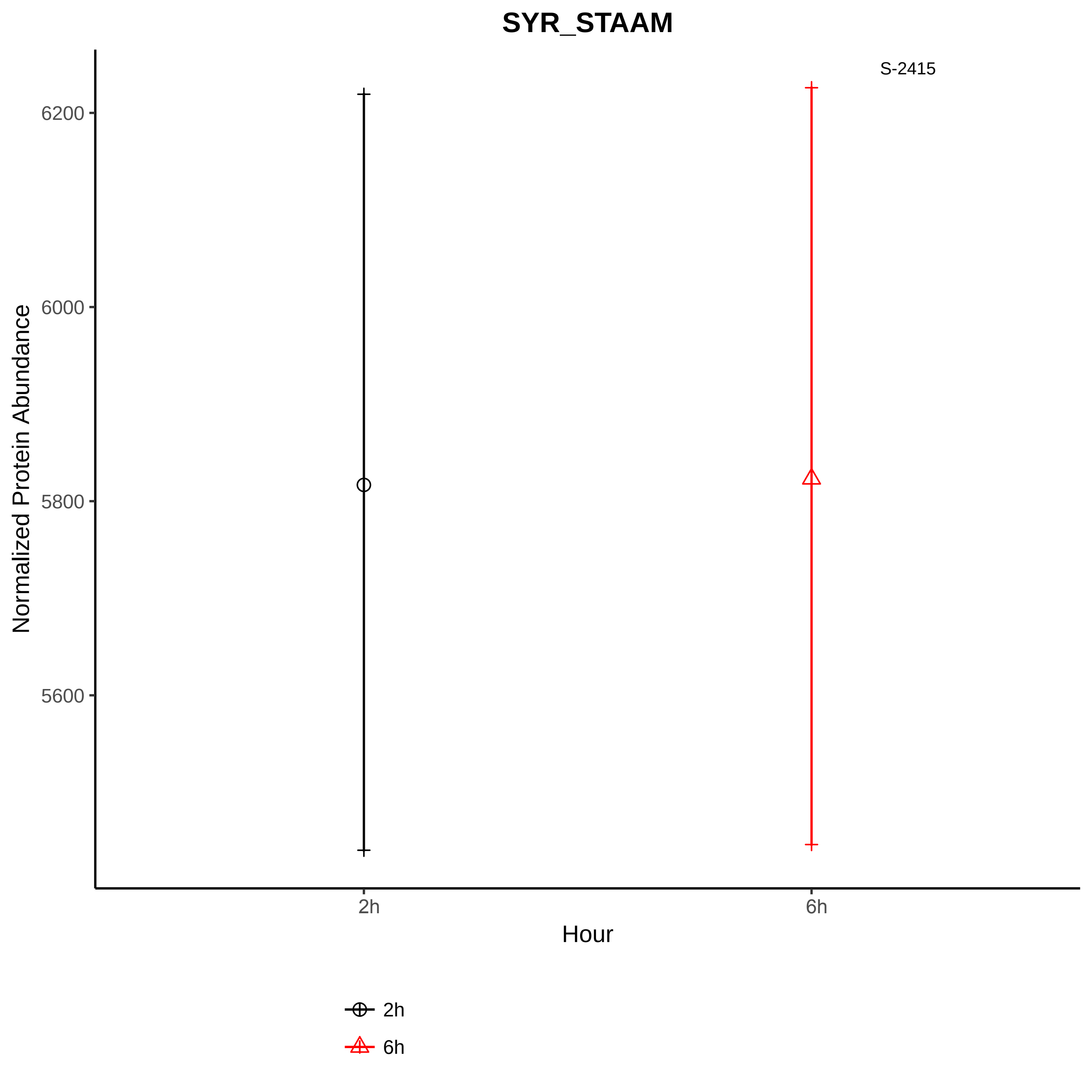
SYR_STAAM
S-2415
Normalized Protein Abundance
6200
6000
5800
5600
2h
6h
Hour
2h
6h

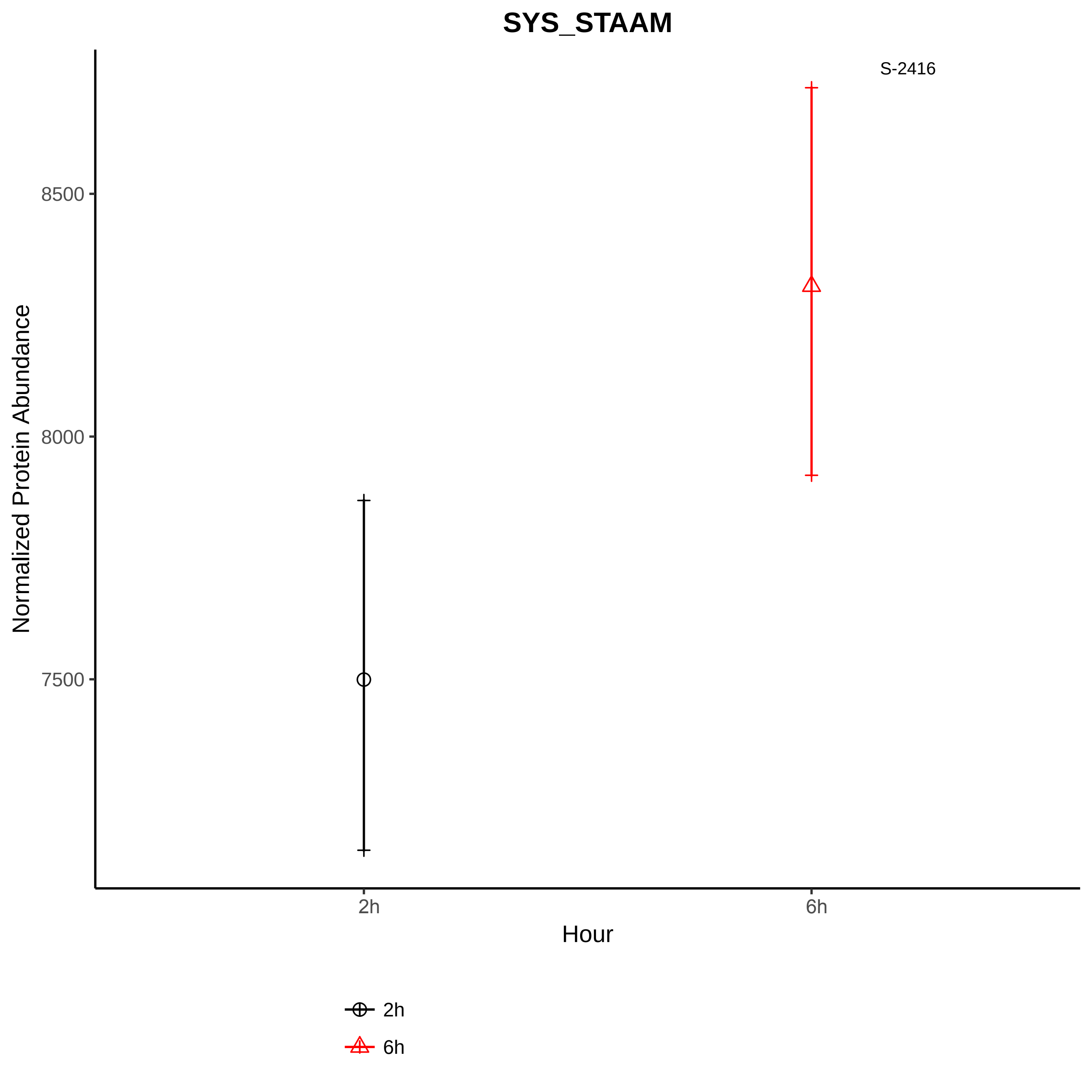
SYS_STAAM
S-2416
8500
8000
7500
Normalized Protein Abundance
2h
6h
Hour
2h
6h

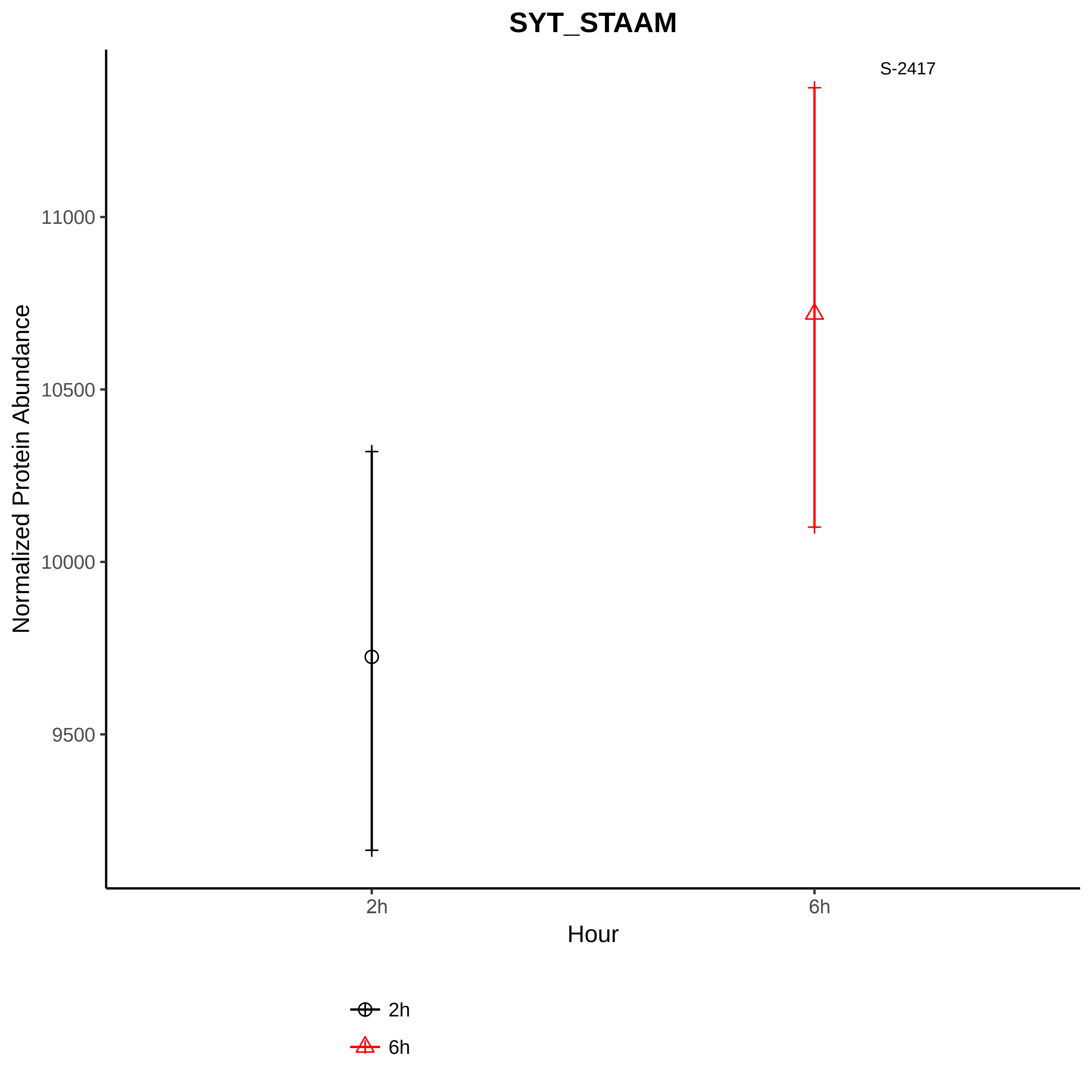

SYT_STAAM
S-2417
Normalized Protein Abundance
11000
10500
10000
9500
2h
6h
Hour
2h
6h

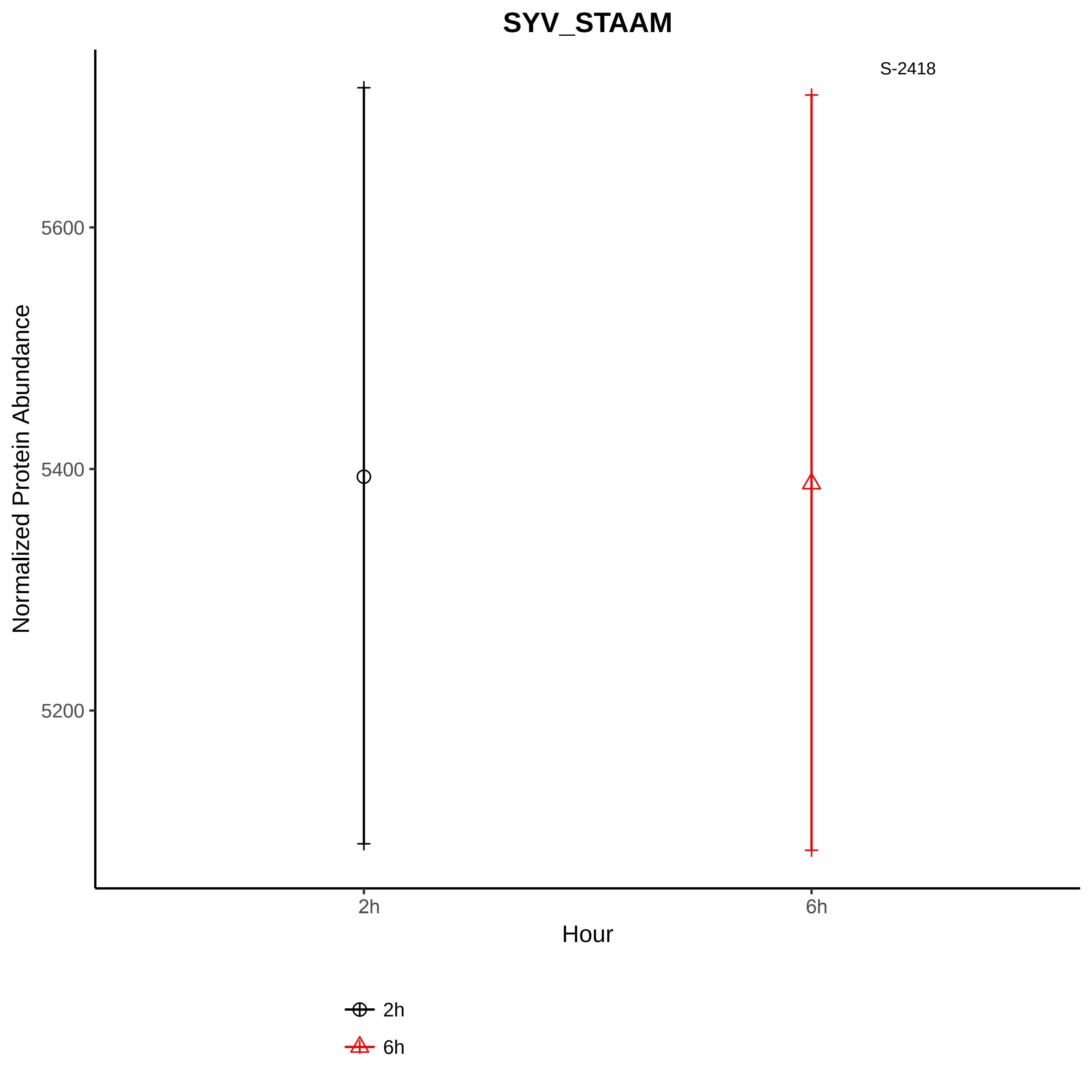

SYV_STAAM
S-2418
Normalized Protein Abundance
5600
5400
5200
2h
6h
Hour
2h
6h

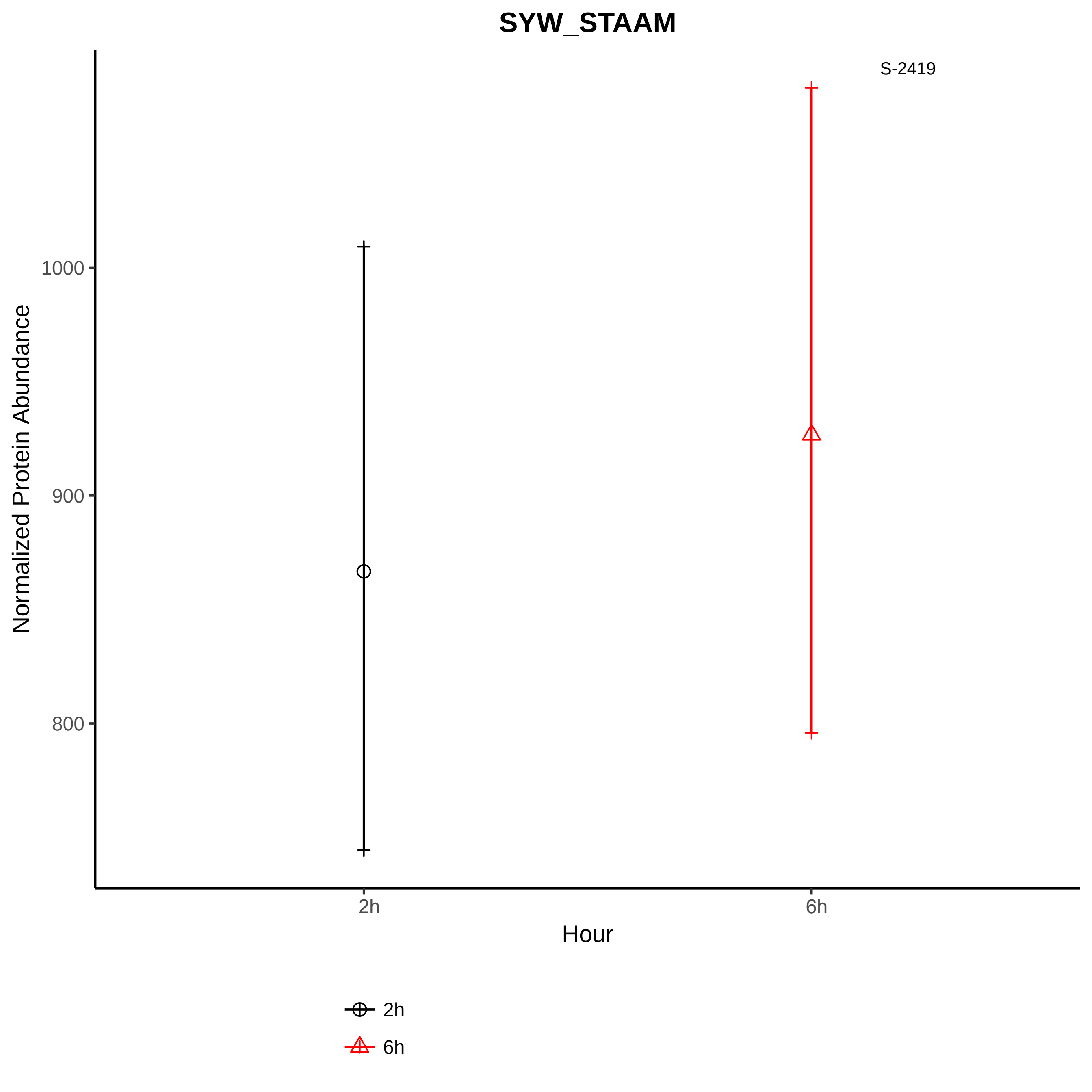

SYW_STAAM
S-2419
Normalized Protein Abundance
1000
900
800
2h
6h
Hour
2h
6h

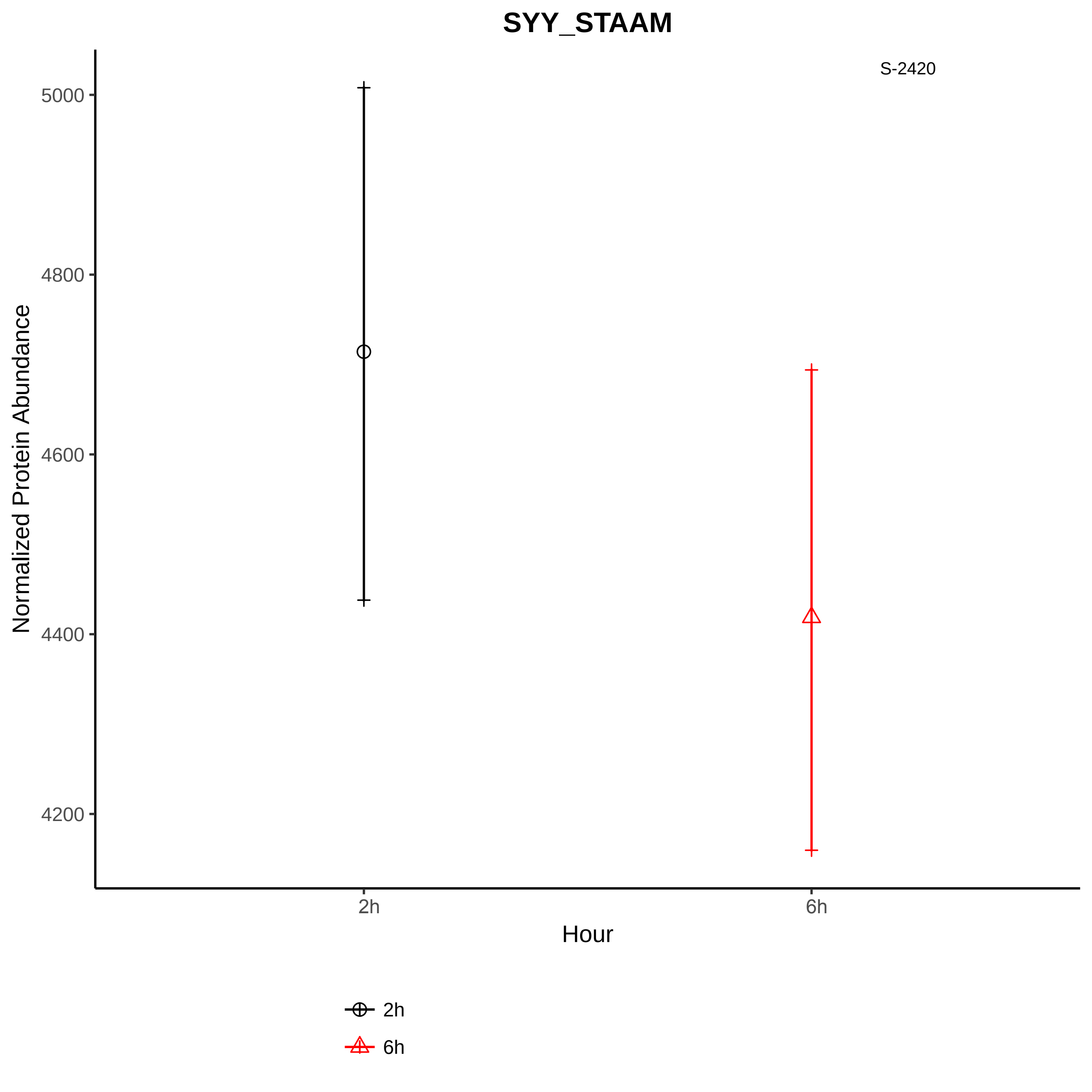

SYY_STAAM
S-2420
Normalized Protein Abundance
5000
4800
4600
4400
4200
2h
6h
Hour
2h
6h

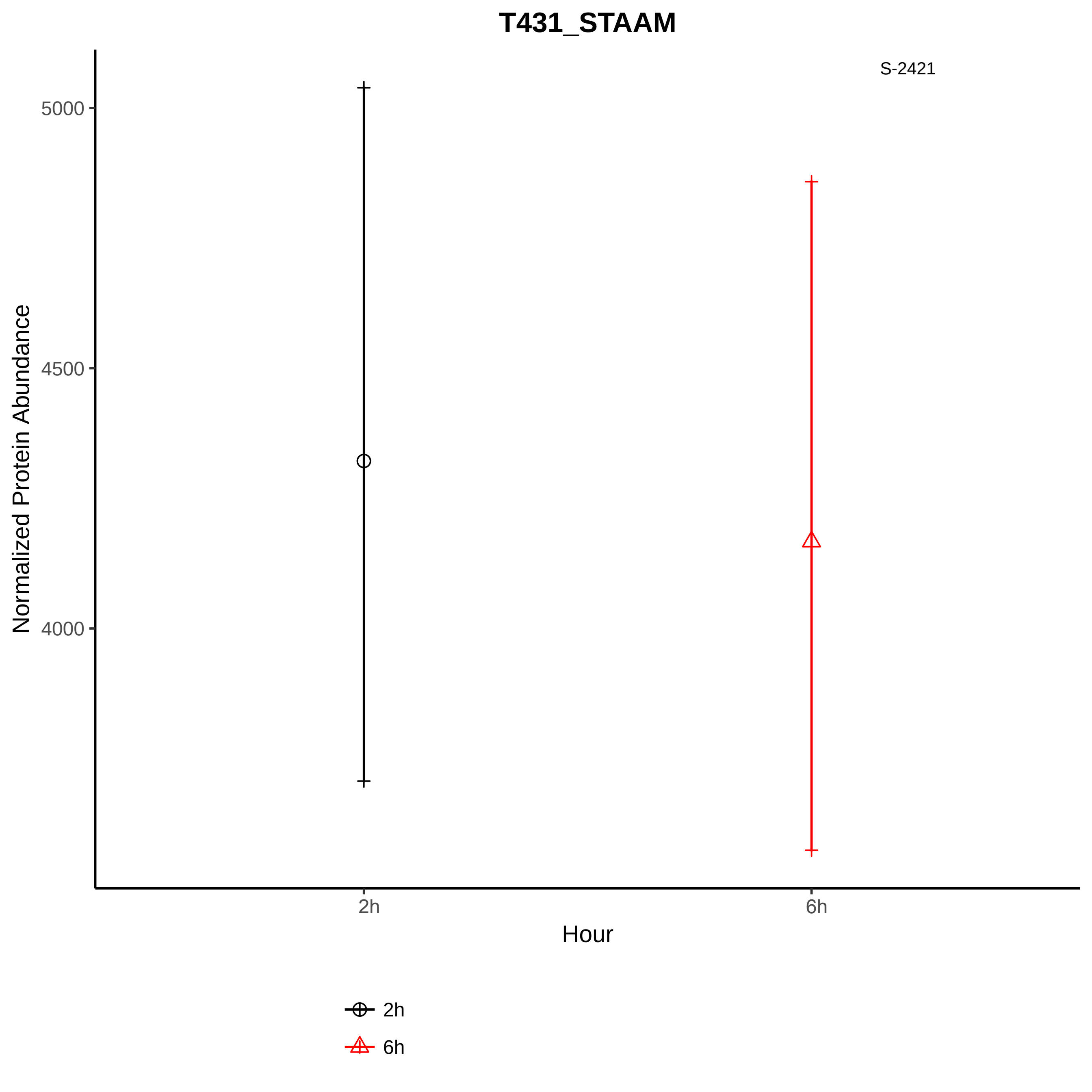
T431_STAAM
S-2421
Normalized Protein Abundance
5000
4500
4000
2h
6h
Hour
2h
6h

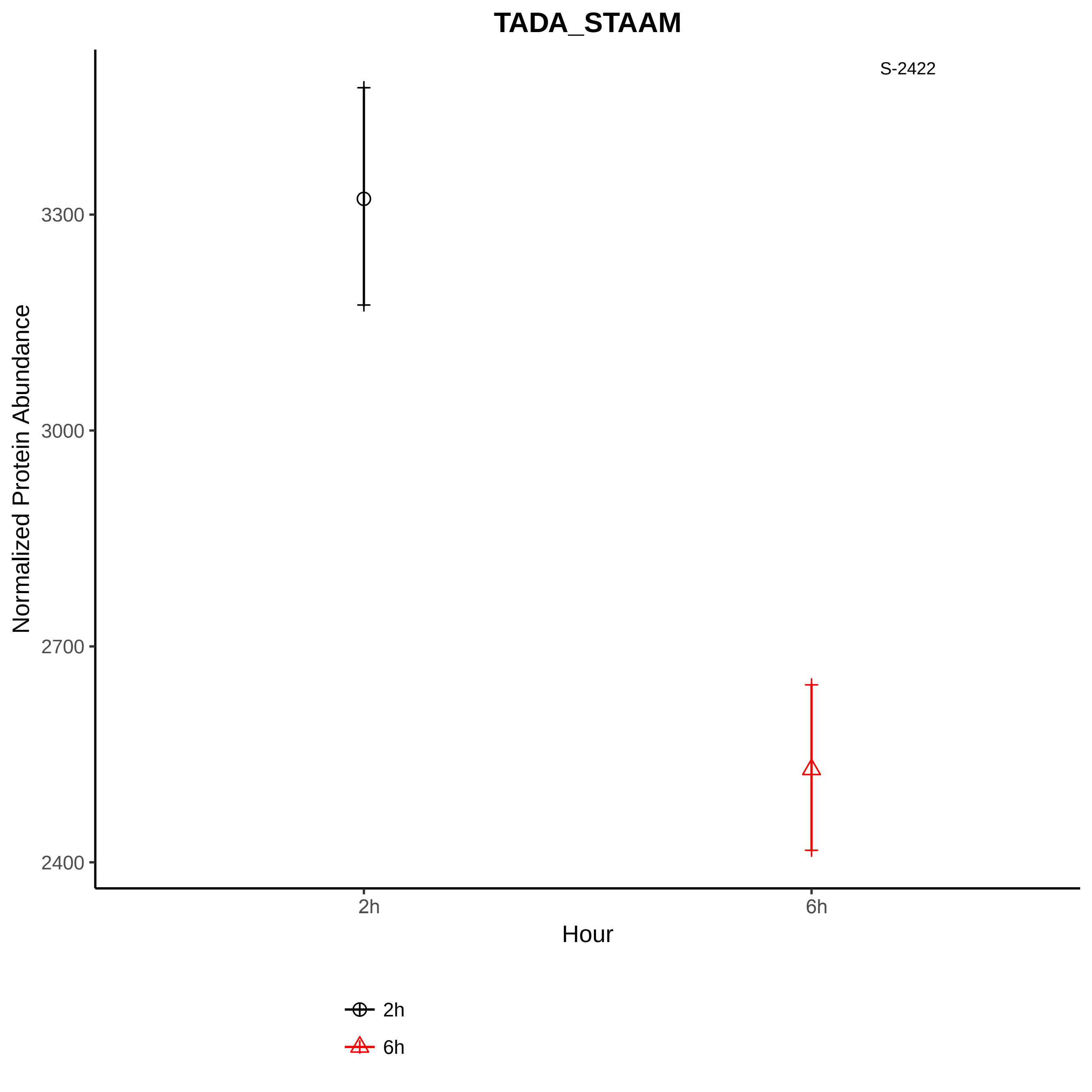

TADA_STAAM
S-2422
Normalized Protein Abundance
3300
3000
2700
2400
2h
6h
Hour
2h
6h

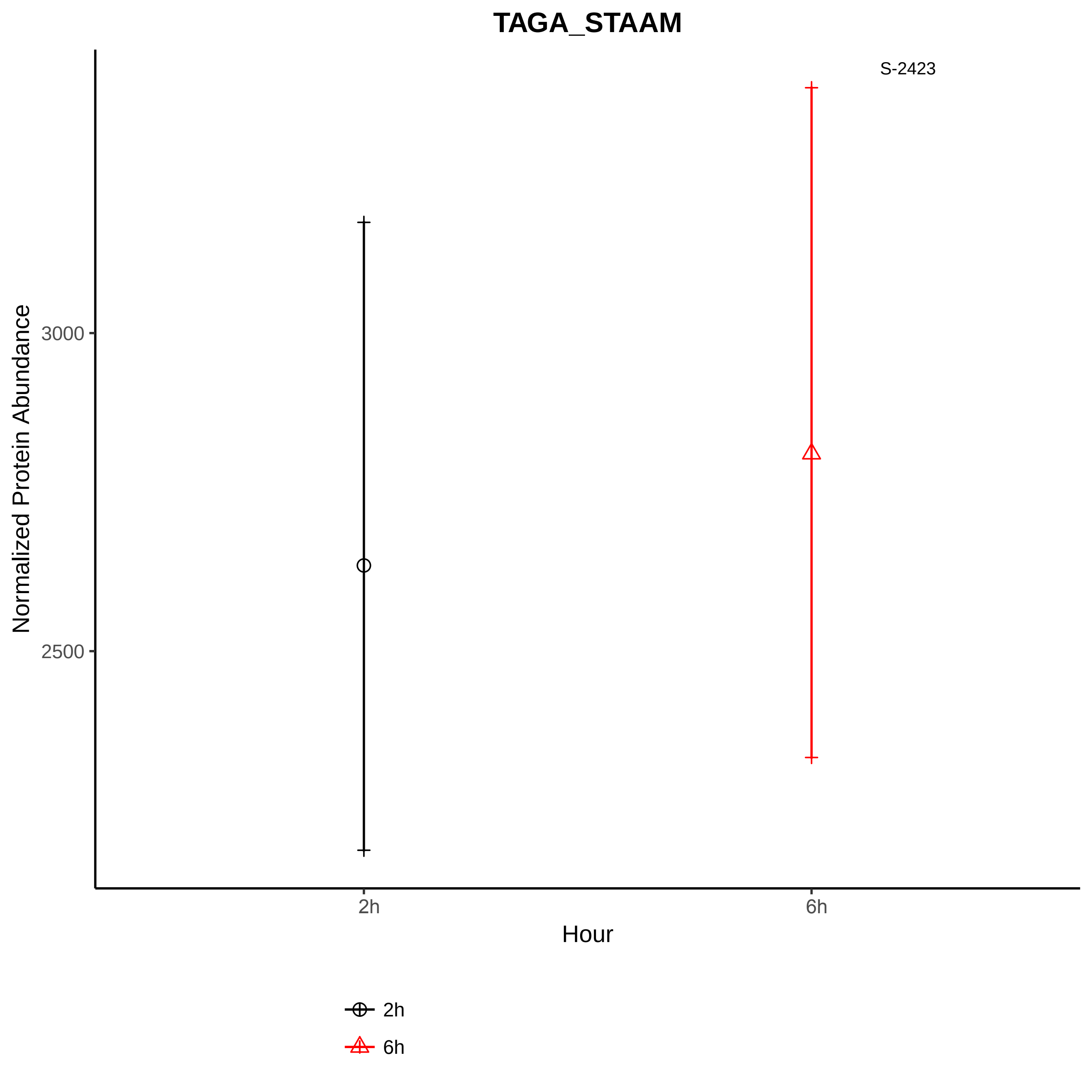
TAGA_STAAM
S-2423
Normalized Protein Abundance
3000
2500
2h
6h
Hour
2h
6h

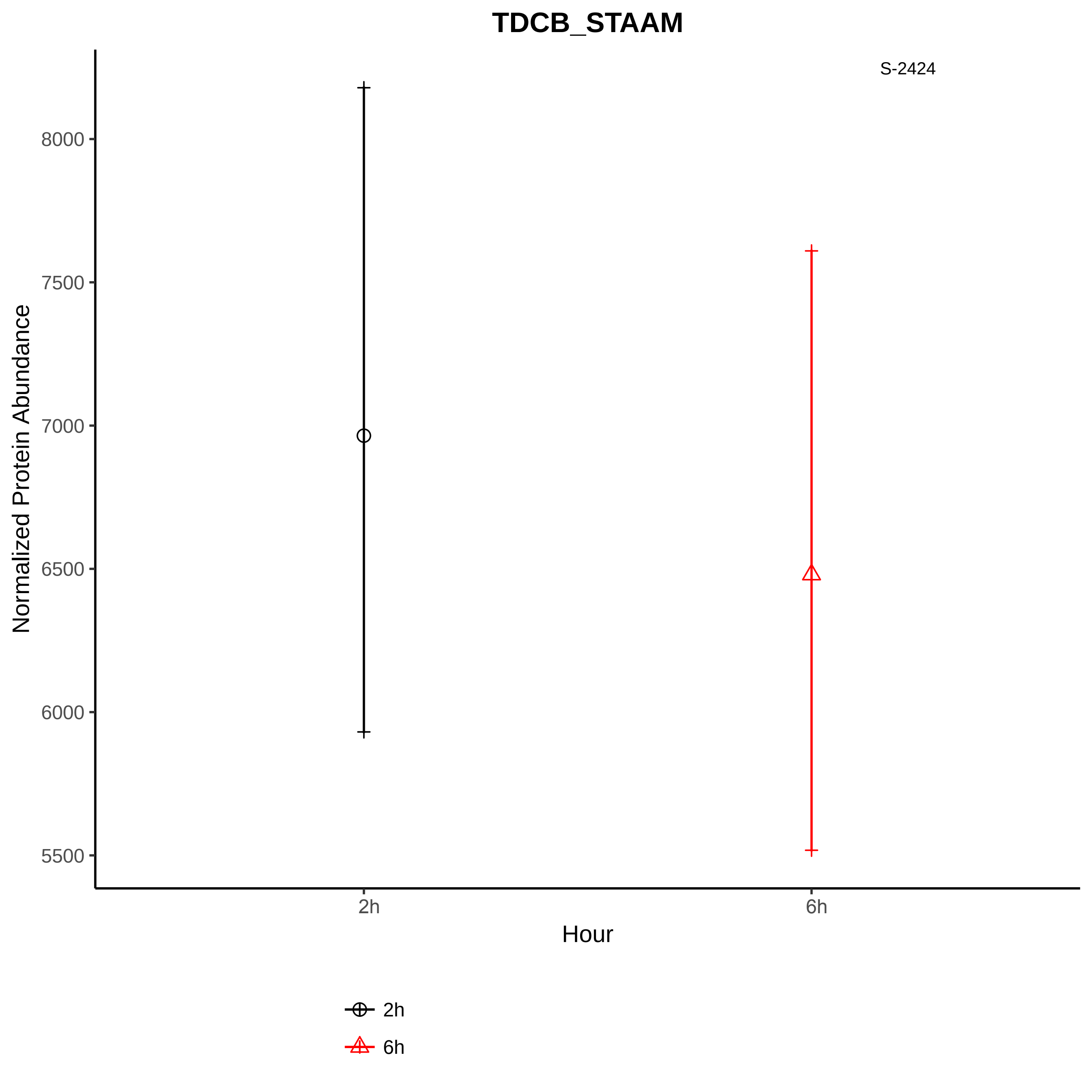

TDCB_STAAM
S-2424
Normalized Protein Abundance
8000
7500
7000
6500
6000
5500
2h
6h
Hour
2h
6h

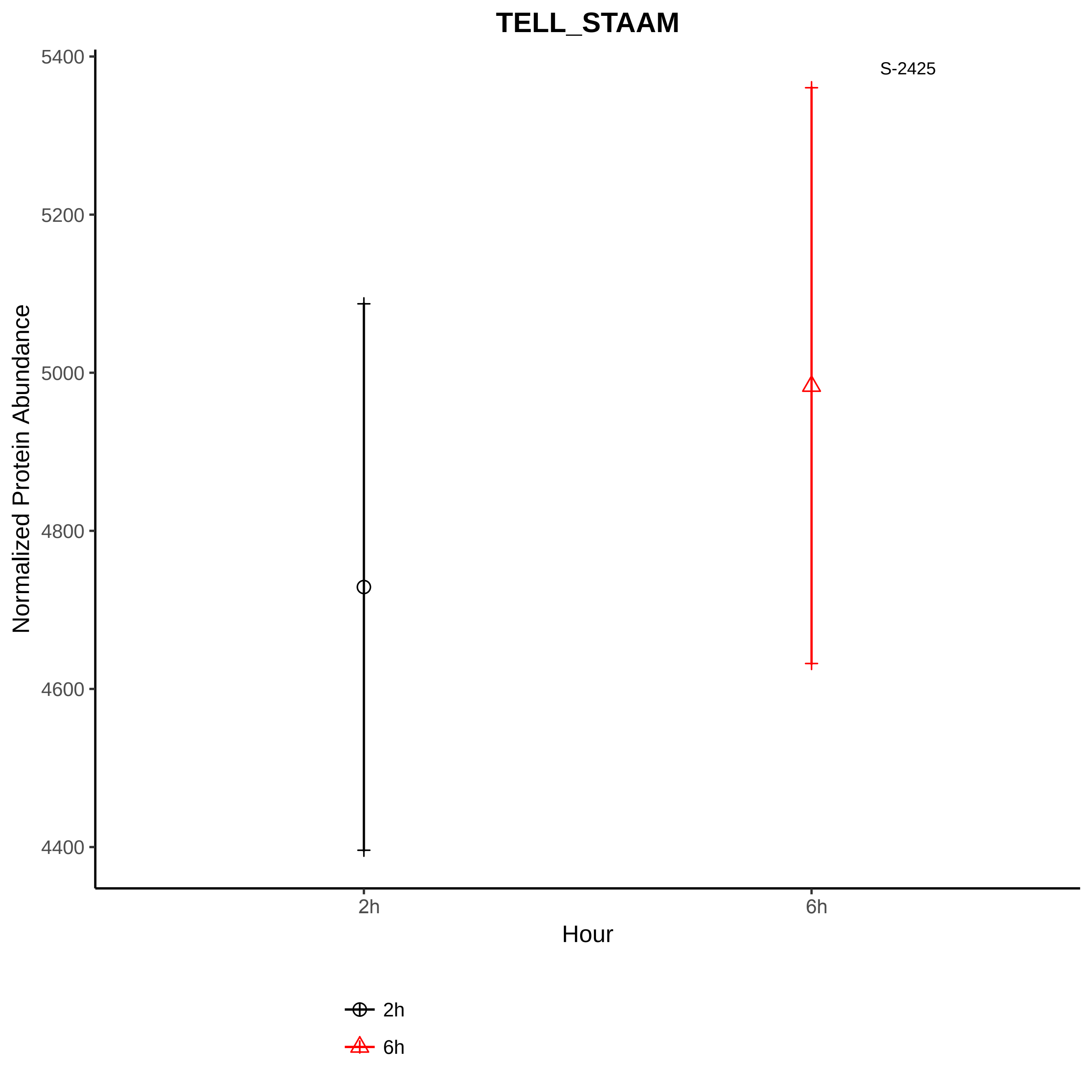

TELL_STAAM
S-2425
Normalized Protein Abundance
5400
5200
5000
4800
4600
4400
2h
6h
Hour
2h
6h

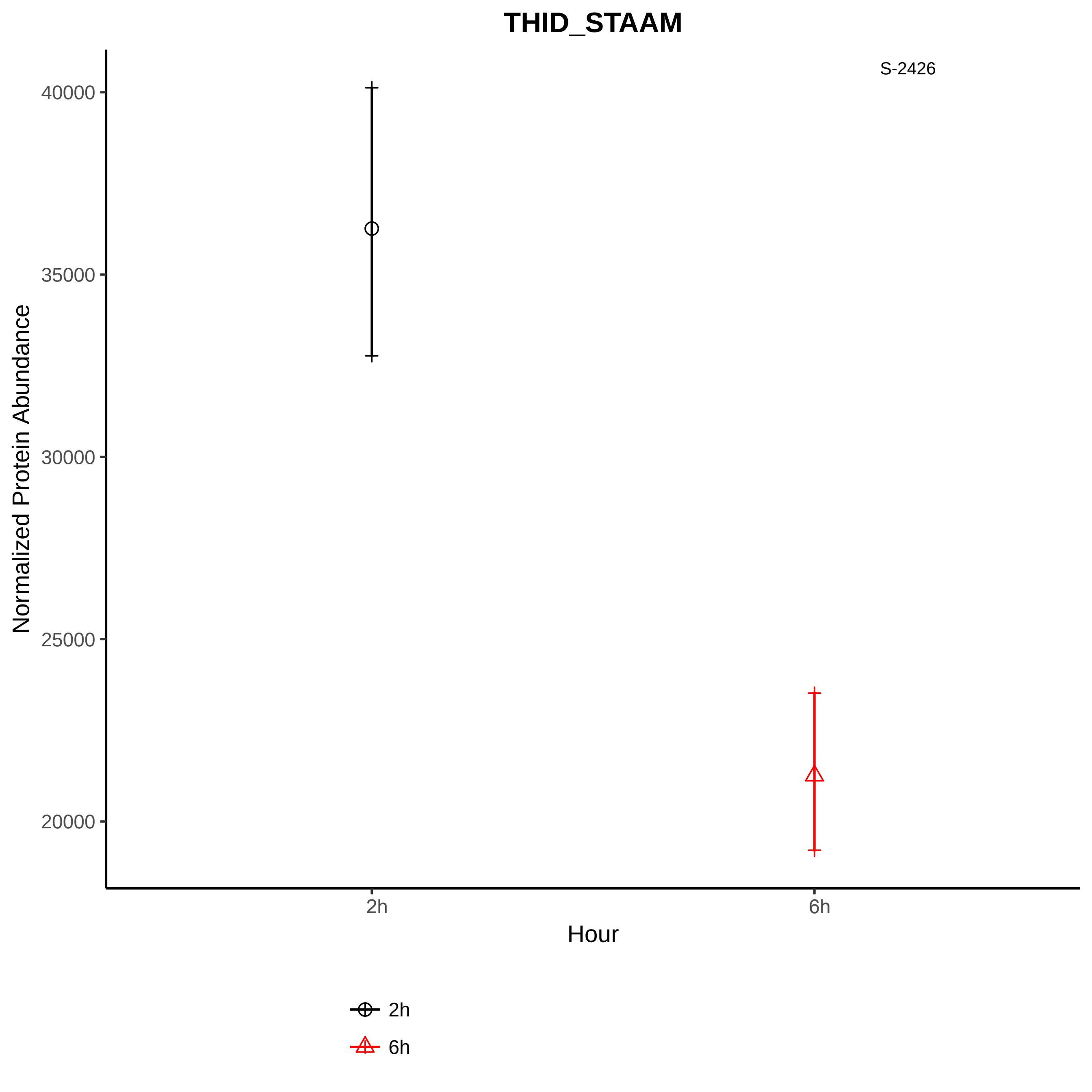

THID_STAAM
S-2426
40000
35000
30000
25000
20000
Normalized Protein Abundance
2h
6h
Hour
2h
6h

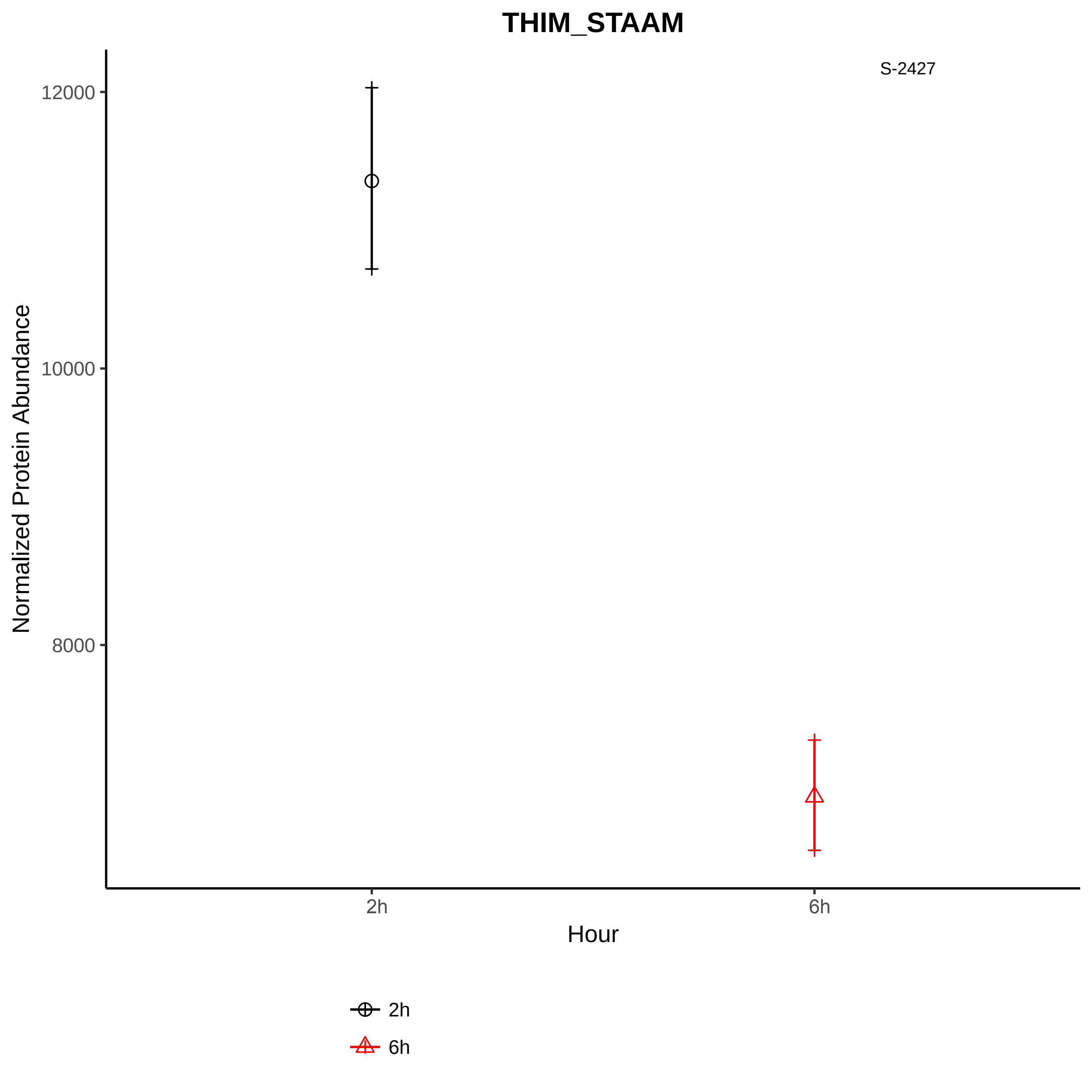

THIM_STAAM
S-2427
12000
10000
8000
Normalized Protein Abundance
2h
6h
Hour
2h
6h

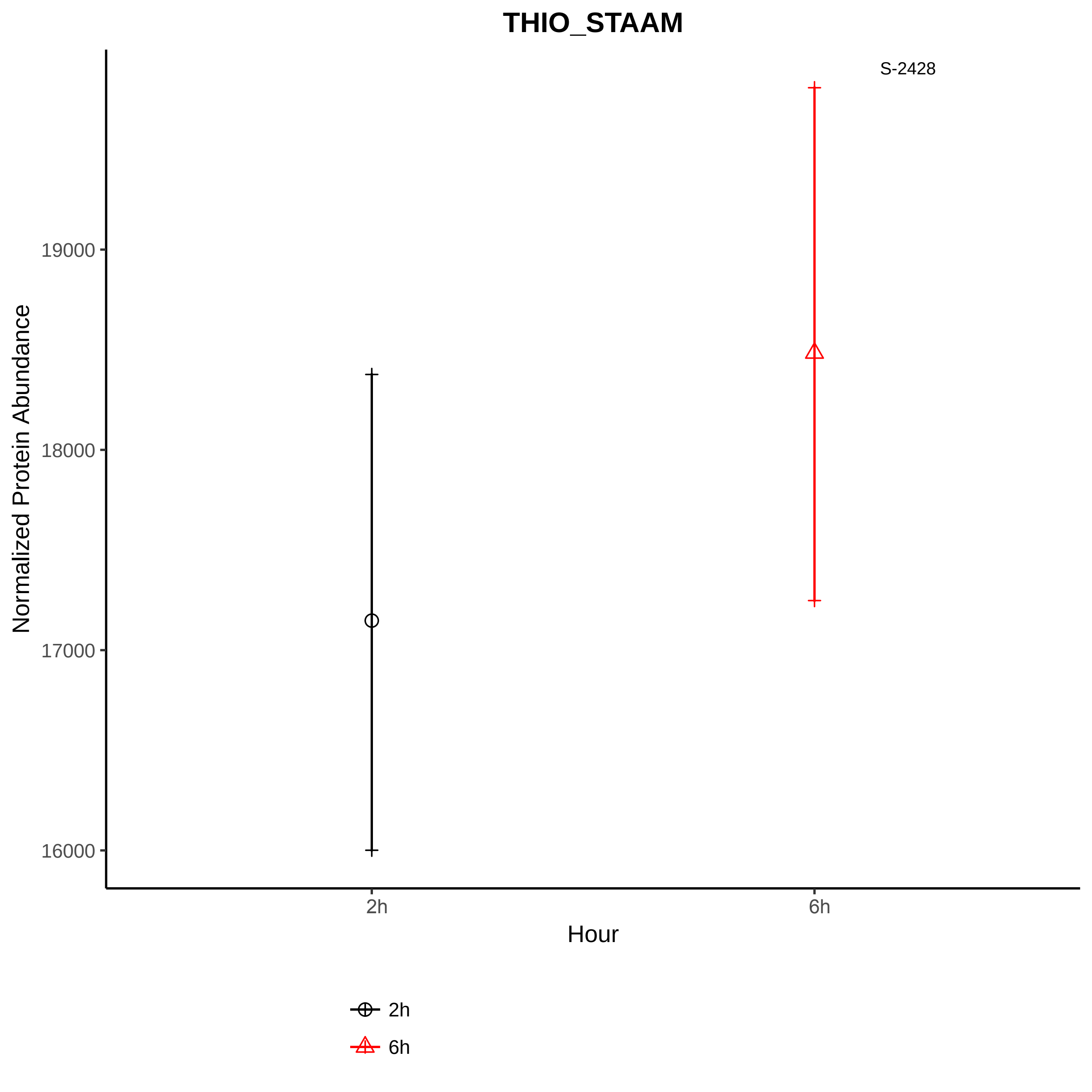

THIO_STAAM
S-2428
Normalized Protein Abundance
19000
18000
17000
16000
2h
6h
Hour
2h
6h

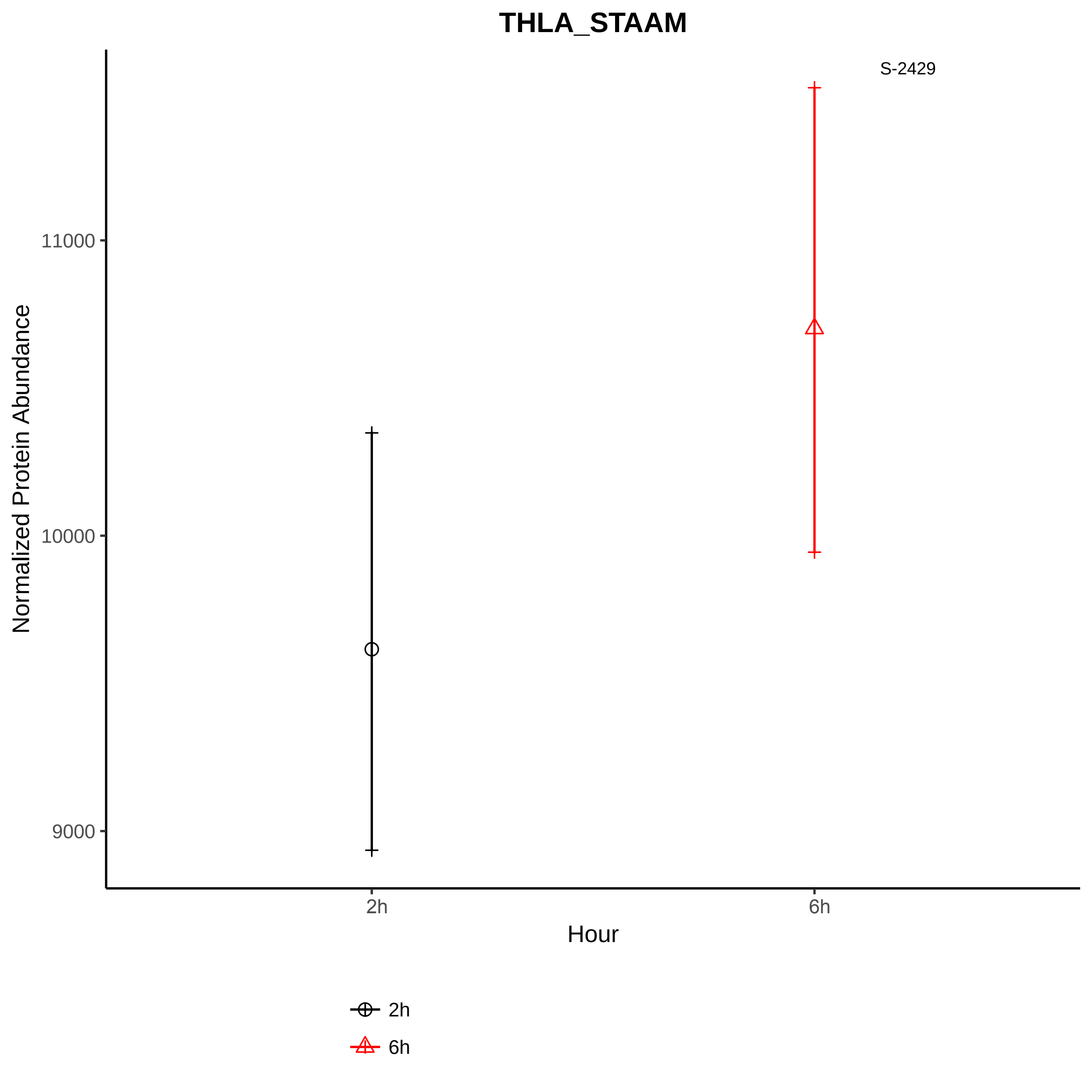
THLA_STAAM
S-2429
Normalized Protein Abundance
11000
10000
9000
2h
6h
Hour
2h
6h

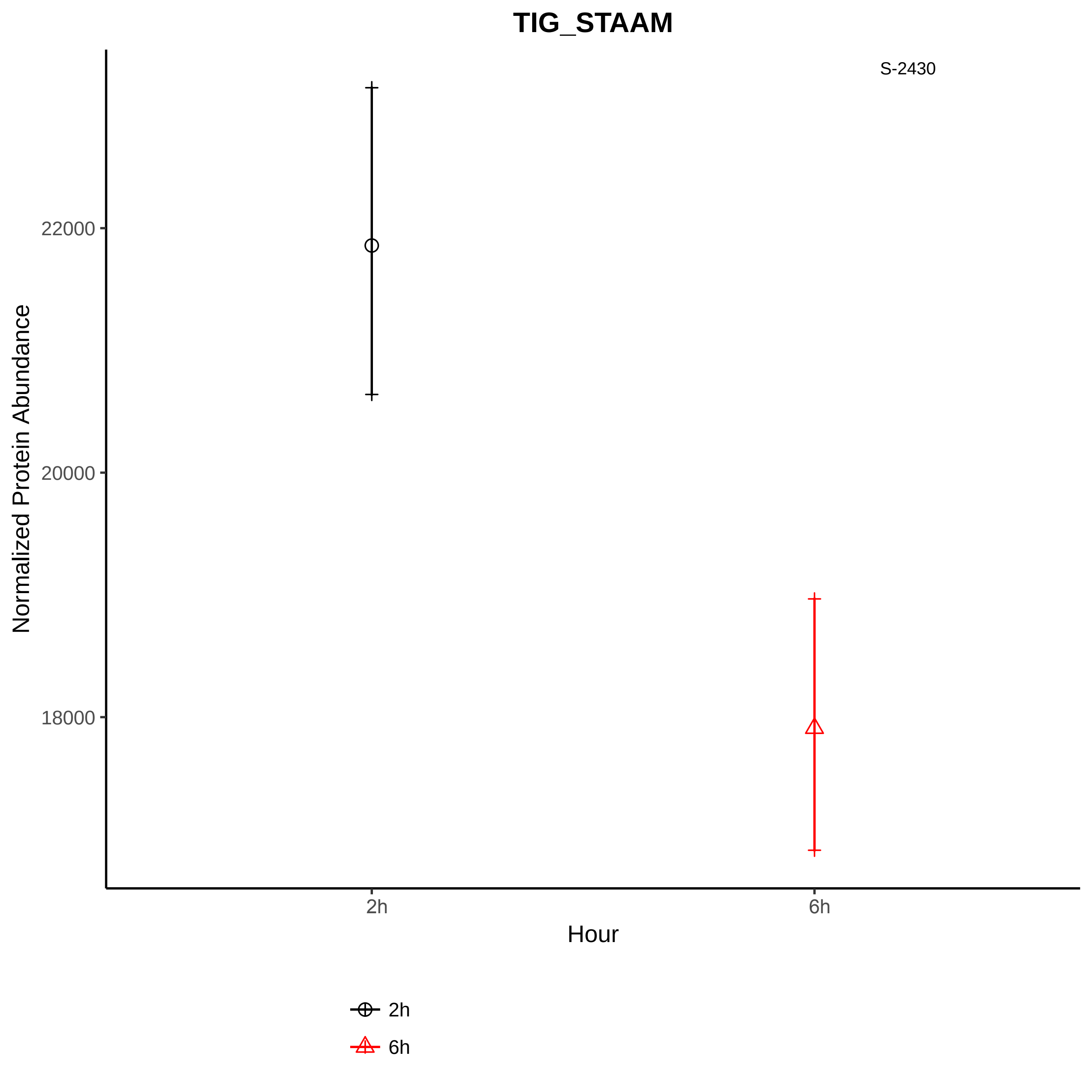

TIG_STAAM
S-2430
22000
20000
18000
Normalized Protein Abundance
2h
6h
Hour
2h
6h

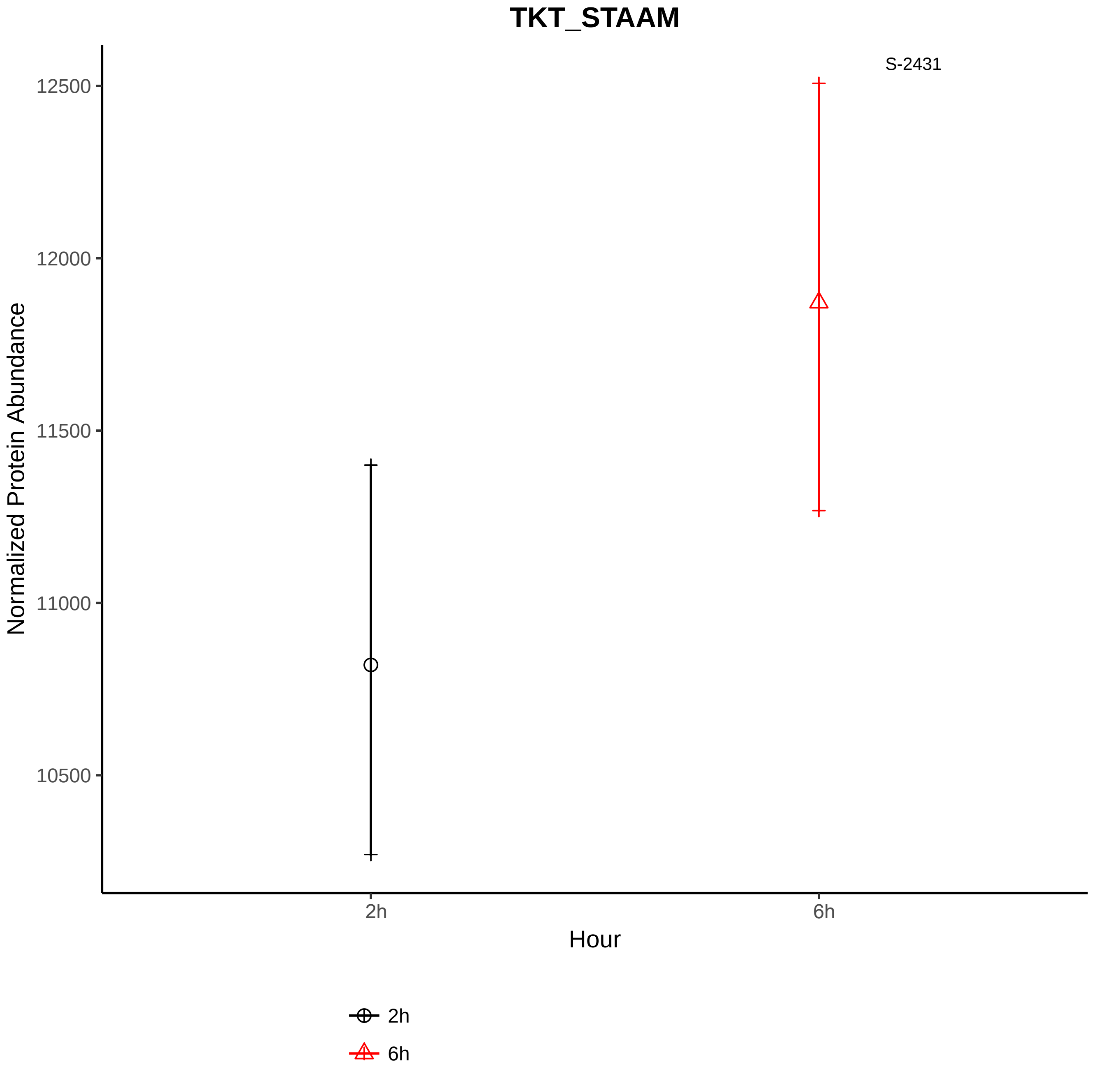

TKT_STAAM
S-2431
Normalized Protein Abundance
12500
12000
11500
11000
10500
2h
6h
Hour
2h
6h

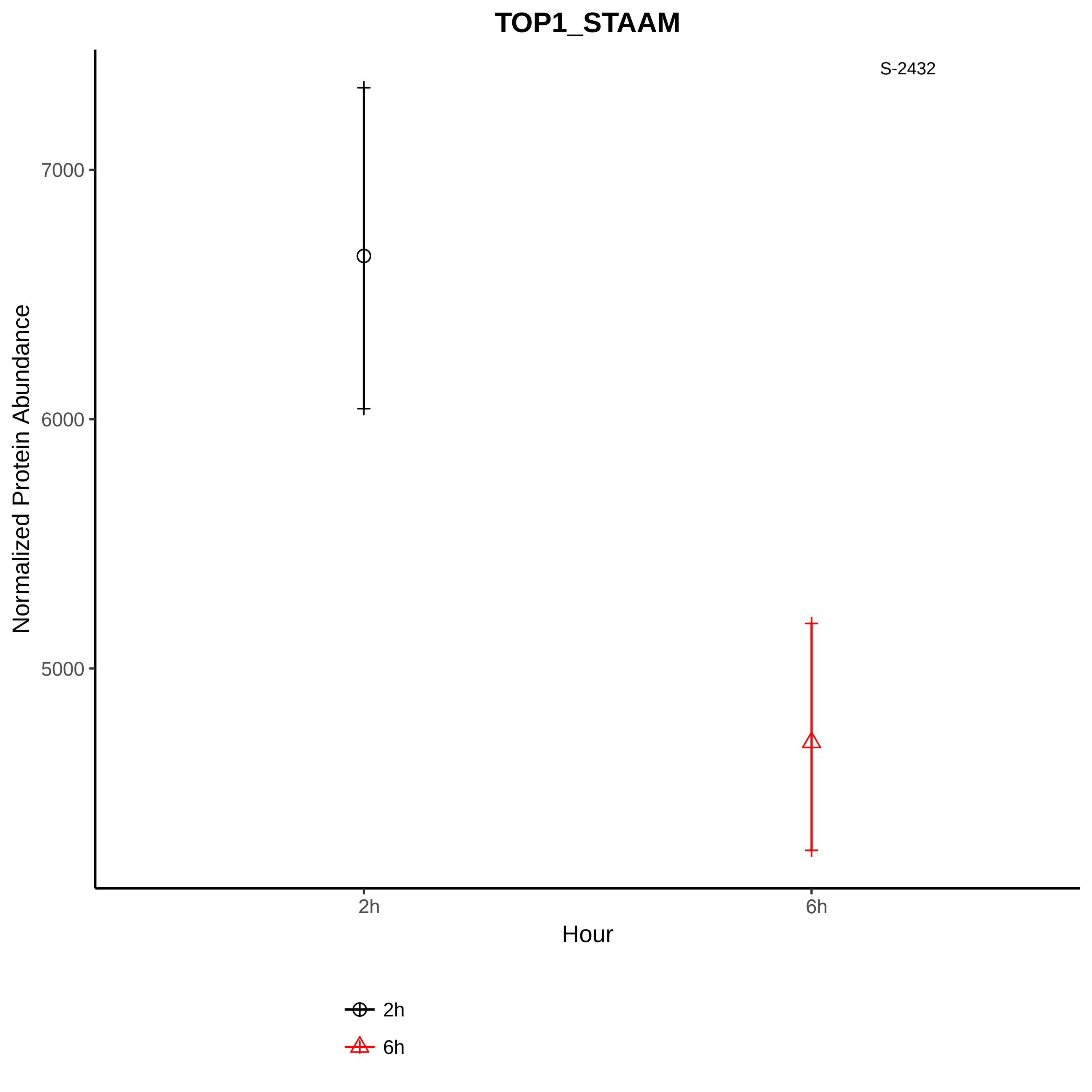
TOP1_STAAM
S-2432
7000
6000
5000
Normalized Protein Abundance
2h
6h
Hour
2h
6h

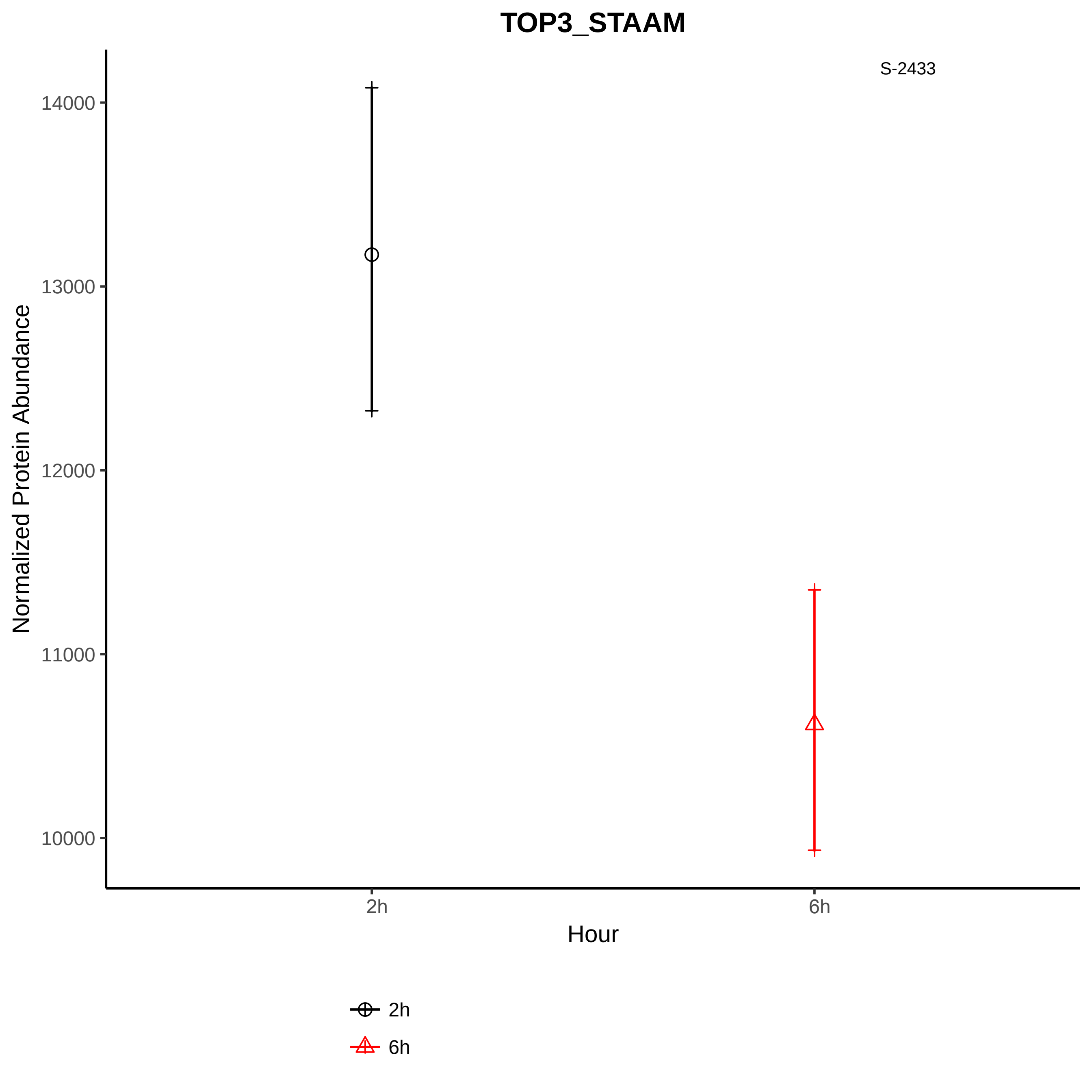

TOP3_STAAM
S-2433
Normalized Protein Abundance
14000
13000
12000
11000
10000
2h
6h
Hour
2h
6h

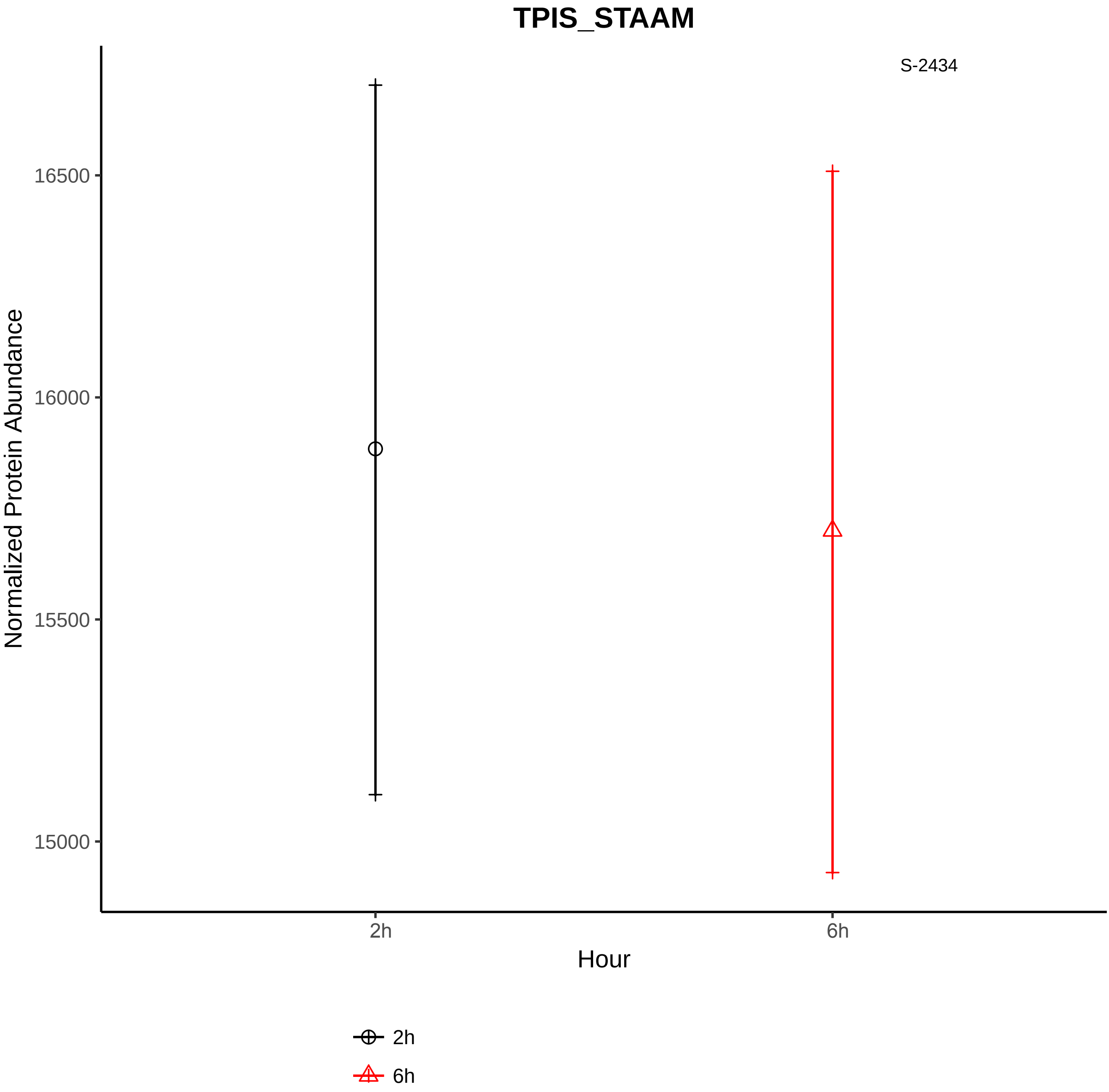
TPIS_STAAM
S-2434
Normalized Protein Abundance
16500
16000
15500
15000
2h
6h
Hour
2h
6h

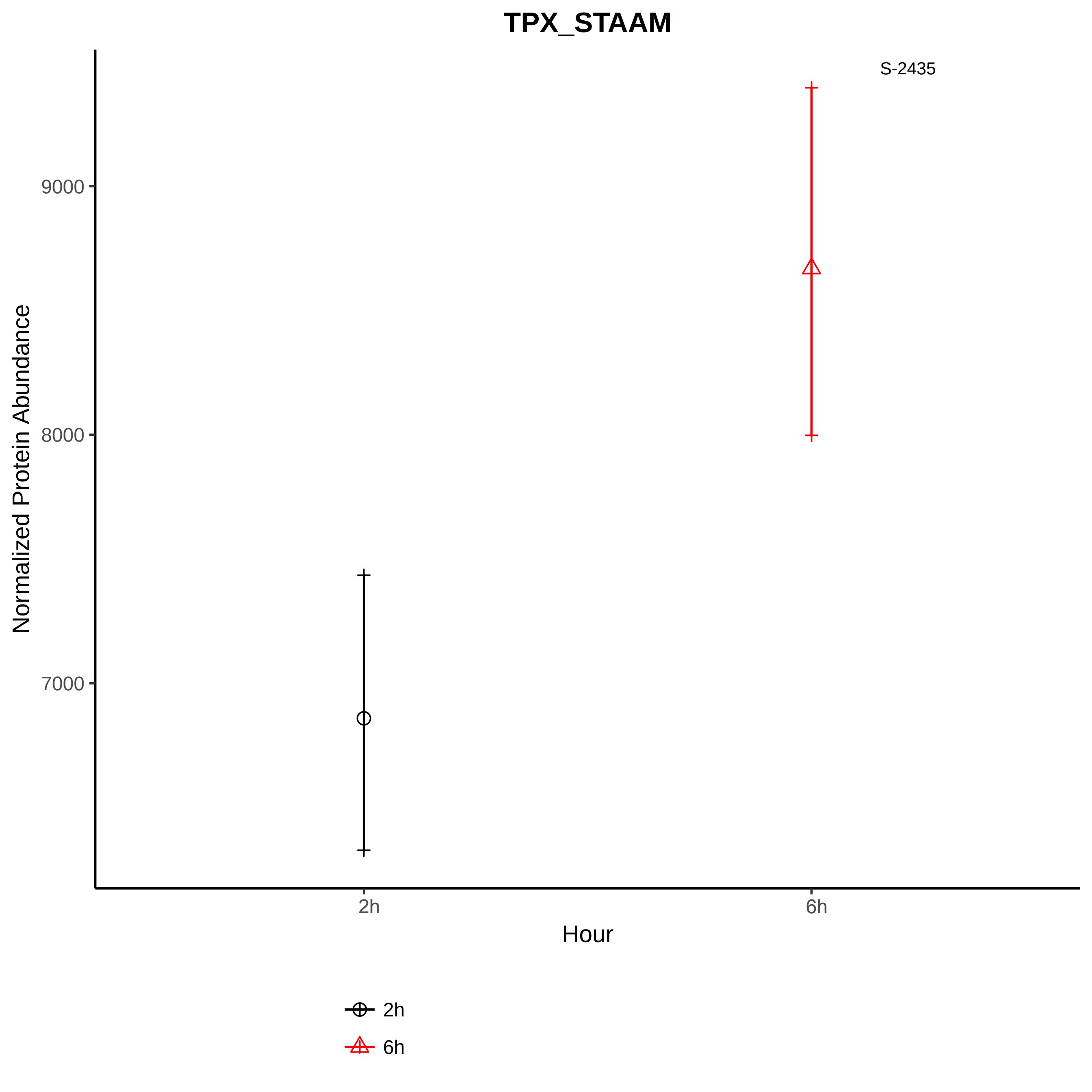

TPX_STAAM
S-2435
Normalized Protein Abundance
9000
8000
7000
2h
6h
Hour
2h
6h

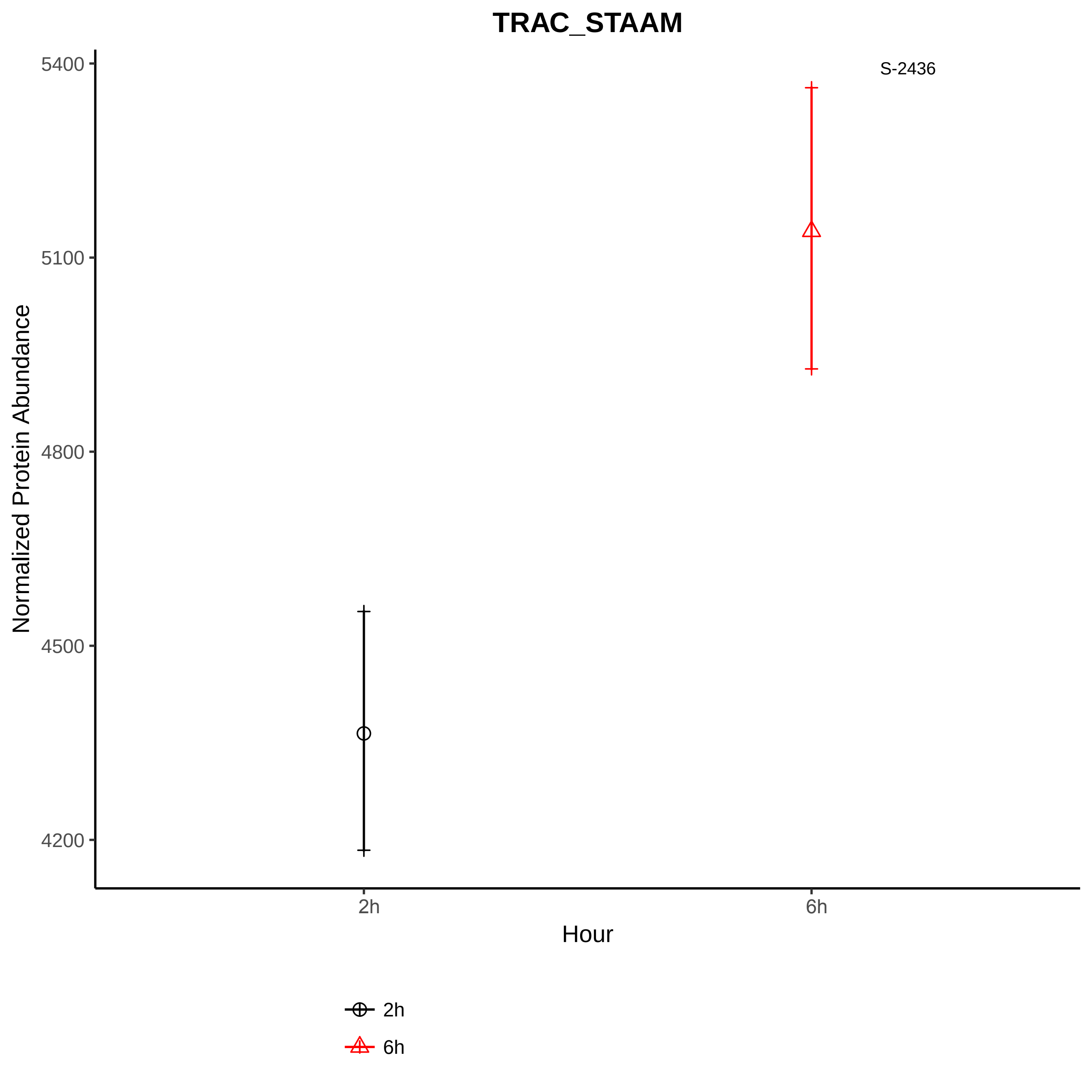
TRAC_STAAM
S-2436
Normalized Protein Abundance
5400
5100
4800
4500
4200
2h
6h
Hour
2h
6h

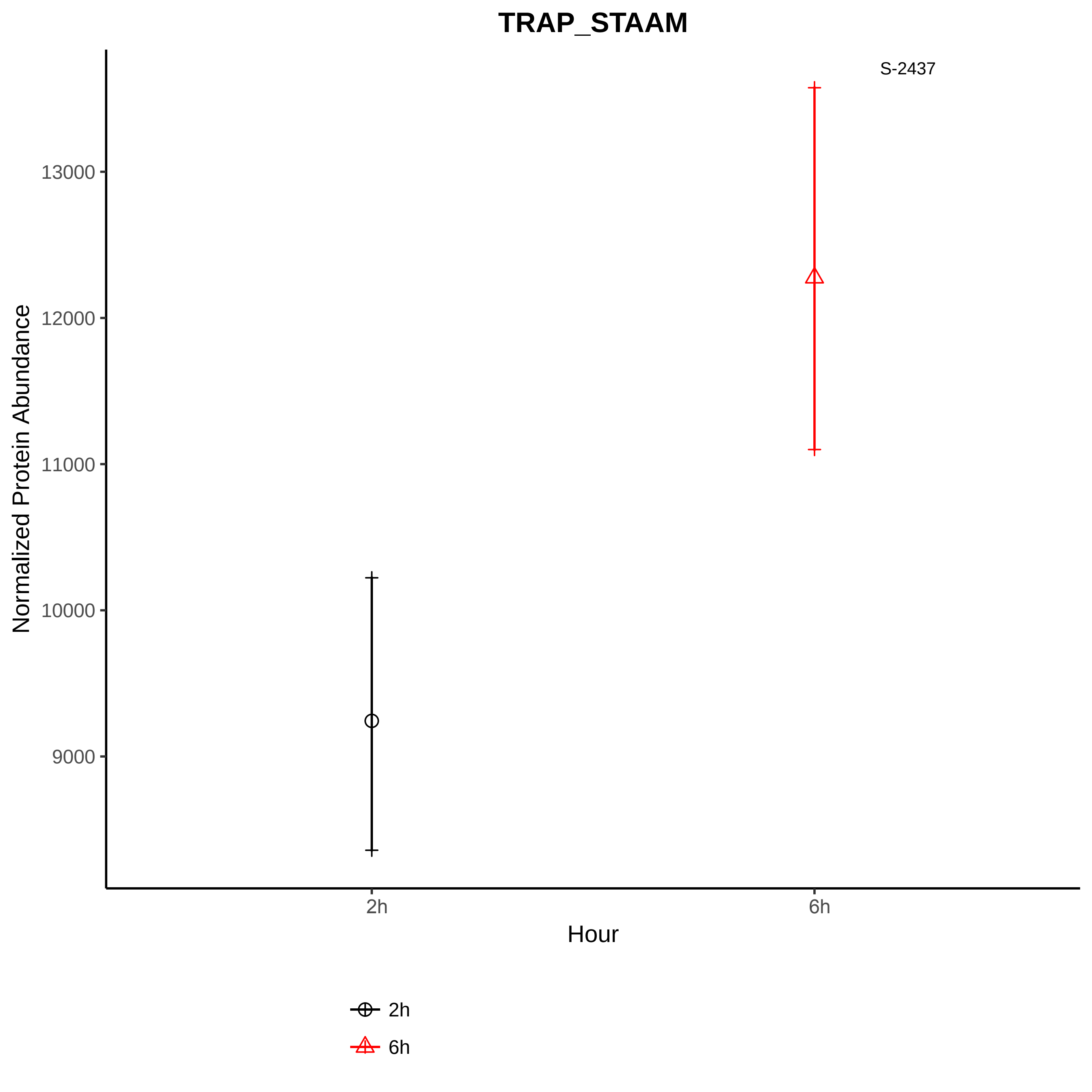

TRAP_STAAM
S-2437
Normalized Protein Abundance
13000
12000
11000
10000
9000
2h
6h
Hour
2h
6h

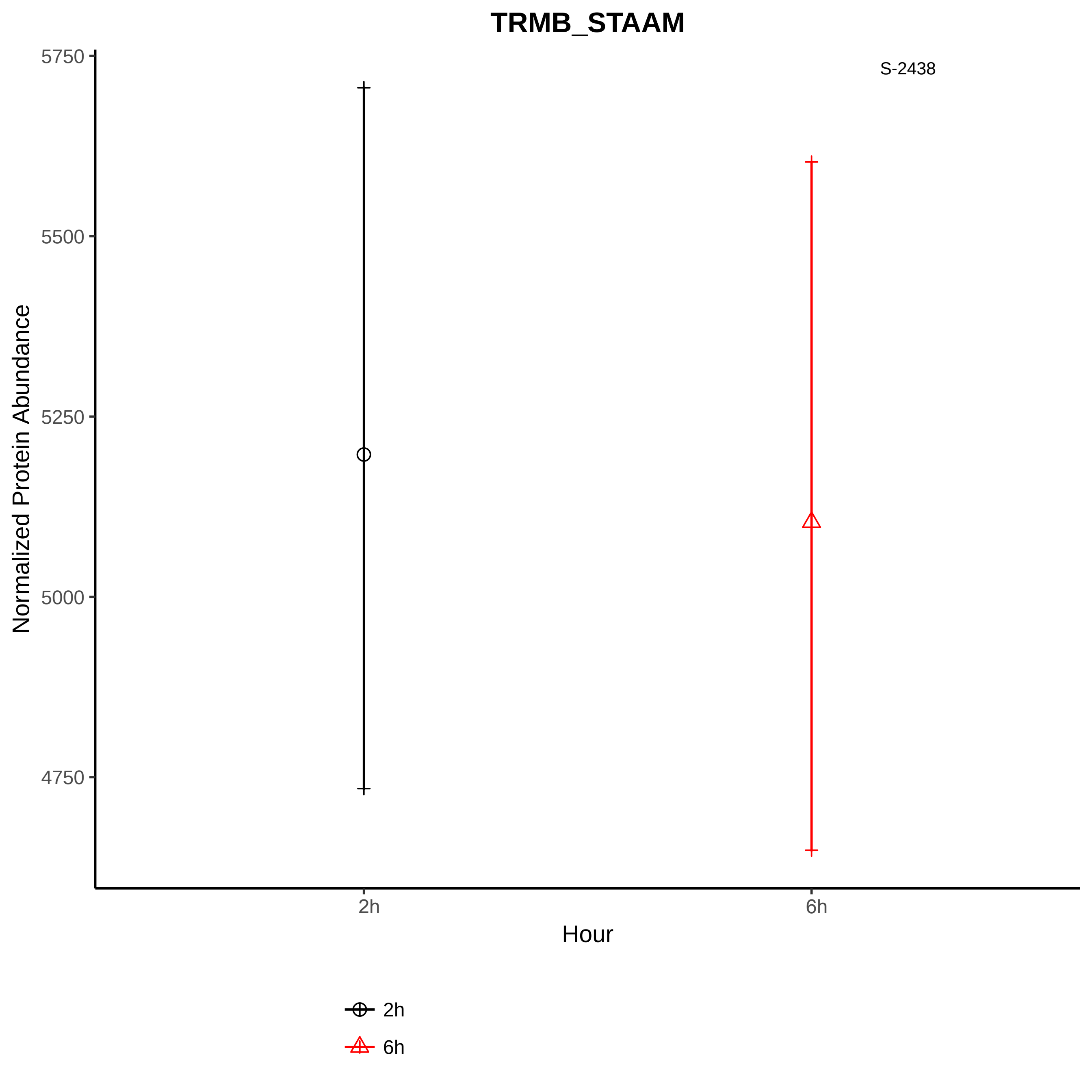

TRMB_STAAM
S-2438
Normalized Protein Abundance
5750
5500
5250
5000
4750
2h
6h
Hour
2h
6h

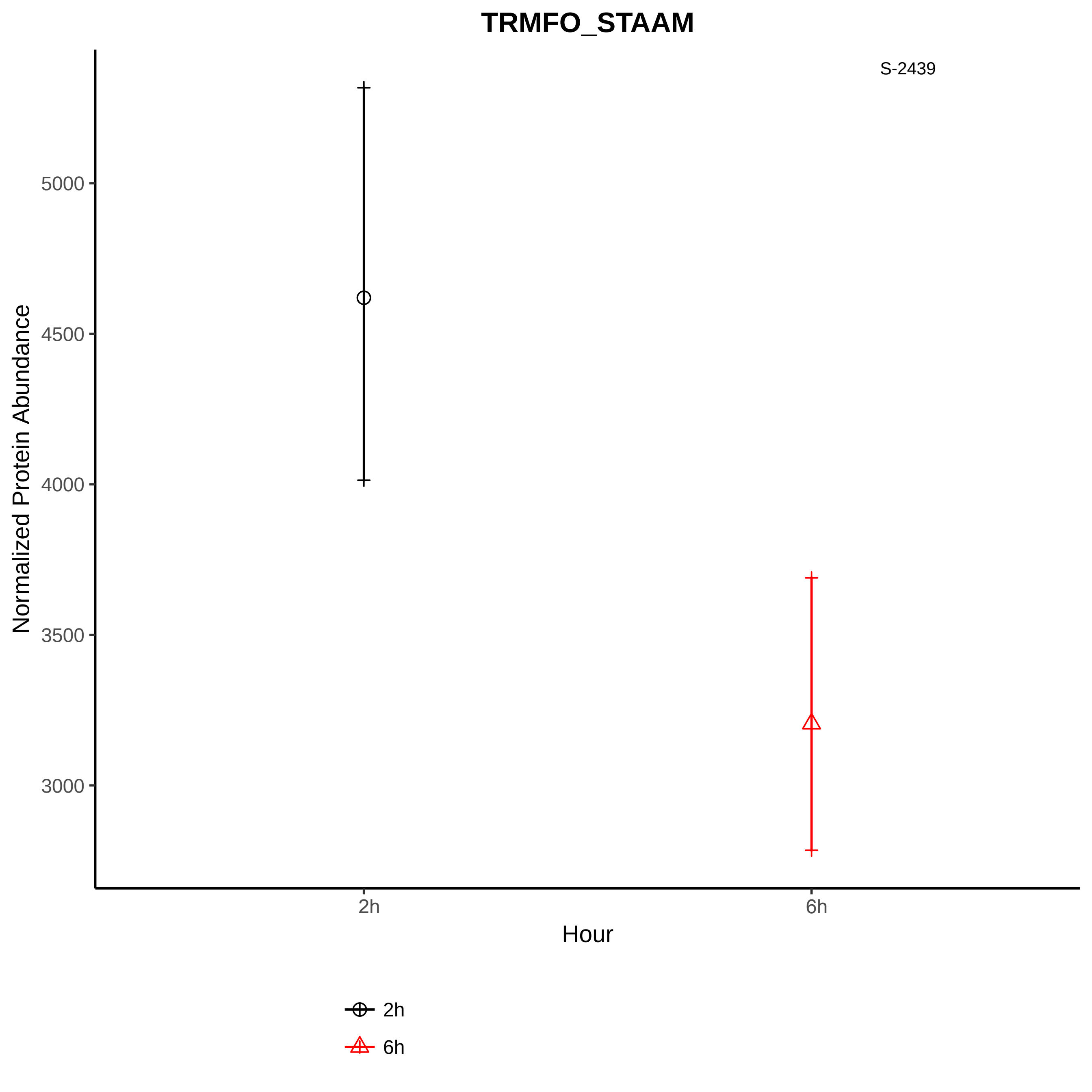

TRMFO_STAAM
S-2439
Normalized Protein Abundance
5000
4500
4000
3500
3000
2h
6h
Hour
2h
6h

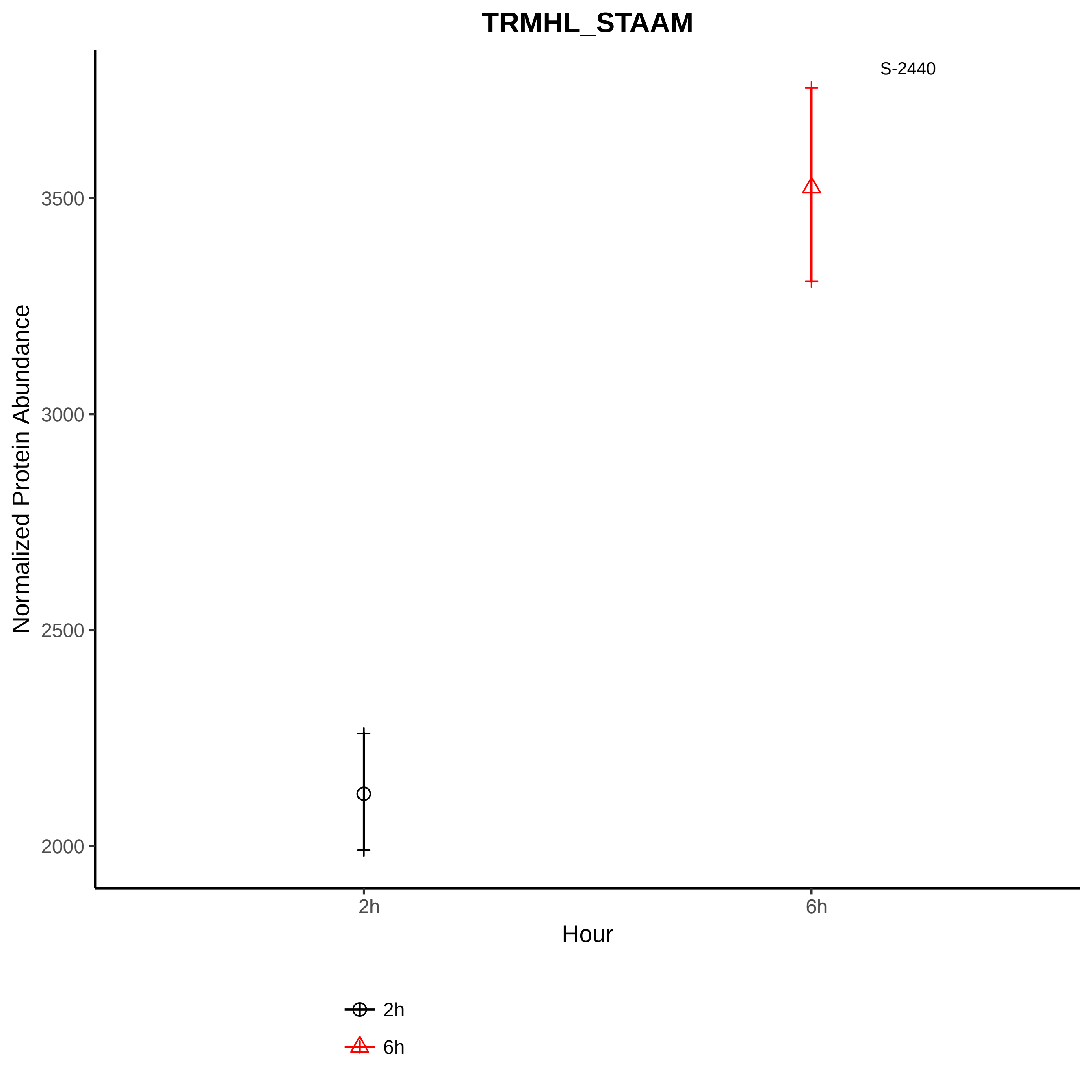

TRMHL_STAAM
S-2440
Normalized Protein Abundance
3500
3000
2500
2000
2h
6h
Hour
2h
6h

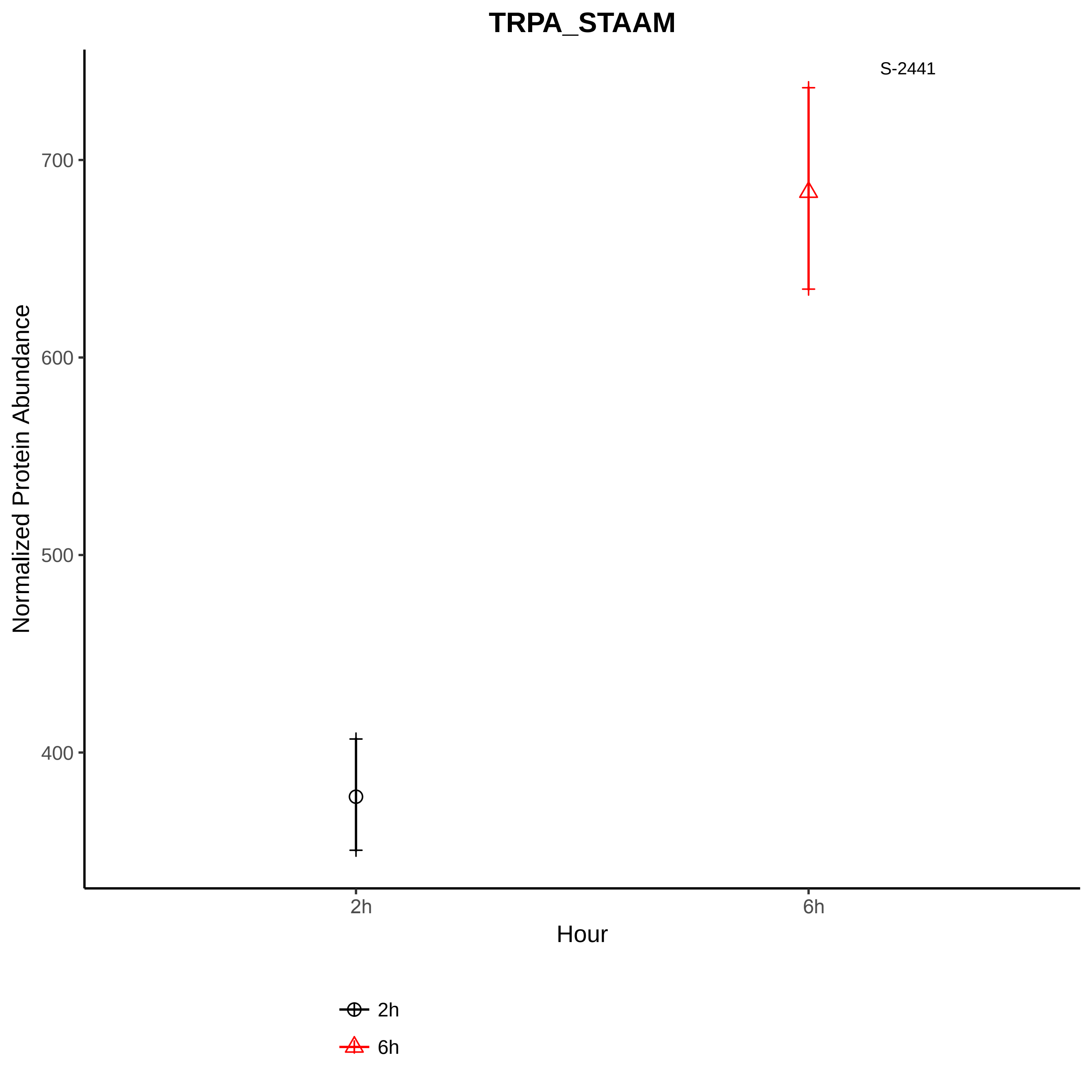
TRPA_STAAM
S-2441
700
600
500
400
Normalized Protein Abundance
2h
6h
Hour
2h
6h

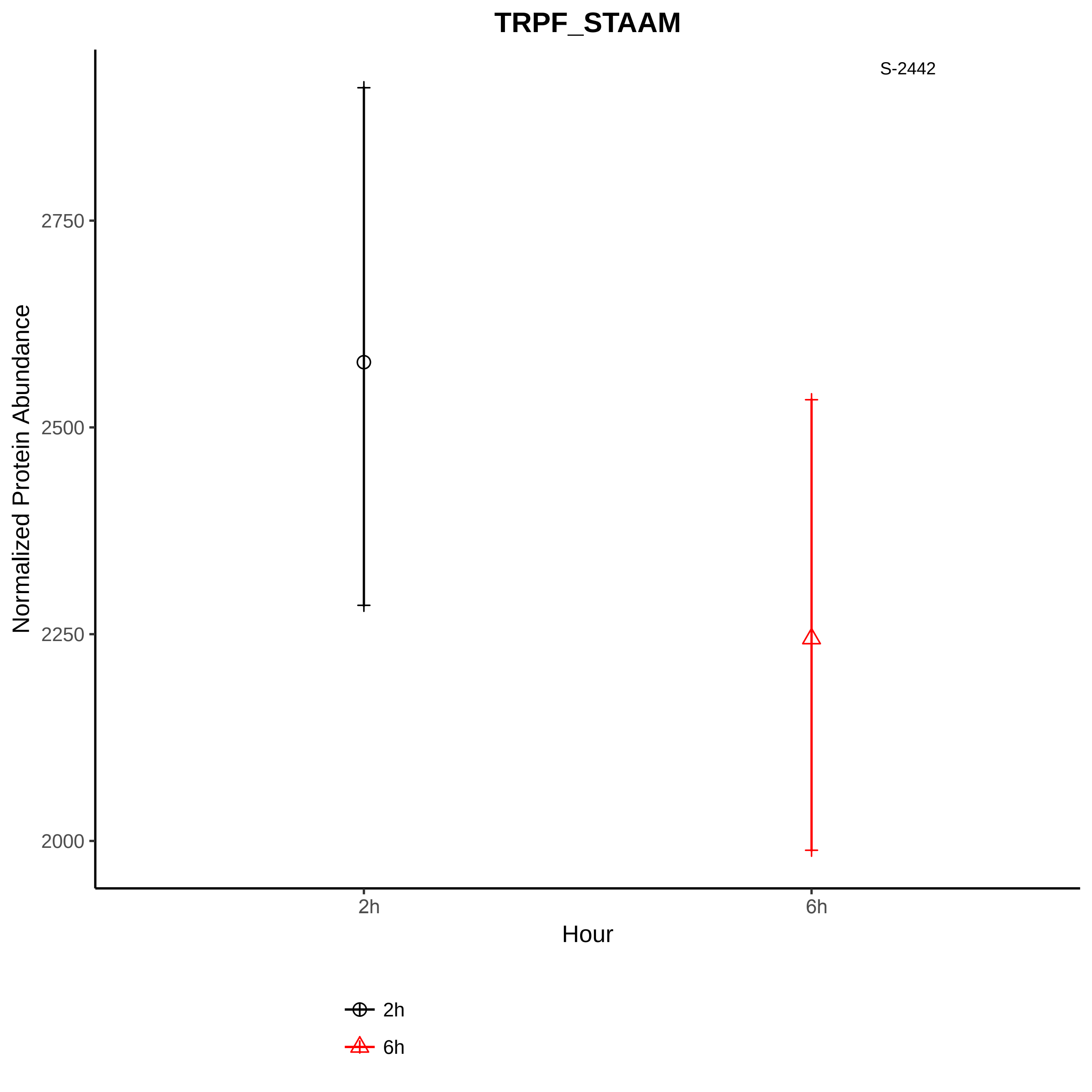
TRPF_STAAM
S-2442
Normalized Protein Abundance
2750
2500
2250
2000
2h
6h
Hour
2h
6h

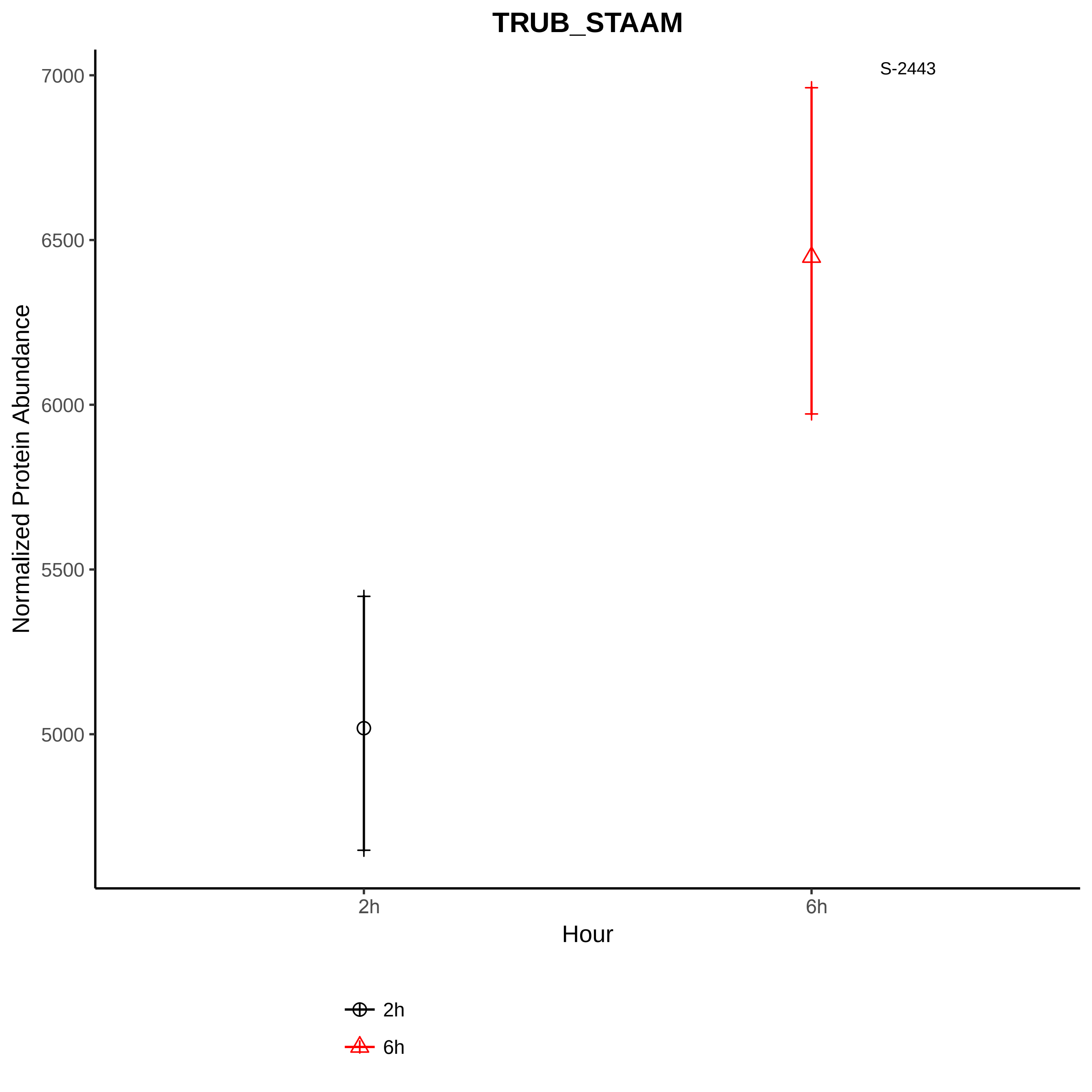
TRUB_STAAM
S-2443
Normalized Protein Abundance
7000
6500
6000
5500
5000
2h
6h
Hour
2h
6h

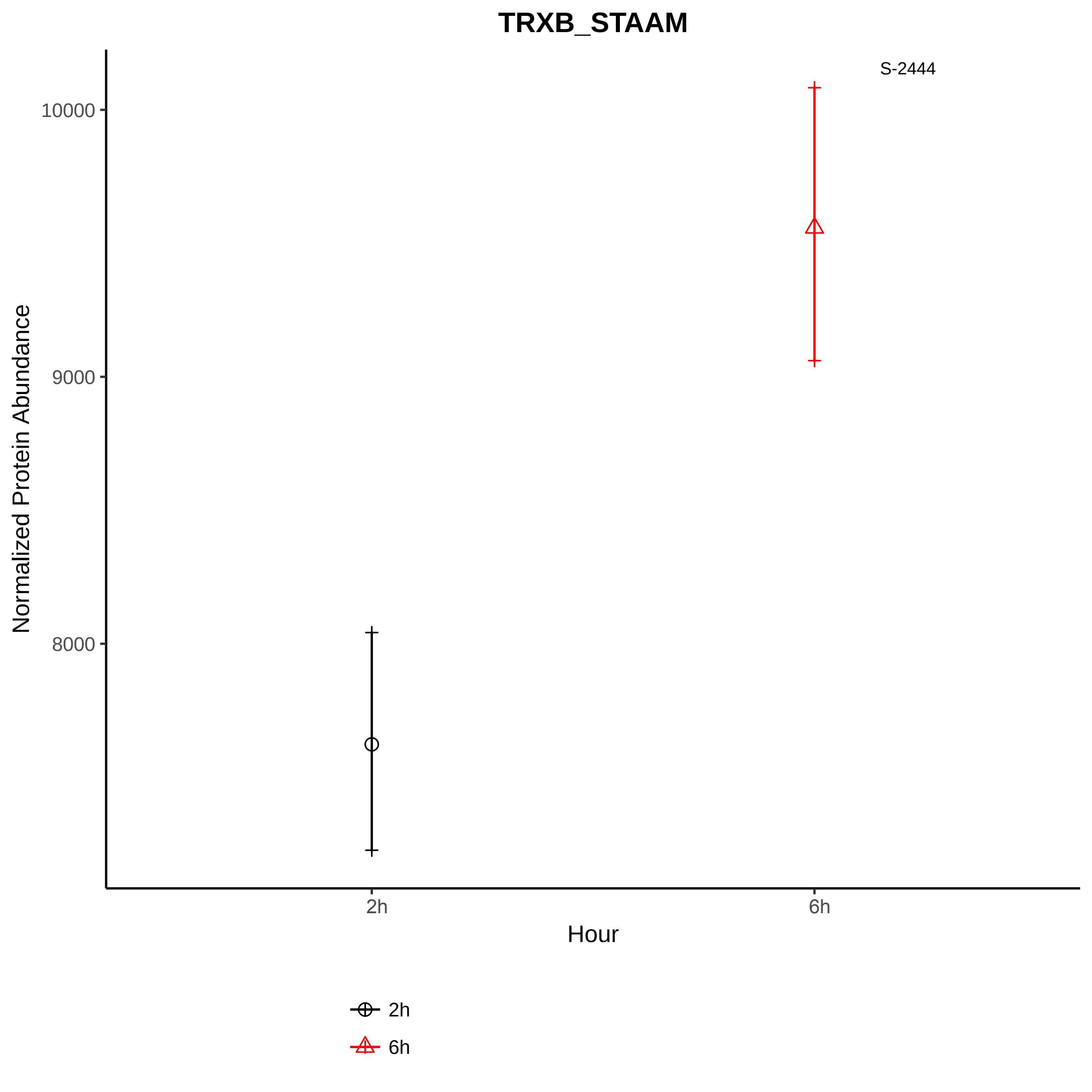

TRXB_STAAM
S-2444
10000
9000
8000
Normalized Protein Abundance
2h
6h
Hour
2h
6h

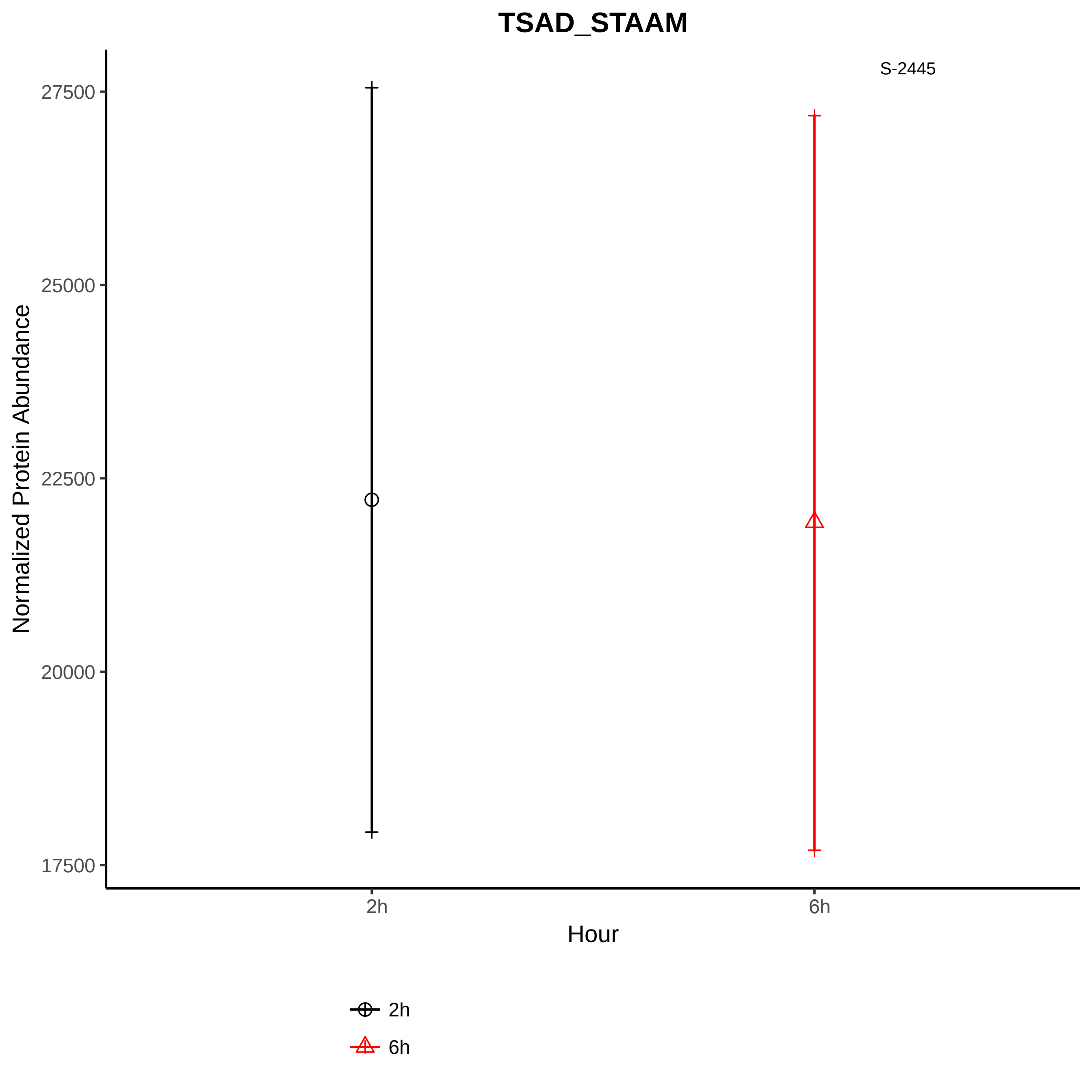

TSAD_STAAM
S-2445
27500
25000
22500
20000
17500
Normalized Protein Abundance
2h
6h
Hour
2h
6h

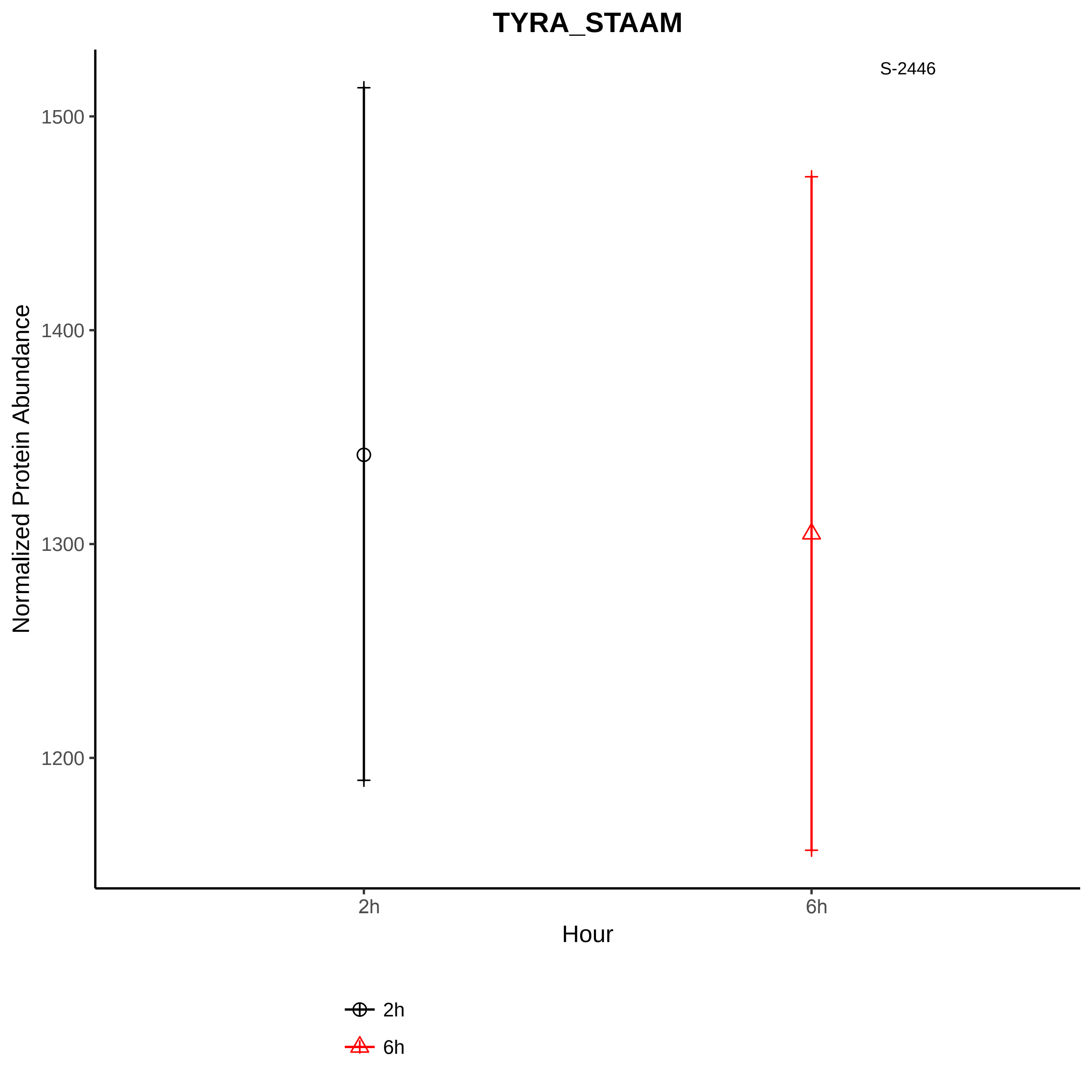

TYRA_STAAM
S-2446
1500
1400
1300
1200
Normalized Protein Abundance
2h
6h
Hour
2h
6h

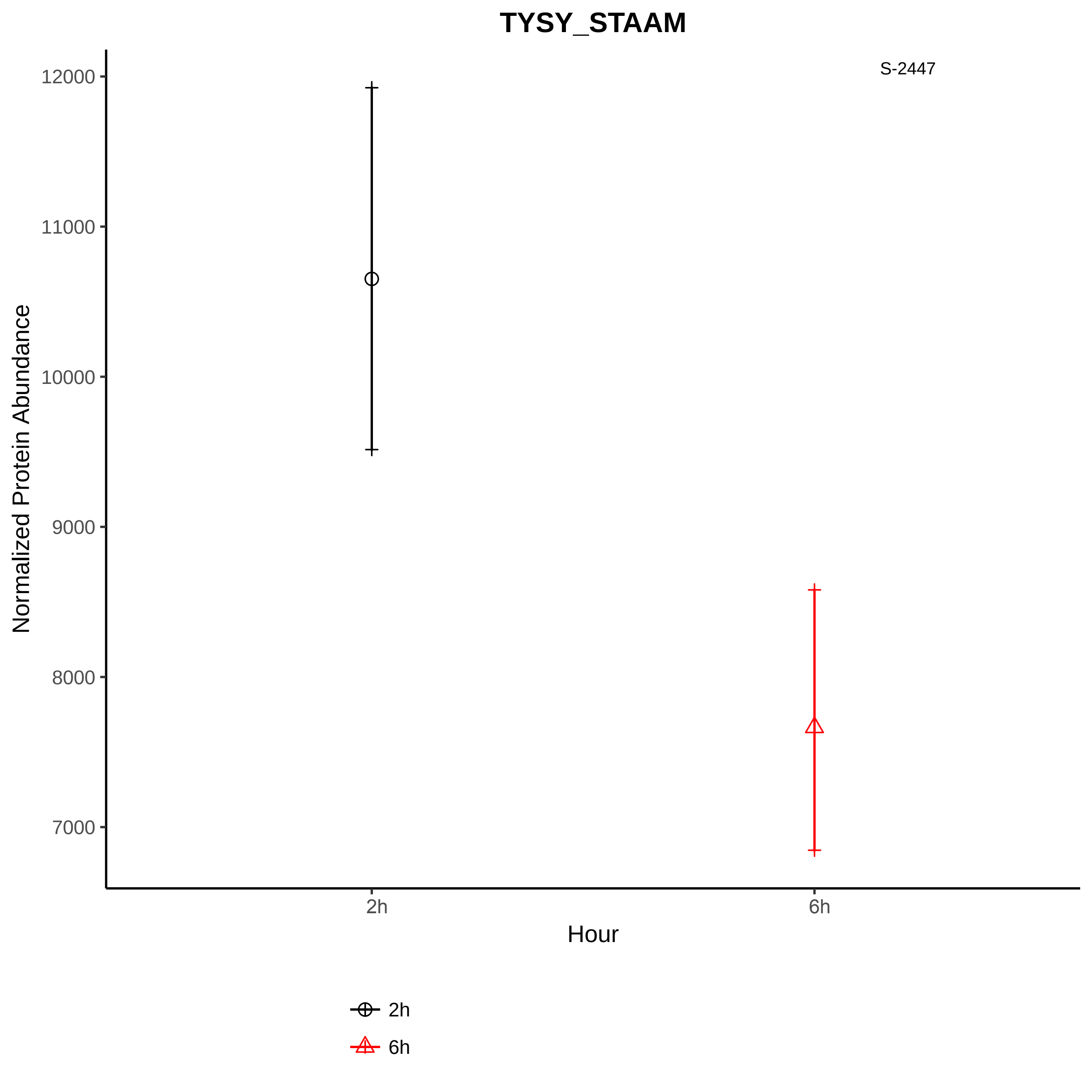

TYSY_STAAM
S-2447
Normalized Protein Abundance
12000
11000
10000
9000
8000
7000
2h
6h
Hour
2h
6h

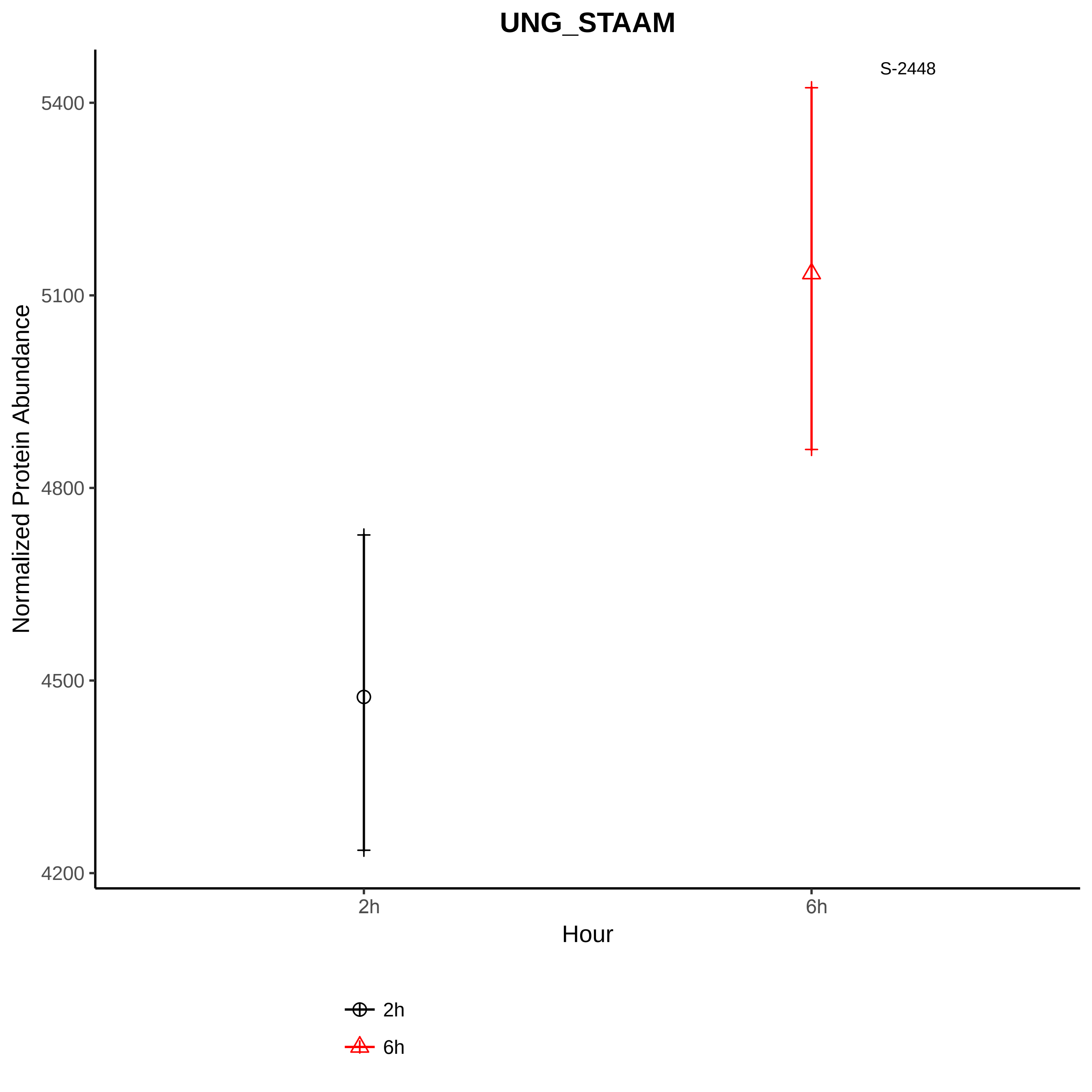

UNG_STAAM
S-2448
Normalized Protein Abundance
5400
5100
4800
4500
4200
2h
6h
Hour
2h
6h

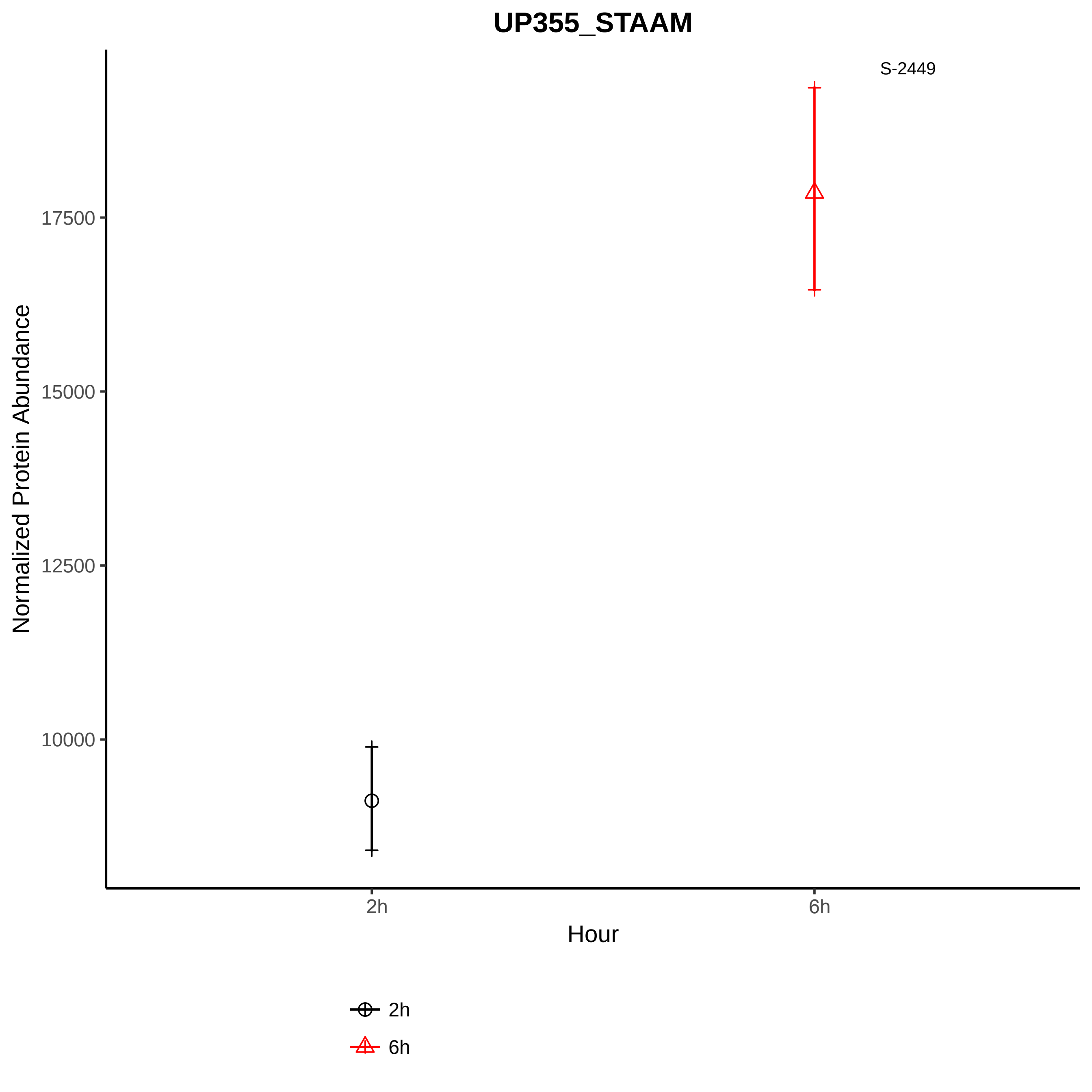

UP355_STAAM
S-2449
Normalized Protein Abundance
17500
15000
12500
10000
2h
6h
Hour
2h
6h

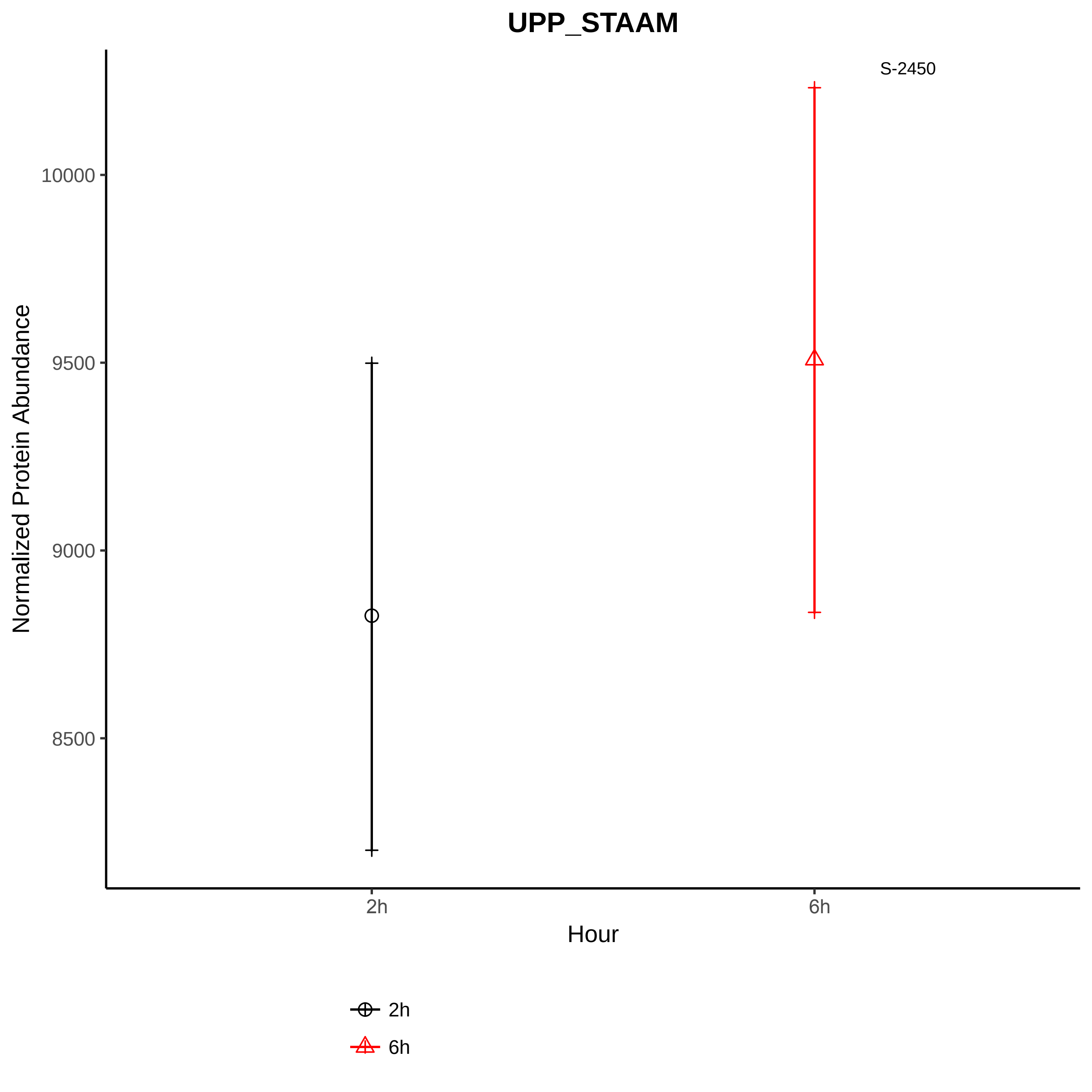

UPP_STAAM
S-2450
Normalized Protein Abundance
10000
9500
9000
8500
2h
6h
Hour
2h
6h

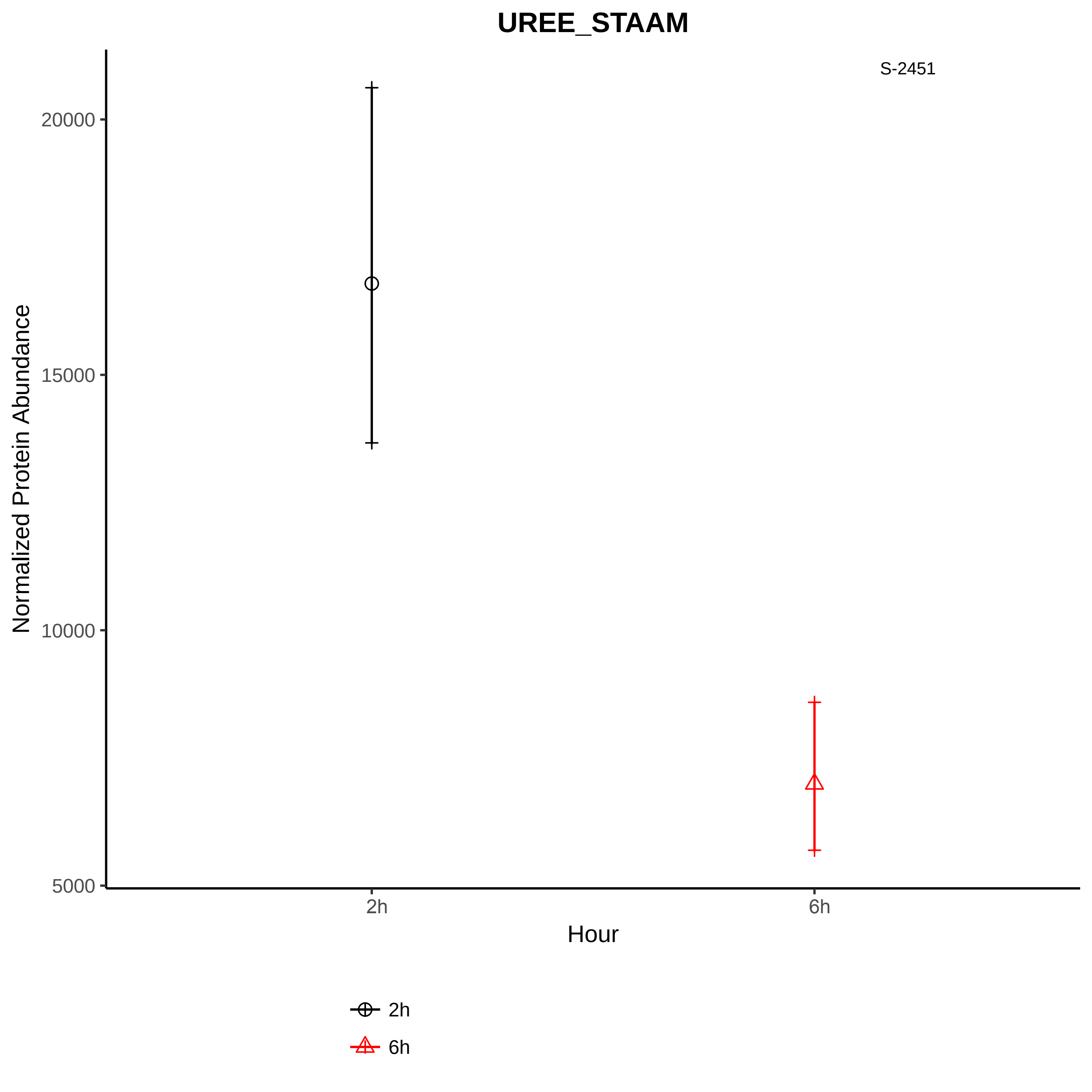
UREE_STAAM
S-2451
20000
15000
10000
5000
Normalized Protein Abundance
2h
6h
Hour
2h
6h

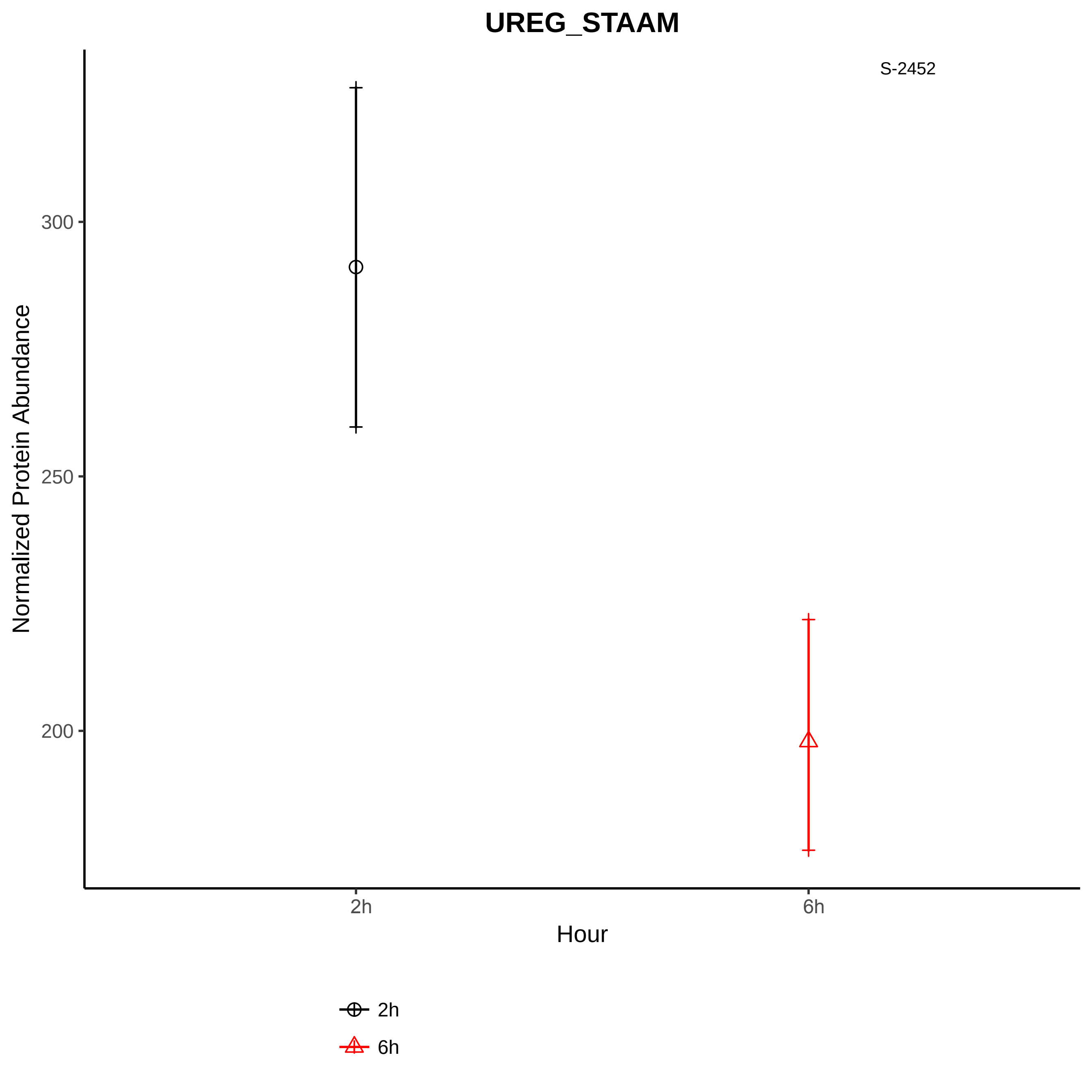
UREG_STAAM
S-2452
Normalized Protein Abundance
300
250
200
2h
6h
Hour
2h
6h

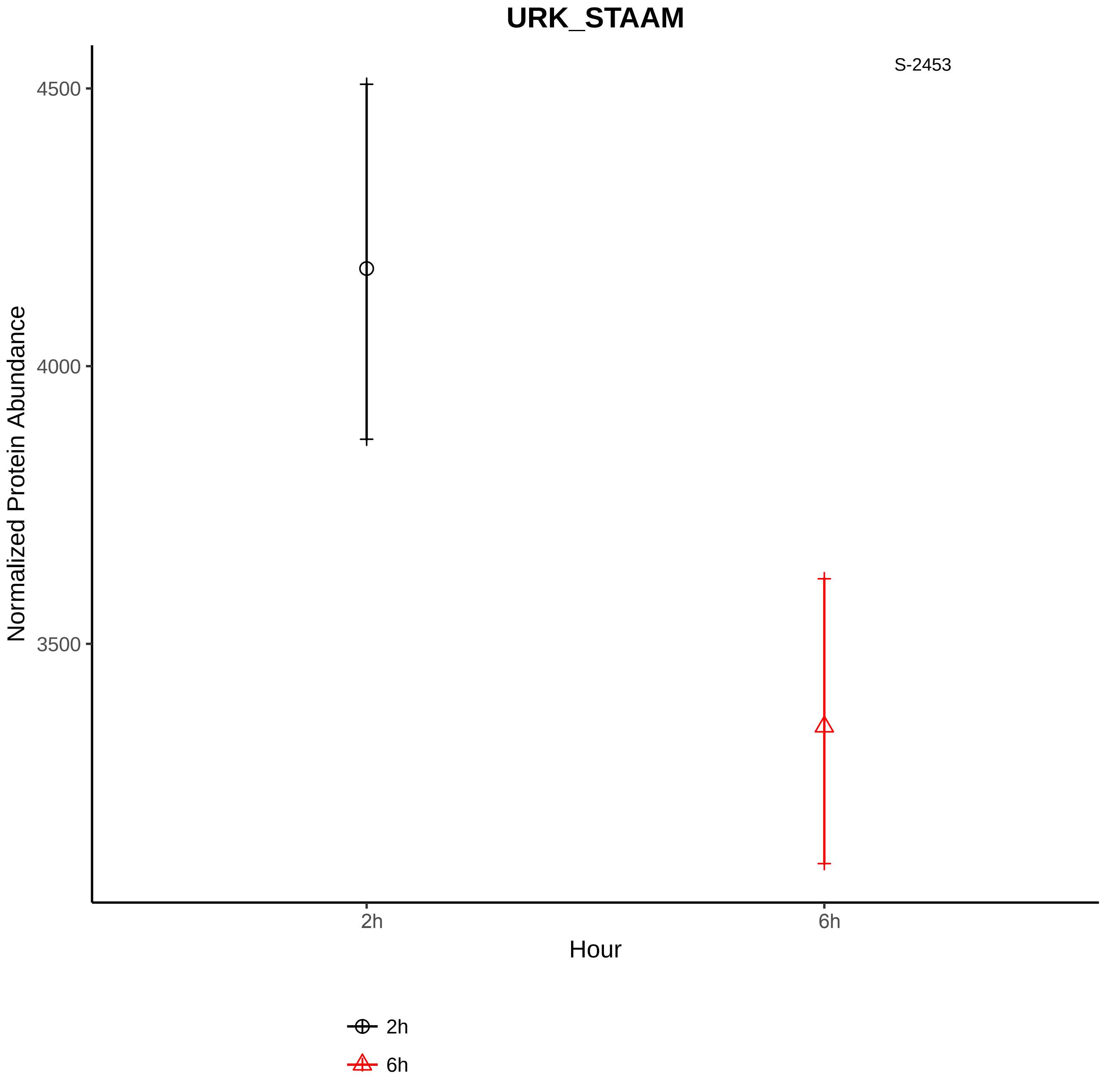
URK_STAAM
S-2453
Normalized Protein Abundance
4500
4000
3500
2h
6h
Hour
2h
6h

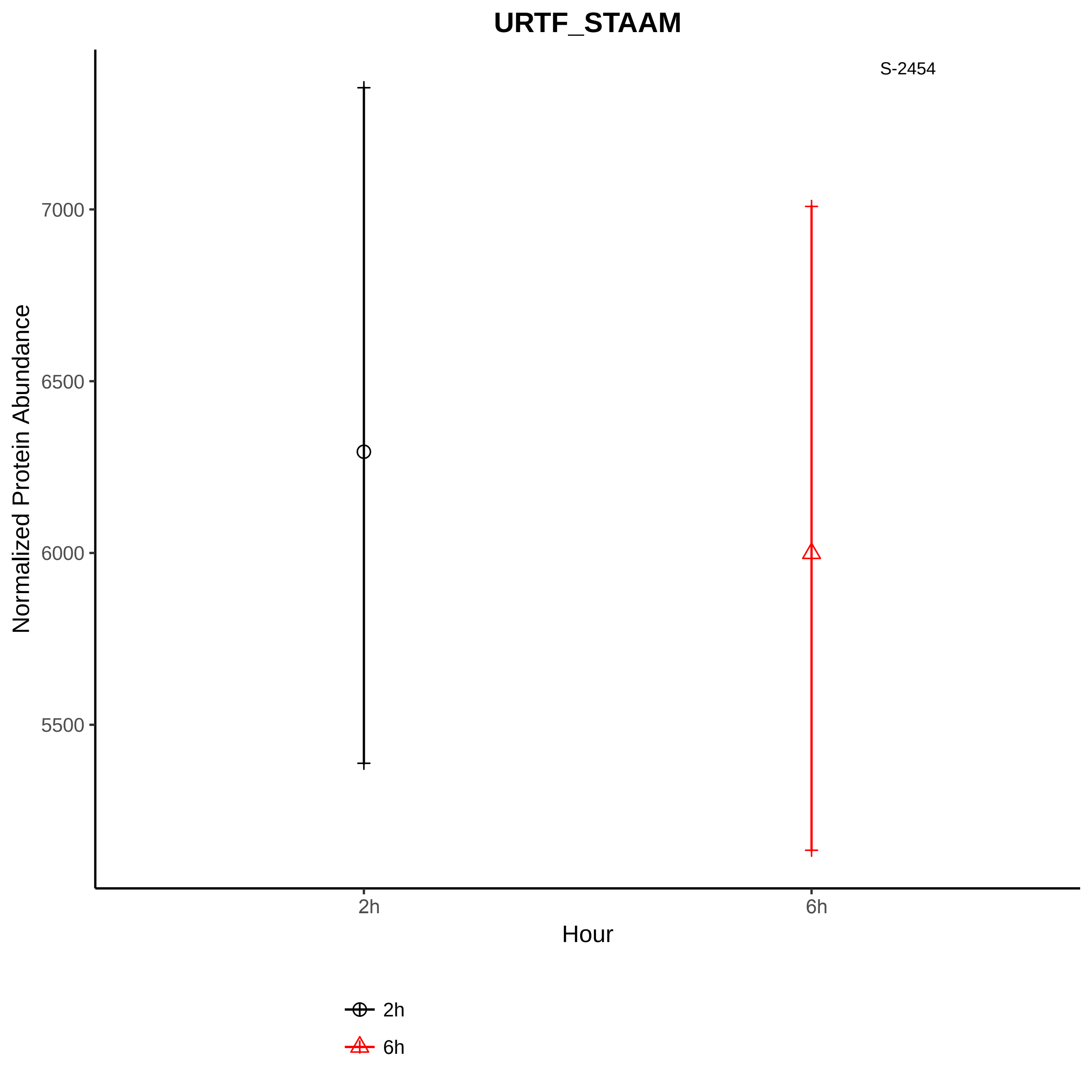

URTF_STAAM
S-2454
Normalized Protein Abundance
7000
6500
6000
5500
2h
6h
Hour
2h
6h

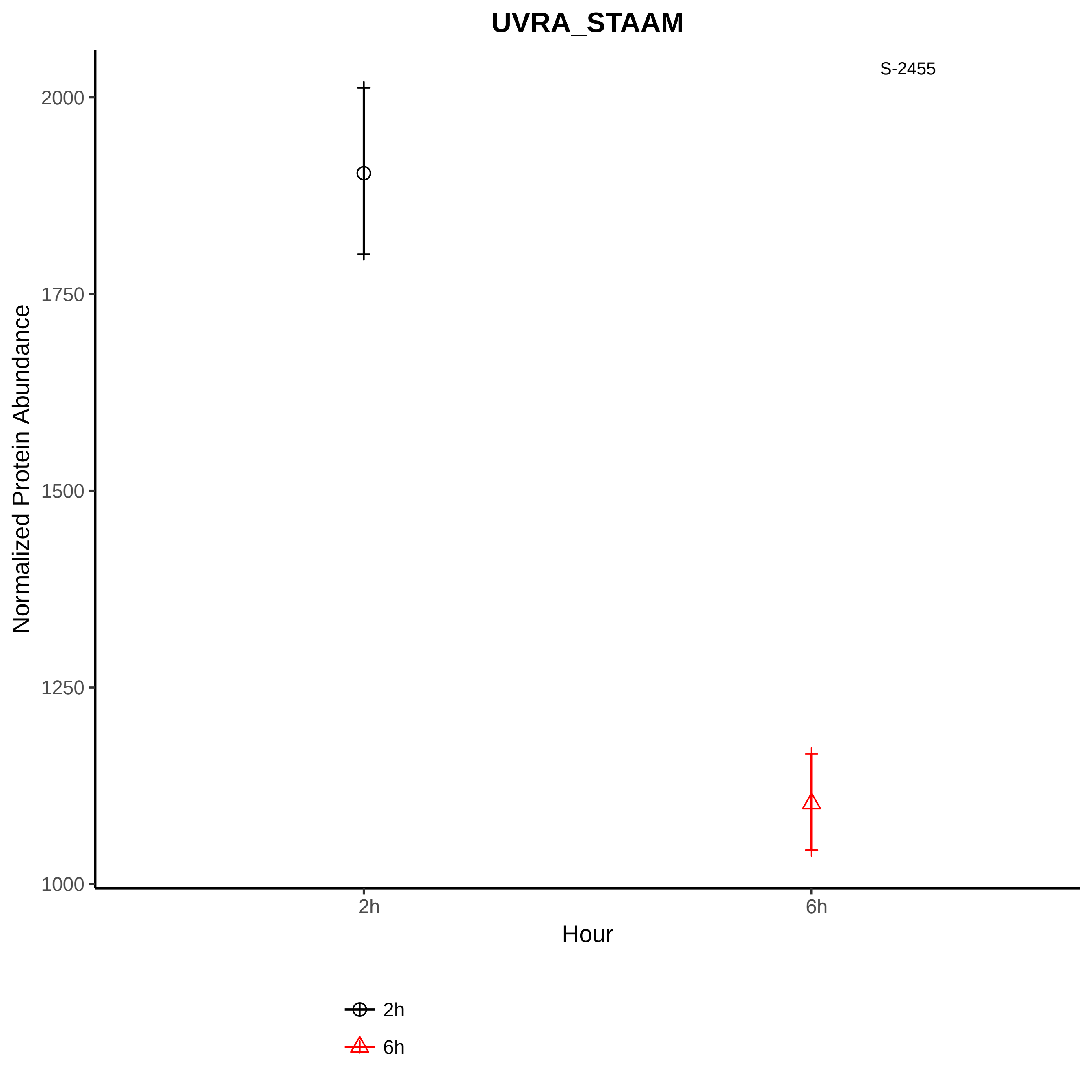
UVRA_STAAM
S-2455
Normalized Protein Abundance
2000
1750
1500
1250
1000
2h
6h
Hour
2h
6h

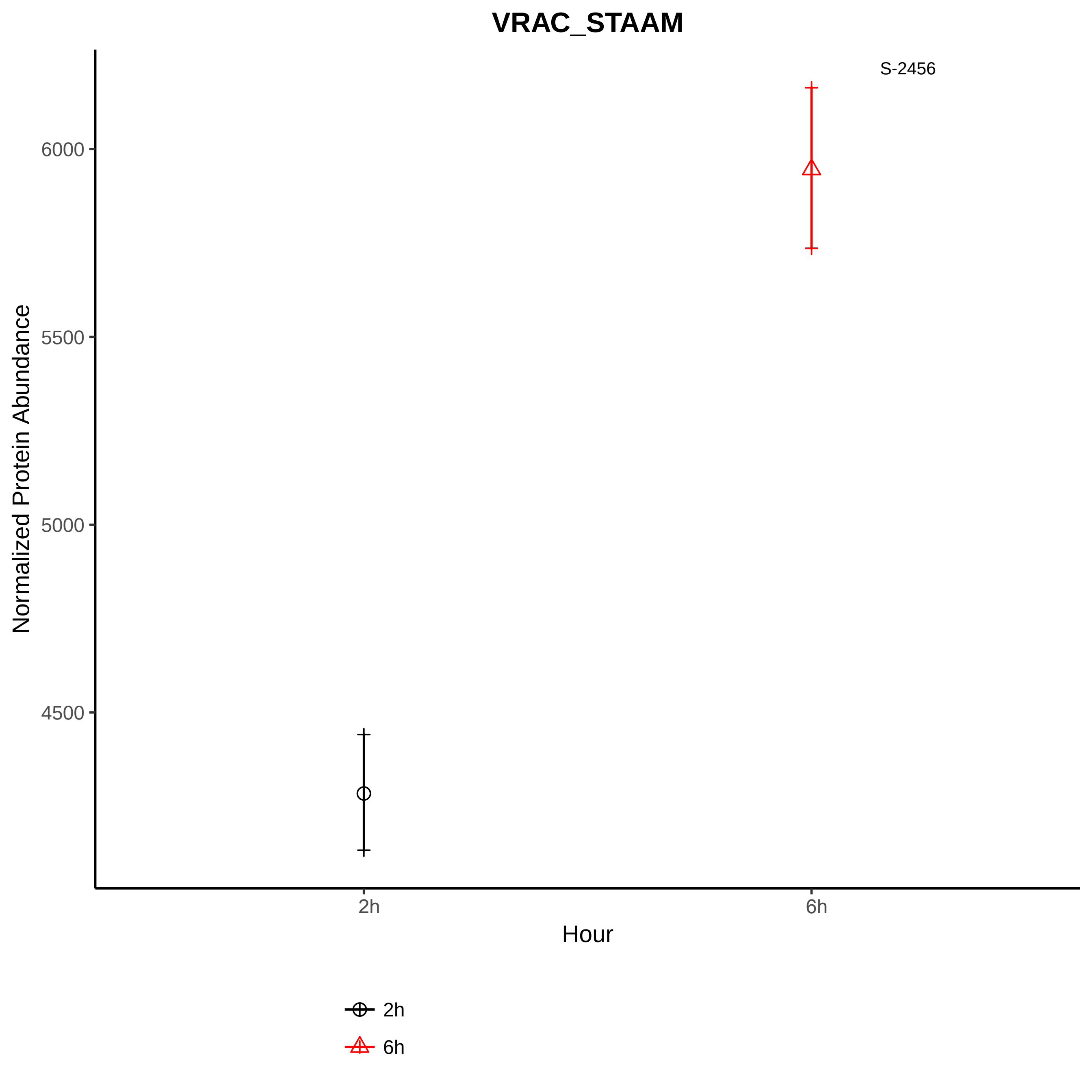

VRAC_STAAM
S-2456
6000
5500
5000
4500
Normalized Protein Abundance
2h
6h
Hour
2h
6h

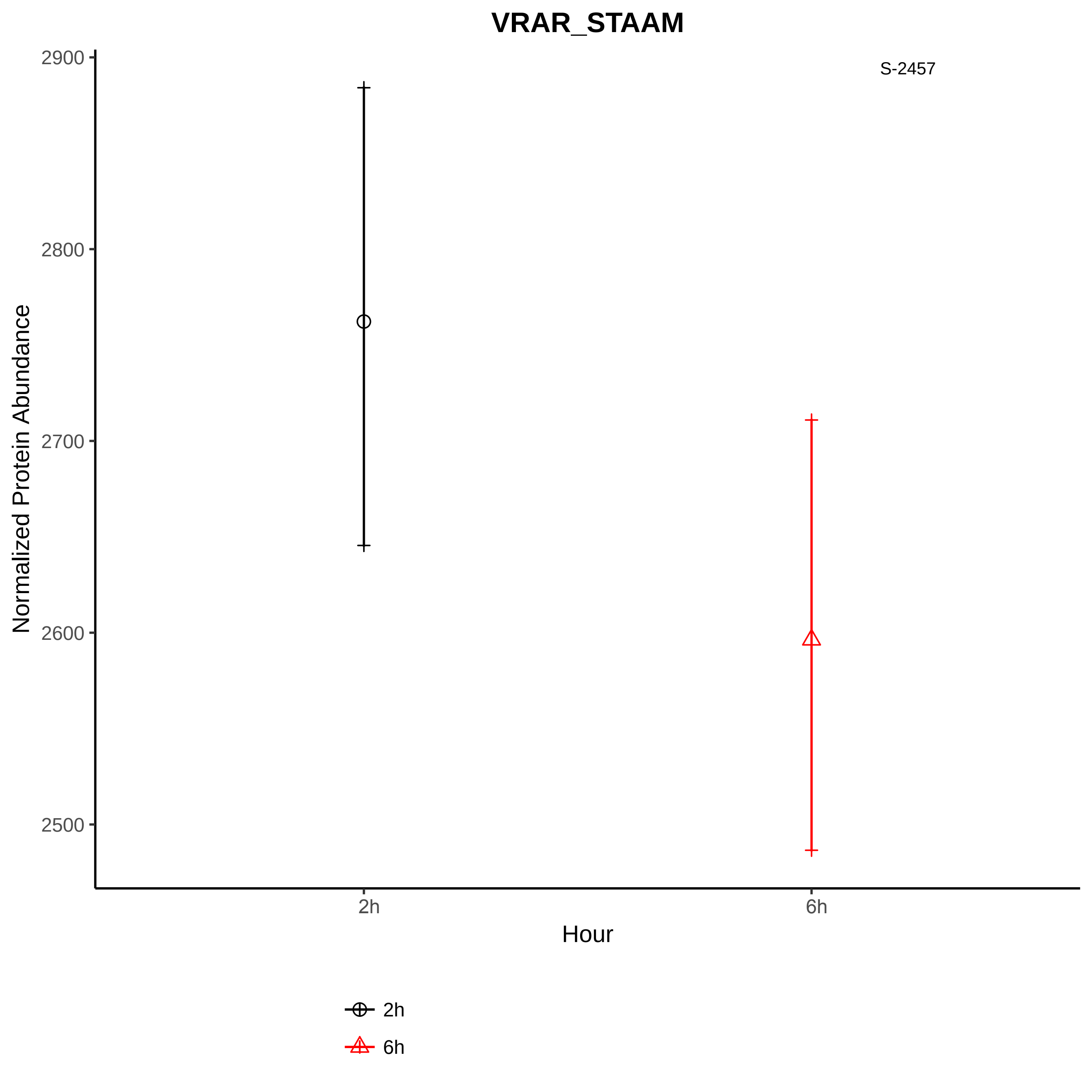

VRAR_STAAM
S-2457
Normalized Protein Abundance
2900
2800
2700
2600
2500
2h
6h
Hour
2h
6h

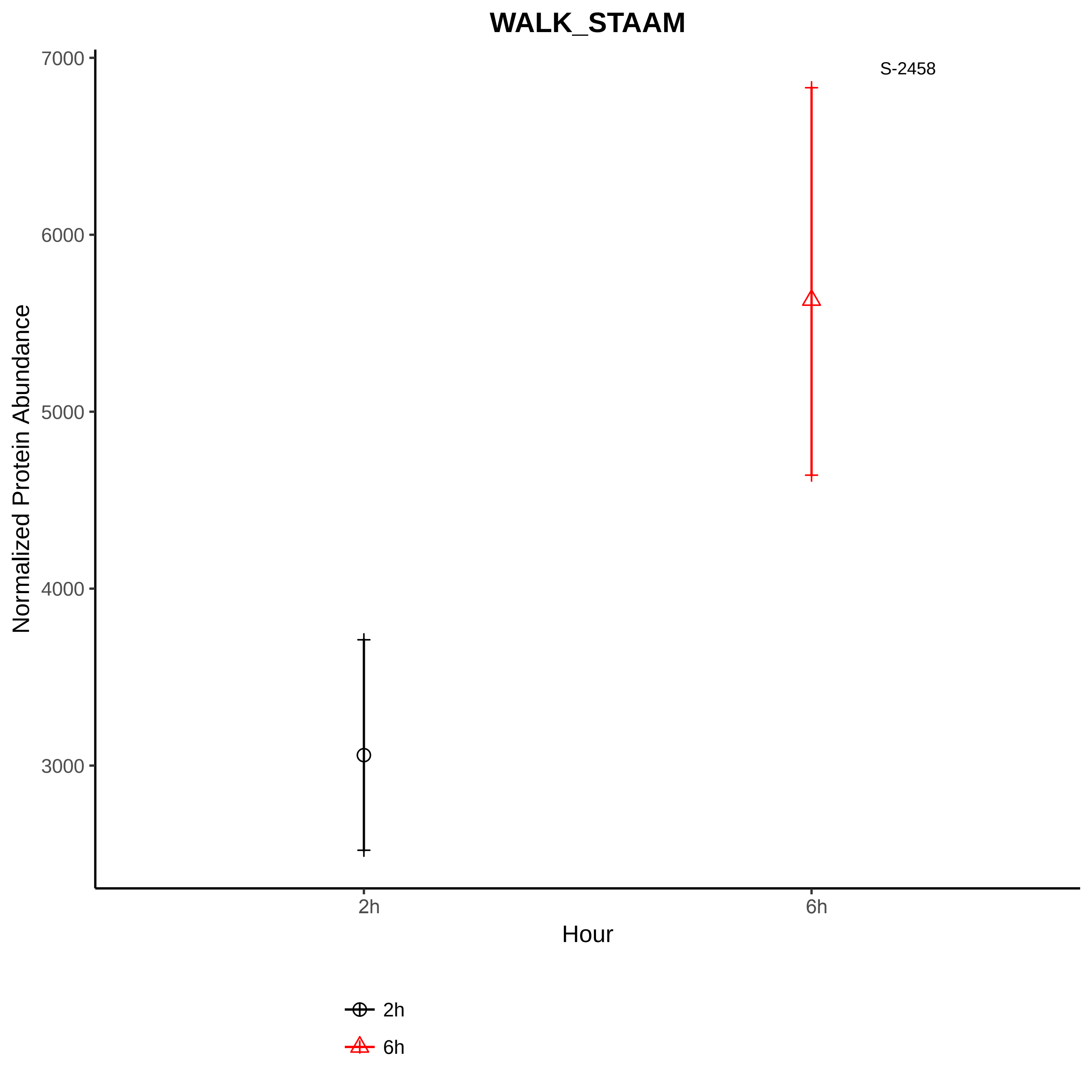

WALK_STAAM
S-2458
Normalized Protein Abundance
7000
6000
5000
4000
3000
2h
6h
Hour
2h
6h

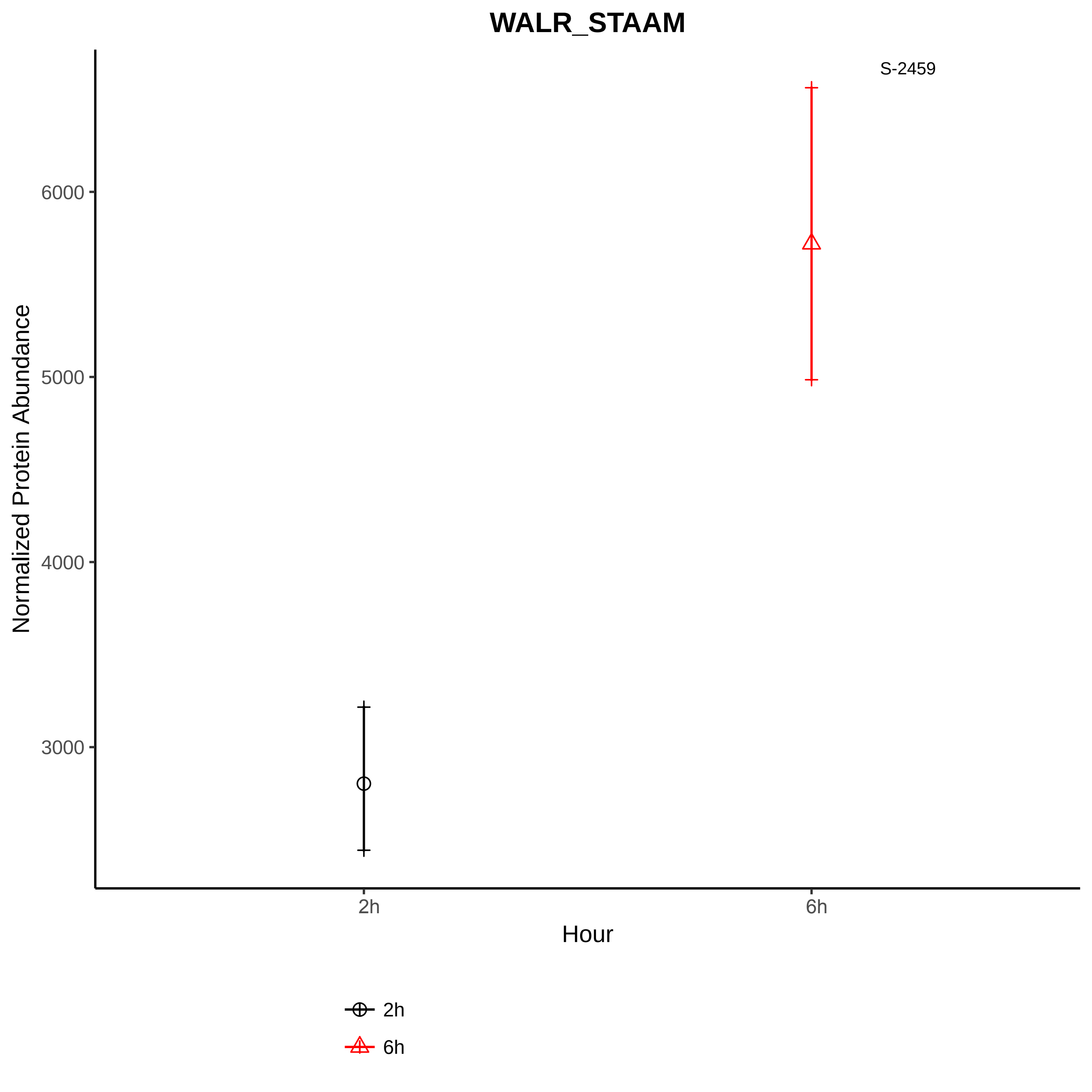

WALR_STAAM
S-2459
Normalized Protein Abundance
6000
5000
4000
3000
2h
6h
Hour
2h
6h

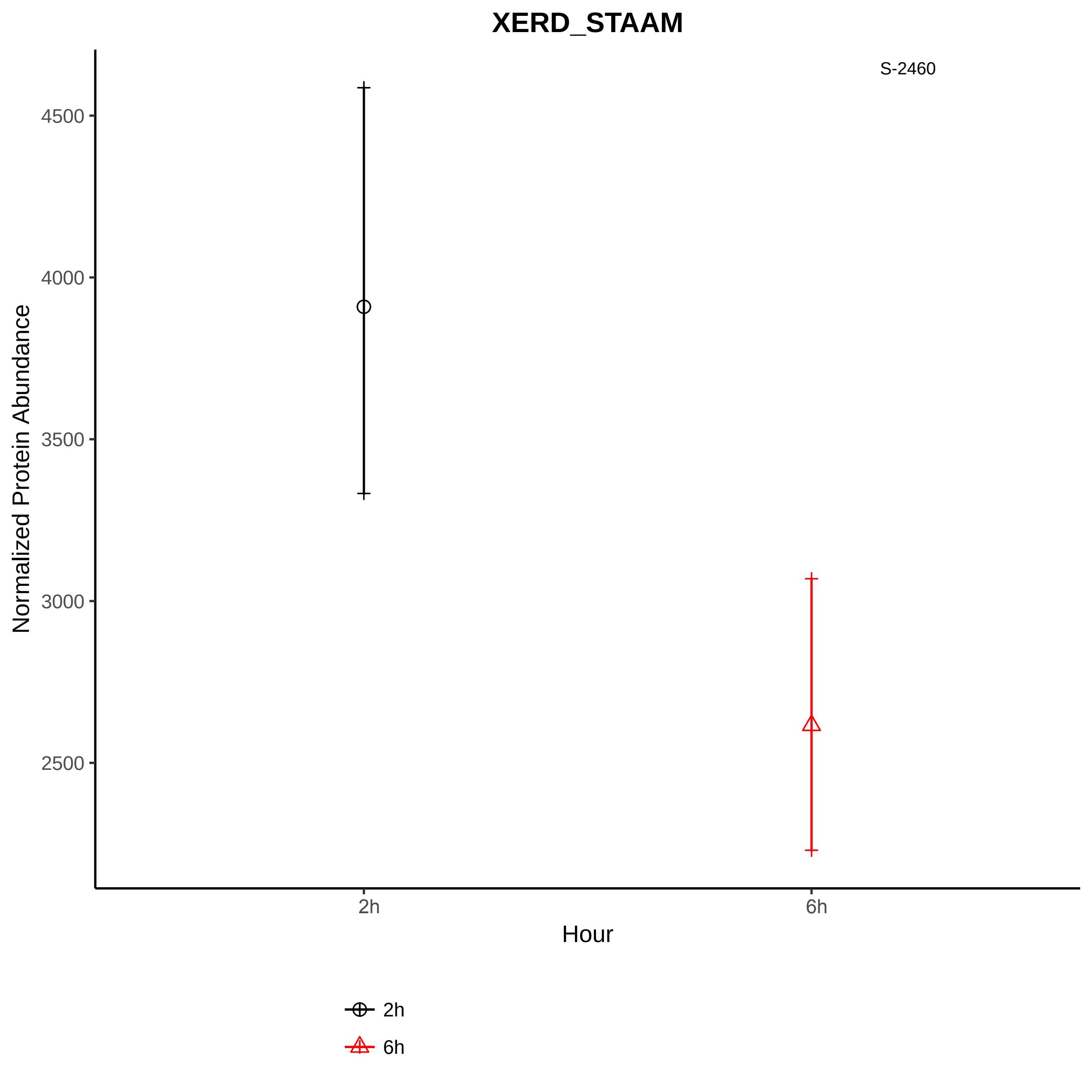

XERD_STAAM
S-2460
4500
4000
3500
3000
2500
Normalized Protein Abundance
2h
6h
Hour
2h
6h

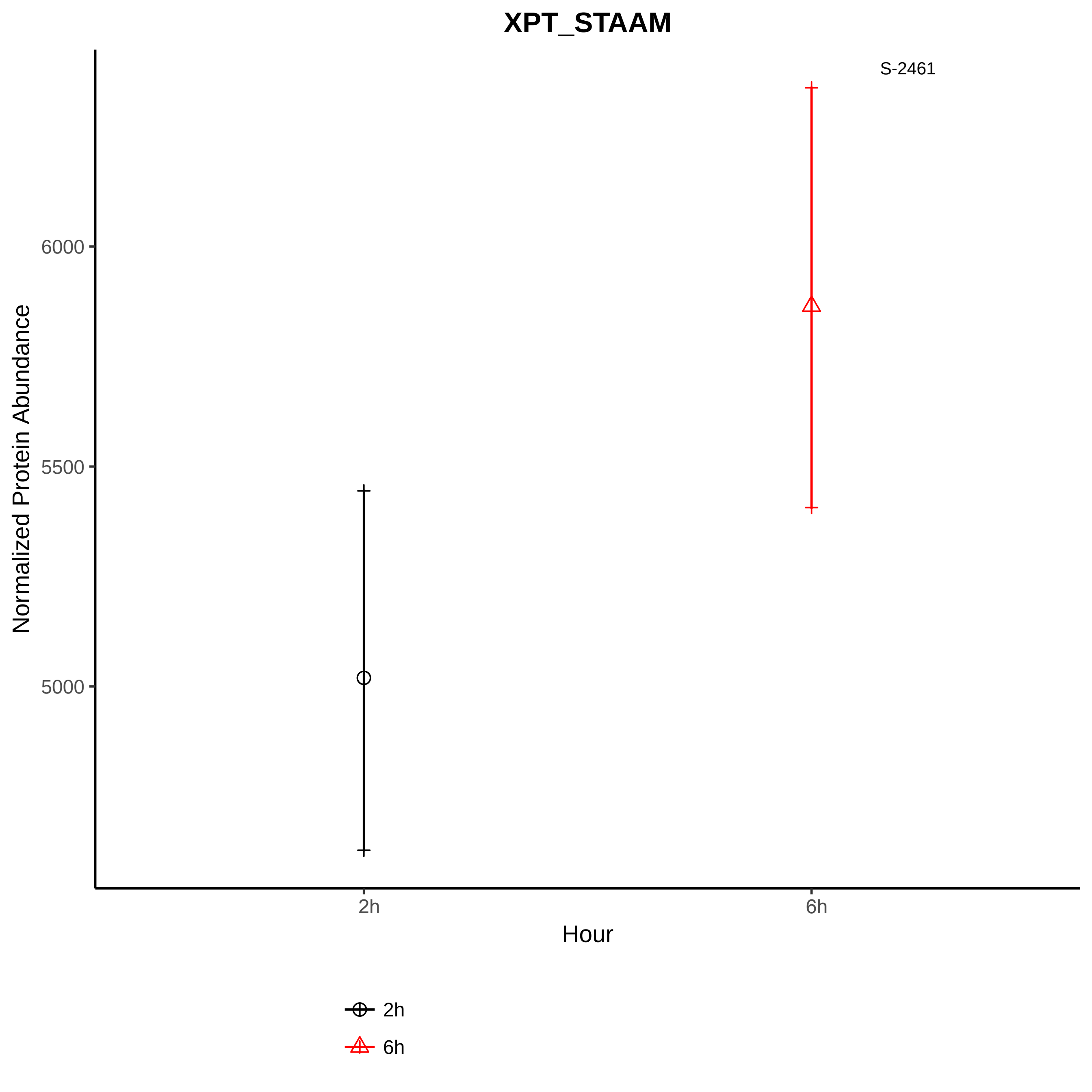

XPT_STAAM
S-2461
6000
5500
5000
Normalized Protein Abundance
2h
6h
Hour
2h
6h

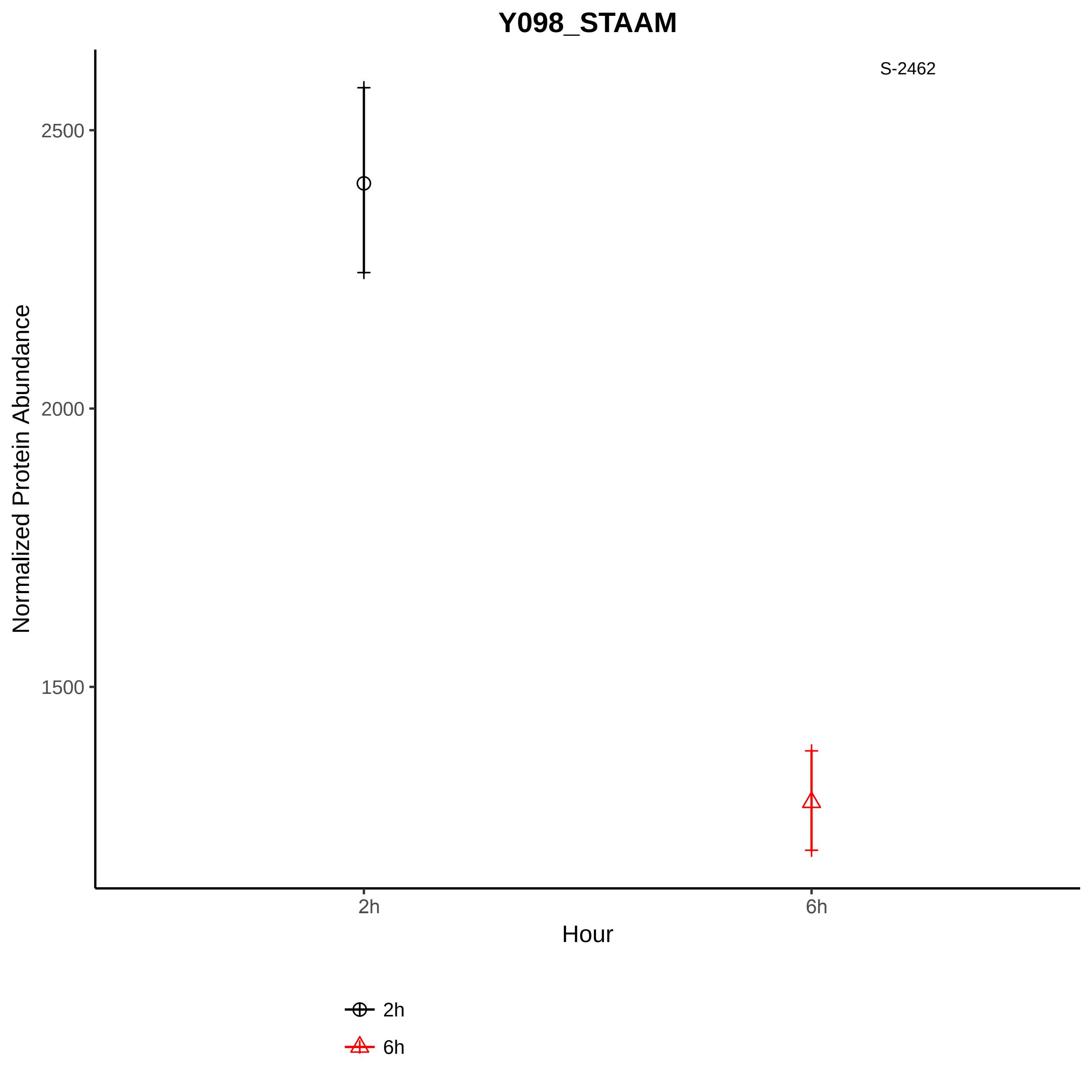

Y098_STAAM
S-2462
2500
2000
1500
Normalized Protein Abundance
2h
6h
Hour
2h
6h

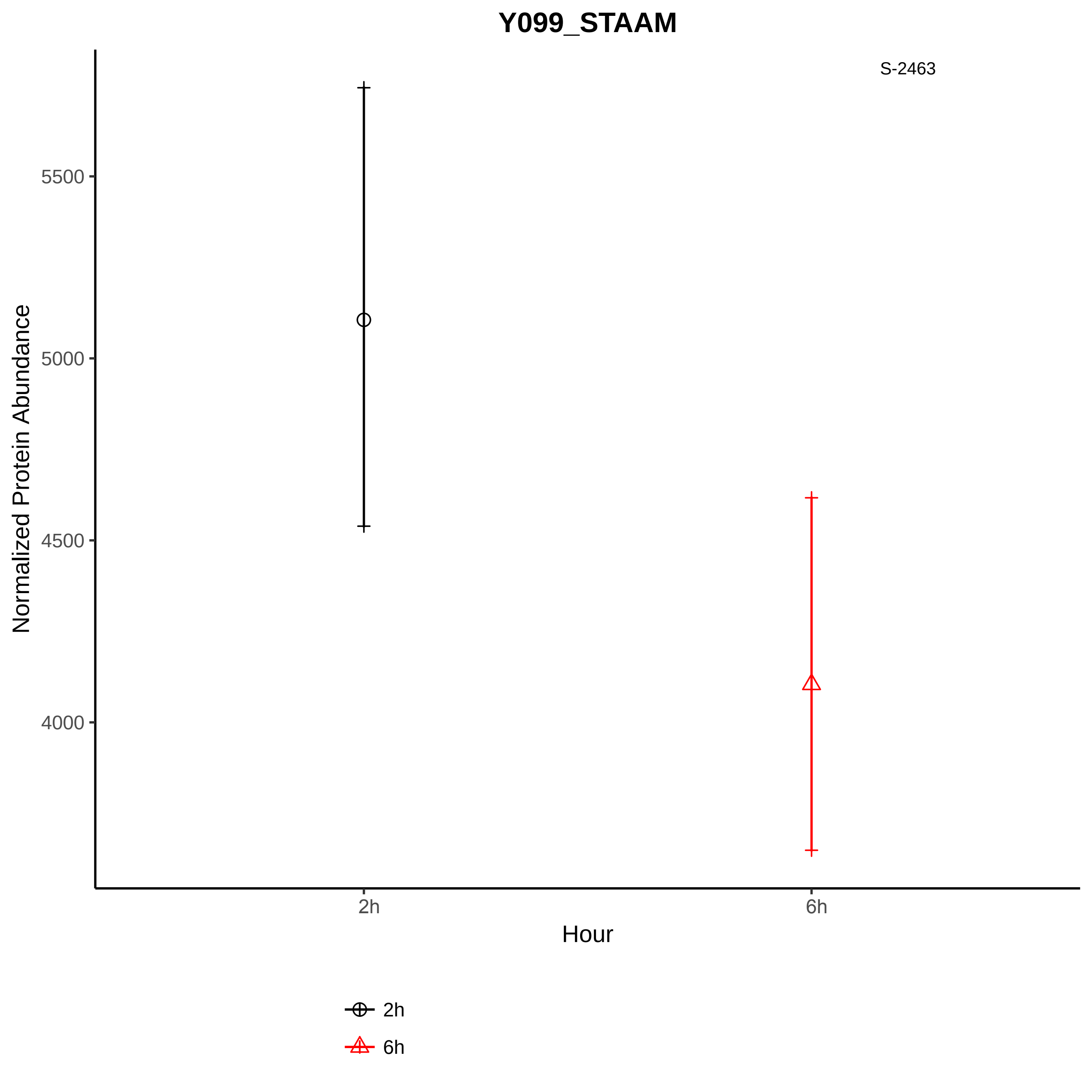

Y099_STAAM
S-2463
Normalized Protein Abundance
5500
5000
4500
4000
2h
6h
Hour
2h
6h

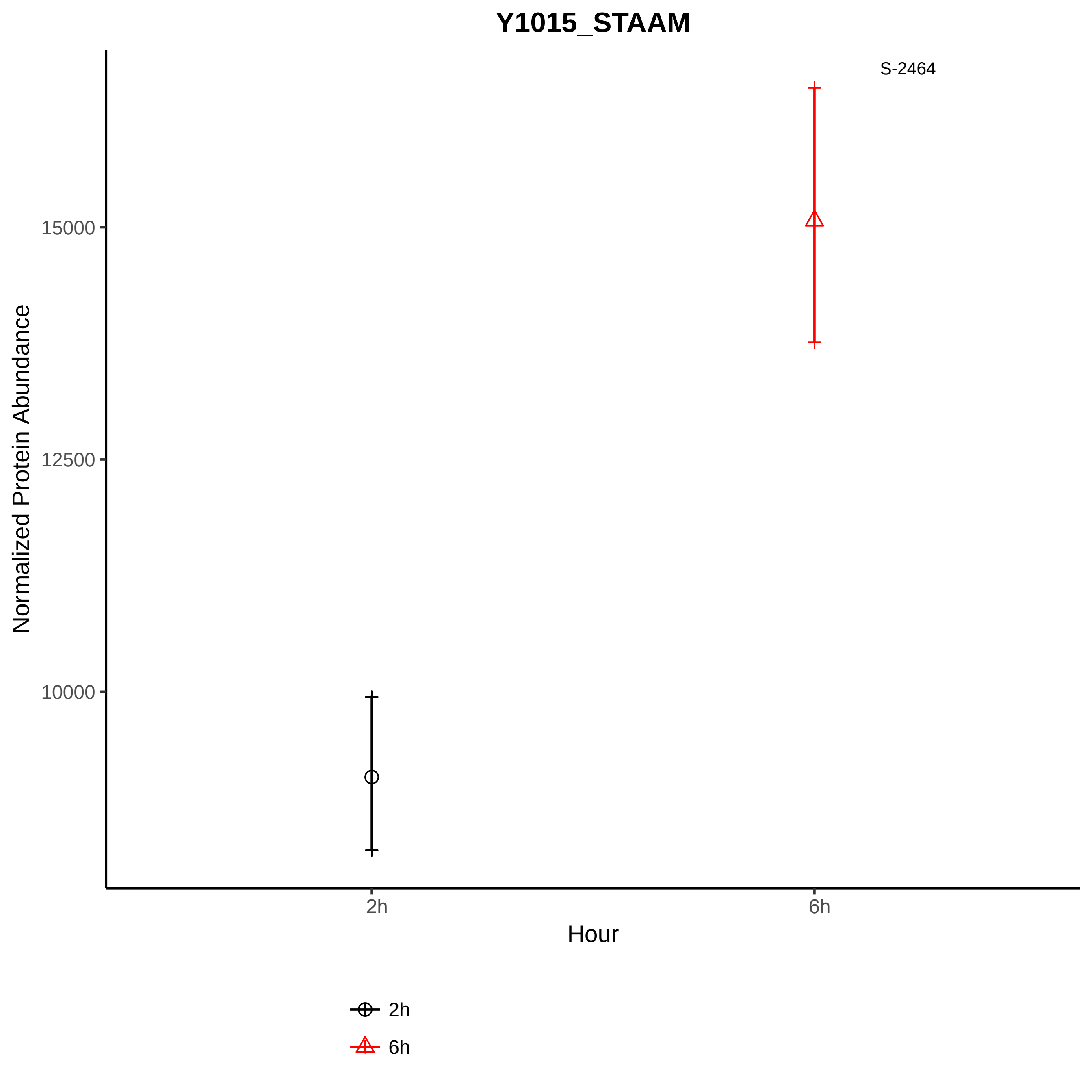

Y1015_STAAM
S-2464
Normalized Protein Abundance
15000
12500
10000
2h
6h
Hour
2h
6h

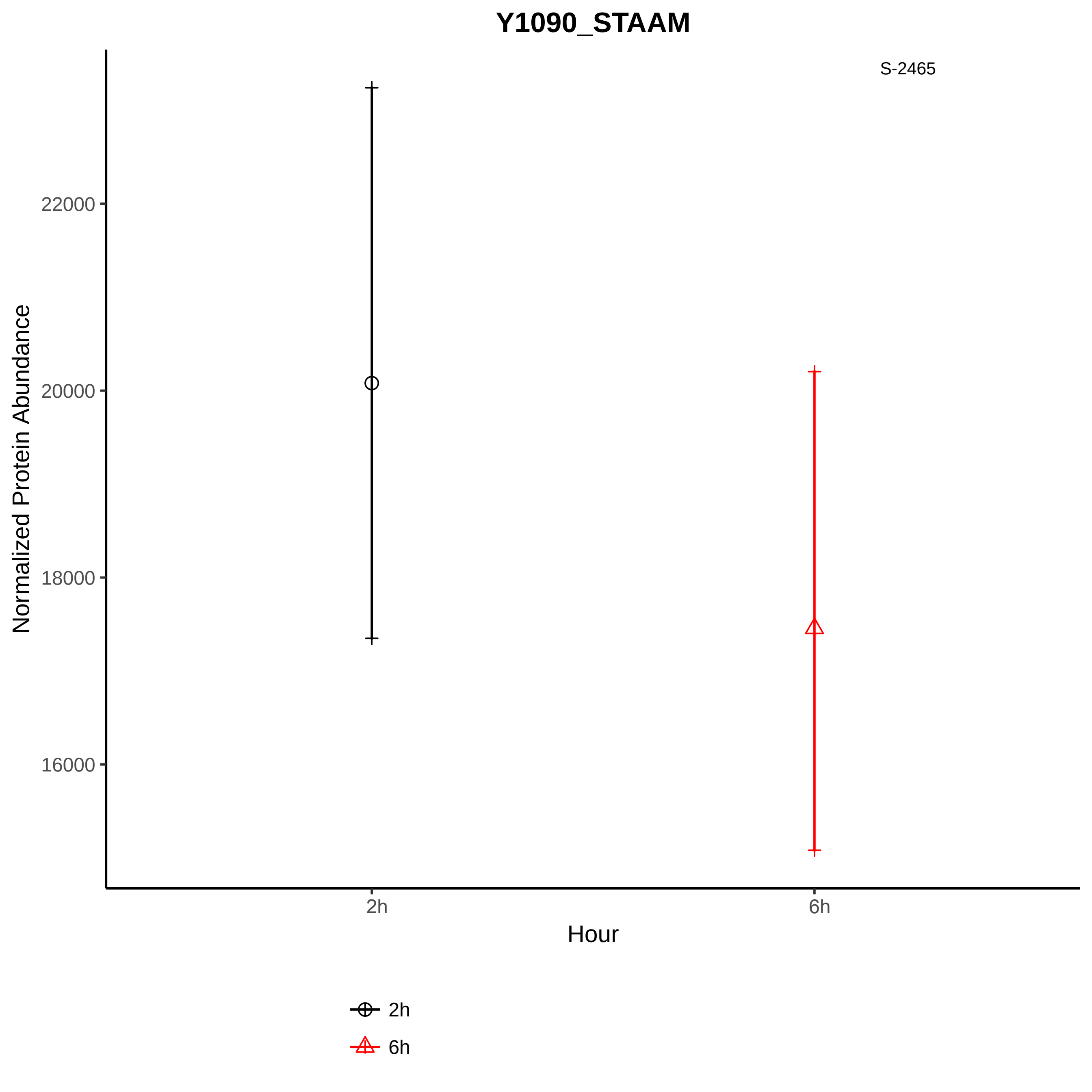

Y1090_STAAM
S-2465
22000
20000
18000
16000
Normalized Protein Abundance
2h
6h
Hour
2h
6h

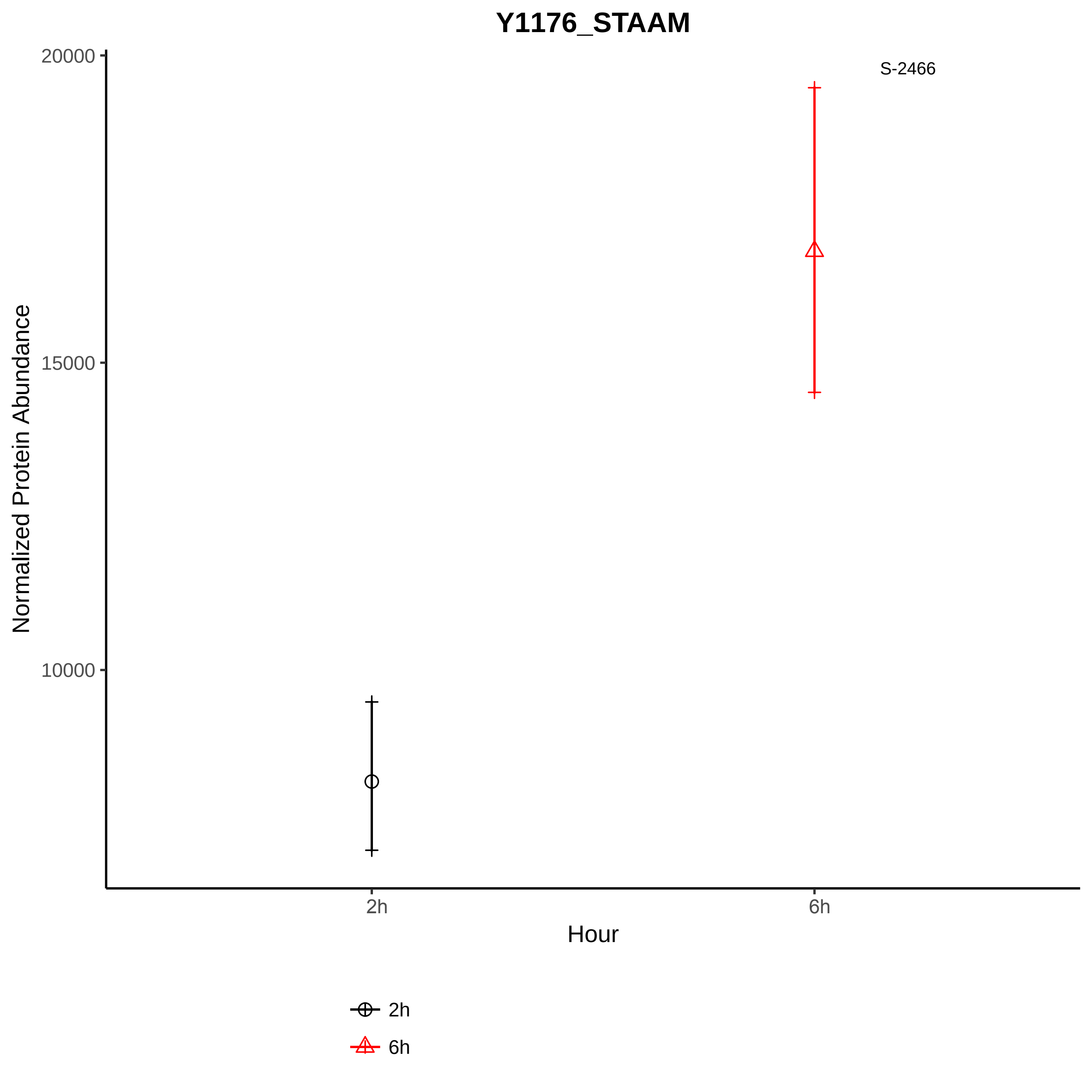
Y1176_STAAM
S-2466
20000
15000
10000
Normalized Protein Abundance
2h
6h
Hour
2h
6h

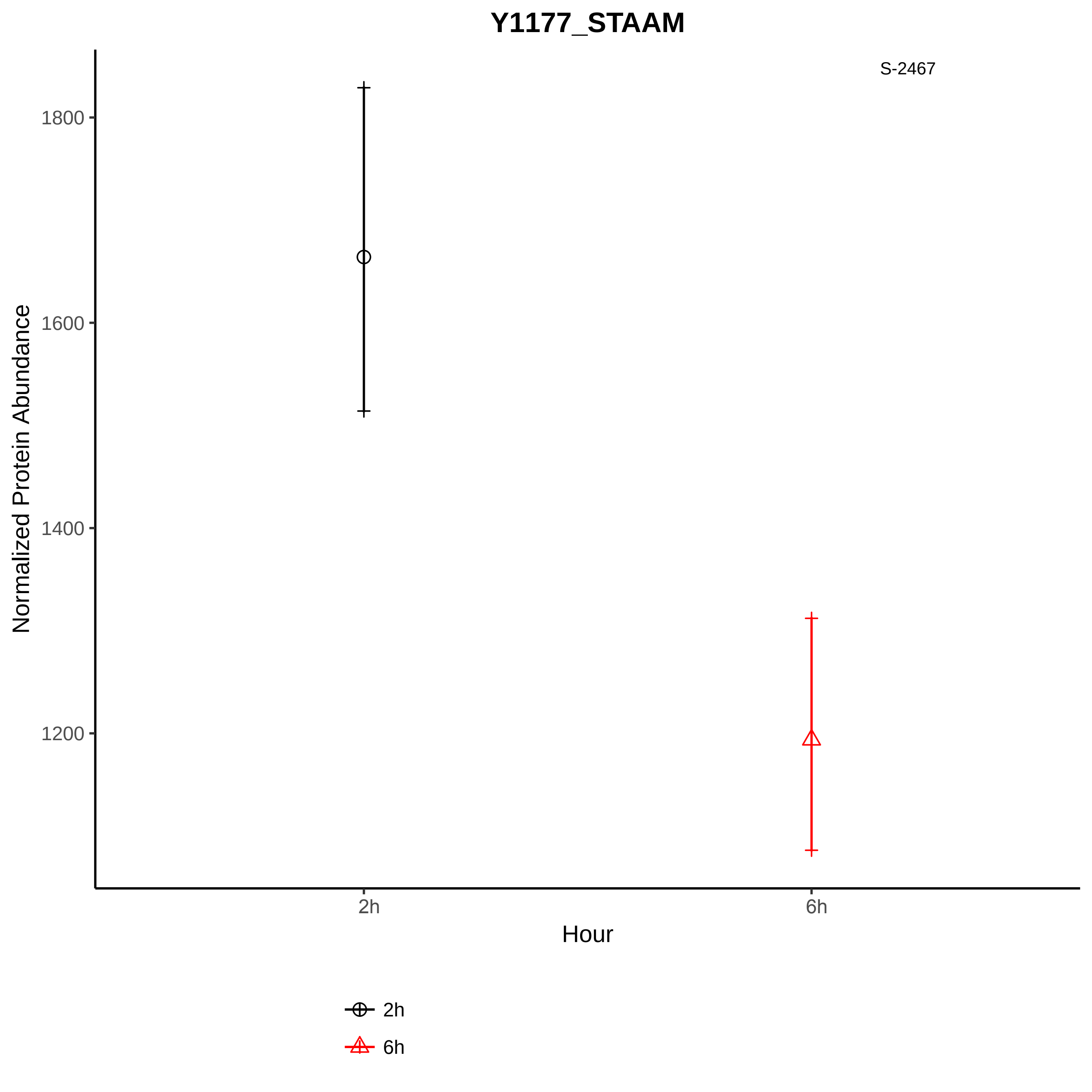

Y1177_STAAM
S-2467
Normalized Protein Abundance
1800
1600
1400
1200
2h
6h
Hour
2h
6h

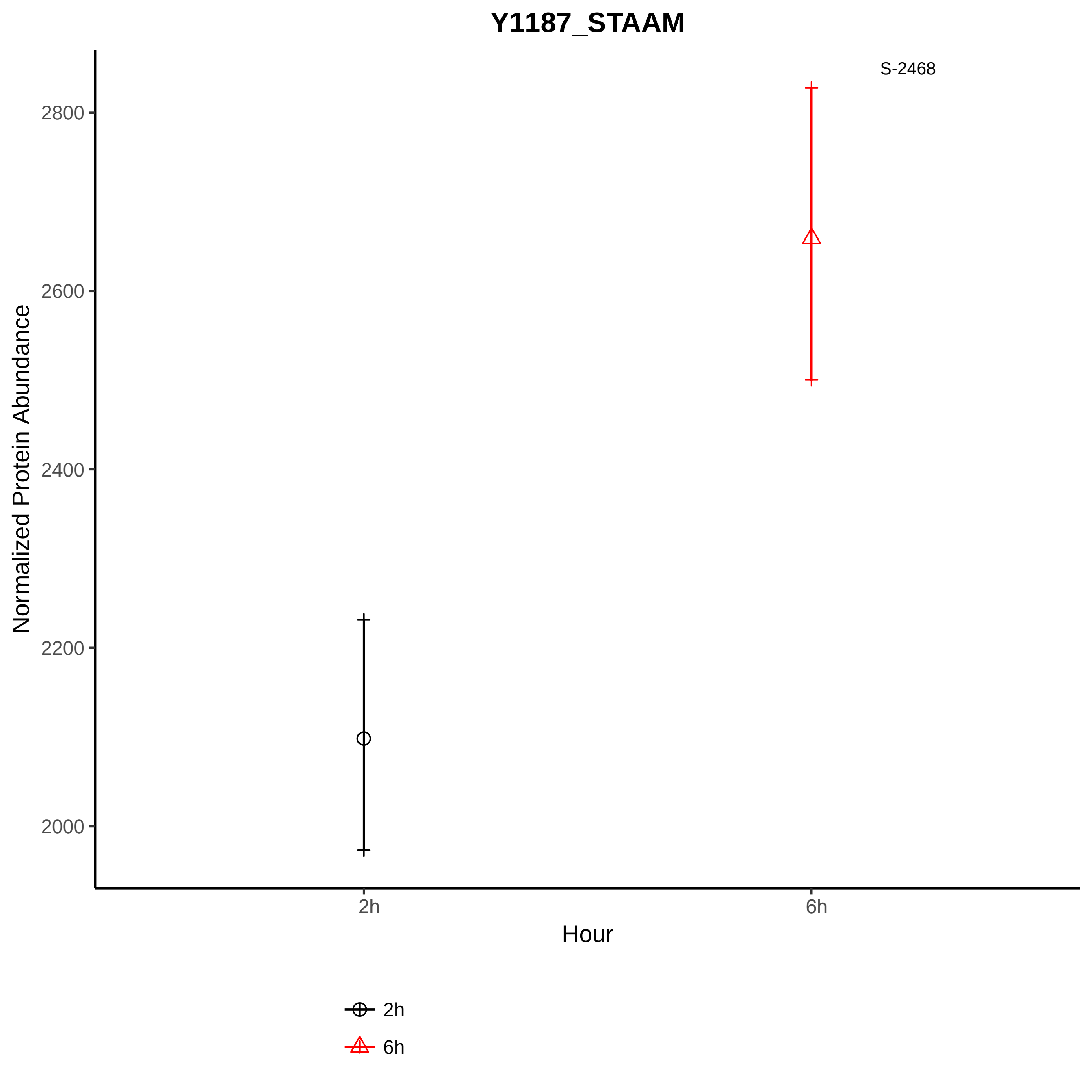

Y1187_STAAM
S-2468
Normalized Protein Abundance
2800
2600
2400
2200
2000
2h
6h
Hour
2h
6h

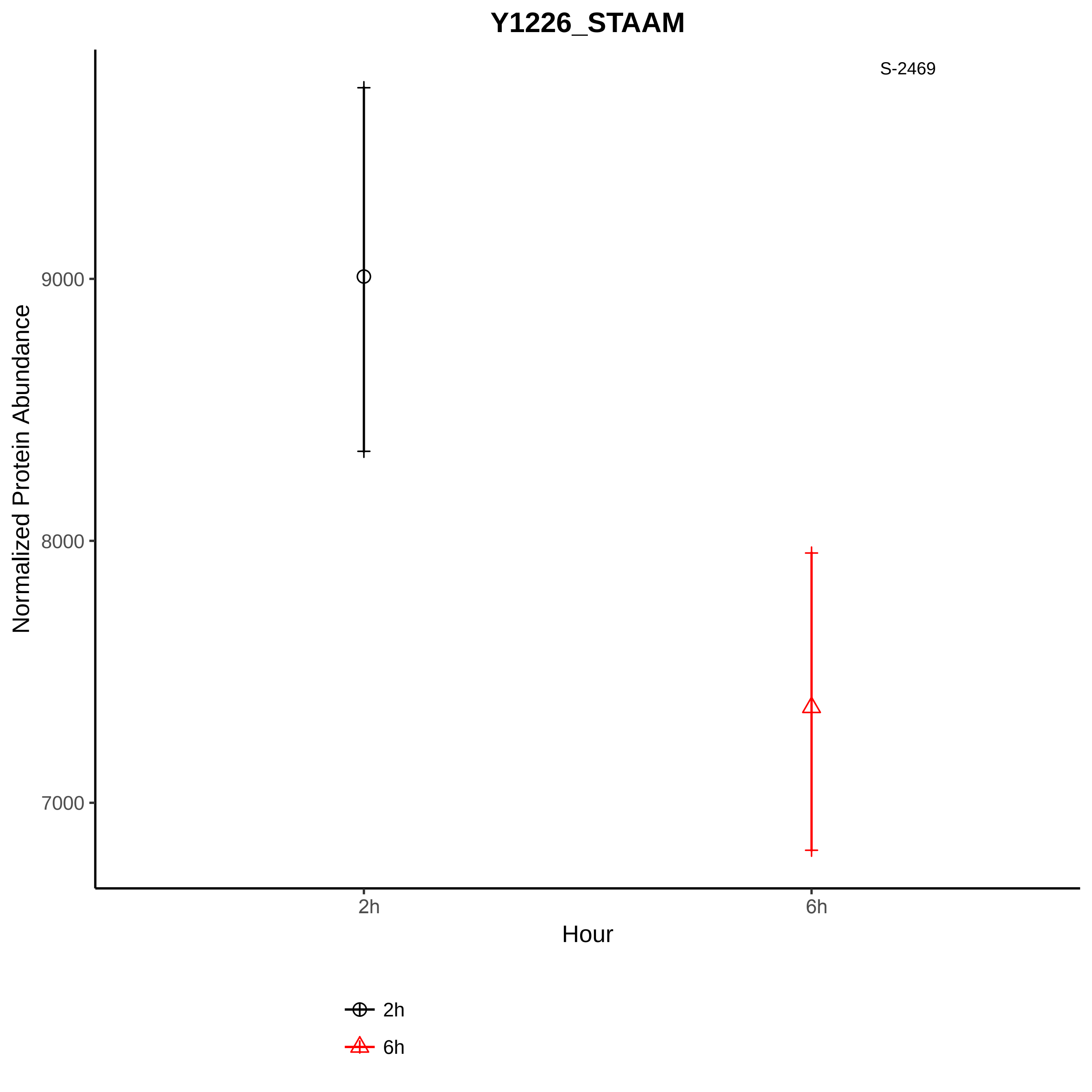

Y1226_STAAM
S-2469
Normalized Protein Abundance
9000
8000
7000
2h
6h
Hour
2h
6h

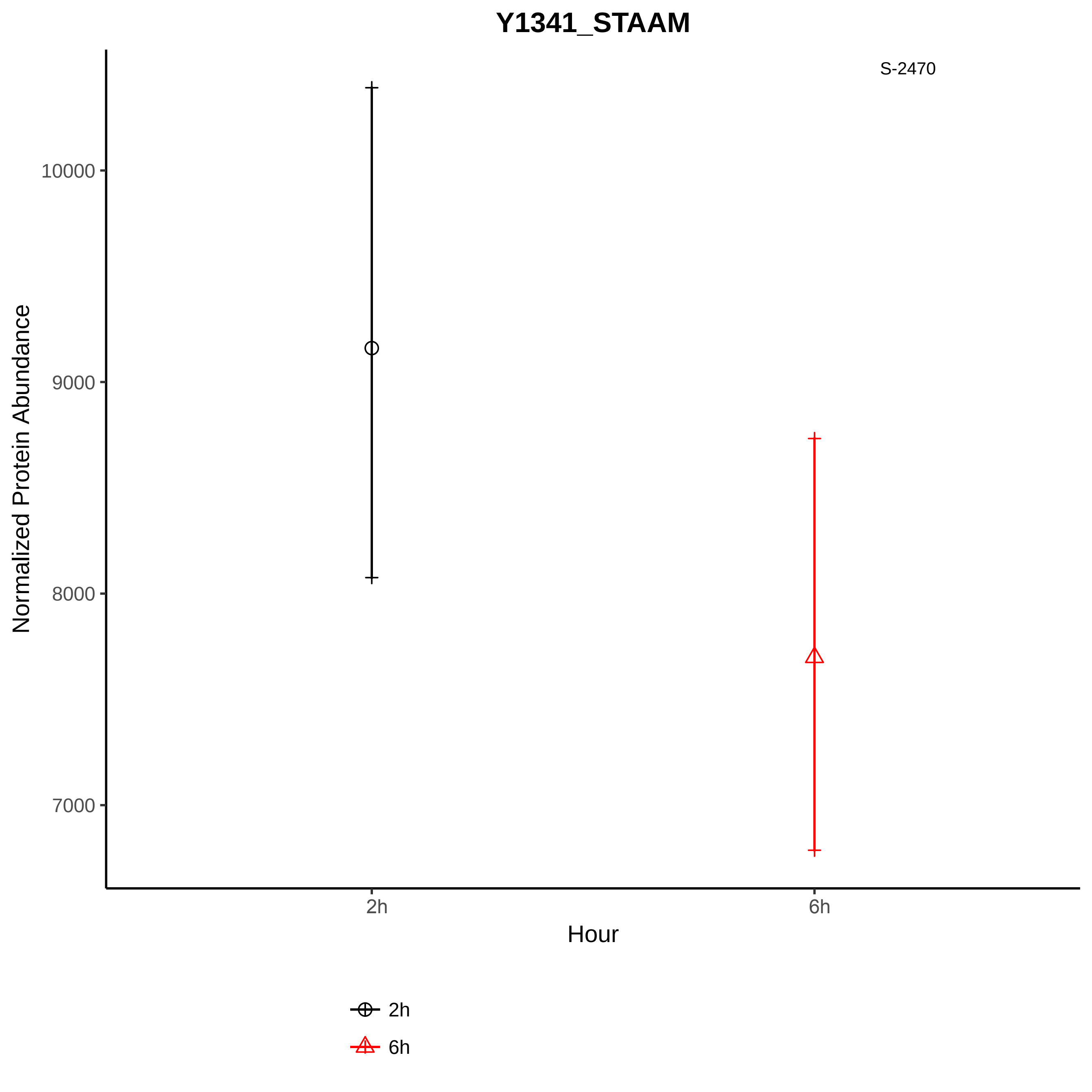

Y1341_STAAM
S-2470
Normalized Protein Abundance
10000
9000
8000
7000
2h
6h
Hour
2h
6h

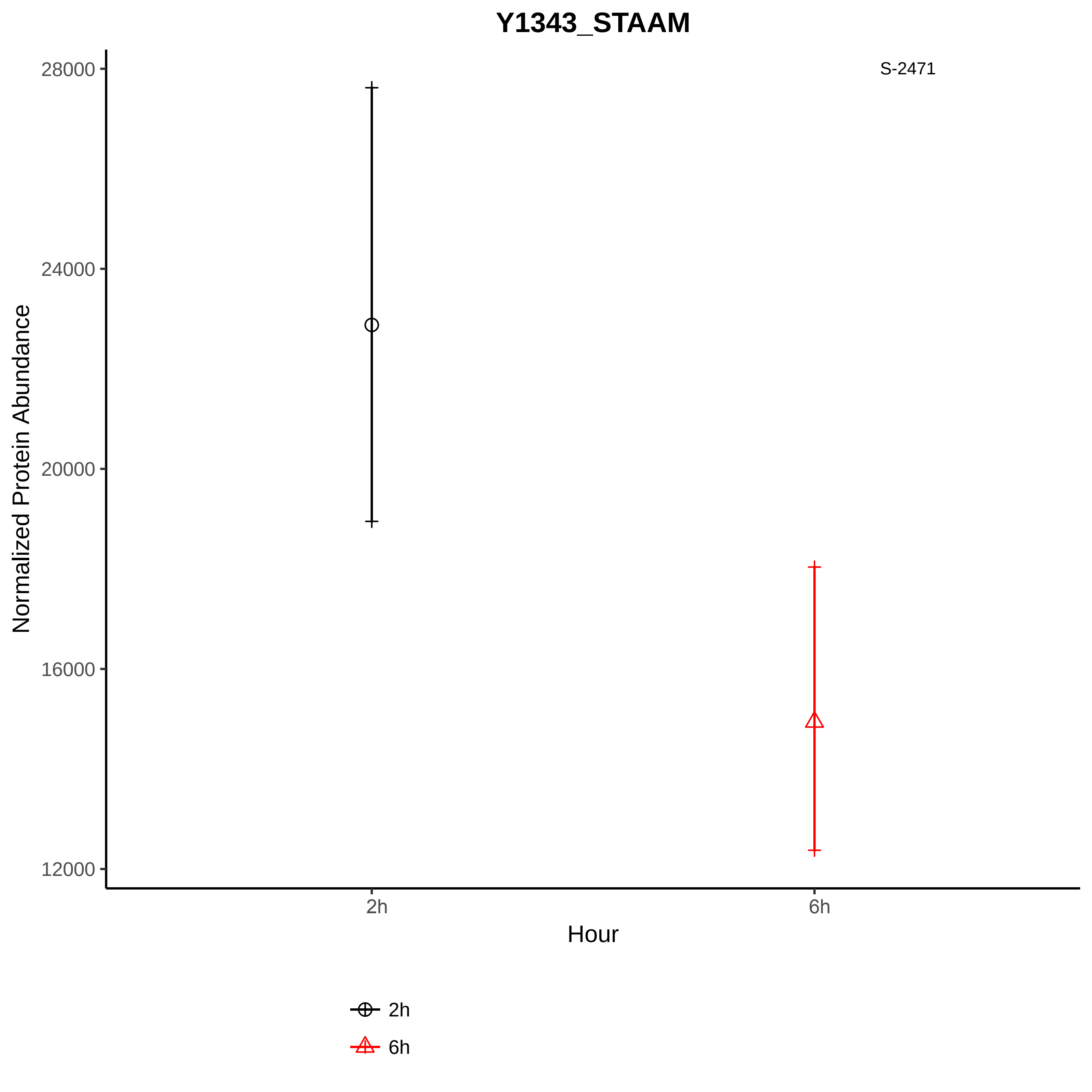

Y1343_STAAM
S-2471
Normalized Protein Abundance
28000
24000
20000
16000
12000
2h
6h
Hour
2h
6h

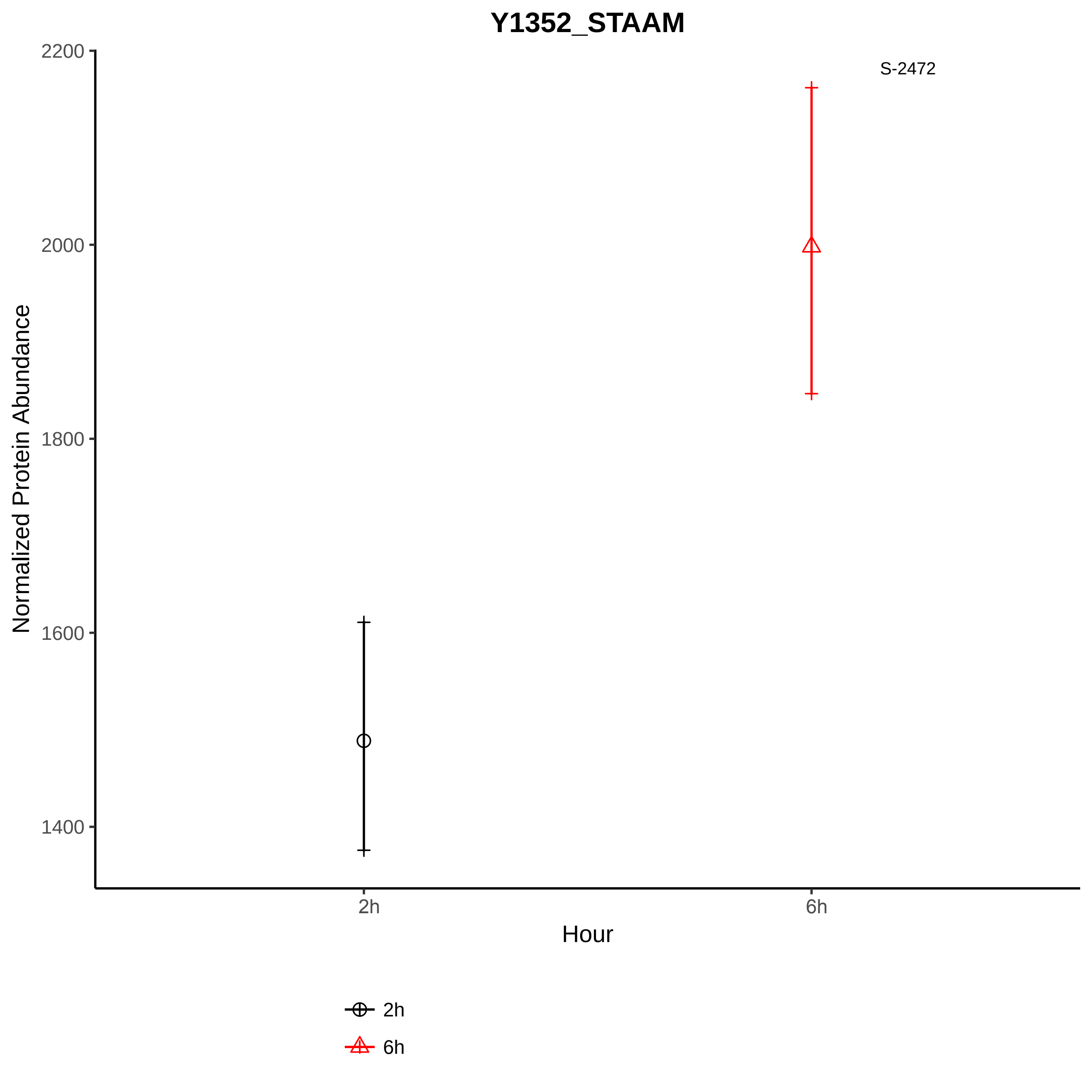

Y1352_STAAM
S-2472
2200
2000
1800
1600
1400
Normalized Protein Abundance
2h
6h
Hour
2h
6h

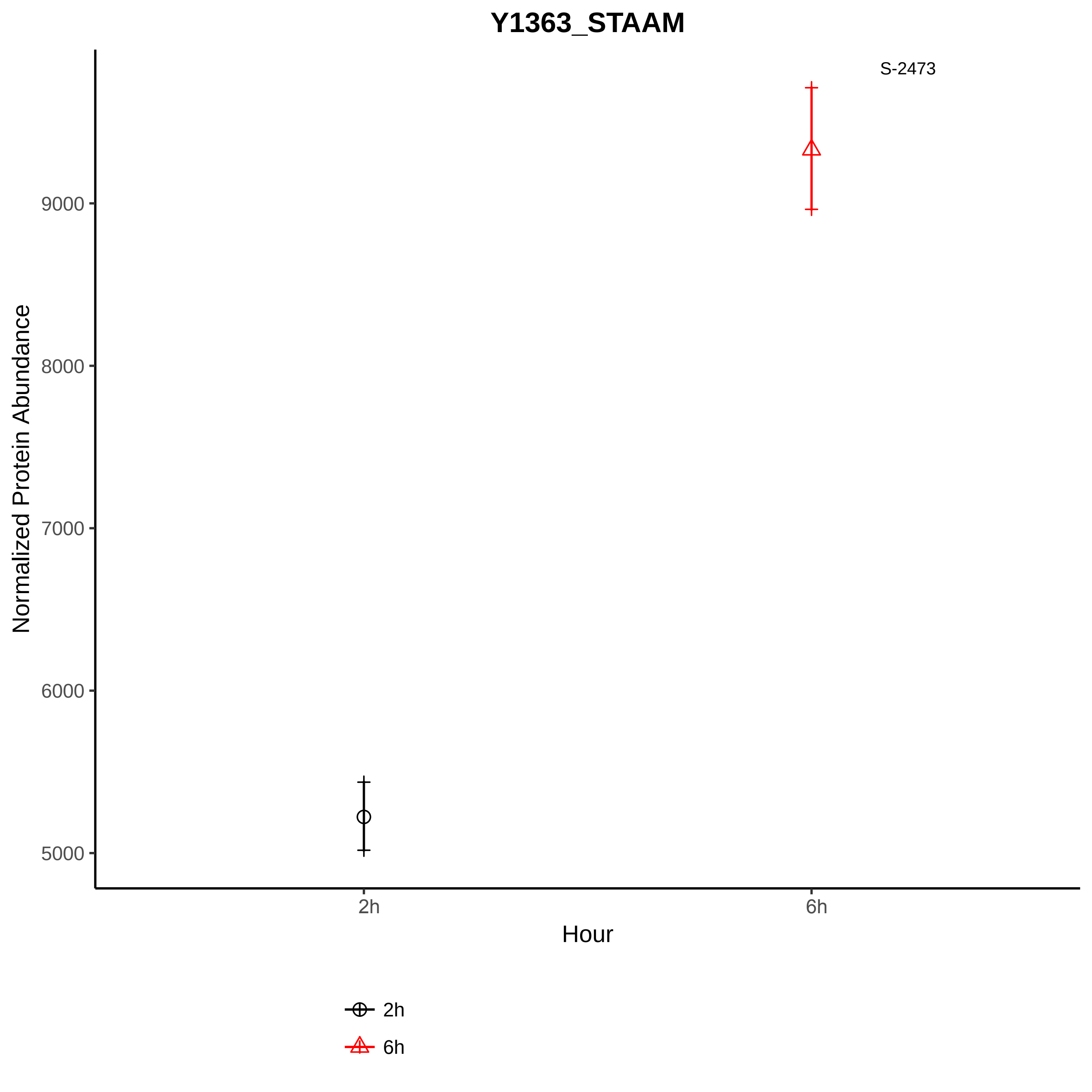

Y1363_STAAM
S-2473
9000
8000
7000
6000
5000
Normalized Protein Abundance
2h
6h
Hour
2h
6h

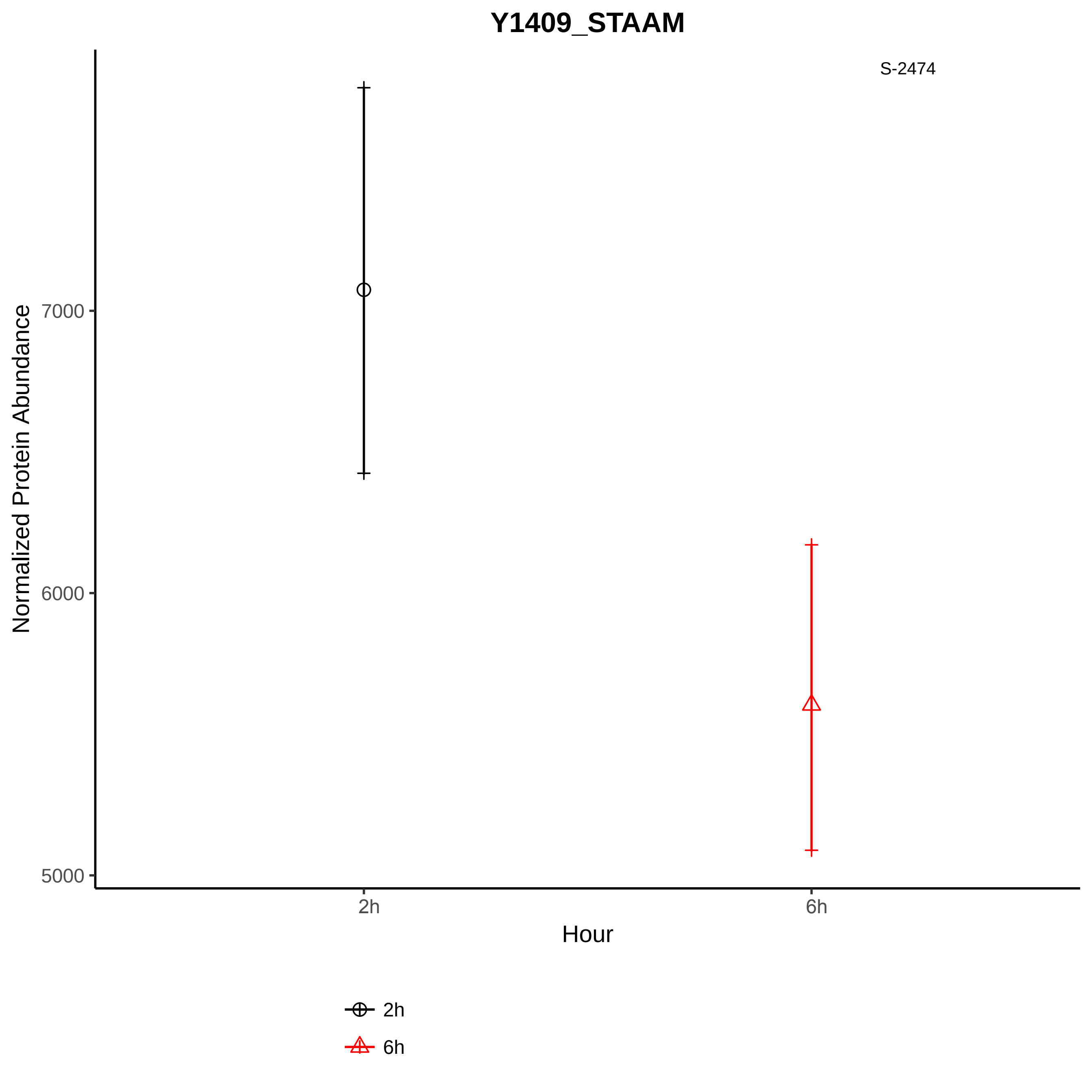

Y1409_STAAM
S-2474
Normalized Protein Abundance
7000
6000
5000
2h
6h
Hour
2h
6h

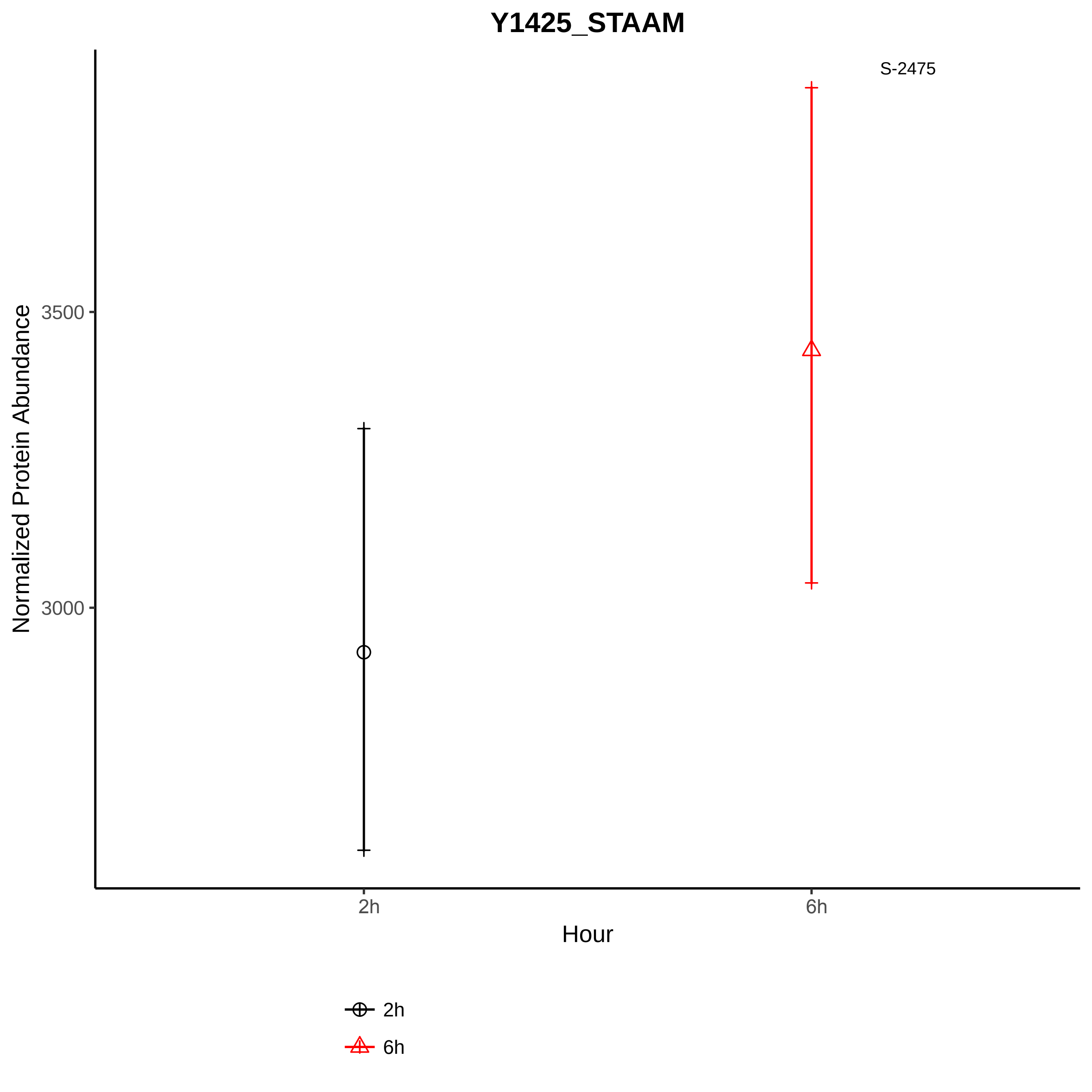

Y1425_STAAM
S-2475
Normalized Protein Abundance
3500
3000
2h
6h
Hour
2h
6h

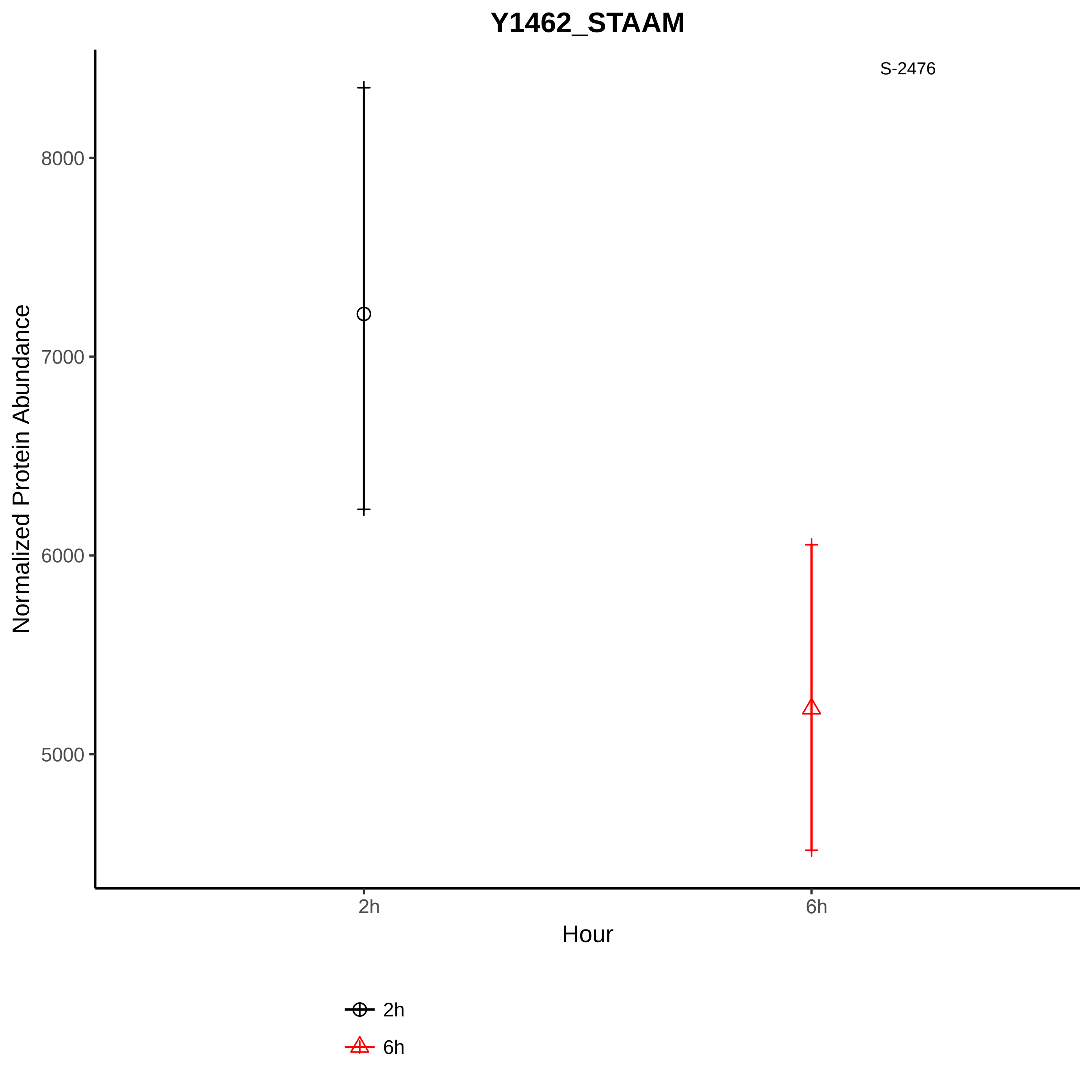

Y1462_STAAM
S-2476
8000
7000
6000
5000
Normalized Protein Abundance
2h
6h
Hour
2h
6h

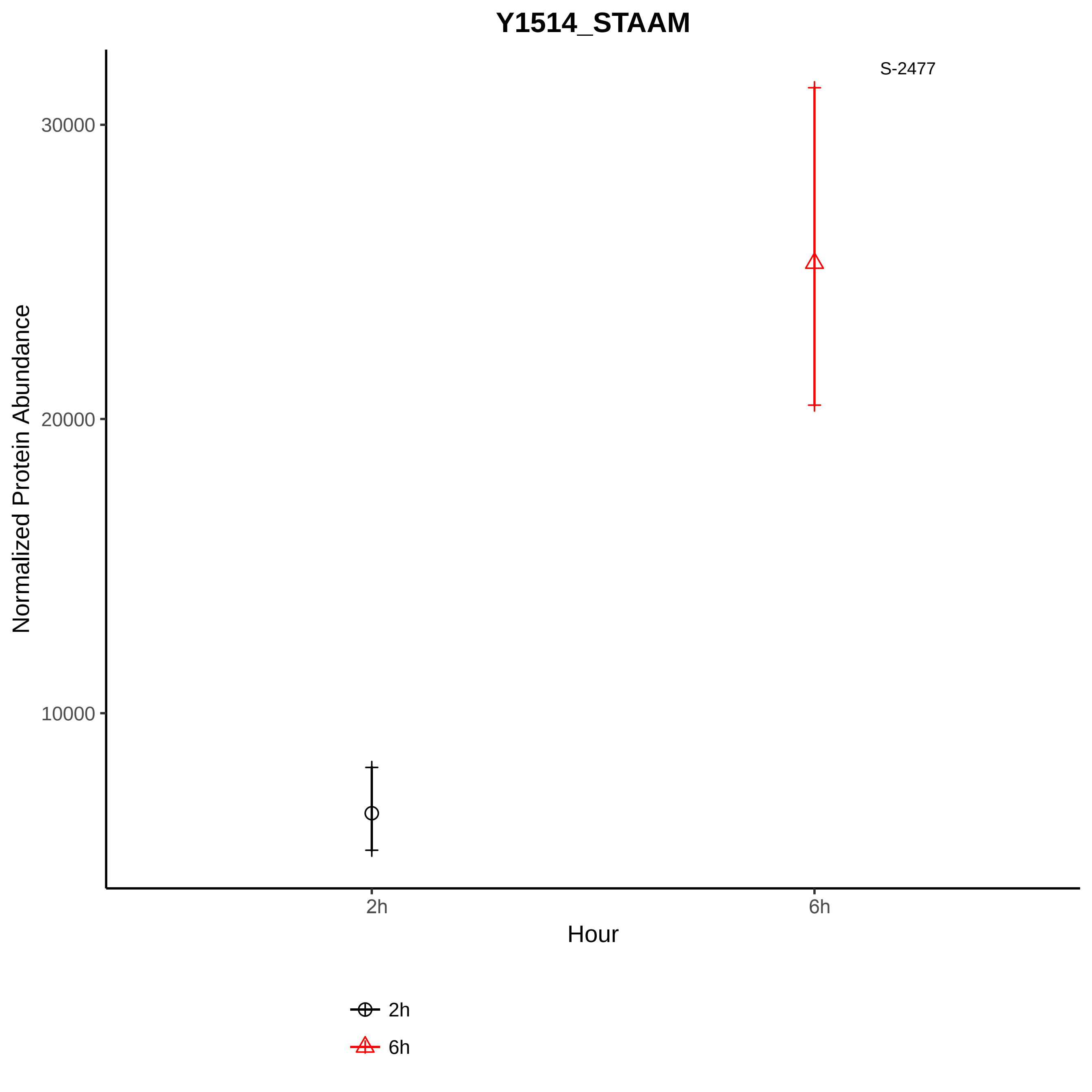

Y1514_STAAM
S-2477
Normalized Protein Abundance
30000
20000
10000
2h
6h
Hour
2h
6h

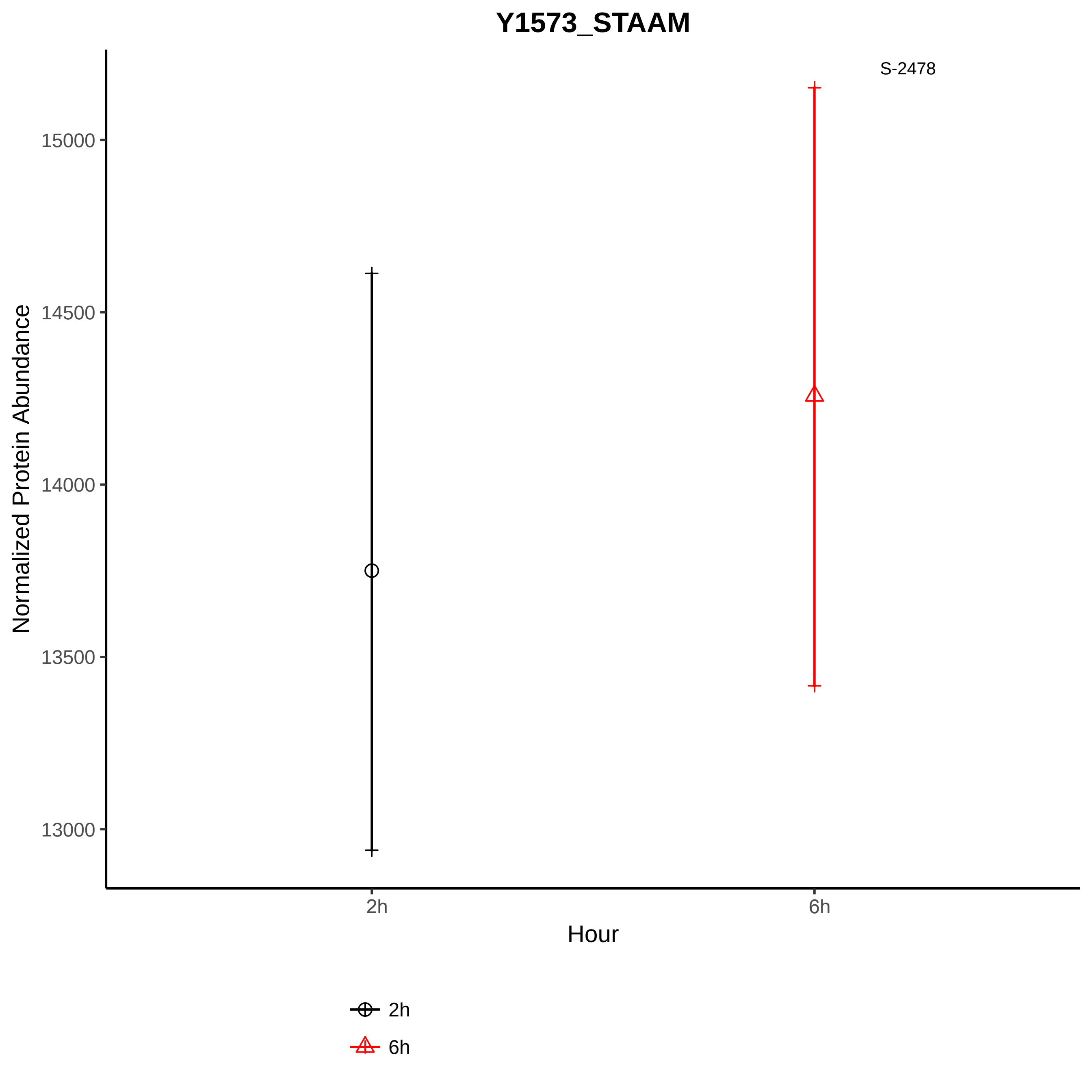
Y1573_STAAM
S-2478
Normalized Protein Abundance
15000
14500
14000
13500
13000
2h
6h
Hour
2h
6h

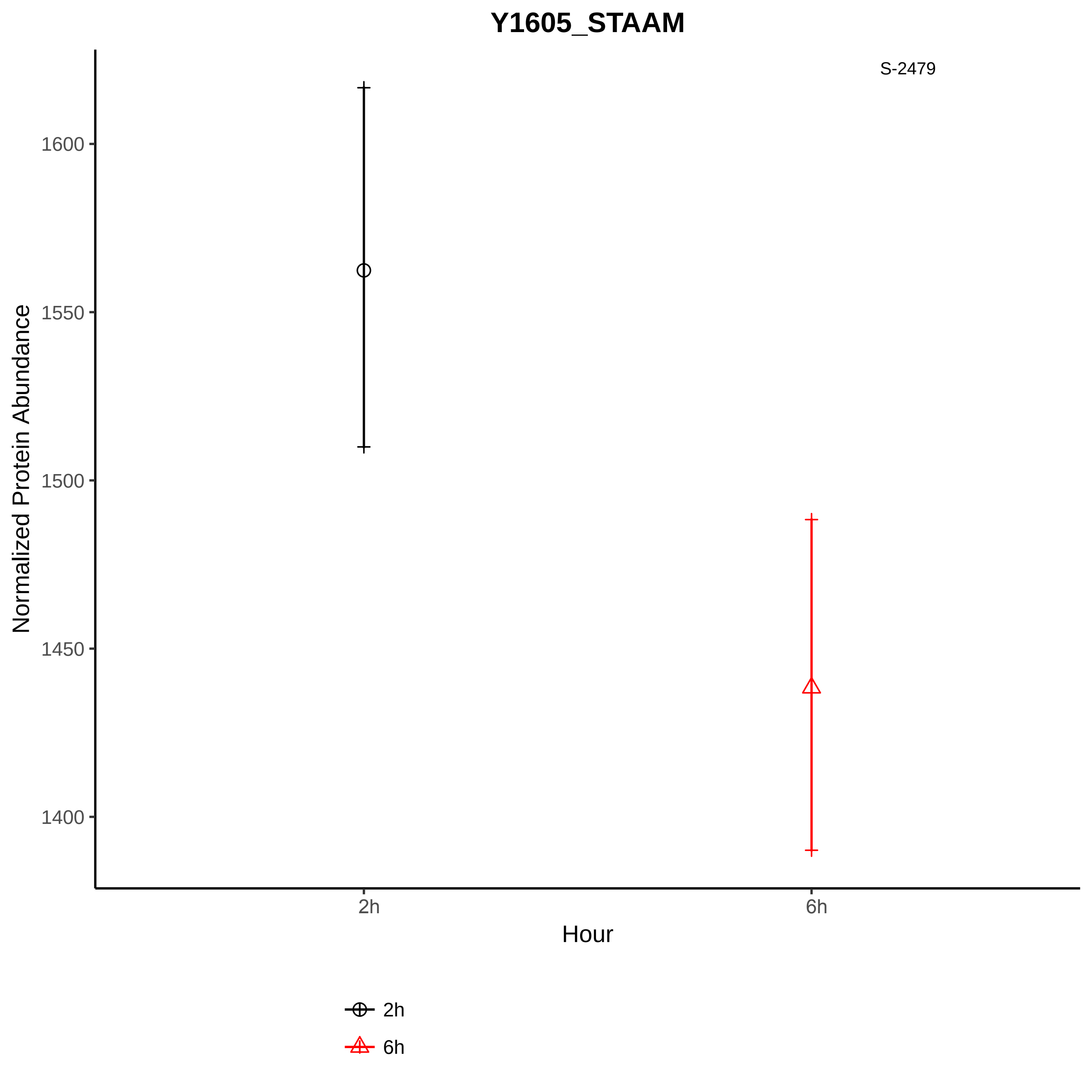

Y1605_STAAM
S-2479
Normalized Protein Abundance
1600
1550
1500
1450
1400
2h
6h
Hour
2h
6h

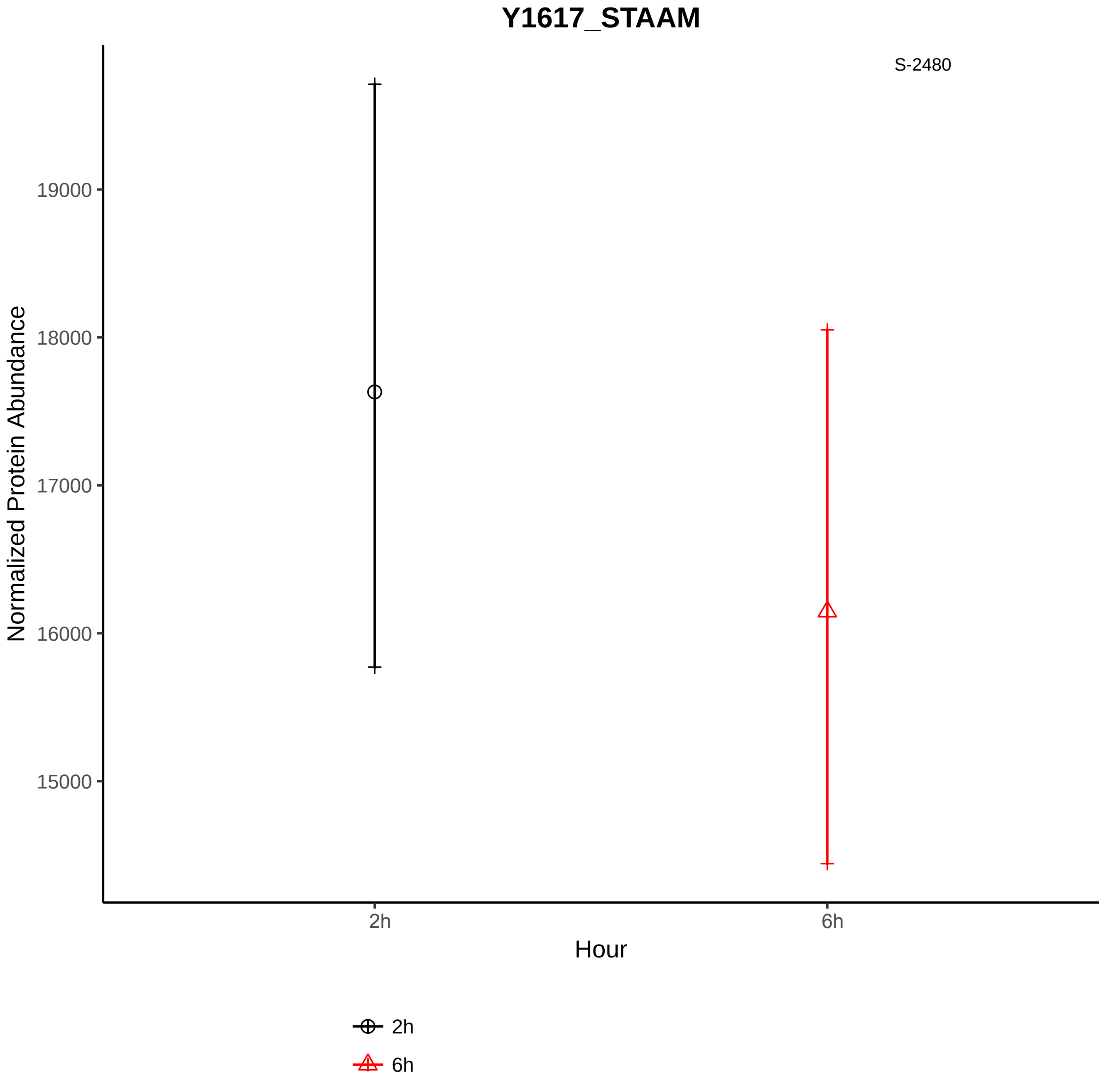
Y1617_STAAM
S-2480
Normalized Protein Abundance
19000
18000
17000
16000
15000
2h
6h
Hour
2h
6h

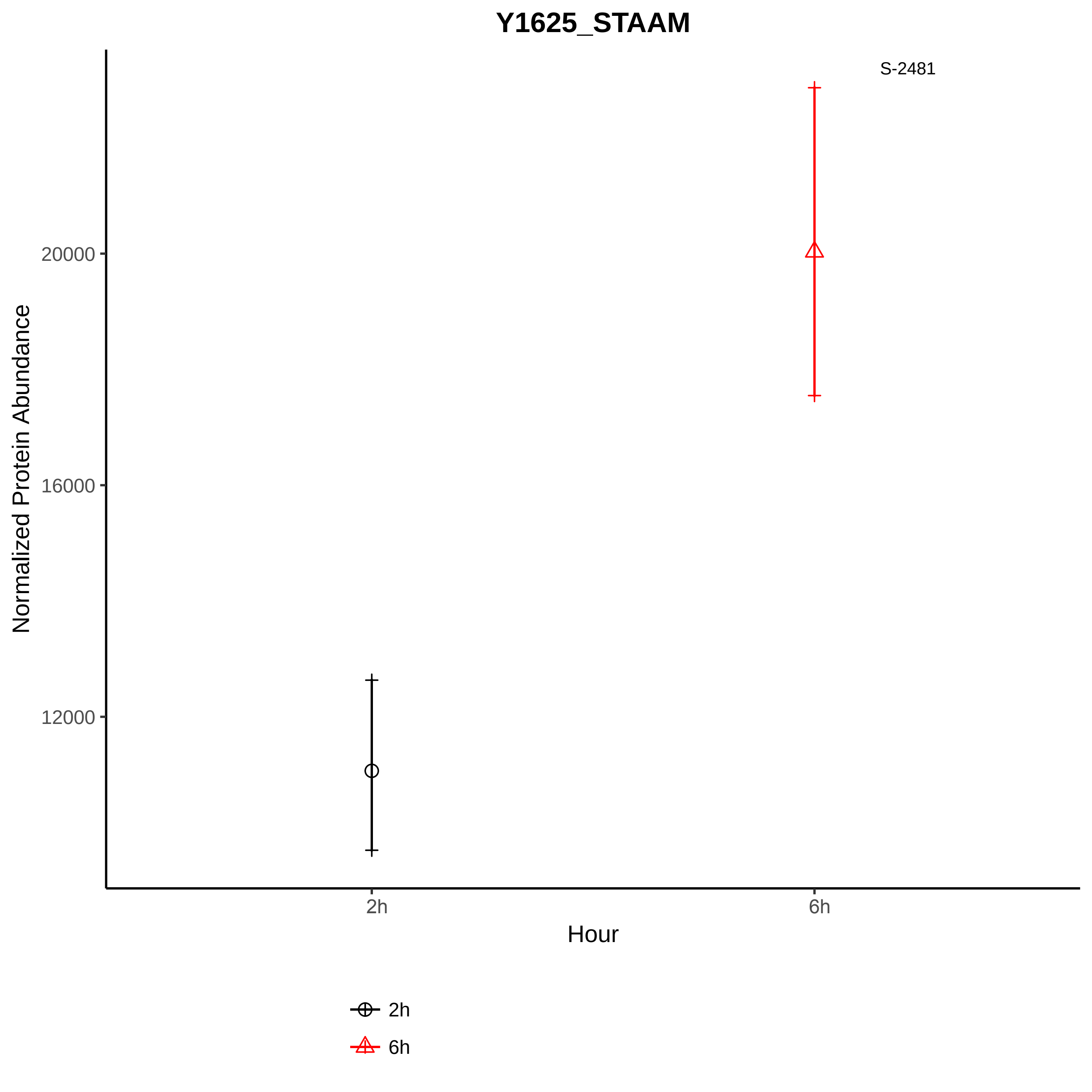

Y1625_STAAM
S-2481
Normalized Protein Abundance
20000
16000
12000
2h
6h
Hour
2h
6h

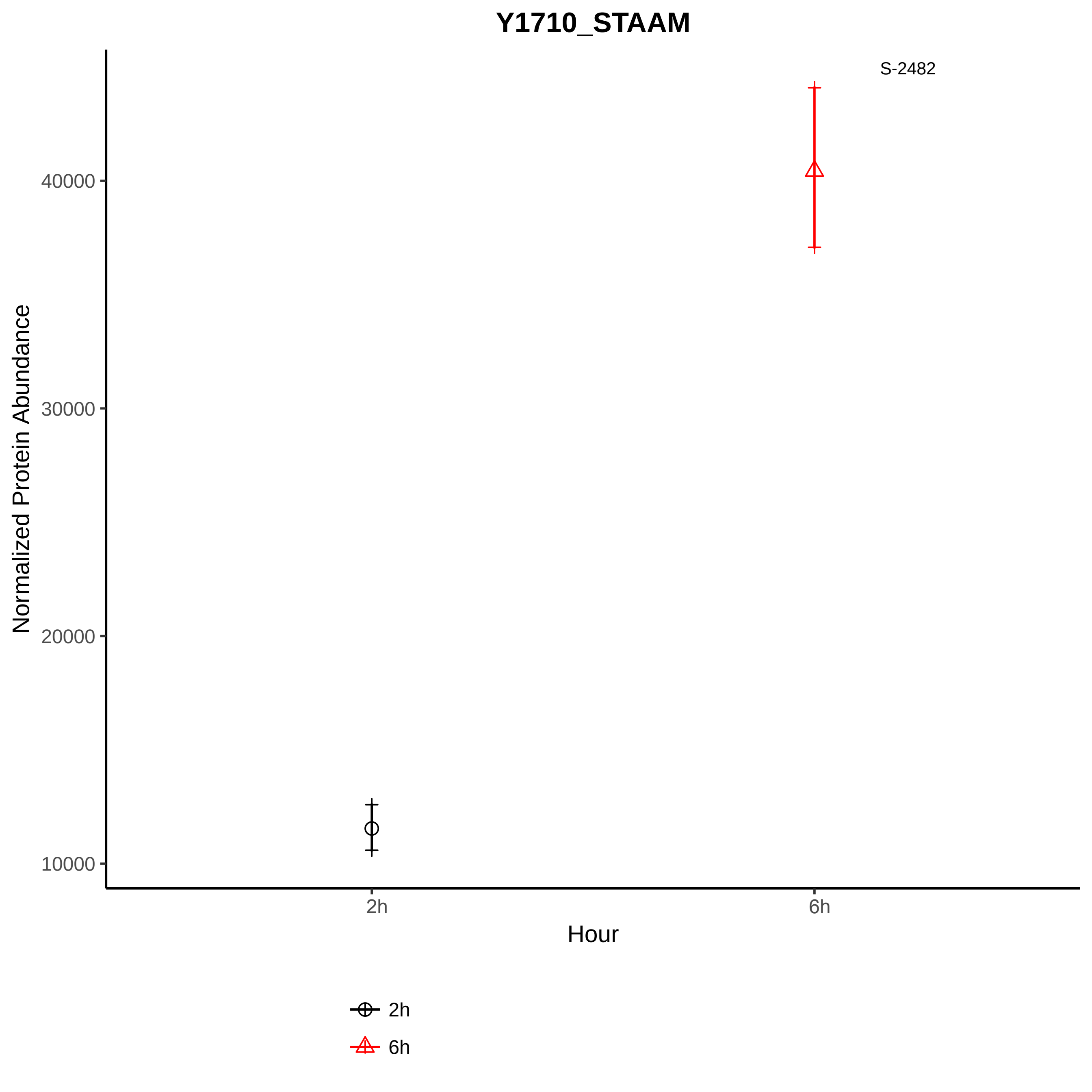

Y1710_STAAM
S-2482
Normalized Protein Abundance
40000
30000
20000
10000
2h
6h
Hour
2h
6h

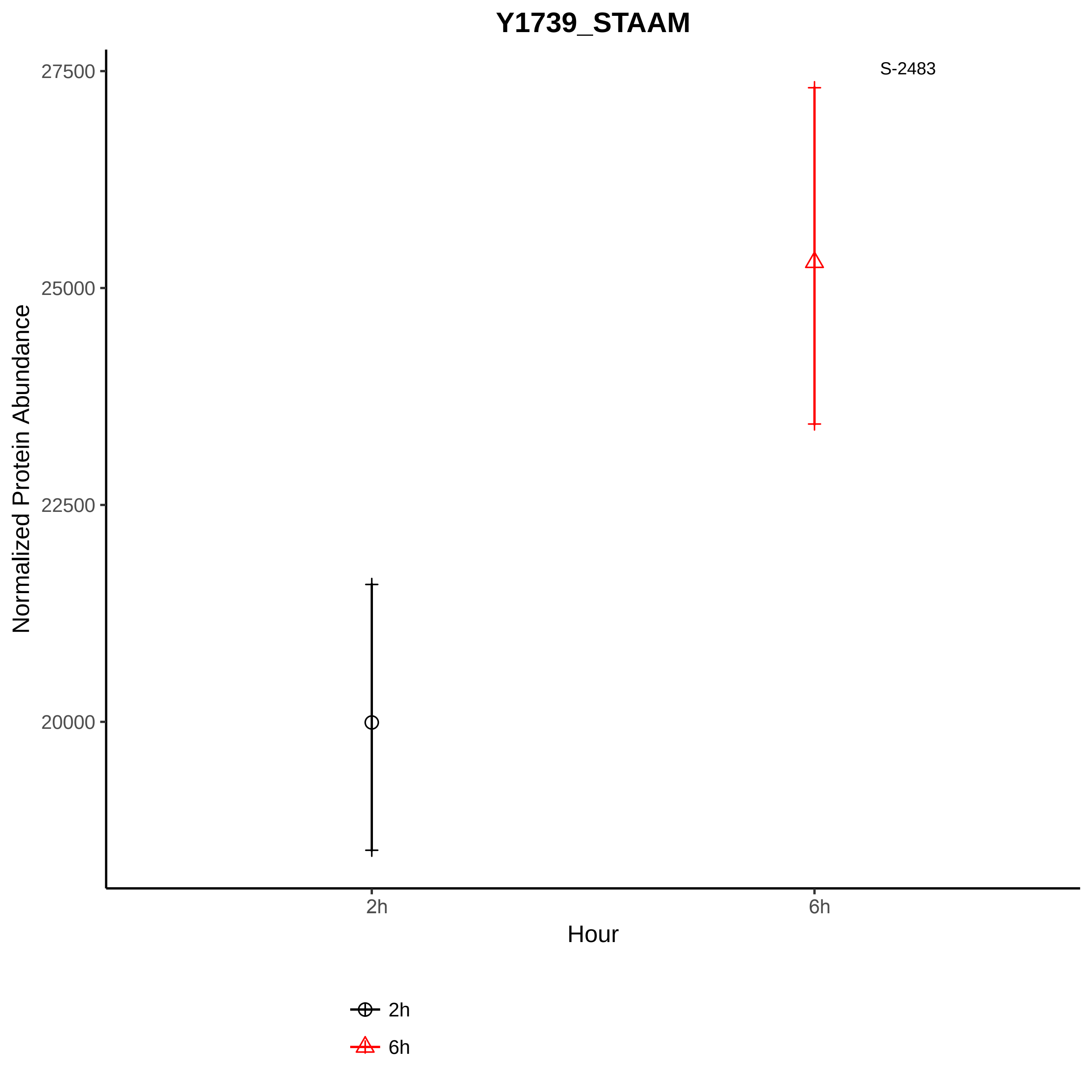

Y1739_STAAM
S-2483
Normalized Protein Abundance
27500
25000
22500
20000
2h
6h
Hour
2h
6h

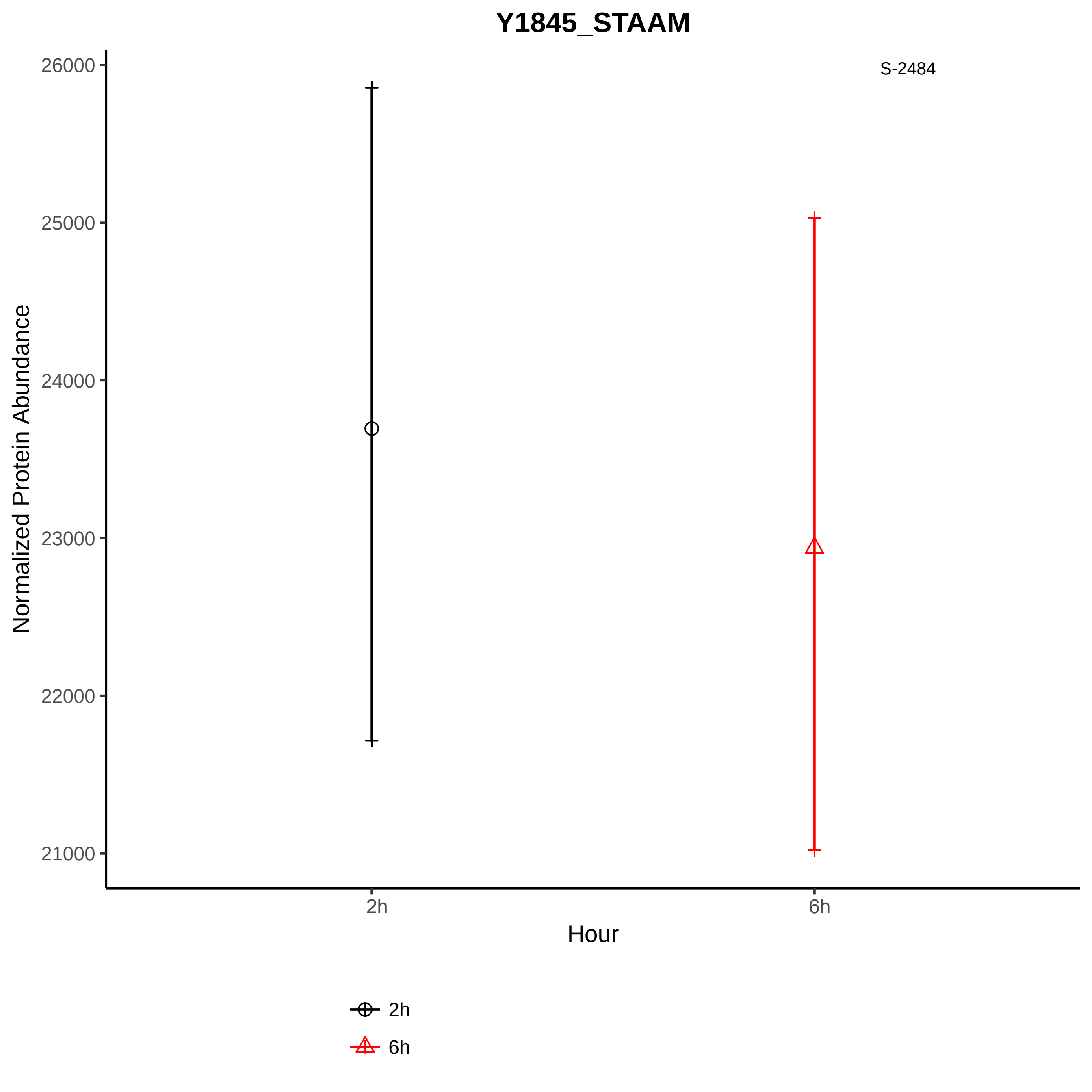

Y1845_STAAM
S-2484
Normalized Protein Abundance
26000
25000
24000
23000
22000
21000
2h
6h
Hour
2h
6h

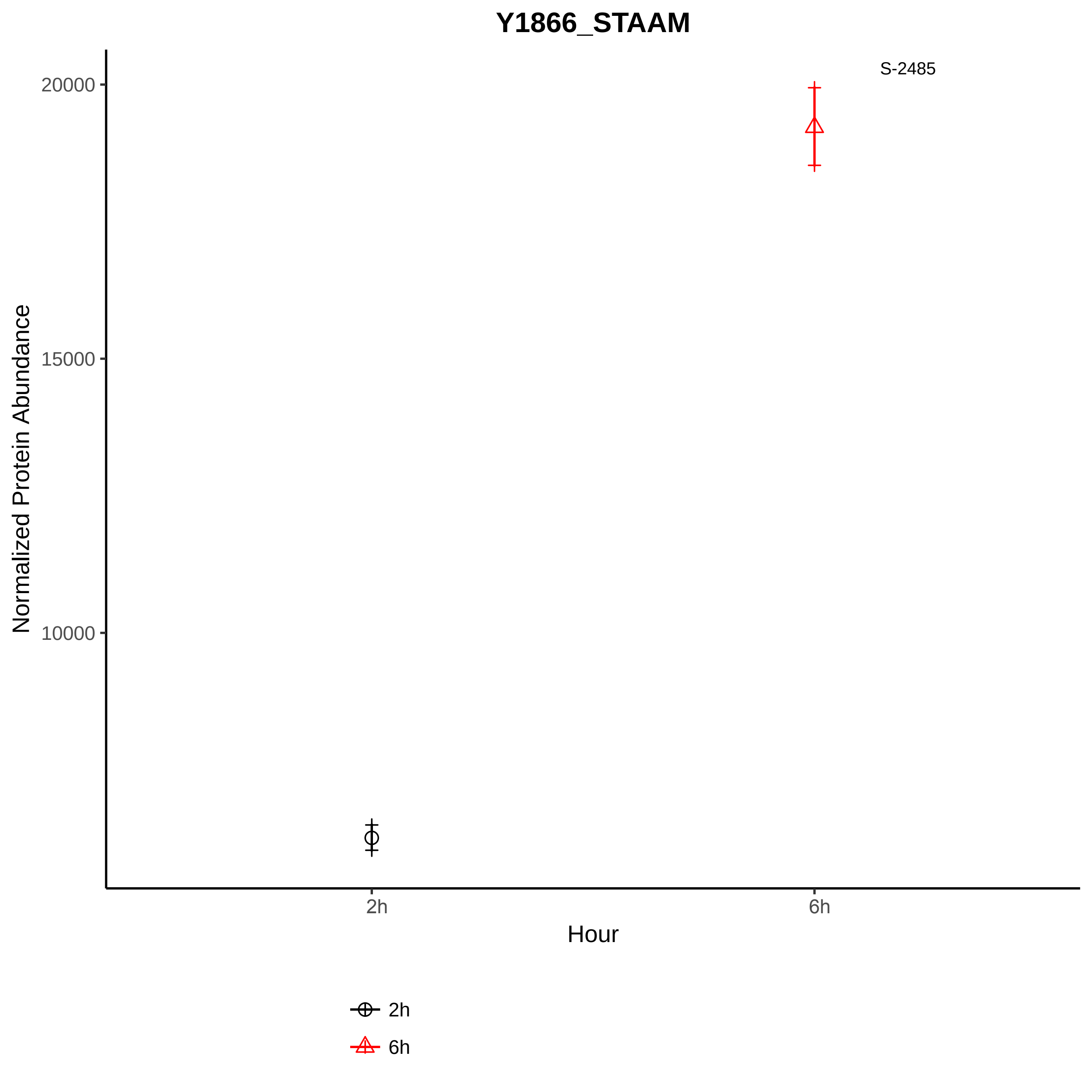

Y1866_STAAM
S-2485
Normalized Protein Abundance
20000
15000
10000
2h
6h
Hour
2h
6h

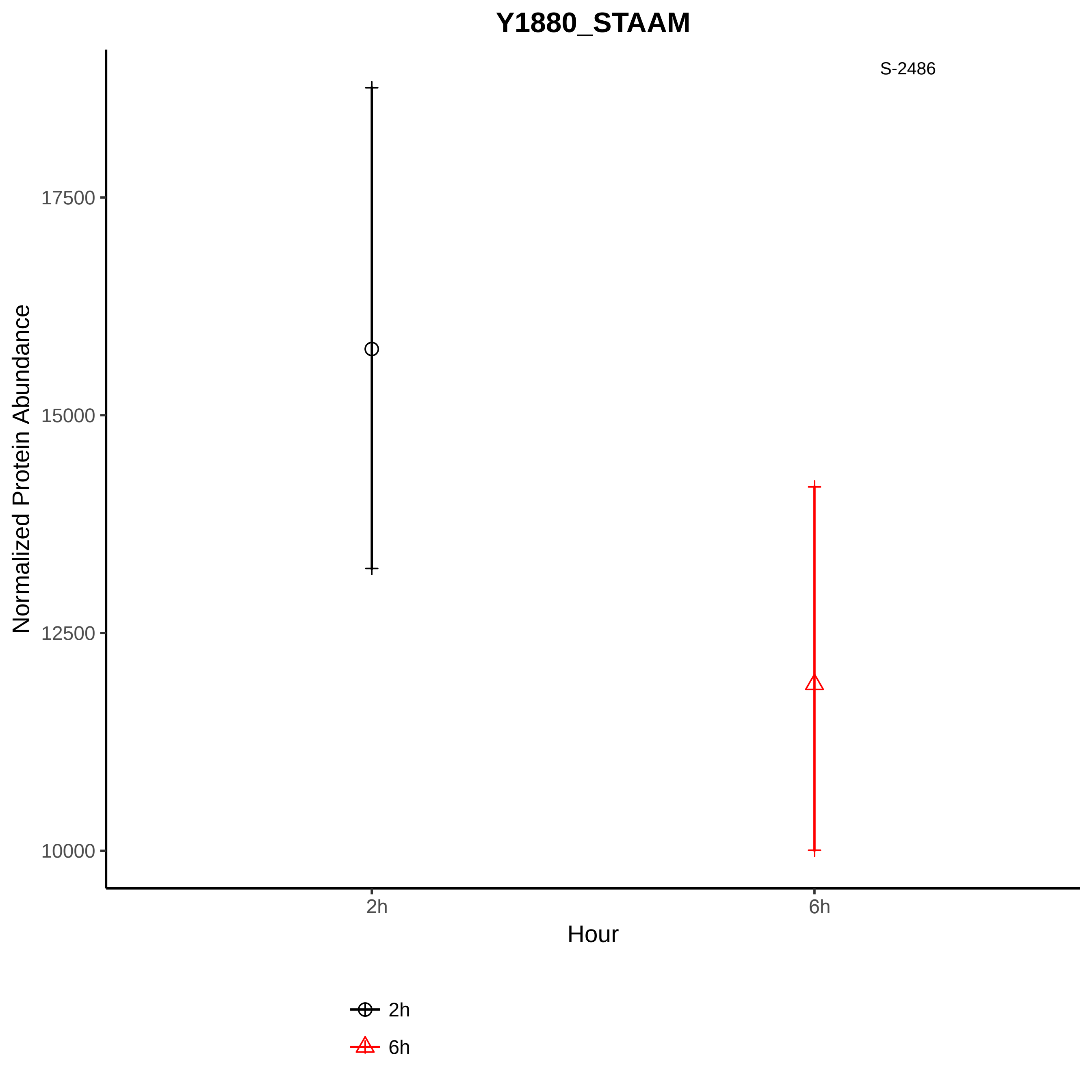

Y1880_STAAM
S-2486
Normalized Protein Abundance
17500
15000
12500
10000
2h
6h
Hour
2h
6h

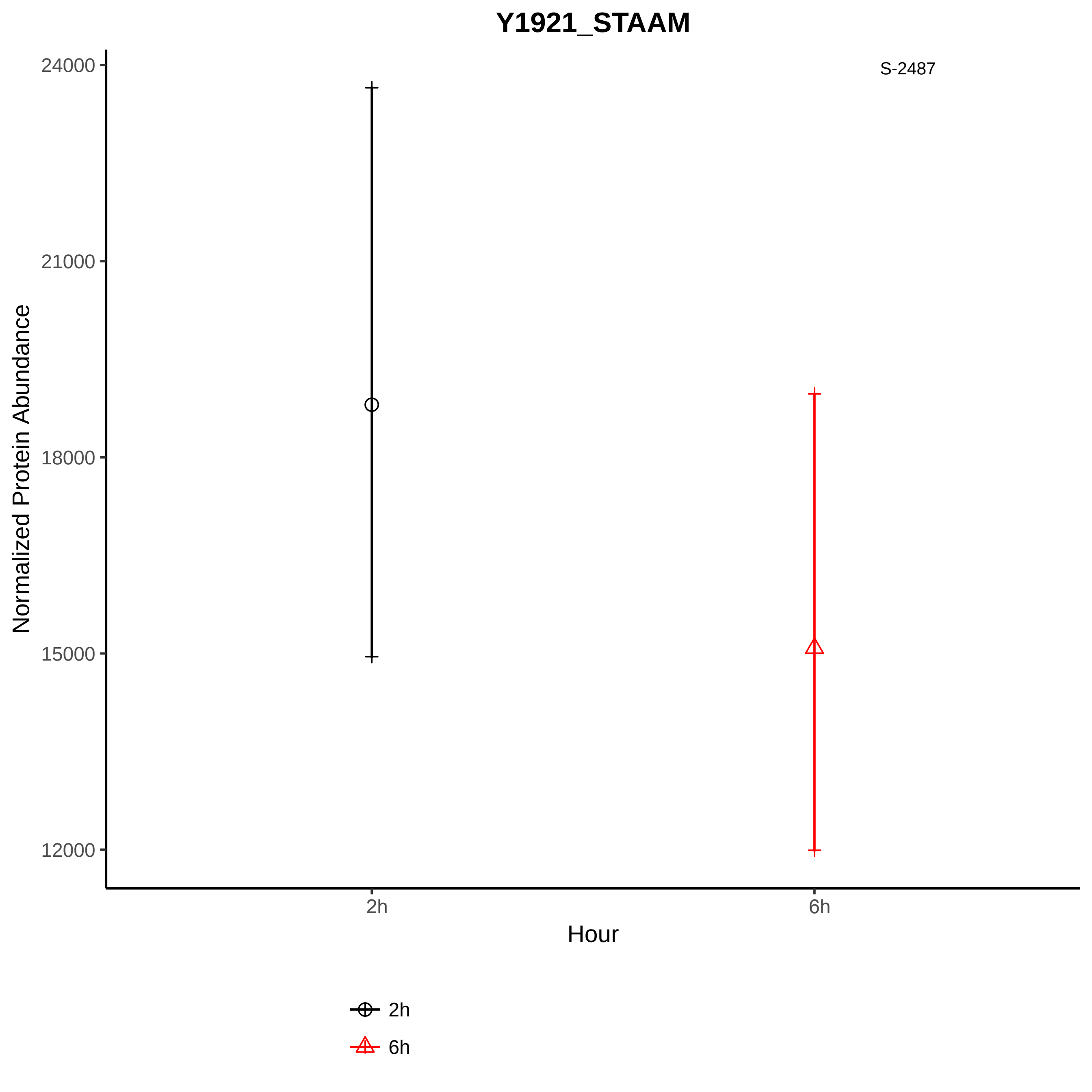

Y1921_STAAM
S-2487
24000
21000
18000
15000
12000
Normalized Protein Abundance
2h
6h
Hour
2h
6h

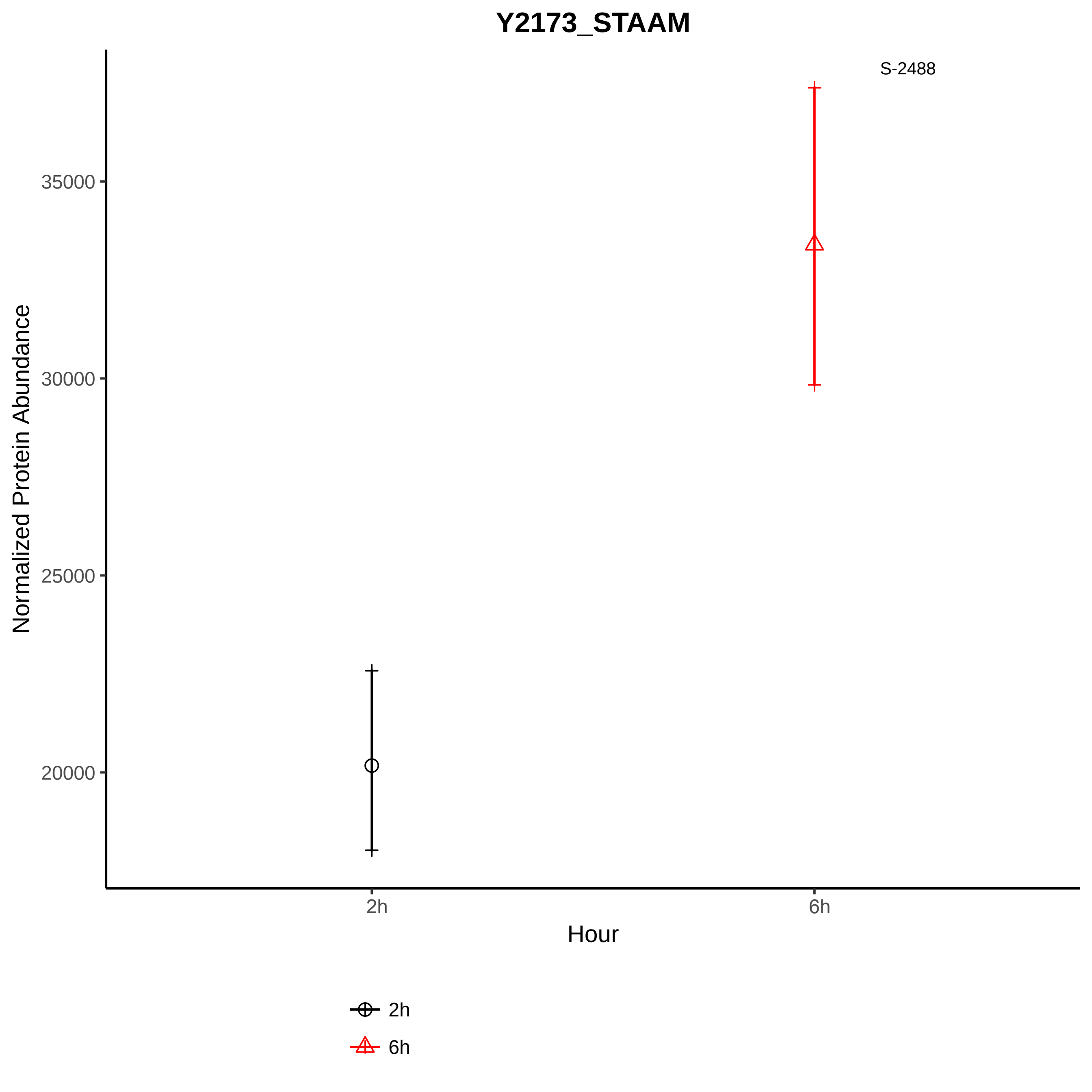

Y2173_STAAM
S-2488
35000
30000
25000
20000
Normalized Protein Abundance
2h
6h
Hour
2h
6h

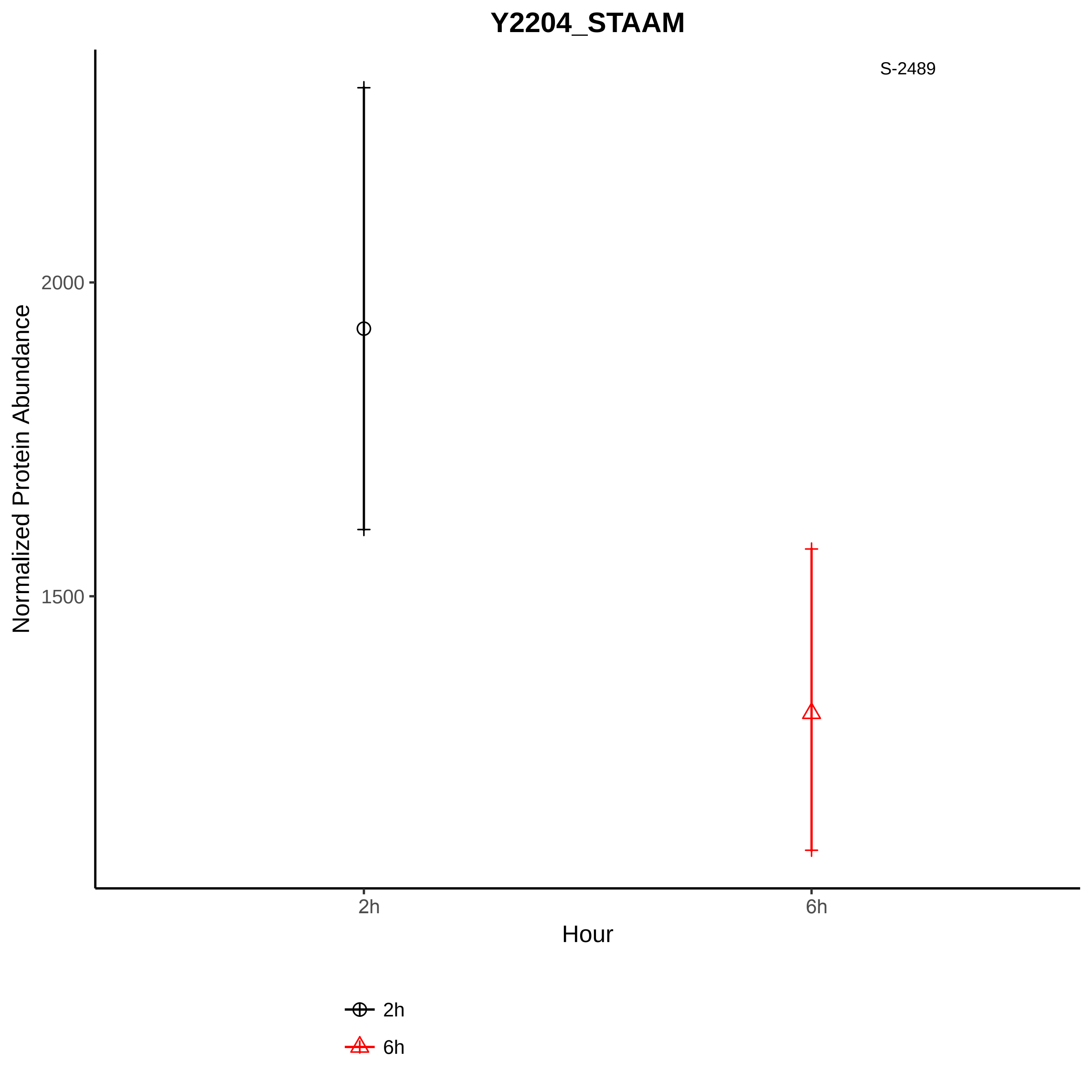

Y2204_STAAM
S-2489
2000
1500
Normalized Protein Abundance
2h
6h
Hour
2h
6h

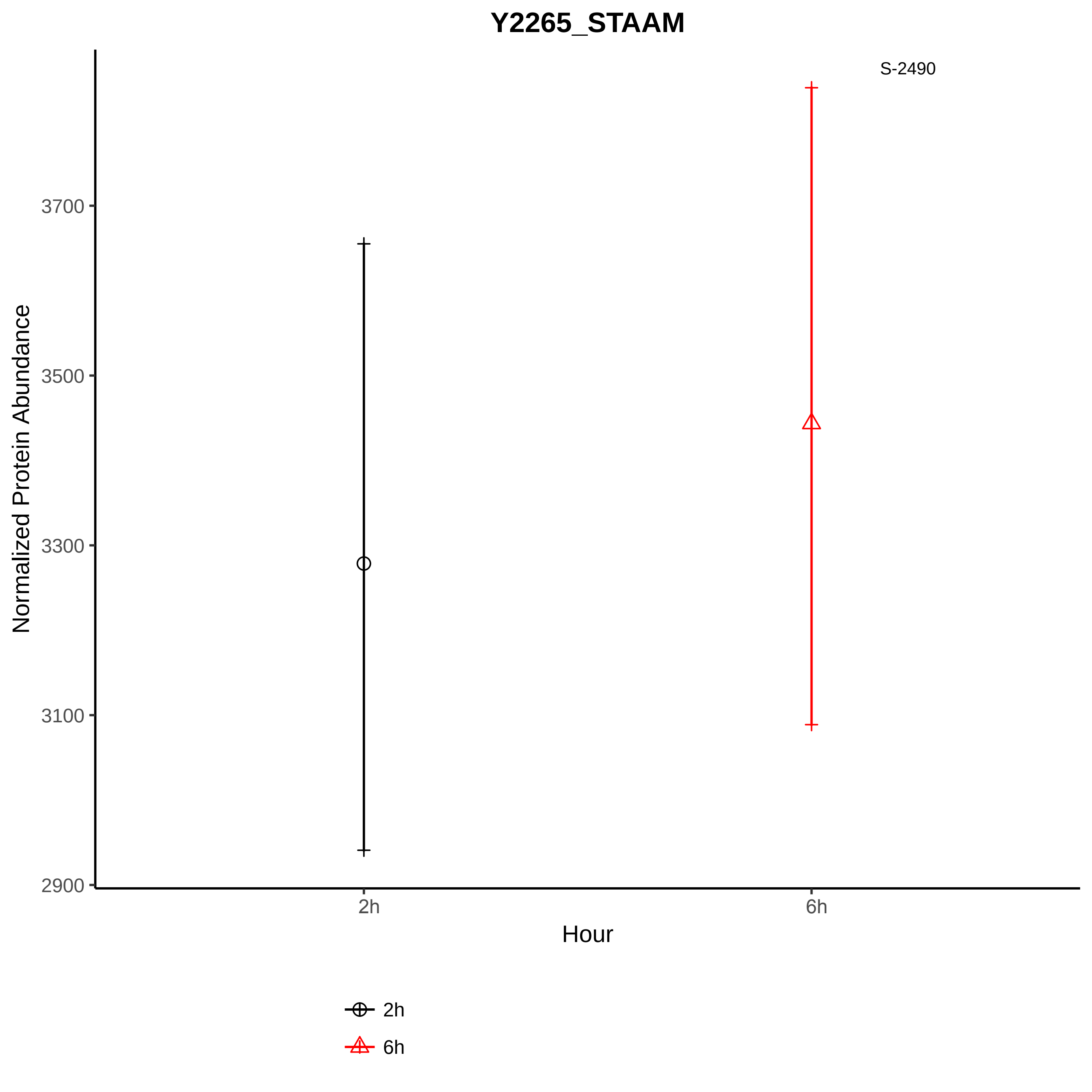

Y2265_STAAM
S-2490
Normalized Protein Abundance
3700
3500
3300
3100
2900
2h
6h
Hour
2h
6h

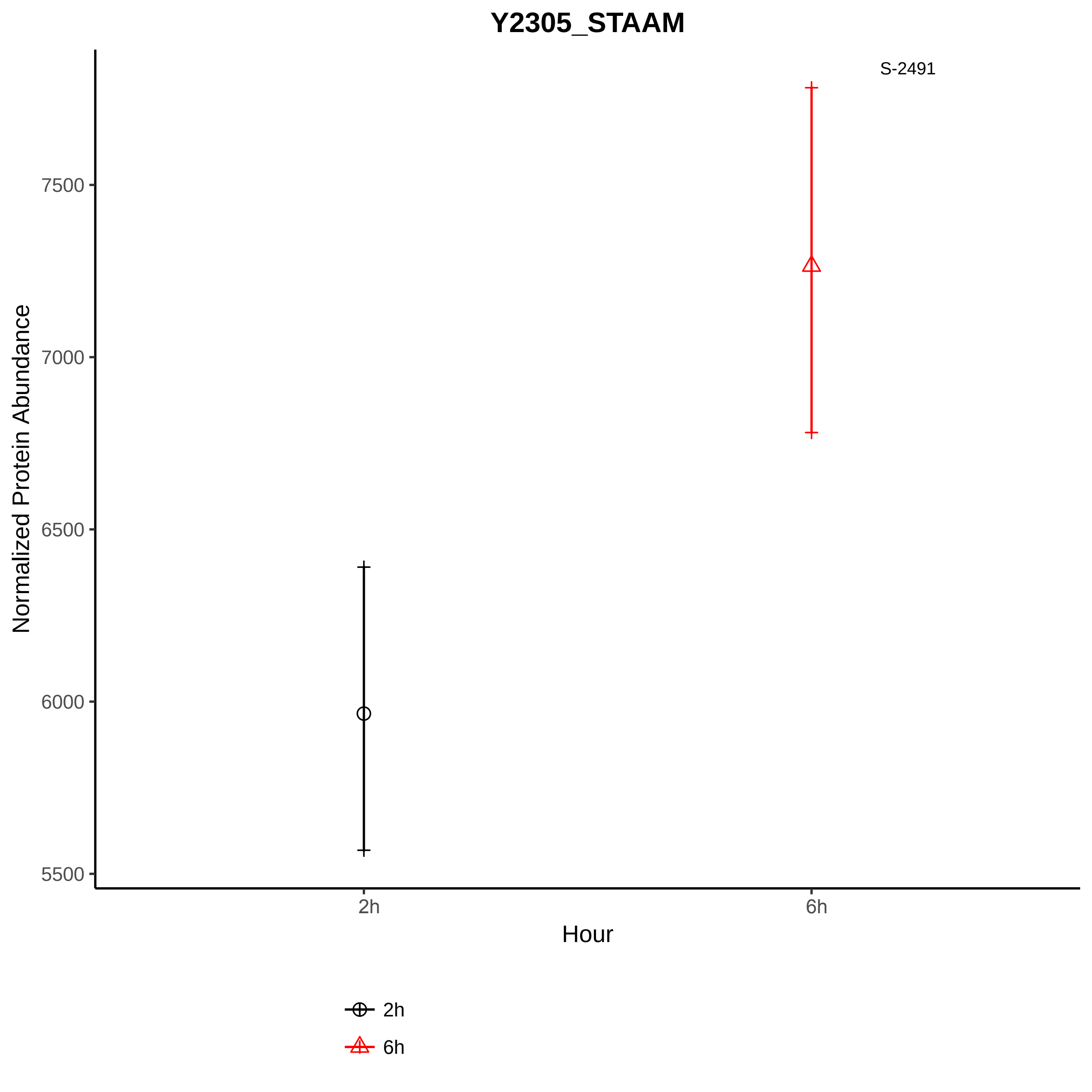

Y2305_STAAM
S-2491
Normalized Protein Abundance
7500
7000
6500
6000
5500
2h
6h
Hour
2h
6h

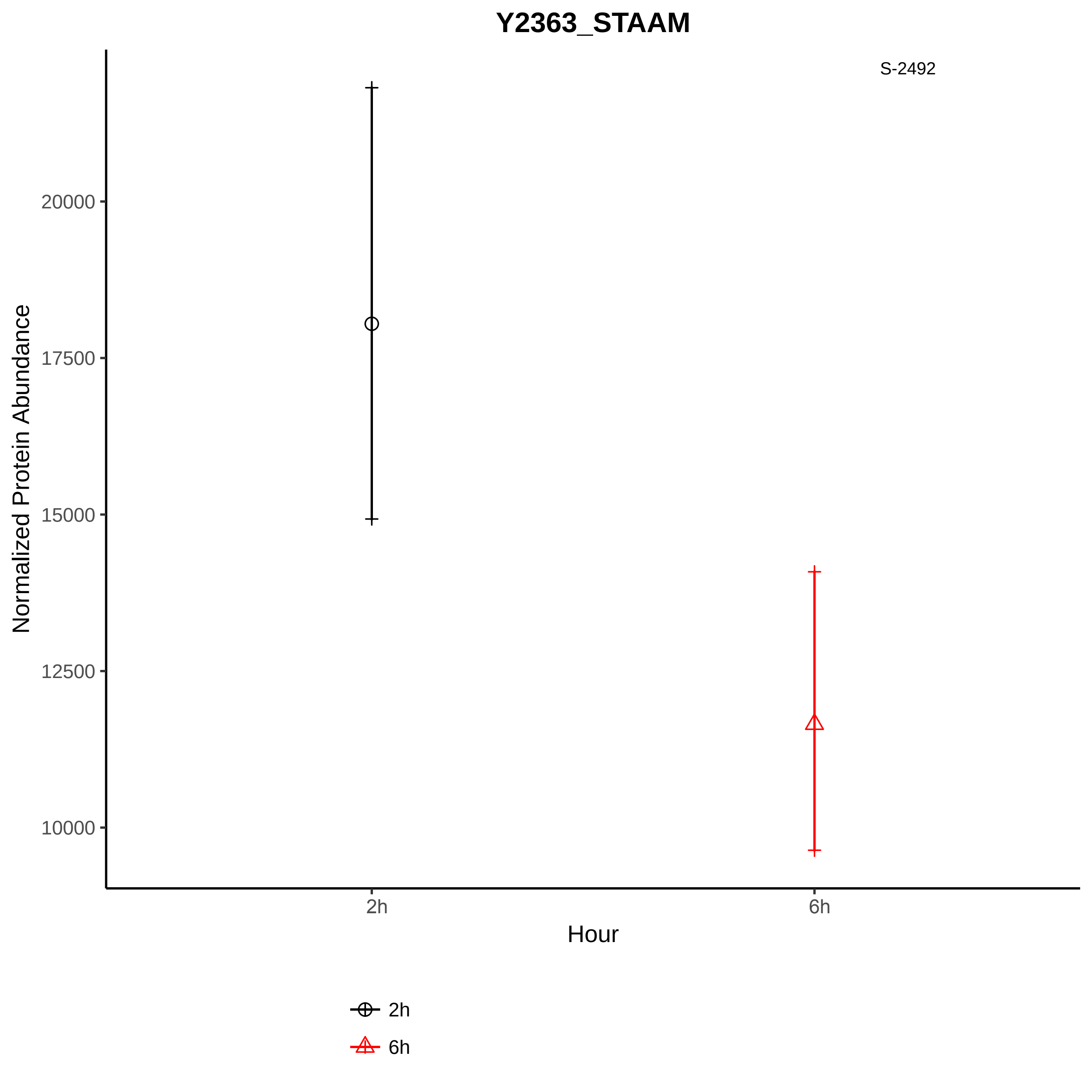

Y2363_STAAM
S-2492
Normalized Protein Abundance
20000
17500
15000
12500
10000
2h
6h
Hour
2h
6h

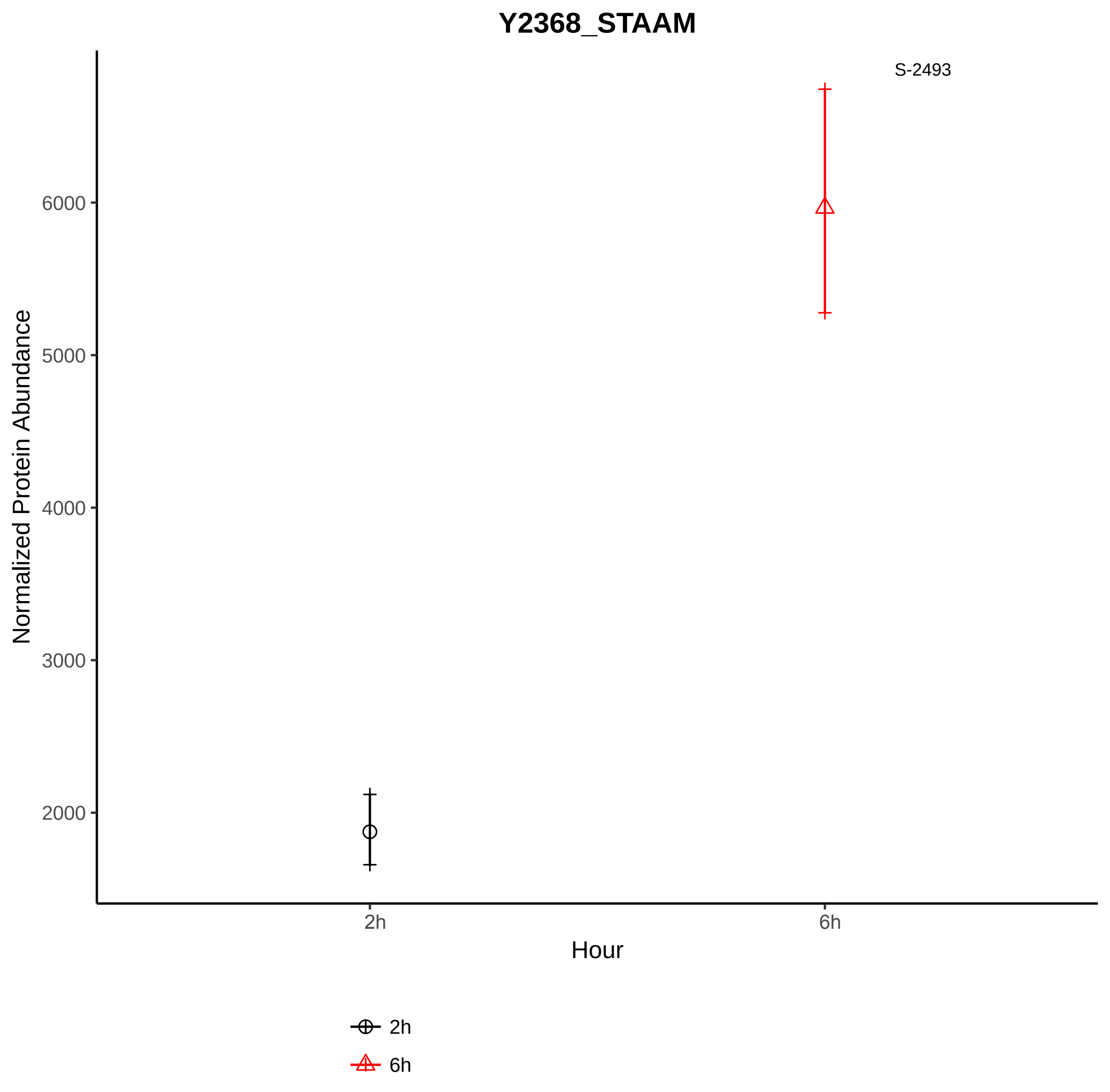
Y2368_STAAM
S-2493
Normalized Protein Abundance
6000
5000
4000
3000
2000
2h
6h
Hour
2h
6h

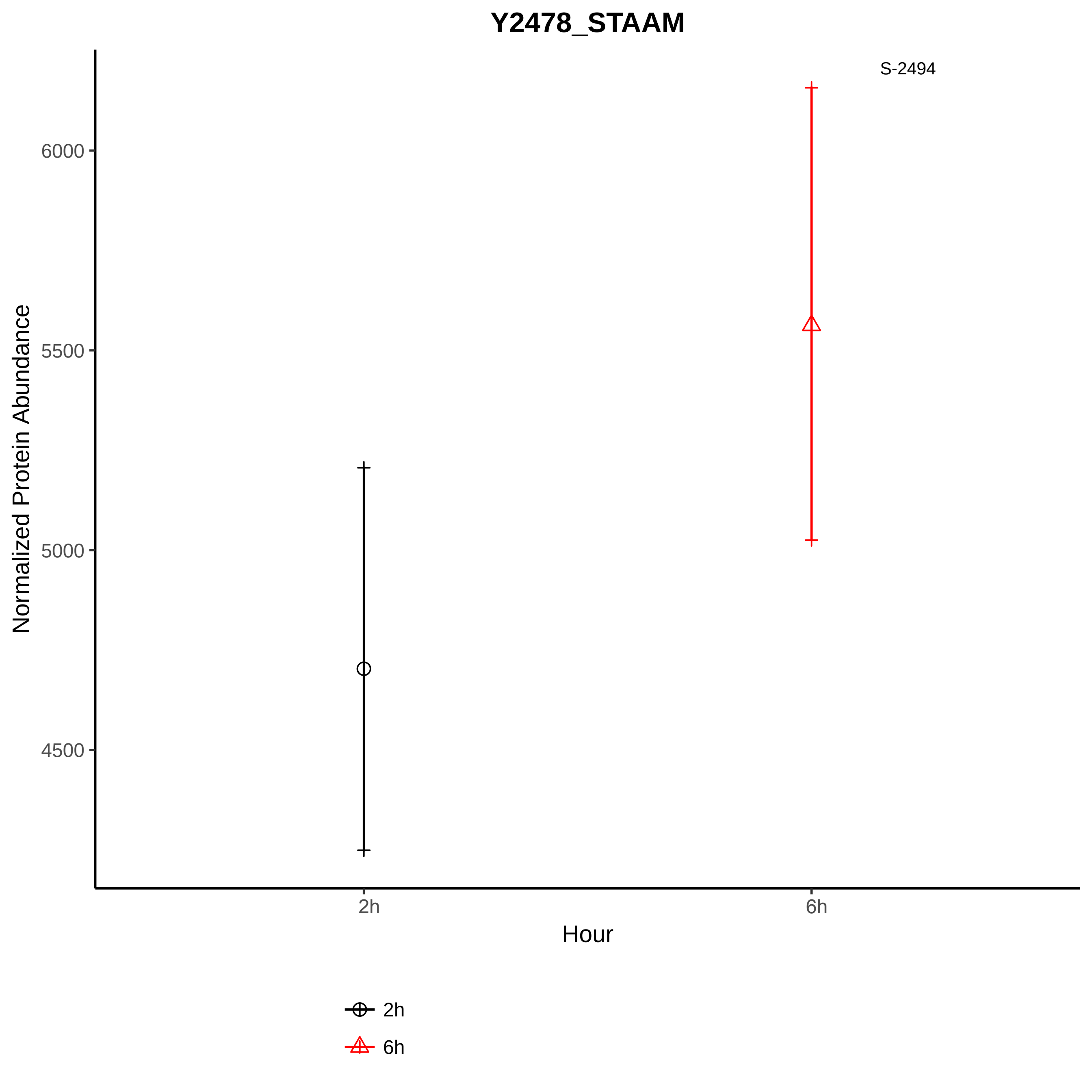

Y2478_STAAM
S-2494
Normalized Protein Abundance
6000
5500
5000
4500
2h
6h
Hour
2h
6h

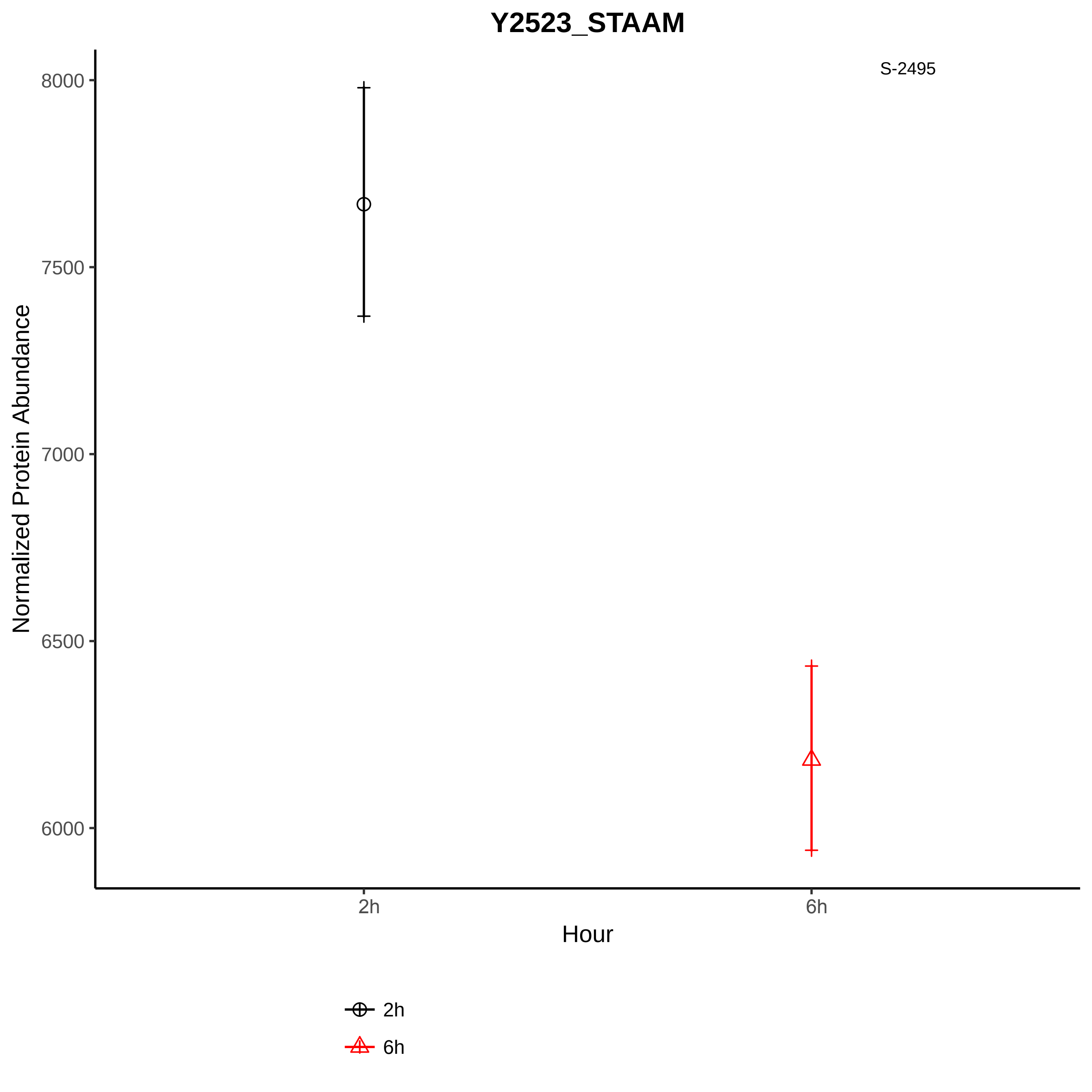
Y2523_STAAM
S-2495
Normalized Protein Abundance
8000
7500
7000
6500
6000
2h
6h
Hour
2h
6h

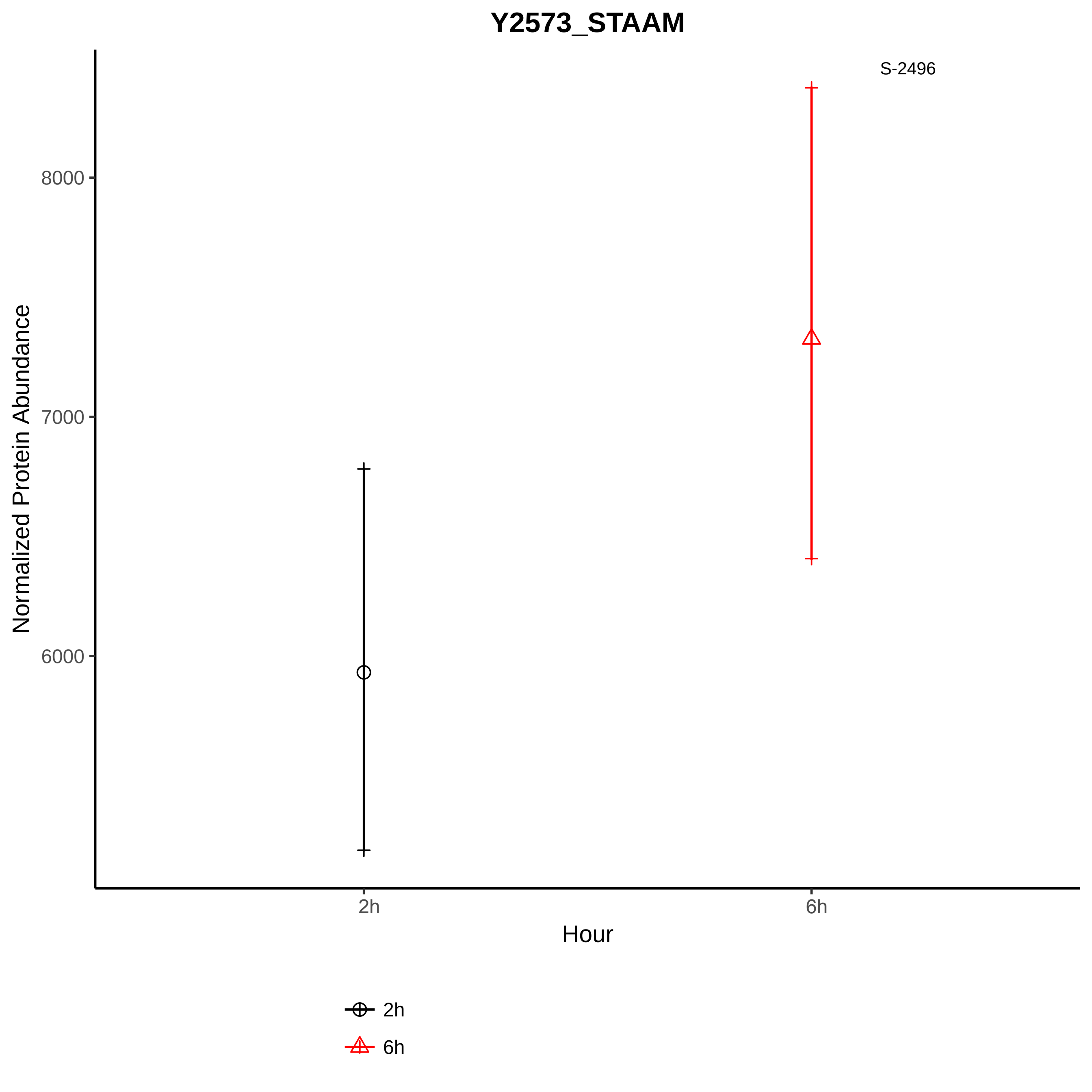

Y2573_STAAM
S-2496
Normalized Protein Abundance
8000
7000
6000
2h
6h
Hour
2h
6h

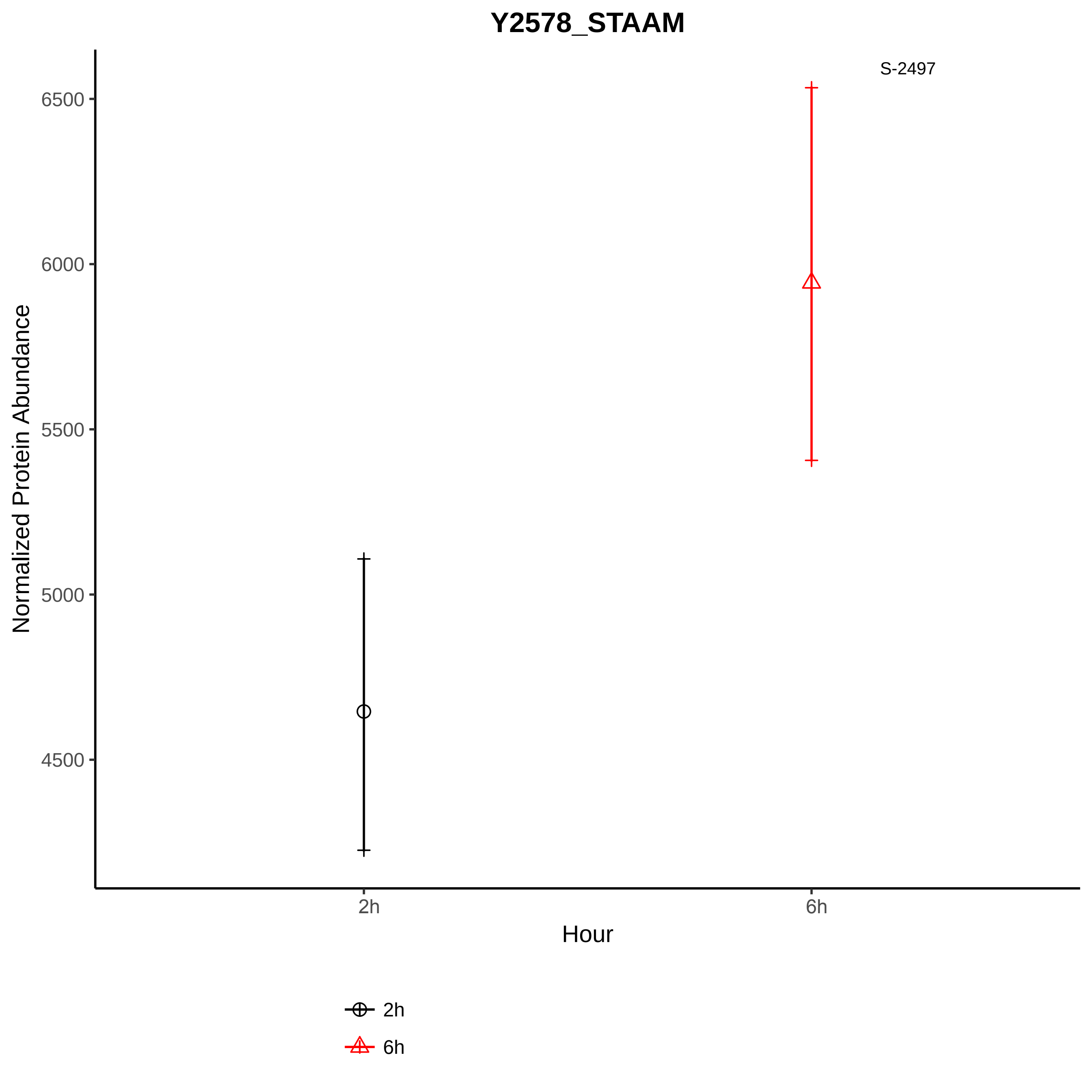

Y2578_STAAM
S-2497
Normalized Protein Abundance
6500
6000
5500
5000
4500
2h
6h
Hour
2h
6h

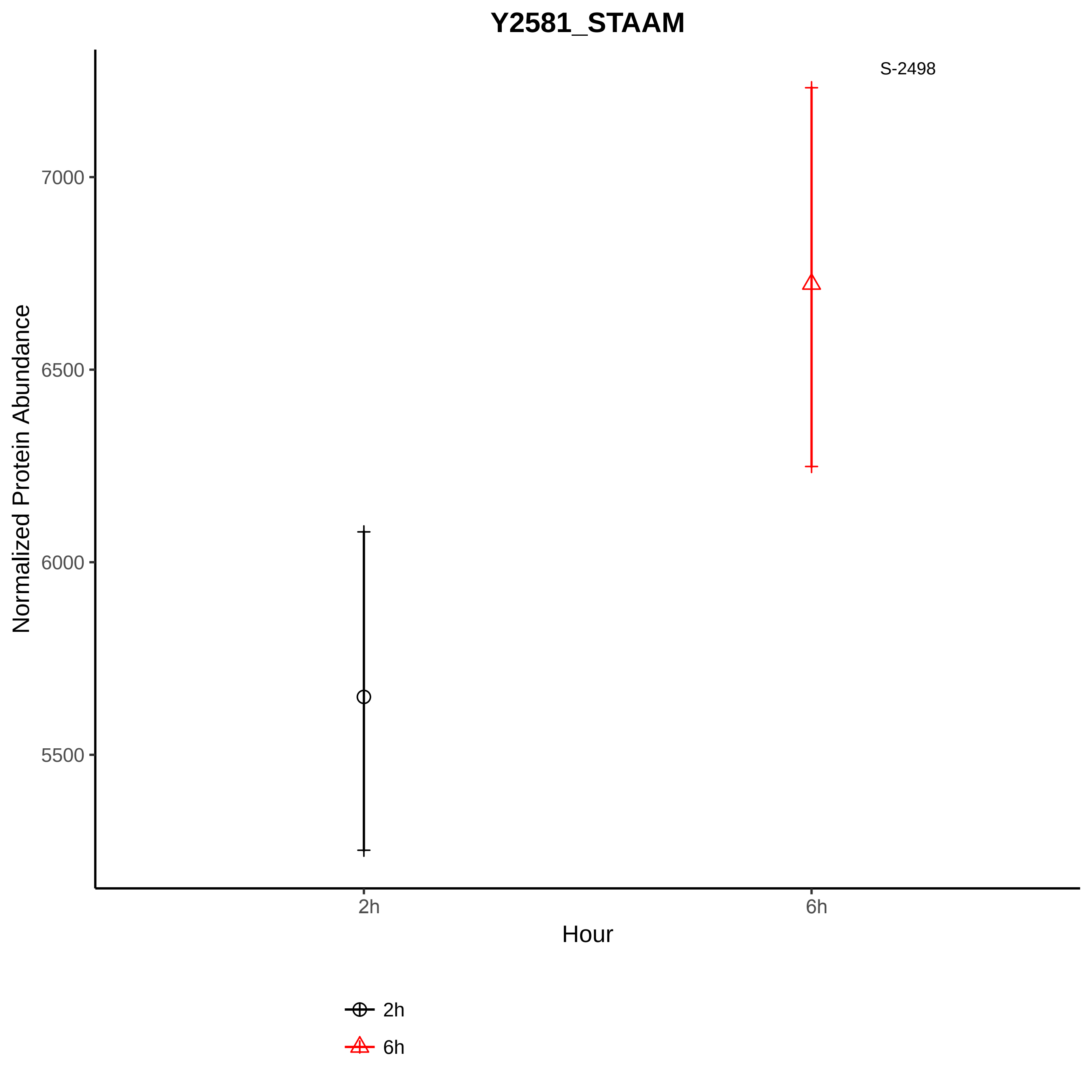
Y2581_STAAM
S-2498
Normalized Protein Abundance
7000
6500
6000
5500
2h
6h
Hour
2h
6h

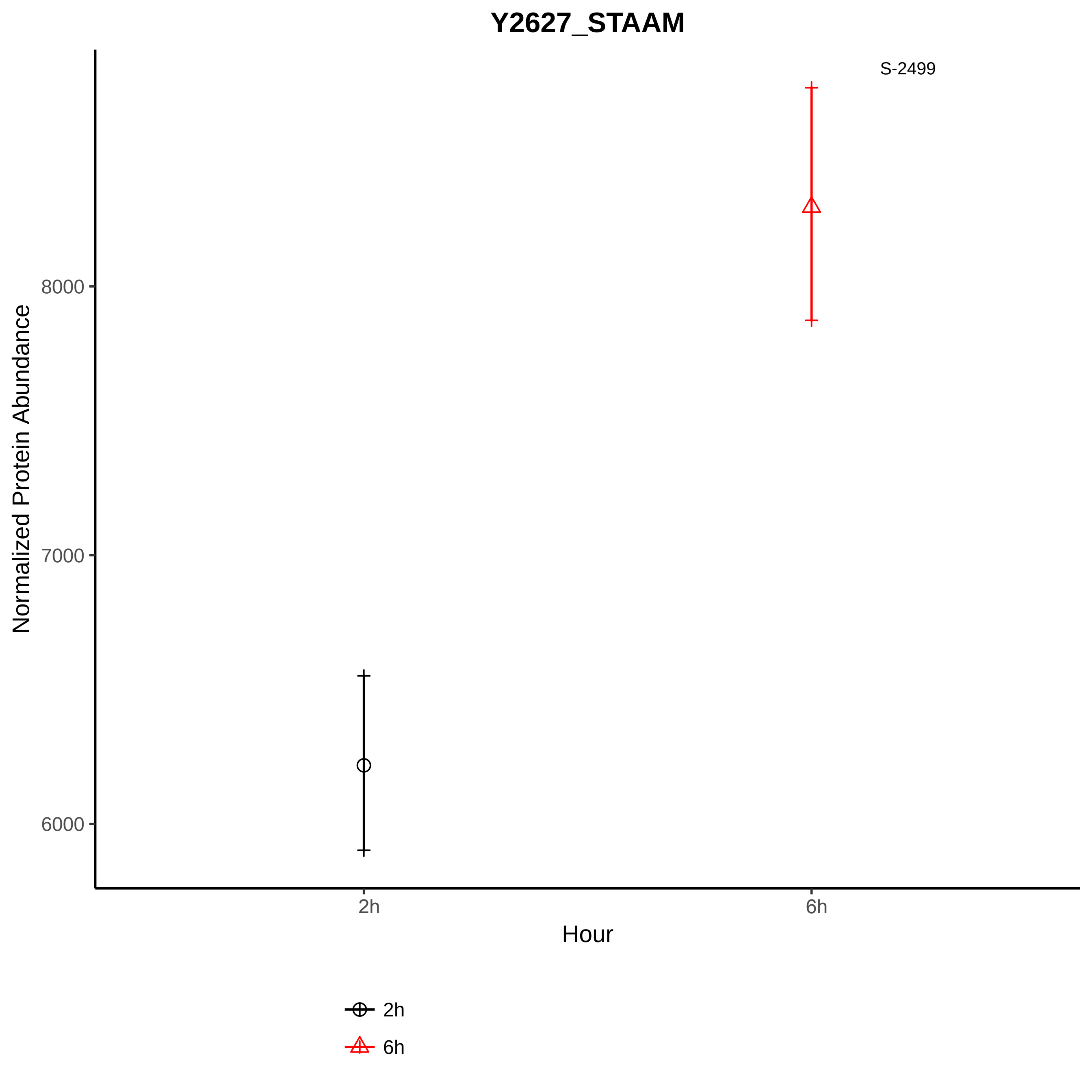

Y2627_STAAM
S-2499
Normalized Protein Abundance
8000
7000
6000
2h
6h
Hour
2h
6h

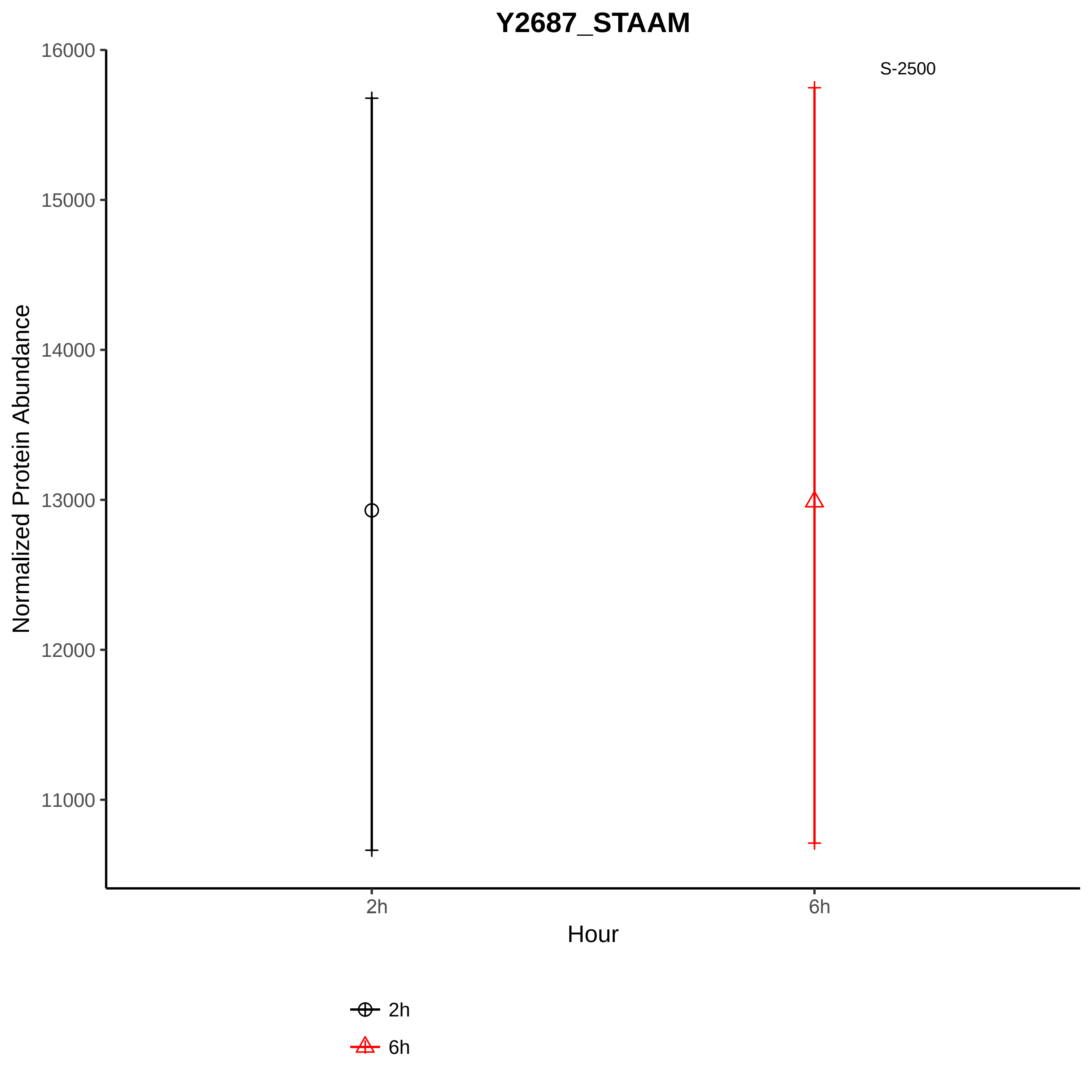

Y2687_STAAM
S-2500
16000
15000
14000
13000
12000
11000
Normalized Protein Abundance
2h
6h
Hour
2h
6h

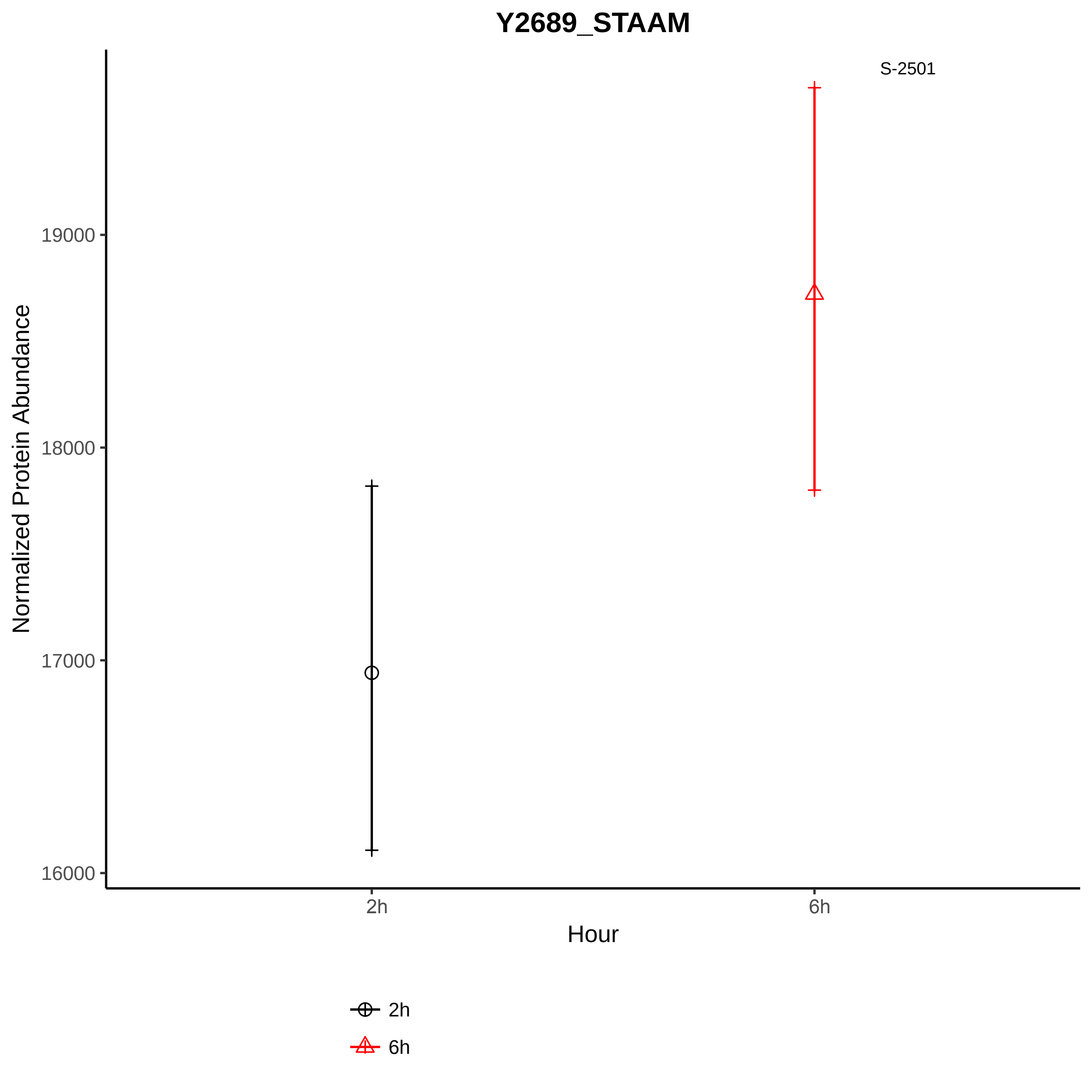

Y2689_STAAM
S-2501
Normalized Protein Abundance
19000
18000
17000
16000
2h
6h
Hour
2h
6h

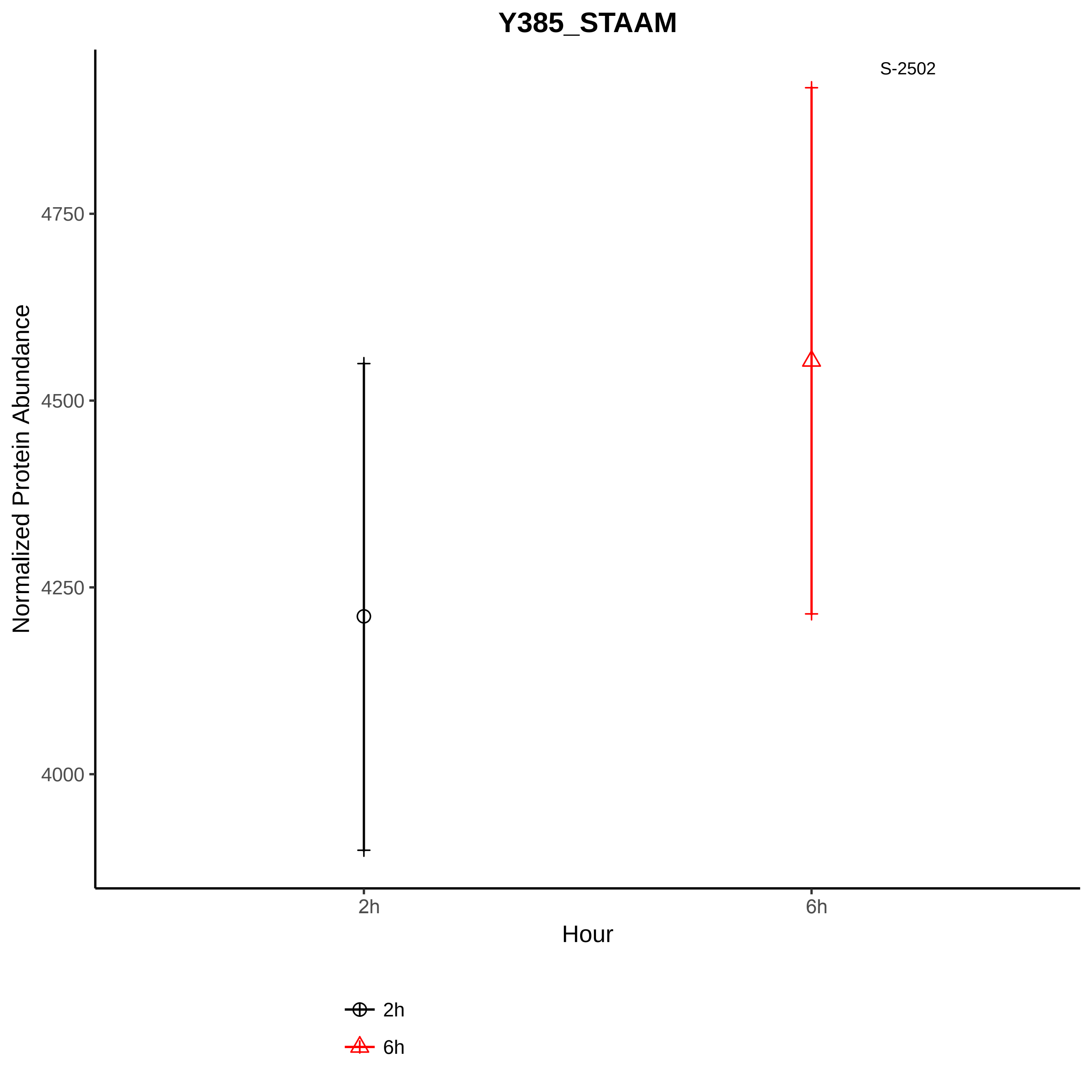

Y385_STAAM
S-2502
Normalized Protein Abundance
4750
4500
4250
4000
2h
6h
Hour
2h
6h

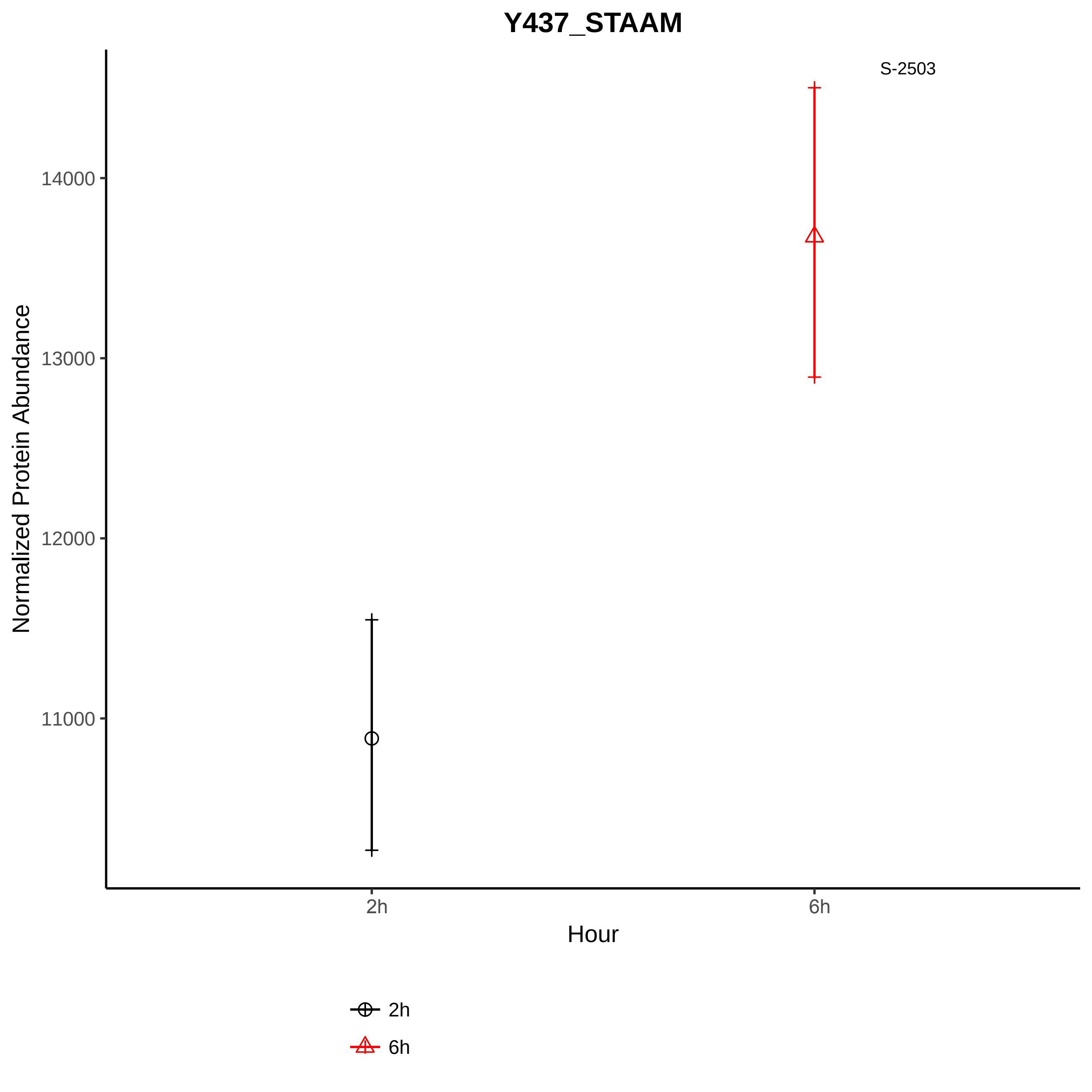

Y437_STAAM
S-2503
Normalized Protein Abundance
14000
13000
12000
11000
2h
6h
Hour
2h
6h

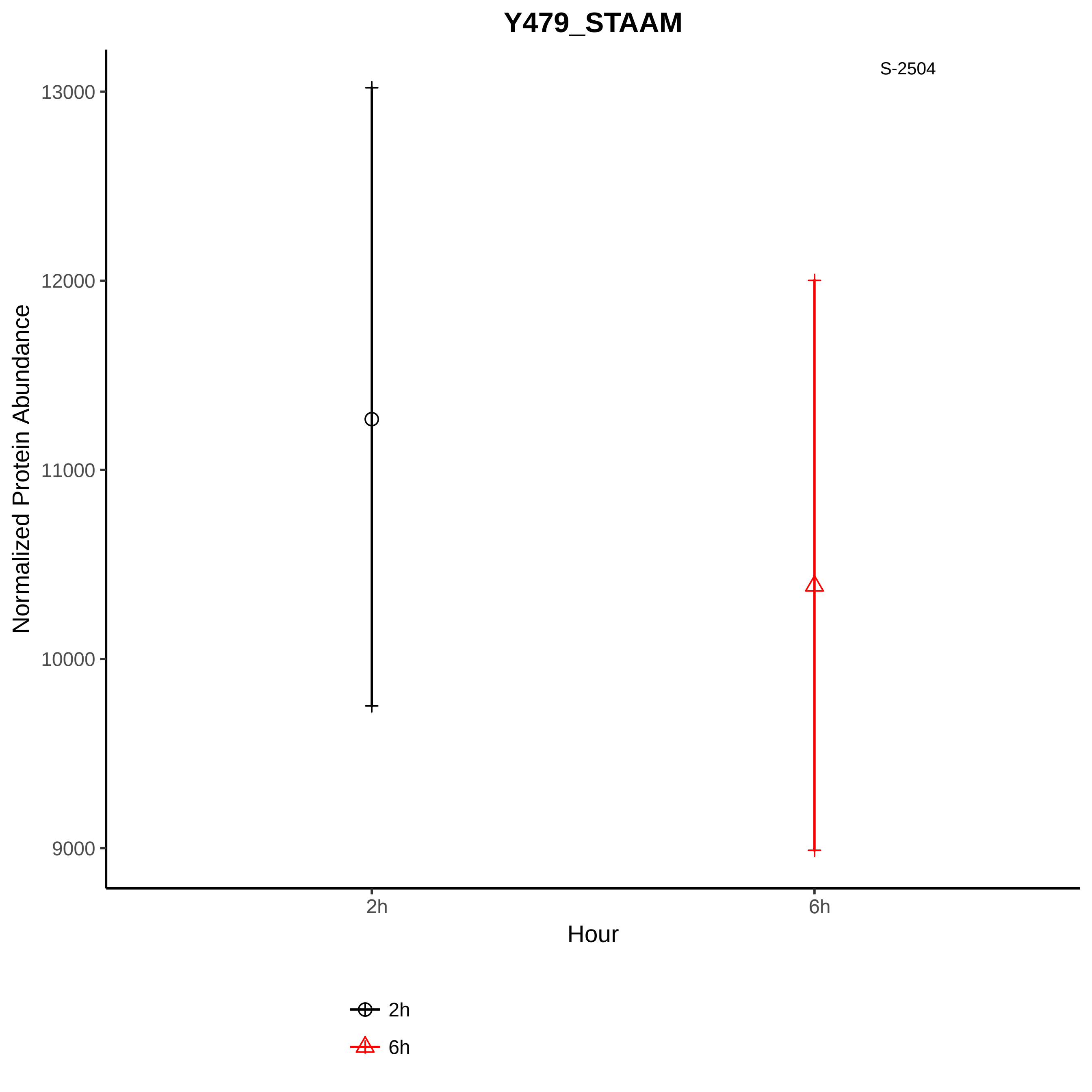

Y479_STAAM
S-2504
Normalized Protein Abundance
13000
12000
11000
10000
9000
2h
6h
Hour
2h
6h

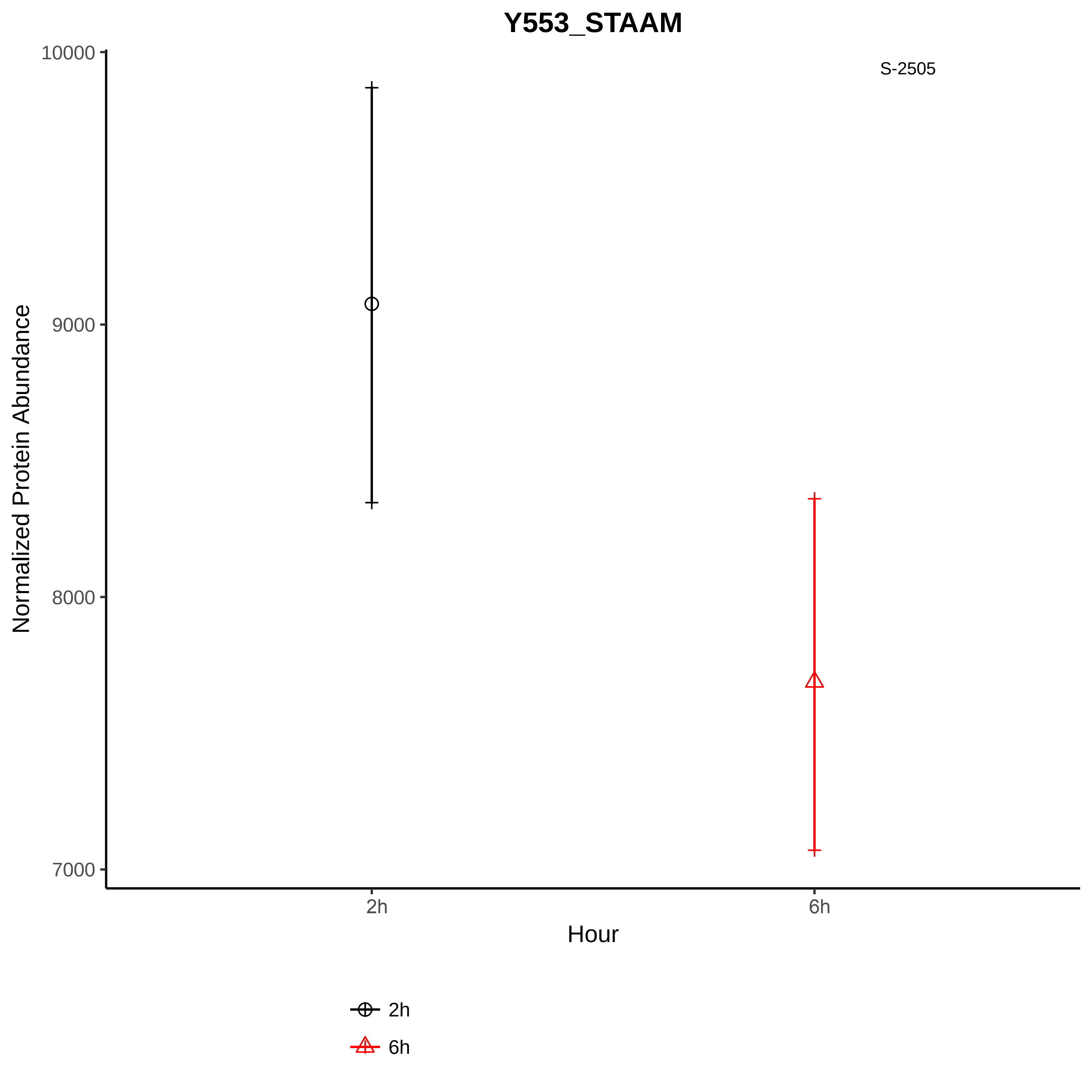

Y553_STAAM
S-2505
10000
9000
8000
7000
Normalized Protein Abundance
2h
6h
Hour
2h
6h

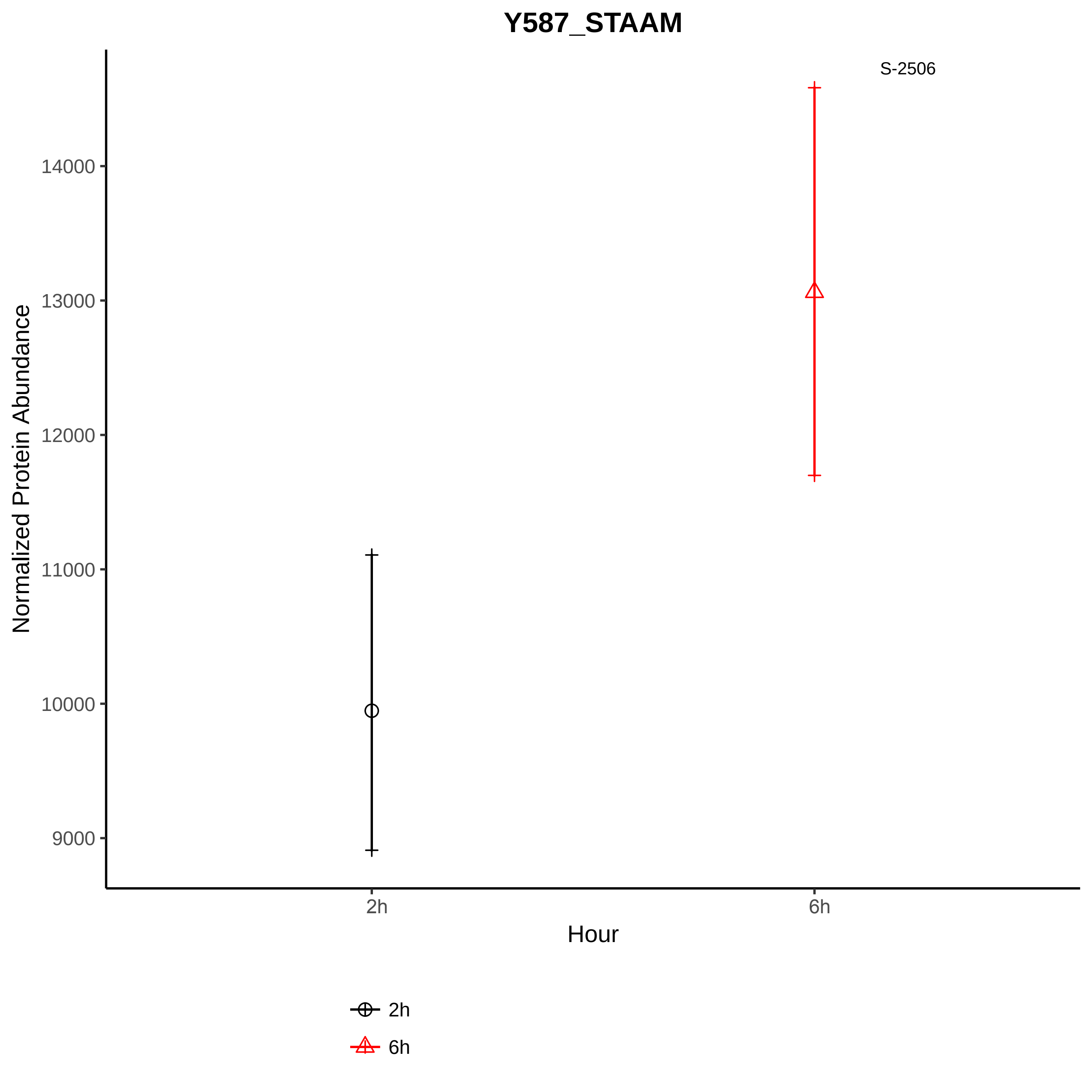

Y587_STAAM
S-2506
Normalized Protein Abundance
14000
13000
12000
11000
10000
9000
2h
6h
Hour
2h
6h

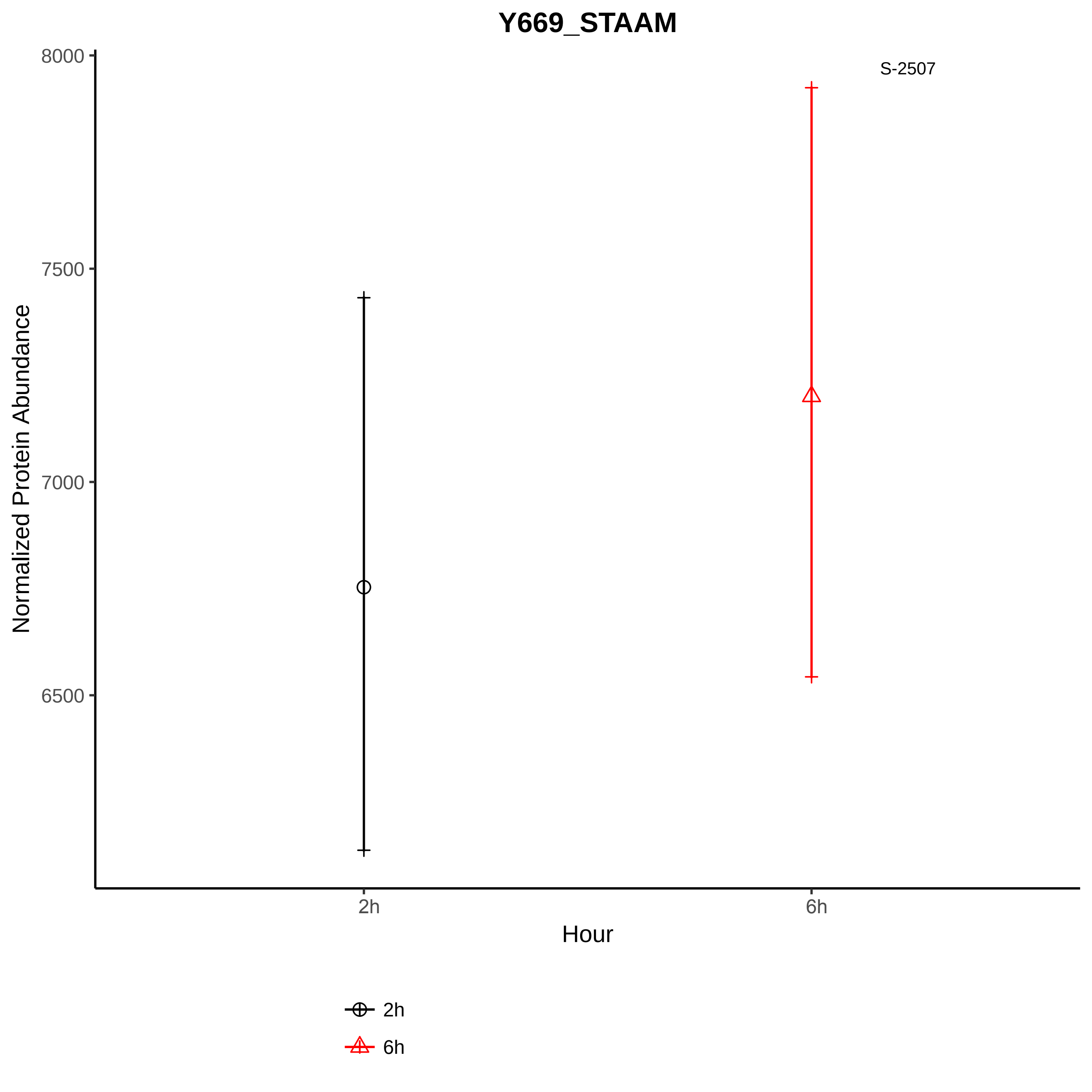

Y669_STAAM
S-2507
8000
7500
7000
6500
Normalized Protein Abundance
2h
6h
Hour
2h
6h

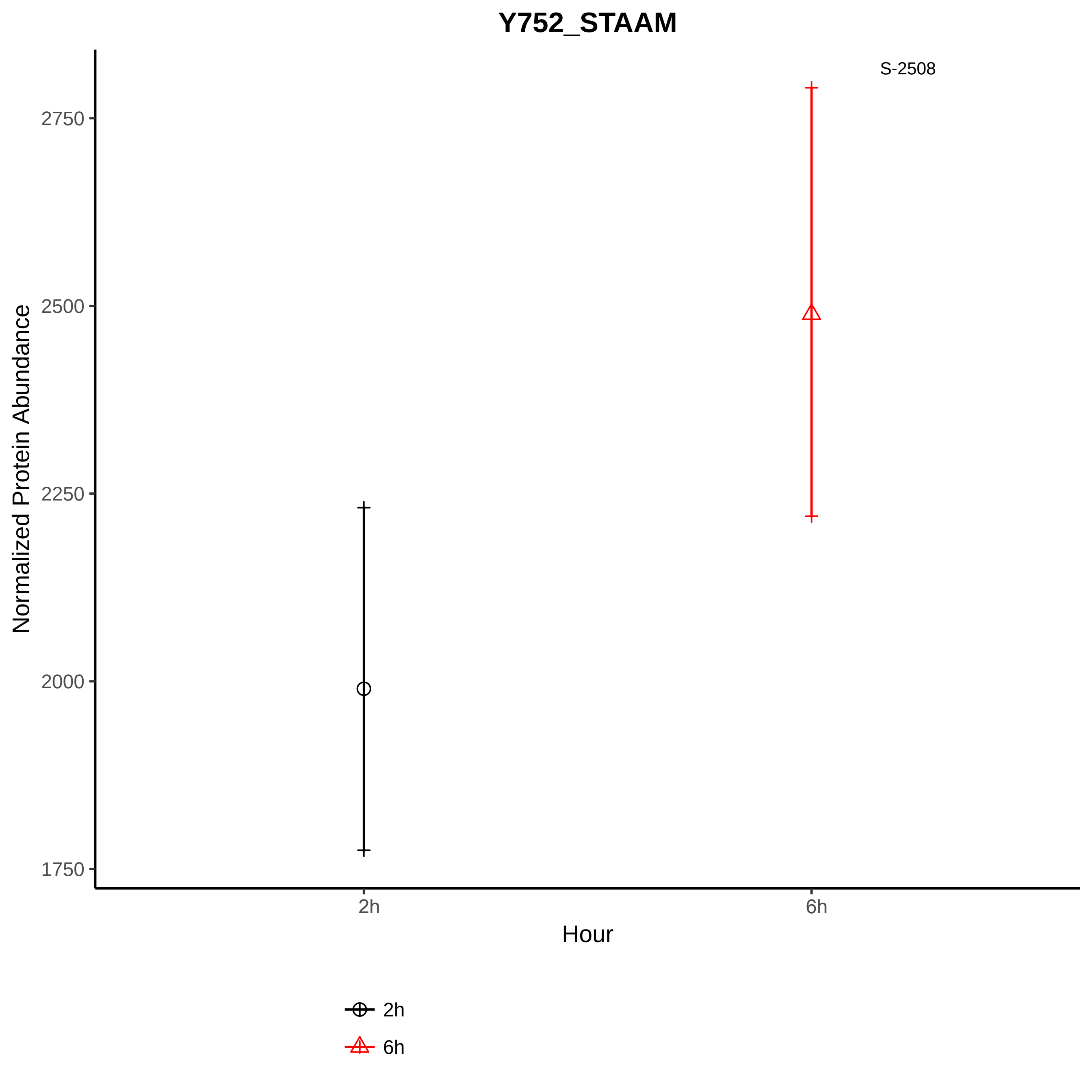

Y752_STAAM
S-2508
2750
2500
2250
2000
1750
Normalized Protein Abundance
2h
6h
Hour
2h
6h

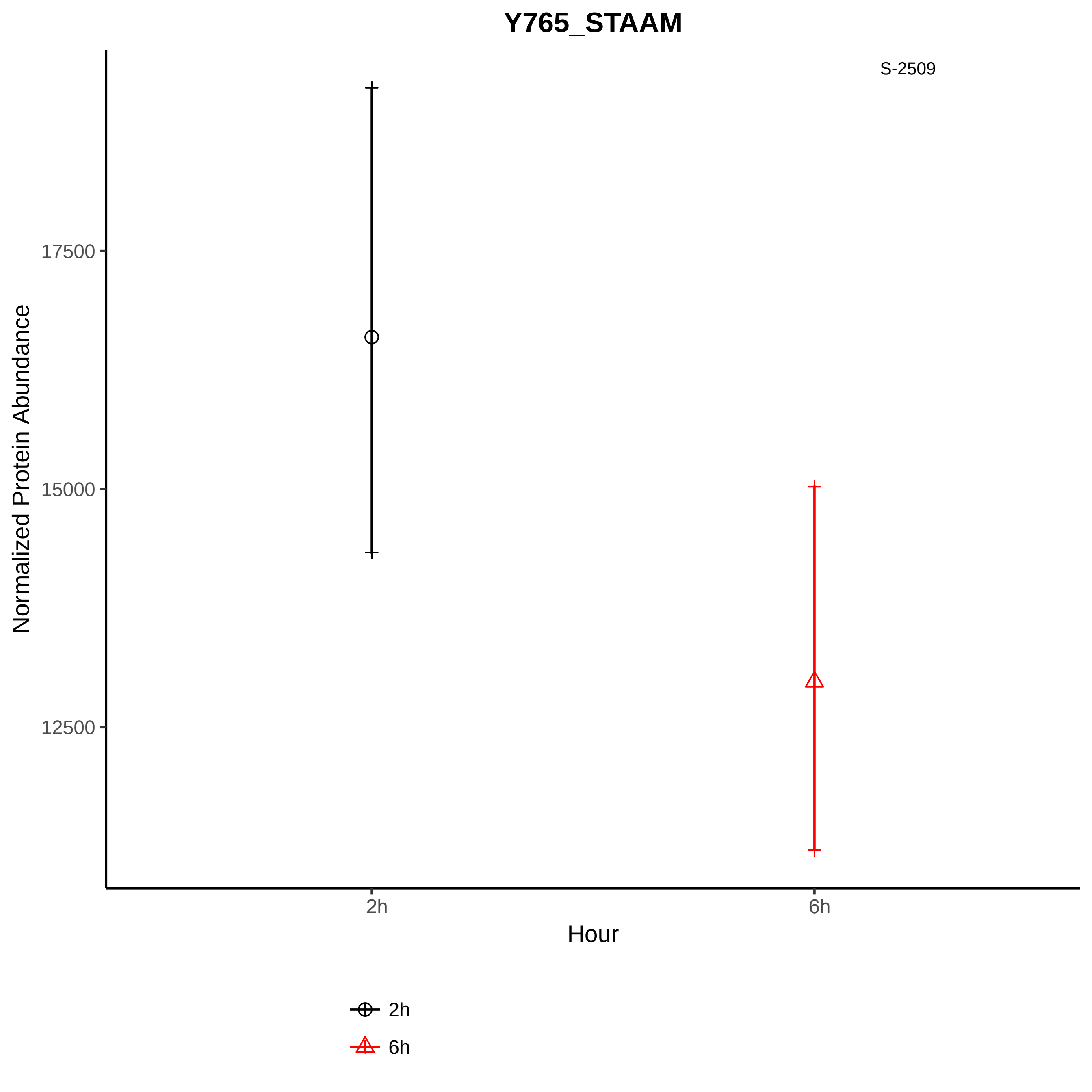
Y765_STAAM
S-2509
Normalized Protein Abundance
17500
15000
12500
2h
6h
Hour
2h
6h

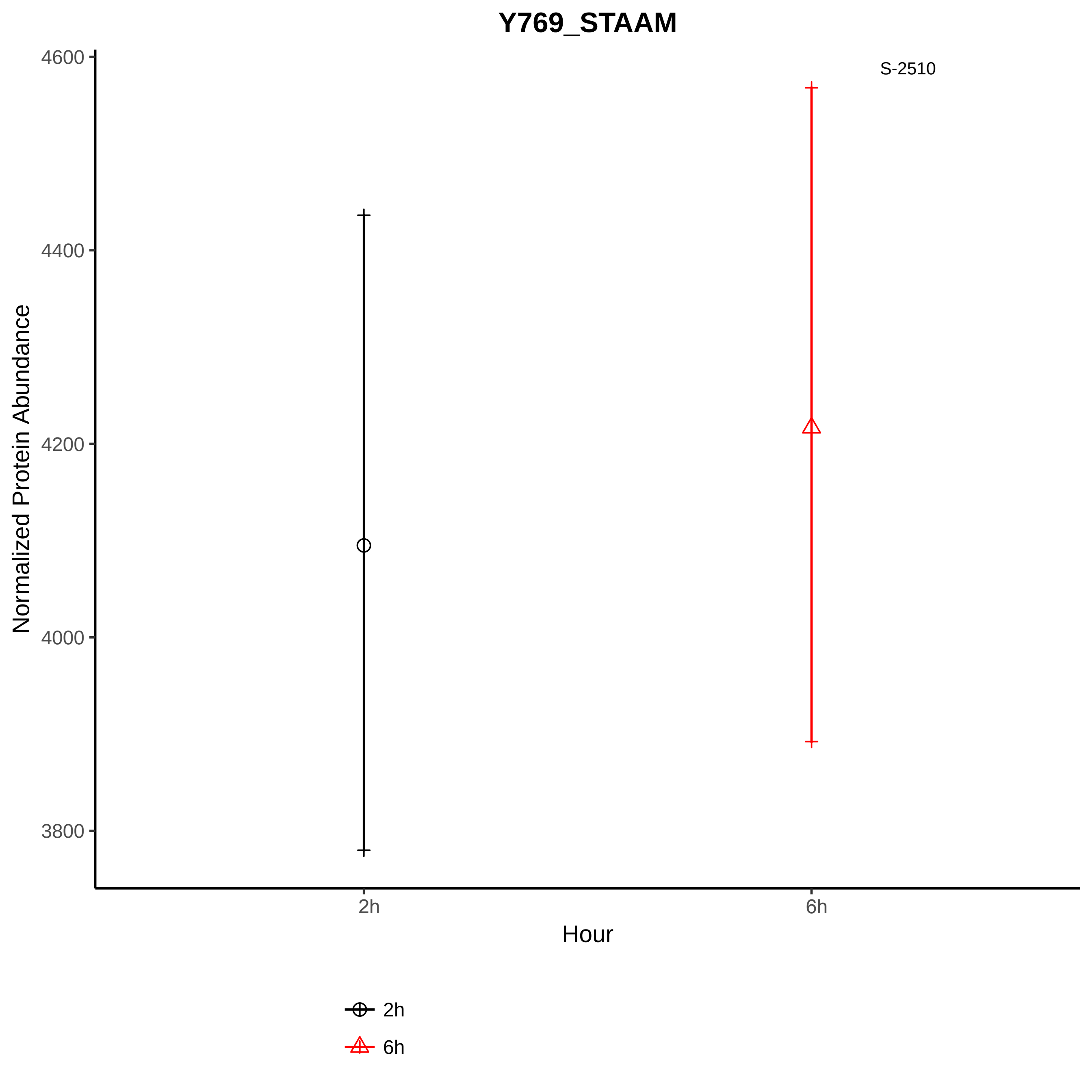
Y769_STAAM
S-2510
4600
4400
4200
4000
3800
Normalized Protein Abundance
2h
6h
Hour
2h
6h

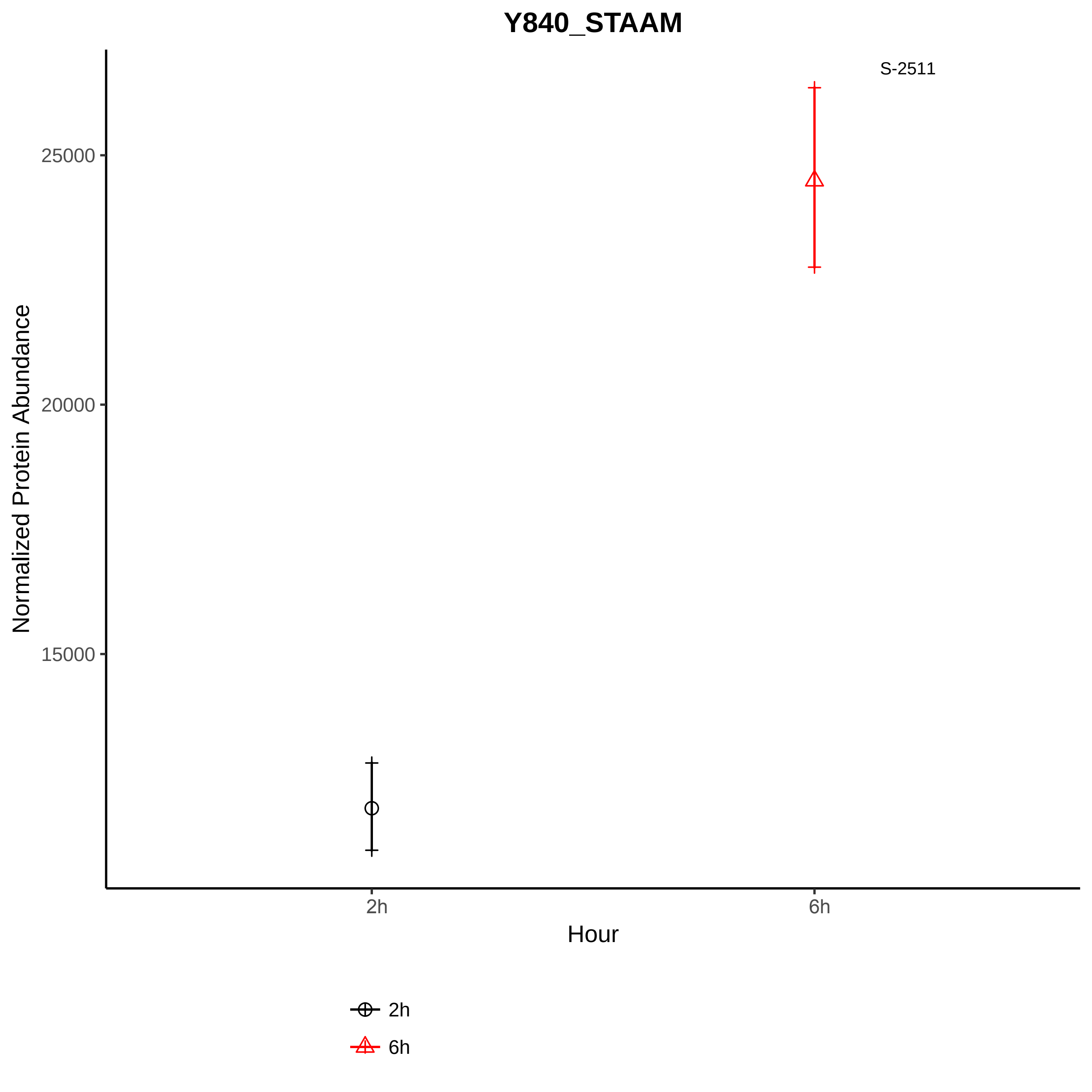

Y840_STAAM
S-2511
25000
20000
15000
Normalized Protein Abundance
2h
6h
Hour
2h
6h

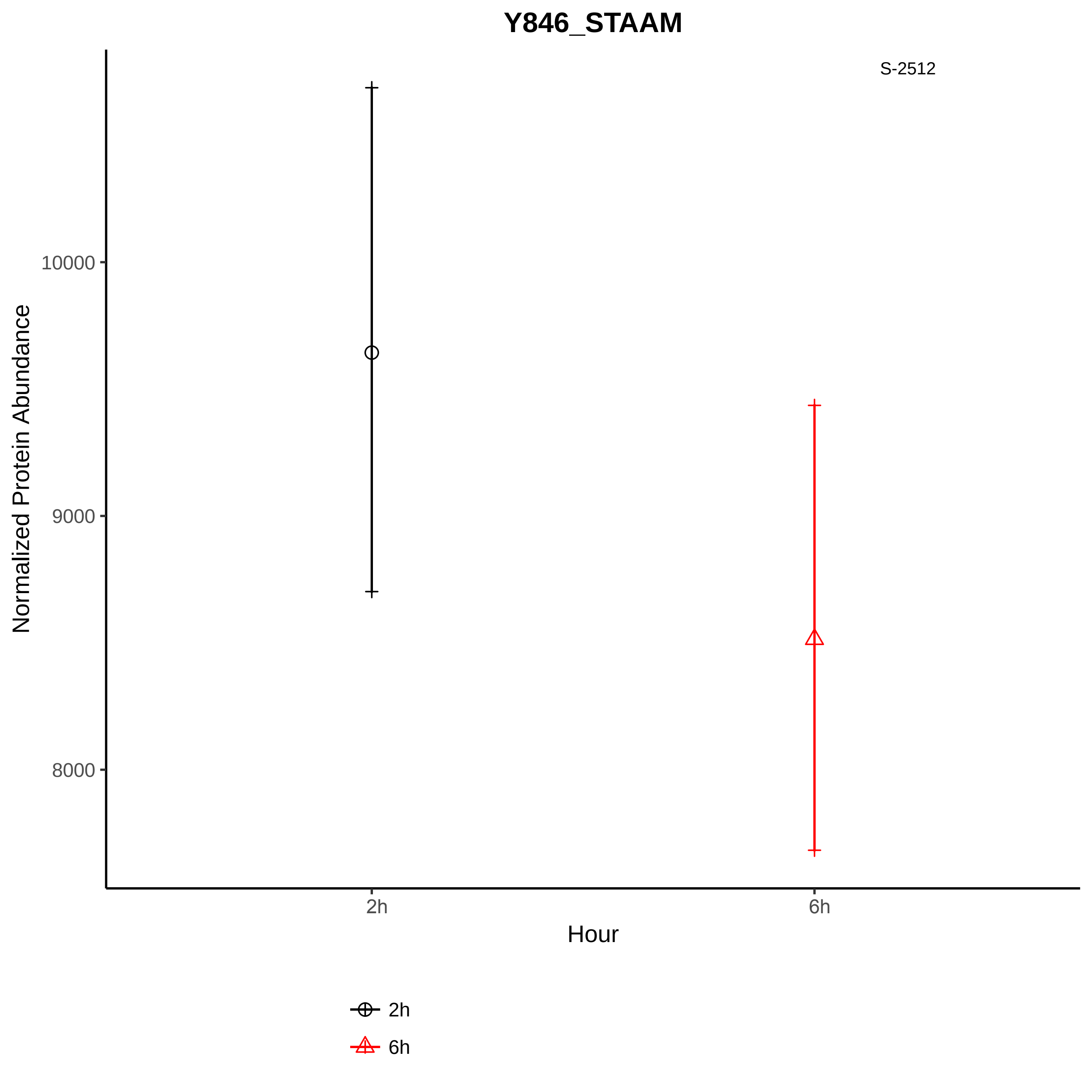
Y846_STAAM
S-2512
Normalized Protein Abundance
10000
9000
8000
2h
6h
Hour
2h
6h

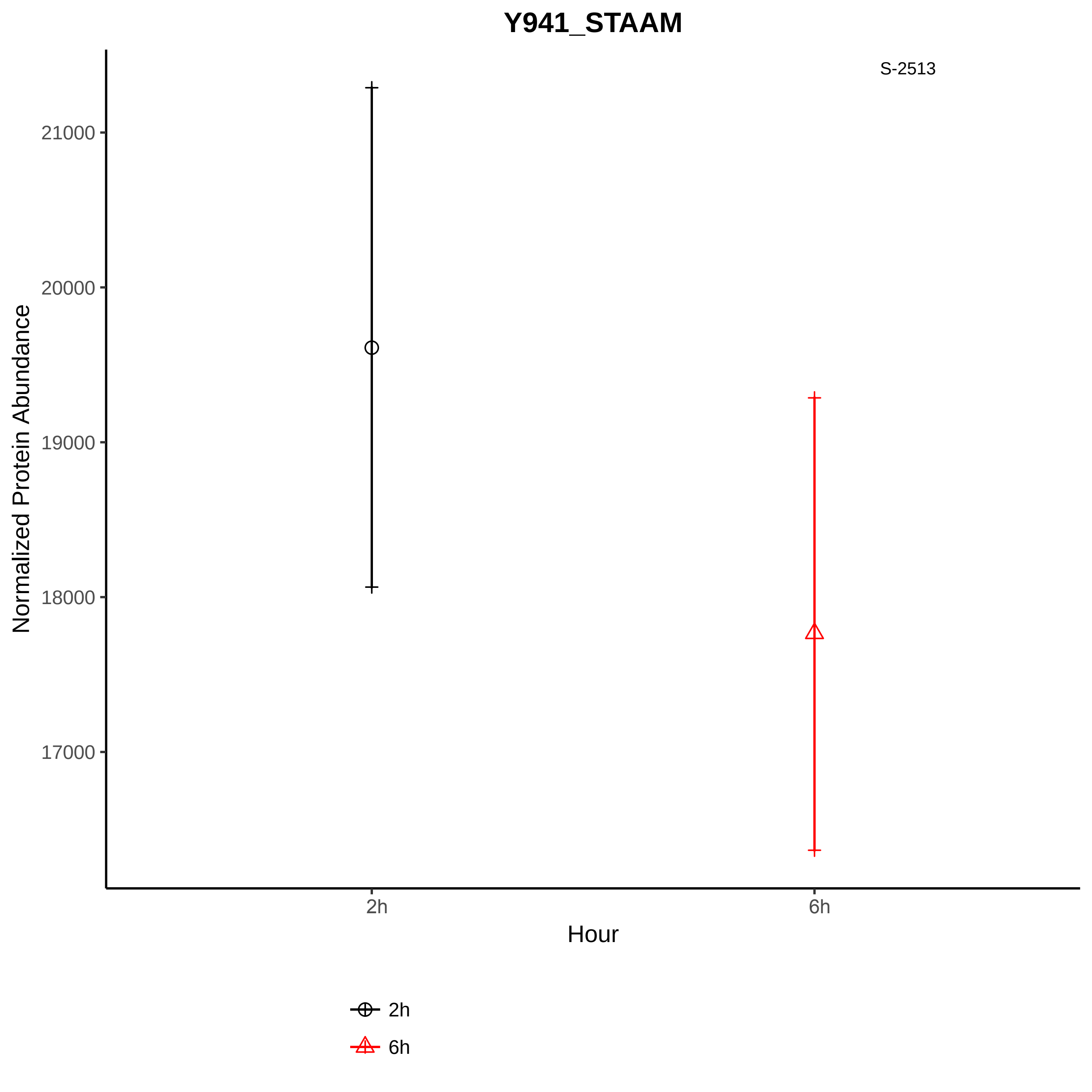
Y941_STAAM
S-2513
21000
20000
19000
18000
17000
Normalized Protein Abundance
2h
6h
Hour
2h
6h

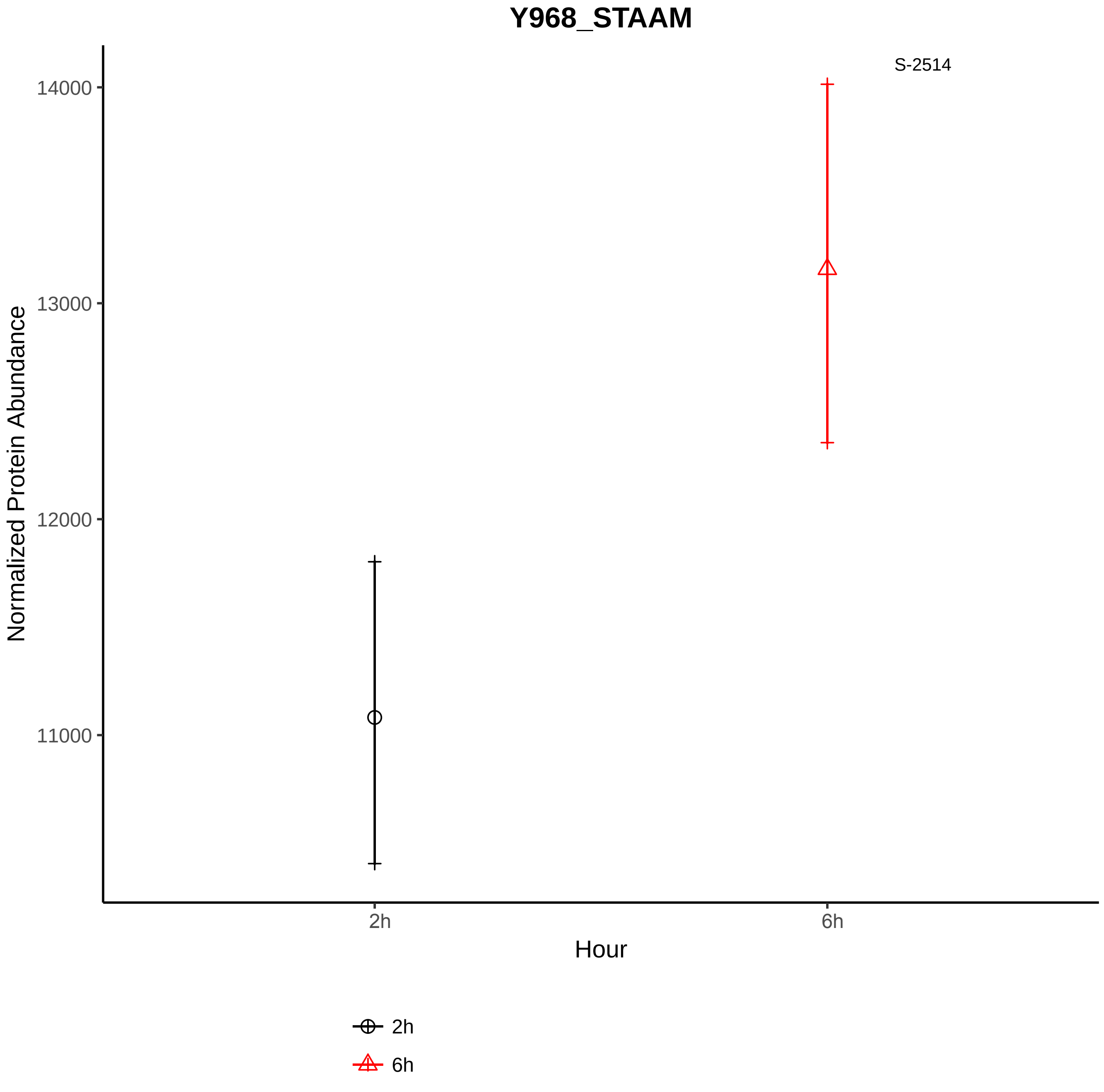
Y968_STAAM
S-2514
14000
13000
12000
11000
Normalized Protein Abundance
2h
6h
Hour
2h
6h

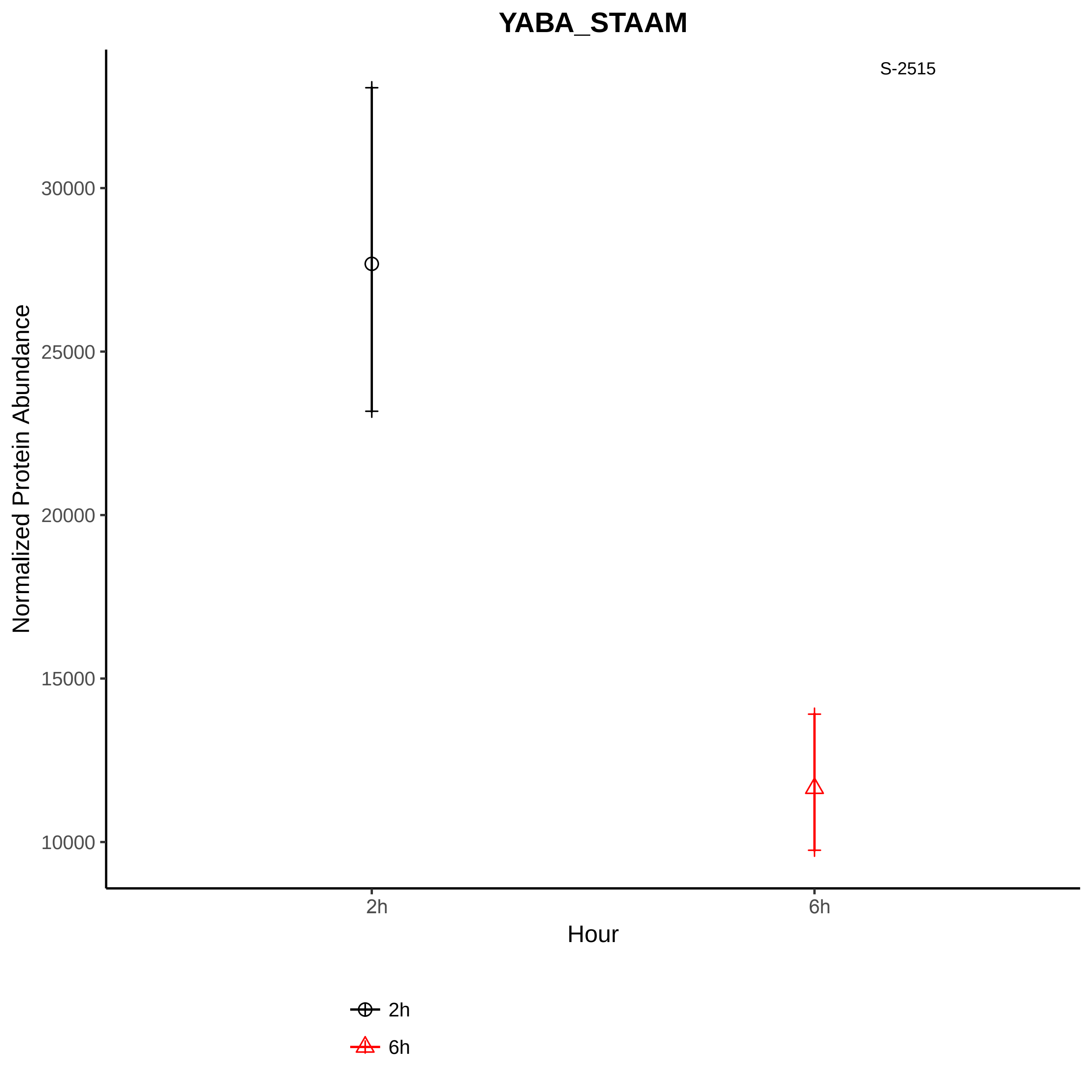

YABA_STAAM
S-2515
Normalized Protein Abundance
30000
25000
20000
15000
10000
2h
6h
Hour
2h
6h

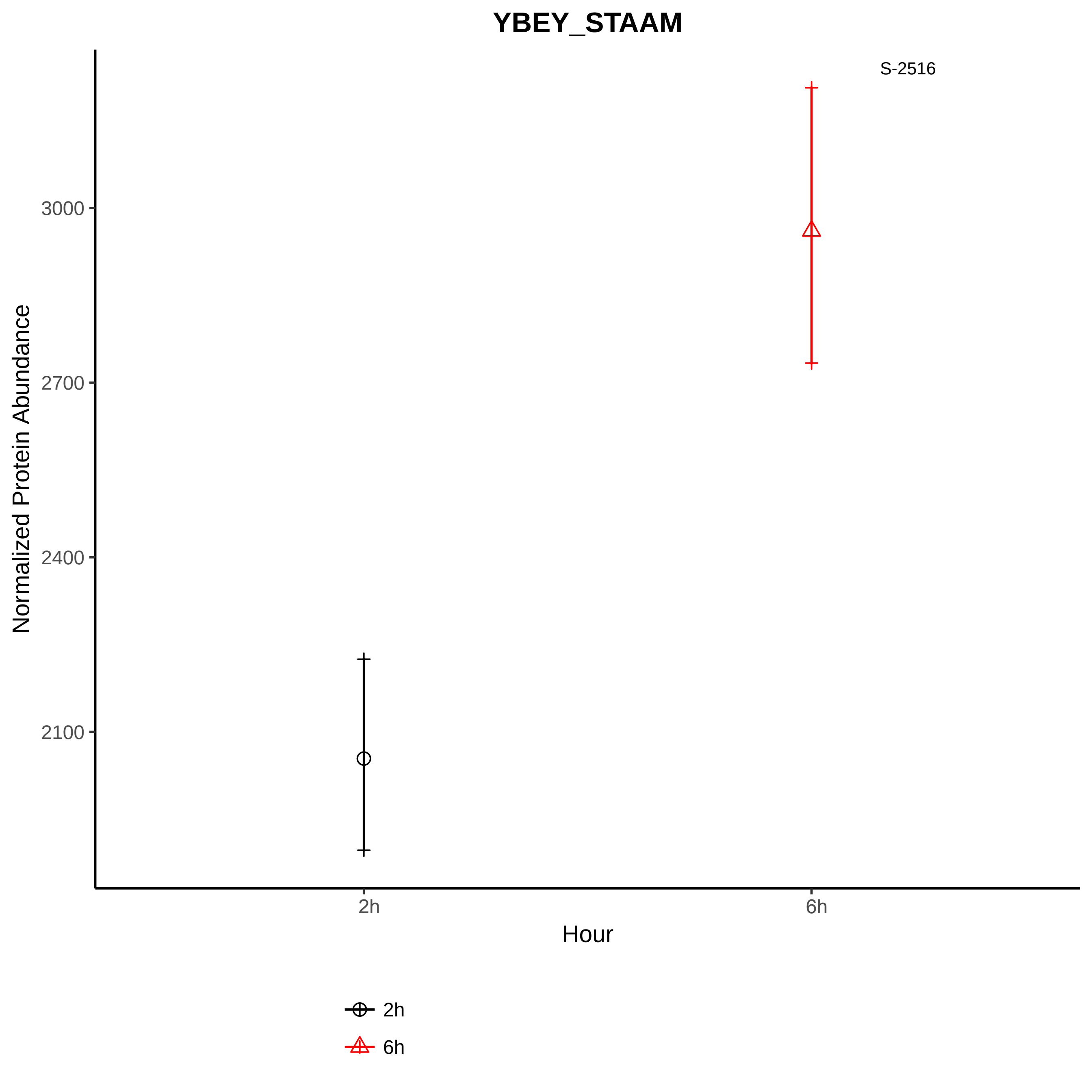

YBEY_STAAM
S-2516
Normalized Protein Abundance
3000
2700
2400
2100
2h
6h
Hour
2h
6h

Figure K.

1-way analysis: m

Pages: S217-S3018

The normalized protein abundance, lower 95% confidence interval, and upper 95% confidence interval were plotted for each protein. These plots were analyzed if the 3-way and 2-way interactions were rejected and a significant main effect (1-way) was identified for media.

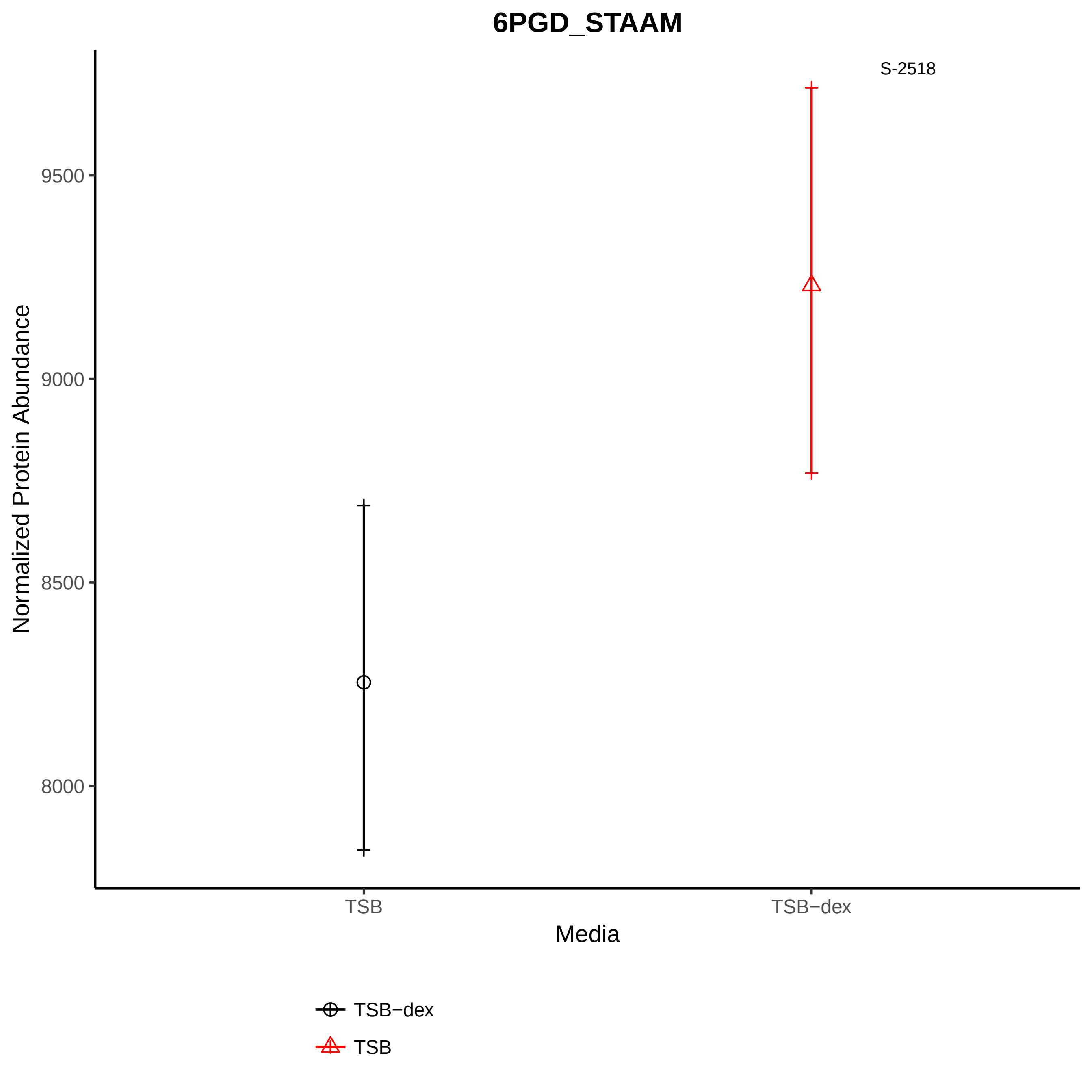
6PGD_STAAM
S-2518
Normalized Protein Abundance
9500
9000
8500
8000
TSB
TSB−dex
Media
TSB−dex
TSB

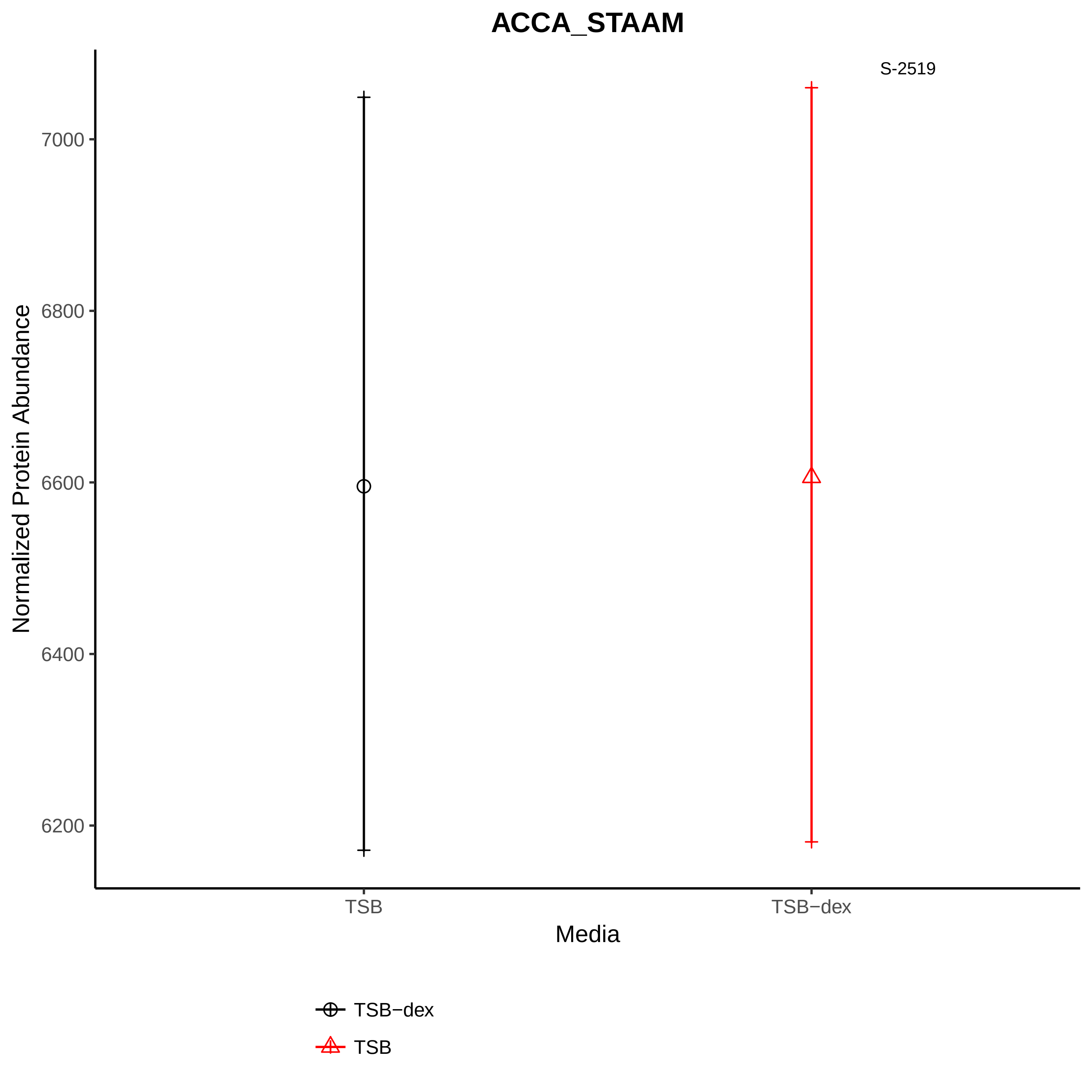
ACCA_STAAM
S-2519
7000
6800
6600
6400
6200
Normalized Protein Abundance
TSB
TSB−dex
Media
TSB−dex
TSB

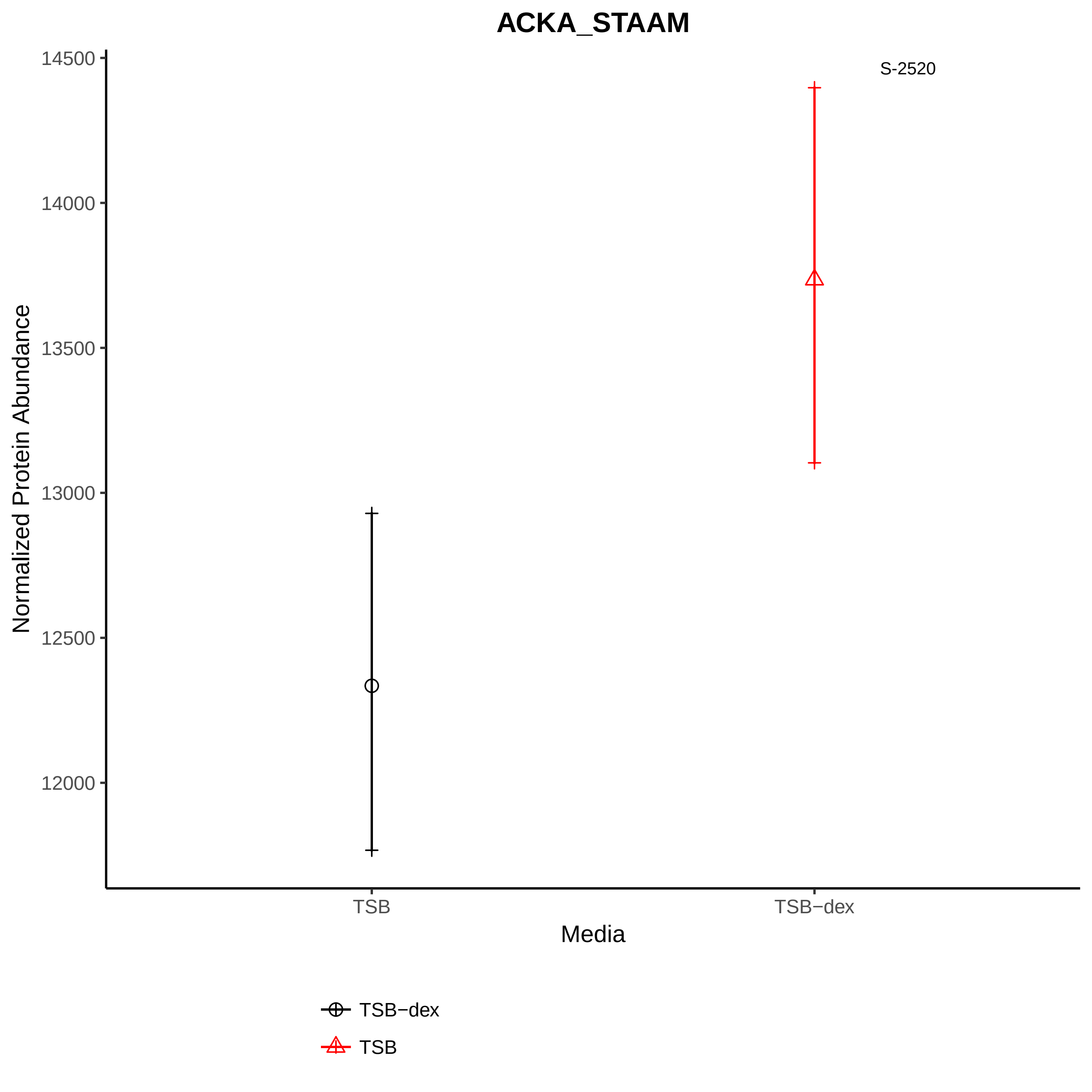

ACKA_STAAM
S-2520
Normalized Protein Abundance
14500
14000
13500
13000
12500
12000
TSB
TSB−dex
Media
TSB−dex
TSB

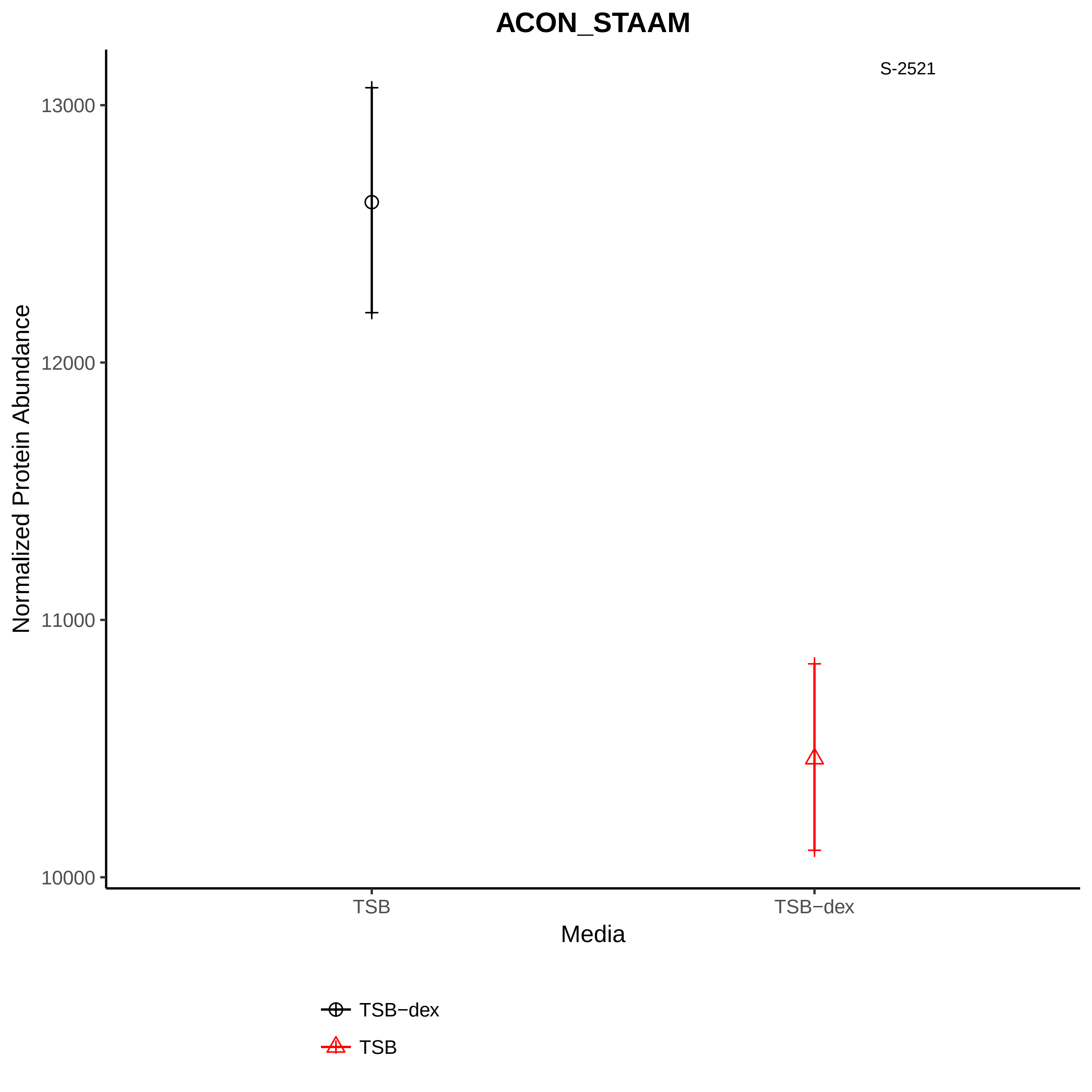

ACON_STAAM
S-2521
Normalized Protein Abundance
13000
12000
11000
10000
TSB
TSB−dex
Media
TSB−dex
TSB

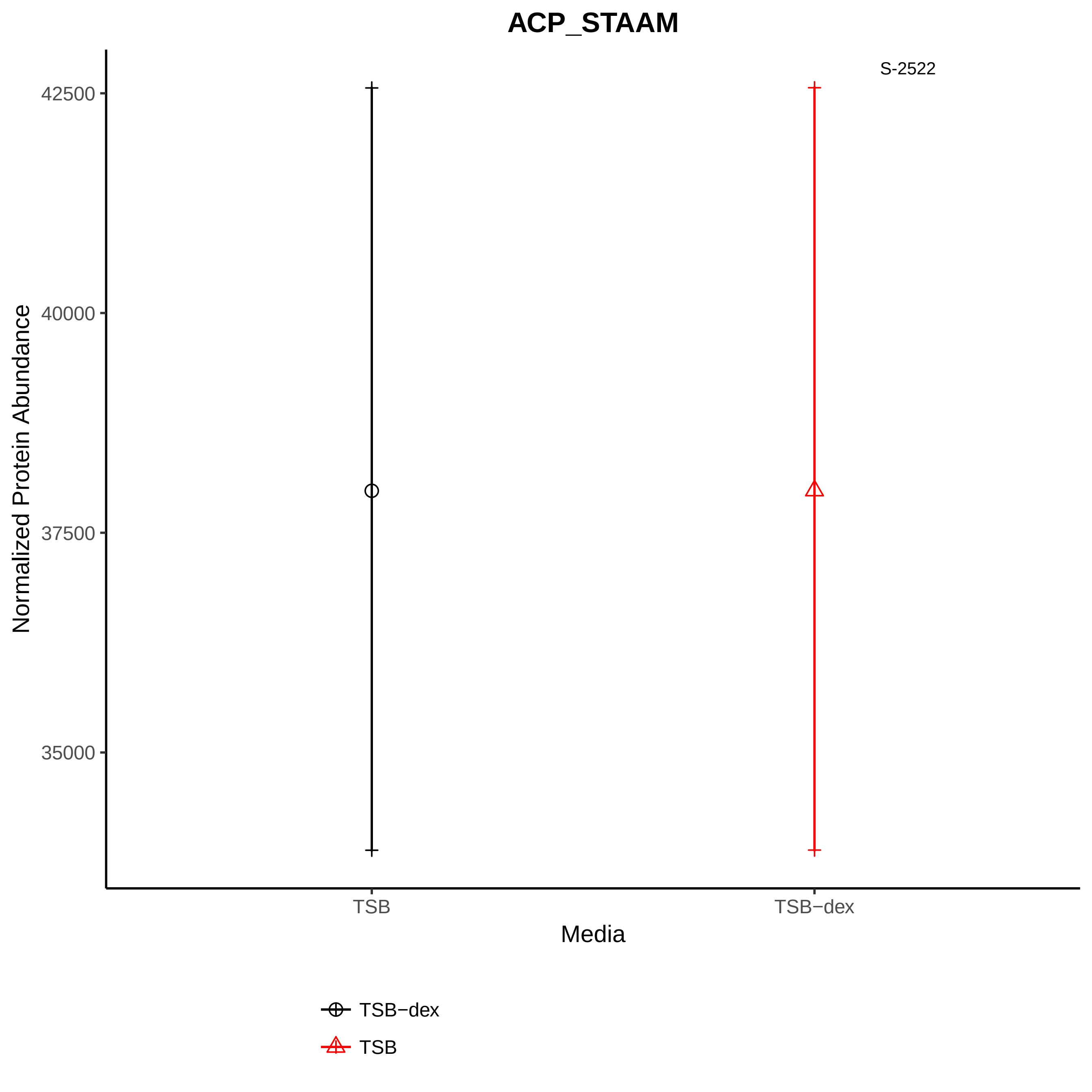

ACP_STAAM
S-2522
Normalized Protein Abundance
42500
40000
37500
35000
TSB
TSB−dex
Media
TSB−dex
TSB

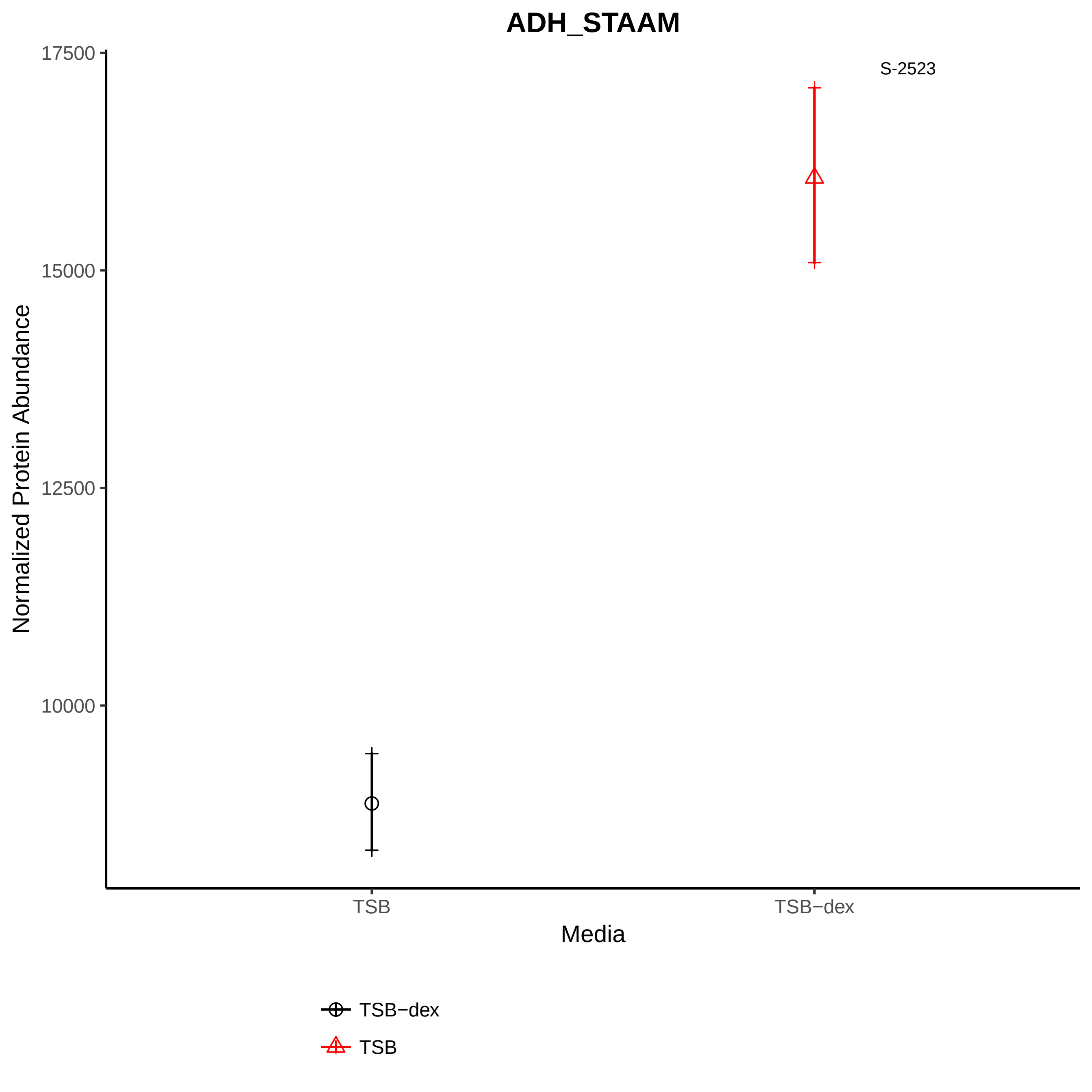
ADH_STAAM
S-2523
17500
15000
12500
10000
Normalized Protein Abundance
TSB
TSB−dex
Media
TSB−dex
TSB

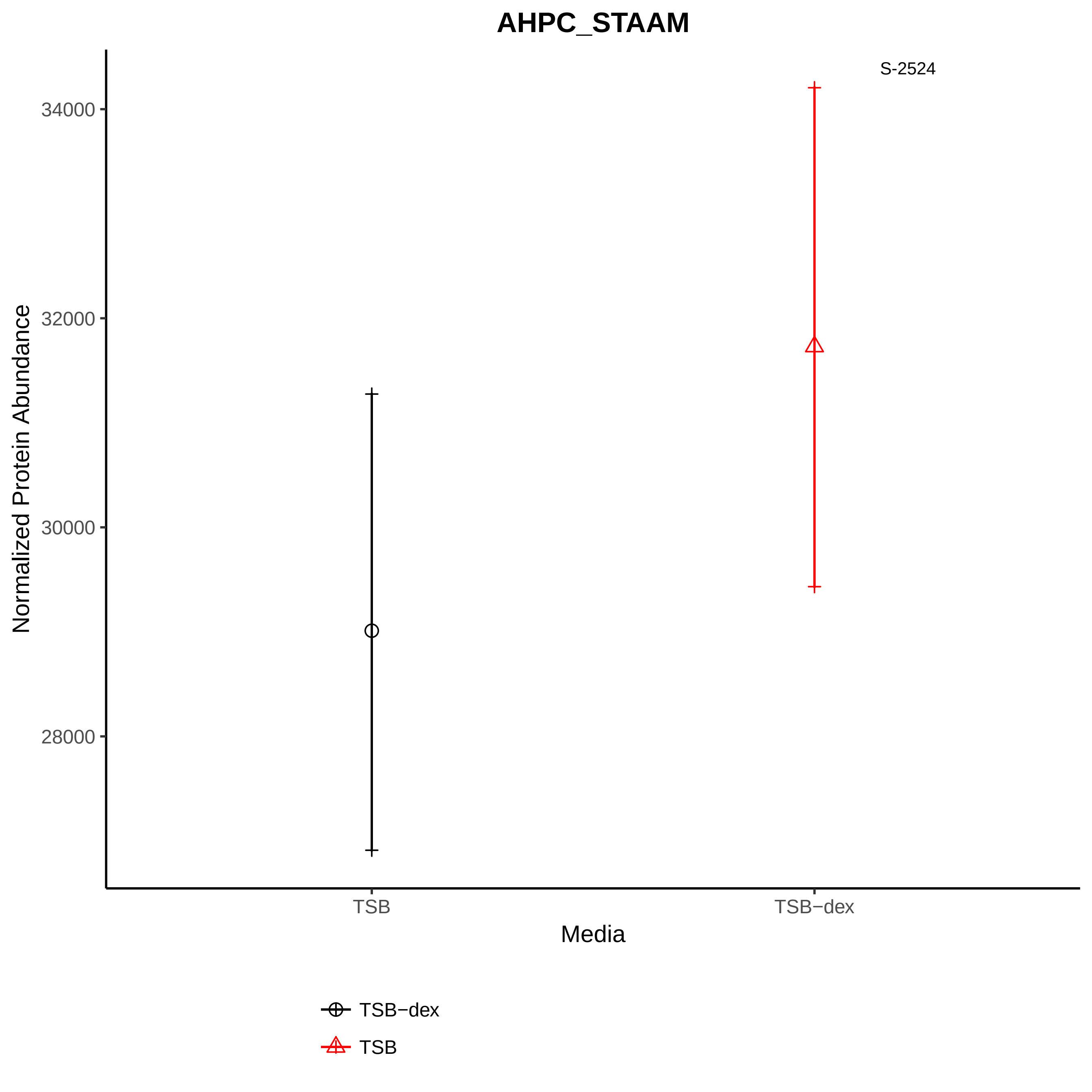

AHPC_STAAM
S-2524
34000
32000
30000
28000
Normalized Protein Abundance
TSB
TSB−dex
Media
TSB−dex
TSB

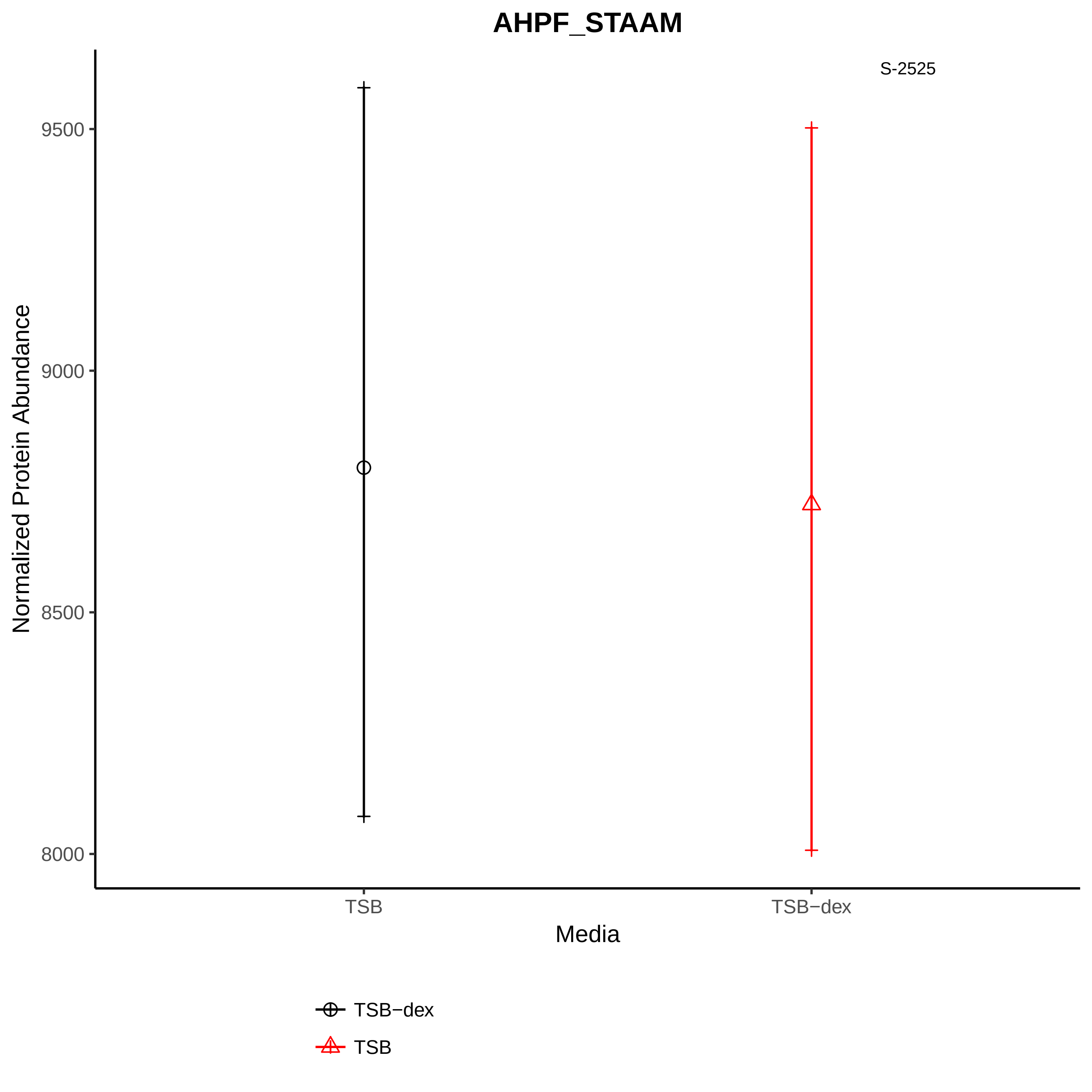

AHPF_STAAM
S-2525
Normalized Protein Abundance
9500
9000
8500
8000
TSB
TSB−dex
Media
TSB−dex
TSB

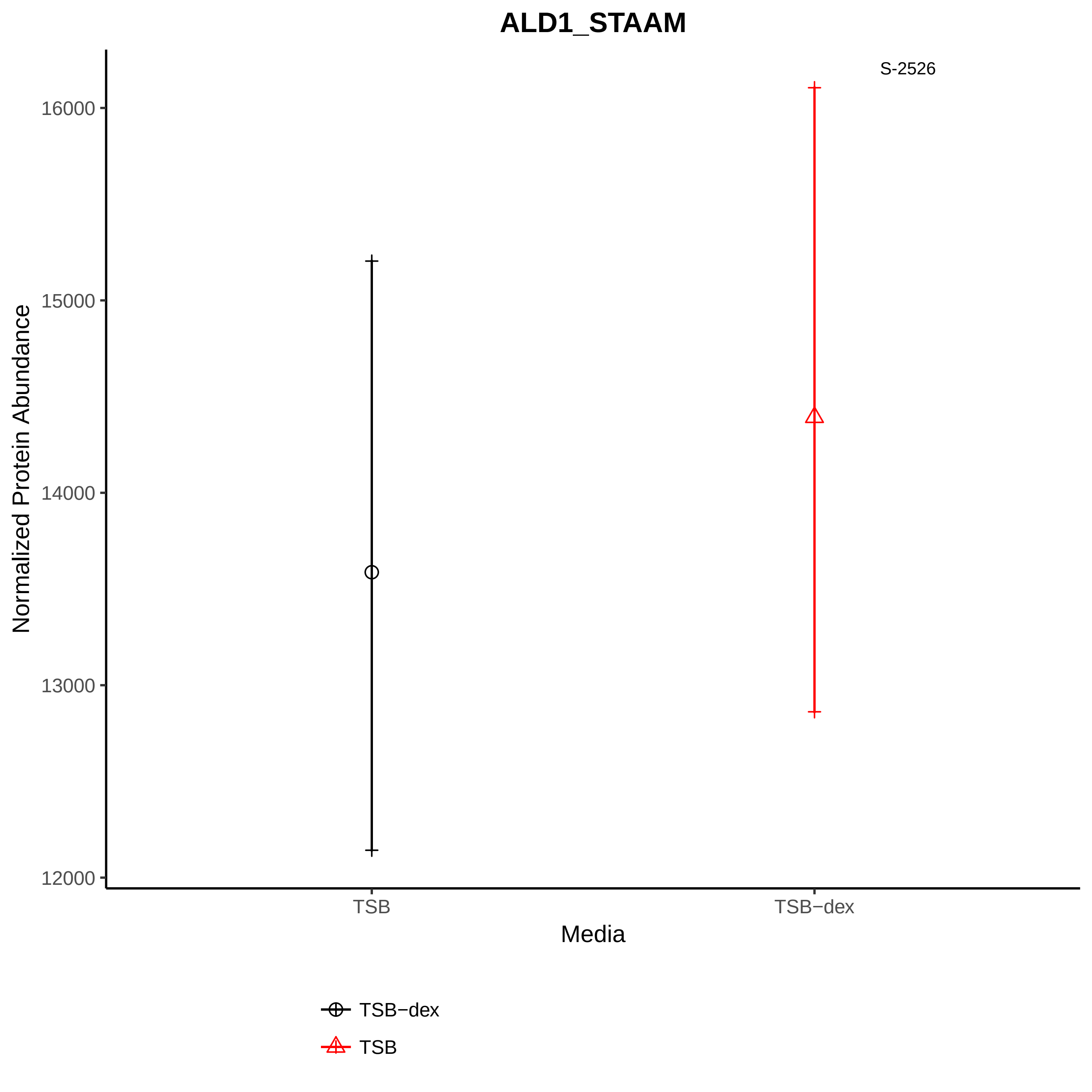
ALD1_STAAM
S-2526
Normalized Protein Abundance
16000
15000
14000
13000
12000
TSB
TSB−dex
Media
TSB−dex
TSB

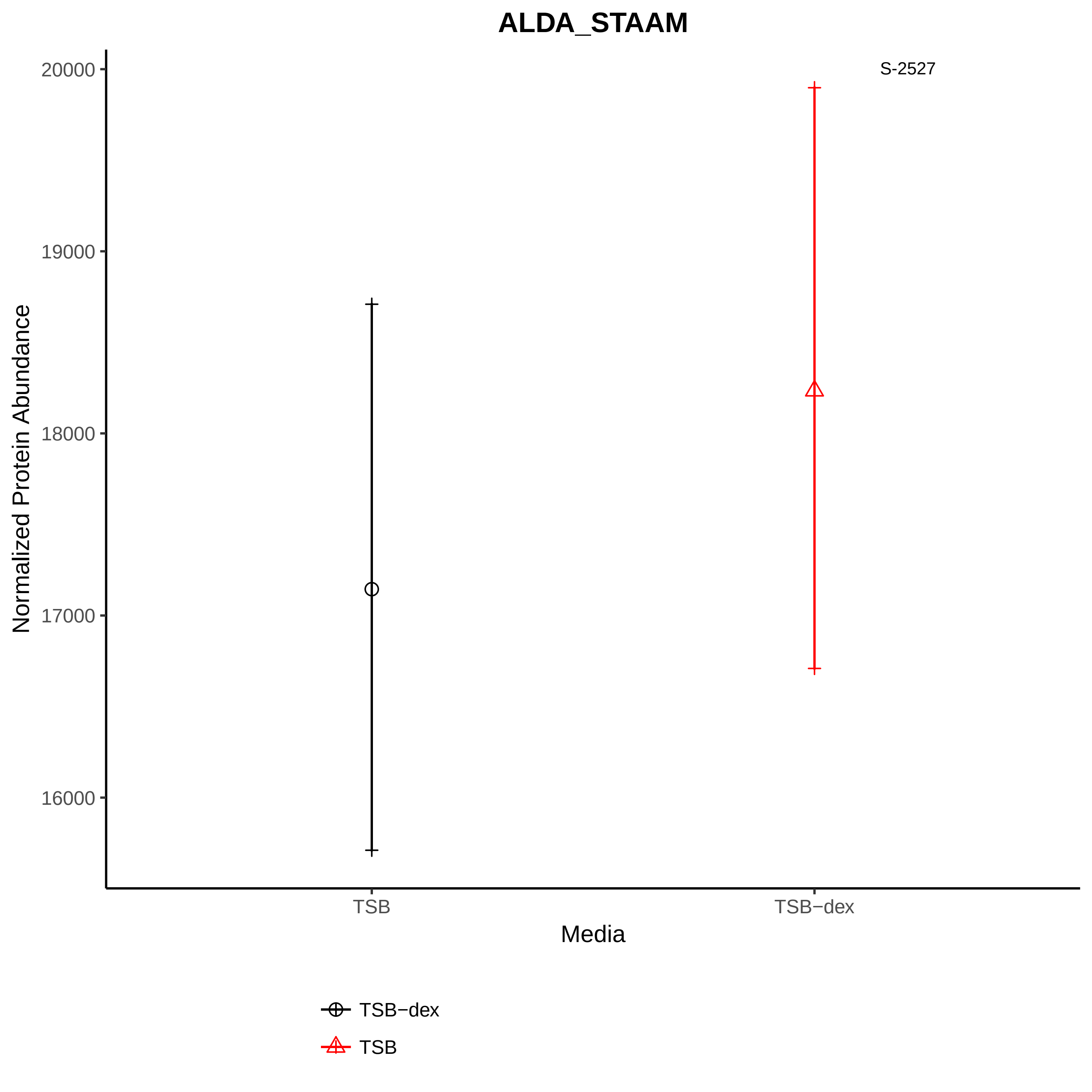
ALDA_STAAM
S-2527
20000
19000
18000
17000
16000
Normalized Protein Abundance
TSB
TSB−dex
Media
TSB−dex
TSB

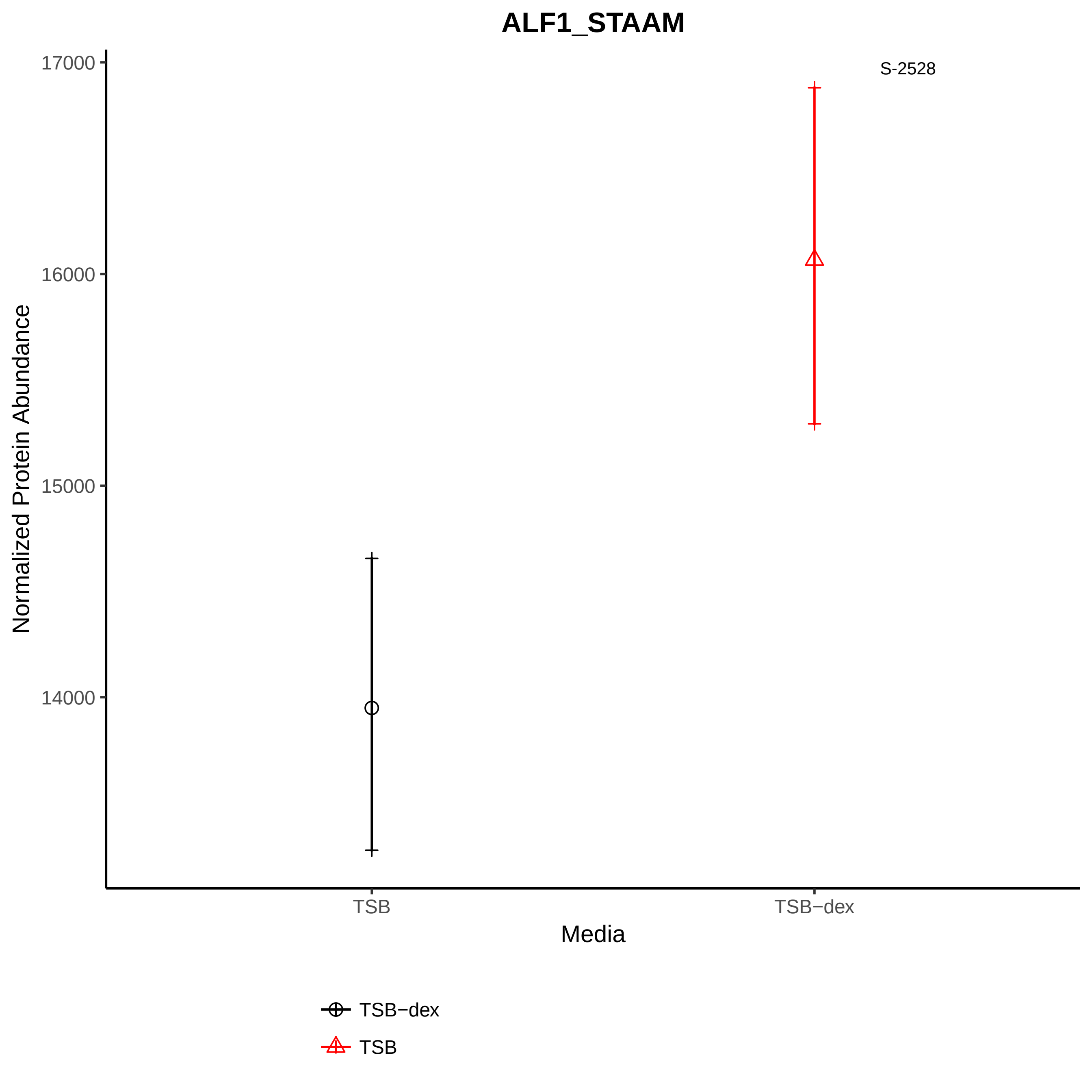
ALF1_STAAM
S-2528
Normalized Protein Abundance
17000
16000
15000
14000
TSB
TSB−dex
Media
TSB−dex
TSB

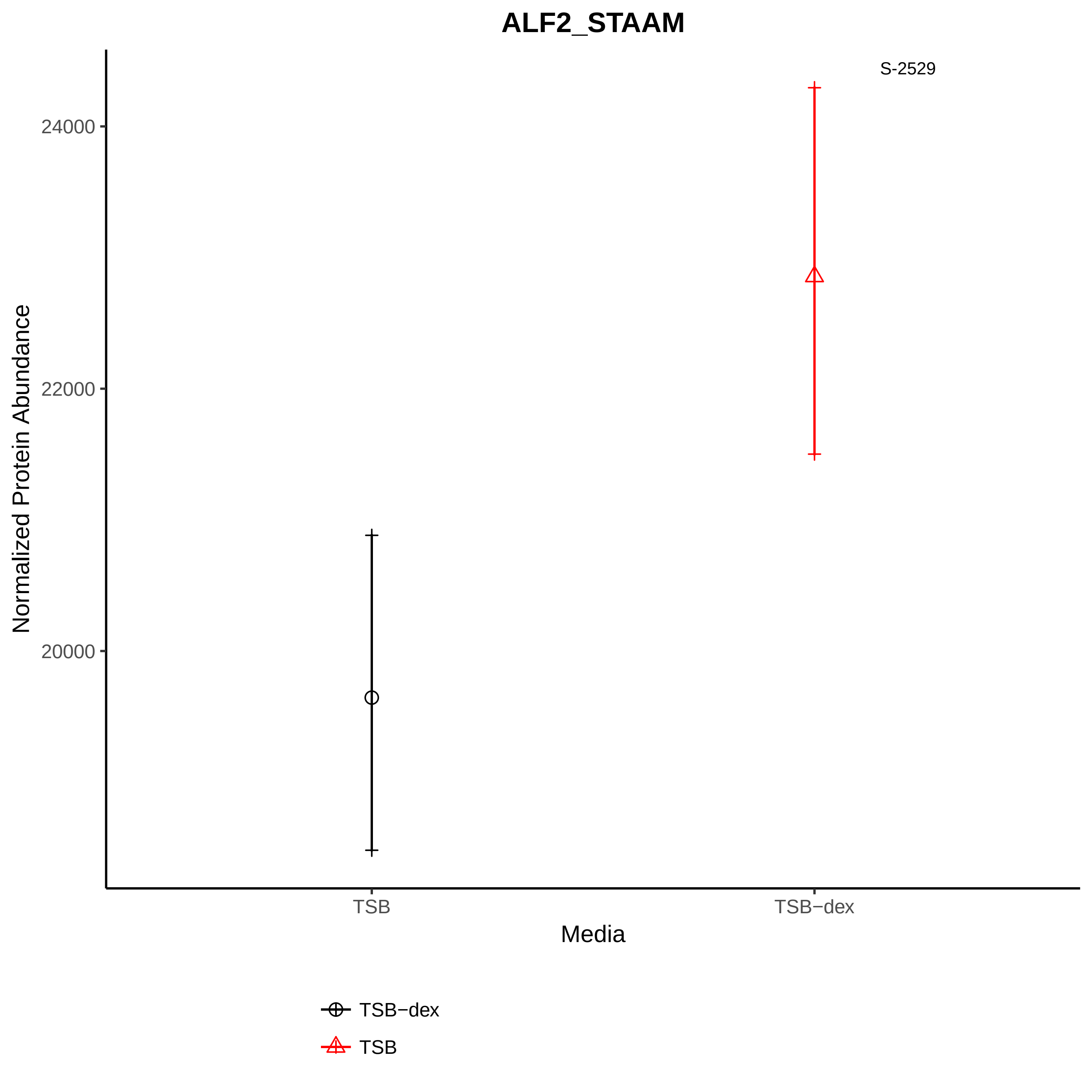

ALF2_STAAM
S-2529
24000
22000
20000
Normalized Protein Abundance
TSB
TSB−dex
Media
TSB−dex
TSB

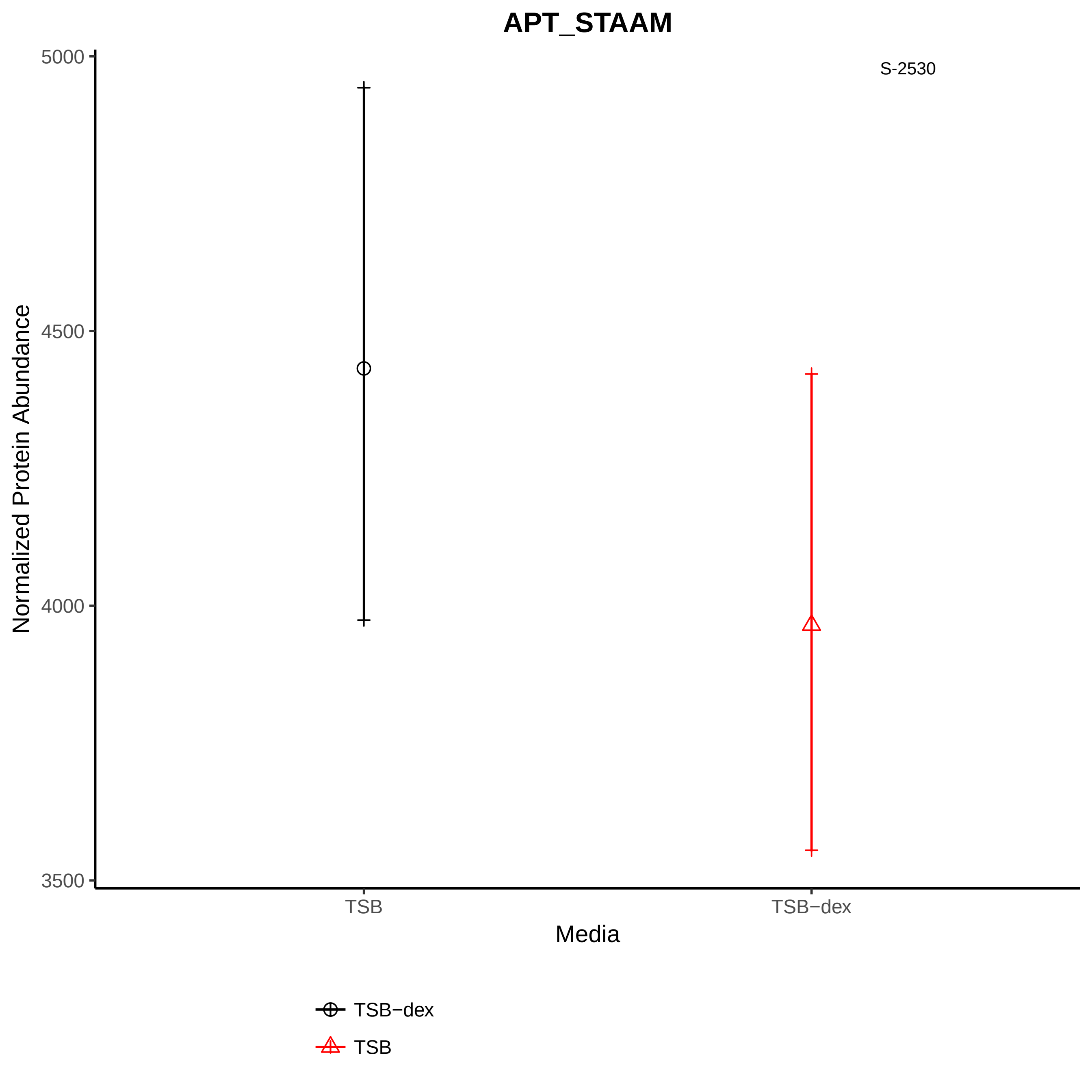

APT_STAAM
S-2530
5000
4500
4000
3500
Normalized Protein Abundance
TSB
TSB−dex
Media
TSB−dex
TSB

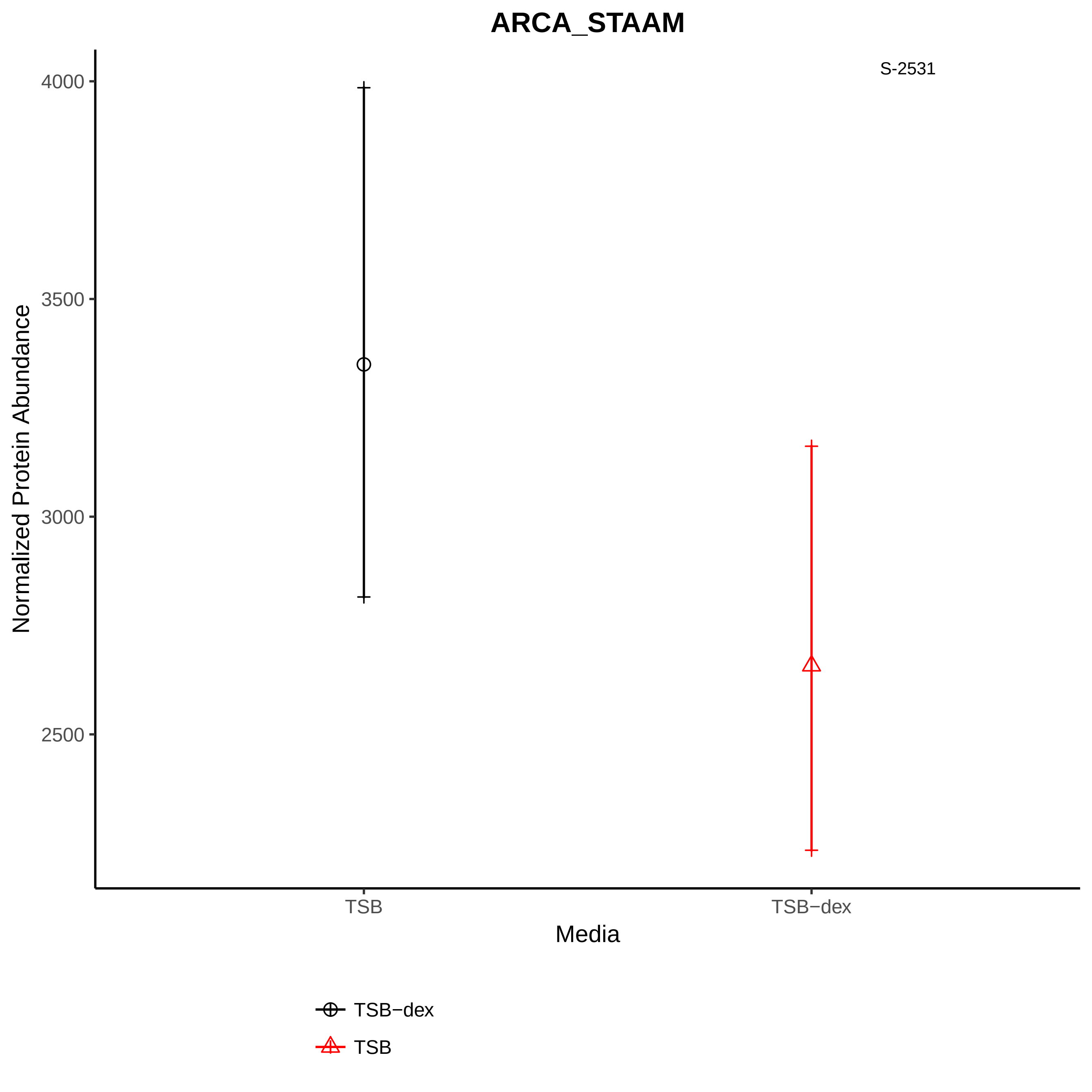
ARCA_STAAM
S-2531
Normalized Protein Abundance
4000
3500
3000
2500
TSB
TSB−dex
Media
TSB−dex
TSB

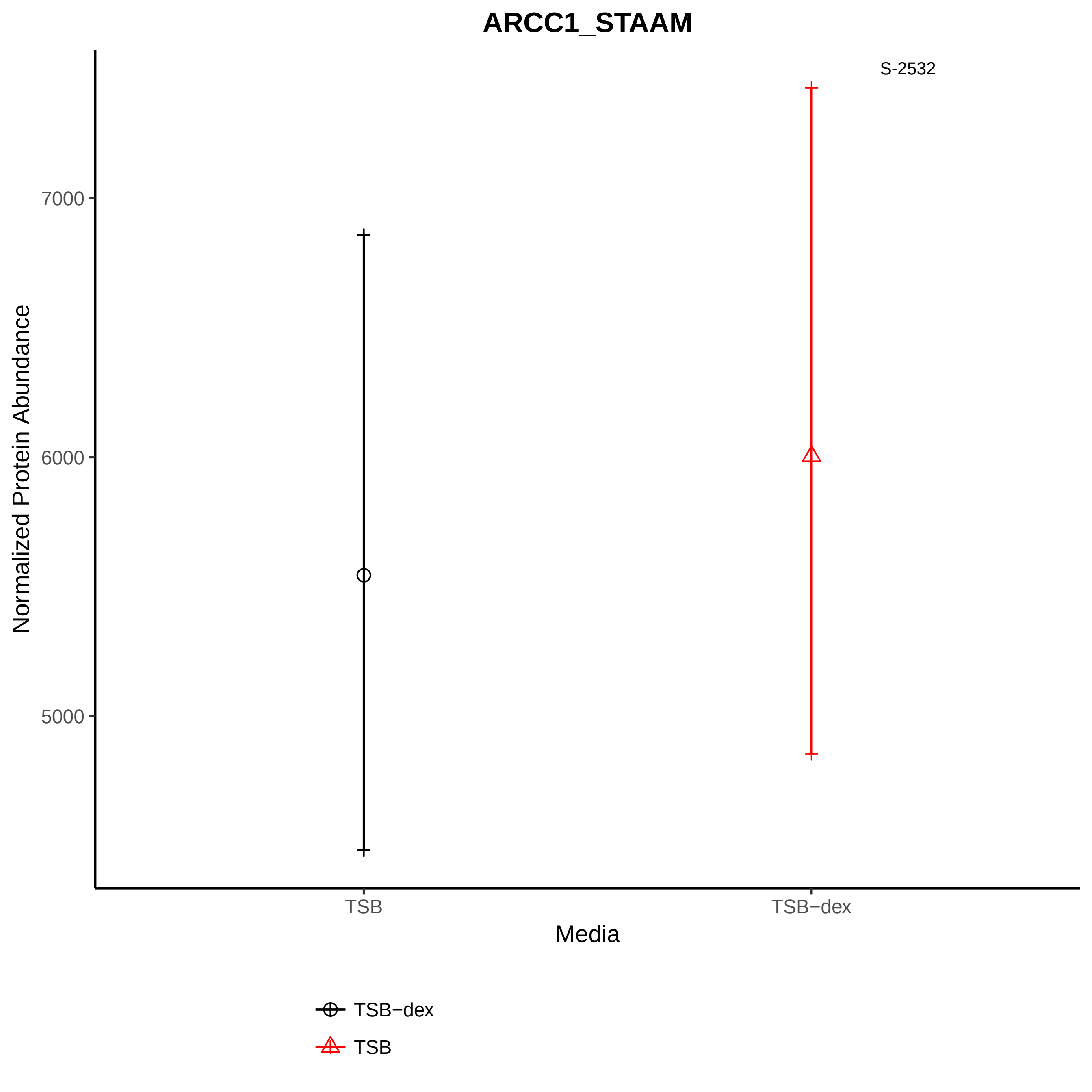

ARCC1_STAAM
S-2532
7000
6000
5000
Normalized Protein Abundance
TSB
TSB−dex
Media
TSB−dex
TSB

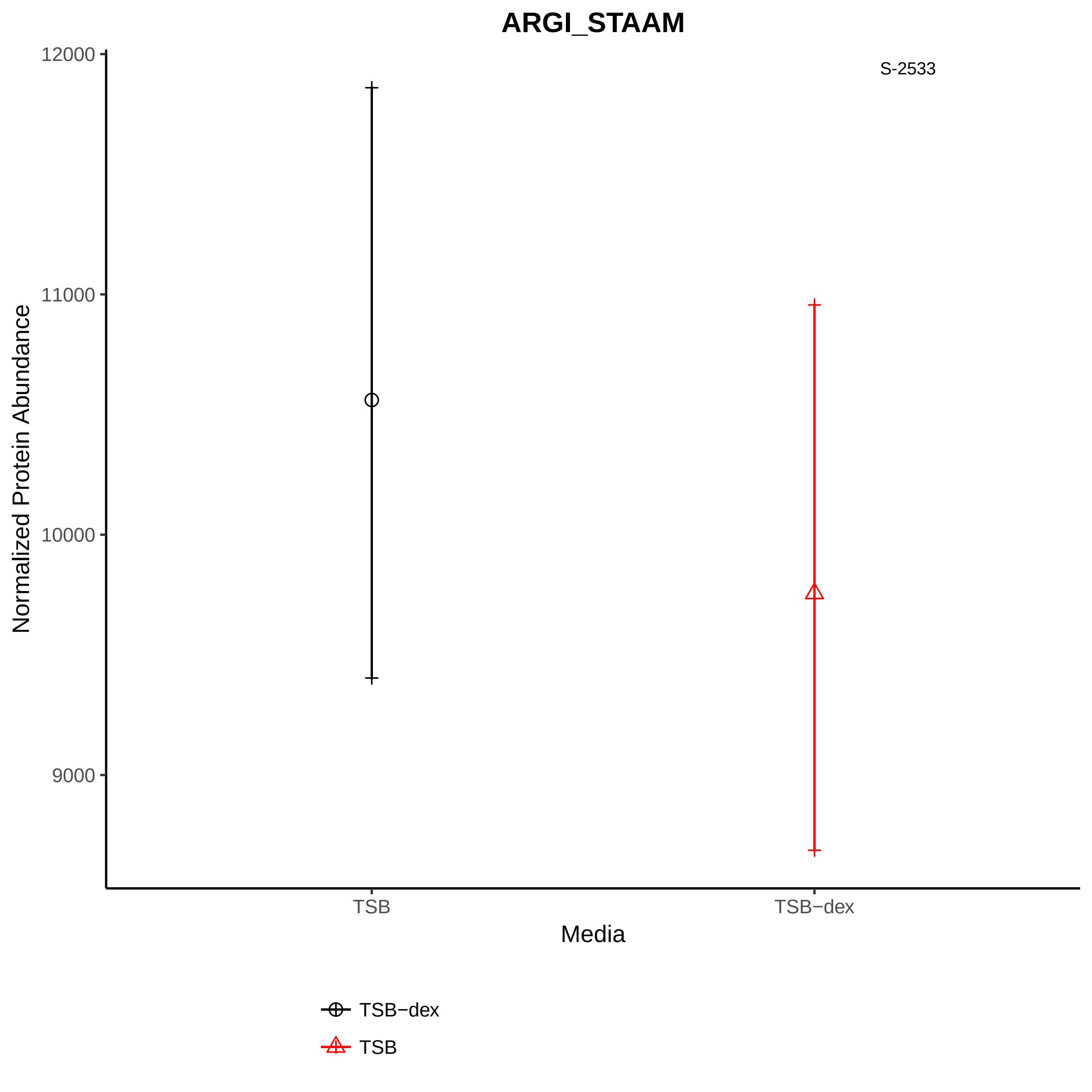

ARGI_STAAM
S-2533
12000
11000
10000
9000
Normalized Protein Abundance
TSB
TSB−dex
Media
TSB−dex
TSB

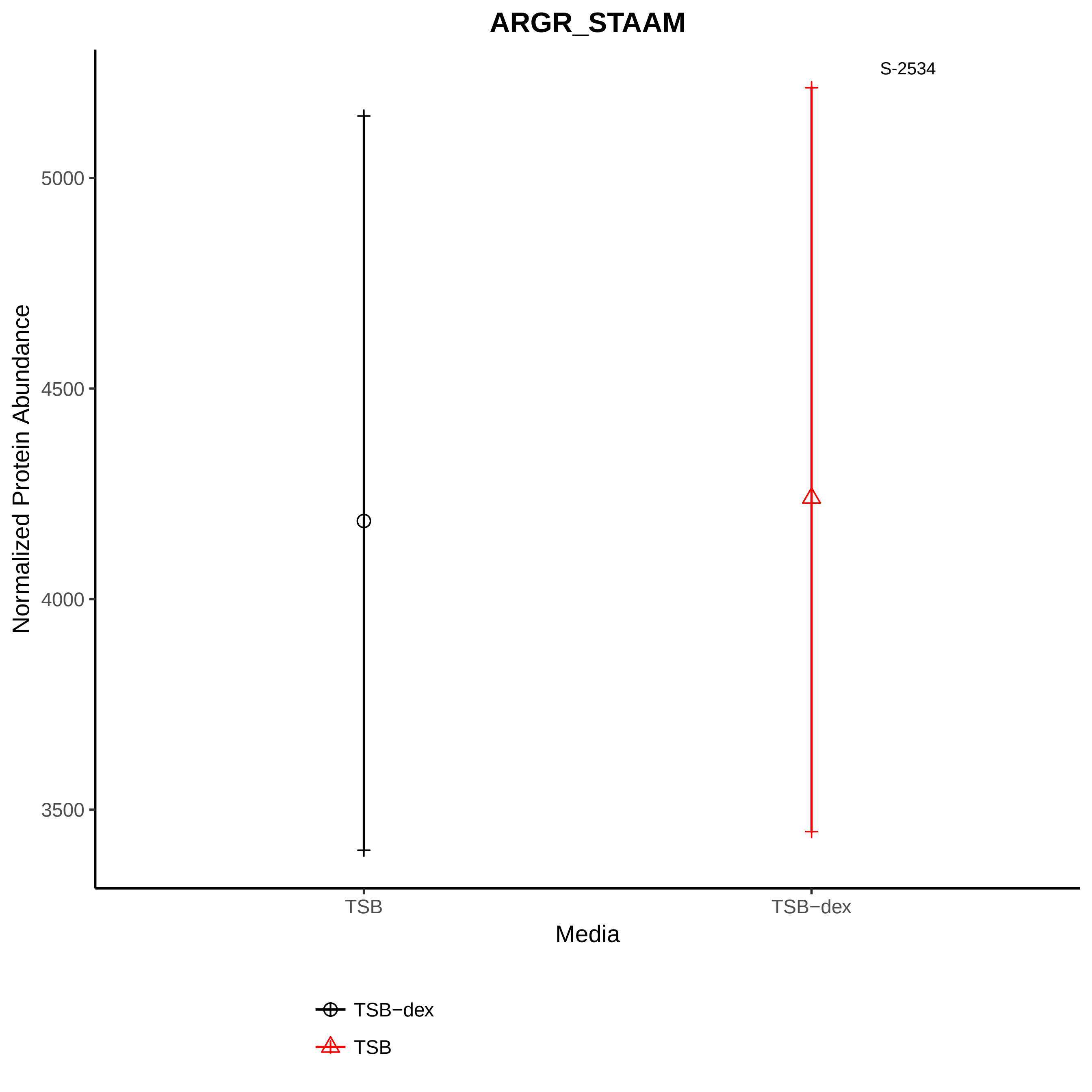
ARGR_STAAM
S-2534
Normalized Protein Abundance
5000
4500
4000
3500
TSB
TSB−dex
Media
TSB−dex
TSB

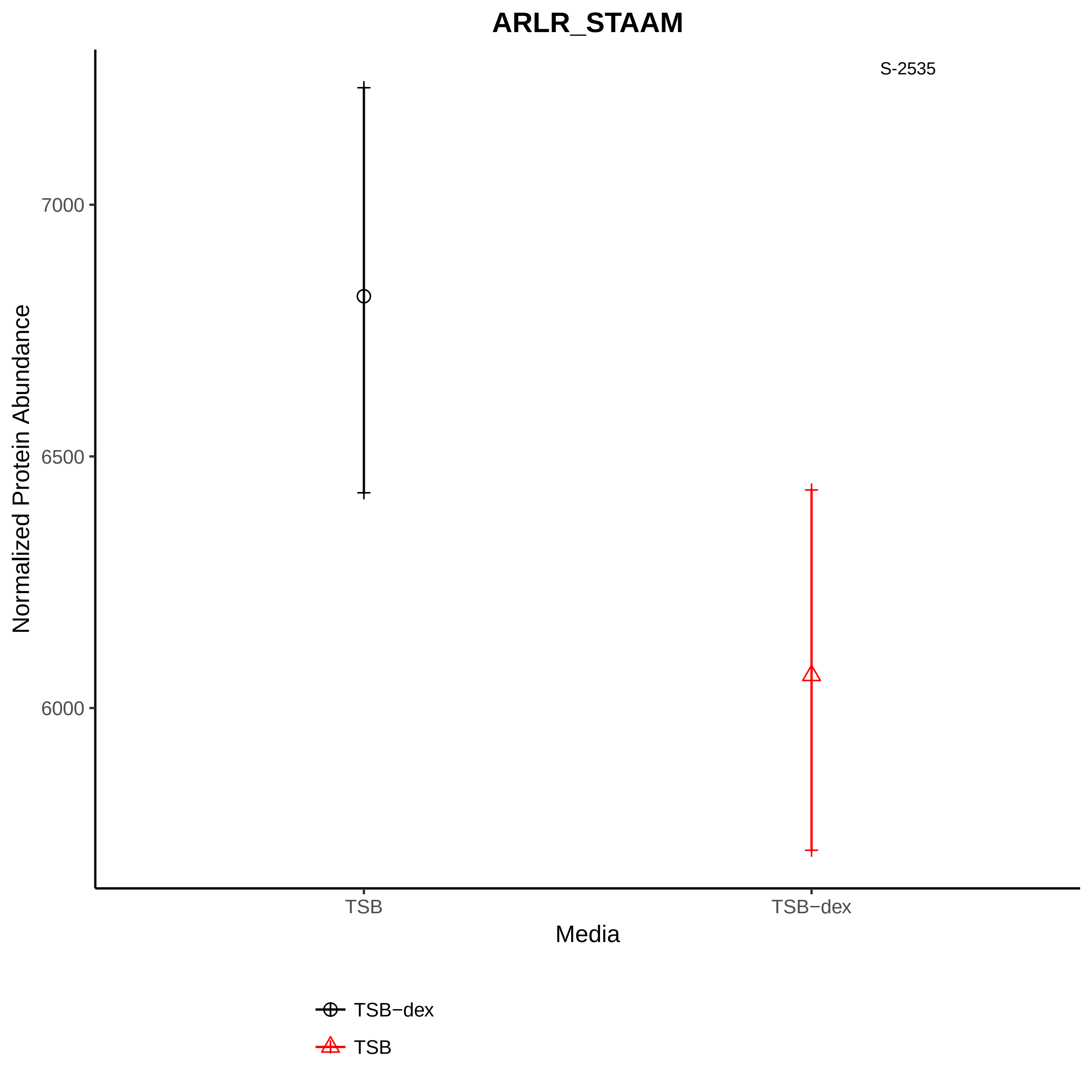
ARLR_STAAM
S-2535
Normalized Protein Abundance
7000
6500
6000
TSB
TSB−dex
Media
TSB−dex
TSB

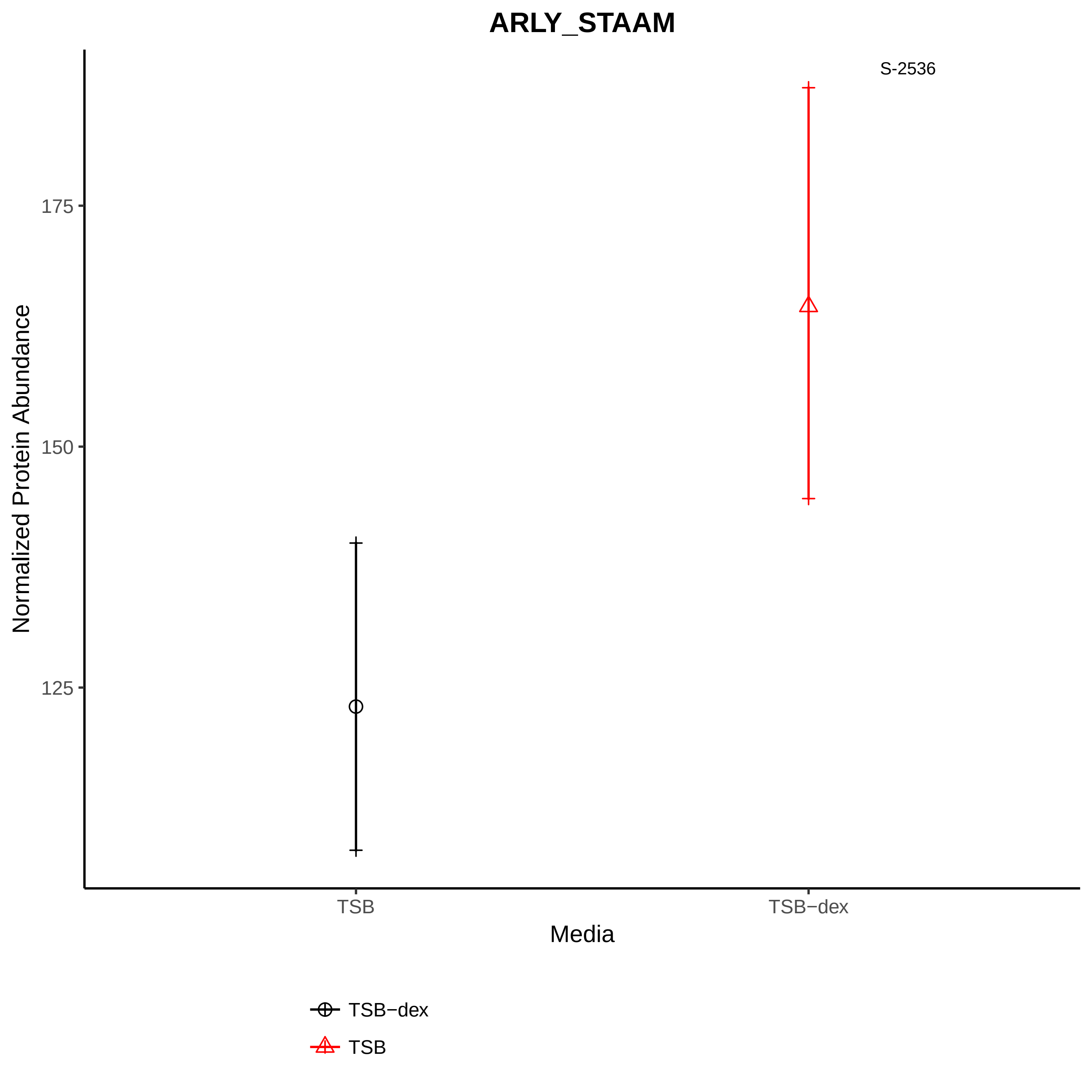

ARLY_STAAM
S-2536
175
150
125
Normalized Protein Abundance
TSB
TSB−dex
Media
TSB−dex
TSB

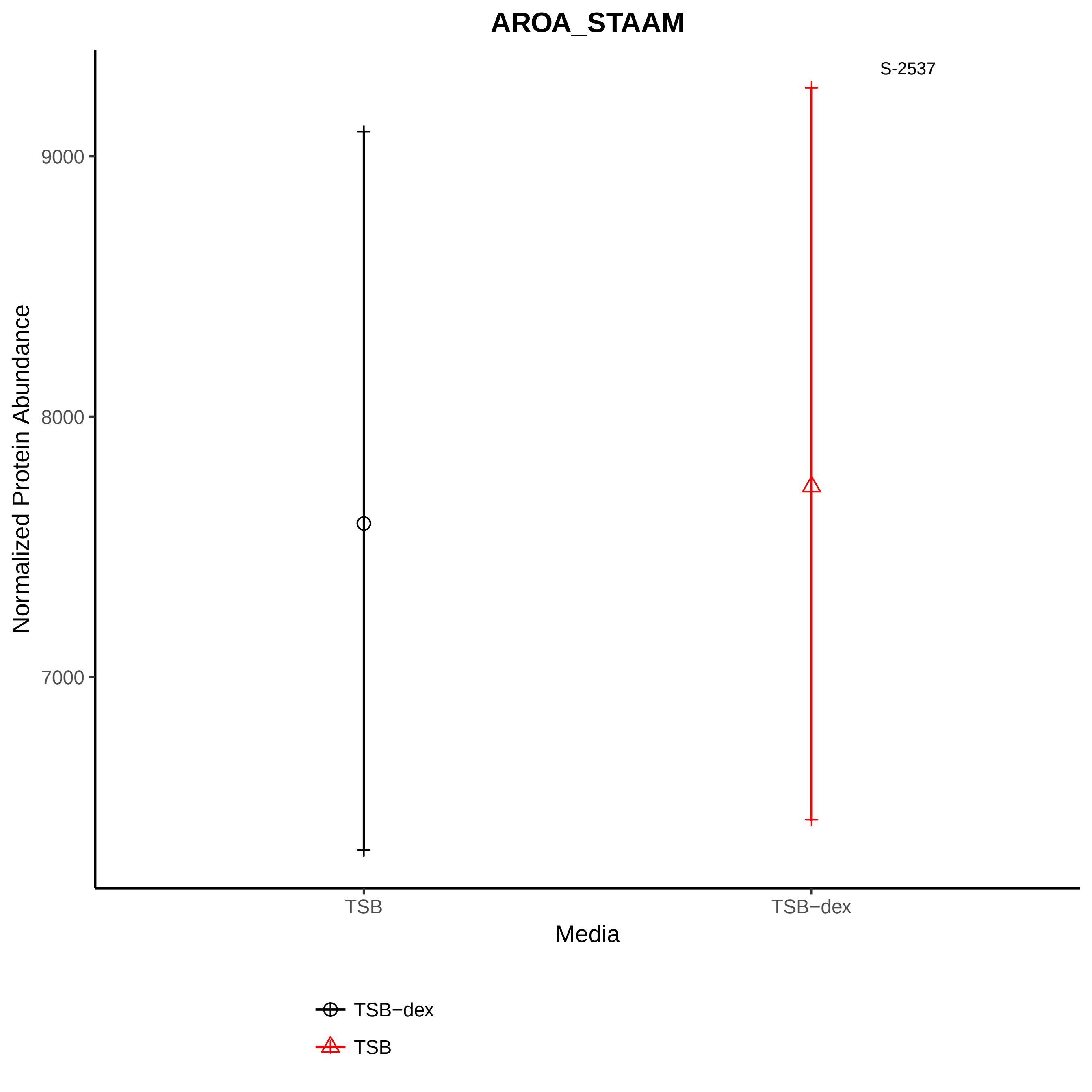

AROA_STAAM
S-2537
9000
8000
7000
Normalized Protein Abundance
TSB
TSB−dex
Media
TSB−dex
TSB

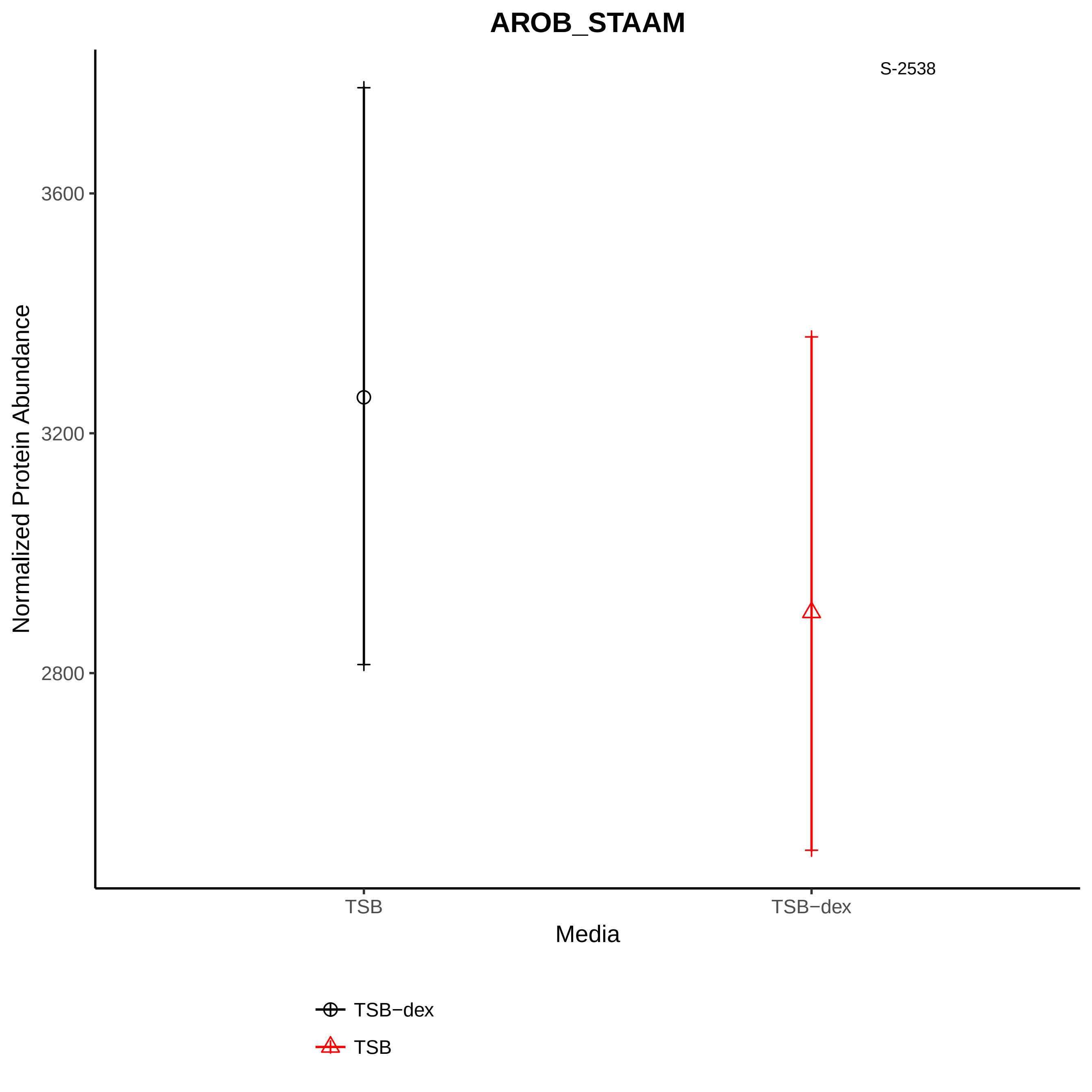

AROB_STAAM
S-2538
Normalized Protein Abundance
3600
3200
2800
TSB
TSB−dex
Media
TSB−dex
TSB

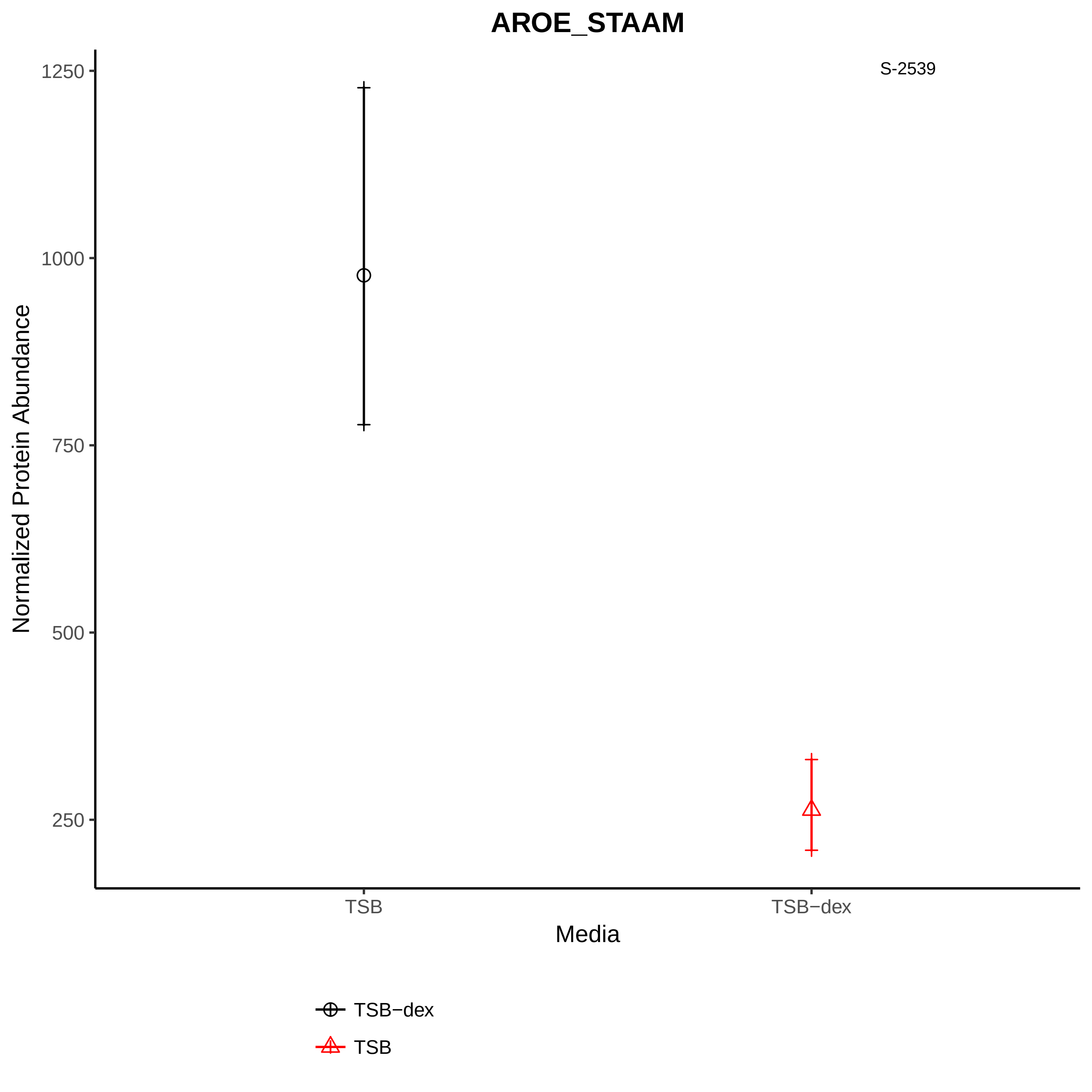

AROE_STAAM
S-2539
1250
1000
750
500
250
Normalized Protein Abundance
TSB
TSB−dex
Media
TSB−dex
TSB

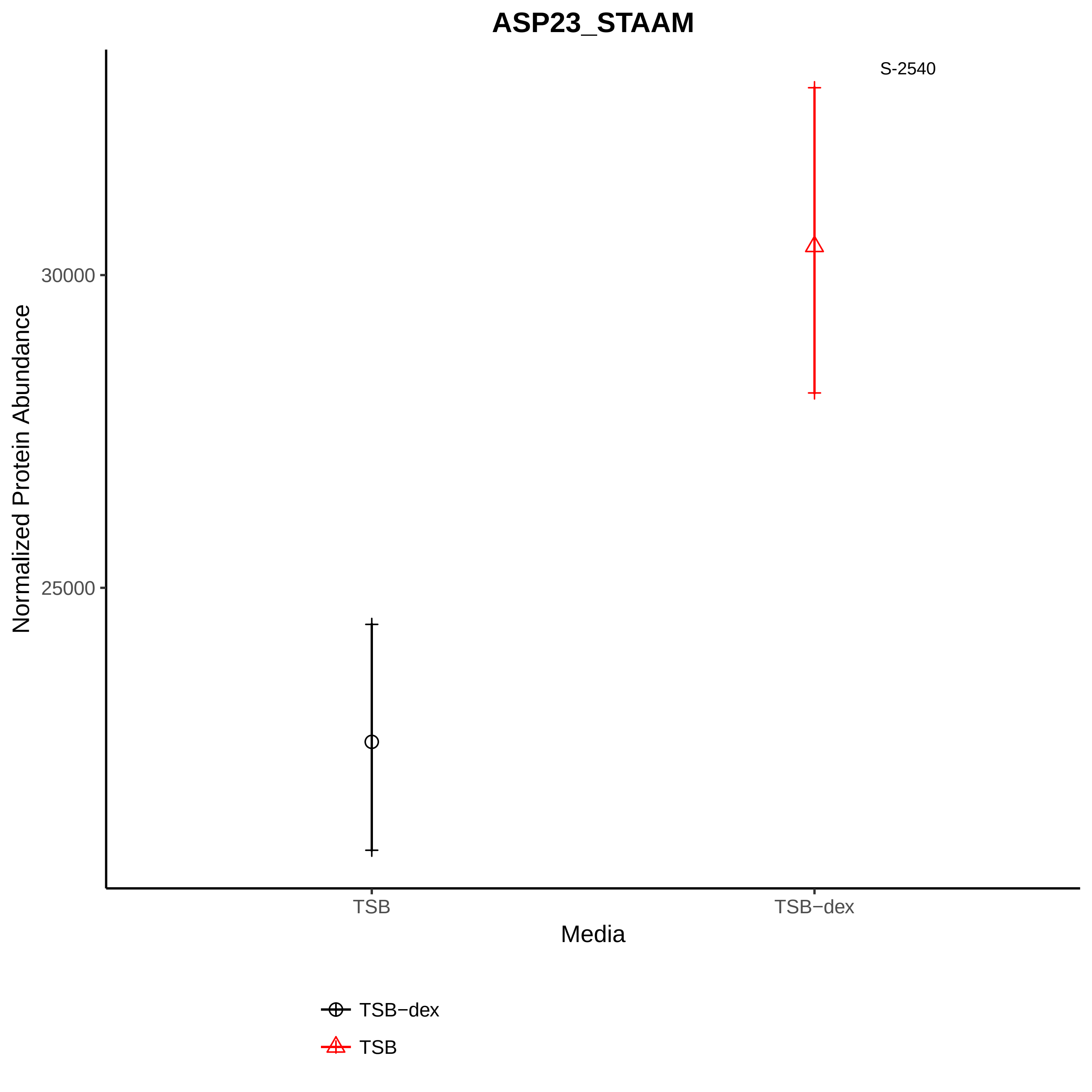

ASP23_STAAM
S-2540
Normalized Protein Abundance
30000
25000
TSB
TSB−dex
Media
TSB−dex
TSB

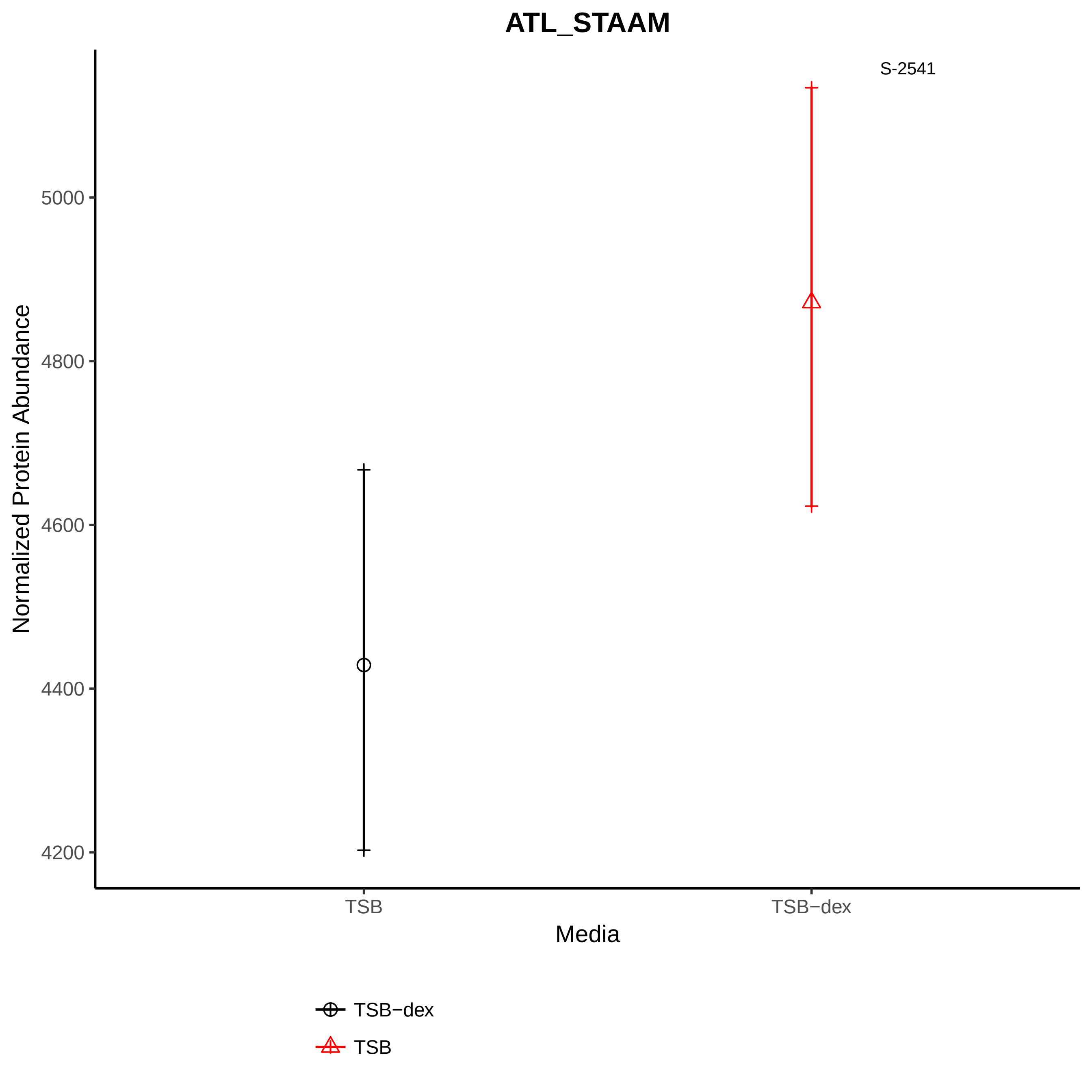

ATL_STAAM
S-2541
Normalized Protein Abundance
5000
4800
4600
4400
4200
TSB
TSB−dex
Media
TSB−dex
TSB

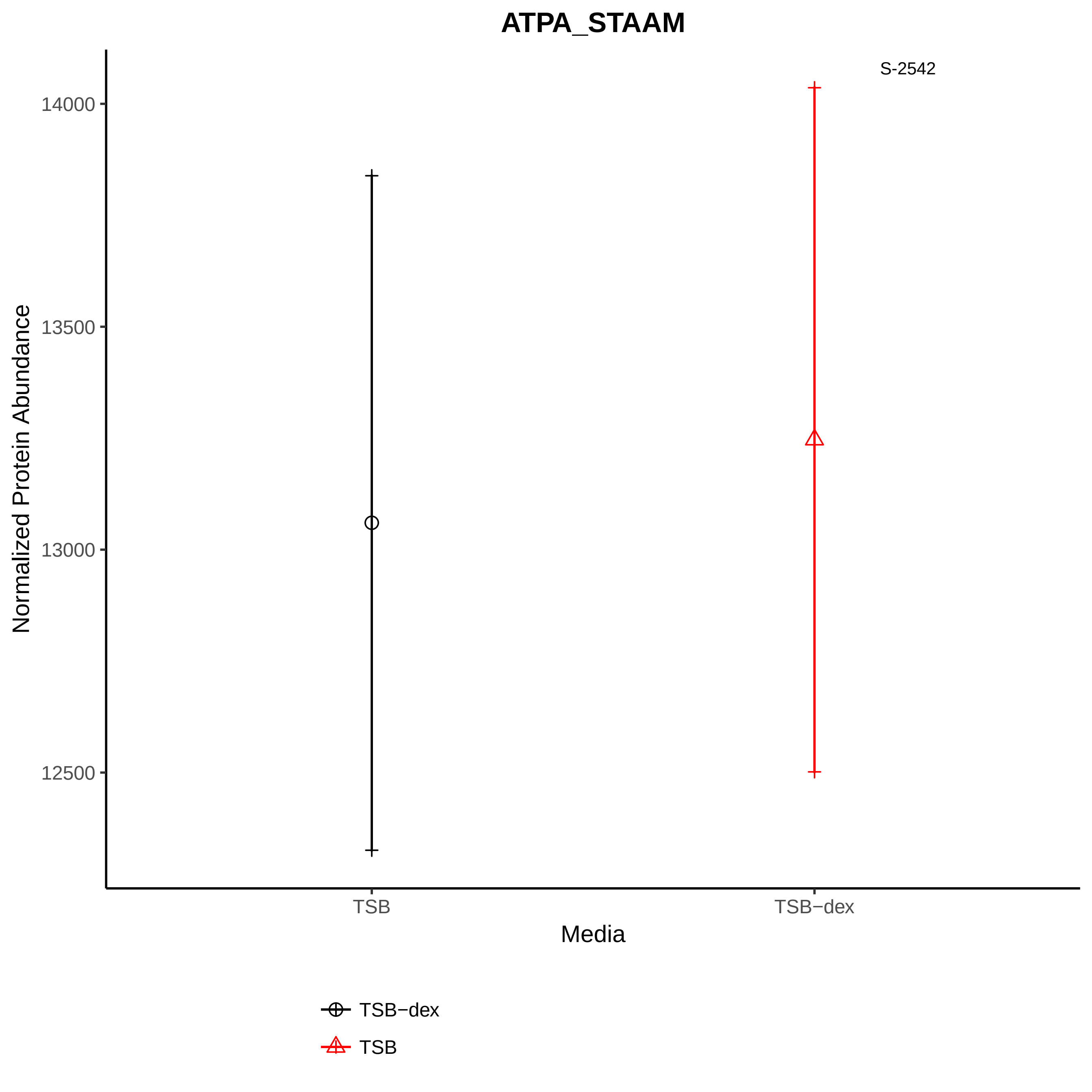

ATPA_STAAM
S-2542
14000
13500
13000
12500
Normalized Protein Abundance
TSB
TSB−dex
Media
TSB−dex
TSB

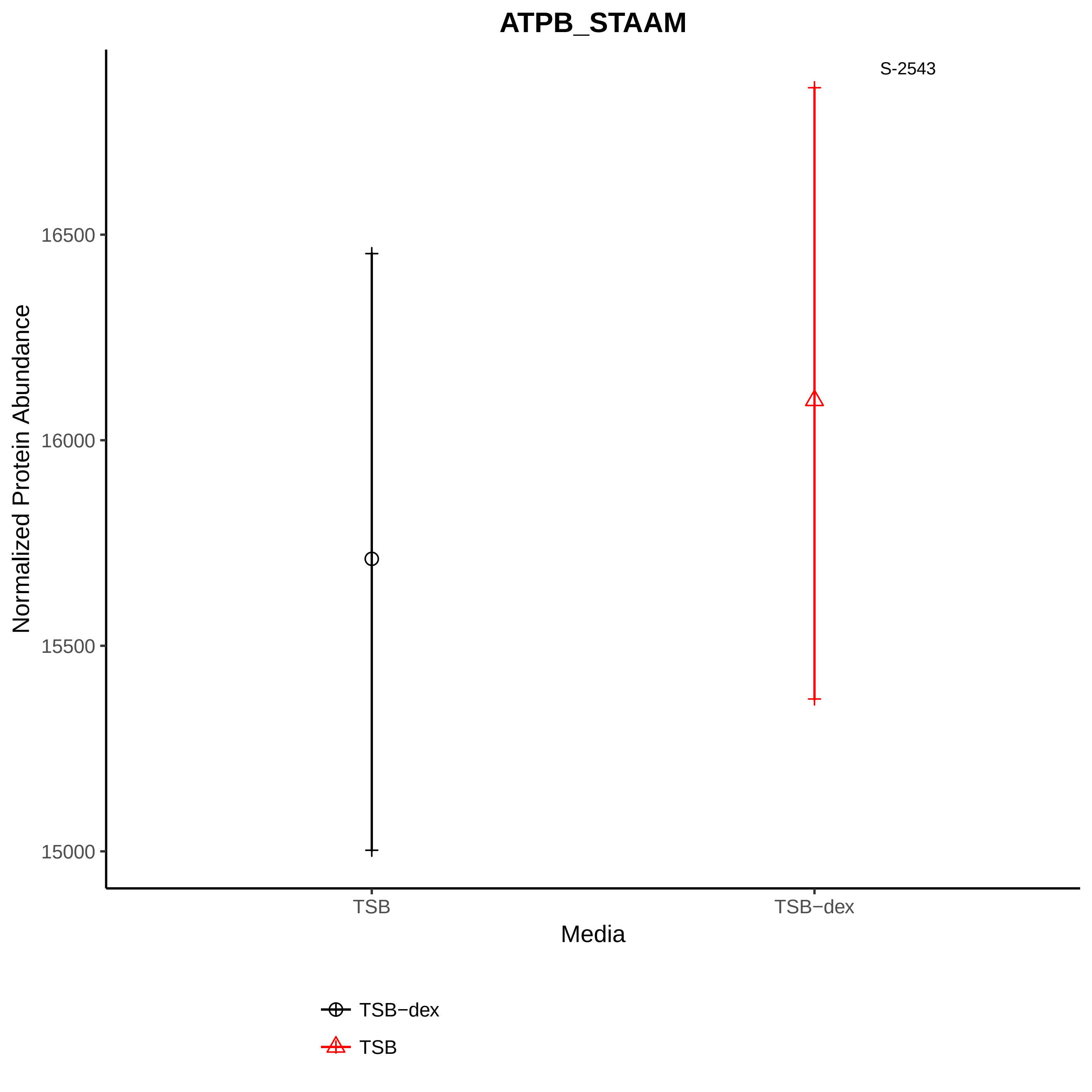

ATPB_STAAM
S-2543
Normalized Protein Abundance
16500
16000
15500
15000
TSB
TSB−dex
Media
TSB−dex
TSB

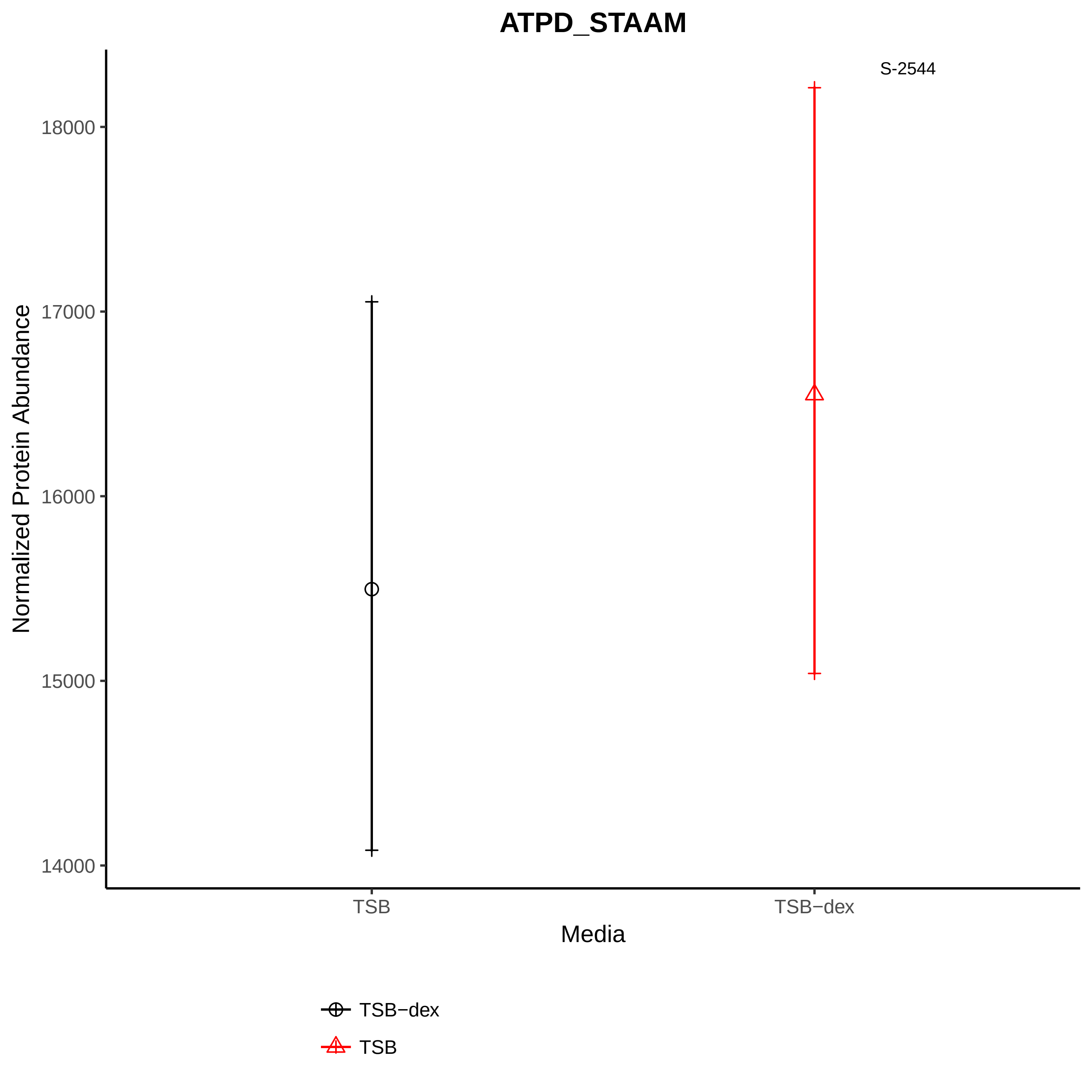

ATPD_STAAM
S-2544
Normalized Protein Abundance
18000
17000
16000
15000
14000
TSB
TSB−dex
Media
TSB−dex
TSB

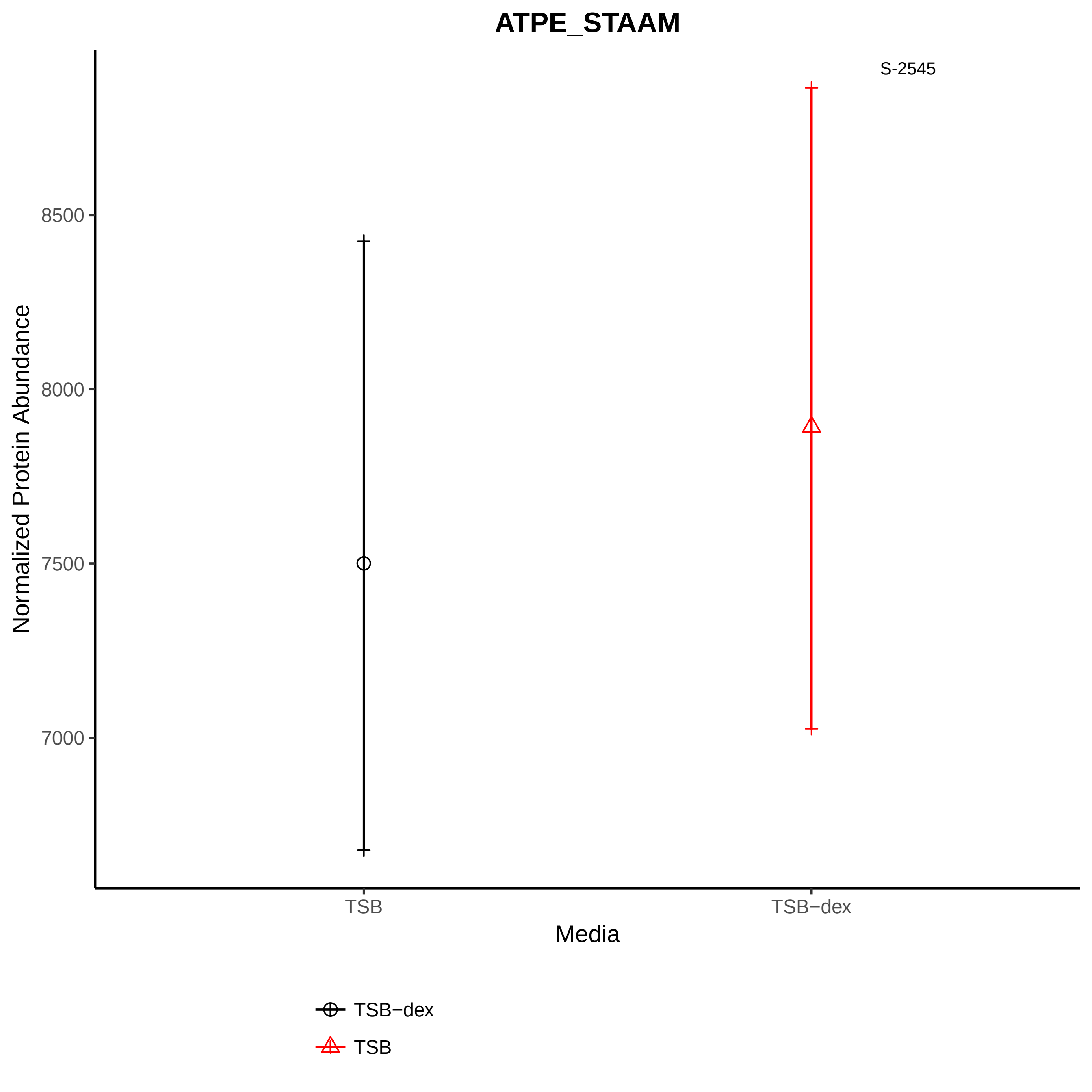

ATPE_STAAM
S-2545
Normalized Protein Abundance
8500
8000
7500
7000
TSB
TSB−dex
Media
TSB−dex
TSB

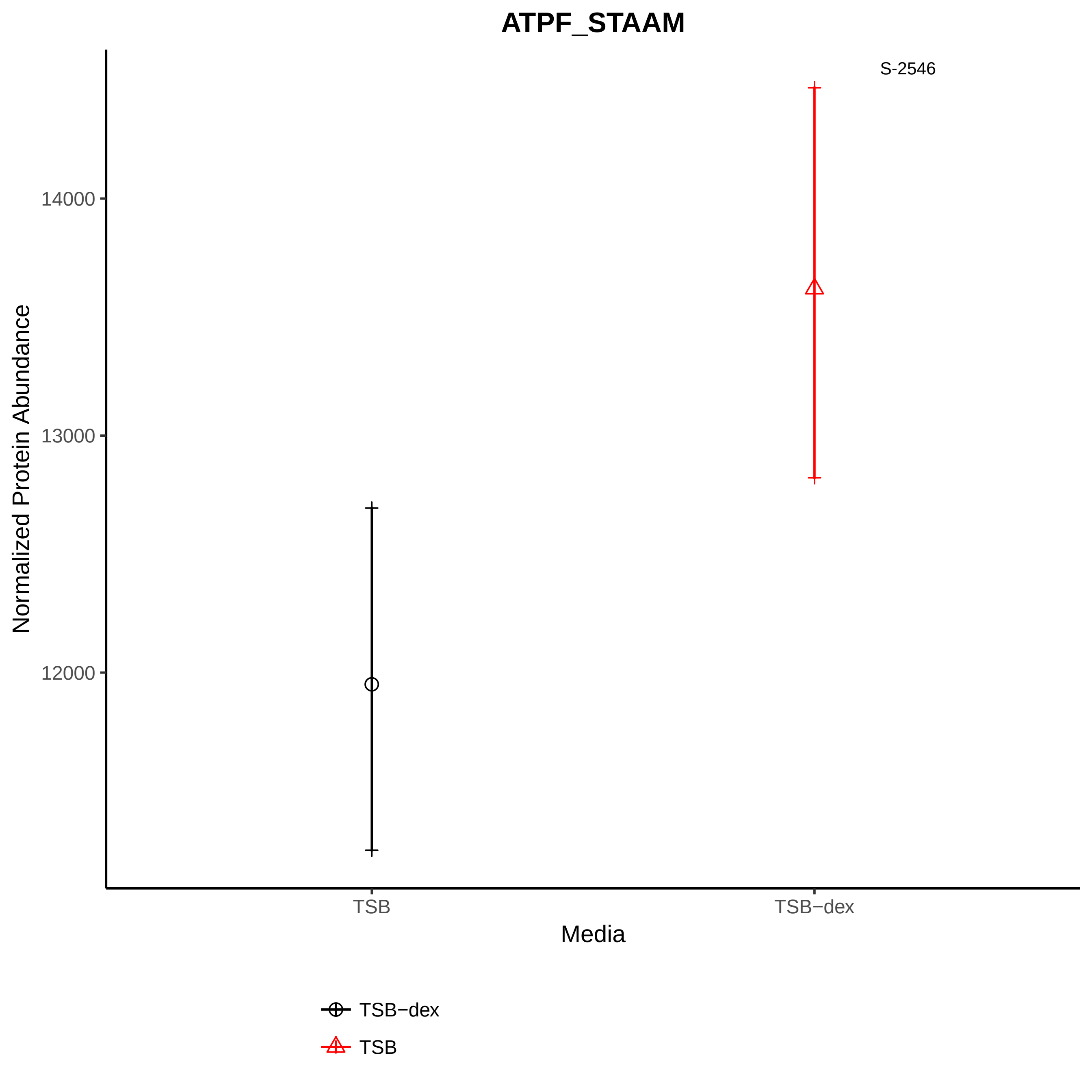
ATPF_STAAM
S-2546
Normalized Protein Abundance
14000
13000
12000
TSB
TSB−dex
Media
TSB−dex
TSB

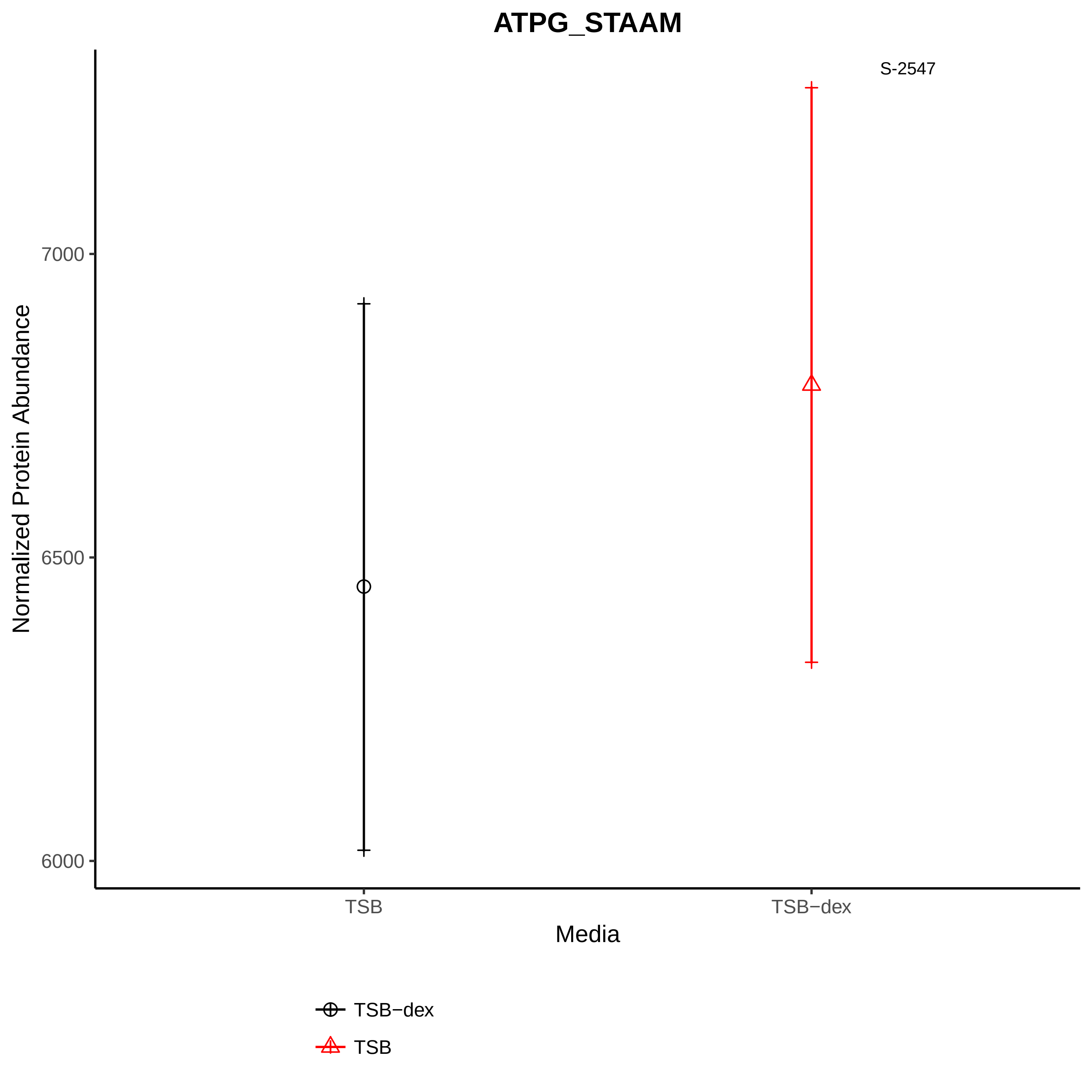

ATPG_STAAM
S-2547
Normalized Protein Abundance
7000
6500
6000
TSB
TSB−dex
Media
TSB−dex
TSB

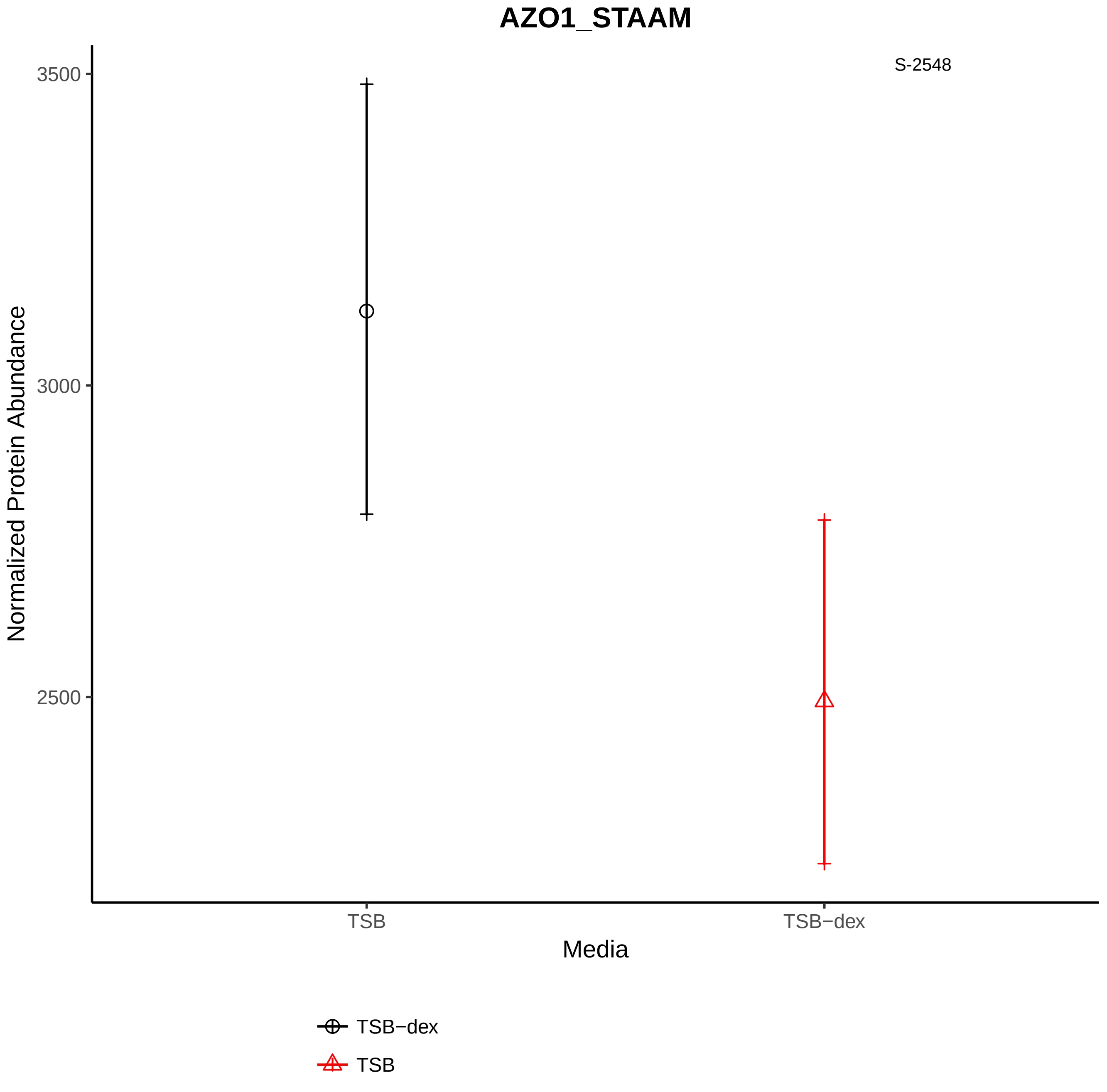

AZO1_STAAM
S-2548
3500
3000
2500
Normalized Protein Abundance
TSB
TSB−dex
Media
TSB−dex
TSB

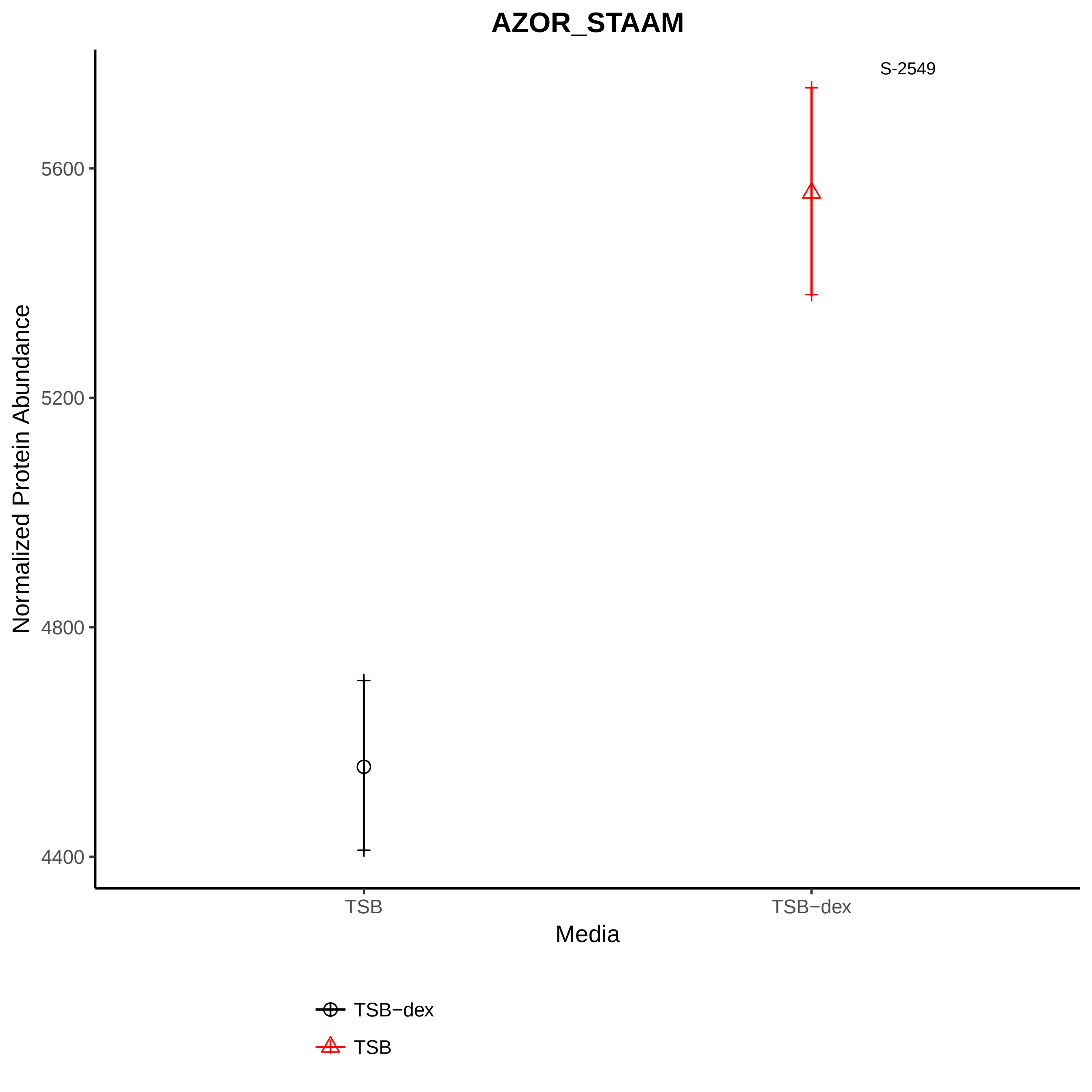

AZOR_STAAM
S-2549
Normalized Protein Abundance
5600
5200
4800
4400
TSB
TSB−dex
Media
TSB−dex
TSB

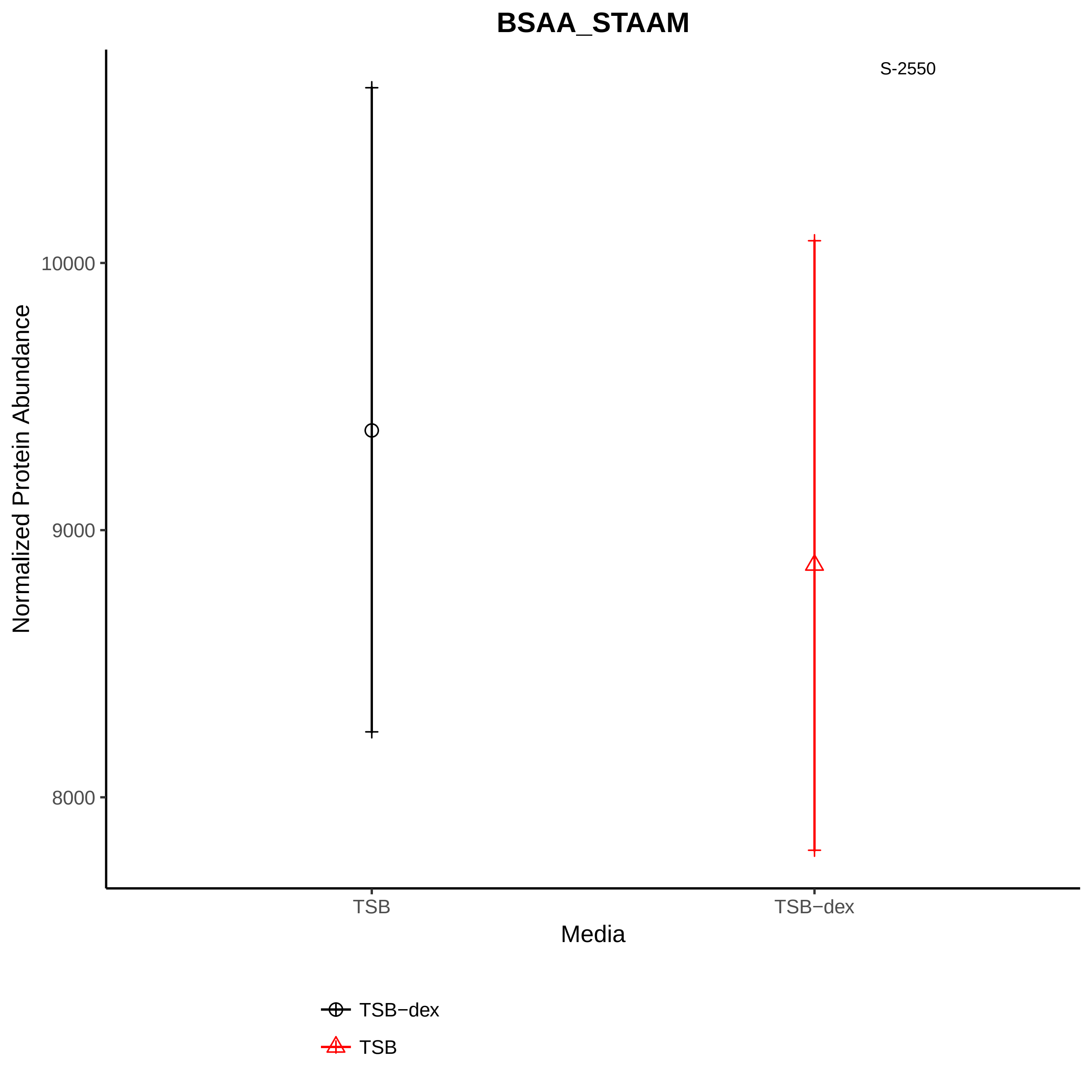

BSAA_STAAM
S-2550
Normalized Protein Abundance
10000
9000
8000
TSB
TSB−dex
Media
TSB−dex
TSB

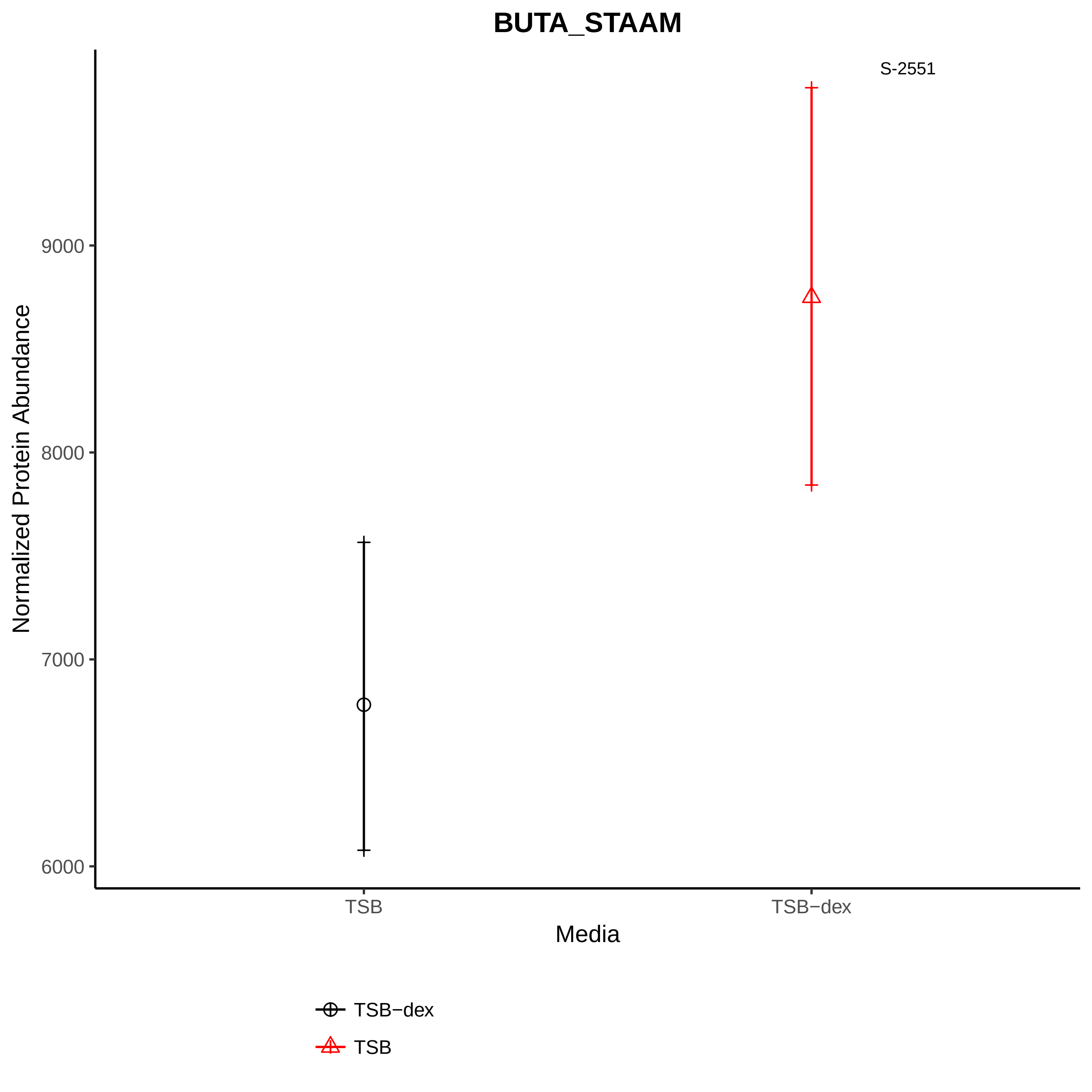

BUTA_STAAM
S-2551
Normalized Protein Abundance
9000
8000
7000
6000
TSB
TSB−dex
Media
TSB−dex
TSB

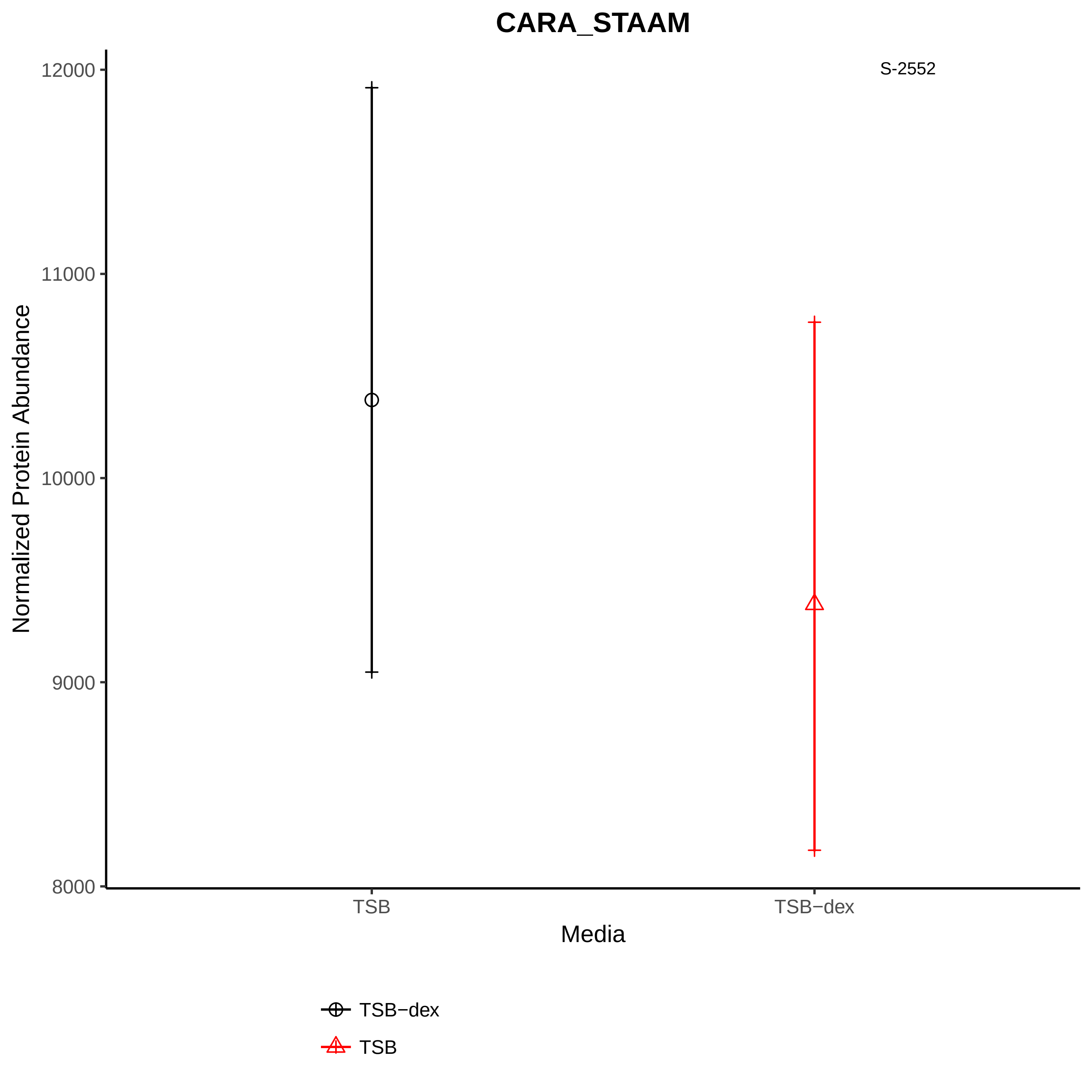

CARA_STAAM
S-2552
Normalized Protein Abundance
12000
11000
10000
9000
8000
TSB
TSB−dex
Media
TSB−dex
TSB

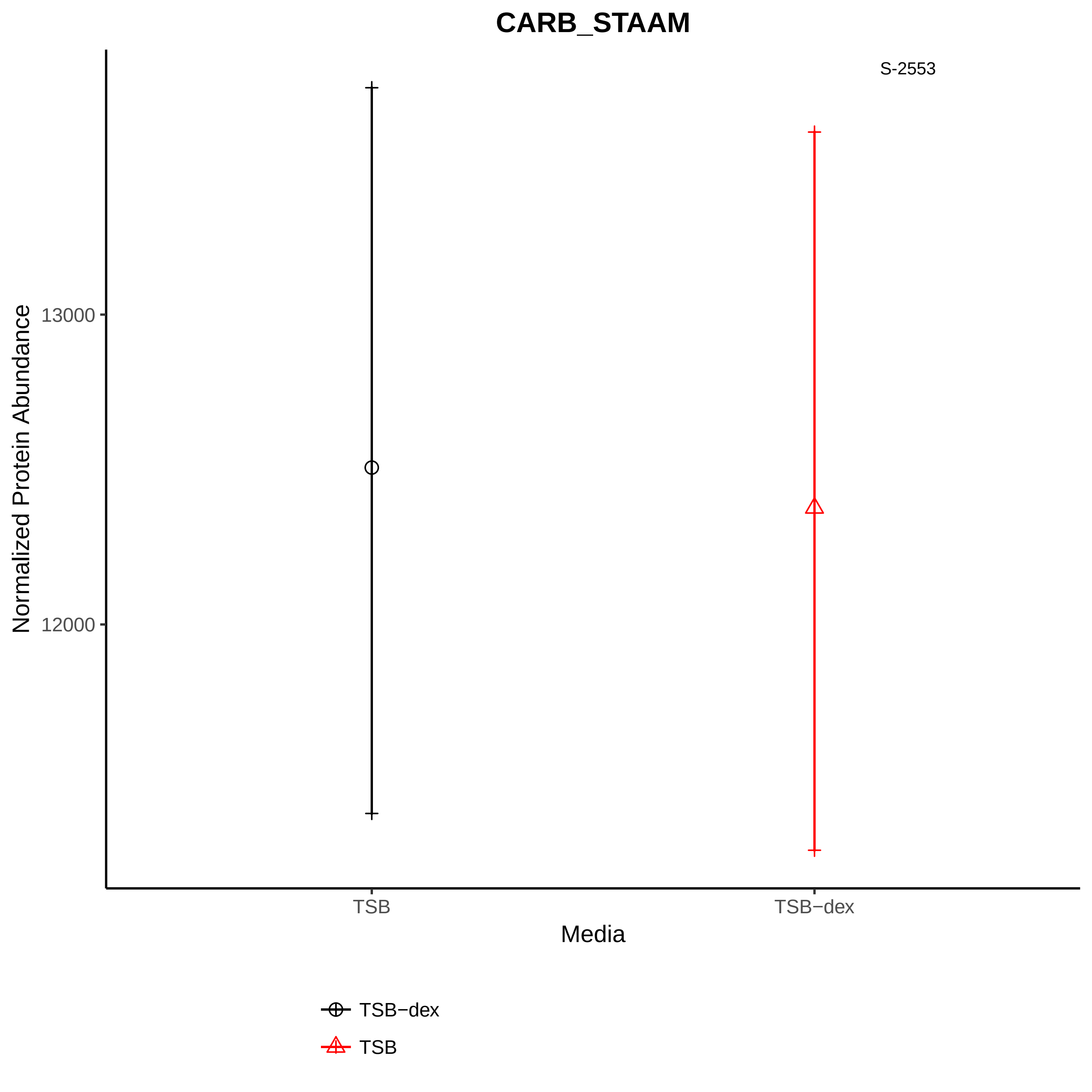

CARB_STAAM
S-2553
13000
12000
Normalized Protein Abundance
TSB
TSB−dex
Media
TSB−dex
TSB

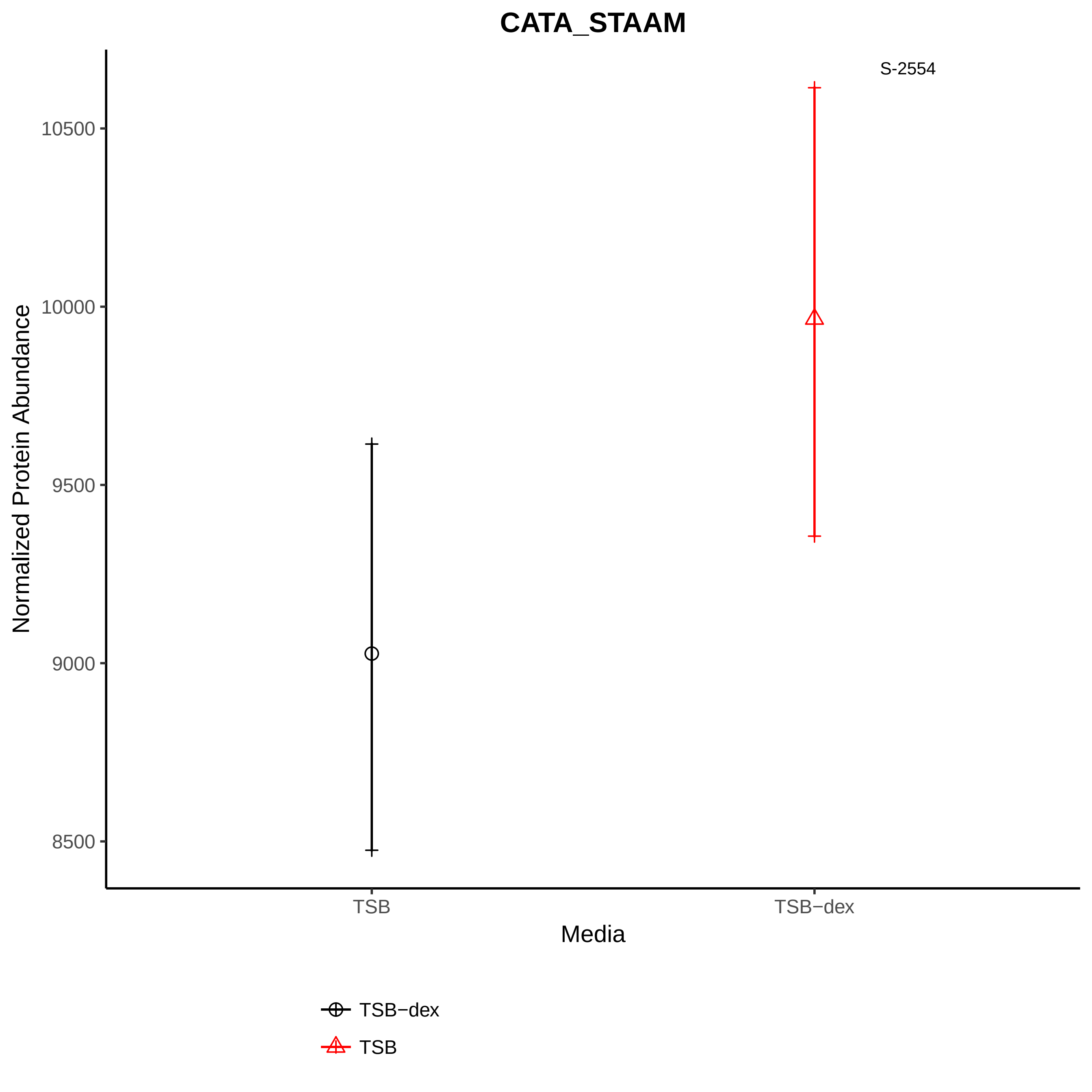

CATA_STAAM
S-2554
Normalized Protein Abundance
10500
10000
9500
9000
8500
TSB
TSB−dex
Media
TSB−dex
TSB

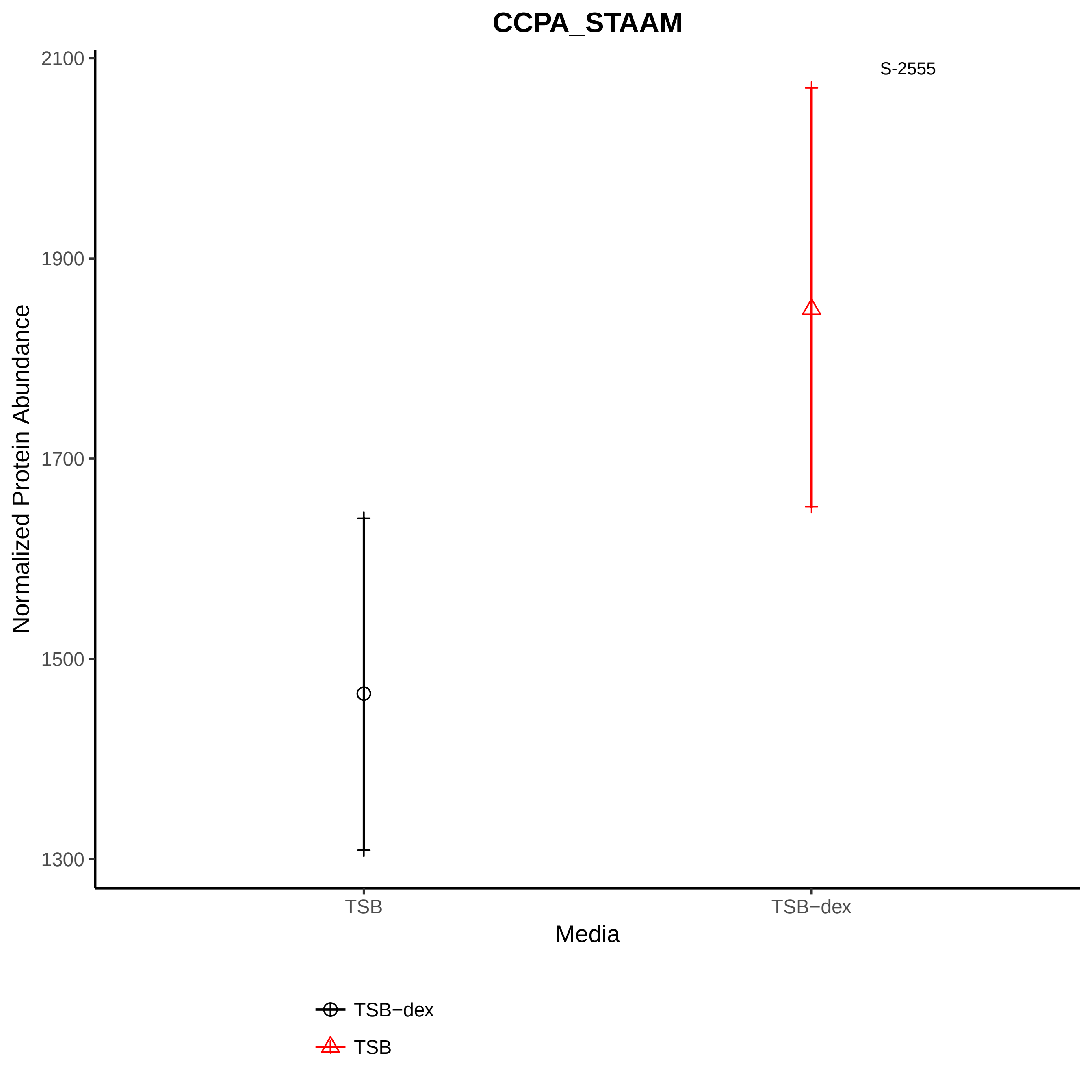

CCPA_STAAM
S-2555
Normalized Protein Abundance
2100
1900
1700
1500
1300
TSB
TSB−dex
Media
TSB−dex
TSB

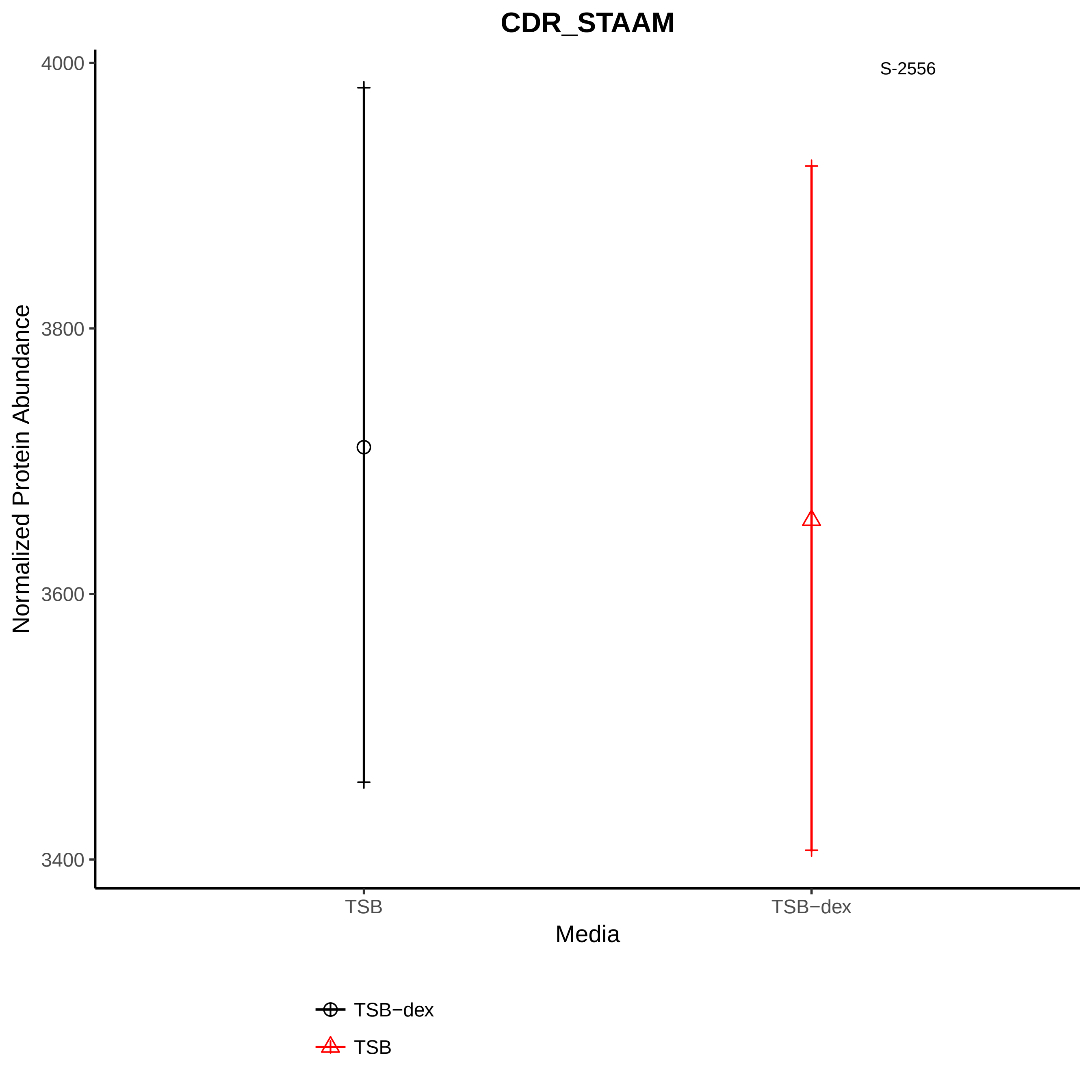
CDR_STAAM
S-2556
4000
3800
3600
3400
Normalized Protein Abundance
TSB
TSB−dex
Media
TSB−dex
TSB

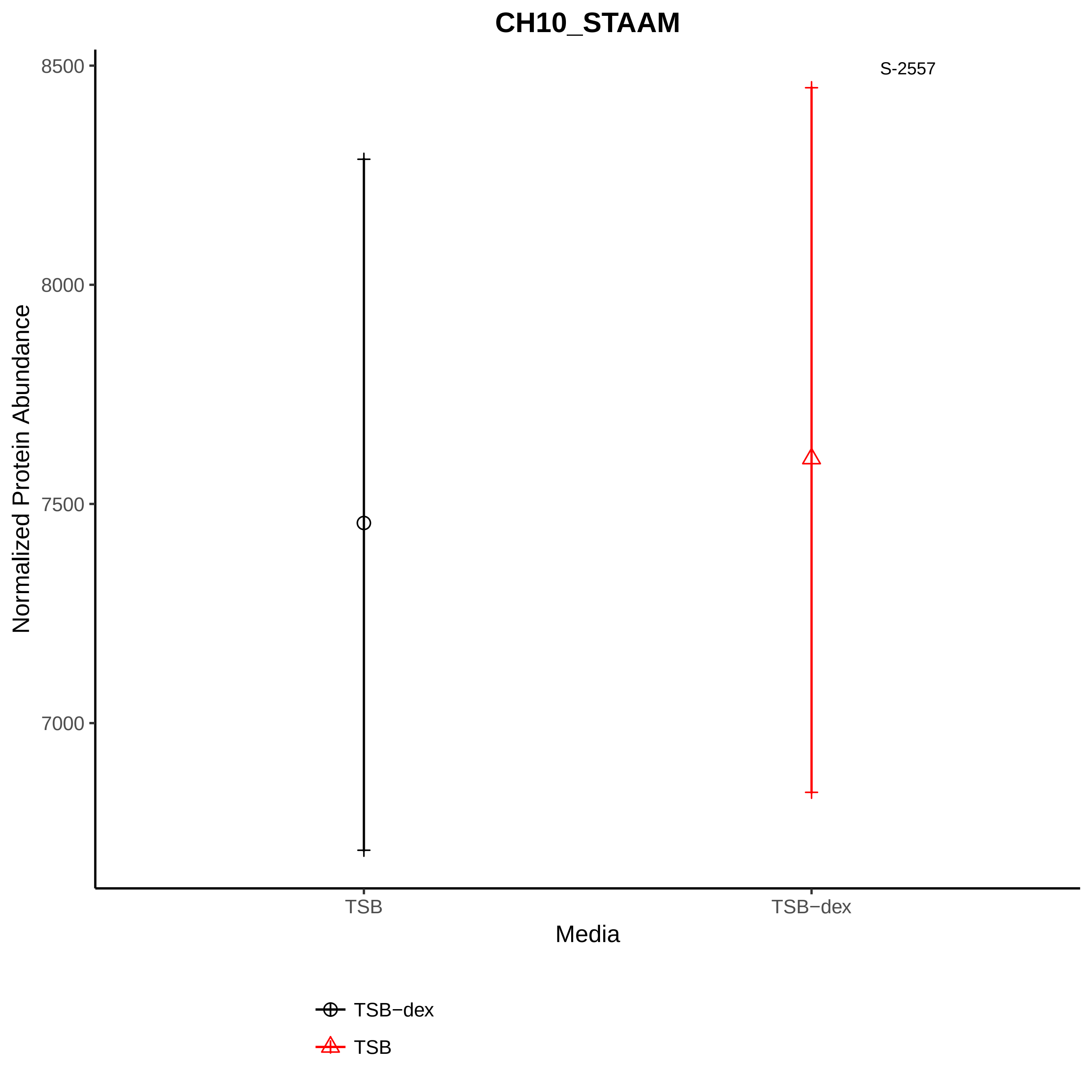
CH10_STAAM
S-2557
8500
8000
7500
7000
Normalized Protein Abundance
TSB
TSB−dex
Media
TSB−dex
TSB

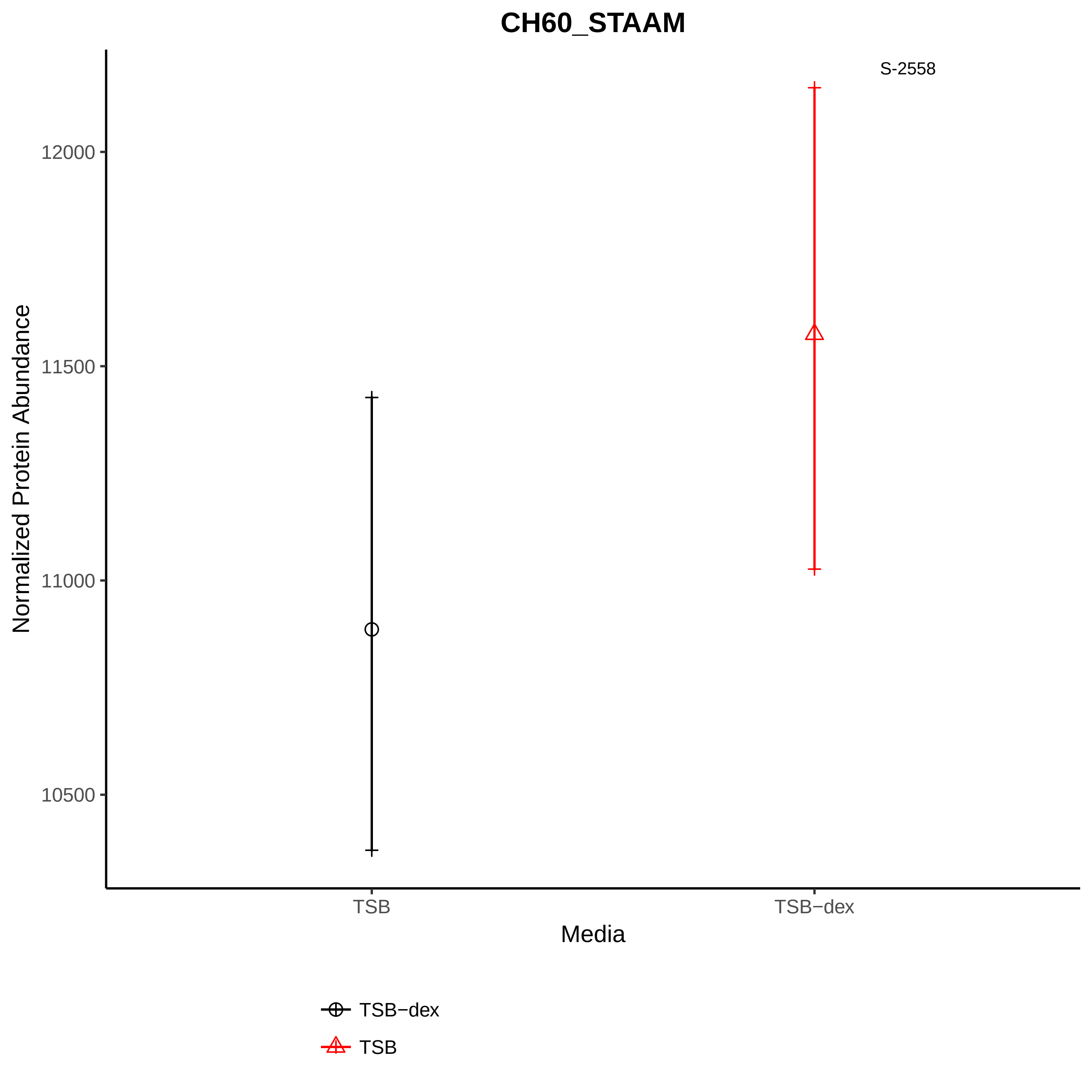
CH60_STAAM
S-2558
12000
11500
11000
10500
Normalized Protein Abundance
TSB
TSB−dex
Media
TSB−dex
TSB

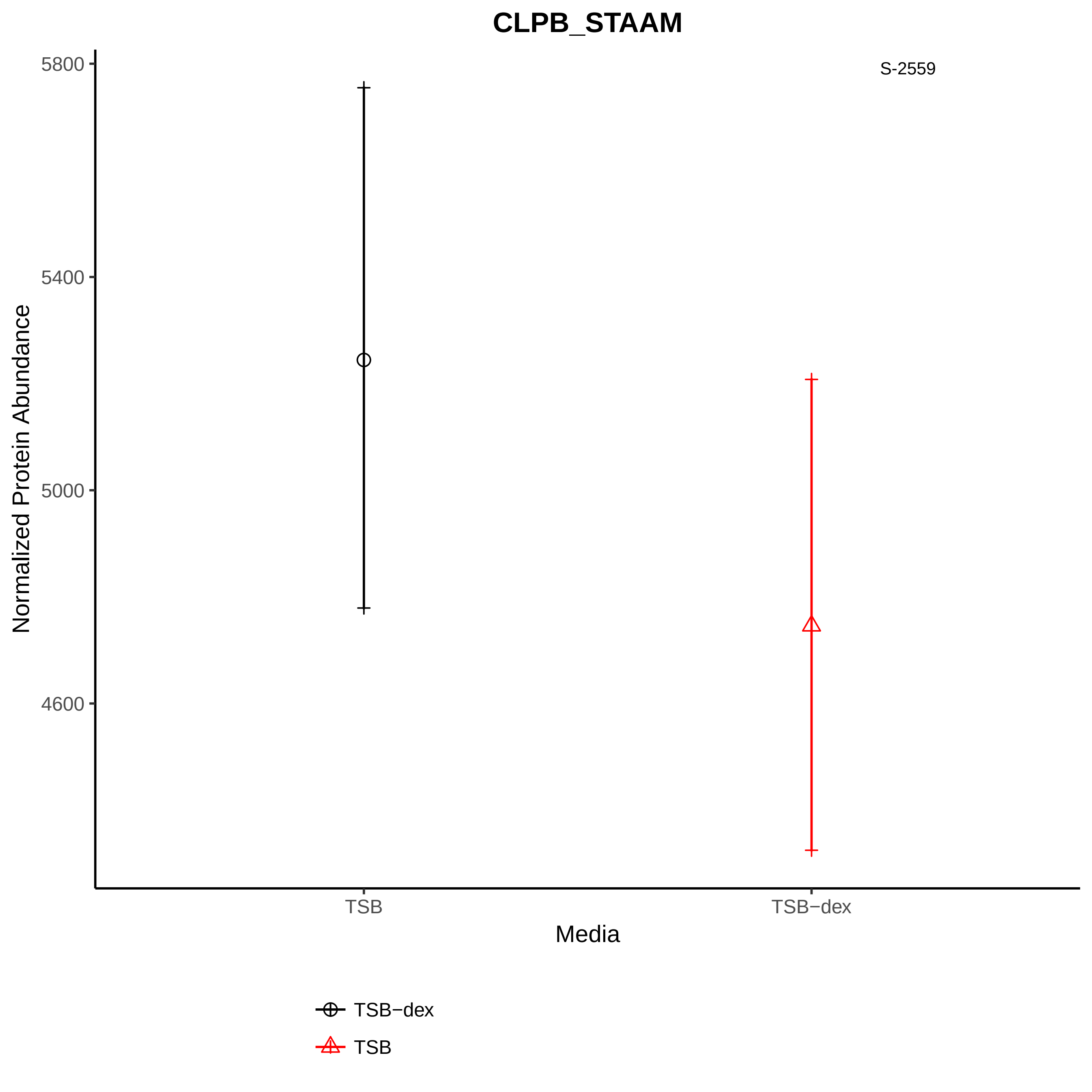

CLPB_STAAM
S-2559
5800
5400
5000
4600
Normalized Protein Abundance
TSB
TSB−dex
Media
TSB−dex
TSB

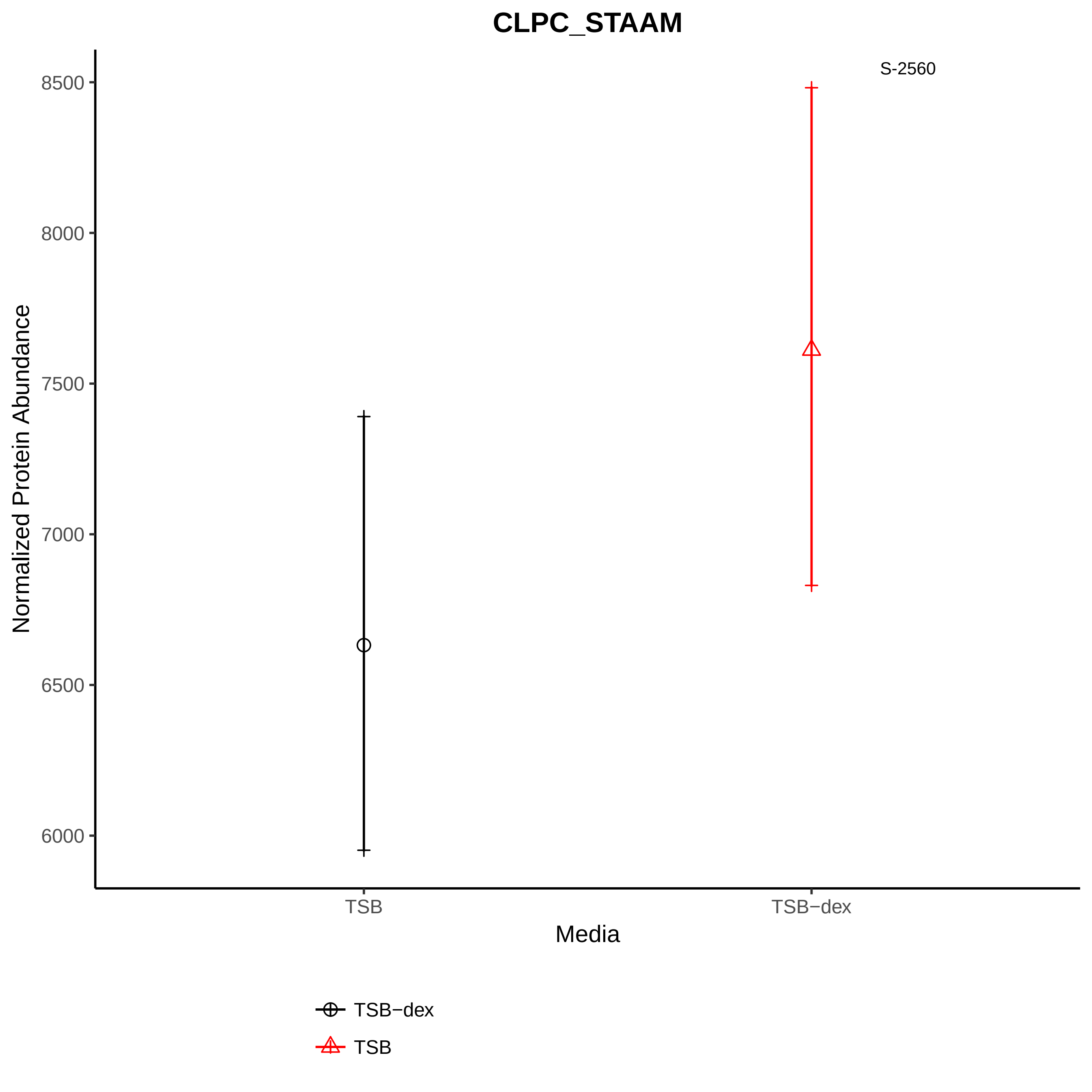

CLPC_STAAM
S-2560
8500
8000
7500
7000
6500
6000
Normalized Protein Abundance
TSB
TSB−dex
Media
TSB−dex
TSB

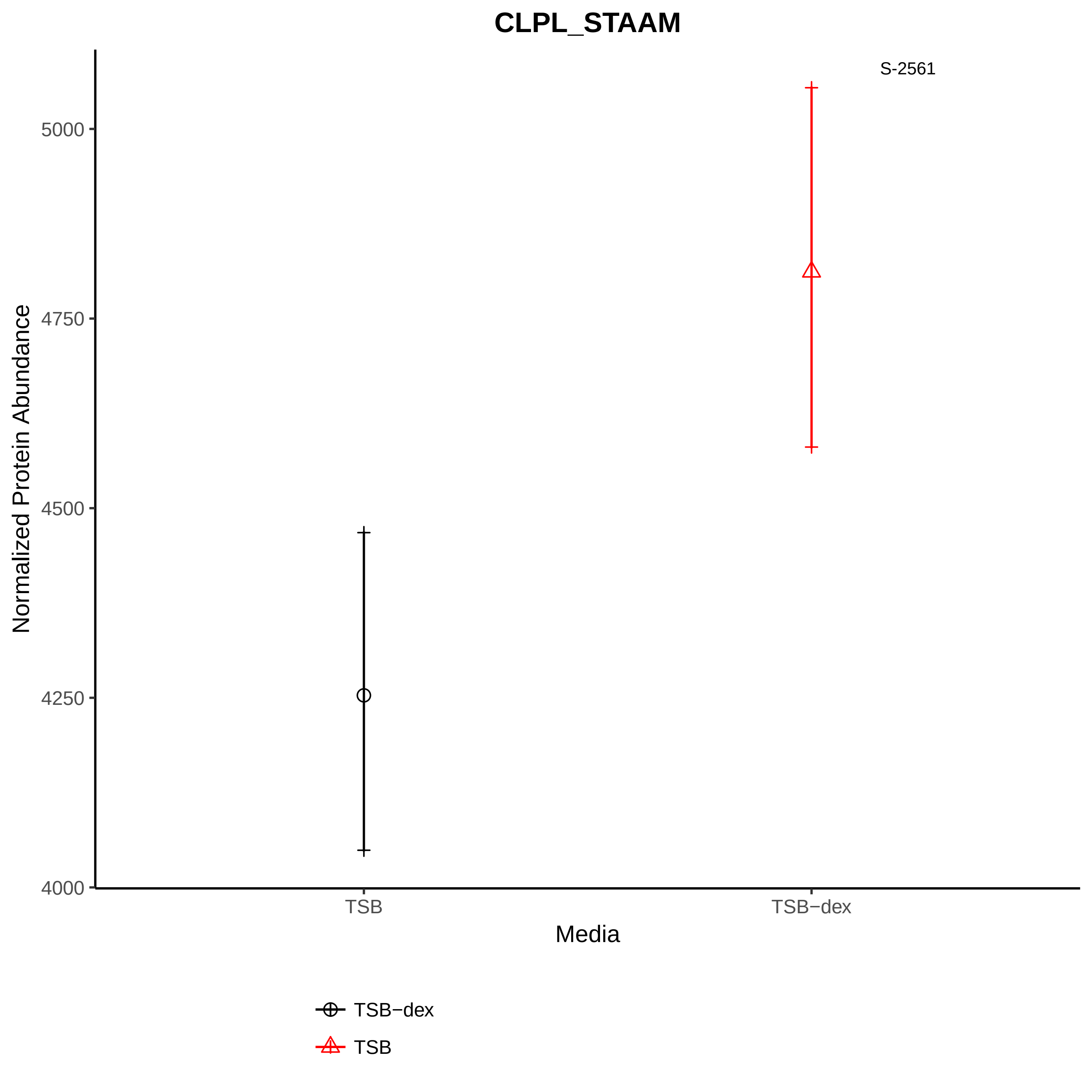

CLPL_STAAM
S-2561
Normalized Protein Abundance
5000
4750
4500
4250
4000
TSB
TSB−dex
Media
TSB−dex
TSB

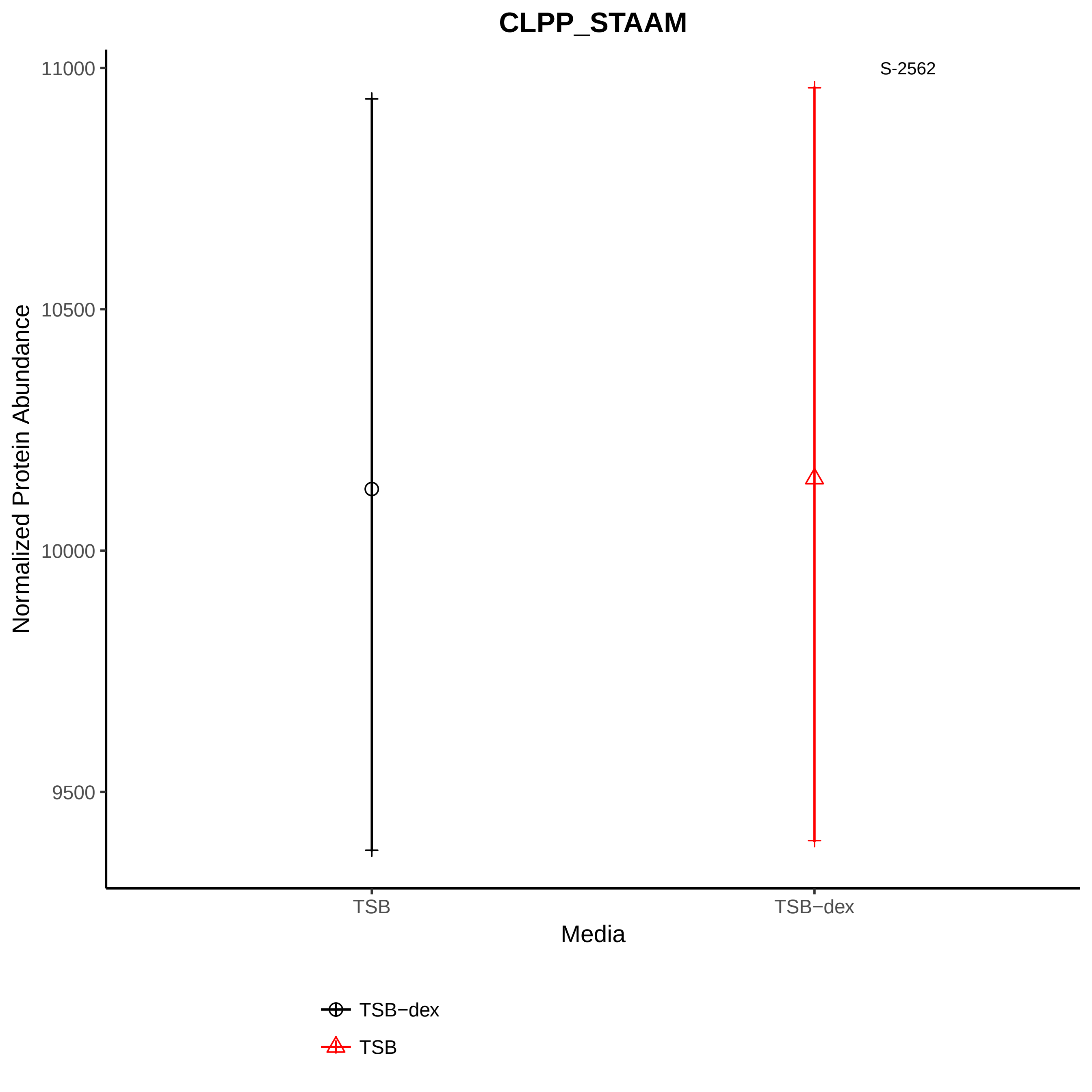
CLPP_STAAM
S-2562
11000
10500
10000
9500
Normalized Protein Abundance
TSB
TSB−dex
Media
TSB−dex
TSB

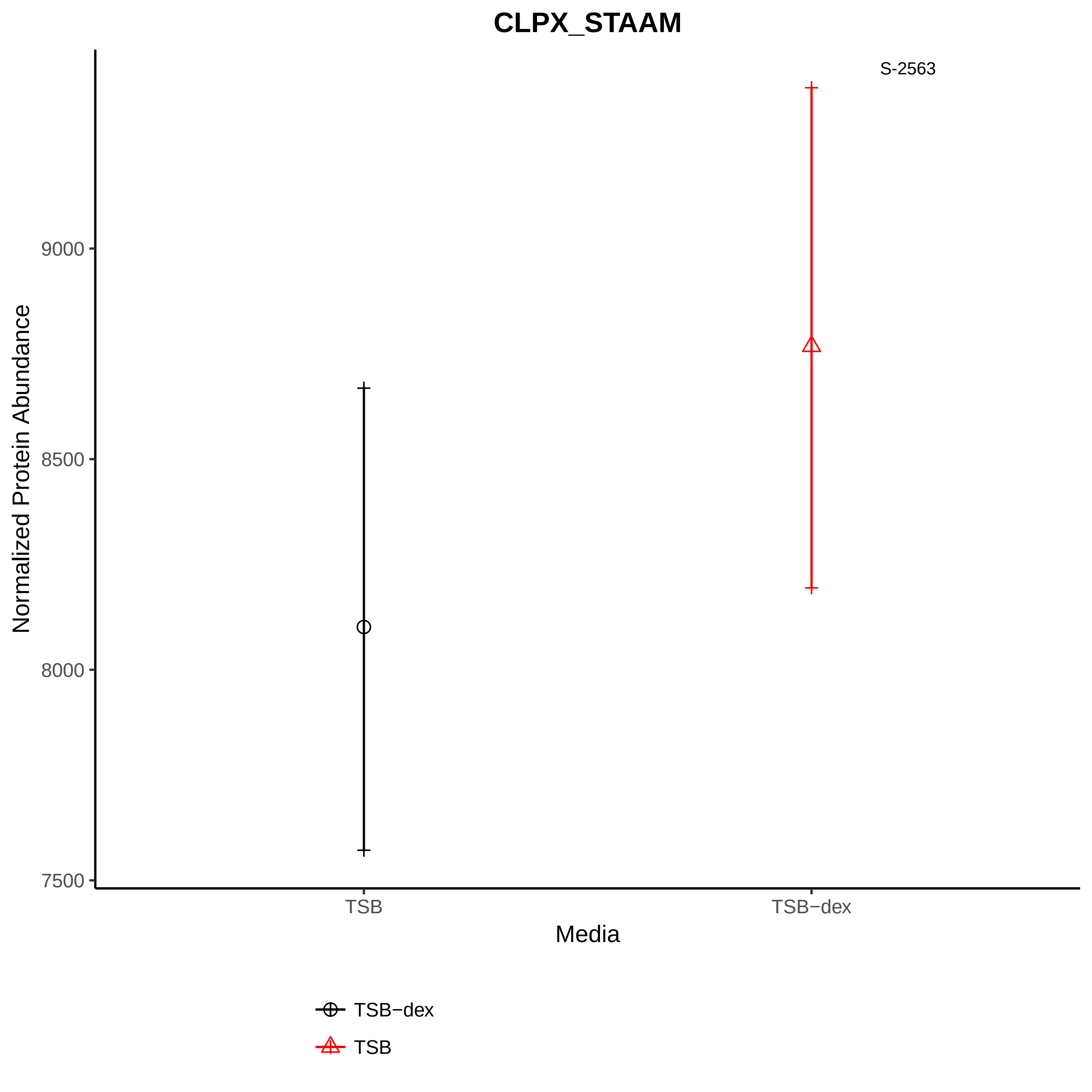
CLPX_STAAM
S-2563
Normalized Protein Abundance
9000
8500
8000
7500
TSB
TSB−dex
Media
TSB−dex
TSB

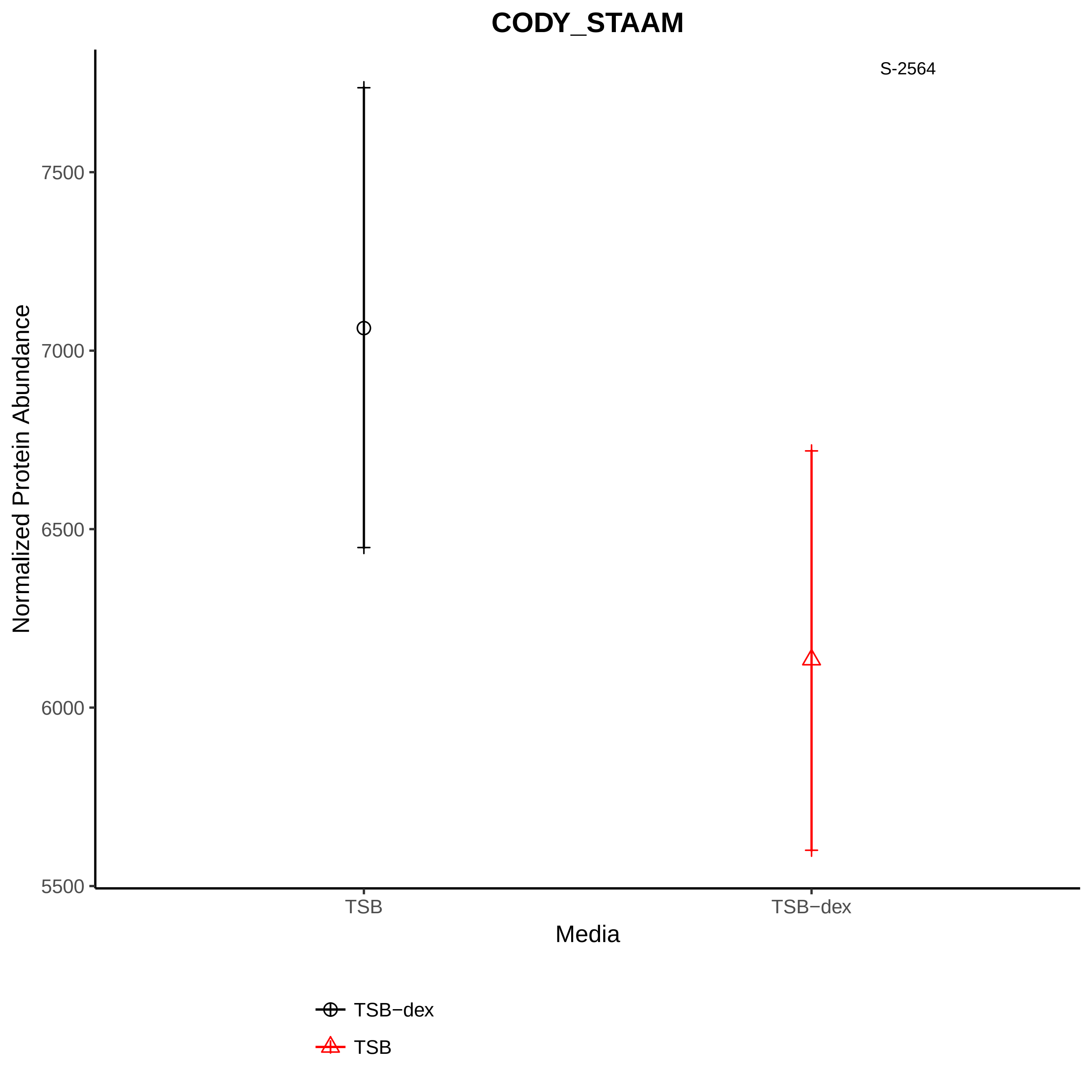

CODY_STAAM
S-2564
Normalized Protein Abundance
7500
7000
6500
6000
5500
TSB
TSB−dex
Media
TSB−dex
TSB

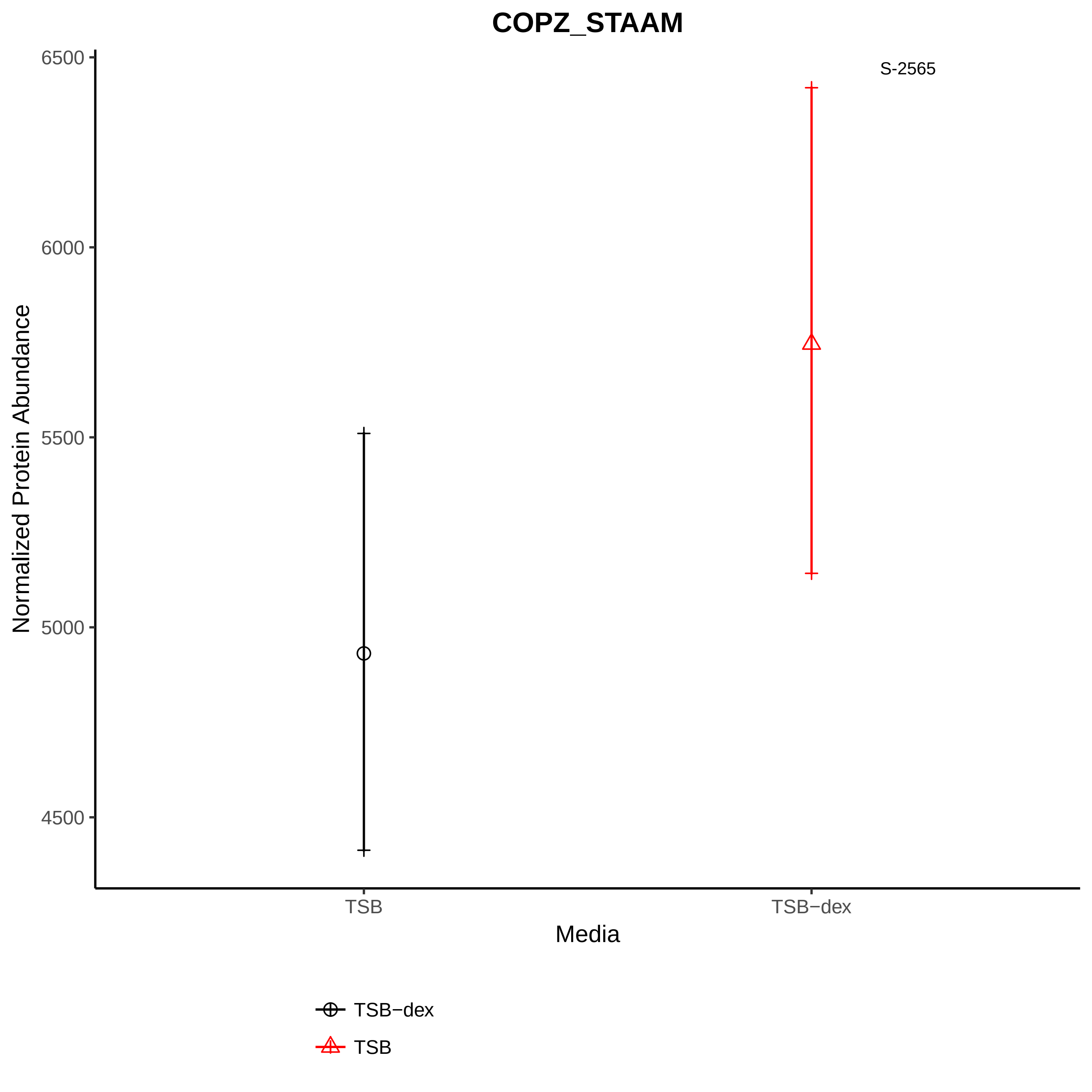

COPZ_STAAM
S-2565
6500
6000
5500
5000
4500
Normalized Protein Abundance
TSB
TSB−dex
Media
TSB−dex
TSB

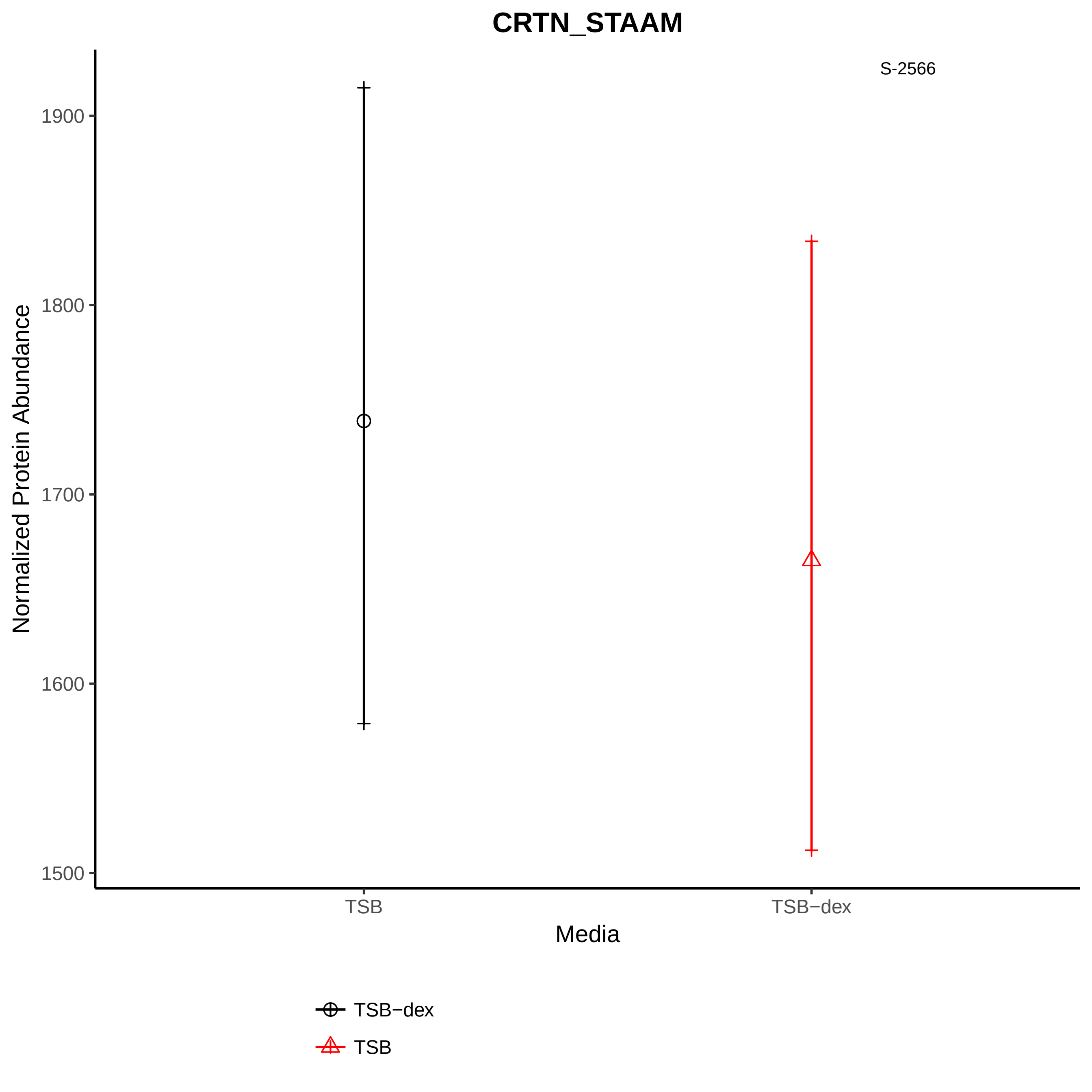
CRTN_STAAM
S-2566
1900
1800
1700
1600
1500
Normalized Protein Abundance
TSB
TSB−dex
Media
TSB−dex
TSB

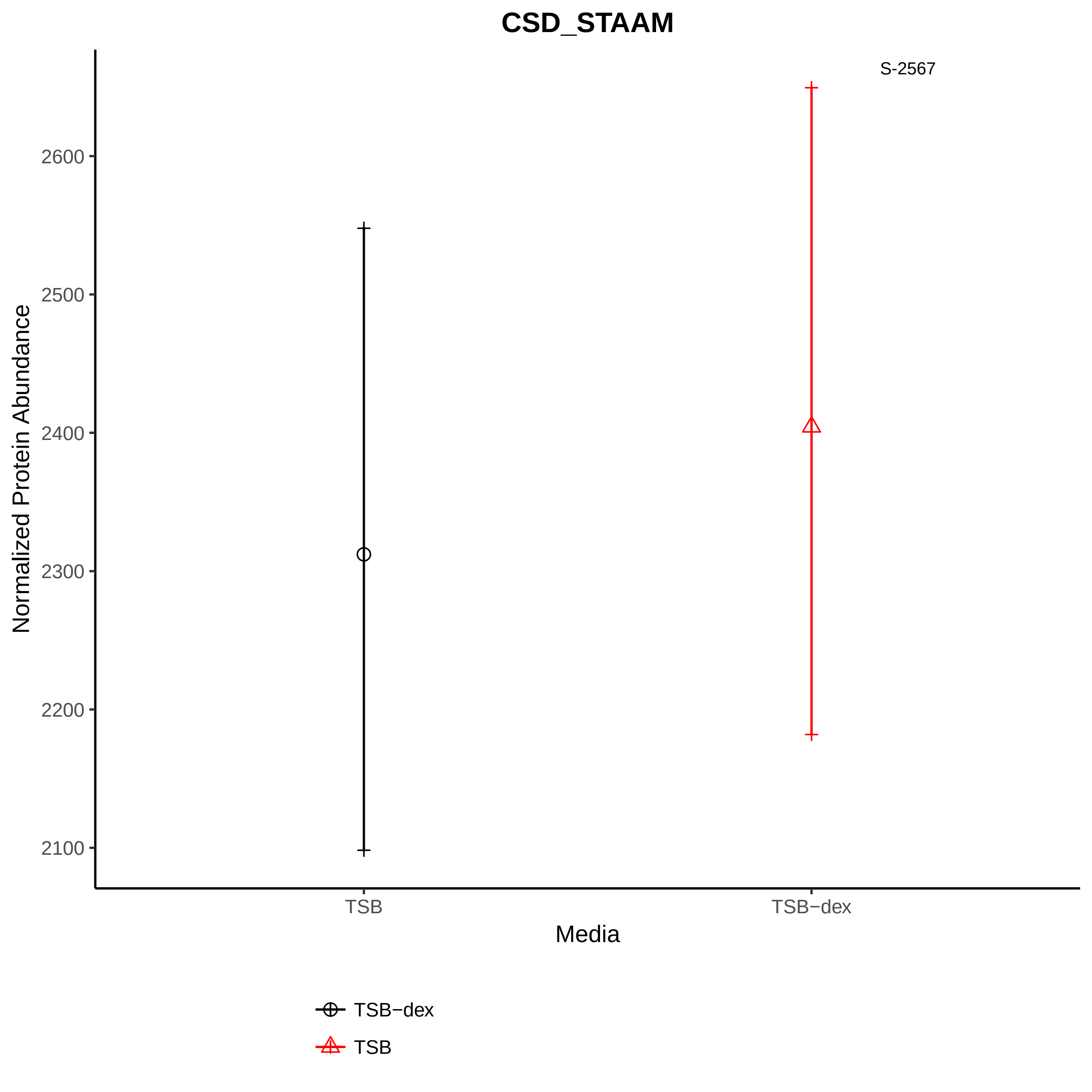

CSD_STAAM
S-2567
Normalized Protein Abundance
2600
2500
2400
2300
2200
2100
TSB
TSB−dex
Media
TSB−dex
TSB

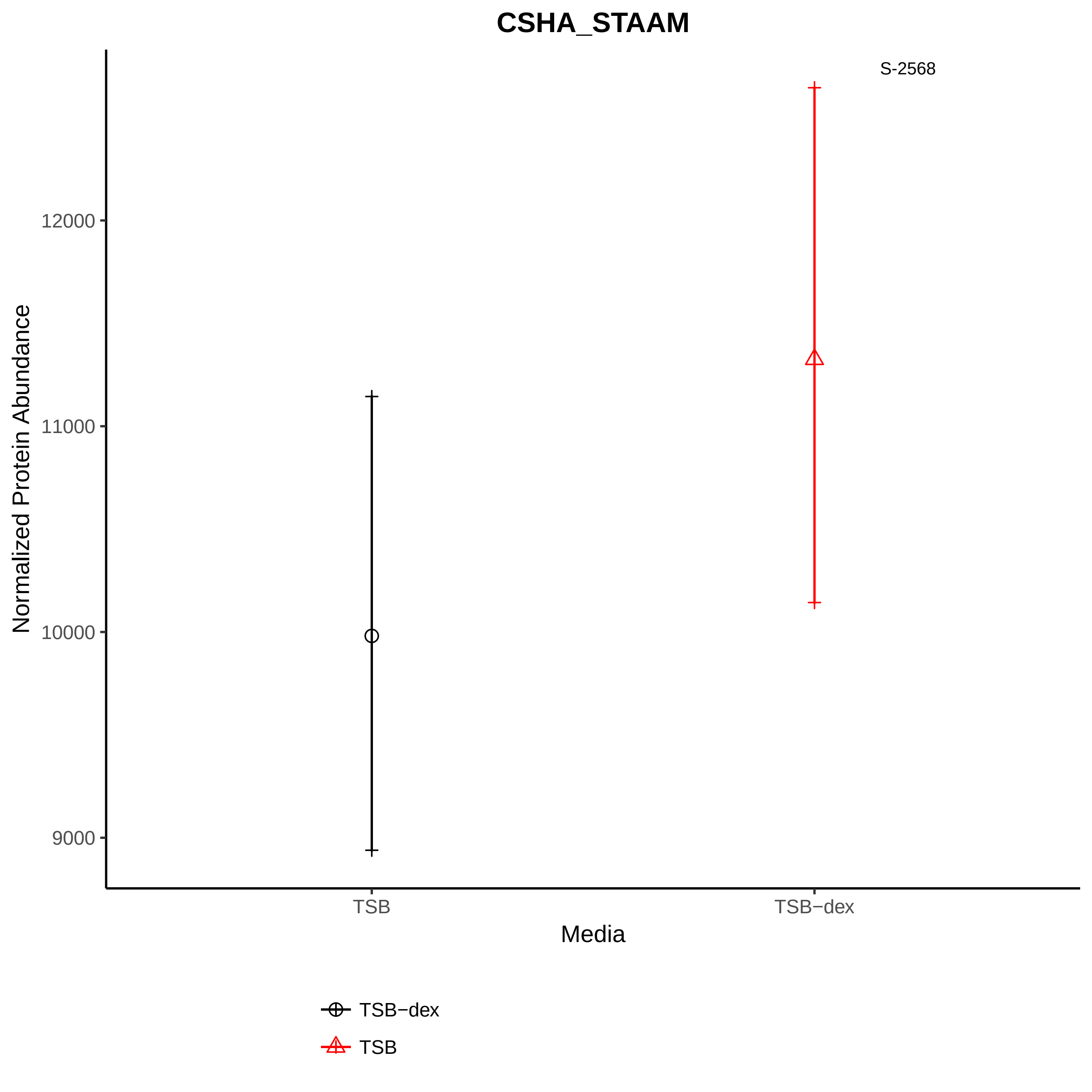

CSHA_STAAM
S-2568
Normalized Protein Abundance
12000
11000
10000
9000
TSB
TSB−dex
Media
TSB−dex
TSB

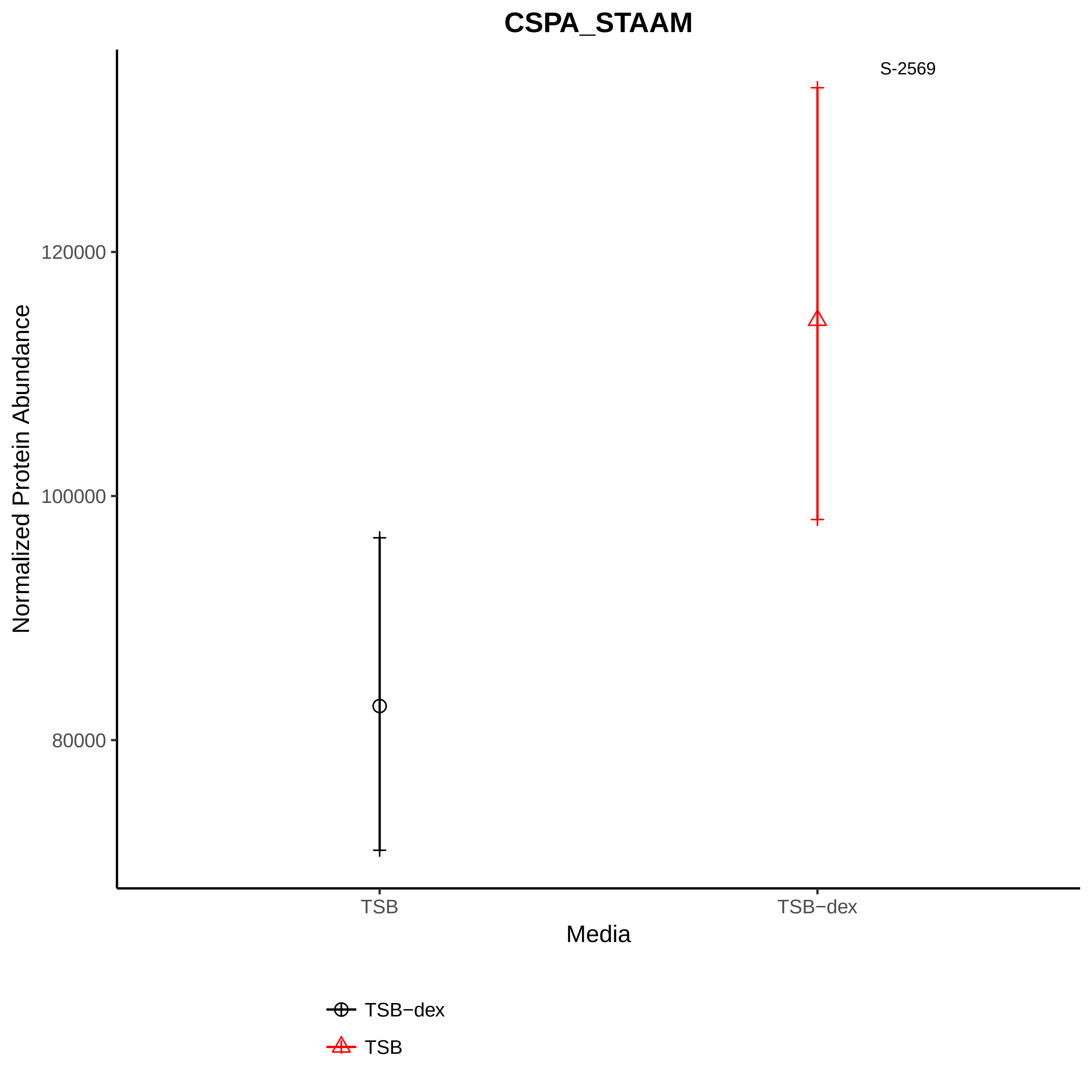

CSPA_STAAM
S-2569
Normalized Protein Abundance
120000
100000
80000
TSB
TSB−dex
Media
TSB−dex
TSB

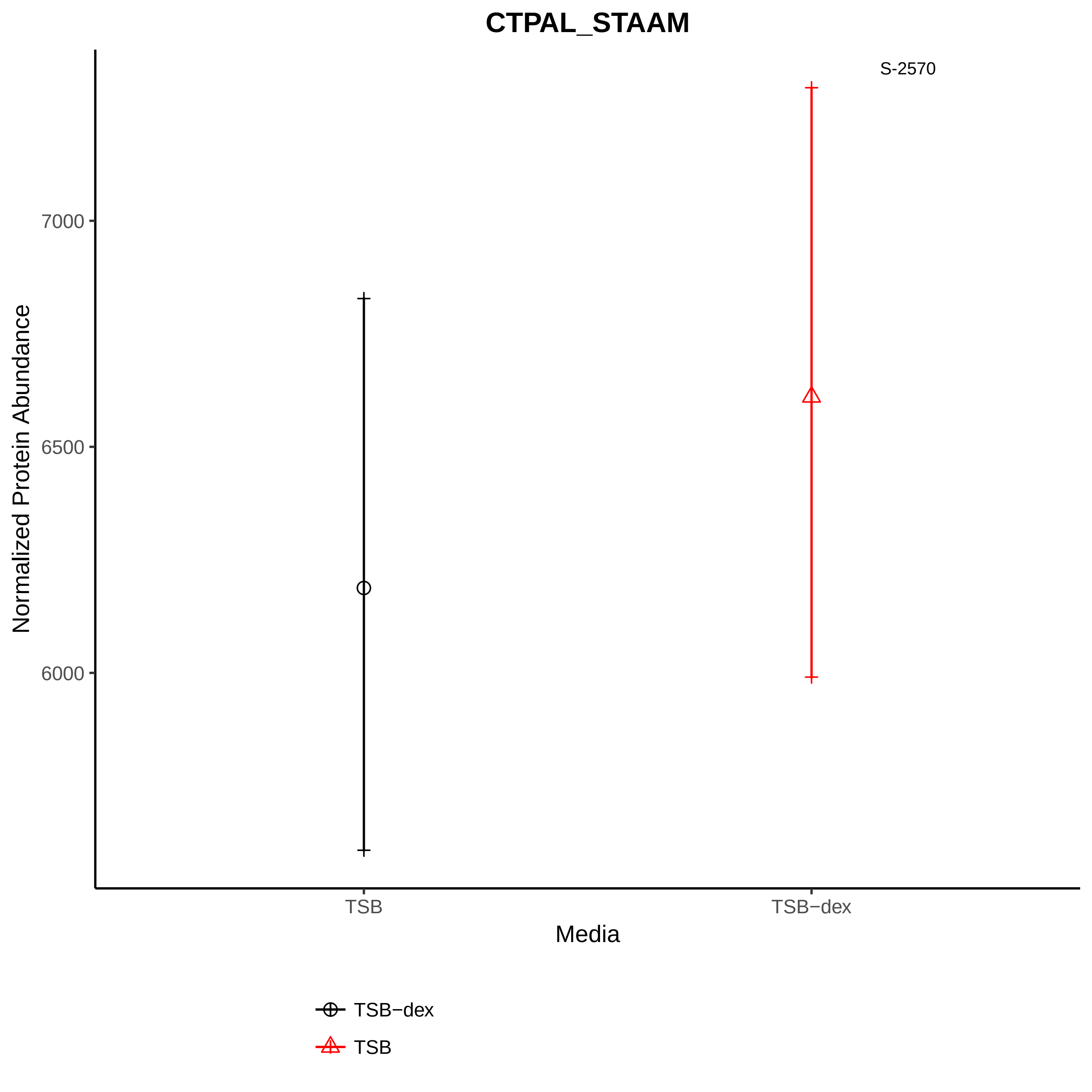

CTPAL_STAAM
S-2570
7000
6500
6000
Normalized Protein Abundance
TSB
TSB−dex
Media
TSB−dex
TSB

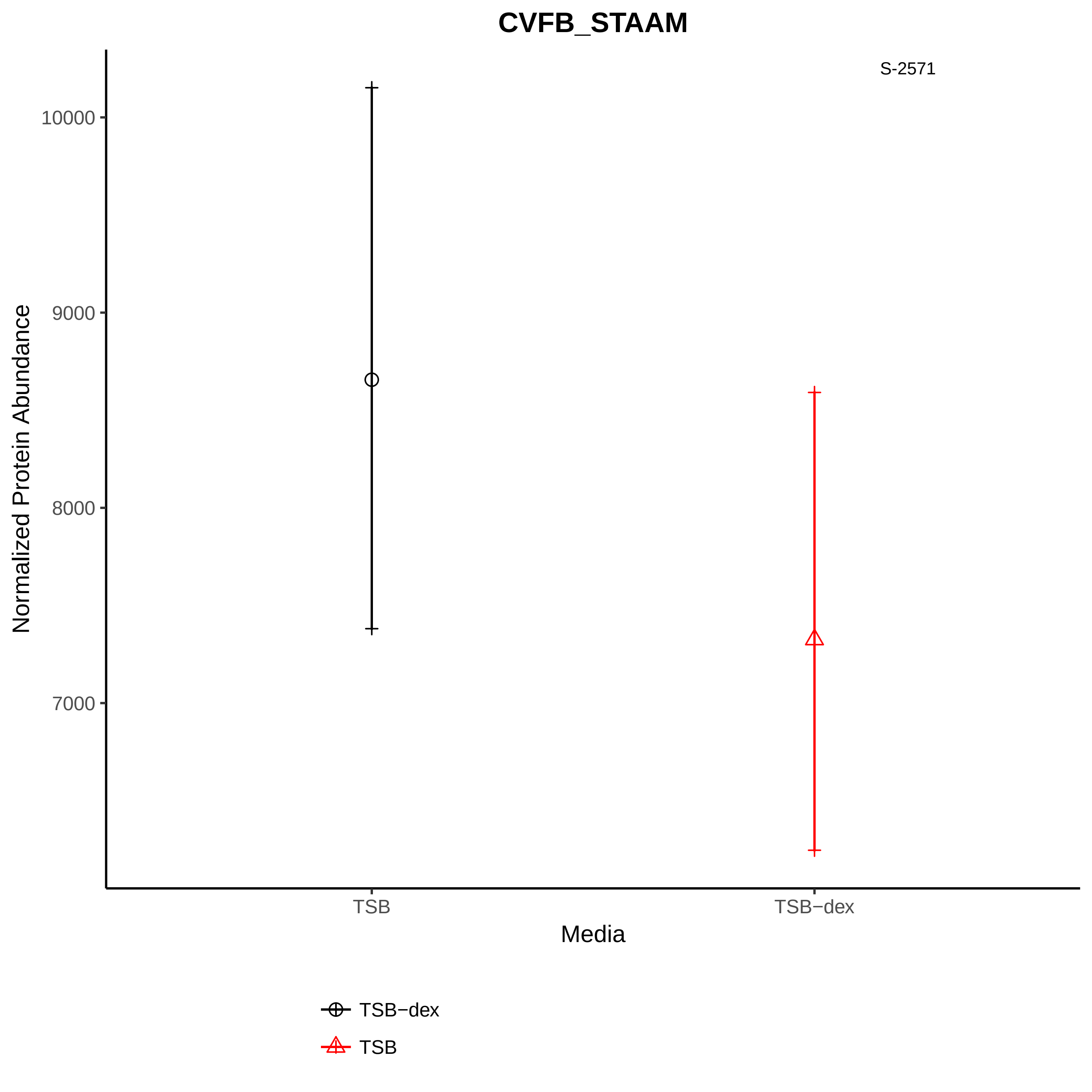
CVFB_STAAM
S-2571
10000
9000
8000
7000
Normalized Protein Abundance
TSB
TSB−dex
Media
TSB−dex
TSB

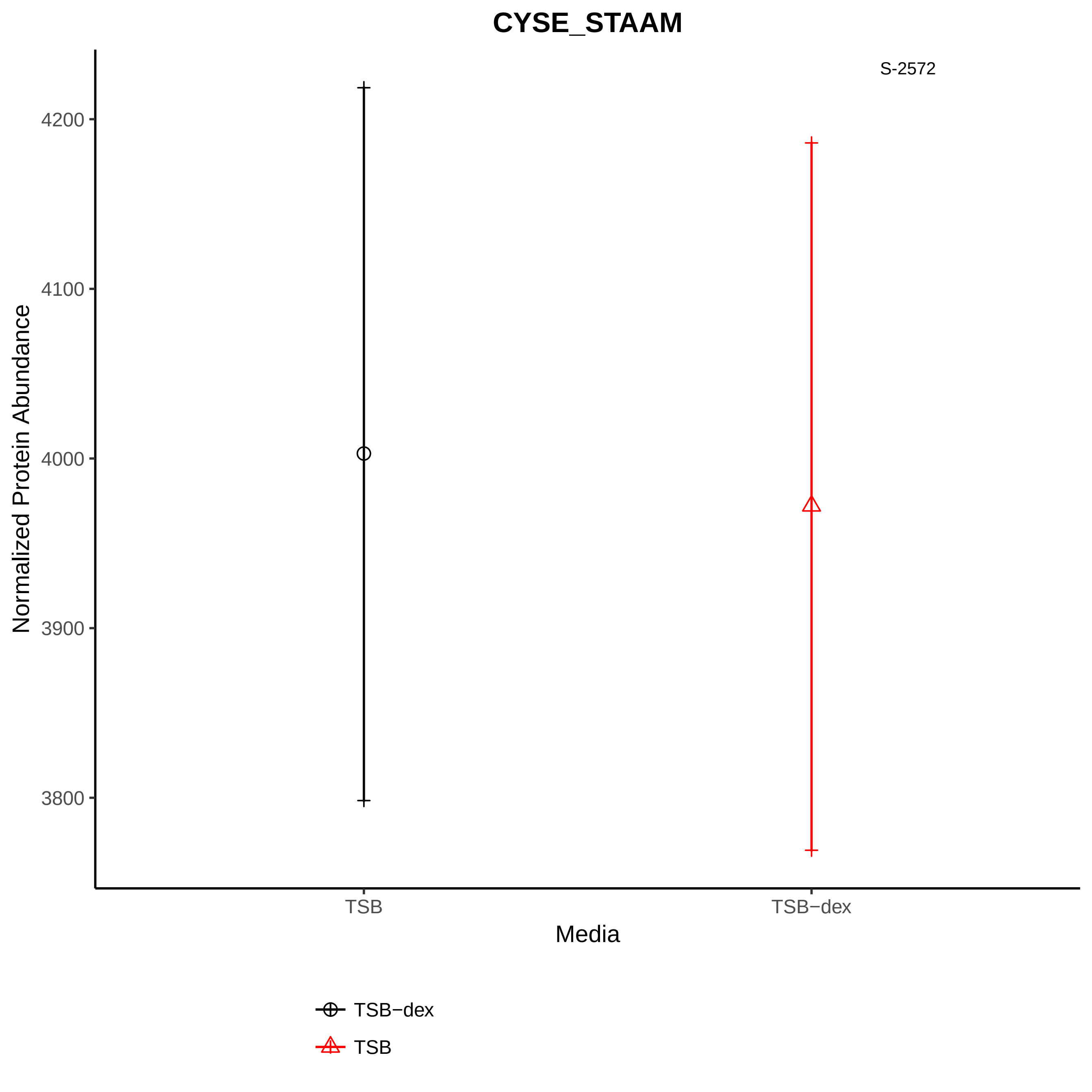

CYSE_STAAM
S-2572
Normalized Protein Abundance
4200
4100
4000
3900
3800
TSB
TSB−dex
Media
TSB−dex
TSB

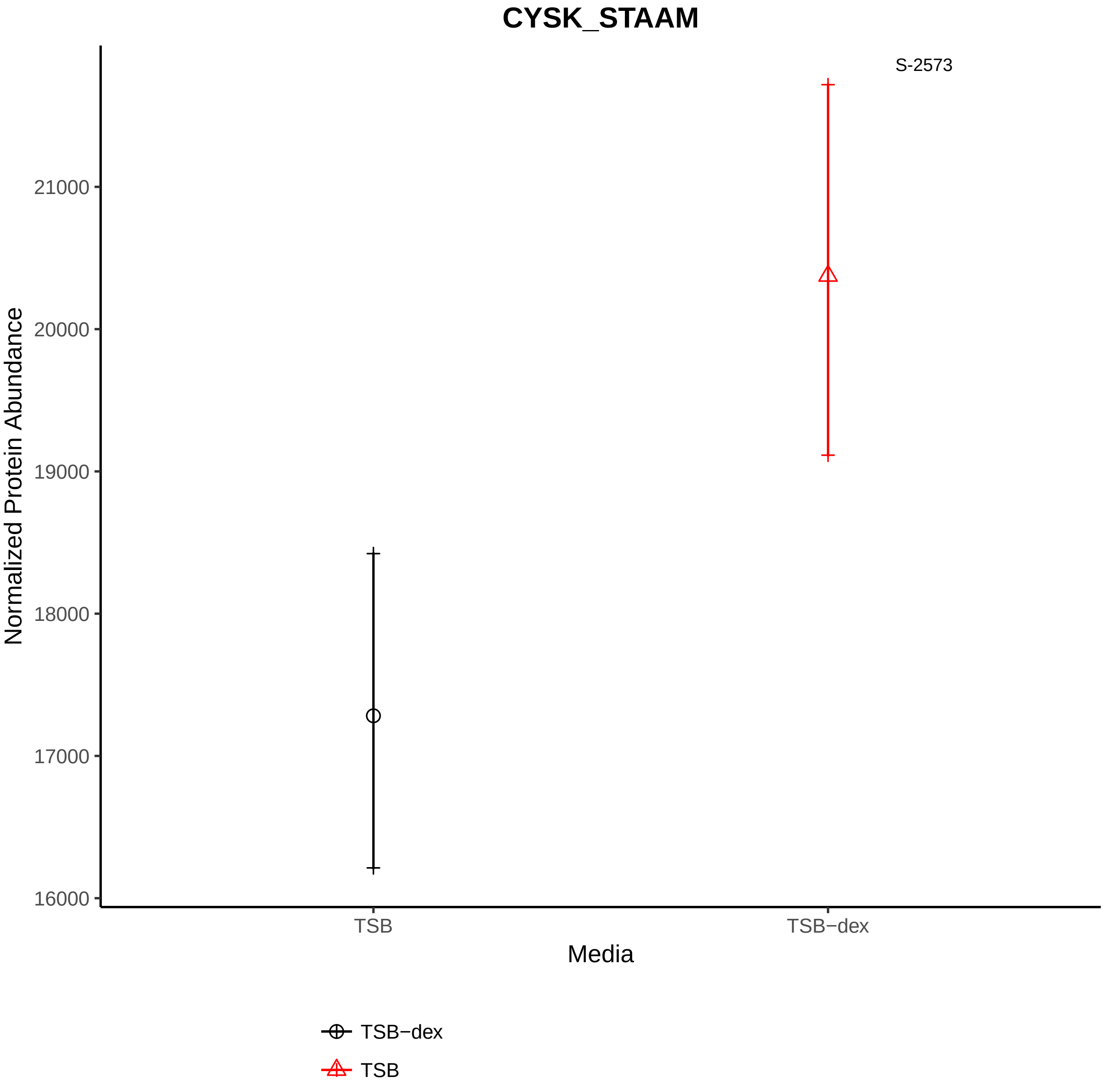

CYSK_STAAM
S-2573
Normalized Protein Abundance
21000
20000
19000
18000
17000
16000
TSB
TSB−dex
Media
TSB−dex
TSB

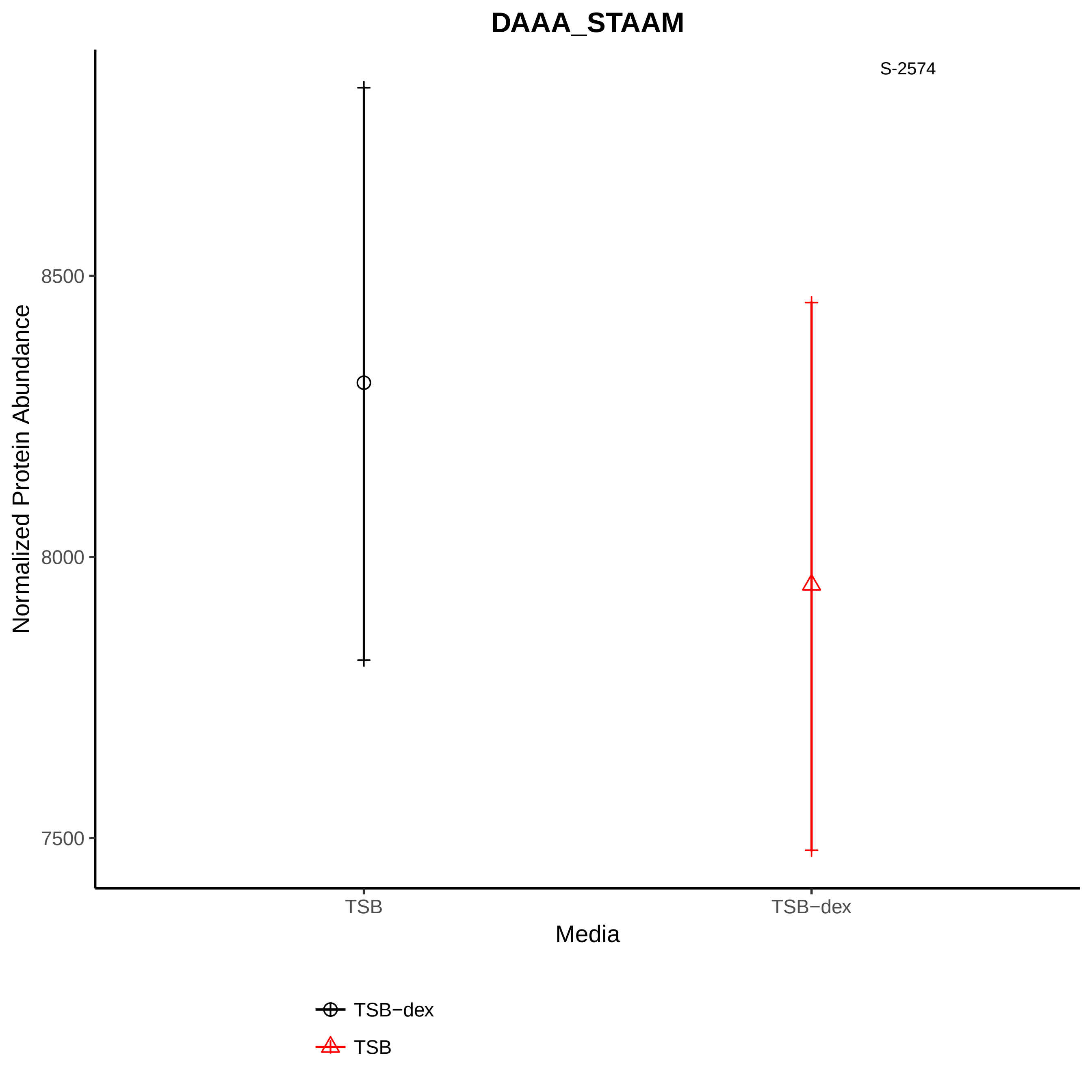

DAAA_STAAM
S-2574
Normalized Protein Abundance
8500
8000
7500
TSB
TSB−dex
Media
TSB−dex
TSB

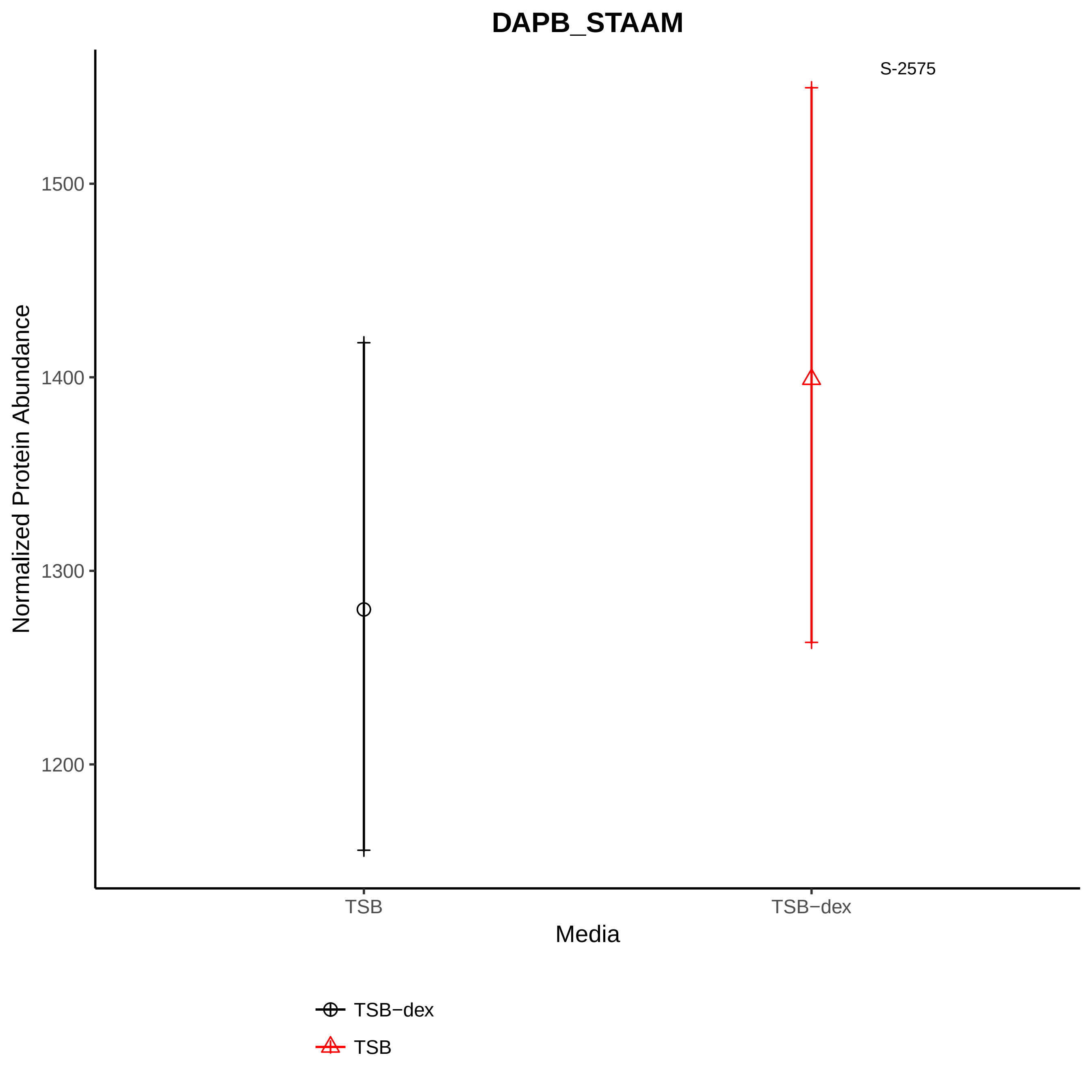
DAPB_STAAM
S-2575
Normalized Protein Abundance
1500
1400
1300
1200
TSB
TSB−dex
Media
TSB−dex
TSB

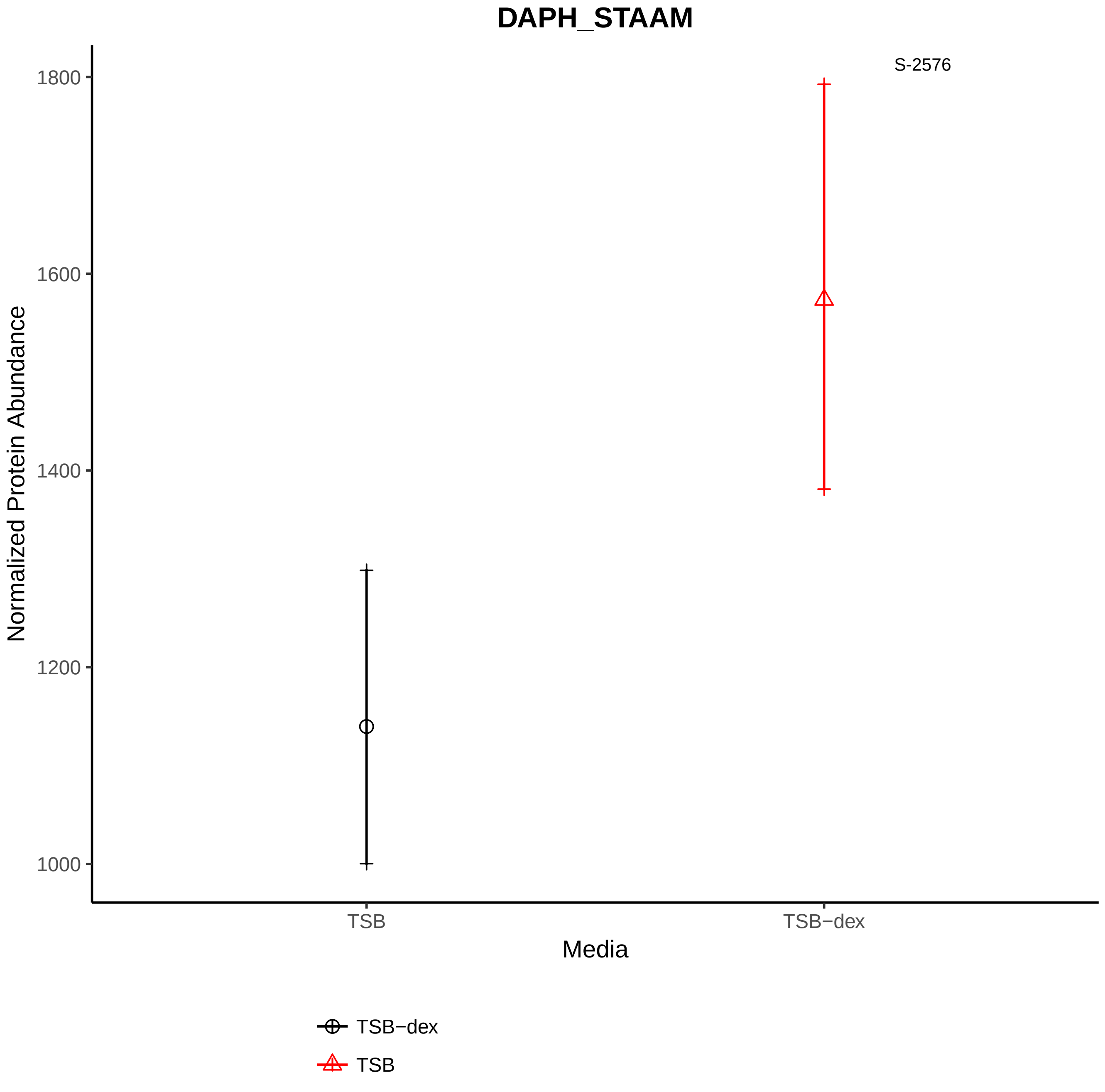
DAPH_STAAM
S-2576
1800
1600
1400
1200
1000
Normalized Protein Abundance
TSB
TSB−dex
Media
TSB−dex
TSB

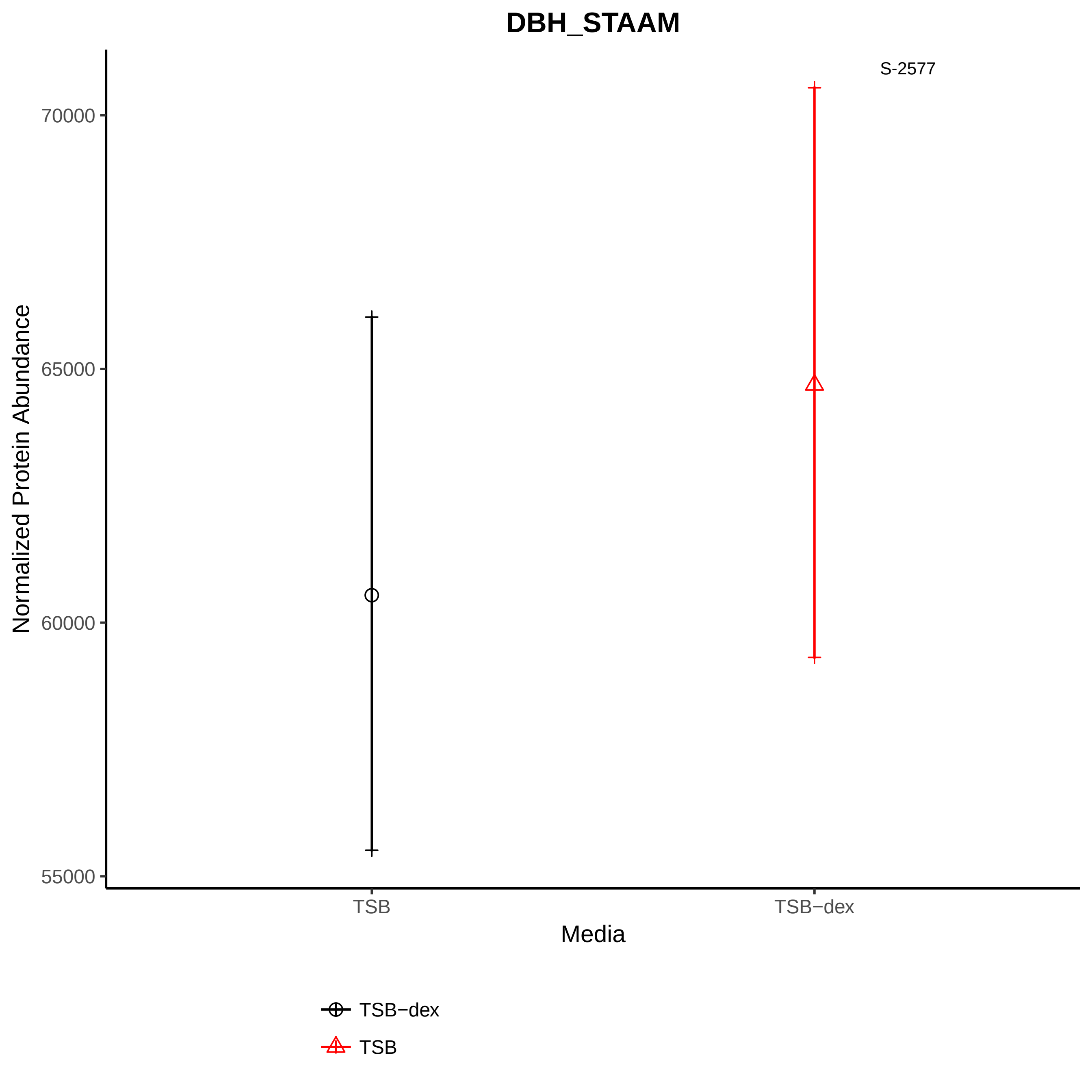

DBH_STAAM
S-2577
Normalized Protein Abundance
70000
65000
60000
55000
TSB
TSB−dex
Media
TSB−dex
TSB

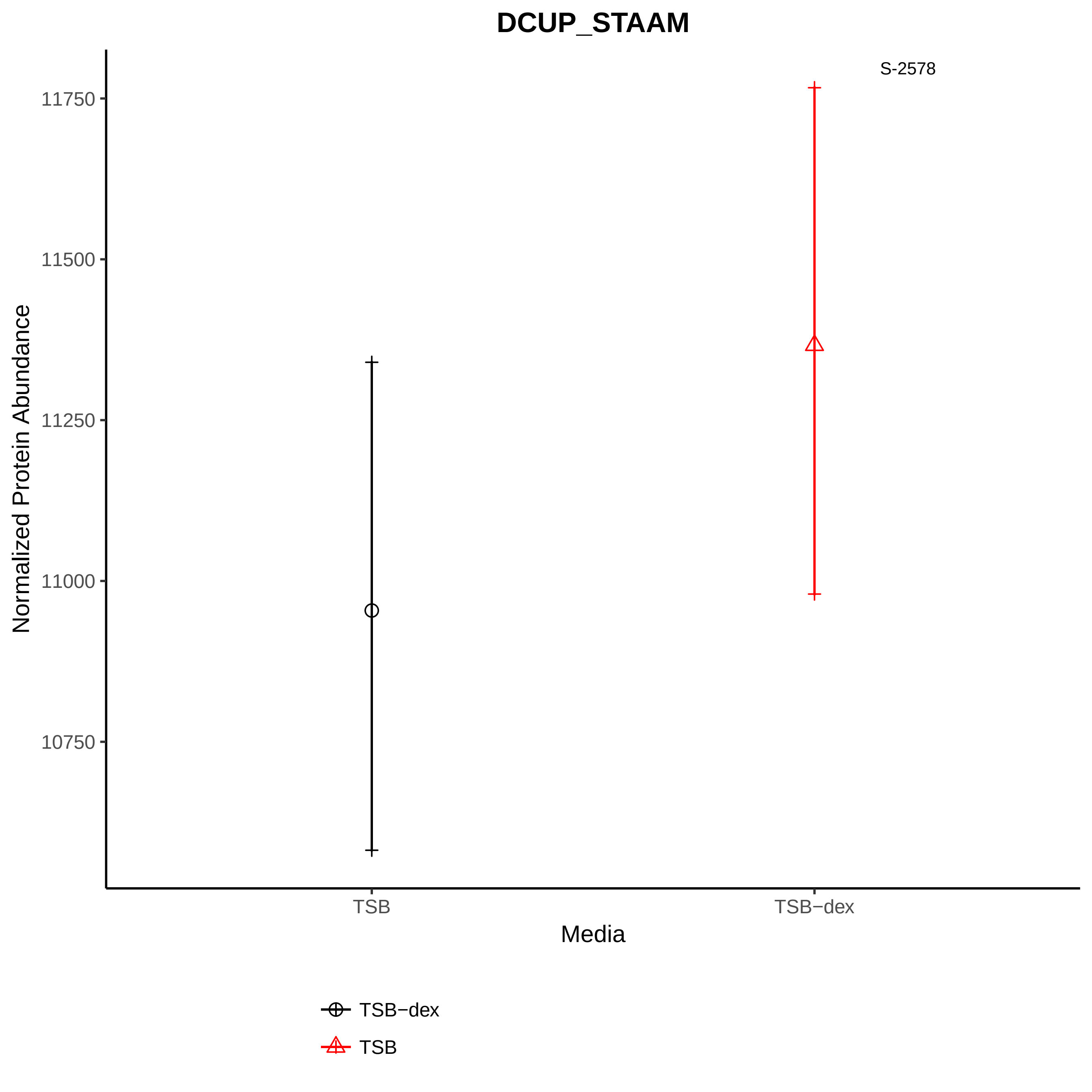
DCUP_STAAM
S-2578
11750
11500
11250
11000
10750
Normalized Protein Abundance
TSB
TSB−dex
Media
TSB−dex
TSB

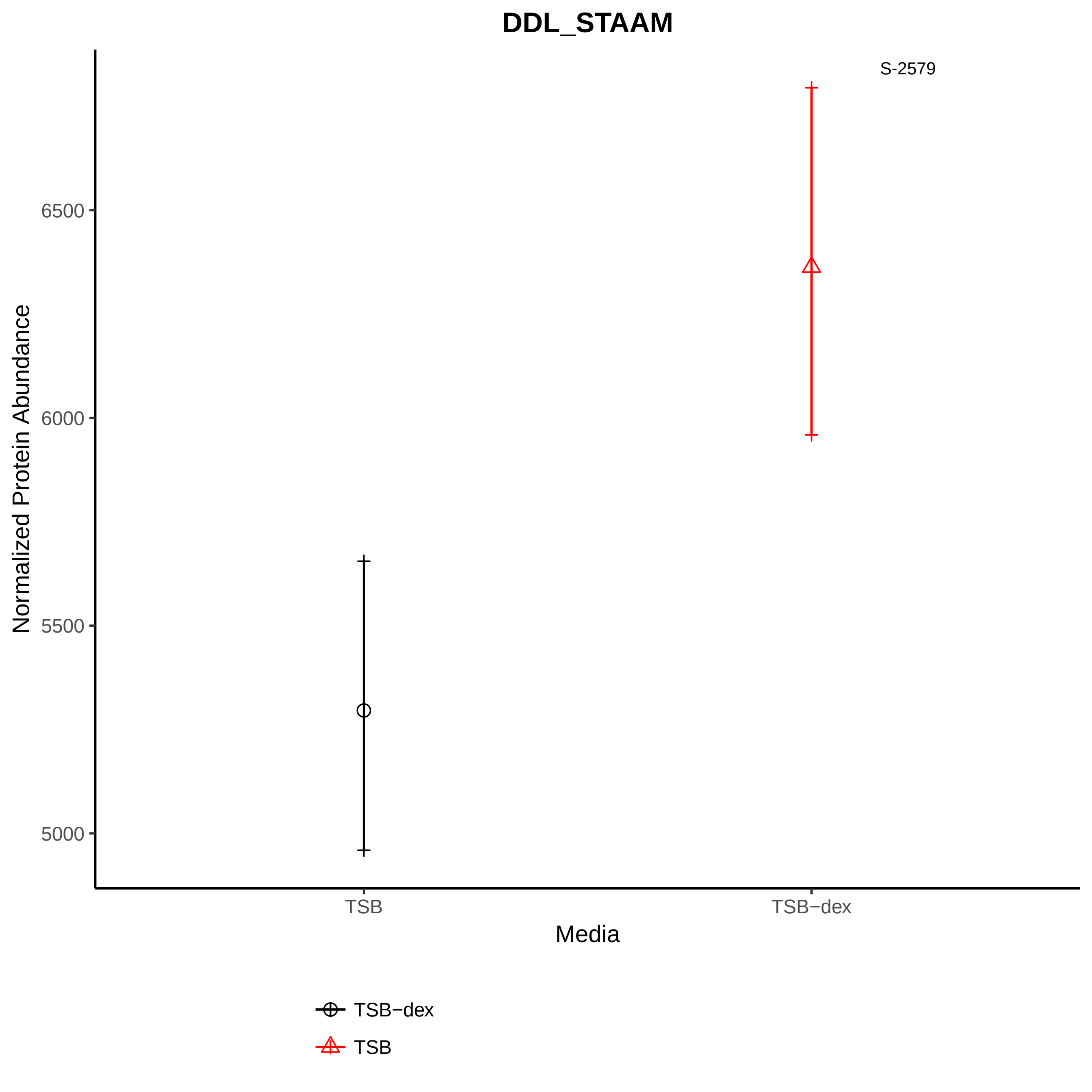

DDL_STAAM
S-2579
Normalized Protein Abundance
6500
6000
5500
5000
TSB
TSB−dex
Media
TSB−dex
TSB

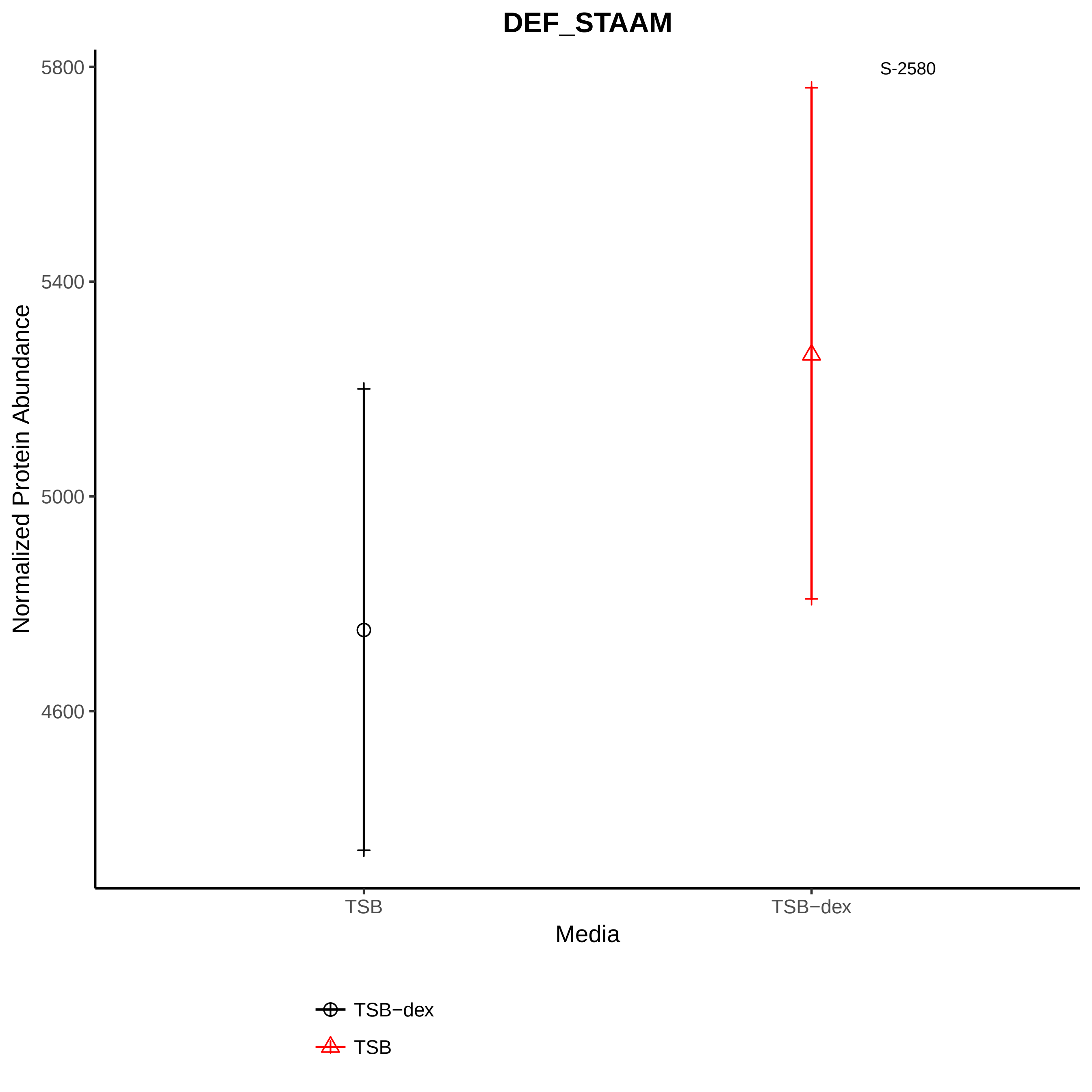
DEF_STAAM
S-2580
Normalized Protein Abundance
5800
5400
5000
4600
TSB
TSB−dex
Media
TSB−dex
TSB

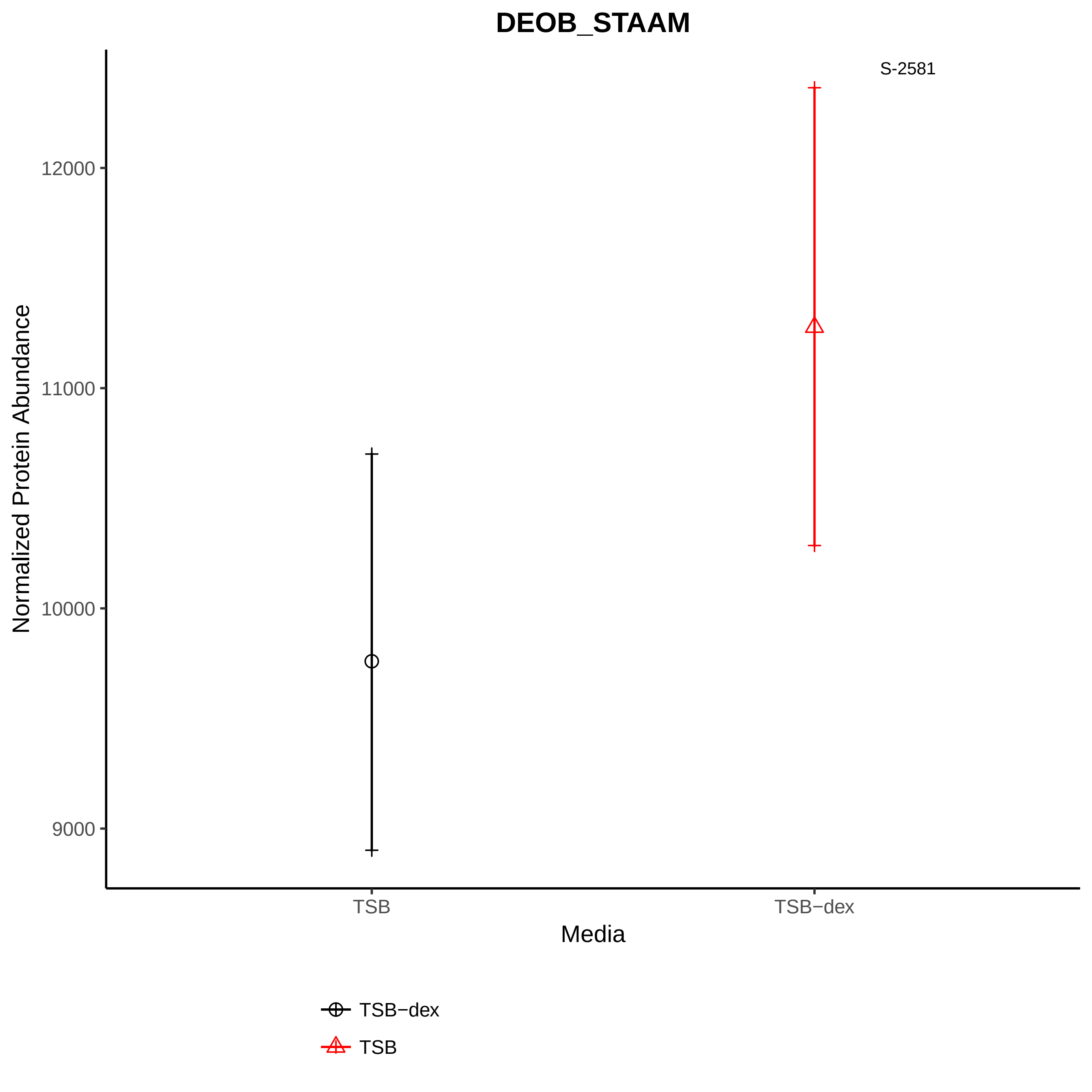
DEOB_STAAM
S-2581
Normalized Protein Abundance
12000
11000
10000
9000
TSB
TSB−dex
Media
TSB−dex
TSB

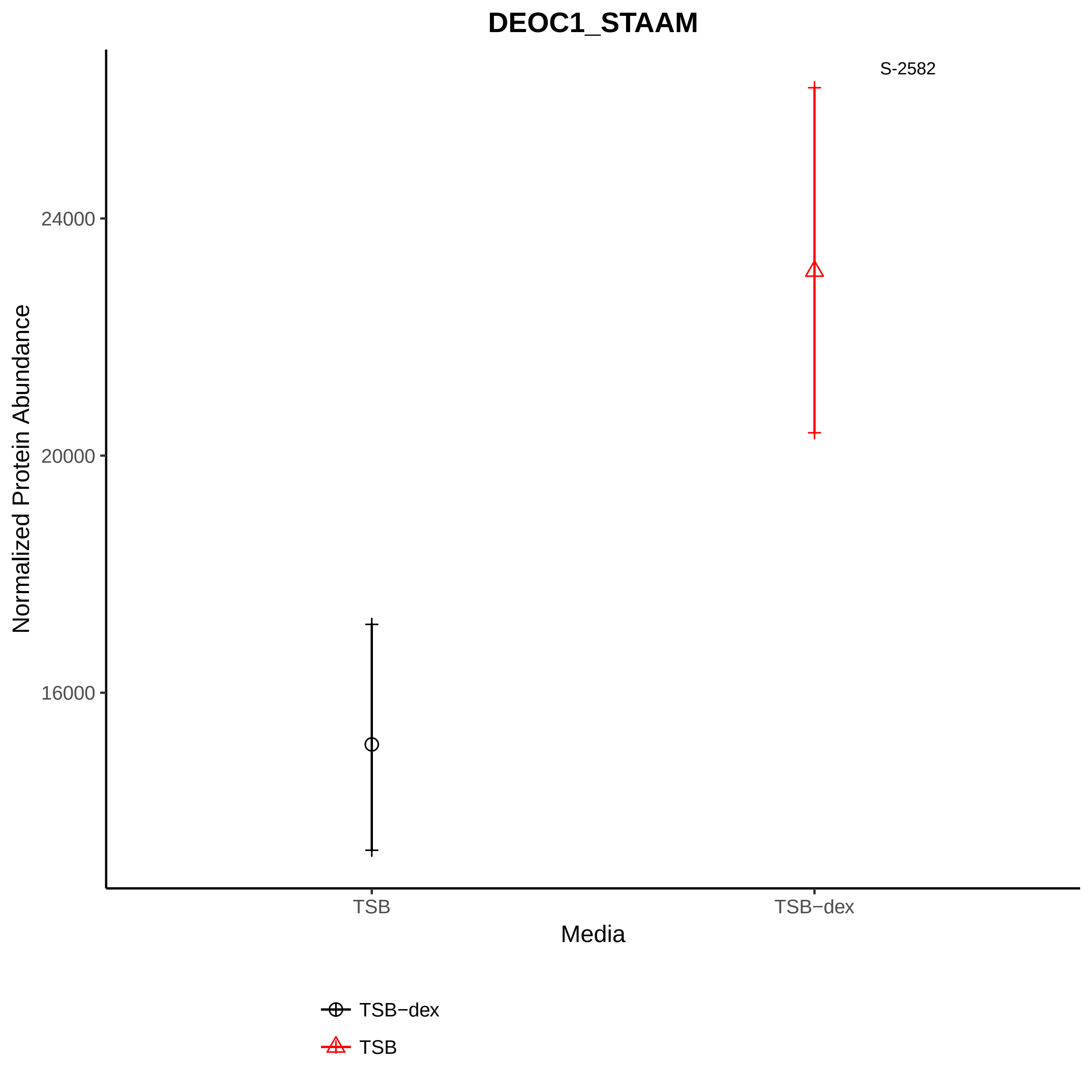

DEOC1_STAAM
S-2582
Normalized Protein Abundance
24000
20000
16000
TSB
TSB−dex
Media
TSB−dex
TSB

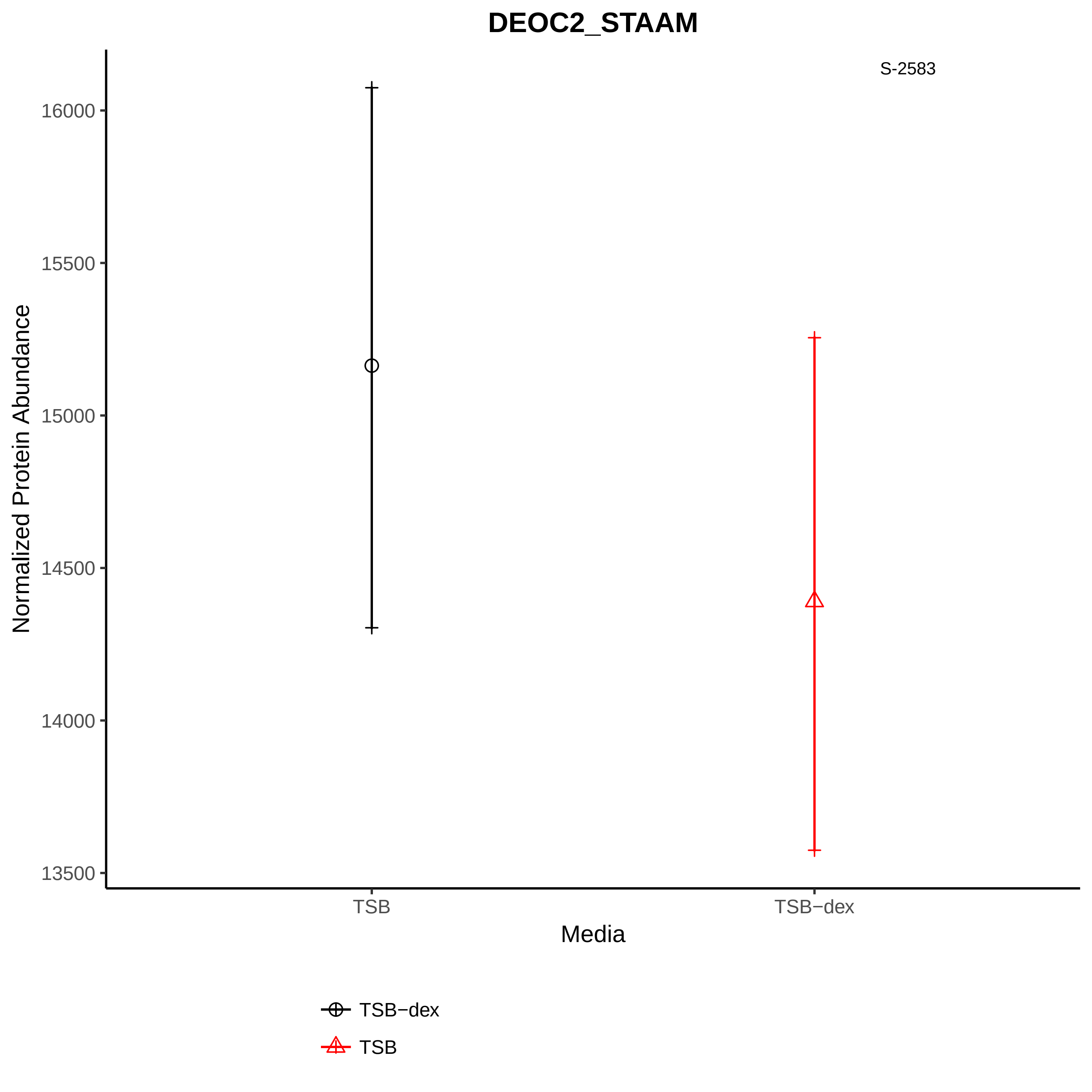

DEOC2_STAAM
S-2583
16000
15500
15000
14500
14000
13500
Normalized Protein Abundance
TSB
TSB−dex
Media
TSB−dex
TSB

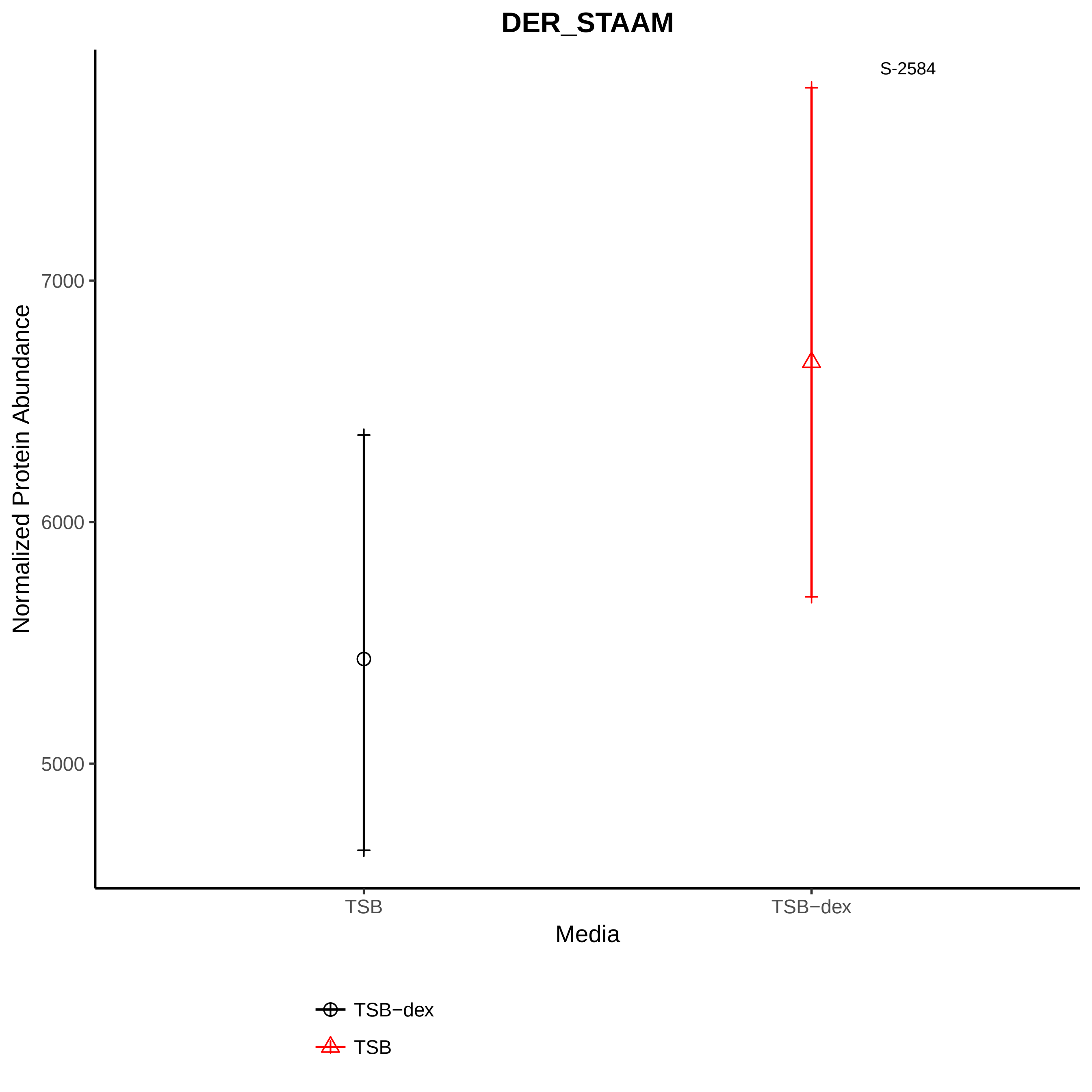

DER_STAAM
S-2584
Normalized Protein Abundance
7000
6000
5000
TSB
TSB−dex
Media
TSB−dex
TSB

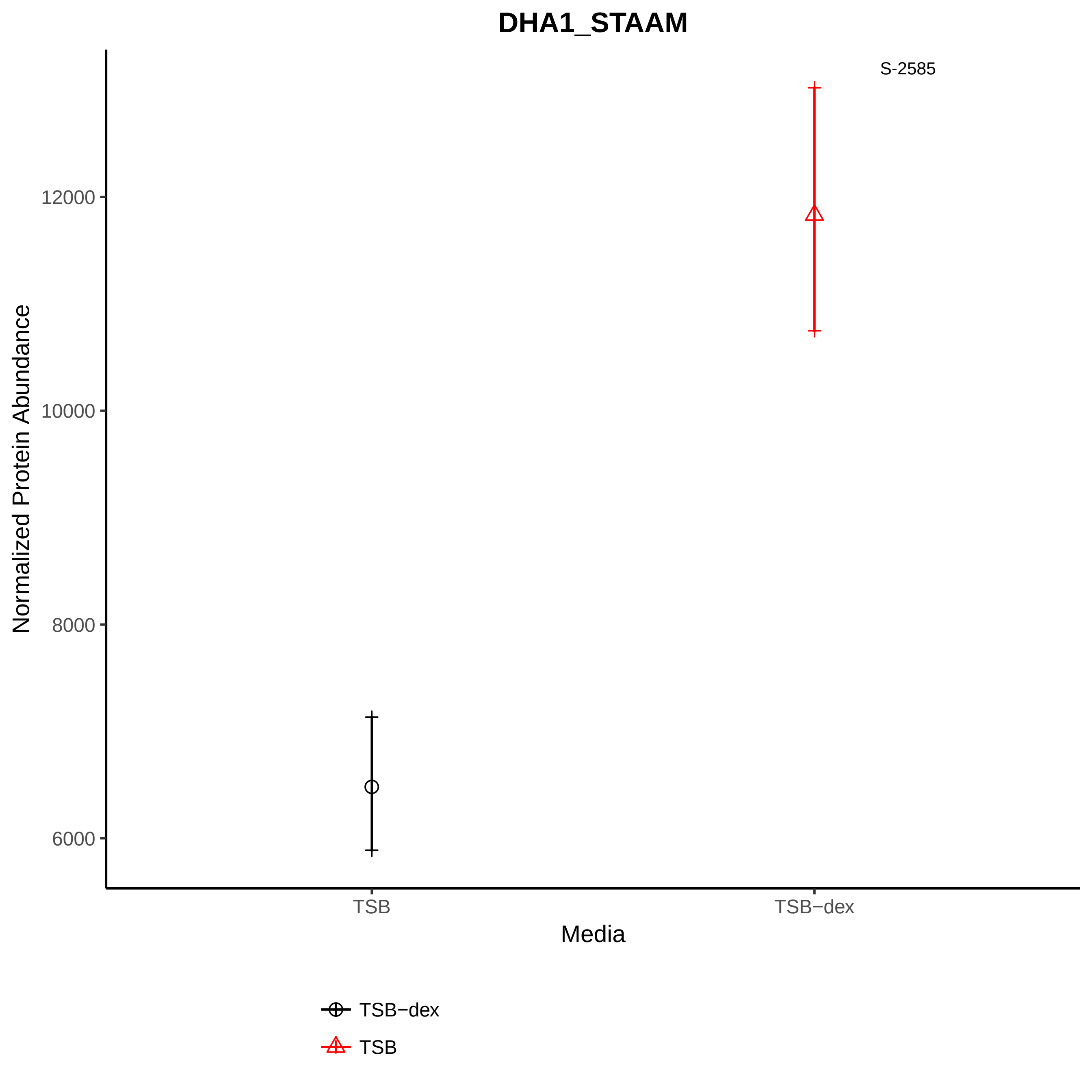
DHA1_STAAM
S-2585
Normalized Protein Abundance
12000
10000
8000
6000
TSB
TSB−dex
Media
TSB−dex
TSB

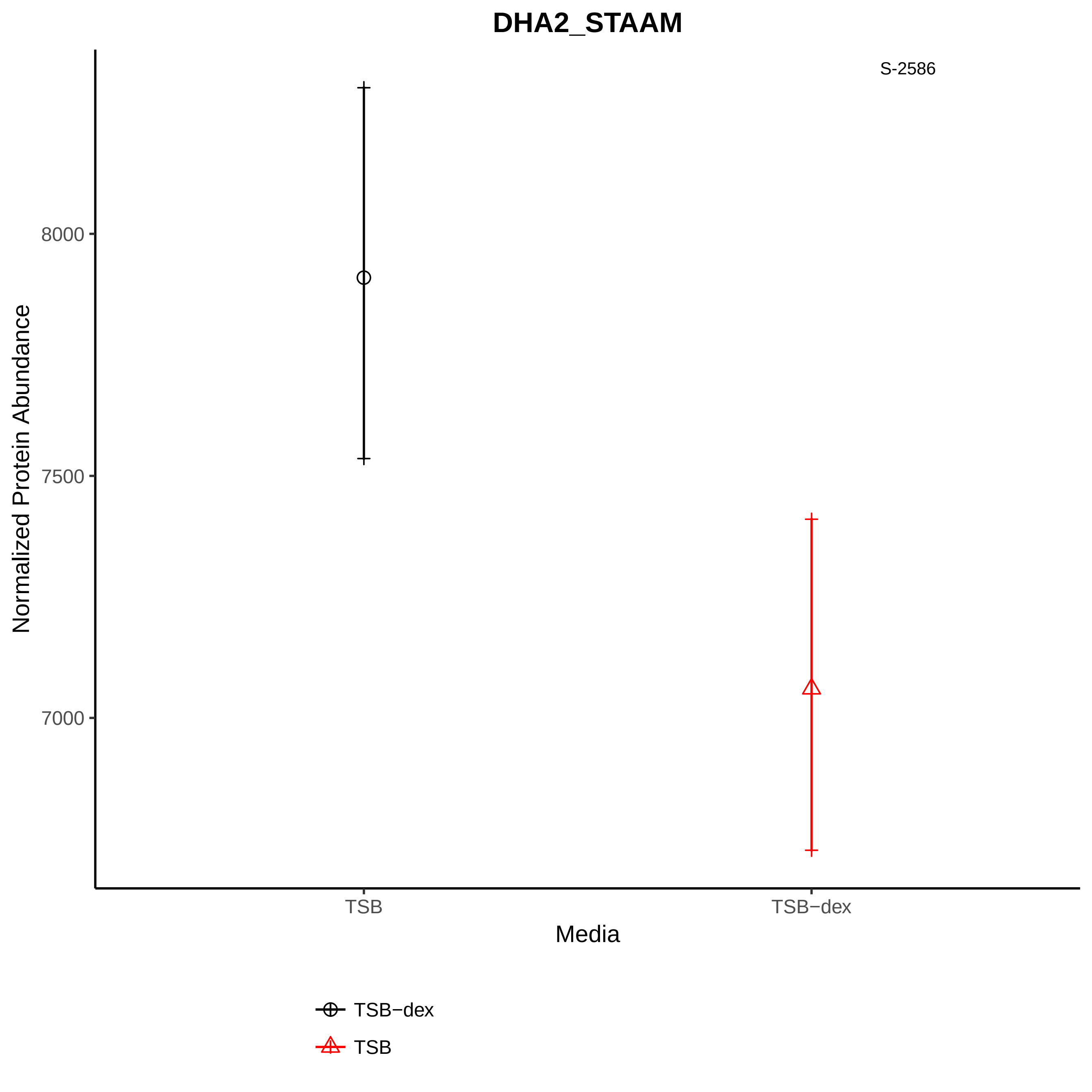

DHA2_STAAM
S-2586
8000
7500
7000
Normalized Protein Abundance
TSB
TSB−dex
Media
TSB−dex
TSB

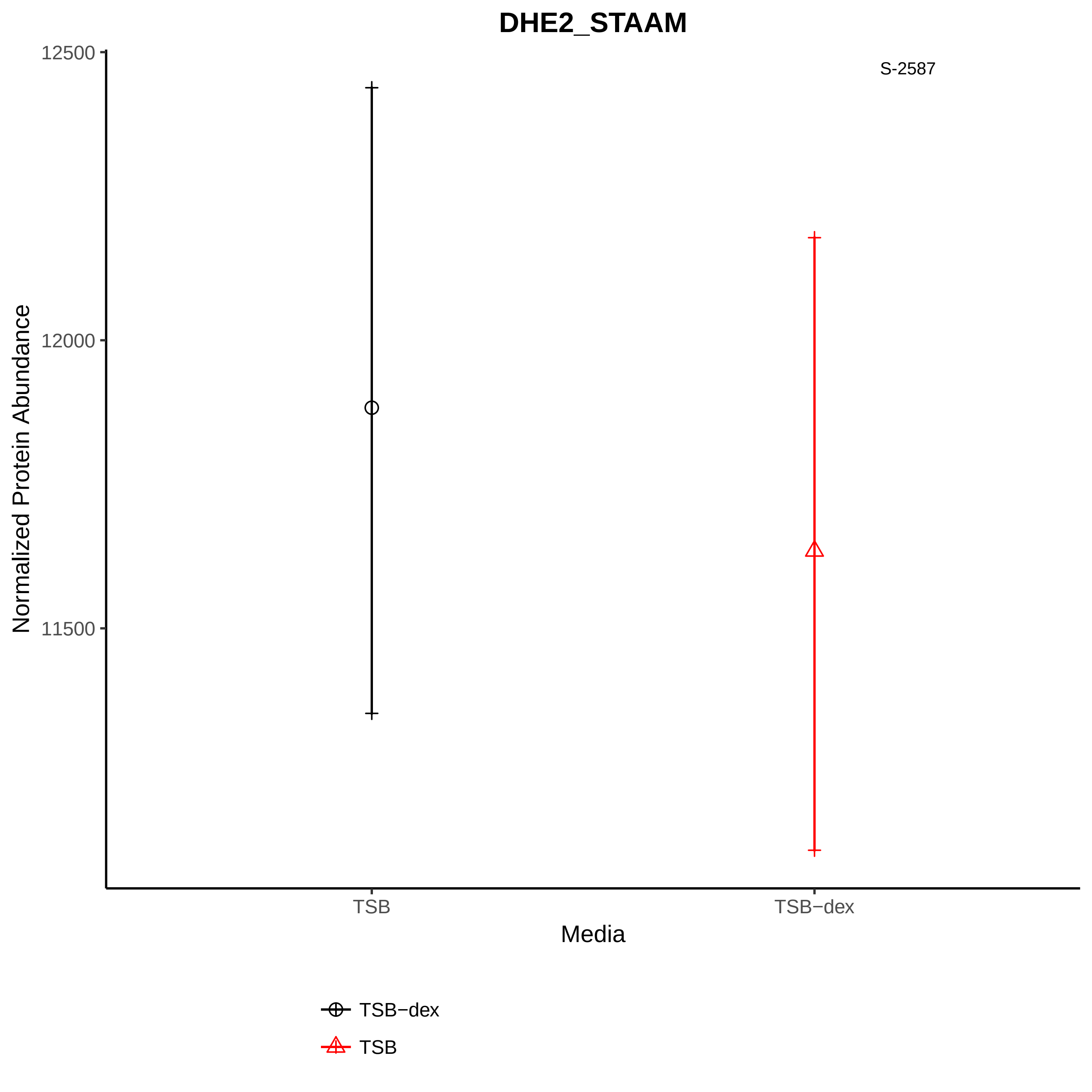

DHE2_STAAM
S-2587
12500
12000
11500
Normalized Protein Abundance
TSB
TSB−dex
Media
TSB−dex
TSB

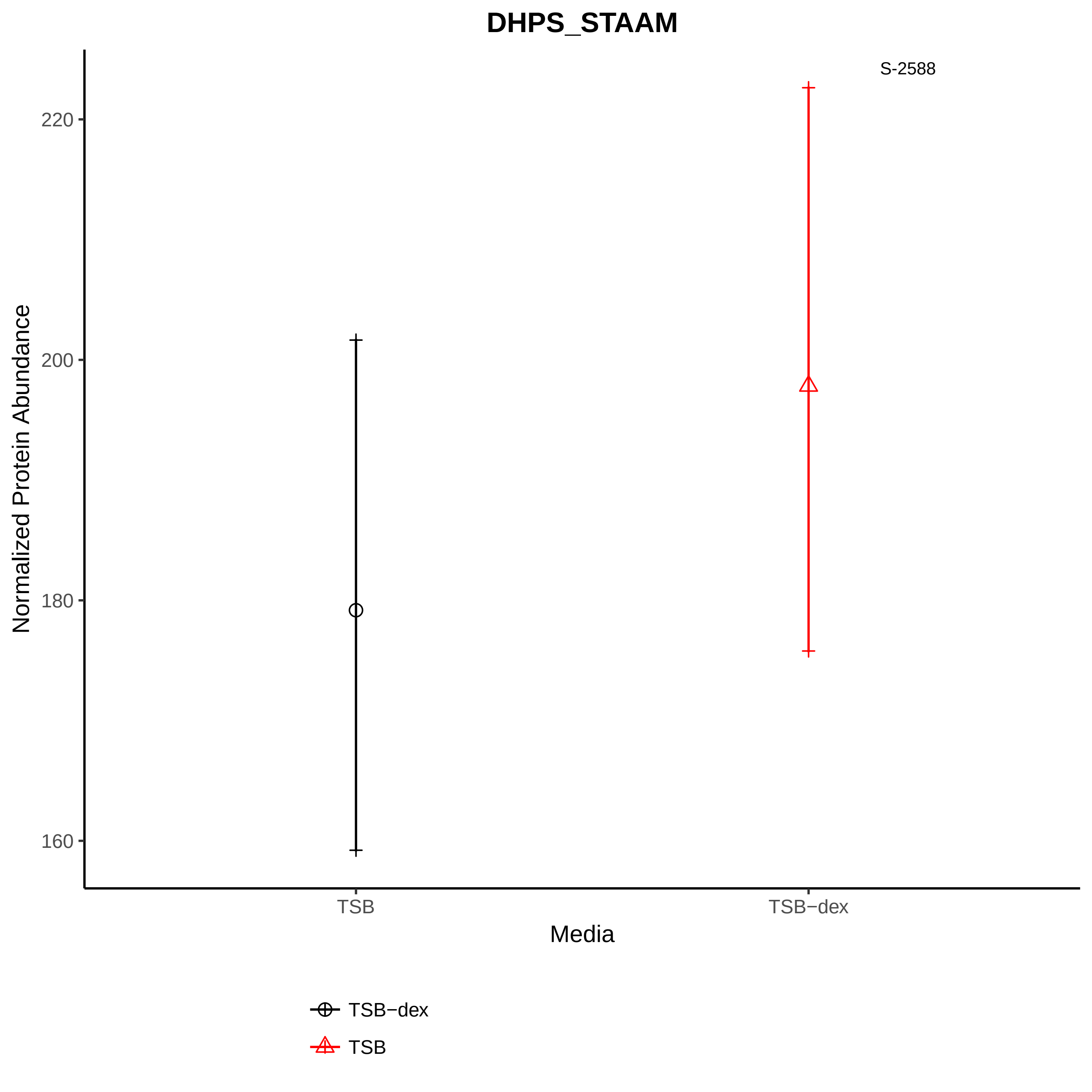

DHPS_STAAM
S-2588
Normalized Protein Abundance
220
200
180
160
TSB
TSB−dex
Media
TSB−dex
TSB

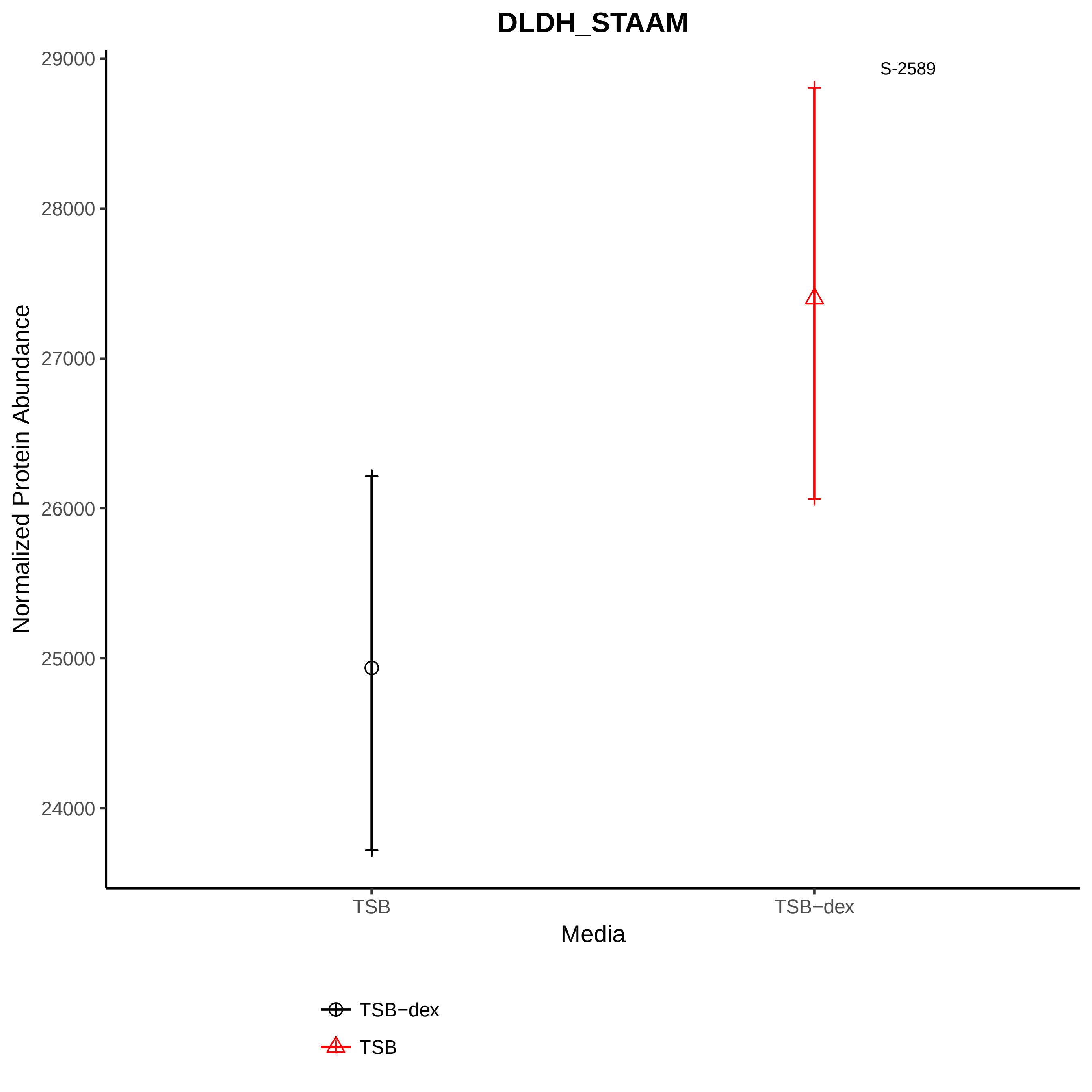

DLDH_STAAM
S-2589
Normalized Protein Abundance
29000
28000
27000
26000
25000
24000
TSB
TSB−dex
Media
TSB−dex
TSB

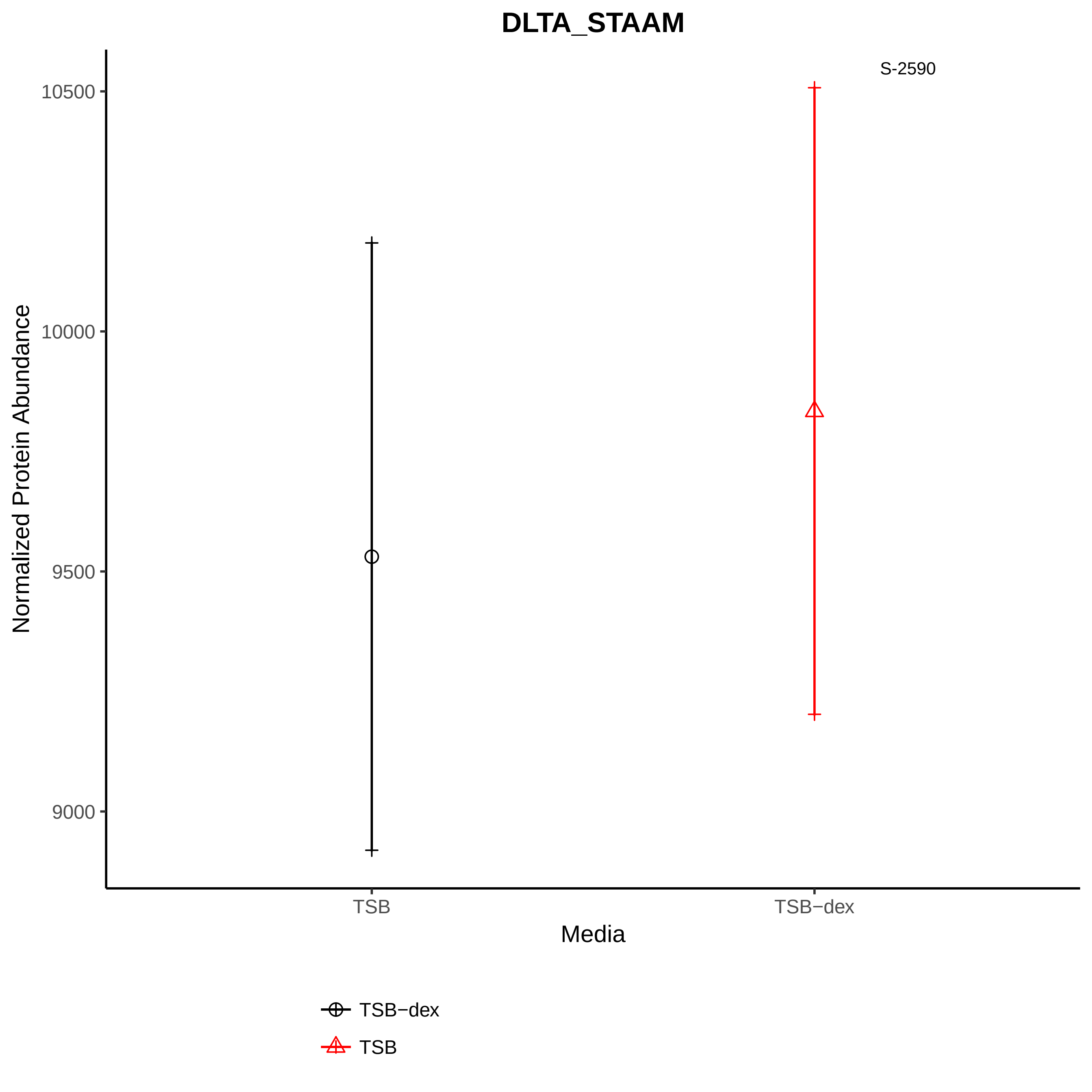

DLTA_STAAM
S-2590
10500
10000
9500
9000
Normalized Protein Abundance
TSB
TSB−dex
Media
TSB−dex
TSB

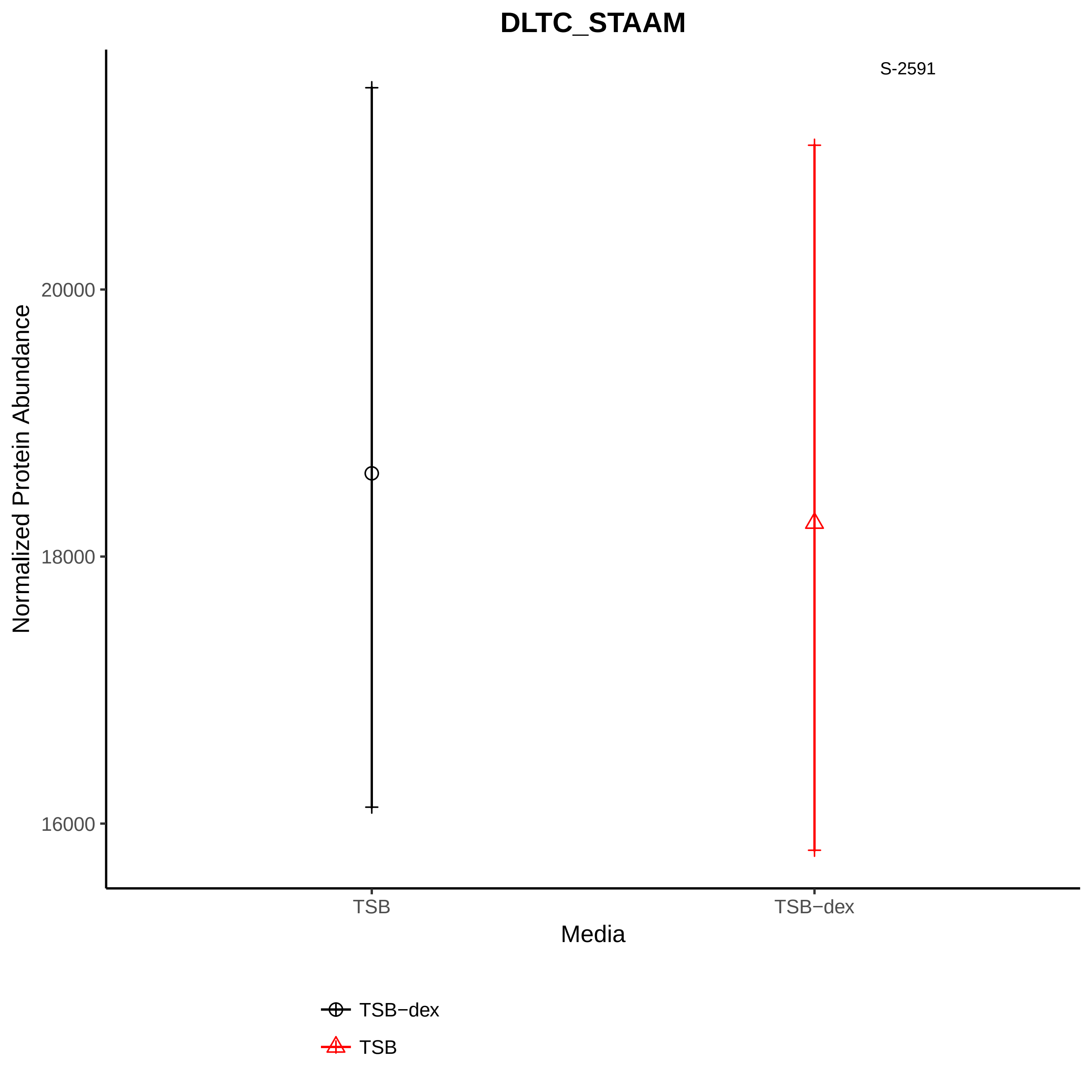
DLTC_STAAM
S-2591
Normalized Protein Abundance
20000
18000
16000
TSB
TSB−dex
Media
TSB−dex
TSB

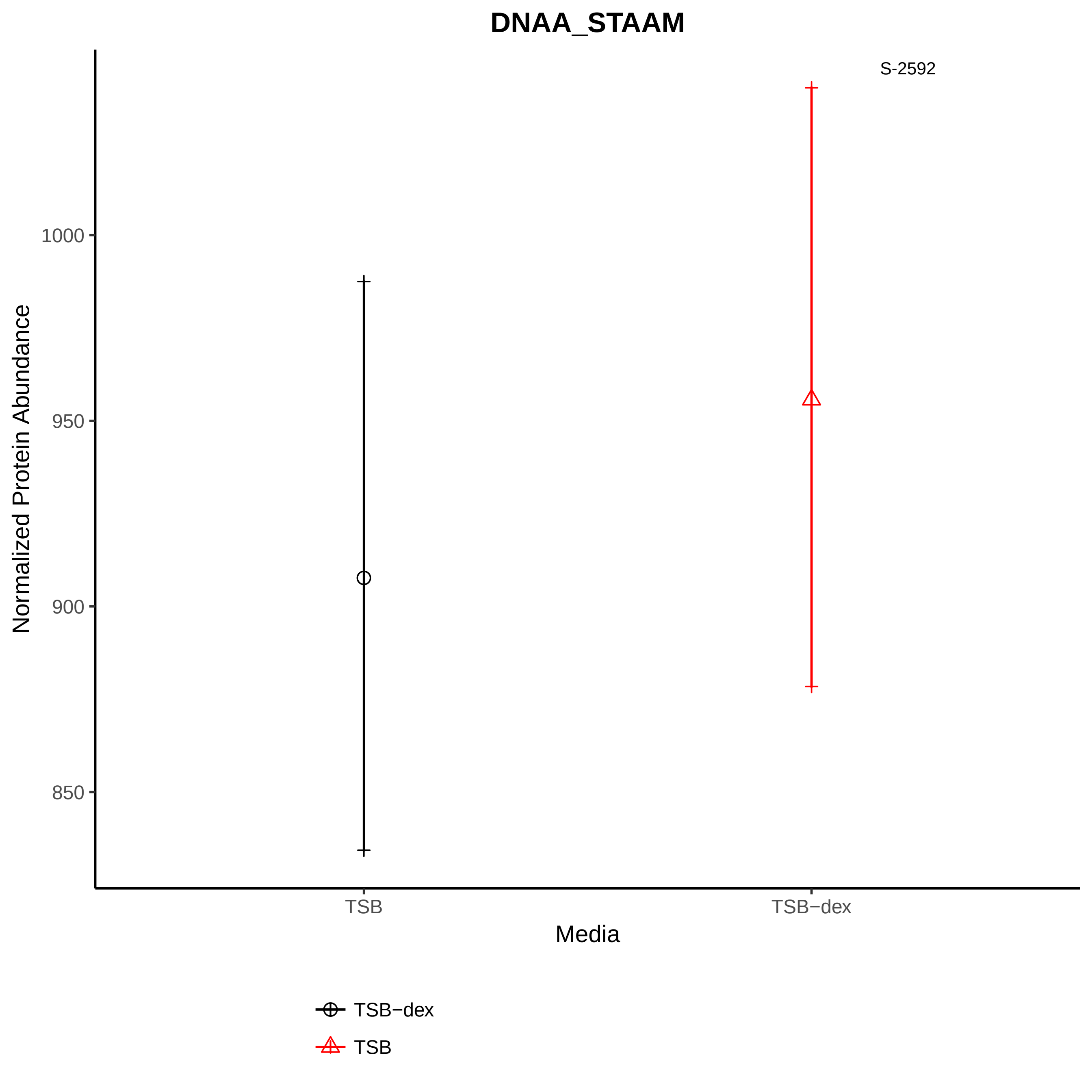

DNAA_STAAM
S-2592
Normalized Protein Abundance
1000
950
900
850
TSB
TSB−dex
Media
TSB−dex
TSB

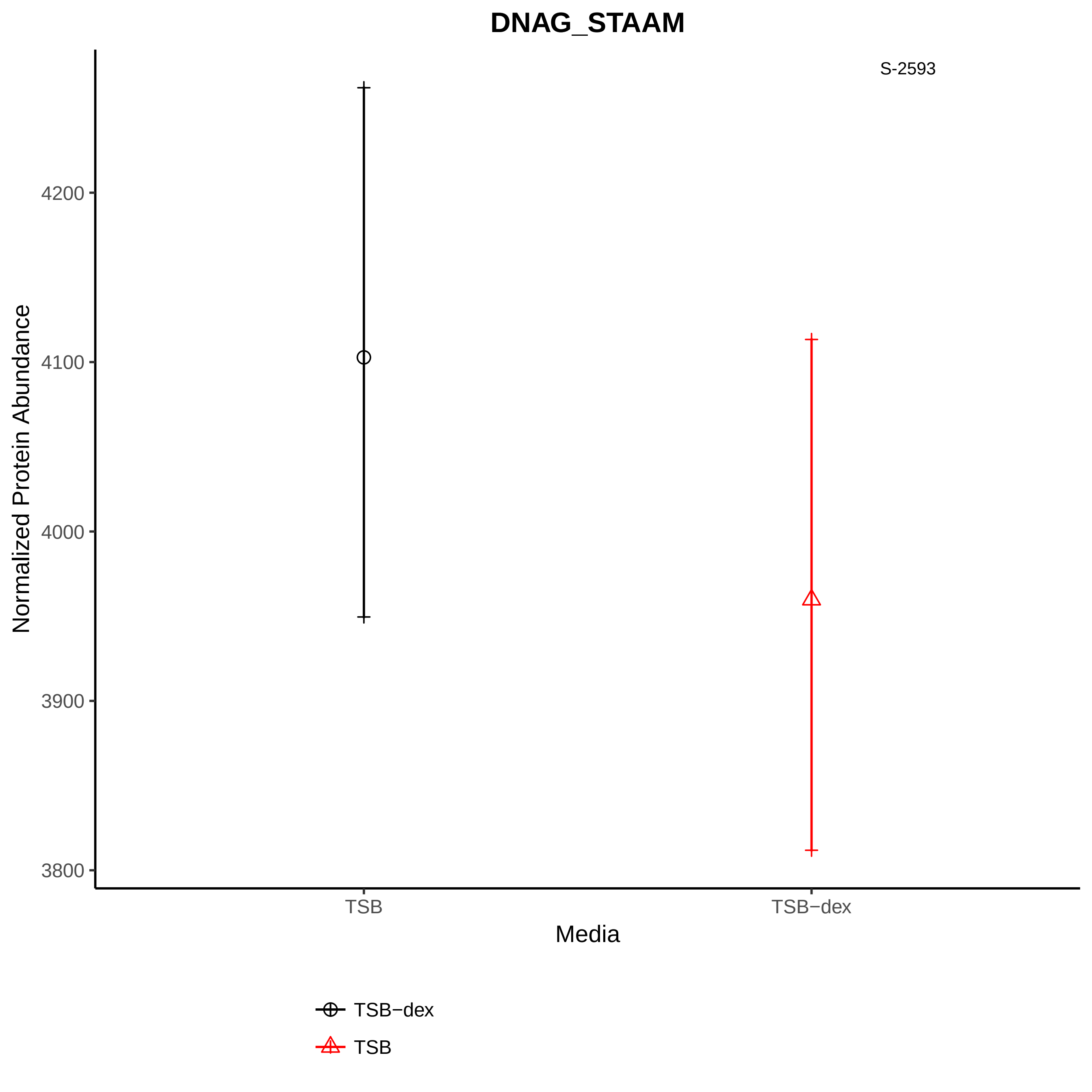
DNAG_STAAM
S-2593
Normalized Protein Abundance
4200
4100
4000
3900
3800
TSB
TSB−dex
Media
TSB−dex
TSB

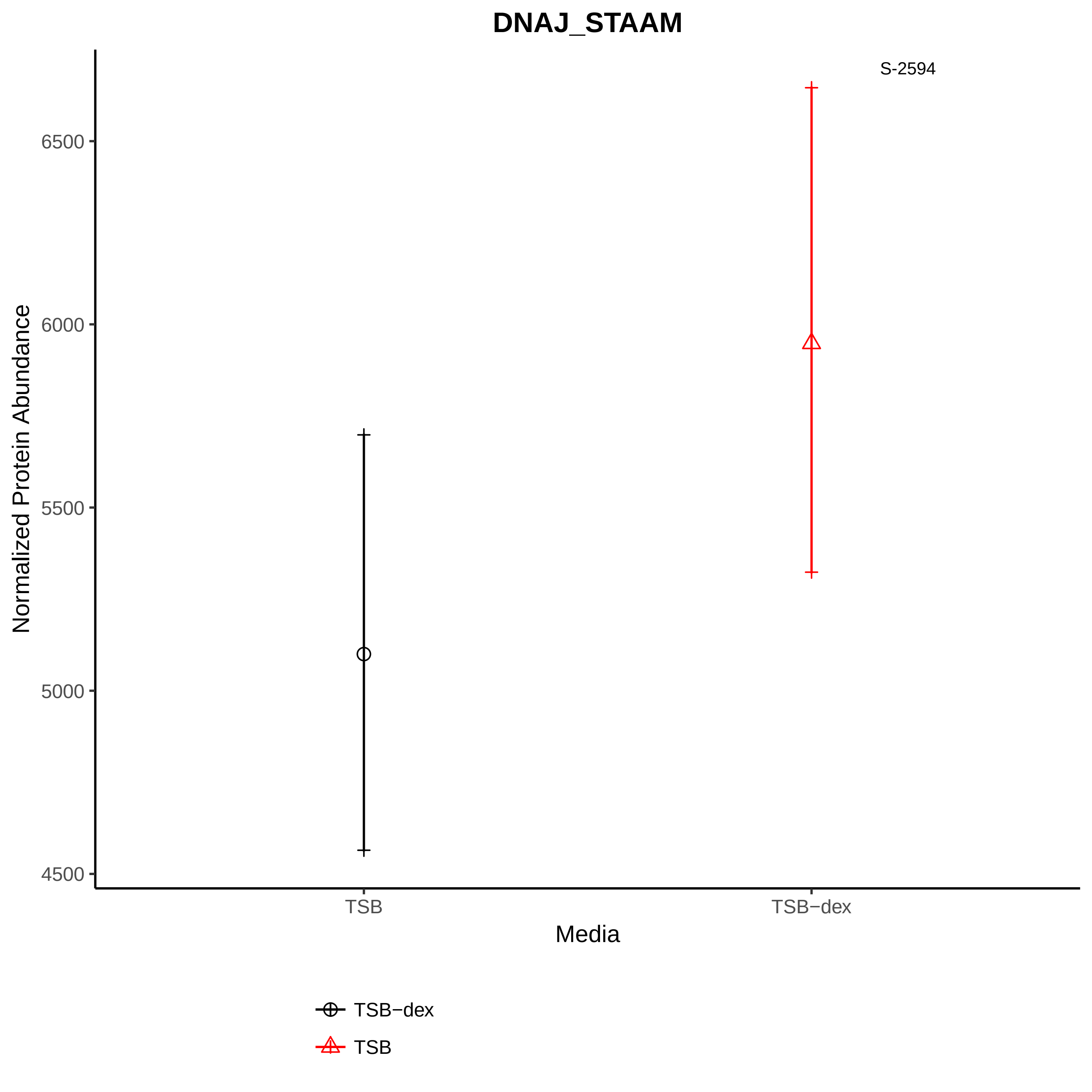
DNAJ_STAAM
S-2594
6500
6000
5500
5000
4500
Normalized Protein Abundance
TSB
TSB−dex
Media
TSB−dex
TSB

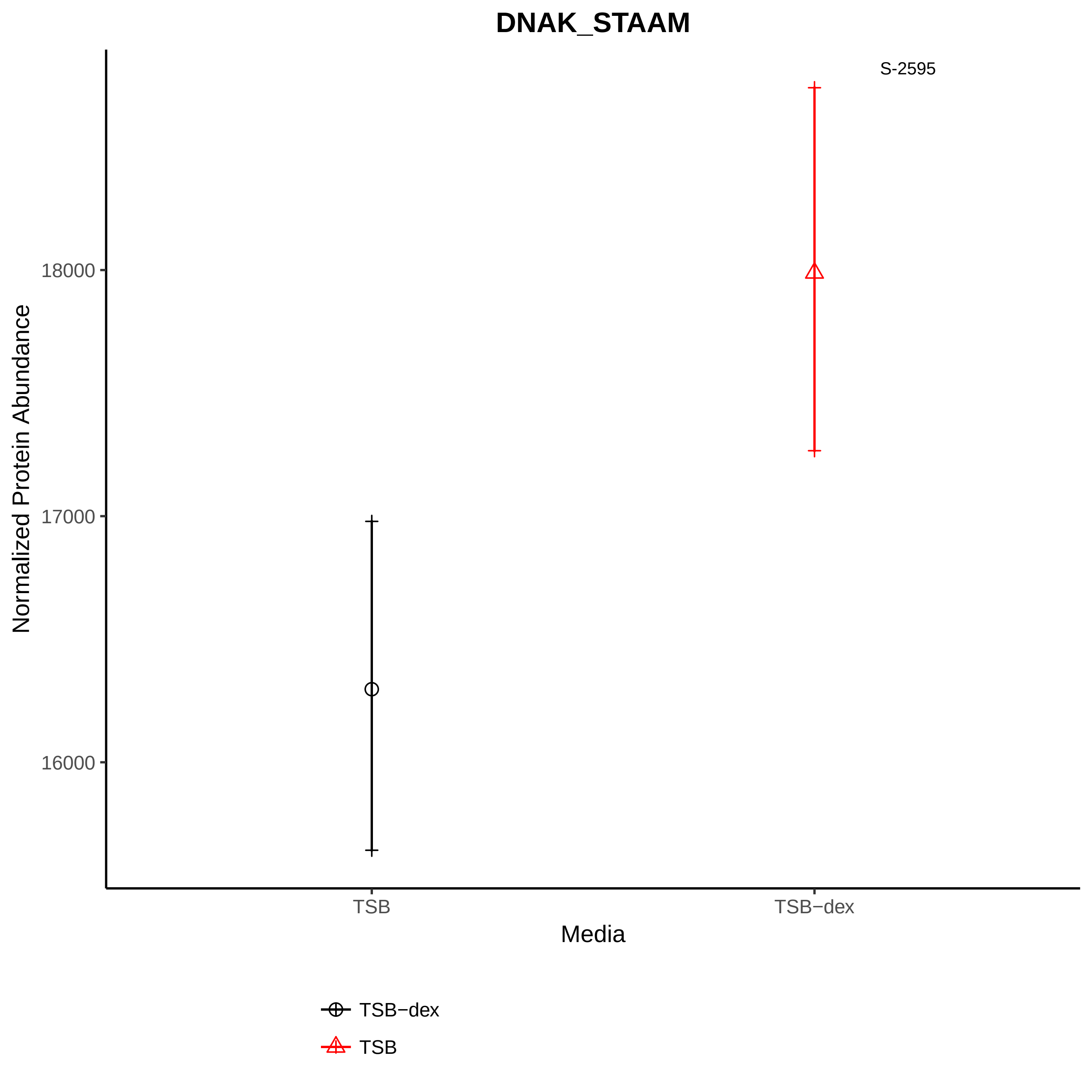
DNAK_STAAM
S-2595
Normalized Protein Abundance
18000
17000
16000
TSB
TSB−dex
Media
TSB−dex
TSB

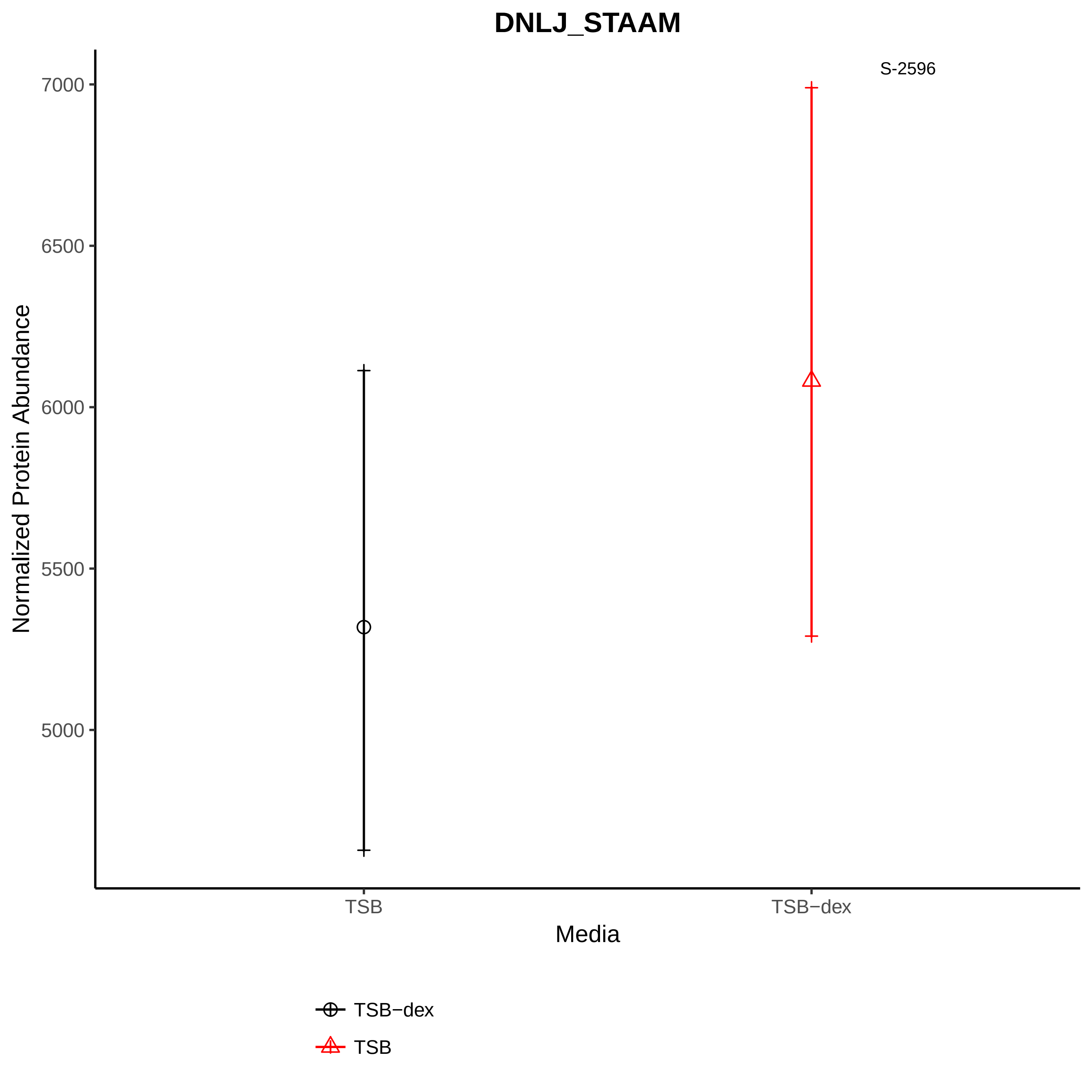
DNLJ_STAAM
S-2596
Normalized Protein Abundance
7000
6500
6000
5500
5000
TSB
TSB−dex
Media
TSB−dex
TSB

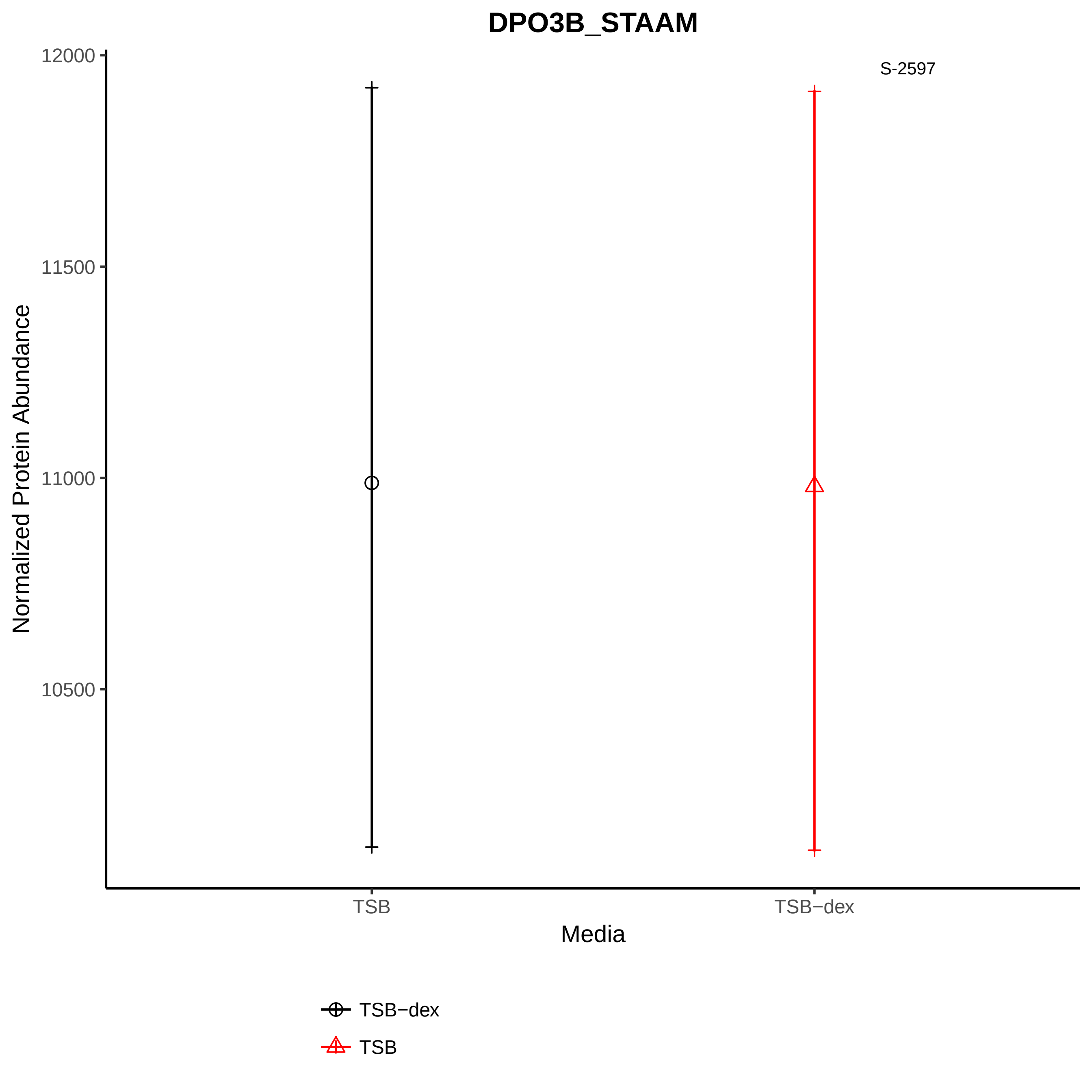

DPO3B_STAAM
S-2597
12000
11500
11000
10500
Normalized Protein Abundance
TSB
TSB−dex
Media
TSB−dex
TSB

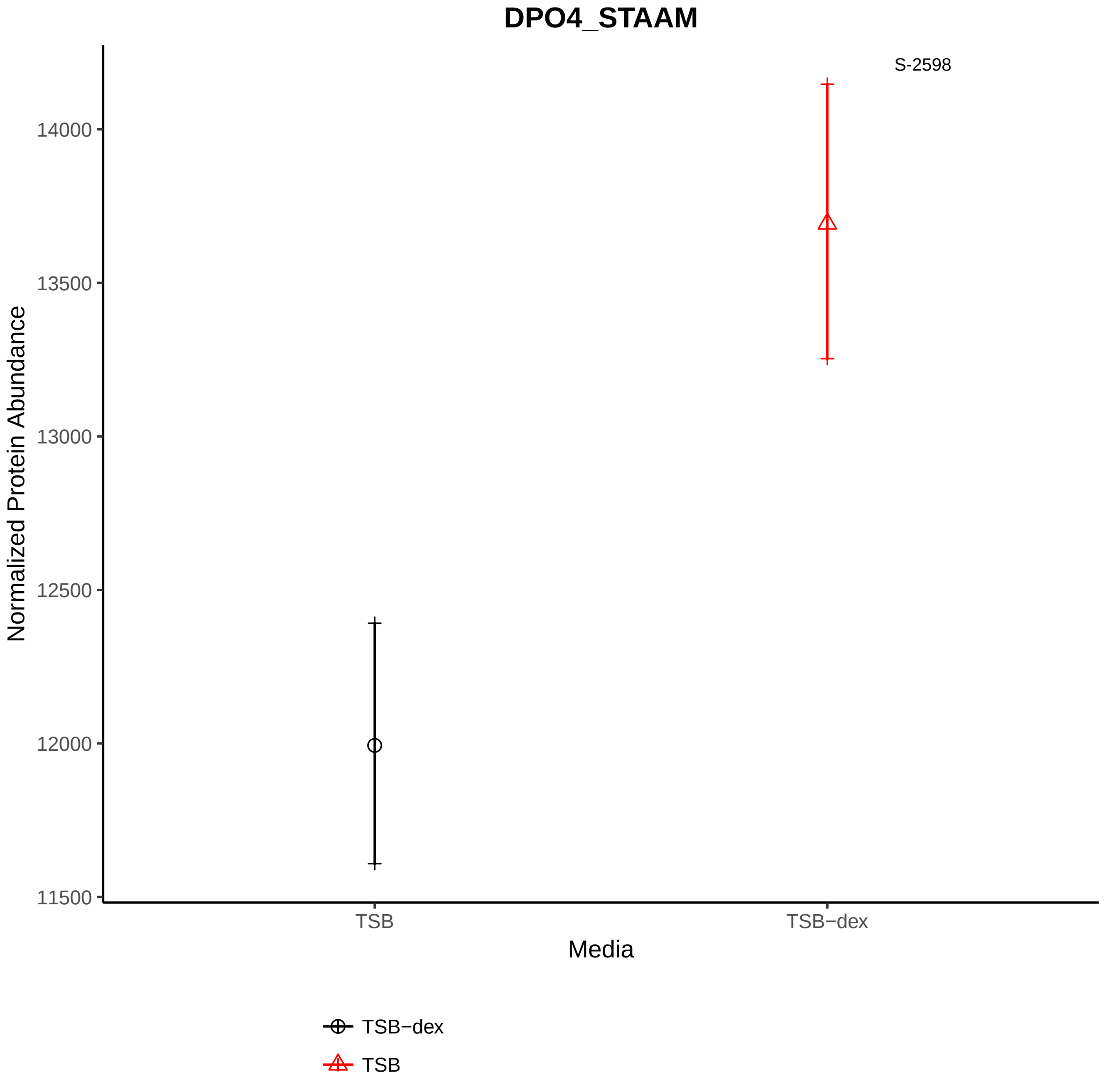

DPO4_STAAM
S-2598
14000
13500
13000
12500
12000
11500
Normalized Protein Abundance
TSB
TSB−dex
Media
TSB−dex
TSB

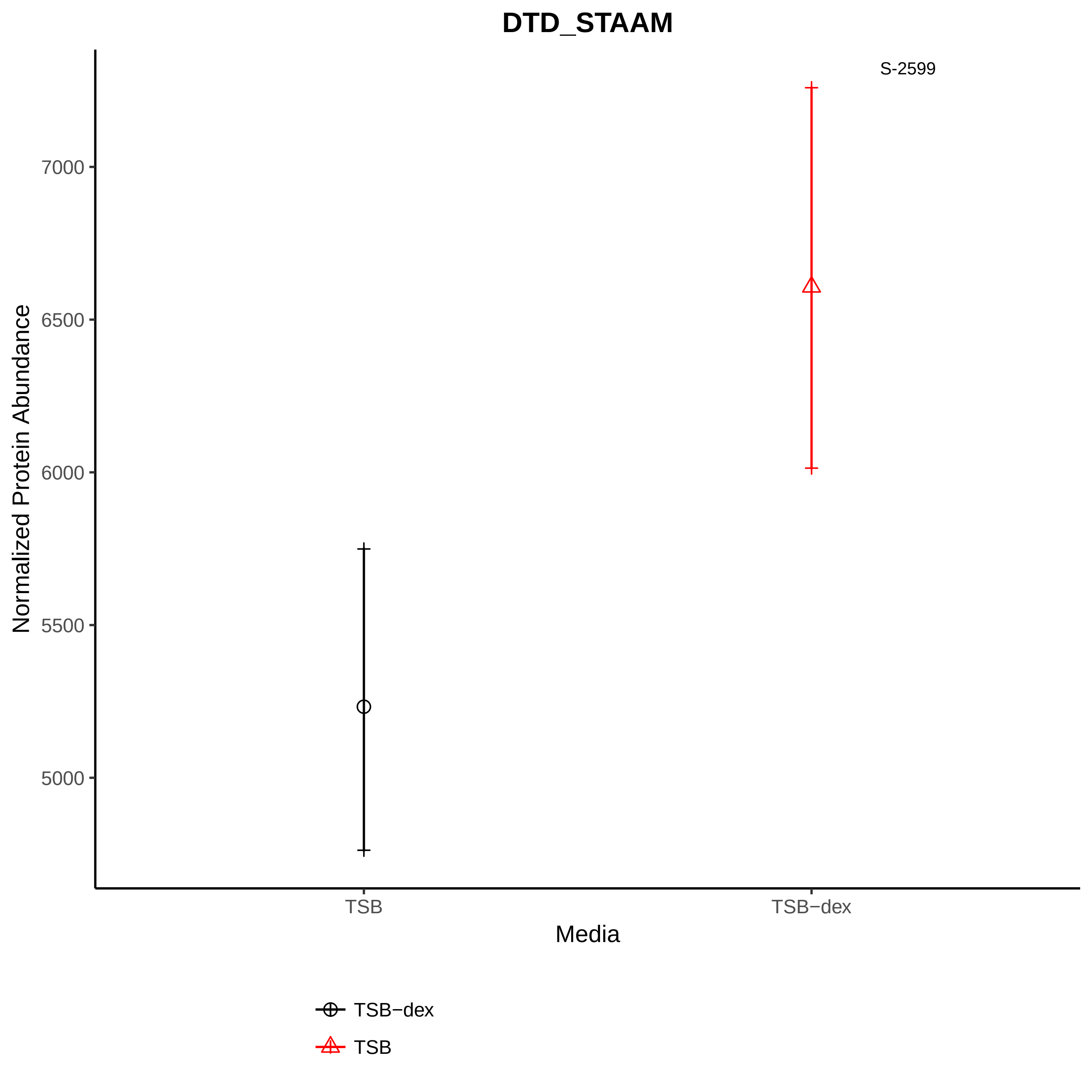
DTD_STAAM
S-2599
Normalized Protein Abundance
7000
6500
6000
5500
5000
TSB
TSB−dex
Media
TSB−dex
TSB

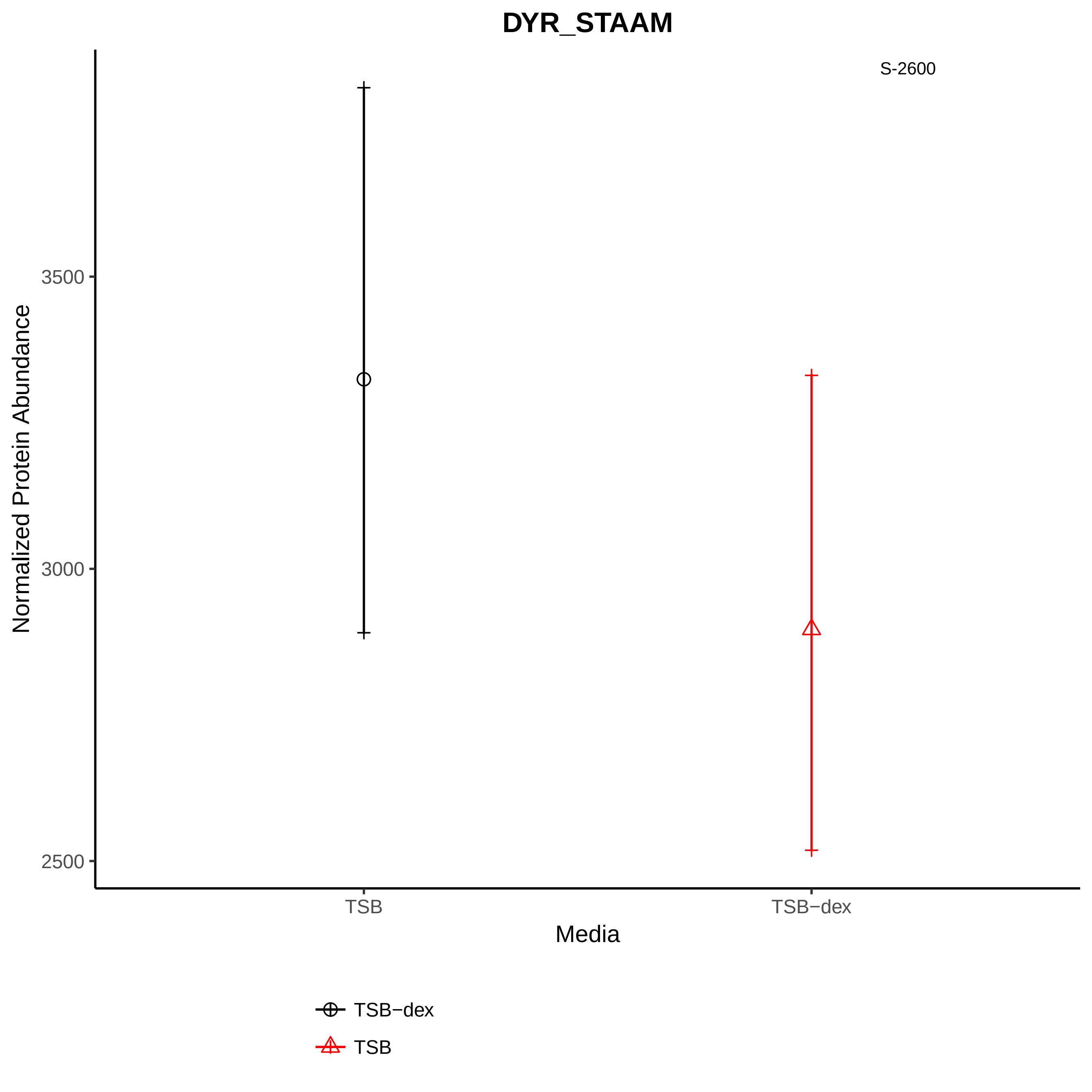
DYR_STAAM
S-2600
Normalized Protein Abundance
3500
3000
2500
TSB
TSB−dex
Media
TSB−dex
TSB

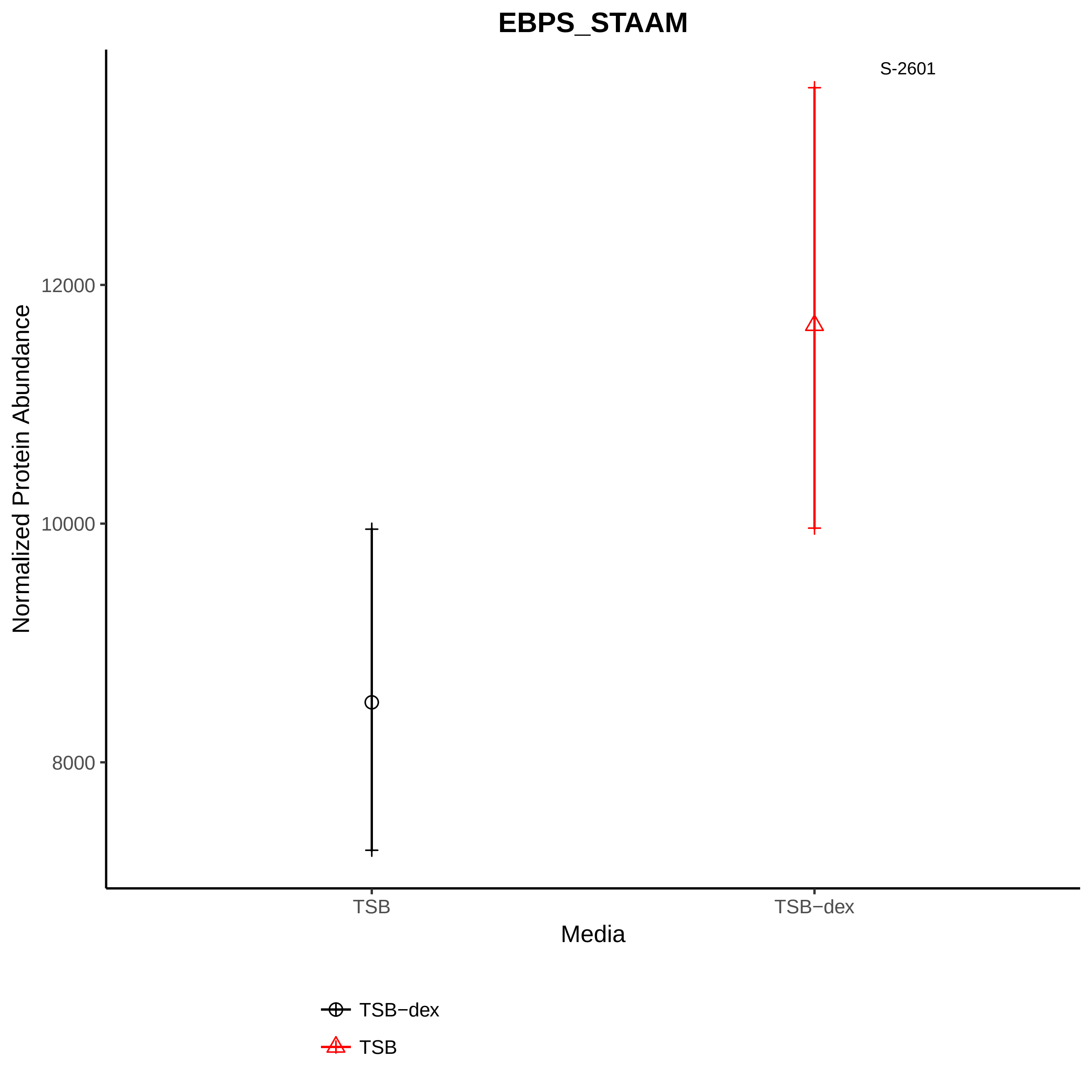

EBPS_STAAM
S-2601
Normalized Protein Abundance
12000
10000
8000
TSB
TSB−dex
Media
TSB−dex
TSB

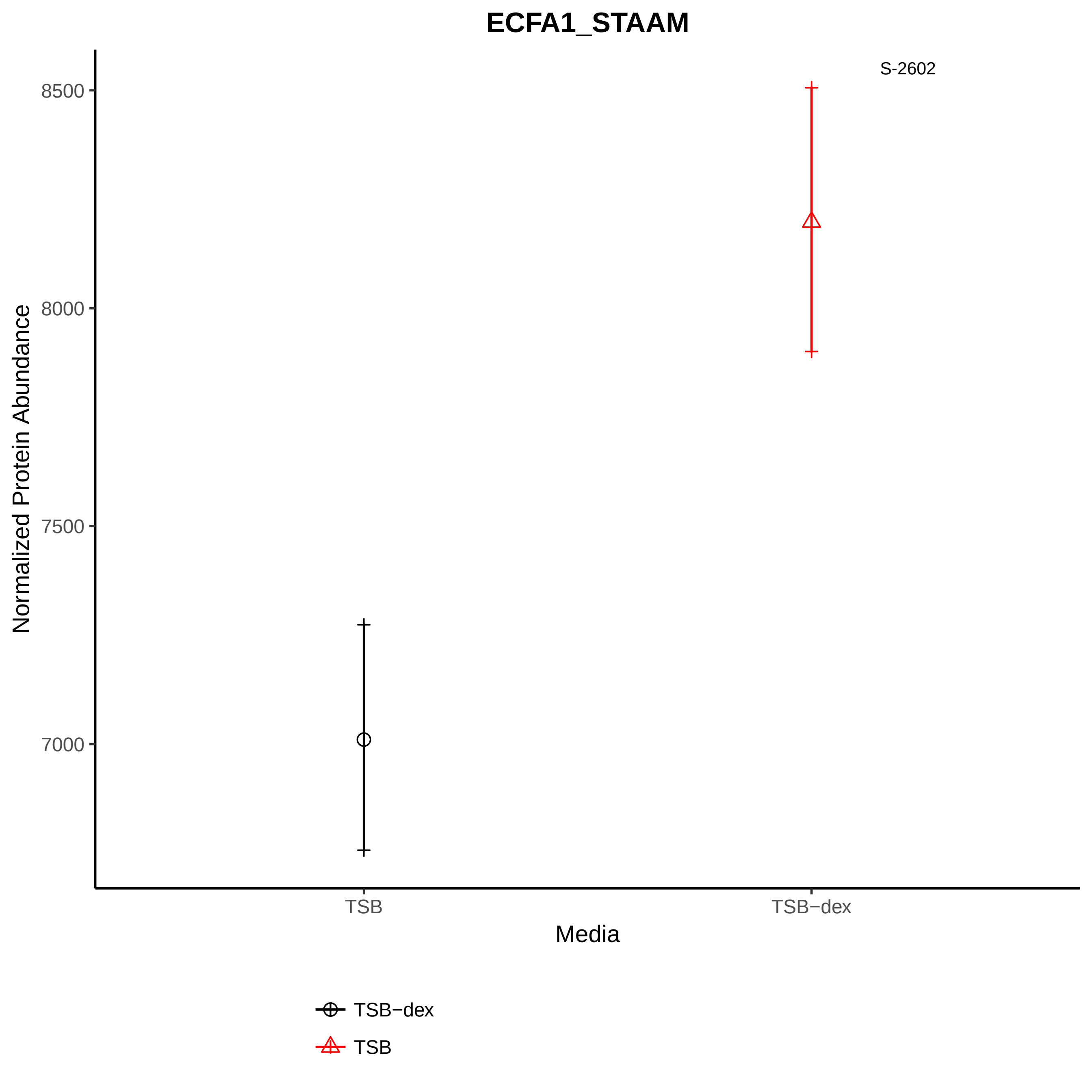
ECFA1_STAAM
S-2602
8500
8000
7500
7000
Normalized Protein Abundance
TSB
TSB−dex
Media
TSB−dex
TSB

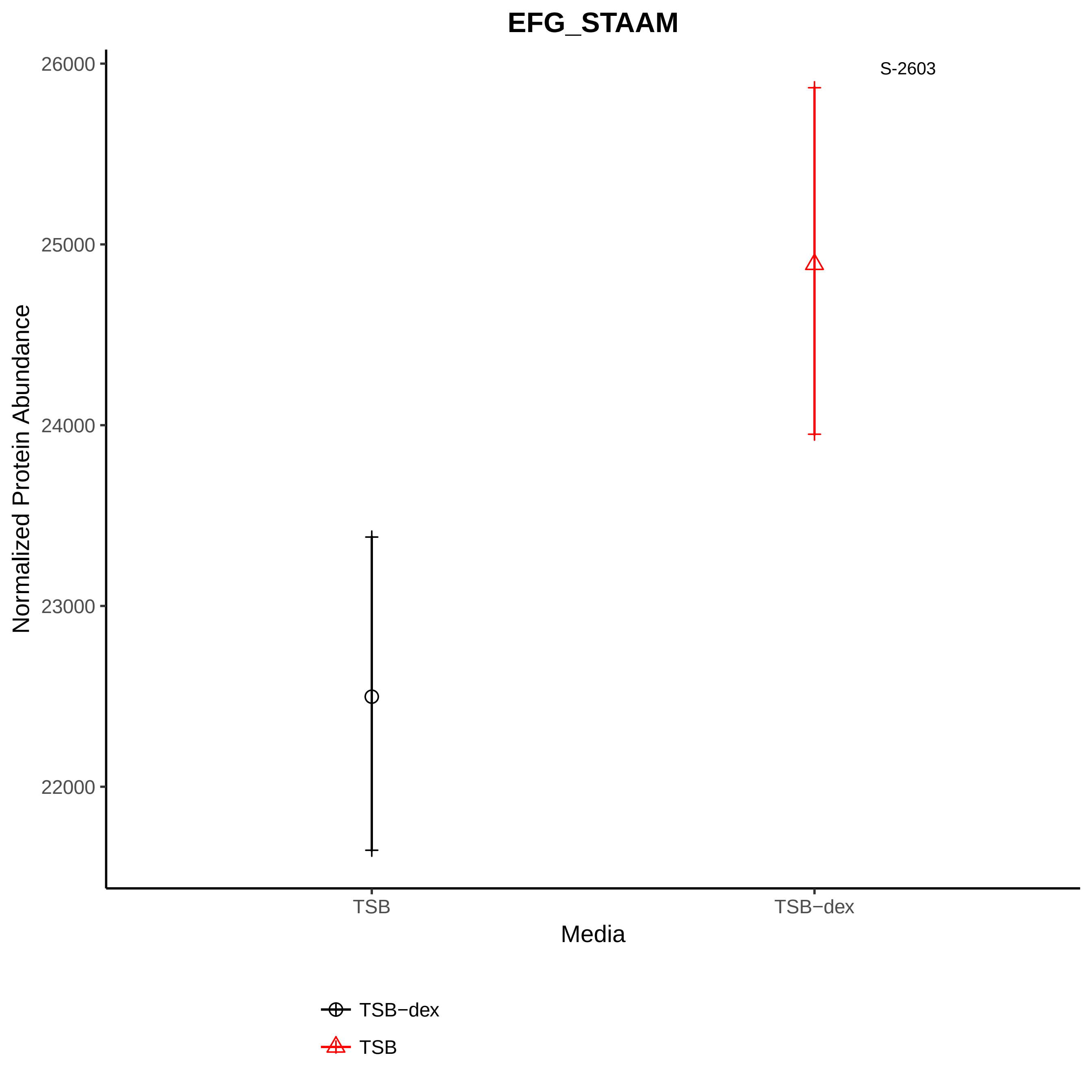
EFG_STAAM
S-2603
26000
25000
24000
23000
22000
Normalized Protein Abundance
TSB
TSB−dex
Media
TSB−dex
TSB

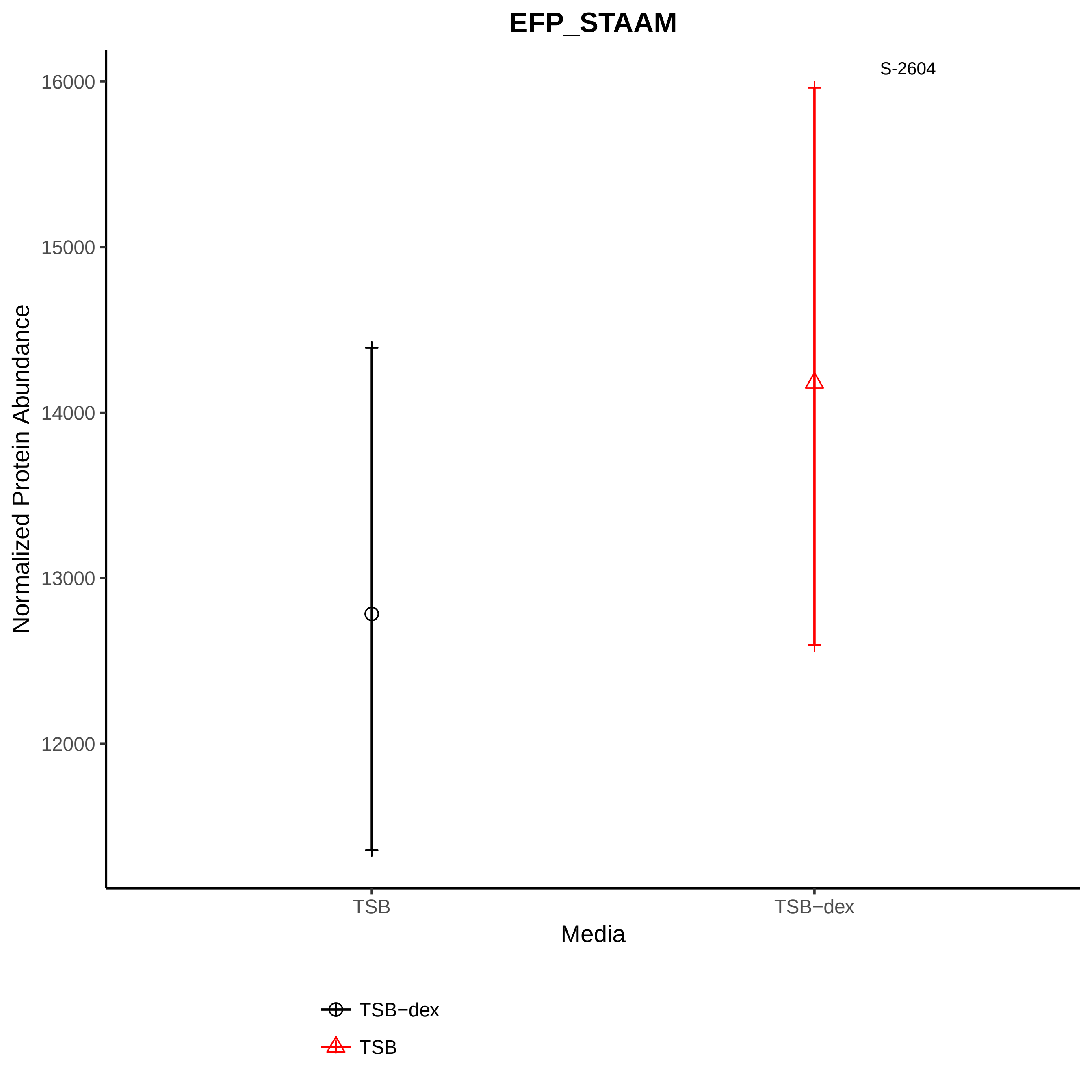
EFP_STAAM
S-2604
16000
15000
14000
13000
12000
Normalized Protein Abundance
TSB
TSB−dex
Media
TSB−dex
TSB

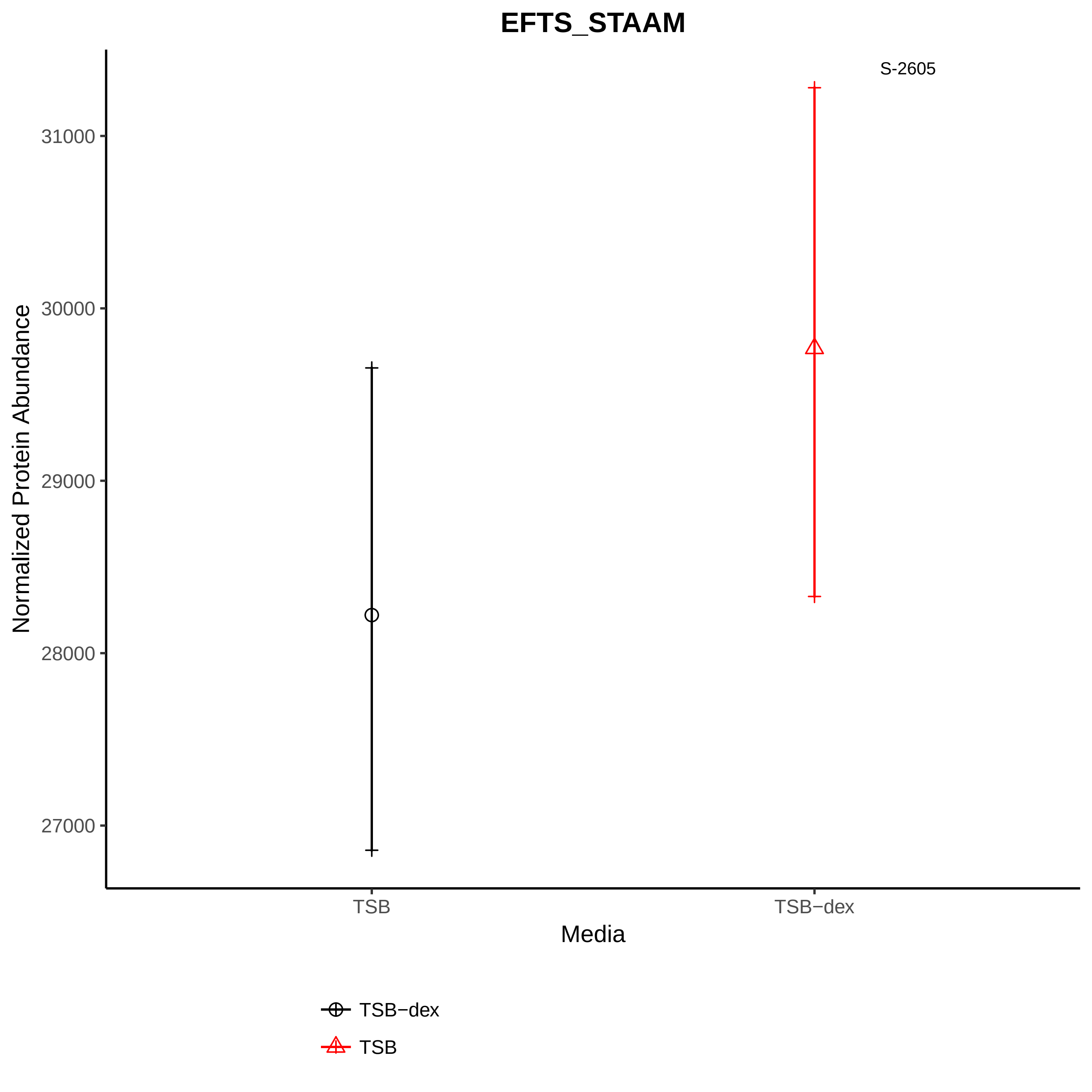
EFTS_STAAM
S-2605
Normalized Protein Abundance
31000
30000
29000
28000
27000
TSB
TSB−dex
Media
TSB−dex
TSB

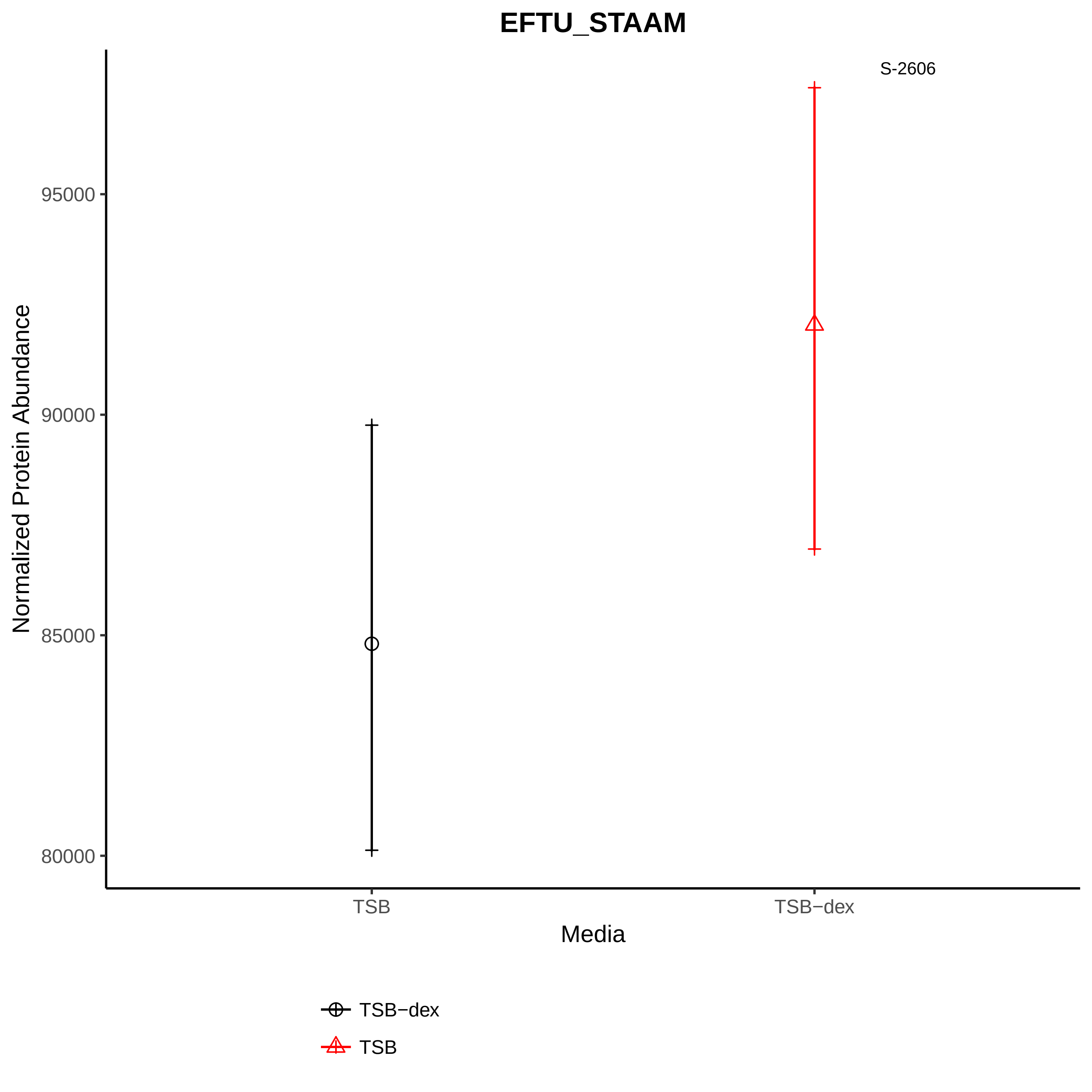

EFTU_STAAM
S-2606
95000
90000
85000
80000
Normalized Protein Abundance
TSB
TSB−dex
Media
TSB−dex
TSB

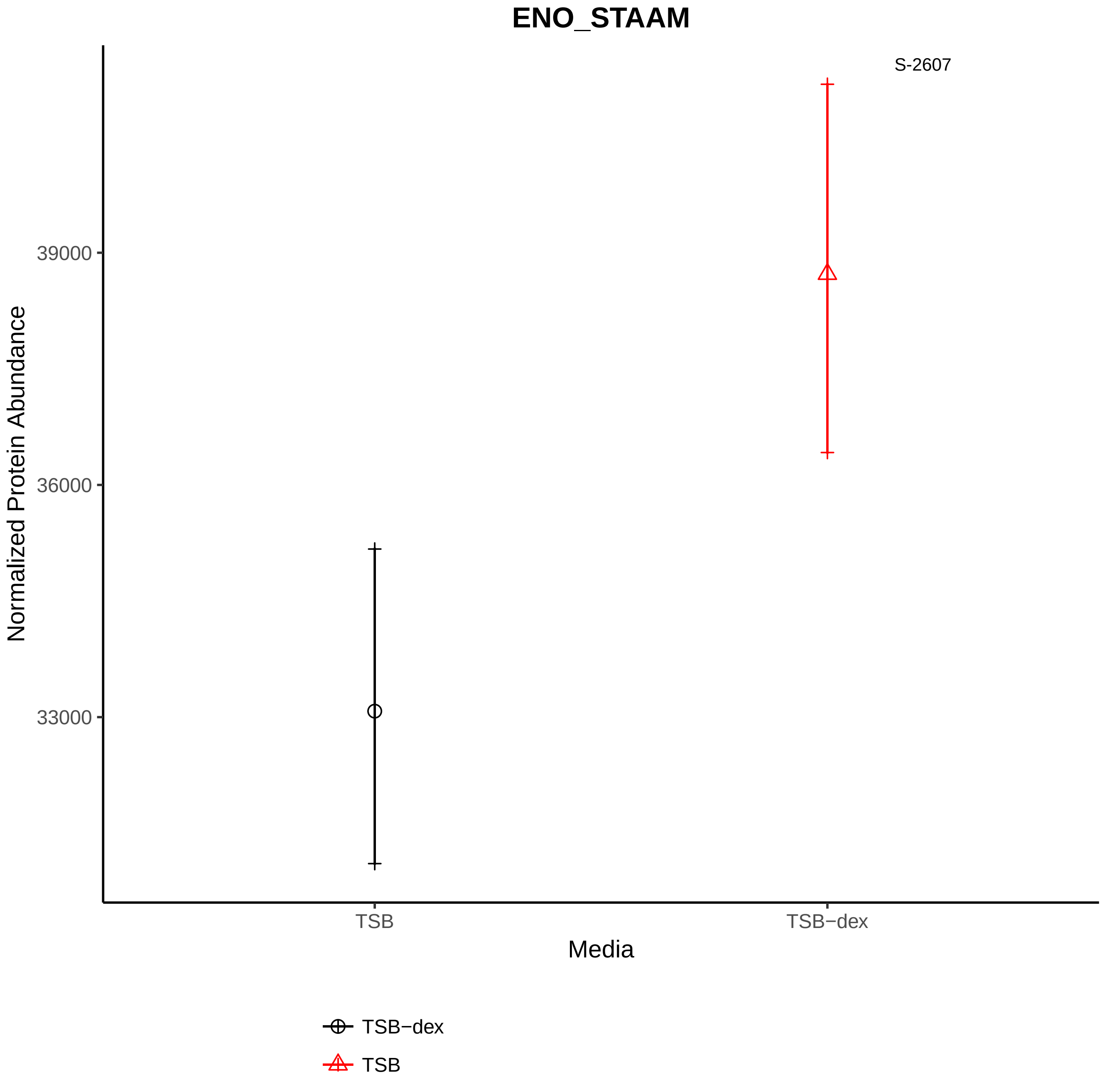
ENO_STAAM
S-2607
39000
36000
33000
Normalized Protein Abundance
TSB
TSB−dex
Media
TSB−dex
TSB

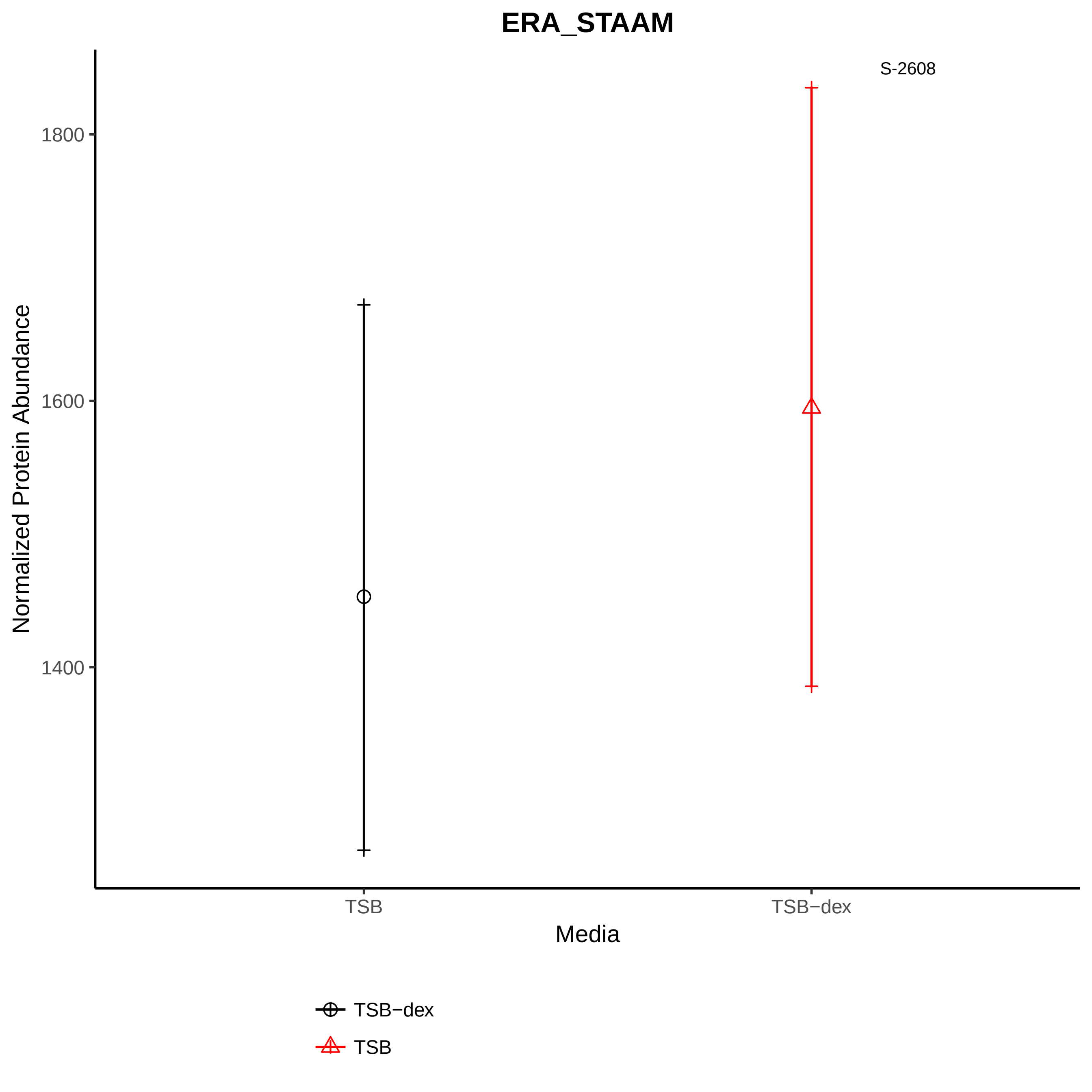
ERA_STAAM
S-2608
Normalized Protein Abundance
1800
1600
1400
TSB
TSB−dex
Media
TSB−dex
TSB

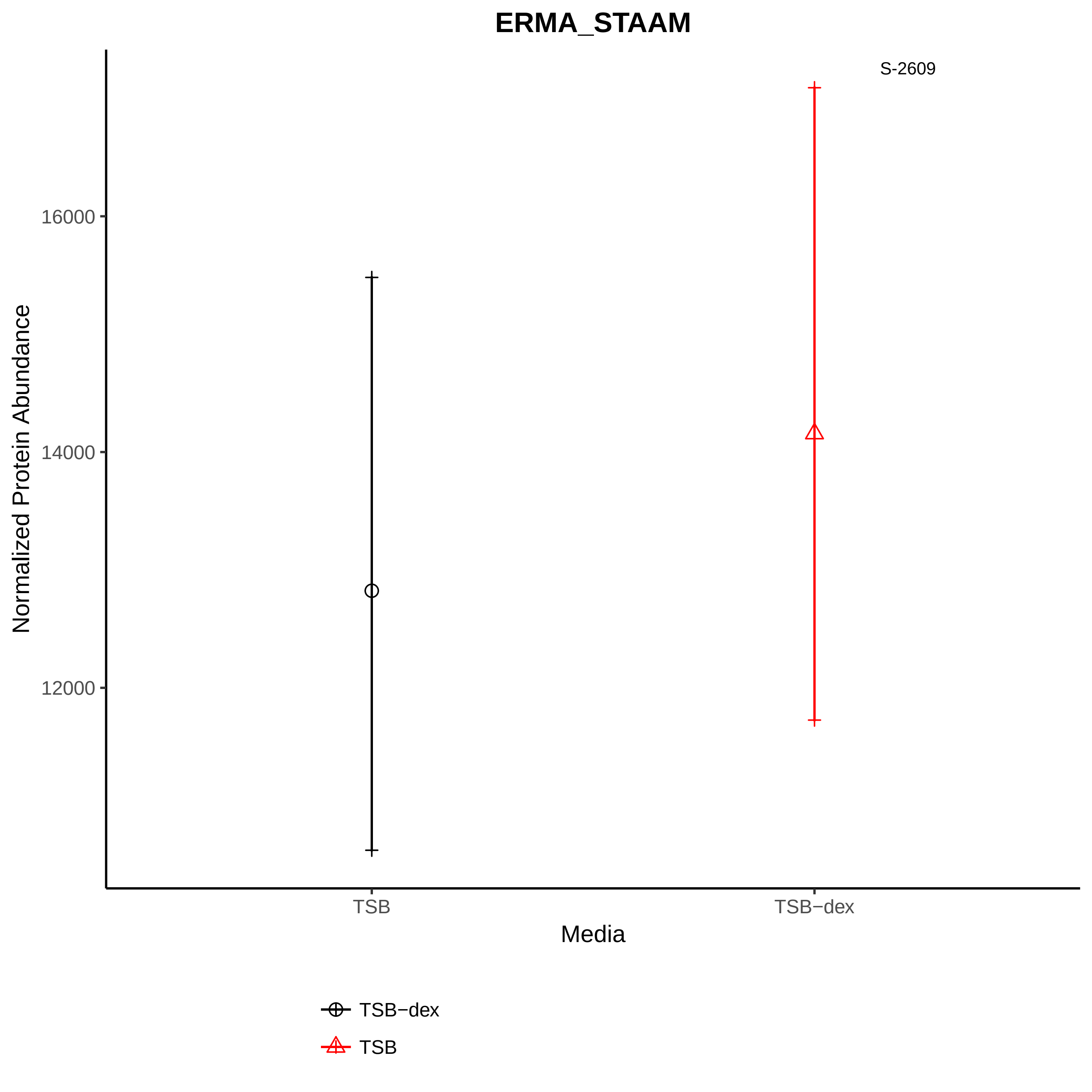
ERMA_STAAM
S-2609
16000
14000
12000
Normalized Protein Abundance
TSB
TSB−dex
Media
TSB−dex
TSB

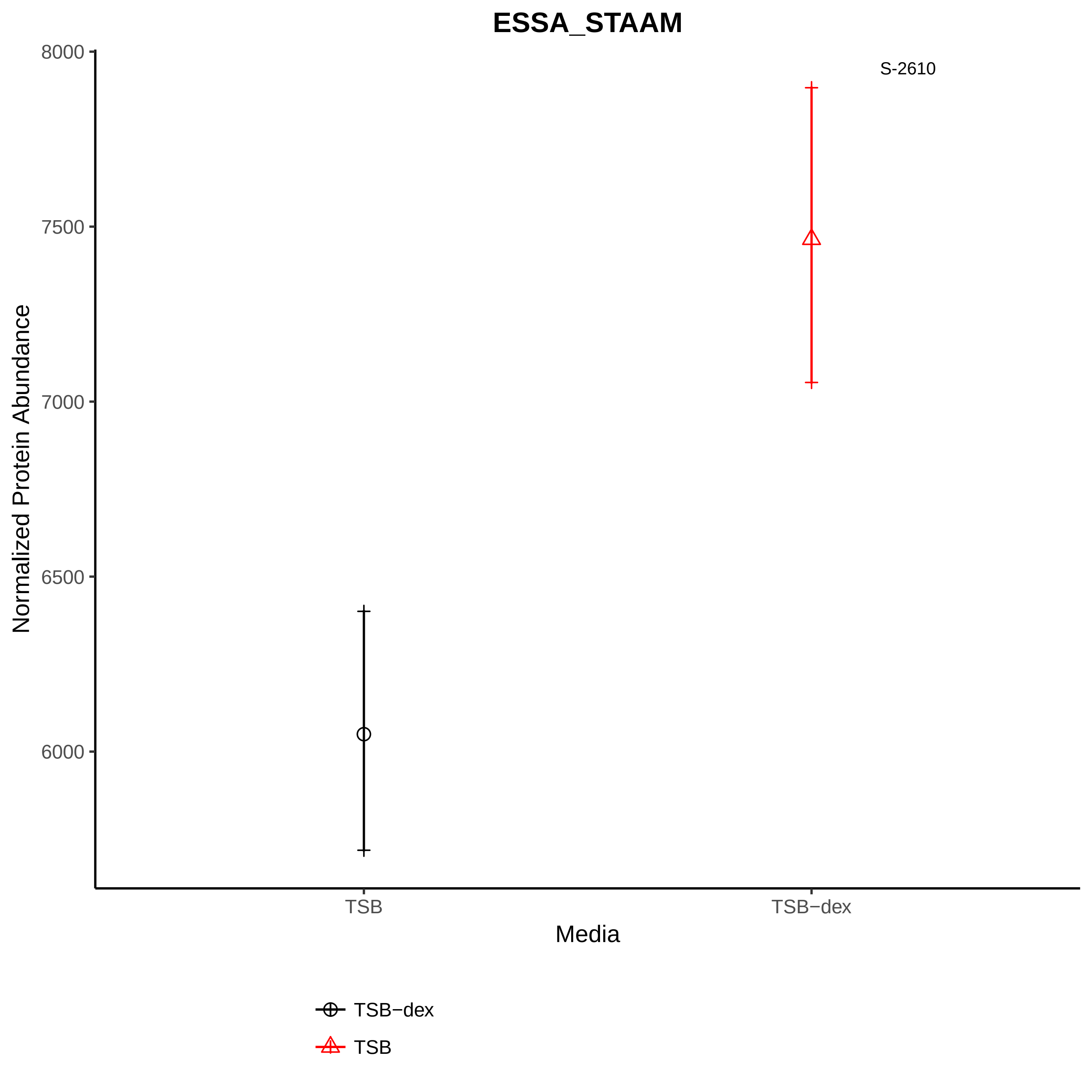

ESSA_STAAM
S-2610
8000
7500
7000
6500
6000
Normalized Protein Abundance
TSB
TSB−dex
Media
TSB−dex
TSB

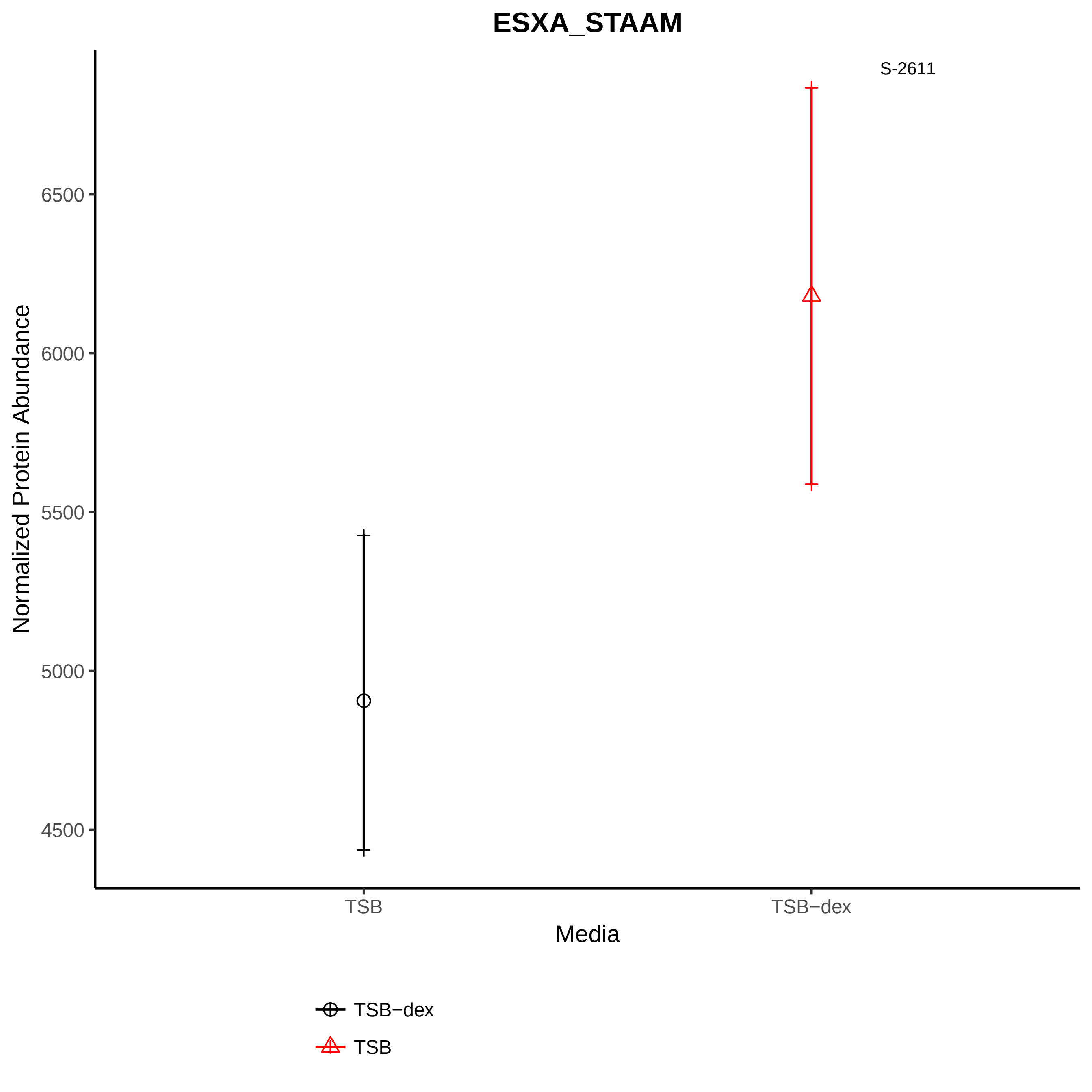
ESXA_STAAM
S-2611
Normalized Protein Abundance
6500
6000
5500
5000
4500
TSB
TSB−dex
Media
TSB−dex
TSB

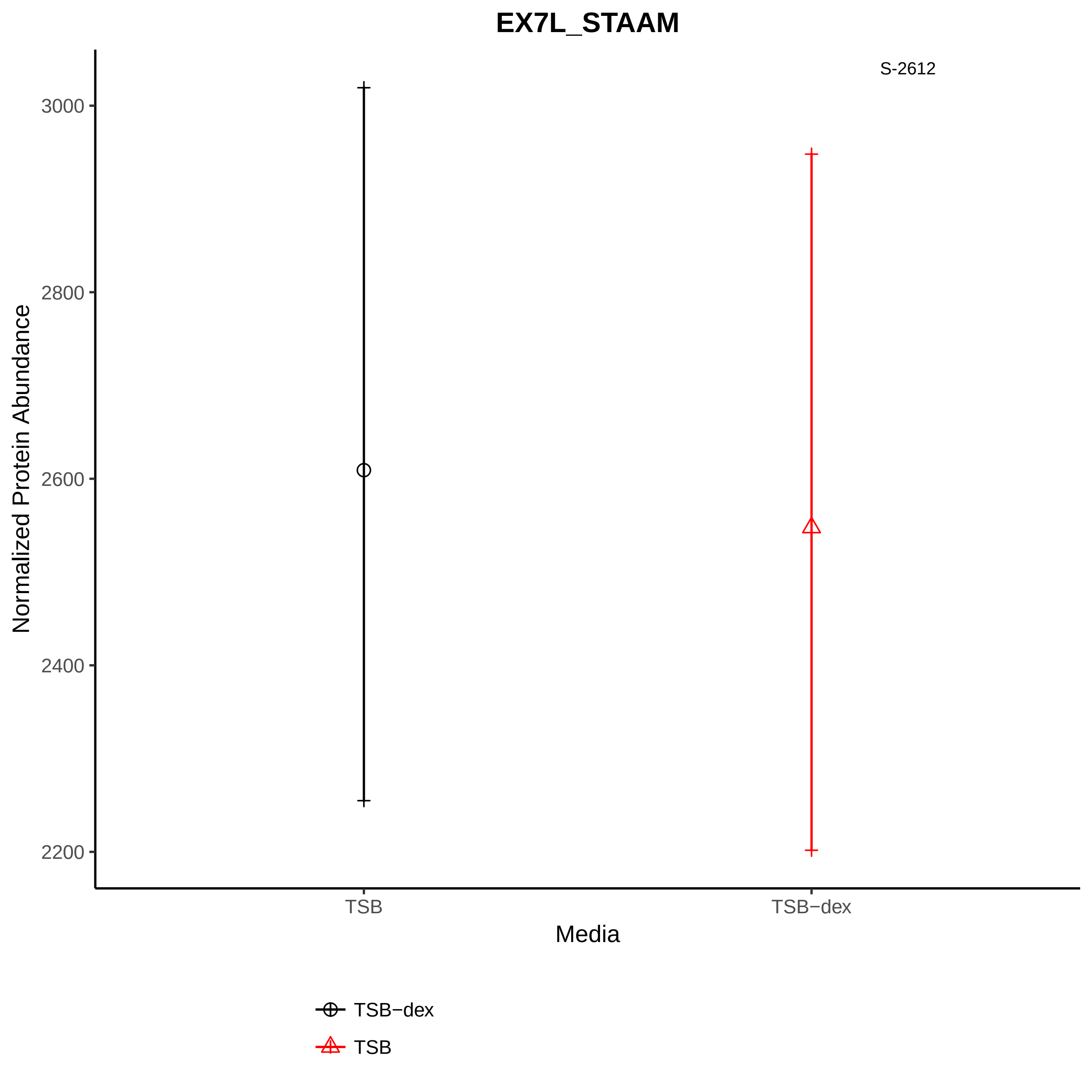

EX7L_STAAM
S-2612
Normalized Protein Abundance
3000
2800
2600
2400
2200
TSB
TSB−dex
Media
TSB−dex
TSB

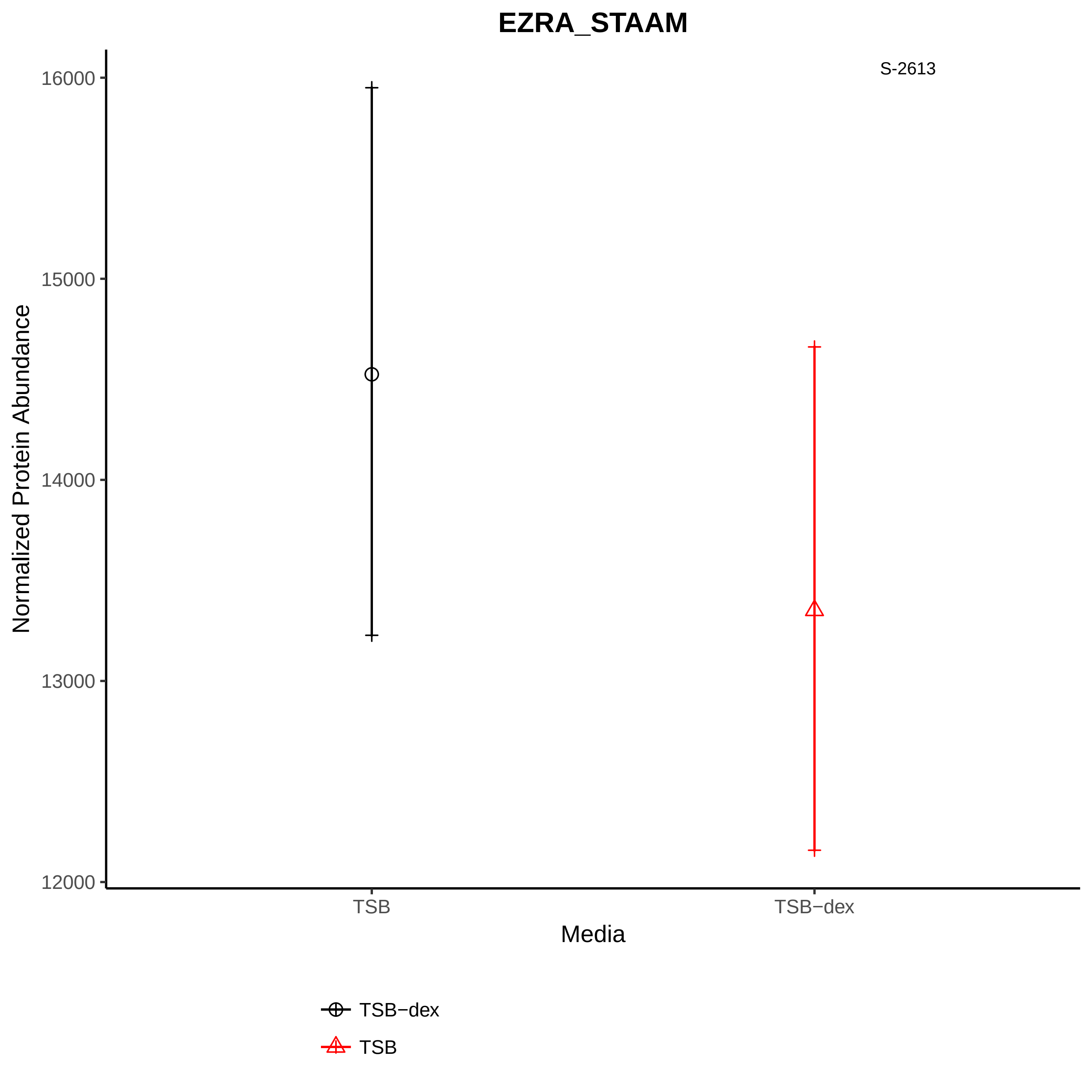

EZRA_STAAM
S-2613
16000
15000
14000
13000
12000
Normalized Protein Abundance
TSB
TSB−dex
Media
TSB−dex
TSB

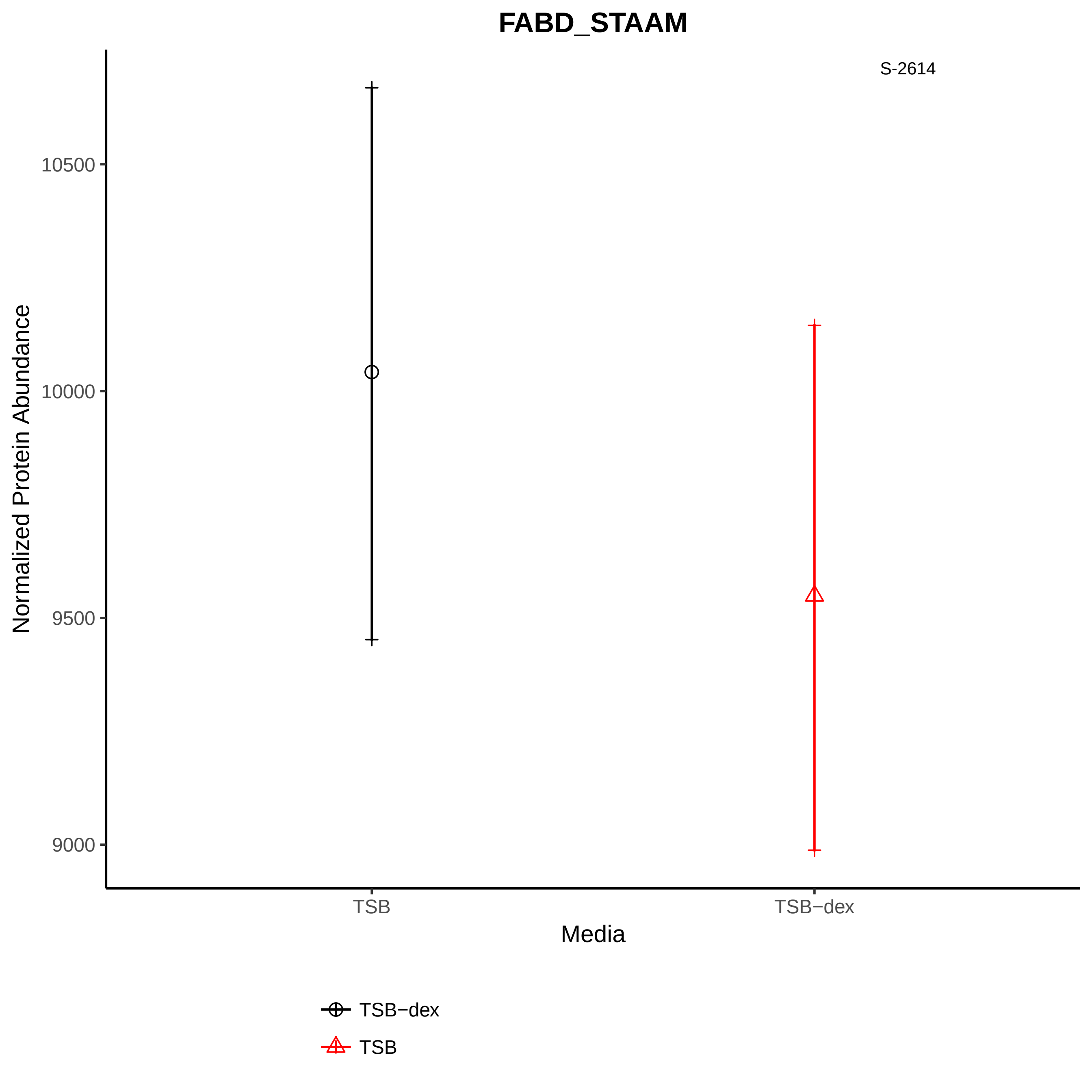

FABD_STAAM
S-2614
Normalized Protein Abundance
10500
10000
9500
9000
TSB
TSB−dex
Media
TSB−dex
TSB

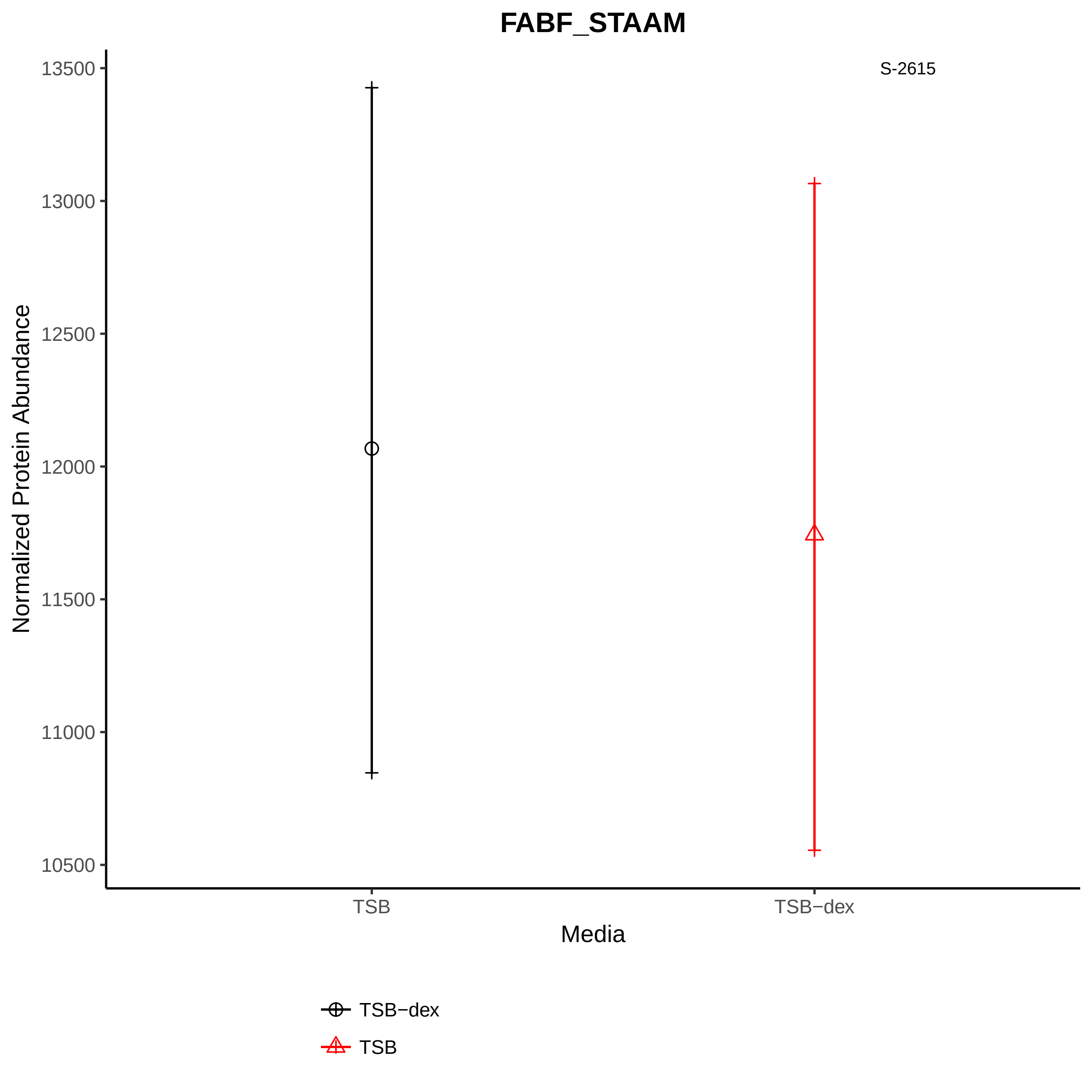
FABF_STAAM
S-2615
Normalized Protein Abundance
13500
13000
12500
12000
11500
11000
10500
TSB
TSB−dex
Media
TSB−dex
TSB

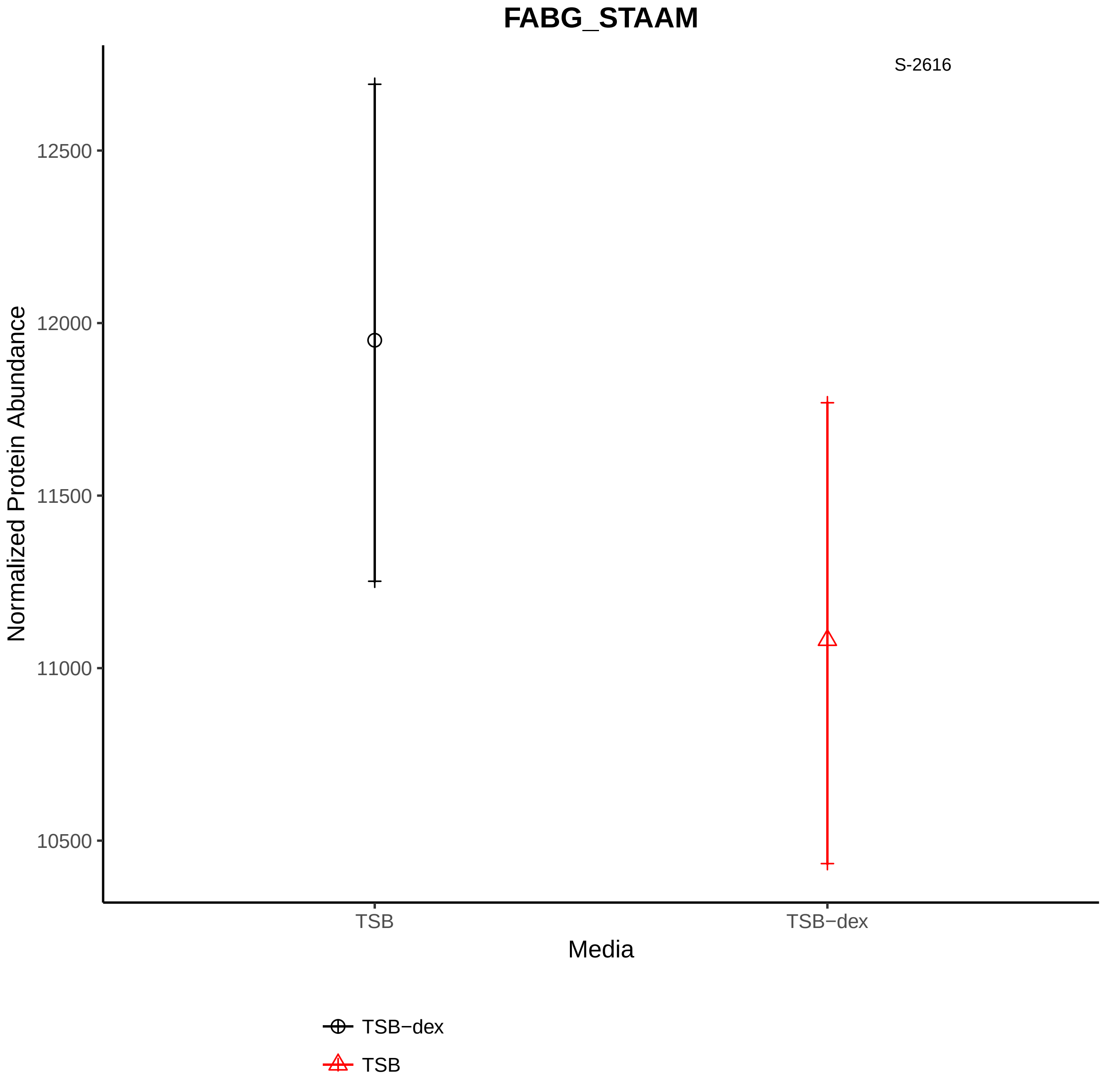

FABG_STAAM
S-2616
Normalized Protein Abundance
12500
12000
11500
11000
10500
TSB
TSB−dex
Media
TSB−dex
TSB

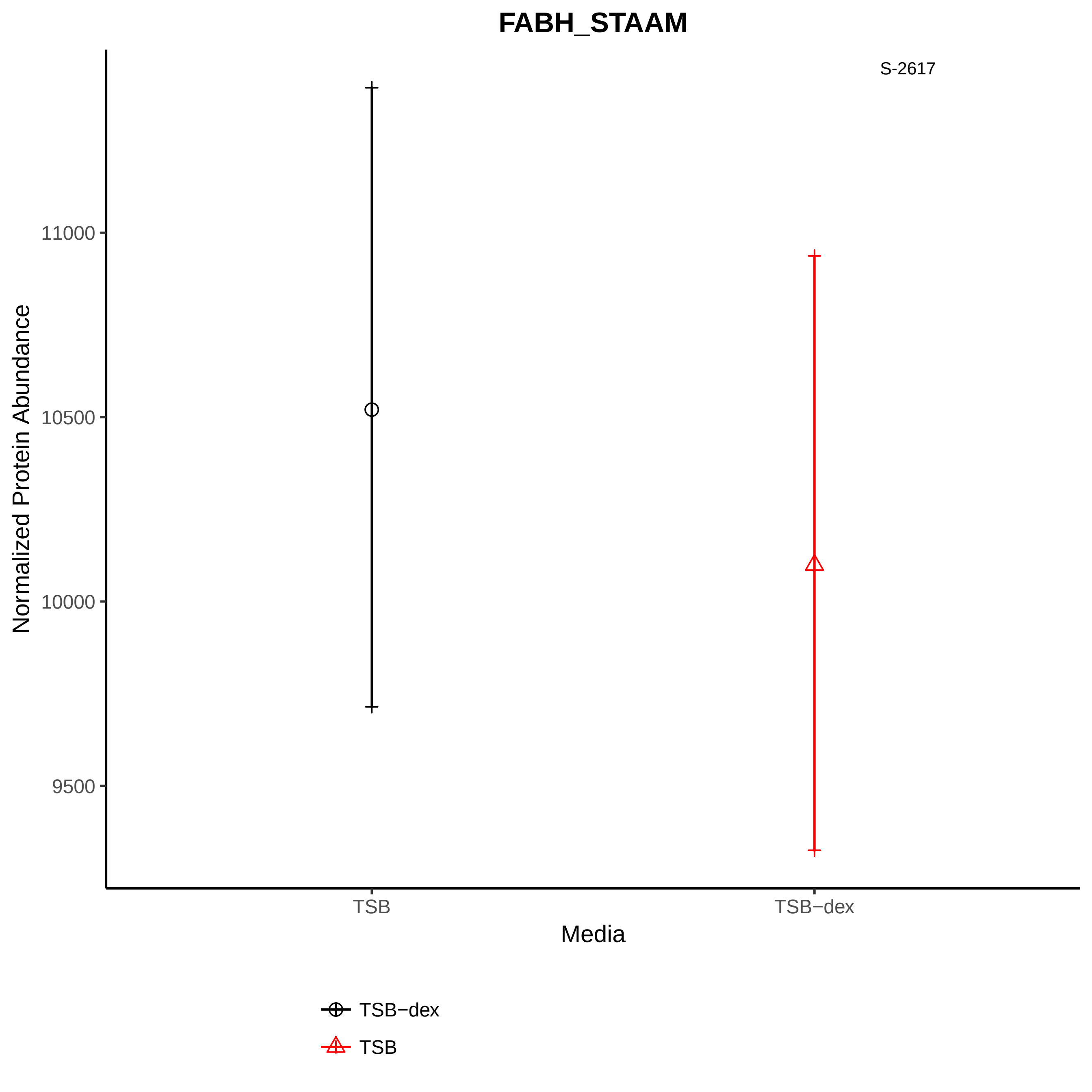

FABH_STAAM
S-2617
Normalized Protein Abundance
11000
10500
10000
9500
TSB
TSB−dex
Media
TSB−dex
TSB

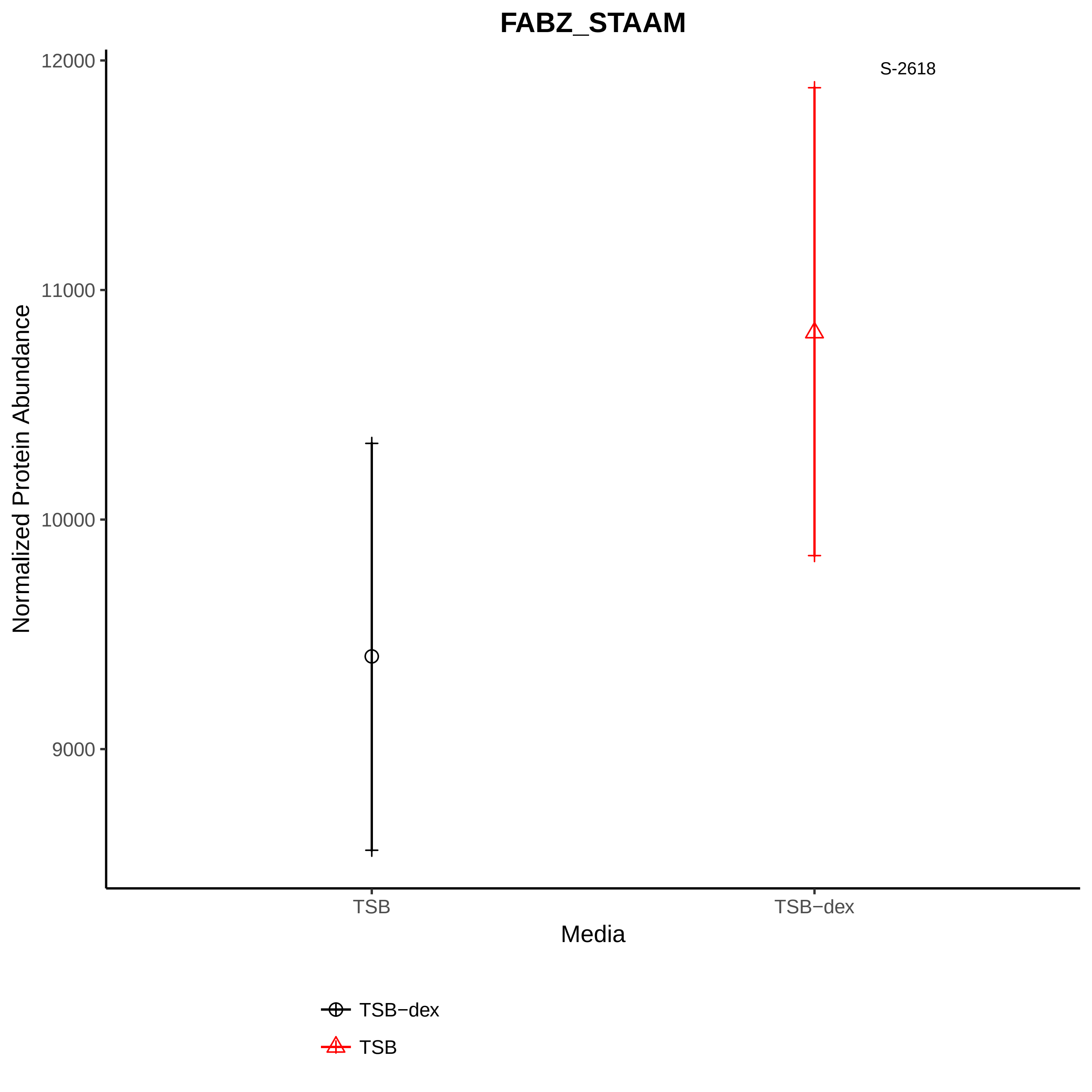

FABZ_STAAM
S-2618
12000
11000
10000
9000
Normalized Protein Abundance
TSB
TSB−dex
Media
TSB−dex
TSB

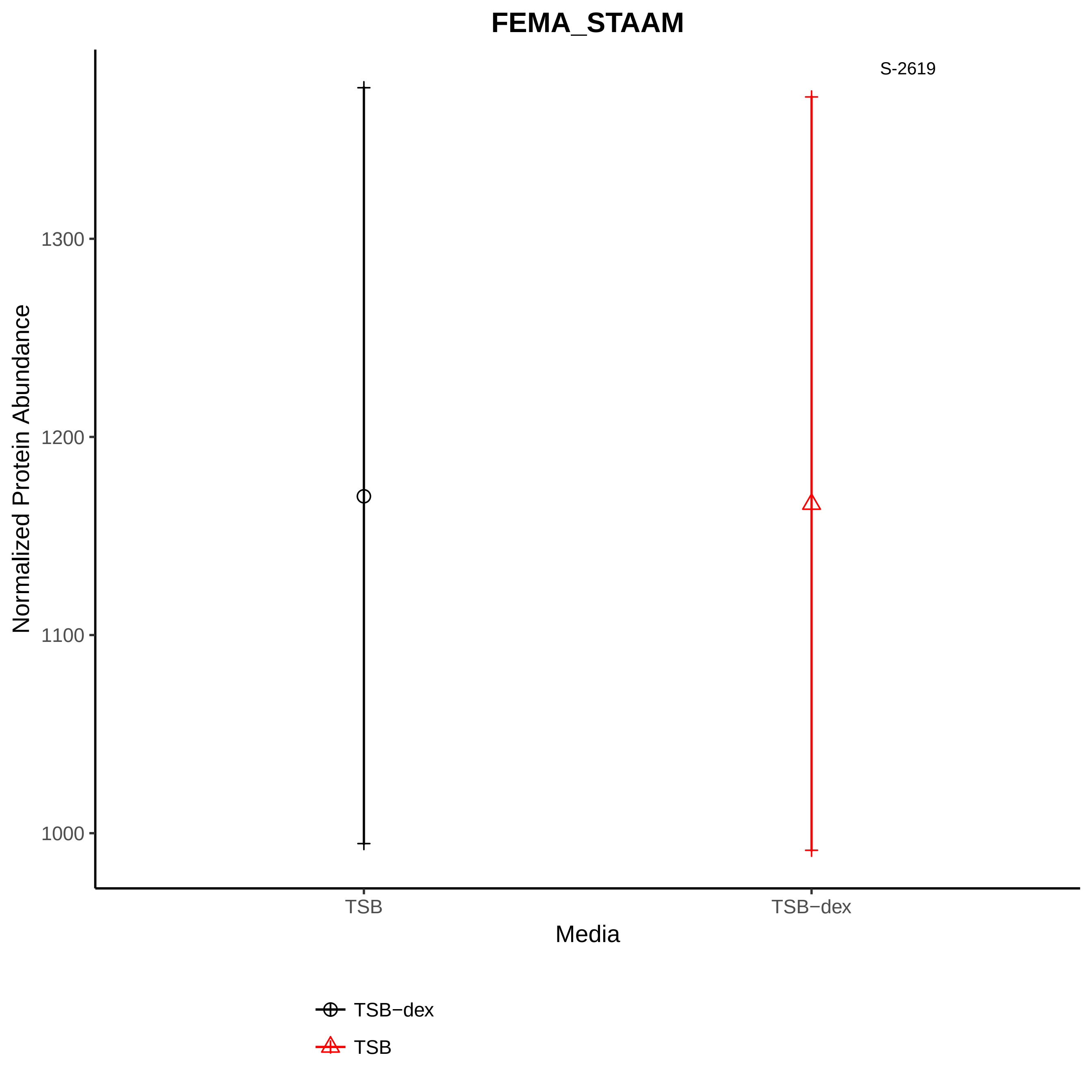
FEMA_STAAM
S-2619
Normalized Protein Abundance
1300
1200
1100
1000
TSB
TSB−dex
Media
TSB−dex
TSB

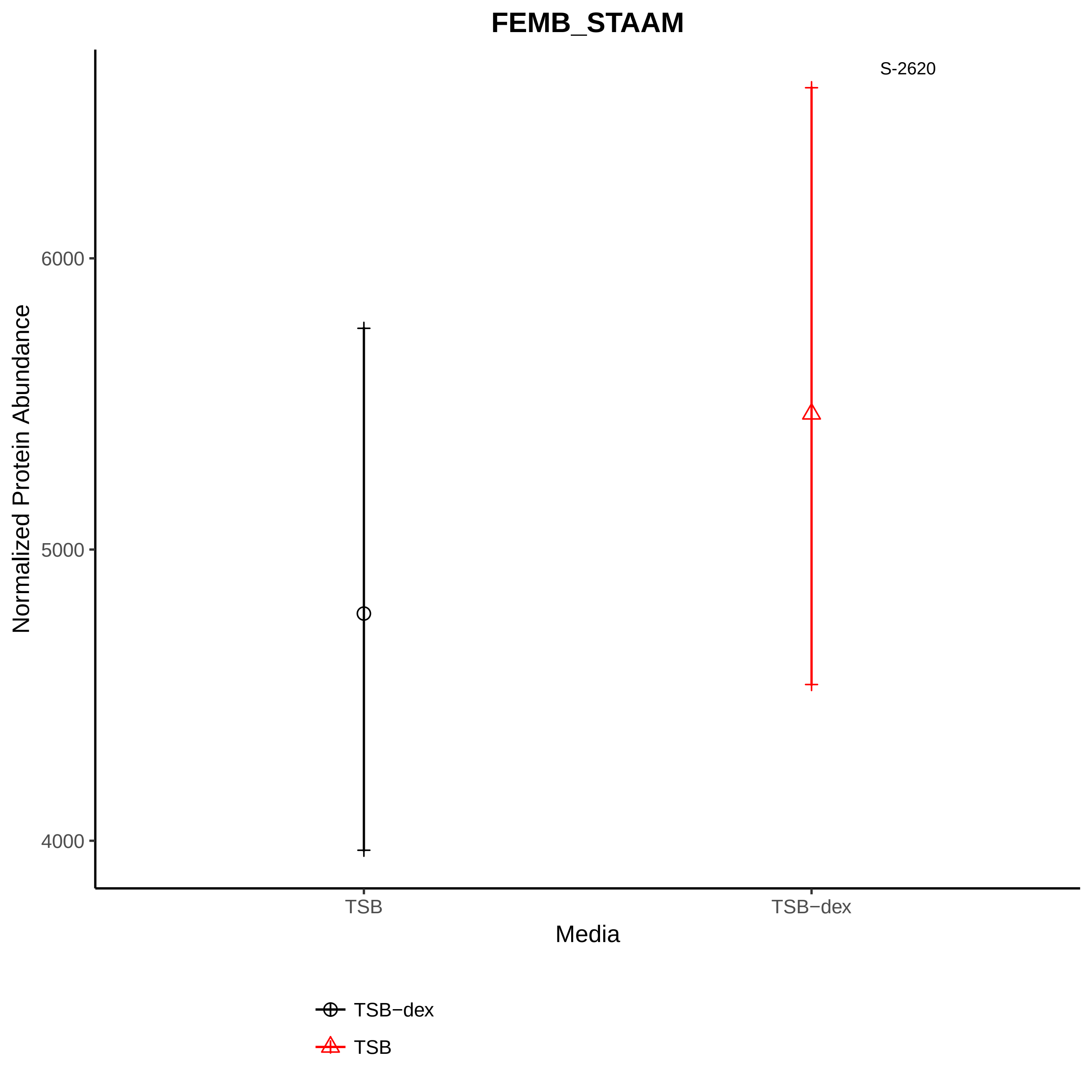

FEMB_STAAM
S-2620
Normalized Protein Abundance
6000
5000
4000
TSB
TSB−dex
Media
TSB−dex
TSB

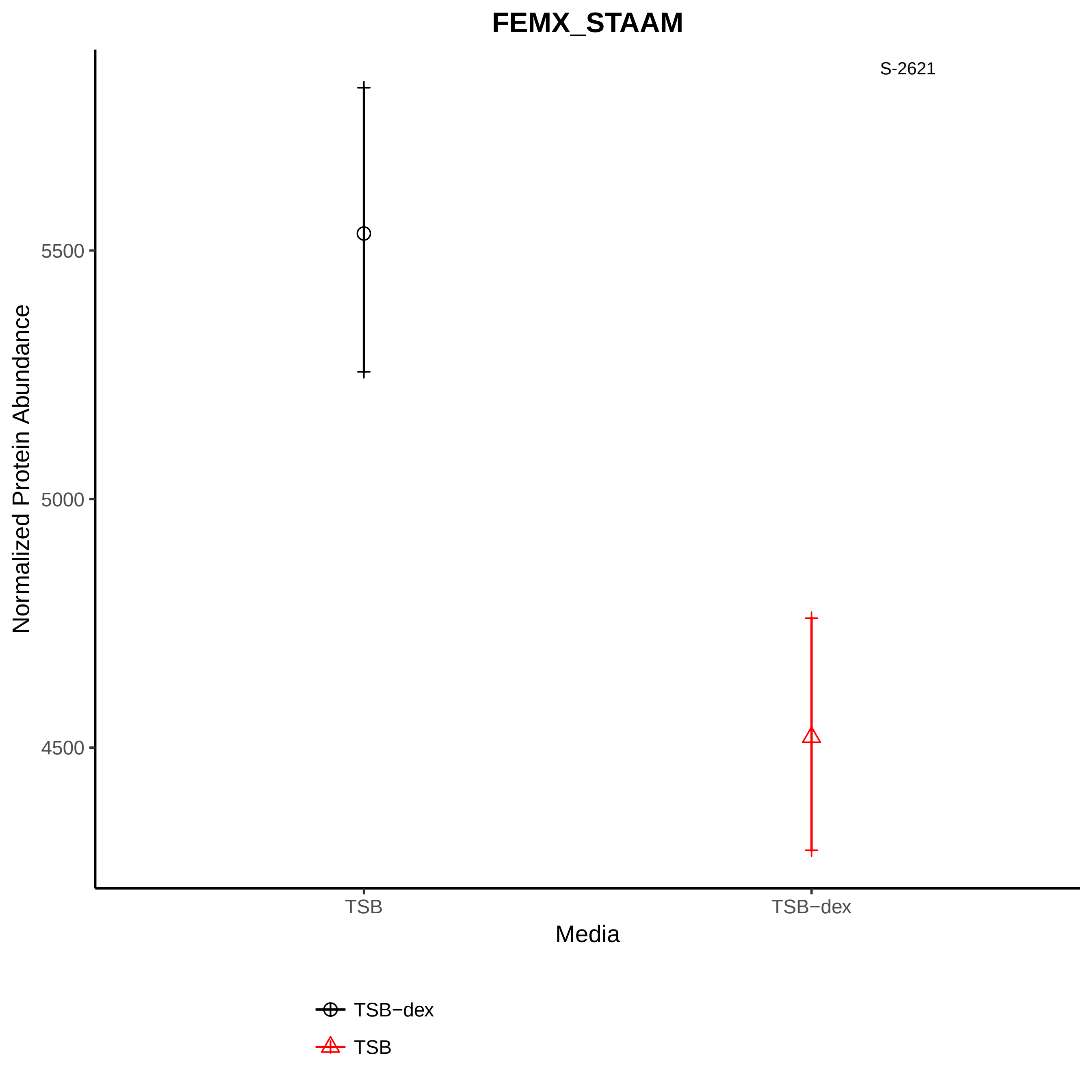
FEMX_STAAM
S-2621
5500
5000
4500
Normalized Protein Abundance
TSB
TSB−dex
Media
TSB−dex
TSB

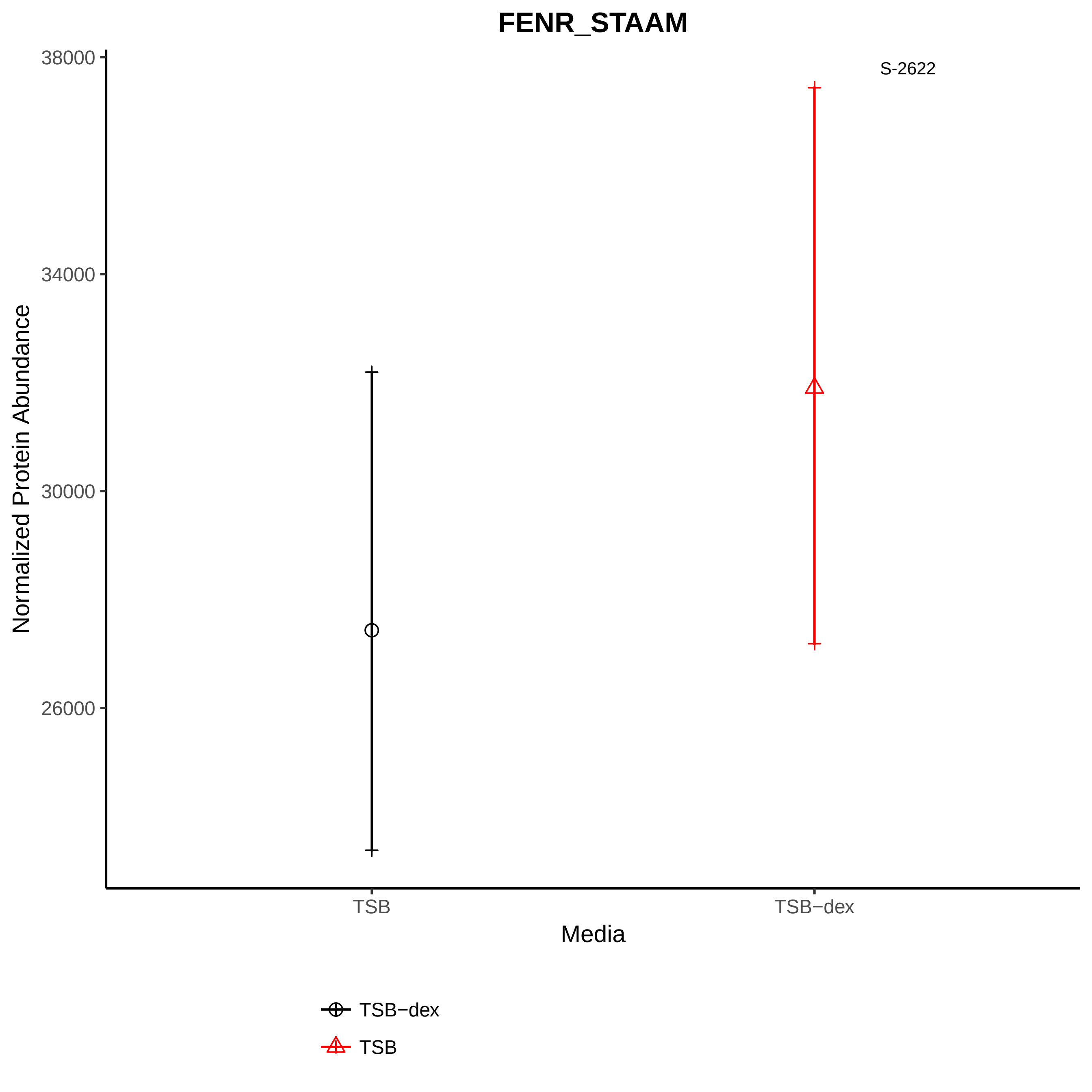

FENR_STAAM
S-2622
38000
34000
30000
26000
Normalized Protein Abundance
TSB
TSB−dex
Media
TSB−dex
TSB

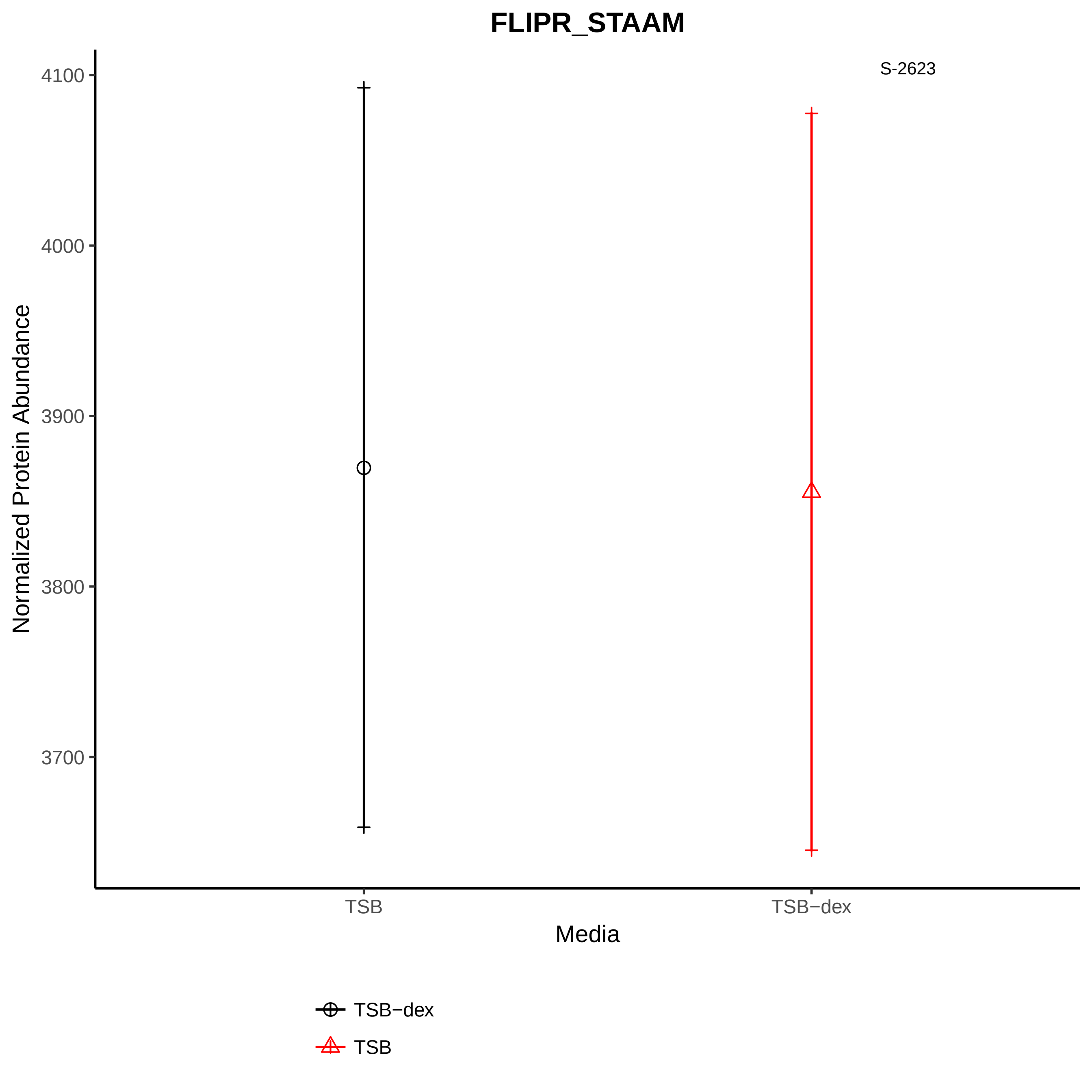
FLIPR_STAAM
S-2623
Normalized Protein Abundance
4100
4000
3900
3800
3700
TSB
TSB−dex
Media
TSB−dex
TSB

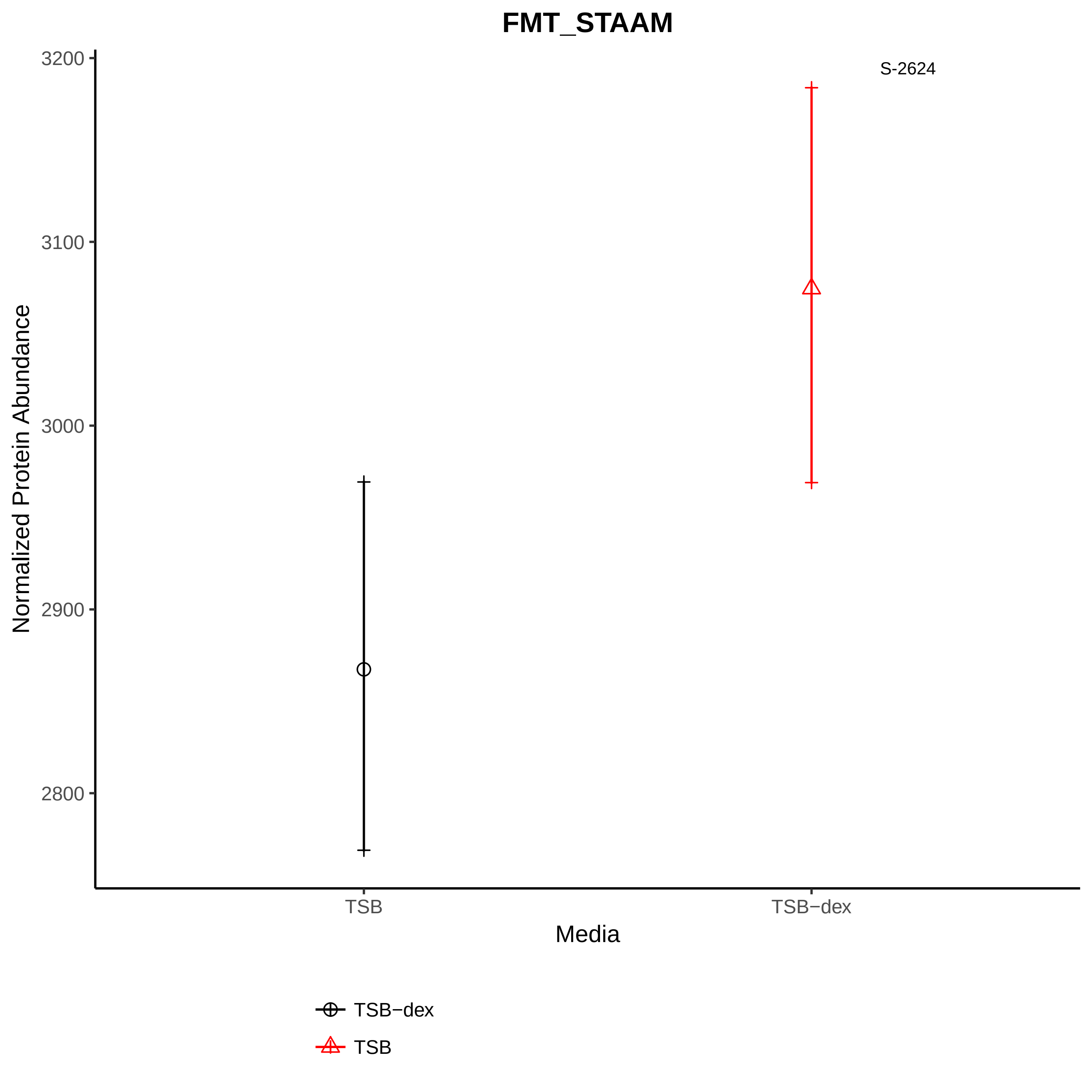
FMT_STAAM
S-2624
3200
3100
3000
2900
2800
Normalized Protein Abundance
TSB
TSB−dex
Media
TSB−dex
TSB

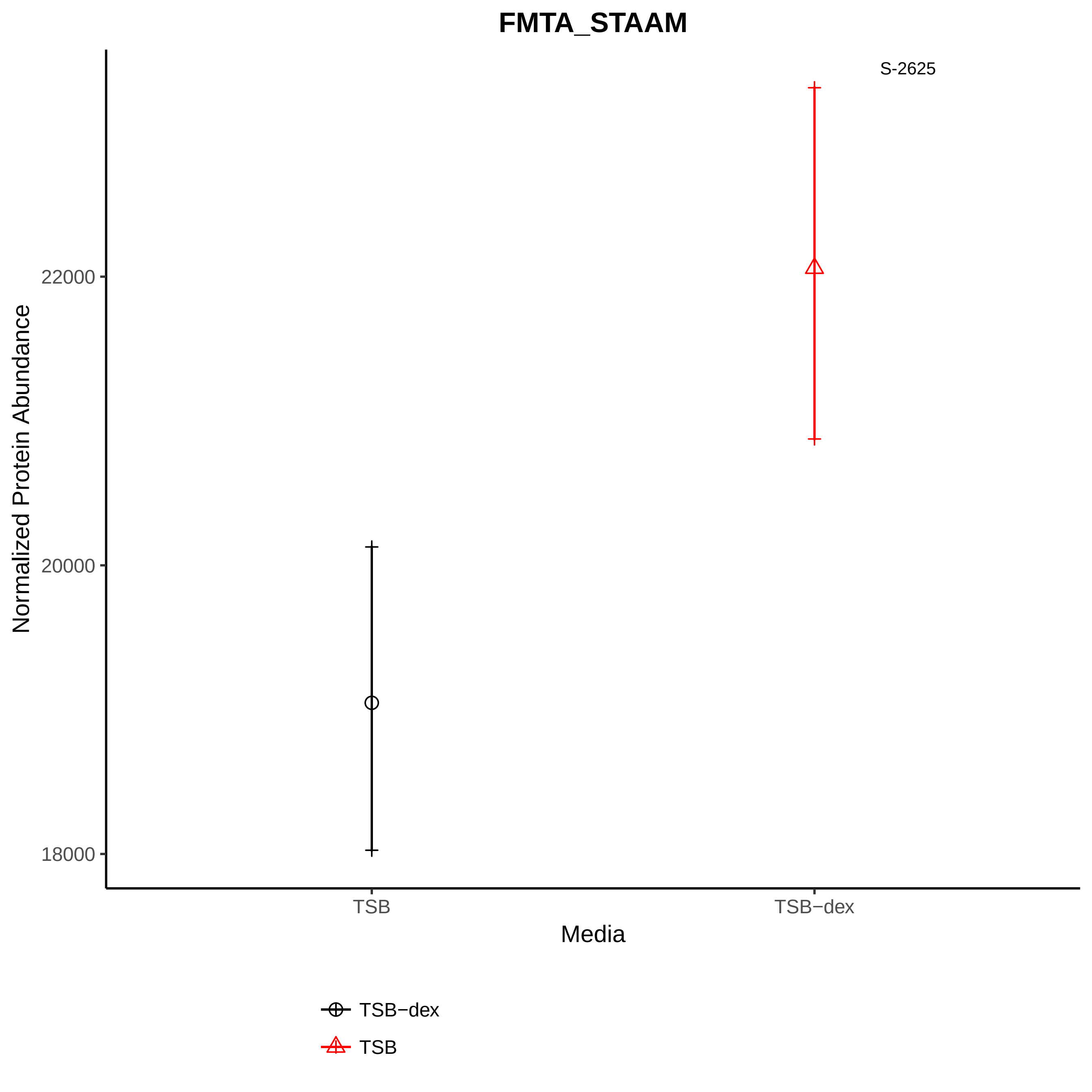
FMTA_STAAM
S-2625
22000
20000
18000
Normalized Protein Abundance
TSB
TSB−dex
Media
TSB−dex
TSB

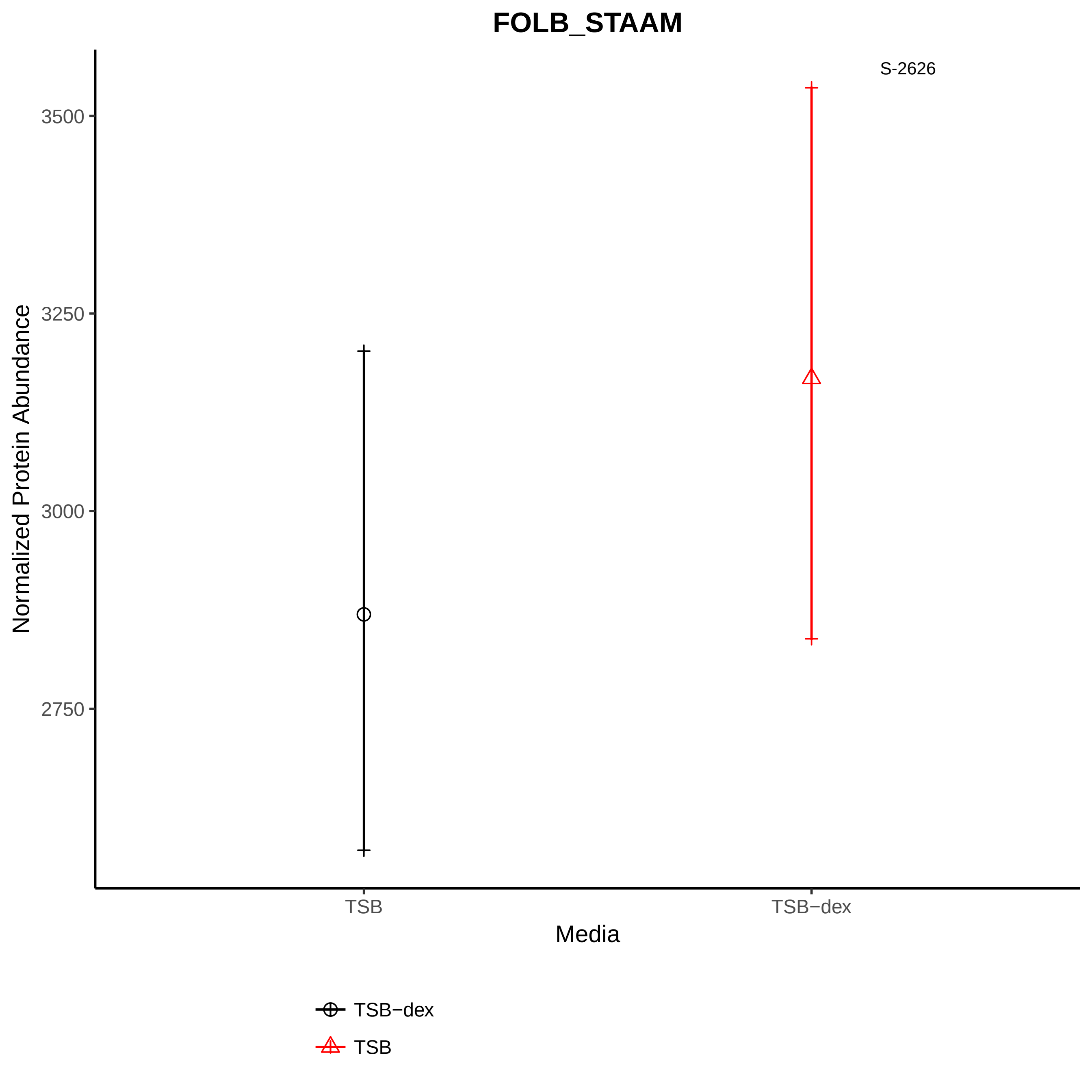
FOLB_STAAM
S-2626
3500
3250
3000
2750
Normalized Protein Abundance
TSB
TSB−dex
Media
TSB−dex
TSB

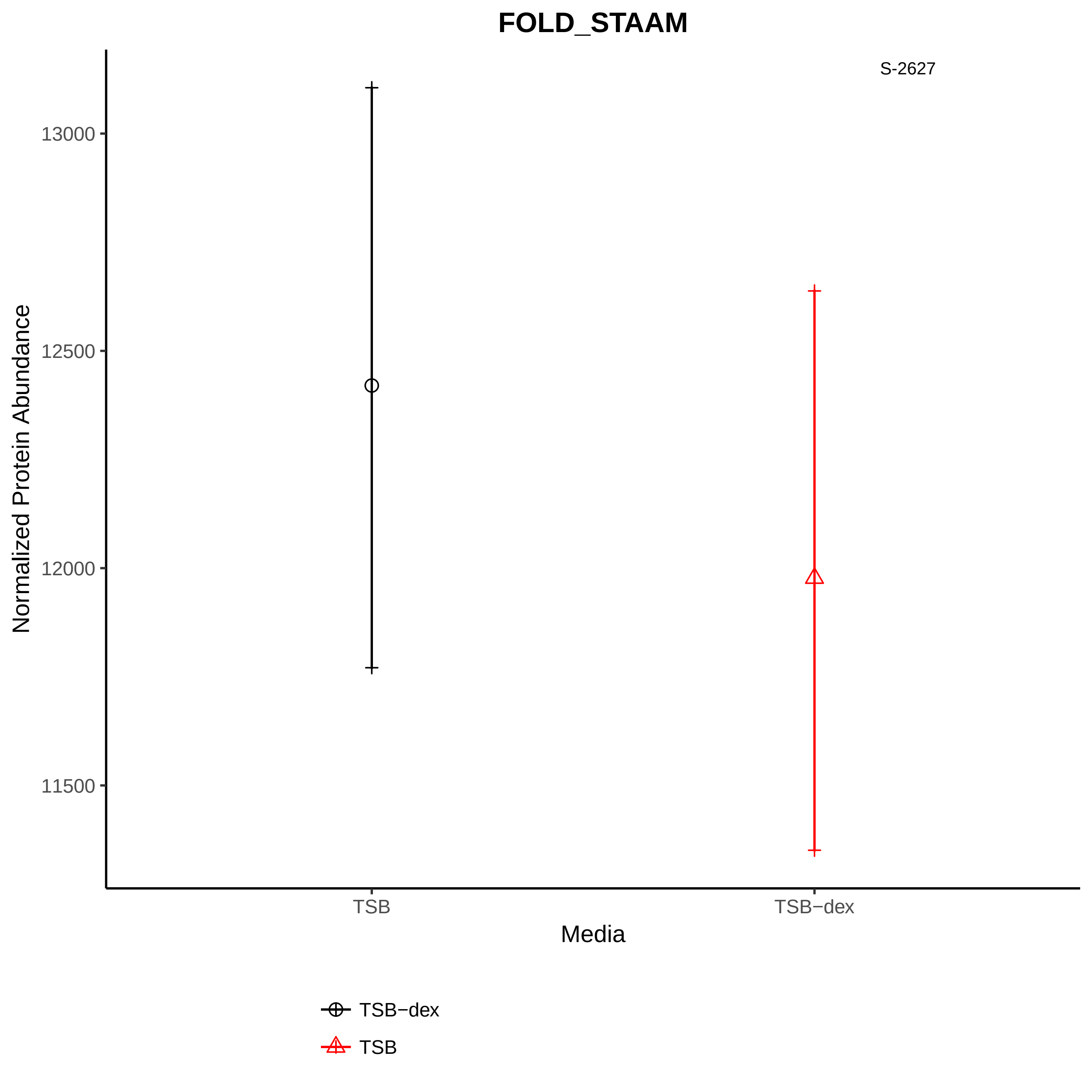

FOLD_STAAM
S-2627
Normalized Protein Abundance
13000
12500
12000
11500
TSB
TSB−dex
Media
TSB−dex
TSB

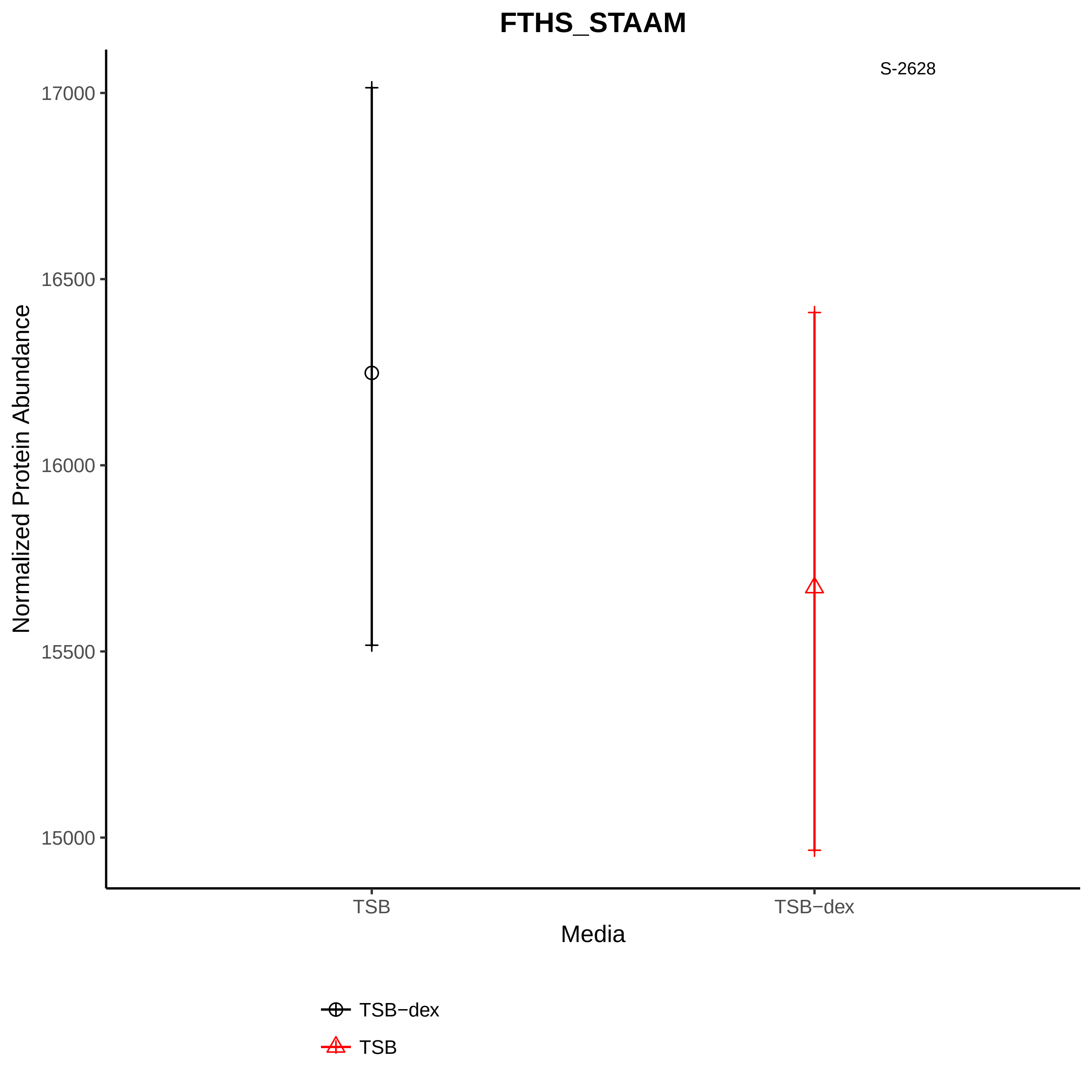

FTHS_STAAM
S-2628
17000
16500
16000
15500
15000
Normalized Protein Abundance
TSB
TSB−dex
Media
TSB−dex
TSB

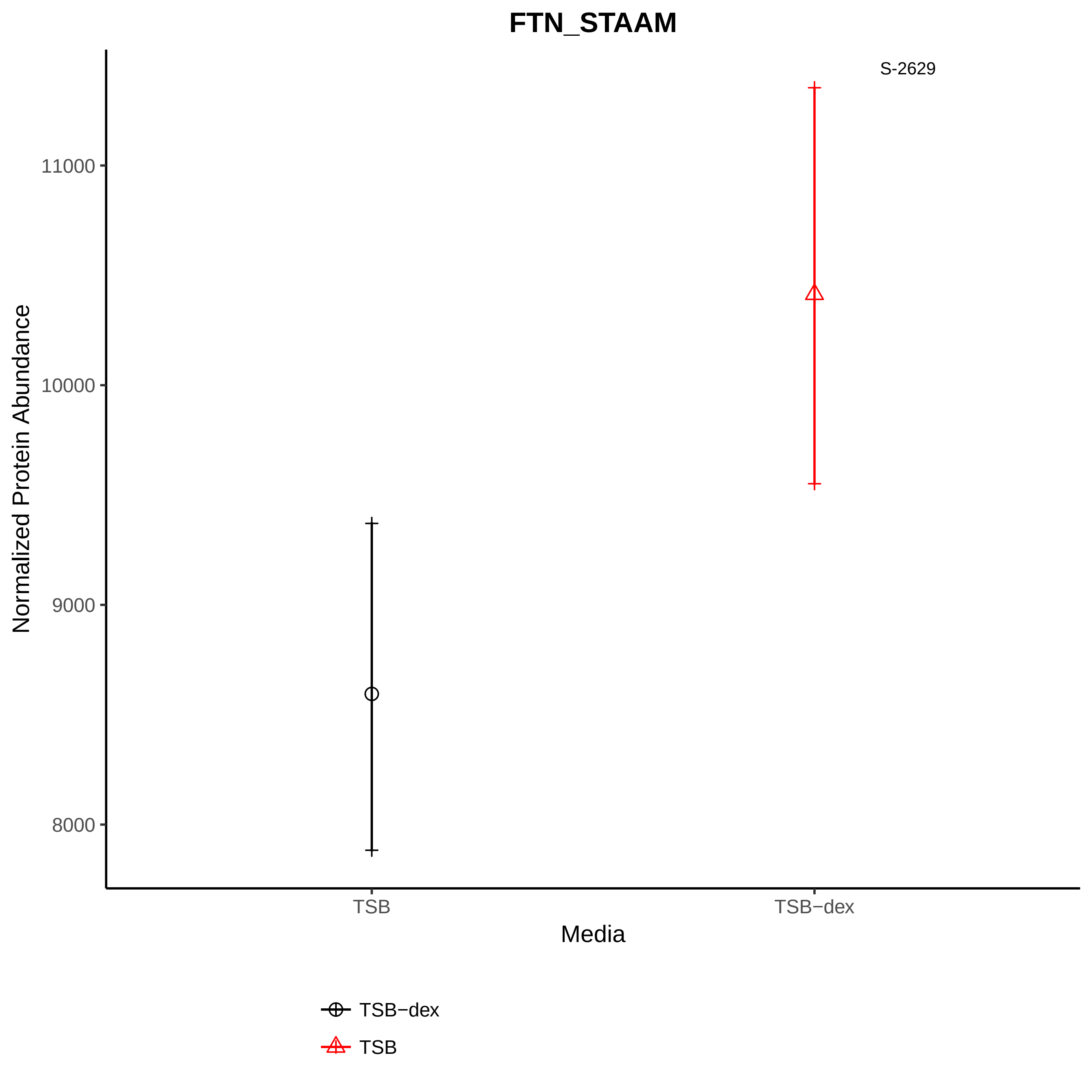
FTN_STAAM
S-2629
Normalized Protein Abundance
11000
10000
9000
8000
TSB
TSB−dex
Media
TSB−dex
TSB

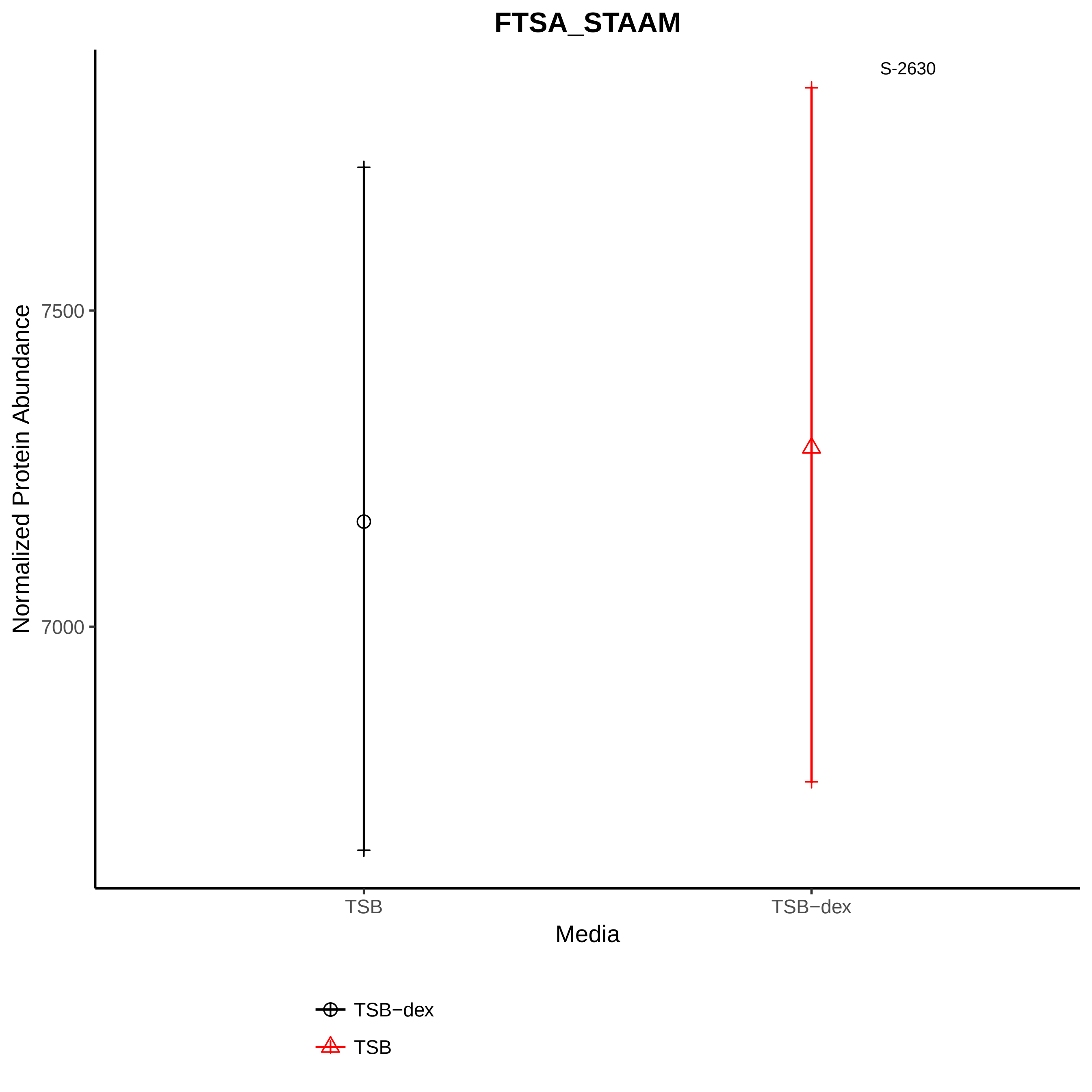
FTSA_STAAM
S-2630
Normalized Protein Abundance
7500
7000
TSB
TSB−dex
Media
TSB−dex
TSB

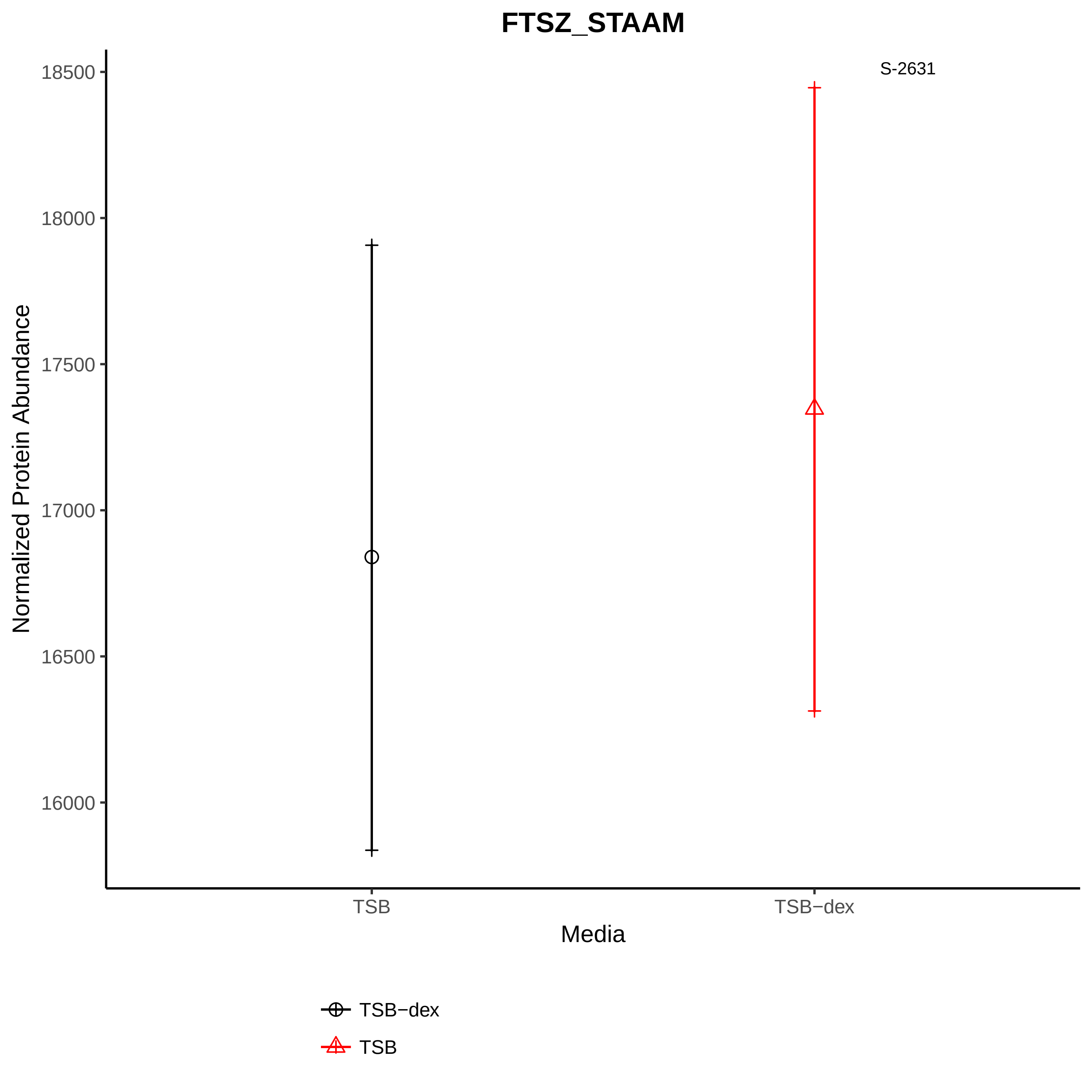
FTSZ_STAAM
S-2631
Normalized Protein Abundance
18500
18000
17500
17000
16500
16000
TSB
TSB−dex
Media
TSB−dex
TSB

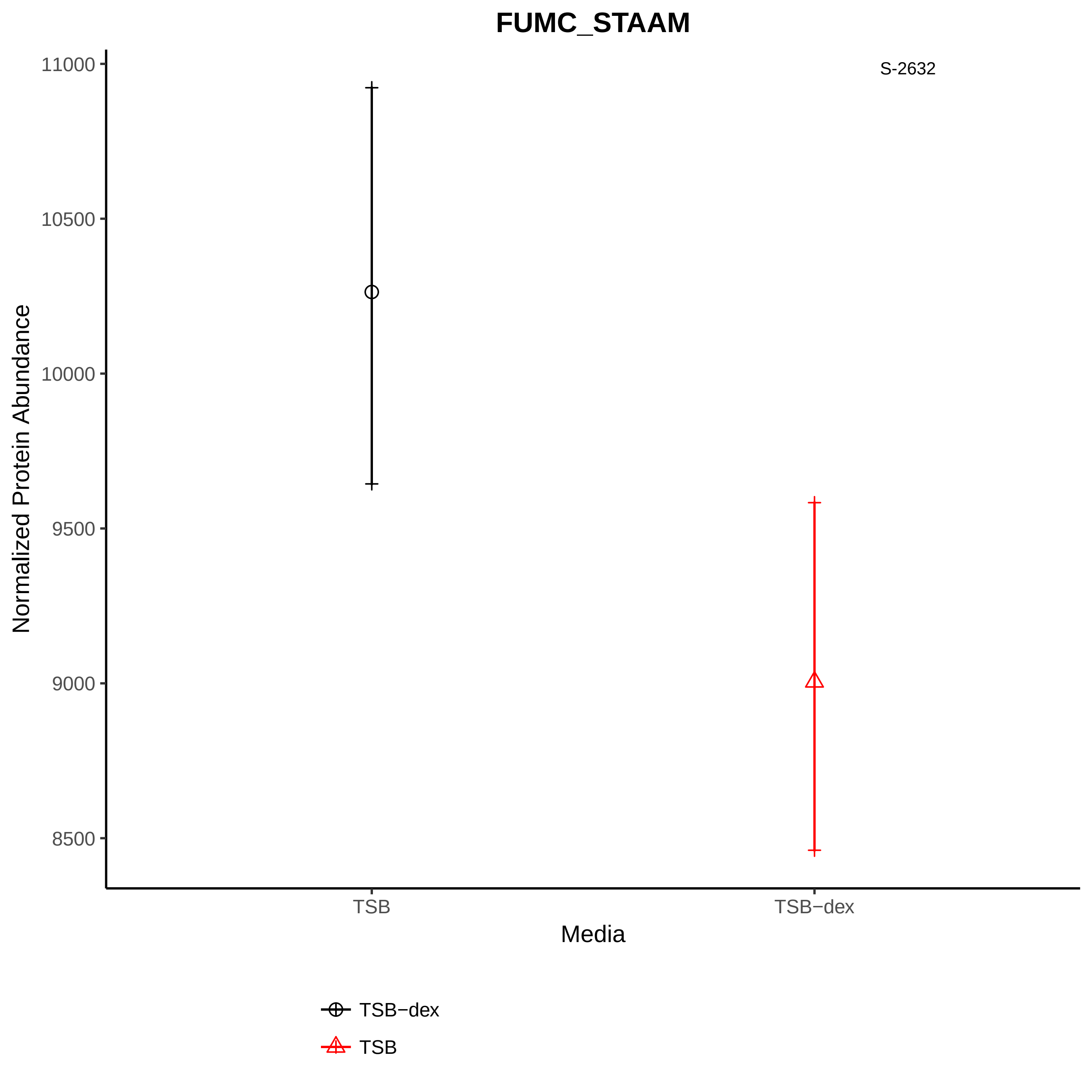

FUMC_STAAM
S-2632
11000
10500
10000
9500
9000
8500
Normalized Protein Abundance
TSB
TSB−dex
Media
TSB−dex
TSB

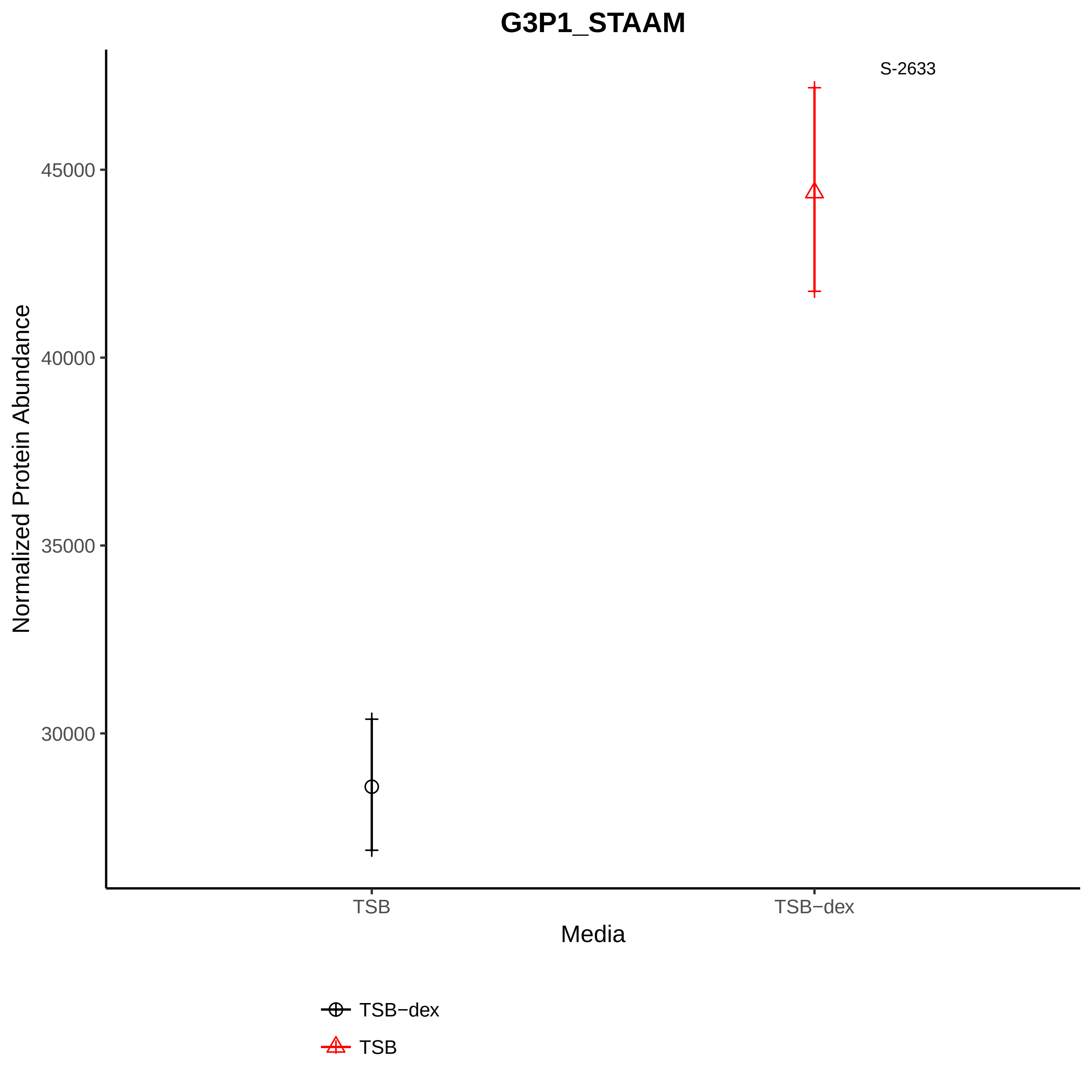

G3P1_STAAM
S-2633
Normalized Protein Abundance
45000
40000
35000
30000
TSB
TSB−dex
Media
TSB−dex
TSB

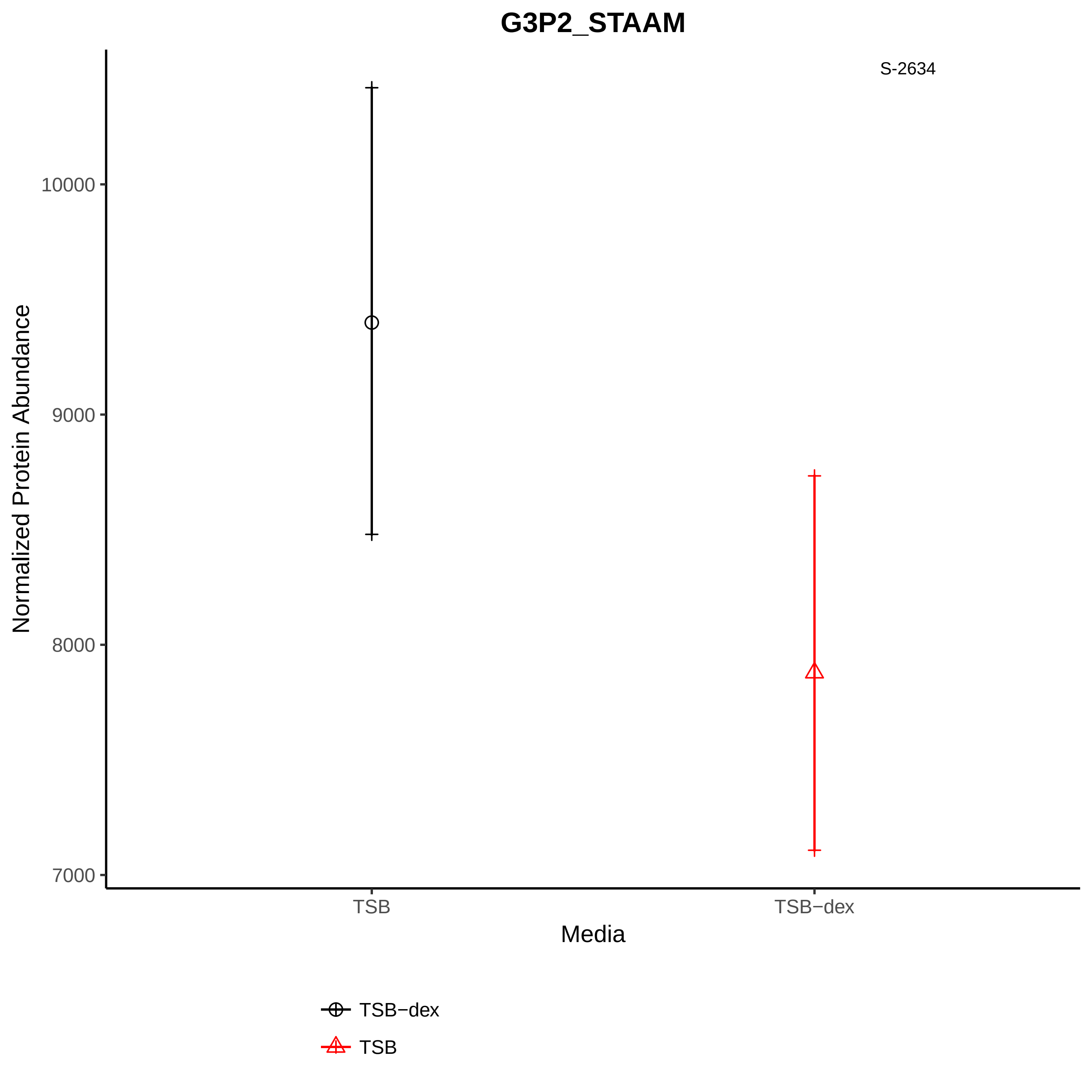

G3P2_STAAM
S-2634
Normalized Protein Abundance
10000
9000
8000
7000
TSB
TSB−dex
Media
TSB−dex
TSB

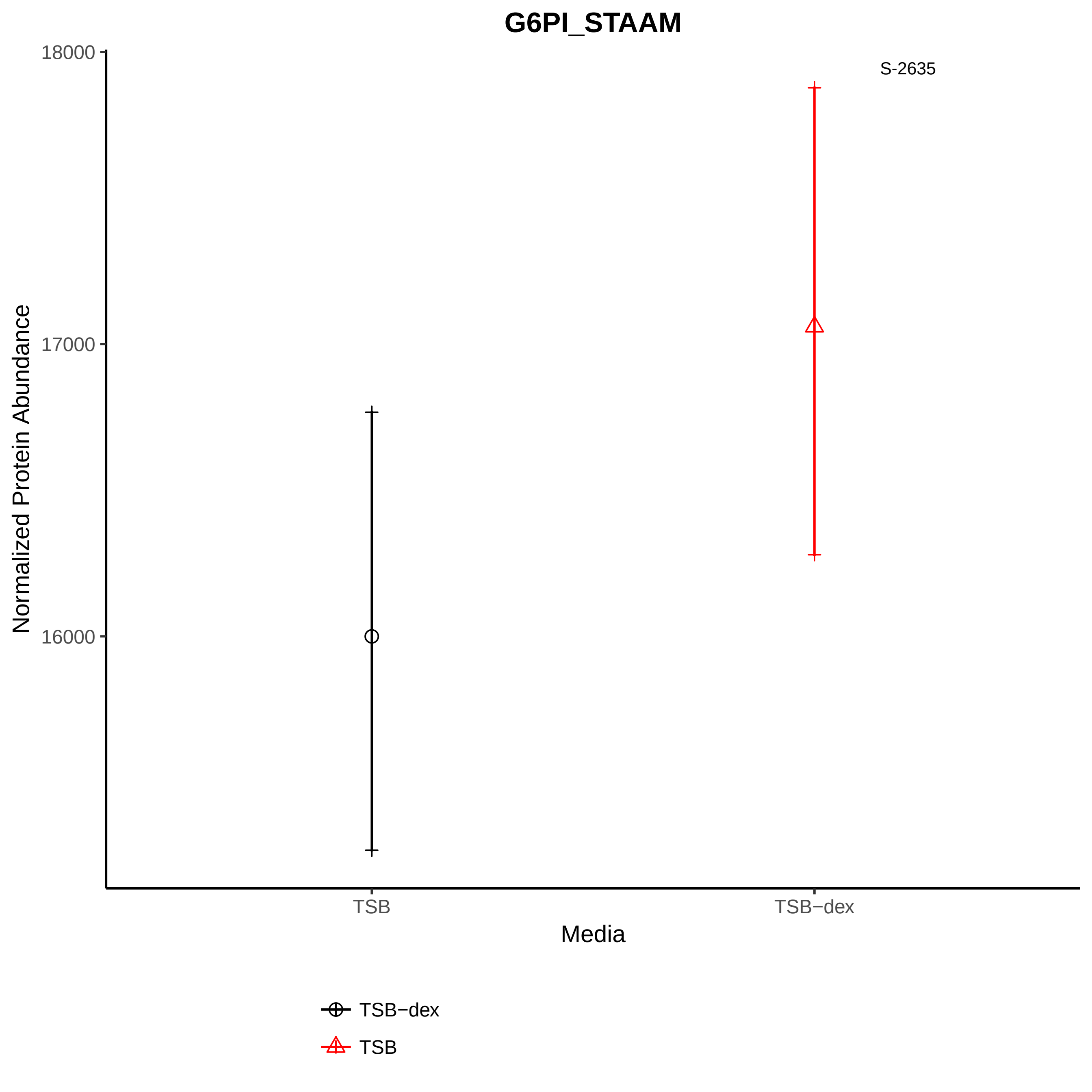
G6PI_STAAM
S-2635
18000
17000
16000
Normalized Protein Abundance
TSB
TSB−dex
Media
TSB−dex
TSB

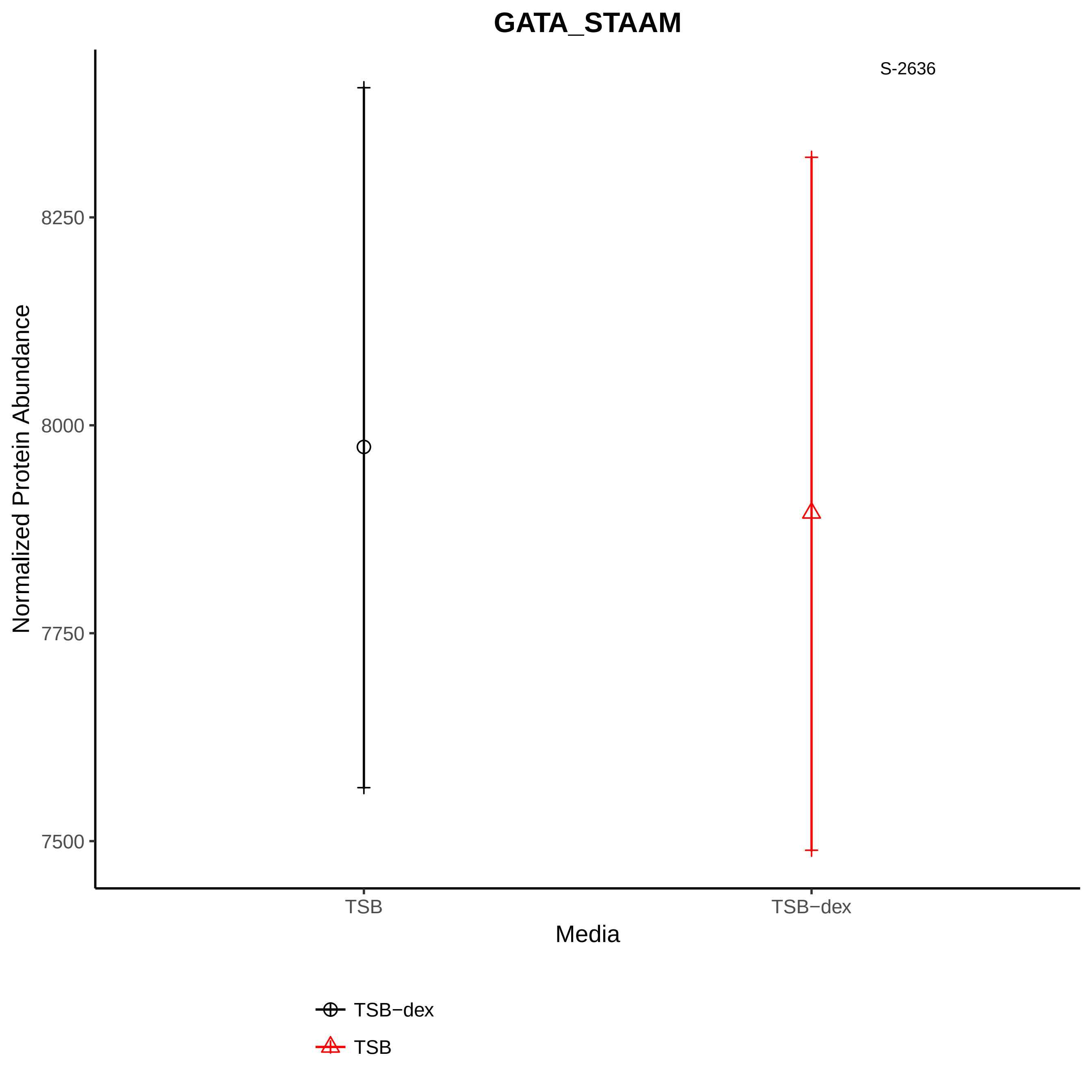

GATA_STAAM
S-2636
Normalized Protein Abundance
8250
8000
7750
7500
TSB
TSB−dex
Media
TSB−dex
TSB

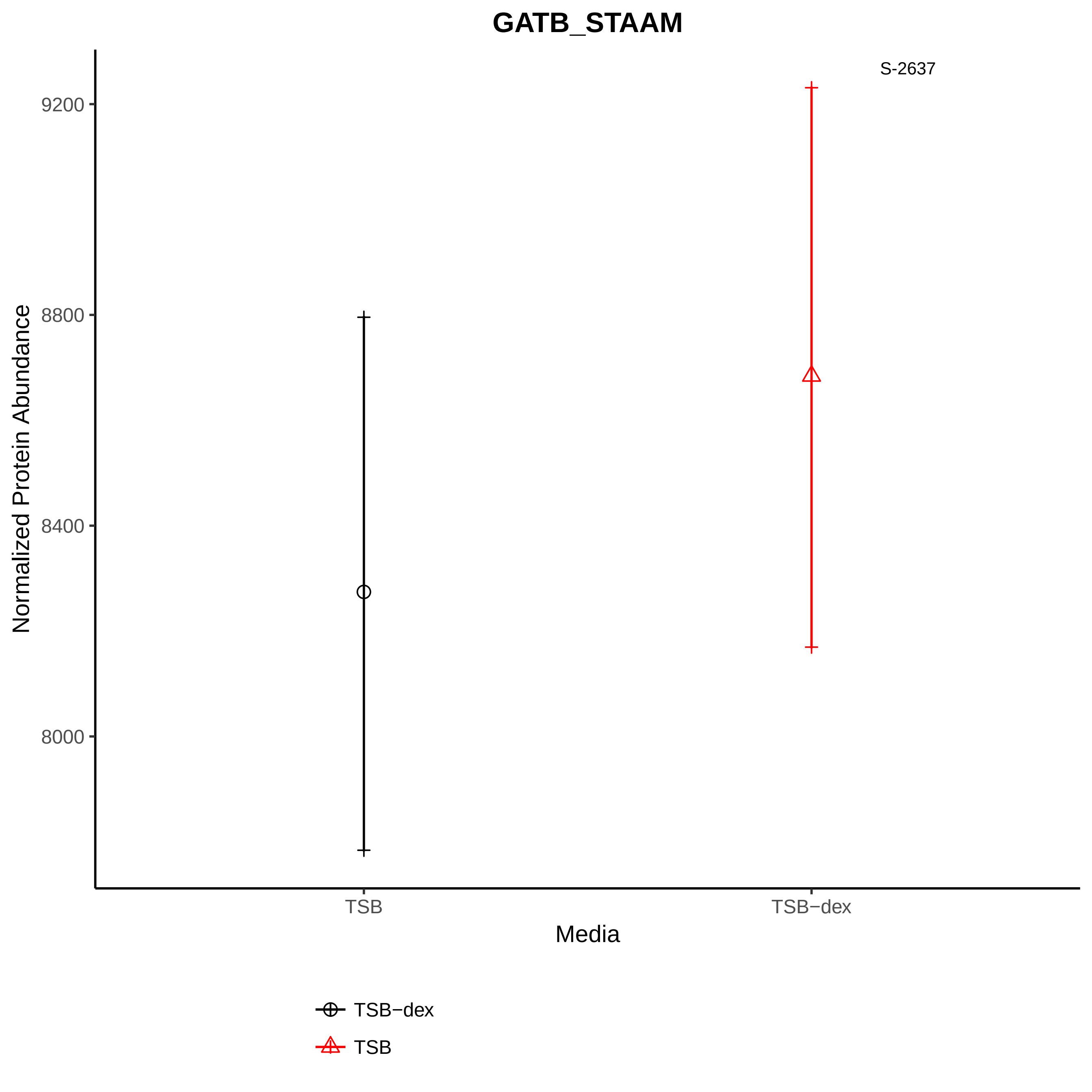
GATB_STAAM
S-2637
Normalized Protein Abundance
9200
8800
8400
8000
TSB
TSB−dex
Media
TSB−dex
TSB

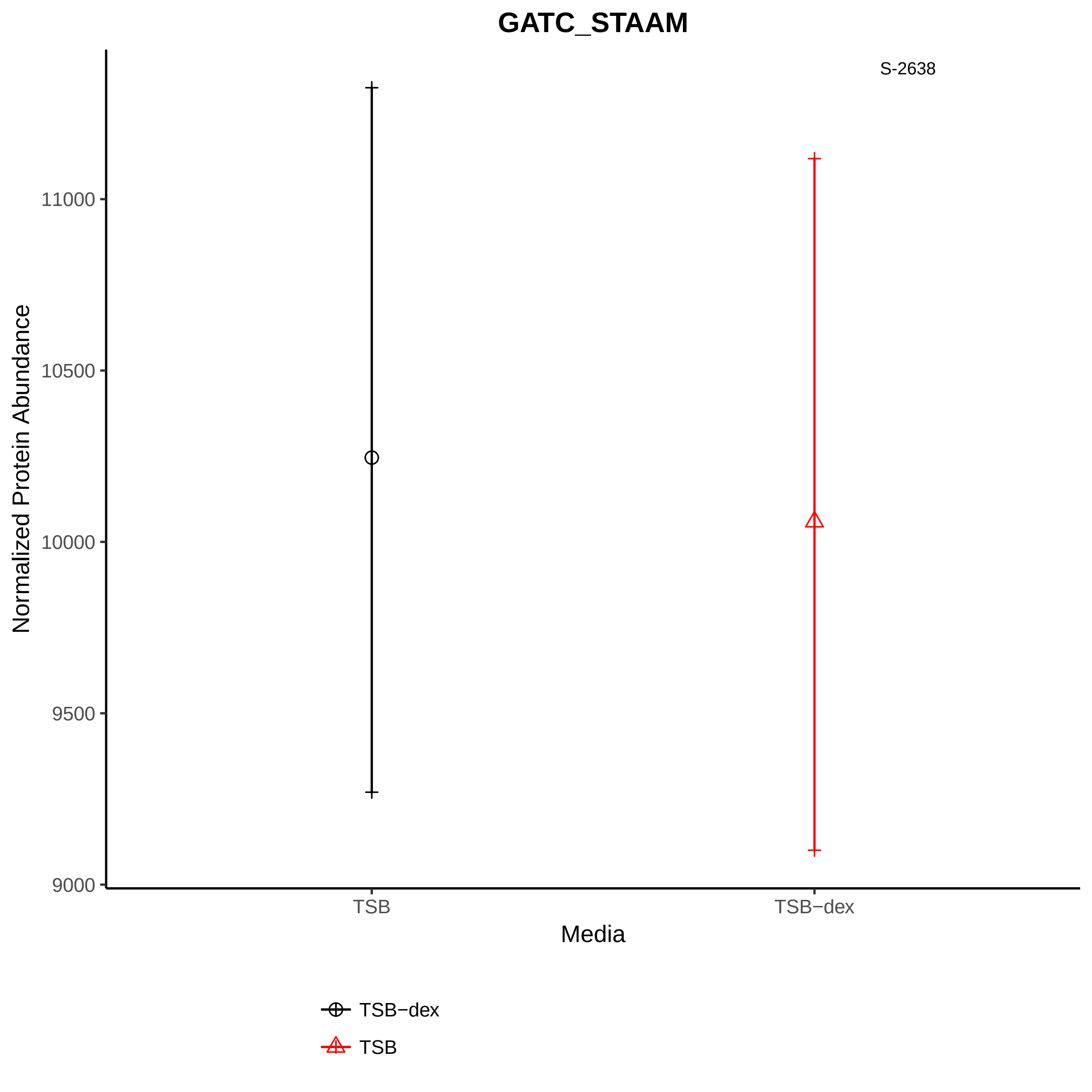

GATC_STAAM
S-2638
Normalized Protein Abundance
11000
10500
10000
9500
9000
TSB
TSB−dex
Media
TSB−dex
TSB

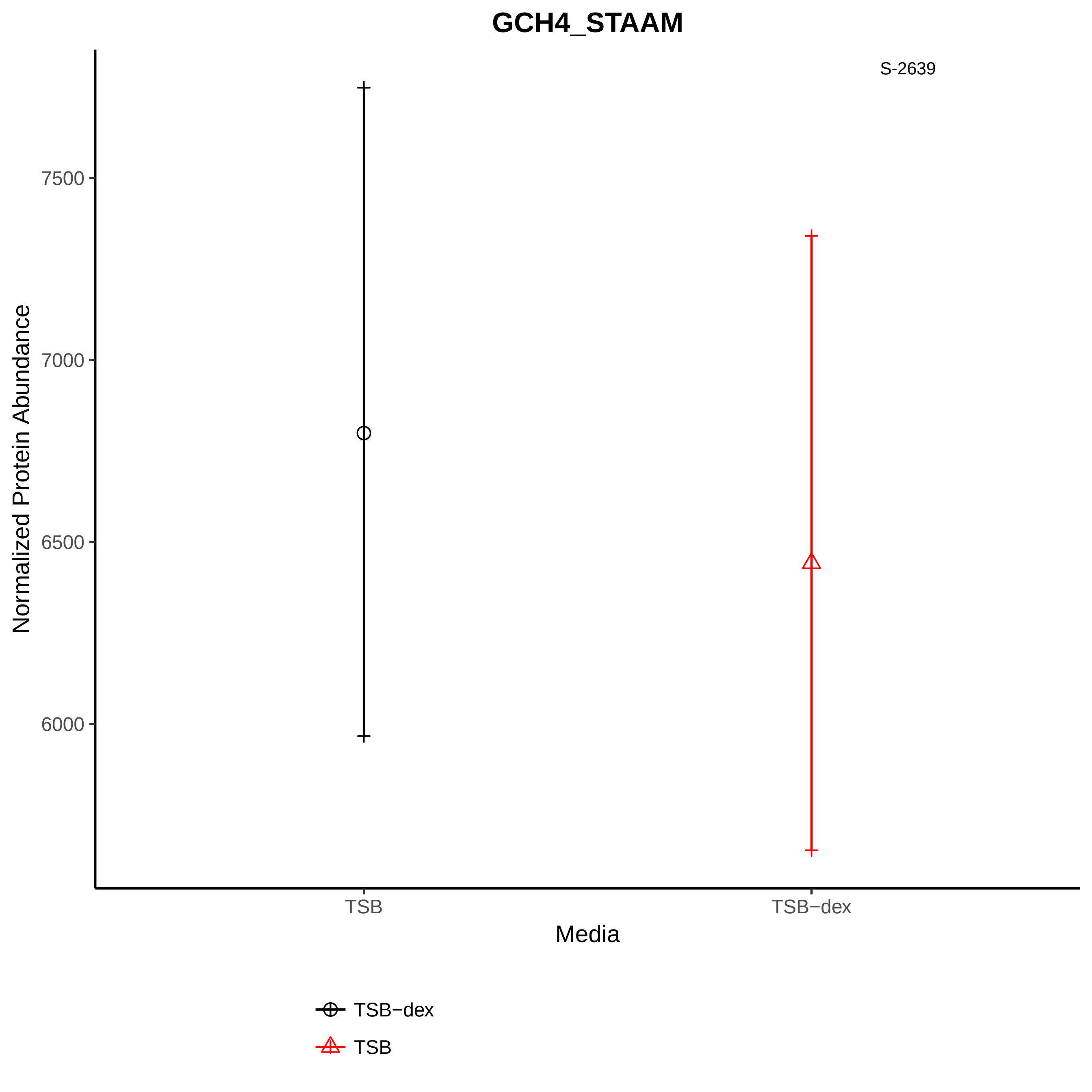

GCH4_STAAM
S-2639
Normalized Protein Abundance
7500
7000
6500
6000
TSB
TSB−dex
Media
TSB−dex
TSB

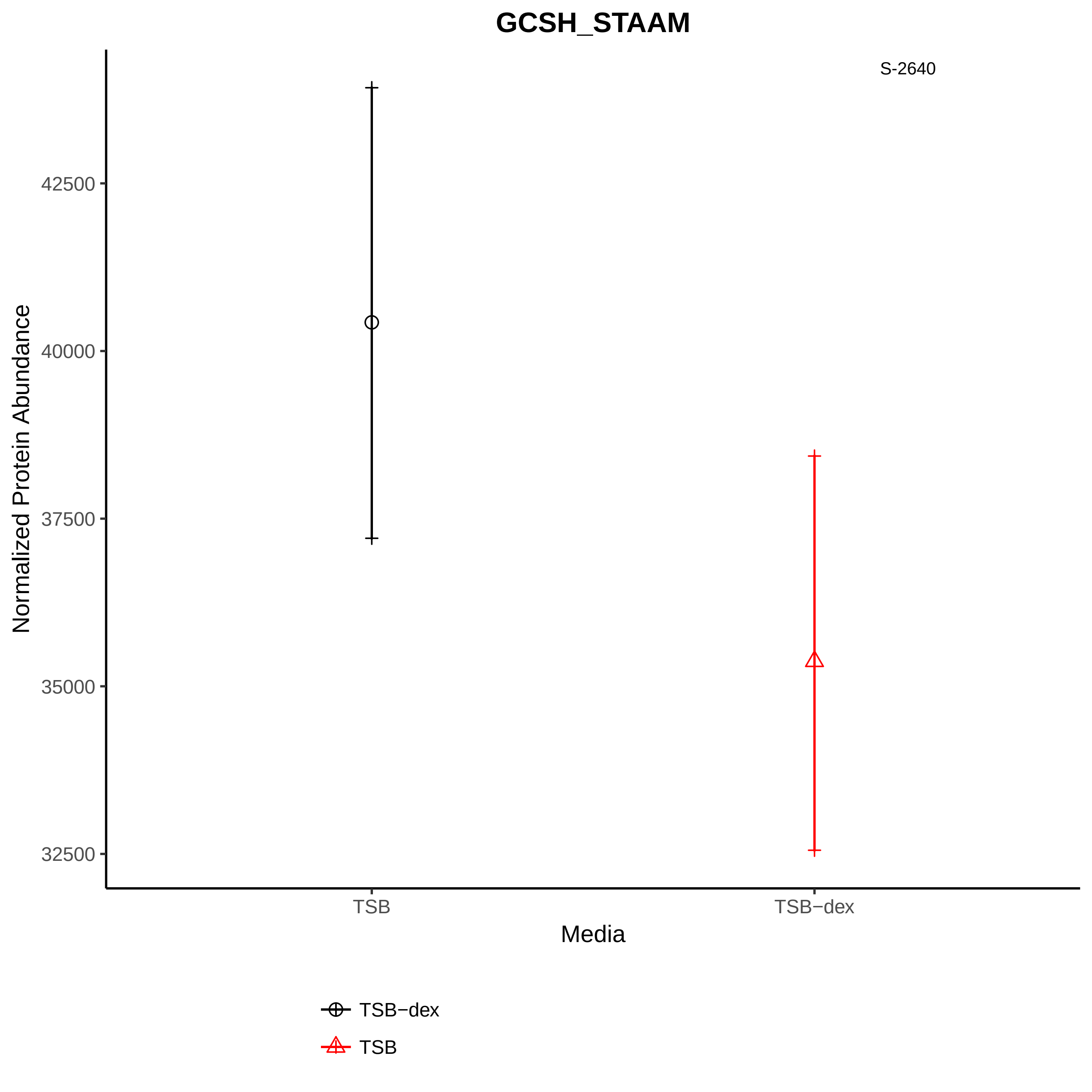
GCSH_STAAM
S-2640
Normalized Protein Abundance
42500
40000
37500
35000
32500
TSB
TSB−dex
Media
TSB−dex
TSB

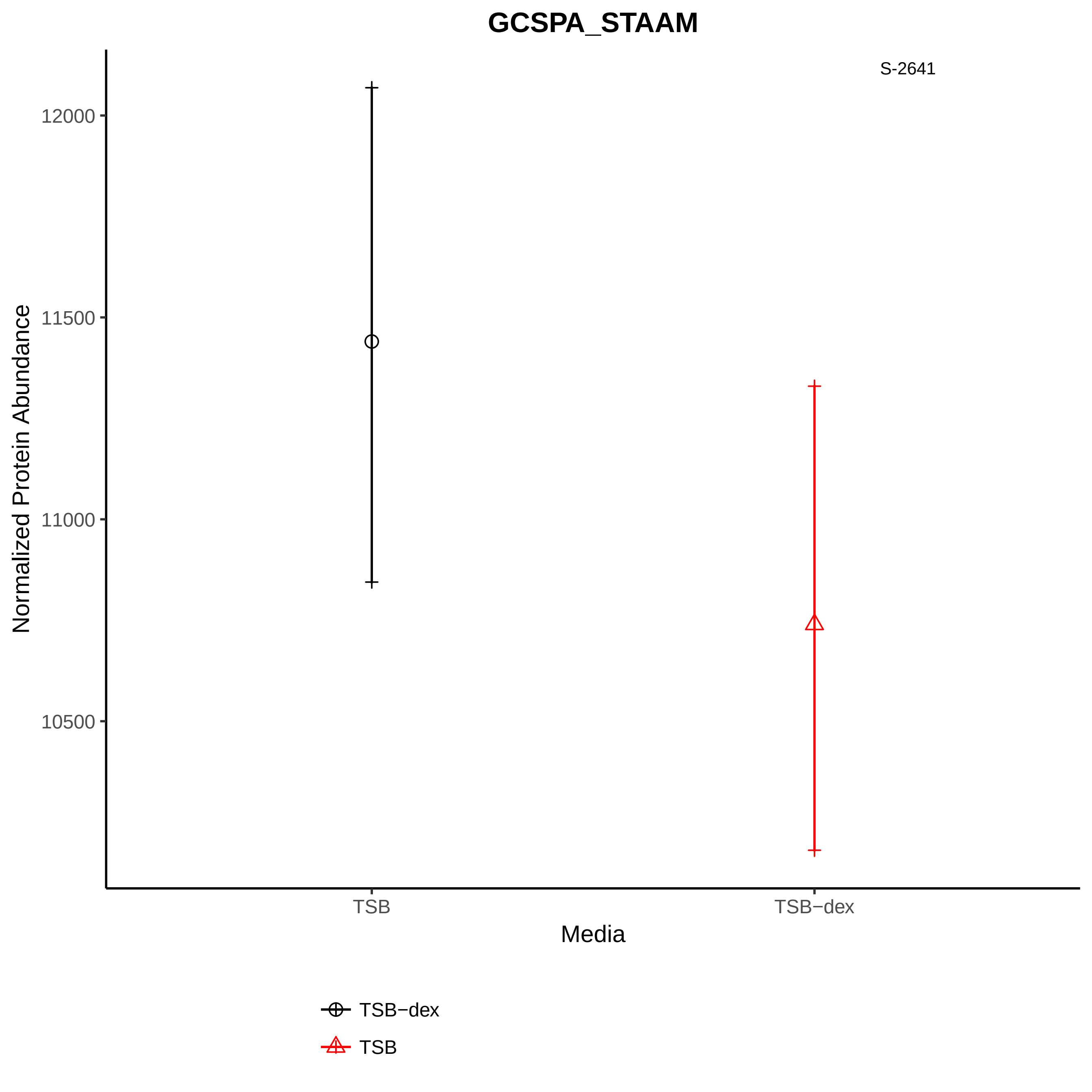
GCSPA_STAAM
S-2641
Normalized Protein Abundance
12000
11500
11000
10500
TSB
TSB−dex
Media
TSB−dex
TSB

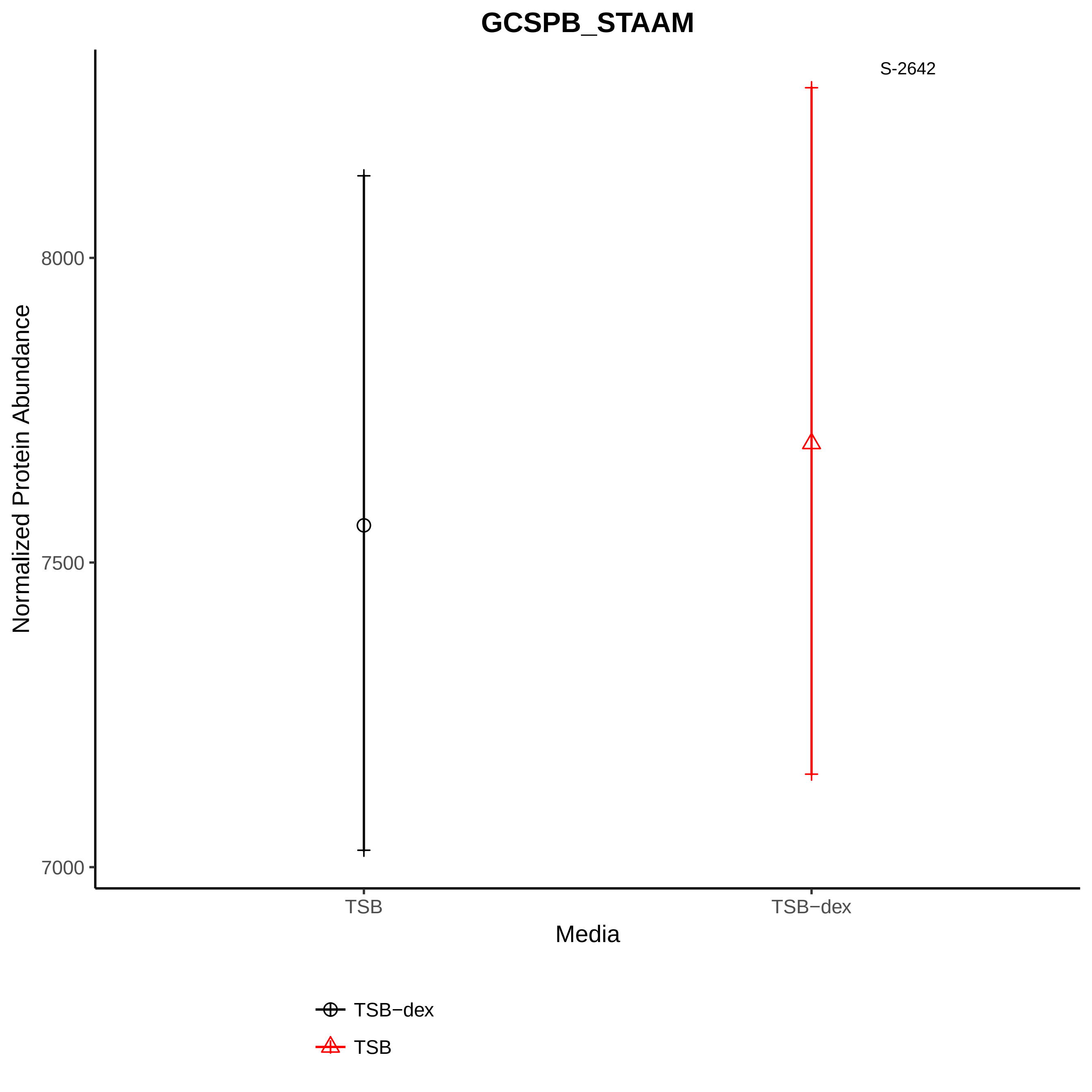
GCSPB_STAAM
S-2642
Normalized Protein Abundance
8000
7500
7000
TSB
TSB−dex
Media
TSB−dex
TSB

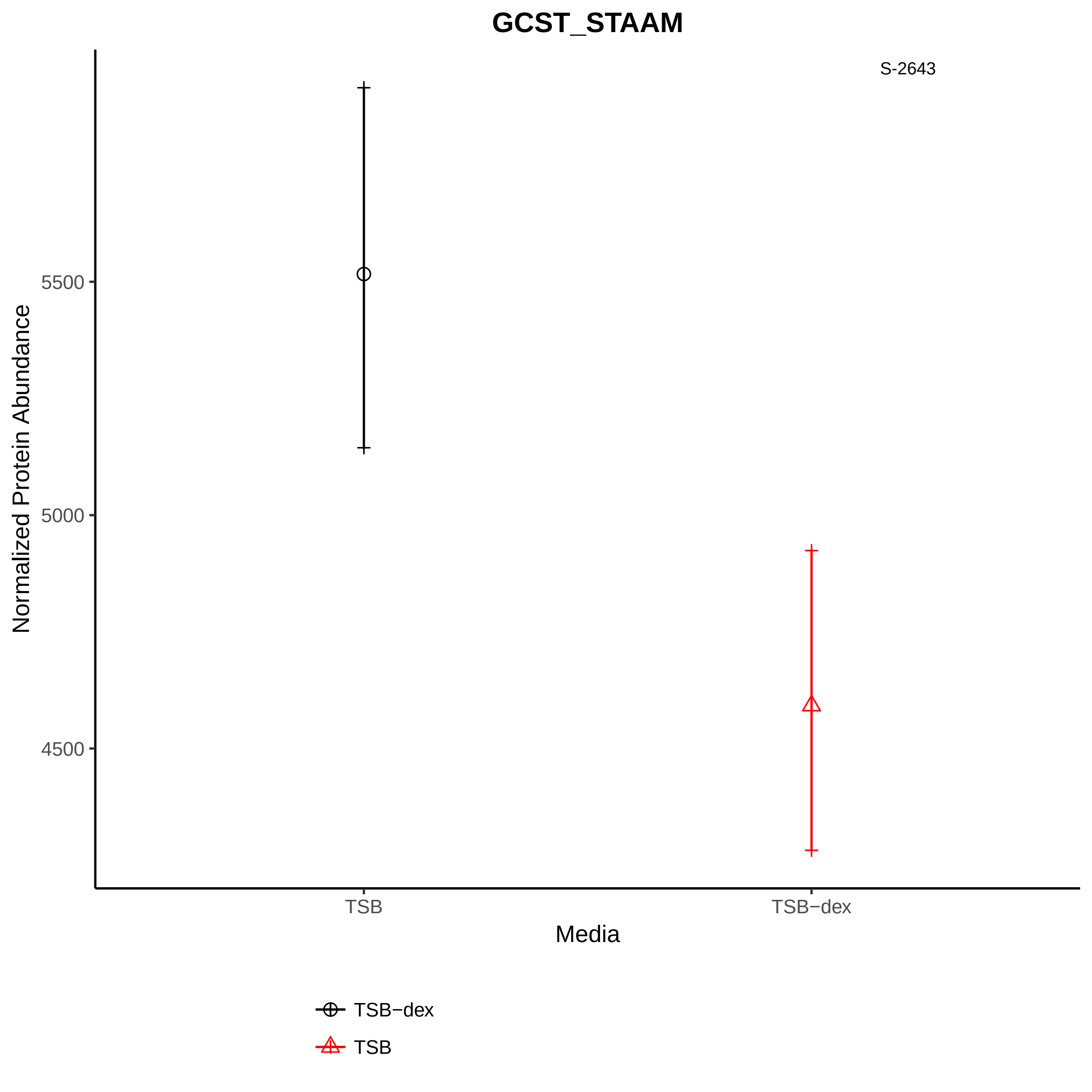

GCST_STAAM
S-2643
Normalized Protein Abundance
5500
5000
4500
TSB
TSB−dex
Media
TSB−dex
TSB

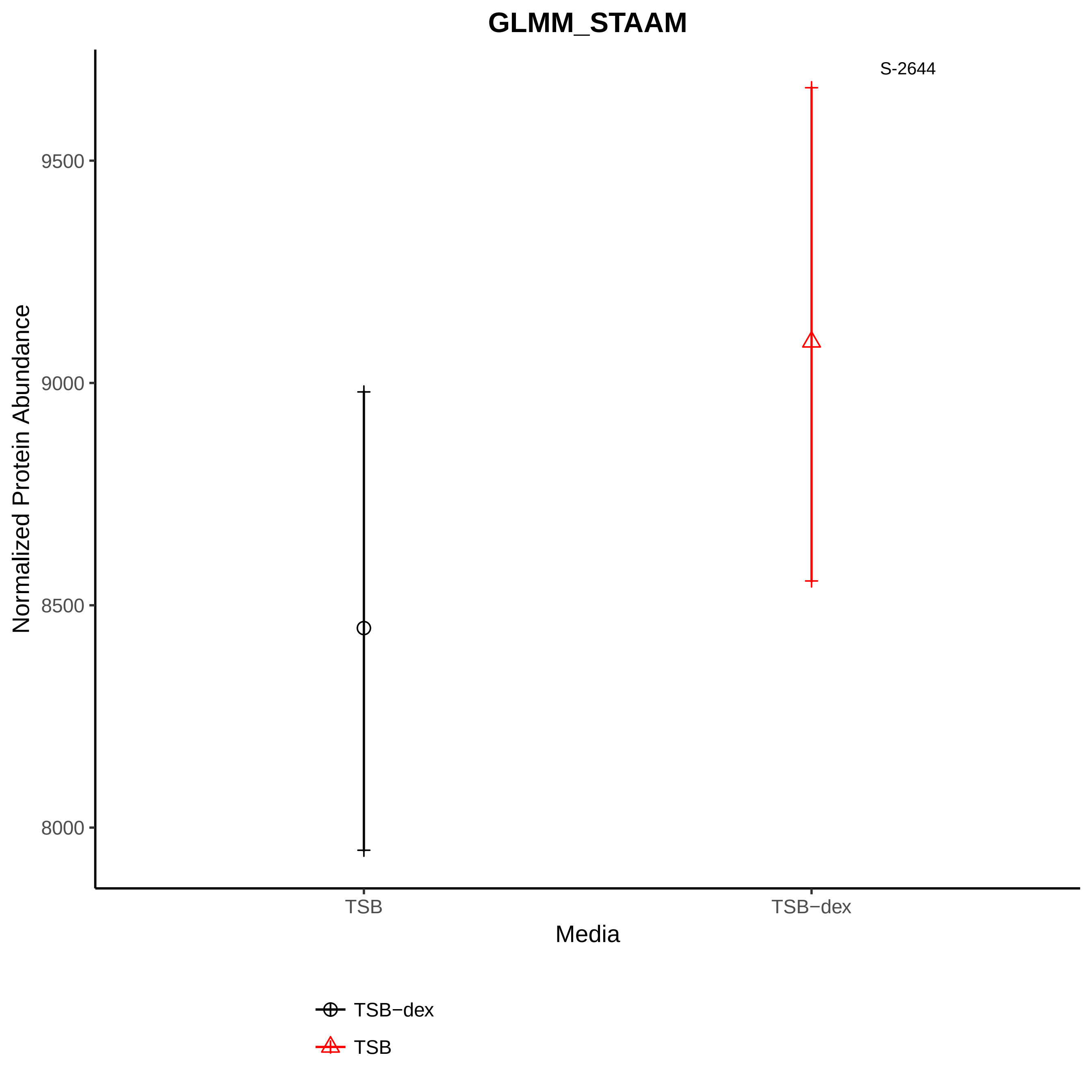

GLMM_STAAM
S-2644
Normalized Protein Abundance
9500
9000
8500
8000
TSB
TSB−dex
Media
TSB−dex
TSB

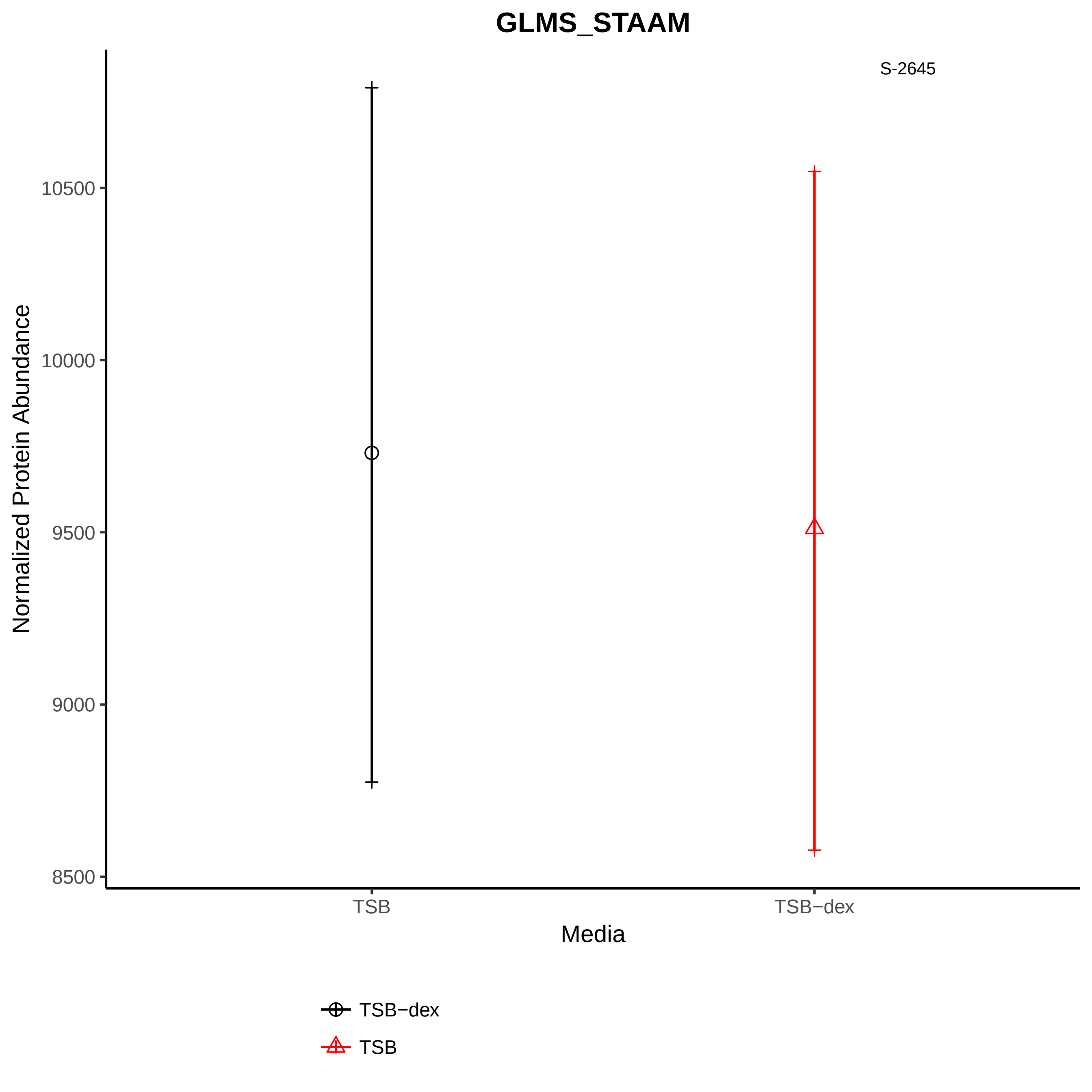

GLMS_STAAM
S-2645
Normalized Protein Abundance
10500
10000
9500
9000
8500
TSB
TSB−dex
Media
TSB−dex
TSB

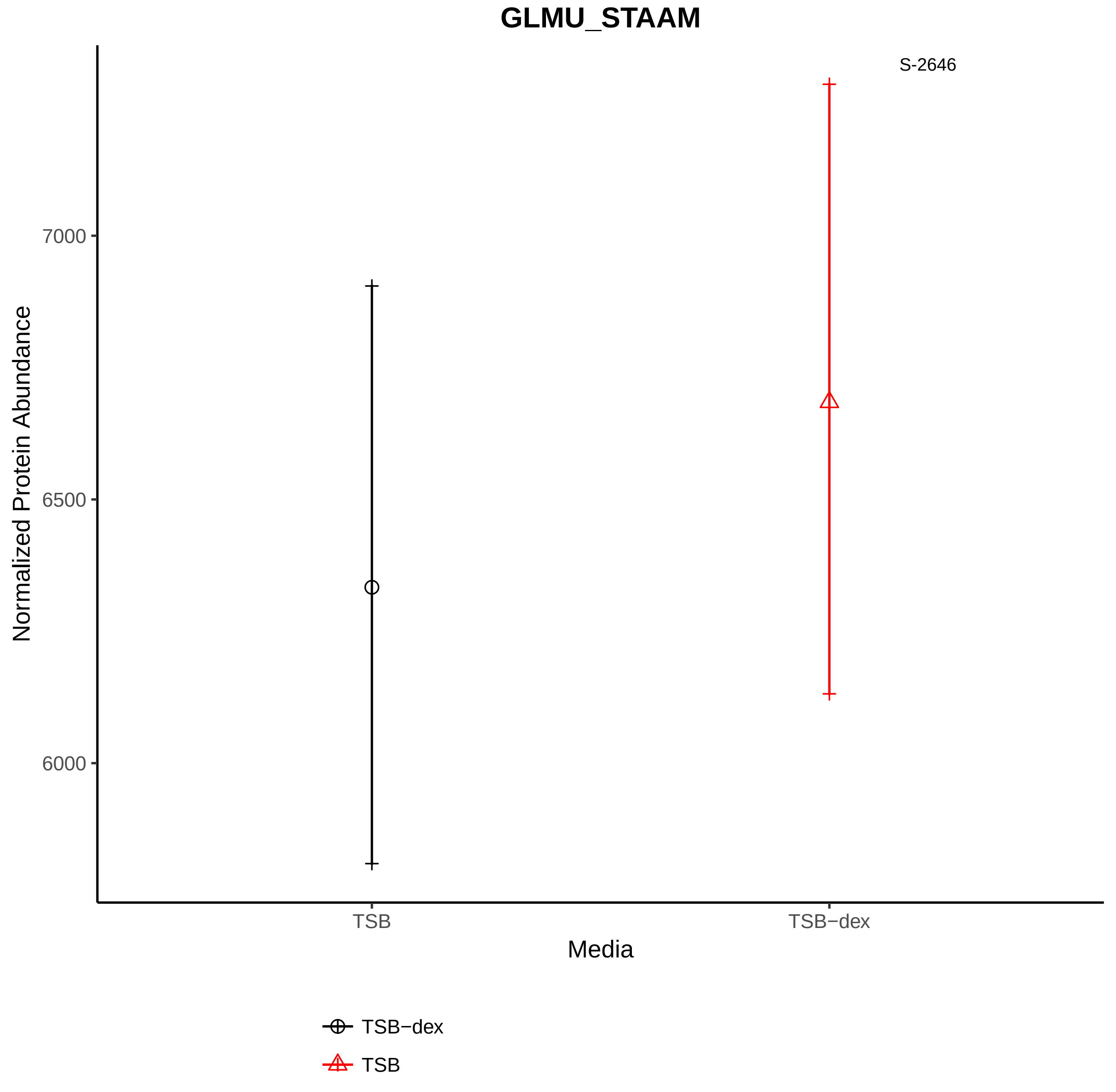

GLMU_STAAM
S-2646
7000
6500
6000
Normalized Protein Abundance
TSB
TSB−dex
Media
TSB−dex
TSB

GLNA_STAAM

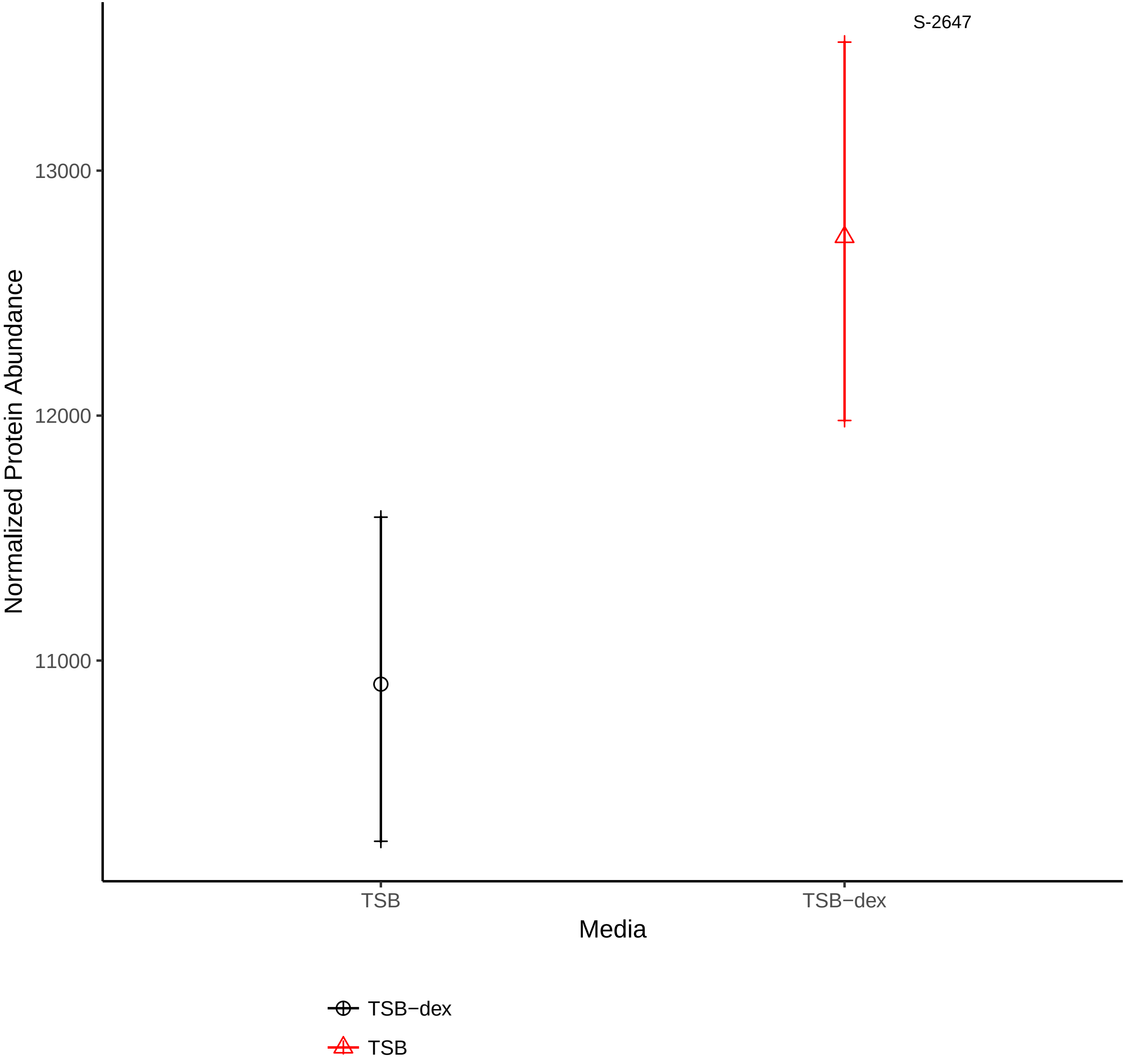

S-2647
13000
12000
11000
Normalized Protein Abundance
TSB
TSB−dex
Media
TSB−dex
TSB

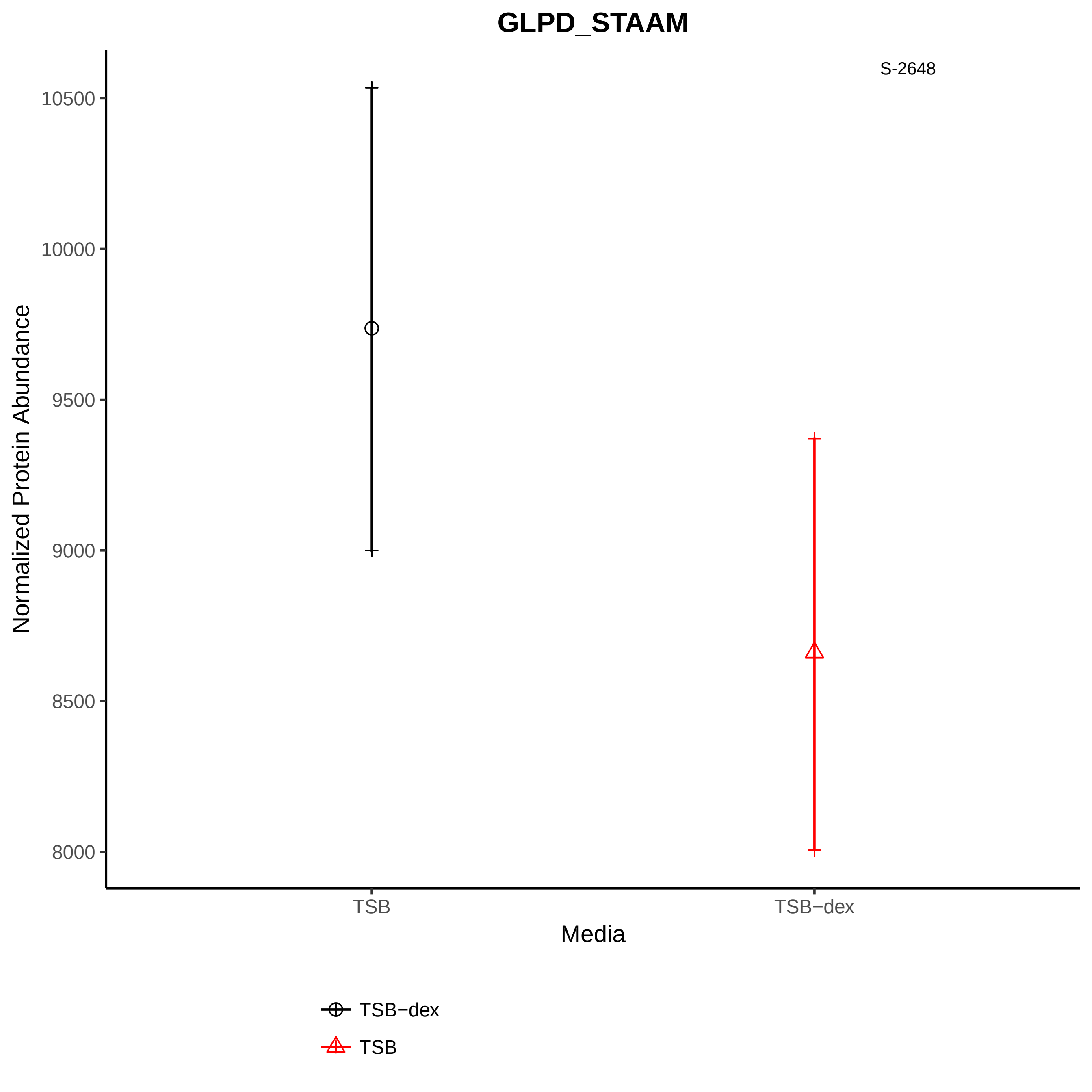

GLPD_STAAM
S-2648
Normalized Protein Abundance
10500
10000
9500
9000
8500
8000
TSB
TSB−dex
Media
TSB−dex
TSB

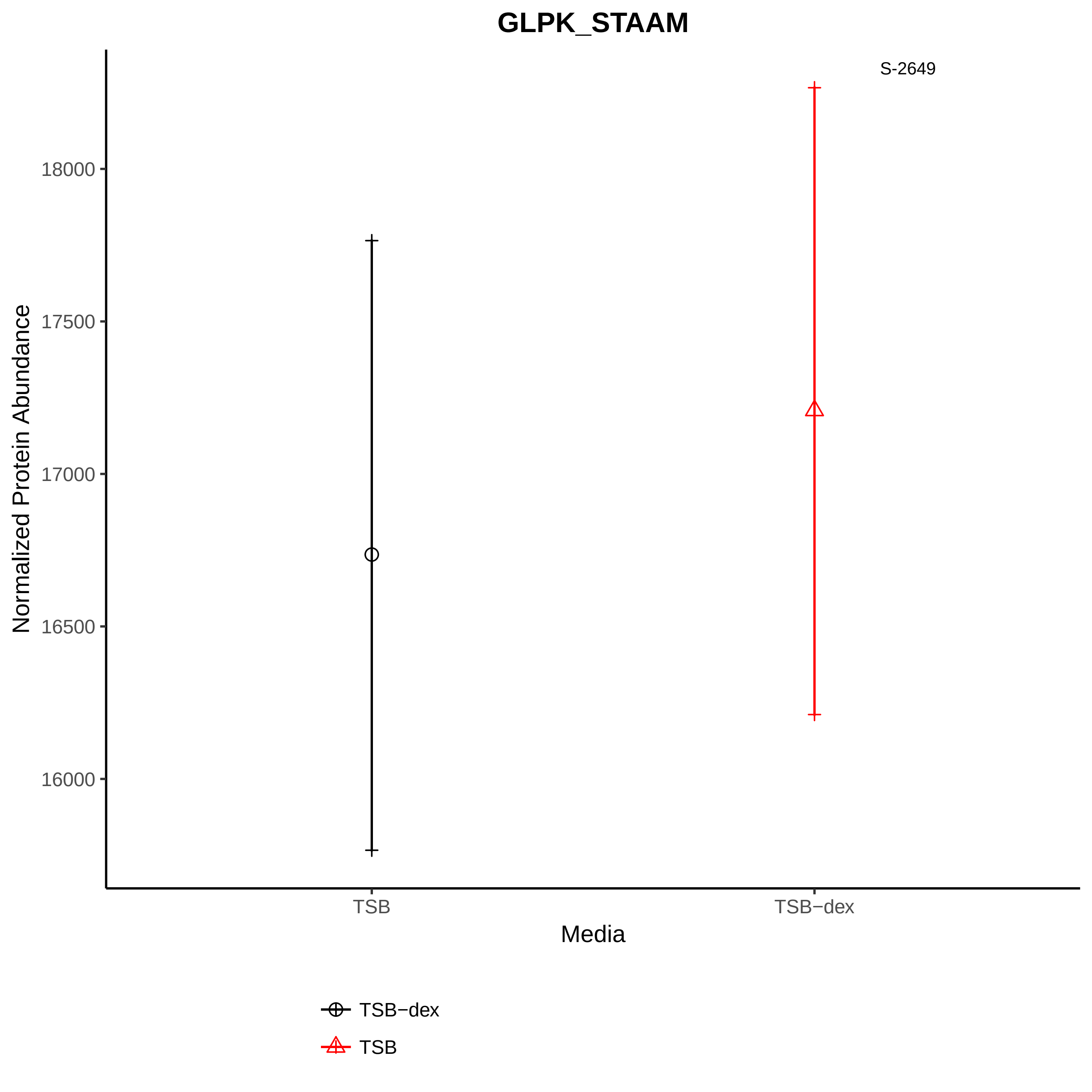

GLPK_STAAM
S-2649
Normalized Protein Abundance
18000
17500
17000
16500
16000
TSB
TSB−dex
Media
TSB−dex
TSB

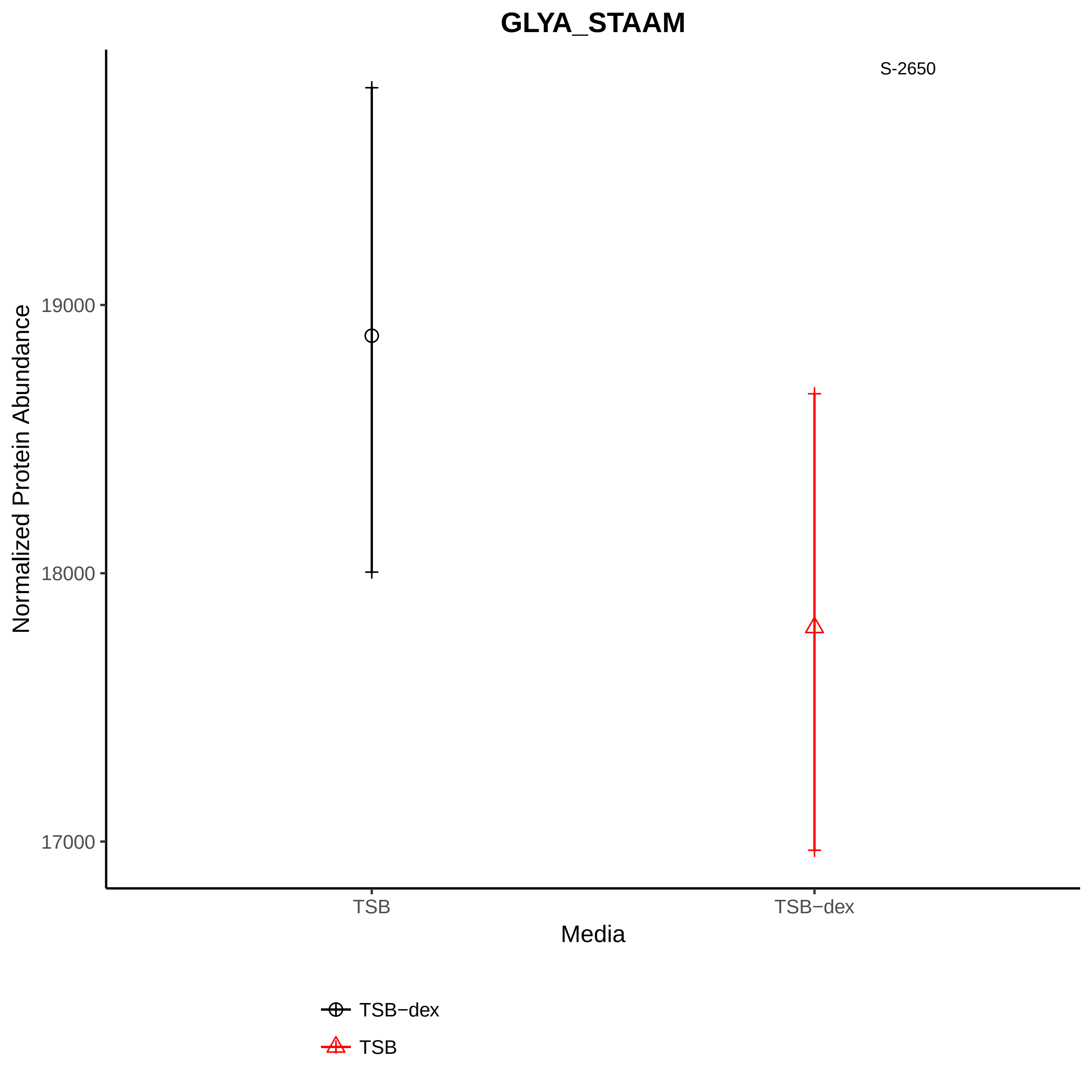

GLYA_STAAM
S-2650
Normalized Protein Abundance
19000
18000
17000
TSB
TSB−dex
Media
TSB−dex
TSB

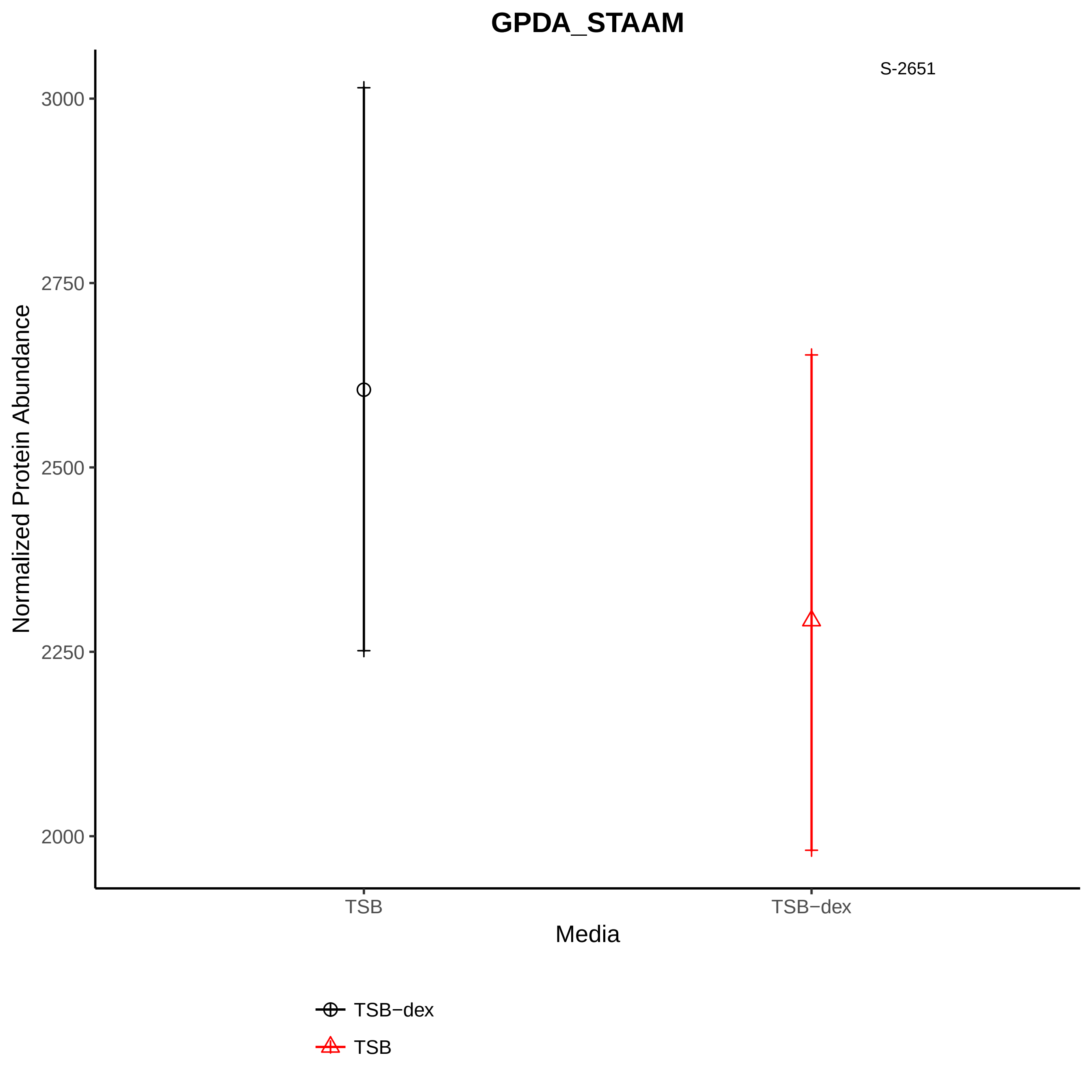

GPDA_STAAM
S-2651
3000
2750
2500
2250
2000
Normalized Protein Abundance
TSB
TSB−dex
Media
TSB−dex
TSB

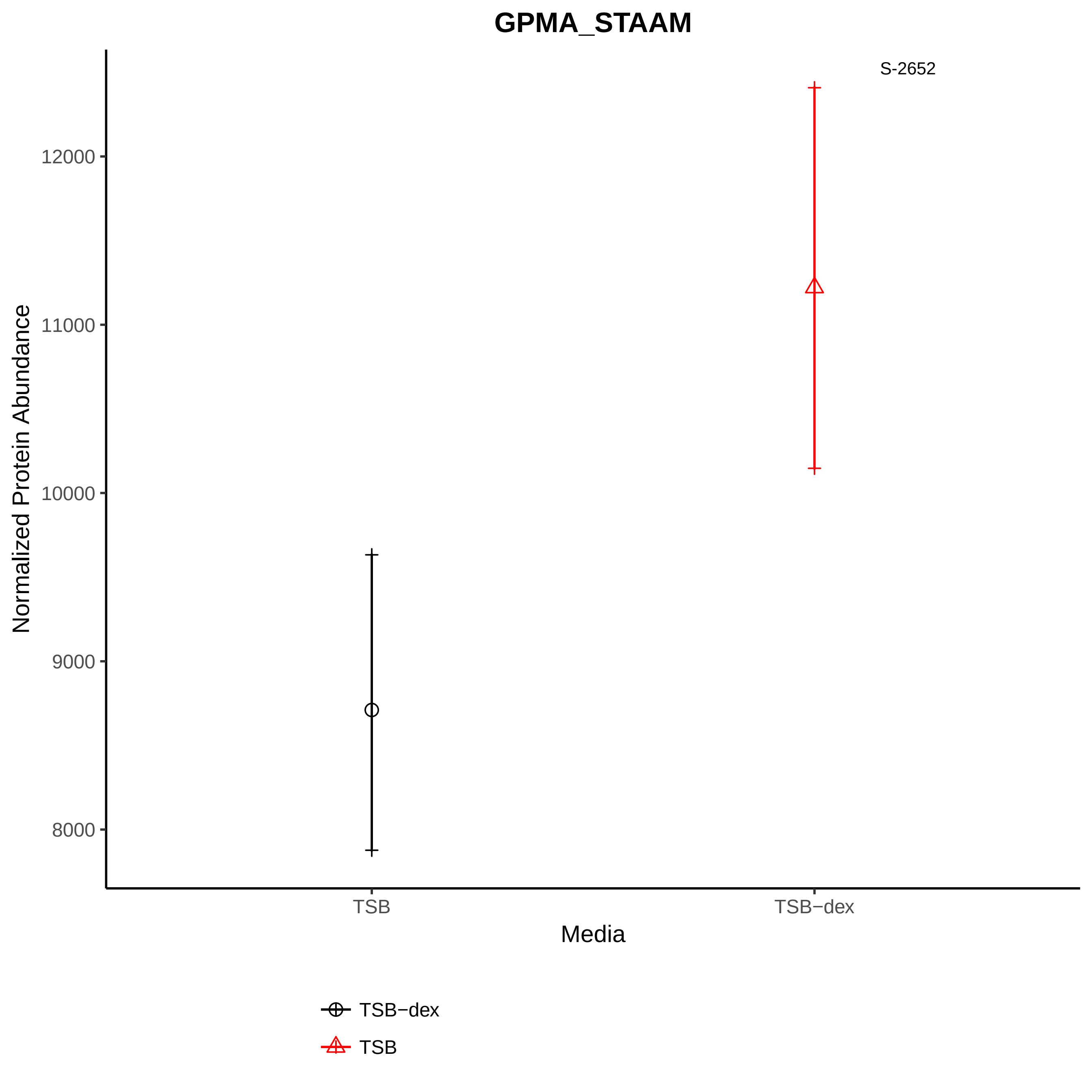

GPMA_STAAM
S-2652
12000
11000
10000
9000
8000
Normalized Protein Abundance
TSB
TSB−dex
Media
TSB−dex
TSB

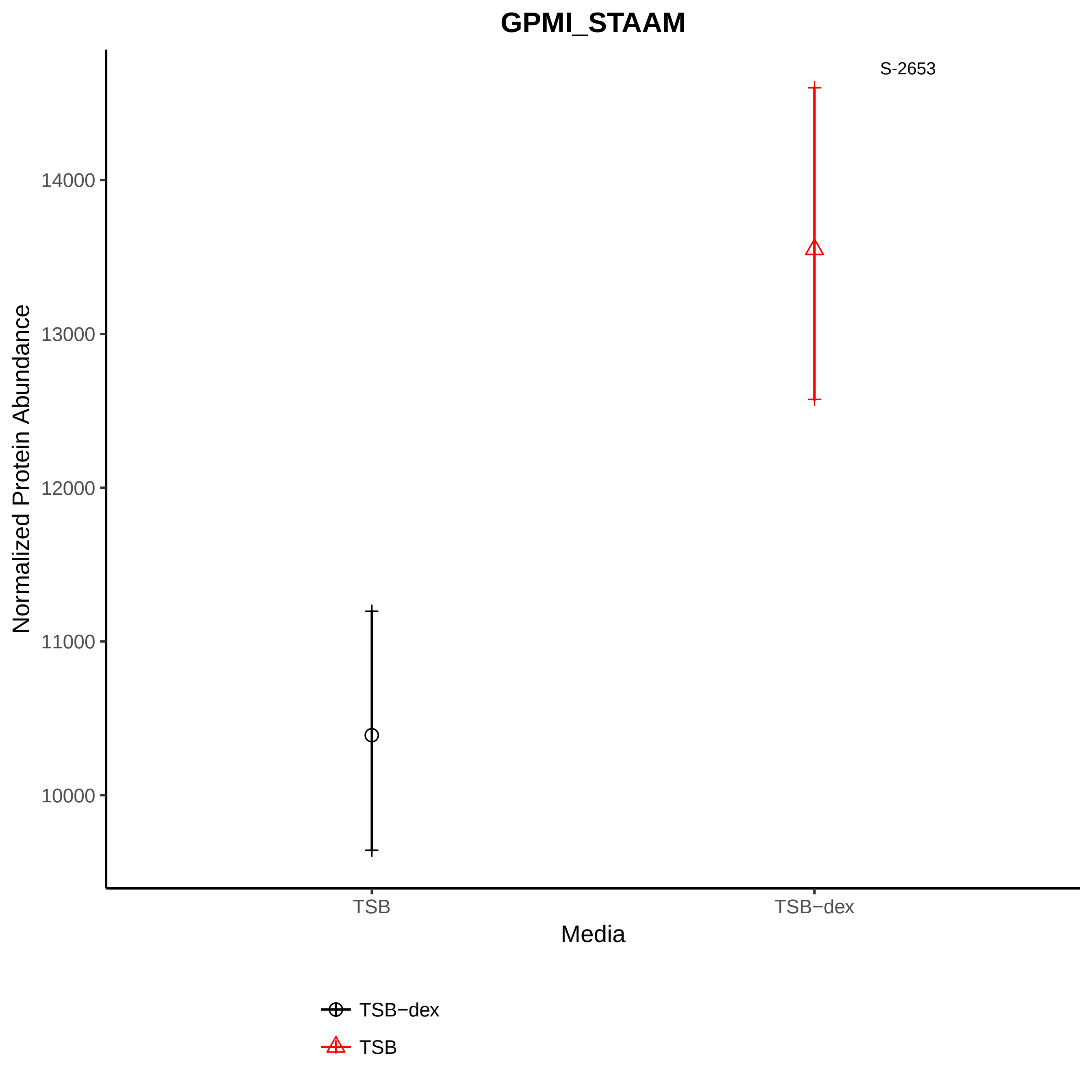

GPMI_STAAM
S-2653
Normalized Protein Abundance
14000
13000
12000
11000
10000
TSB
TSB−dex
Media
TSB−dex
TSB

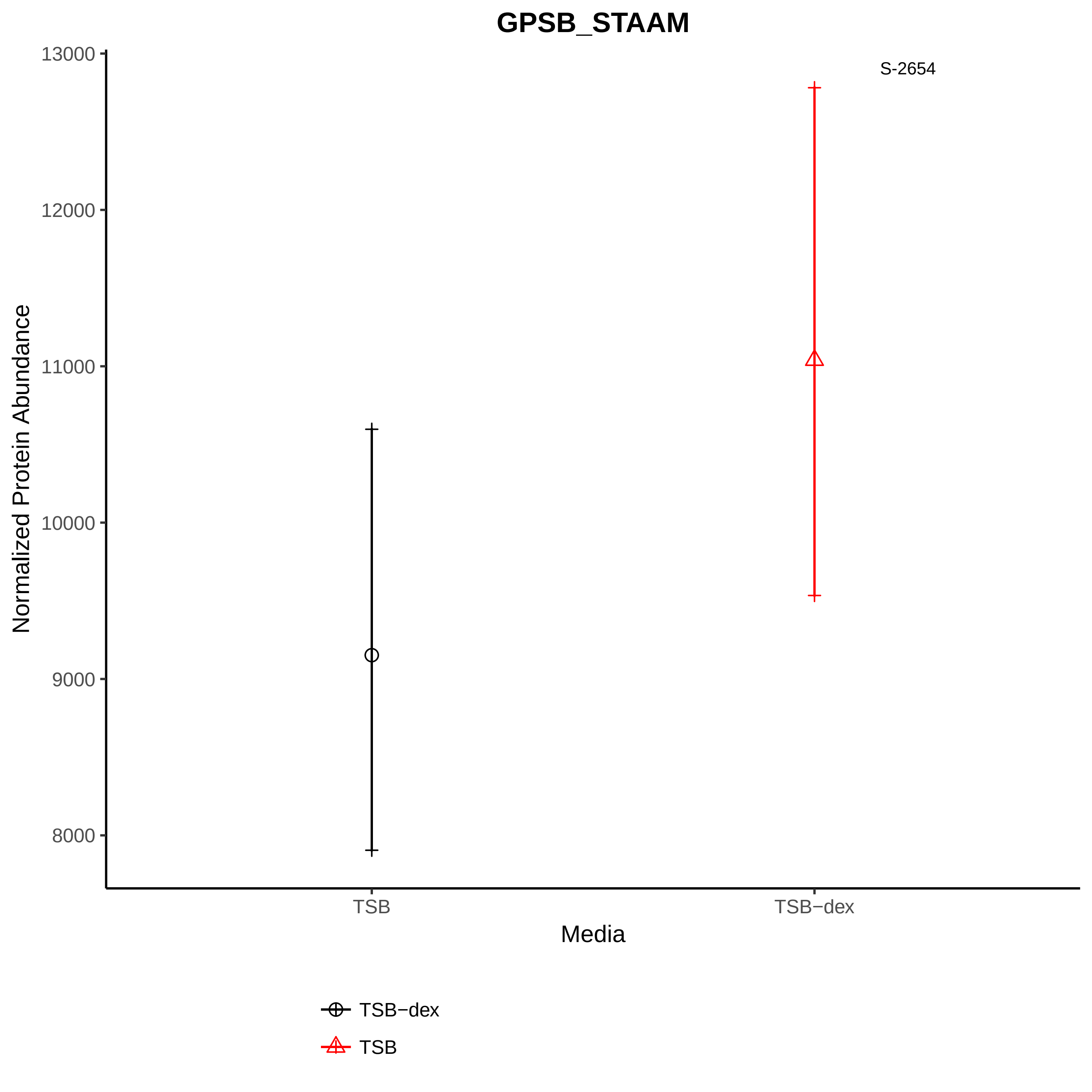
GPSB_STAAM
S-2654
13000
12000
11000
10000
9000
8000
Normalized Protein Abundance
TSB
TSB−dex
Media
TSB−dex
TSB

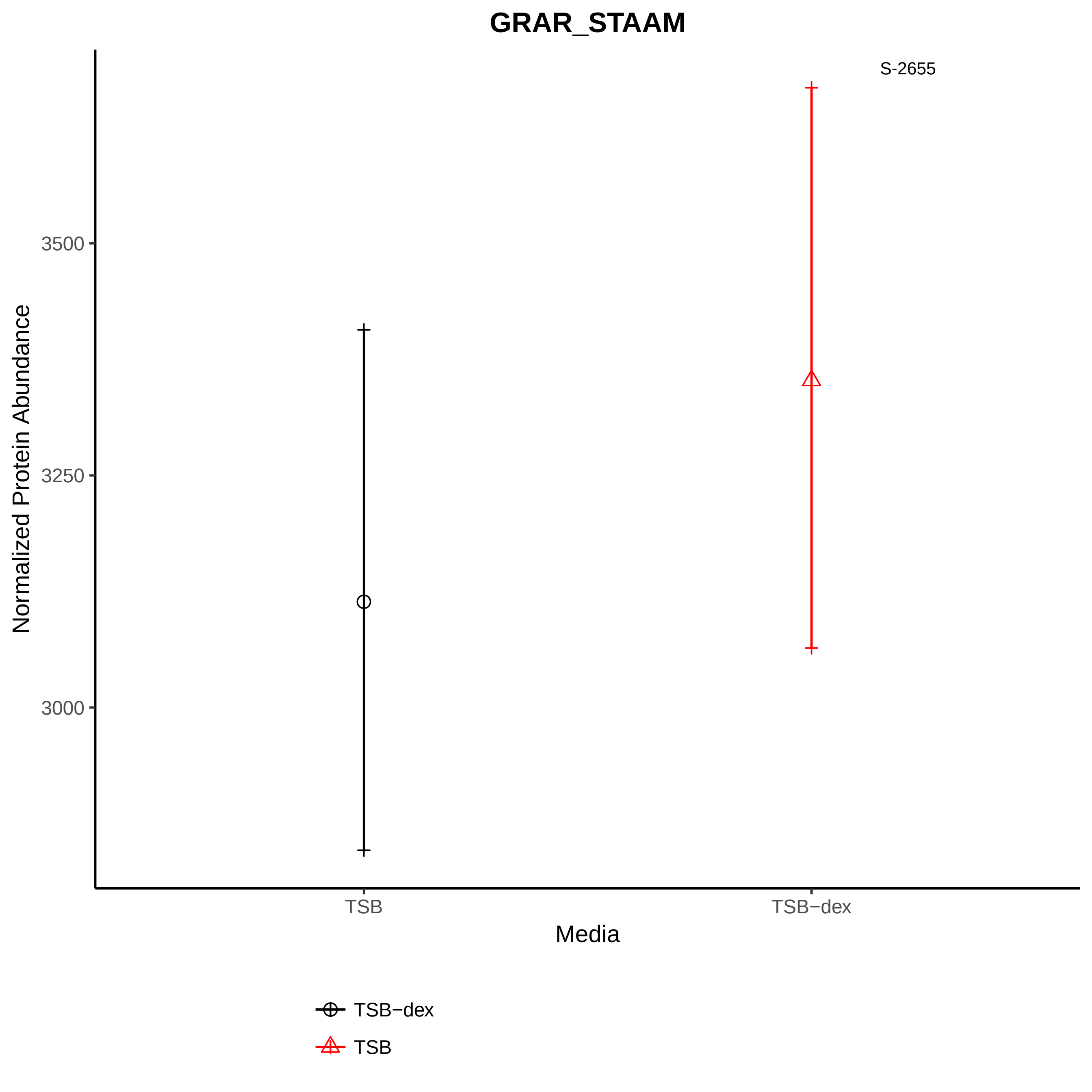

GRAR_STAAM
S-2655
Normalized Protein Abundance
3500
3250
3000
TSB
TSB−dex
Media
TSB−dex
TSB

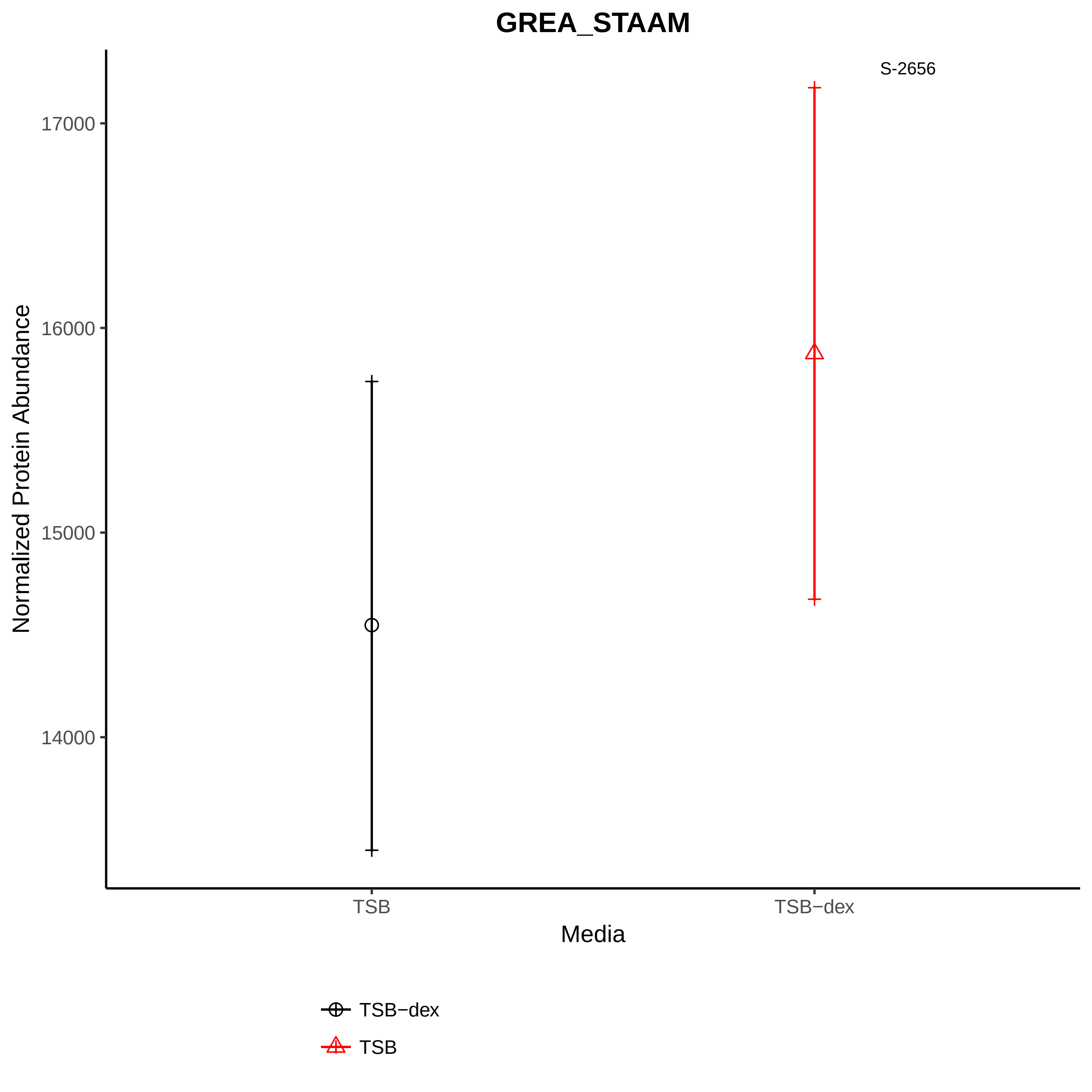

GREA_STAAM
S-2656
17000
16000
15000
14000
Normalized Protein Abundance
TSB
TSB−dex
Media
TSB−dex
TSB

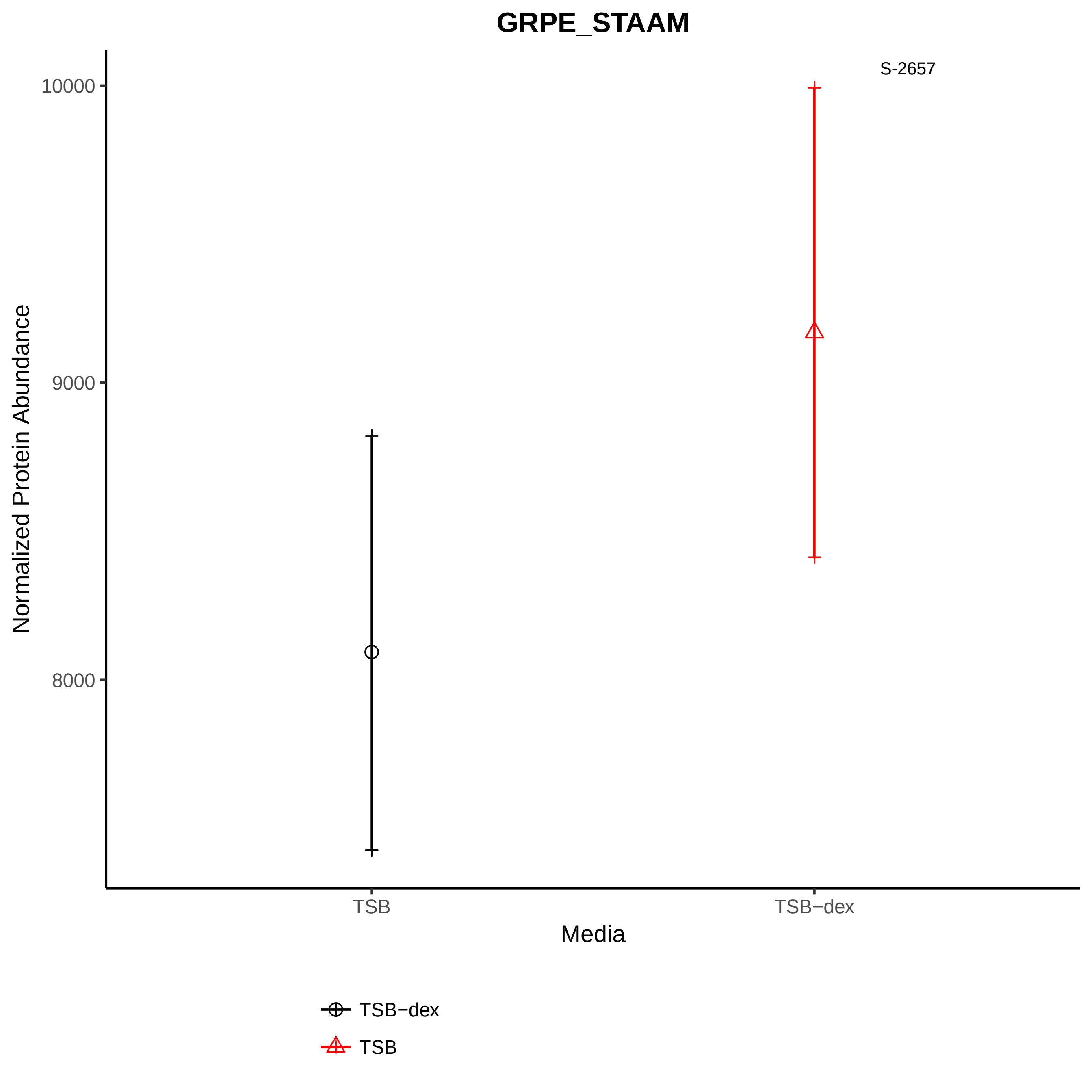

GRPE_STAAM
S-2657
10000
9000
8000
Normalized Protein Abundance
TSB
TSB−dex
Media
TSB−dex
TSB

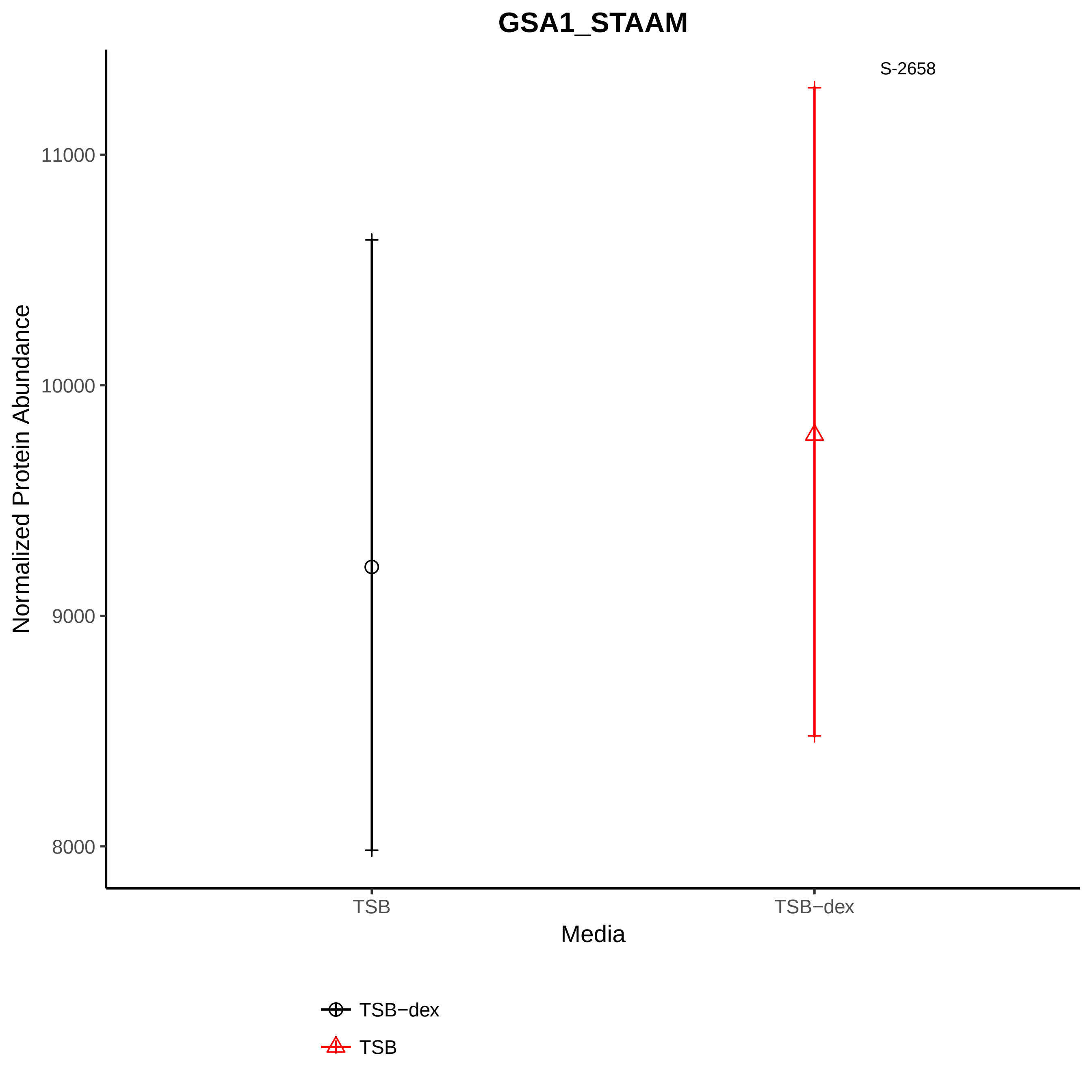
GSA1_STAAM
S-2658
Normalized Protein Abundance
11000
10000
9000
8000
TSB
TSB−dex
Media
TSB−dex
TSB

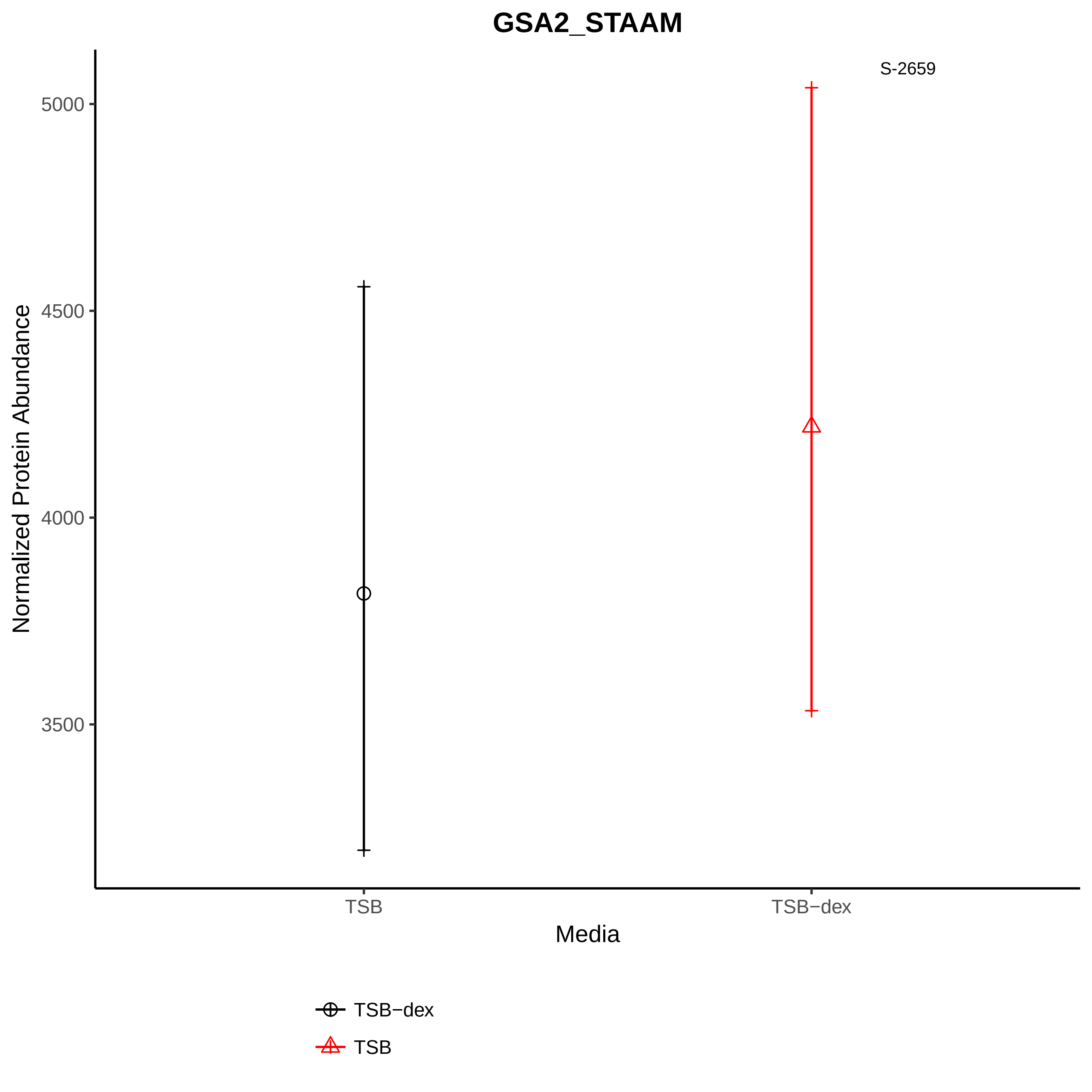

GSA2_STAAM
S-2659
5000
4500
4000
3500
Normalized Protein Abundance
TSB
TSB−dex
Media
TSB−dex
TSB

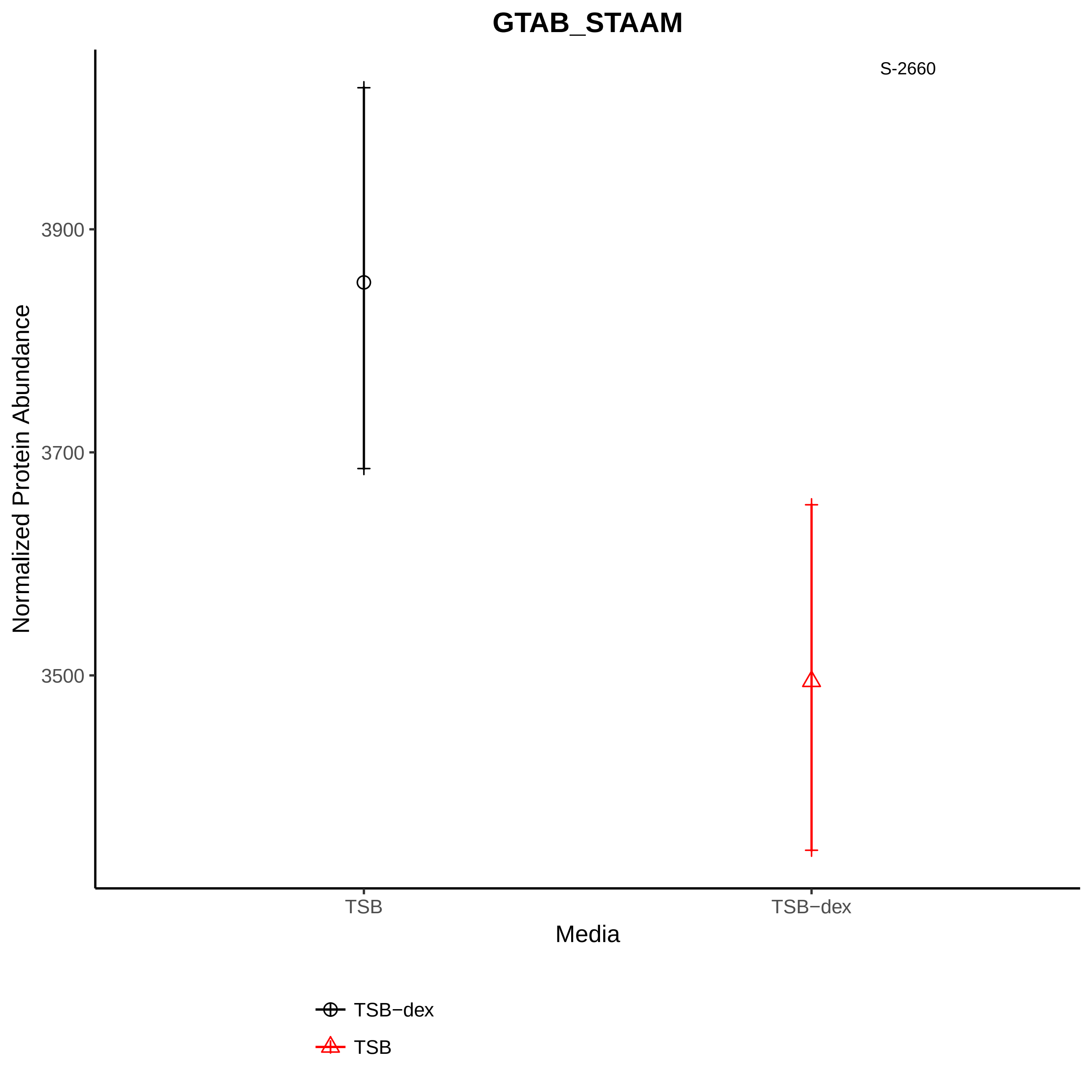

GTAB_STAAM
S-2660
Normalized Protein Abundance
3900
3700
3500
TSB
TSB−dex
Media
TSB−dex
TSB

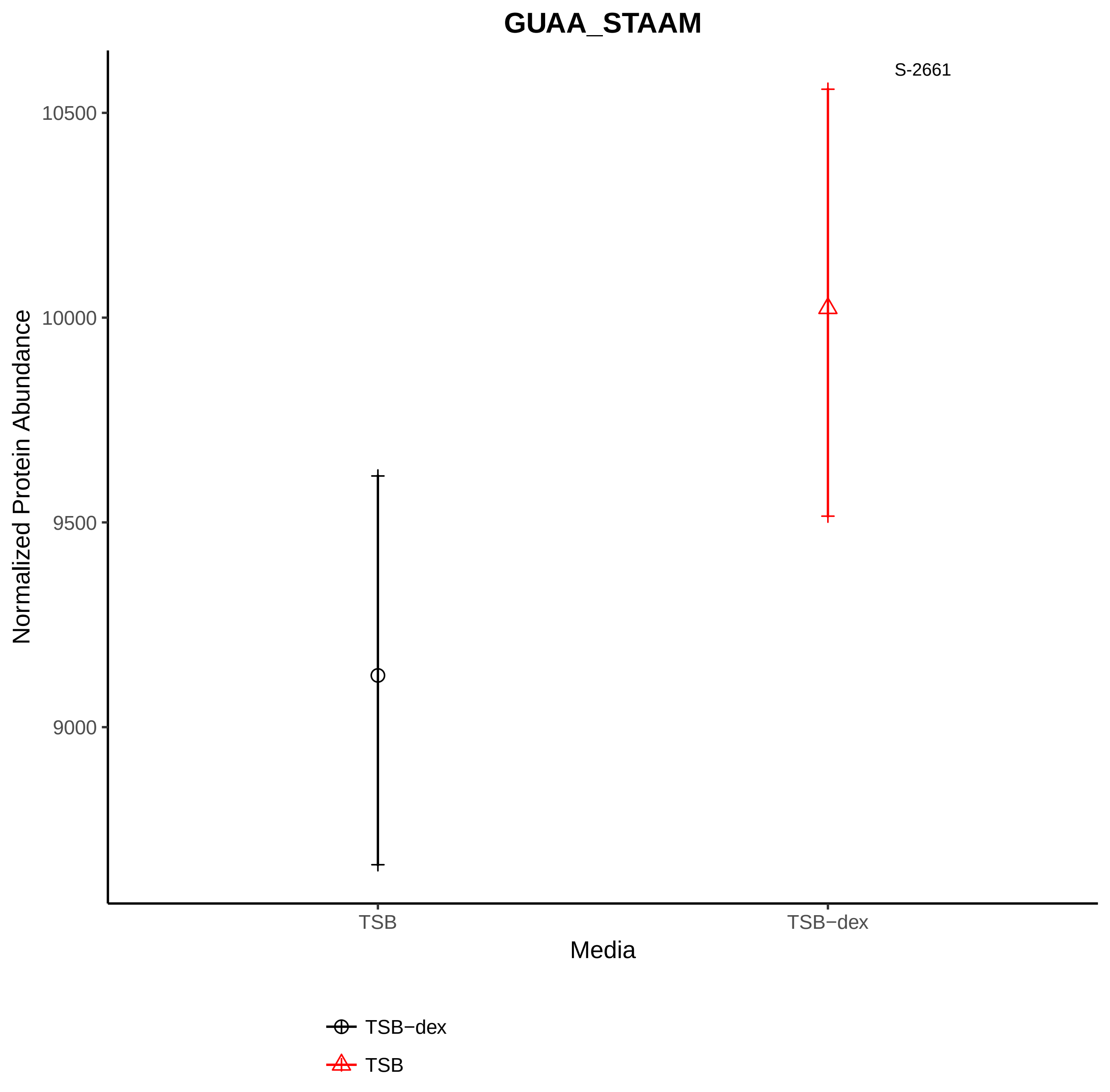
GUAA_STAAM
S-2661
10500
10000
9500
9000
Normalized Protein Abundance
TSB
TSB−dex
Media
TSB−dex
TSB

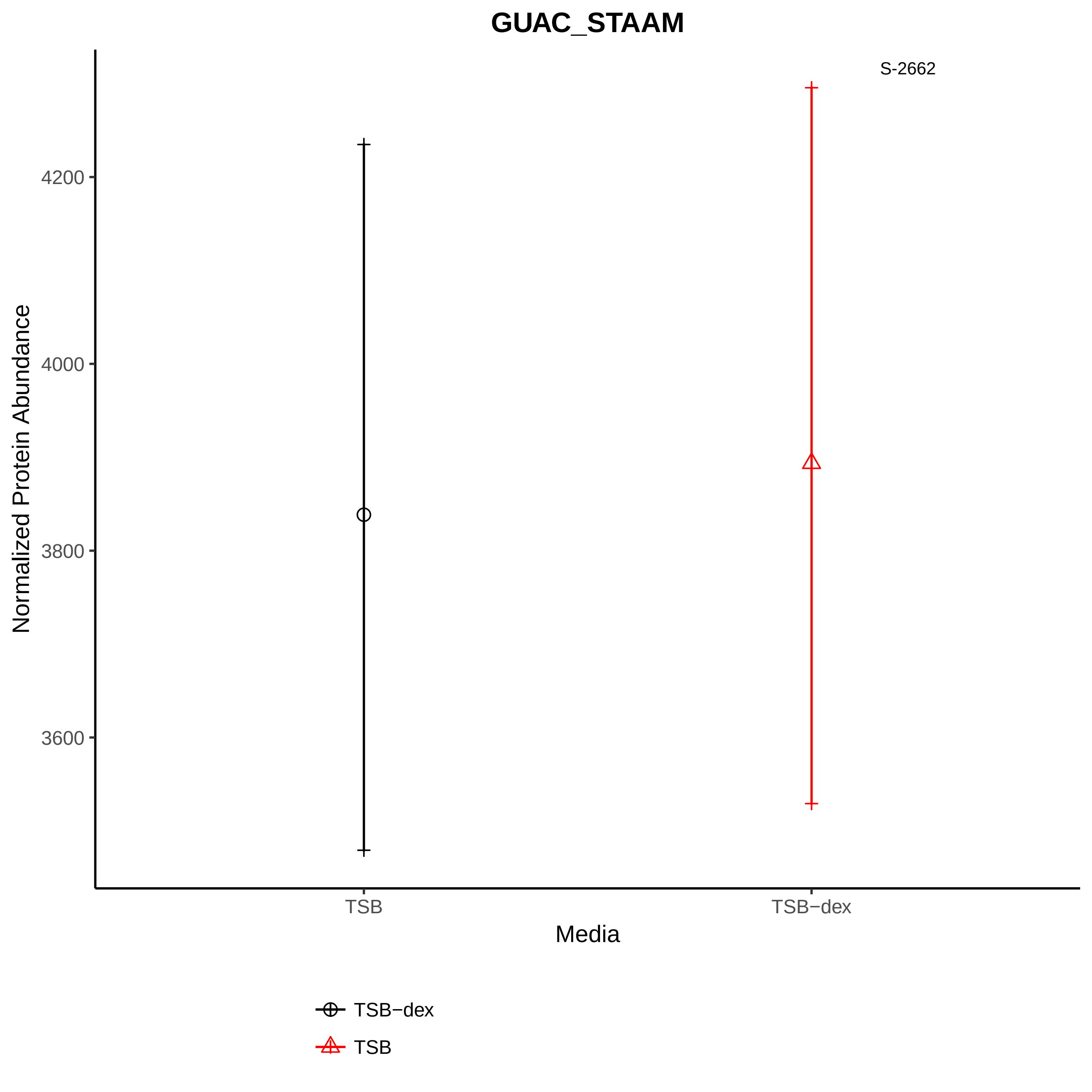
GUAC_STAAM
S-2662
Normalized Protein Abundance
4200
4000
3800
3600
TSB
TSB−dex
Media
TSB−dex
TSB

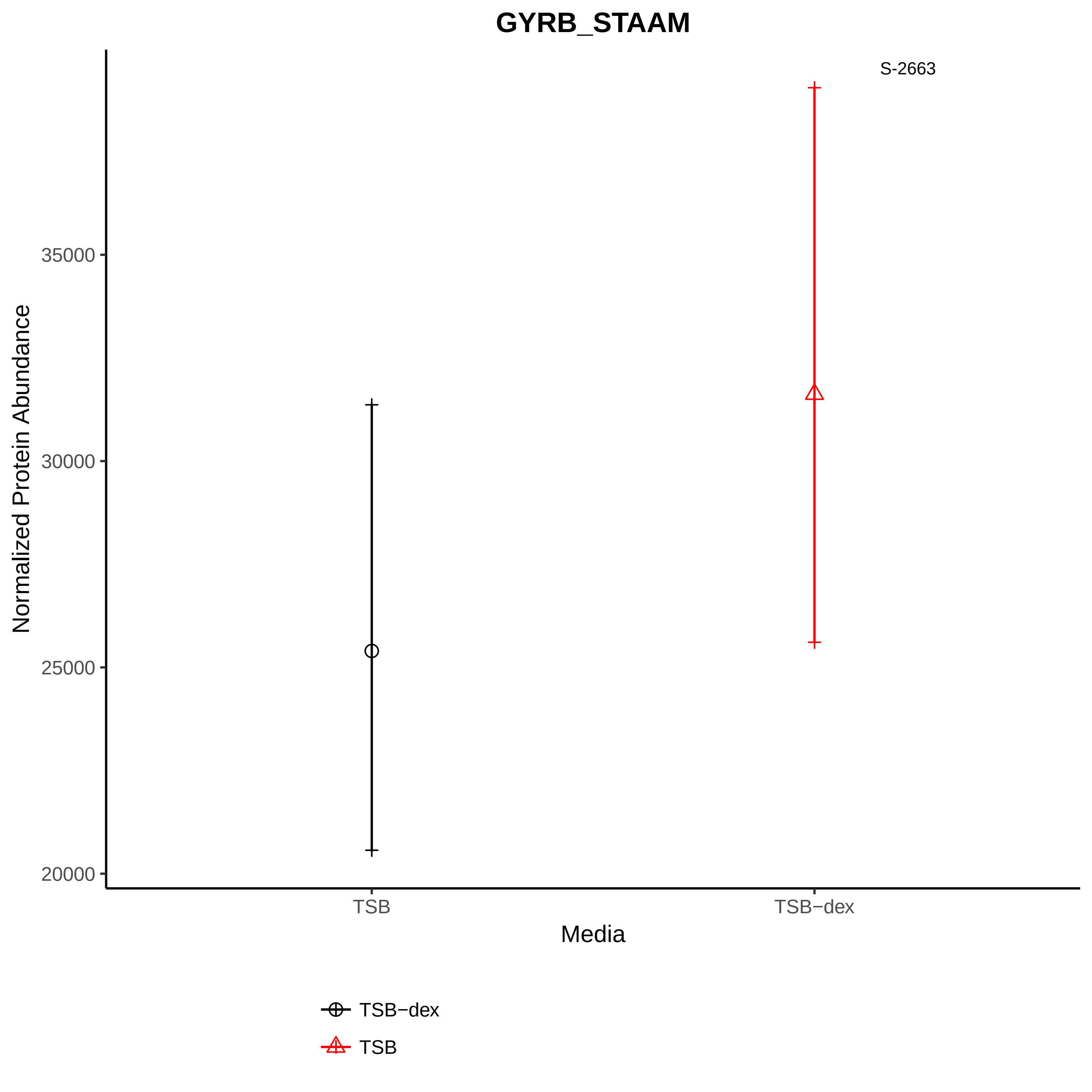
GYRB_STAAM
S-2663
Normalized Protein Abundance
35000
30000
25000
20000
TSB
TSB−dex
Media
TSB−dex
TSB

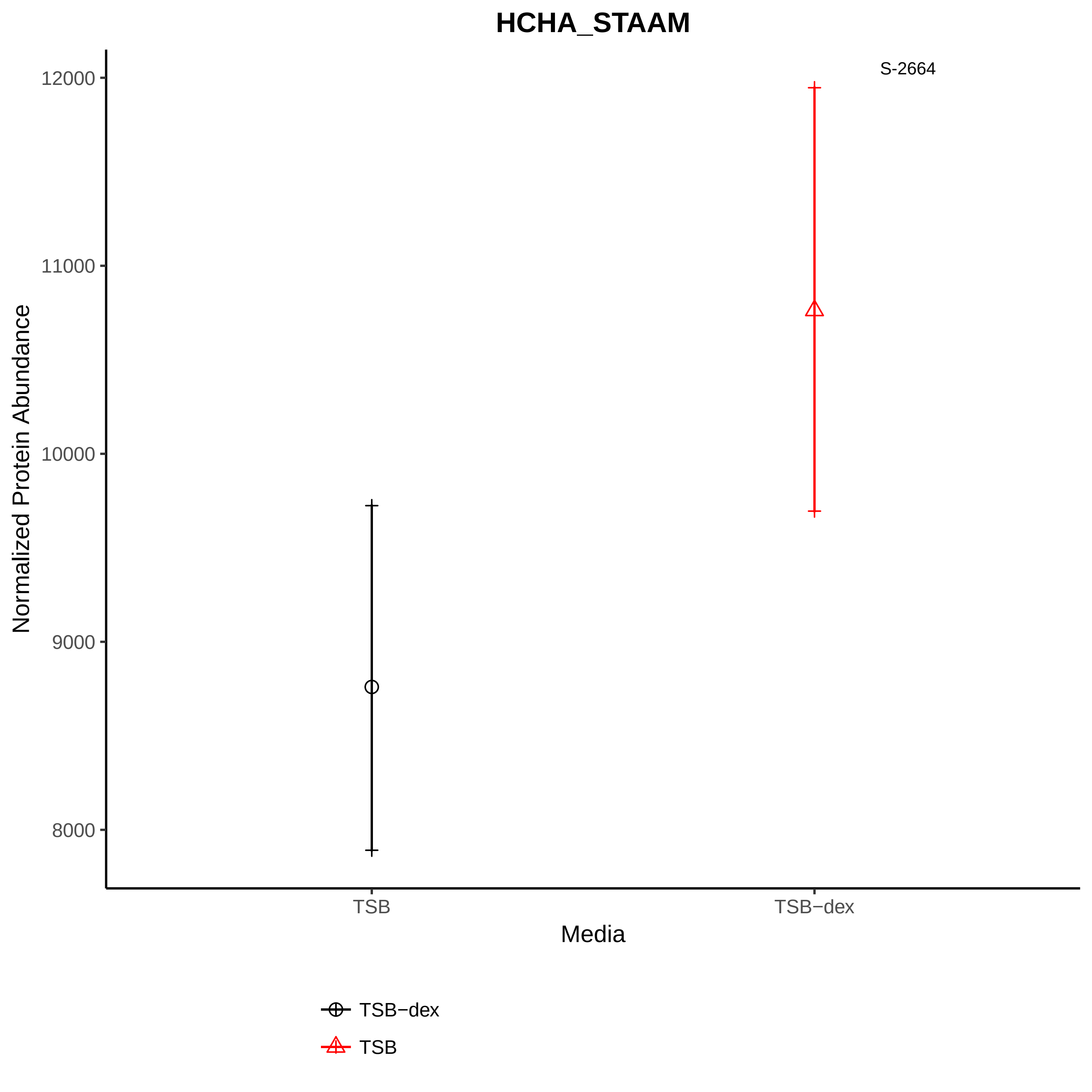

HCHA_STAAM
S-2664
Normalized Protein Abundance
12000
11000
10000
9000
8000
TSB
TSB−dex
Media
TSB−dex
TSB

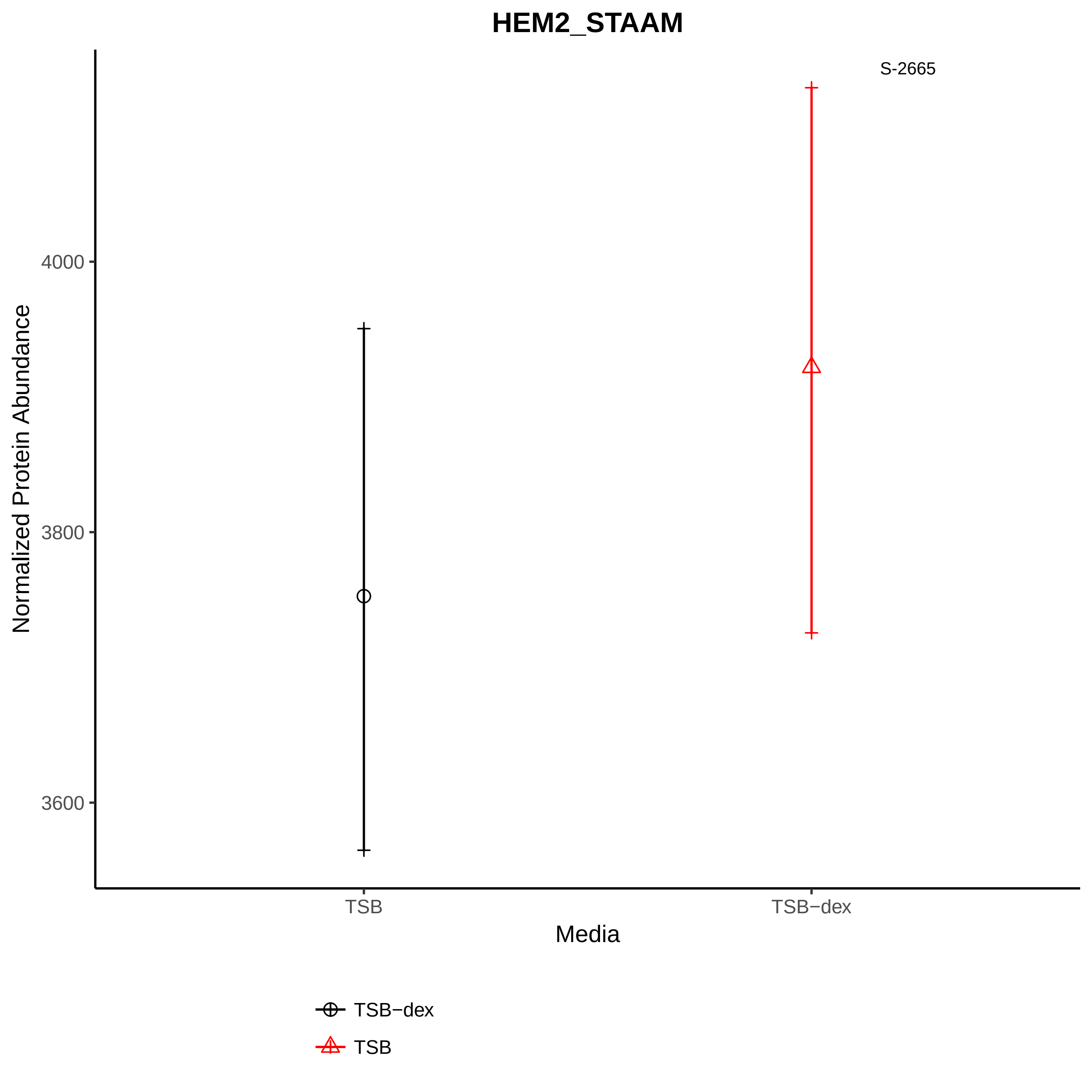
HEM2_STAAM
S-2665
Normalized Protein Abundance
4000
3800
3600
TSB
TSB−dex
Media
TSB−dex
TSB

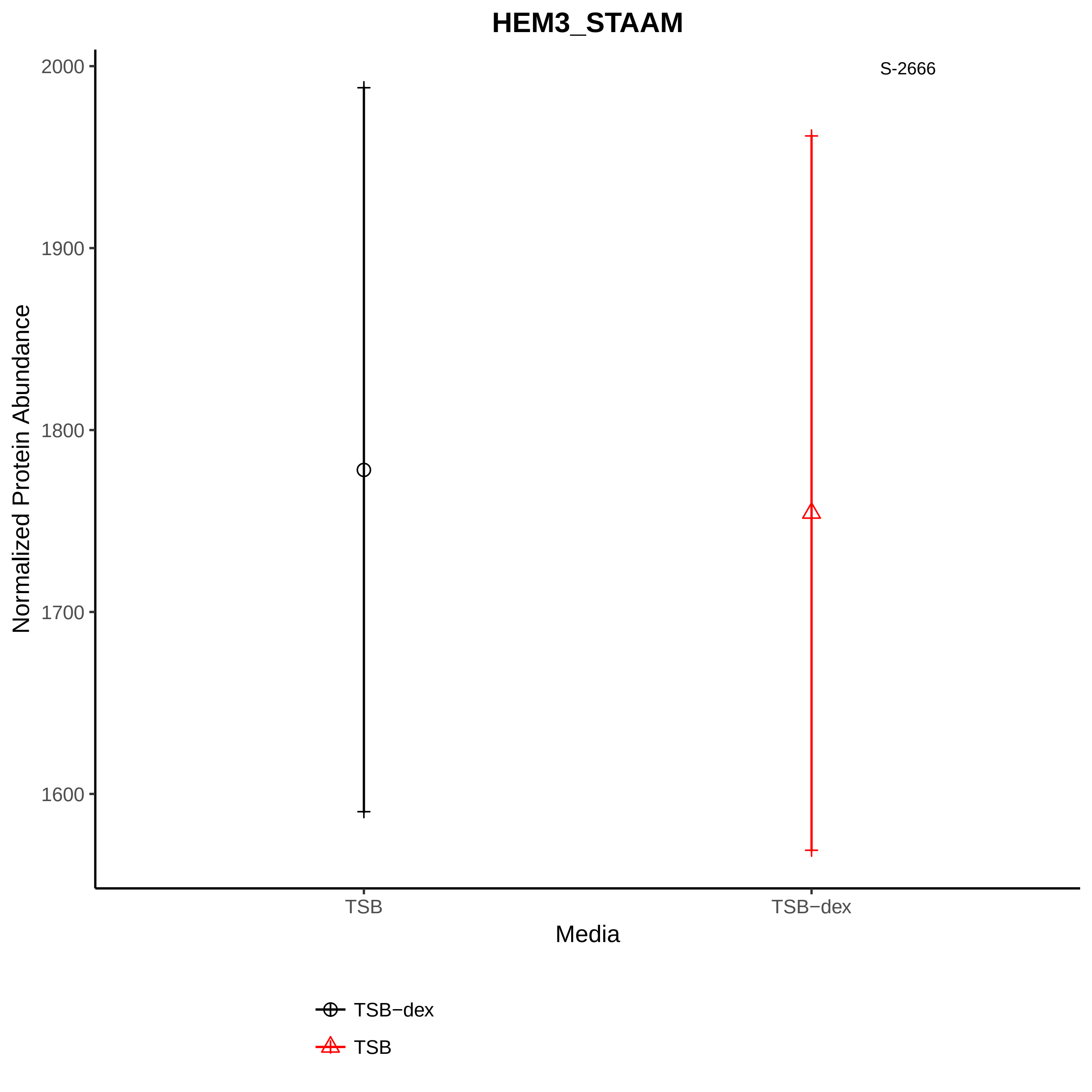
HEM3_STAAM
S-2666
2000
1900
1800
1700
1600
Normalized Protein Abundance
TSB
TSB−dex
Media
TSB−dex
TSB

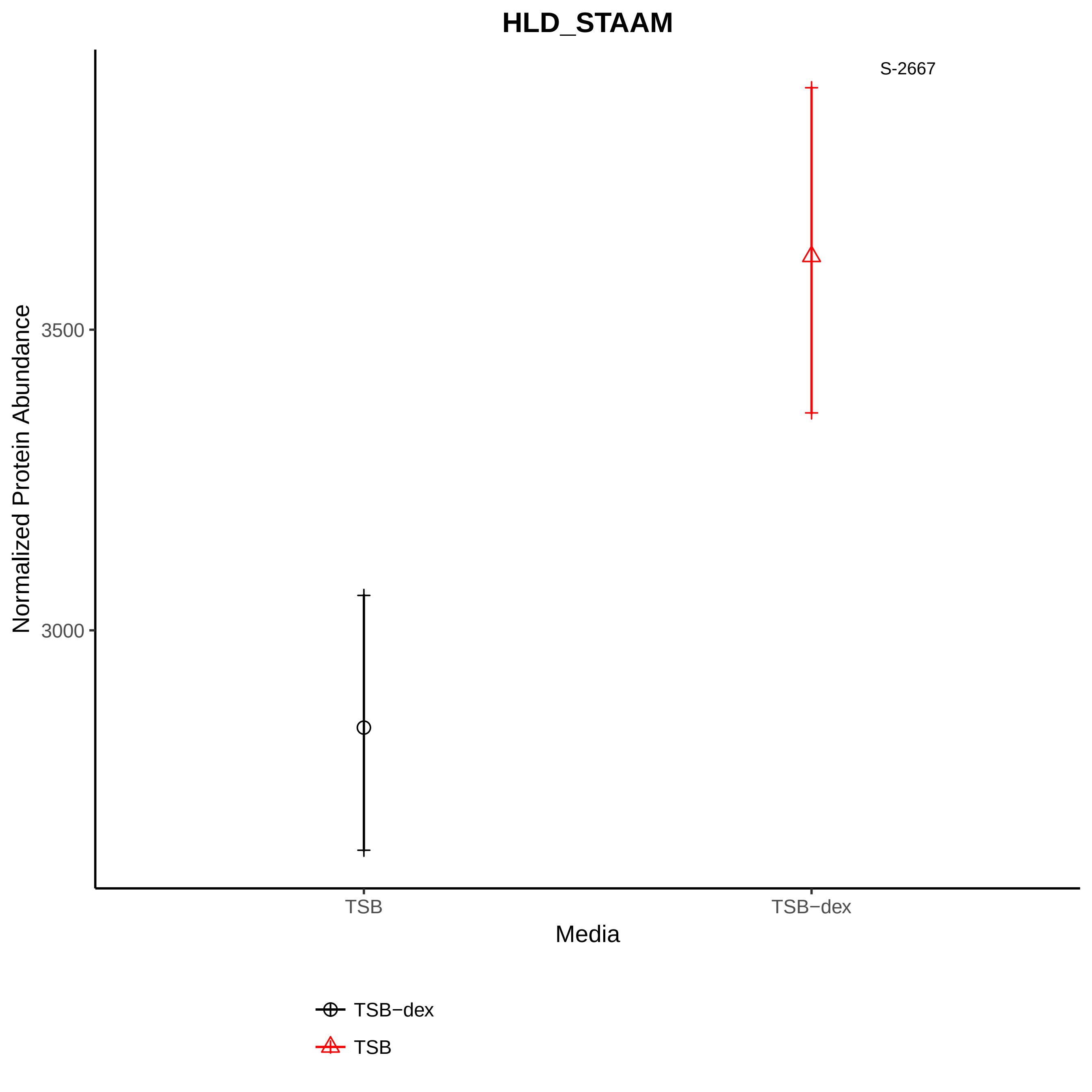
HLD_STAAM
S-2667
Normalized Protein Abundance
3500
3000
TSB
TSB−dex
Media
TSB−dex
TSB

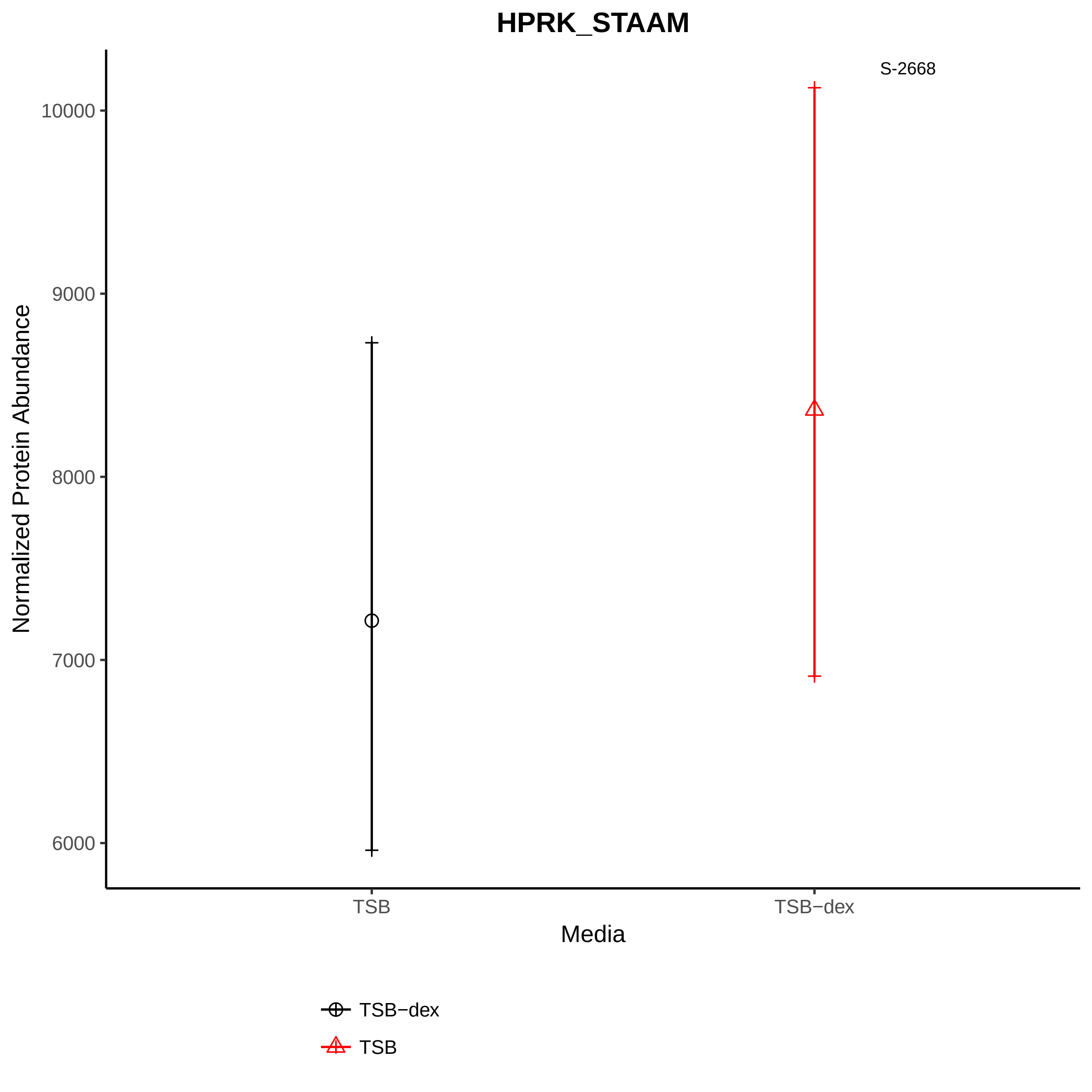

HPRK_STAAM
S-2668
Normalized Protein Abundance
10000
9000
8000
7000
6000
TSB
TSB−dex
Media
TSB−dex
TSB

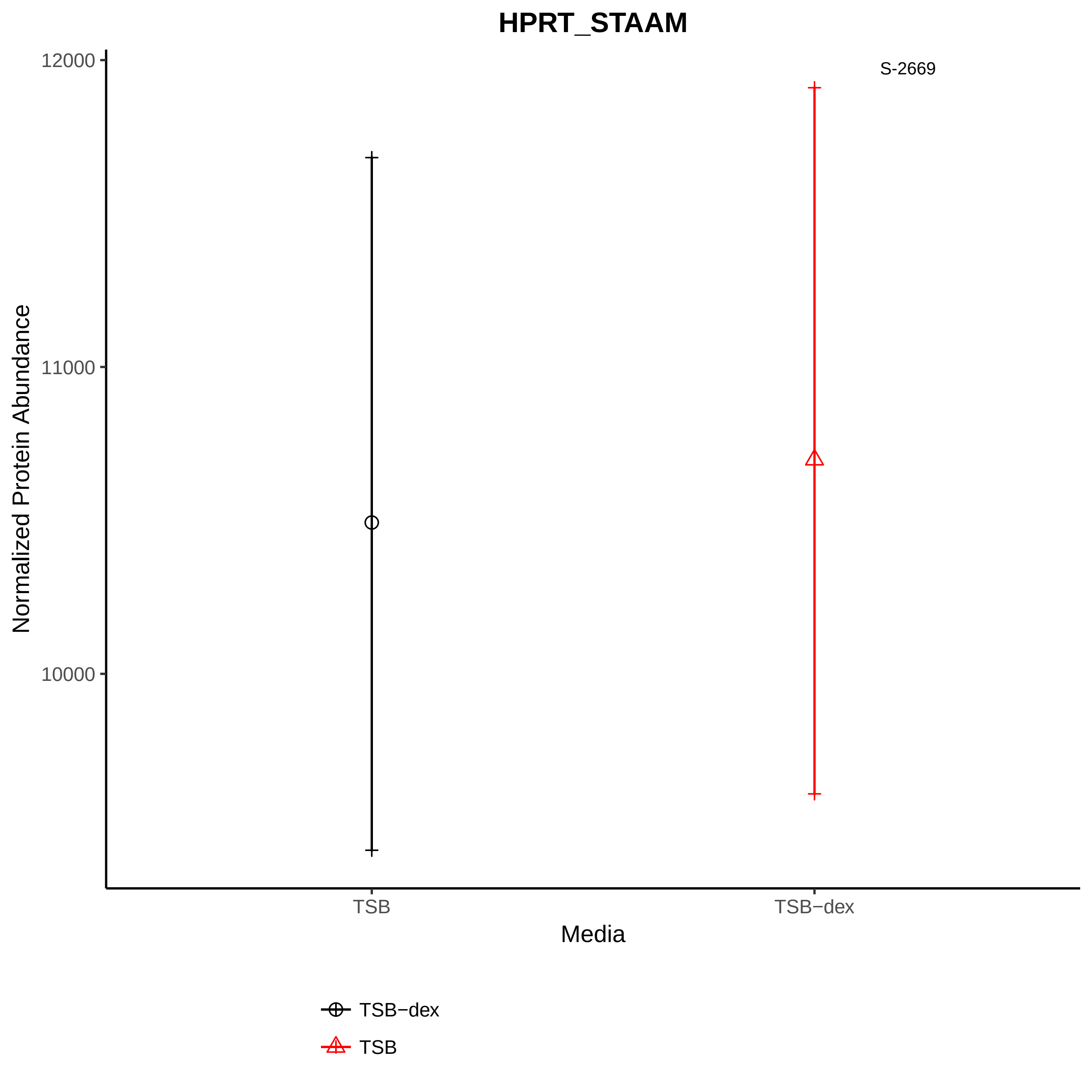

HPRT_STAAM
S-2669
12000
11000
10000
Normalized Protein Abundance
TSB
TSB−dex
Media
TSB−dex
TSB

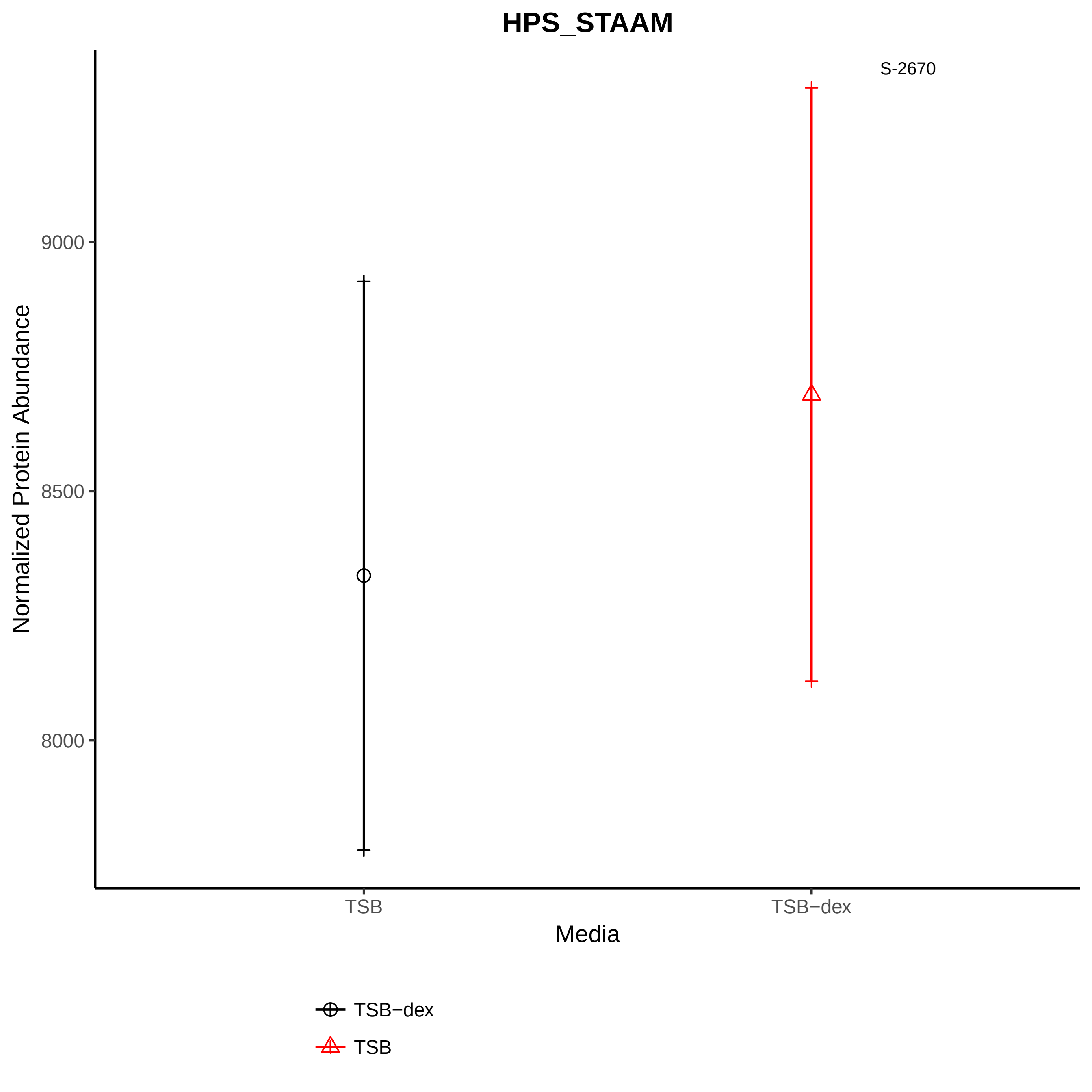

HPS_STAAM
S-2670
9000
8500
8000
Normalized Protein Abundance
TSB
TSB−dex
Media
TSB−dex
TSB

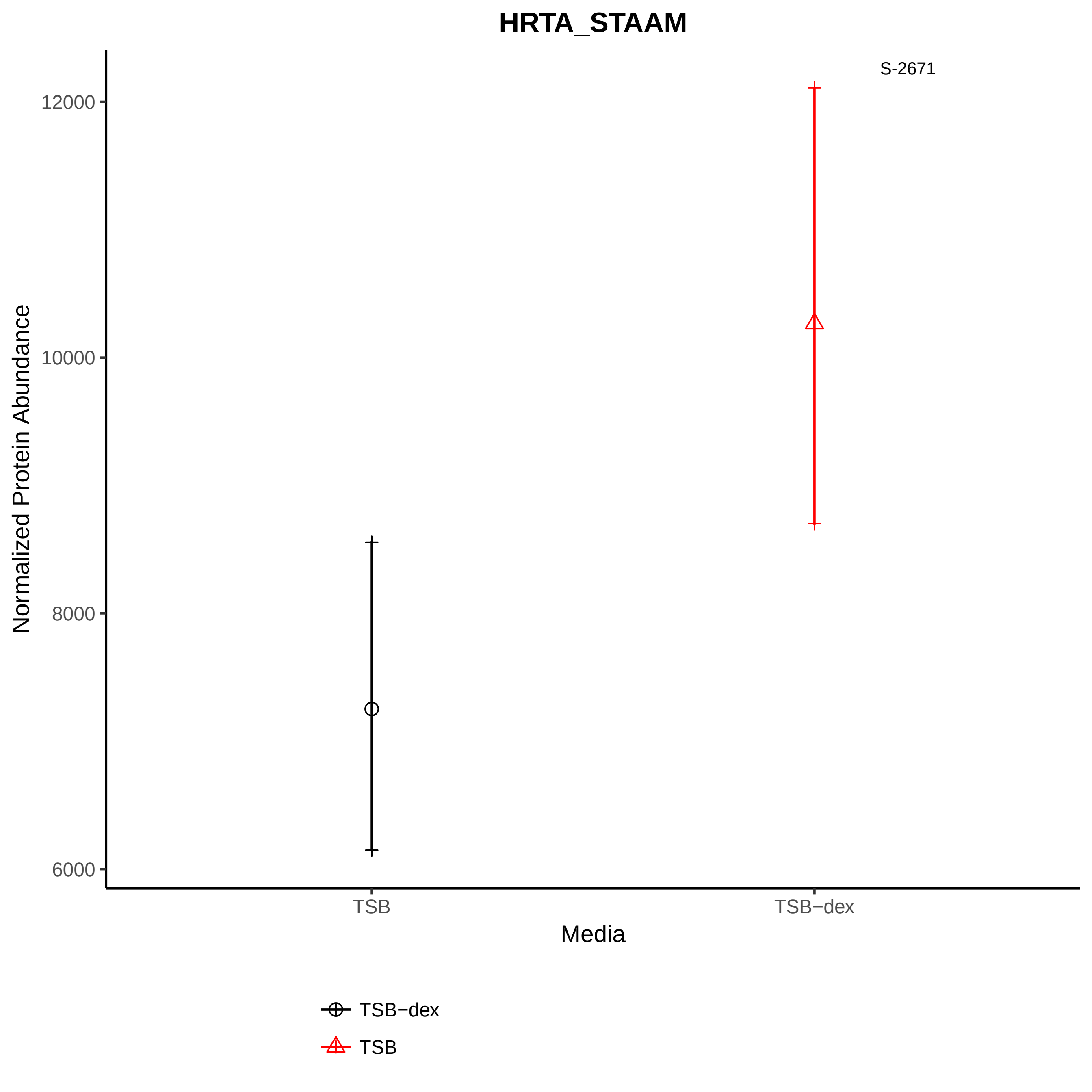
HRTA_STAAM
S-2671
Normalized Protein Abundance
12000
10000
8000
6000
TSB
TSB−dex
Media
TSB−dex
TSB

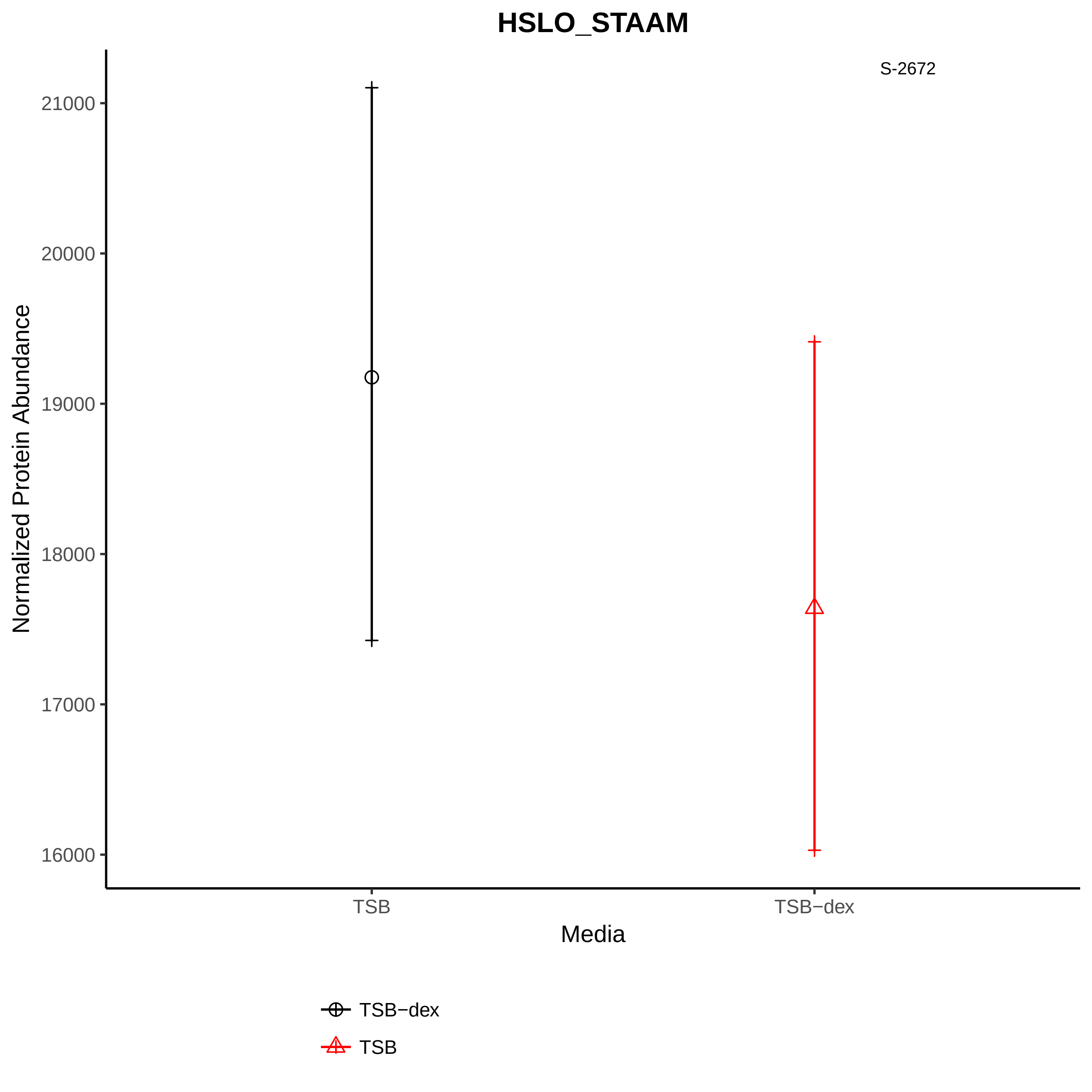

HSLO_STAAM
S-2672
Normalized Protein Abundance
21000
20000
19000
18000
17000
16000
TSB
TSB−dex
Media
TSB−dex
TSB

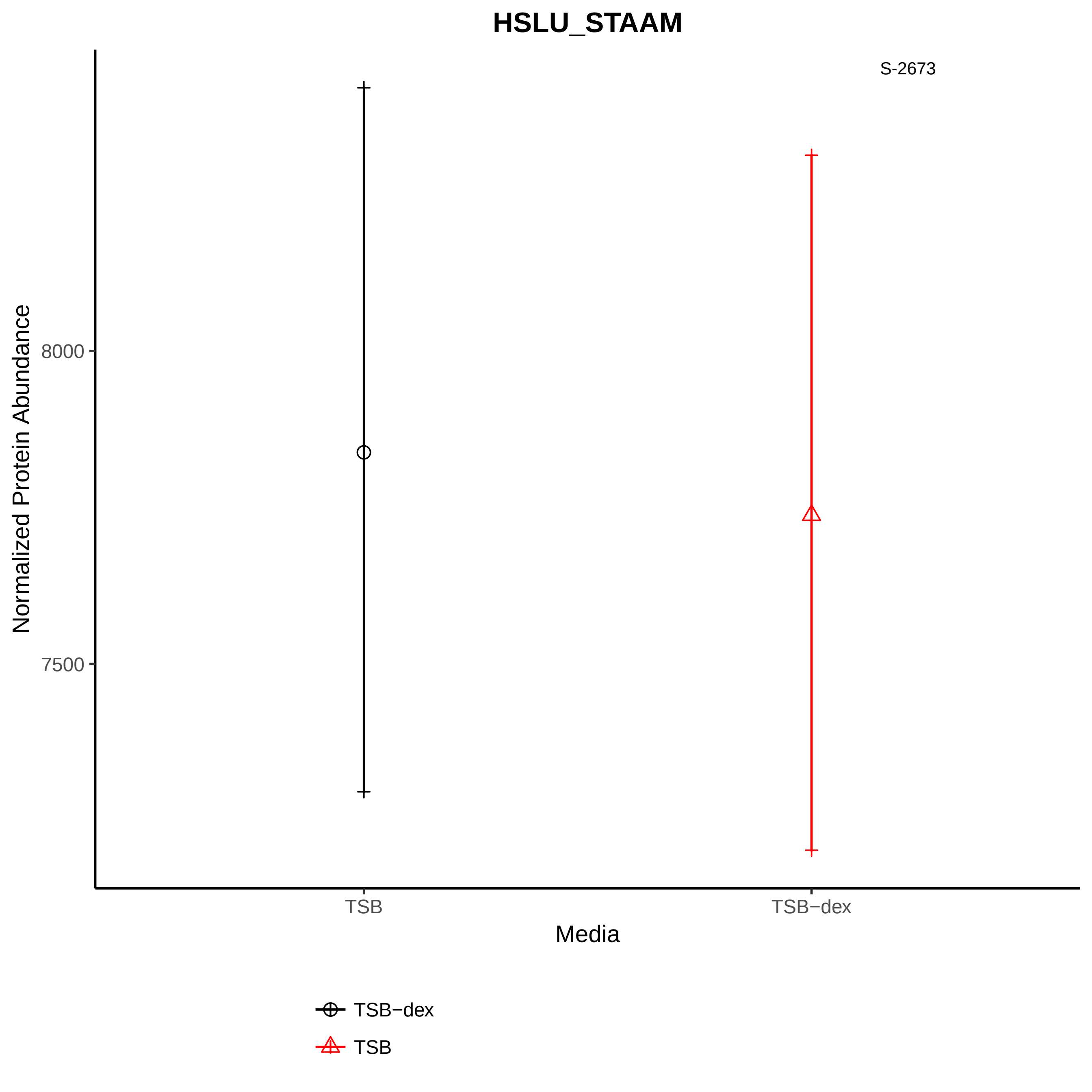

HSLU_STAAM
S-2673
Normalized Protein Abundance
8000
7500
TSB
TSB−dex
Media
TSB−dex
TSB

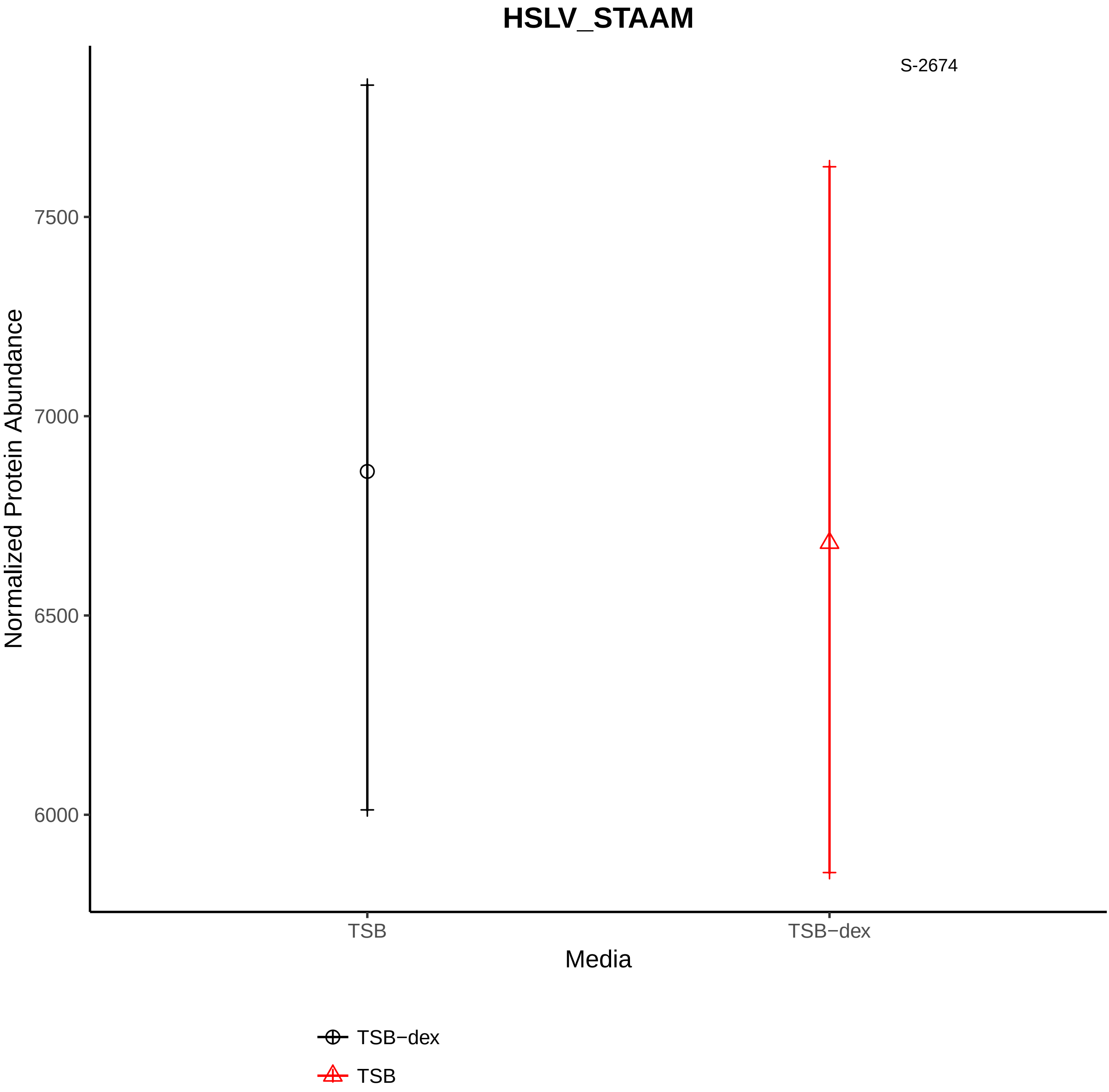
HSLV_STAAM
S-2674
Normalized Protein Abundance
7500
7000
6500
6000
TSB
TSB−dex
Media
TSB−dex
TSB

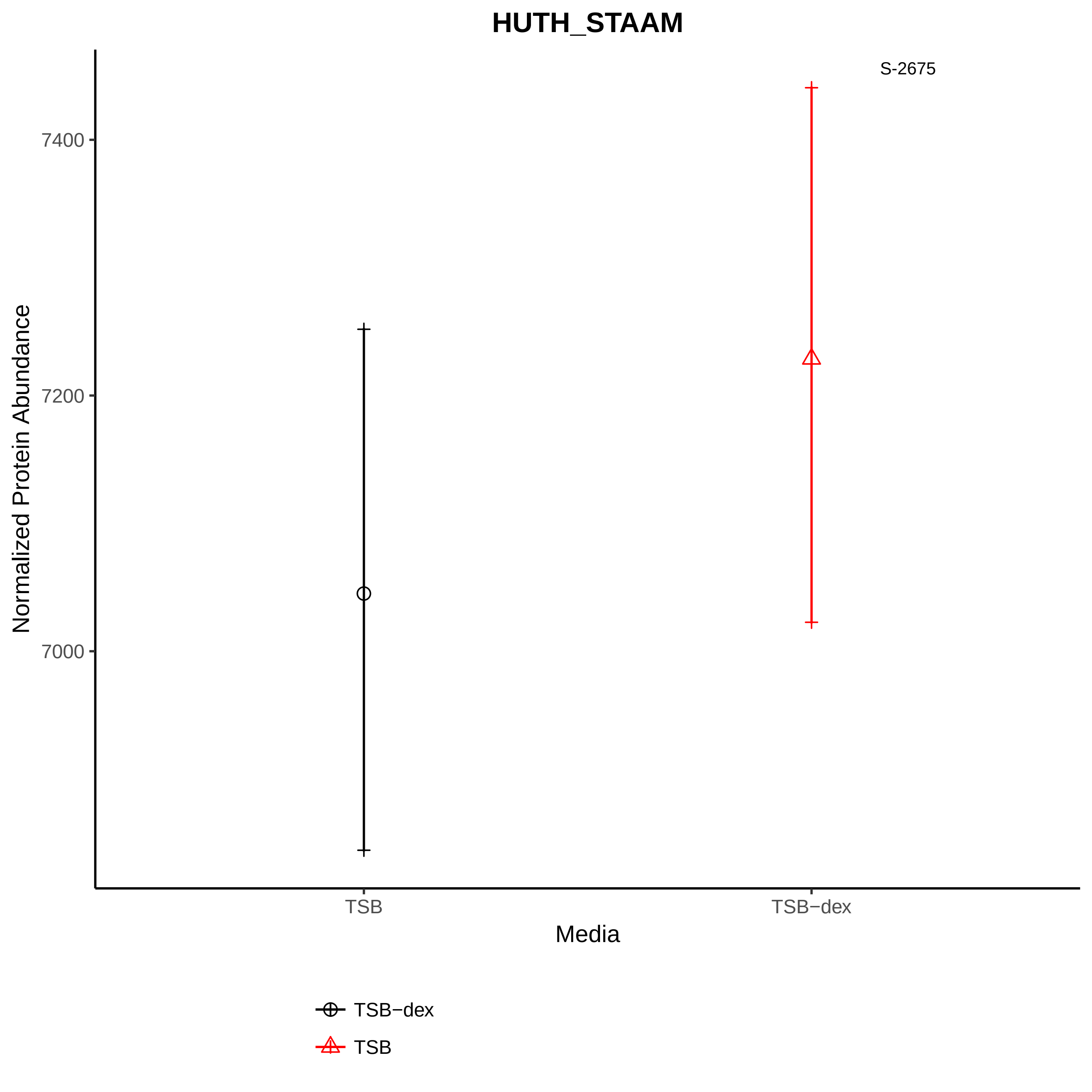

HUTH_STAAM
S-2675
7400
7200
7000
Normalized Protein Abundance
TSB
TSB−dex
Media
TSB−dex
TSB

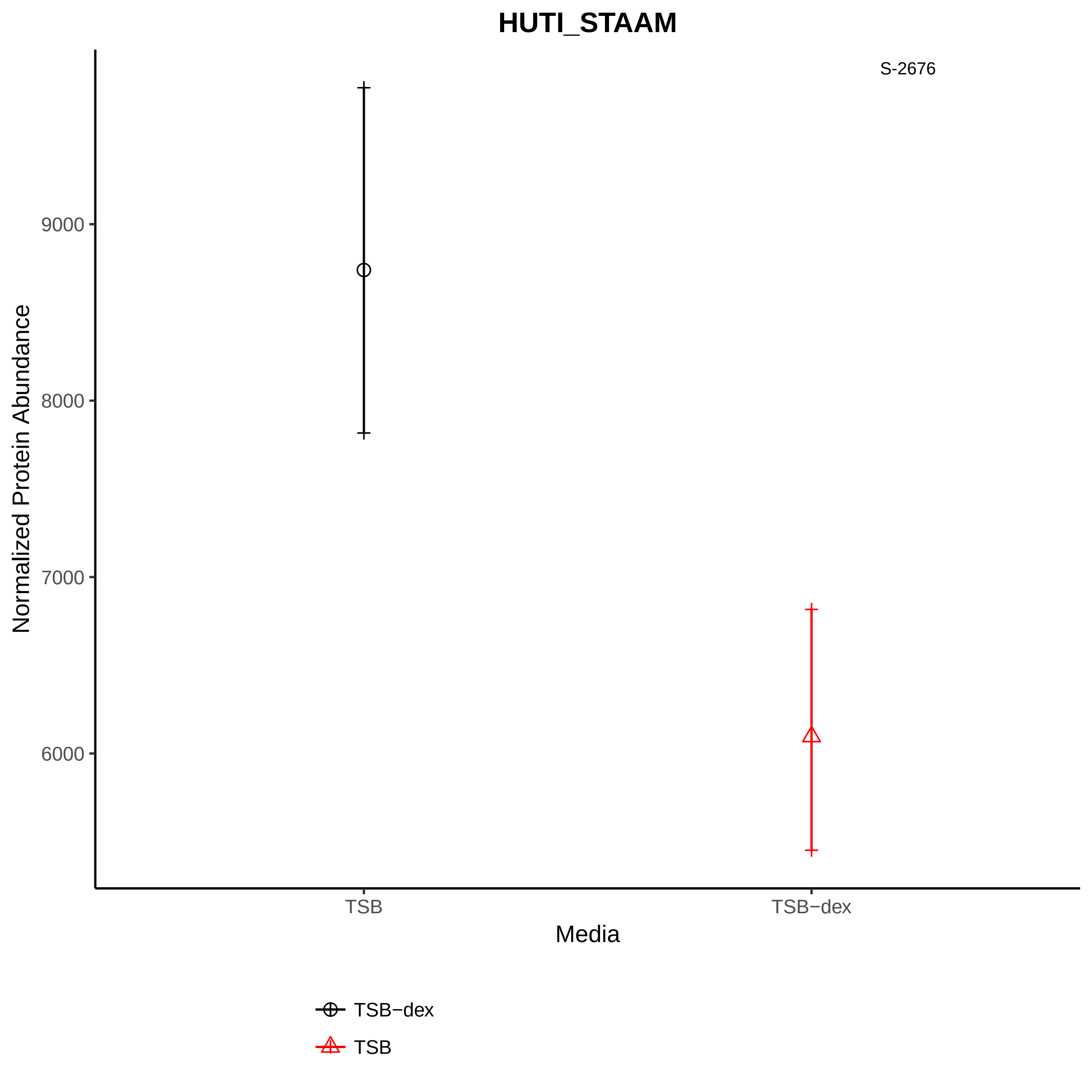

HUTI_STAAM
S-2676
9000
8000
7000
6000
Normalized Protein Abundance
TSB
TSB−dex
Media
TSB−dex
TSB

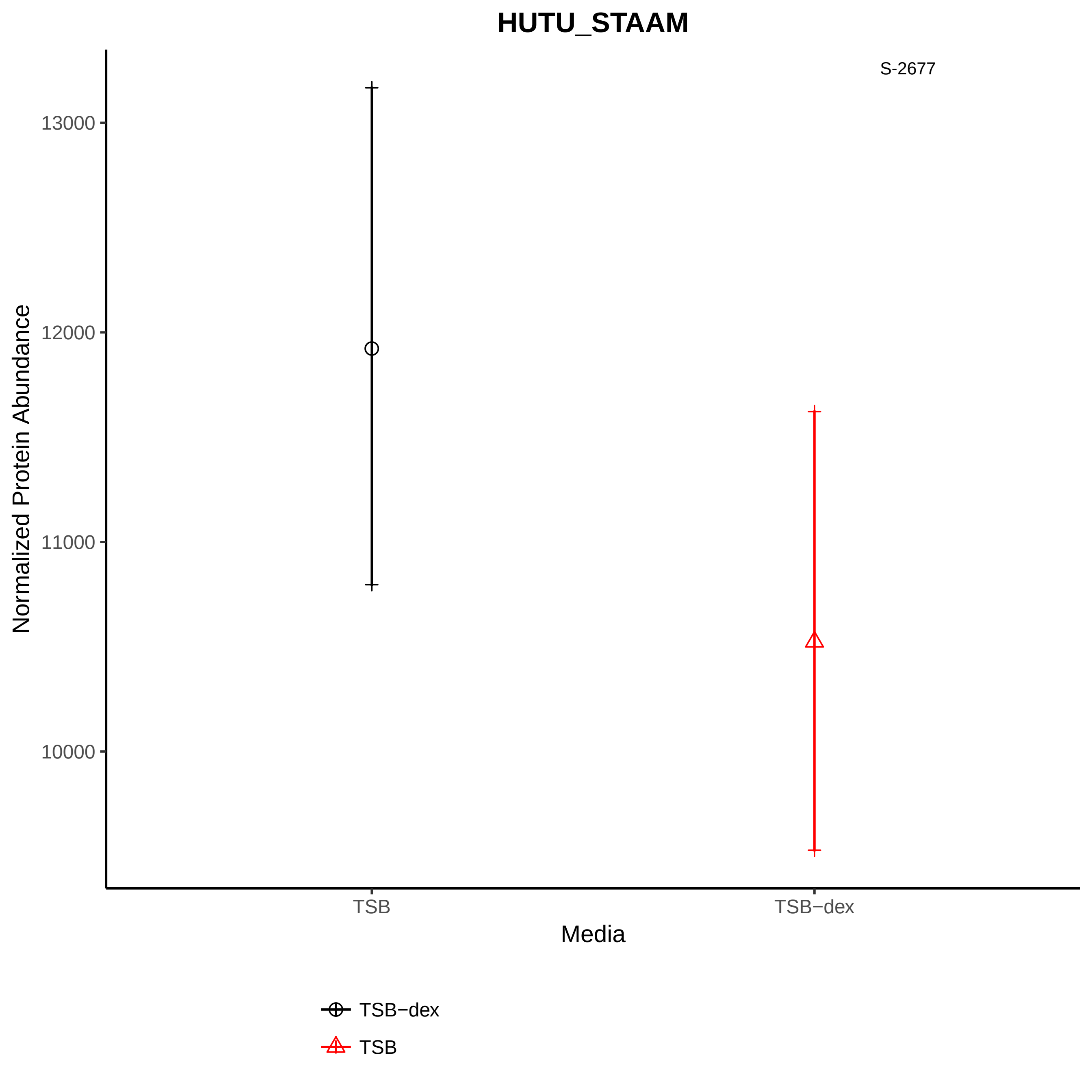

HUTU_STAAM
S-2677
13000
12000
11000
10000
Normalized Protein Abundance
TSB
TSB−dex
Media
TSB−dex
TSB

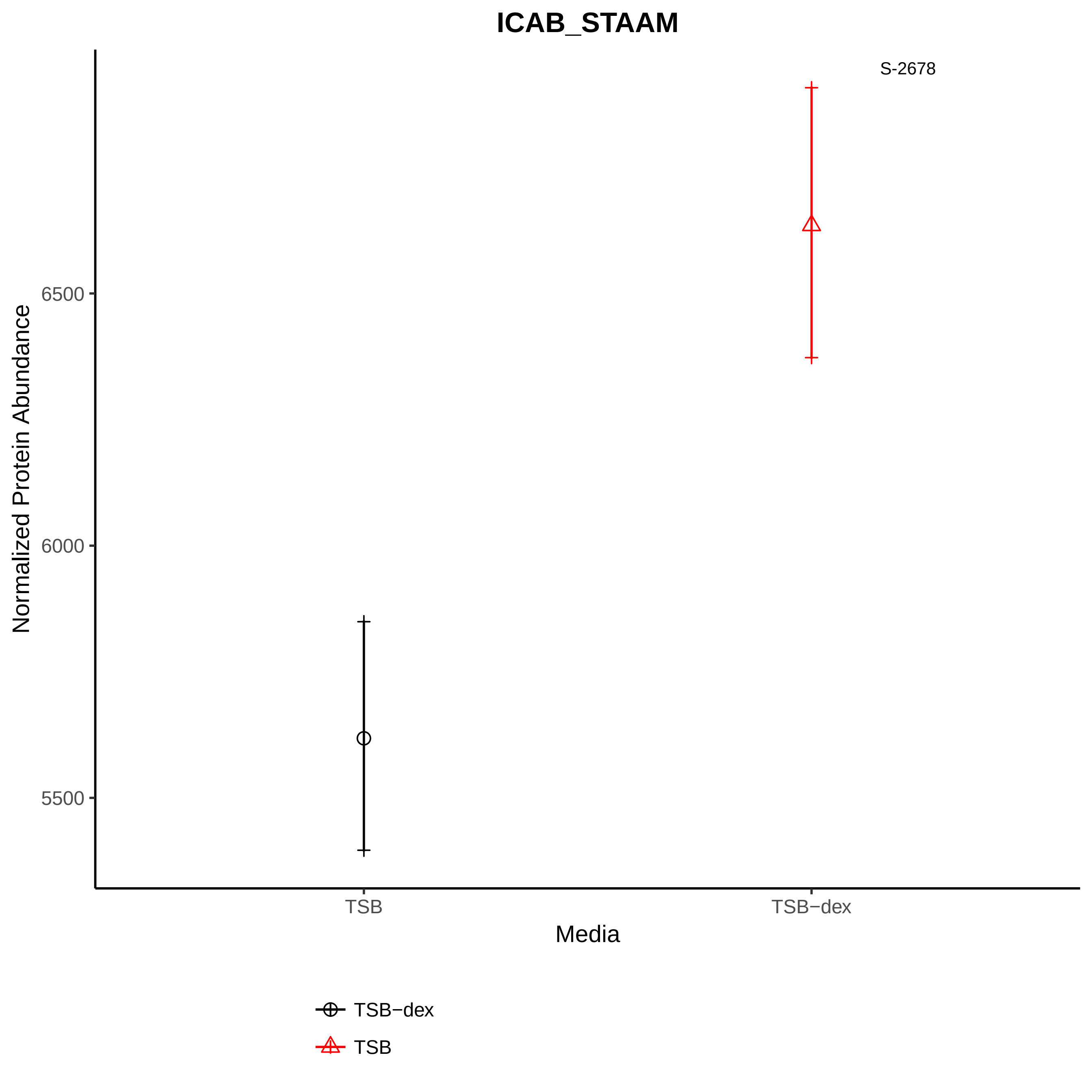
ICAB_STAAM
S-2678
Normalized Protein Abundance
6500
6000
5500
TSB
TSB−dex
Media
TSB−dex
TSB

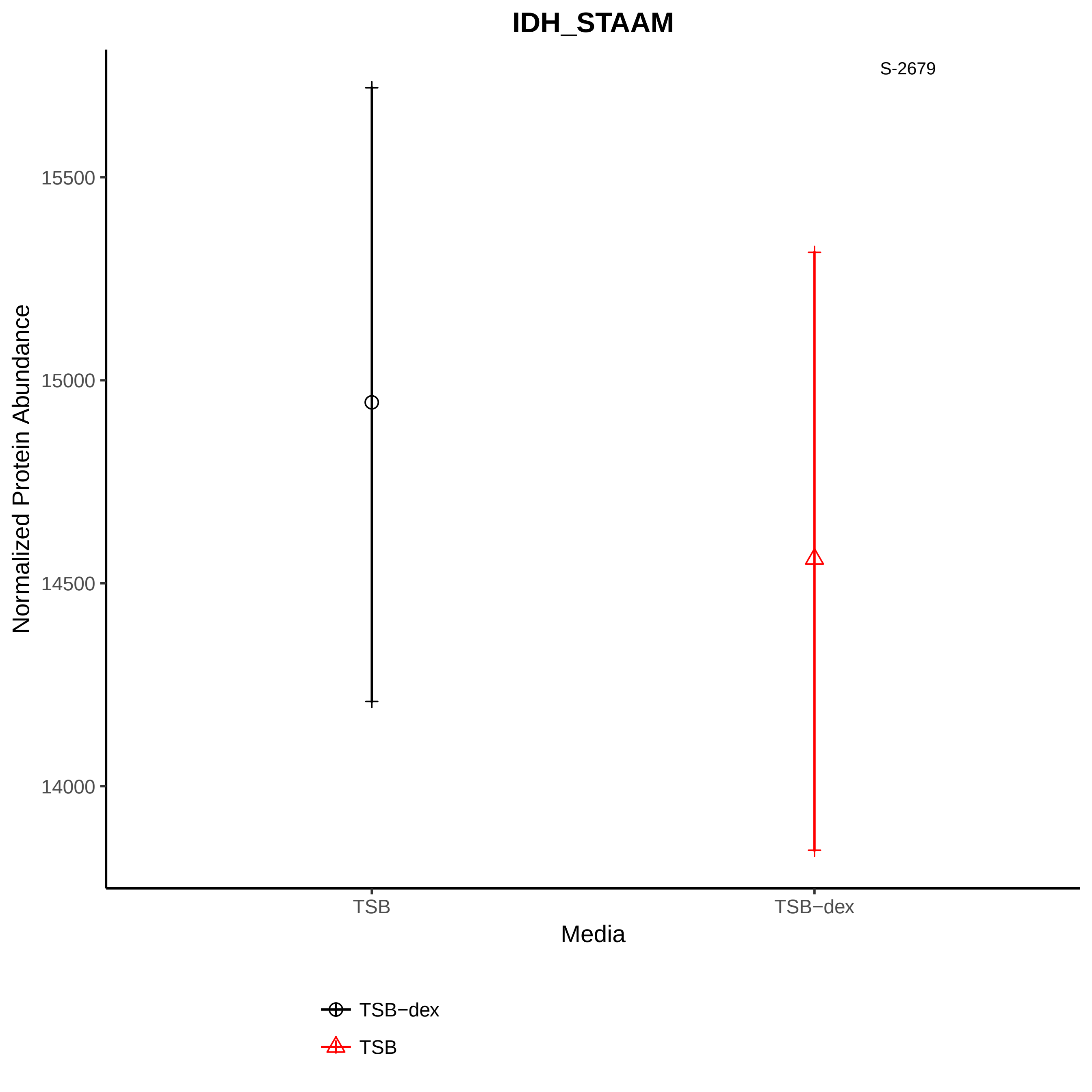
IDH_STAAM
S-2679
Normalized Protein Abundance
15500
15000
14500
14000
TSB
TSB−dex
Media
TSB−dex
TSB

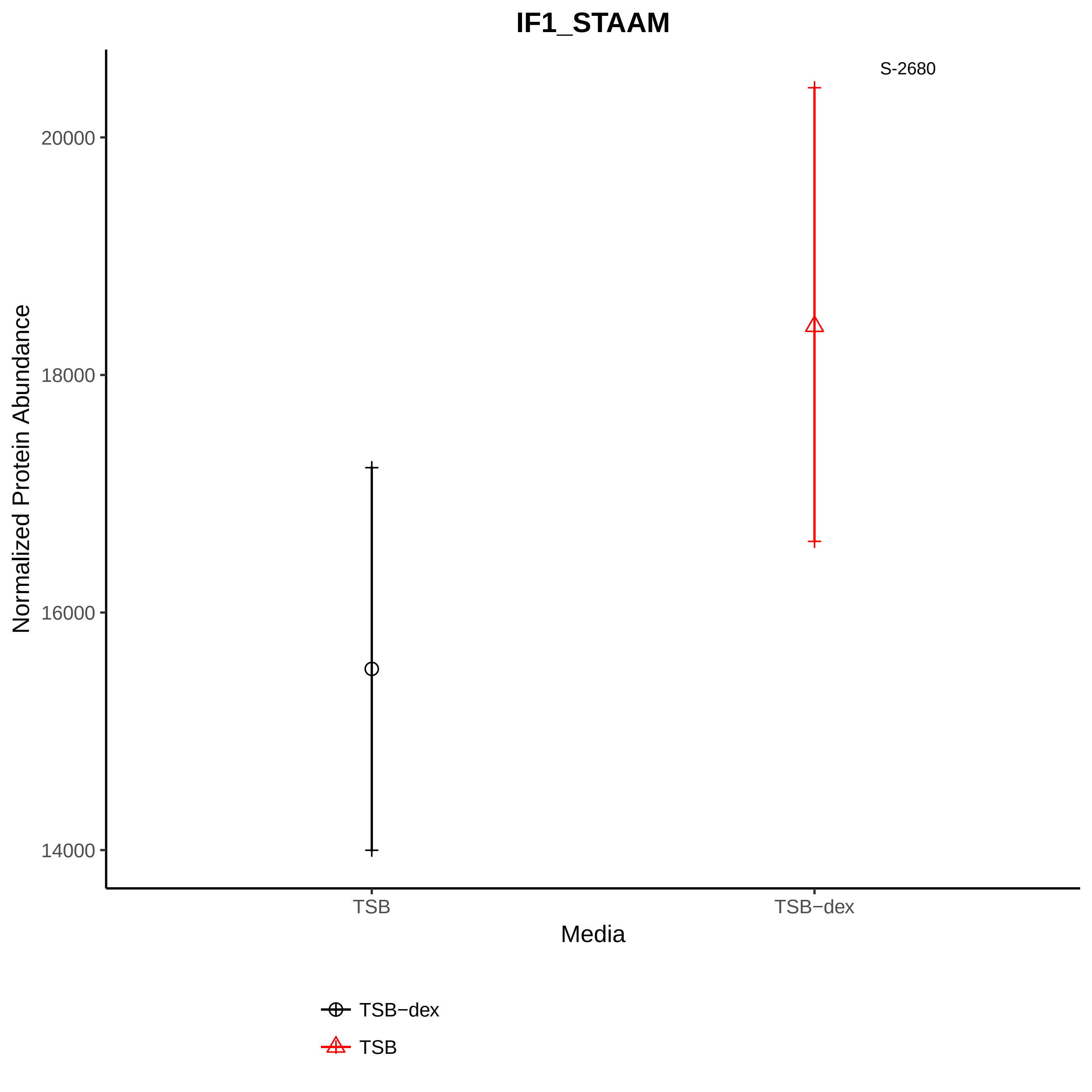
IF1_STAAM
S-2680
Normalized Protein Abundance
20000
18000
16000
14000
TSB
TSB−dex
Media
TSB−dex
TSB

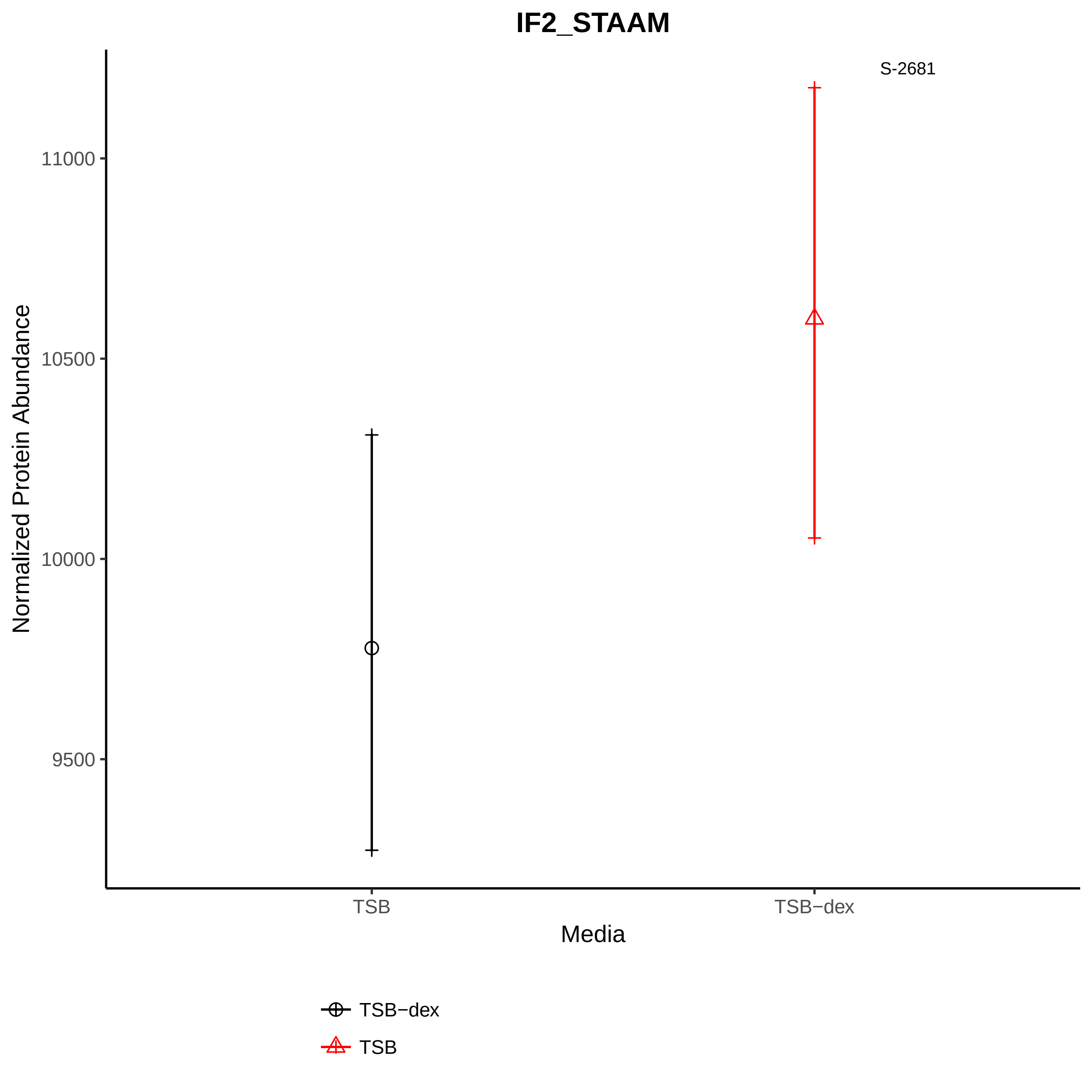

IF2_STAAM
S-2681
11000
10500
10000
9500
Normalized Protein Abundance
TSB
TSB−dex
Media
TSB−dex
TSB

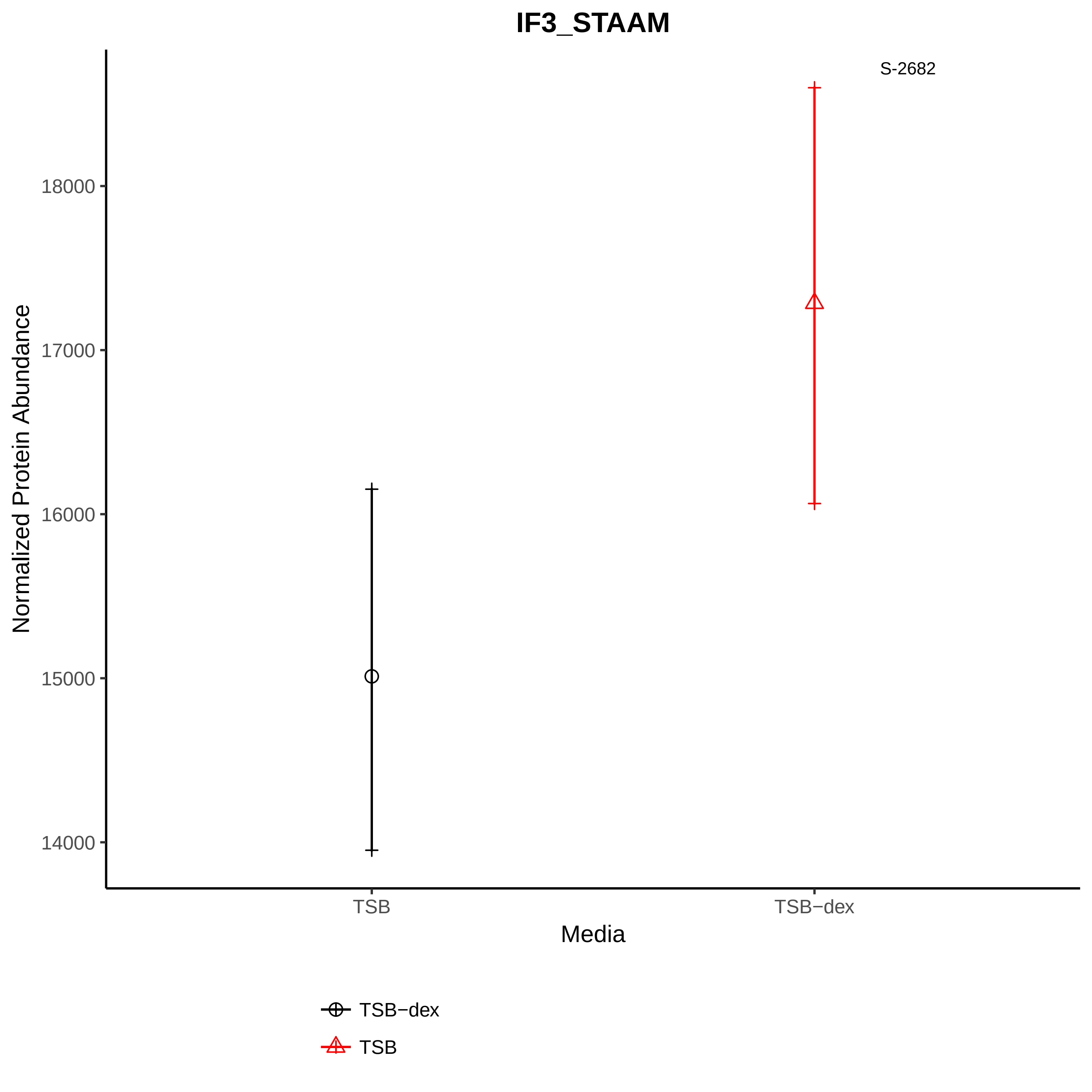
IF3_STAAM
S-2682
Normalized Protein Abundance
18000
17000
16000
15000
14000
TSB
TSB−dex
Media
TSB−dex
TSB

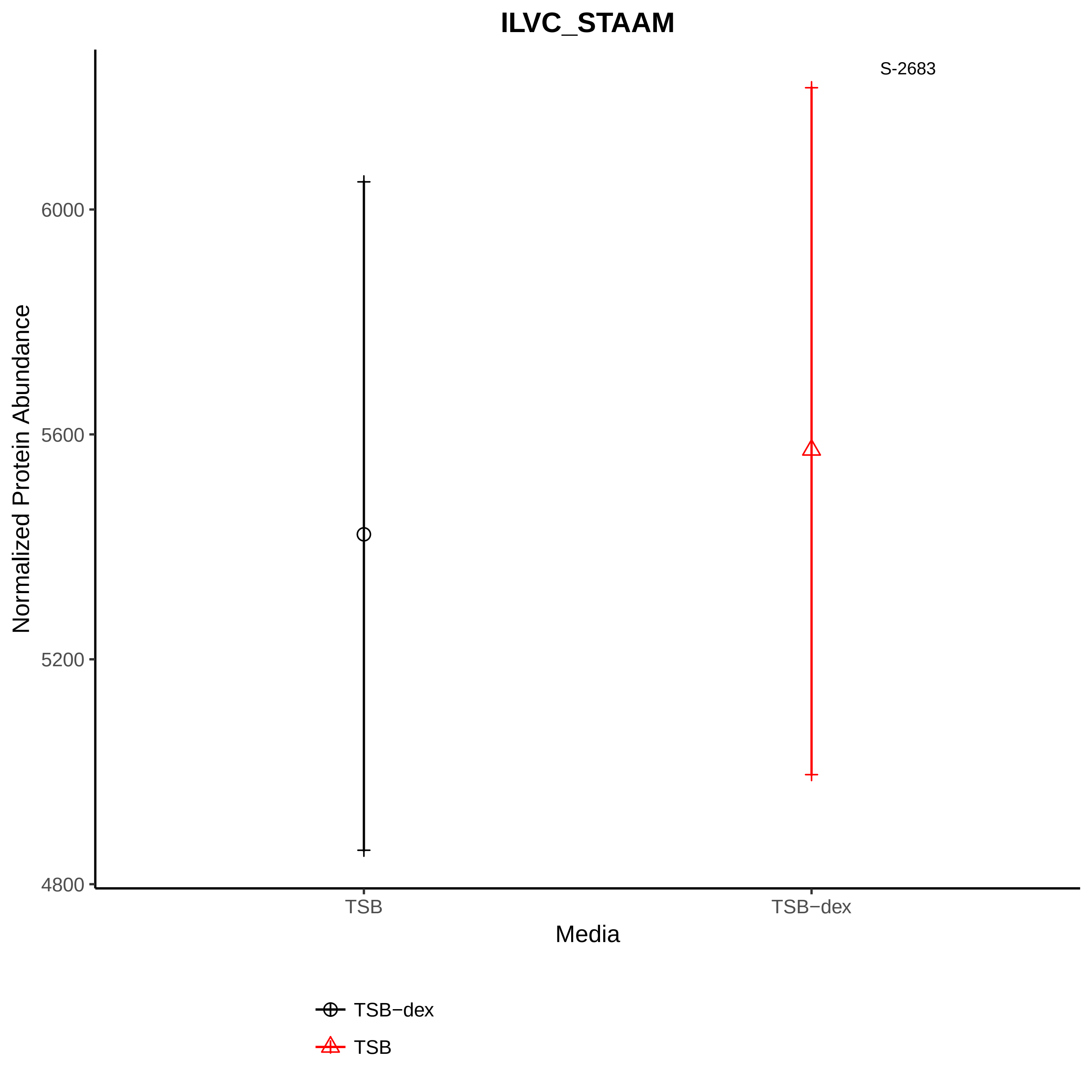
ILVC_STAAM
S-2683
Normalized Protein Abundance
6000
5600
5200
4800
TSB
TSB−dex
Media
TSB−dex
TSB

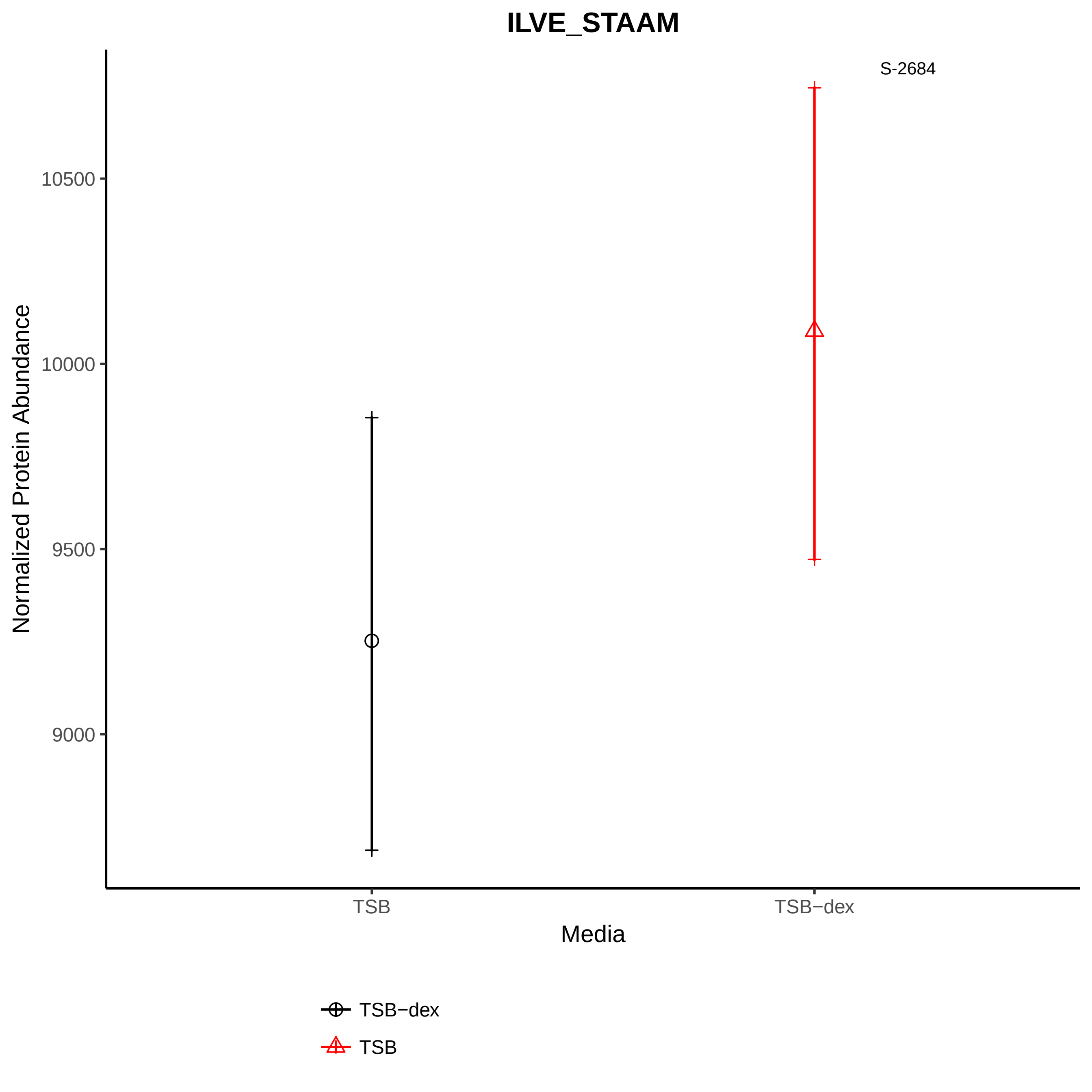

ILVE_STAAM
S-2684
Normalized Protein Abundance
10500
10000
9500
9000
TSB
TSB−dex
Media
TSB−dex
TSB

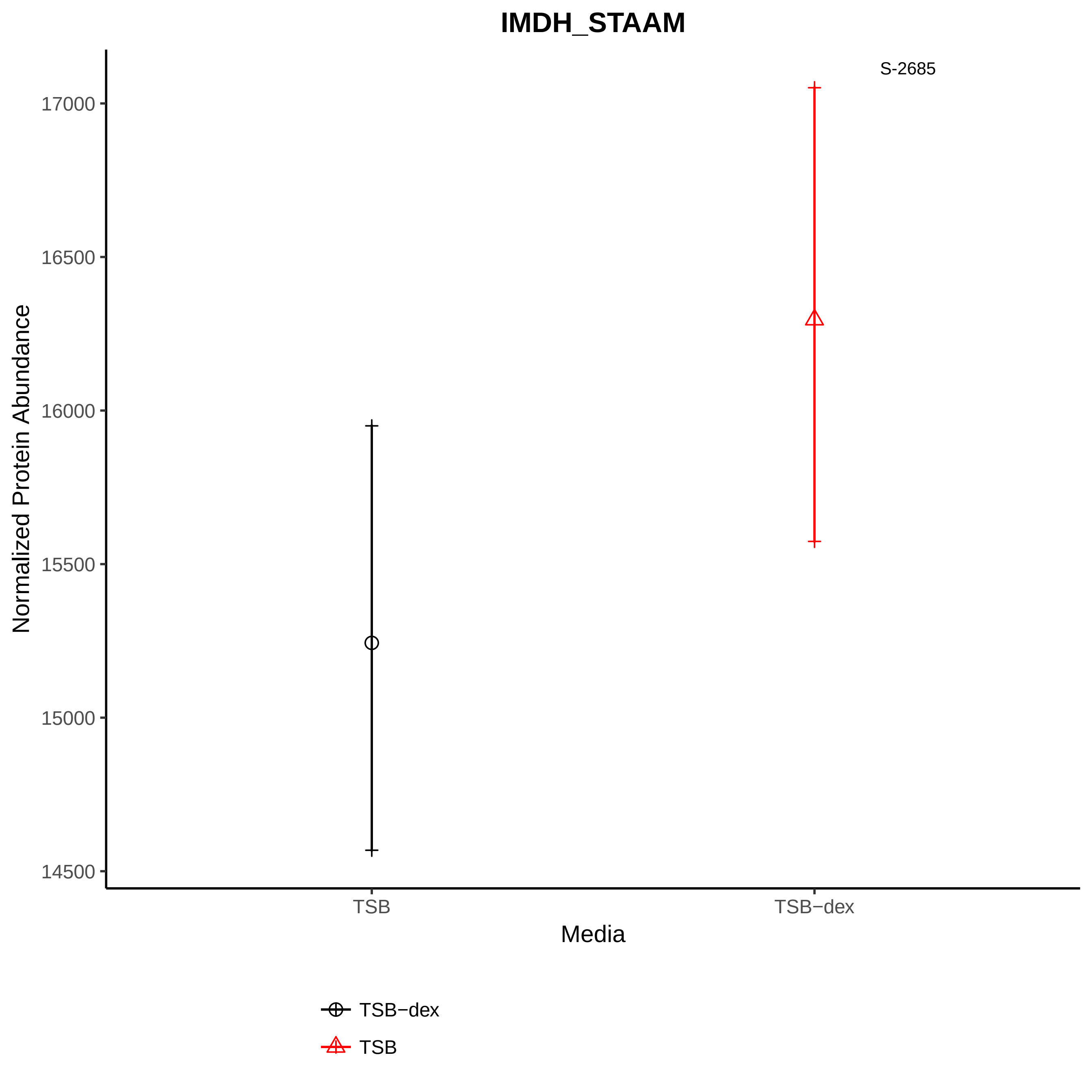

IMDH_STAAM
S-2685
Normalized Protein Abundance
17000
16500
16000
15500
15000
14500
TSB
TSB−dex
Media
TSB−dex
TSB

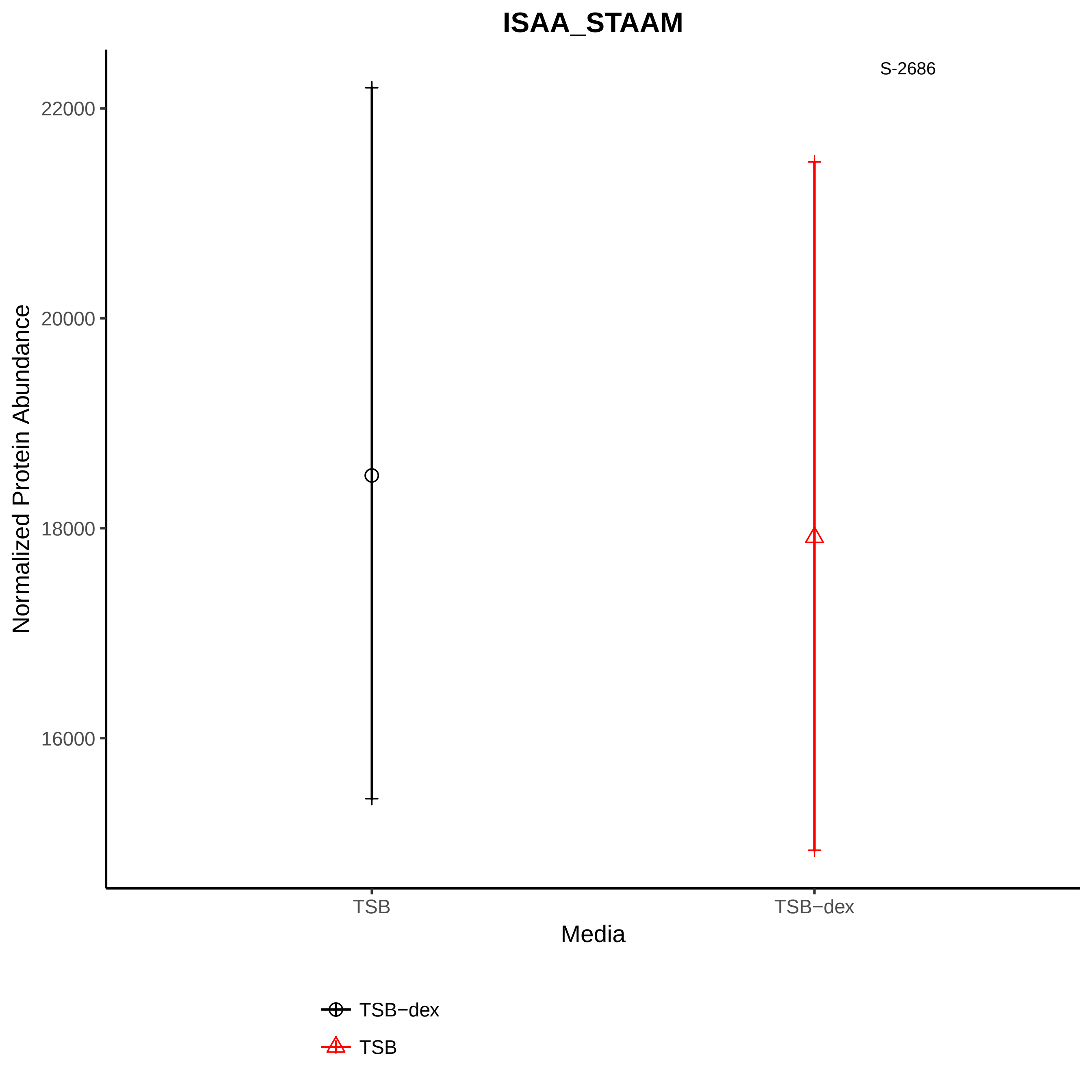

ISAA_STAAM
S-2686
22000
20000
18000
16000
Normalized Protein Abundance
TSB
TSB−dex
Media
TSB−dex
TSB

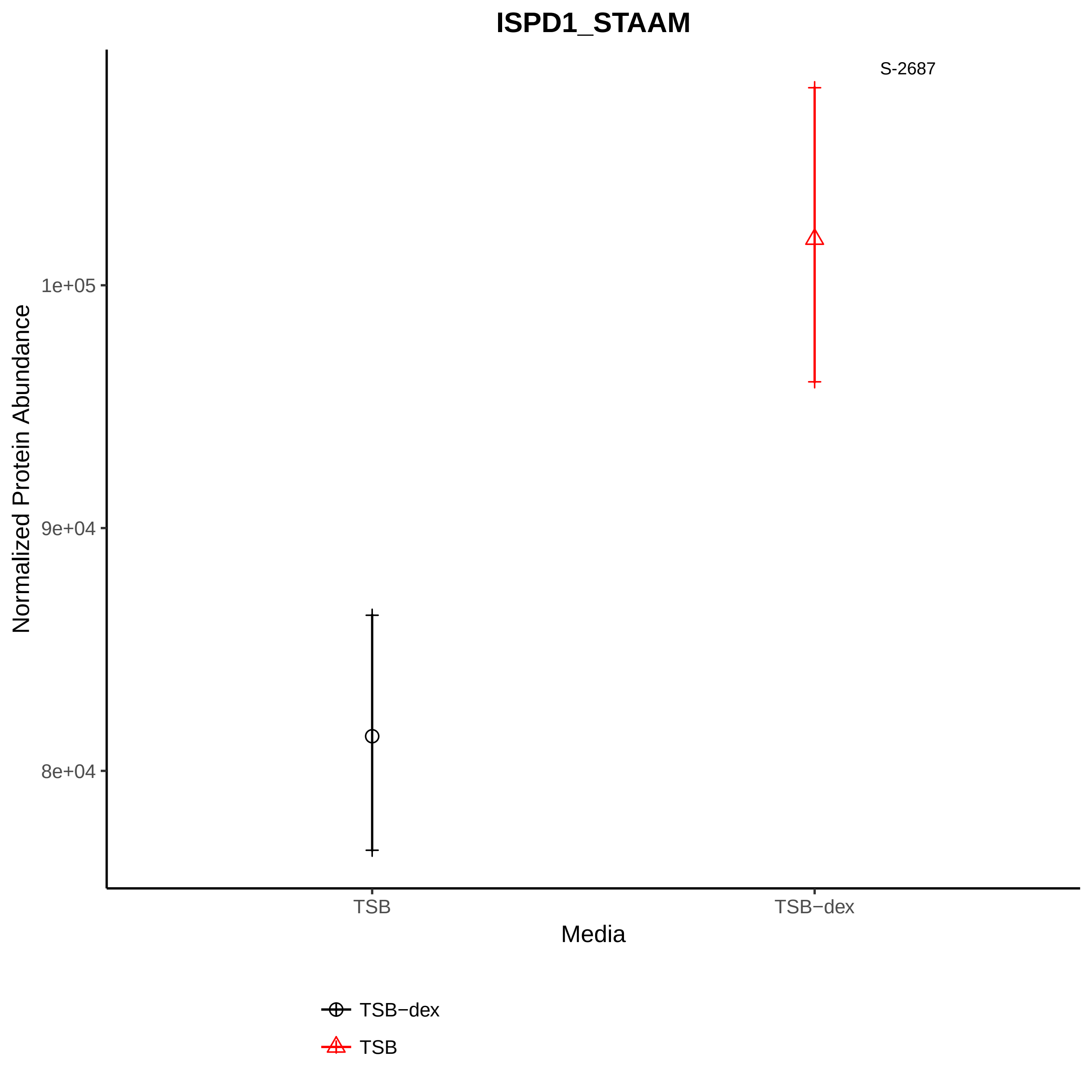

ISPD1_STAAM
S-2687
Normalized Protein Abundance
1e+05
9e+04
8e+04
TSB
TSB−dex
Media
TSB−dex
TSB

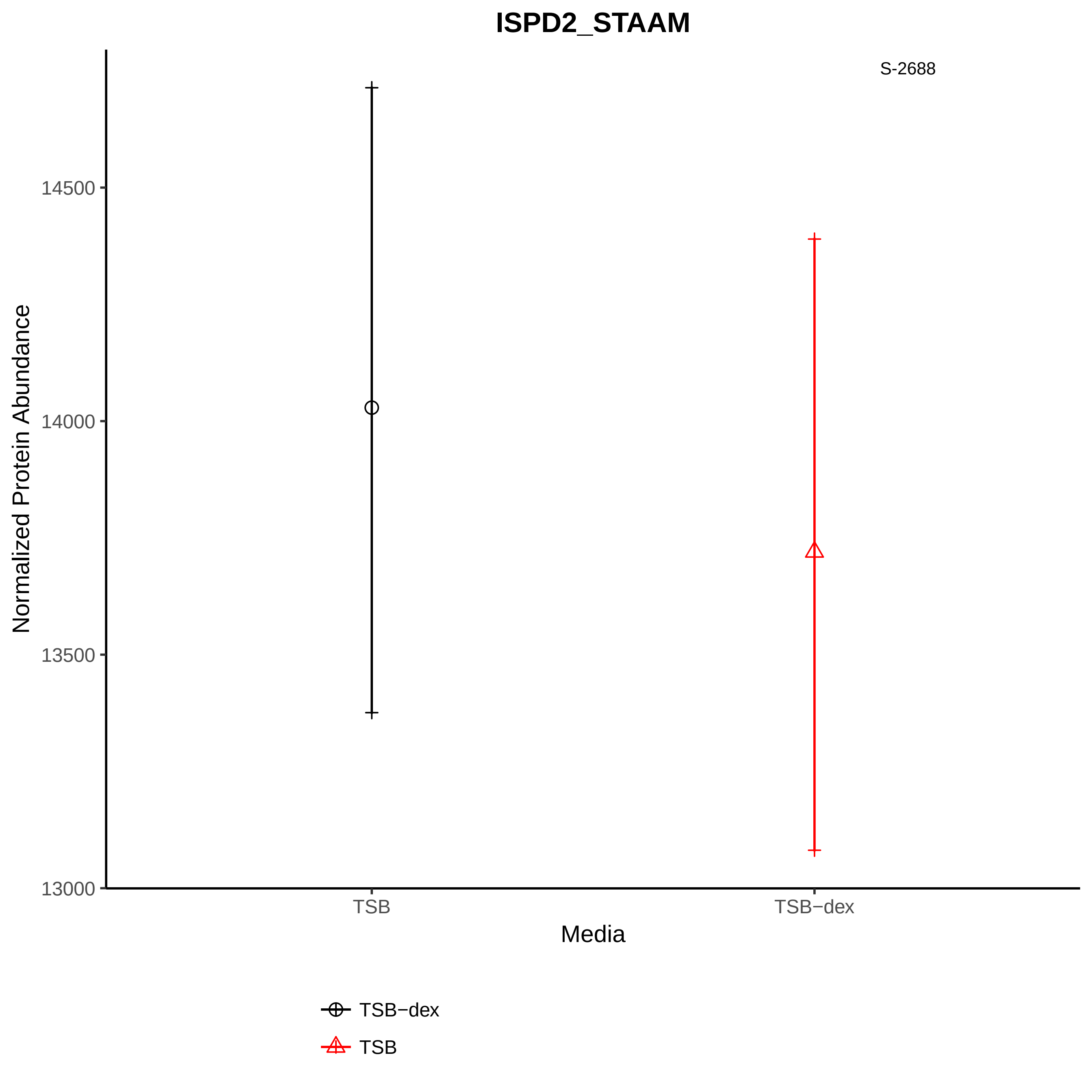

ISPD2_STAAM
S-2688
Normalized Protein Abundance
14500
14000
13500
13000
TSB
TSB−dex
Media
TSB−dex
TSB

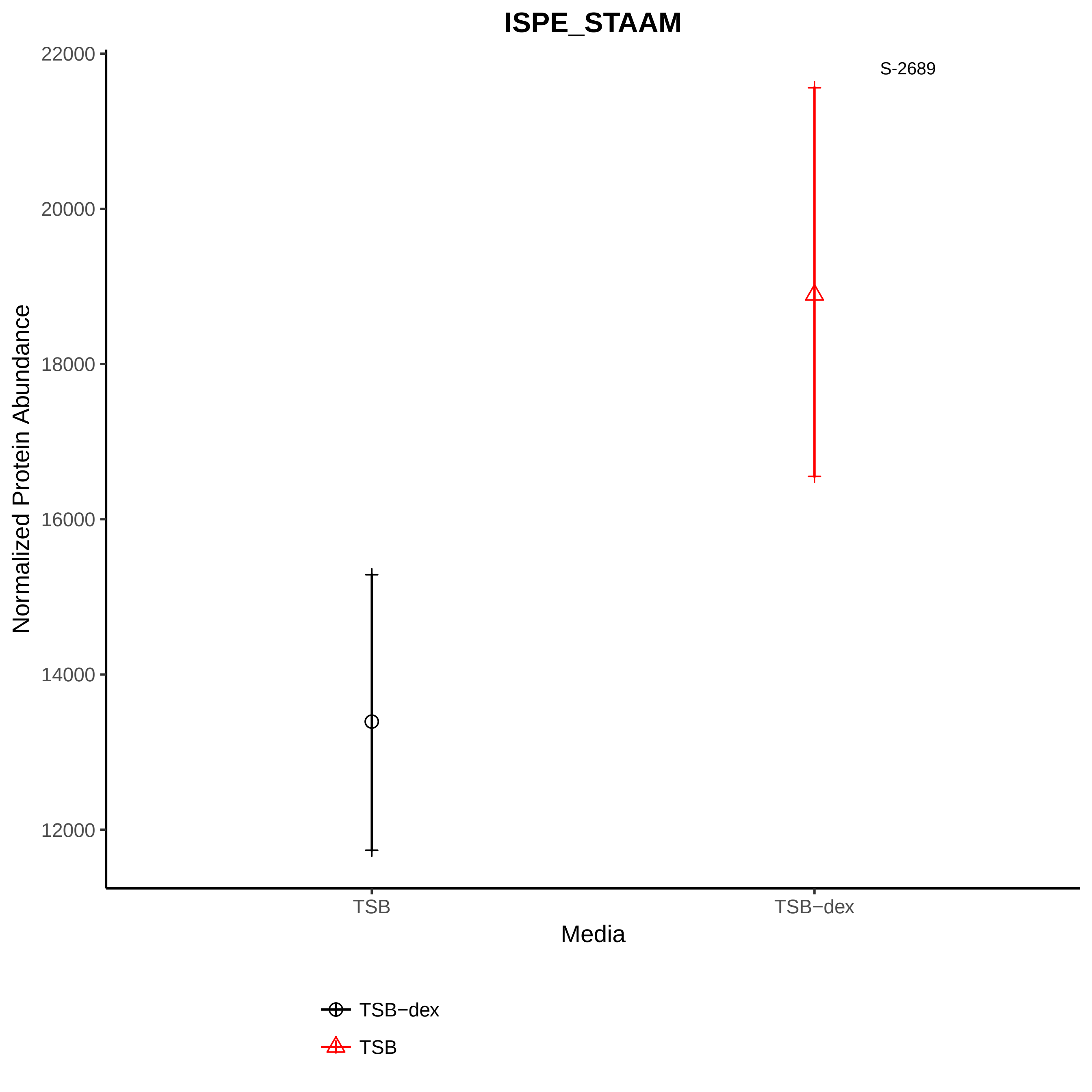
ISPE_STAAM
S-2689
Normalized Protein Abundance
22000
20000
18000
16000
14000
12000
TSB
TSB−dex
Media
TSB−dex
TSB

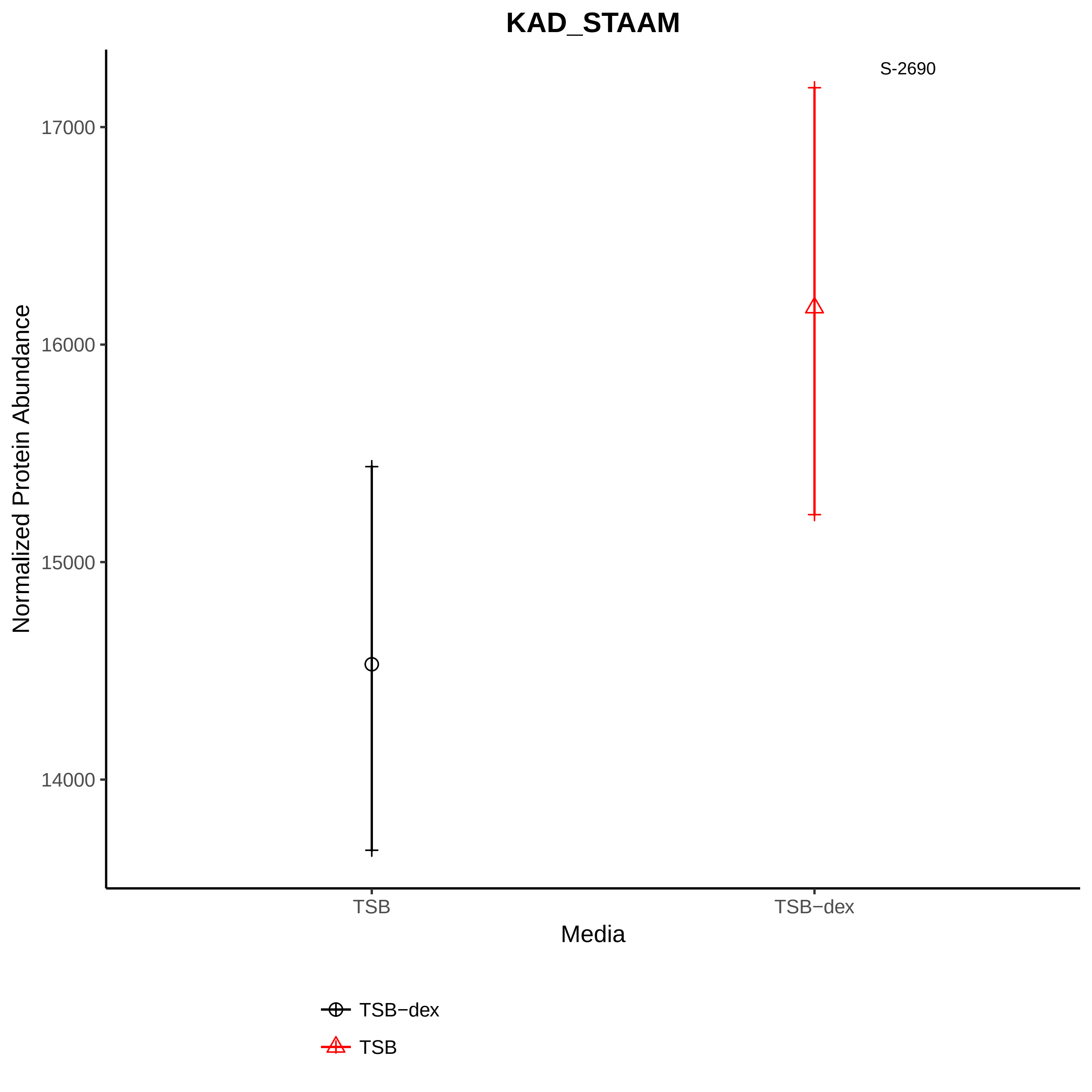

KAD_STAAM
S-2690
Normalized Protein Abundance
17000
16000
15000
14000
TSB
TSB−dex
Media
TSB−dex
TSB

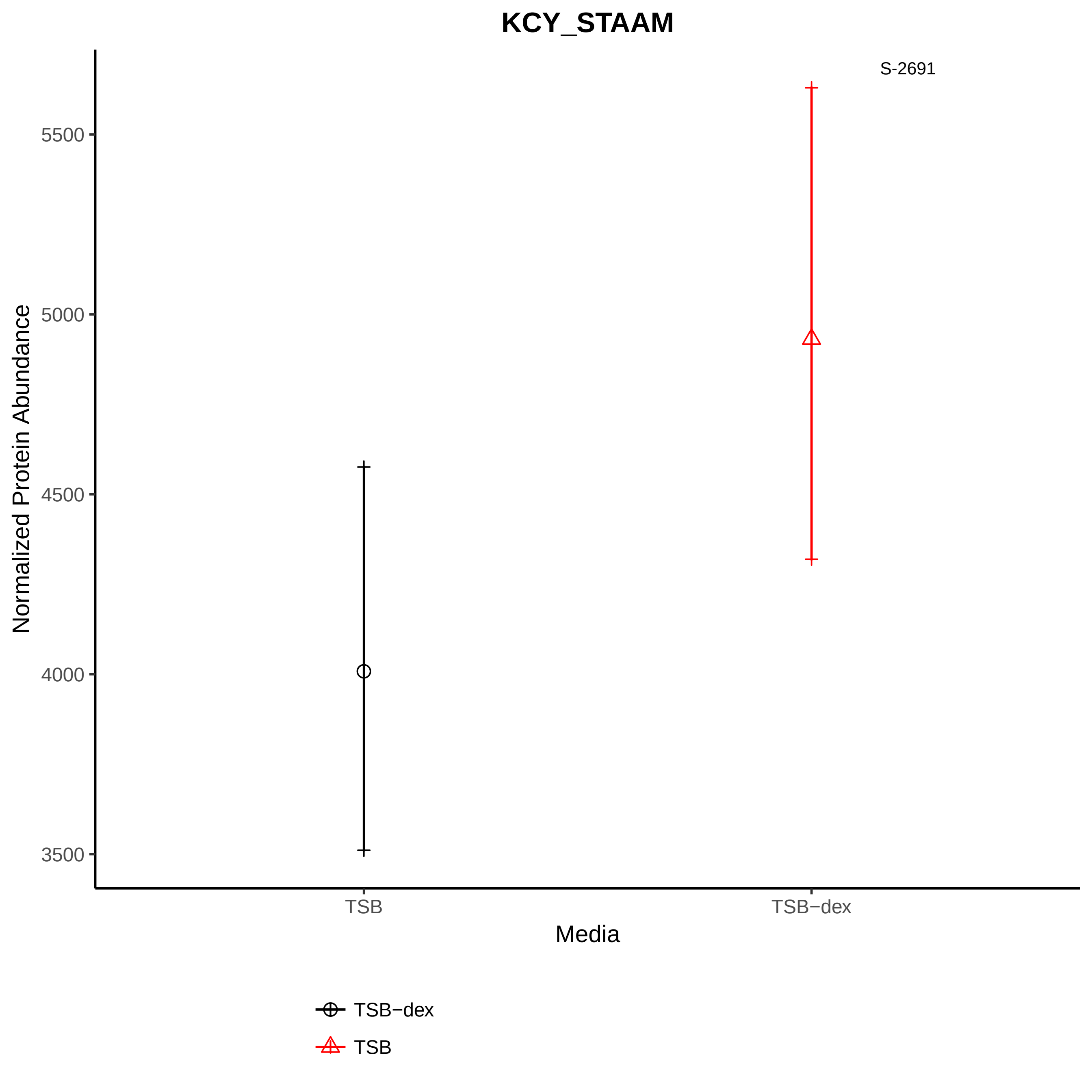

KCY_STAAM
S-2691
5500
5000
4500
4000
3500
Normalized Protein Abundance
TSB
TSB−dex
Media
TSB−dex
TSB

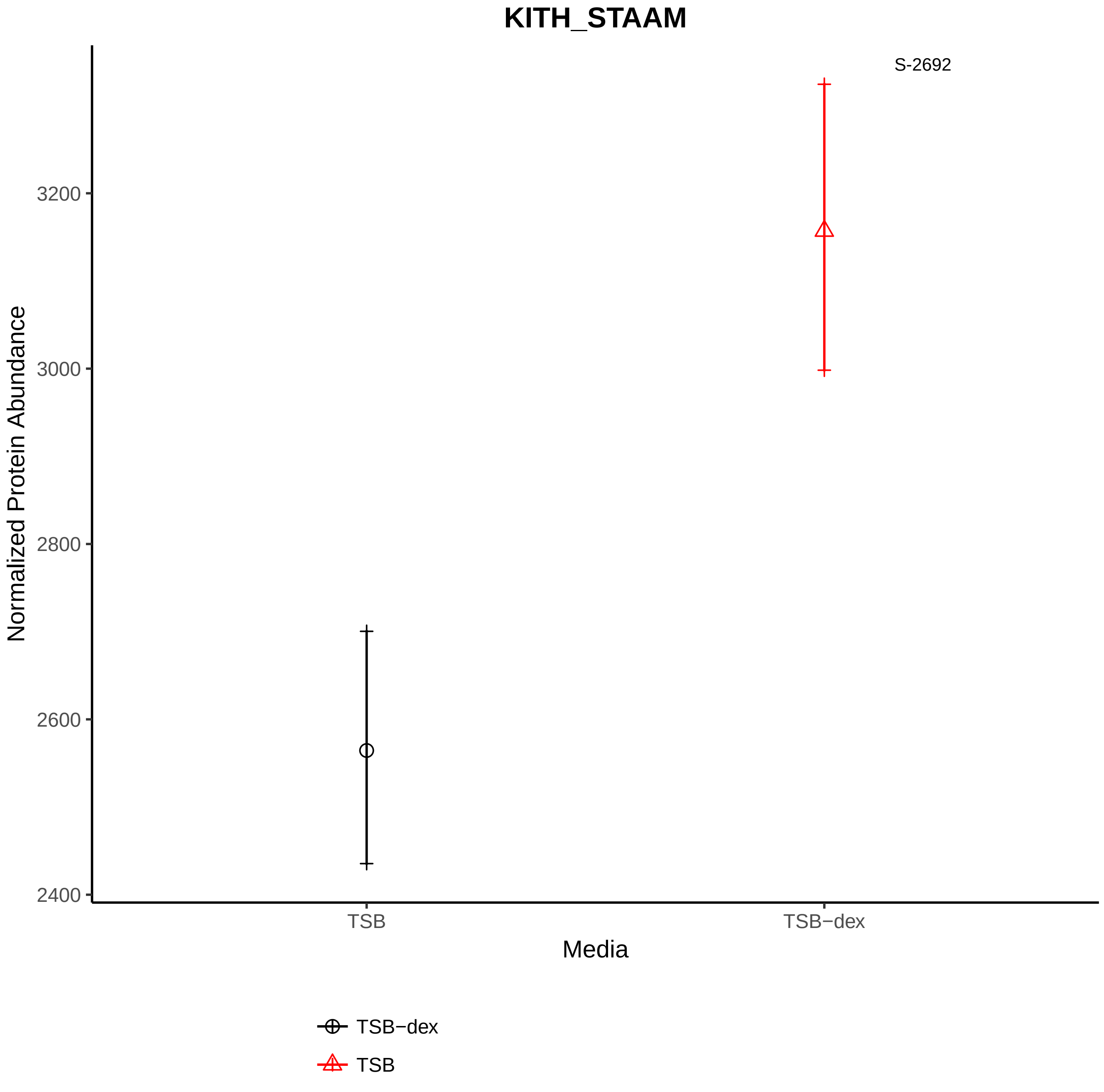

KITH_STAAM
S-2692
Normalized Protein Abundance
3200
3000
2800
2600
2400
TSB
TSB−dex
Media
TSB−dex
TSB

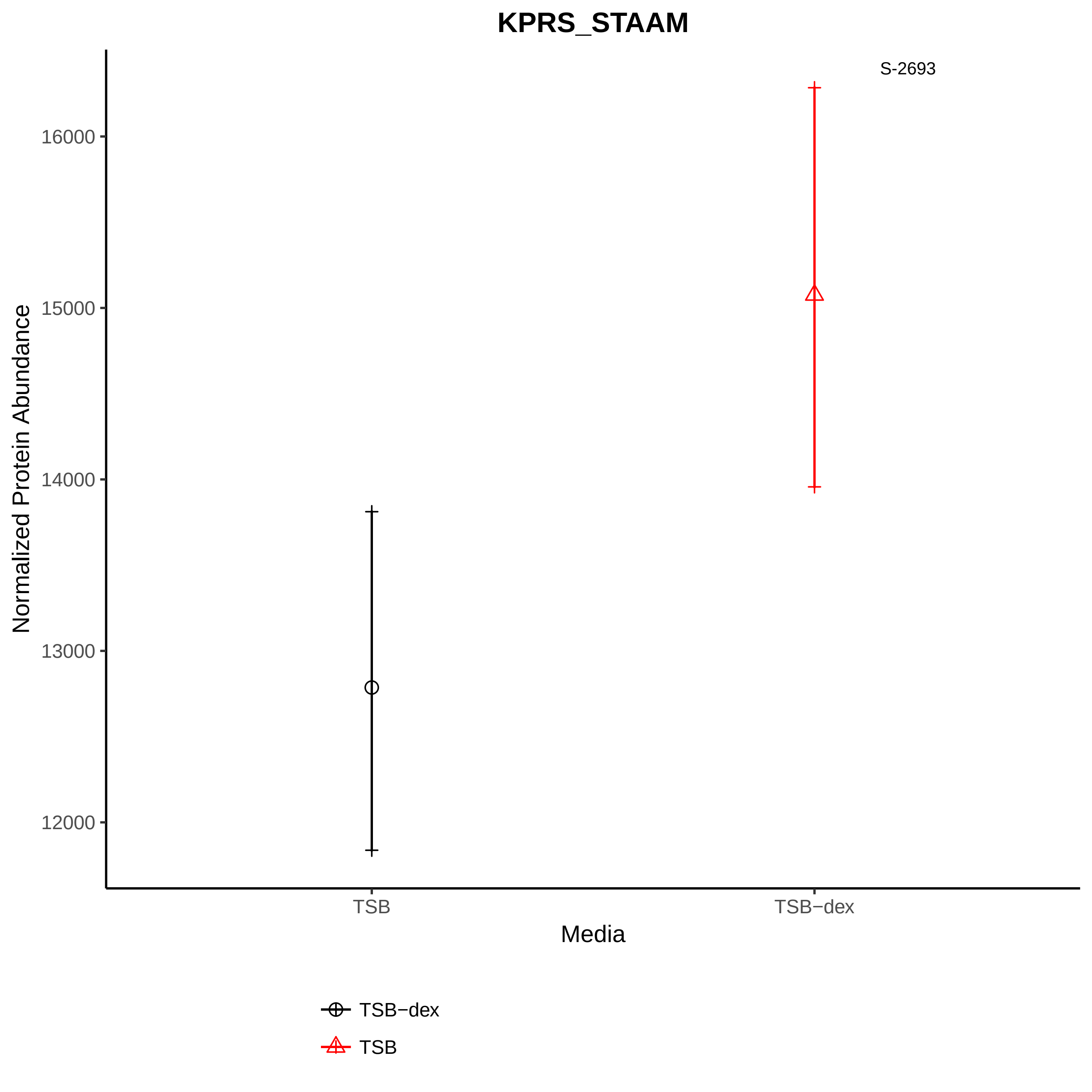

KPRS_STAAM
S-2693
Normalized Protein Abundance
16000
15000
14000
13000
12000
TSB
TSB−dex
Media
TSB−dex
TSB

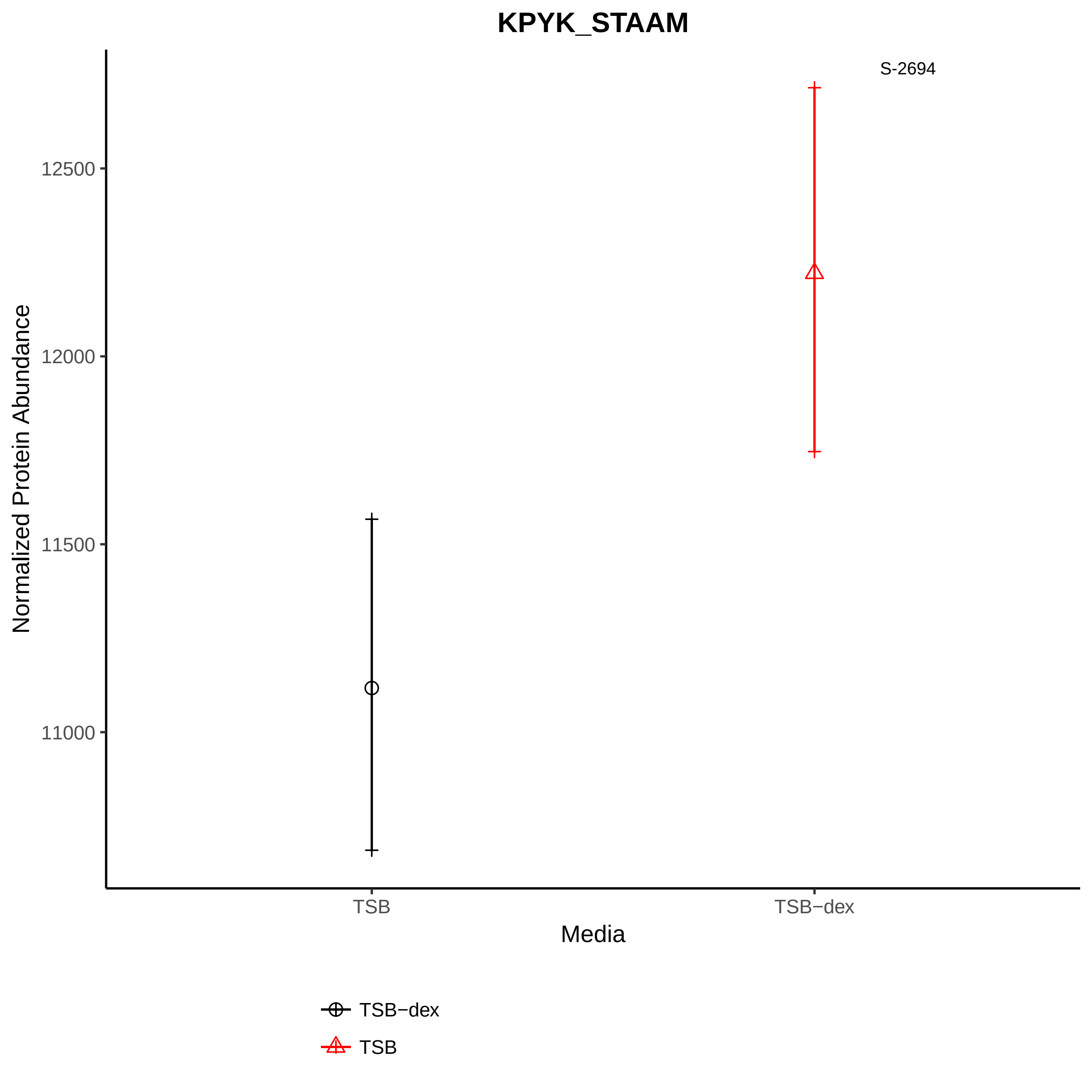
KPYK_STAAM
S-2694
12500
12000
11500
11000
Normalized Protein Abundance
TSB
TSB−dex
Media
TSB−dex
TSB

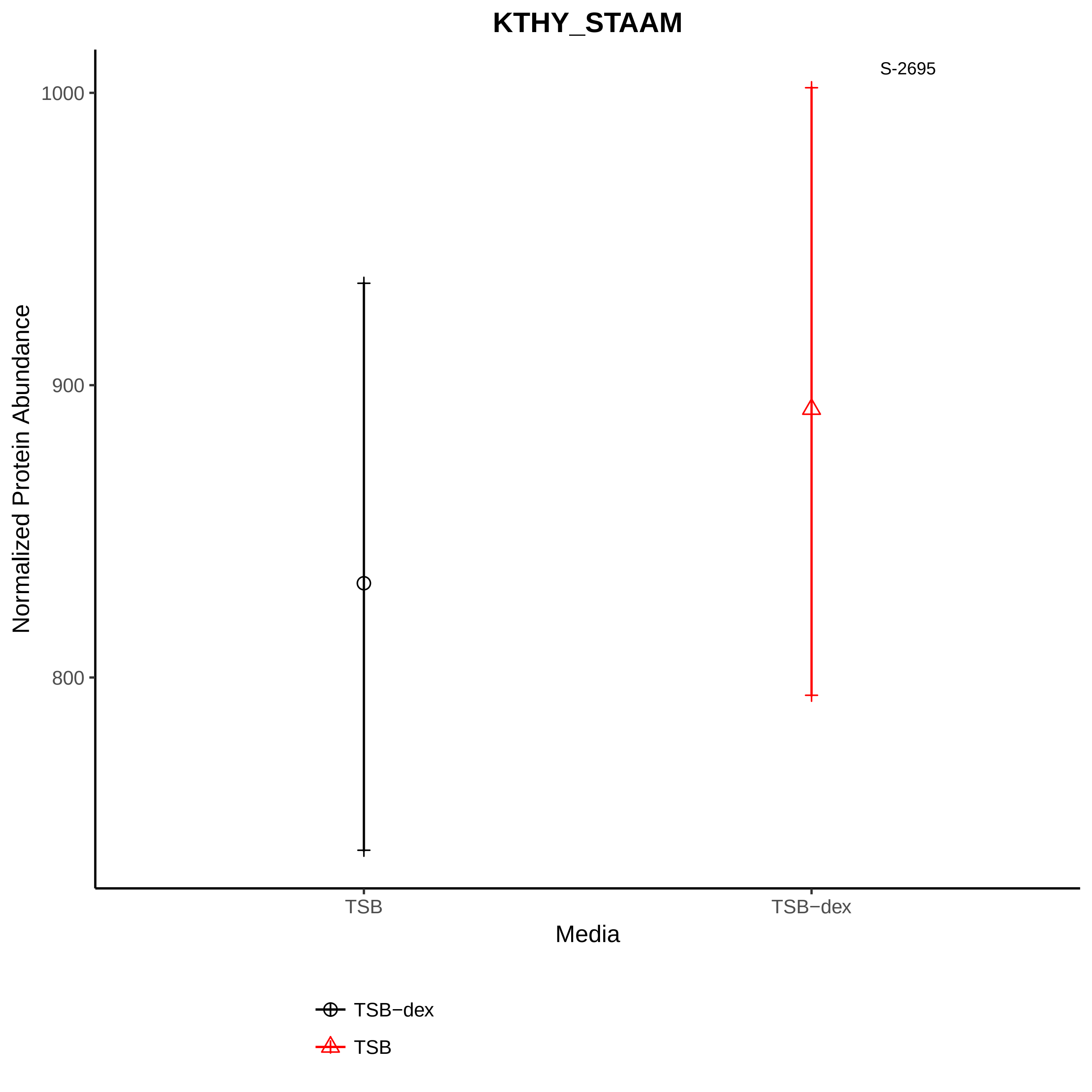

KTHY_STAAM
S-2695
1000
900
800
Normalized Protein Abundance
TSB
TSB−dex
Media
TSB−dex
TSB

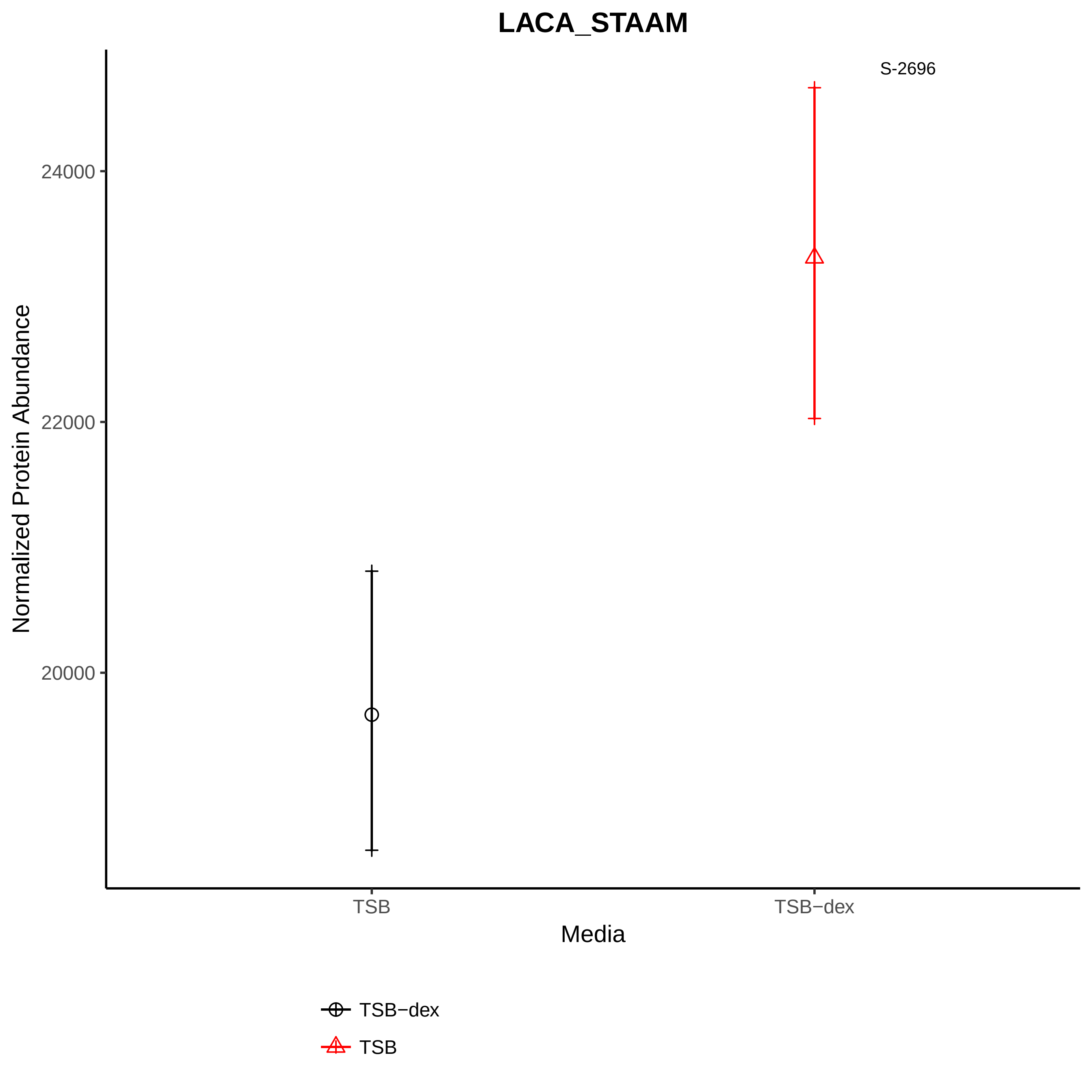

LACA_STAAM
S-2696
24000
22000
20000
Normalized Protein Abundance
TSB
TSB−dex
Media
TSB−dex
TSB

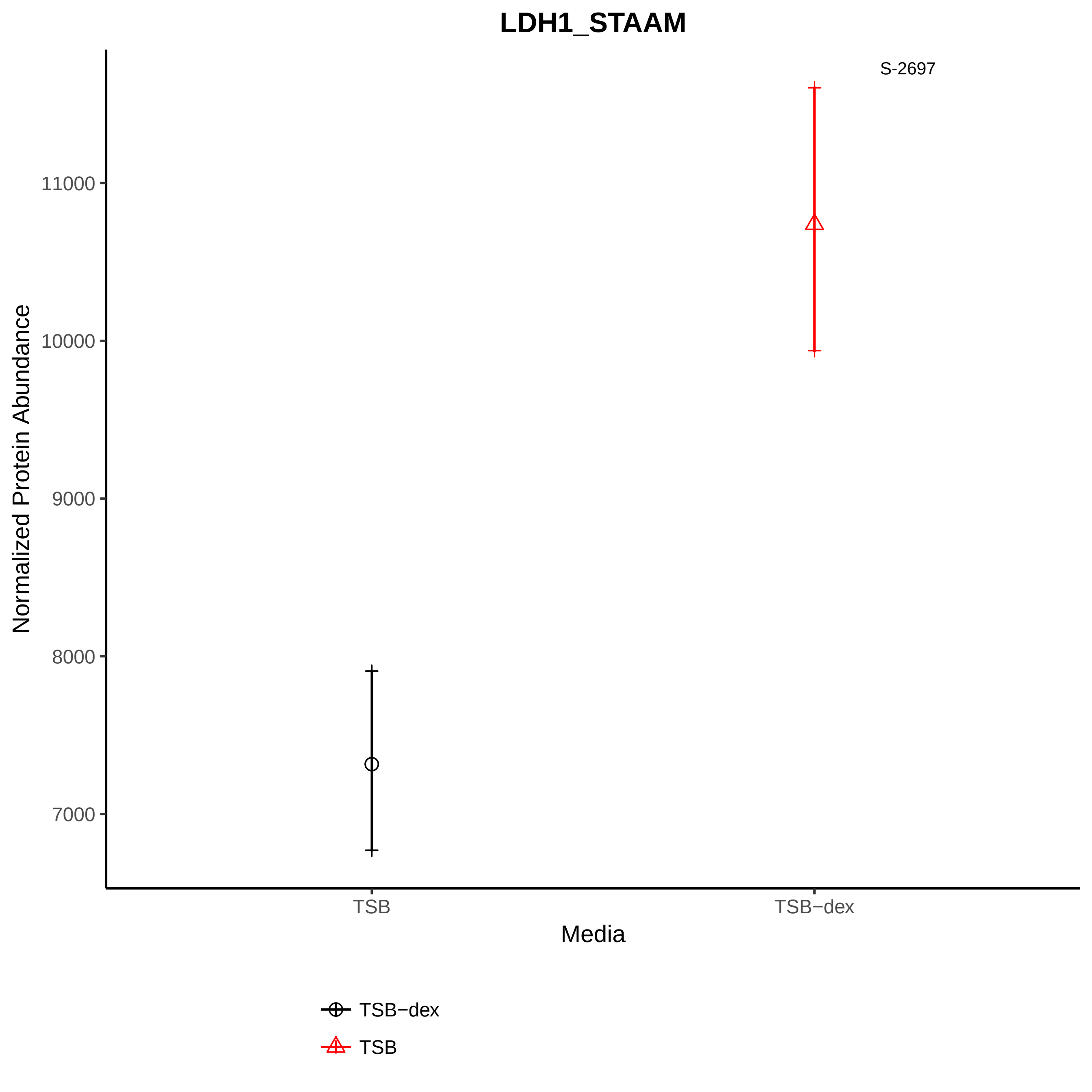

LDH1_STAAM
S-2697
Normalized Protein Abundance
11000
10000
9000
8000
7000
TSB
TSB−dex
Media
TSB−dex
TSB

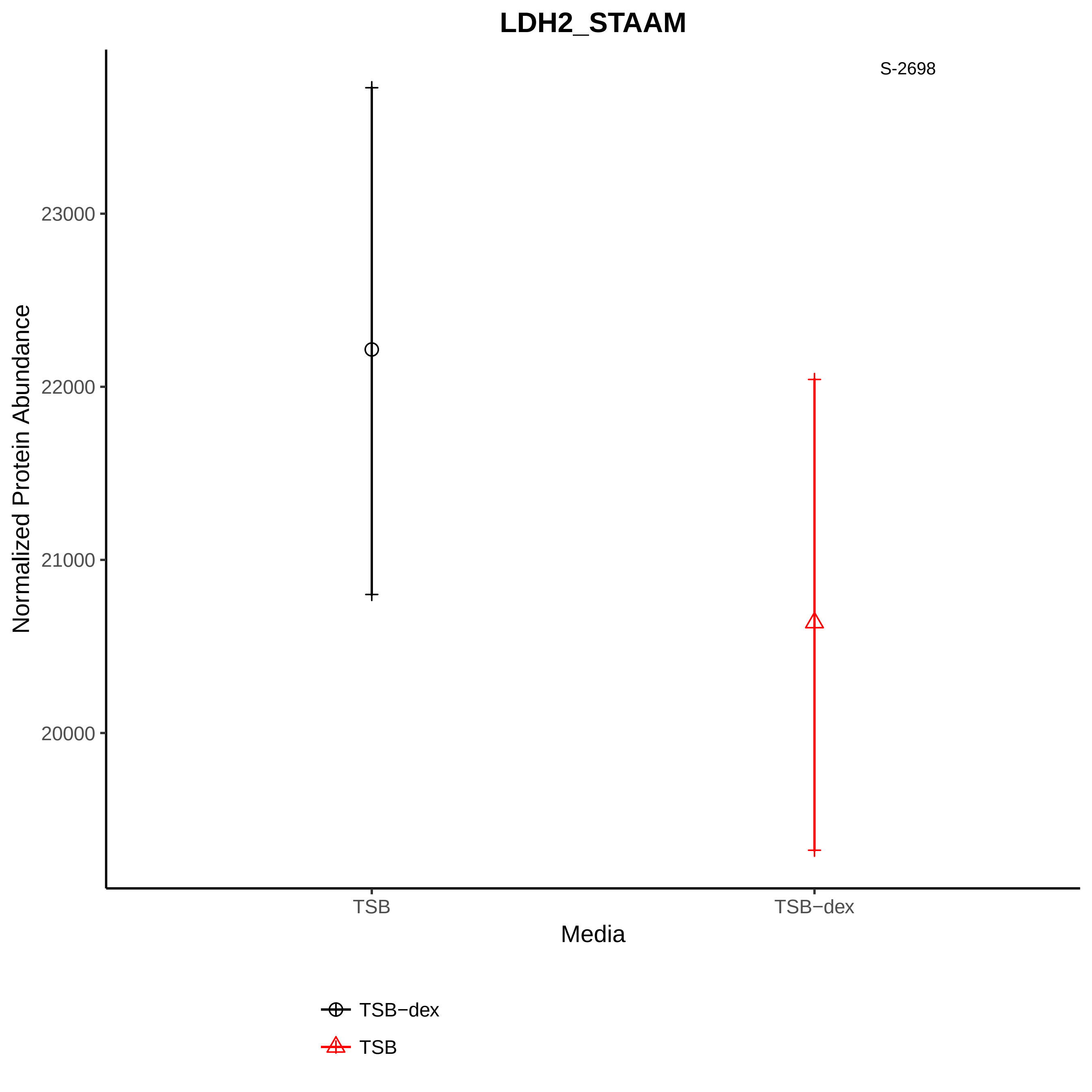

LDH2_STAAM
S-2698
Normalized Protein Abundance
23000
22000
21000
20000
TSB
TSB−dex
Media
TSB−dex
TSB

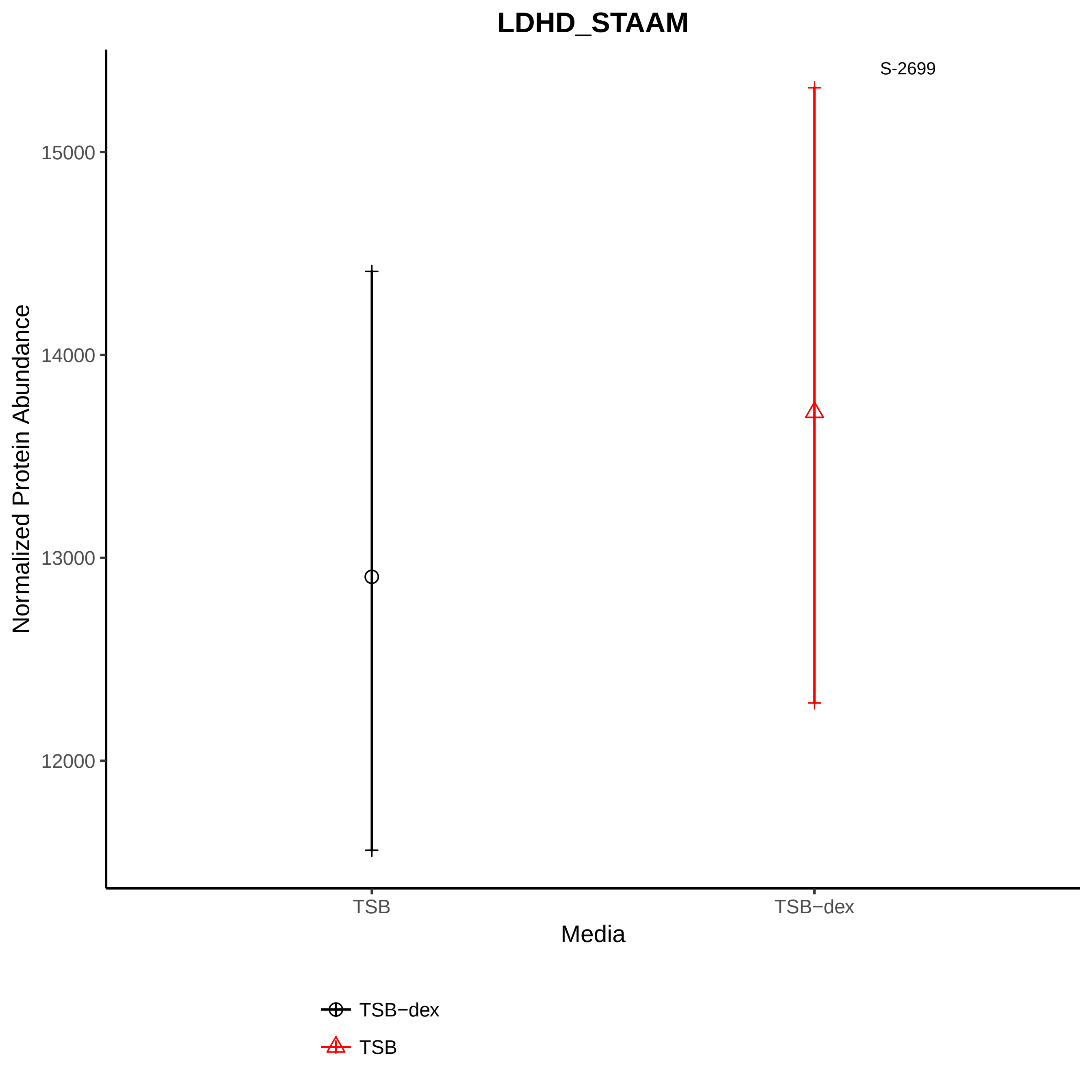

LDHD_STAAM
S-2699
Normalized Protein Abundance
15000
14000
13000
12000
TSB
TSB−dex
Media
TSB−dex
TSB

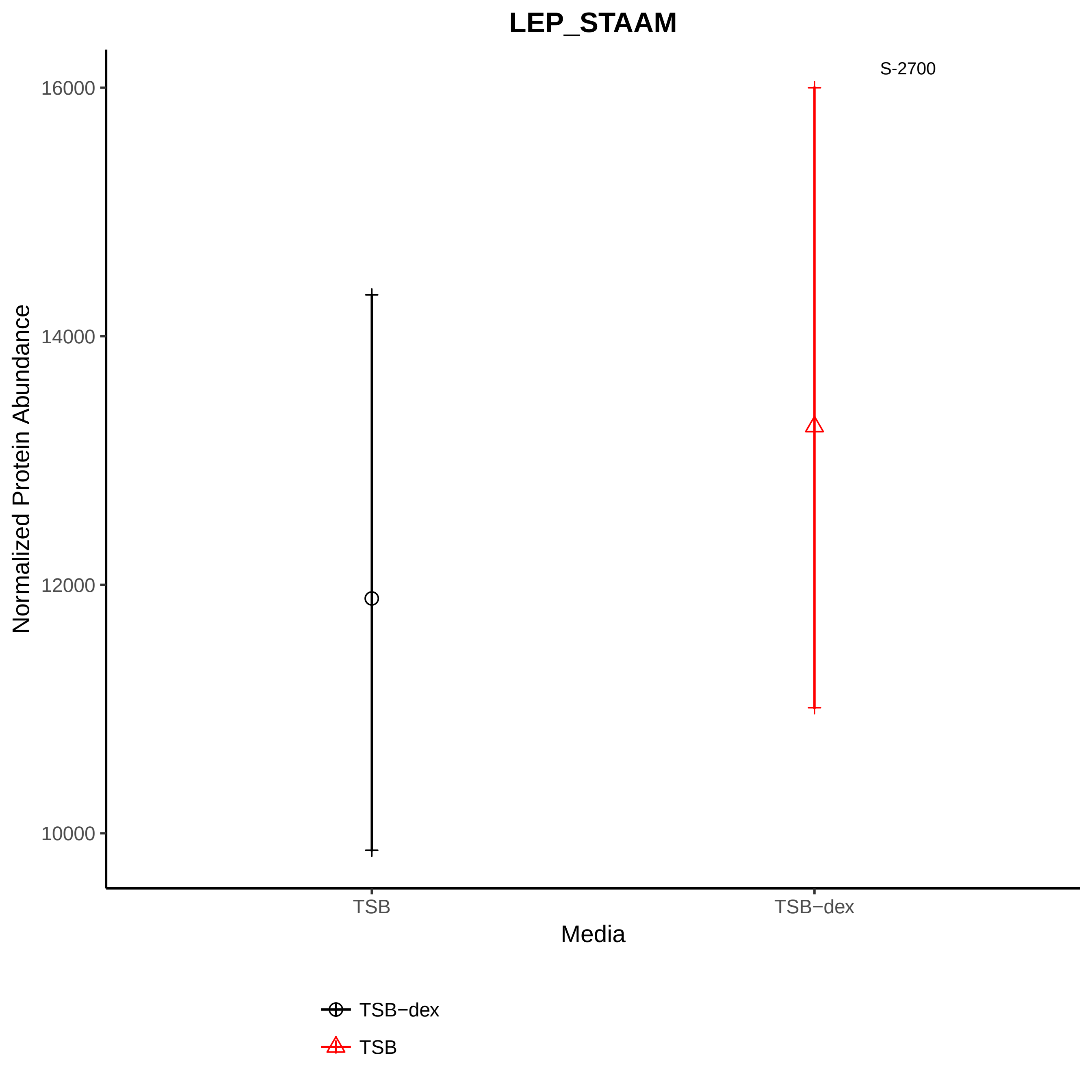

LEP_STAAM
S-2700
16000
14000
12000
10000
Normalized Protein Abundance
TSB
TSB−dex
Media
TSB−dex
TSB

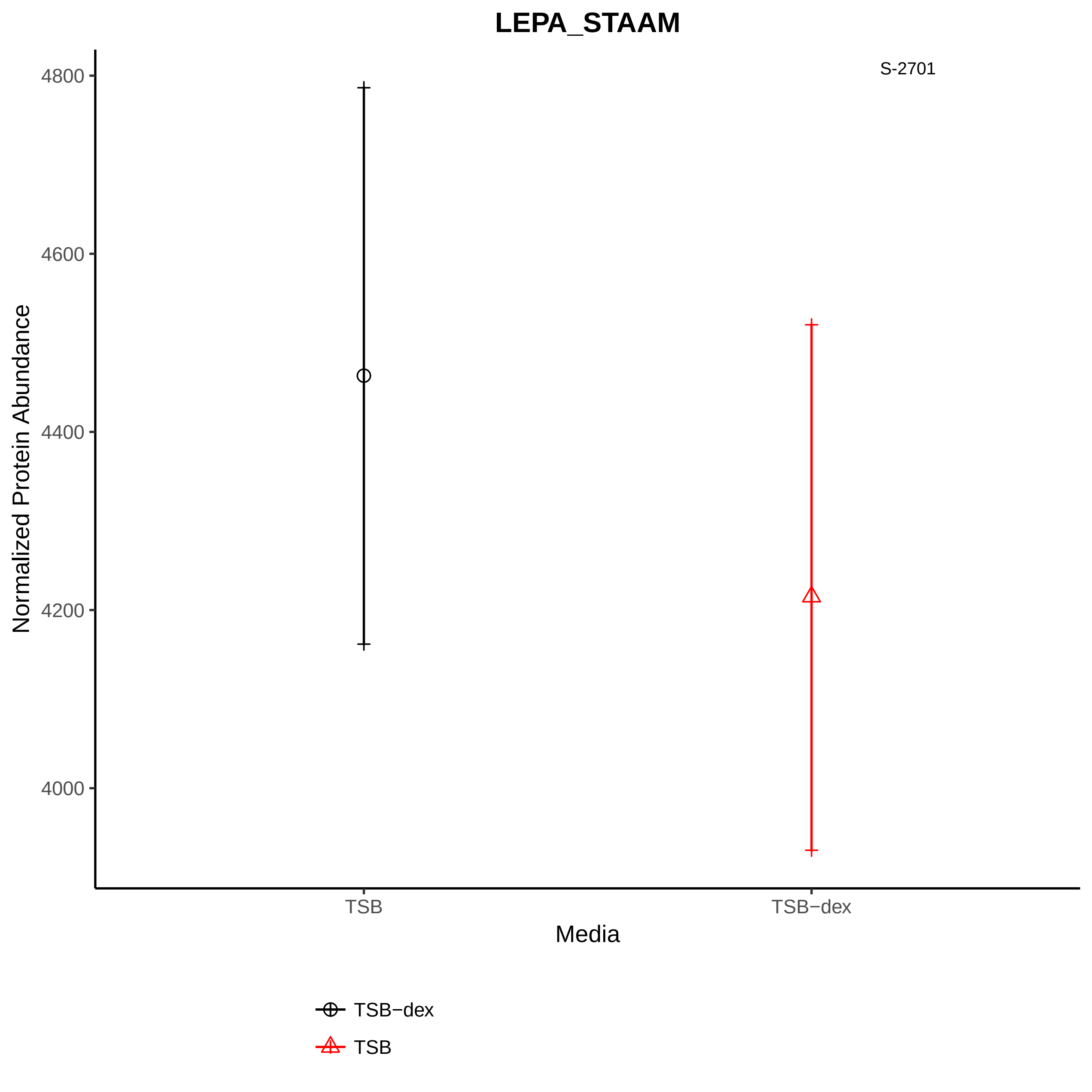

LEPA_STAAM
S-2701
4800
4600
4400
4200
4000
Normalized Protein Abundance
TSB
TSB−dex
Media
TSB−dex
TSB

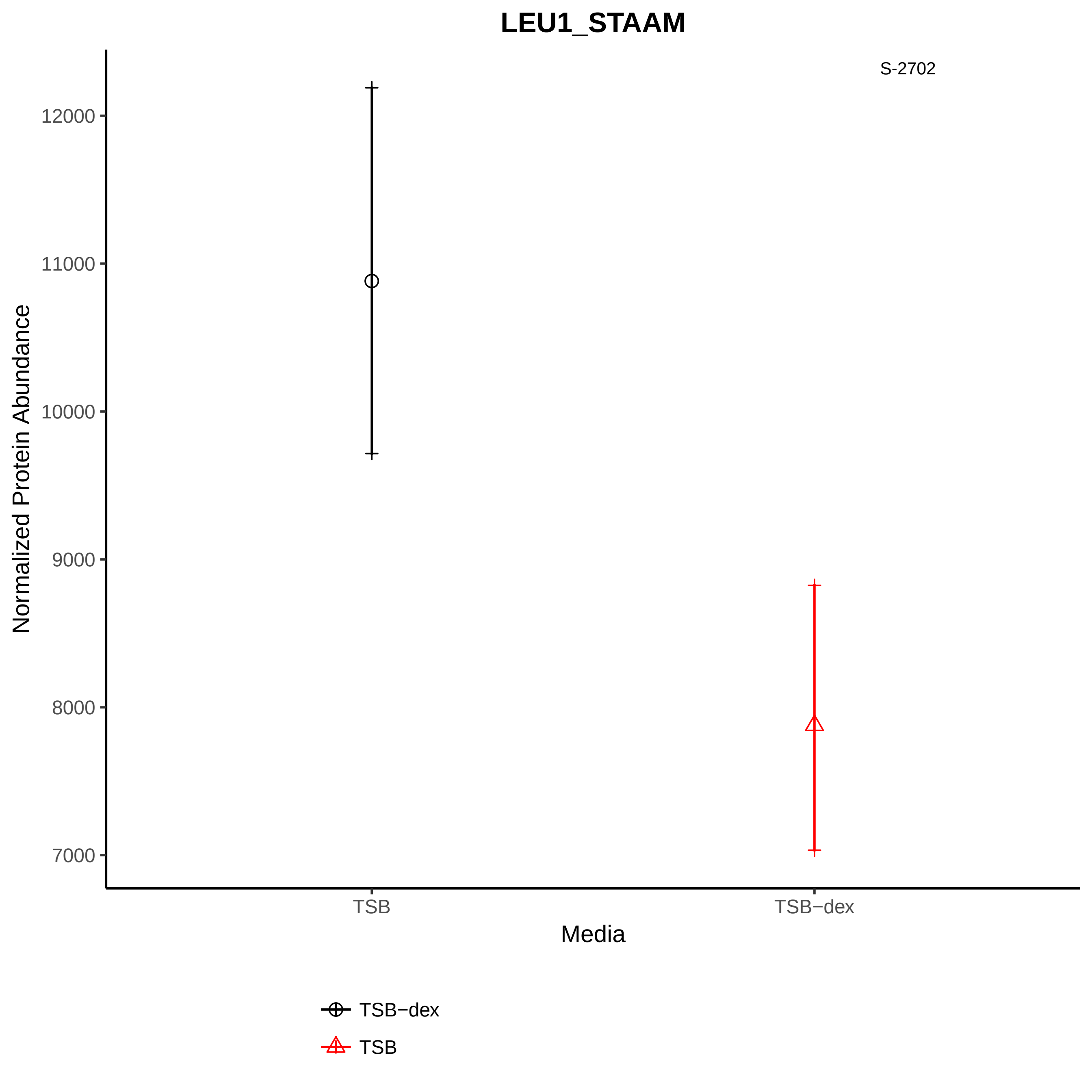
LEU1_STAAM
S-2702
Normalized Protein Abundance
12000
11000
10000
9000
8000
7000
TSB
TSB−dex
Media
TSB−dex
TSB

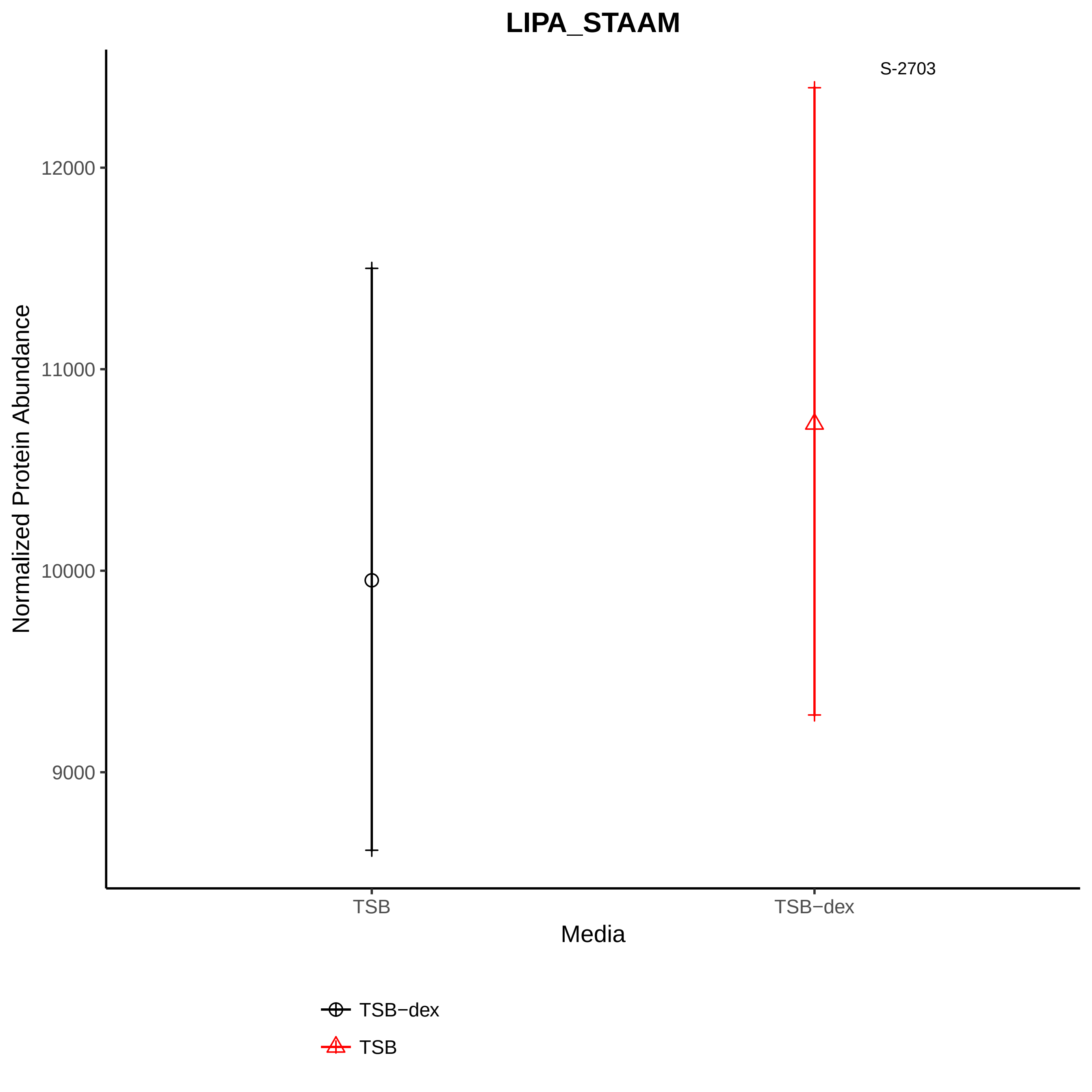

LIPA_STAAM
S-2703
Normalized Protein Abundance
12000
11000
10000
9000
TSB
TSB−dex
Media
TSB−dex
TSB

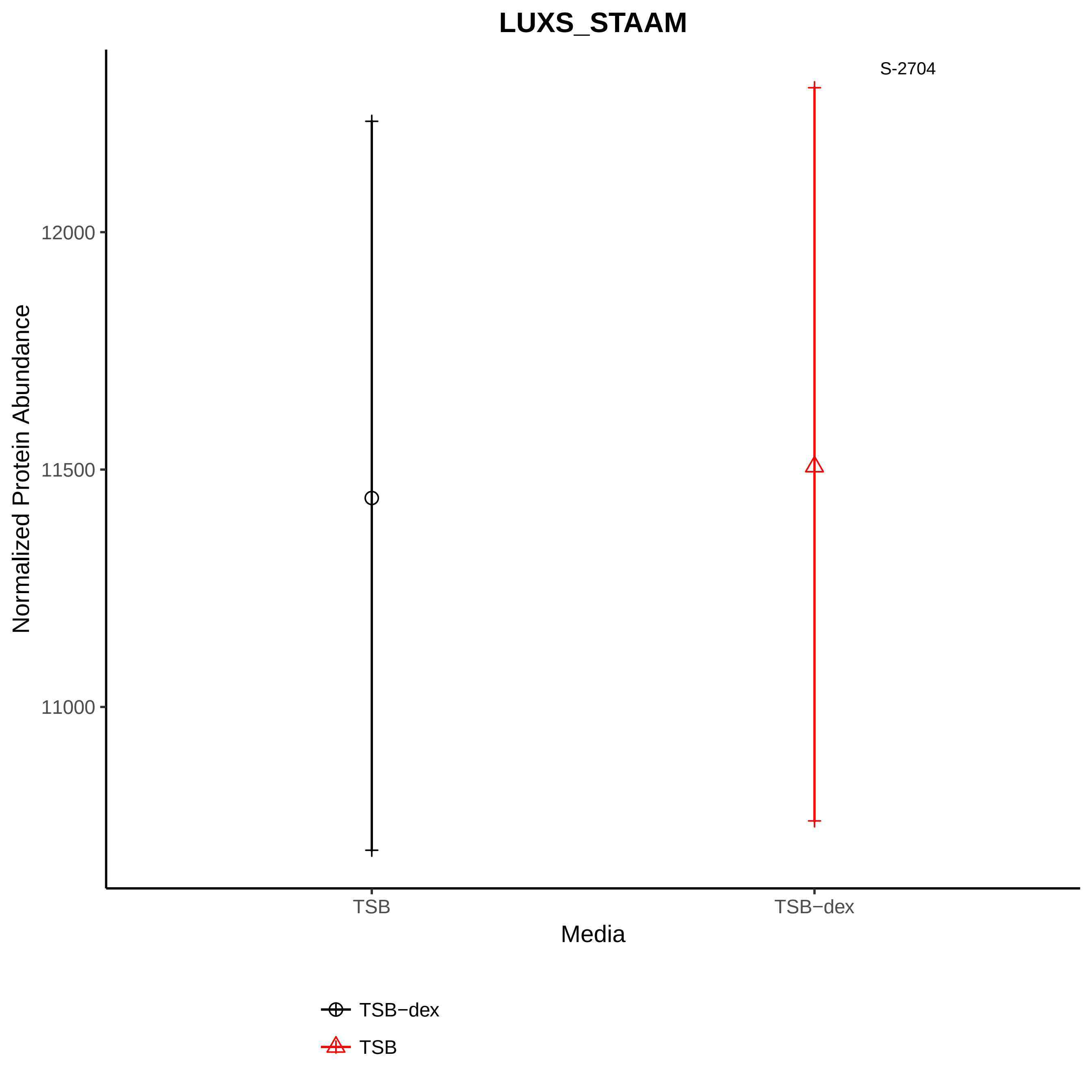

LUXS_STAAM
S-2704
Normalized Protein Abundance
12000
11500
11000
TSB
TSB−dex
Media
TSB−dex
TSB

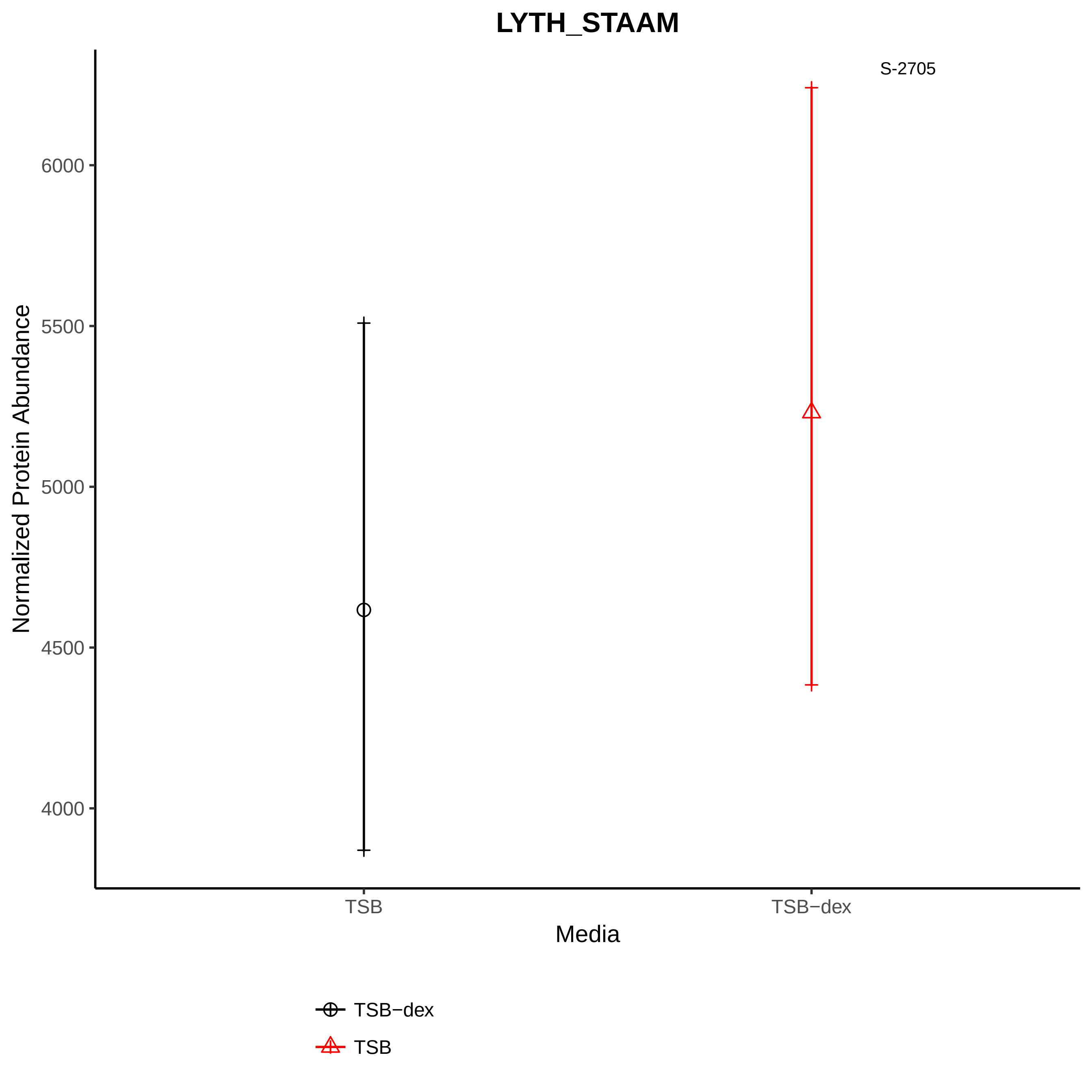

LYTH_STAAM
S-2705
6000
5500
5000
4500
4000
Normalized Protein Abundance
TSB
TSB−dex
Media
TSB−dex
TSB

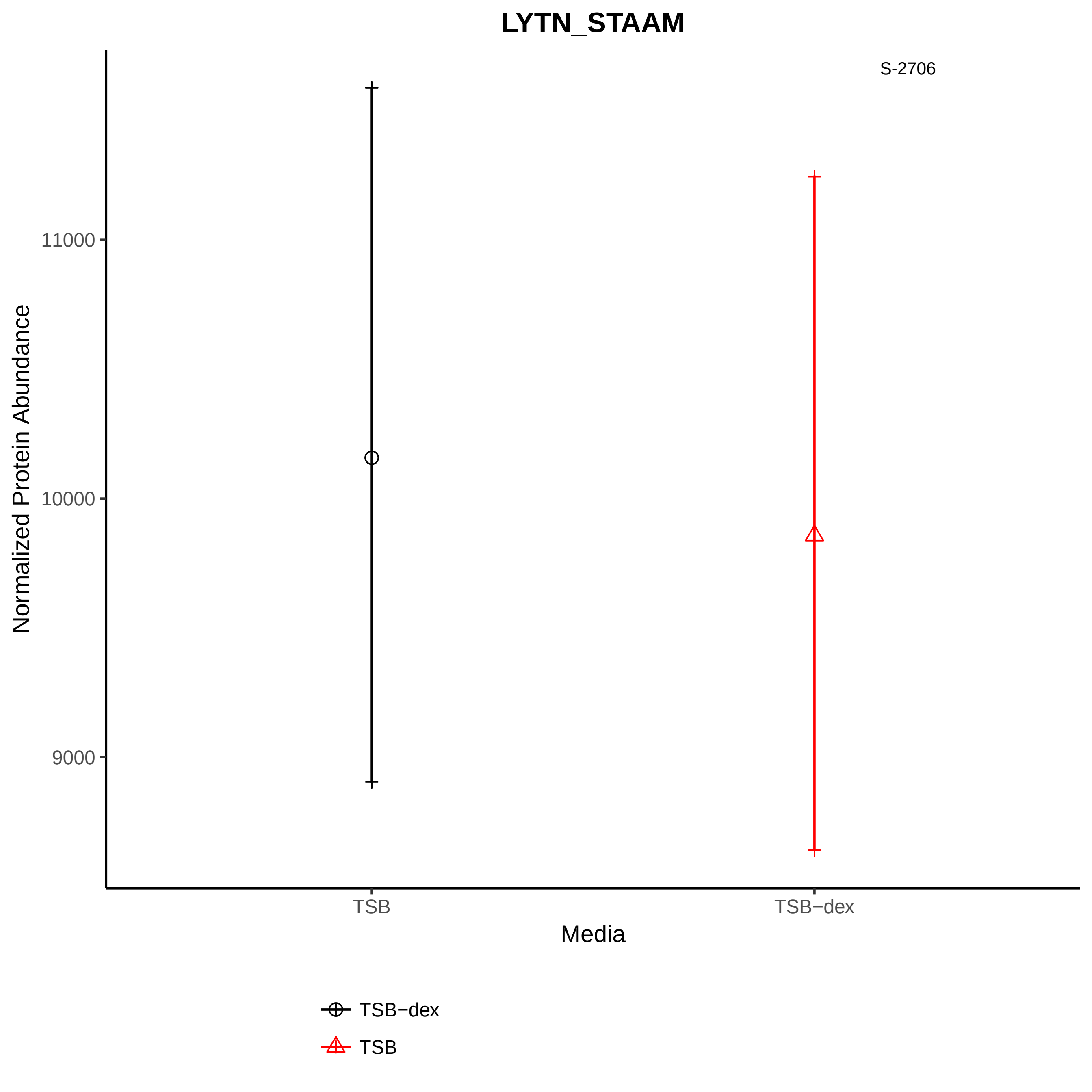

LYTN_STAAM
S-2706
Normalized Protein Abundance
11000
10000
9000
TSB
TSB−dex
Media
TSB−dex
TSB

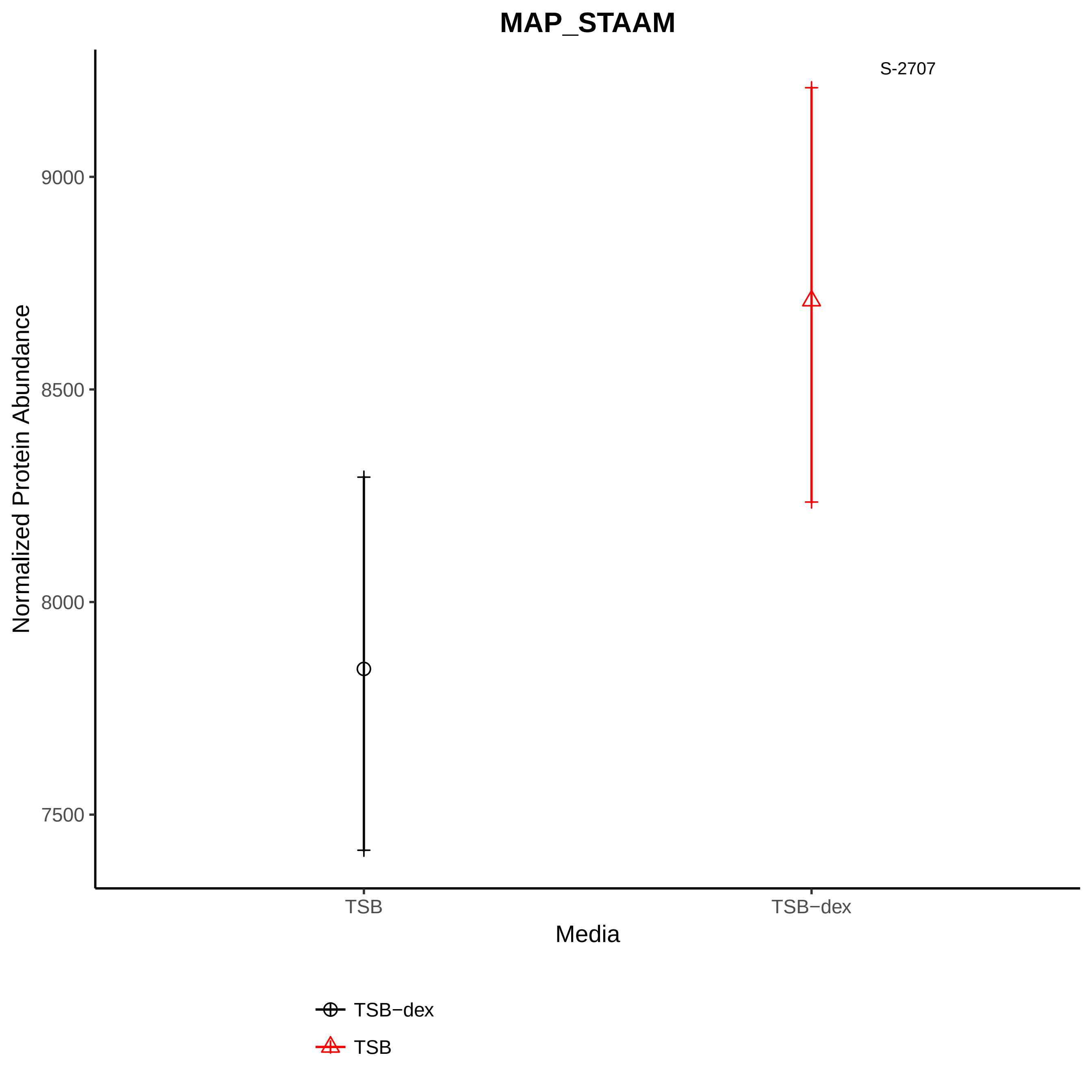

MAP_STAAM
S-2707
Normalized Protein Abundance
9000
8500
8000
7500
TSB
TSB−dex
Media
TSB−dex
TSB

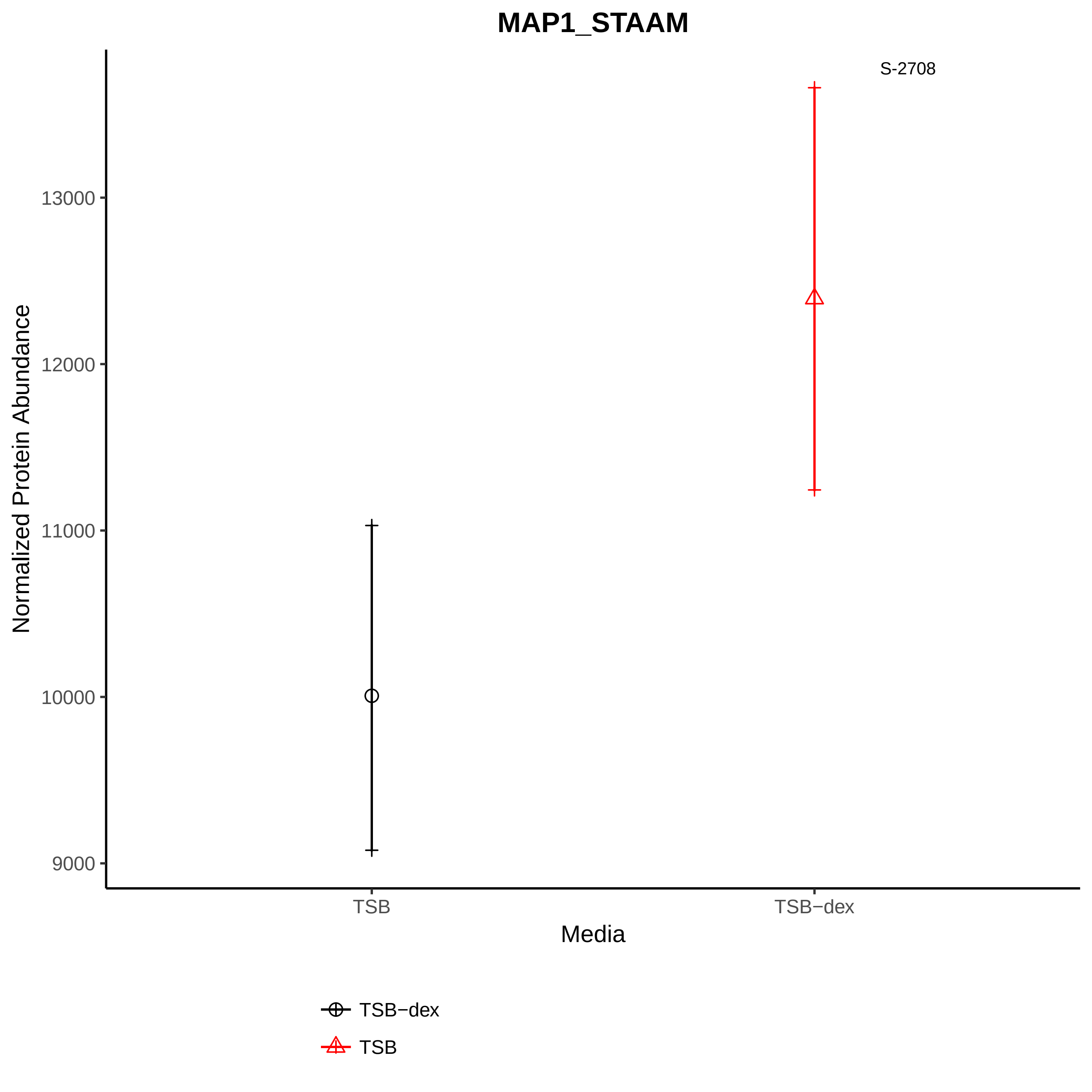
MAP1_STAAM
S-2708
Normalized Protein Abundance
13000
12000
11000
10000
9000
TSB
TSB−dex
Media
TSB−dex
TSB

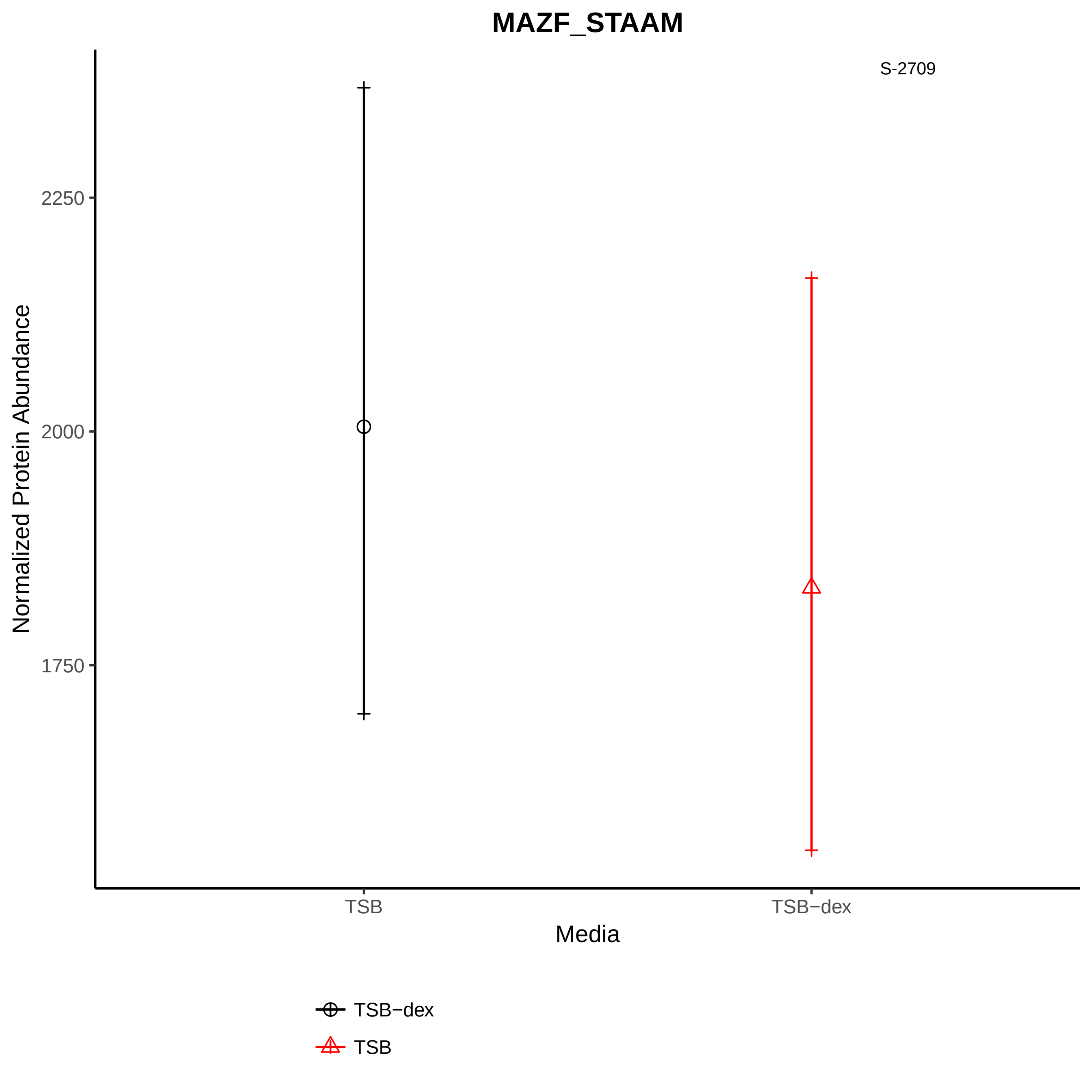
MAZF_STAAM
S-2709
2250
2000
1750
Normalized Protein Abundance
TSB
TSB−dex
Media
TSB−dex
TSB

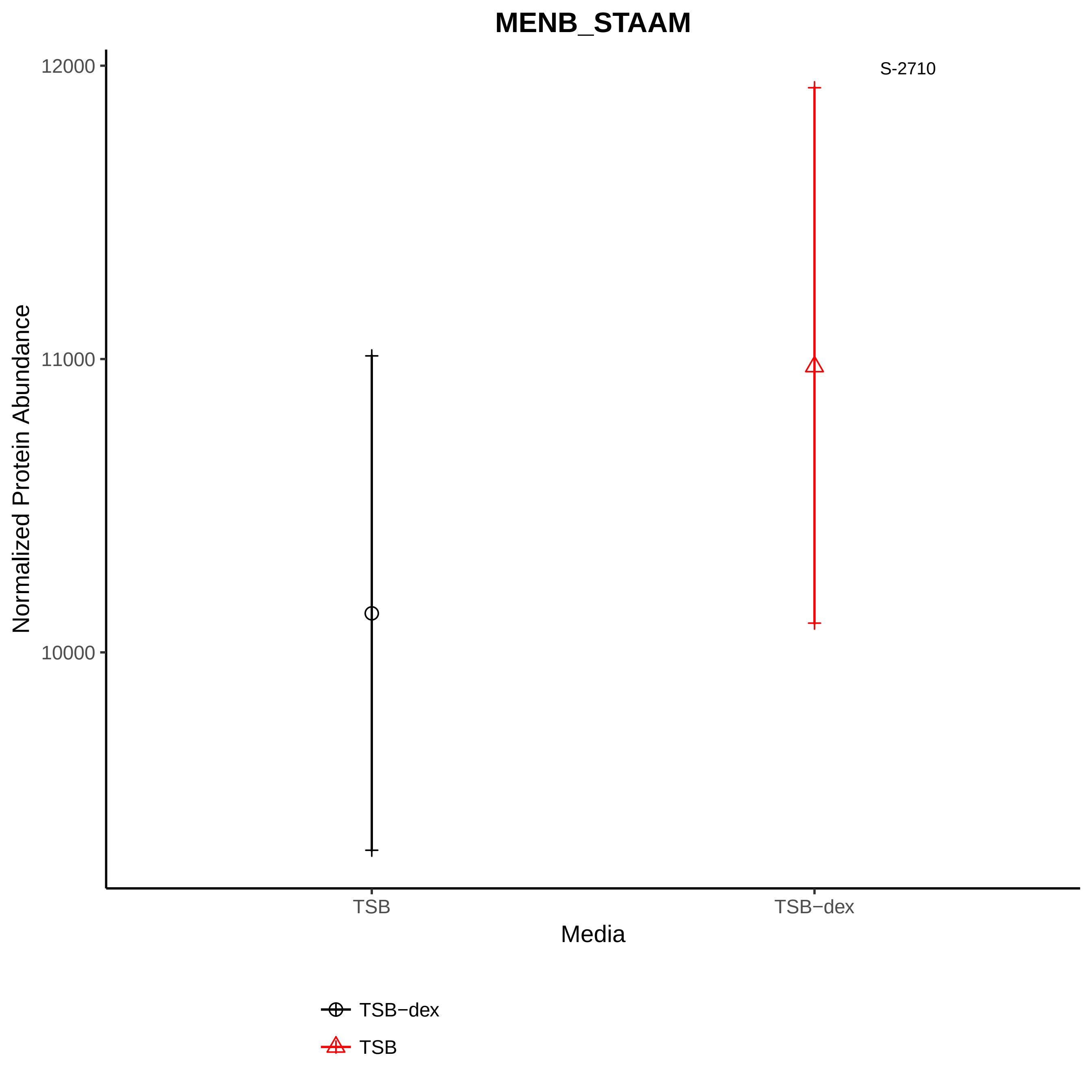

MENB_STAAM
S-2710
12000
11000
10000
Normalized Protein Abundance
TSB
TSB−dex
Media
TSB−dex
TSB

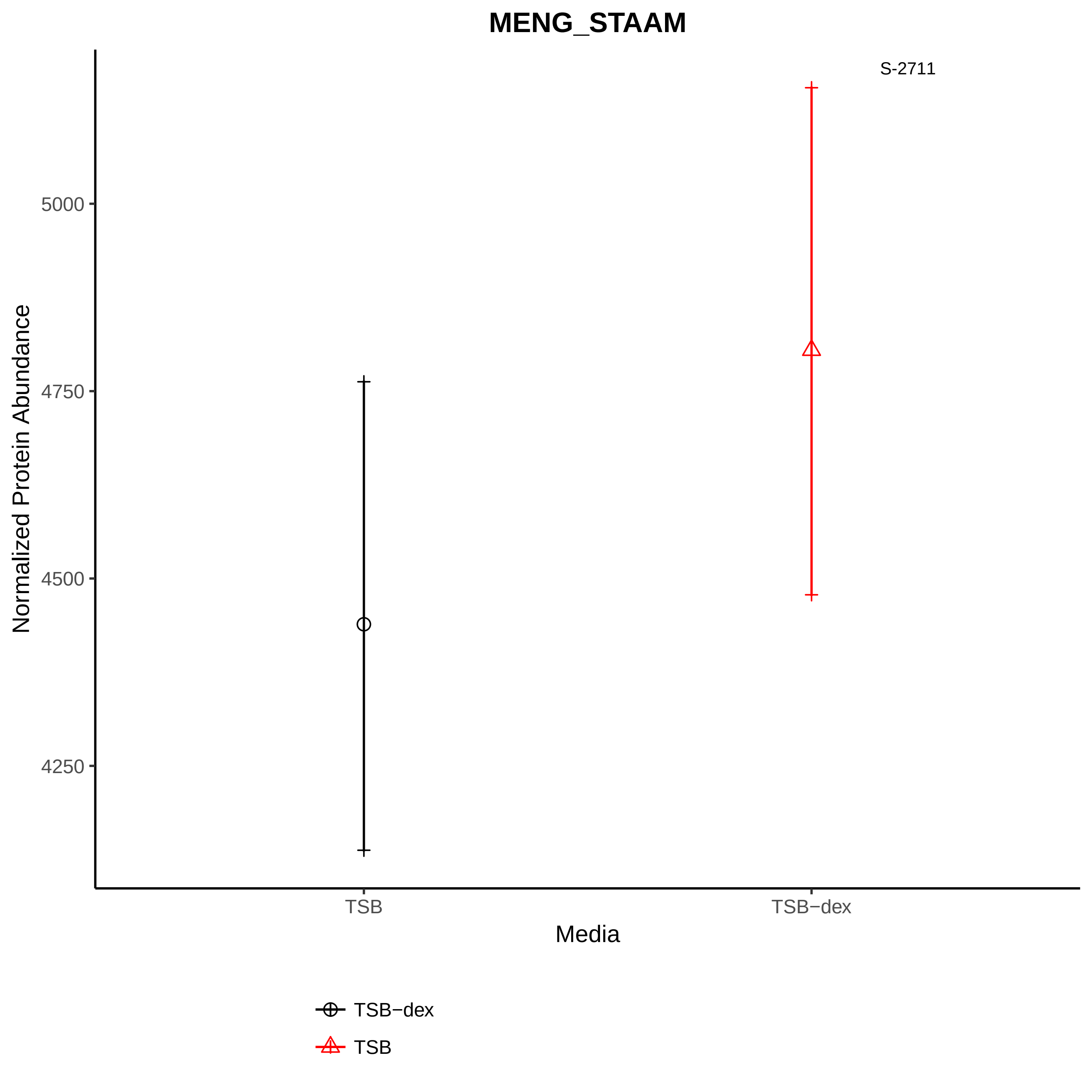

MENG_STAAM
S-2711
5000
4750
4500
4250
Normalized Protein Abundance
TSB
TSB−dex
Media
TSB−dex
TSB

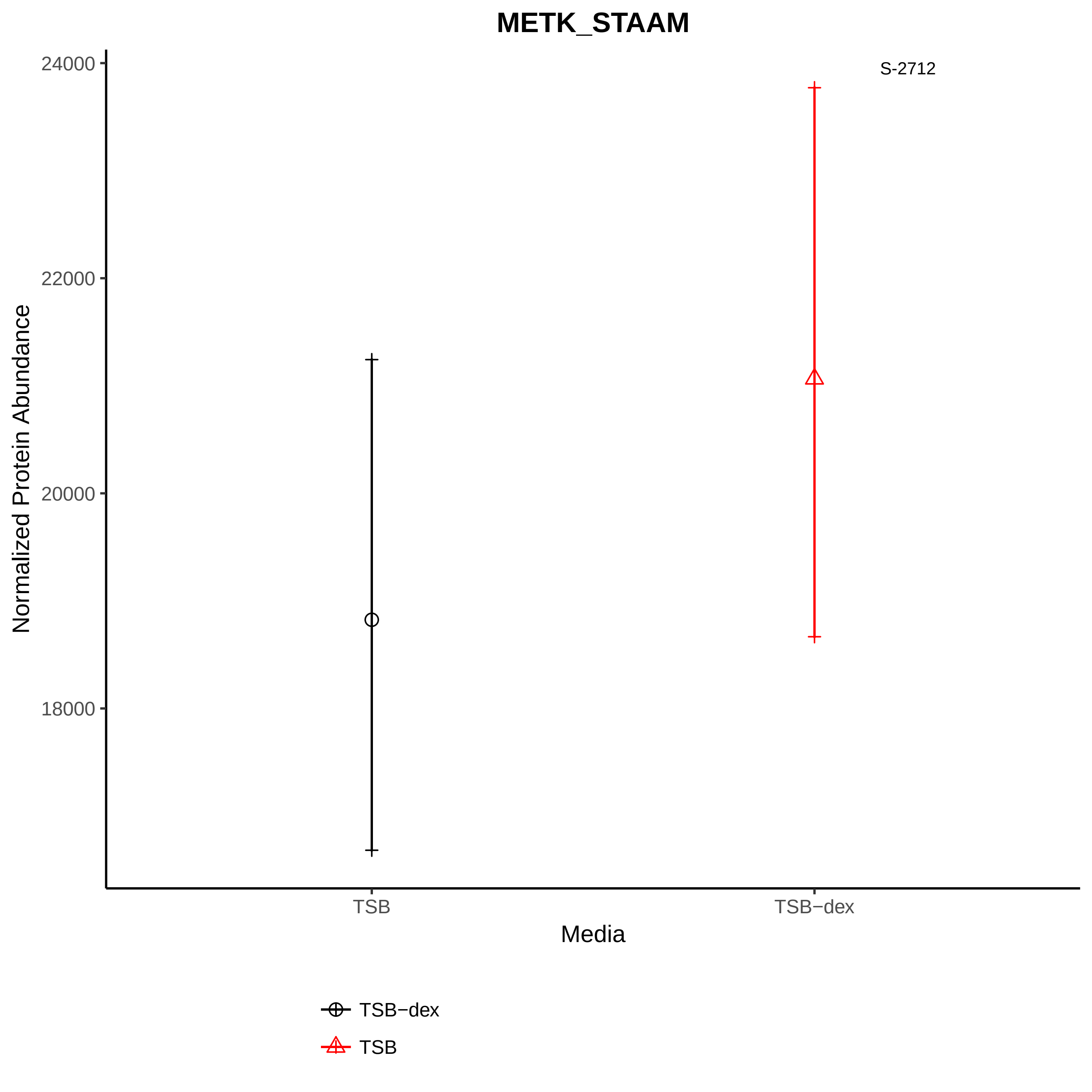

METK_STAAM
S-2712
Normalized Protein Abundance
24000
22000
20000
18000
TSB
TSB−dex
Media
TSB−dex
TSB

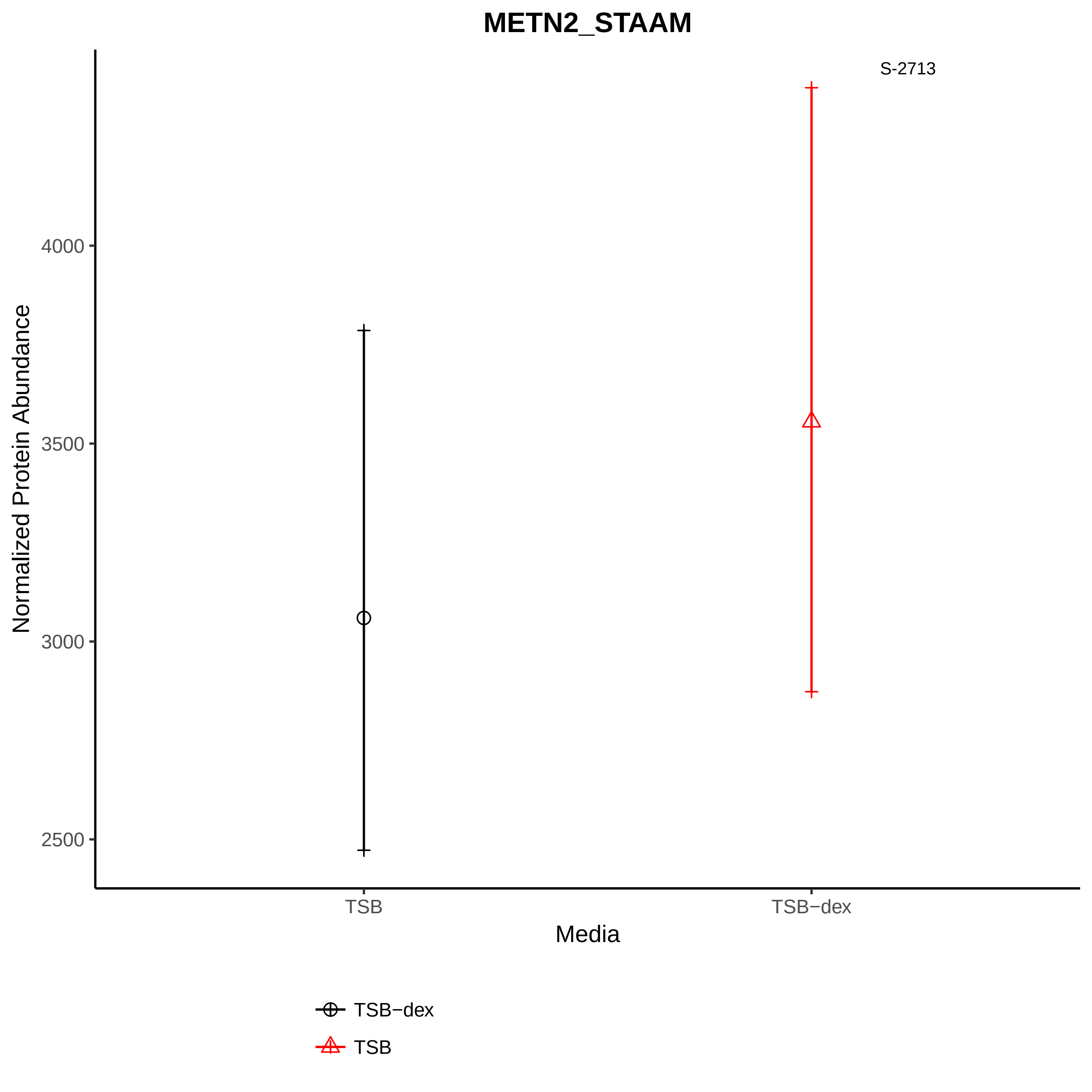

METN2_STAAM
S-2713
Normalized Protein Abundance
4000
3500
3000
2500
TSB
TSB−dex
Media
TSB−dex
TSB

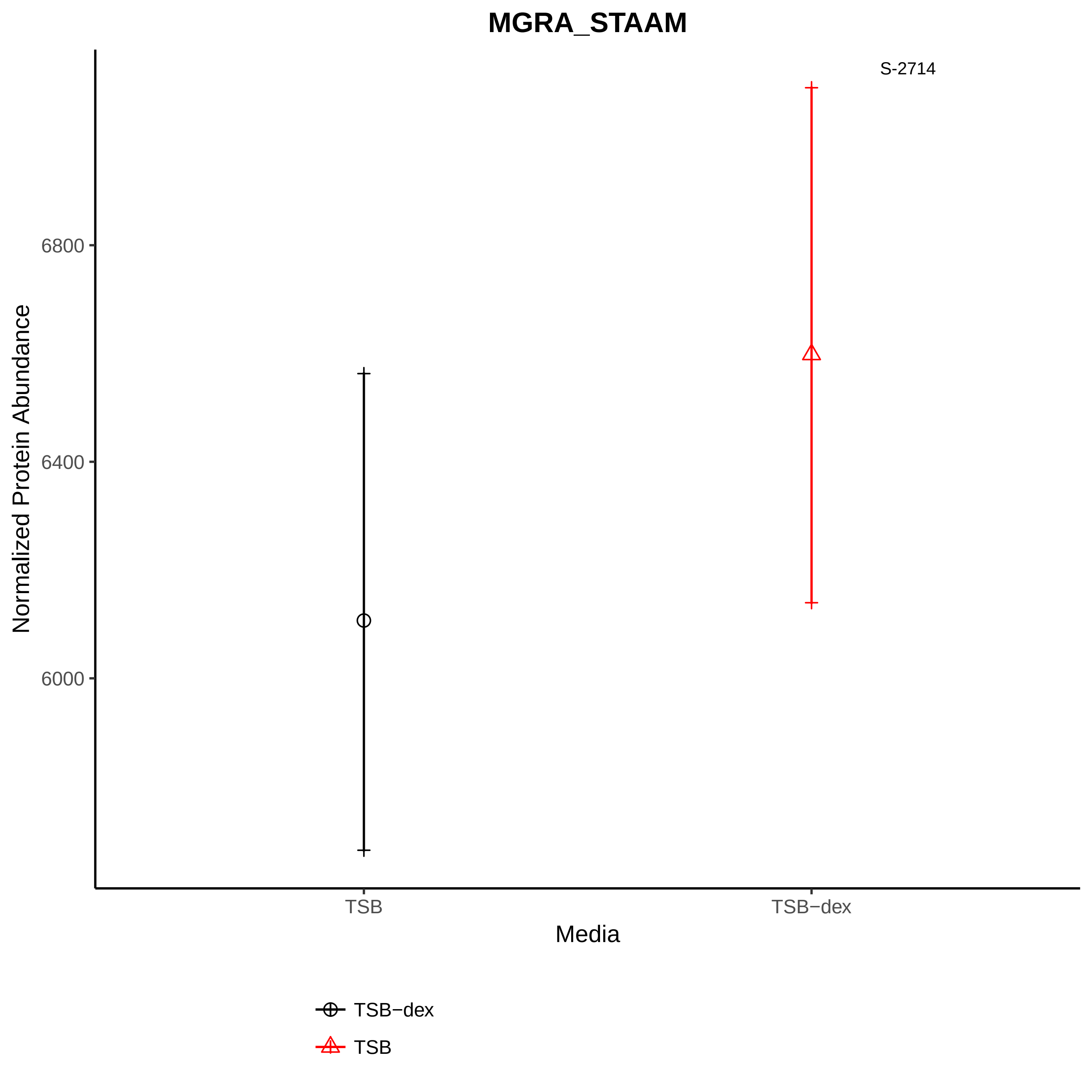

MGRA_STAAM
S-2714
Normalized Protein Abundance
6800
6400
6000
TSB
TSB−dex
Media
TSB−dex
TSB

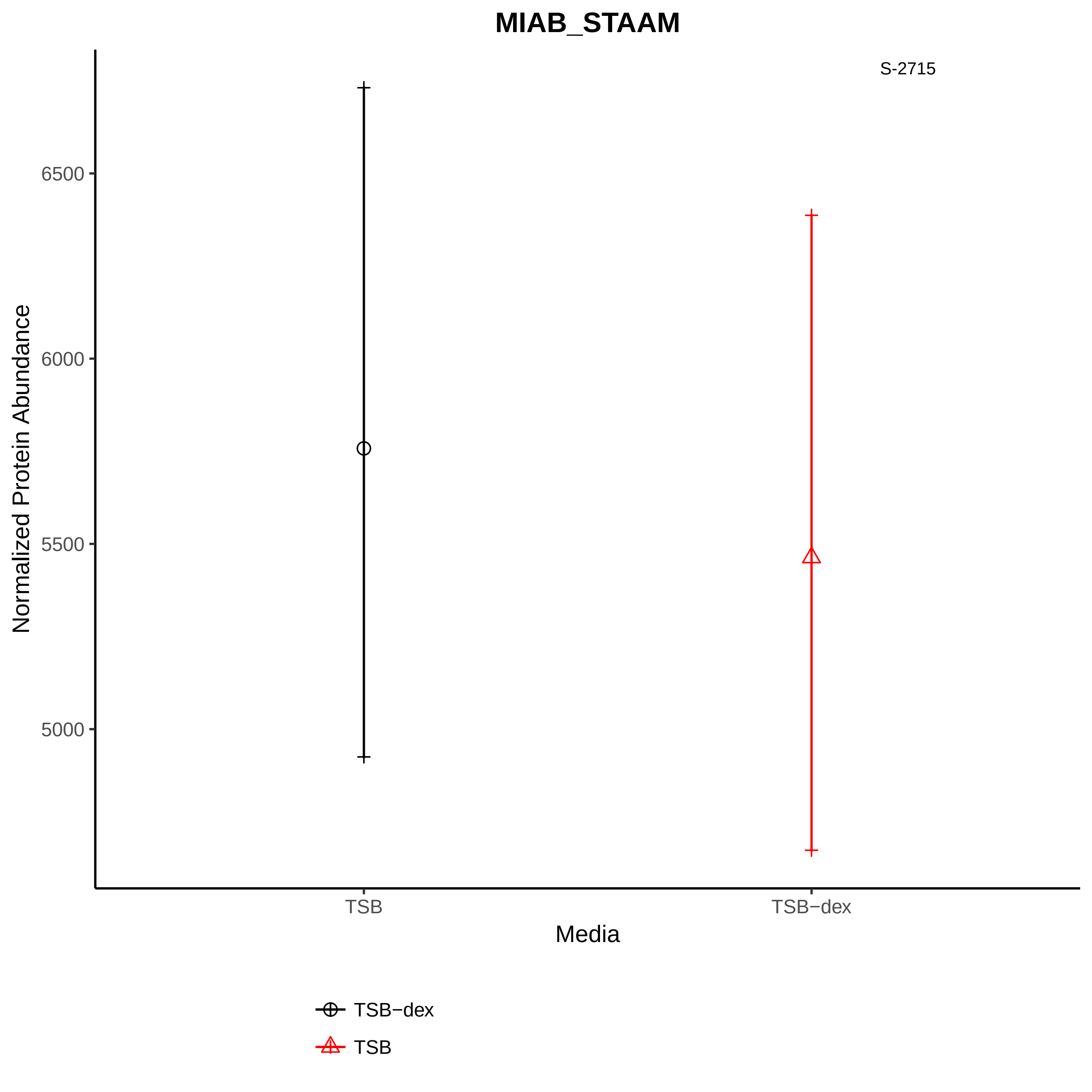

MIAB_STAAM
S-2715
6500
6000
5500
5000
Normalized Protein Abundance
TSB
TSB−dex
Media
TSB−dex
TSB

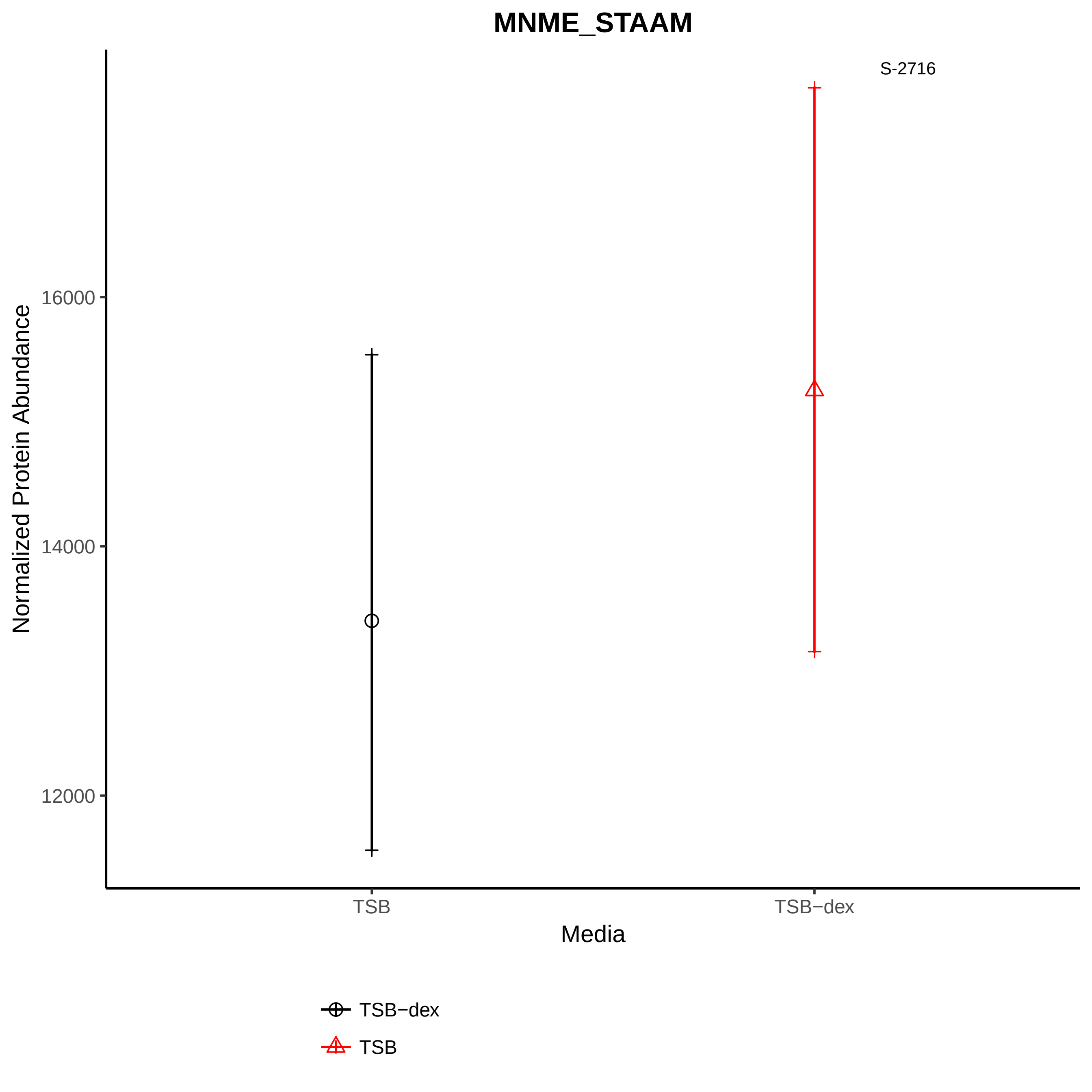

MNME_STAAM
S-2716
Normalized Protein Abundance
16000
14000
12000
TSB
TSB−dex
Media
TSB−dex
TSB

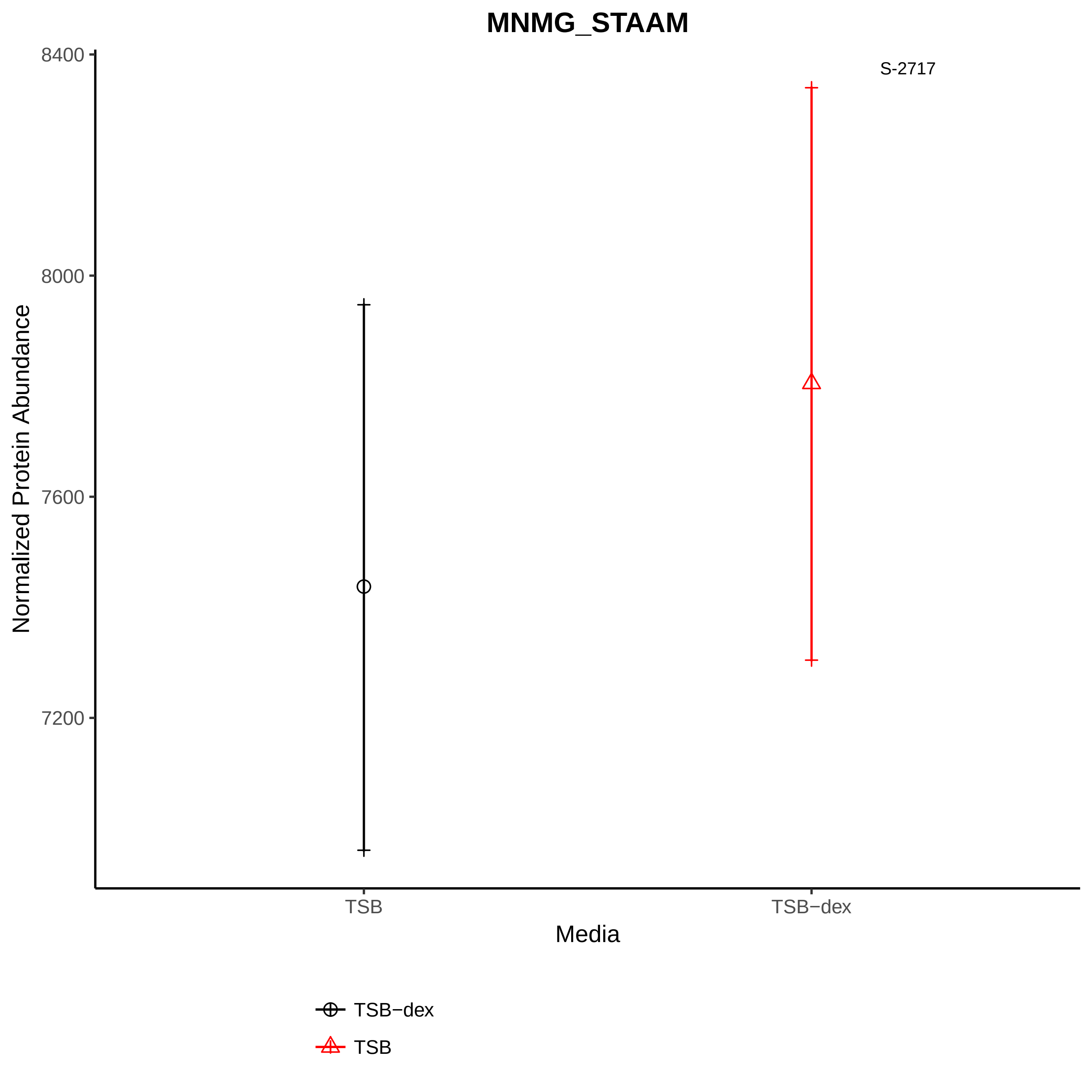

MNMG_STAAM
S-2717
8400
8000
7600
7200
Normalized Protein Abundance
TSB
TSB−dex
Media
TSB−dex
TSB

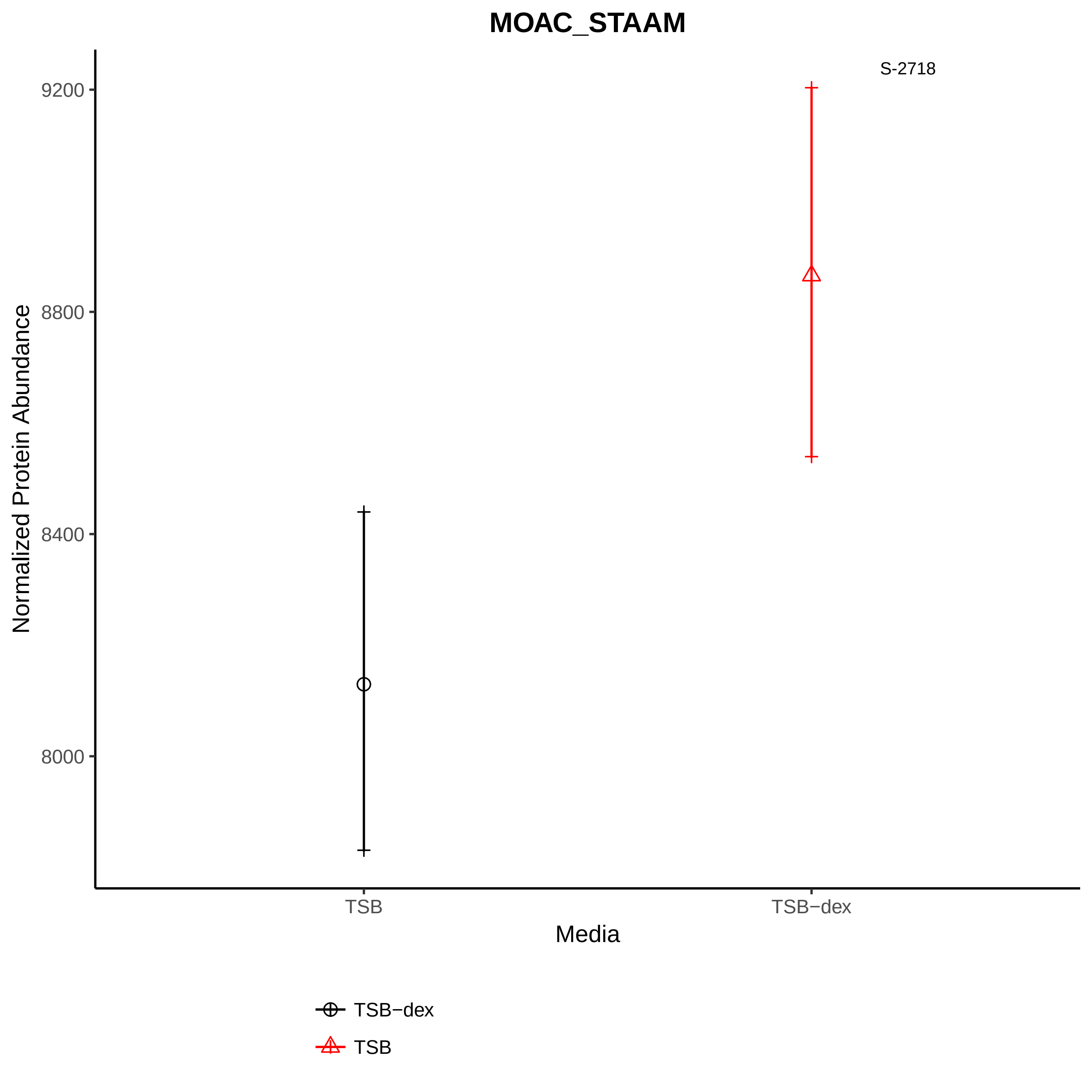
MOAC_STAAM
S-2718
9200
8800
8400
8000
Normalized Protein Abundance
TSB
TSB−dex
Media
TSB−dex
TSB

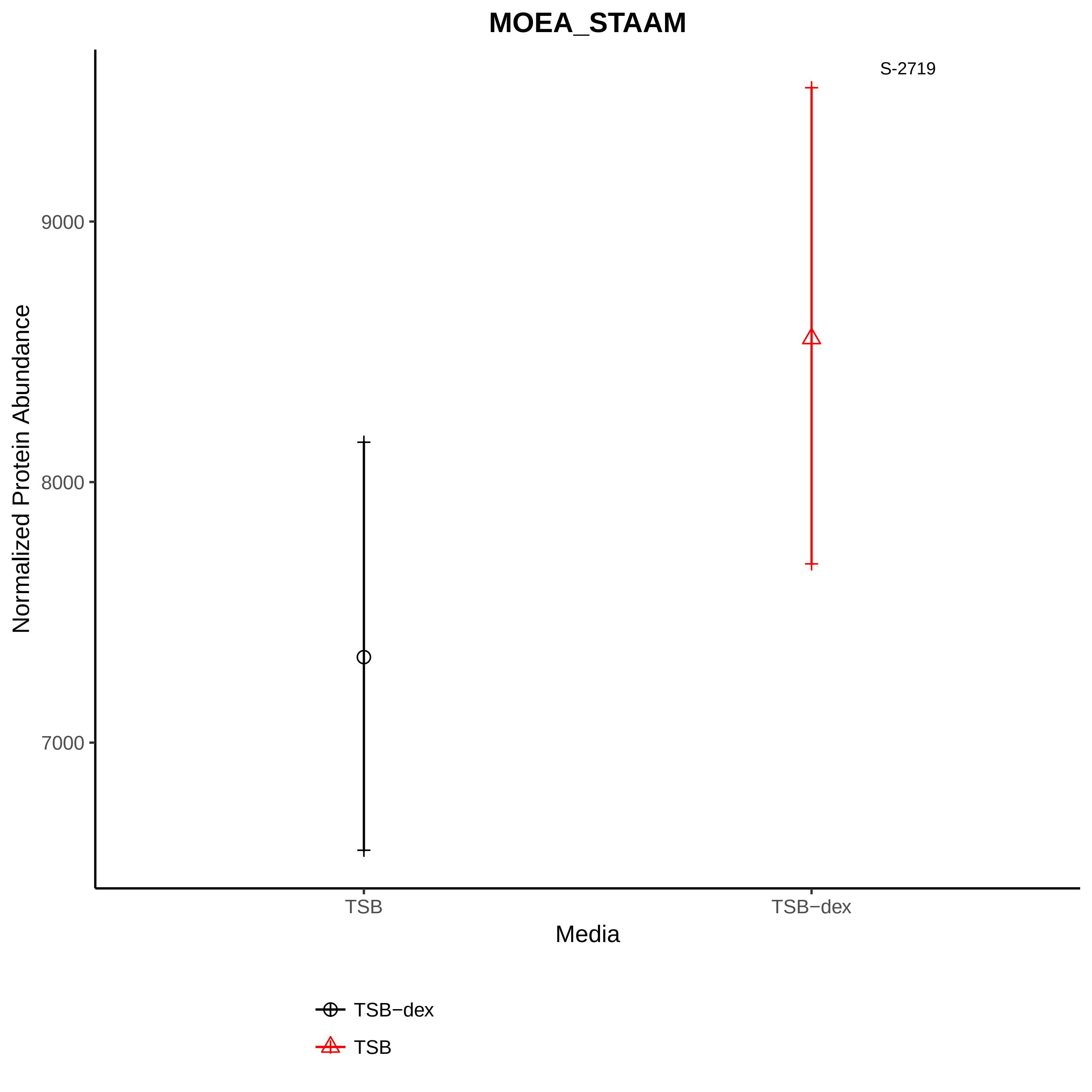

MOEA_STAAM
S-2719
Normalized Protein Abundance
9000
8000
7000
TSB
TSB−dex
Media
TSB−dex
TSB

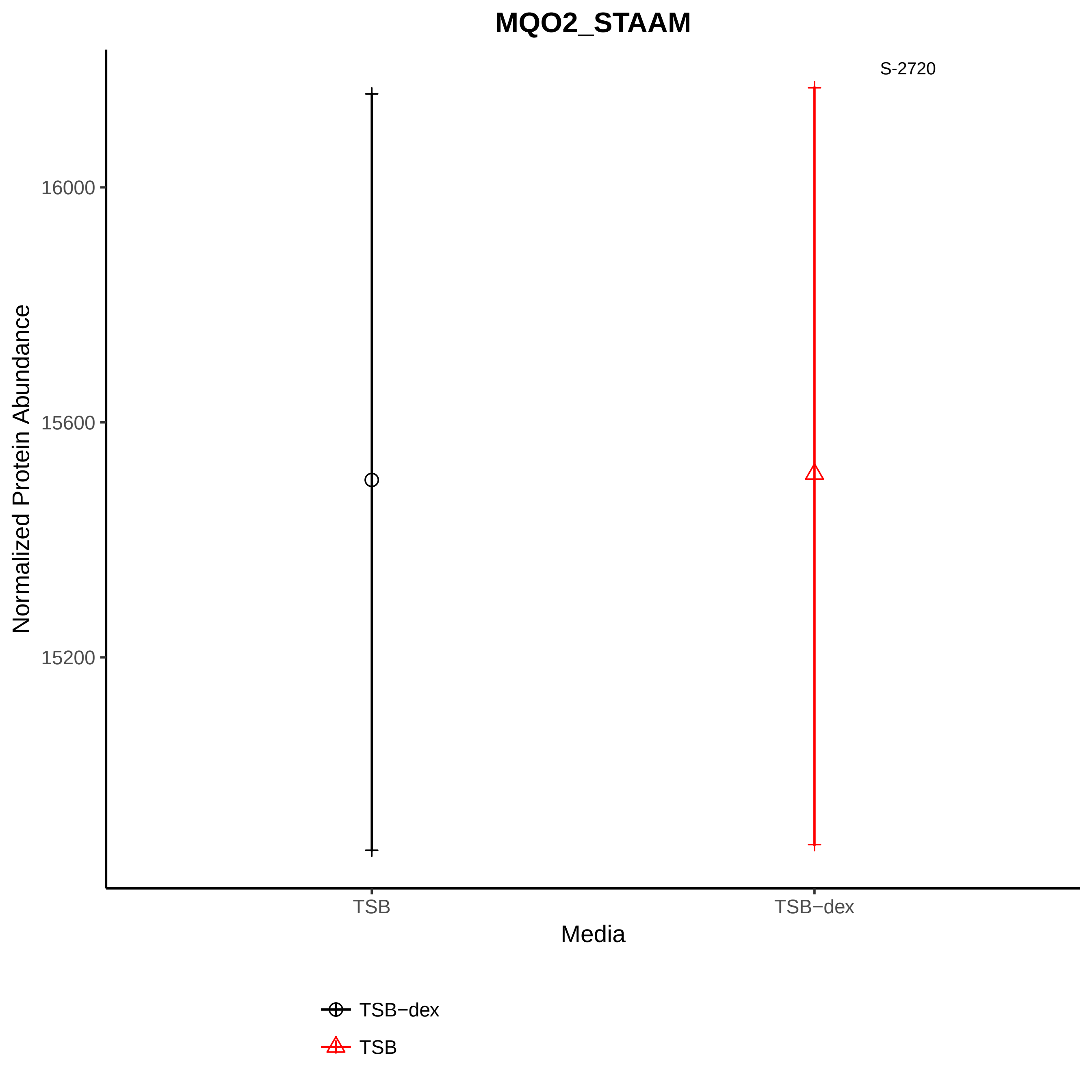
MQO2_STAAM
S-2720
Normalized Protein Abundance
16000
15600
15200
TSB
TSB−dex
Media
TSB−dex
TSB

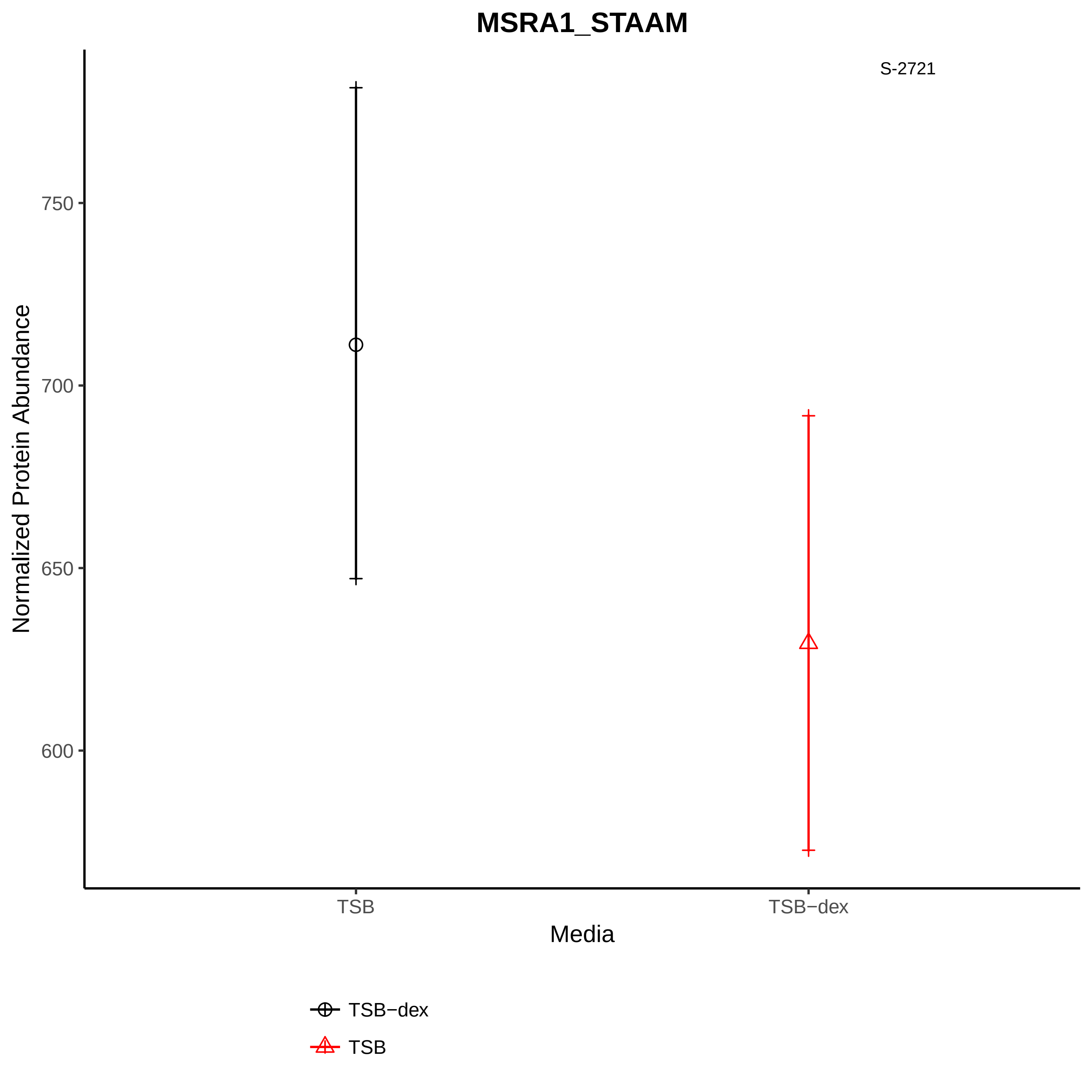
MSRA1_STAAM
S-2721
Normalized Protein Abundance
750
700
650
600
TSB
TSB−dex
Media
TSB−dex
TSB

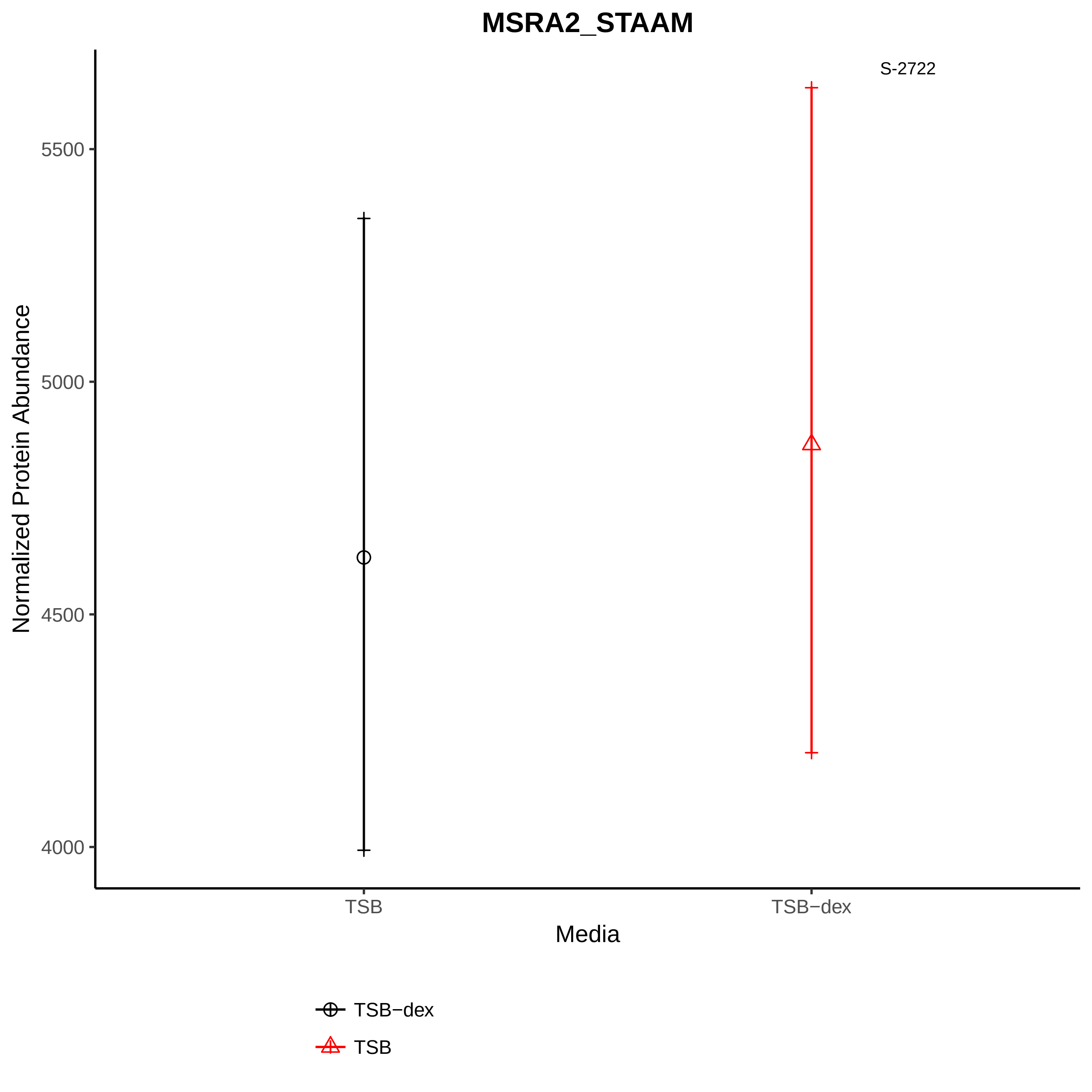
MSRA2_STAAM
S-2722
5500
5000
4500
4000
Normalized Protein Abundance
TSB
TSB−dex
Media
TSB−dex
TSB

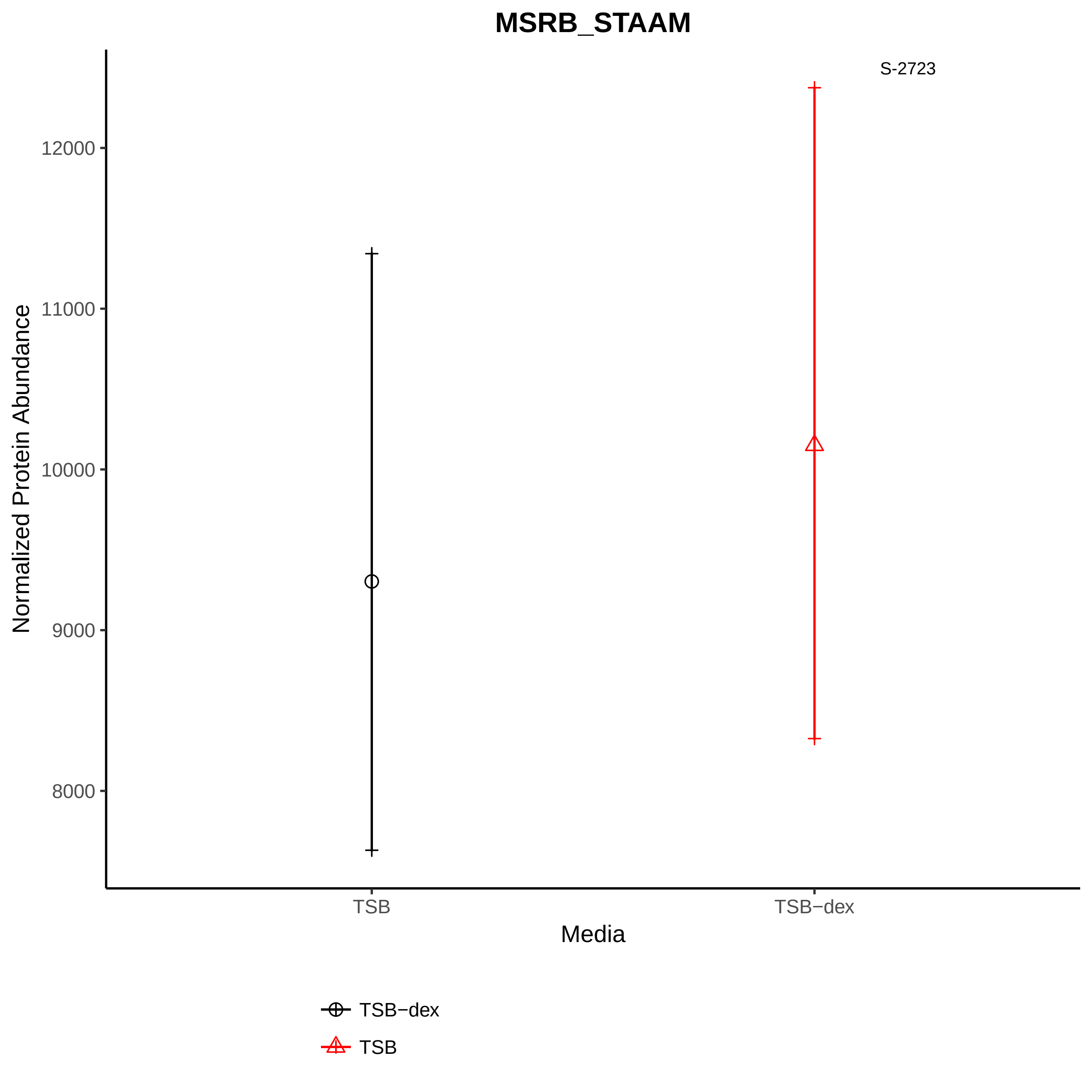

MSRB_STAAM
S-2723
12000
11000
10000
9000
8000
Normalized Protein Abundance
TSB
TSB−dex
Media
TSB−dex
TSB

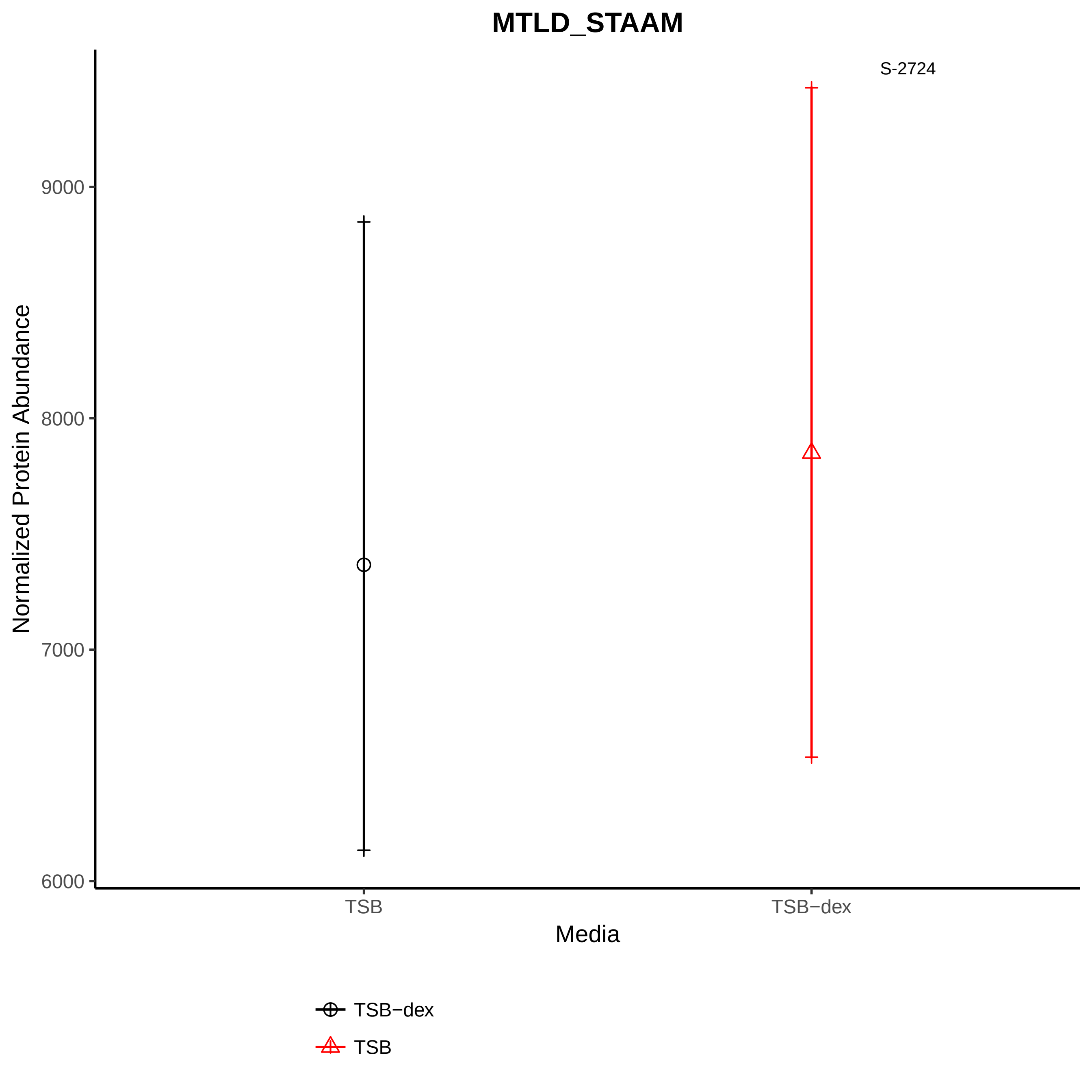
MTLD_STAAM
S-2724
Normalized Protein Abundance
9000
8000
7000
6000
TSB
TSB−dex
Media
TSB−dex
TSB

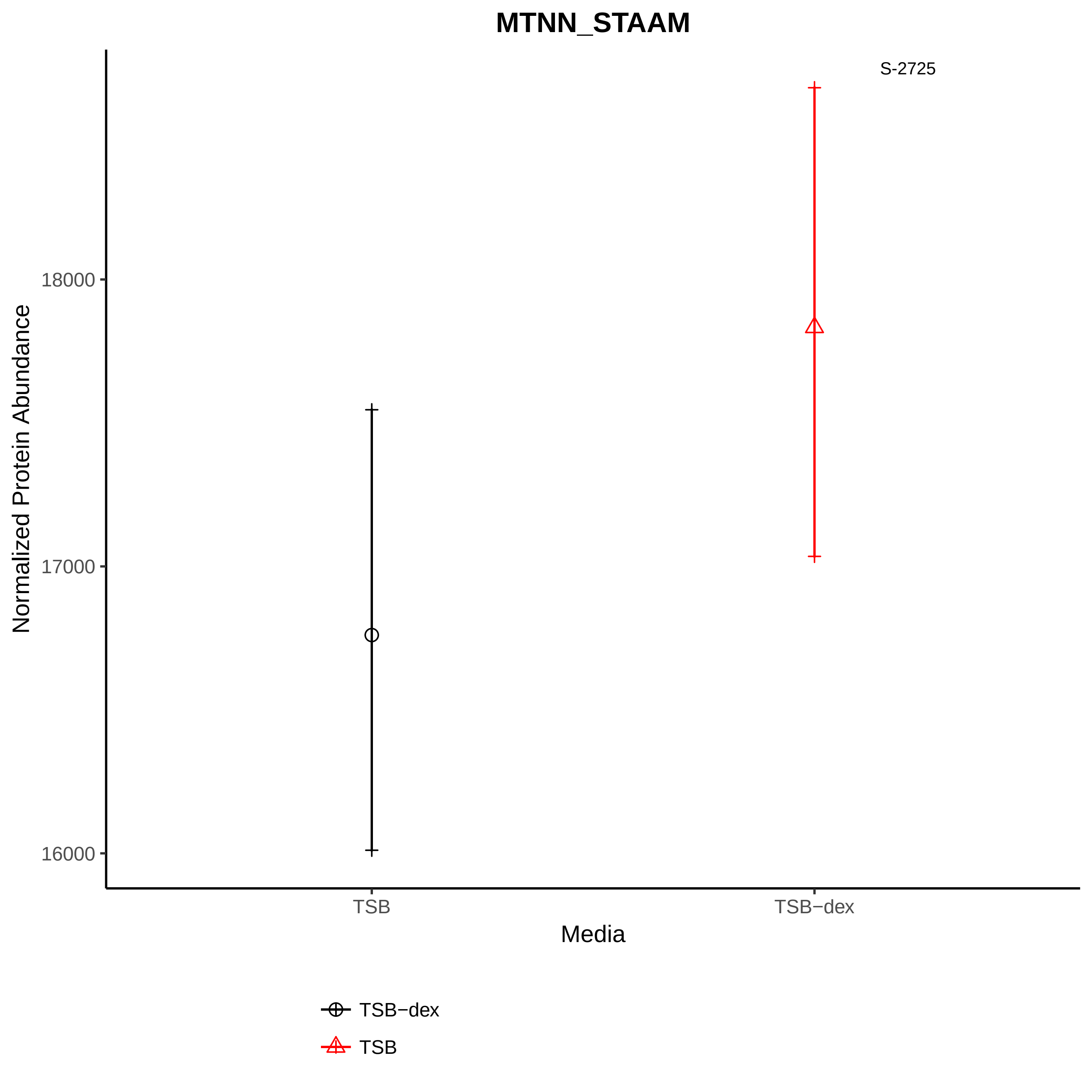

MTNN_STAAM
S-2725
Normalized Protein Abundance
18000
17000
16000
TSB
TSB−dex
Media
TSB−dex
TSB

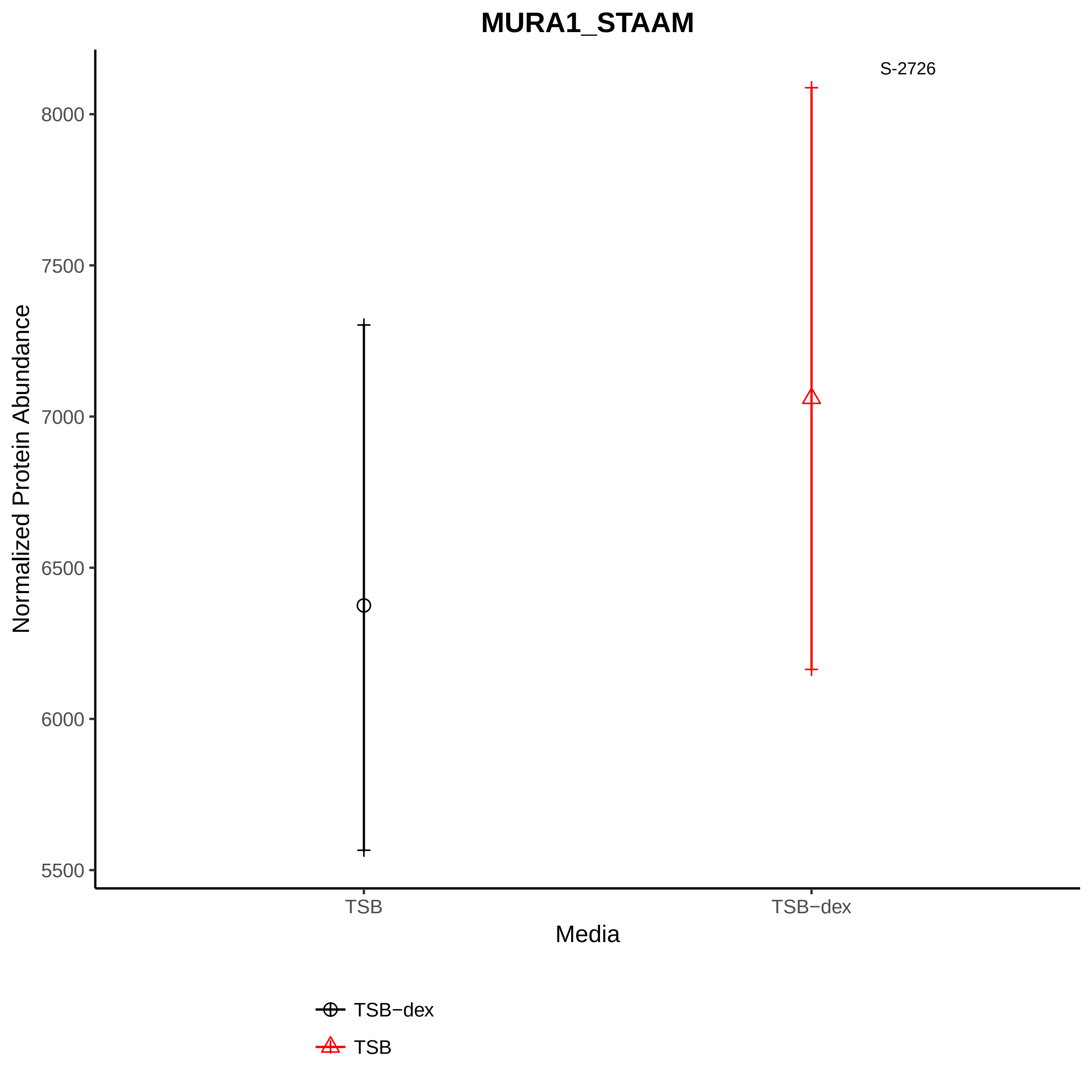
MURA1_STAAM
S-2726
Normalized Protein Abundance
8000
7500
7000
6500
6000
5500
TSB
TSB−dex
Media
TSB−dex
TSB

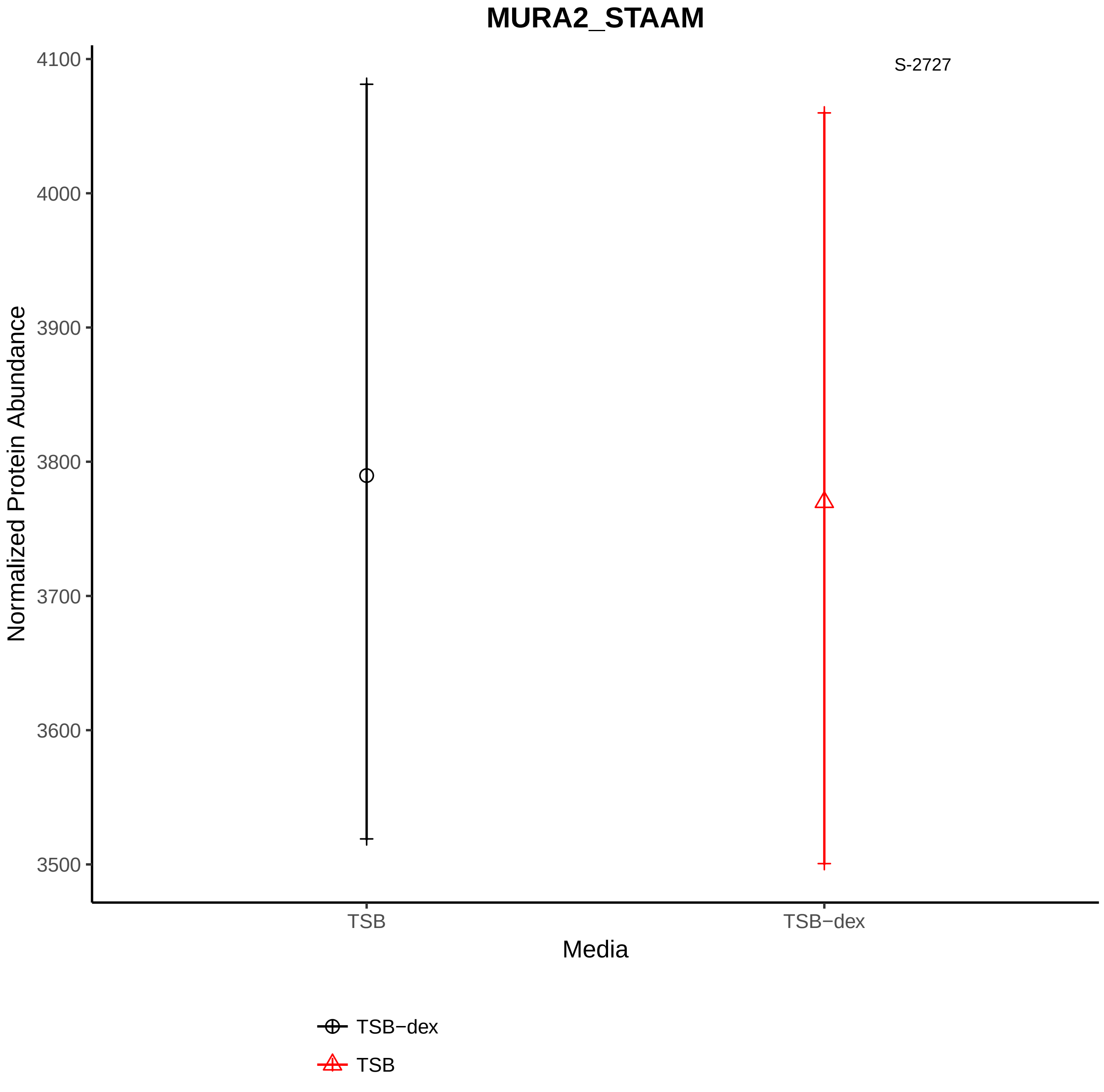
MURA2_STAAM
S-2727
Normalized Protein Abundance
4100
4000
3900
3800
3700
3600
3500
TSB
TSB−dex
Media
TSB−dex
TSB

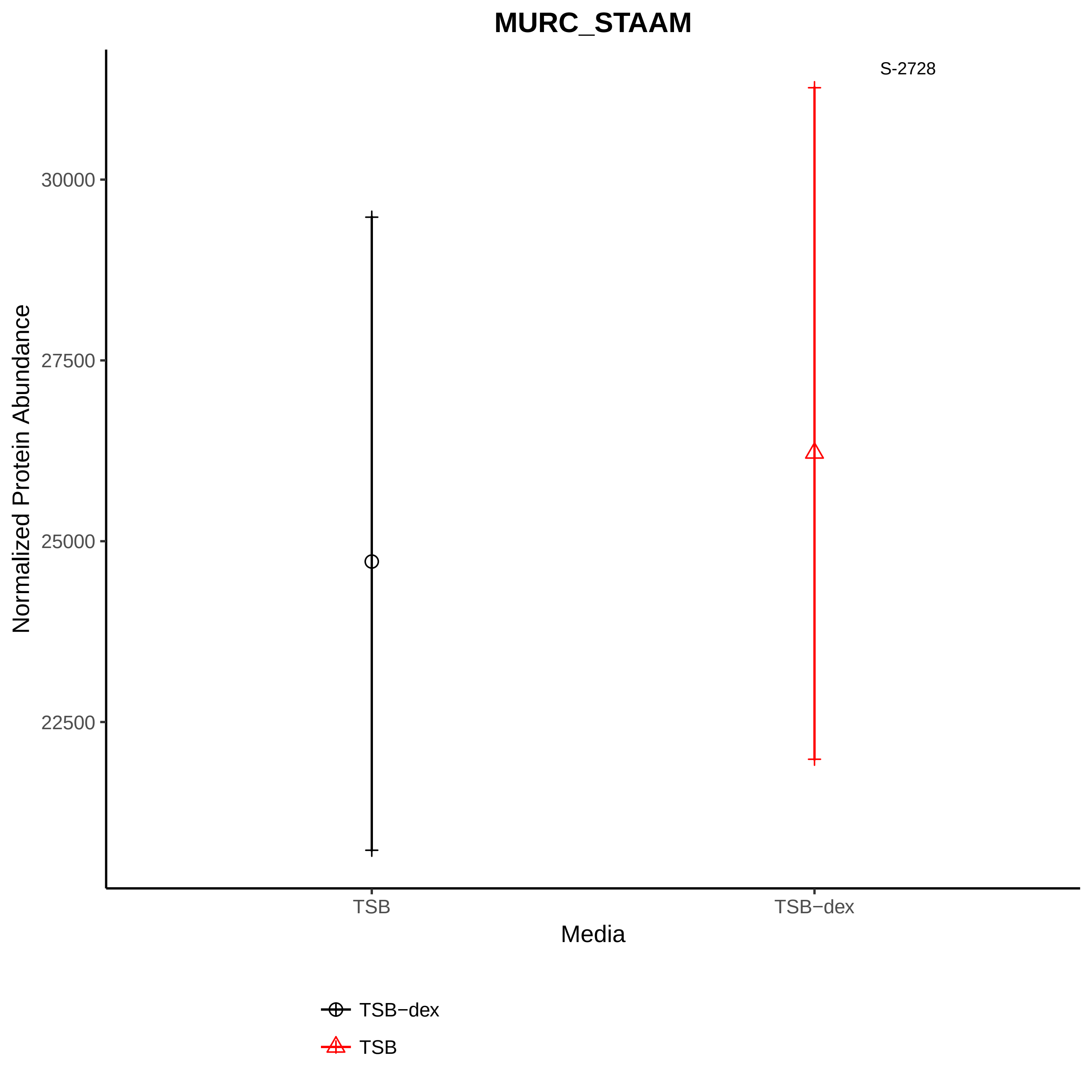

MURC_STAAM
S-2728
Normalized Protein Abundance
30000
27500
25000
22500
TSB
TSB−dex
Media
TSB−dex
TSB

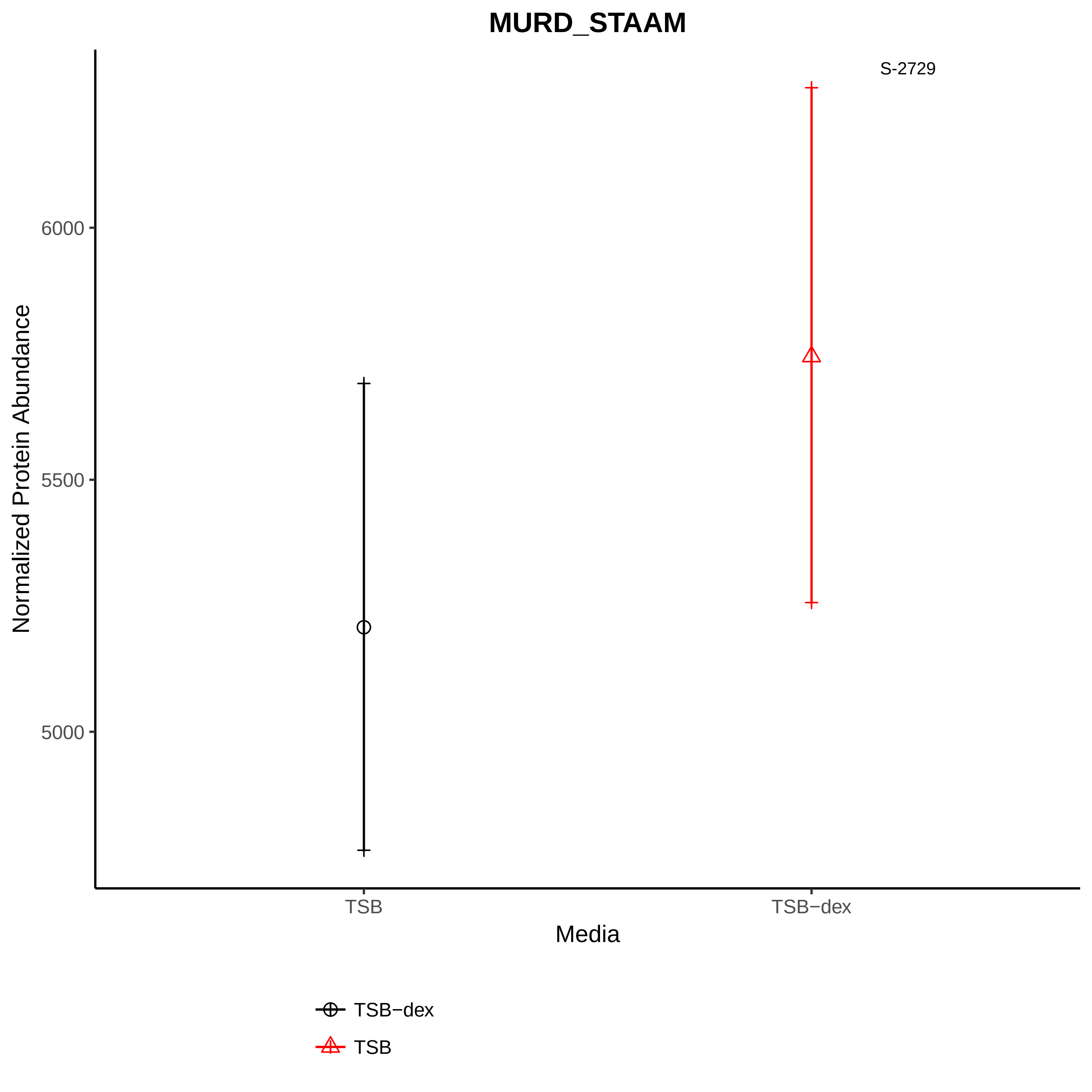

MURD_STAAM
S-2729
6000
5500
5000
Normalized Protein Abundance
TSB
TSB−dex
Media
TSB−dex
TSB

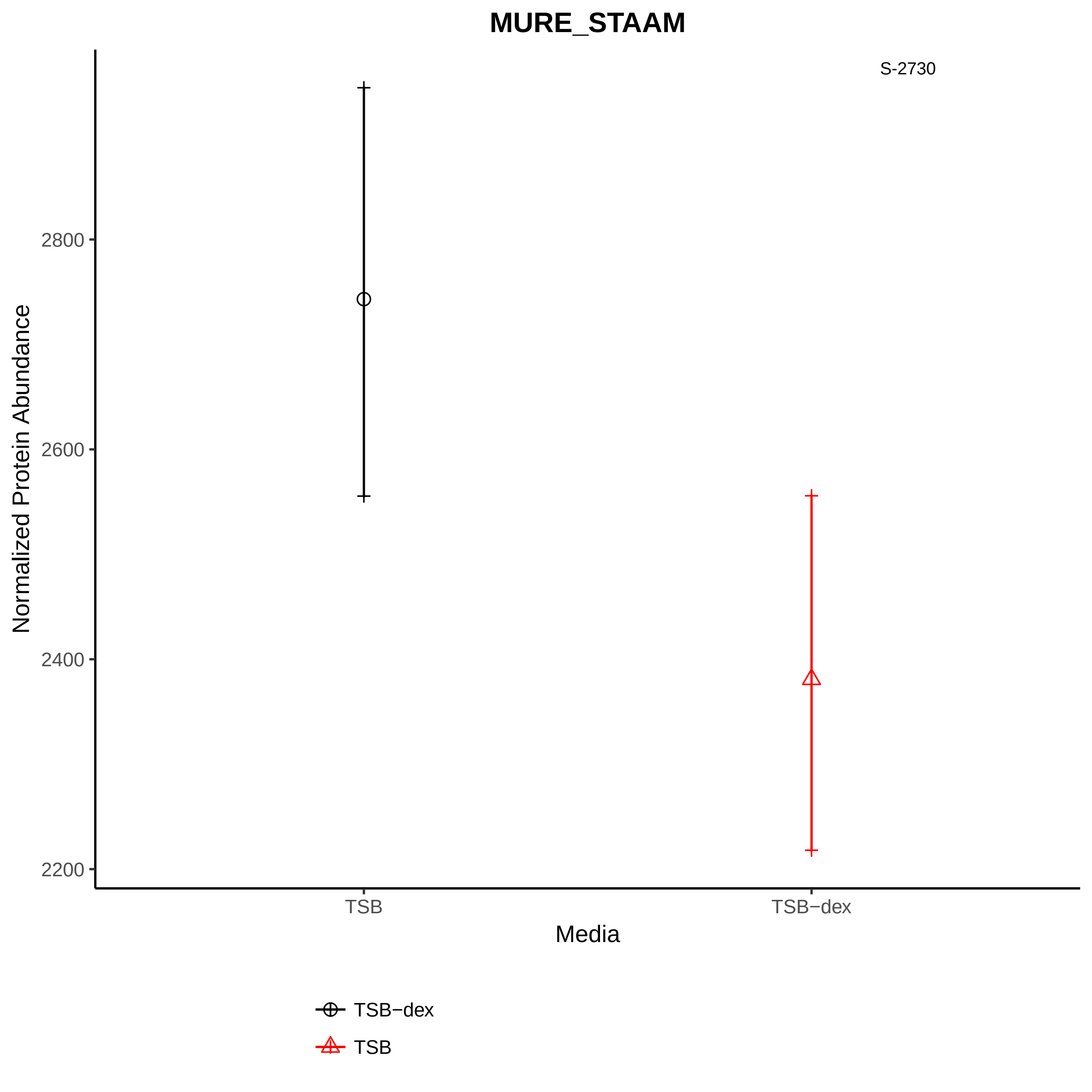

MURE_STAAM
S-2730
Normalized Protein Abundance
2800
2600
2400
2200
TSB
TSB−dex
Media
TSB−dex
TSB

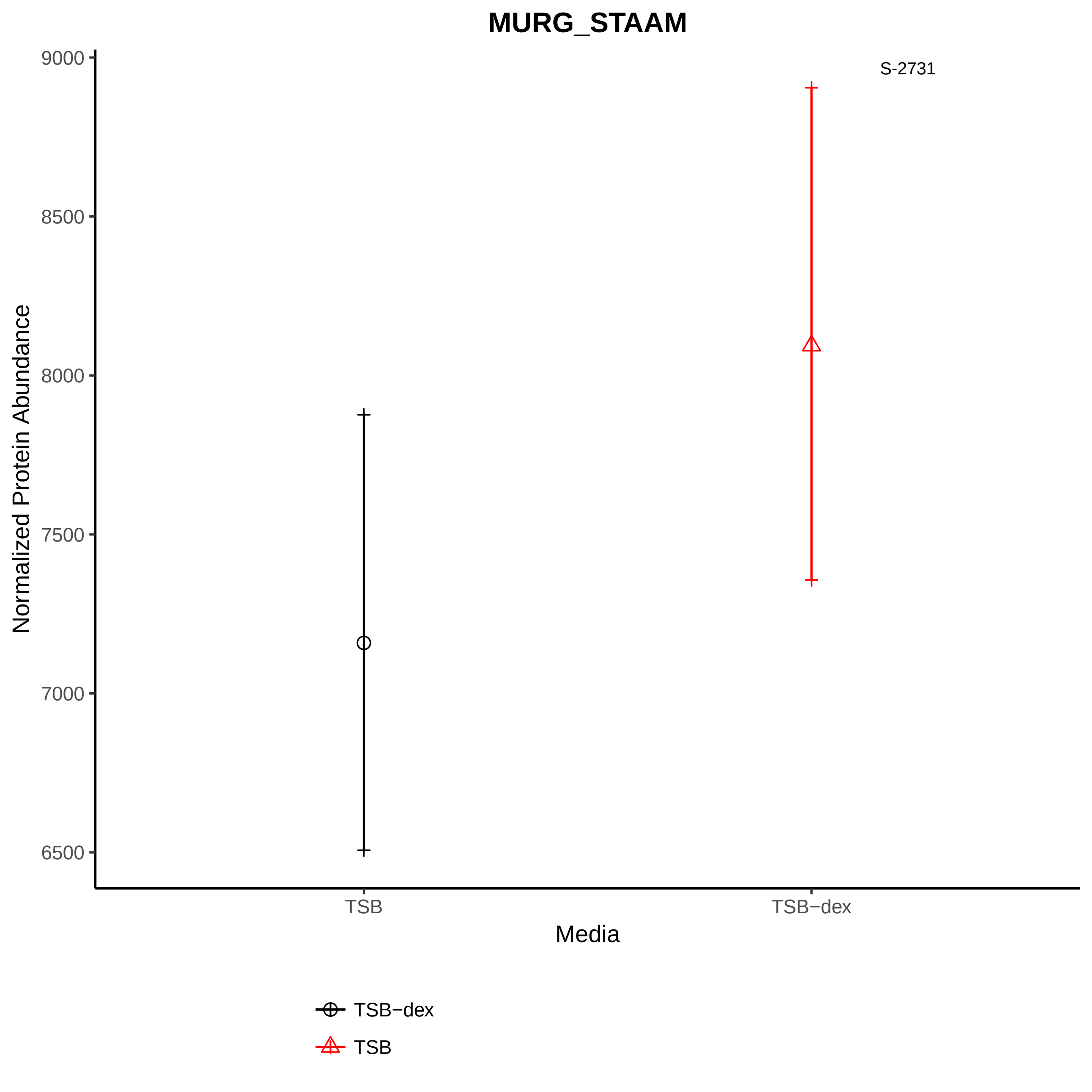

MURG_STAAM
S-2731
9000
8500
8000
7500
7000
6500
Normalized Protein Abundance
TSB
TSB−dex
Media
TSB−dex
TSB

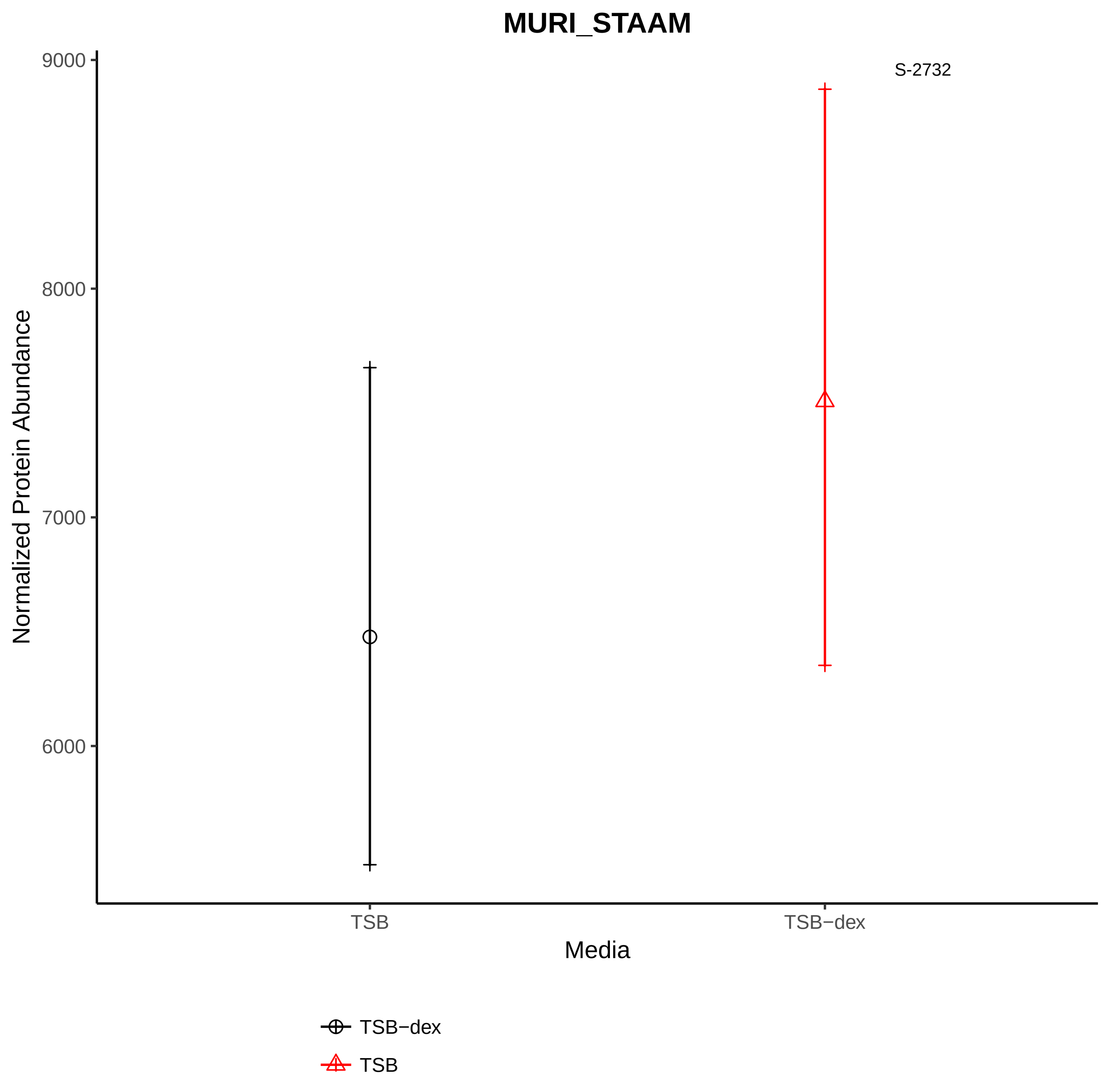
MURI_STAAM
S-2732
9000
8000
7000
6000
Normalized Protein Abundance
TSB
TSB−dex
Media
TSB−dex
TSB

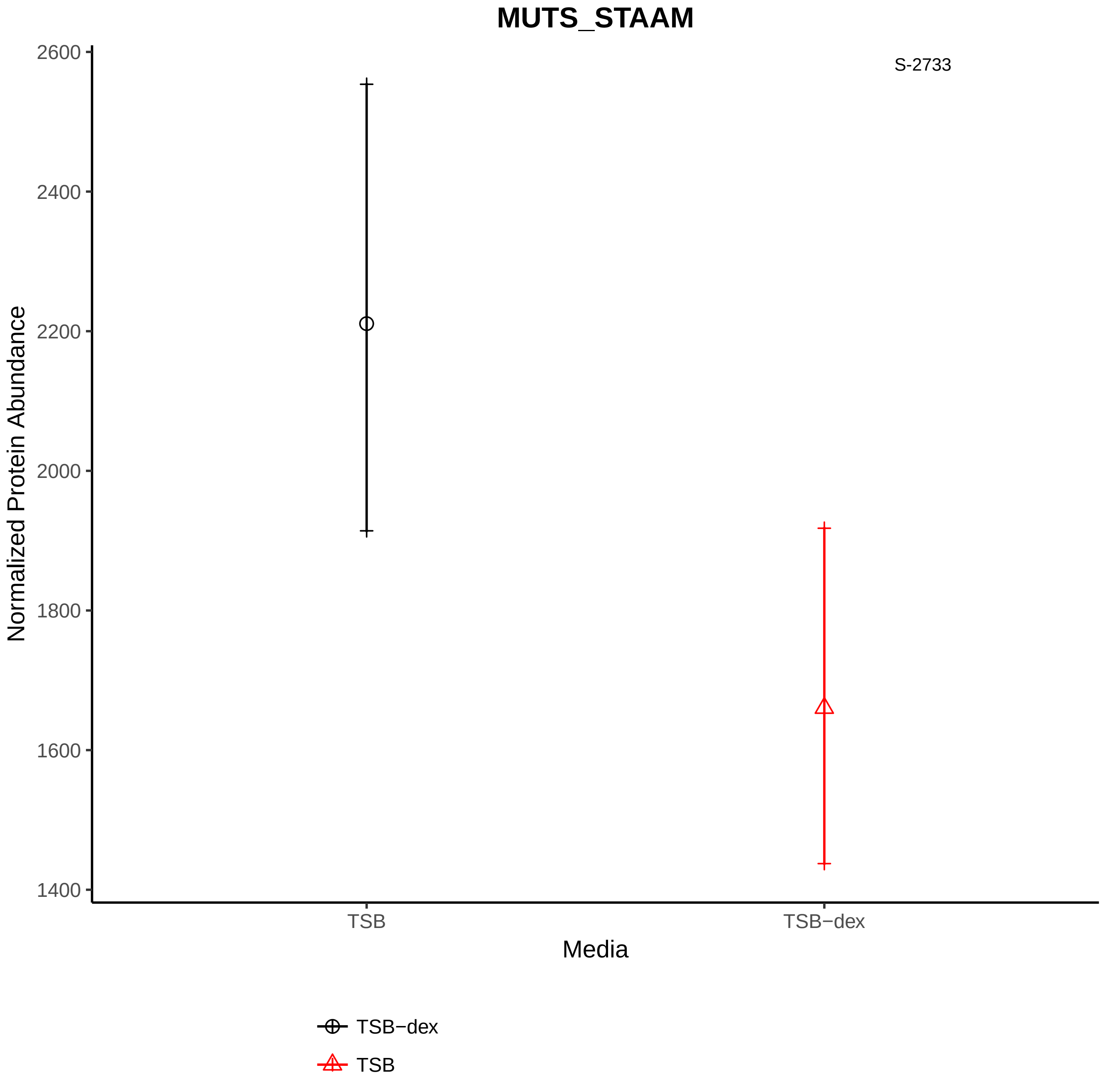

MUTS_STAAM
S-2733
Normalized Protein Abundance
2600
2400
2200
2000
1800
1600
1400
TSB
TSB−dex
Media
TSB−dex
TSB

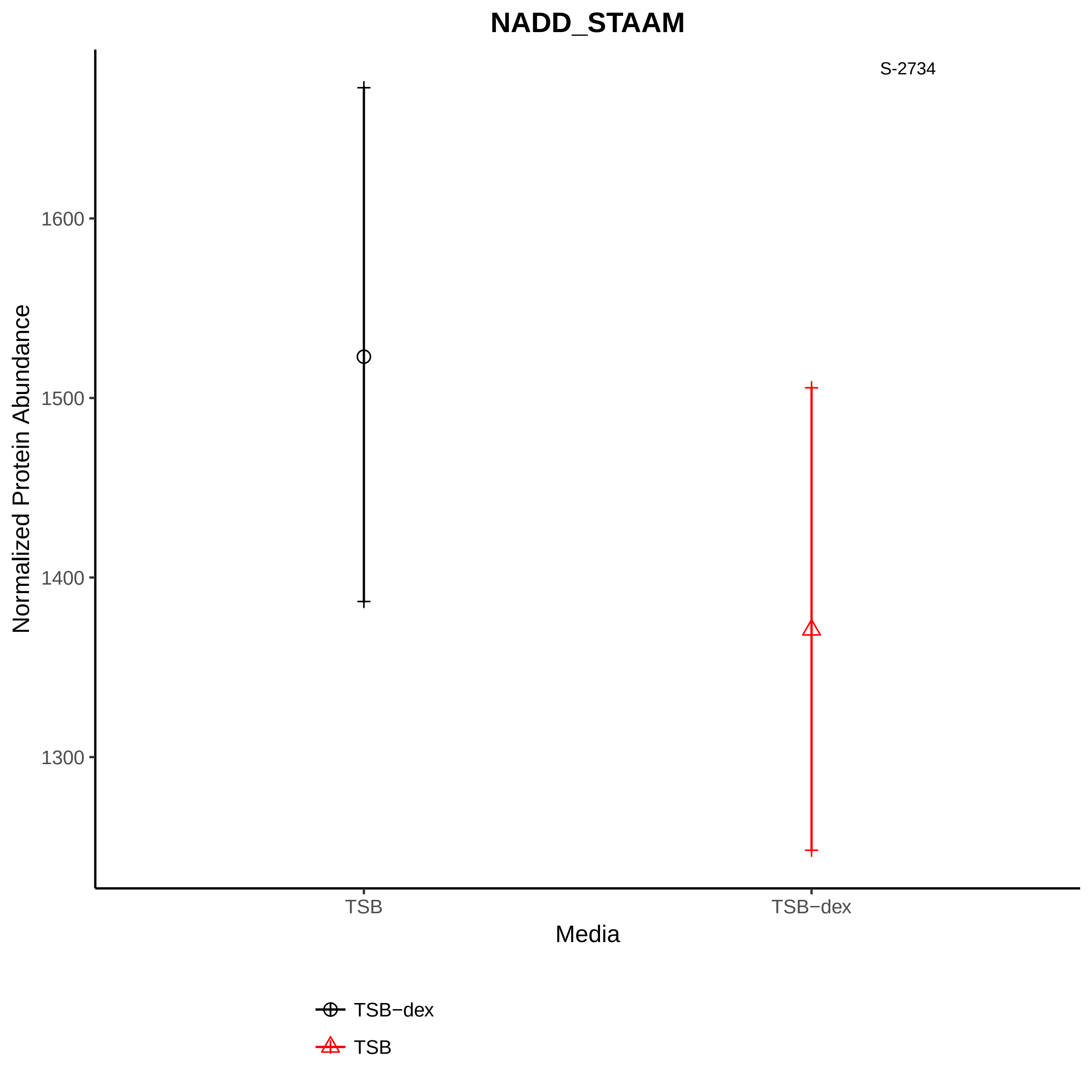
NADD_STAAM
S-2734
Normalized Protein Abundance
1600
1500
1400
1300
TSB
TSB−dex
Media
TSB−dex
TSB

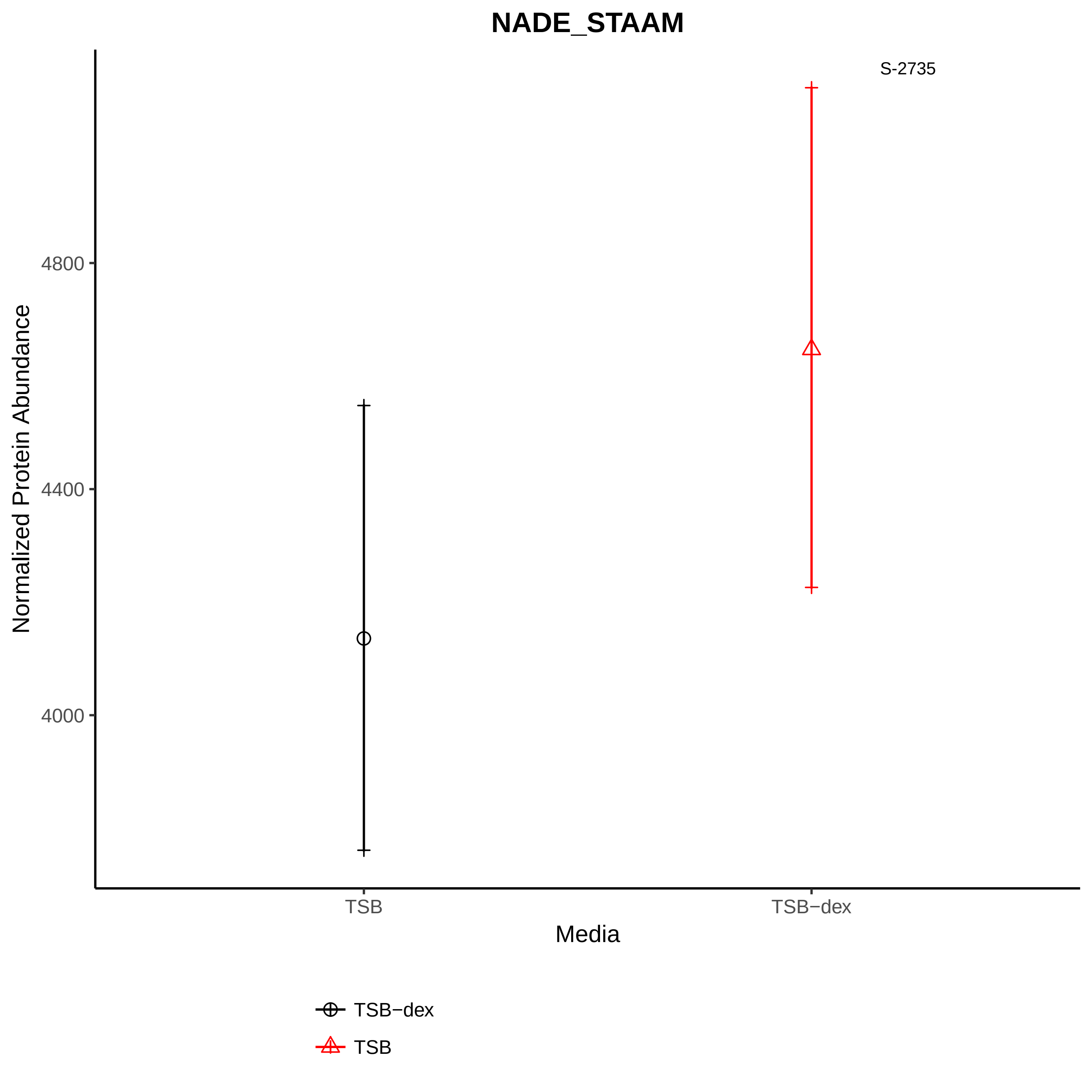

NADE_STAAM
S-2735
4800
4400
4000
Normalized Protein Abundance
TSB
TSB−dex
Media
TSB−dex
TSB

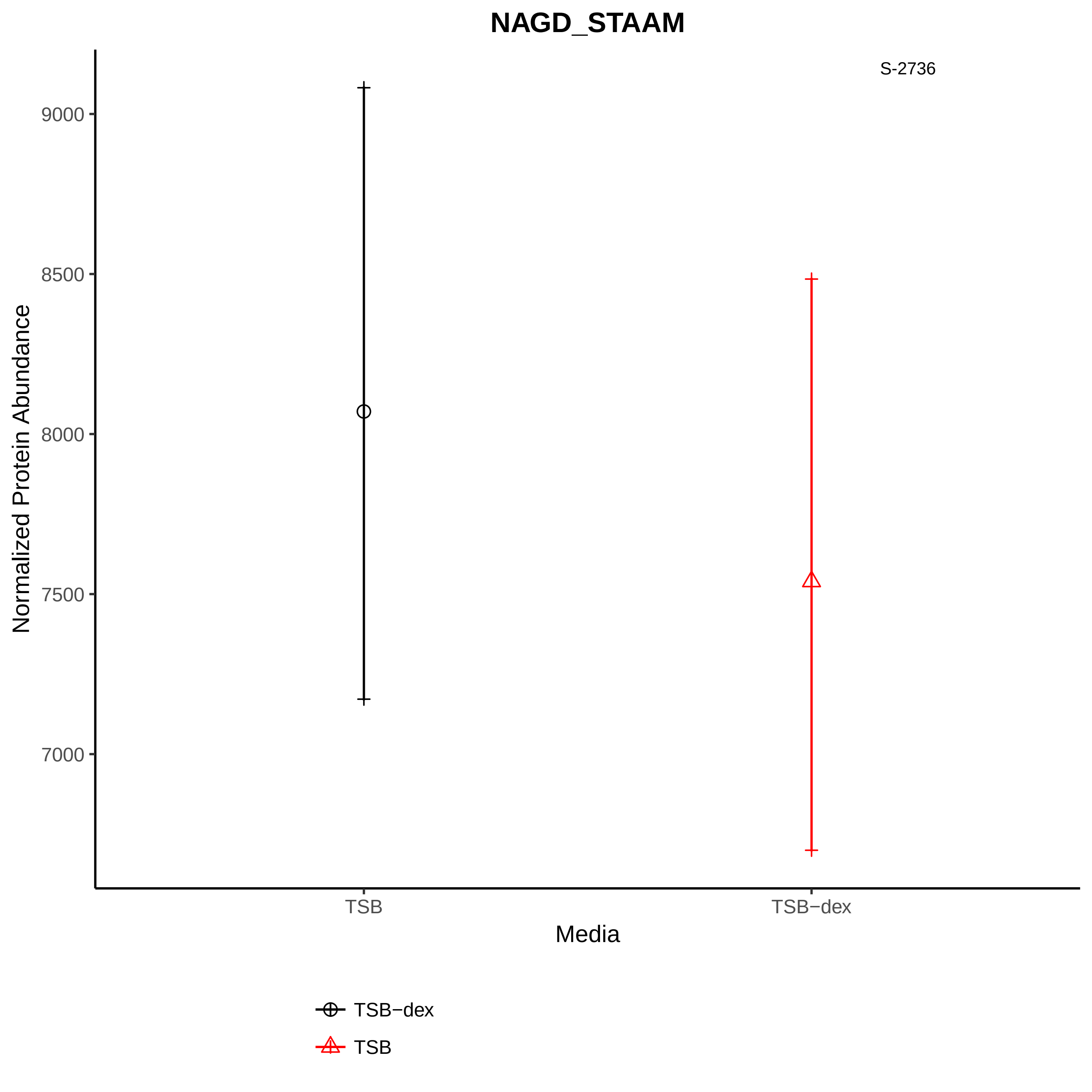

NAGD_STAAM
S-2736
9000
8500
8000
7500
7000
Normalized Protein Abundance
TSB
TSB−dex
Media
TSB−dex
TSB

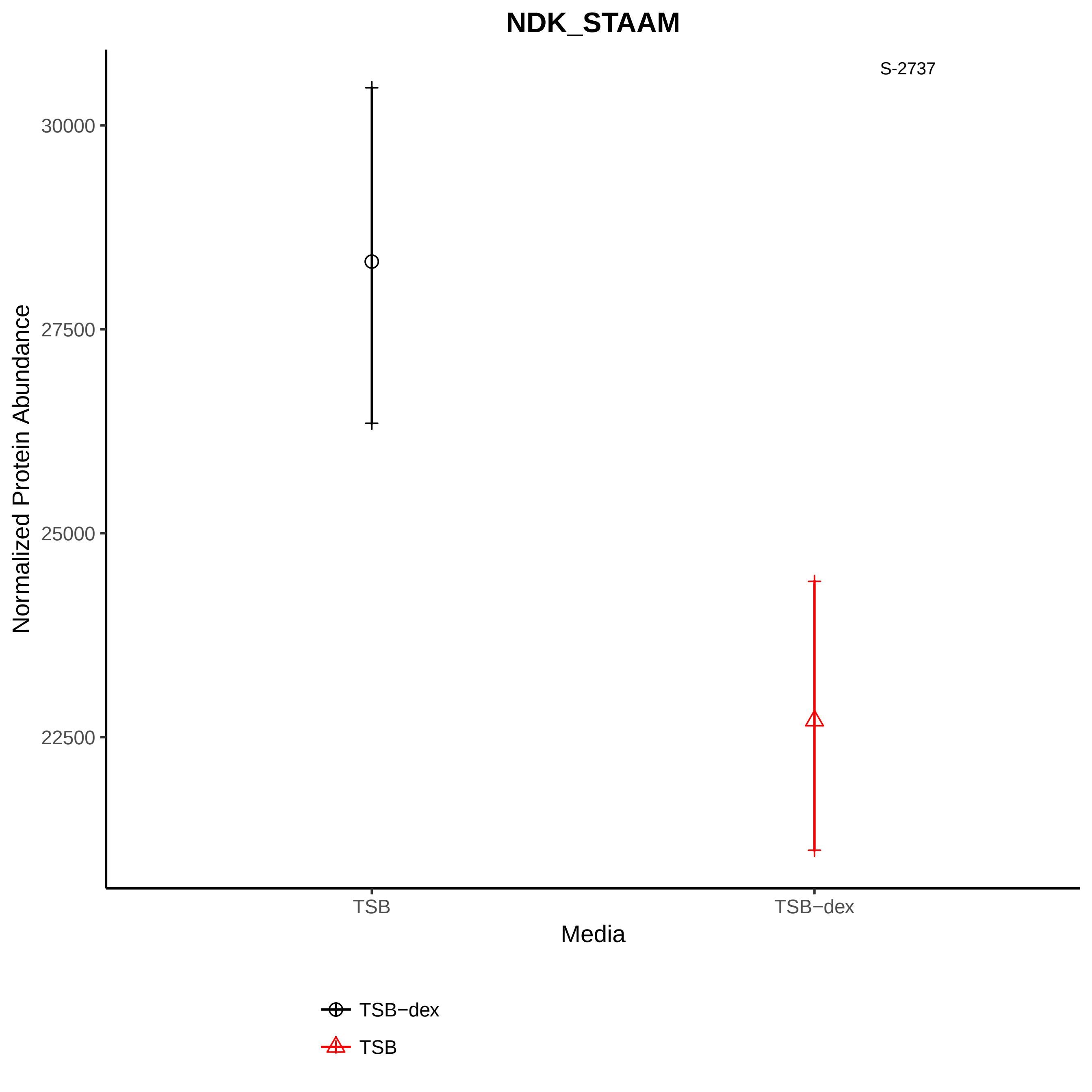

NDK_STAAM
S-2737
Normalized Protein Abundance
30000
27500
25000
22500
TSB
TSB−dex
Media
TSB−dex
TSB

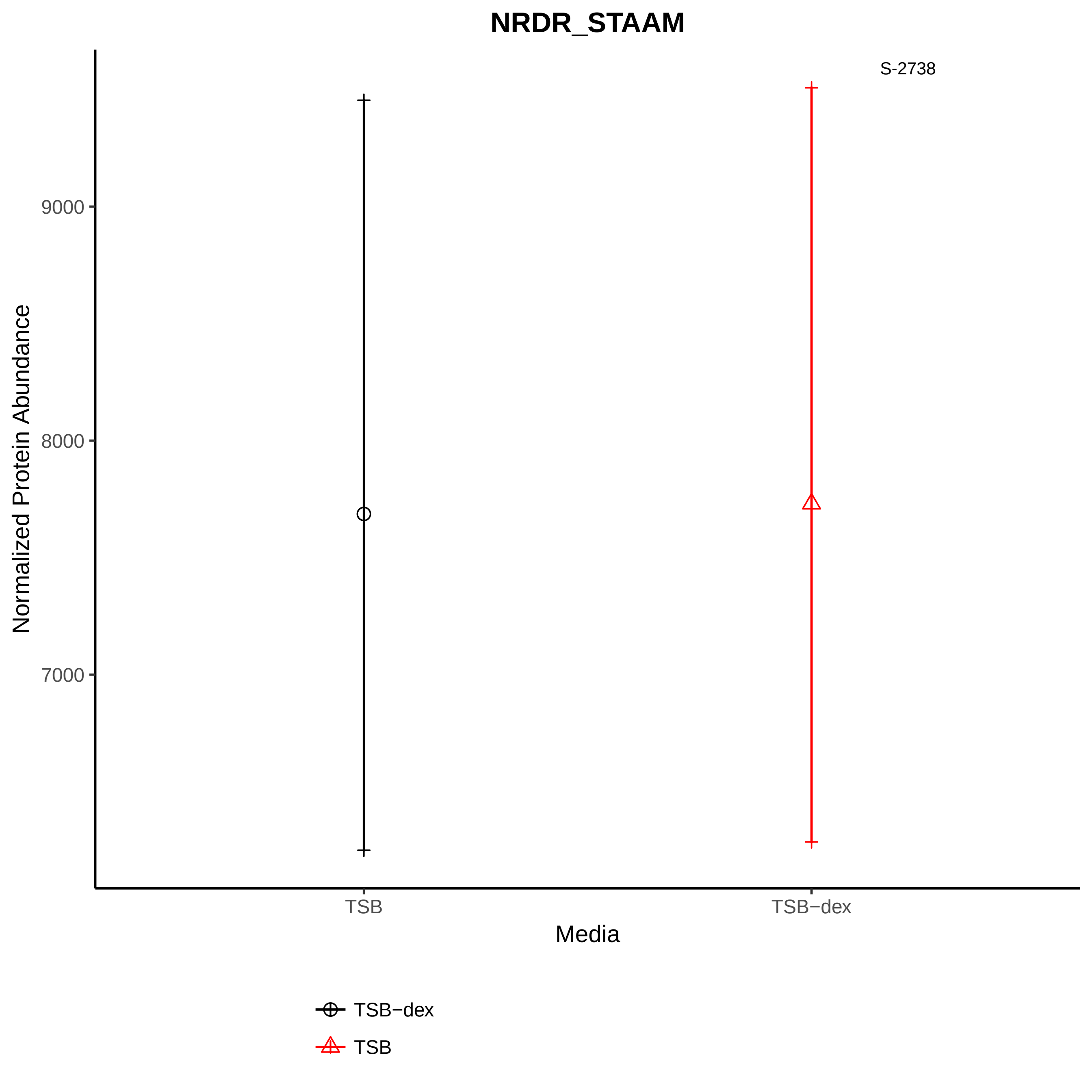

NRDR_STAAM
S-2738
Normalized Protein Abundance
9000
8000
7000
TSB
TSB−dex
Media
TSB−dex
TSB

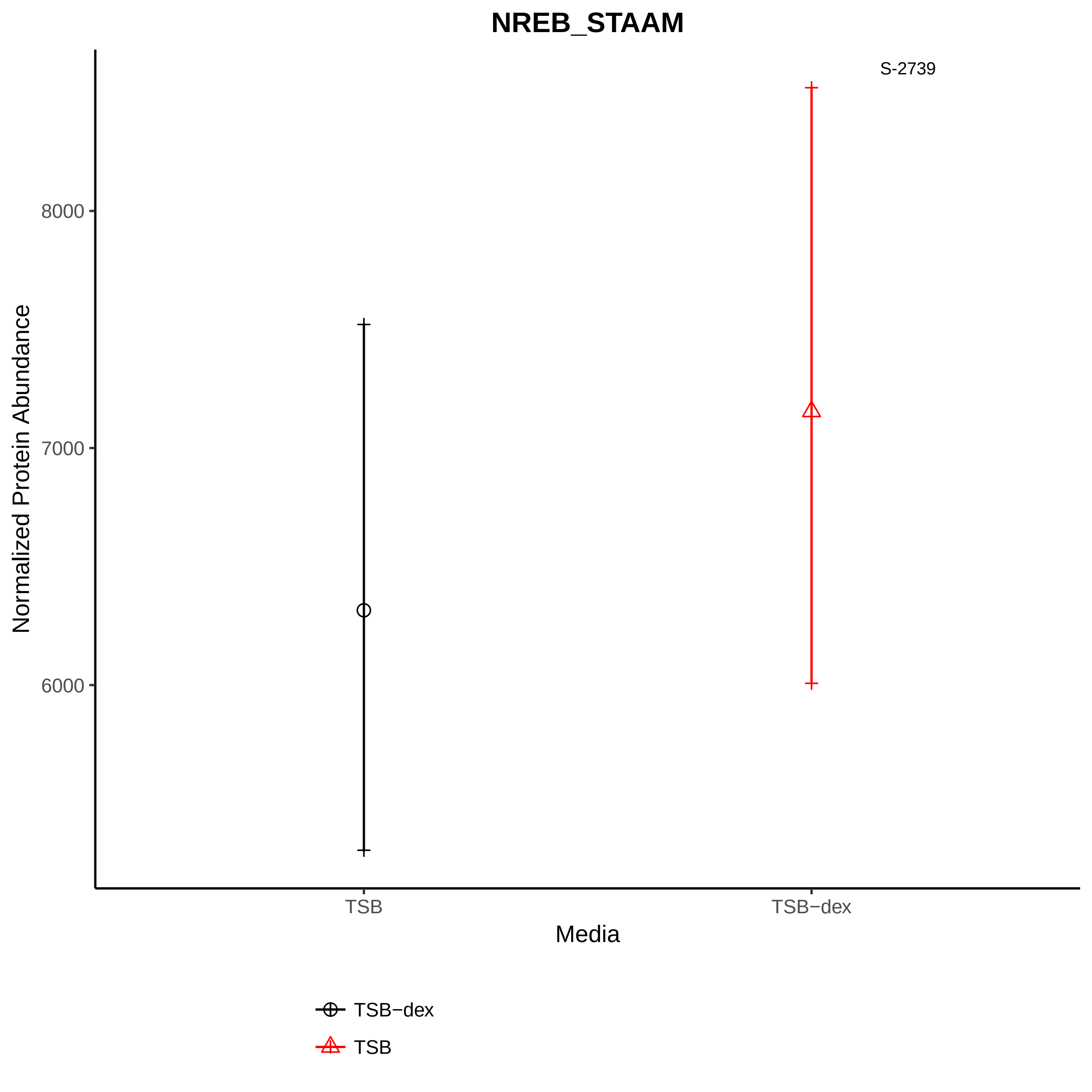

NREB_STAAM
S-2739
Normalized Protein Abundance
8000
7000
6000
TSB
TSB−dex
Media
TSB−dex
TSB

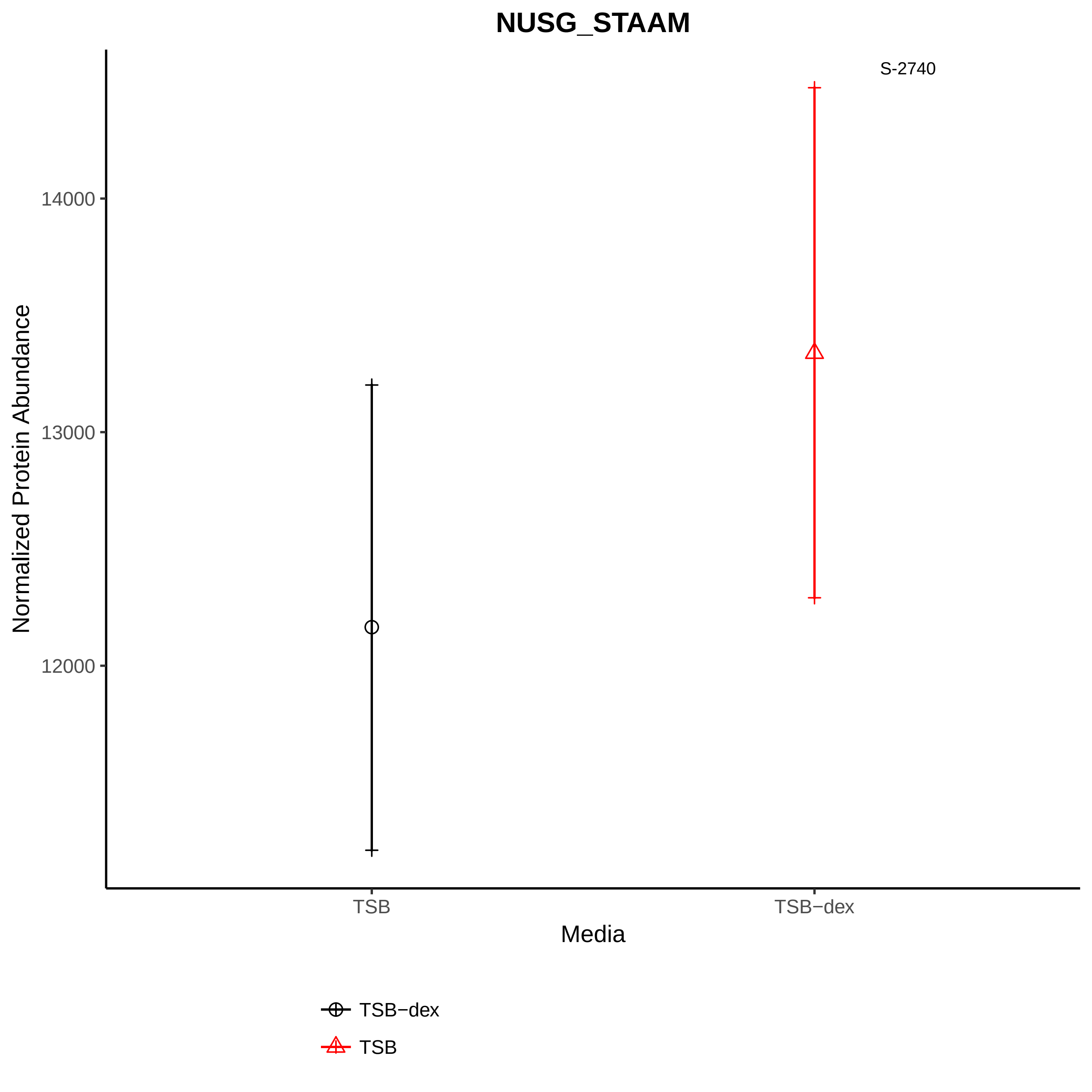

NUSG_STAAM
S-2740
14000
13000
12000
Normalized Protein Abundance
TSB
TSB−dex
Media
TSB−dex
TSB

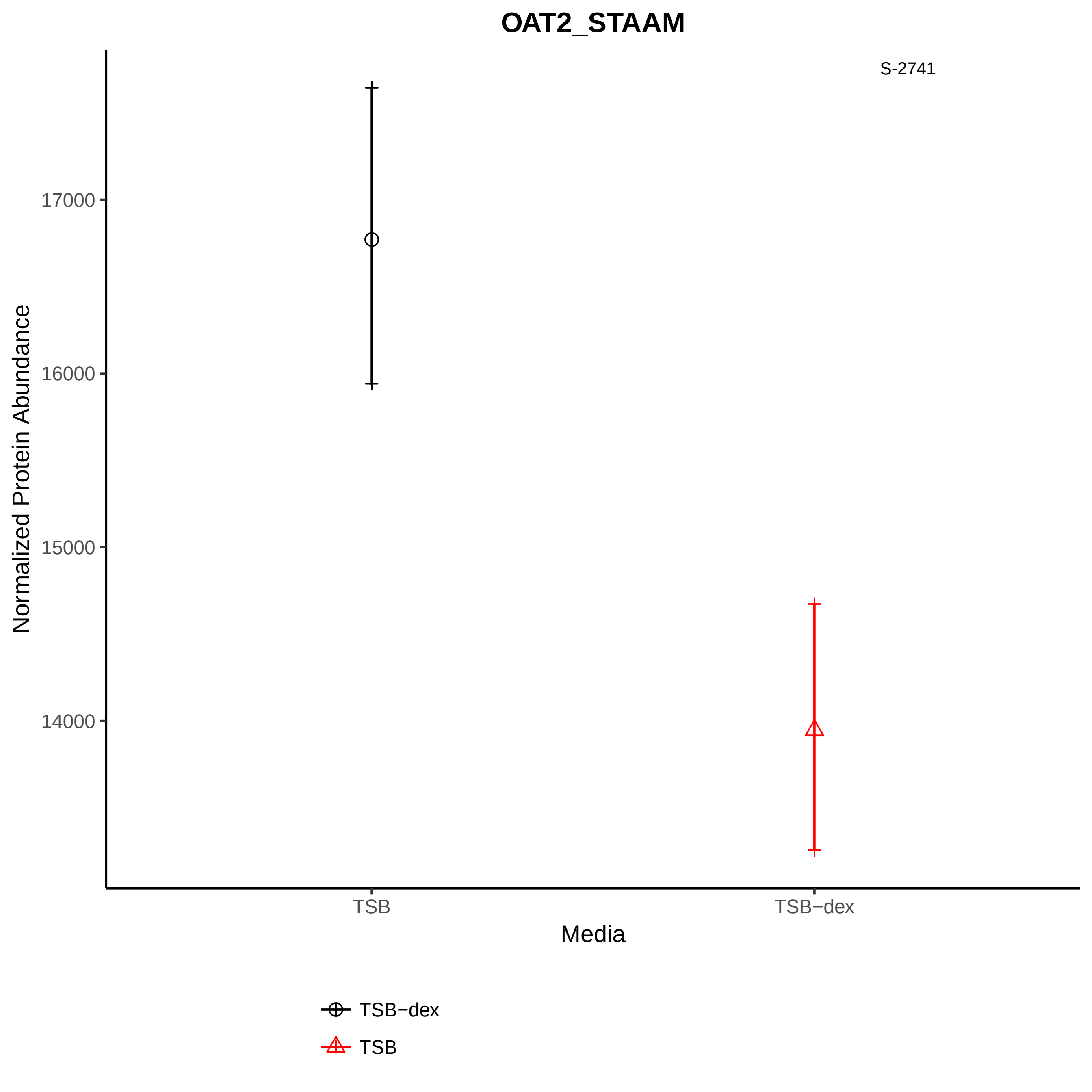

OAT2_STAAM
S-2741
Normalized Protein Abundance
17000
16000
15000
14000
TSB
TSB−dex
Media
TSB−dex
TSB

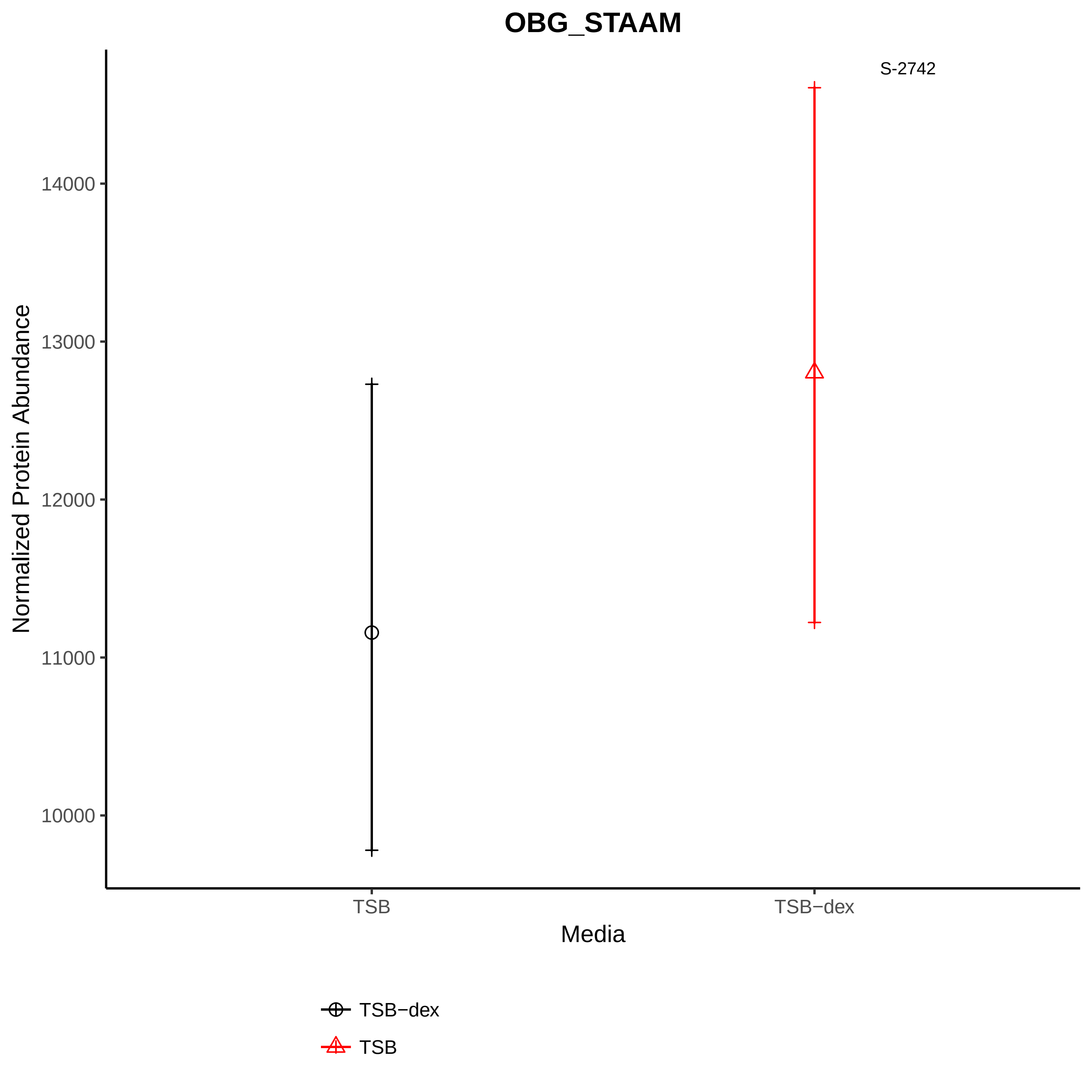

OBG_STAAM
S-2742
Normalized Protein Abundance
14000
13000
12000
11000
10000
TSB
TSB−dex
Media
TSB−dex
TSB

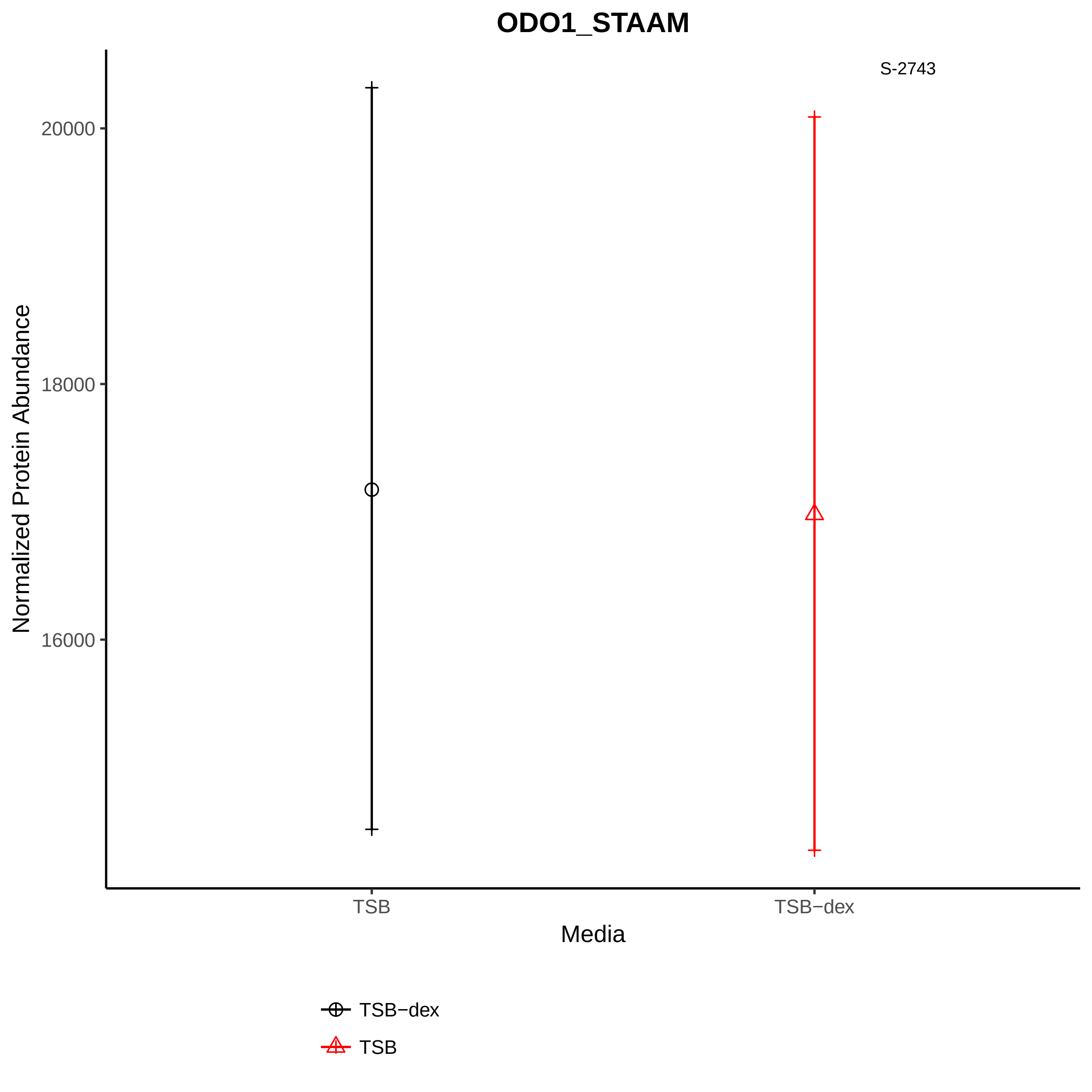

ODO1_STAAM
S-2743
20000
18000
16000
Normalized Protein Abundance
TSB
TSB−dex
Media
TSB−dex
TSB

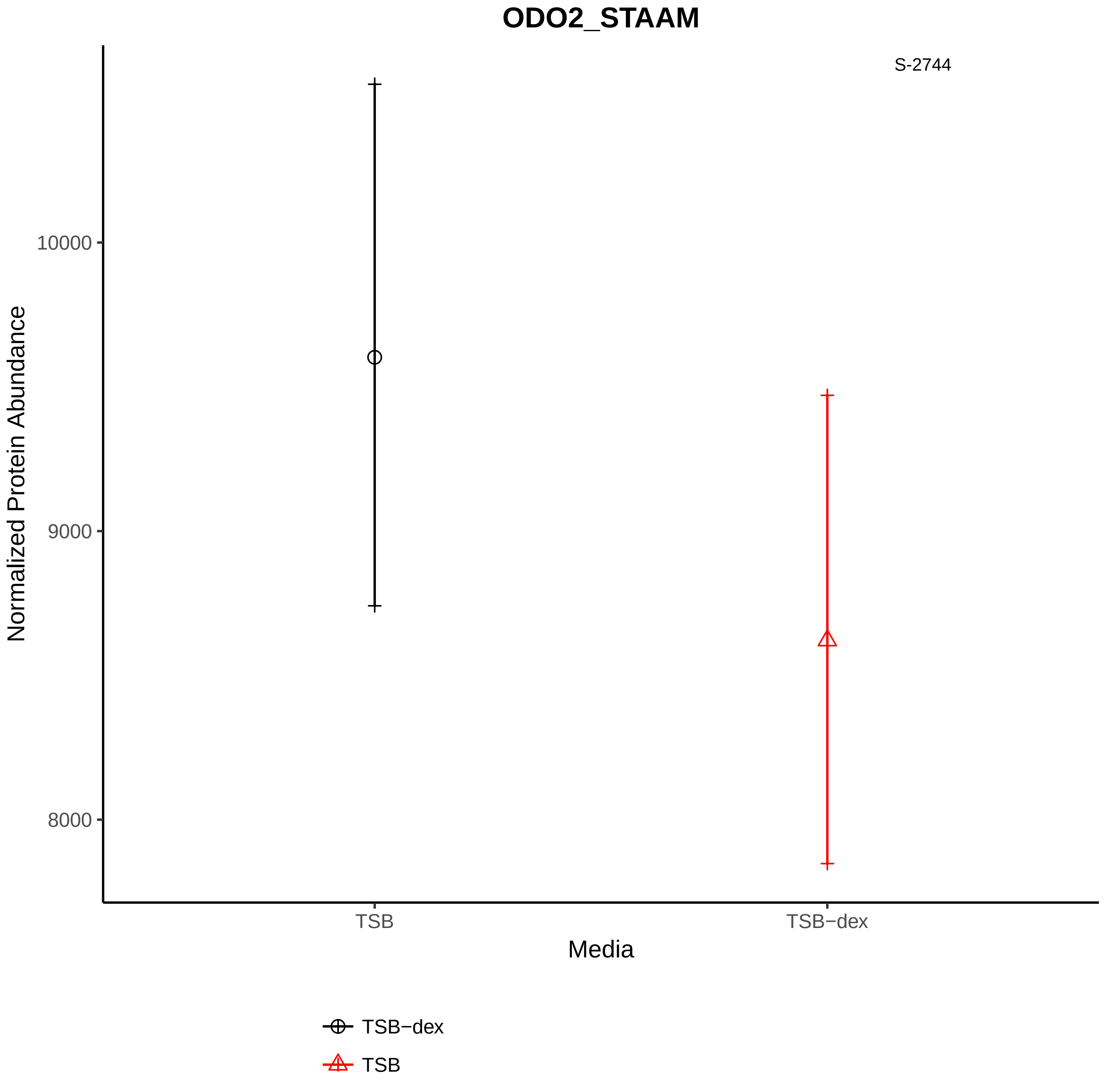

ODO2_STAAM
S-2744
Normalized Protein Abundance
10000
9000
8000
TSB
TSB−dex
Media
TSB−dex
TSB

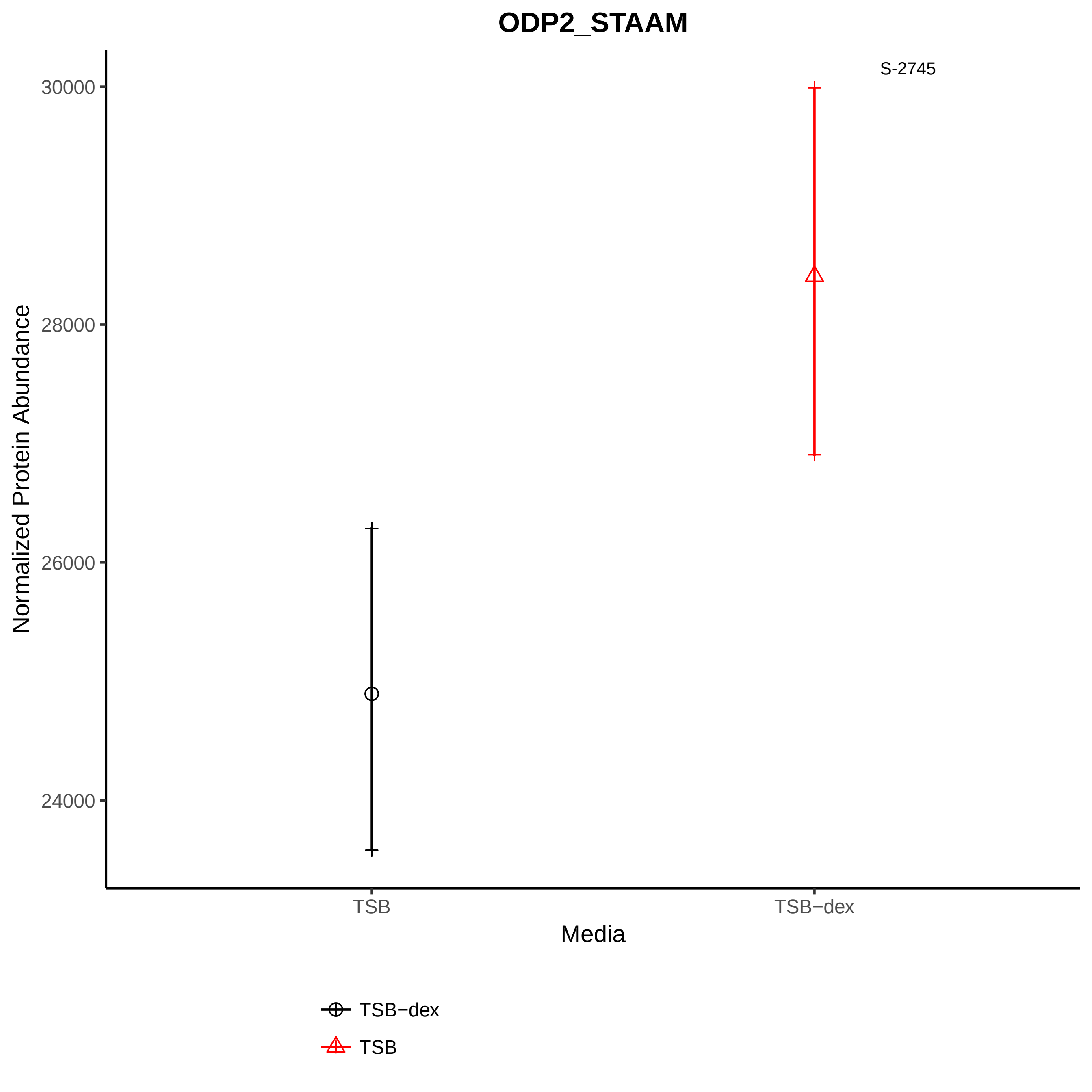
ODP2_STAAM
S-2745
Normalized Protein Abundance
30000
28000
26000
24000
TSB
TSB−dex
Media
TSB−dex
TSB

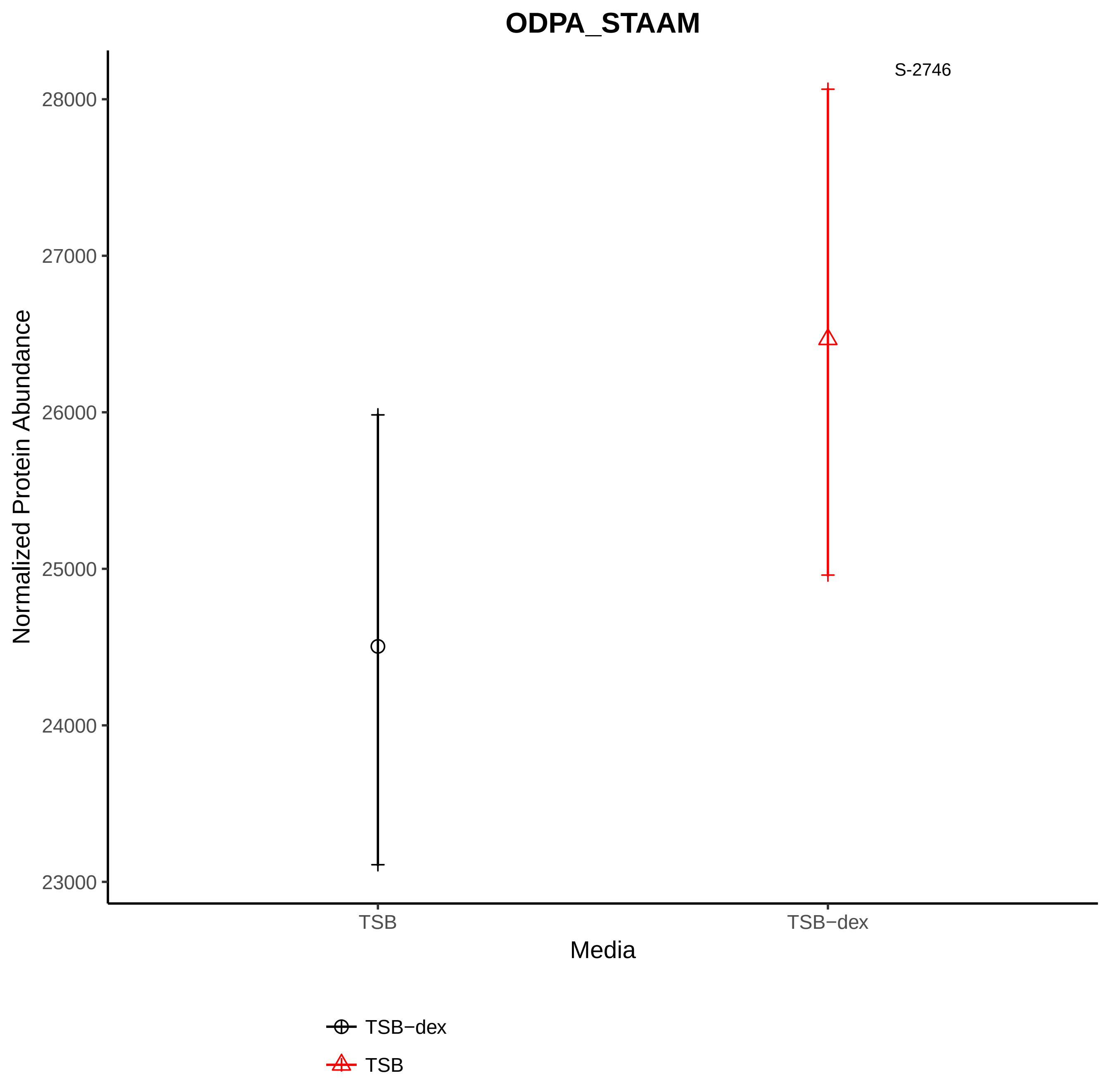
ODPA_STAAM
S-2746
Normalized Protein Abundance
28000
27000
26000
25000
24000
23000
TSB
TSB−dex
Media
TSB−dex
TSB

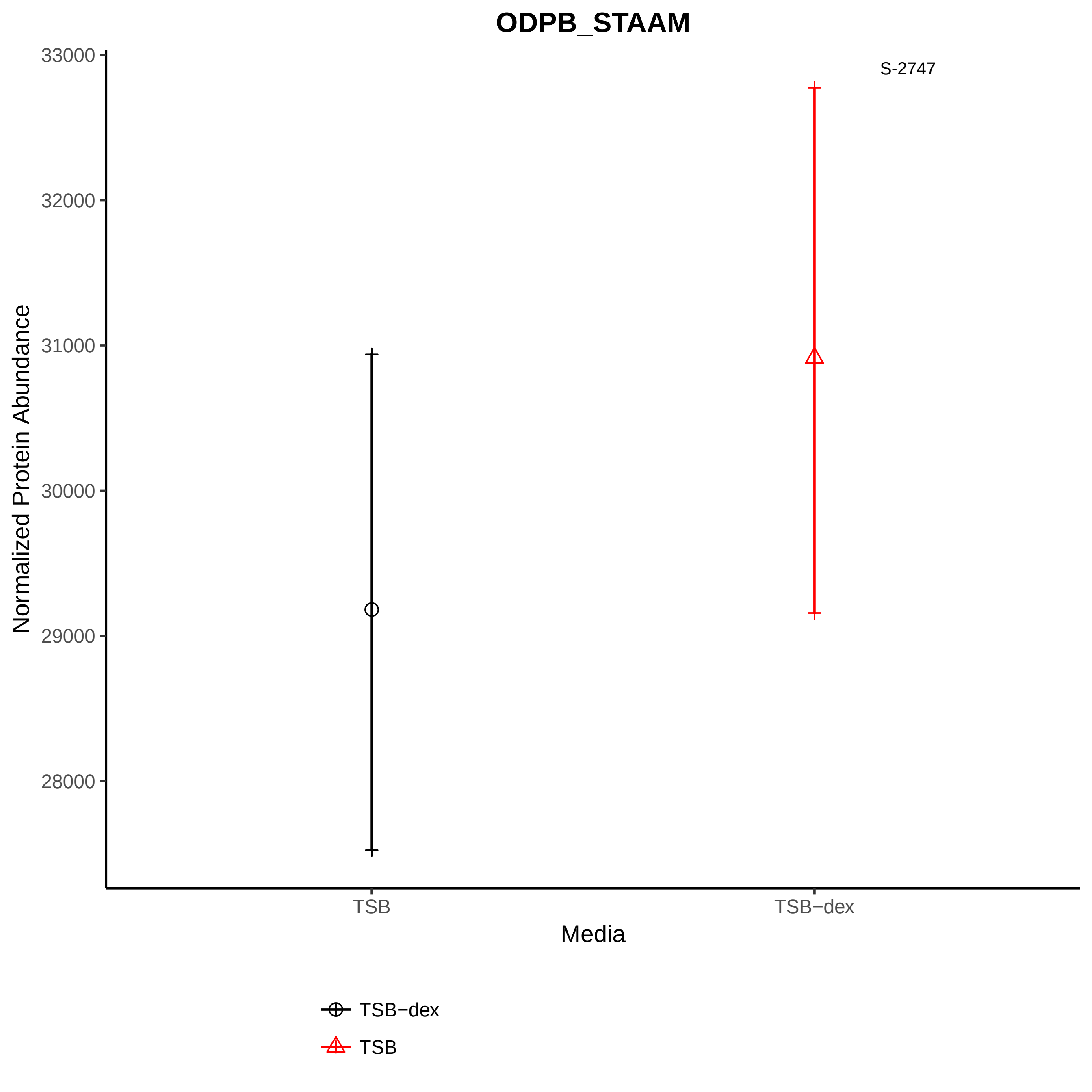
ODPB_STAAM
S-2747
Normalized Protein Abundance
33000
32000
31000
30000
29000
28000
TSB
TSB−dex
Media
TSB−dex
TSB

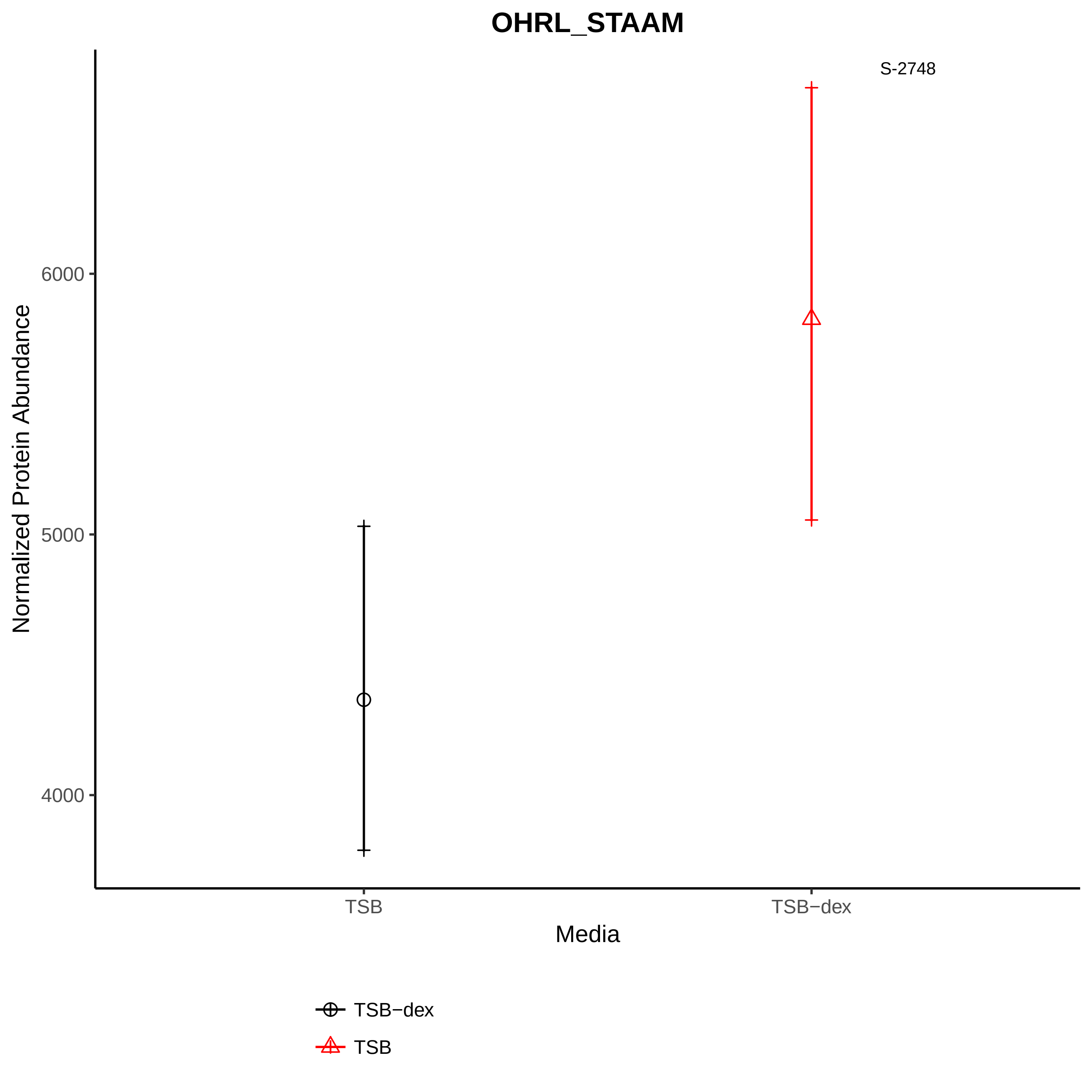

OHRL_STAAM
S-2748
Normalized Protein Abundance
6000
5000
4000
TSB
TSB−dex
Media
TSB−dex
TSB

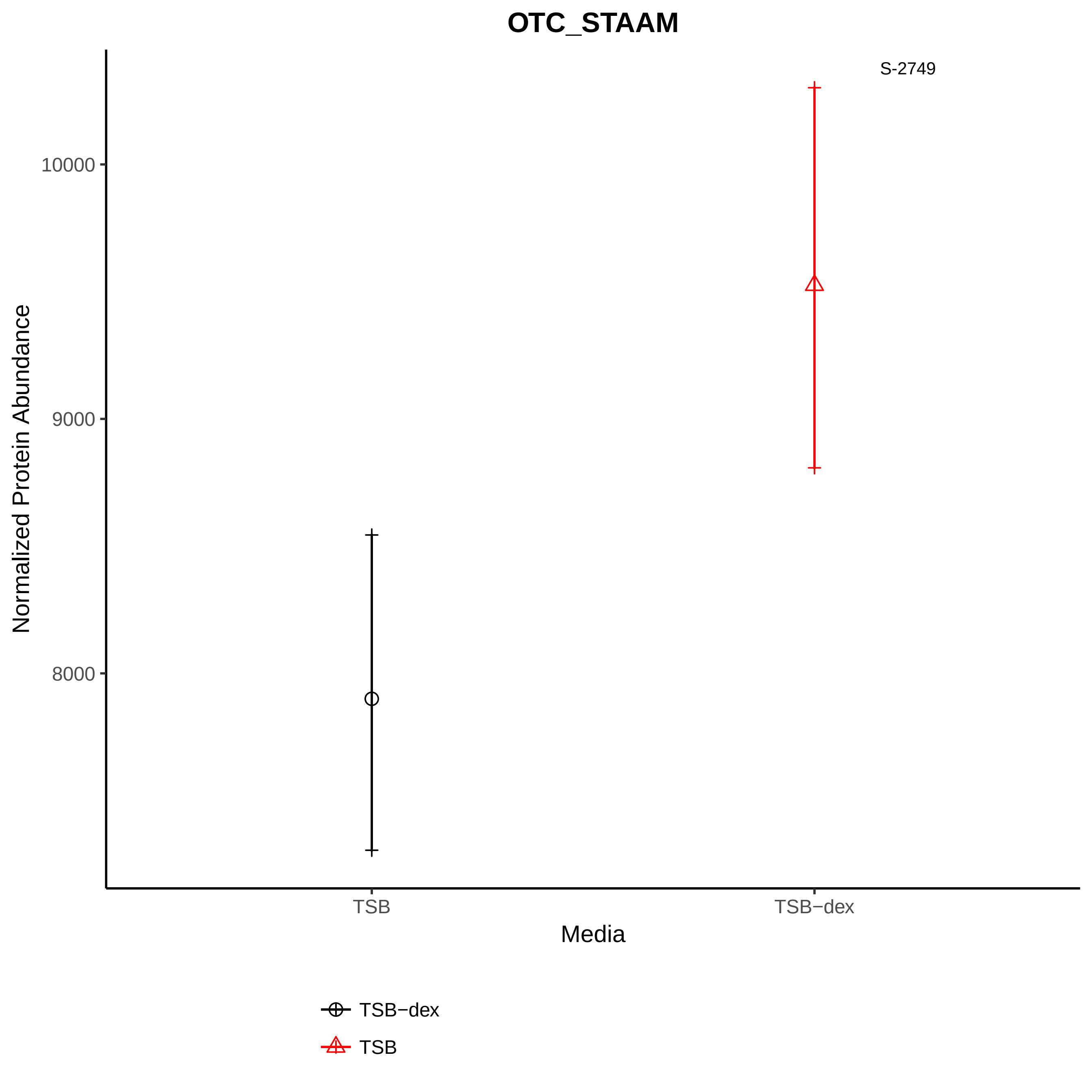

OTC_STAAM
S-2749
10000
9000
8000
Normalized Protein Abundance
TSB
TSB−dex
Media
TSB−dex
TSB

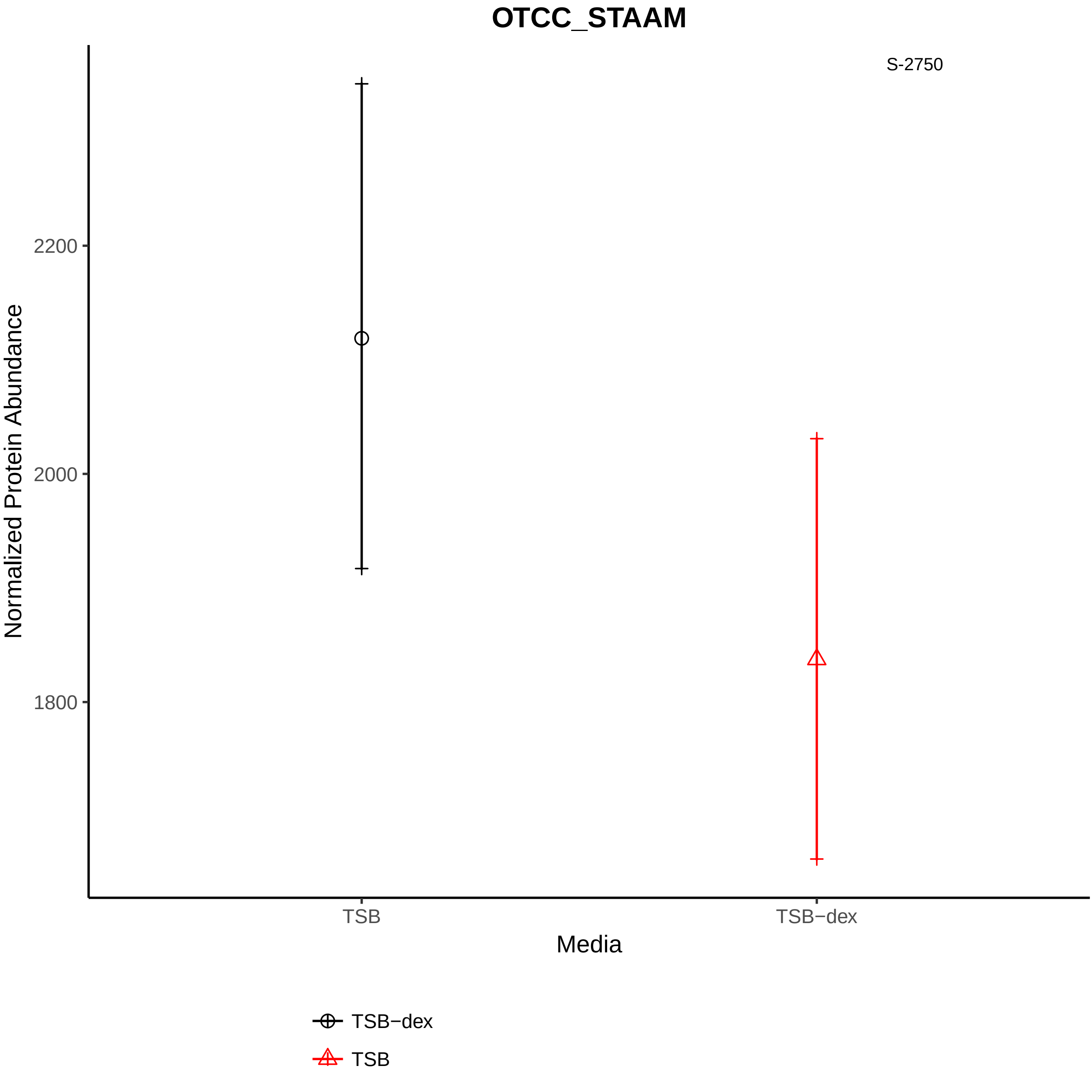

OTCC_STAAM
S-2750
Normalized Protein Abundance
2200
2000
1800
TSB
TSB−dex
Media
TSB−dex
TSB

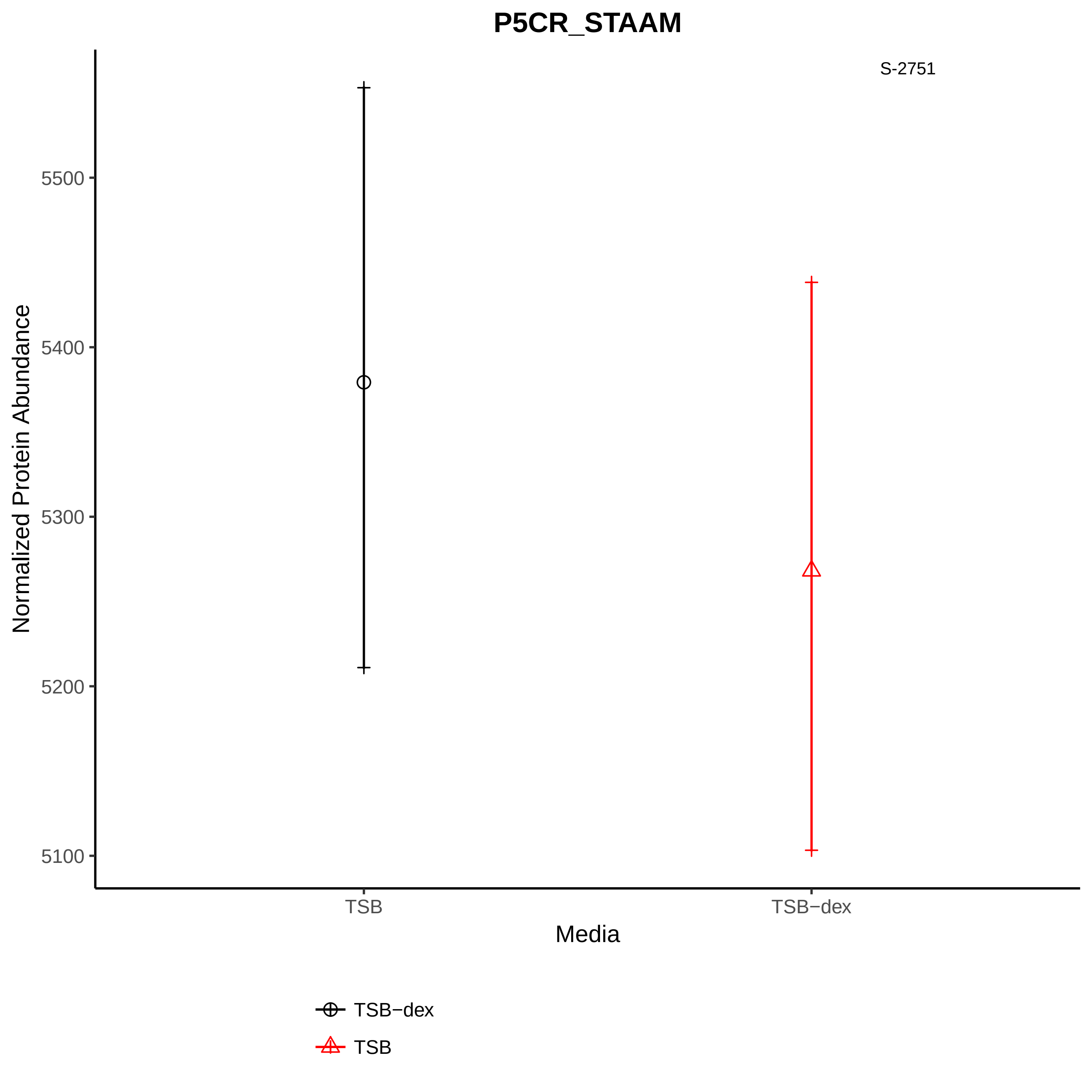

P5CR_STAAM
S-2751
Normalized Protein Abundance
5500
5400
5300
5200
5100
TSB
TSB−dex
Media
TSB−dex
TSB

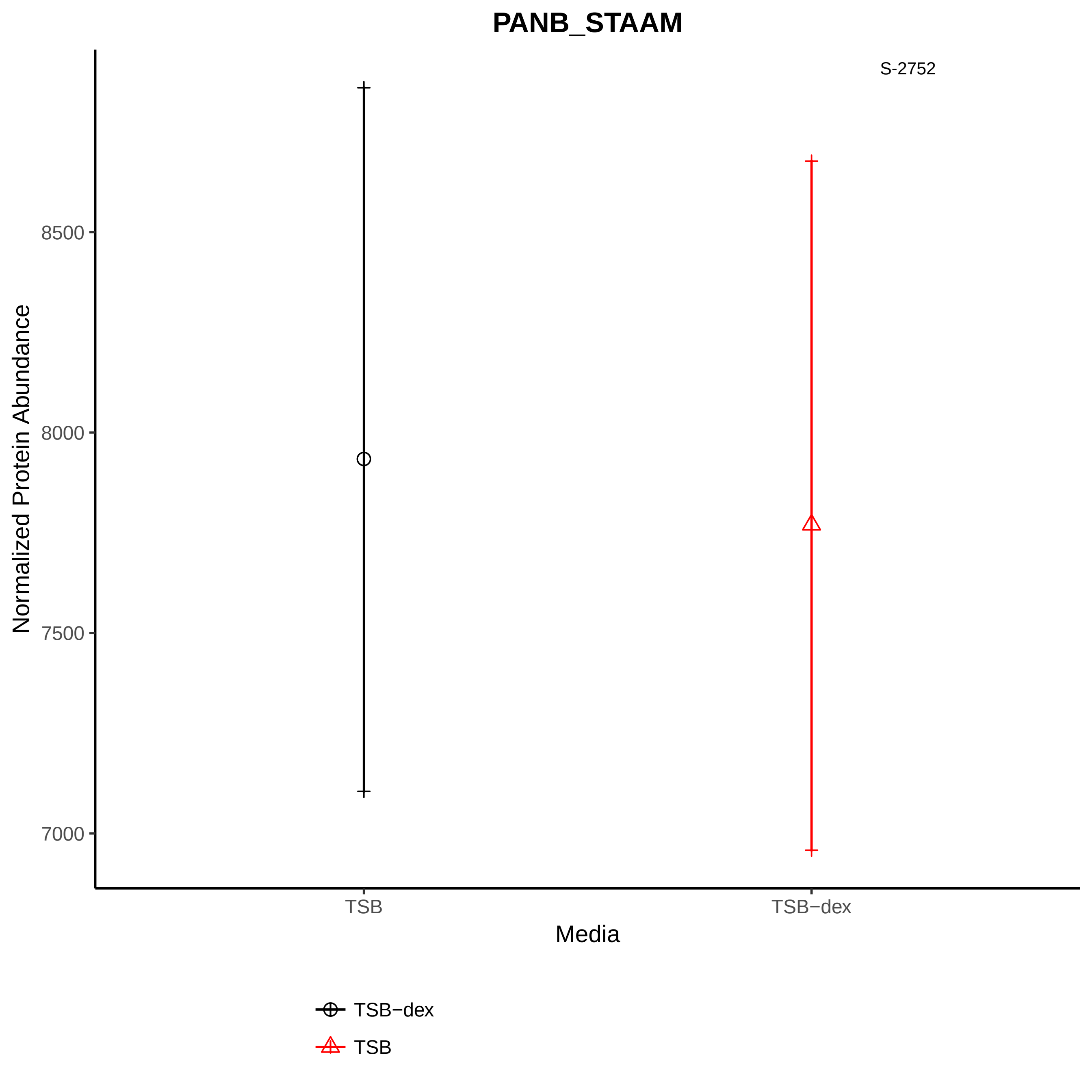

PANB_STAAM
S-2752
Normalized Protein Abundance
8500
8000
7500
7000
TSB
TSB−dex
Media
TSB−dex
TSB

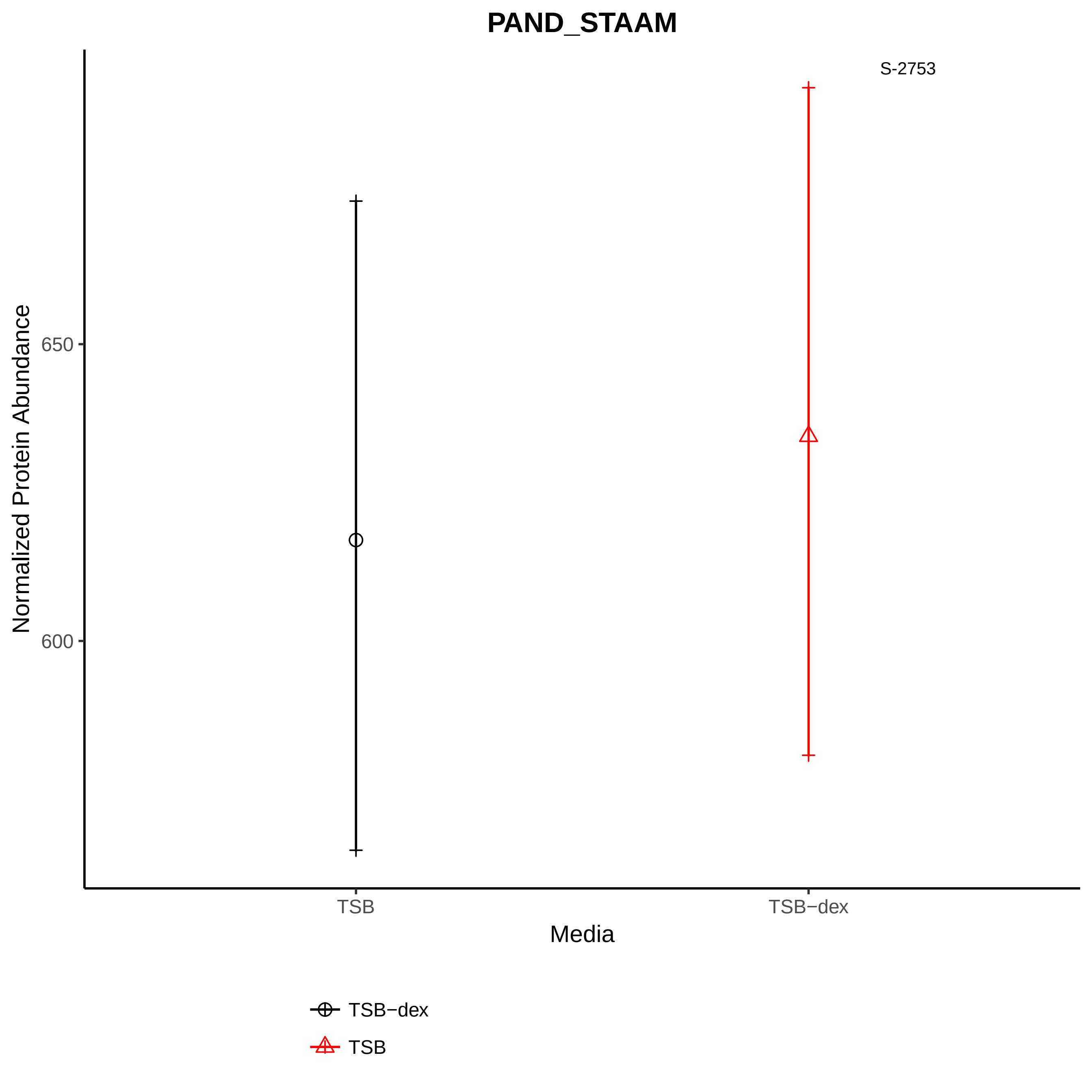

PAND_STAAM
S-2753
Normalized Protein Abundance
650
600
TSB
TSB−dex
Media
TSB−dex
TSB

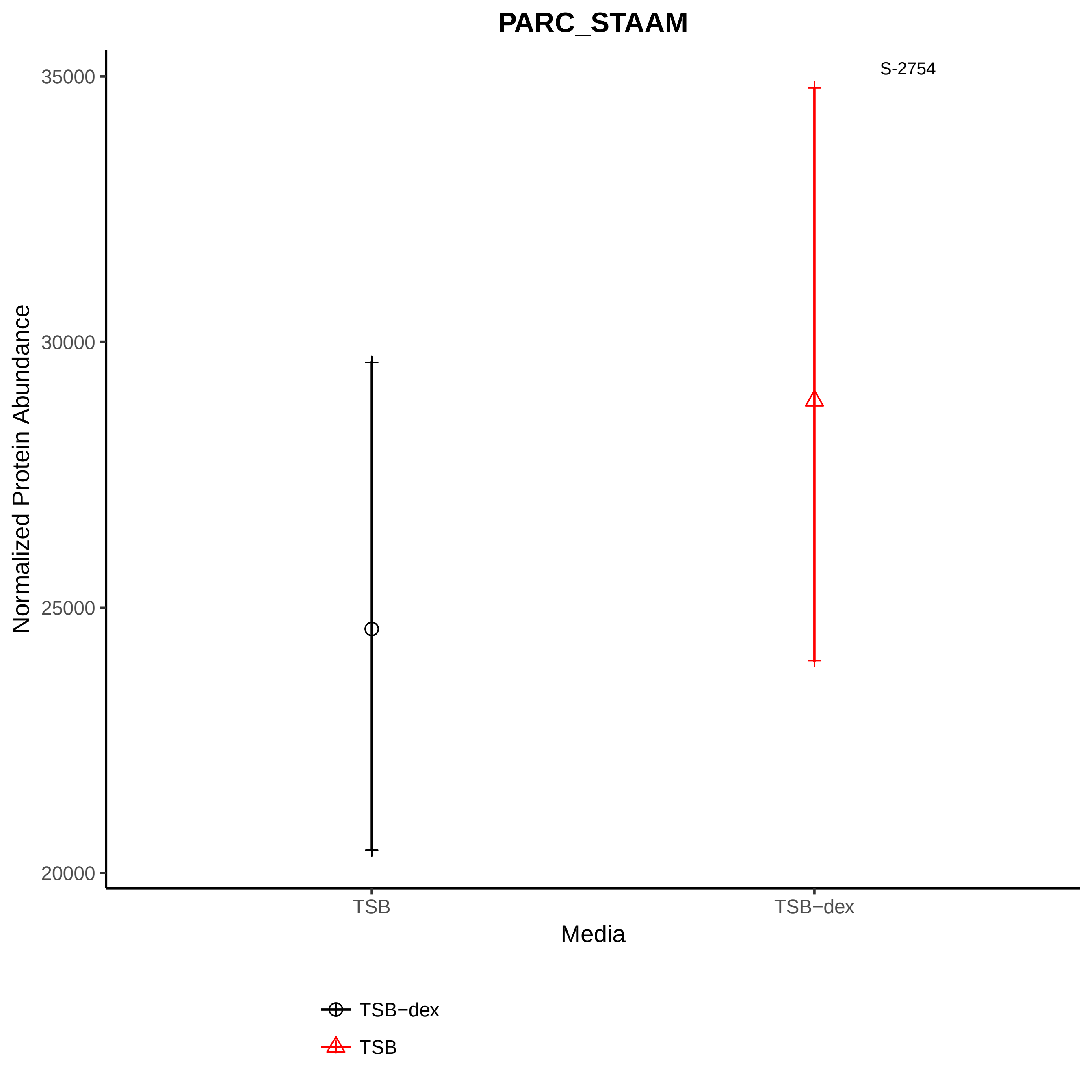
PARC_STAAM
S-2754
Normalized Protein Abundance
35000
30000
25000
20000
TSB
TSB−dex
Media
TSB−dex
TSB

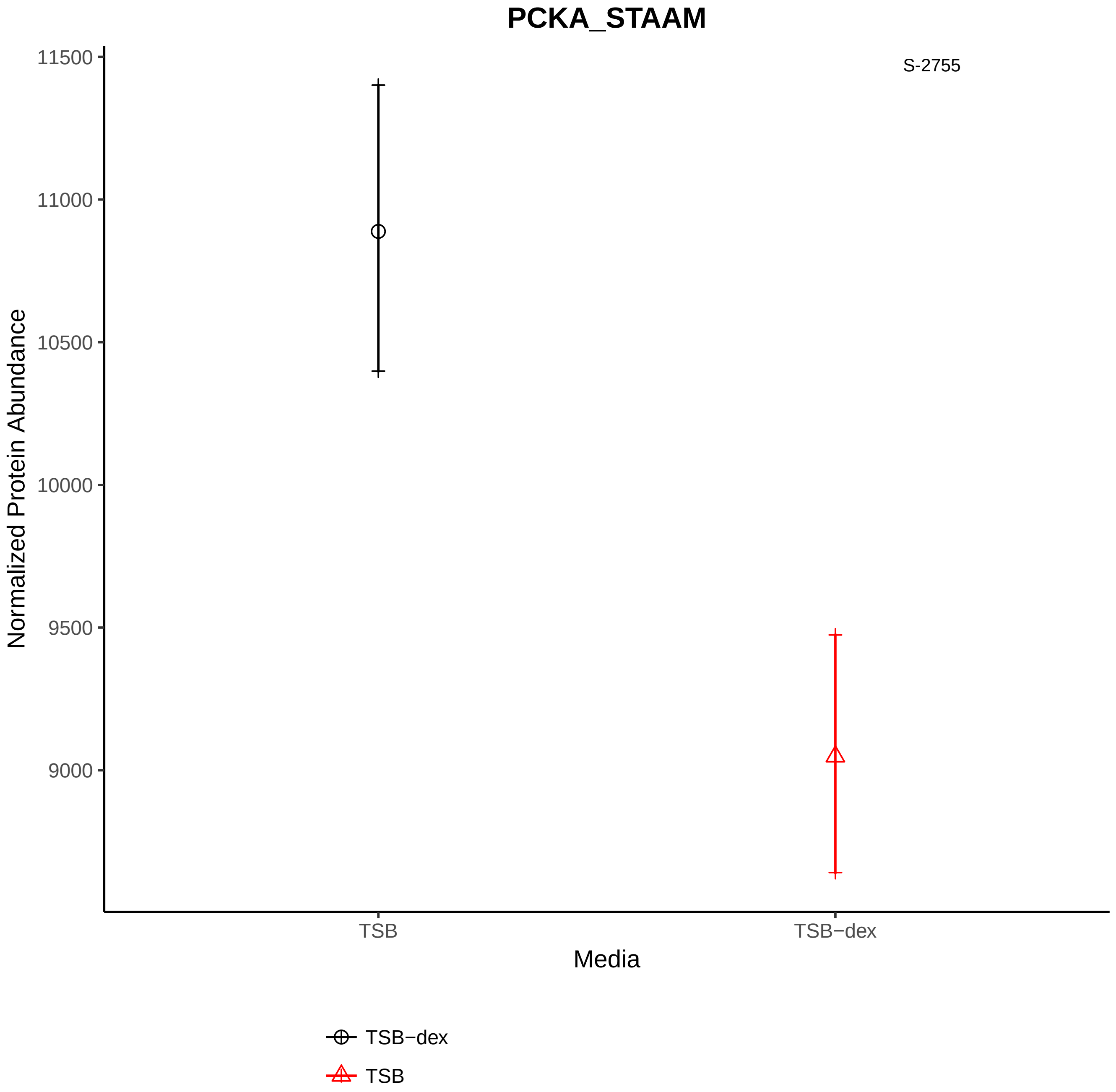
PCKA_STAAM
S-2755
11500
11000
10500
10000
9500
9000
Normalized Protein Abundance
TSB
TSB−dex
Media
TSB−dex
TSB

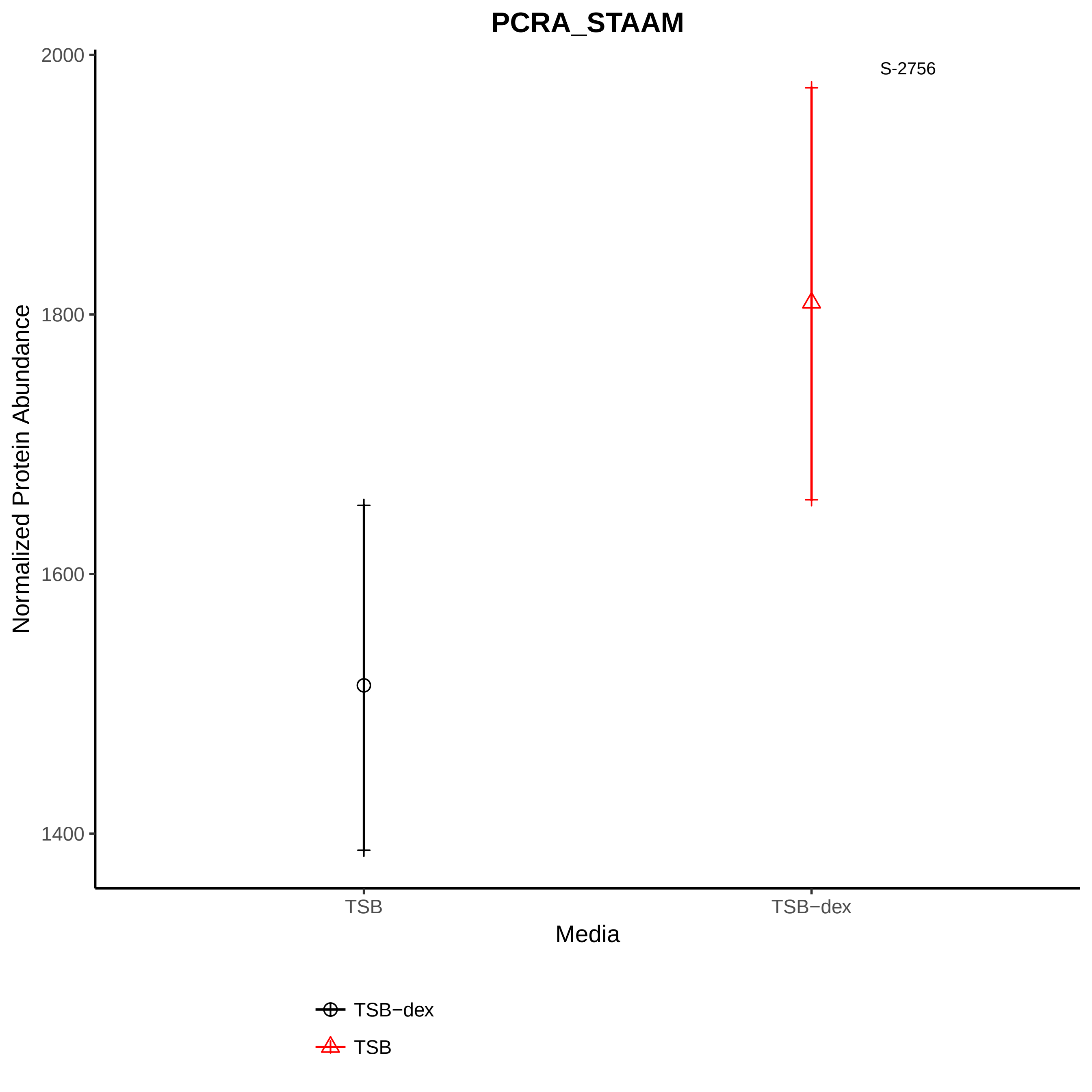
PCRA_STAAM
S-2756
2000
1800
1600
1400
Normalized Protein Abundance
TSB
TSB−dex
Media
TSB−dex
TSB

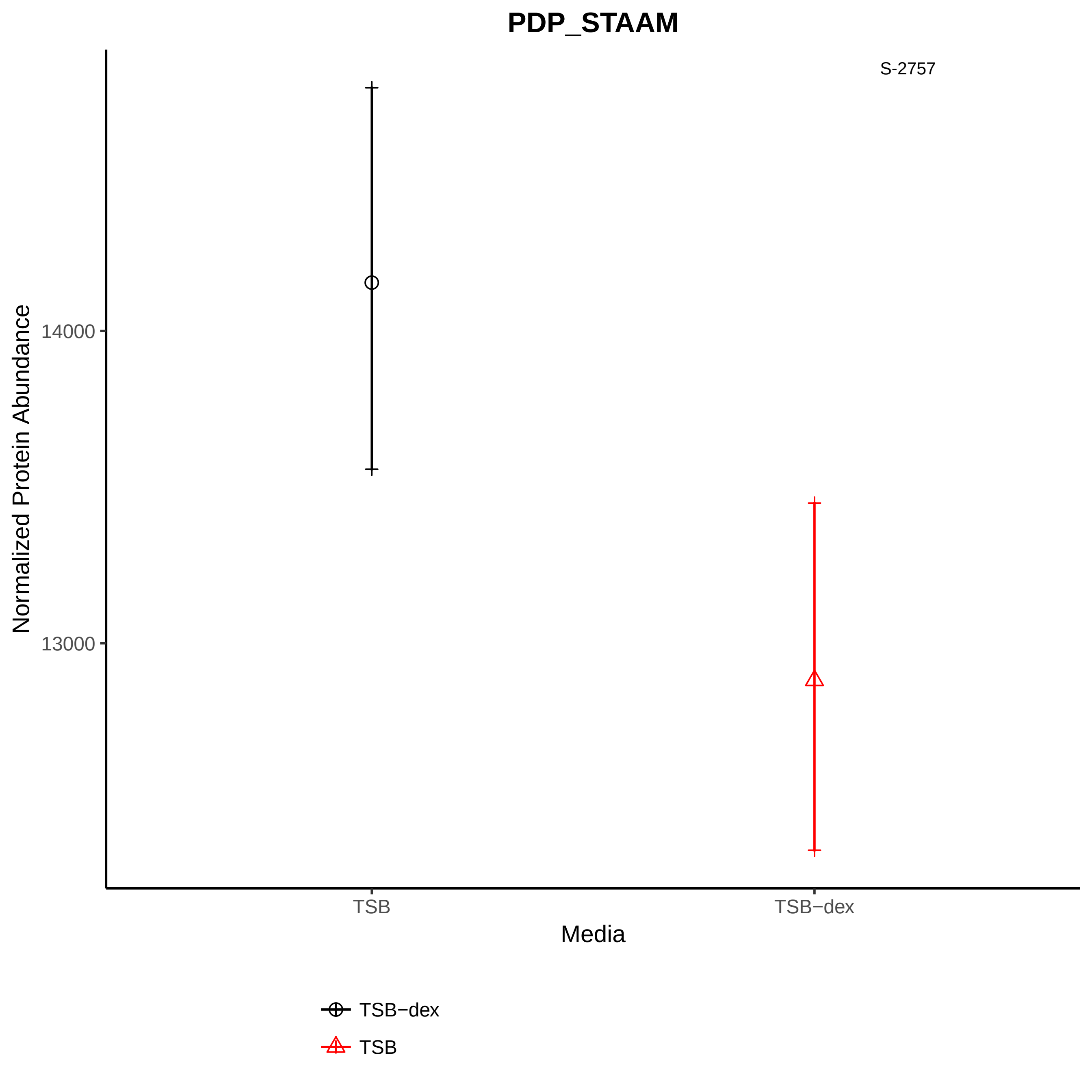
PDP_STAAM
S-2757
Normalized Protein Abundance
14000
13000
TSB
TSB−dex
Media
TSB−dex
TSB

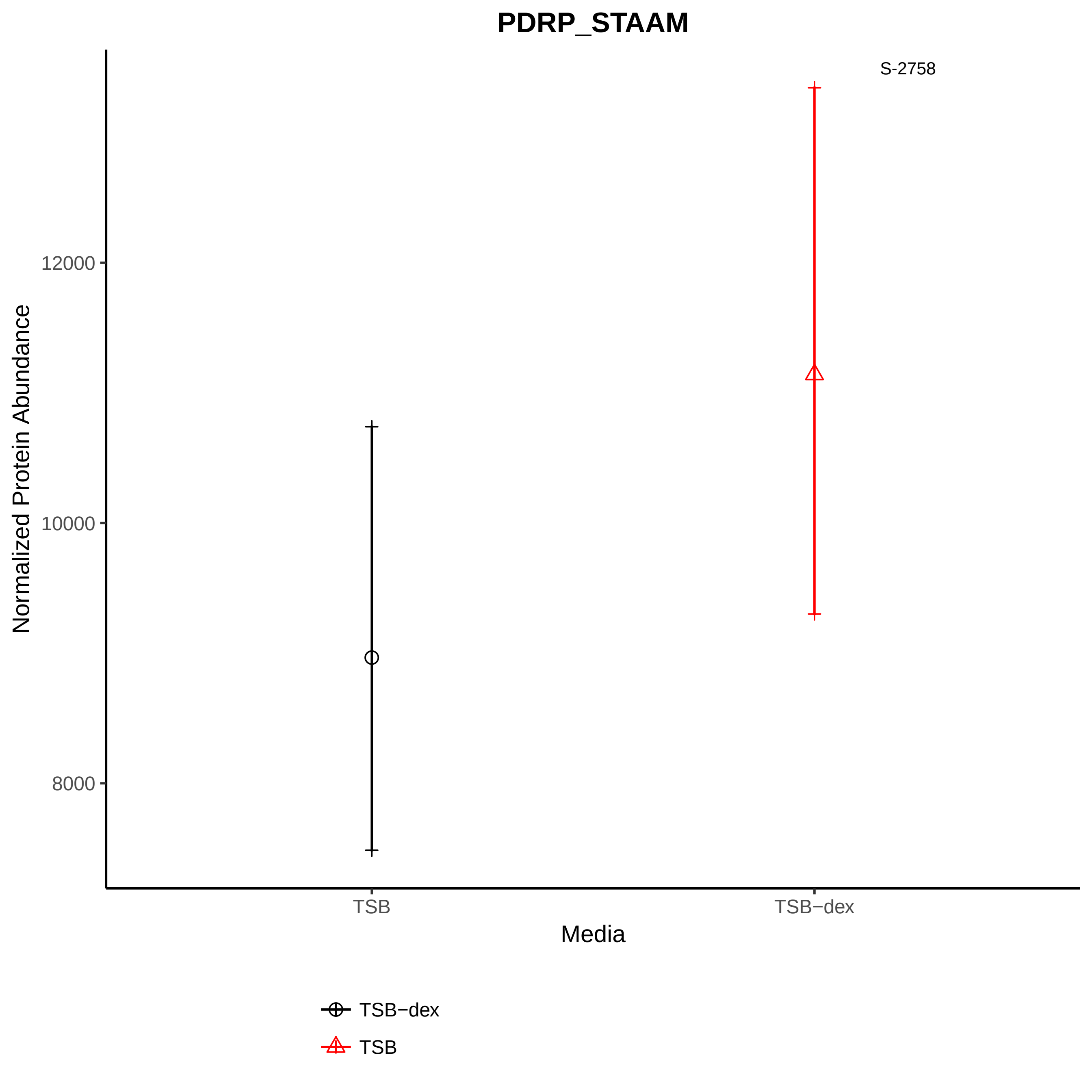
PDRP_STAAM
S-2758
Normalized Protein Abundance
12000
10000
8000
TSB
TSB−dex
Media
TSB−dex
TSB

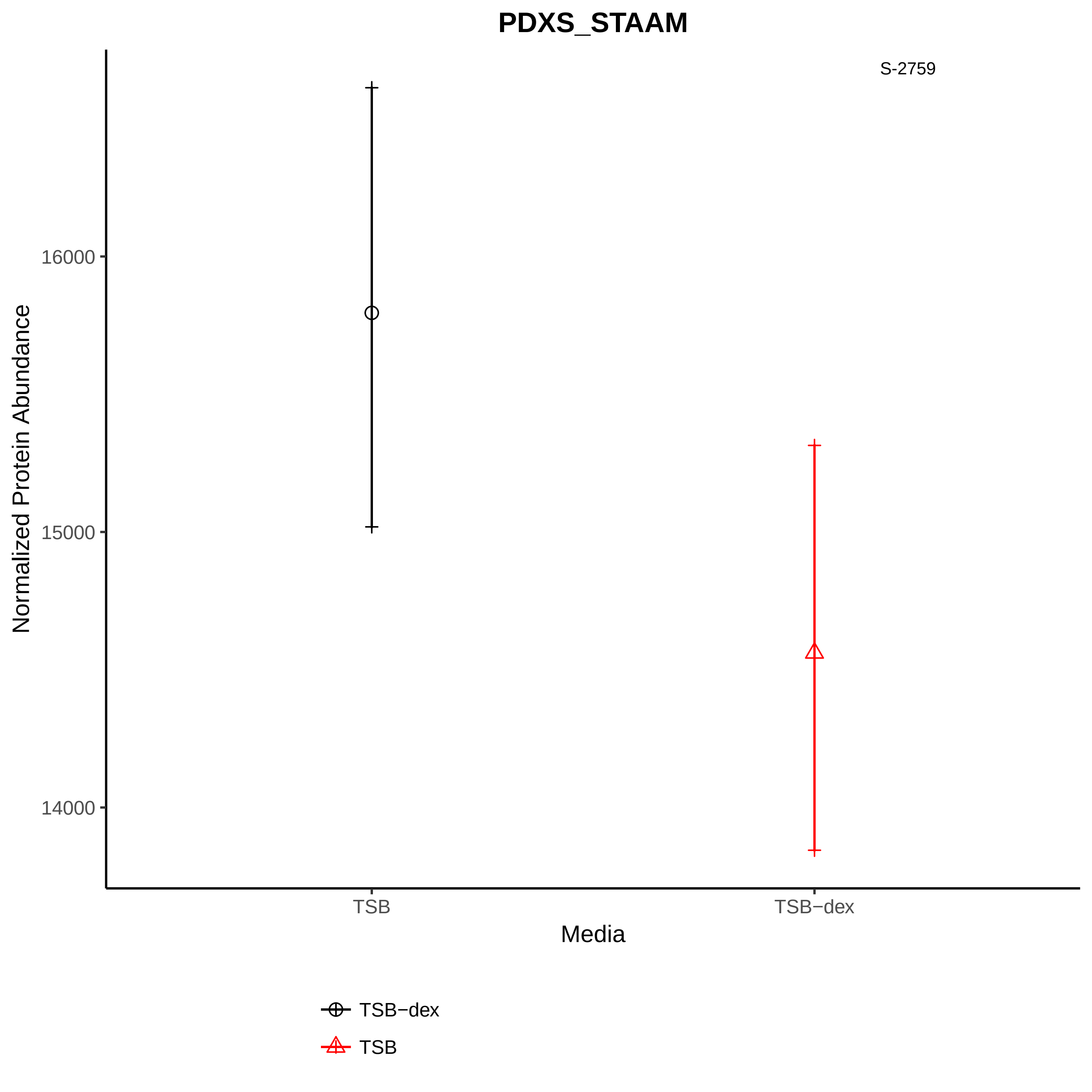

PDXS_STAAM
S-2759
Normalized Protein Abundance
16000
15000
14000
TSB
TSB−dex
Media
TSB−dex
TSB

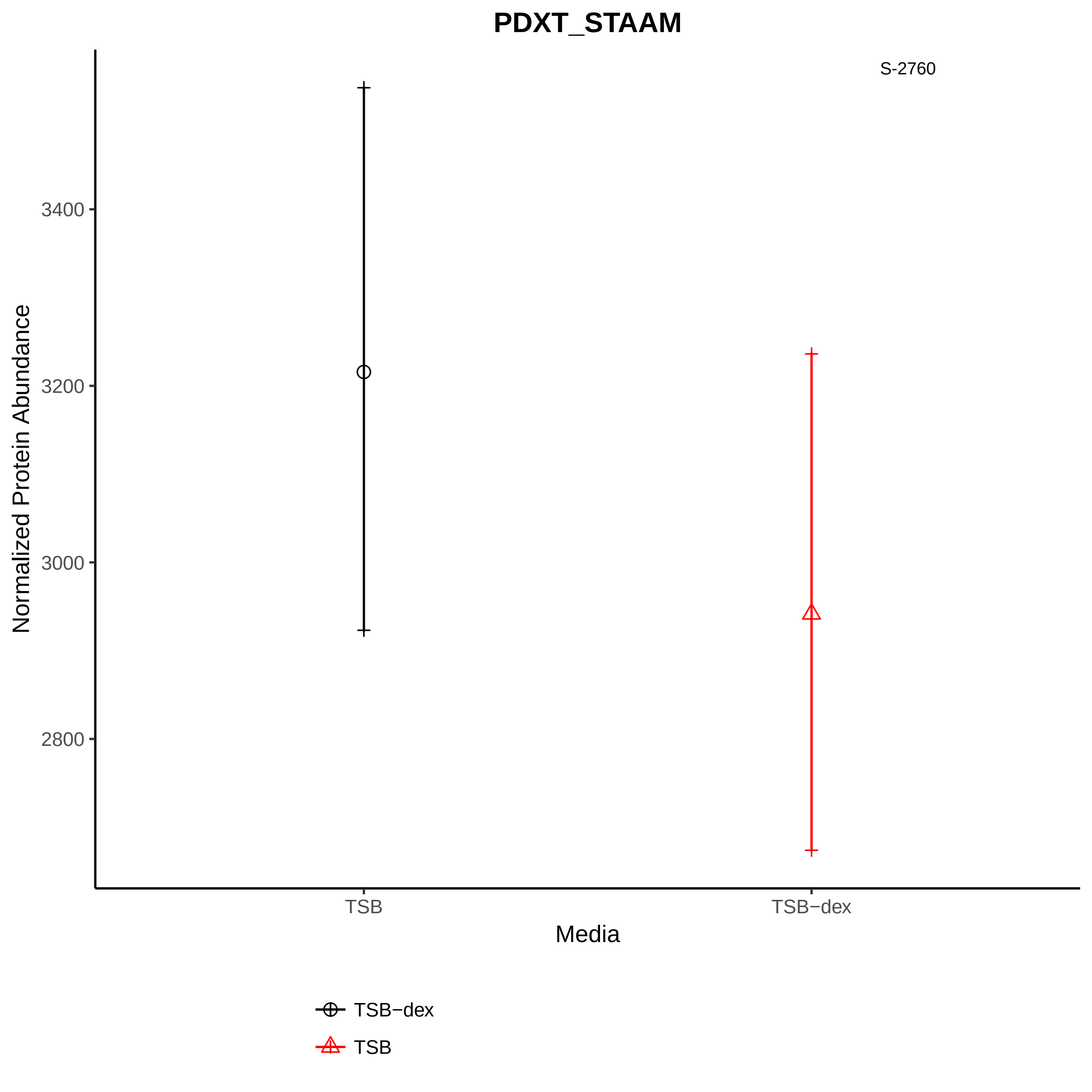

PDXT_STAAM
S-2760
Normalized Protein Abundance
3400
3200
3000
2800
TSB
TSB−dex
Media
TSB−dex
TSB

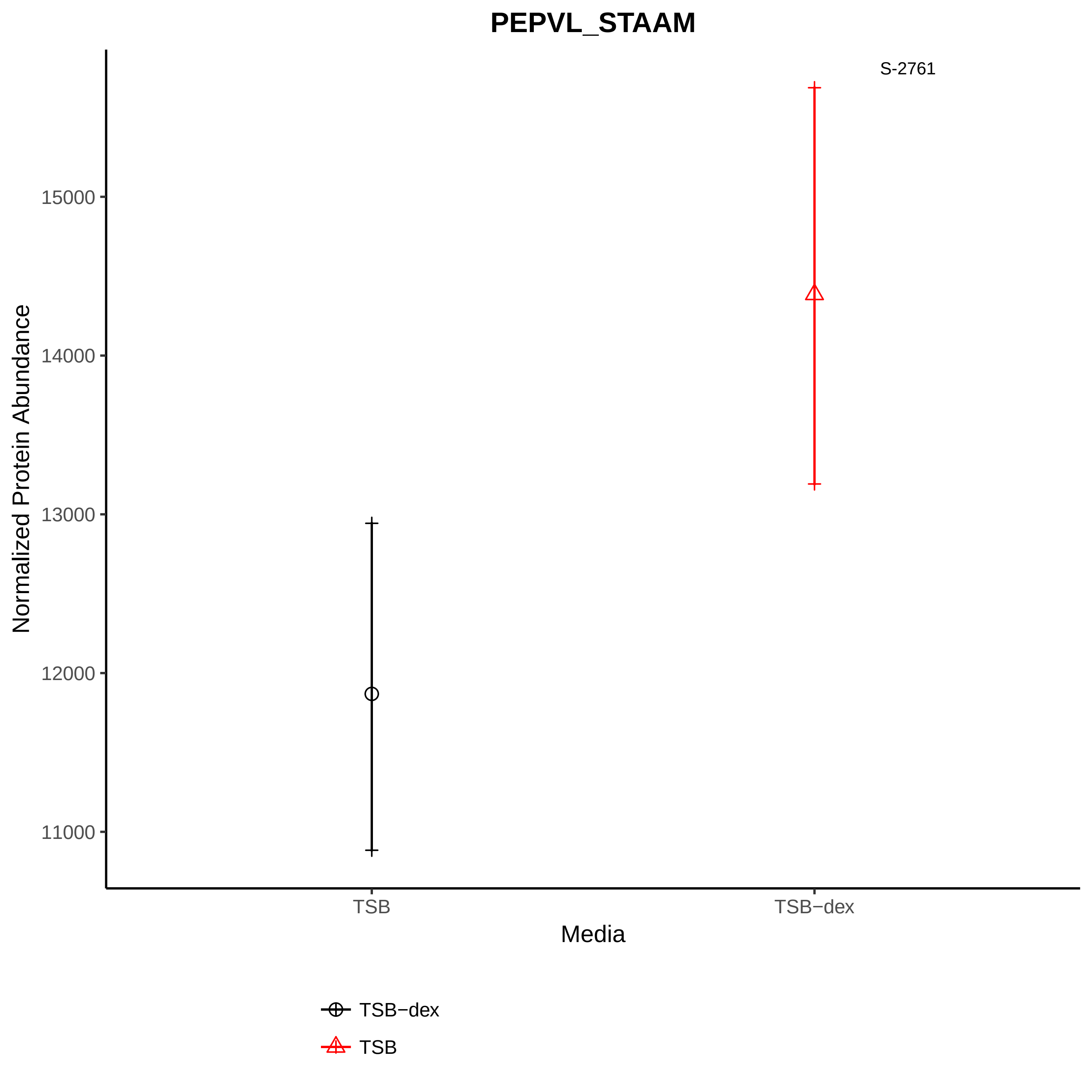

PEPVL_STAAM
S-2761
Normalized Protein Abundance
15000
14000
13000
12000
11000
TSB
TSB−dex
Media
TSB−dex
TSB

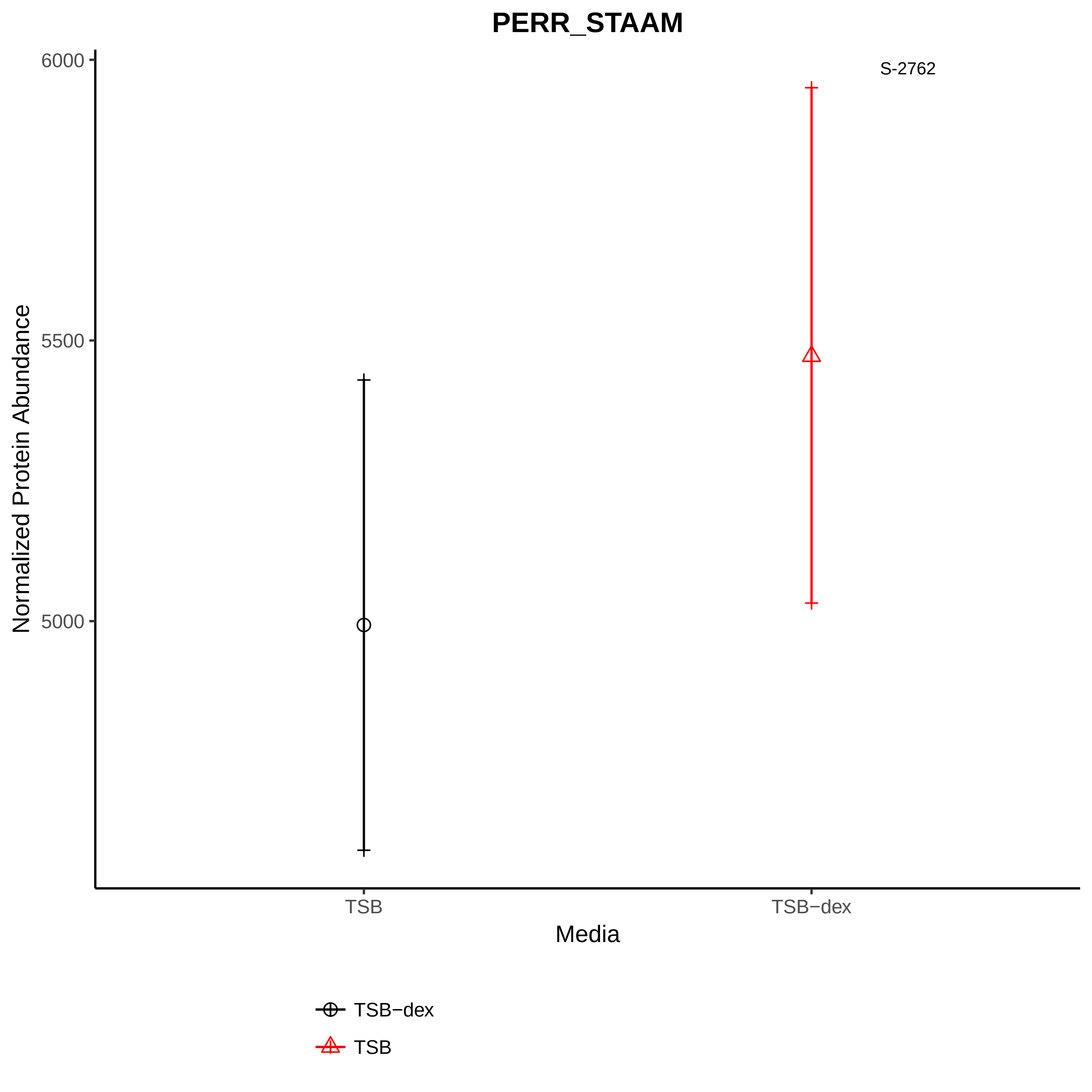

PERR_STAAM
S-2762
6000
5500
5000
Normalized Protein Abundance
TSB
TSB−dex
Media
TSB−dex
TSB

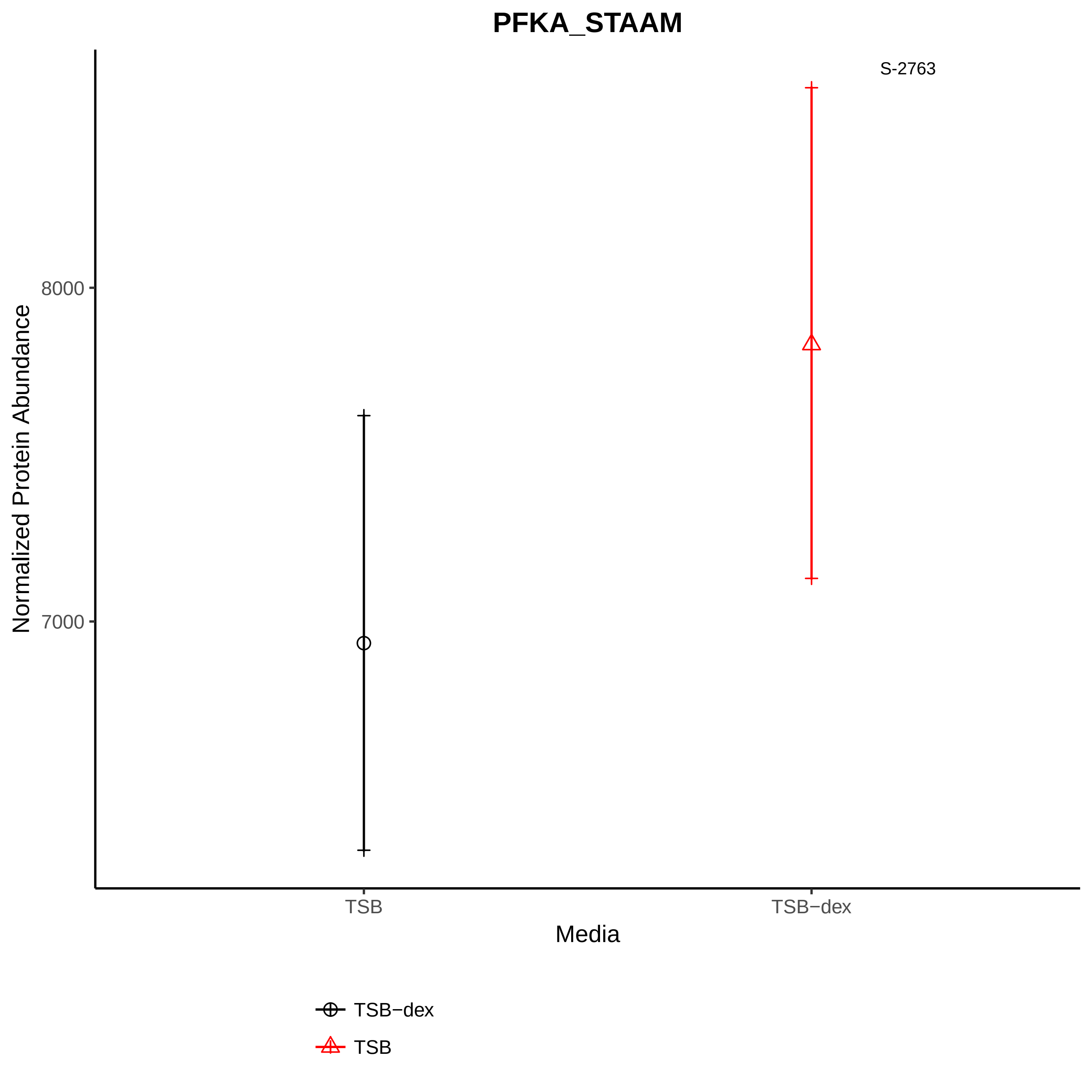

PFKA_STAAM
S-2763
8000
7000
Normalized Protein Abundance
TSB
TSB−dex
Media
TSB−dex
TSB

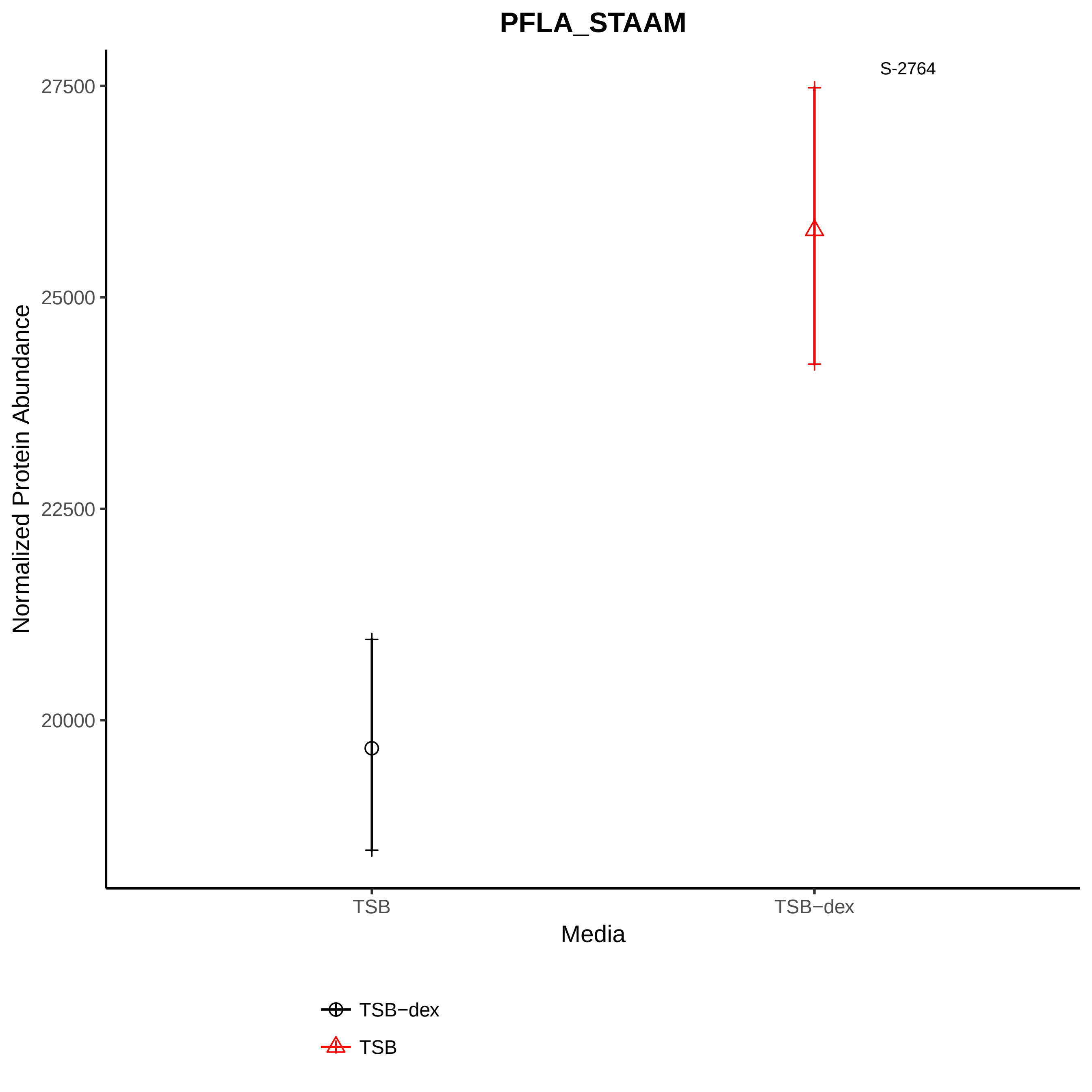

PFLA_STAAM
S-2764
27500
25000
22500
20000
Normalized Protein Abundance
TSB
TSB−dex
Media
TSB−dex
TSB

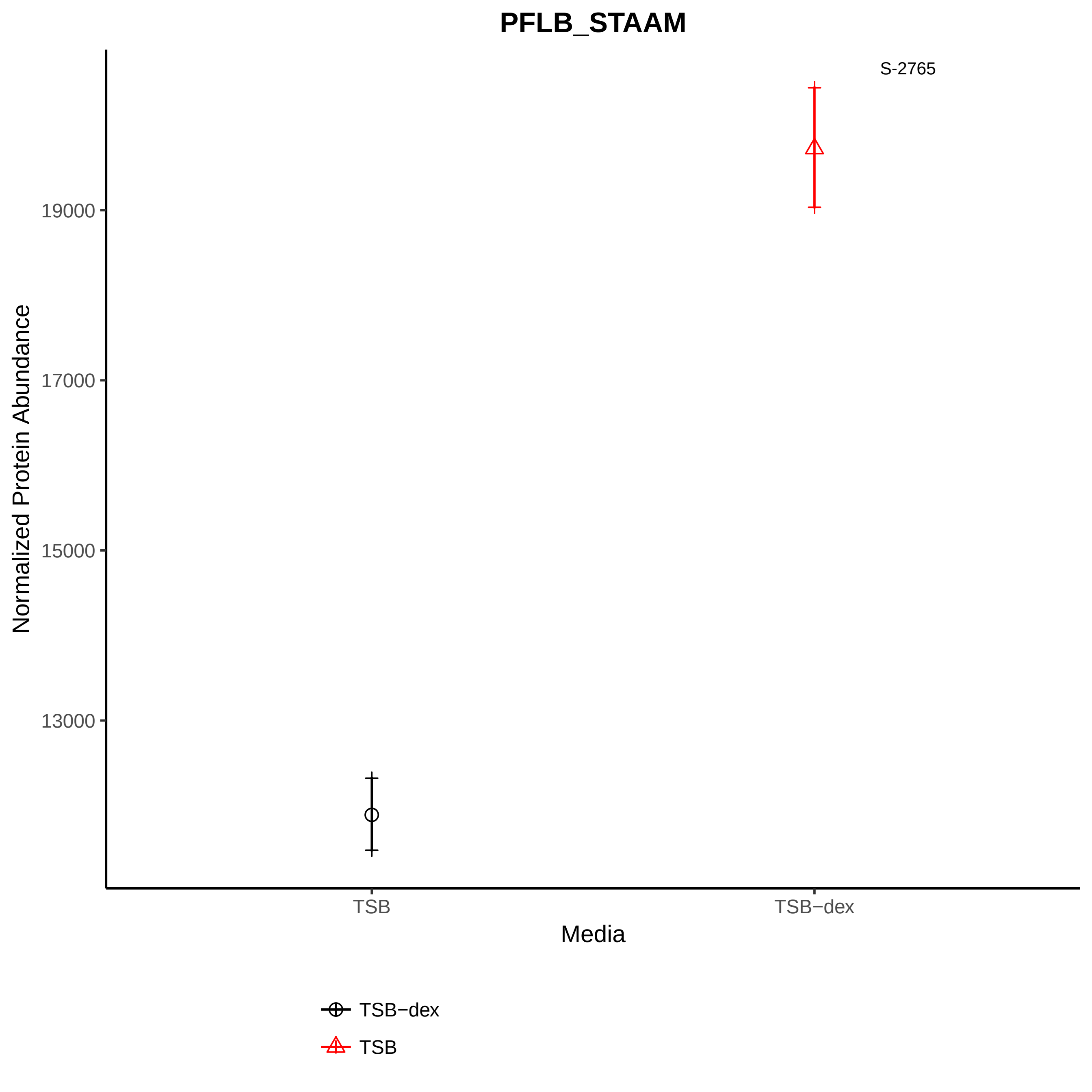

PFLB_STAAM
S-2765
Normalized Protein Abundance
19000
17000
15000
13000
TSB
TSB−dex
Media
TSB−dex
TSB

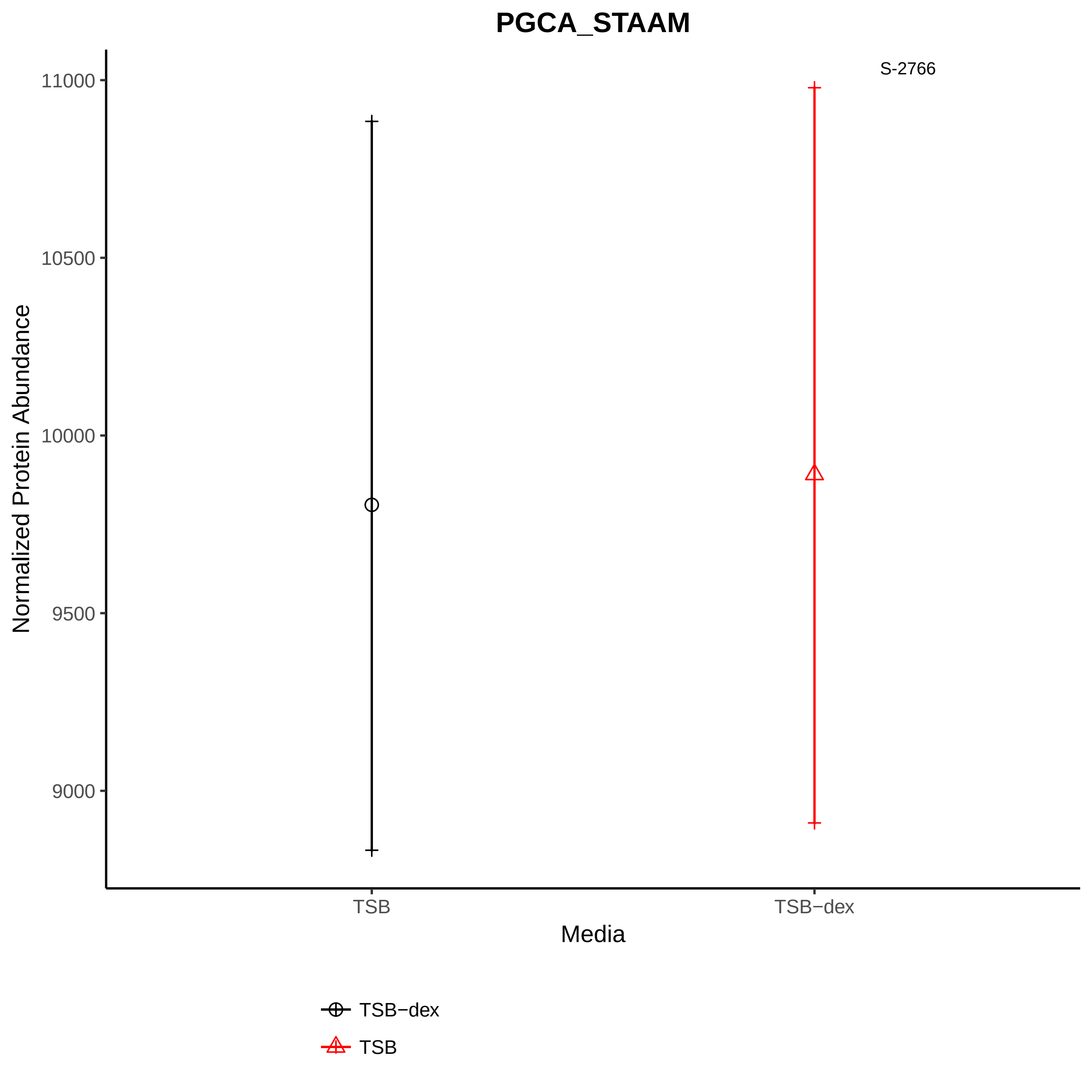
PGCA_STAAM
S-2766
Normalized Protein Abundance
11000
10500
10000
9500
9000
TSB
TSB−dex
Media
TSB−dex
TSB

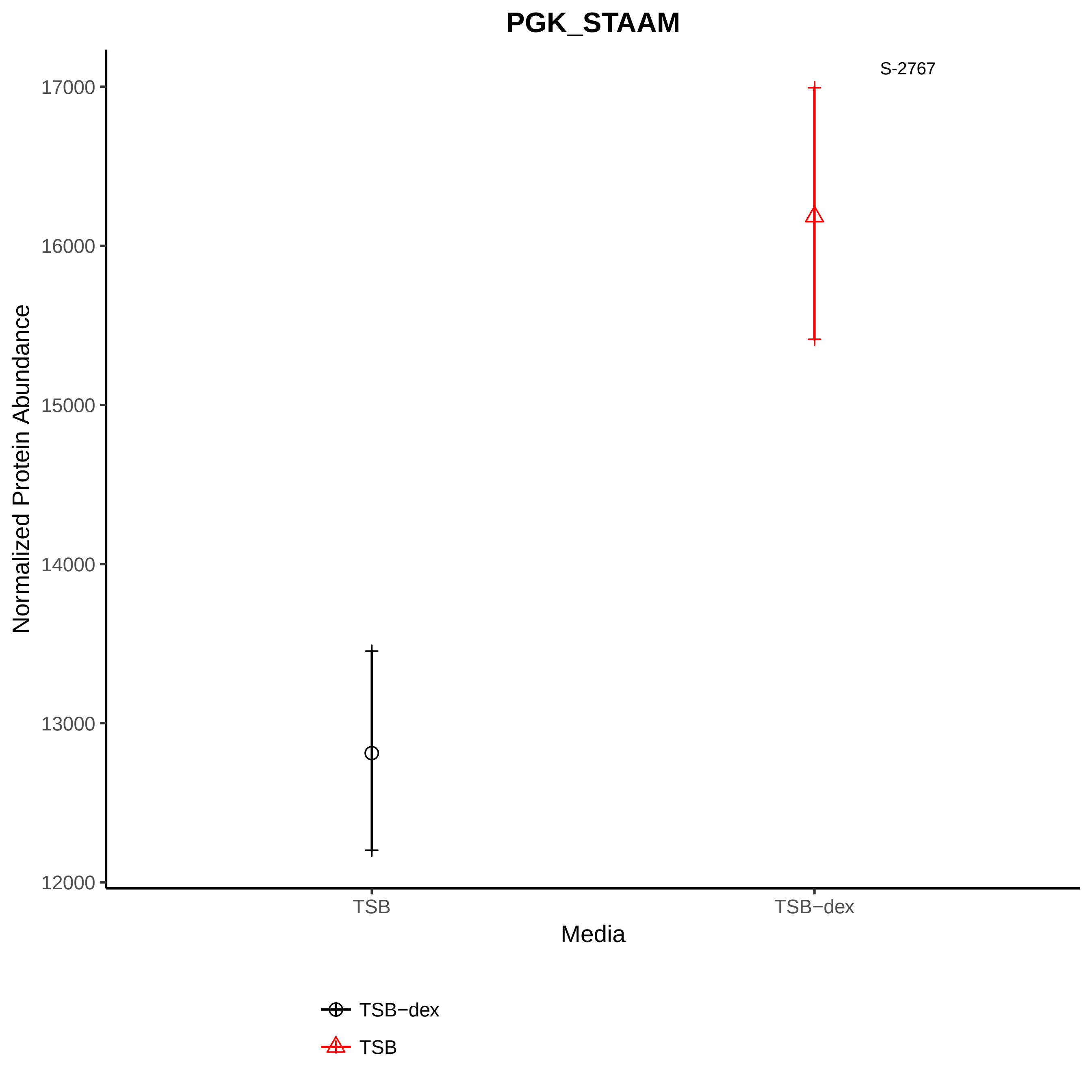

PGK_STAAM
S-2767
17000
16000
15000
14000
13000
12000
Normalized Protein Abundance
TSB
TSB−dex
Media
TSB−dex
TSB

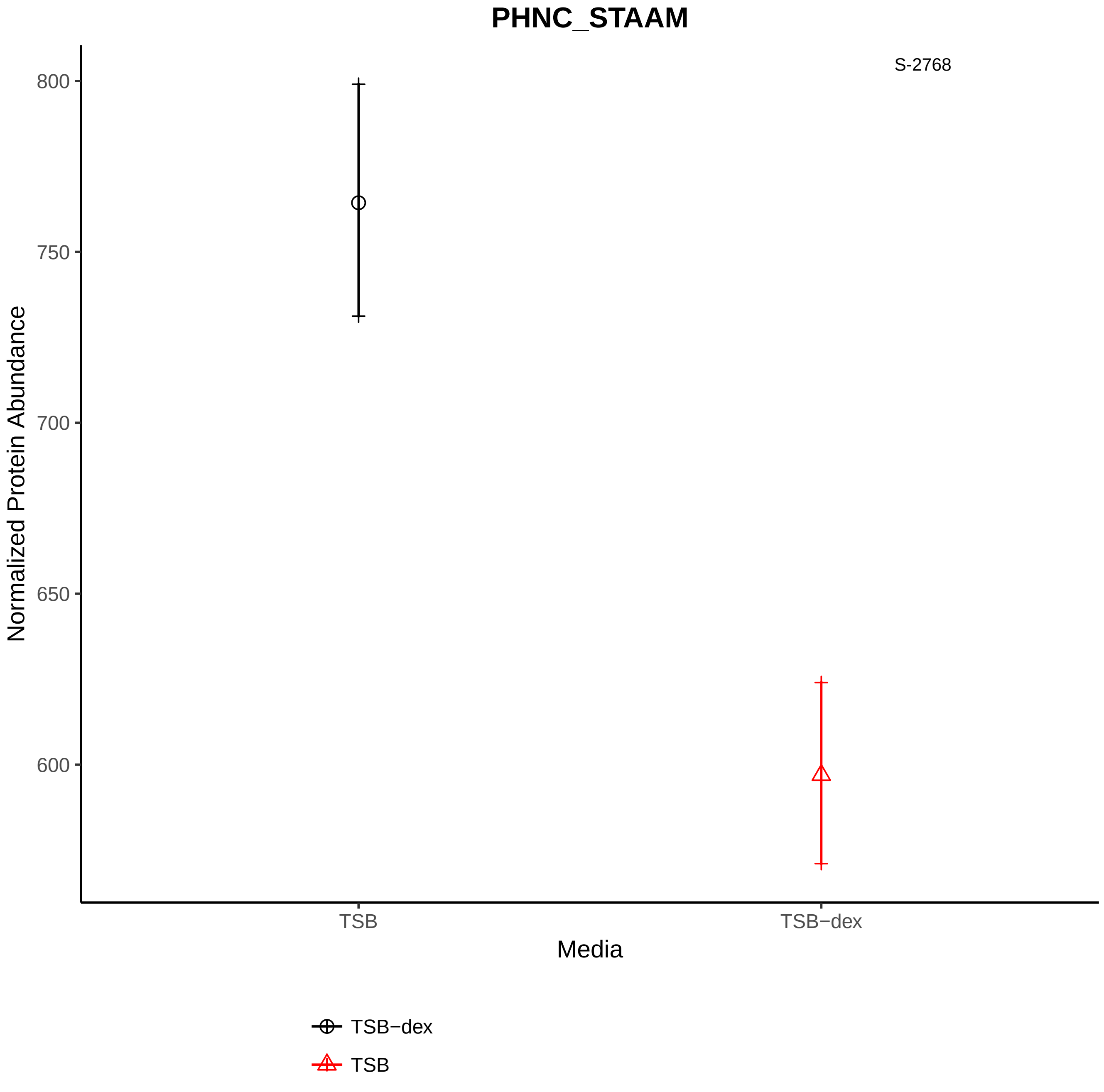
PHNC_STAAM
S-2768
Normalized Protein Abundance
800
750
700
650
600
TSB
TSB−dex
Media
TSB−dex
TSB

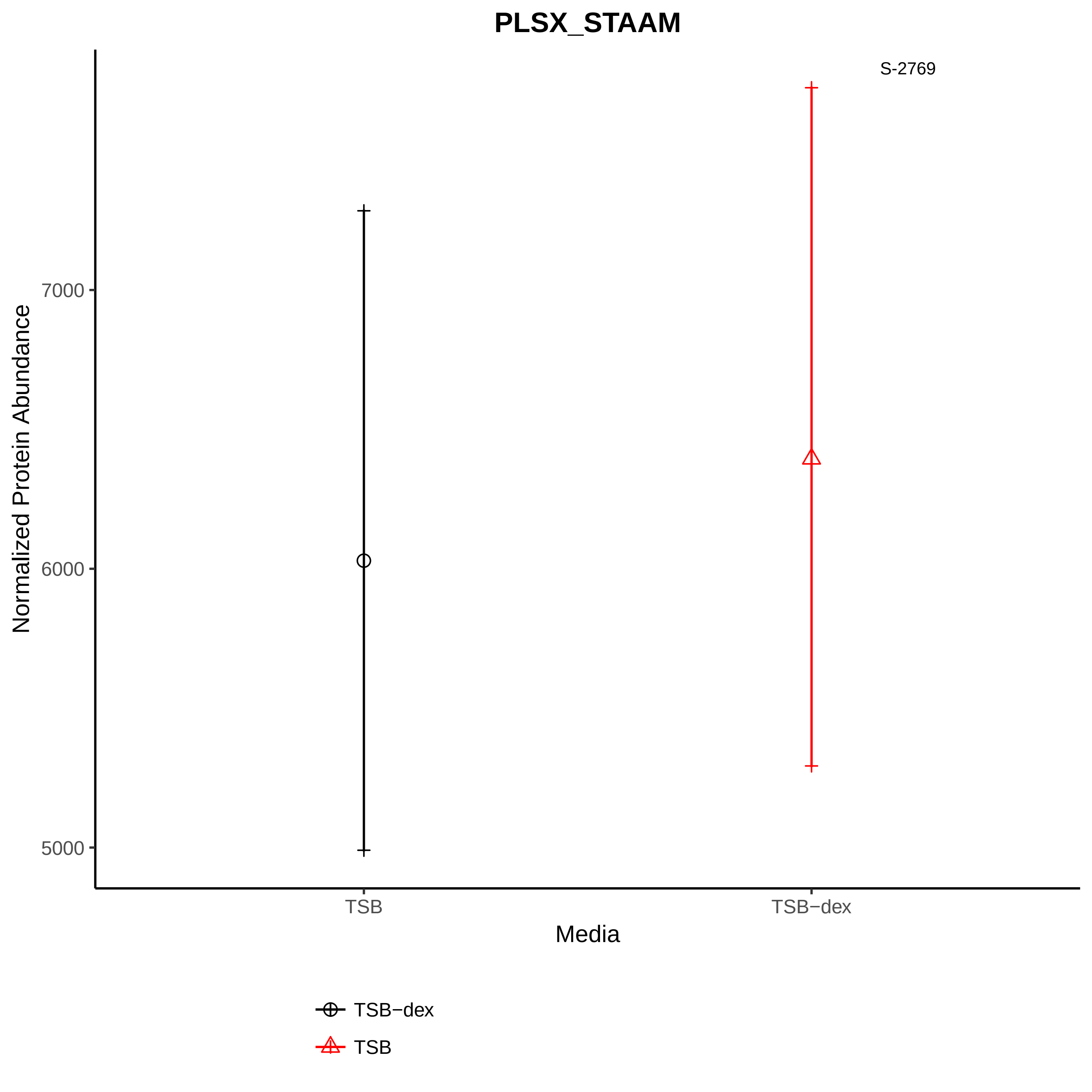

PLSX_STAAM
S-2769
Normalized Protein Abundance
7000
6000
5000
TSB
TSB−dex
Media
TSB−dex
TSB

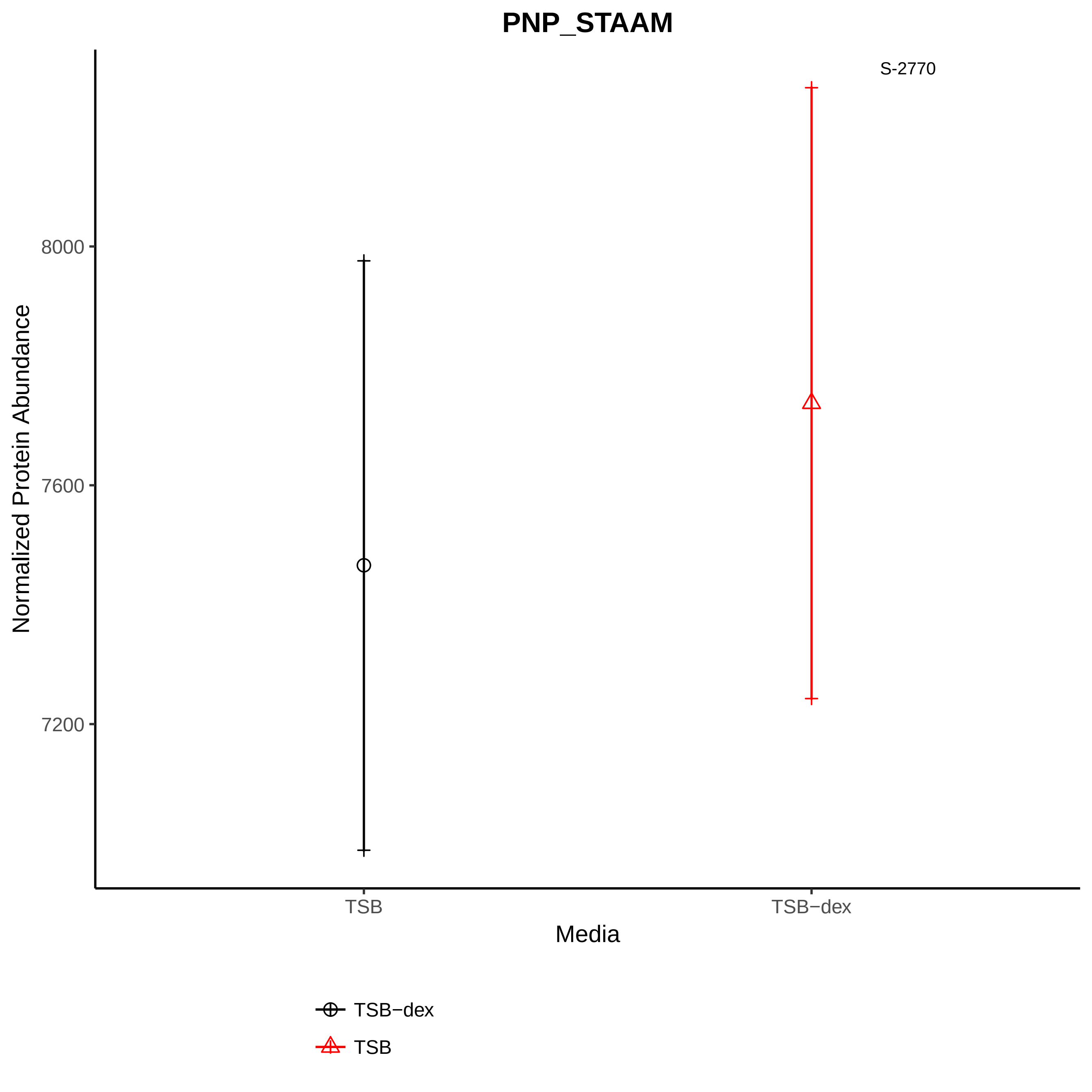
PNP_STAAM
Normalized Protein Abundance
8000
7600
7200
TSB
TSB−dex
Media
TSB−dex
TSB

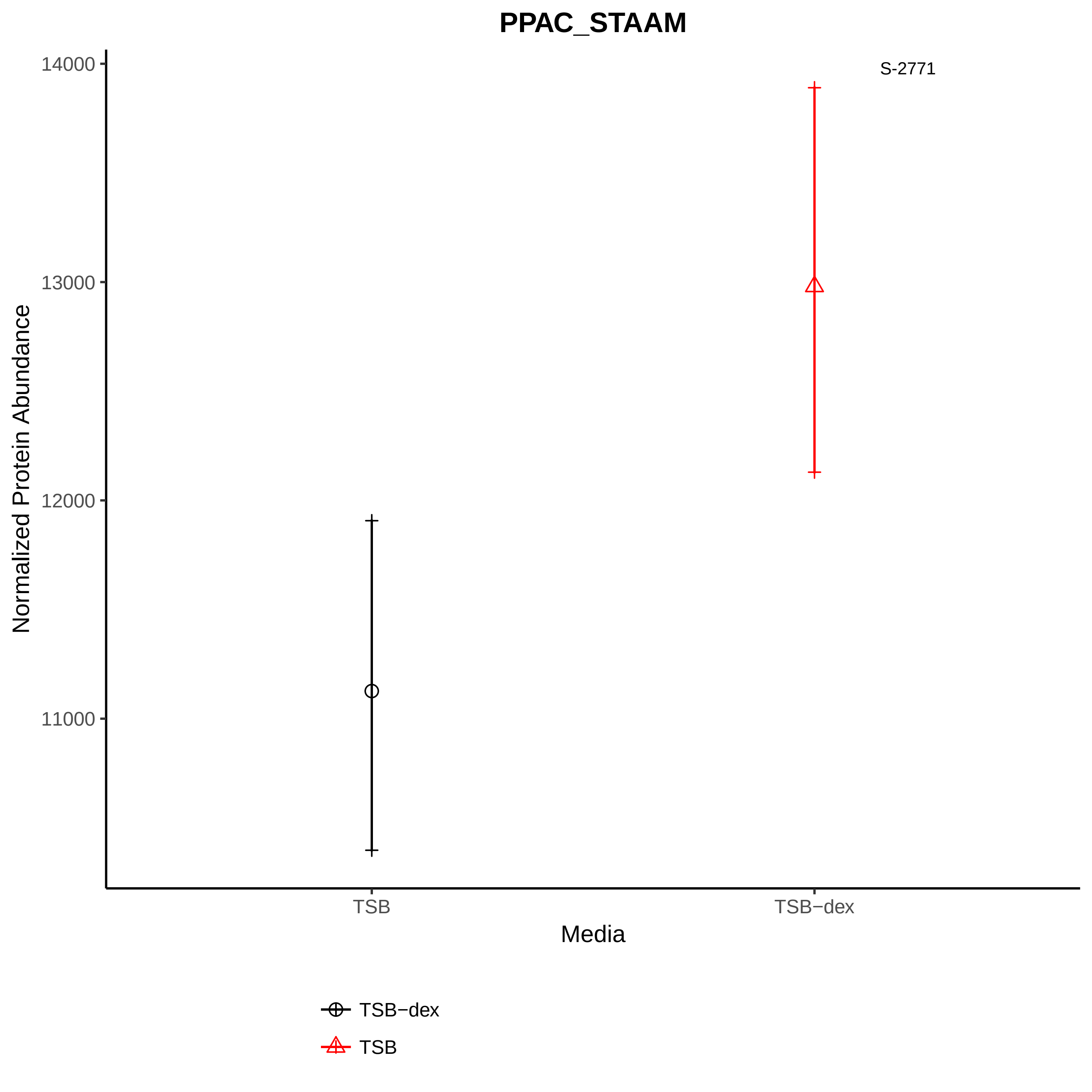

PPAC_STAAM
S-2771
Normalized Protein Abundance
14000
13000
12000
11000
TSB
TSB−dex
Media
TSB−dex
TSB

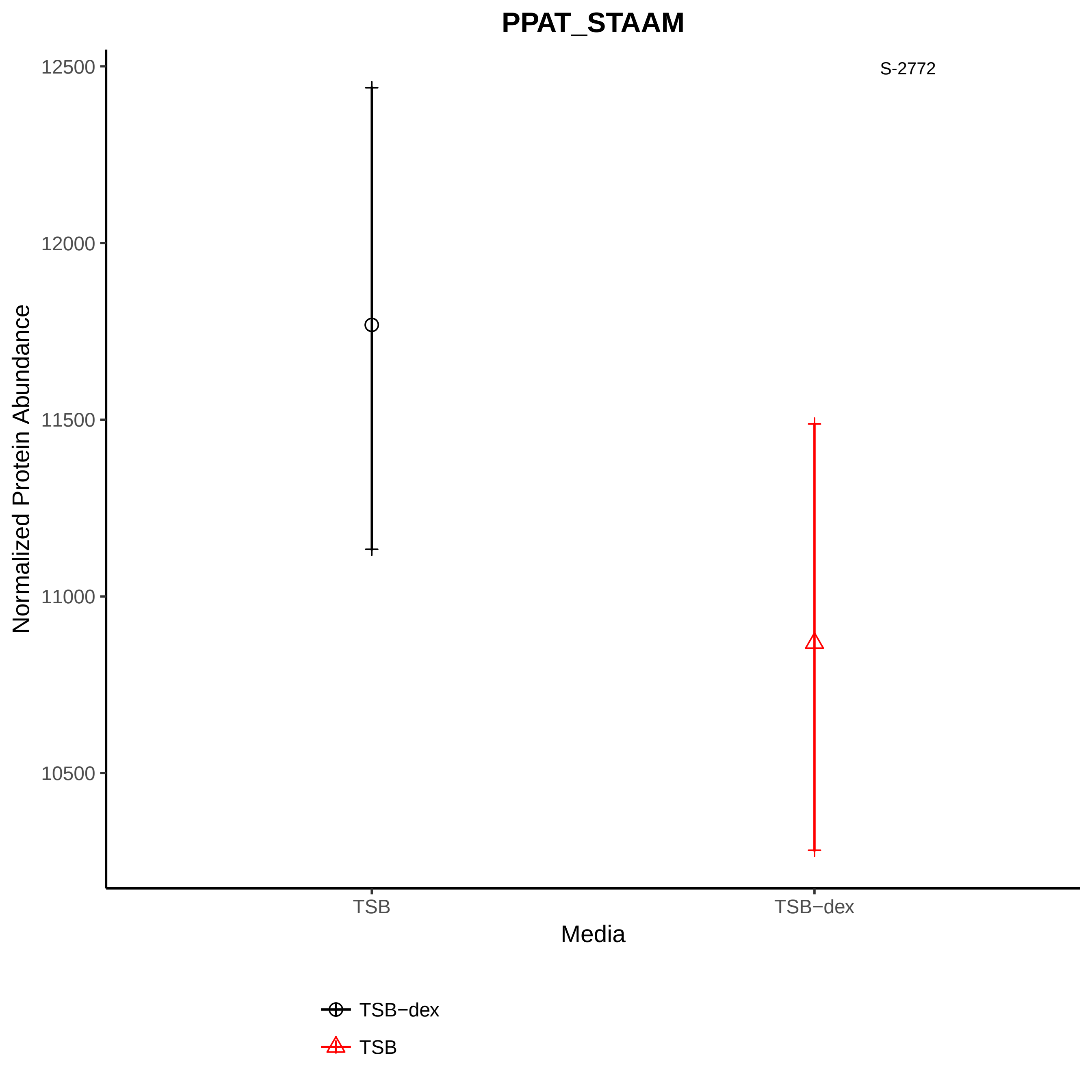

PPAT_STAAM
S-2772
12500
12000
11500
11000
10500
Normalized Protein Abundance
TSB
TSB−dex
Media
TSB−dex
TSB

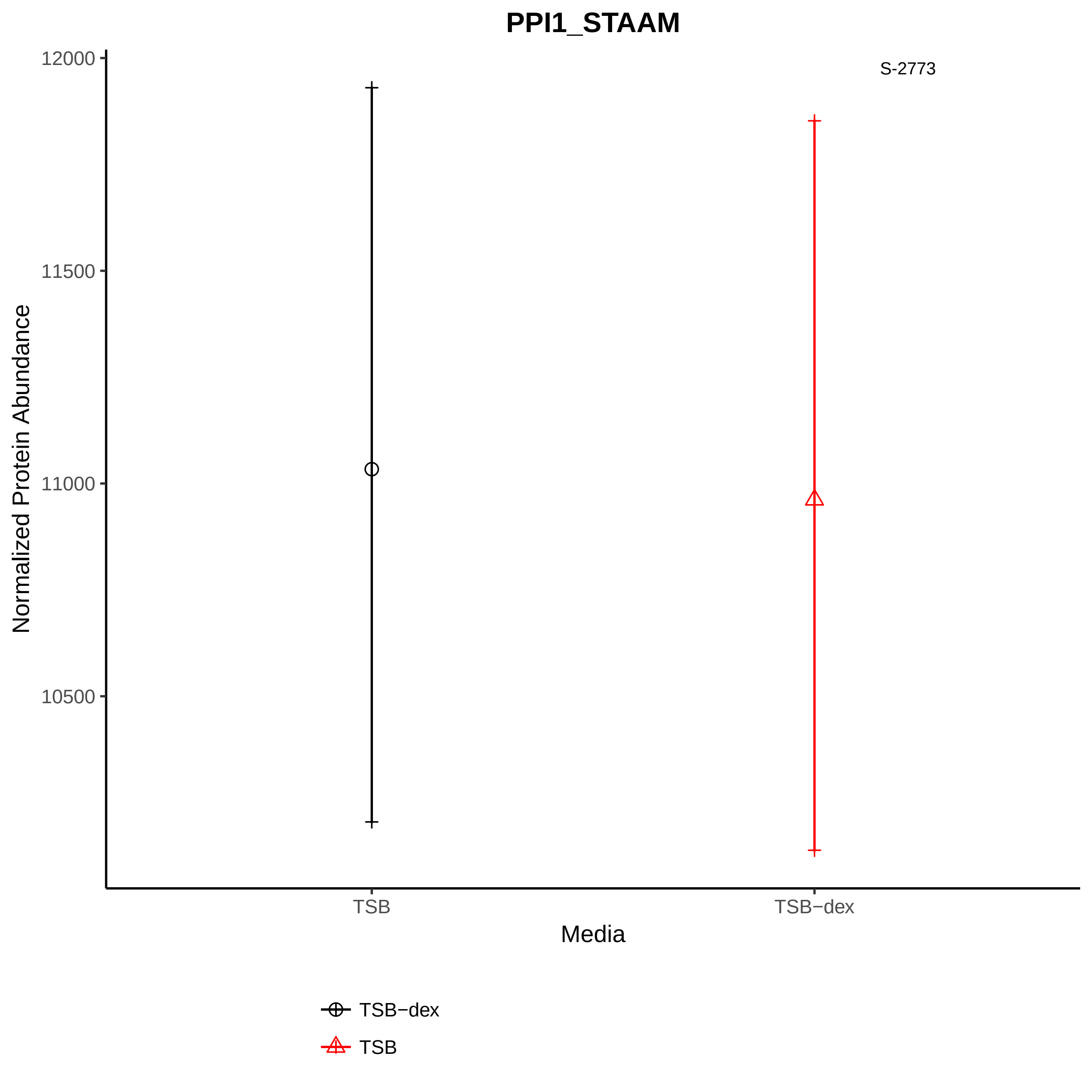

PPI1_STAAM
S-2773
Normalized Protein Abundance
12000
11500
11000
10500
TSB
TSB−dex
Media
TSB−dex
TSB

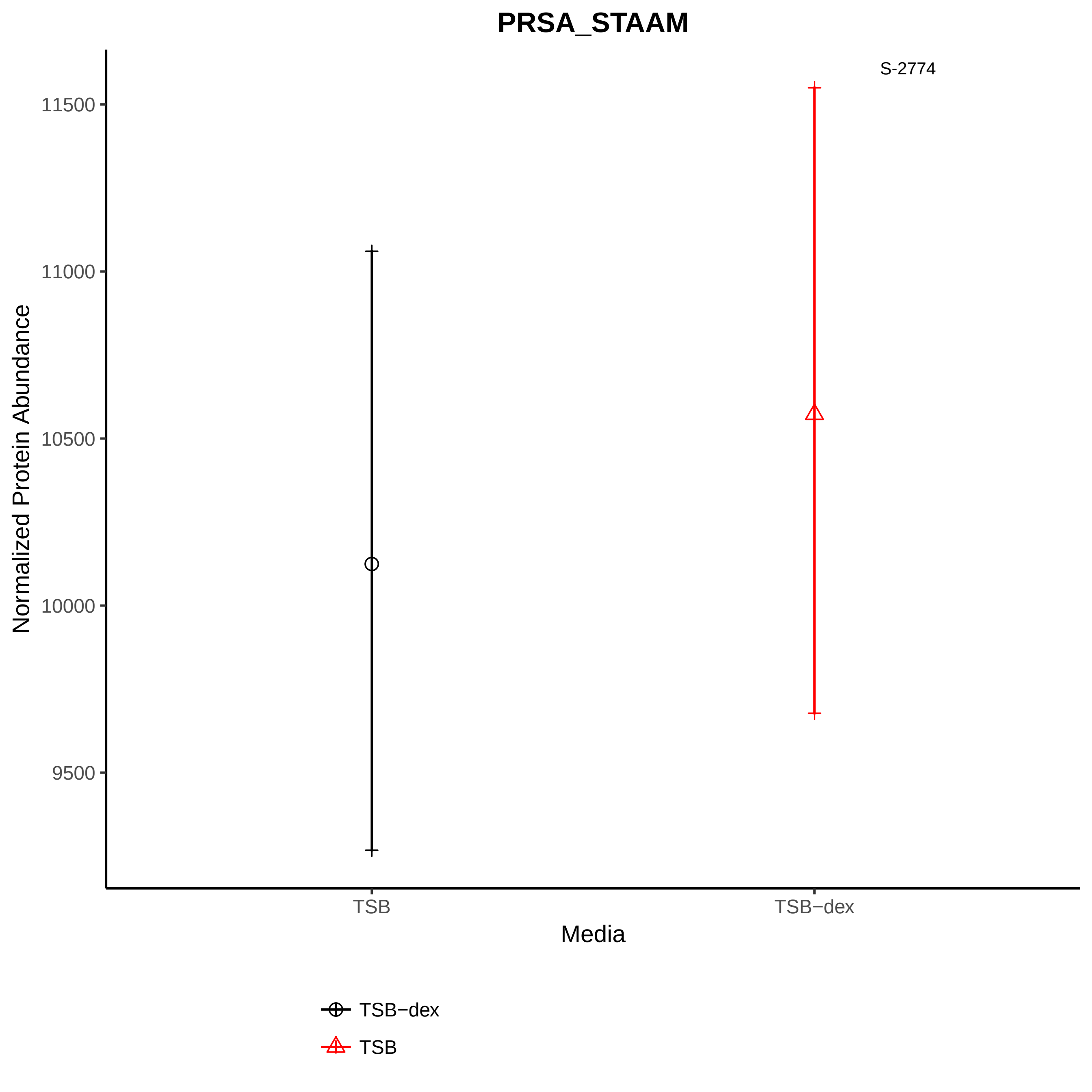

PRSA_STAAM
S-2774
Normalized Protein Abundance
11500
11000
10500
10000
9500
TSB
TSB−dex
Media
TSB−dex
TSB

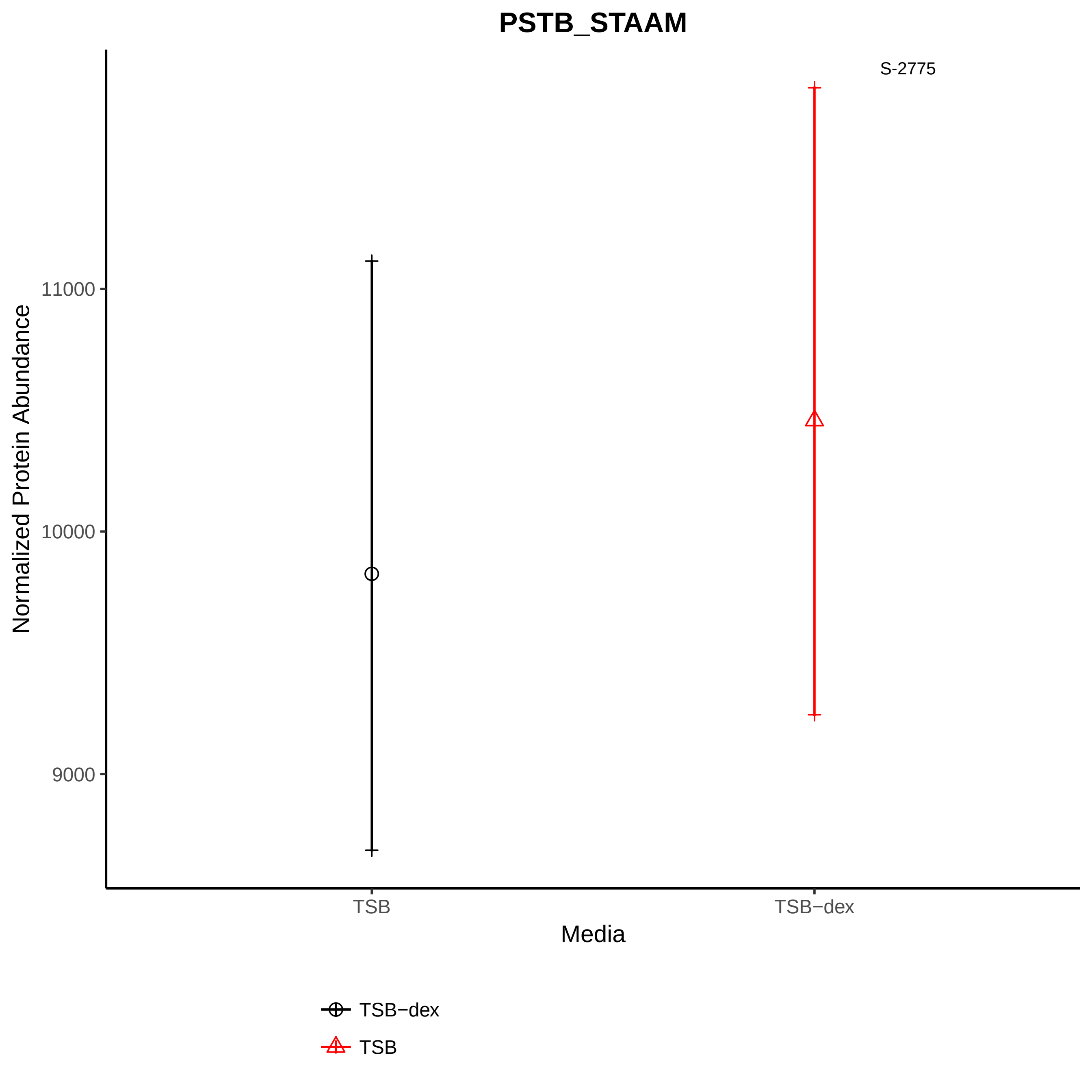
PSTB_STAAM
S-2775
Normalized Protein Abundance
11000
10000
9000
TSB
TSB−dex
Media
TSB−dex
TSB

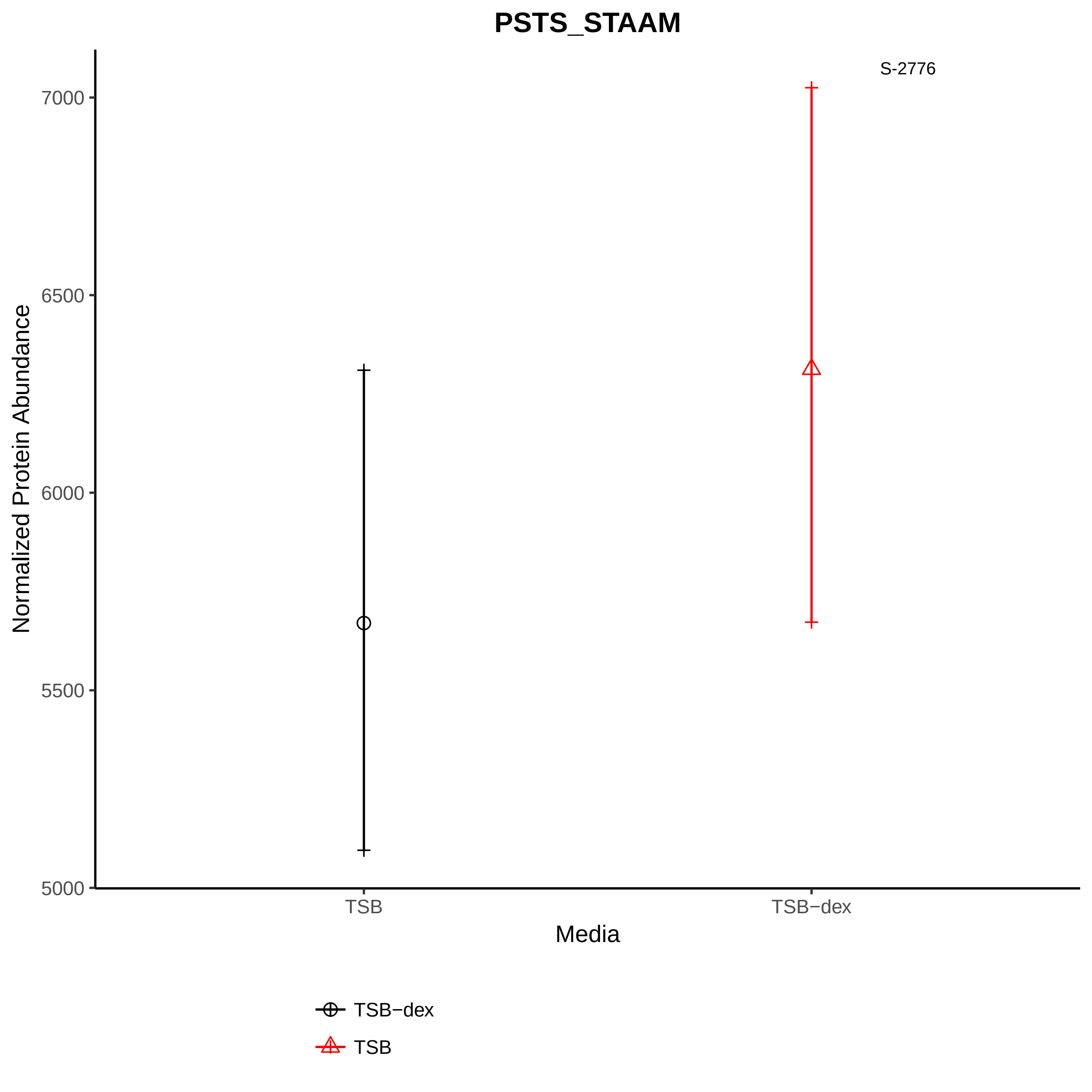
PSTS_STAAM
S-2776
Normalized Protein Abundance
7000
6500
6000
5500
5000
TSB
TSB−dex
Media
TSB−dex
TSB

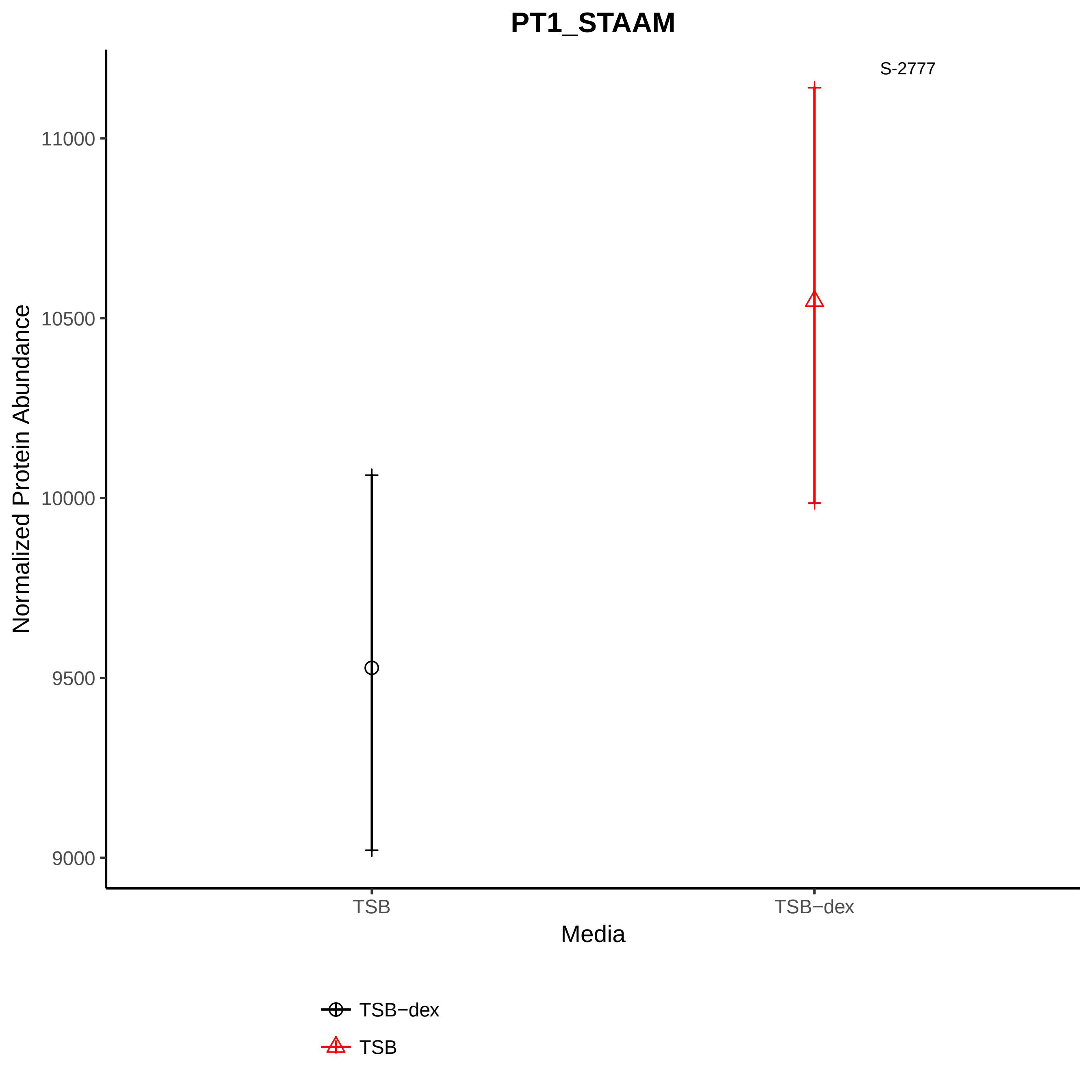

PT1_STAAM
S-2777
Normalized Protein Abundance
11000
10500
10000
9500
9000
TSB
TSB−dex
Media
TSB−dex
TSB

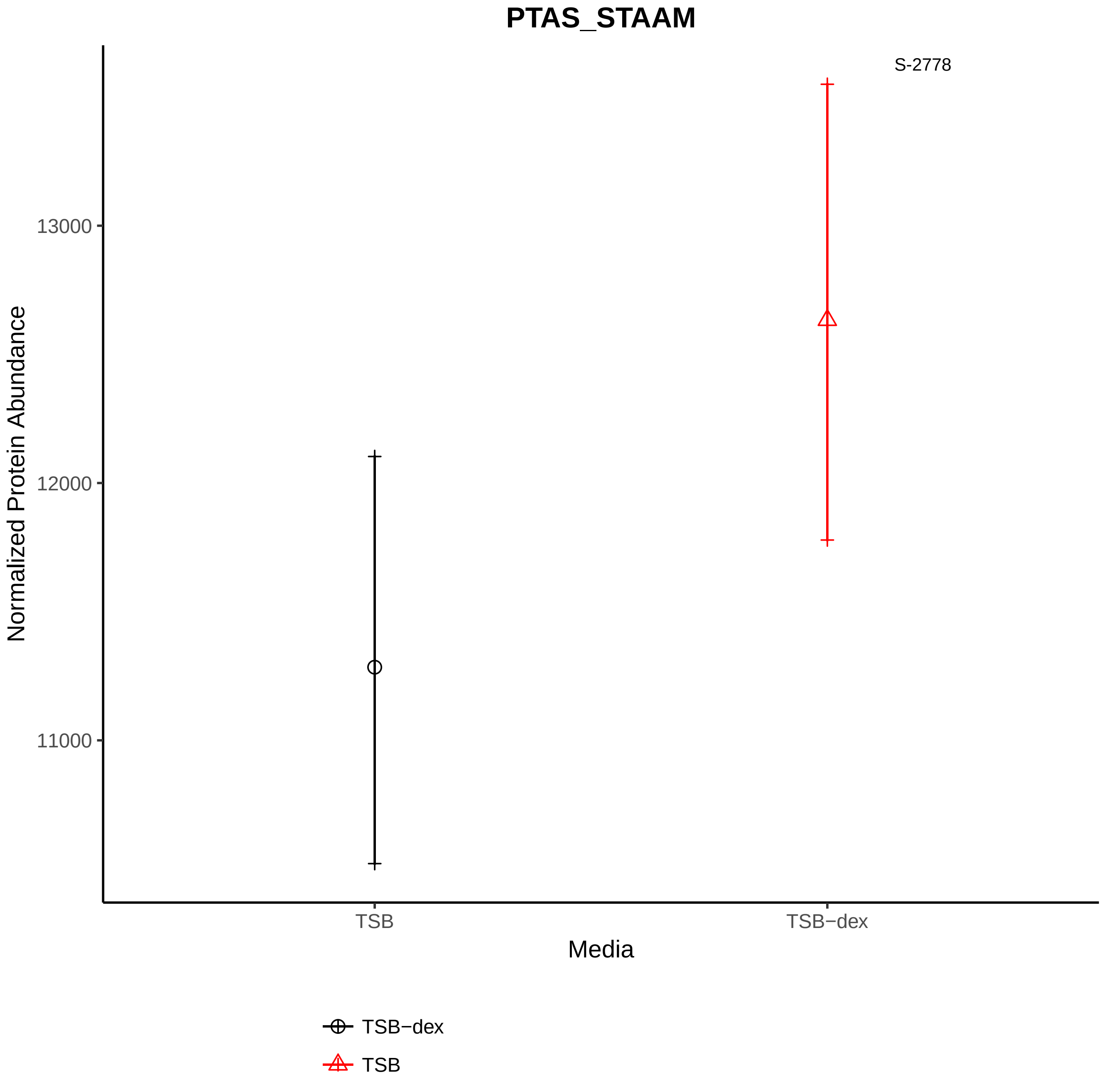
PTAS_STAAM
S-2778
13000
12000
11000
Normalized Protein Abundance
TSB
TSB−dex
Media
TSB−dex
TSB

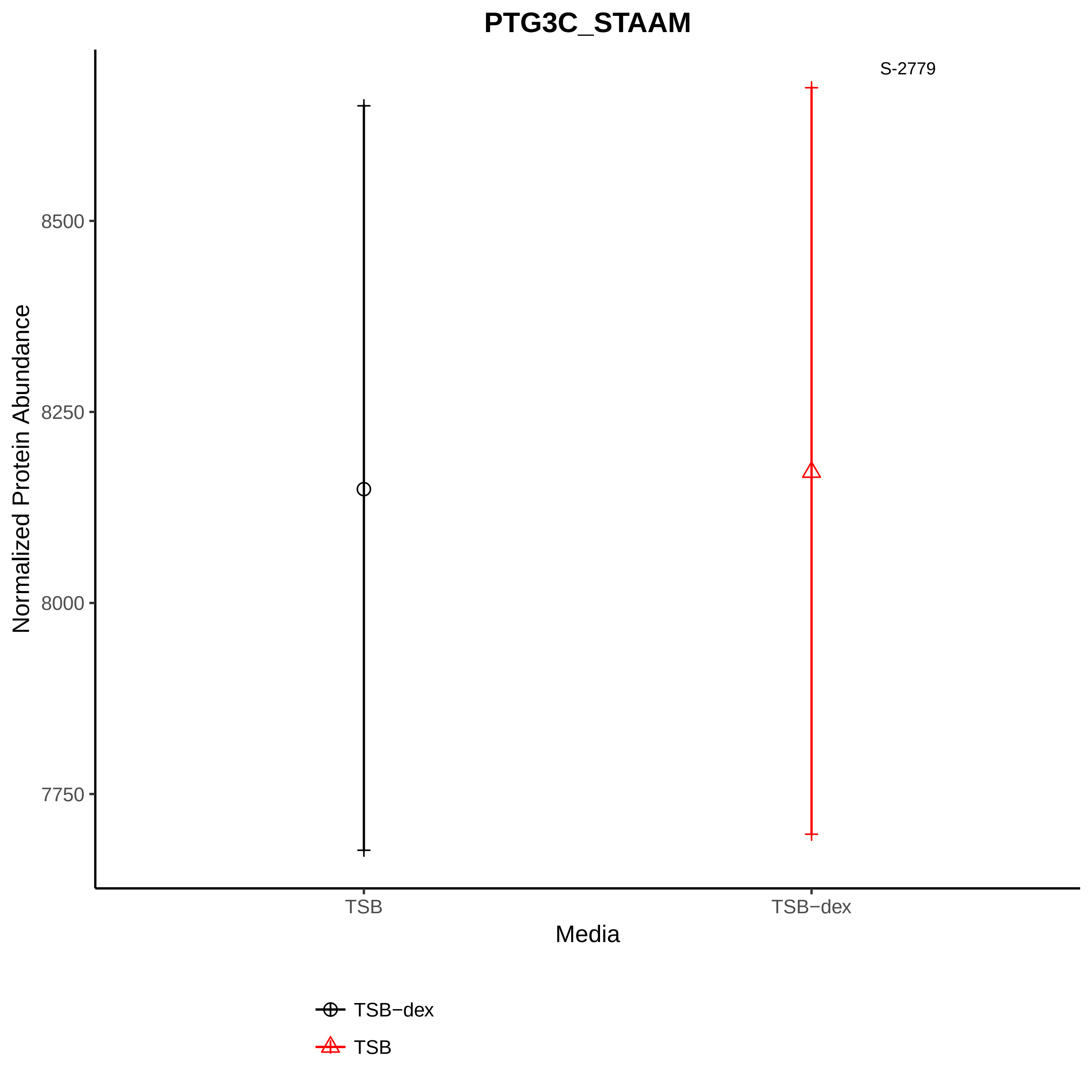
PTG3C_STAAM
S-2779
Normalized Protein Abundance
8500
8250
8000
7750
TSB
TSB−dex
Media
TSB−dex
TSB

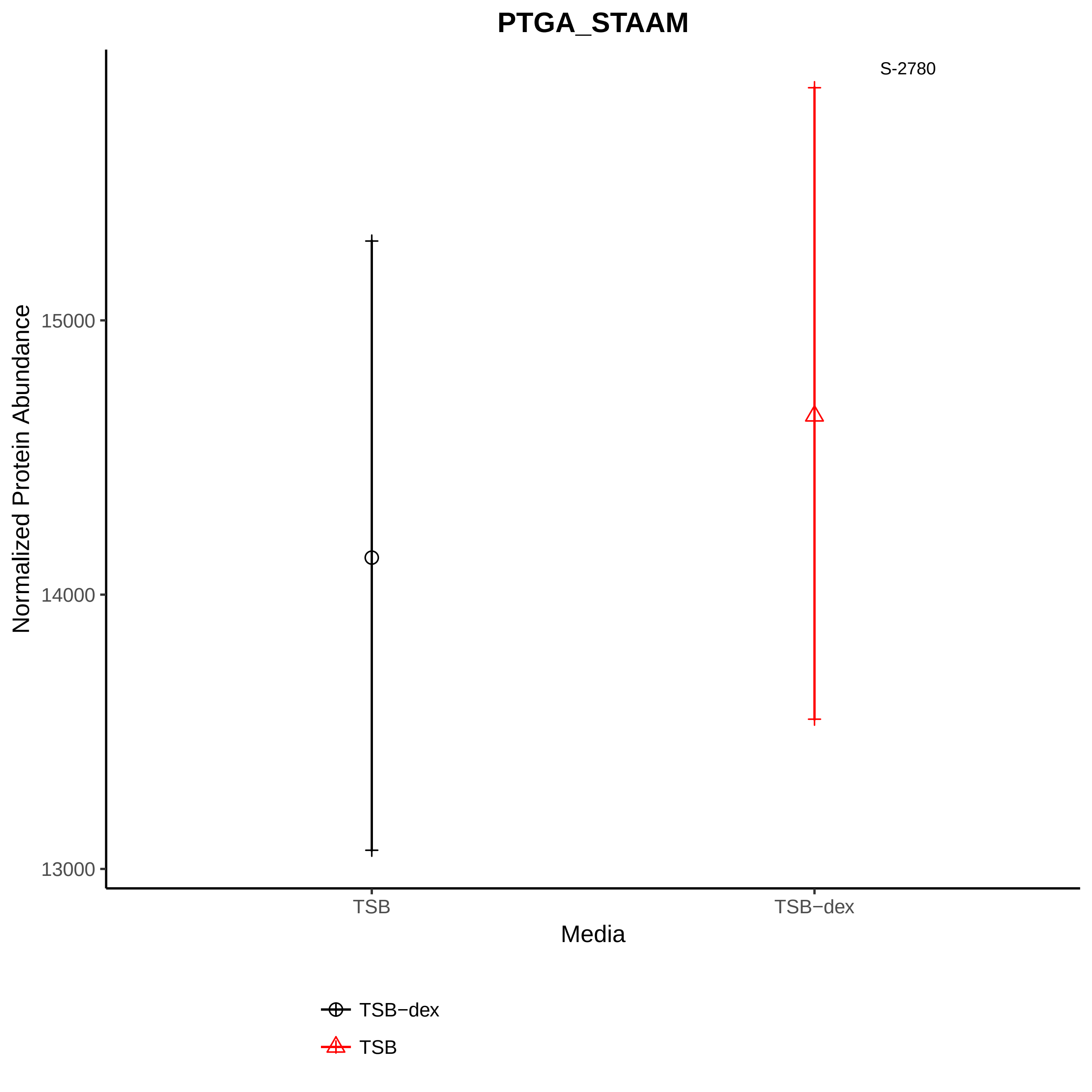
PTGA_STAAM
S-2780
Normalized Protein Abundance
15000
14000
13000
TSB
TSB−dex
Media
TSB−dex
TSB

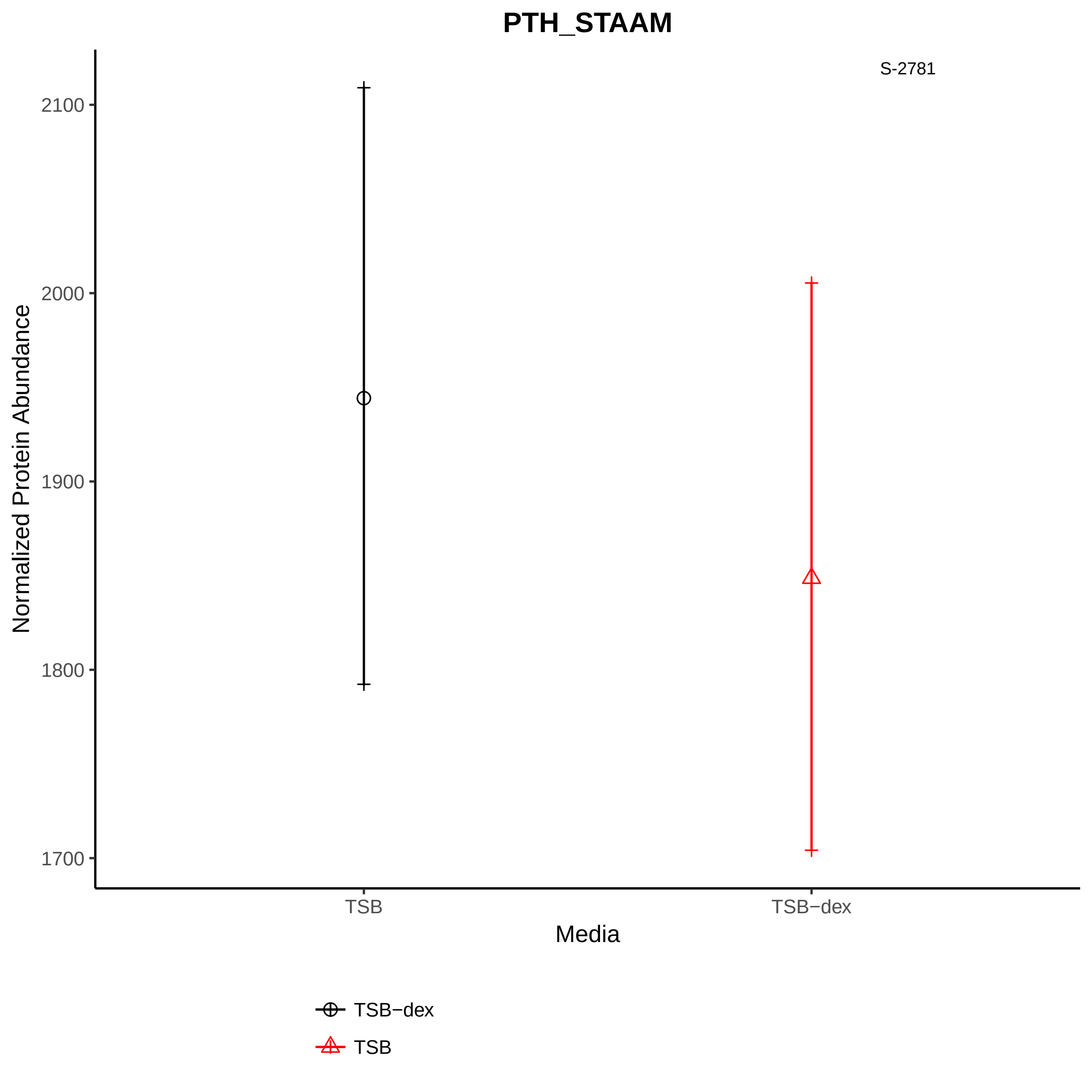
PTH_STAAM
S-2781
Normalized Protein Abundance
2100
2000
1900
1800
1700
TSB
TSB−dex
Media
TSB−dex
TSB

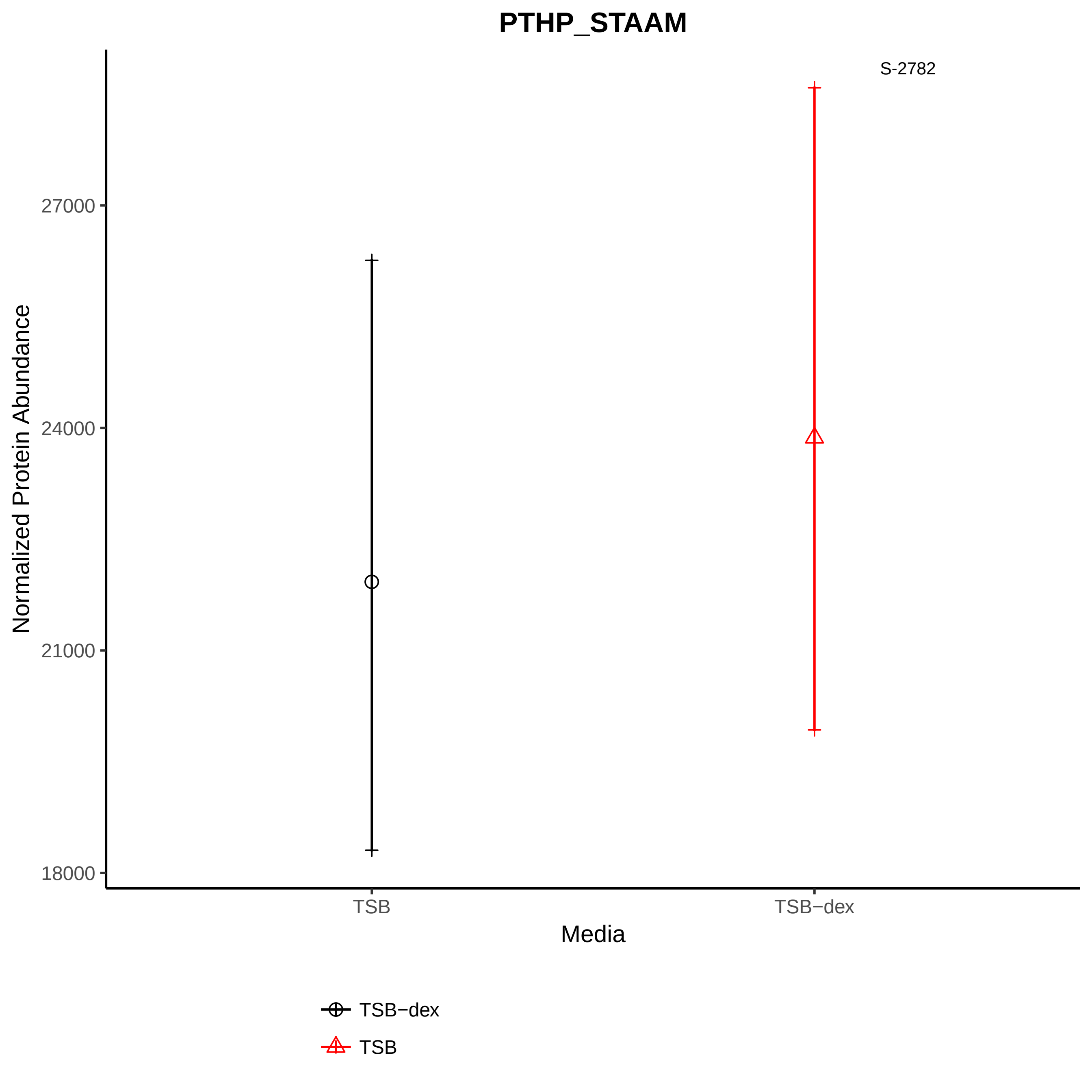

PTHP_STAAM
S-2782
Normalized Protein Abundance
27000
24000
21000
18000
TSB
TSB−dex
Media
TSB−dex
TSB

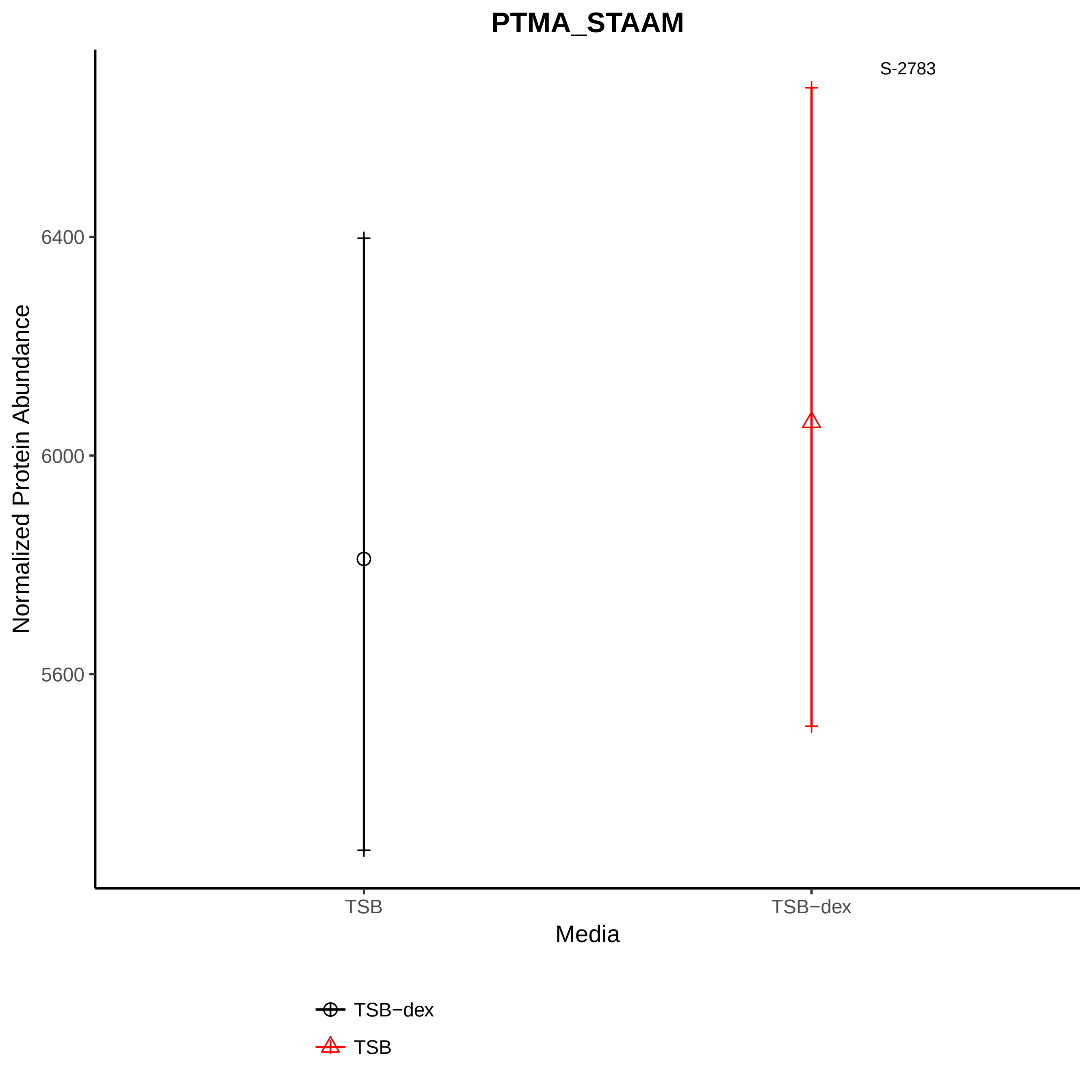

PTMA_STAAM
S-2783
6400
6000
5600
Normalized Protein Abundance
TSB
TSB−dex
Media
TSB−dex
TSB

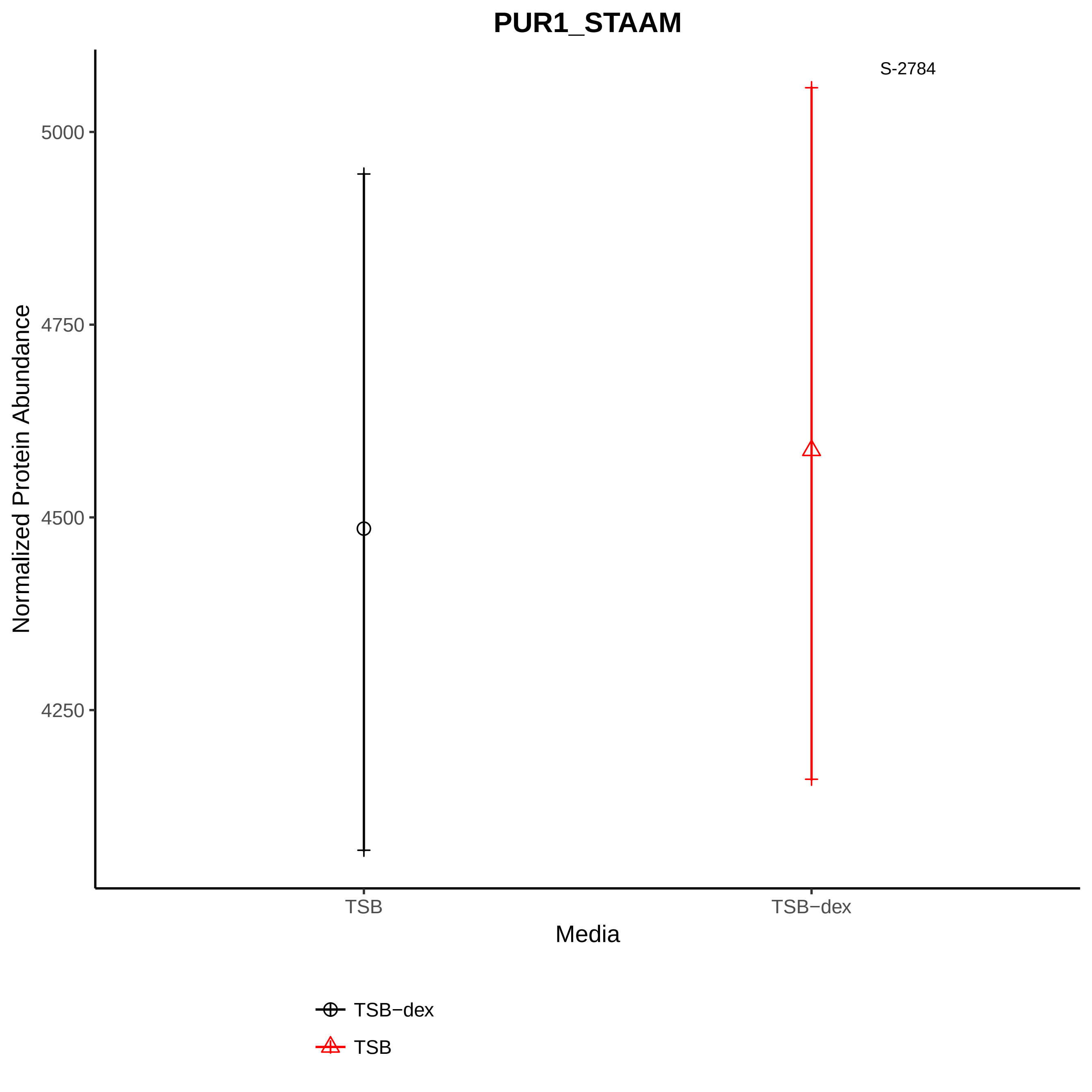

PUR1_STAAM
S-2784
Normalized Protein Abundance
5000
4750
4500
4250
TSB
TSB−dex
Media
TSB−dex
TSB

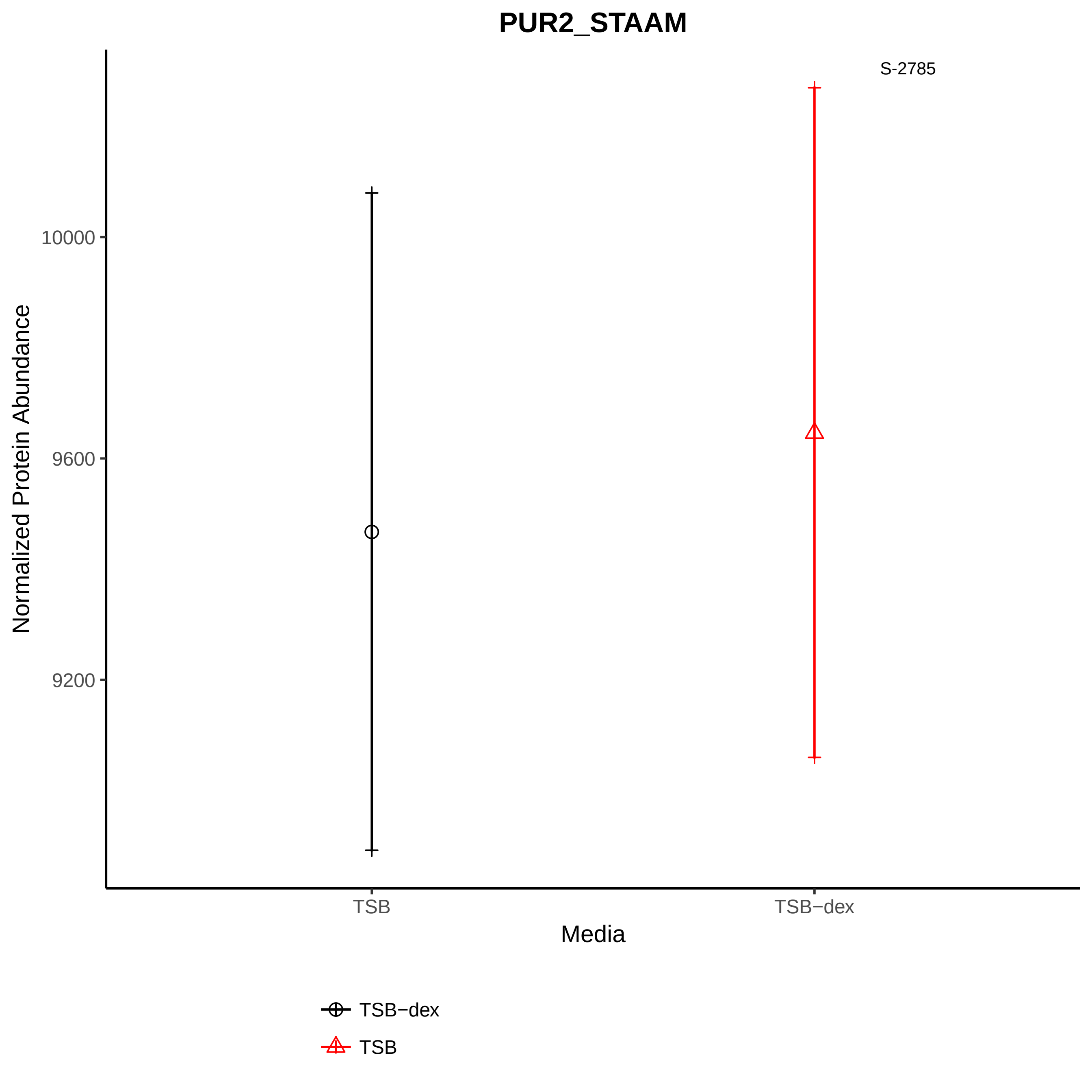

PUR2_STAAM
S-2785
Normalized Protein Abundance
10000
9600
9200
TSB
TSB−dex
Media
TSB−dex
TSB

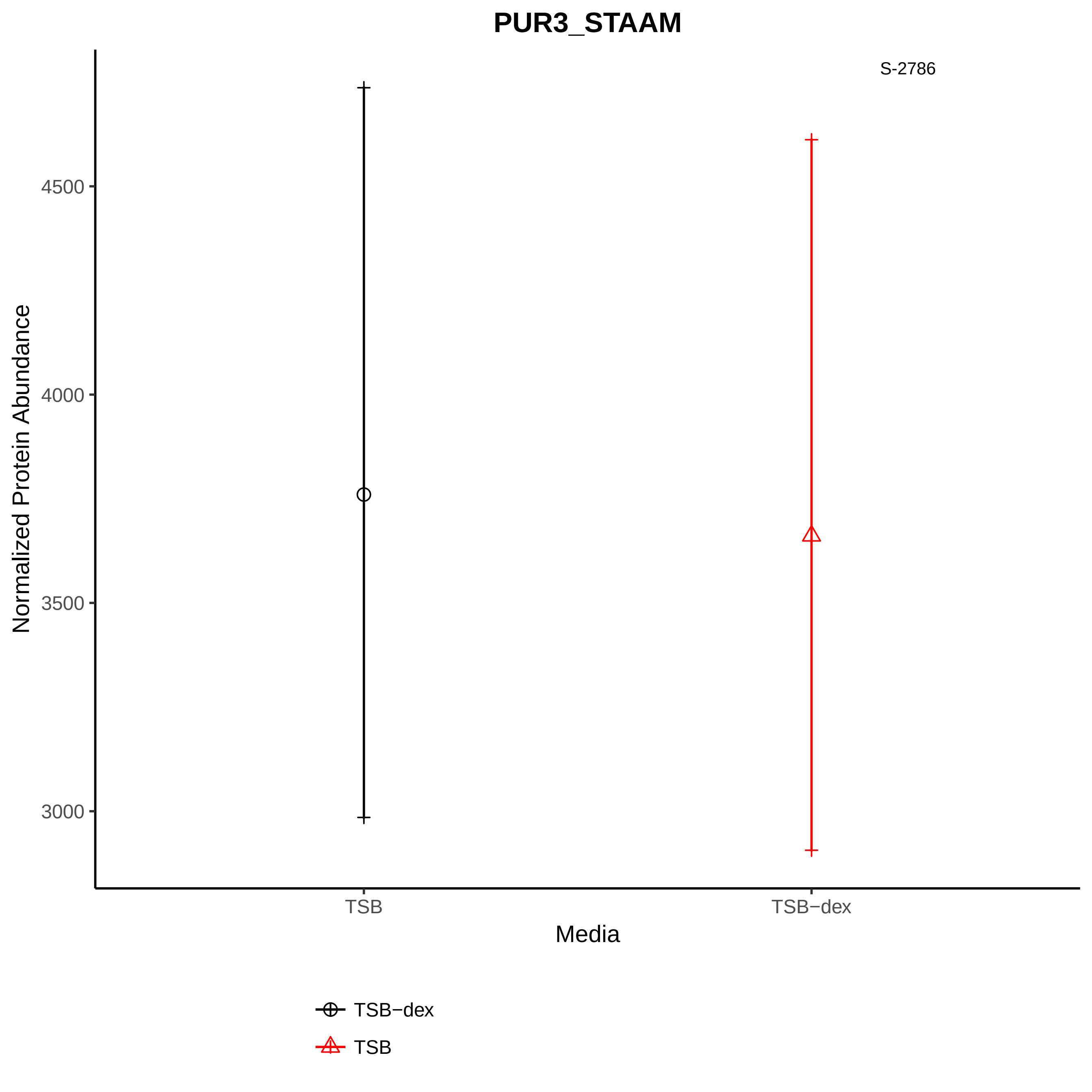

PUR3_STAAM
S-2786
Normalized Protein Abundance
4500
4000
3500
3000
TSB
TSB−dex
Media
TSB−dex
TSB

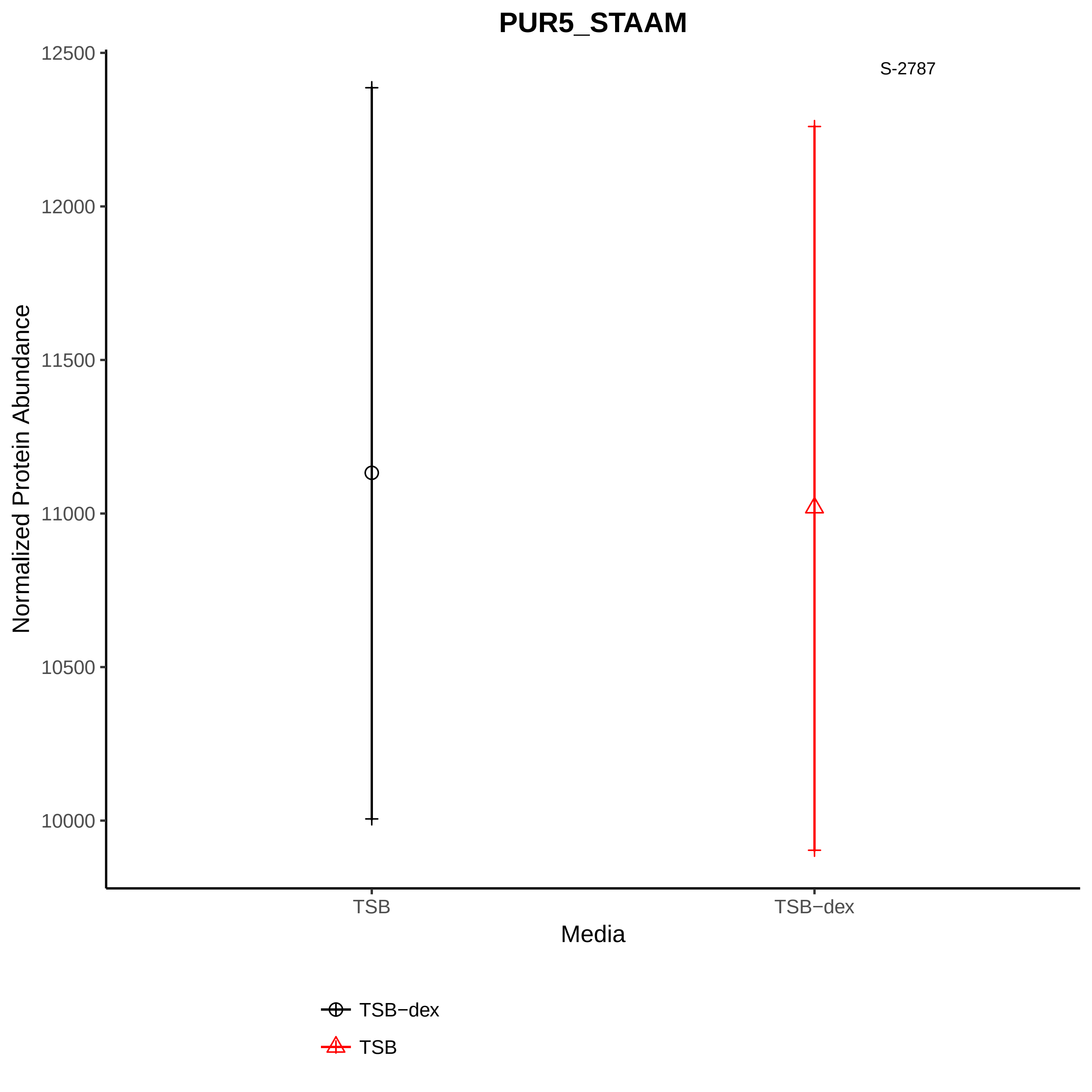

PUR5_STAAM
S-2787
Normalized Protein Abundance
12500
12000
11500
11000
10500
10000
TSB
TSB−dex
Media
TSB−dex
TSB

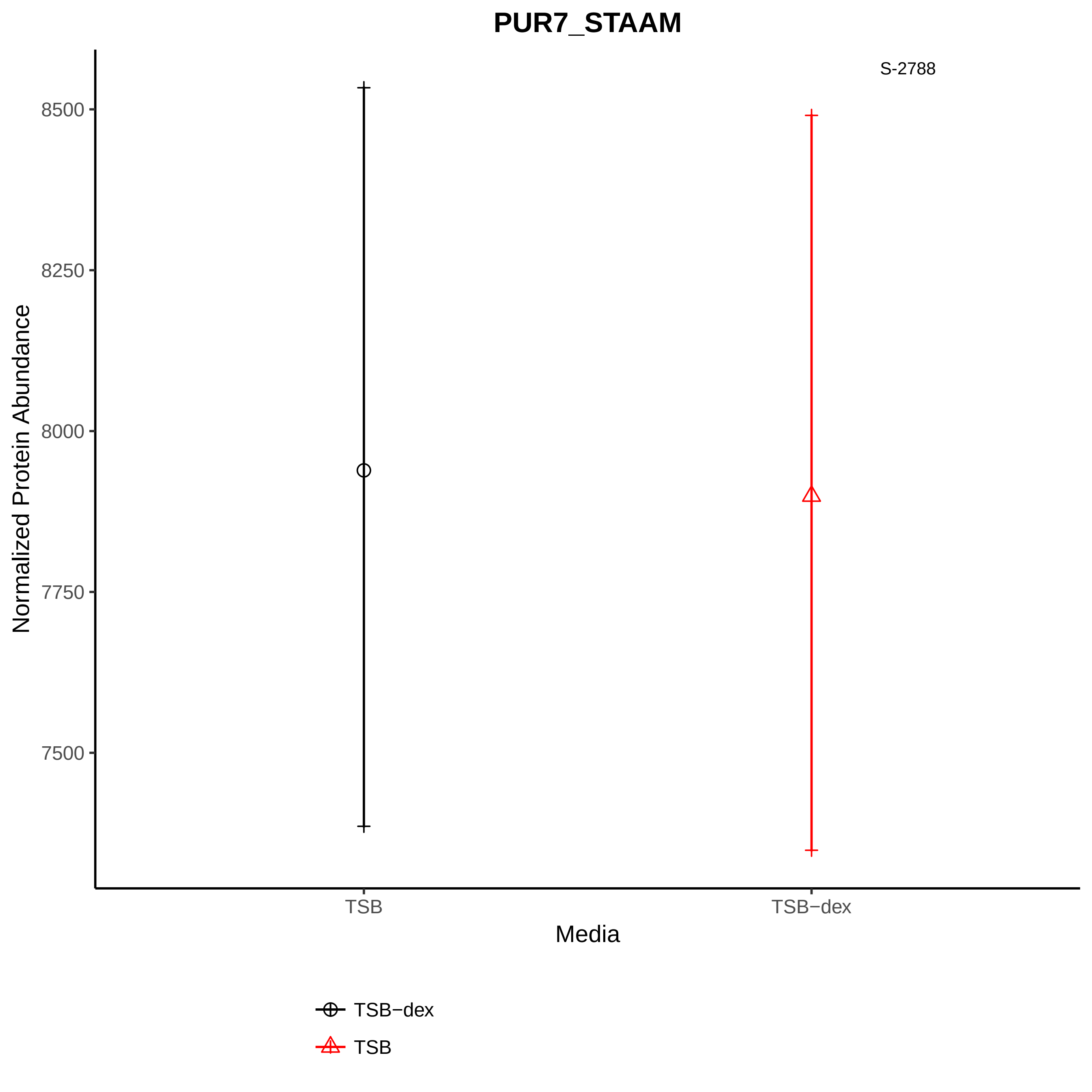
PUR7_STAAM
S-2788
Normalized Protein Abundance
8500
8250
8000
7750
7500
TSB
TSB−dex
Media
TSB−dex
TSB

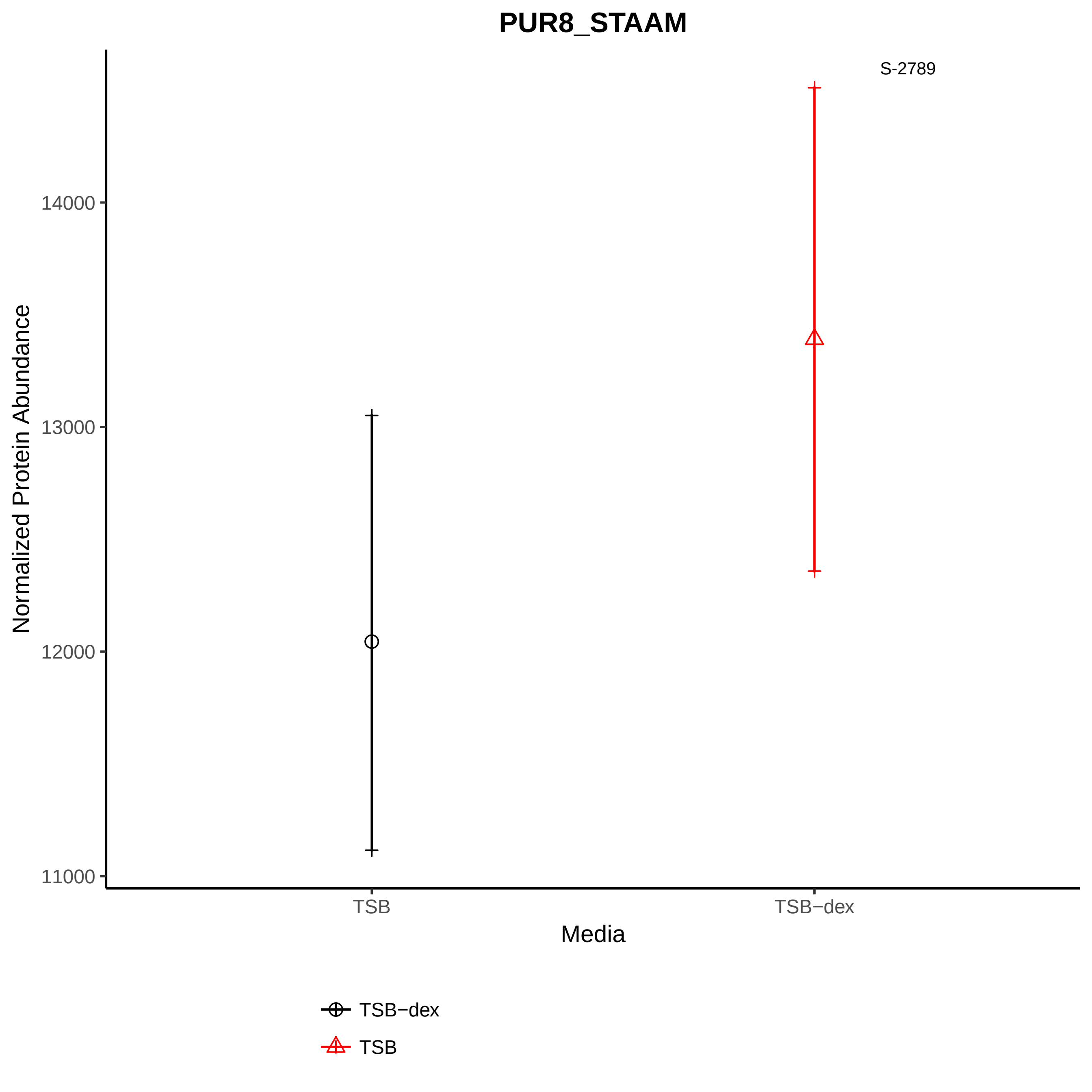
PUR8_STAAM
S-2789
Normalized Protein Abundance
14000
13000
12000
11000
TSB
TSB−dex
Media
TSB−dex
TSB

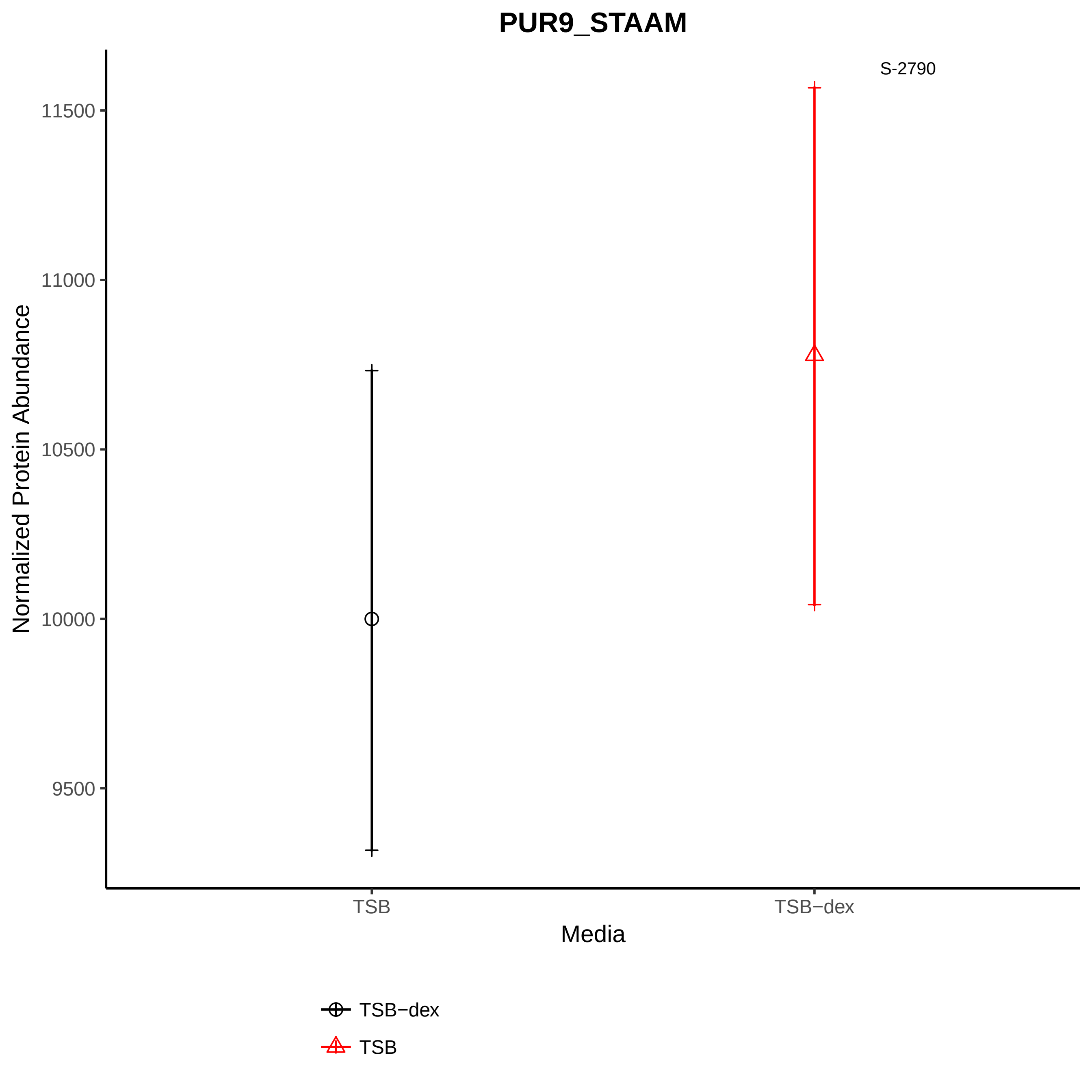

PUR9_STAAM
S-2790
Normalized Protein Abundance
11500
11000
10500
10000
9500
TSB
TSB−dex
Media
TSB−dex
TSB

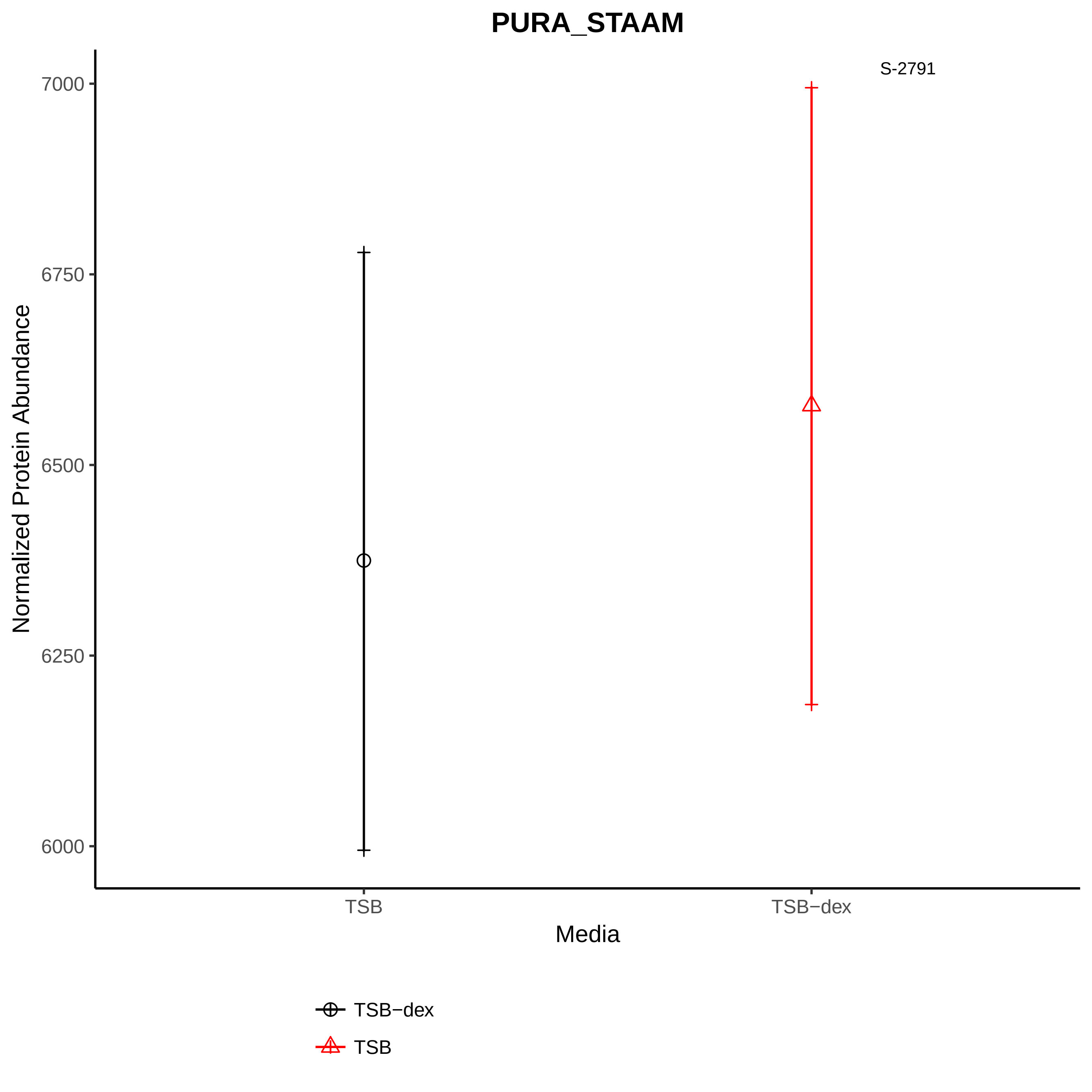

PURA_STAAM
S-2791
7000
6750
6500
6250
6000
Normalized Protein Abundance
TSB
TSB−dex
Media
TSB−dex
TSB

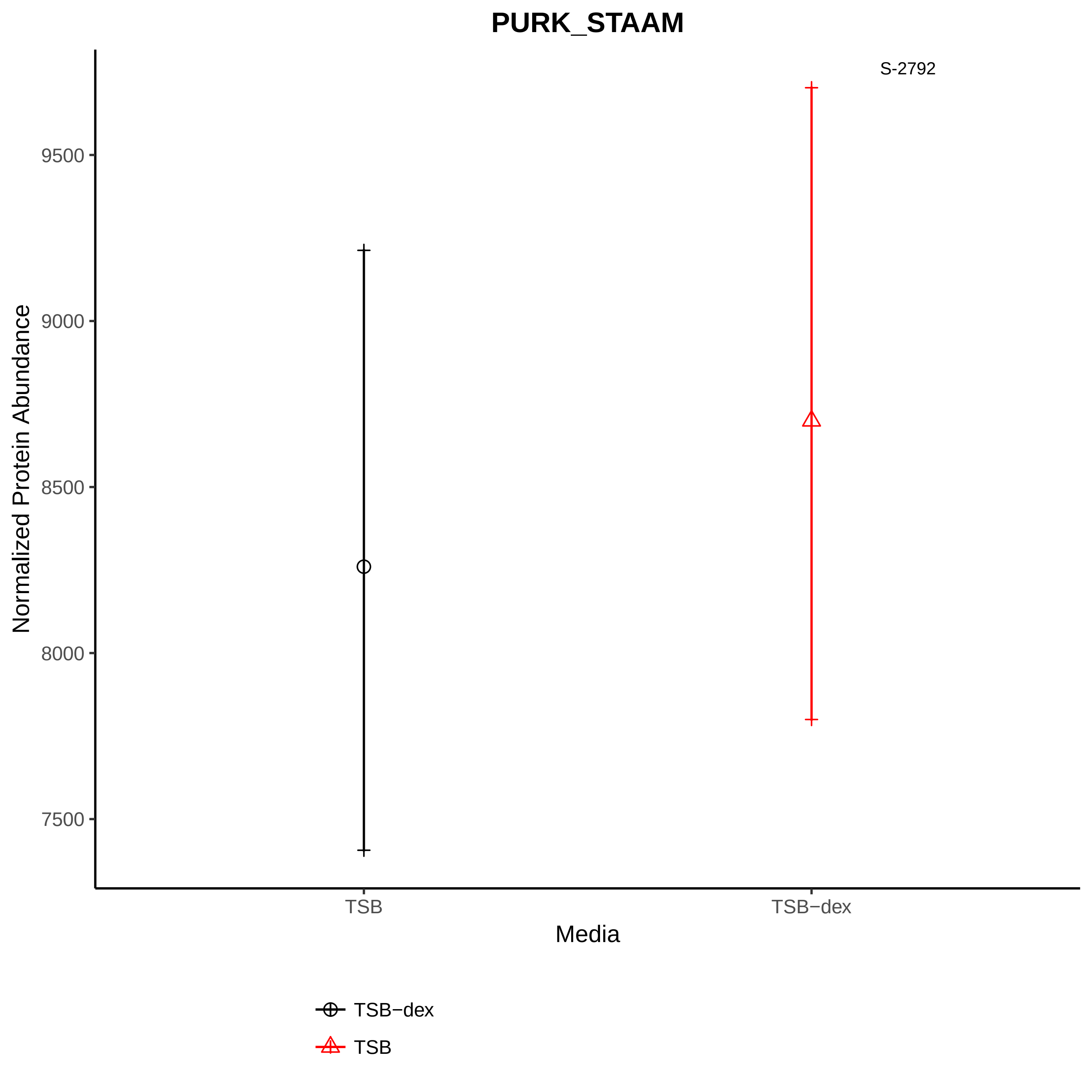

PURK_STAAM
S-2792
Normalized Protein Abundance
9500
9000
8500
8000
7500
TSB
TSB−dex
Media
TSB−dex
TSB

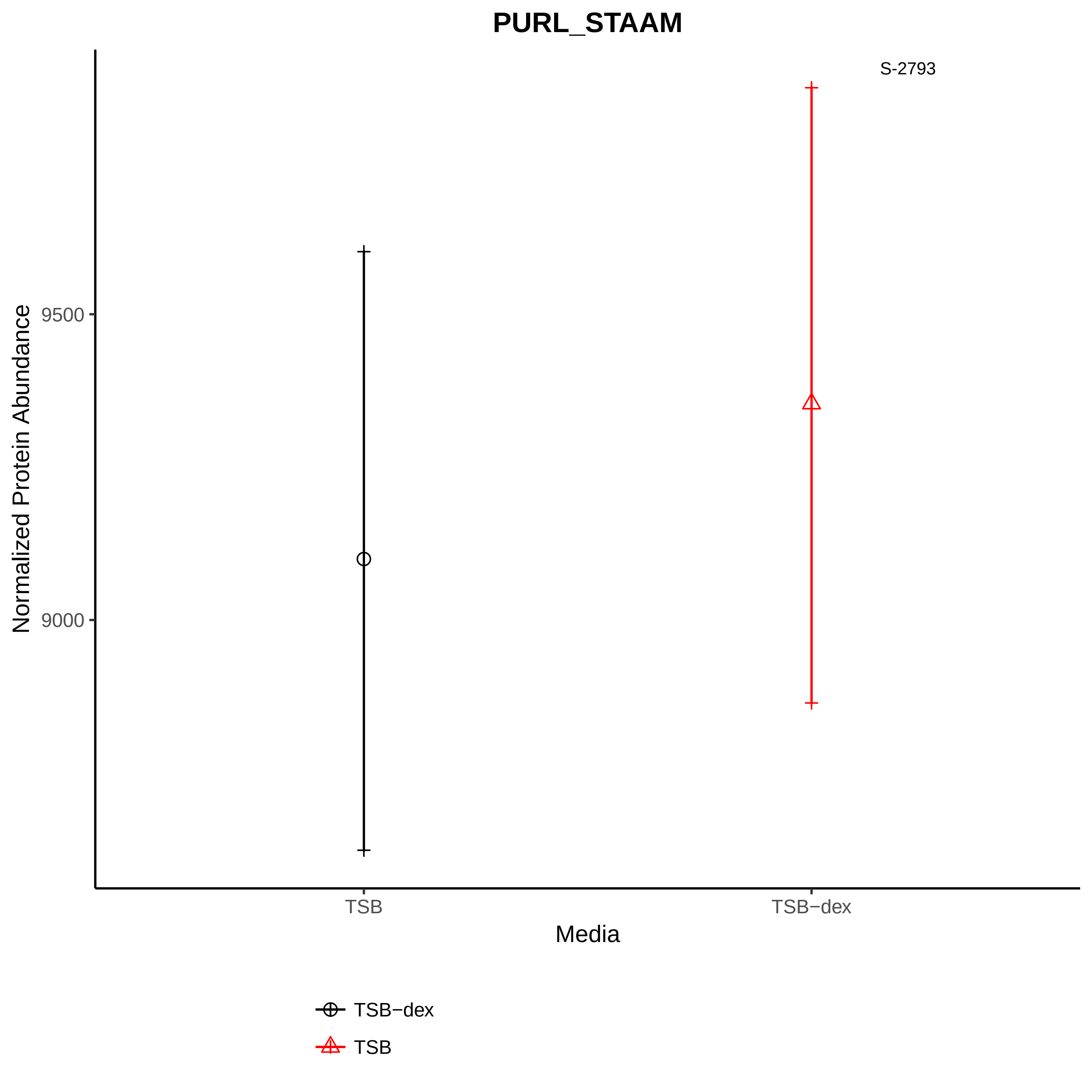

PURL_STAAM
S-2793
Normalized Protein Abundance
9500
9000
TSB
TSB−dex
Media
TSB−dex
TSB

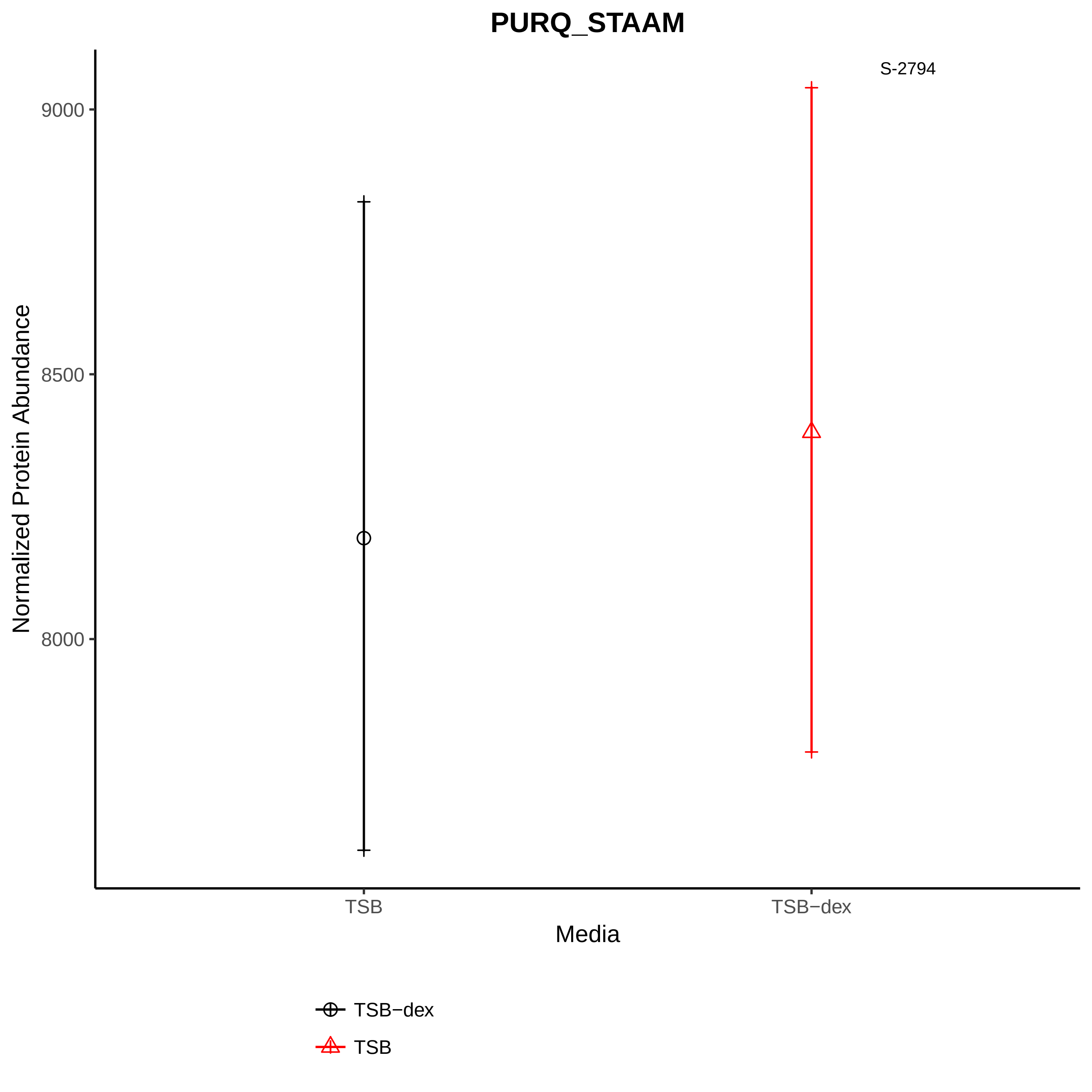
PURQ_STAAM
S-2794
9000
8500
8000
Normalized Protein Abundance
TSB
TSB−dex
Media
TSB−dex
TSB

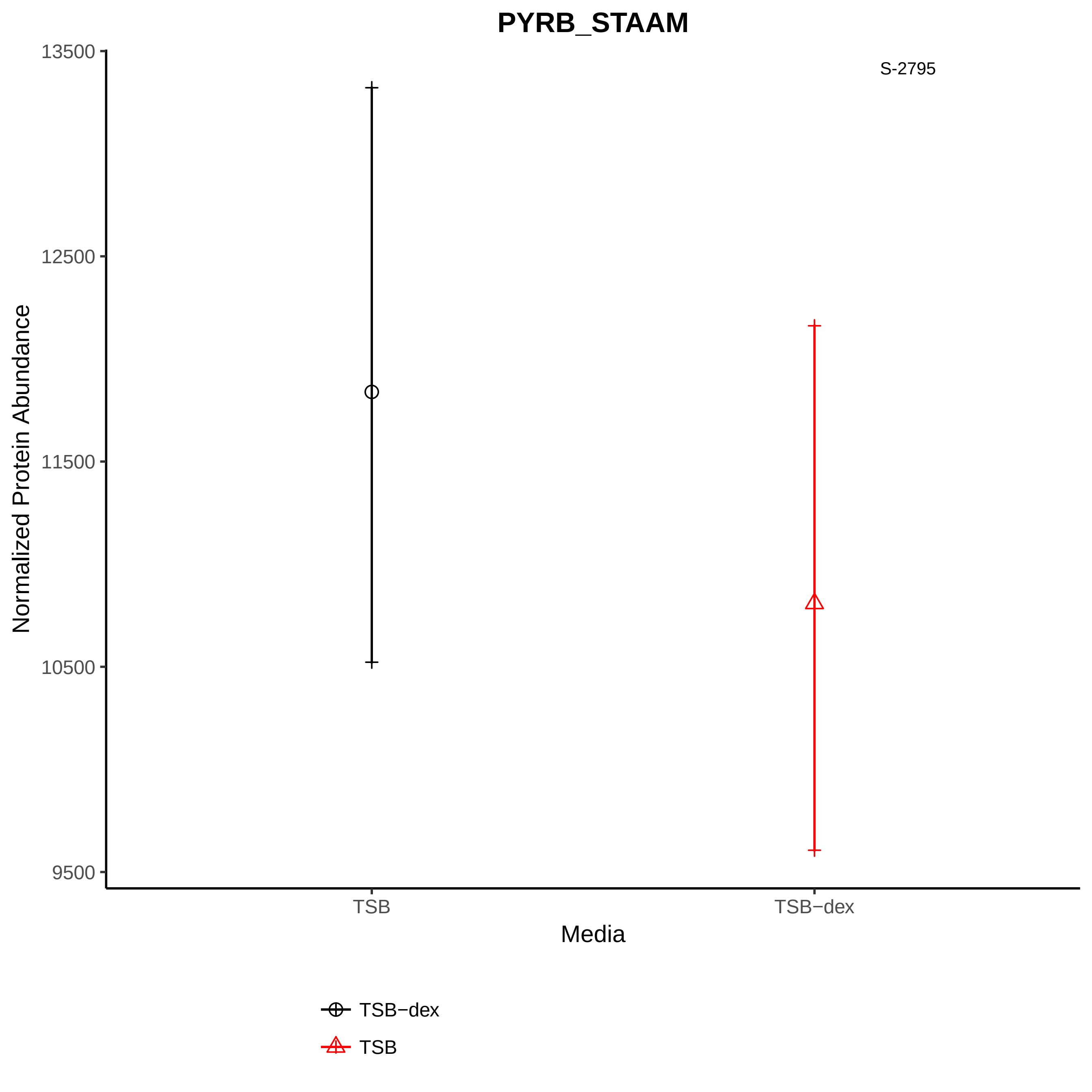
PYRB_STAAM
S-2795
Normalized Protein Abundance
13500
12500
11500
10500
9500
TSB
TSB−dex
Media
TSB−dex
TSB

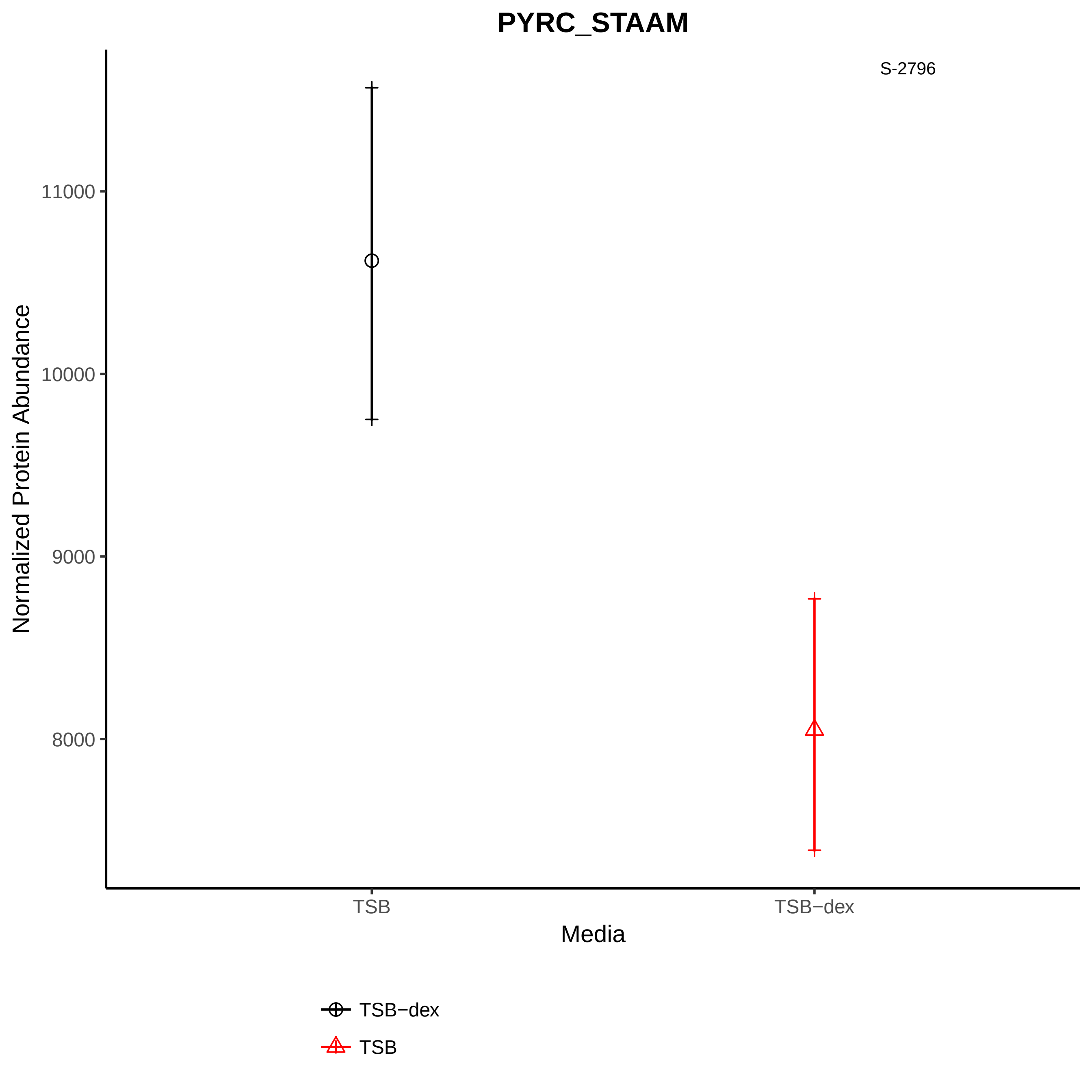

PYRC_STAAM
S-2796
Normalized Protein Abundance
11000
10000
9000
8000
TSB
TSB−dex
Media
TSB−dex
TSB

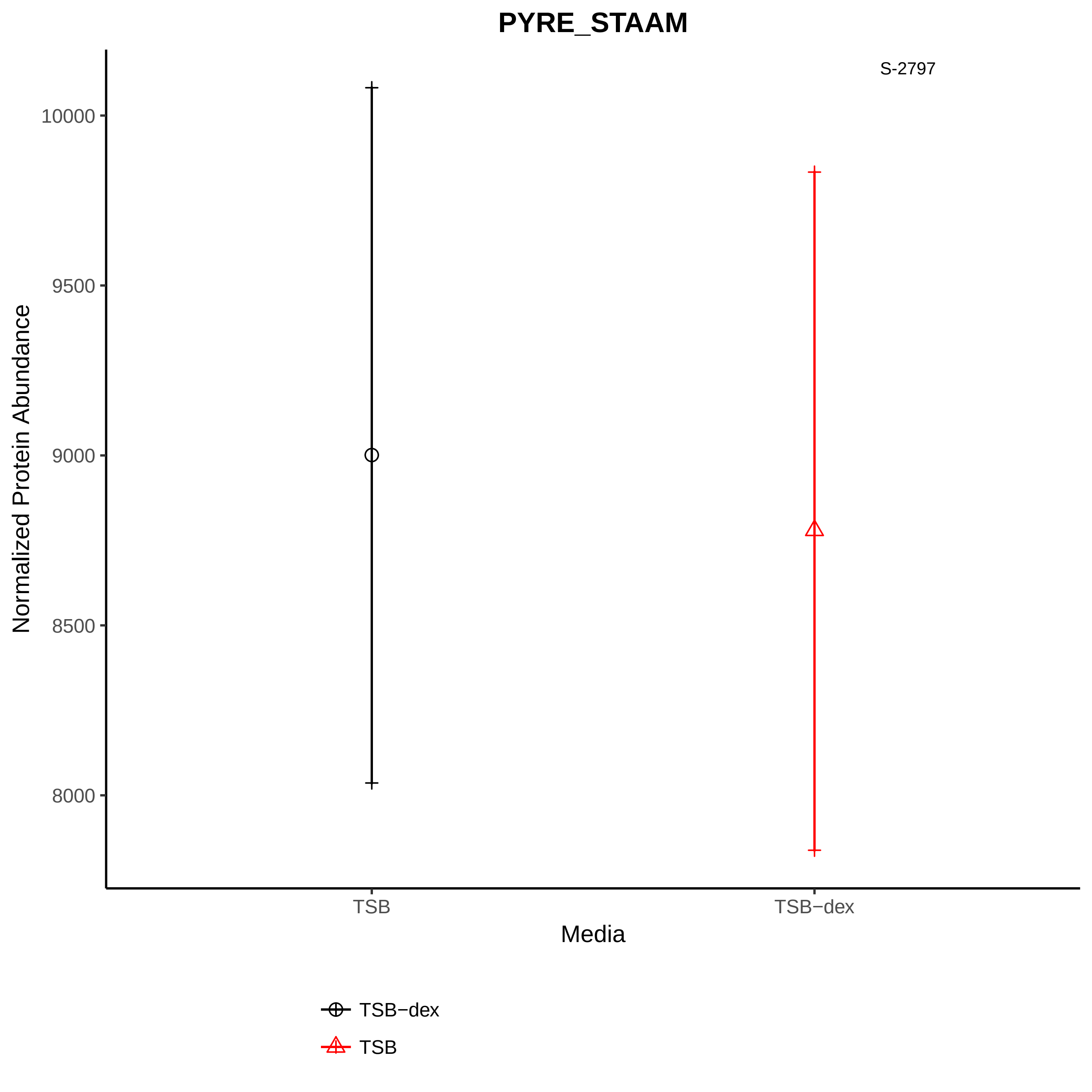

PYRE_STAAM
S-2797
10000
9500
9000
8500
8000
Normalized Protein Abundance
TSB
TSB−dex
Media
TSB−dex
TSB

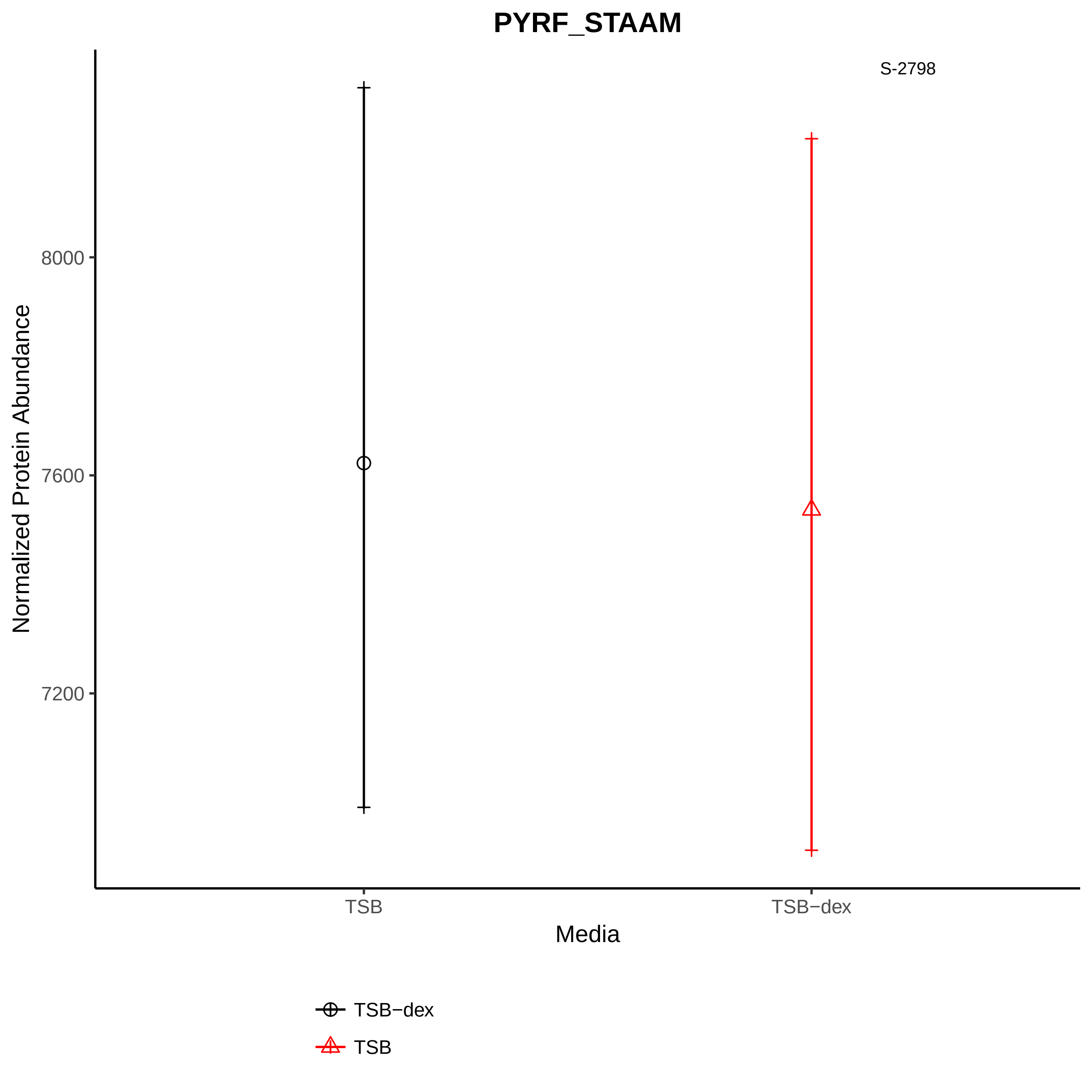

PYRF_STAAM
S-2798
8000
7600
7200
Normalized Protein Abundance
TSB
TSB−dex
Media
TSB−dex
TSB

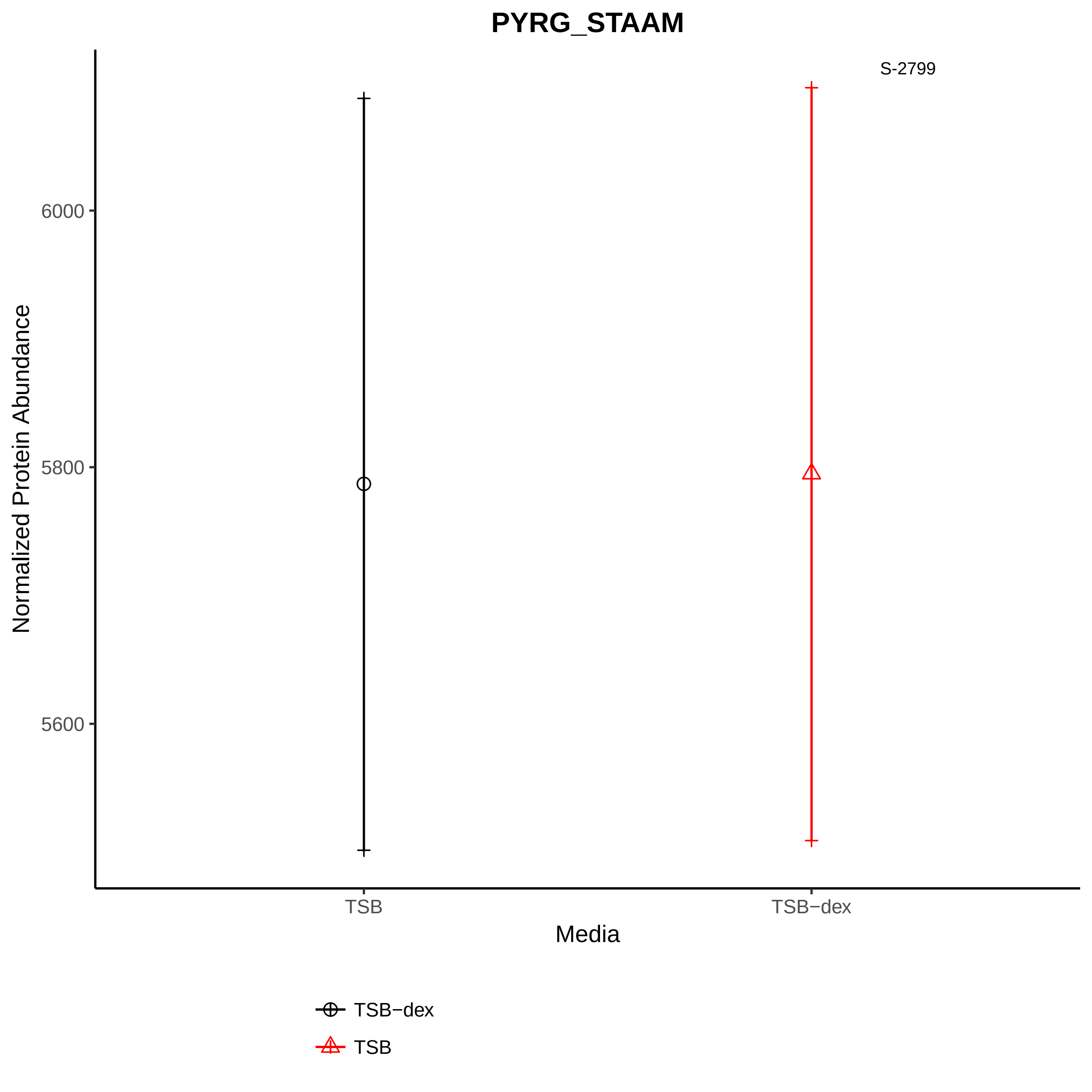
PYRG_STAAM
S-2799
6000
5800
5600
Normalized Protein Abundance
TSB
TSB−dex
Media
TSB−dex
TSB

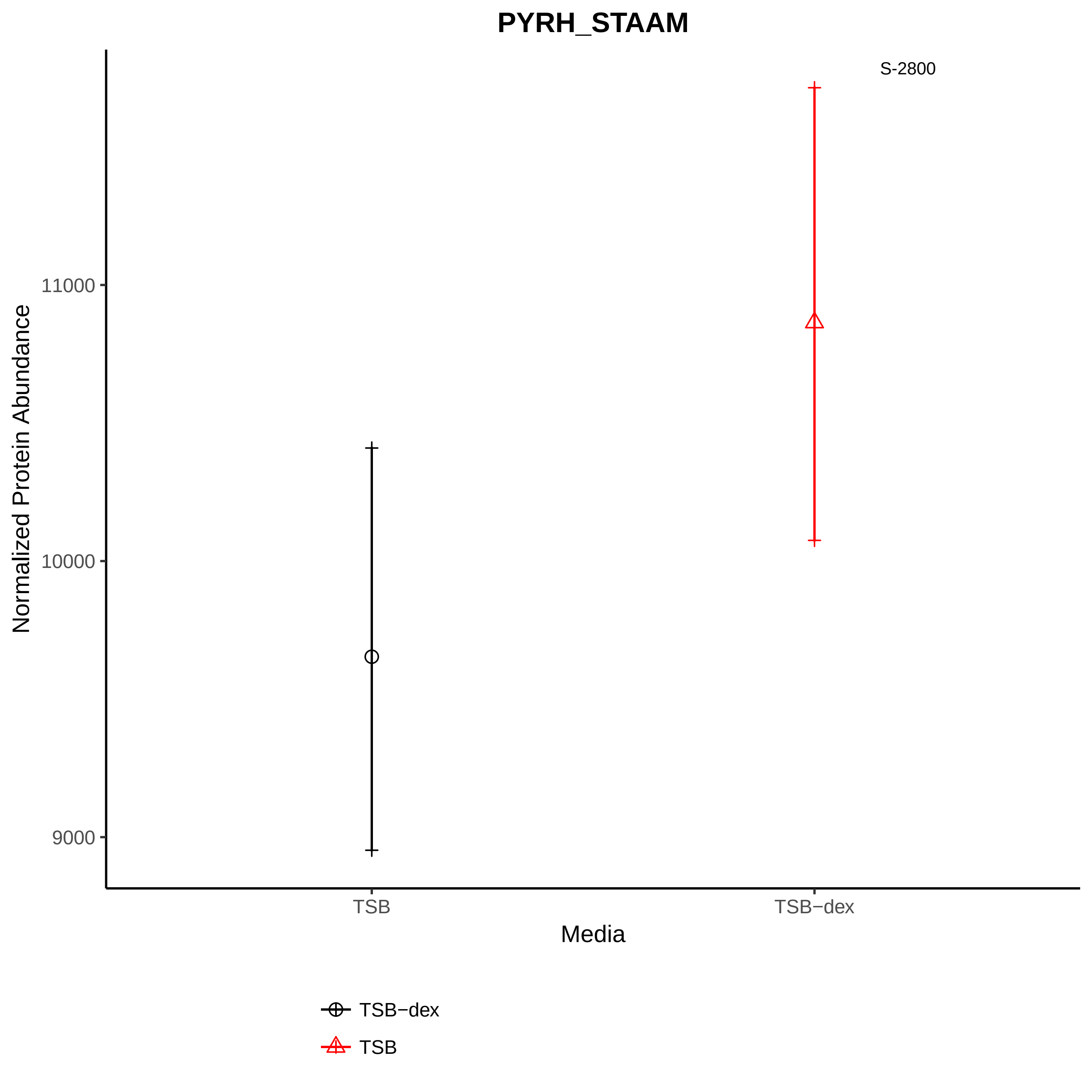
PYRH_STAAM
S-2800
11000
10000
9000
Normalized Protein Abundance
TSB
TSB−dex
Media
TSB−dex
TSB

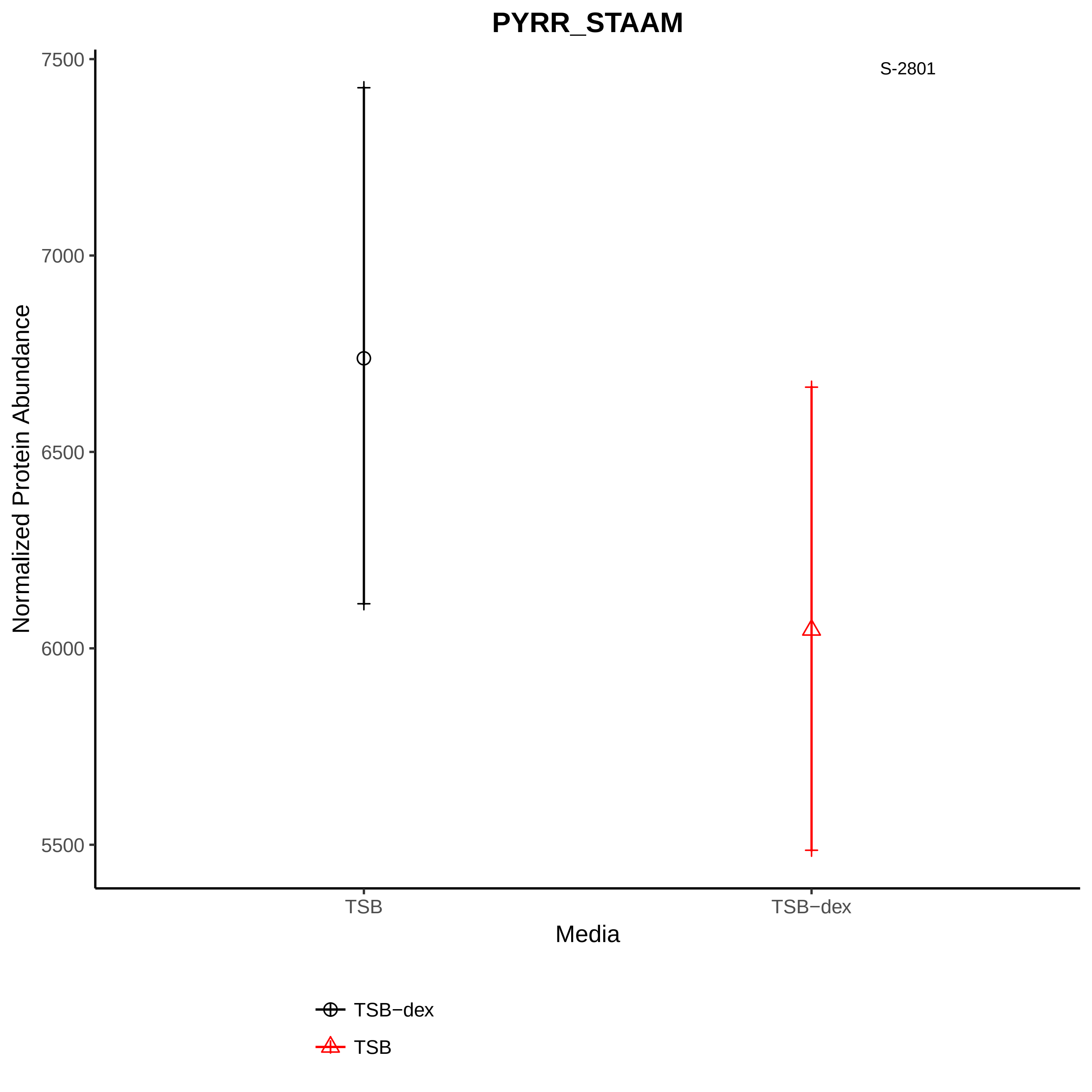

PYRR_STAAM
S-2801
Normalized Protein Abundance
7500
7000
6500
6000
5500
TSB
TSB−dex
Media
TSB−dex
TSB

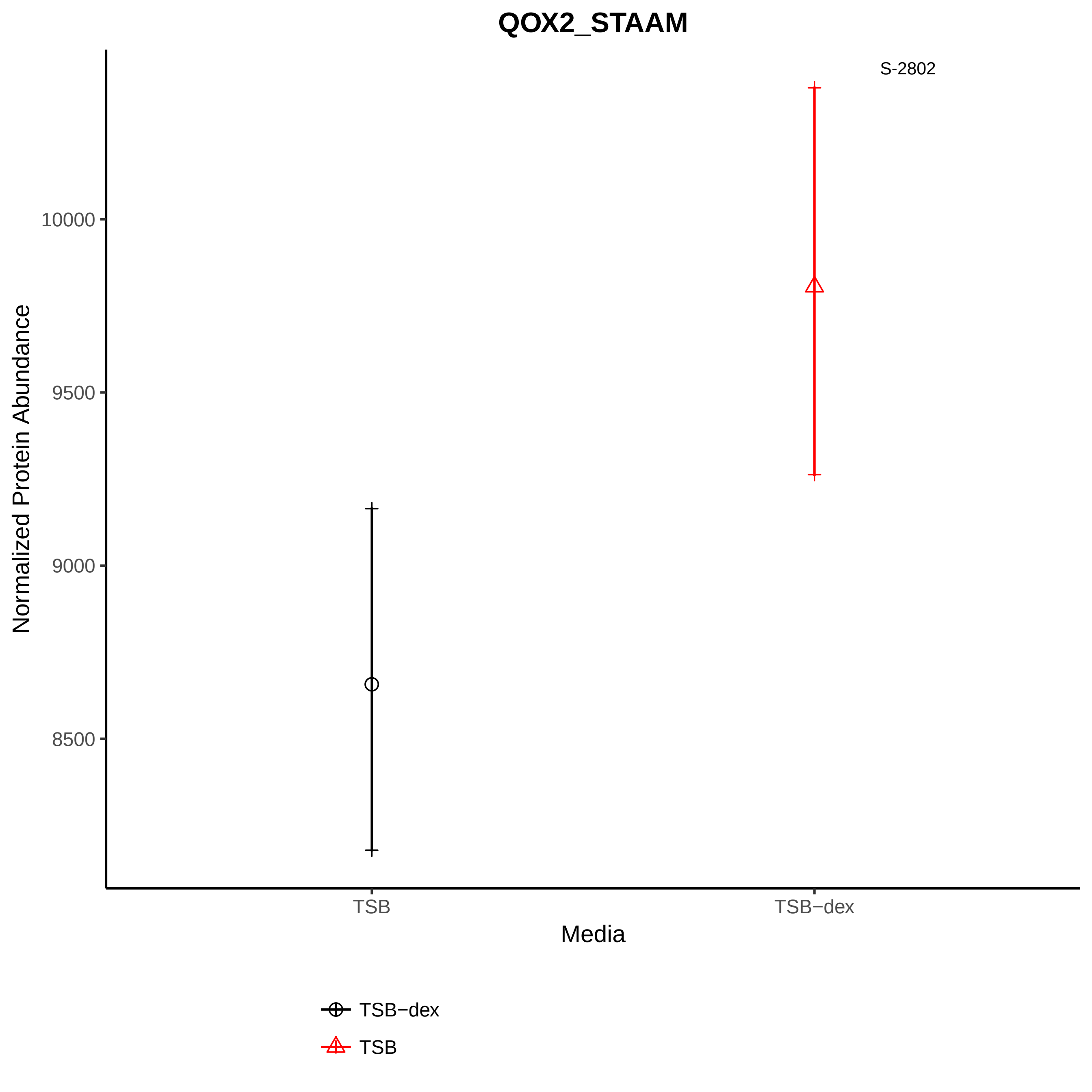

QOX2_STAAM
S-2802
10000
9500
9000
8500
Normalized Protein Abundance
TSB
TSB−dex
Media
TSB−dex
TSB

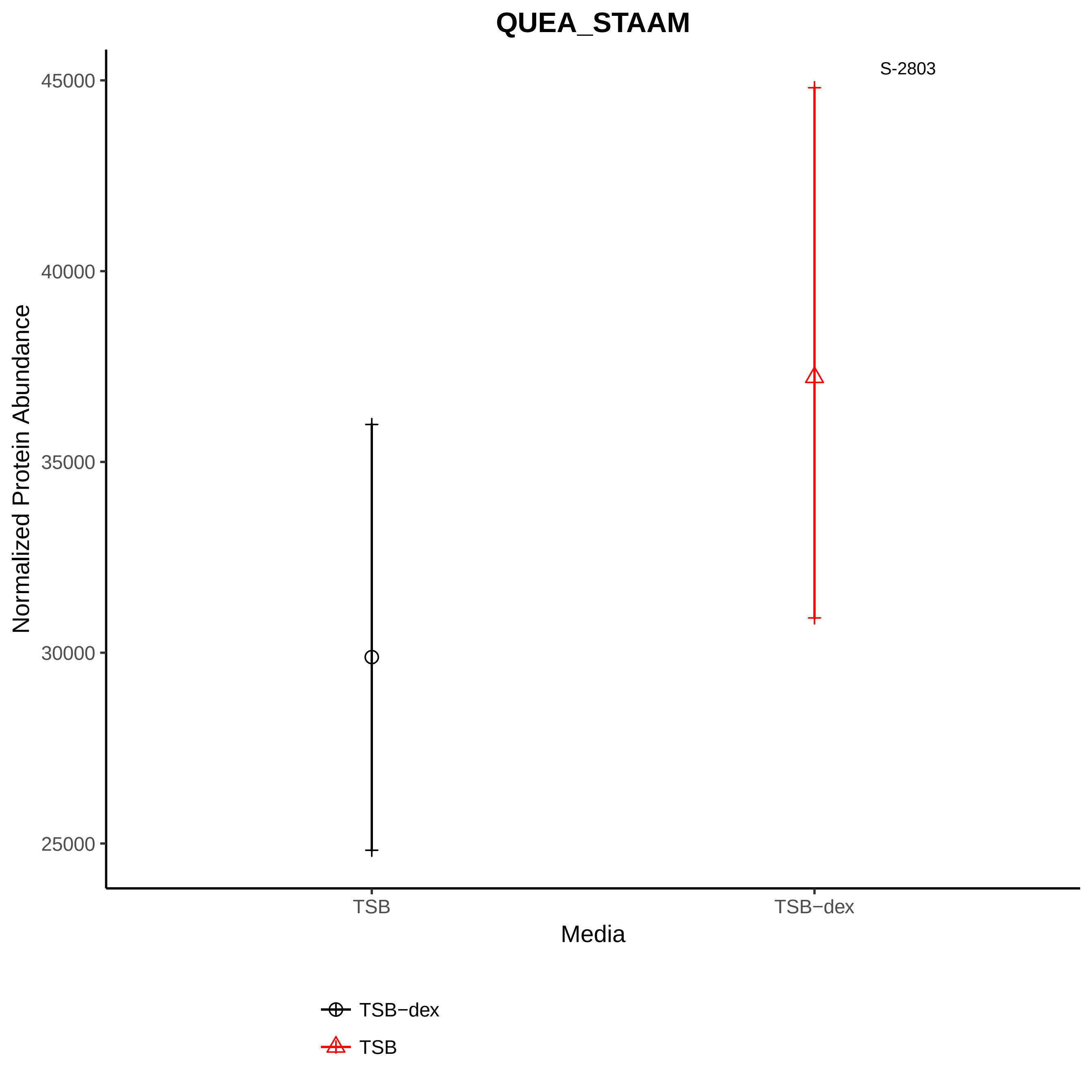
QUEA_STAAM
S-2803
Normalized Protein Abundance
45000
40000
35000
30000
25000
TSB
TSB−dex
Media
TSB−dex
TSB

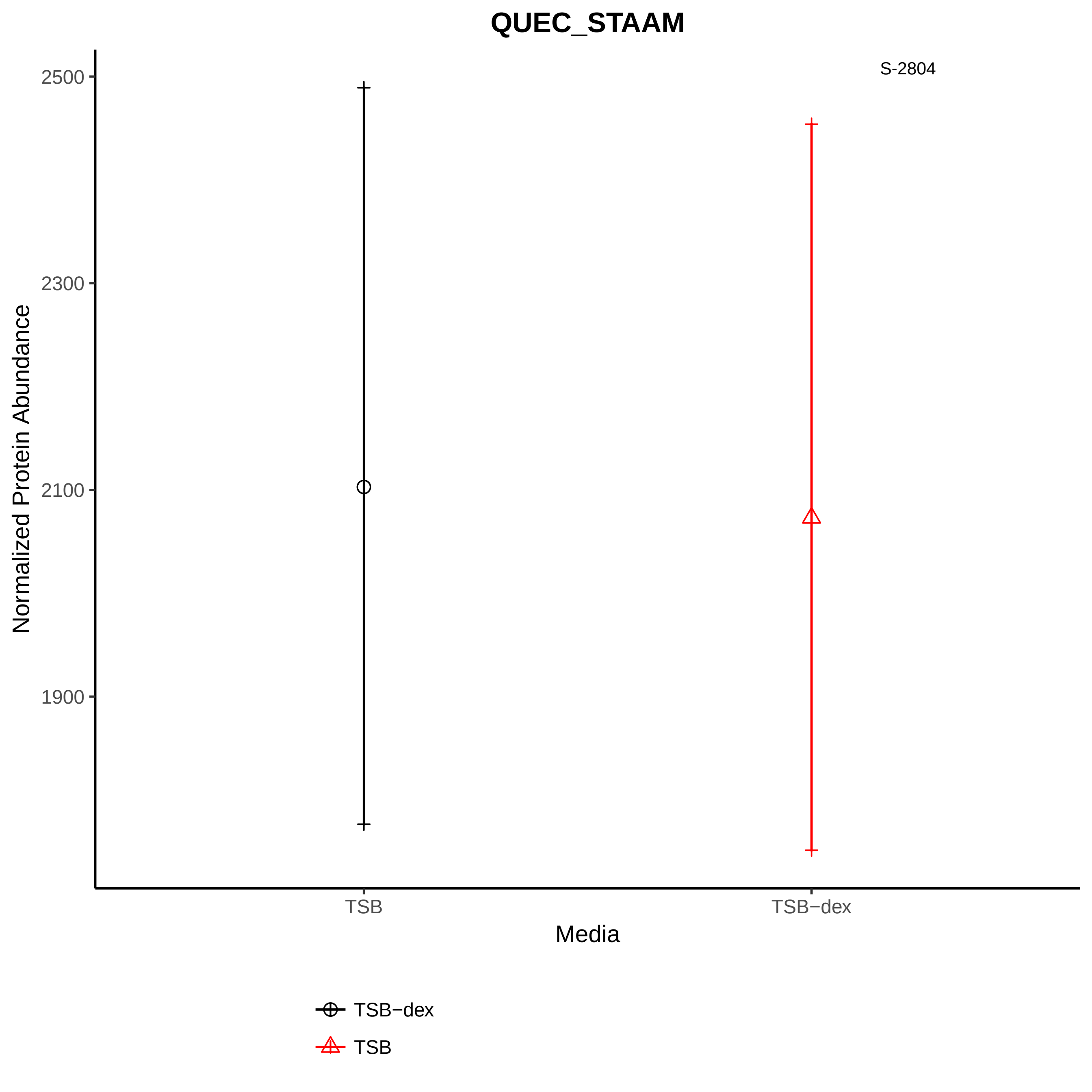

QUEC_STAAM
S-2804
2500
2300
2100
1900
Normalized Protein Abundance
TSB
TSB−dex
Media
TSB−dex
TSB

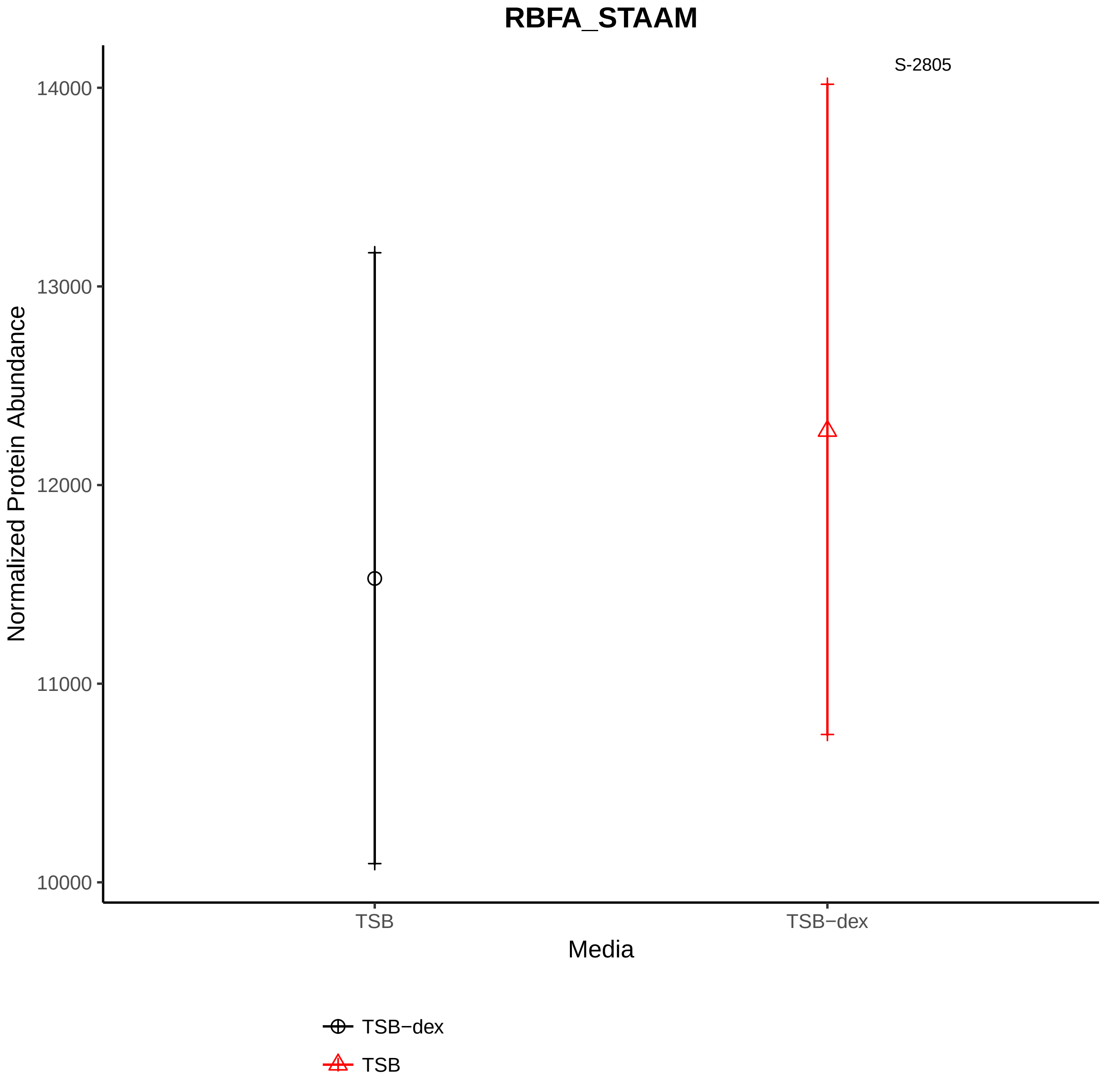

RBFA_STAAM
S-2805
Normalized Protein Abundance
14000
13000
12000
11000
10000
TSB
TSB−dex
Media
TSB−dex
TSB

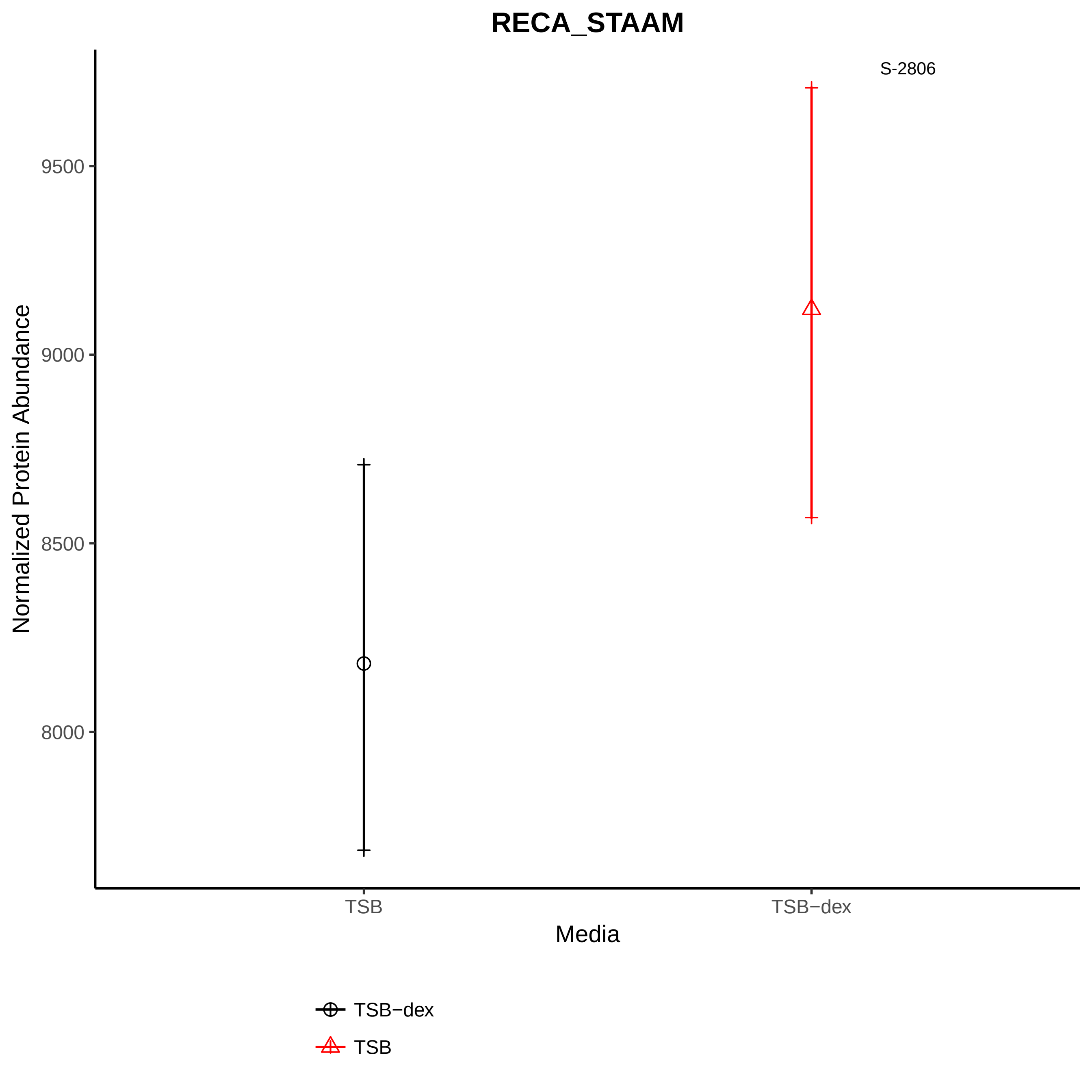
RECA_STAAM
S-2806
9500
9000
8500
8000
Normalized Protein Abundance
TSB
TSB−dex
Media
TSB−dex
TSB

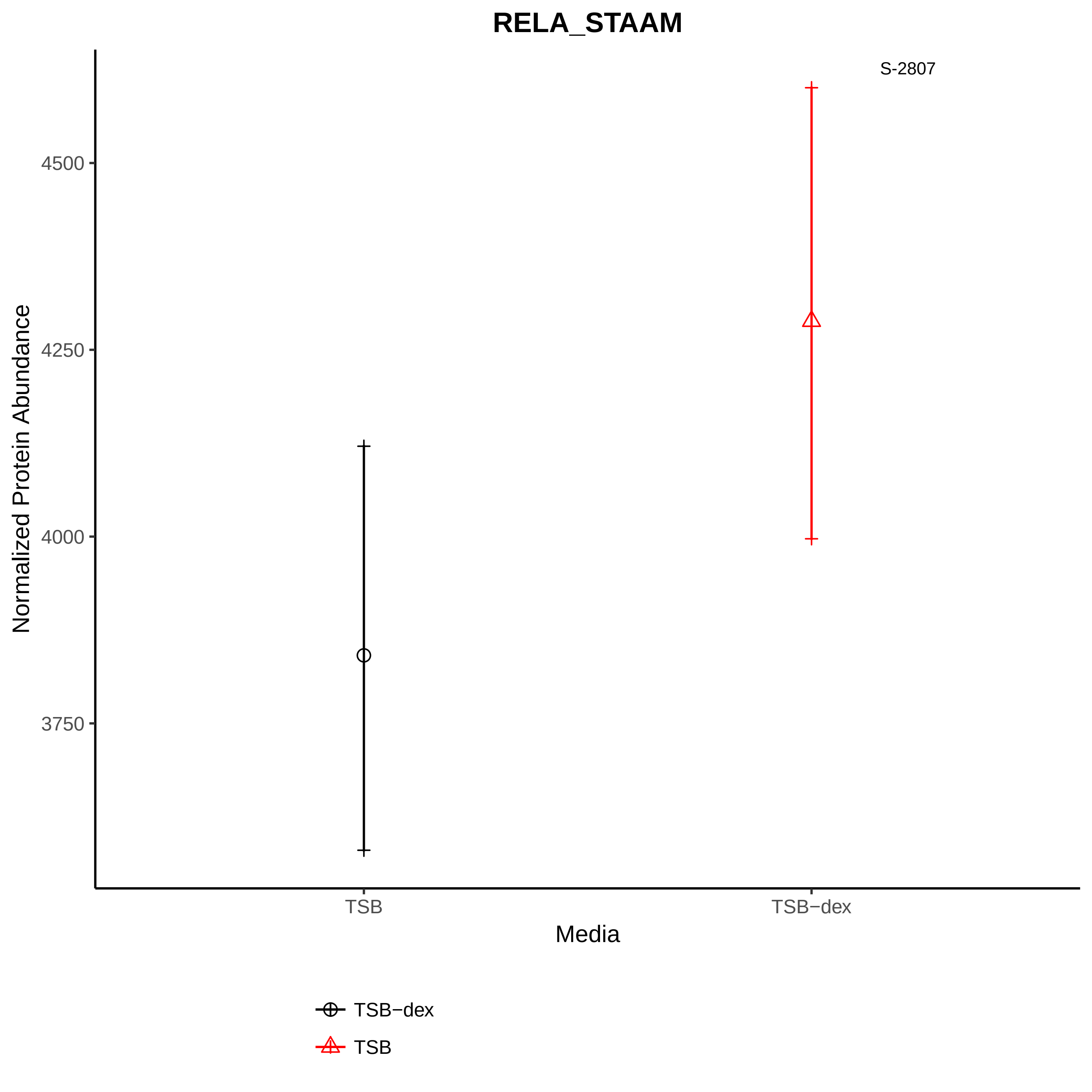

RELA_STAAM
S-2807
4500
4250
4000
3750
Normalized Protein Abundance
TSB
TSB−dex
Media
TSB−dex
TSB

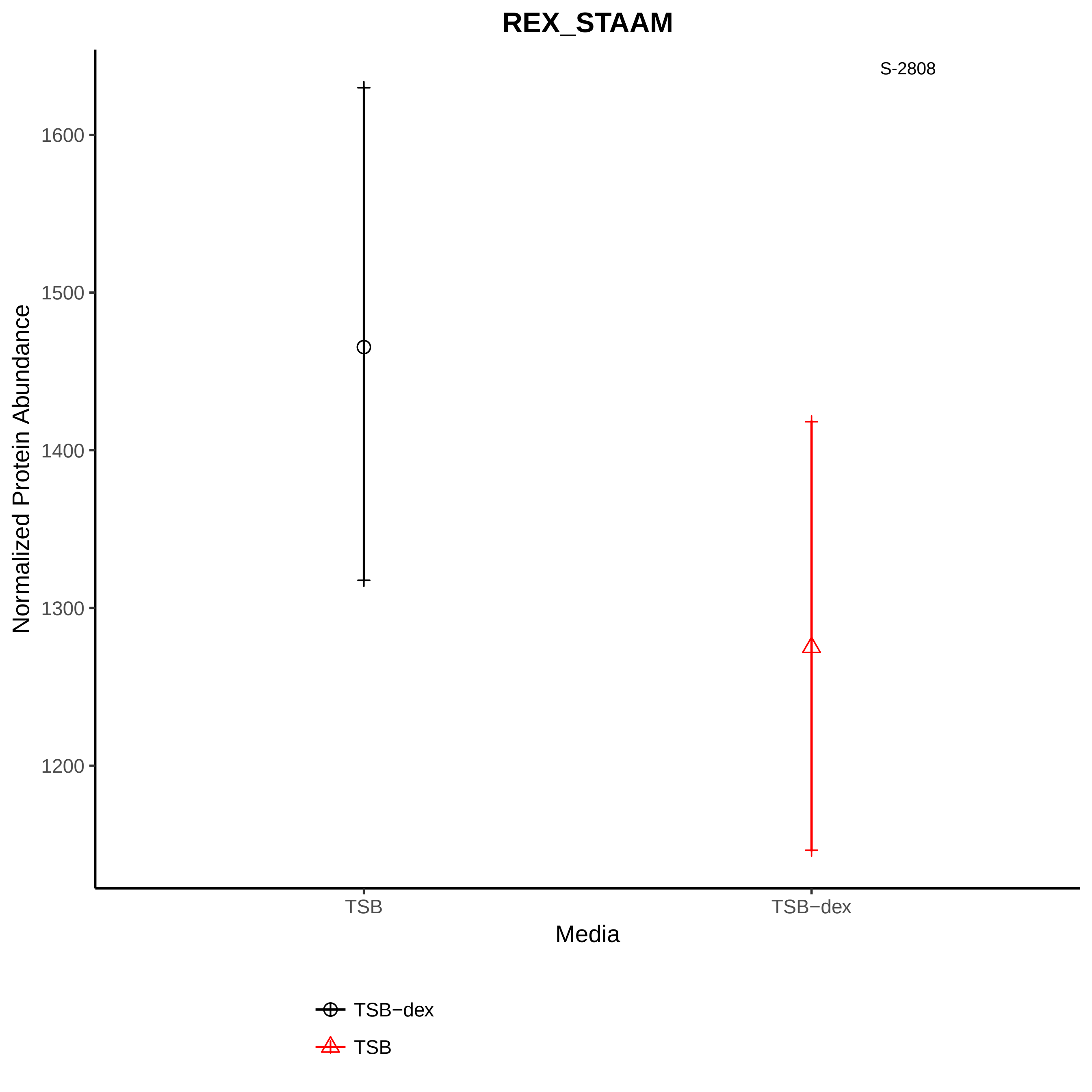
REX_STAAM
S-2808
Normalized Protein Abundance
1600
1500
1400
1300
1200
TSB
TSB−dex
Media
TSB−dex
TSB

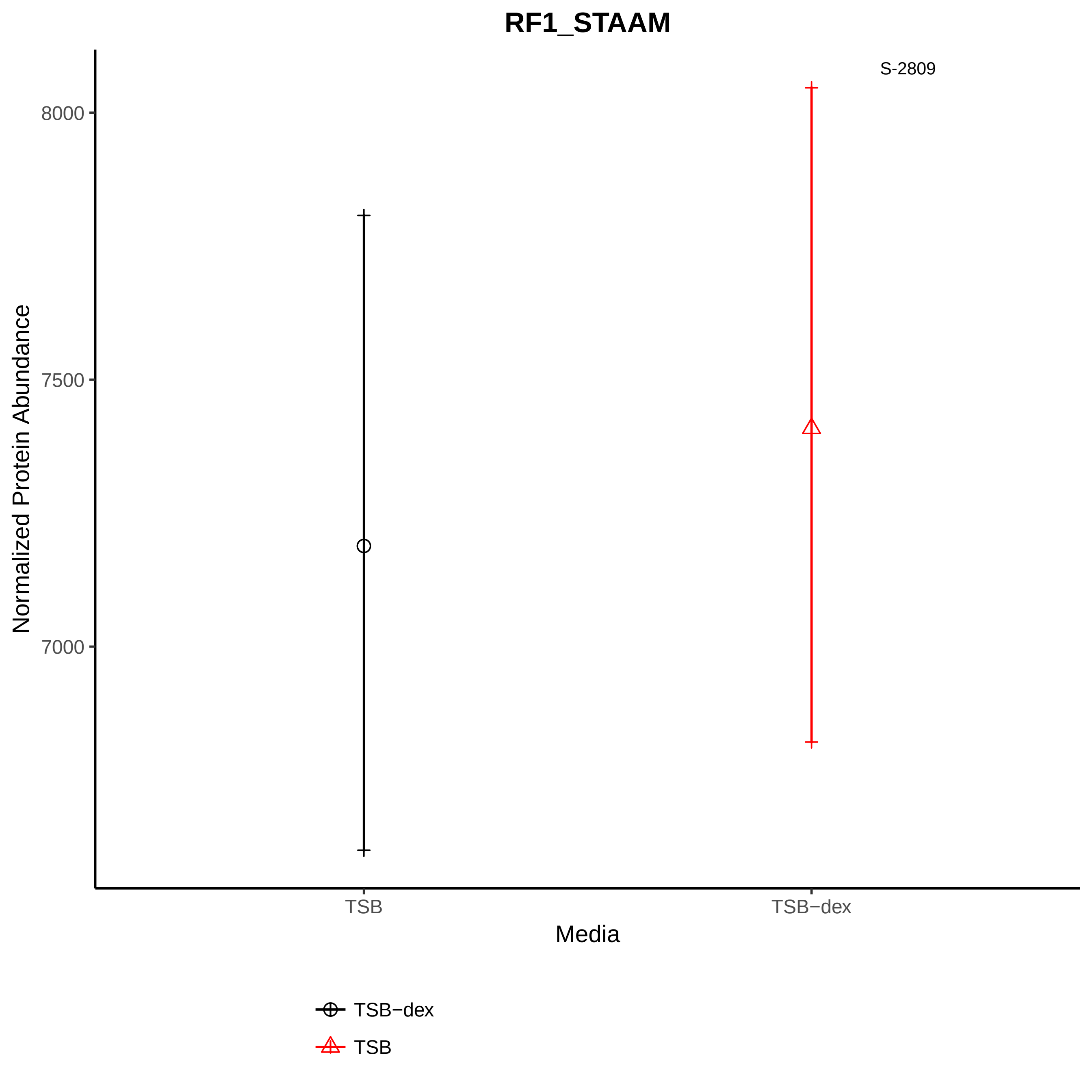
RF1_STAAM
S-2809
8000
7500
7000
Normalized Protein Abundance
TSB
TSB−dex
Media
TSB−dex
TSB

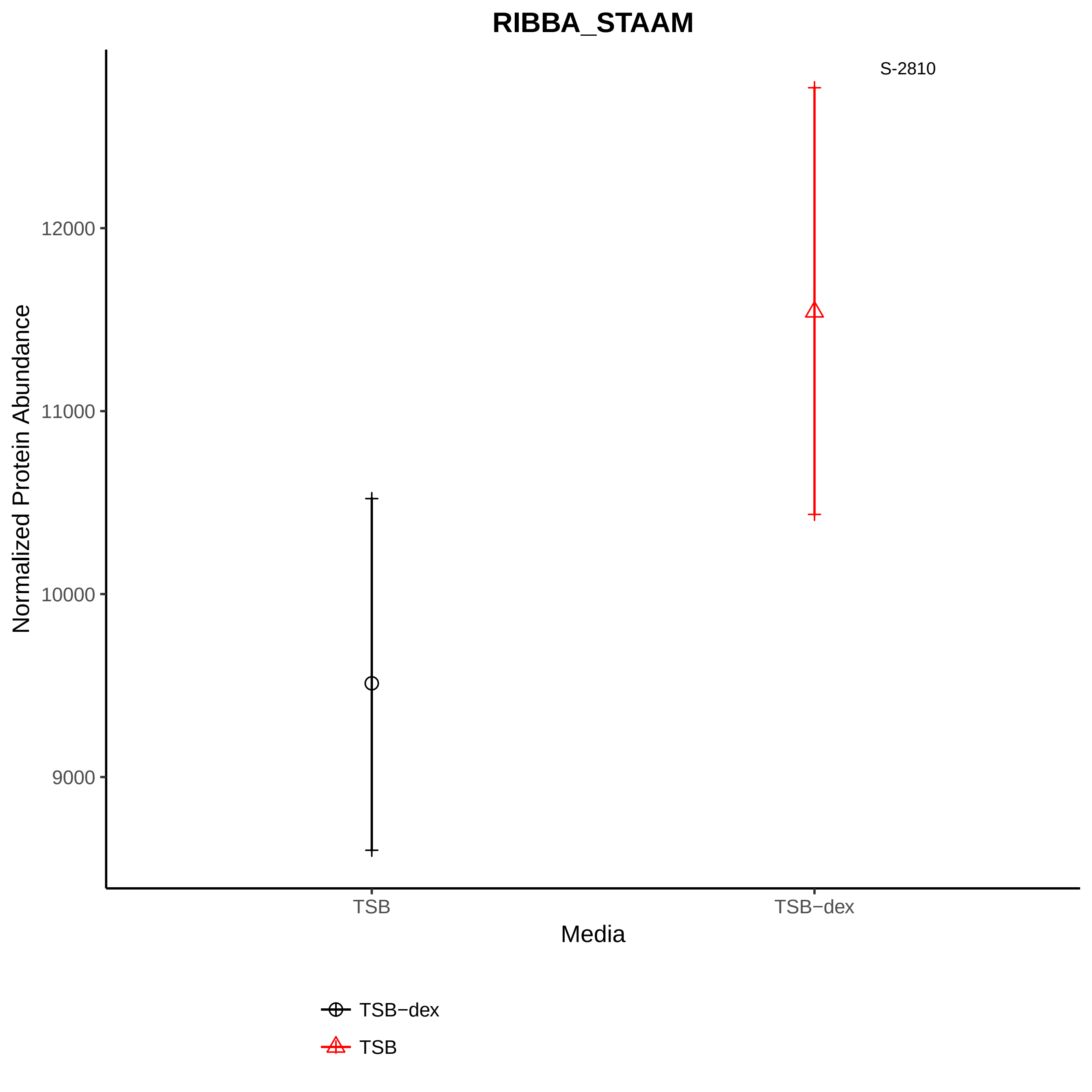
RIBBA_STAAM
S-2810
Normalized Protein Abundance
12000
11000
10000
9000
TSB
TSB−dex
Media
TSB−dex
TSB

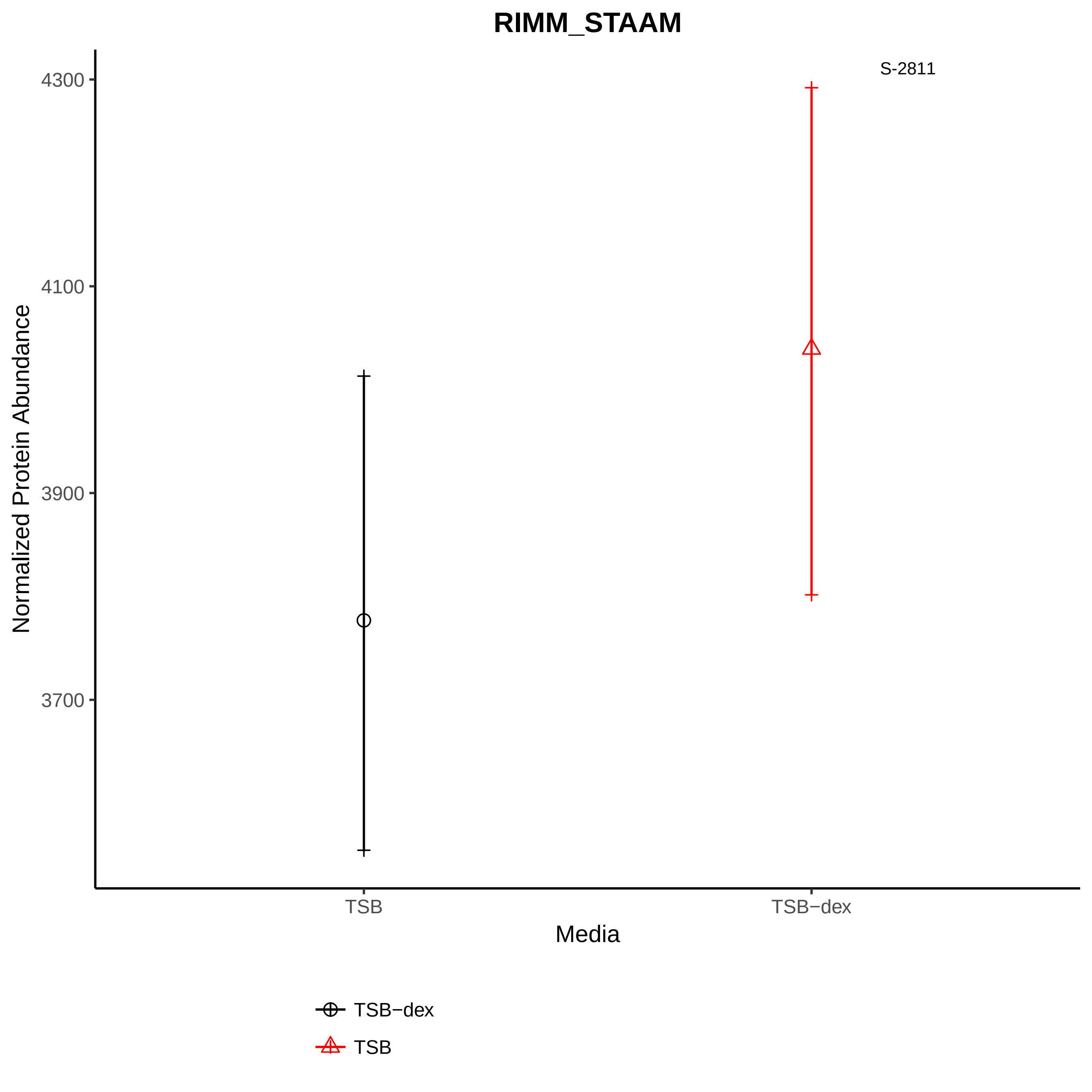

RIMM_STAAM
S-2811
4300
4100
3900
3700
Normalized Protein Abundance
TSB
TSB−dex
Media
TSB−dex
TSB

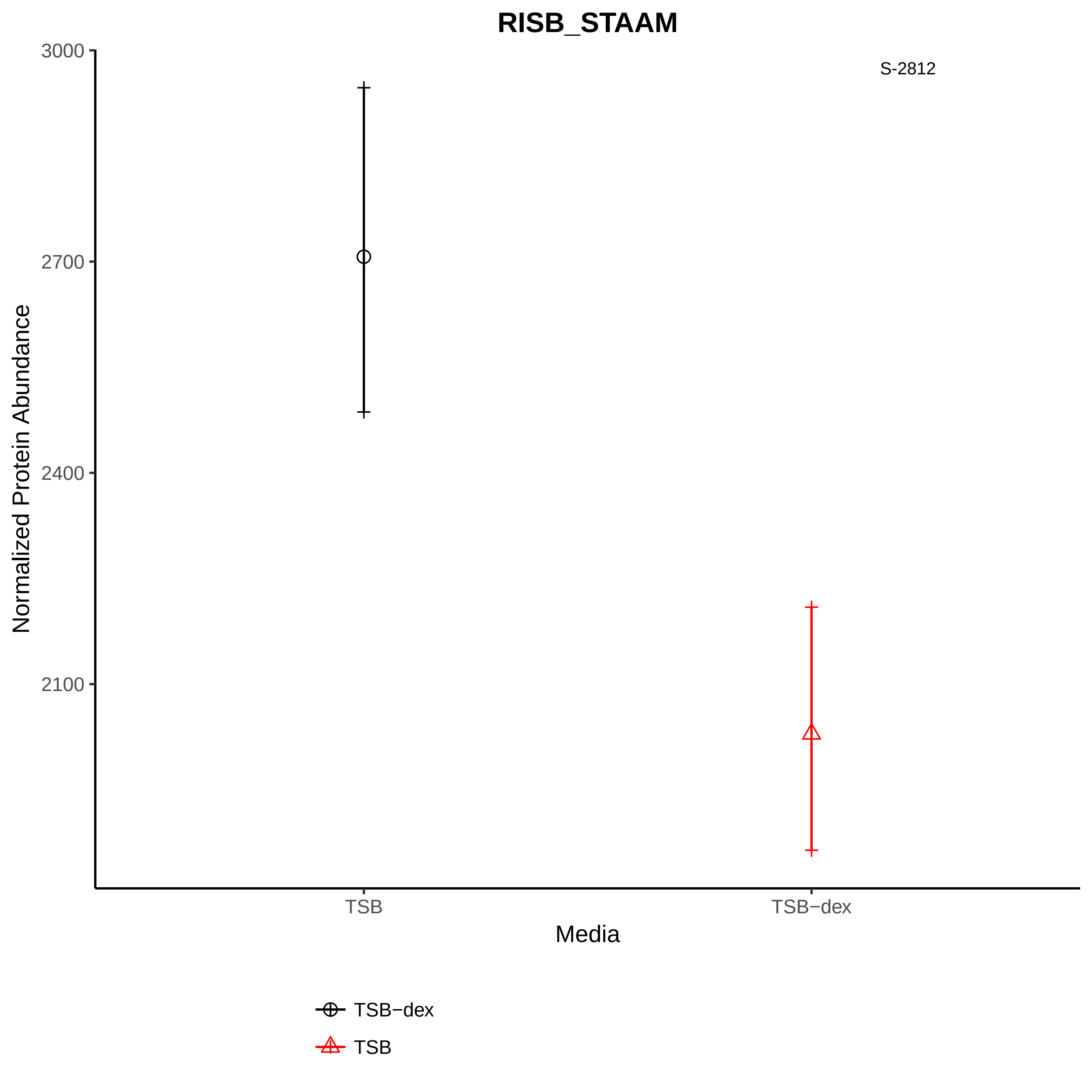

RISB_STAAM
S-2812
3000
2700
2400
2100
Normalized Protein Abundance
TSB
TSB−dex
Media
TSB−dex
TSB

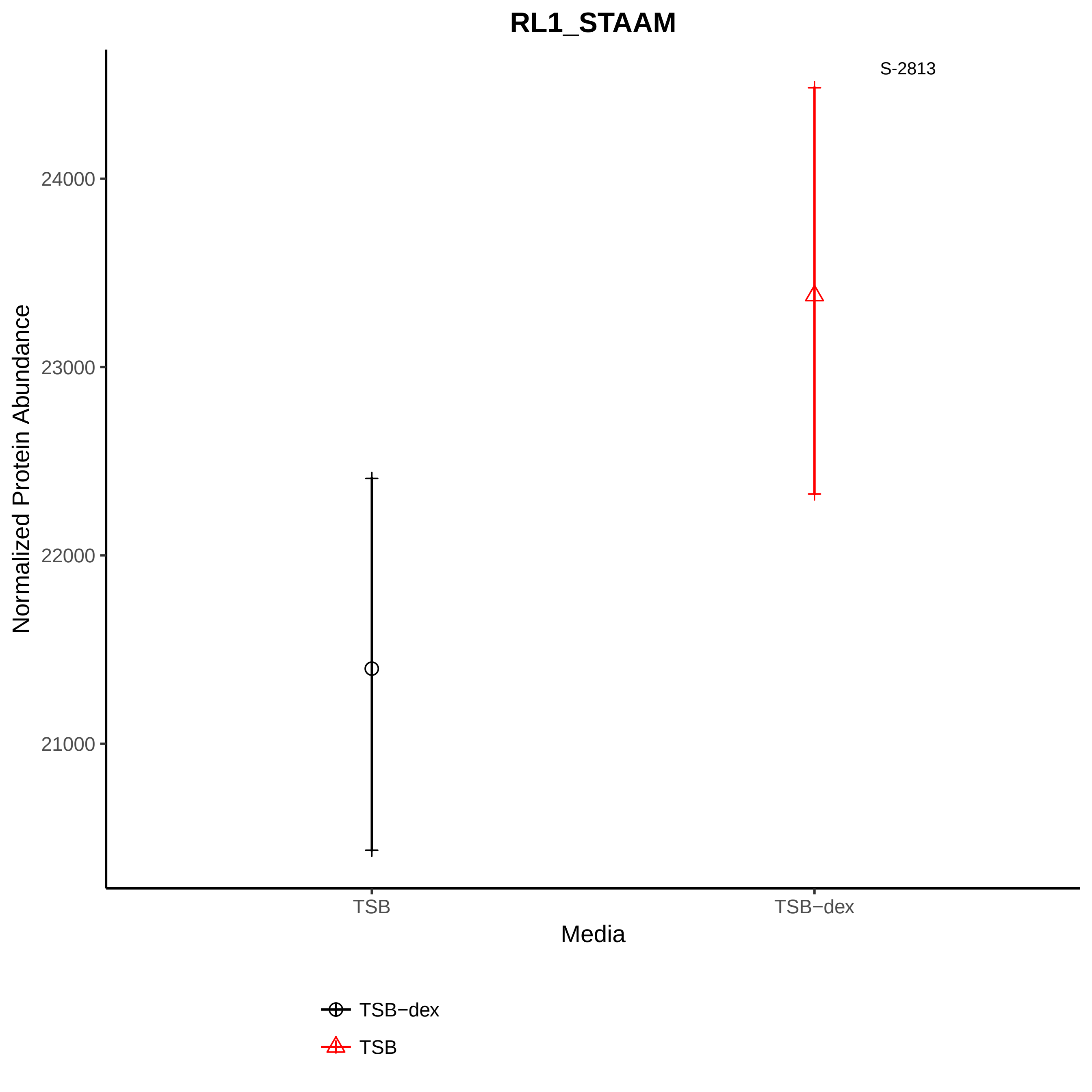

RL1_STAAM
S-2813
24000
23000
22000
21000
Normalized Protein Abundance
TSB
TSB−dex
Media
TSB−dex
TSB

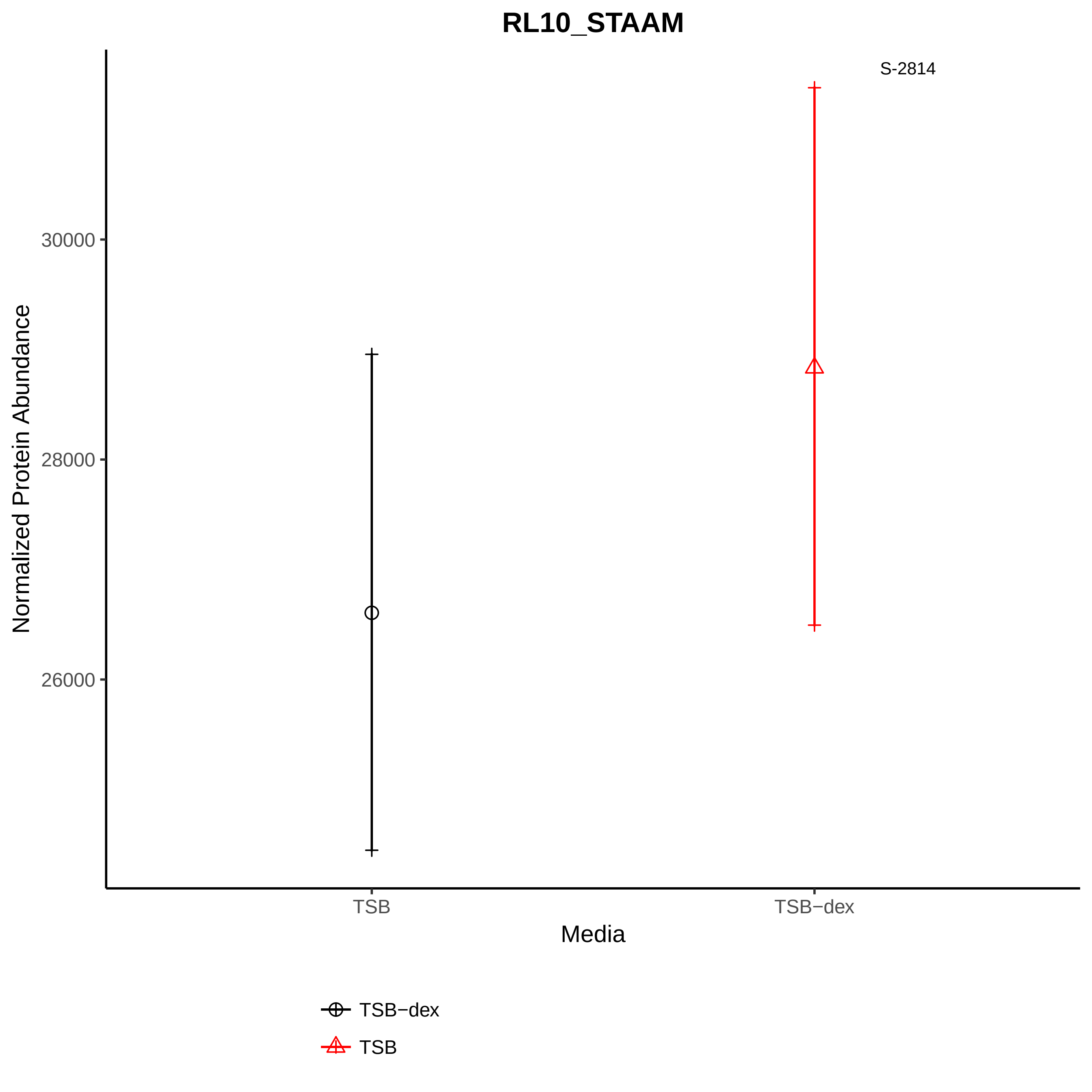

RL10_STAAM
S-2814
30000
28000
26000
Normalized Protein Abundance
TSB
TSB−dex
Media
TSB−dex
TSB

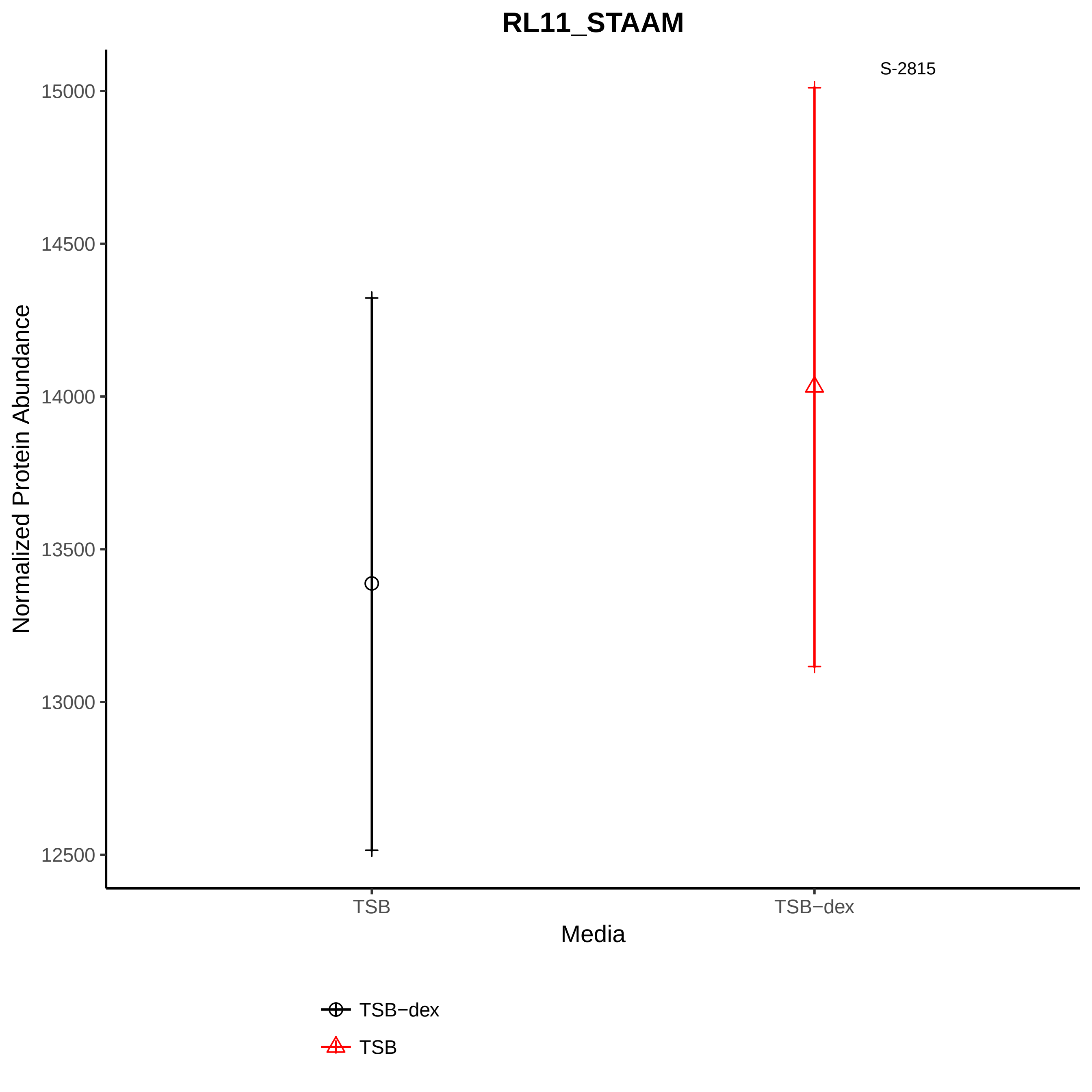

RL11_STAAM
S-2815
15000
14500
14000
13500
13000
12500
Normalized Protein Abundance
TSB
TSB−dex
Media
TSB−dex
TSB

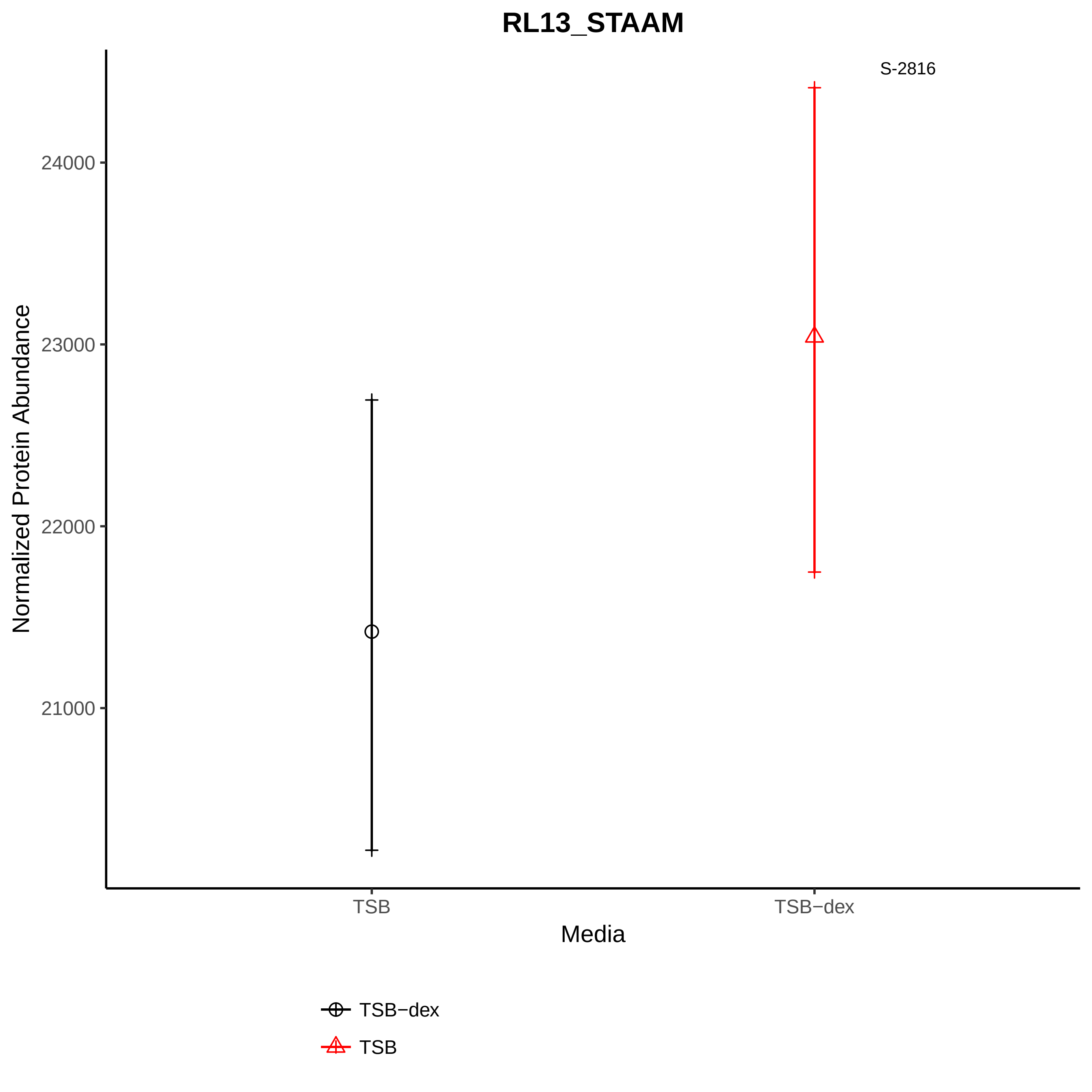
RL13_STAAM
S-2816
Normalized Protein Abundance
24000
23000
22000
21000
TSB
TSB−dex
Media
TSB−dex
TSB

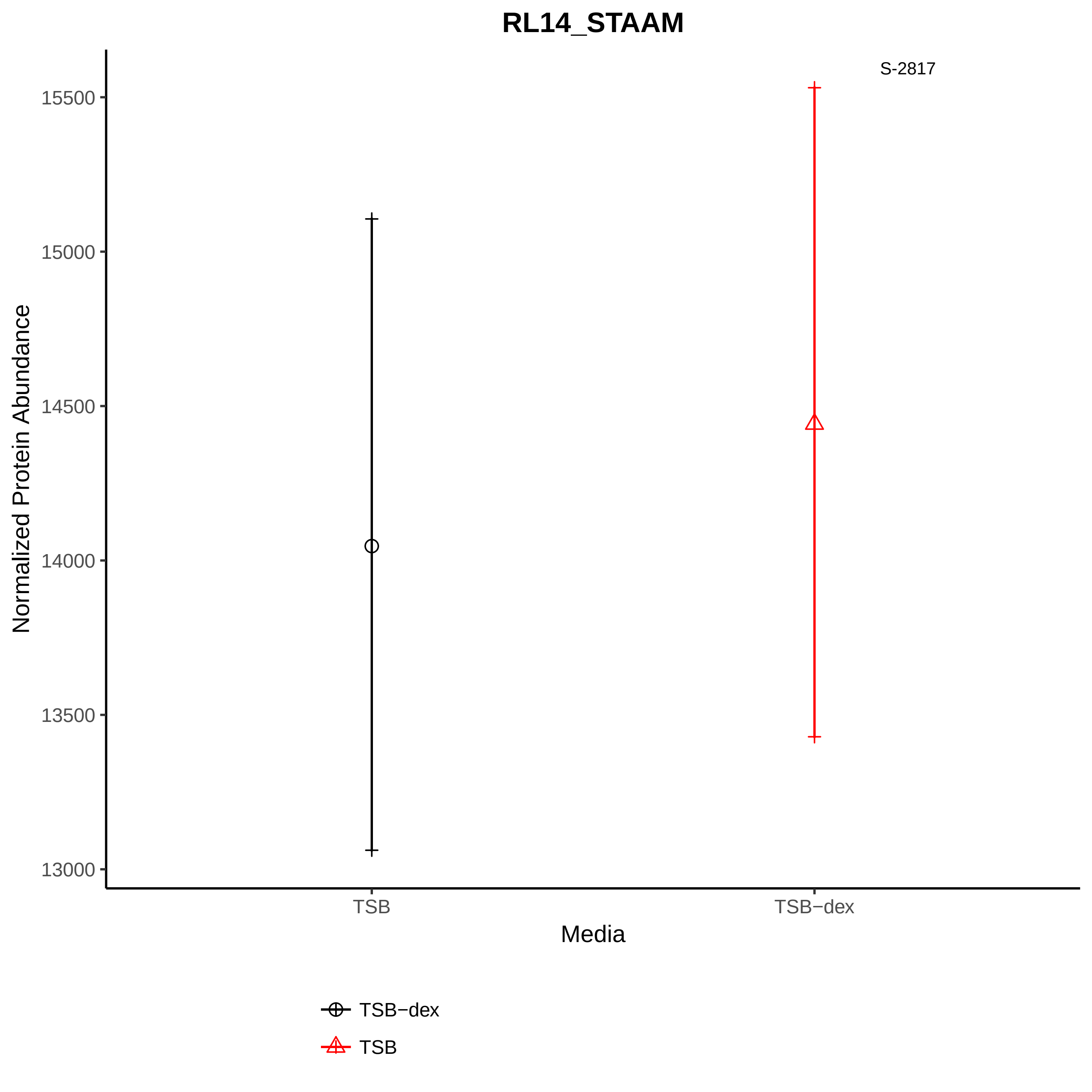

RL14_STAAM
S-2817
15500
15000
14500
14000
13500
13000
Normalized Protein Abundance
TSB
TSB−dex
Media
TSB−dex
TSB

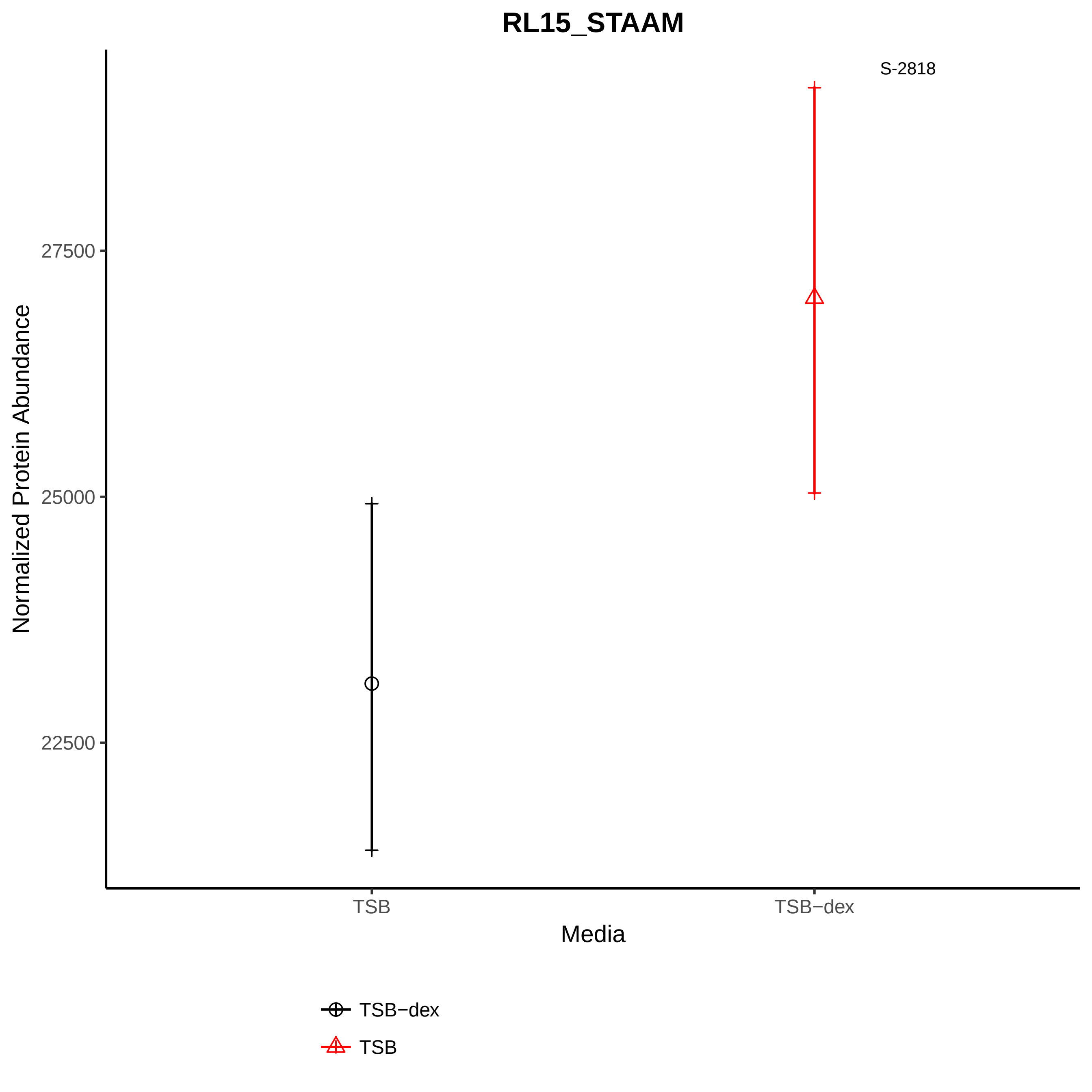
RL15_STAAM
S-2818
27500
25000
22500
Normalized Protein Abundance
TSB
TSB−dex
Media
TSB−dex
TSB

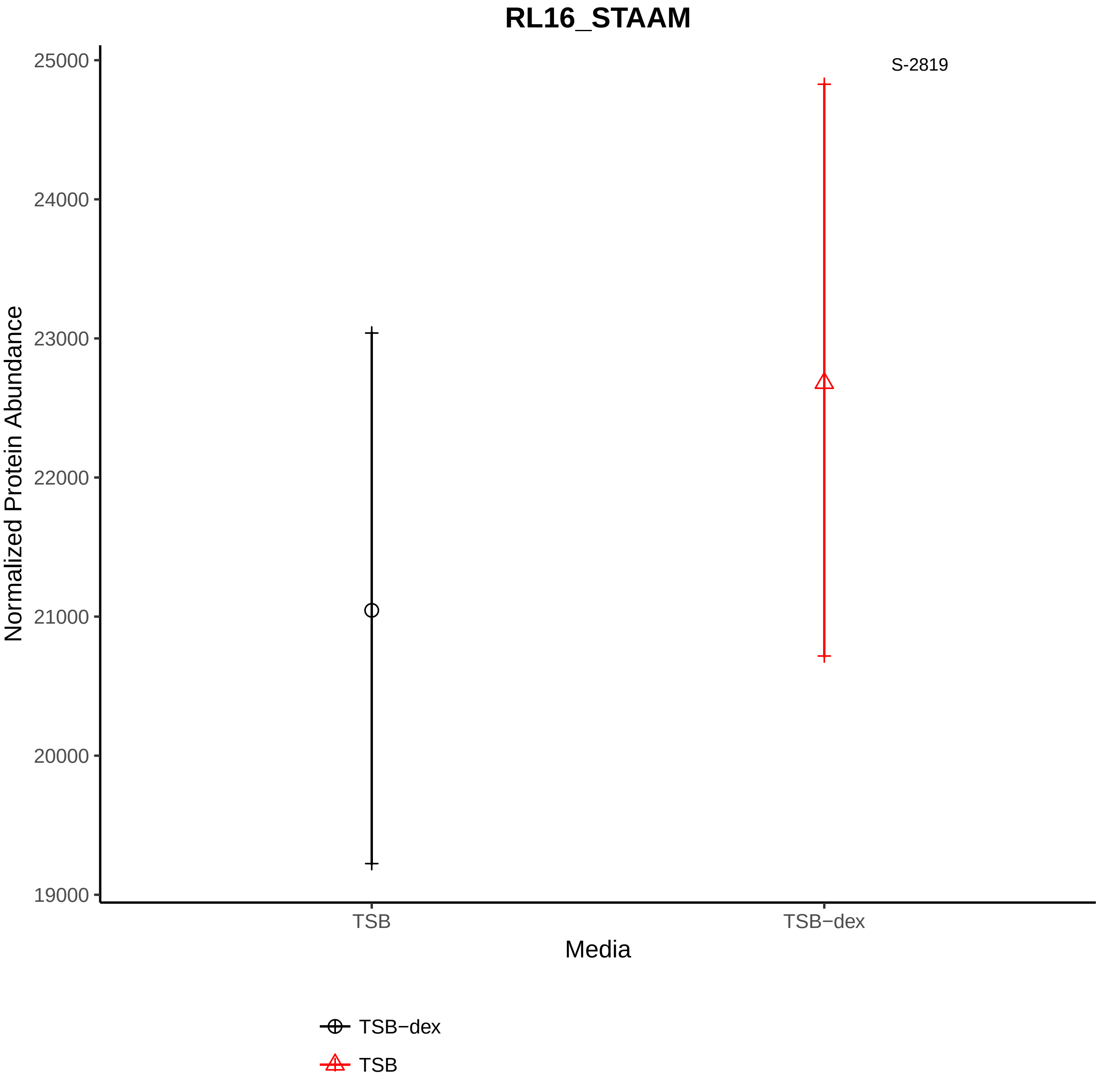

RL16_STAAM
S-2819
Normalized Protein Abundance
25000
24000
23000
22000
21000
20000
19000
TSB
TSB−dex
Media
TSB−dex
TSB

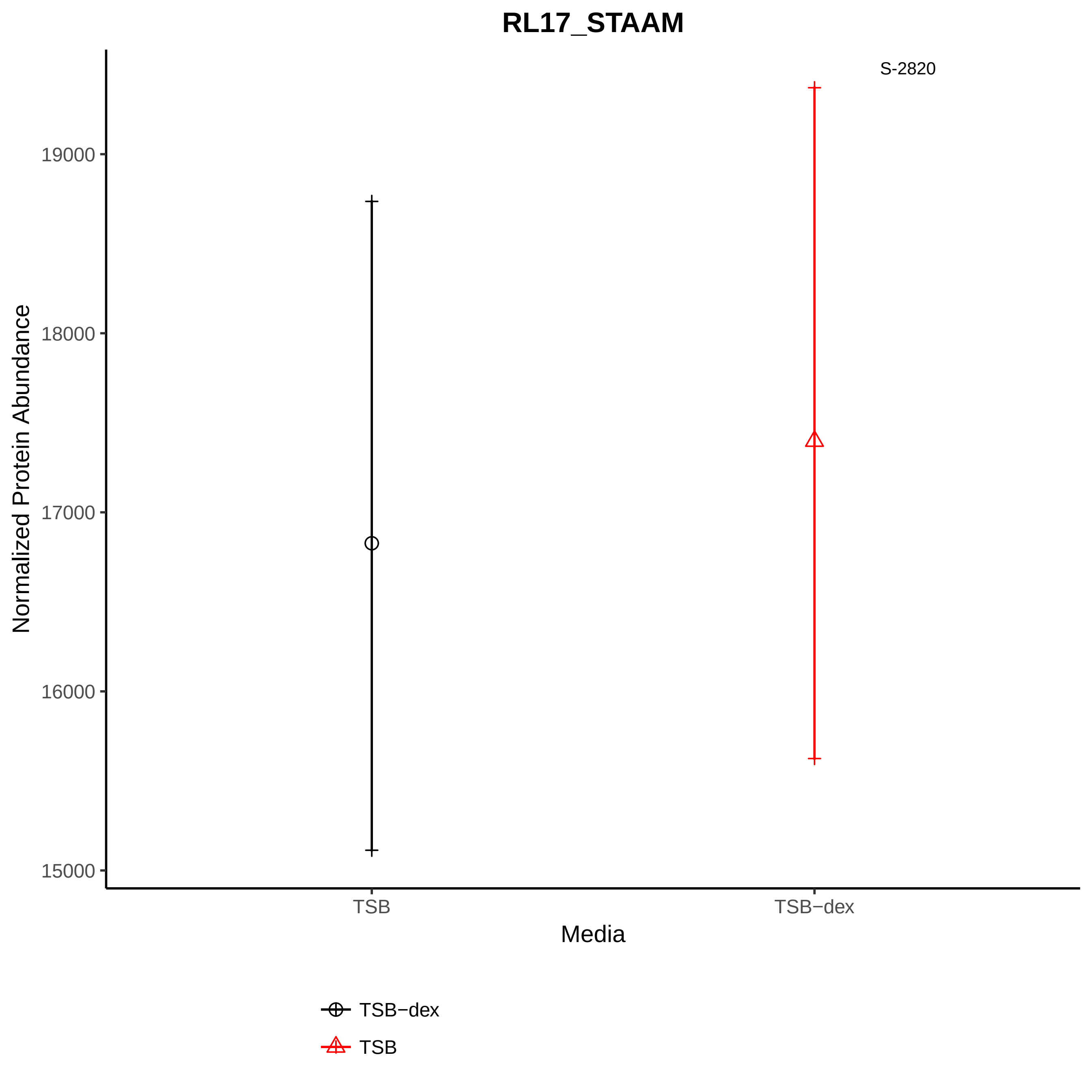

RL17_STAAM
S-2820
Normalized Protein Abundance
19000
18000
17000
16000
15000
TSB
TSB−dex
Media
TSB−dex
TSB

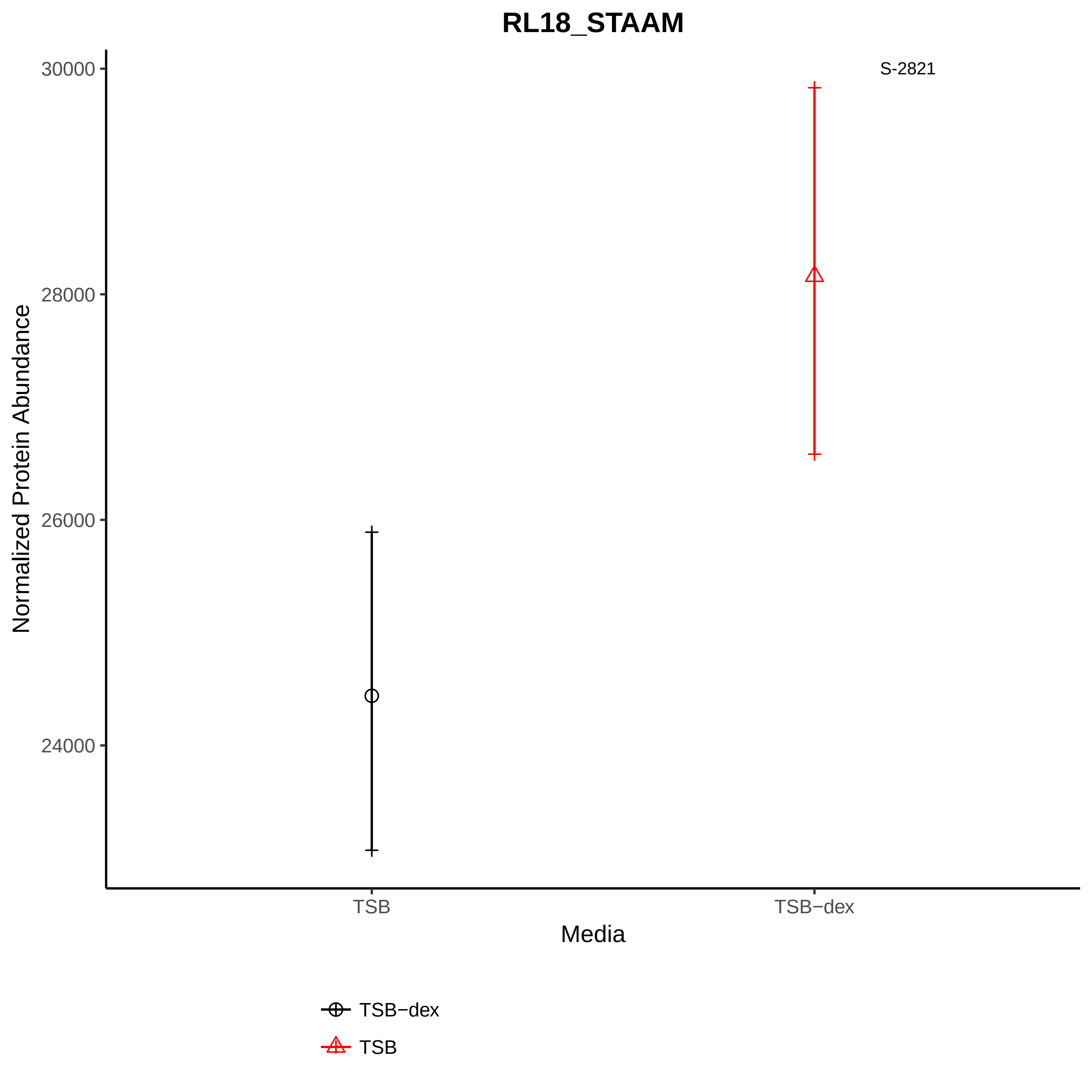

RL18_STAAM
S-2821
30000
28000
26000
24000
Normalized Protein Abundance
TSB
TSB−dex
Media
TSB−dex
TSB

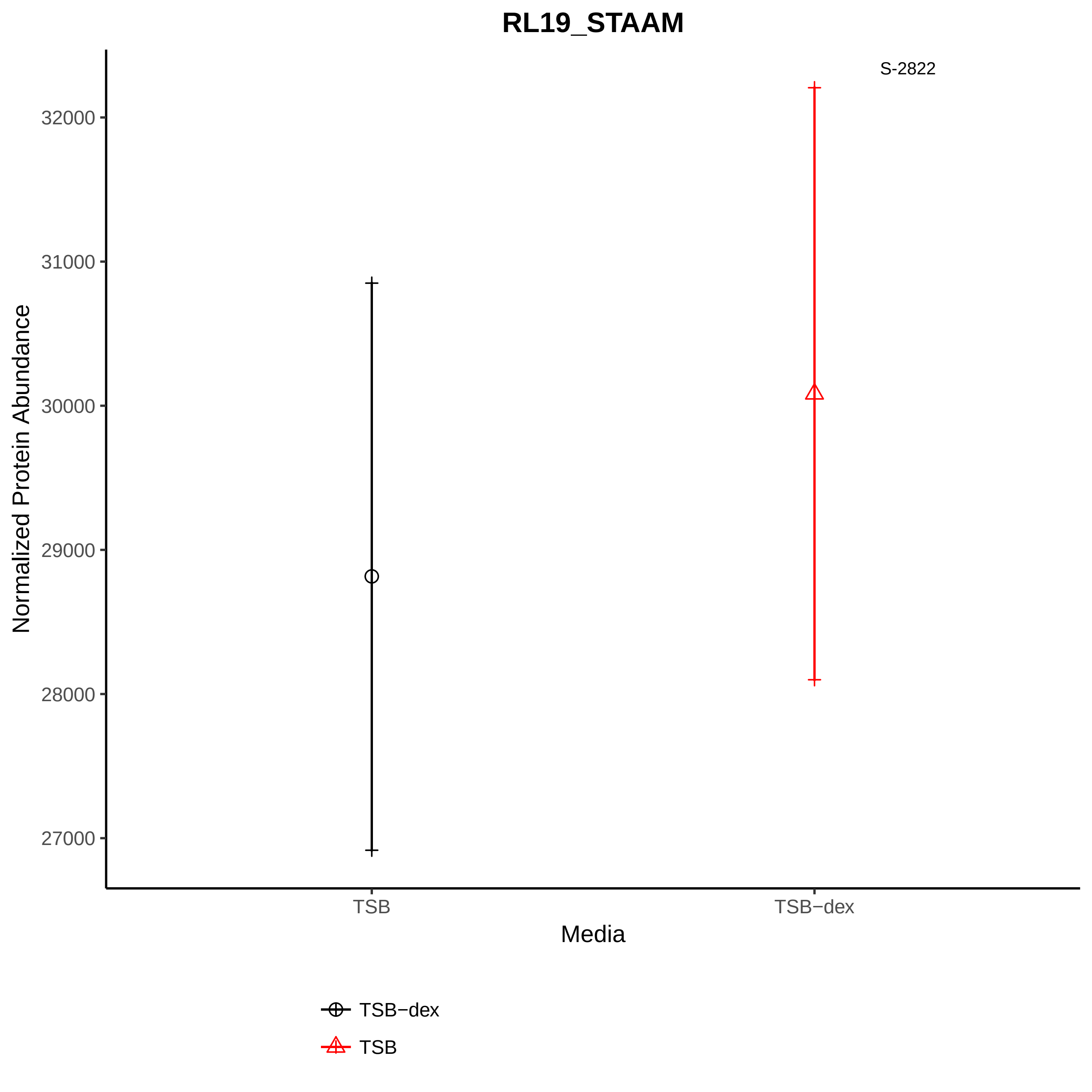
RL19_STAAM
S-2822
Normalized Protein Abundance
32000
31000
30000
29000
28000
27000
TSB
TSB−dex
Media
TSB−dex
TSB

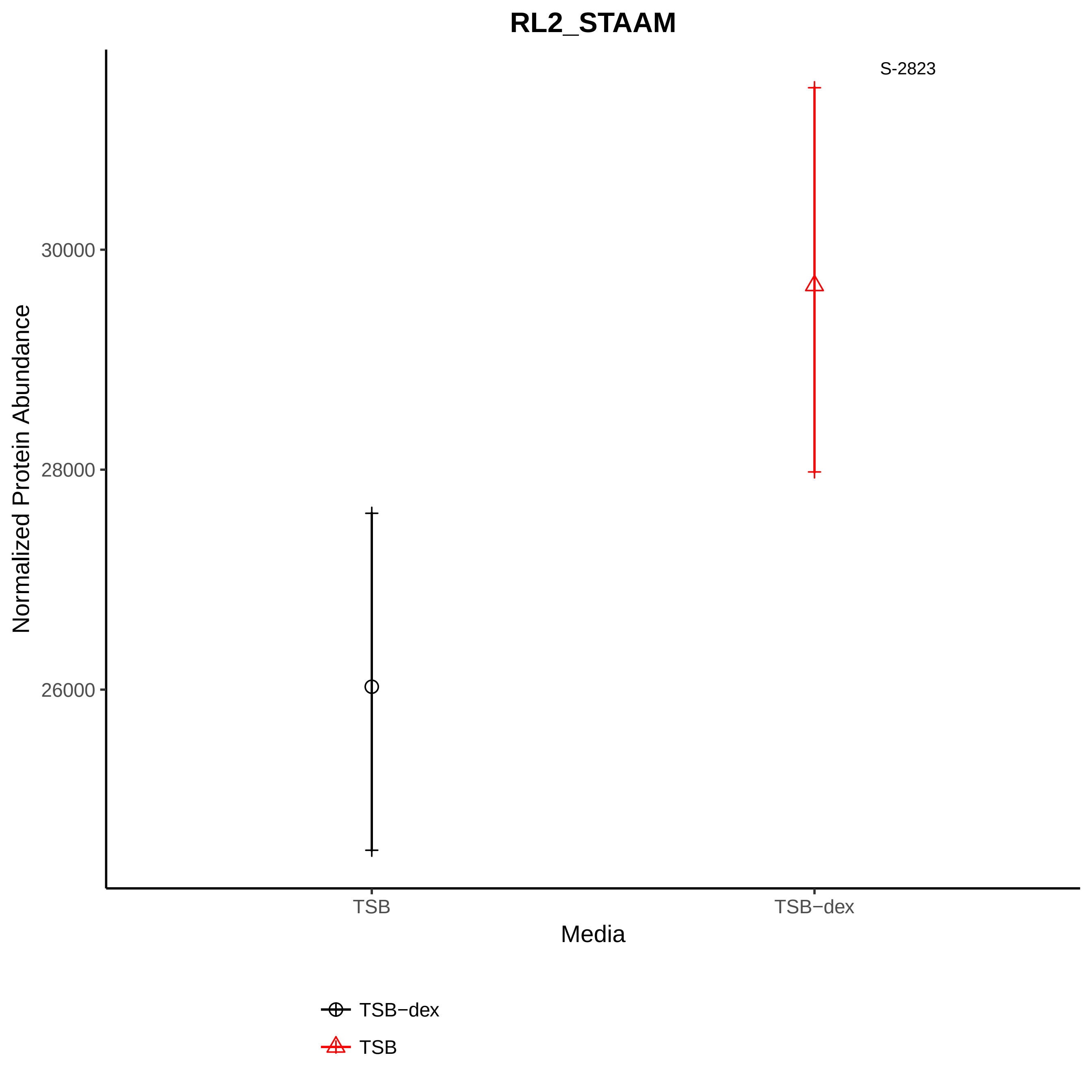

RL2_STAAM
S-2823
Normalized Protein Abundance
30000
28000
26000
TSB
TSB−dex
Media
TSB−dex
TSB

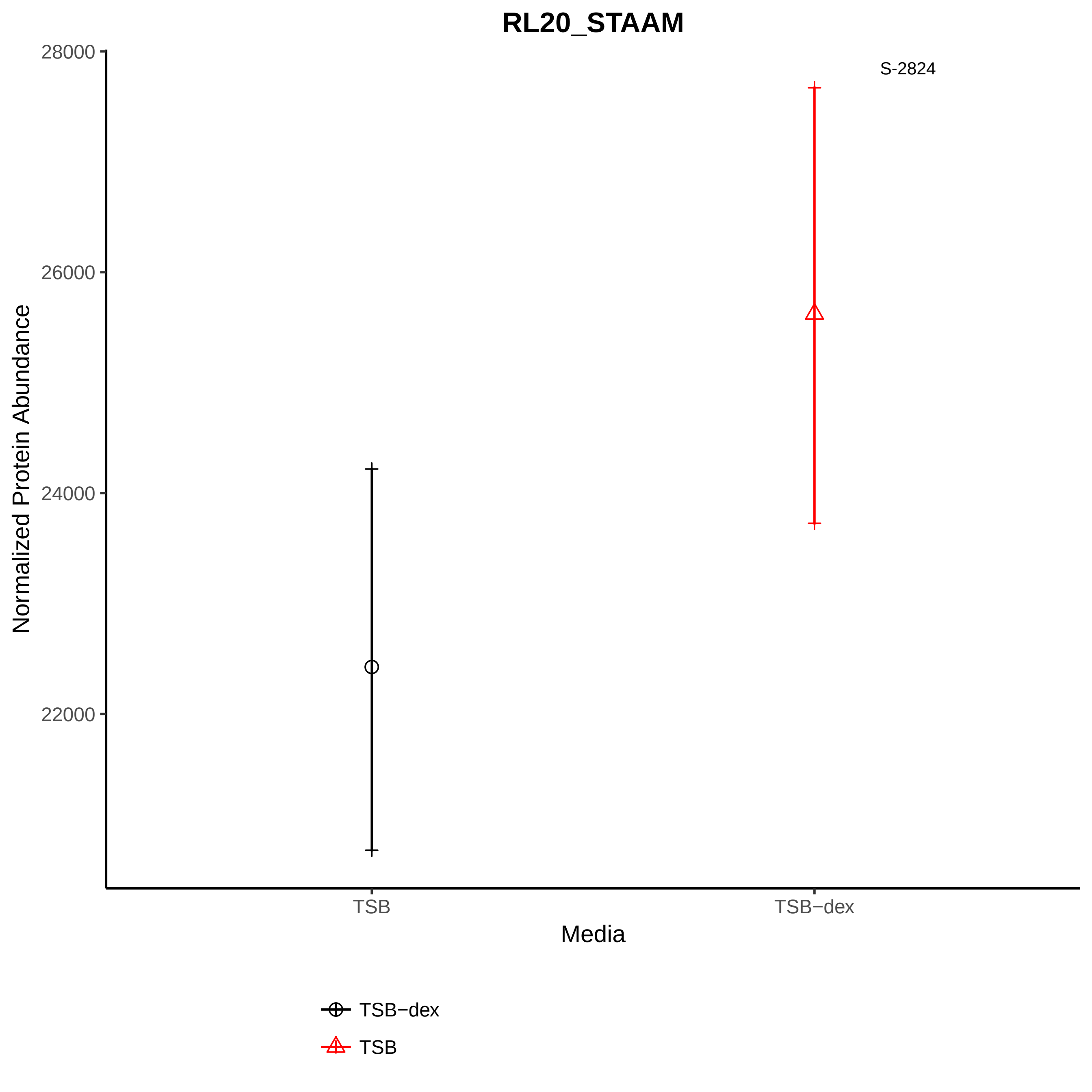
RL20_STAAM
S-2824
28000
26000
24000
22000
Normalized Protein Abundance
TSB
TSB−dex
Media
TSB−dex
TSB

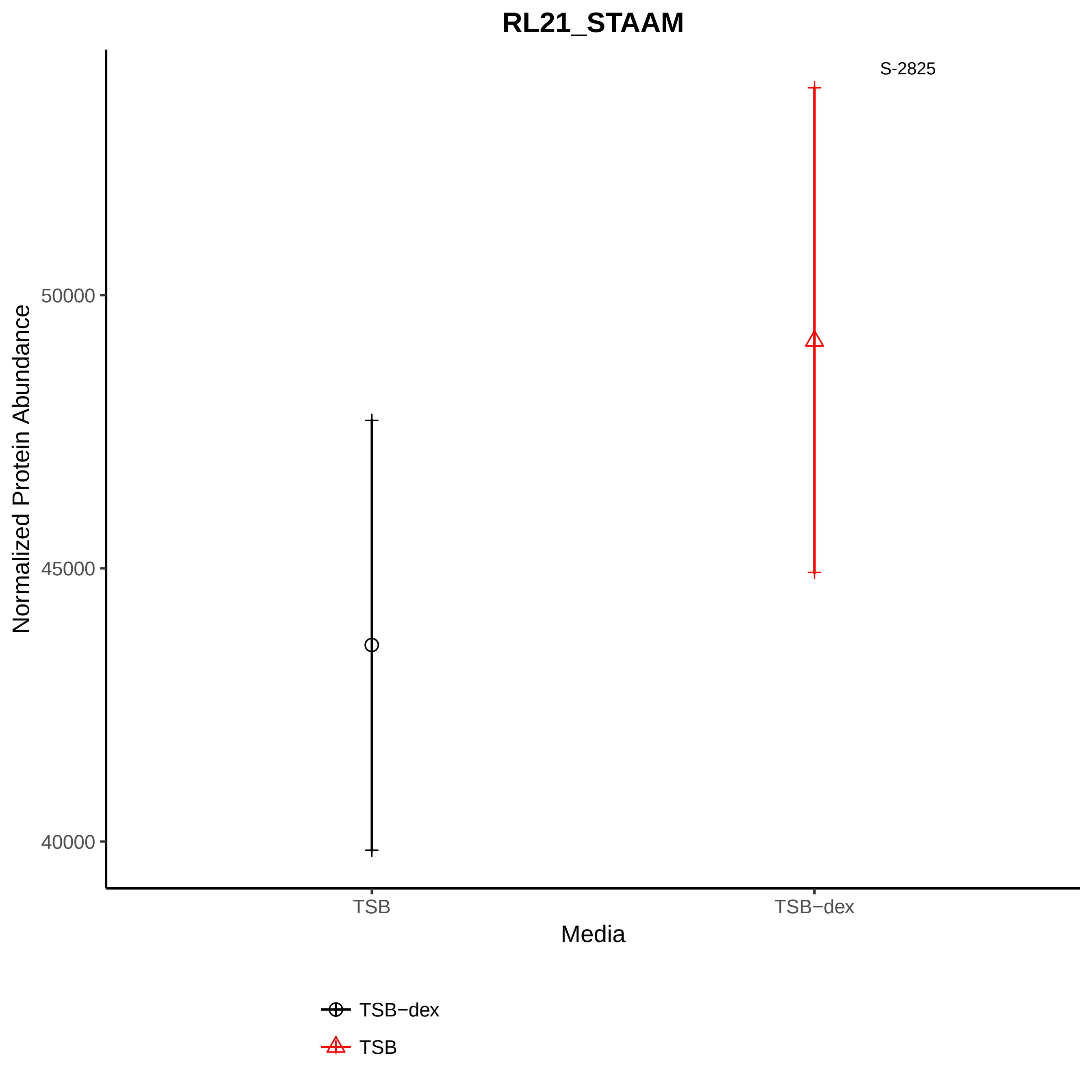

RL21_STAAM
S-2825
Normalized Protein Abundance
50000
45000
40000
TSB
TSB−dex
Media
TSB−dex
TSB

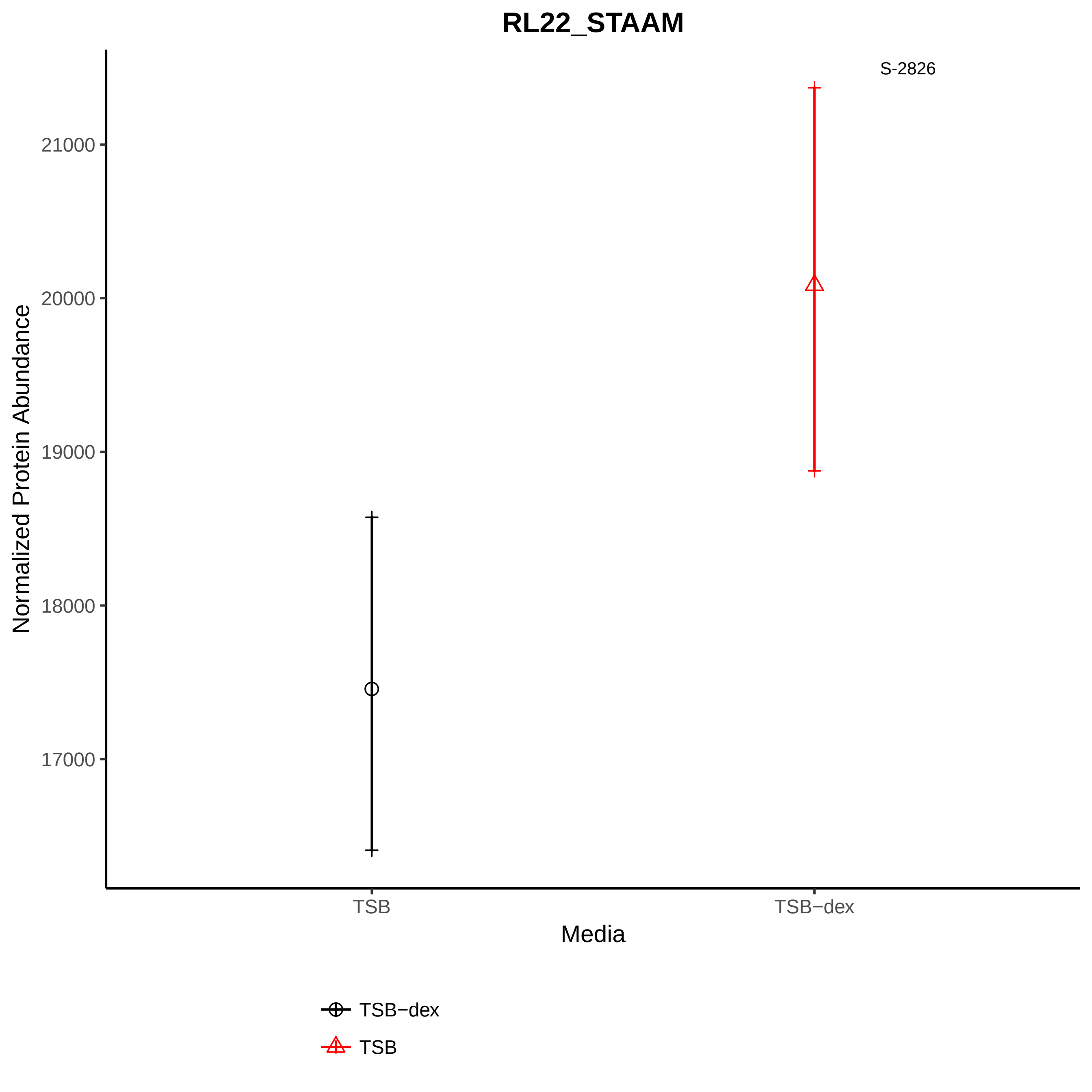
RL22_STAAM
S-2826
Normalized Protein Abundance
21000
20000
19000
18000
17000
TSB
TSB−dex
Media
TSB−dex
TSB

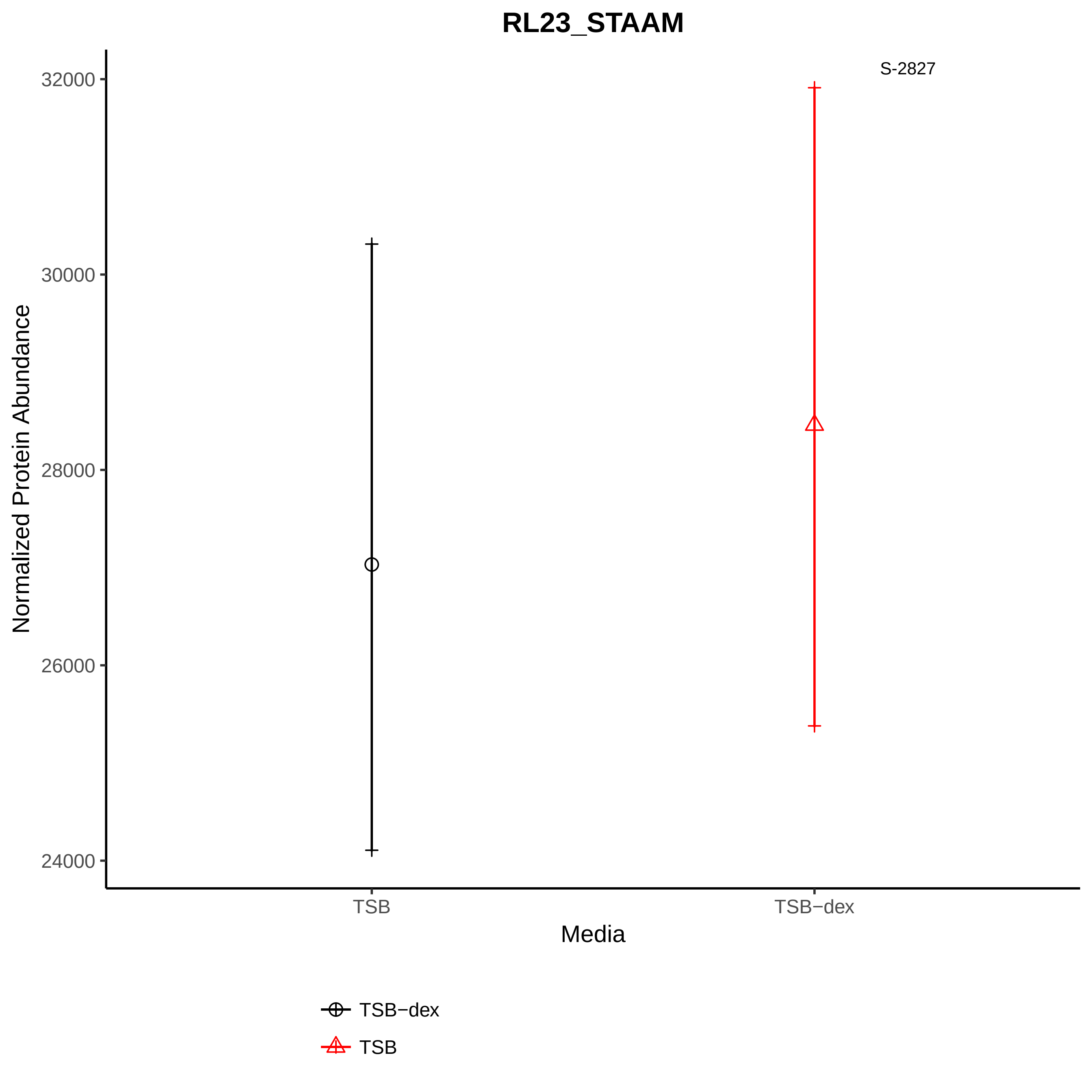

RL23_STAAM
S-2827
Normalized Protein Abundance
32000
30000
28000
26000
24000
TSB
TSB−dex
Media
TSB−dex
TSB

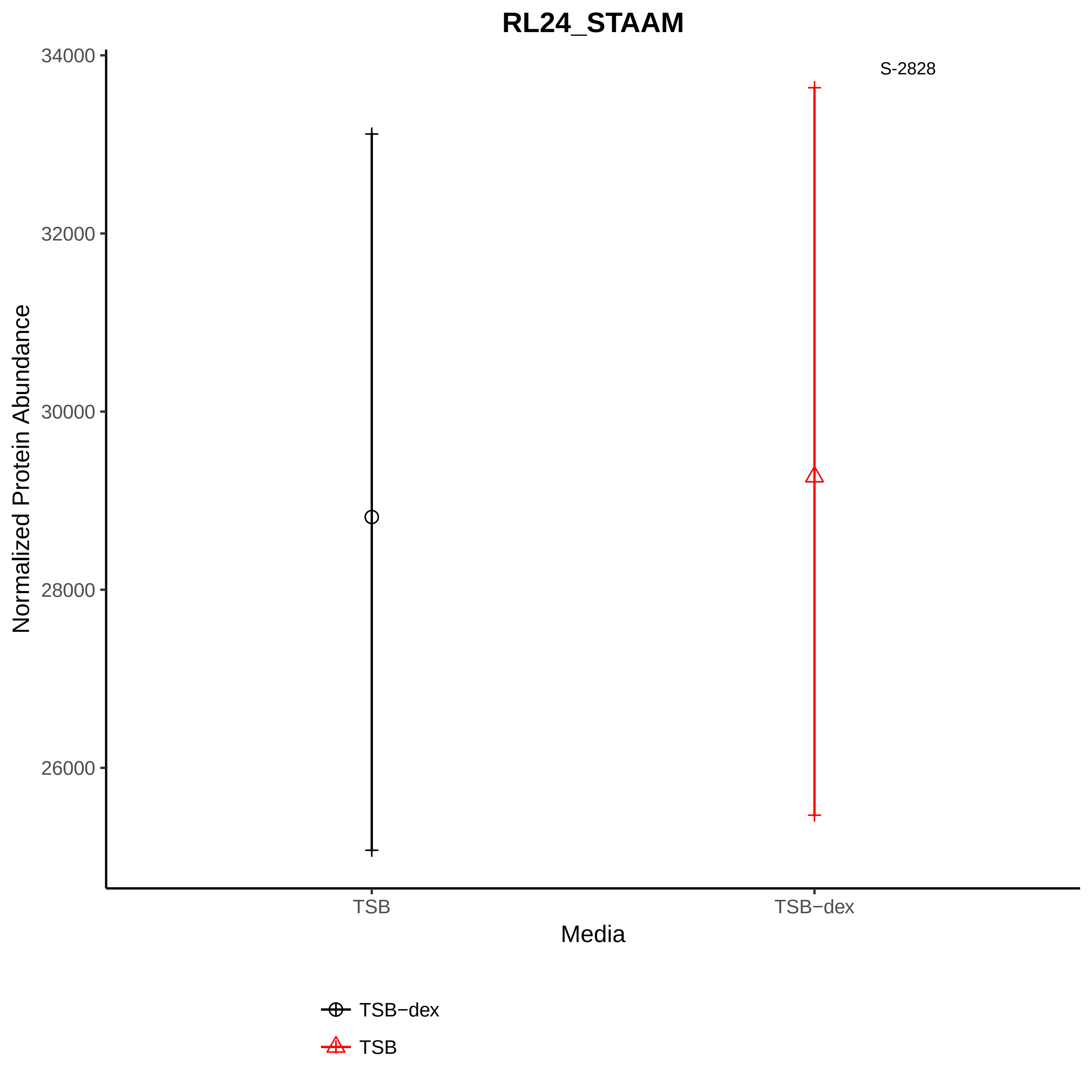

RL24_STAAM
S-2828
34000
32000
30000
28000
26000
Normalized Protein Abundance
TSB
TSB−dex
Media
TSB−dex
TSB

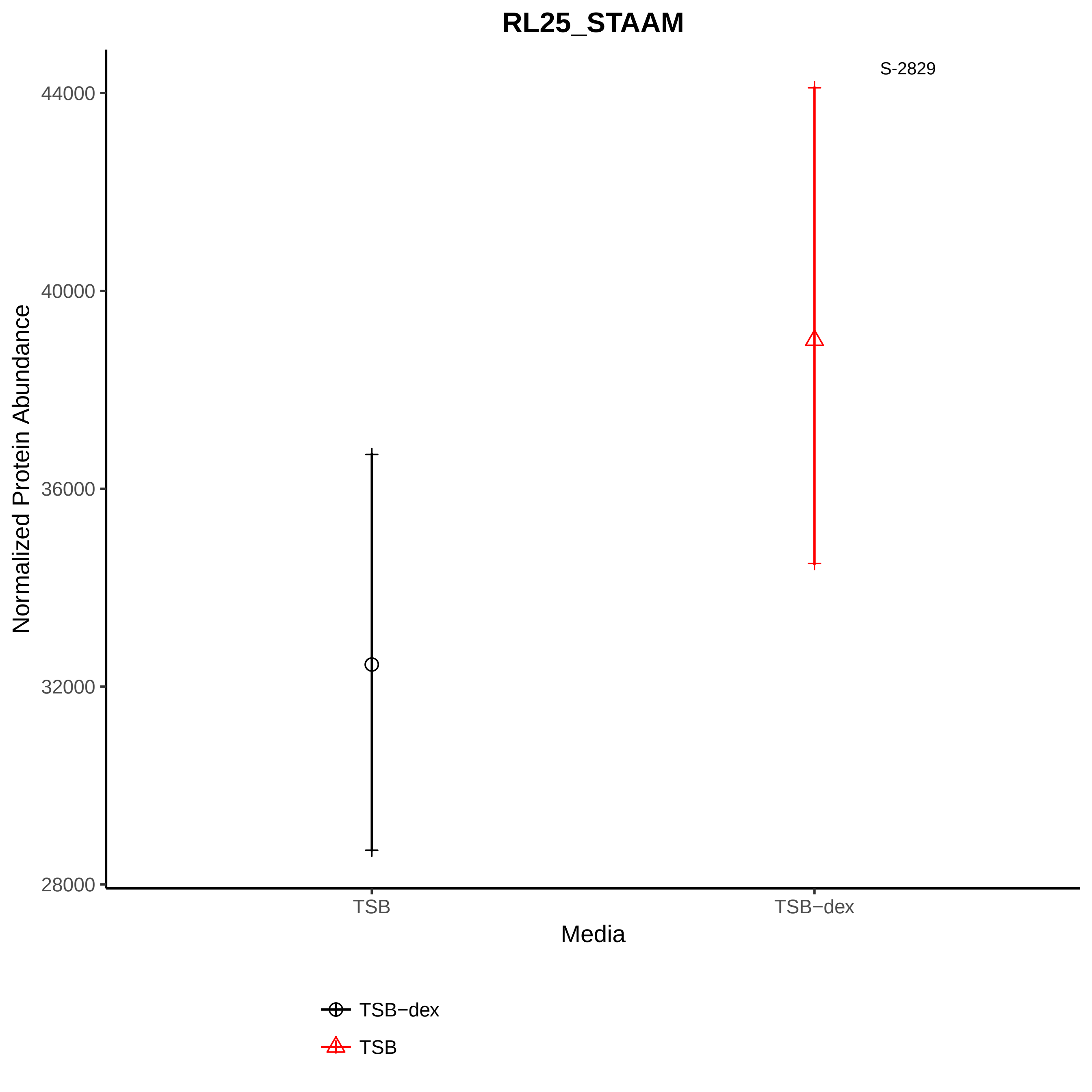
RL25_STAAM
S-2829
44000
40000
36000
32000
28000
Normalized Protein Abundance
TSB
TSB−dex
Media
TSB−dex
TSB

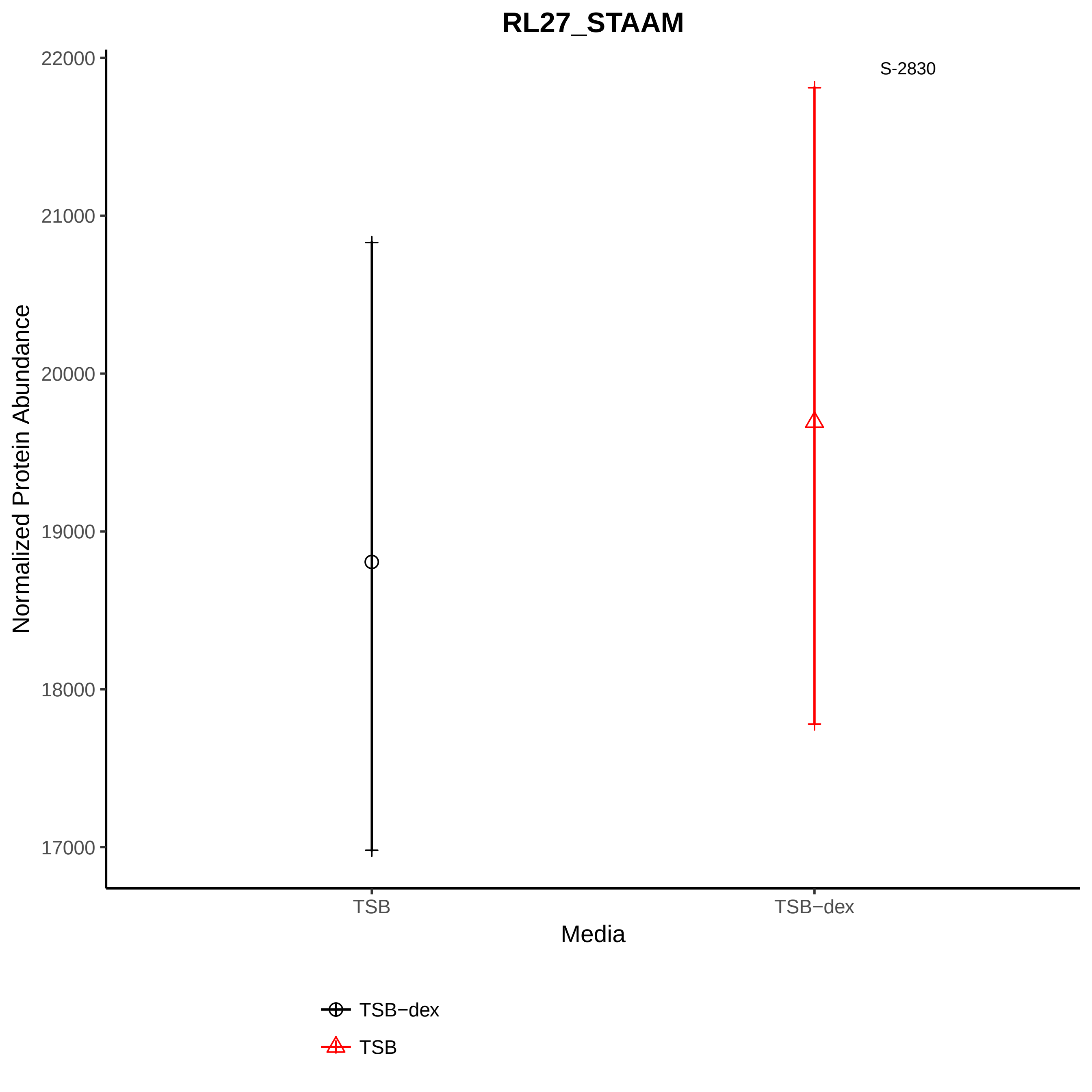
RL27_STAAM
S-2830
22000
21000
20000
19000
18000
17000
Normalized Protein Abundance
TSB
TSB−dex
Media
TSB−dex
TSB

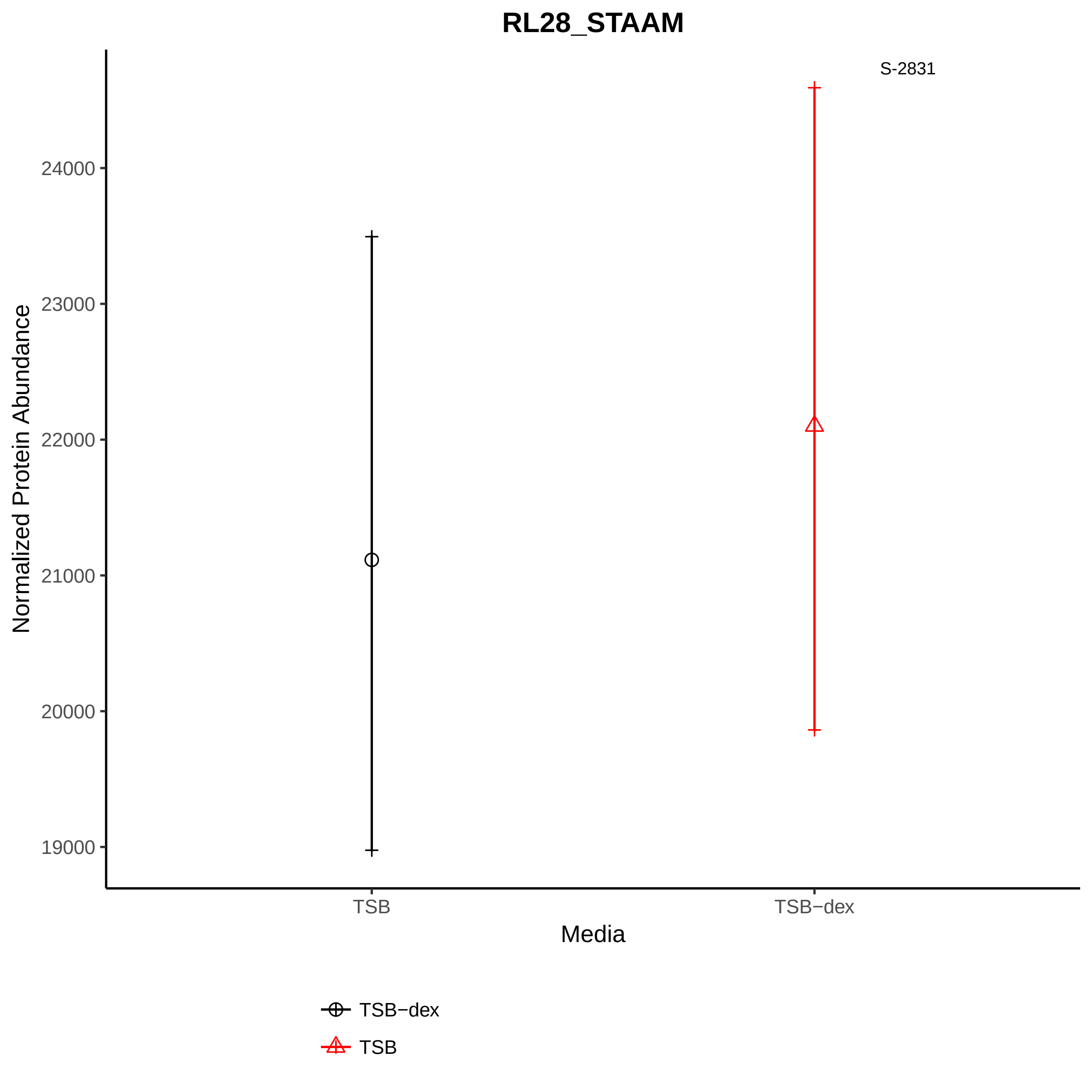

RL28_STAAM
S-2831
Normalized Protein Abundance
24000
23000
22000
21000
20000
19000
TSB
TSB−dex
Media
TSB−dex
TSB

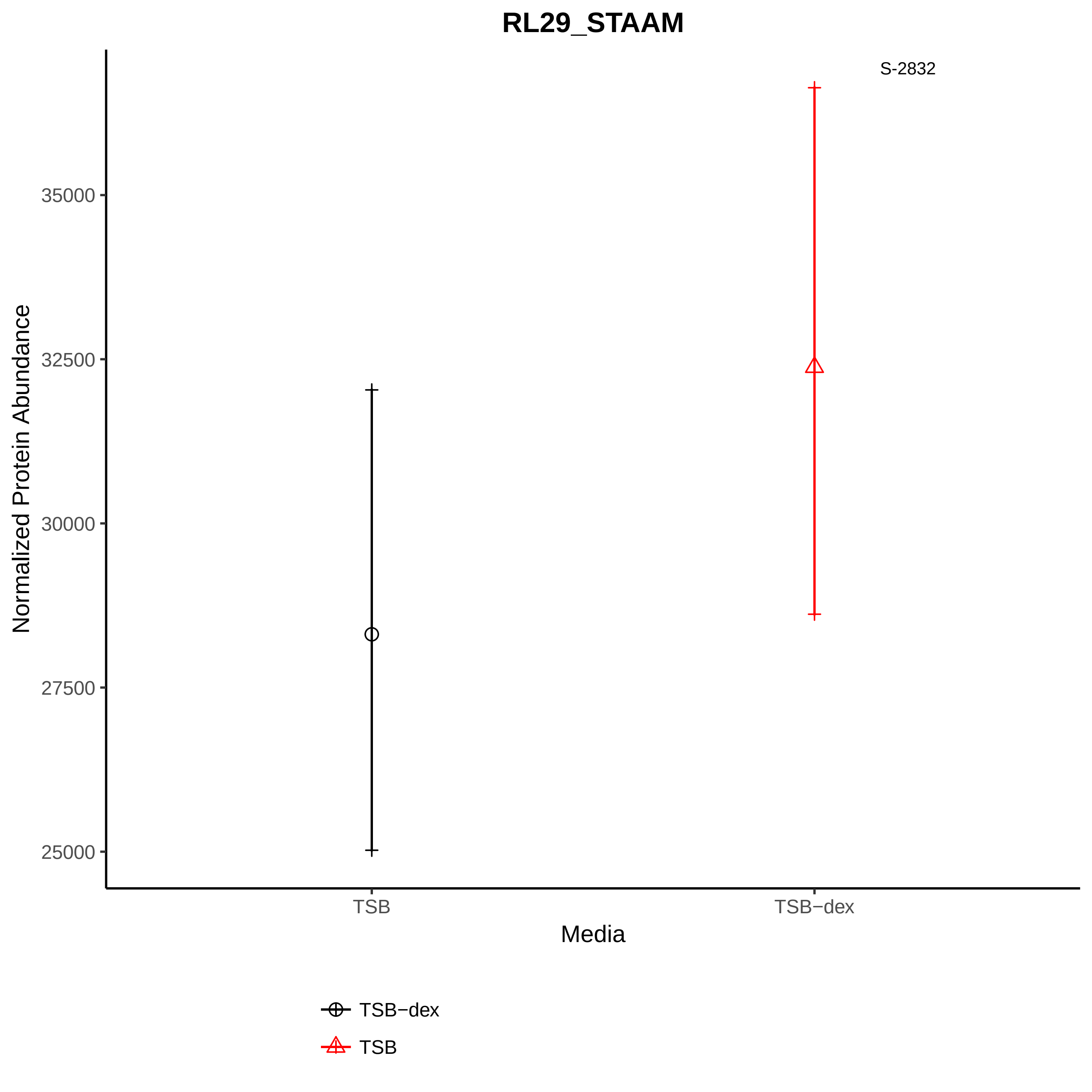

RL29_STAAM
S-2832
Normalized Protein Abundance
35000
32500
30000
27500
25000
TSB
TSB−dex
Media
TSB−dex
TSB

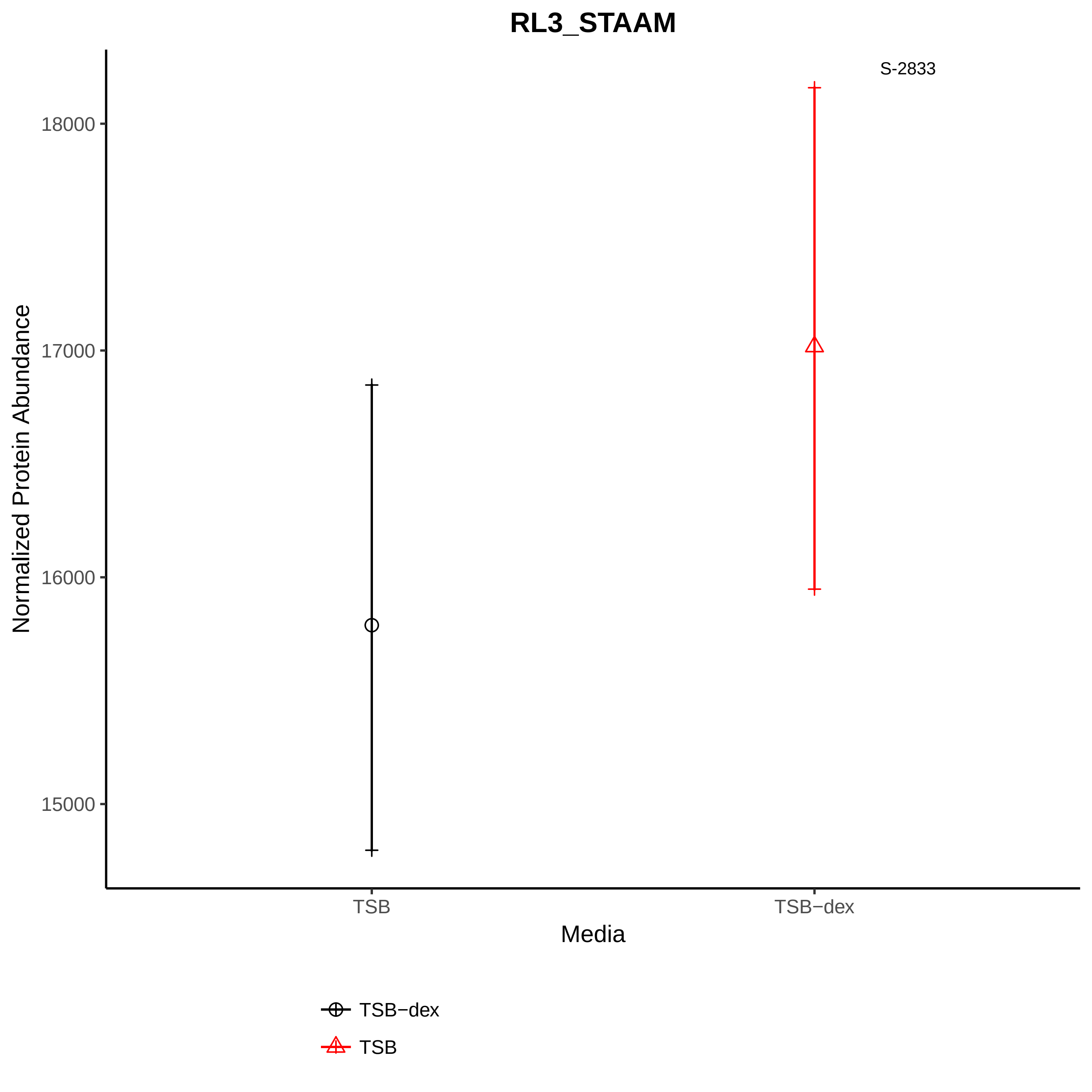

RL3_STAAM
S-2833
Normalized Protein Abundance
18000
17000
16000
15000
TSB
TSB−dex
Media
TSB−dex
TSB

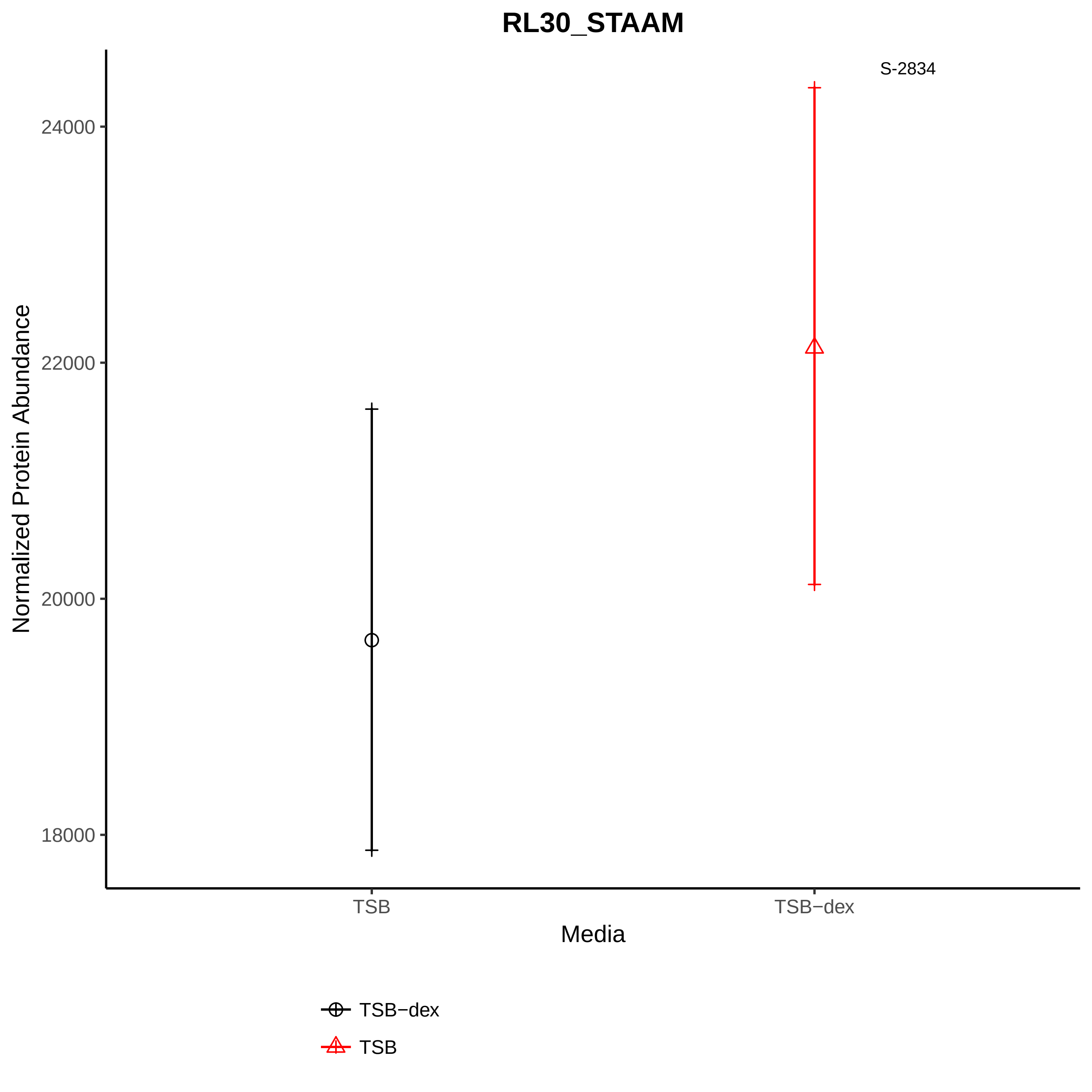

RL30_STAAM
S-2834
24000
22000
20000
18000
Normalized Protein Abundance
TSB
TSB−dex
Media
TSB−dex
TSB

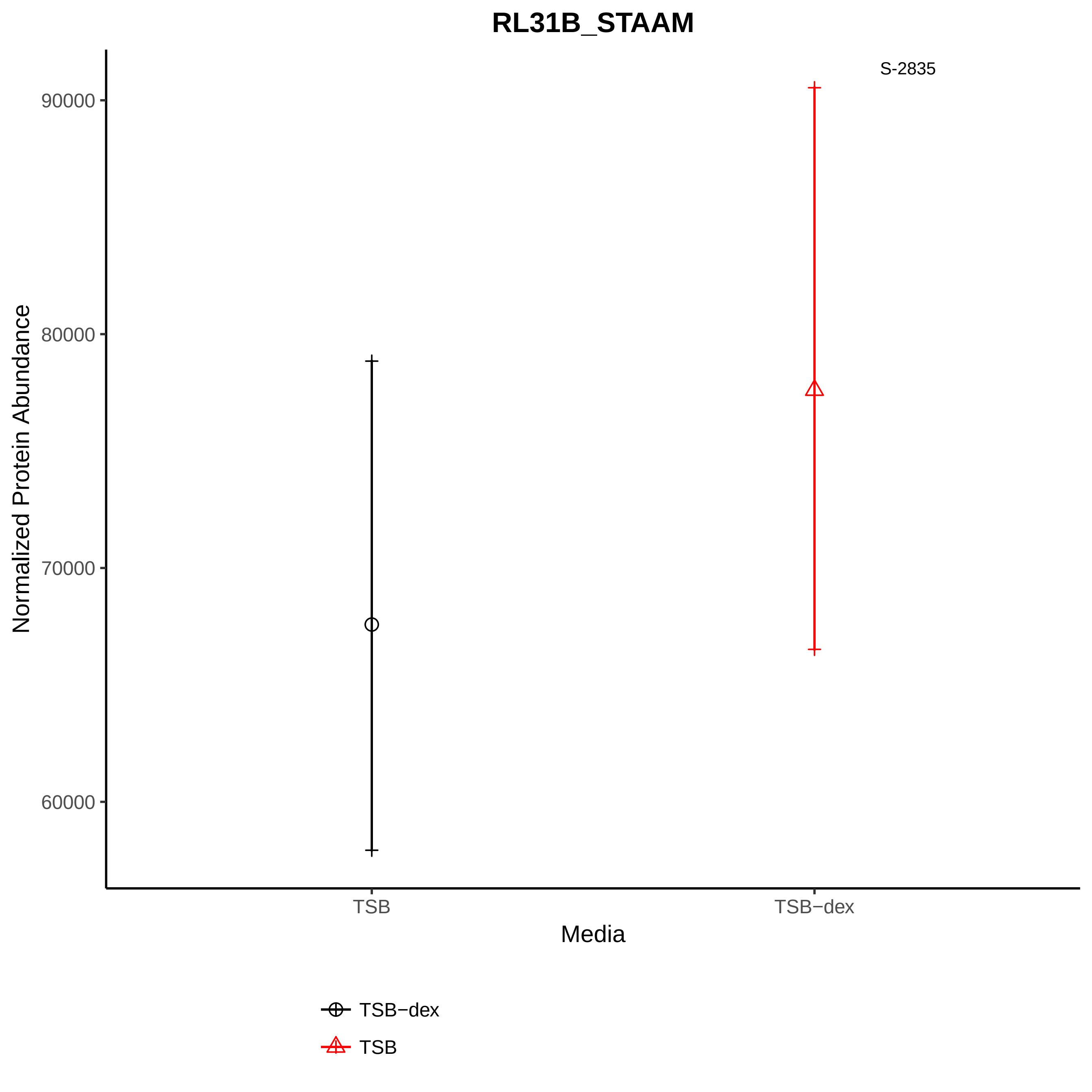

RL31B_STAAM
S-2835
90000
80000
70000
60000
Normalized Protein Abundance
TSB
TSB−dex
Media
TSB−dex
TSB

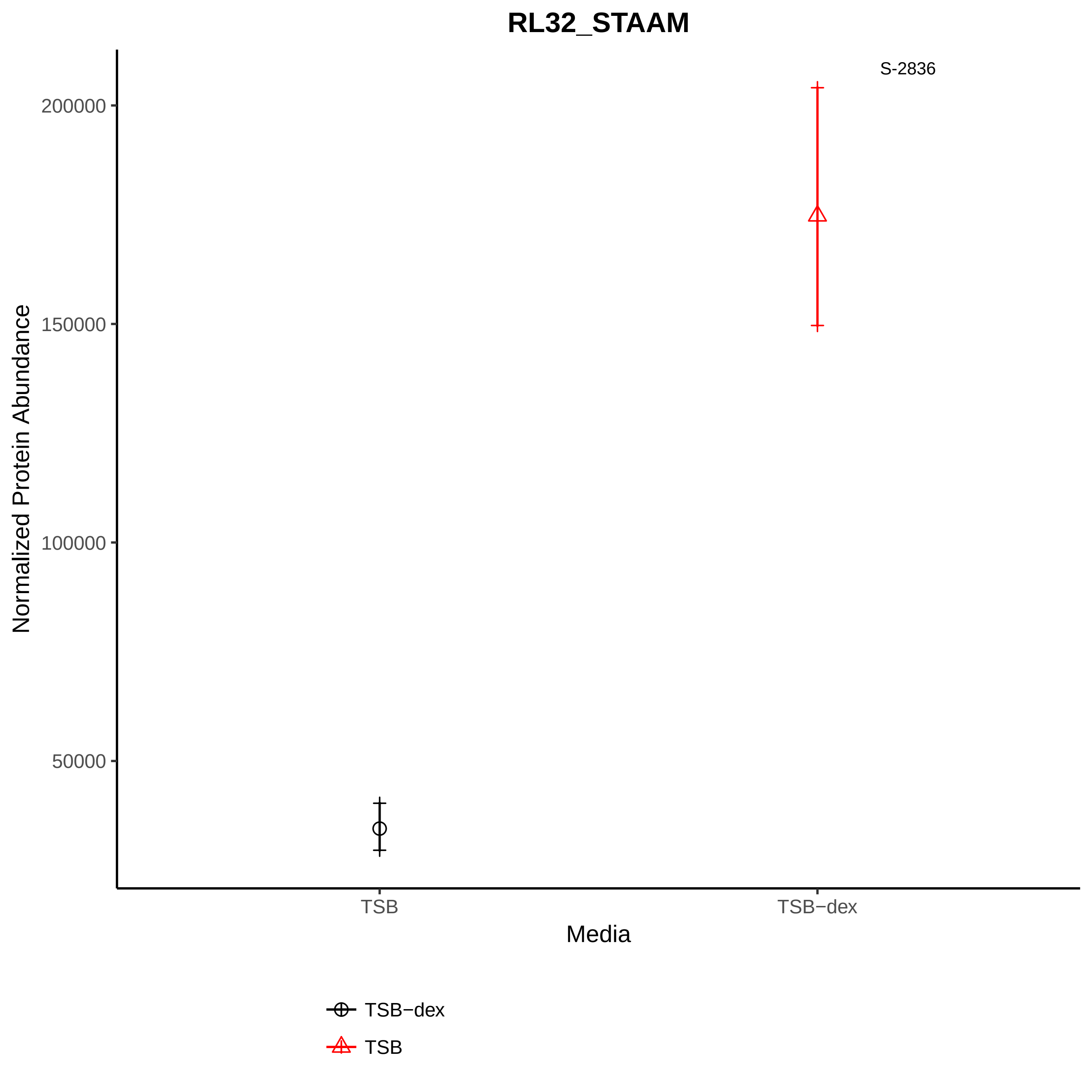
RL32_STAAM
S-2836
200000
150000
100000
50000
Normalized Protein Abundance
TSB
TSB−dex
Media
TSB−dex
TSB

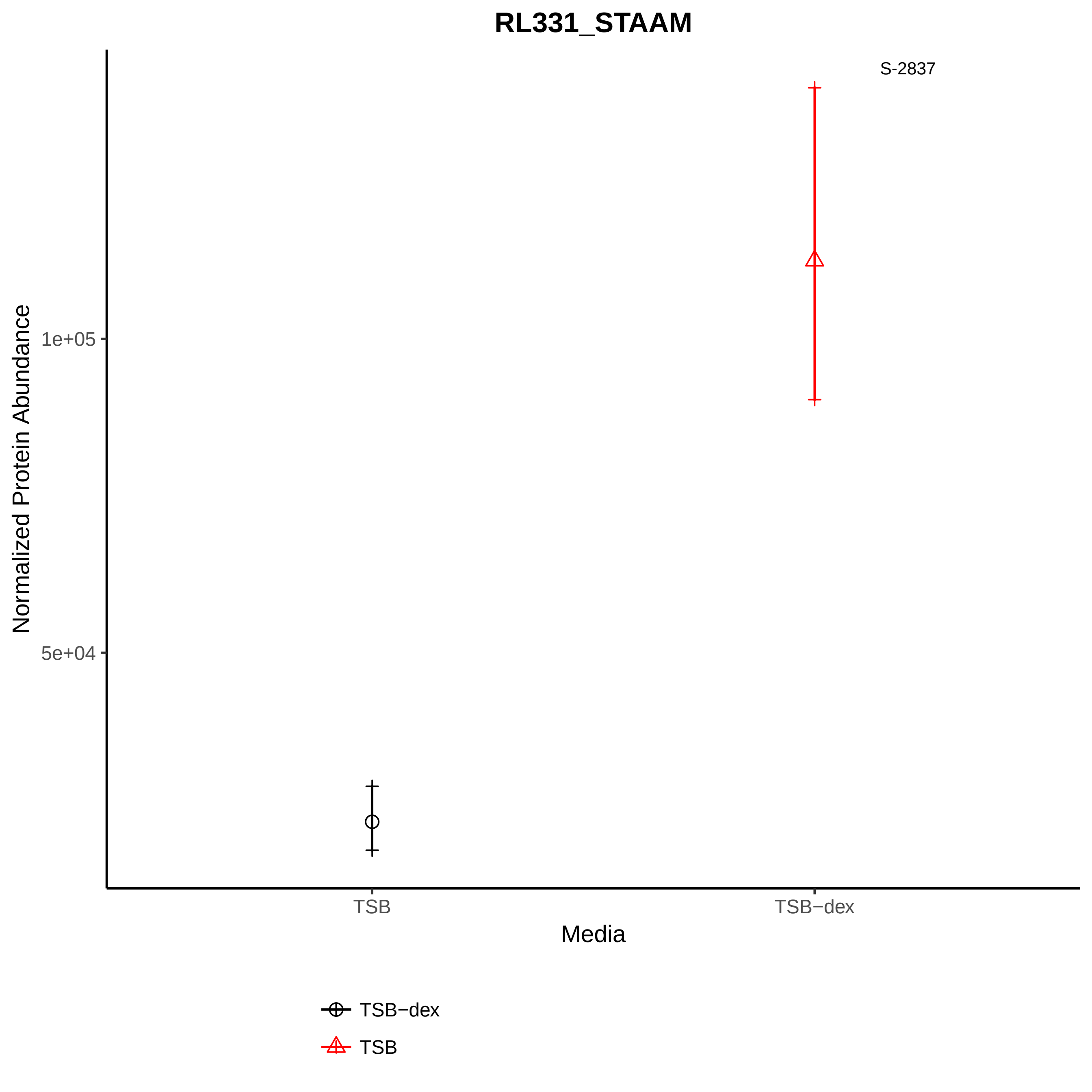
RL331_STAAM
S-2837
Normalized Protein Abundance
1e+05
5e+04
TSB
TSB−dex
Media
TSB−dex
TSB

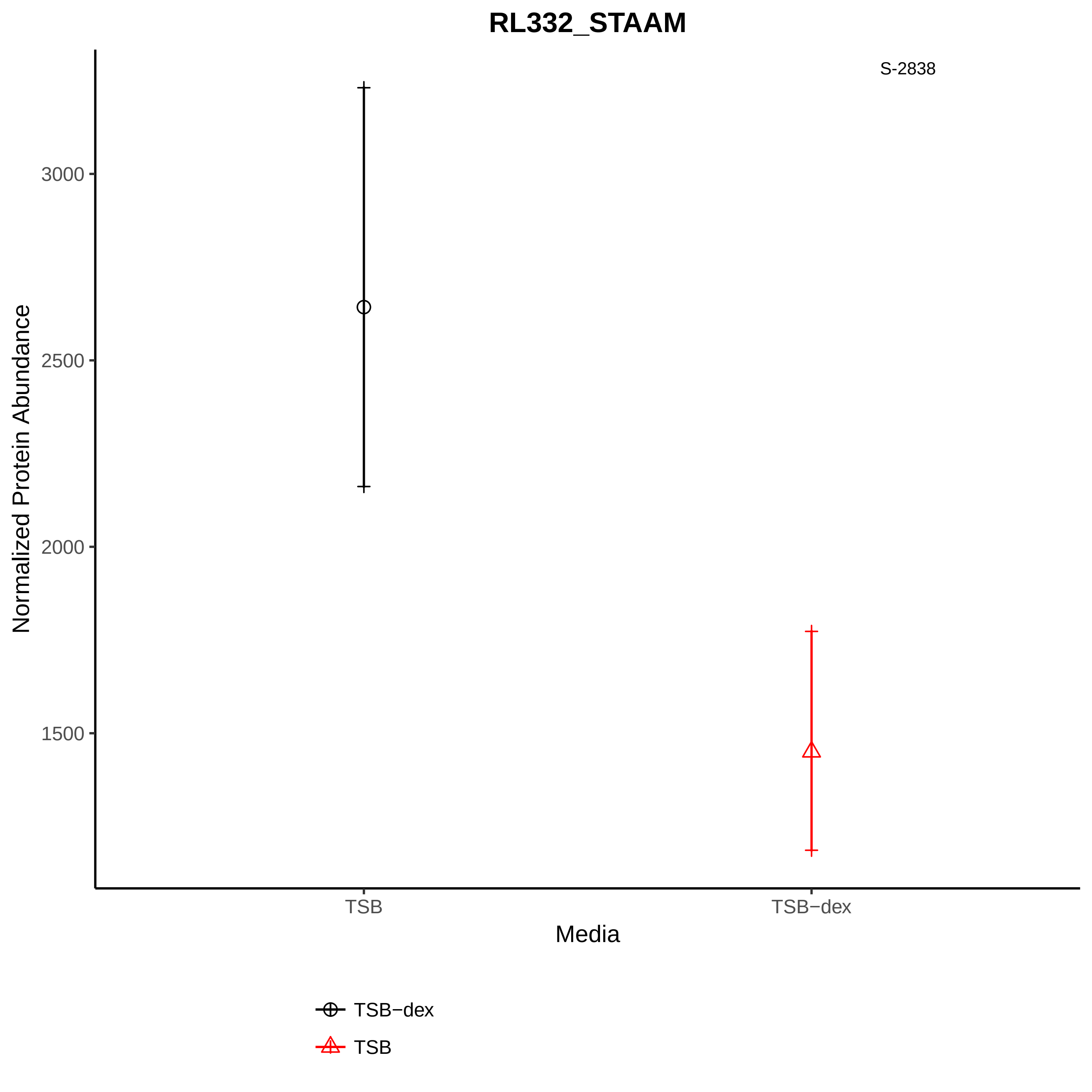

RL332_STAAM
S-2838
Normalized Protein Abundance
3000
2500
2000
1500
TSB
TSB−dex
Media
TSB−dex
TSB

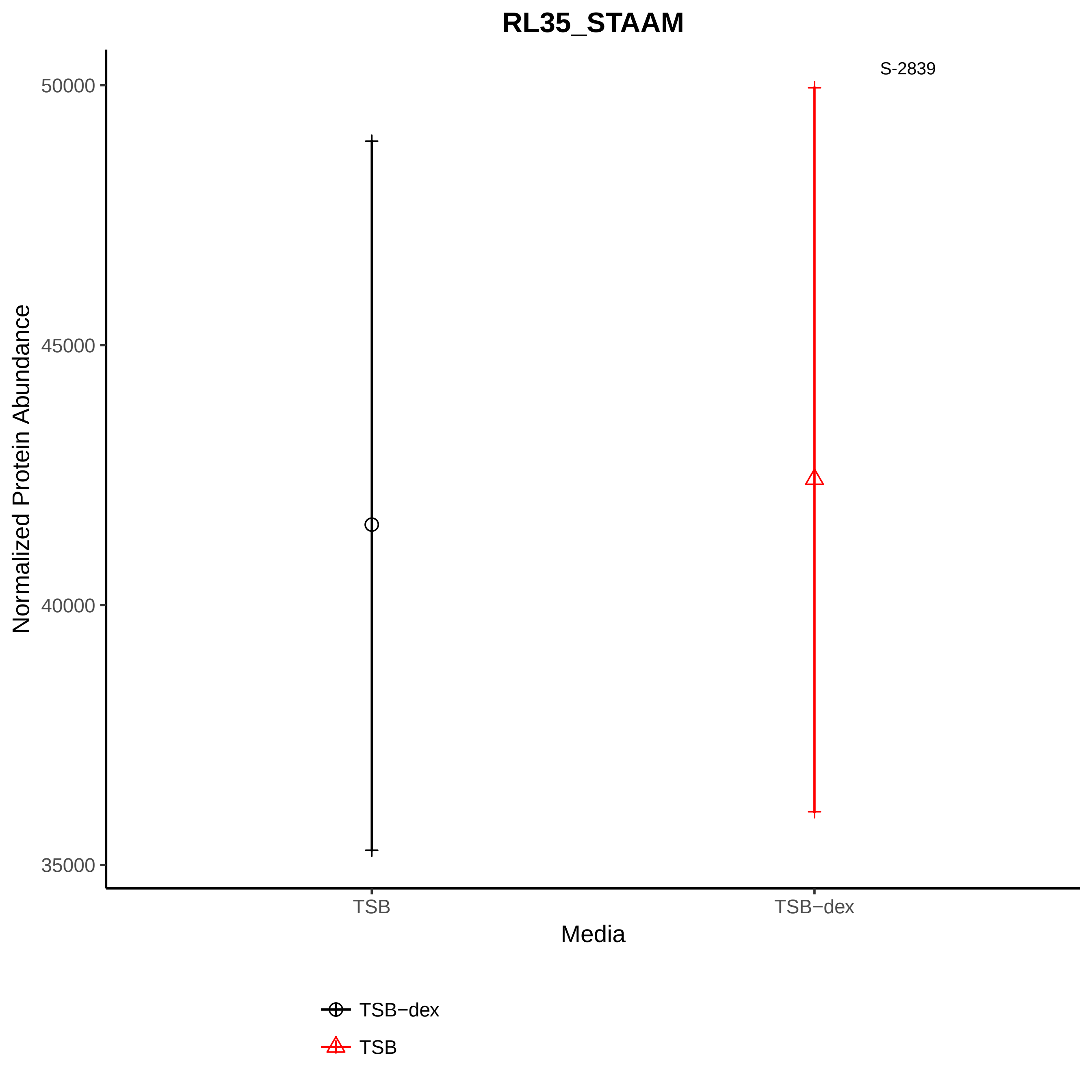

RL35_STAAM
S-2839
50000
45000
40000
35000
Normalized Protein Abundance
TSB
TSB−dex
Media
TSB−dex
TSB

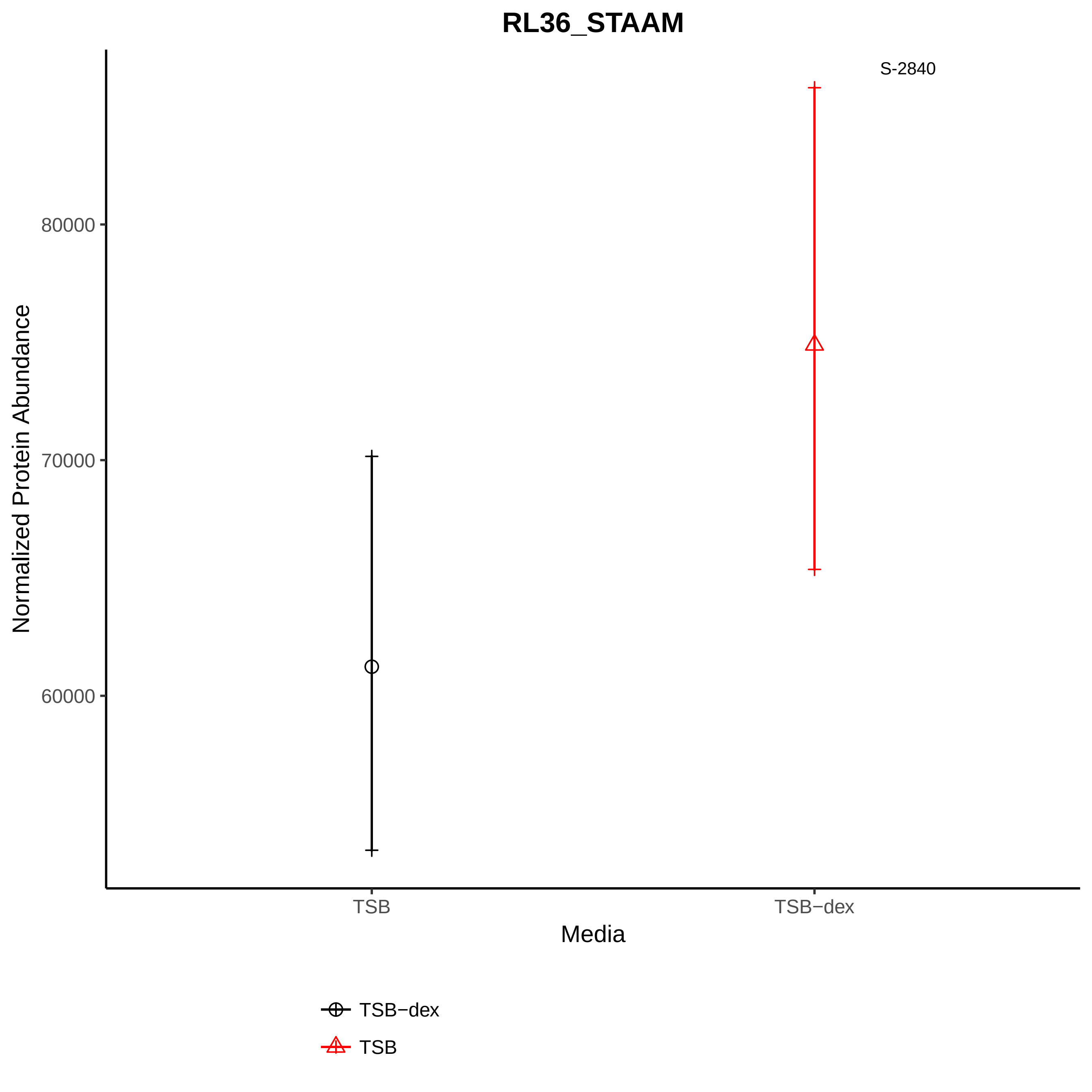
RL36_STAAM
S-2840
Normalized Protein Abundance
80000
70000
60000
TSB
TSB−dex
Media
TSB−dex
TSB

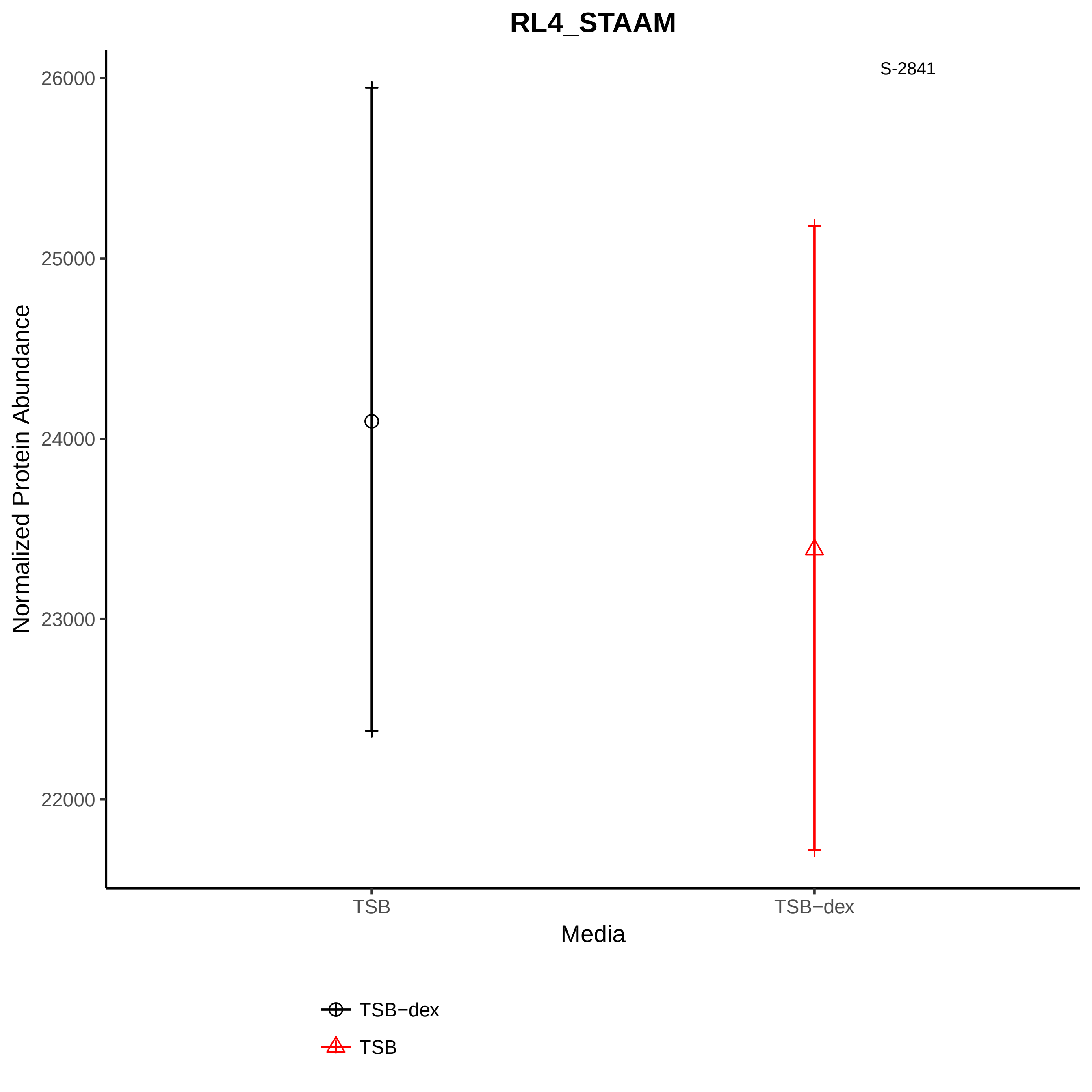
RL4_STAAM
S-2841
Normalized Protein Abundance
26000
25000
24000
23000
22000
TSB
TSB−dex
Media
TSB−dex
TSB

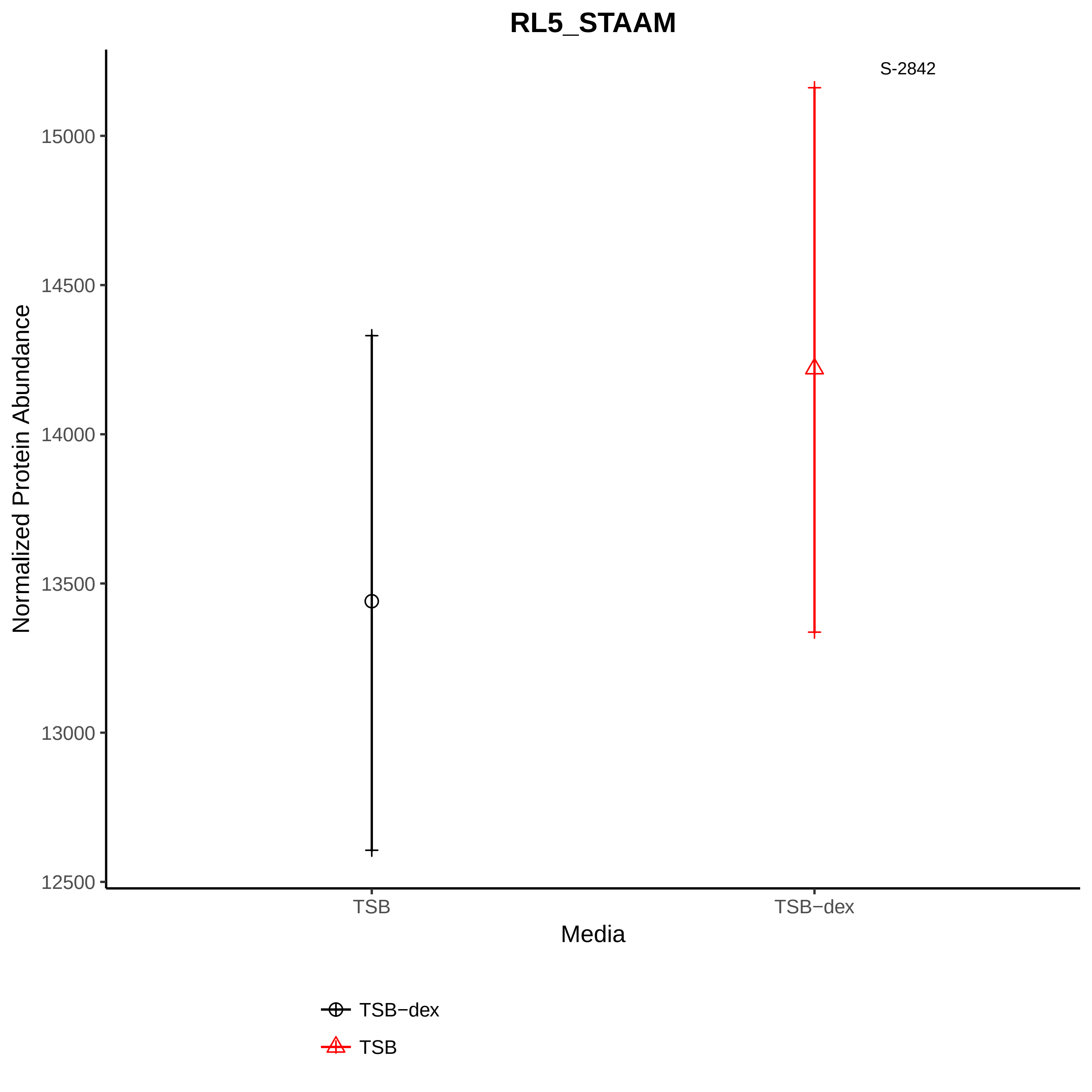

RL5_STAAM
S-2842
Normalized Protein Abundance
15000
14500
14000
13500
13000
12500
TSB
TSB−dex
Media
TSB−dex
TSB

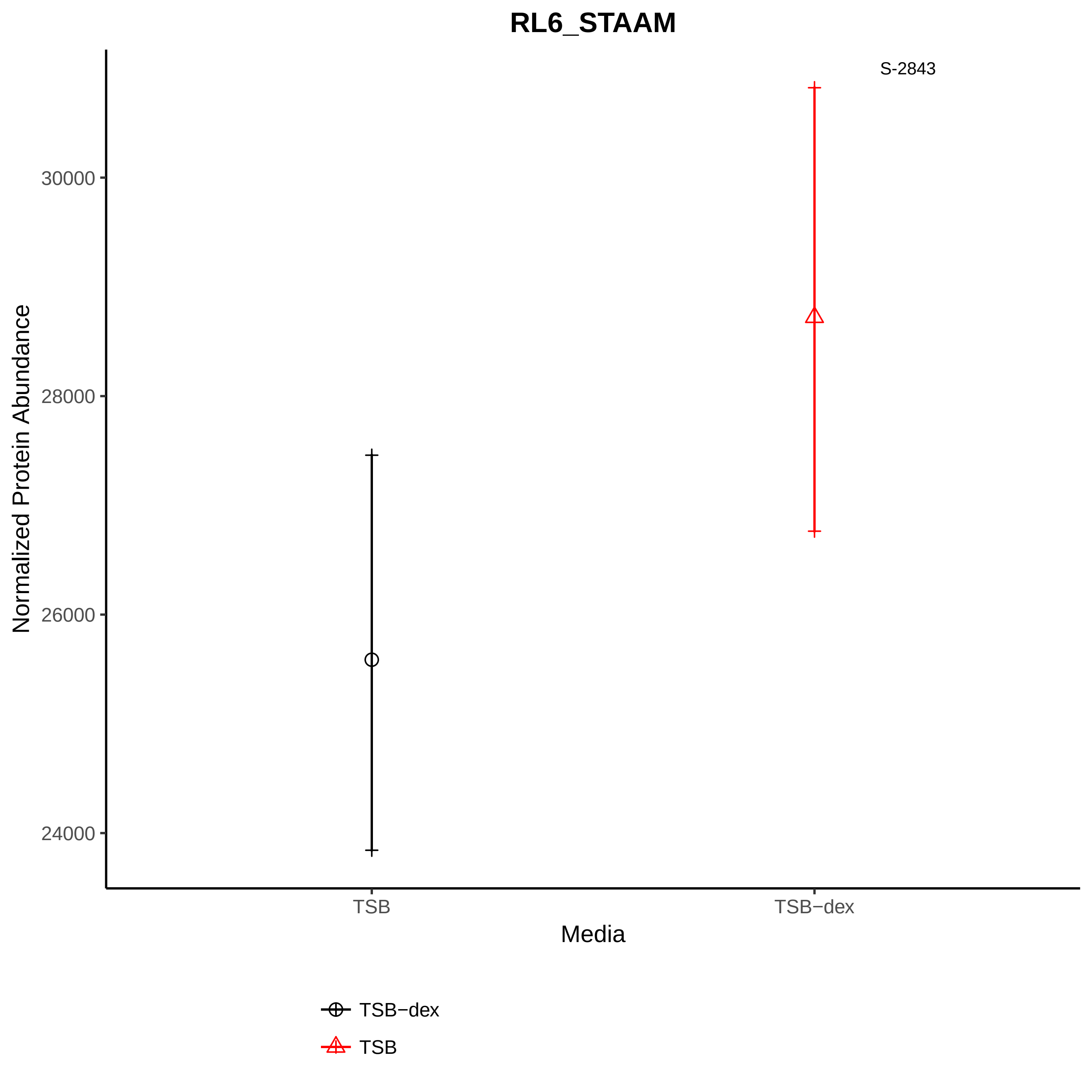

RL6_STAAM
S-2843
Normalized Protein Abundance
30000
28000
26000
24000
TSB
TSB−dex
Media
TSB−dex
TSB

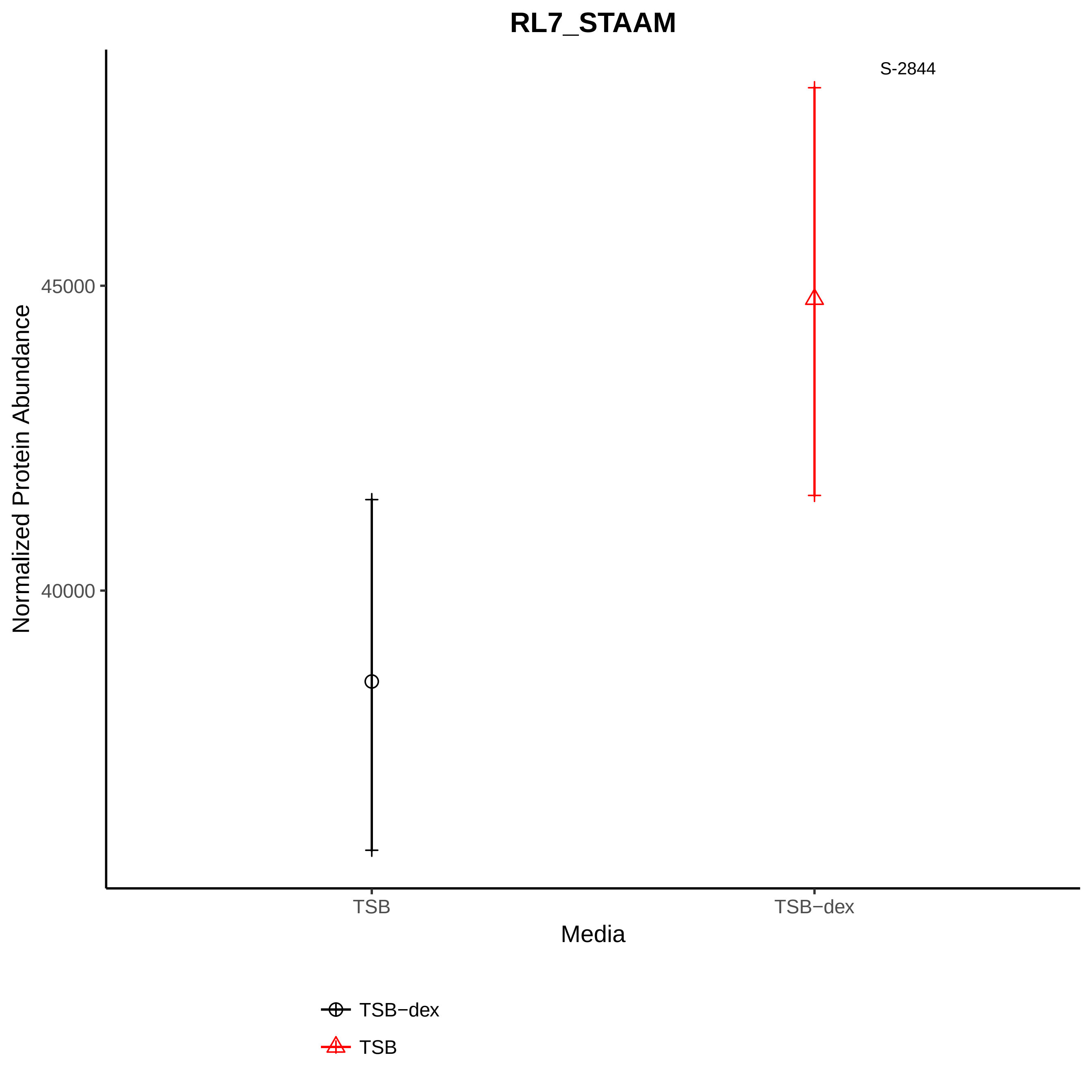

RL7_STAAM
S-2844
Normalized Protein Abundance
45000
40000
TSB
TSB−dex
Media
TSB−dex
TSB

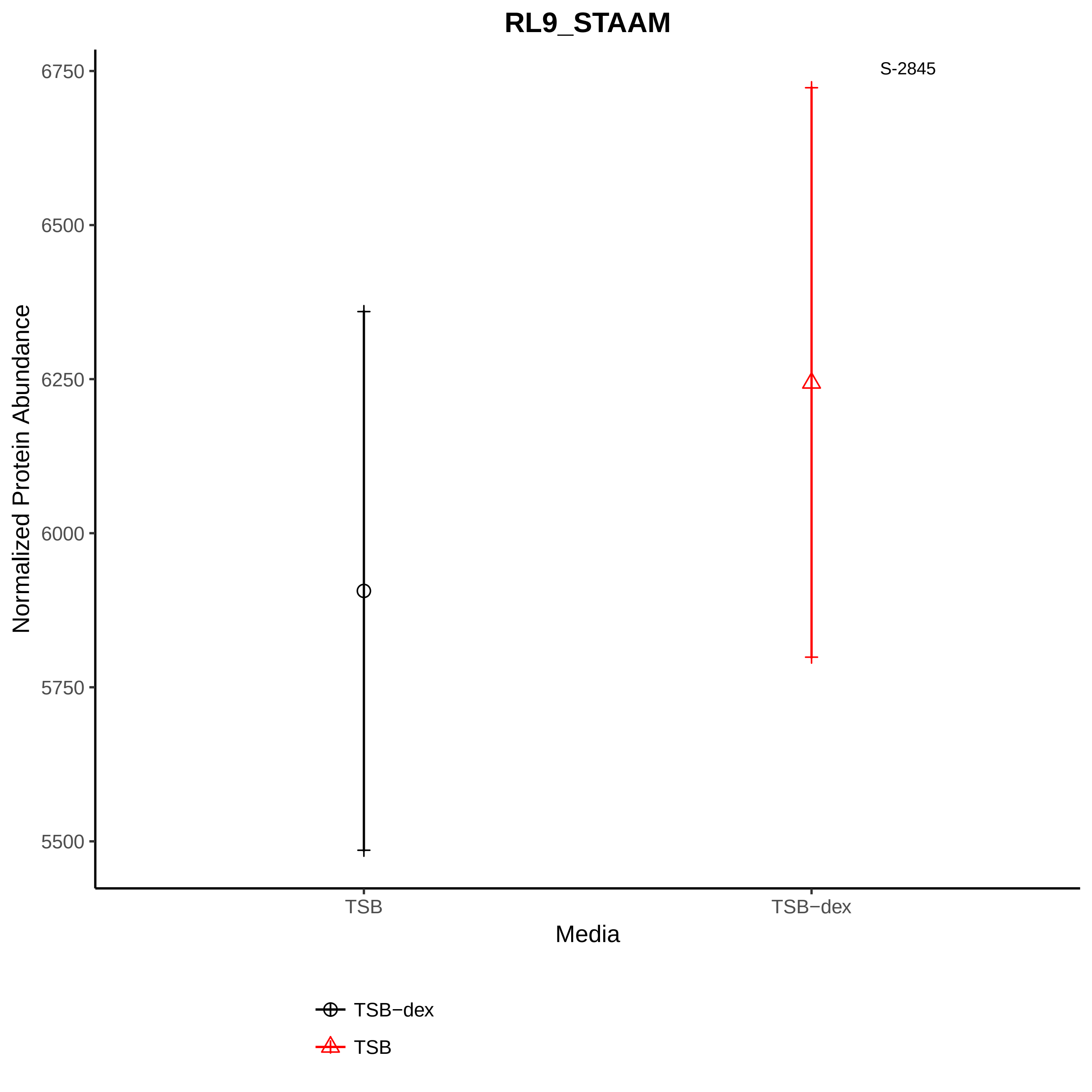
RL9_STAAM
S-2845
Normalized Protein Abundance
6750
6500
6250
6000
5750
5500
TSB
TSB−dex
Media
TSB−dex
TSB

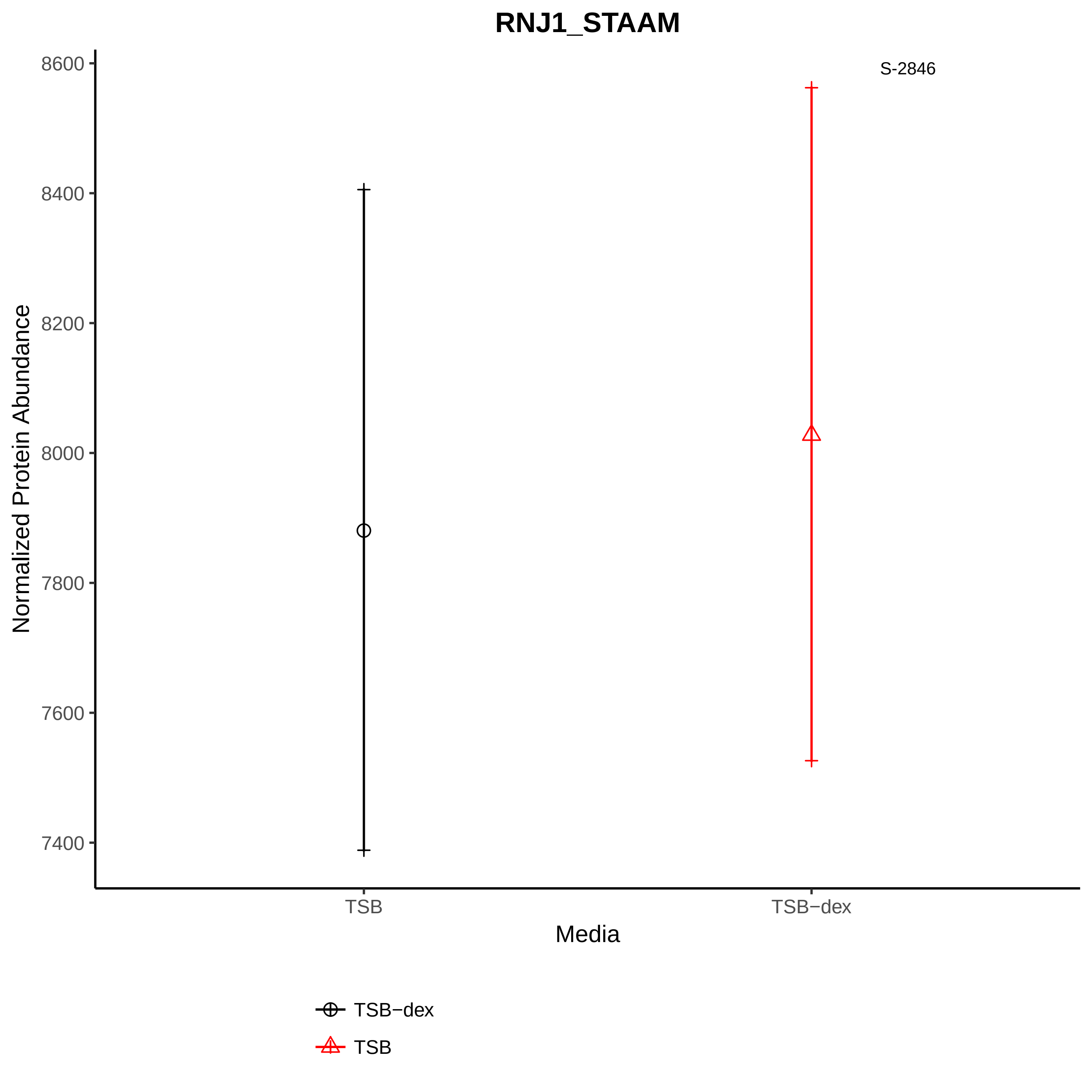

RNJ1_STAAM
S-2846
Normalized Protein Abundance
8600
8400
8200
8000
7800
7600
7400
TSB
TSB−dex
Media
TSB−dex
TSB

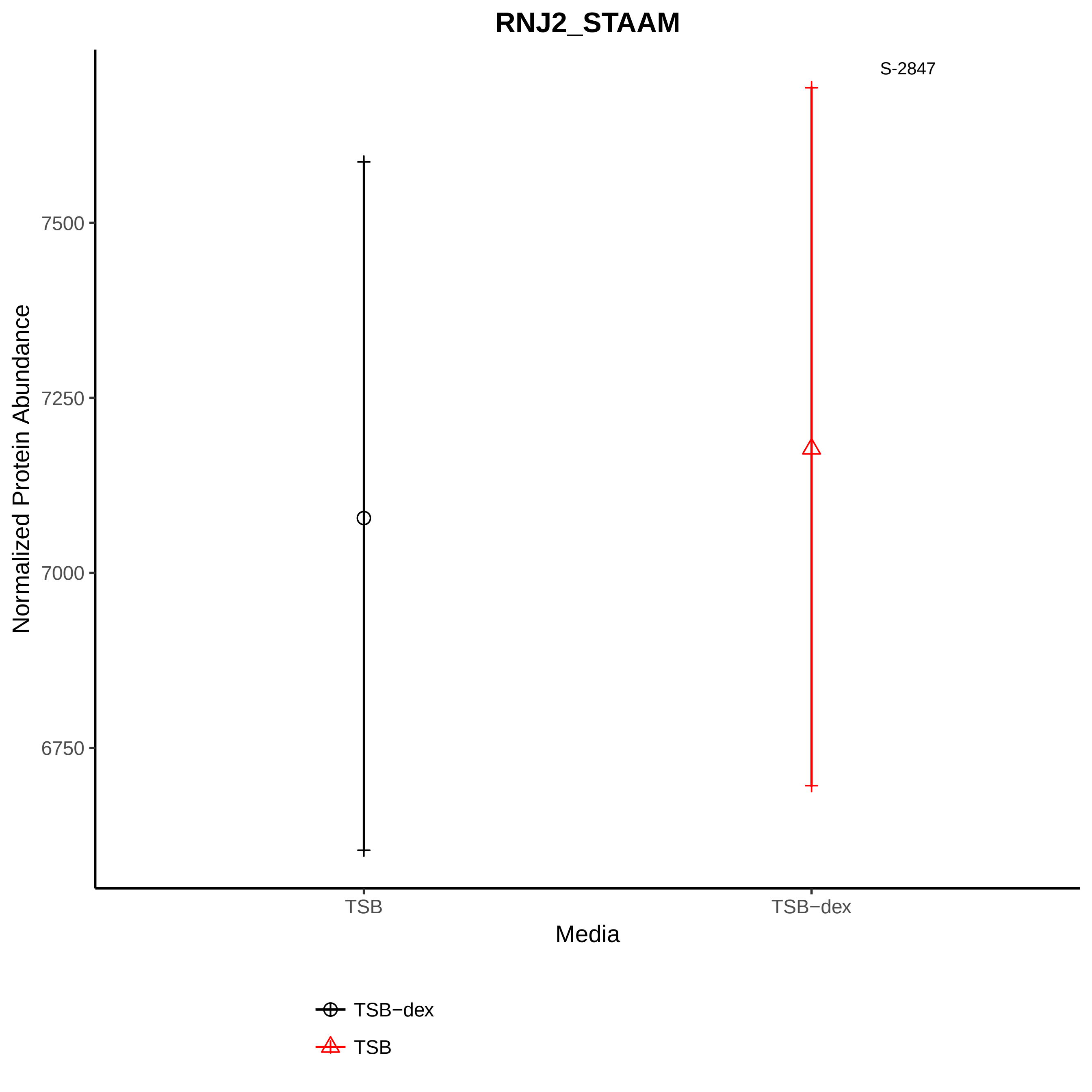

RNJ2_STAAM
S-2847
Normalized Protein Abundance
7500
7250
7000
6750
TSB
TSB−dex
Media
TSB−dex
TSB

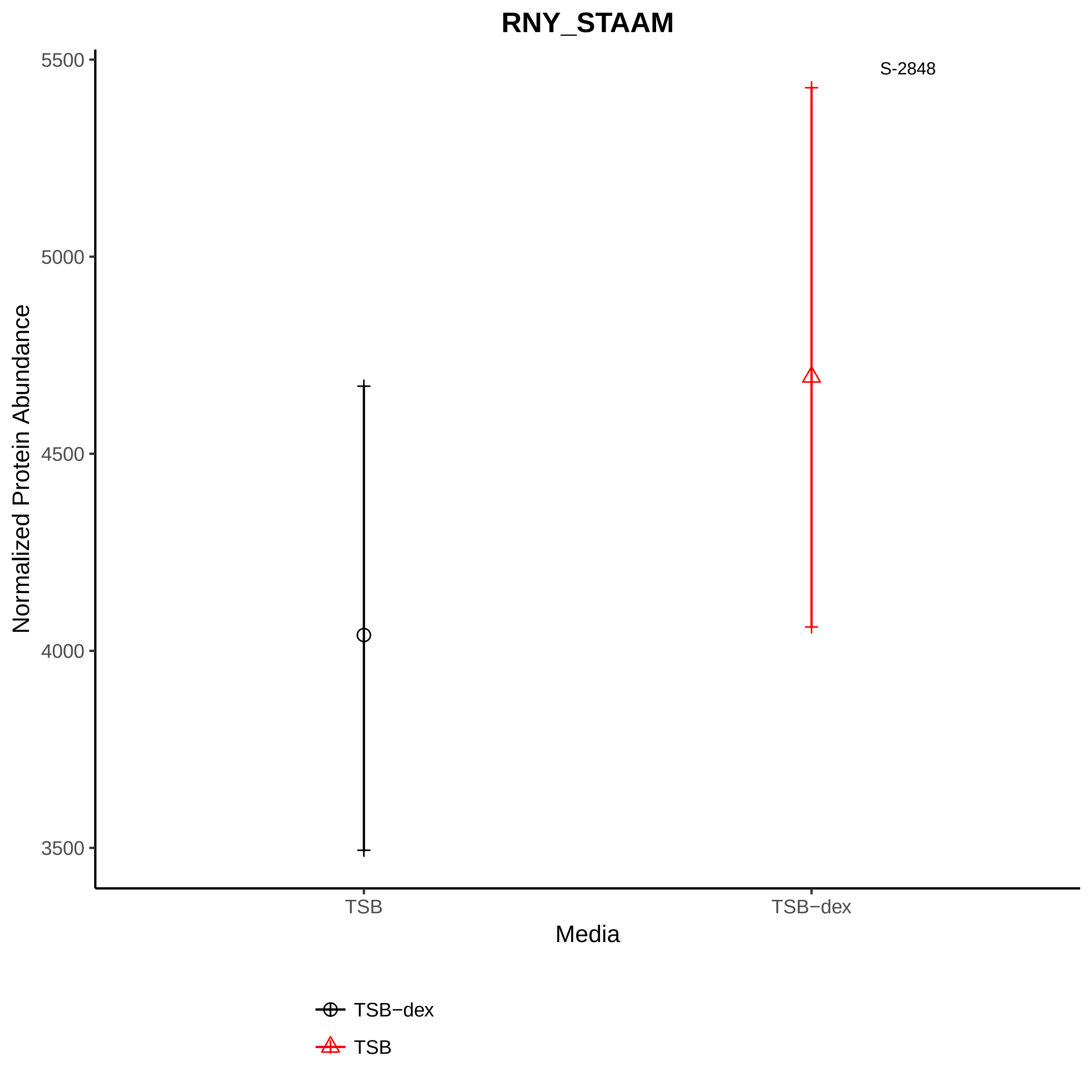

RNY_STAAM
S-2848
Normalized Protein Abundance
5500
5000
4500
4000
3500
TSB
TSB−dex
Media
TSB−dex
TSB

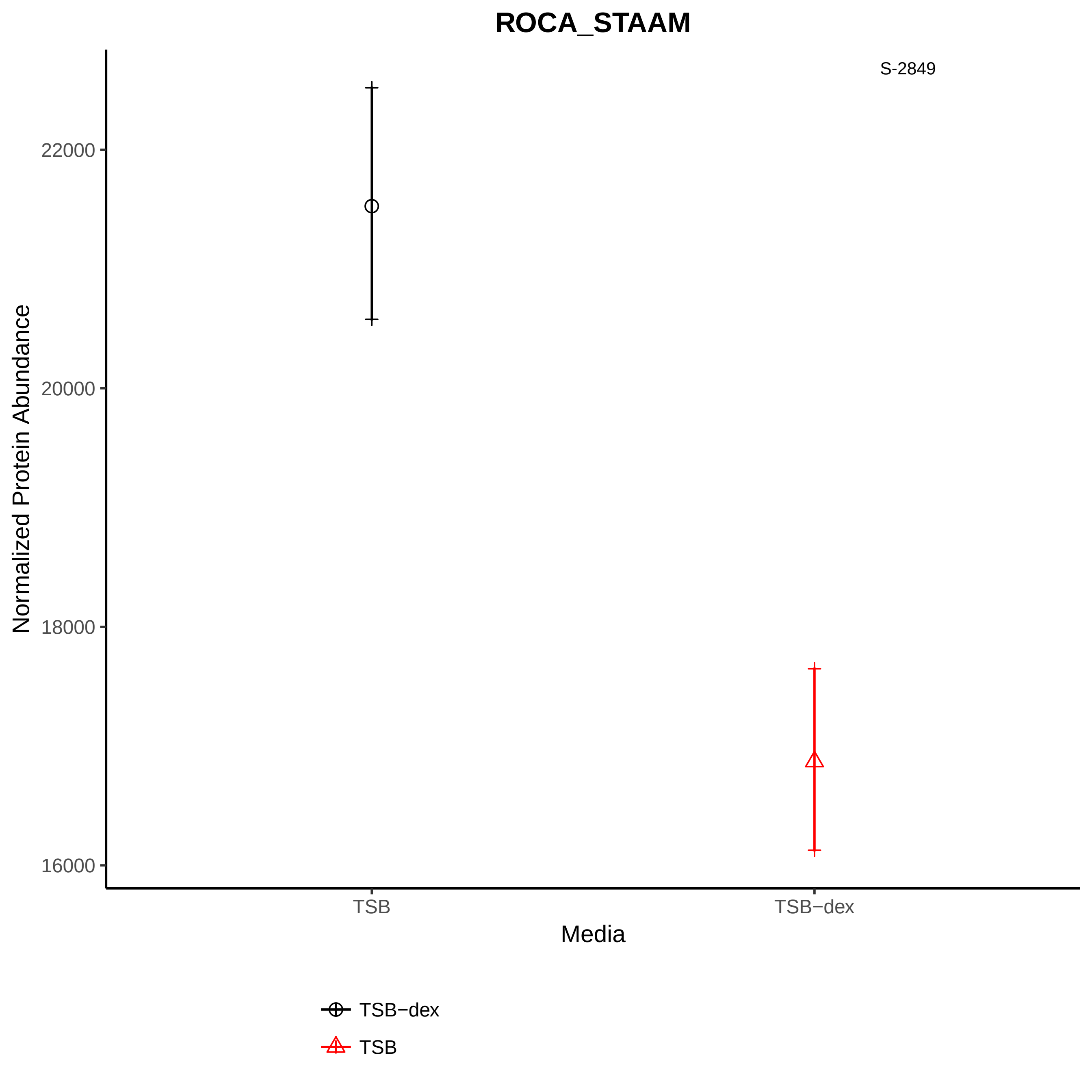

ROCA_STAAM
S-2849
22000
20000
18000
16000
Normalized Protein Abundance
TSB
TSB−dex
Media
TSB−dex
TSB

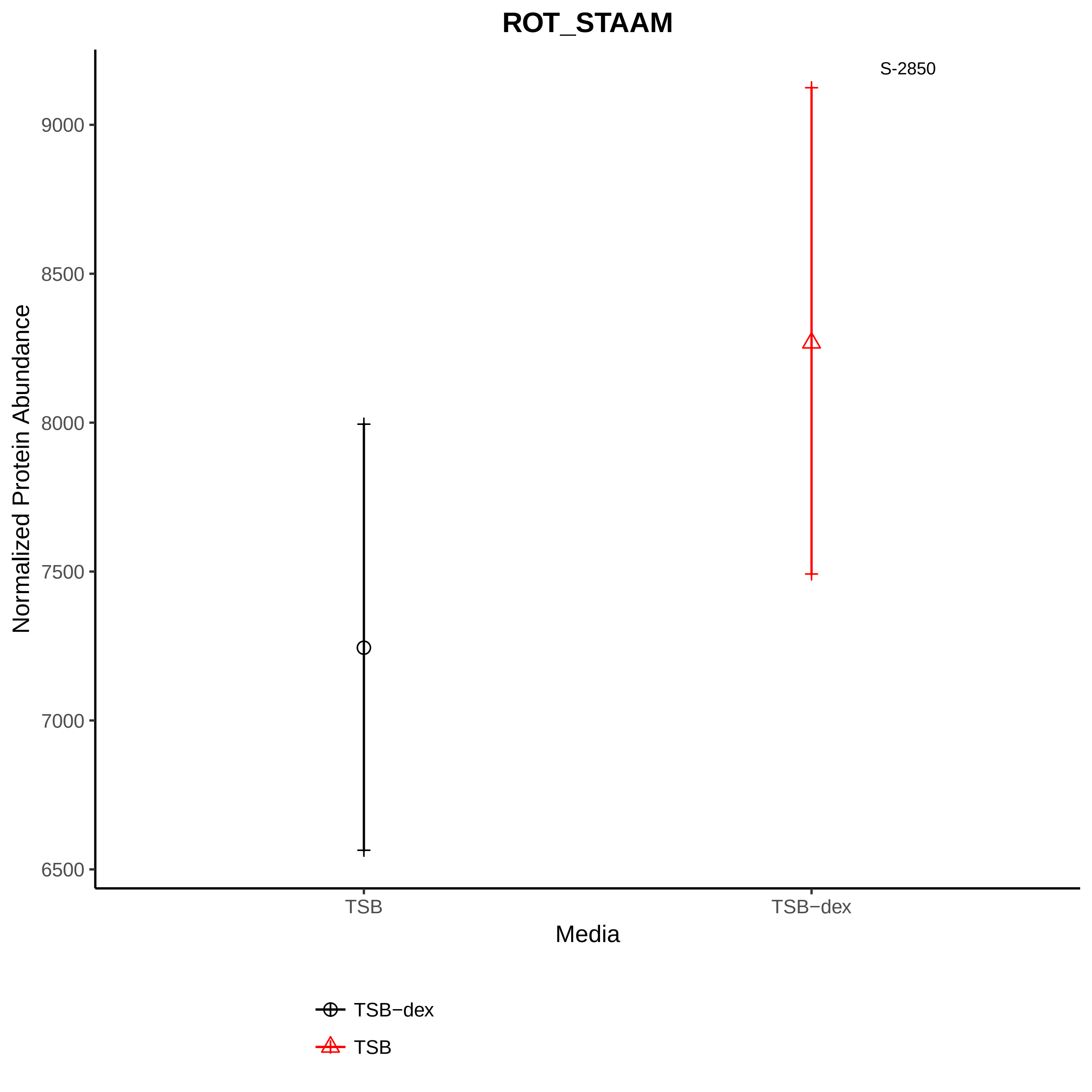

ROT_STAAM
S-2850
Normalized Protein Abundance
9000
8500
8000
7500
7000
6500
TSB
TSB−dex
Media
TSB−dex
TSB

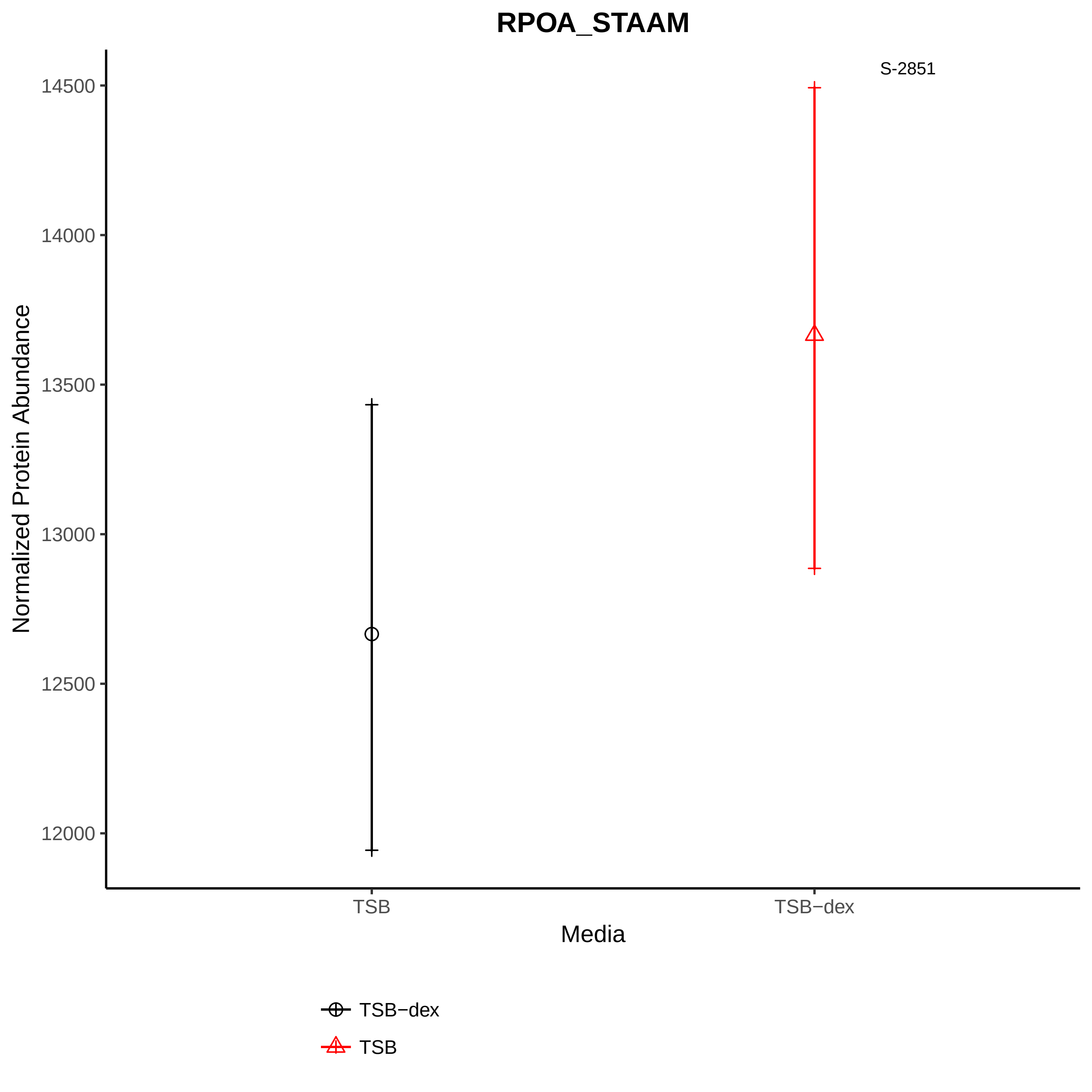

RPOA_STAAM
S-2851
14500
14000
13500
13000
12500
12000
Normalized Protein Abundance
TSB
TSB−dex
Media
TSB−dex
TSB

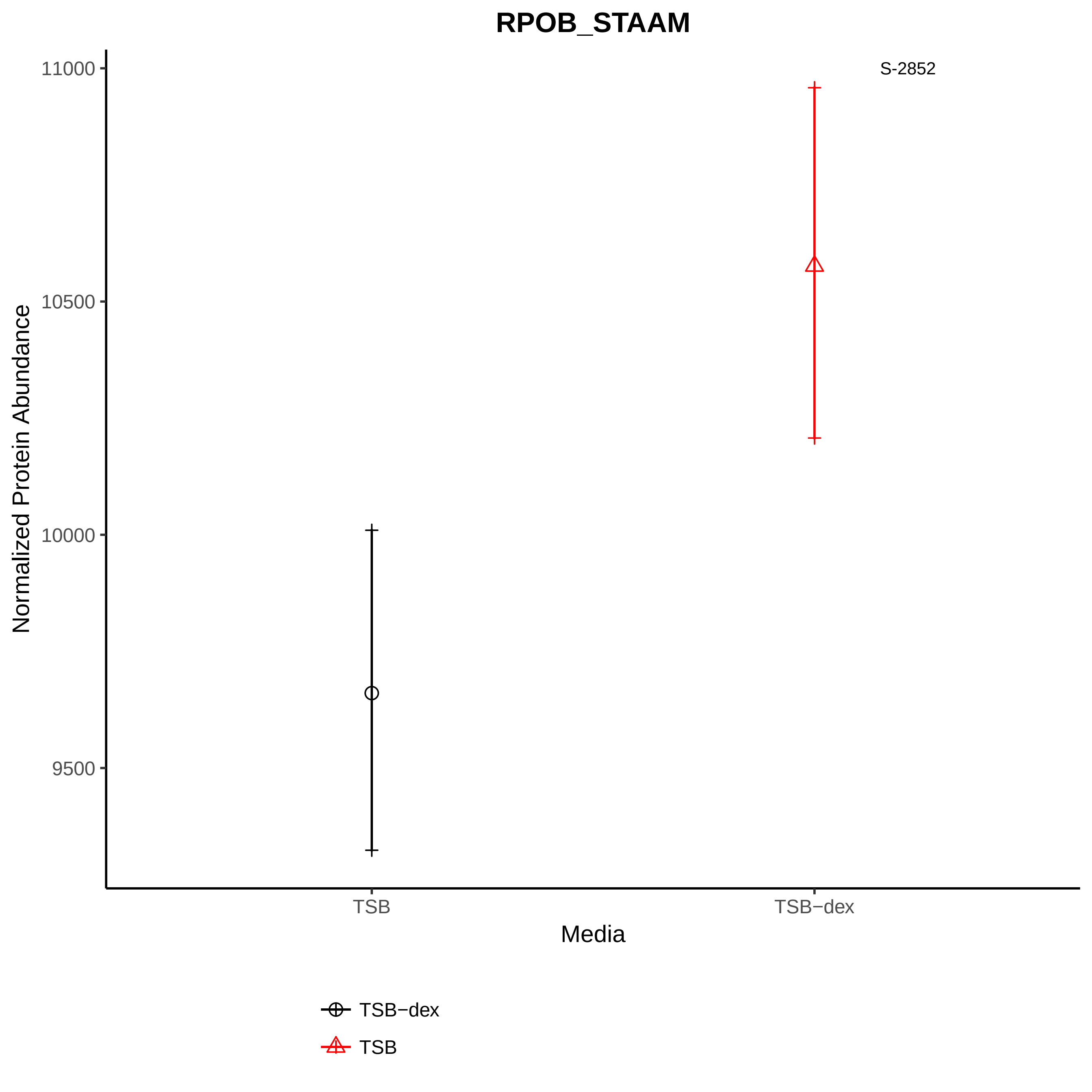
RPOB_STAAM
S-2852
11000
10500
10000
9500
Normalized Protein Abundance
TSB
TSB−dex
Media
TSB−dex
TSB

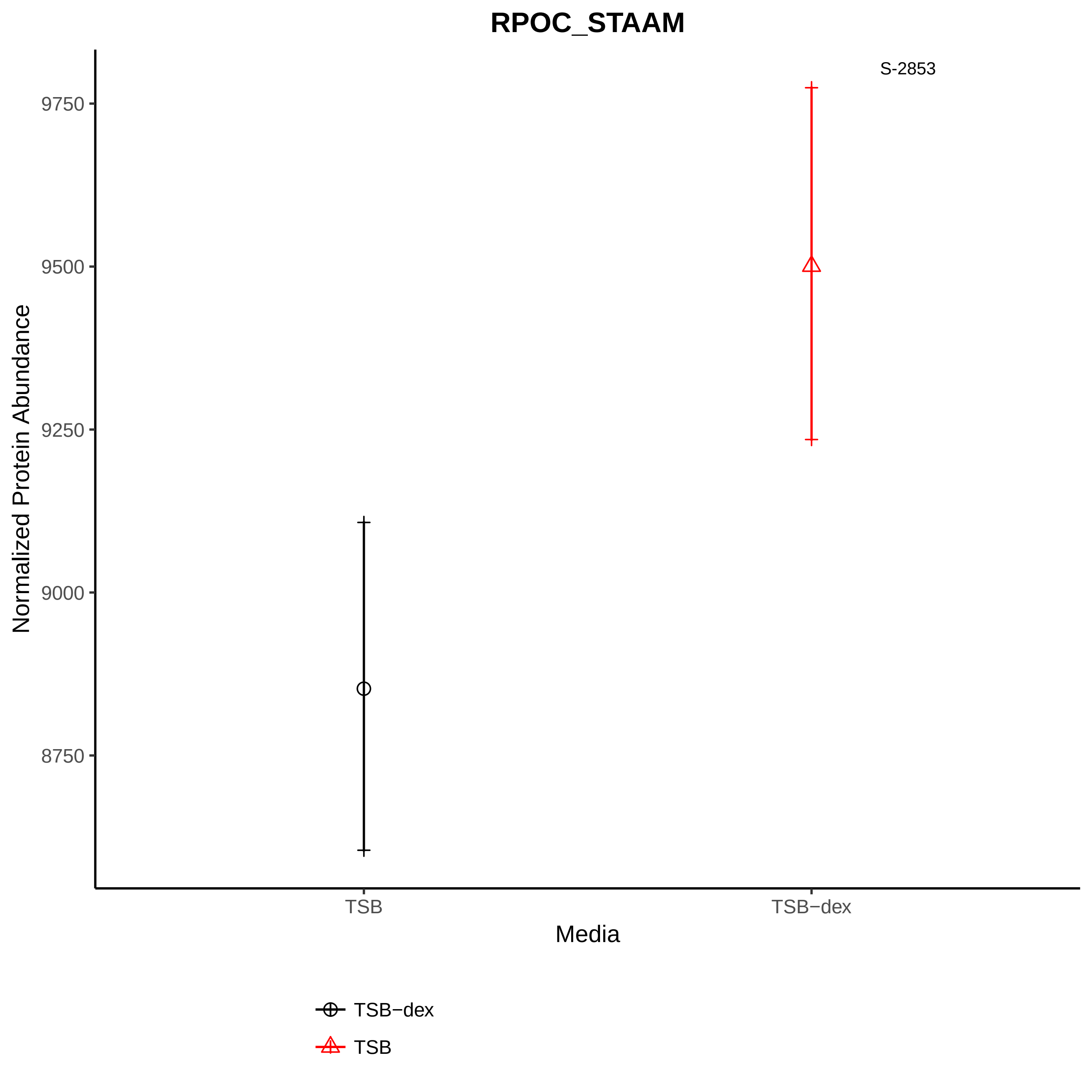
RPOC_STAAM
S-2853
Normalized Protein Abundance
9750
9500
9250
9000
8750
TSB
TSB−dex
Media
TSB−dex
TSB

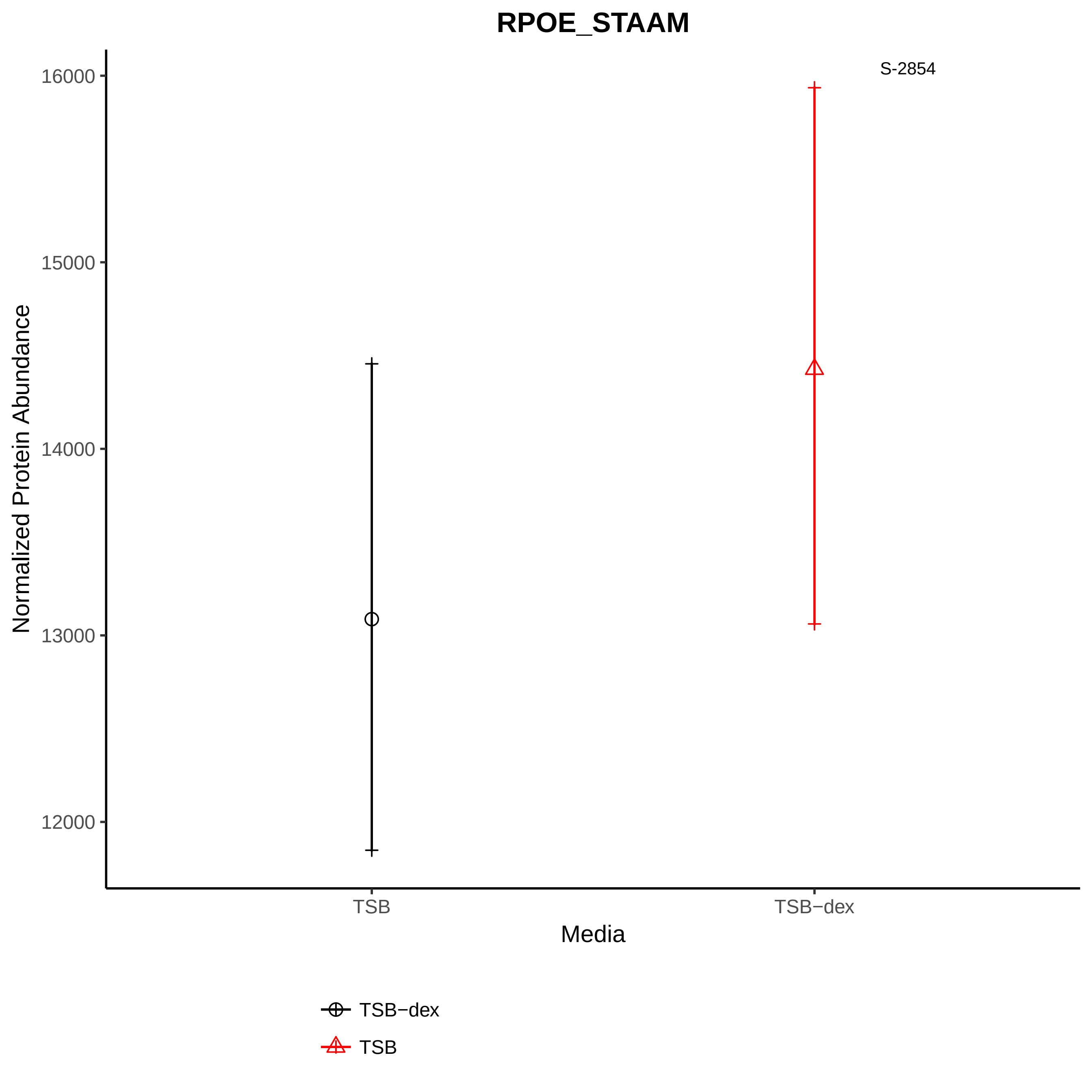

RPOE_STAAM
S-2854
Normalized Protein Abundance
16000
15000
14000
13000
12000
TSB
TSB−dex
Media
TSB−dex
TSB

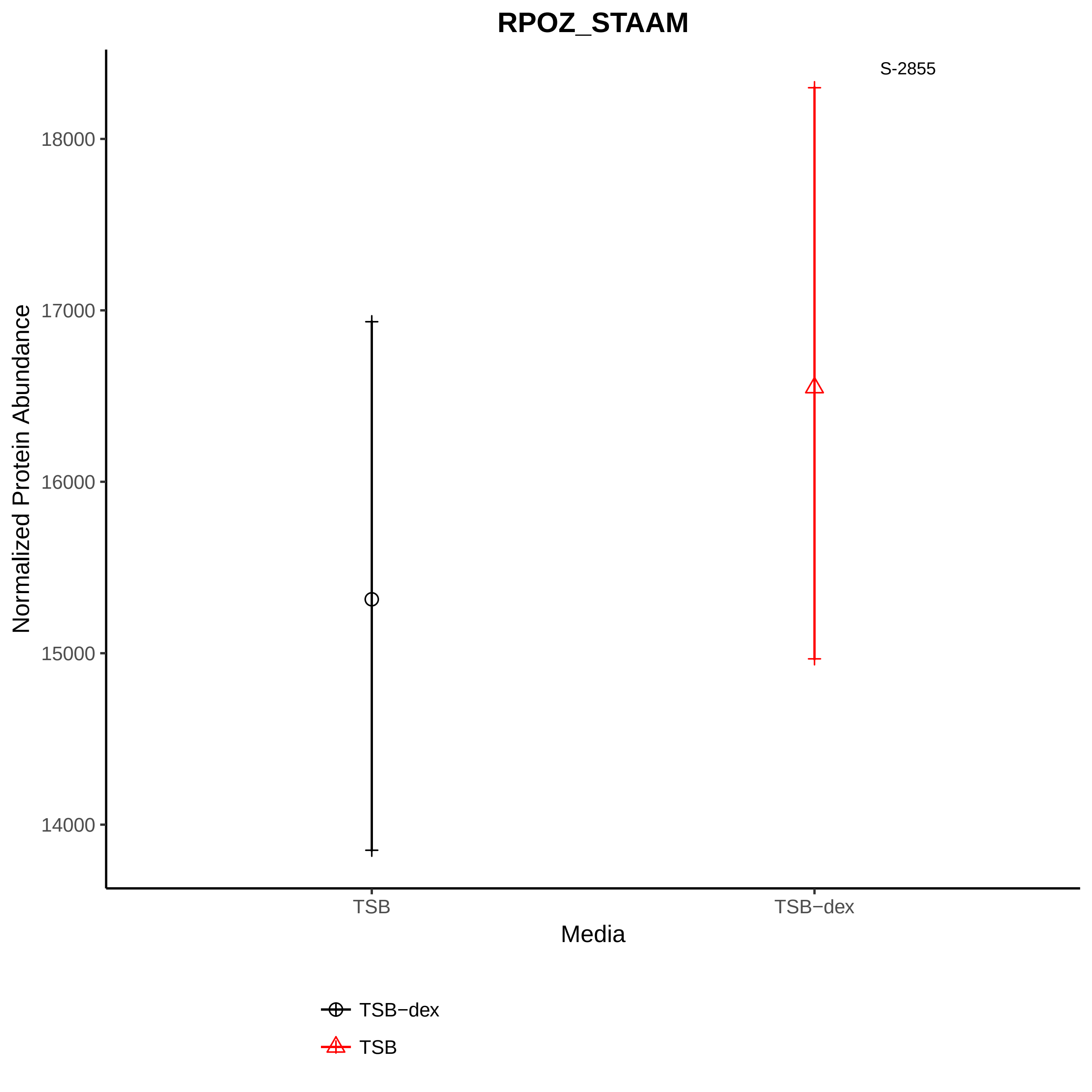
RPOZ_STAAM
S-2855
Normalized Protein Abundance
18000
17000
16000
15000
14000
TSB
TSB−dex
Media
TSB−dex
TSB

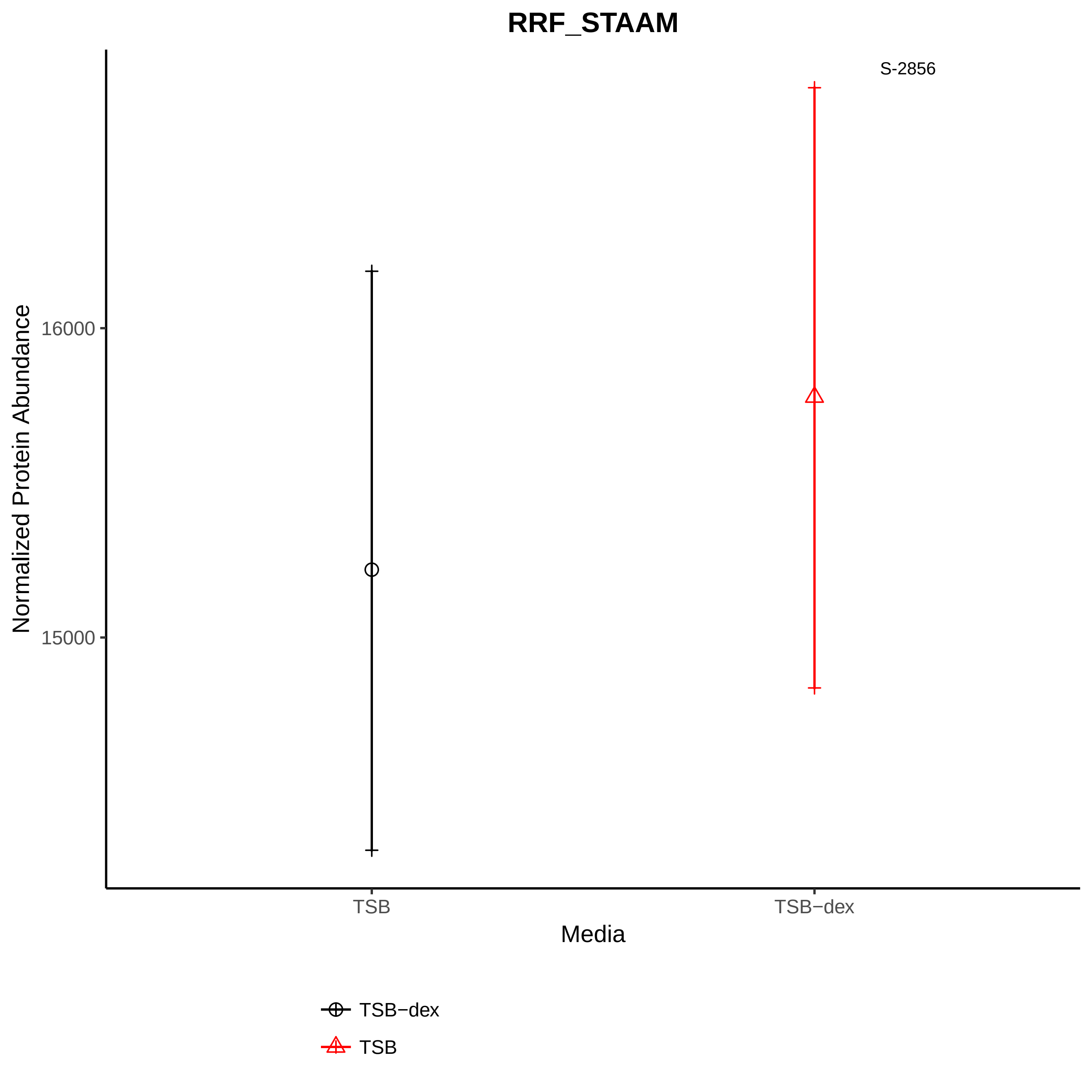

RRF_STAAM
S-2856
Normalized Protein Abundance
16000
15000
TSB
TSB−dex
Media
TSB−dex
TSB

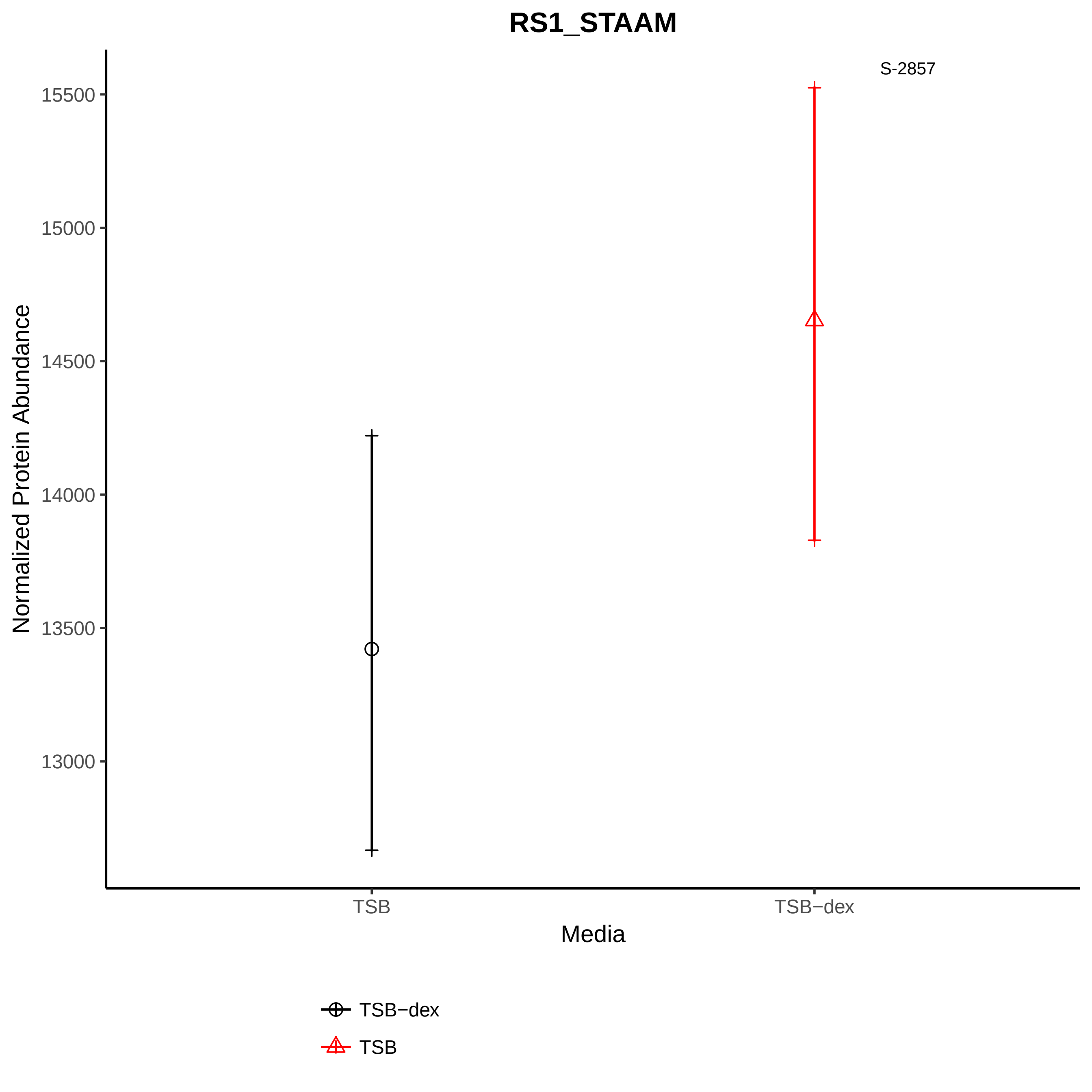

RS1_STAAM
S-2857
Normalized Protein Abundance
15500
15000
14500
14000
13500
13000
TSB
TSB−dex
Media
TSB−dex
TSB

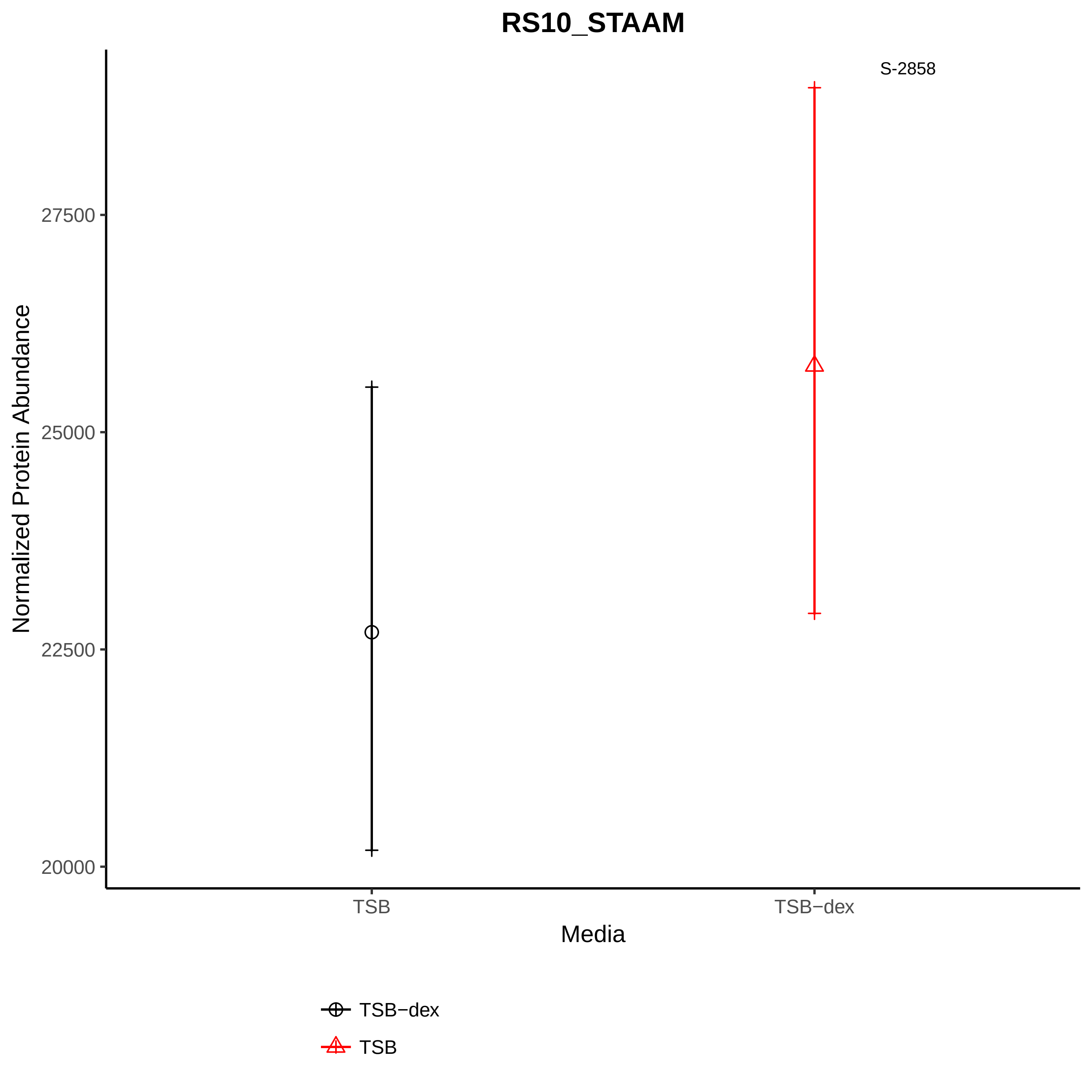
RS10_STAAM
S-2858
Normalized Protein Abundance
27500
25000
22500
20000
TSB
TSB−dex
Media
TSB−dex
TSB

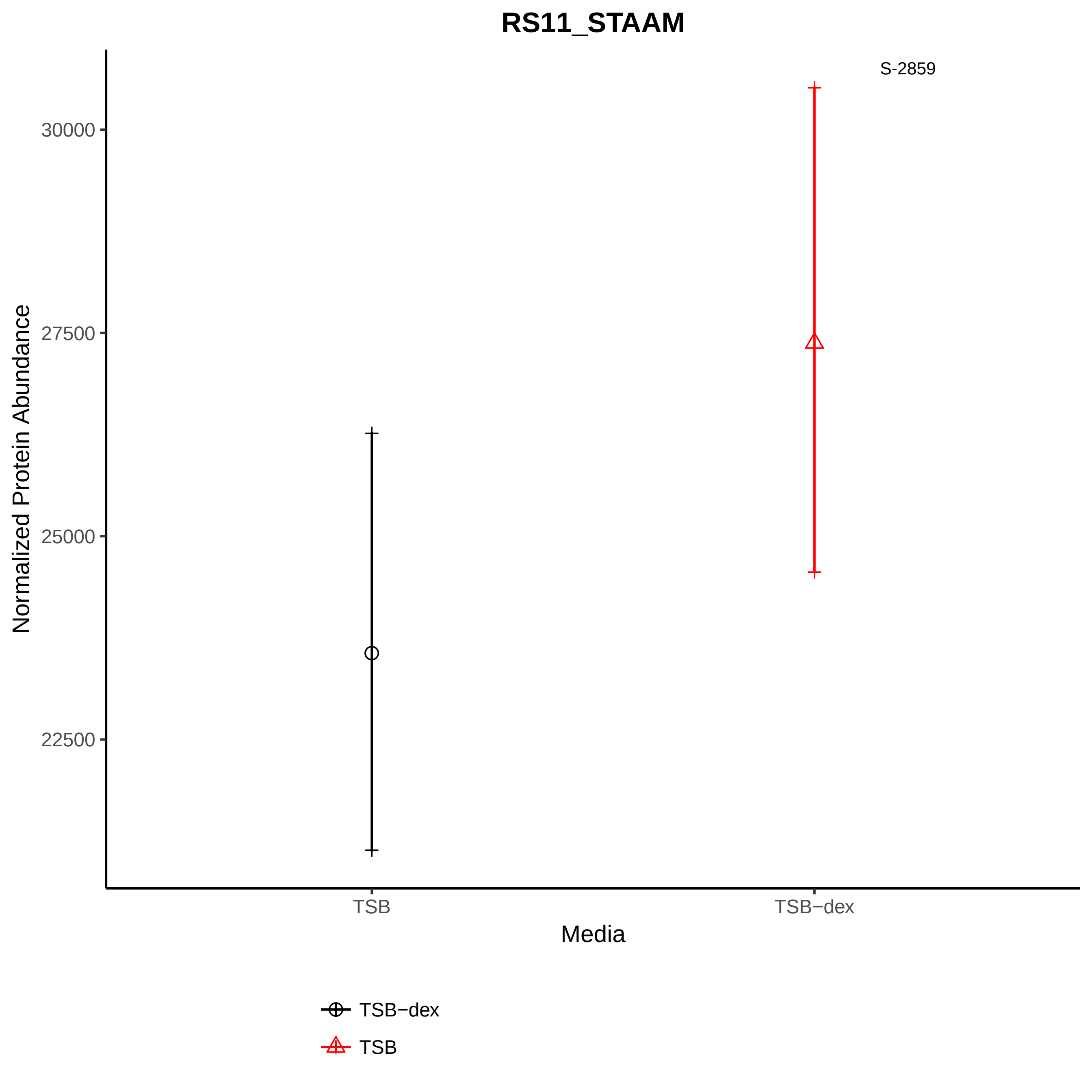

RS11_STAAM
S-2859
30000
27500
25000
22500
Normalized Protein Abundance
TSB
TSB−dex
Media
TSB−dex
TSB

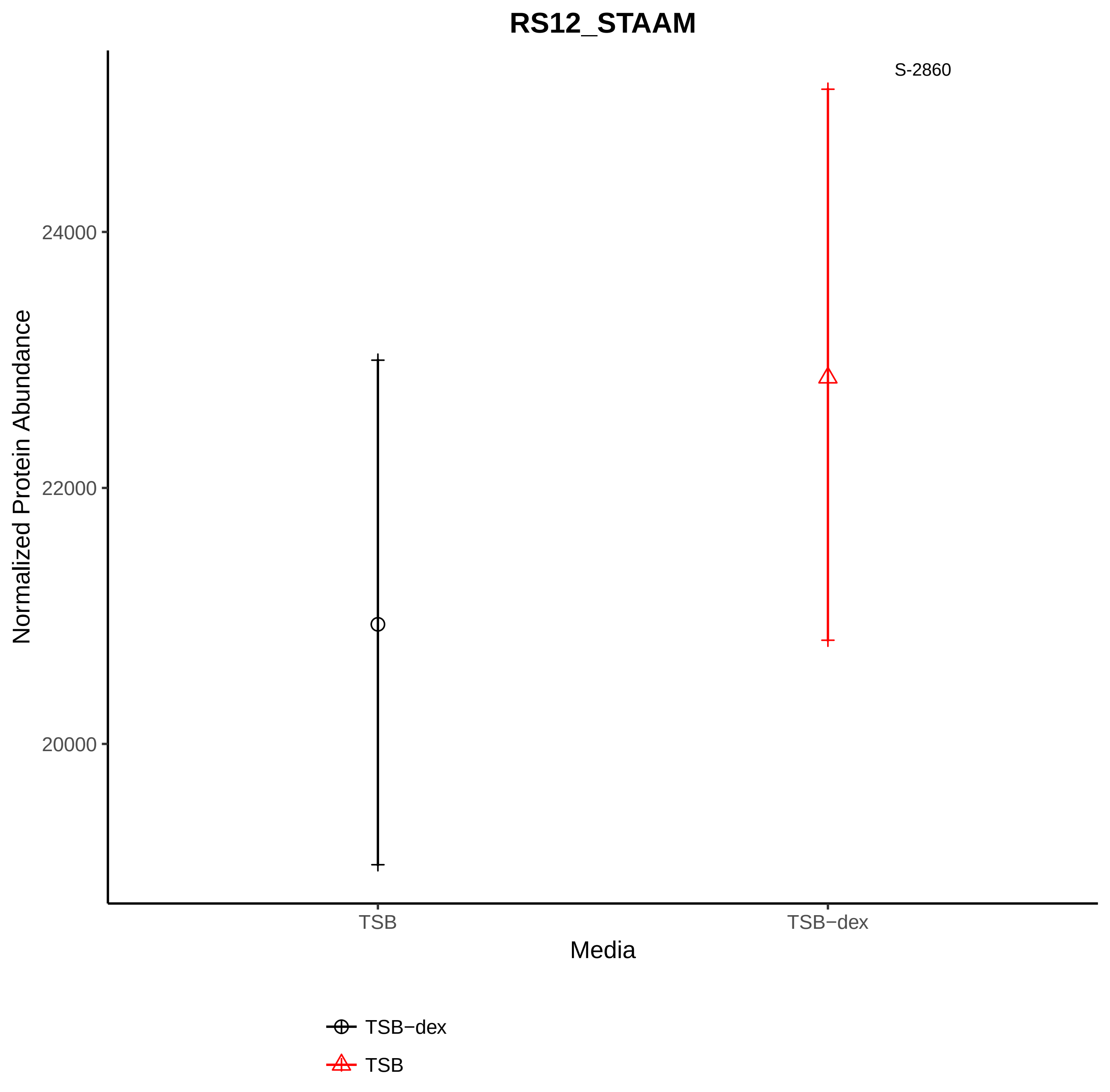

RS12_STAAM
S-2860
Normalized Protein Abundance
24000
22000
20000
TSB
TSB−dex
Media
TSB−dex
TSB

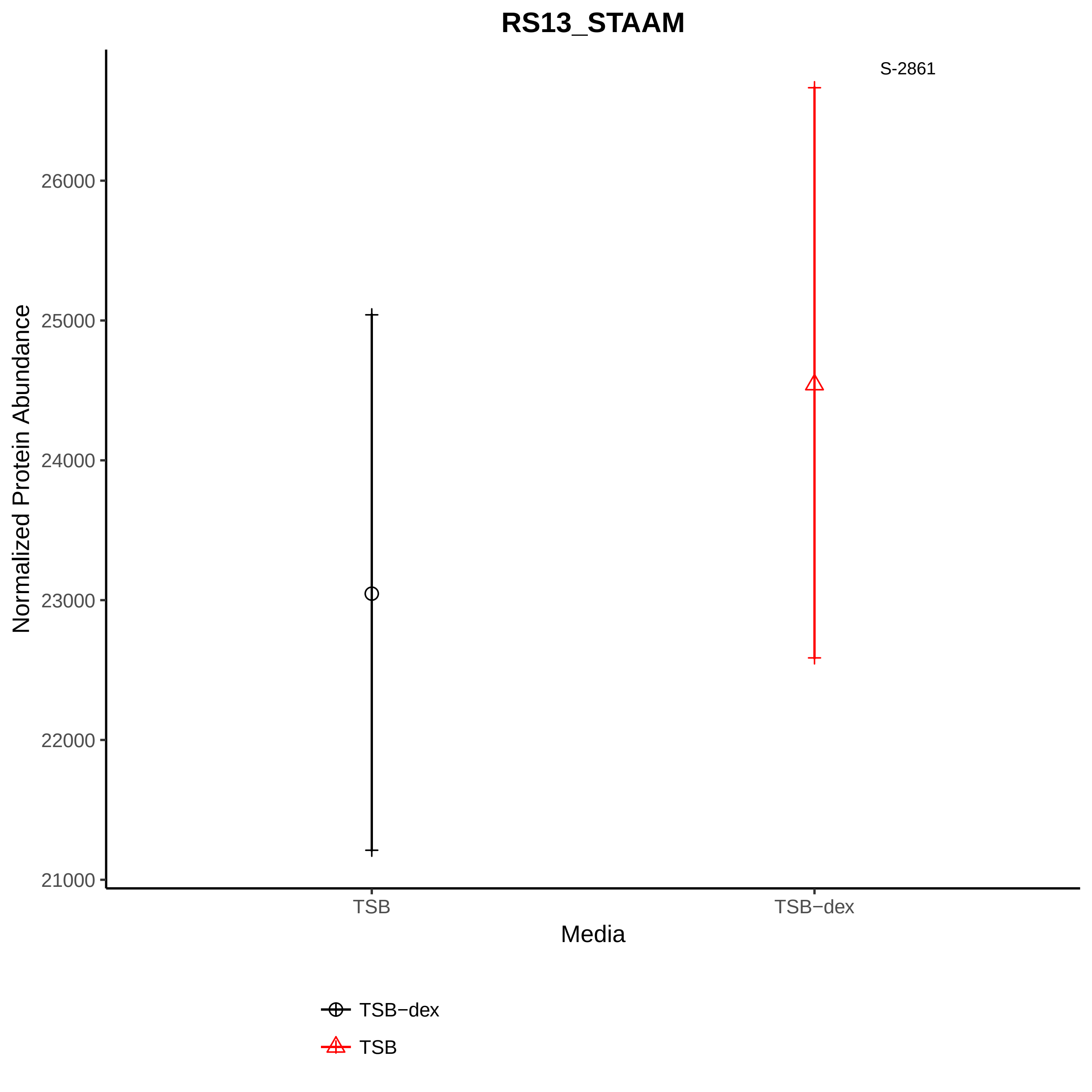

RS13_STAAM
S-2861
Normalized Protein Abundance
26000
25000
24000
23000
22000
21000
TSB
TSB−dex
Media
TSB−dex
TSB

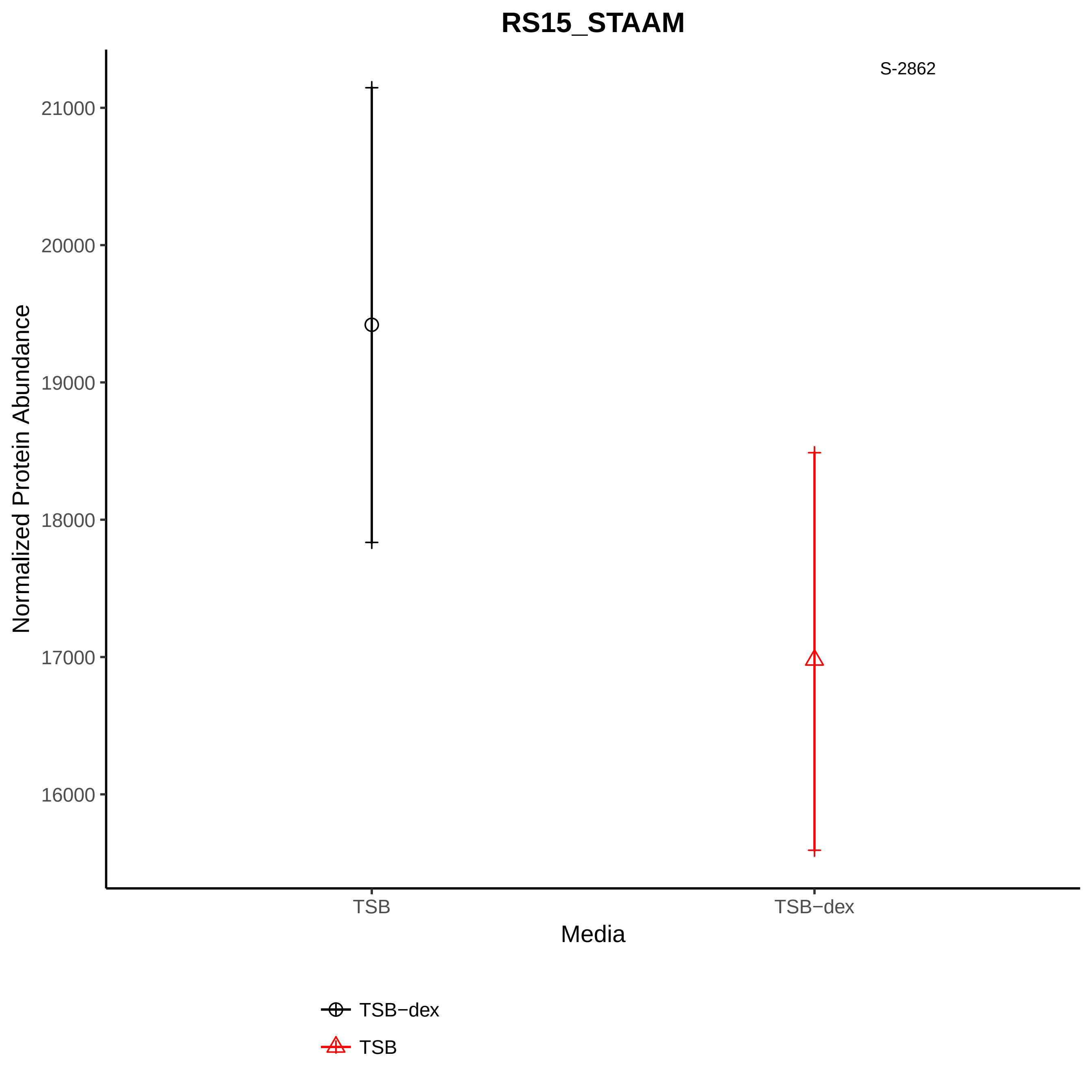
RS15_STAAM
S-2862
Normalized Protein Abundance
21000
20000
19000
18000
17000
16000
TSB
TSB−dex
Media
TSB−dex
TSB

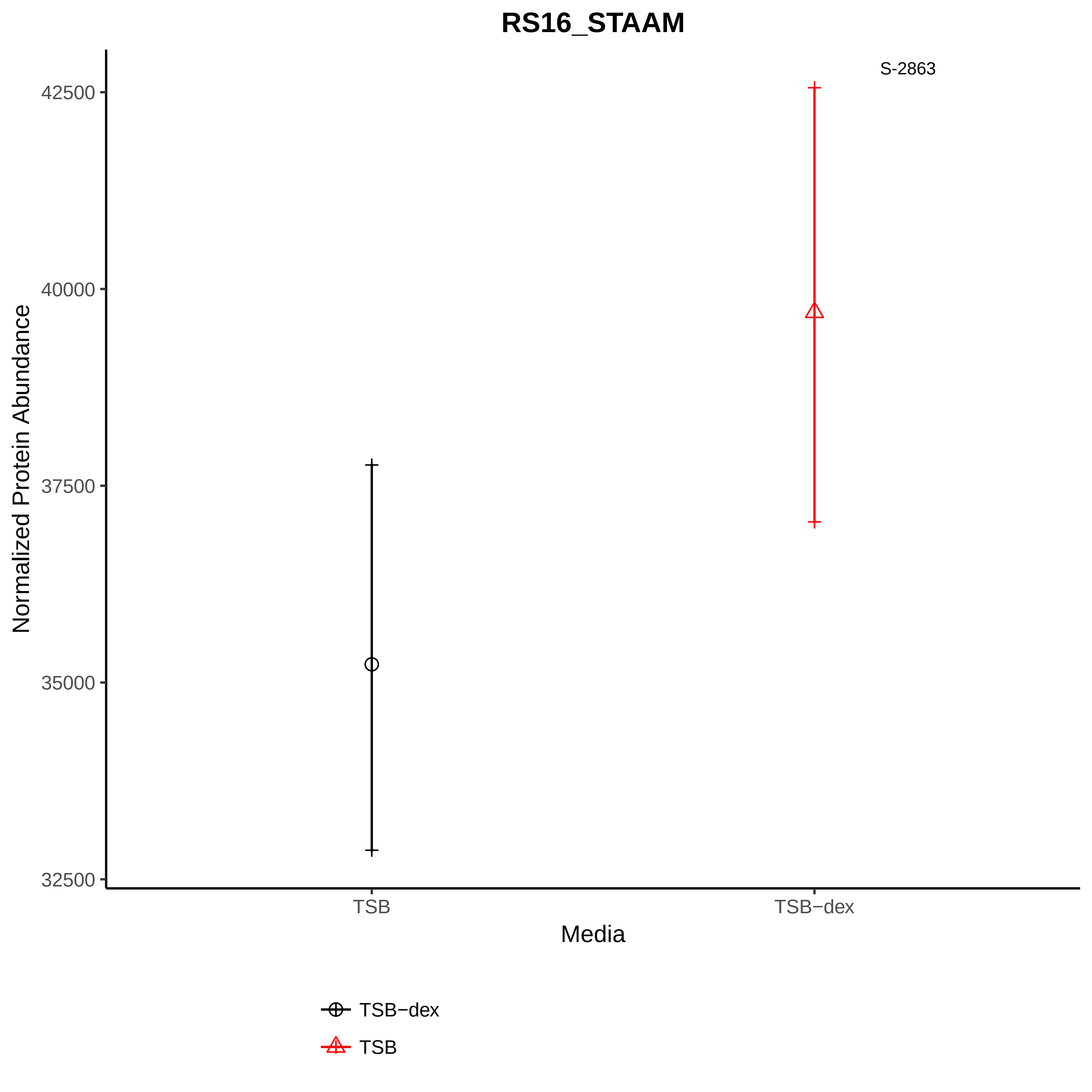

RS16_STAAM
S-2863
42500
40000
37500
35000
32500
Normalized Protein Abundance
TSB
TSB−dex
Media
TSB−dex
TSB

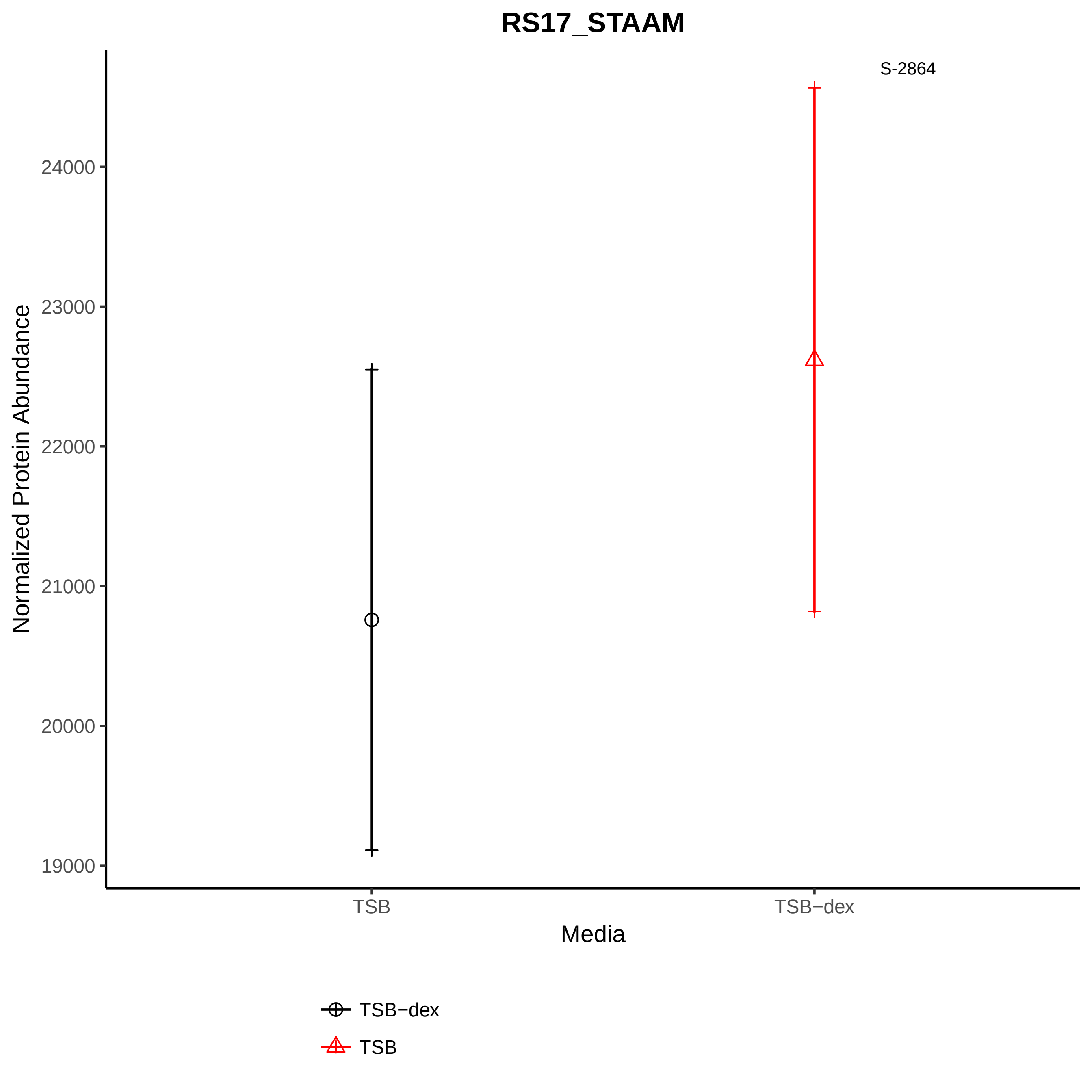

RS17_STAAM
S-2864
Normalized Protein Abundance
24000
23000
22000
21000
20000
19000
TSB
TSB−dex
Media
TSB−dex
TSB

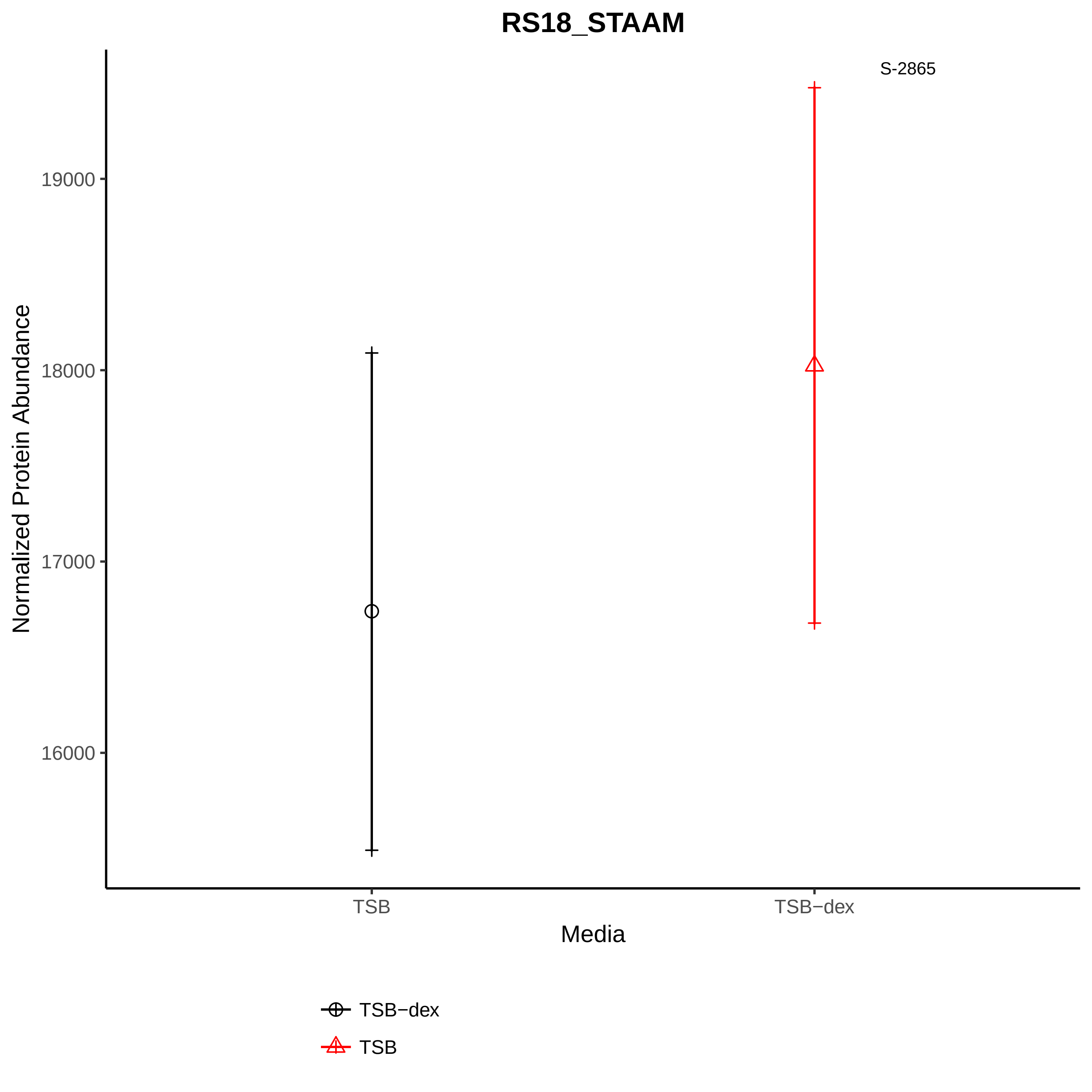

RS18_STAAM
S-2865
19000
18000
17000
16000
Normalized Protein Abundance
TSB
TSB−dex
Media
TSB−dex
TSB

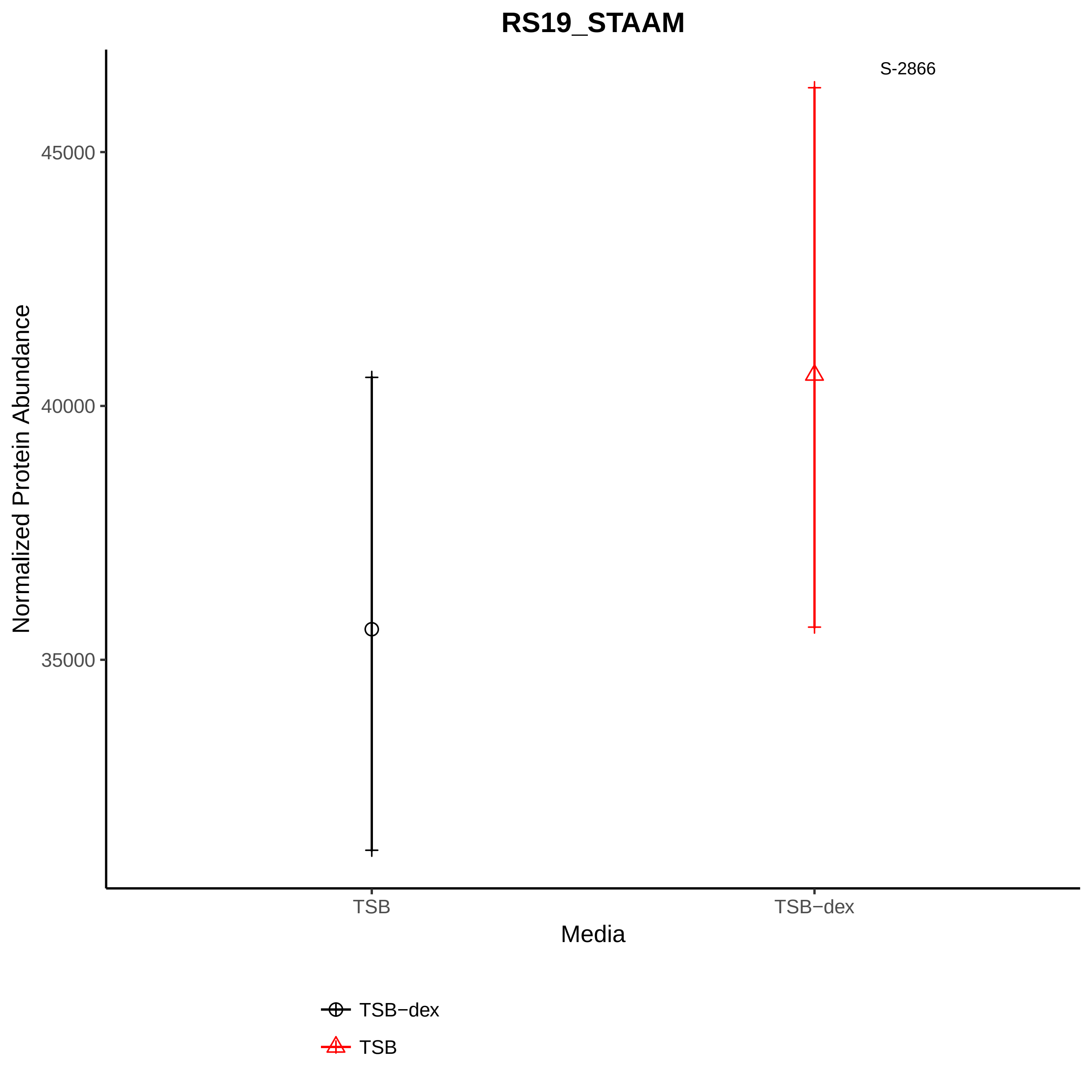

RS19_STAAM
S-2866
45000
40000
35000
Normalized Protein Abundance
TSB
TSB−dex
Media
TSB−dex
TSB

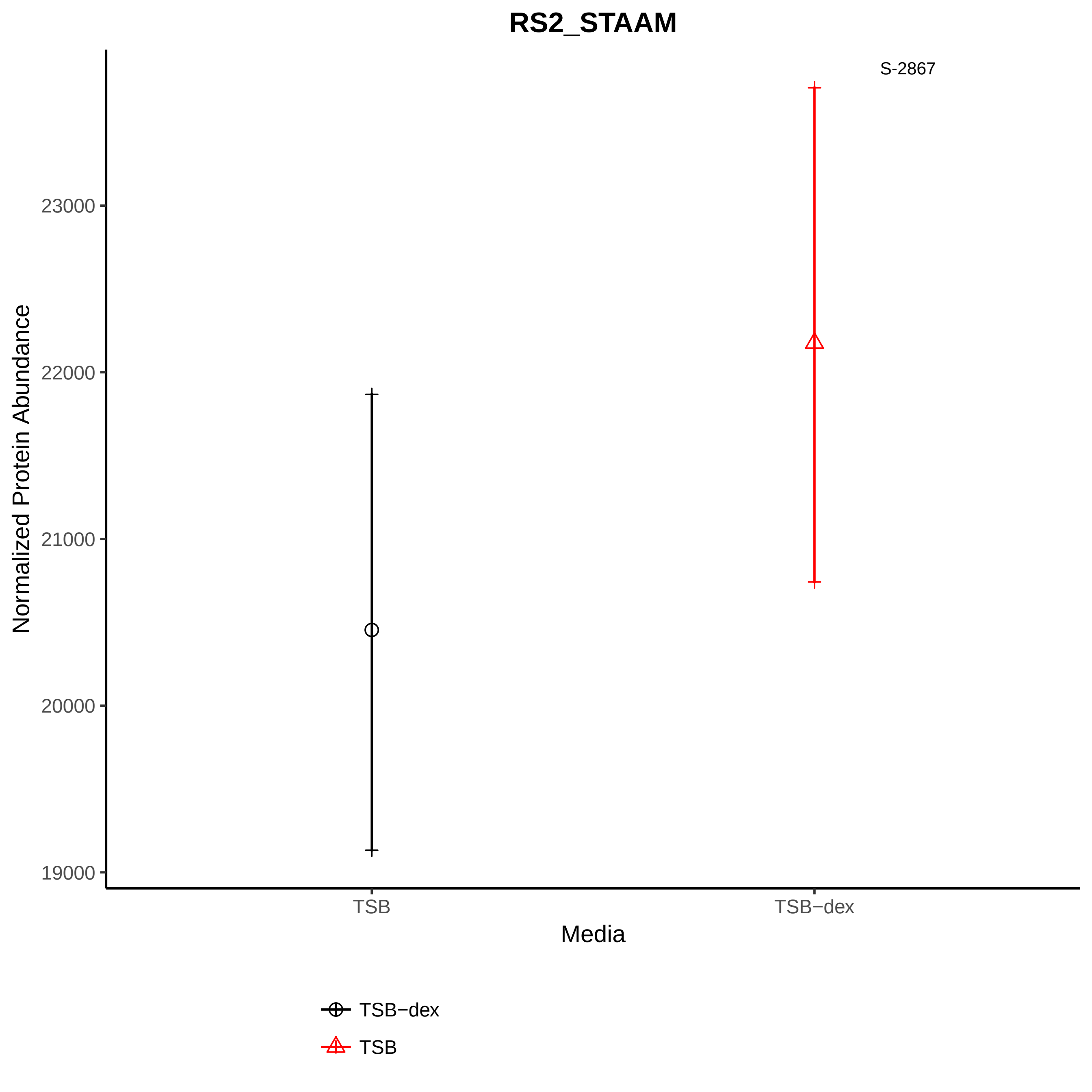

RS2_STAAM
S-2867
23000
22000
21000
20000
19000
Normalized Protein Abundance
TSB
TSB−dex
Media
TSB−dex
TSB

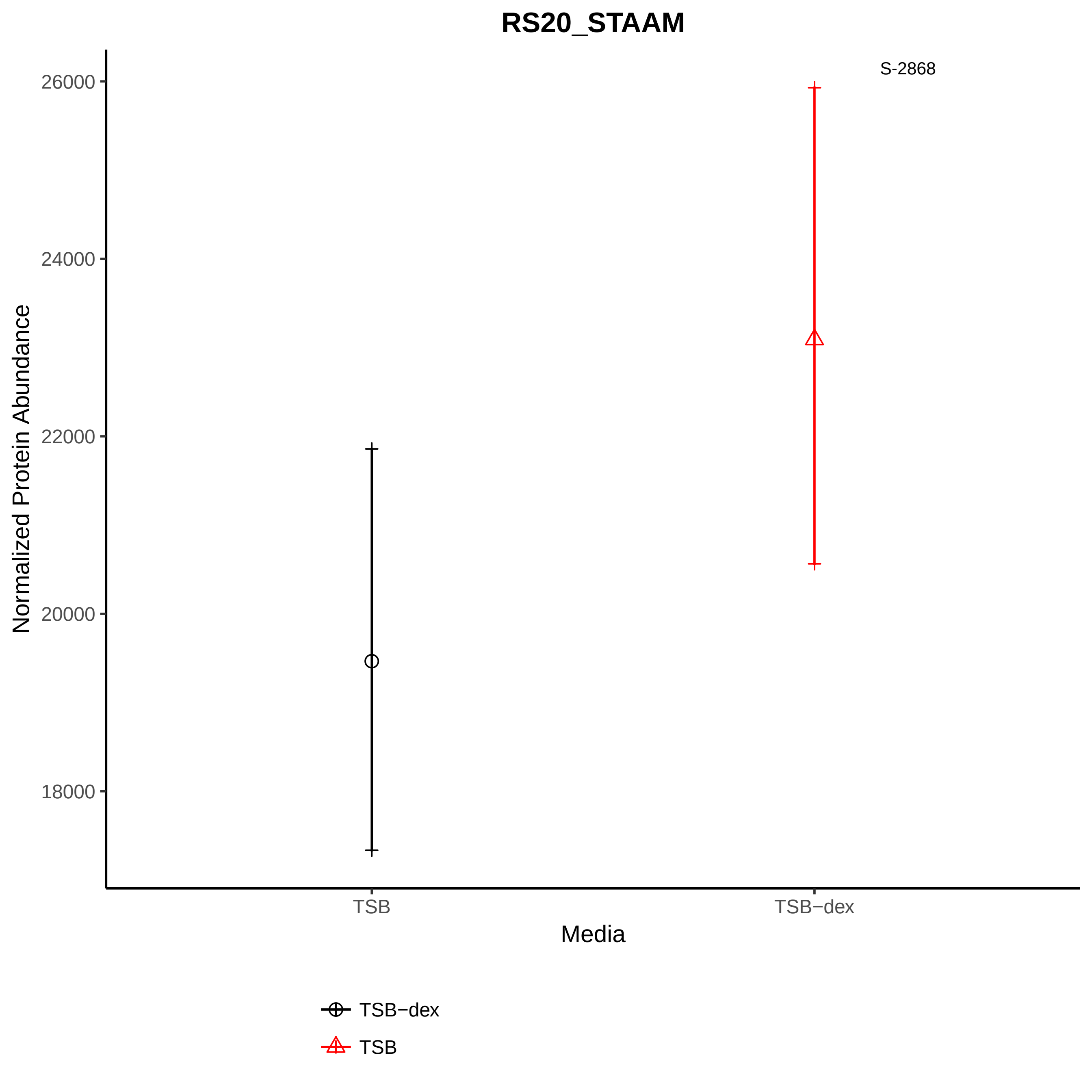

RS20_STAAM
S-2868
Normalized Protein Abundance
26000
24000
22000
20000
18000
TSB
TSB−dex
Media
TSB−dex
TSB

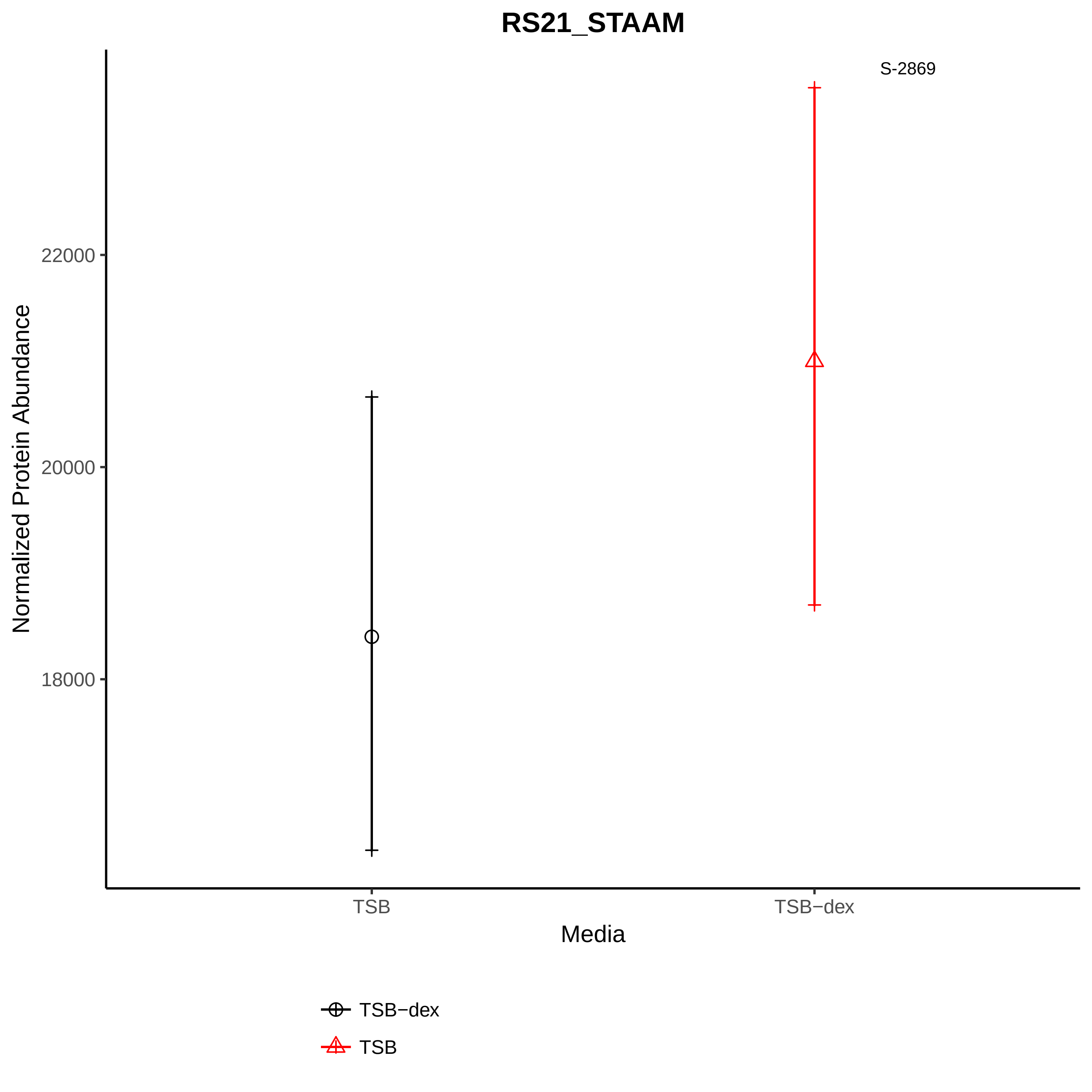

RS21_STAAM
S-2869
22000
20000
18000
Normalized Protein Abundance
TSB
TSB−dex
Media
TSB−dex
TSB

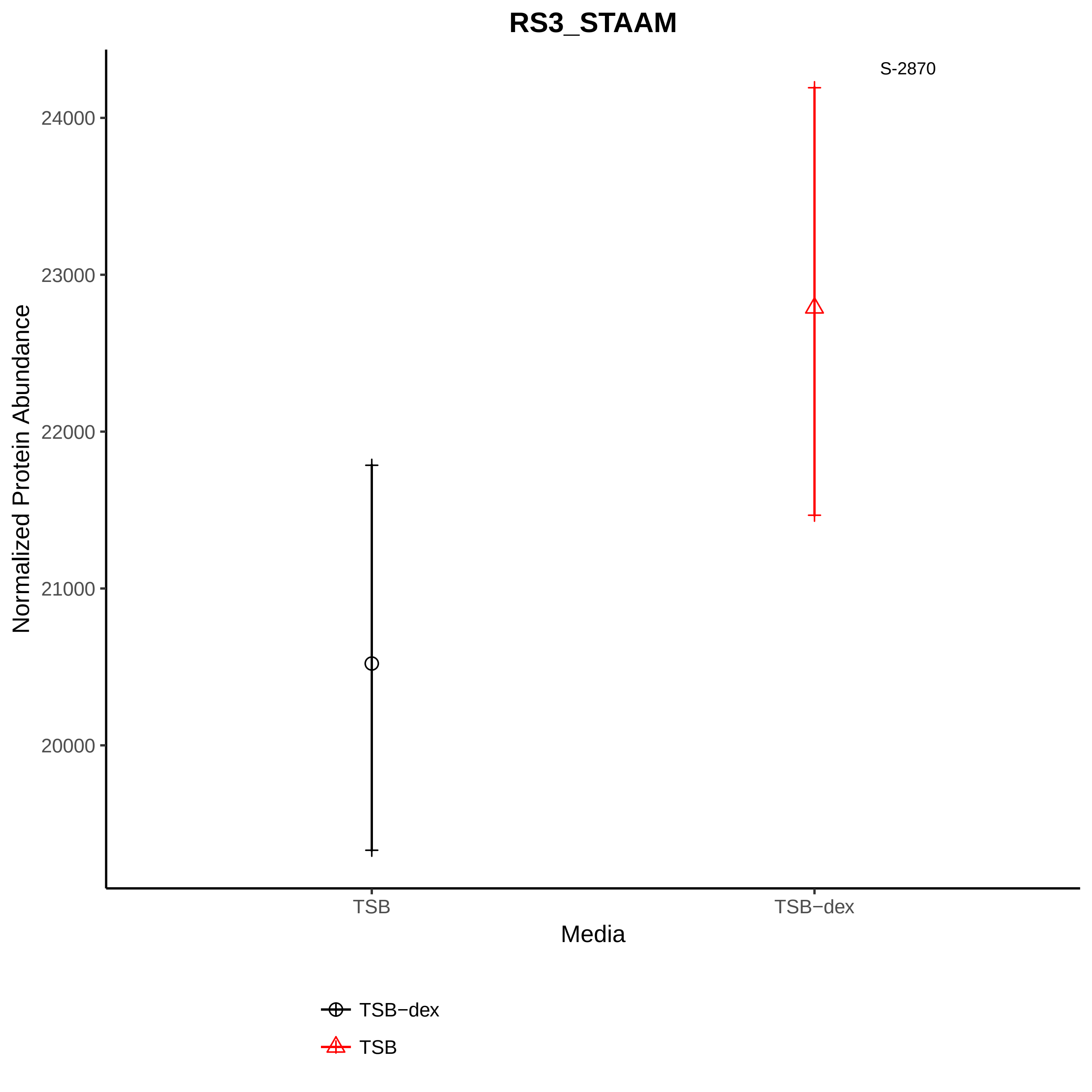
RS3_STAAM
S-2870
24000
23000
22000
21000
20000
Normalized Protein Abundance
TSB
TSB−dex
Media
TSB−dex
TSB

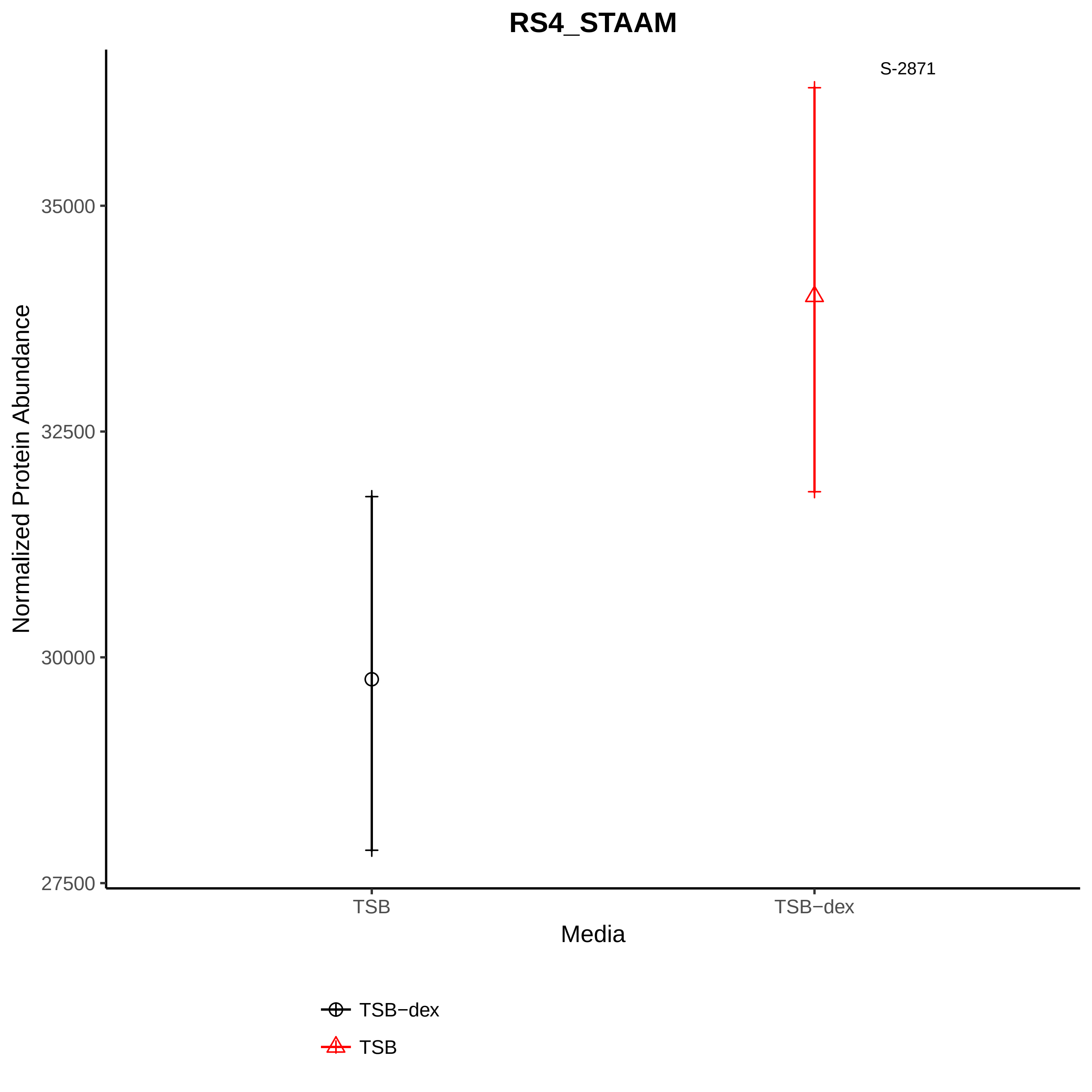

RS4_STAAM
S-2871
Normalized Protein Abundance
35000
32500
30000
27500
TSB
TSB−dex
Media
TSB−dex
TSB

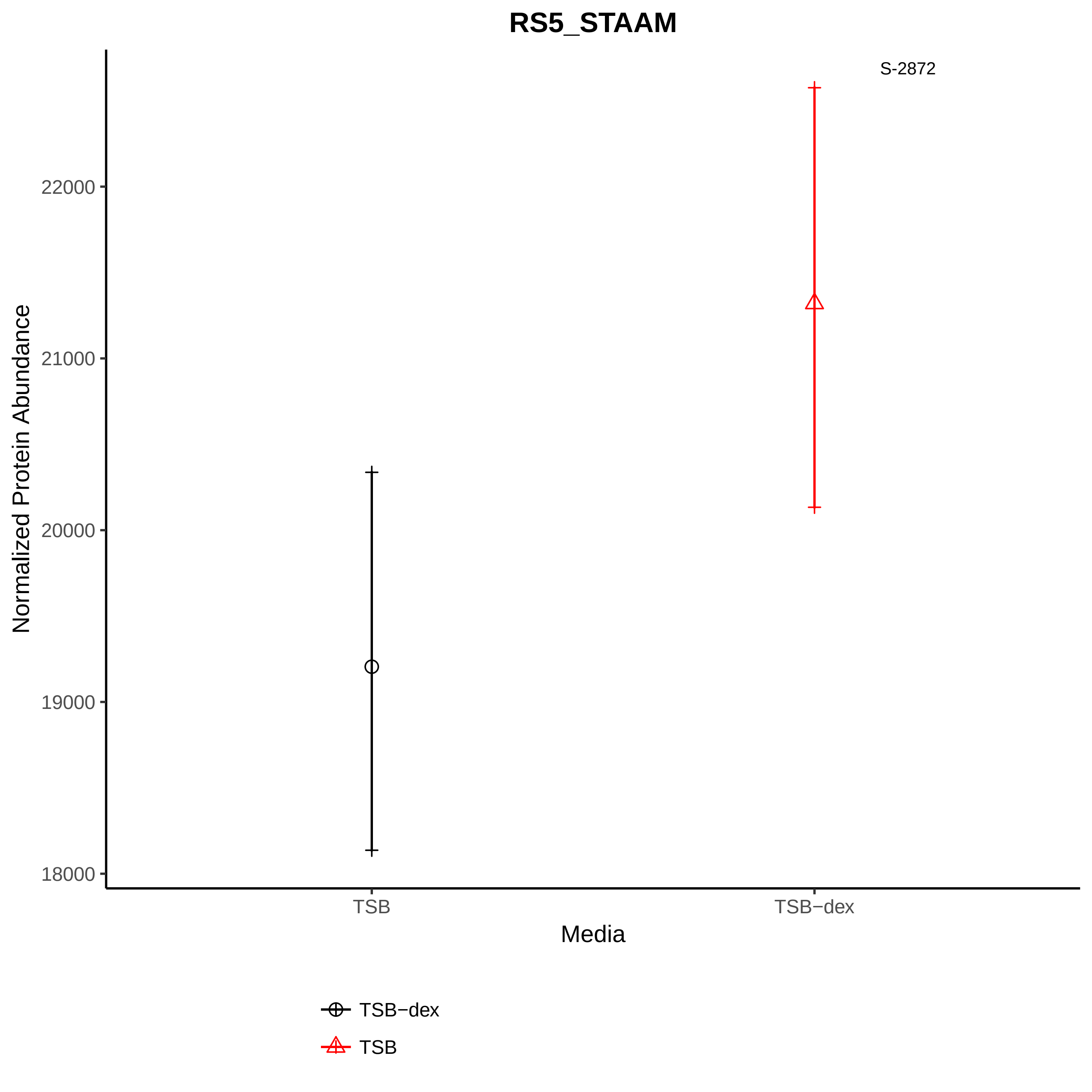
RS5_STAAM
S-2872
Normalized Protein Abundance
22000
21000
20000
19000
18000
TSB
TSB−dex
Media
TSB−dex
TSB

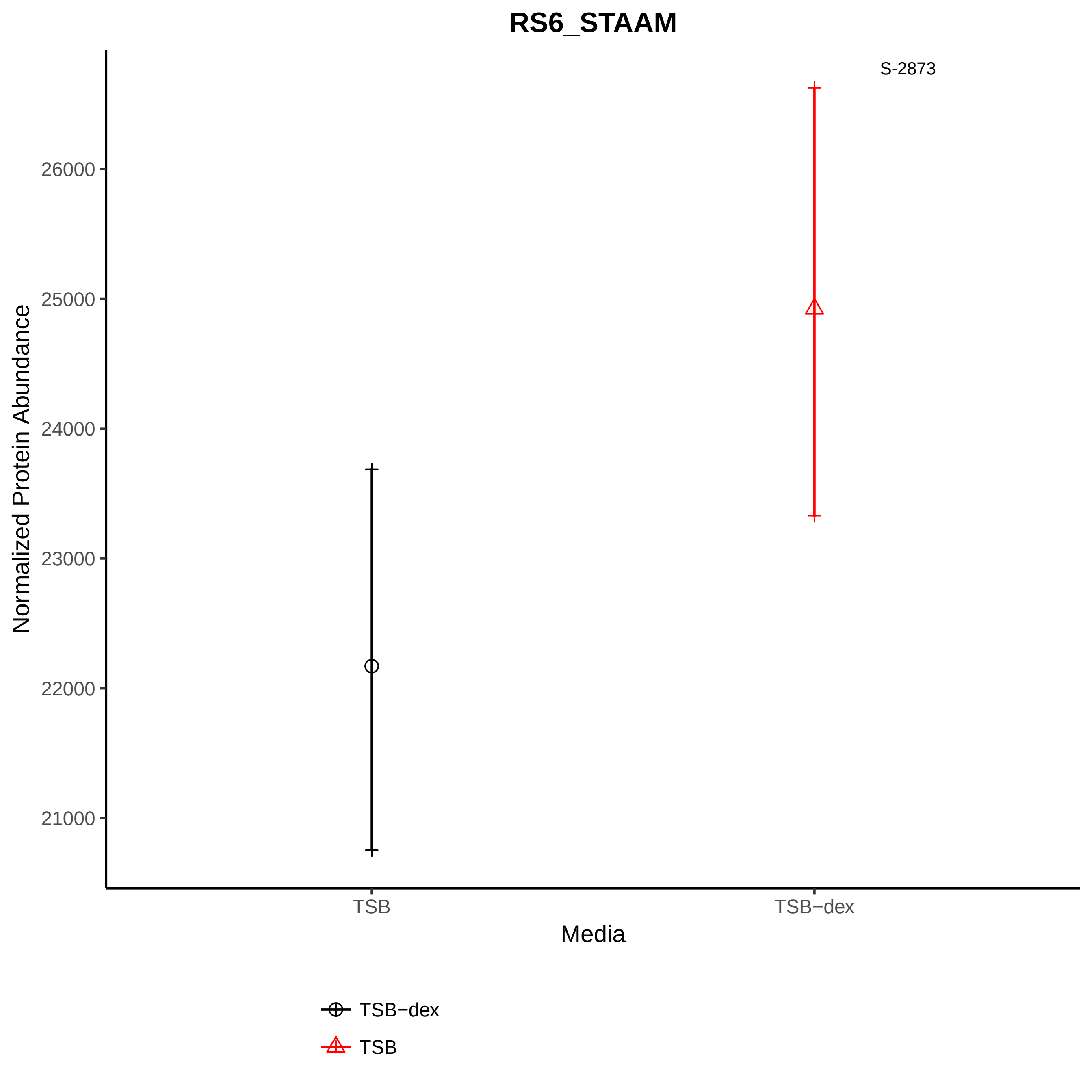

RS6_STAAM
S-2873
Normalized Protein Abundance
26000
25000
24000
23000
22000
21000
TSB
TSB−dex
Media
TSB−dex
TSB

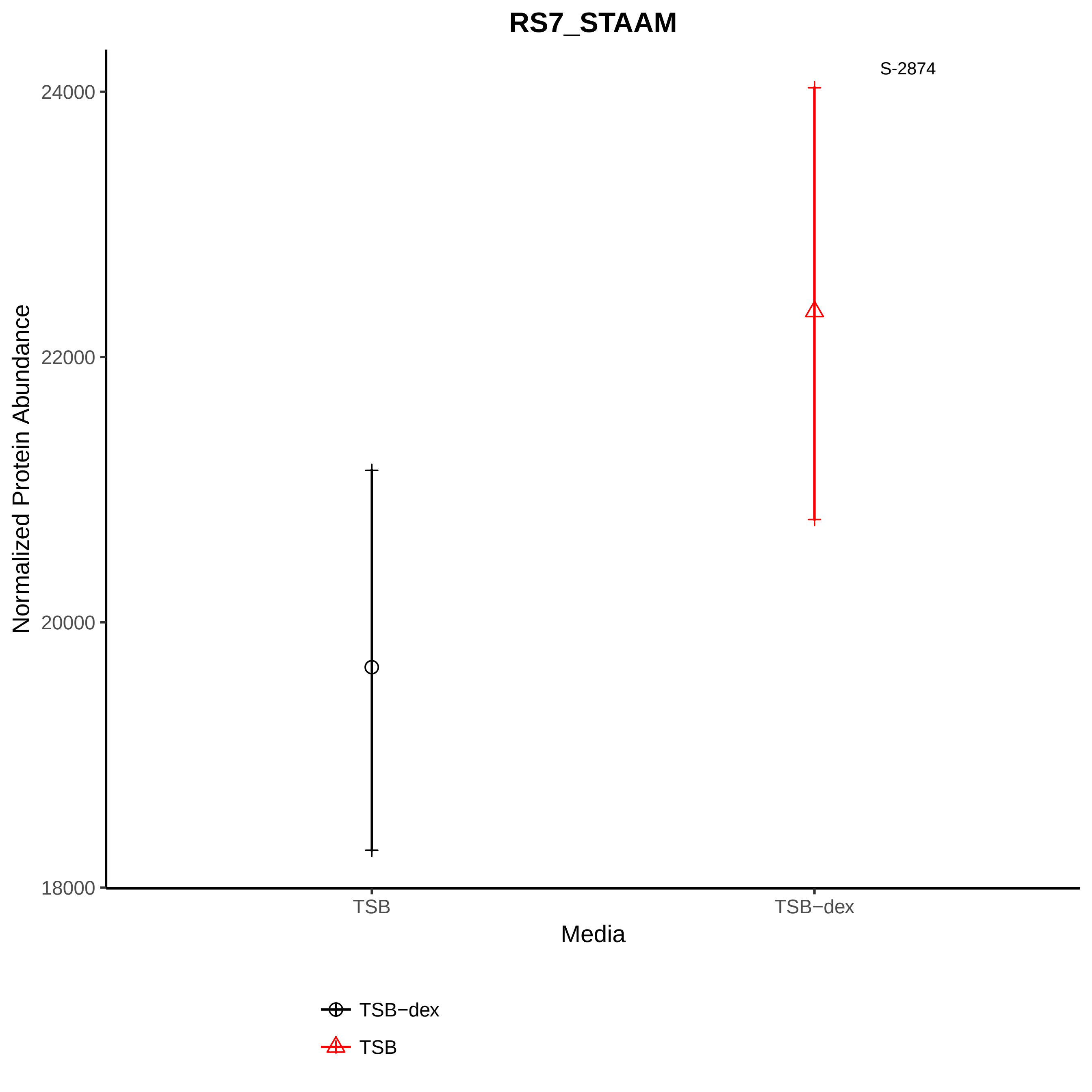

RS7_STAAM
S-2874
Normalized Protein Abundance
24000
22000
20000
18000
TSB
TSB−dex
Media
TSB−dex
TSB

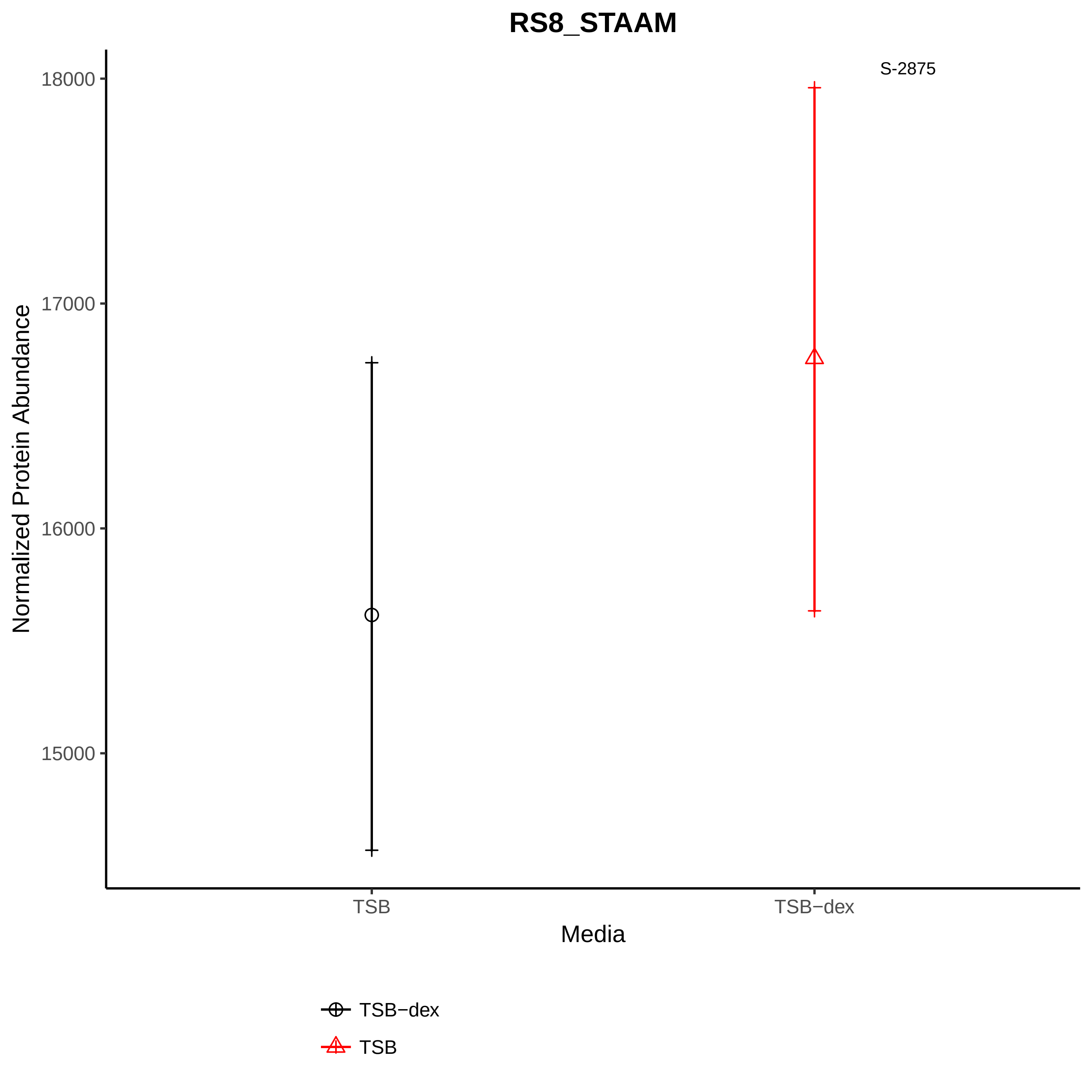

RS8_STAAM
S-2875
18000
17000
16000
15000
Normalized Protein Abundance
TSB
TSB−dex
Media
TSB−dex
TSB

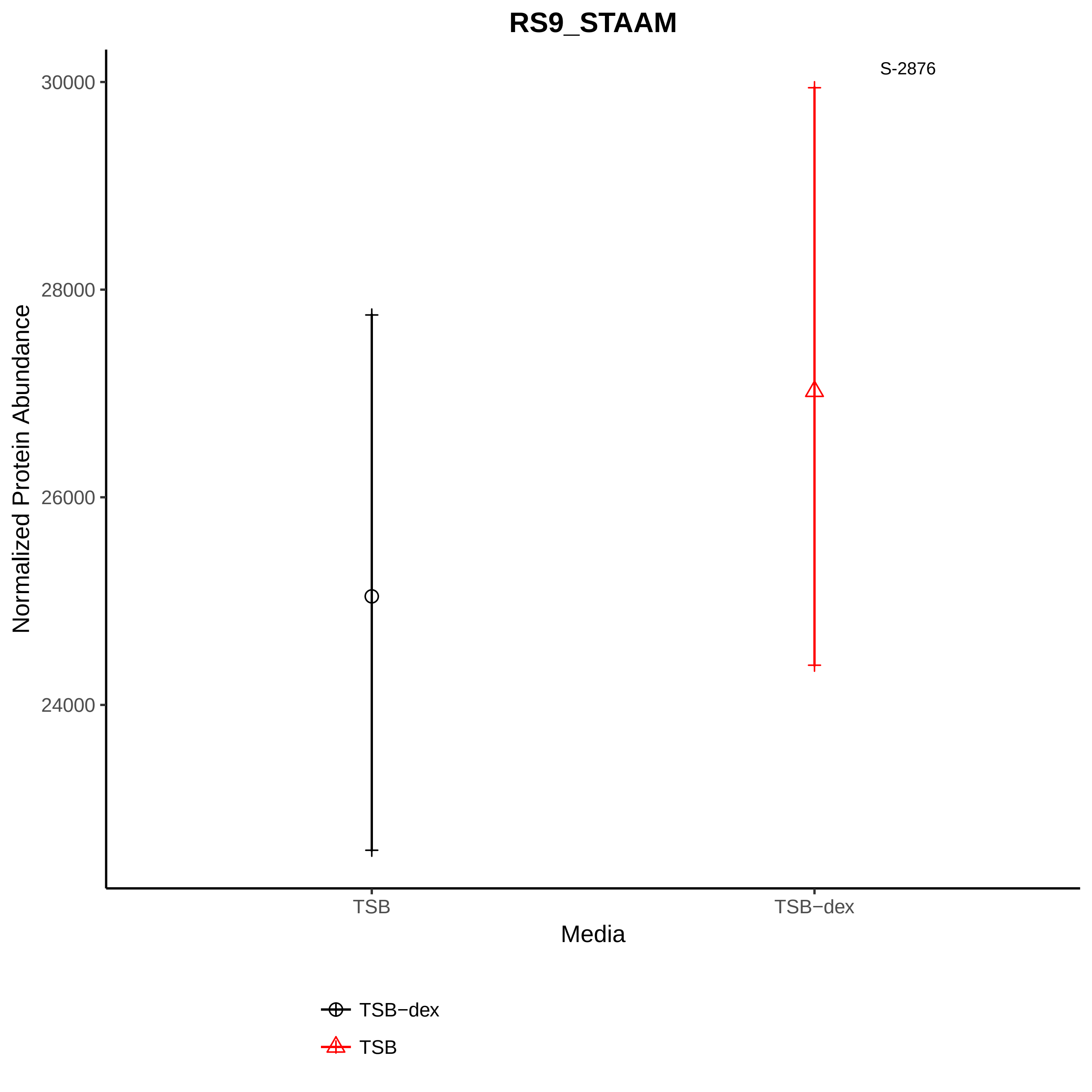

RS9_STAAM
S-2876
30000
28000
26000
24000
Normalized Protein Abundance
TSB
TSB−dex
Media
TSB−dex
TSB

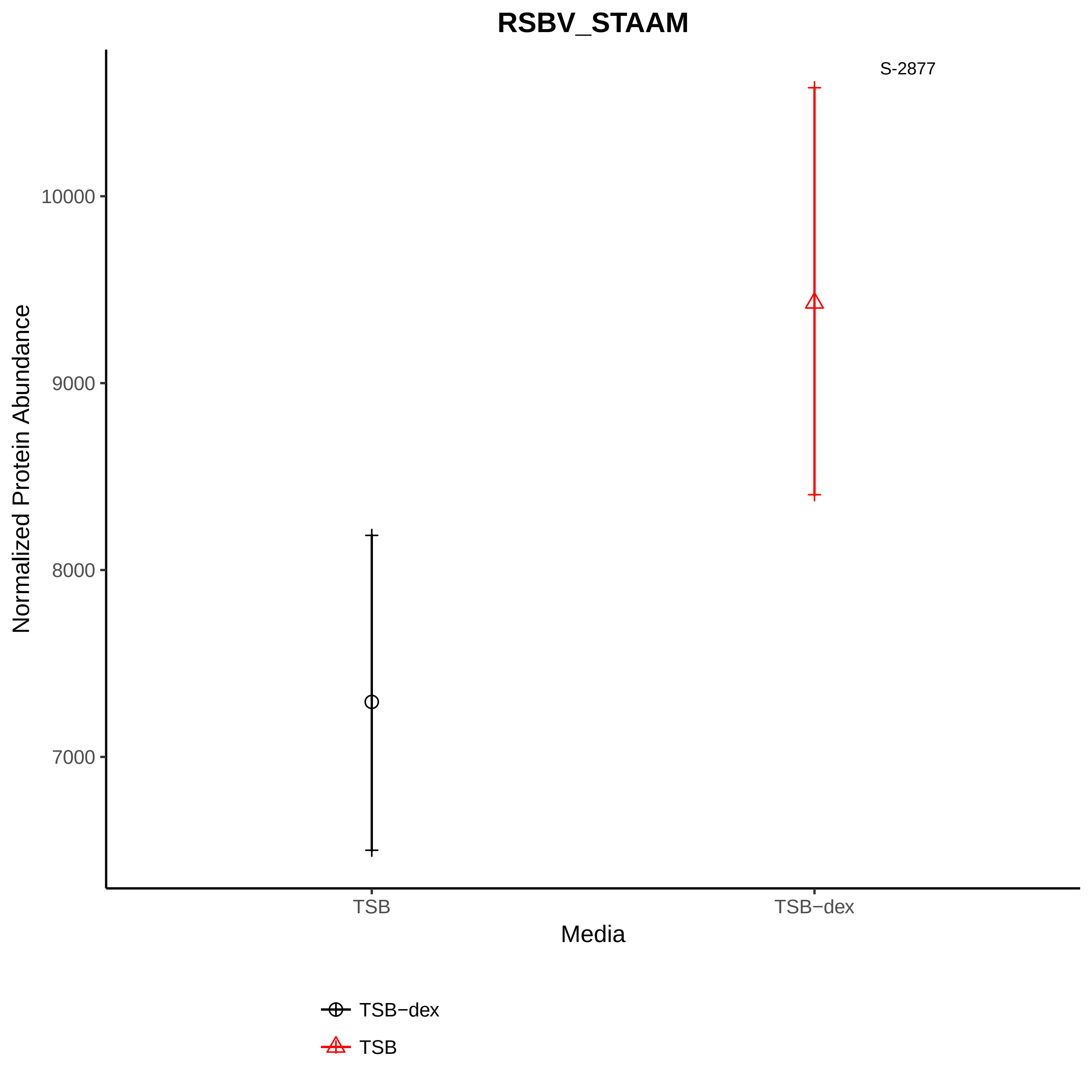

RSBV_STAAM
S-2877
Normalized Protein Abundance
10000
9000
8000
7000
TSB
TSB−dex
Media
TSB−dex
TSB

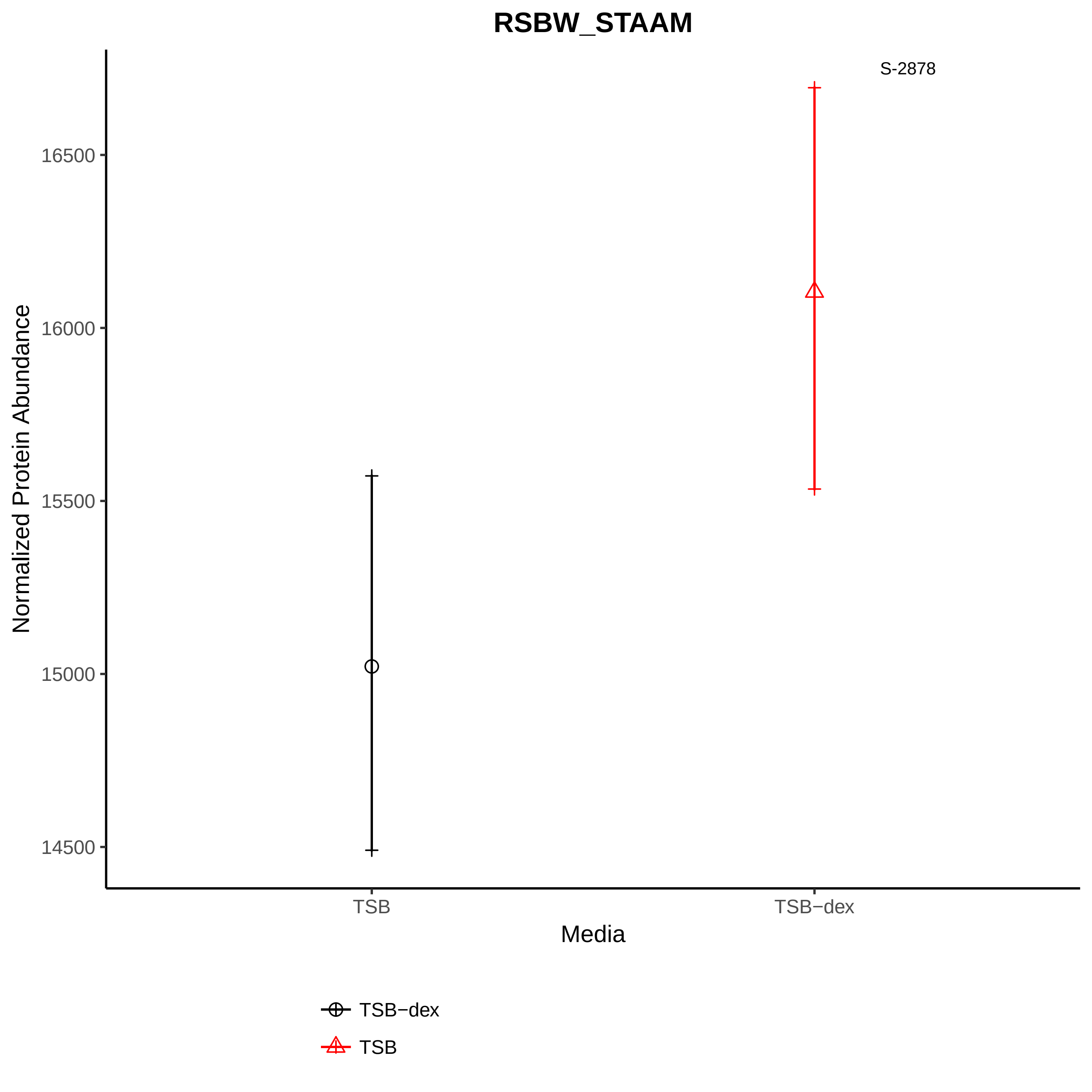
RSBW_STAAM
S-2878
Normalized Protein Abundance
16500
16000
15500
15000
14500
TSB
TSB−dex
Media
TSB−dex
TSB

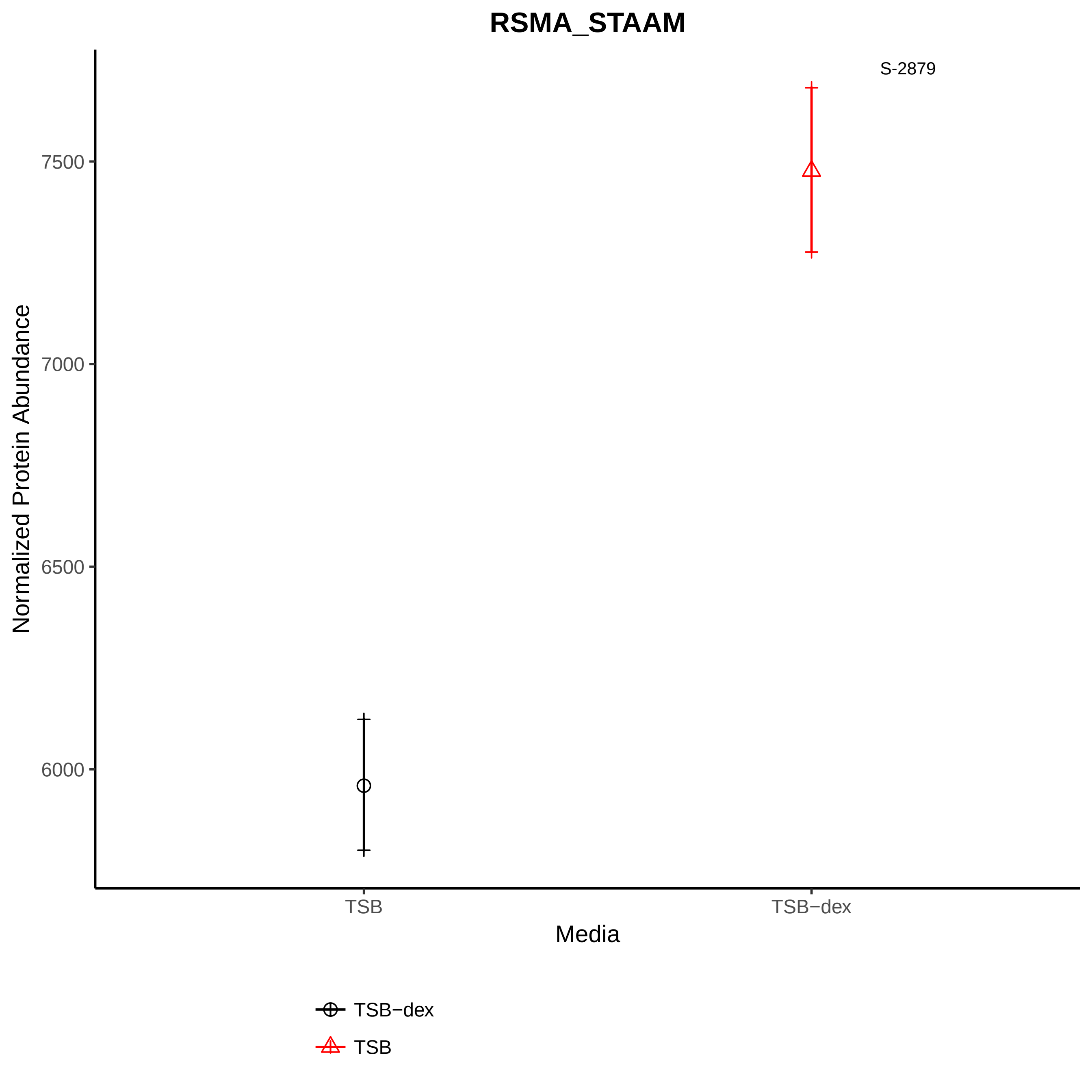

RSMA_STAAM
S-2879
Normalized Protein Abundance
7500
7000
6500
6000
TSB
TSB−dex
Media
TSB−dex
TSB

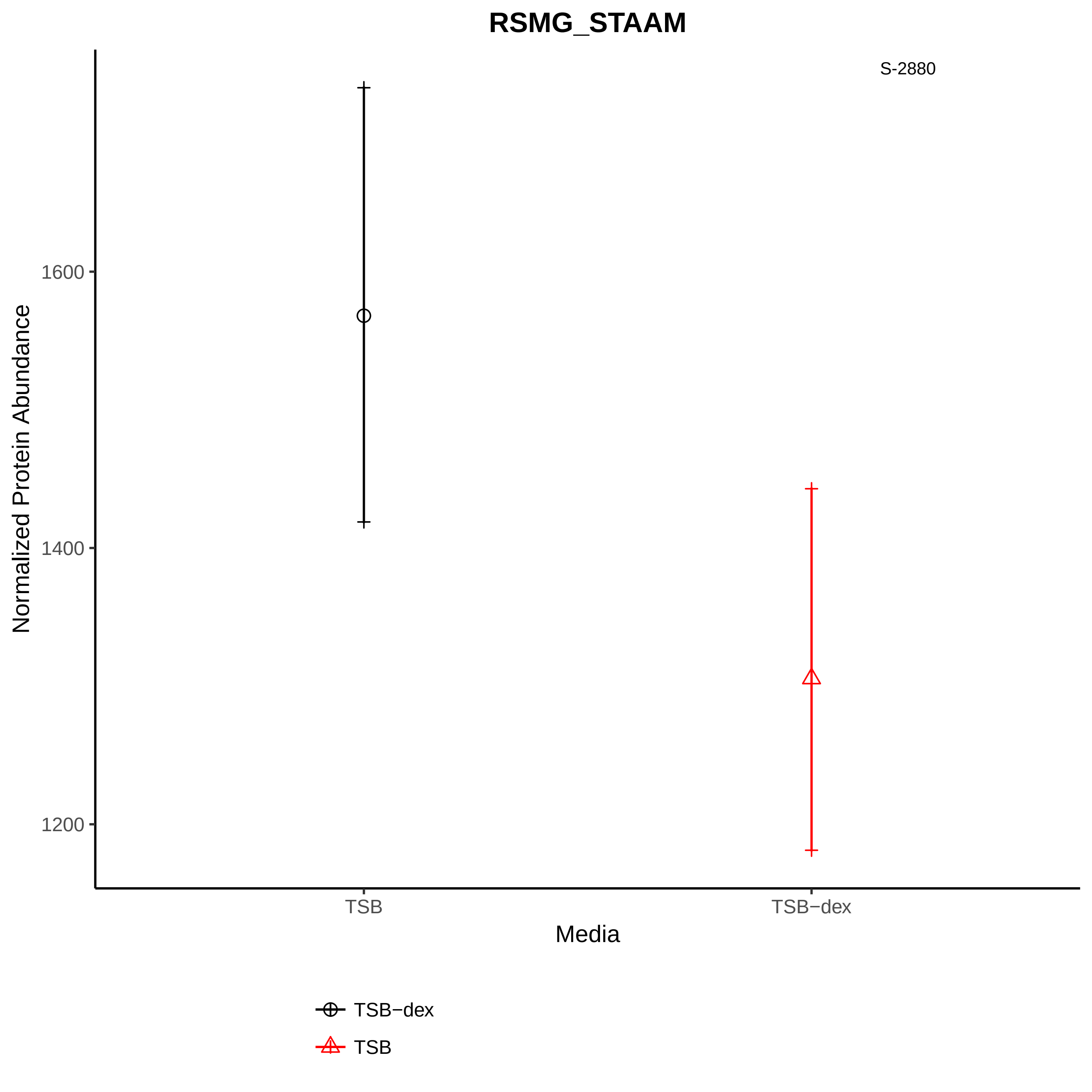
RSMG_STAAM
S-2880
1600
1400
1200
Normalized Protein Abundance
TSB
TSB−dex
Media
TSB−dex
TSB

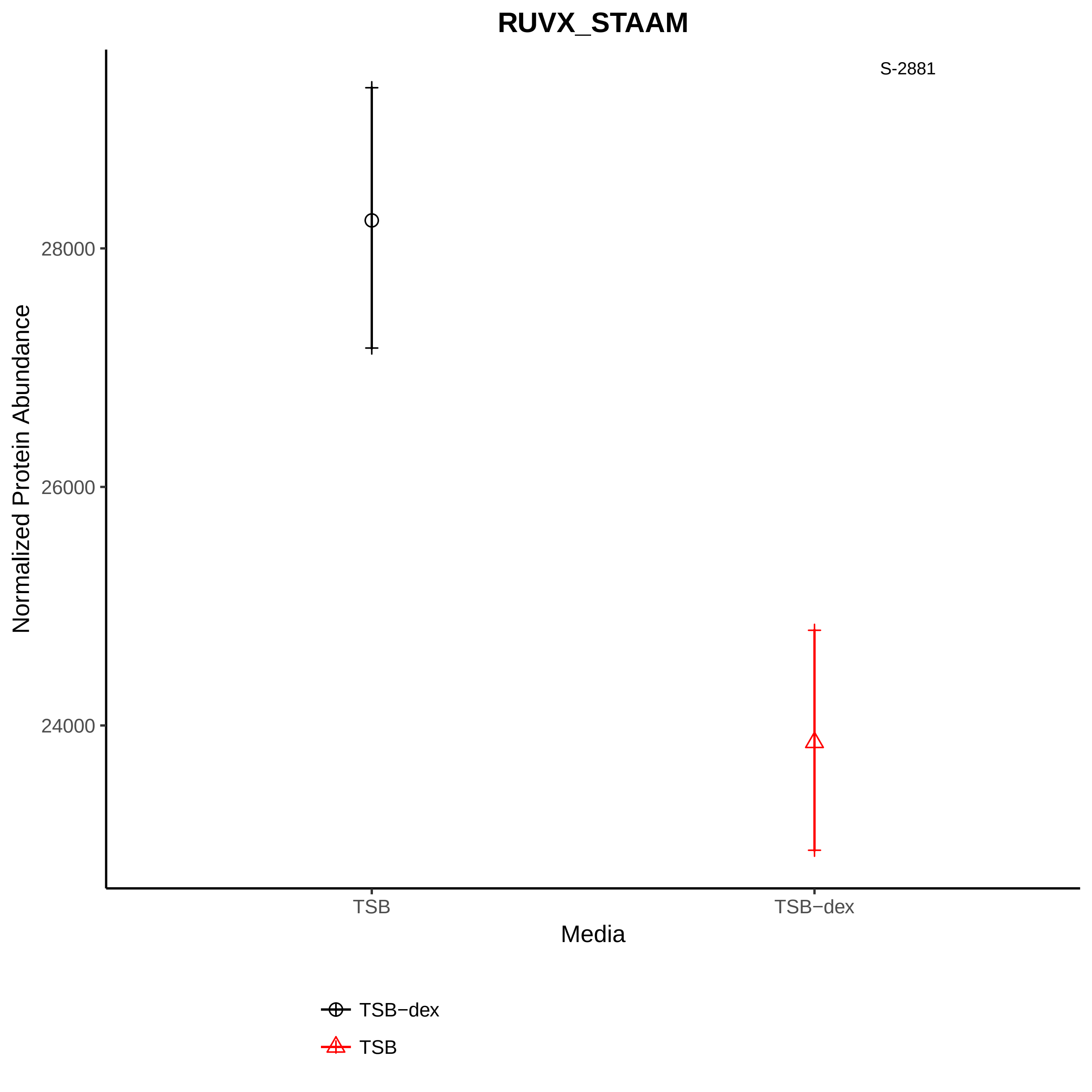

RUVX_STAAM
S-2881
Normalized Protein Abundance
28000
26000
24000
TSB
TSB−dex
Media
TSB−dex
TSB

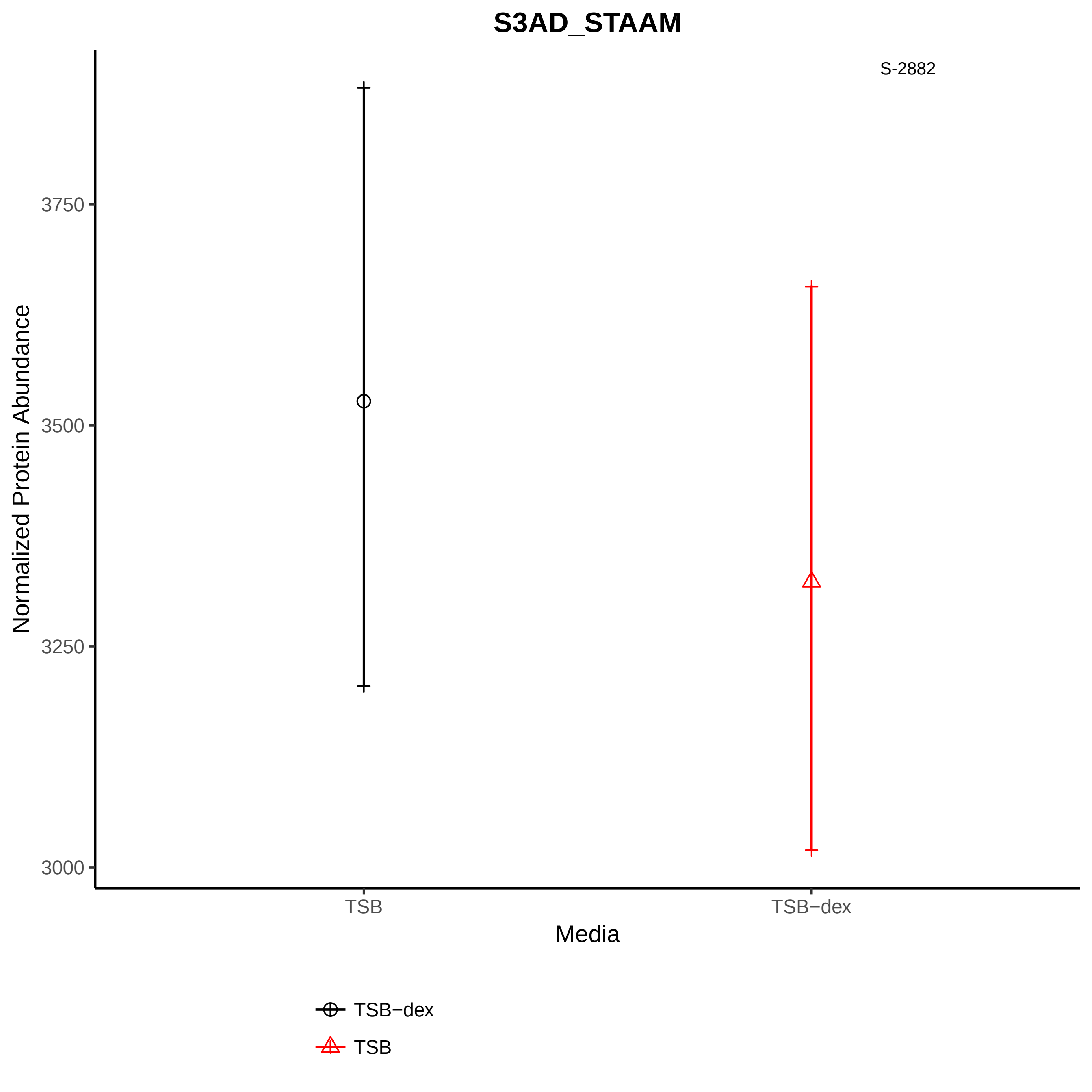

S3AD_STAAM
S-2882
Normalized Protein Abundance
3750
3500
3250
3000
TSB
TSB−dex
Media
TSB−dex
TSB

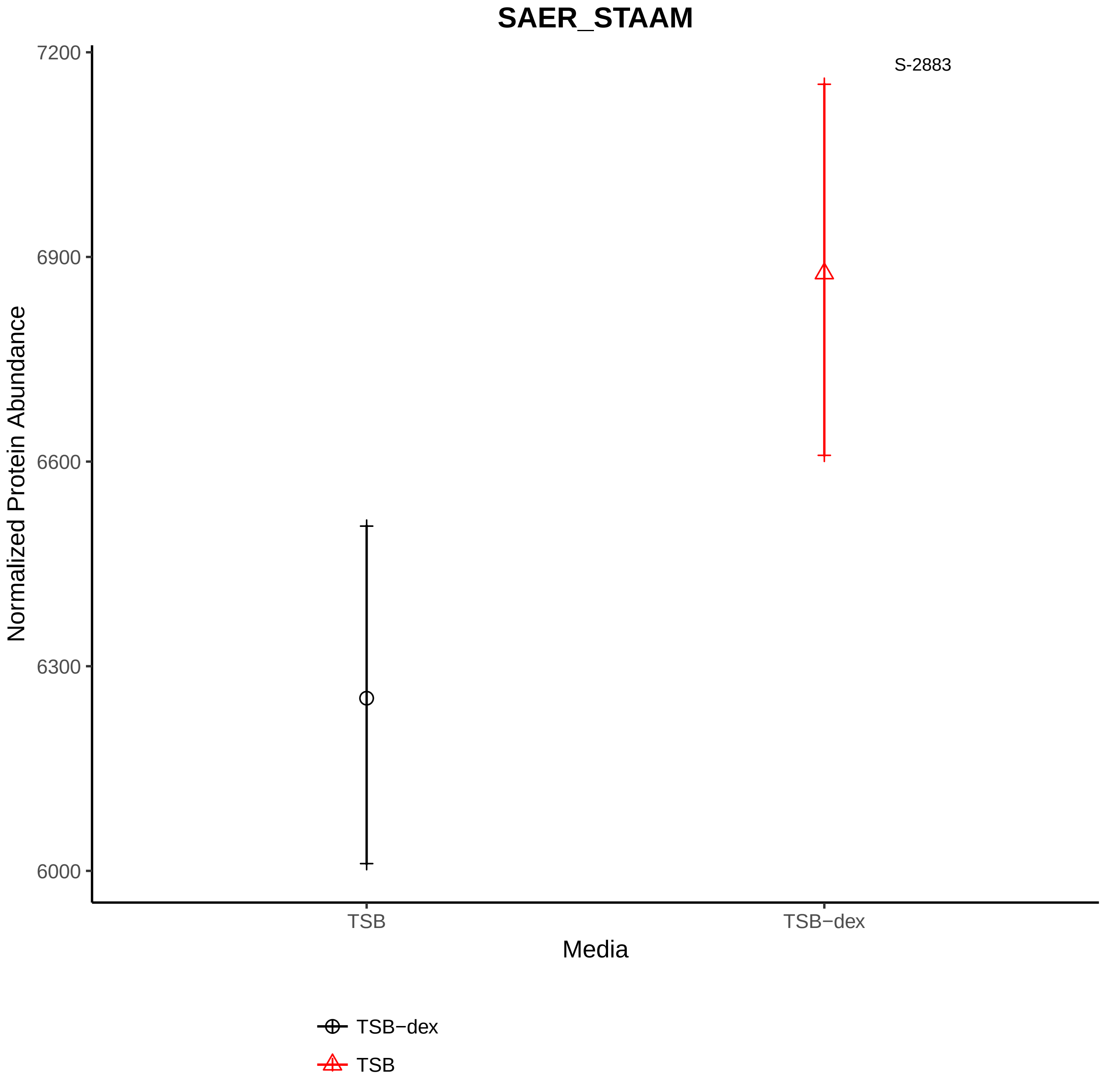

SAER_STAAM
S-2883
Normalized Protein Abundance
7200
6900
6600
6300
6000
TSB
TSB−dex
Media
TSB−dex
TSB

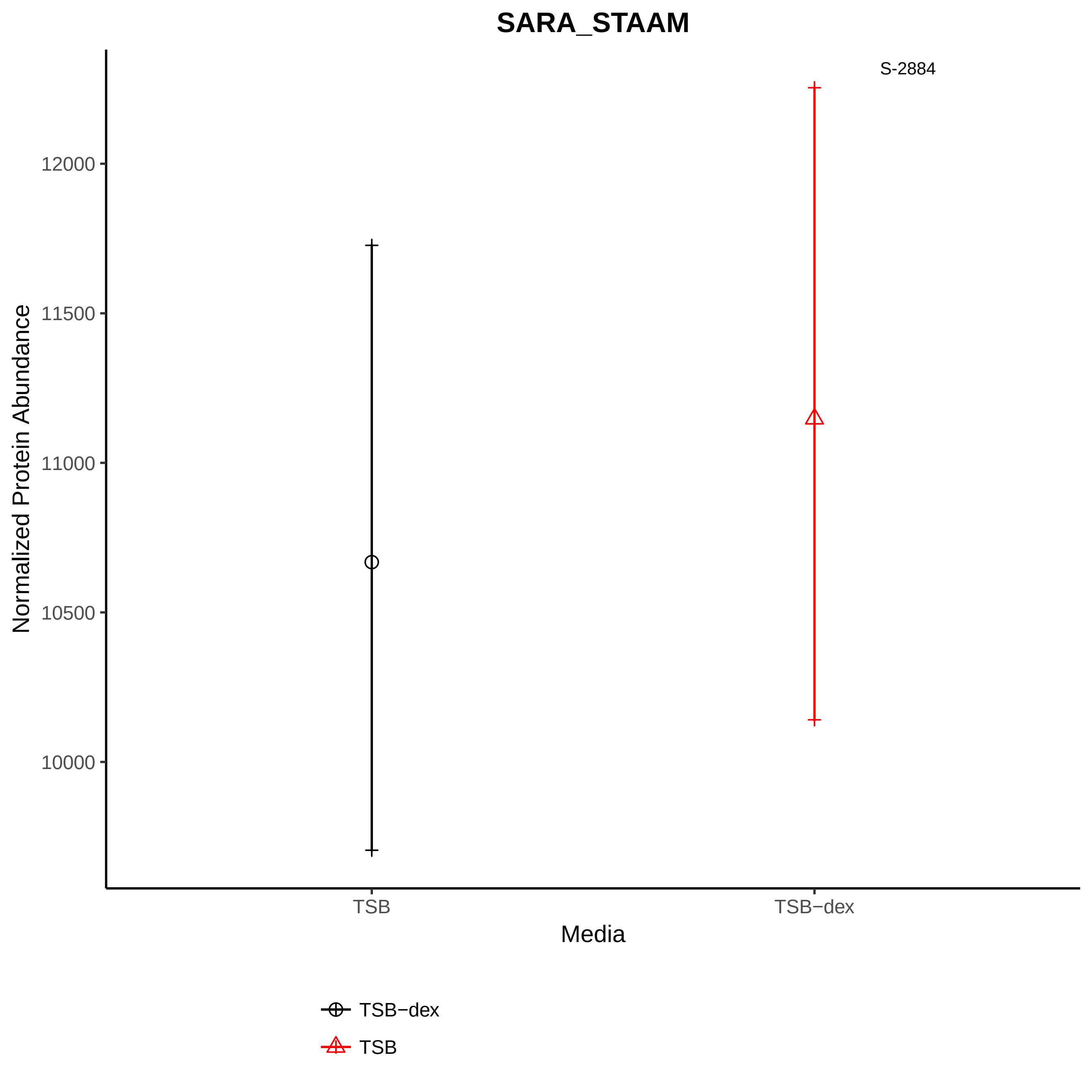
SARA_STAAM
S-2884
Normalized Protein Abundance
12000
11500
11000
10500
10000
TSB
TSB−dex
Media
TSB−dex
TSB

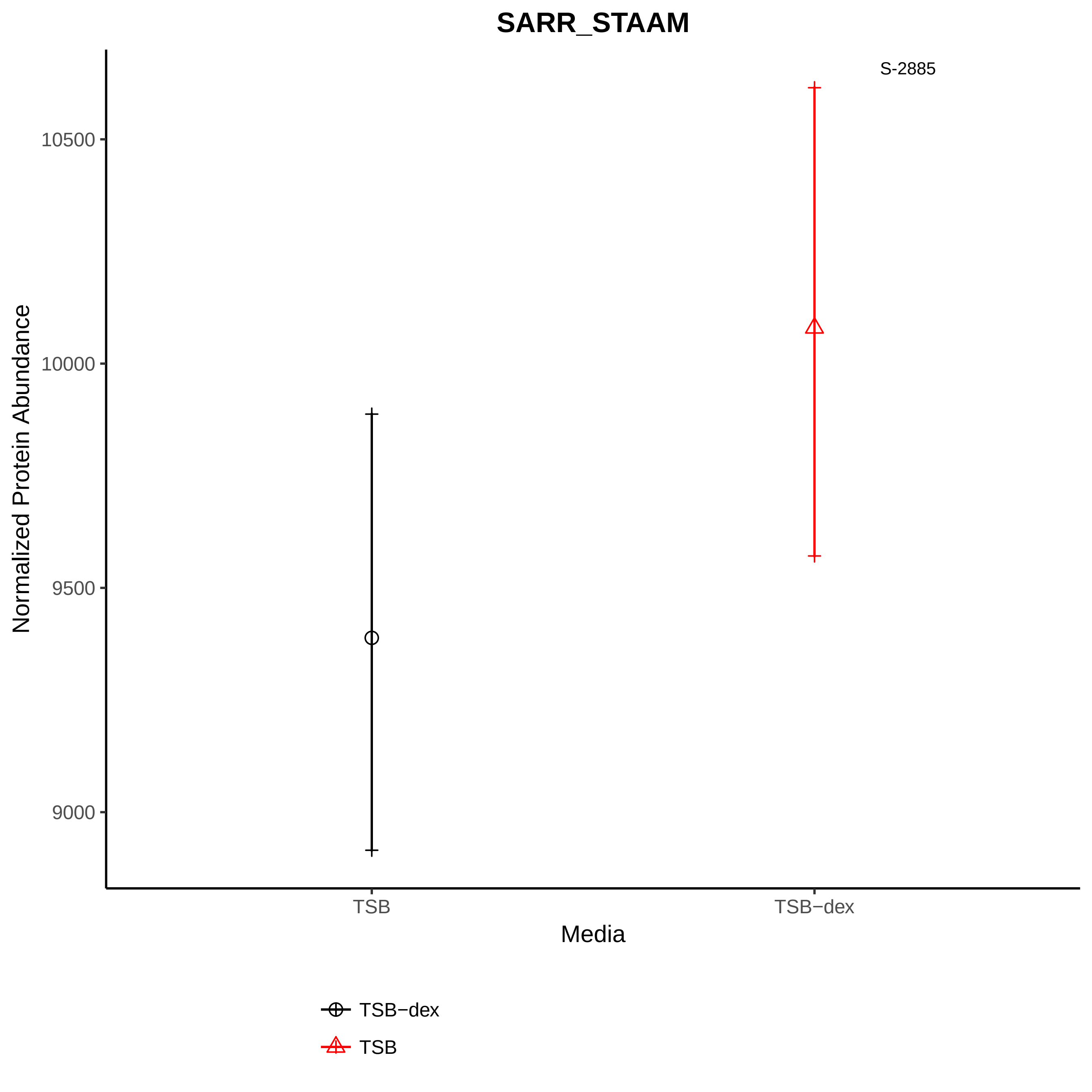

SARR_STAAM
S-2885
Normalized Protein Abundance
10500
10000
9500
9000
TSB
TSB−dex
Media
TSB−dex
TSB

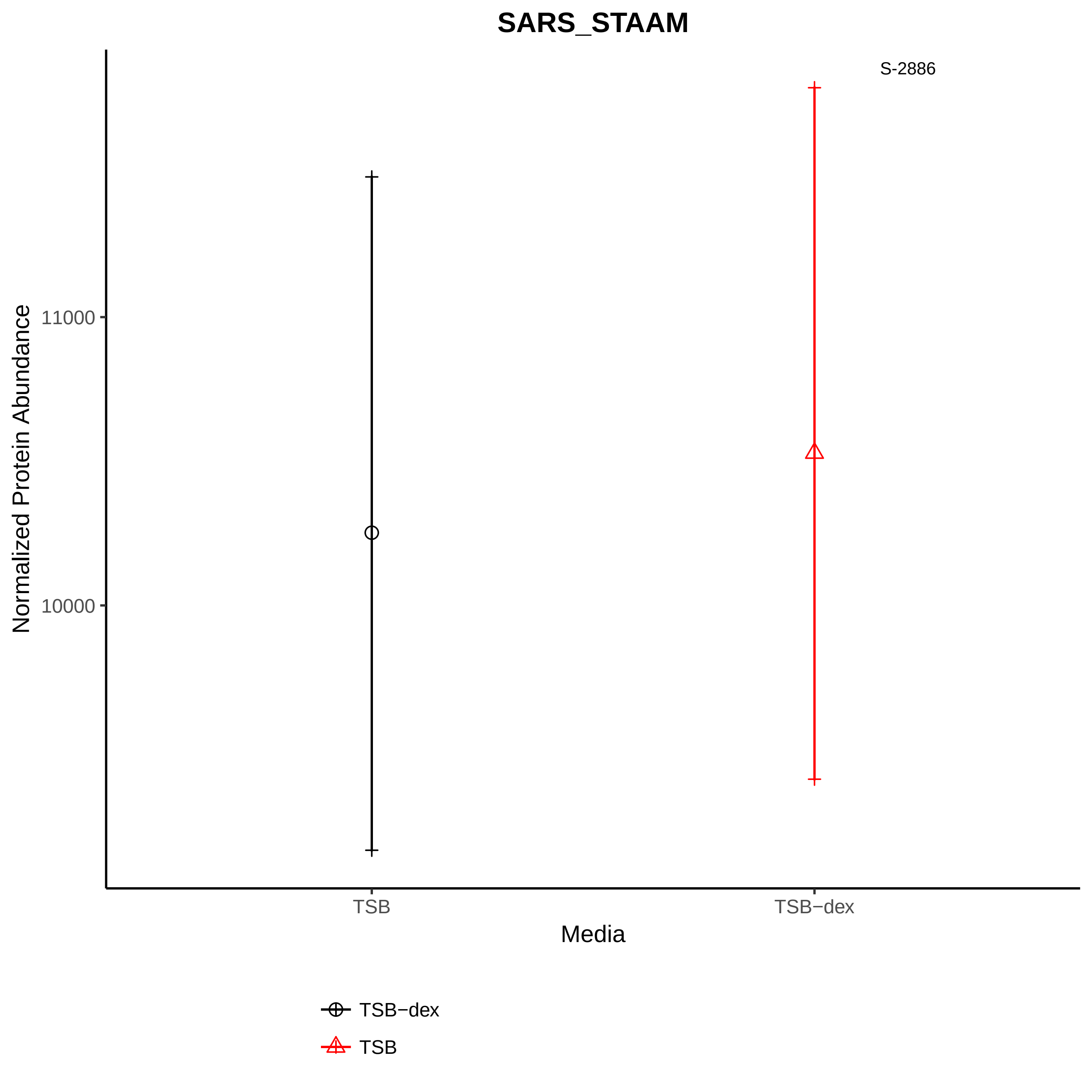

SARS_STAAM
S-2886
Normalized Protein Abundance
11000
10000
TSB
TSB−dex
Media
TSB−dex
TSB

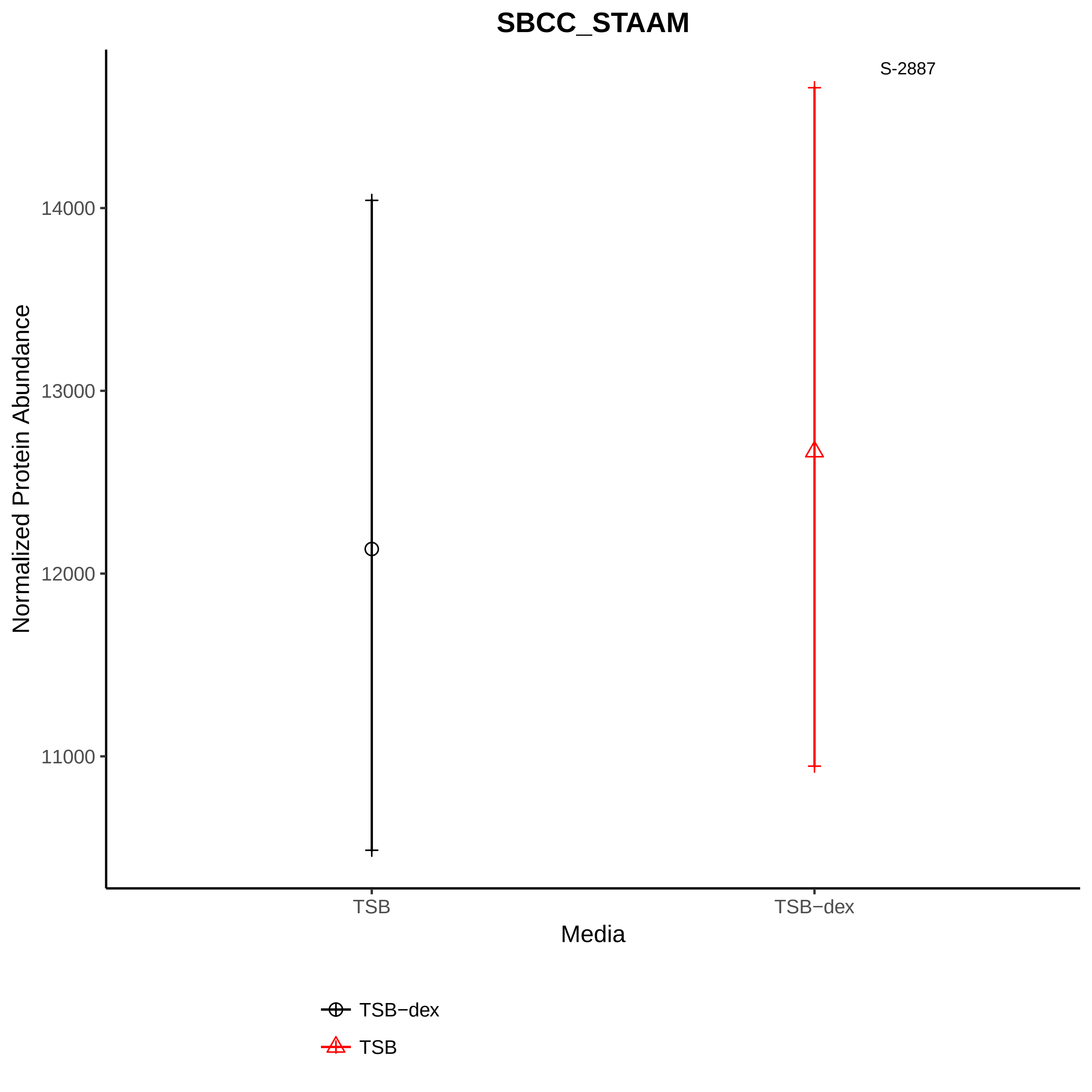
SBCC_STAAM
S-2887
Normalized Protein Abundance
14000
13000
12000
11000
TSB
TSB−dex
Media
TSB−dex
TSB

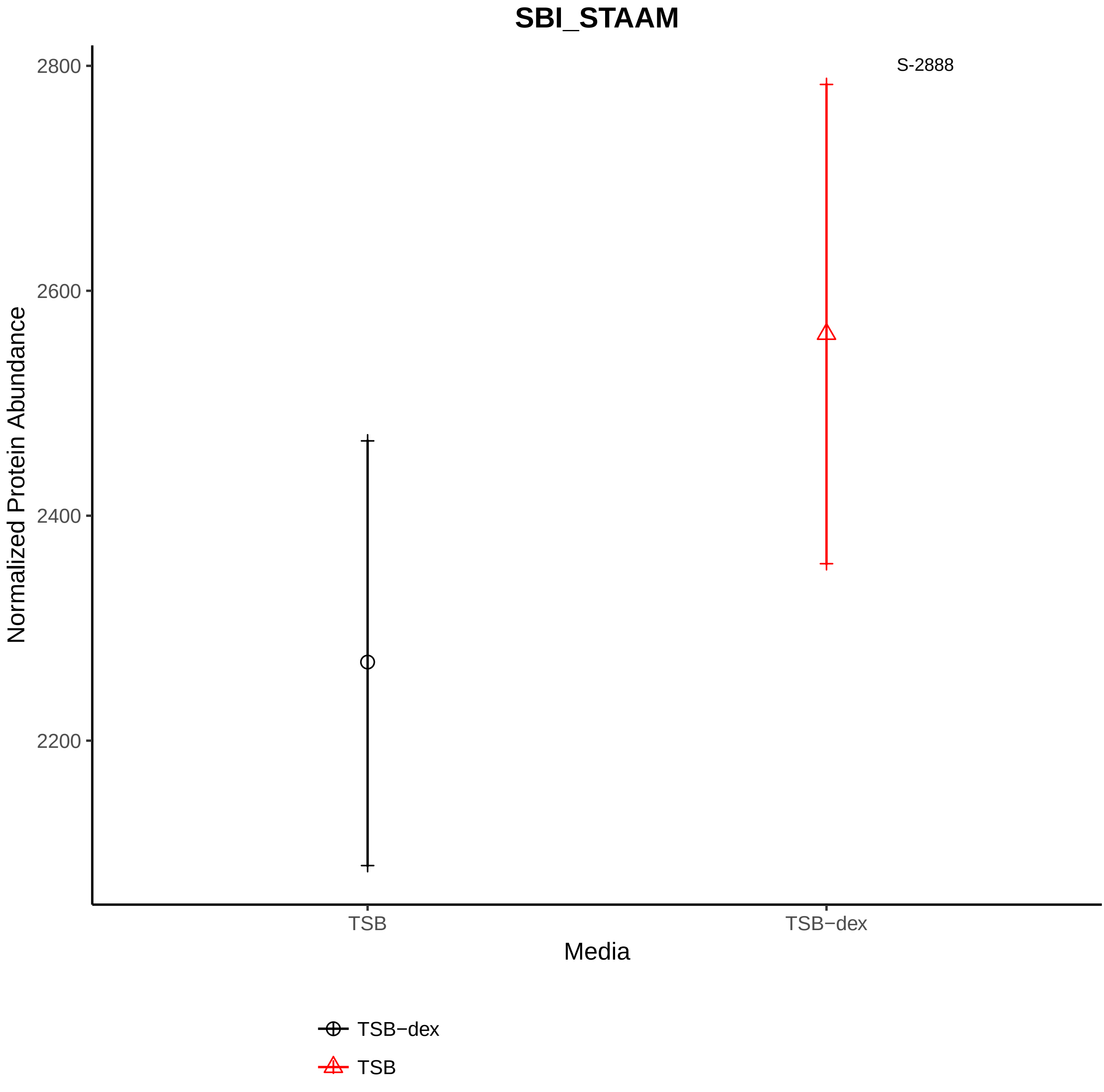

SBI_STAAM
S-2888
2800
2600
2400
2200
Normalized Protein Abundance
TSB
TSB−dex
Media
TSB−dex
TSB

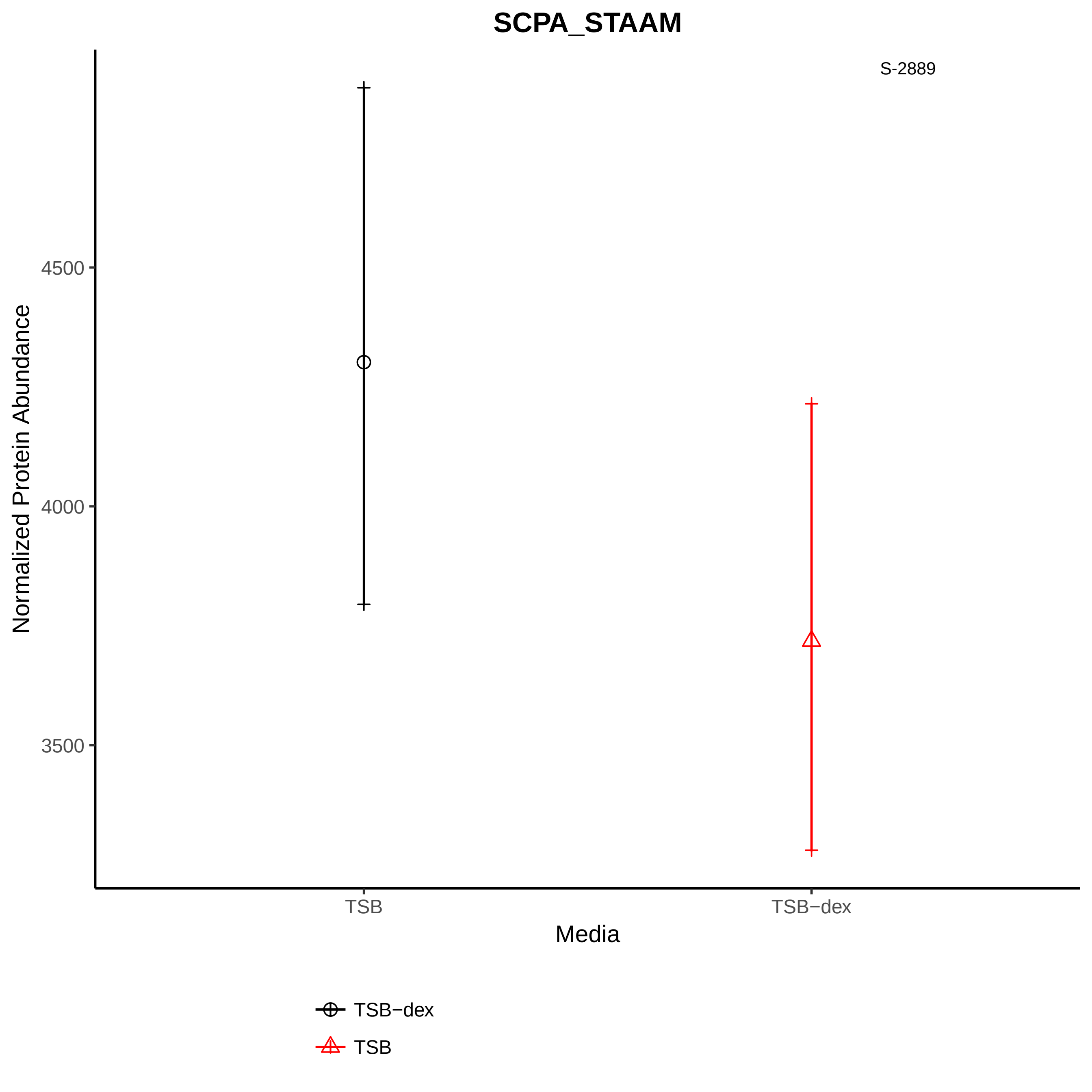

SCPA_STAAM
S-2889
Normalized Protein Abundance
4500
4000
3500
TSB
TSB−dex
Media
TSB−dex
TSB

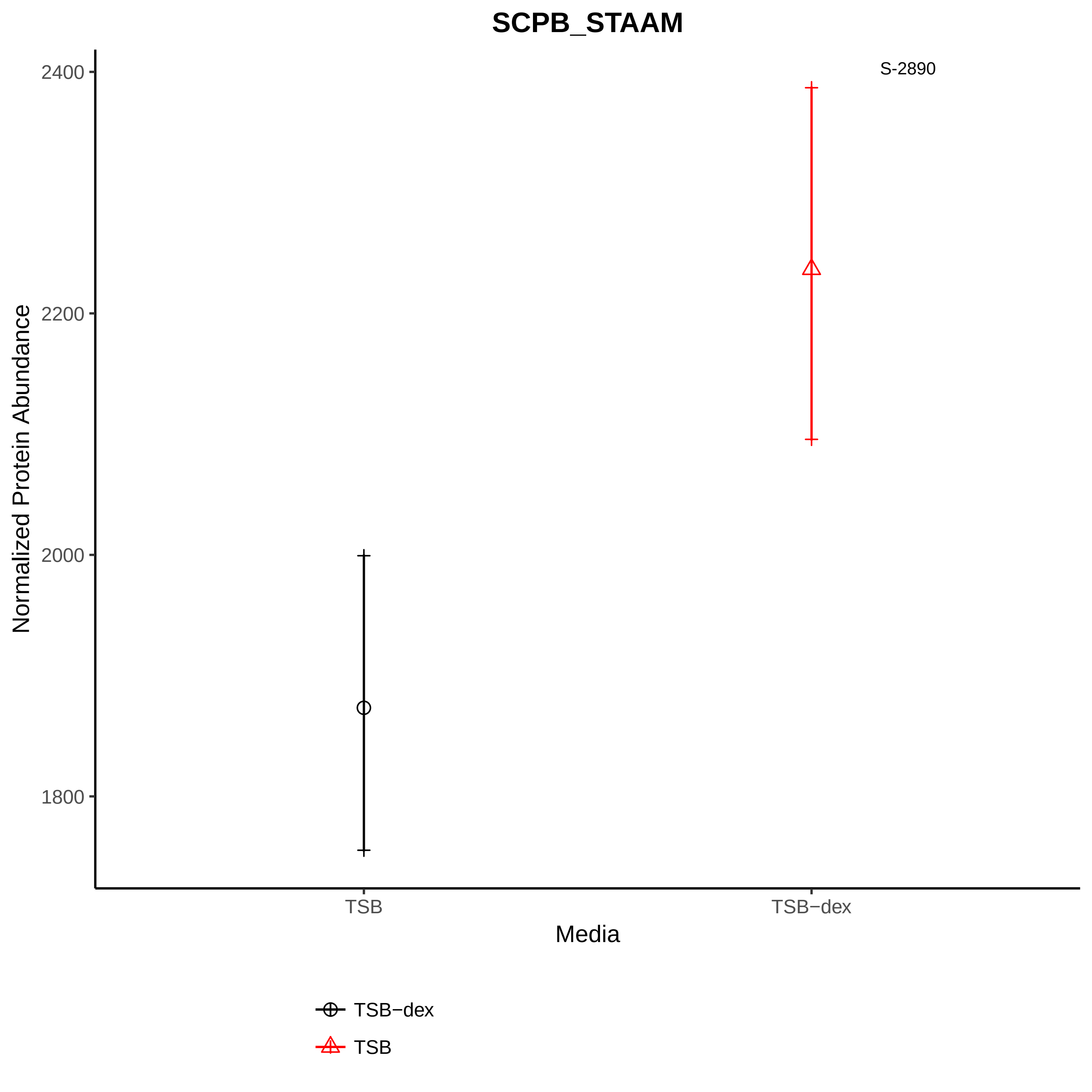
SCPB_STAAM
S-2890
2400
2200
2000
1800
Normalized Protein Abundance
TSB
TSB−dex
Media
TSB−dex
TSB

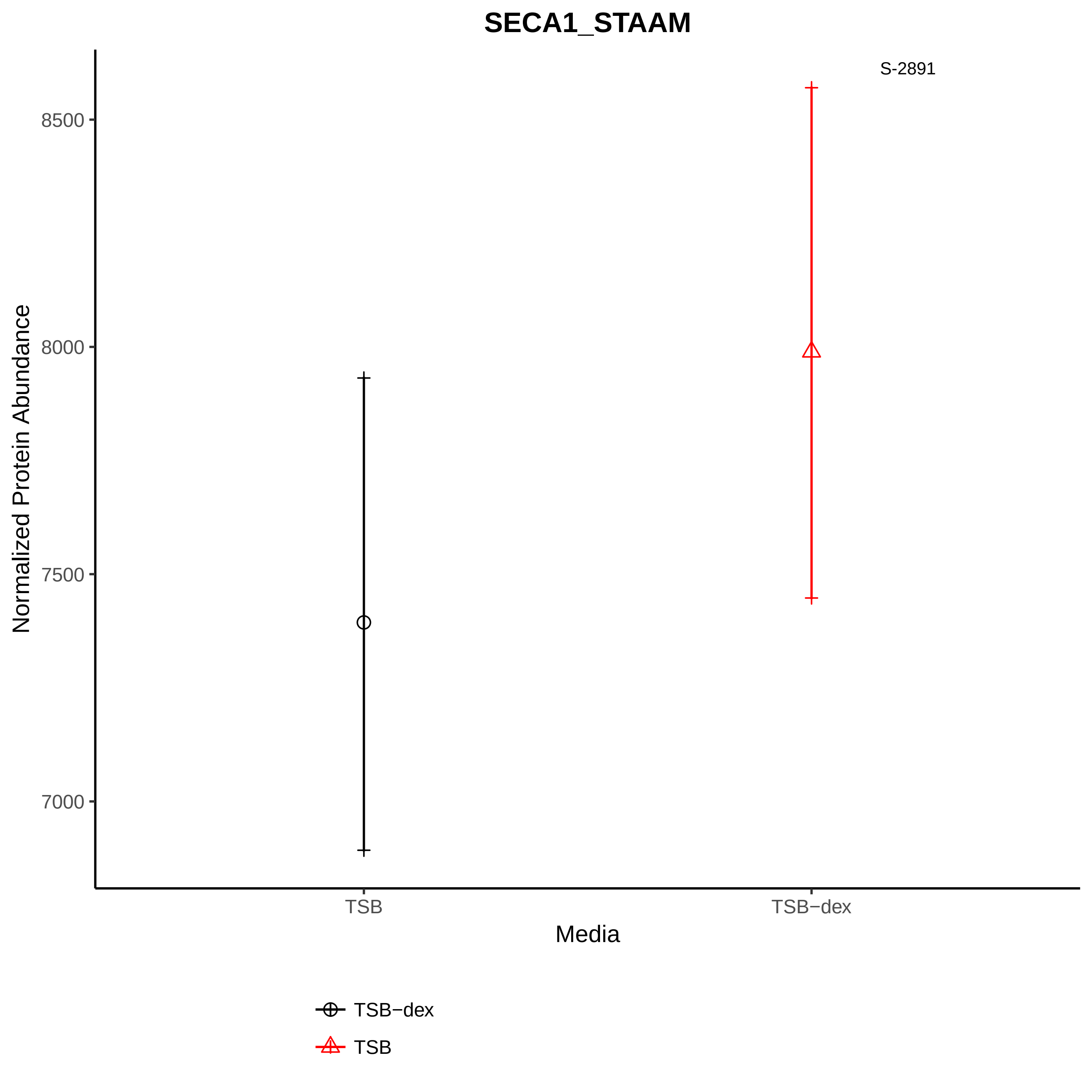
SECA1_STAAM
S-2891
Normalized Protein Abundance
8500
8000
7500
7000
TSB
TSB−dex
Media
TSB−dex
TSB

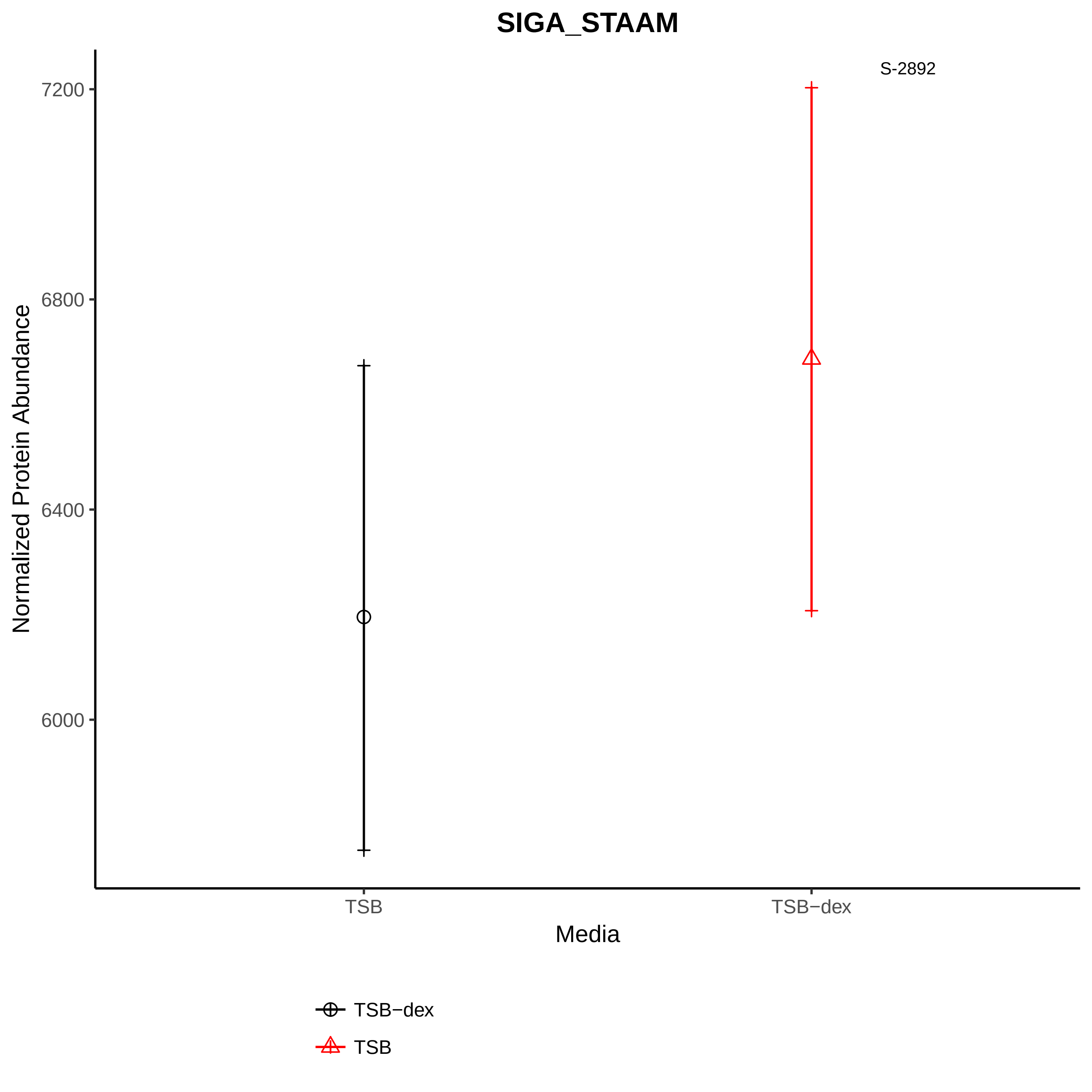

SIGA_STAAM
S-2892
7200
6800
6400
6000
Normalized Protein Abundance
TSB
TSB−dex
Media
TSB−dex
TSB

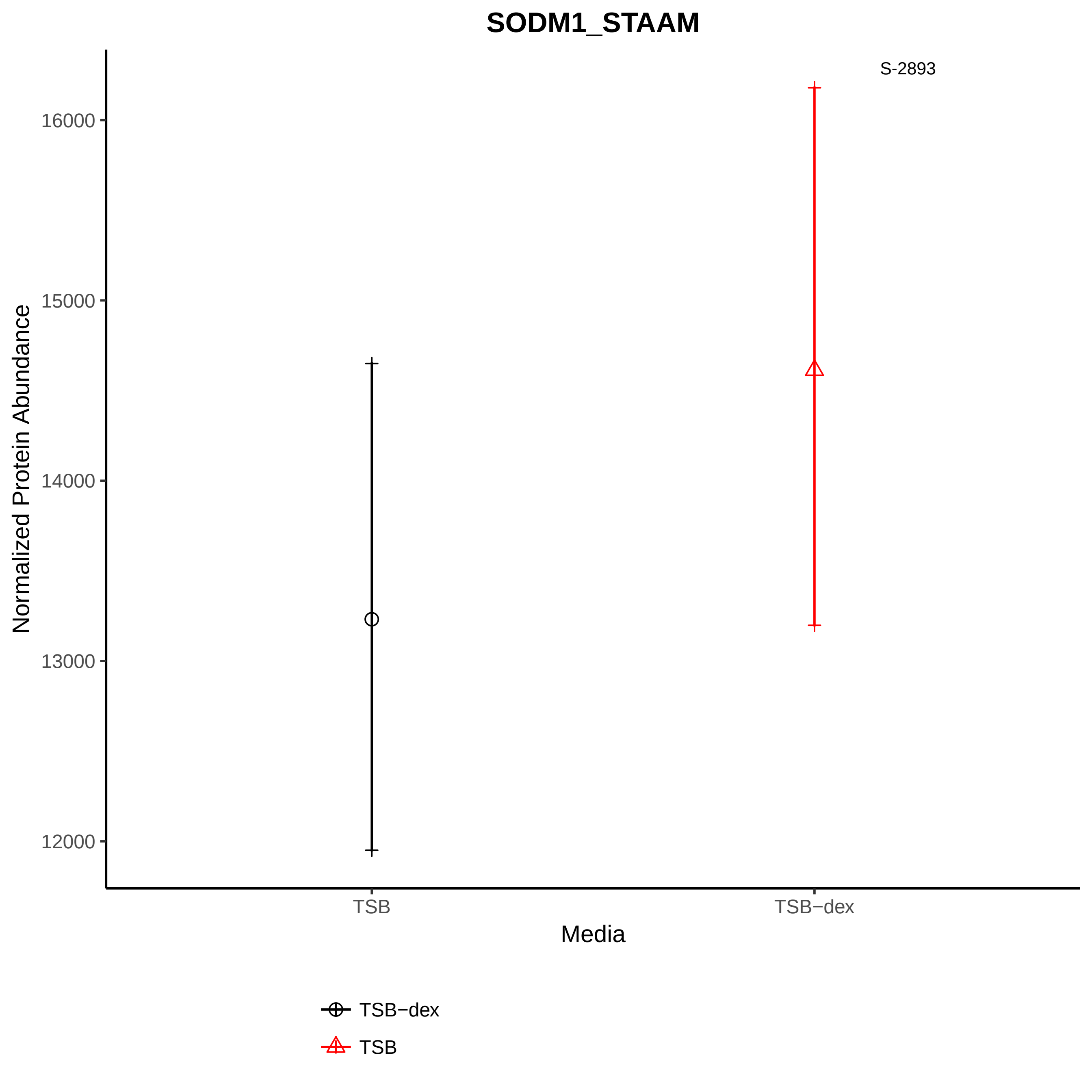
SODM1_STAAM
S-2893
Normalized Protein Abundance
16000
15000
14000
13000
12000
TSB
TSB−dex
Media
TSB−dex
TSB

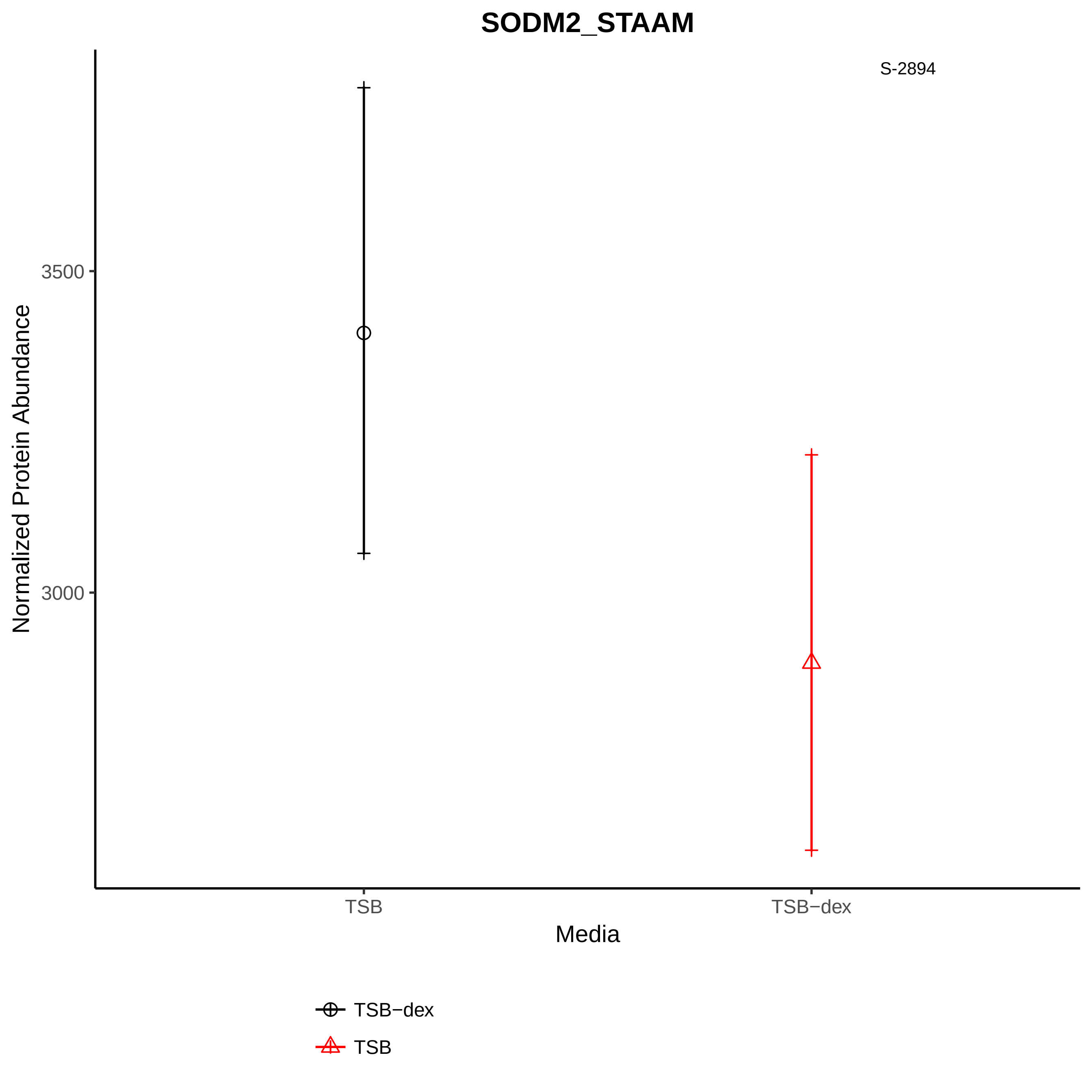

SODM2_STAAM
S-2894
Normalized Protein Abundance
3500
3000
TSB
TSB−dex
Media
TSB−dex
TSB

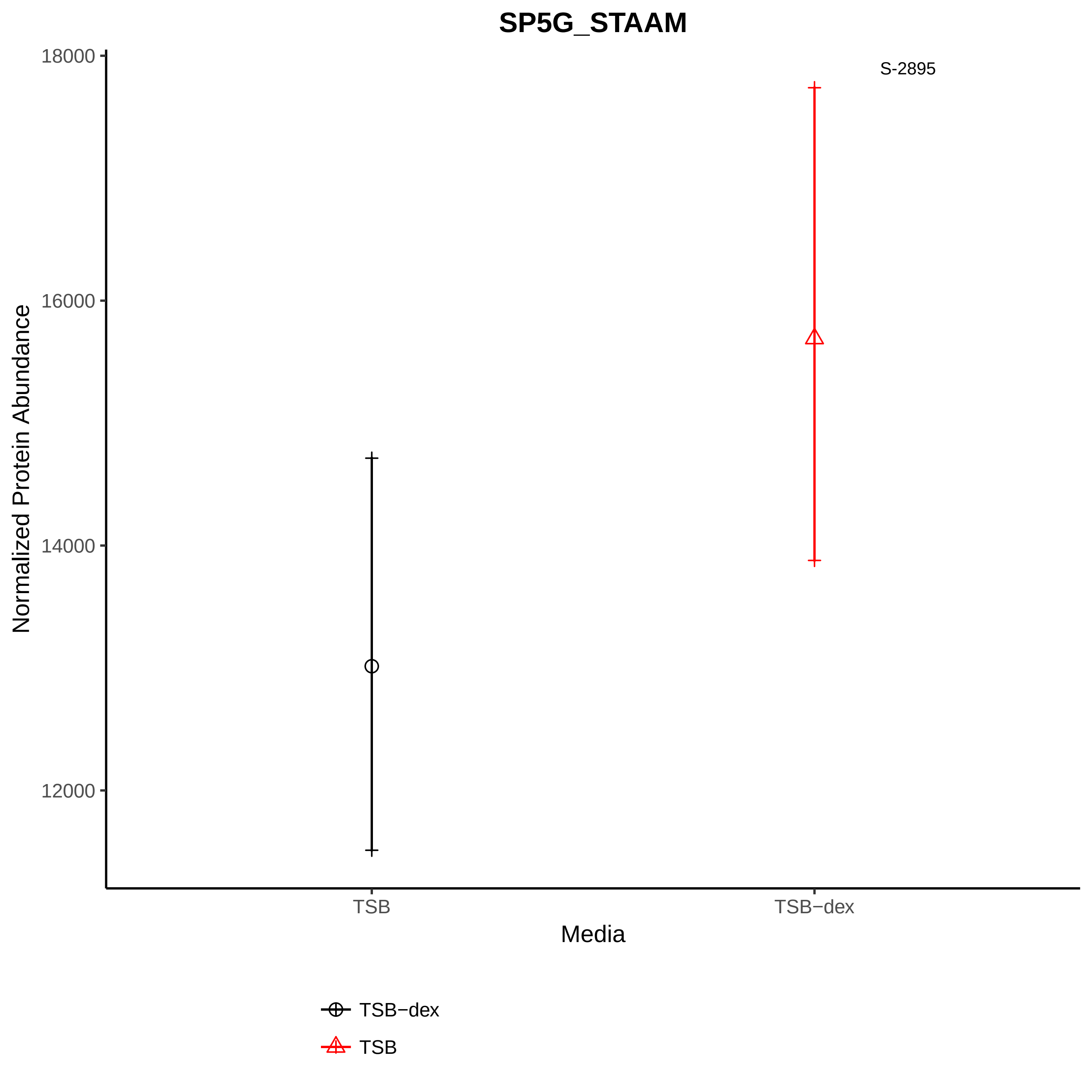
SP5G_STAAM
S-2895
Normalized Protein Abundance
18000
16000
14000
12000
TSB
TSB−dex
Media
TSB−dex
TSB

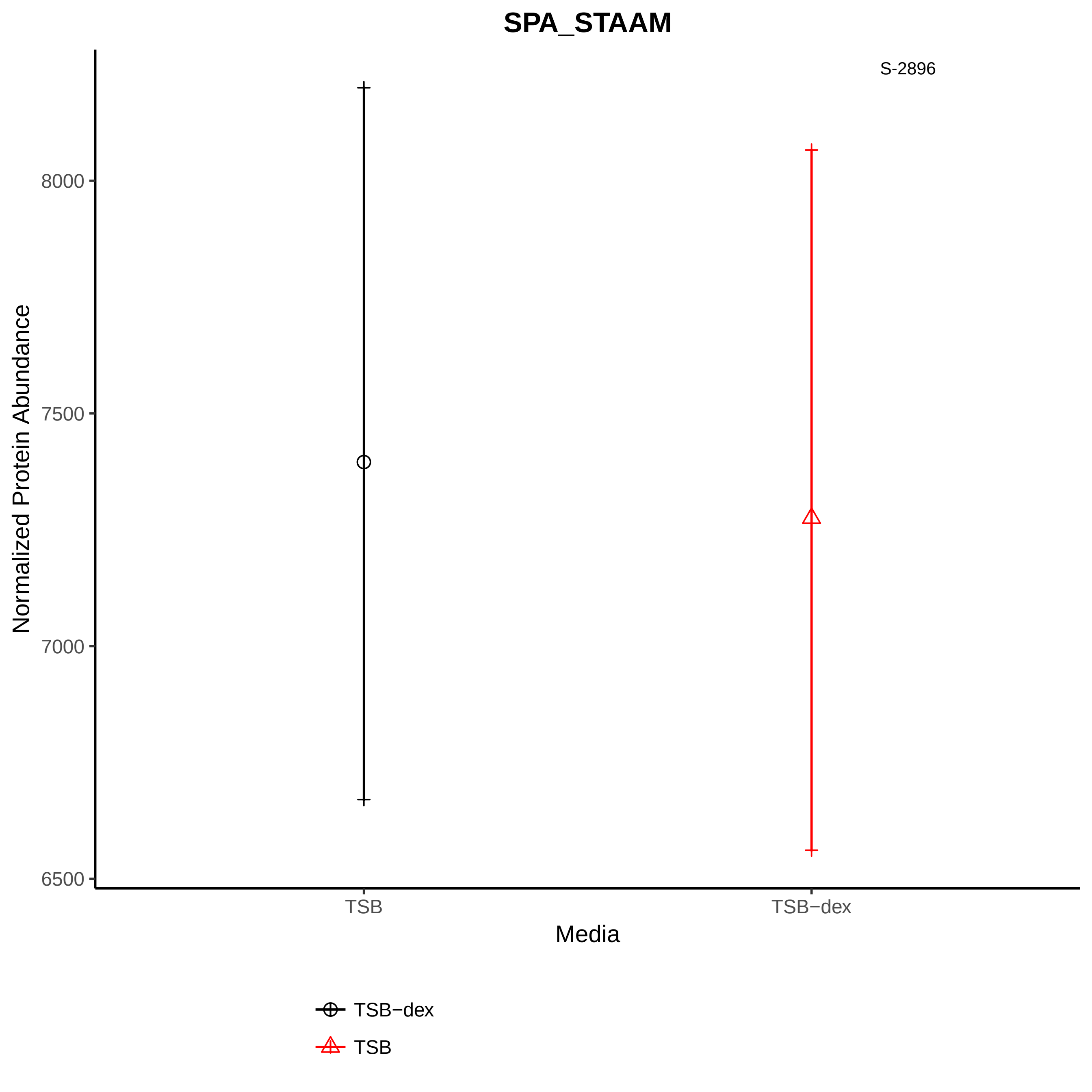

SPA_STAAM
S-2896
Normalized Protein Abundance
8000
7500
7000
6500
TSB
TSB−dex
Media
TSB−dex
TSB

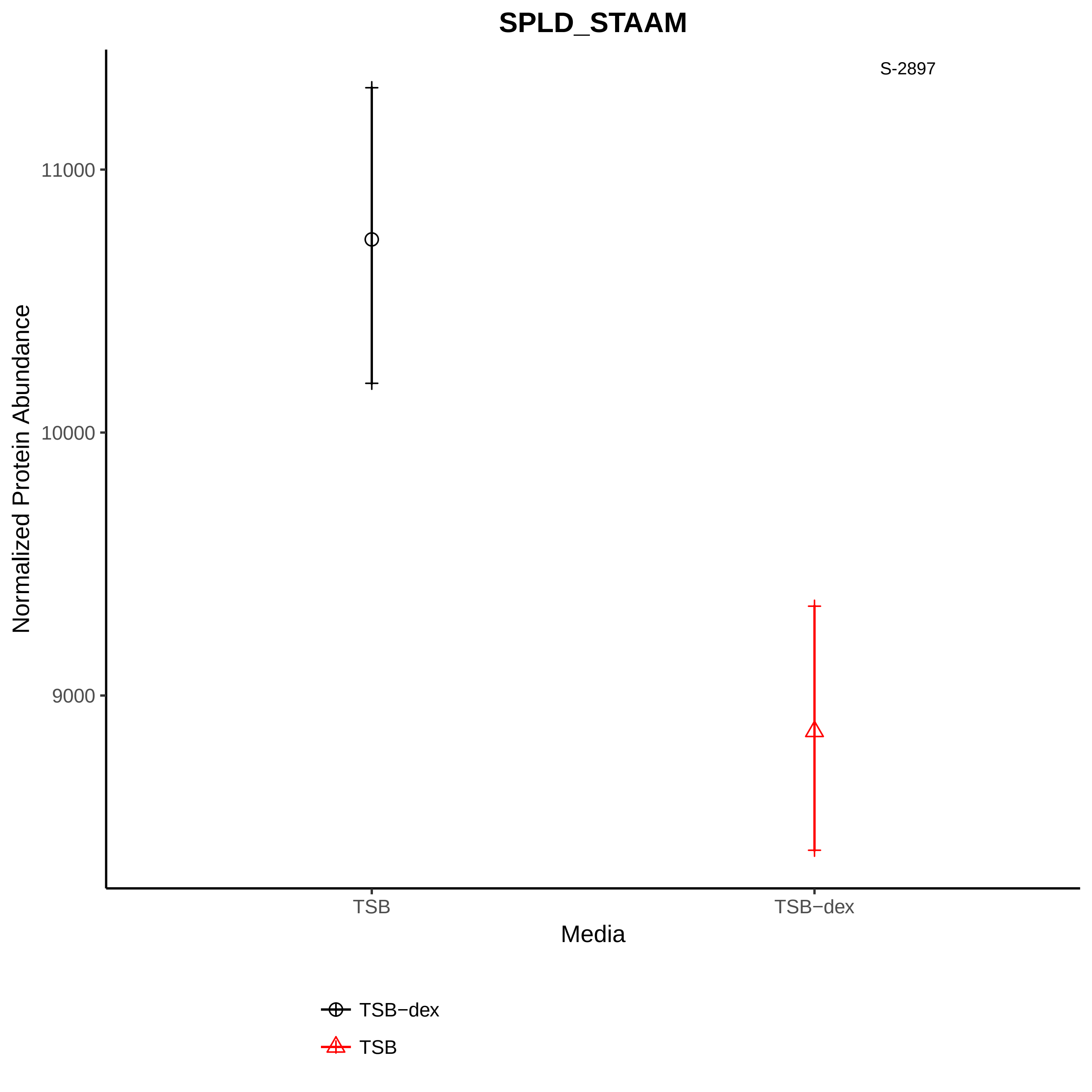

SPLD_STAAM
S-2897
11000
10000
9000
Normalized Protein Abundance
TSB
TSB−dex
Media
TSB−dex
TSB

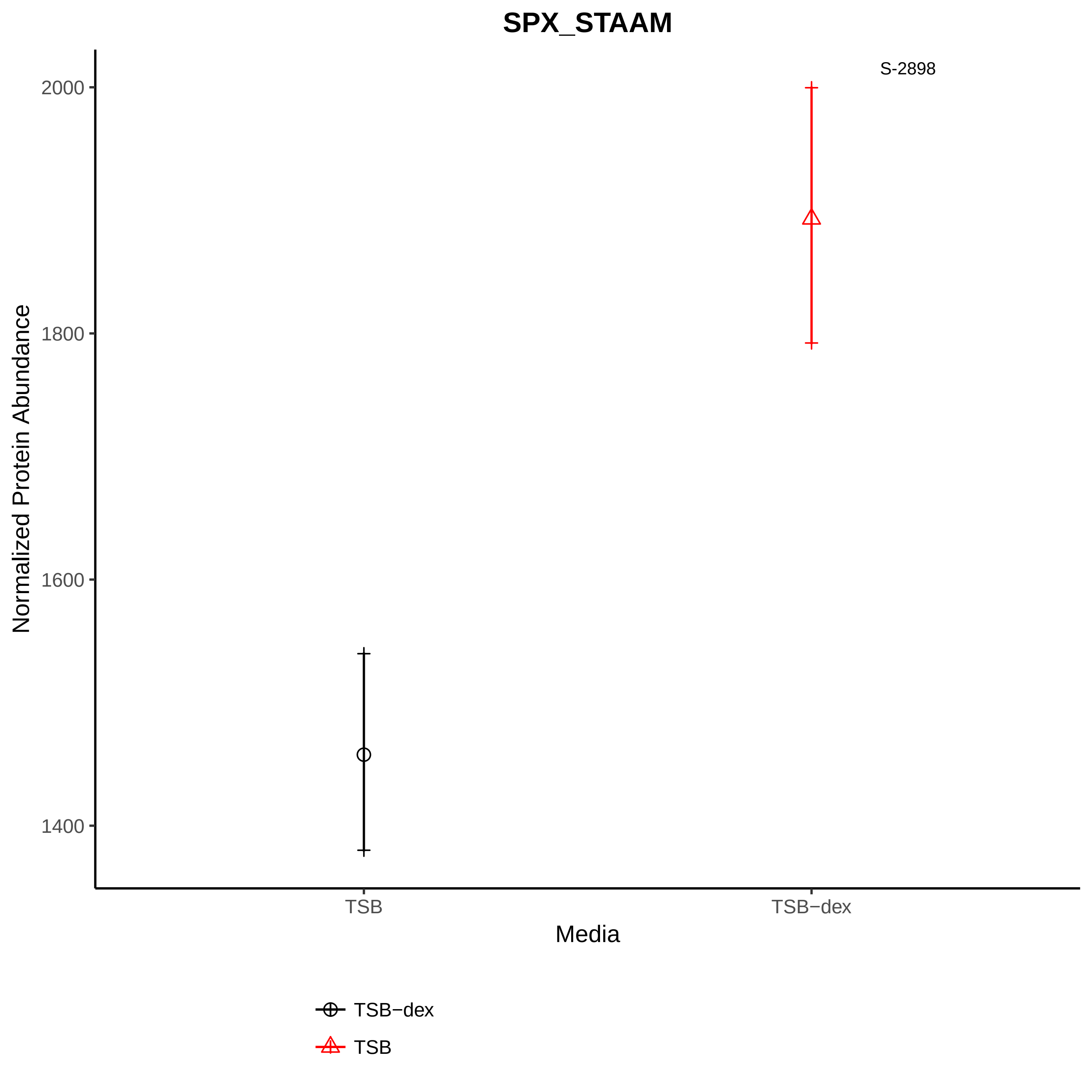

SPX_STAAM
S-2898
2000
1800
1600
1400
Normalized Protein Abundance
TSB
TSB−dex
Media
TSB−dex
TSB

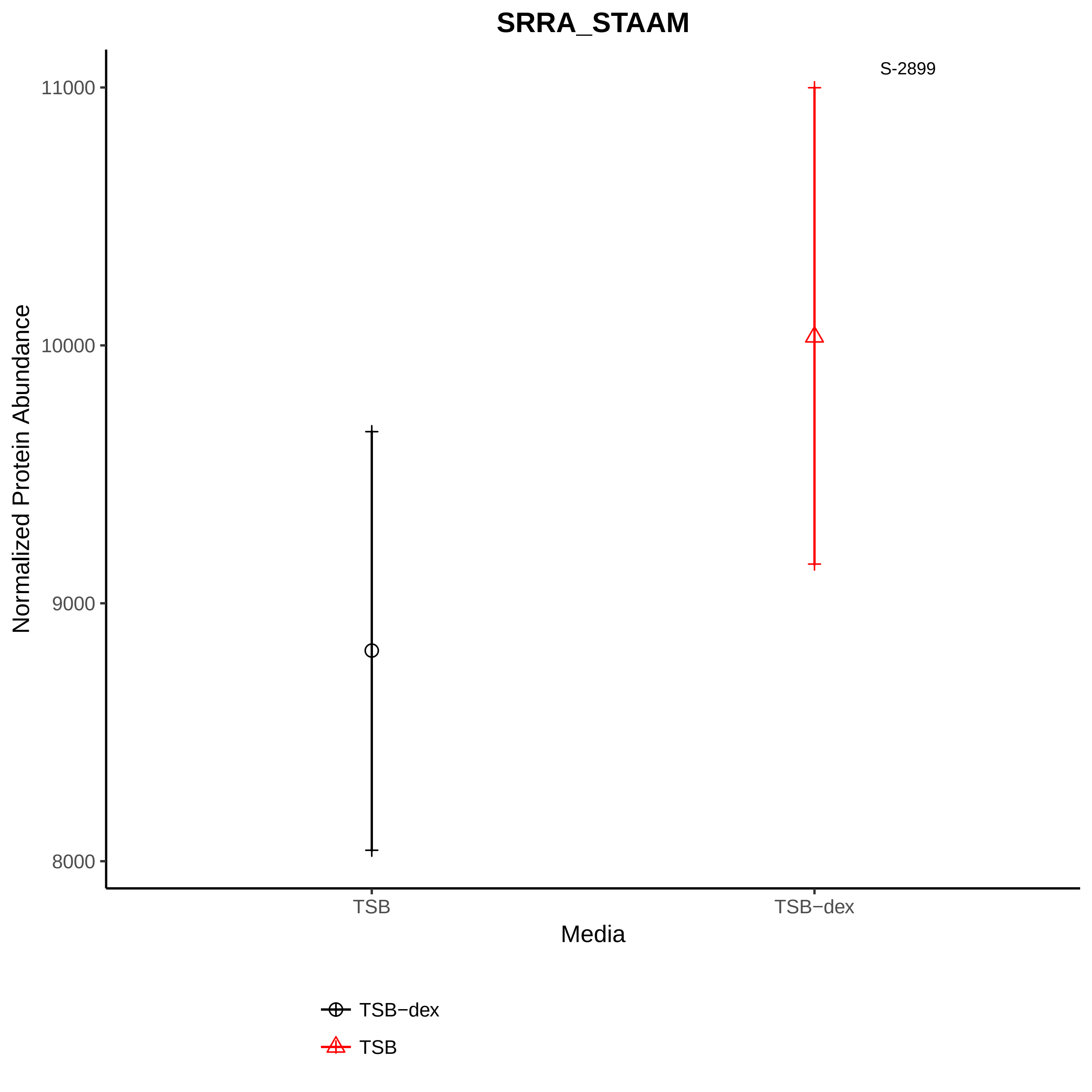

SRRA_STAAM
S-2899
Normalized Protein Abundance
11000
10000
9000
8000
TSB
TSB−dex
Media
TSB−dex
TSB

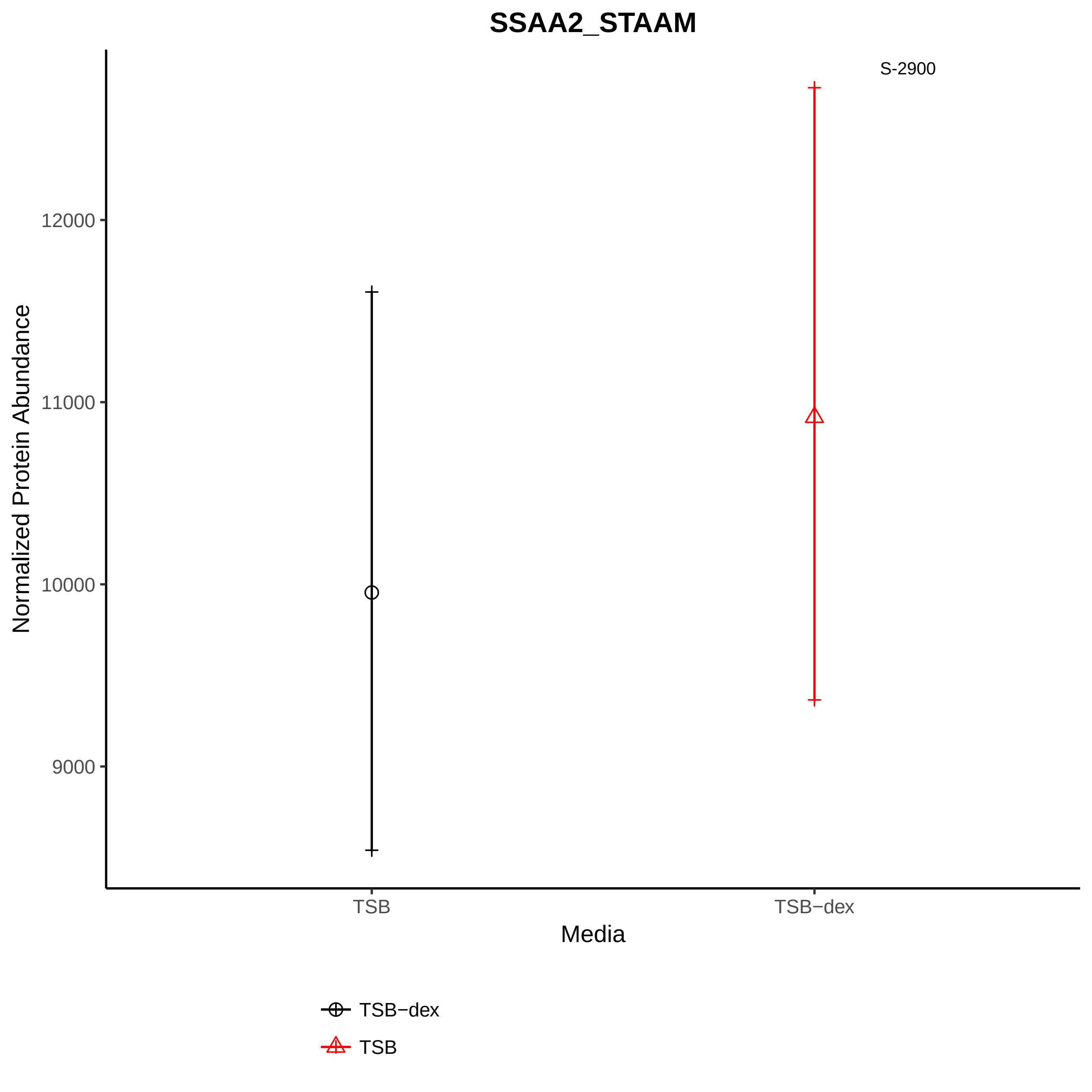
SSAA2_STAAM
S-2900
Normalized Protein Abundance
12000
11000
10000
9000
TSB
TSB−dex
Media
TSB−dex
TSB

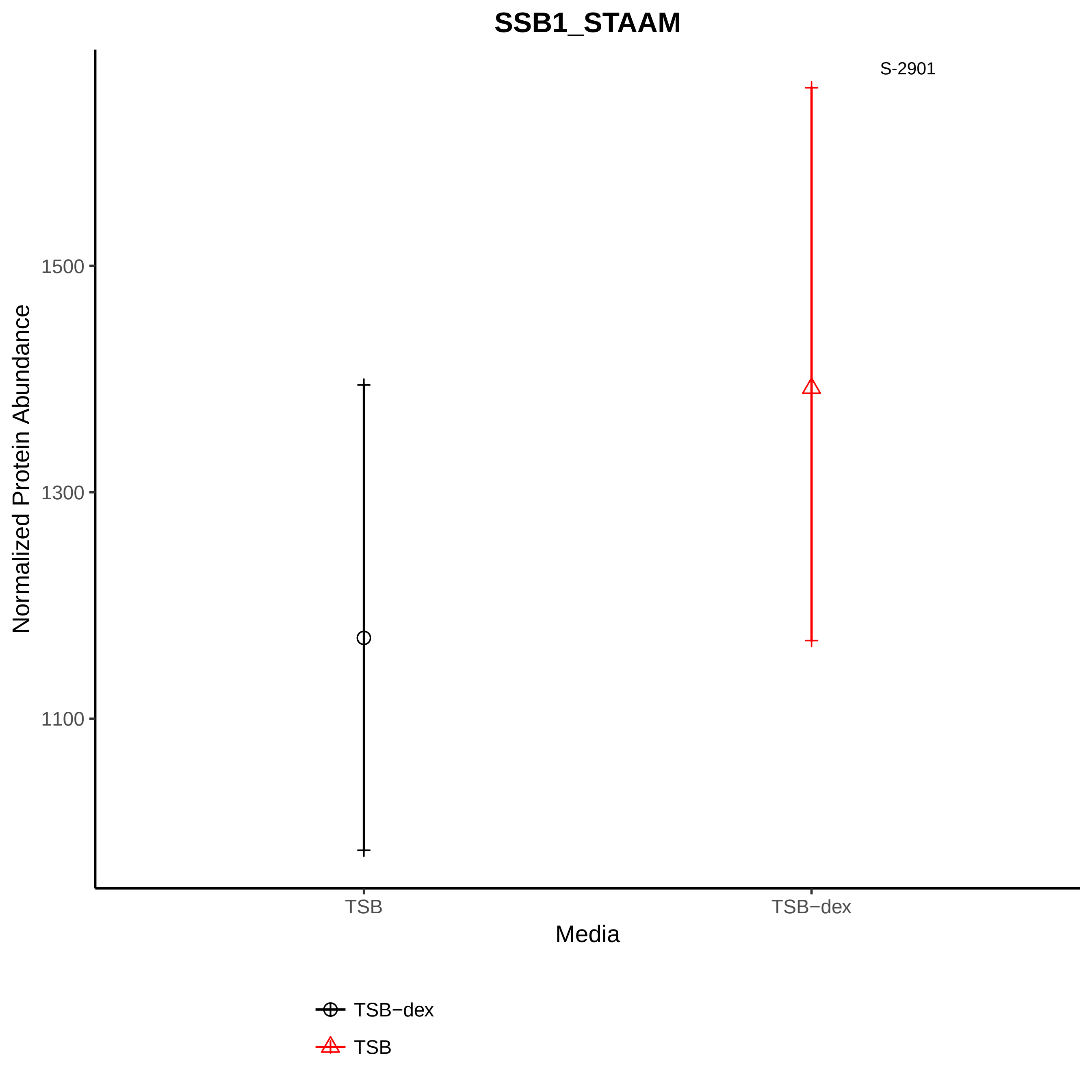

SSB1_STAAM
S-2901
Normalized Protein Abundance
1500
1300
1100
TSB
TSB−dex
Media
TSB−dex
TSB

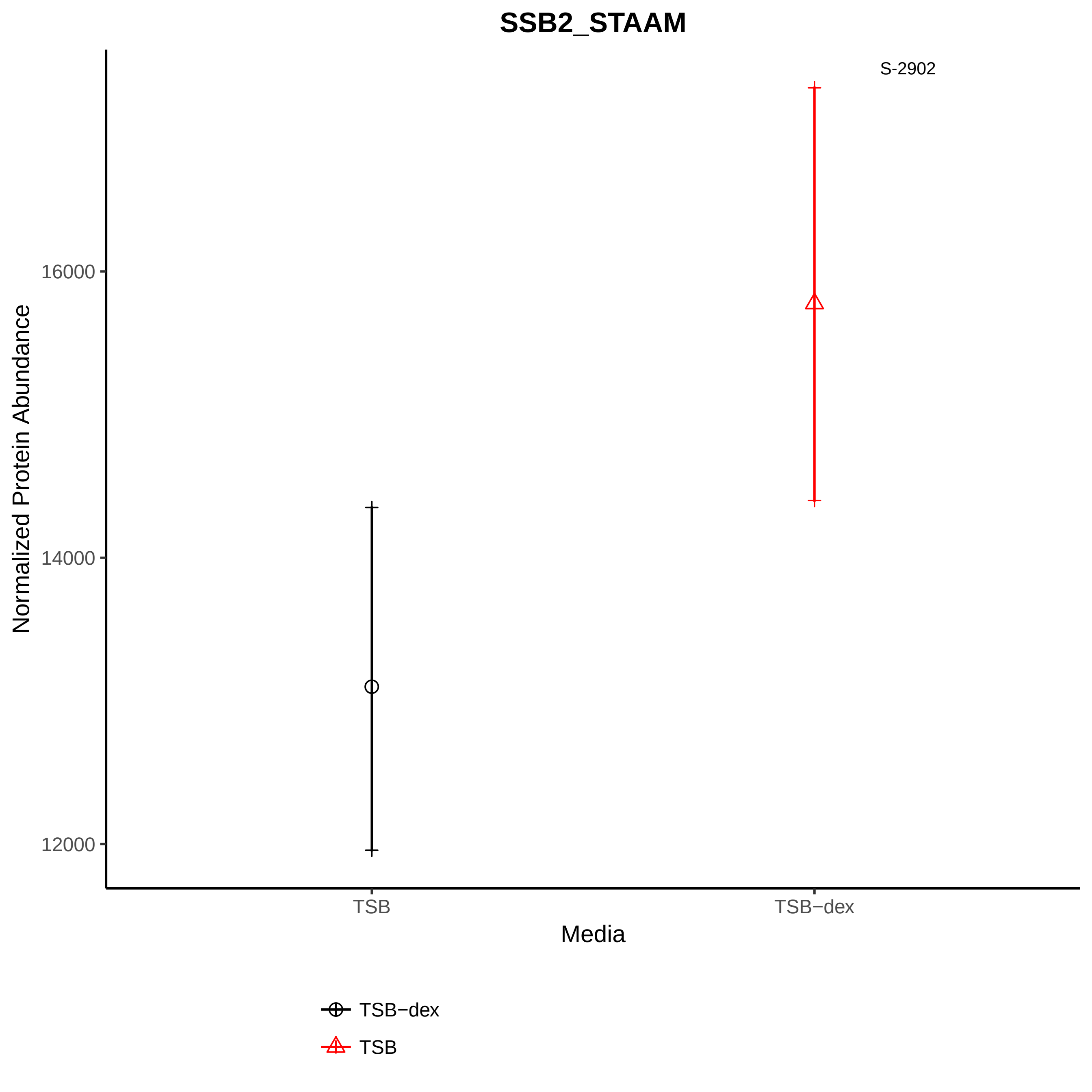
SSB2_STAAM
S-2902
Normalized Protein Abundance
16000
14000
12000
TSB
TSB−dex
Media
TSB−dex
TSB

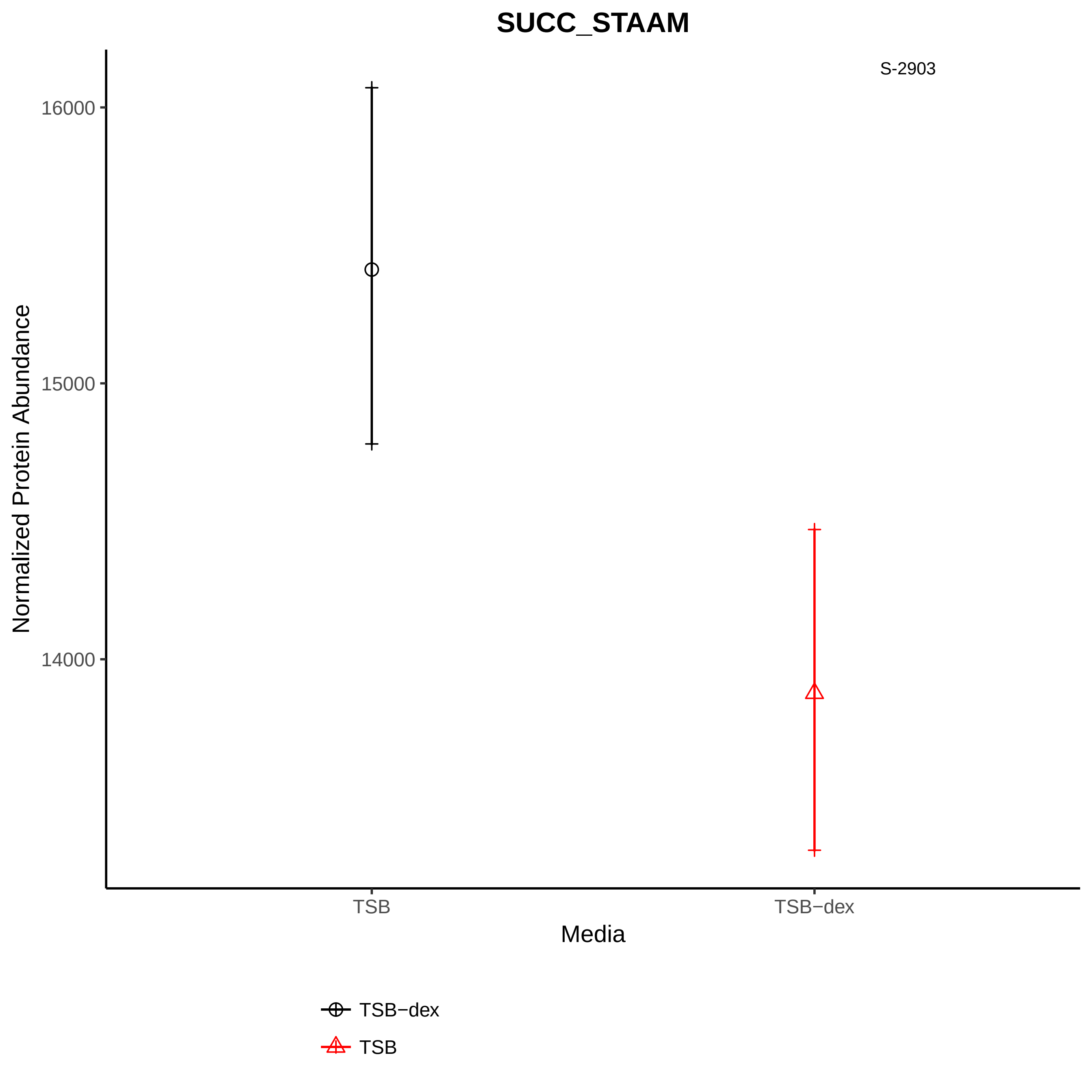

SUCC_STAAM
S-2903
16000
15000
14000
Normalized Protein Abundance
TSB
TSB−dex
Media
TSB−dex
TSB

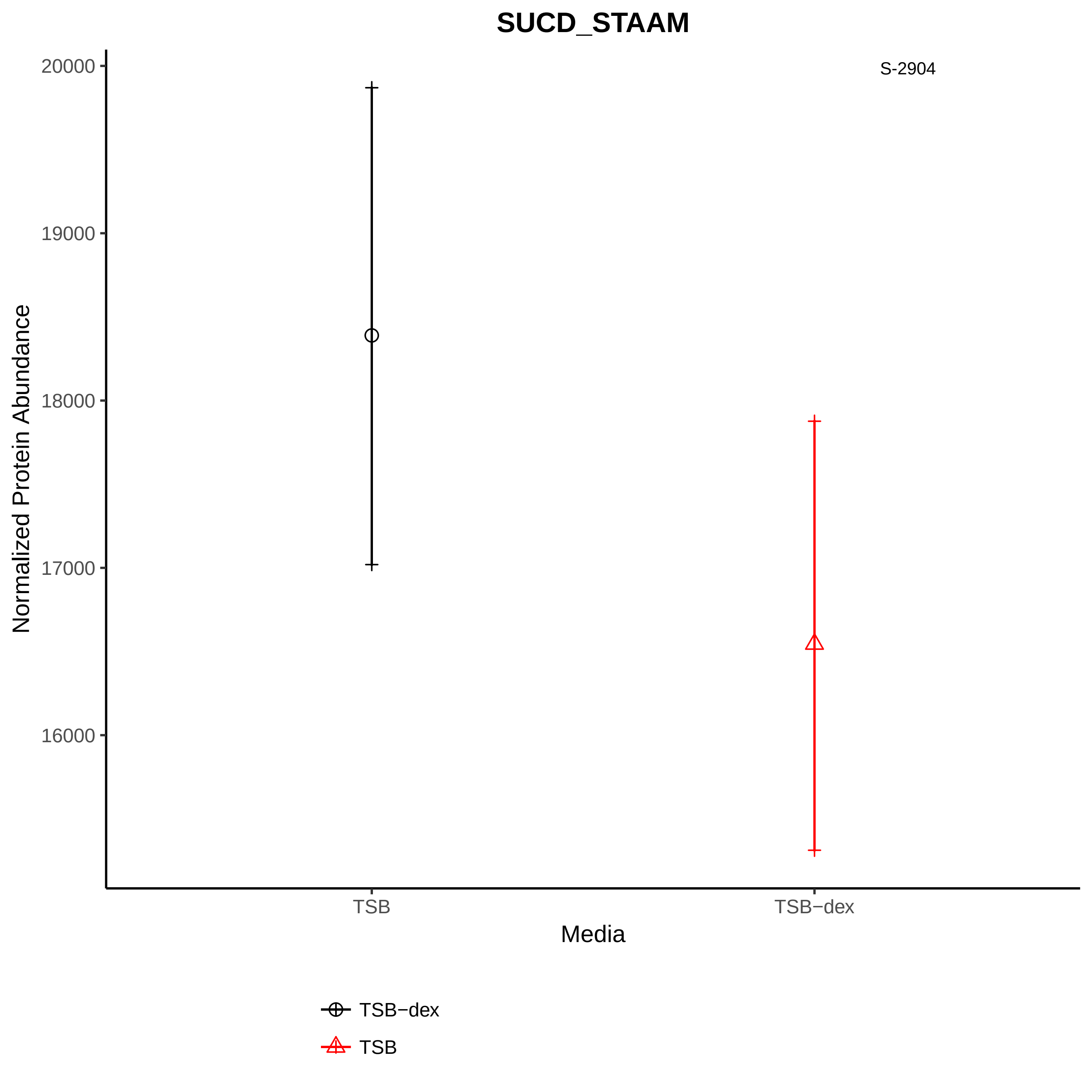

SUCD_STAAM
S-2904
20000
19000
18000
17000
16000
Normalized Protein Abundance
TSB
TSB−dex
Media
TSB−dex
TSB

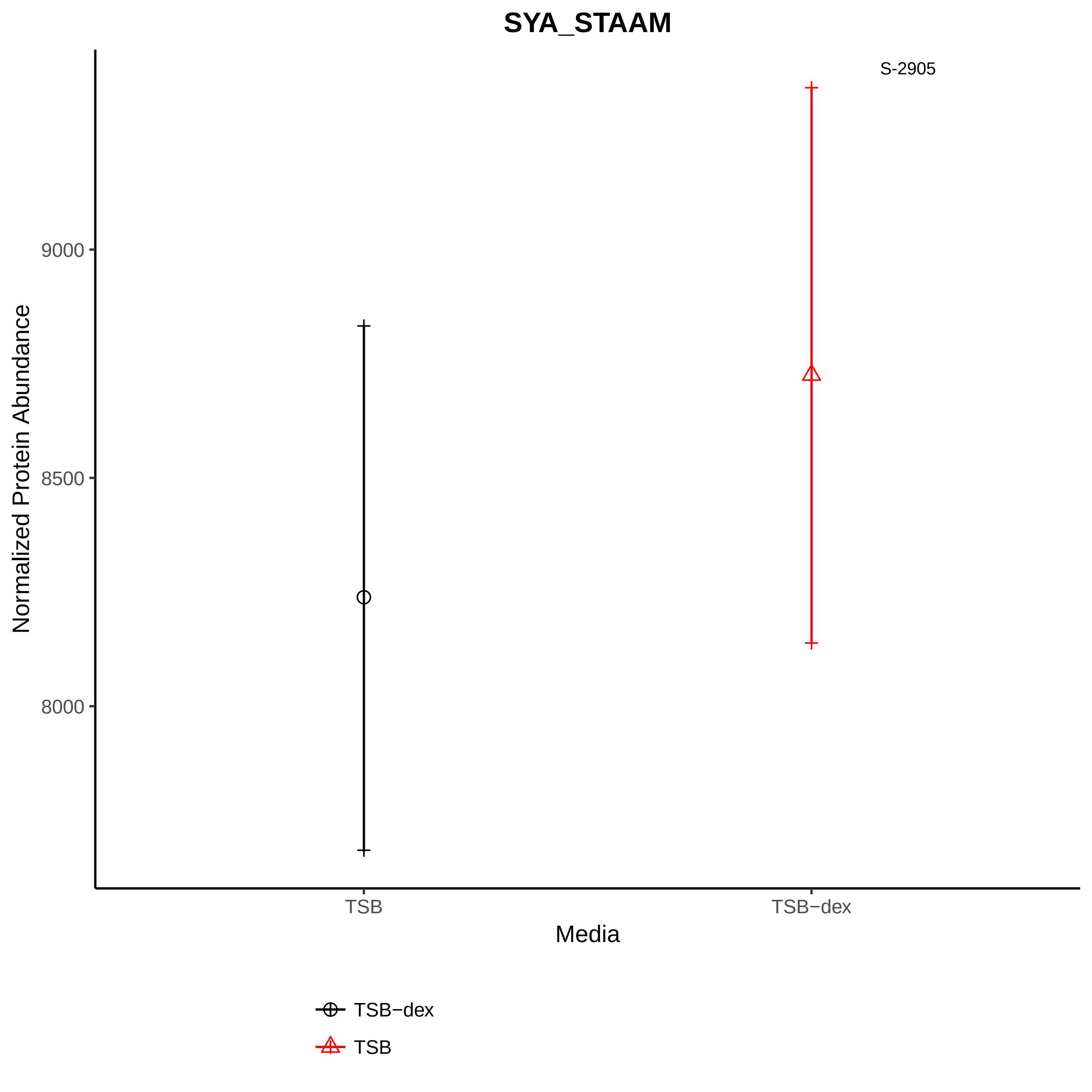
SYA_STAAM
S-2905
Normalized Protein Abundance
9000
8500
8000
TSB
TSB−dex
Media
TSB−dex
TSB

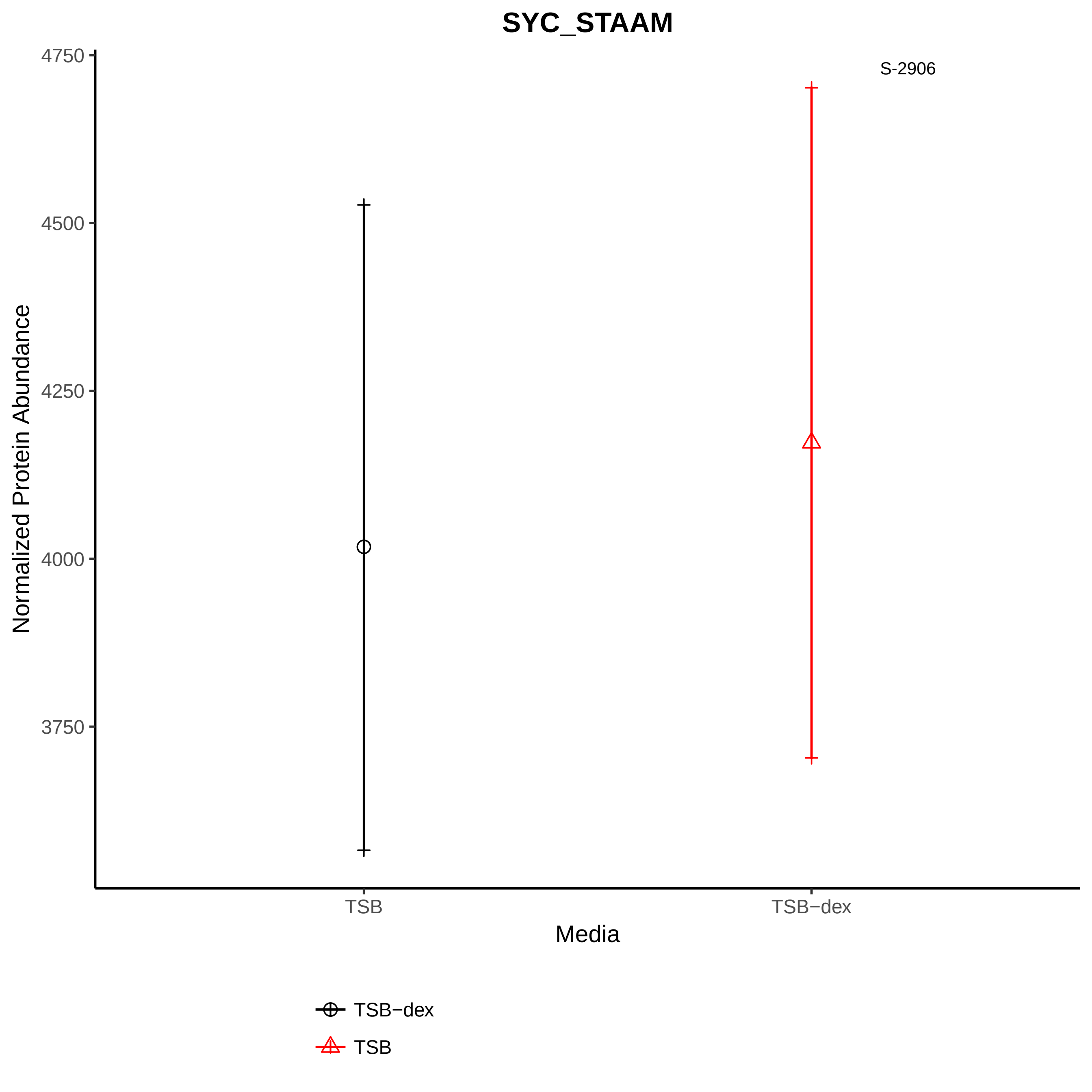

SYC_STAAM
S-2906
4750
4500
4250
4000
3750
Normalized Protein Abundance
TSB
TSB−dex
Media
TSB−dex
TSB

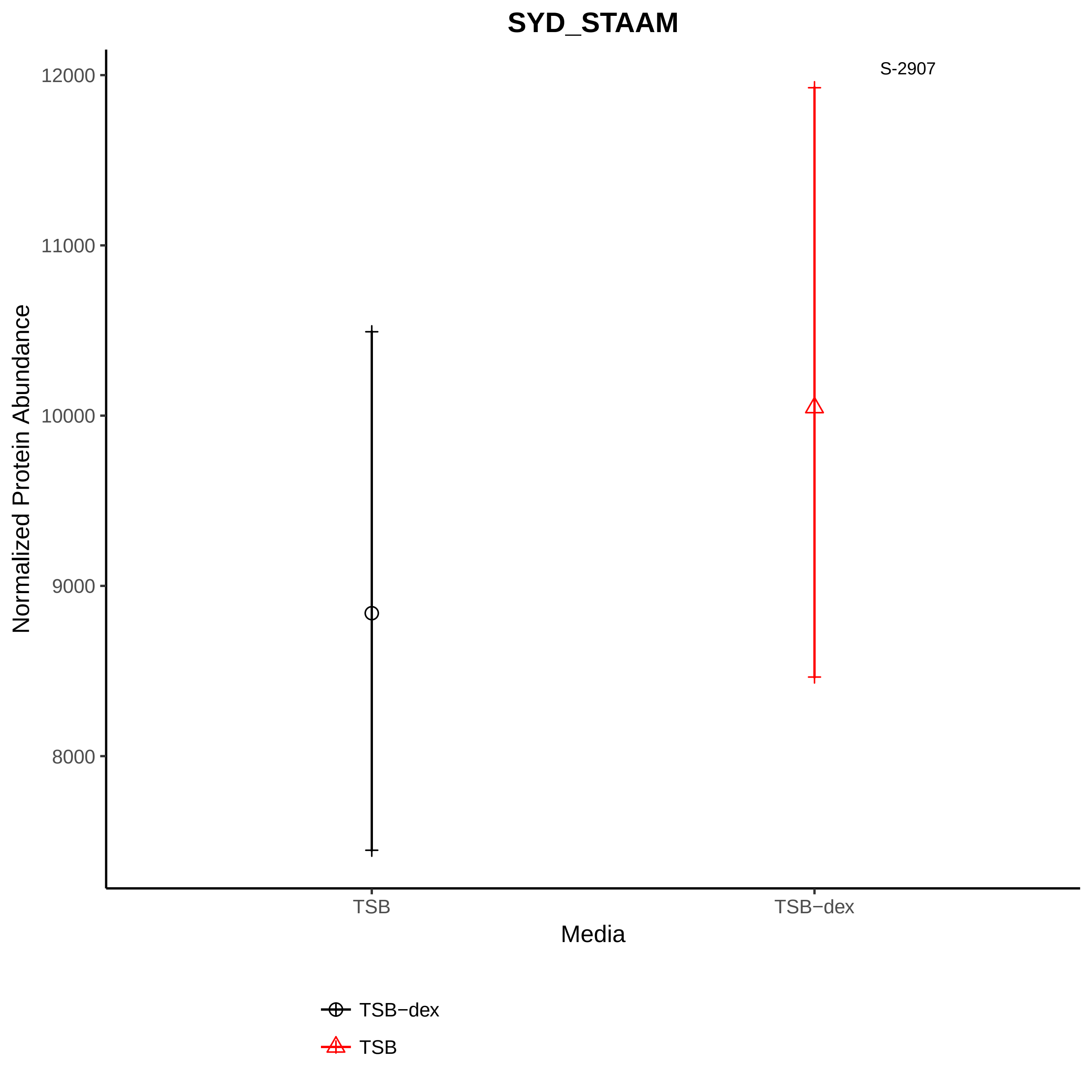

SYD_STAAM
S-2907
12000
11000
10000
9000
8000
Normalized Protein Abundance
TSB
TSB−dex
Media
TSB−dex
TSB

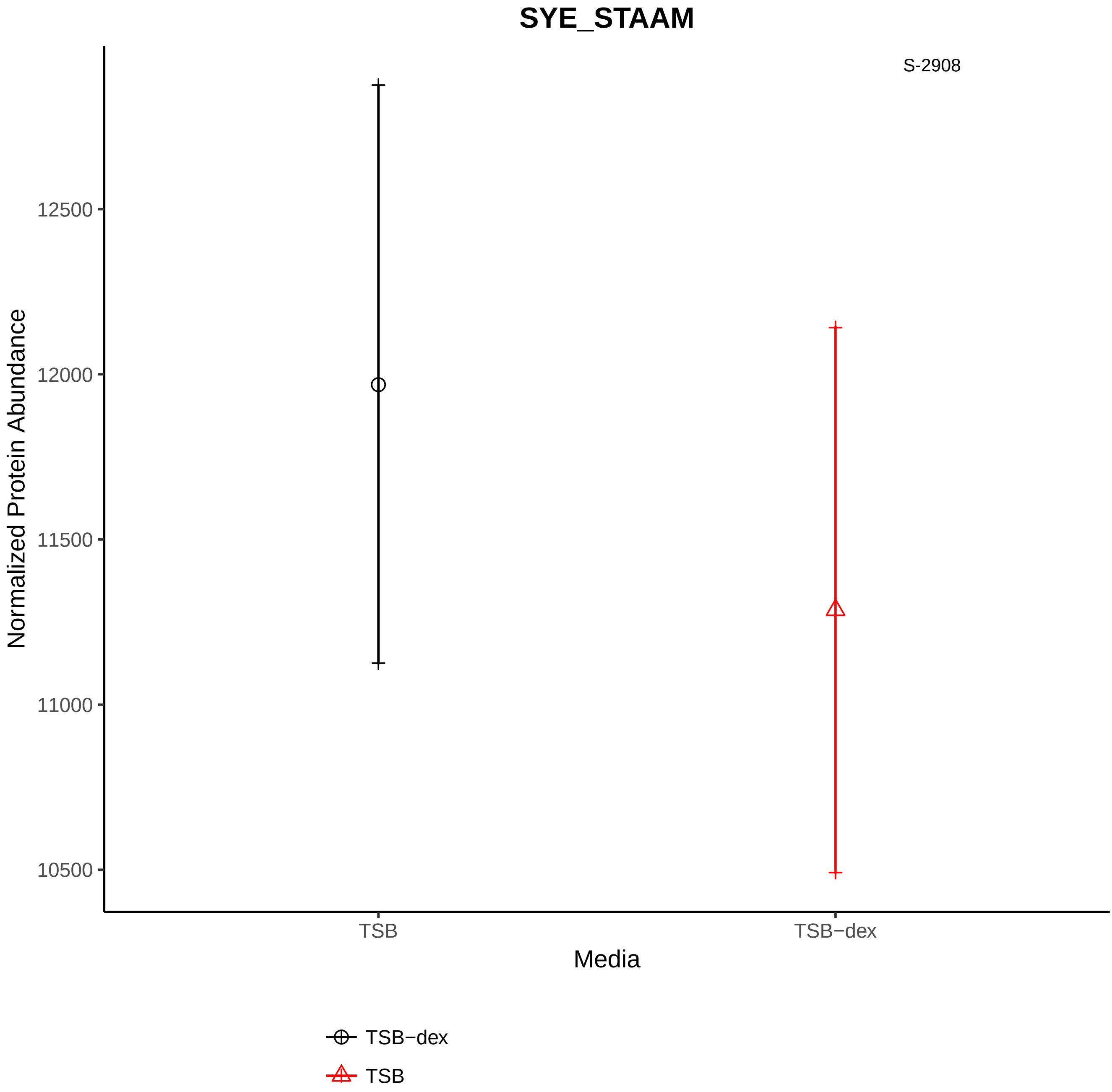

SYE_STAAM
S-2908
12500
12000
11500
11000
10500
Normalized Protein Abundance
TSB
TSB−dex
Media
TSB−dex
TSB

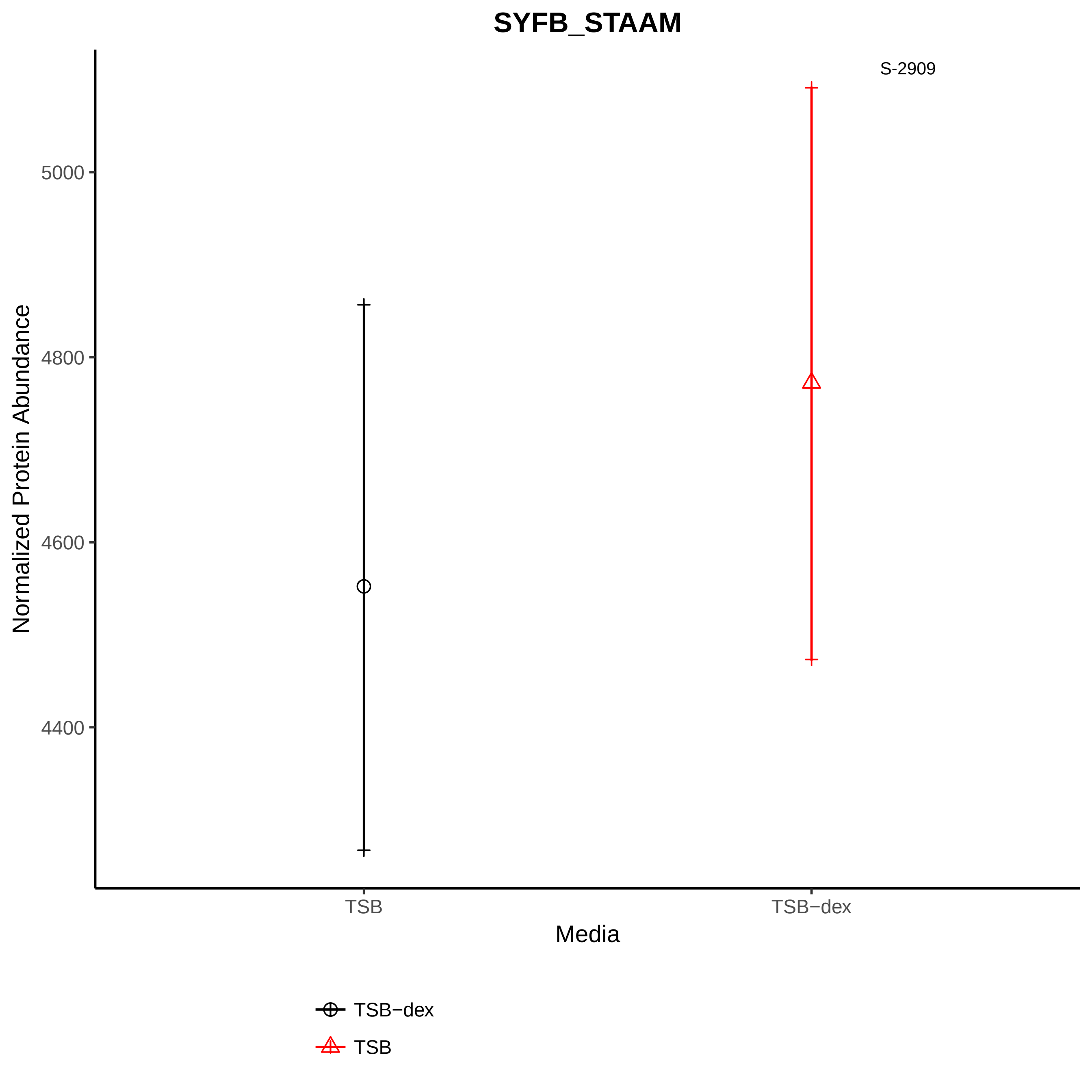

SYFB_STAAM
S-2909
Normalized Protein Abundance
5000
4800
4600
4400
TSB
TSB−dex
Media
TSB−dex
TSB

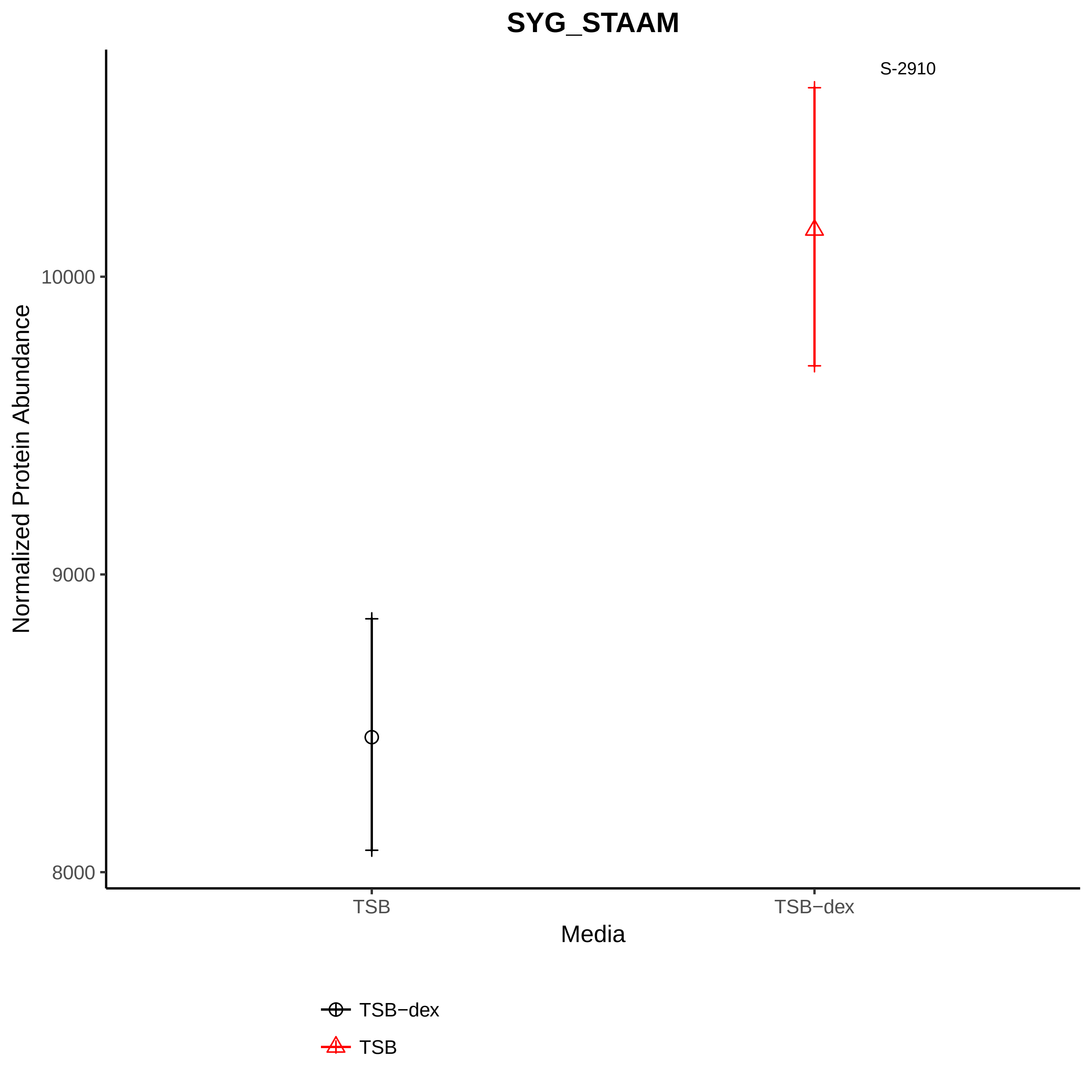
SYG_STAAM
S-2910
Normalized Protein Abundance
10000
9000
8000
TSB
TSB−dex
Media
TSB−dex
TSB

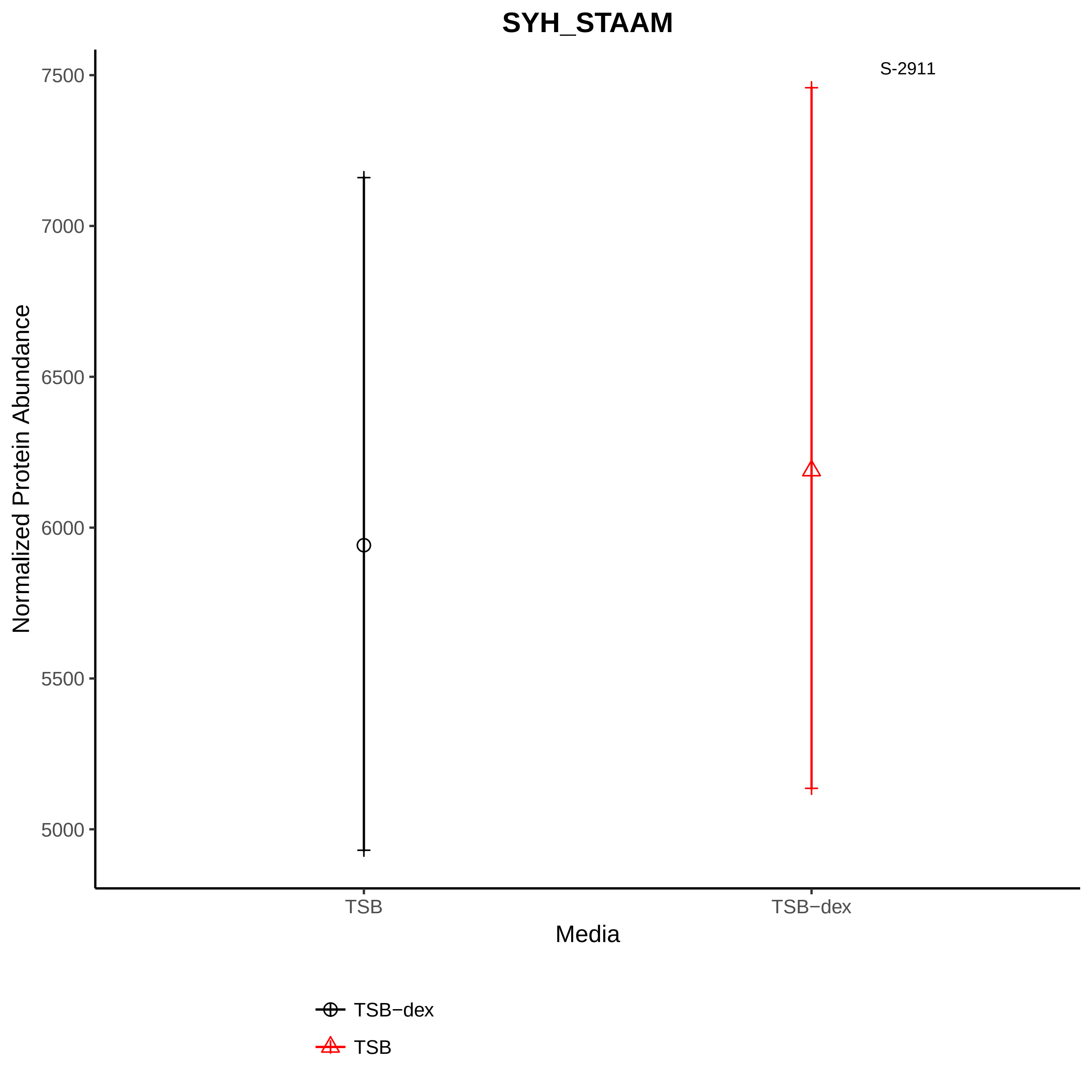

SYH_STAAM
S-2911
Normalized Protein Abundance
7500
7000
6500
6000
5500
5000
TSB
TSB−dex
Media
TSB−dex
TSB

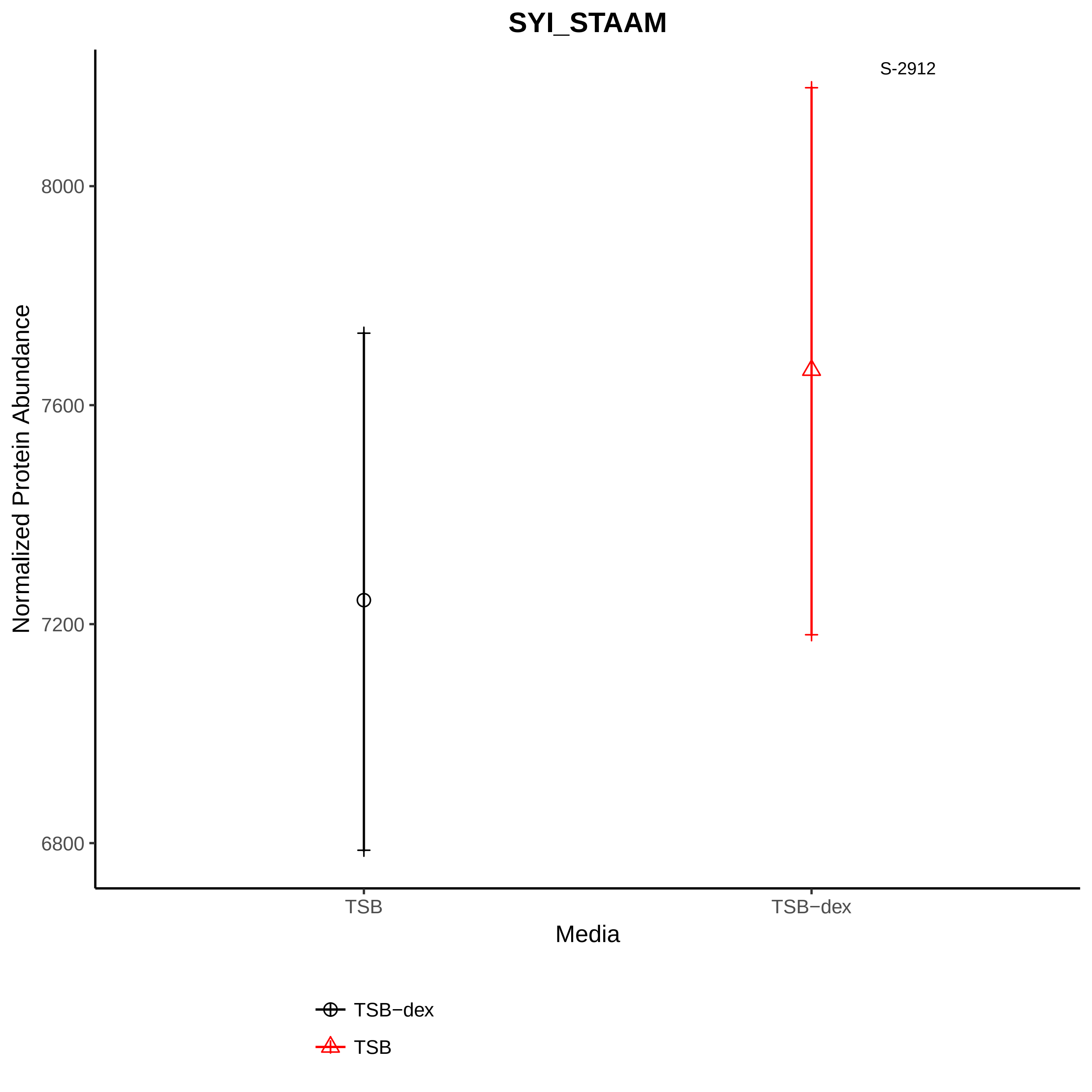
SYI_STAAM
S-2912
Normalized Protein Abundance
8000
7600
7200
6800
TSB
TSB−dex
Media
TSB−dex
TSB

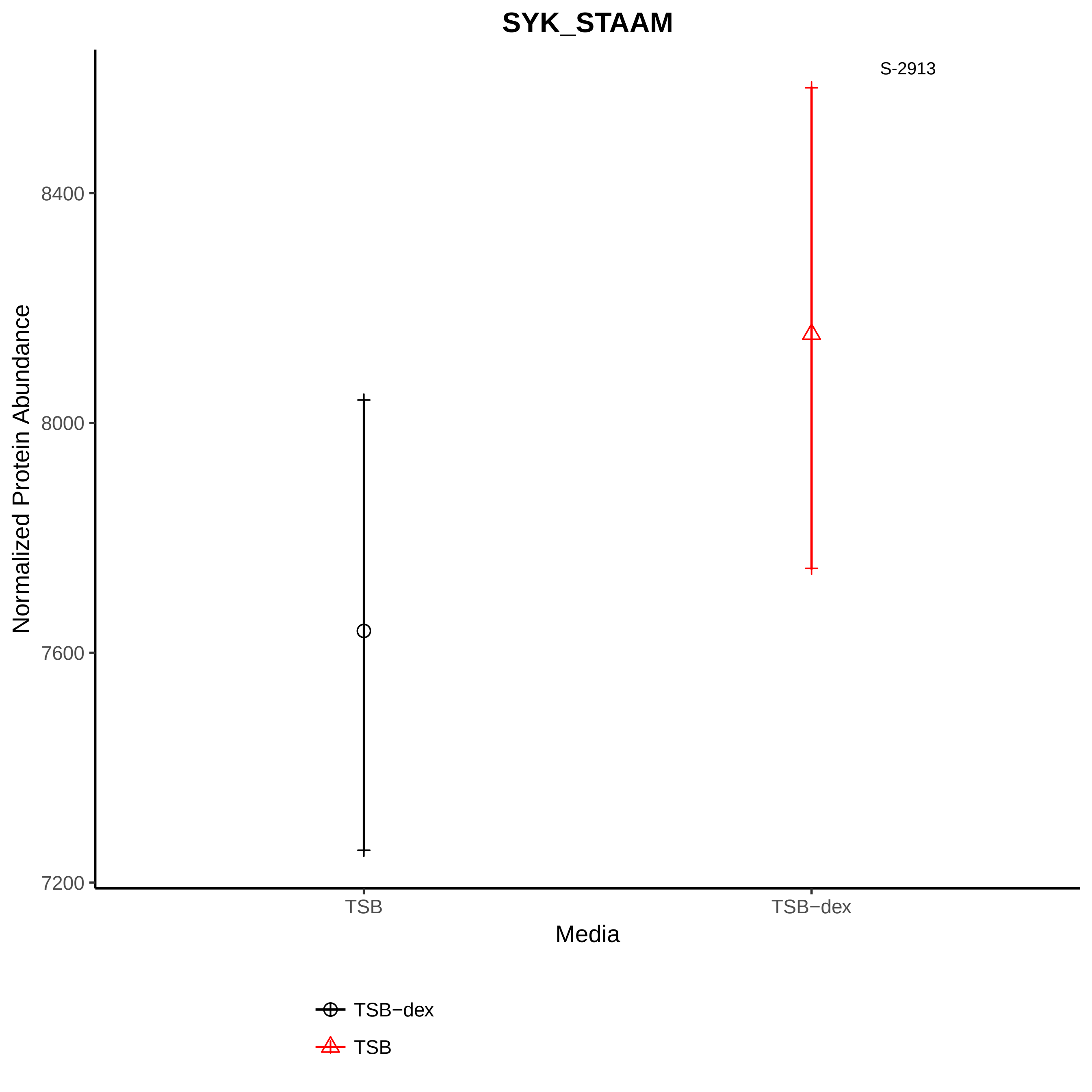

SYK_STAAM
S-2913
8400
8000
7600
7200
Normalized Protein Abundance
TSB
TSB−dex
Media
TSB−dex
TSB

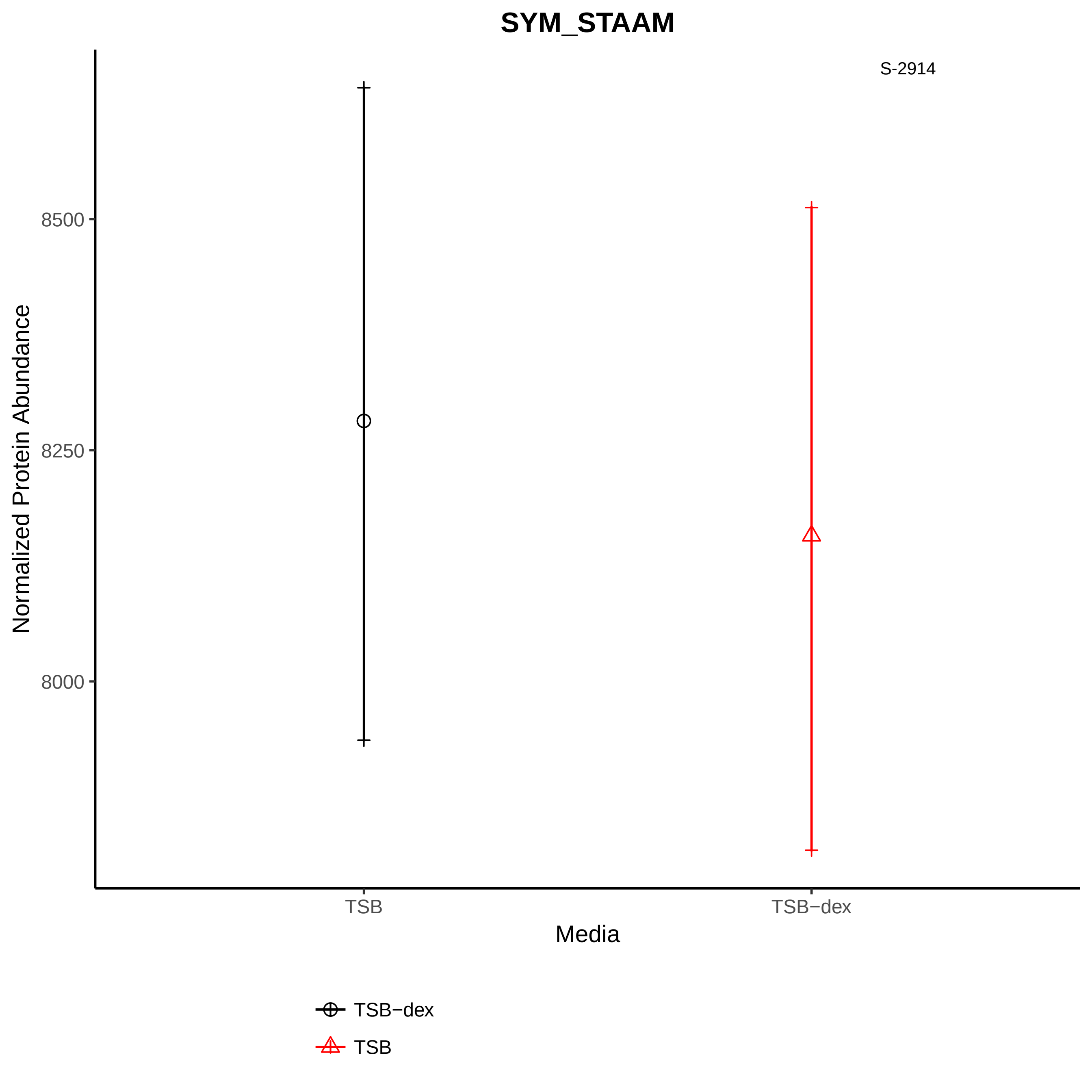

SYM_STAAM
S-2914
Normalized Protein Abundance
8500
8250
8000
TSB
TSB−dex
Media
TSB−dex
TSB

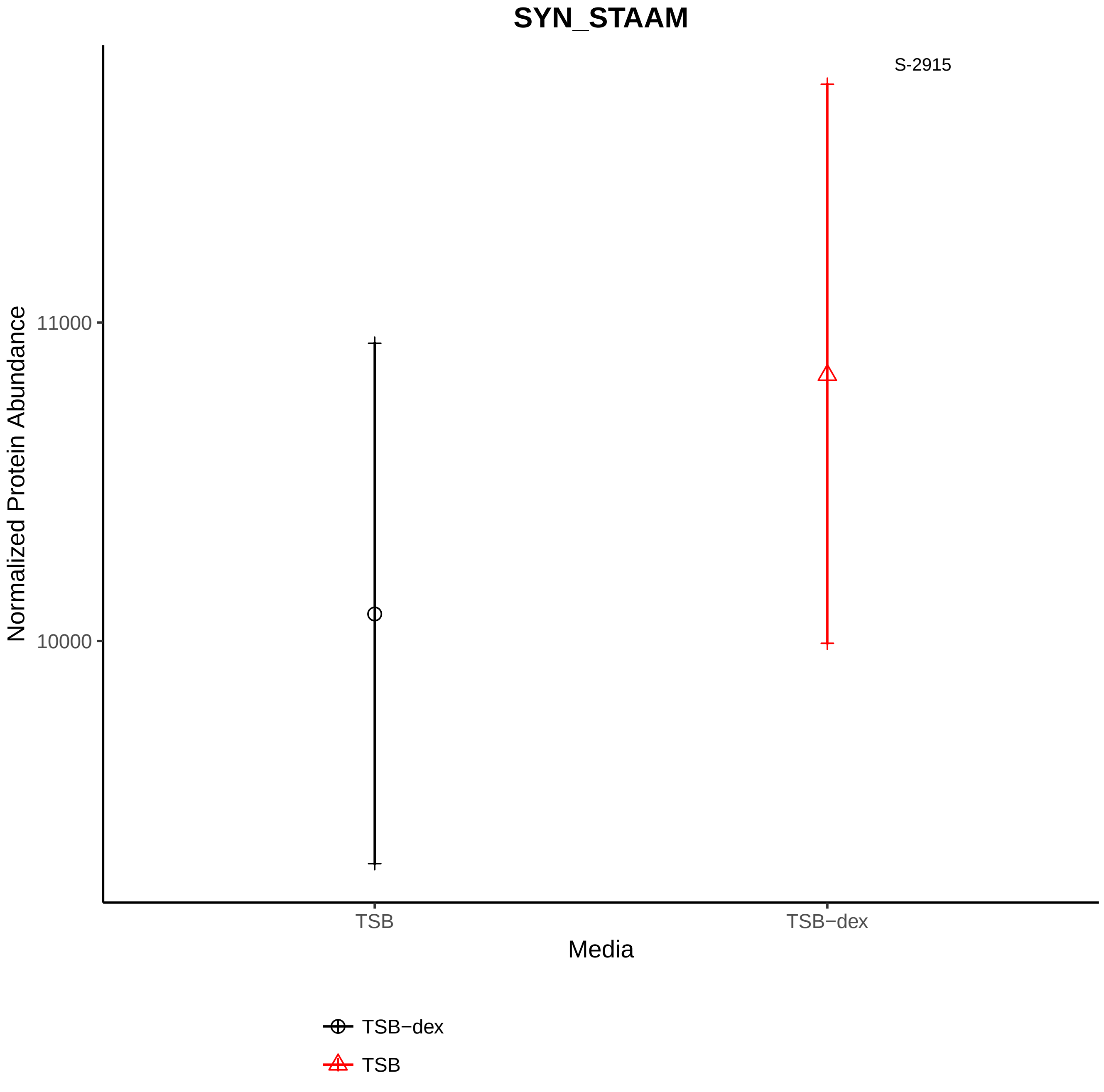

SYN_STAAM
S-2915
Normalized Protein Abundance
11000
10000
TSB
TSB−dex
Media
TSB−dex
TSB

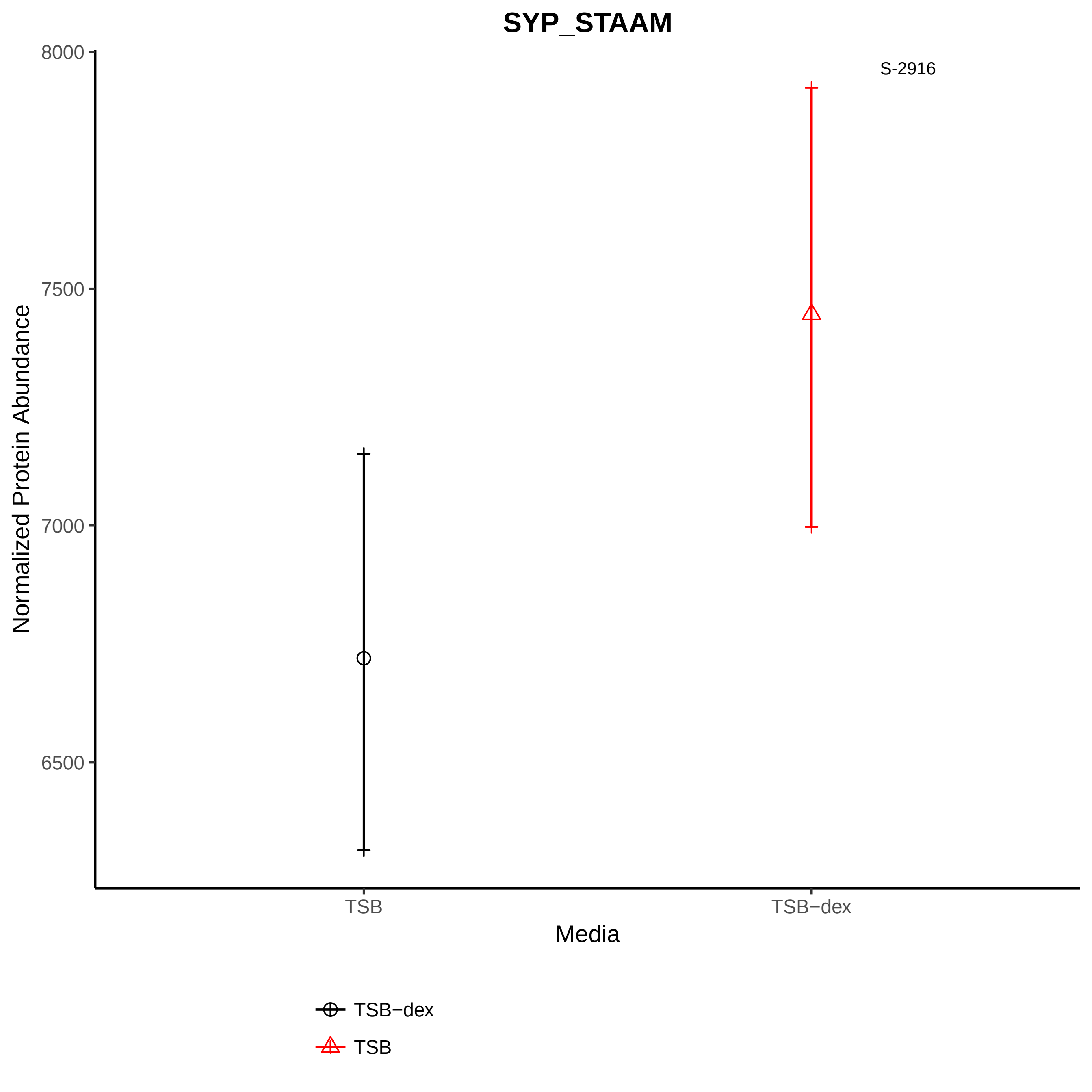

SYP_STAAM
S-2916
8000
7500
7000
6500
Normalized Protein Abundance
TSB
TSB−dex
Media
TSB−dex
TSB

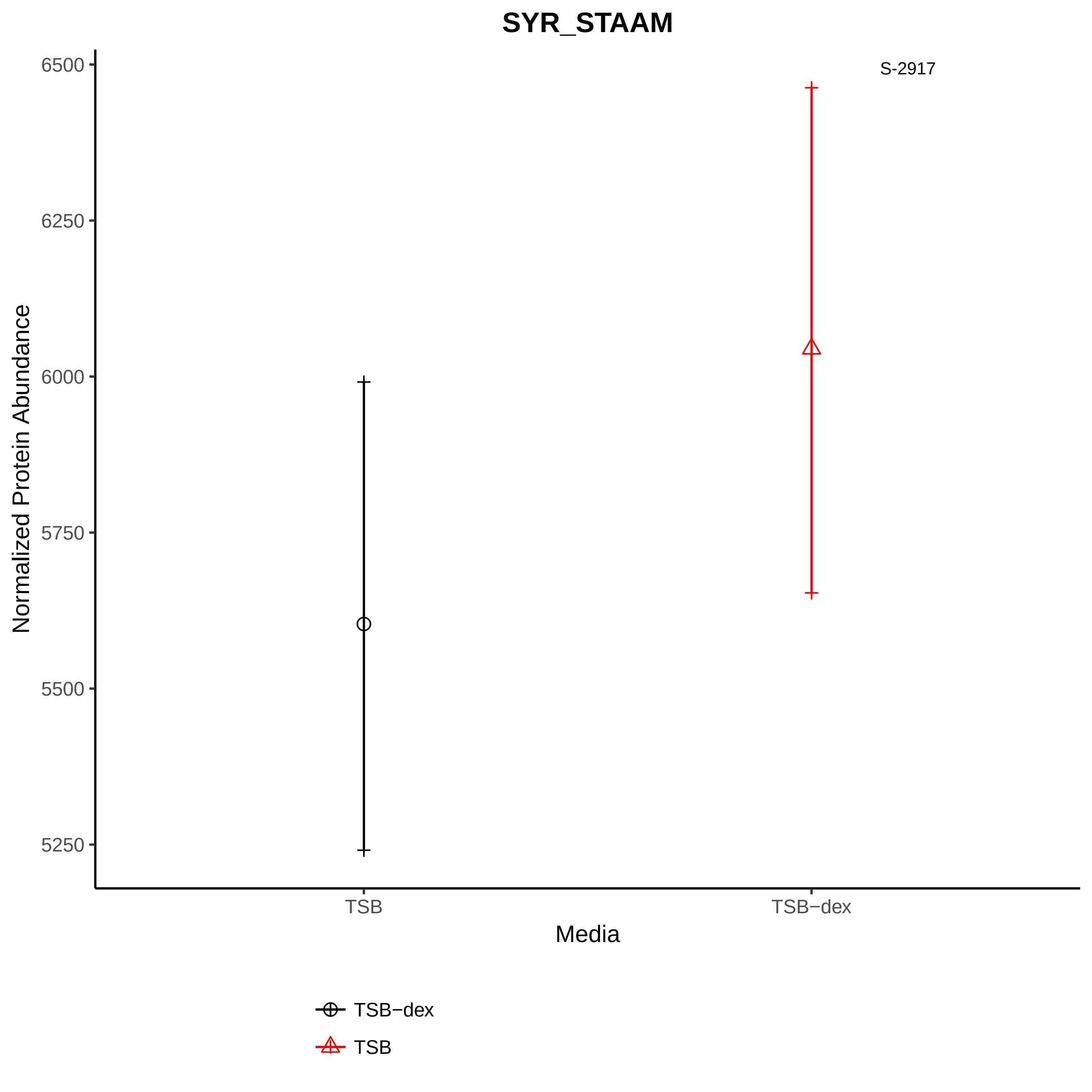

SYR_STAAM
S-2917
6500
6250
6000
5750
5500
5250
Normalized Protein Abundance
TSB
TSB−dex
Media
TSB−dex
TSB

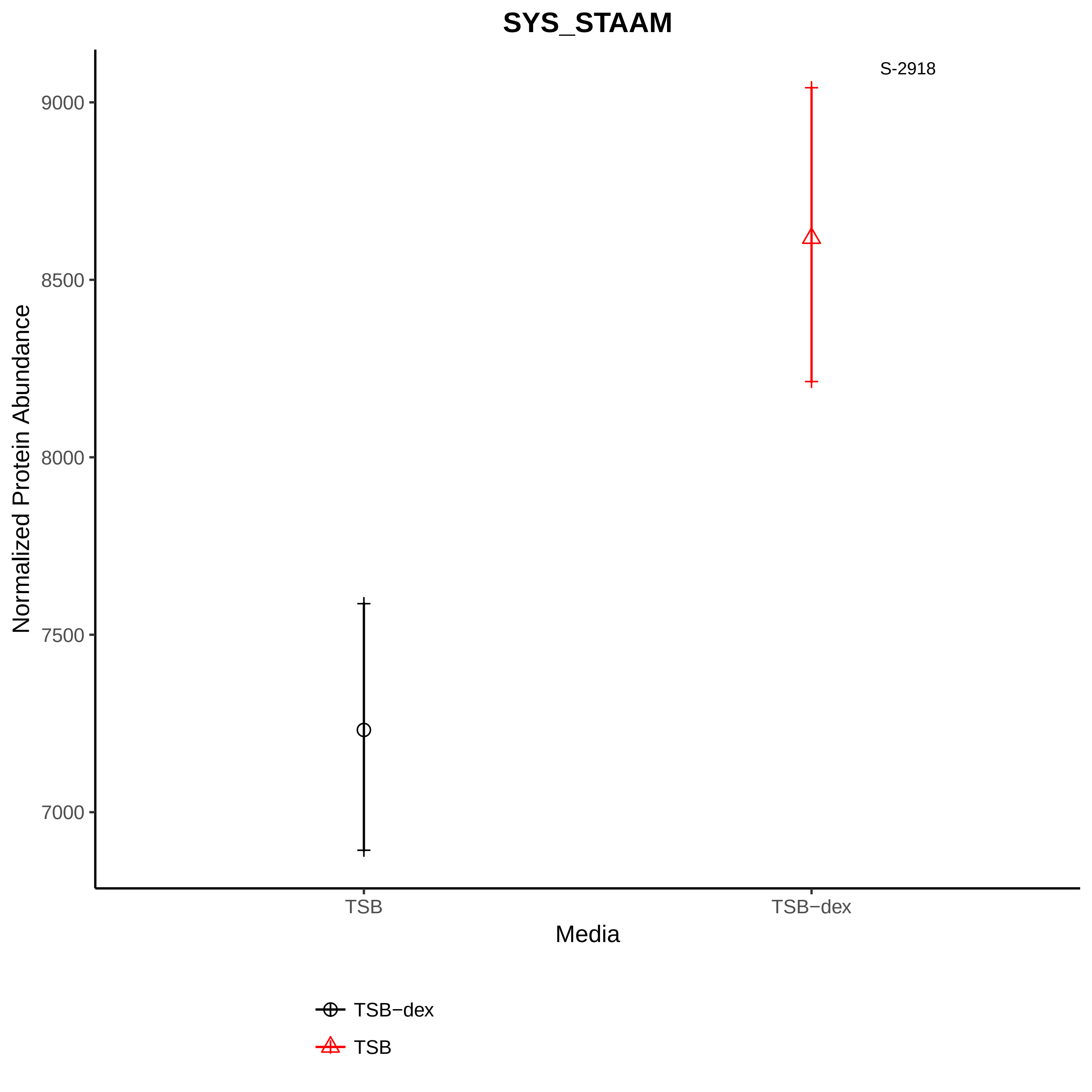
SYS_STAAM
S-2918
9000
8500
8000
7500
7000
Normalized Protein Abundance
TSB
TSB−dex
Media
TSB−dex
TSB

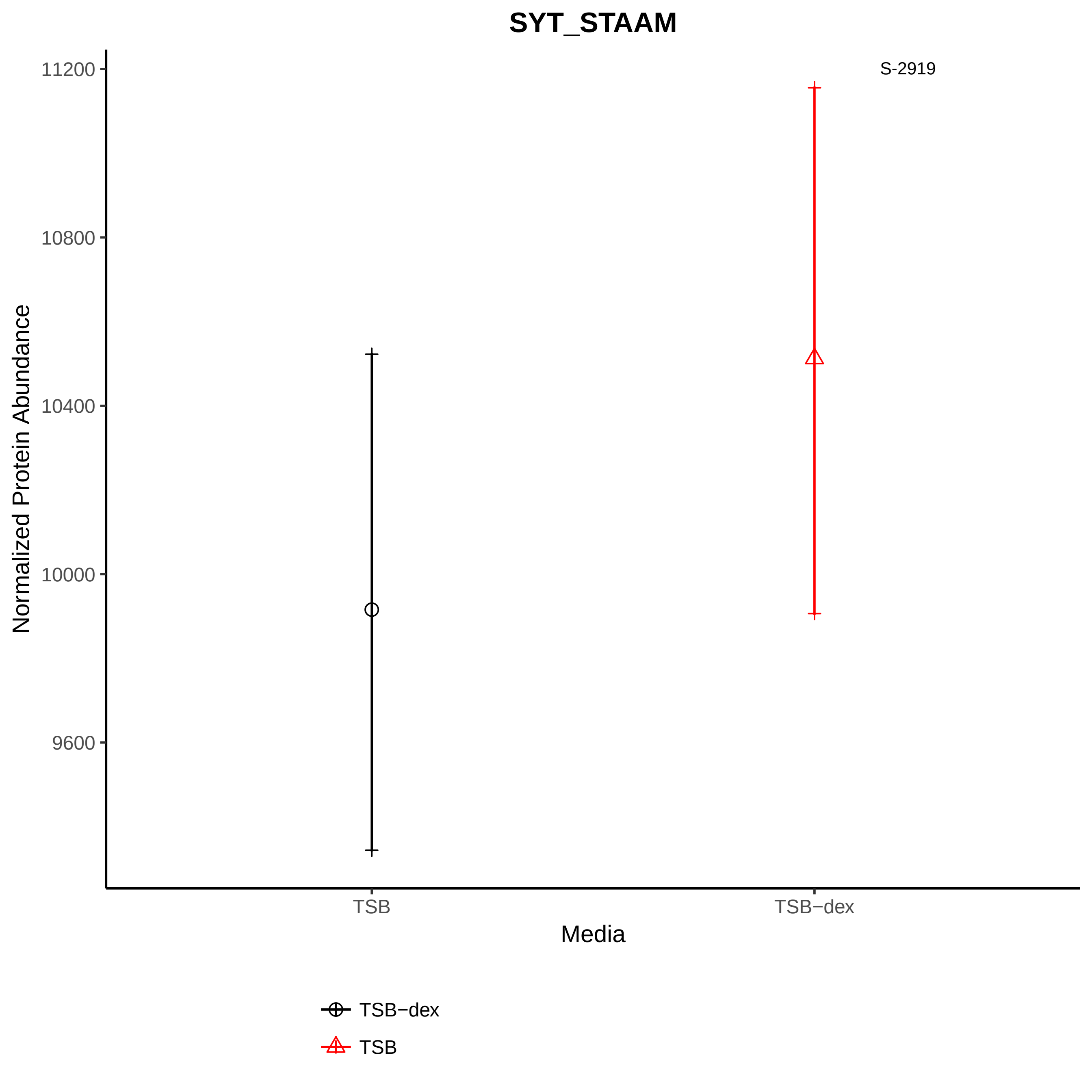
SYT_STAAM
S-2919
Normalized Protein Abundance
11200
10800
10400
10000
9600
TSB
TSB−dex
Media
TSB−dex
TSB

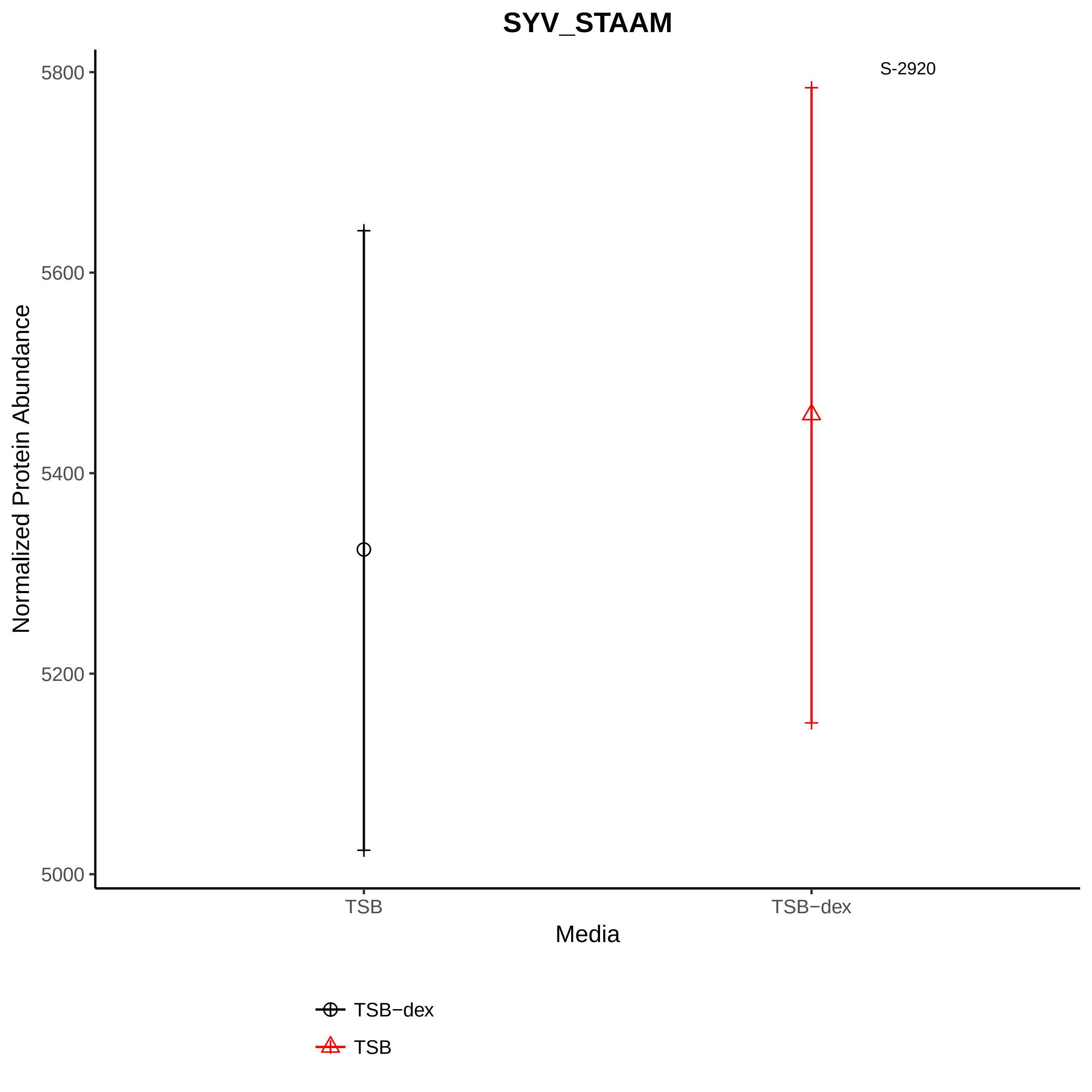

SYV_STAAM
S-2920
5800
5600
5400
5200
5000
Normalized Protein Abundance
TSB
TSB−dex
Media
TSB−dex
TSB

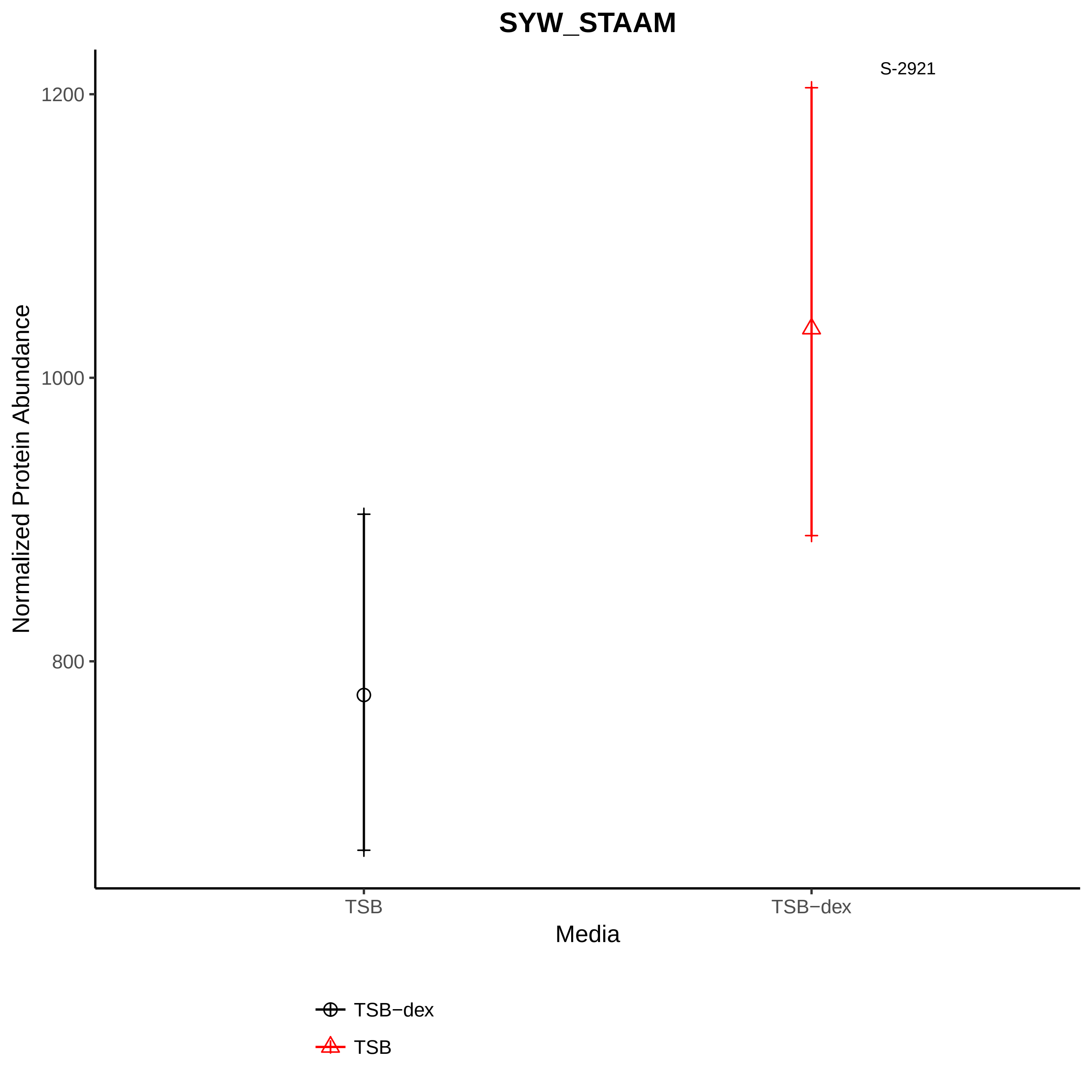
SYW_STAAM
S-2921
Normalized Protein Abundance
1200
1000
800
TSB
TSB−dex
Media
TSB−dex
TSB

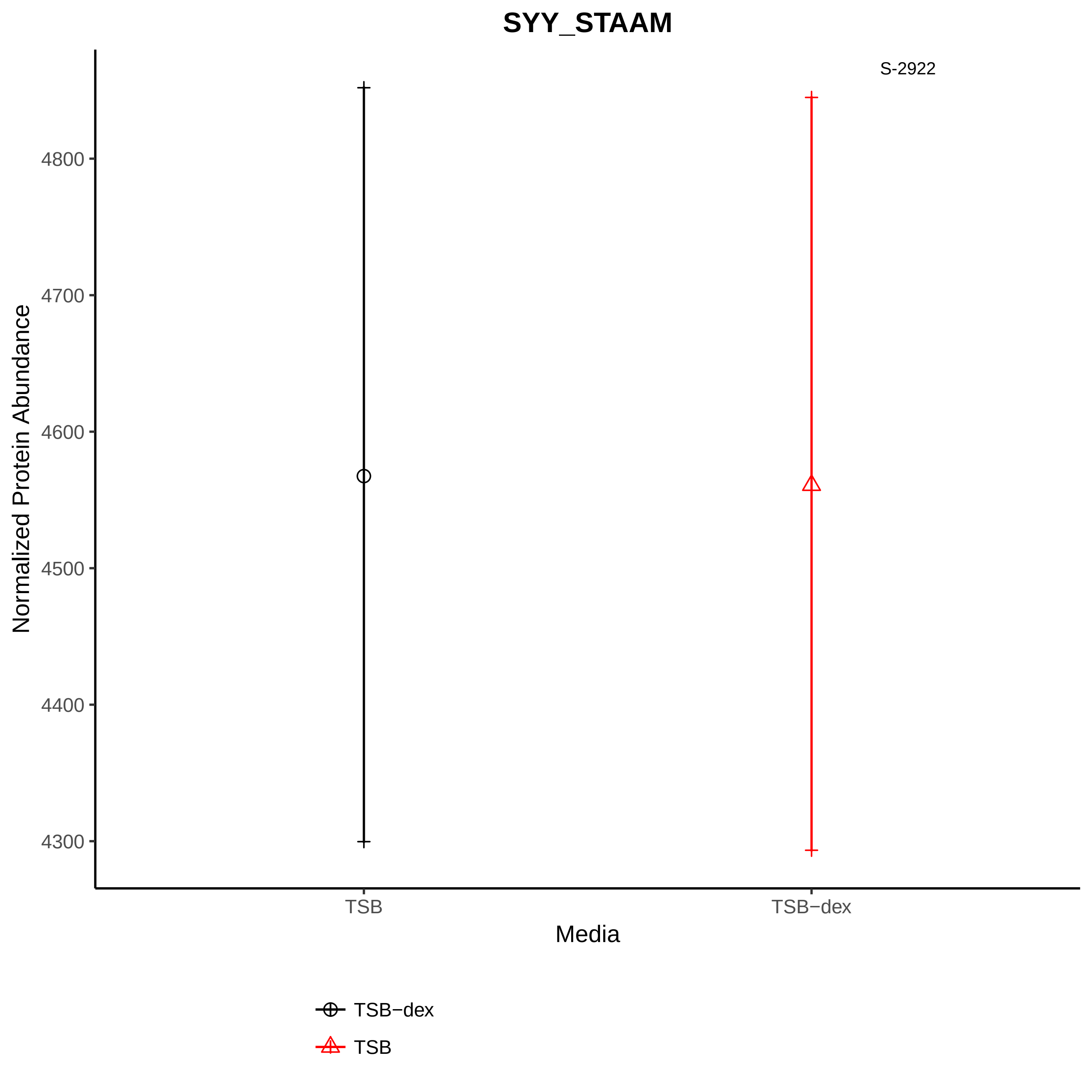

SYY_STAAM
S-2922
Normalized Protein Abundance
4800
4700
4600
4500
4400
4300
TSB
TSB−dex
Media
TSB−dex
TSB

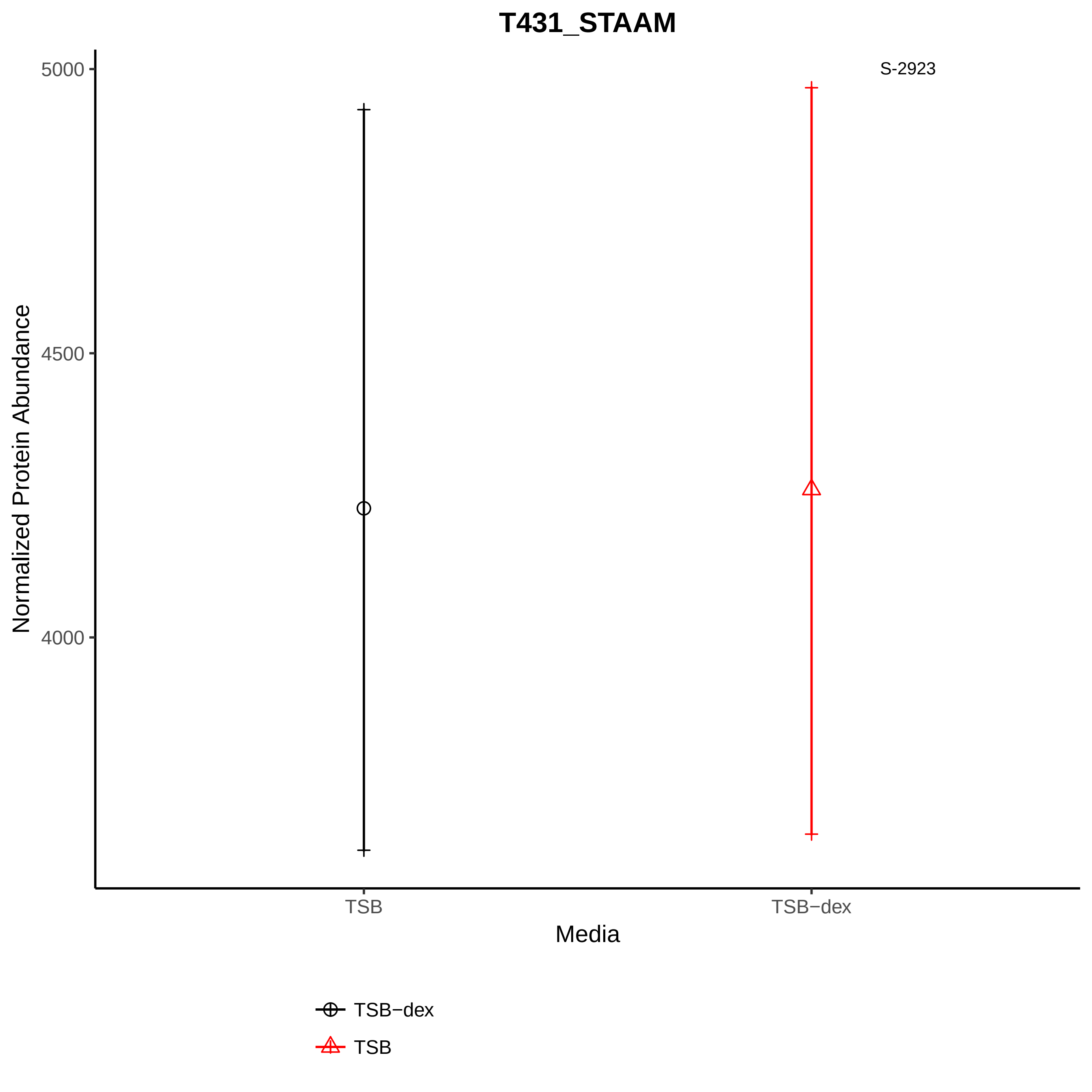

T431_STAAM
S-2923
5000
4500
4000
Normalized Protein Abundance
TSB
TSB−dex
Media
TSB−dex
TSB

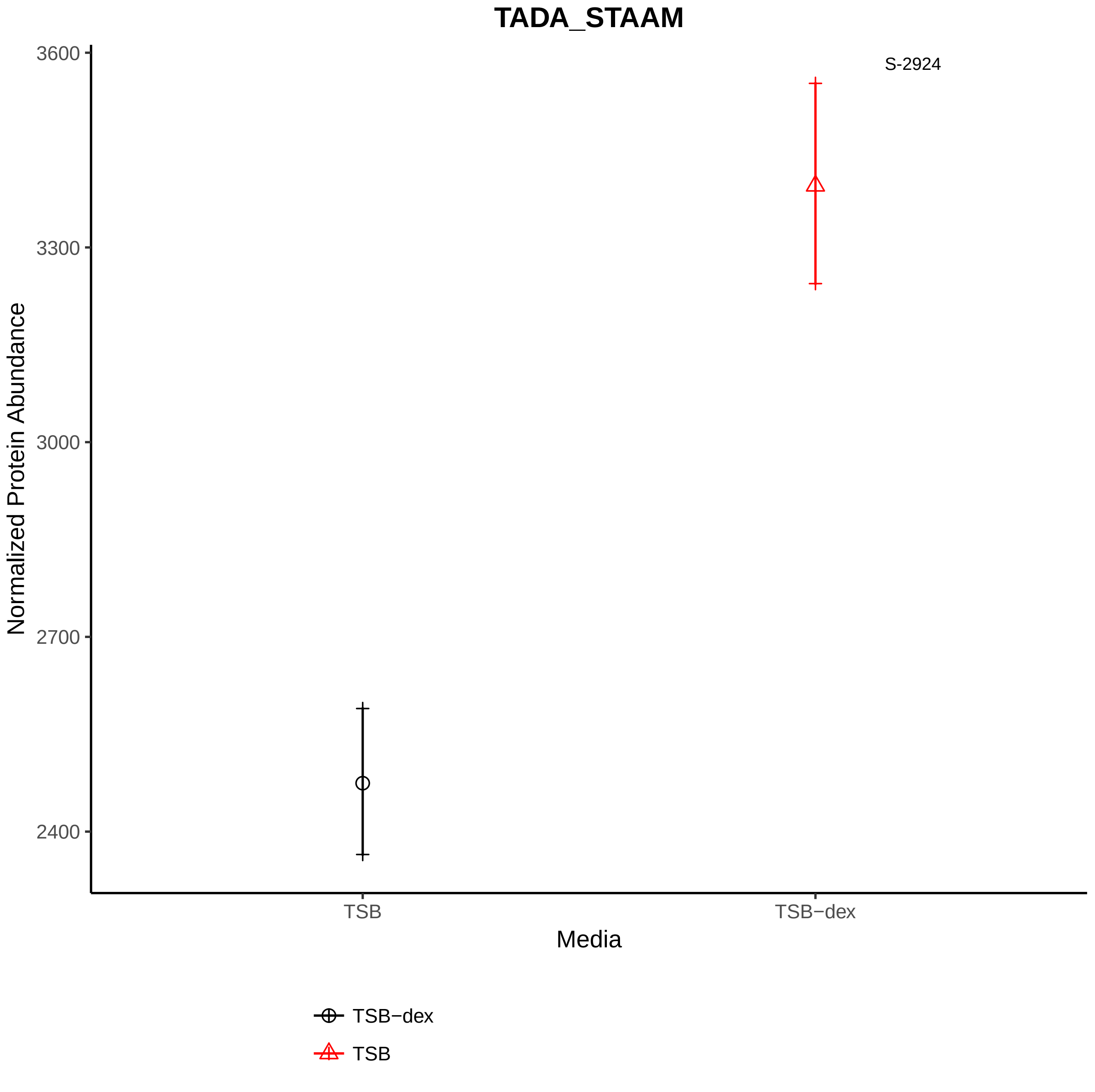

TADA_STAAM
S-2924
3600
3300
3000
2700
2400
Normalized Protein Abundance
TSB
TSB−dex
Media
TSB−dex
TSB

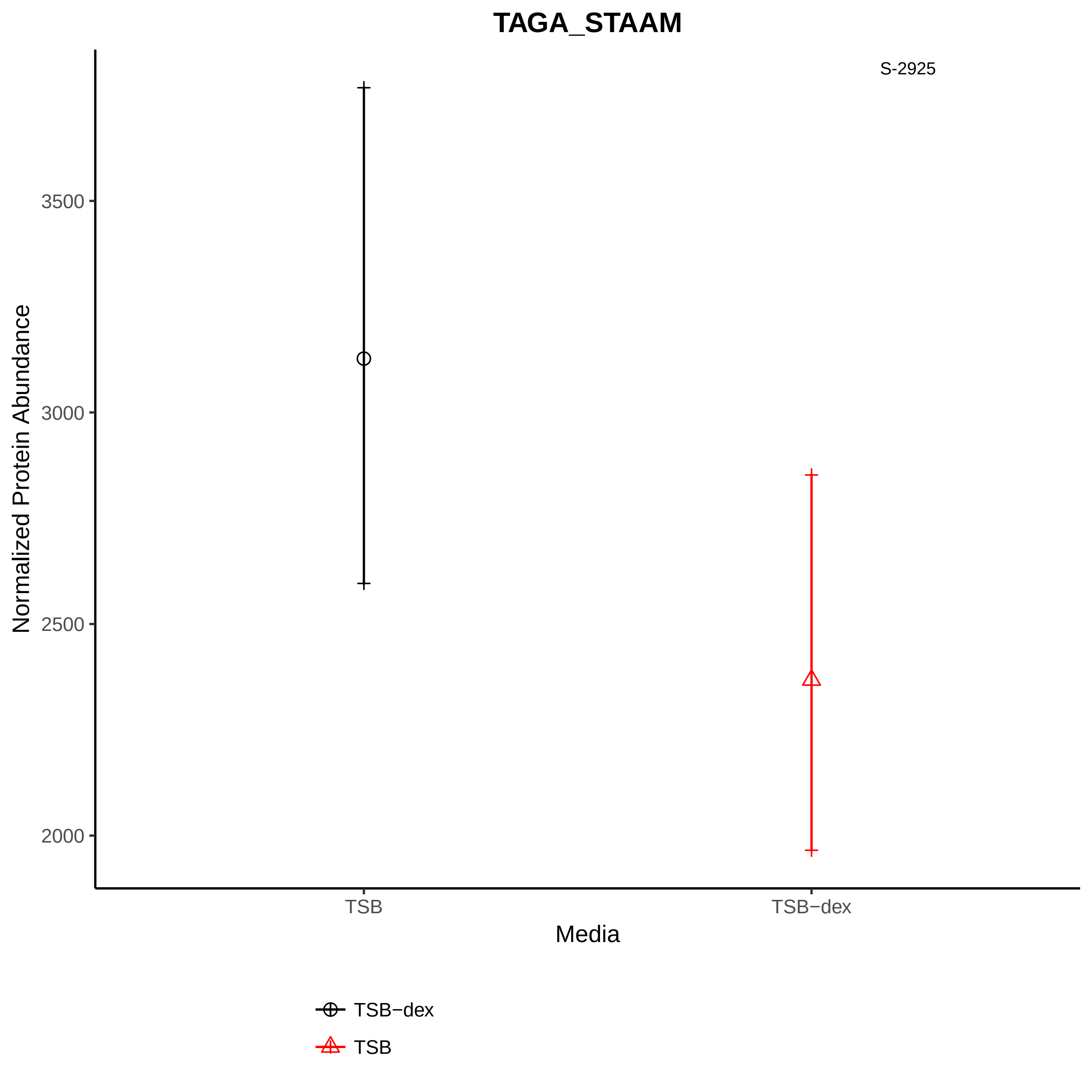
TAGA_STAAM
S-2925
Normalized Protein Abundance
3500
3000
2500
2000
TSB
TSB−dex
Media
TSB−dex
TSB

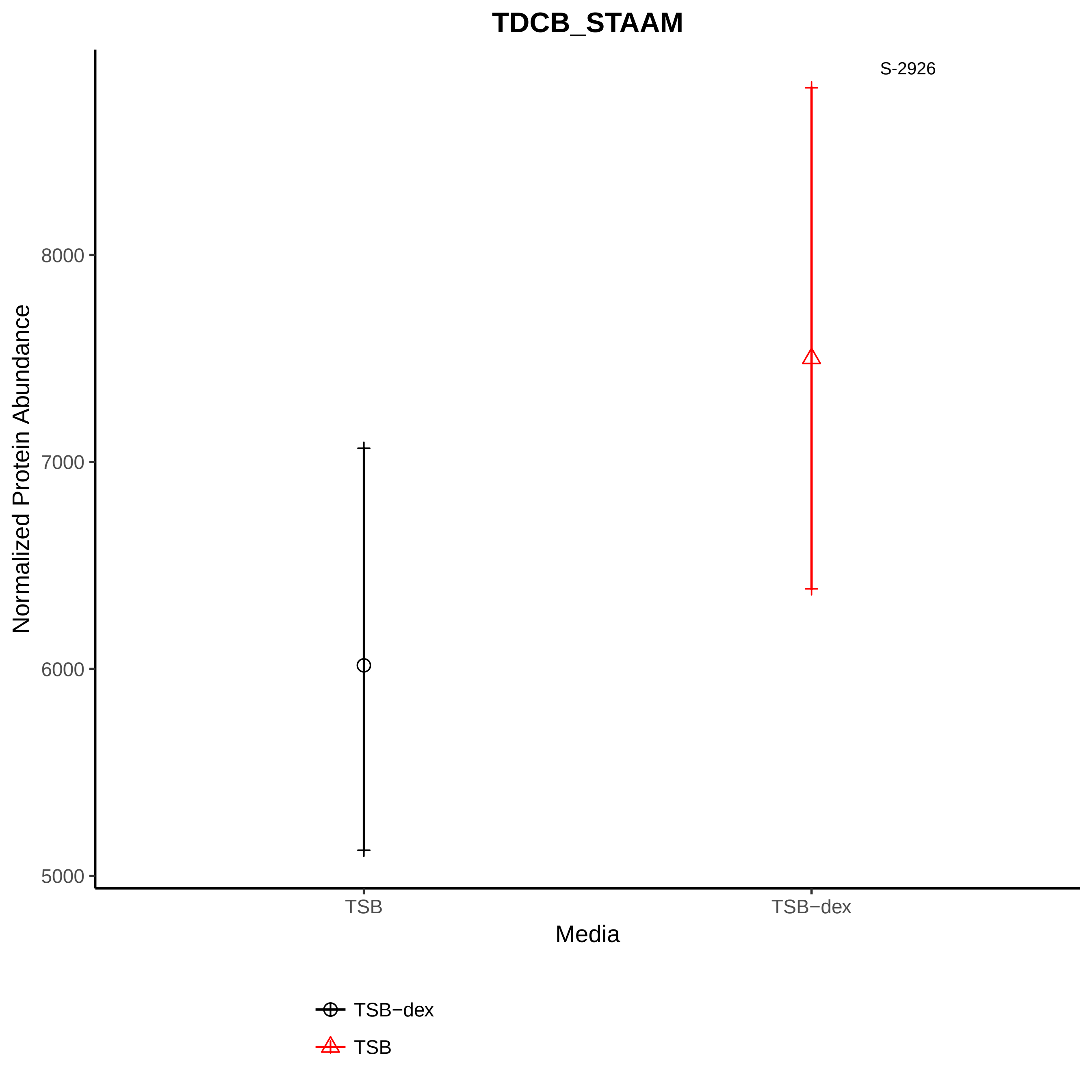

TDCB_STAAM
S-2926
Normalized Protein Abundance
8000
7000
6000
5000
TSB
TSB−dex
Media
TSB−dex
TSB

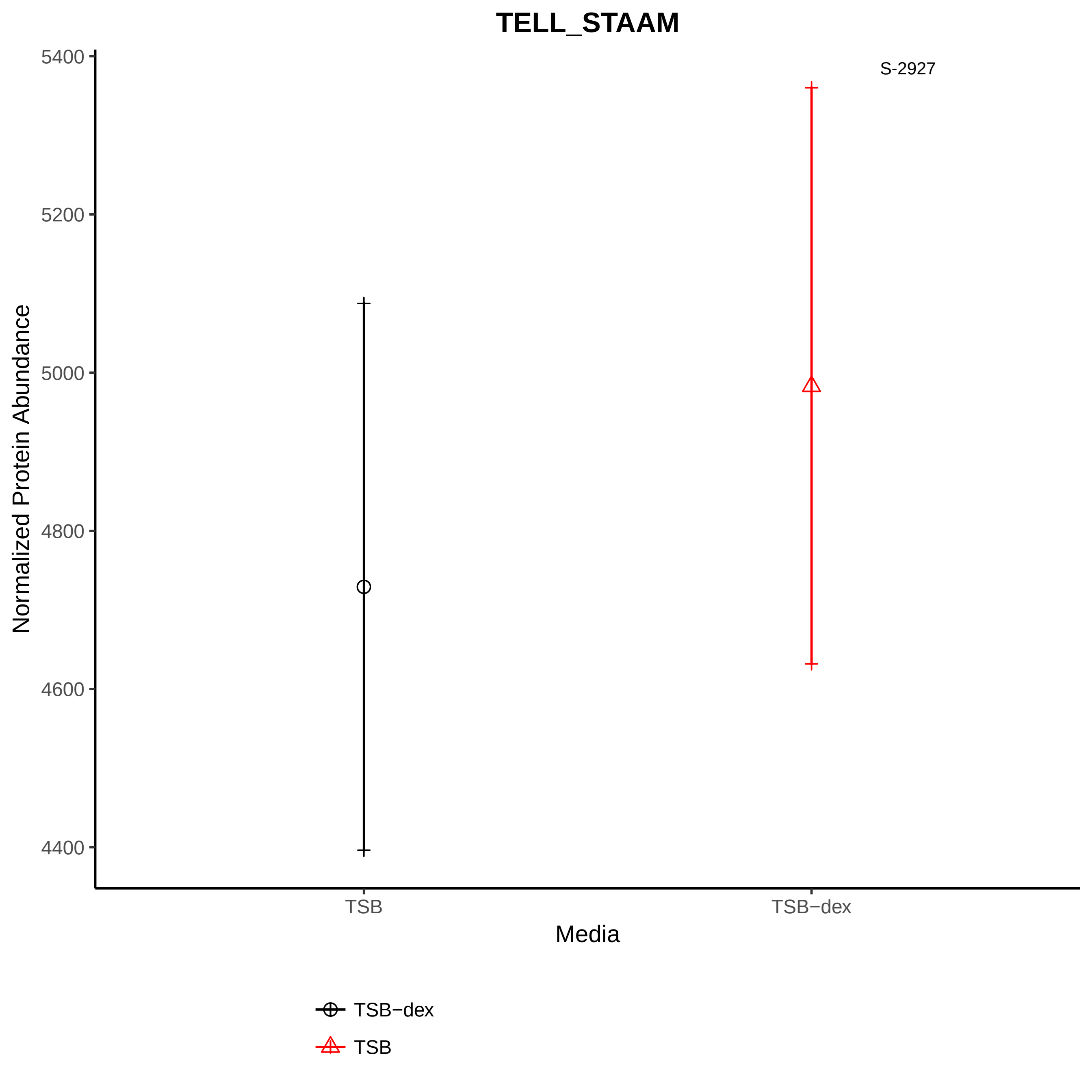
TELL_STAAM
S-2927
Normalized Protein Abundance
5400
5200
5000
4800
4600
4400
TSB
TSB−dex
Media
TSB−dex
TSB

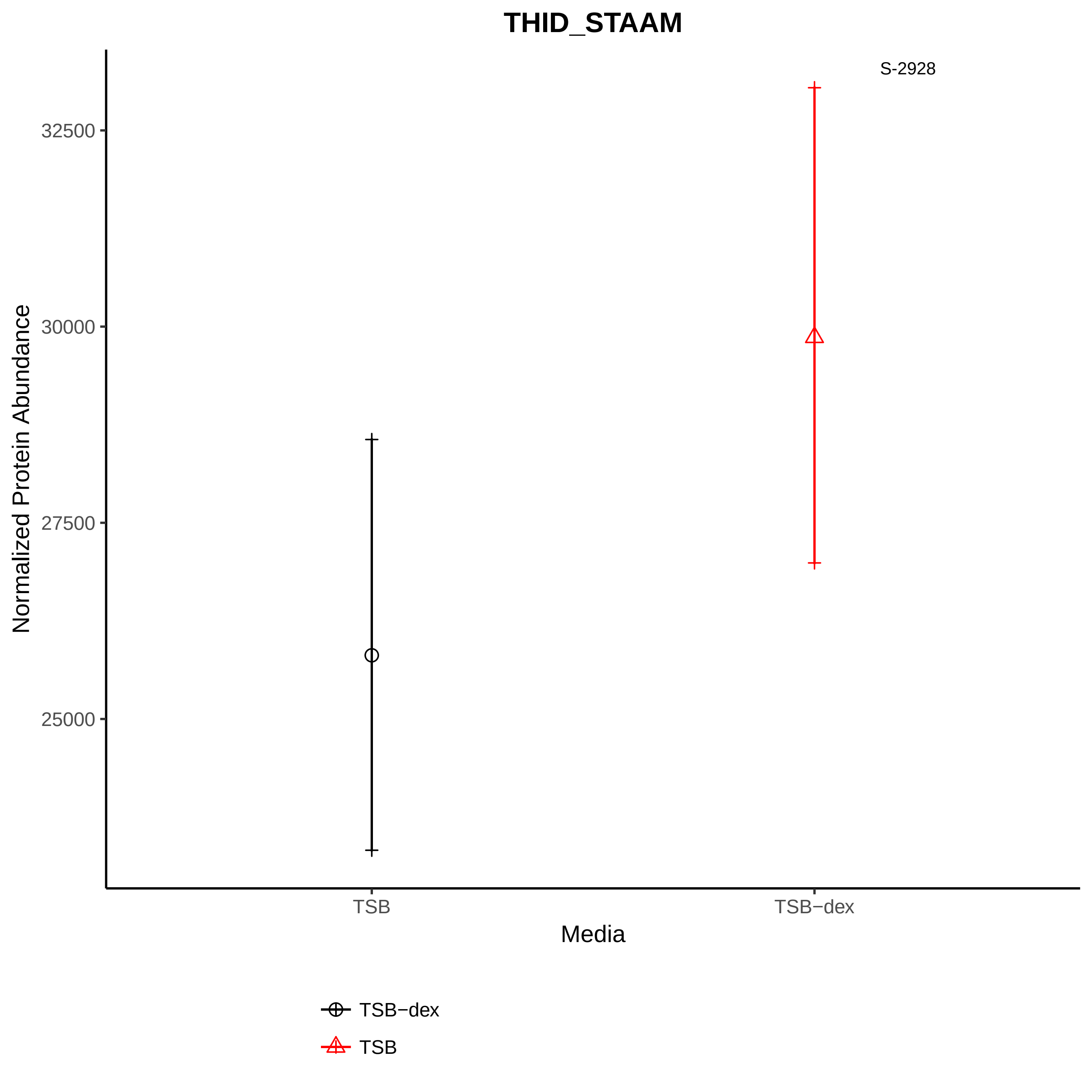

THID_STAAM
S-2928
32500
30000
27500
25000
Normalized Protein Abundance
TSB
TSB−dex
Media
TSB−dex
TSB

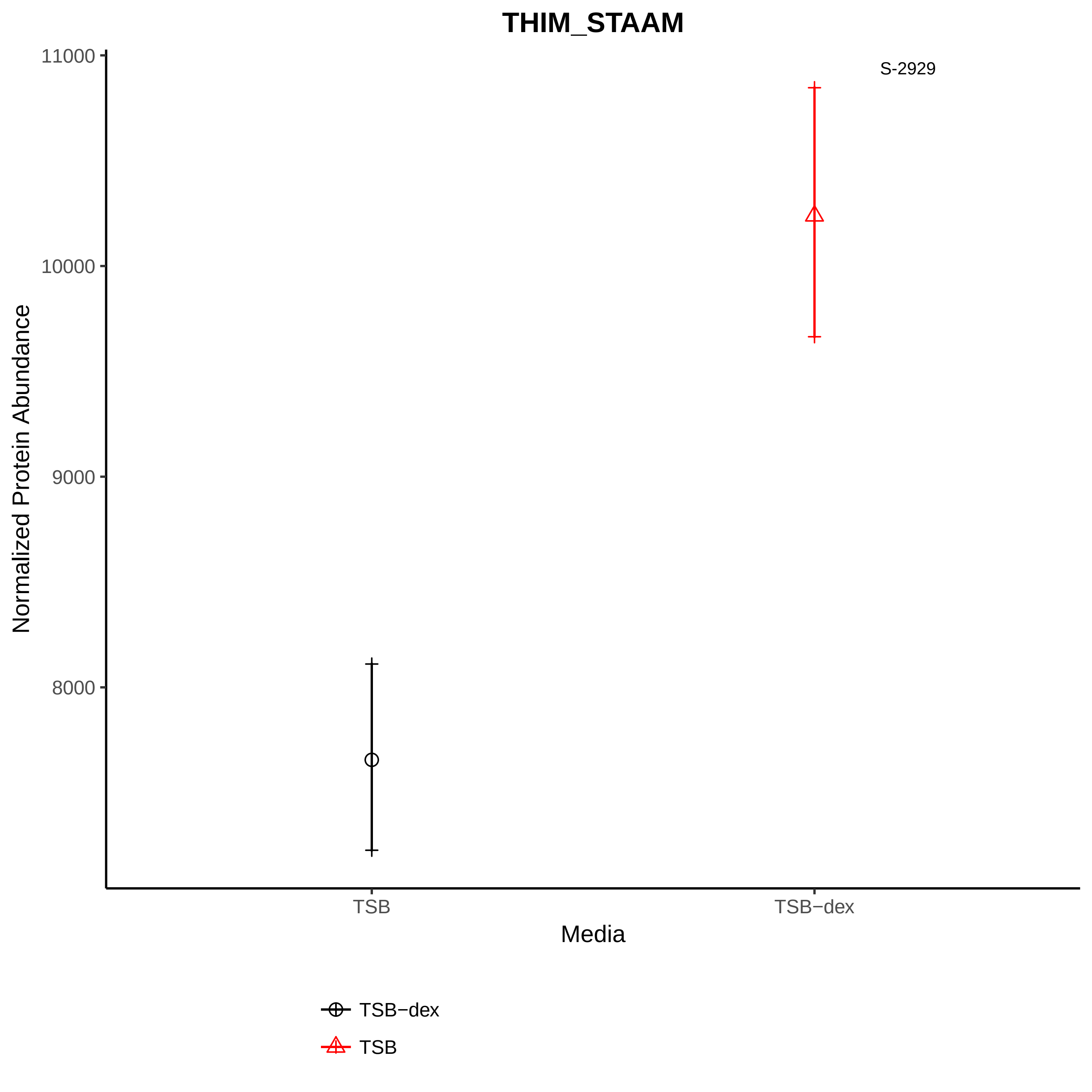
THIM_STAAM
S-2929
11000
10000
9000
8000
Normalized Protein Abundance
TSB
TSB−dex
Media
TSB−dex
TSB

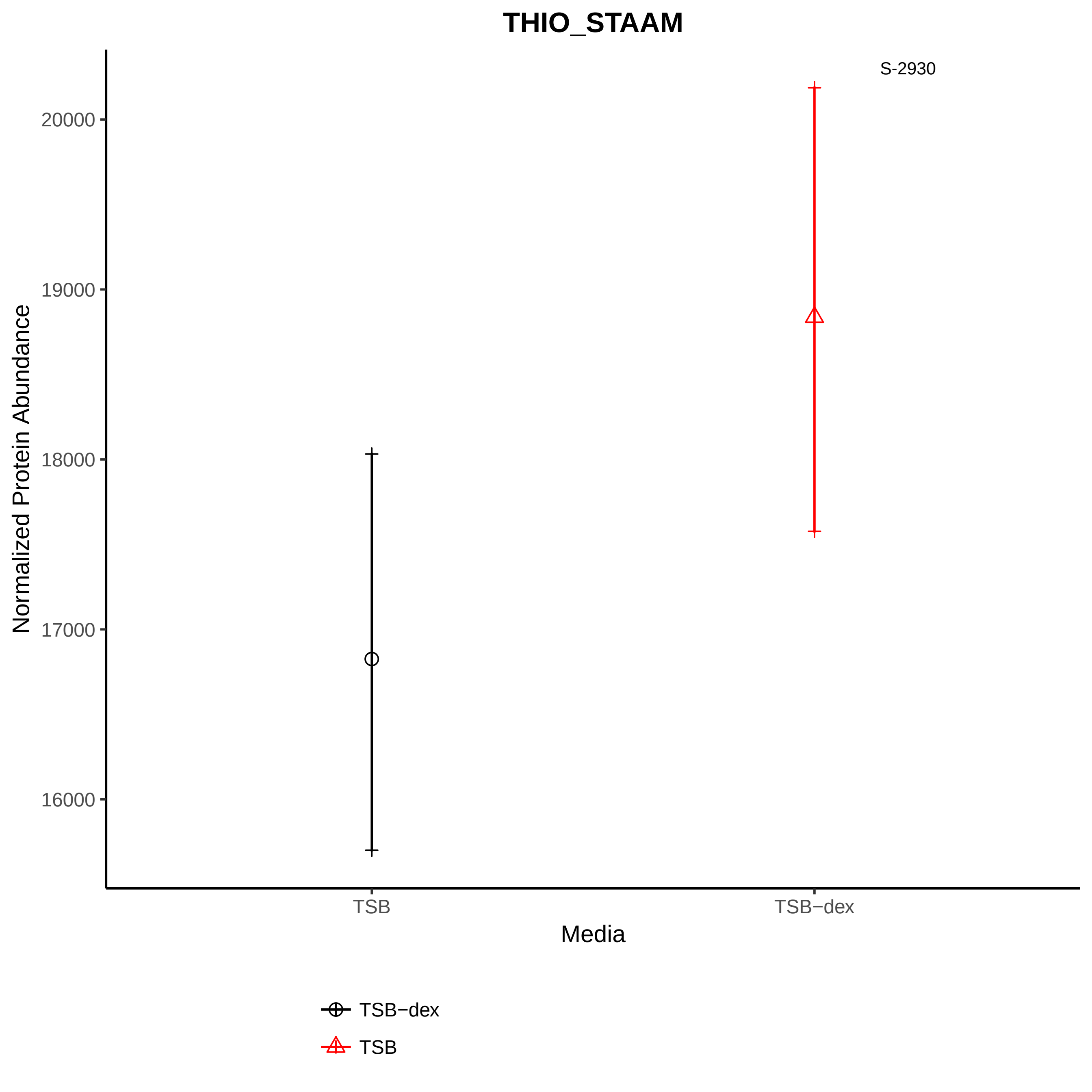

THIO_STAAM
S-2930
Normalized Protein Abundance
20000
19000
18000
17000
16000
TSB
TSB−dex
Media
TSB−dex
TSB

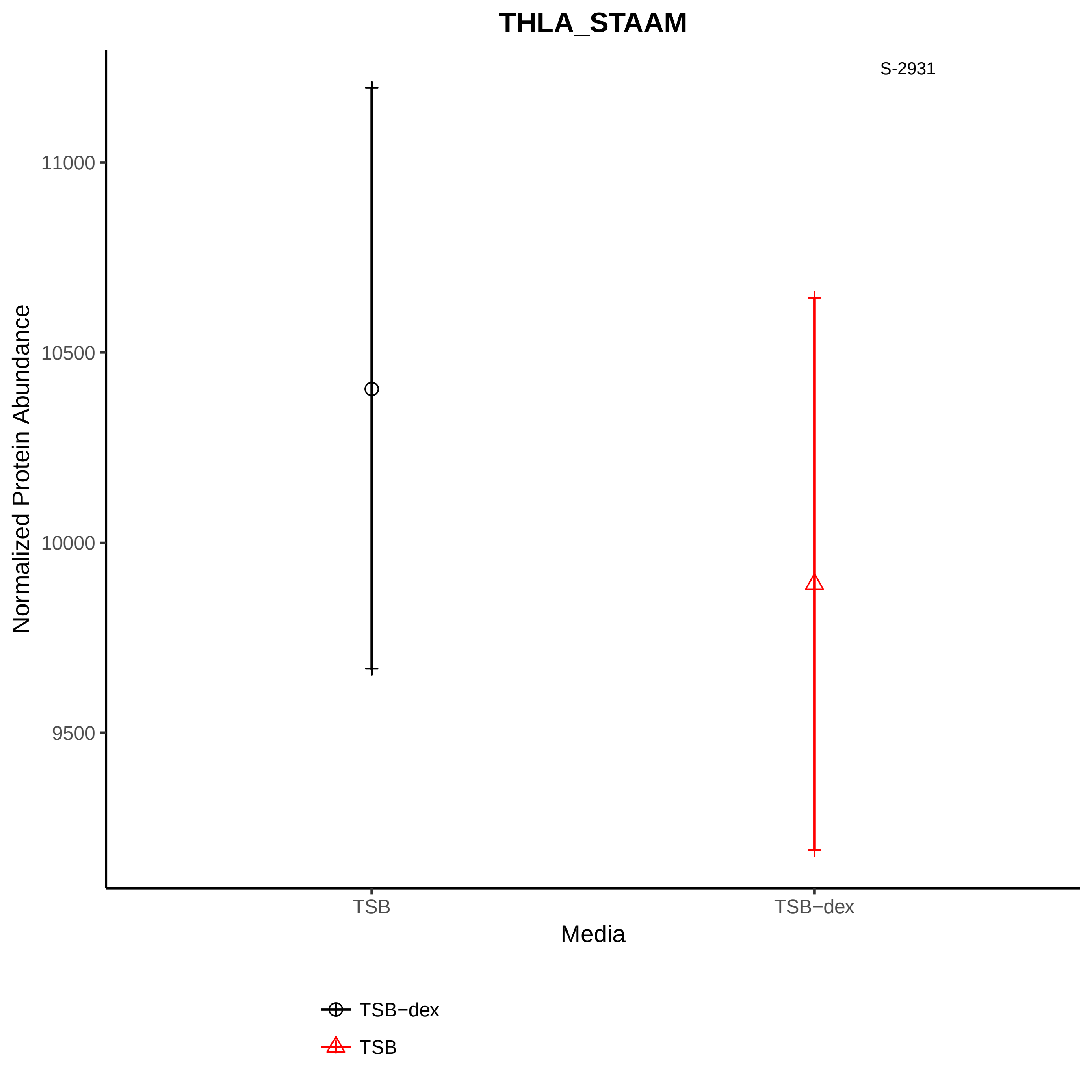
THLA_STAAM
S-2931
Normalized Protein Abundance
11000
10500
10000
9500
TSB
TSB−dex
Media
TSB−dex
TSB

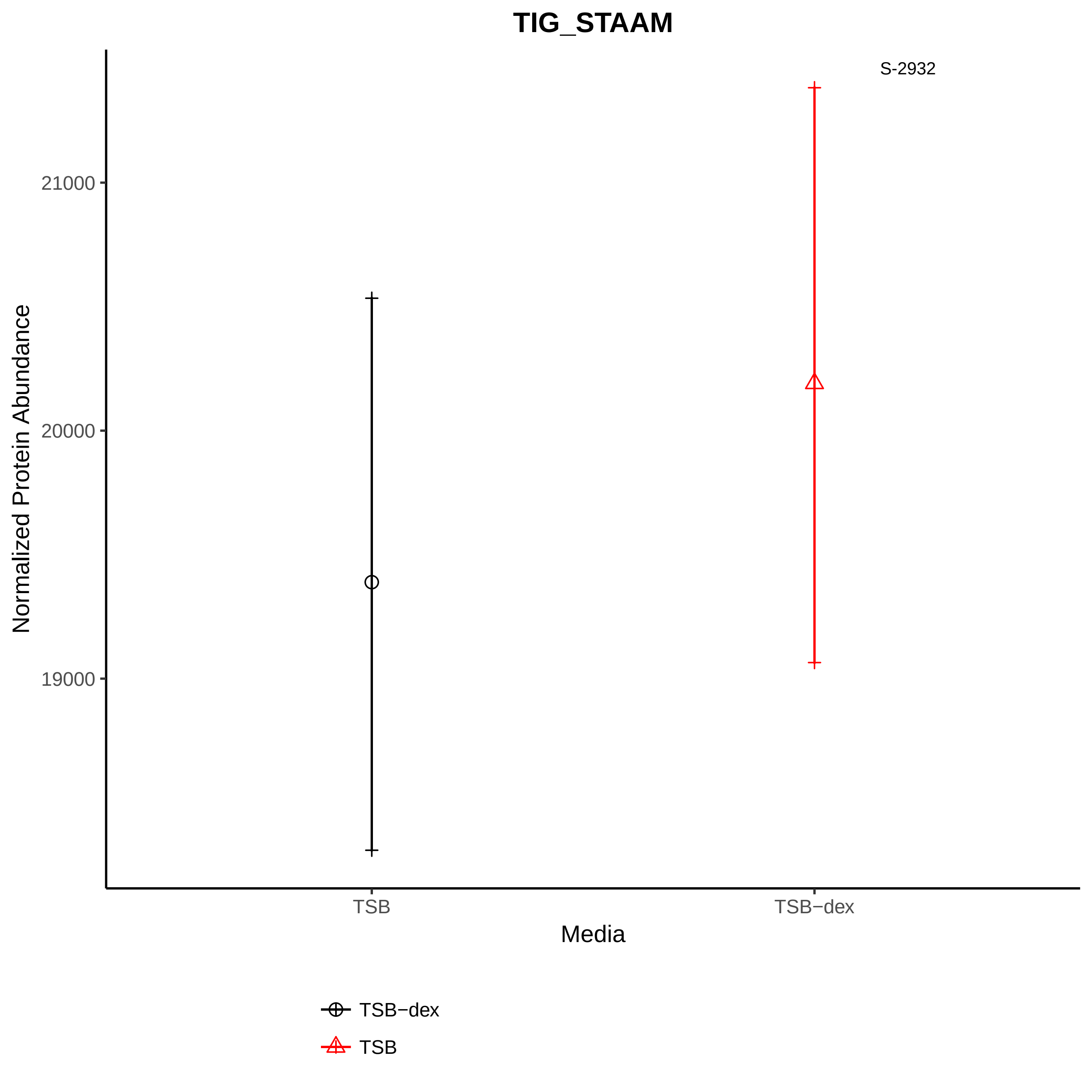

TIG_STAAM
S-2932
21000
20000
19000
Normalized Protein Abundance
TSB
TSB−dex
Media
TSB−dex
TSB

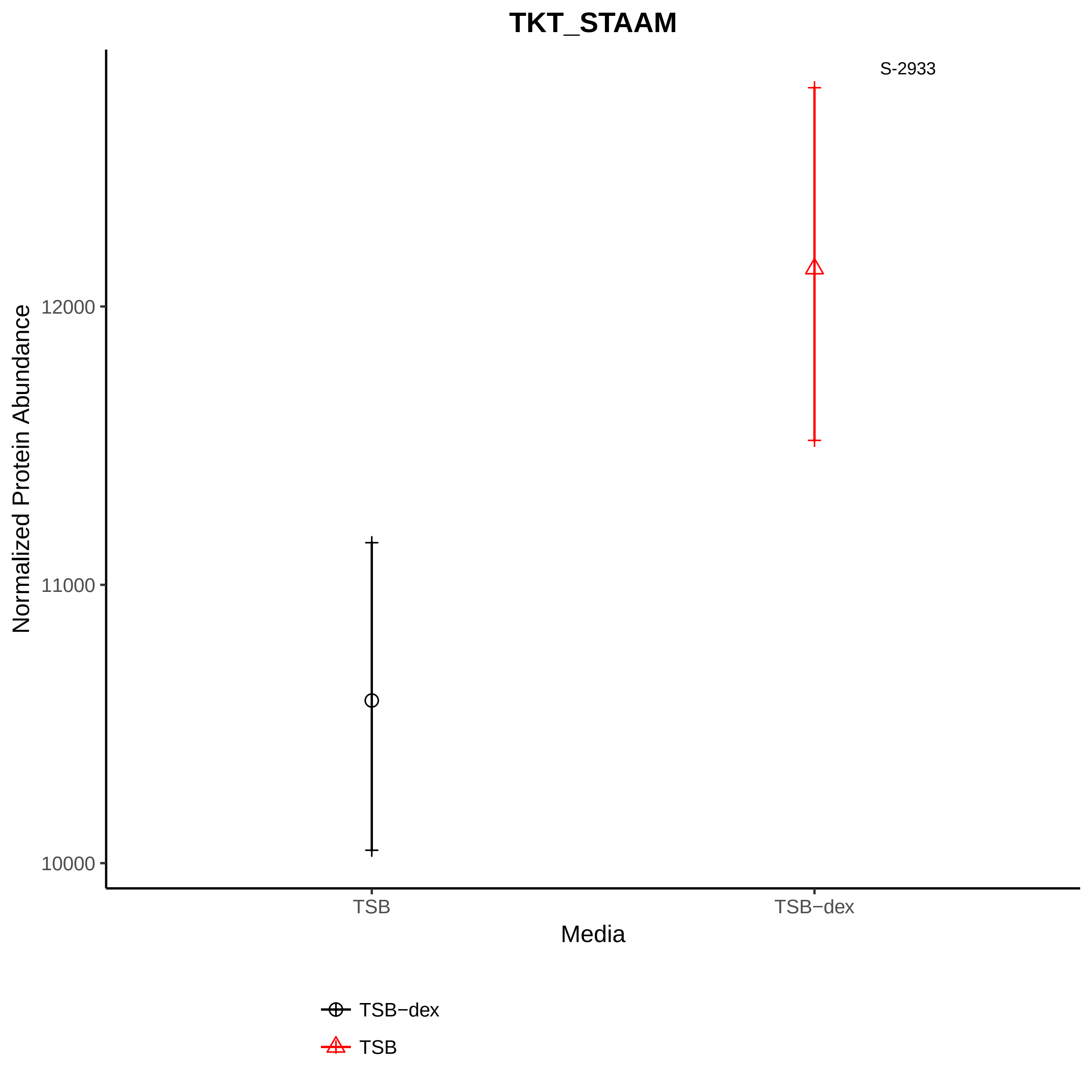

TKT_STAAM
S-2933
Normalized Protein Abundance
12000
11000
10000
TSB
TSB−dex
Media
TSB−dex
TSB

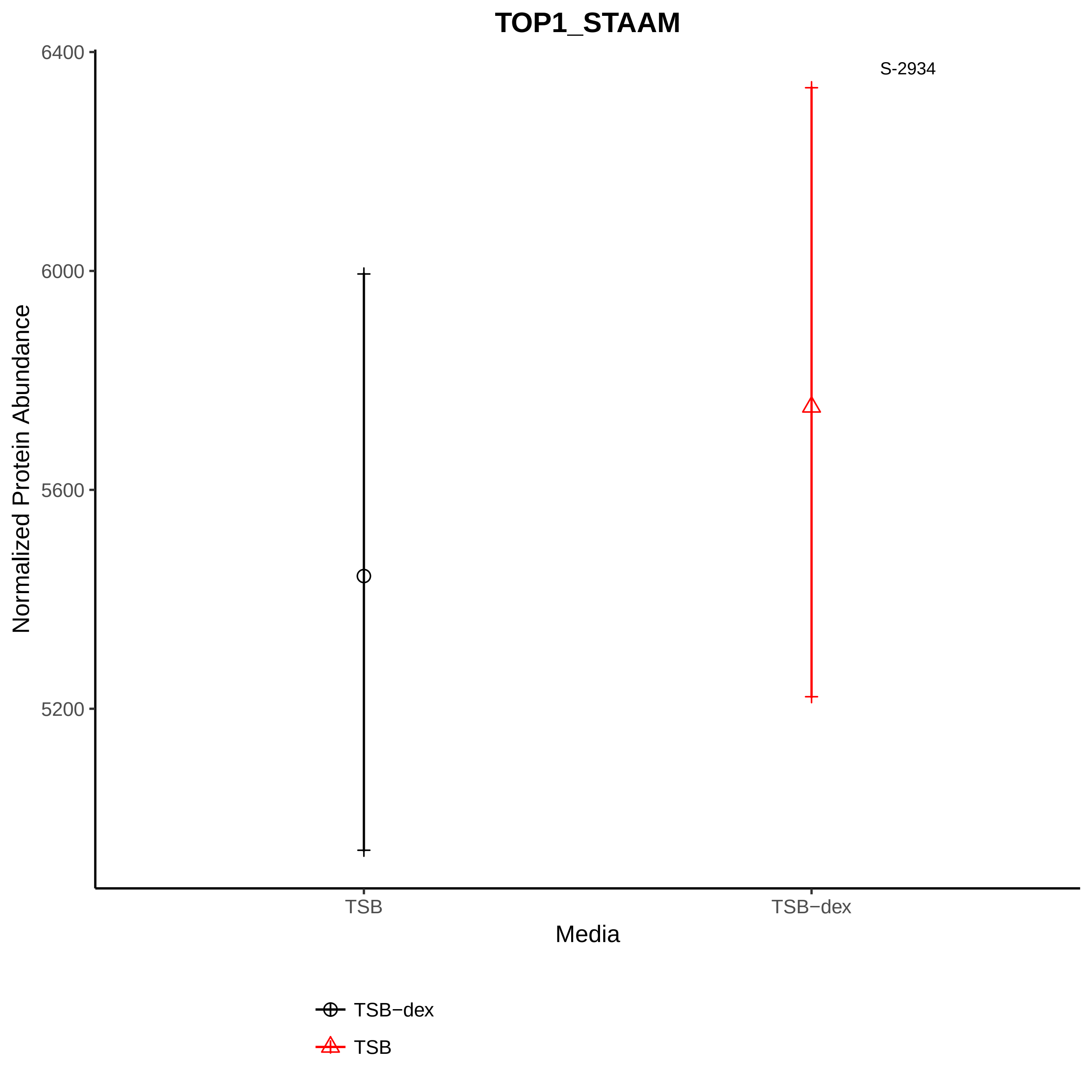

TOP1_STAAM
S-2934
6400
6000
5600
5200
Normalized Protein Abundance
TSB
TSB−dex
Media
TSB−dex
TSB

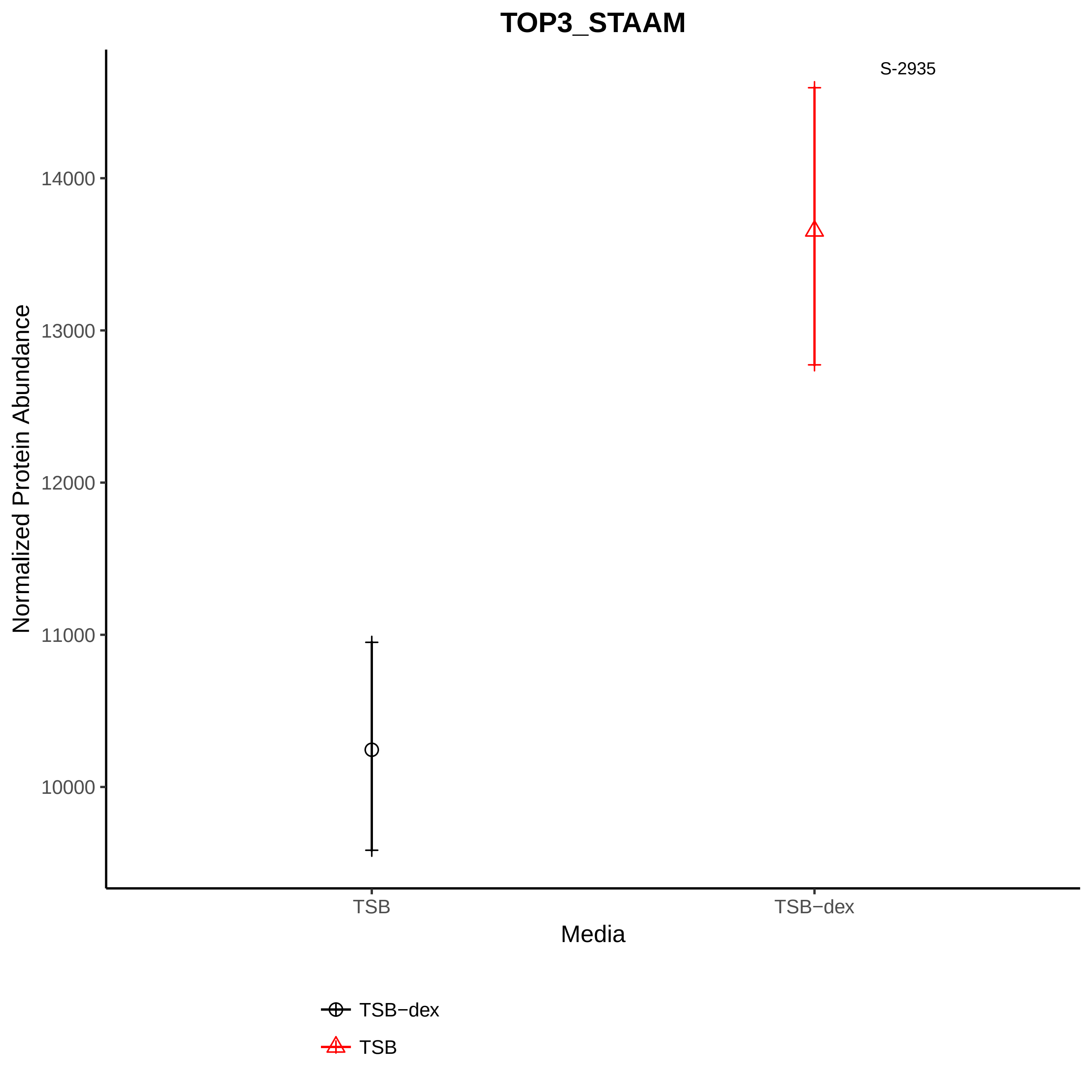
TOP3_STAAM
S-2935
Normalized Protein Abundance
14000
13000
12000
11000
10000
TSB
TSB−dex
Media
TSB−dex
TSB

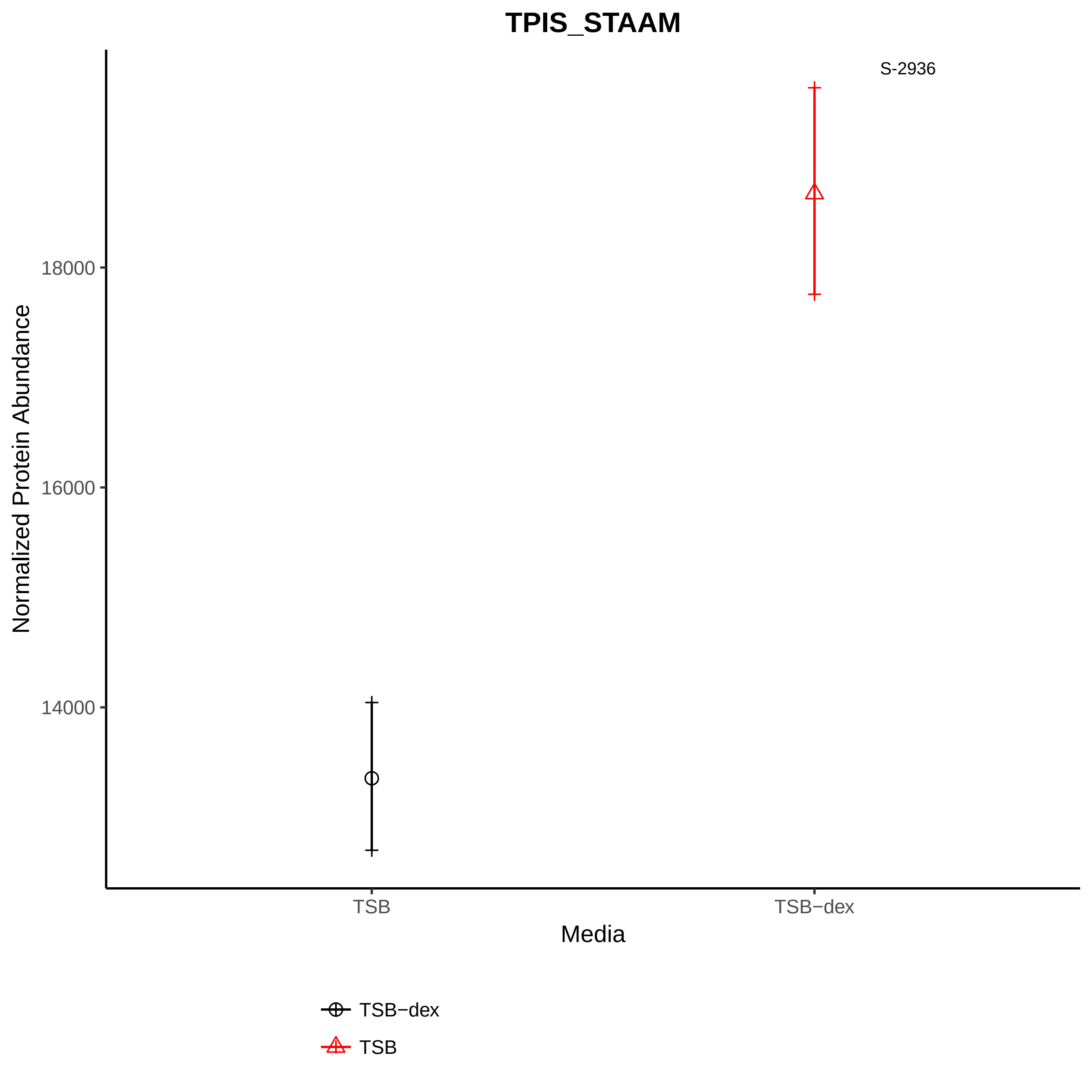
TPIS_STAAM
S-2936
Normalized Protein Abundance
18000
16000
14000
TSB
TSB−dex
Media
TSB−dex
TSB

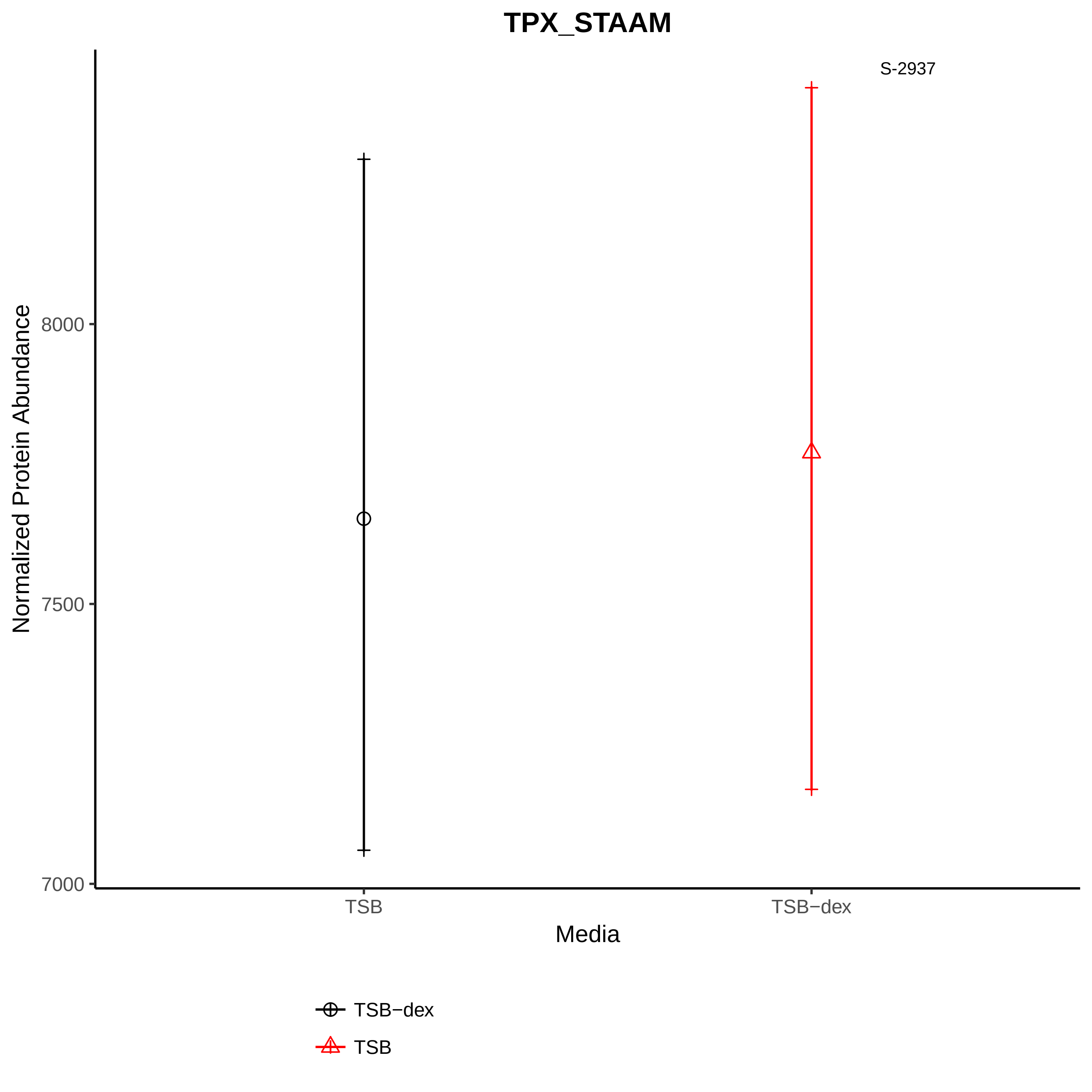

TPX_STAAM
S-2937
Normalized Protein Abundance
8000
7500
7000
TSB
TSB−dex
Media
TSB−dex
TSB

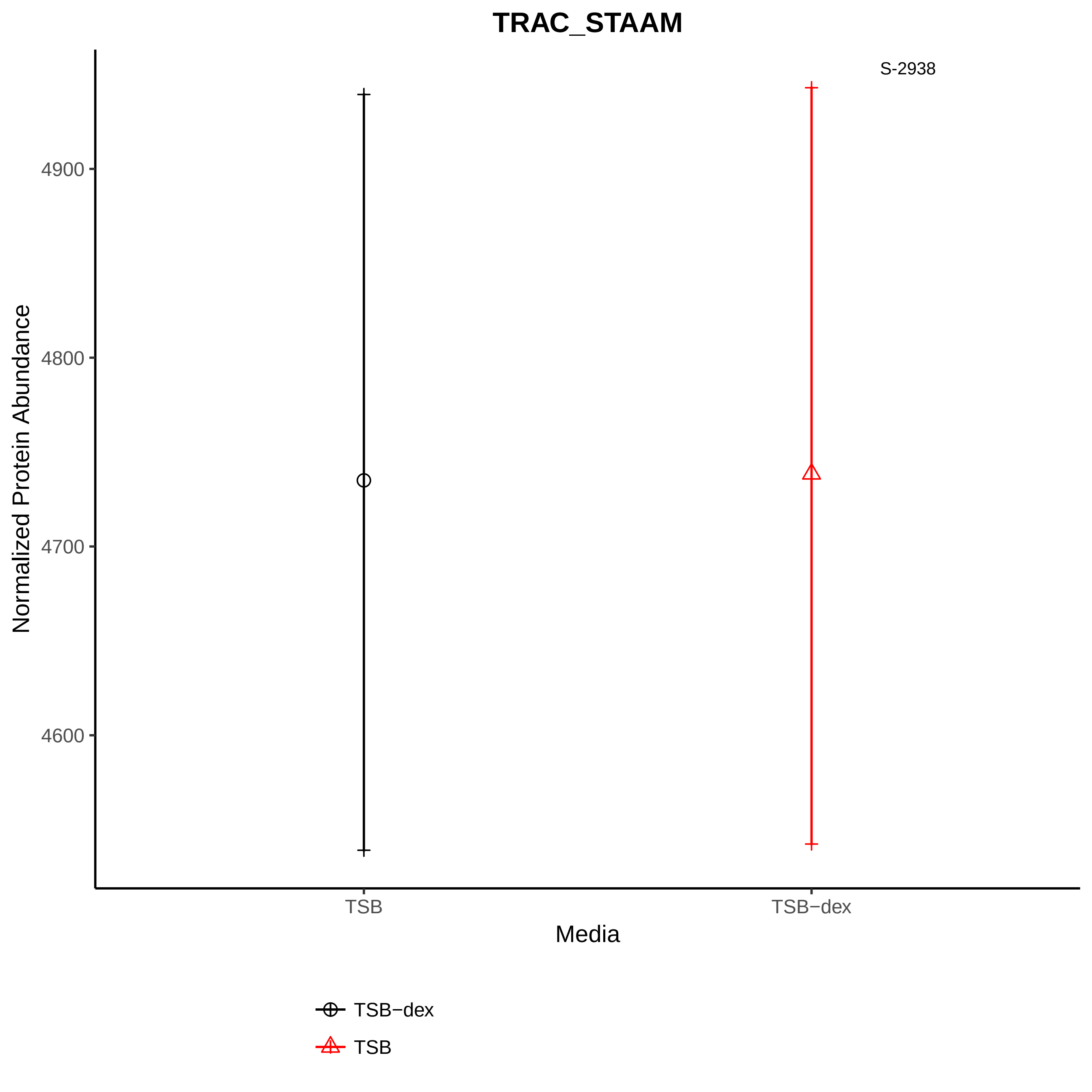

TRAC_STAAM
S-2938
Normalized Protein Abundance
4900
4800
4700
4600
TSB
TSB−dex
Media
TSB−dex
TSB

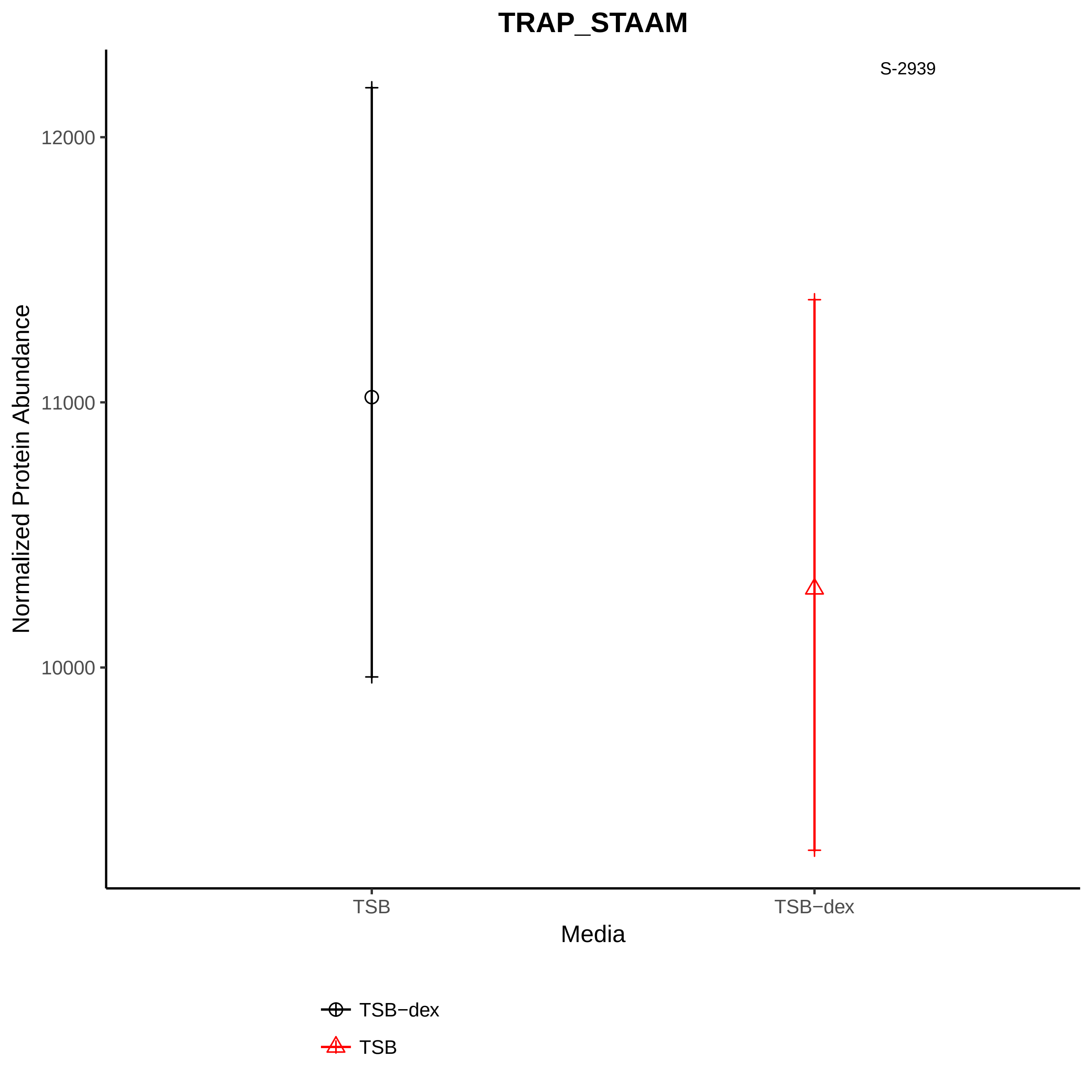
TRAP_STAAM
S-2939
Normalized Protein Abundance
12000
11000
10000
TSB
TSB−dex
Media
TSB−dex
TSB

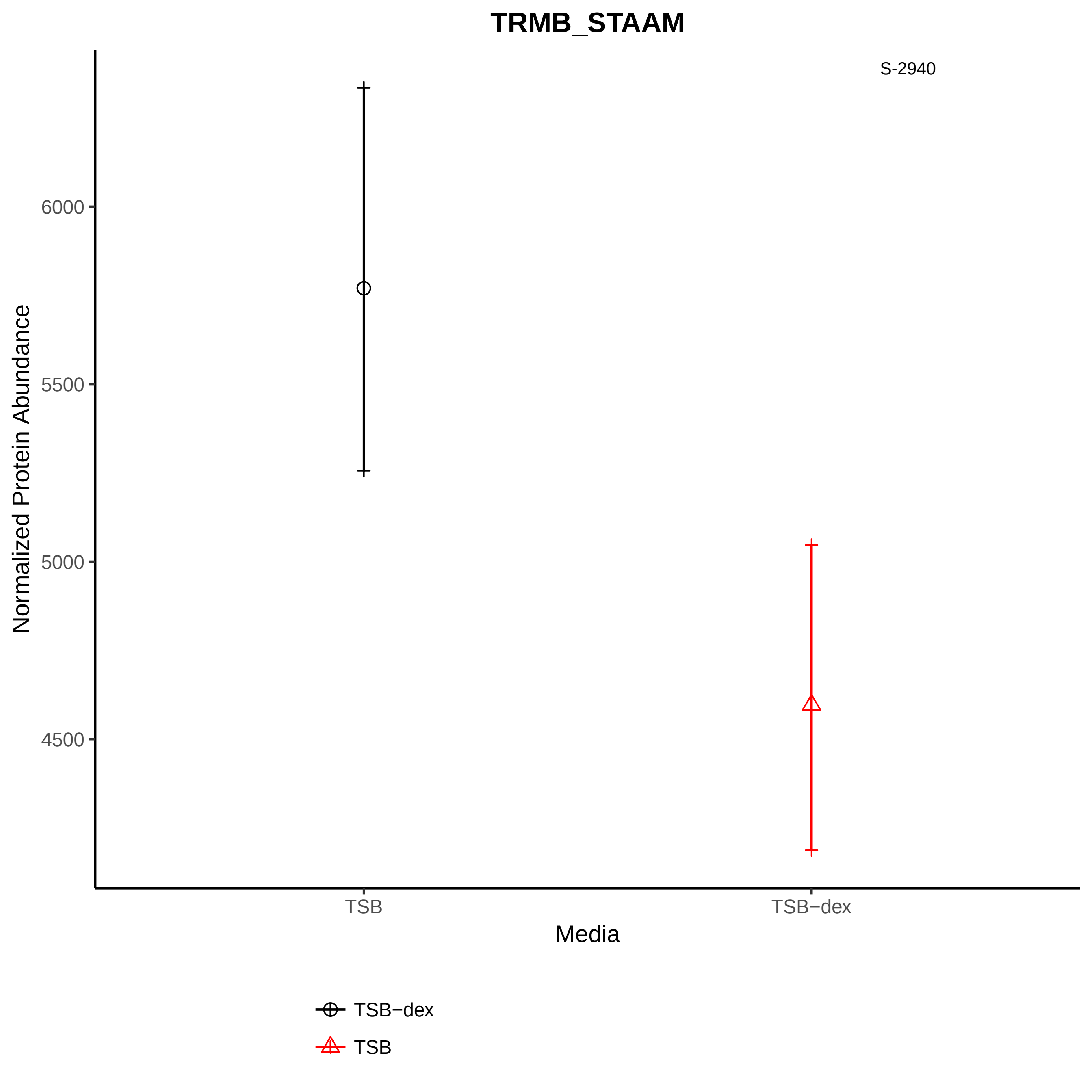
TRMB_STAAM
S-2940
Normalized Protein Abundance
6000
5500
5000
4500
TSB
TSB−dex
Media
TSB−dex
TSB

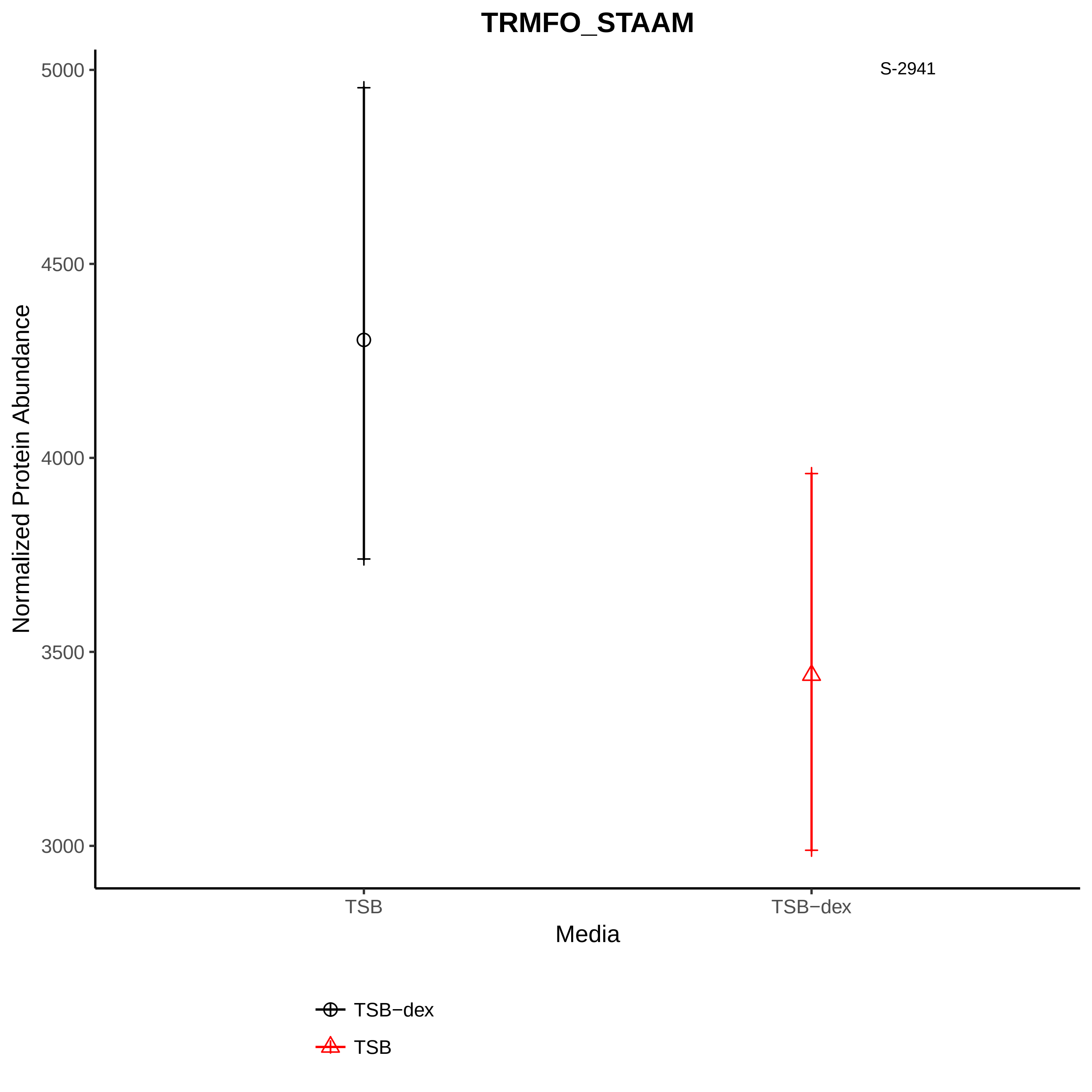
TRMFO_STAAM
S-2941
Normalized Protein Abundance
5000
4500
4000
3500
3000
TSB
TSB−dex
Media
TSB−dex
TSB

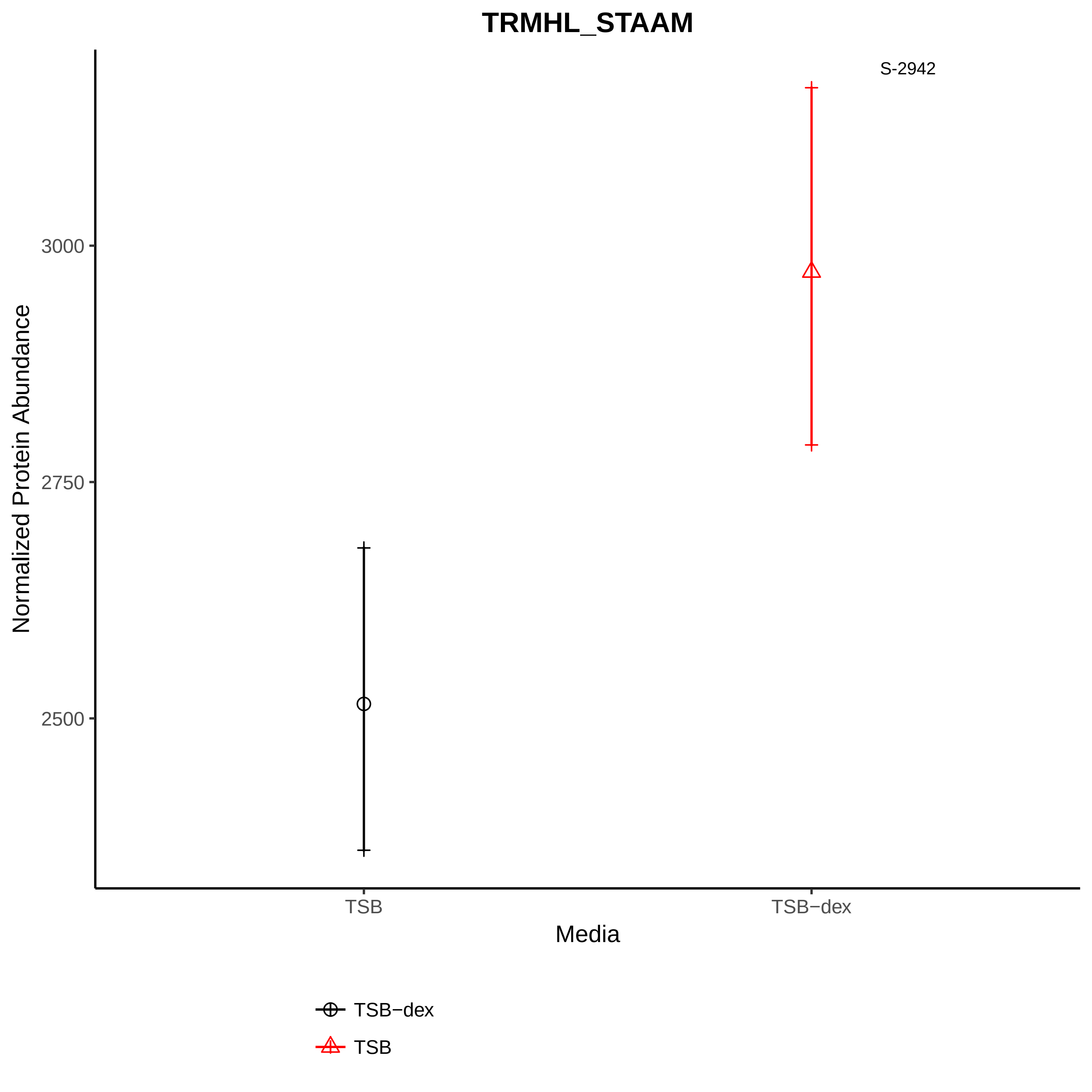

TRMHL_STAAM
S-2942
3000
2750
2500
Normalized Protein Abundance
TSB
TSB−dex
Media
TSB−dex
TSB

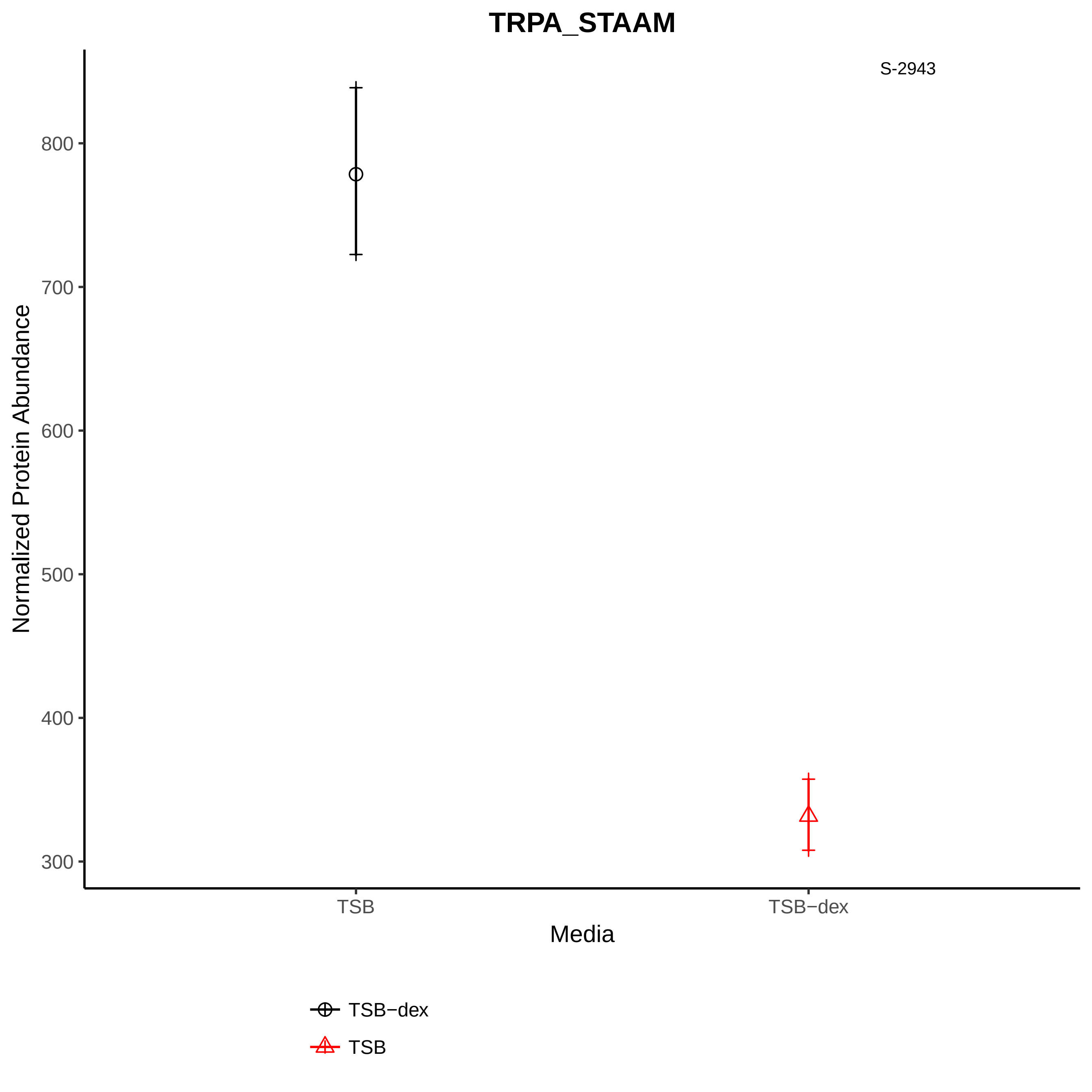

TRPA_STAAM
S-2943
Normalized Protein Abundance
800
700
600
500
400
300
TSB
TSB−dex
Media
TSB−dex
TSB

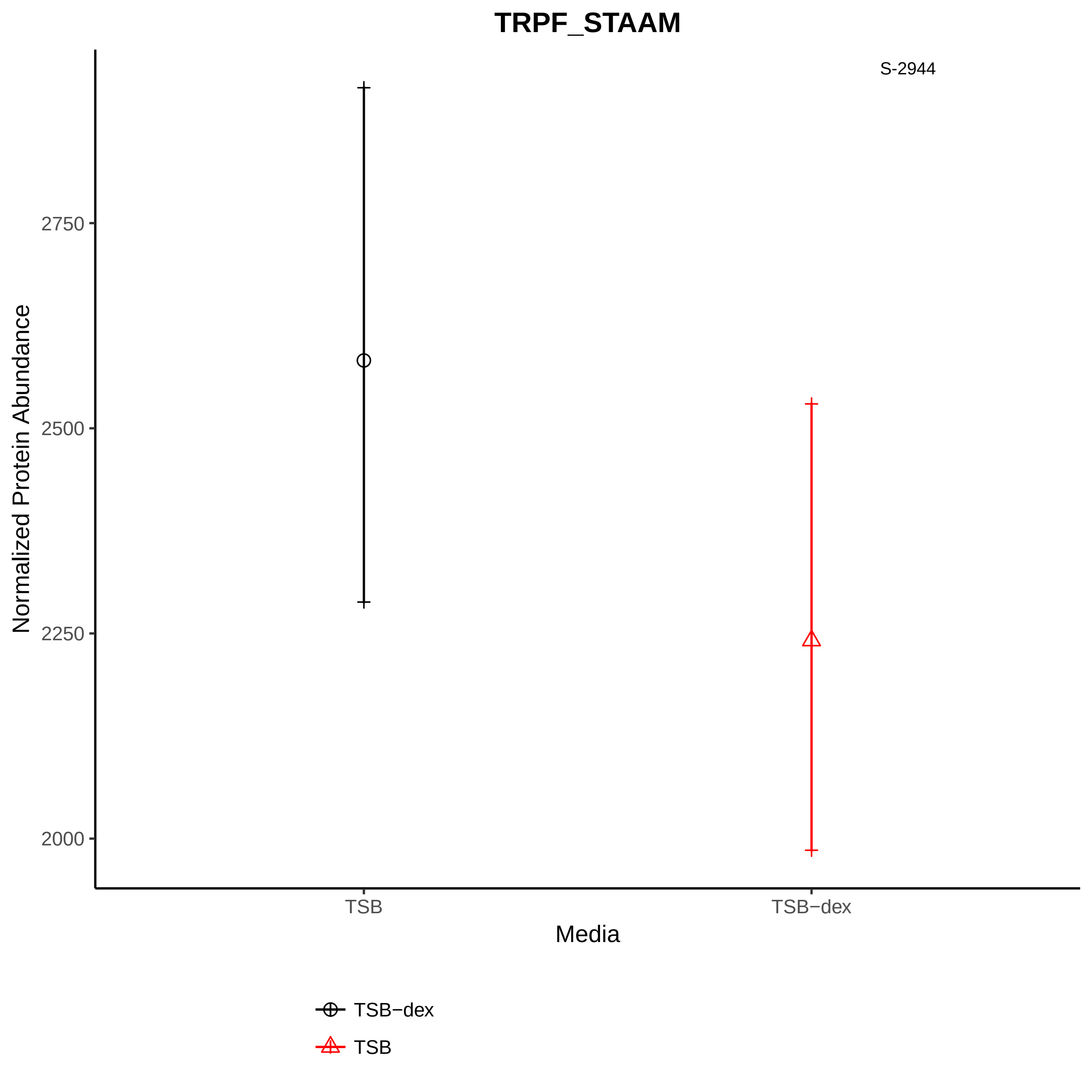

TRPF_STAAM
S-2944
Normalized Protein Abundance
2750
2500
2250
2000
TSB
TSB−dex
Media
TSB−dex
TSB

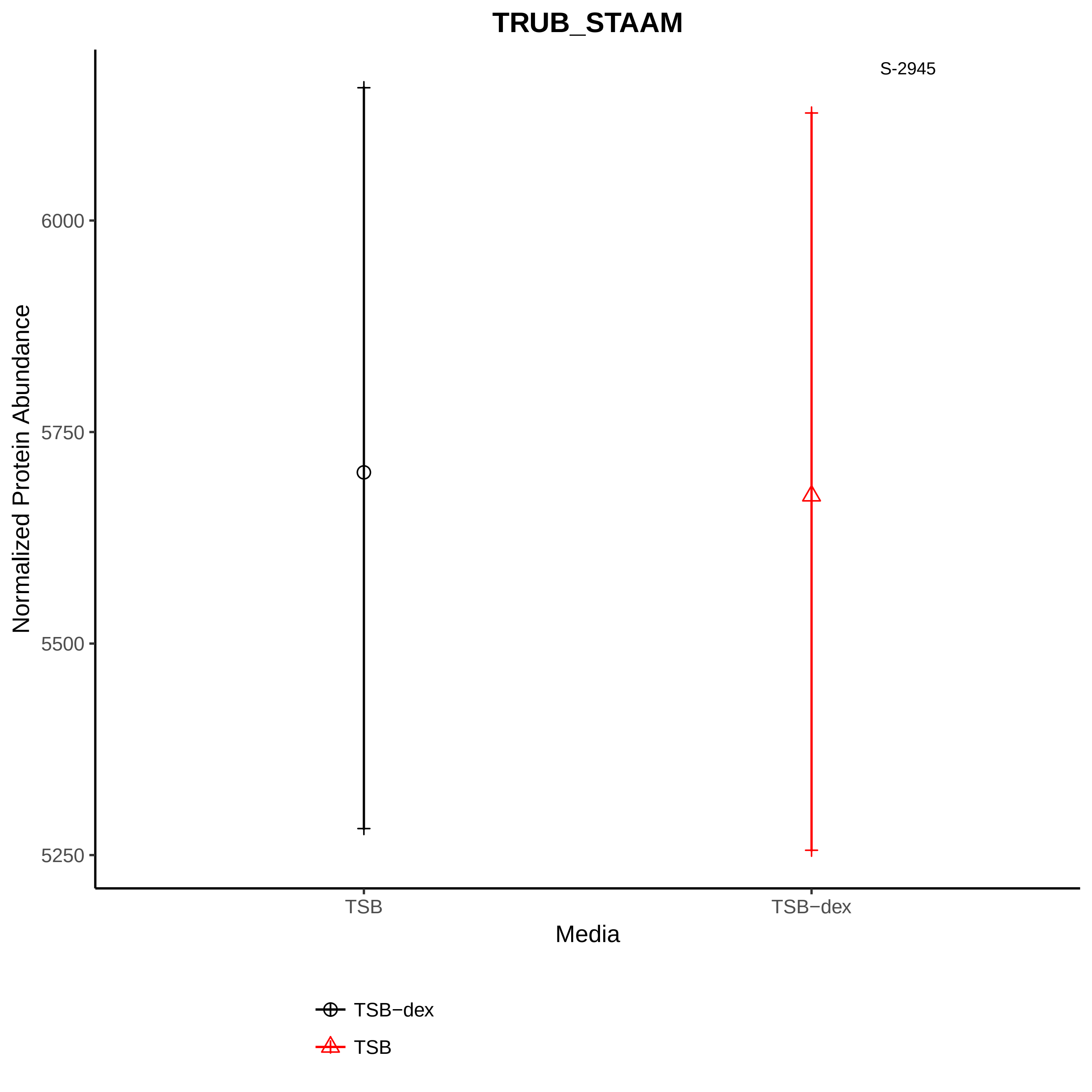

TRUB_STAAM
S-2945
Normalized Protein Abundance
6000
5750
5500
5250
TSB
TSB−dex
Media
TSB−dex
TSB

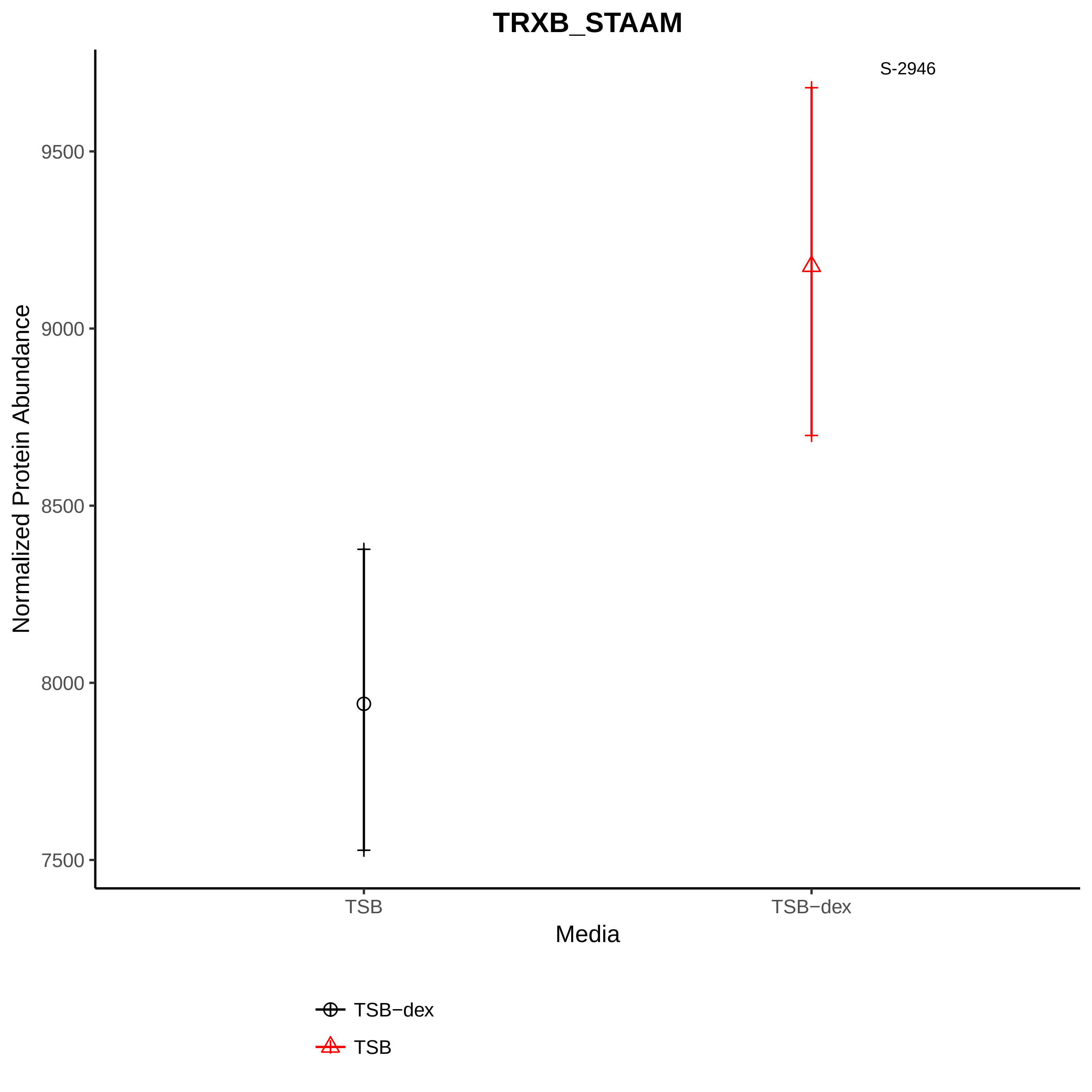

TRXB_STAAM
S-2946
Normalized Protein Abundance
9500
9000
8500
8000
7500
TSB
TSB−dex
Media
TSB−dex
TSB

TSAD_STAAM

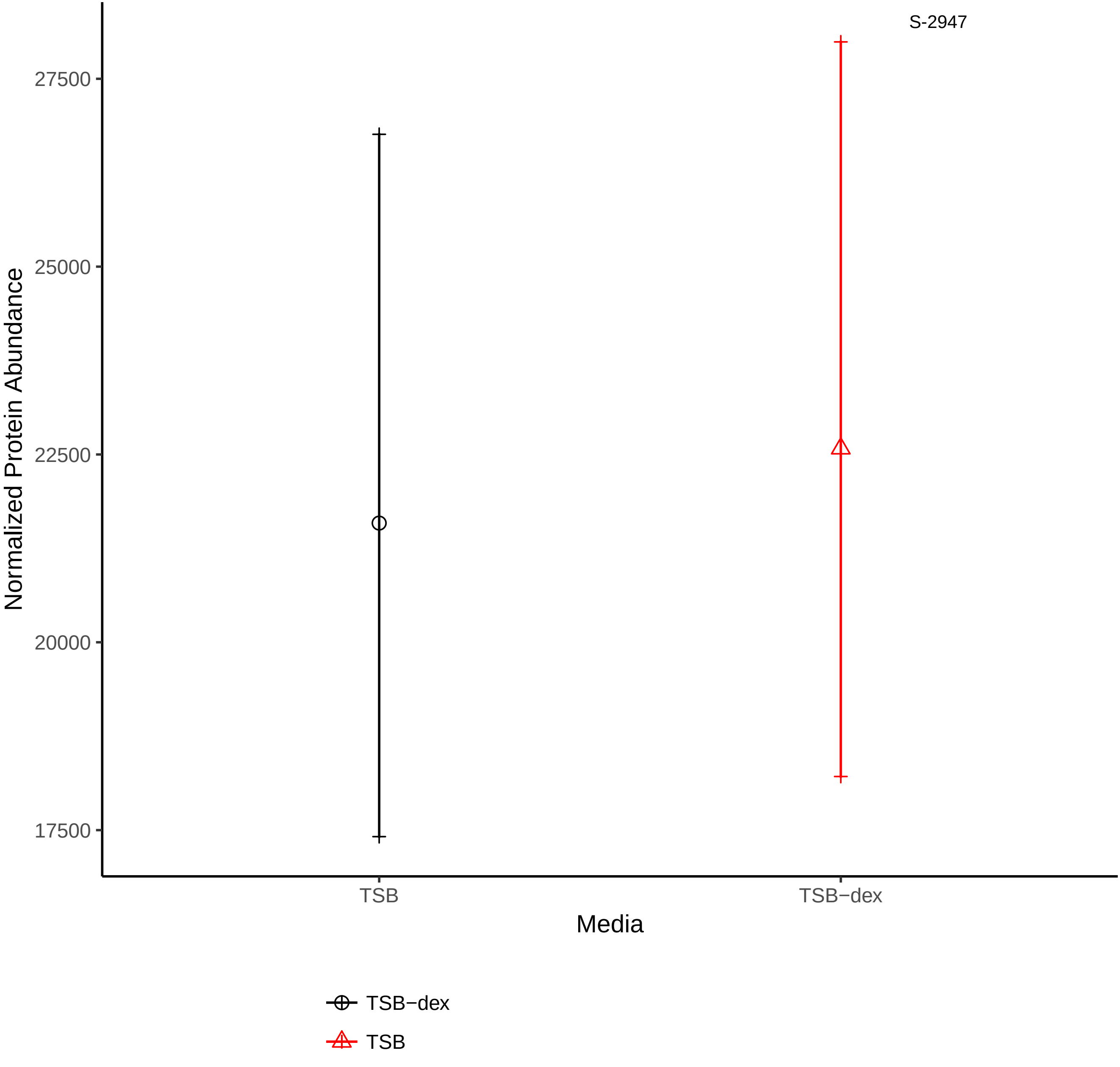

S-2947
27500
25000
22500
20000
17500
Normalized Protein Abundance
TSB
TSB−dex
Media
TSB−dex
TSB

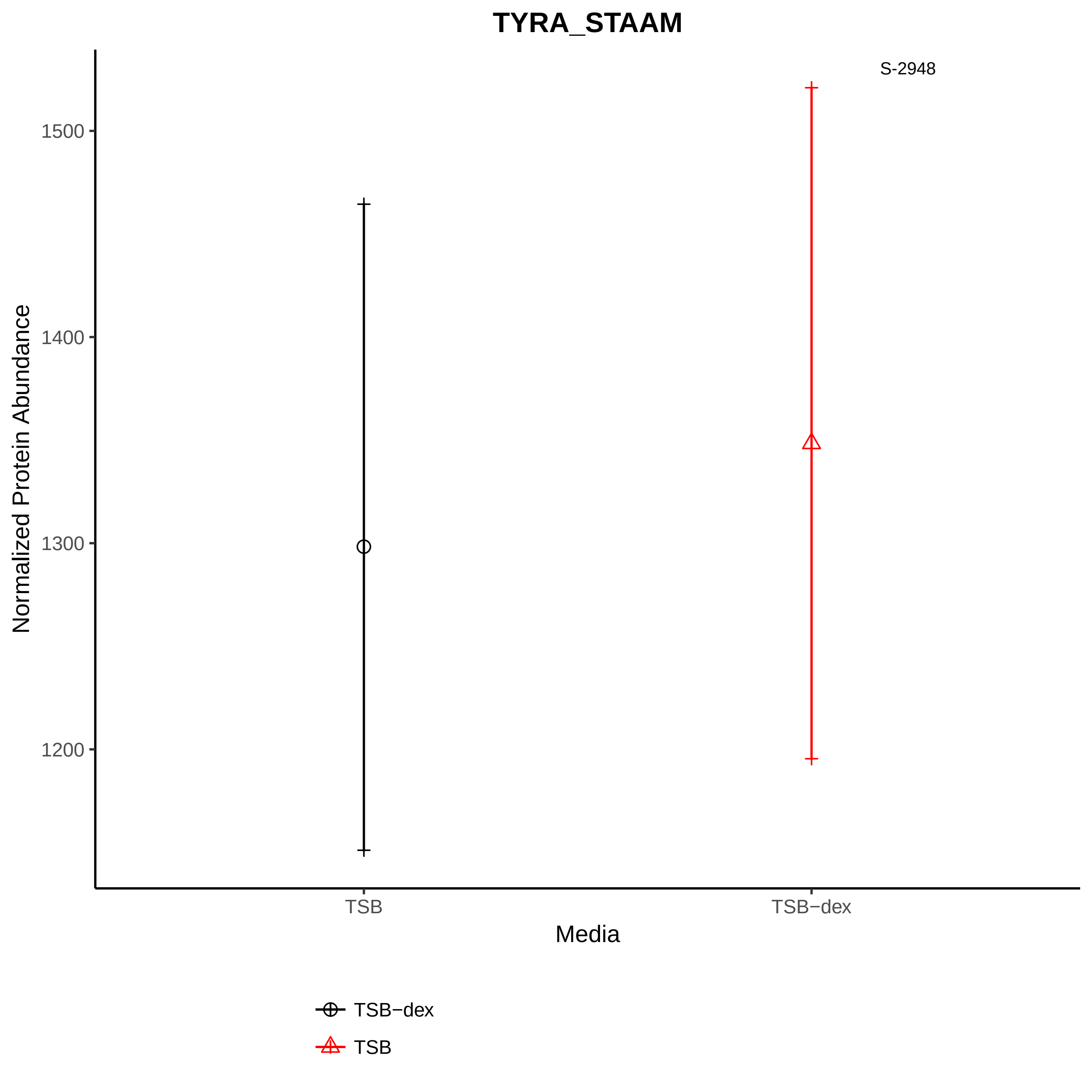
TYRA_STAAM
S-2948
Normalized Protein Abundance
1500
1400
1300
1200
TSB
TSB−dex
Media
TSB−dex
TSB

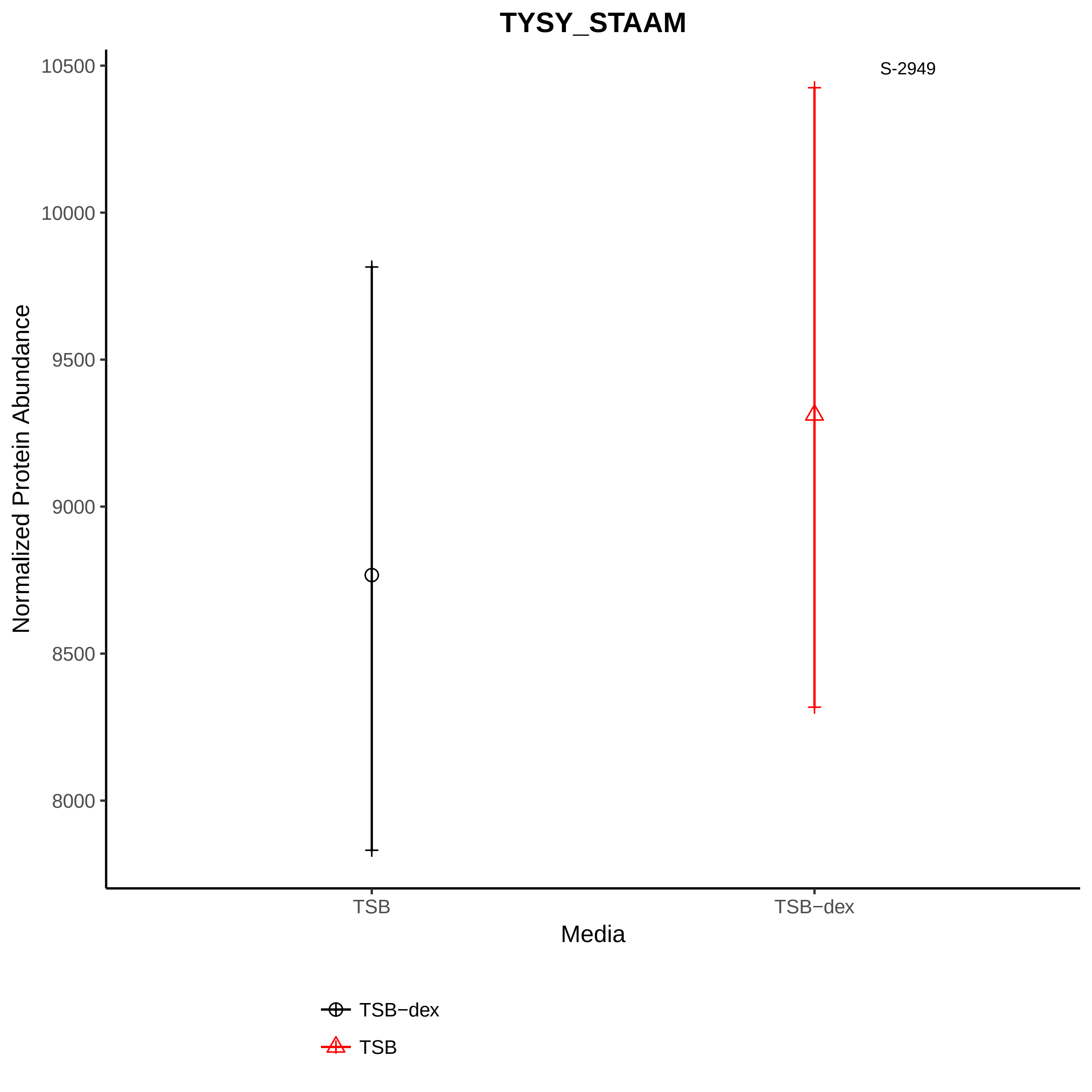

TYSY_STAAM
S-2949
Normalized Protein Abundance
10500
10000
9500
9000
8500
8000
TSB
TSB−dex
Media
TSB−dex
TSB

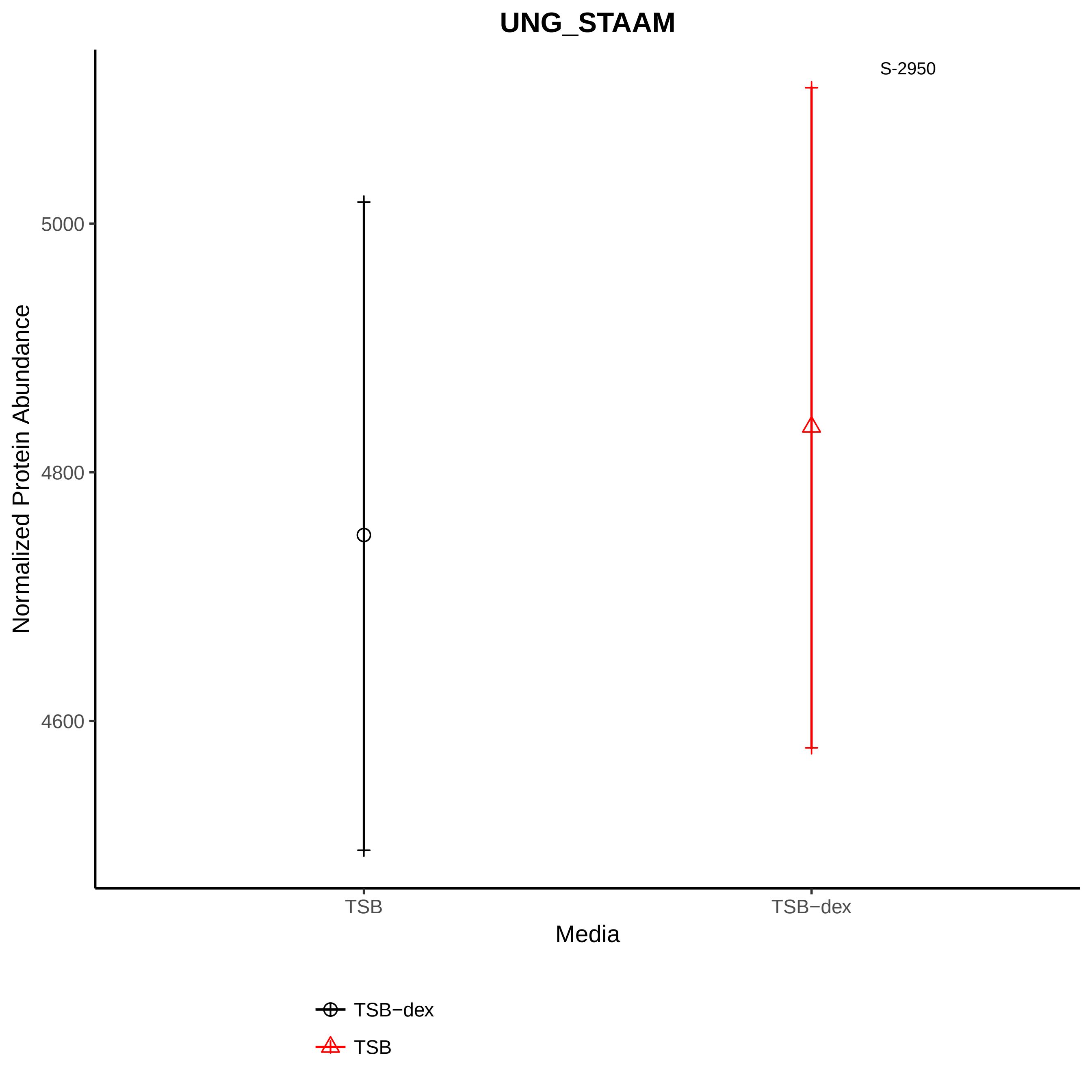

UNG_STAAM
S-2950
5000
4800
4600
Normalized Protein Abundance
TSB
TSB−dex
Media
TSB−dex
TSB

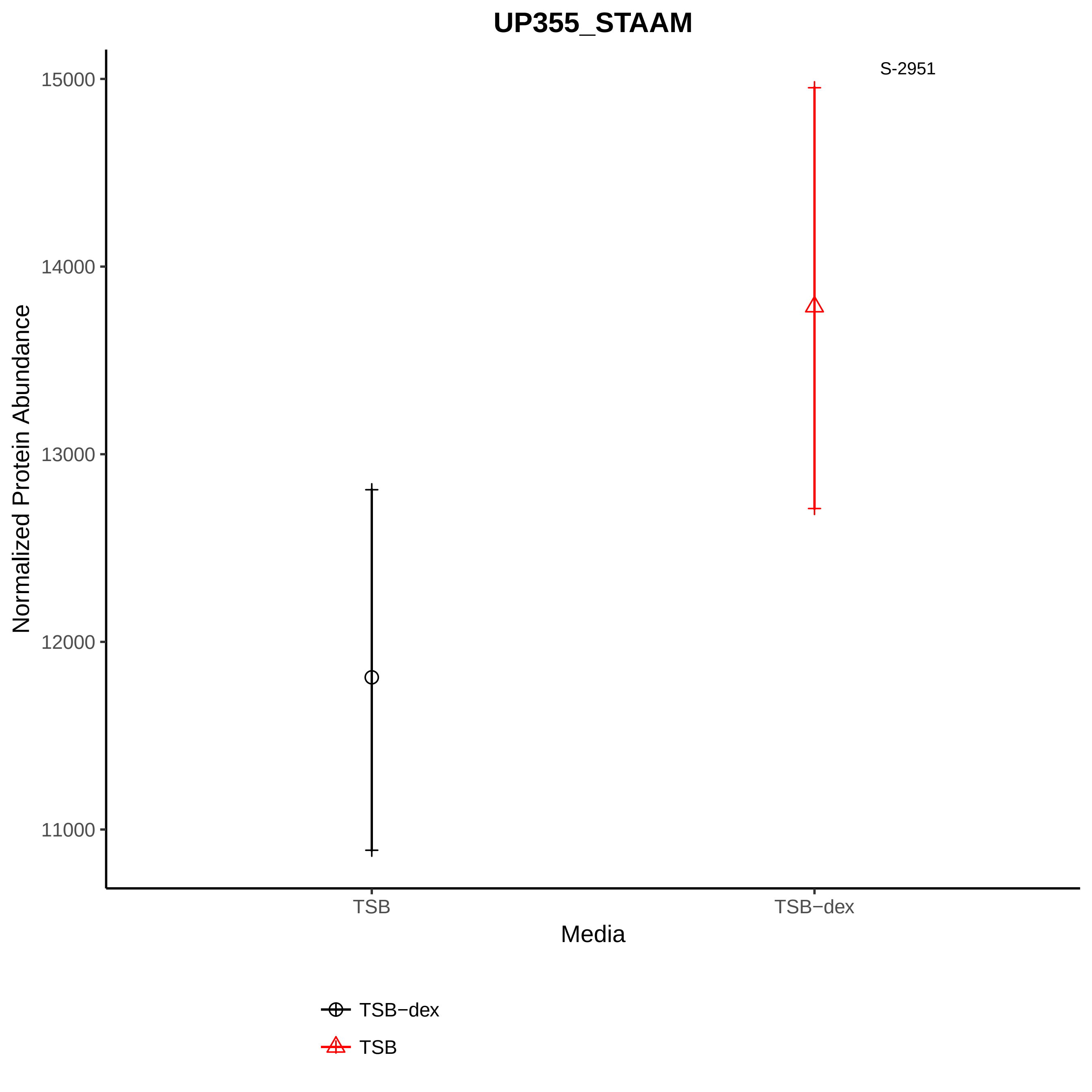

UP355_STAAM
S-2951
Normalized Protein Abundance
15000
14000
13000
12000
11000
TSB
TSB−dex
Media
TSB−dex
TSB

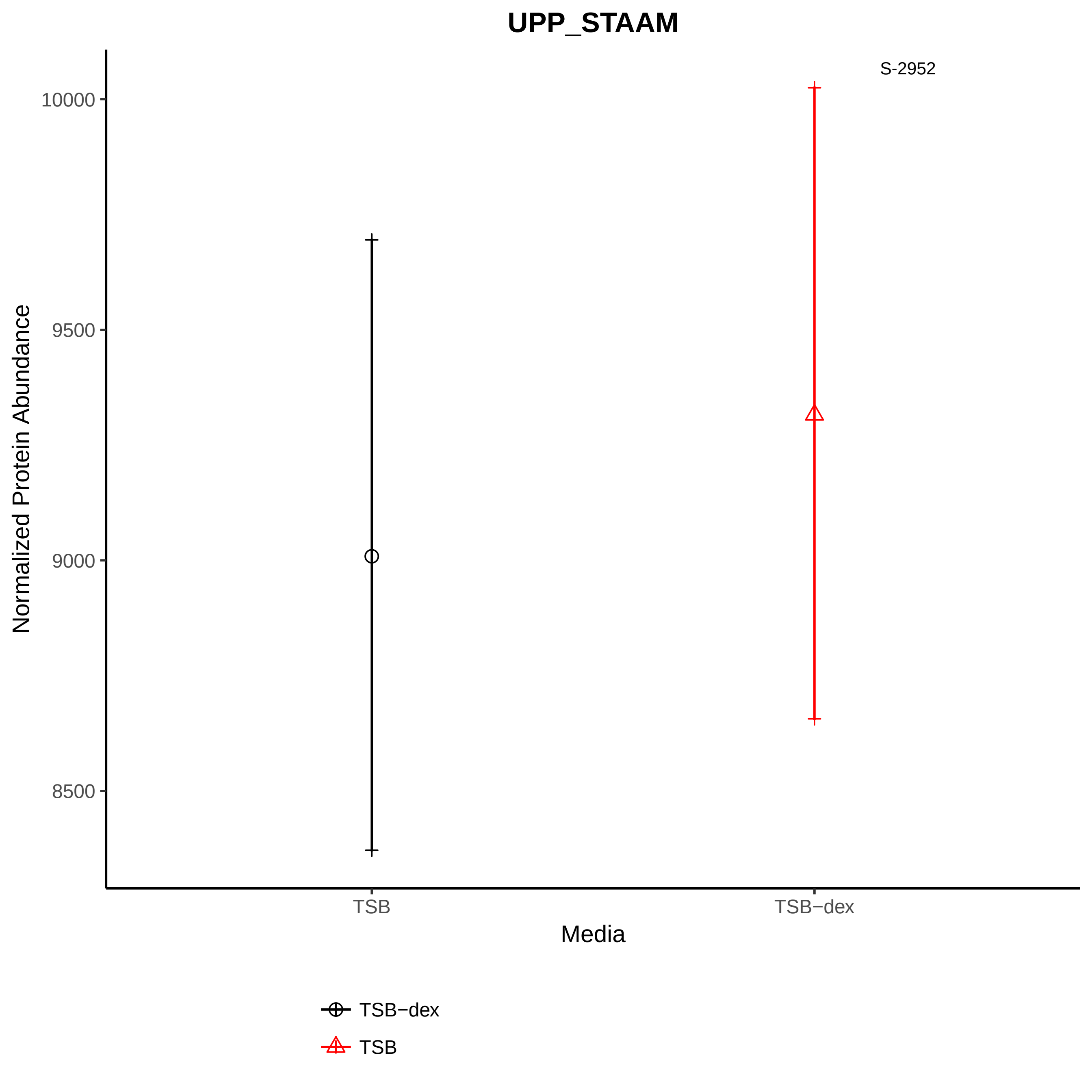

UPP_STAAM
S-2952
Normalized Protein Abundance
10000
9500
9000
8500
TSB
TSB−dex
Media
TSB−dex
TSB

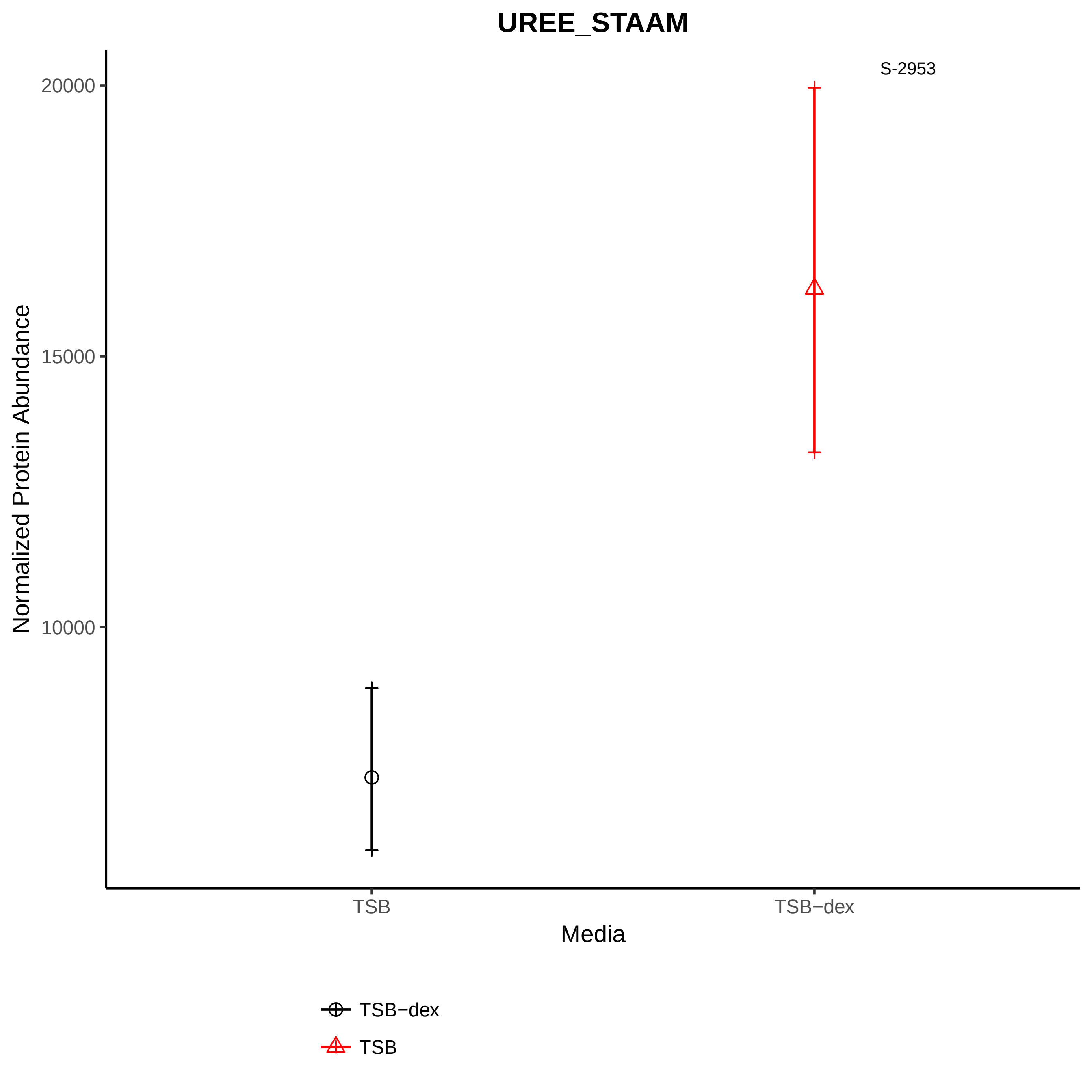
UREE_STAAM
S-2953
20000
15000
10000
Normalized Protein Abundance
TSB
TSB−dex
Media
TSB−dex
TSB

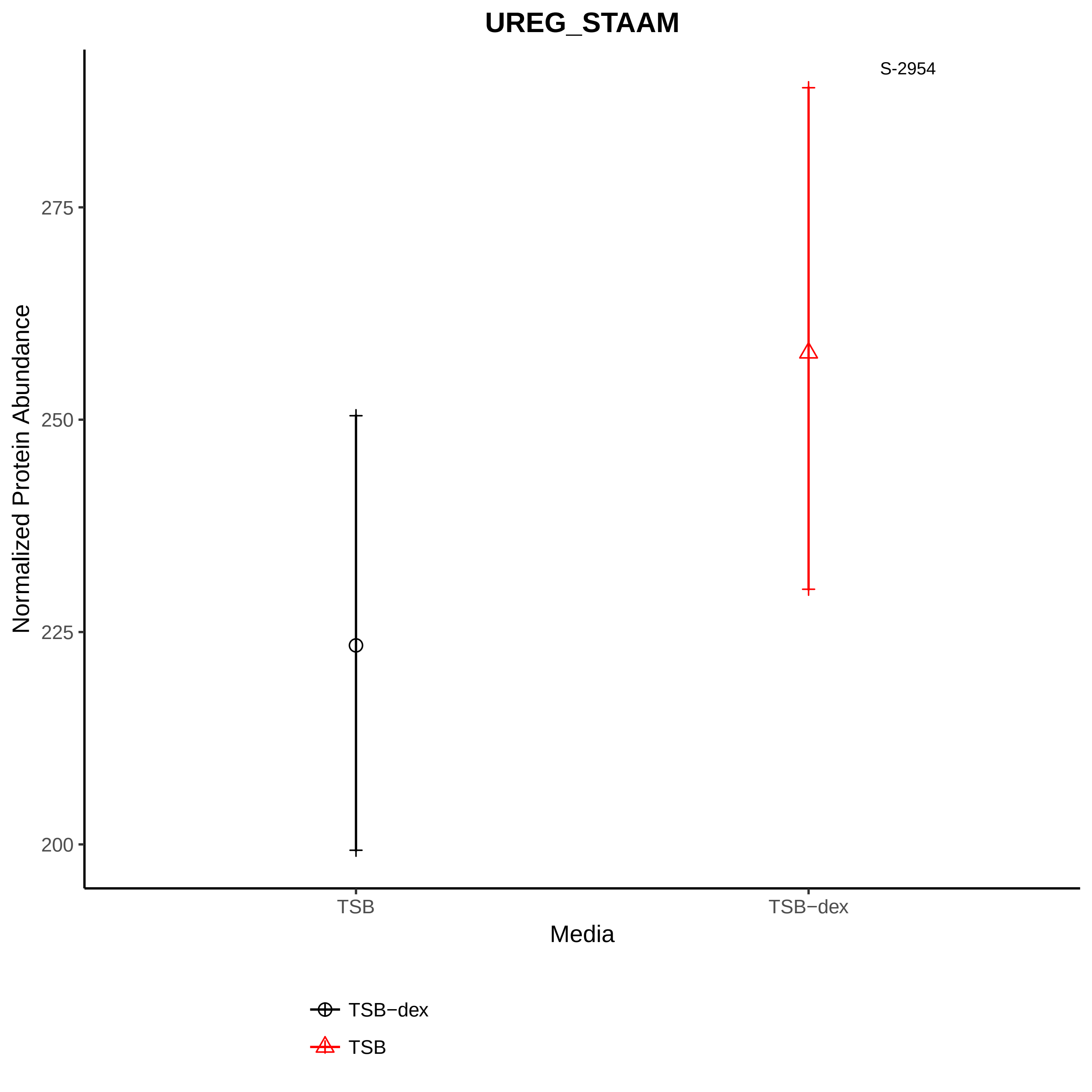

UREG_STAAM
S-2954
Normalized Protein Abundance
275
250
225
200
TSB
TSB−dex
Media
TSB−dex
TSB

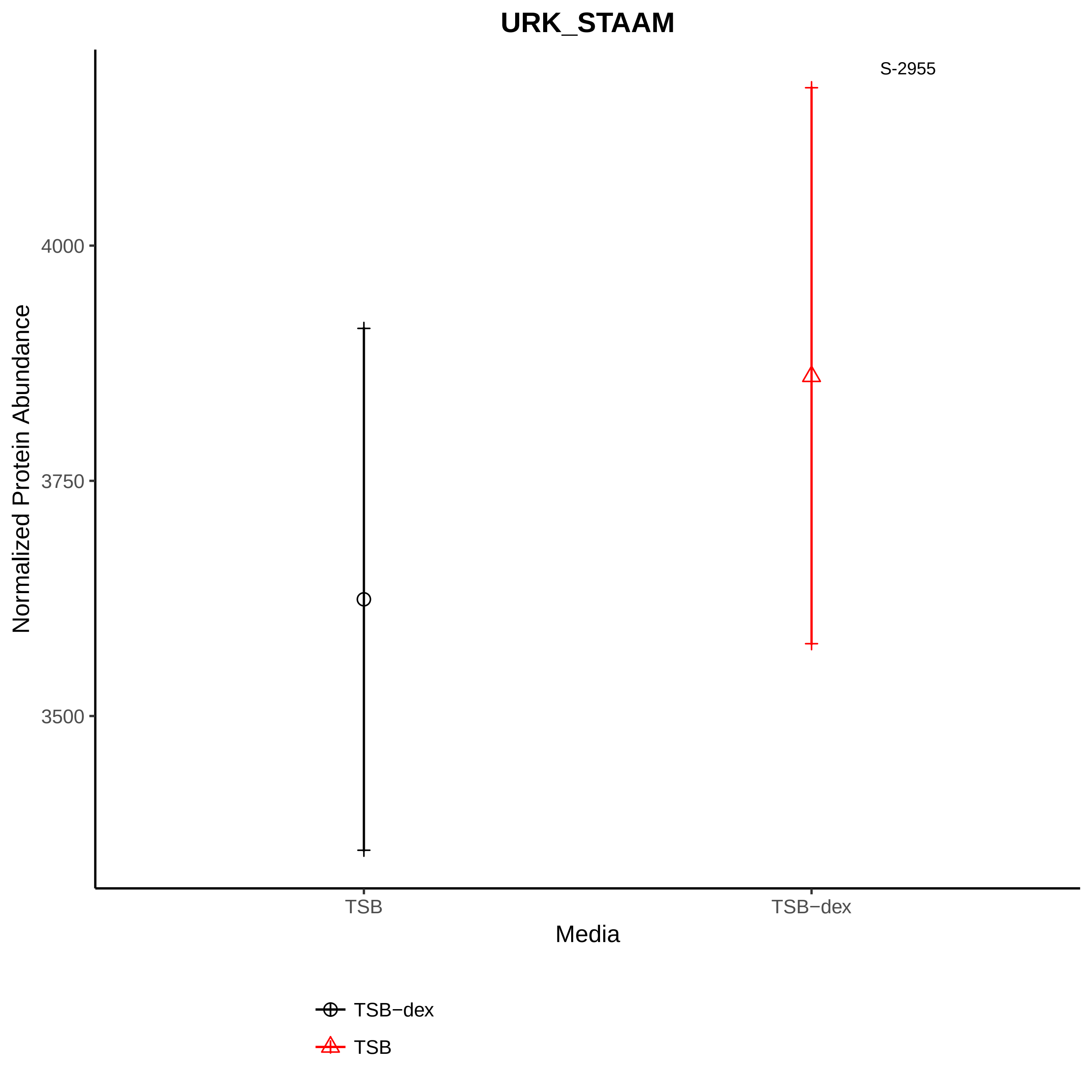
URK_STAAM
S-2955
Normalized Protein Abundance
4000
3750
3500
TSB
TSB−dex
Media
TSB−dex
TSB

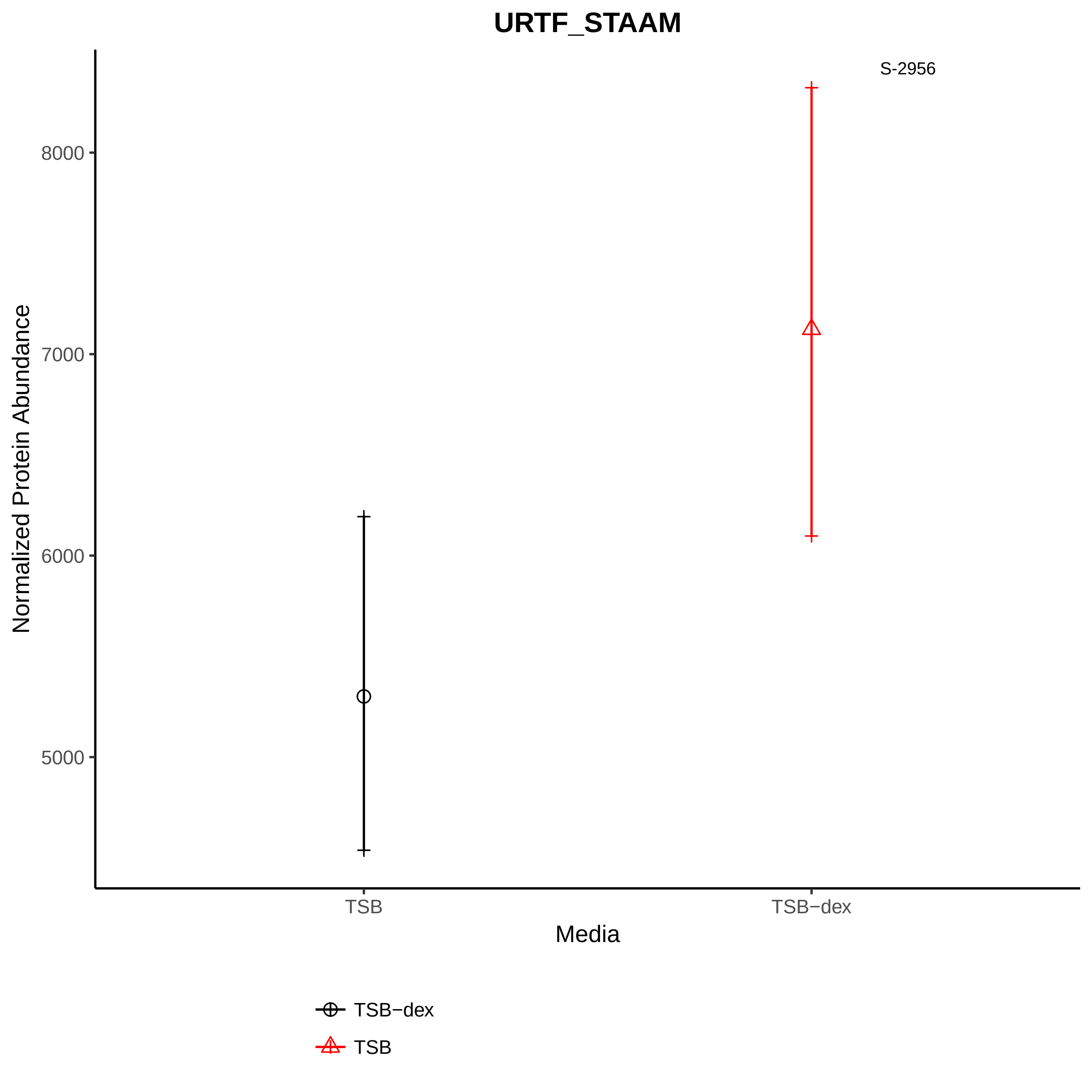

URTF_STAAM
S-2956
8000
7000
6000
5000
Normalized Protein Abundance
TSB
TSB−dex
Media
TSB−dex
TSB

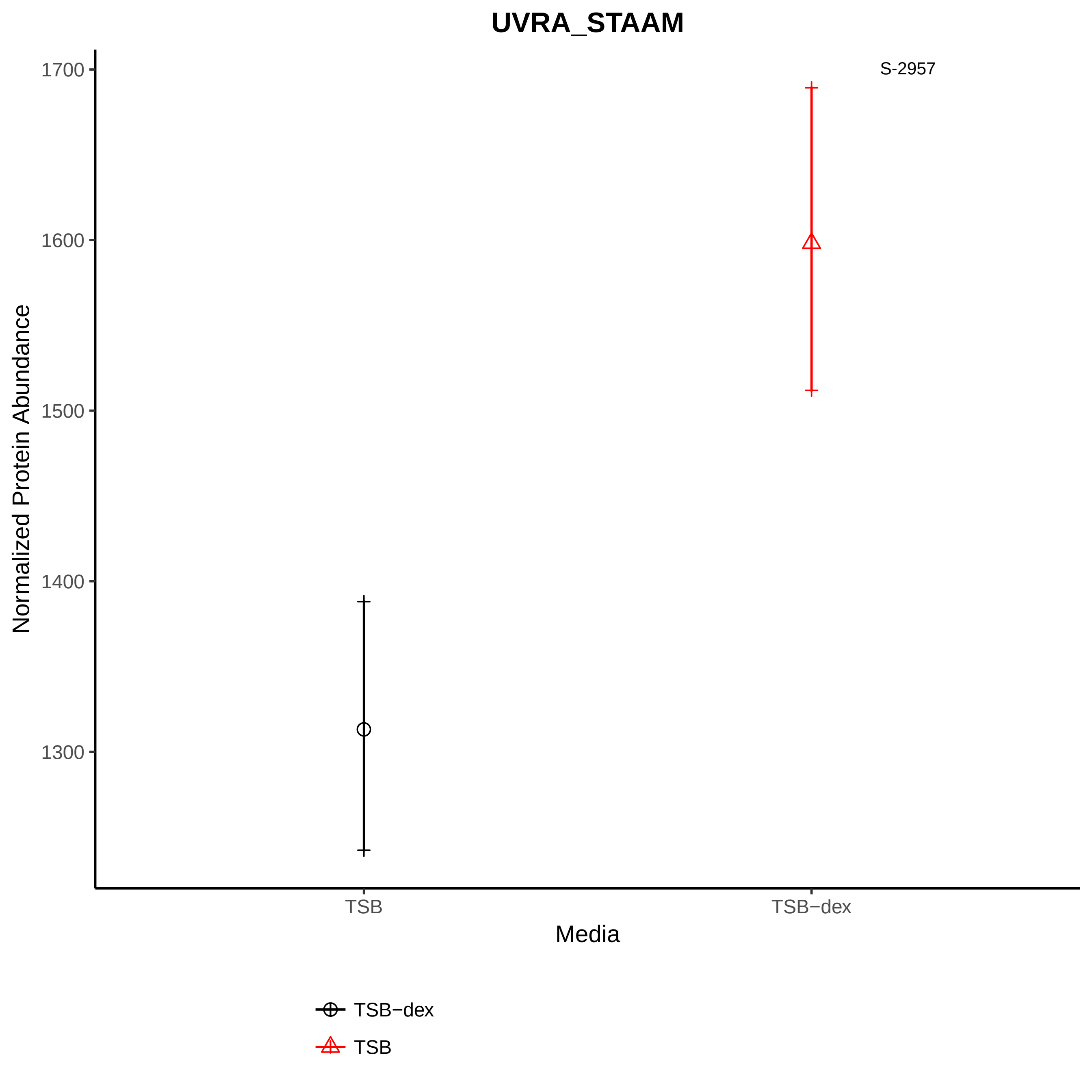

UVRA_STAAM
S-2957
1700
1600
1500
1400
1300
Normalized Protein Abundance
TSB
TSB−dex
Media
TSB−dex
TSB

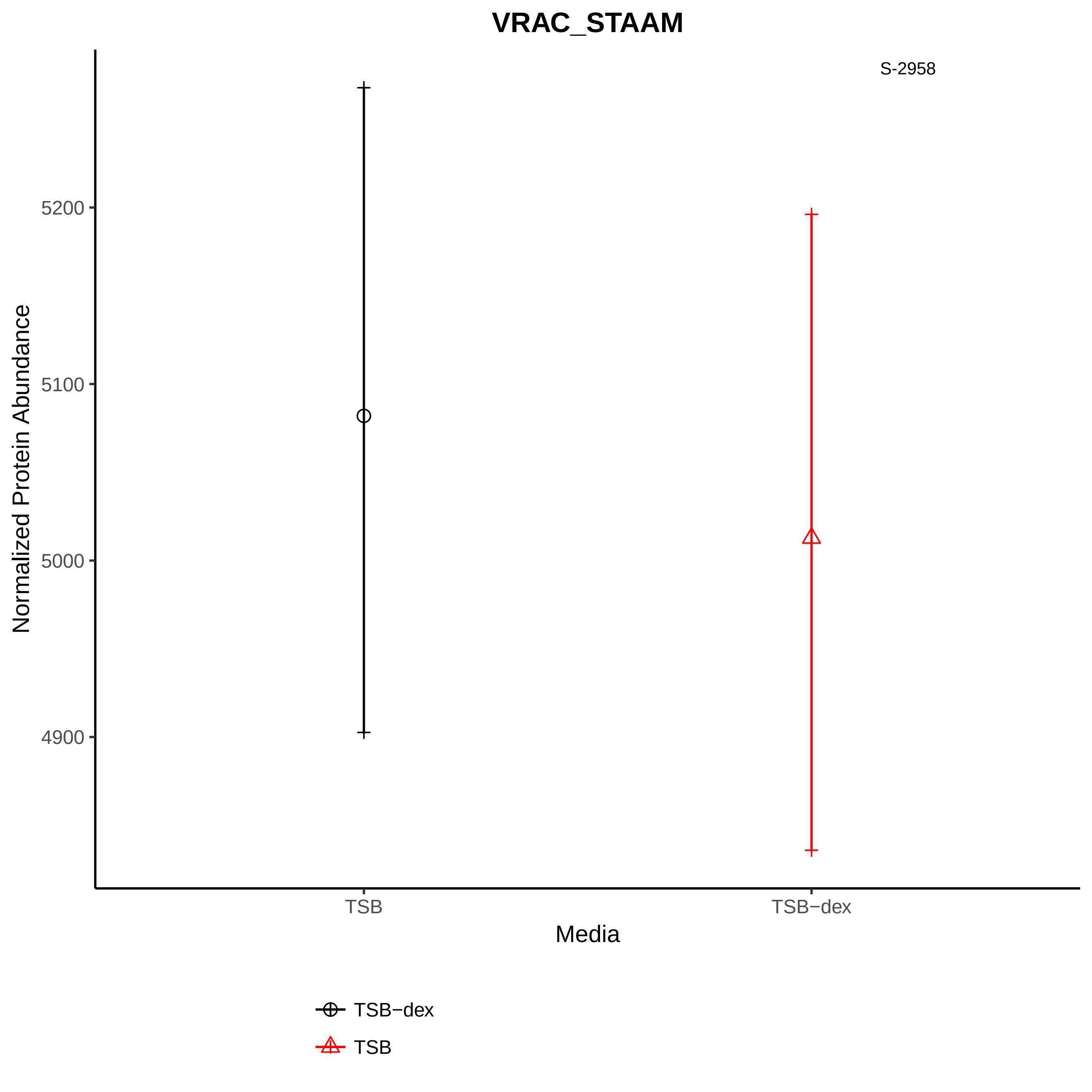
VRAC_STAAM
S-2958
Normalized Protein Abundance
5200
5100
5000
4900
TSB
TSB−dex
Media
TSB−dex
TSB

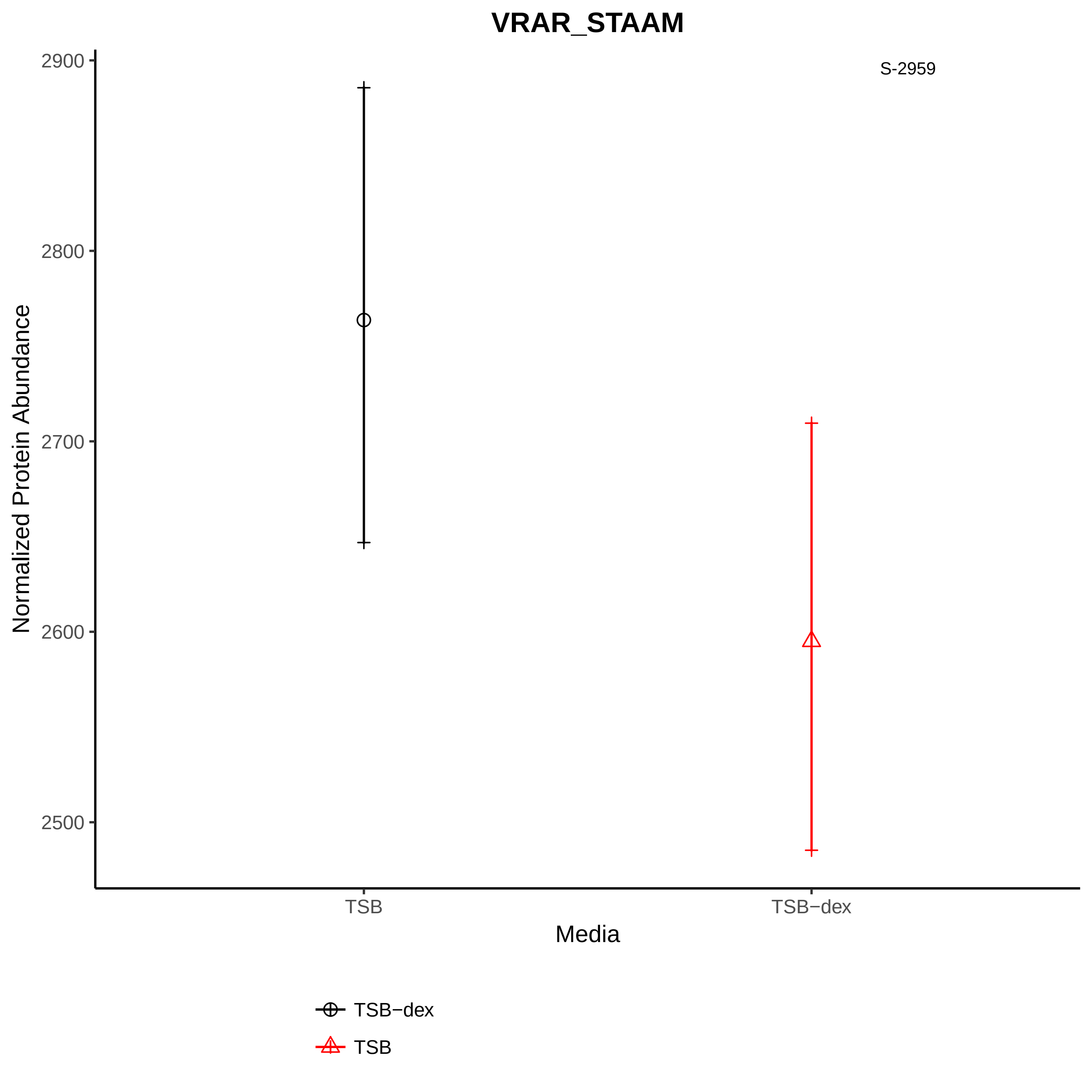

VRAR_STAAM
S-2959
Normalized Protein Abundance
2900
2800
2700
2600
2500
TSB
TSB−dex
Media
TSB−dex
TSB

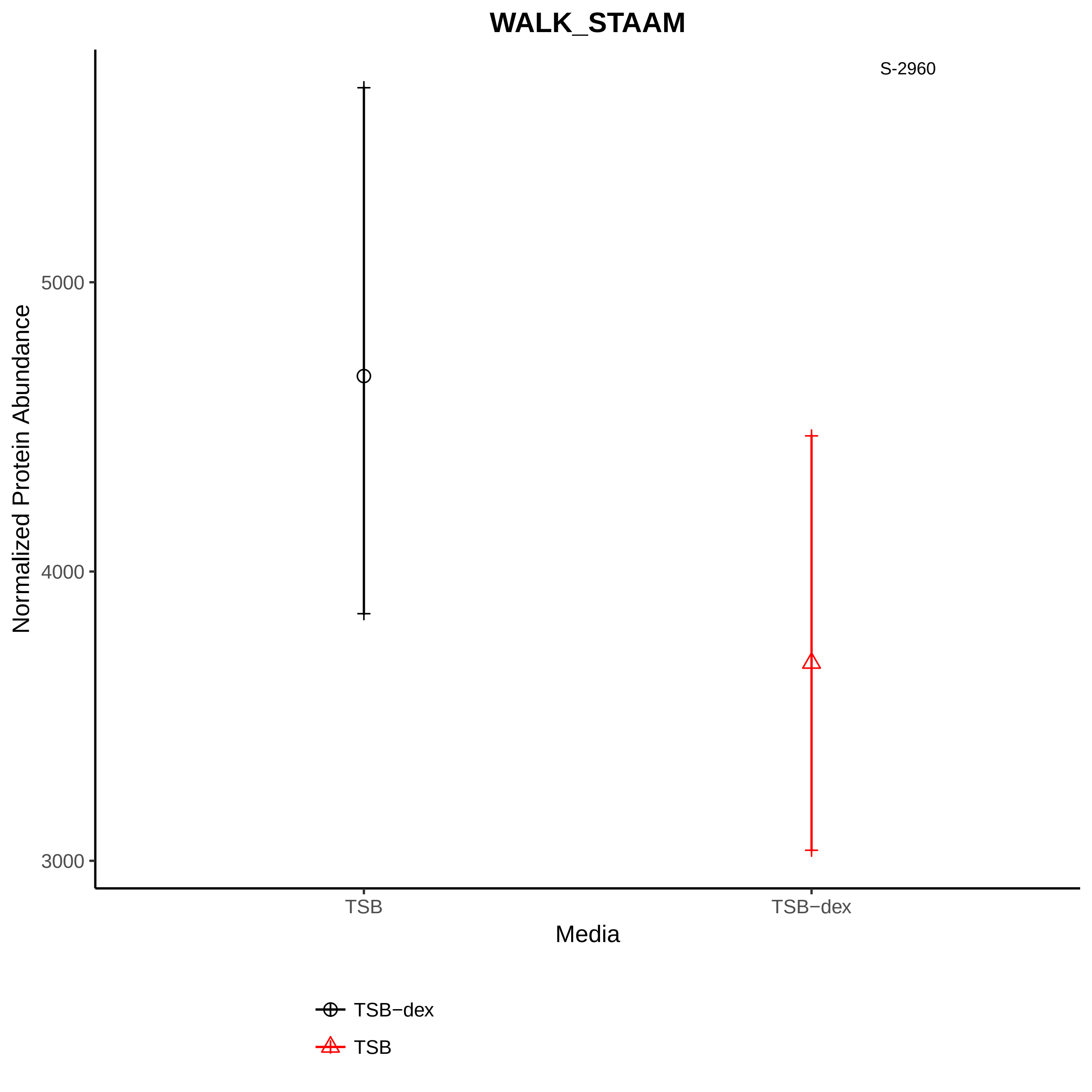

WALK_STAAM
S-2960
Normalized Protein Abundance
5000
4000
3000
TSB
TSB−dex
Media
TSB−dex
TSB

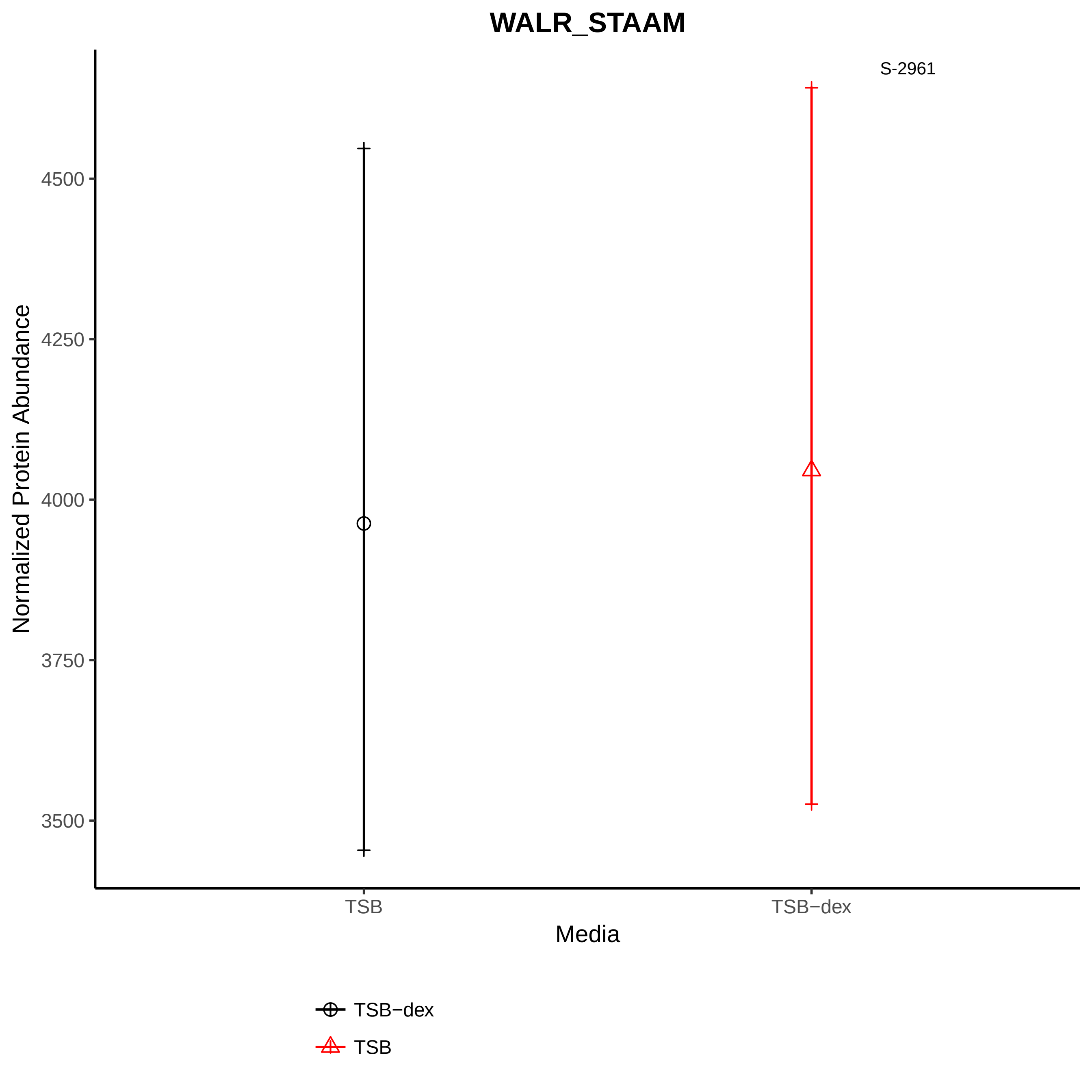
WALR_STAAM
S-2961
Normalized Protein Abundance
4500
4250
4000
3750
3500
TSB
TSB−dex
Media
TSB−dex
TSB

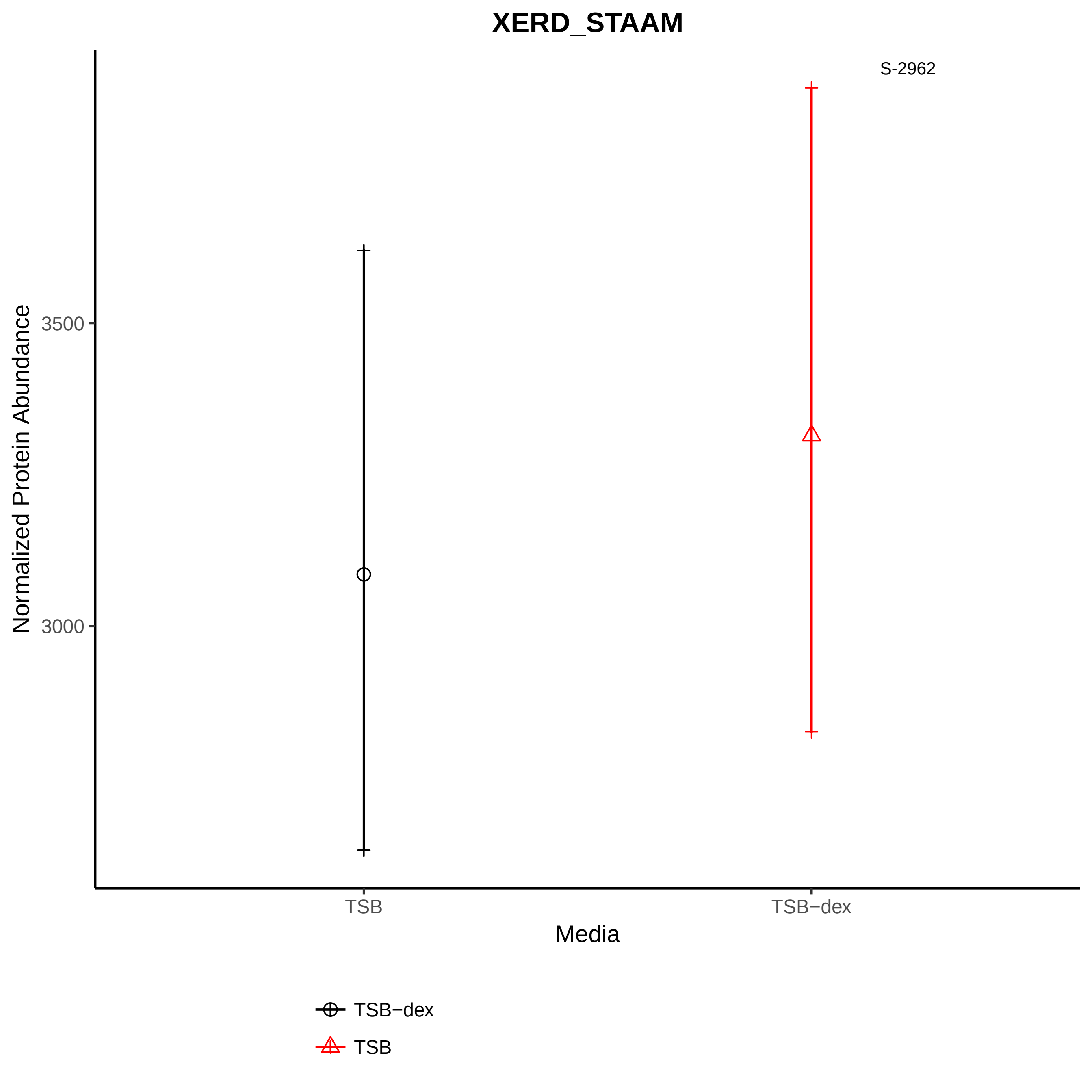

XERD_STAAM
S-2962
3500
3000
Normalized Protein Abundance
TSB
TSB−dex
Media
TSB−dex
TSB

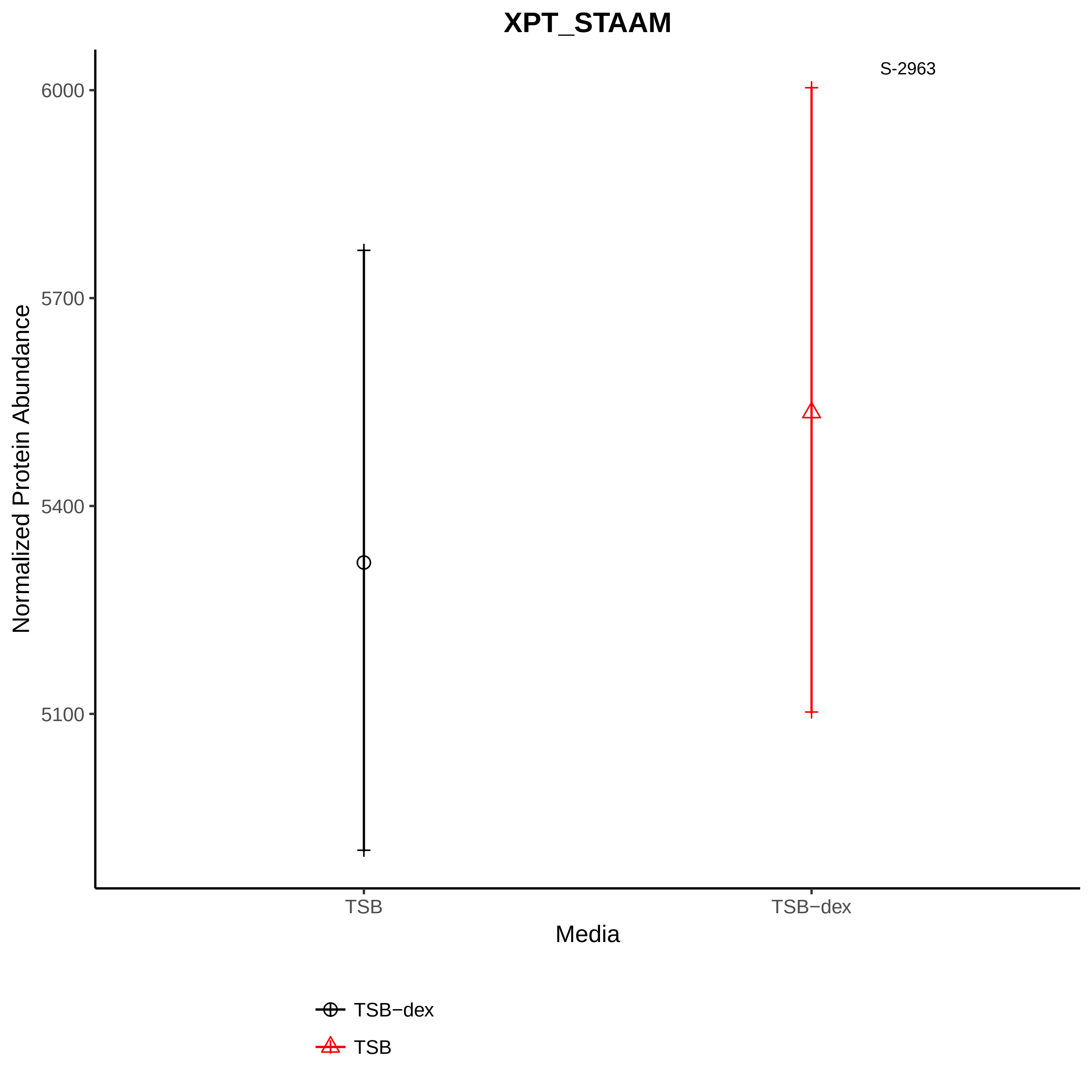

XPT_STAAM
S-2963
6000
5700
5400
5100
Normalized Protein Abundance
TSB
TSB−dex
Media
TSB−dex
TSB

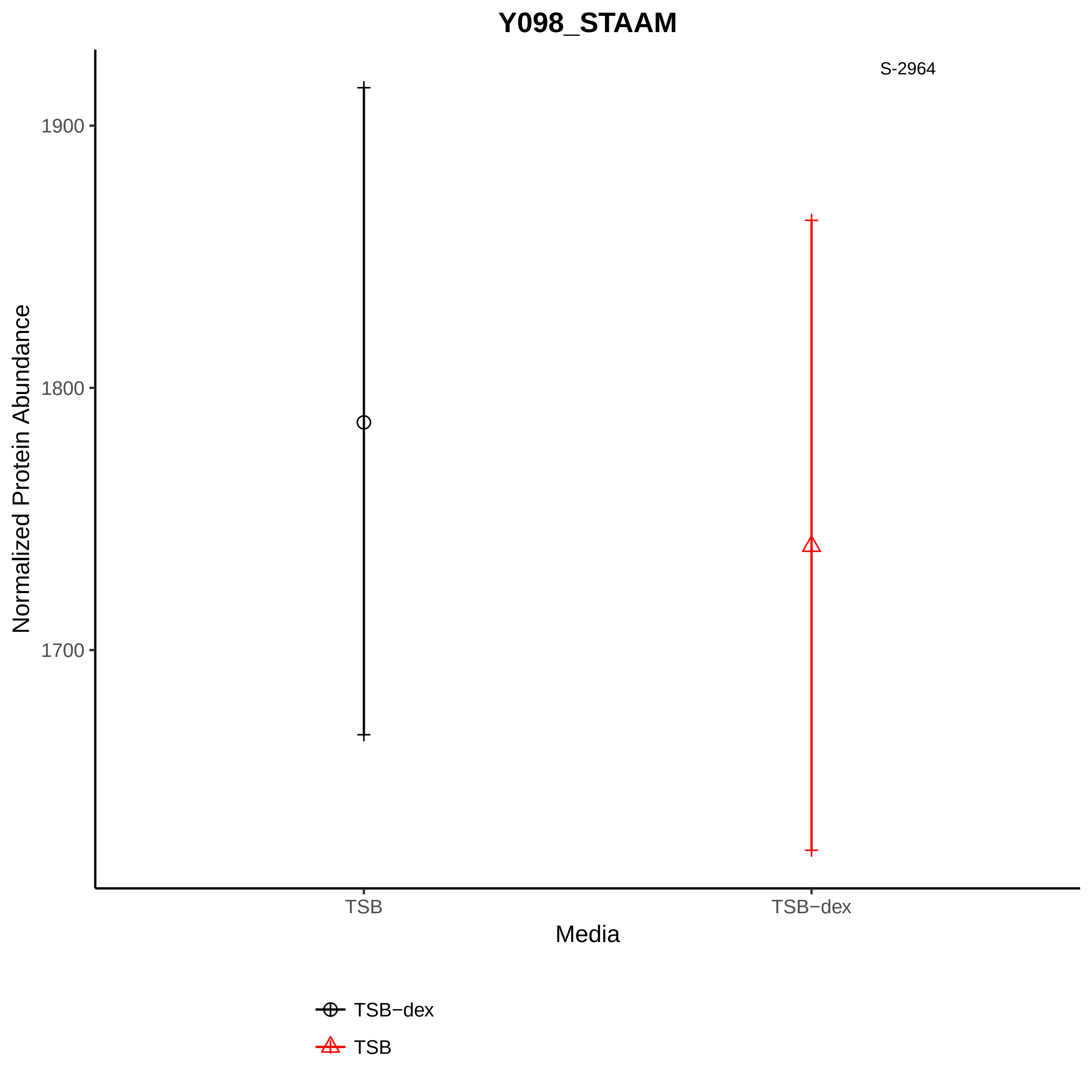

Y098_STAAM
S-2964
1900
1800
1700
Normalized Protein Abundance
TSB
TSB−dex
Media
TSB−dex
TSB

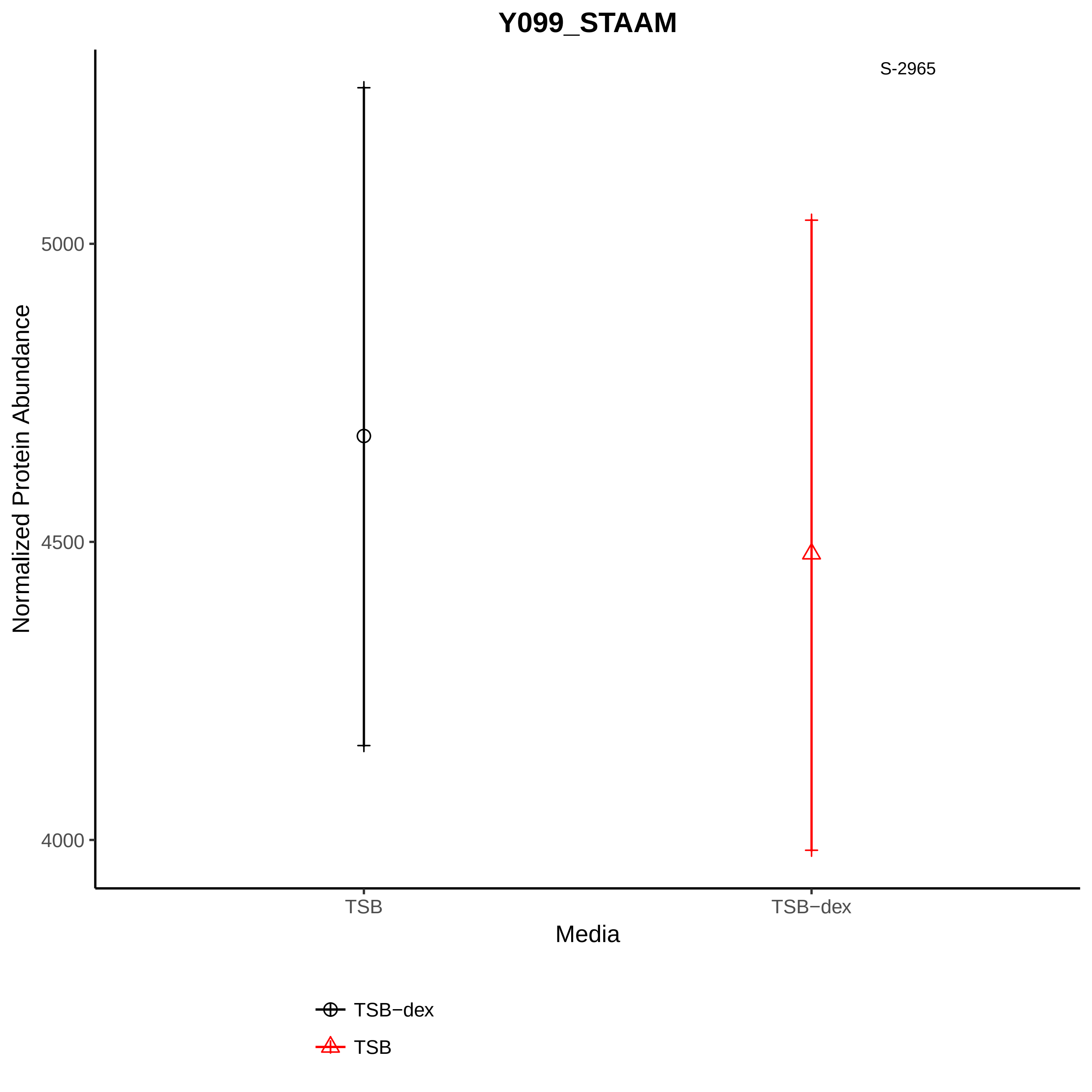

Y099_STAAM
S-2965
5000
4500
4000
Normalized Protein Abundance
TSB
TSB−dex
Media
TSB−dex
TSB

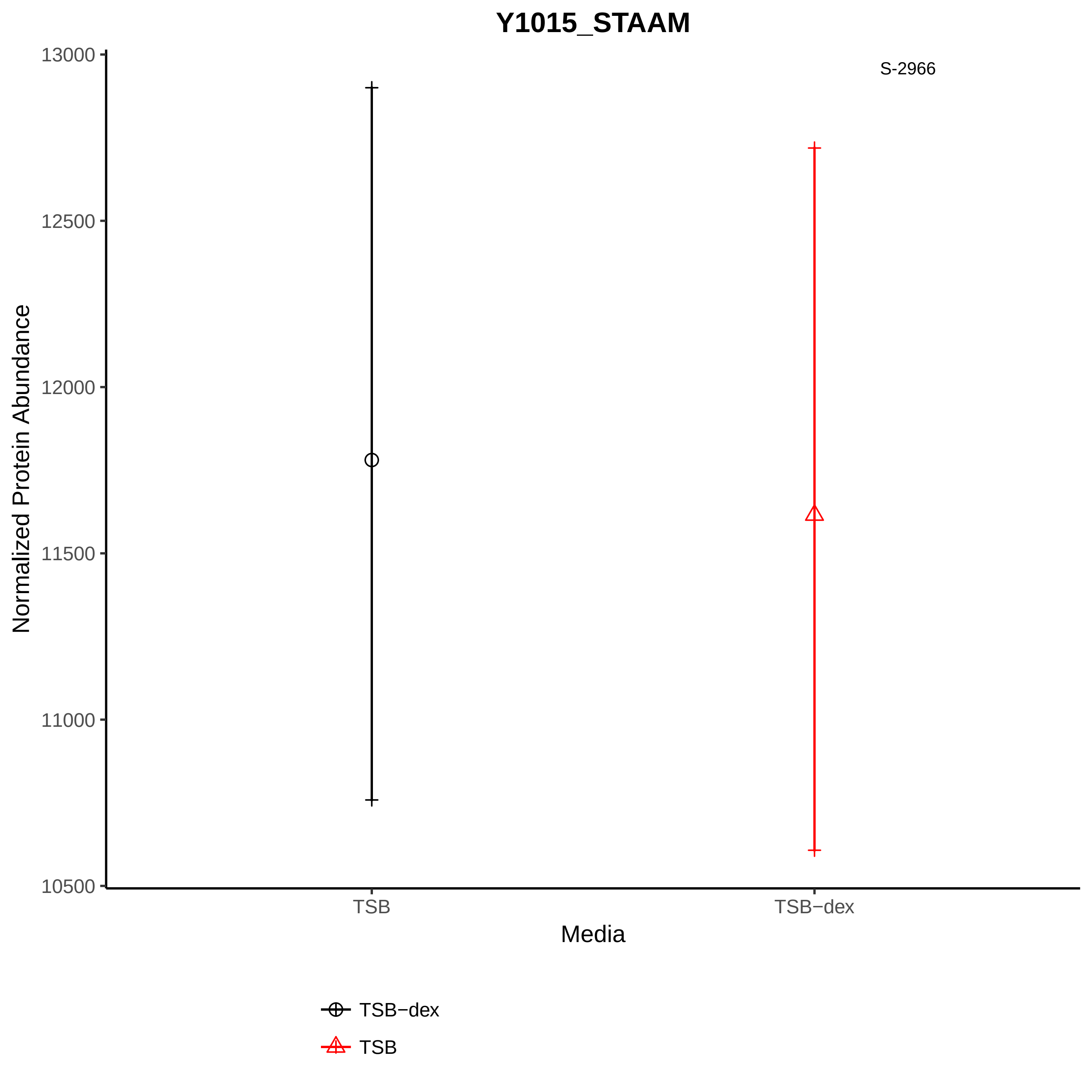

Y1015_STAAM
S-2966
Normalized Protein Abundance
13000
12500
12000
11500
11000
10500
TSB
TSB−dex
Media
TSB−dex
TSB

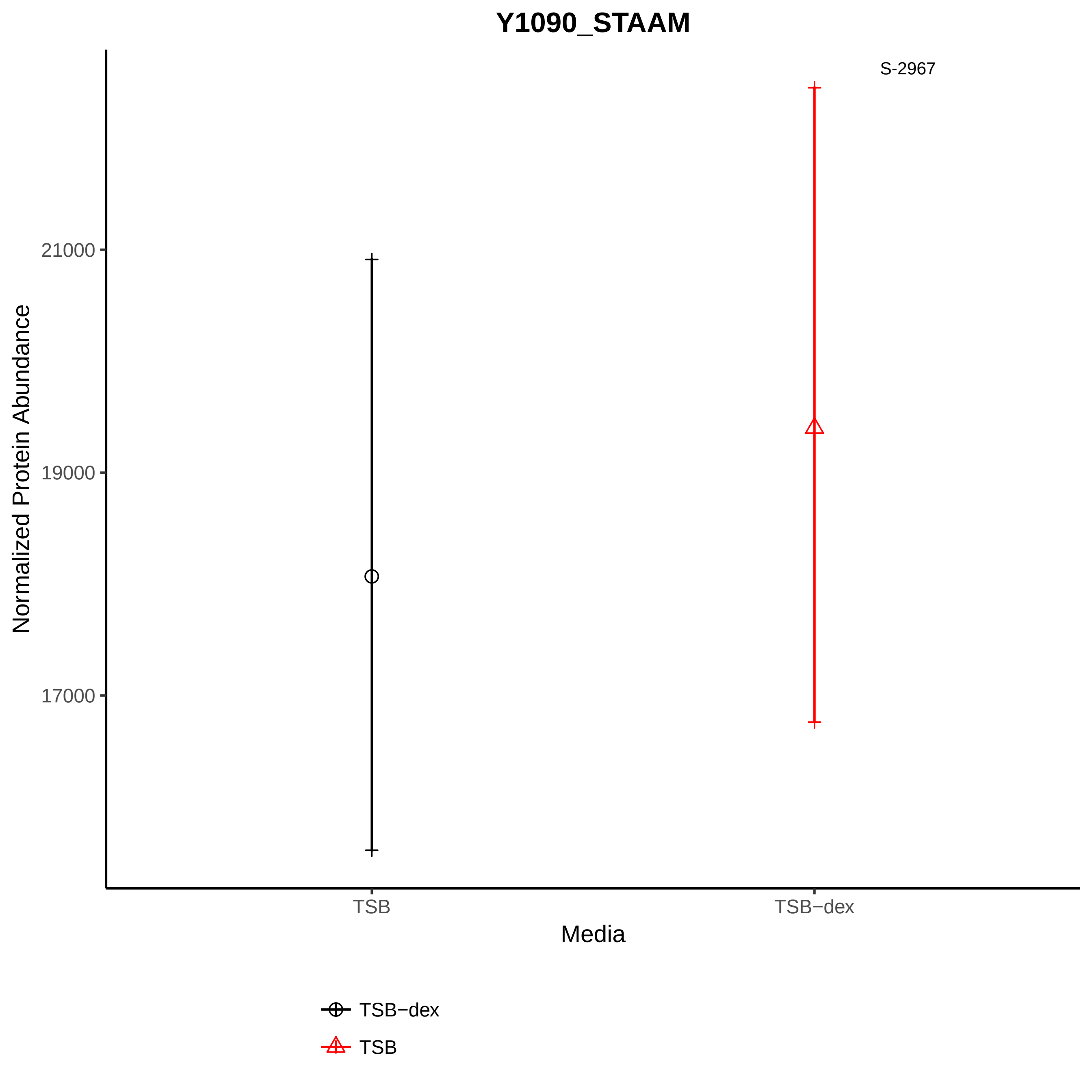

Y1090_STAAM
S-2967
Normalized Protein Abundance
21000
19000
17000
TSB
TSB−dex
Media
TSB−dex
TSB

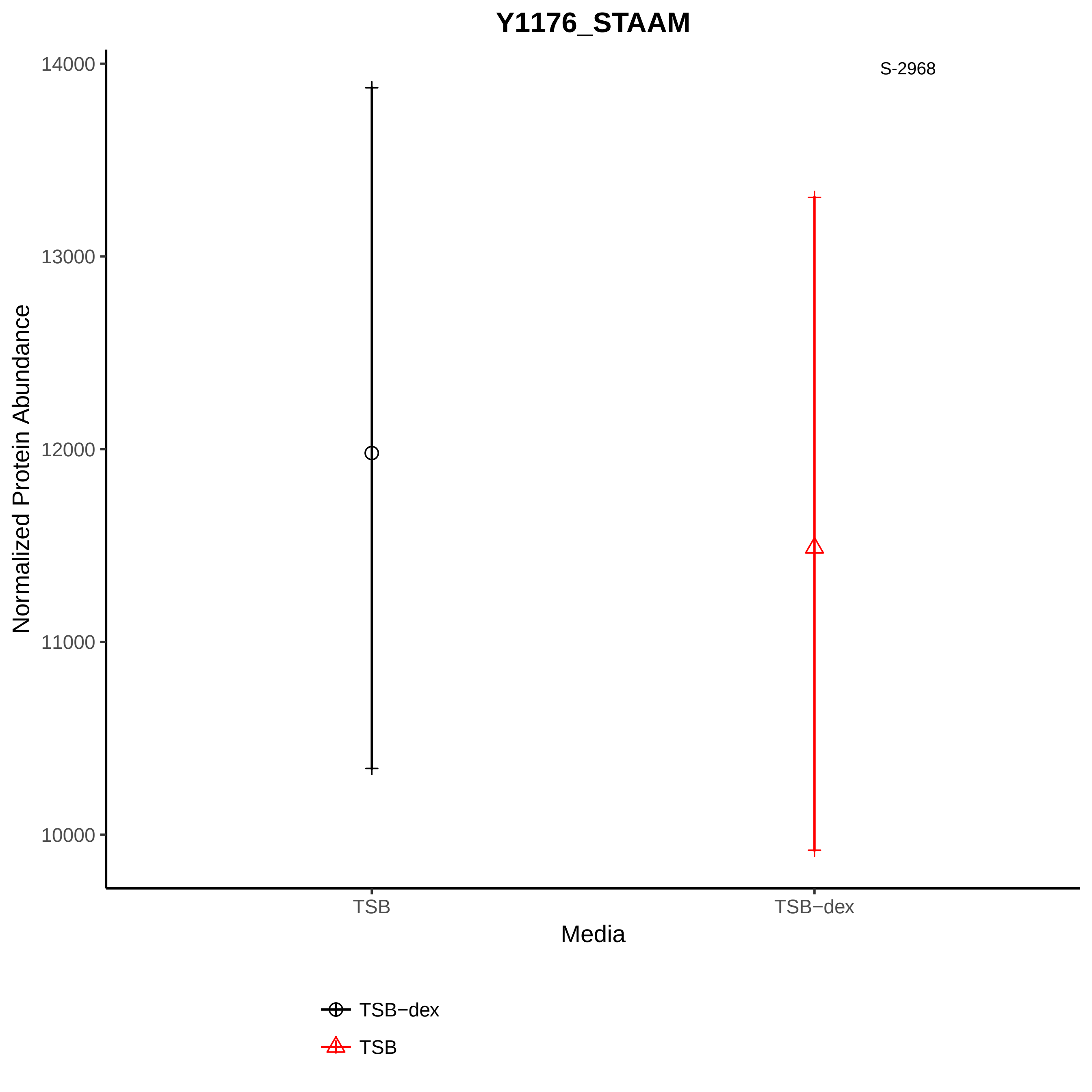

Y1176_STAAM
S-2968
Normalized Protein Abundance
14000
13000
12000
11000
10000
TSB
TSB−dex
Media
TSB−dex
TSB

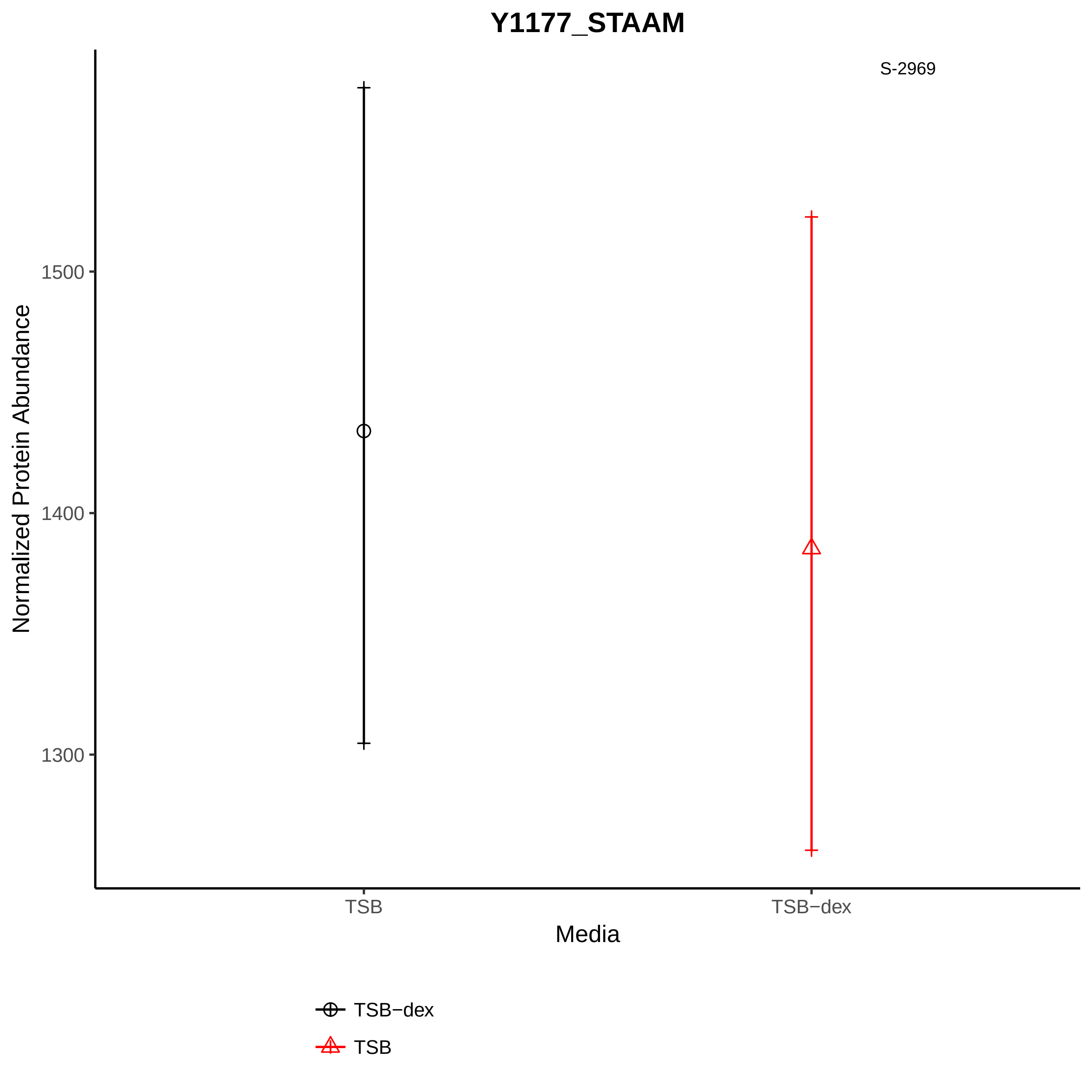

Y1177_STAAM
S-2969
Normalized Protein Abundance
1500
1400
1300
TSB
TSB−dex
Media
TSB−dex
TSB

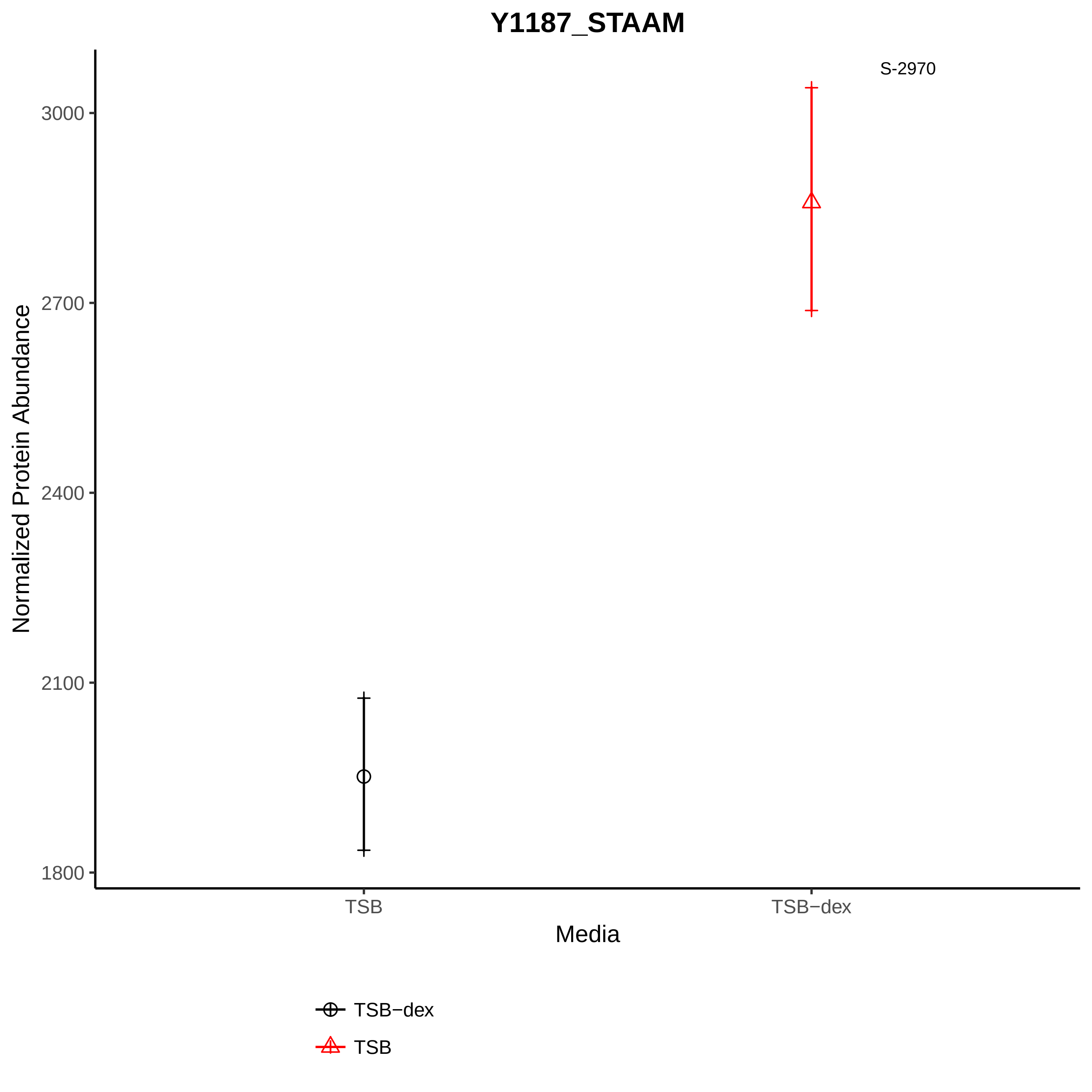

Y1187_STAAM
S-2970
Normalized Protein Abundance
3000
2700
2400
2100
1800
TSB
TSB−dex
Media
TSB−dex
TSB

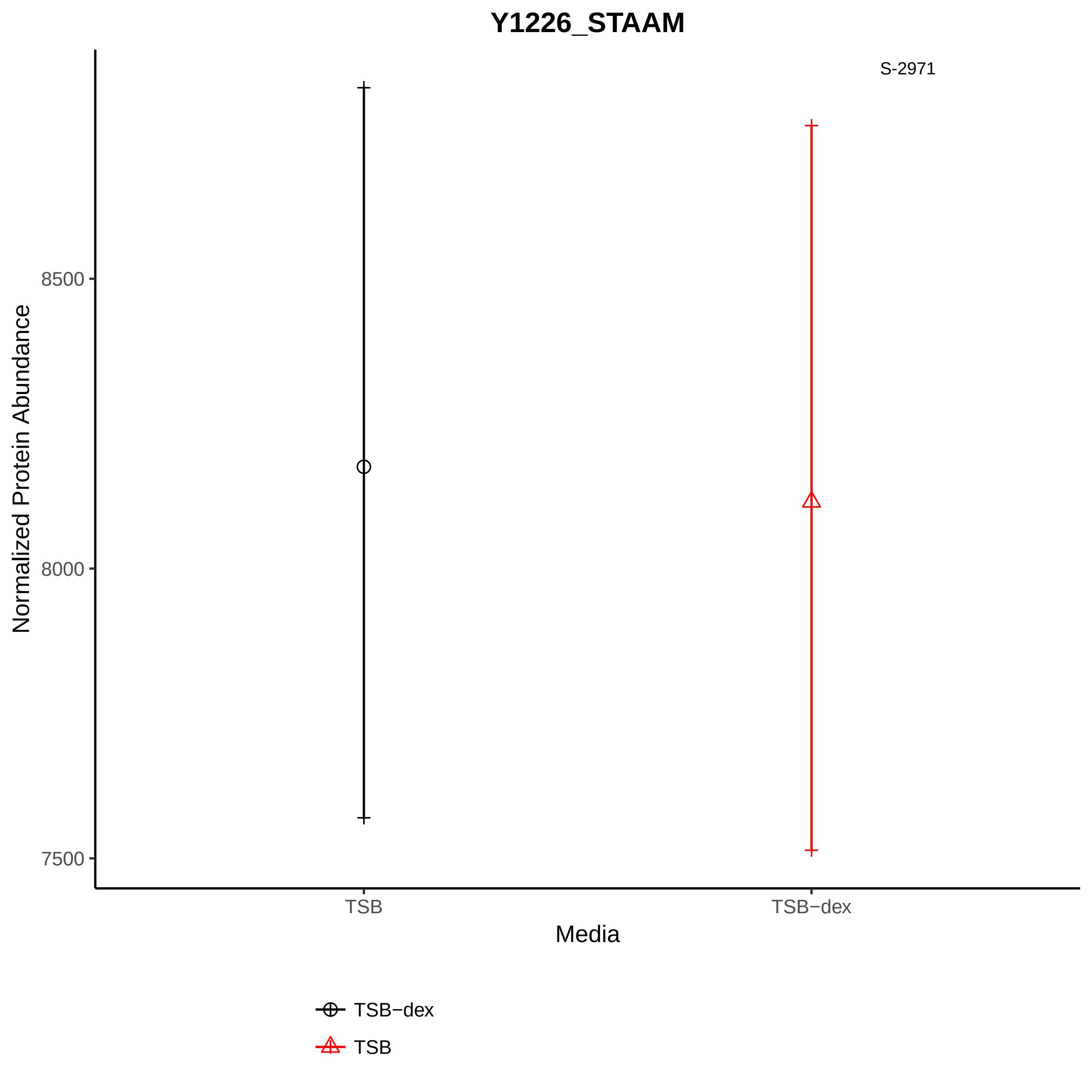

Y1226_STAAM
S-2971
Normalized Protein Abundance
8500
8000
7500
TSB
TSB−dex
Media
TSB−dex
TSB

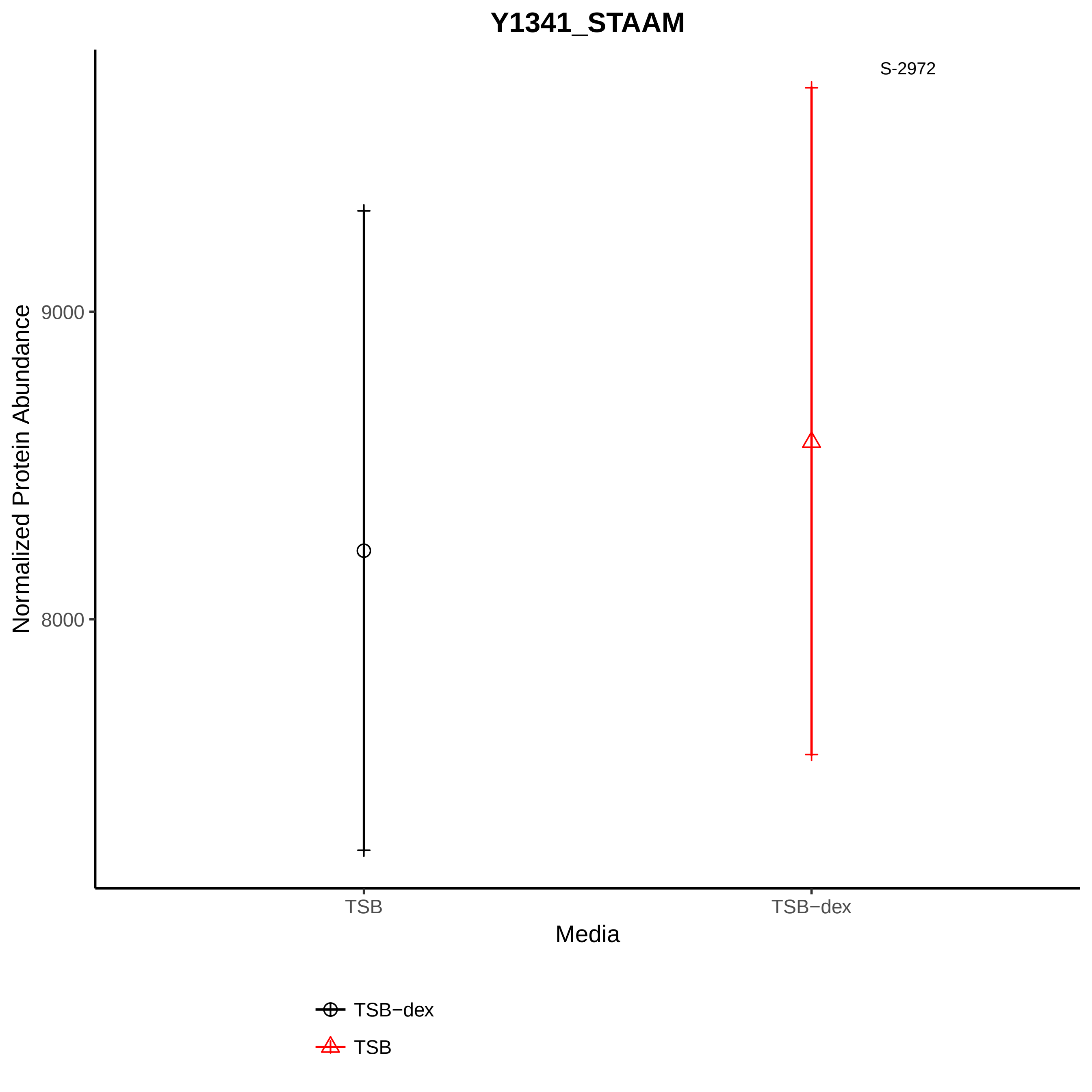

Y1341_STAAM
S-2972
9000
8000
Normalized Protein Abundance
TSB
TSB−dex
Media
TSB−dex
TSB

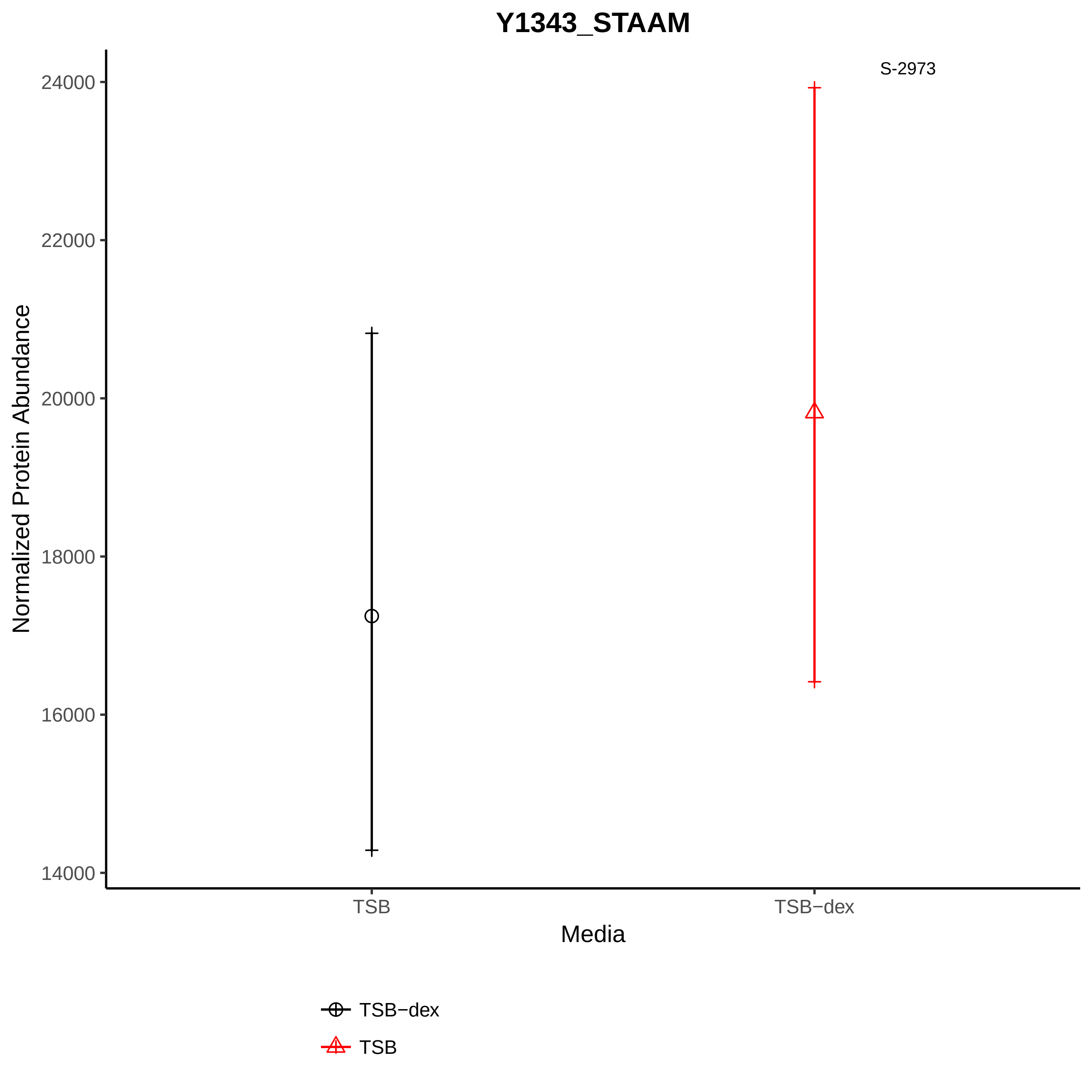

Y1343_STAAM
S-2973
24000
22000
20000
18000
16000
14000
Normalized Protein Abundance
TSB
TSB−dex
Media
TSB−dex
TSB

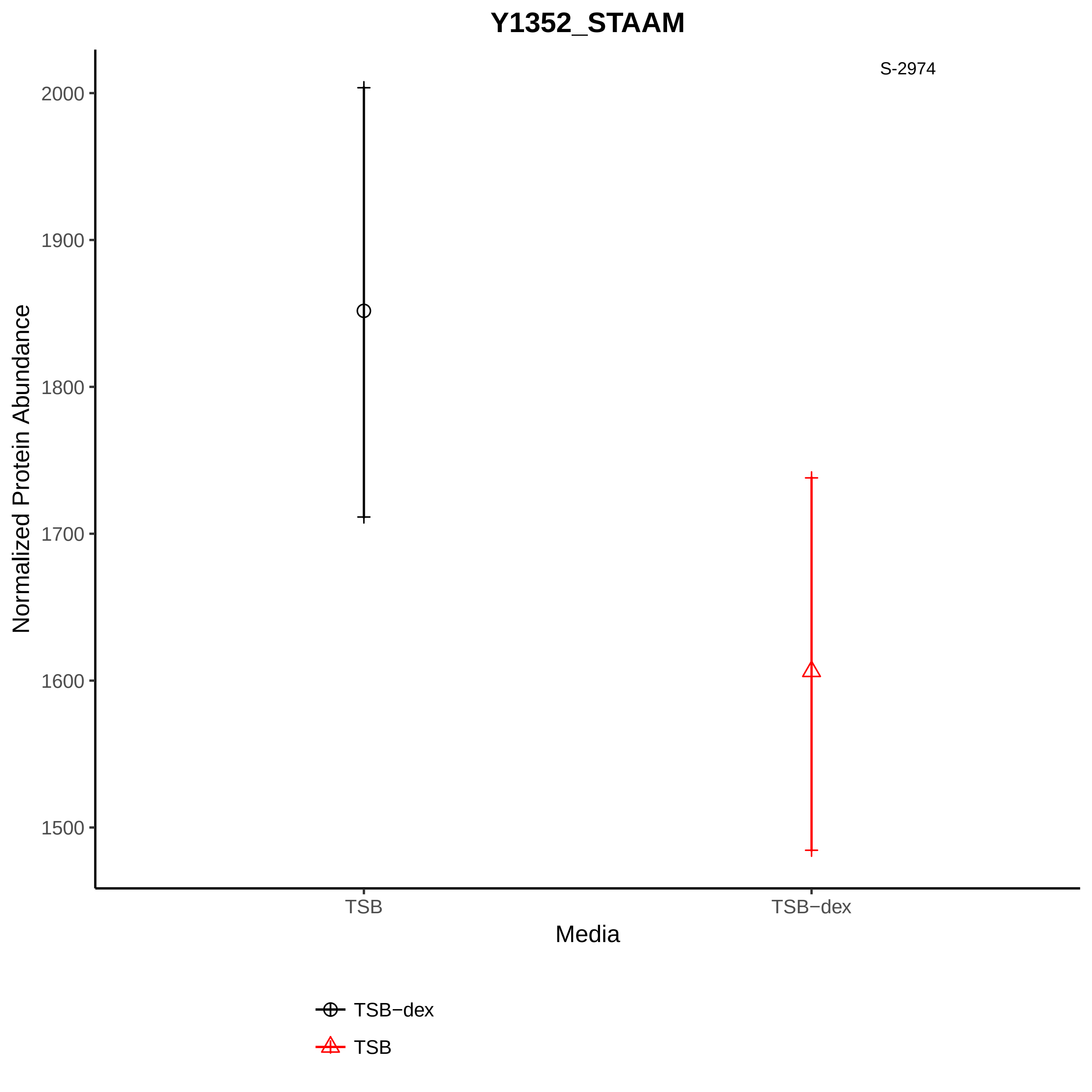

Y1352_STAAM
S-2974
Normalized Protein Abundance
2000
1900
1800
1700
1600
1500
TSB
TSB−dex
Media
TSB−dex
TSB

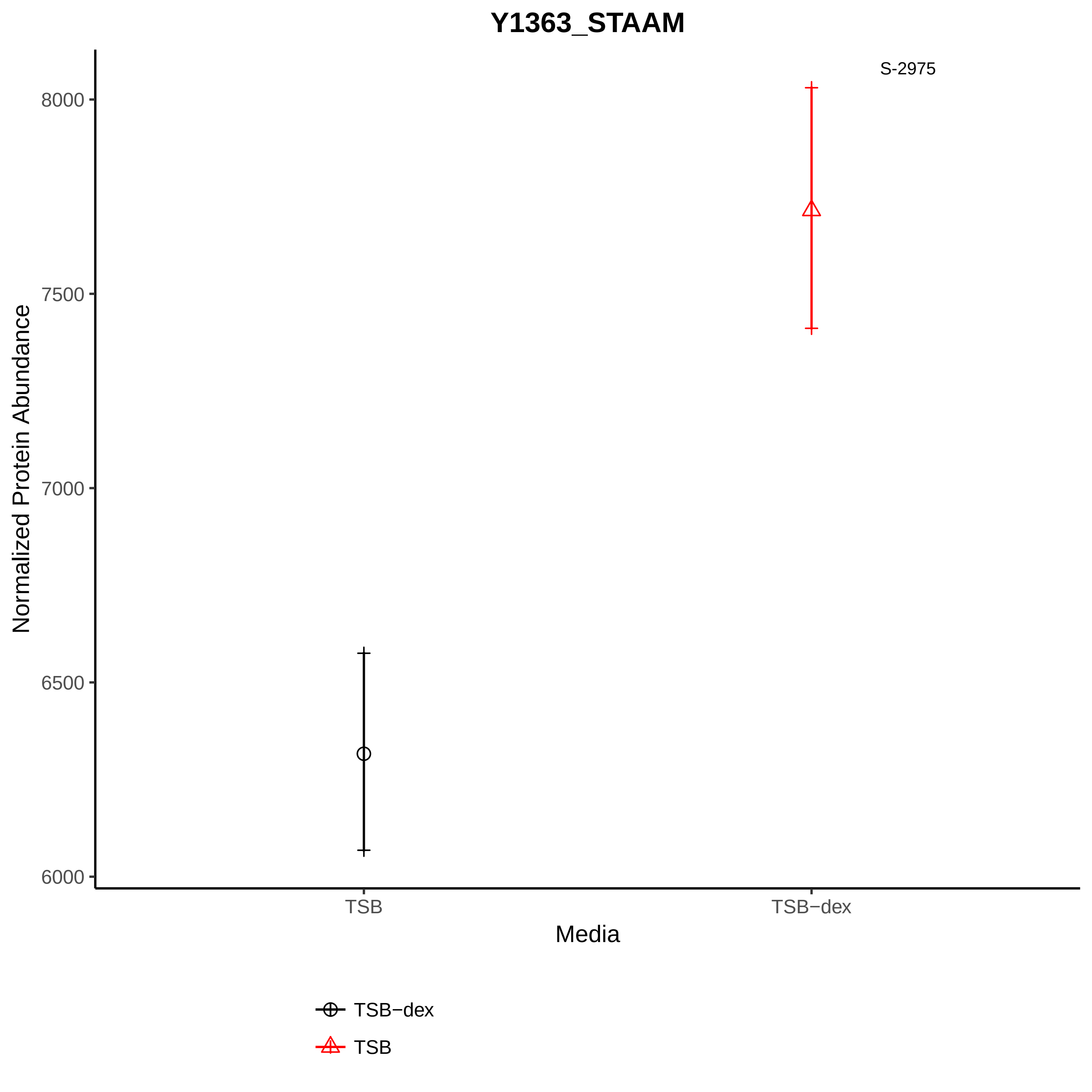

Y1363_STAAM
S-2975
Normalized Protein Abundance
8000
7500
7000
6500
6000
TSB
TSB−dex
Media
TSB−dex
TSB

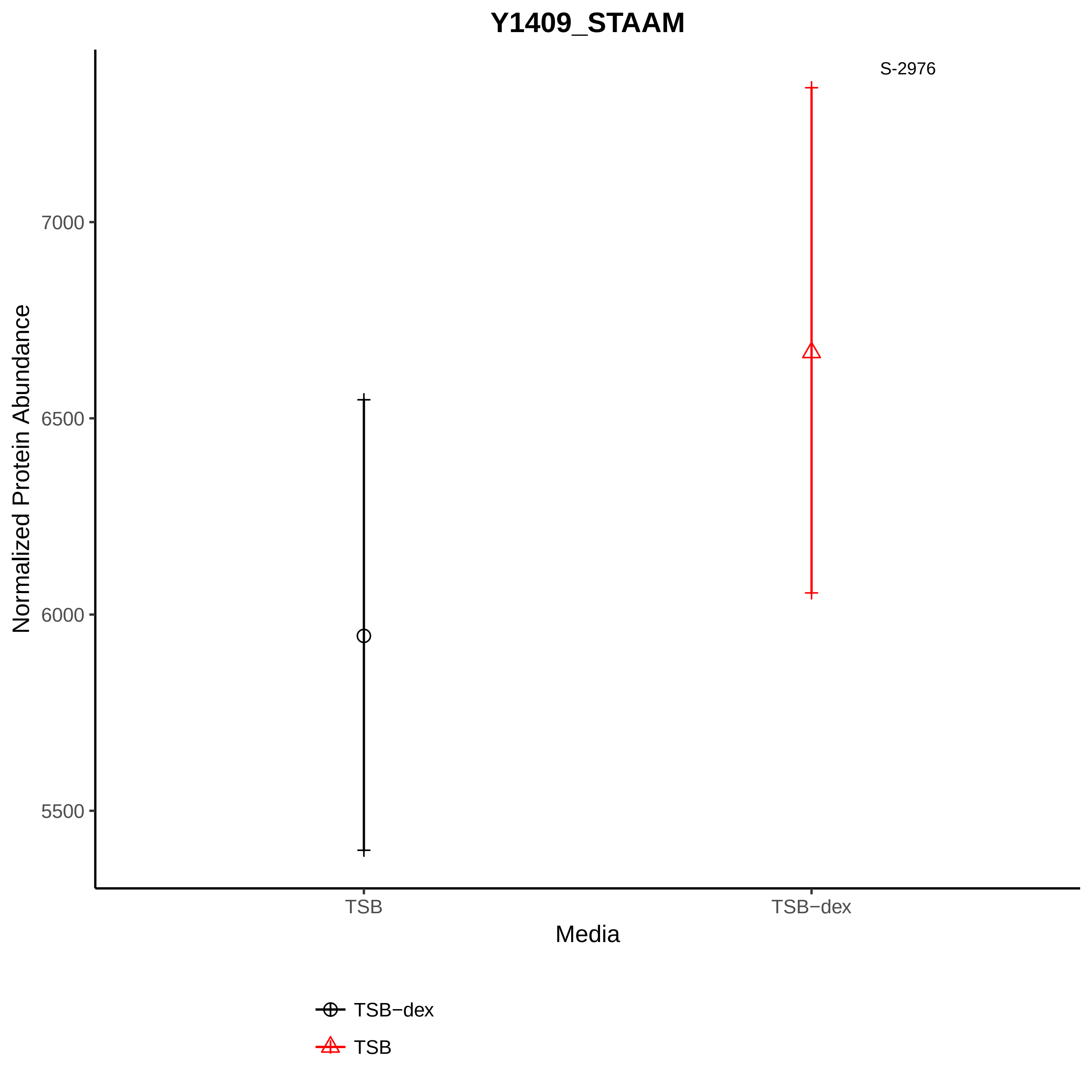

Y1409_STAAM
S-2976
Normalized Protein Abundance
7000
6500
6000
5500
TSB
TSB−dex
Media
TSB−dex
TSB

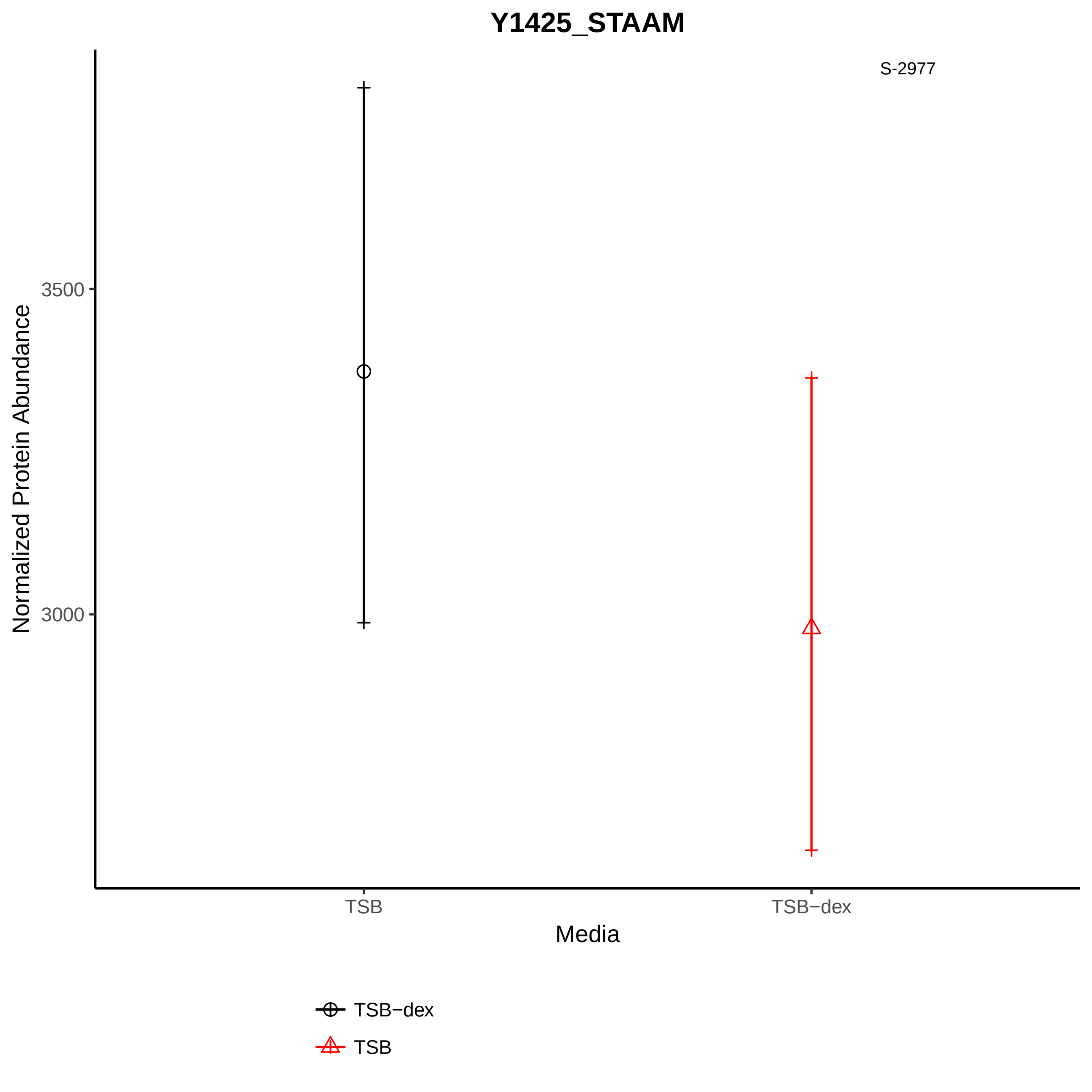
Y1425_STAAM
S-2977
Normalized Protein Abundance
3500
3000
TSB
TSB−dex
Media
TSB−dex
TSB

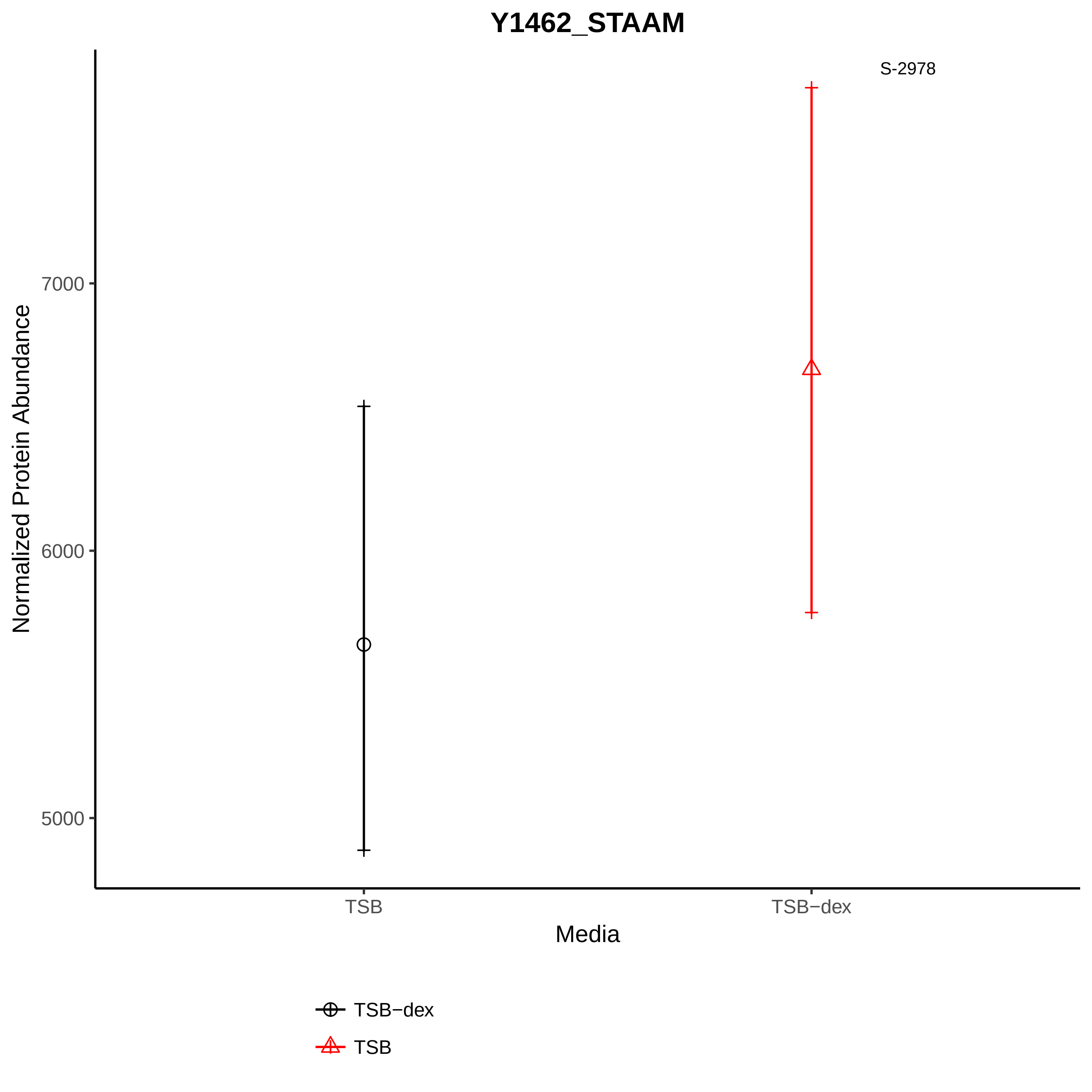
Y1462_STAAM
S-2978
Normalized Protein Abundance
7000
6000
5000
TSB
TSB−dex
Media
TSB−dex
TSB

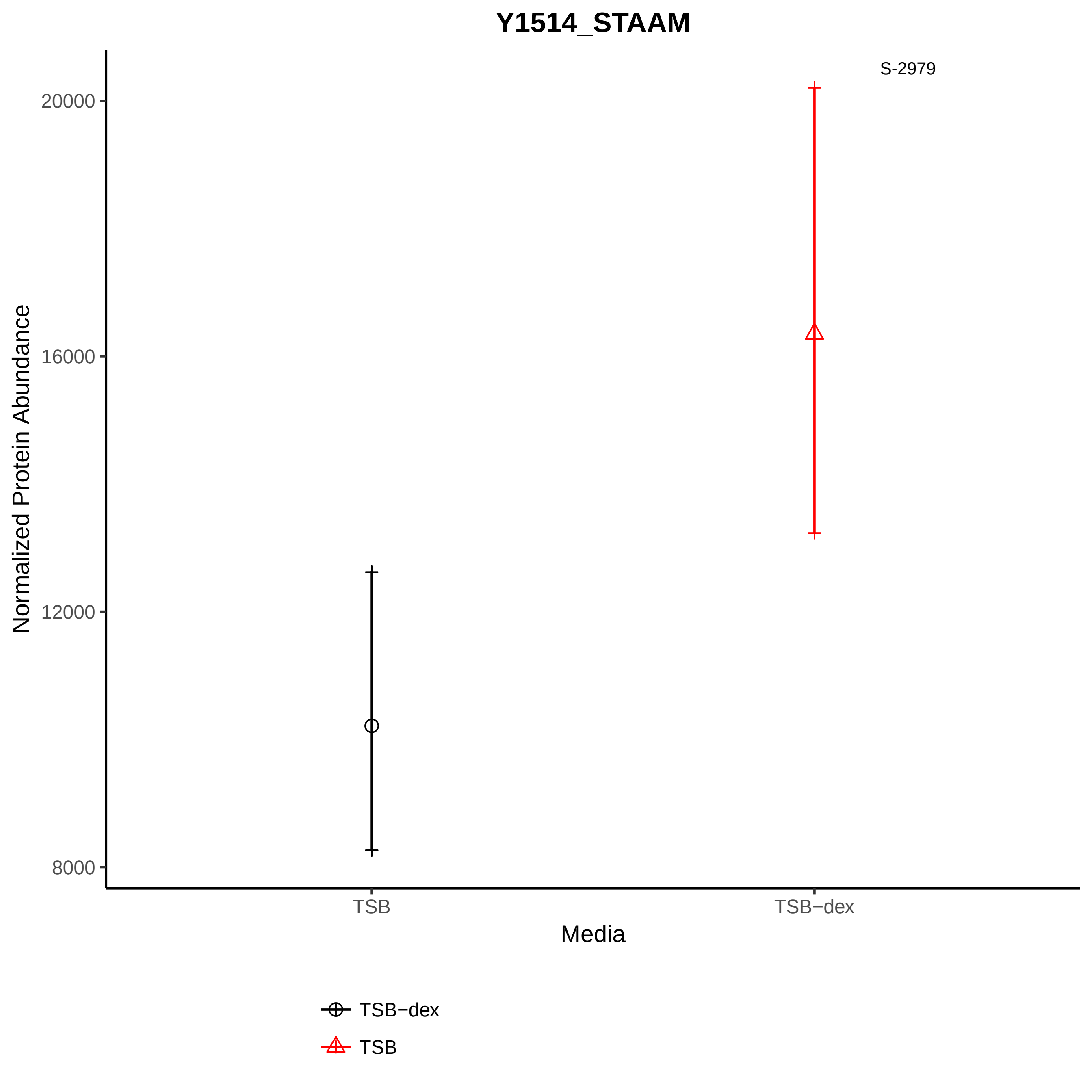

Y1514_STAAM
S-2979
Normalized Protein Abundance
20000
16000
12000
8000
TSB
TSB−dex
Media
TSB−dex
TSB

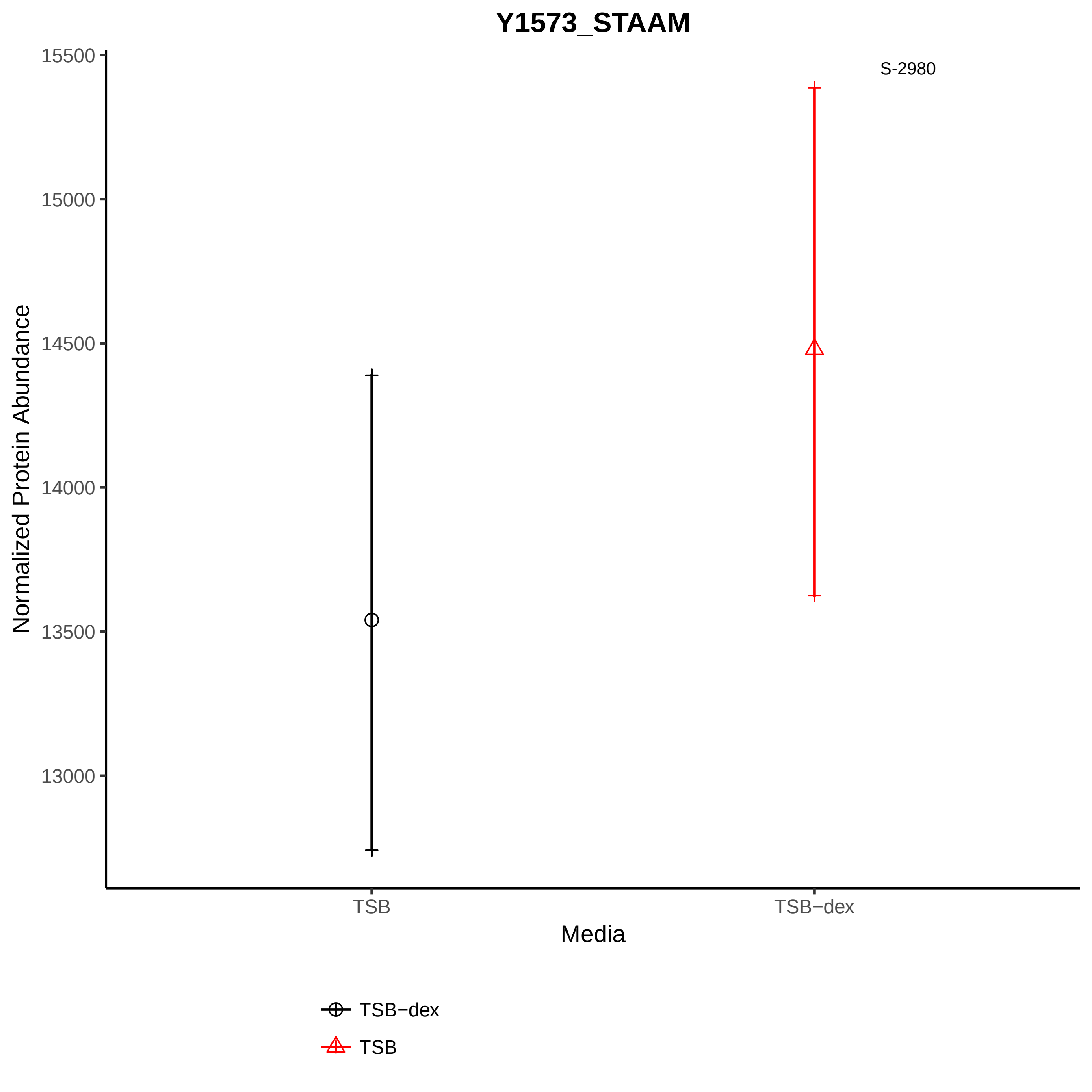

Y1573_STAAM
S-2980
Normalized Protein Abundance
15500
15000
14500
14000
13500
13000
TSB
TSB−dex
Media
TSB−dex
TSB

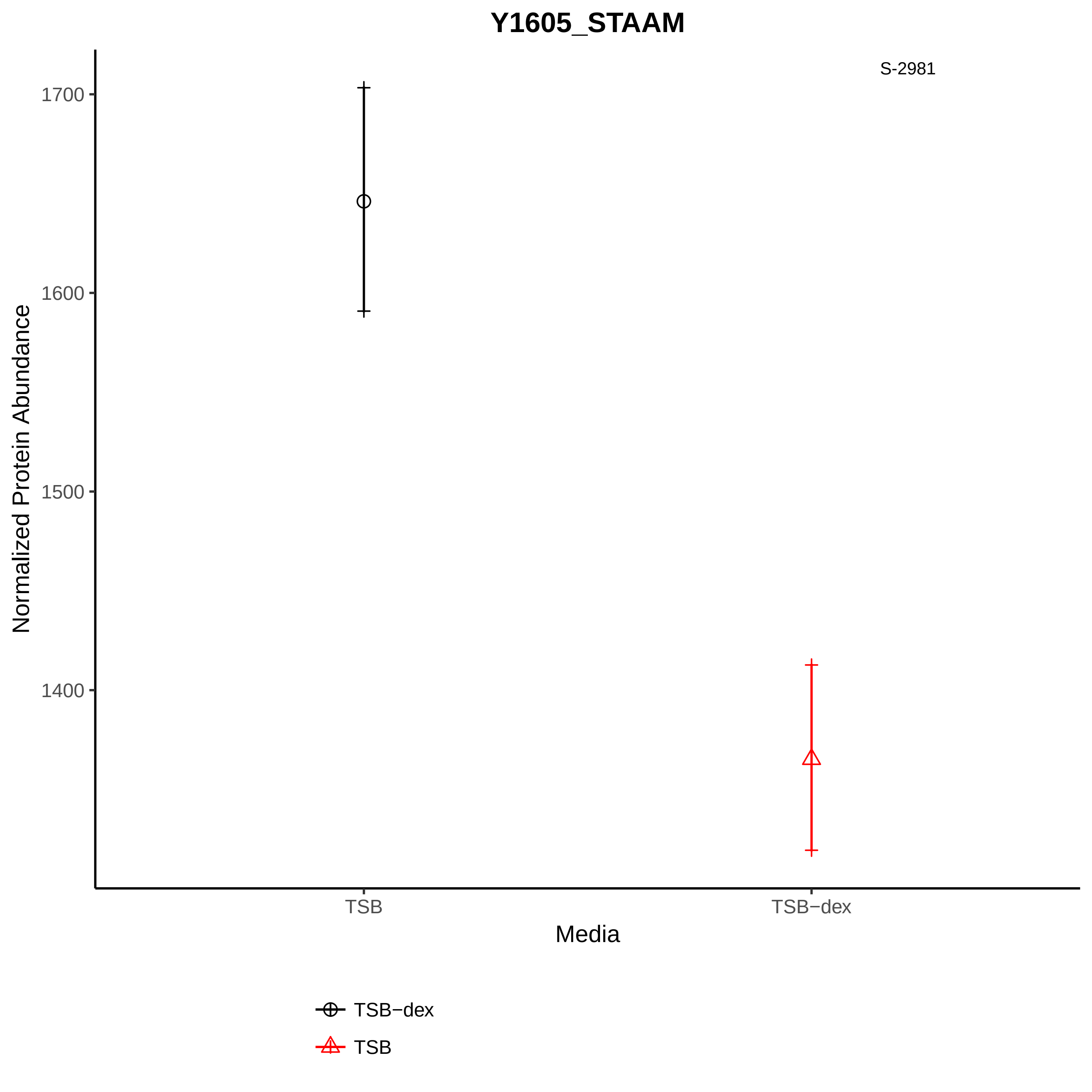
Y1605_STAAM
S-2981
1700
1600
1500
1400
Normalized Protein Abundance
TSB
TSB−dex
Media
TSB−dex
TSB

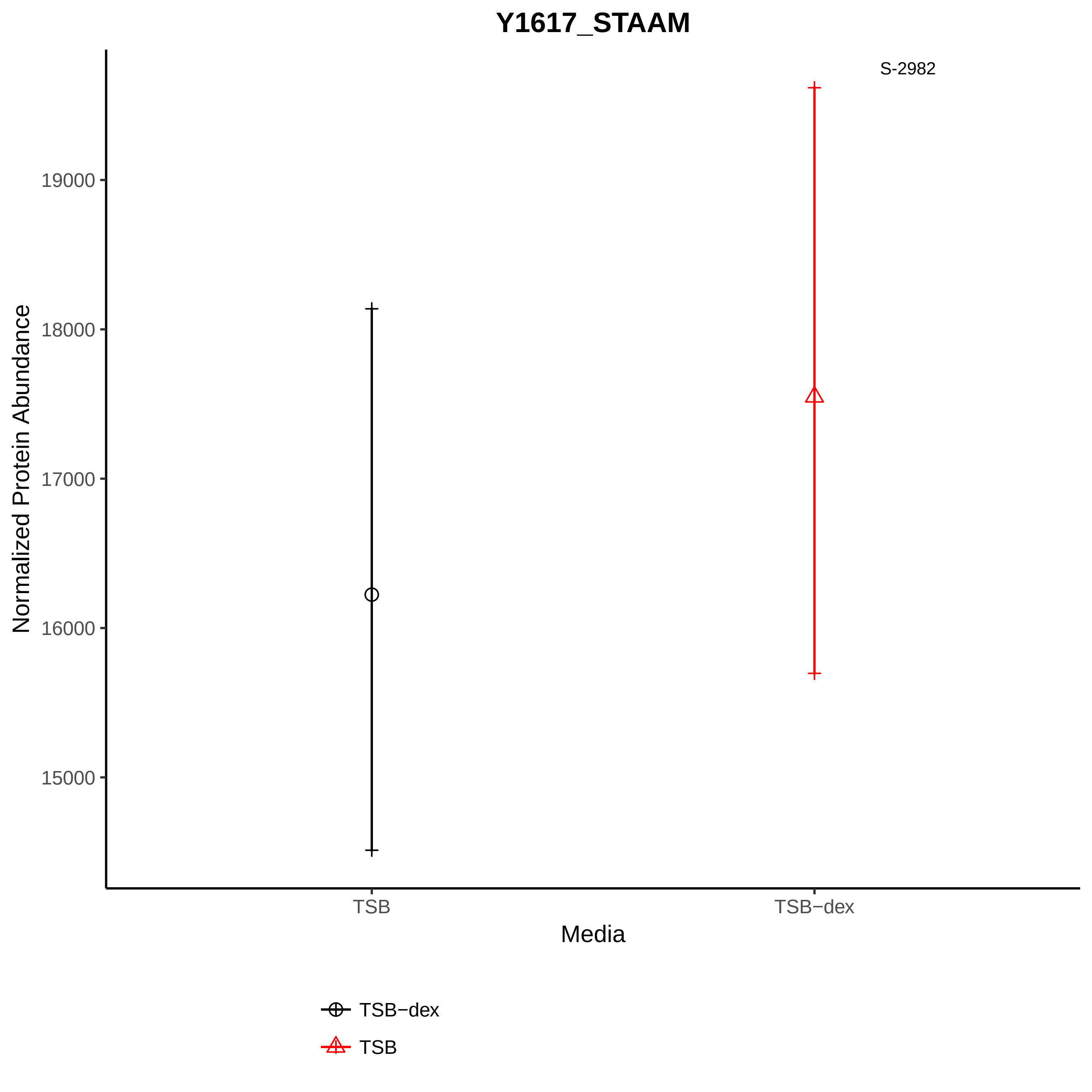

Y1617_STAAM
S-2982
19000
18000
17000
16000
15000
Normalized Protein Abundance
TSB
TSB−dex
Media
TSB−dex
TSB

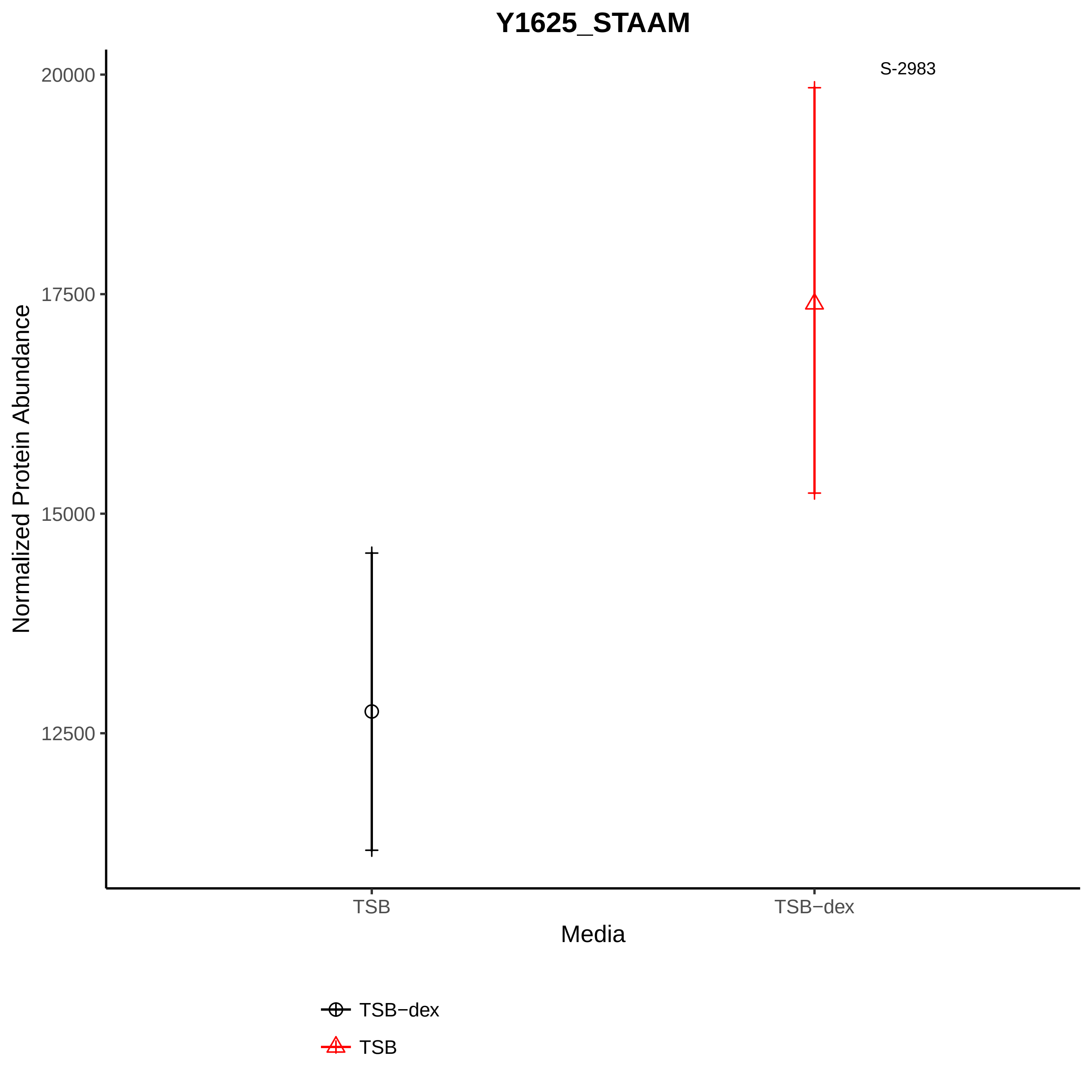

Y1625_STAAM
S-2983
20000
17500
15000
12500
Normalized Protein Abundance
TSB
TSB−dex
Media
TSB−dex
TSB

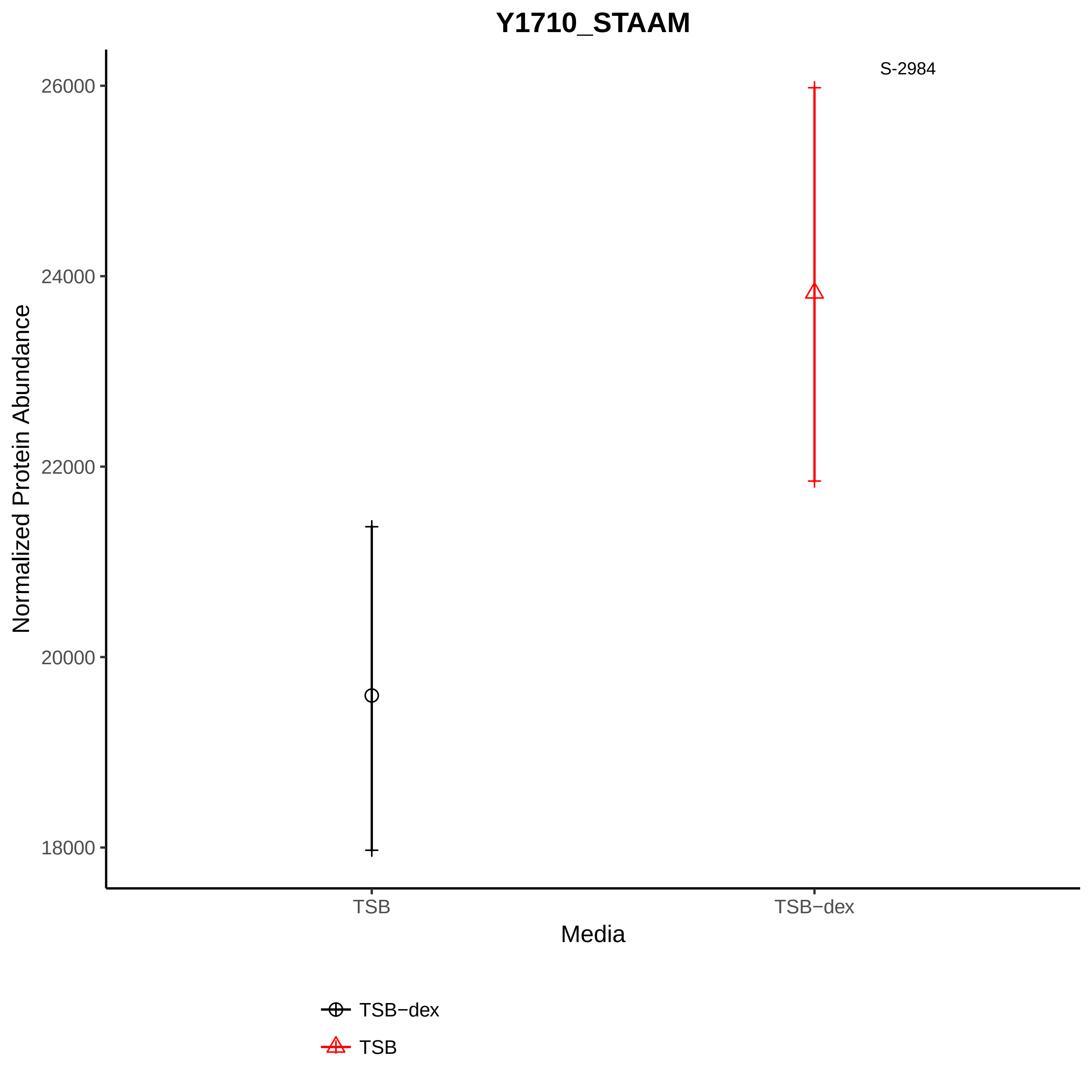

Y1710_STAAM
S-2984
26000
24000
22000
20000
18000
Normalized Protein Abundance
TSB
TSB−dex
Media
TSB−dex
TSB

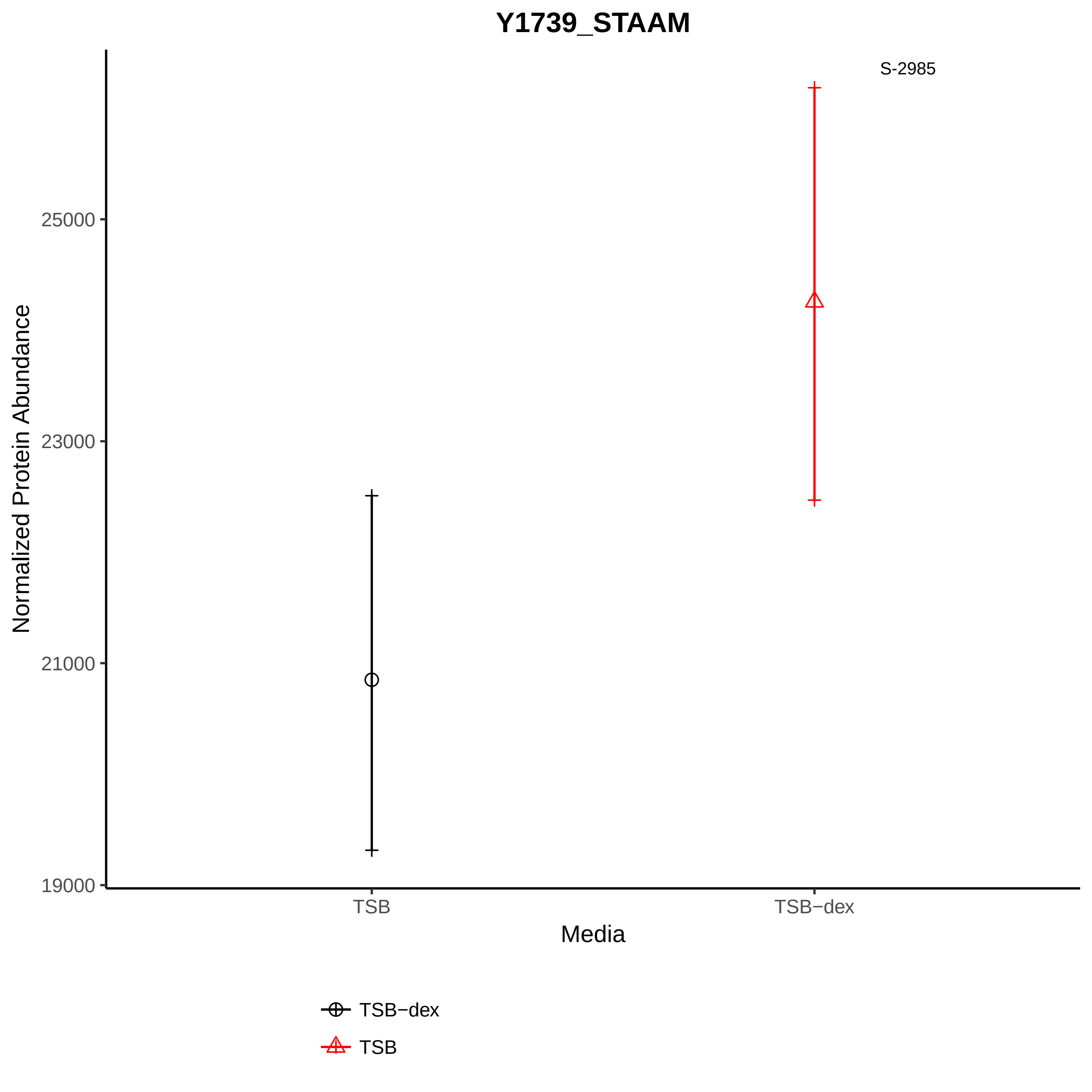

Y1739_STAAM
S-2985
Normalized Protein Abundance
25000
23000
21000
19000
TSB
TSB−dex
Media
TSB−dex
TSB

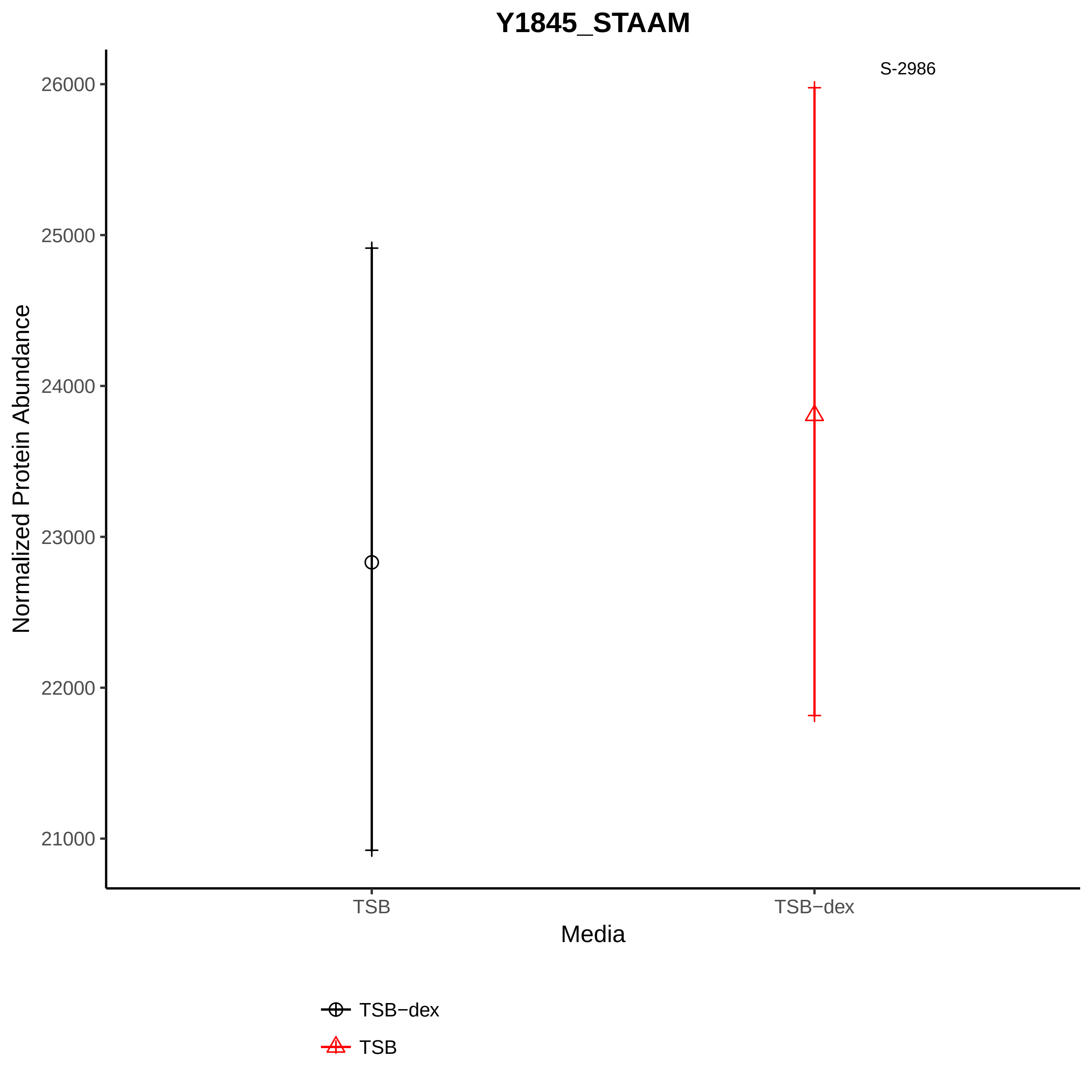

Y1845_STAAM
S-2986
Normalized Protein Abundance
26000
25000
24000
23000
22000
21000
TSB
TSB−dex
Media
TSB−dex
TSB

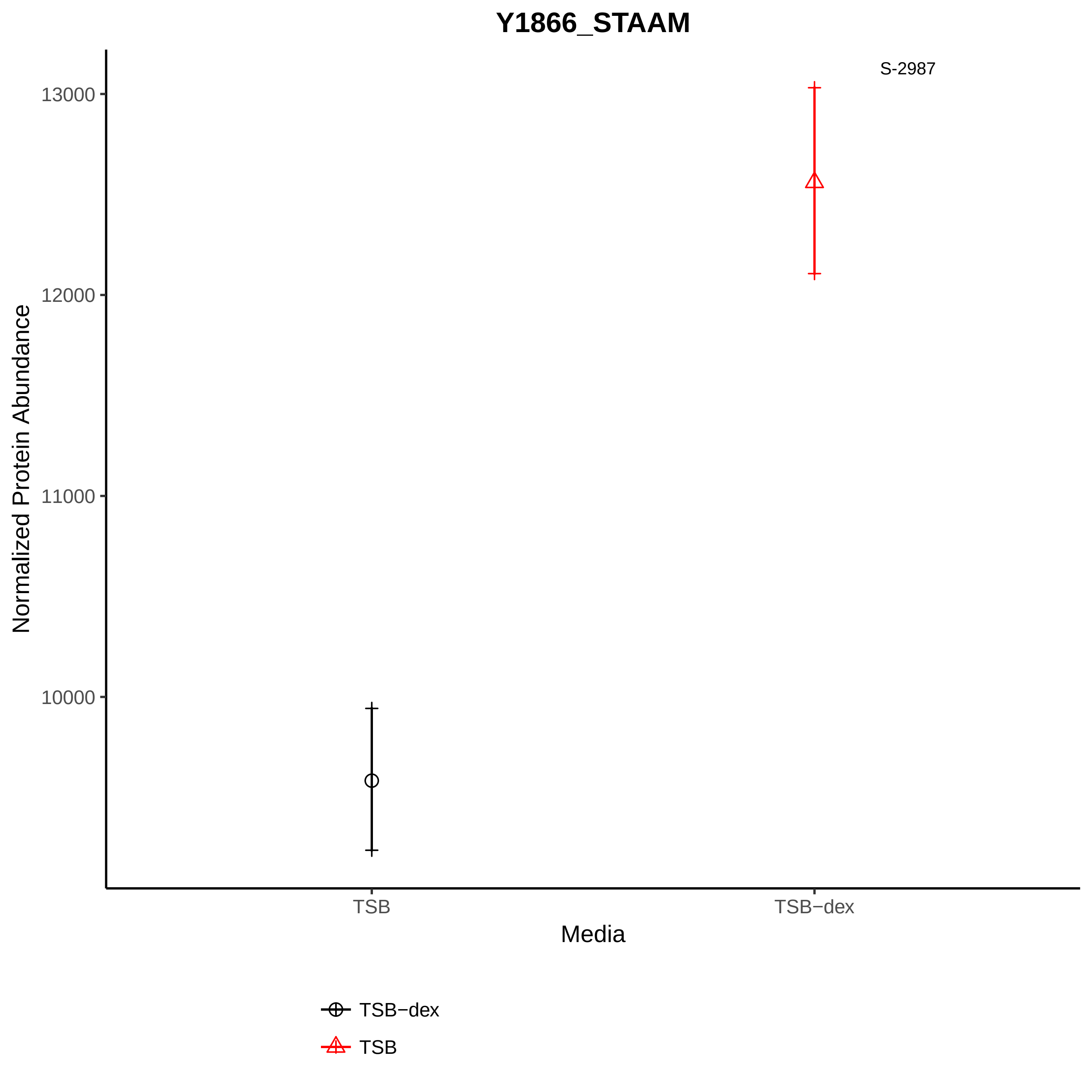

Y1866_STAAM
S-2987
Normalized Protein Abundance
13000
12000
11000
10000
TSB
TSB−dex
Media
TSB−dex
TSB

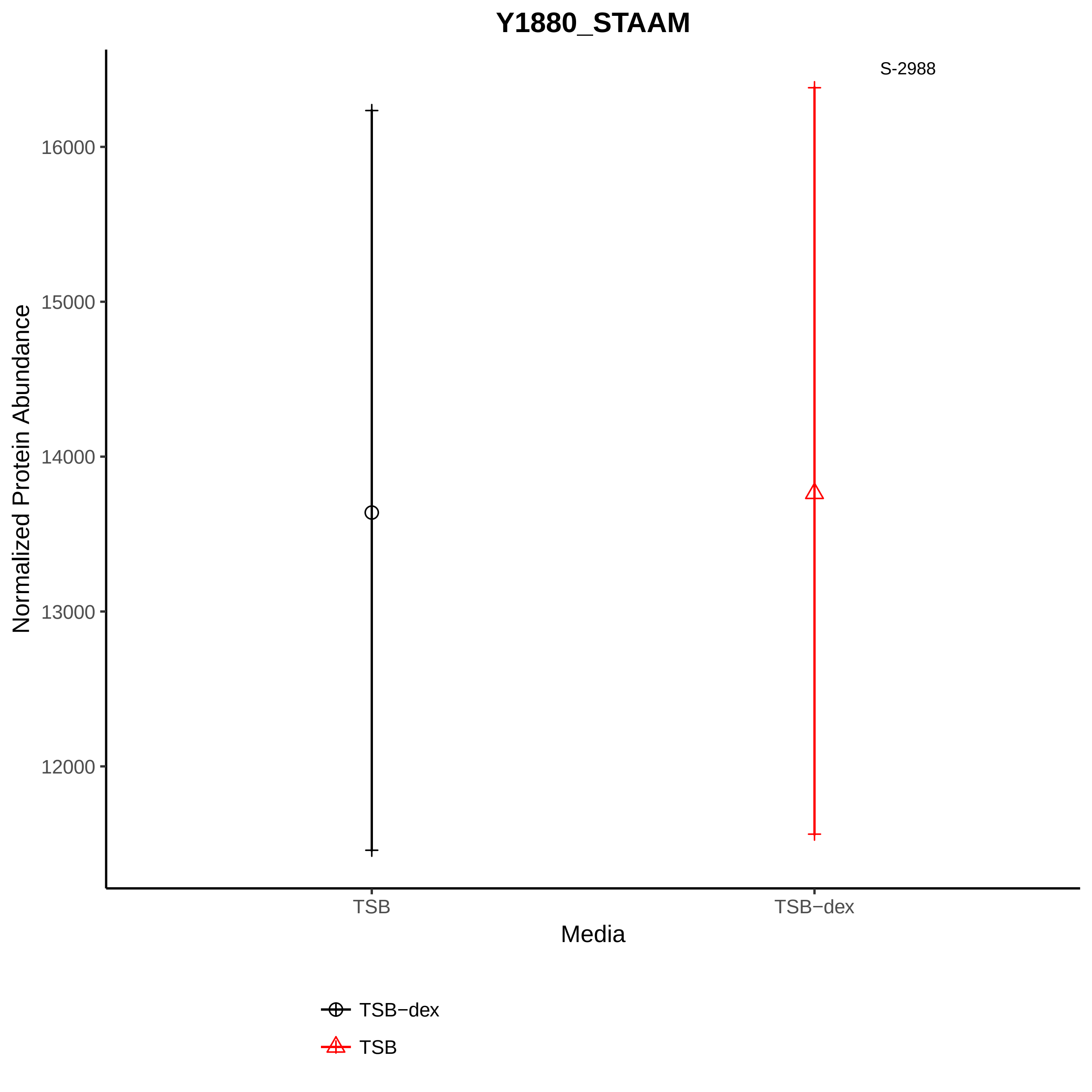

Y1880_STAAM
S-2988
Normalized Protein Abundance
16000
15000
14000
13000
12000
TSB
TSB−dex
Media
TSB−dex
TSB

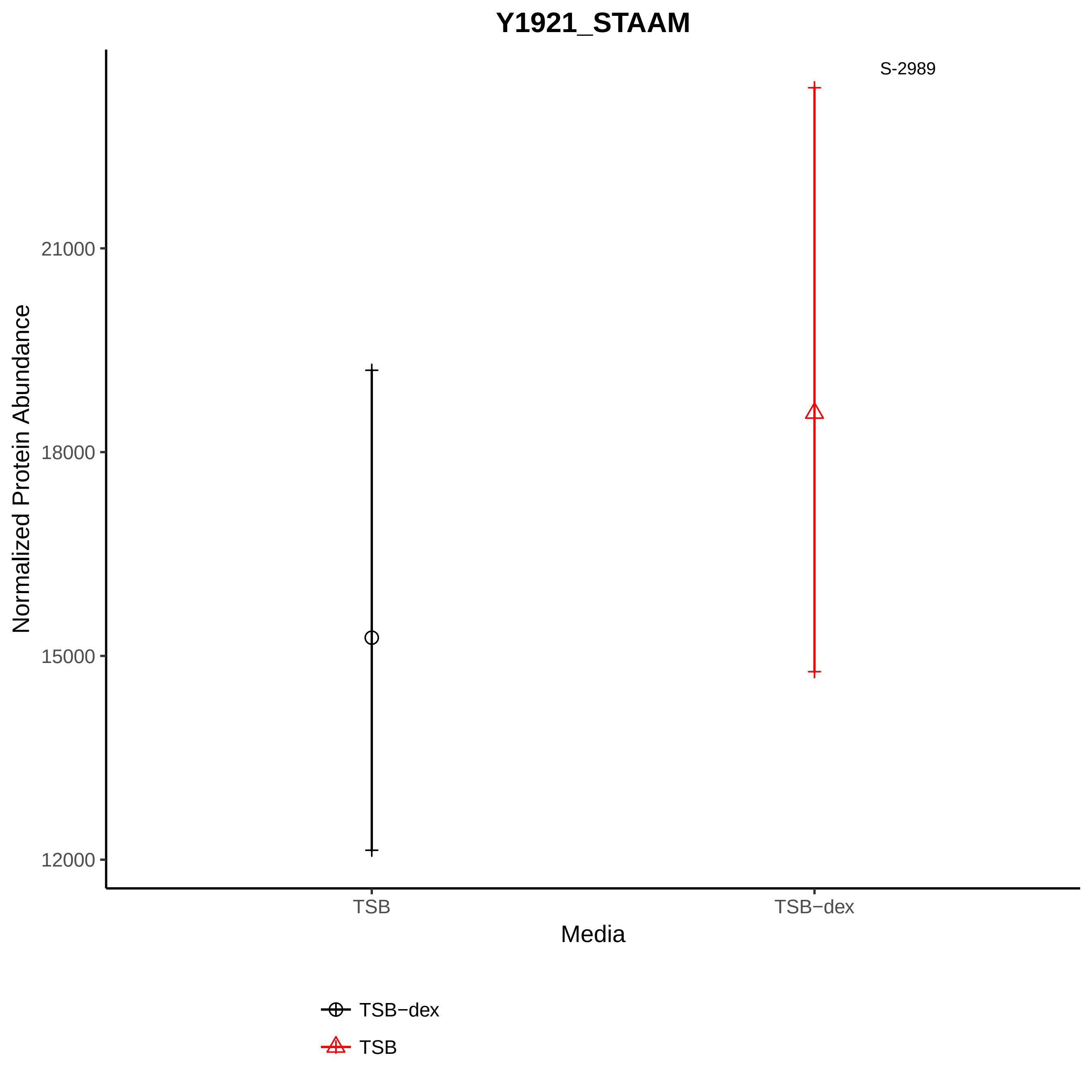

Y1921_STAAM
S-2989
Normalized Protein Abundance
21000
18000
15000
12000
TSB
TSB−dex
Media
TSB−dex
TSB

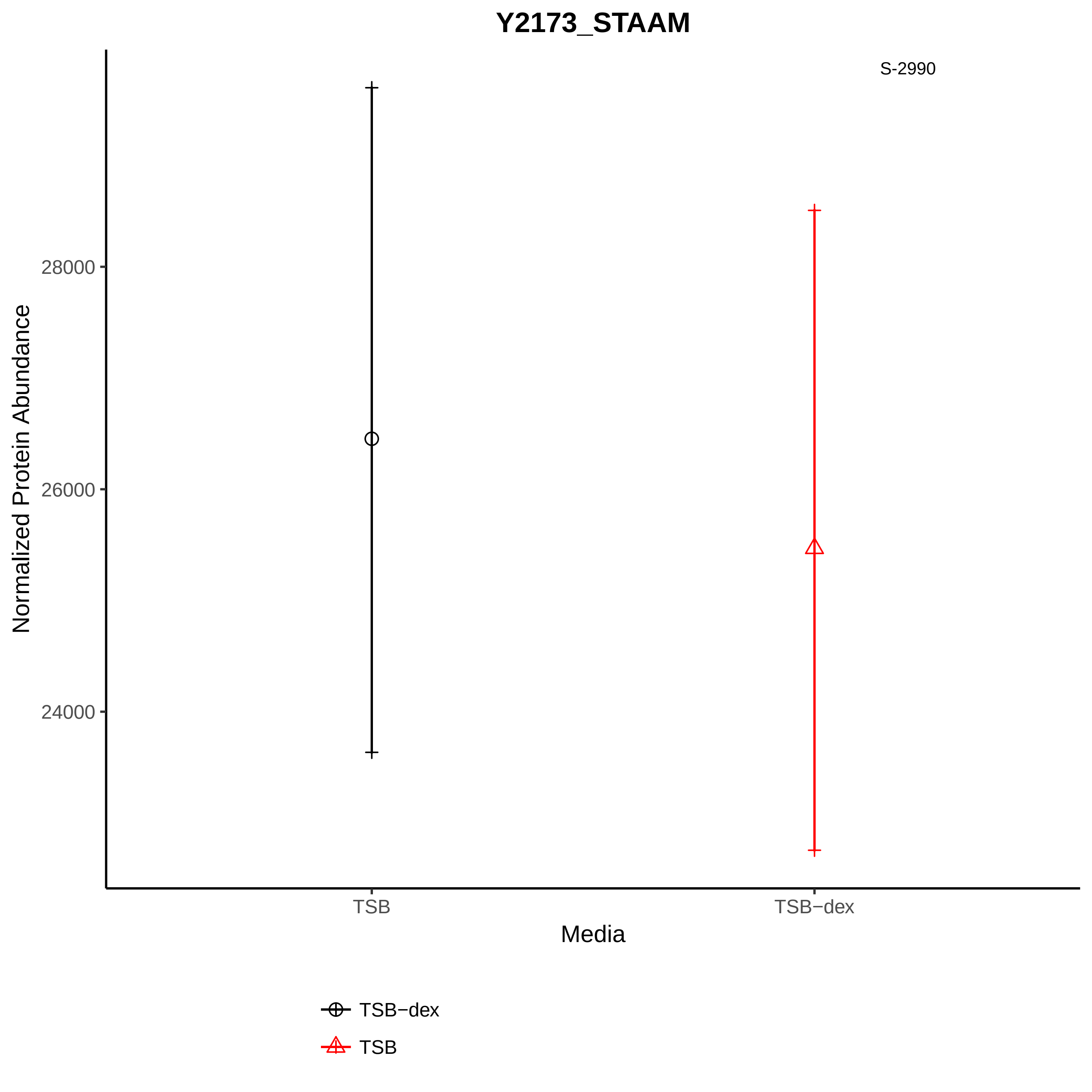
Y2173_STAAM
S-2990
28000
26000
24000
Normalized Protein Abundance
TSB
TSB−dex
Media
TSB−dex
TSB

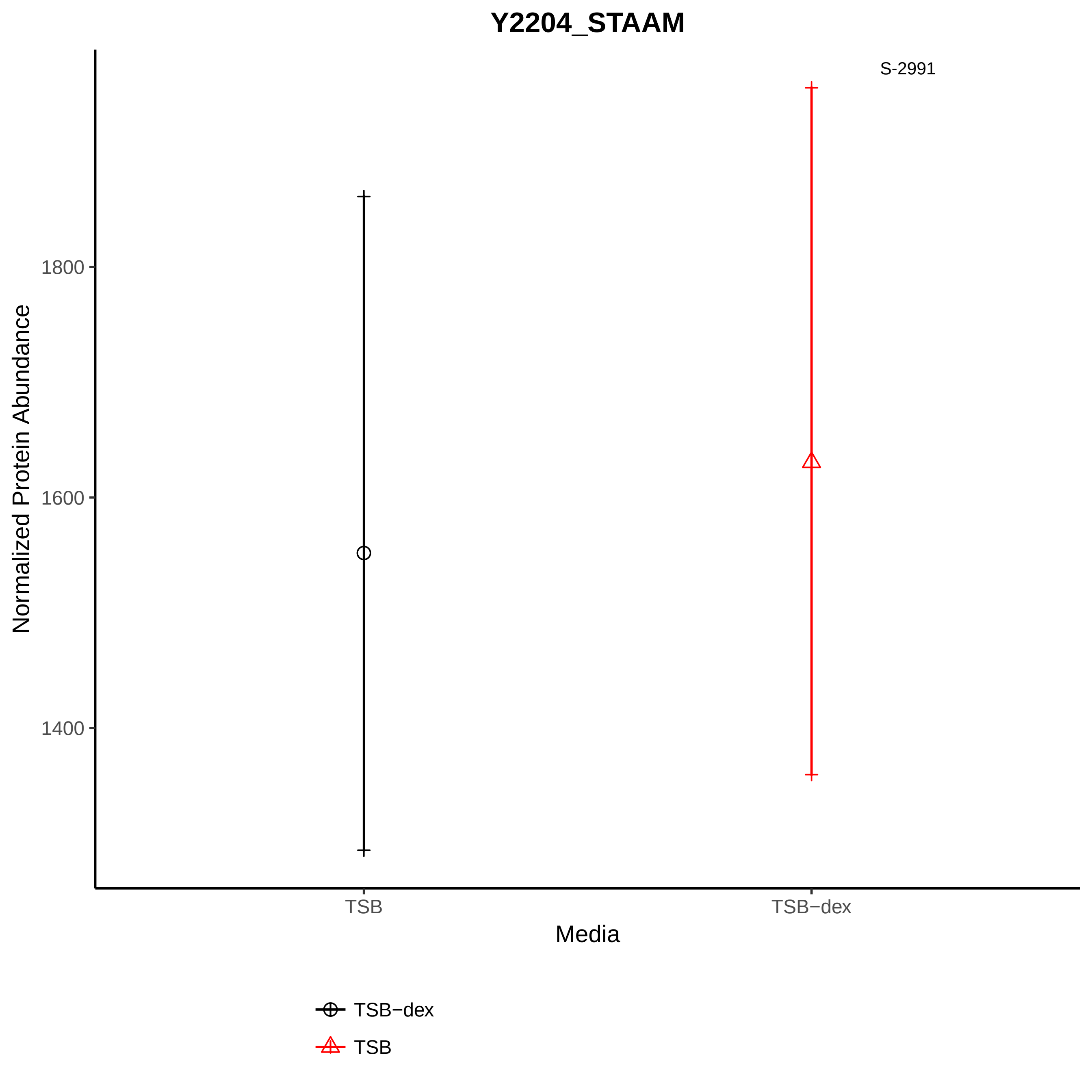

Y2204_STAAM
S-2991
Normalized Protein Abundance
1800
1600
1400
TSB
TSB−dex
Media
TSB−dex
TSB

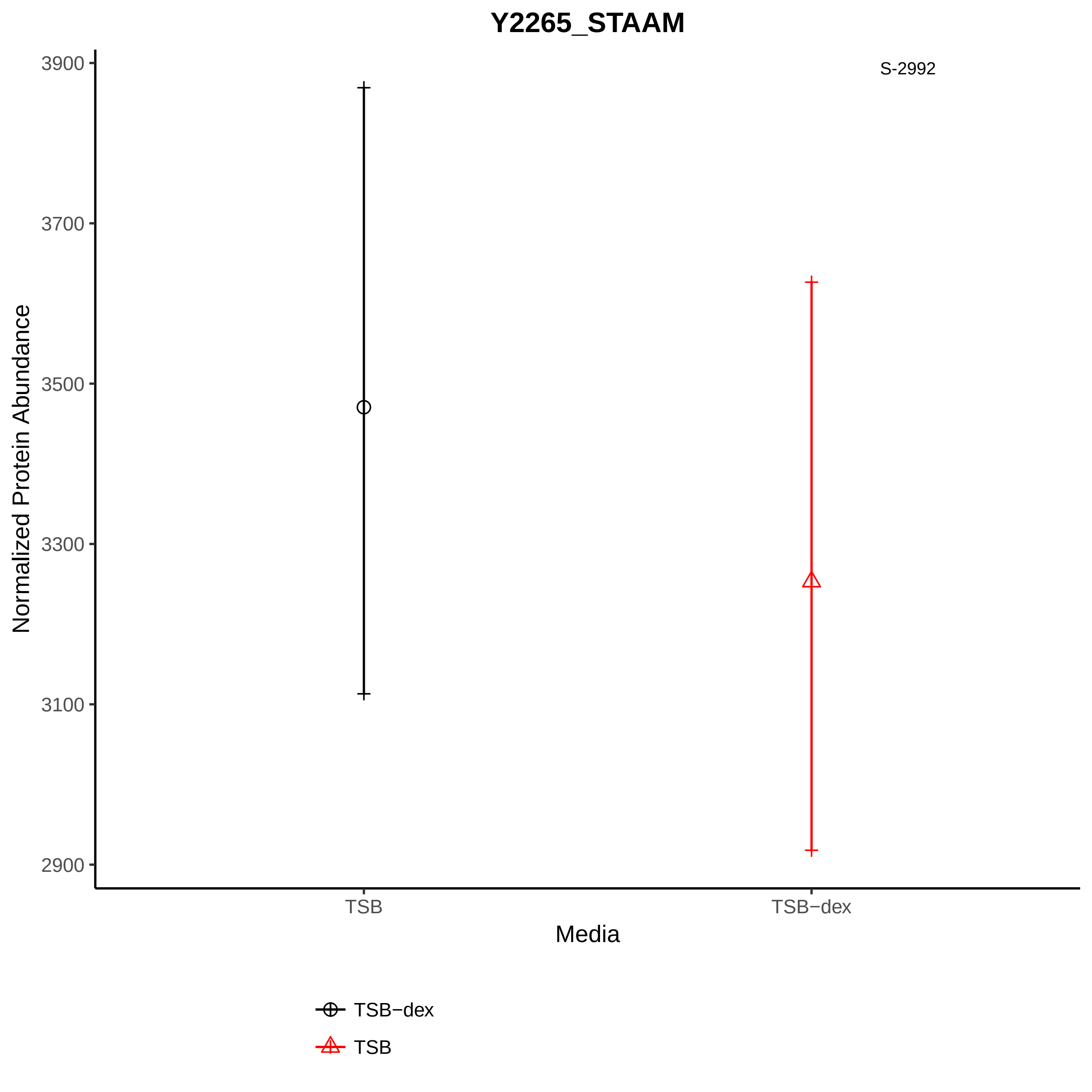
Y2265_STAAM
S-2992
3900
3700
3500
3300
3100
2900
Normalized Protein Abundance
TSB
TSB−dex
Media
TSB−dex
TSB

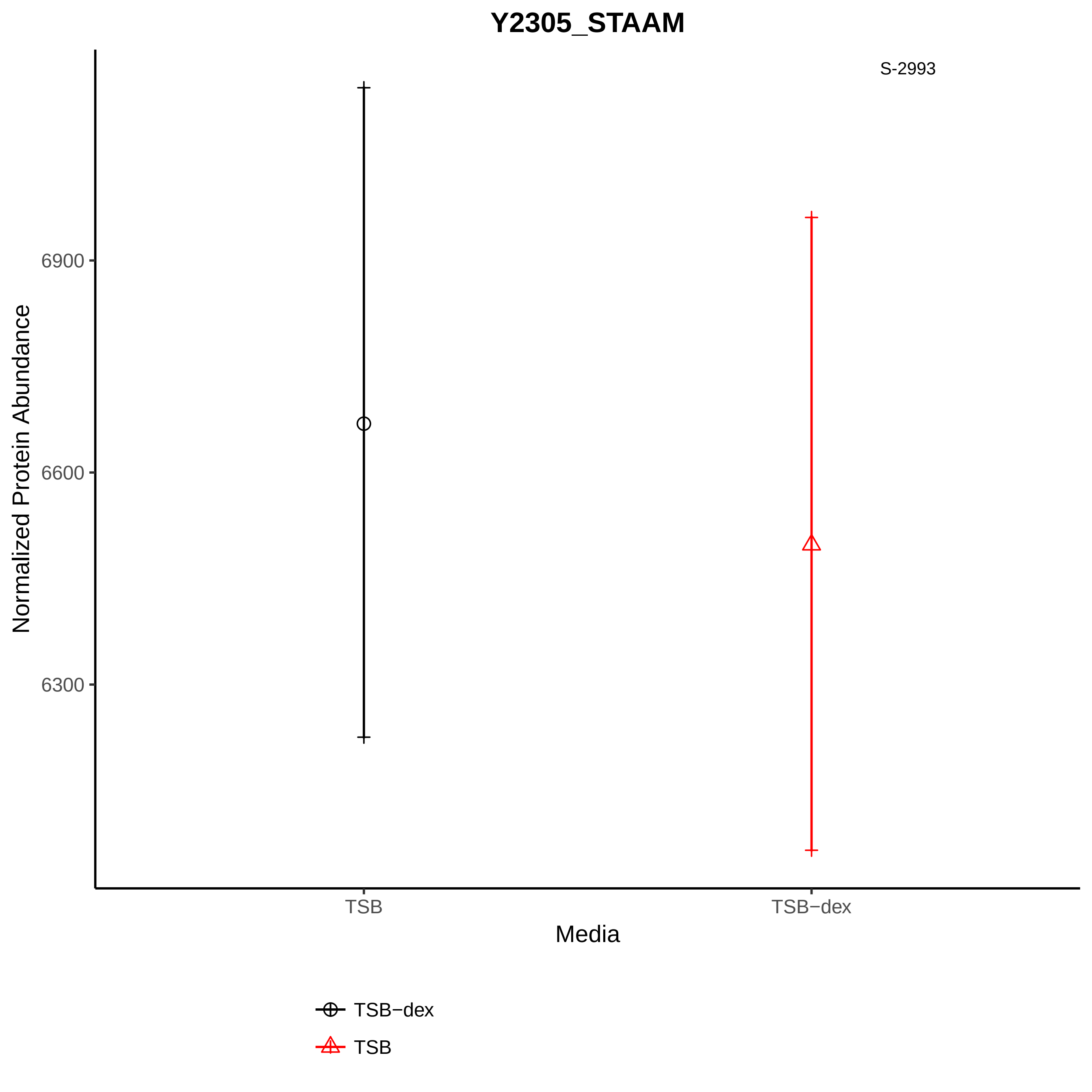

Y2305_STAAM
S-2993
6900
6600
6300
Normalized Protein Abundance
TSB
TSB−dex
Media
TSB−dex
TSB

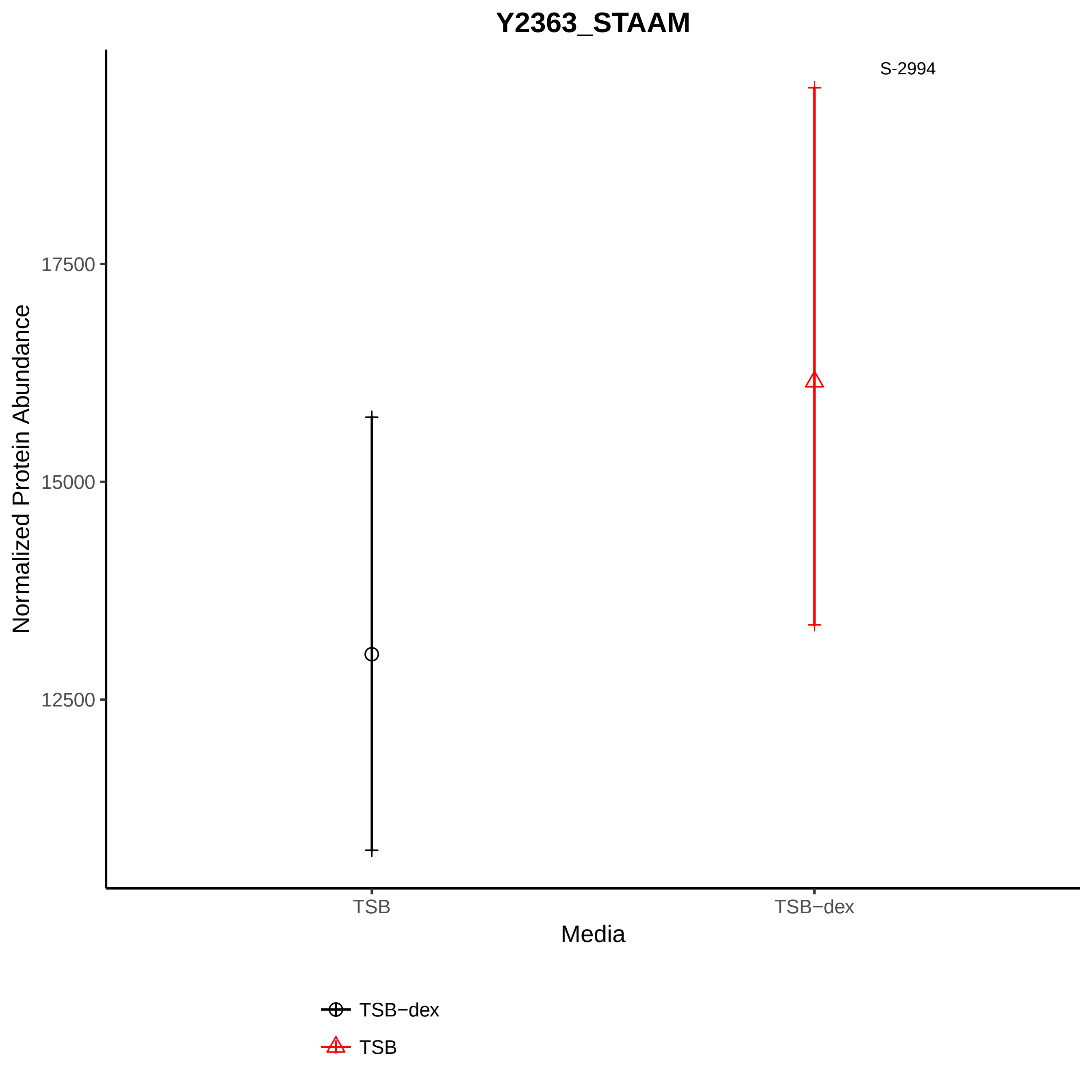

Y2363_STAAM
S-2994
17500
15000
12500
Normalized Protein Abundance
TSB
TSB−dex
Media
TSB−dex
TSB

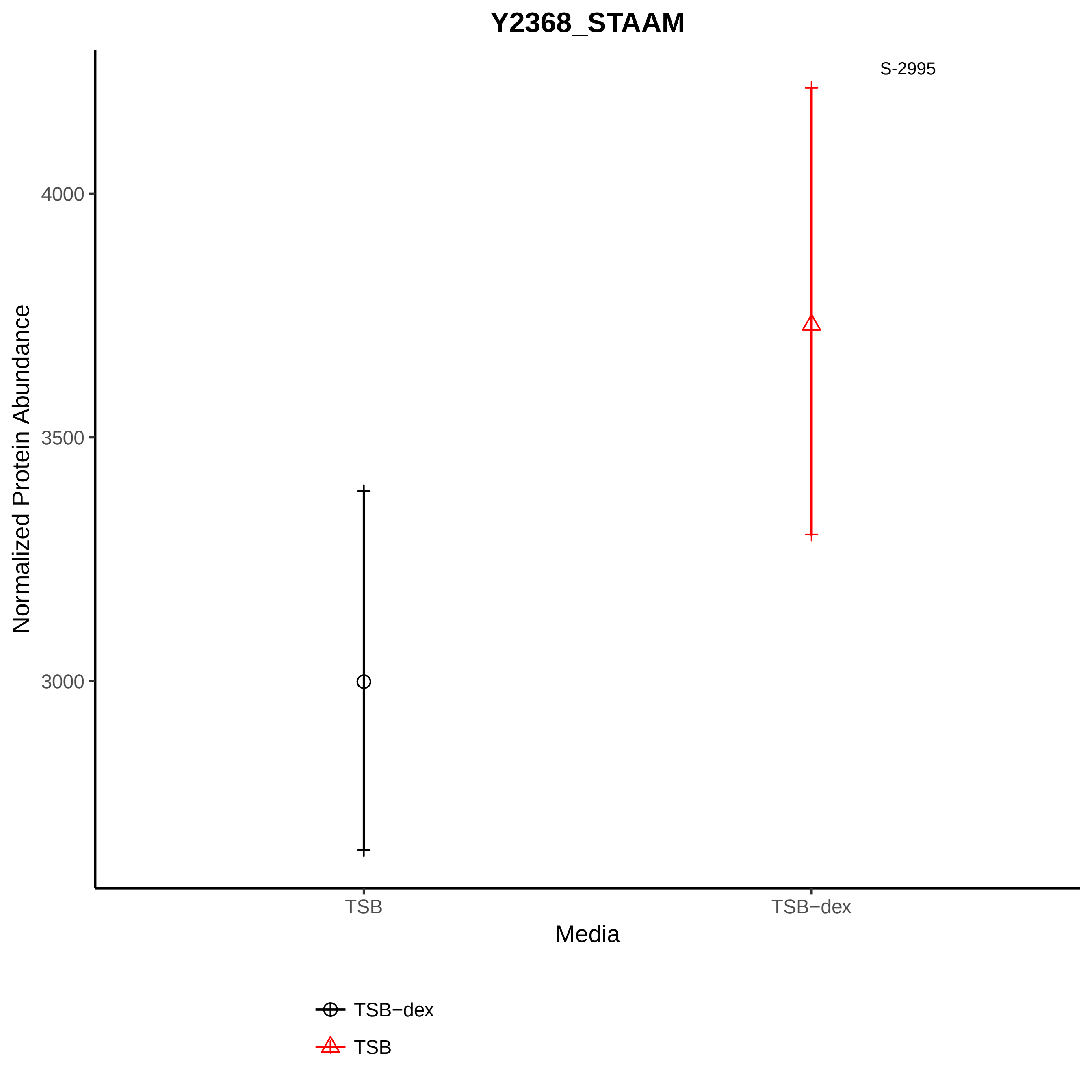

Y2368_STAAM
S-2995
4000
3500
3000
Normalized Protein Abundance
TSB
TSB−dex
Media
TSB−dex
TSB

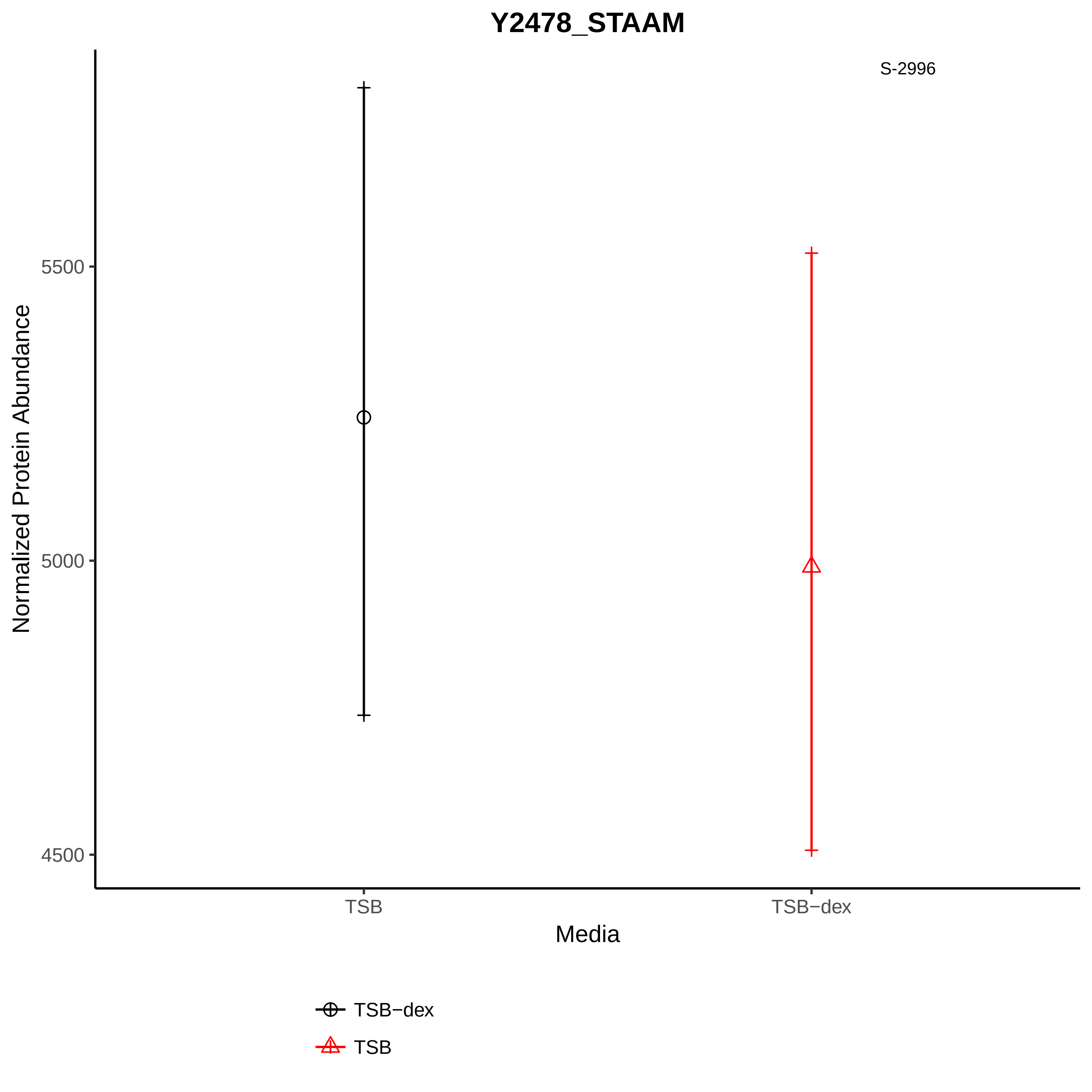

Y2478_STAAM
S-2996
Normalized Protein Abundance
5500
5000
4500
TSB
TSB−dex
Media
TSB−dex
TSB

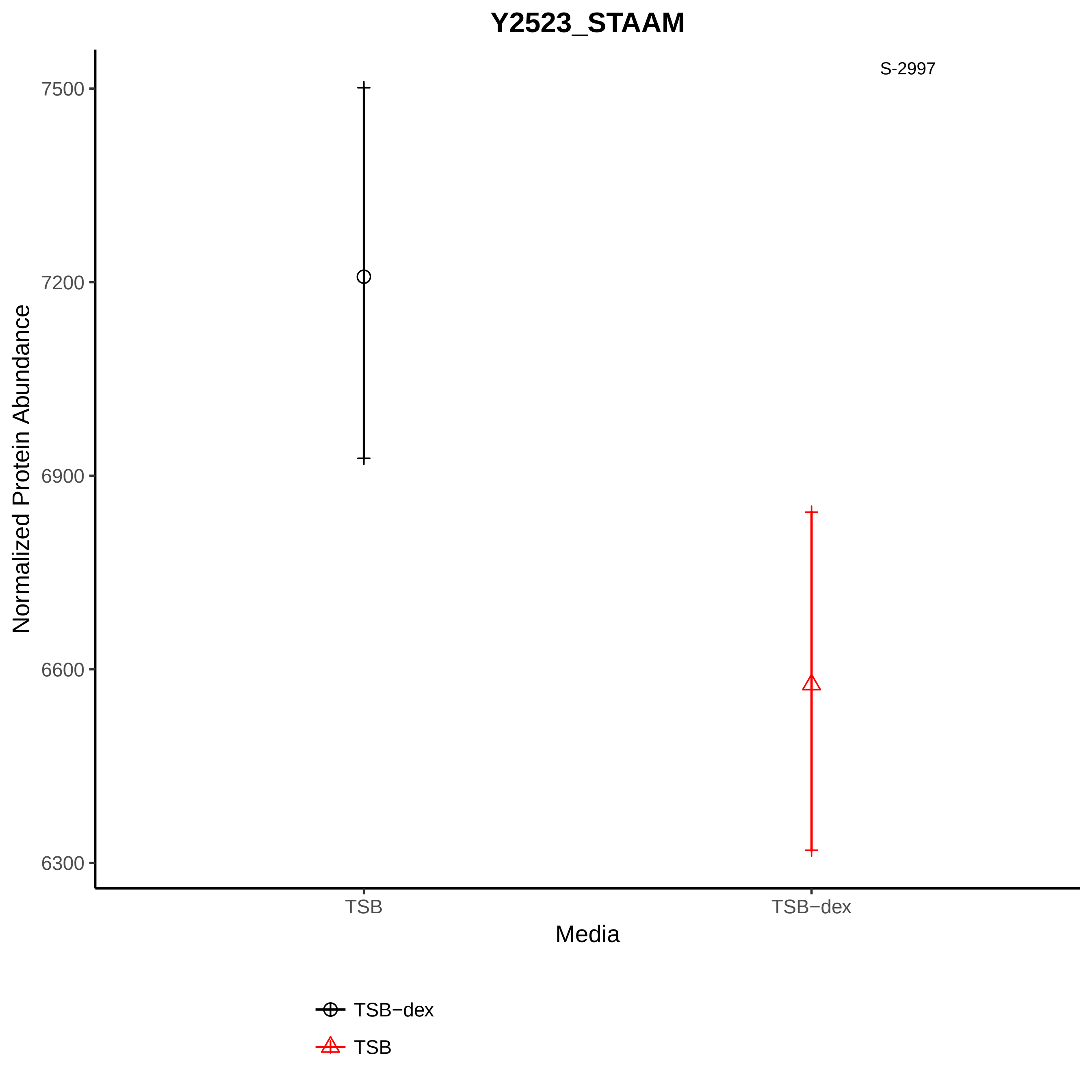

Y2523_STAAM
S-2997
Normalized Protein Abundance
7500
7200
6900
6600
6300
TSB
TSB−dex
Media
TSB−dex
TSB

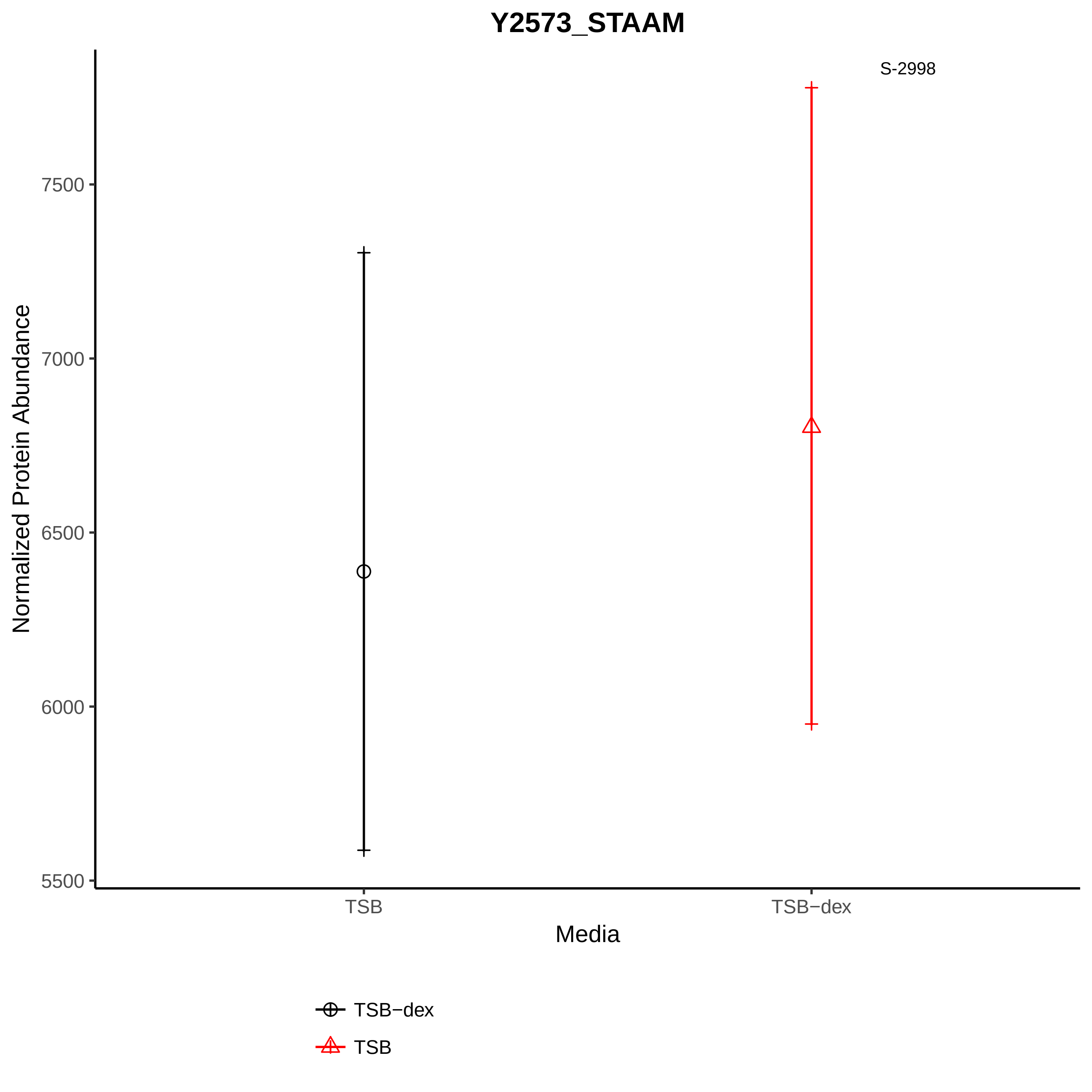

Y2573_STAAM
S-2998
Normalized Protein Abundance
7500
7000
6500
6000
5500
TSB
TSB−dex
Media
TSB−dex
TSB

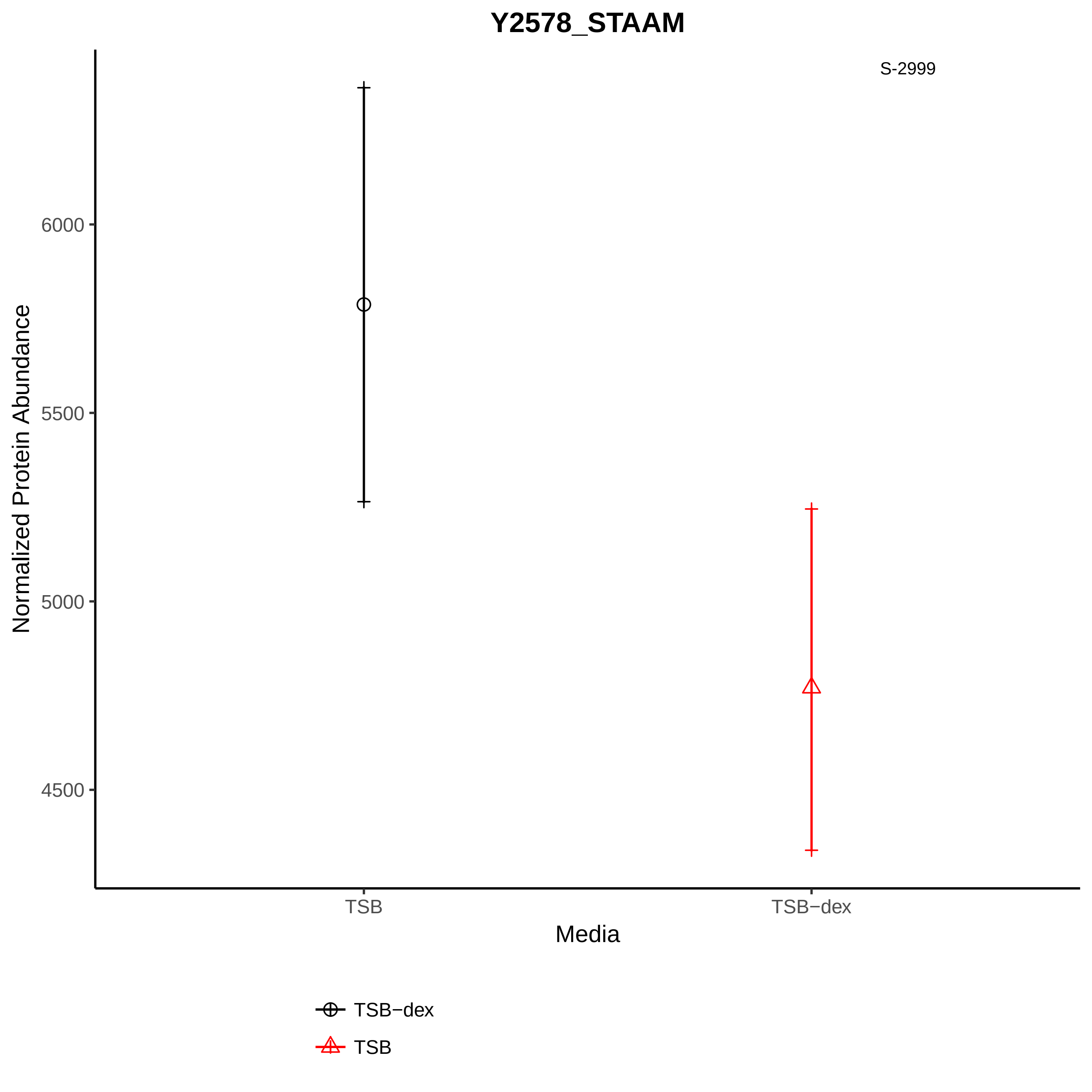

Y2578_STAAM
S-2999
Normalized Protein Abundance
6000
5500
5000
4500
TSB
TSB−dex
Media
TSB−dex
TSB

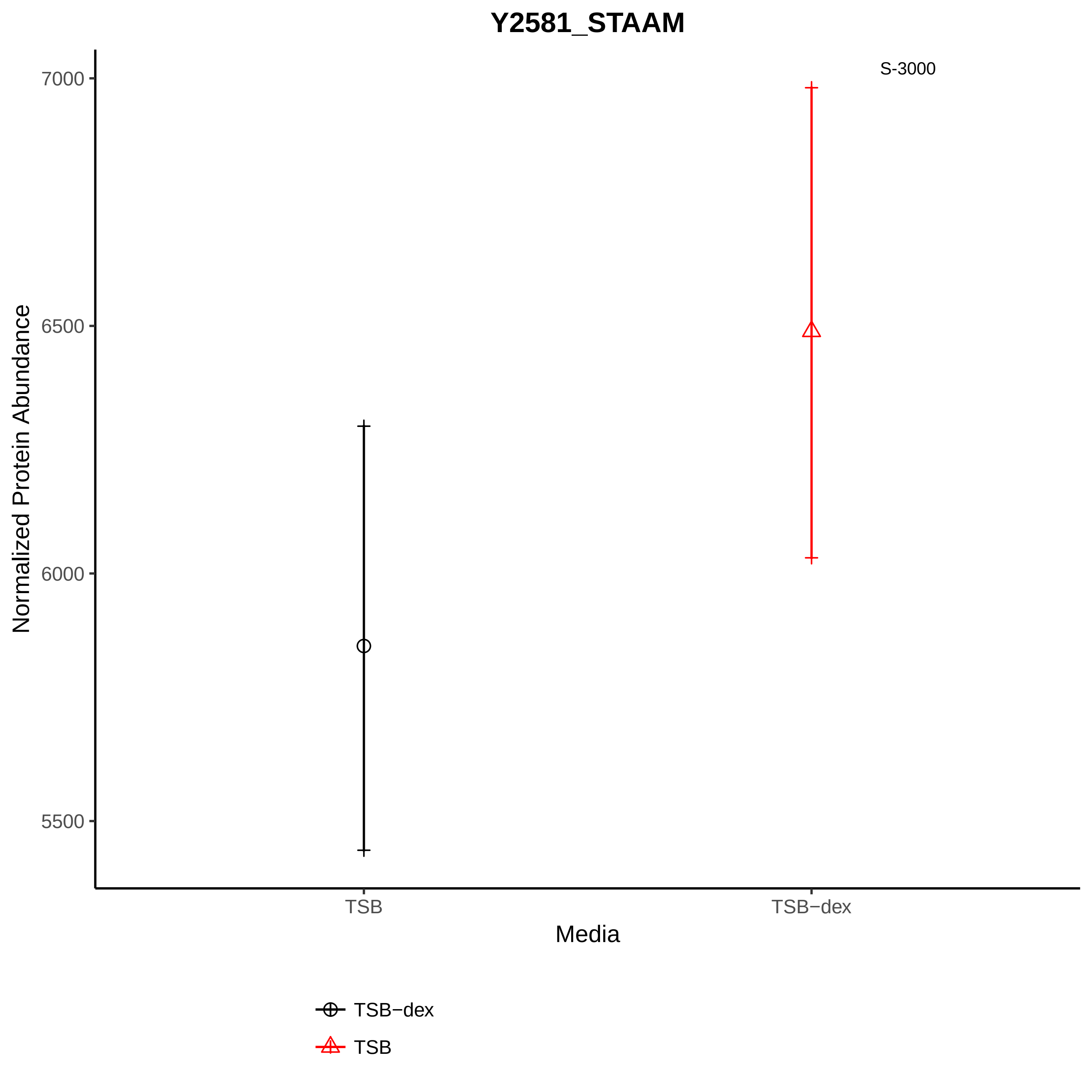

Y2581_STAAM
S-3000
Normalized Protein Abundance
7000
6500
6000
5500
TSB
TSB−dex
Media
TSB−dex
TSB

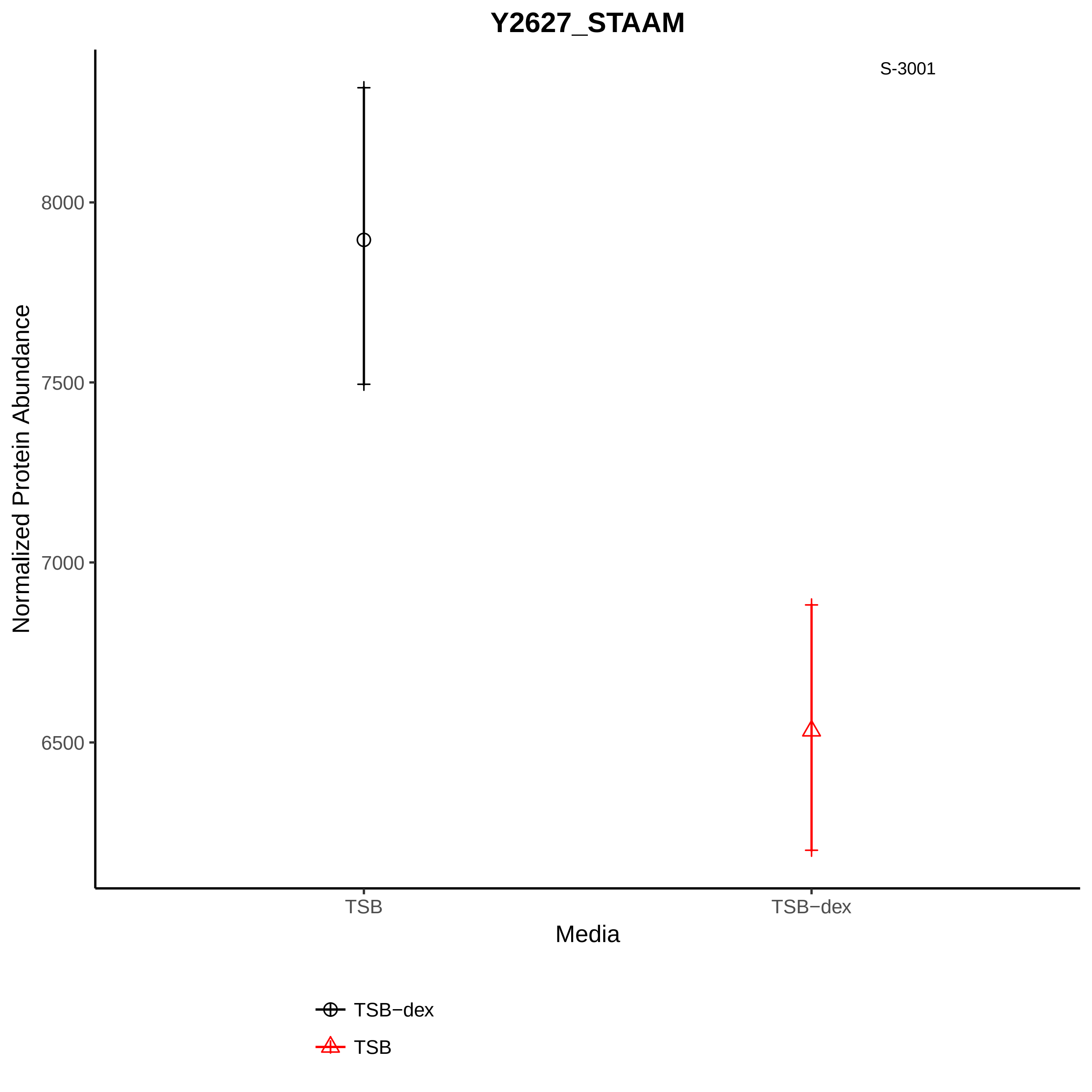
Y2627_STAAM
S-3001
Normalized Protein Abundance
8000
7500
7000
6500
TSB
TSB−dex
Media
TSB−dex
TSB

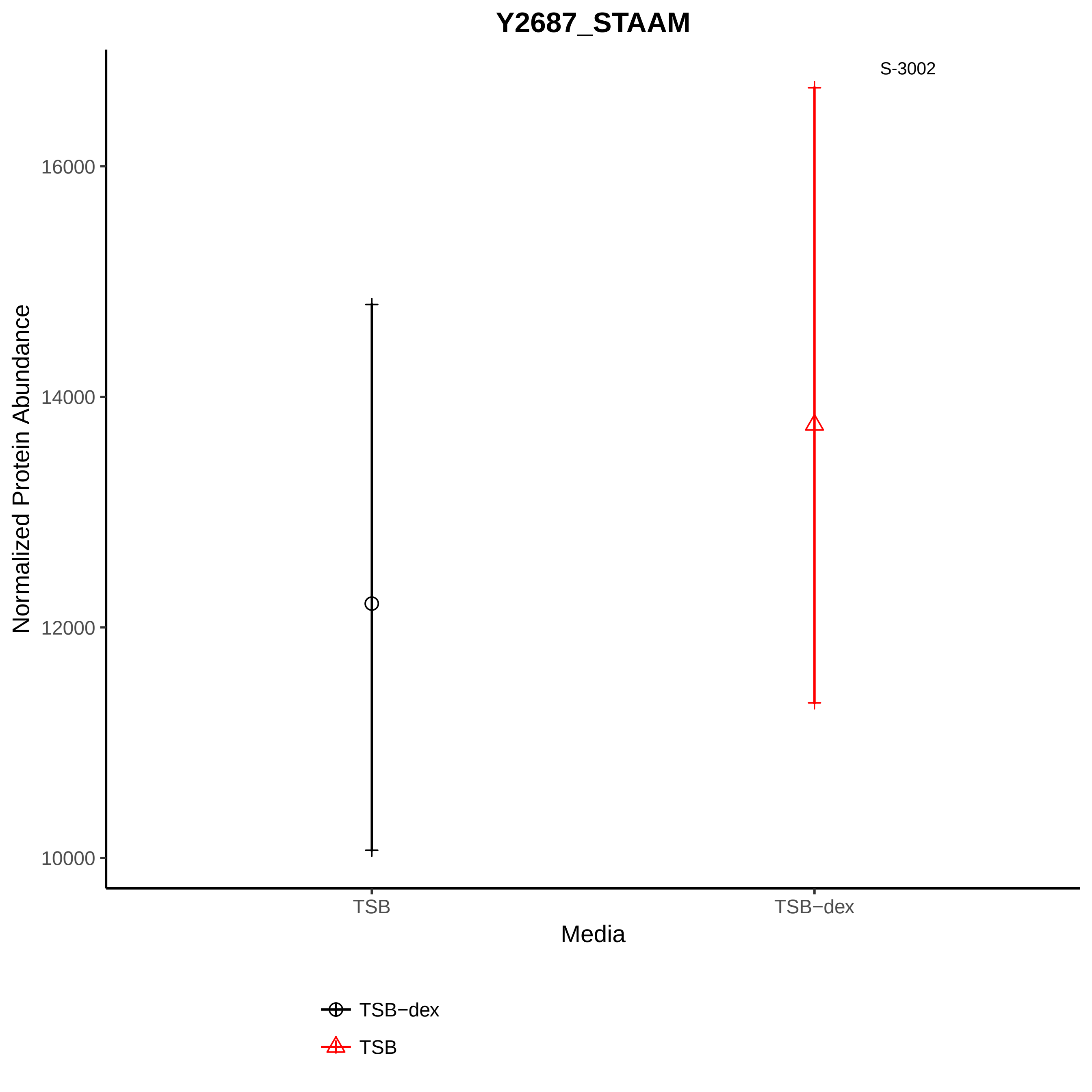

Y2687_STAAM
S-3002
Normalized Protein Abundance
16000
14000
12000
10000
TSB
TSB−dex
Media
TSB−dex
TSB

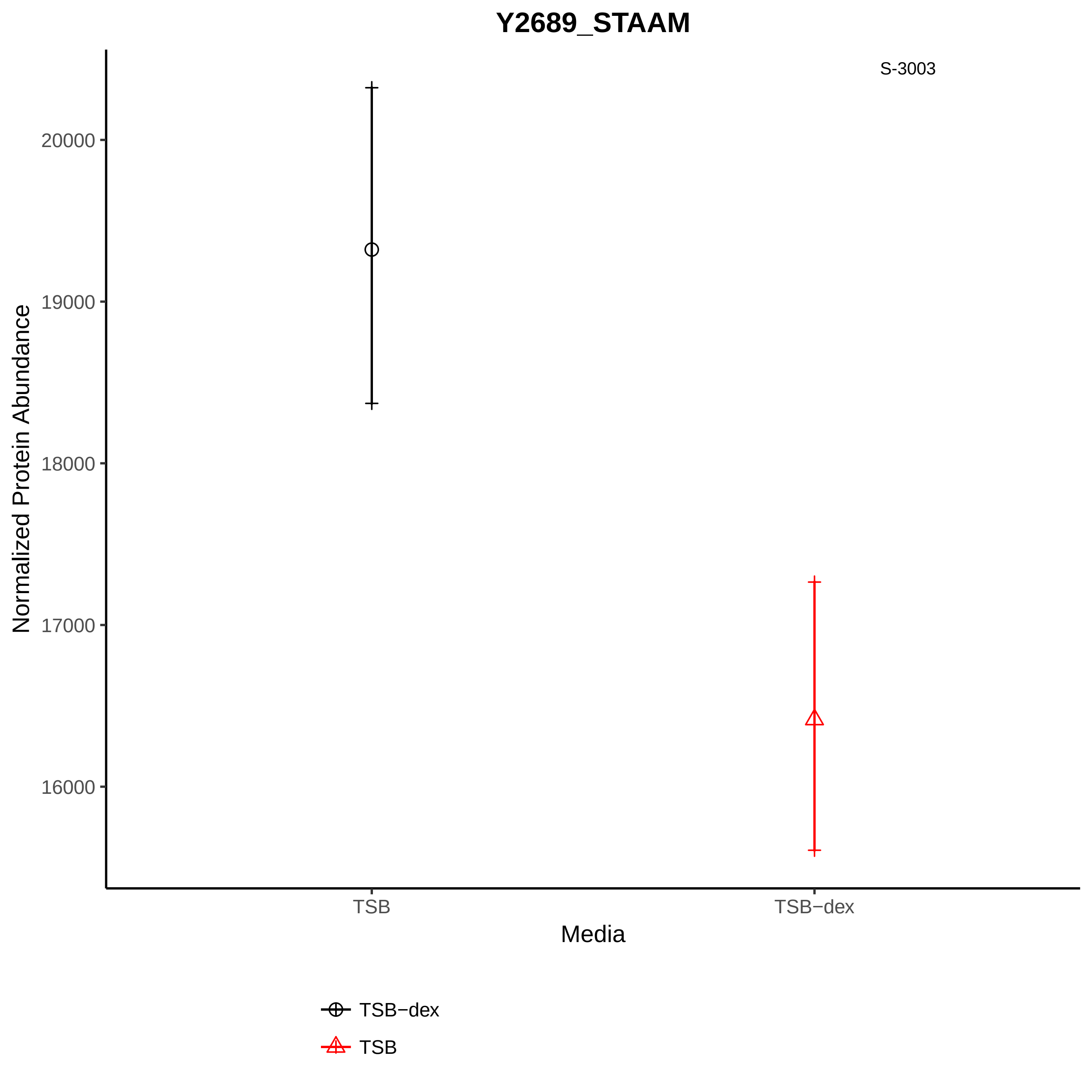
Y2689_STAAM
S-3003
Normalized Protein Abundance
20000
19000
18000
17000
16000
TSB
TSB−dex
Media
TSB−dex
TSB

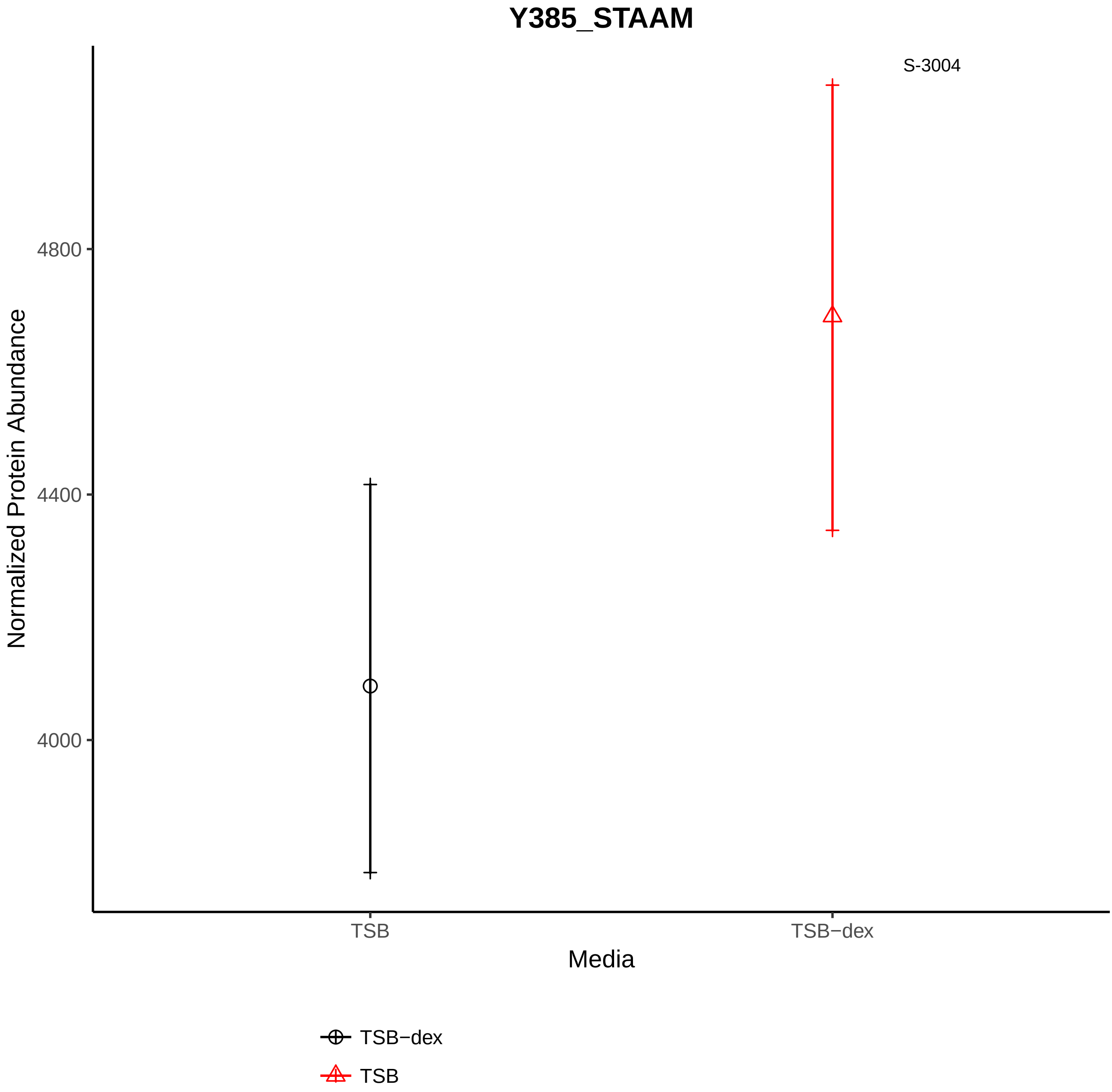

Y385_STAAM
S-3004
Normalized Protein Abundance
4800
4400
4000
TSB
TSB−dex
Media
TSB−dex
TSB

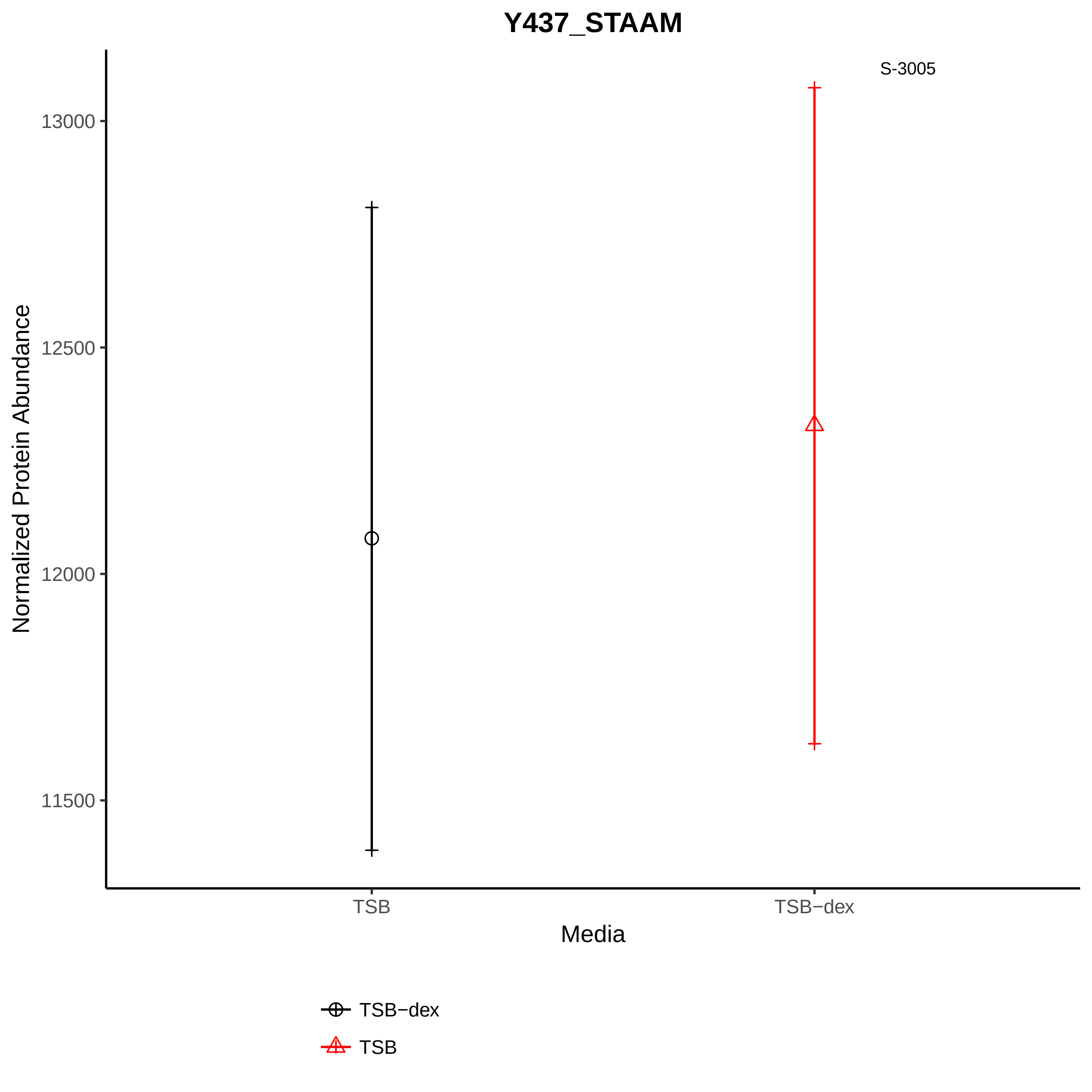
Y437_STAAM
S-3005
Normalized Protein Abundance
13000
12500
12000
11500
TSB
TSB−dex
Media
TSB−dex
TSB

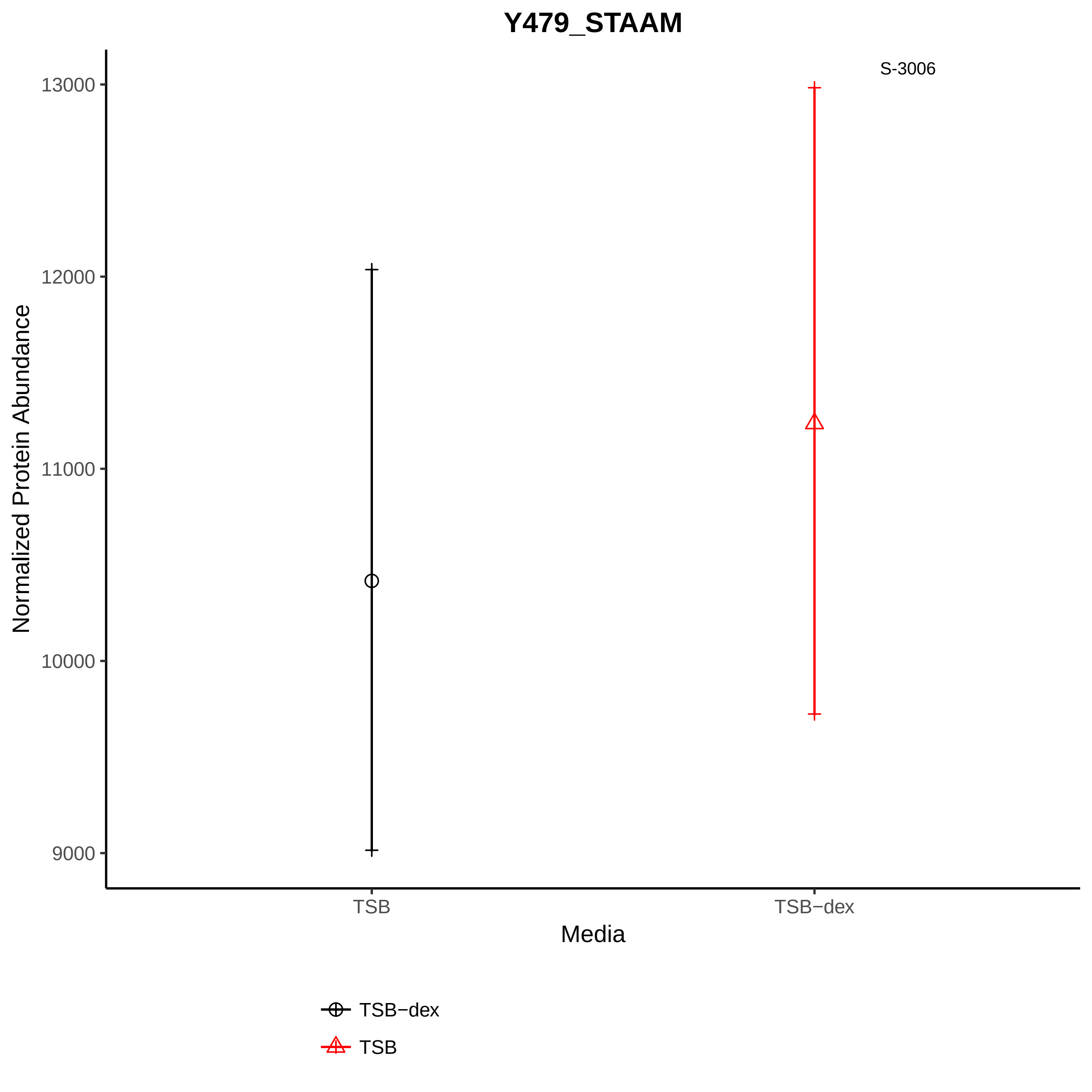

Y479_STAAM
S-3006
13000
12000
11000
10000
9000
Normalized Protein Abundance
TSB
TSB−dex
Media
TSB−dex
TSB

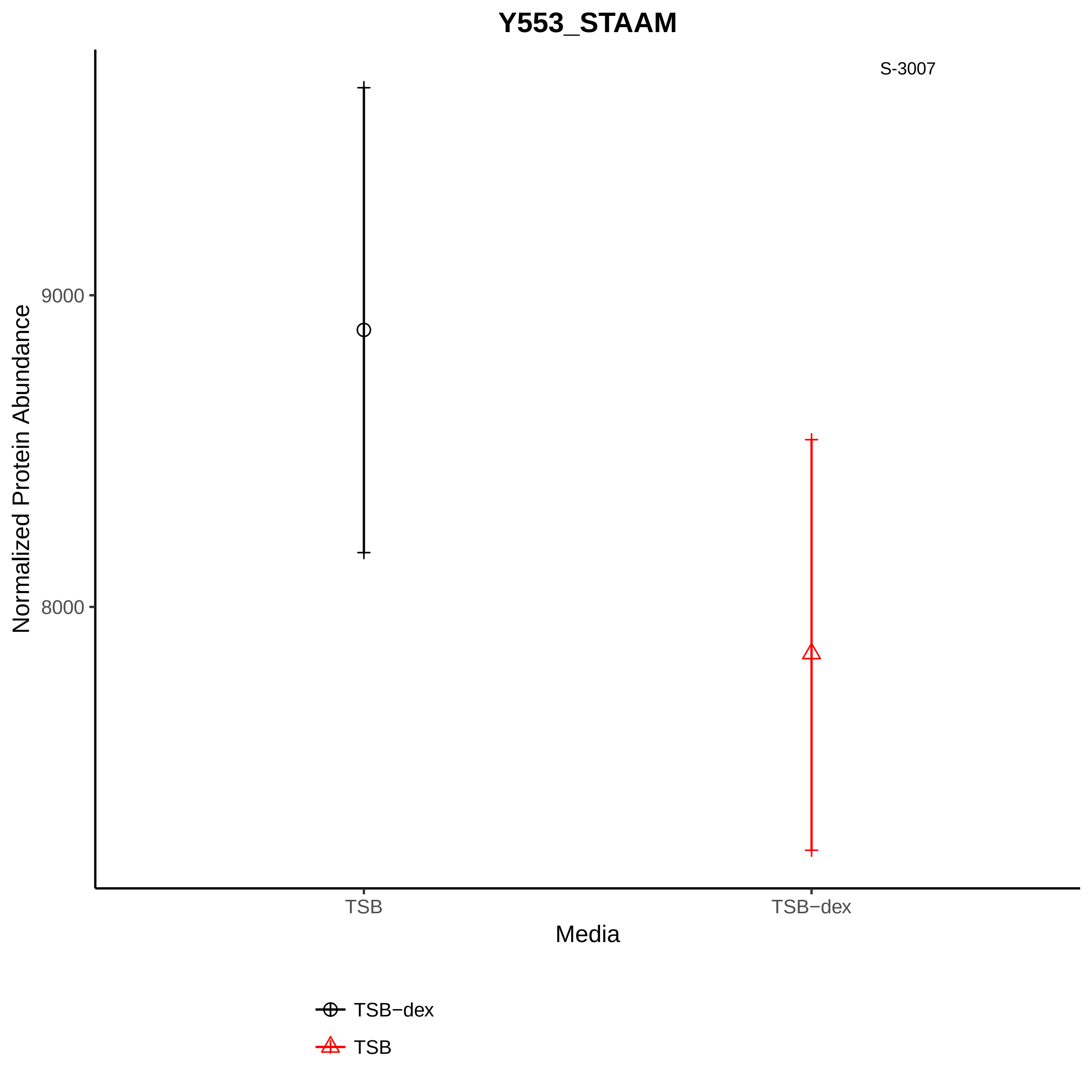

Y553_STAAM
S-3007
Normalized Protein Abundance
9000
8000
TSB
TSB−dex
Media
TSB−dex
TSB

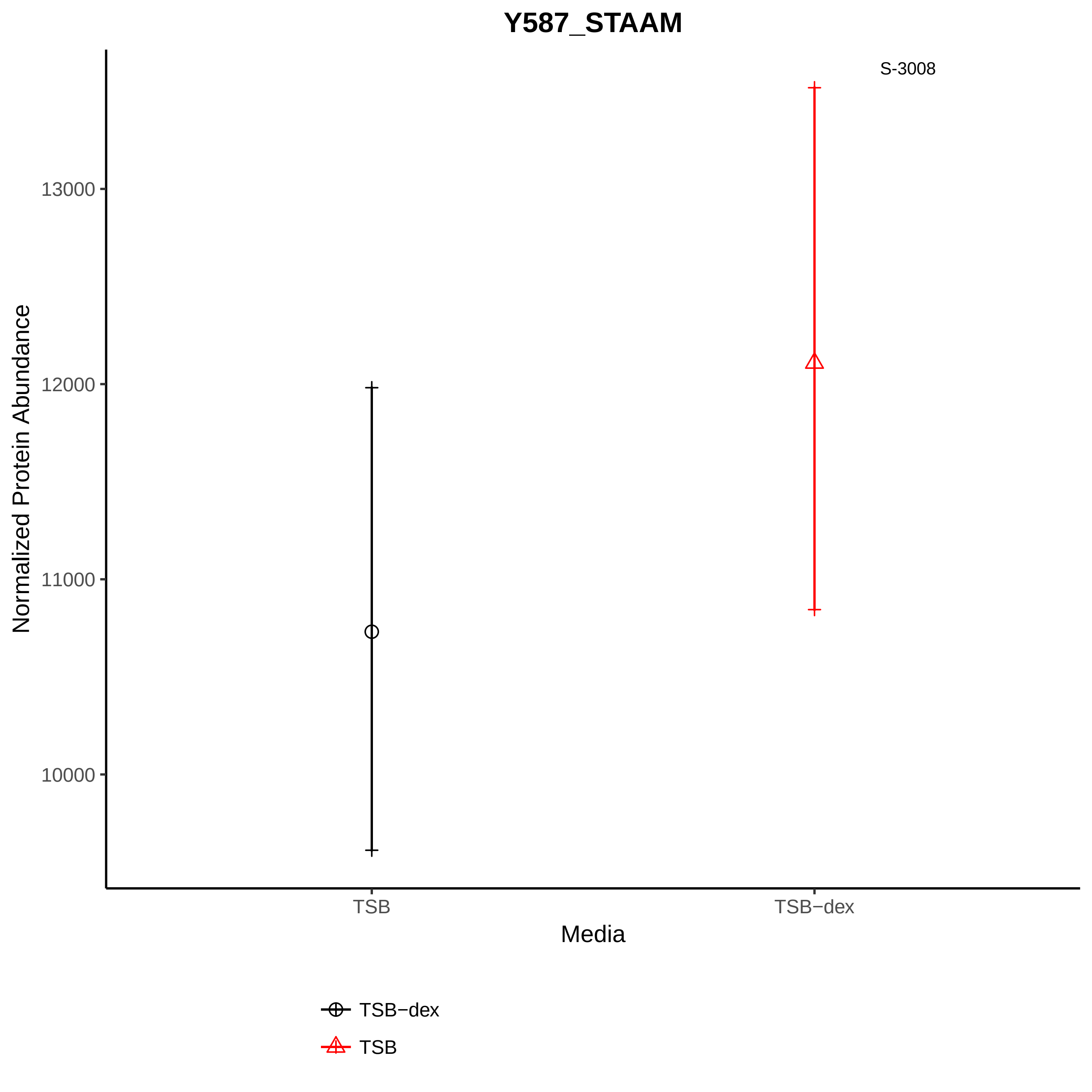

Y587_STAAM
S-3008
13000
12000
11000
10000
Normalized Protein Abundance
TSB
TSB−dex
Media
TSB−dex
TSB

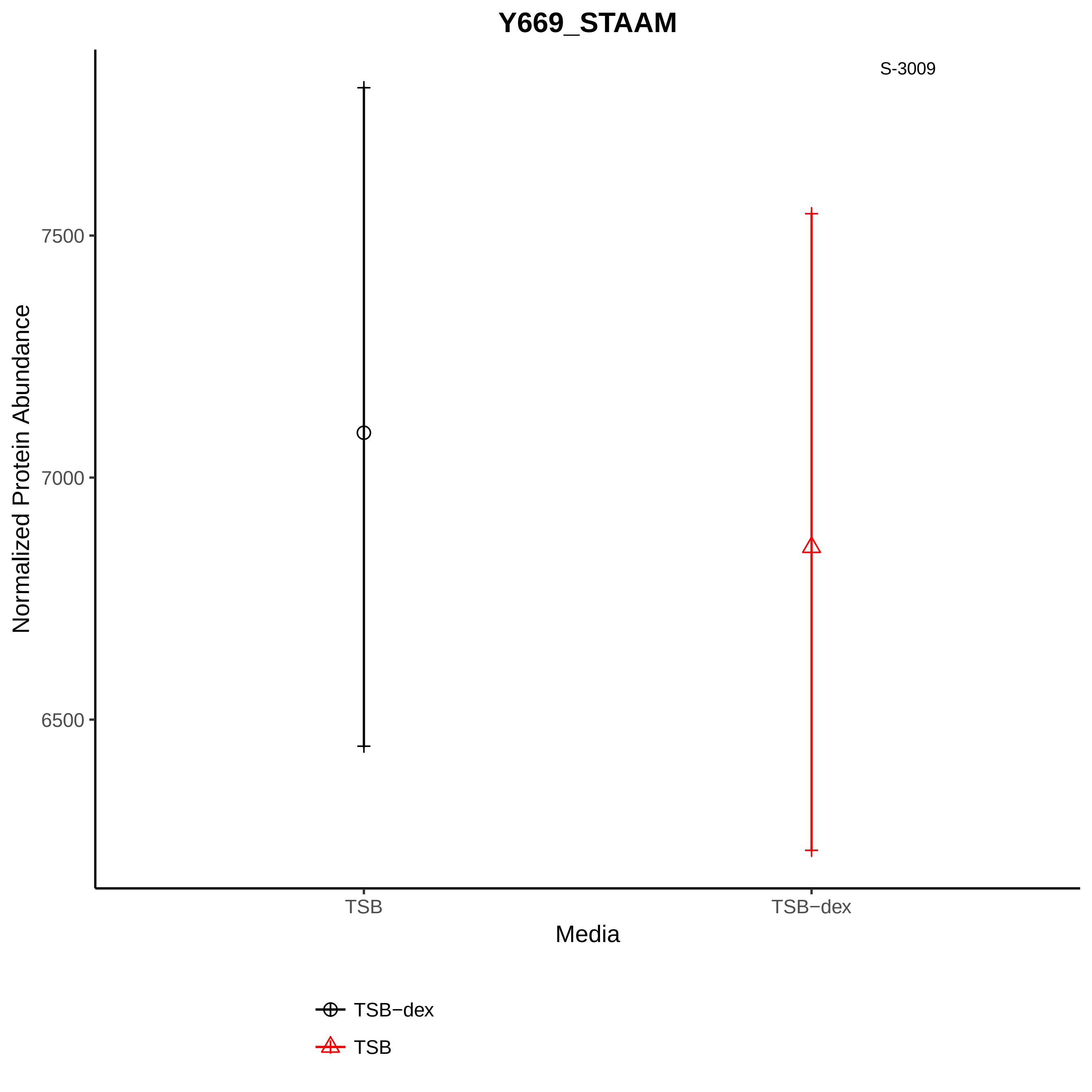
Y669_STAAM
S-3009
Normalized Protein Abundance
7500
7000
6500
TSB
TSB−dex
Media
TSB−dex
TSB

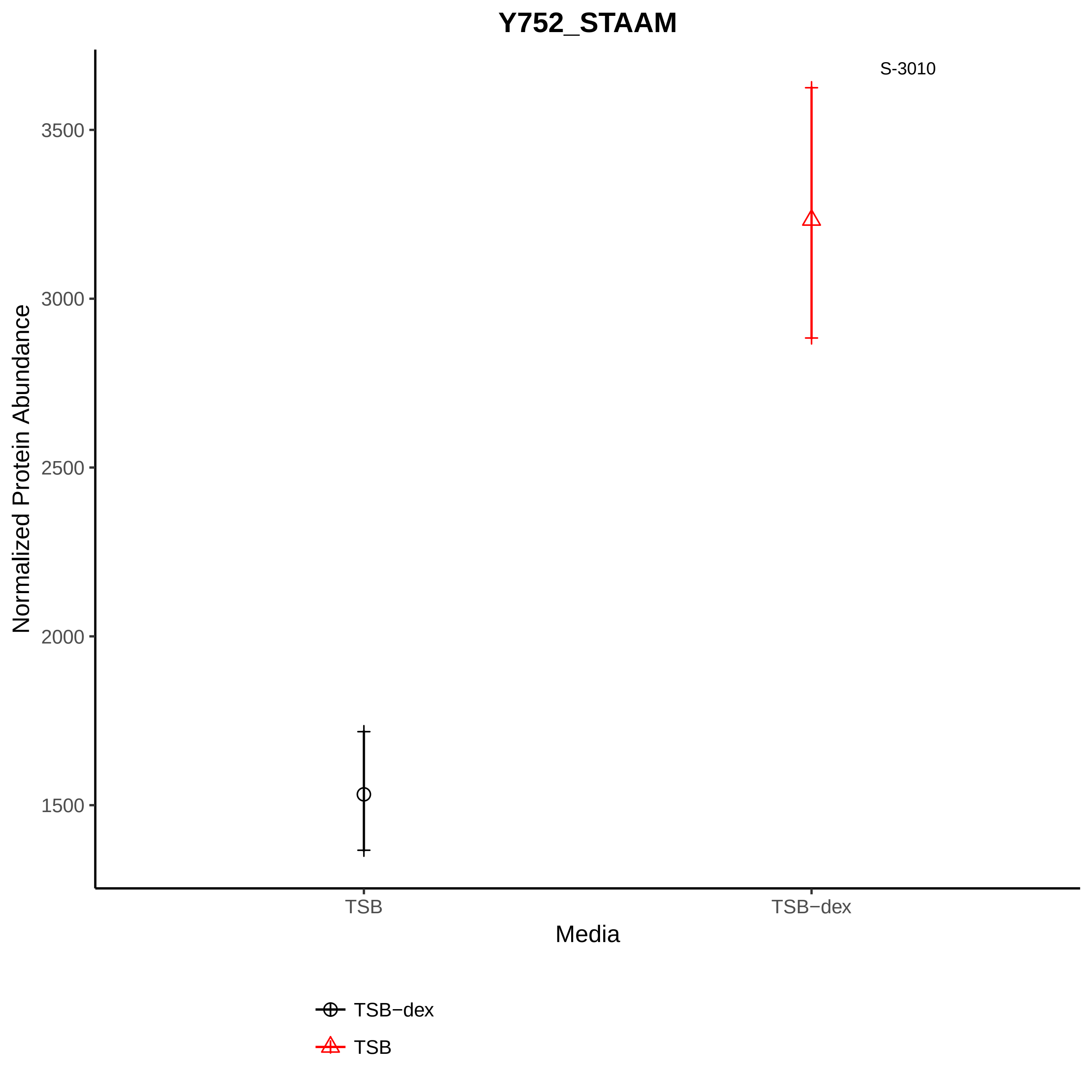

Y752_STAAM
S-3010
Normalized Protein Abundance
3500
3000
2500
2000
1500
TSB
TSB−dex
Media
TSB−dex
TSB

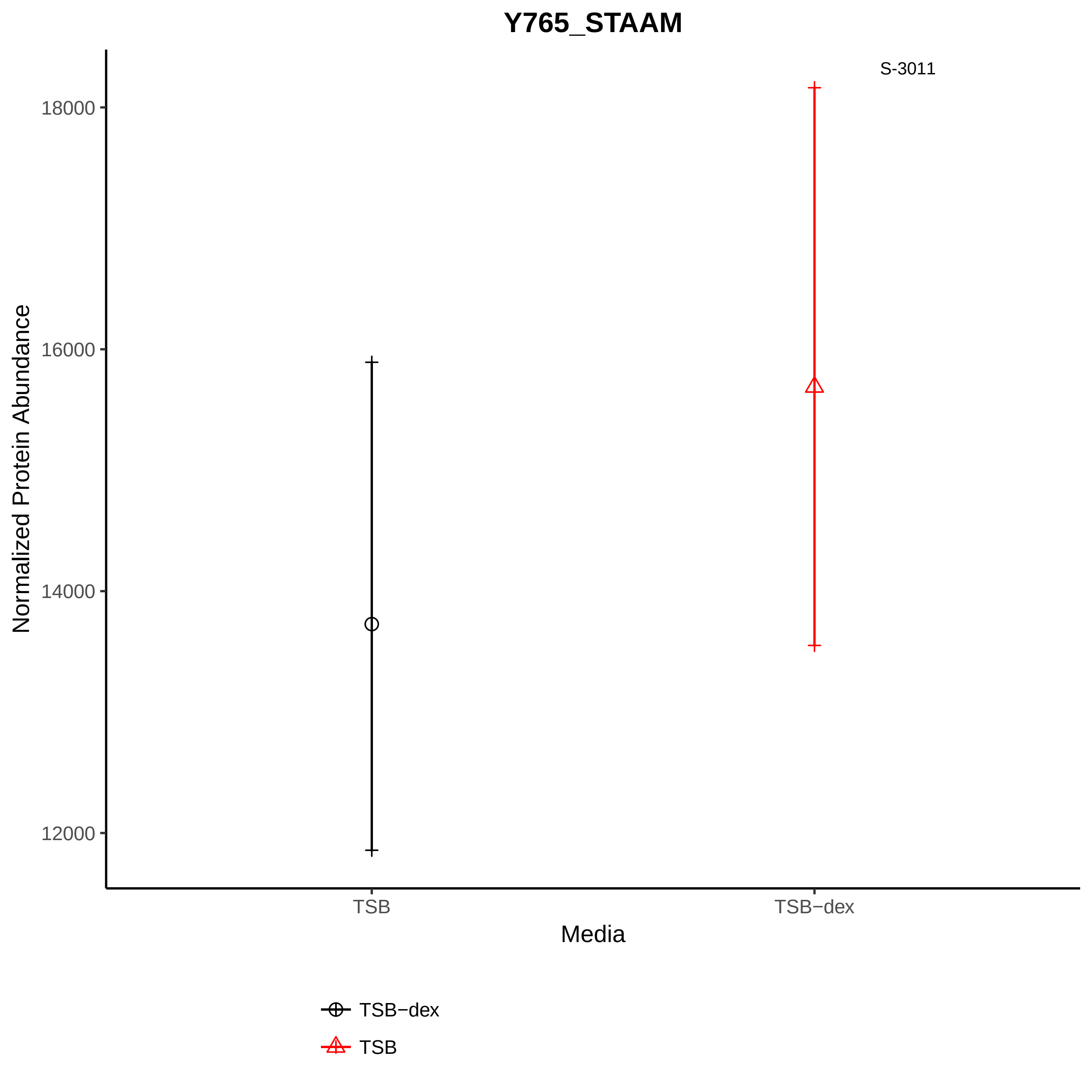

Y765_STAAM
S-3011
Normalized Protein Abundance
18000
16000
14000
12000
TSB
TSB−dex
Media
TSB−dex
TSB

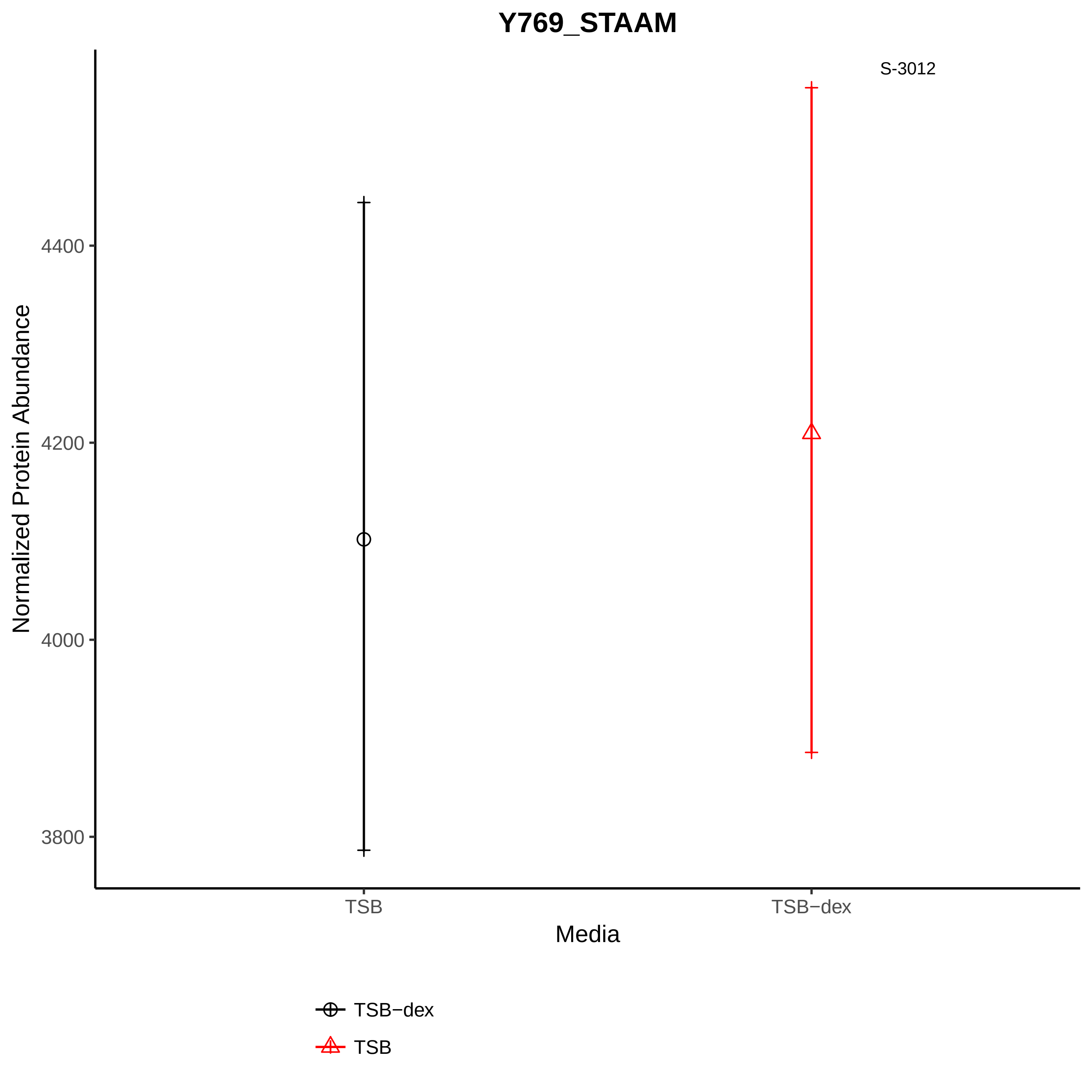
Y769_STAAM
S-3012
Normalized Protein Abundance
4400
4200
4000
3800
TSB
TSB−dex
Media
TSB−dex
TSB

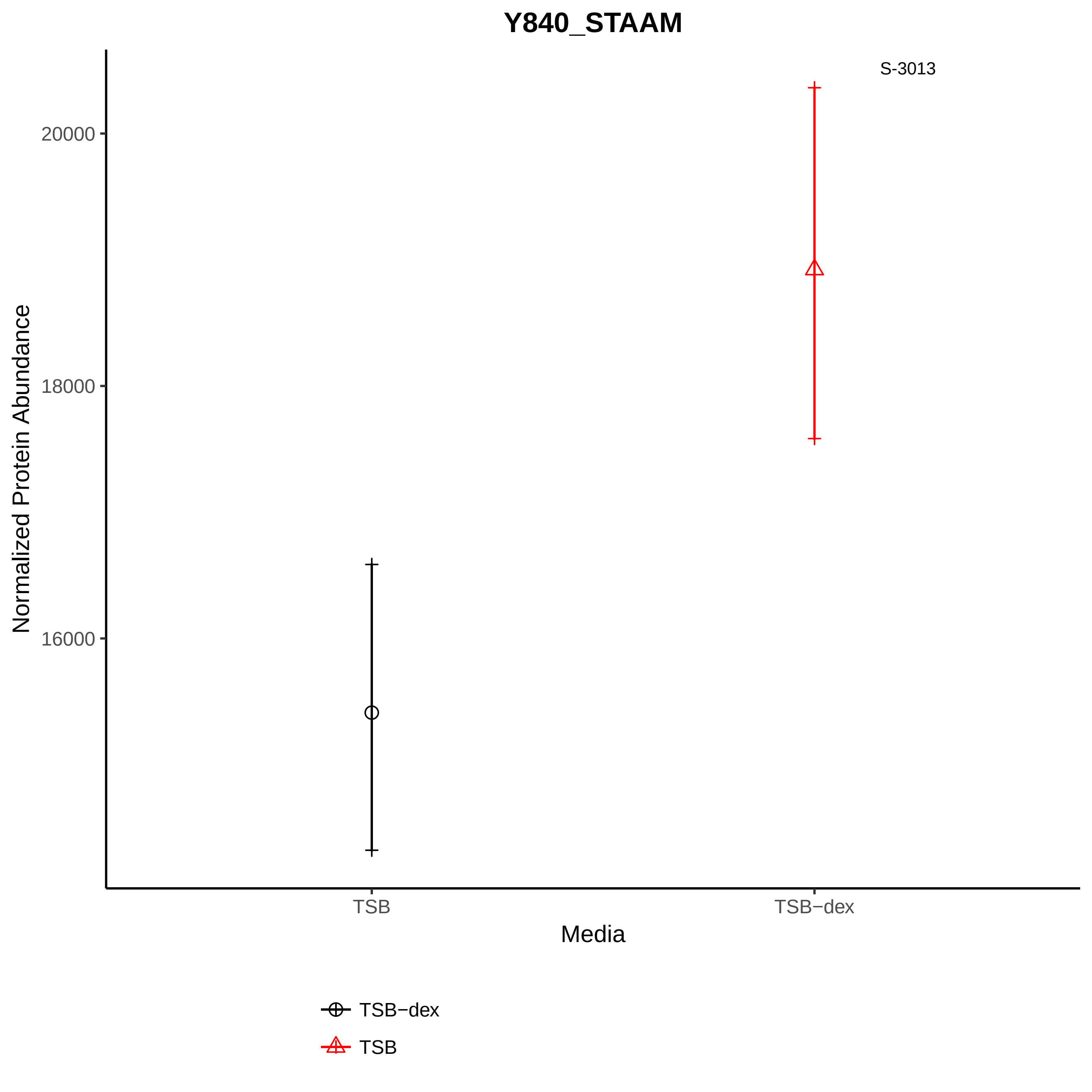
Y840_STAAM
S-3013
20000
18000
16000
Normalized Protein Abundance
TSB
TSB−dex
Media
TSB−dex
TSB

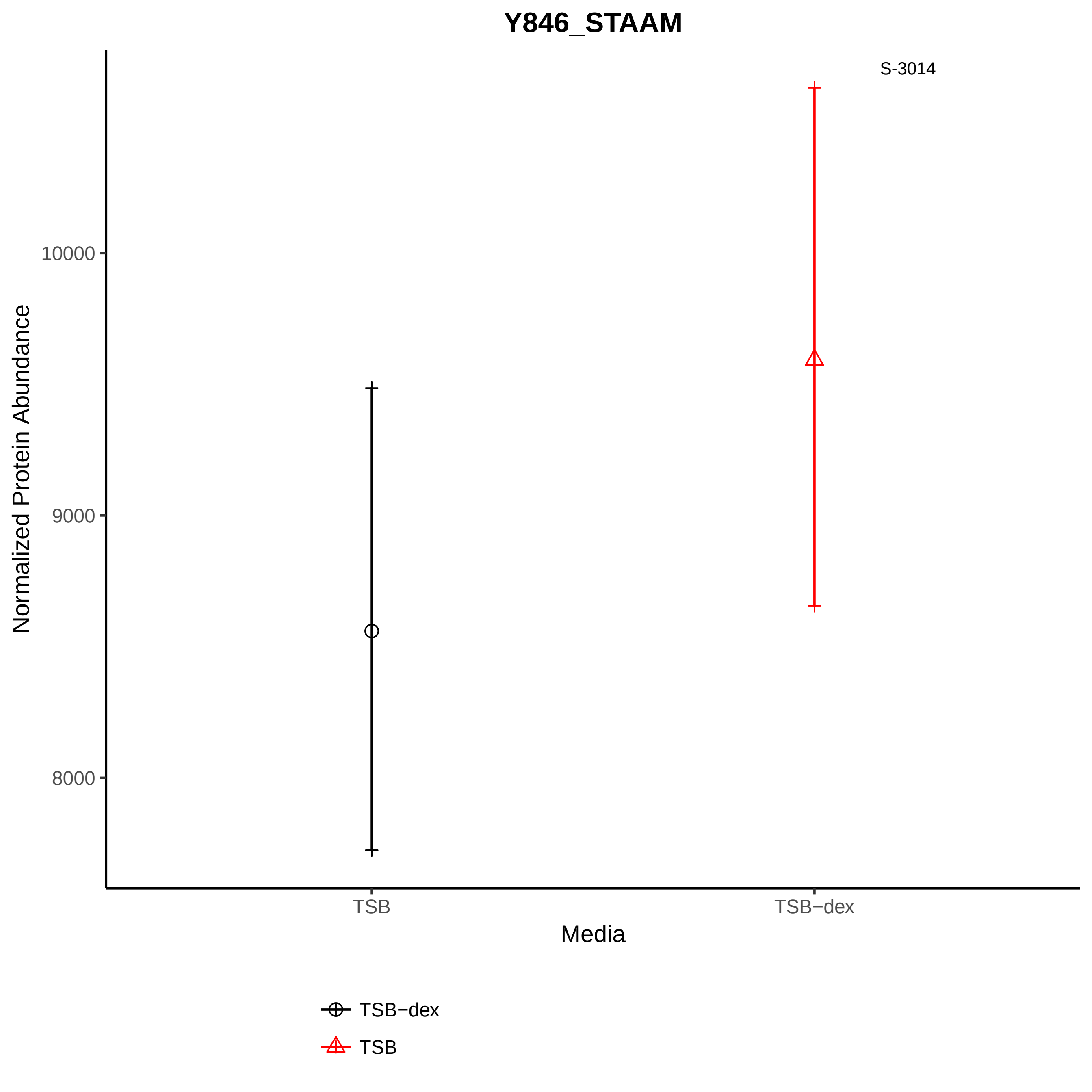
Y846_STAAM
S-3014
Normalized Protein Abundance
10000
9000
8000
TSB
TSB−dex
Media
TSB−dex
TSB

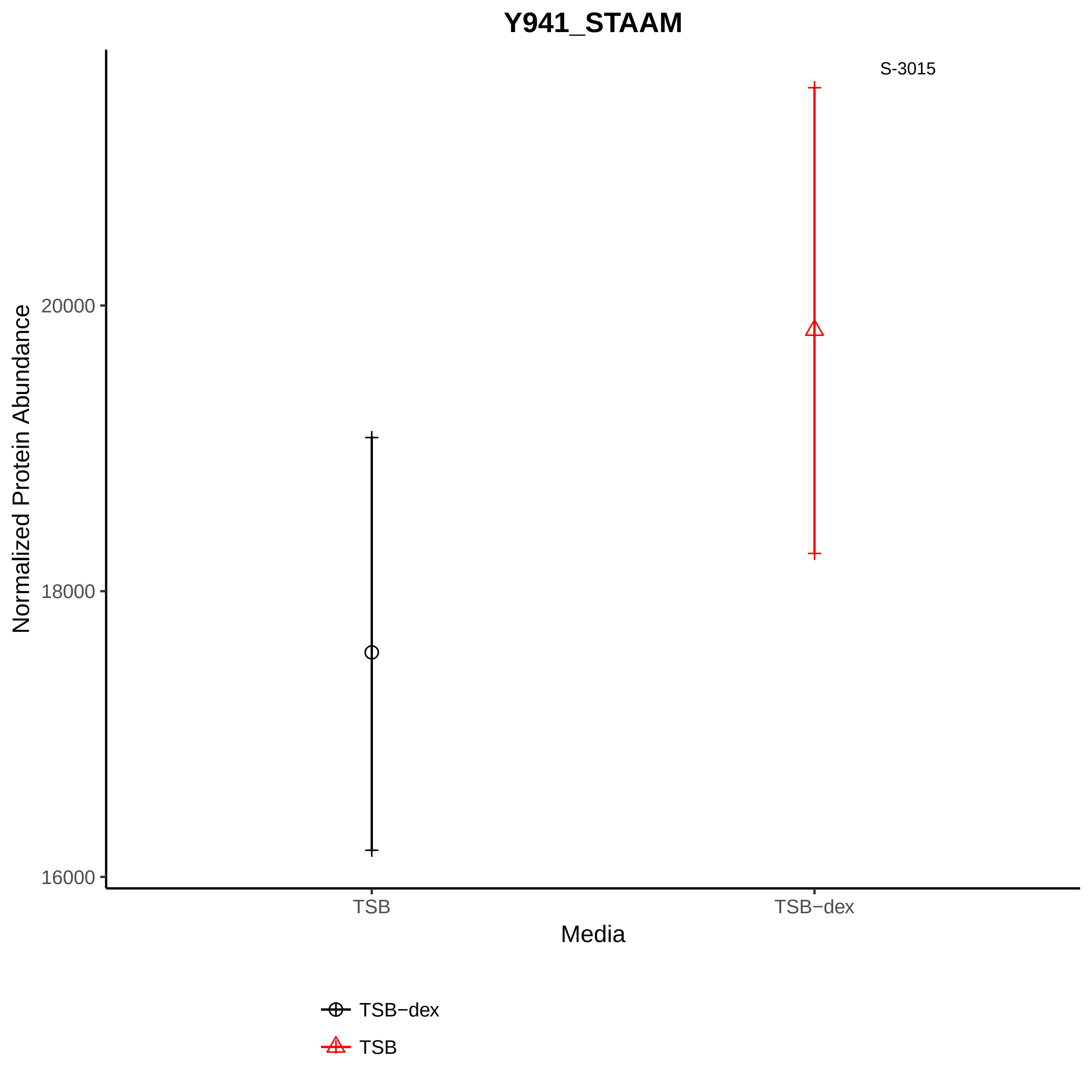

Y941_STAAM
S-3015
Normalized Protein Abundance
20000
18000
16000
TSB
TSB−dex
Media
TSB−dex
TSB

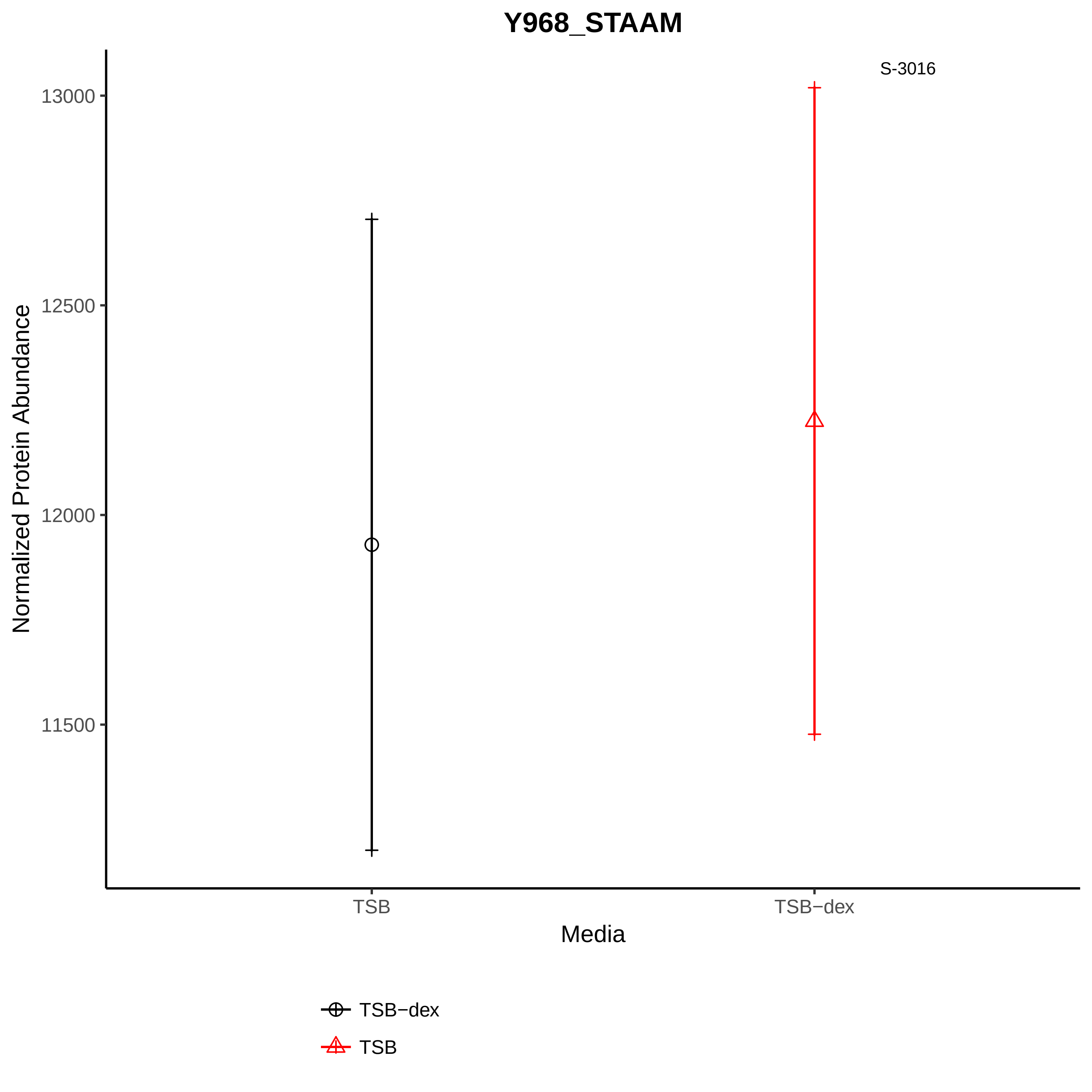

Y968_STAAM
S-3016
Normalized Protein Abundance
13000
12500
12000
11500
TSB
TSB−dex
Media
TSB−dex
TSB

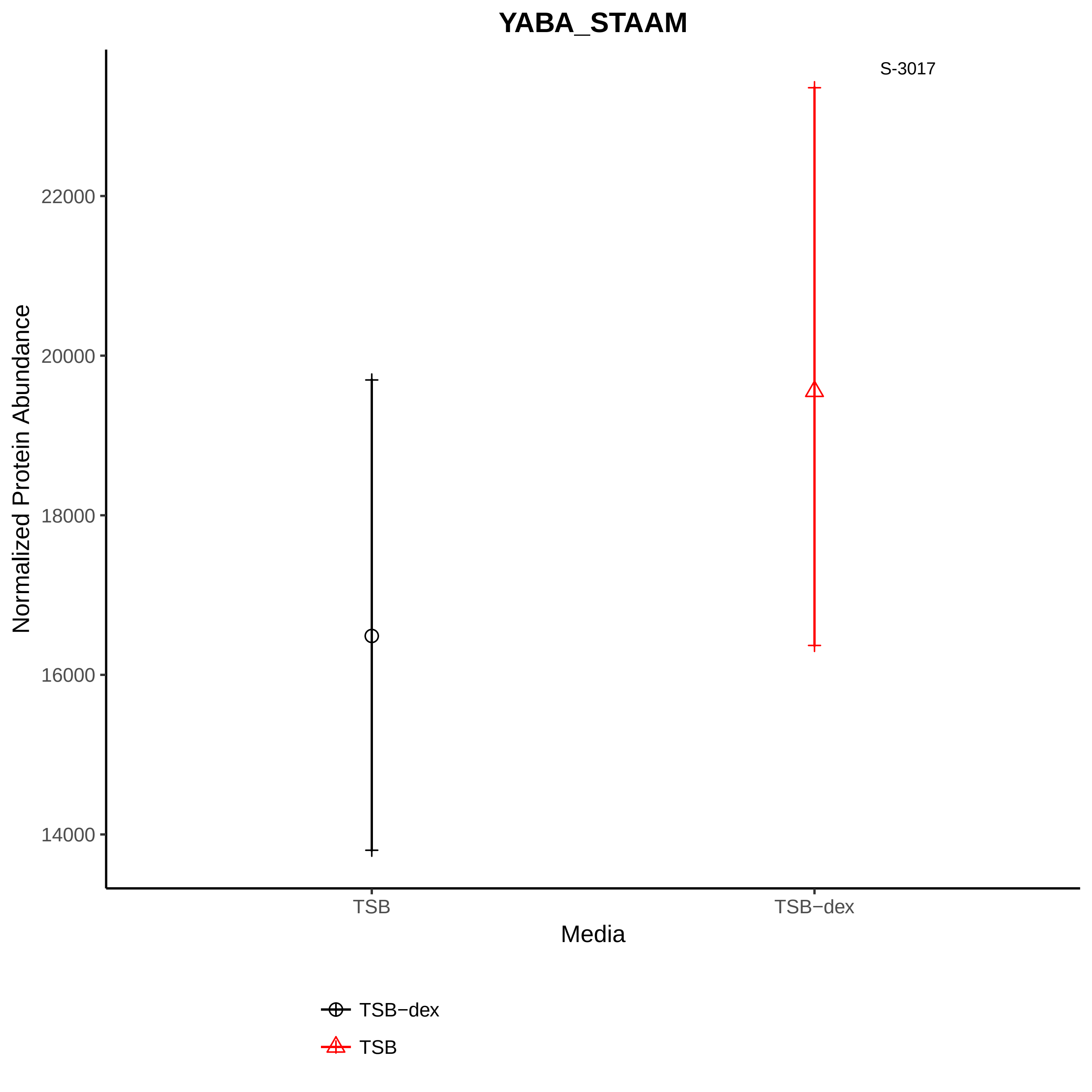
YABA_STAAM
S-3017
Normalized Protein Abundance
22000
20000
18000
16000
14000
TSB
TSB−dex
Media
TSB−dex
TSB

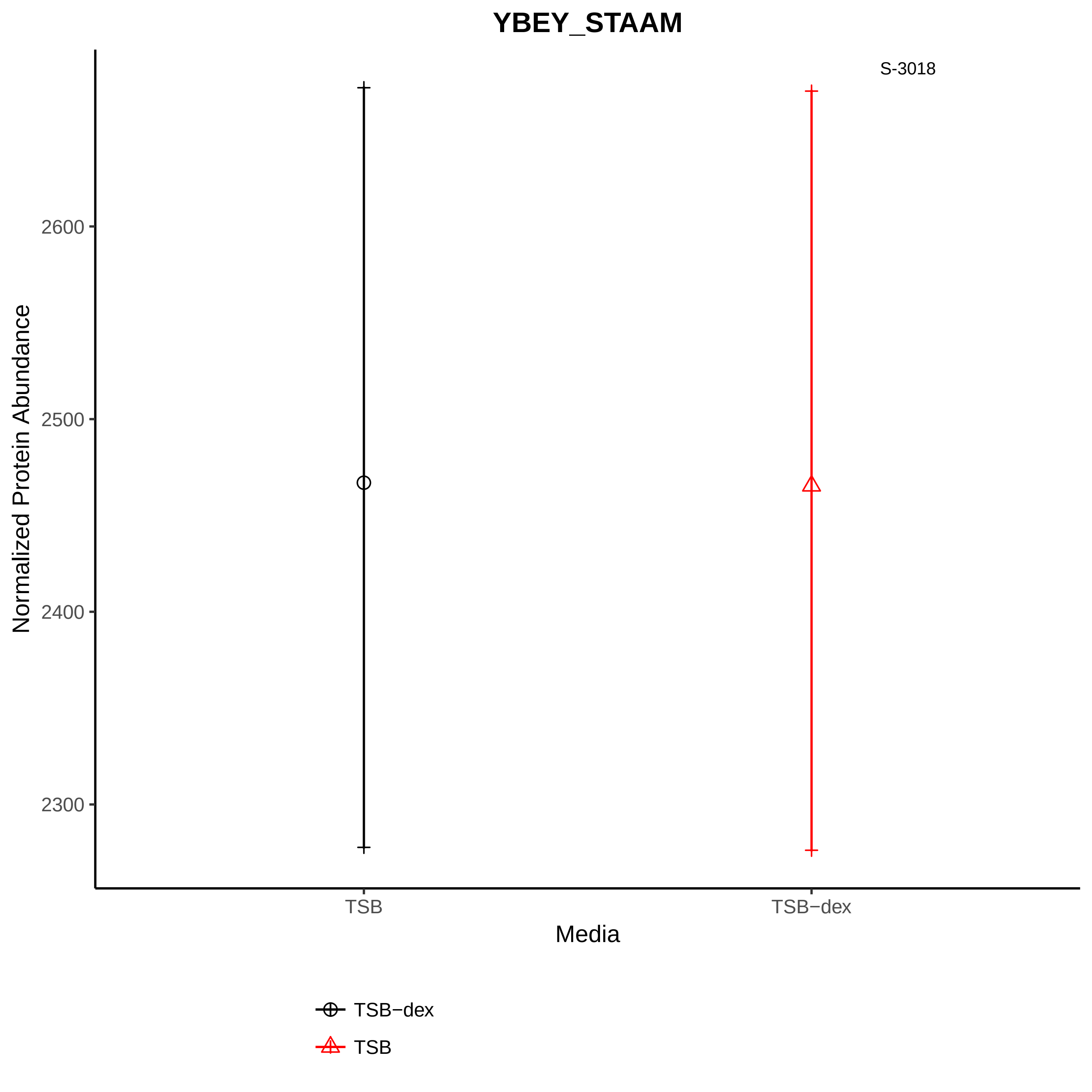

YBEY_STAAM
S-3018
Normalized Protein Abundance
2600
2500
2400
2300
TSB
TSB−dex
Media
TSB−dex
TSB

Figure L.

1-way analysis: s

Pages: S3019-S3520

The normalized protein abundance, lower 95% confidence interval, and upper 95% confidence interval were plotted for each protein. These plots were analyzed if the 3-way and 2-way interactions were rejected and a significant main effect (1-way) was identified for strain.

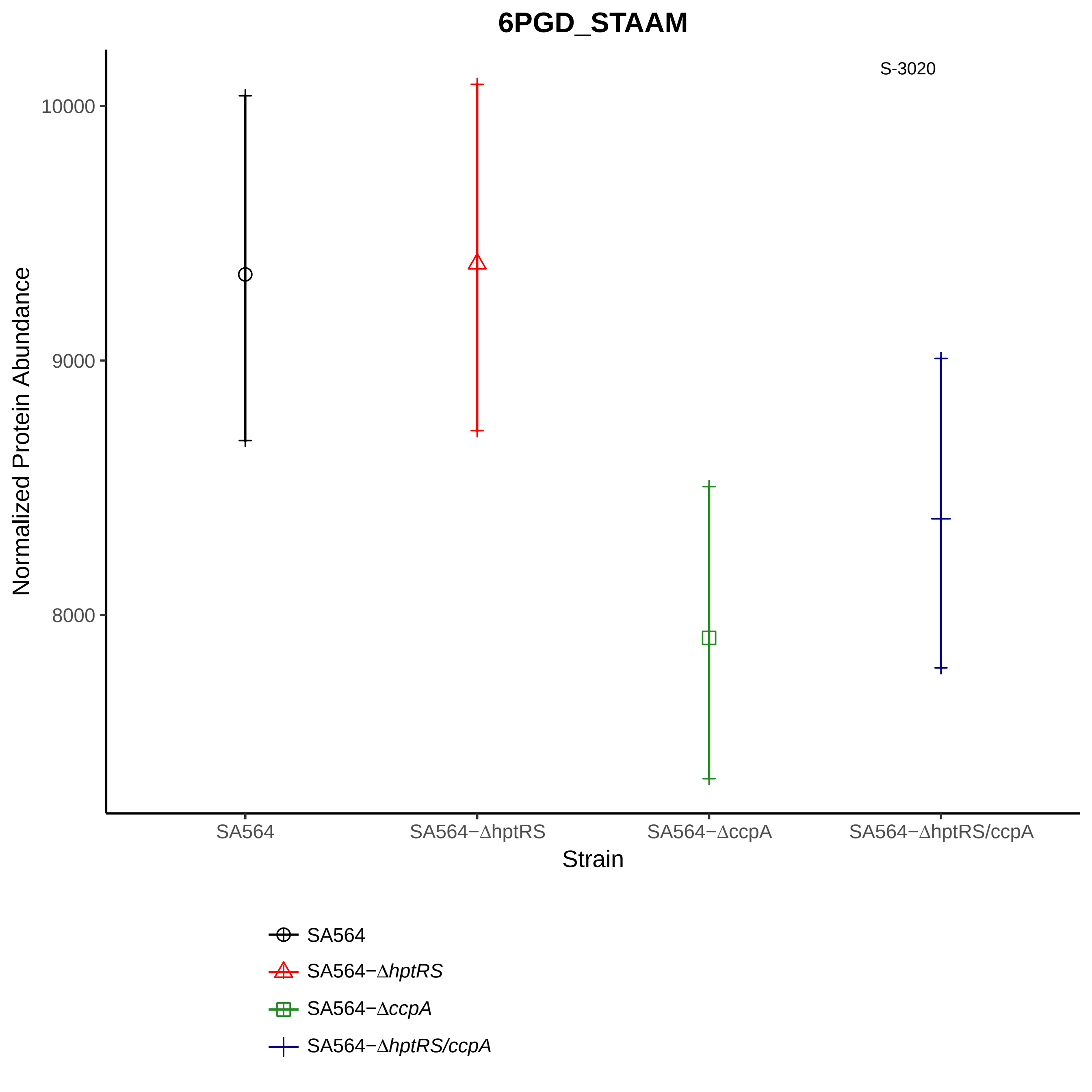

6PGD_STAAM
S-3020
10000
9000
8000
Normalized Protein Abundance
SA564
SA564−ΔhptRS
SA564−ΔccpA
SA564−ΔhptRS/ccpA
Strain
SA564
SA564−ΔhptRS
SA564−ΔccpA
SA564−ΔhptRS/ccpA

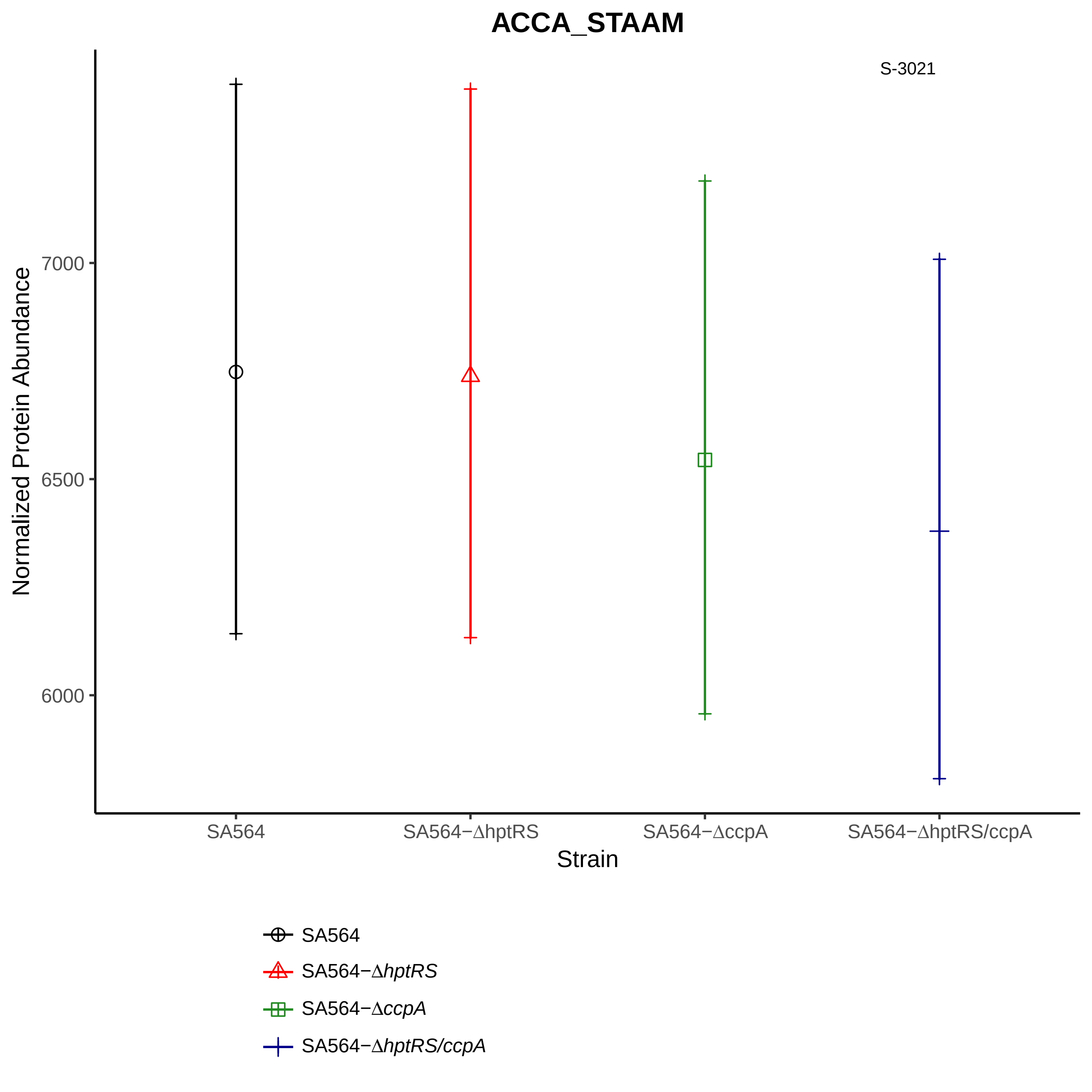
ACCA_STAAM
S-3021
Normalized Protein Abundance
7000
6500
6000
SA564
SA564−ΔhptRS
SA564−ΔccpA
SA564−ΔhptRS/ccpA
Strain
SA564
SA564−ΔhptRS
SA564−ΔccpA
SA564−ΔhptRS/ccpA

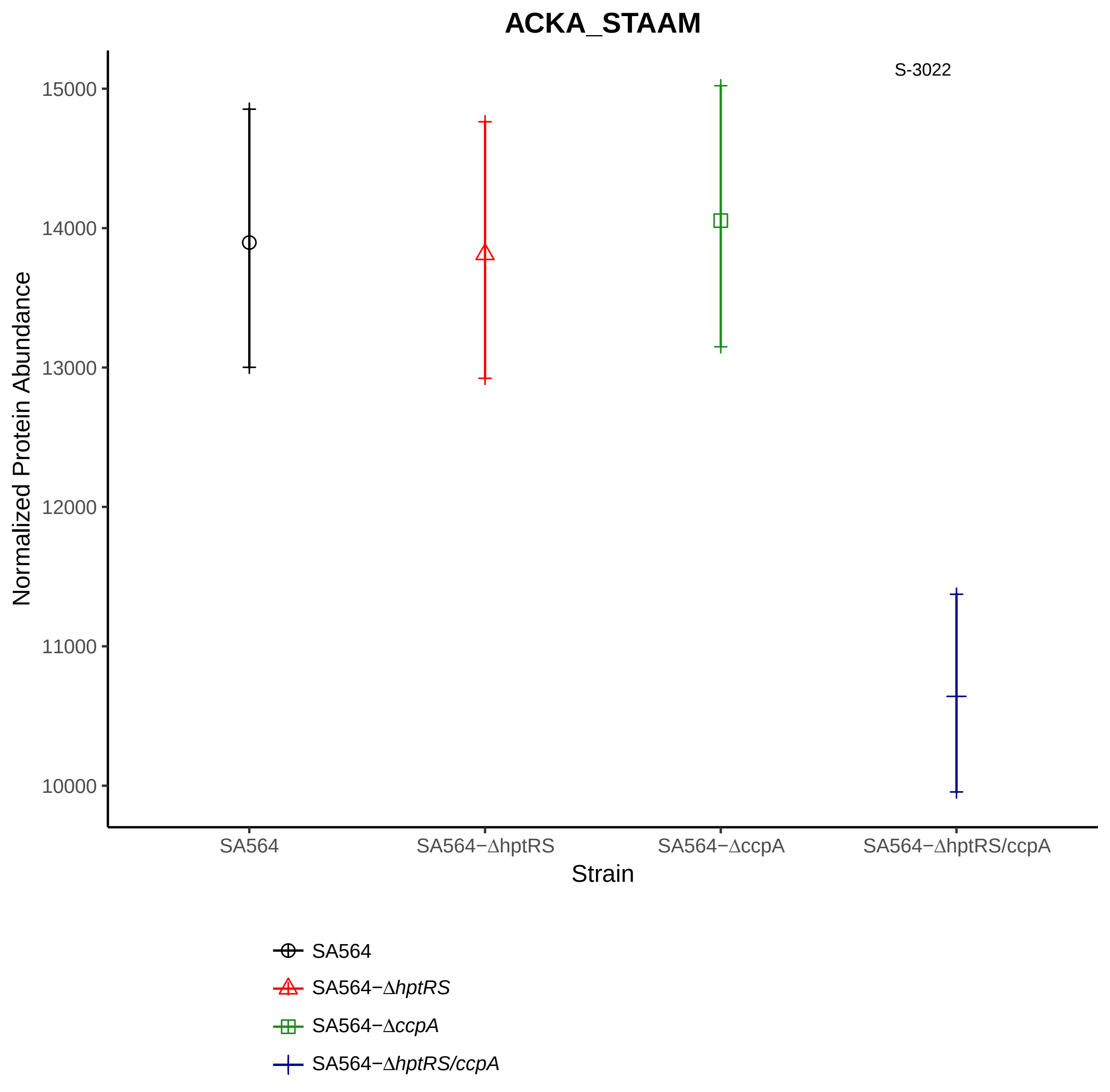

ACKA_STAAM
S-3022
15000
14000
13000
12000
11000
10000
Normalized Protein Abundance
SA564
SA564−ΔhptRS
SA564−ΔccpA
SA564−ΔhptRS/ccpA
Strain
SA564
SA564−Δ*hptRS*
SA564−Δ*ccpA*
SA564−Δ*hptRS/ccpA*

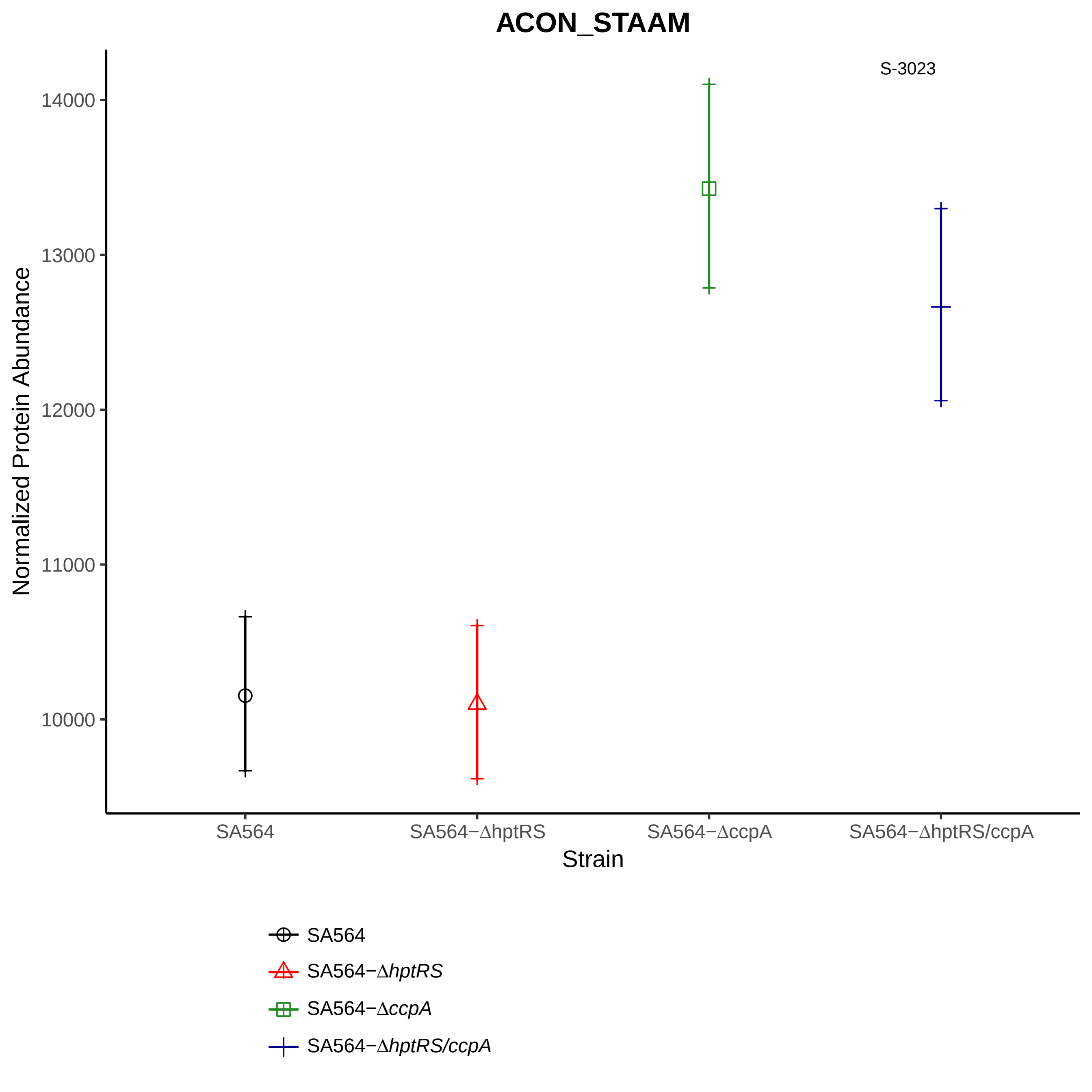

ACON_STAAM
S-3023
Normalized Protein Abundance
14000
13000
12000
11000
10000
SA564
SA564−ΔhptRS
SA564−ΔccpA
SA564−ΔhptRS/ccpA
Strain
SA564
SA564−ΔhptRS
SA564−ΔccpA
SA564−ΔhptRS/ccpA

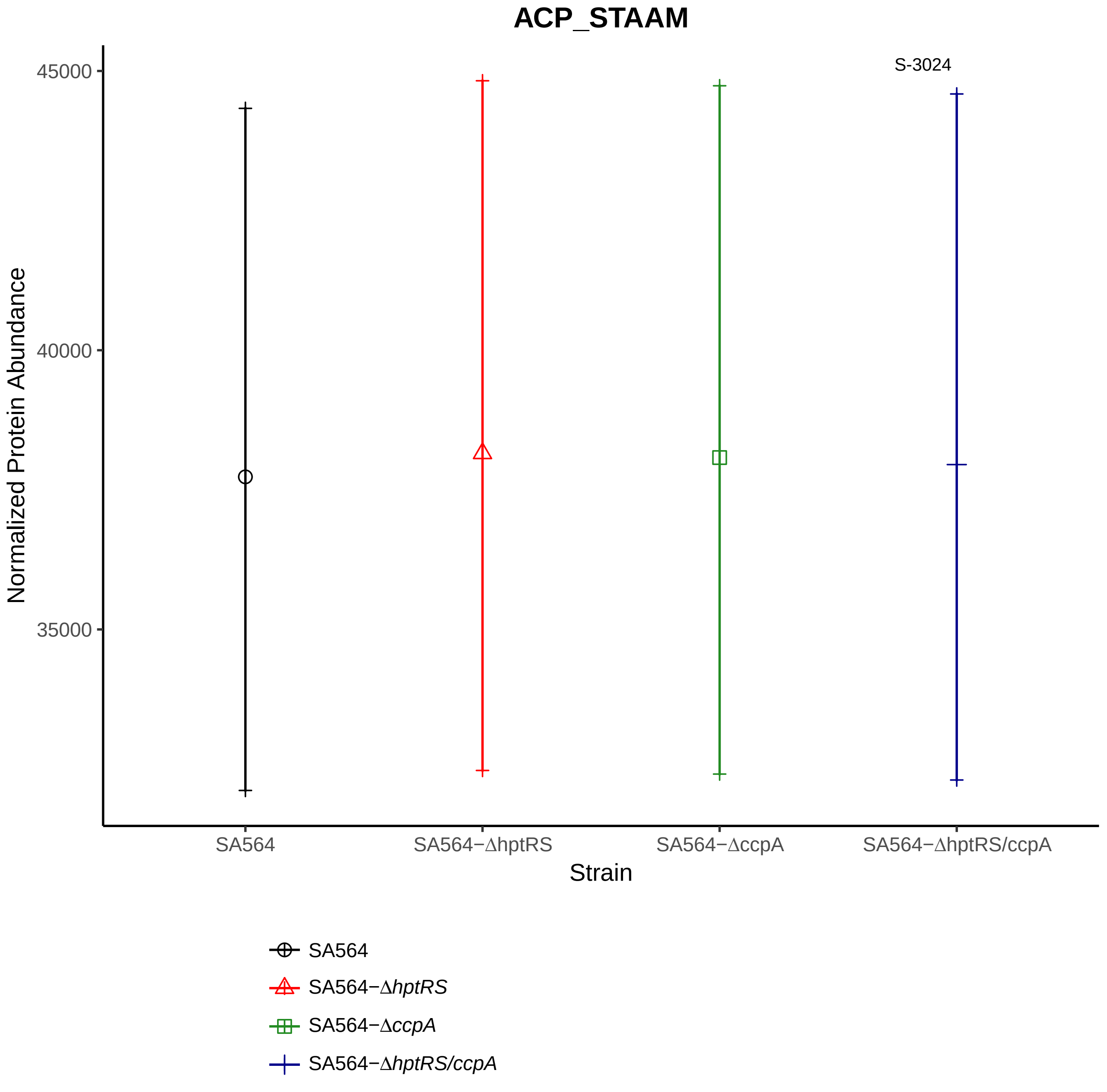
ACP_STAAM
S-3024
Normalized Protein Abundance
45000
40000
35000
SA564
SA564−ΔhptRS
SA564−ΔccpA
SA564−ΔhptRS/ccpA
Strain
SA564
SA564−ΔhptRS
SA564−ΔccpA
SA564−ΔhptRS/ccpA

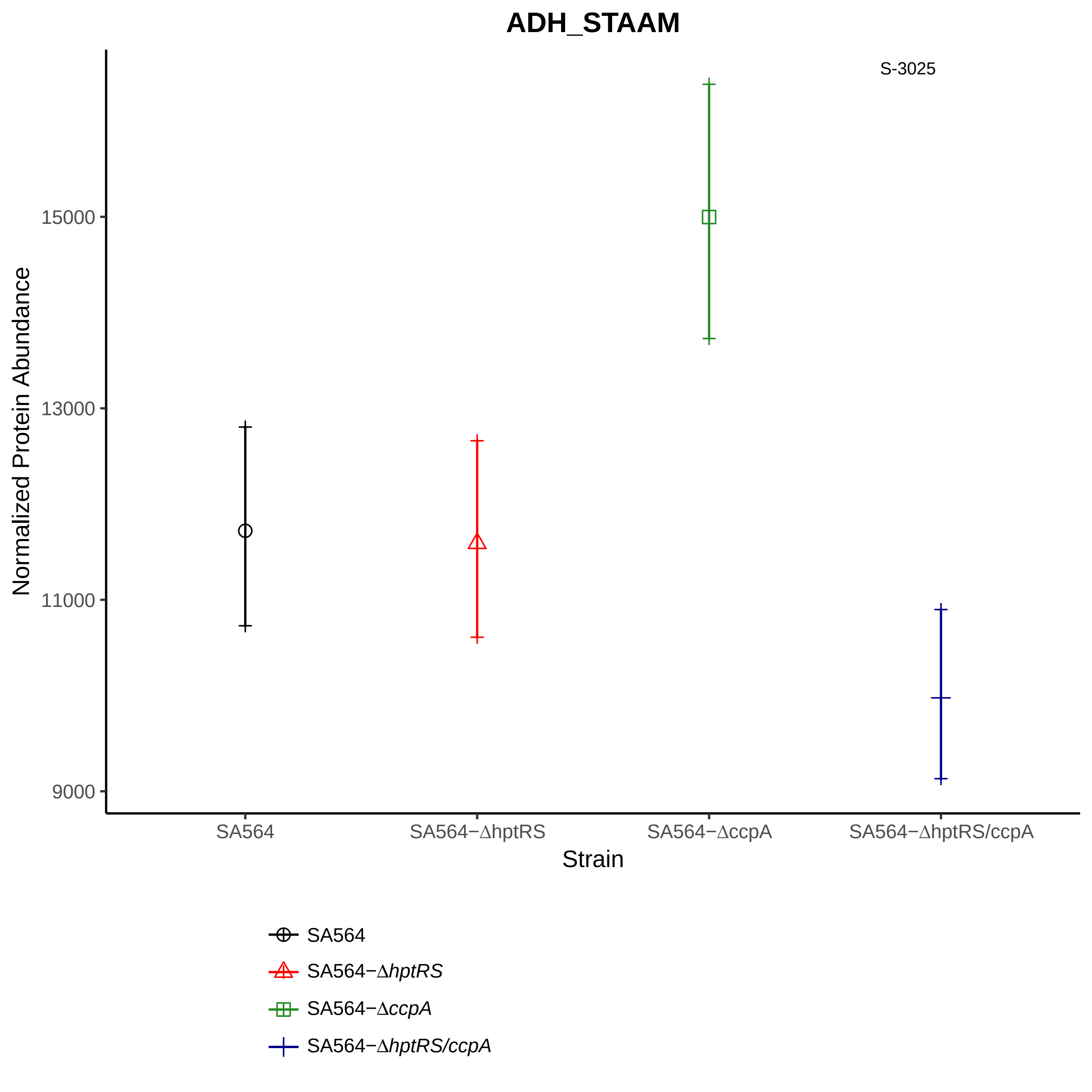

ADH_STAAM
S-3025
Normalized Protein Abundance
15000
13000
11000
9000
SA564
SA564−ΔhptRS
SA564−ΔccpA
SA564−ΔhptRS/ccpA
Strain
SA564
SA564−ΔhptRS
SA564−ΔccpA
SA564−ΔhptRS/ccpA

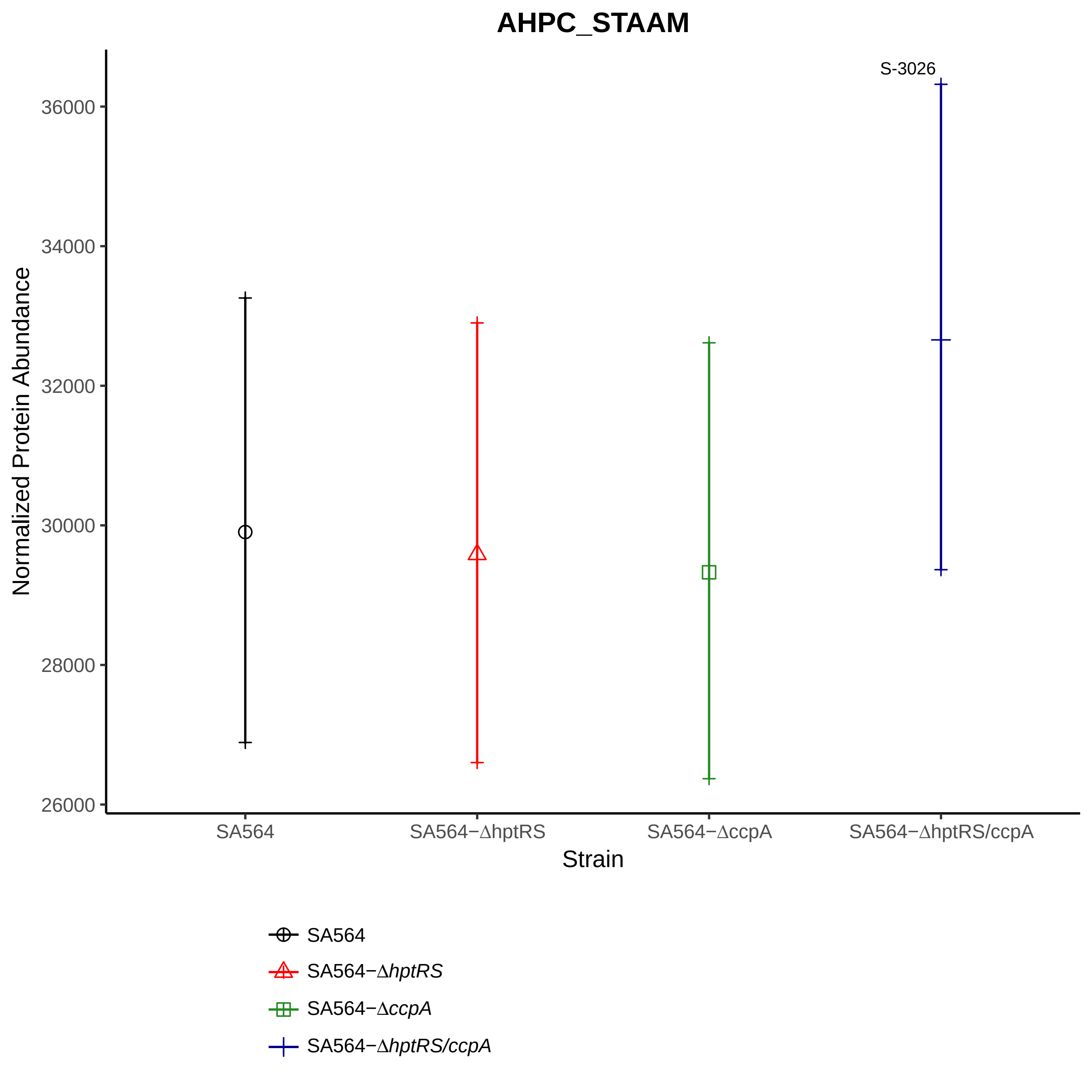
AHPC_STAAM
S-3026
Normalized Protein Abundance
36000
34000
32000
30000
28000
26000
SA564
SA564−ΔhptRS
SA564−ΔccpA
SA564−ΔhptRS/ccpA
Strain
SA564
SA564−ΔhptRS
SA564−ΔccpA
SA564−ΔhptRS/ccpA

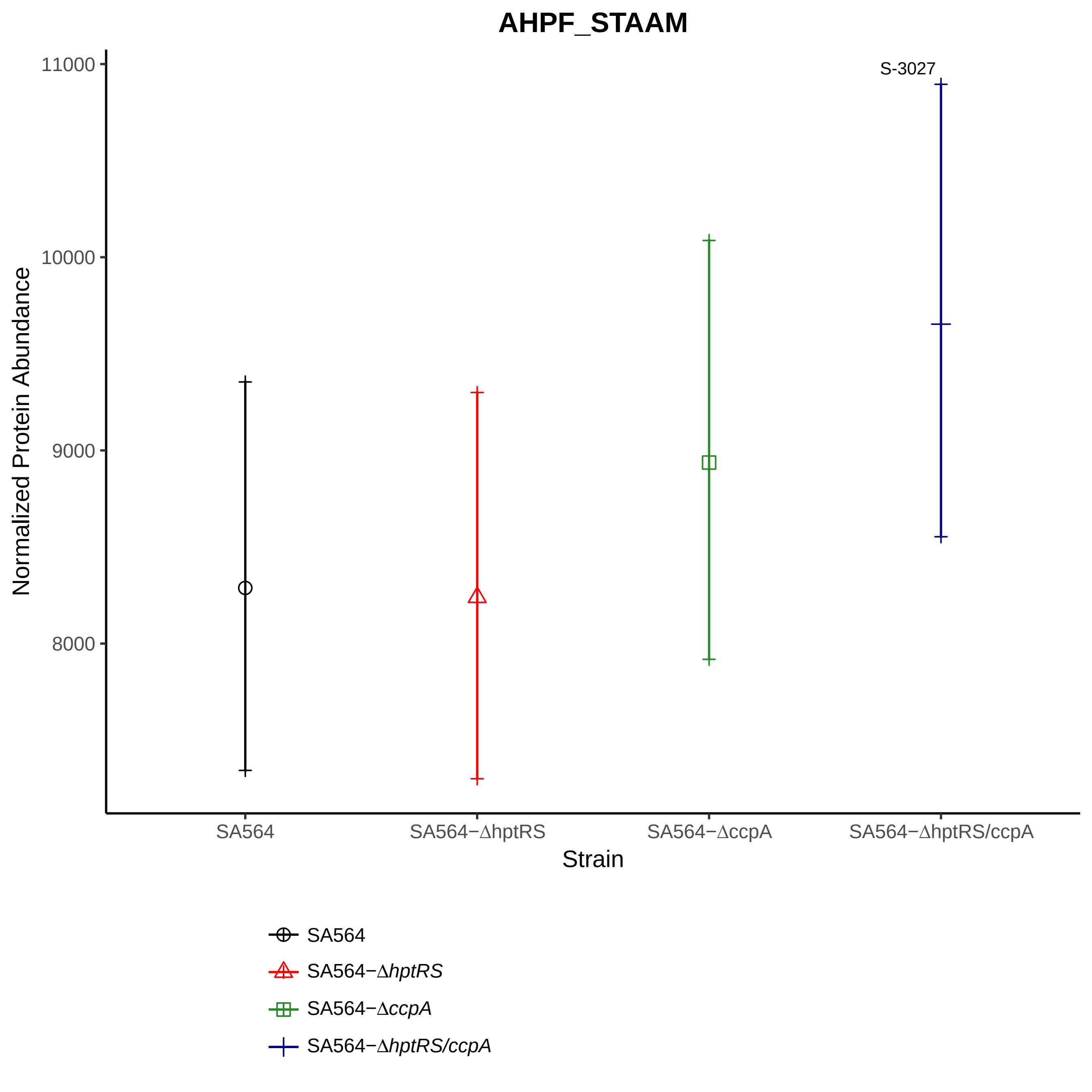
AHPF_STAAM
S-3027
11000
10000
9000
8000
Normalized Protein Abundance
SA564
SA564−ΔhptRS
SA564−ΔccpA
SA564−ΔhptRS/ccpA
Strain
SA564
SA564−ΔhptRS
SA564−ΔccpA
SA564−ΔhptRS/ccpA

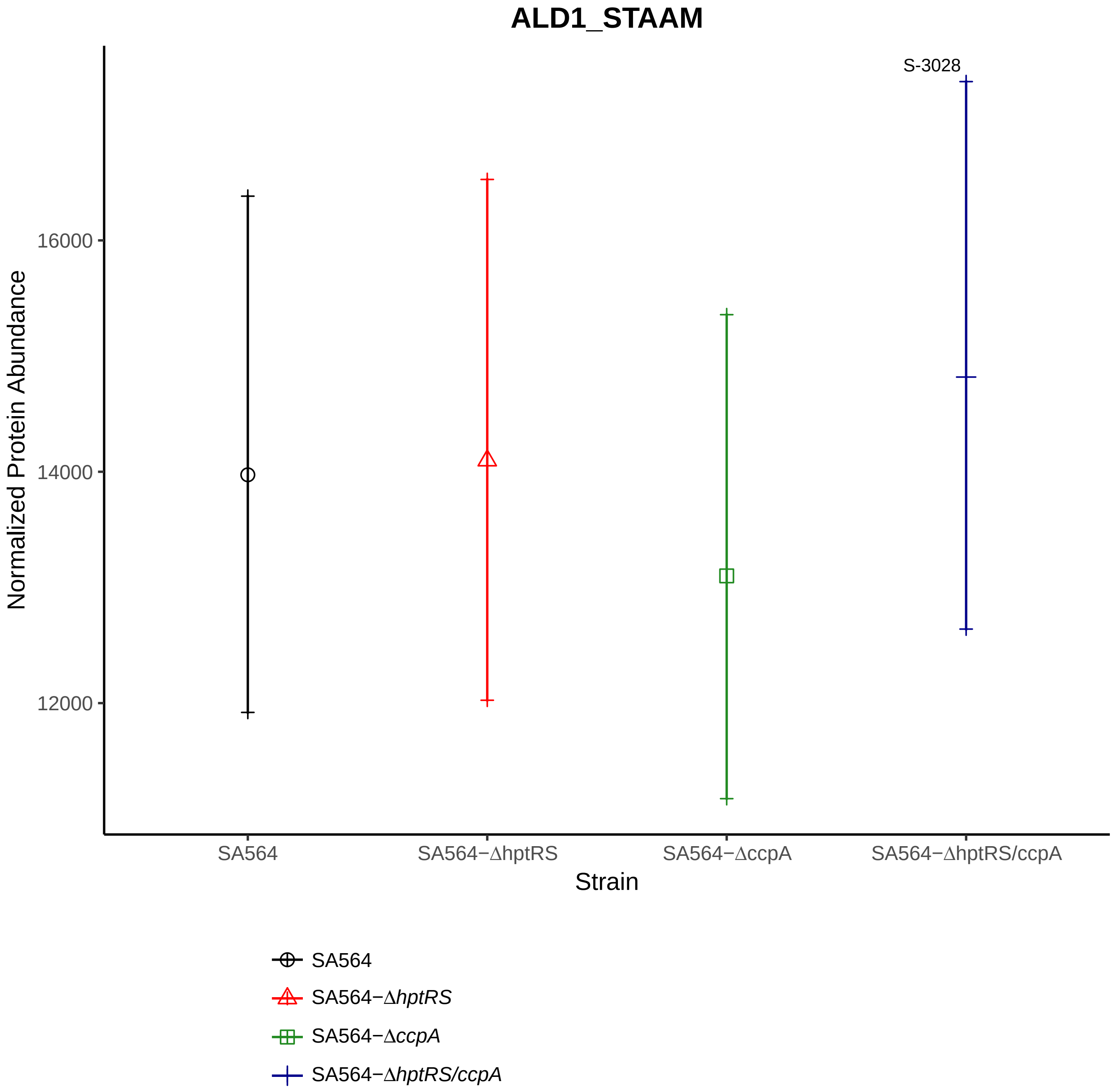

ALD1_STAAM
S-3028
16000
14000
12000
Normalized Protein Abundance
SA564
SA564−ΔhptRS
SA564−ΔccpA
SA564−ΔhptRS/ccpA
Strain
SA564
SA564−Δ*hptRS*
SA564−Δ*ccpA*
SA564−Δ*hptRS/ccpA*

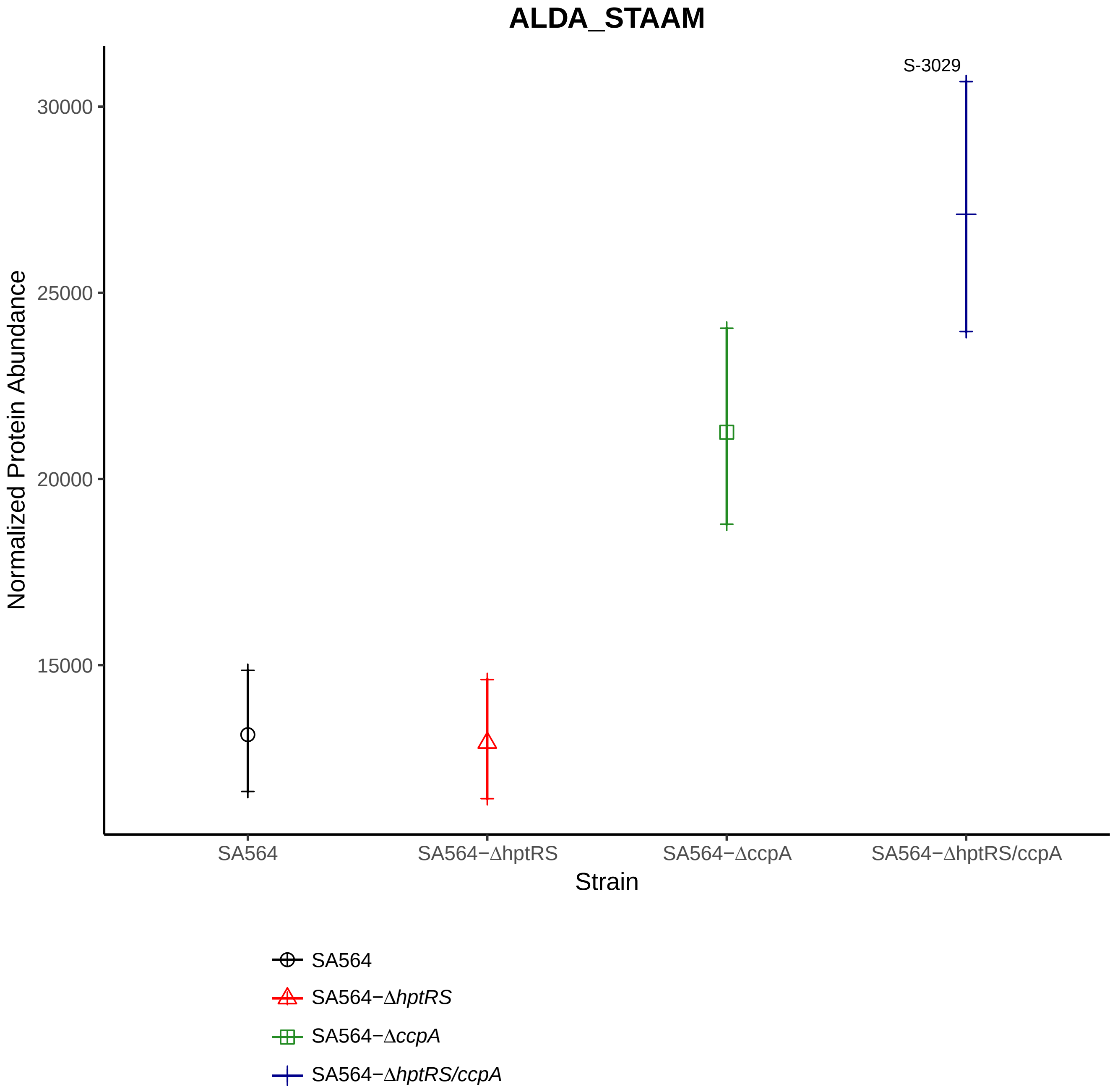
ALDA_STAAM
S-3029
30000
25000
20000
15000
Normalized Protein Abundance
SA564
SA564−ΔhptRS
SA564−ΔccpA
SA564−ΔhptRS/ccpA
Strain
SA564
SA564−ΔhptRS
SA564−ΔccpA
SA564−ΔhptRS/ccpA

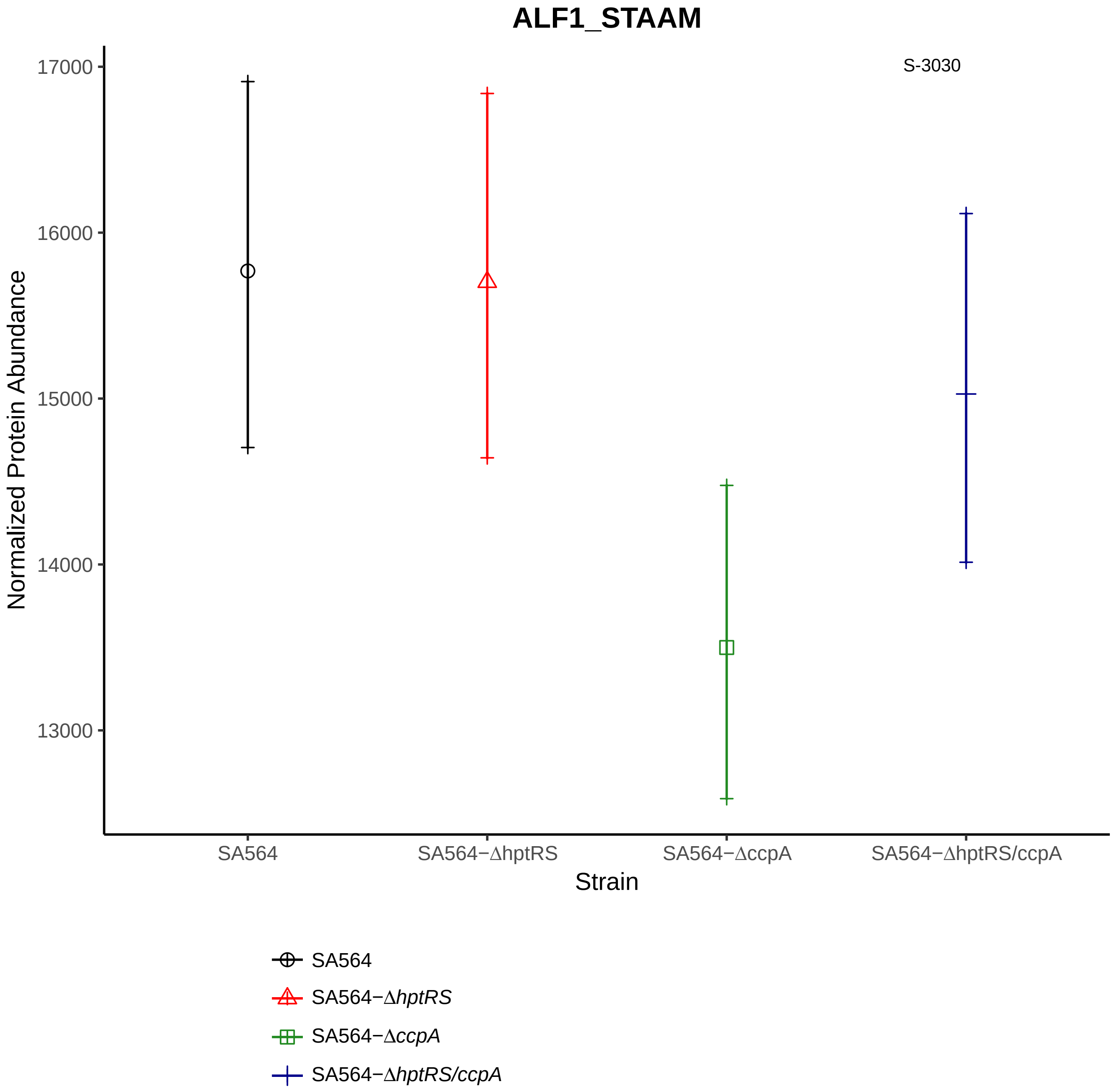
ALF1_STAAM
S-3030
Normalized Protein Abundance
17000
16000
15000
14000
13000
SA564
SA564−ΔhptRS
SA564−ΔccpA
SA564−ΔhptRS/ccpA
Strain
SA564
SA564−ΔhptRS
SA564−ΔccpA
SA564−ΔhptRS/ccpA

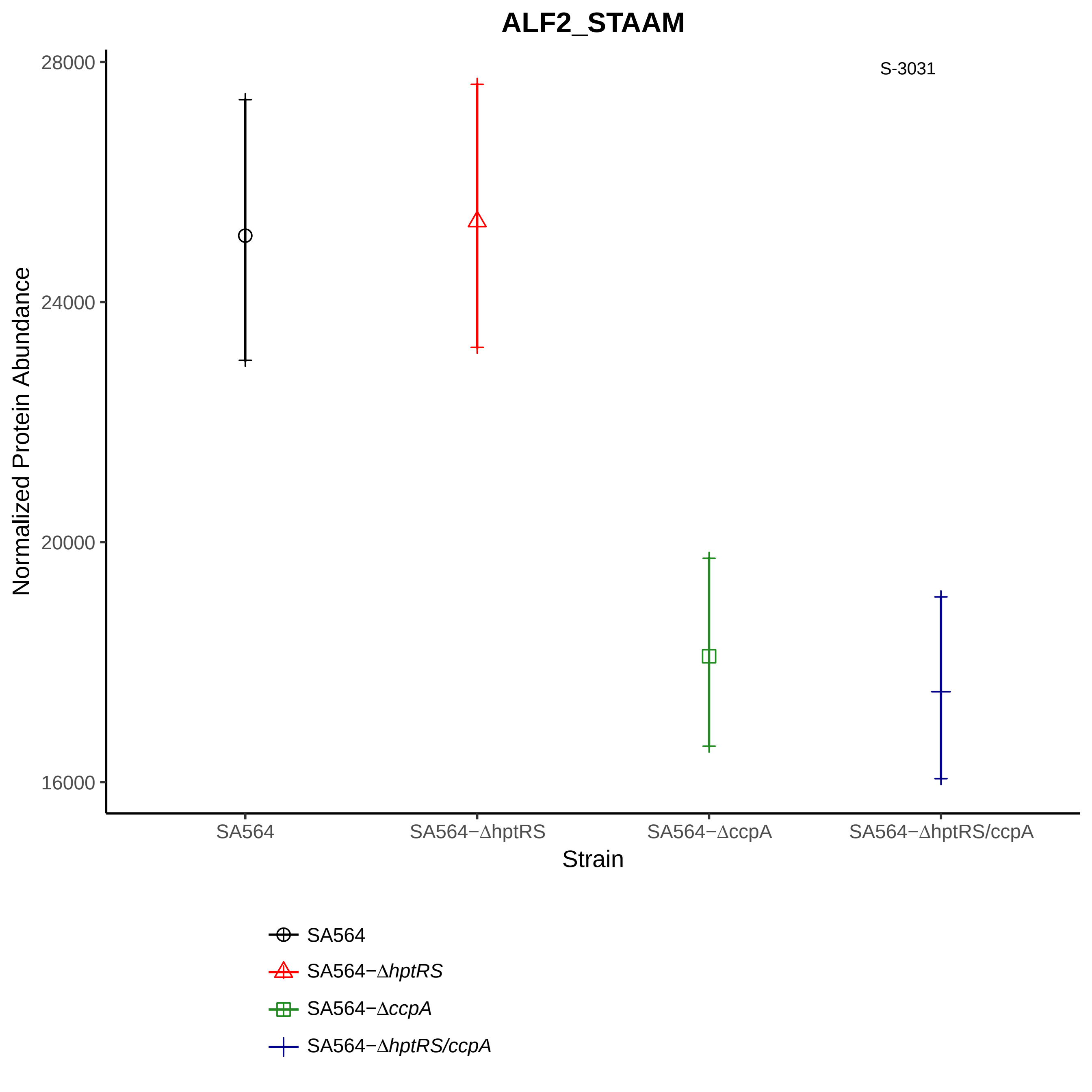

ALF2_STAAM
S-3031
28000
24000
20000
16000
Normalized Protein Abundance
SA564
SA564−ΔhptRS
SA564−ΔccpA
SA564−ΔhptRS/ccpA
Strain
SA564
SA564−ΔhptRS
SA564−ΔccpA
SA564−ΔhptRS/ccpA

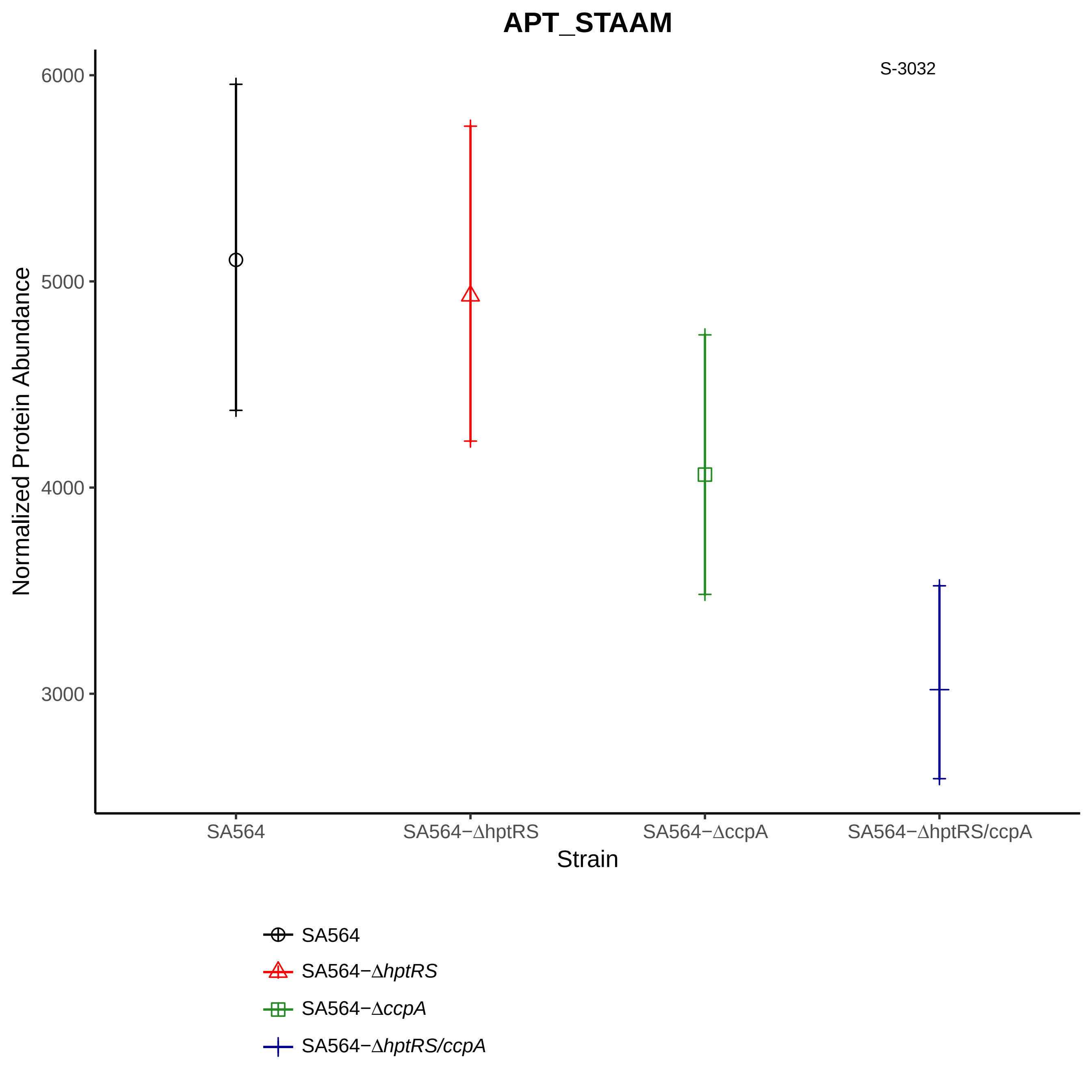

APT_STAAM
S-3032
Normalized Protein Abundance
6000
5000
4000
3000
SA564
SA564−ΔhptRS
SA564−ΔccpA
SA564−ΔhptRS/ccpA
Strain
SA564
SA564−ΔhptRS
SA564−ΔccpA
SA564−ΔhptRS/ccpA

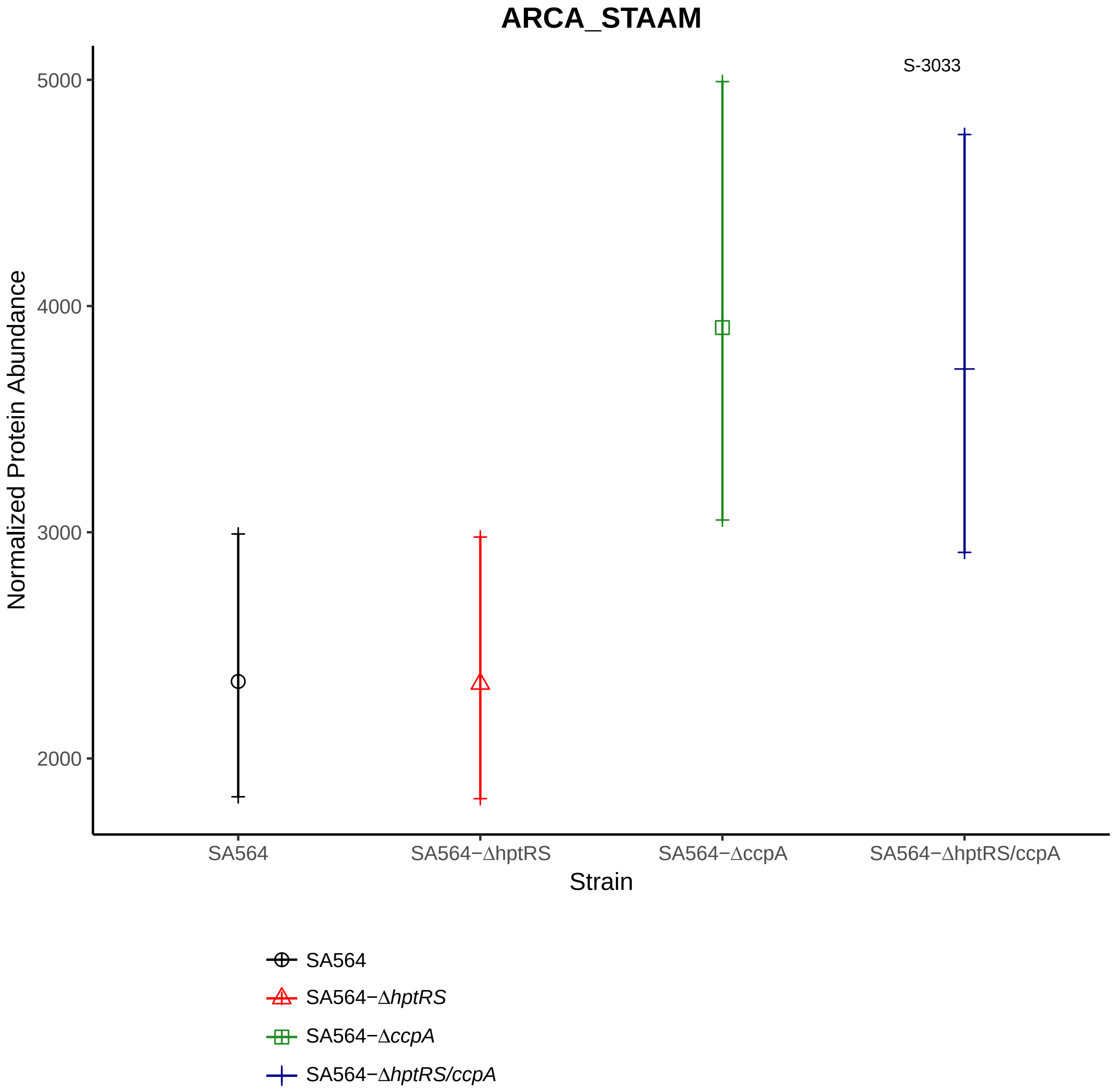

ARCA_STAAM
S-3033
Normalized Protein Abundance
5000
4000
3000
2000
SA564
SA564−ΔhptRS
SA564−ΔccpA
SA564−ΔhptRS/ccpA
Strain
SA564
SA564−ΔhptRS
SA564−ΔccpA
SA564−ΔhptRS/ccpA

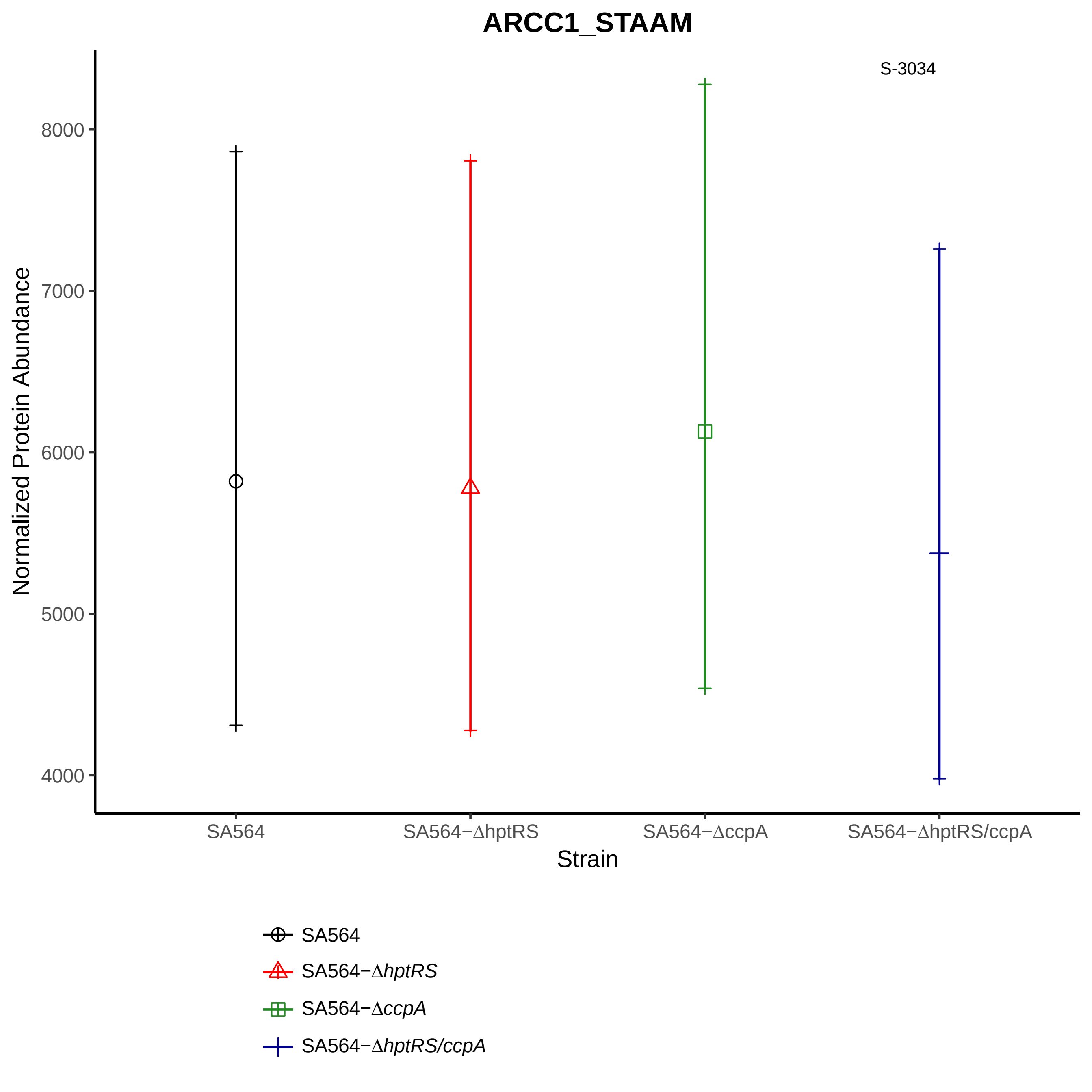

ARCC1_STAAM
S-3034
Normalized Protein Abundance
8000
7000
6000
5000
4000
SA564
SA564−ΔhptRS
SA564−ΔccpA
SA564−ΔhptRS/ccpA
Strain
SA564
SA564−ΔhptRS
SA564−ΔccpA
SA564−ΔhptRS/ccpA

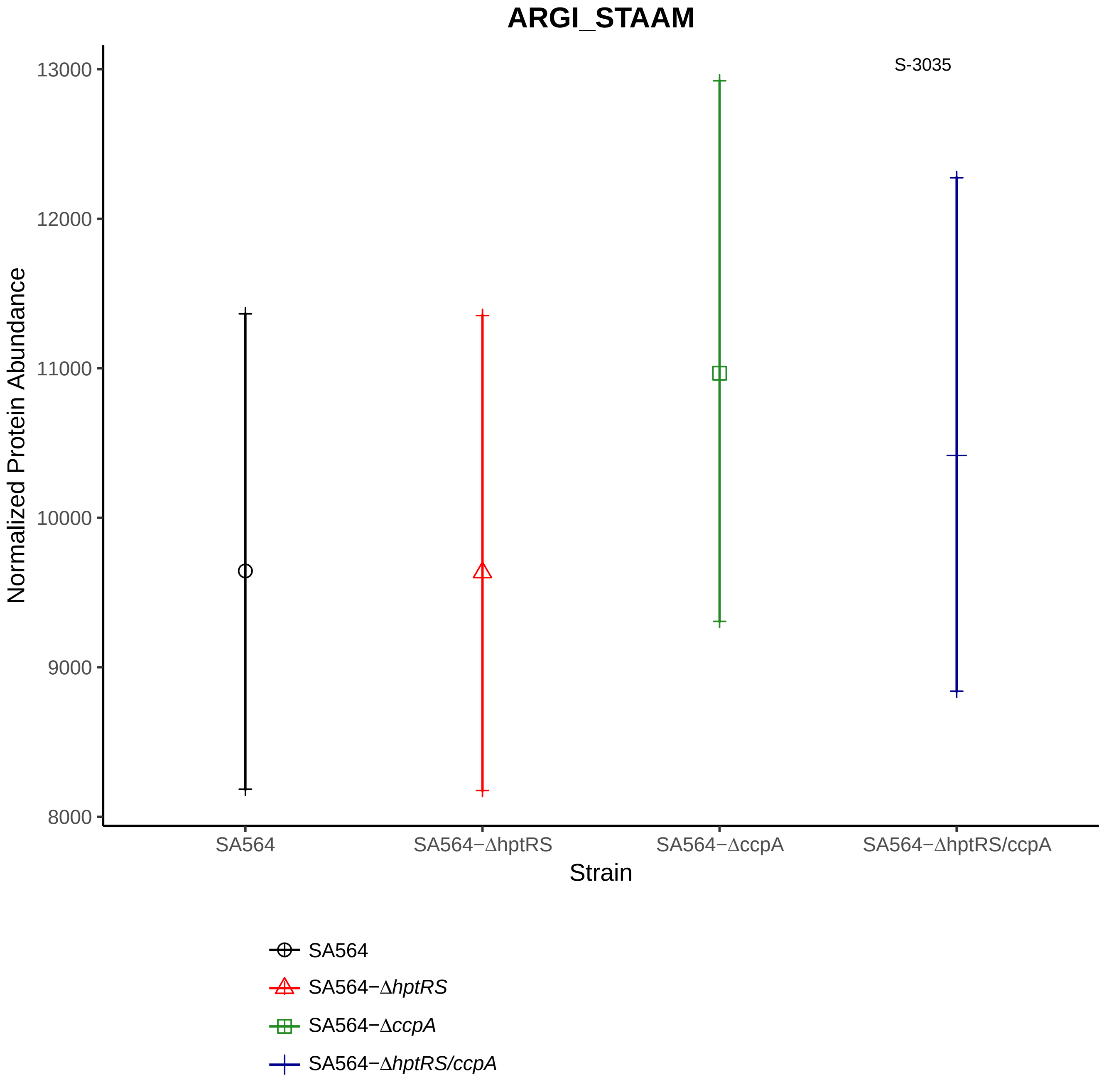
ARGI_STAAM
S-3035
Normalized Protein Abundance
13000
12000
11000
10000
9000
8000
SA564
SA564−ΔhptRS
SA564−ΔccpA
SA564−ΔhptRS/ccpA
Strain
SA564
SA564−ΔhptRS
SA564−ΔccpA
SA564−ΔhptRS/ccpA

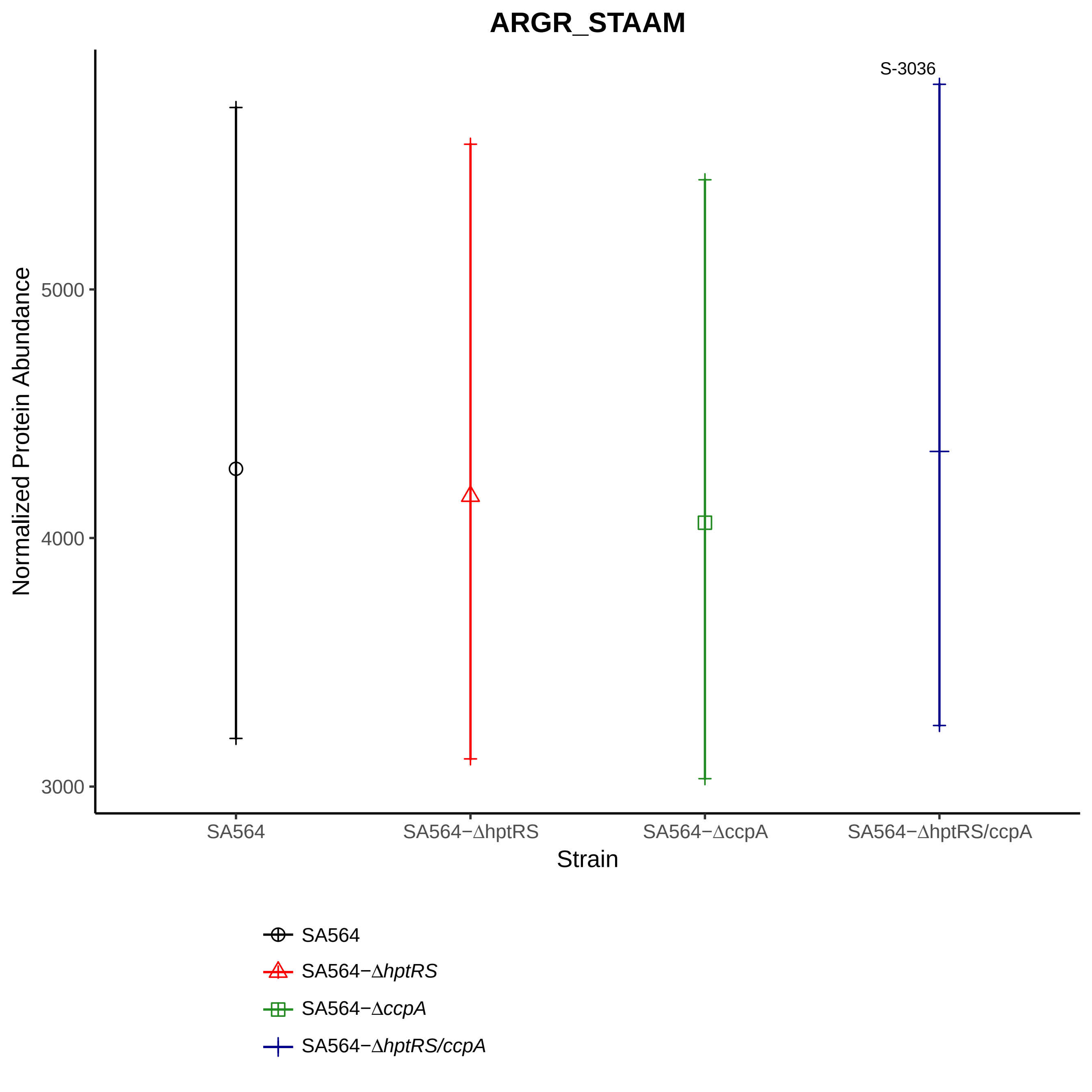

ARGR_STAAM
S-3036
Normalized Protein Abundance
5000
4000
3000
SA564
SA564−ΔhptRS
SA564−ΔccpA
SA564−ΔhptRS/ccpA
Strain
SA564
SA564−ΔhptRS
SA564−ΔccpA
SA564−ΔhptRS/ccpA

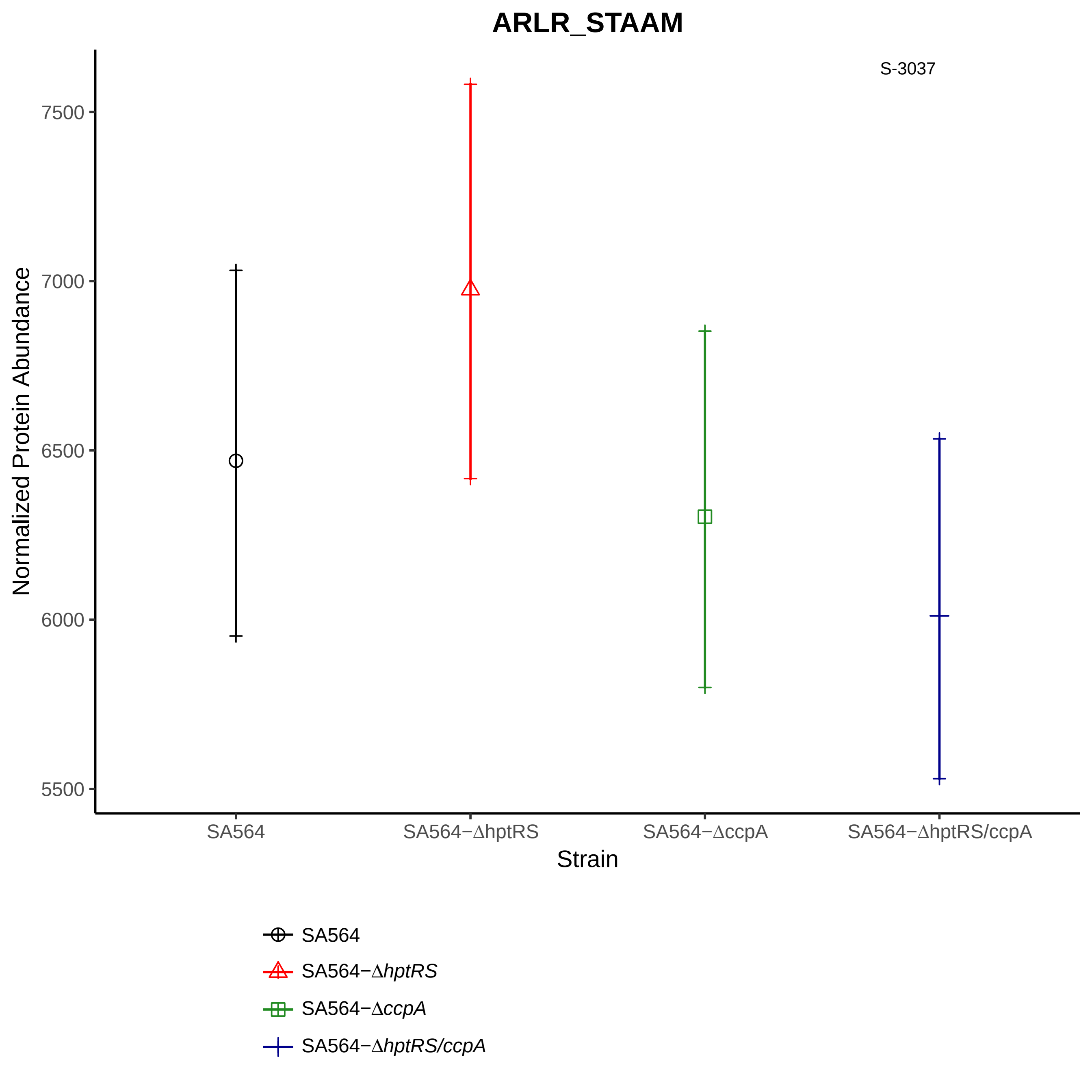
ARLR_STAAM
S-3037
Normalized Protein Abundance
7500
7000
6500
6000
5500
SA564
SA564−ΔhptRS
SA564−ΔccpA
SA564−ΔhptRS/ccpA
Strain
SA564
SA564−ΔhptRS
SA564−ΔccpA
SA564−ΔhptRS/ccpA

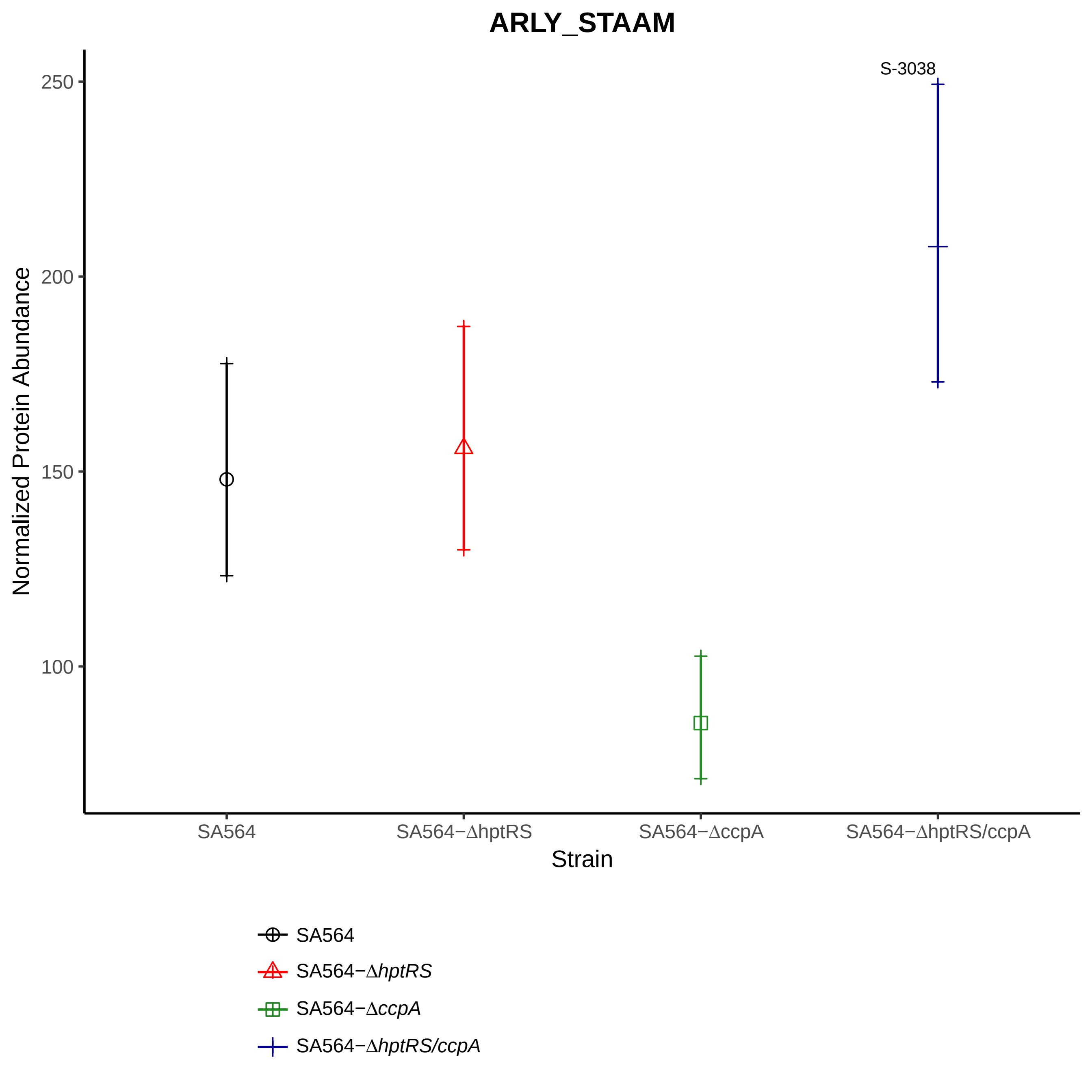
ARLY_STAAM
S-3038
Normalized Protein Abundance
250
200
150
100
SA564
SA564−ΔhptRS
SA564−ΔccpA
SA564−ΔhptRS/ccpA
Strain
SA564
SA564−Δ*hptRS*
SA564−Δ*ccpA*
SA564−Δ*hptRS/ccpA*

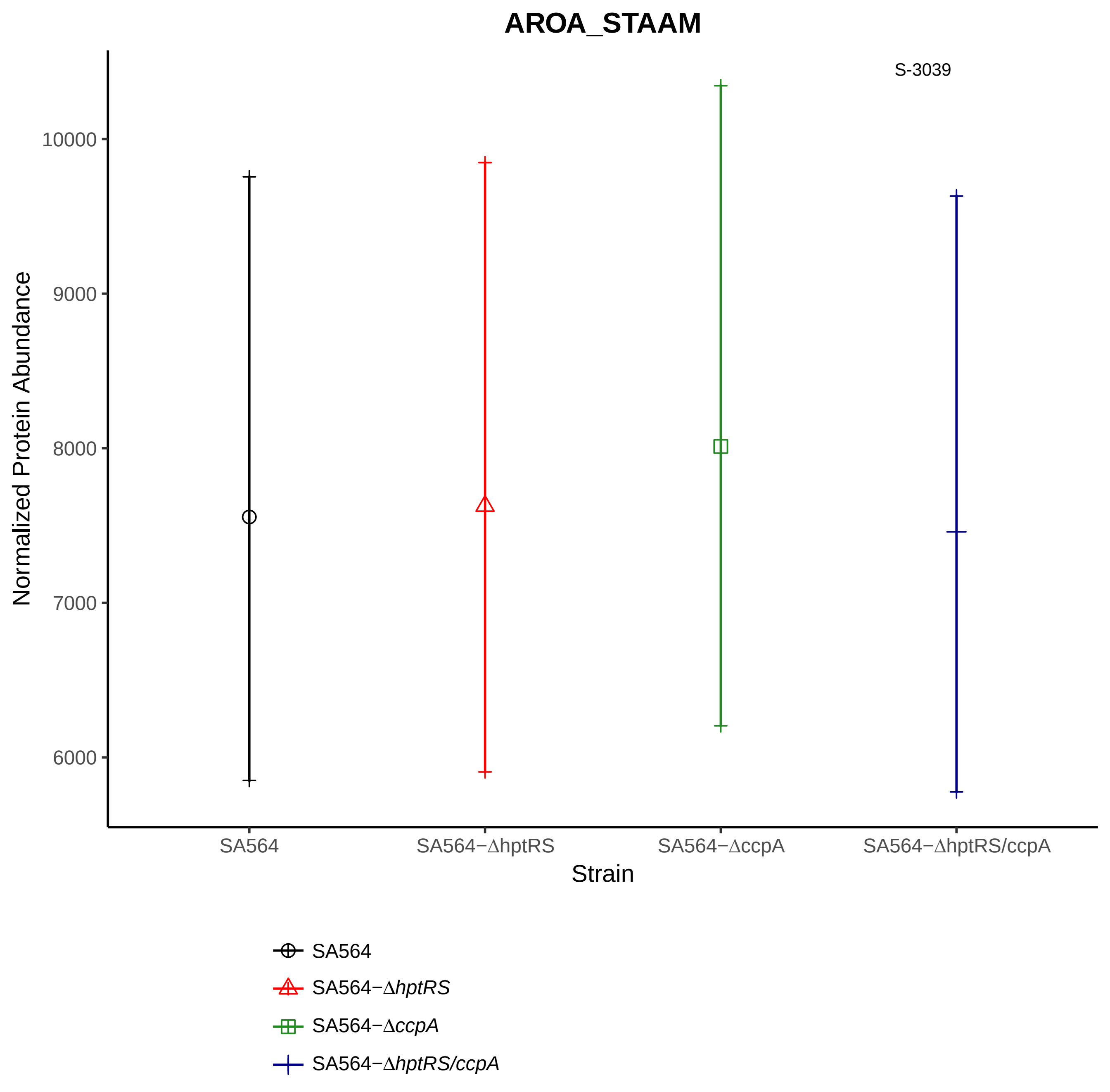
AROA_STAAM
S-3039
Normalized Protein Abundance
10000
9000
8000
7000
6000
SA564
SA564−ΔhptRS
SA564−ΔccpA
SA564−ΔhptRS/ccpA
Strain
SA564
SA564−ΔhptRS
SA564−ΔccpA
SA564−ΔhptRS/ccpA

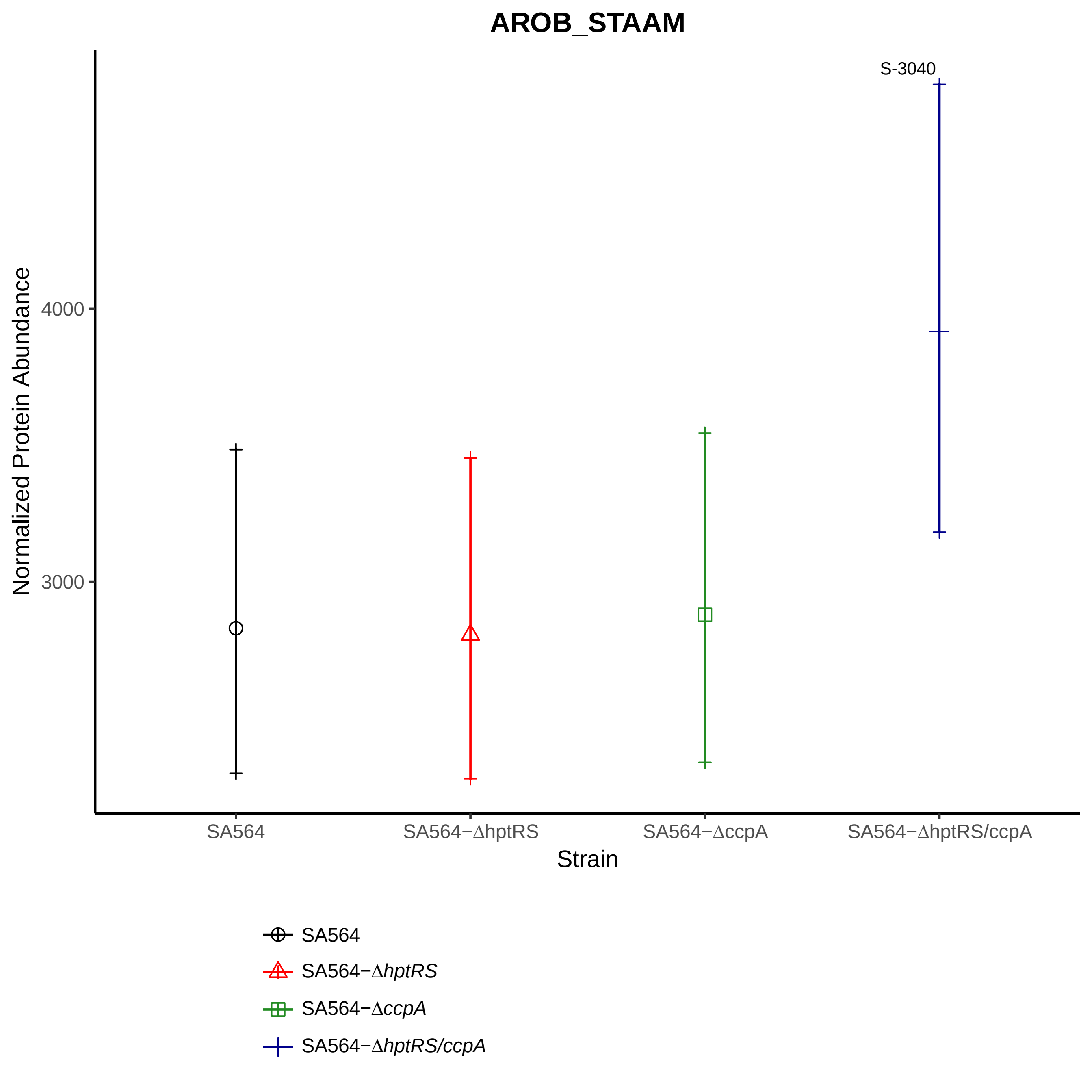

AROB_STAAM
S-3040
Normalized Protein Abundance
4000
3000
SA564
SA564−ΔhptRS
SA564−ΔccpA
SA564−ΔhptRS/ccpA
Strain
SA564
SA564−ΔhptRS
SA564−ΔccpA
SA564−ΔhptRS/ccpA

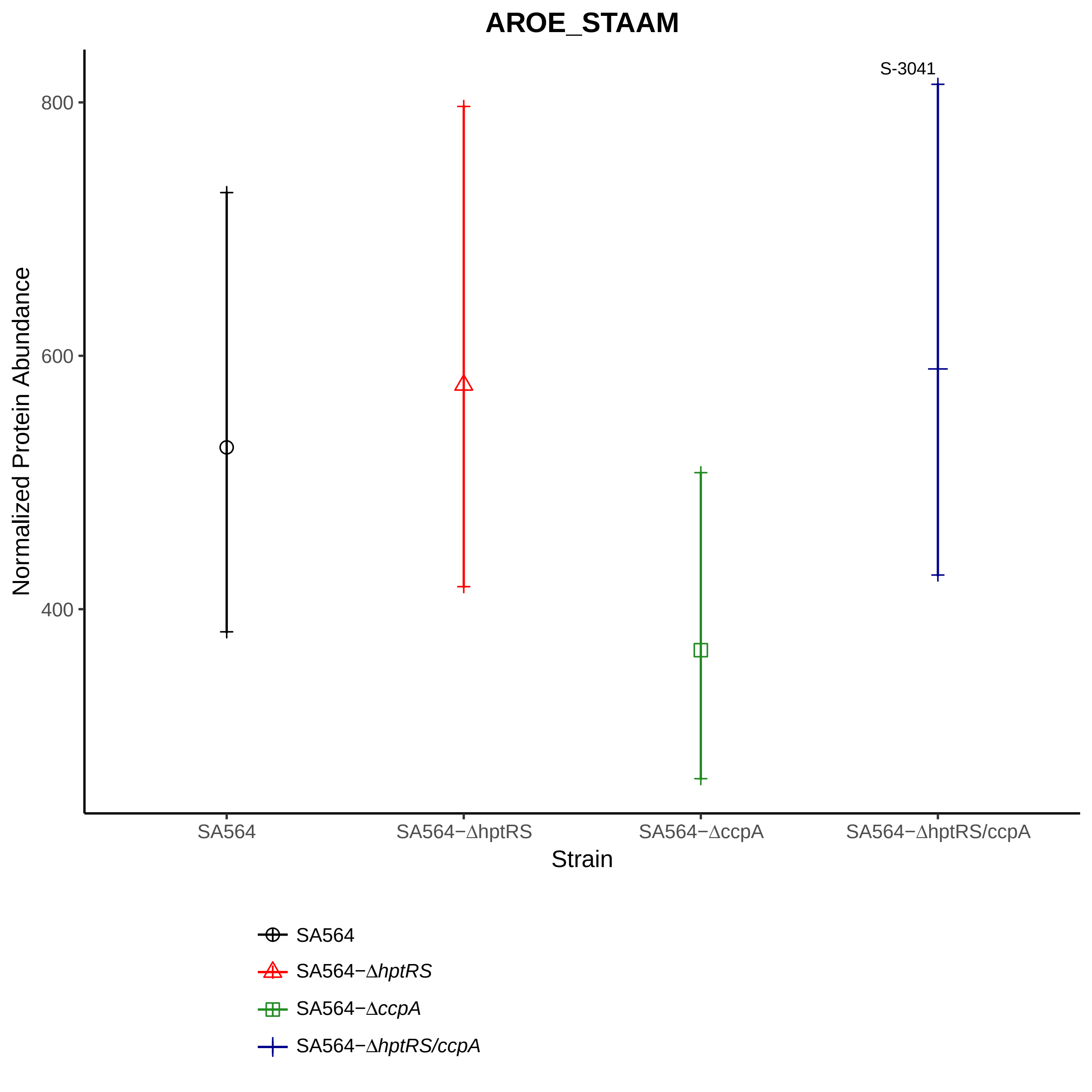

AROE_STAAM
S-3041
800
600
400
Normalized Protein Abundance
SA564
SA564−ΔhptRS
SA564−ΔccpA
SA564−ΔhptRS/ccpA
Strain
SA564
SA564−ΔhptRS
SA564−ΔccpA
SA564−ΔhptRS/ccpA

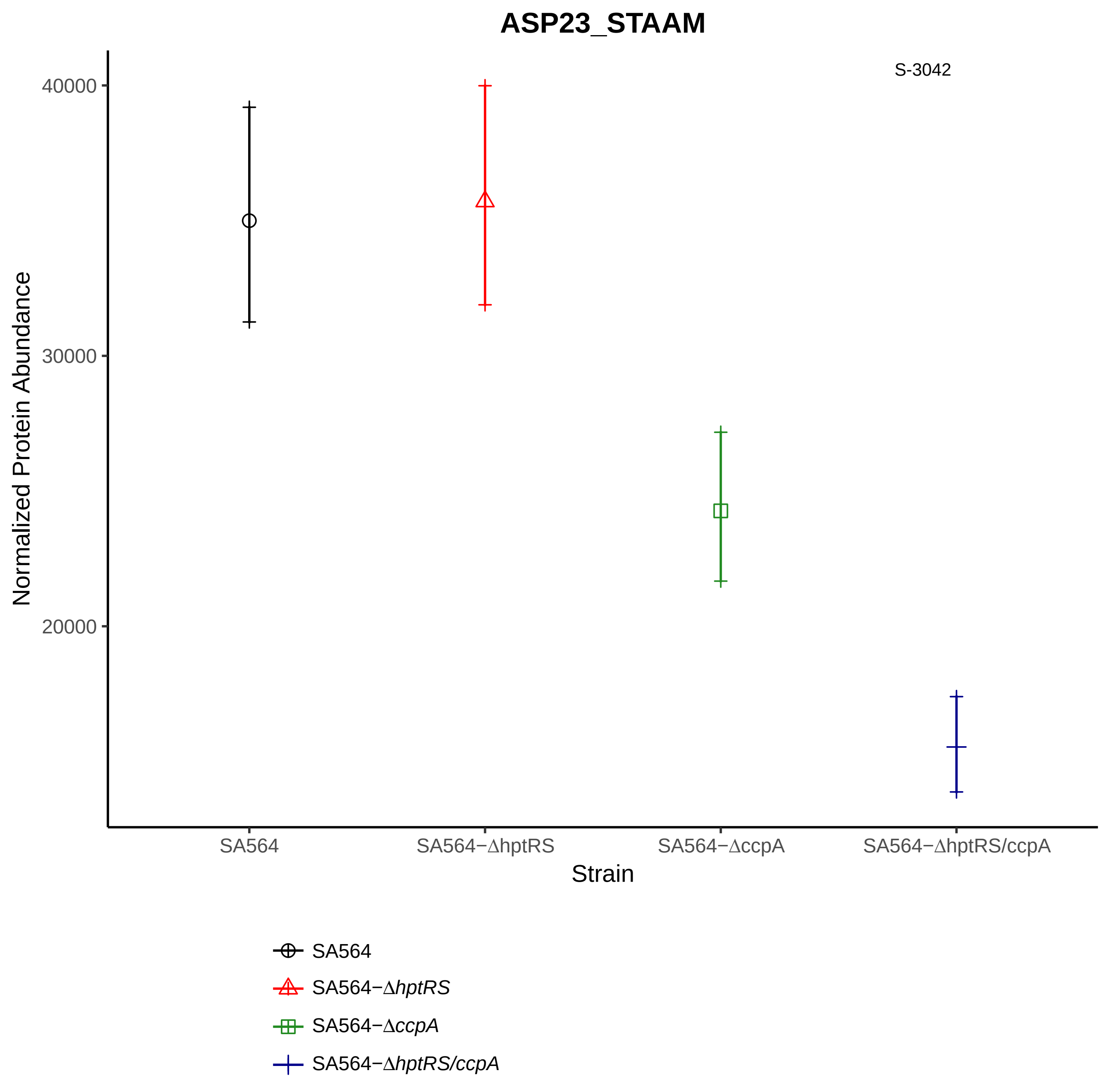

ASP23_STAAM
S-3042
40000
30000
20000
Normalized Protein Abundance
SA564
SA564−ΔhptRS
SA564−ΔccpA
SA564−ΔhptRS/ccpA
Strain
SA564
SA564−ΔhptRS
SA564−ΔccpA
SA564−ΔhptRS/ccpA

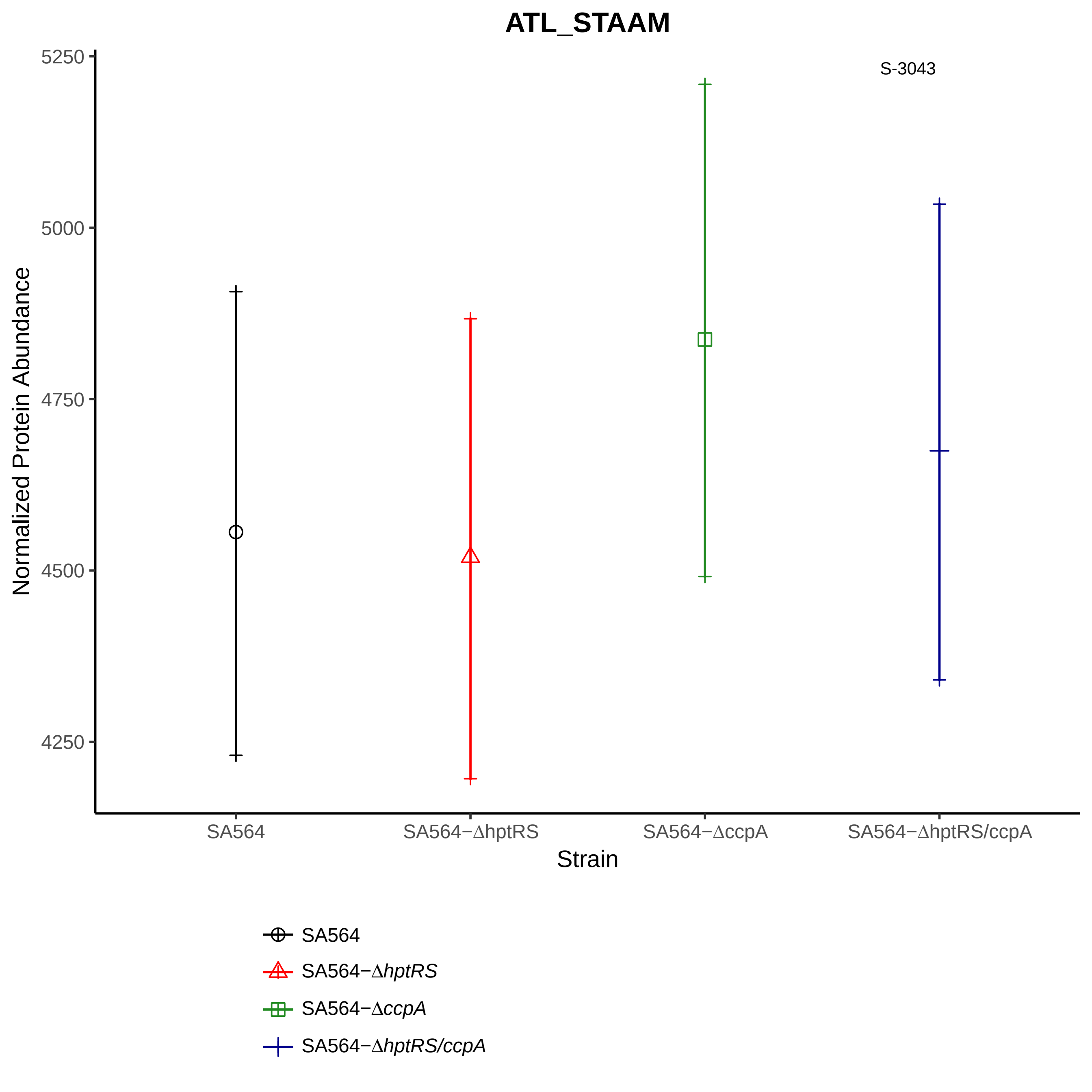

ATL_STAAM
S-3043
Normalized Protein Abundance
5250
5000
4750
4500
4250
SA564
SA564−ΔhptRS
SA564−ΔccpA
SA564−ΔhptRS/ccpA
Strain
SA564
SA564−Δ*hptRS*
SA564−Δ*ccpA*
SA564−Δ*hptRS/ccpA*

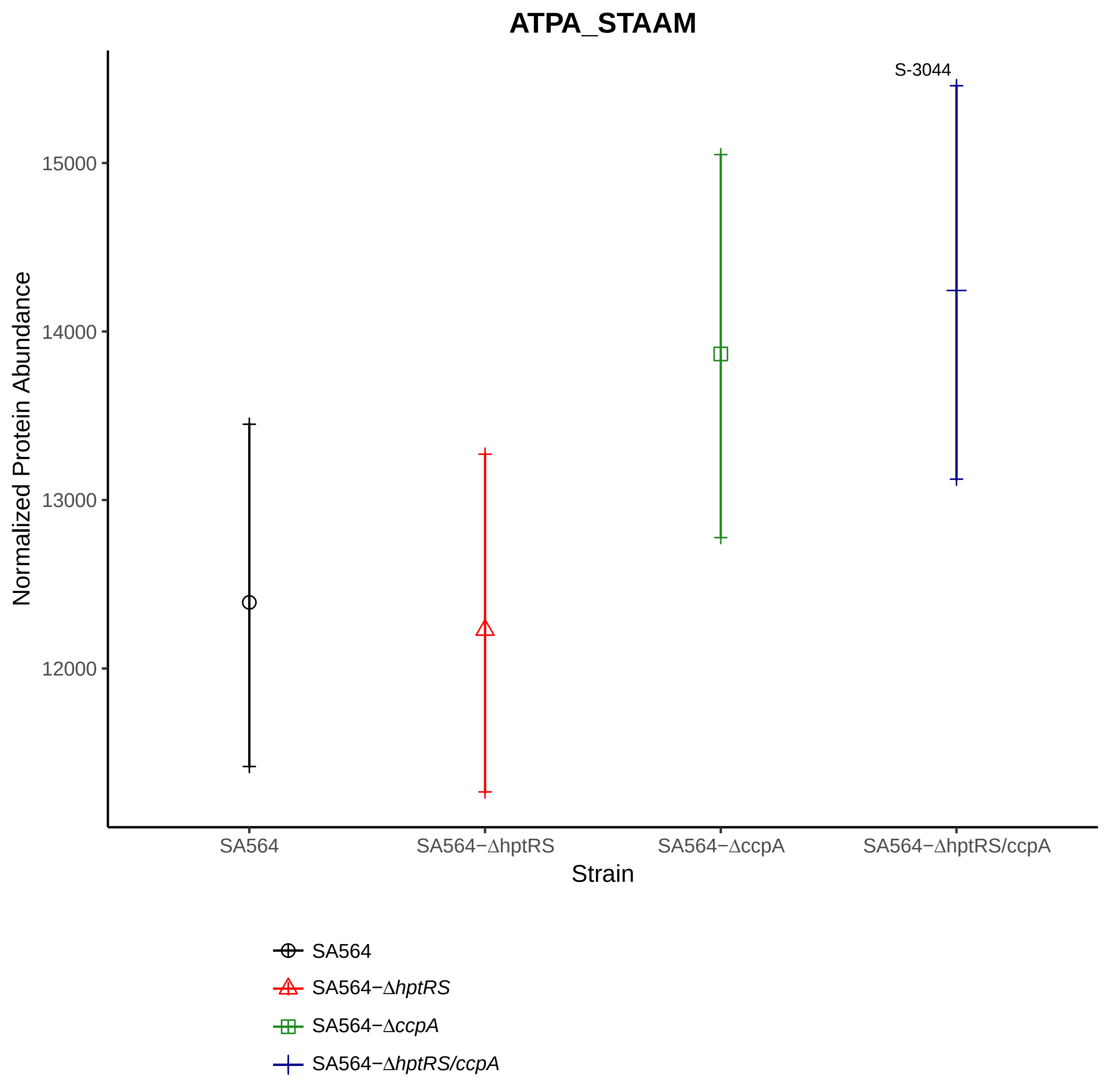

ATPA_STAAM
S-3044
Normalized Protein Abundance
15000
14000
13000
12000
SA564
SA564−ΔhptRS
SA564−ΔccpA
SA564−ΔhptRS/ccpA
Strain
SA564
SA564−Δ*hptRS*
SA564−Δ*ccpA*
SA564−Δ*hptRS/ccpA*

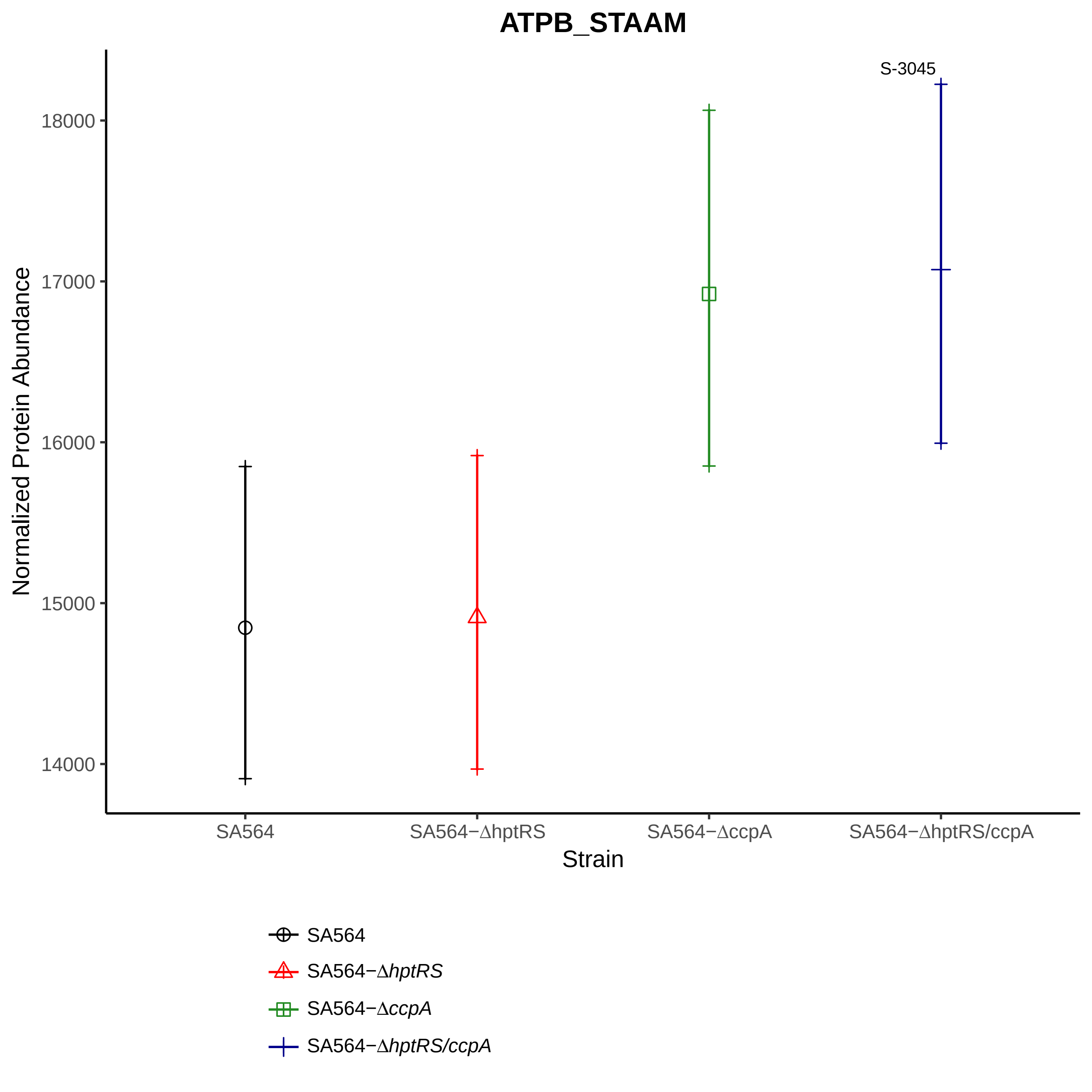

ATPB_STAAM
S-3045
Normalized Protein Abundance
18000
17000
16000
15000
14000
SA564
SA564−ΔhptRS
SA564−ΔccpA
SA564−ΔhptRS/ccpA
Strain
SA564
SA564−ΔhptRS
SA564−ΔccpA
SA564−ΔhptRS/ccpA

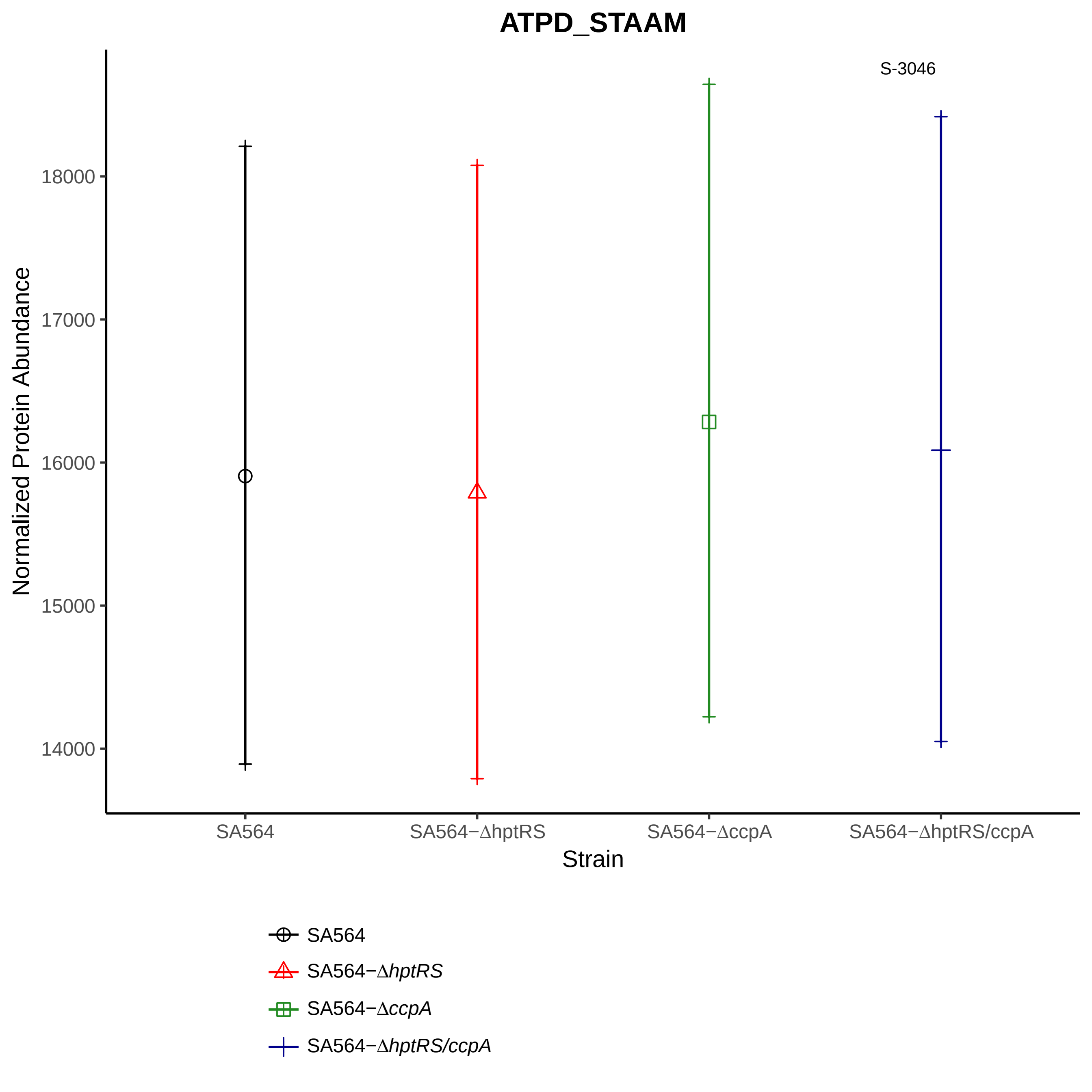

ATPD_STAAM
S-3046
Normalized Protein Abundance
18000
17000
16000
15000
14000
SA564
SA564−ΔhptRS
SA564−ΔccpA
SA564−ΔhptRS/ccpA
Strain
SA564
SA564−ΔhptRS
SA564−ΔccpA
SA564−ΔhptRS/ccpA

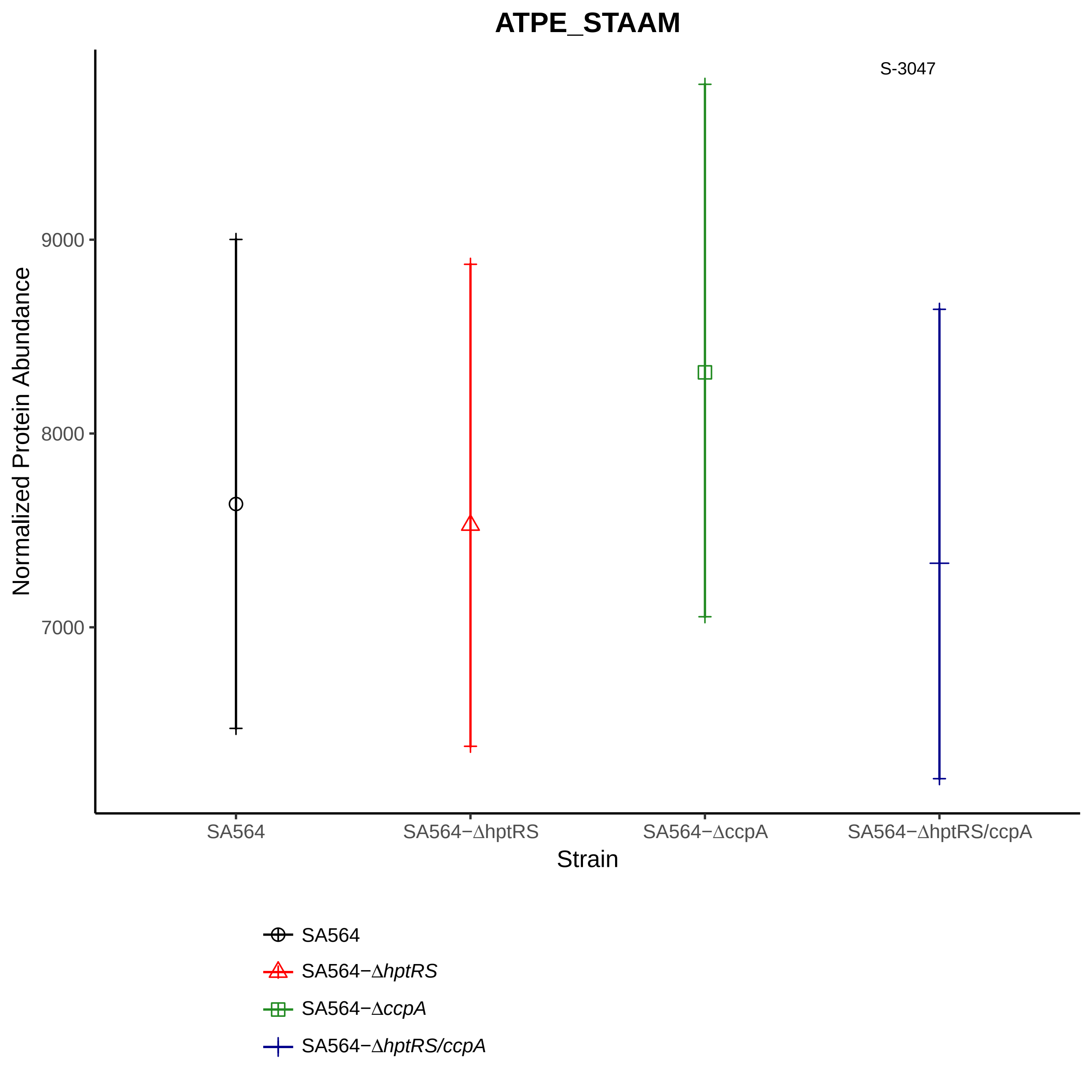
ATPE_STAAM
S-3047
Normalized Protein Abundance
9000
8000
7000
SA564
SA564−ΔhptRS
SA564−ΔccpA
SA564−ΔhptRS/ccpA
Strain
SA564
SA564−Δ*hptRS*
SA564−Δ*ccpA*
SA564−Δ*hptRS/ccpA*

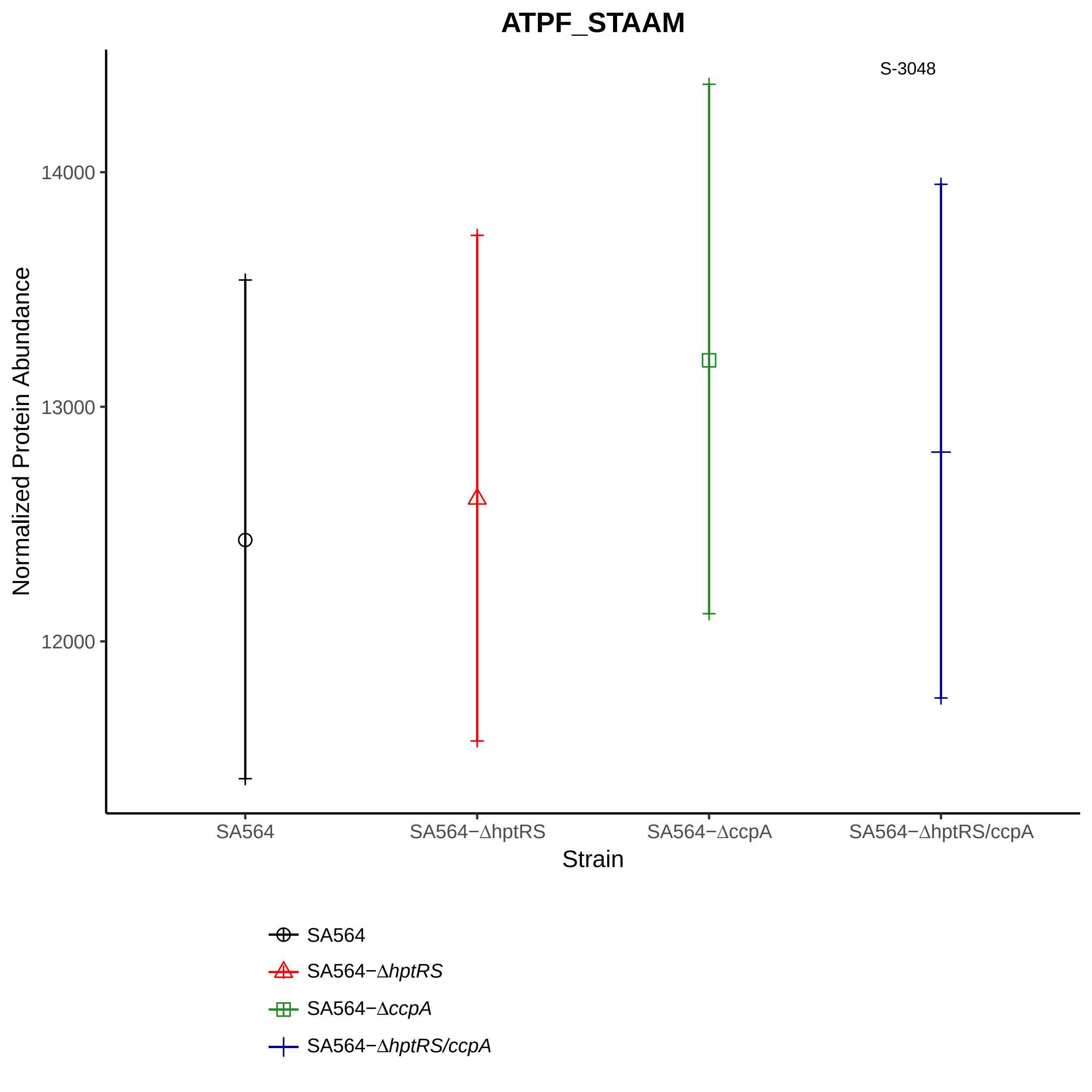

ATPF_STAAM
S-3048
14000
13000
12000
Normalized Protein Abundance
SA564
SA564−ΔhptRS
SA564−ΔccpA
SA564−ΔhptRS/ccpA
Strain
SA564
SA564−ΔhptRS
SA564−ΔccpA
SA564−ΔhptRS/ccpA

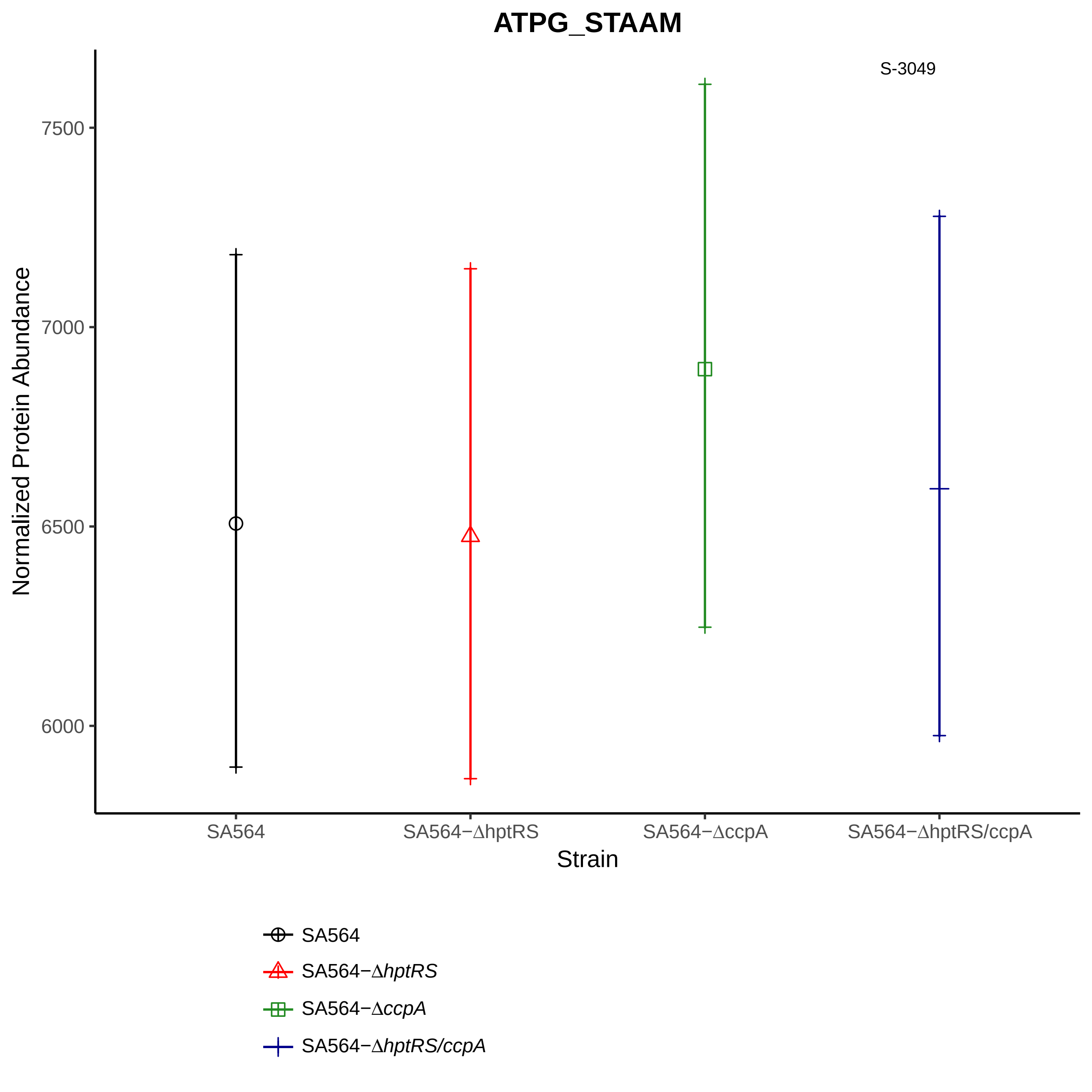
ATPG_STAAM
S-3049
Normalized Protein Abundance
7500
7000
6500
6000
SA564
SA564−ΔhptRS
SA564−ΔccpA
SA564−ΔhptRS/ccpA
Strain
SA564
SA564−ΔhptRS
SA564−ΔccpA
SA564−ΔhptRS/ccpA

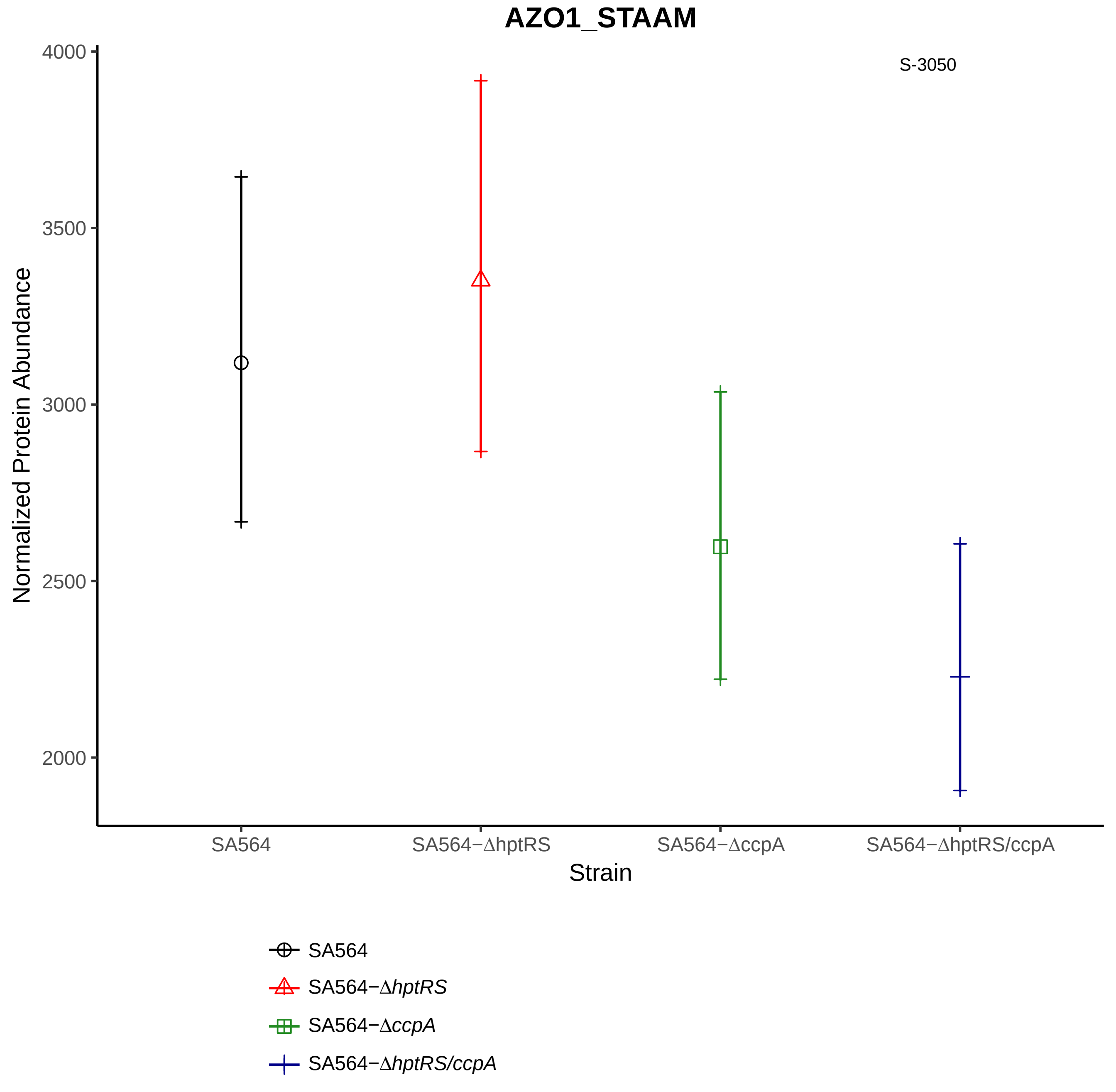
AZO1_STAAM
S-3050
Normalized Protein Abundance
4000
3500
3000
2500
2000
SA564
SA564−ΔhptRS
SA564−ΔccpA
SA564−ΔhptRS/ccpA
Strain
SA564
SA564−ΔhptRS
SA564−ΔccpA
SA564−ΔhptRS/ccpA

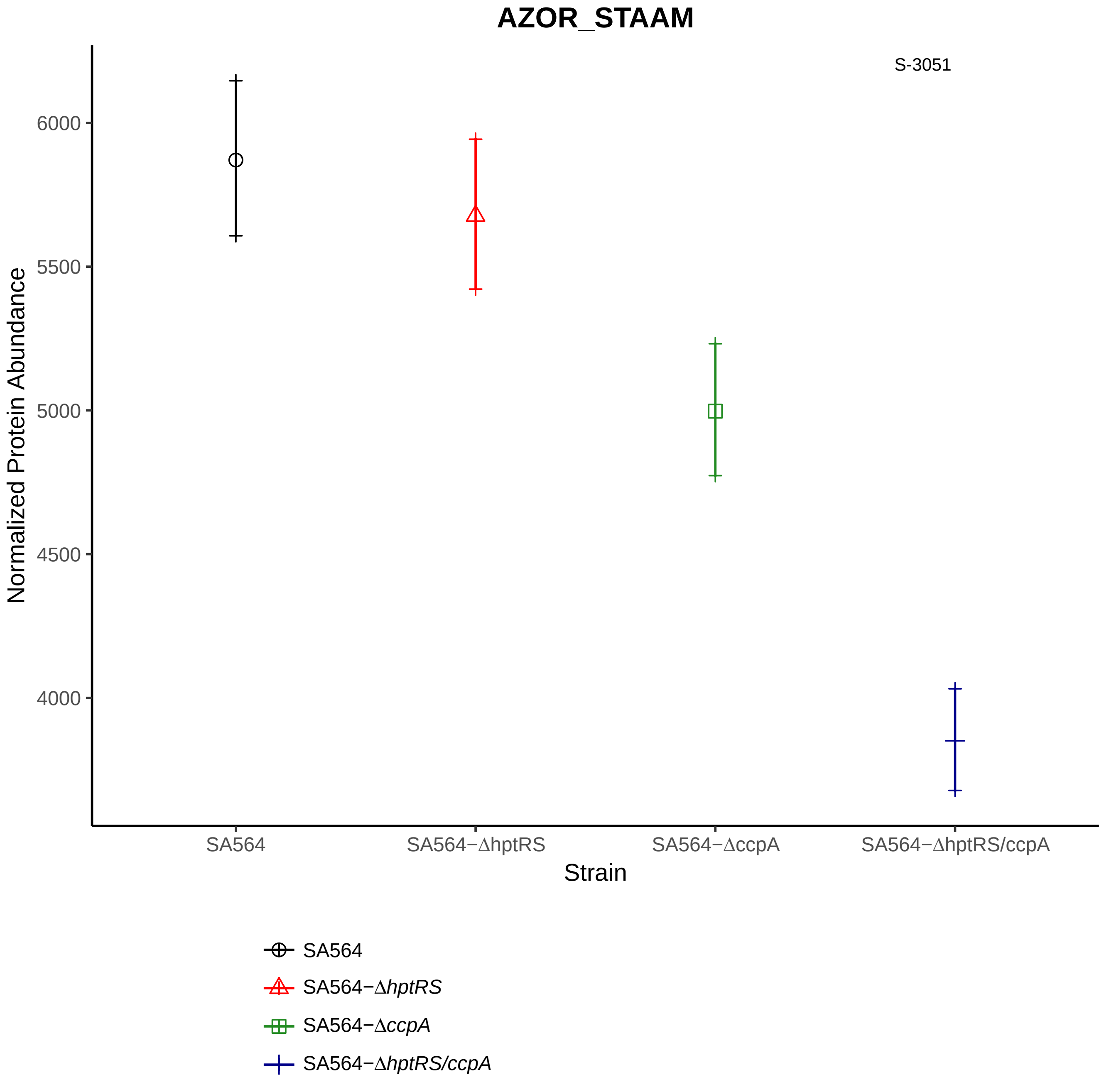
AZOR_STAAM
S-3051
Normalized Protein Abundance
6000
5500
5000
4500
4000
SA564
SA564−ΔhptRS
SA564−ΔccpA
SA564−ΔhptRS/ccpA
Strain
SA564
SA564−ΔhptRS
SA564−ΔccpA
SA564−ΔhptRS/ccpA

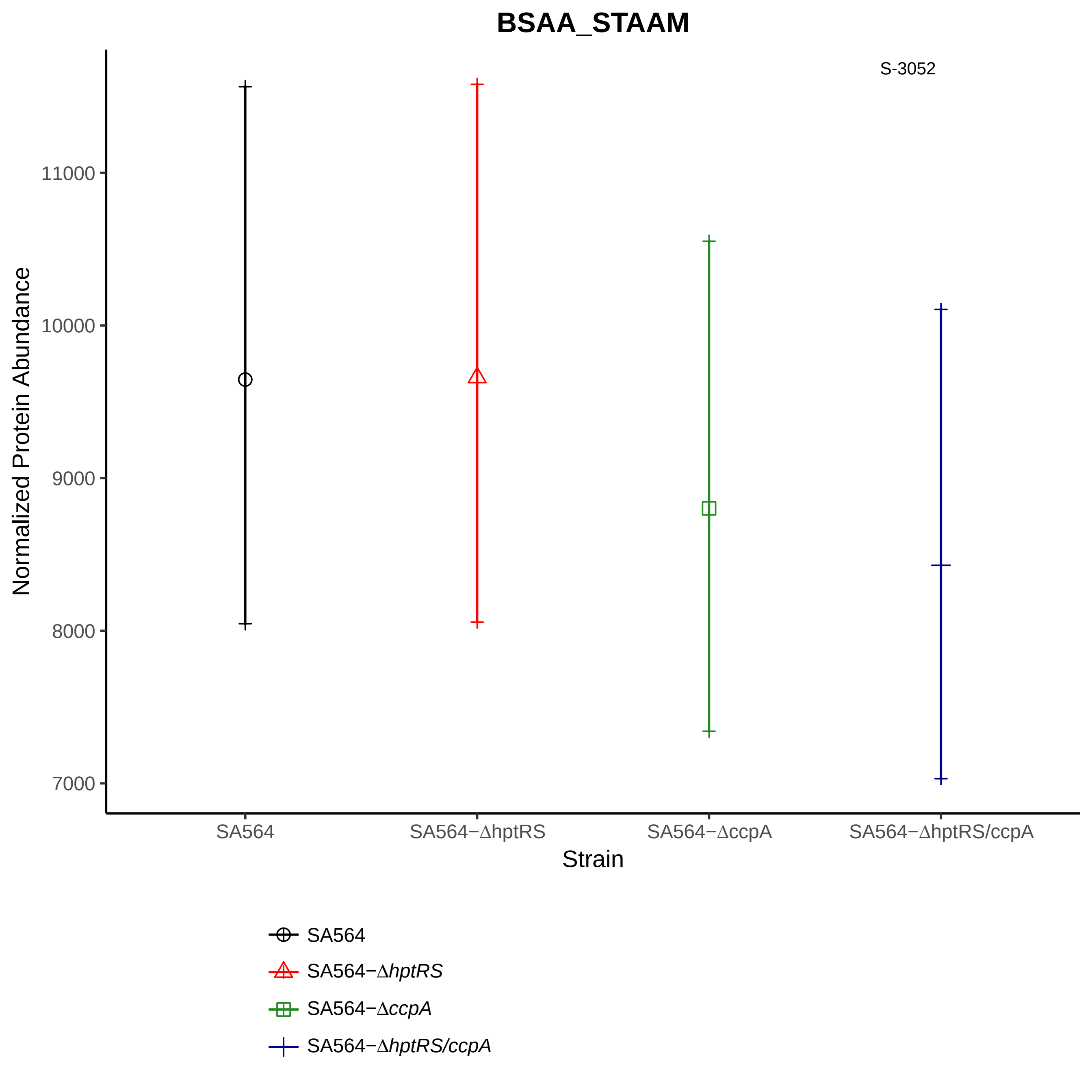
BSAA_STAAM
S-3052
Normalized Protein Abundance
11000
10000
9000
8000
7000
SA564
SA564−ΔhptRS
SA564−ΔccpA
SA564−ΔhptRS/ccpA
Strain
SA564
SA564−ΔhptRS
SA564−ΔccpA
SA564−ΔhptRS/ccpA

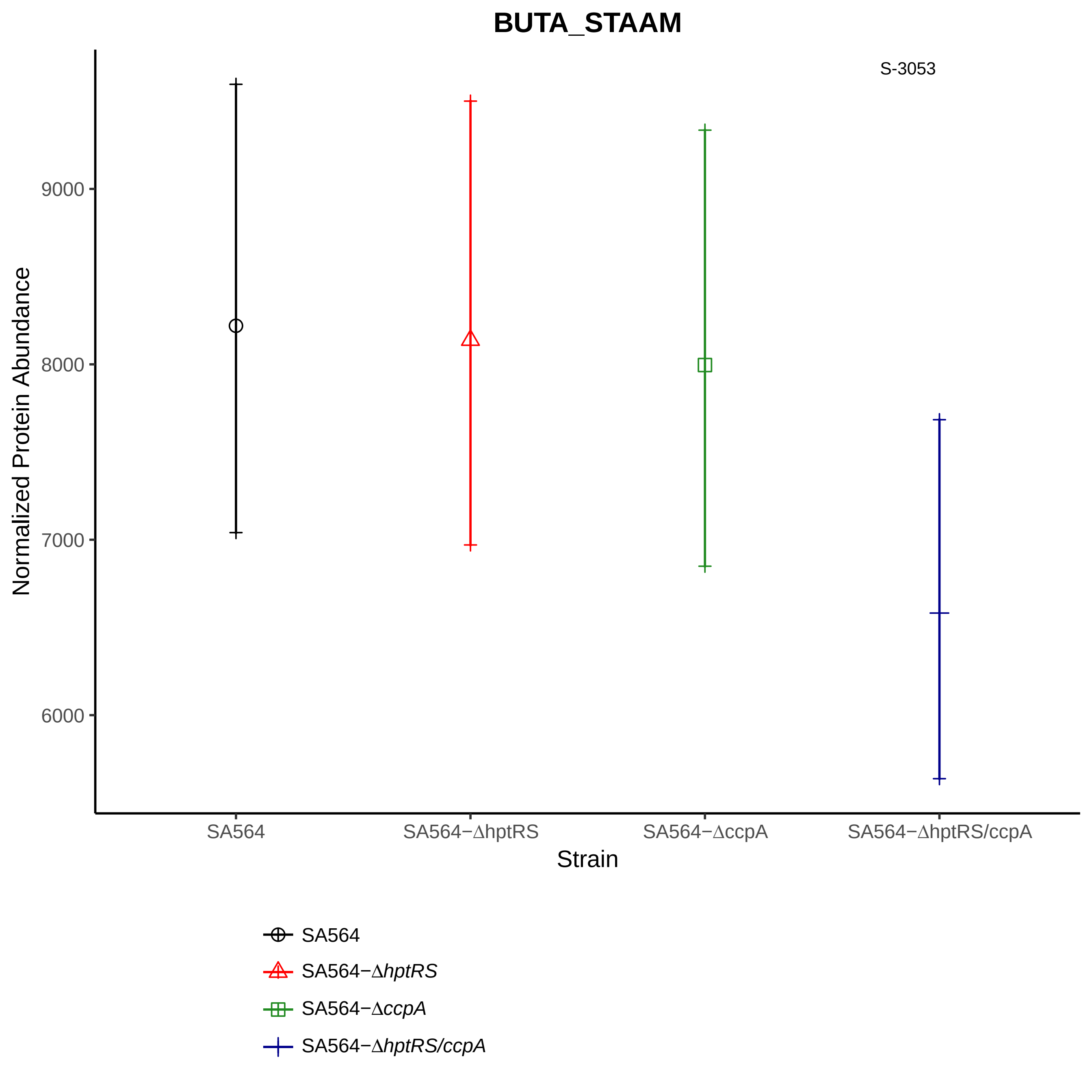
BUTA_STAAM
S-3053
Normalized Protein Abundance
9000
8000
7000
6000
SA564
SA564−ΔhptRS
SA564−ΔccpA
SA564−ΔhptRS/ccpA
Strain
SA564
SA564−ΔhptRS
SA564−ΔccpA
SA564−ΔhptRS/ccpA

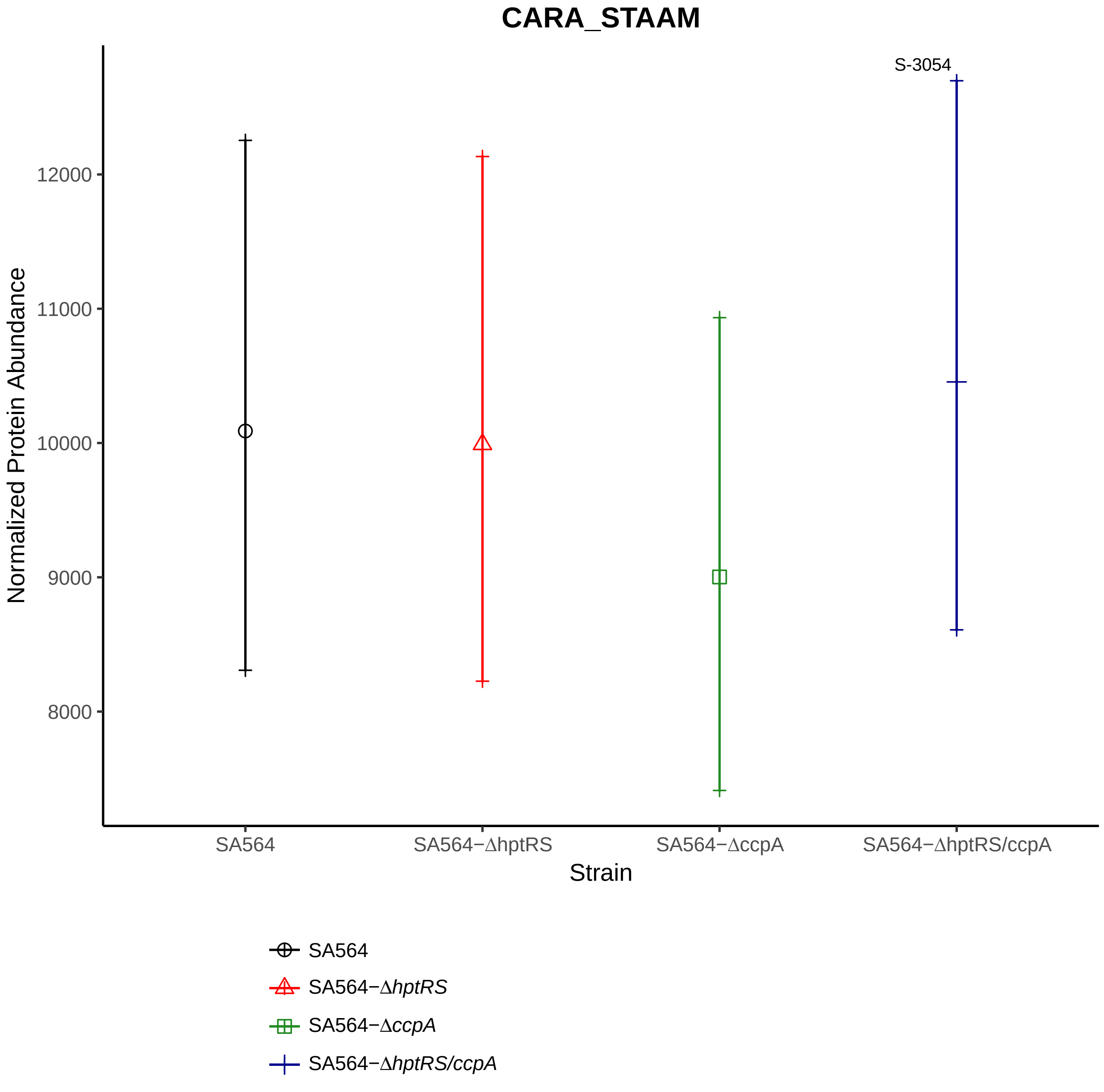

CARA_STAAM
S-3054
Normalized Protein Abundance
12000
11000
10000
9000
8000
SA564
SA564−ΔhptRS
SA564−ΔccpA
SA564−ΔhptRS/ccpA
Strain
SA564
SA564−ΔhptRS
SA564−ΔccpA
SA564−ΔhptRS/ccpA

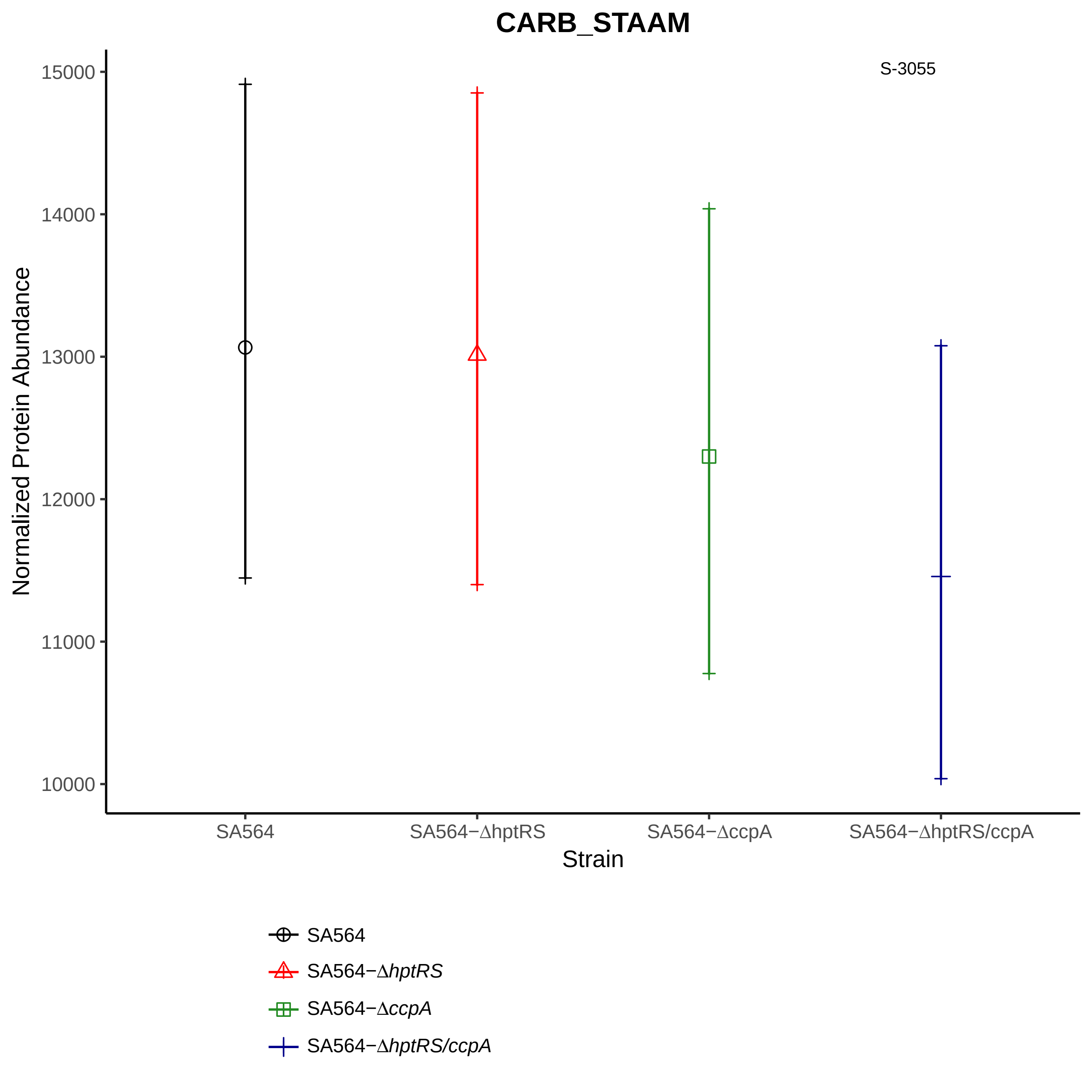

CARB_STAAM
S-3055
Normalized Protein Abundance
15000
14000
13000
12000
11000
10000
SA564
SA564−ΔhptRS
SA564−ΔccpA
SA564−ΔhptRS/ccpA
Strain
SA564
SA564−Δ*hptRS*
SA564−Δ*ccpA*
SA564−Δ*hptRS/ccpA*

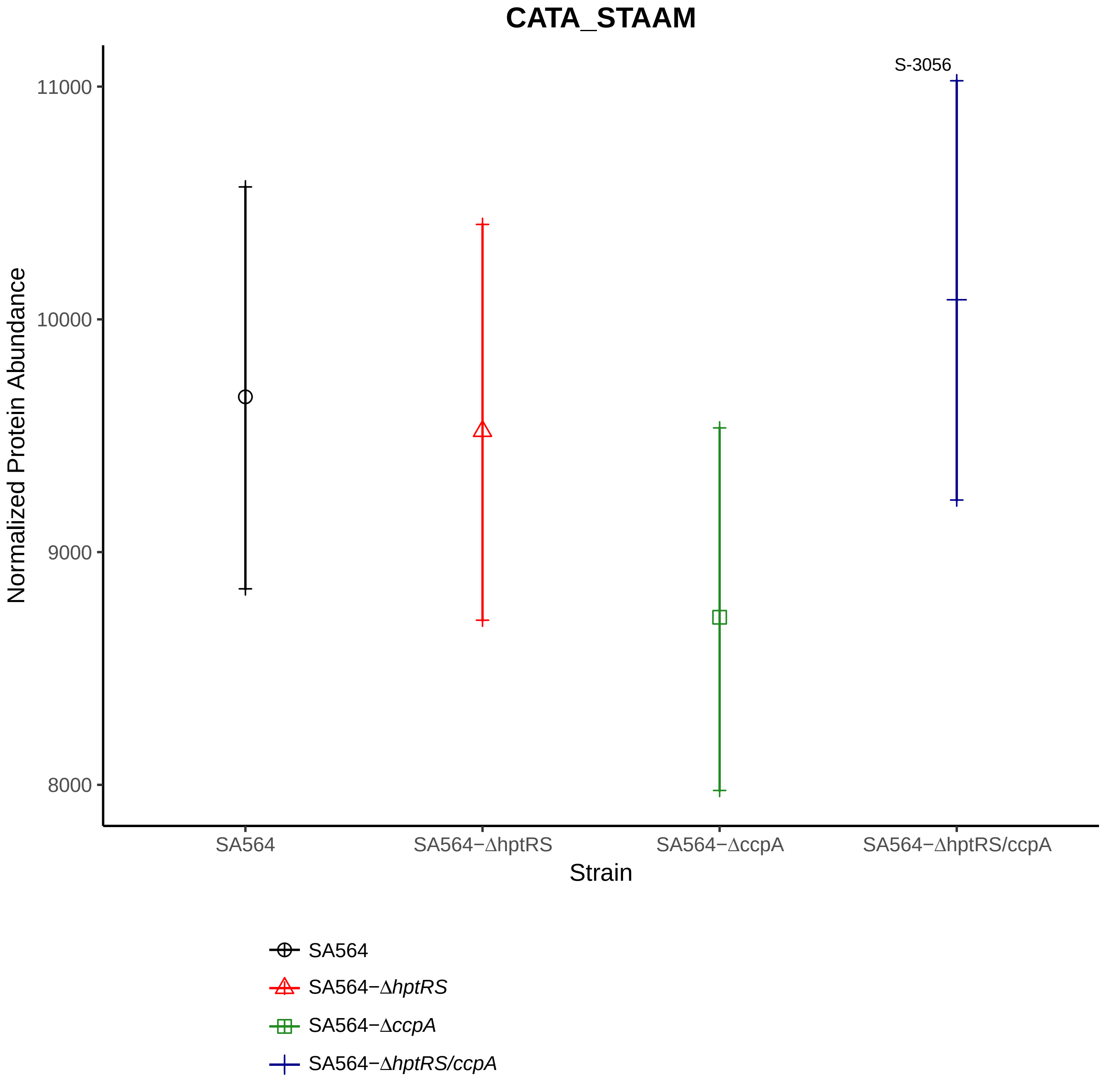
CATA_STAAM
S-3056
Normalized Protein Abundance
11000
10000
9000
8000
SA564
SA564−ΔhptRS
SA564−ΔccpA
SA564−ΔhptRS/ccpA
Strain
SA564
SA564−ΔhptRS
SA564−ΔccpA
SA564−ΔhptRS/ccpA

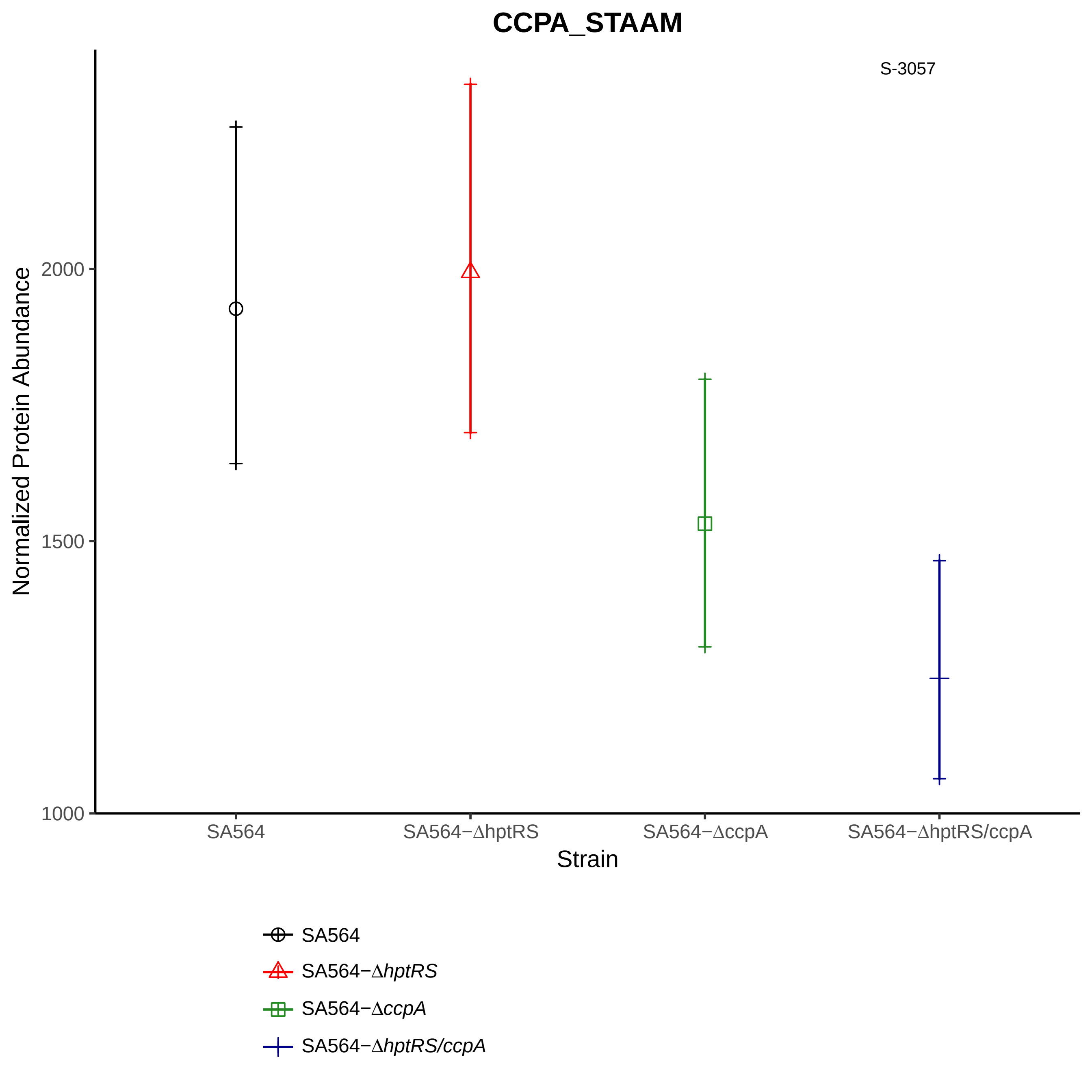
CCPA_STAAM
S-3057
Normalized Protein Abundance
2000
1500
1000
SA564
SA564−ΔhptRS
SA564−ΔccpA
SA564−ΔhptRS/ccpA
Strain
SA564
SA564−ΔhptRS
SA564−ΔccpA
SA564−ΔhptRS/ccpA

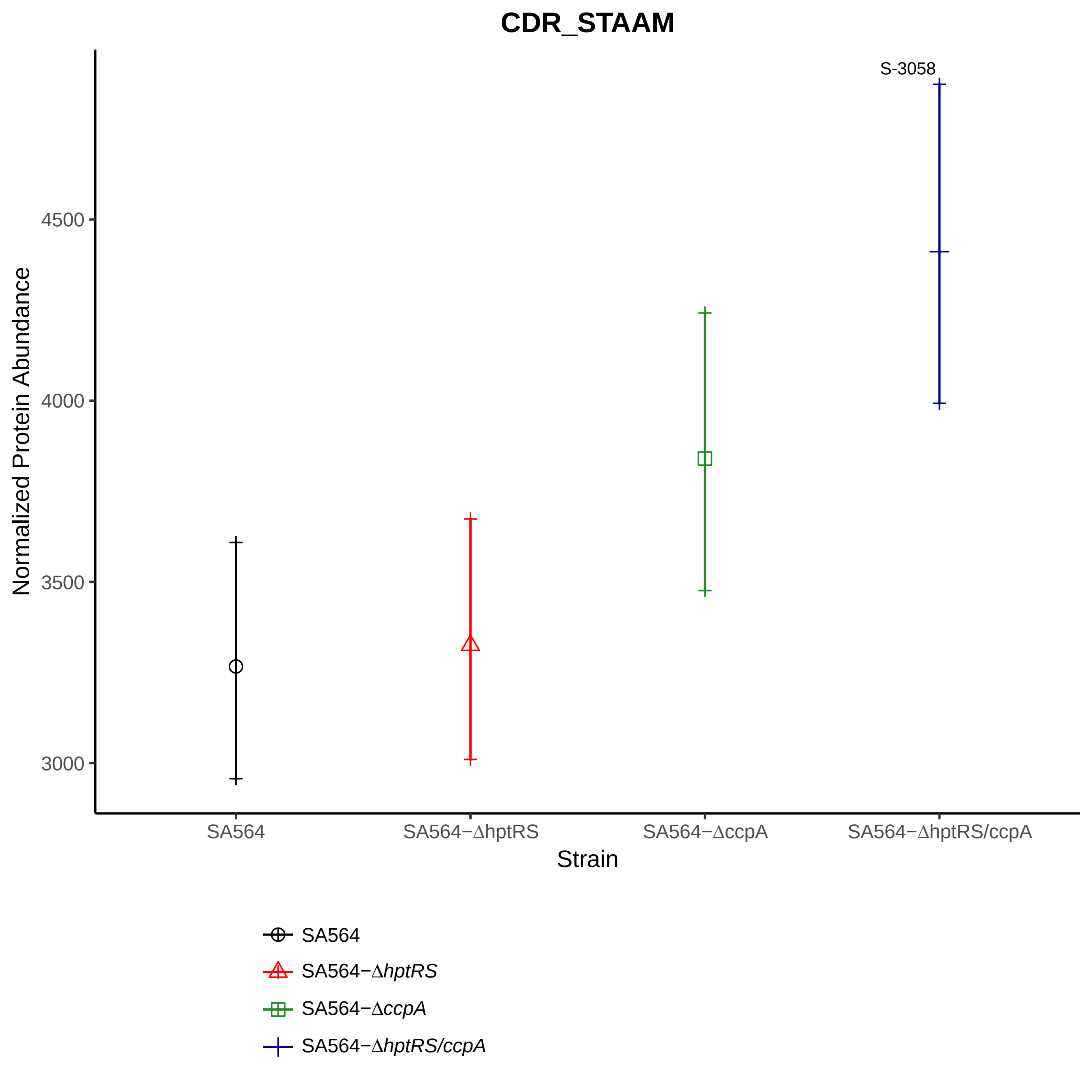
CDR_STAAM
S-3058
Normalized Protein Abundance
4500
4000
3500
3000
SA564
SA564−ΔhptRS
SA564−ΔccpA
SA564−ΔhptRS/ccpA
Strain
SA564
SA564−ΔhptRS
SA564−ΔccpA
SA564−ΔhptRS/ccpA

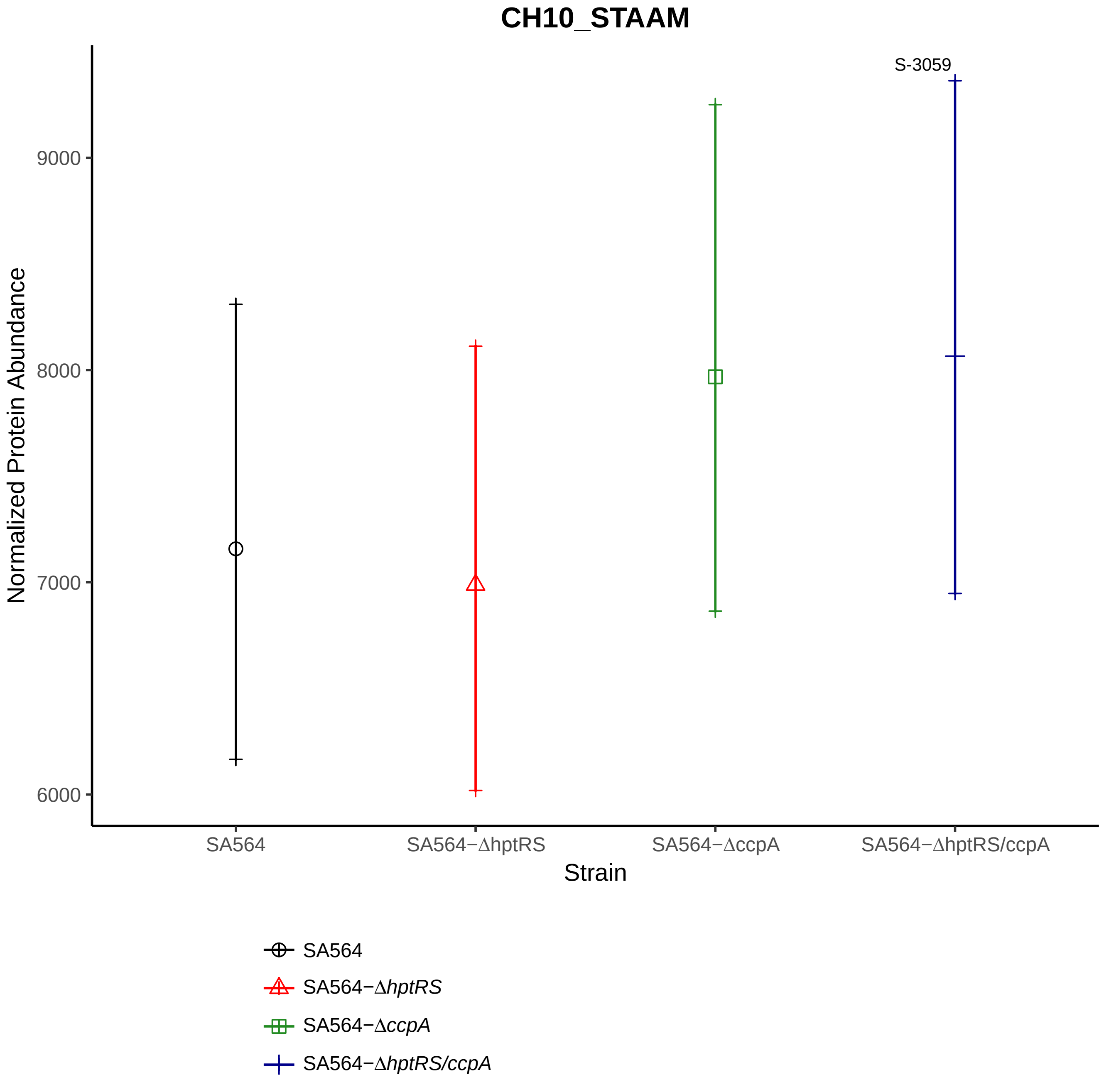

CH10_STAAM
S-3059
Normalized Protein Abundance
9000
8000
7000
6000
SA564
SA564−ΔhptRS
SA564−ΔccpA
SA564−ΔhptRS/ccpA
Strain
SA564
SA564−ΔhptRS
SA564−ΔccpA
SA564−ΔhptRS/ccpA

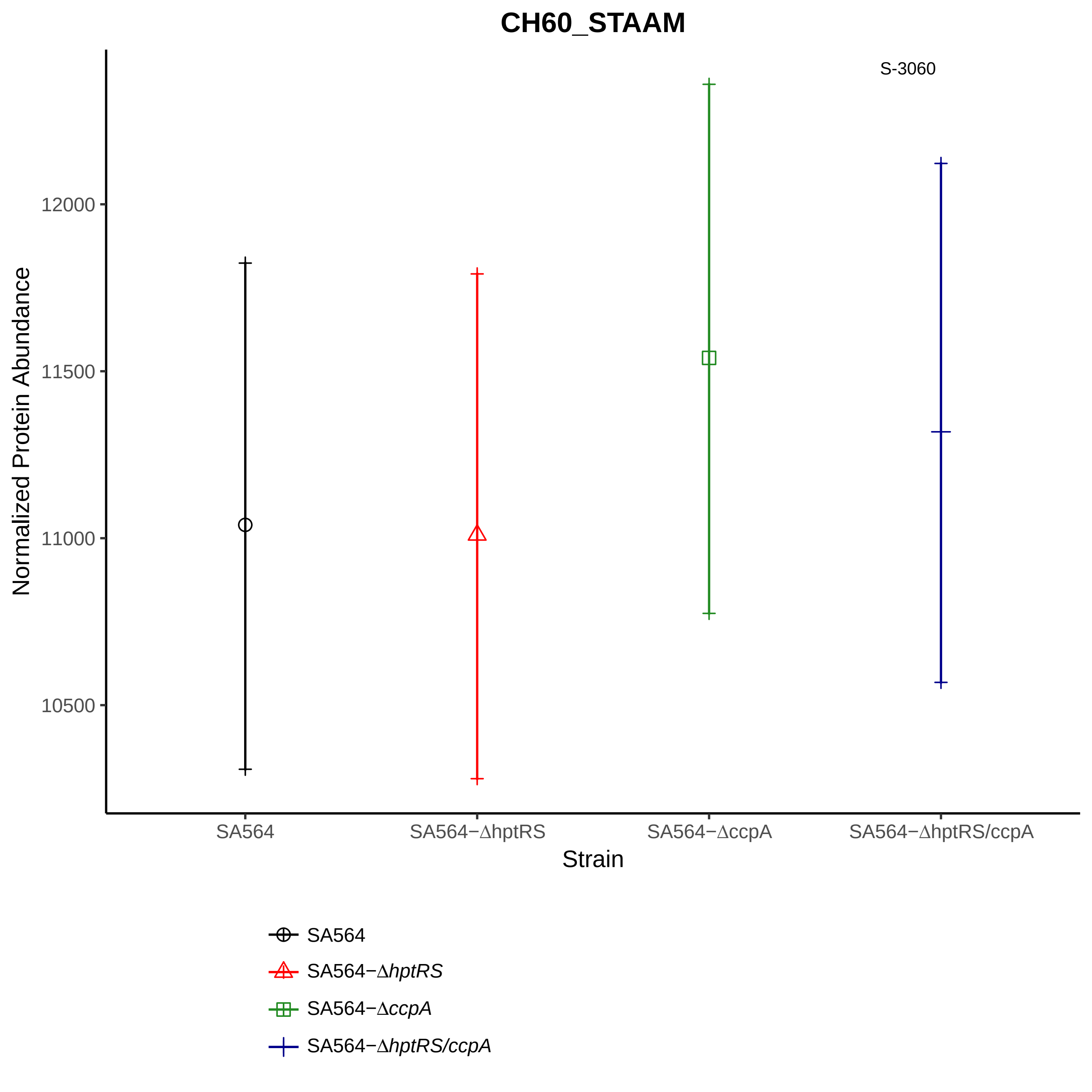
CH60_STAAM
S-3060
Normalized Protein Abundance
12000
11500
11000
10500
SA564
SA564−ΔhptRS
SA564−ΔccpA
SA564−ΔhptRS/ccpA
Strain
SA564
SA564−ΔhptRS
SA564−ΔccpA
SA564−ΔhptRS/ccpA

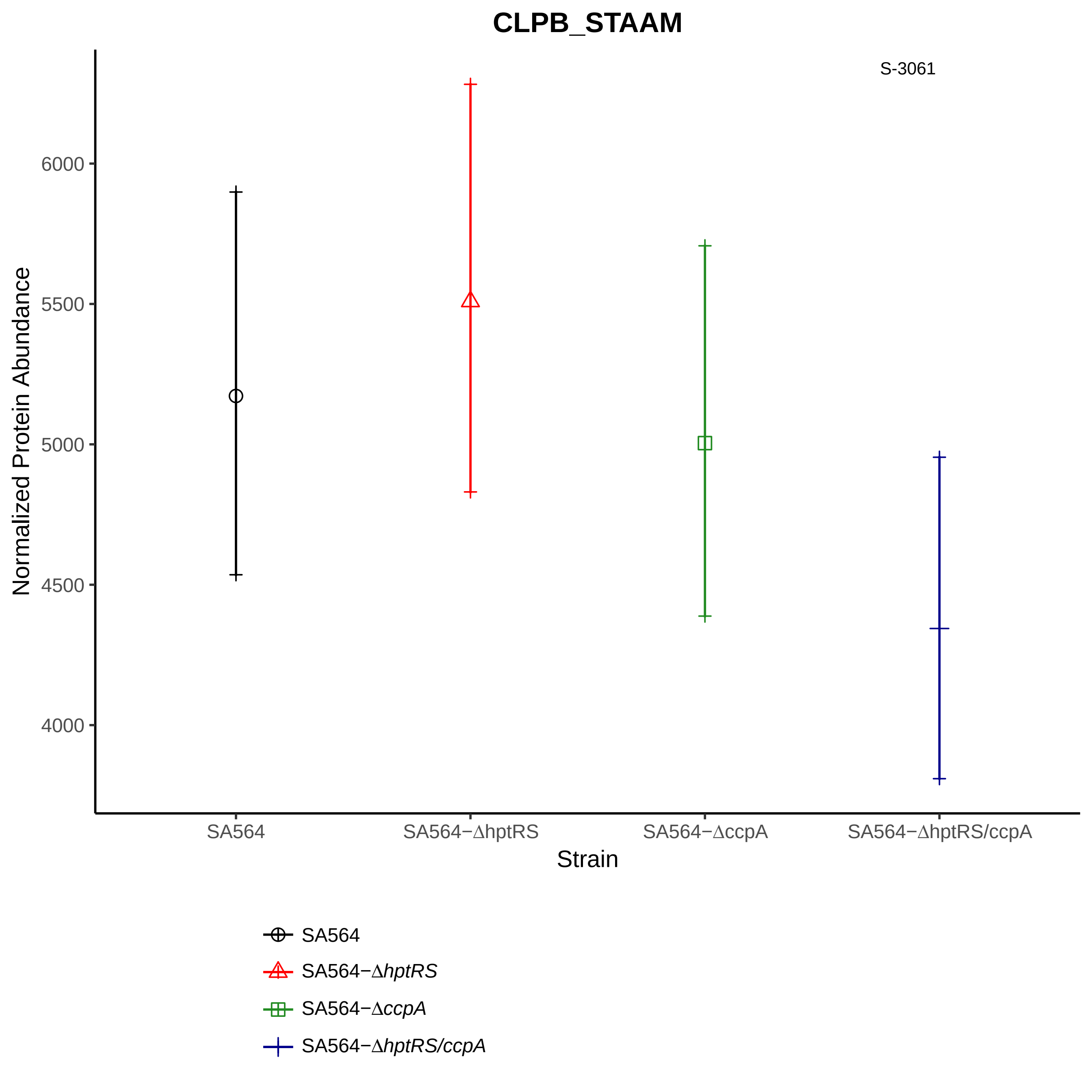
CLPB_STAAM
S-3061
Normalized Protein Abundance
6000
5500
5000
4500
4000
SA564
SA564−ΔhptRS
SA564−ΔccpA
SA564−ΔhptRS/ccpA
Strain
SA564
SA564−ΔhptRS
SA564−ΔccpA
SA564−ΔhptRS/ccpA

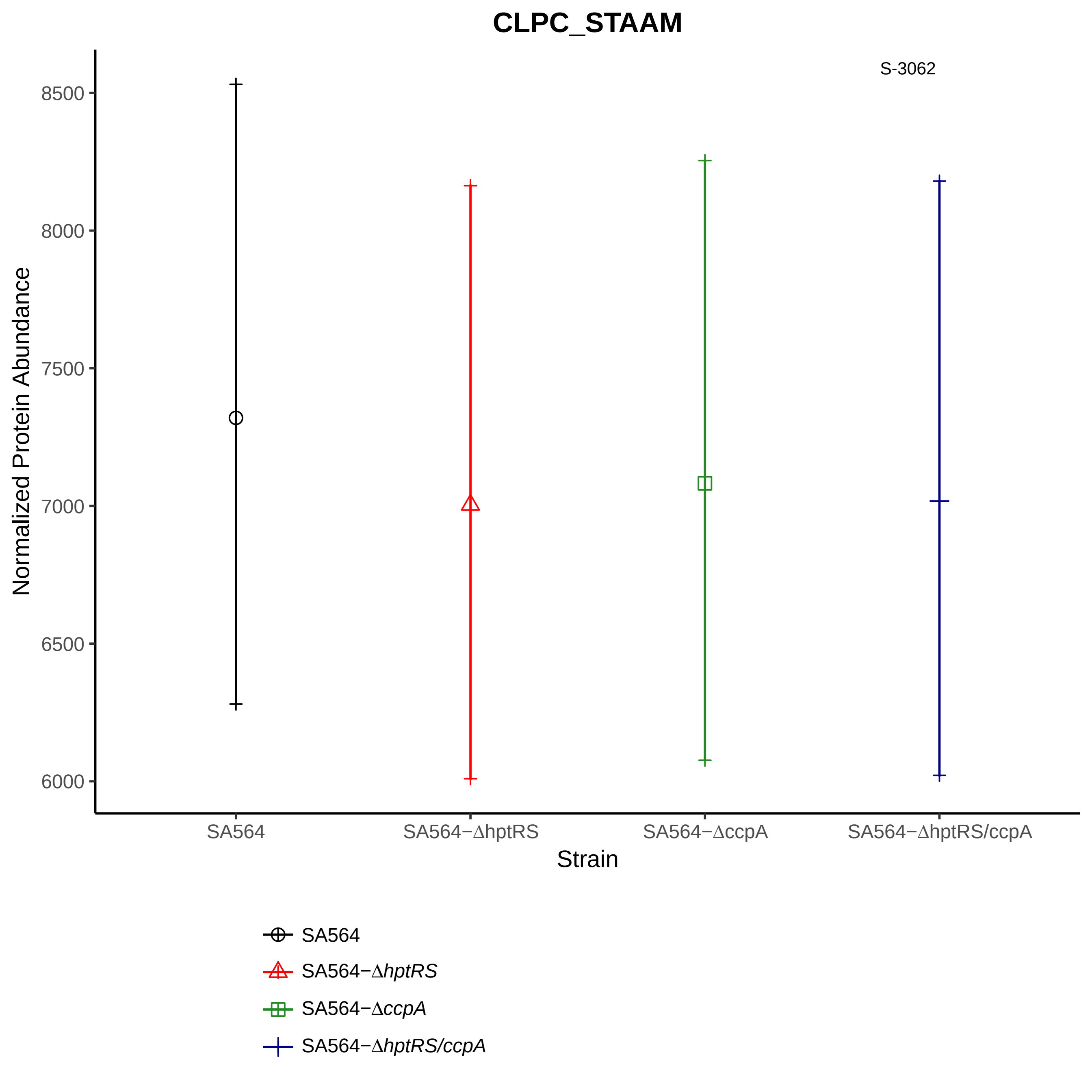

CLPC_STAAM
S-3062
Normalized Protein Abundance
8500
8000
7500
7000
6500
6000
SA564
SA564−ΔhptRS
SA564−ΔccpA
SA564−ΔhptRS/ccpA
Strain
SA564
SA564−Δ*hptRS*
SA564−Δ*ccpA*
SA564−Δ*hptRS/ccpA*

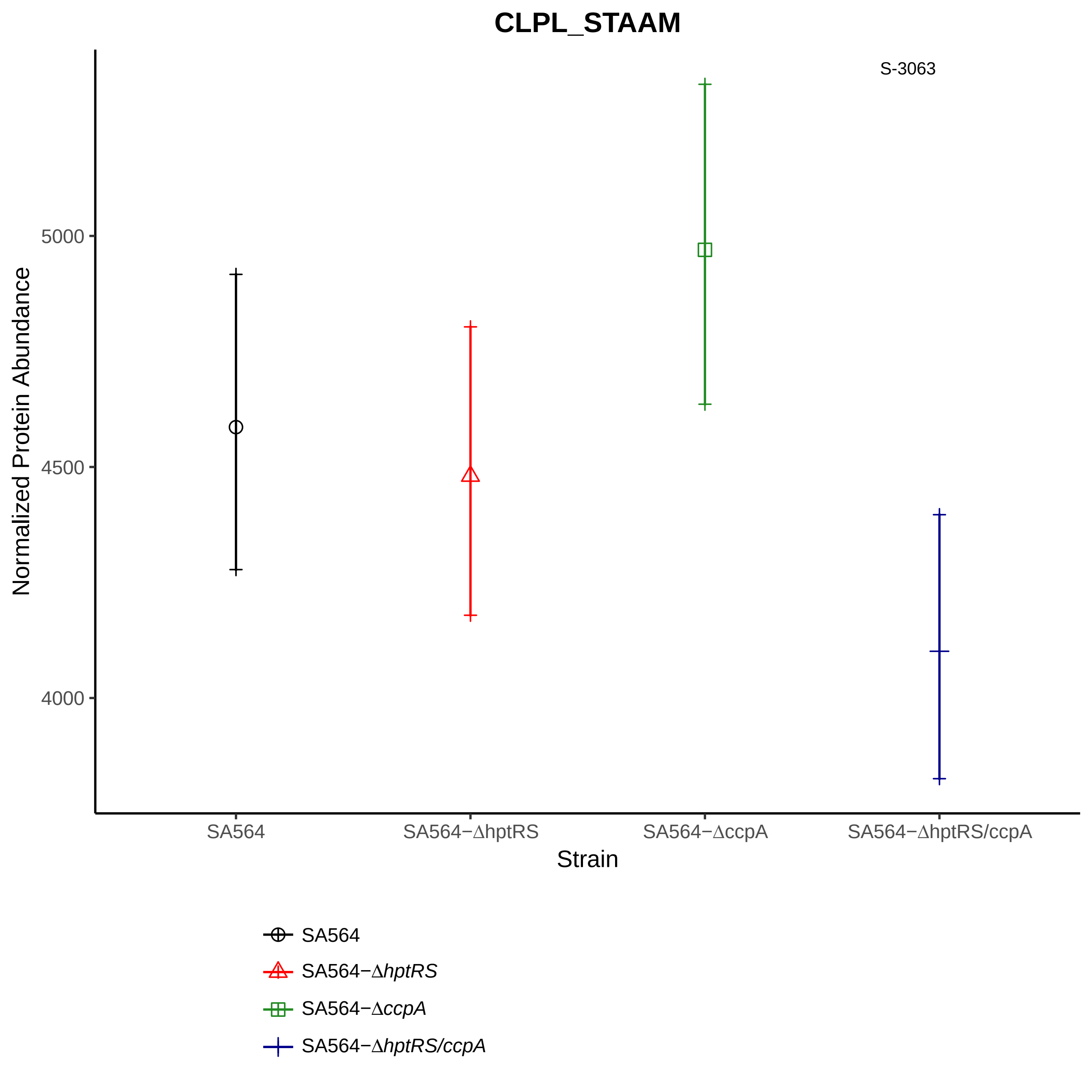

CLPL_STAAM
S-3063
Normalized Protein Abundance
5000
4500
4000
SA564
SA564−ΔhptRS
SA564−ΔccpA
SA564−ΔhptRS/ccpA
Strain
SA564
SA564−Δ*hptRS*
SA564−Δ*ccpA*
SA564−Δ*hptRS/ccpA*

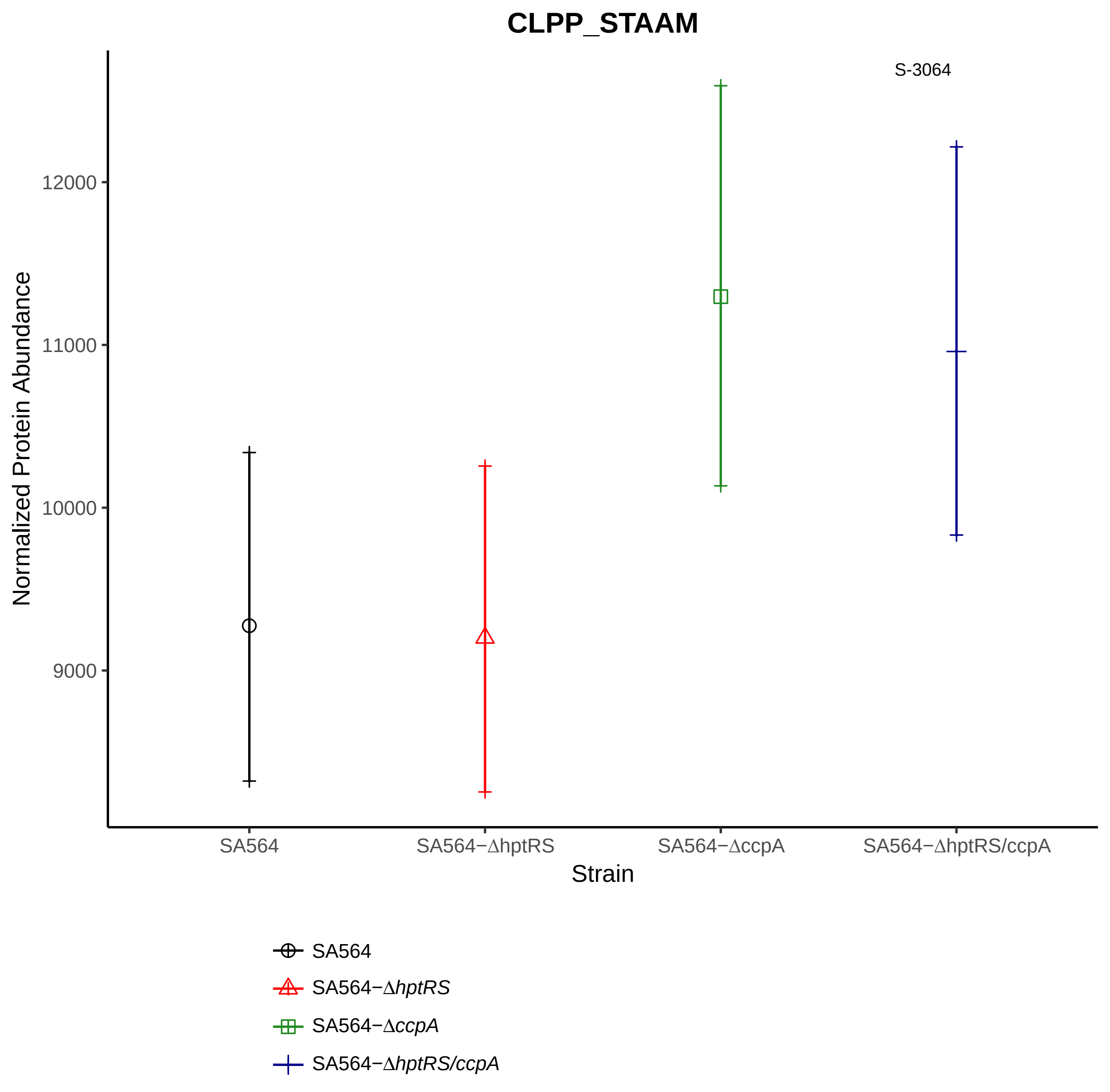

CLPP_STAAM
S-3064
Normalized Protein Abundance
12000
11000
10000
9000
SA564
SA564−ΔhptRS
SA564−ΔccpA
SA564−ΔhptRS/ccpA
Strain
SA564
SA564−ΔhptRS
SA564−ΔccpA
SA564−ΔhptRS/ccpA

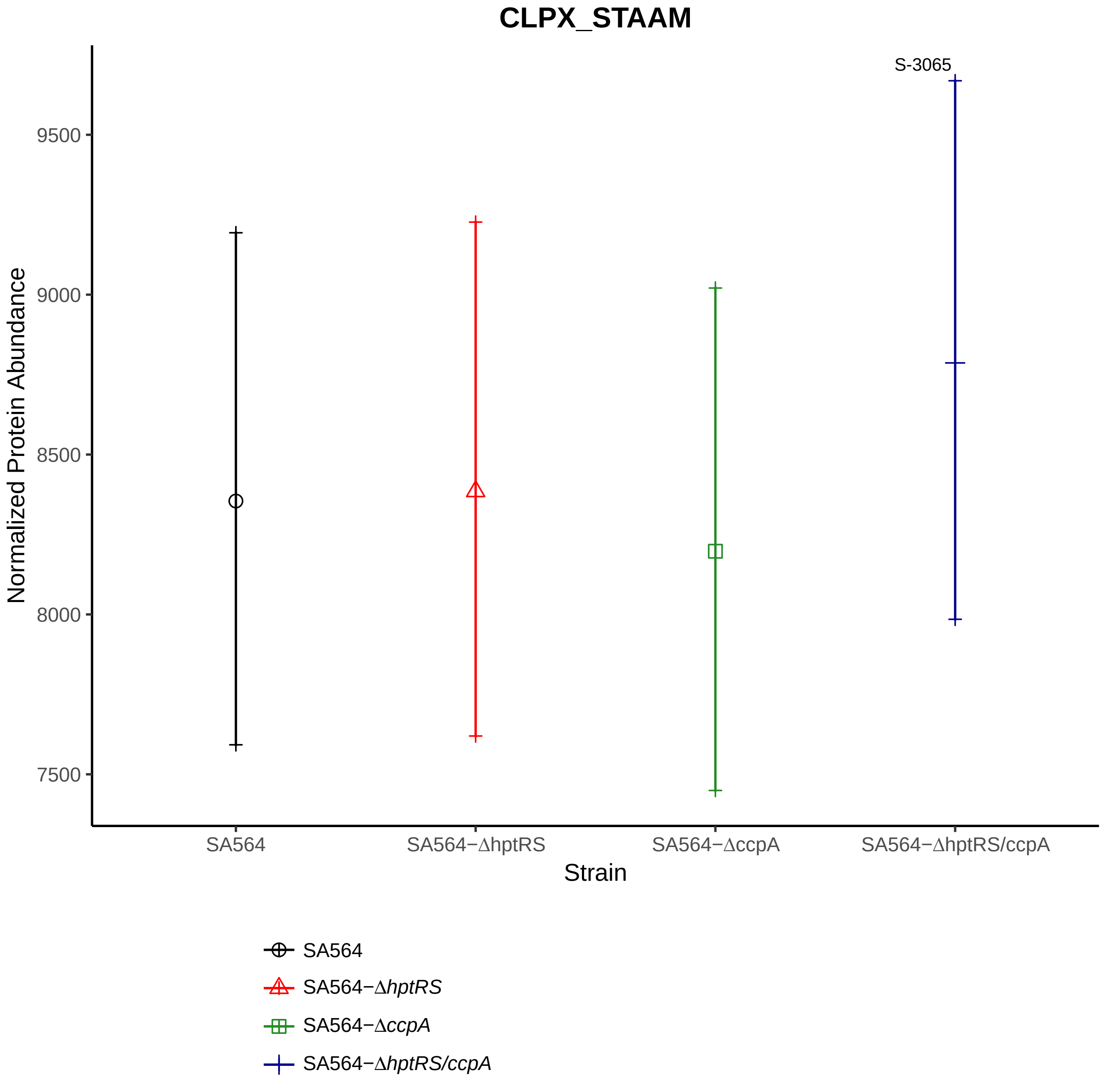

CLPX_STAAM
S-3065
9500
9000
8500
8000
7500
Normalized Protein Abundance
SA564
SA564−ΔhptRS
SA564−ΔccpA
SA564−ΔhptRS/ccpA
Strain
SA564
SA564−Δ*hptRS*
SA564−Δ*ccpA*
SA564−Δ*hptRS/ccpA*

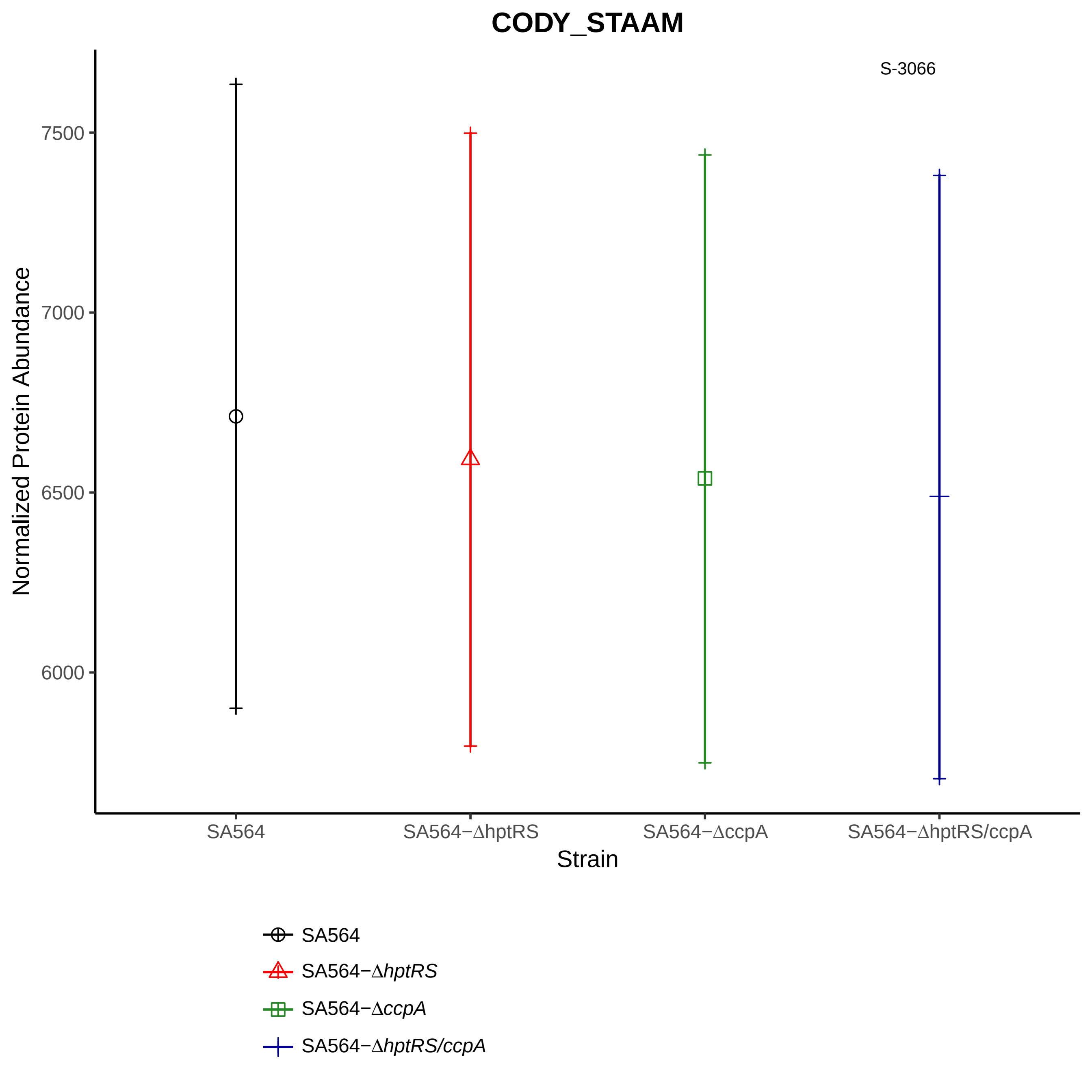

CODY_STAAM
S-3066
Normalized Protein Abundance
7500
7000
6500
6000
SA564
SA564−ΔhptRS
SA564−ΔccpA
SA564−ΔhptRS/ccpA
Strain
SA564
SA564−ΔhptRS
SA564−ΔccpA
SA564−ΔhptRS/ccpA

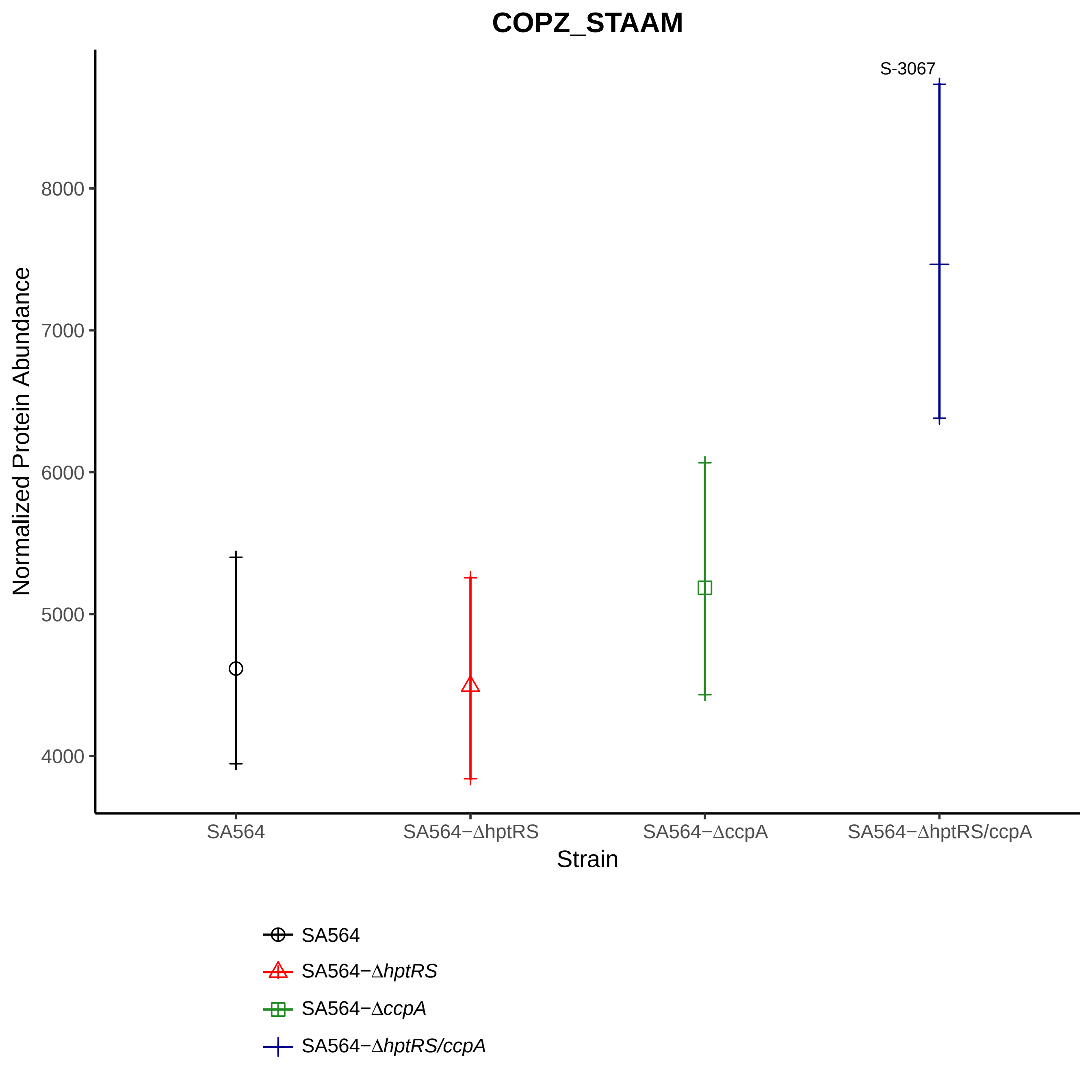

COPZ_STAAM
S-3067
Normalized Protein Abundance
8000
7000
6000
5000
4000
SA564
SA564−ΔhptRS
SA564−ΔccpA
SA564−ΔhptRS/ccpA
Strain
SA564
SA564−ΔhptRS
SA564−ΔccpA
SA564−ΔhptRS/ccpA

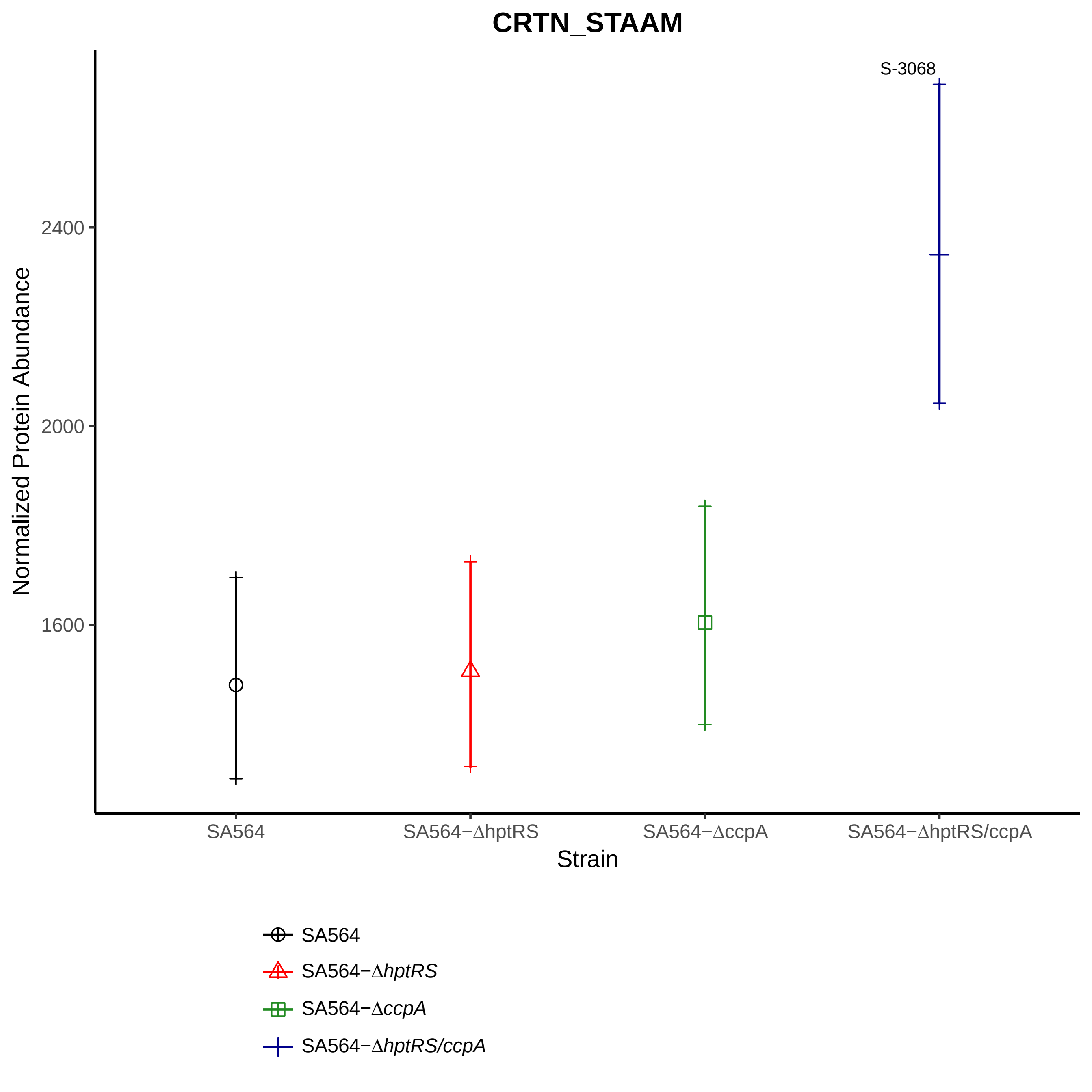

CRTN_STAAM
S-3068
2400
2000
1600
Normalized Protein Abundance
SA564
SA564−ΔhptRS
SA564−ΔccpA
SA564−ΔhptRS/ccpA
Strain
SA564
SA564−ΔhptRS
SA564−ΔccpA
SA564−ΔhptRS/ccpA

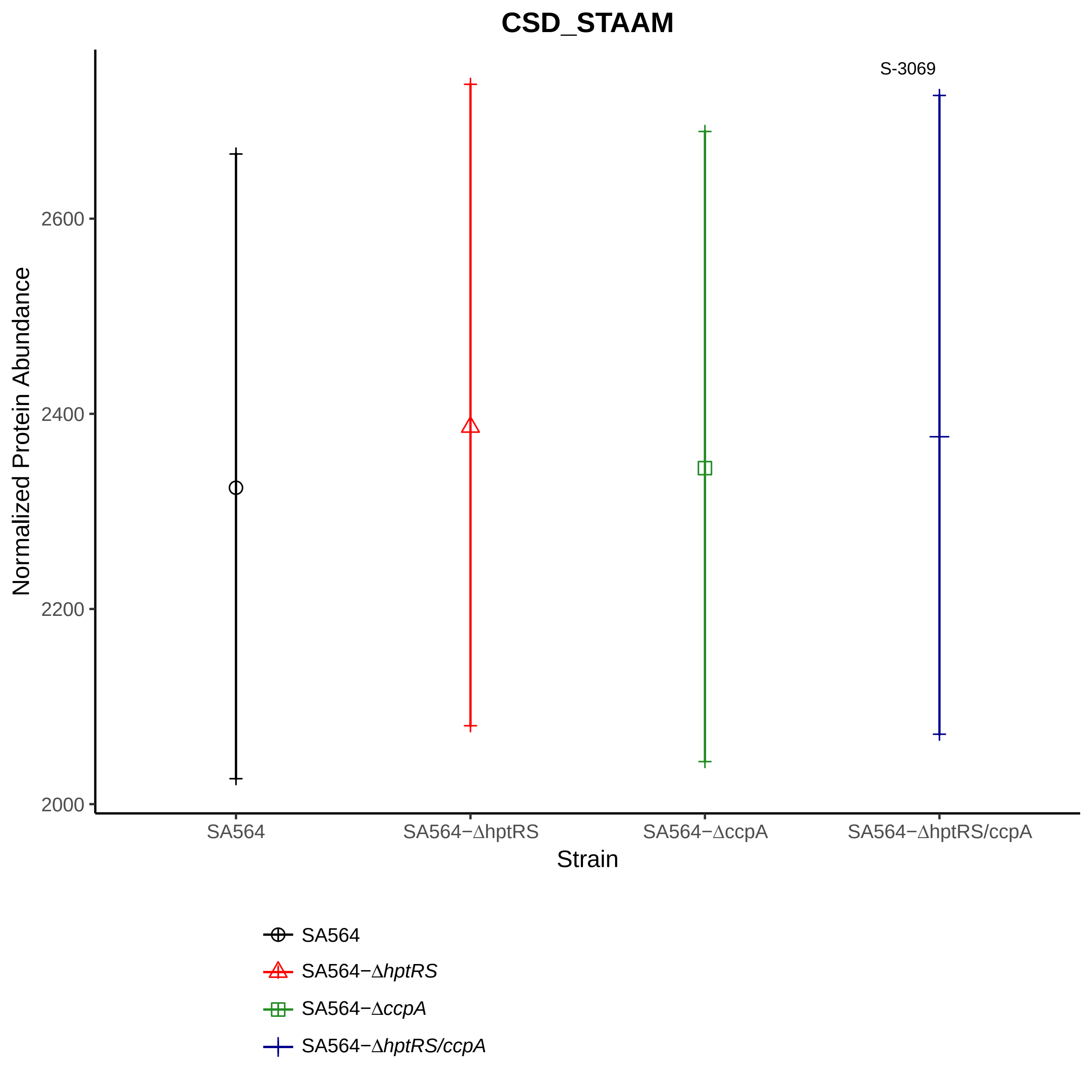

CSD_STAAM
S-3069
Normalized Protein Abundance
2600
2400
2200
2000
SA564
SA564−ΔhptRS
SA564−ΔccpA
SA564−ΔhptRS/ccpA
Strain
SA564
SA564−ΔhptRS
SA564−ΔccpA
SA564−ΔhptRS/ccpA

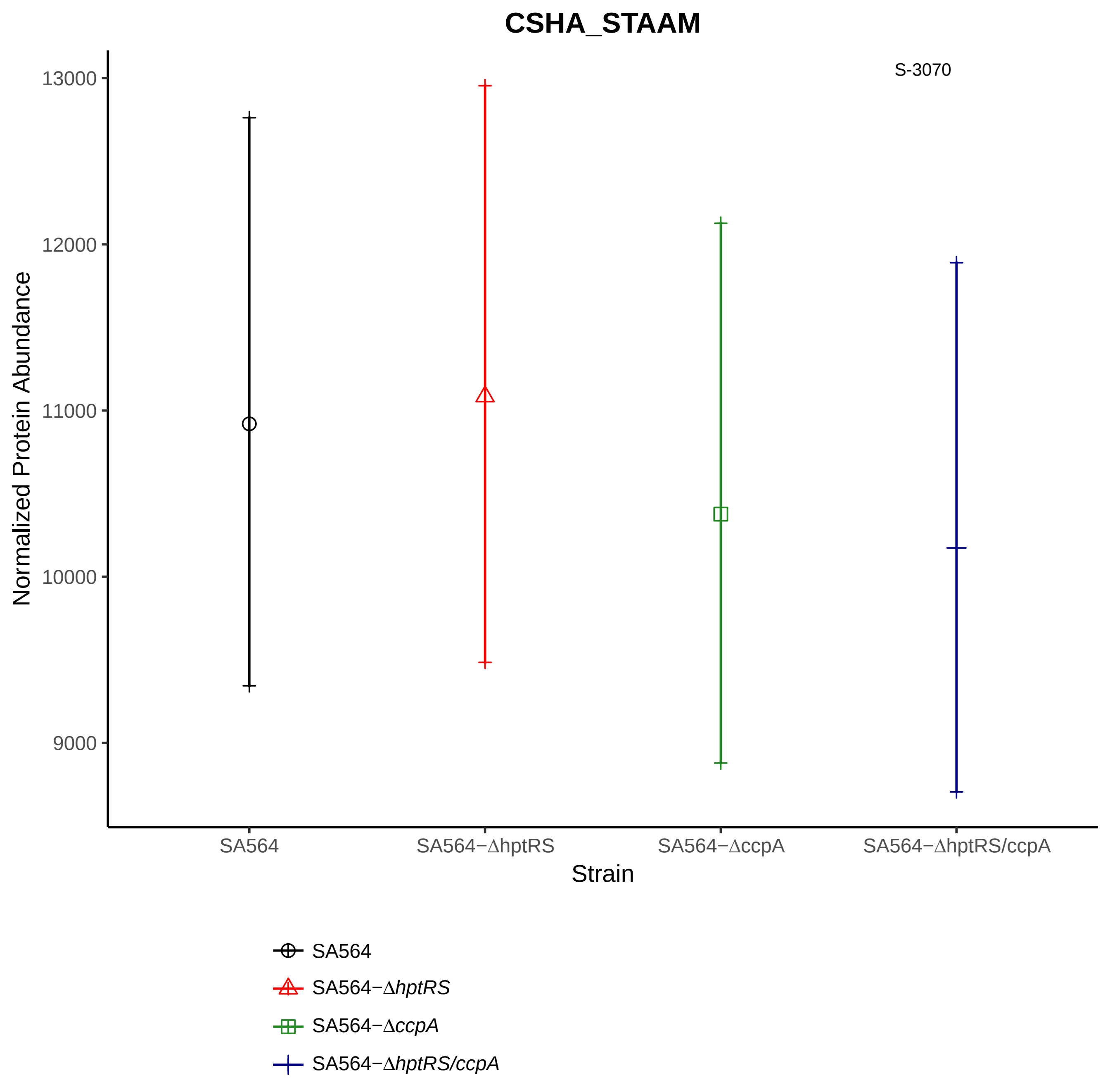

CSHA_STAAM
S-3070
Normalized Protein Abundance
13000
12000
11000
10000
9000
SA564
SA564−ΔhptRS
SA564−ΔccpA
SA564−ΔhptRS/ccpA
Strain
SA564
SA564−ΔhptRS
SA564−ΔccpA
SA564−ΔhptRS/ccpA

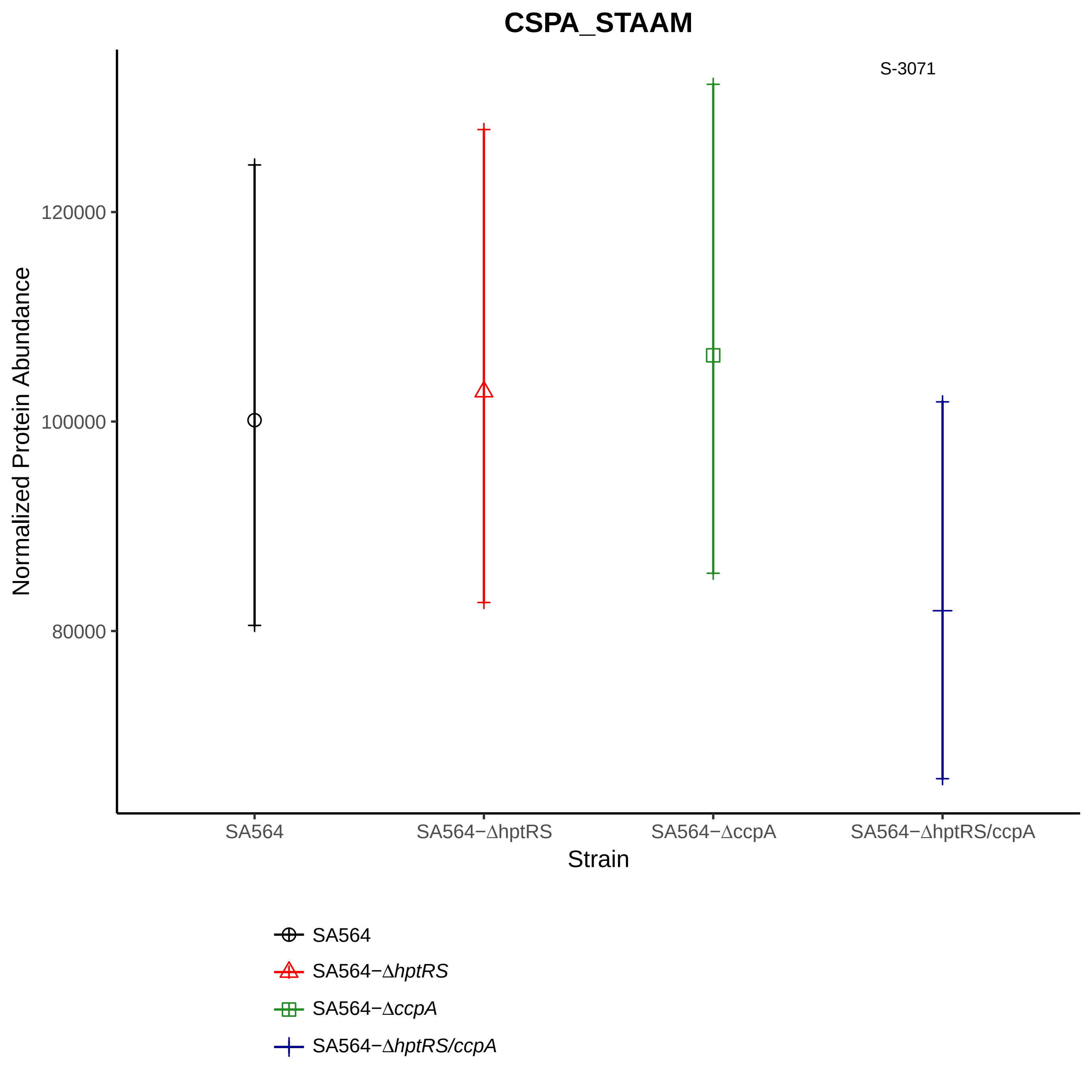

CSPA_STAAM
S-3071
Normalized Protein Abundance
120000
100000
80000
SA564
SA564−ΔhptRS
SA564−ΔccpA
SA564−ΔhptRS/ccpA
Strain
SA564
SA564−ΔhptRS
SA564−ΔccpA
SA564−ΔhptRS/ccpA

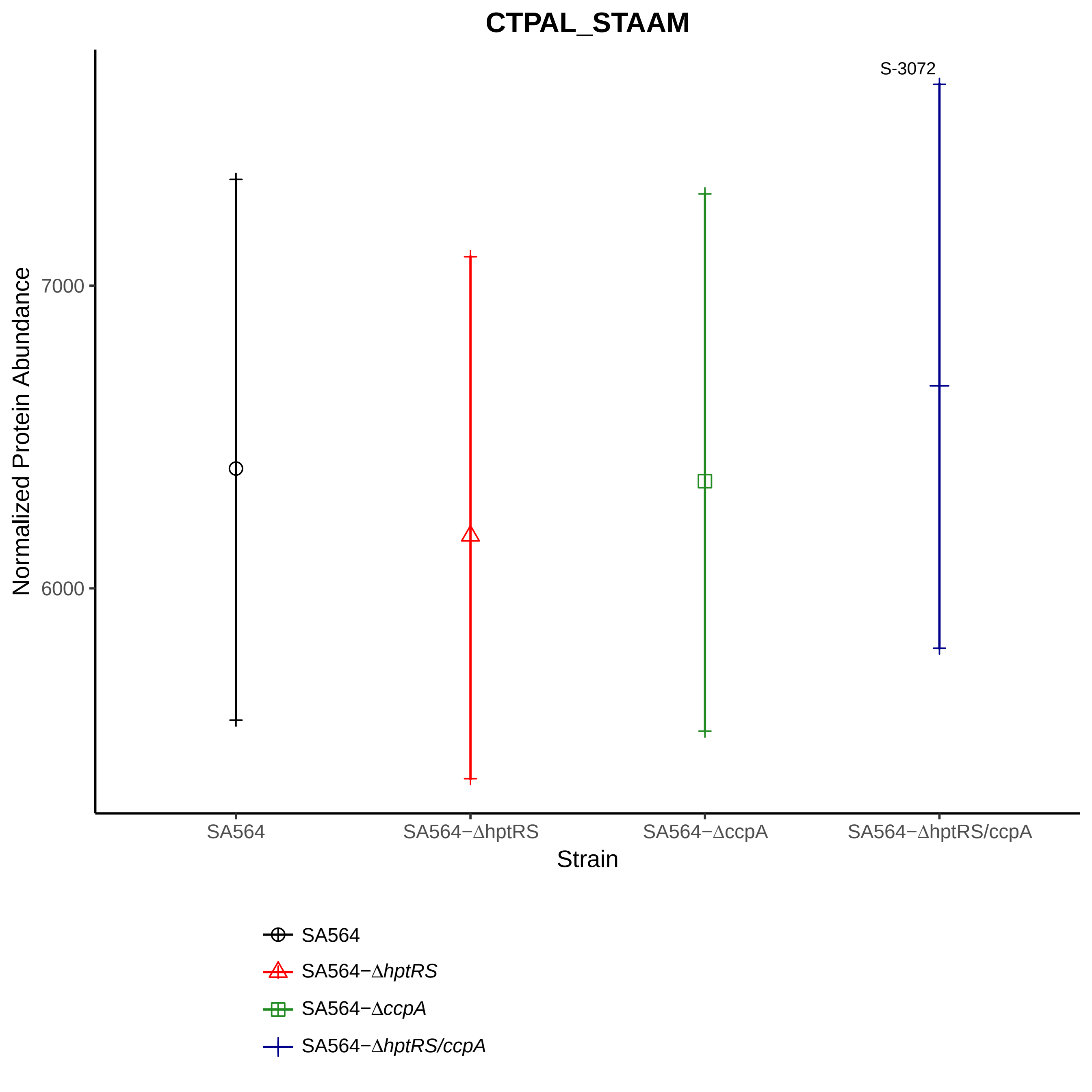
CTPAL_STAAM
S-3072
Normalized Protein Abundance
7000
6000
SA564
SA564−ΔhptRS
SA564−ΔccpA
SA564−ΔhptRS/ccpA
Strain
SA564
SA564−ΔhptRS
SA564−ΔccpA
SA564−ΔhptRS/ccpA

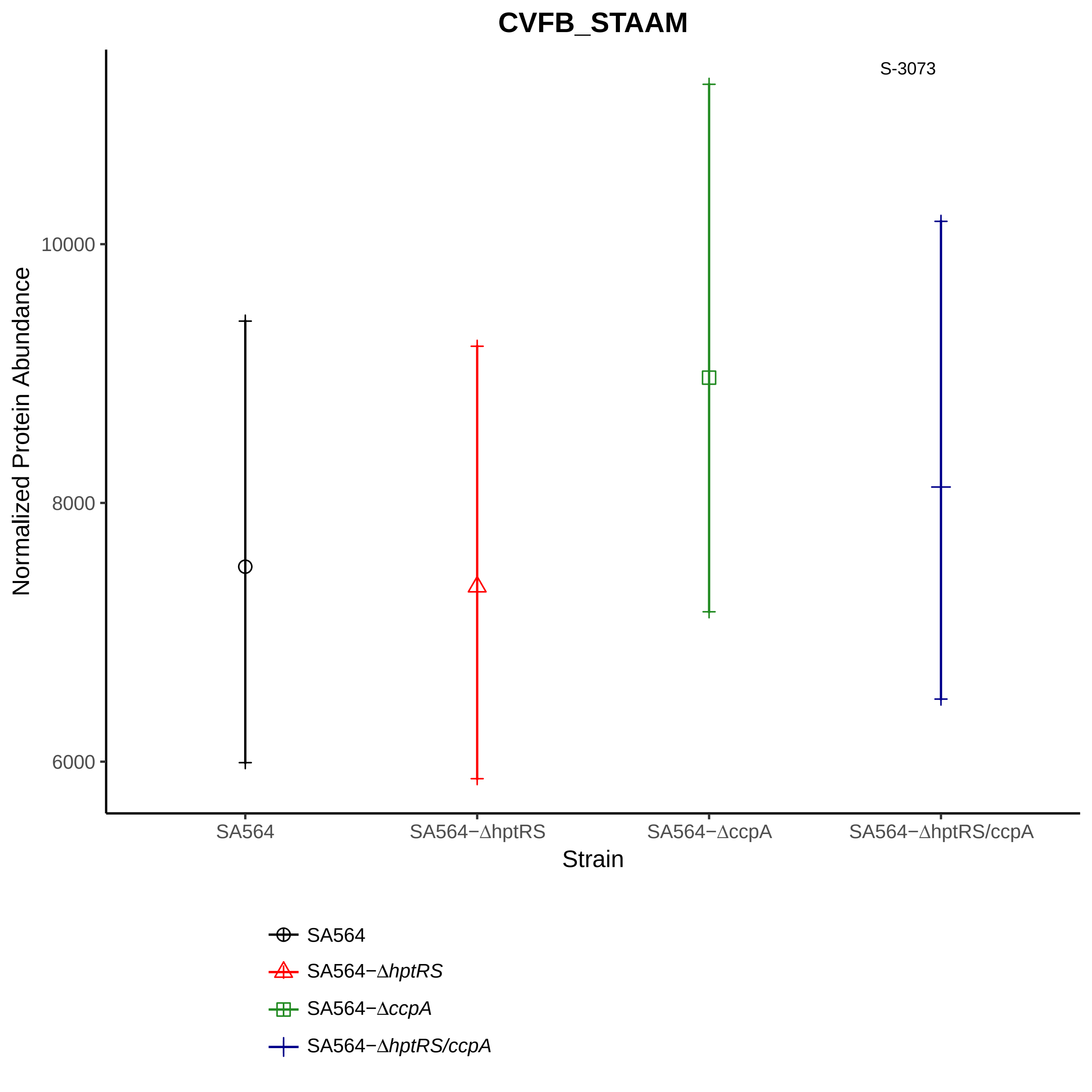
CVFB_STAAM
S-3073
Normalized Protein Abundance
10000
8000
6000
SA564
SA564−ΔhptRS
SA564−ΔccpA
SA564−ΔhptRS/ccpA
Strain
SA564
SA564−ΔhptRS
SA564−ΔccpA
SA564−ΔhptRS/ccpA

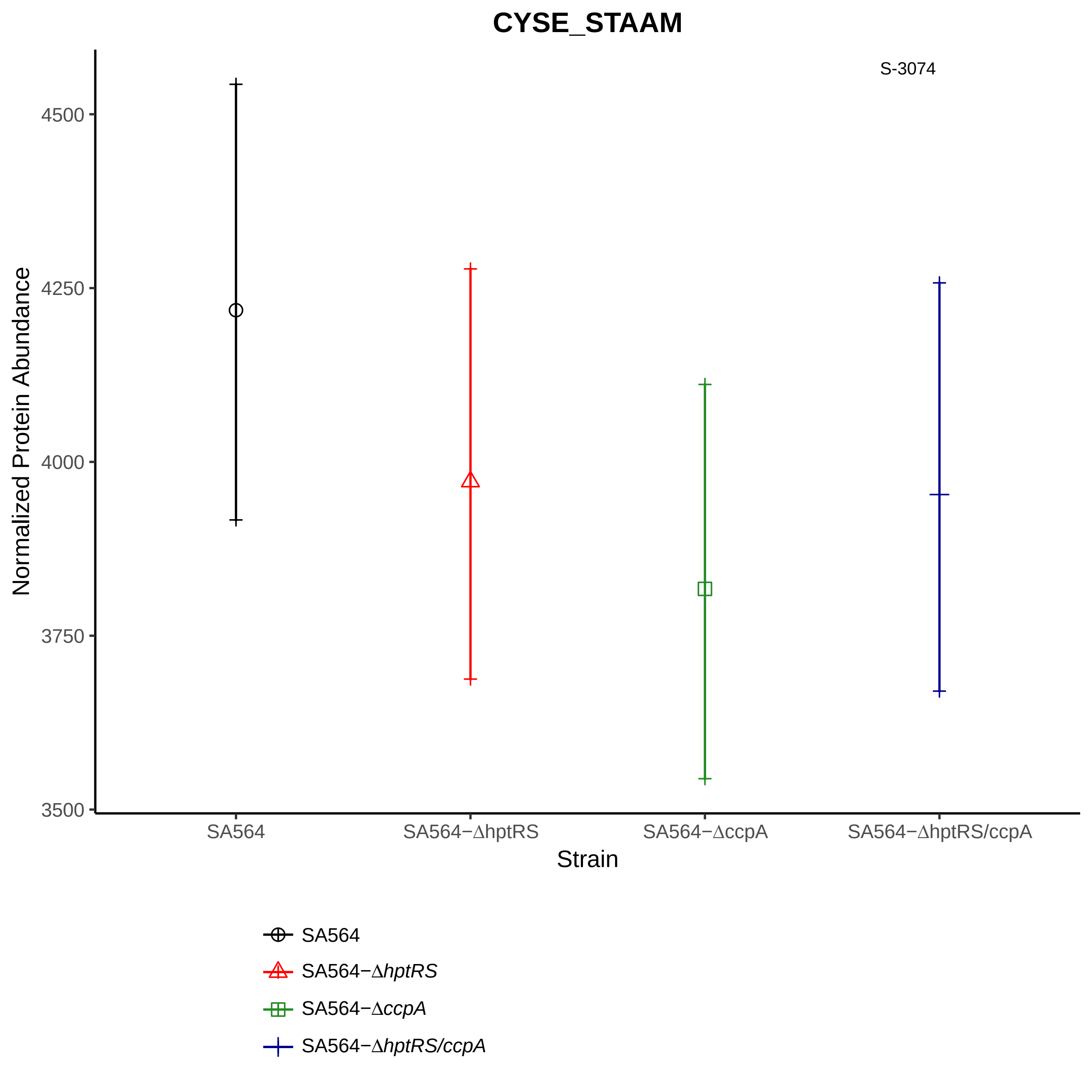
CYSE_STAAM
S-3074
Normalized Protein Abundance
4500
4250
4000
3750
3500
SA564
SA564−ΔhptRS
SA564−ΔccpA
SA564−ΔhptRS/ccpA
Strain
SA564
SA564−ΔhptRS
SA564−ΔccpA
SA564−ΔhptRS/ccpA

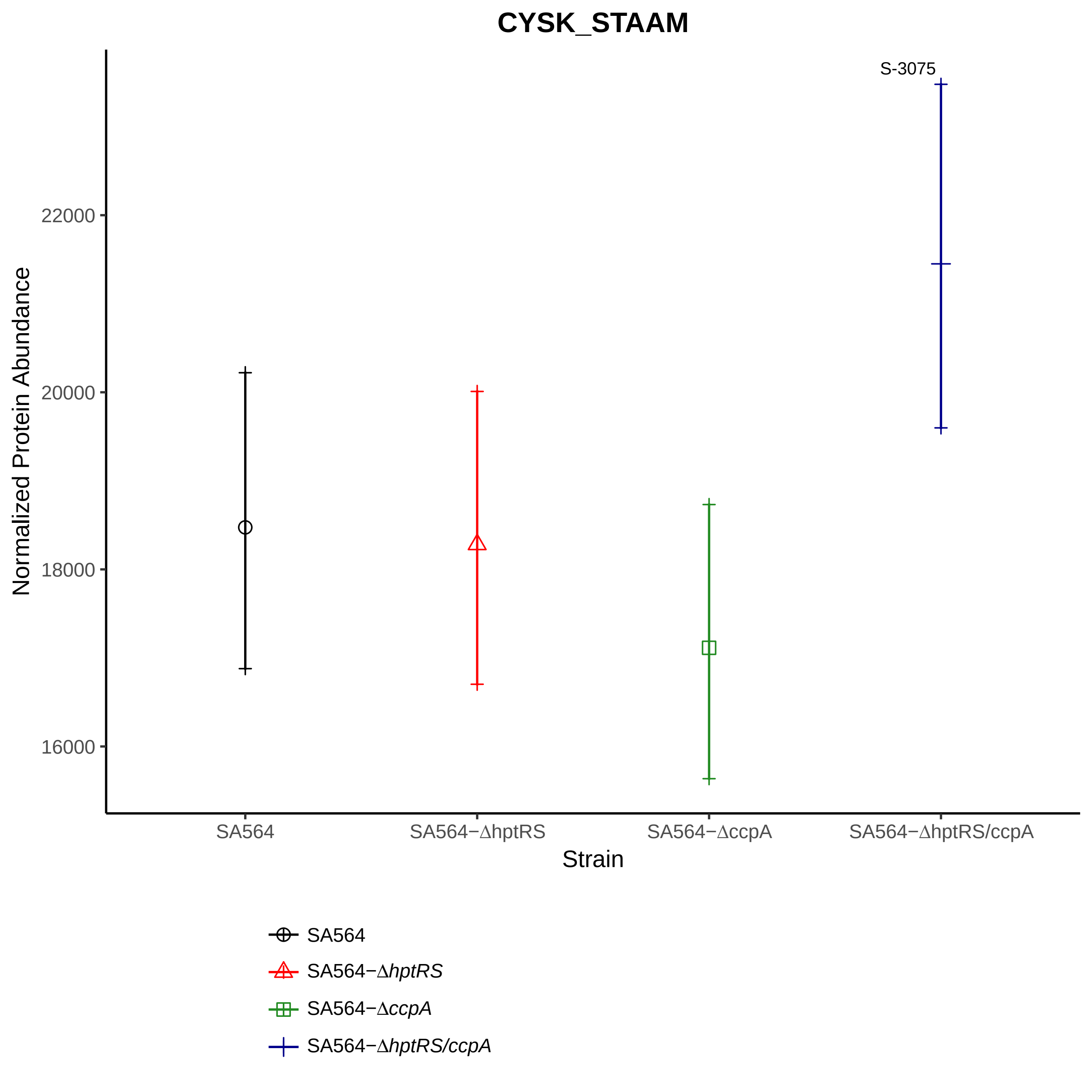
CYSK_STAAM
S-3075
Normalized Protein Abundance
22000
20000
18000
16000
SA564
SA564−ΔhptRS
SA564−ΔccpA
SA564−ΔhptRS/ccpA
Strain
SA564
SA564−ΔhptRS
SA564−ΔccpA
SA564−ΔhptRS/ccpA

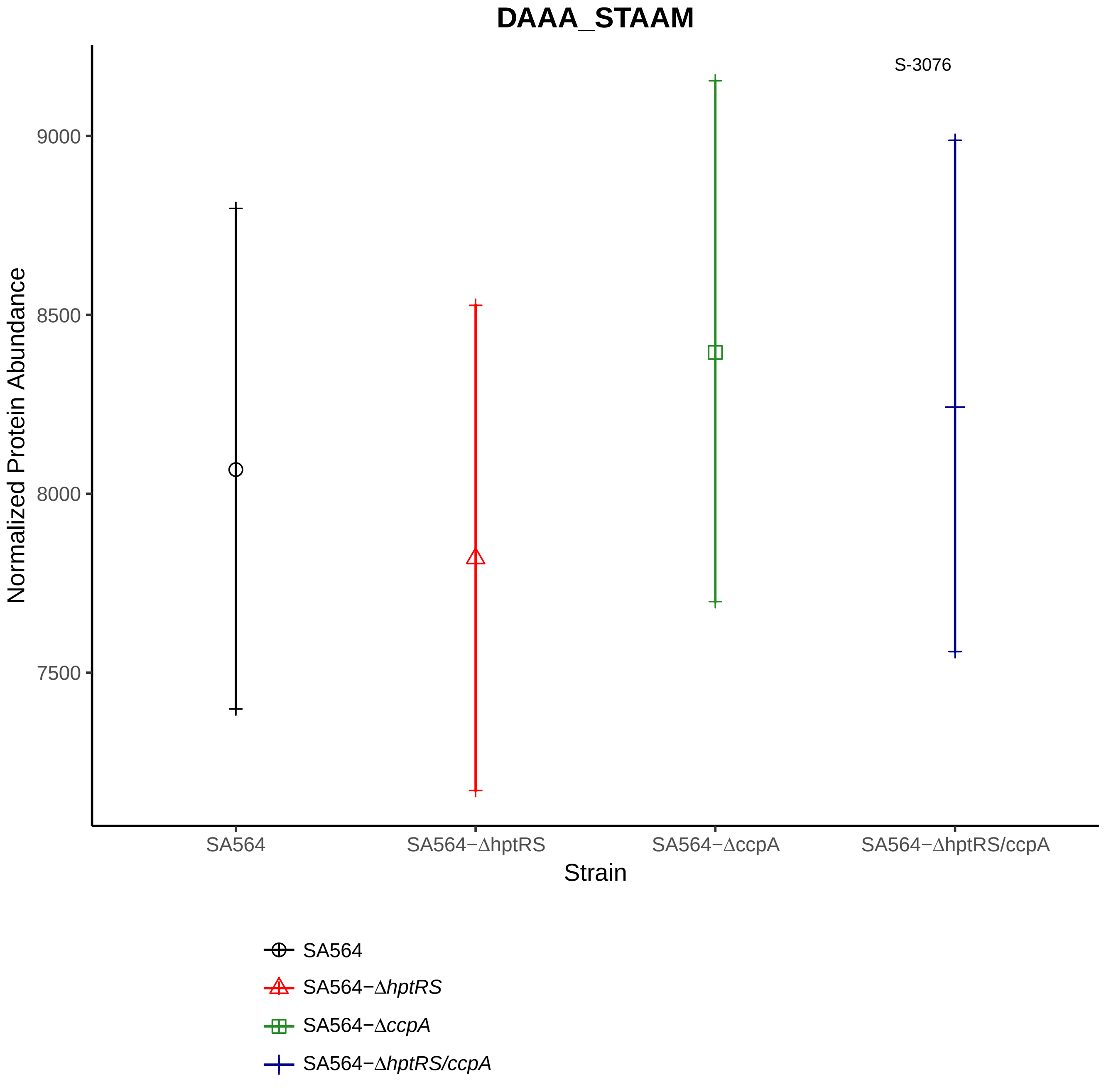

DAAA_STAAM
S-3076
Normalized Protein Abundance
9000
8500
8000
7500
SA564
SA564−ΔhptRS
SA564−ΔccpA
SA564−ΔhptRS/ccpA
Strain
SA564
SA564−ΔhptRS
SA564−ΔccpA
SA564−ΔhptRS/ccpA

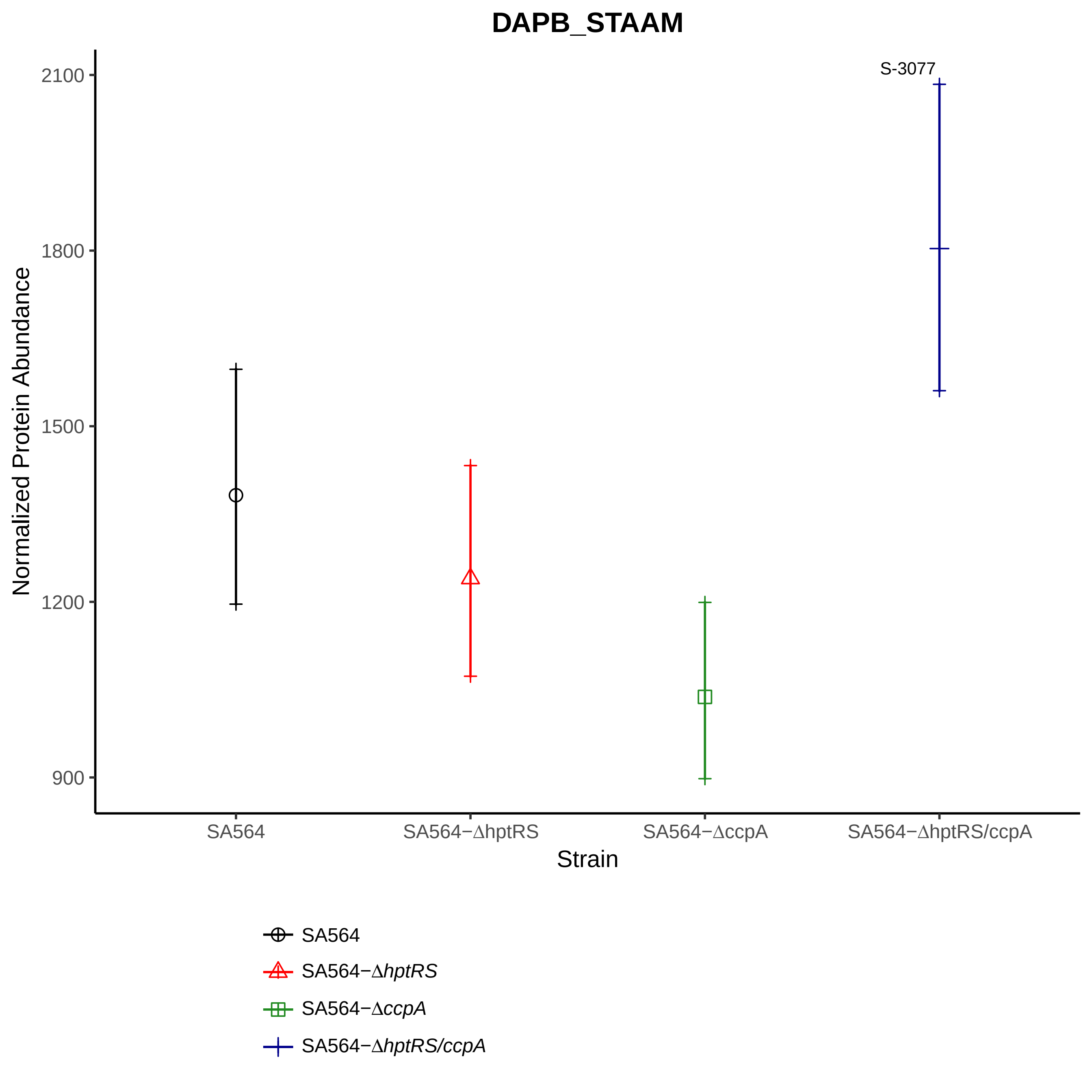

DAPB_STAAM
S-3077
2100
1800
1500
1200
900
Normalized Protein Abundance
SA564
SA564−ΔhptRS
SA564−ΔccpA
SA564−ΔhptRS/ccpA
Strain
SA564
SA564−Δ*hptRS*
SA564−Δ*ccpA*
SA564−Δ*hptRS/ccpA*

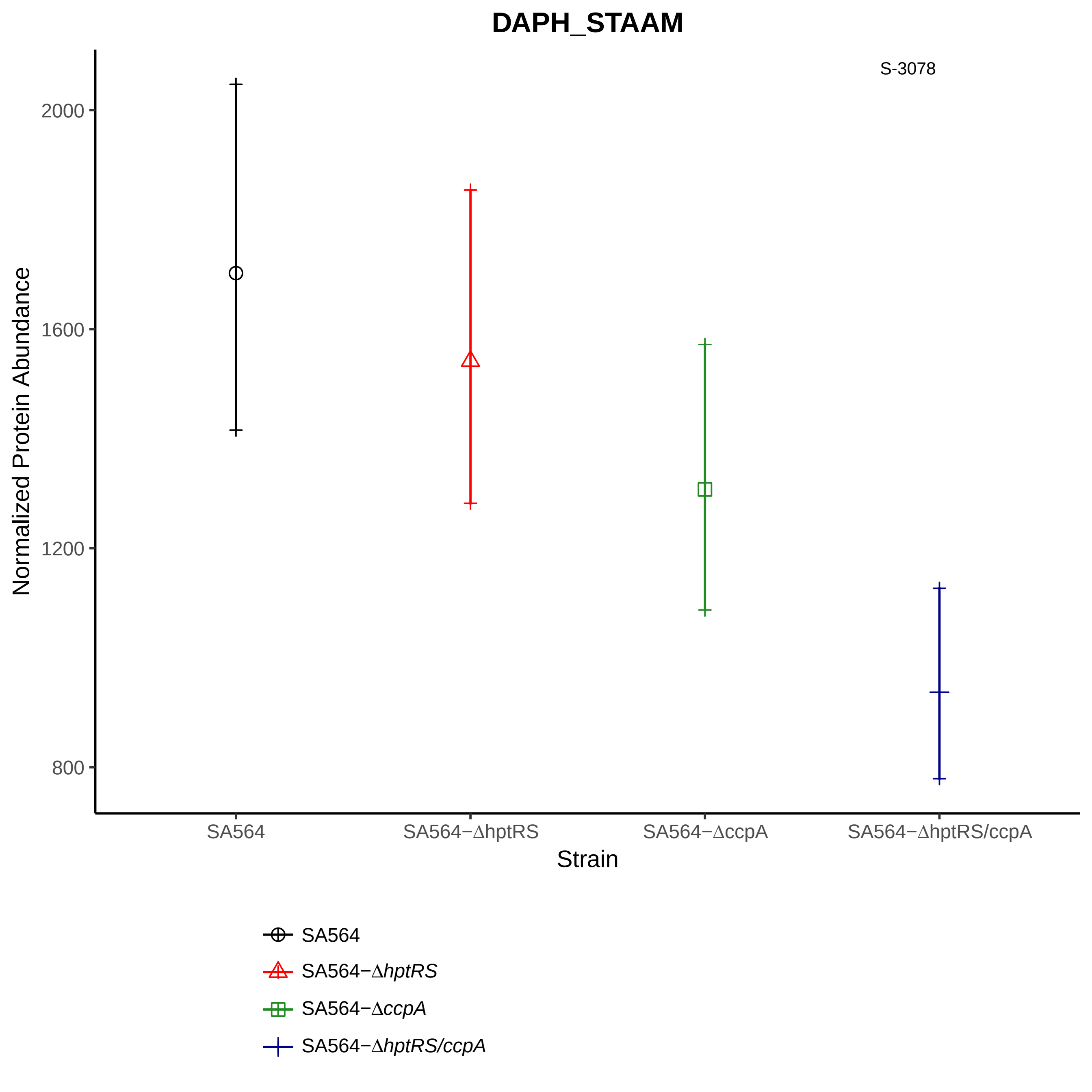
DAPH_STAAM
S-3078
Normalized Protein Abundance
2000
1600
1200
800
SA564
SA564−ΔhptRS
SA564−ΔccpA
SA564−ΔhptRS/ccpA
Strain
SA564
SA564−ΔhptRS
SA564−ΔccpA
SA564−ΔhptRS/ccpA

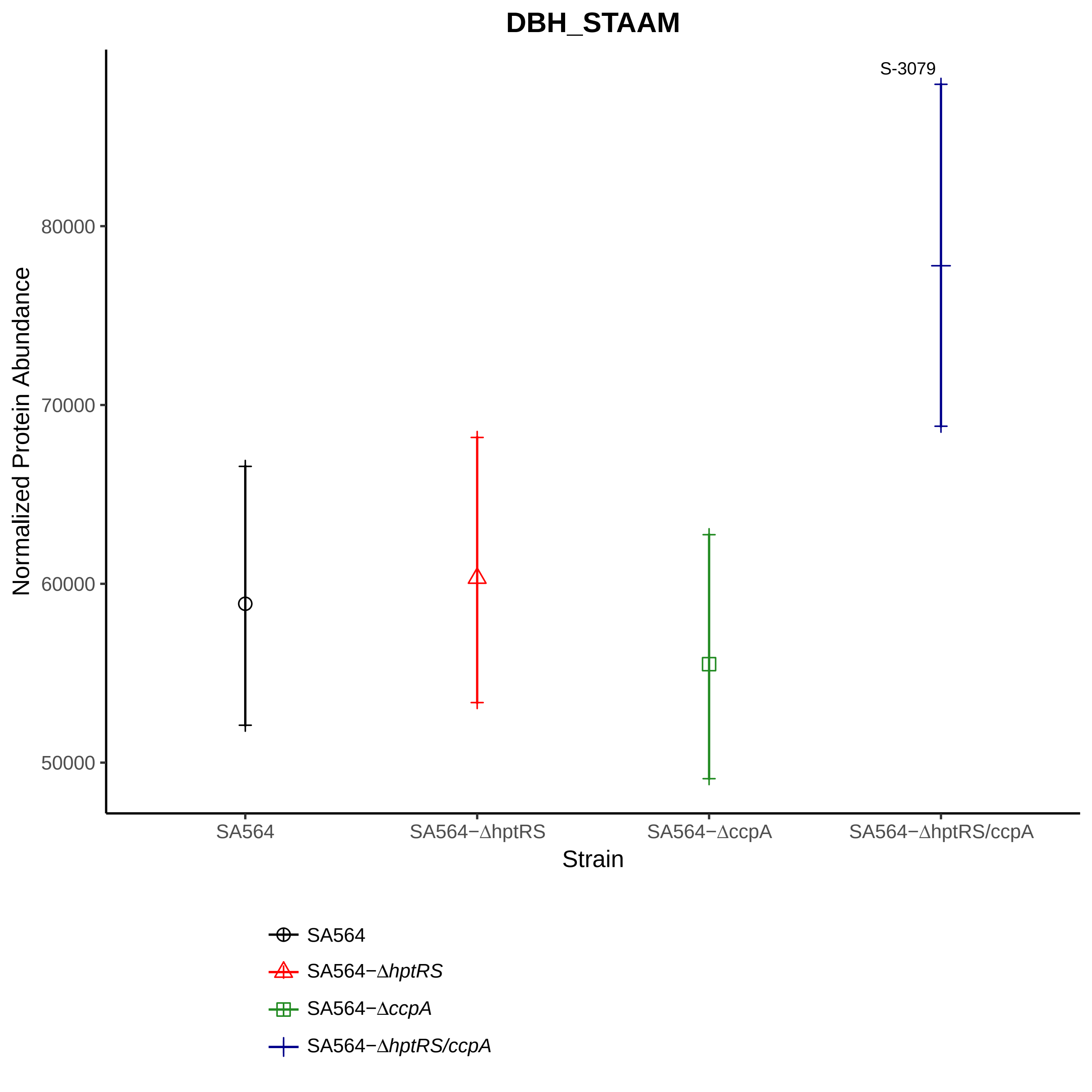

DBH_STAAM
S-3079
Normalized Protein Abundance
80000
70000
60000
50000
SA564
SA564−ΔhptRS
SA564−ΔccpA
SA564−ΔhptRS/ccpA
Strain
SA564
SA564−ΔhptRS
SA564−ΔccpA
SA564−ΔhptRS/ccpA

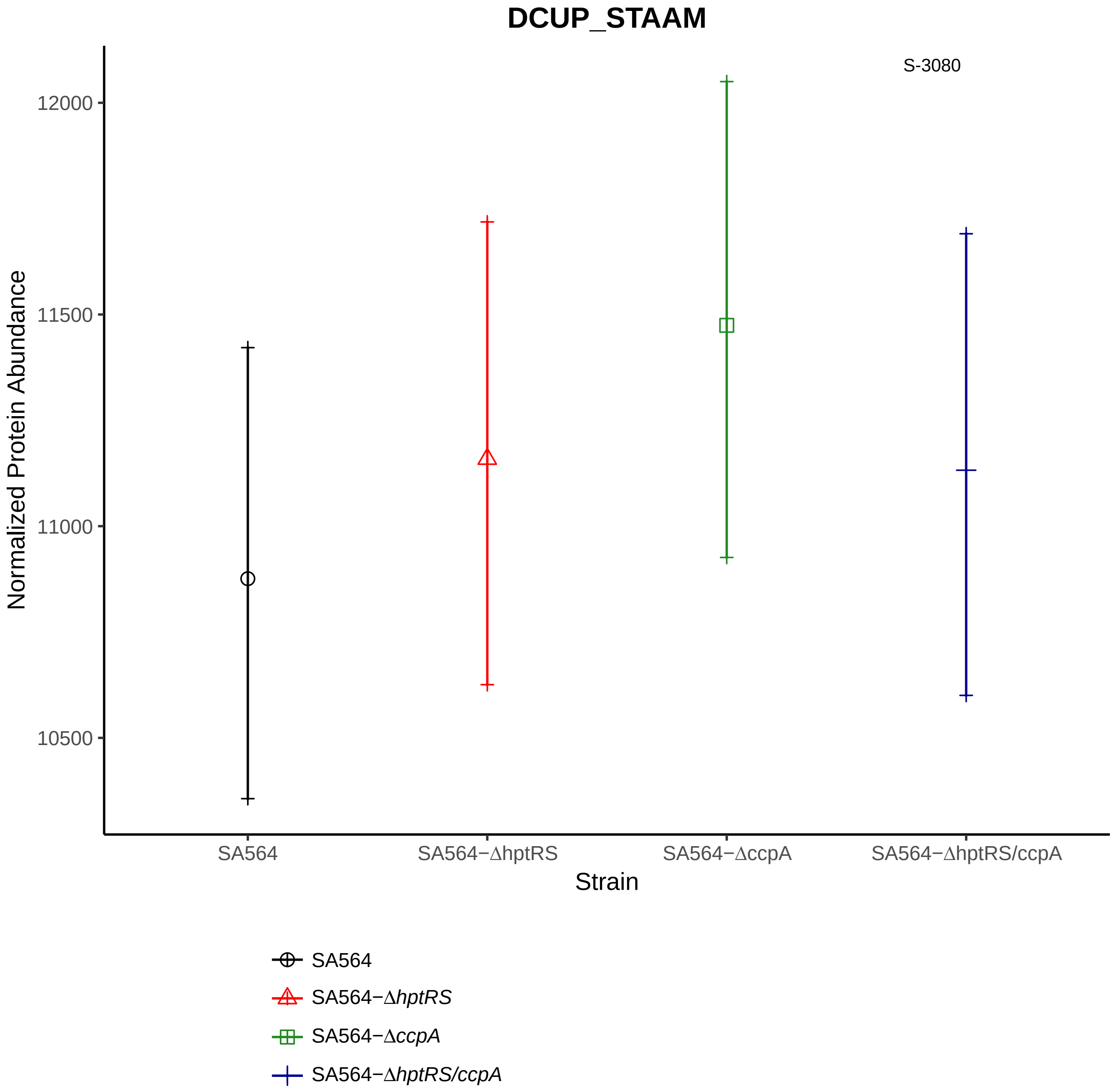
DCUP_STAAM
S-3080
Normalized Protein Abundance
12000
11500
11000
10500
SA564
SA564−ΔhptRS
SA564−ΔccpA
SA564−ΔhptRS/ccpA
Strain
SA564
SA564−ΔhptRS
SA564−ΔccpA
SA564−ΔhptRS/ccpA

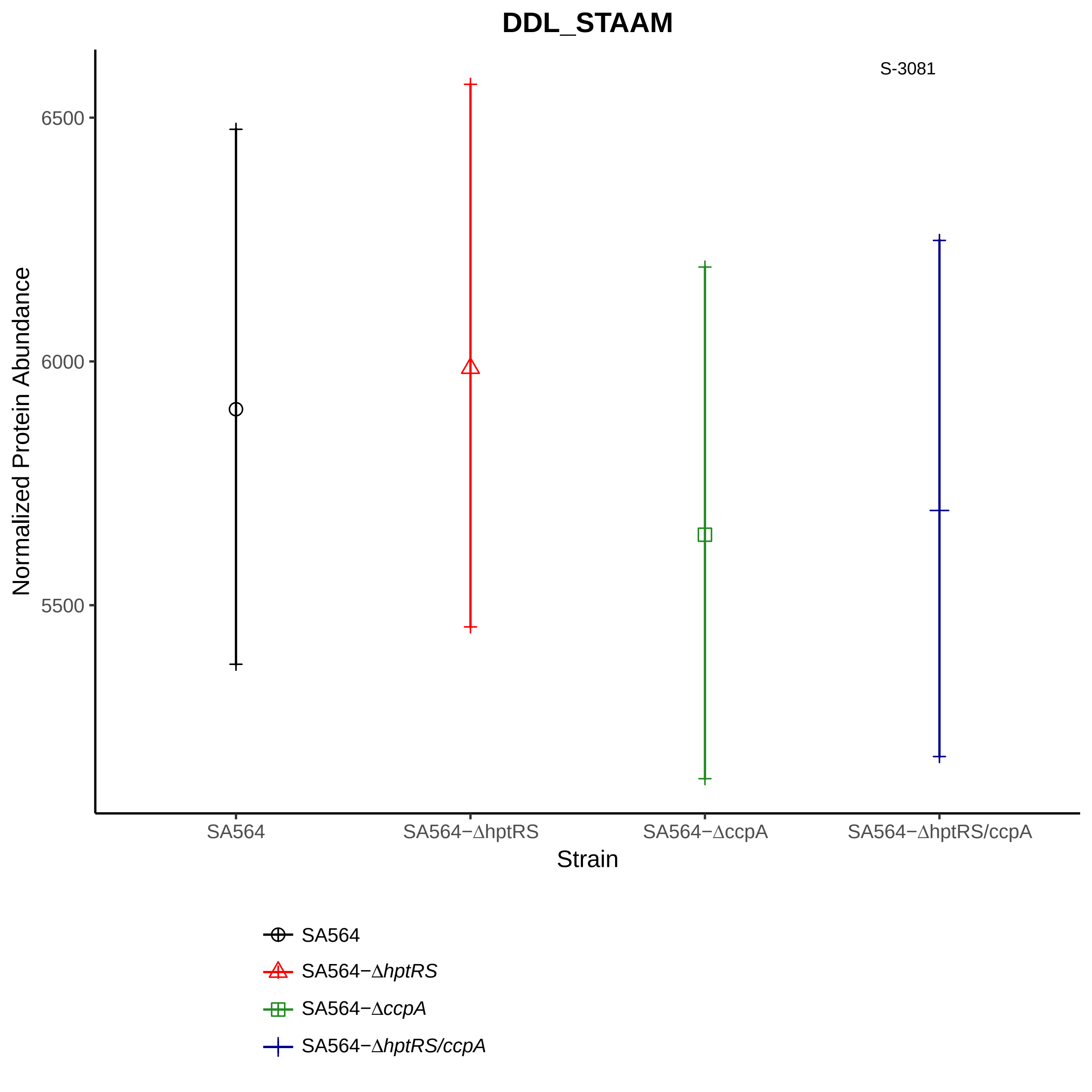

DDL_STAAM
S-3081
6500
6000
5500
Normalized Protein Abundance
SA564
SA564−ΔhptRS
SA564−ΔccpA
SA564−ΔhptRS/ccpA
Strain
SA564
SA564−ΔhptRS
SA564−ΔccpA
SA564−ΔhptRS/ccpA

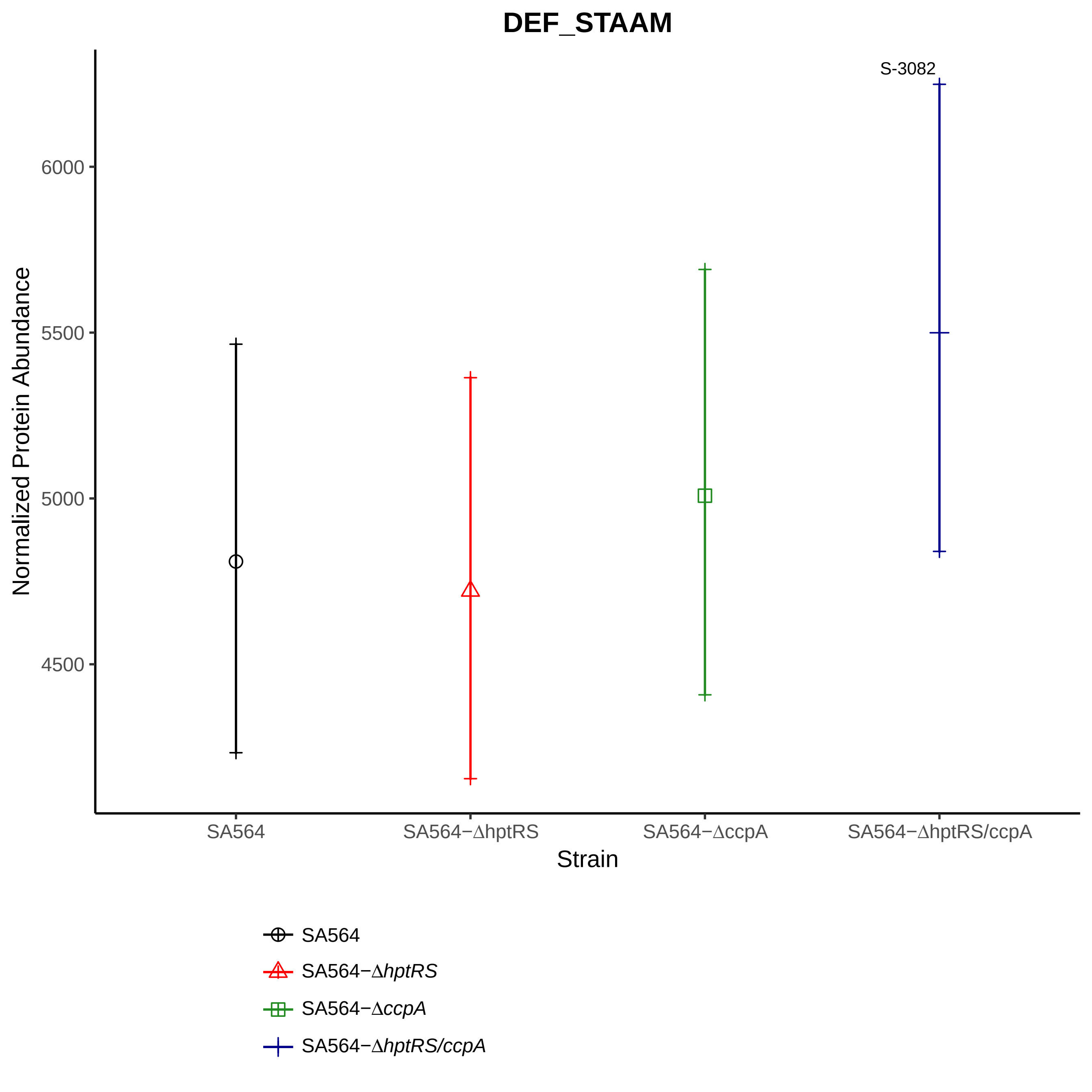
DEF_STAAM
S-3082
6000
5500
5000
4500
Normalized Protein Abundance
SA564
SA564−ΔhptRS
SA564−ΔccpA
SA564−ΔhptRS/ccpA
Strain
SA564
SA564−ΔhptRS
SA564−ΔccpA
SA564−ΔhptRS/ccpA

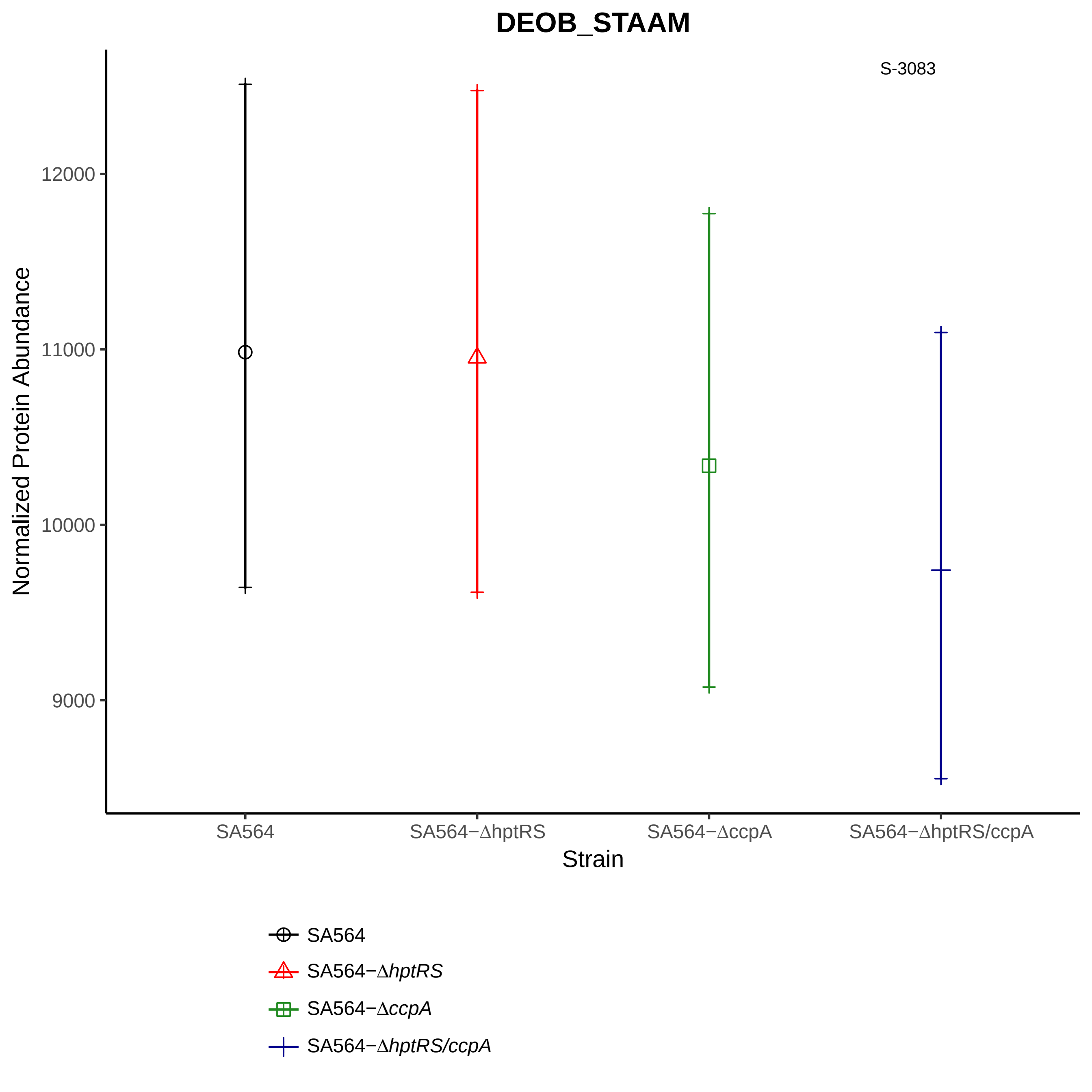
DEOB_STAAM
S-3083
Normalized Protein Abundance
12000
11000
10000
9000
SA564
SA564−ΔhptRS
SA564−ΔccpA
SA564−ΔhptRS/ccpA
Strain
SA564
SA564−ΔhptRS
SA564−ΔccpA
SA564−ΔhptRS/ccpA

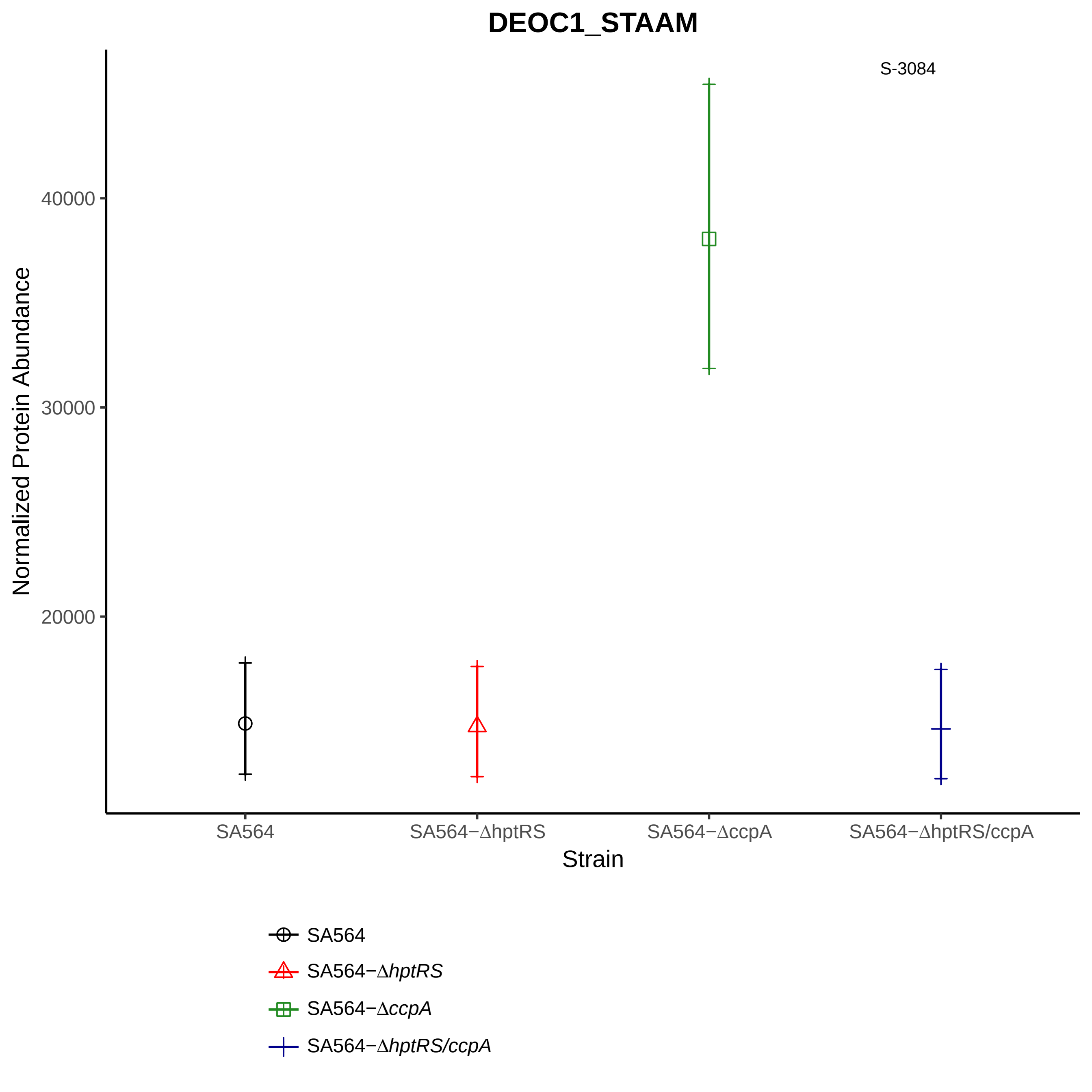
DEOC1_STAAM
S-3084
Normalized Protein Abundance
40000
30000
20000
SA564
SA564−ΔhptRS
SA564−ΔccpA
SA564−ΔhptRS/ccpA
Strain
SA564
SA564−ΔhptRS
SA564−ΔccpA
SA564−ΔhptRS/ccpA

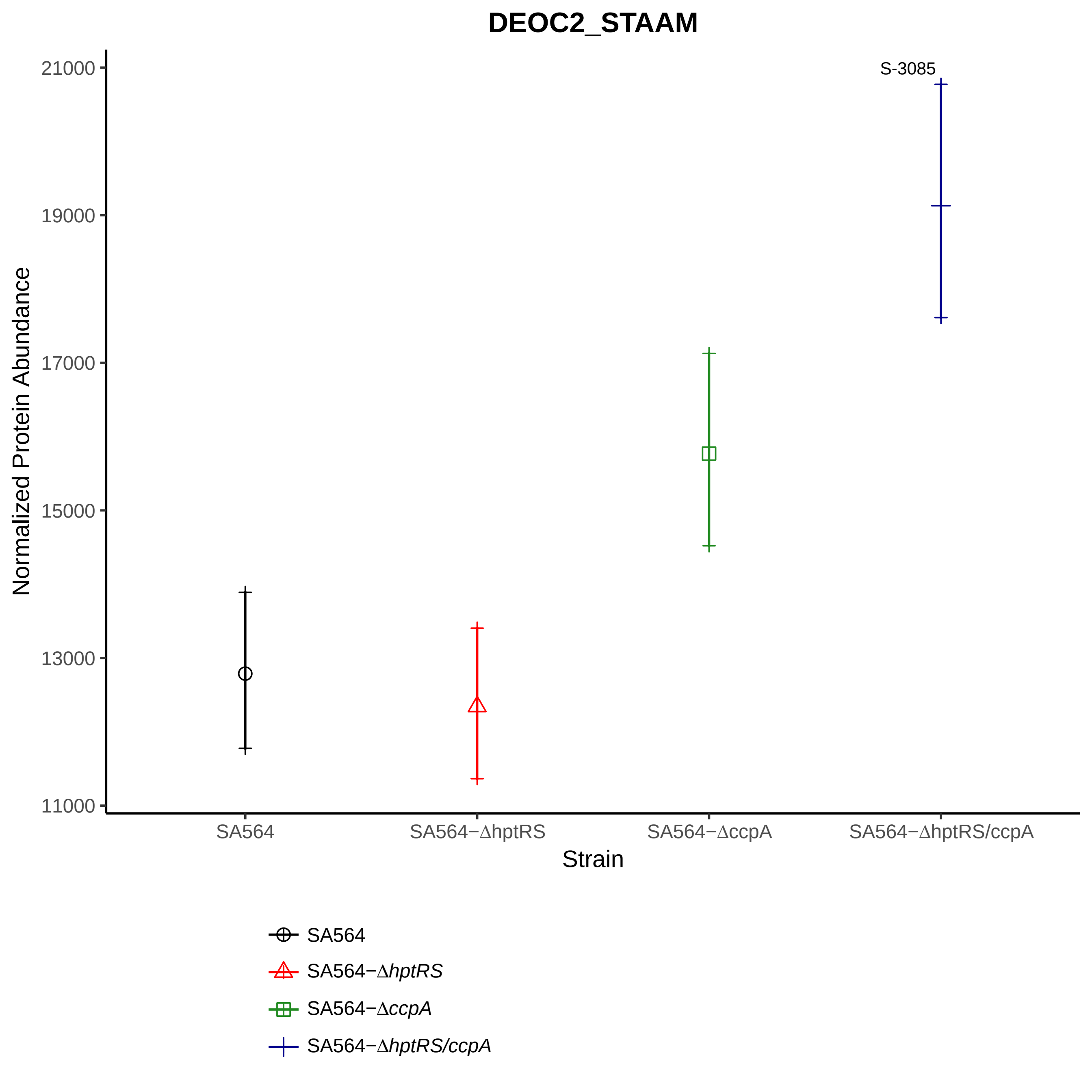

DEOC2_STAAM
S-3085
Normalized Protein Abundance
21000
19000
17000
15000
13000
11000
SA564
SA564−ΔhptRS
SA564−ΔccpA
SA564−ΔhptRS/ccpA
Strain
SA564
SA564−ΔhptRS
SA564−ΔccpA
SA564−ΔhptRS/ccpA

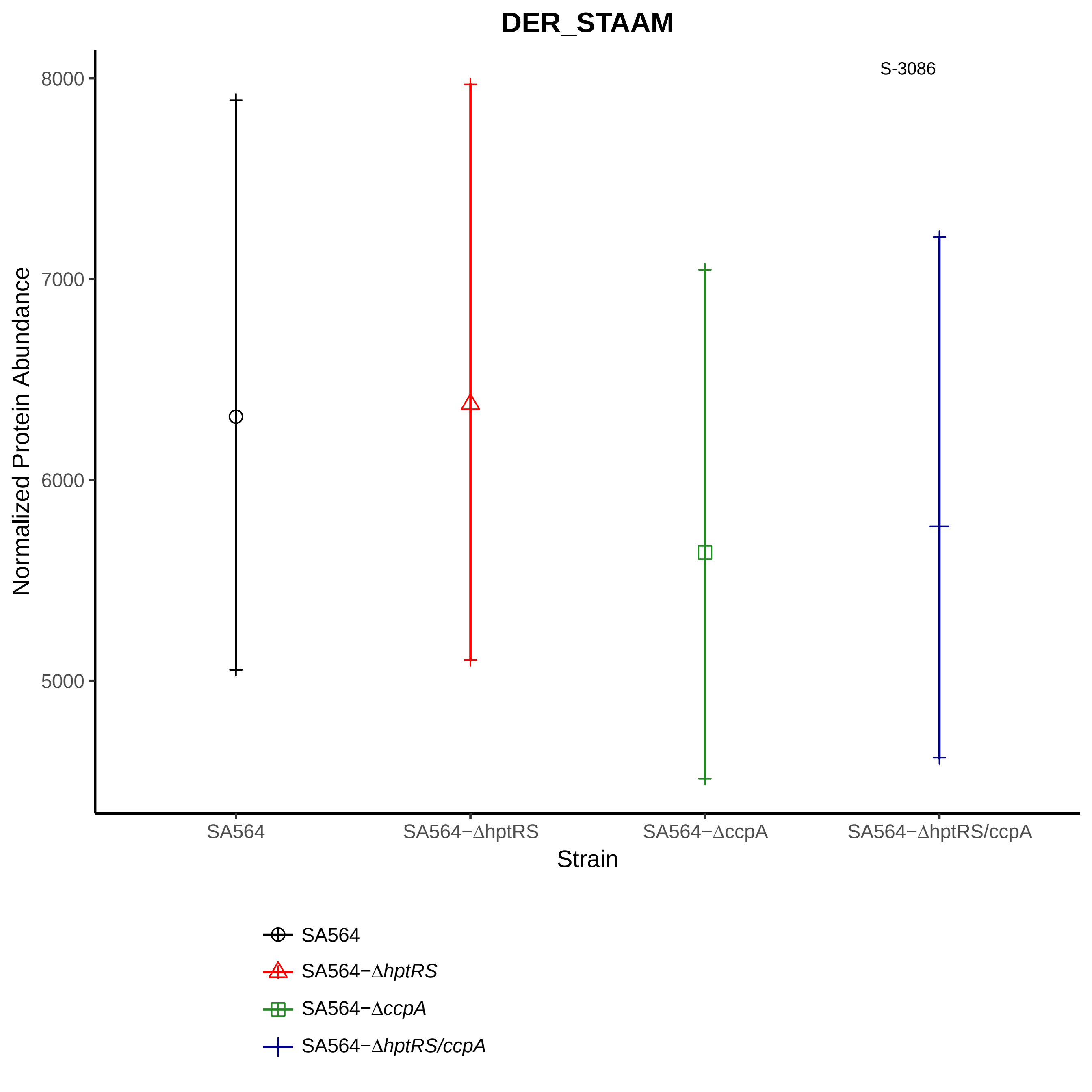

DER_STAAM
S-3086
Normalized Protein Abundance
8000
7000
6000
5000
SA564
SA564−ΔhptRS
SA564−ΔccpA
SA564−ΔhptRS/ccpA
Strain
SA564
SA564−ΔhptRS
SA564−ΔccpA
SA564−ΔhptRS/ccpA

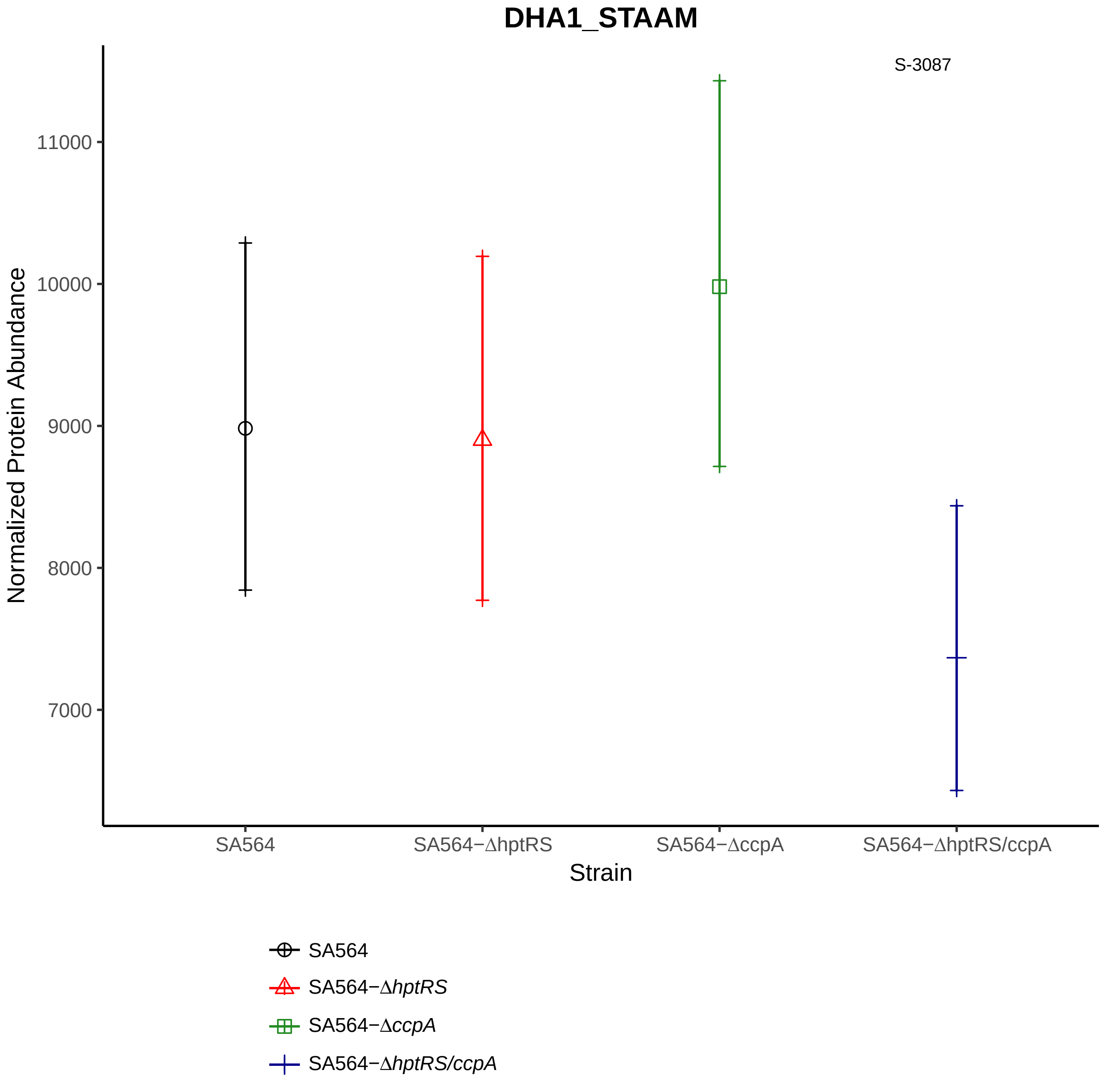

DHA1_STAAM
S-3087
Normalized Protein Abundance
11000
10000
9000
8000
7000
SA564
SA564−ΔhptRS
SA564−ΔccpA
SA564−ΔhptRS/ccpA
Strain
SA564
SA564−ΔhptRS
SA564−ΔccpA
SA564−ΔhptRS/ccpA

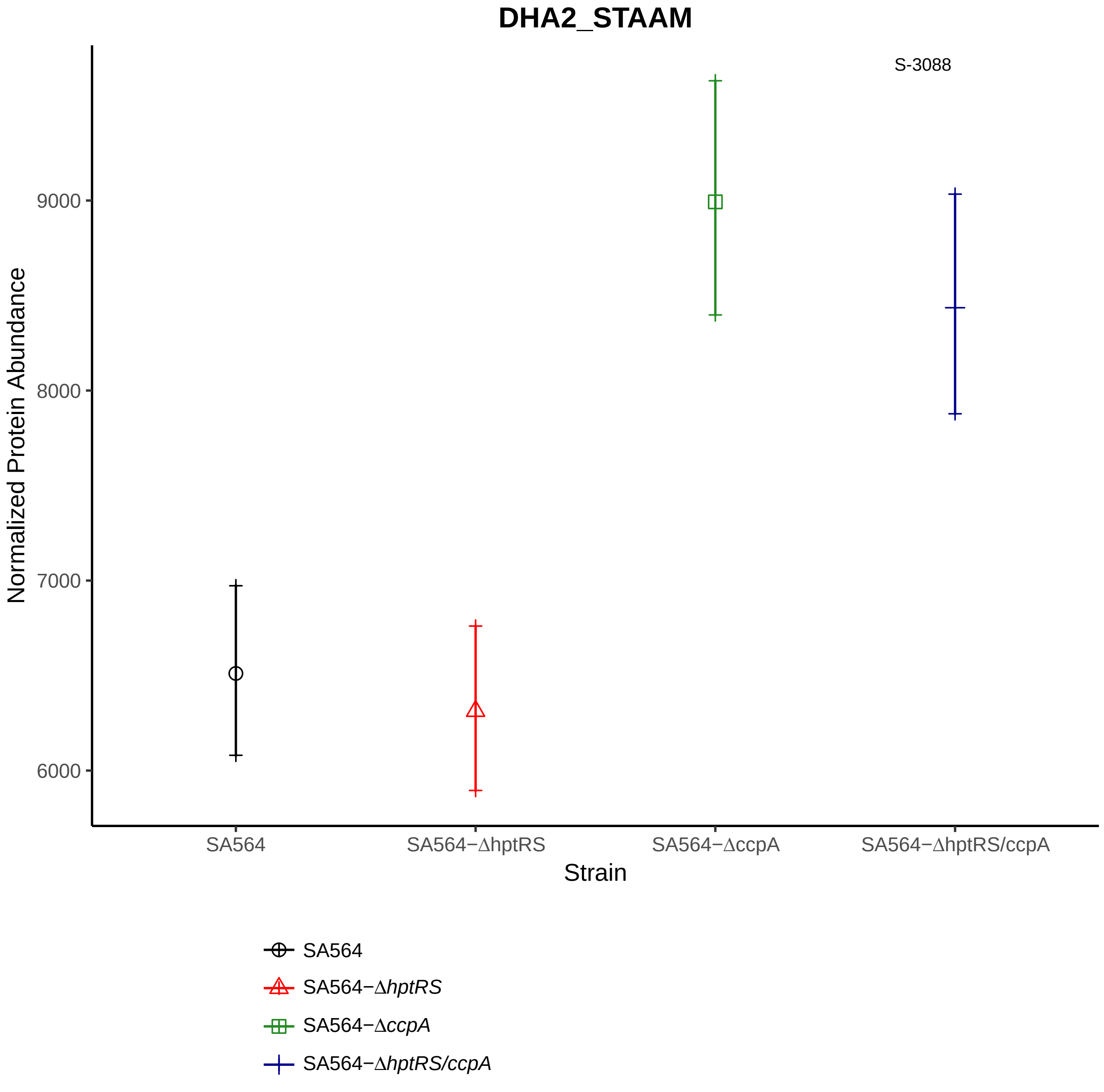

DHA2_STAAM
S-3088
Normalized Protein Abundance
9000
8000
7000
6000
SA564
SA564−ΔhptRS
SA564−ΔccpA
SA564−ΔhptRS/ccpA
Strain
SA564
SA564−ΔhptRS
SA564−ΔccpA
SA564−ΔhptRS/ccpA

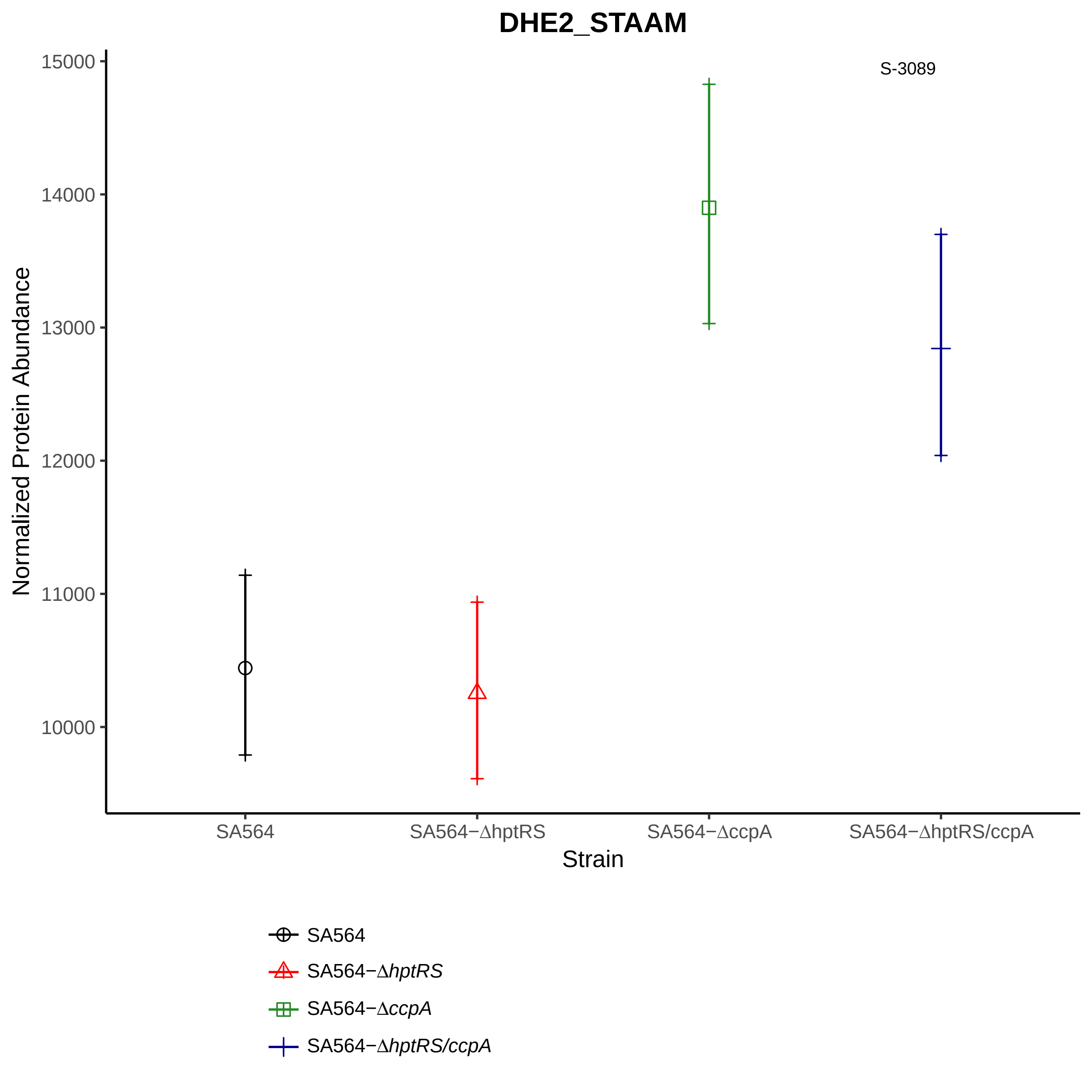

DHE2_STAAM
S-3089
Normalized Protein Abundance
15000
14000
13000
12000
11000
10000
SA564
SA564−ΔhptRS
SA564−ΔccpA
SA564−ΔhptRS/ccpA
Strain
SA564
SA564−Δ*hptRS*
SA564−Δ*ccpA*
SA564−Δ*hptRS/ccpA*

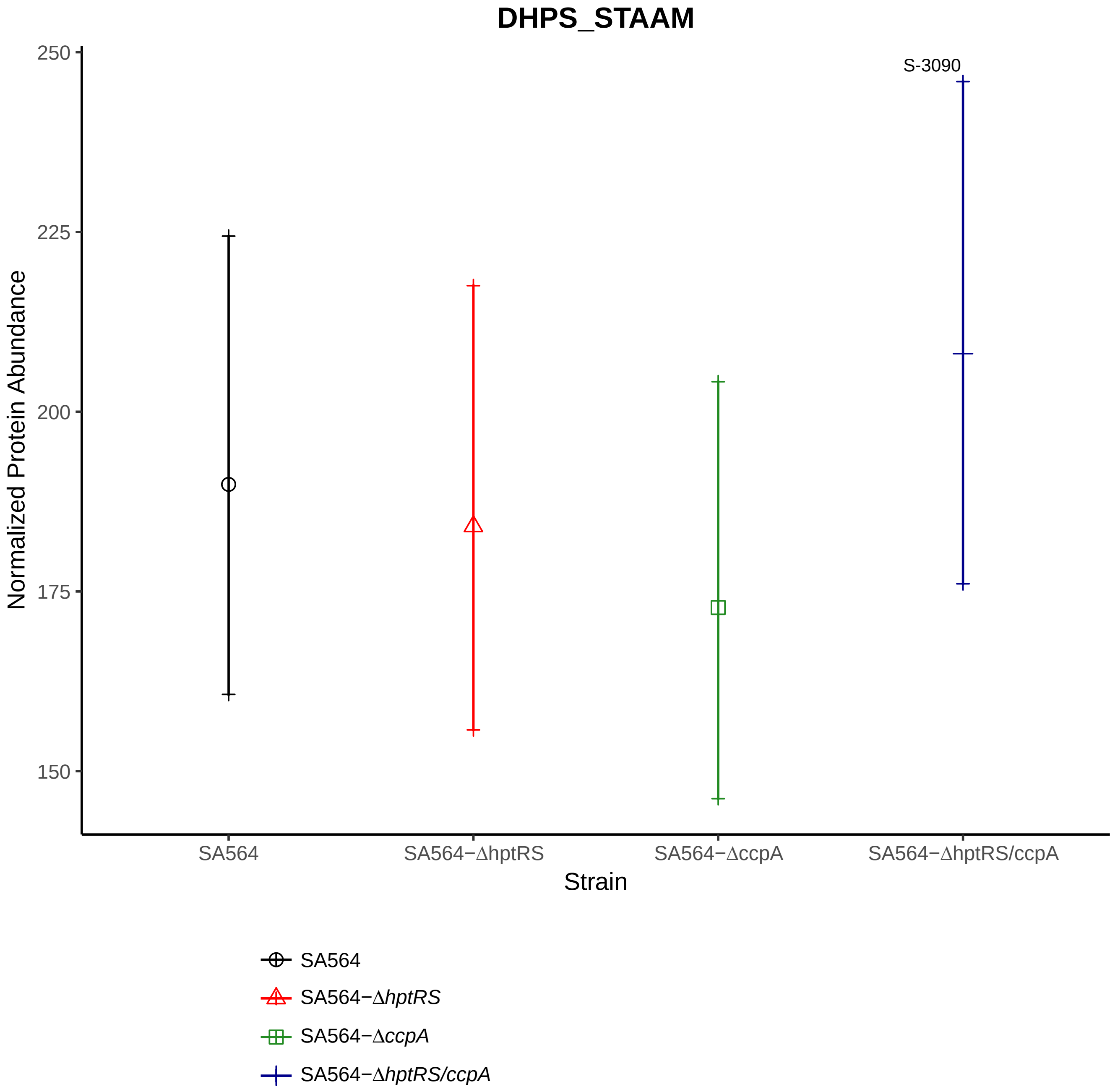

DHPS_STAAM
S-3090
250
225
200
175
150
Normalized Protein Abundance
SA564
SA564−ΔhptRS
SA564−ΔccpA
SA564−ΔhptRS/ccpA
Strain
SA564
SA564−ΔhptRS
SA564−ΔccpA
SA564−ΔhptRS/ccpA

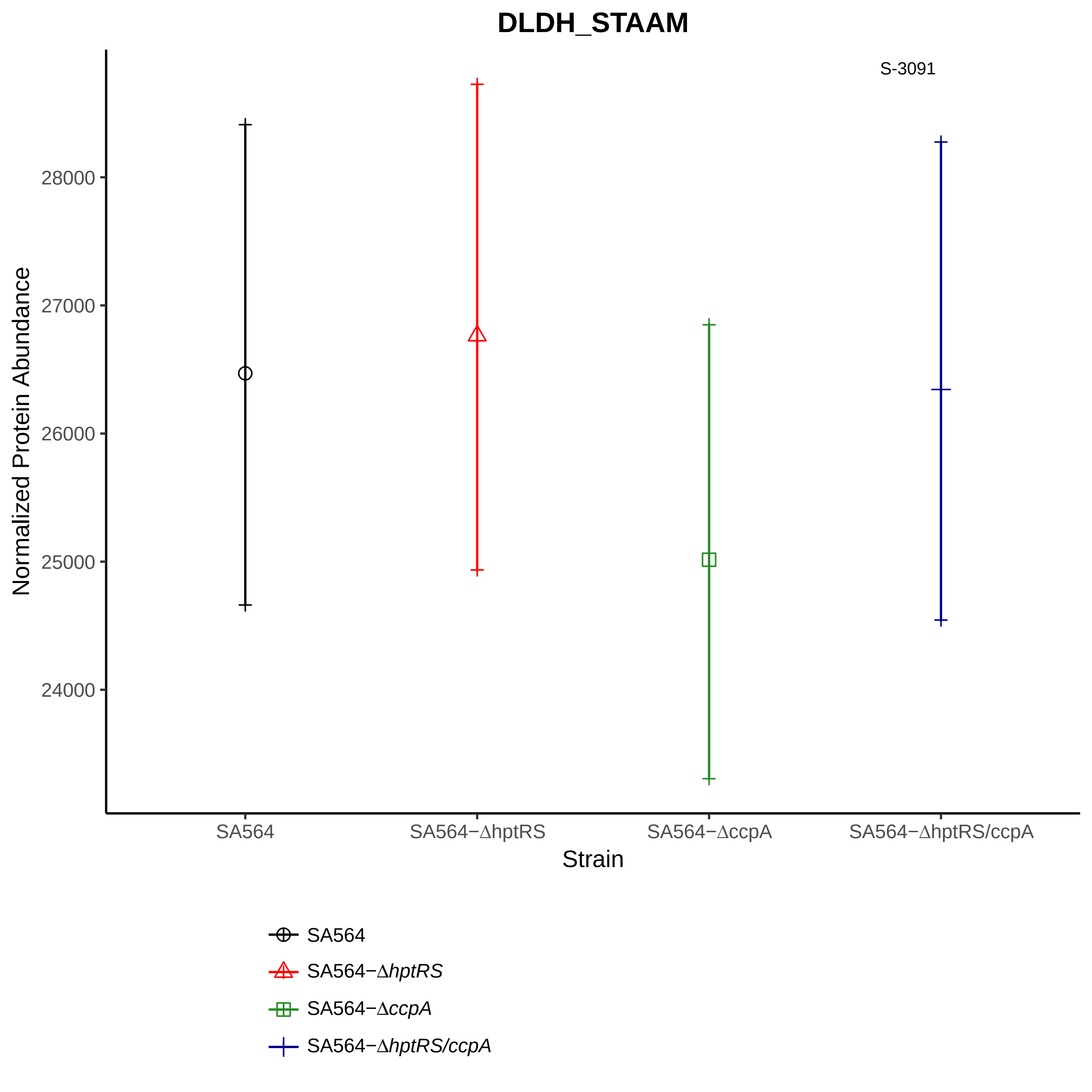
DLDH_STAAM
S-3091
Normalized Protein Abundance
28000
27000
26000
25000
24000
SA564
SA564−ΔhptRS
SA564−ΔccpA
SA564−ΔhptRS/ccpA
Strain
SA564
SA564−ΔhptRS
SA564−ΔccpA
SA564−ΔhptRS/ccpA

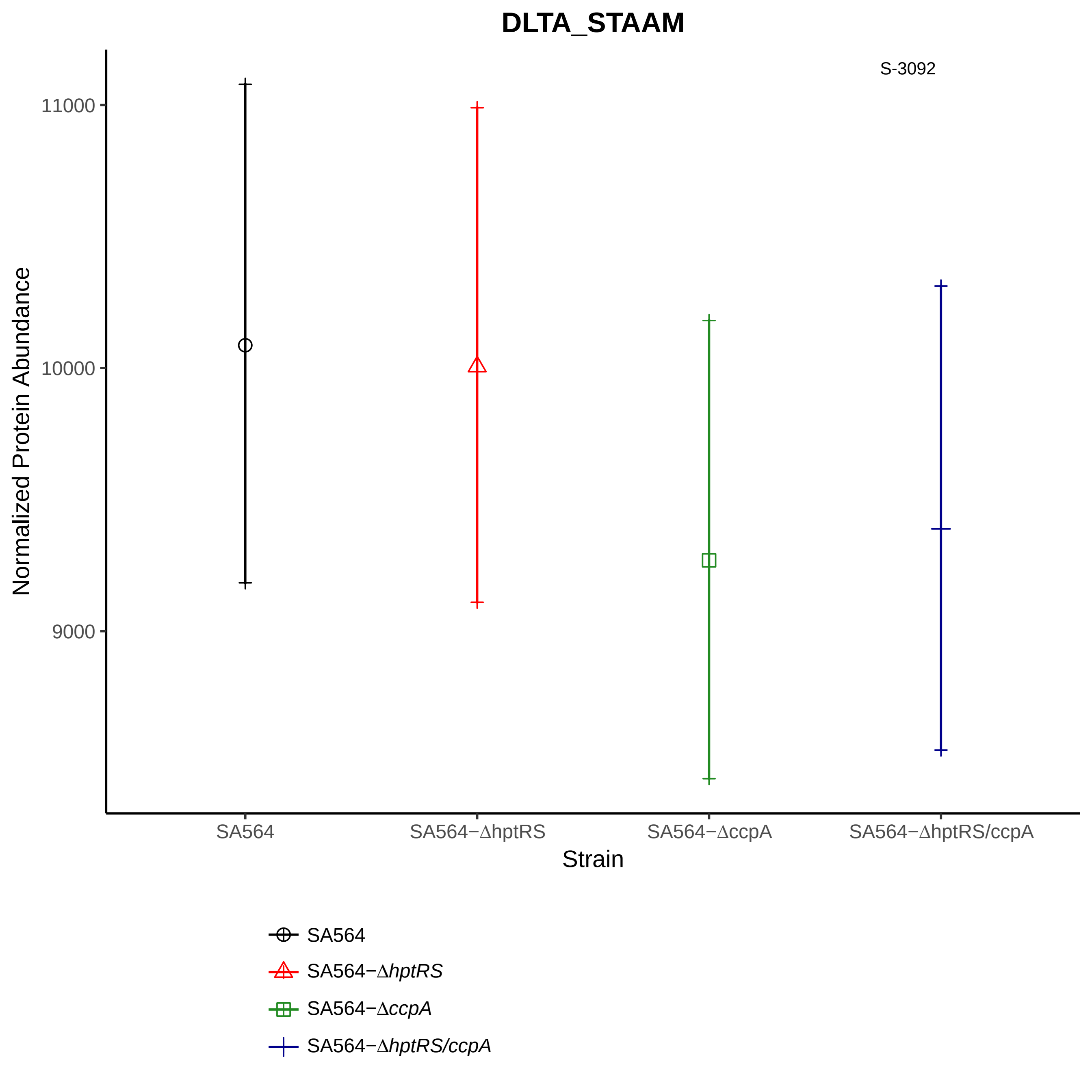

DLTA_STAAM
S-3092
Normalized Protein Abundance
11000
10000
9000
SA564
SA564−ΔhptRS
SA564−ΔccpA
SA564−ΔhptRS/ccpA
Strain
SA564
SA564−ΔhptRS
SA564−ΔccpA
SA564−ΔhptRS/ccpA

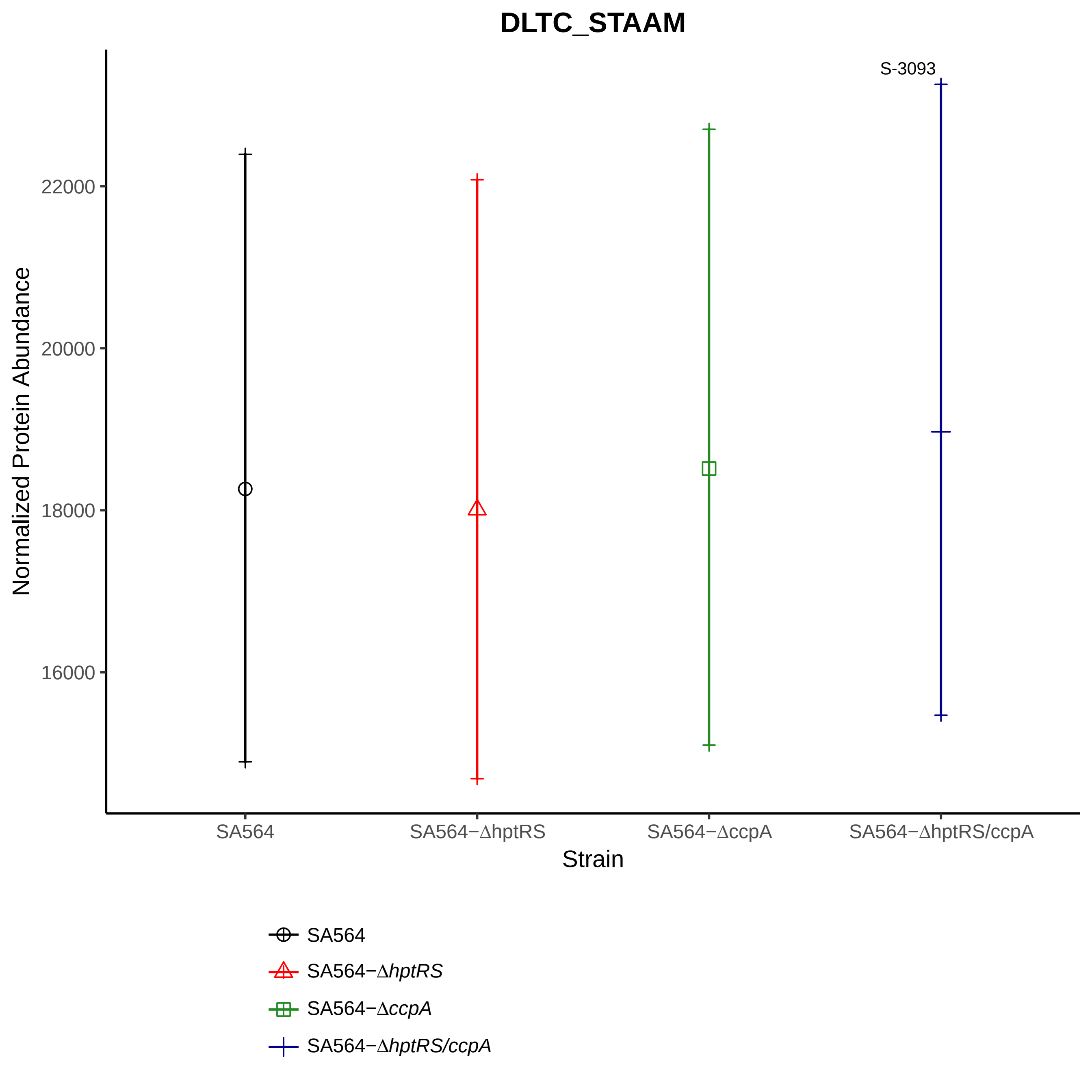

DLTC_STAAM
S-3093
22000
20000
18000
16000
Normalized Protein Abundance
SA564
SA564−ΔhptRS
SA564−ΔccpA
SA564−ΔhptRS/ccpA
Strain
SA564
SA564−Δ*hptRS*
SA564−Δ*ccpA*
SA564−Δ*hptRS/ccpA*

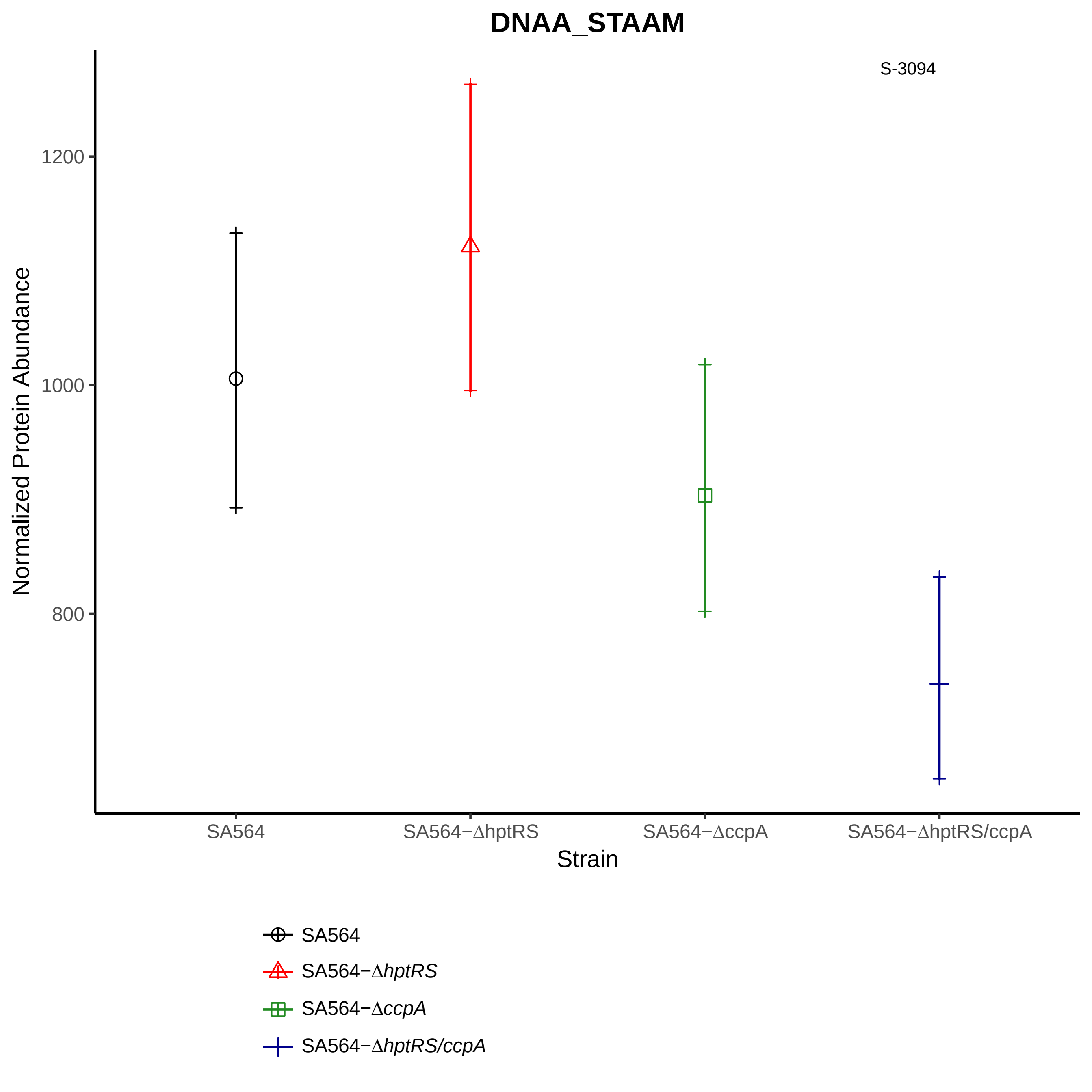

DNAA_STAAM
S-3094
Normalized Protein Abundance
1200
1000
800
SA564
SA564−ΔhptRS
SA564−ΔccpA
SA564−ΔhptRS/ccpA
Strain
SA564
SA564−ΔhptRS
SA564−ΔccpA
SA564−ΔhptRS/ccpA

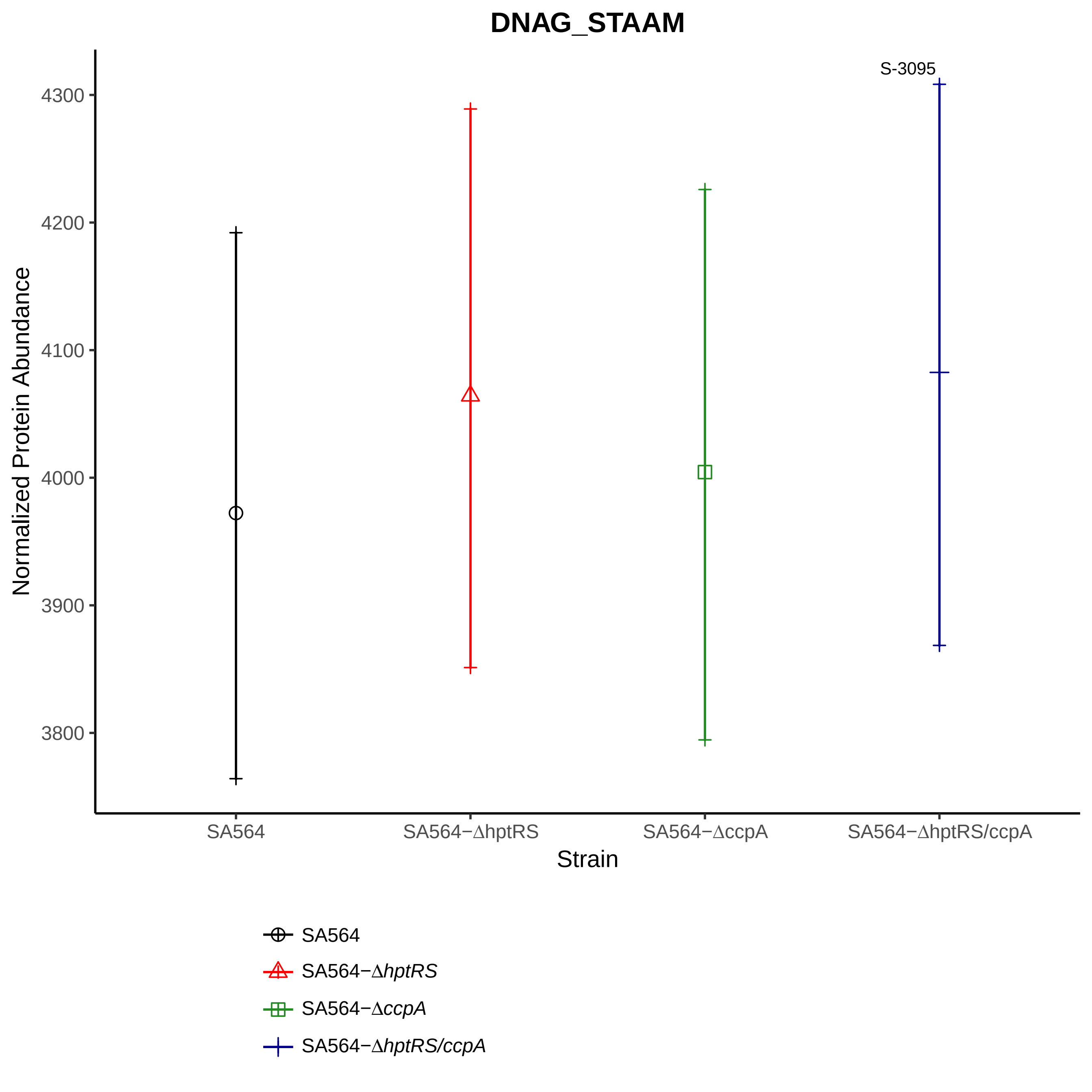
DNAG_STAAM
S-3095
Normalized Protein Abundance
4300
4200
4100
4000
3900
3800
SA564
SA564−ΔhptRS
SA564−ΔccpA
SA564−ΔhptRS/ccpA
Strain
SA564
SA564−ΔhptRS
SA564−ΔccpA
SA564−ΔhptRS/ccpA

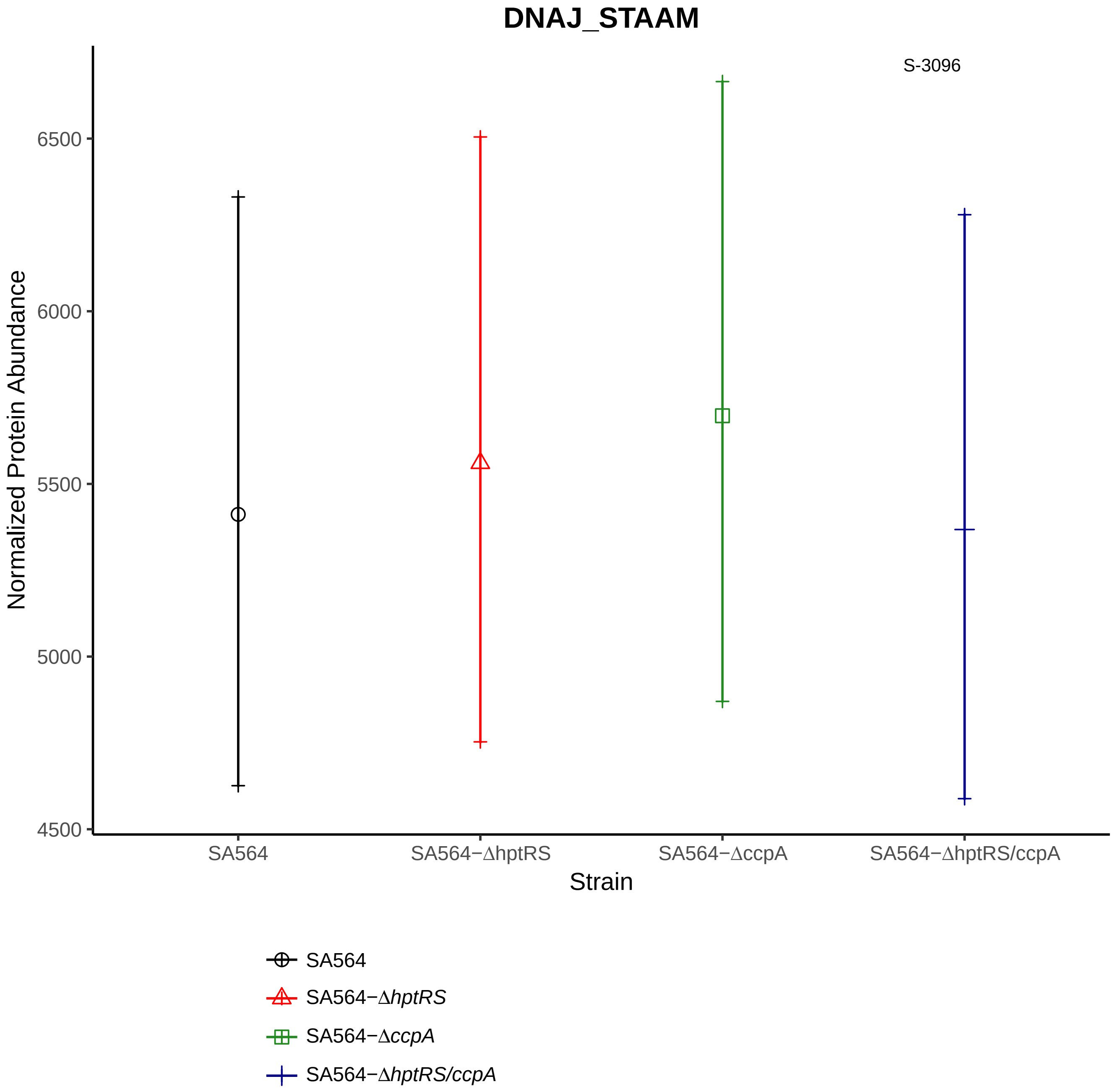

DNAJ_STAAM
S-3096
Normalized Protein Abundance
6500
6000
5500
5000
4500
SA564
SA564−ΔhptRS
SA564−ΔccpA
SA564−ΔhptRS/ccpA
Strain
SA564
SA564−ΔhptRS
SA564−ΔccpA
SA564−ΔhptRS/ccpA

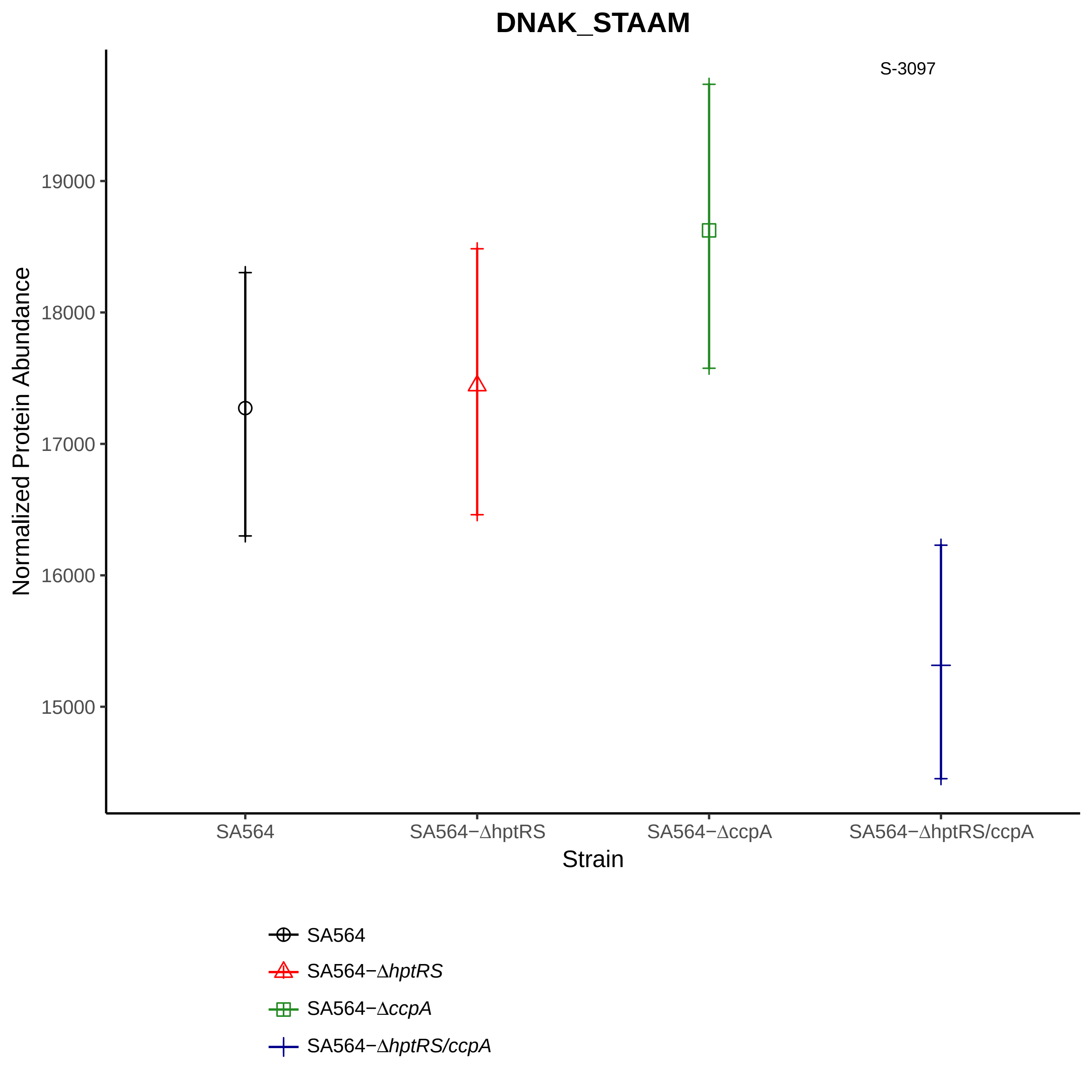

DNAK_STAAM
S-3097
Normalized Protein Abundance
19000
18000
17000
16000
15000
SA564
SA564−ΔhptRS
SA564−ΔccpA
SA564−ΔhptRS/ccpA
Strain
SA564
SA564−ΔhptRS
SA564−ΔccpA
SA564−ΔhptRS/ccpA

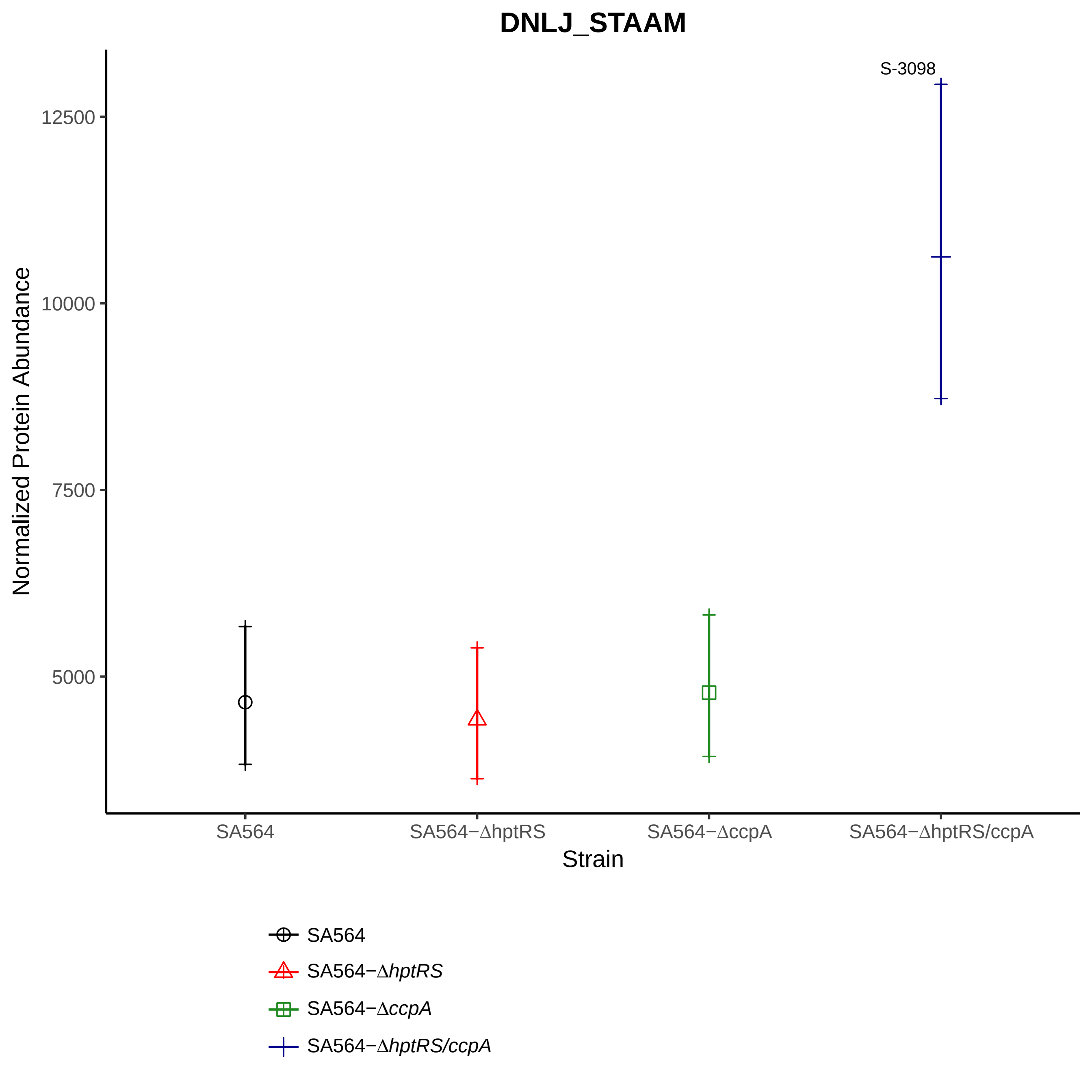

DNLJ_STAAM
S-3098
12500
10000
7500
5000
Normalized Protein Abundance
SA564
SA564−ΔhptRS
SA564−ΔccpA
SA564−ΔhptRS/ccpA
Strain
SA564
SA564−ΔhptRS
SA564−ΔccpA
SA564−ΔhptRS/ccpA

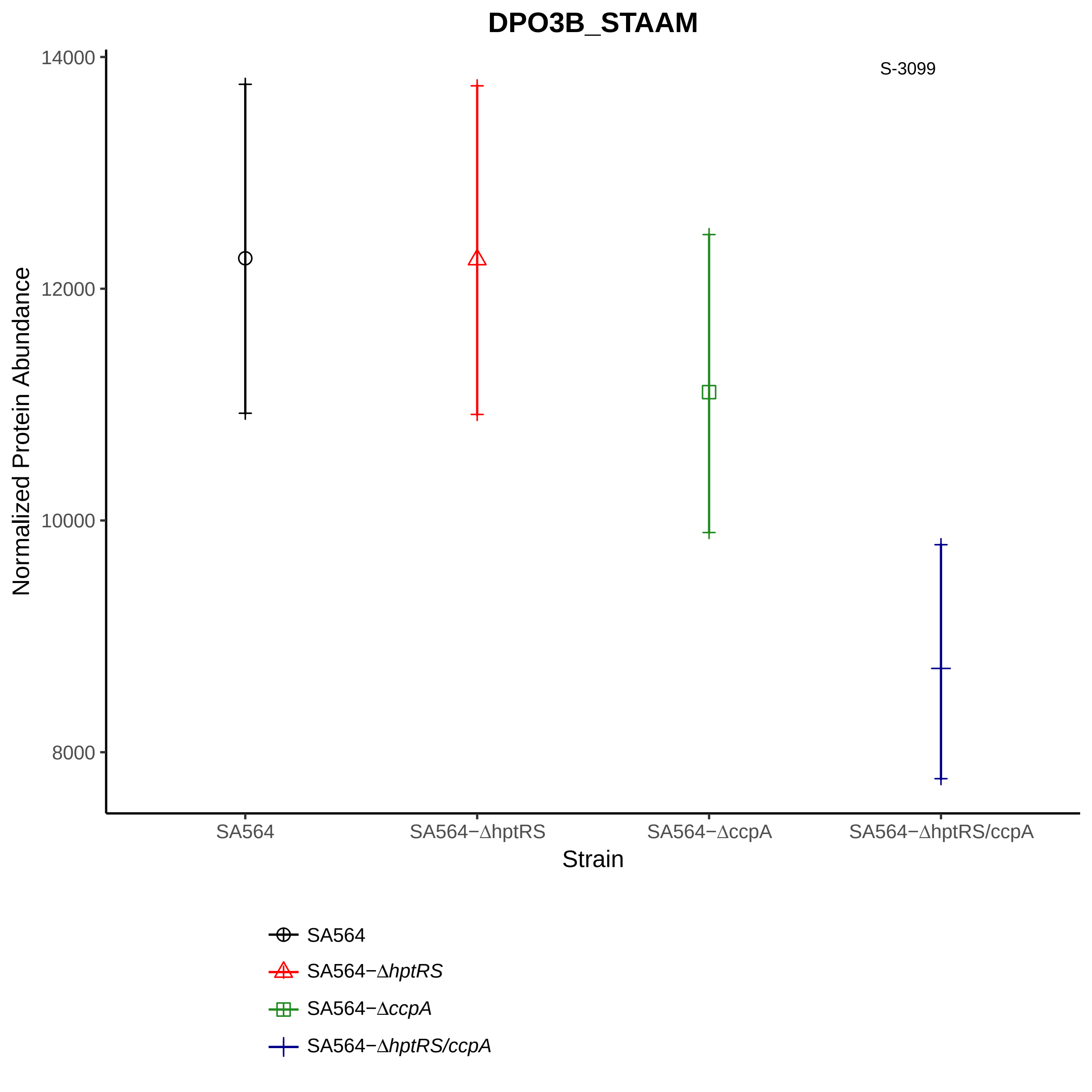

DPO3B_STAAM
S-3099
14000
12000
10000
8000
Normalized Protein Abundance
SA564
SA564−ΔhptRS
SA564−ΔccpA
SA564−ΔhptRS/ccpA
Strain
SA564
SA564−Δ*hptRS*
SA564−Δ*ccpA*
SA564−Δ*hptRS/ccpA*

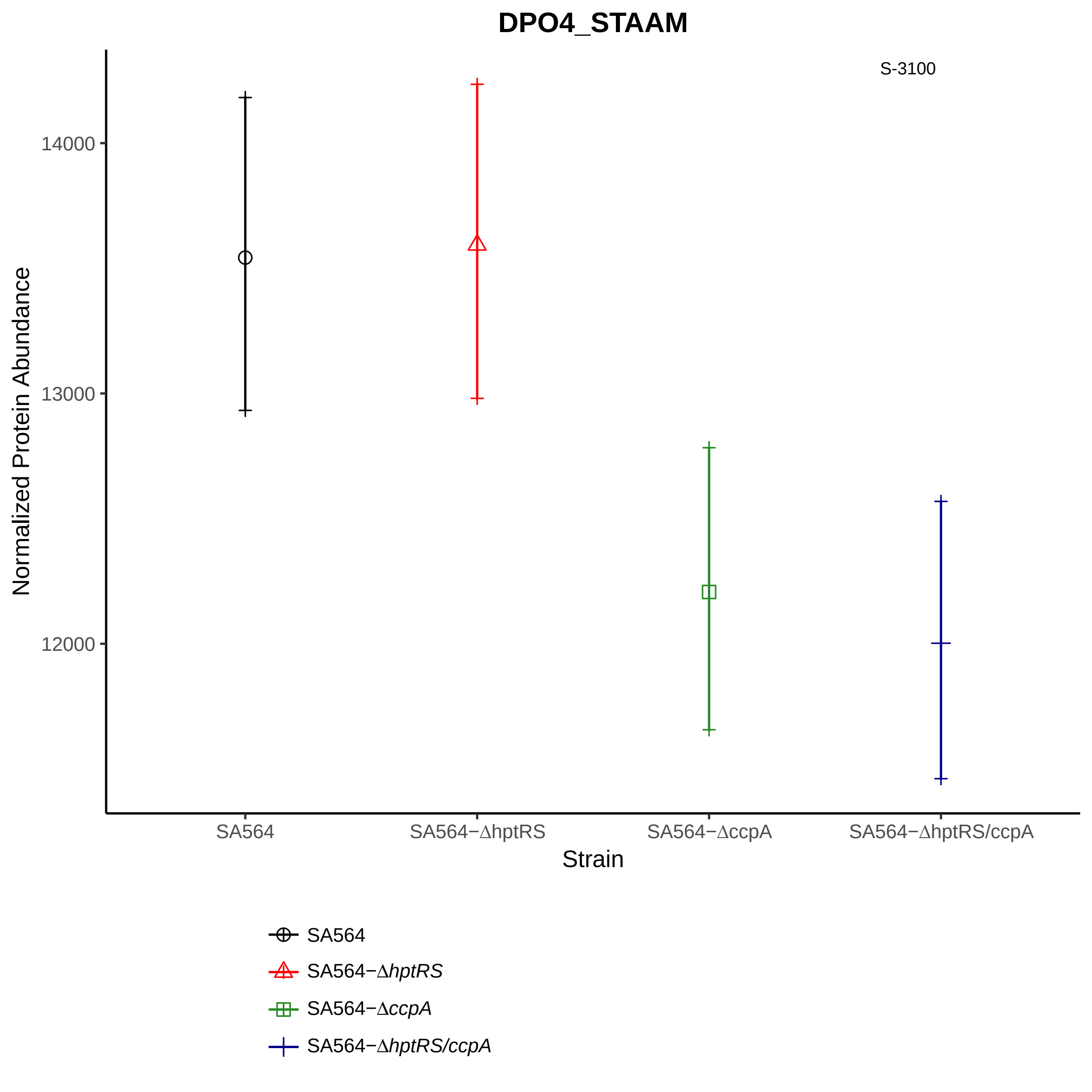

DPO4_STAAM
S-3100
Normalized Protein Abundance
14000
13000
12000
SA564
SA564−ΔhptRS
SA564−ΔccpA
SA564−ΔhptRS/ccpA
Strain
SA564
SA564−ΔhptRS
SA564−ΔccpA
SA564−ΔhptRS/ccpA

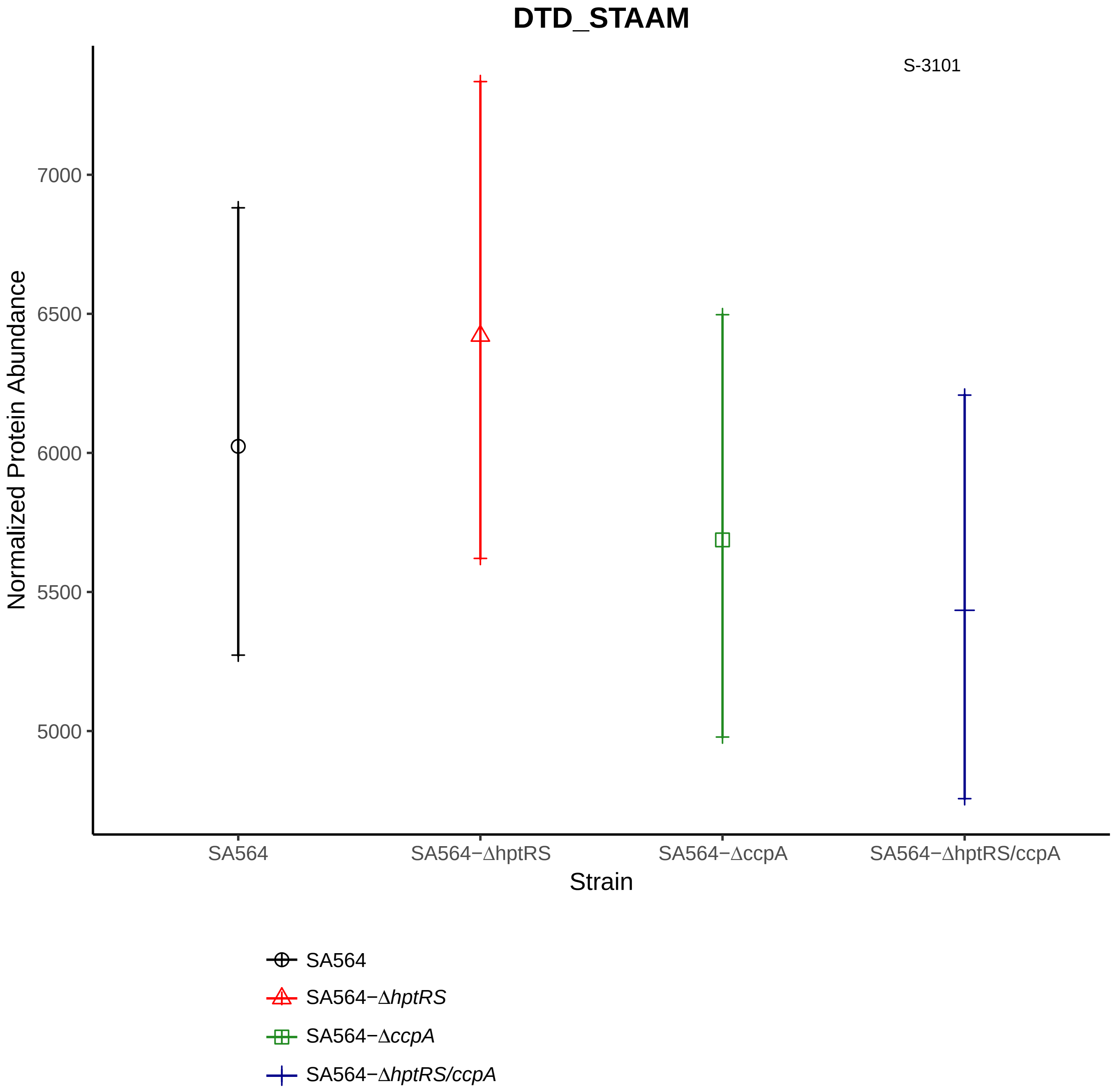

DTD_STAAM
S-3101
Normalized Protein Abundance
7000
6500
6000
5500
5000
SA564
SA564−ΔhptRS
SA564−ΔccpA
SA564−ΔhptRS/ccpA
Strain
SA564
SA564−ΔhptRS
SA564−ΔccpA
SA564−ΔhptRS/ccpA

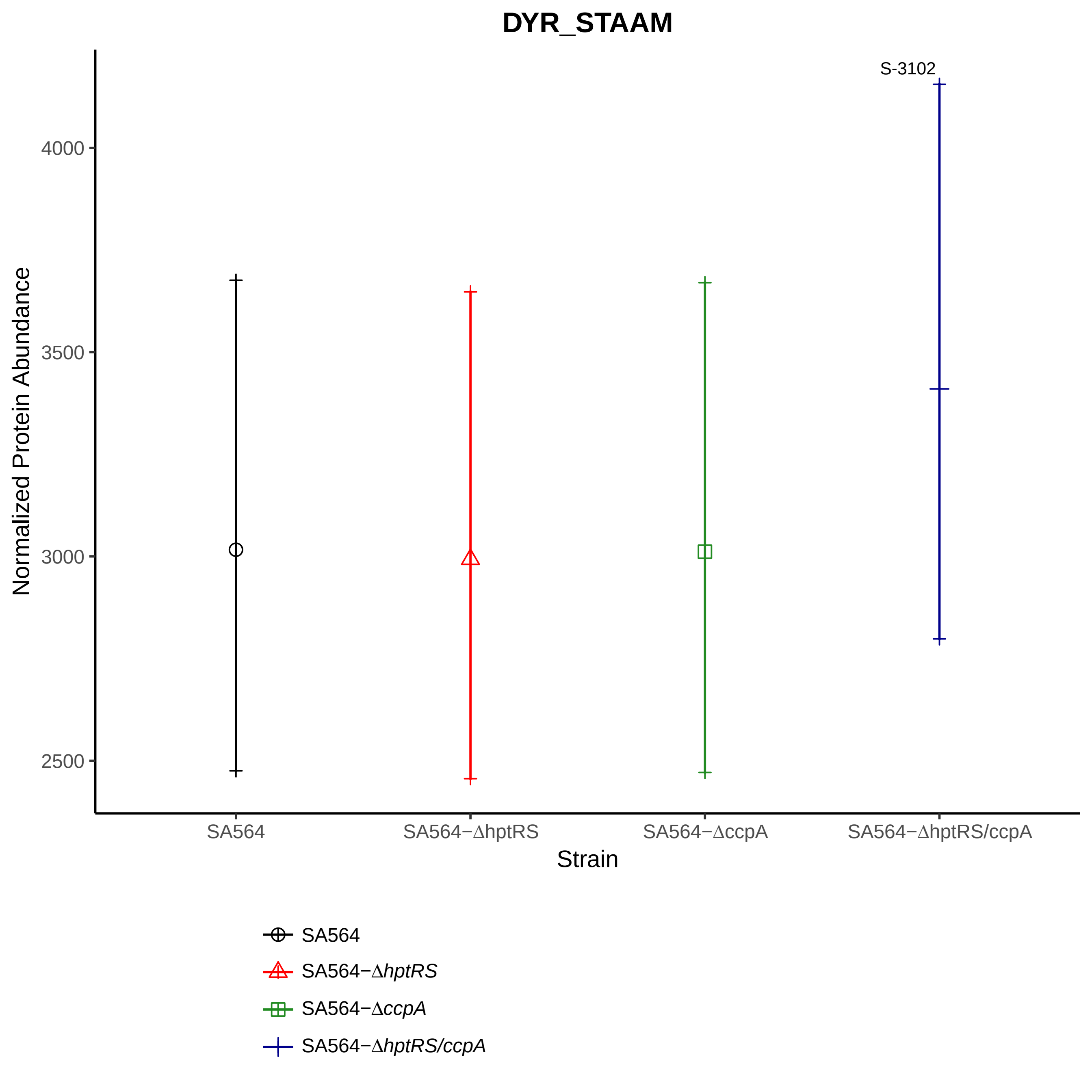
DYR_STAAM
S-3102
4000
3500
3000
2500
Normalized Protein Abundance
SA564
SA564−ΔhptRS
SA564−ΔccpA
SA564−ΔhptRS/ccpA
Strain
SA564
SA564−ΔhptRS
SA564−ΔccpA
SA564−ΔhptRS/ccpA

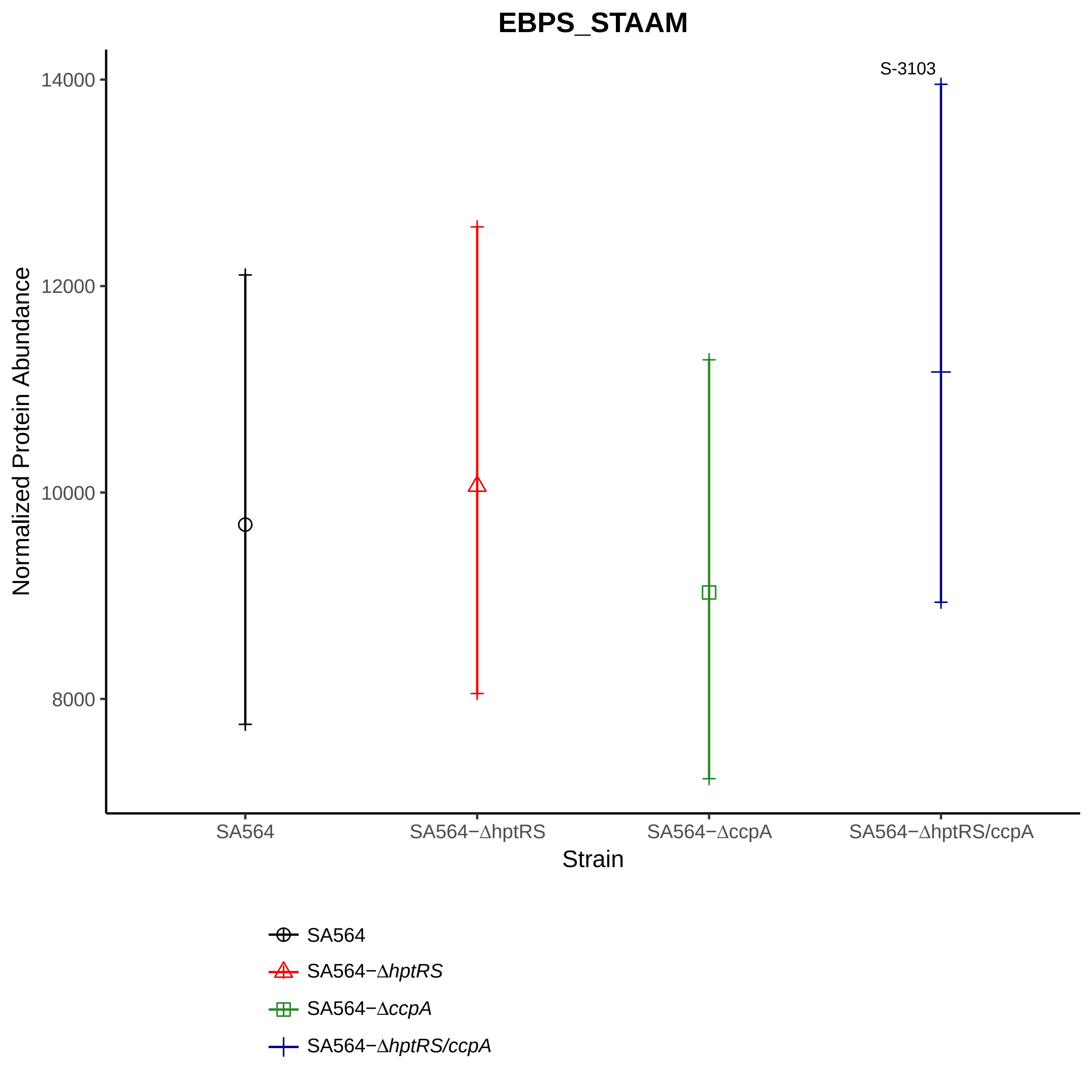

EBPS_STAAM
S-3103
Normalized Protein Abundance
14000
12000
10000
8000
SA564
SA564−ΔhptRS
SA564−ΔccpA
SA564−ΔhptRS/ccpA
Strain
SA564
SA564−ΔhptRS
SA564−ΔccpA
SA564−ΔhptRS/ccpA

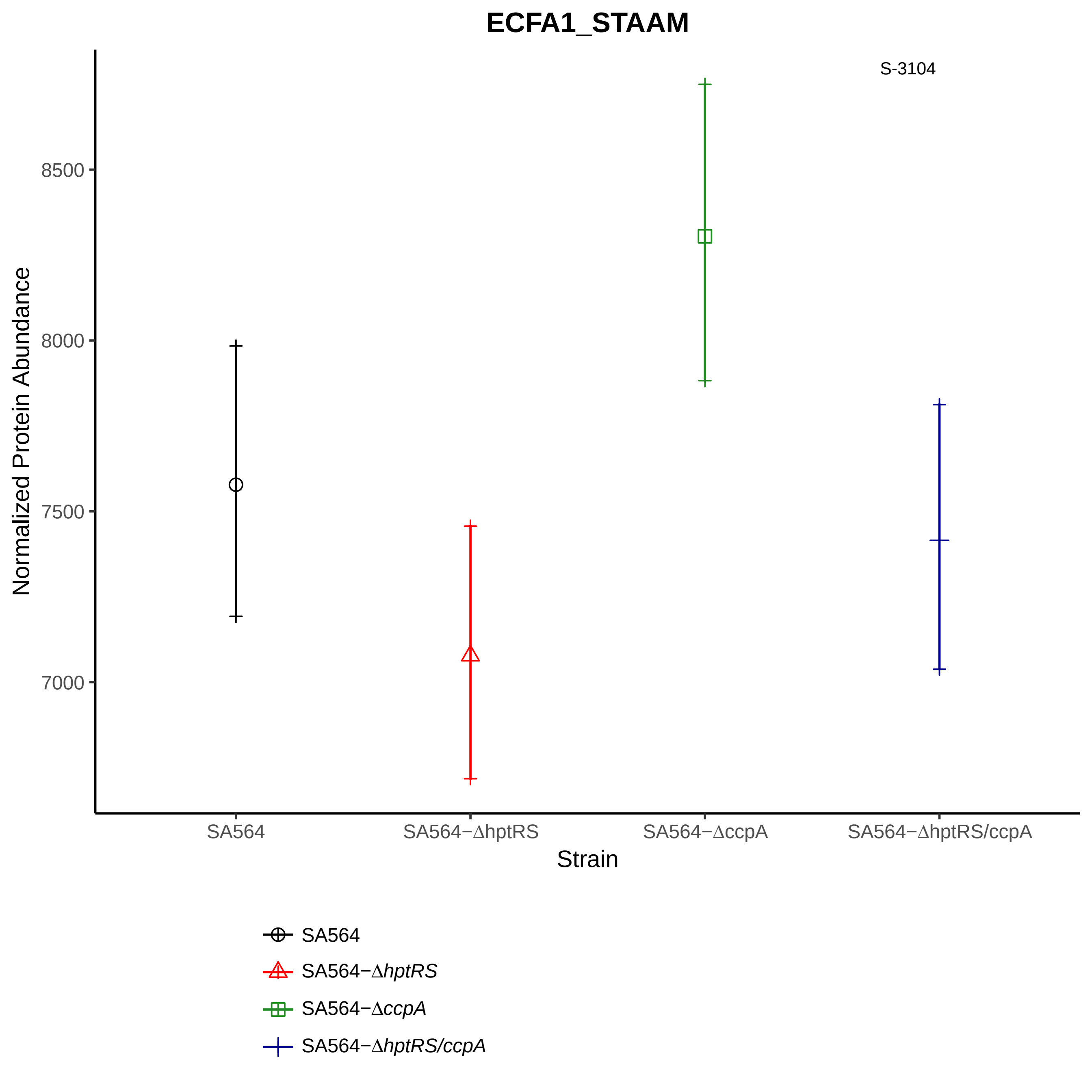

ECFA1_STAAM
S-3104
Normalized Protein Abundance
8500
8000
7500
7000
SA564
SA564−ΔhptRS
SA564−ΔccpA
SA564−ΔhptRS/ccpA
Strain
SA564
SA564−ΔhptRS
SA564−ΔccpA
SA564−ΔhptRS/ccpA

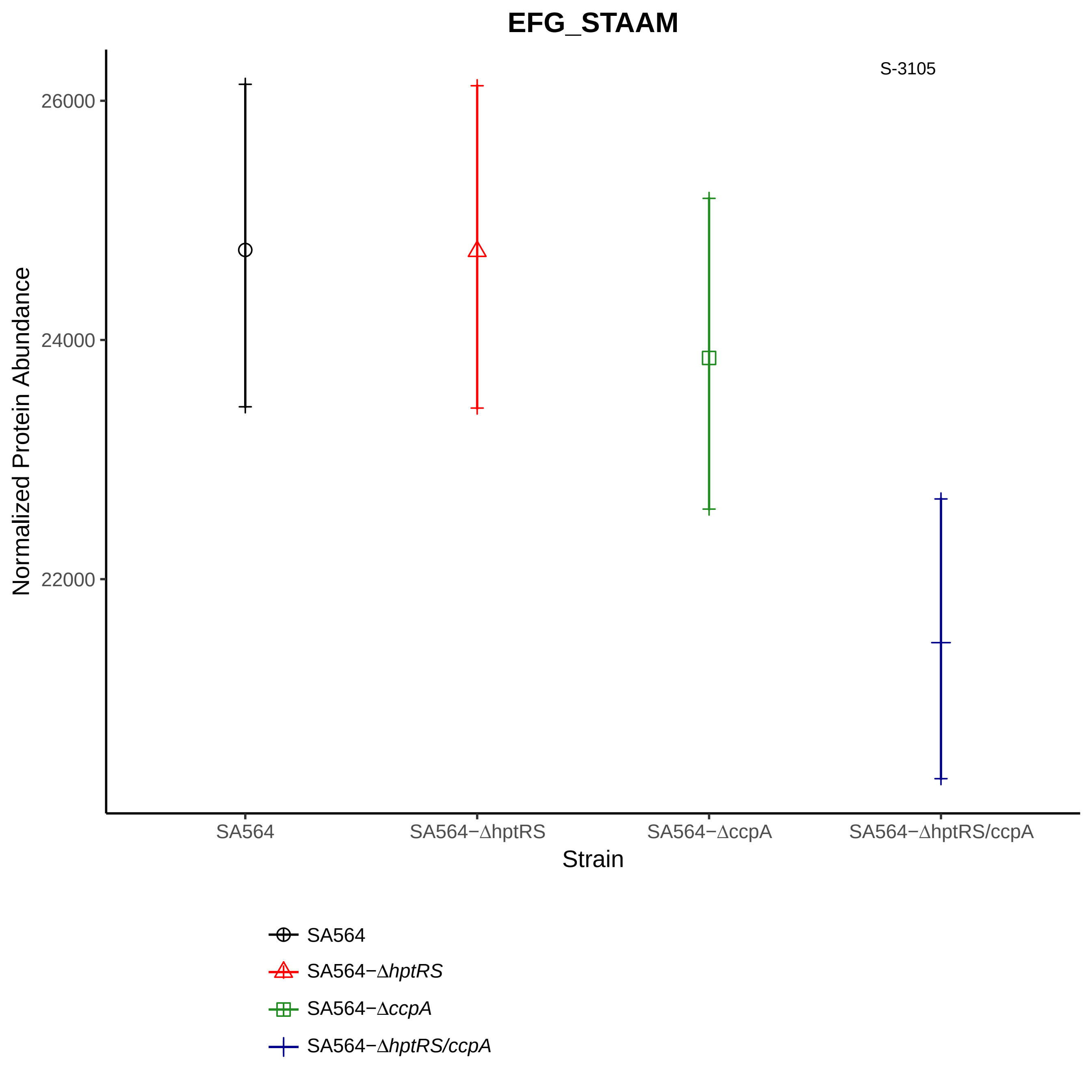

EFG_STAAM
S-3105
26000
24000
22000
Normalized Protein Abundance
SA564
SA564−ΔhptRS
SA564−ΔccpA
SA564−ΔhptRS/ccpA
Strain
SA564
SA564−ΔhptRS
SA564−ΔccpA
SA564−ΔhptRS/ccpA

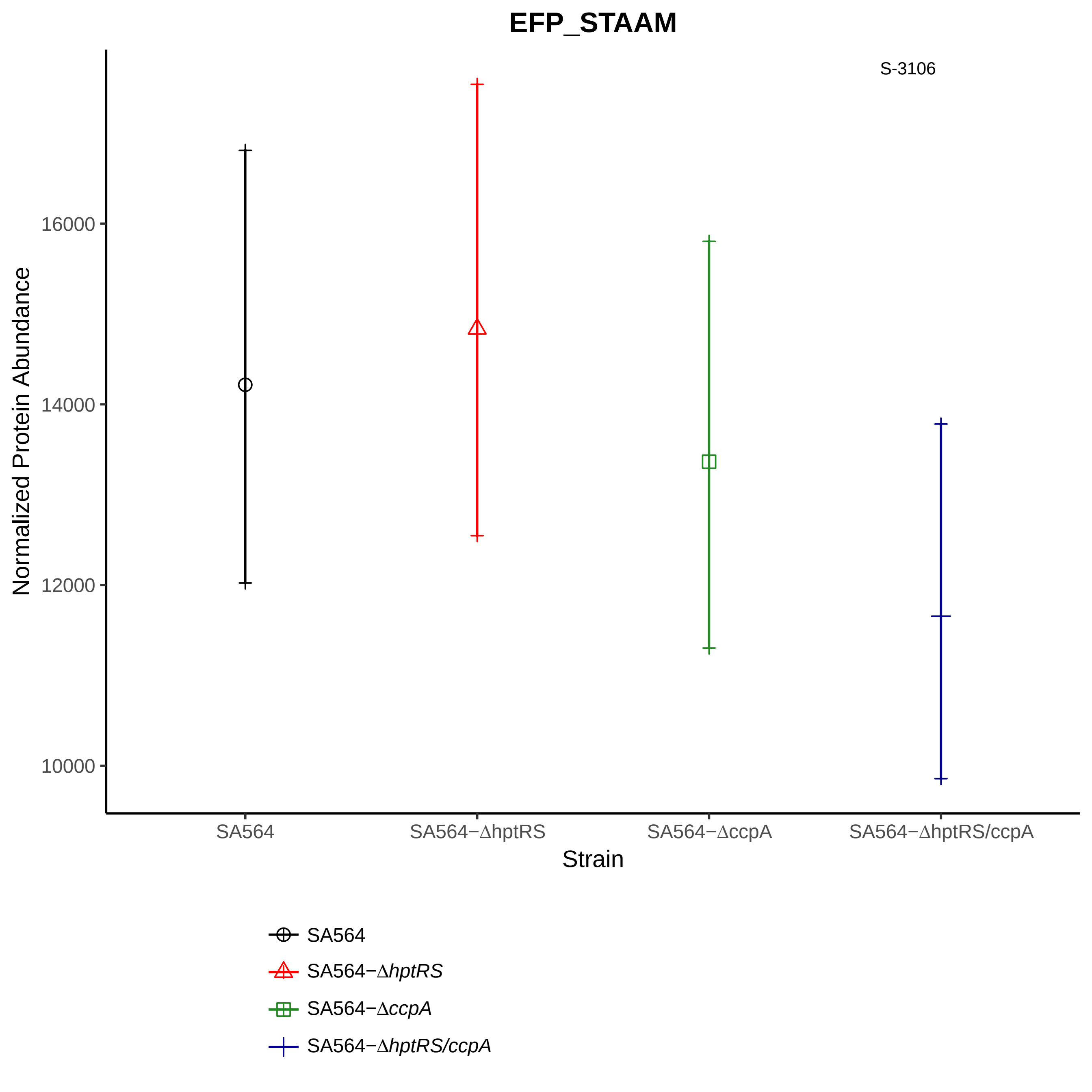
EFP_STAAM
S-3106
Normalized Protein Abundance
16000
14000
12000
10000
SA564
SA564−ΔhptRS
SA564−ΔccpA
SA564−ΔhptRS/ccpA
Strain
SA564
SA564−ΔhptRS
SA564−ΔccpA
SA564−ΔhptRS/ccpA

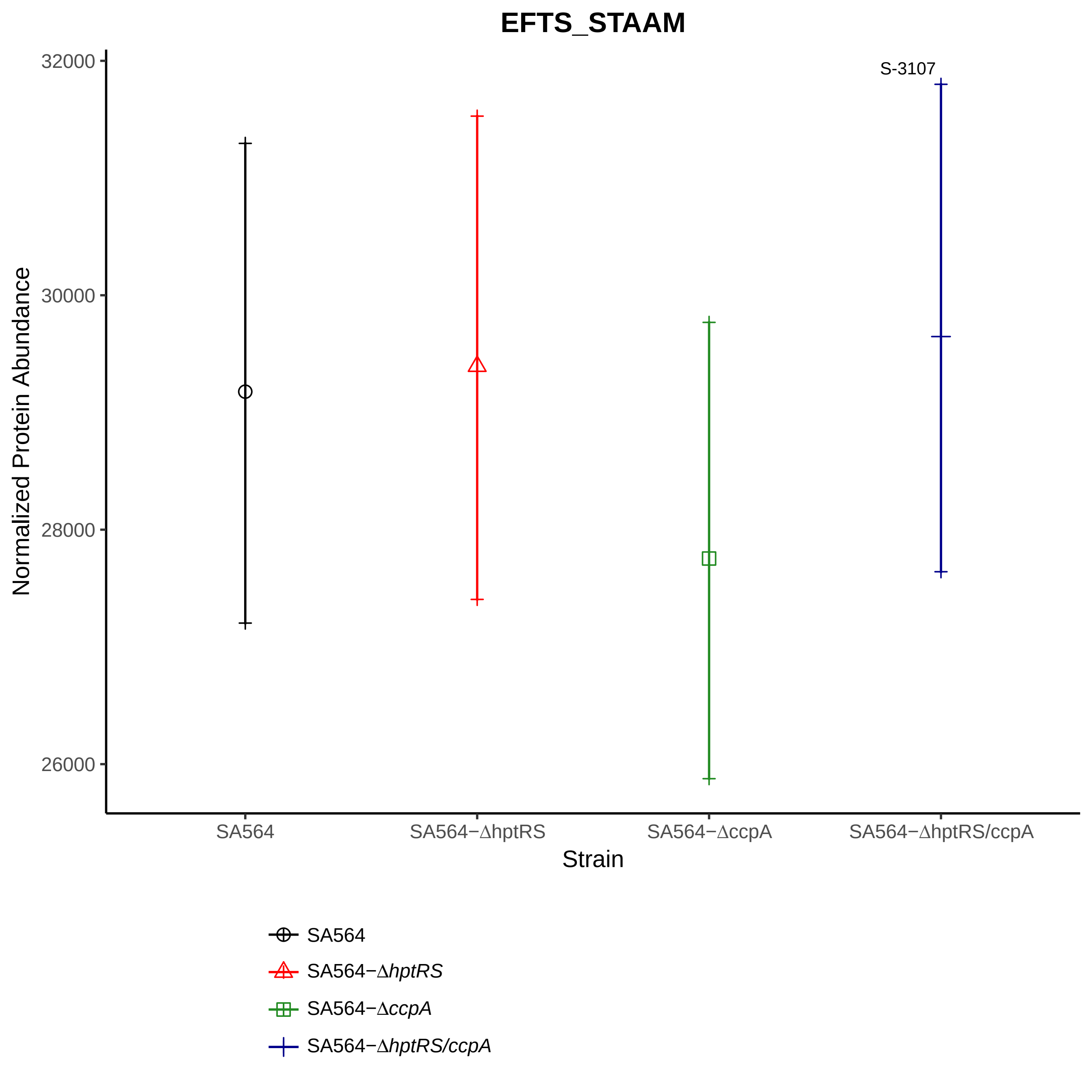

EFTS_STAAM
S-3107
32000
30000
28000
26000
Normalized Protein Abundance
SA564
SA564−ΔhptRS
SA564−ΔccpA
SA564−ΔhptRS/ccpA
Strain
SA564
SA564−ΔhptRS
SA564−ΔccpA
SA564−ΔhptRS/ccpA

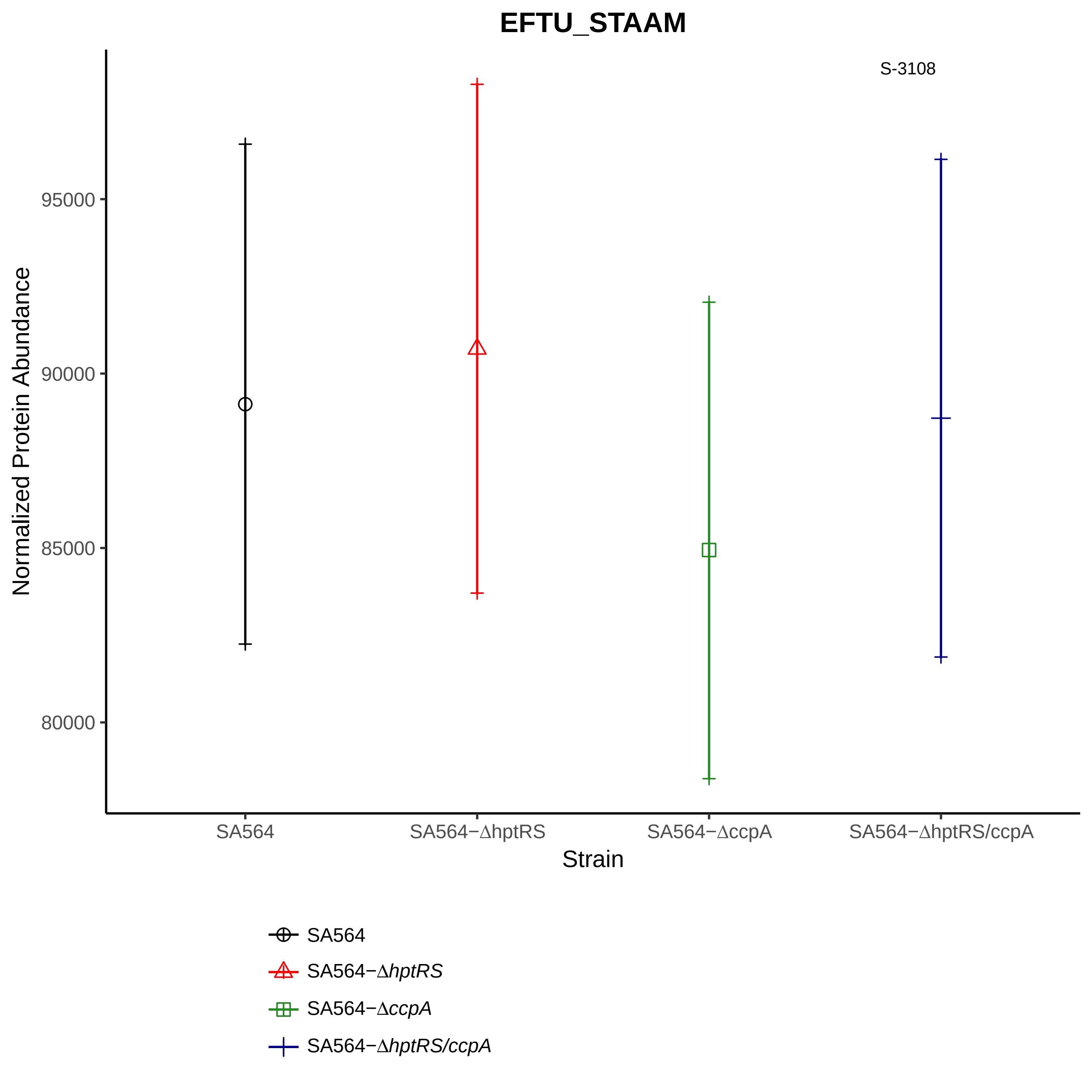
EFTU_STAAM
S-3108
Normalized Protein Abundance
95000
90000
85000
80000
SA564
SA564−ΔhptRS
SA564−ΔccpA
SA564−ΔhptRS/ccpA
Strain
SA564
SA564−ΔhptRS
SA564−ΔccpA
SA564−ΔhptRS/ccpA

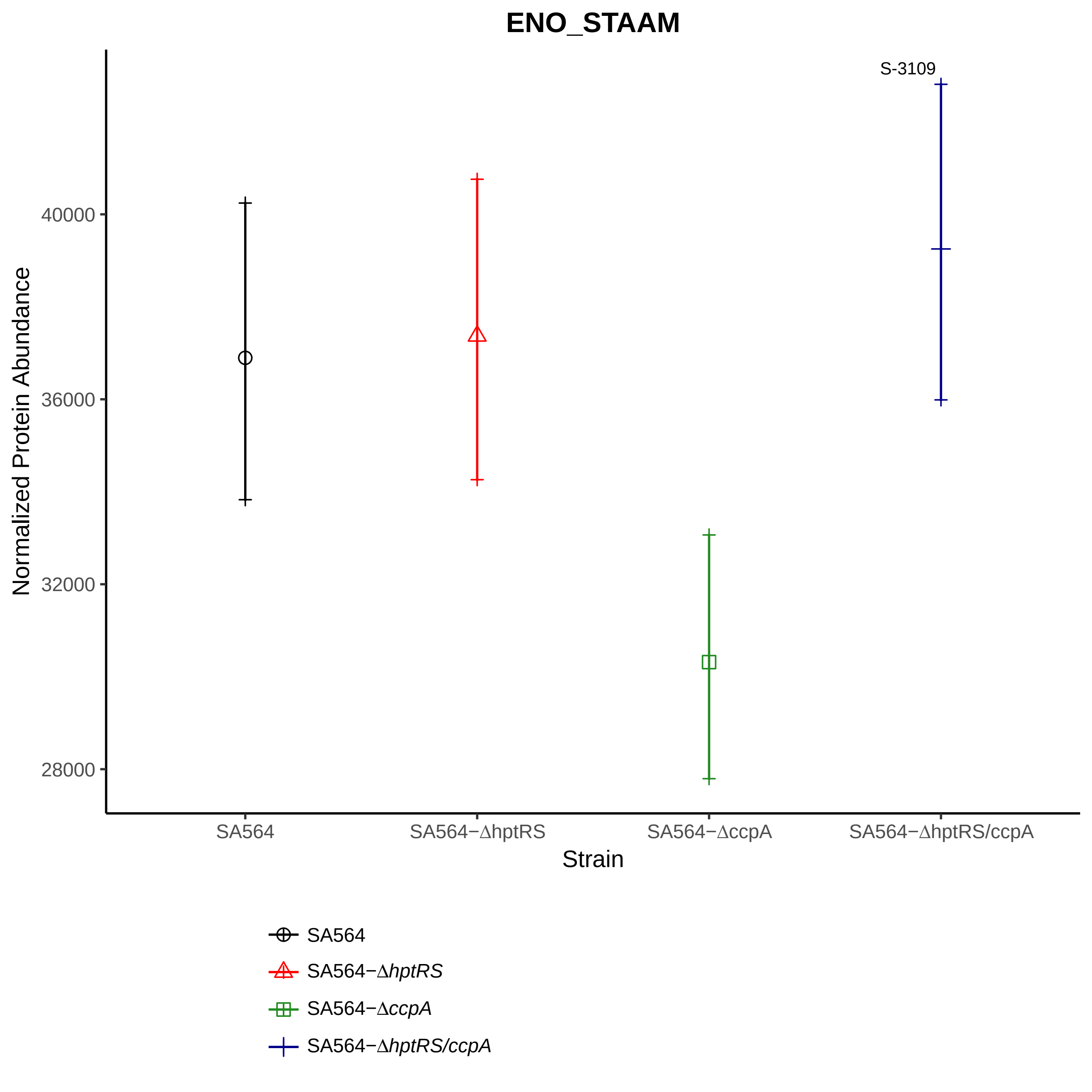

ENO_STAAM
S-3109
Normalized Protein Abundance
40000
36000
32000
28000
SA564
SA564−ΔhptRS
SA564−ΔccpA
SA564−ΔhptRS/ccpA
Strain
SA564
SA564−ΔhptRS
SA564−ΔccpA
SA564−ΔhptRS/ccpA

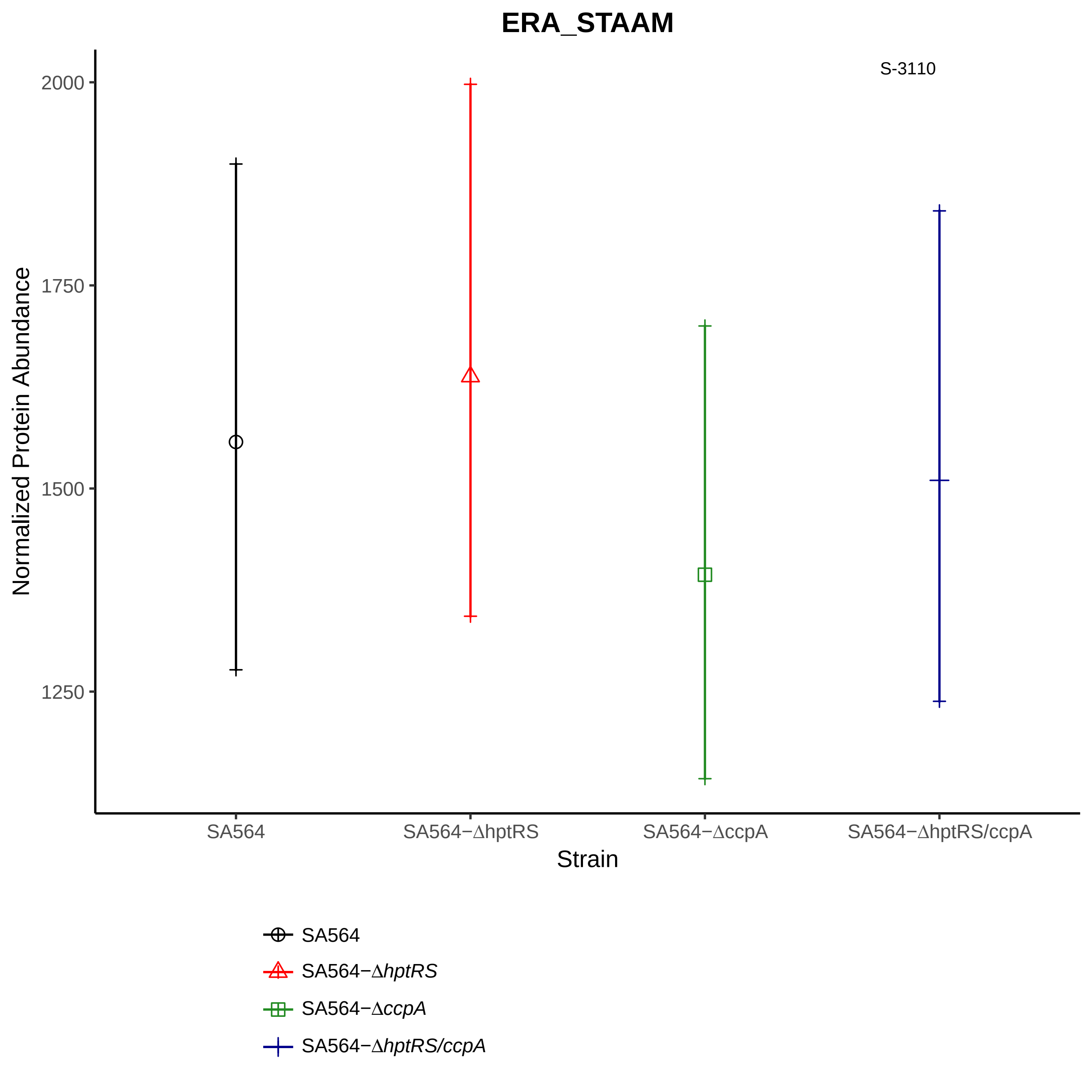

ERA_STAAM
S-3110
2000
1750
1500
1250
Normalized Protein Abundance
SA564
SA564−ΔhptRS
SA564−ΔccpA
SA564−ΔhptRS/ccpA
Strain
SA564
SA564−ΔhptRS
SA564−ΔccpA
SA564−ΔhptRS/ccpA

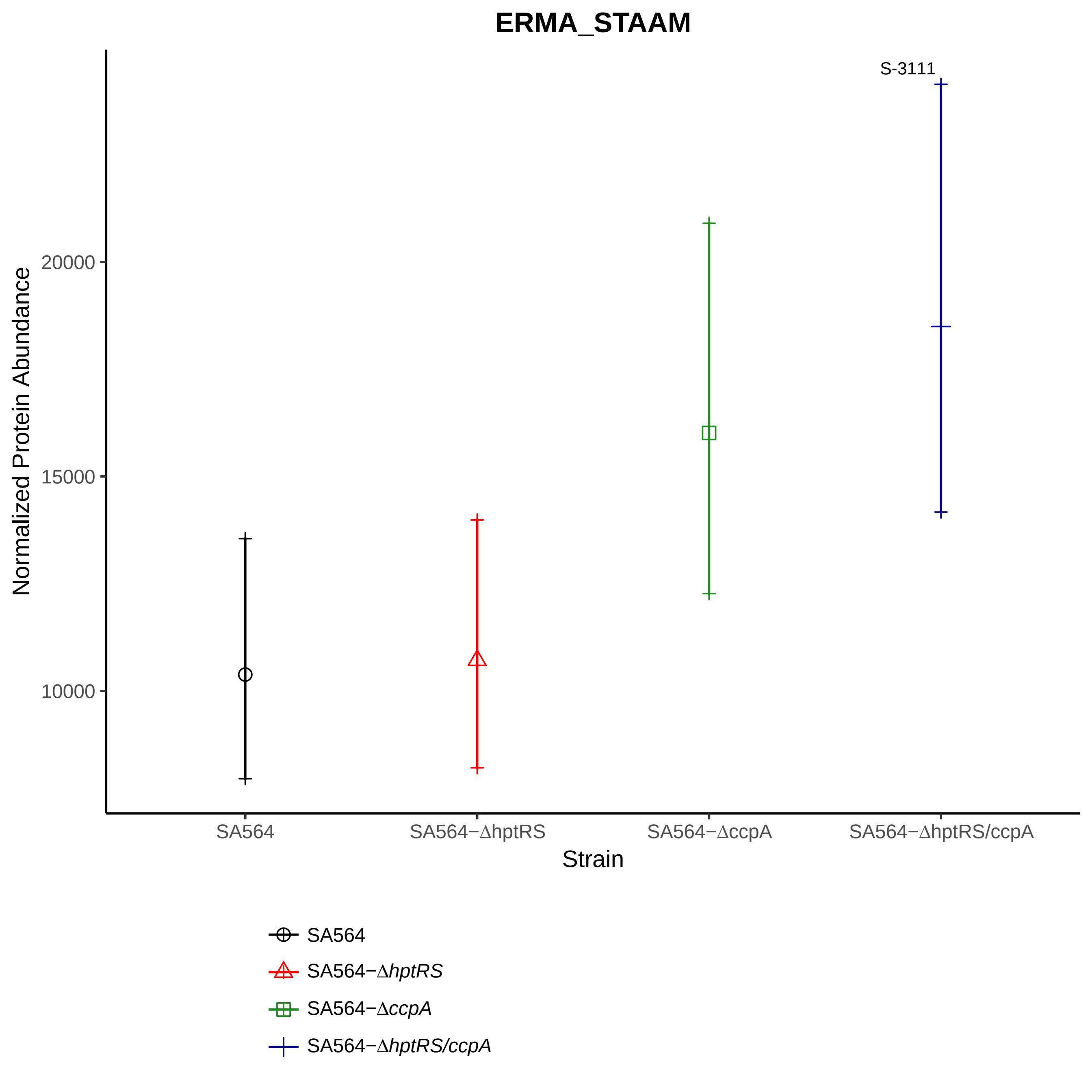

ERMA_STAAM
S-3111
Normalized Protein Abundance
20000
15000
10000
SA564
SA564−ΔhptRS
SA564−ΔccpA
SA564−ΔhptRS/ccpA
Strain
SA564
SA564−ΔhptRS
SA564−ΔccpA
SA564−ΔhptRS/ccpA

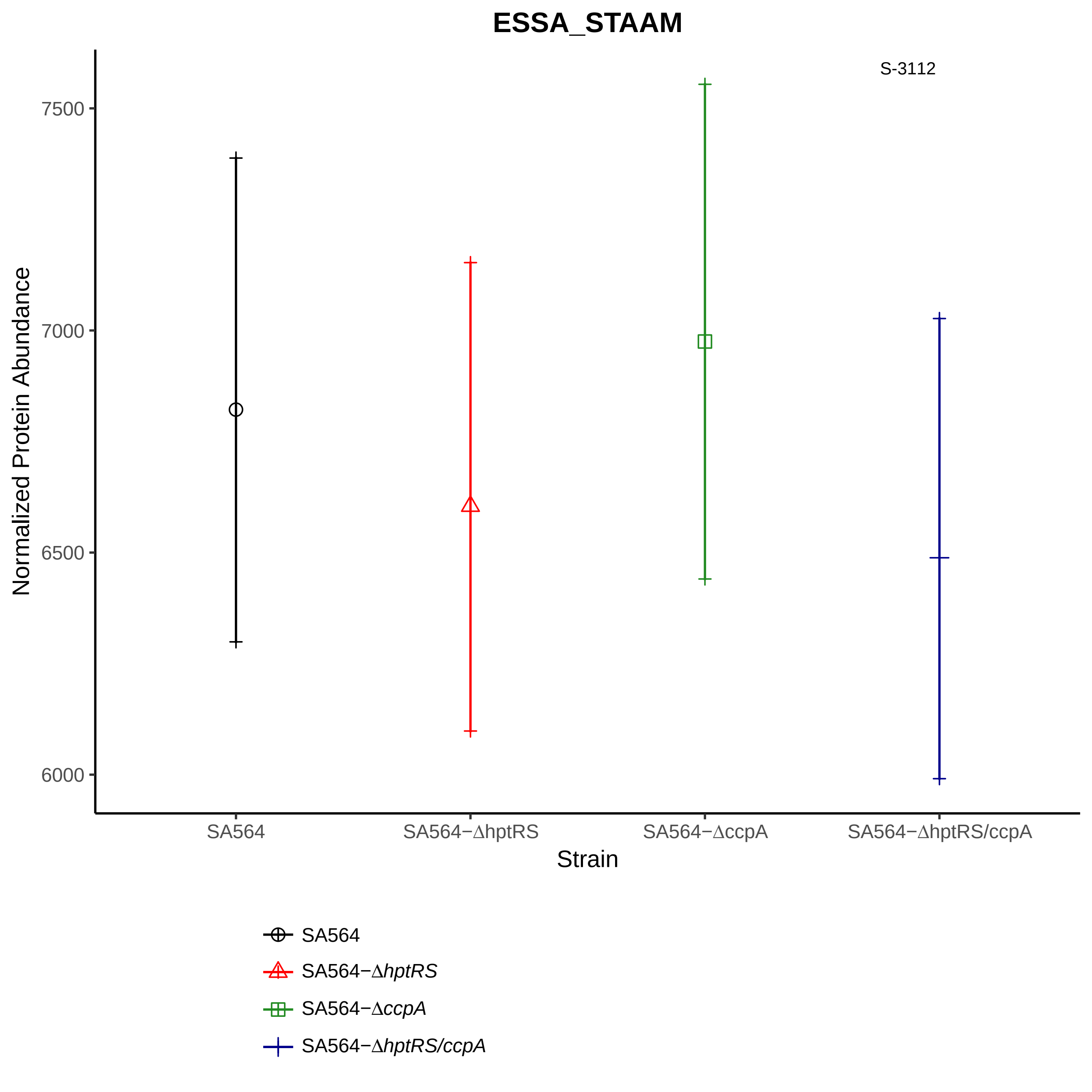

ESSA_STAAM
S-3112
Normalized Protein Abundance
7500
7000
6500
6000
SA564
SA564−ΔhptRS
SA564−ΔccpA
SA564−ΔhptRS/ccpA
Strain
SA564
SA564−Δ*hptRS*
SA564−Δ*ccpA*
SA564−Δ*hptRS/ccpA*

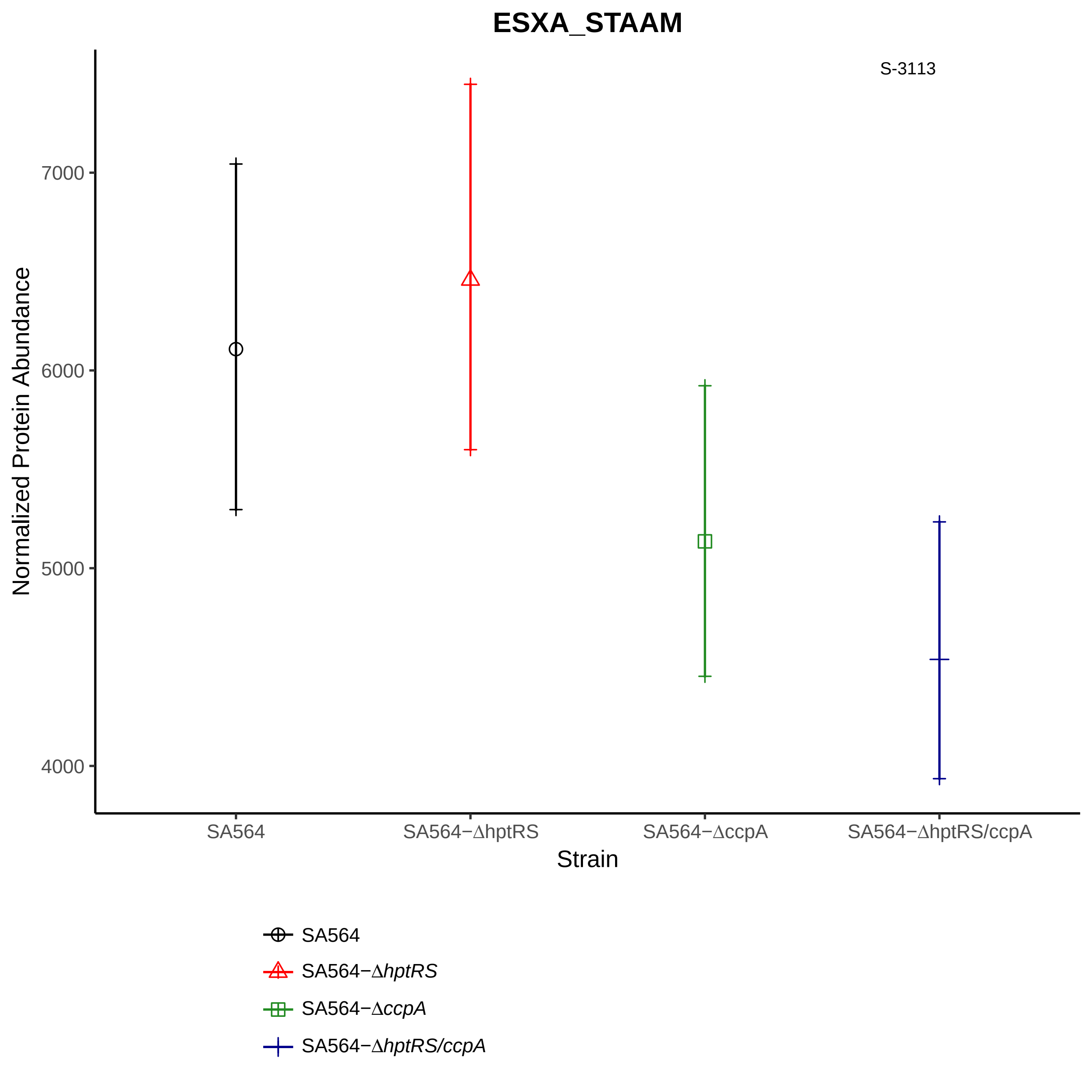

ESXA_STAAM
S-3113
Normalized Protein Abundance
7000
6000
5000
4000
SA564
SA564−ΔhptRS
SA564−ΔccpA
SA564−ΔhptRS/ccpA
Strain
SA564
SA564−ΔhptRS
SA564−ΔccpA
SA564−ΔhptRS/ccpA

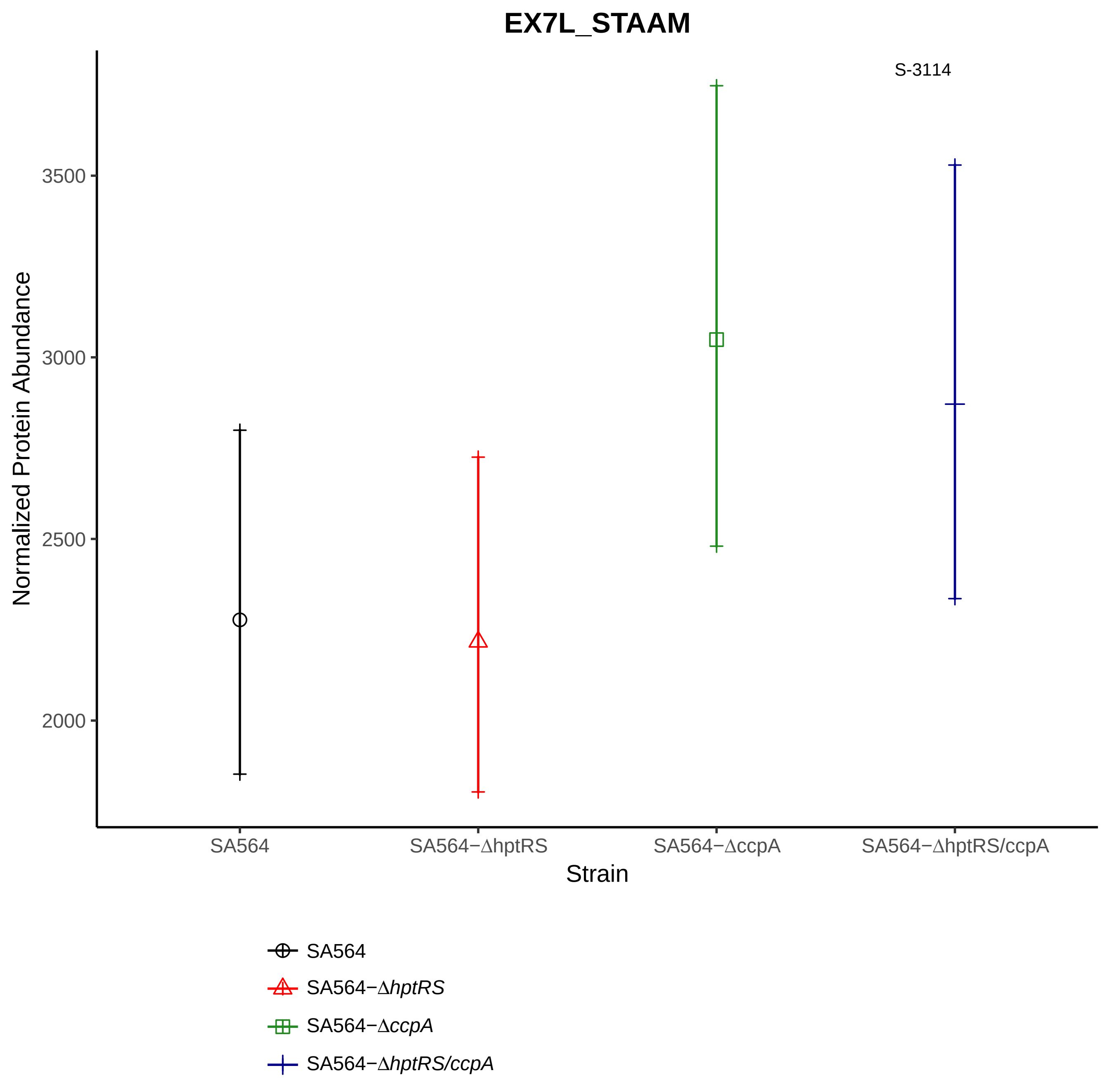
EX7L_STAAM
S-3114
Normalized Protein Abundance
3500
3000
2500
2000
SA564
SA564−ΔhptRS
SA564−ΔccpA
SA564−ΔhptRS/ccpA
Strain
SA564
SA564−ΔhptRS
SA564−ΔccpA
SA564−ΔhptRS/ccpA

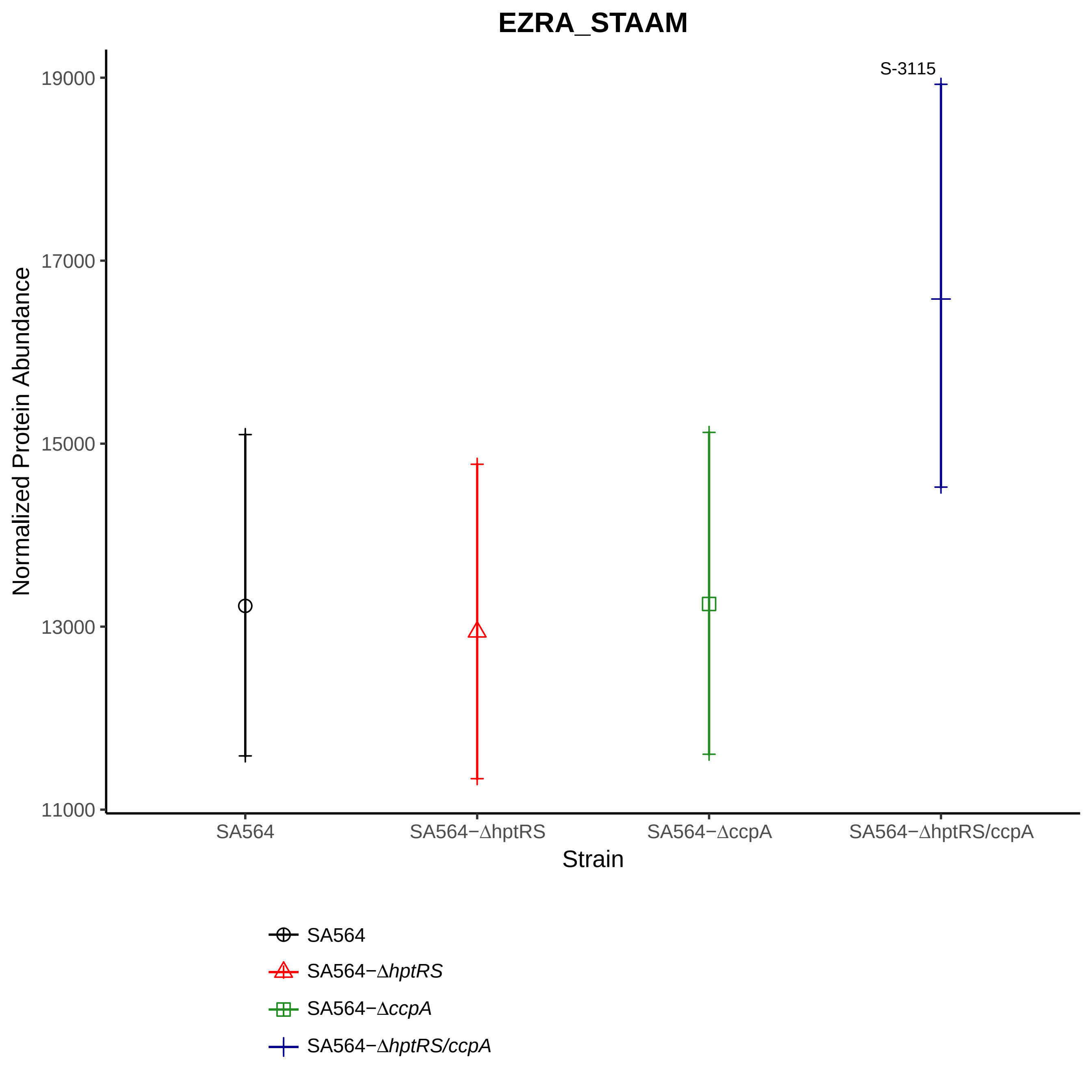

EZRA_STAAM
S-3115
19000
17000
15000
13000
11000
Normalized Protein Abundance
SA564
SA564−ΔhptRS
SA564−ΔccpA
SA564−ΔhptRS/ccpA
Strain
SA564
SA564−Δ*hptRS*
SA564−Δ*ccpA*
SA564−Δ*hptRS/ccpA*

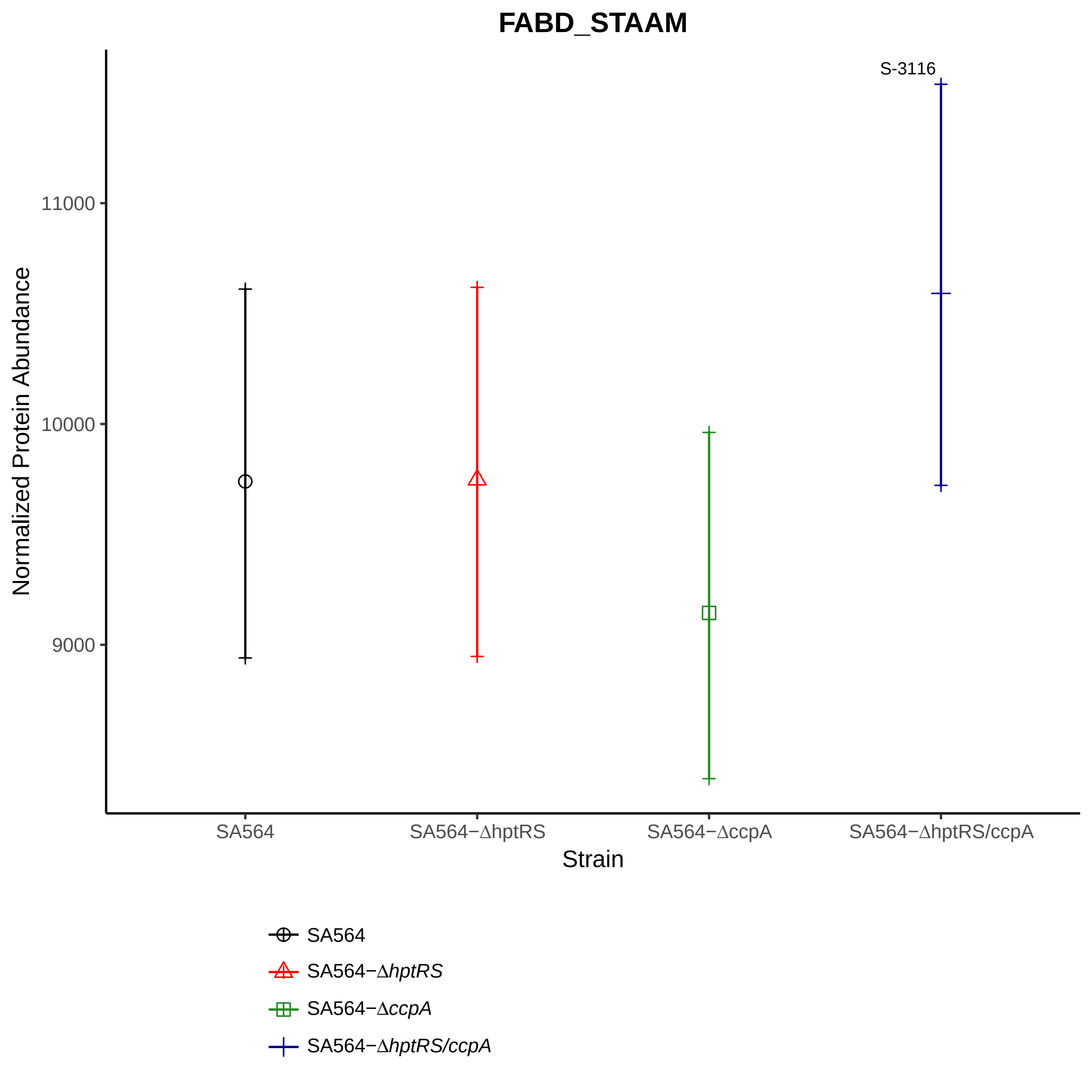

FABD_STAAM
S-3116
Normalized Protein Abundance
11000
10000
9000
SA564
SA564−ΔhptRS
SA564−ΔccpA
SA564−ΔhptRS/ccpA
Strain
SA564
SA564−ΔhptRS
SA564−ΔccpA
SA564−ΔhptRS/ccpA

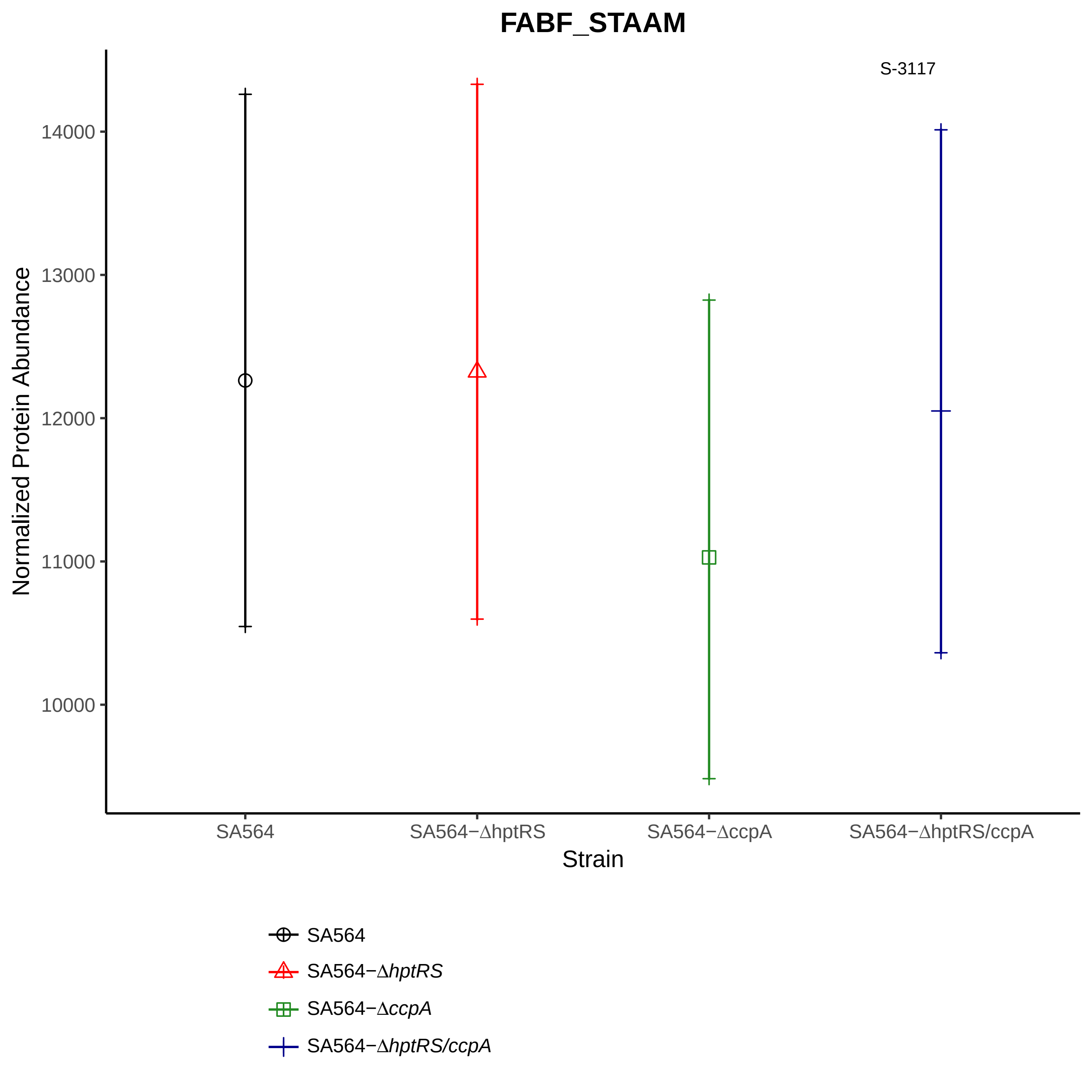

FABF_STAAM
S-3117
Normalized Protein Abundance
14000
13000
12000
11000
10000
SA564
SA564−ΔhptRS
SA564−ΔccpA
SA564−ΔhptRS/ccpA
Strain
SA564
SA564−ΔhptRS
SA564−ΔccpA
SA564−ΔhptRS/ccpA

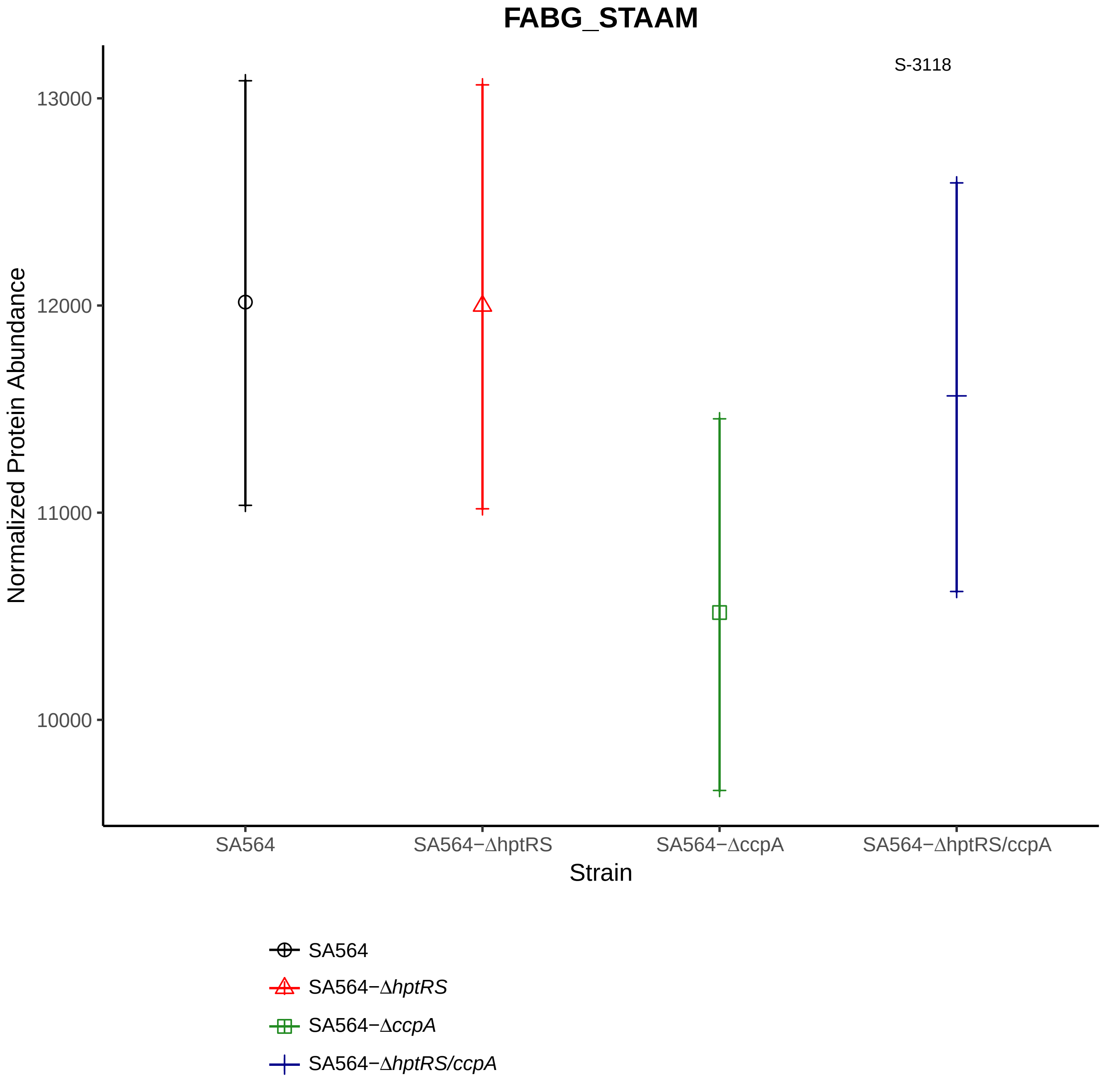

FABG_STAAM
S-3118
Normalized Protein Abundance
13000
12000
11000
10000
SA564
SA564−ΔhptRS
SA564−ΔccpA
SA564−ΔhptRS/ccpA
Strain
SA564
SA564−ΔhptRS
SA564−ΔccpA
SA564−ΔhptRS/ccpA

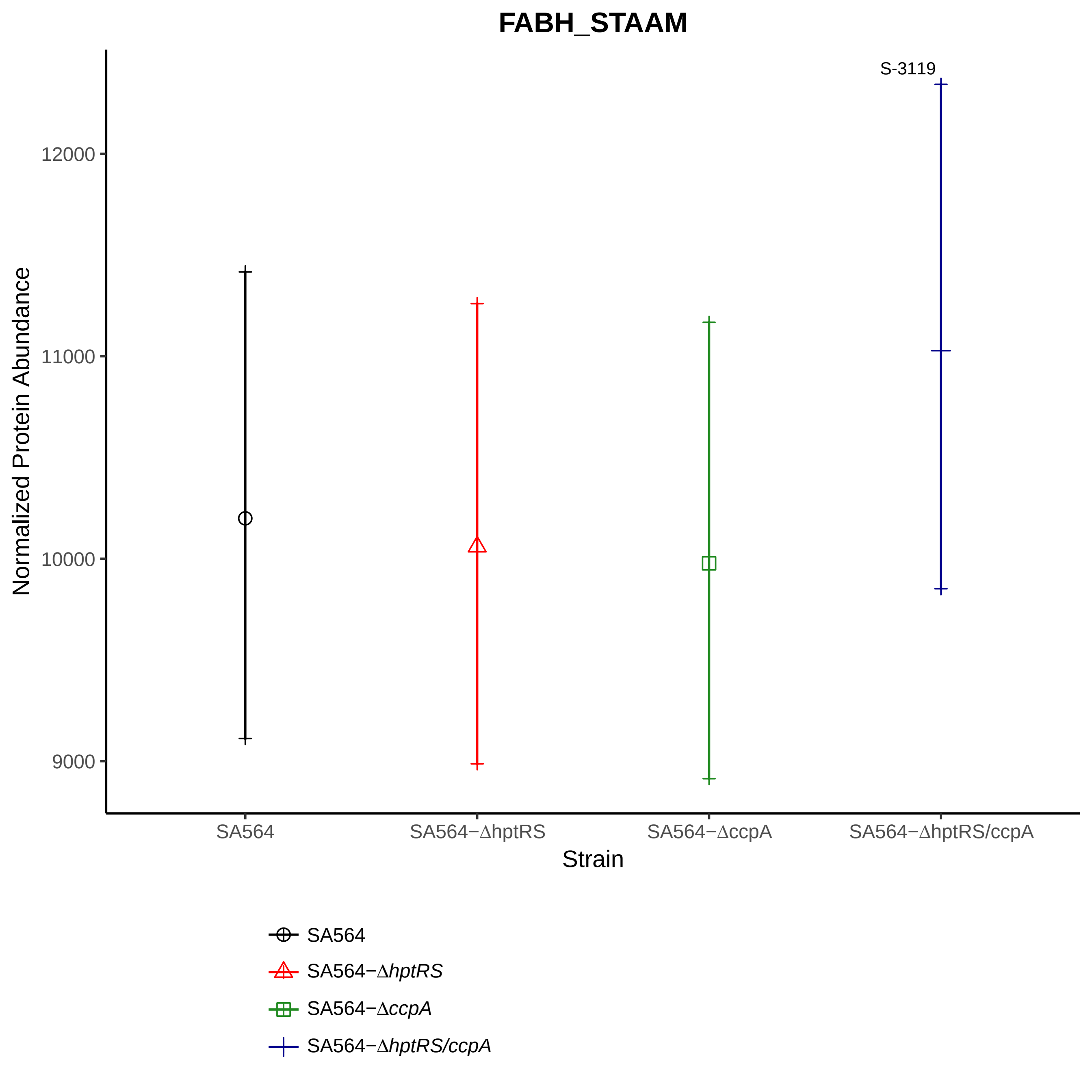

FABH_STAAM
S-3119
Normalized Protein Abundance
12000
11000
10000
9000
SA564
SA564−ΔhptRS
SA564−ΔccpA
SA564−ΔhptRS/ccpA
Strain
SA564
SA564−ΔhptRS
SA564−ΔccpA
SA564−ΔhptRS/ccpA

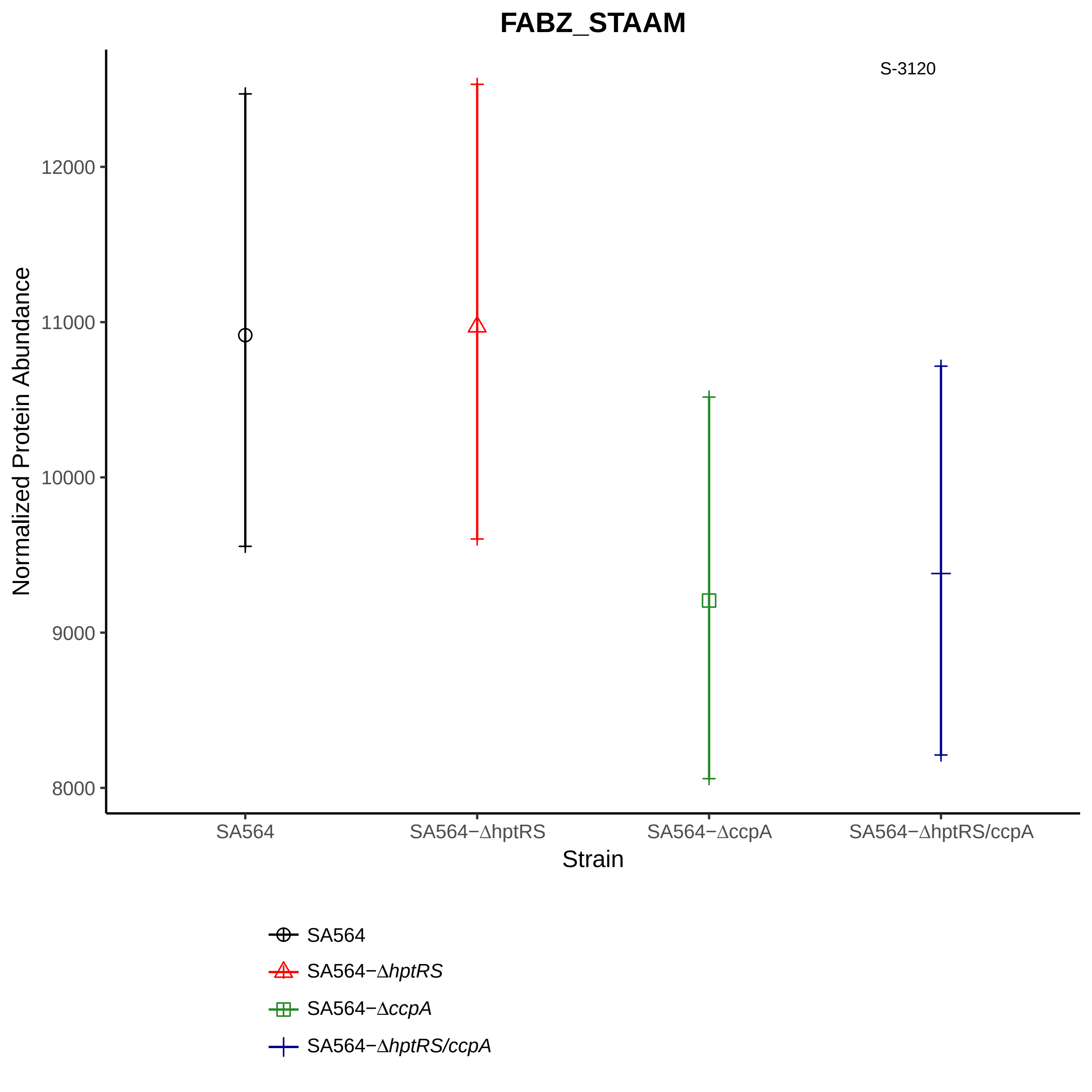

FABZ_STAAM
S-3120
Normalized Protein Abundance
12000
11000
10000
9000
8000
SA564
SA564−ΔhptRS
SA564−ΔccpA
SA564−ΔhptRS/ccpA
Strain
SA564
SA564−ΔhptRS
SA564−ΔccpA
SA564−ΔhptRS/ccpA

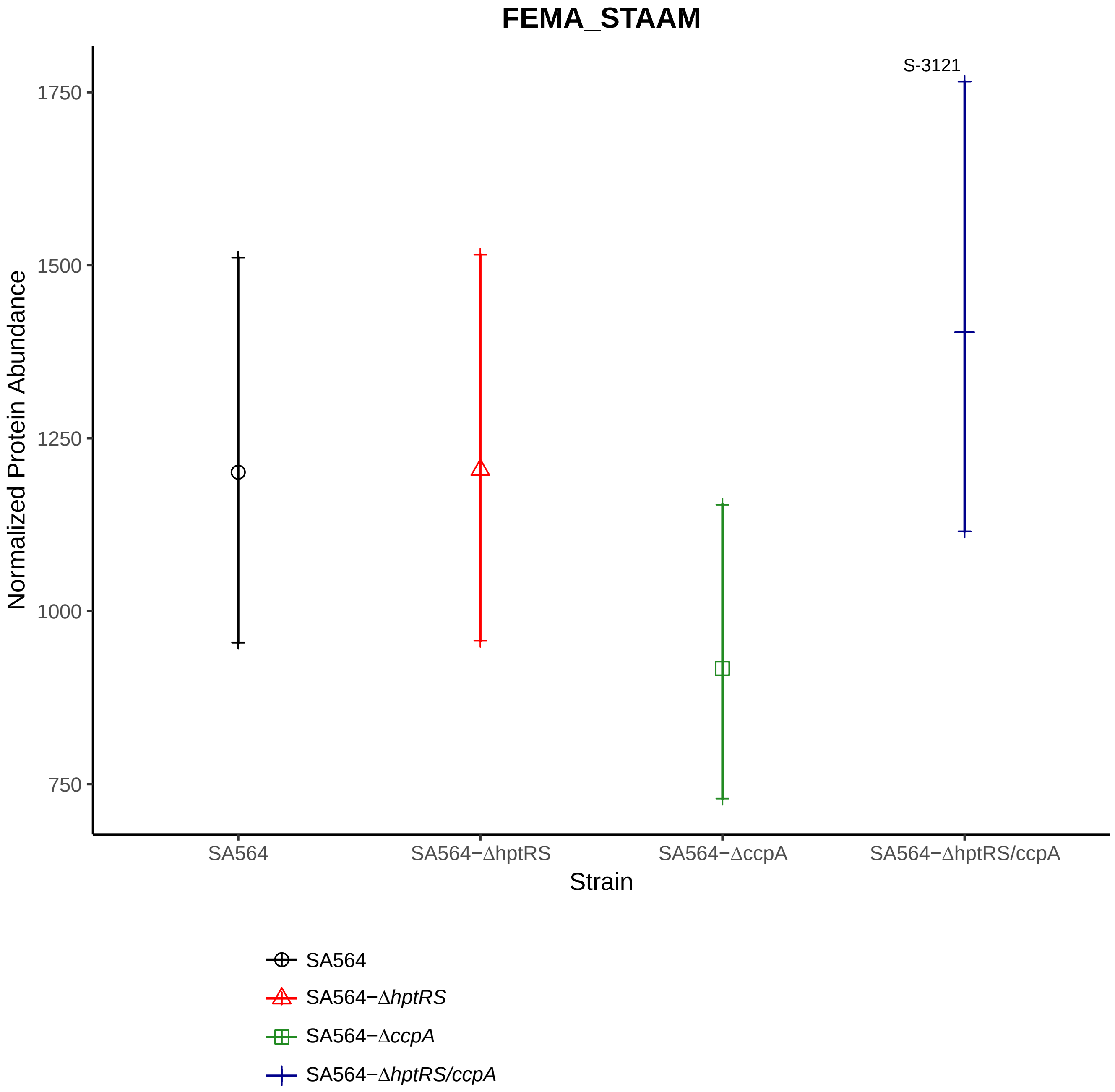
FEMA_STAAM
S-3121
1750
1500
1250
1000
750
Normalized Protein Abundance
SA564
SA564−ΔhptRS
SA564−ΔccpA
SA564−ΔhptRS/ccpA
Strain
SA564
SA564−Δ*hptRS*
SA564−Δ*ccpA*
SA564−Δ*hptRS/ccpA*

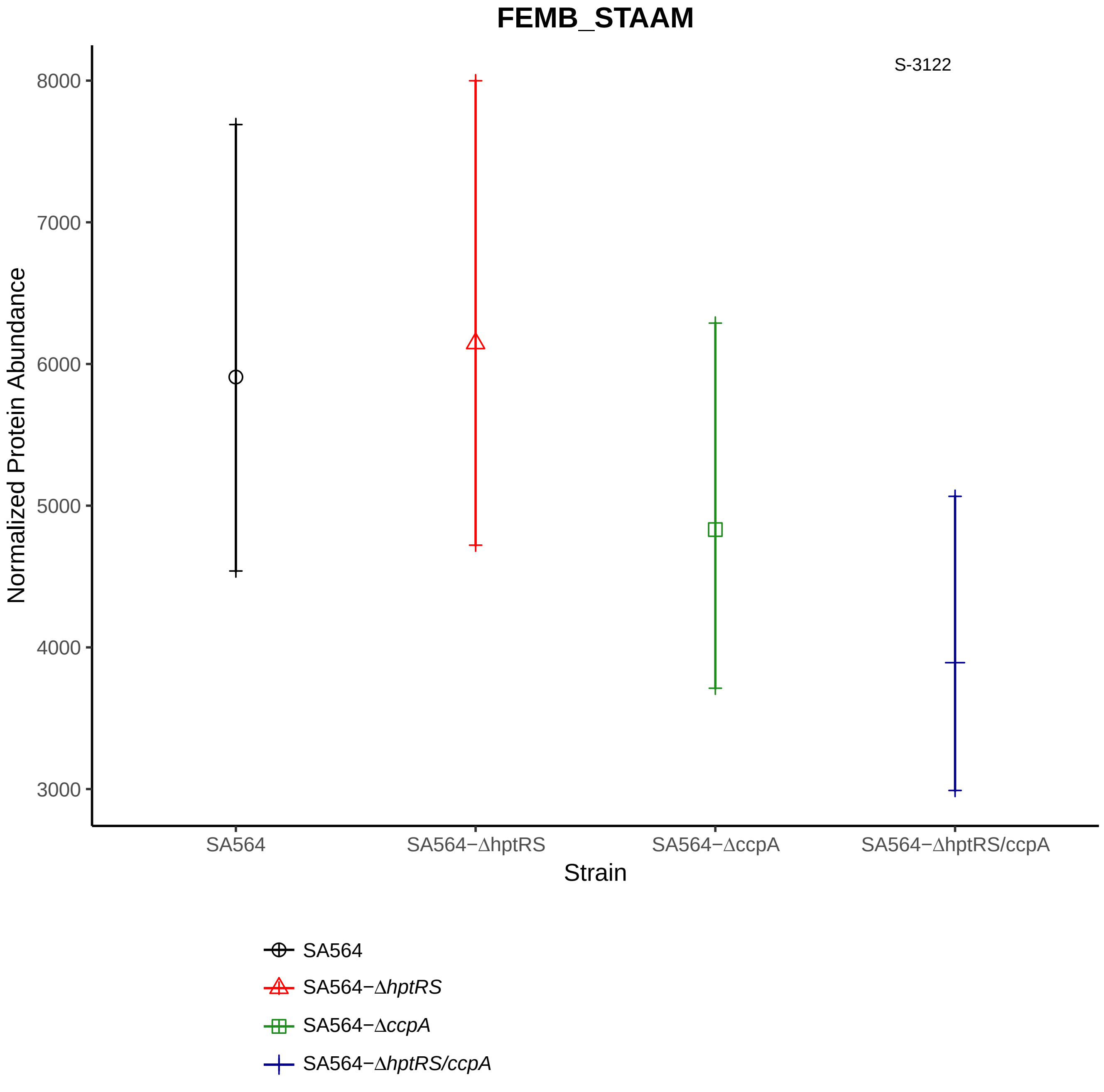
FEMB_STAAM
S-3122
Normalized Protein Abundance
8000
7000
6000
5000
4000
3000
SA564
SA564−ΔhptRS
SA564−ΔccpA
SA564−ΔhptRS/ccpA
Strain
SA564
SA564−ΔhptRS
SA564−ΔccpA
SA564−ΔhptRS/ccpA

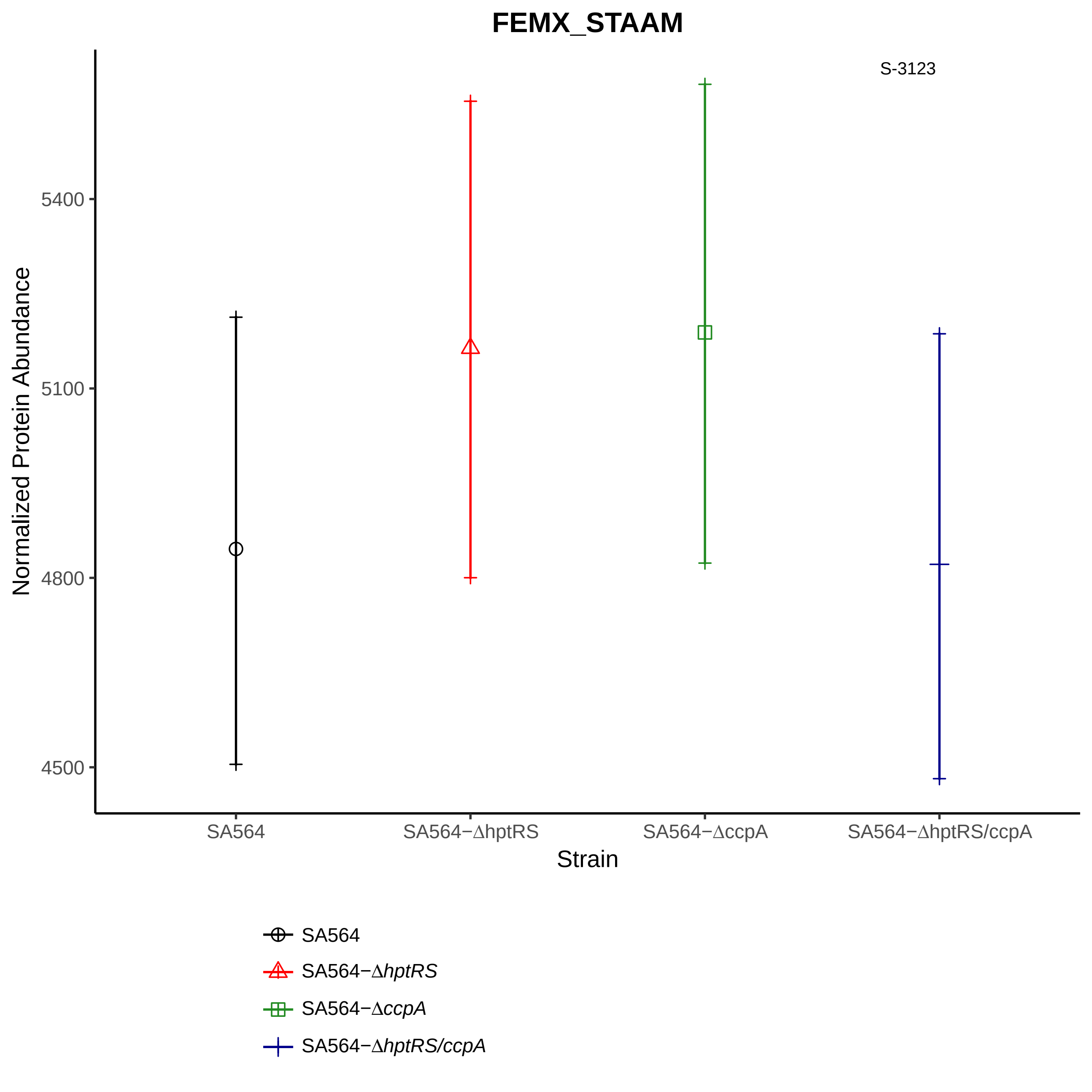

FEMX_STAAM
S-3123
Normalized Protein Abundance
5400
5100
4800
4500
SA564
SA564−ΔhptRS
SA564−ΔccpA
SA564−ΔhptRS/ccpA
Strain
SA564
SA564−ΔhptRS
SA564−ΔccpA
SA564−ΔhptRS/ccpA

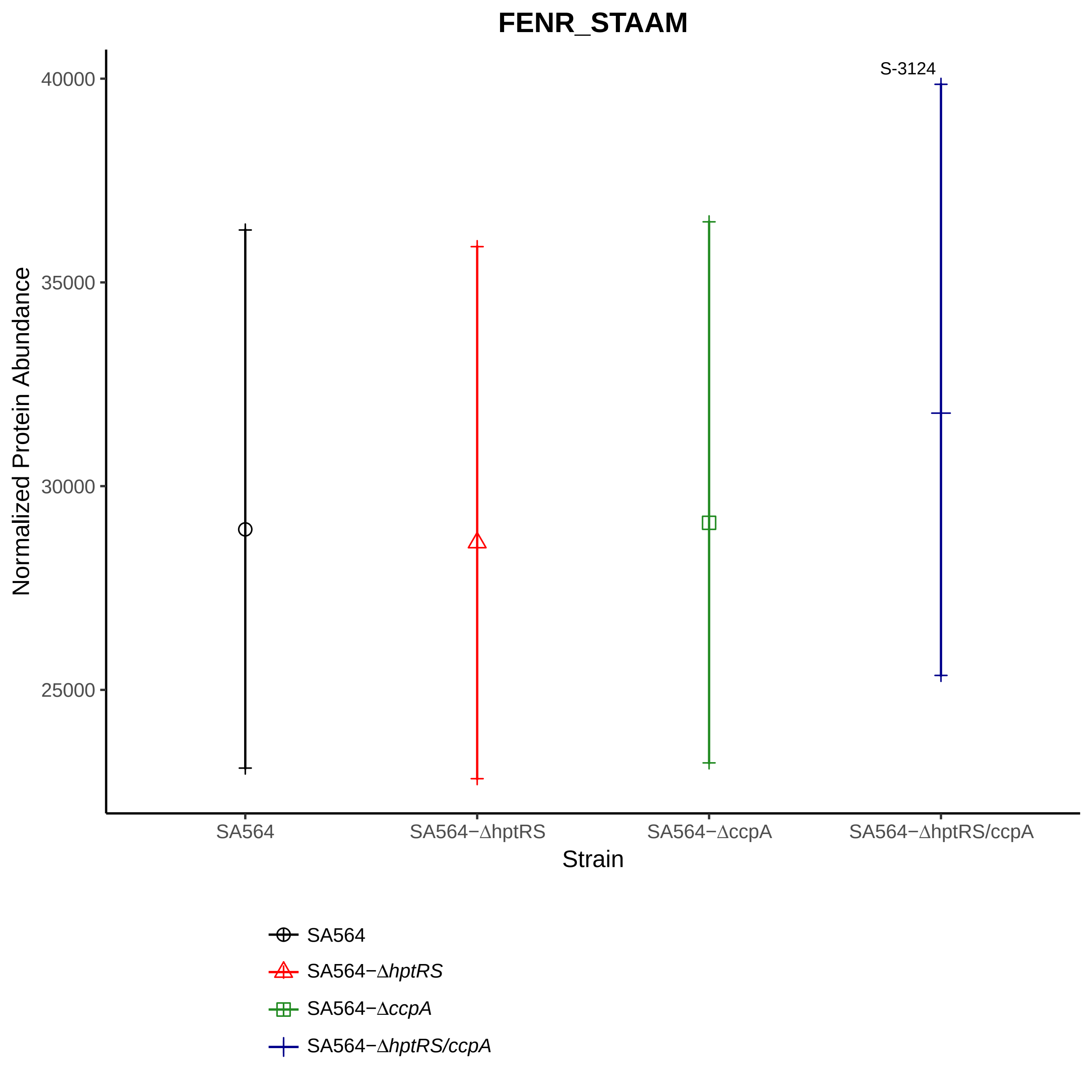

FENR_STAAM
S-3124
Normalized Protein Abundance
40000
35000
30000
25000
SA564
SA564−ΔhptRS
SA564−ΔccpA
SA564−ΔhptRS/ccpA
Strain
SA564
SA564−ΔhptRS
SA564−ΔccpA
SA564−ΔhptRS/ccpA

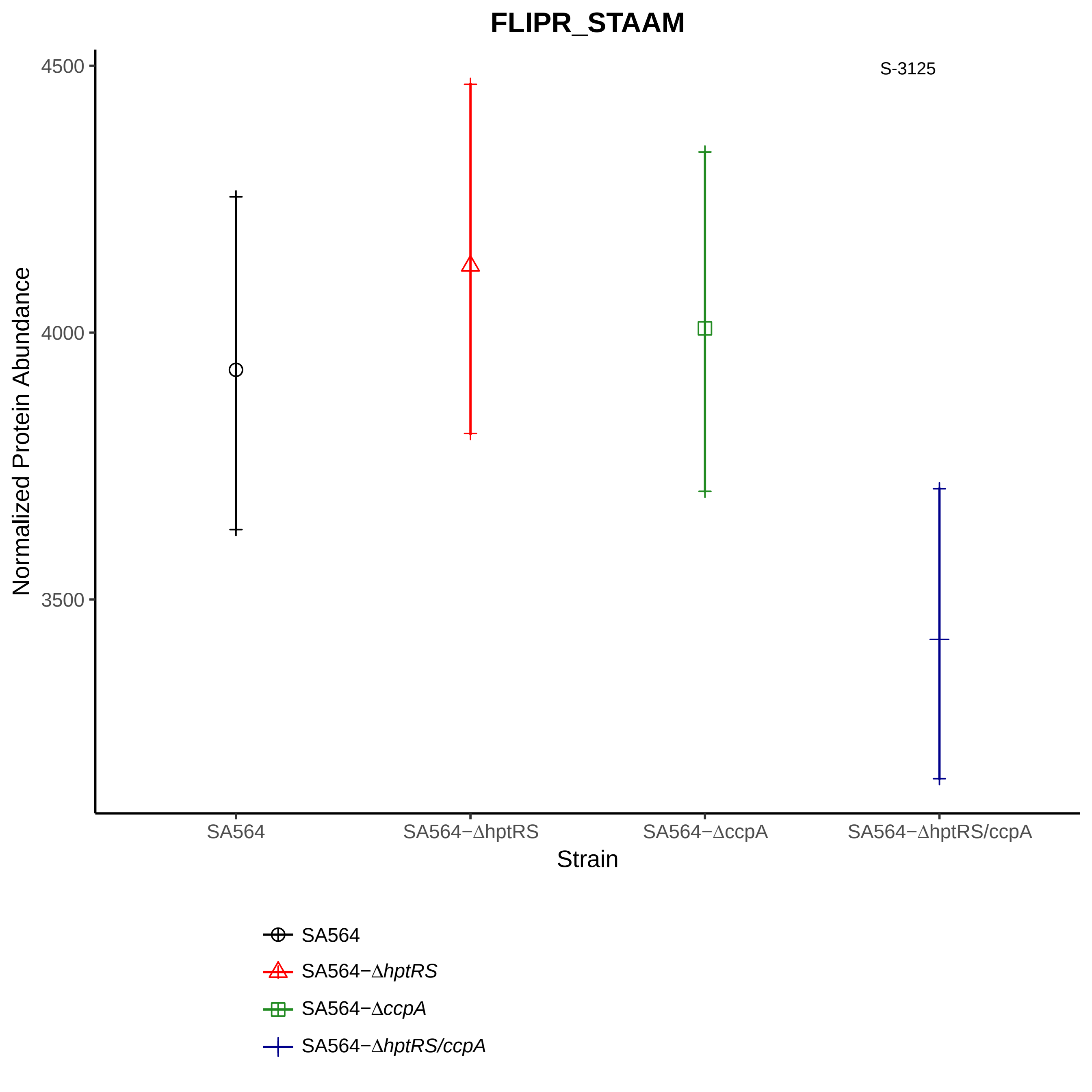
FLIPR_STAAM
S-3125
Normalized Protein Abundance
4500
4000
3500
Strain
SA564
SA564−ΔhptRS
SA564−ΔccpA
SA564−ΔhptRS/ccpA
SA564
SA564−ΔhptRS
SA564−ΔccpA
SA564−ΔhptRS/ccpA

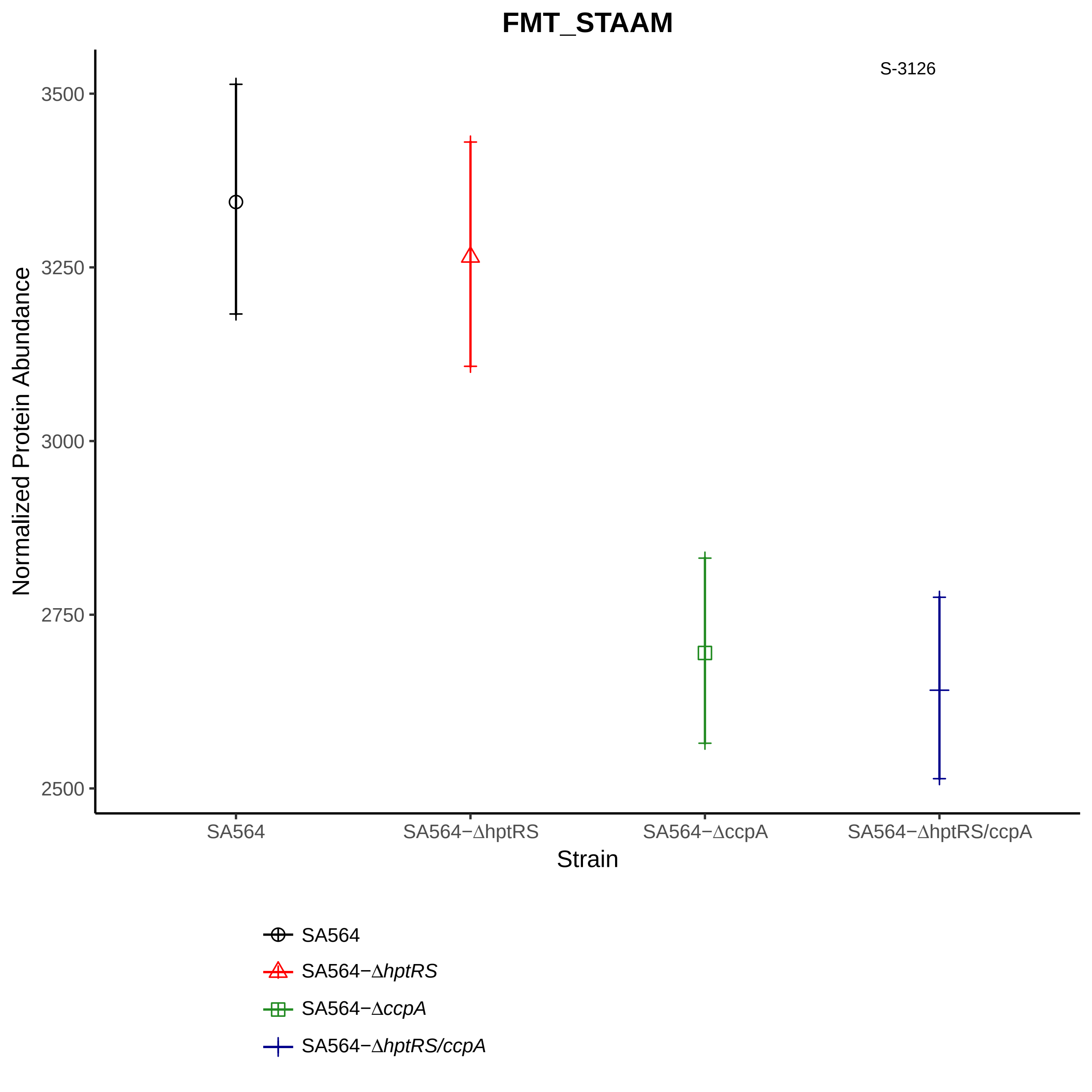

FMT_STAAM
S-3126
Normalized Protein Abundance
3500
3250
3000
2750
2500
SA564
SA564−ΔhptRS
SA564−ΔccpA
SA564−ΔhptRS/ccpA
Strain
SA564
SA564−ΔhptRS
SA564−ΔccpA
SA564−ΔhptRS/ccpA

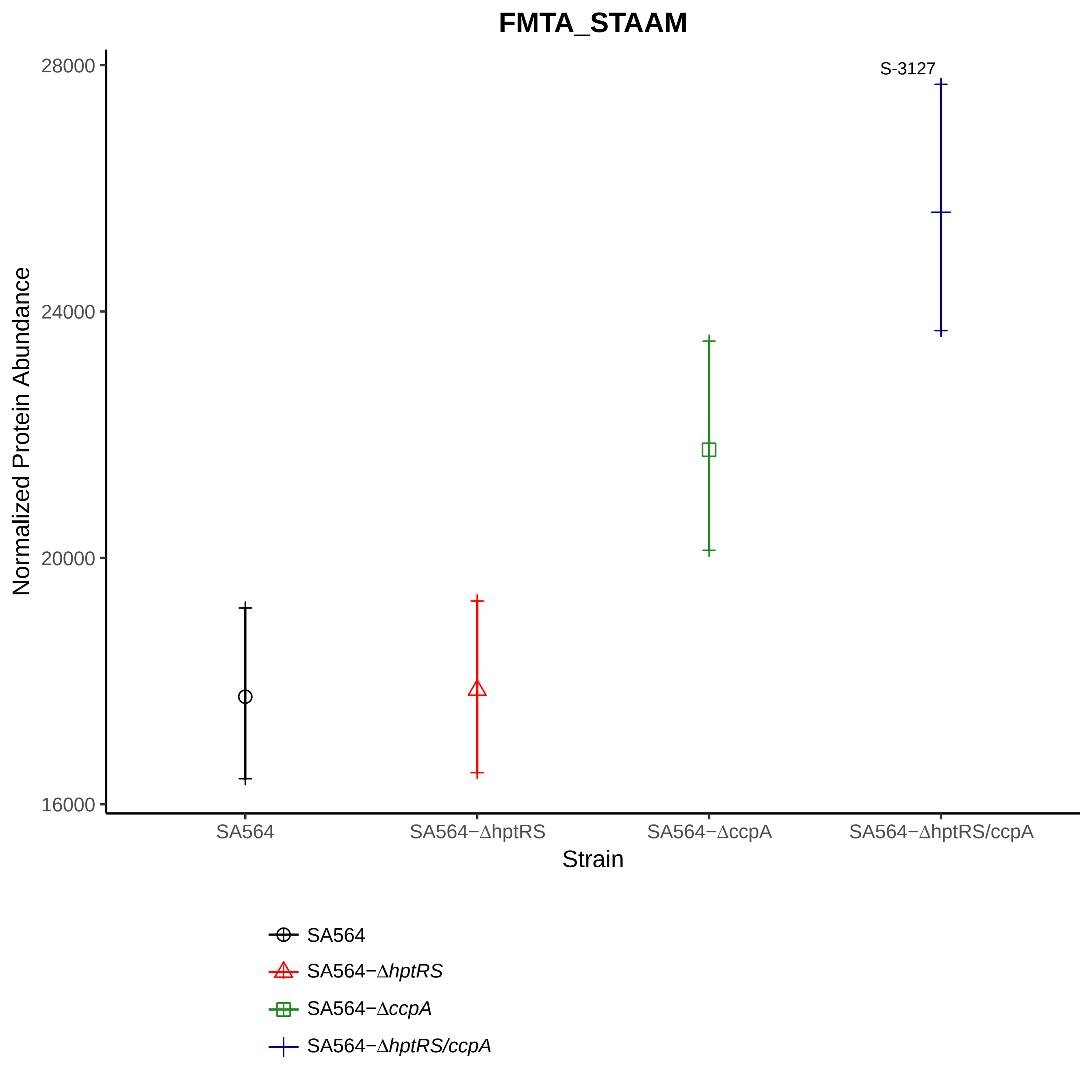

FMTA_STAAM
S-3127
28000
24000
20000
16000
Normalized Protein Abundance
SA564
SA564−ΔhptRS
SA564−ΔccpA
SA564−ΔhptRS/ccpA
Strain
SA564
SA564−Δ*hptRS*
SA564−Δ*ccpA*
SA564−Δ*hptRS/ccpA*

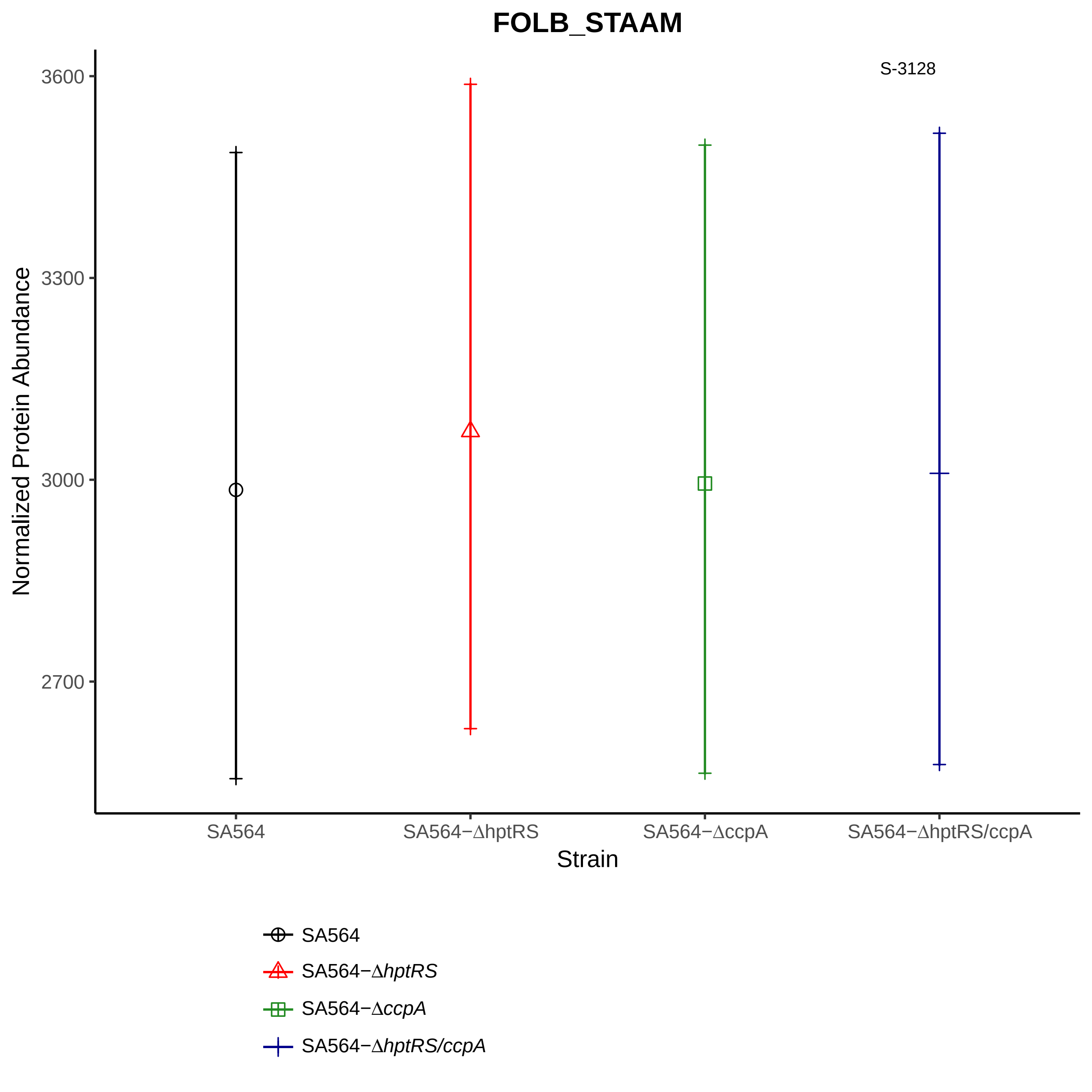

FOLB_STAAM
S-3128
Normalized Protein Abundance
3600
3300
3000
2700
SA564
SA564−ΔhptRS
SA564−ΔccpA
SA564−ΔhptRS/ccpA
Strain
SA564
SA564−ΔhptRS
SA564−ΔccpA
SA564−ΔhptRS/ccpA

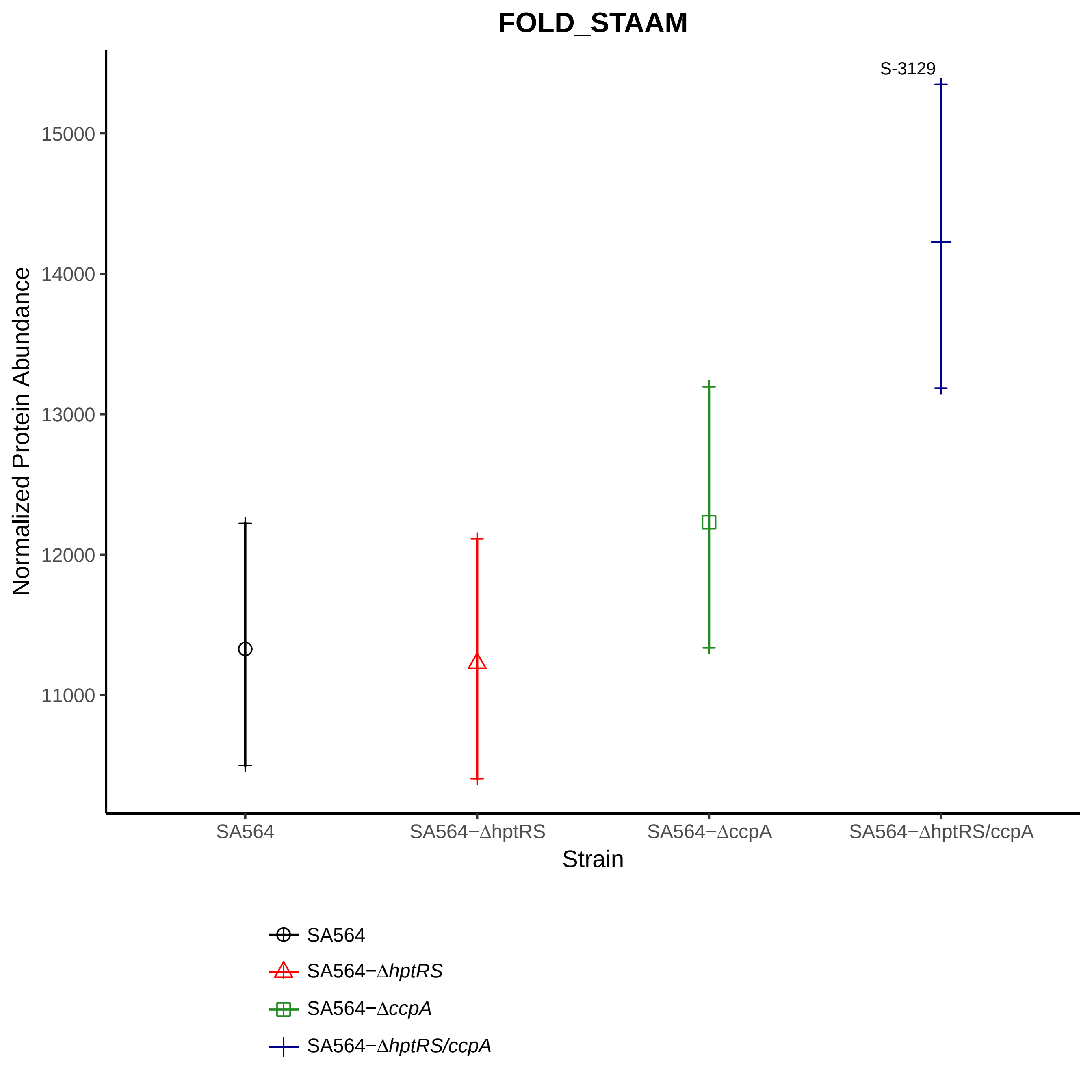

FOLD_STAAM
S-3129
15000
14000
13000
12000
11000
Normalized Protein Abundance
SA564
SA564−ΔhptRS
SA564−ΔccpA
SA564−ΔhptRS/ccpA
Strain
SA564
SA564−ΔhptRS
SA564−ΔccpA
SA564−ΔhptRS/ccpA

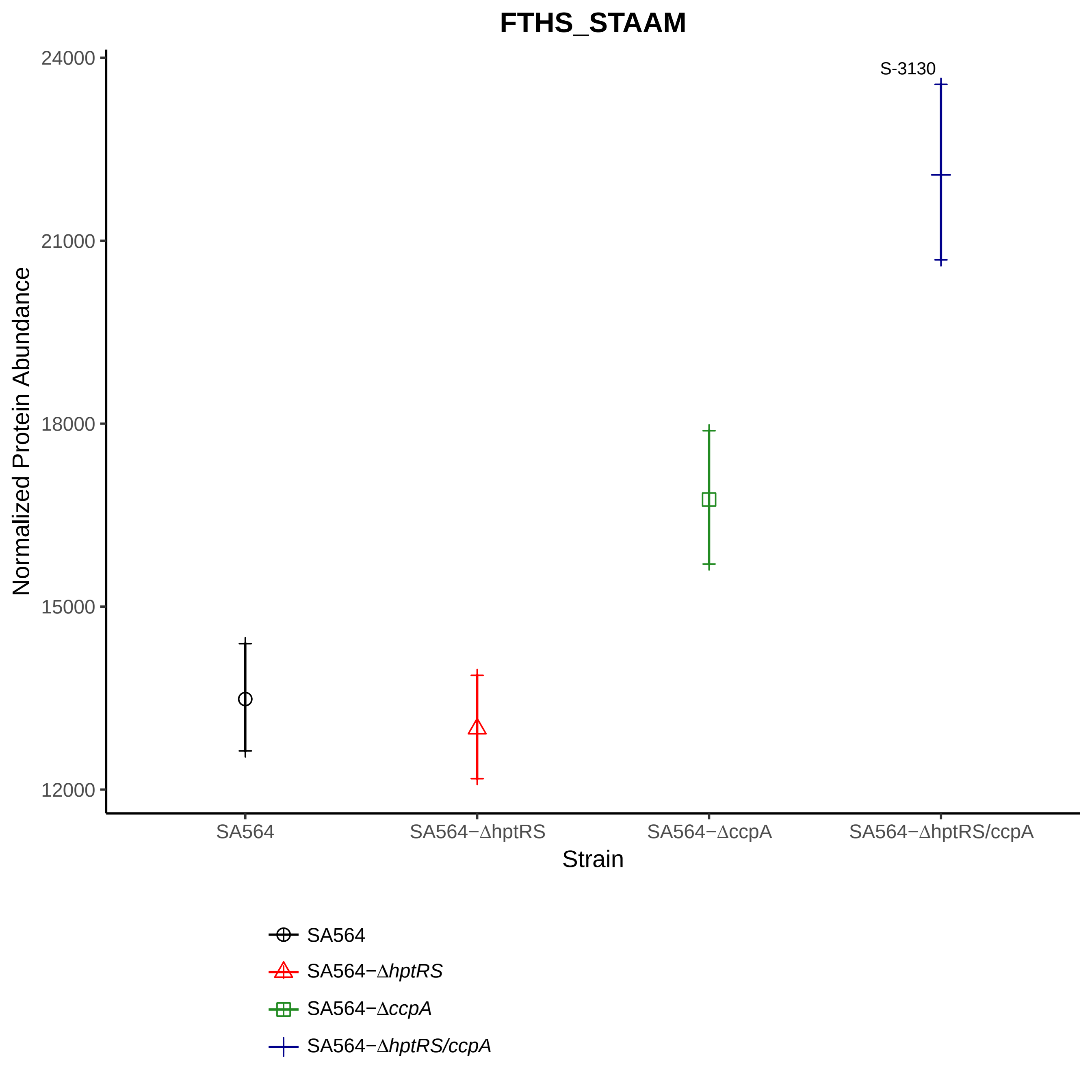

FTHS_STAAM
S-3130
Normalized Protein Abundance
24000
21000
18000
15000
12000
SA564
SA564−ΔhptRS
SA564−ΔccpA
SA564−ΔhptRS/ccpA
Strain
SA564
SA564−Δ*hptRS*
SA564−Δ*ccpA*
SA564−Δ*hptRS/ccpA*

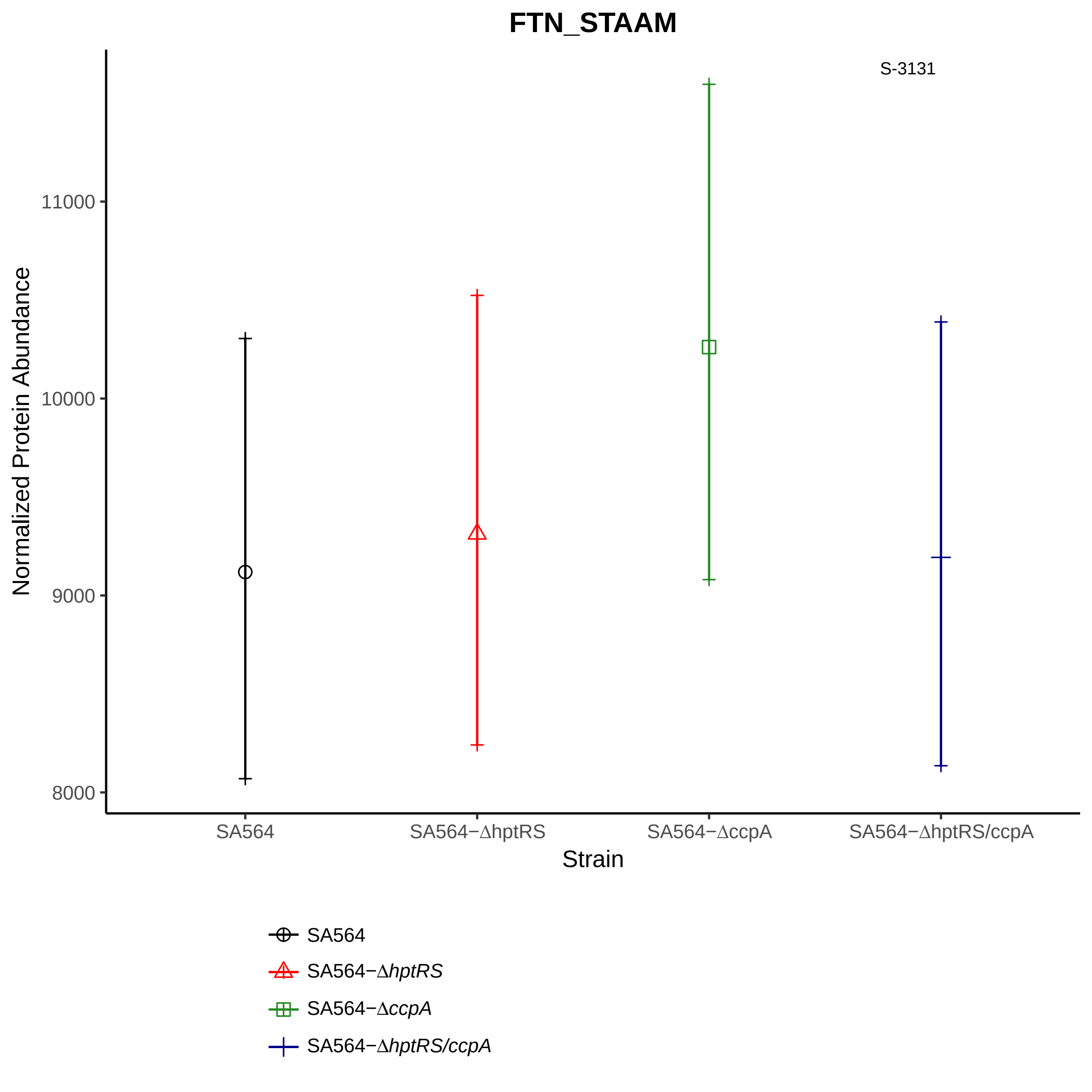

FTN_STAAM
S-3131
Normalized Protein Abundance
11000
10000
9000
8000
SA564
SA564−ΔhptRS
SA564−ΔccpA
SA564−ΔhptRS/ccpA
Strain
SA564
SA564−Δ*hptRS*
SA564−Δ*ccpA*
SA564−Δ*hptRS/ccpA*

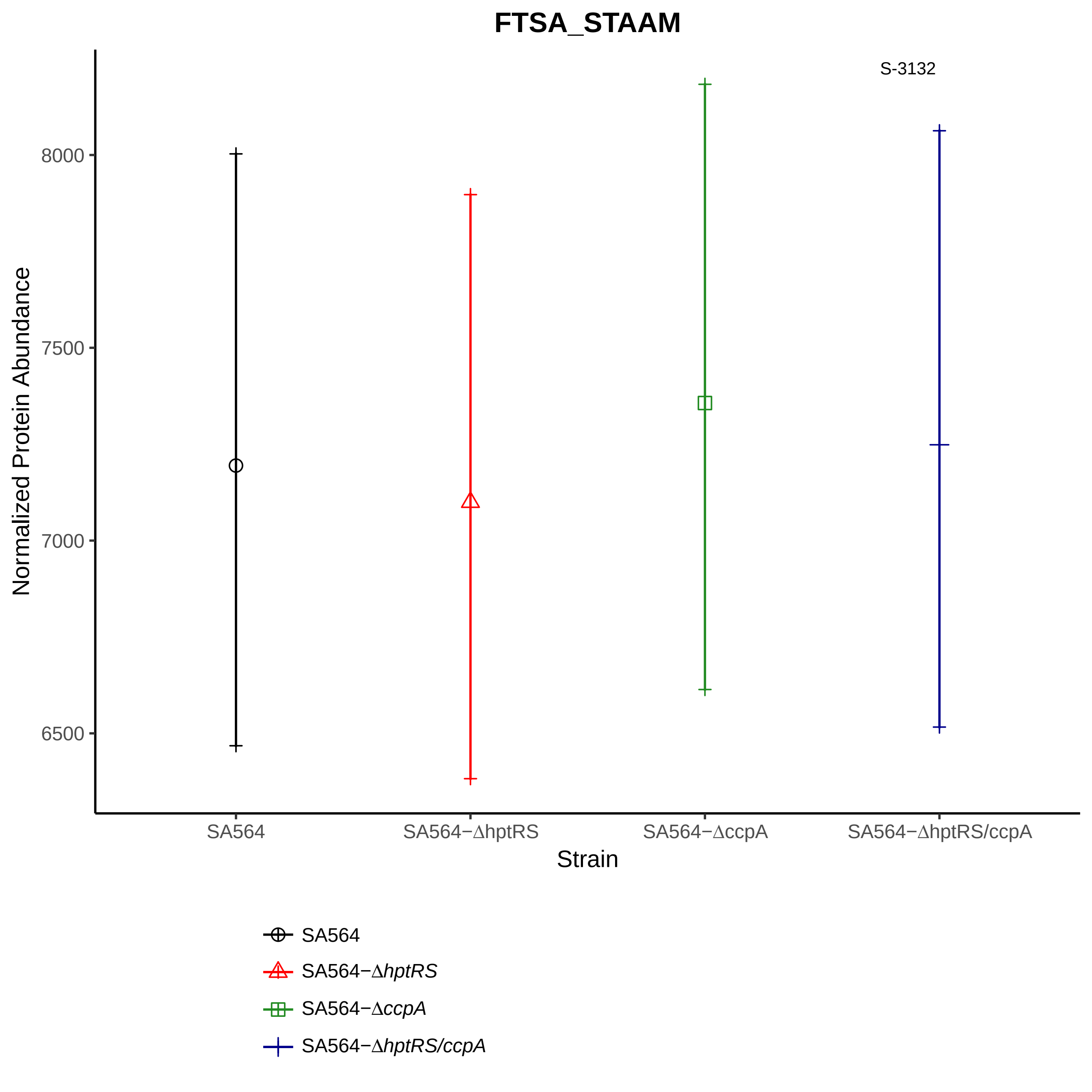

FTSA_STAAM
S-3132
Normalized Protein Abundance
8000
7500
7000
6500
SA564
SA564−ΔhptRS
SA564−ΔccpA
SA564−ΔhptRS/ccpA
Strain
SA564
SA564−ΔhptRS
SA564−ΔccpA
SA564−ΔhptRS/ccpA

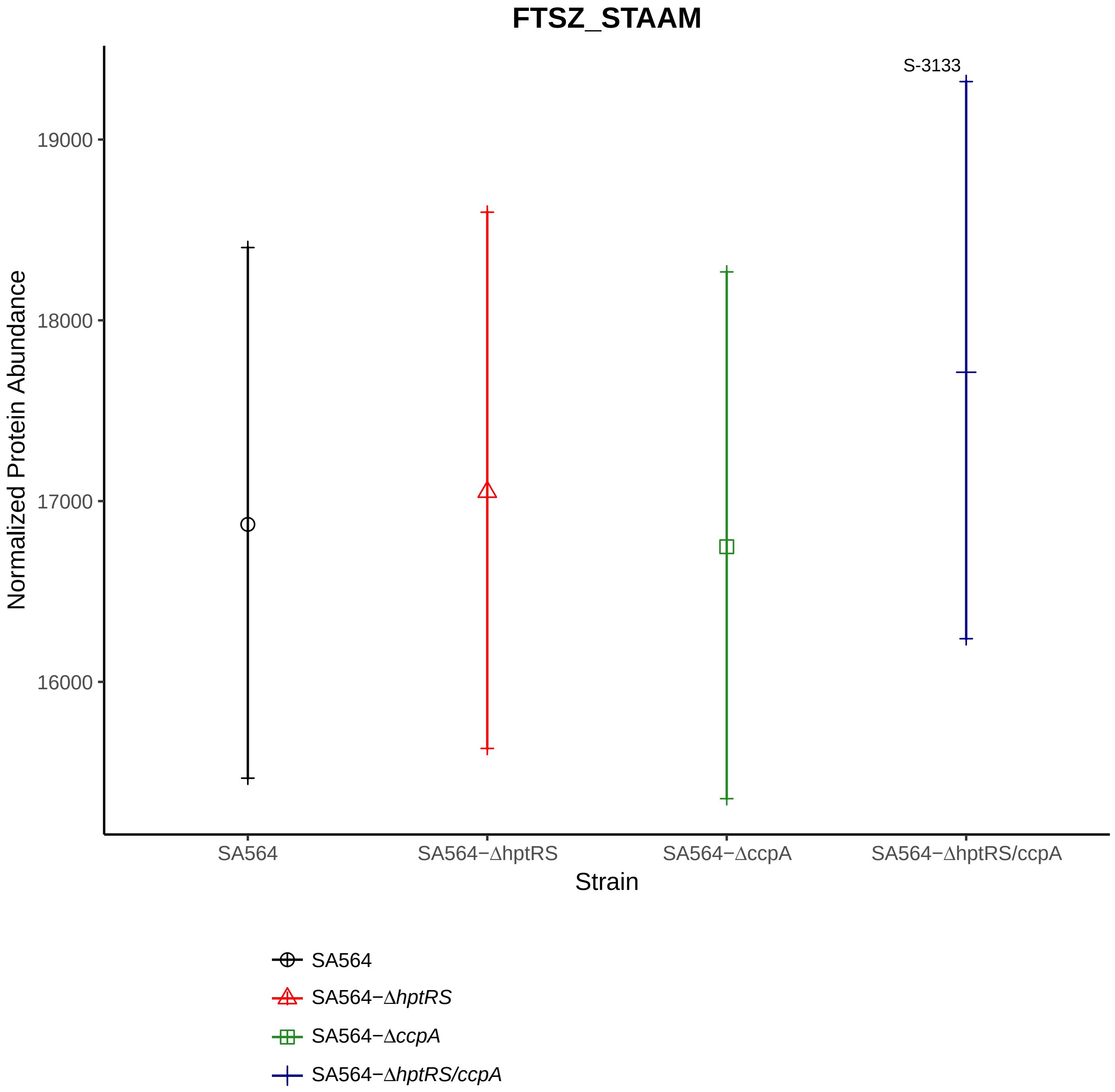

FTSZ_STAAM
S-3133
19000
18000
17000
16000
Normalized Protein Abundance
SA564
SA564−ΔhptRS
SA564−ΔccpA
SA564−ΔhptRS/ccpA
Strain
SA564
SA564−Δ*hptRS*
SA564−Δ*ccpA*
SA564−Δ*hptRS/ccpA*

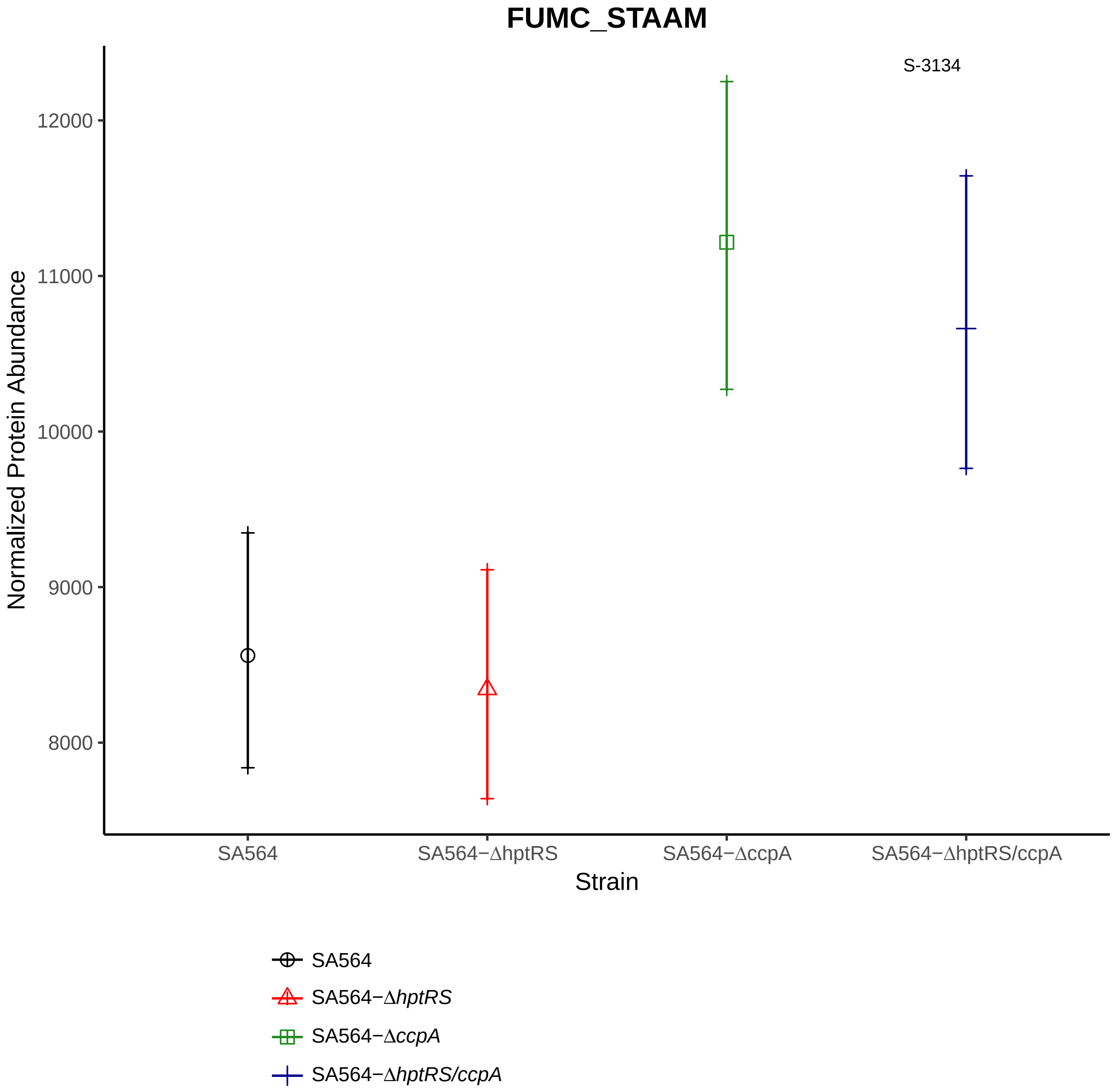
FUMC_STAAM
S-3134
Normalized Protein Abundance
12000
11000
10000
9000
8000
SA564
SA564−ΔhptRS
SA564−ΔccpA
SA564−ΔhptRS/ccpA
Strain
SA564
SA564−ΔhptRS
SA564−ΔccpA
SA564−ΔhptRS/ccpA

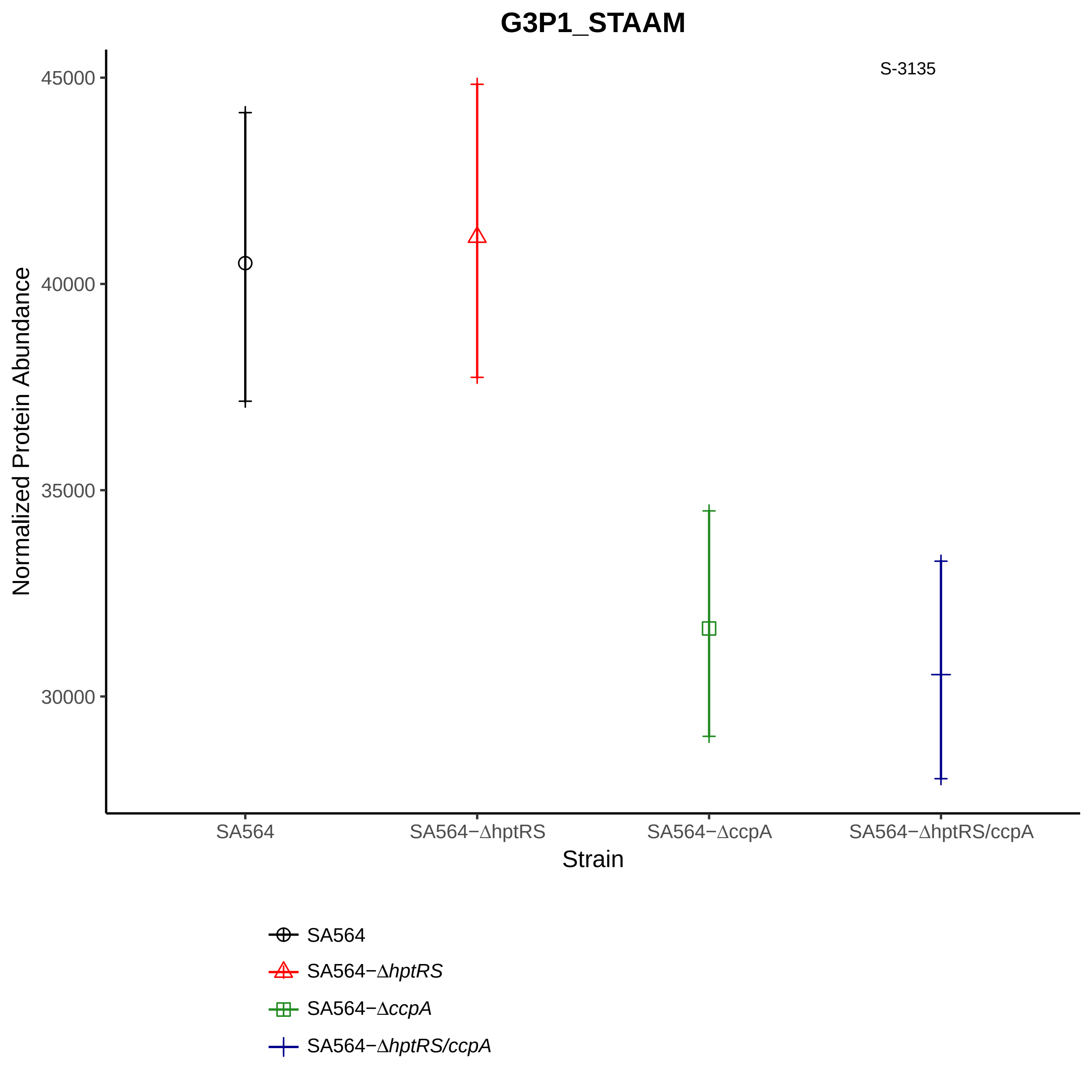

G3P1_STAAM
S-3135
Normalized Protein Abundance
45000
40000
35000
30000
SA564
SA564−ΔhptRS
SA564−ΔccpA
SA564−ΔhptRS/ccpA
Strain
SA564
SA564−ΔhptRS
SA564−ΔccpA
SA564−ΔhptRS/ccpA

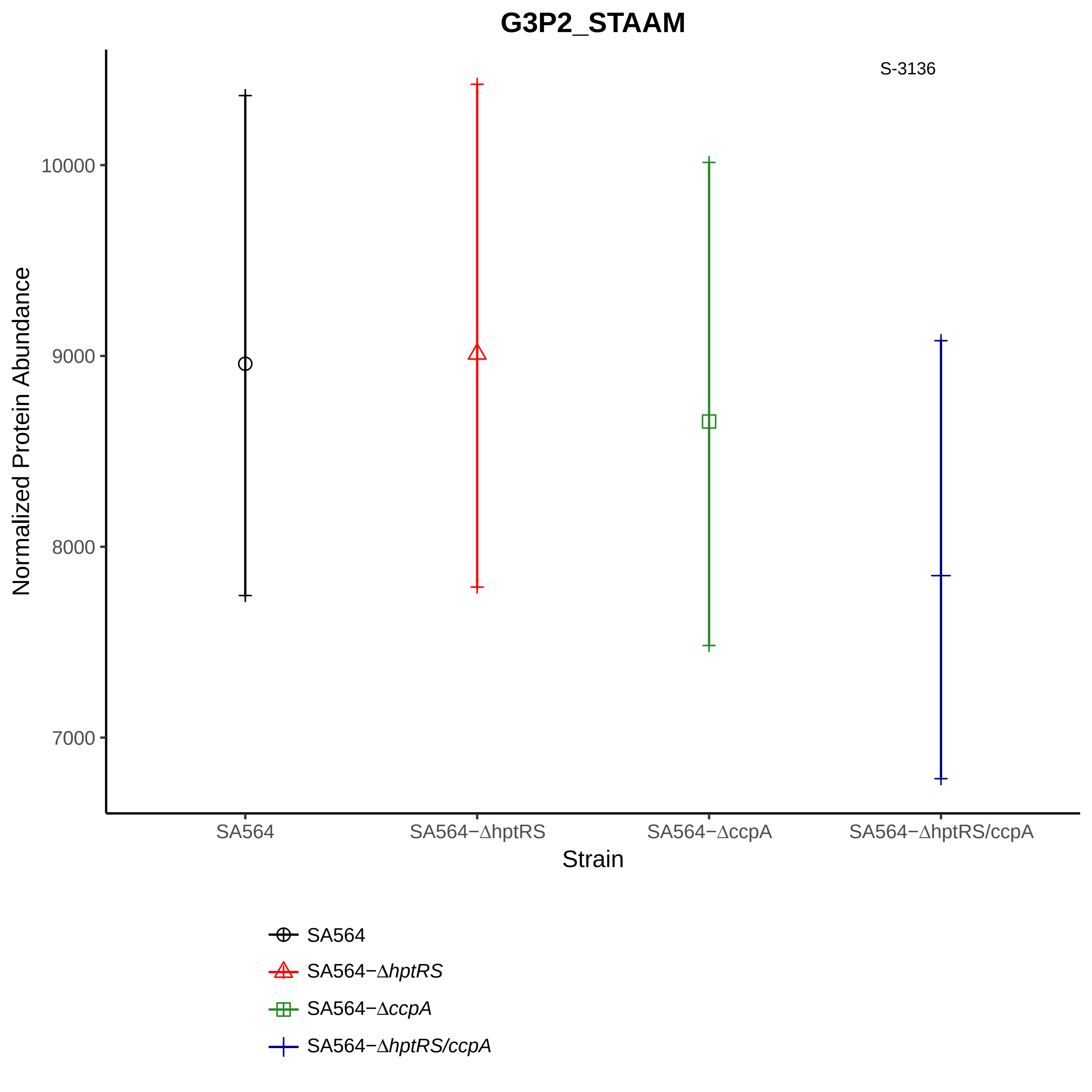
G3P2_STAAM
S-3136
Normalized Protein Abundance
10000
9000
8000
7000
SA564
SA564−ΔhptRS
SA564−ΔccpA
SA564−ΔhptRS/ccpA
Strain
SA564
SA564−ΔhptRS
SA564−ΔccpA
SA564−ΔhptRS/ccpA

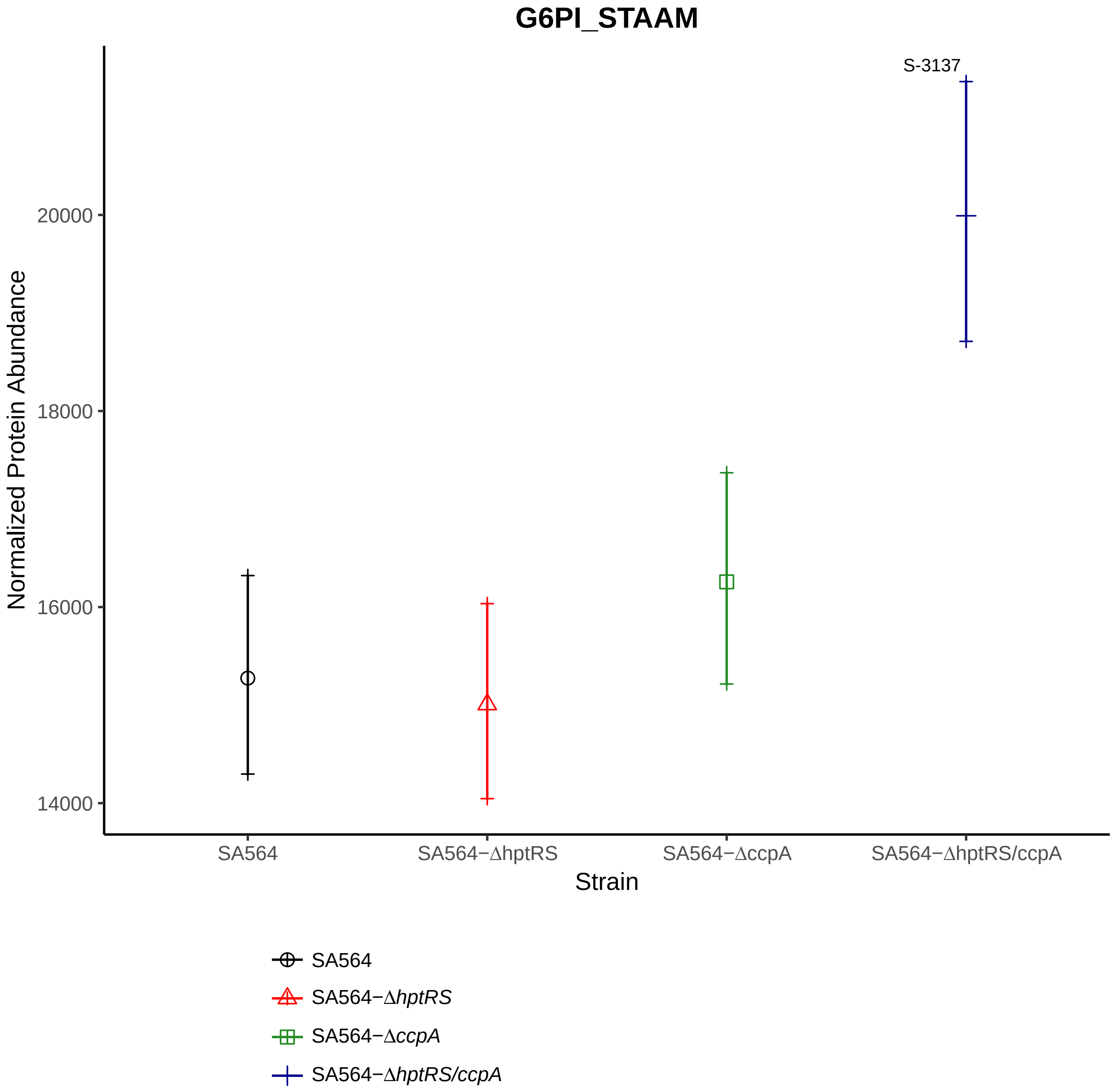
G6PI_STAAM
S-3137
Normalized Protein Abundance
20000
18000
16000
14000
SA564
SA564−ΔhptRS
SA564−ΔccpA
SA564−ΔhptRS/ccpA
Strain
SA564
SA564−ΔhptRS
SA564−ΔccpA
SA564−ΔhptRS/ccpA

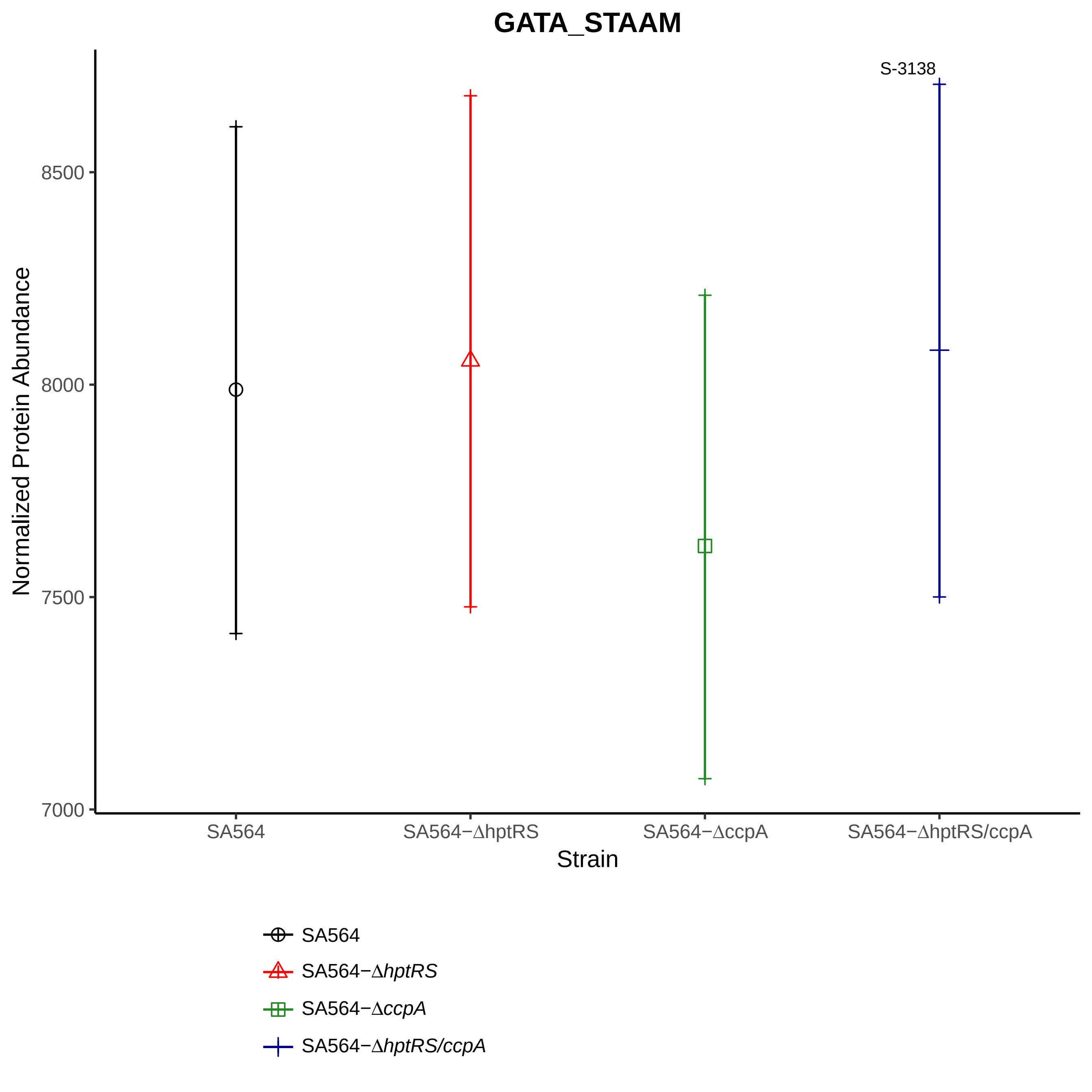

GATA_STAAM
S-3138
Normalized Protein Abundance
8500
8000
7500
7000
SA564
SA564−ΔhptRS
SA564−ΔccpA
SA564−ΔhptRS/ccpA
Strain
SA564
SA564−ΔhptRS
SA564−ΔccpA
SA564−ΔhptRS/ccpA

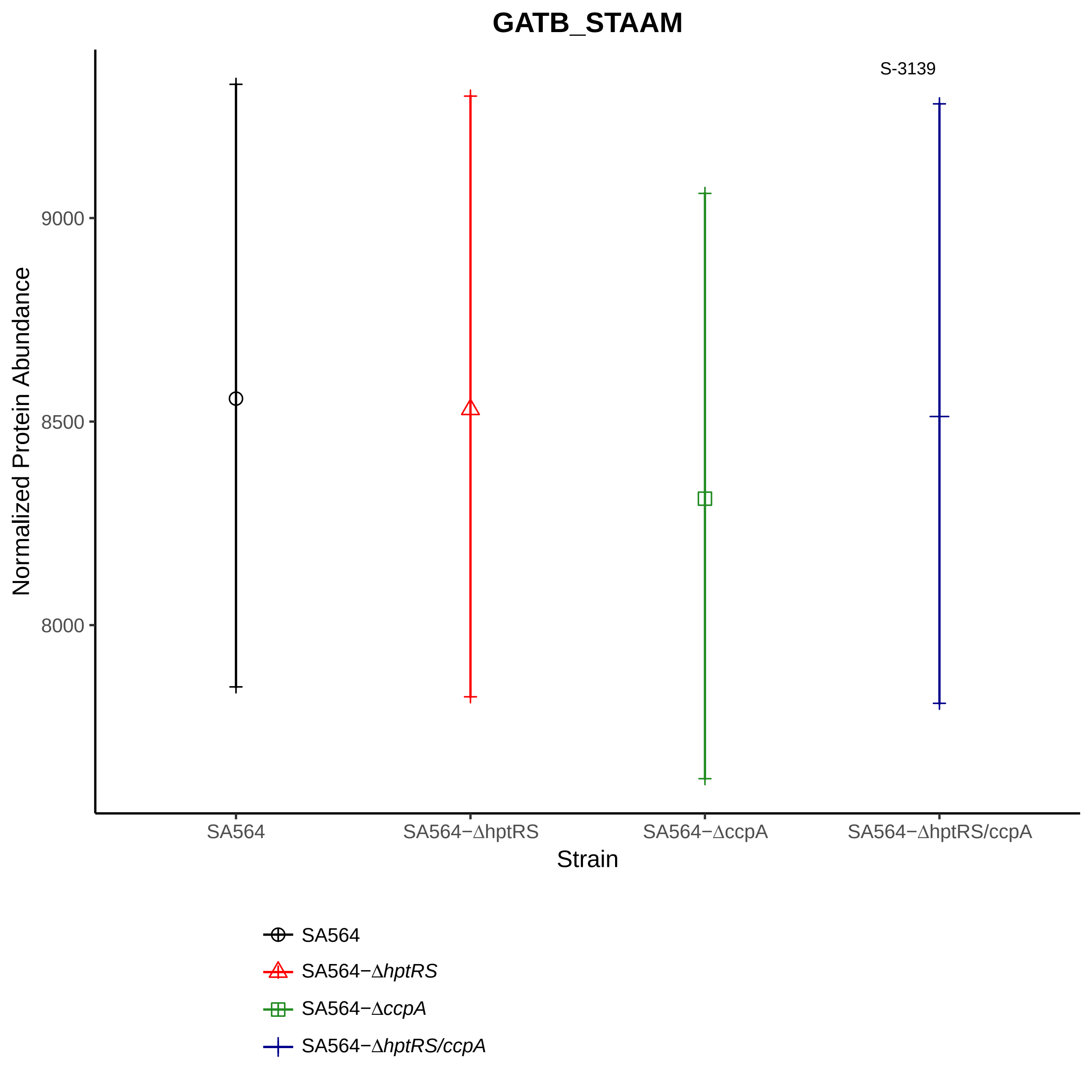

GATB_STAAM
S-3139
9000
8500
8000
Normalized Protein Abundance
SA564
SA564−ΔhptRS
SA564−ΔccpA
SA564−ΔhptRS/ccpA
Strain
SA564
SA564−ΔhptRS
SA564−ΔccpA
SA564−ΔhptRS/ccpA

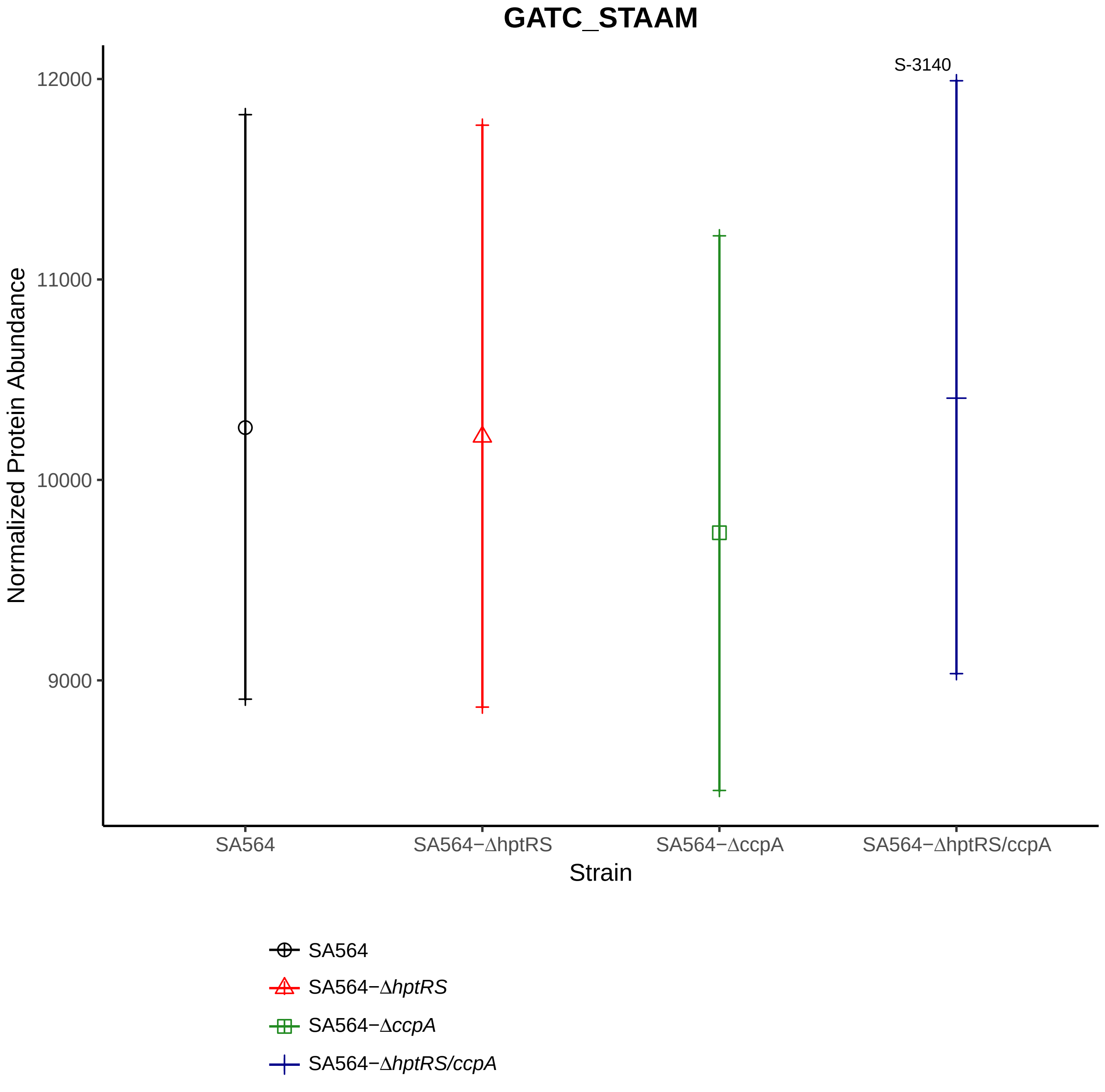

GATC_STAAM
S-3140
12000
11000
10000
9000
Normalized Protein Abundance
SA564
SA564−ΔhptRS
SA564−ΔccpA
SA564−ΔhptRS/ccpA
Strain
SA564
SA564−ΔhptRS
SA564−ΔccpA
SA564−ΔhptRS/ccpA

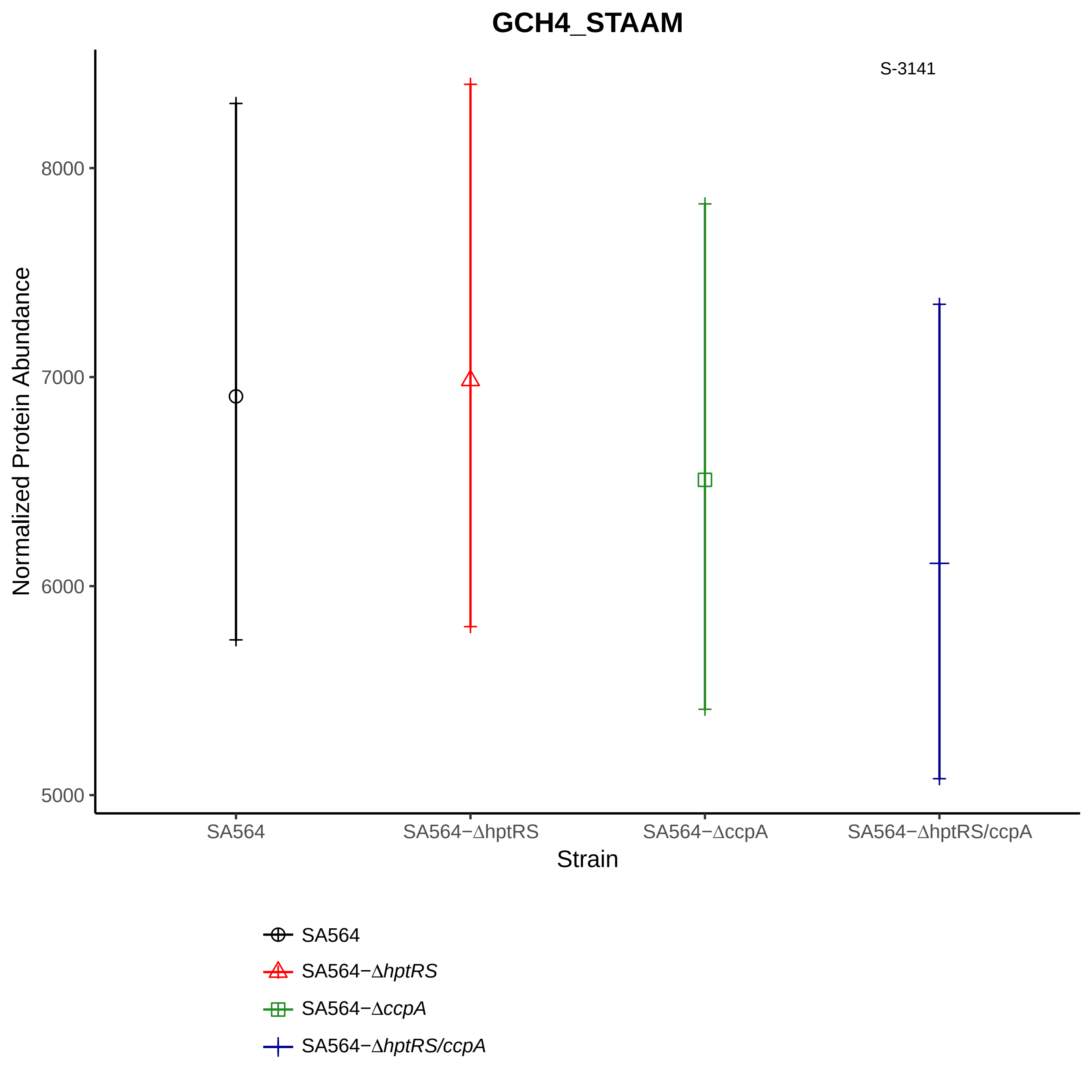

GCH4_STAAM
S-3141
Normalized Protein Abundance
8000
7000
6000
5000
SA564
SA564−ΔhptRS
SA564−ΔccpA
SA564−ΔhptRS/ccpA
Strain
SA564
SA564−ΔhptRS
SA564−ΔccpA
SA564−ΔhptRS/ccpA

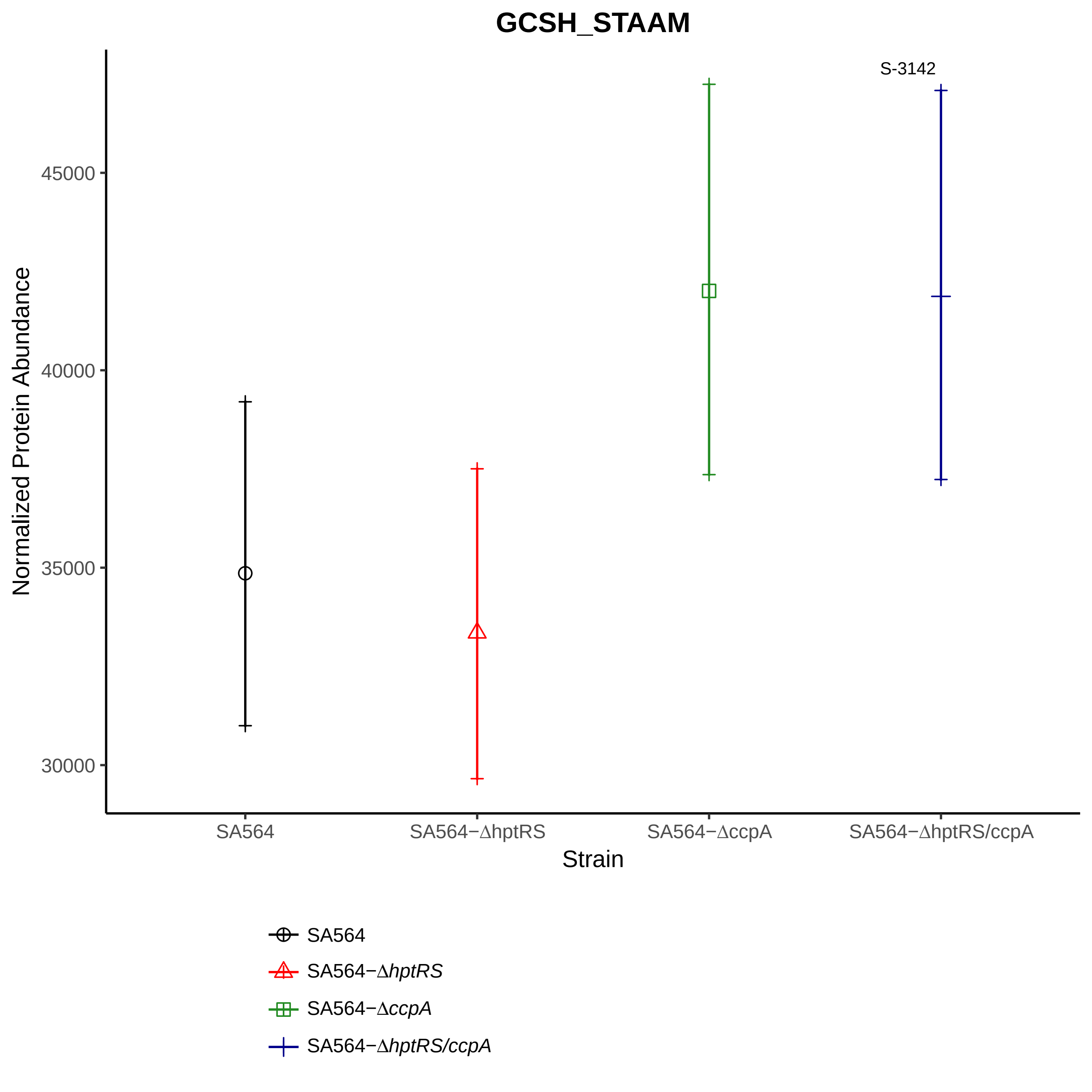

GCSH_STAAM
S-3142
Normalized Protein Abundance
45000
40000
35000
30000
SA564
SA564−ΔhptRS
SA564−ΔccpA
SA564−ΔhptRS/ccpA
Strain
SA564
SA564−ΔhptRS
SA564−ΔccpA
SA564−ΔhptRS/ccpA

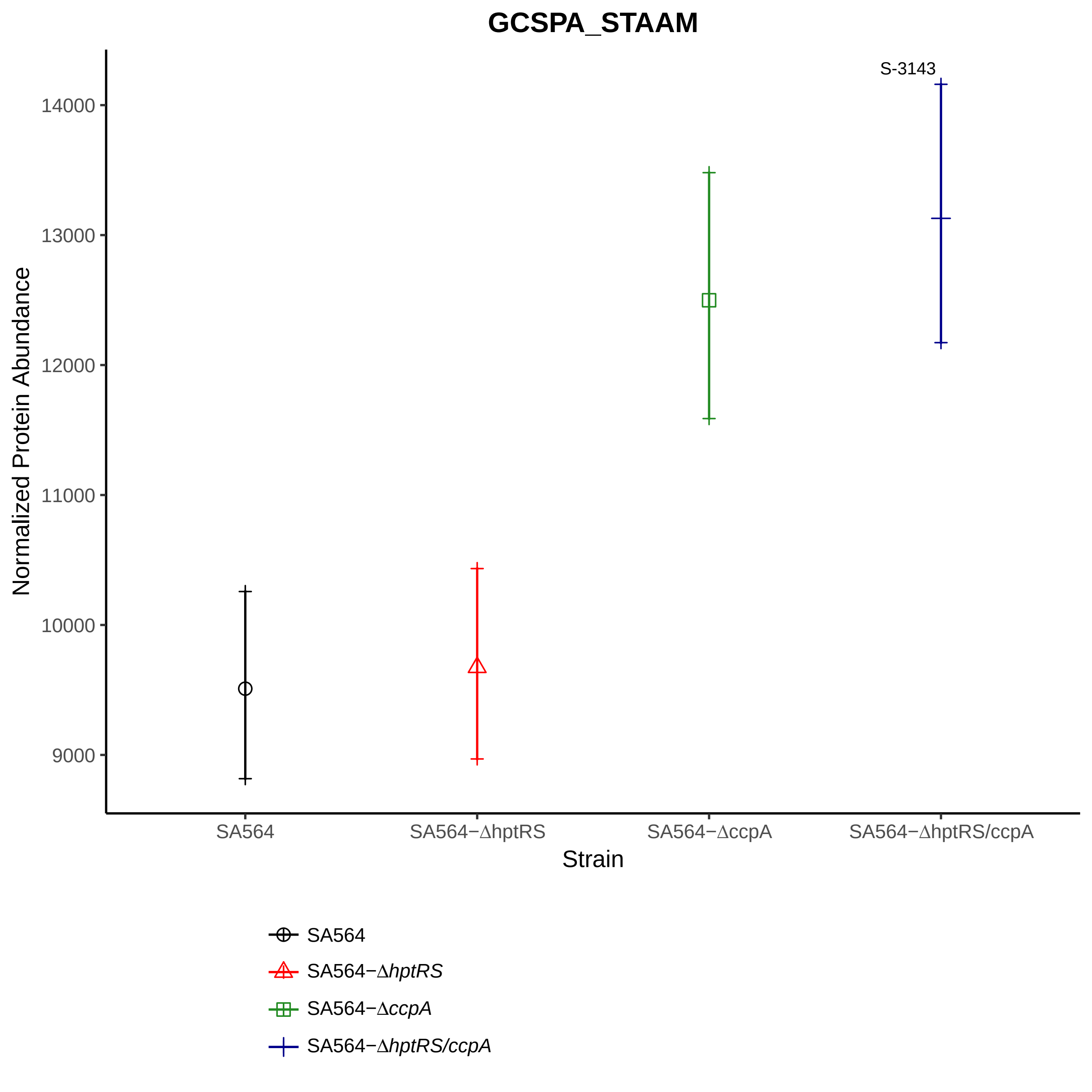

GCSPA_STAAM
S-3143
Normalized Protein Abundance
14000
13000
12000
11000
10000
9000
SA564
SA564−ΔhptRS
SA564−ΔccpA
SA564−ΔhptRS/ccpA
Strain
SA564
SA564−ΔhptRS
SA564−ΔccpA
SA564−ΔhptRS/ccpA

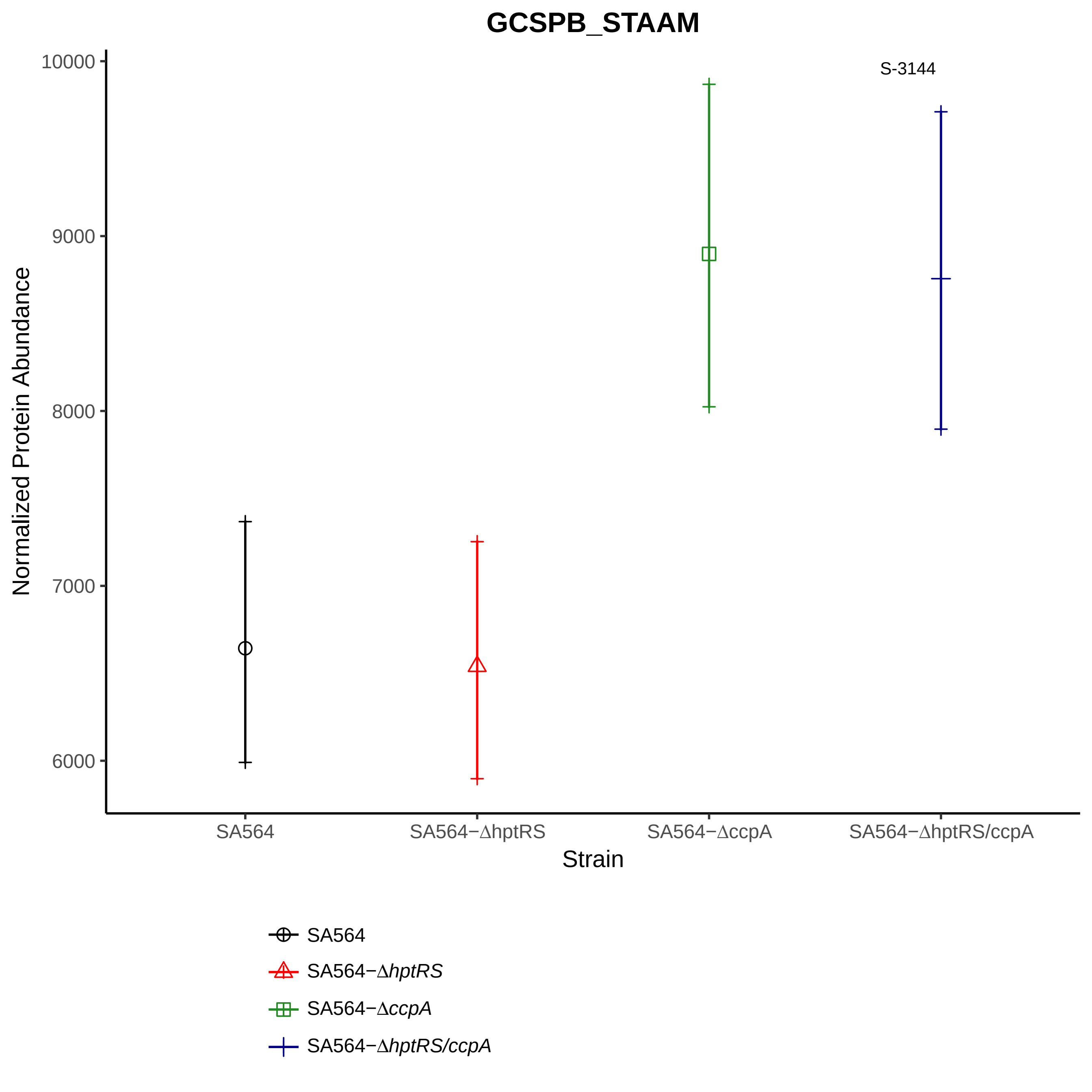
GCSPB_STAAM
S-3144
Normalized Protein Abundance
10000
9000
8000
7000
6000
SA564
SA564−ΔhptRS
SA564−ΔccpA
SA564−ΔhptRS/ccpA
Strain
SA564
SA564−ΔhptRS
SA564−ΔccpA
SA564−ΔhptRS/ccpA

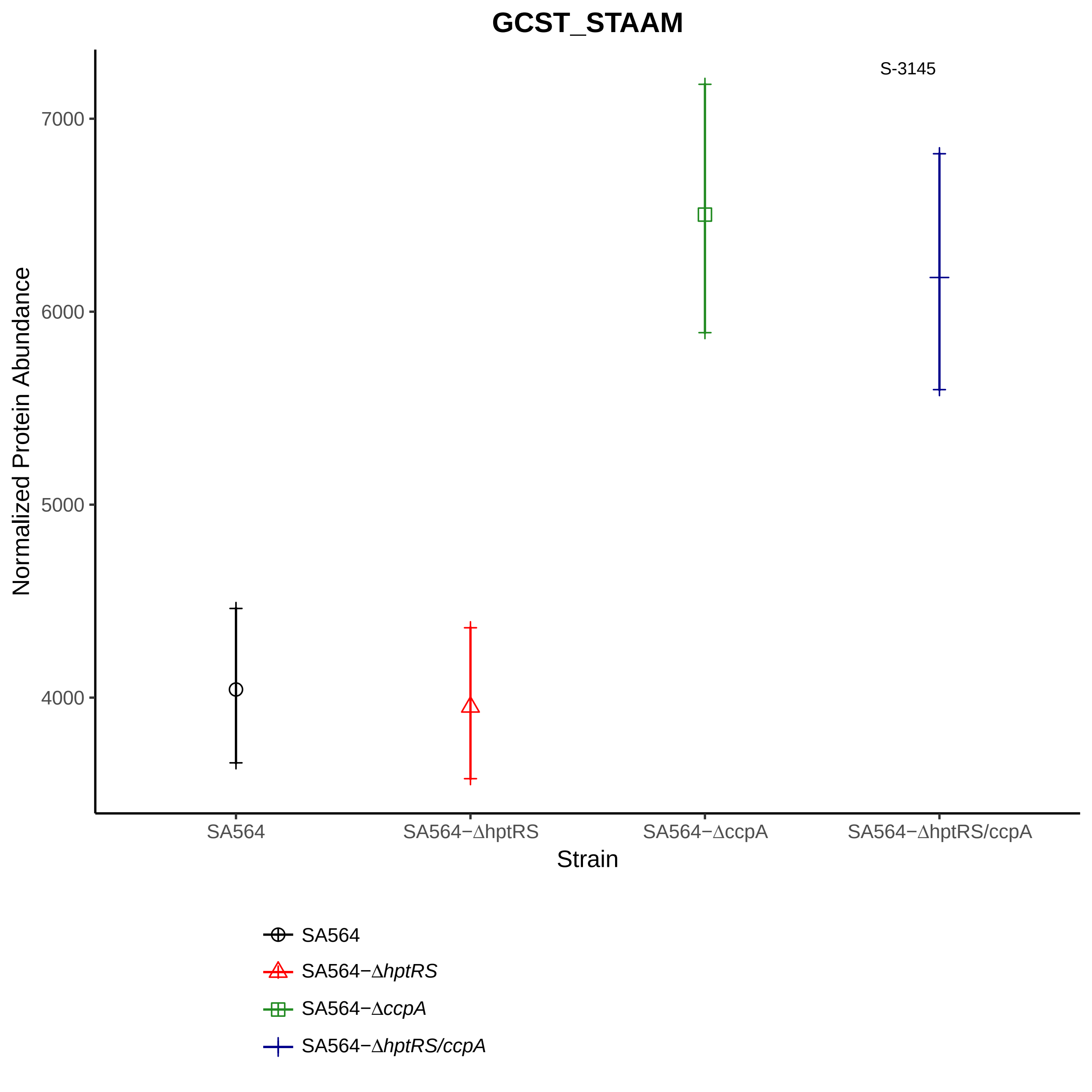
GCST_STAAM
S-3145
Normalized Protein Abundance
7000
6000
5000
4000
SA564
SA564−ΔhptRS
SA564−ΔccpA
SA564−ΔhptRS/ccpA
Strain
SA564
SA564−ΔhptRS
SA564−ΔccpA
SA564−ΔhptRS/ccpA

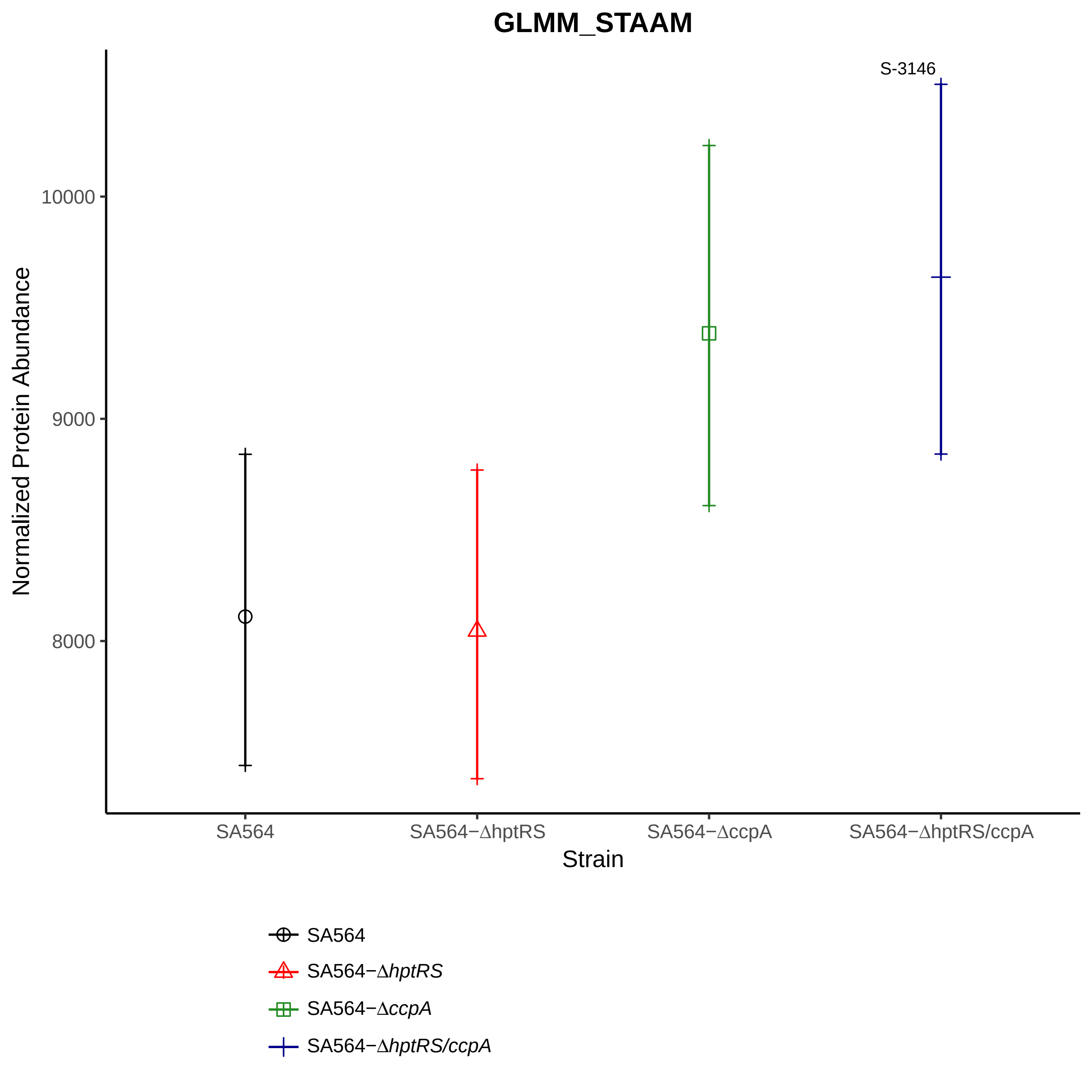

GLMM_STAAM
S-3146
Normalized Protein Abundance
10000
9000
8000
SA564
SA564−ΔhptRS
SA564−ΔccpA
SA564−ΔhptRS/ccpA
Strain
SA564
SA564−ΔhptRS
SA564−ΔccpA
SA564−ΔhptRS/ccpA

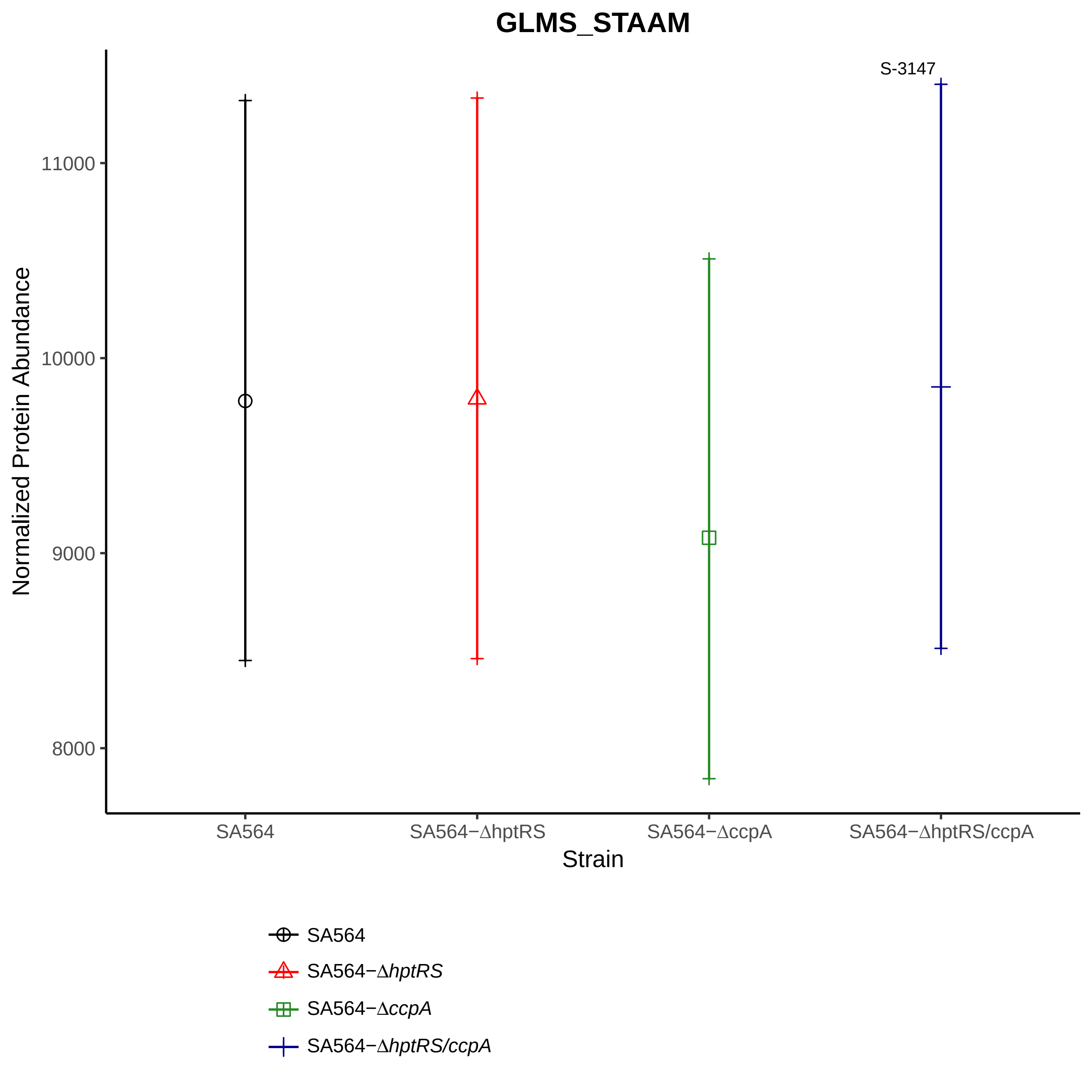

GLMS_STAAM
S-3147
Normalized Protein Abundance
11000
10000
9000
8000
SA564
SA564−ΔhptRS
SA564−ΔccpA
SA564−ΔhptRS/ccpA
Strain
SA564
SA564−ΔhptRS
SA564−ΔccpA
SA564−ΔhptRS/ccpA

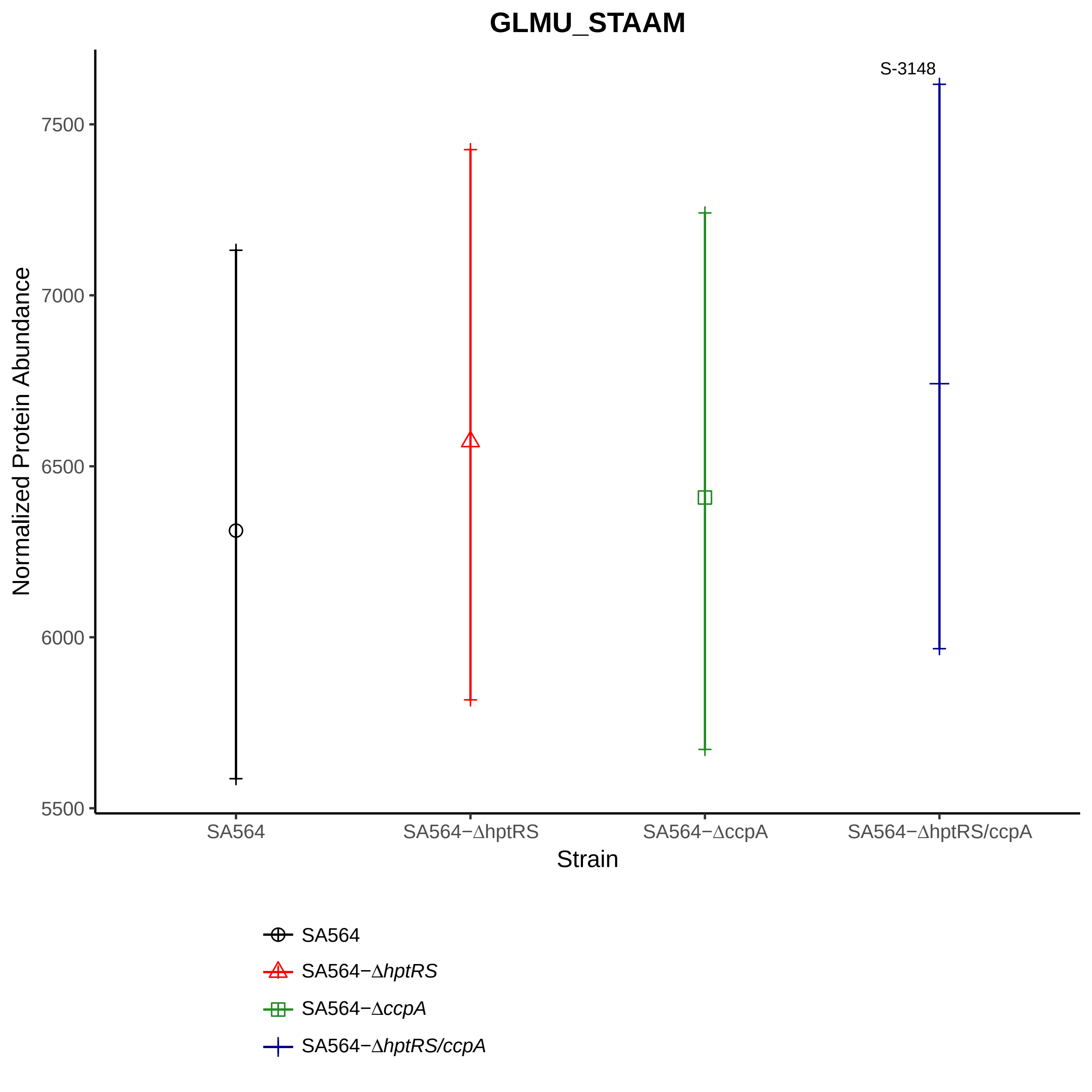
GLMU_STAAM
S-3148
Normalized Protein Abundance
7500
7000
6500
6000
5500
SA564
SA564−ΔhptRS
SA564−ΔccpA
SA564−ΔhptRS/ccpA
Strain
SA564
SA564−ΔhptRS
SA564−ΔccpA
SA564−ΔhptRS/ccpA

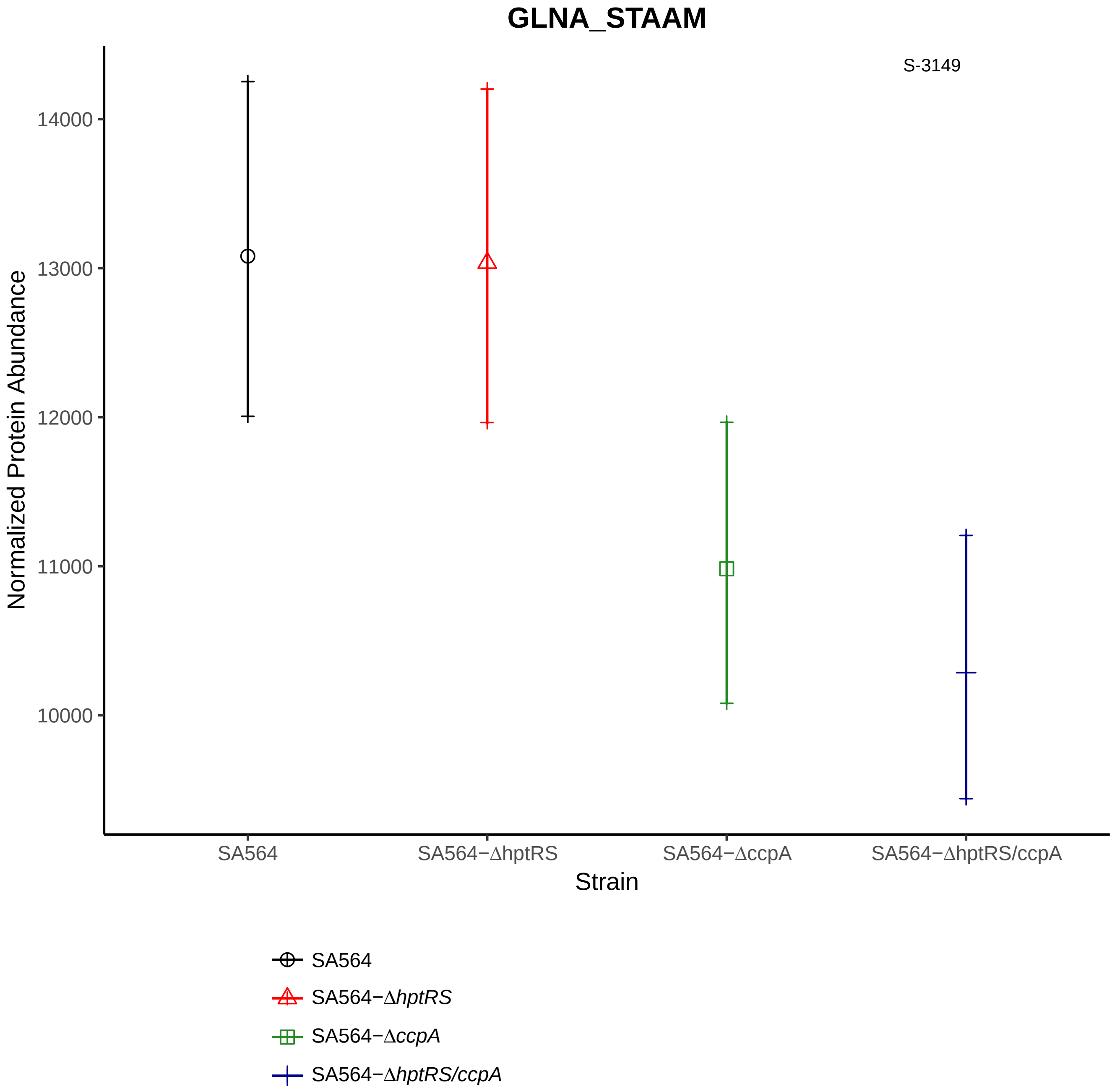
GLNA_STAAM
S-3149
Normalized Protein Abundance
14000
13000
12000
11000
10000
SA564
SA564−ΔhptRS
SA564−ΔccpA
SA564−ΔhptRS/ccpA
Strain
SA564
SA564−ΔhptRS
SA564−ΔccpA
SA564−ΔhptRS/ccpA

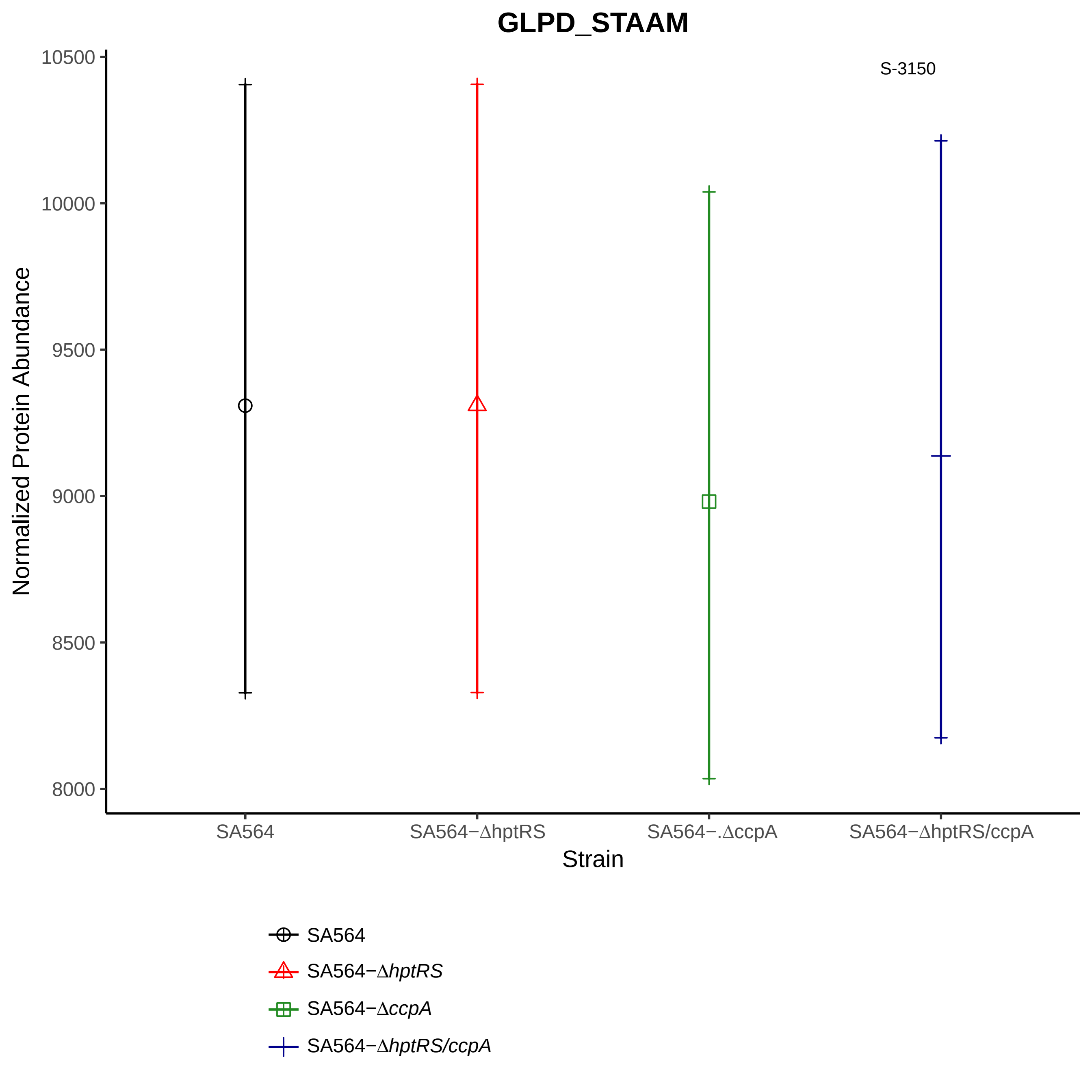

GLPD_STAAM
S-3150
10500
10000
9500
9000
8500
8000
Normalized Protein Abundance
SA564
SA564−ΔhptRS
SA564−.ΔccpA
SA564−ΔhptRS/ccpA
Strain
SA564
SA564−Δ*hptRS*
SA564−Δ*ccpA*
SA564−Δ*hptRS/ccpA*

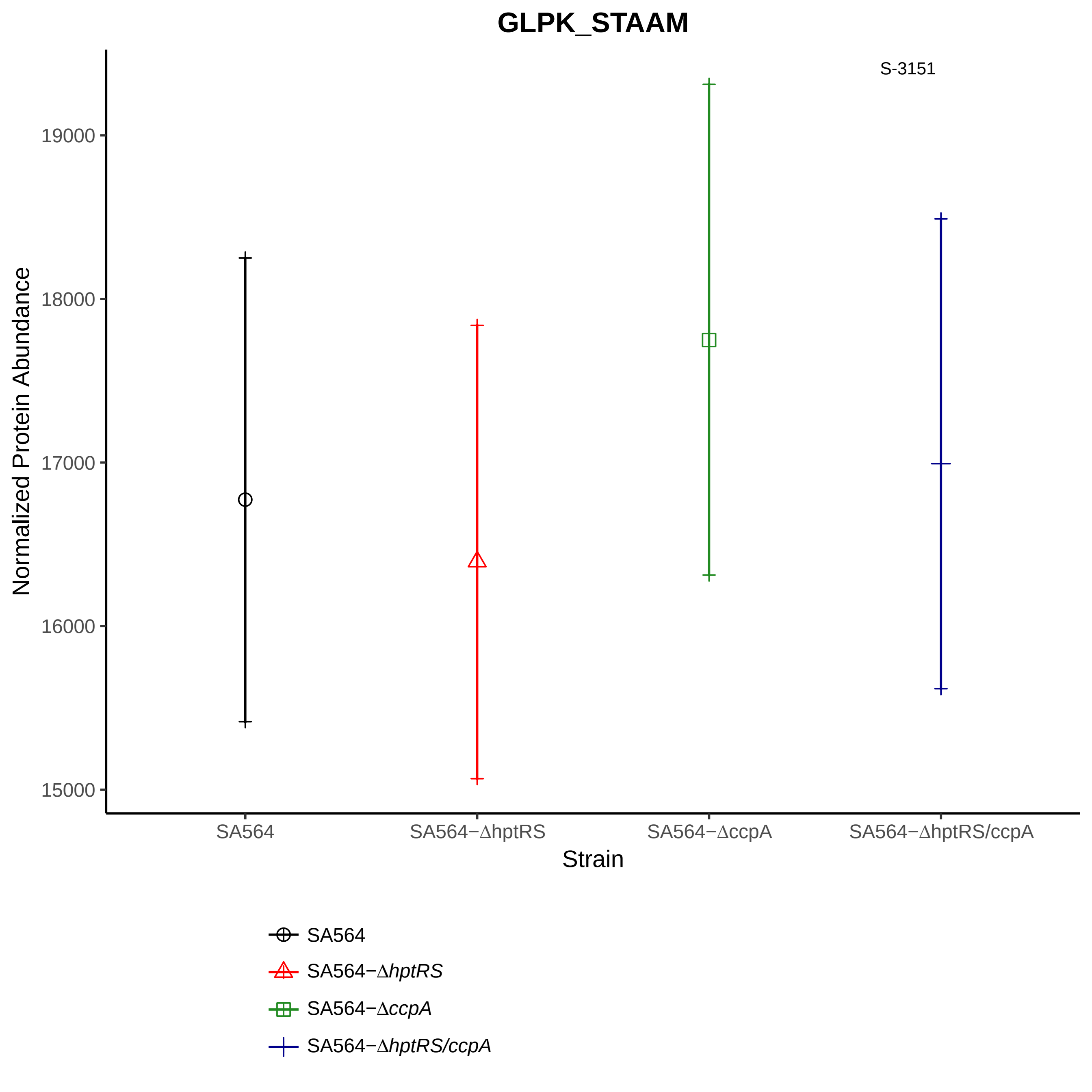

GLPK_STAAM
S-3151
Normalized Protein Abundance
19000
18000
17000
16000
15000
SA564
SA564−ΔhptRS
SA564−ΔccpA
SA564−ΔhptRS/ccpA
Strain
SA564
SA564−Δ*hptRS*
SA564−Δ*ccpA*
SA564−Δ*hptRS/ccpA*

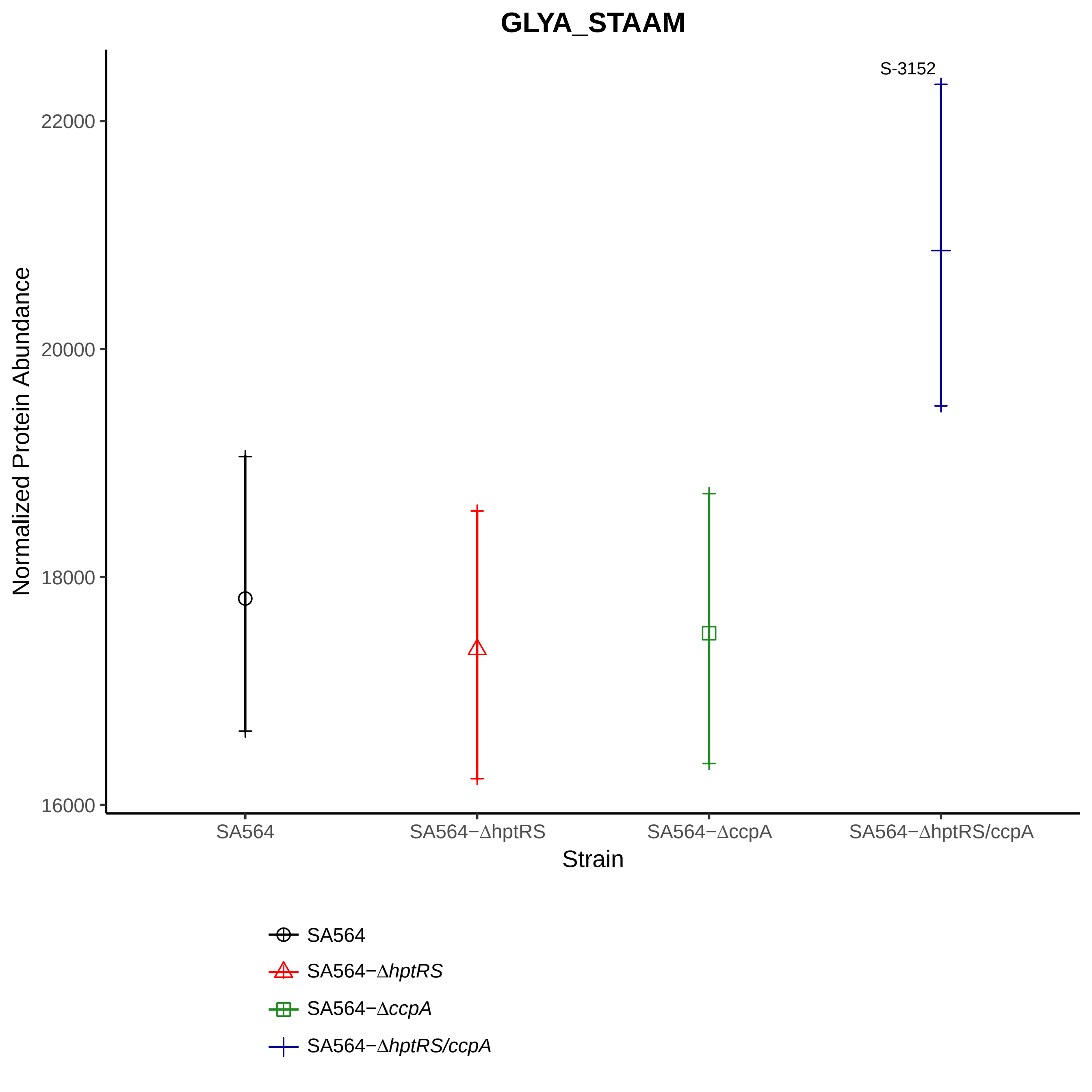

GLYA_STAAM
S-3152
Normalized Protein Abundance
22000
20000
18000
16000
SA564
SA564−ΔhptRS
SA564−ΔccpA
SA564−ΔhptRS/ccpA
Strain
SA564
SA564−ΔhptRS
SA564−ΔccpA
SA564−ΔhptRS/ccpA

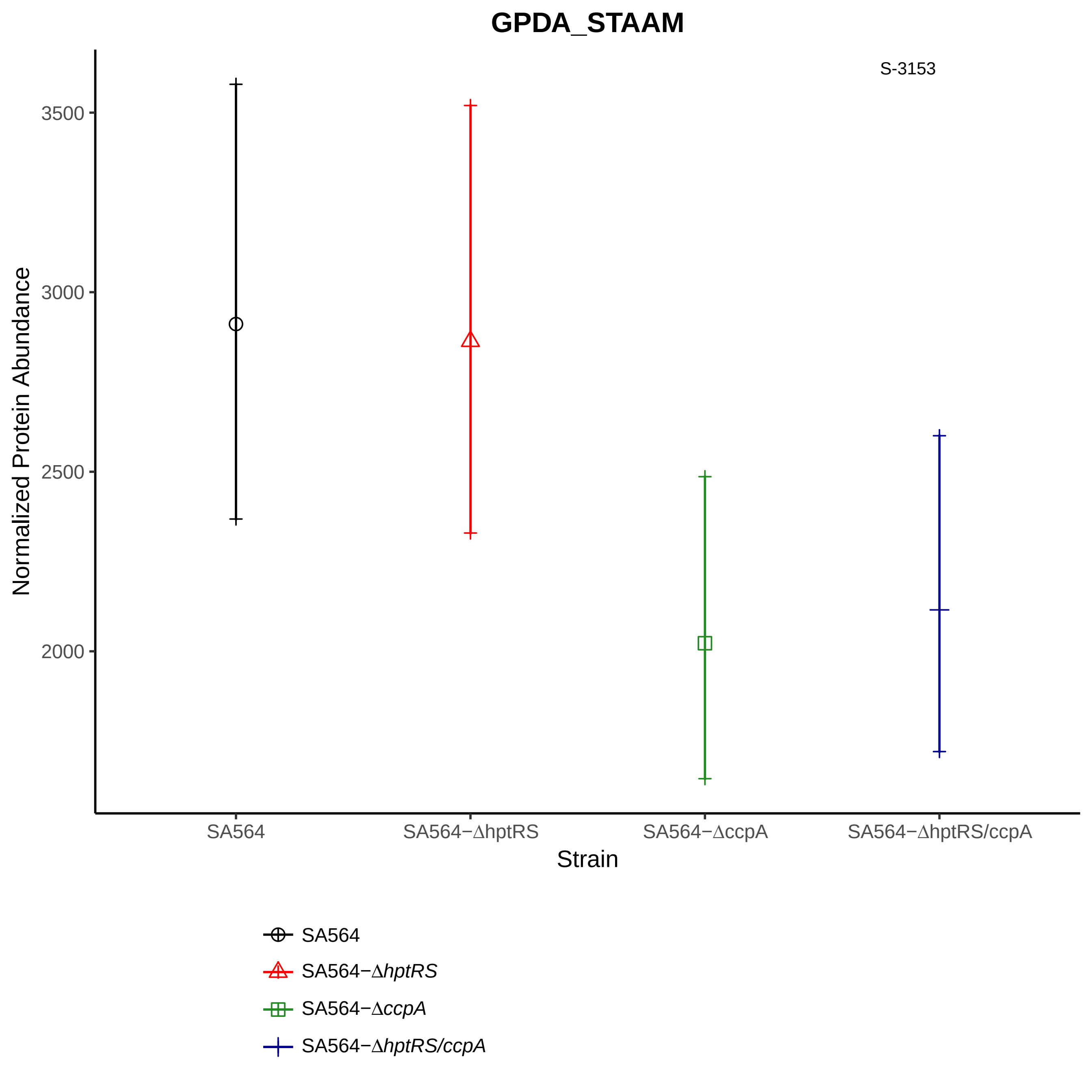

GPDA_STAAM
S-3153
Normalized Protein Abundance
3500
3000
2500
2000
SA564
SA564−ΔhptRS
SA564−ΔccpA
SA564−ΔhptRS/ccpA
Strain
SA564
SA564−ΔhptRS
SA564−ΔccpA
SA564−ΔhptRS/ccpA

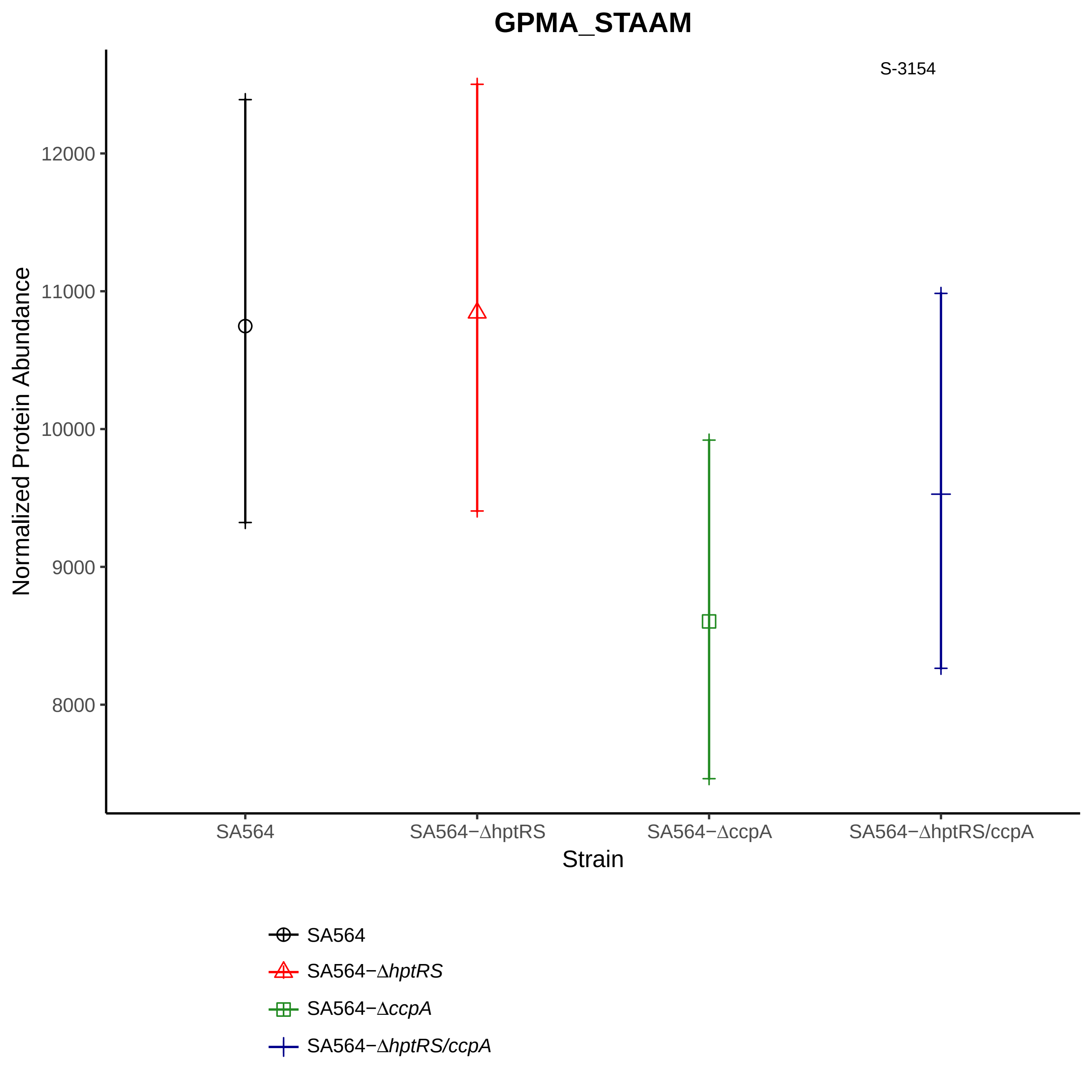
GPMA_STAAM
S-3154
Normalized Protein Abundance
12000
11000
10000
9000
8000
SA564
SA564−ΔhptRS
SA564−ΔccpA
SA564−ΔhptRS/ccpA
Strain
SA564
SA564−ΔhptRS
SA564−ΔccpA
SA564−ΔhptRS/ccpA

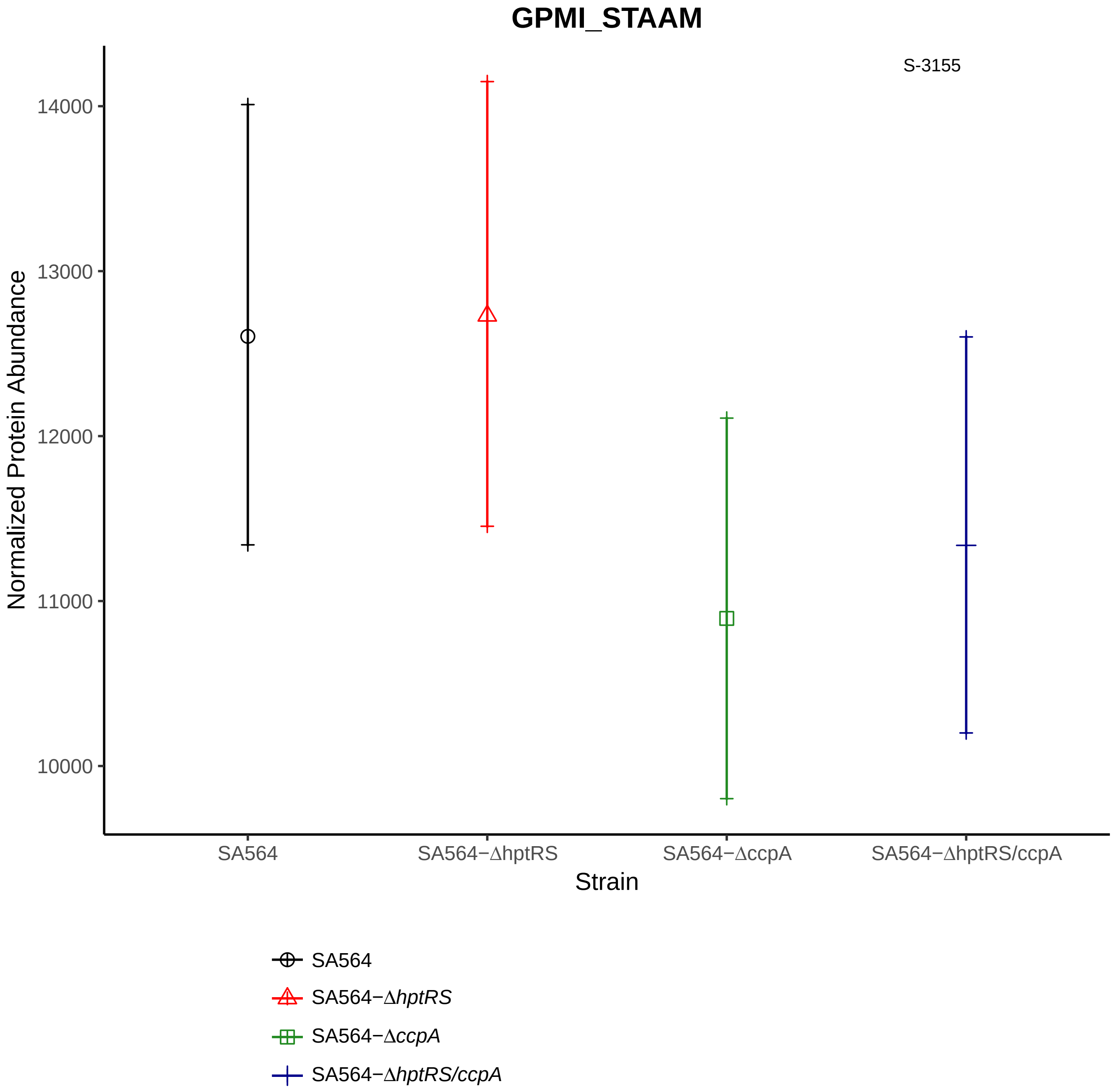

GPMI_STAAM
S-3155
Normalized Protein Abundance
14000
13000
12000
11000
10000
SA564
SA564−ΔhptRS
SA564−ΔccpA
SA564−ΔhptRS/ccpA
Strain
SA564
SA564−ΔhptRS
SA564−ΔccpA
SA564−ΔhptRS/ccpA

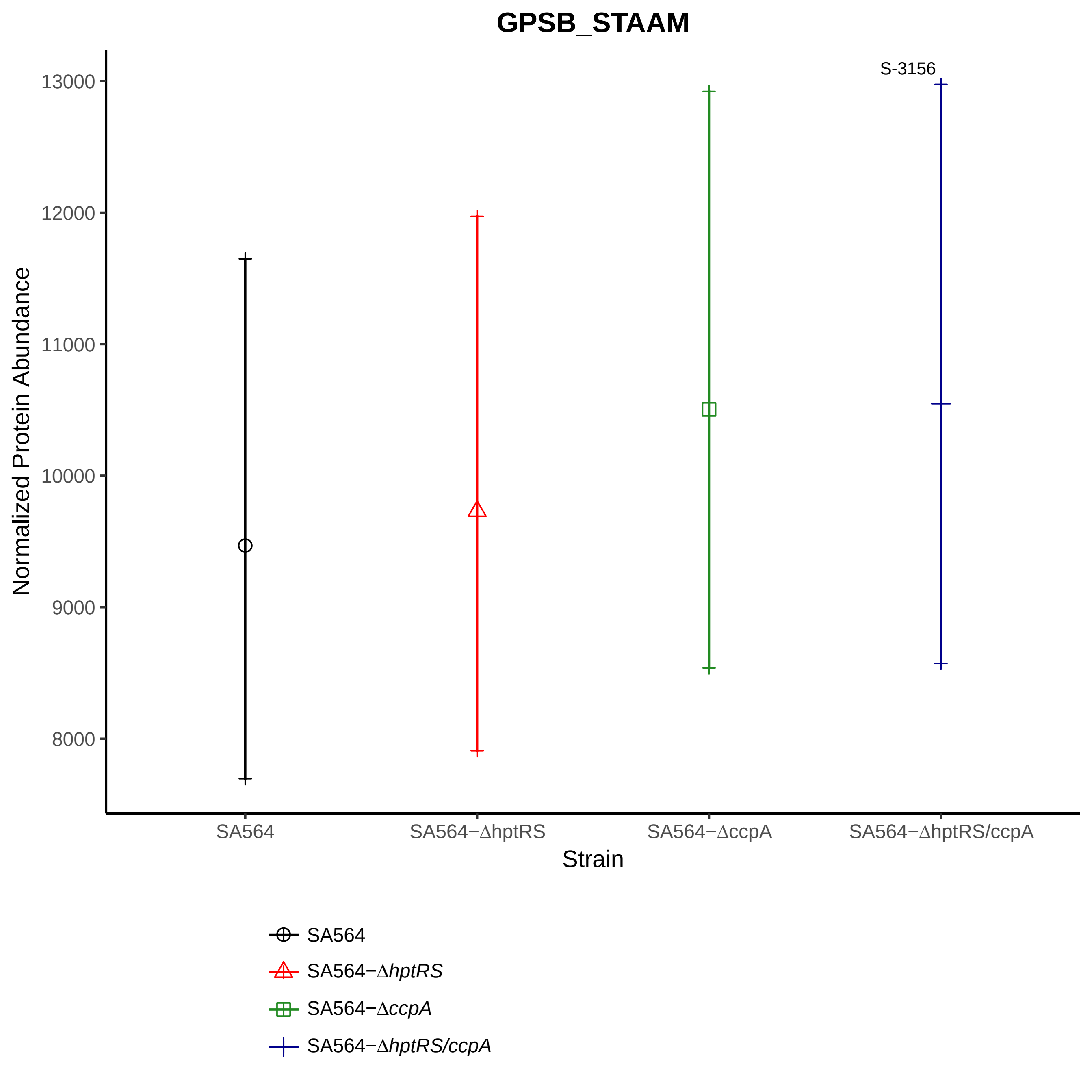

GPSB_STAAM
S-3156
13000
12000
11000
10000
9000
8000
Normalized Protein Abundance
SA564
SA564−ΔhptRS
SA564−ΔccpA
SA564−ΔhptRS/ccpA
Strain
SA564
SA564−Δ*hptRS*
SA564−Δ*ccpA*
SA564−Δ*hptRS/ccpA*

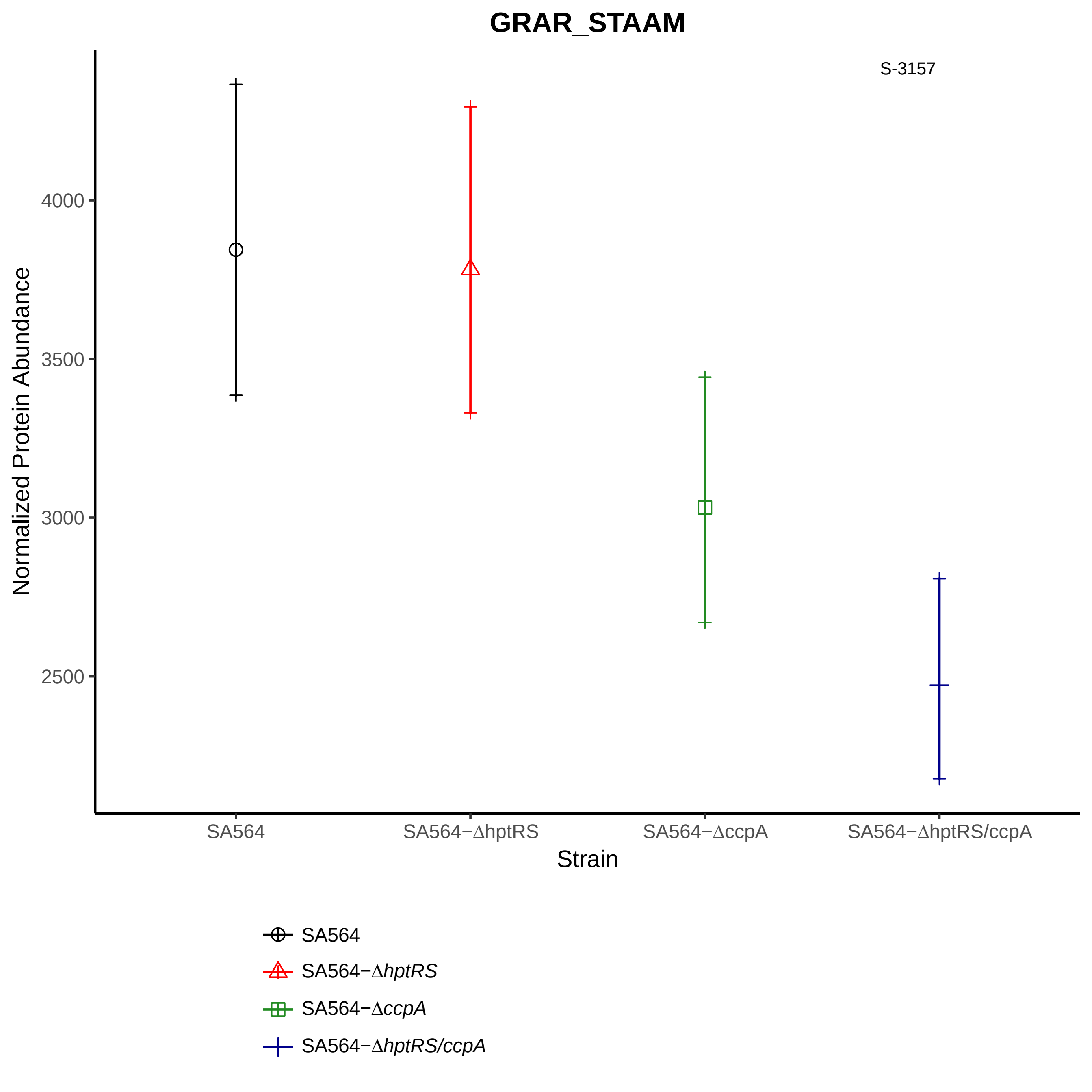

GRAR_STAAM
S-3157
Normalized Protein Abundance
4000
3500
3000
2500
SA564
SA564−ΔhptRS
SA564−ΔccpA
SA564−ΔhptRS/ccpA
Strain
SA564
SA564−ΔhptRS
SA564−ΔccpA
SA564−ΔhptRS/ccpA

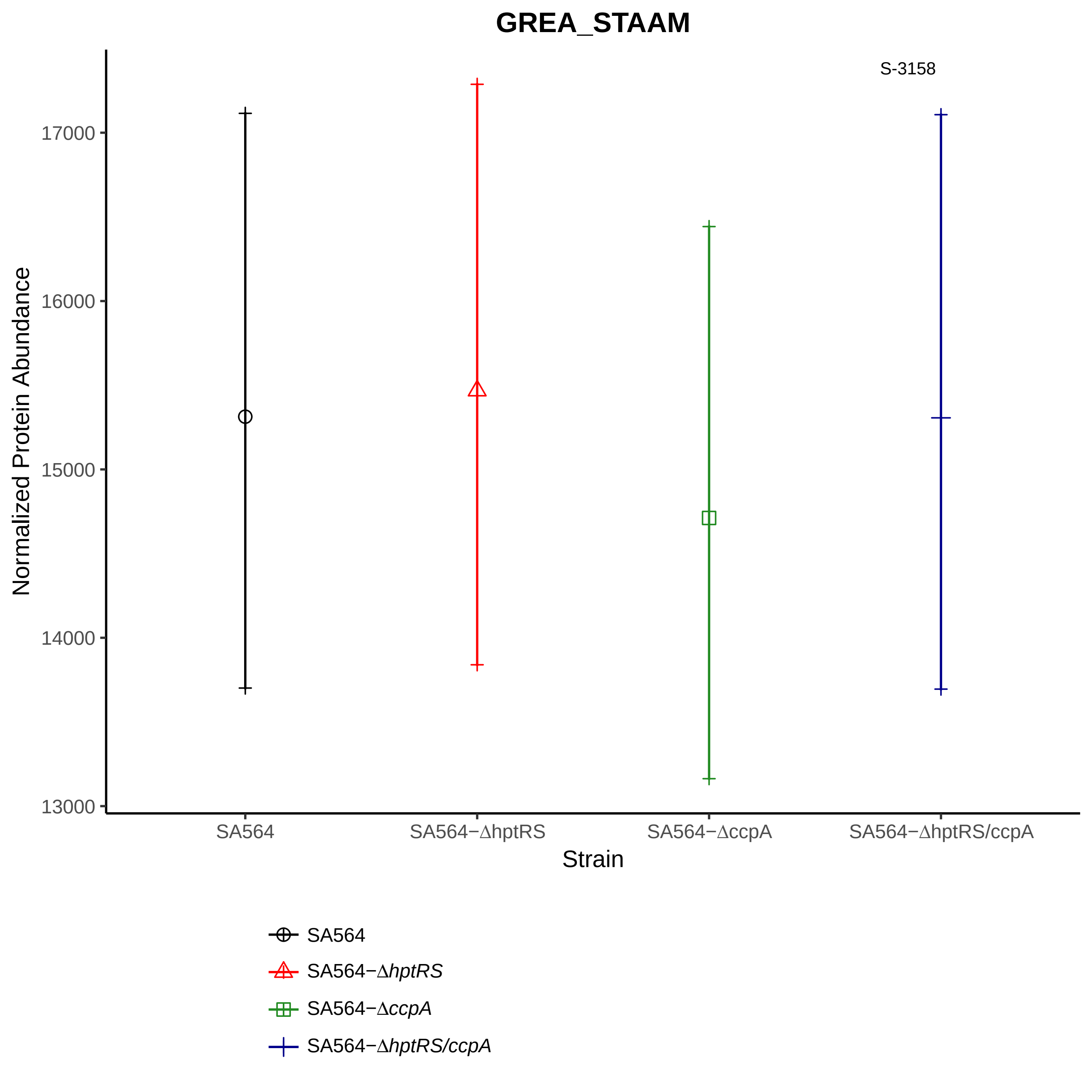

GREA_STAAM
S-3158
Normalized Protein Abundance
17000
16000
15000
14000
13000
SA564
SA564−ΔhptRS
SA564−ΔccpA
SA564−ΔhptRS/ccpA
Strain
SA564
SA564−ΔhptRS
SA564−ΔccpA
SA564−ΔhptRS/ccpA

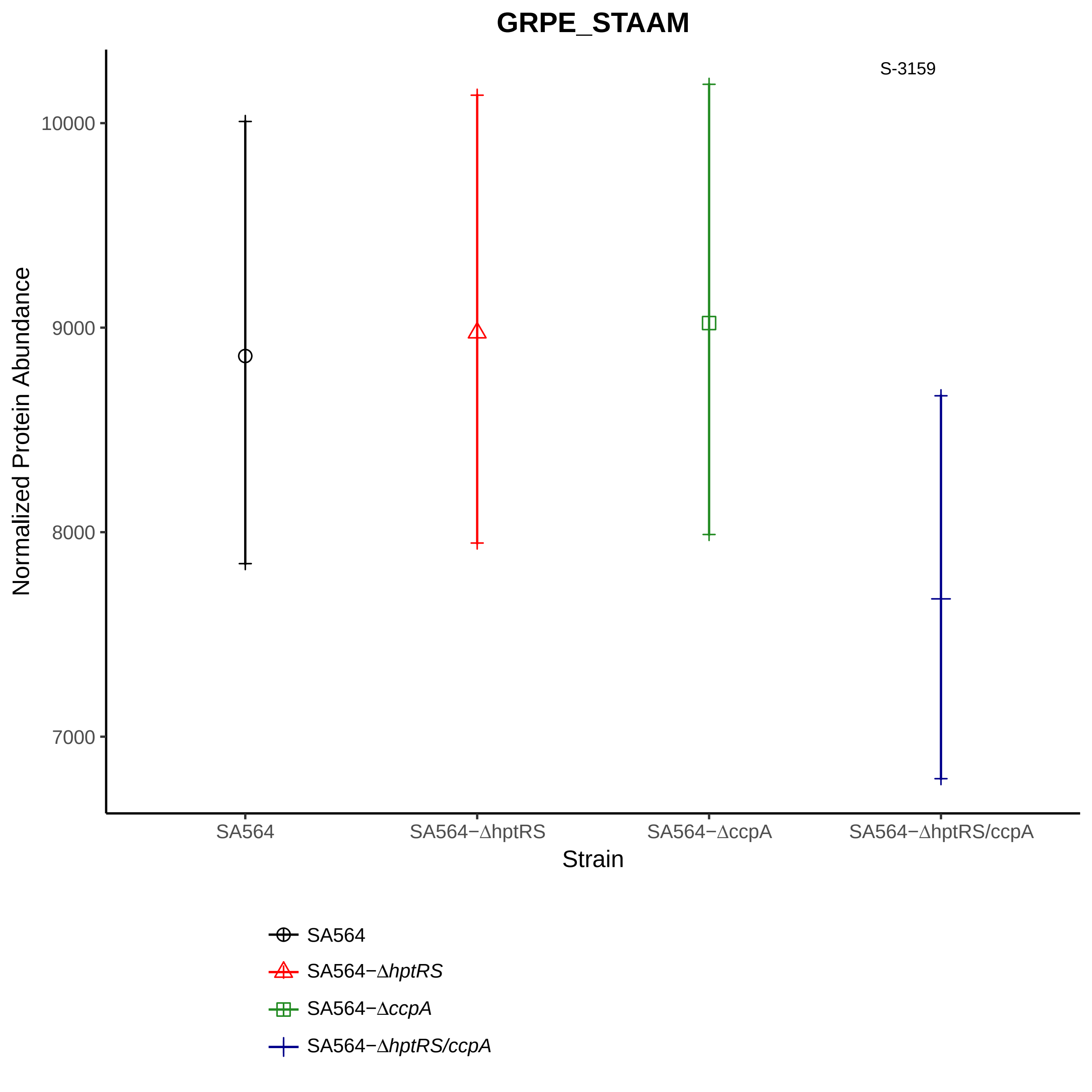

GRPE_STAAM
S-3159
Normalized Protein Abundance
10000
9000
8000
7000
SA564
SA564−ΔhptRS
SA564−ΔccpA
SA564−ΔhptRS/ccpA
Strain
SA564
SA564−ΔhptRS
SA564−ΔccpA
SA564−ΔhptRS/ccpA

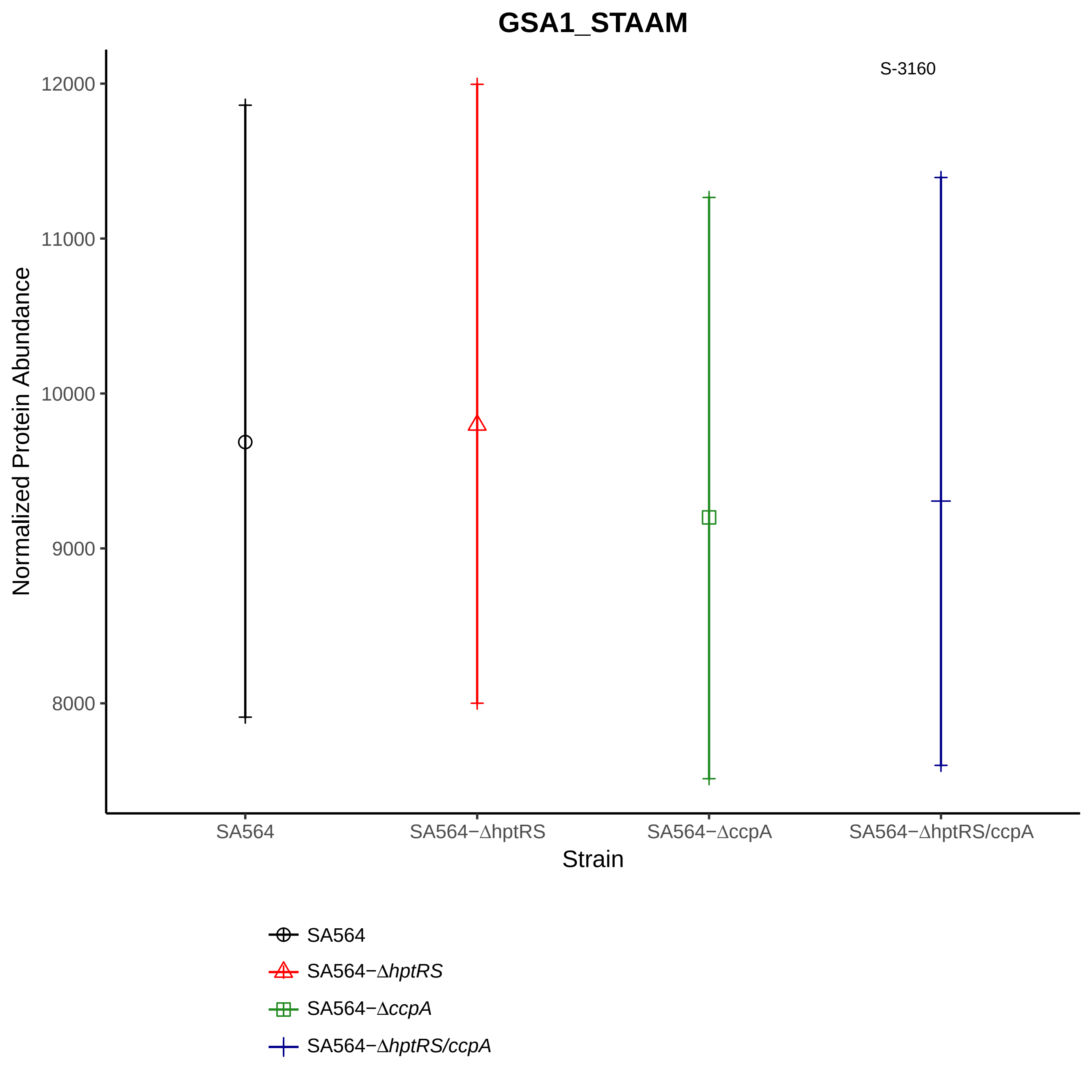
GSA1_STAAM
S-3160
Normalized Protein Abundance
12000
11000
10000
9000
8000
SA564
SA564−ΔhptRS
SA564−ΔccpA
SA564−ΔhptRS/ccpA
Strain
SA564
SA564−ΔhptRS
SA564−ΔccpA
SA564−ΔhptRS/ccpA

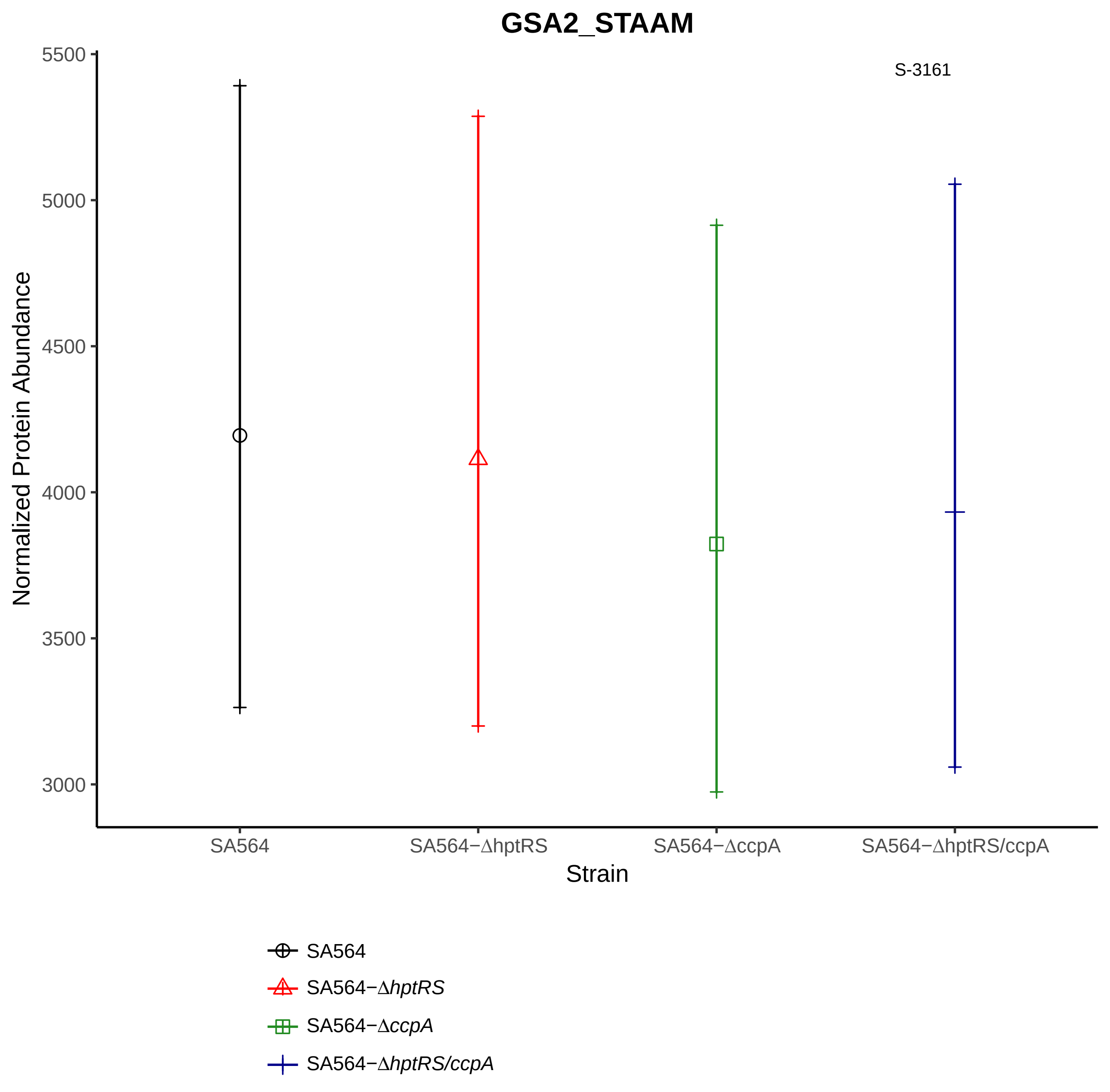

GSA2_STAAM
S-3161
Normalized Protein Abundance
5500
5000
4500
4000
3500
3000
SA564
SA564−ΔhptRS
SA564−ΔccpA
SA564−ΔhptRS/ccpA
Strain
SA564
SA564−ΔhptRS
SA564−ΔccpA
SA564−ΔhptRS/ccpA

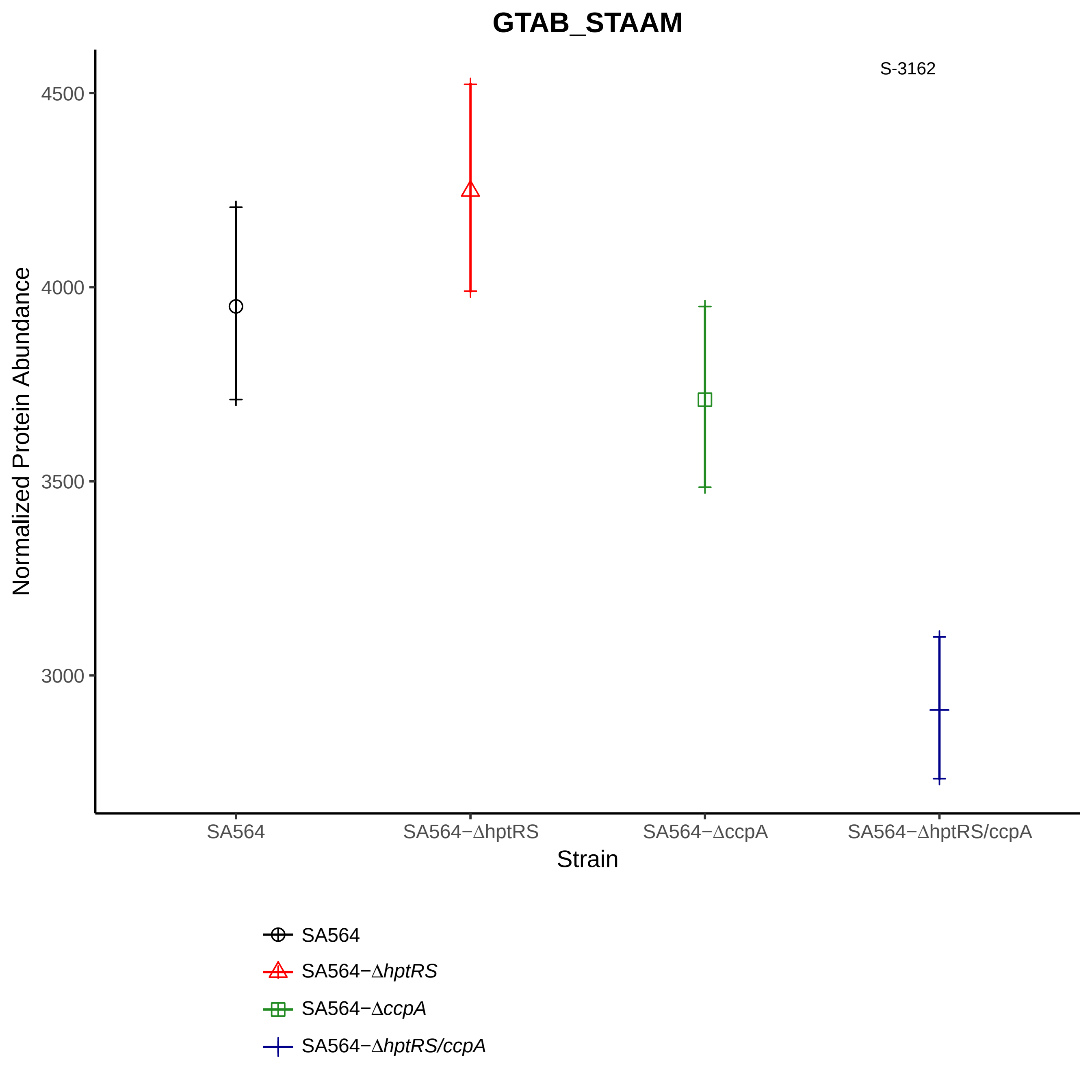
GTAB_STAAM
S-3162
Normalized Protein Abundance
4500
4000
3500
3000
SA564
SA564−ΔhptRS
SA564−ΔccpA
SA564−ΔhptRS/ccpA
Strain
SA564
SA564−ΔhptRS
SA564−ΔccpA
SA564−ΔhptRS/ccpA

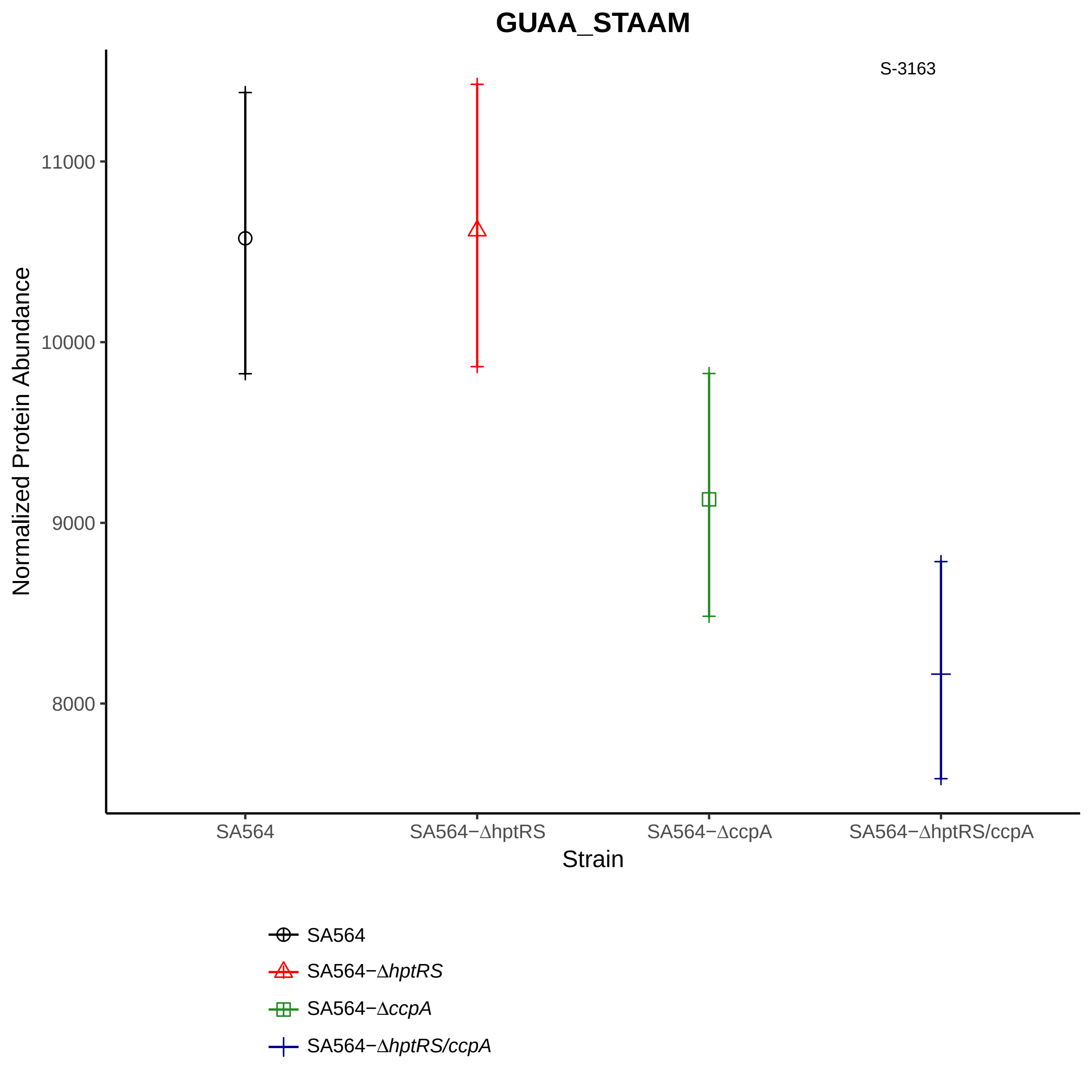

GUAA_STAAM
S-3163
Normalized Protein Abundance
11000
10000
9000
8000
SA564
SA564−ΔhptRS
SA564−ΔccpA
SA564−ΔhptRS/ccpA
Strain
SA564
SA564−Δ*hptRS*
SA564−Δ*ccpA*
SA564−Δ*hptRS/ccpA*

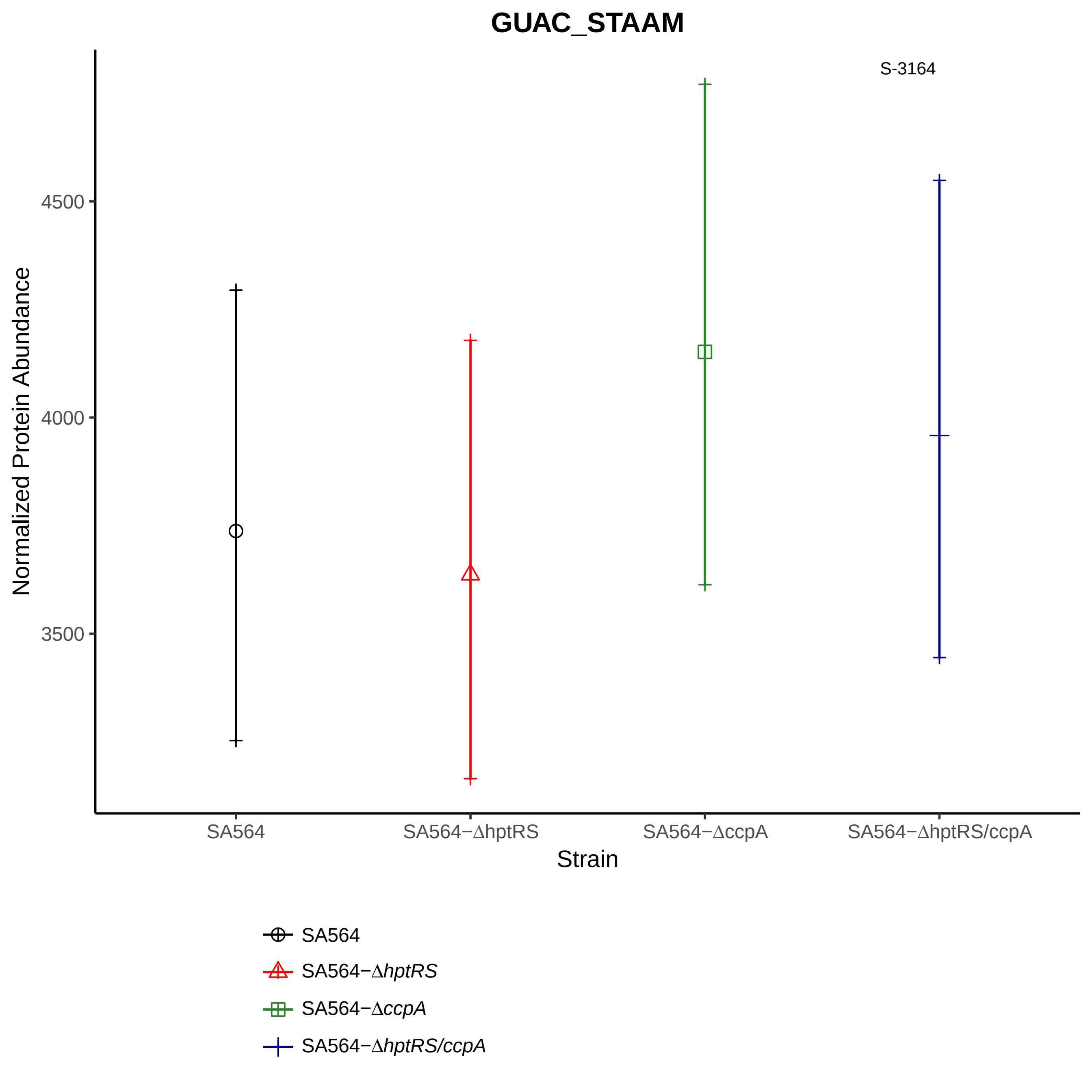

GUAC_STAAM
S-3164
Normalized Protein Abundance
4500
4000
3500
SA564
SA564−ΔhptRS
SA564−ΔccpA
SA564−ΔhptRS/ccpA
Strain
SA564
SA564−ΔhptRS
SA564−ΔccpA
SA564−ΔhptRS/ccpA

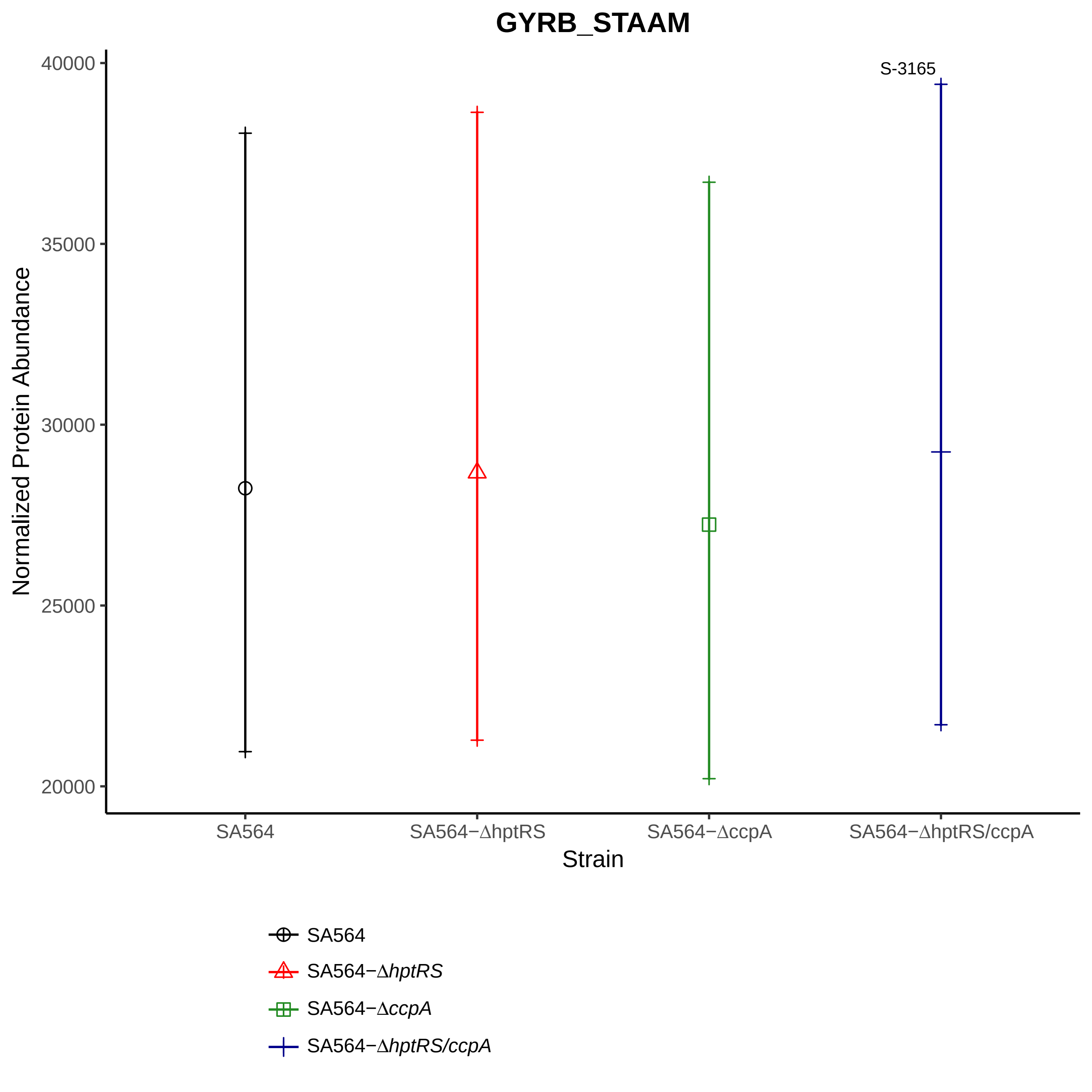

GYRB_STAAM
S-3165
40000
35000
30000
25000
20000
Normalized Protein Abundance
SA564
SA564−ΔhptRS
SA564−ΔccpA
SA564−ΔhptRS/ccpA
Strain
SA564
SA564−ΔhptRS
SA564−ΔccpA
SA564−ΔhptRS/ccpA

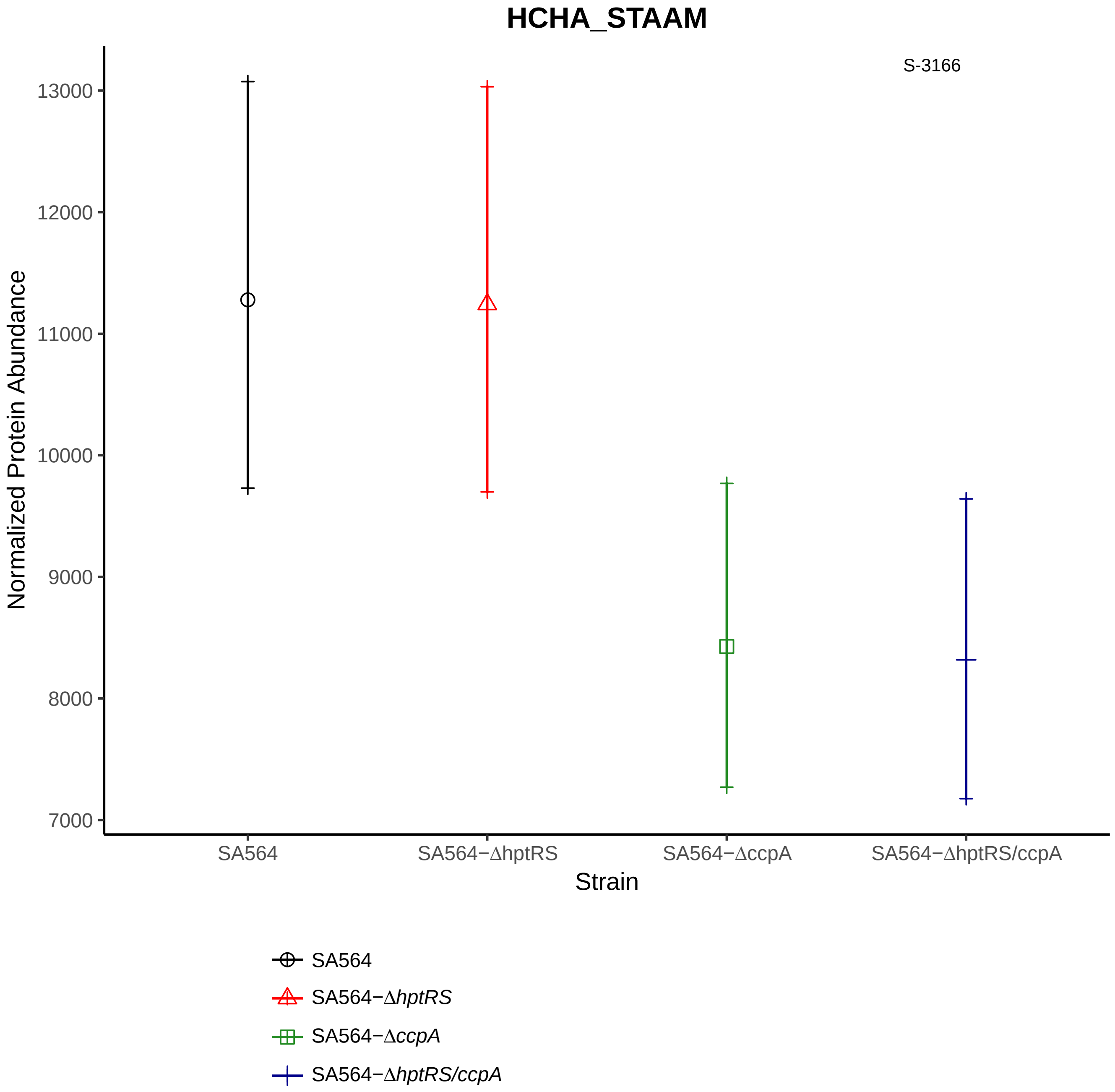

HCHA_STAAM
S-3166
Normalized Protein Abundance
13000
12000
11000
10000
9000
8000
7000
SA564
SA564−ΔhptRS
SA564−ΔccpA
SA564−ΔhptRS/ccpA
Strain
SA564
SA564−ΔhptRS
SA564−ΔccpA
SA564−ΔhptRS/ccpA

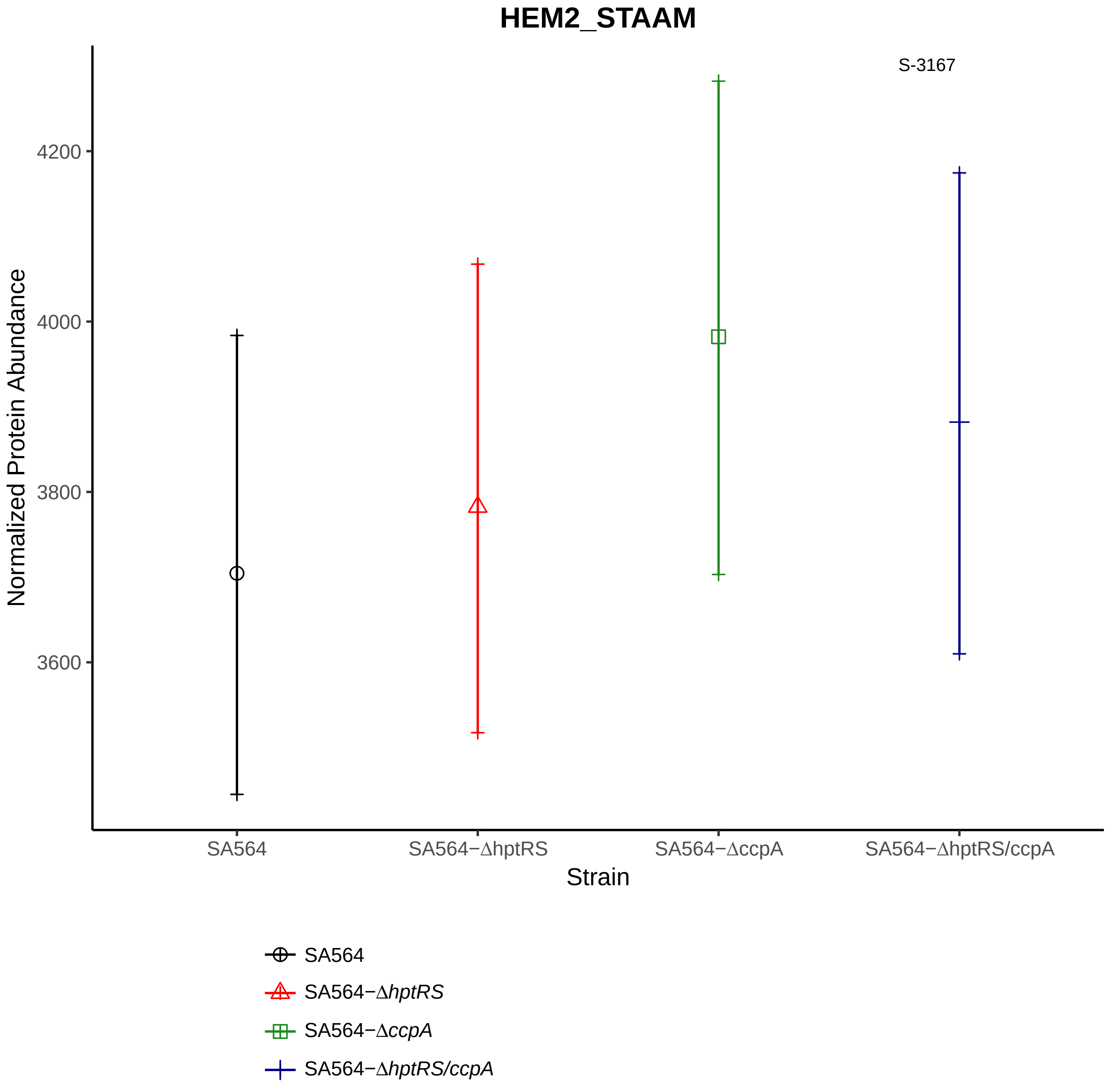

HEM2_STAAM
S-3167
Normalized Protein Abundance
4200
4000
3800
3600
SA564
SA564−ΔhptRS
SA564−ΔccpA
SA564−ΔhptRS/ccpA
Strain
SA564
SA564−ΔhptRS
SA564−ΔccpA
SA564−ΔhptRS/ccpA

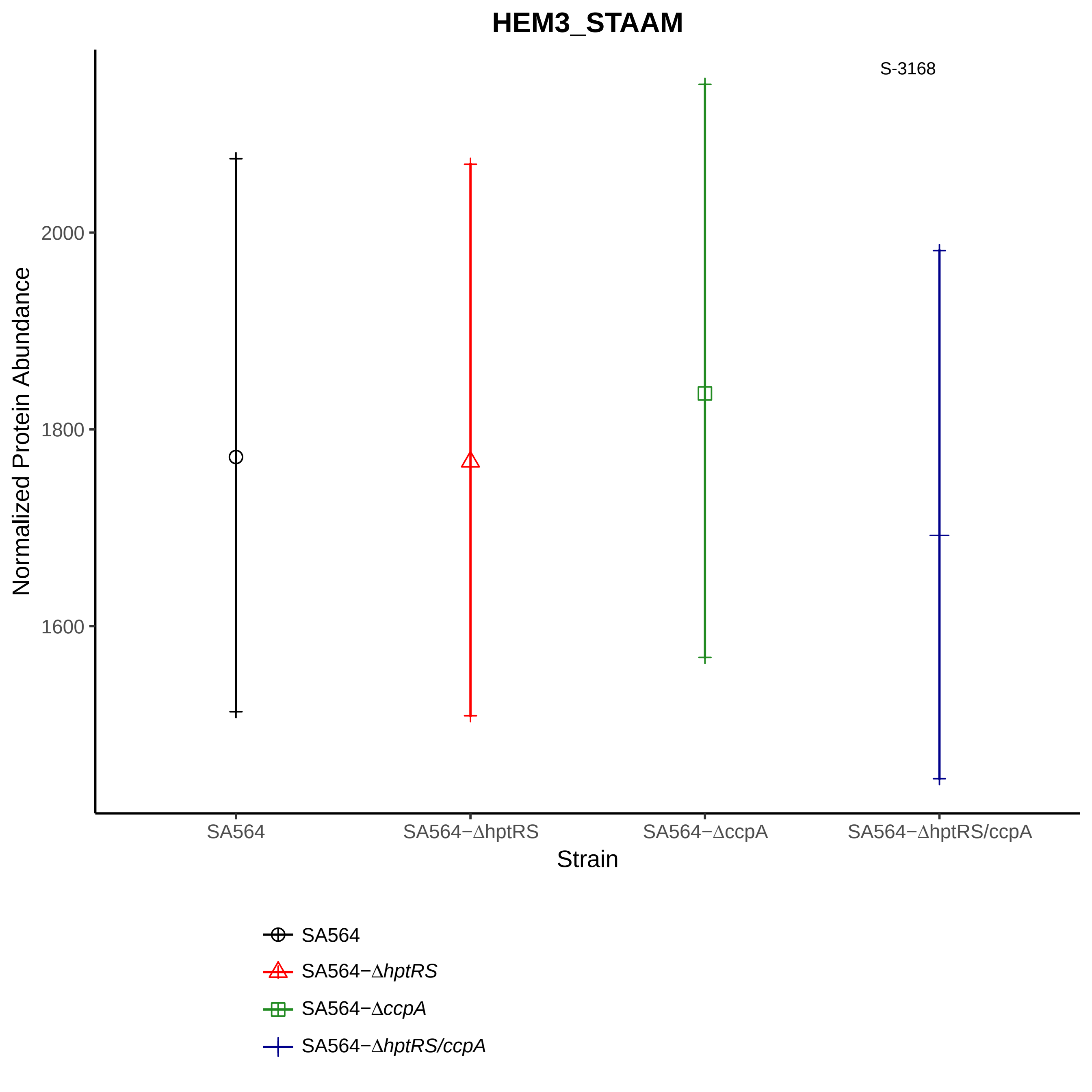

HEM3_STAAM
S-3168
Normalized Protein Abundance
2000
1800
1600
SA564
SA564−ΔhptRS
SA564−ΔccpA
SA564−ΔhptRS/ccpA
Strain
SA564
SA564−ΔhptRS
SA564−ΔccpA
SA564−ΔhptRS/ccpA

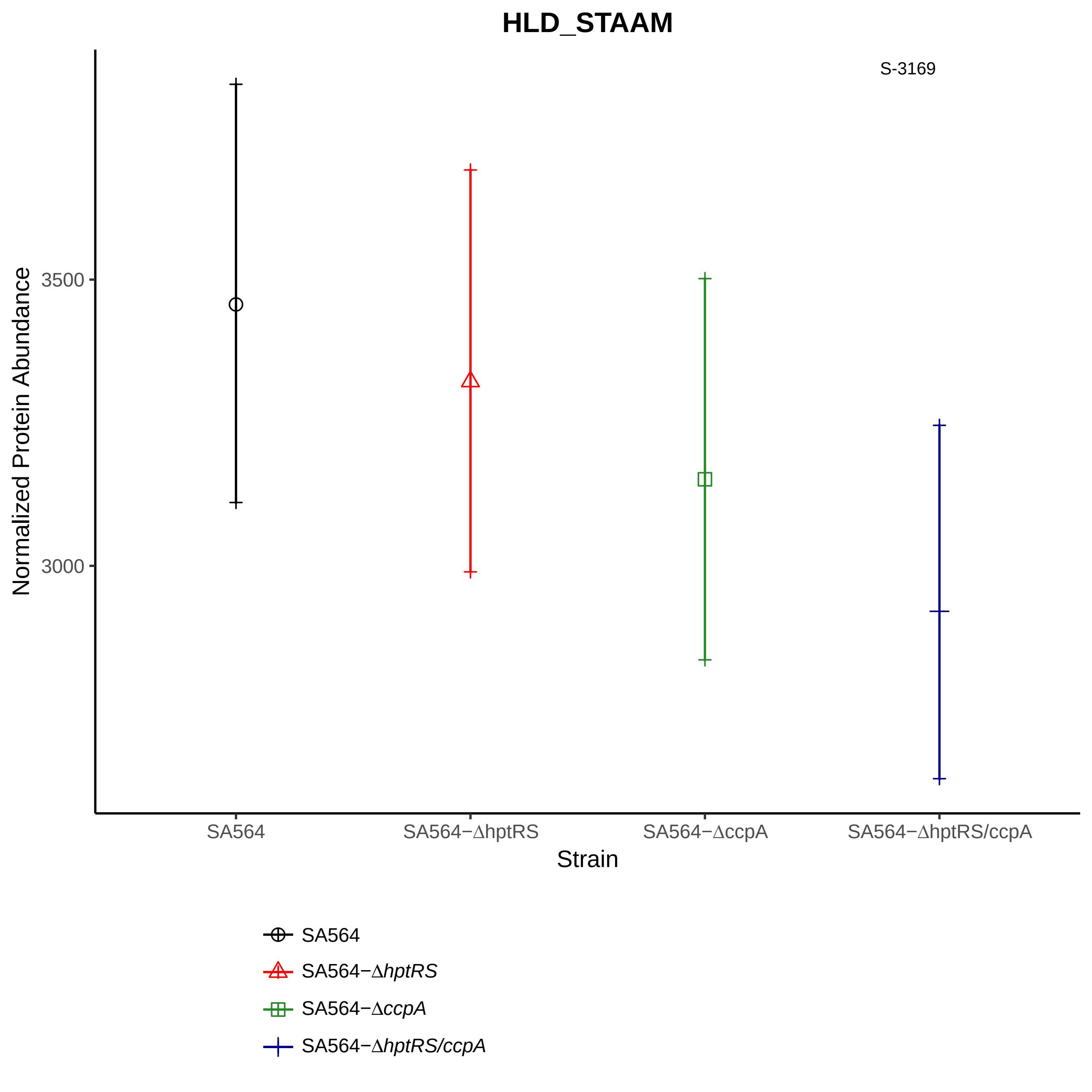

HLD_STAAM
S-3169
Normalized Protein Abundance
3500
3000
SA564
SA564−ΔhptRS
SA564−ΔccpA
SA564−ΔhptRS/ccpA
Strain
SA564
SA564−ΔhptRS
SA564−ΔccpA
SA564−ΔhptRS/ccpA

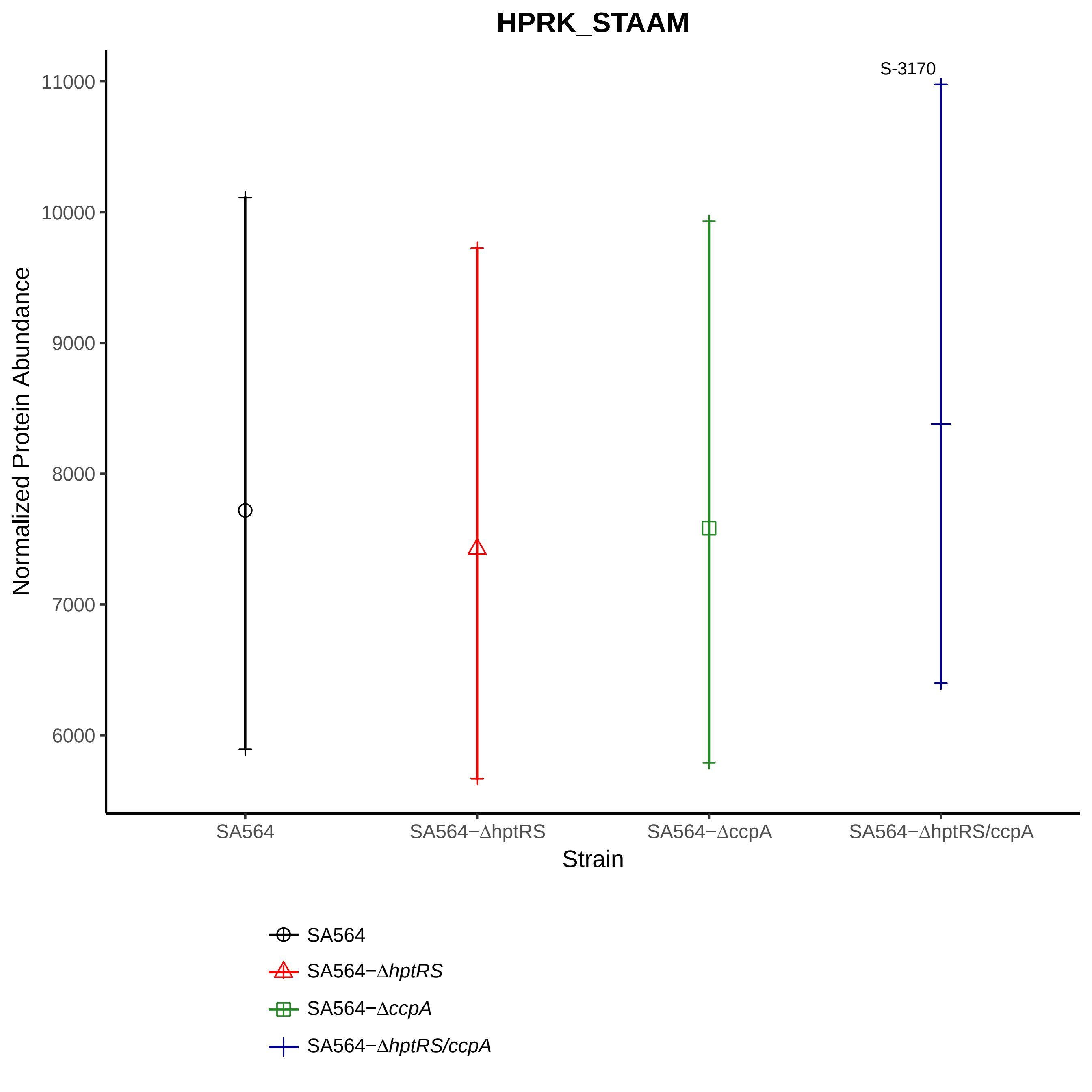

HPRK_STAAM
S-3170
11000
10000
9000
8000
7000
6000
Normalized Protein Abundance
SA564
SA564−ΔhptRS
SA564−ΔccpA
SA564−ΔhptRS/ccpA
Strain
SA564
SA564−Δ*hptRS*
SA564−Δ*ccpA*
SA564−Δ*hptRS/ccpA*

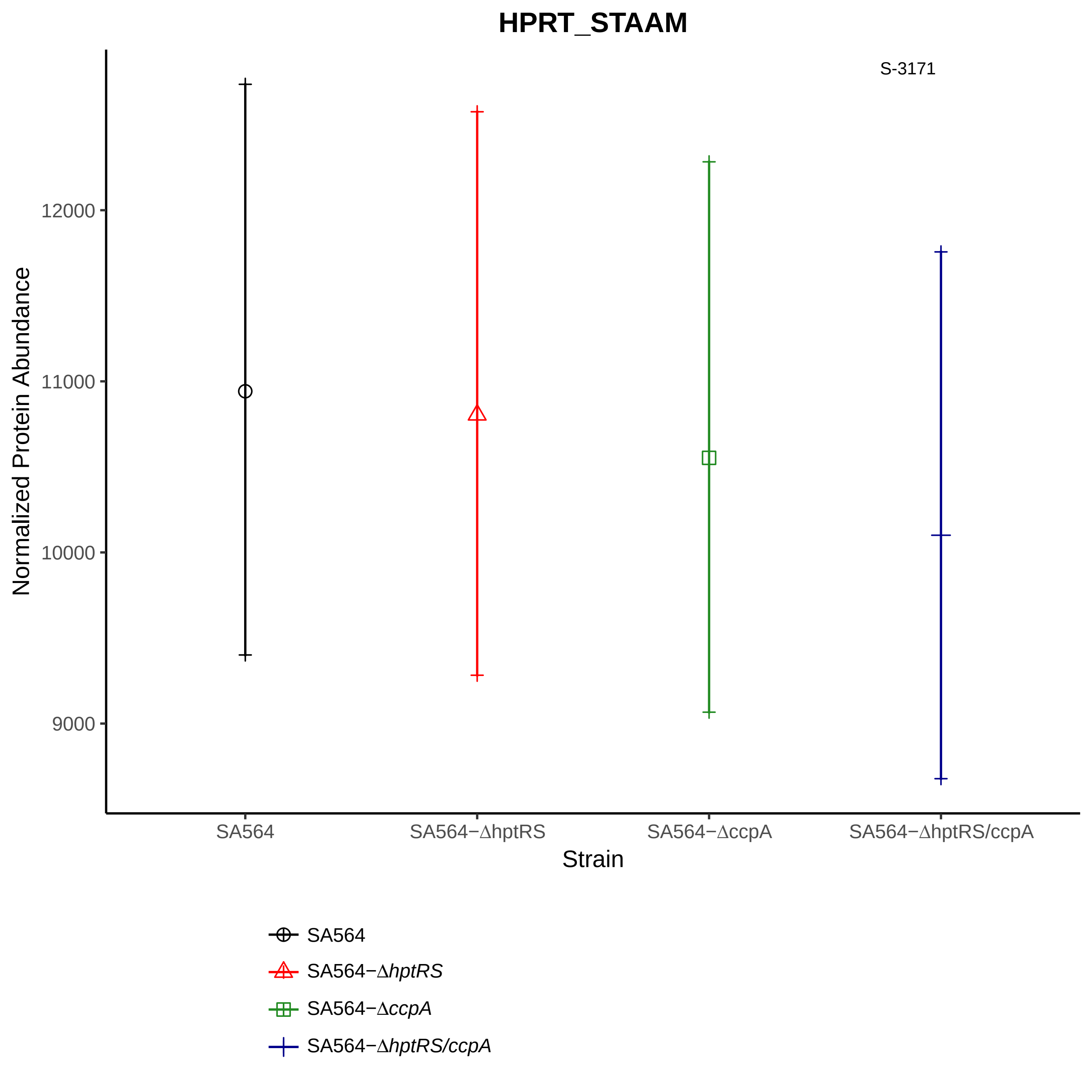

HPRT_STAAM
S-3171
Normalized Protein Abundance
12000
11000
10000
9000
SA564
SA564−ΔhptRS
SA564−ΔccpA
SA564−ΔhptRS/ccpA
Strain
SA564
SA564−Δ*hptRS*
SA564−Δ*ccpA*
SA564−Δ*hptRS/ccpA*

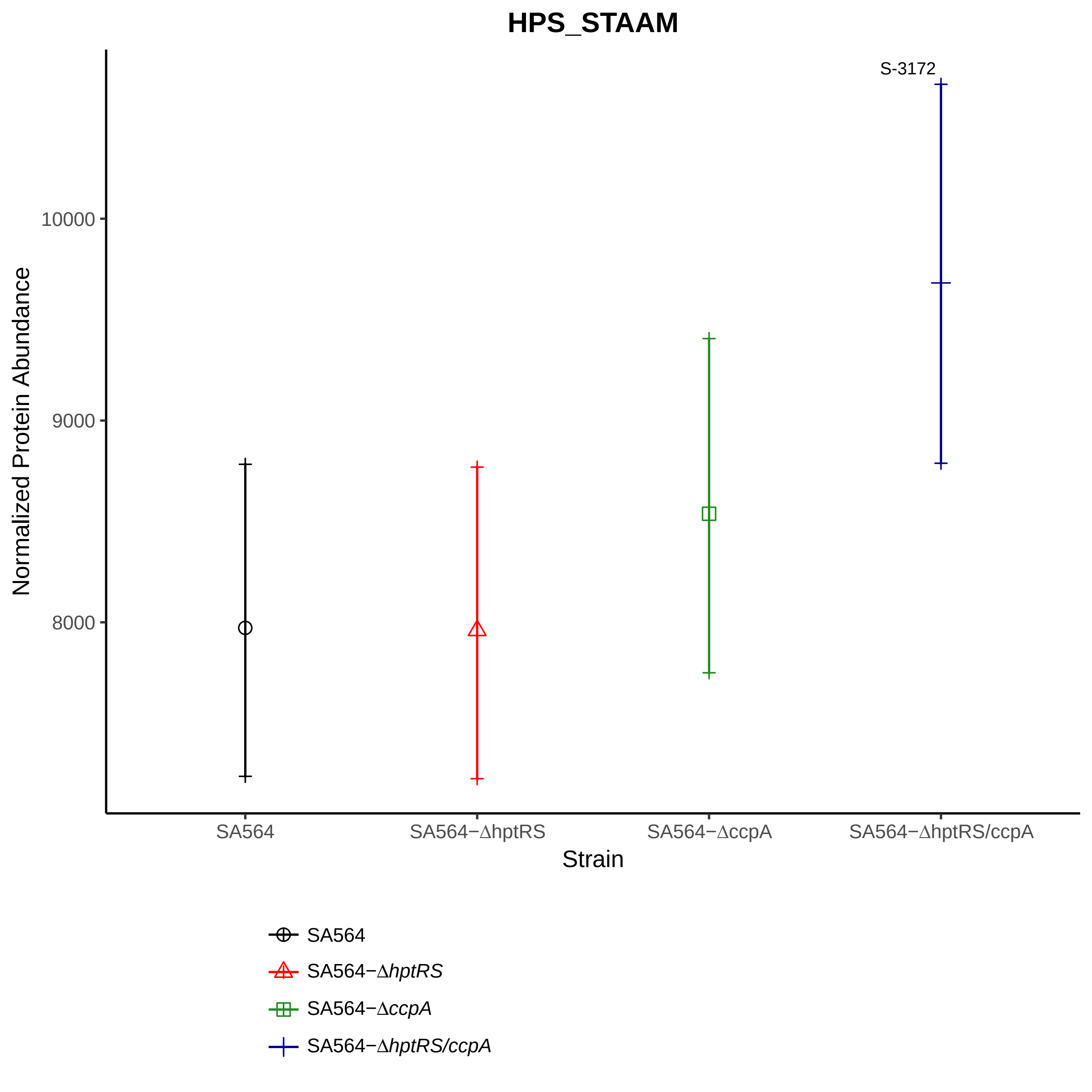
HPS_STAAM
S-3172
10000
9000
8000
Normalized Protein Abundance
SA564
SA564−ΔhptRS
SA564−ΔccpA
SA564−ΔhptRS/ccpA
Strain
SA564
SA564−ΔhptRS
SA564−ΔccpA
SA564−ΔhptRS/ccpA

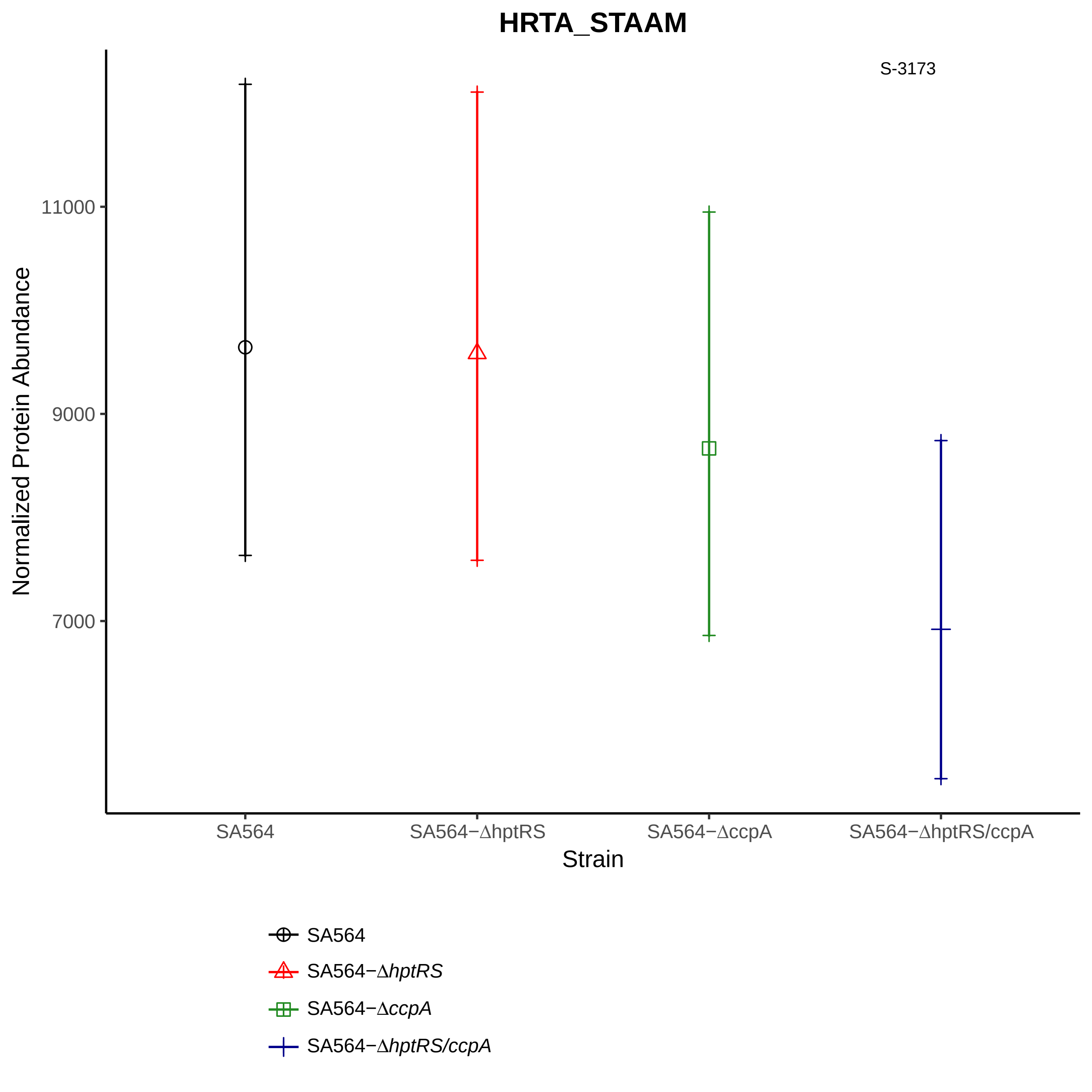

HRTA_STAAM
S-3173
Normalized Protein Abundance
11000
9000
7000
SA564
SA564−ΔhptRS
SA564−ΔccpA
SA564−ΔhptRS/ccpA
Strain
SA564
SA564−Δ*hptRS*
SA564−Δ*ccpA*
SA564−Δ*hptRS/ccpA*

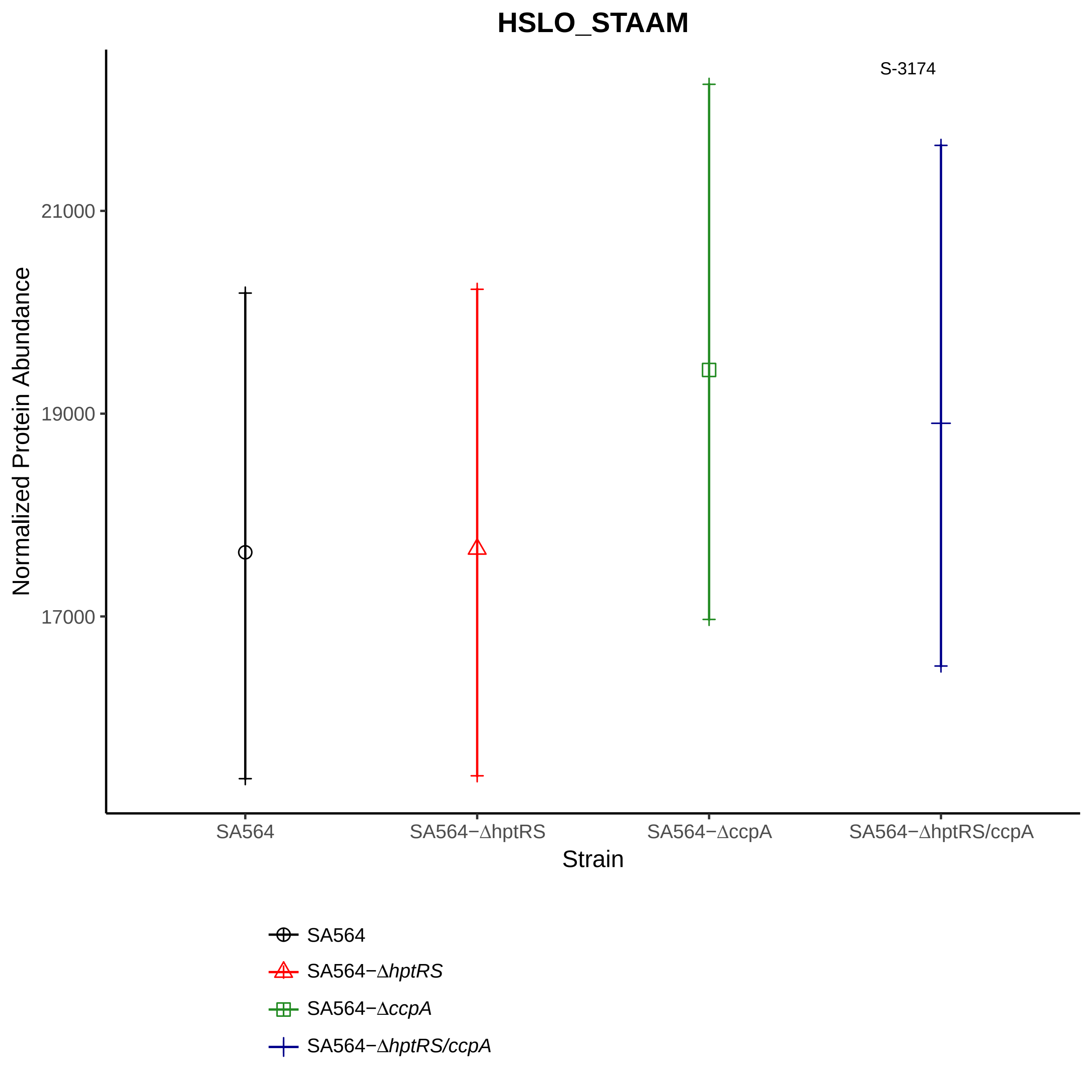

HSLO_STAAM
S-3174
Normalized Protein Abundance
21000
19000
17000
SA564
SA564−ΔhptRS
SA564−ΔccpA
SA564−ΔhptRS/ccpA
Strain
SA564
SA564−ΔhptRS
SA564−ΔccpA
SA564−ΔhptRS/ccpA

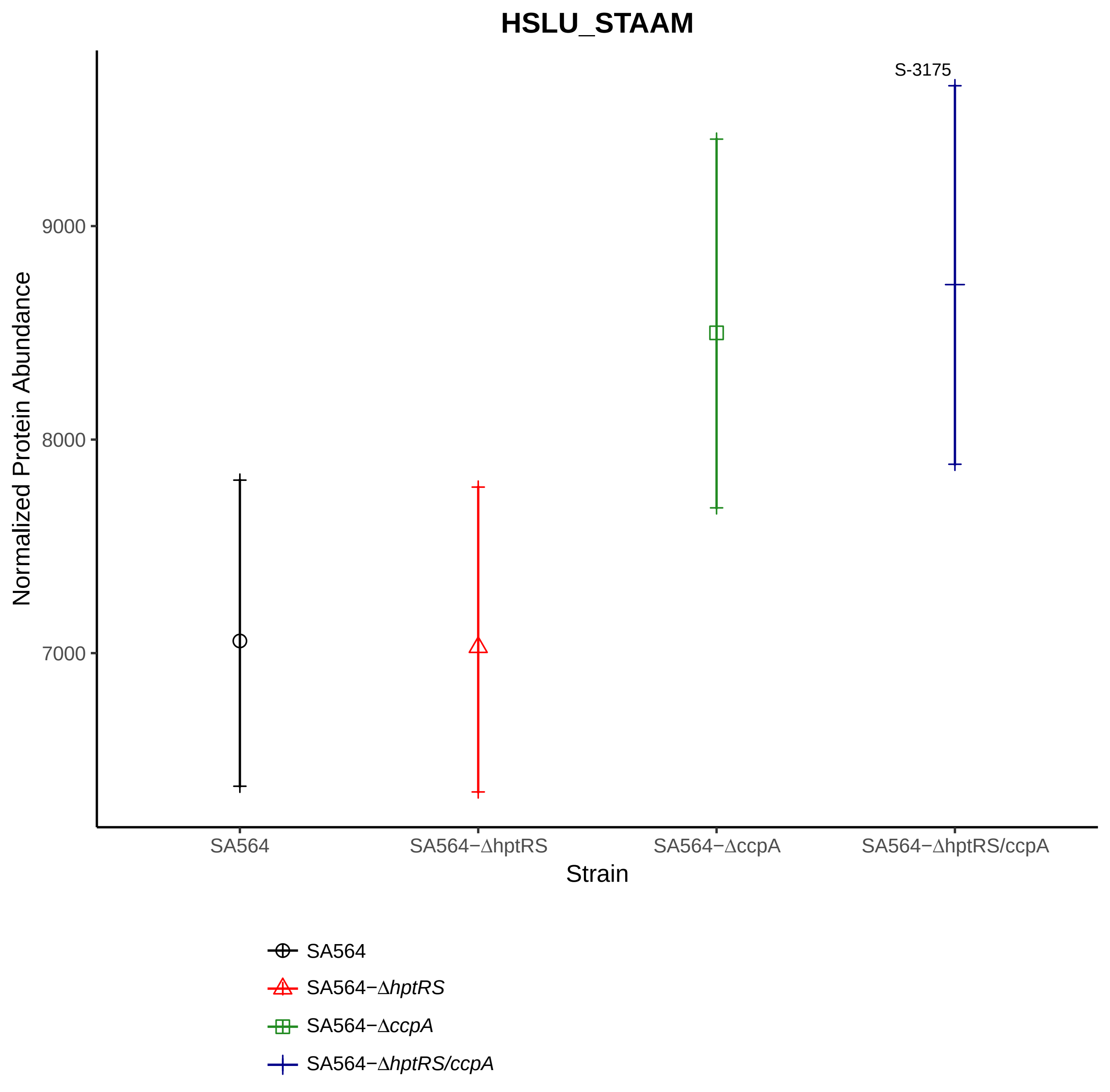
HSLU_STAAM
S-3175
Normalized Protein Abundance
9000
8000
7000
SA564
SA564−ΔhptRS
SA564−ΔccpA
SA564−ΔhptRS/ccpA
Strain
SA564
SA564−ΔhptRS
SA564−ΔccpA
SA564−ΔhptRS/ccpA

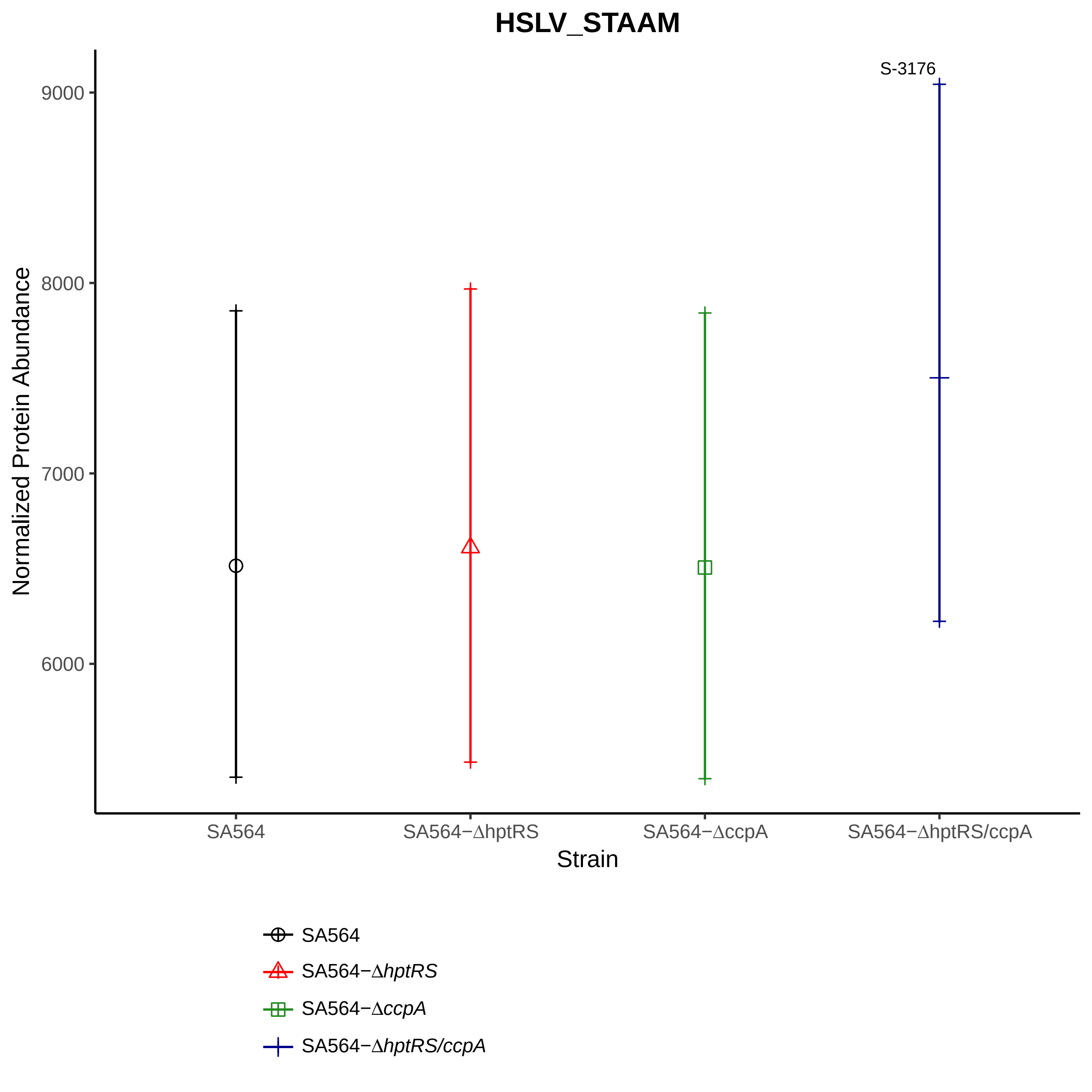

HSLV_STAAM
S-3176
9000
8000
7000
6000
Normalized Protein Abundance
SA564
SA564−ΔhptRS
SA564−ΔccpA
SA564−ΔhptRS/ccpA
Strain
SA564
SA564−Δ*hptRS*
SA564−Δ*ccpA*
SA564−Δ*hptRS/ccpA*

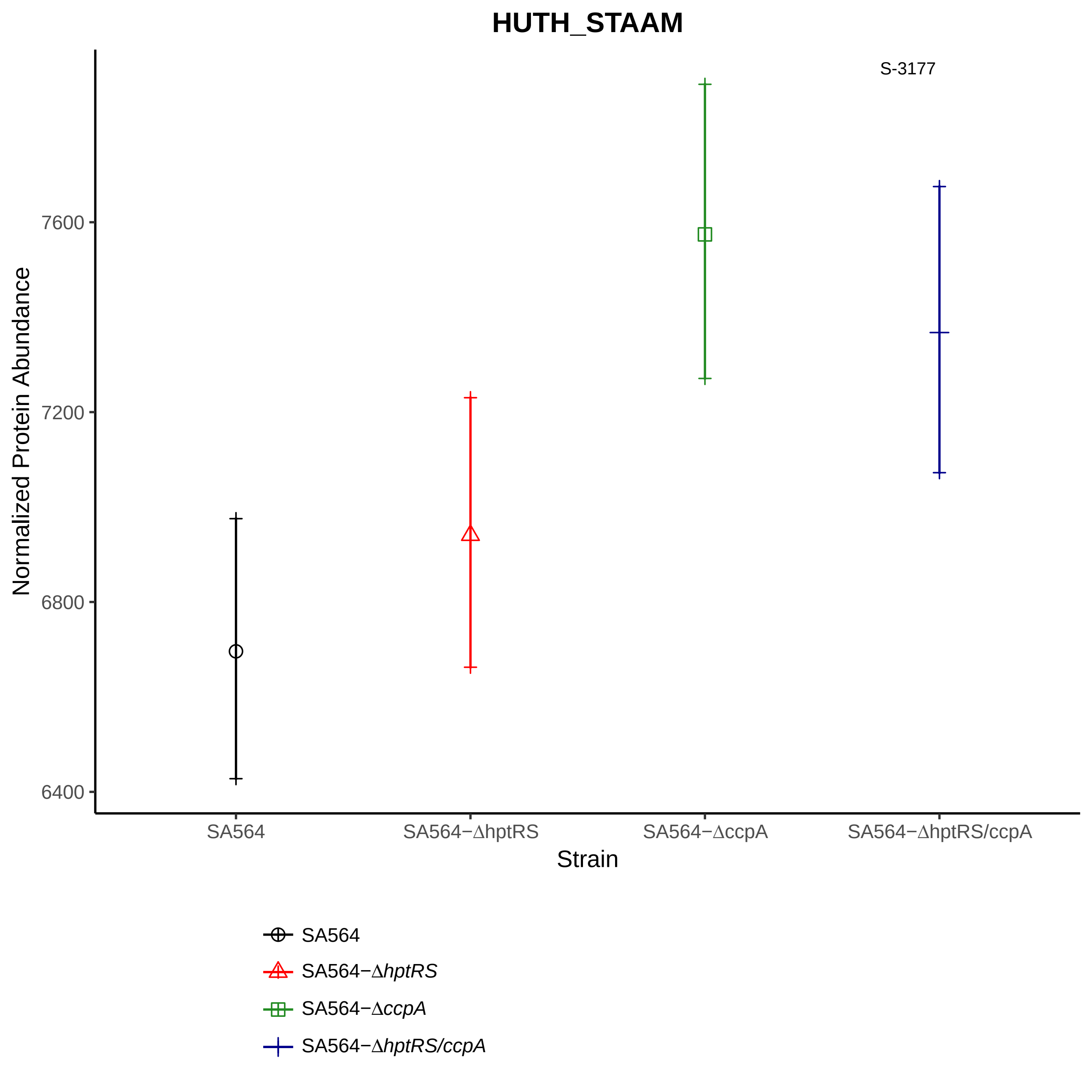
HUTH_STAAM
S-3177
Normalized Protein Abundance
7600
7200
6800
6400
SA564
SA564−ΔhptRS
SA564−ΔccpA
SA564−ΔhptRS/ccpA
Strain
SA564
SA564−ΔhptRS
SA564−ΔccpA
SA564−ΔhptRS/ccpA

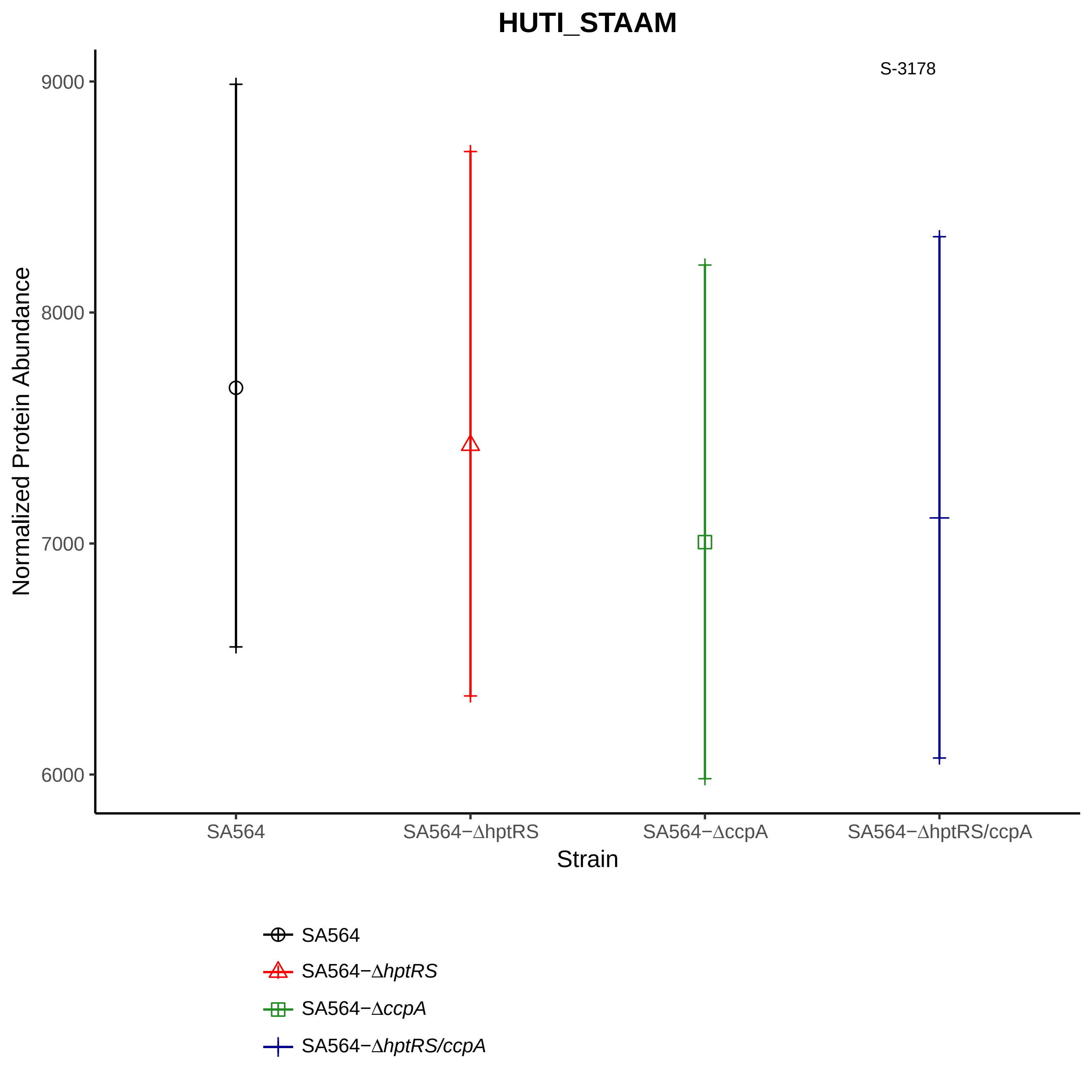
HUTI_STAAM
S-3178
Normalized Protein Abundance
9000
8000
7000
6000
SA564
SA564−ΔhptRS
SA564−ΔccpA
SA564−ΔhptRS/ccpA
Strain
SA564
SA564−ΔhptRS
SA564−ΔccpA
SA564−ΔhptRS/ccpA

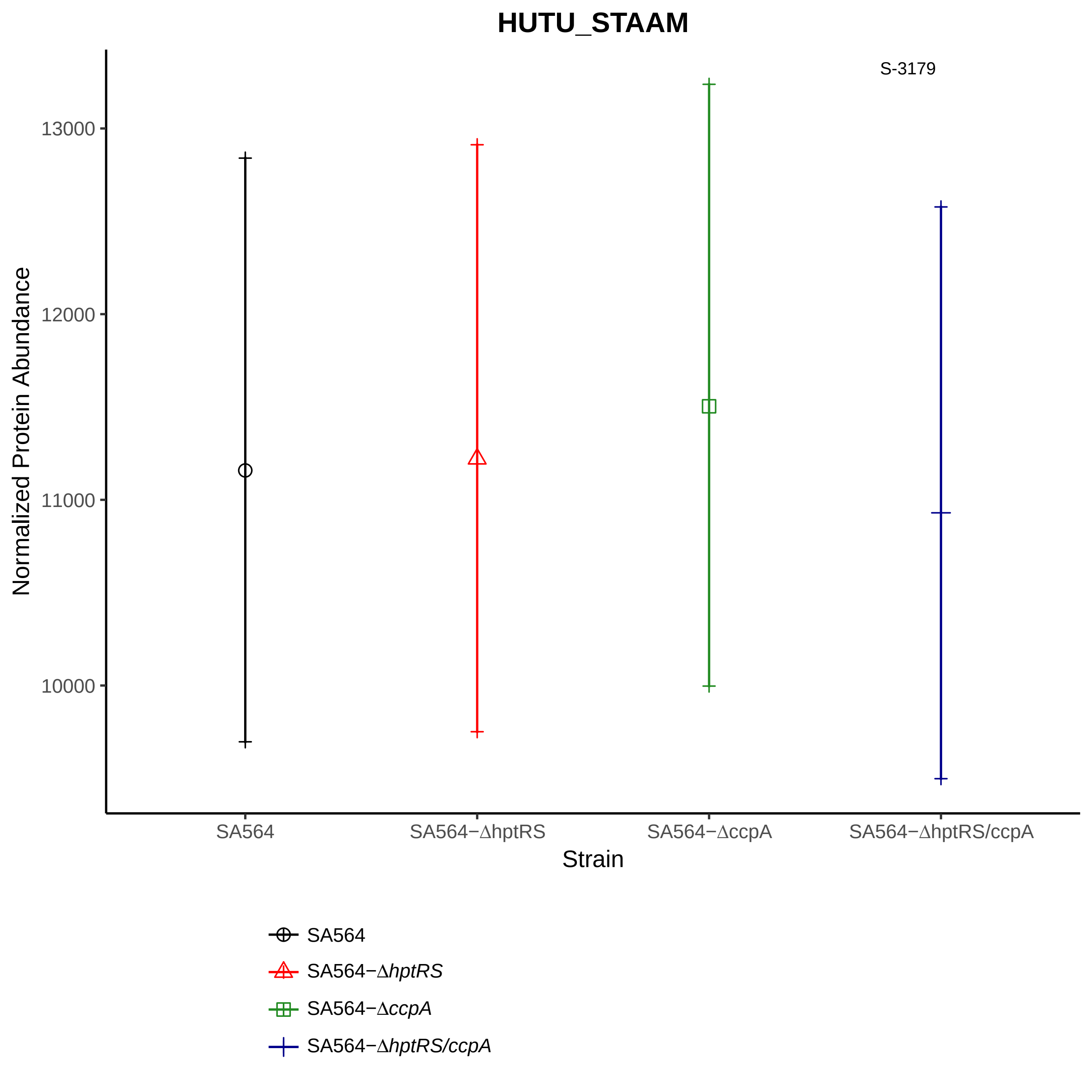

HUTU_STAAM
S-3179
Normalized Protein Abundance
13000
12000
11000
10000
SA564
SA564−ΔhptRS
SA564−ΔccpA
SA564−ΔhptRS/ccpA
Strain
SA564
SA564−ΔhptRS
SA564−ΔccpA
SA564−ΔhptRS/ccpA

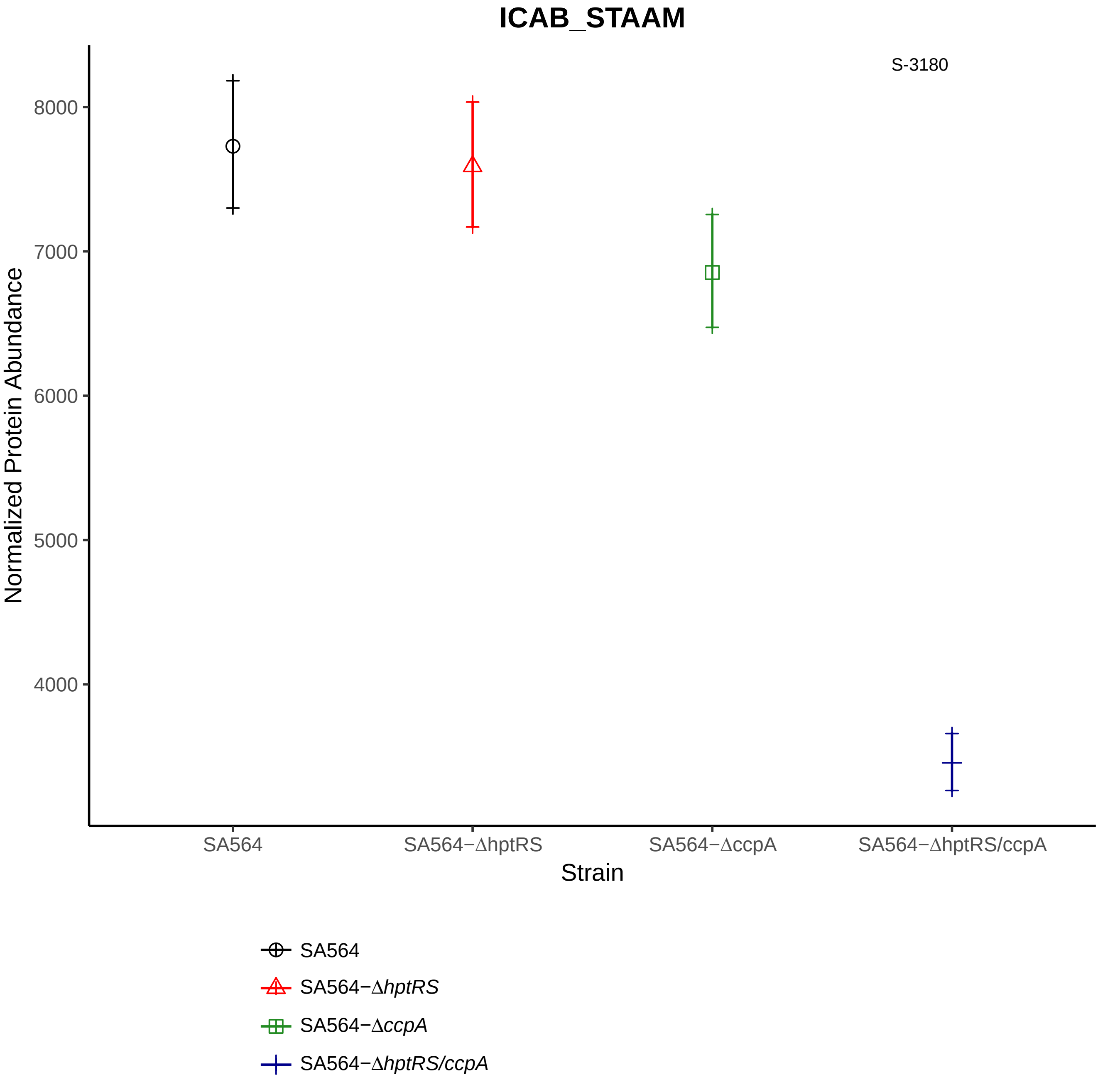

ICAB_STAAM
S-3180
Normalized Protein Abundance
8000
7000
6000
5000
4000
SA564
SA564−ΔhptRS
SA564−ΔccpA
SA564−ΔhptRS/ccpA
Strain
SA564
SA564−ΔhptRS
SA564−ΔccpA
SA564−ΔhptRS/ccpA

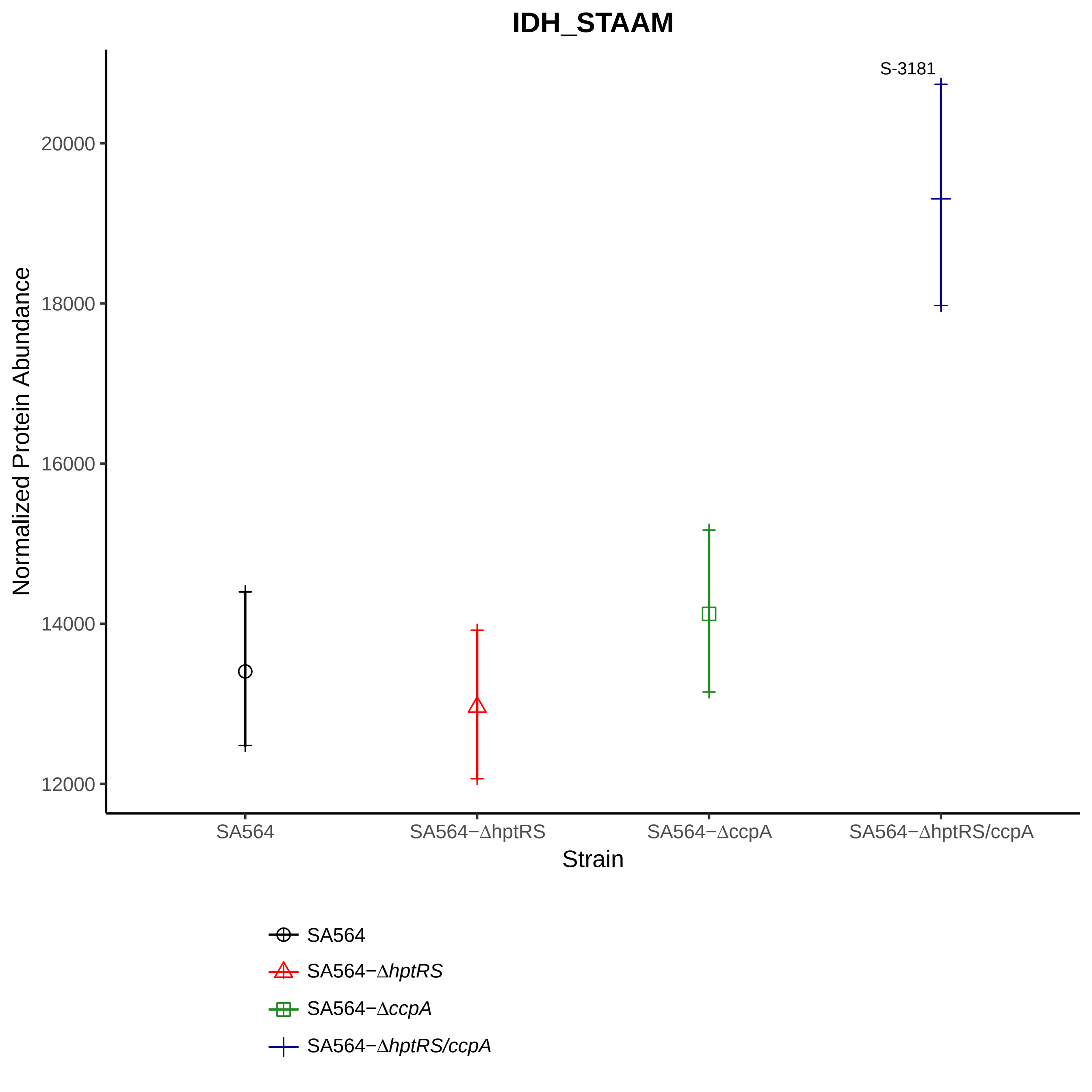

IDH_STAAM
S-3181
Normalized Protein Abundance
20000
18000
16000
14000
12000
SA564
SA564−ΔhptRS
SA564−ΔccpA
SA564−ΔhptRS/ccpA
Strain
SA564
SA564−ΔhptRS
SA564−ΔccpA
SA564−ΔhptRS/ccpA

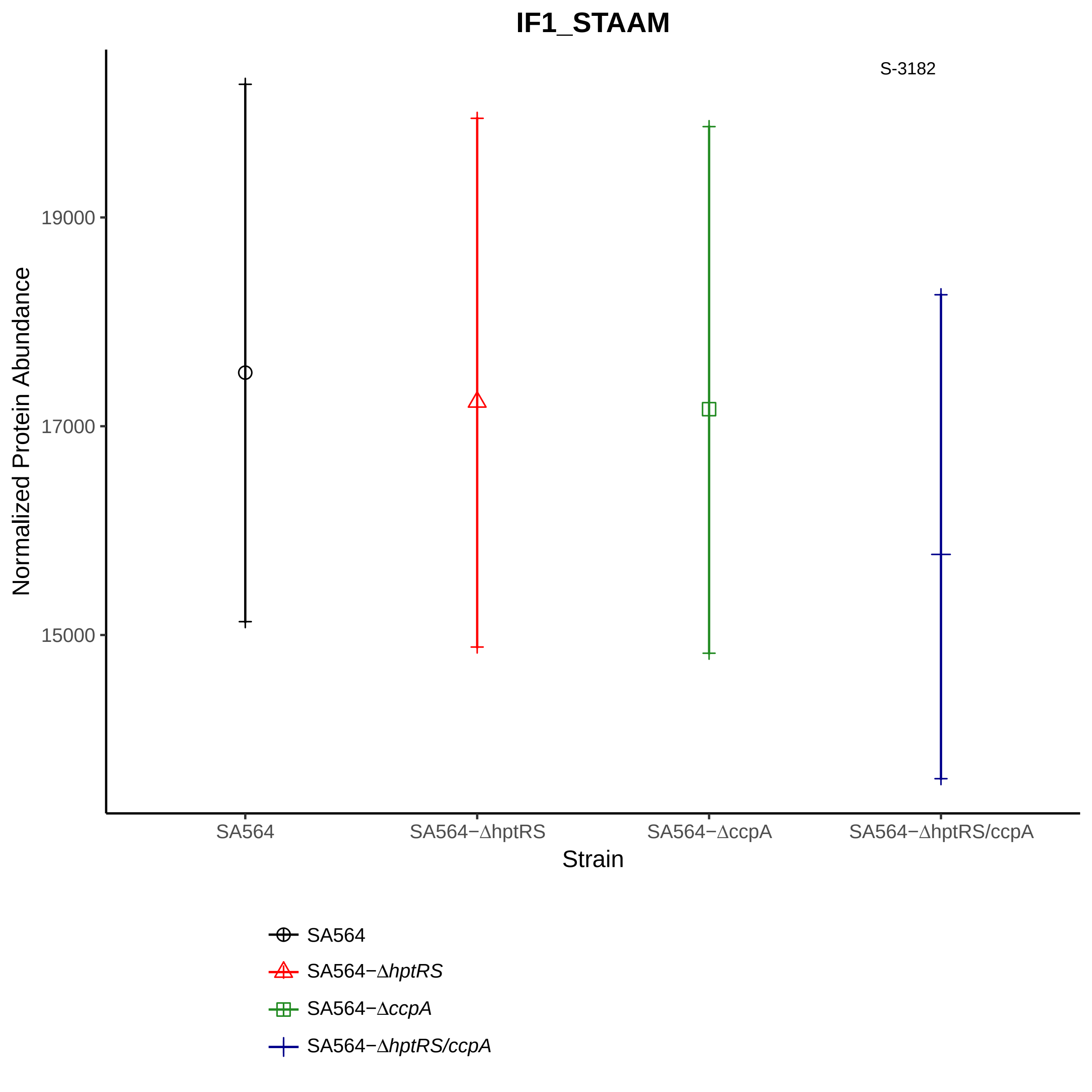

IF1_STAAM
S-3182
Normalized Protein Abundance
19000
17000
15000
SA564
SA564−ΔhptRS
SA564−ΔccpA
SA564−ΔhptRS/ccpA
Strain
SA564
SA564−ΔhptRS
SA564−ΔccpA
SA564−ΔhptRS/ccpA

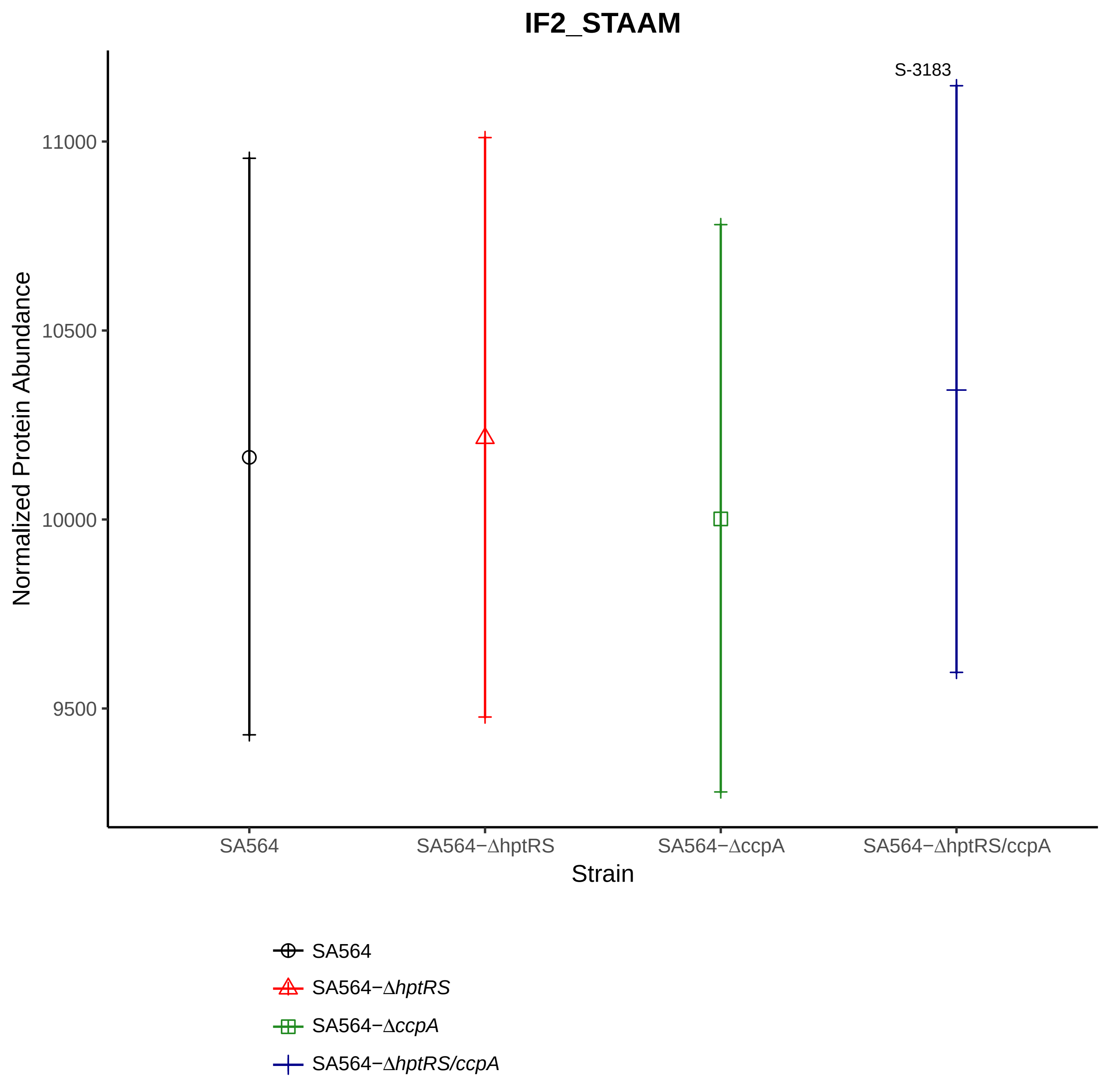

IF2_STAAM
S-3183
11000
10500
10000
9500
Normalized Protein Abundance
SA564
SA564−ΔhptRS
SA564−ΔccpA
SA564−ΔhptRS/ccpA
Strain
SA564
SA564−ΔhptRS
SA564−ΔccpA
SA564−ΔhptRS/ccpA

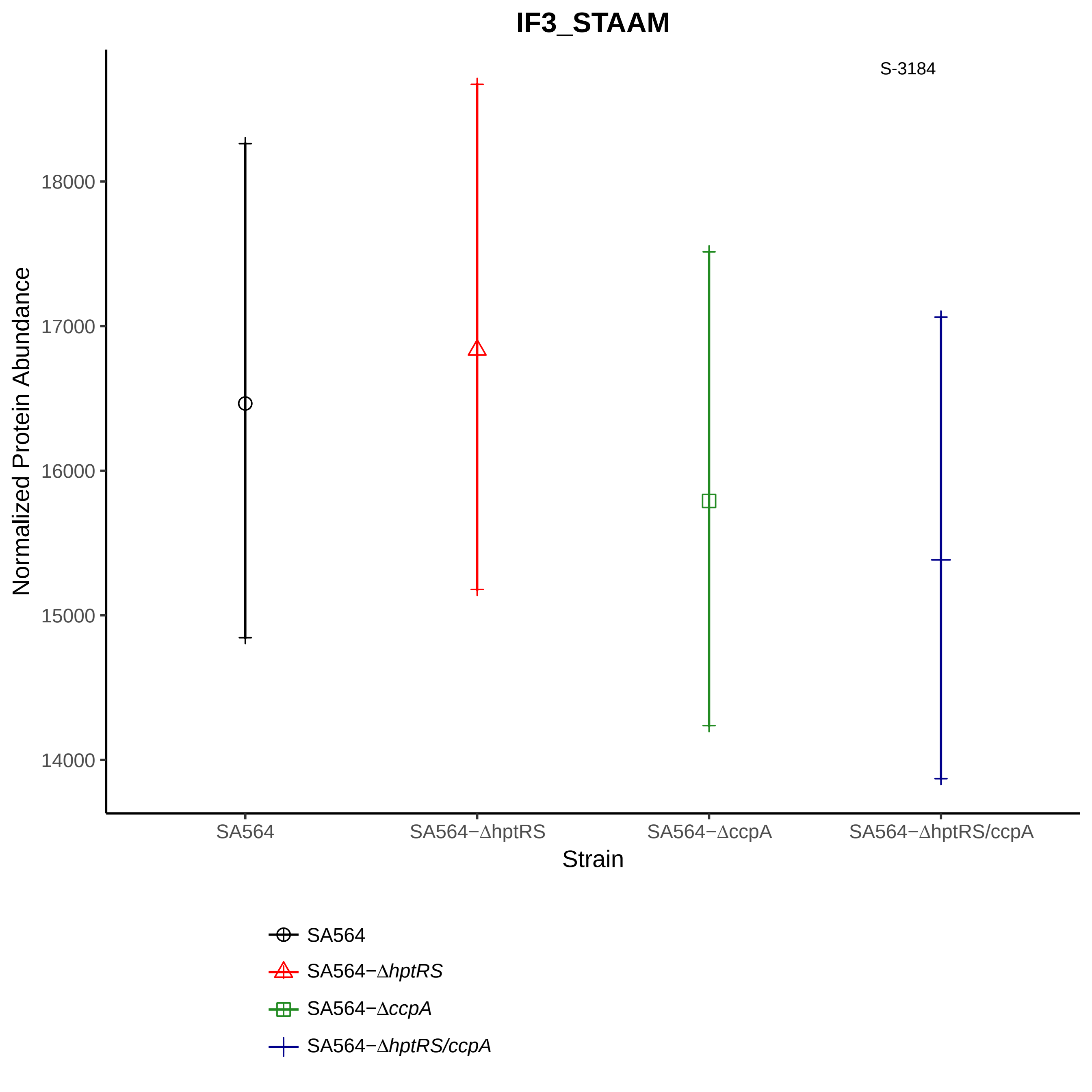
IF3_STAAM
S-3184
Normalized Protein Abundance
18000
17000
16000
15000
14000
SA564
SA564−ΔhptRS
SA564−ΔccpA
SA564−ΔhptRS/ccpA
Strain
SA564
SA564−ΔhptRS
SA564−ΔccpA
SA564−ΔhptRS/ccpA

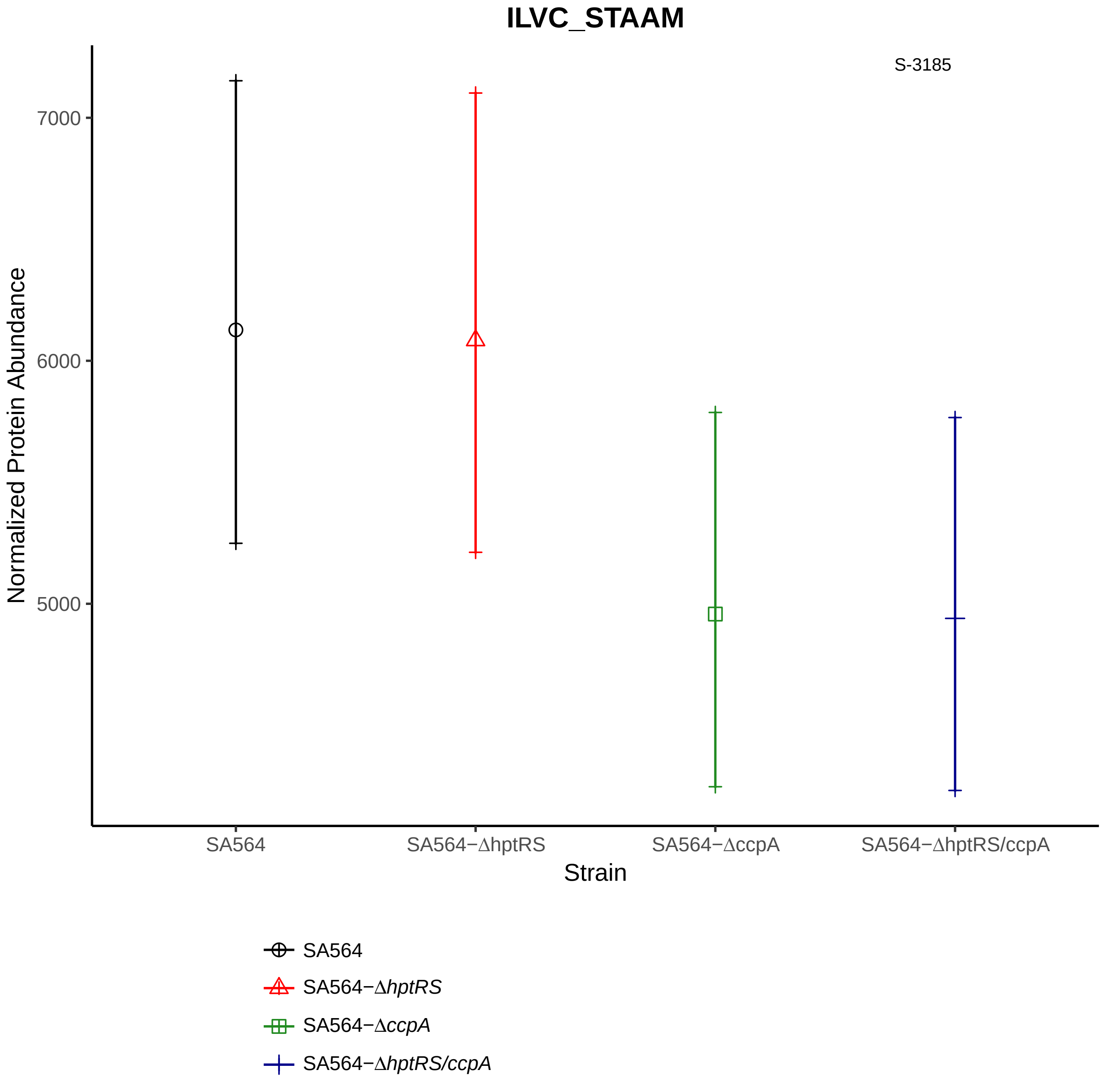

ILVC_STAAM
S-3185
Normalized Protein Abundance
7000
6000
5000
SA564
SA564−ΔhptRS
SA564−ΔccpA
SA564−ΔhptRS/ccpA
Strain
SA564
SA564−ΔhptRS
SA564−ΔccpA
SA564−ΔhptRS/ccpA

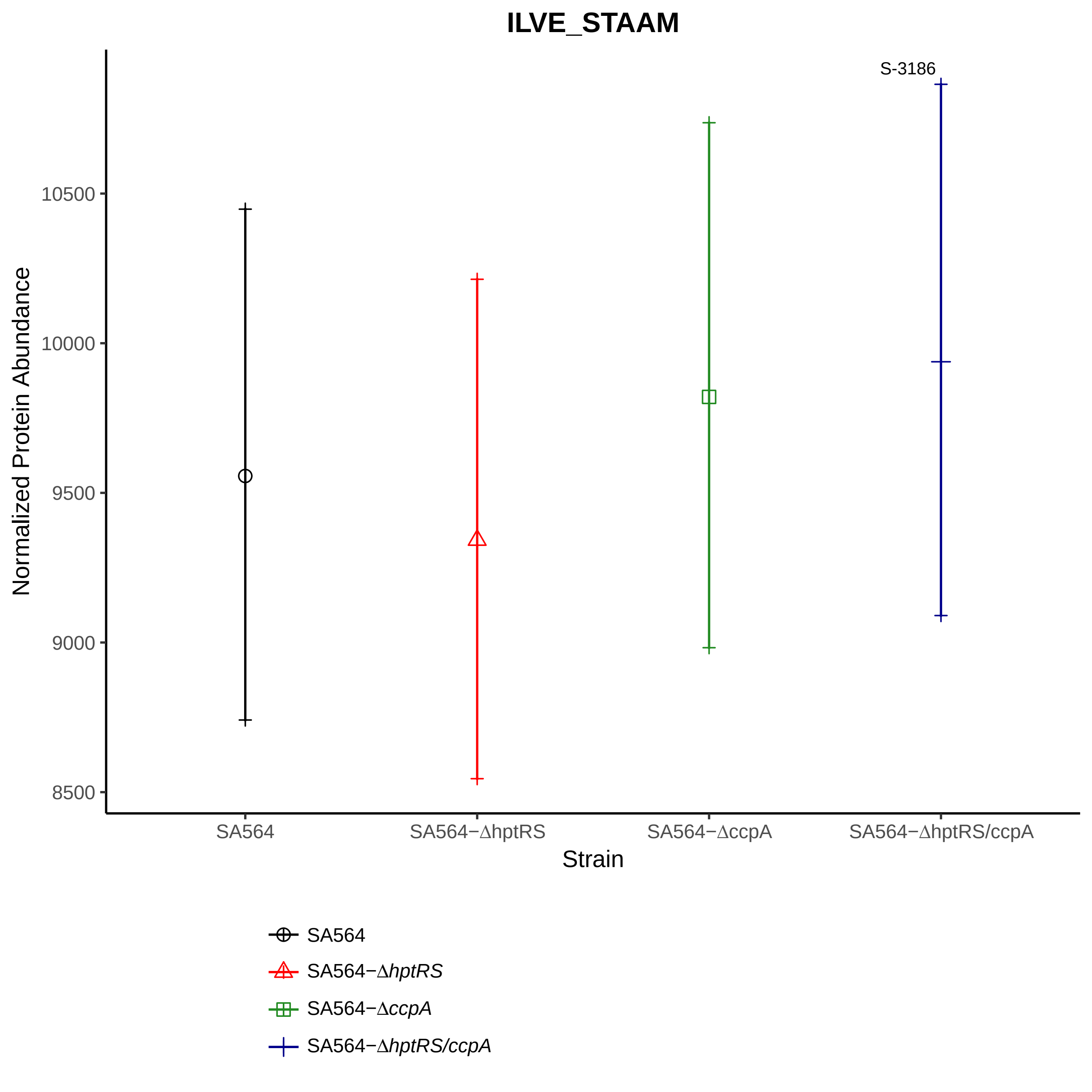

ILVE_STAAM
S-3186
Normalized Protein Abundance
10500
10000
9500
9000
8500
SA564
SA564−ΔhptRS
SA564−ΔccpA
SA564−ΔhptRS/ccpA
Strain
SA564
SA564−ΔhptRS
SA564−ΔccpA
SA564−ΔhptRS/ccpA

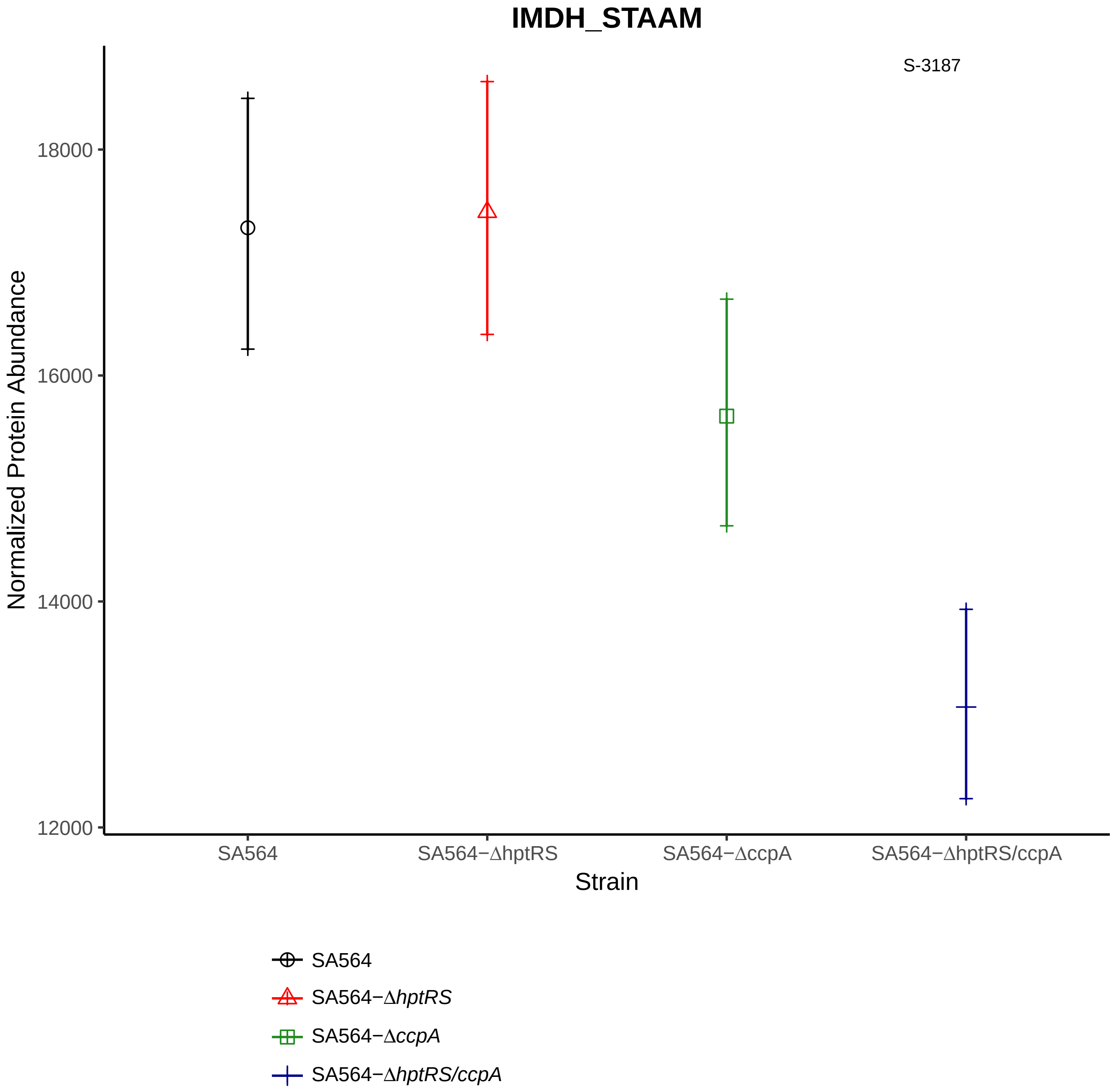
IMDH_STAAM
S-3187
Normalized Protein Abundance
18000
16000
14000
12000
SA564
SA564−ΔhptRS
SA564−ΔccpA
SA564−ΔhptRS/ccpA
Strain
SA564
SA564−ΔhptRS
SA564−ΔccpA
SA564−ΔhptRS/ccpA

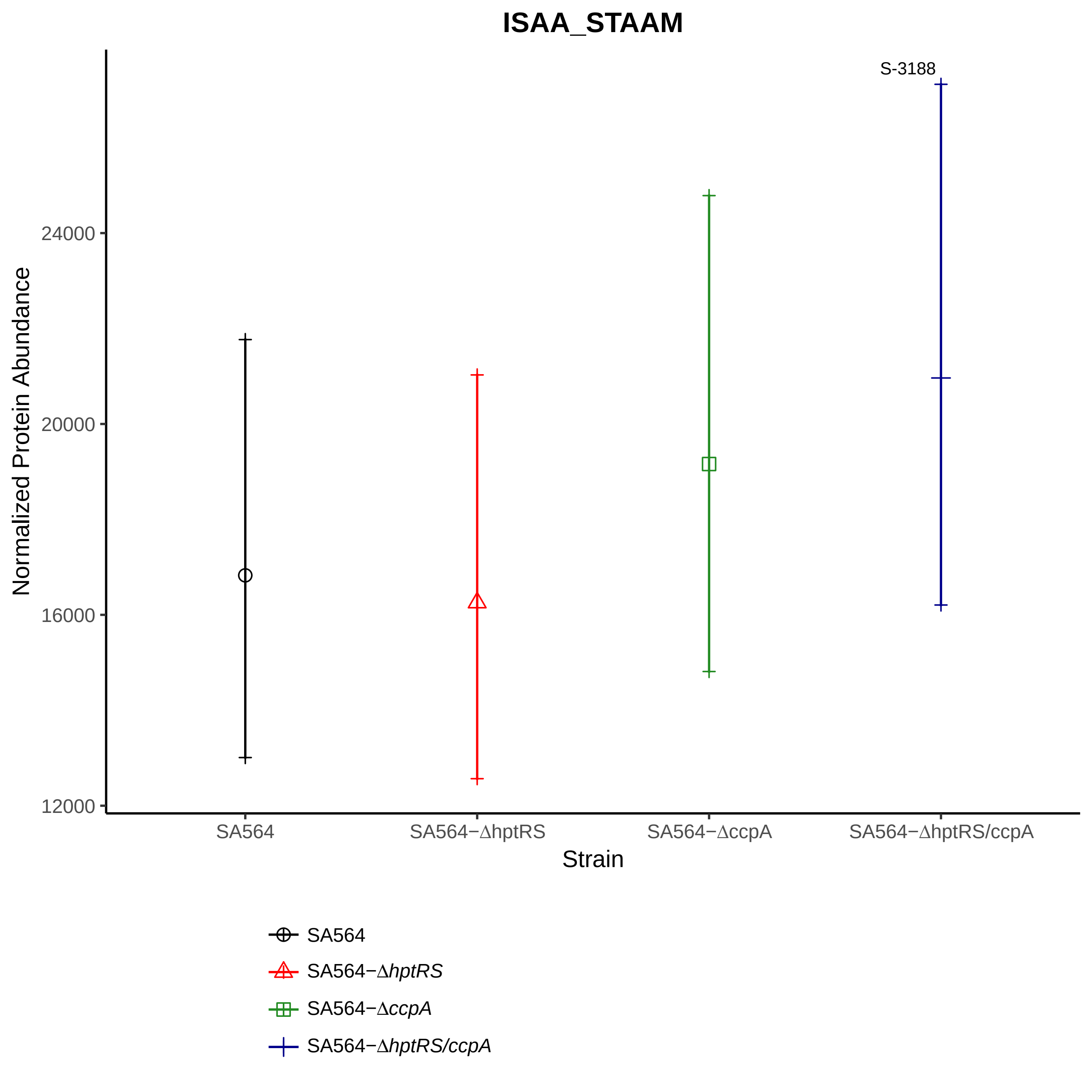
ISAA_STAAM
S-3188
Normalized Protein Abundance
24000
20000
16000
12000
SA564
SA564−ΔhptRS
SA564−ΔccpA
SA564−ΔhptRS/ccpA
Strain
SA564
SA564−ΔhptRS
SA564−ΔccpA
SA564−ΔhptRS/ccpA

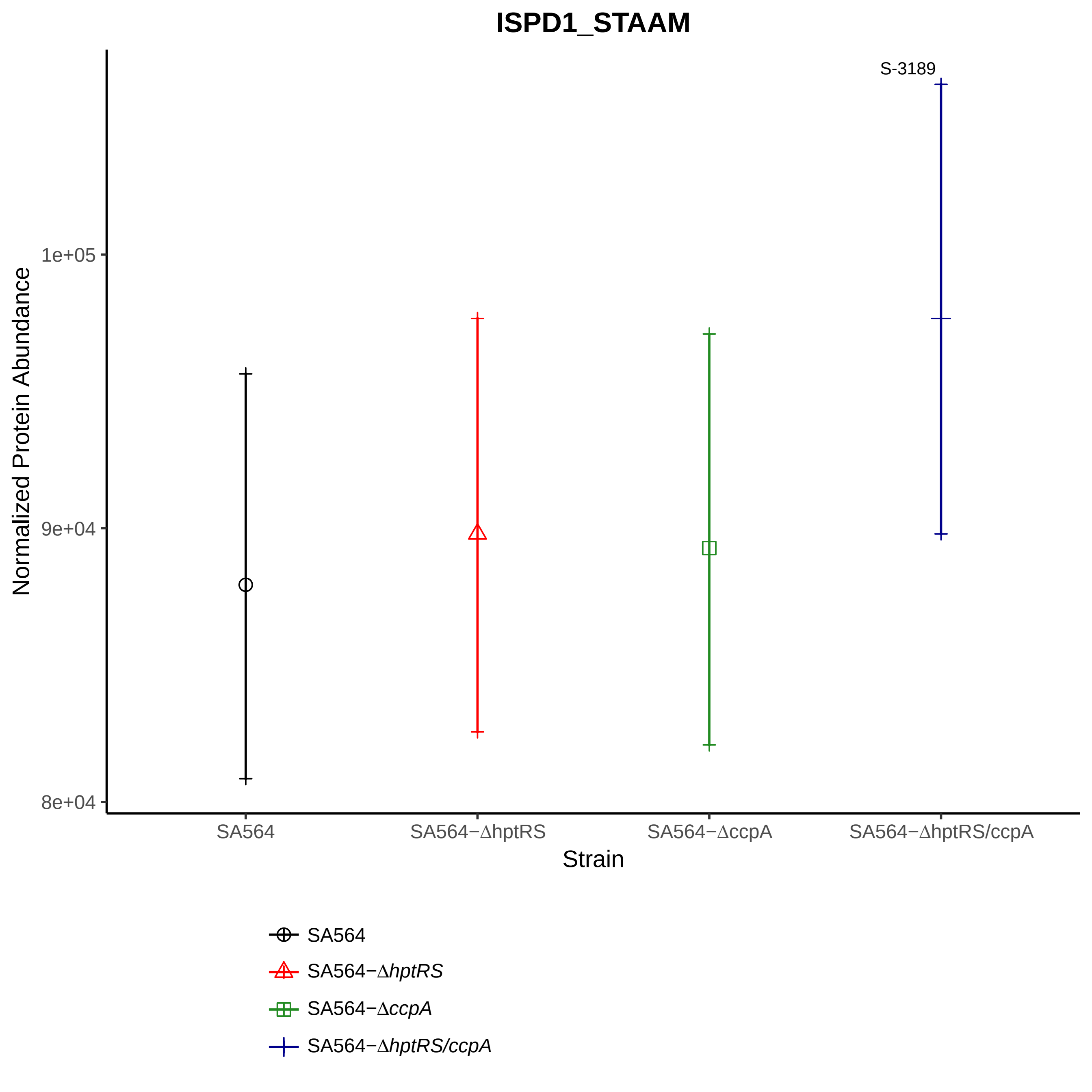

ISPD1_STAAM
S-3189
Normalized Protein Abundance
1e+05
9e+04
8e+04
SA564
SA564−ΔhptRS
SA564−ΔccpA
SA564−ΔhptRS/ccpA
Strain
SA564
SA564−ΔhptRS
SA564−ΔccpA
SA564−ΔhptRS/ccpA

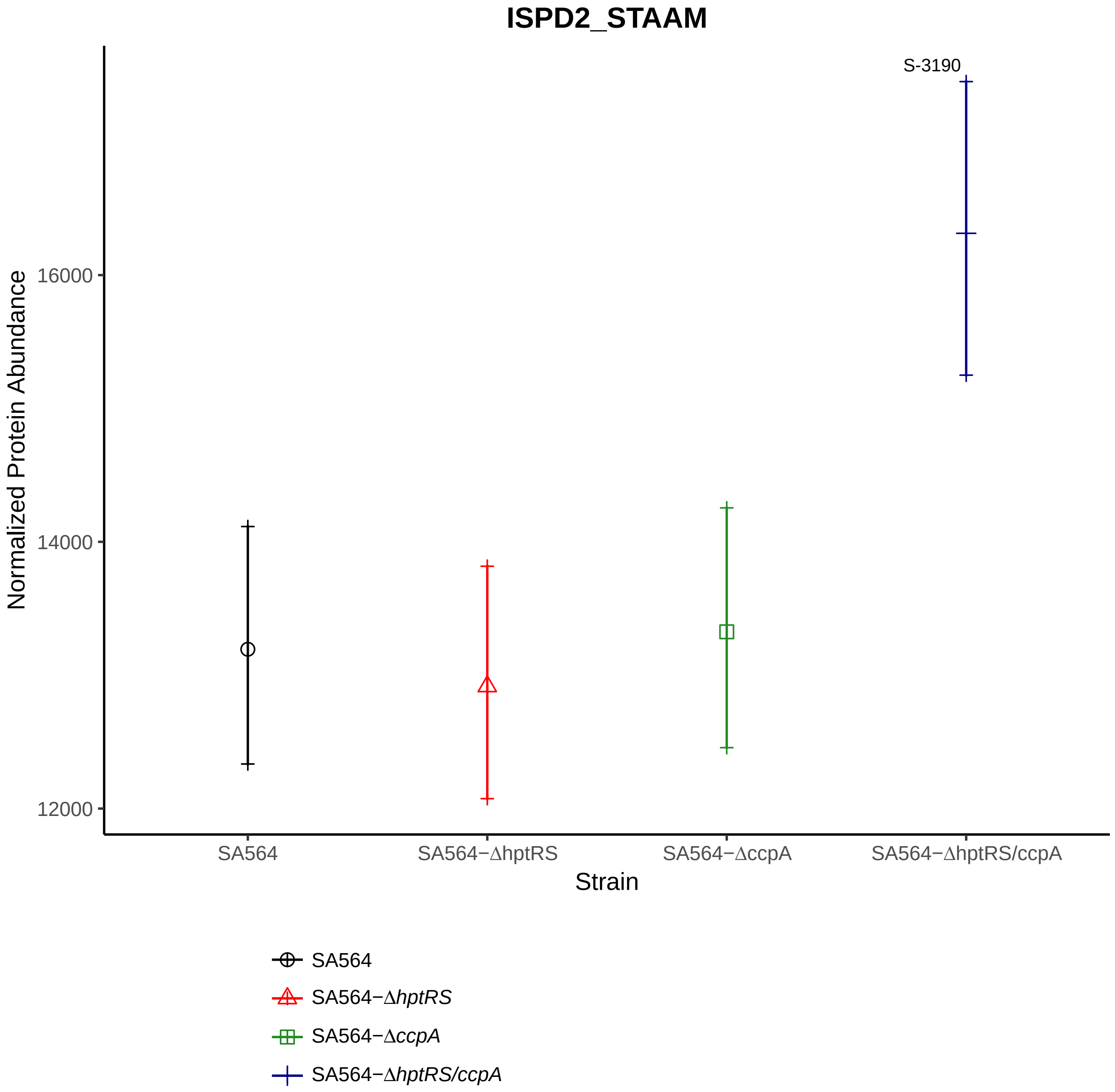
ISPD2_STAAM
S-3190
Normalized Protein Abundance
16000
14000
12000
SA564
SA564−ΔhptRS
SA564−ΔccpA
SA564−ΔhptRS/ccpA
Strain
SA564
SA564−ΔhptRS
SA564−ΔccpA
SA564−ΔhptRS/ccpA

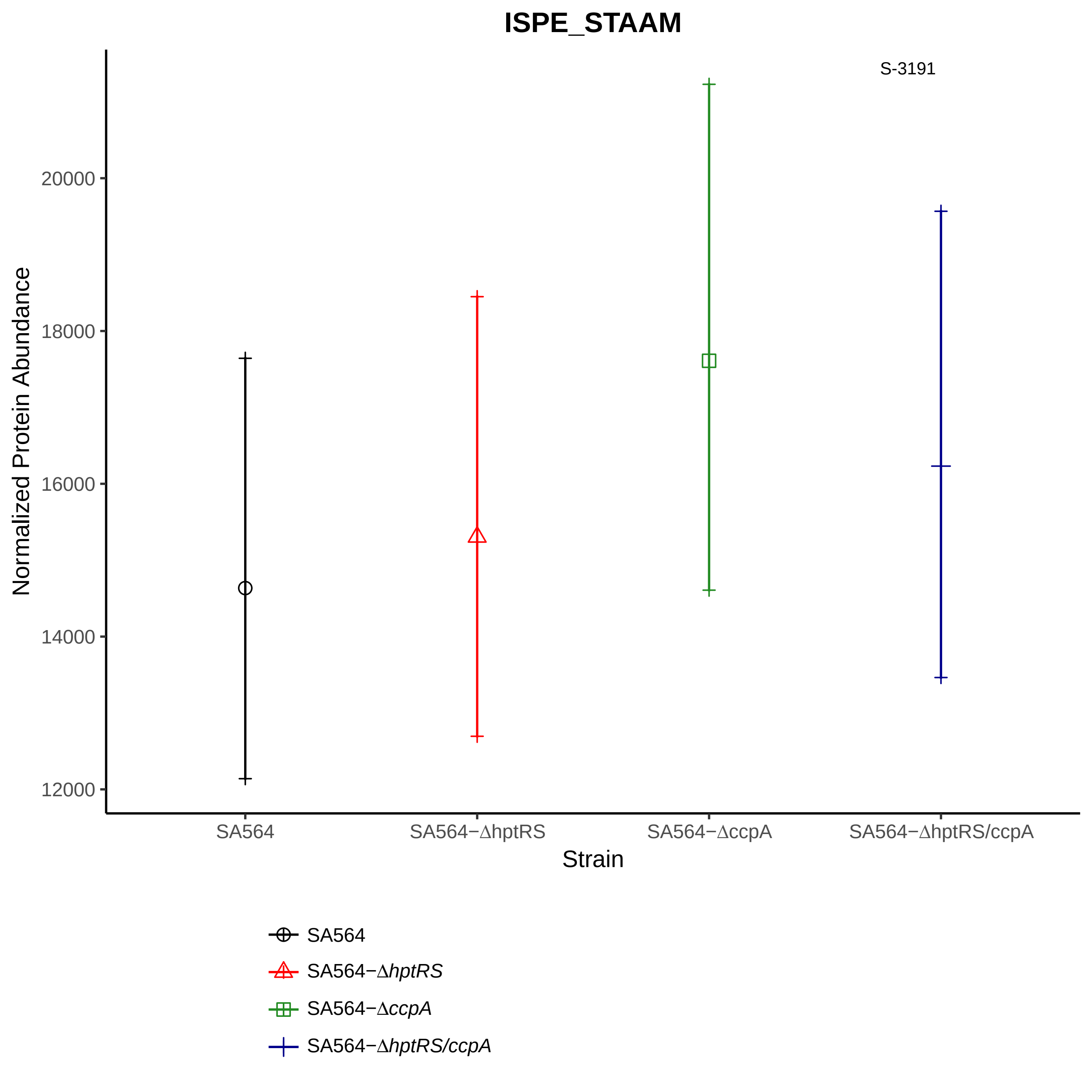

ISPE_STAAM
S-3191
Normalized Protein Abundance
20000
18000
16000
14000
12000
SA564
SA564−ΔhptRS
SA564−ΔccpA
SA564−ΔhptRS/ccpA
Strain
SA564
SA564−ΔhptRS
SA564−ΔccpA
SA564−ΔhptRS/ccpA

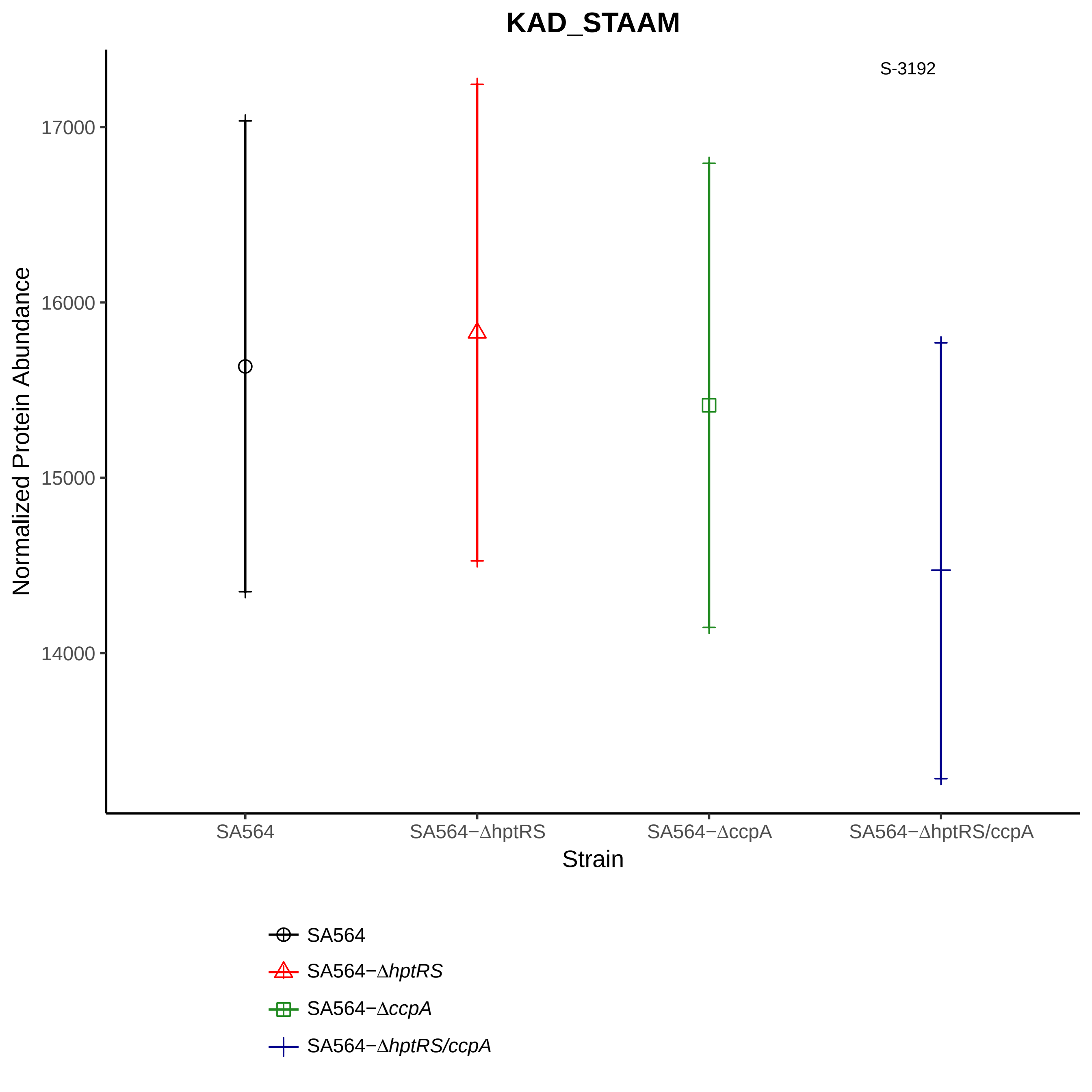
KAD_STAAM
S-3192
Normalized Protein Abundance
17000
16000
15000
14000
SA564
SA564−ΔhptRS
SA564−ΔccpA
SA564−ΔhptRS/ccpA
Strain
SA564
SA564−ΔhptRS
SA564−ΔccpA
SA564−ΔhptRS/ccpA

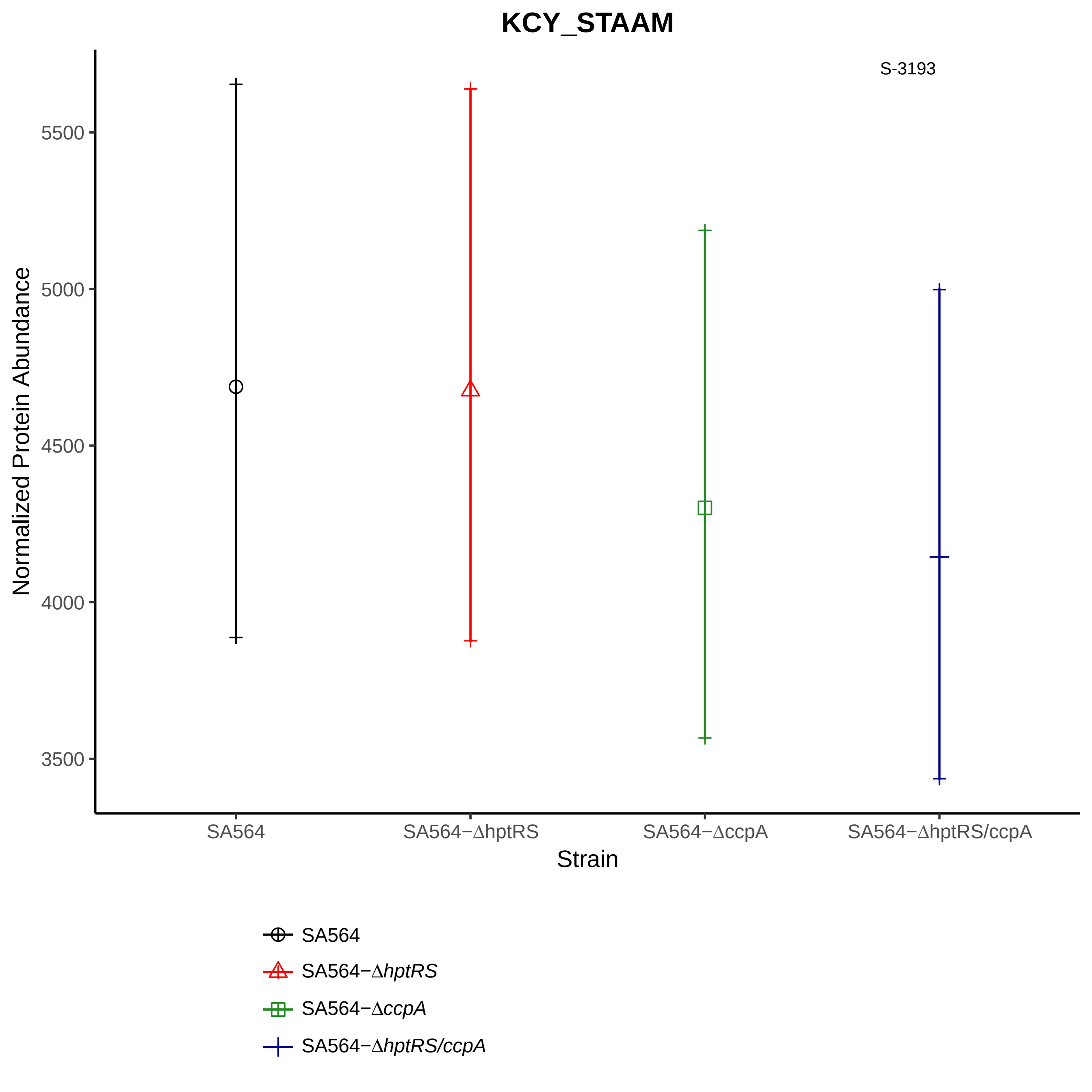
KCY_STAAM
S-3193
Normalized Protein Abundance
5500
5000
4500
4000
3500
SA564
SA564−ΔhptRS
SA564−ΔccpA
SA564−ΔhptRS/ccpA
Strain
SA564
SA564−Δ*hptRS*
SA564−Δ*ccpA*
SA564−Δ*hptRS/ccpA*

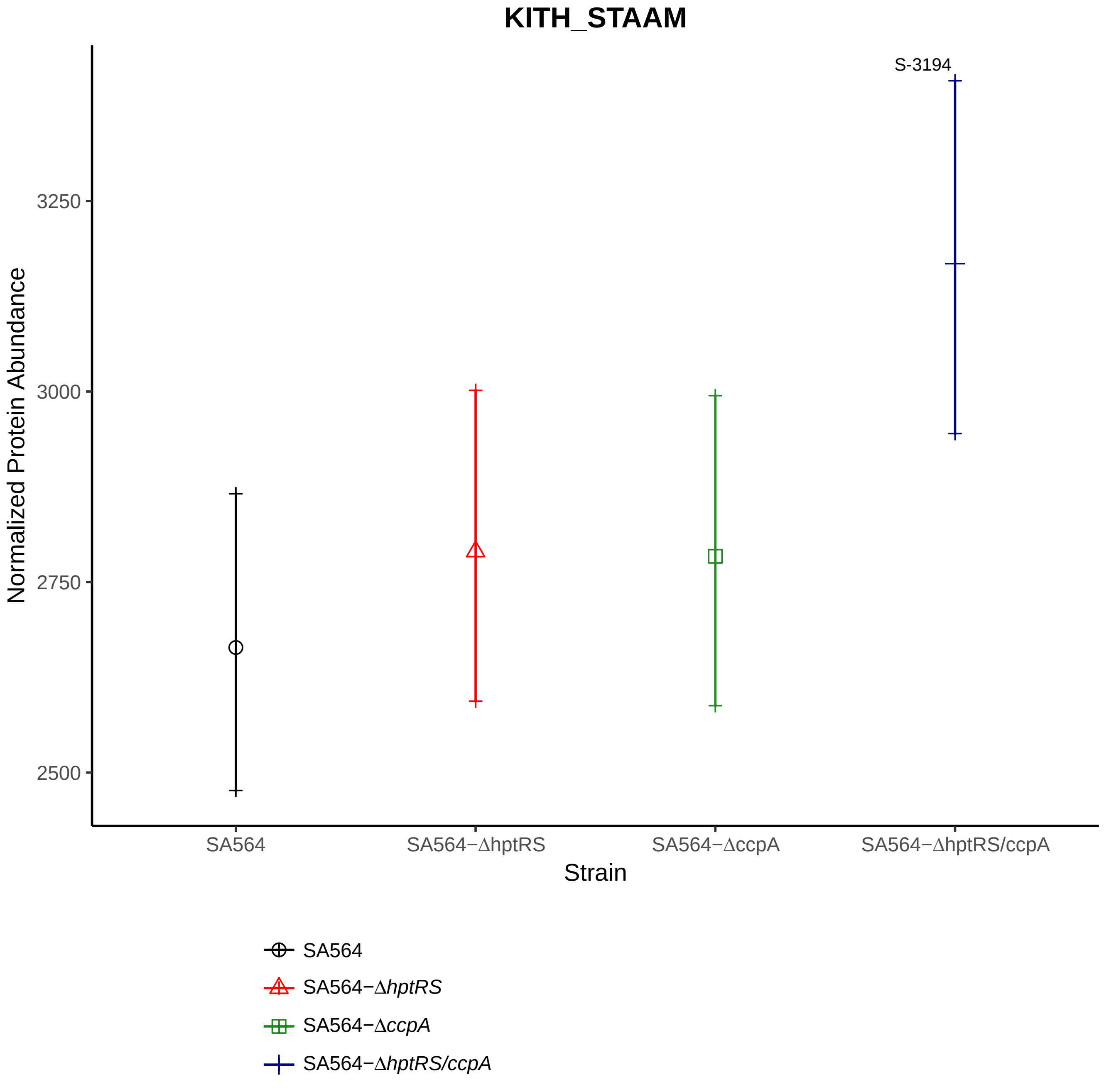

KITH_STAAM
S-3194
Normalized Protein Abundance
3250
3000
2750
2500
SA564
SA564−ΔhptRS
SA564−ΔccpA
SA564−ΔhptRS/ccpA
Strain
SA564
SA564−ΔhptRS
SA564−ΔccpA
SA564−ΔhptRS/ccpA

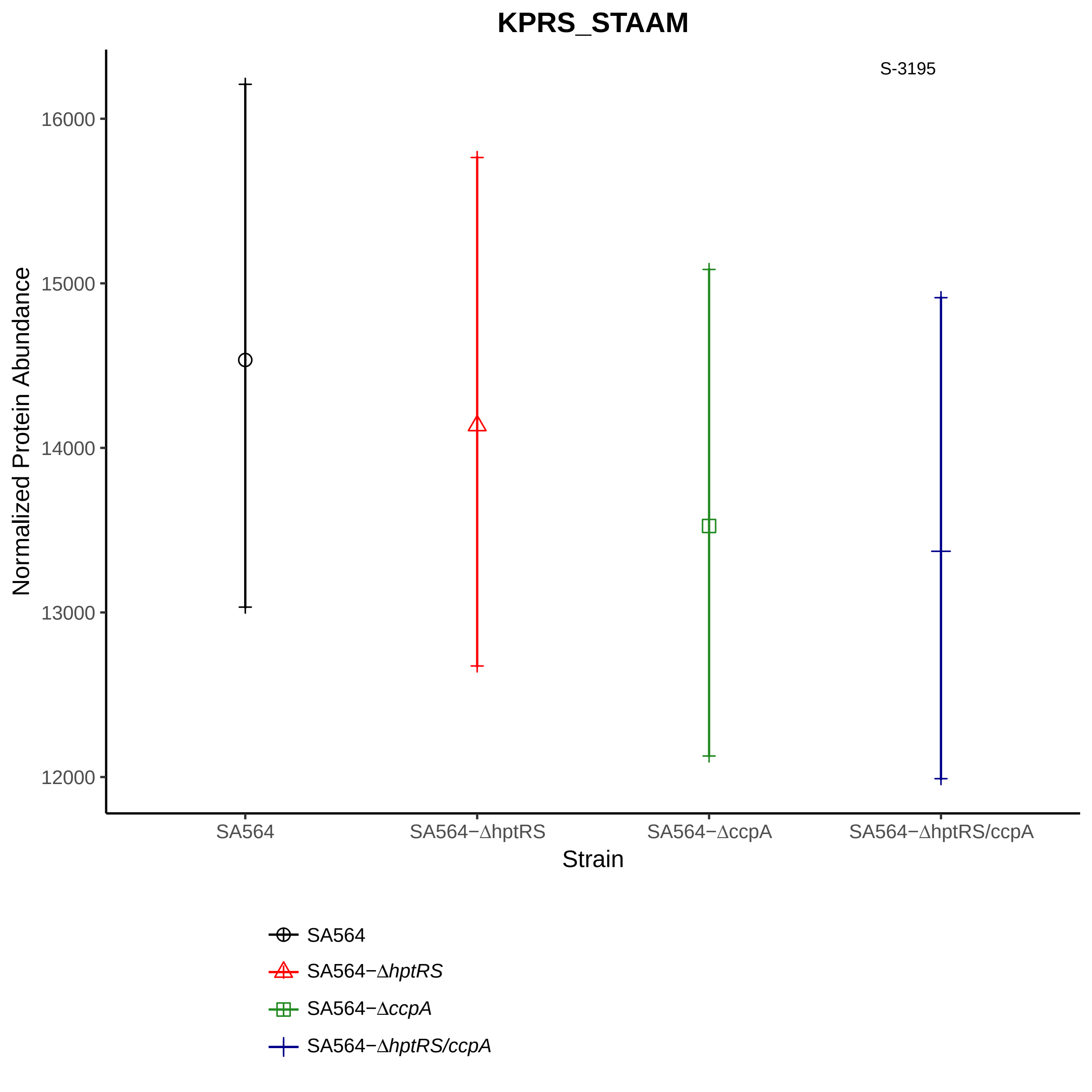

KPRS_STAAM
S-3195
Normalized Protein Abundance
16000
15000
14000
13000
12000
SA564
SA564−ΔhptRS
SA564−ΔccpA
SA564−ΔhptRS/ccpA
Strain
SA564
SA564−ΔhptRS
SA564−ΔccpA
SA564−ΔhptRS/ccpA

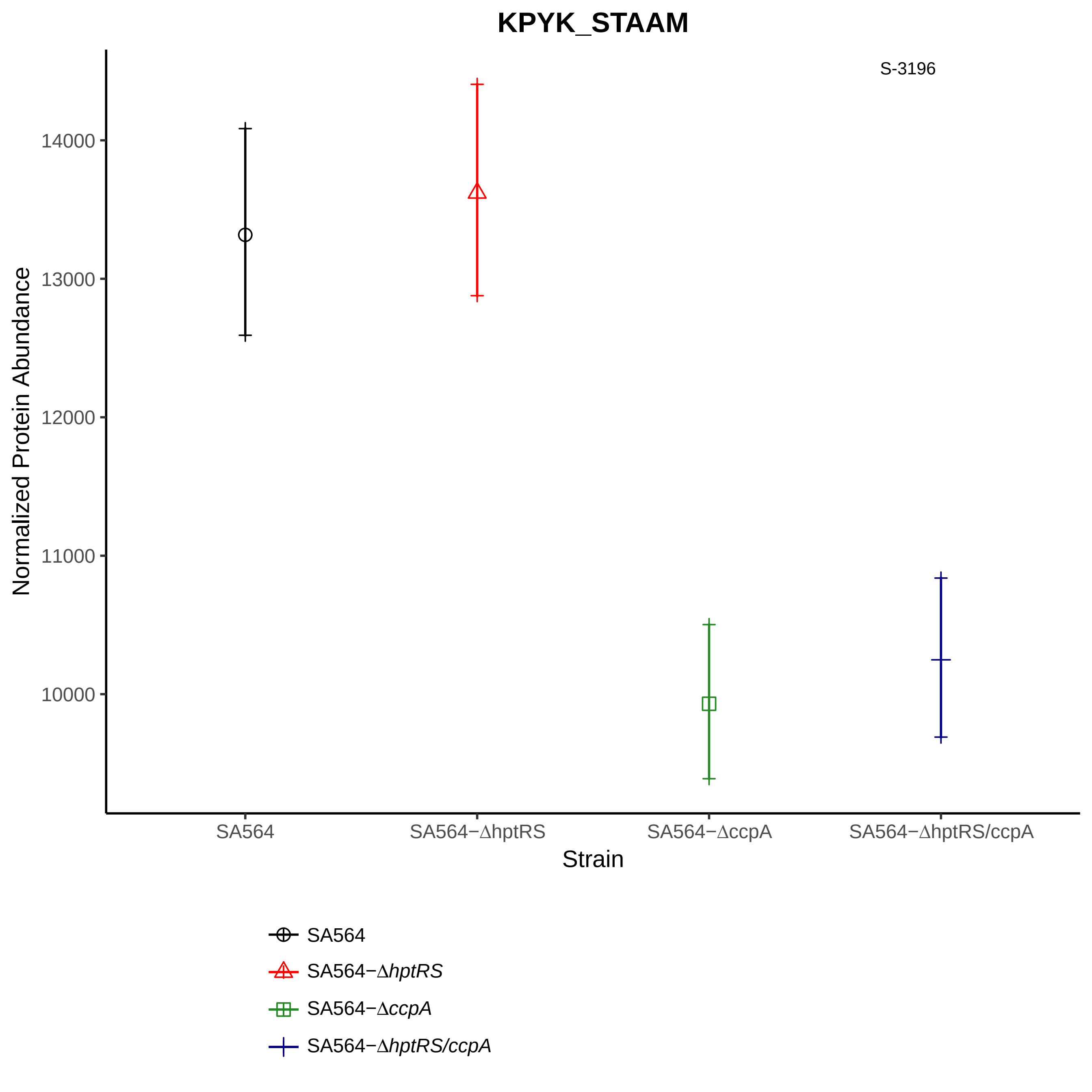

KPYK_STAAM
S-3196
Normalized Protein Abundance
14000
13000
12000
11000
10000
SA564
SA564−ΔhptRS
SA564−ΔccpA
SA564−ΔhptRS/ccpA
Strain
SA564
SA564−ΔhptRS
SA564−ΔccpA
SA564−ΔhptRS/ccpA

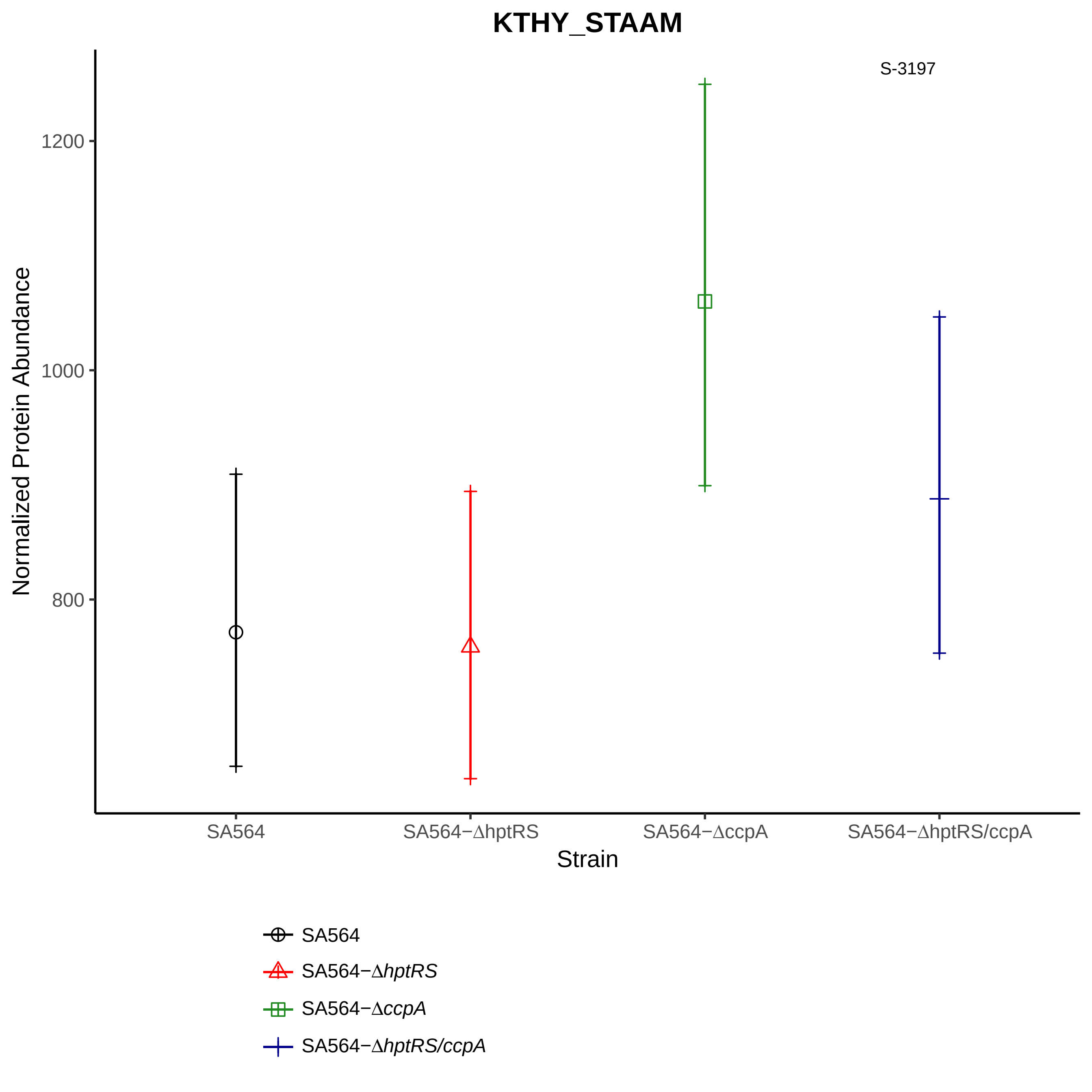
KTHY_STAAM
S-3197
Normalized Protein Abundance
1200
1000
800
SA564
SA564−ΔhptRS
SA564−ΔccpA
SA564−ΔhptRS/ccpA
Strain
SA564
SA564−ΔhptRS
SA564−ΔccpA
SA564−ΔhptRS/ccpA

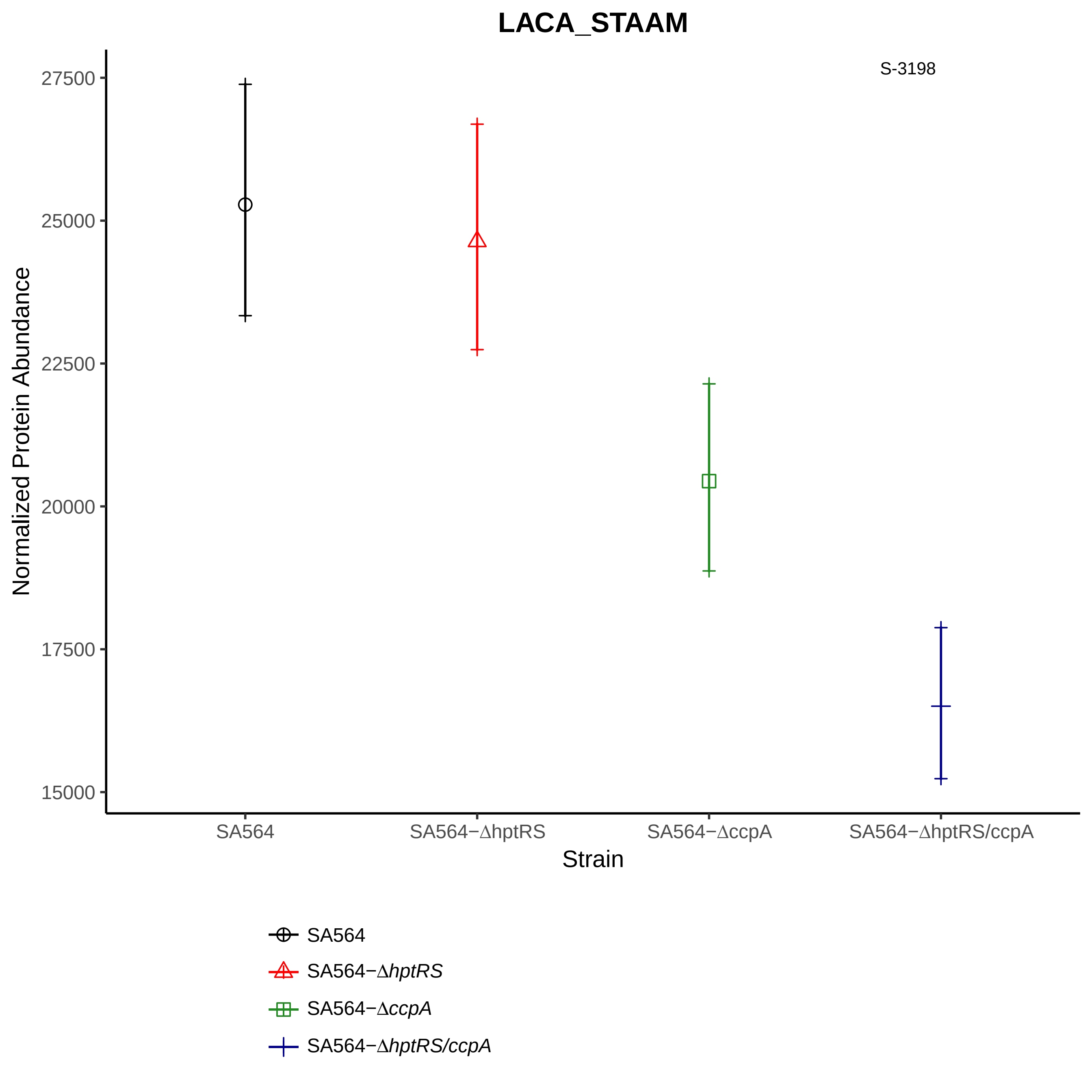

LACA_STAAM
S-3198
Normalized Protein Abundance
27500
25000
22500
20000
17500
15000
SA564
SA564−ΔhptRS
SA564−ΔccpA
SA564−ΔhptRS/ccpA
Strain
SA564
SA564−ΔhptRS
SA564−ΔccpA
SA564−ΔhptRS/ccpA

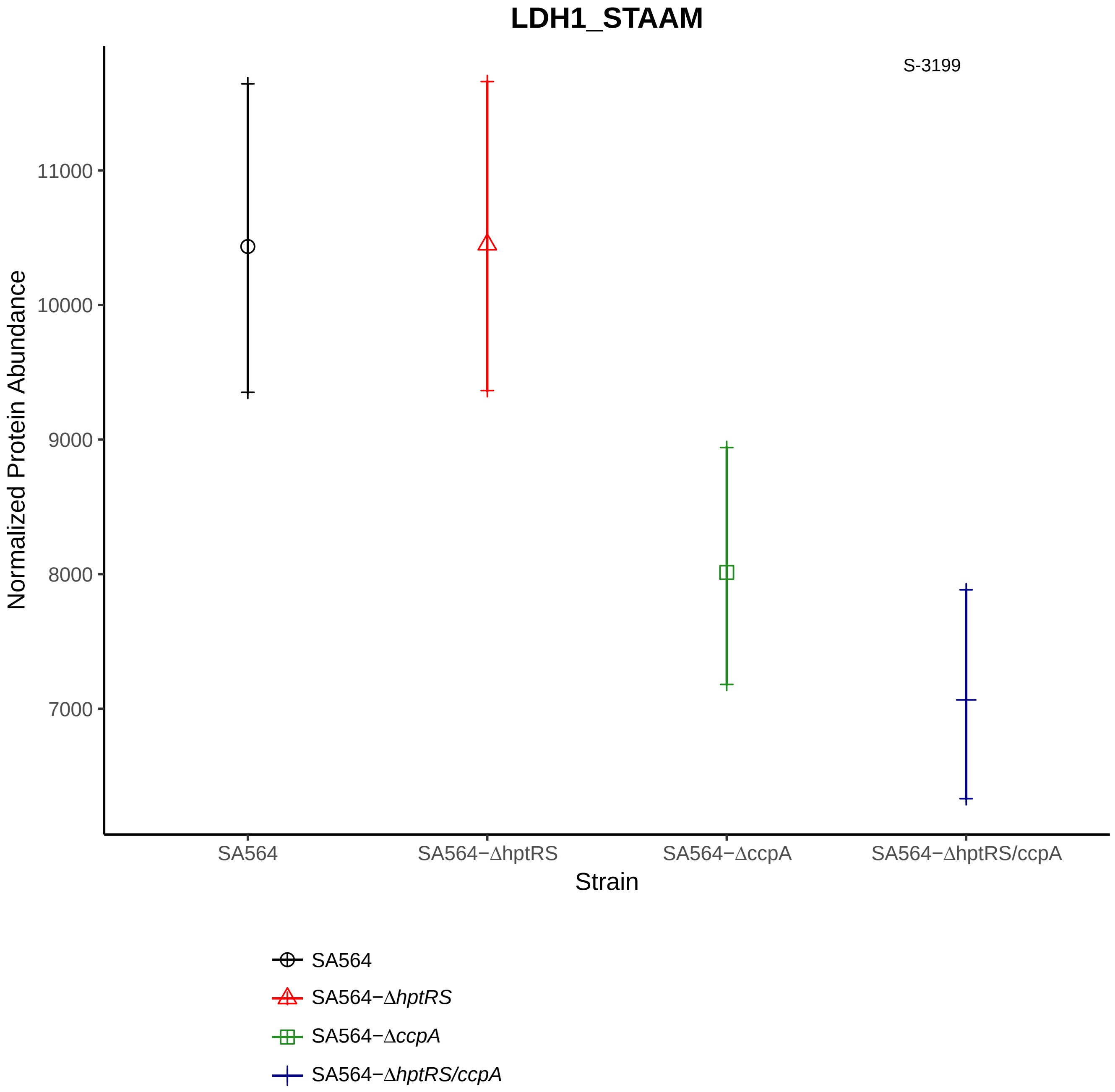

LDH1_STAAM
S-3199
Normalized Protein Abundance
11000
10000
9000
8000
7000
SA564
SA564−ΔhptRS
SA564−ΔccpA
SA564−ΔhptRS/ccpA
Strain
SA564
SA564−Δ*hptRS*
SA564−Δ*ccpA*
SA564−Δ*hptRS/ccpA*

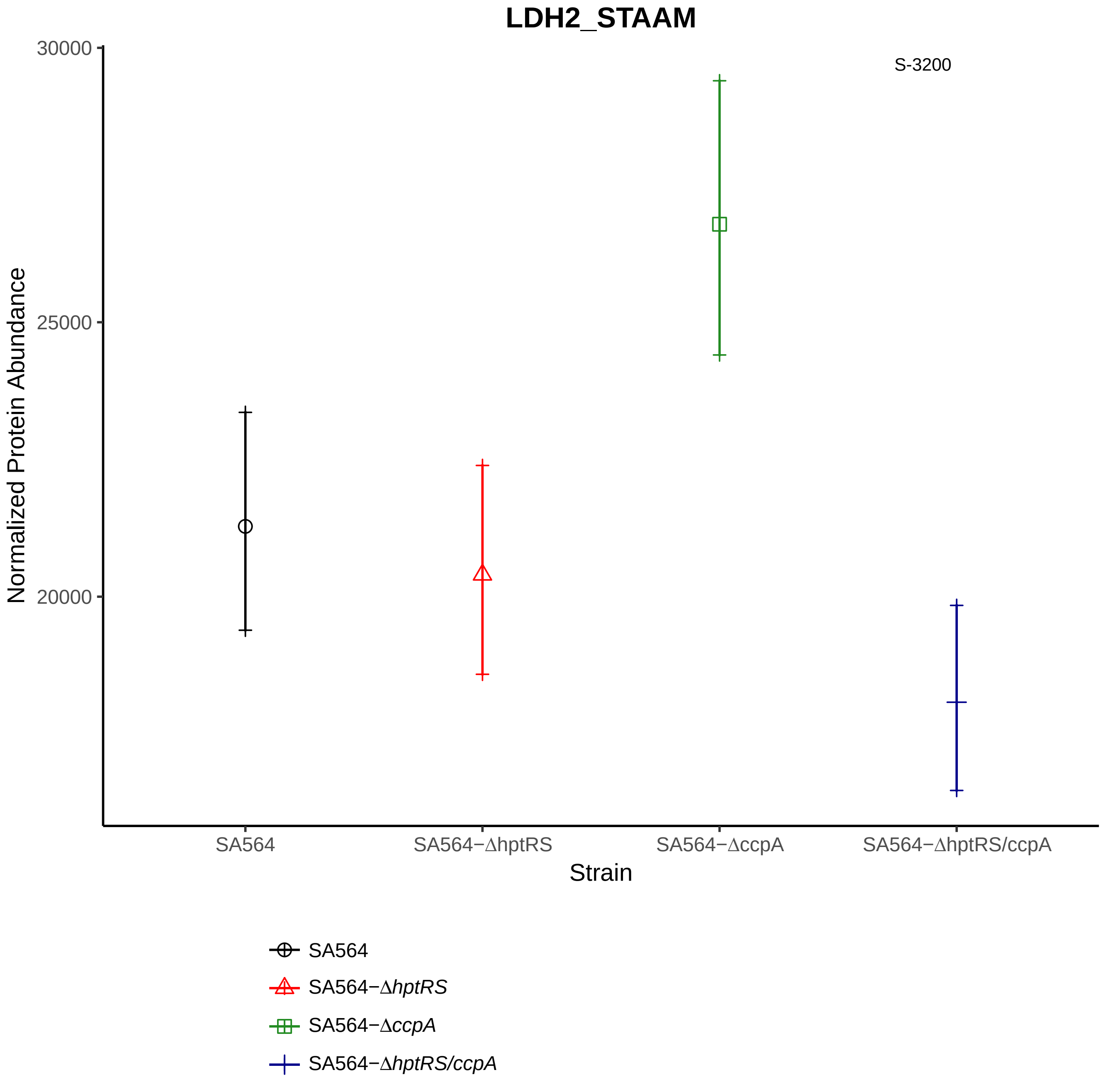
LDH2_STAAM
S-3200
Normalized Protein Abundance
30000
25000
20000
SA564
SA564−ΔhptRS
SA564−ΔccpA
SA564−ΔhptRS/ccpA
Strain
SA564
SA564−ΔhptRS
SA564−ΔccpA
SA564−ΔhptRS/ccpA

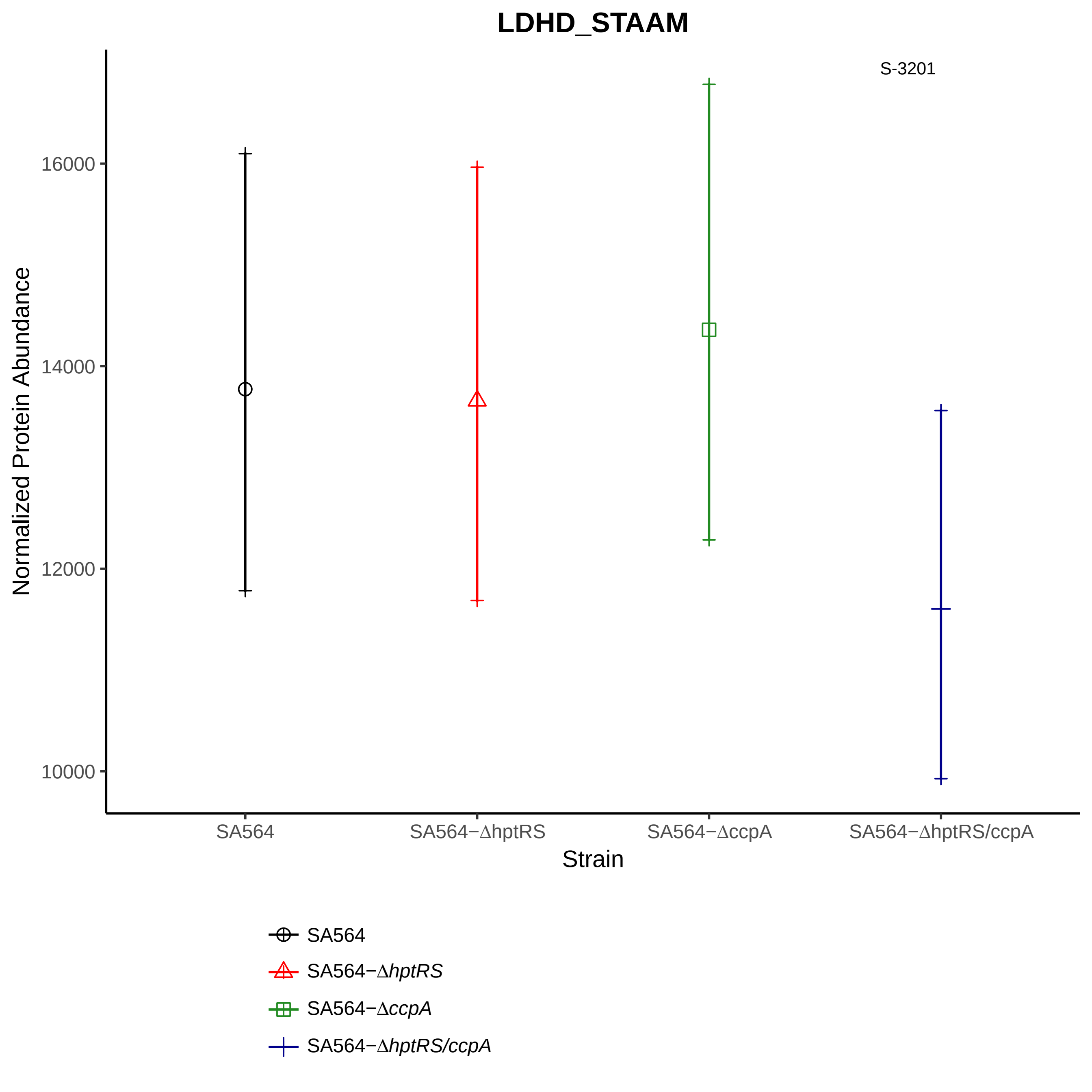

LDHD_STAAM
S-3201
Normalized Protein Abundance
16000
14000
12000
10000
SA564
SA564−ΔhptRS
SA564−ΔccpA
SA564−ΔhptRS/ccpA
Strain
SA564
SA564−Δ*hptRS*
SA564−Δ*ccpA*
SA564−Δ*hptRS/ccpA*

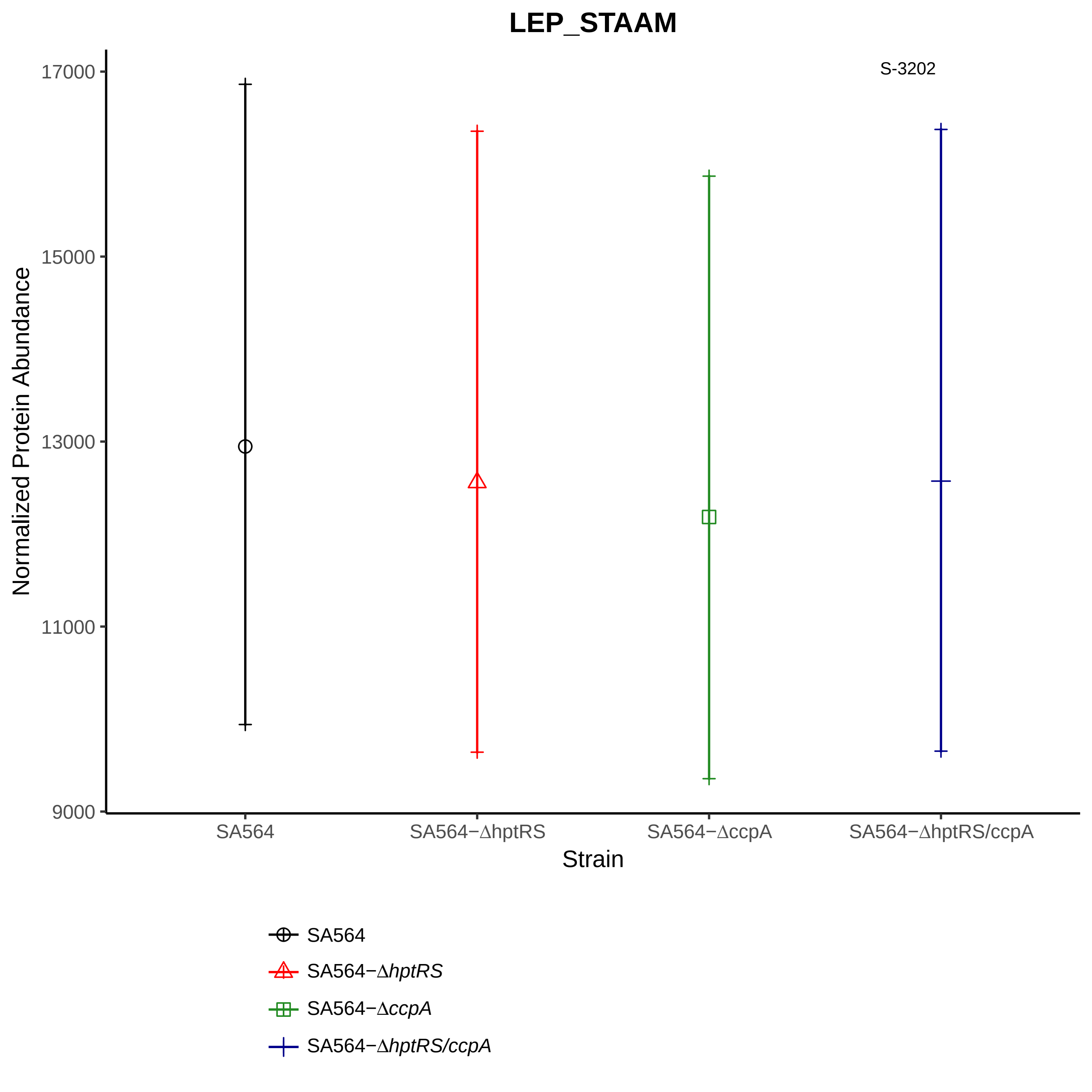

LEP_STAAM
S-3202
17000
15000
13000
11000
9000
Normalized Protein Abundance
SA564
SA564−ΔhptRS
SA564−ΔccpA
SA564−ΔhptRS/ccpA
Strain
SA564
SA564−Δ*hptRS*
SA564−Δ*ccpA*
SA564−Δ*hptRS/ccpA*

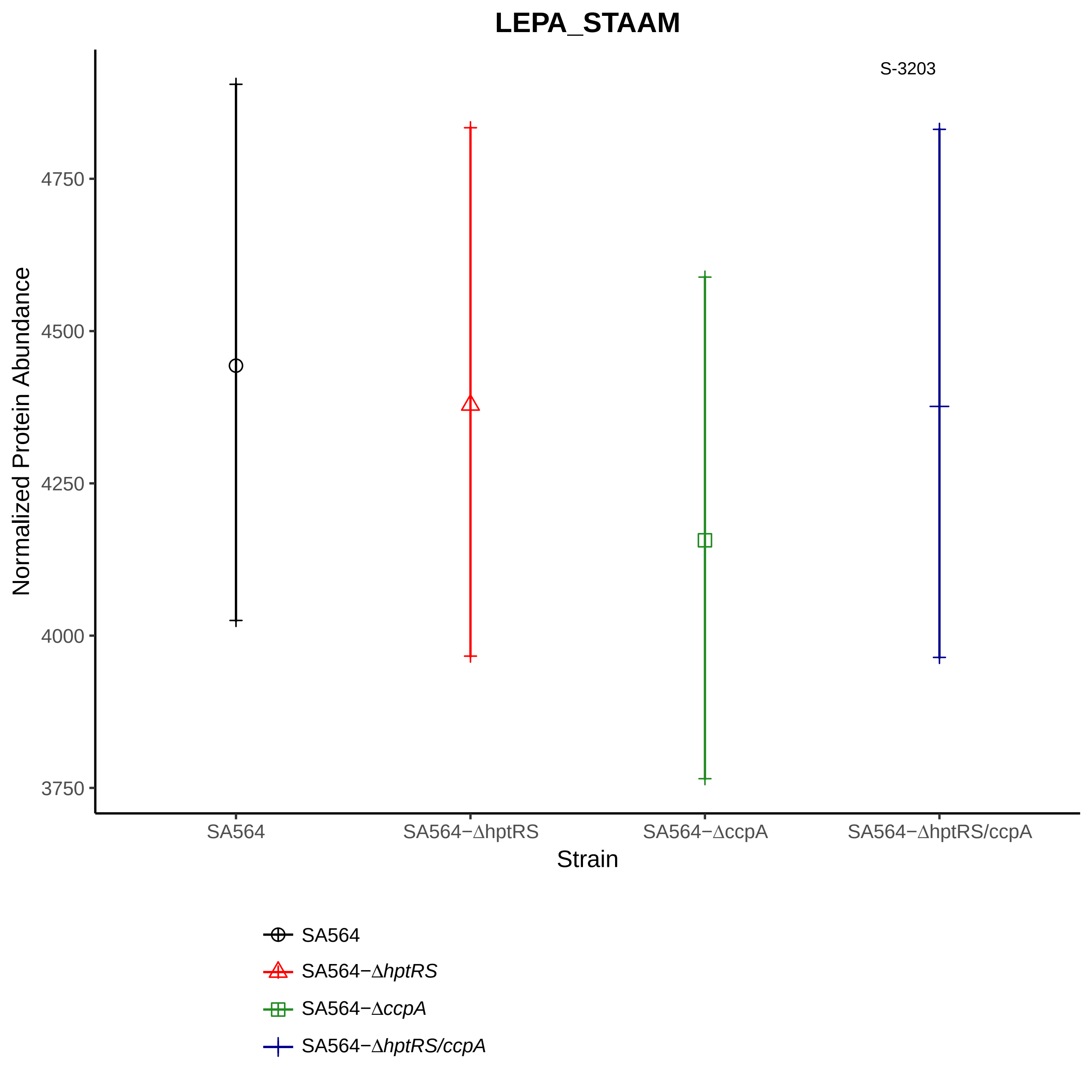

LEPA_STAAM
S-3203
Normalized Protein Abundance
4750
4500
4250
4000
3750
SA564
SA564−ΔhptRS
SA564−ΔccpA
SA564−ΔhptRS/ccpA
Strain
SA564
SA564−ΔhptRS
SA564−ΔccpA
SA564−ΔhptRS/ccpA

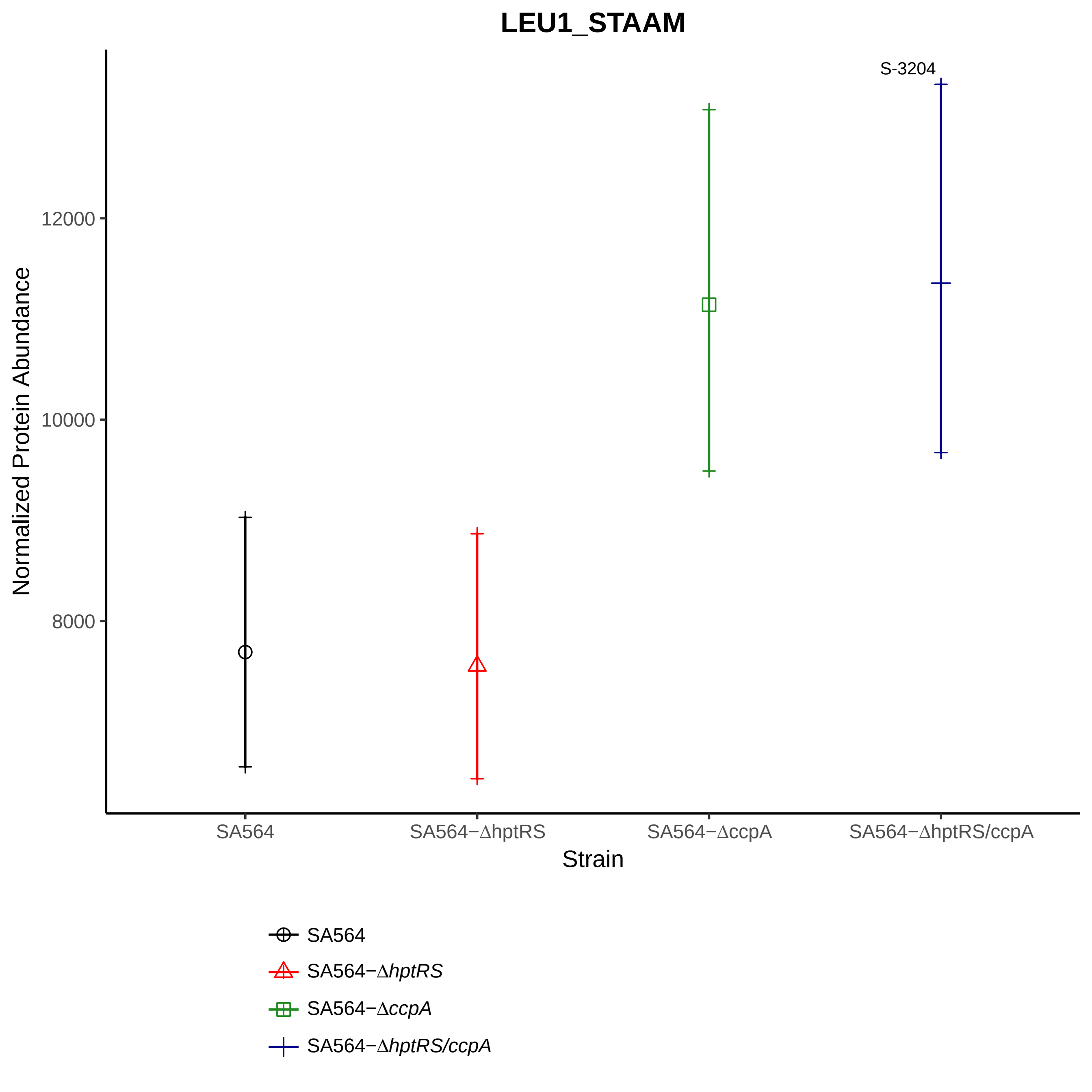

LEU1_STAAM
S-3204
Normalized Protein Abundance
12000
10000
8000
SA564
SA564−ΔhptRS
SA564−ΔccpA
SA564−ΔhptRS/ccpA
Strain
SA564
SA564−ΔhptRS
SA564−ΔccpA
SA564−ΔhptRS/ccpA

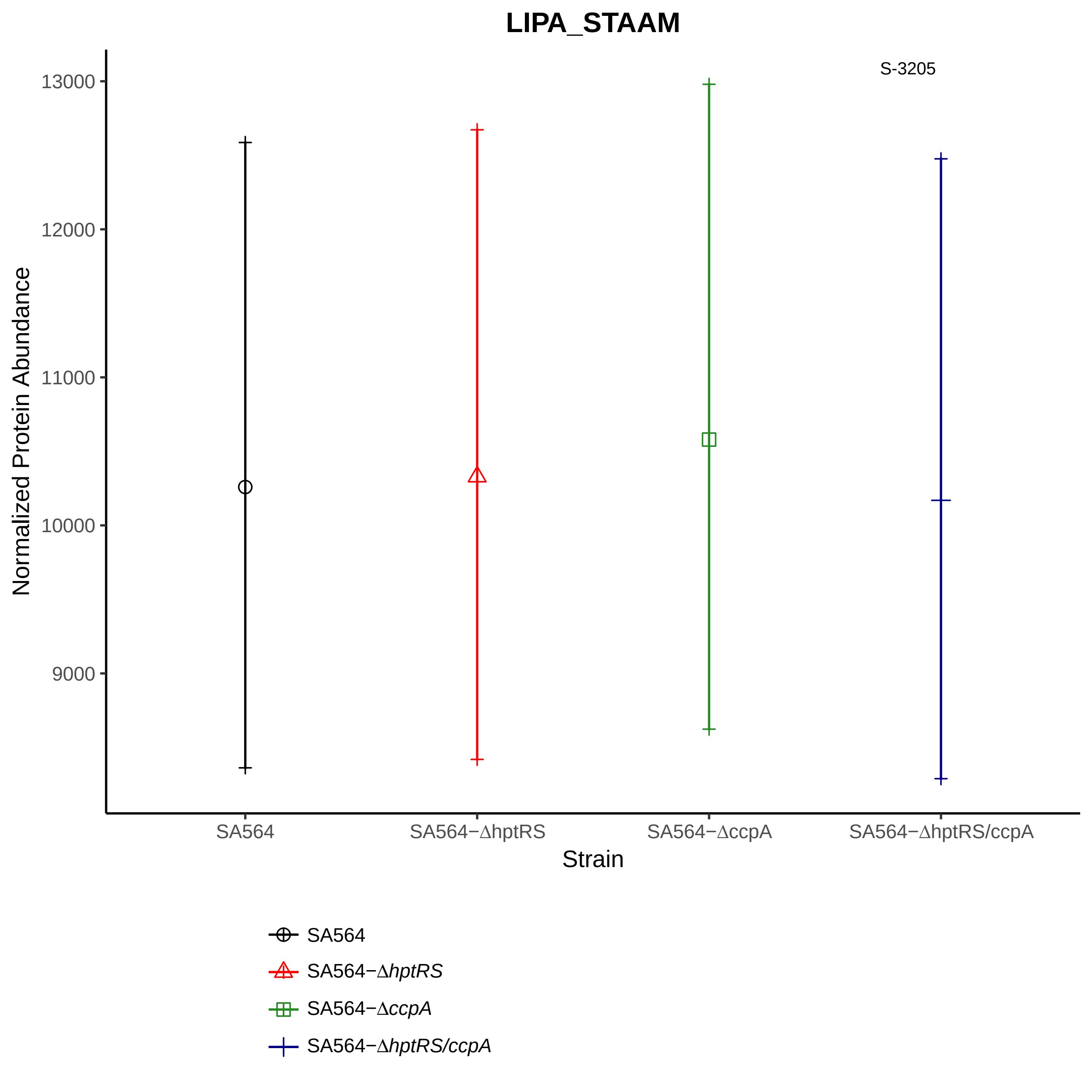
LIPA_STAAM
S-3205
Normalized Protein Abundance
13000
12000
11000
10000
9000
SA564
SA564−ΔhptRS
SA564−ΔccpA
SA564−ΔhptRS/ccpA
Strain
SA564
SA564−ΔhptRS
SA564−ΔccpA
SA564−ΔhptRS/ccpA

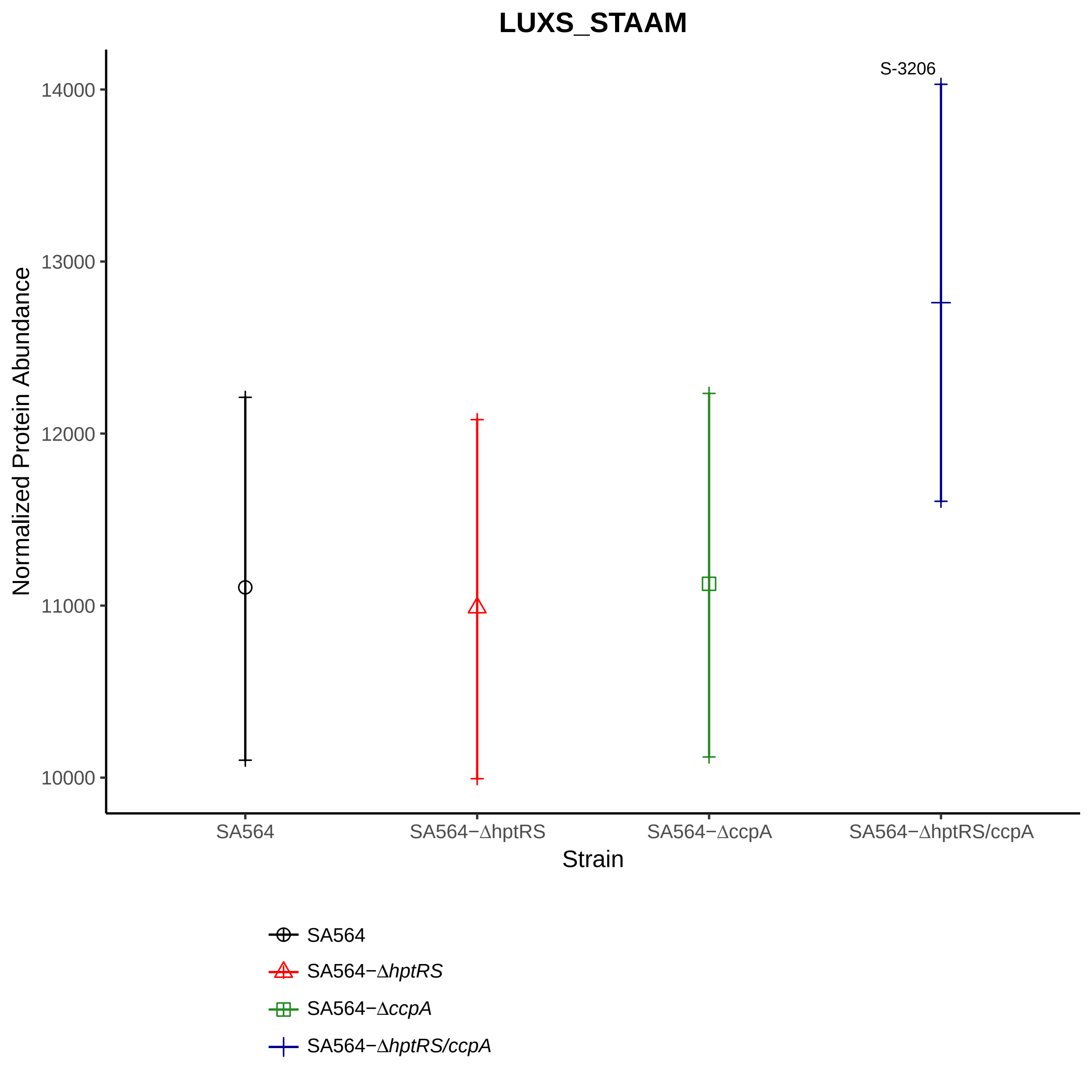

LUXS_STAAM
S-3206
Normalized Protein Abundance
14000
13000
12000
11000
10000
SA564
SA564−ΔhptRS
SA564−ΔccpA
SA564−ΔhptRS/ccpA
Strain
SA564
SA564−Δ*hptRS*
SA564−Δ*ccpA*
SA564−Δ*hptRS/ccpA*

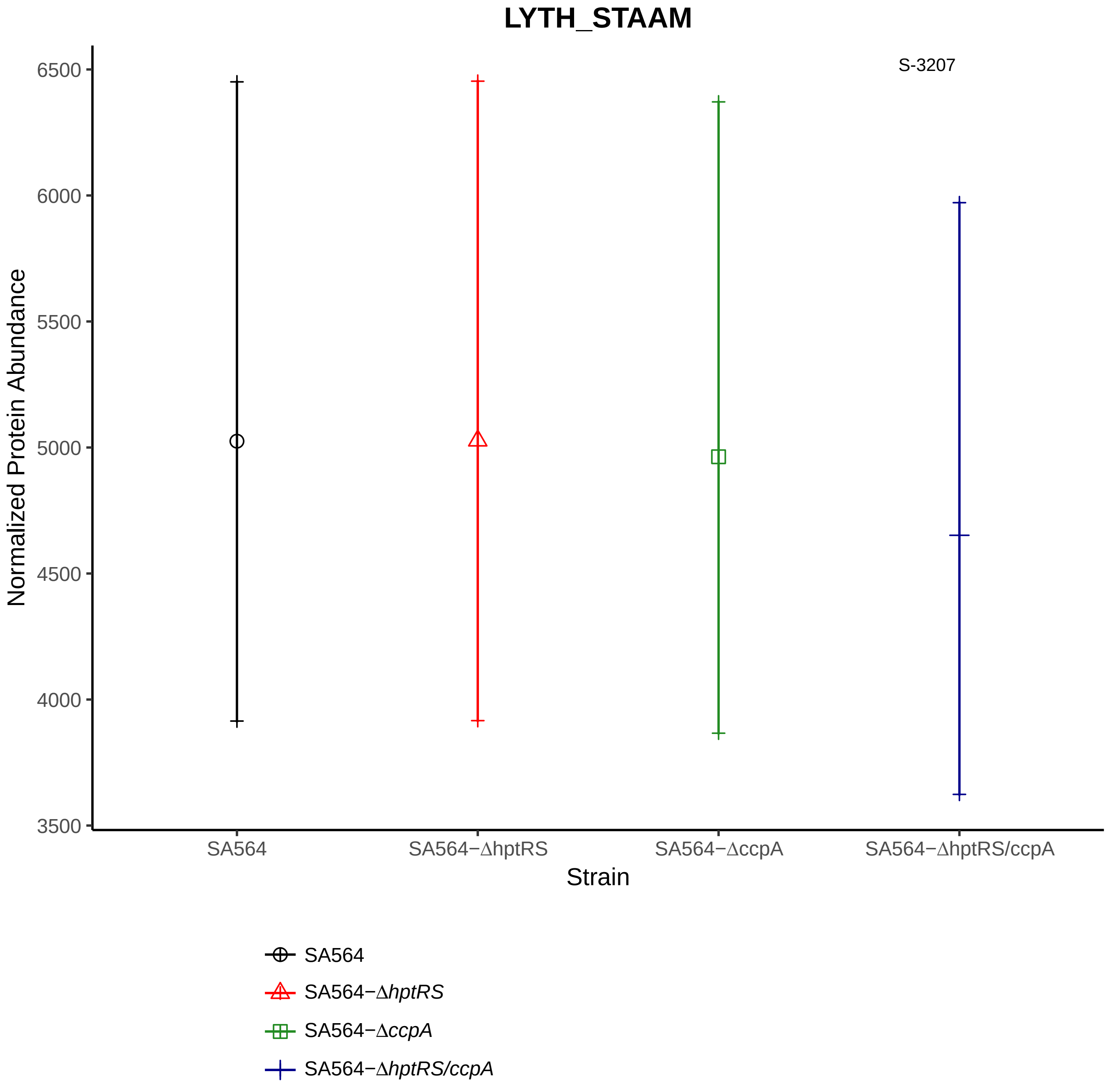

LYTH_STAAM
S-3207
Normalized Protein Abundance
6500
6000
5500
5000
4500
4000
3500
SA564
SA564−ΔhptRS
SA564−ΔccpA
SA564−ΔhptRS/ccpA
Strain
SA564
SA564−ΔhptRS
SA564−ΔccpA
SA564−ΔhptRS/ccpA

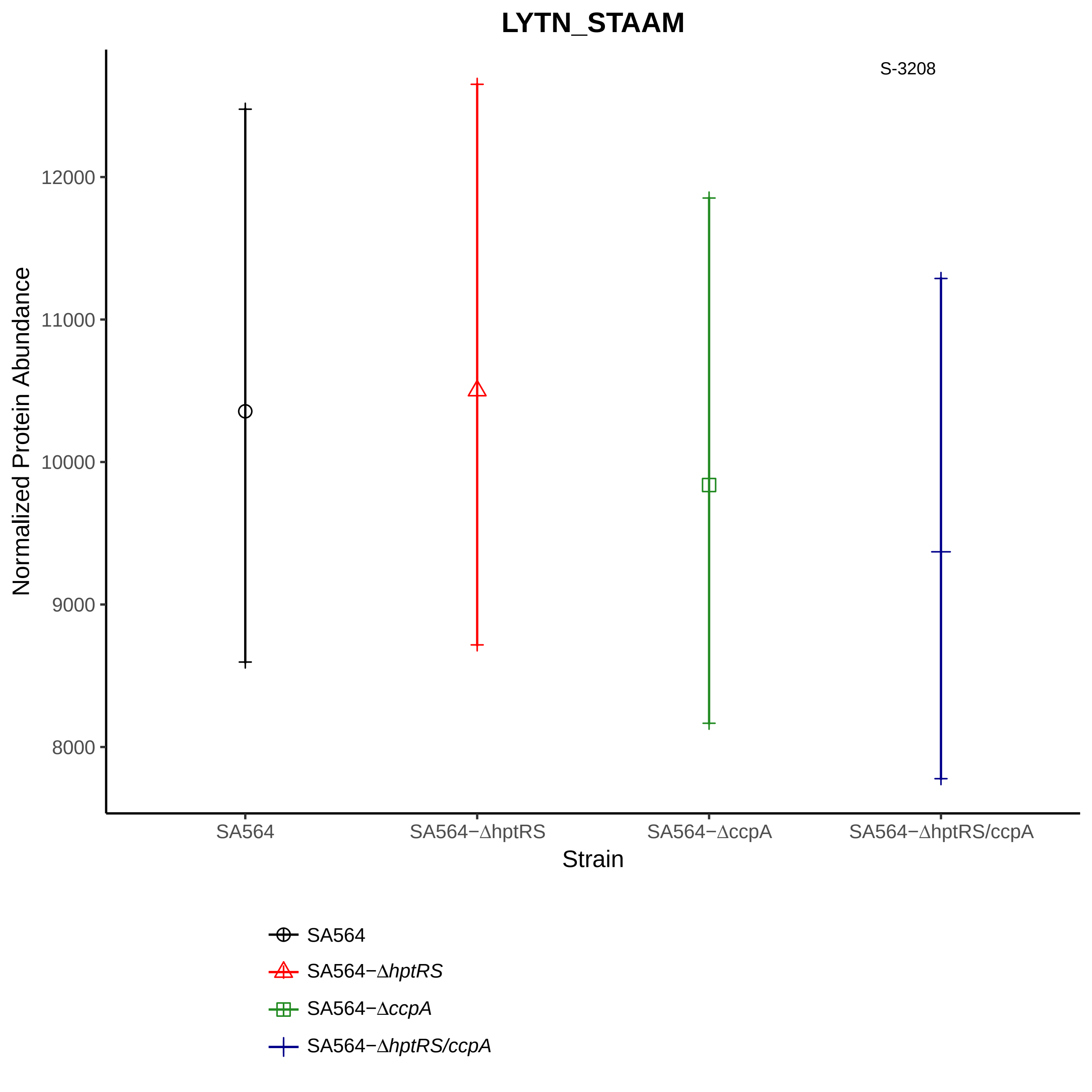

LYTN_STAAM
S-3208
Normalized Protein Abundance
12000
11000
10000
9000
8000
SA564
SA564−ΔhptRS
SA564−ΔccpA
SA564−ΔhptRS/ccpA
Strain
SA564
SA564−ΔhptRS
SA564−ΔccpA
SA564−ΔhptRS/ccpA

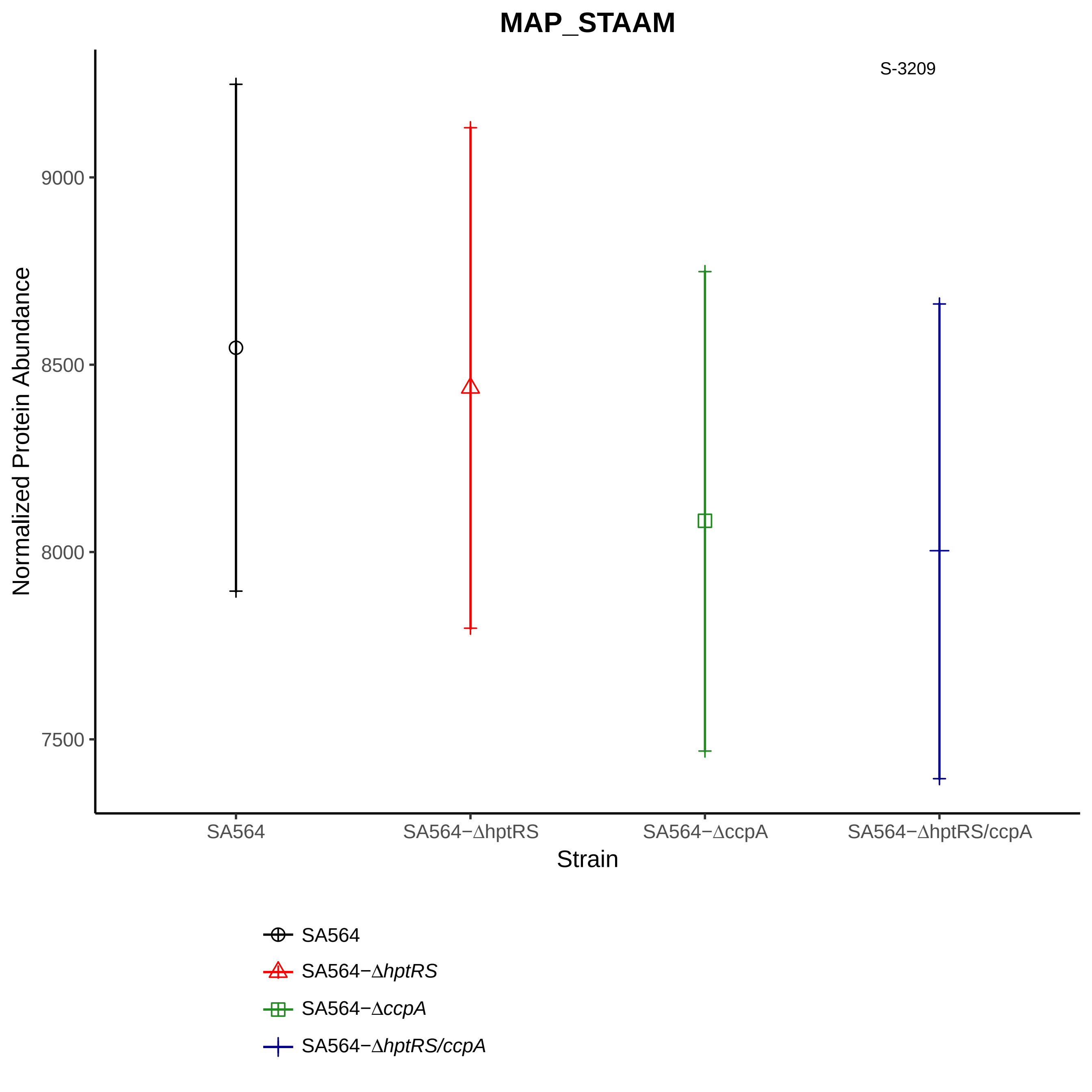

MAP_STAAM
S-3209
Normalized Protein Abundance
9000
8500
8000
7500
SA564
SA564−ΔhptRS
SA564−ΔccpA
SA564−ΔhptRS/ccpA
Strain
SA564
SA564−ΔhptRS
SA564−ΔccpA
SA564−ΔhptRS/ccpA

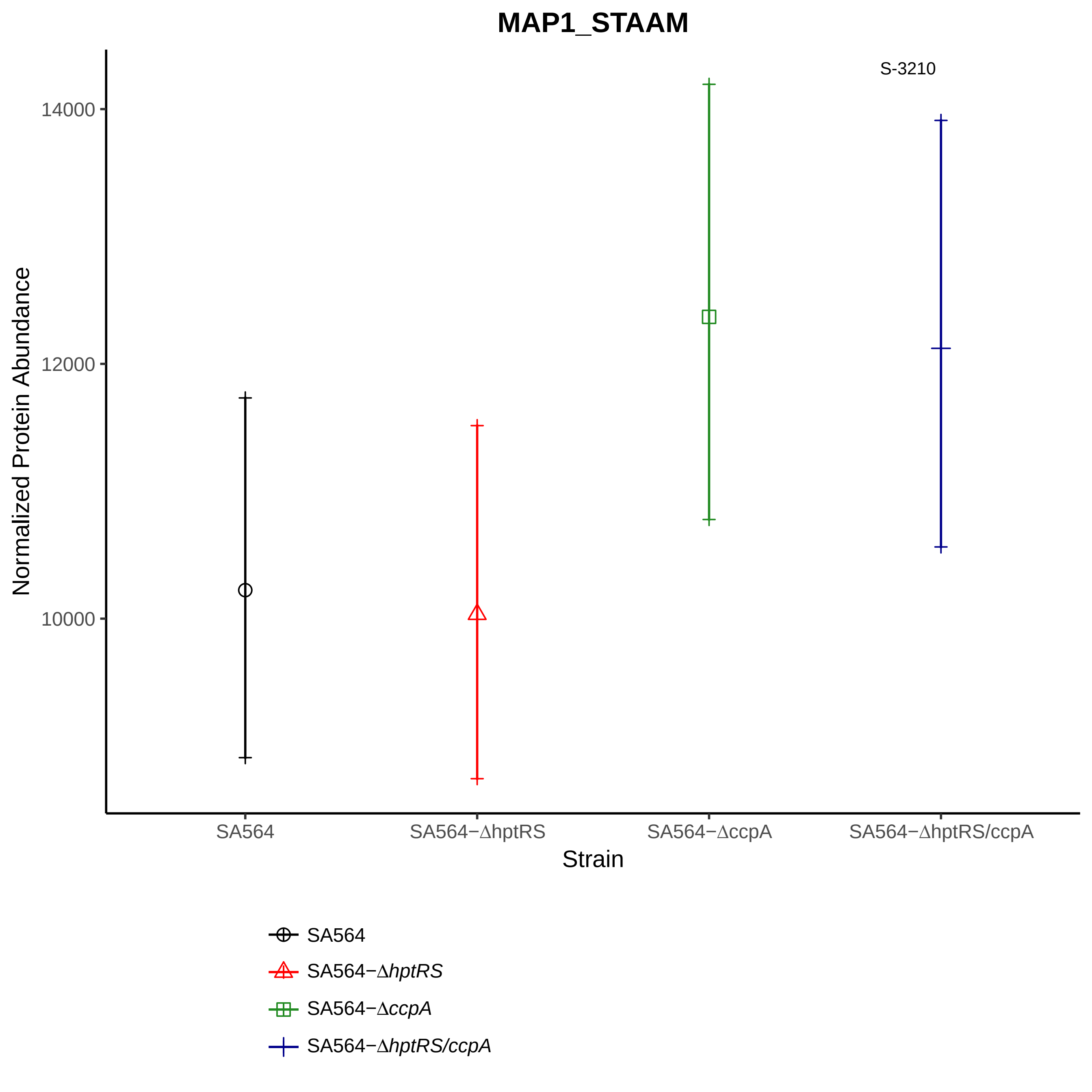

MAP1_STAAM
S-3210
Normalized Protein Abundance
14000
12000
10000
SA564
SA564−ΔhptRS
SA564−ΔccpA
SA564−ΔhptRS/ccpA
Strain
SA564
SA564−ΔhptRS
SA564−ΔccpA
SA564−ΔhptRS/ccpA

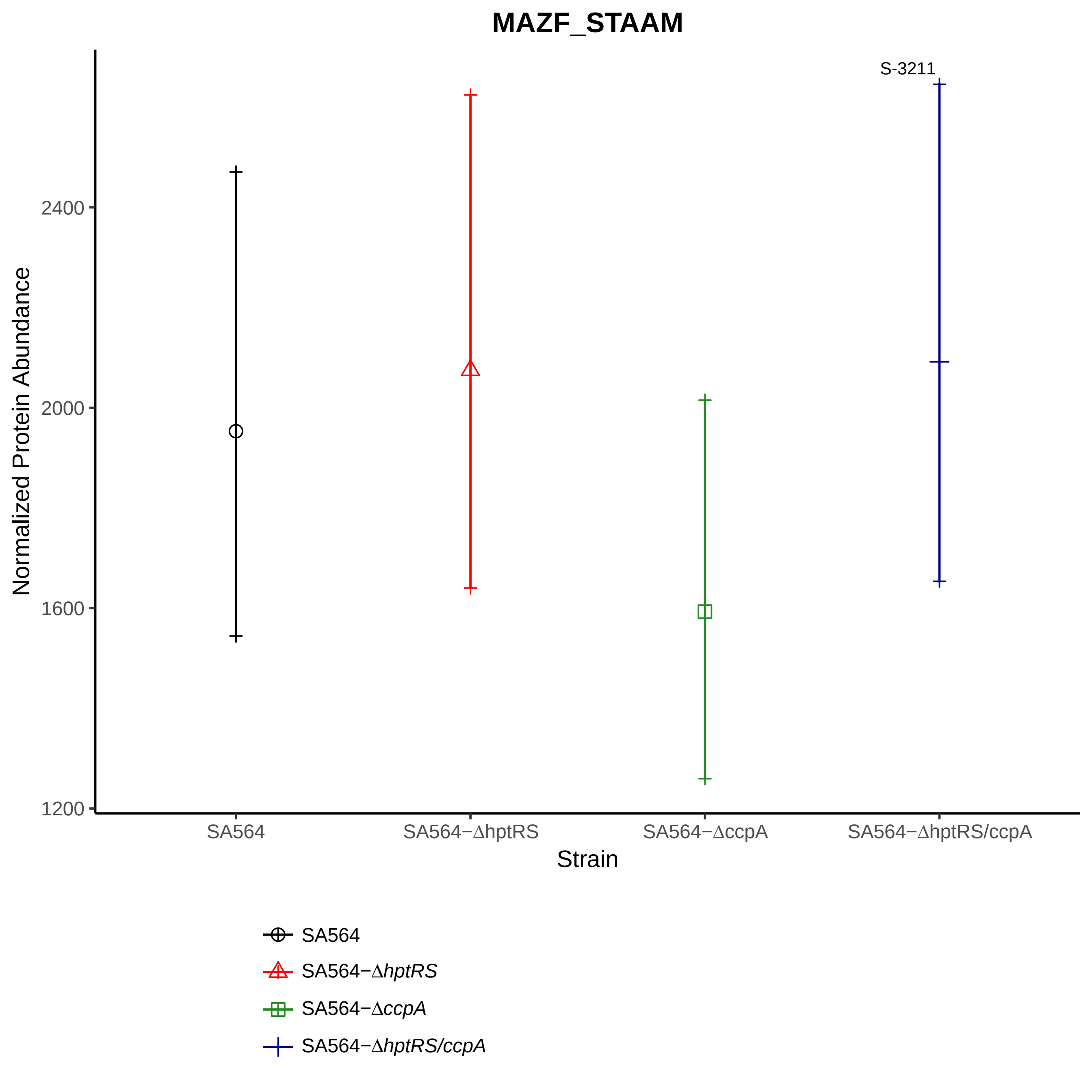

MAZF_STAAM
S-3211
Normalized Protein Abundance
2400
2000
1600
1200
SA564
SA564−ΔhptRS
SA564−ΔccpA
SA564−ΔhptRS/ccpA
Strain
SA564
SA564−ΔhptRS
SA564−ΔccpA
SA564−ΔhptRS/ccpA

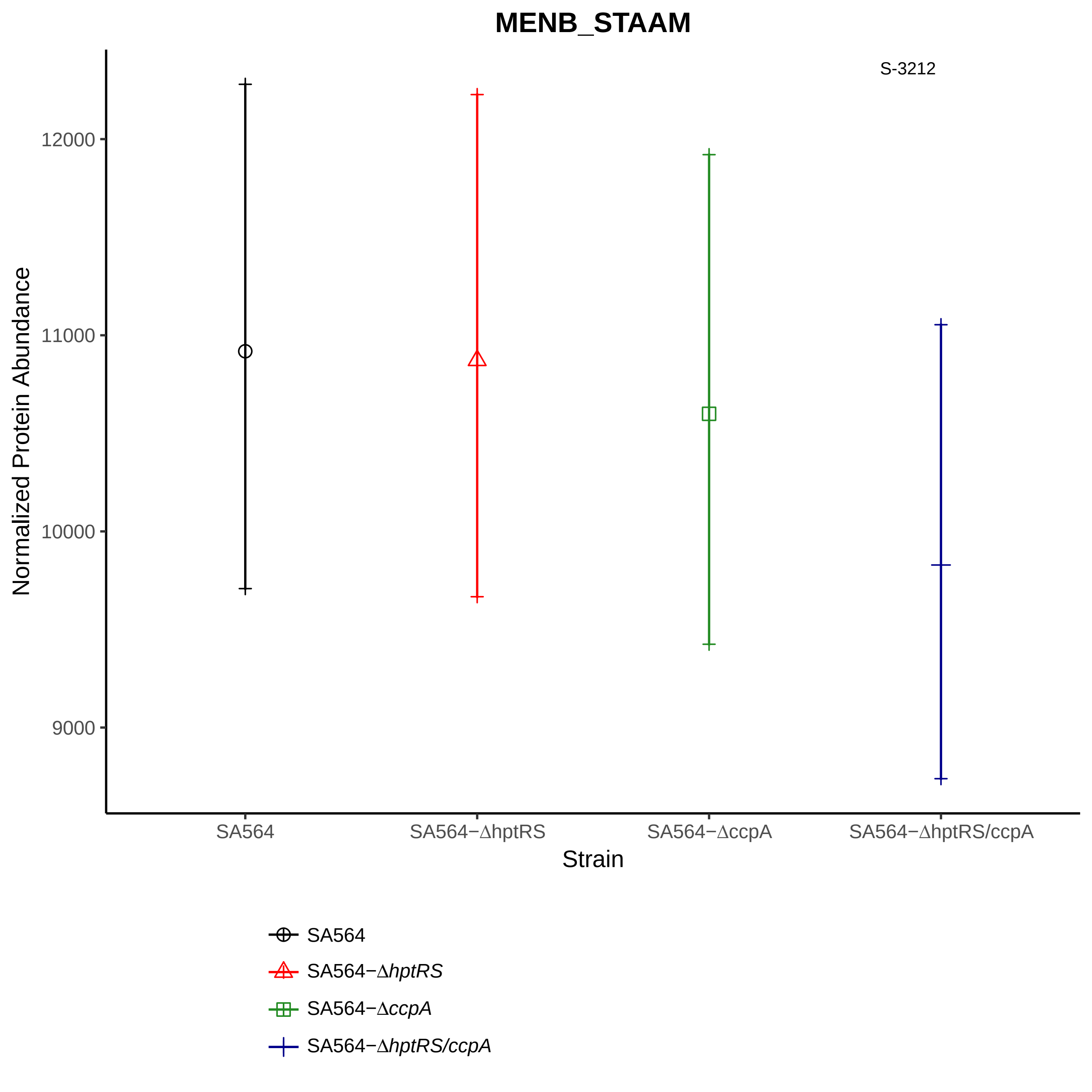
MENB_STAAM
S-3212
Normalized Protein Abundance
12000
11000
10000
9000
SA564
SA564−ΔhptRS
SA564−ΔccpA
SA564−ΔhptRS/ccpA
Strain
SA564
SA564−ΔhptRS
SA564−ΔccpA
SA564−ΔhptRS/ccpA

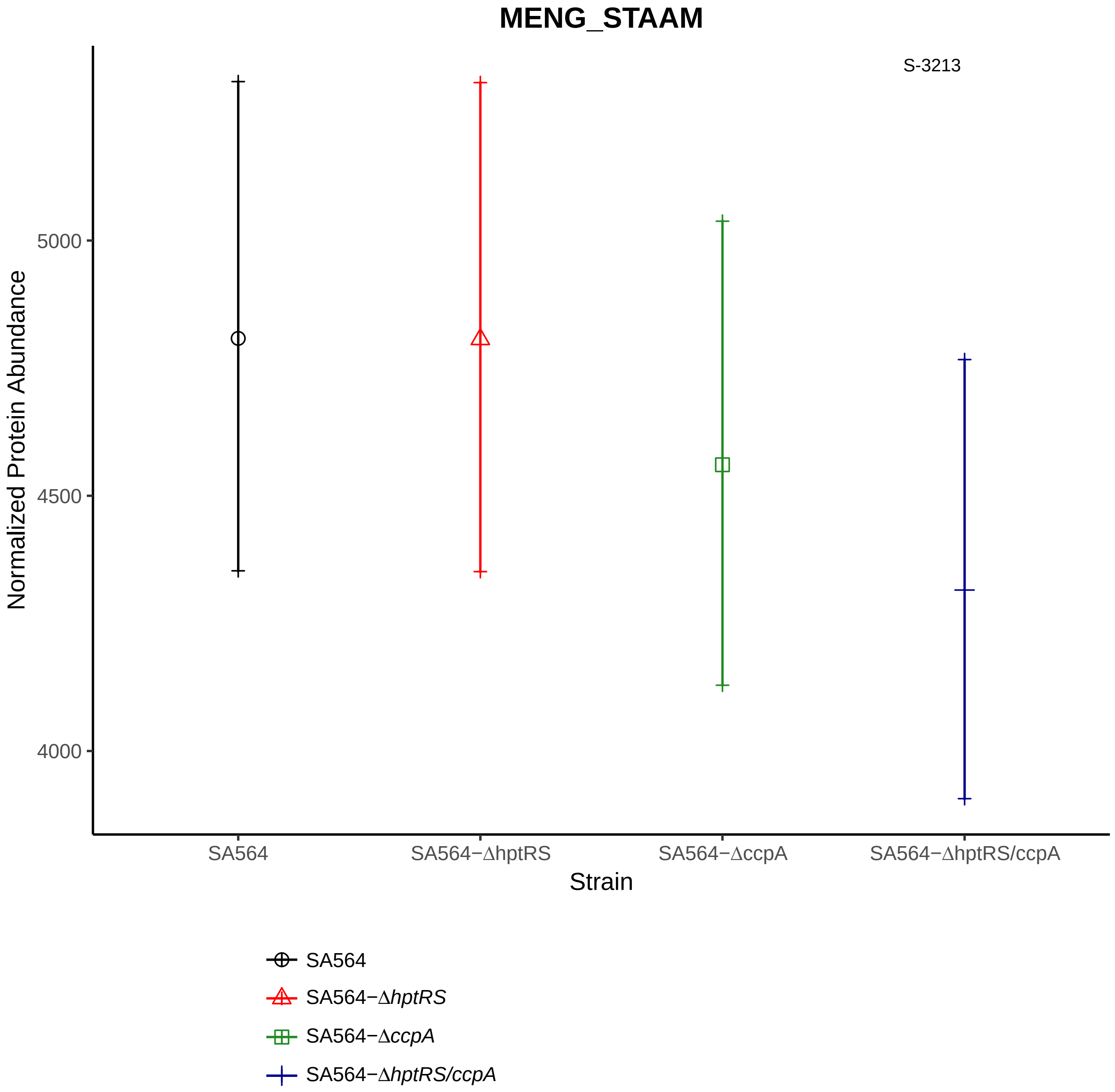

MENG_STAAM
S-3213
Normalized Protein Abundance
5000
4500
4000
SA564
SA564−ΔhptRS
SA564−ΔccpA
SA564−ΔhptRS/ccpA
Strain
SA564
SA564−ΔhptRS
SA564−ΔccpA
SA564−ΔhptRS/ccpA

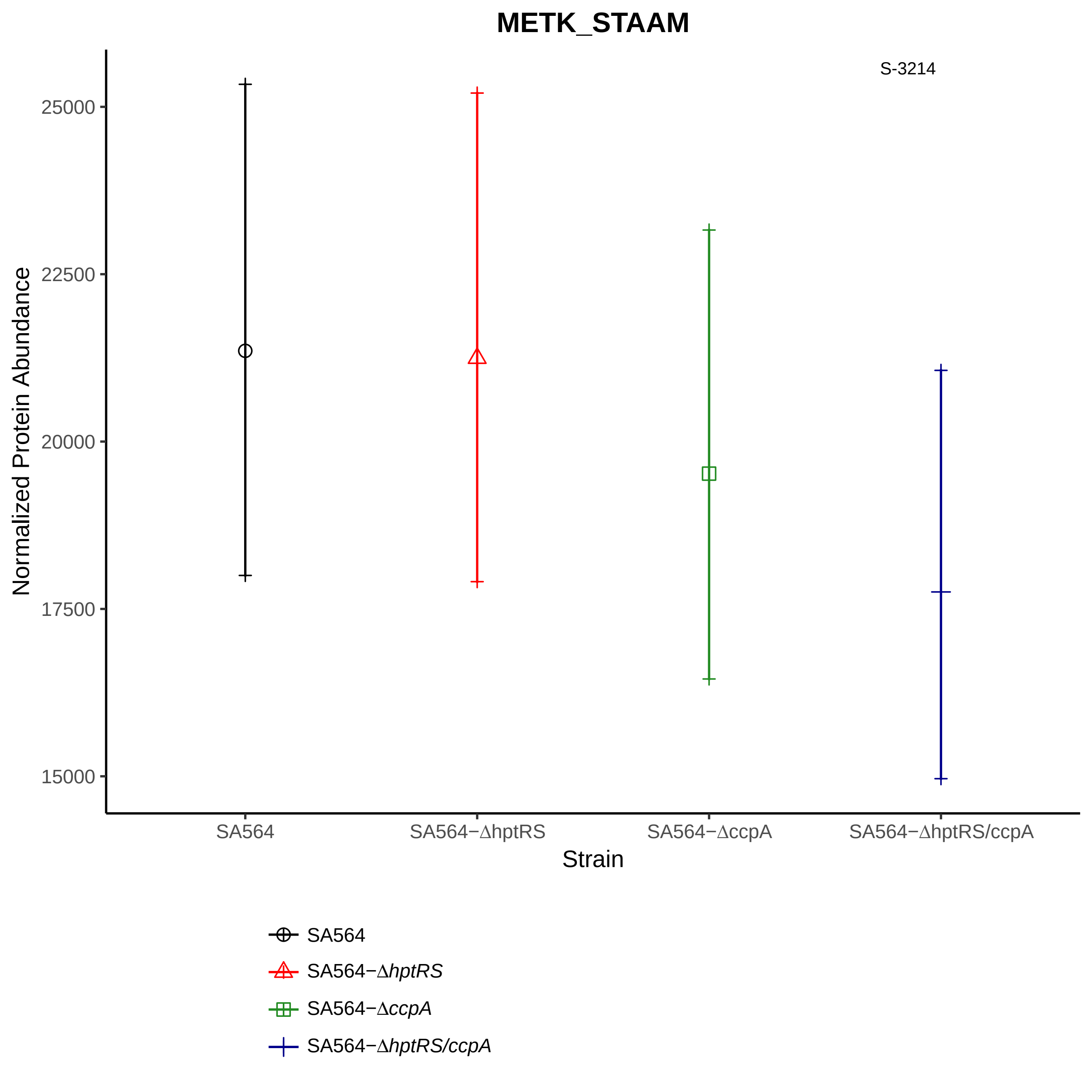

METK_STAAM
S-3214
Normalized Protein Abundance
25000
22500
20000
17500
15000
SA564
SA564−ΔhptRS
SA564−ΔccpA
SA564−ΔhptRS/ccpA
Strain
SA564
SA564−ΔhptRS
SA564−ΔccpA
SA564−ΔhptRS/ccpA

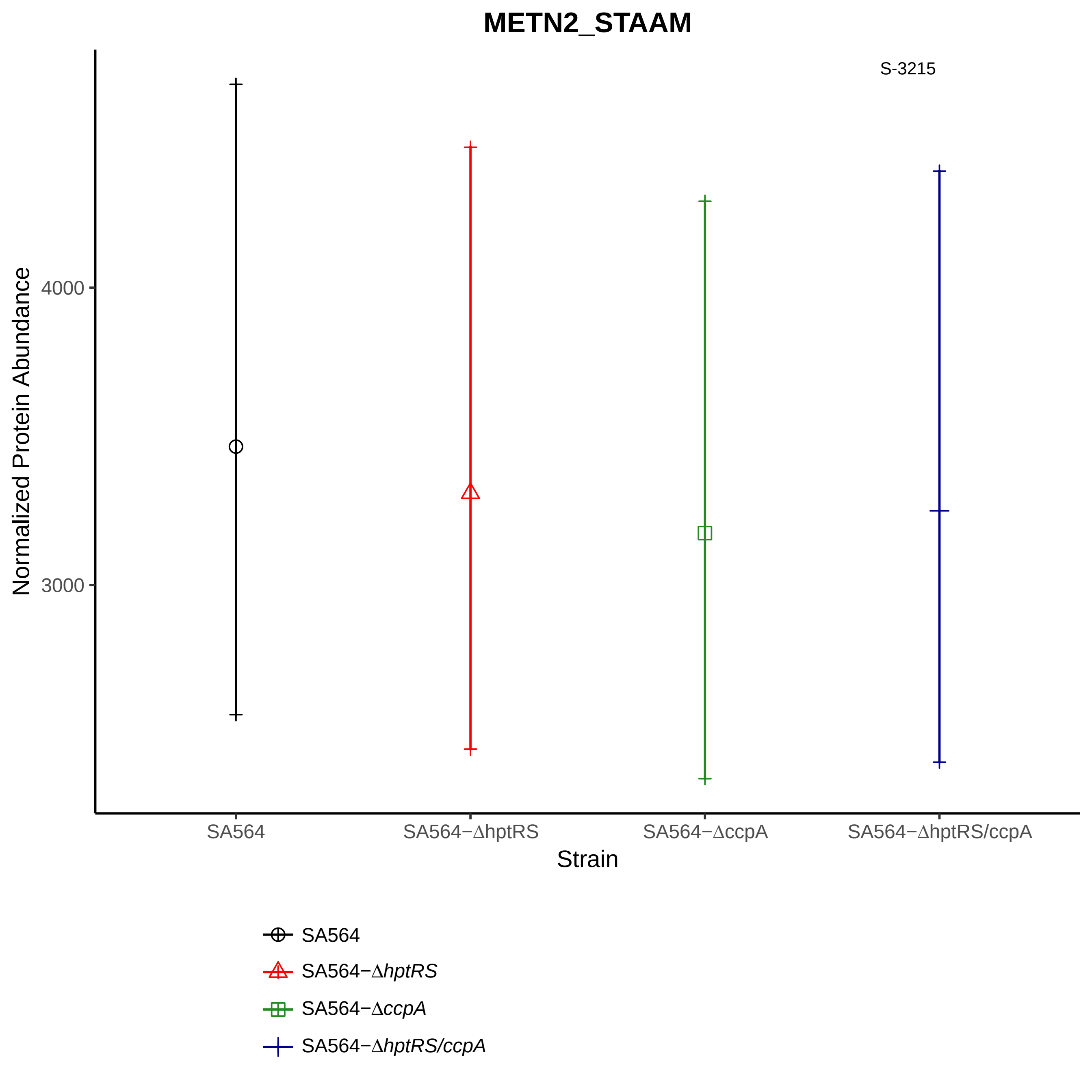

METN2_STAAM
S-3215
Normalized Protein Abundance
4000
3000
SA564
SA564−ΔhptRS
SA564−ΔccpA
SA564−ΔhptRS/ccpA
Strain
SA564
SA564−ΔhptRS
SA564−ΔccpA
SA564−ΔhptRS/ccpA

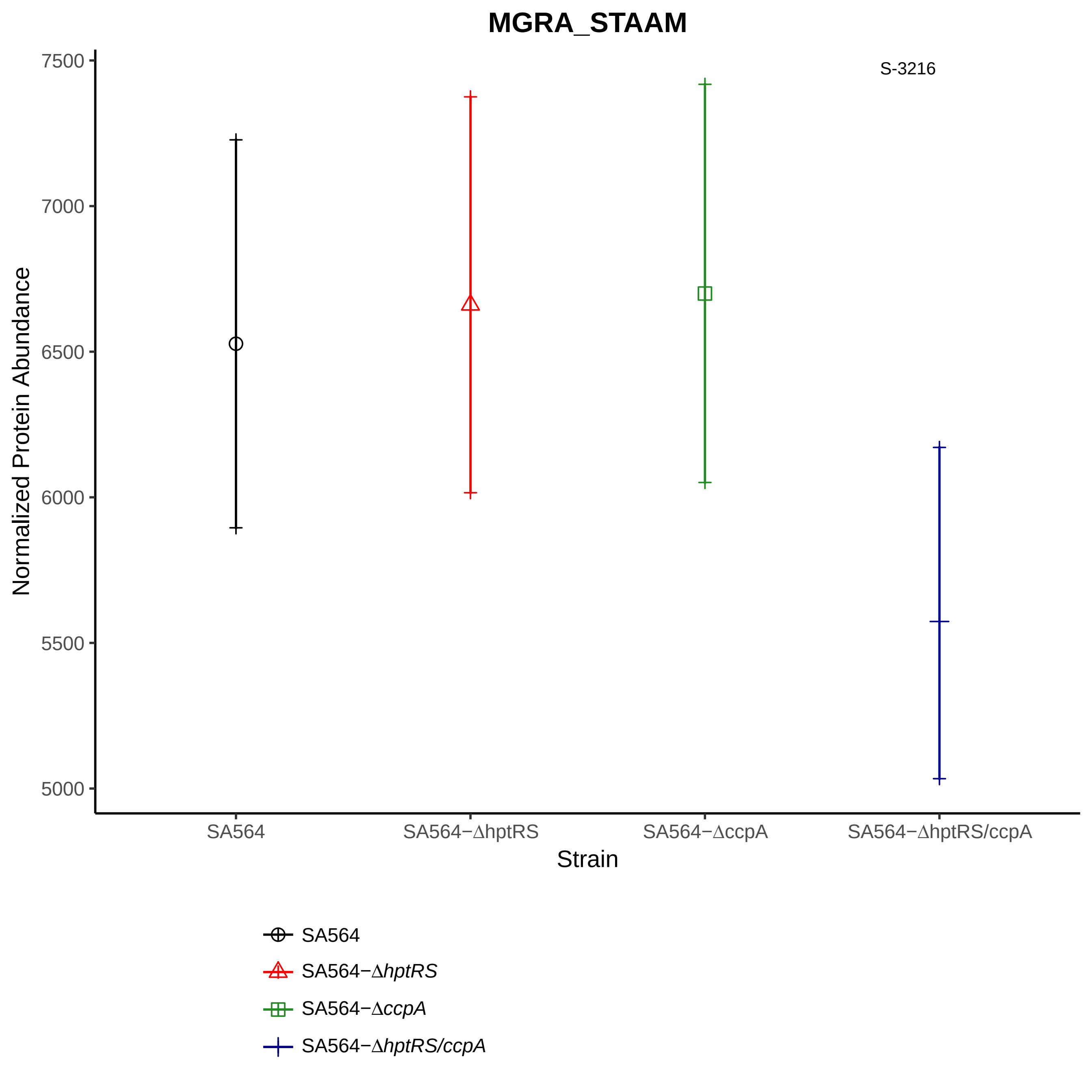
MGRA_STAAM
S-3216
Normalized Protein Abundance
7500
7000
6500
6000
5500
5000
SA564
SA564−ΔhptRS
SA564−ΔccpA
SA564−ΔhptRS/ccpA
Strain
SA564
SA564−ΔhptRS
SA564−ΔccpA
SA564−ΔhptRS/ccpA

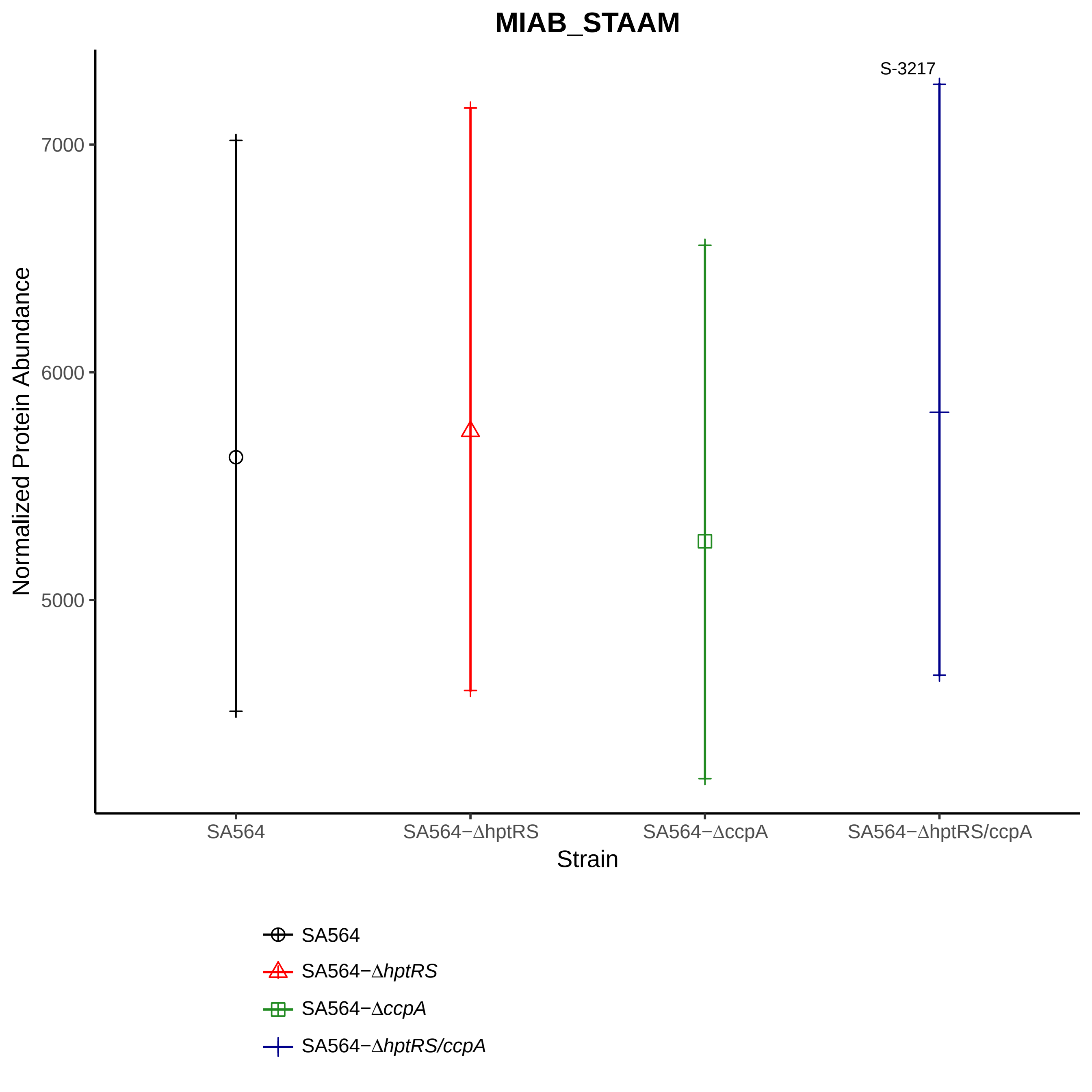

MIAB_STAAM
S-3217
Normalized Protein Abundance
7000
6000
5000
SA564
SA564−ΔhptRS
SA564−ΔccpA
SA564−ΔhptRS/ccpA
Strain
SA564
SA564−ΔhptRS
SA564−ΔccpA
SA564−ΔhptRS/ccpA

MNME_STAAM

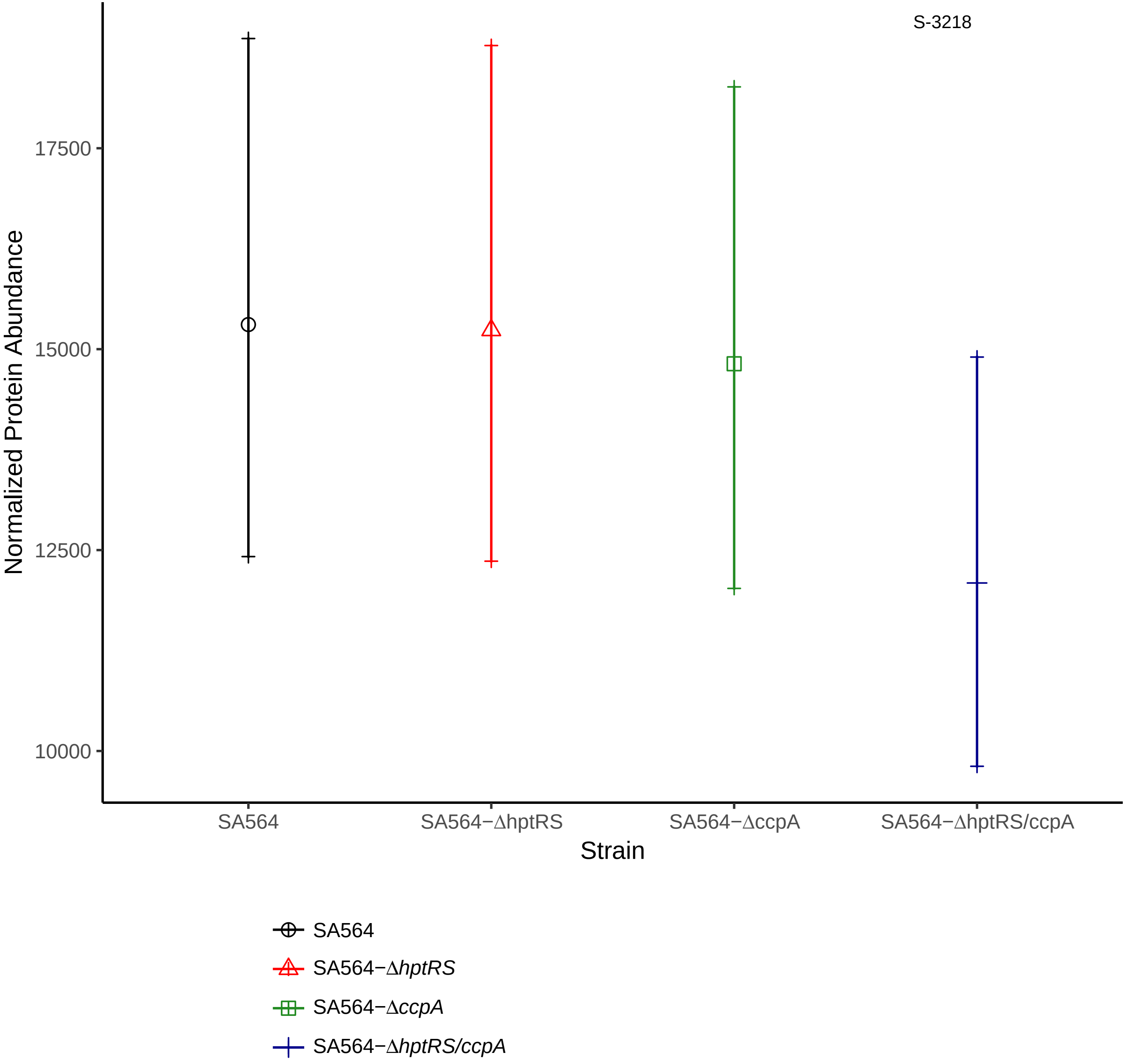

S-3218
17500
15000
12500
10000
Normalized Protein Abundance
SA564
SA564−ΔhptRS
SA564−ΔccpA
SA564−ΔhptRS/ccpA
Strain
SA564
SA564−ΔhptRS
SA564−ΔccpA
SA564−ΔhptRS/ccpA

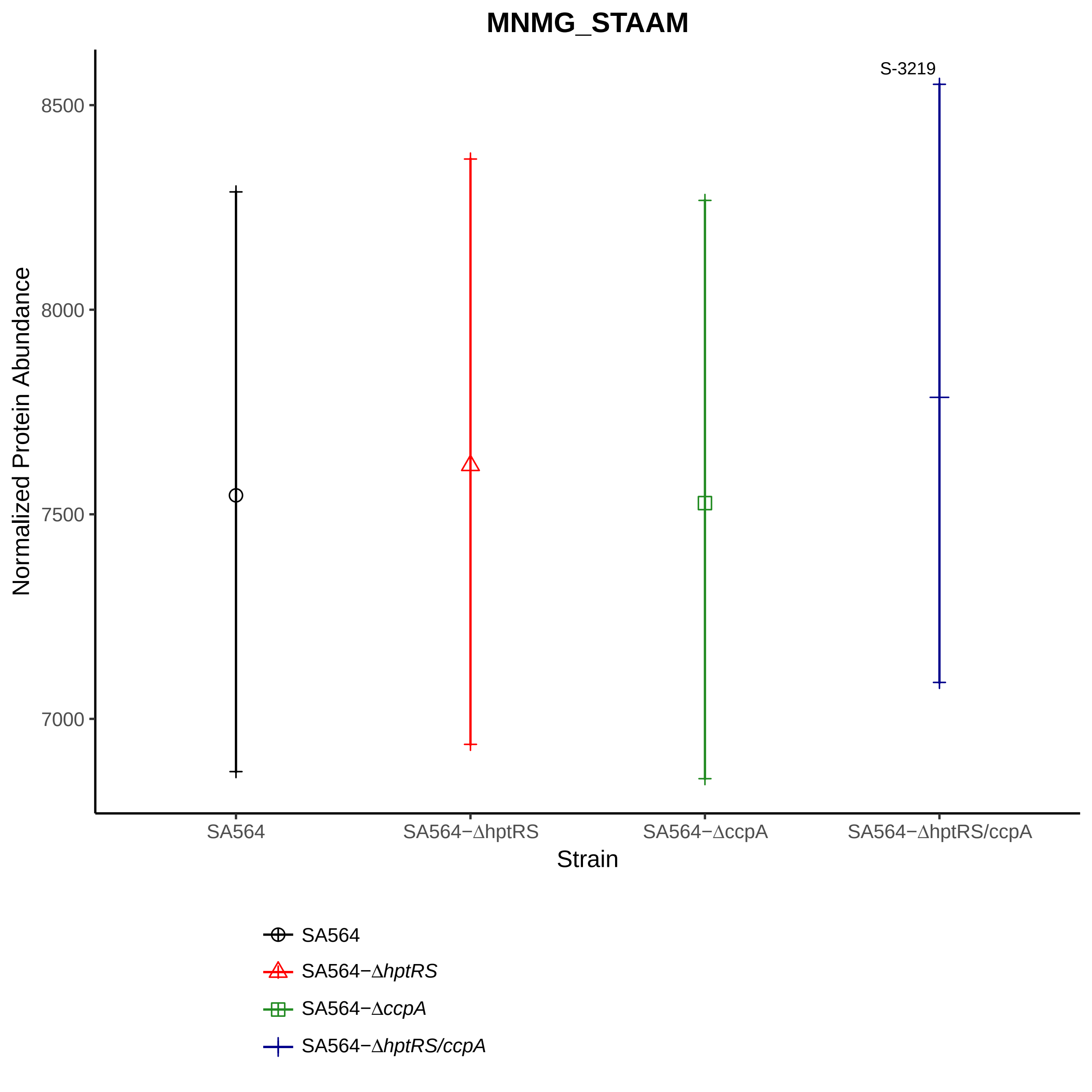

MNMG_STAAM
S-3219
8500
8000
7500
7000
Normalized Protein Abundance
SA564
SA564−ΔhptRS
SA564−ΔccpA
SA564−ΔhptRS/ccpA
Strain
SA564
SA564−ΔhptRS
SA564−ΔccpA
SA564−ΔhptRS/ccpA

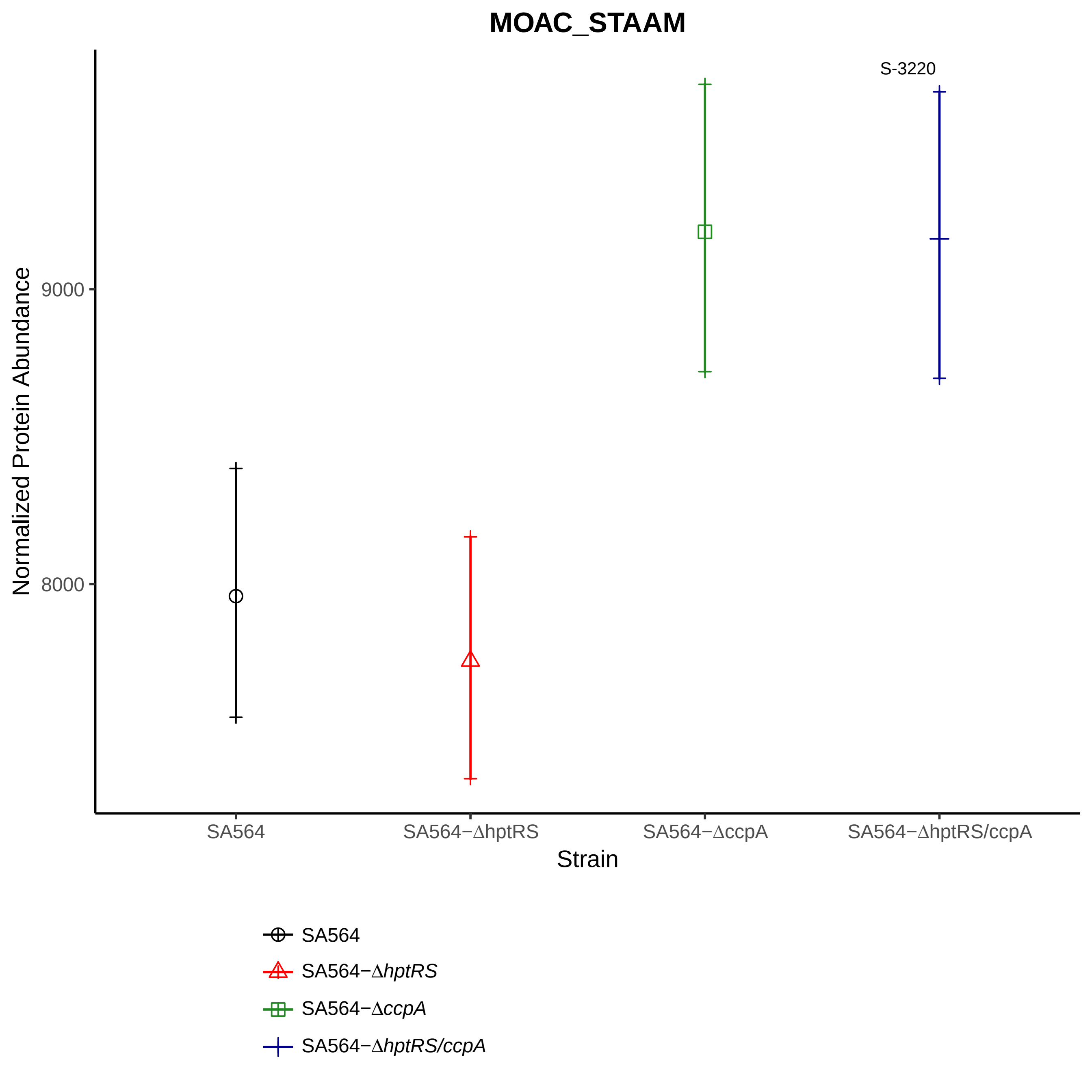
MOAC_STAAM
S-3220
Normalized Protein Abundance
9000
8000
SA564
SA564−ΔhptRS
SA564−ΔccpA
SA564−ΔhptRS/ccpA
Strain
SA564
SA564−ΔhptRS
SA564−ΔccpA
SA564−ΔhptRS/ccpA

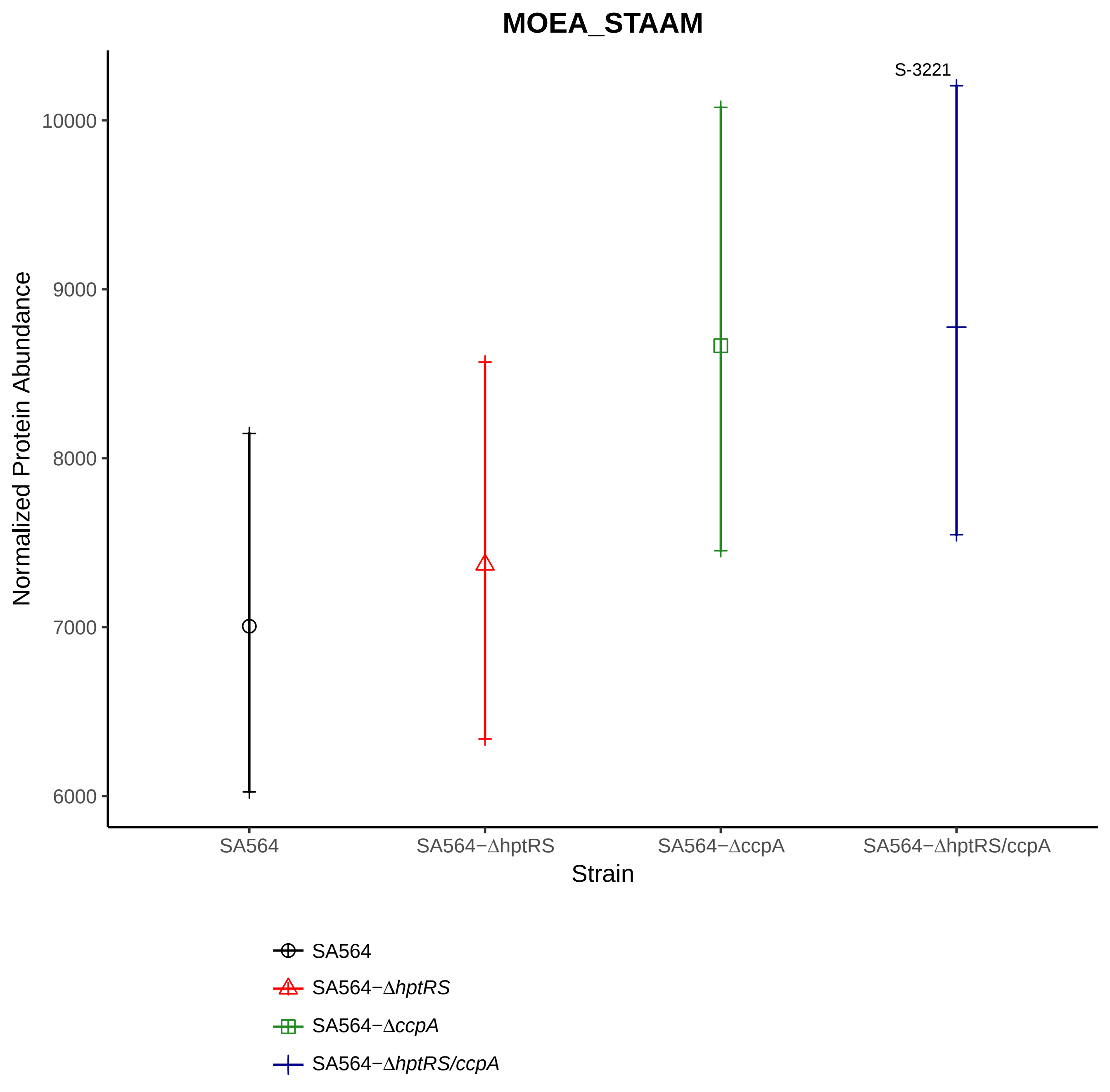
MOEA_STAAM
S-3221
10000
9000
8000
7000
6000
Normalized Protein Abundance
SA564
SA564−ΔhptRS
SA564−ΔccpA
SA564−ΔhptRS/ccpA
Strain
SA564
SA564−Δ*hptRS*
SA564−Δ*ccpA*
SA564−Δ*hptRS/ccpA*

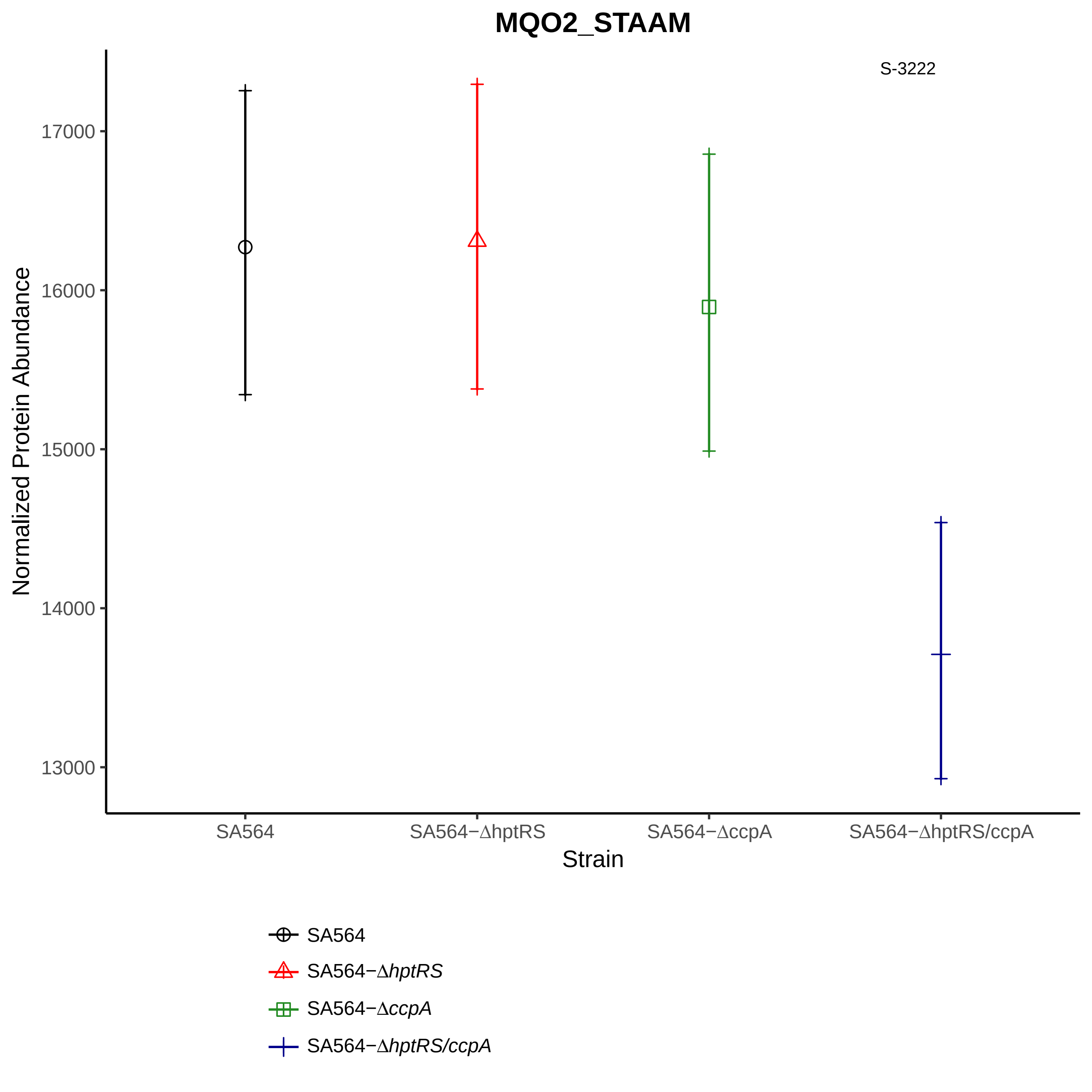

MQO2_STAAM
S-3222
Normalized Protein Abundance
17000
16000
15000
14000
13000
SA564
SA564−ΔhptRS
SA564−ΔccpA
SA564−ΔhptRS/ccpA
Strain
SA564
SA564−Δ*hptRS*
SA564−Δ*ccpA*
SA564−Δ*hptRS/ccpA*

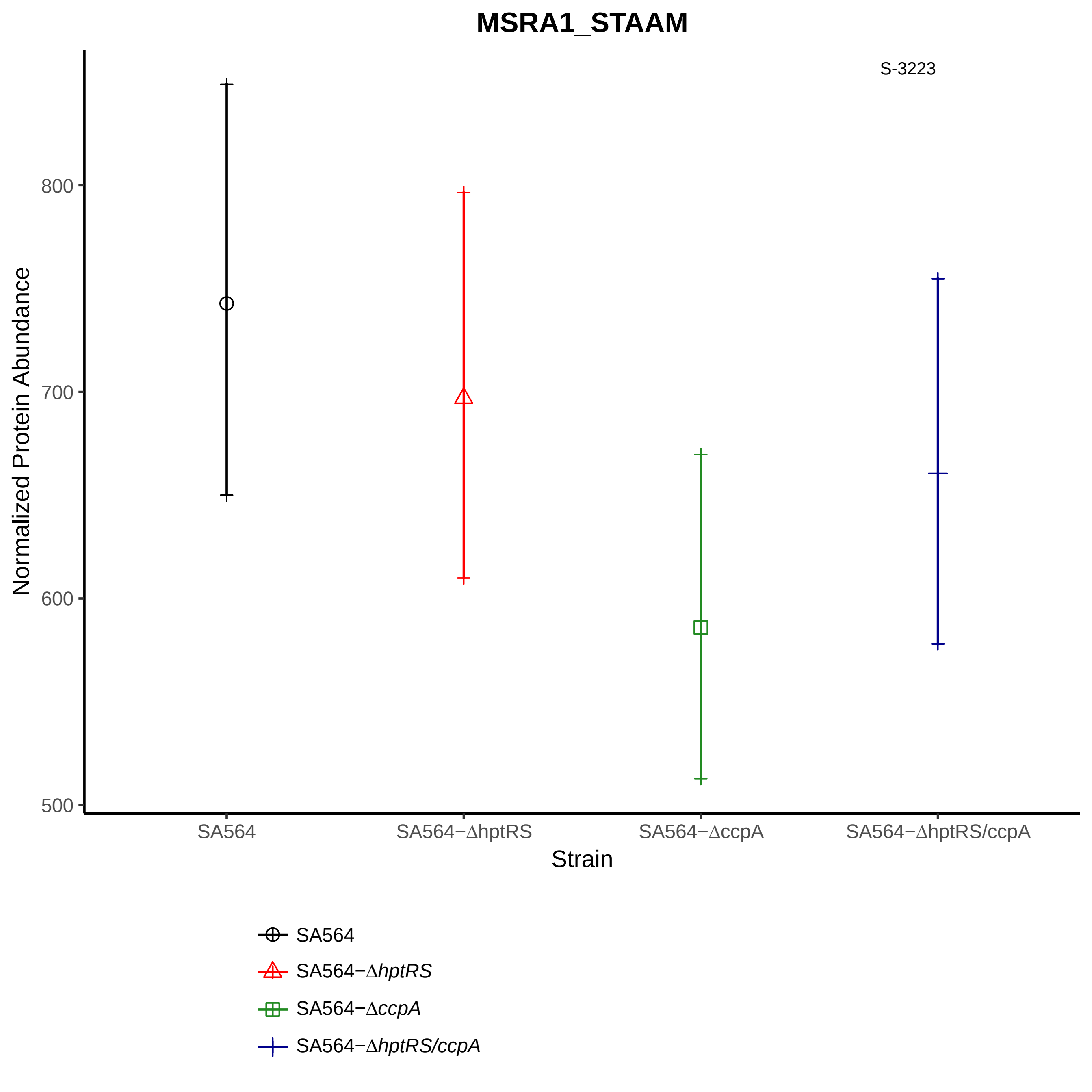
MSRA1_STAAM
S-3223
Normalized Protein Abundance
800
700
600
500
SA564
SA564−ΔhptRS
SA564−ΔccpA
SA564−ΔhptRS/ccpA
Strain
SA564
SA564−ΔhptRS
SA564−ΔccpA
SA564−ΔhptRS/ccpA

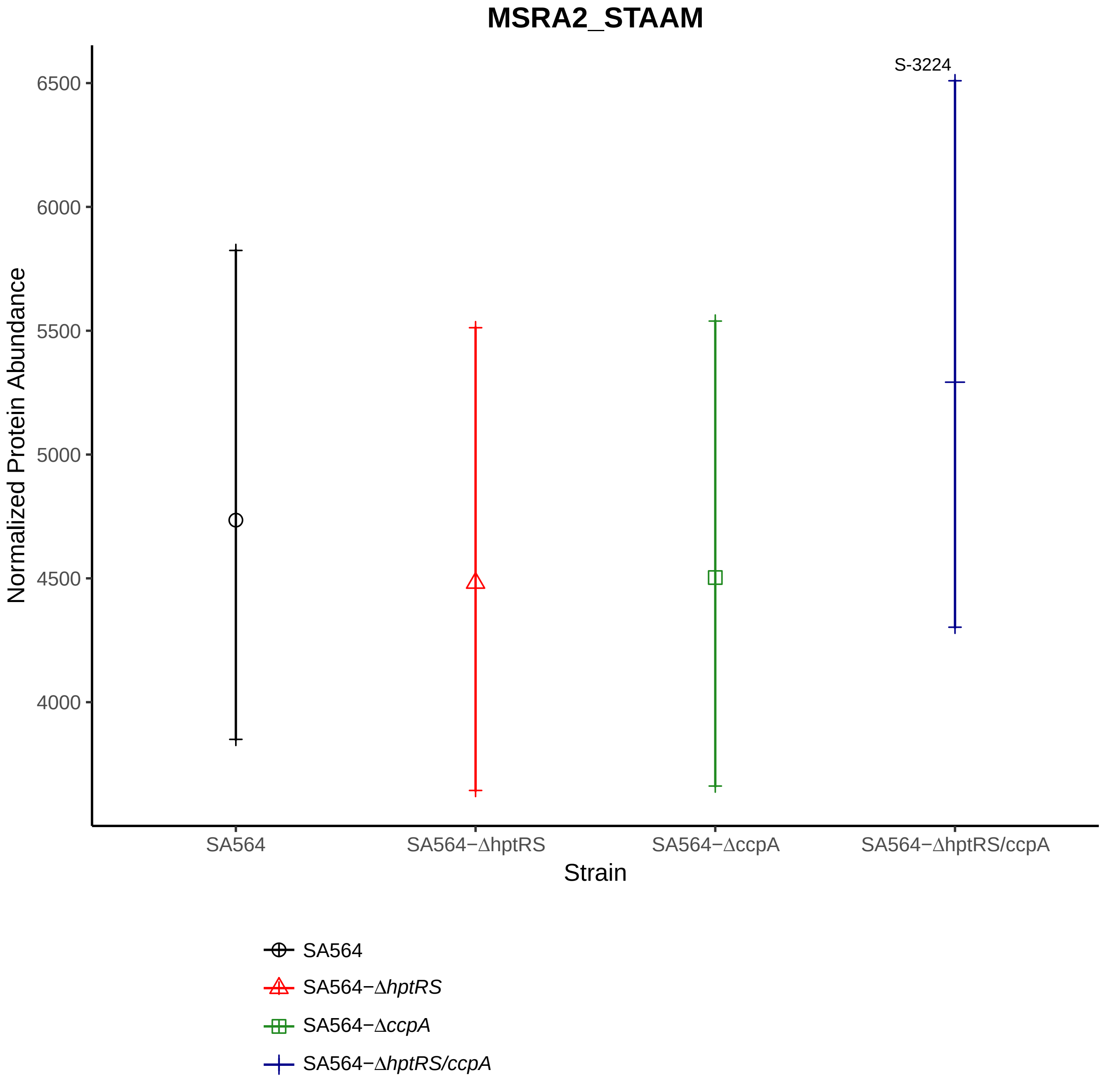

MSRA2_STAAM
S-3224
6500
6000
5500
5000
4500
4000
Normalized Protein Abundance
SA564
SA564−ΔhptRS
SA564−ΔccpA
SA564−ΔhptRS/ccpA
Strain
SA564
SA564−ΔhptRS
SA564−ΔccpA
SA564−ΔhptRS/ccpA

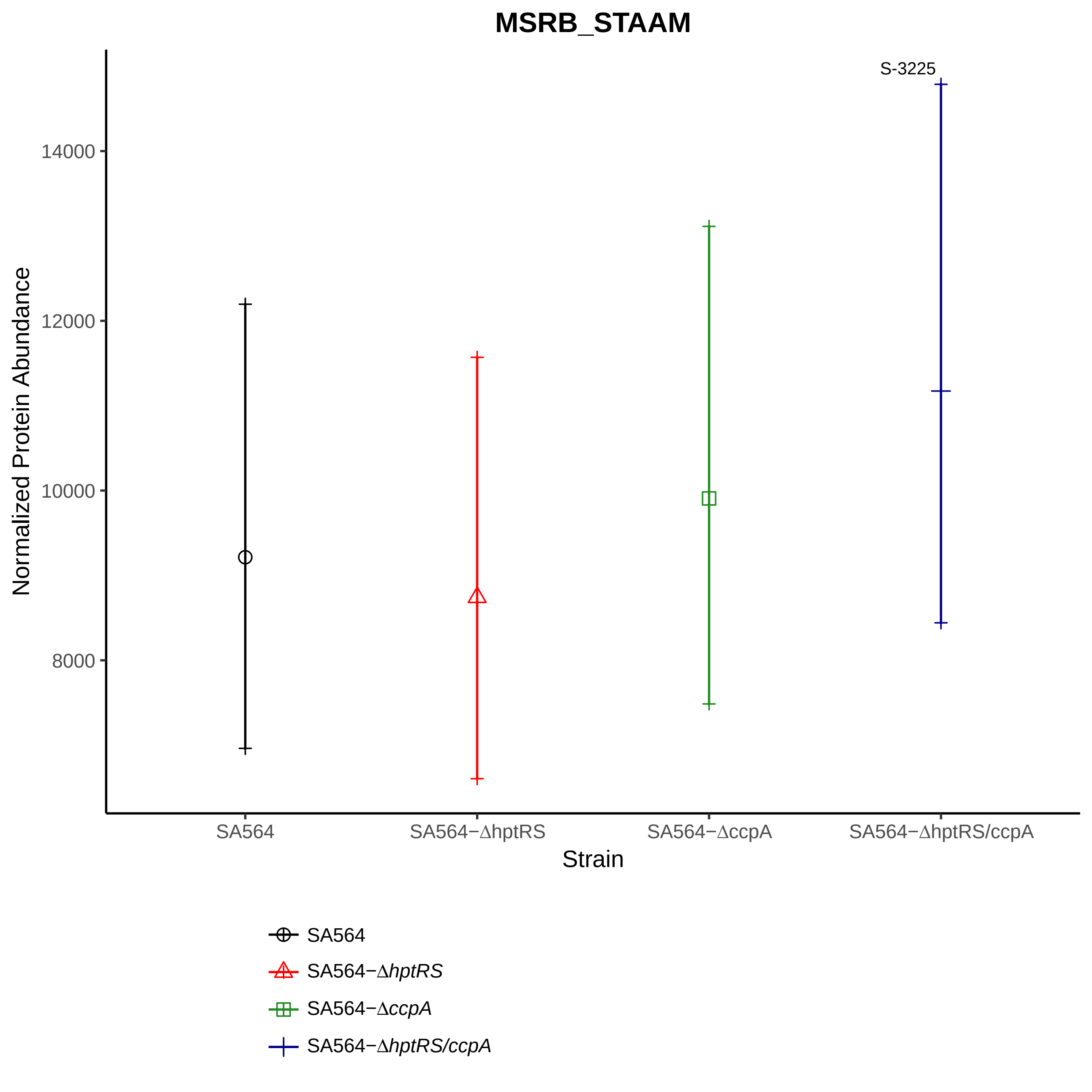

MSRB_STAAM
S-3225
Normalized Protein Abundance
14000
12000
10000
8000
SA564
SA564−ΔhptRS
SA564−ΔccpA
SA564−ΔhptRS/ccpA
Strain
SA564
SA564−ΔhptRS
SA564−ΔccpA
SA564−ΔhptRS/ccpA

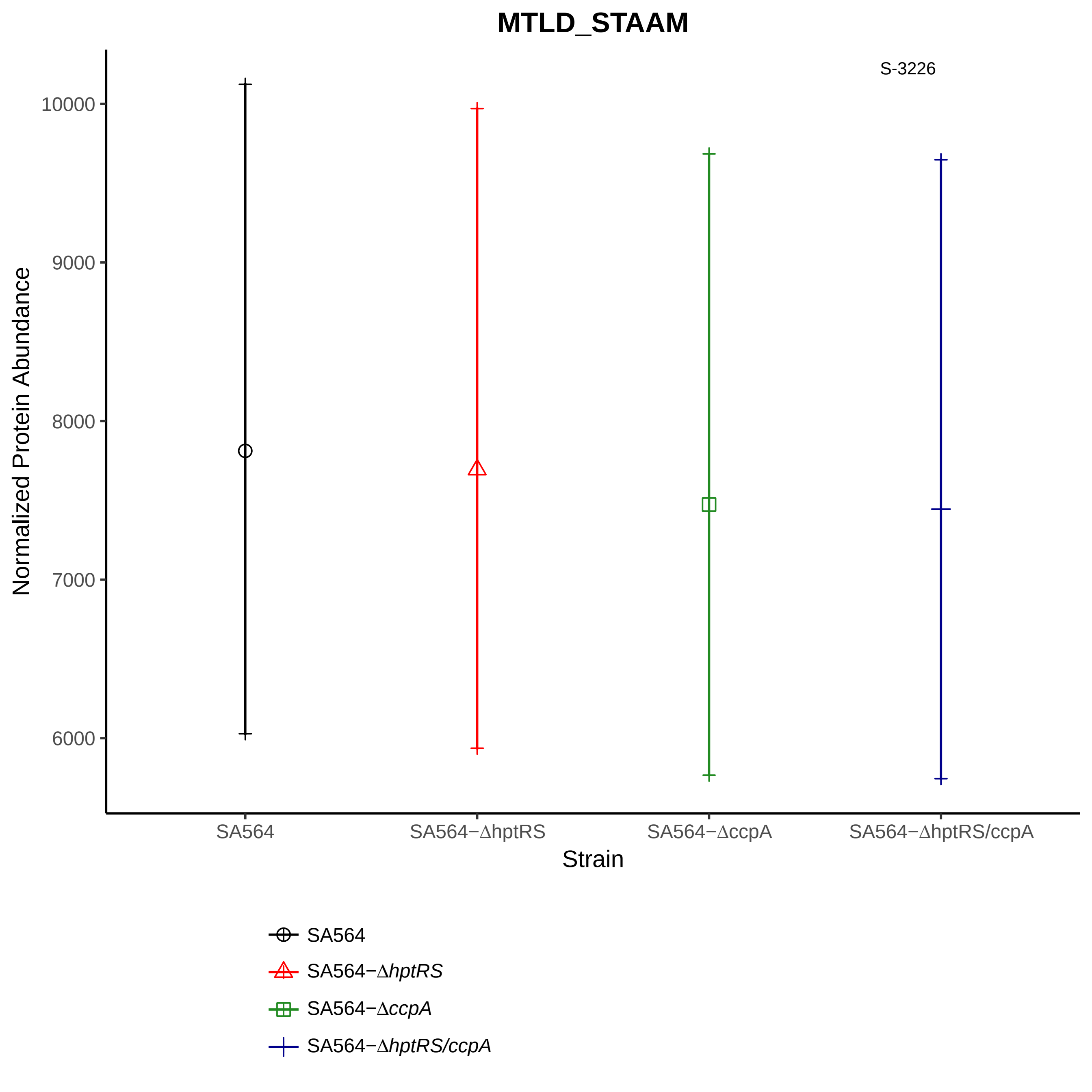

MTLD_STAAM
S-3226
Normalized Protein Abundance
10000
9000
8000
7000
6000
SA564
SA564−ΔhptRS
SA564−ΔccpA
SA564−ΔhptRS/ccpA
Strain
SA564
SA564−Δ*hptRS*
SA564−Δ*ccpA*
SA564−Δ*hptRS/ccpA*

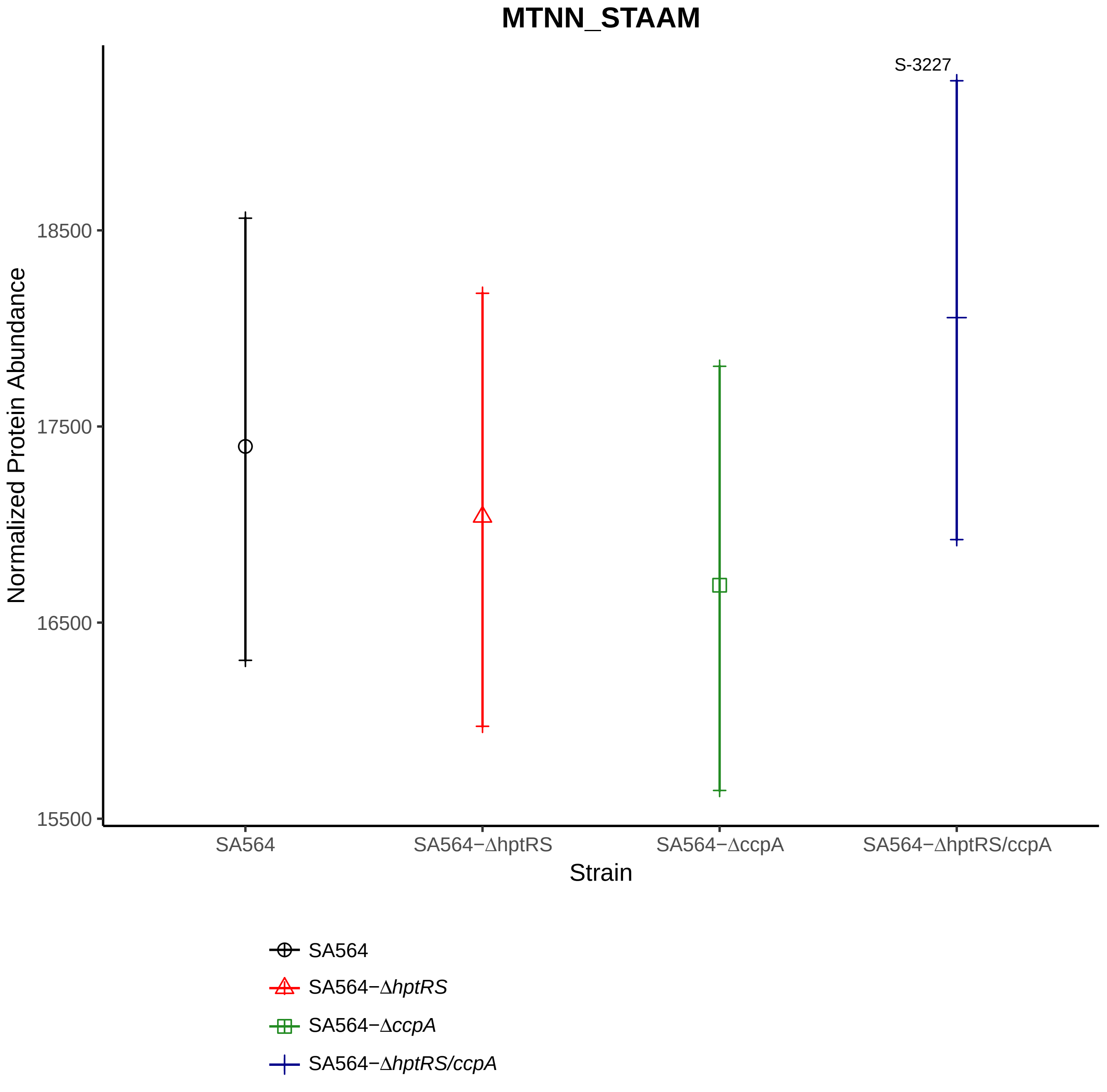

MTNN_STAAM
S-3227
Normalized Protein Abundance
18500
17500
16500
15500
SA564
SA564−ΔhptRS
SA564−ΔccpA
SA564−ΔhptRS/ccpA
Strain
SA564
SA564−ΔhptRS
SA564−ΔccpA
SA564−ΔhptRS/ccpA

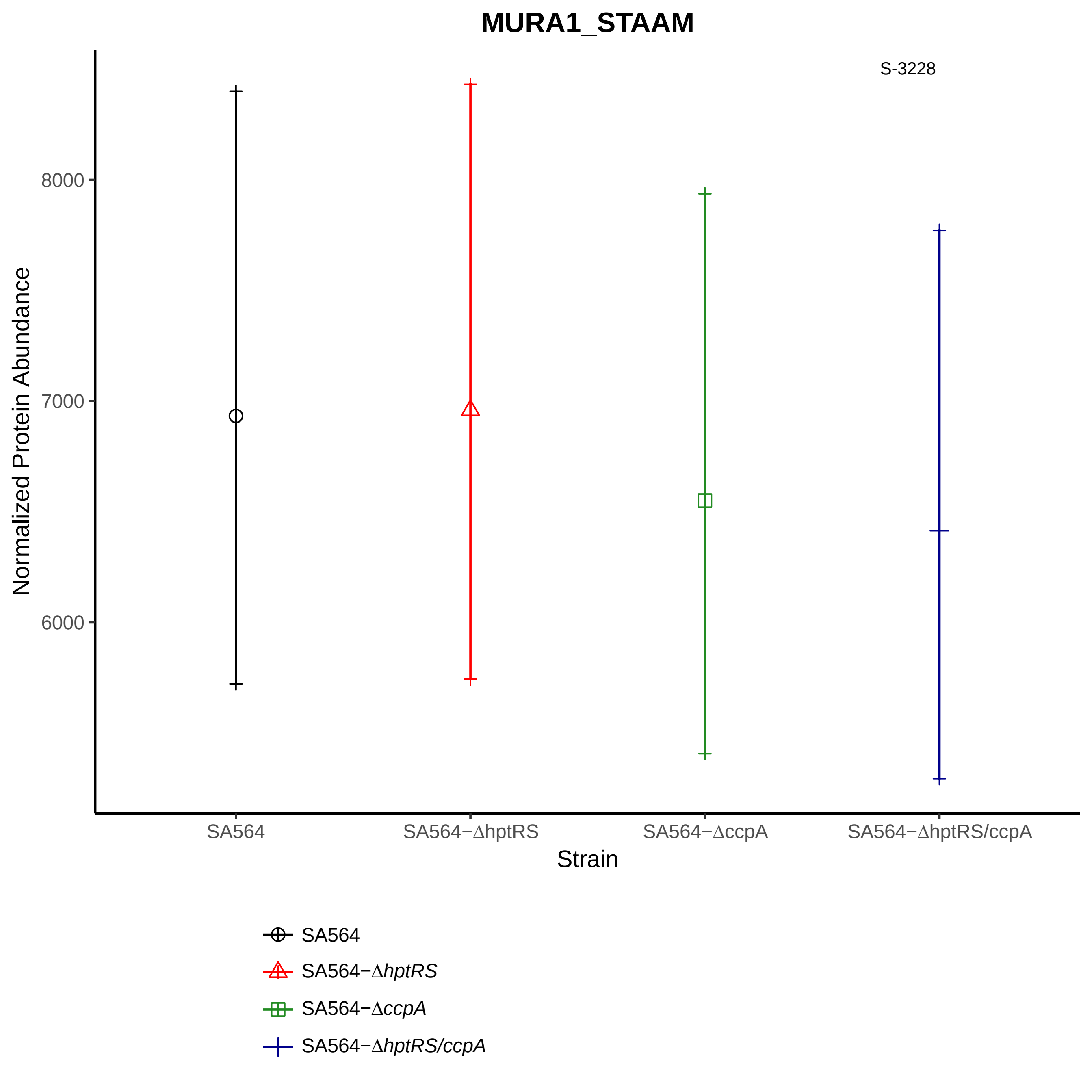
MURA1_STAAM
S-3228
Normalized Protein Abundance
8000
7000
6000
SA564
SA564−ΔhptRS
SA564−ΔccpA
SA564−ΔhptRS/ccpA
Strain
SA564
SA564−ΔhptRS
SA564−ΔccpA
SA564−ΔhptRS/ccpA

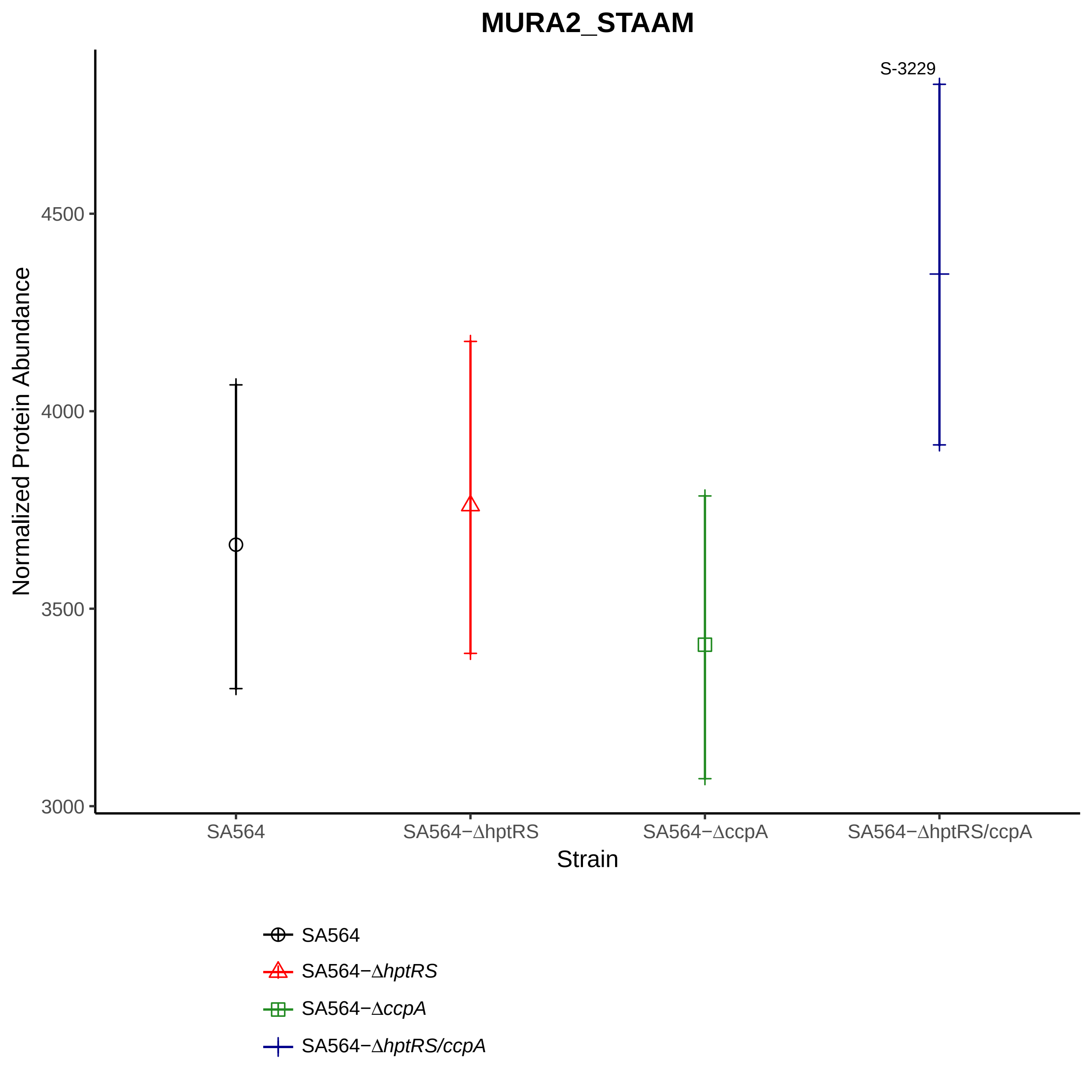
MURA2_STAAM
S-3229
Normalized Protein Abundance
4500
4000
3500
3000
SA564
SA564−ΔhptRS
SA564−ΔccpA
SA564−ΔhptRS/ccpA
Strain
SA564
SA564−ΔhptRS
SA564−ΔccpA
SA564−ΔhptRS/ccpA

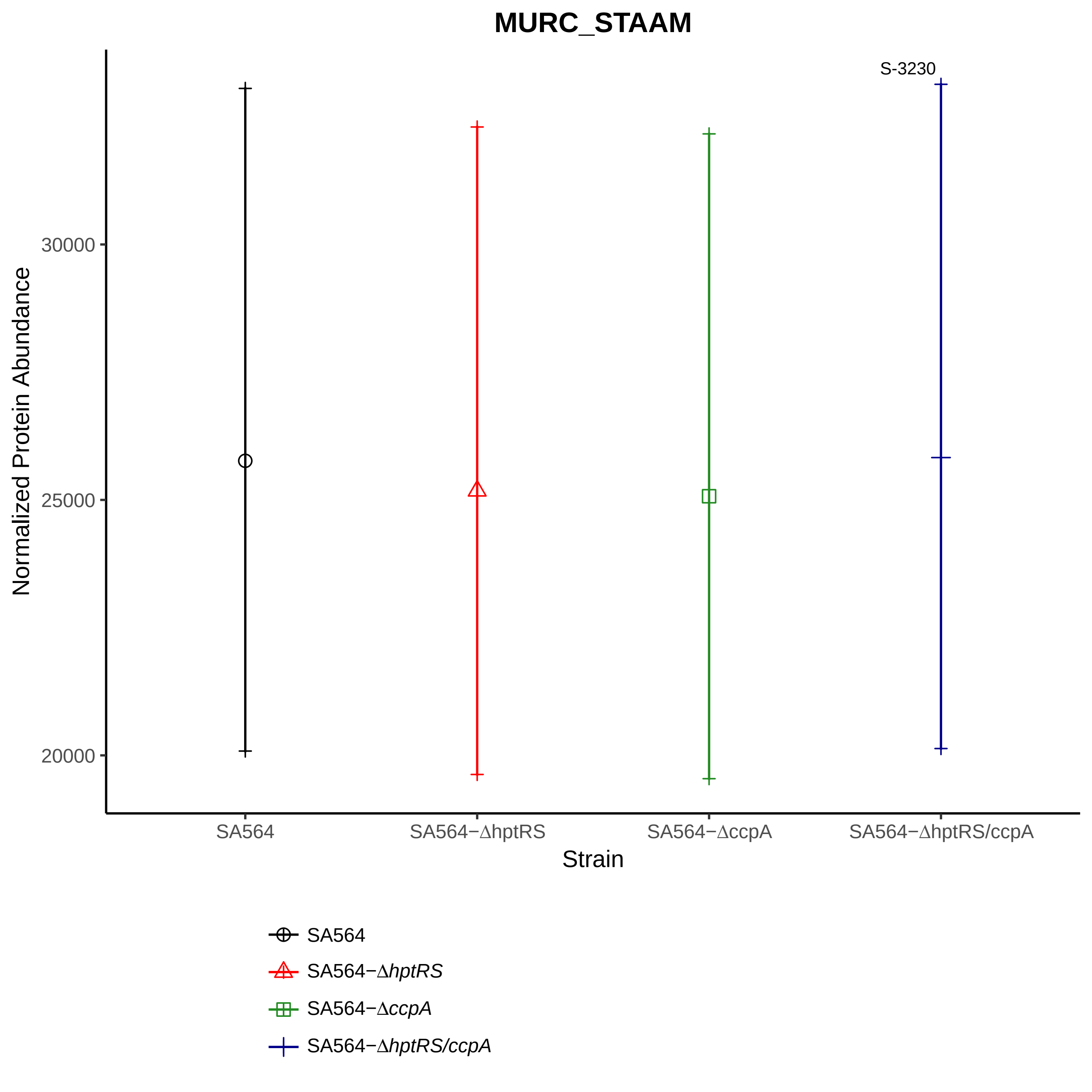

MURC_STAAM
S-3230
Normalized Protein Abundance
30000
25000
20000
SA564
SA564−ΔhptRS
SA564−ΔccpA
SA564−ΔhptRS/ccpA
Strain
SA564
SA564−Δ*hptRS*
SA564−Δ*ccpA*
SA564−Δ*hptRS/ccpA*

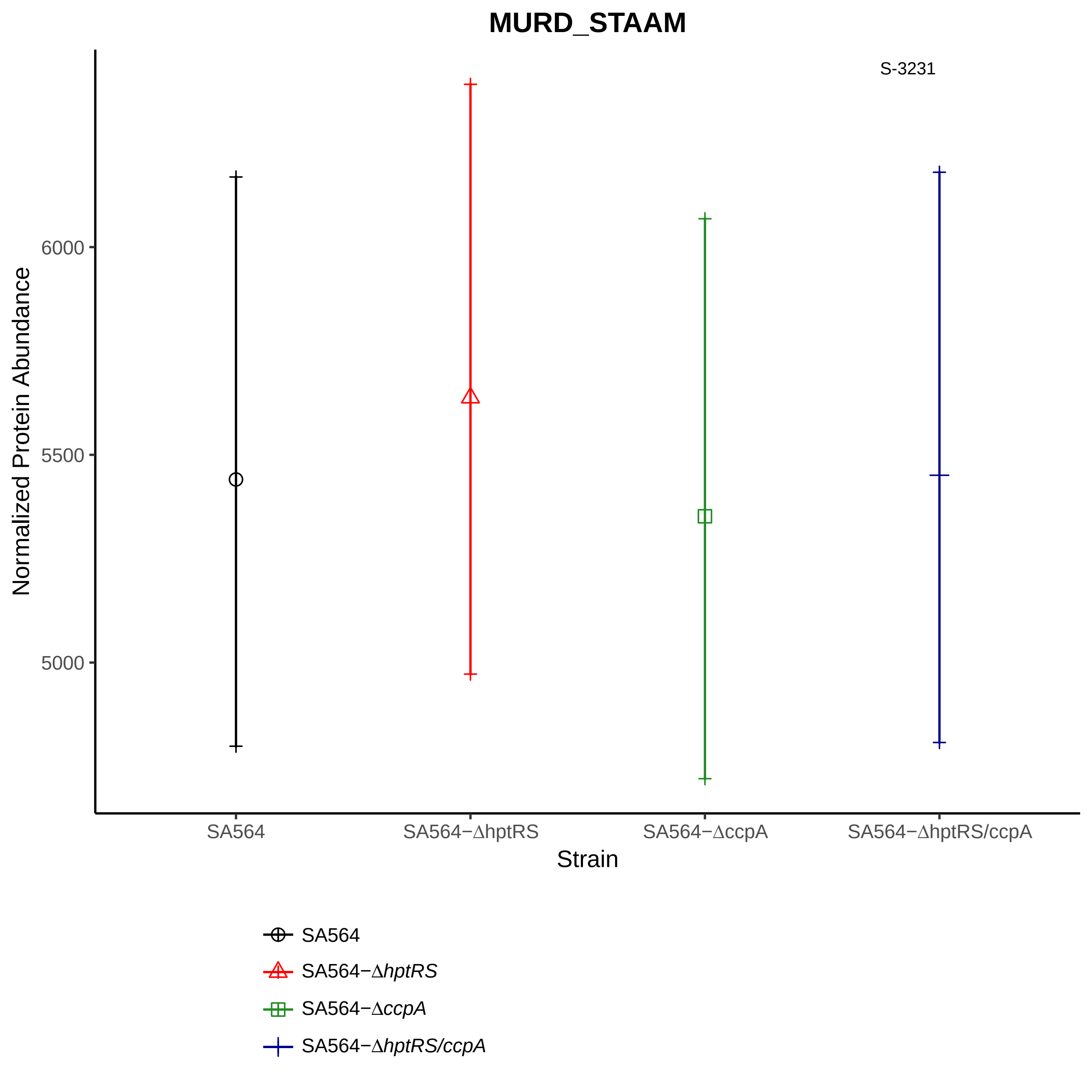

MURD_STAAM
S-3231
Normalized Protein Abundance
6000
5500
5000
SA564
SA564−ΔhptRS
SA564−ΔccpA
SA564−ΔhptRS/ccpA
Strain
SA564
SA564−ΔhptRS
SA564−ΔccpA
SA564−ΔhptRS/ccpA

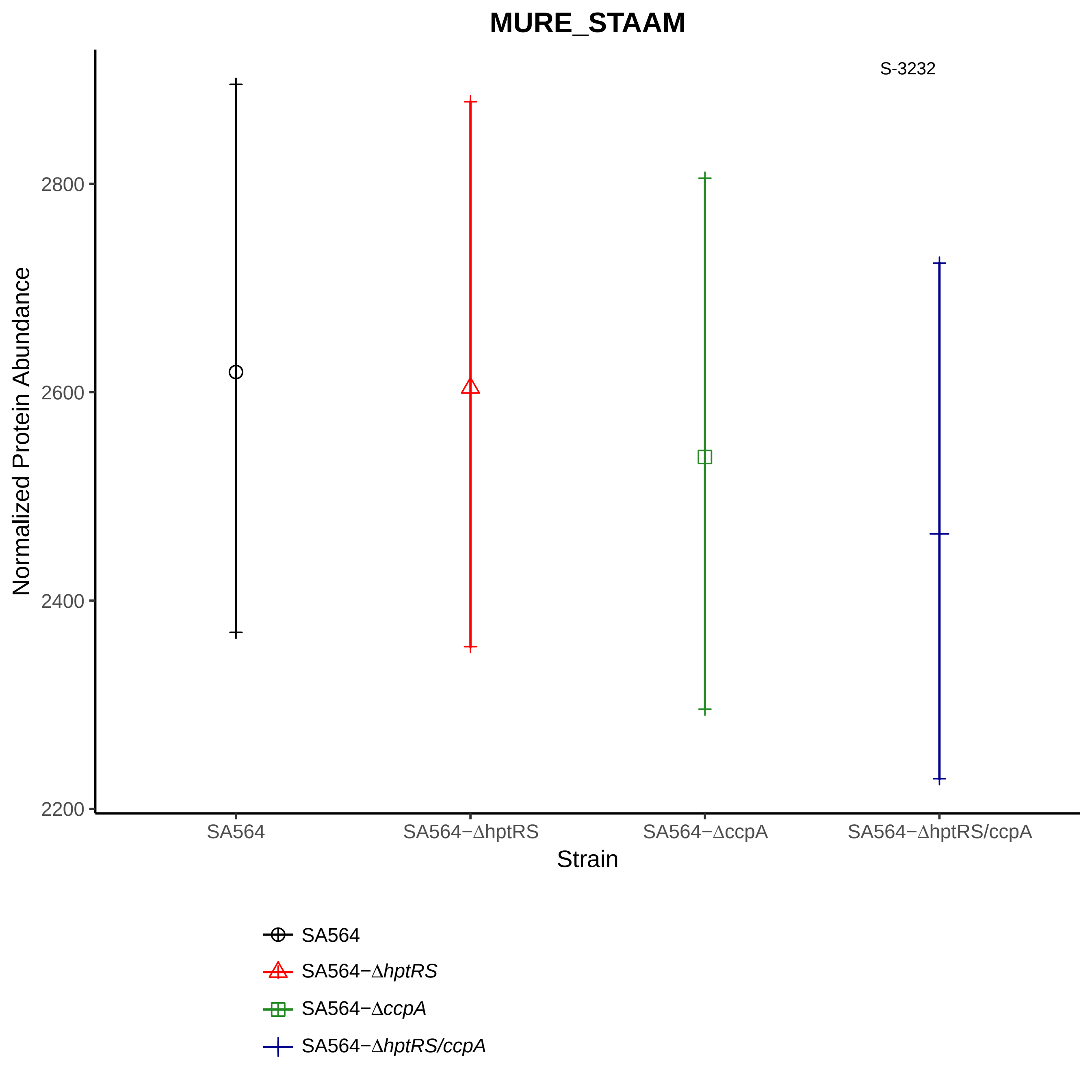
MURE_STAAM
S-3232
Normalized Protein Abundance
2200
2400
2600
2800
SA564
SA564−ΔhptRS
SA564−ΔccpA
SA564−ΔhptRS/ccpA
Strain
SA564
SA564−ΔhptRS
SA564−ΔccpA
SA564−ΔhptRS/ccpA

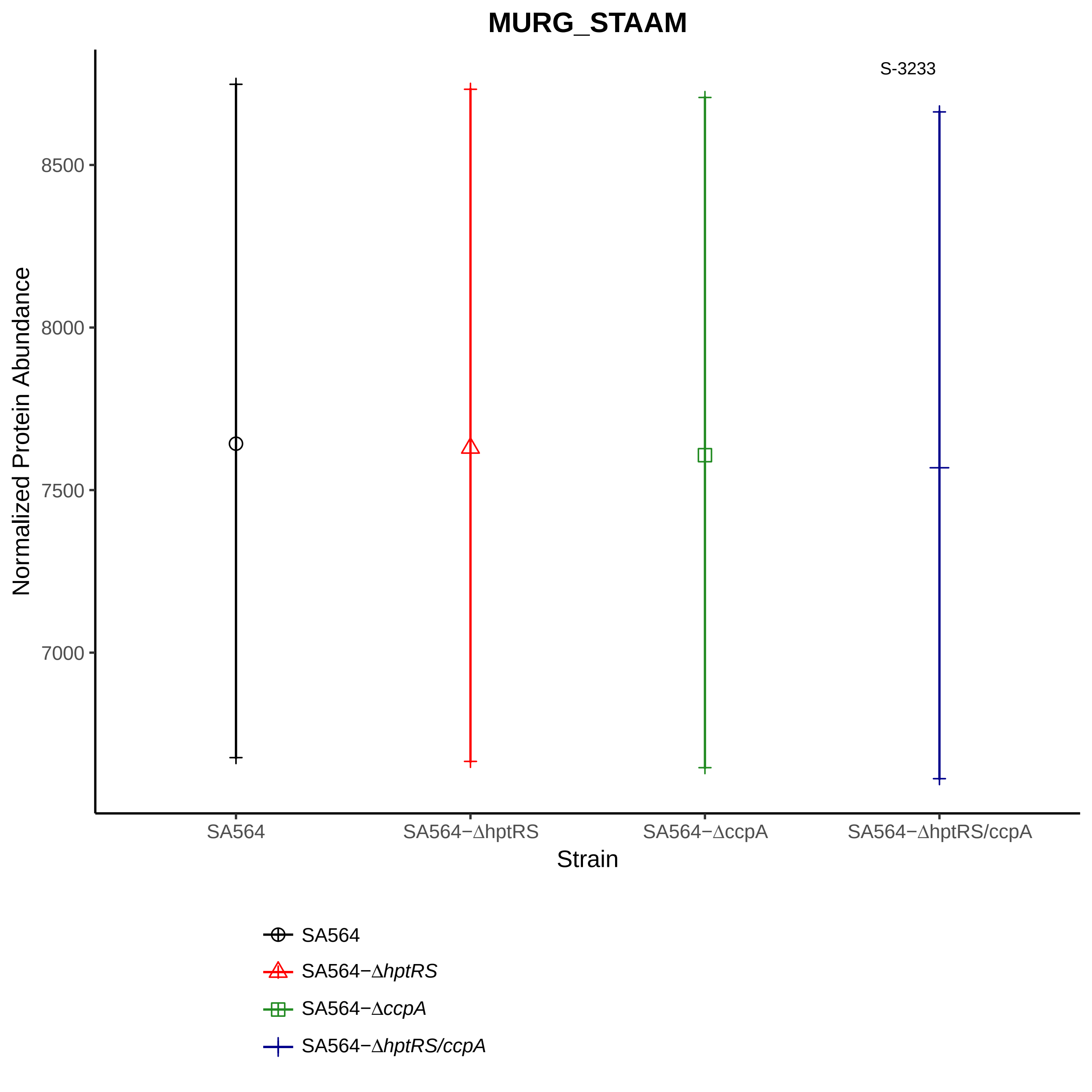

MURG_STAAM
S-3233
Normalized Protein Abundance
8500
8000
7500
7000
SA564
SA564−ΔhptRS
SA564−ΔccpA
SA564−ΔhptRS/ccpA
Strain
SA564
SA564−ΔhptRS
SA564−ΔccpA
SA564−ΔhptRS/ccpA

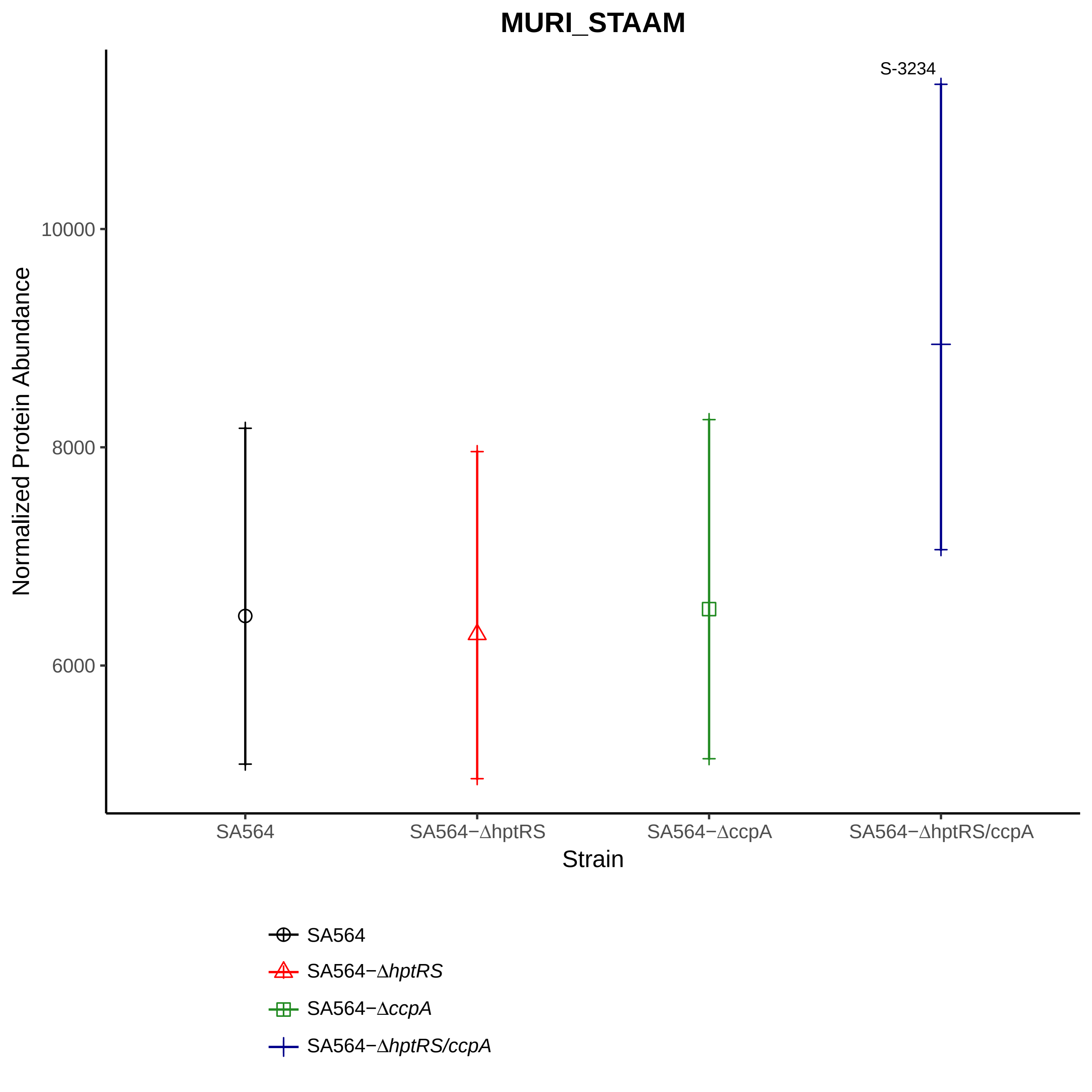

MURI_STAAM
S-3234
Normalized Protein Abundance
10000
8000
6000
SA564
SA564−ΔhptRS
SA564−ΔccpA
SA564−ΔhptRS/ccpA
Strain
SA564
SA564−Δ*hptRS*
SA564−Δ*ccpA*
SA564−Δ*hptRS/ccpA*

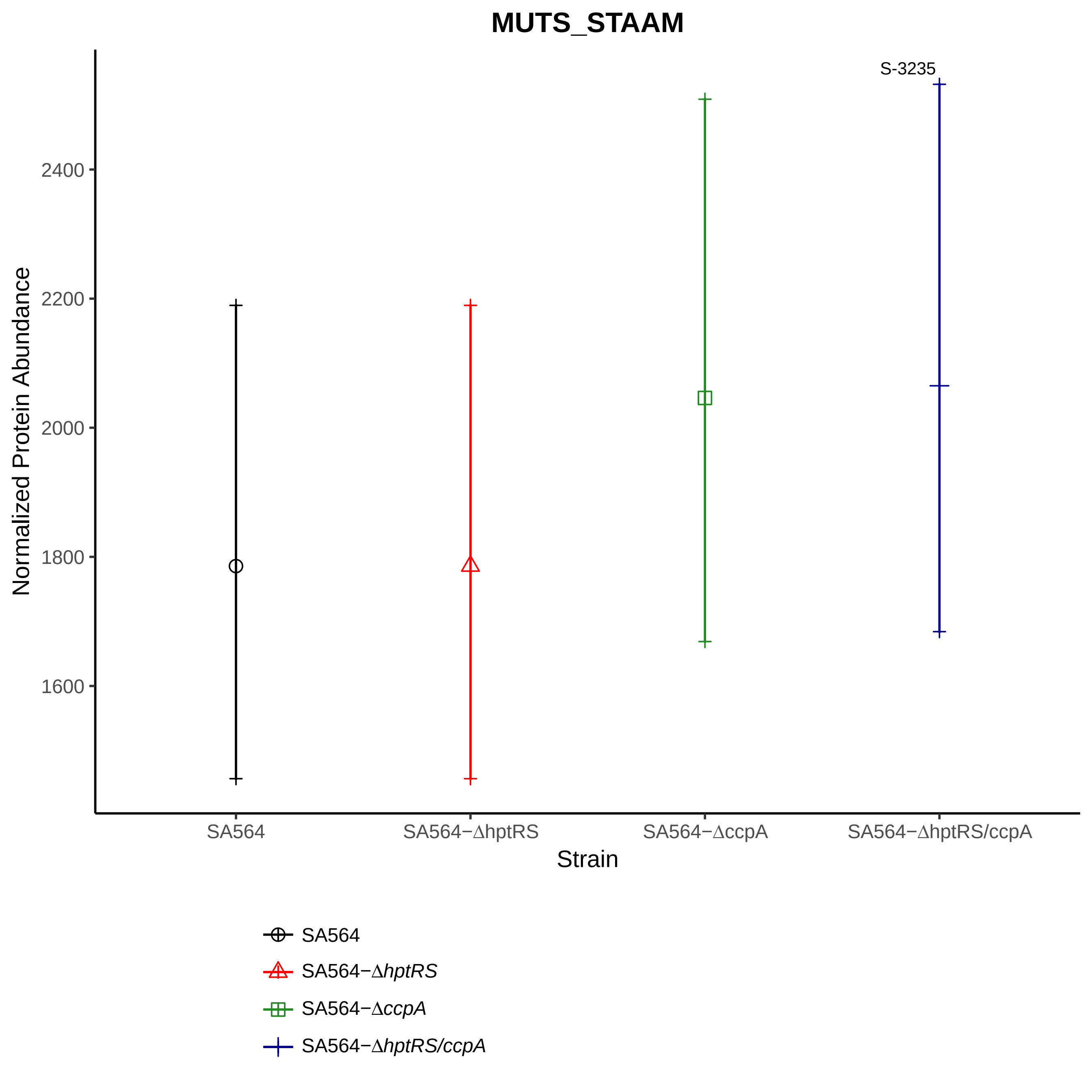

MUTS_STAAM
S-3235
Normalized Protein Abundance
2400
2200
2000
1800
1600
SA564
SA564−ΔhptRS
SA564−ΔccpA
SA564−ΔhptRS/ccpA
Strain
SA564
SA564−ΔhptRS
SA564−ΔccpA
SA564−ΔhptRS/ccpA

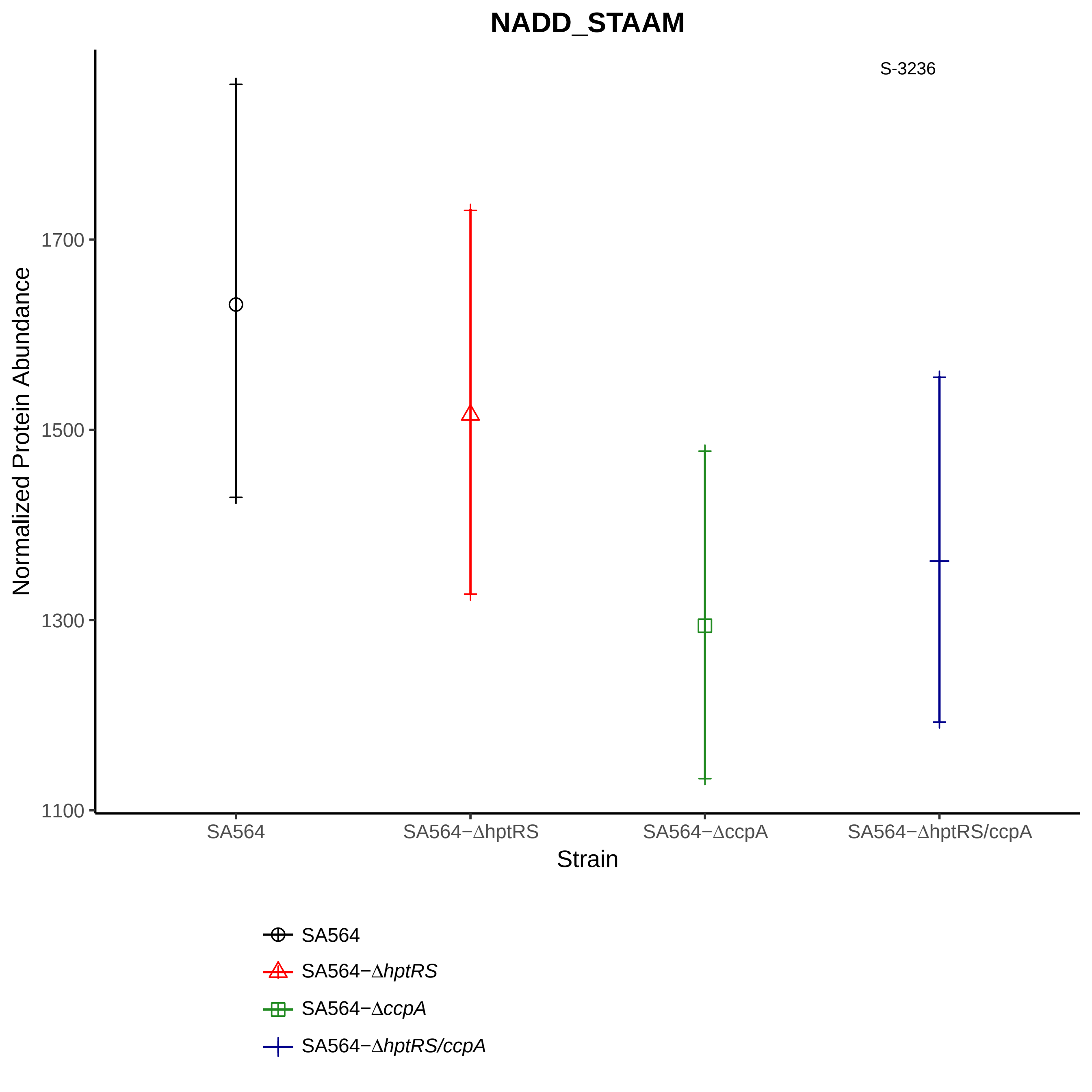
NADD_STAAM
S-3236
Normalized Protein Abundance
1700
1500
1300
1100
SA564
SA564−ΔhptRS
SA564−ΔccpA
SA564−ΔhptRS/ccpA
Strain
SA564
SA564−ΔhptRS
SA564−ΔccpA
SA564−ΔhptRS/ccpA

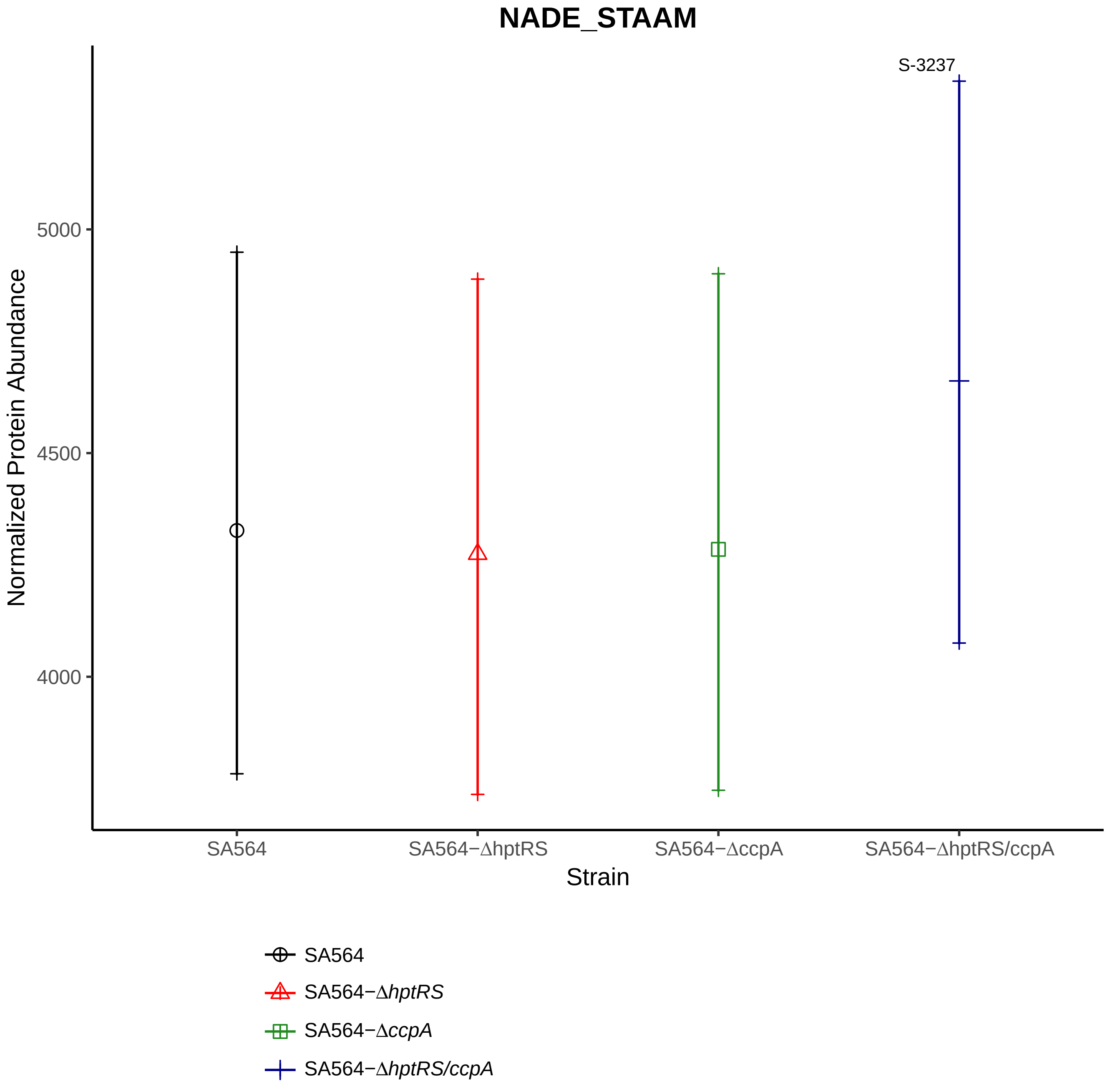
NADE_STAAM
S-3237
5000
4500
4000
Normalized Protein Abundance
SA564
SA564−ΔhptRS
SA564−ΔccpA
SA564−ΔhptRS/ccpA
Strain
SA564
SA564−ΔhptRS
SA564−ΔccpA
SA564−ΔhptRS/ccpA

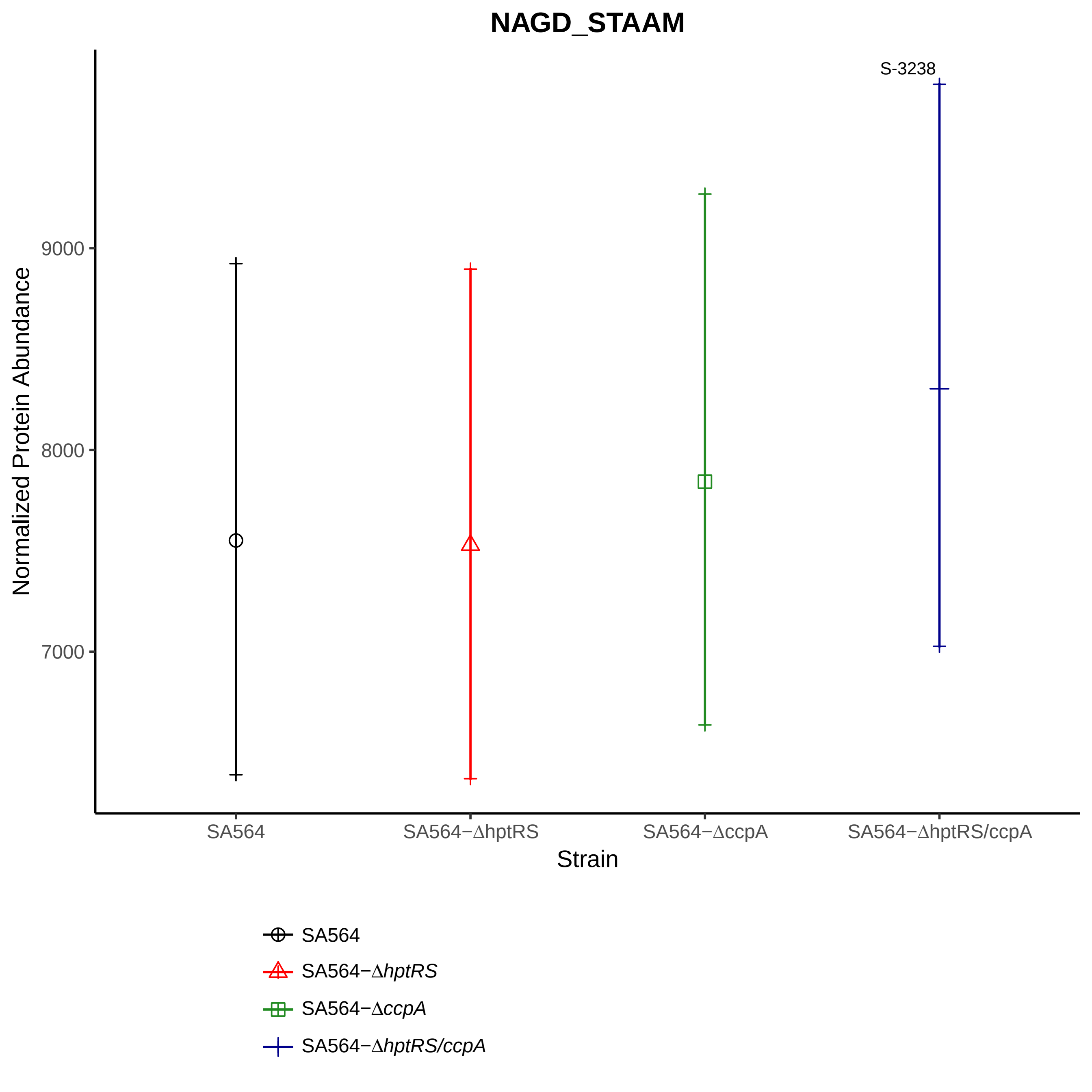

NAGD_STAAM
S-3238
Normalized Protein Abundance
9000
8000
7000
SA564
SA564−ΔhptRS
SA564−ΔccpA
SA564−ΔhptRS/ccpA
Strain
SA564
SA564−ΔhptRS
SA564−ΔccpA
SA564−ΔhptRS/ccpA

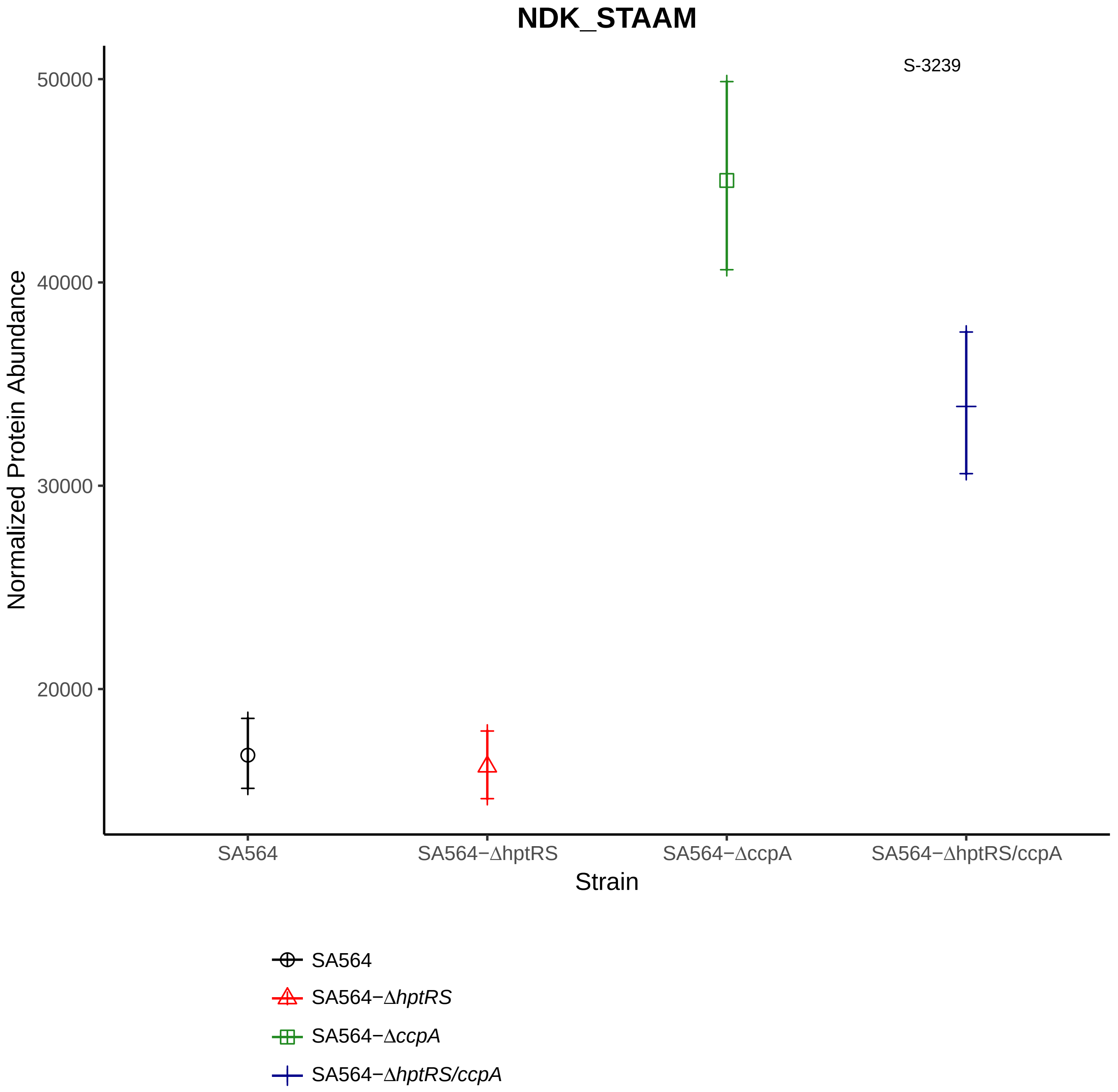

NDK_STAAM
S-3239
Normalized Protein Abundance
50000
40000
30000
20000
SA564
SA564−ΔhptRS
SA564−ΔccpA
SA564−ΔhptRS/ccpA
Strain
SA564
SA564−ΔhptRS
SA564−ΔccpA
SA564−ΔhptRS/ccpA

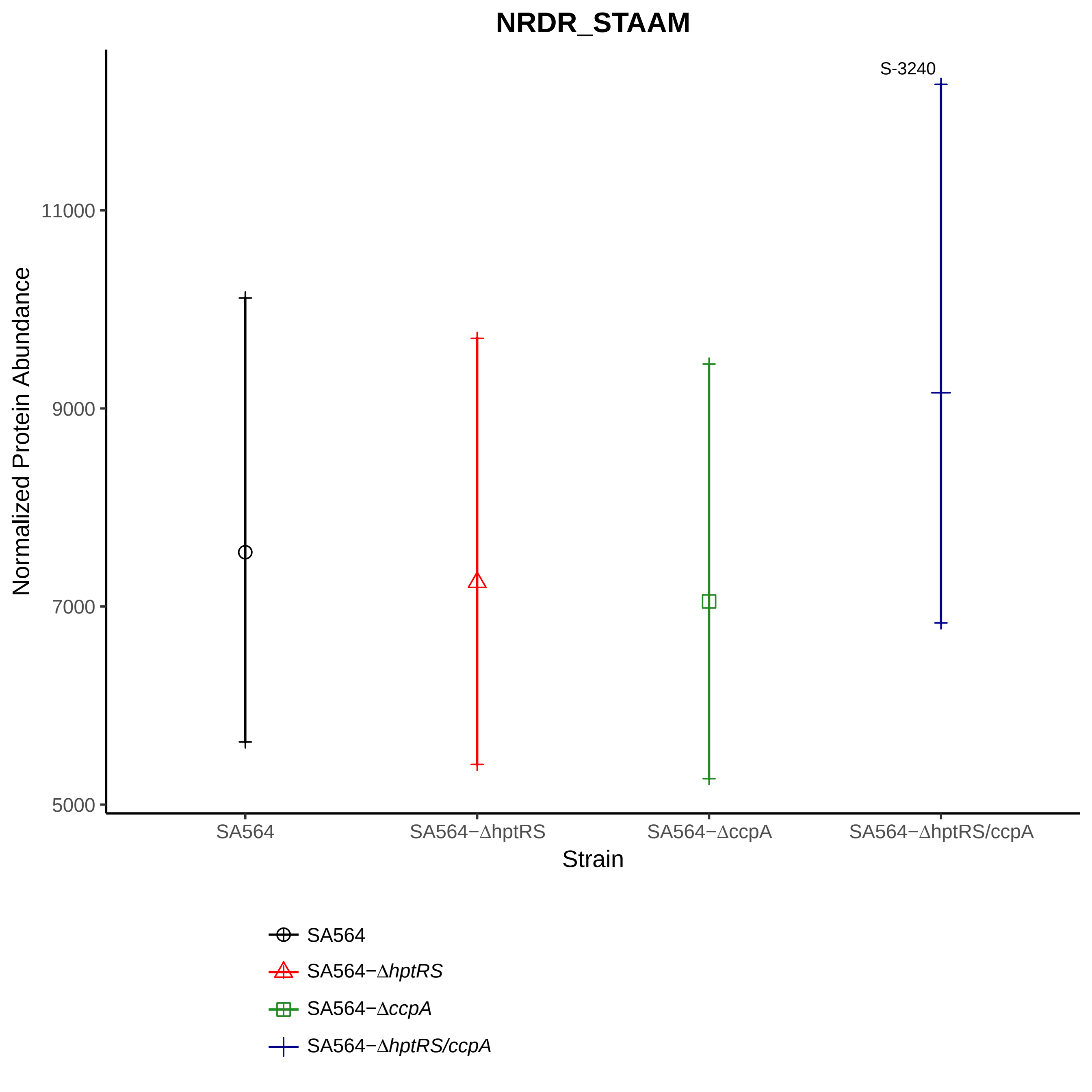
NRDR_STAAM
S-3240
Normalized Protein Abundance
11000
9000
7000
5000
SA564
SA564−ΔhptRS
SA564−ΔccpA
SA564−ΔhptRS/ccpA
Strain
SA564
SA564−ΔhptRS
SA564−ΔccpA
SA564−ΔhptRS/ccpA

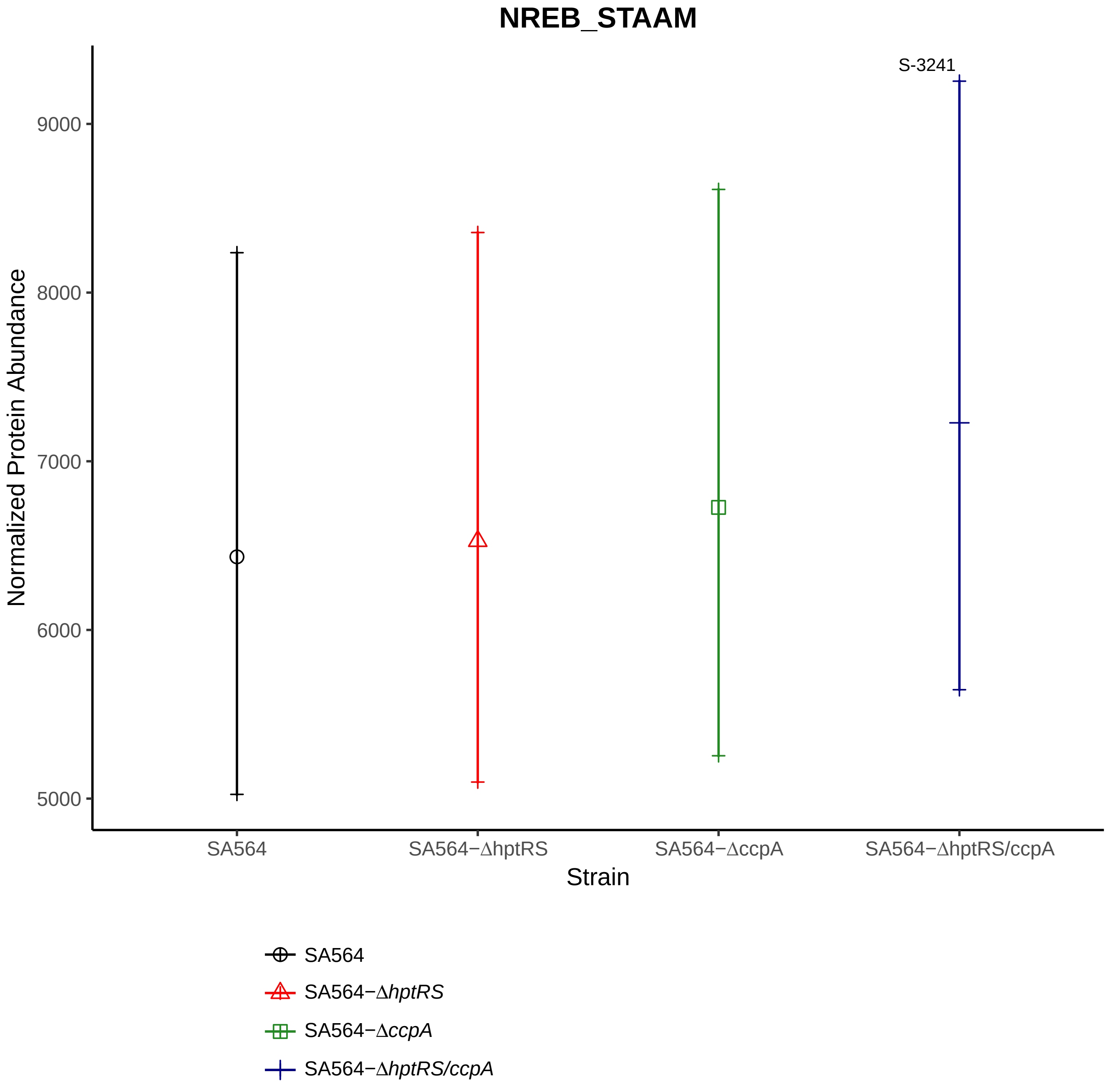

NREB_STAAM
S-3241
9000
8000
7000
6000
5000
Normalized Protein Abundance
SA564
SA564−ΔhptRS
SA564−ΔccpA
SA564−ΔhptRS/ccpA
Strain
SA564
SA564−ΔhptRS
SA564−ΔccpA
SA564−ΔhptRS/ccpA

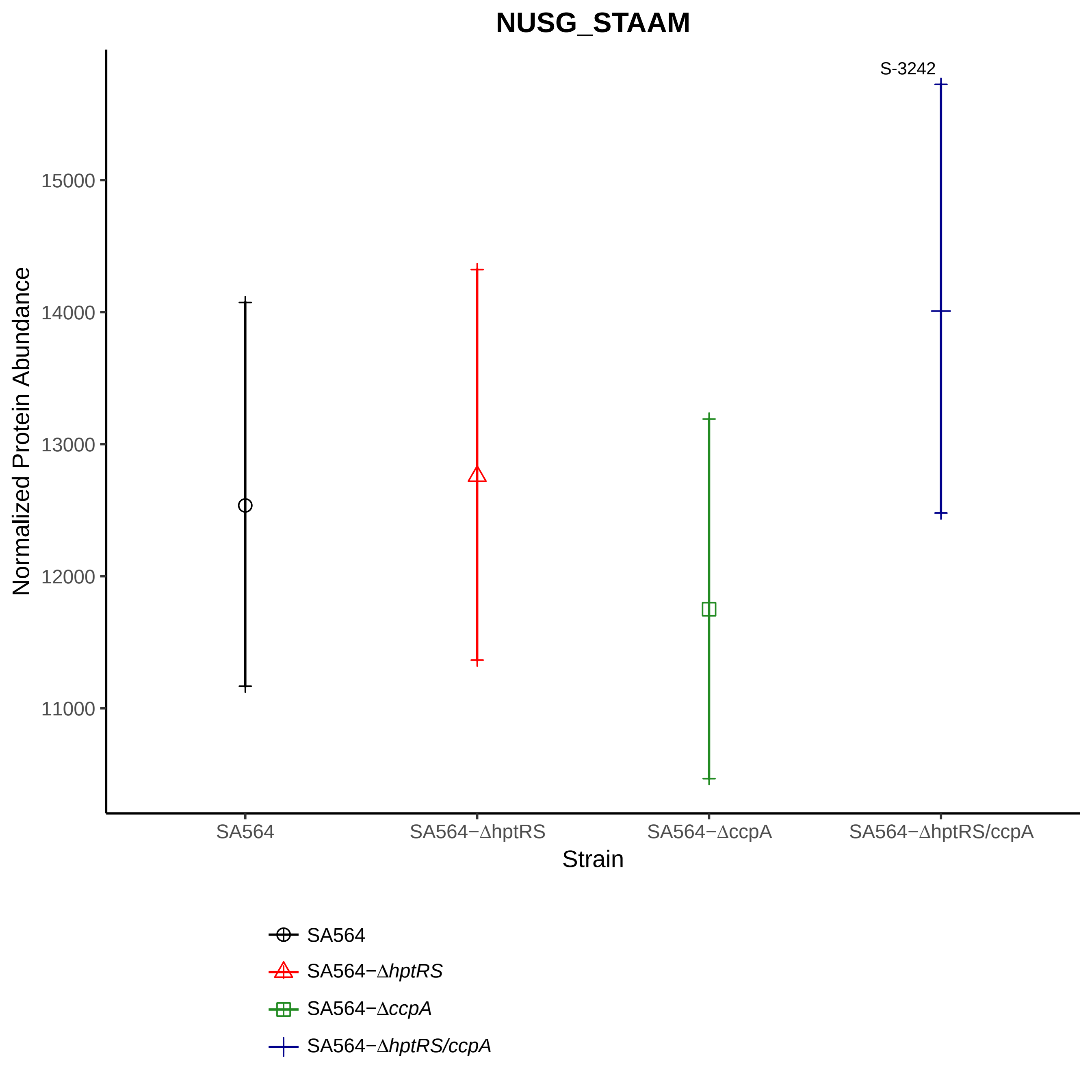

NUSG_STAAM
S-3242
Normalized Protein Abundance
15000
14000
13000
12000
11000
SA564
SA564−ΔhptRS
SA564−ΔccpA
SA564−ΔhptRS/ccpA
Strain
SA564
SA564−ΔhptRS
SA564−ΔccpA
SA564−ΔhptRS/ccpA

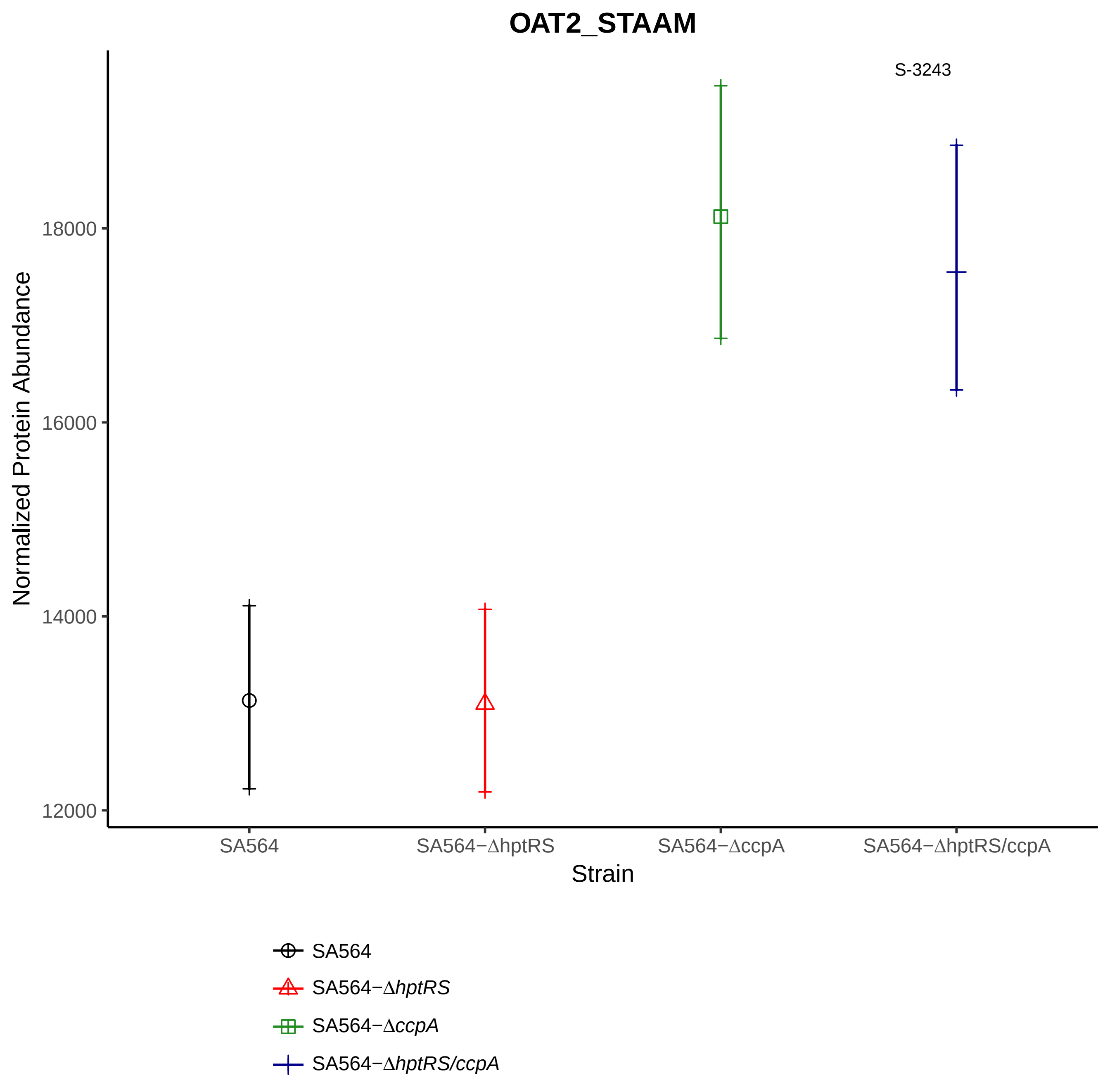

OAT2_STAAM
S-3243
Normalized Protein Abundance
18000
16000
14000
12000
SA564
SA564−ΔhptRS
SA564−ΔccpA
SA564−ΔhptRS/ccpA
Strain
SA564
SA564−ΔhptRS
SA564−ΔccpA
SA564−ΔhptRS/ccpA

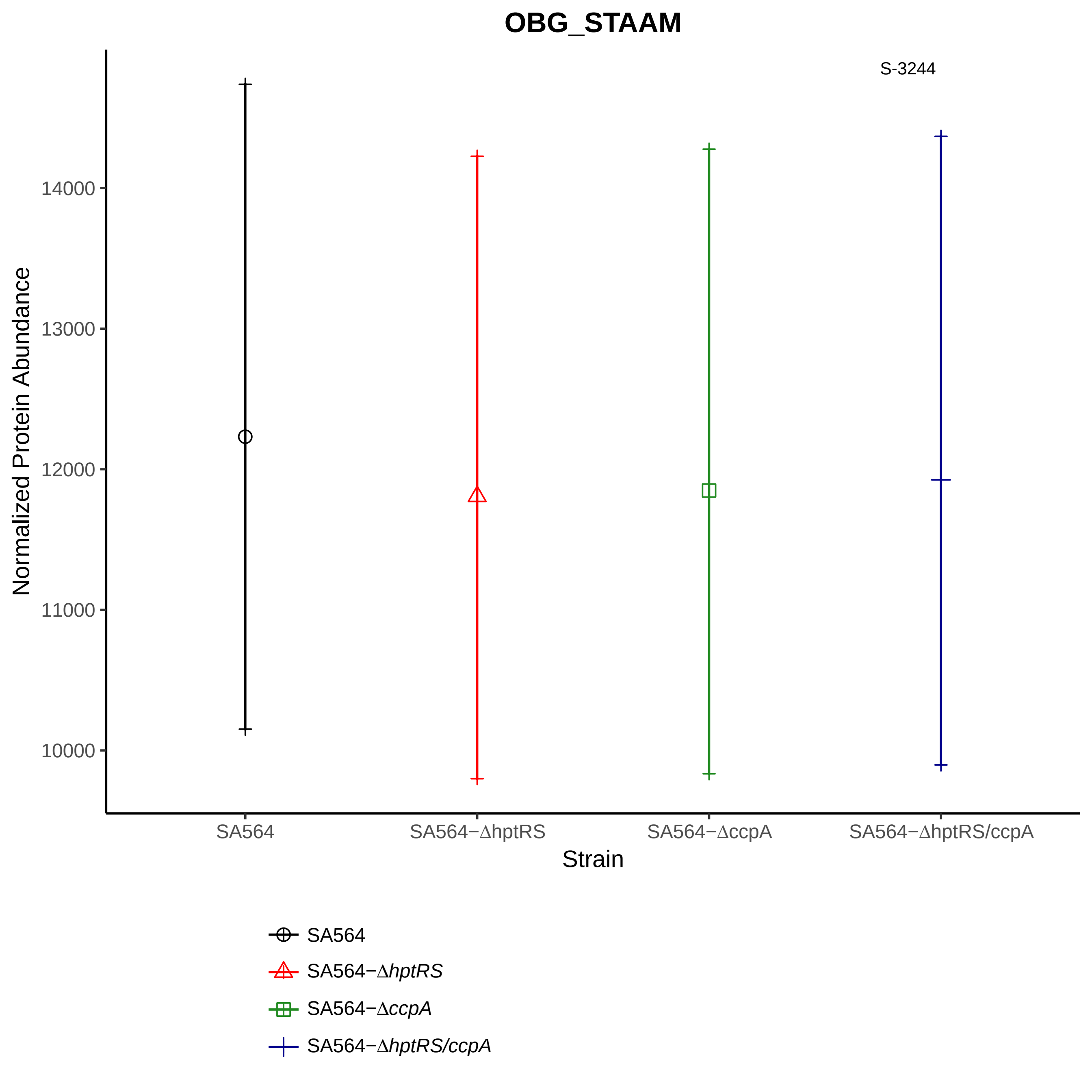

OBG_STAAM
S-3244
Normalized Protein Abundance
14000
13000
12000
11000
10000
SA564
SA564−ΔhptRS
SA564−ΔccpA
SA564−ΔhptRS/ccpA
Strain
SA564
SA564−Δ*hptRS*
SA564−Δ*ccpA*
SA564−Δ*hptRS/ccpA*

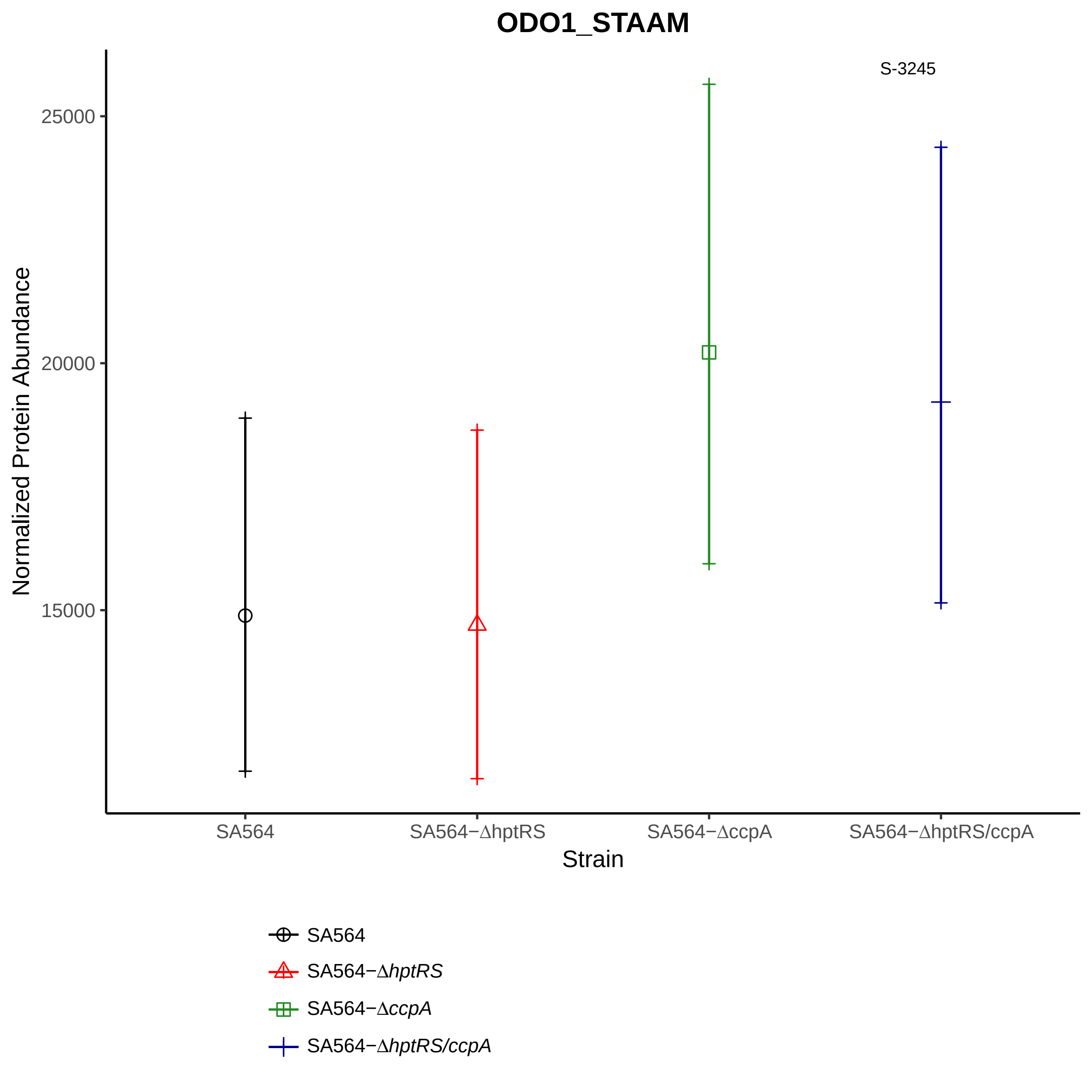
ODO1_STAAM
S-3245
Normalized Protein Abundance
25000
20000
15000
SA564
SA564−ΔhptRS
SA564−ΔccpA
SA564−ΔhptRS/ccpA
Strain
SA564
SA564−ΔhptRS
SA564−ΔccpA
SA564−ΔhptRS/ccpA

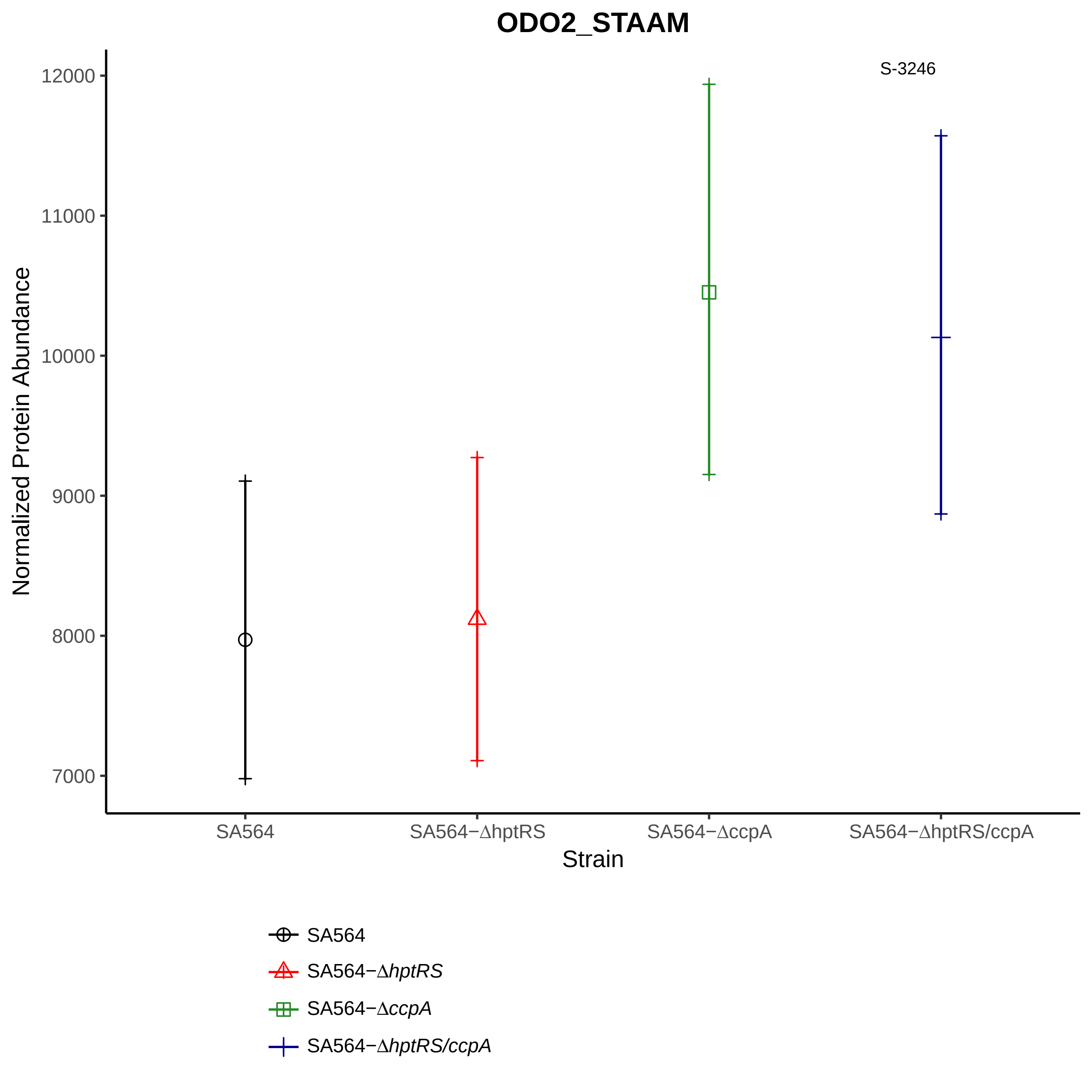

ODO2_STAAM
S-3246
Normalized Protein Abundance
12000
11000
10000
9000
8000
7000
SA564
SA564−ΔhptRS
SA564−ΔccpA
SA564−ΔhptRS/ccpA
Strain
SA564
SA564−ΔhptRS
SA564−ΔccpA
SA564−ΔhptRS/ccpA

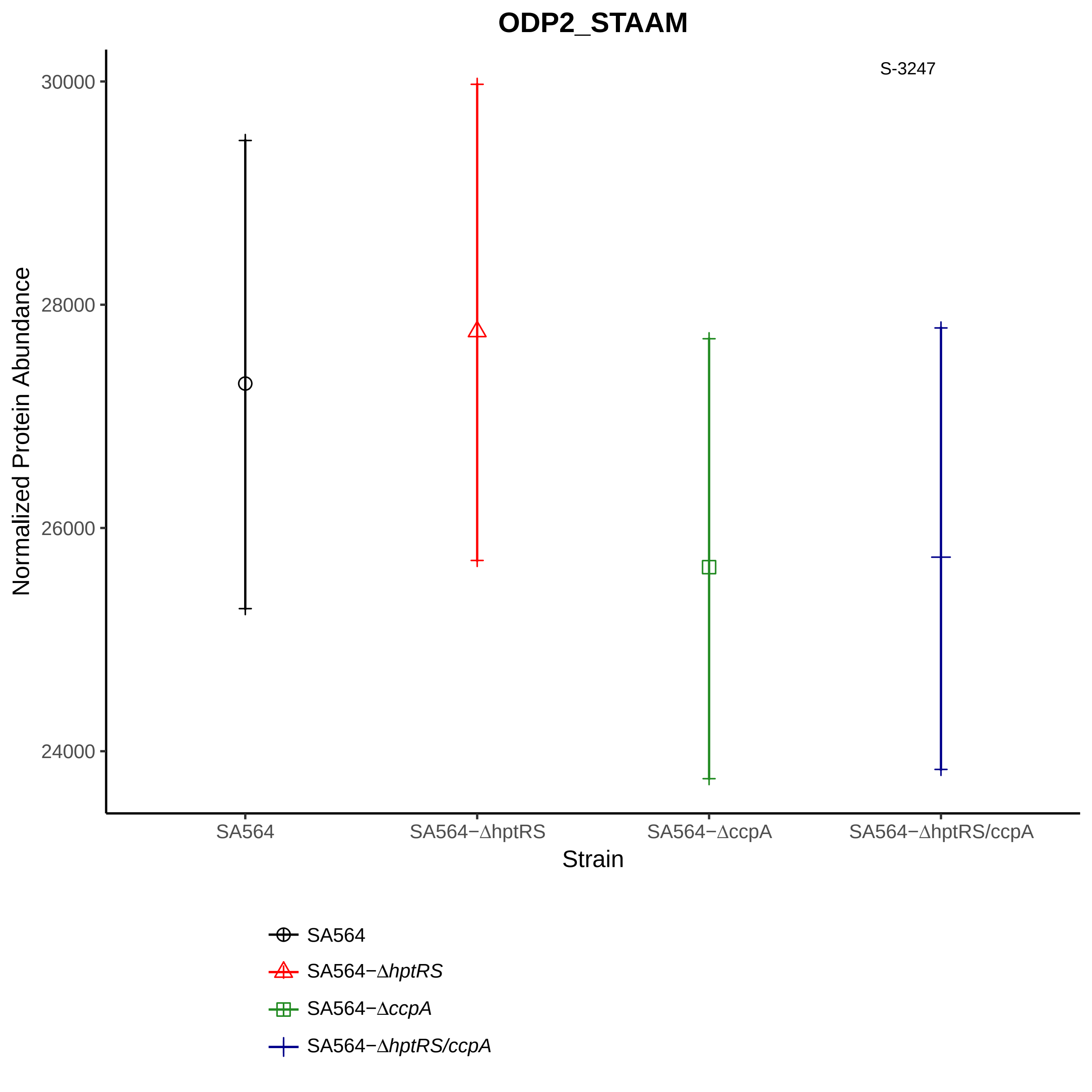

ODP2_STAAM
S-3247
30000
28000
26000
24000
Normalized Protein Abundance
SA564
SA564−ΔhptRS
SA564−ΔccpA
SA564−ΔhptRS/ccpA
Strain
SA564
SA564−Δ*hptRS*
SA564−Δ*ccpA*
SA564−Δ*hptRS/ccpA*

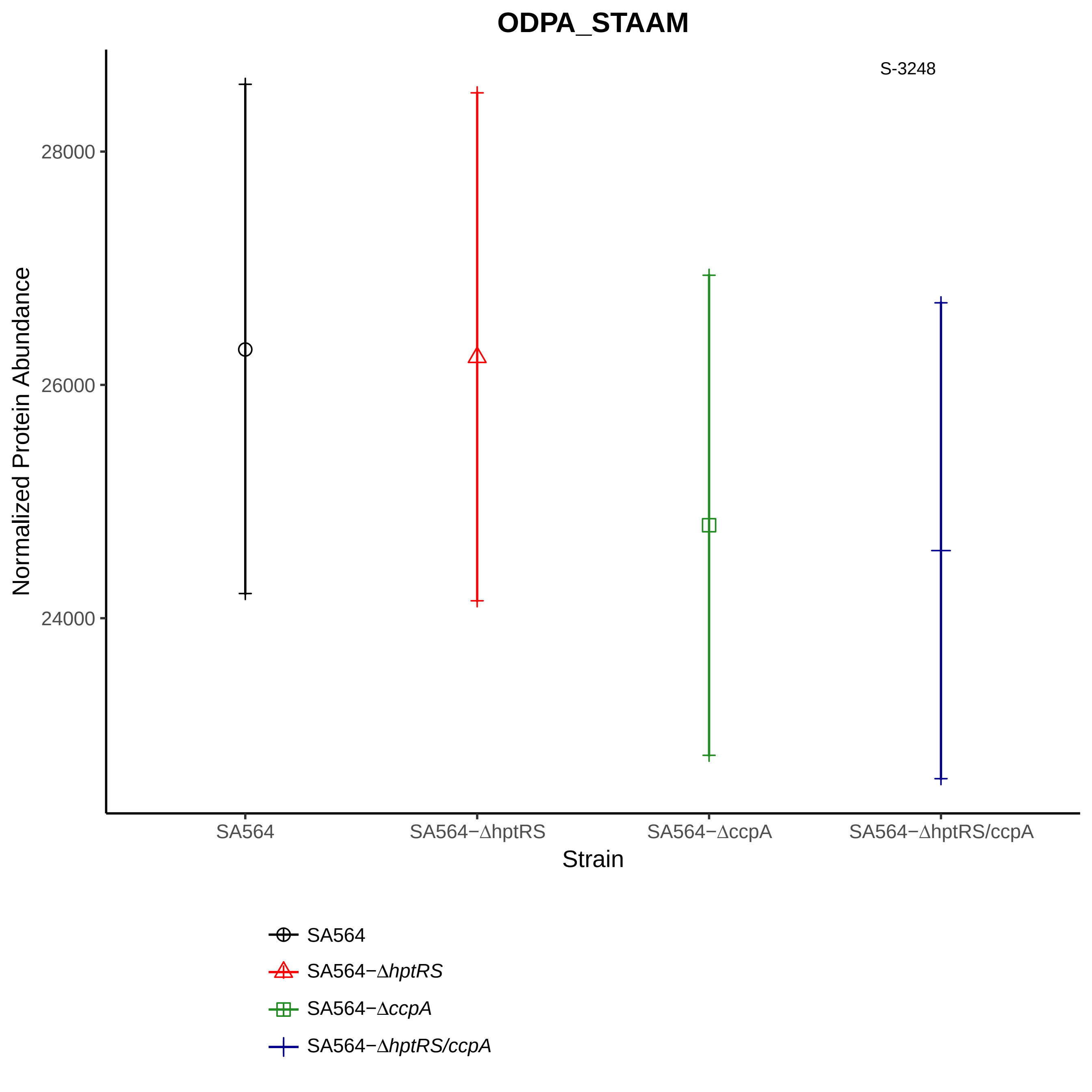

ODPA_STAAM
S-3248
Normalized Protein Abundance
28000
26000
24000
SA564
SA564−ΔhptRS
SA564−ΔccpA
SA564−ΔhptRS/ccpA
Strain
SA564
SA564−Δ*hptRS*
SA564−Δ*ccpA*
SA564−Δ*hptRS/ccpA*

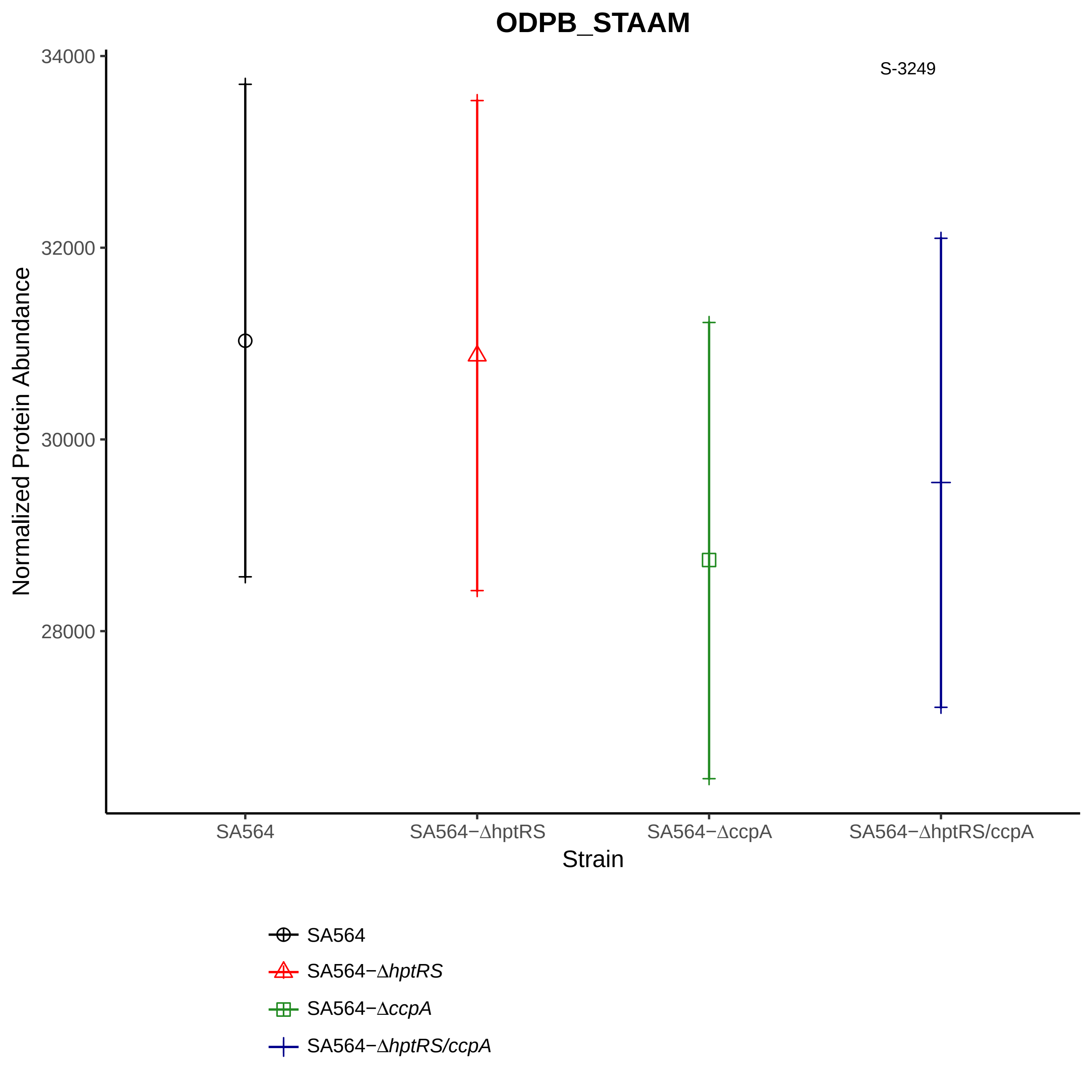

ODPB_STAAM
S-3249
Normalized Protein Abundance
34000
32000
30000
28000
SA564
SA564−ΔhptRS
SA564−ΔccpA
SA564−ΔhptRS/ccpA
Strain
SA564
SA564−ΔhptRS
SA564−ΔccpA
SA564−ΔhptRS/ccpA

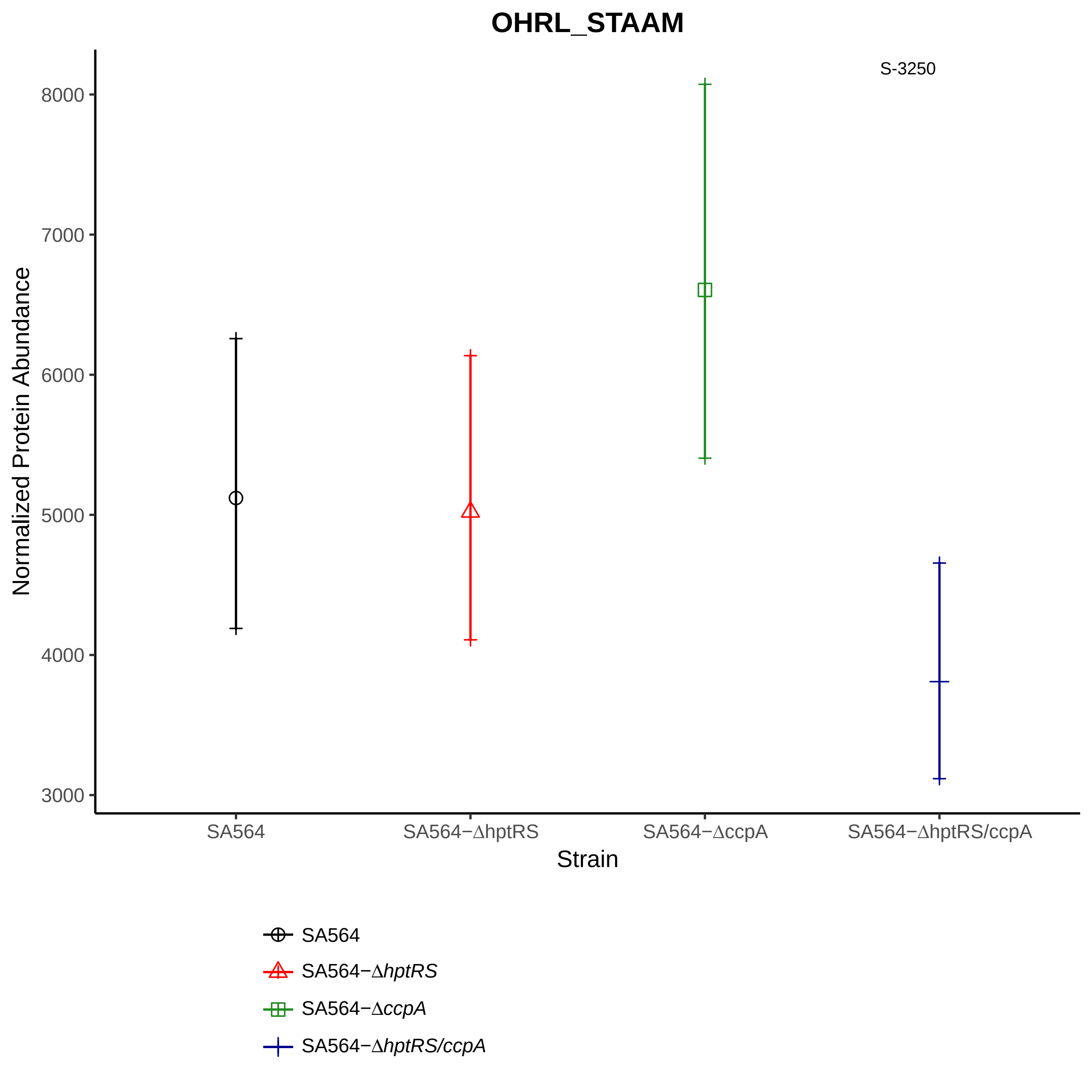

OHRL_STAAM
S-3250
Normalized Protein Abundance
8000
7000
6000
5000
4000
3000
SA564
SA564−ΔhptRS
SA564−ΔccpA
SA564−ΔhptRS/ccpA
Strain
SA564
SA564−ΔhptRS
SA564−ΔccpA
SA564−ΔhptRS/ccpA

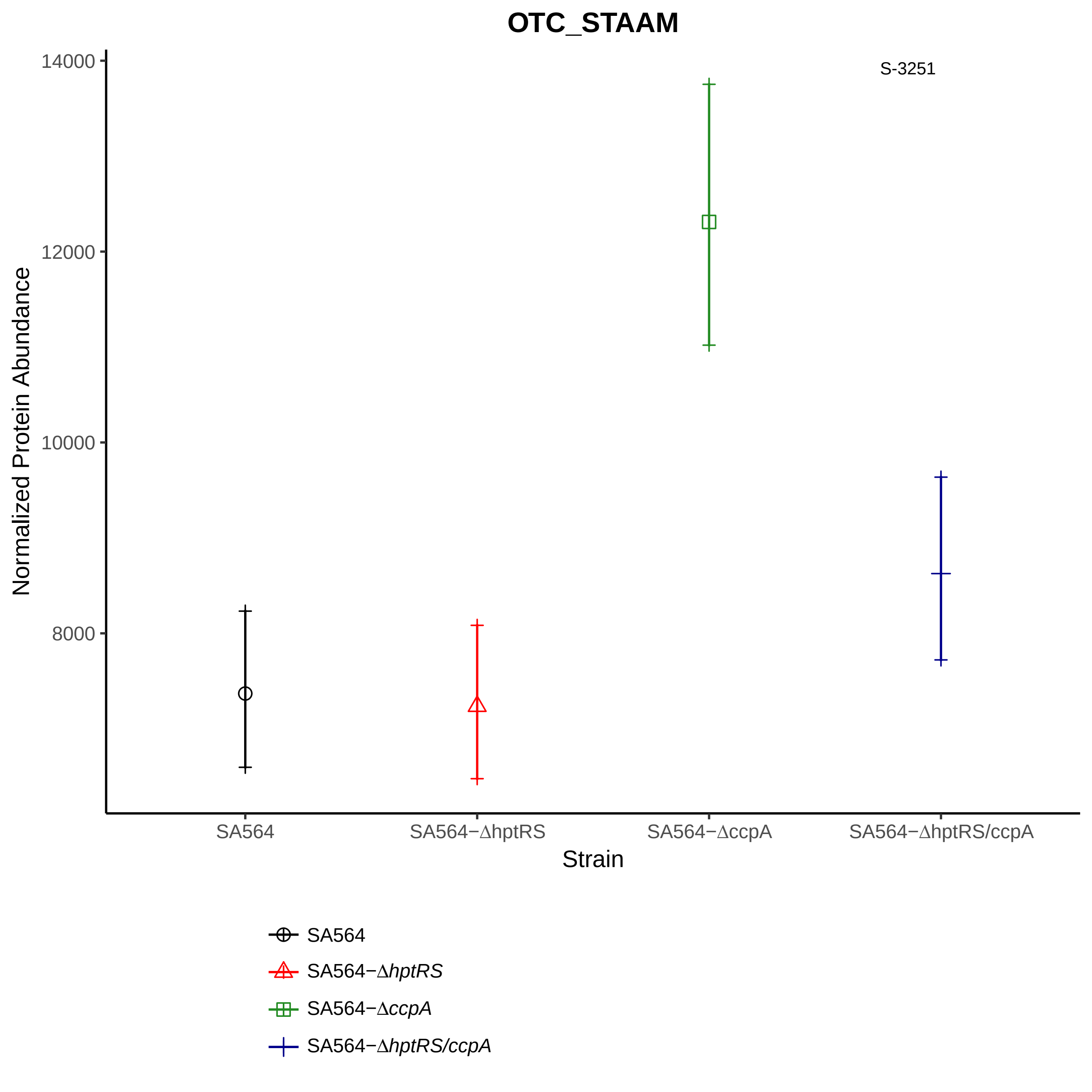

OTC_STAAM
S-3251
Normalized Protein Abundance
14000
12000
10000
8000
SA564
SA564−ΔhptRS
SA564−ΔccpA
SA564−ΔhptRS/ccpA
Strain
SA564
SA564−ΔhptRS
SA564−ΔccpA
SA564−ΔhptRS/ccpA

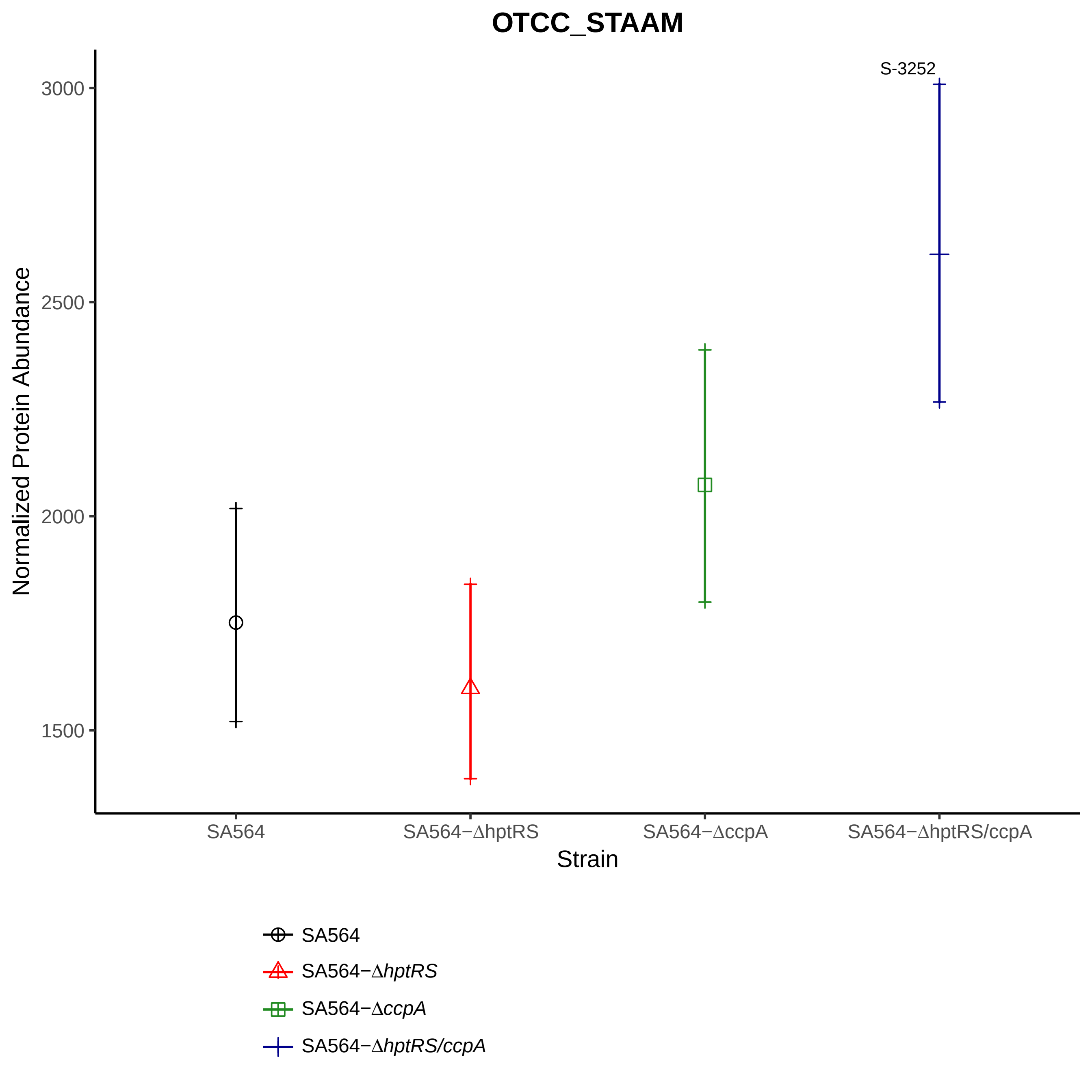
OTCC_STAAM
S-3252
3000
2500
2000
1500
Normalized Protein Abundance
SA564
SA564−ΔhptRS
SA564−ΔccpA
SA564−ΔhptRS/ccpA
Strain
SA564
SA564−Δ*hptRS*
SA564−Δ*ccpA*
SA564−Δ*hptRS/ccpA*

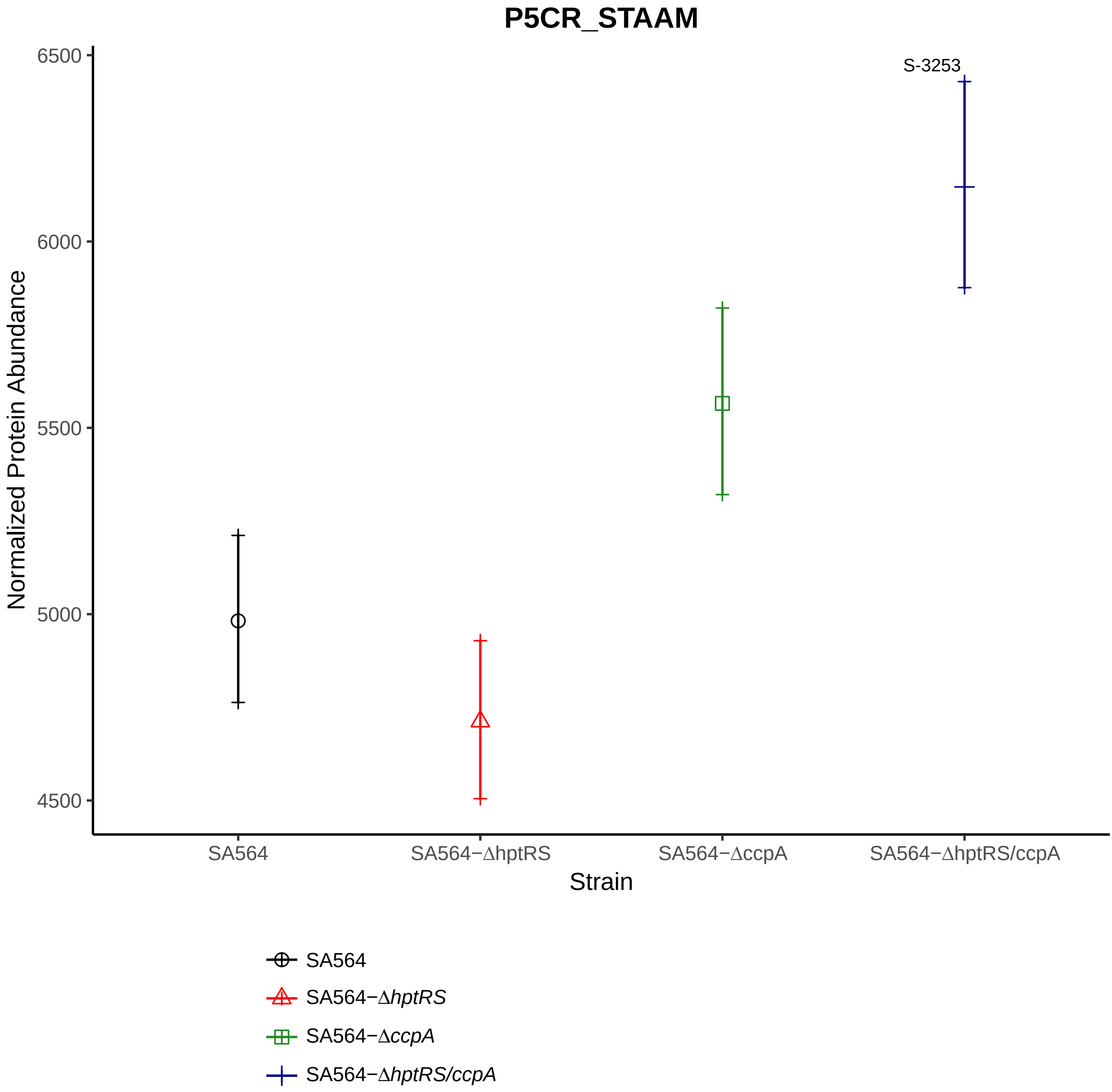

P5CR_STAAM
S-3253
6500
6000
5500
5000
4500
Normalized Protein Abundance
SA564
SA564−ΔhptRS
SA564−ΔccpA
SA564−ΔhptRS/ccpA
Strain
SA564
SA564−Δ*hptRS*
SA564−Δ*ccpA*
SA564−Δ*hptRS/ccpA*

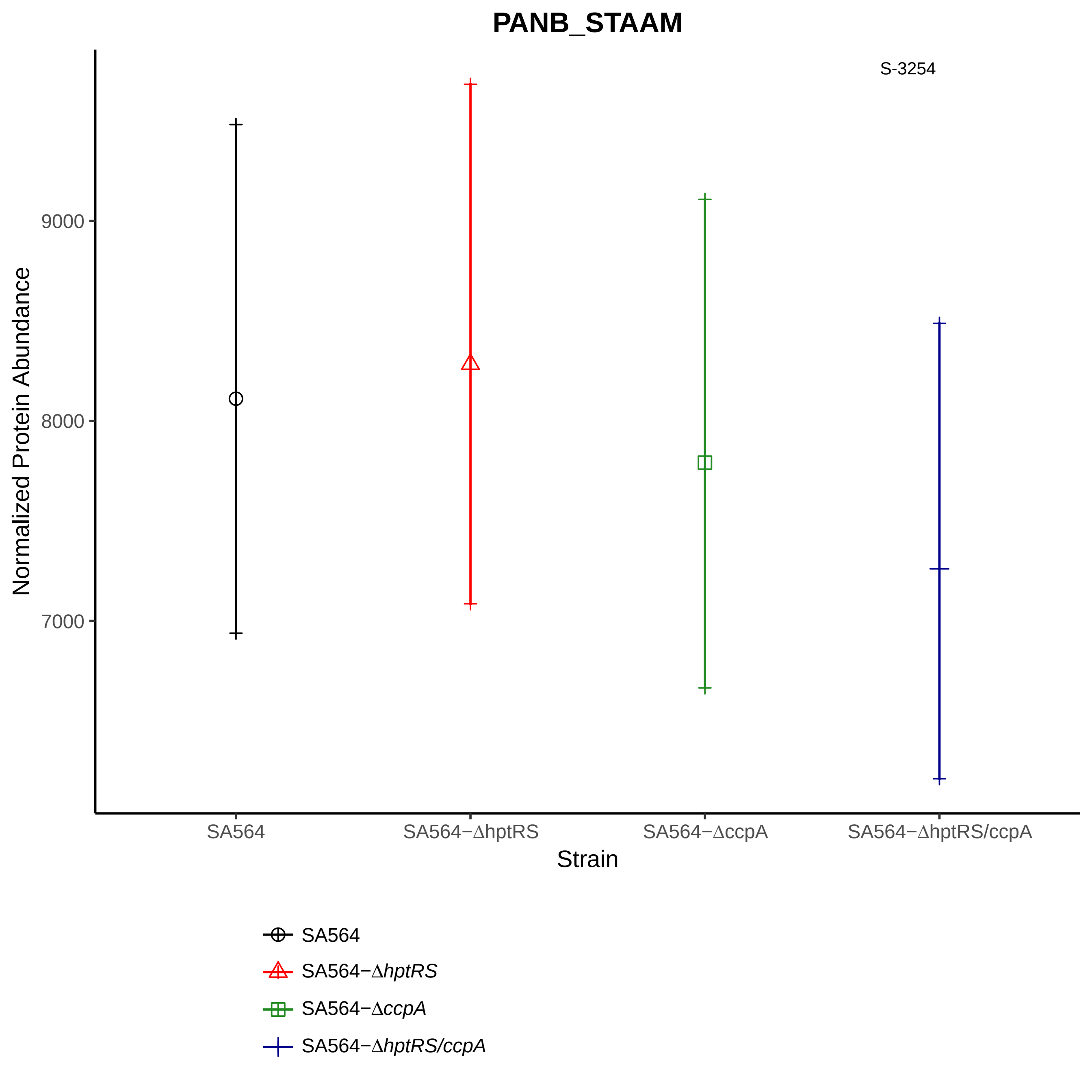

PANB_STAAM
S-3254
Normalized Protein Abundance
9000
8000
7000
SA564
SA564−ΔhptRS
SA564−ΔccpA
SA564−ΔhptRS/ccpA
Strain
SA564
SA564−Δ*hptRS*
SA564−Δ*ccpA*
SA564−Δ*hptRS/ccpA*

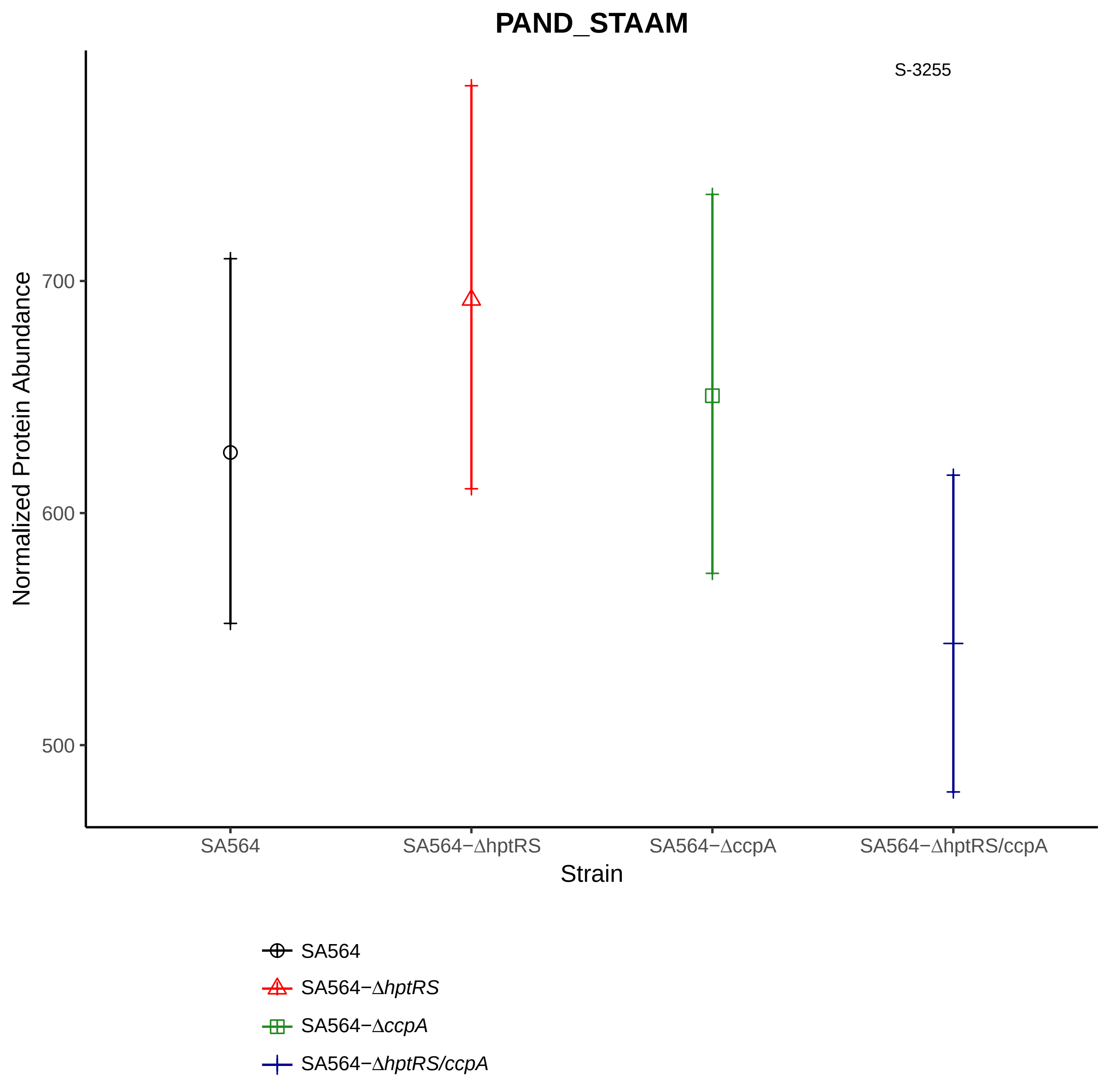

PAND_STAAM
S-3255
Normalized Protein Abundance
700
600
500
SA564
SA564−ΔhptRS
SA564−ΔccpA
SA564−ΔhptRS/ccpA
Strain
SA564
SA564−ΔhptRS
SA564−ΔccpA
SA564−ΔhptRS/ccpA

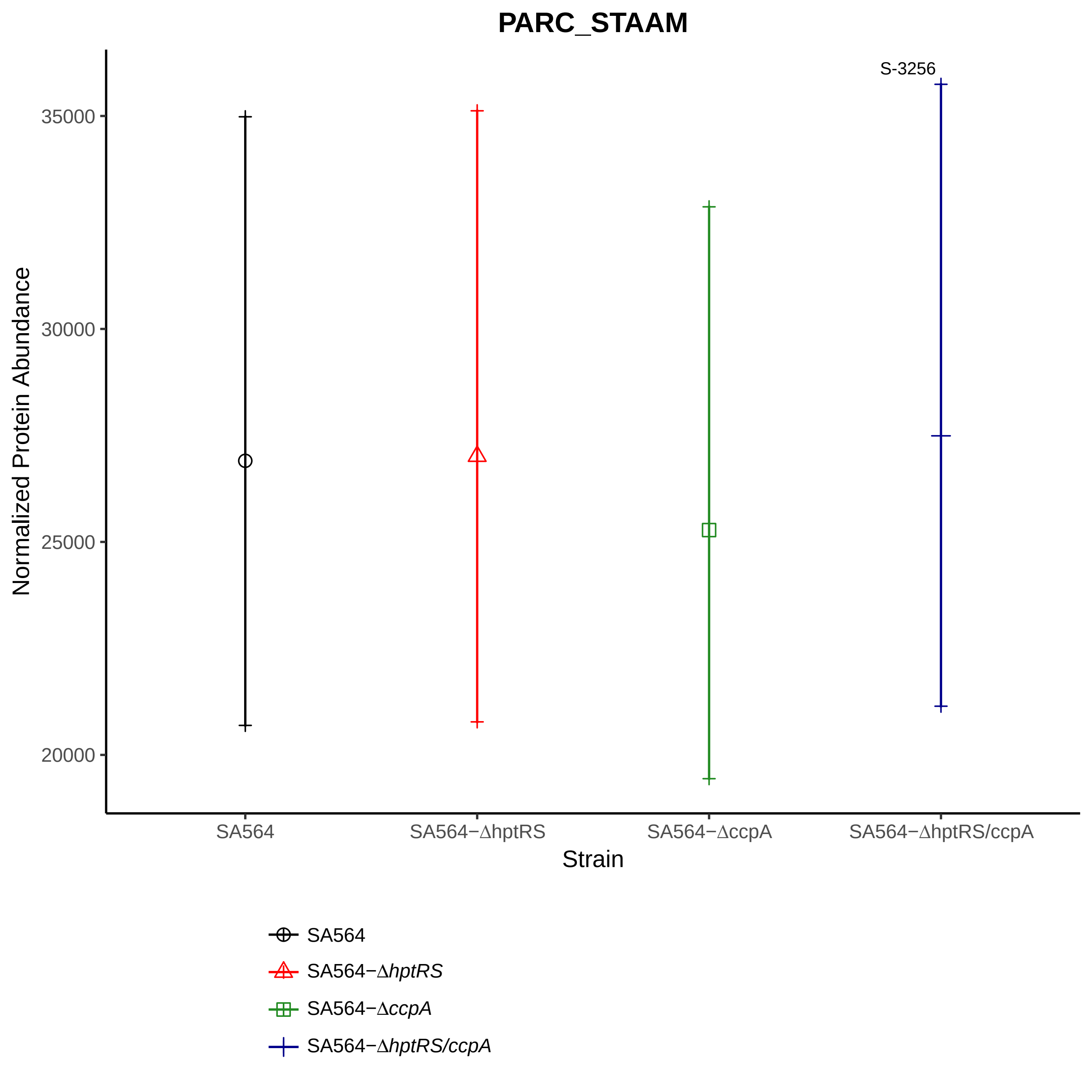

PARC_STAAM
S-3256
35000
30000
25000
20000
Normalized Protein Abundance
SA564
SA564−ΔhptRS
SA564−ΔccpA
SA564−ΔhptRS/ccpA
Strain
SA564
SA564−Δ*hptRS*
SA564−Δ*ccpA*
SA564−Δ*hptRS/ccpA*

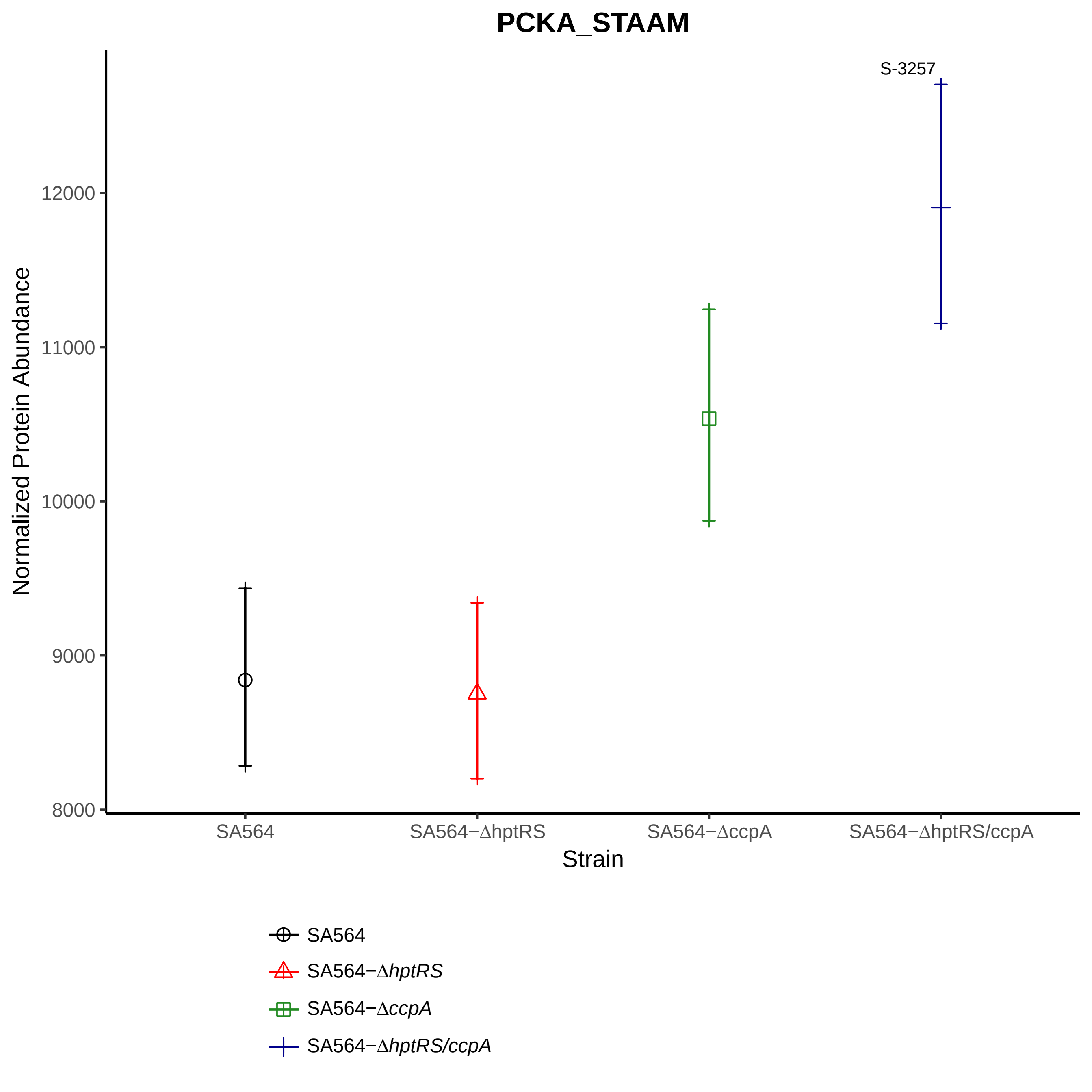

PCKA_STAAM
S-3257
Normalized Protein Abundance
12000
11000
10000
9000
8000
SA564
SA564−ΔhptRS
SA564−ΔccpA
SA564−ΔhptRS/ccpA
Strain
SA564
SA564−Δ*hptRS*
SA564−Δ*ccpA*
SA564−Δ*hptRS/ccpA*

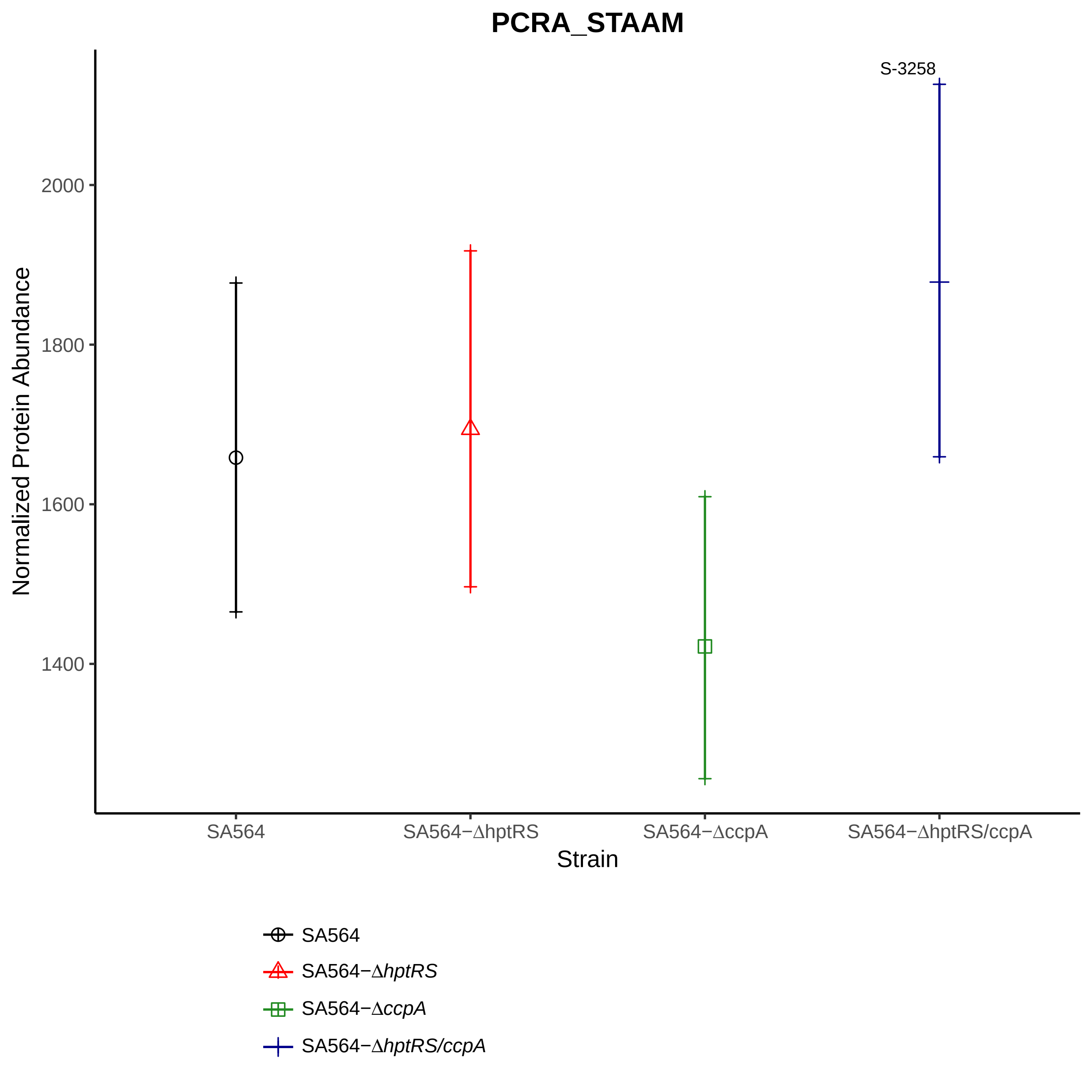
PCRA_STAAM
S-3258
Normalized Protein Abundance
2000
1800
1600
1400
SA564
SA564−ΔhptRS
SA564−ΔccpA
SA564−ΔhptRS/ccpA
Strain
SA564
SA564−ΔhptRS
SA564−ΔccpA
SA564−ΔhptRS/ccpA

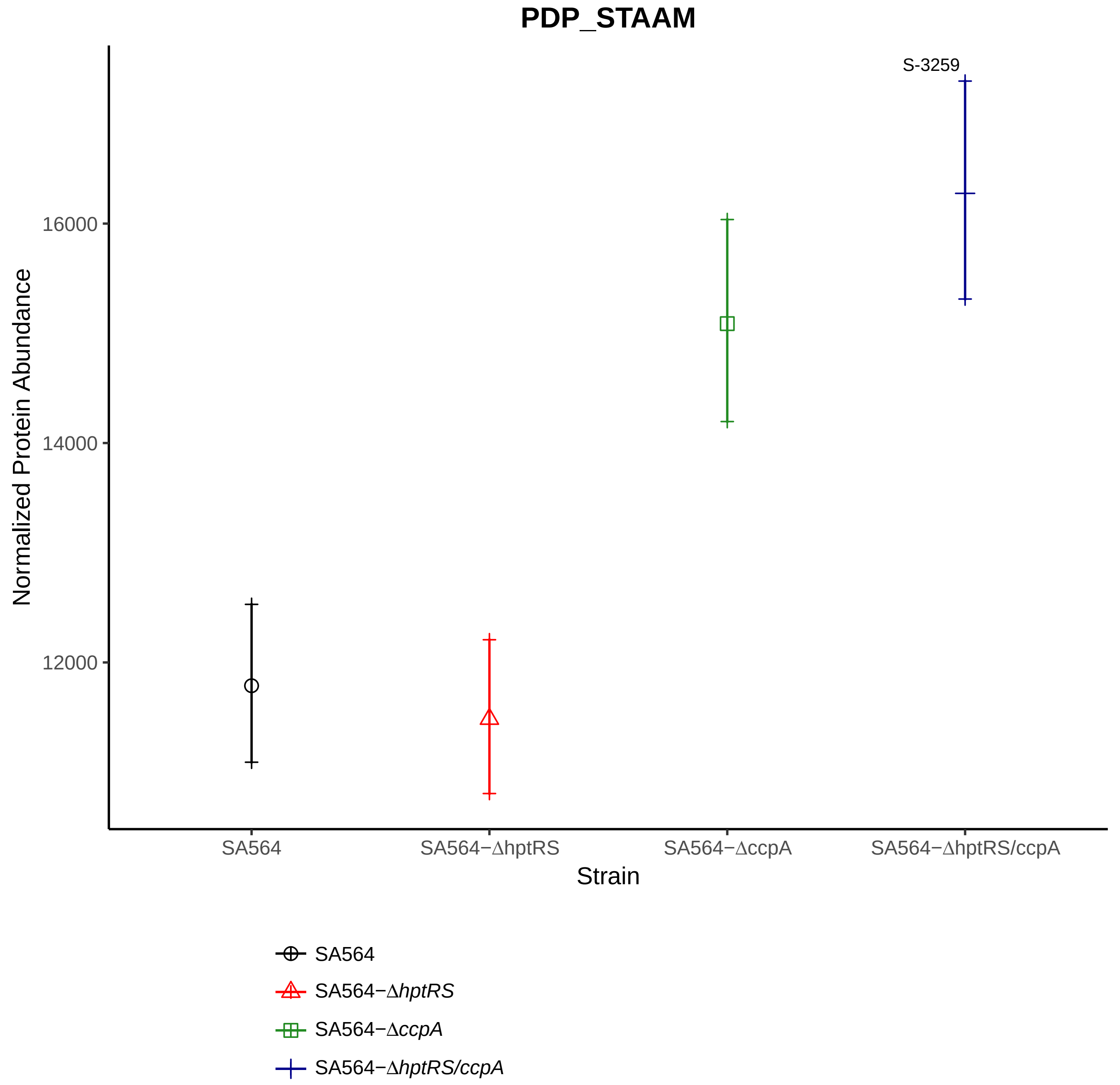

PDP_STAAM
S-3259
Normalized Protein Abundance
16000
14000
12000
SA564
SA564−ΔhptRS
SA564−ΔccpA
SA564−ΔhptRS/ccpA
Strain
SA564
SA564−ΔhptRS
SA564−ΔccpA
SA564−ΔhptRS/ccpA

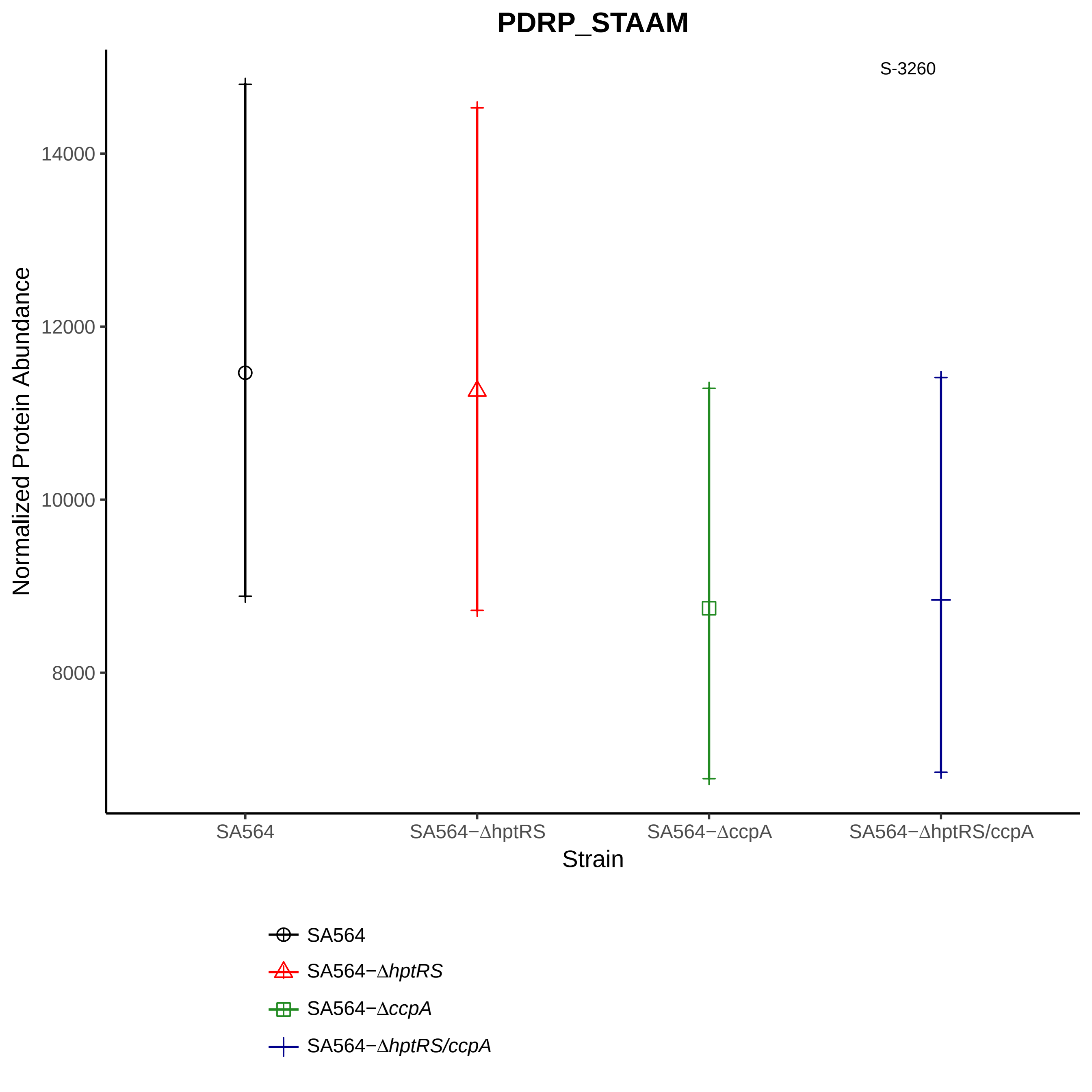

PDRP_STAAM
S-3260
Normalized Protein Abundance
14000
12000
10000
8000
SA564
SA564−ΔhptRS
SA564−ΔccpA
SA564−ΔhptRS/ccpA
Strain
SA564
SA564−ΔhptRS
SA564−ΔccpA
SA564−ΔhptRS/ccpA

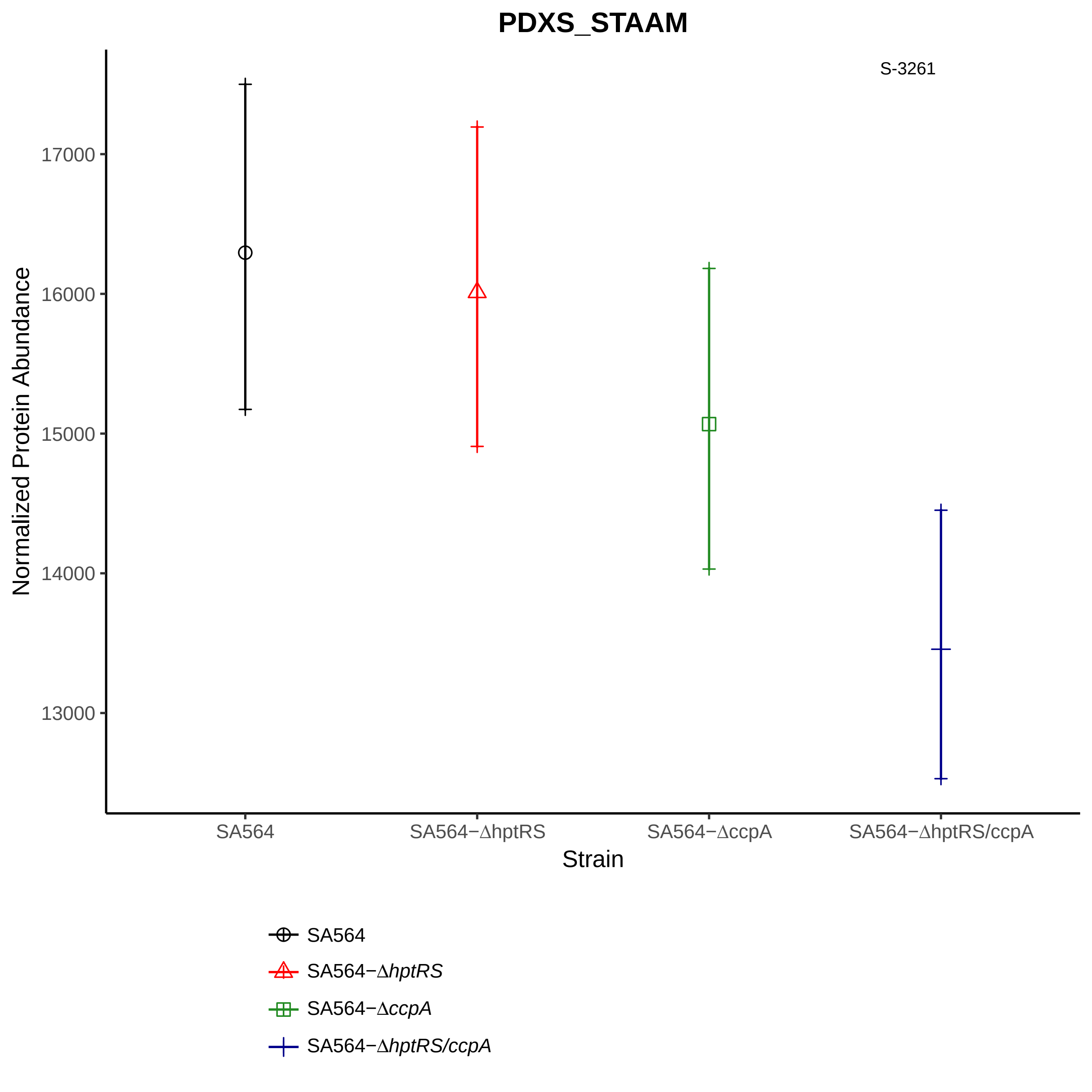

PDXS_STAAM
S-3261
Normalized Protein Abundance
17000
16000
15000
14000
13000
SA564
SA564−ΔhptRS
SA564−ΔccpA
SA564−ΔhptRS/ccpA
Strain
SA564
SA564−Δ*hptRS*
SA564−Δ*ccpA*
SA564−Δ*hptRS/ccpA*

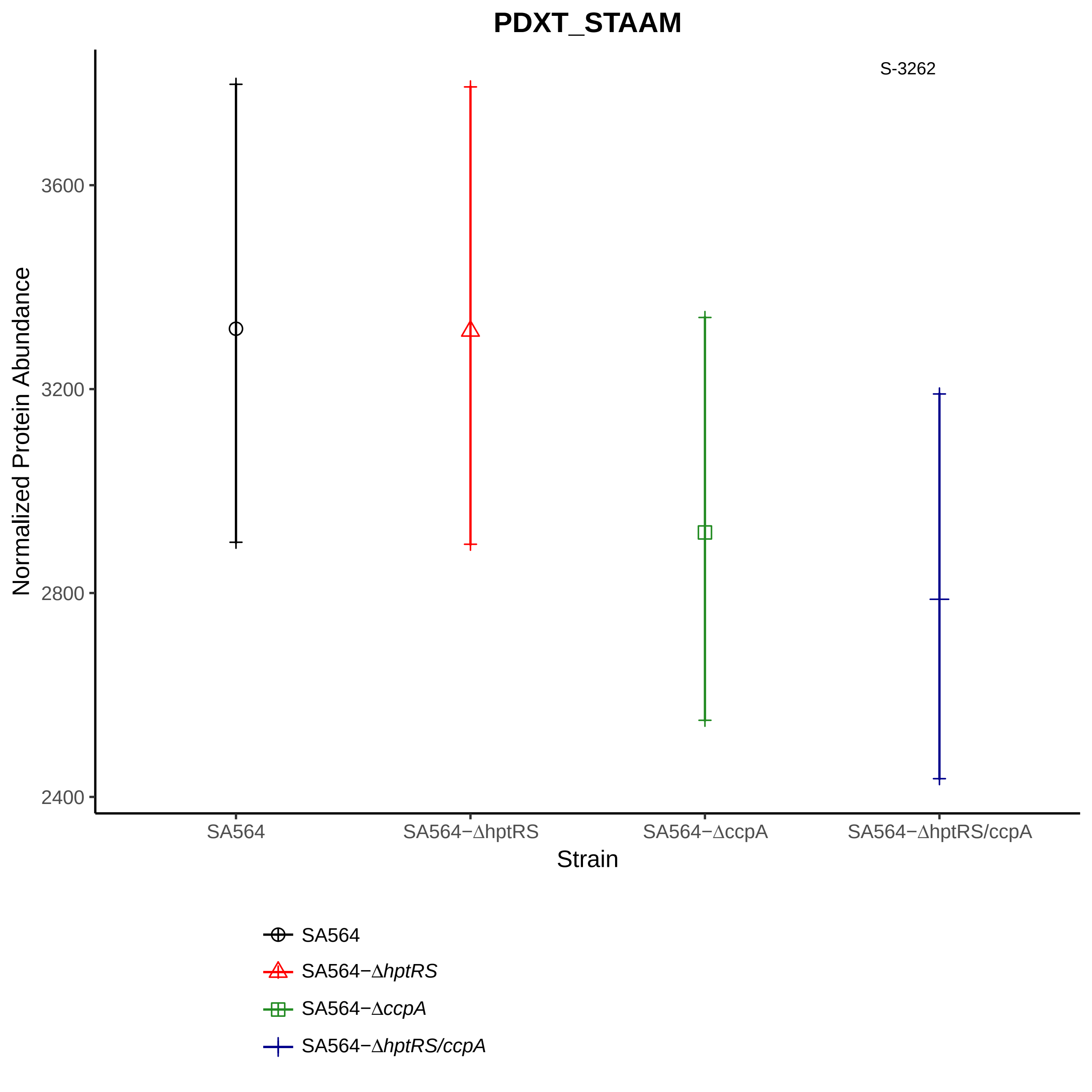

PDXT_STAAM
S-3262
Normalized Protein Abundance
3600
3200
2800
2400
SA564
SA564−ΔhptRS
SA564−ΔccpA
SA564−ΔhptRS/ccpA
Strain
SA564
SA564−ΔhptRS
SA564−ΔccpA
SA564−ΔhptRS/ccpA

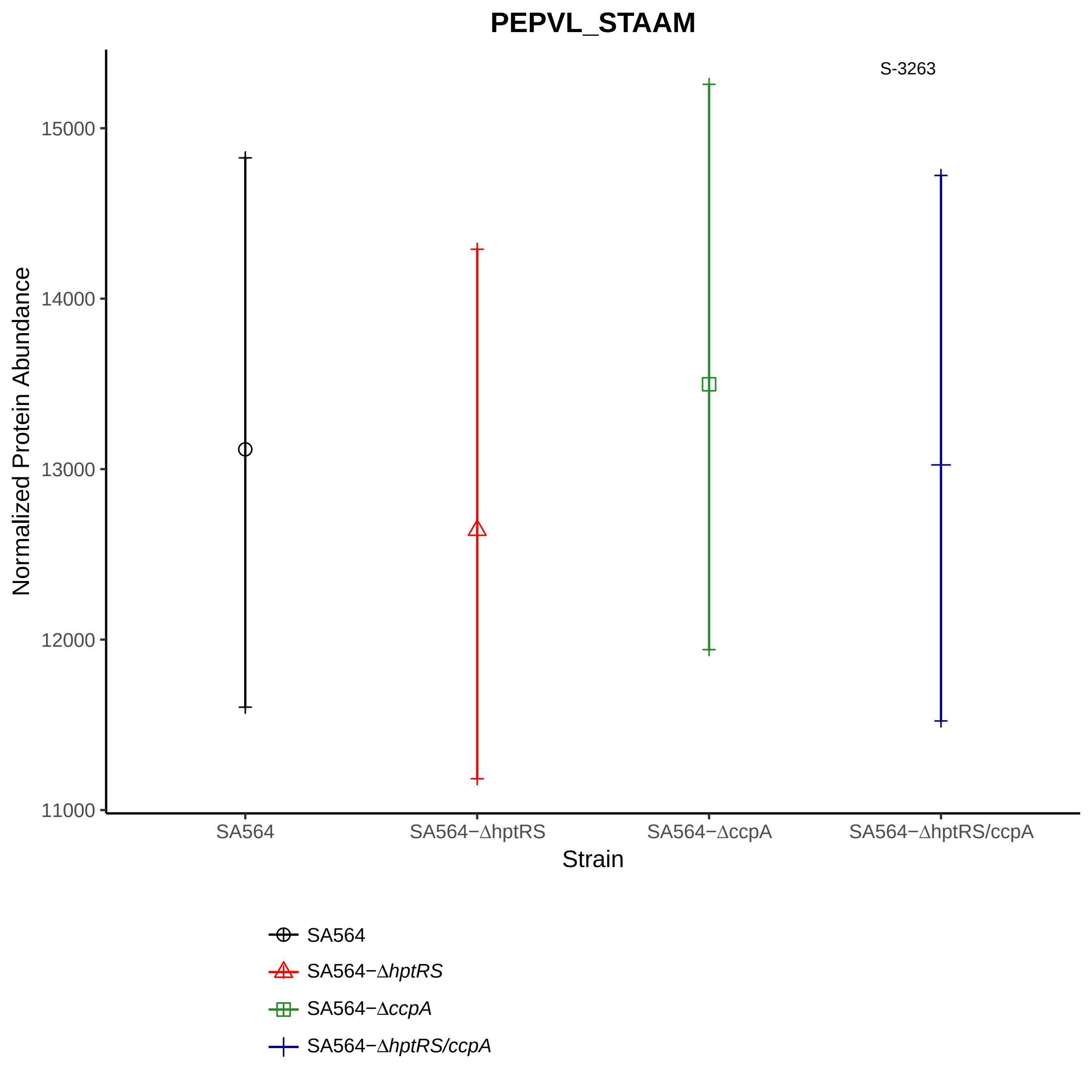

PEPVL_STAAM
S-3263
Normalized Protein Abundance
15000
14000
13000
12000
11000
SA564
SA564−ΔhptRS
SA564−ΔccpA
SA564−ΔhptRS/ccpA
Strain
SA564
SA564−ΔhptRS
SA564−ΔccpA
SA564−ΔhptRS/ccpA

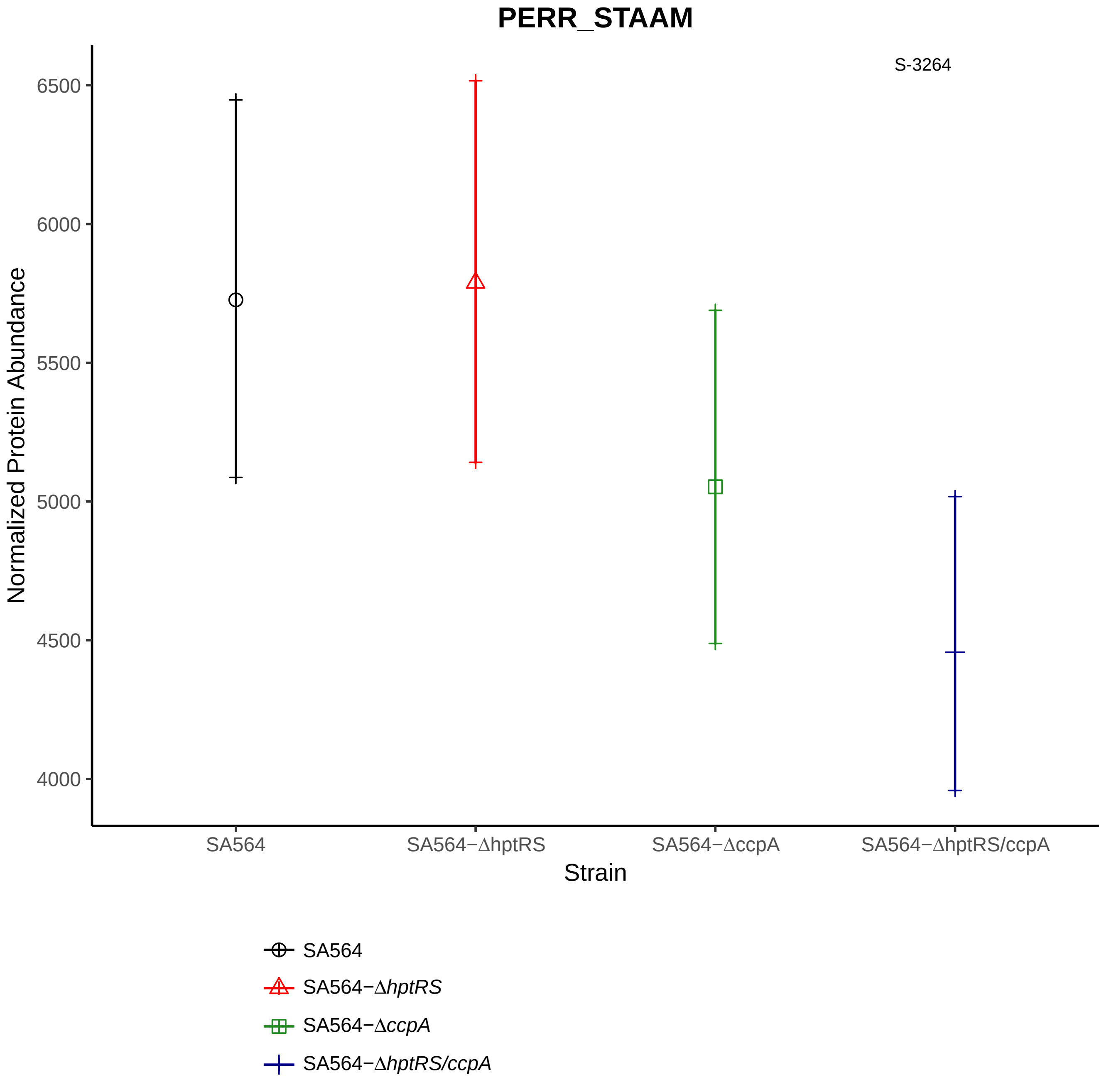
PERR_STAAM
S-3264
6500
6000
5500
5000
4500
4000
Normalized Protein Abundance
SA564
SA564−ΔhptRS
SA564−ΔccpA
SA564−ΔhptRS/ccpA
Strain
SA564
SA564−ΔhptRS
SA564−ΔccpA
SA564−ΔhptRS/ccpA

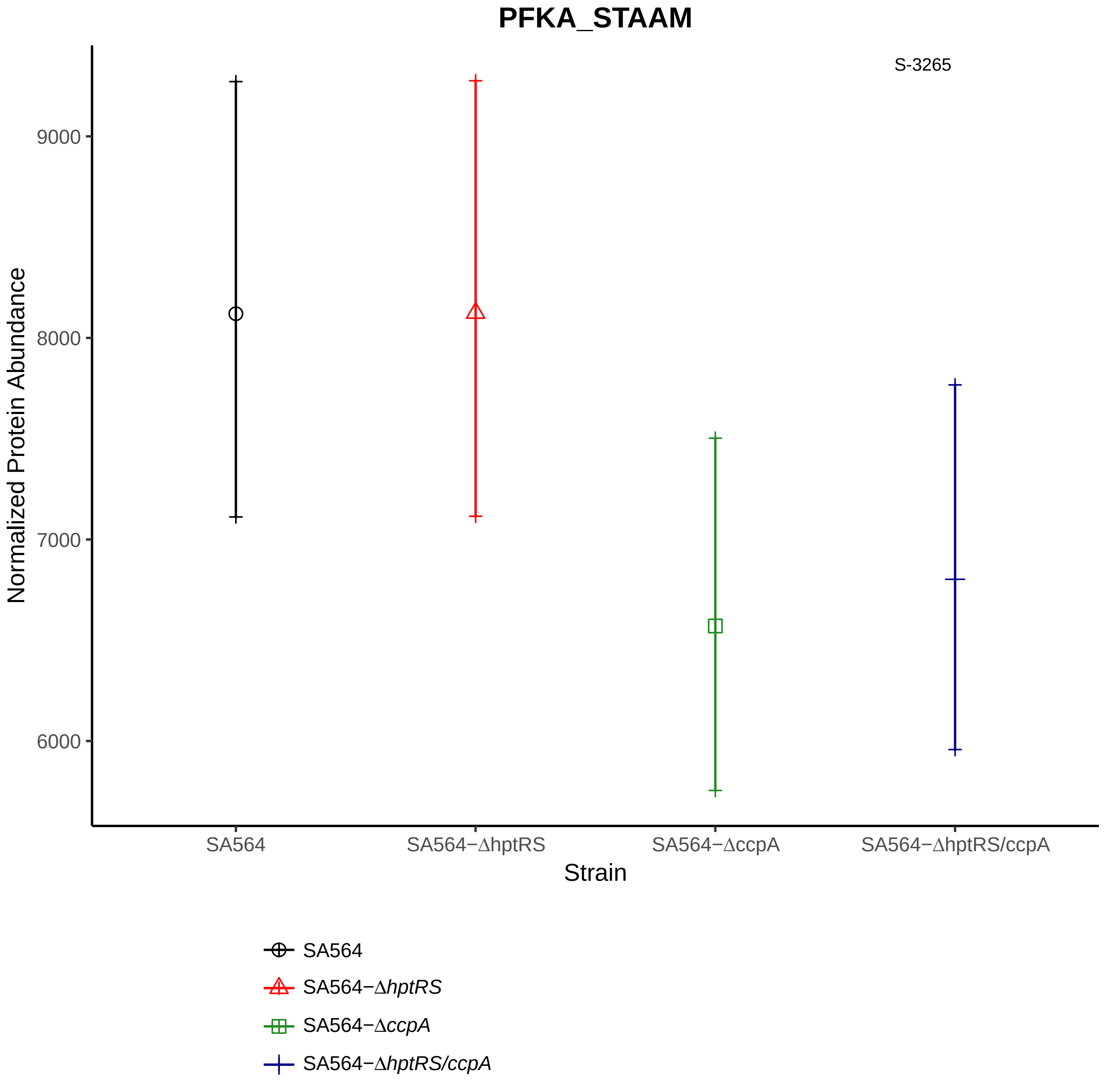
PFKA_STAAM
S-3265
Normalized Protein Abundance
9000
8000
7000
6000
SA564
SA564−ΔhptRS
SA564−ΔccpA
SA564−ΔhptRS/ccpA
Strain
SA564
SA564−ΔhptRS
SA564−ΔccpA
SA564−ΔhptRS/ccpA

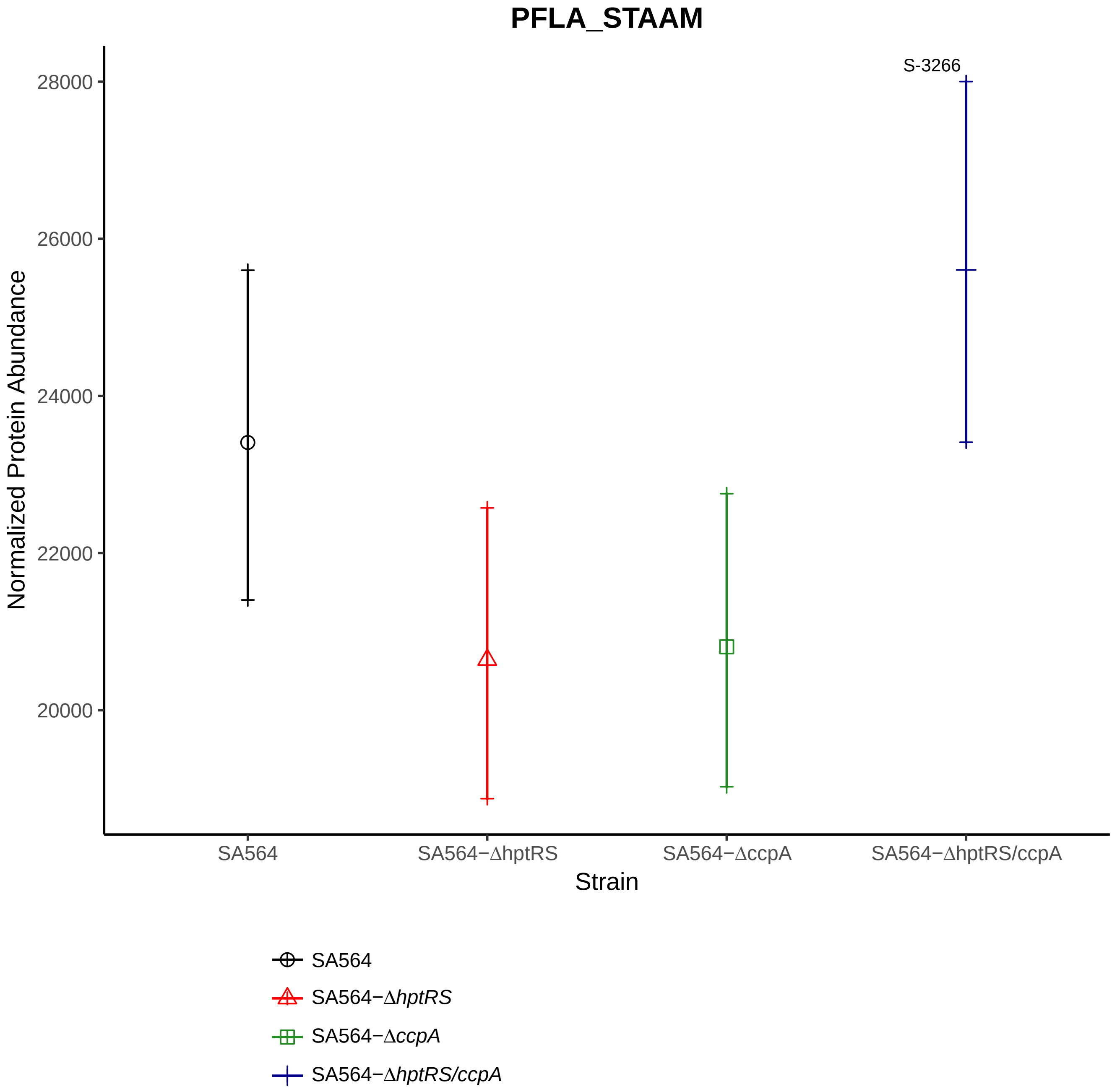

PFLA_STAAM
S-3266
28000
26000
24000
22000
20000
Normalized Protein Abundance
SA564
SA564−ΔhptRS
SA564−ΔccpA
SA564−ΔhptRS/ccpA
Strain
SA564
SA564−Δ*hptRS*
SA564−Δ*ccpA*
SA564−Δ*hptRS/ccpA*

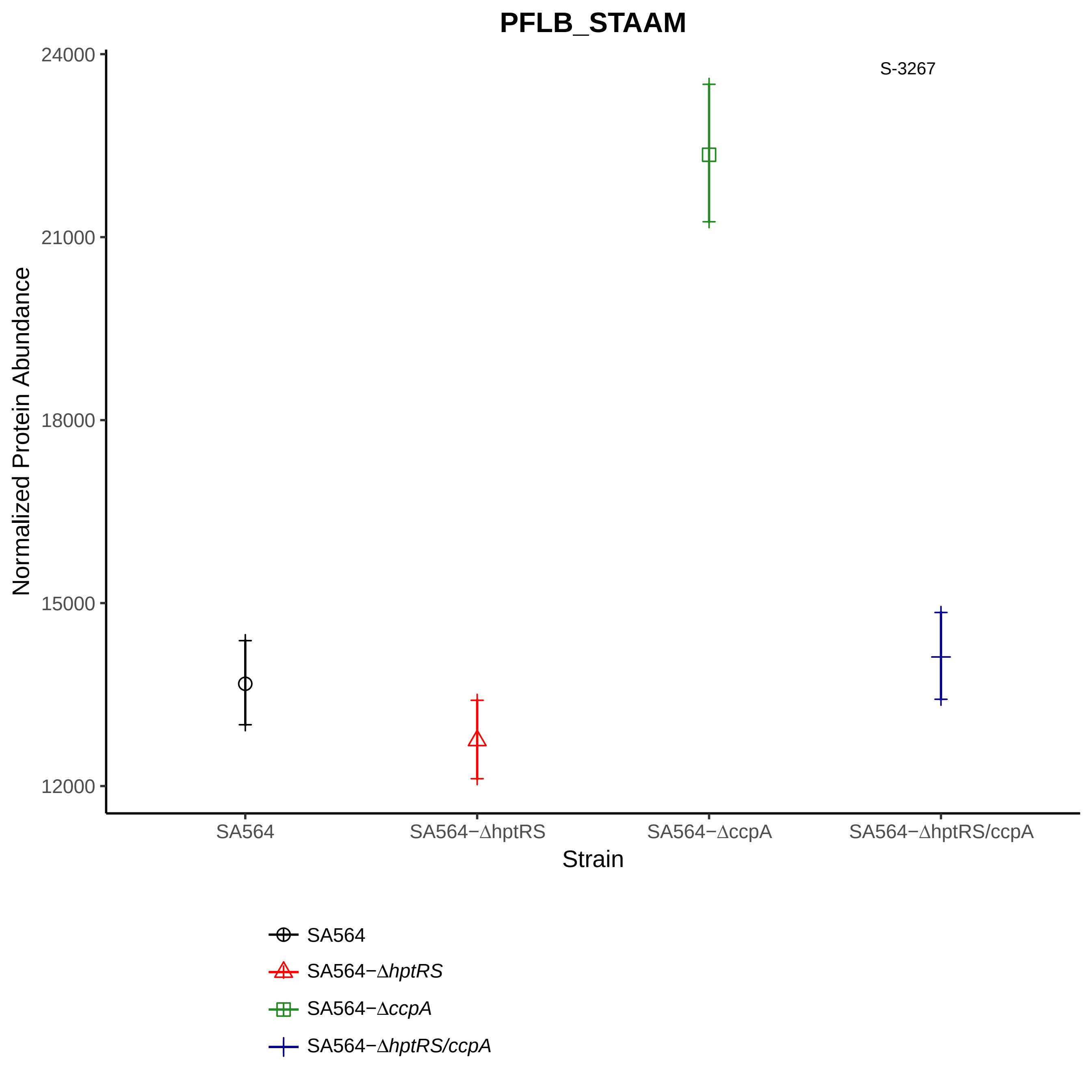
PFLB_STAAM
S-3267
Normalized Protein Abundance
24000
21000
18000
15000
12000
SA564
SA564−ΔhptRS
SA564−ΔccpA
SA564−ΔhptRS/ccpA
Strain
SA564
SA564−ΔhptRS
SA564−ΔccpA
SA564−ΔhptRS/ccpA

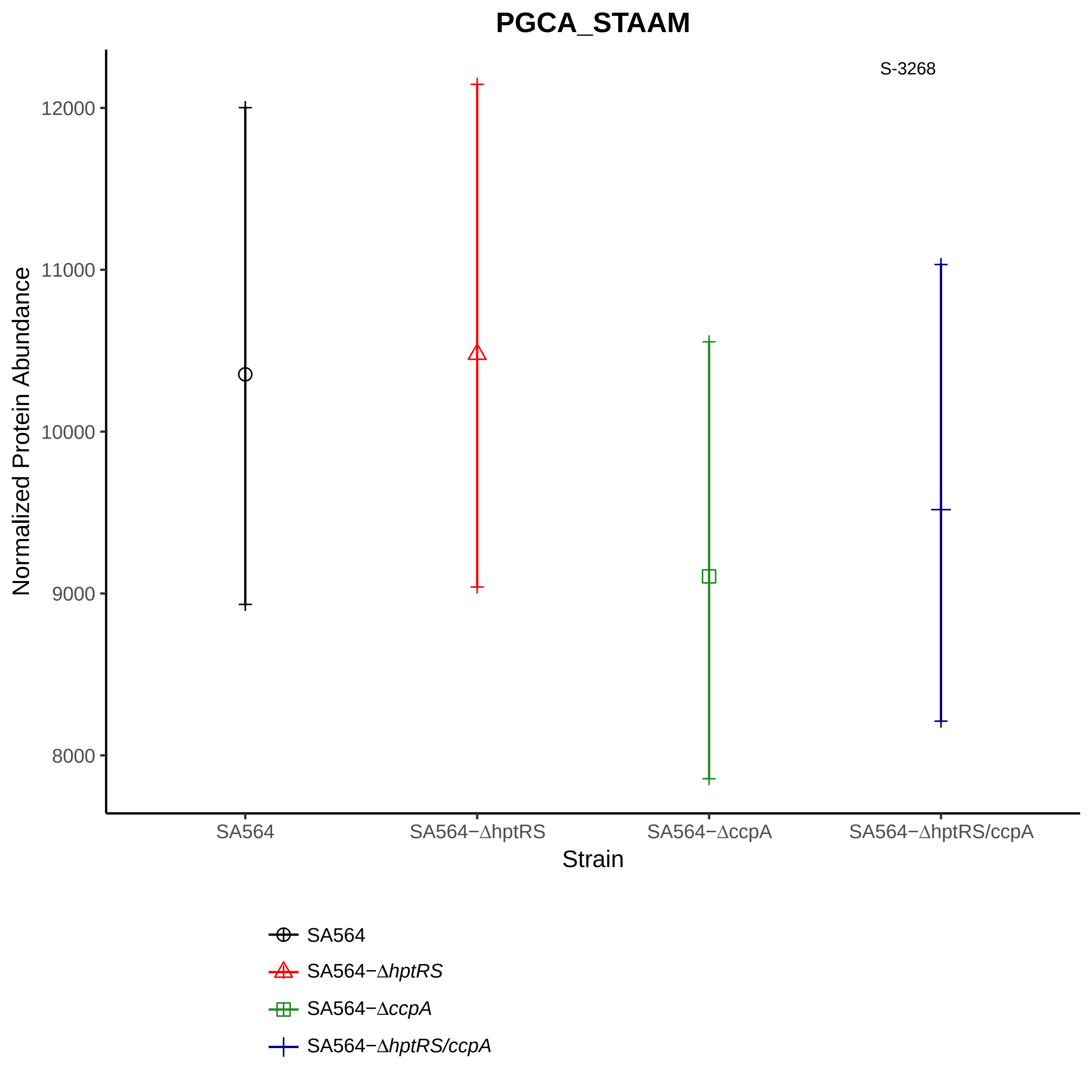
PGCA_STAAM
S-3268
Normalized Protein Abundance
12000
11000
10000
9000
8000
SA564
SA564−ΔhptRS
SA564−ΔccpA
SA564−ΔhptRS/ccpA
Strain
SA564
SA564−ΔhptRS
SA564−ΔccpA
SA564−ΔhptRS/ccpA

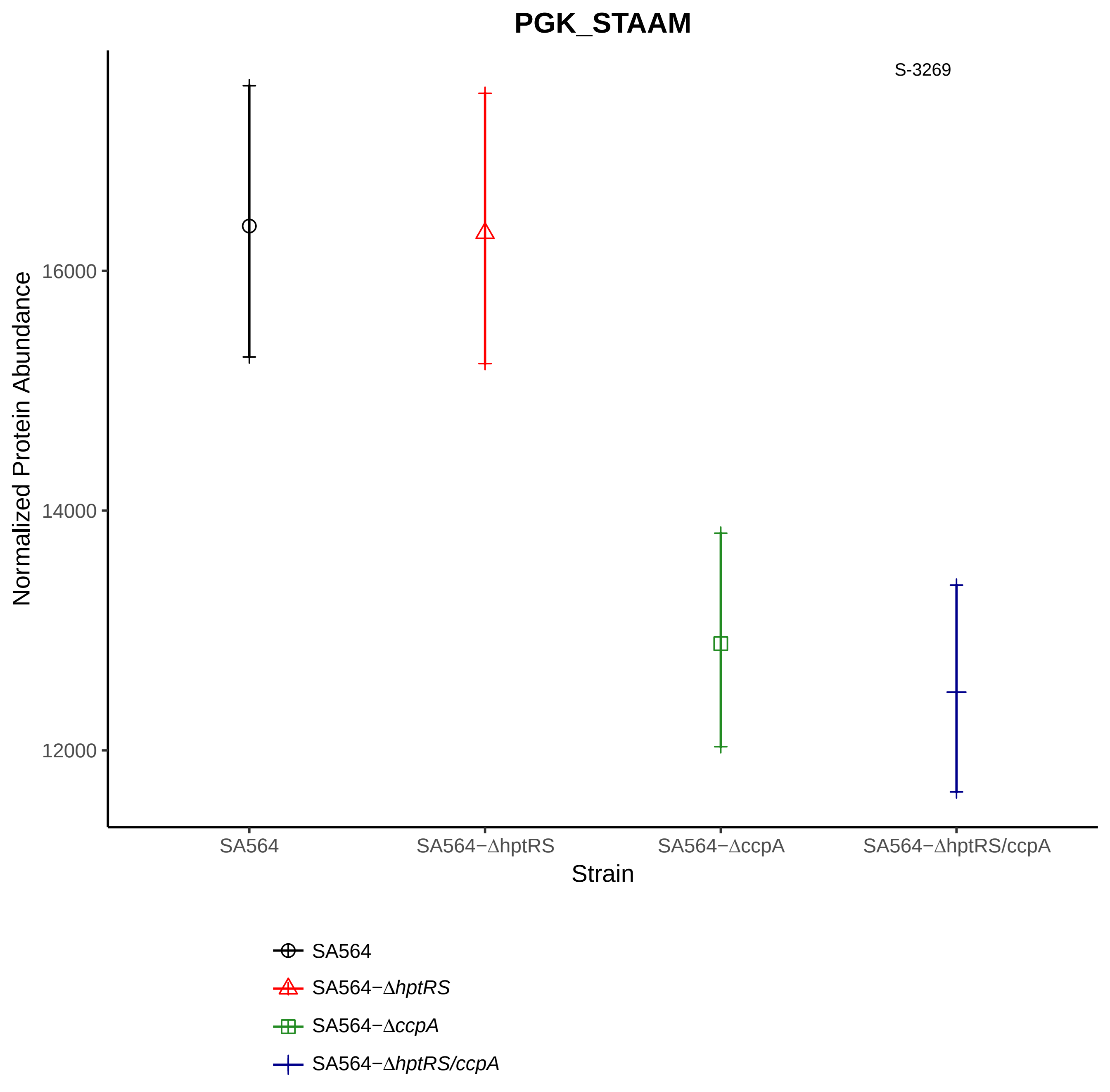

PGK_STAAM
S-3269
Normalized Protein Abundance
16000
14000
12000
SA564
SA564−ΔhptRS
SA564−ΔccpA
SA564−ΔhptRS/ccpA
Strain
SA564
SA564−Δ*hptRS*
SA564−Δ*ccpA*
SA564−Δ*hptRS/ccpA*

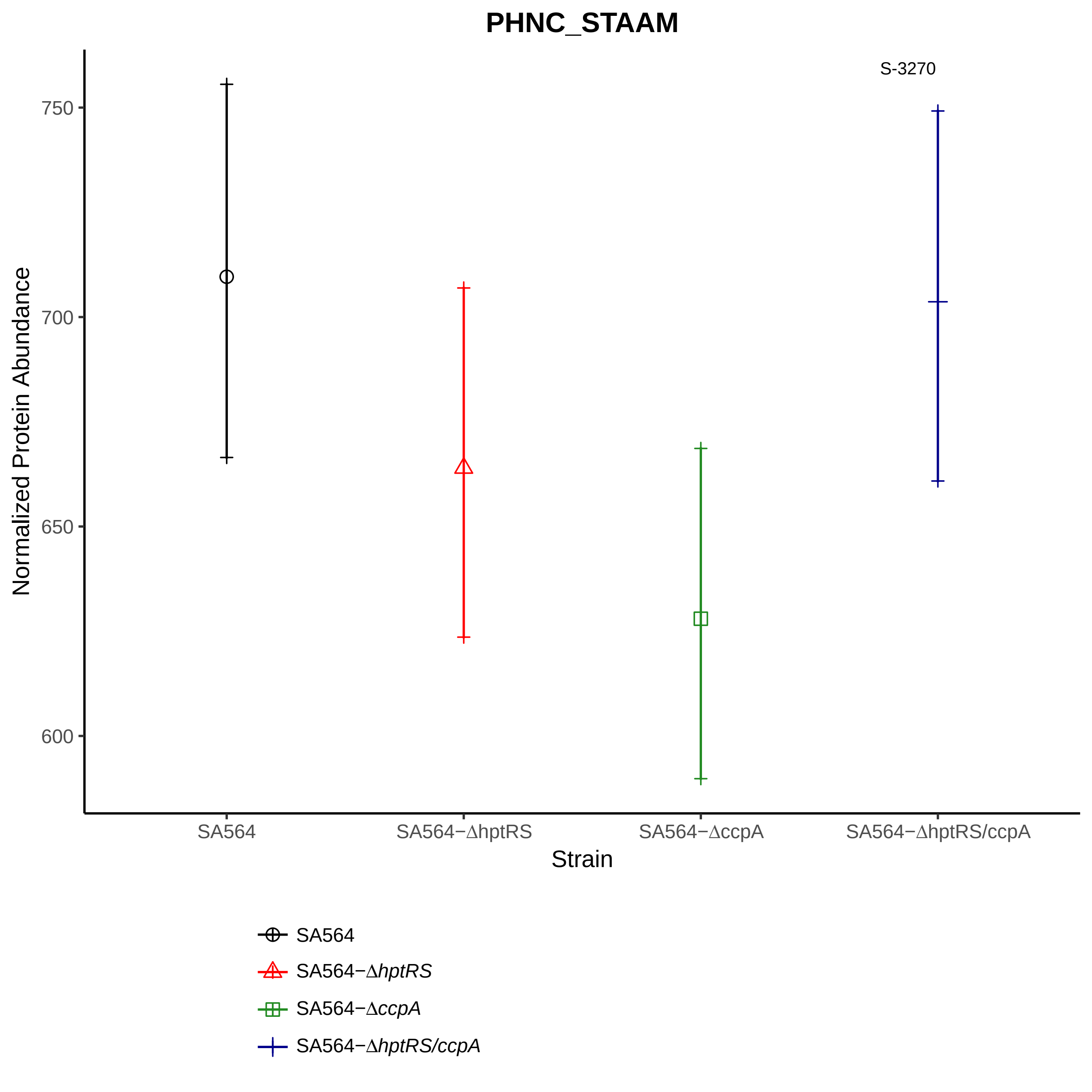
PHNC_STAAM
S-3270
Normalized Protein Abundance
750
700
650
600
SA564
SA564−ΔhptRS
SA564−ΔccpA
SA564−ΔhptRS/ccpA
Strain
SA564
SA564−ΔhptRS
SA564−ΔccpA
SA564−ΔhptRS/ccpA

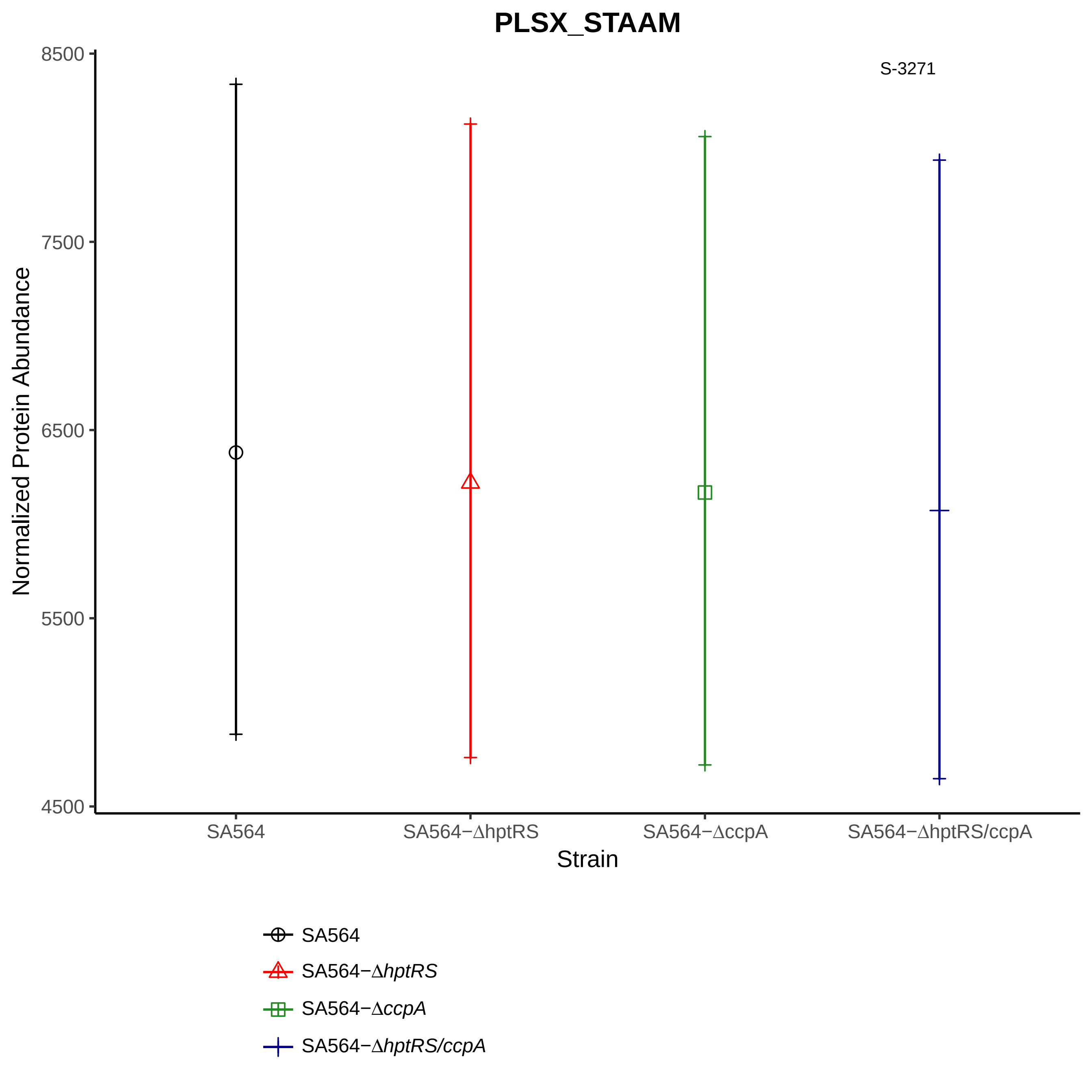
PLSX_STAAM
S-3271
8500
7500
6500
5500
4500
Normalized Protein Abundance
SA564
SA564−ΔhptRS
SA564−ΔccpA
SA564−ΔhptRS/ccpA
Strain
SA564
SA564−ΔhptRS
SA564−ΔccpA
SA564−ΔhptRS/ccpA

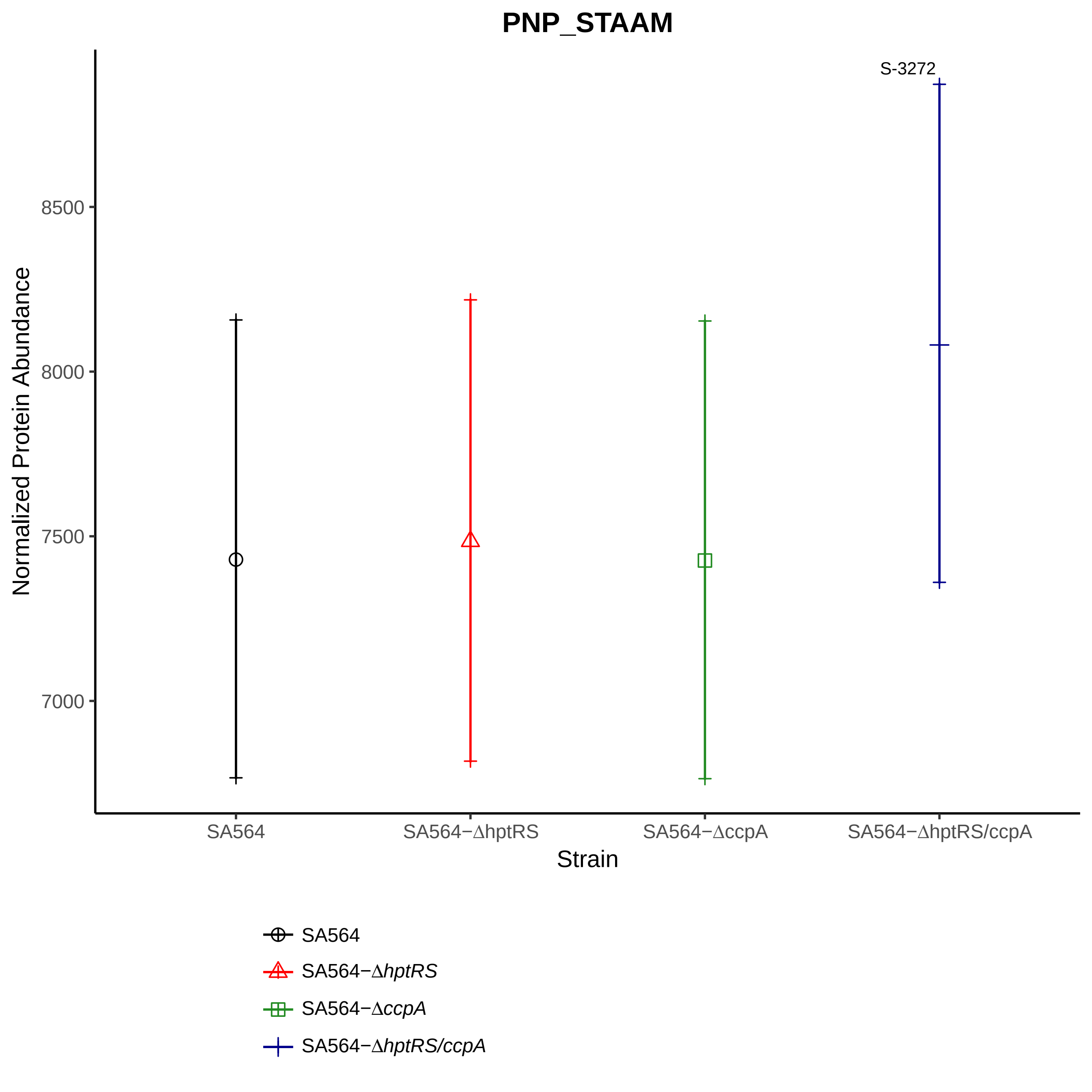
PNP_STAAM
S-3272
8500
8000
7500
7000
Normalized Protein Abundance
SA564
SA564−ΔhptRS
SA564−ΔccpA
SA564−ΔhptRS/ccpA
Strain
SA564
SA564−ΔhptRS
SA564−ΔccpA
SA564−ΔhptRS/ccpA

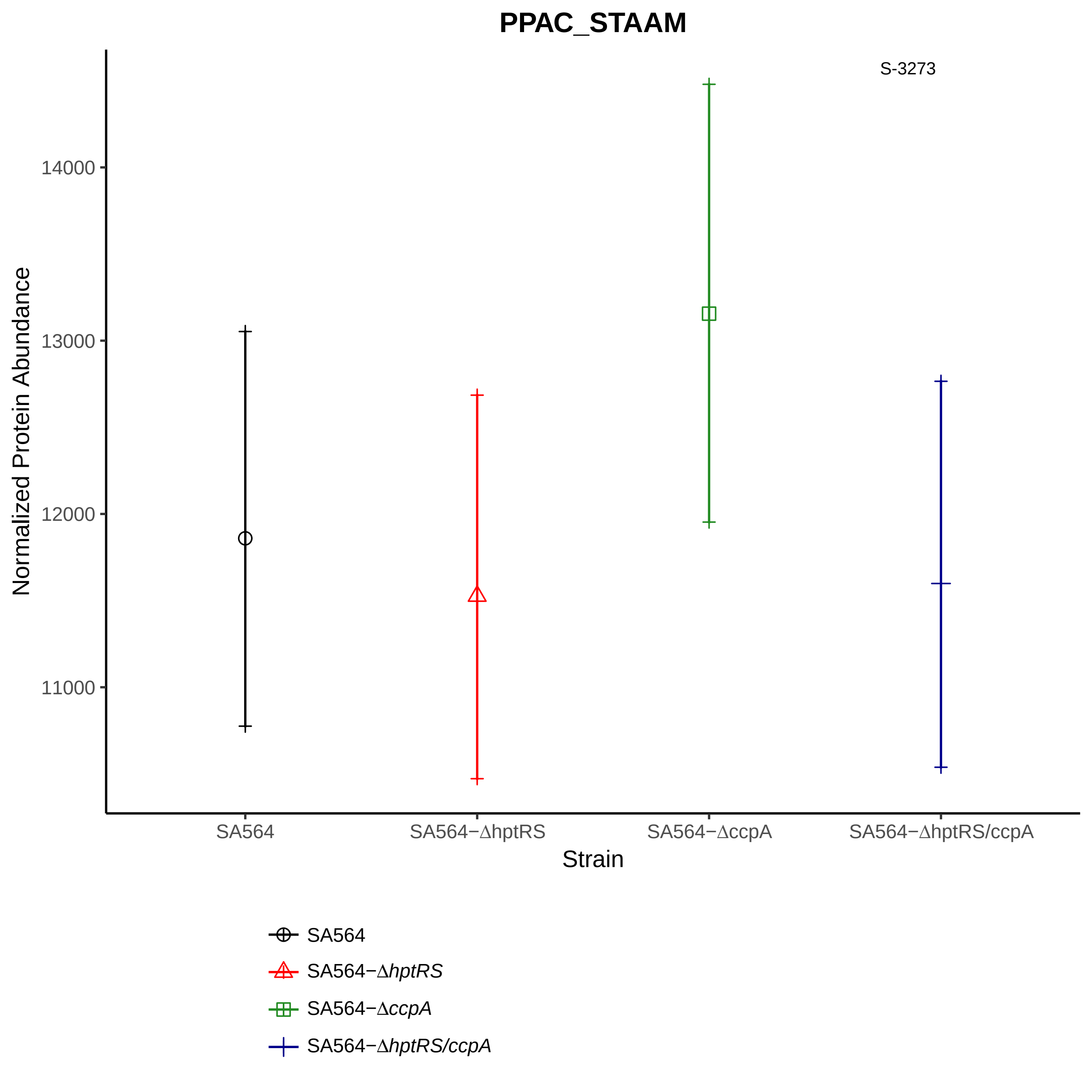
PPAC_STAAM
S-3273
Normalized Protein Abundance
14000
13000
12000
11000
SA564
SA564−ΔhptRS
SA564−ΔccpA
SA564−ΔhptRS/ccpA
Strain
SA564
SA564−ΔhptRS
SA564−ΔccpA
SA564−ΔhptRS/ccpA

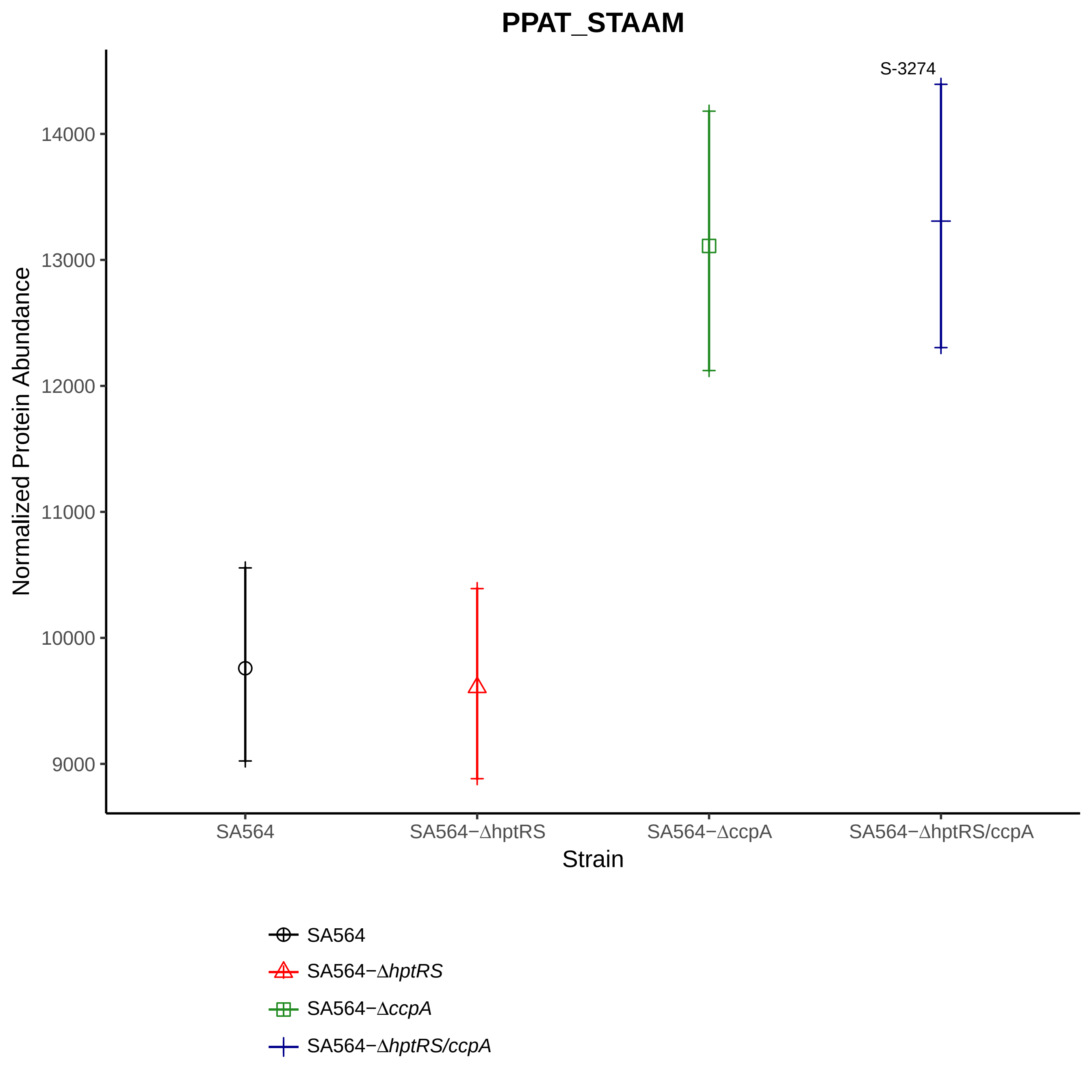
PPAT_STAAM
S-3274
Normalized Protein Abundance
14000
13000
12000
11000
10000
9000
SA564
SA564−ΔhptRS
SA564−ΔccpA
SA564−ΔhptRS/ccpA
Strain
SA564
SA564−ΔhptRS
SA564−ΔccpA
SA564−ΔhptRS/ccpA

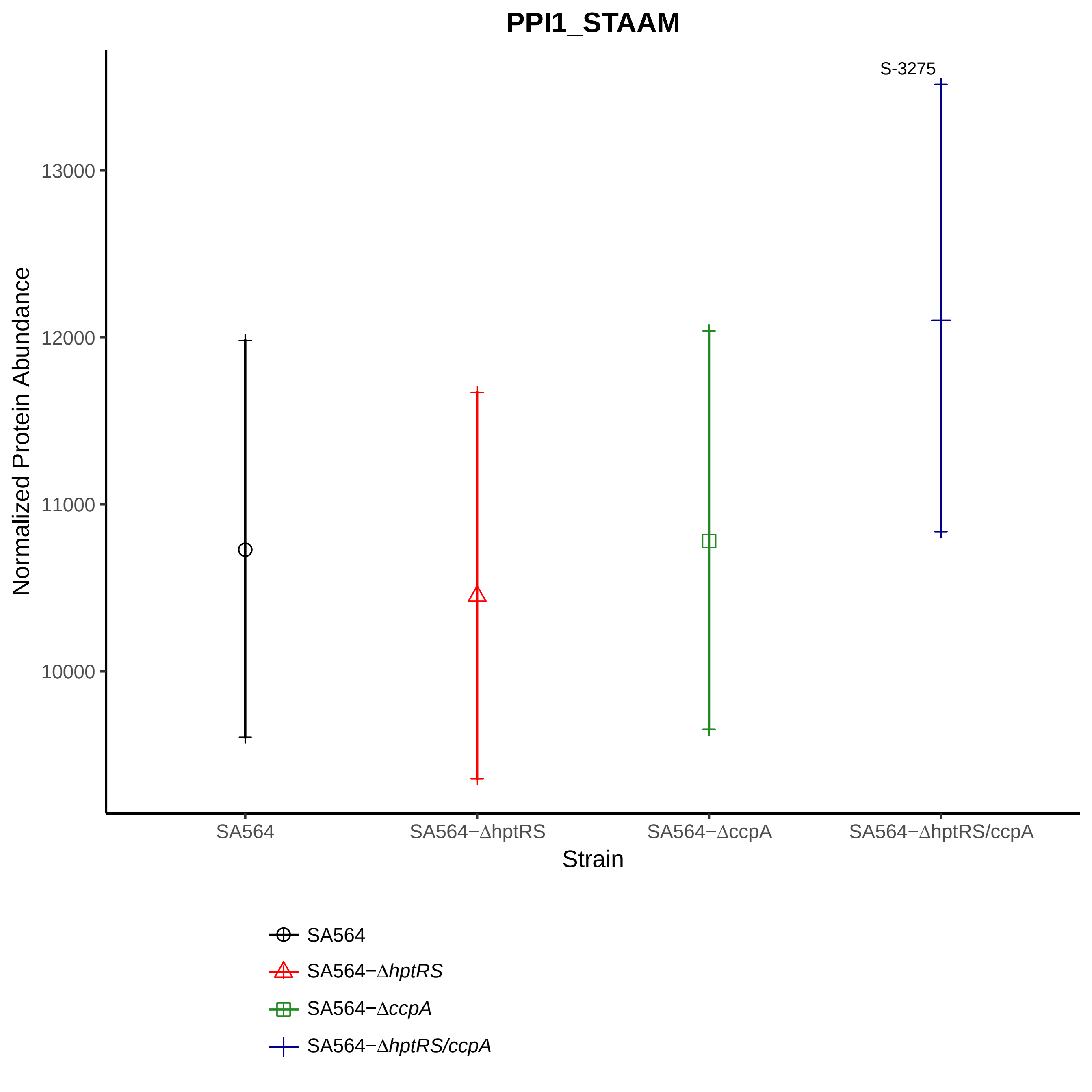

PPI1_STAAM
S-3275
Normalized Protein Abundance
13000
12000
11000
10000
SA564
SA564−ΔhptRS
SA564−ΔccpA
SA564−ΔhptRS/ccpA
Strain
SA564
SA564−ΔhptRS
SA564−ΔccpA
SA564−ΔhptRS/ccpA

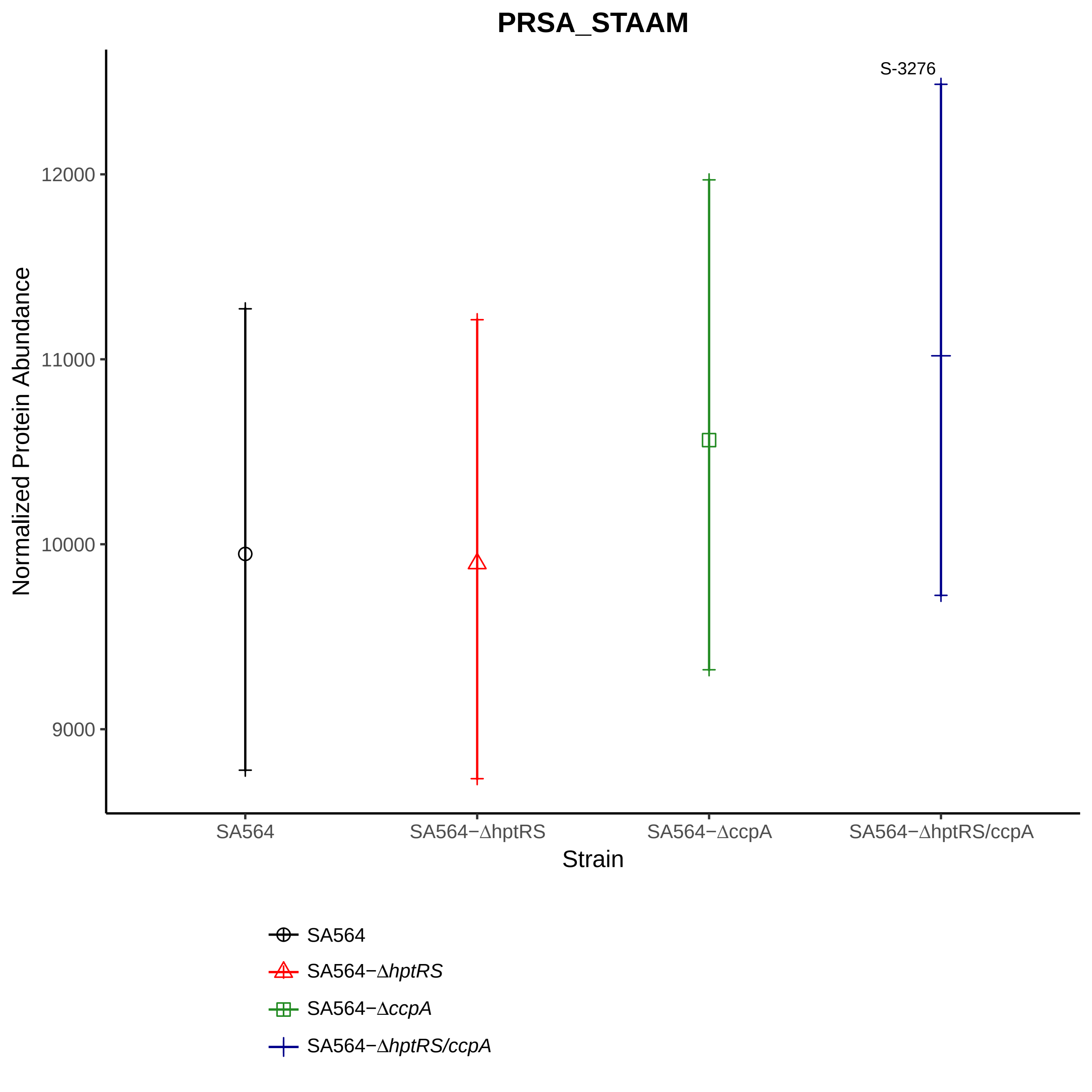

PRSA_STAAM
S-3276
12000
11000
10000
9000
Normalized Protein Abundance
SA564
SA564−ΔhptRS
SA564−ΔccpA
SA564−ΔhptRS/ccpA
Strain
SA564
SA564−ΔhptRS
SA564−ΔccpA
SA564−ΔhptRS/ccpA

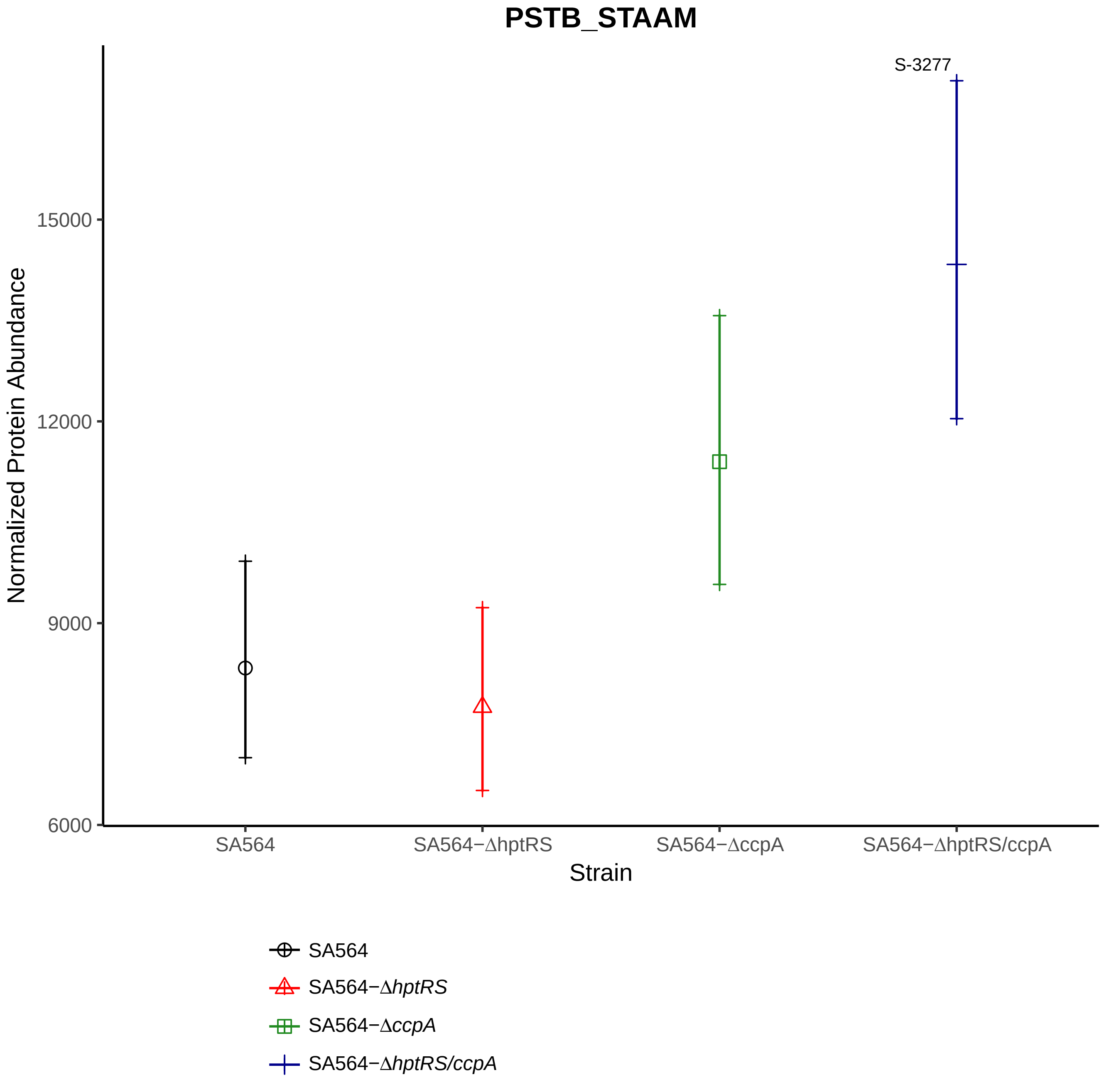

PSTB_STAAM
S-3277
Normalized Protein Abundance
15000
12000
9000
6000
SA564
SA564−ΔhptRS
SA564−ΔccpA
SA564−ΔhptRS/ccpA
Strain
SA564
SA564−Δ*hptRS*
SA564−Δ*ccpA*
SA564−Δ*hptRS/ccpA*

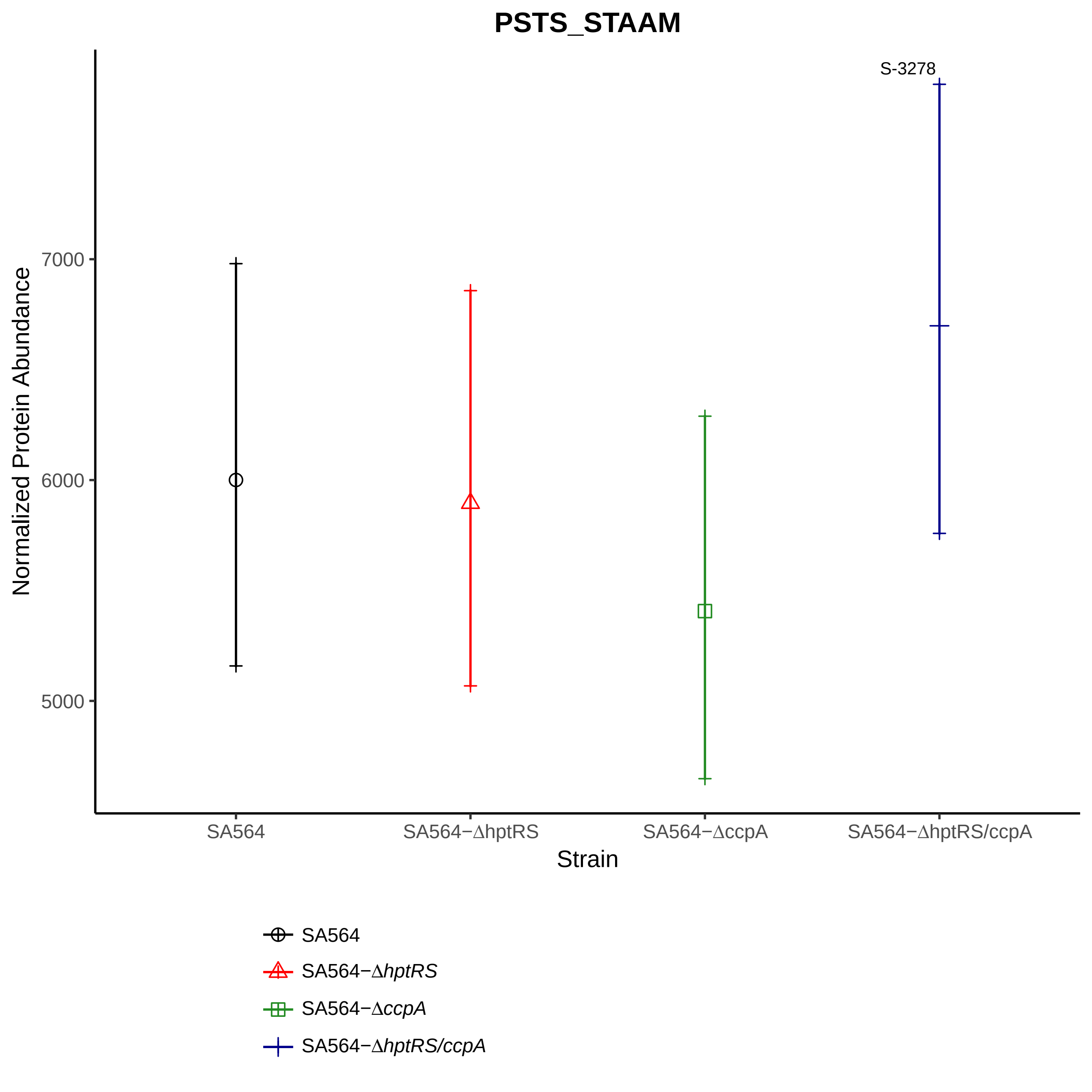

PSTS_STAAM
S-3278
7000
6000
5000
Normalized Protein Abundance
SA564
SA564−ΔhptRS
SA564−ΔccpA
SA564−ΔhptRS/ccpA
Strain
SA564
SA564−Δ*hptRS*
SA564−Δ*ccpA*
SA564−Δ*hptRS/ccpA*

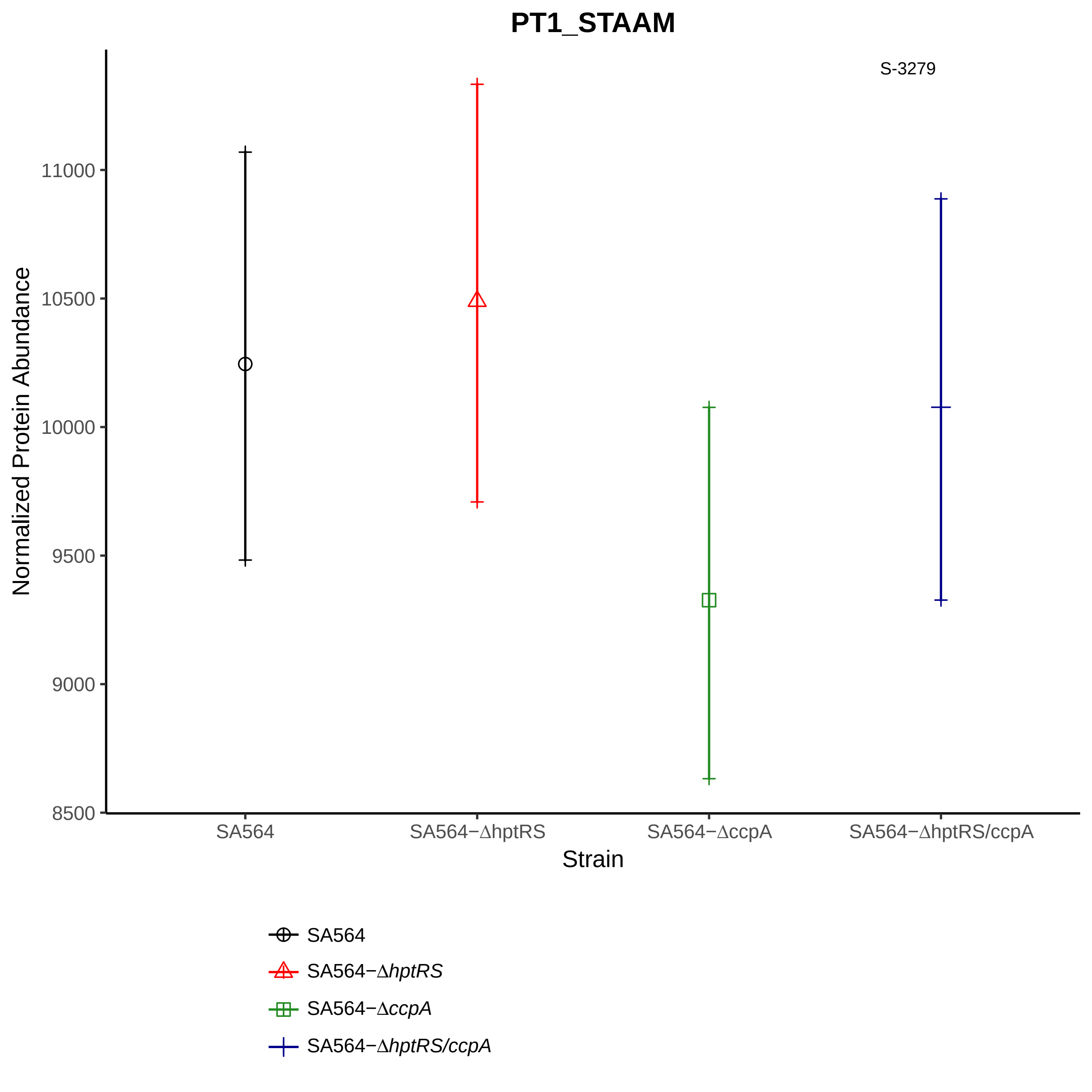

PT1_STAAM
S-3279
Normalized Protein Abundance
11000
10500
10000
9500
9000
8500
SA564
SA564−ΔhptRS
SA564−ΔccpA
SA564−ΔhptRS/ccpA
Strain
SA564
SA564−Δ*hptRS*
SA564−Δ*ccpA*
SA564−Δ*hptRS/ccpA*

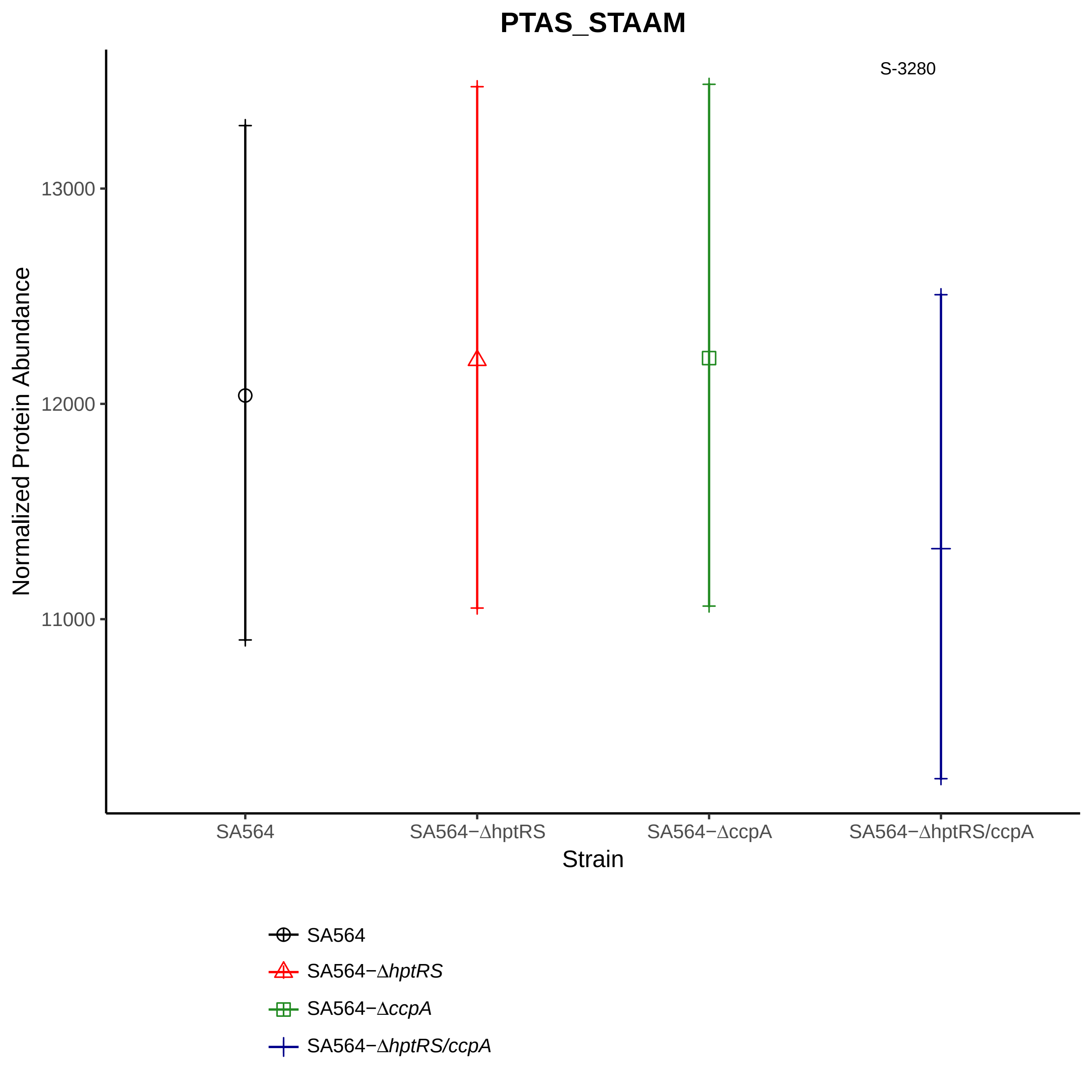

PTAS_STAAM
S-3280
Normalized Protein Abundance
13000
12000
11000
SA564
SA564−ΔhptRS
SA564−ΔccpA
SA564−ΔhptRS/ccpA
Strain
SA564
SA564−ΔhptRS
SA564−ΔccpA
SA564−ΔhptRS/ccpA

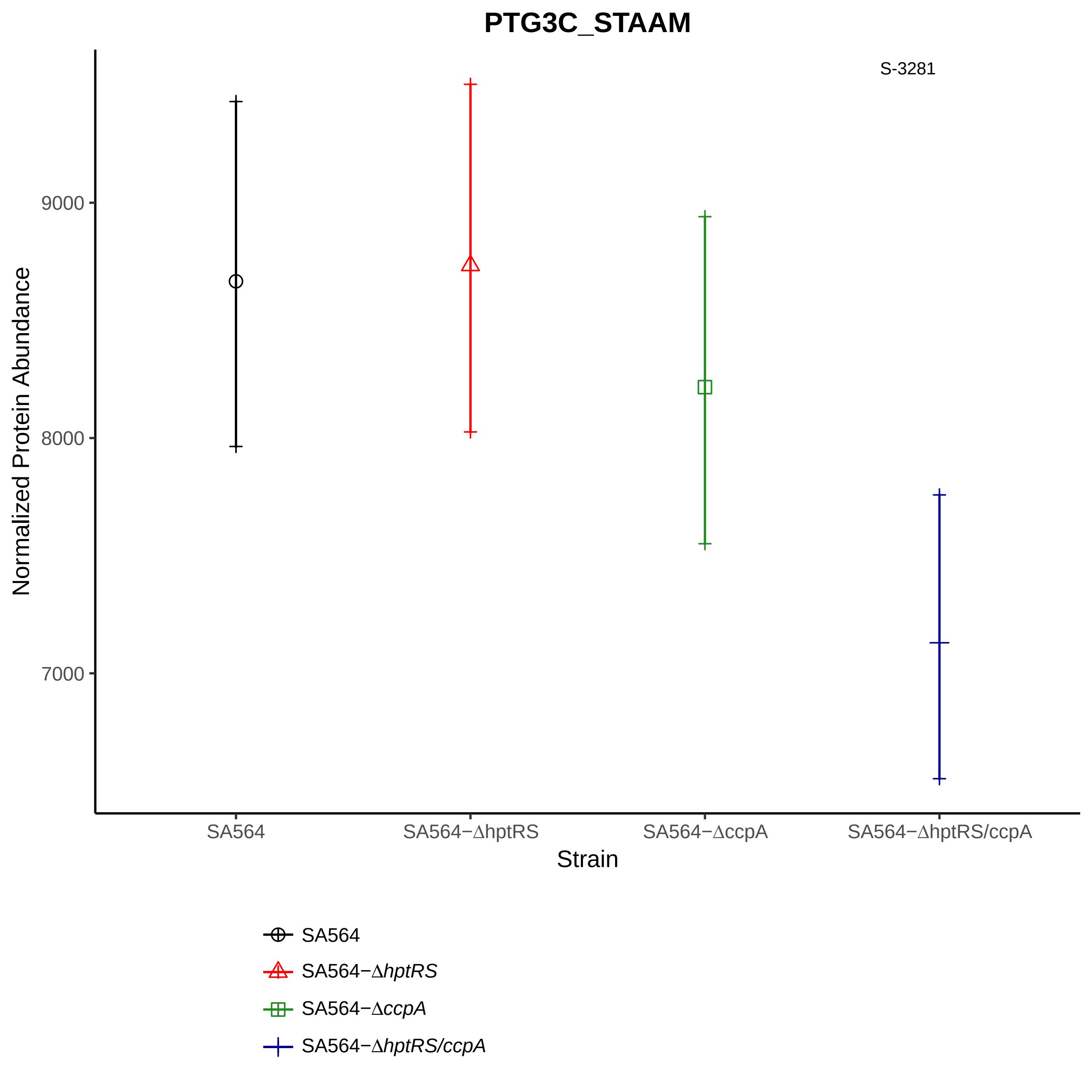

PTG3C_STAAM
S-3281
Normalized Protein Abundance
9000
8000
7000
SA564
SA564−ΔhptRS
SA564−ΔccpA
SA564−ΔhptRS/ccpA
Strain
SA564
SA564−Δ*hptRS*
SA564−Δ*ccpA*
SA564−Δ*hptRS/ccpA*

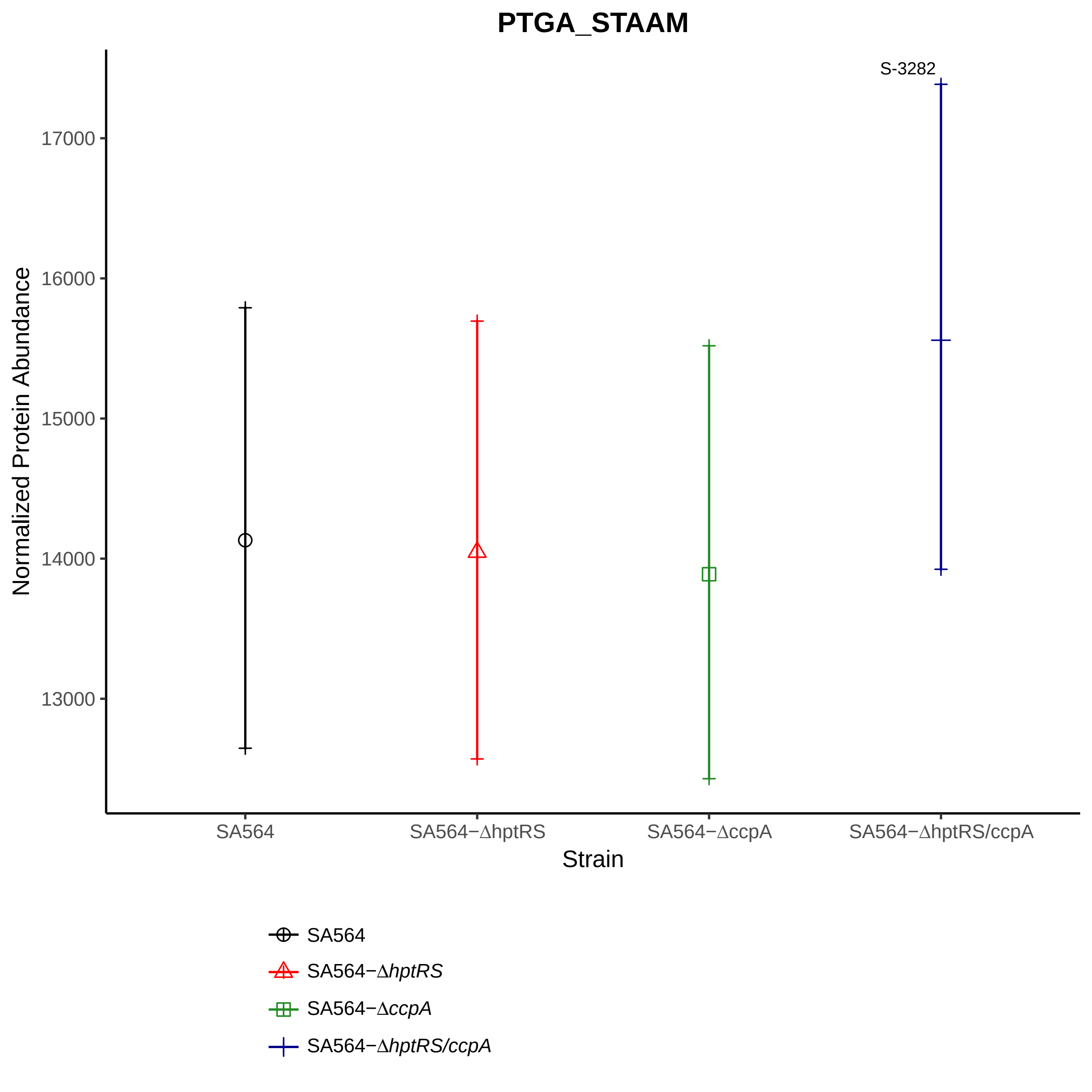

PTGA_STAAM
S-3282
17000
16000
15000
14000
13000
Normalized Protein Abundance
SA564
SA564−ΔhptRS
SA564−ΔccpA
SA564−ΔhptRS/ccpA
Strain
SA564
SA564−Δ*hptRS*
SA564−Δ*ccpA*
SA564−Δ*hptRS/ccpA*

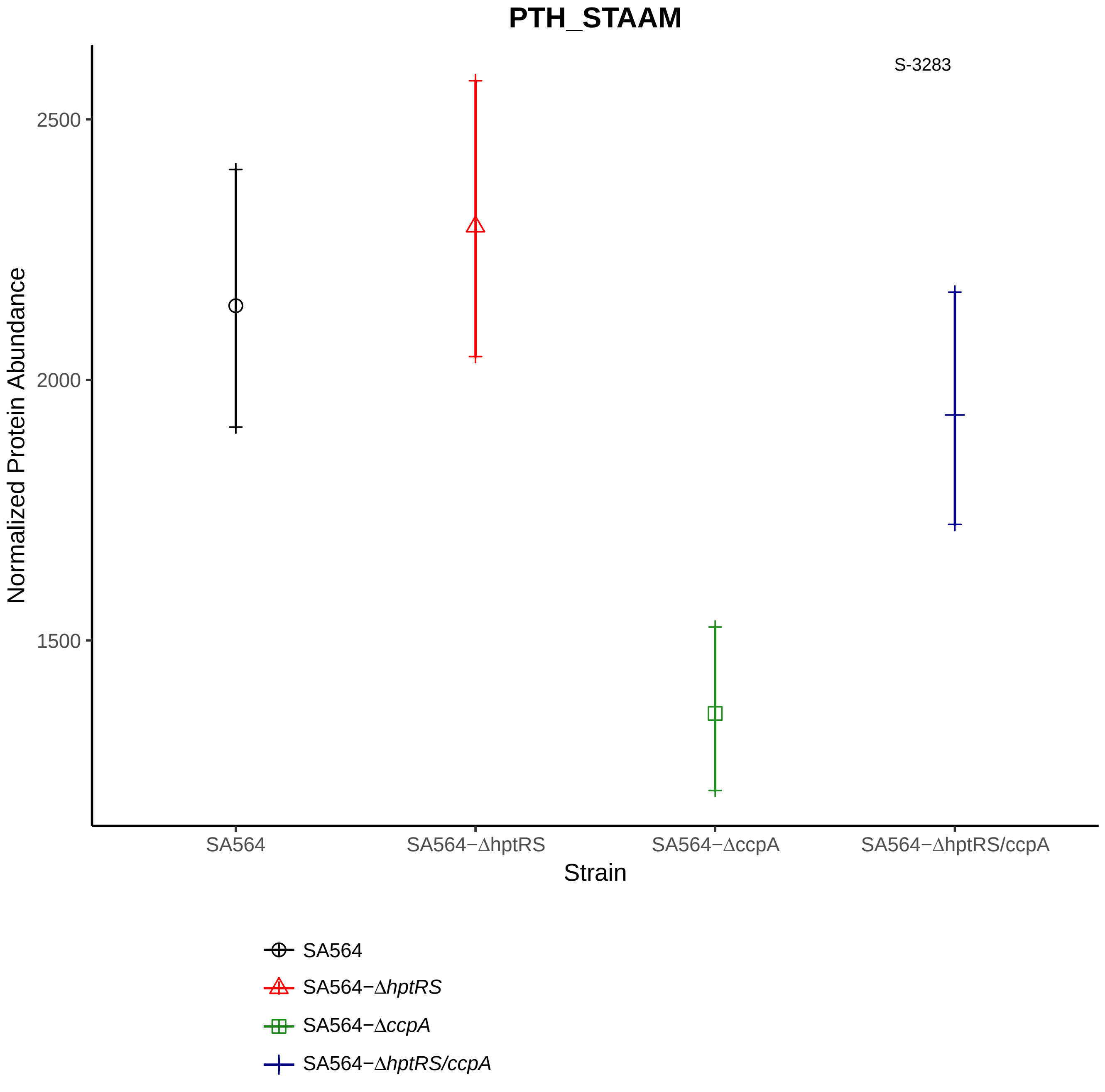

PTH_STAAM
S-3283
2500
2000
1500
Normalized Protein Abundance
SA564
SA564−ΔhptRS
SA564−ΔccpA
SA564−ΔhptRS/ccpA
Strain
SA564
SA564−Δ*hptRS*
SA564−Δ*ccpA*
SA564−Δ*hptRS/ccpA*

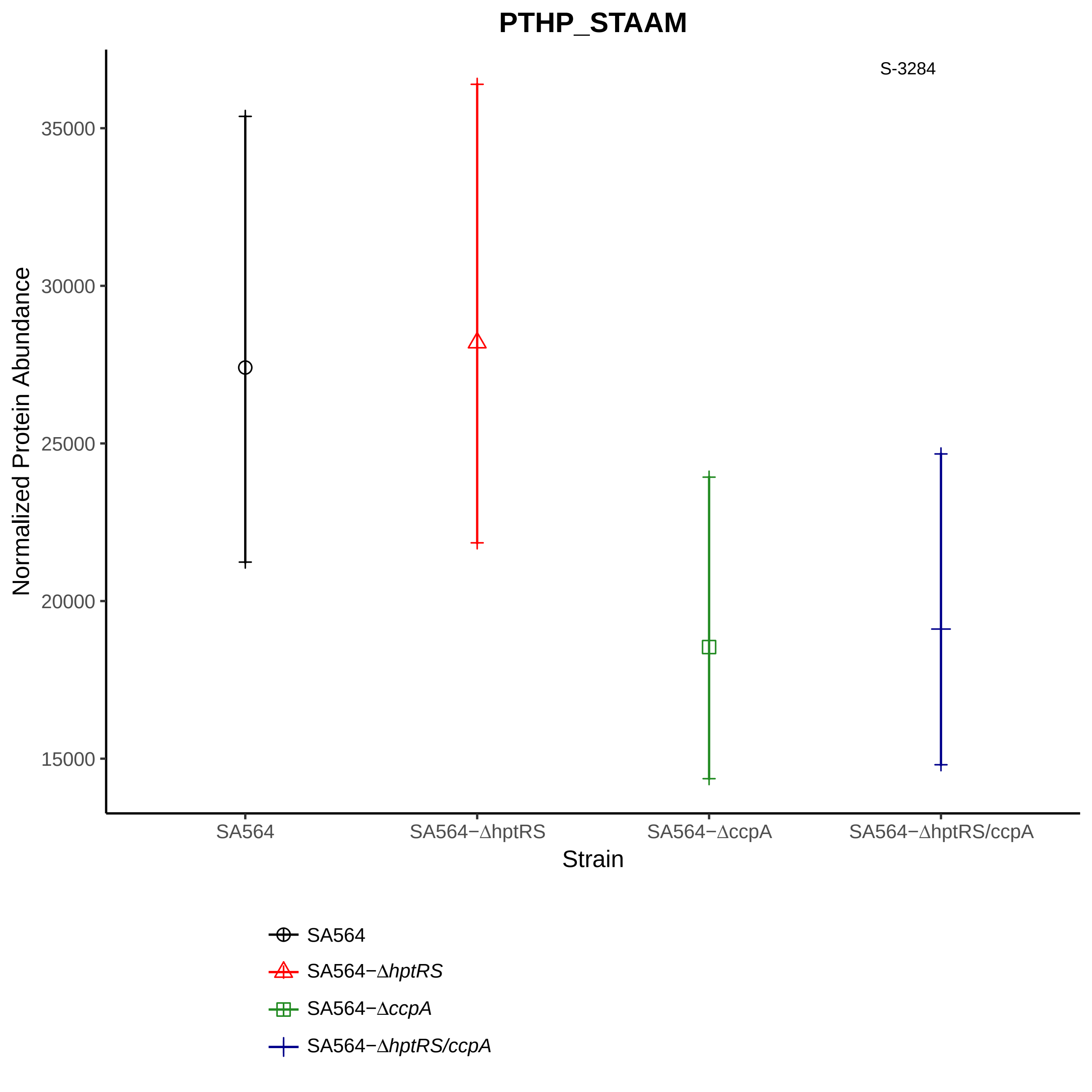

PTHP_STAAM
S-3284
Normalized Protein Abundance
35000
30000
25000
20000
15000
SA564
SA564−ΔhptRS
SA564−ΔccpA
SA564−ΔhptRS/ccpA
Strain
SA564
SA564−ΔhptRS
SA564−ΔccpA
SA564−ΔhptRS/ccpA

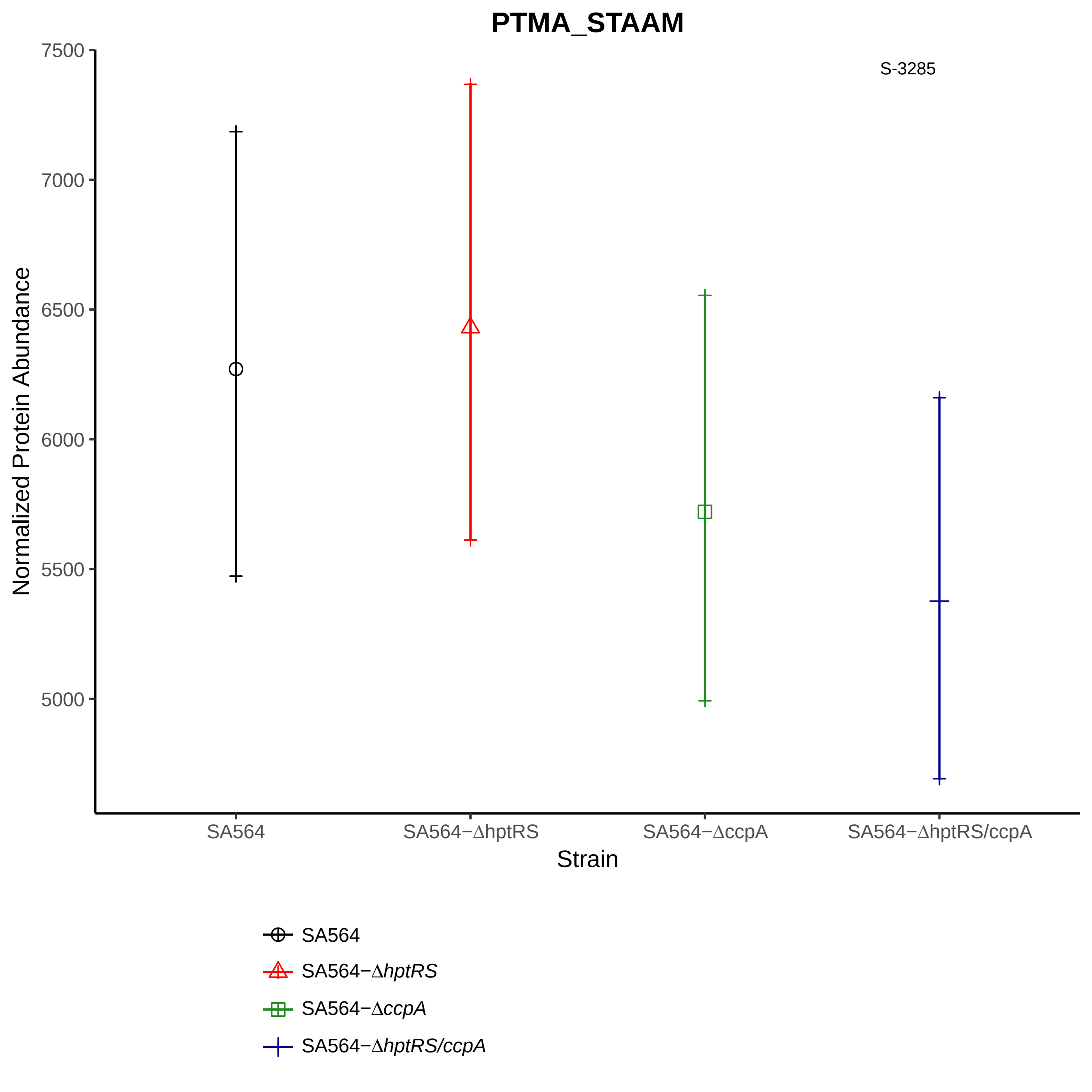

PTMA_STAAM
S-3285
Normalized Protein Abundance
7500
7000
6500
6000
5500
5000
SA564
SA564−ΔhptRS
SA564−ΔccpA
SA564−ΔhptRS/ccpA
Strain
SA564
SA564−Δ*hptRS*
SA564−Δ*ccpA*
SA564−Δ*hptRS/ccpA*

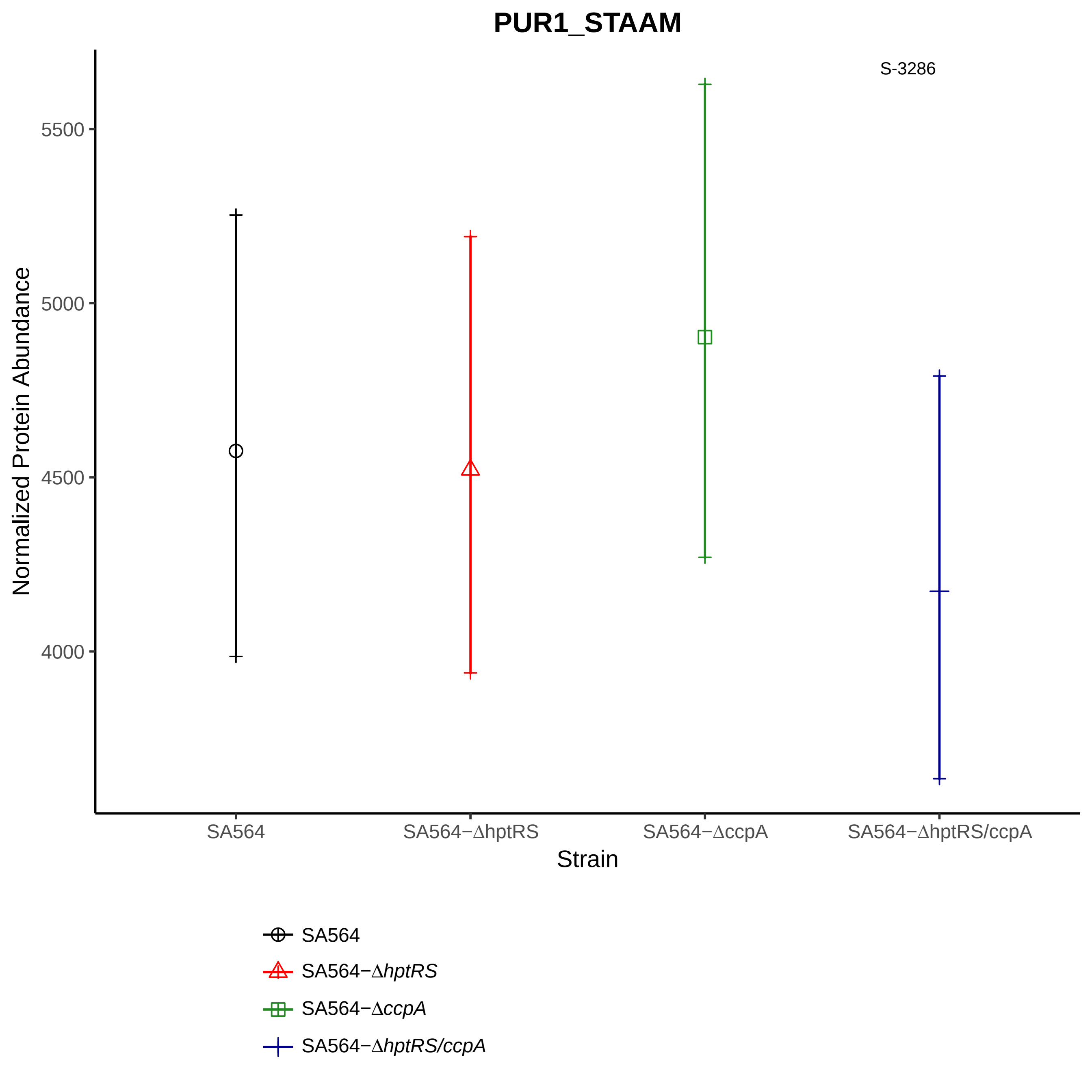
PUR1_STAAM
S-3286
Normalized Protein Abundance
5500
5000
4500
4000
SA564
SA564−ΔhptRS
SA564−ΔccpA
SA564−ΔhptRS/ccpA
Strain
SA564
SA564−ΔhptRS
SA564−ΔccpA
SA564−ΔhptRS/ccpA

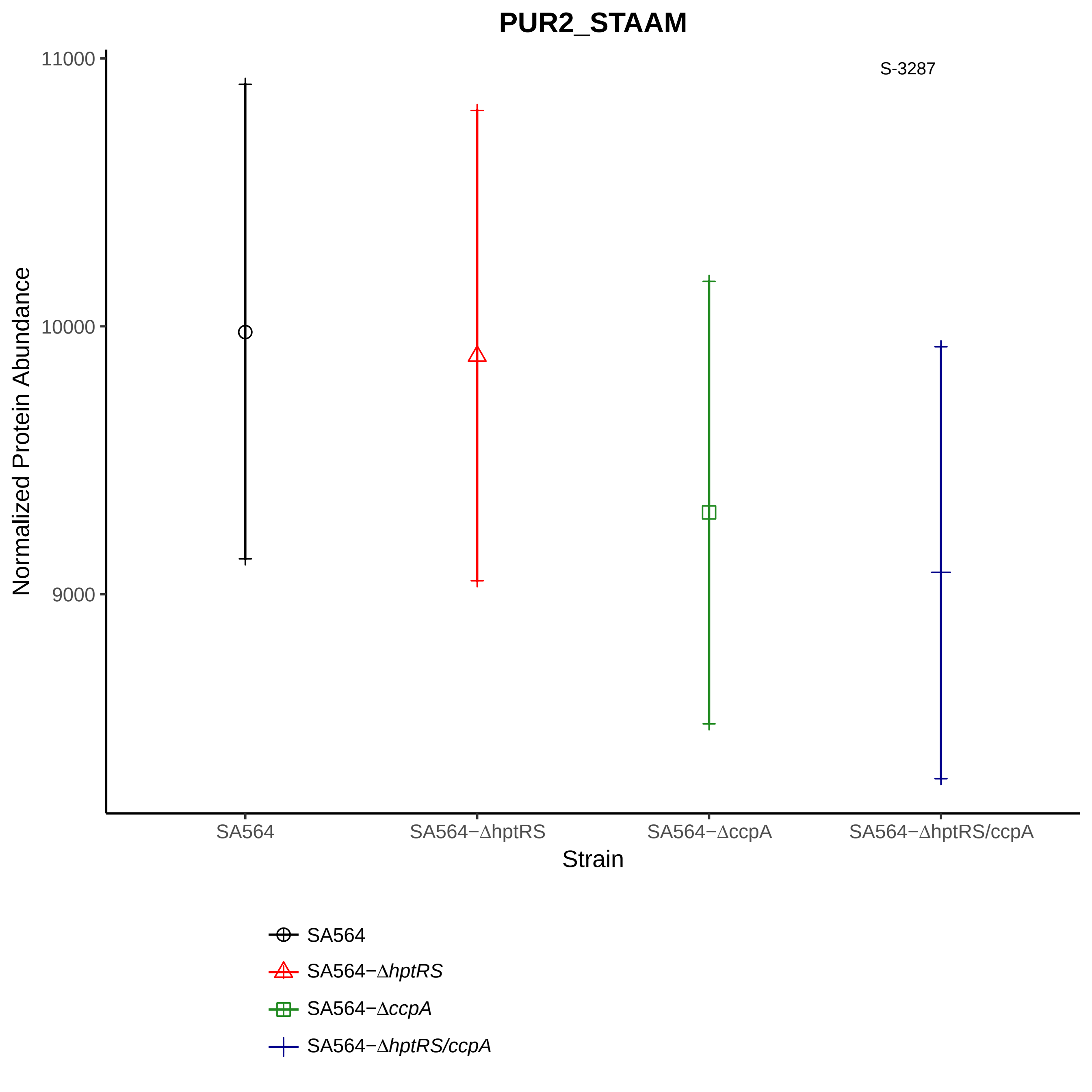

PUR2_STAAM
S-3287
Normalized Protein Abundance
11000
10000
9000
SA564
SA564−ΔhptRS
SA564−ΔccpA
SA564−ΔhptRS/ccpA
Strain
SA564
SA564−ΔhptRS
SA564−ΔccpA
SA564−ΔhptRS/ccpA

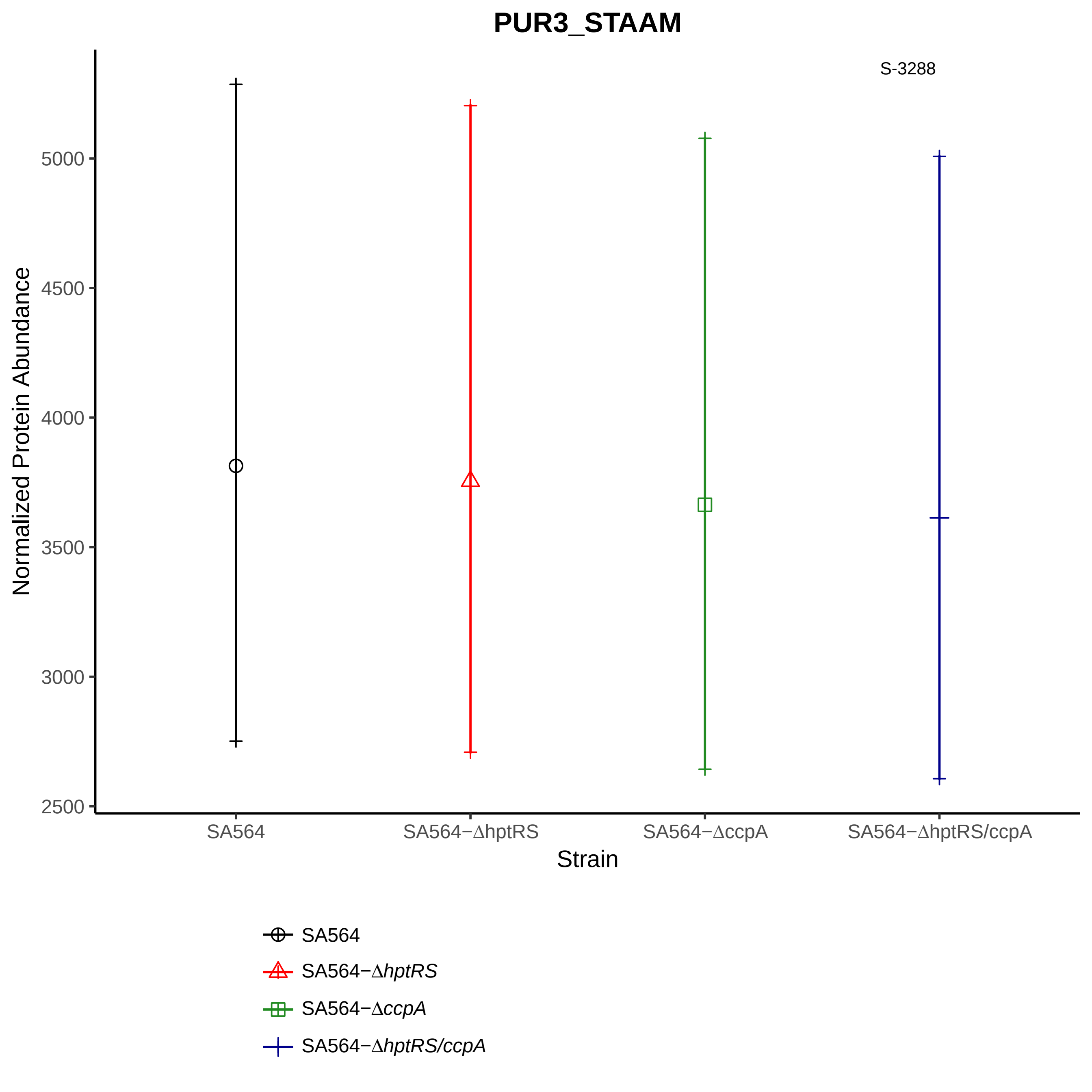

PUR3_STAAM
S-3288
Normalized Protein Abundance
5000
4500
4000
3500
3000
2500
SA564
SA564−ΔhptRS
SA564−ΔccpA
SA564−ΔhptRS/ccpA
Strain
SA564
SA564−ΔhptRS
SA564−ΔccpA
SA564−ΔhptRS/ccpA

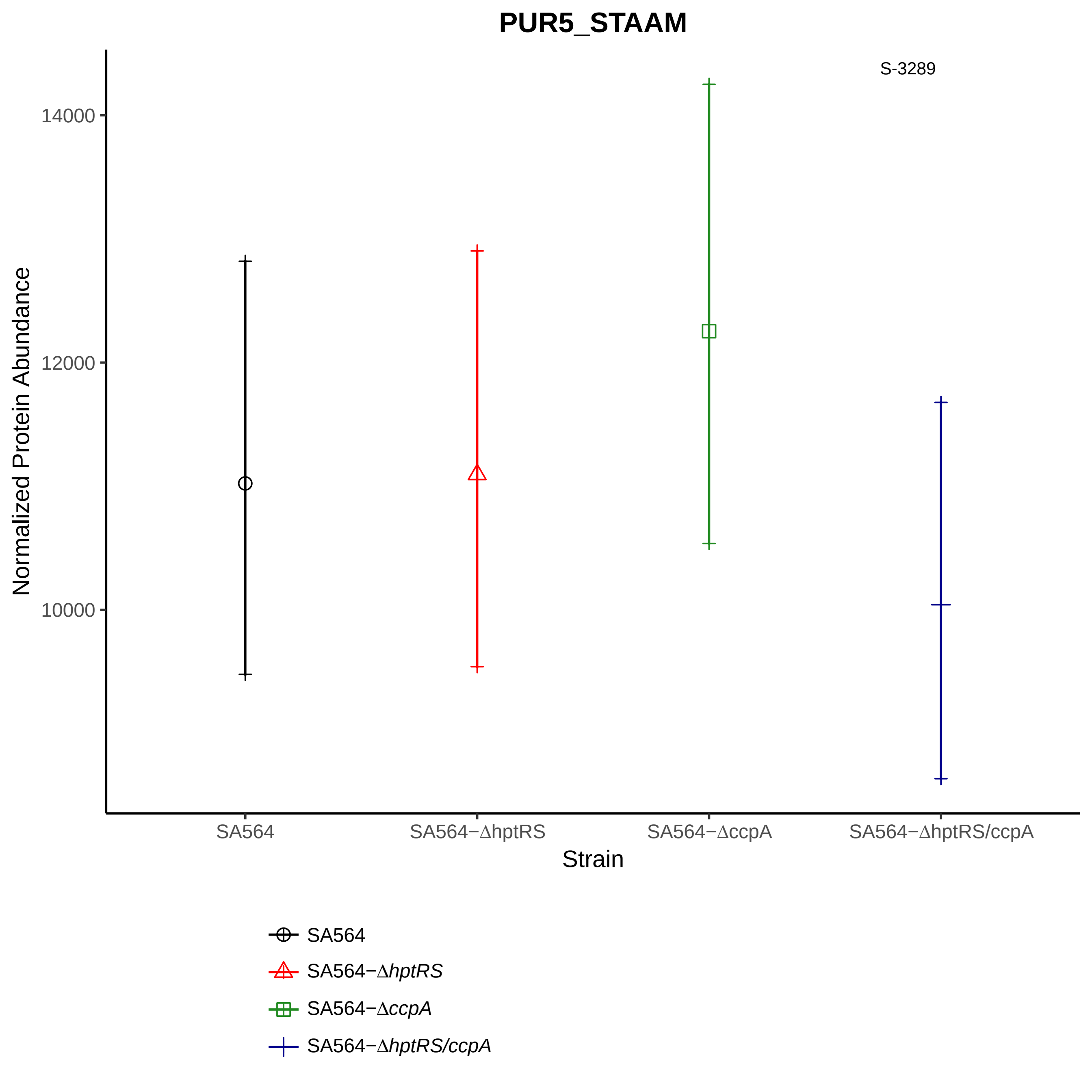

PUR5_STAAM
S-3289
Normalized Protein Abundance
14000
12000
10000
SA564
SA564−ΔhptRS
SA564−ΔccpA
SA564−ΔhptRS/ccpA
Strain
SA564
SA564−ΔhptRS
SA564−ΔccpA
SA564−ΔhptRS/ccpA

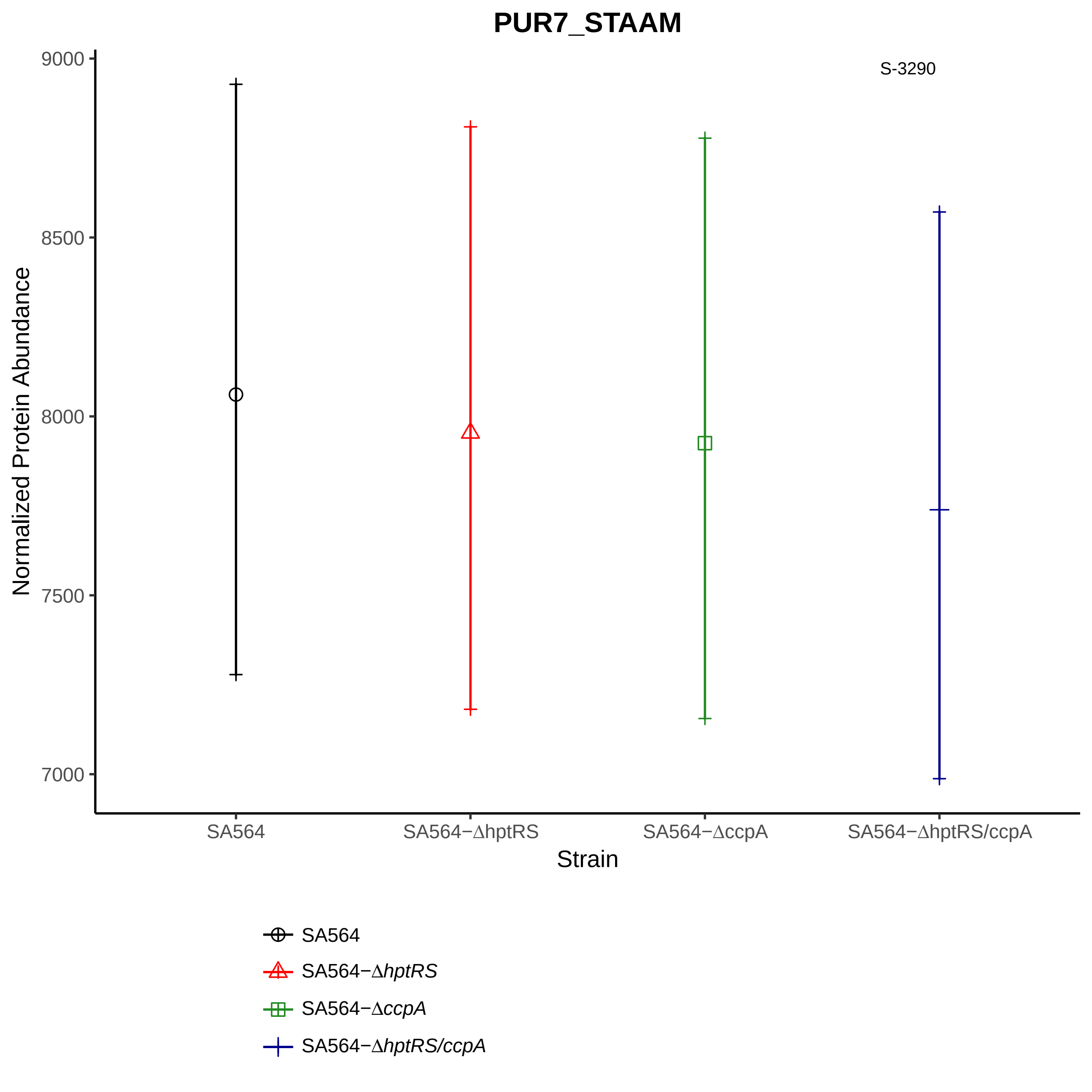

PUR7_STAAM
S-3290
Normalized Protein Abundance
9000
8500
8000
7500
7000
SA564
SA564−ΔhptRS
SA564−ΔccpA
SA564−ΔhptRS/ccpA
Strain
SA564
SA564−Δ*hptRS*
SA564−Δ*ccpA*
SA564−Δ*hptRS/ccpA*

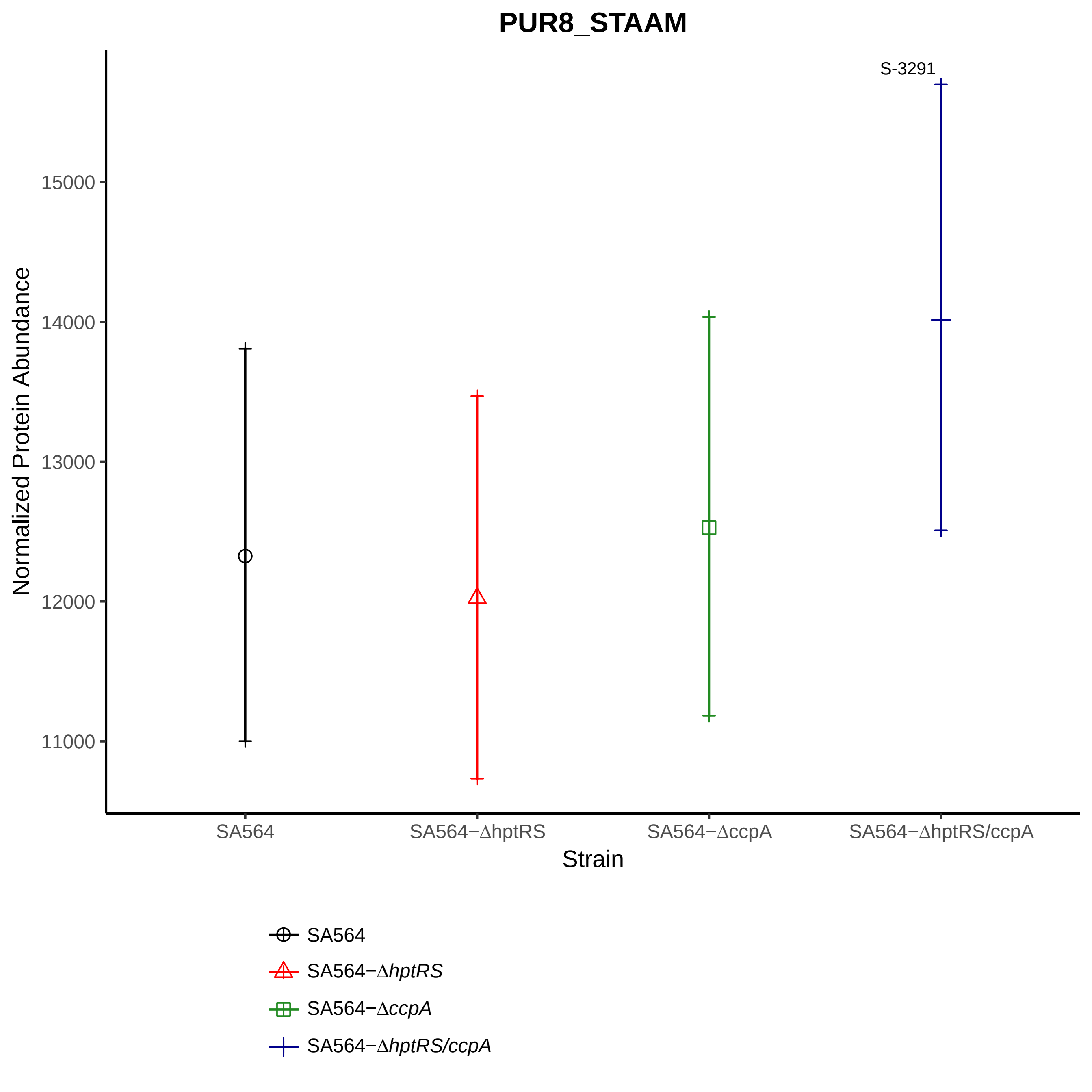

PUR8_STAAM
S-3291
15000
14000
13000
12000
11000
Normalized Protein Abundance
SA564
SA564−ΔhptRS
SA564−ΔccpA
SA564−ΔhptRS/ccpA
Strain
SA564
SA564−Δ*hptRS*
SA564−Δ*ccpA*
SA564−Δ*hptRS/ccpA*

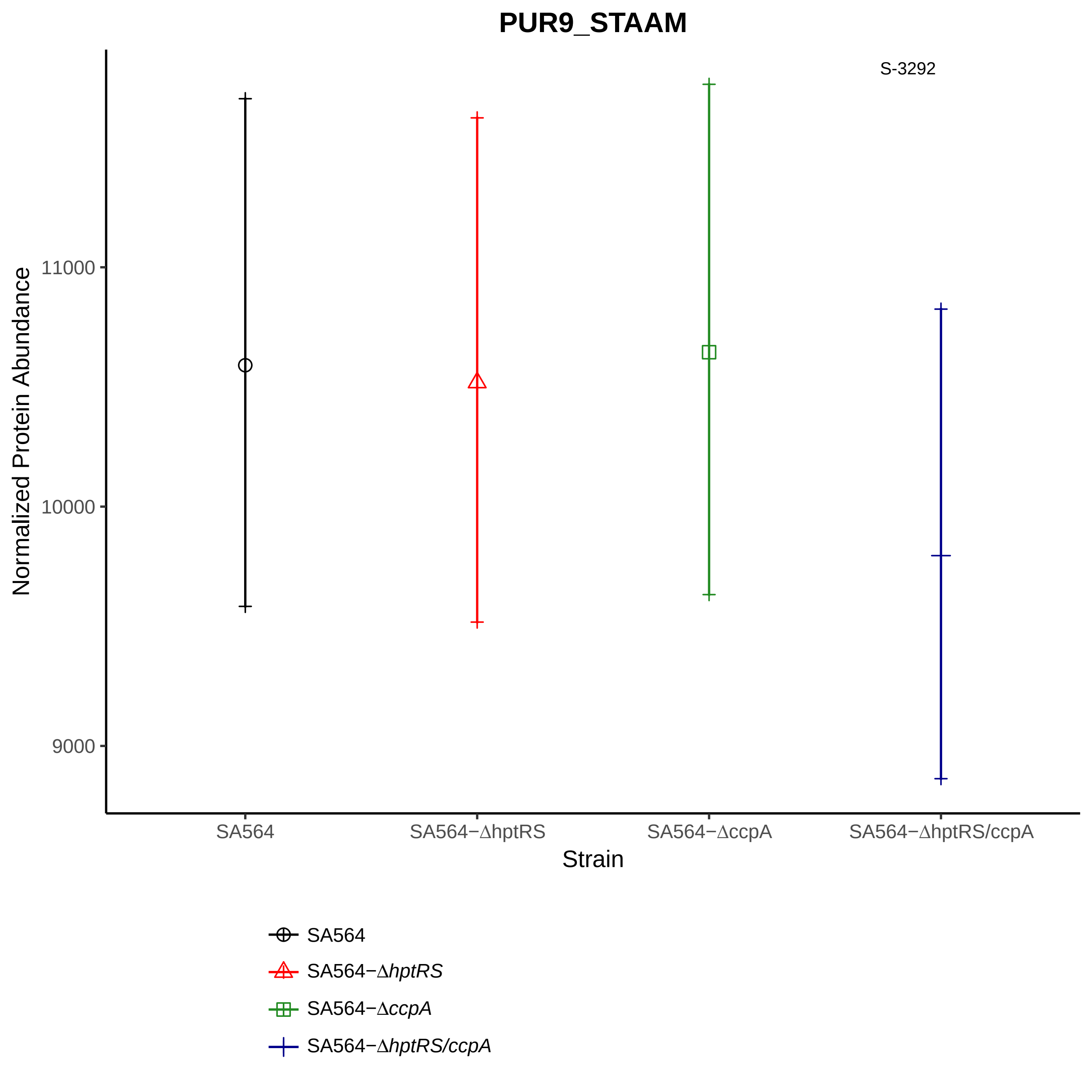
PUR9_STAAM
S-3292
Normalized Protein Abundance
11000
10000
9000
SA564
SA564−ΔhptRS
SA564−ΔccpA
SA564−ΔhptRS/ccpA
Strain
SA564
SA564−ΔhptRS
SA564−ΔccpA
SA564−ΔhptRS/ccpA

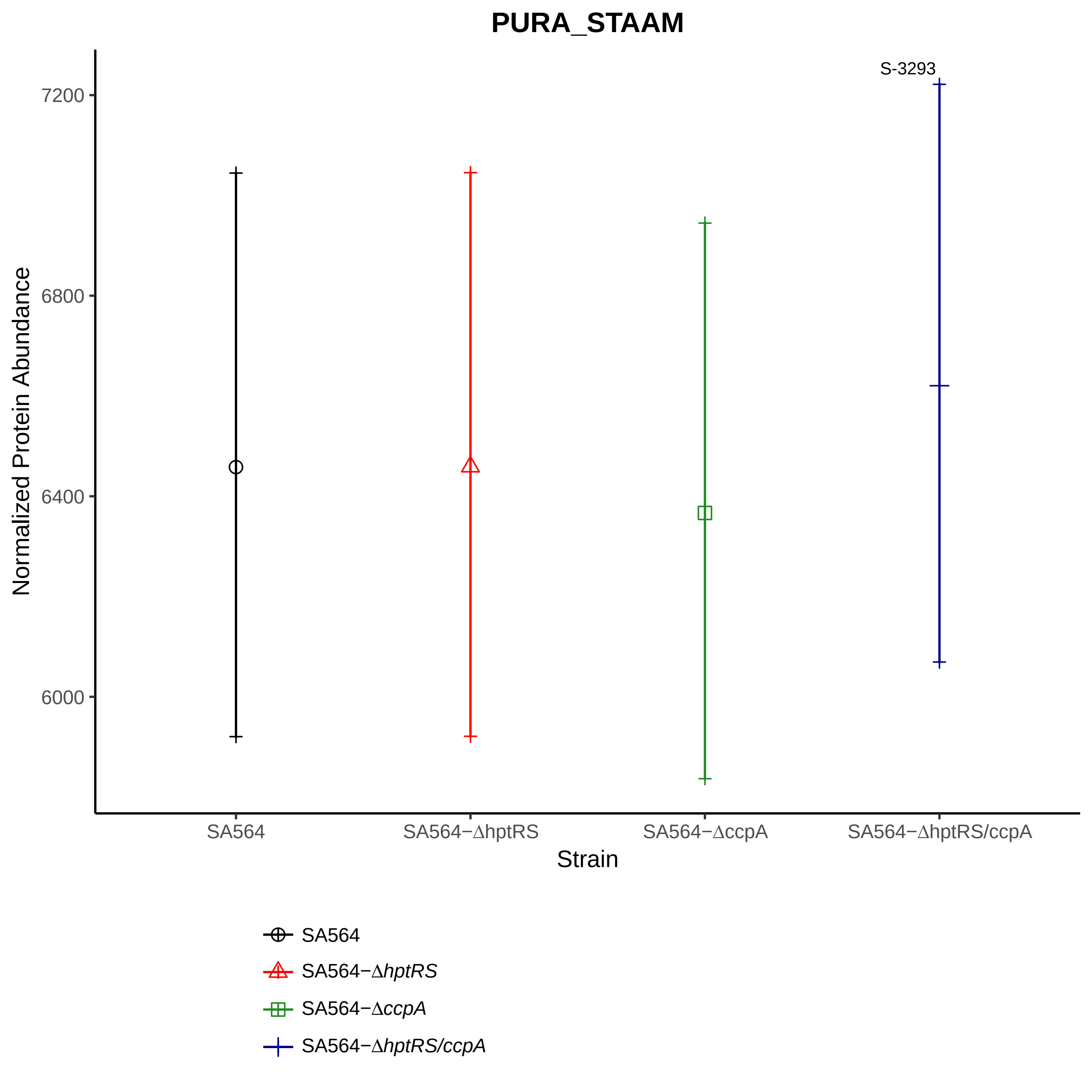
PURA_STAAM
S-3293
Normalized Protein Abundance
7200
6800
6400
6000
SA564
SA564−ΔhptRS
SA564−ΔccpA
SA564−ΔhptRS/ccpA
Strain
SA564
SA564−ΔhptRS
SA564−ΔccpA
SA564−ΔhptRS/ccpA

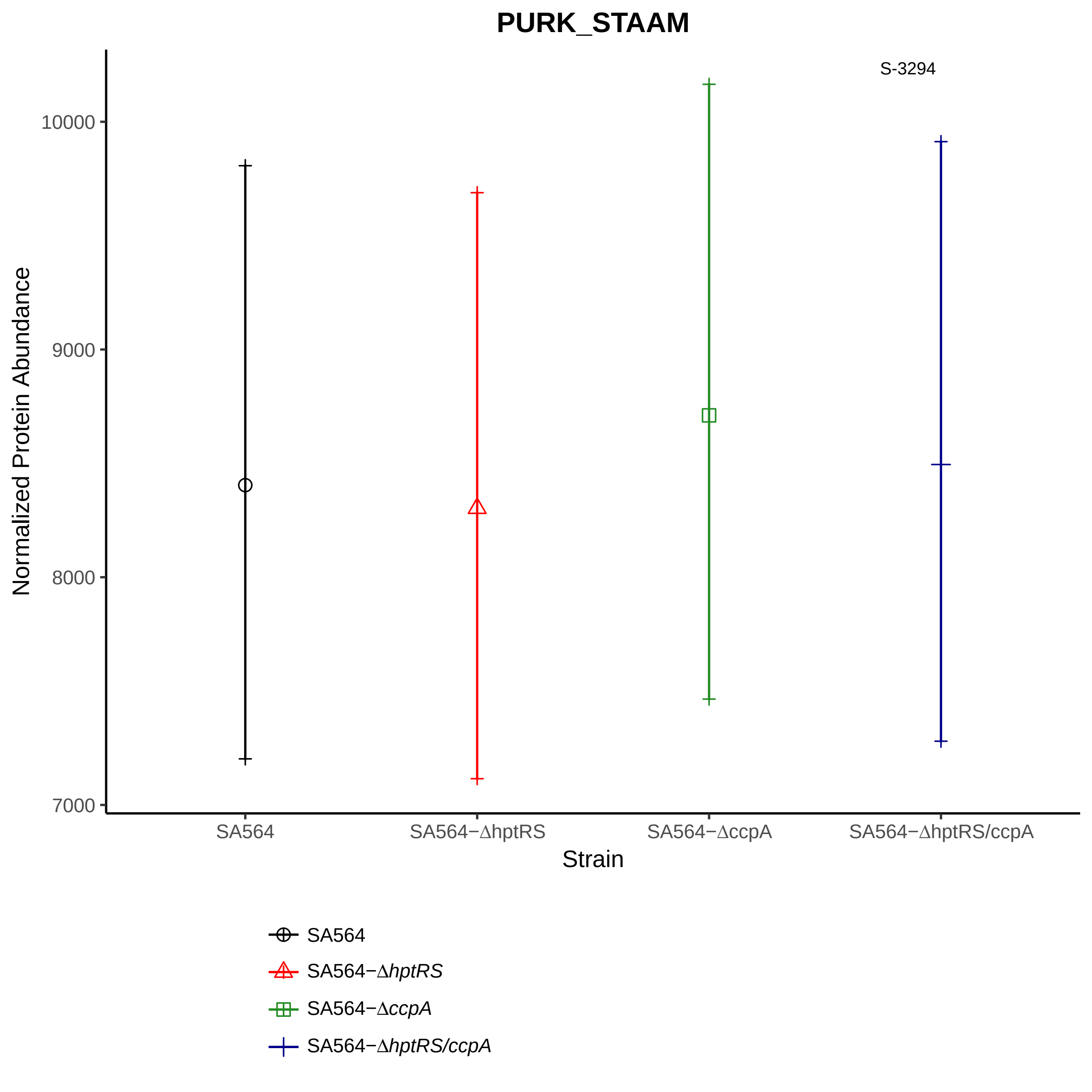
PURK_STAAM
S-3294
Normalized Protein Abundance
10000
9000
8000
7000
SA564
SA564−ΔhptRS
SA564−ΔccpA
SA564−ΔhptRS/ccpA
Strain
SA564
SA564−ΔhptRS
SA564−ΔccpA
SA564−ΔhptRS/ccpA

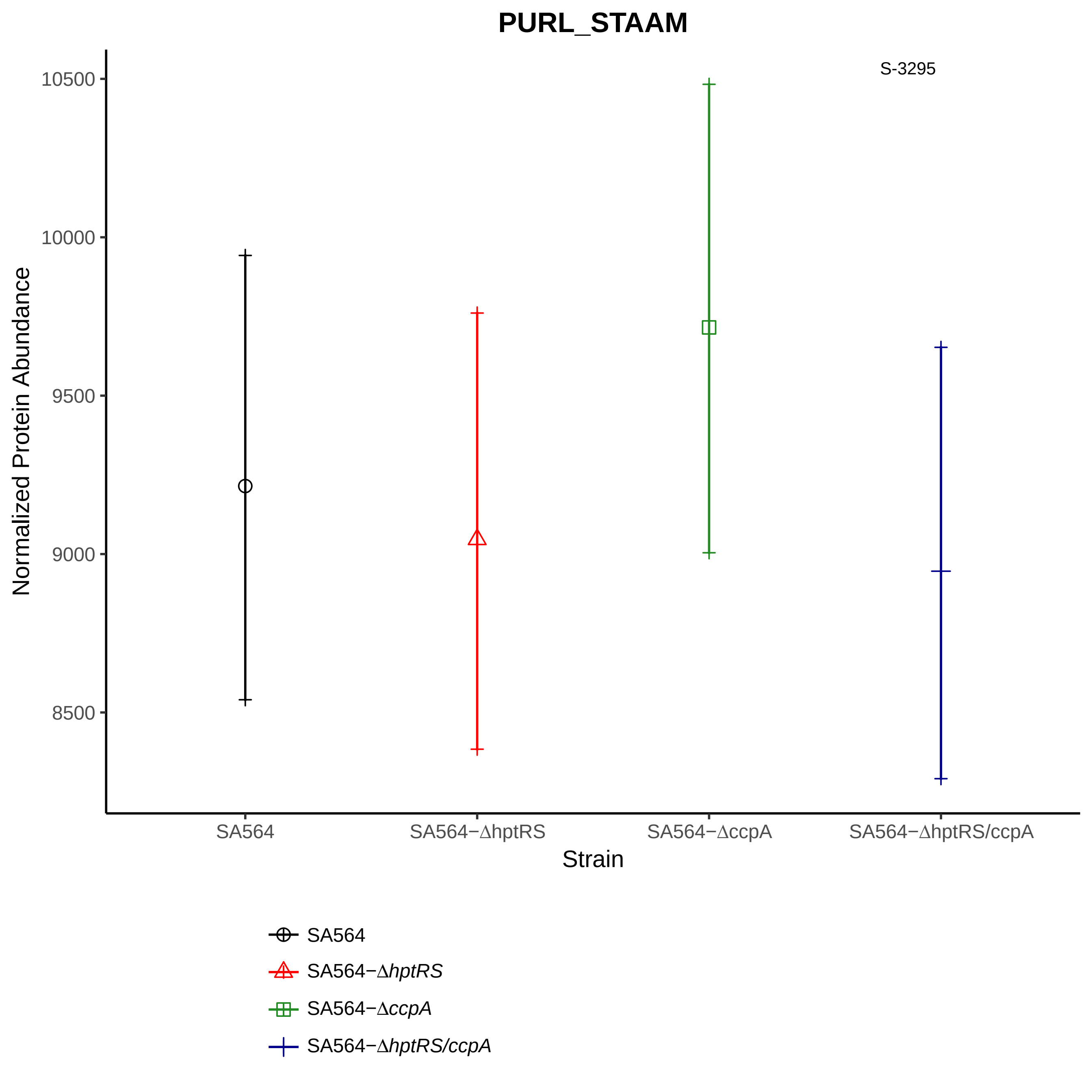

PURL_STAAM
S-3295
Normalized Protein Abundance
10500
10000
9500
9000
8500
SA564
SA564−ΔhptRS
SA564−ΔccpA
SA564−ΔhptRS/ccpA
Strain
SA564
SA564−ΔhptRS
SA564−ΔccpA
SA564−ΔhptRS/ccpA

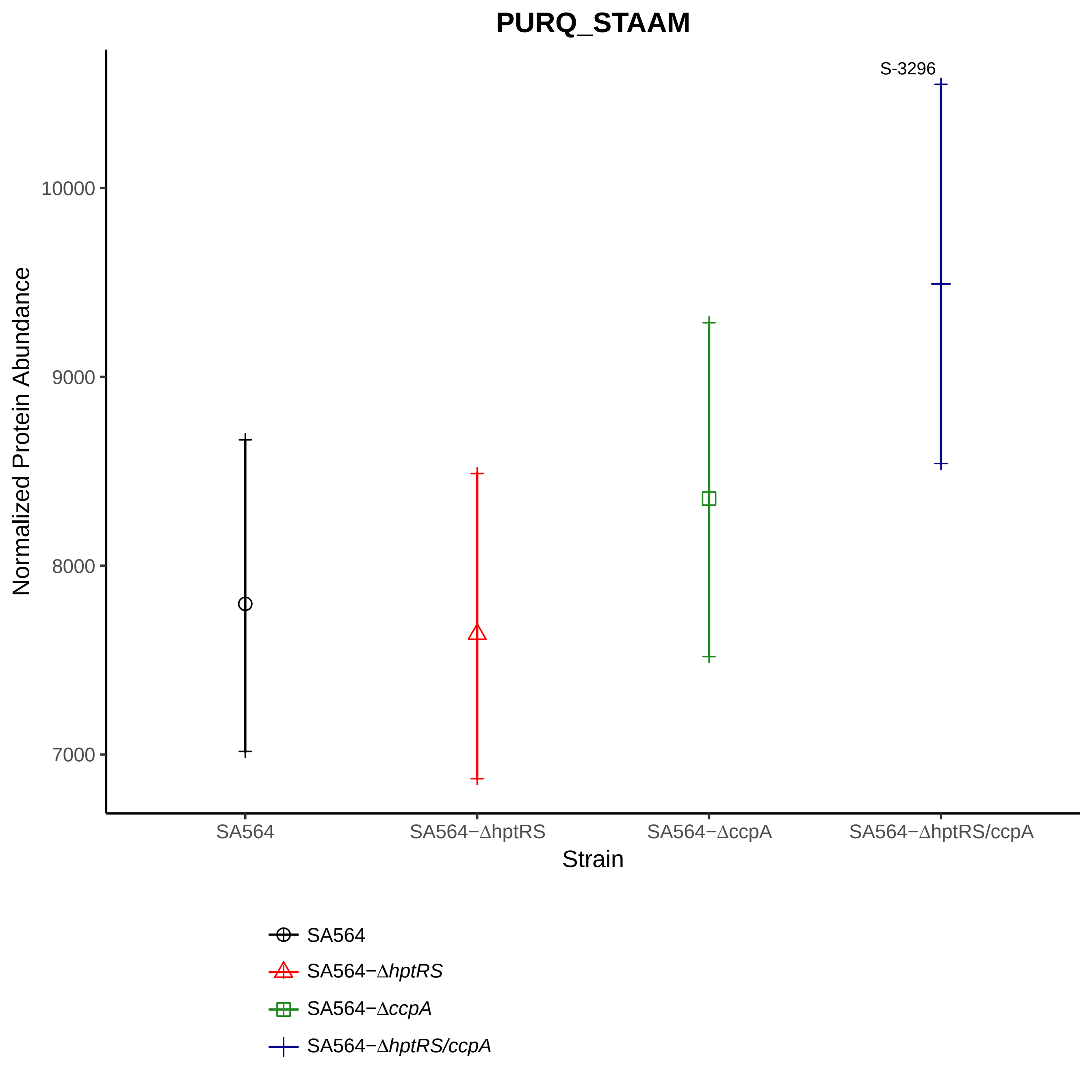

PURQ_STAAM
S-3296
Normalized Protein Abundance
10000
9000
8000
7000
SA564
SA564−ΔhptRS
SA564−ΔccpA
SA564−ΔhptRS/ccpA
Strain
SA564
SA564−Δ*hptRS*
SA564−Δ*ccpA*
SA564−Δ*hptRS/ccpA*

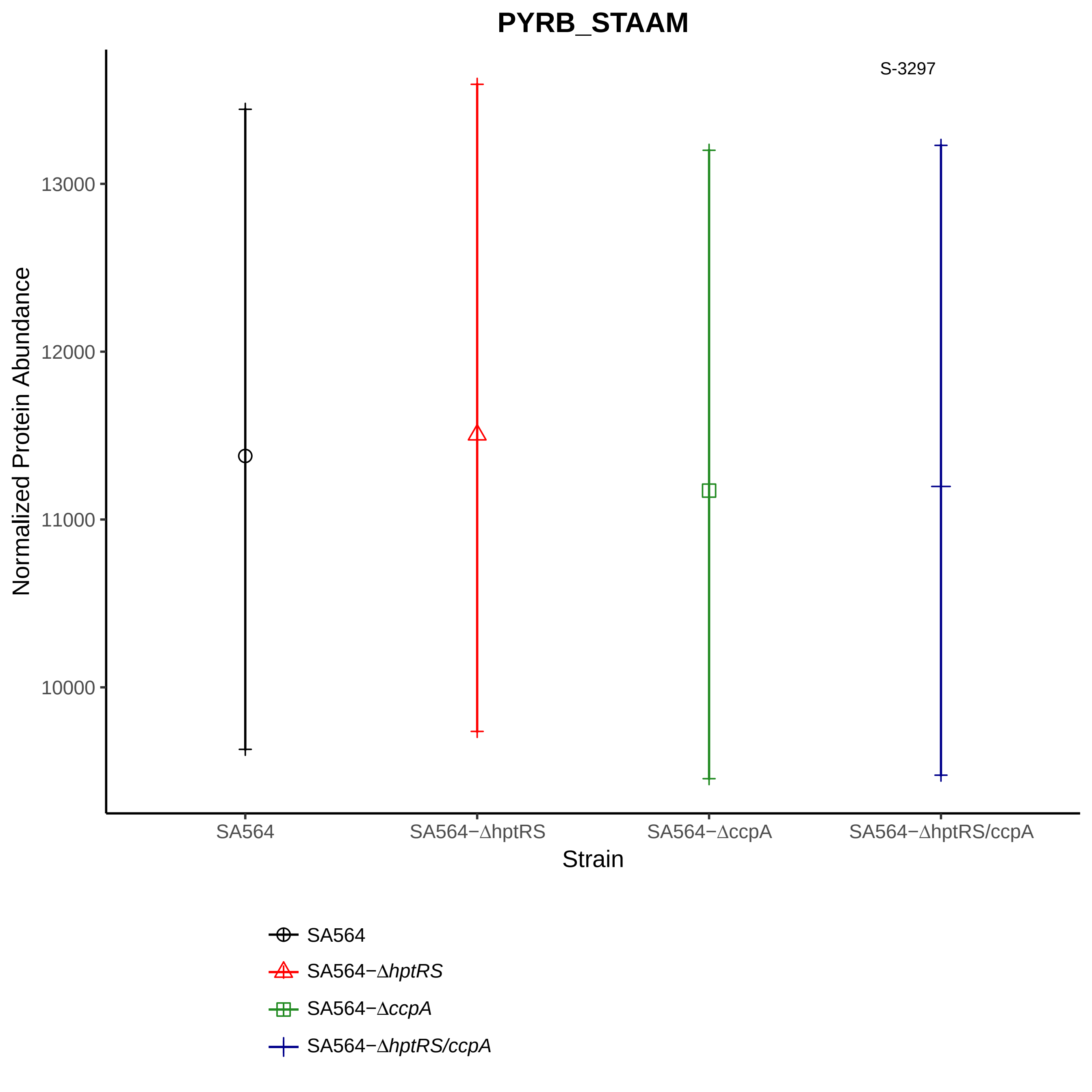

PYRB_STAAM
S-3297
Normalized Protein Abundance
13000
12000
11000
10000
SA564
SA564−ΔhptRS
SA564−ΔccpA
SA564−ΔhptRS/ccpA
Strain
SA564
SA564−ΔhptRS
SA564−ΔccpA
SA564−ΔhptRS/ccpA

PYRC_STAAM

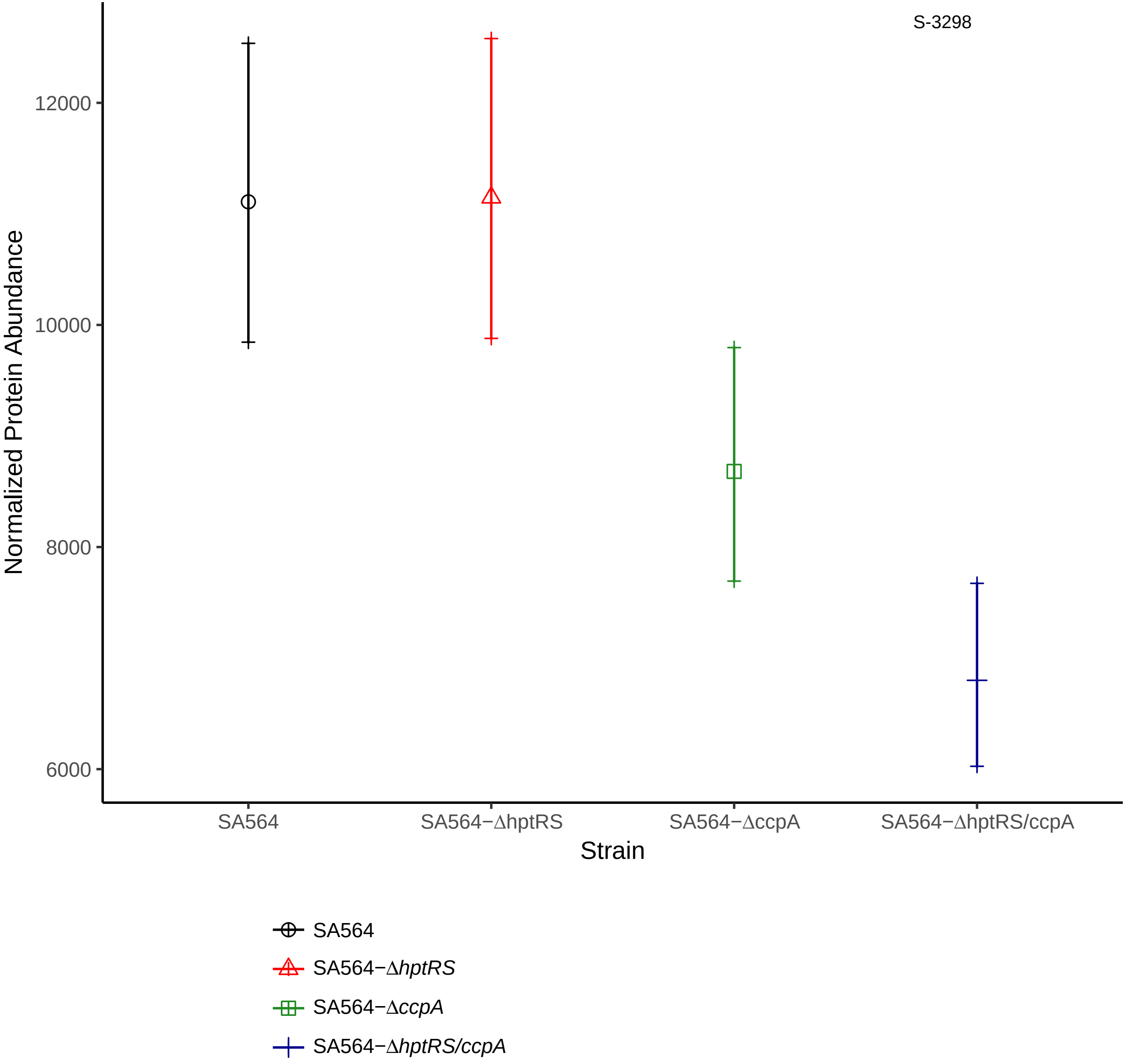

S-3298
Normalized Protein Abundance
12000
10000
8000
6000
SA564
SA564−ΔhptRS
SA564−ΔccpA
SA564−ΔhptRS/ccpA
Strain
SA564
SA564−Δ*hptRS*
SA564−Δ*ccpA*
SA564−Δ*hptRS/ccpA*

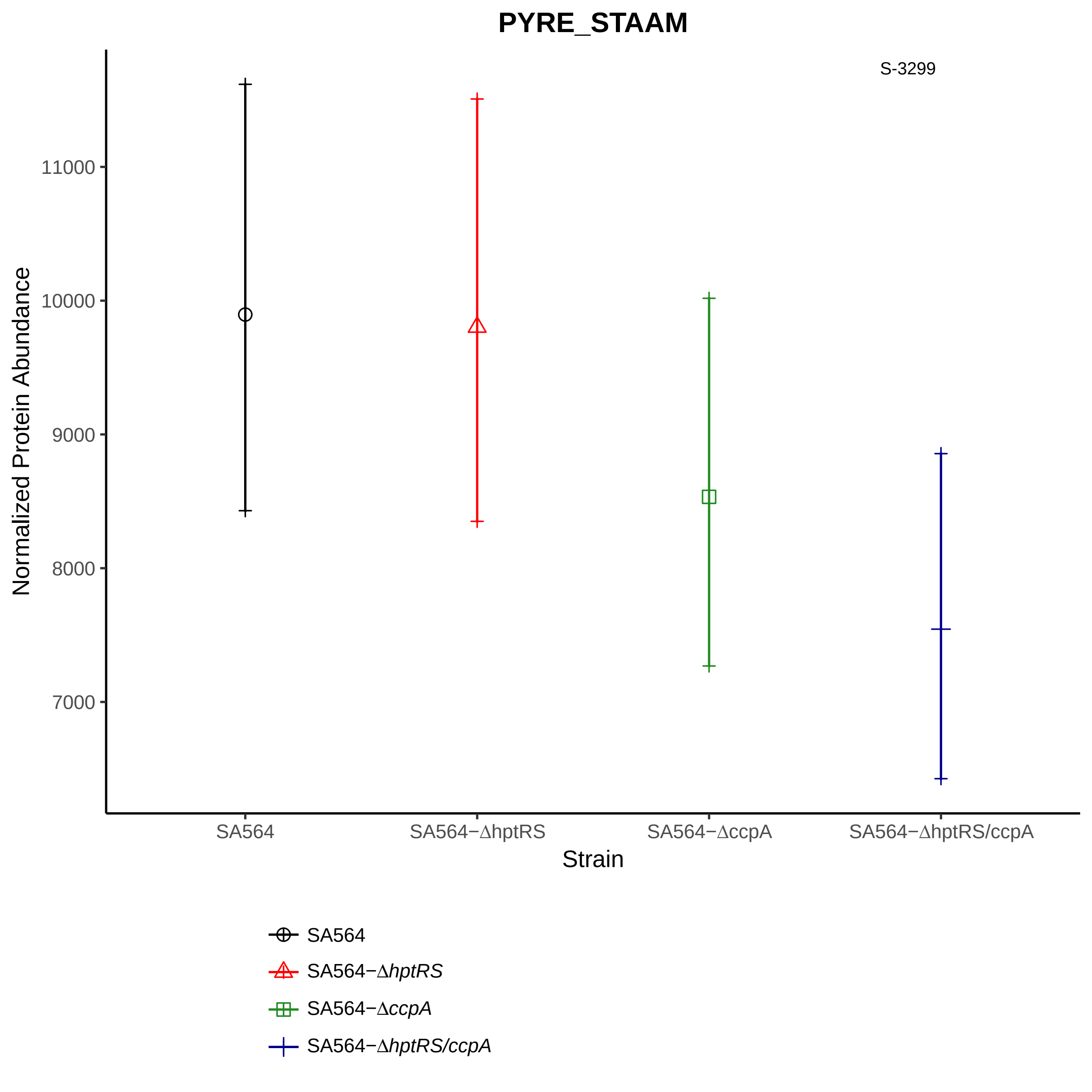
PYRE_STAAM
S-3299
Normalized Protein Abundance
11000
10000
9000
8000
7000
SA564
SA564−ΔhptRS
SA564−ΔccpA
SA564−ΔhptRS/ccpA
Strain
SA564
SA564−ΔhptRS
SA564−ΔccpA
SA564−ΔhptRS/ccpA

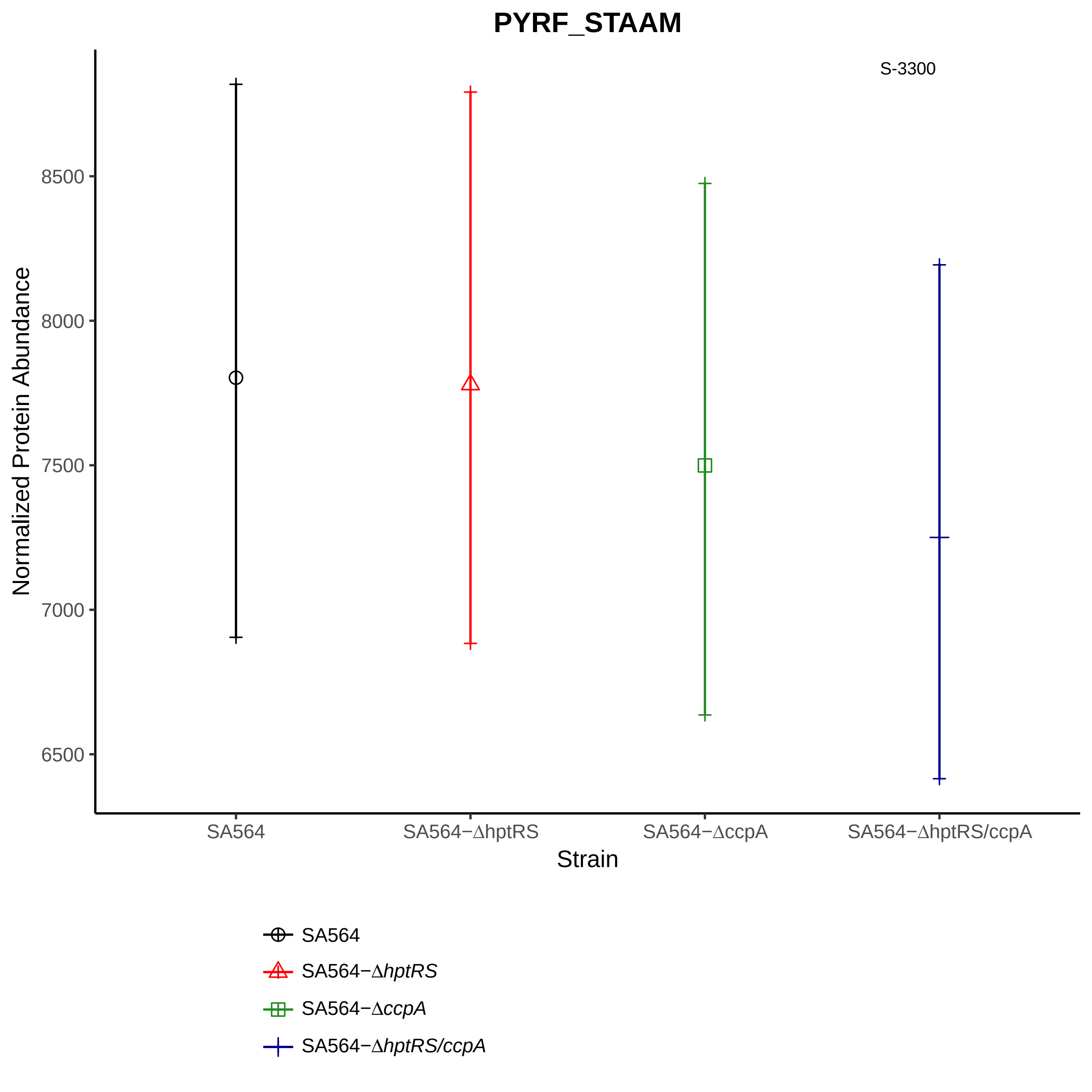

PYRF_STAAM
S-3300
Normalized Protein Abundance
8500
8000
7500
7000
6500
SA564
SA564−ΔhptRS
SA564−ΔccpA
SA564−ΔhptRS/ccpA
Strain
SA564
SA564−ΔhptRS
SA564−ΔccpA
SA564−ΔhptRS/ccpA

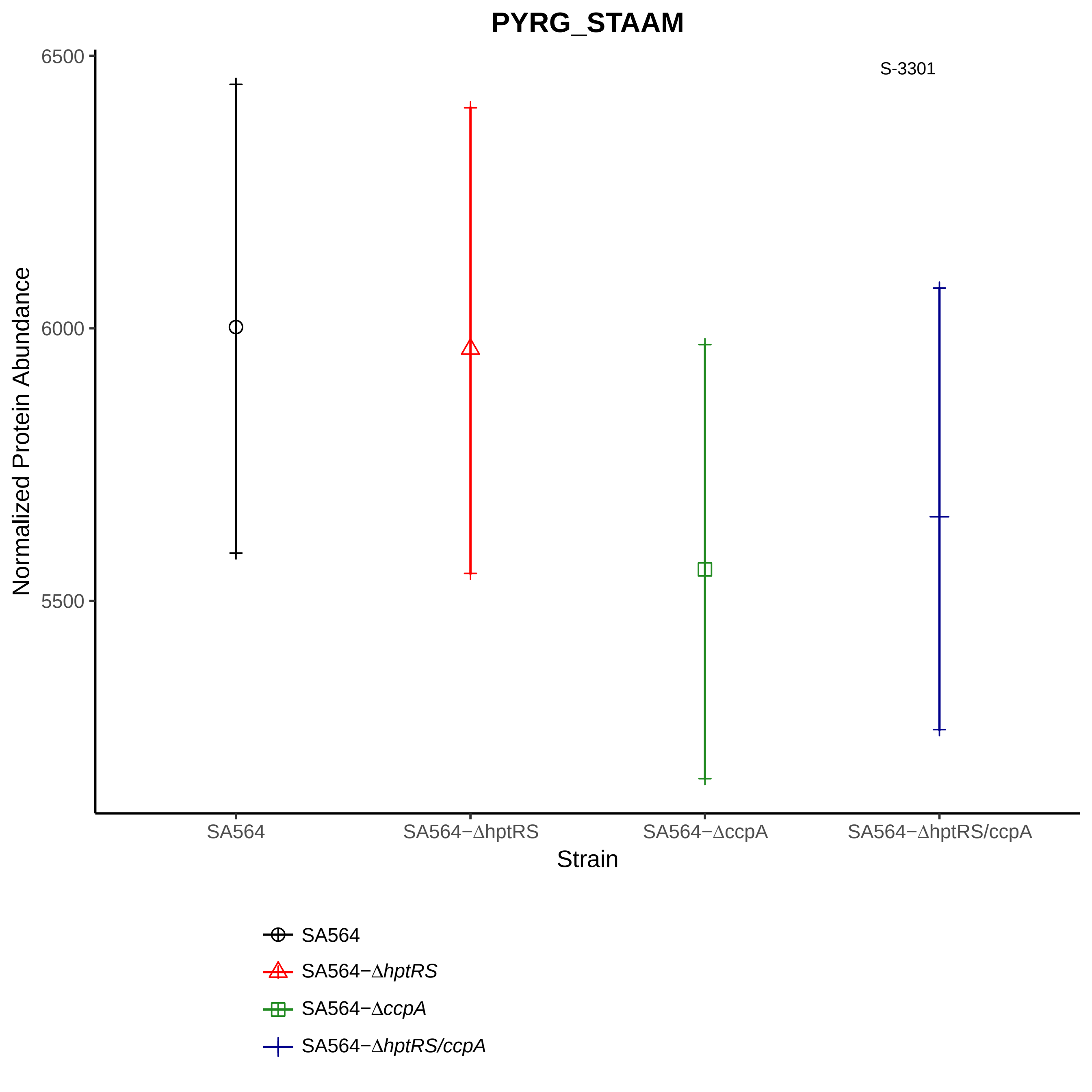

PYRG_STAAM
S-3301
6500
6000
5500
Normalized Protein Abundance
SA564
SA564−ΔhptRS
SA564−ΔccpA
SA564−ΔhptRS/ccpA
Strain
SA564
SA564−ΔhptRS
SA564−ΔccpA
SA564−ΔhptRS/ccpA

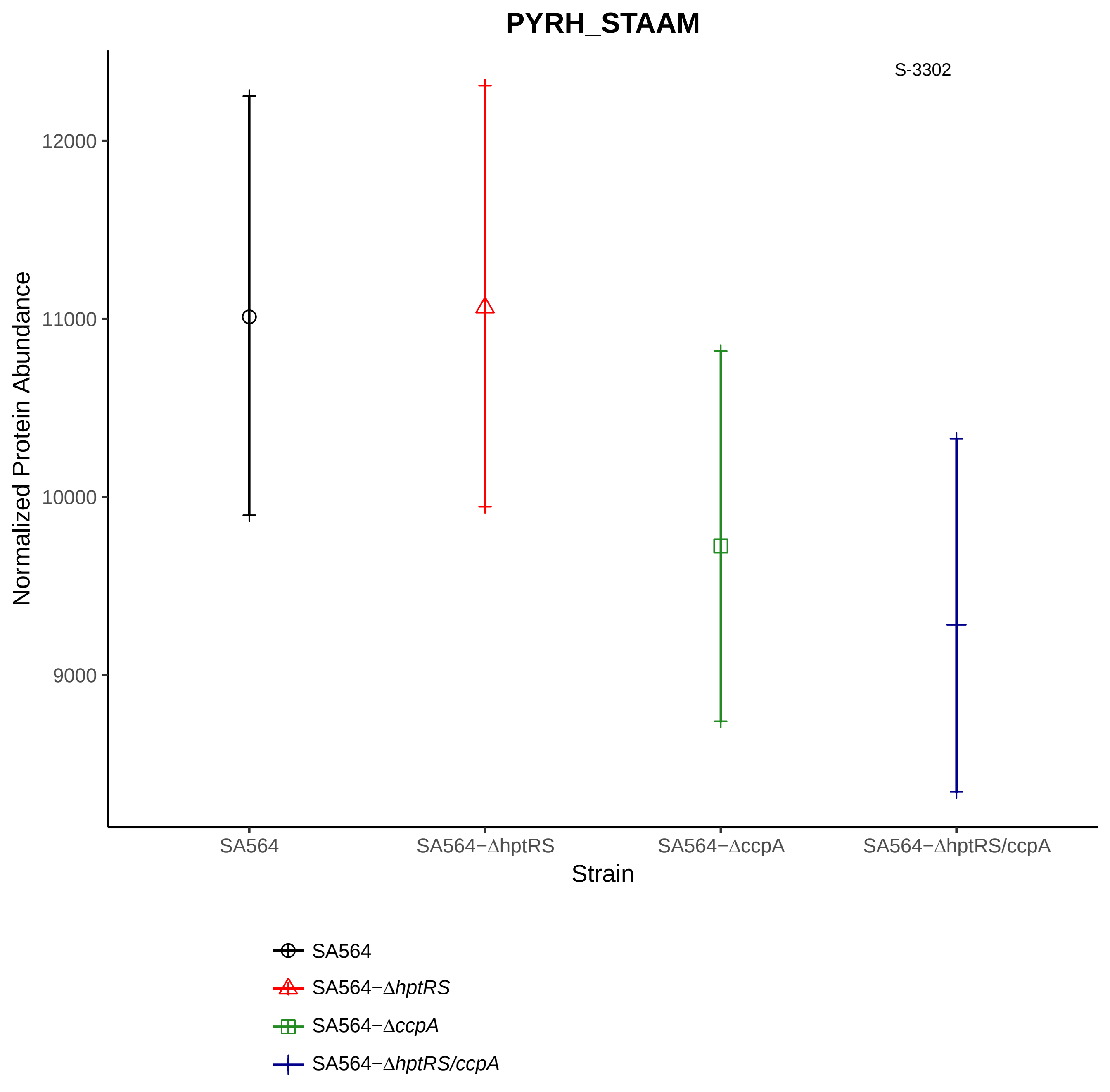

PYRH_STAAM
S-3302
Normalized Protein Abundance
12000
11000
10000
9000
SA564
SA564−ΔhptRS
SA564−ΔccpA
SA564−ΔhptRS/ccpA
Strain
SA564
SA564−Δ*hptRS*
SA564−Δ*ccpA*
SA564−Δ*hptRS/ccpA*

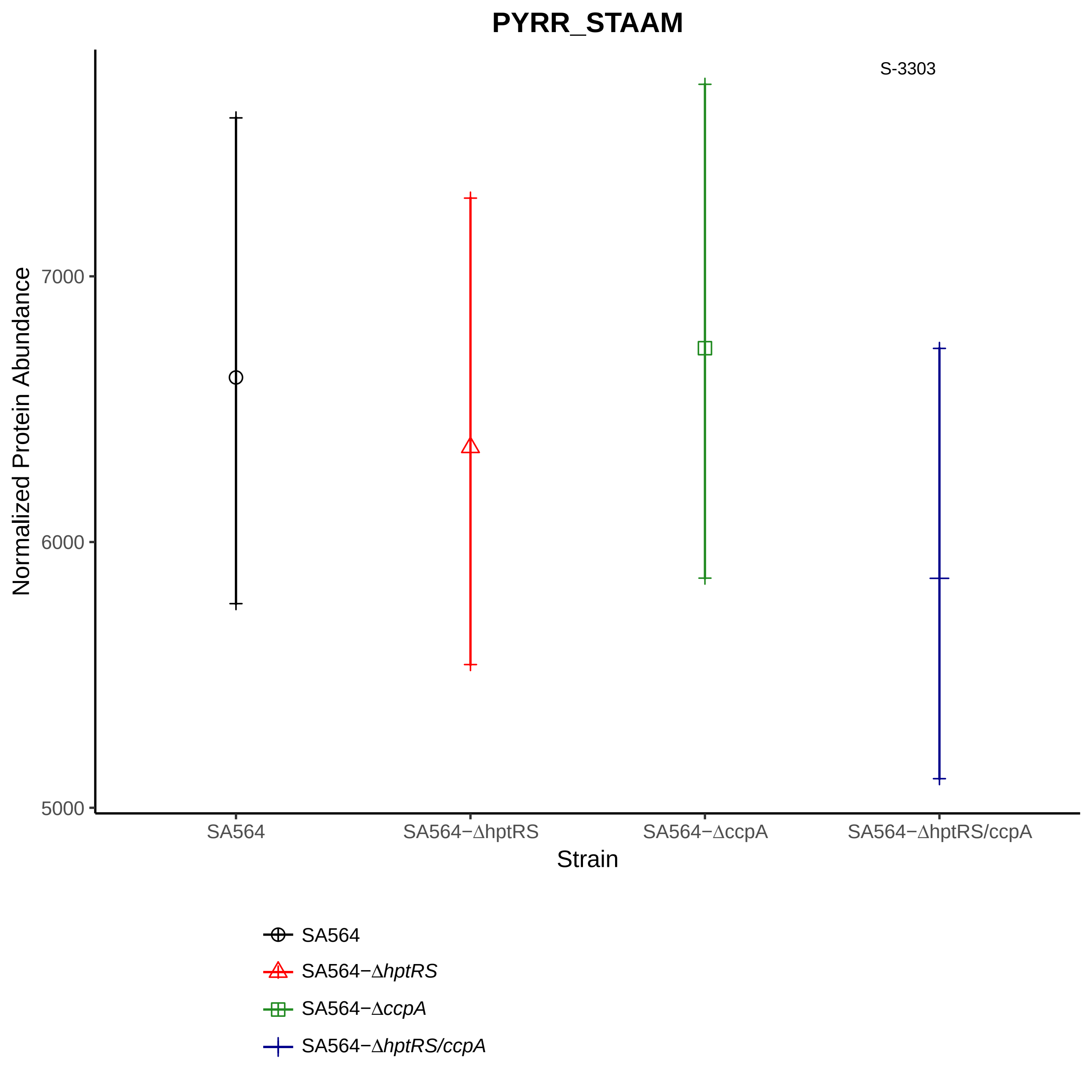
PYRR_STAAM
S-3303
Normalized Protein Abundance
7000
6000
5000
SA564
SA564−ΔhptRS
SA564−ΔccpA
SA564−ΔhptRS/ccpA
Strain
SA564
SA564−ΔhptRS
SA564−ΔccpA
SA564−ΔhptRS/ccpA

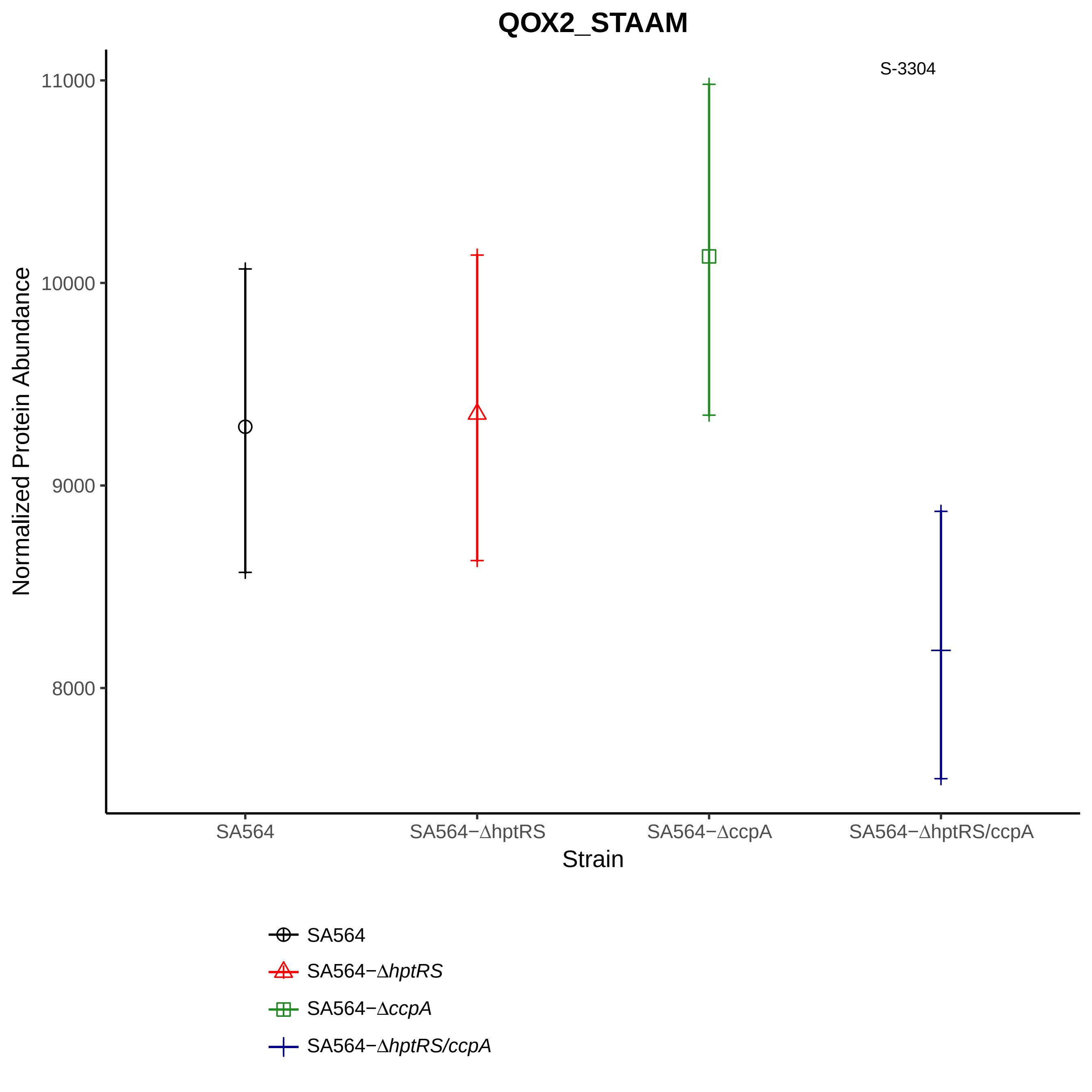
QOX2_STAAM
S-3304
Normalized Protein Abundance
11000
10000
9000
8000
SA564
SA564−ΔhptRS
SA564−ΔccpA
SA564−ΔhptRS/ccpA
Strain
SA564
SA564−ΔhptRS
SA564−ΔccpA
SA564−ΔhptRS/ccpA

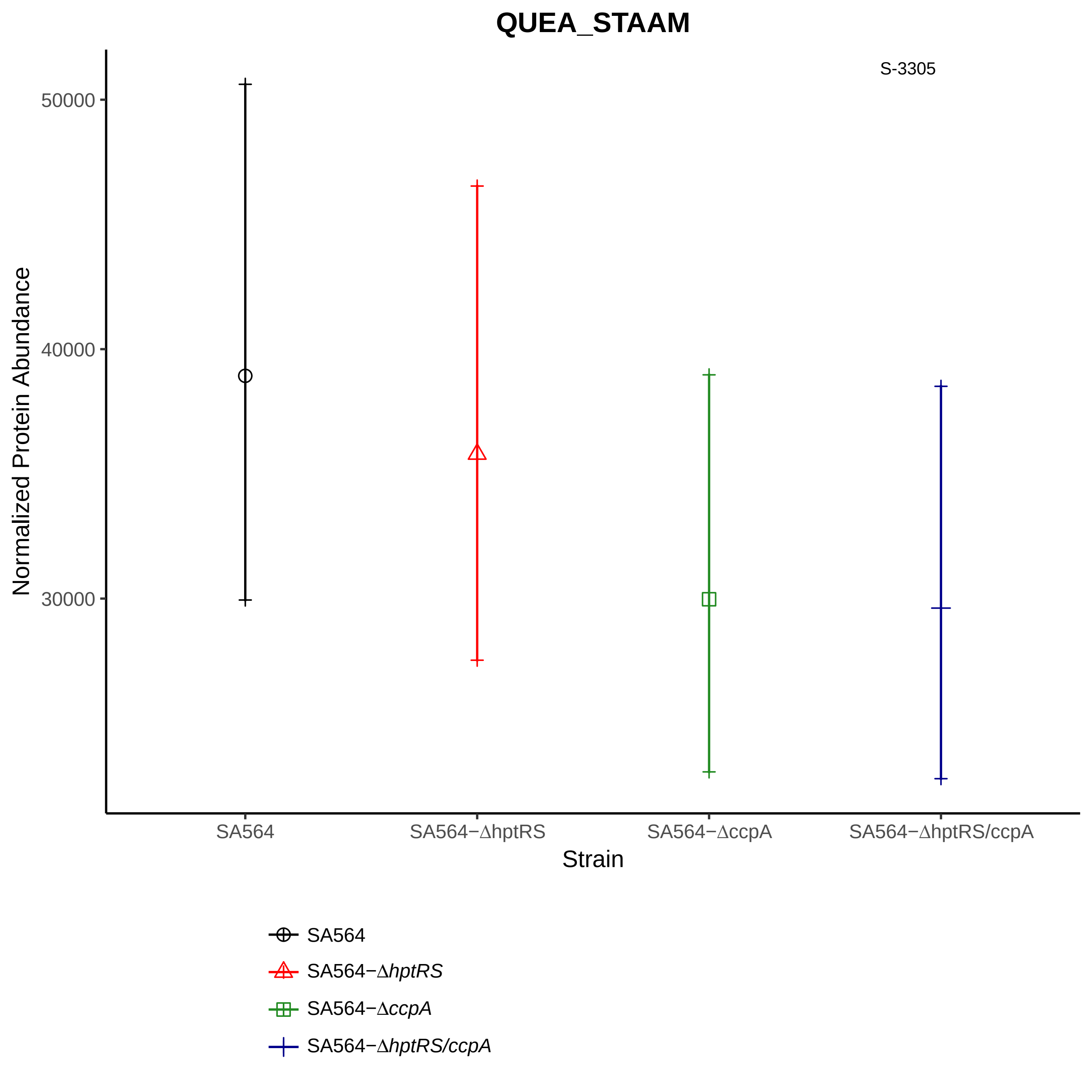
QUEA_STAAM
S-3305
Normalized Protein Abundance
50000
40000
30000
SA564
SA564−ΔhptRS
SA564−ΔccpA
SA564−ΔhptRS/ccpA
Strain
SA564
SA564−ΔhptRS
SA564−ΔccpA
SA564−ΔhptRS/ccpA

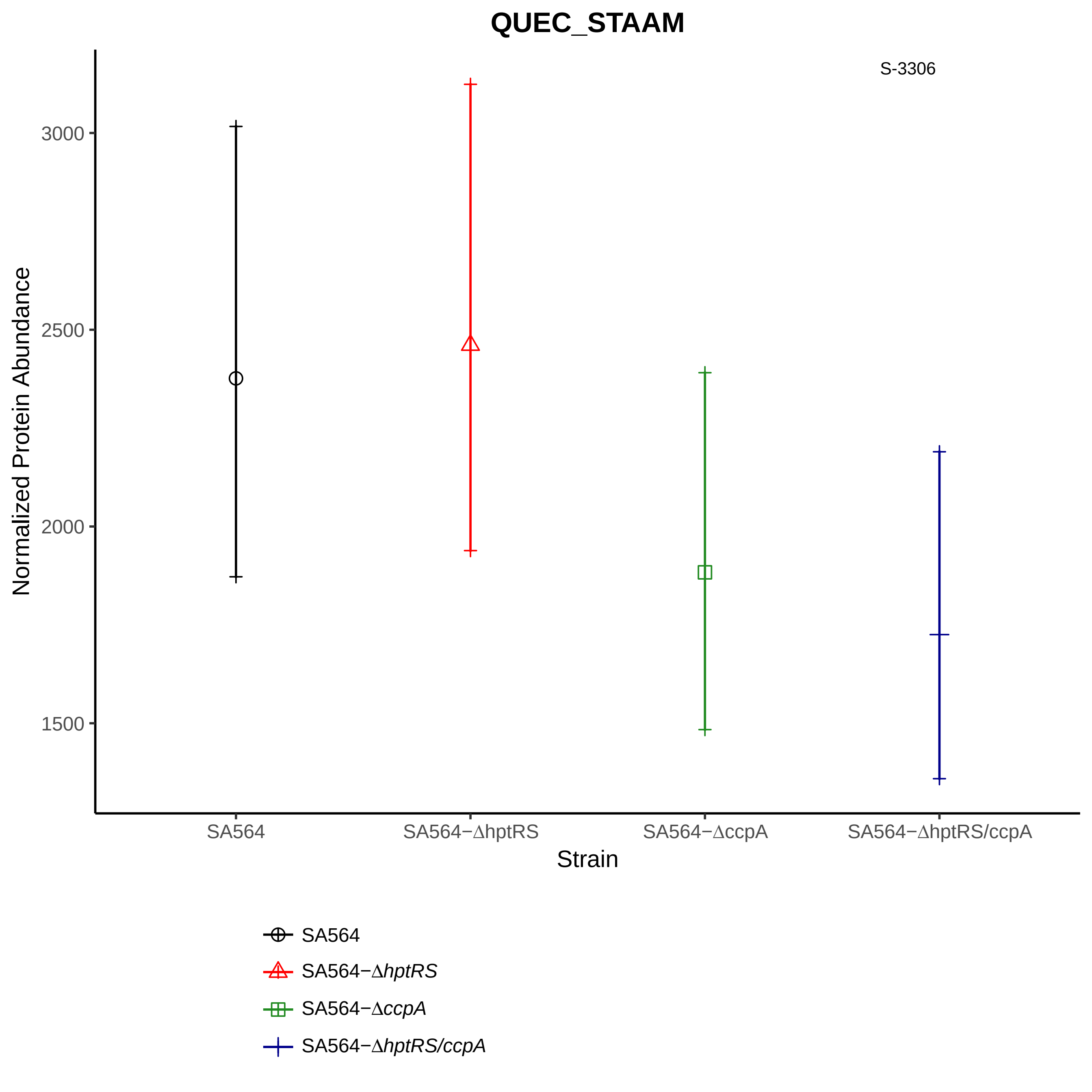
QUEC_STAAM
S-3306
Normalized Protein Abundance
3000
2500
2000
1500
SA564
SA564−ΔhptRS
SA564−ΔccpA
SA564−ΔhptRS/ccpA
Strain
SA564
SA564−ΔhptRS
SA564−ΔccpA
SA564−ΔhptRS/ccpA

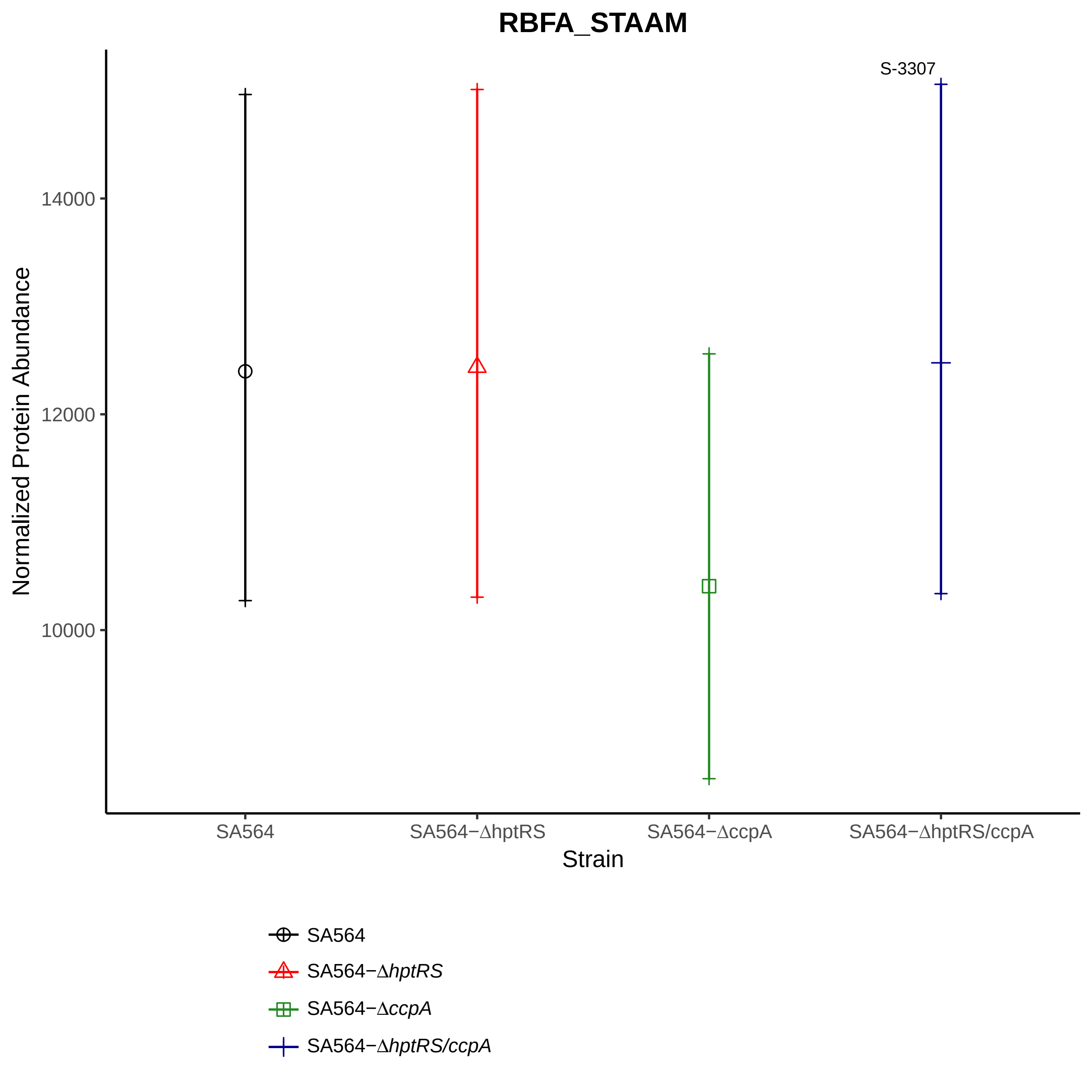

RBFA_STAAM
S-3307
14000
12000
10000
Normalized Protein Abundance
SA564
SA564−ΔhptRS
SA564−ΔccpA
SA564−ΔhptRS/ccpA
Strain
SA564
SA564−ΔhptRS
SA564−ΔccpA
SA564−ΔhptRS/ccpA

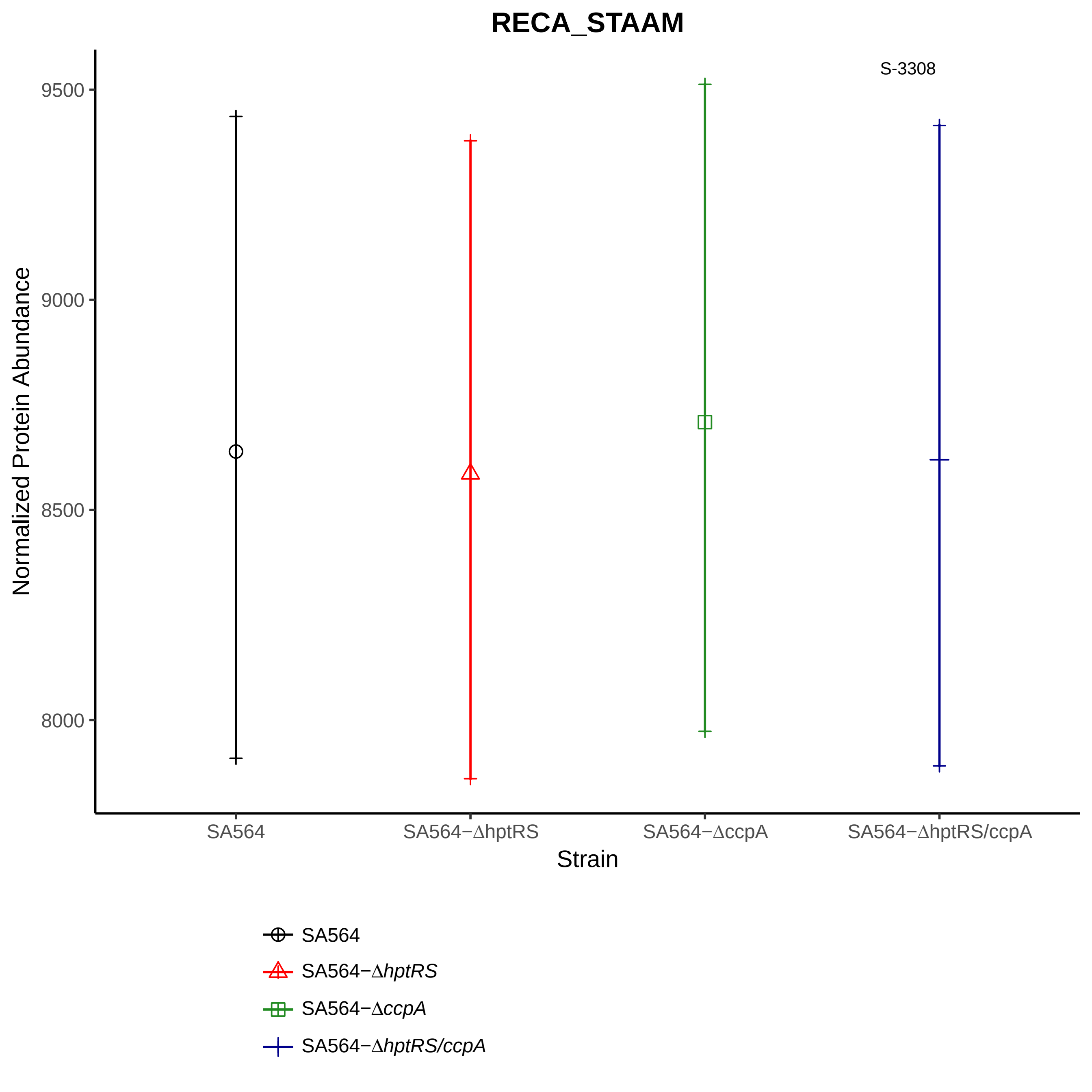

RECA_STAAM
S-3308
Normalized Protein Abundance
9500
9000
8500
8000
SA564
SA564−ΔhptRS
SA564−ΔccpA
SA564−ΔhptRS/ccpA
Strain
SA564
SA564−ΔhptRS
SA564−ΔccpA
SA564−ΔhptRS/ccpA

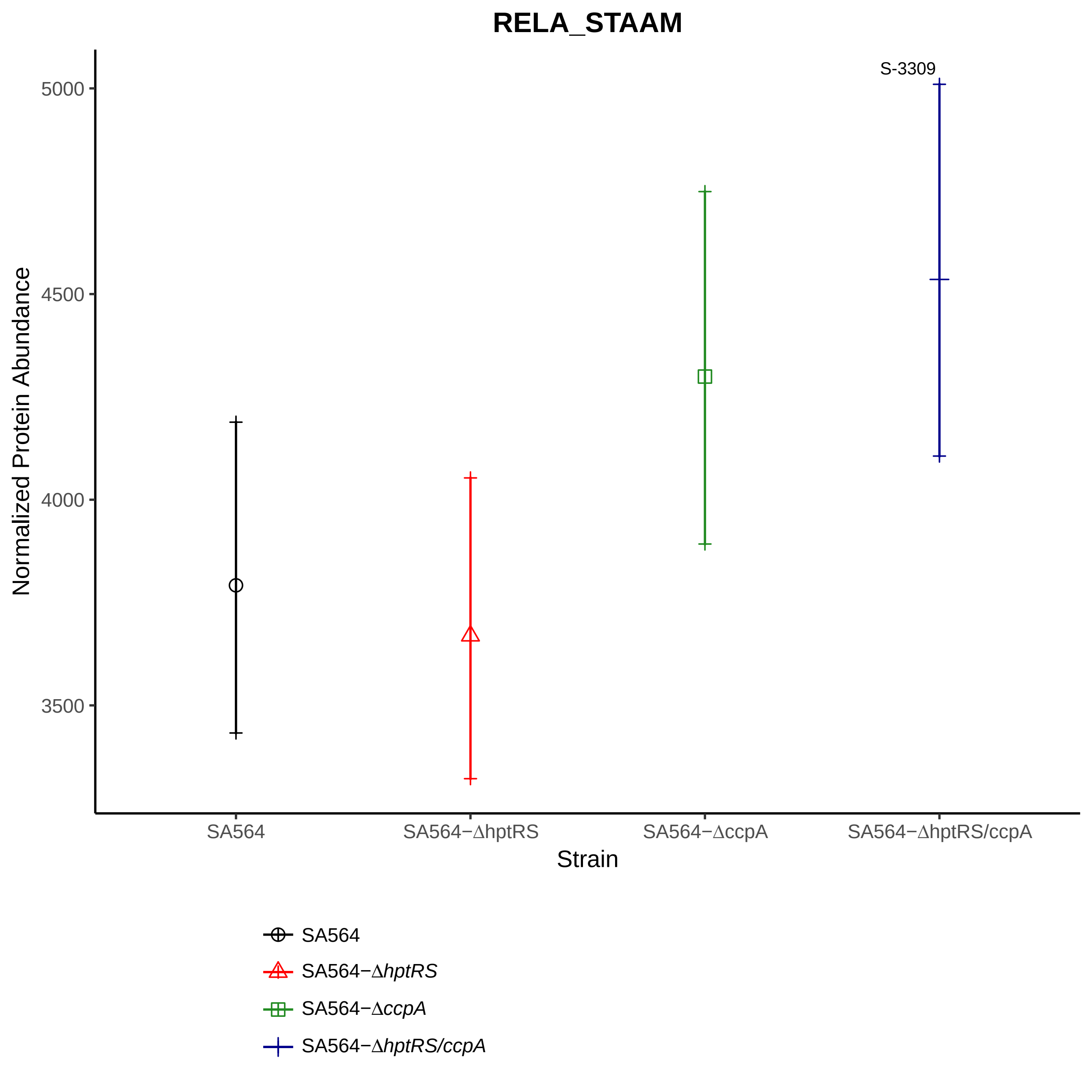

RELA_STAAM
S-3309
5000
4500
4000
3500
Normalized Protein Abundance
SA564
SA564−ΔhptRS
SA564−ΔccpA
SA564−ΔhptRS/ccpA
Strain
SA564
SA564−ΔhptRS
SA564−ΔccpA
SA564−ΔhptRS/ccpA

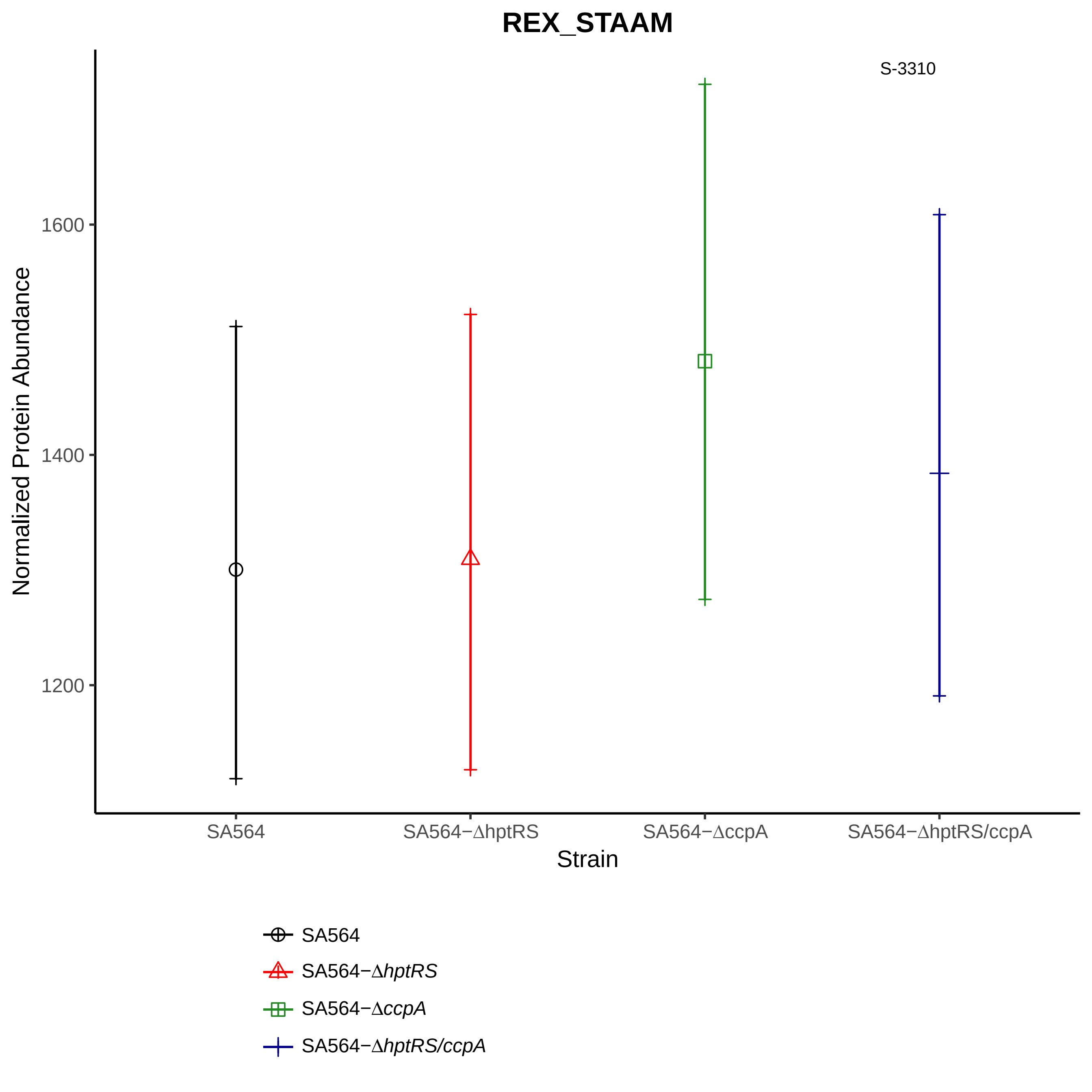
REX_STAAM
S-3310
1600
1400
1200
Normalized Protein Abundance
SA564
SA564−ΔhptRS
SA564−ΔccpA
SA564−ΔhptRS/ccpA
Strain
SA564
SA564−ΔhptRS
SA564−ΔccpA
SA564−ΔhptRS/ccpA

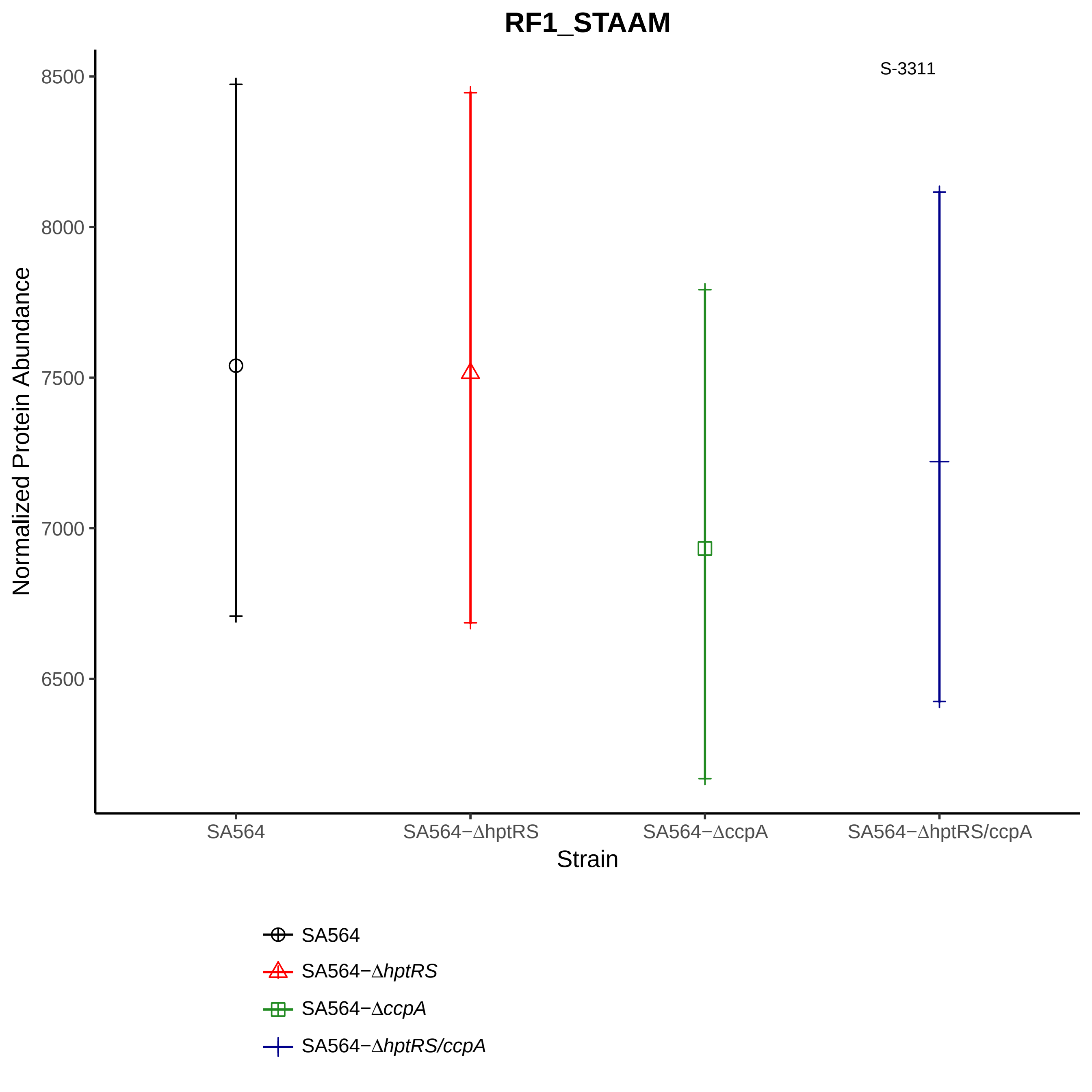

RF1_STAAM
S-3311
Normalized Protein Abundance
8500
8000
7500
7000
6500
SA564
SA564−ΔhptRS
SA564−ΔccpA
SA564−ΔhptRS/ccpA
Strain
SA564
SA564−ΔhptRS
SA564−ΔccpA
SA564−ΔhptRS/ccpA

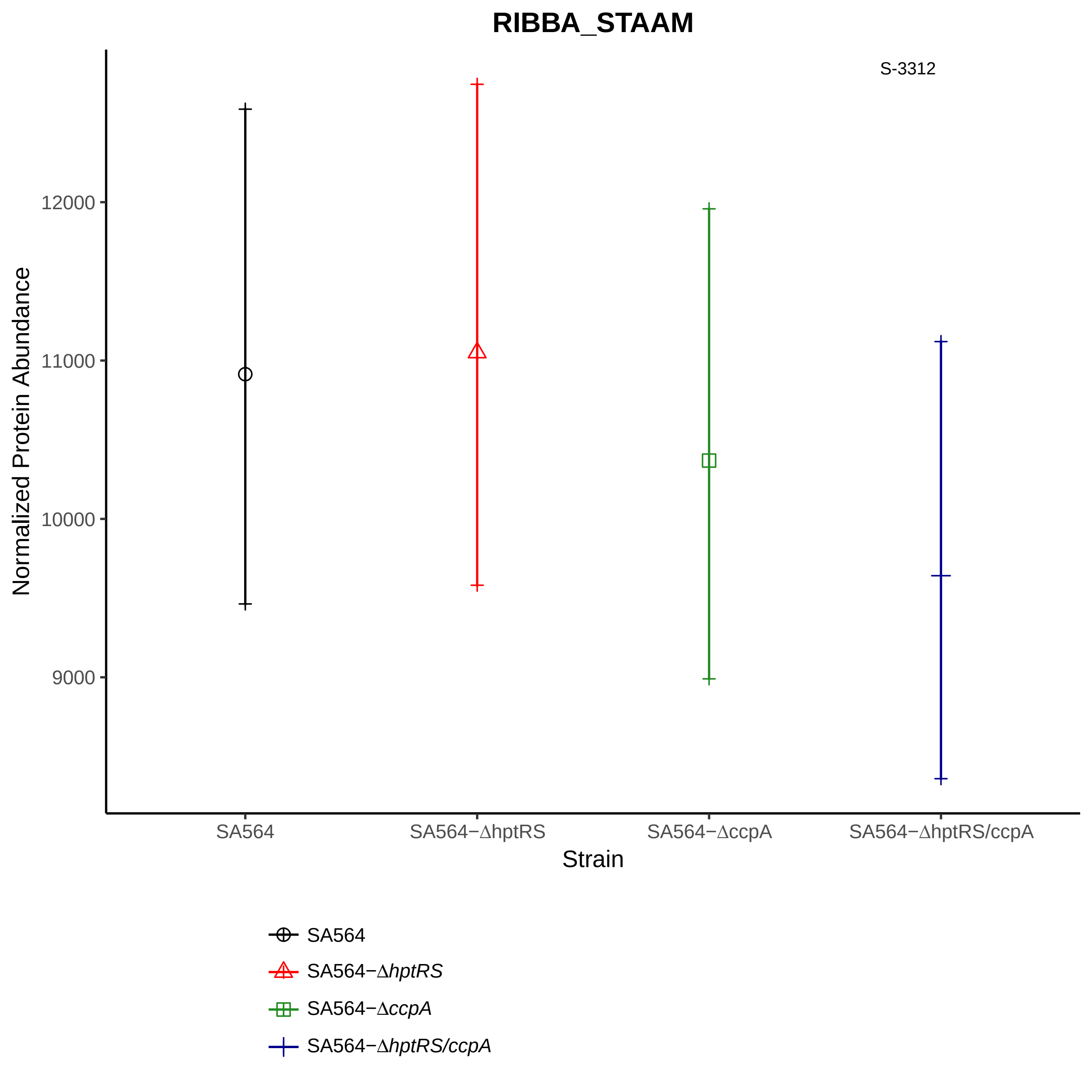

RIBBA_STAAM
S-3312
12000
11000
10000
9000
Normalized Protein Abundance
SA564
SA564−ΔhptRS
SA564−ΔccpA
SA564−ΔhptRS/ccpA
Strain
SA564
SA564−ΔhptRS
SA564−ΔccpA
SA564−ΔhptRS/ccpA

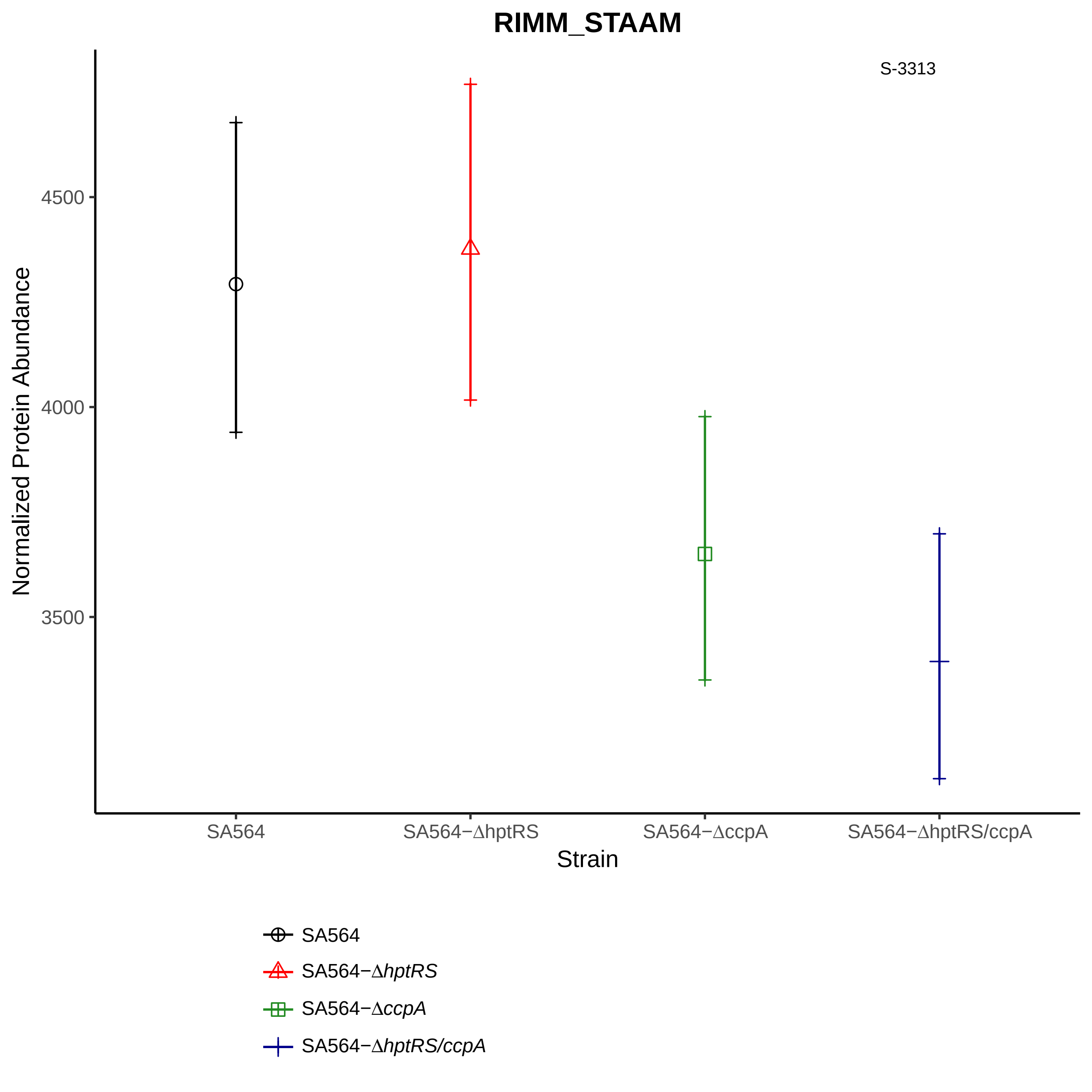

RIMM_STAAM
S-3313
Normalized Protein Abundance
4500
4000
3500
SA564
SA564−ΔhptRS
SA564−ΔccpA
SA564−ΔhptRS/ccpA
Strain
SA564
SA564−ΔhptRS
SA564−ΔccpA
SA564−ΔhptRS/ccpA

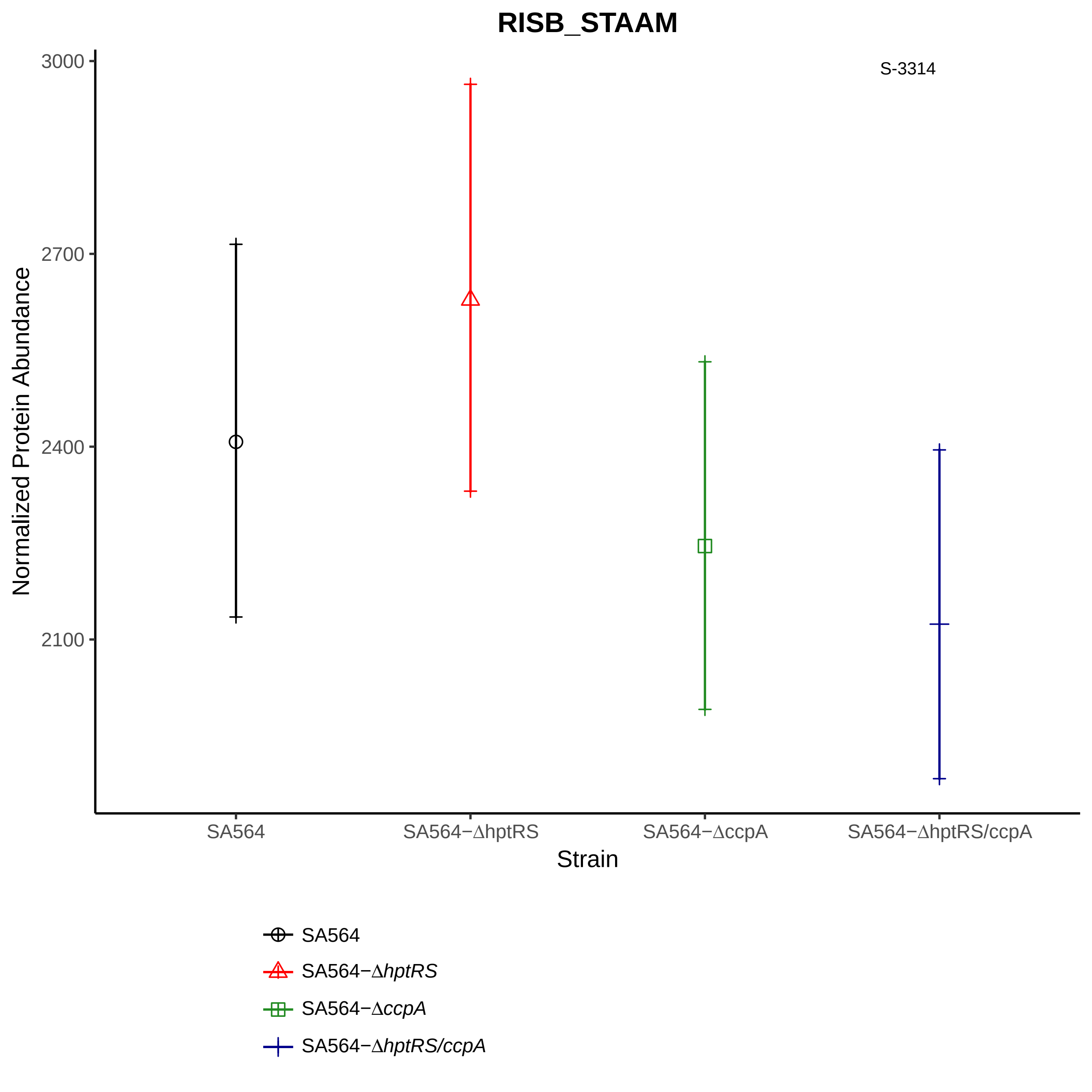

RISB_STAAM
S-3314
Normalized Protein Abundance
3000
2700
2400
2100
SA564
SA564−ΔhptRS
SA564−ΔccpA
SA564−ΔhptRS/ccpA
Strain
SA564
SA564−ΔhptRS
SA564−ΔccpA
SA564−ΔhptRS/ccpA

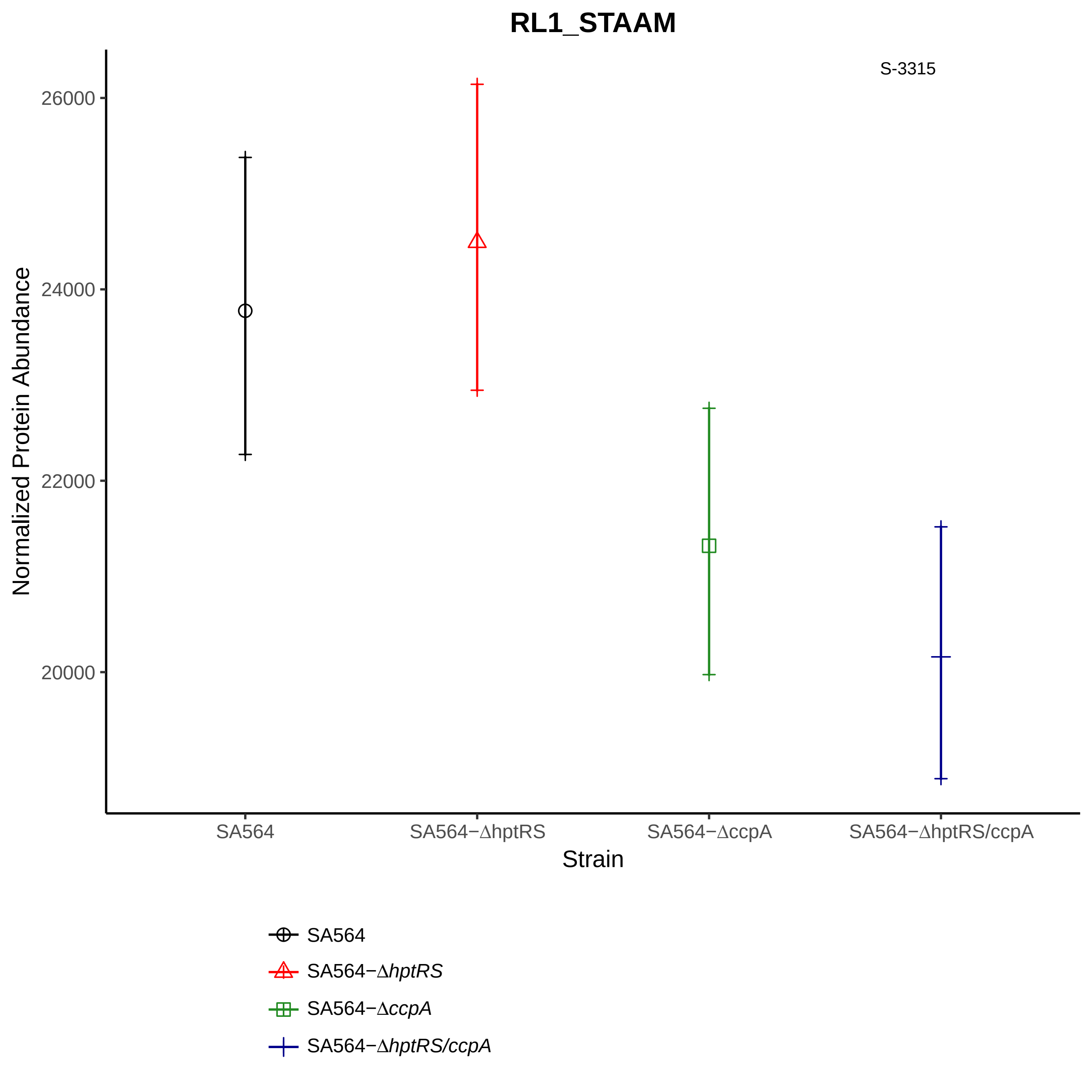

RL1_STAAM
S-3315
Normalized Protein Abundance
26000
24000
22000
20000
SA564
SA564−ΔhptRS
SA564−ΔccpA
SA564−ΔhptRS/ccpA
Strain
SA564
SA564−ΔhptRS
SA564−ΔccpA
SA564−ΔhptRS/ccpA

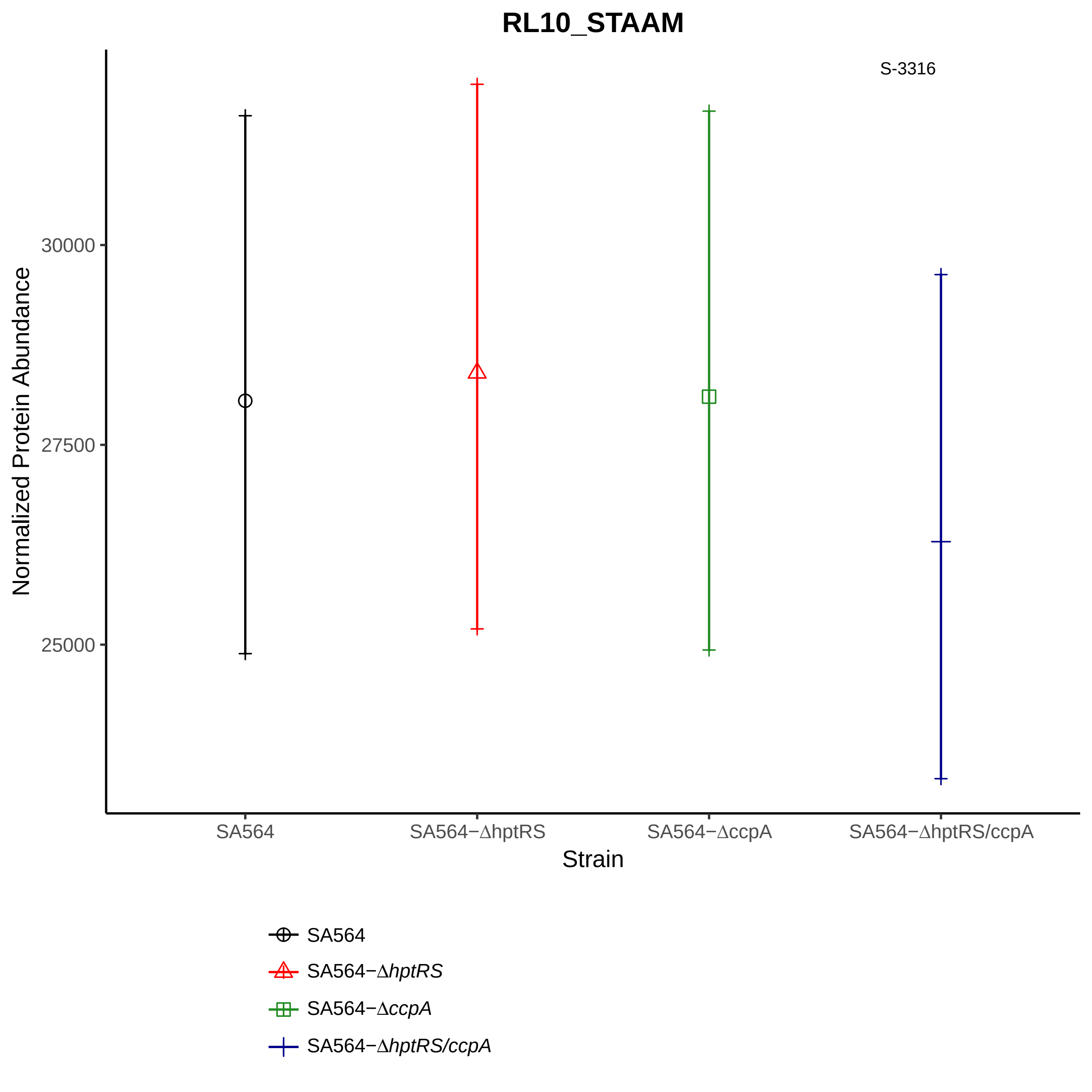

RL10_STAAM
S-3316
Normalized Protein Abundance
30000
27500
25000
SA564
SA564−ΔhptRS
SA564−ΔccpA
SA564−ΔhptRS/ccpA
Strain
SA564
SA564−Δ*hptRS*
SA564−Δ*ccpA*
SA564−Δ*hptRS/ccpA*

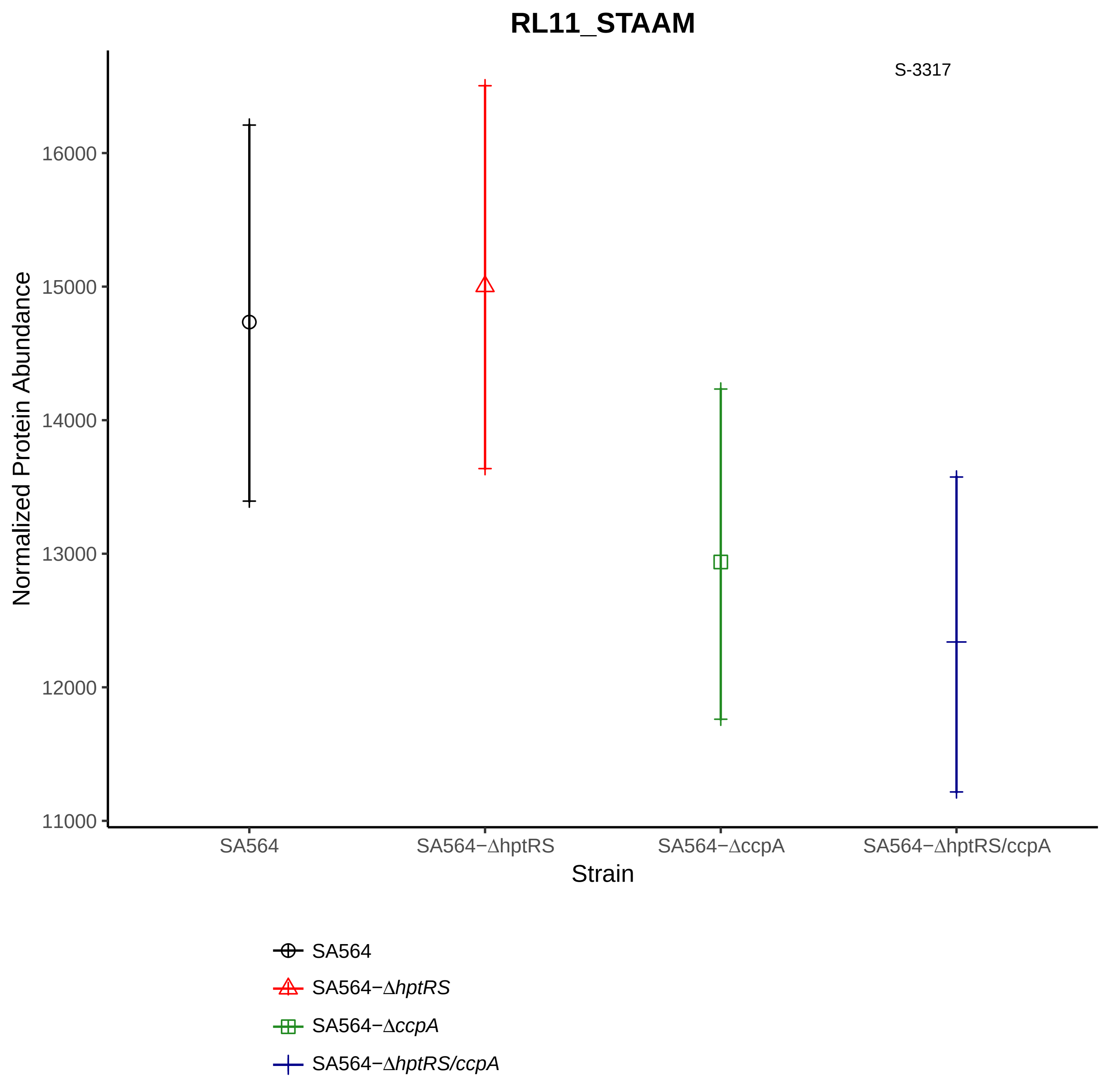

RL11_STAAM
S-3317
Normalized Protein Abundance
16000
15000
14000
13000
12000
11000
SA564
SA564−ΔhptRS
SA564−ΔccpA
SA564−ΔhptRS/ccpA
Strain
SA564
SA564−Δ*hptRS*
SA564−Δ*ccpA*
SA564−Δ*hptRS/ccpA*

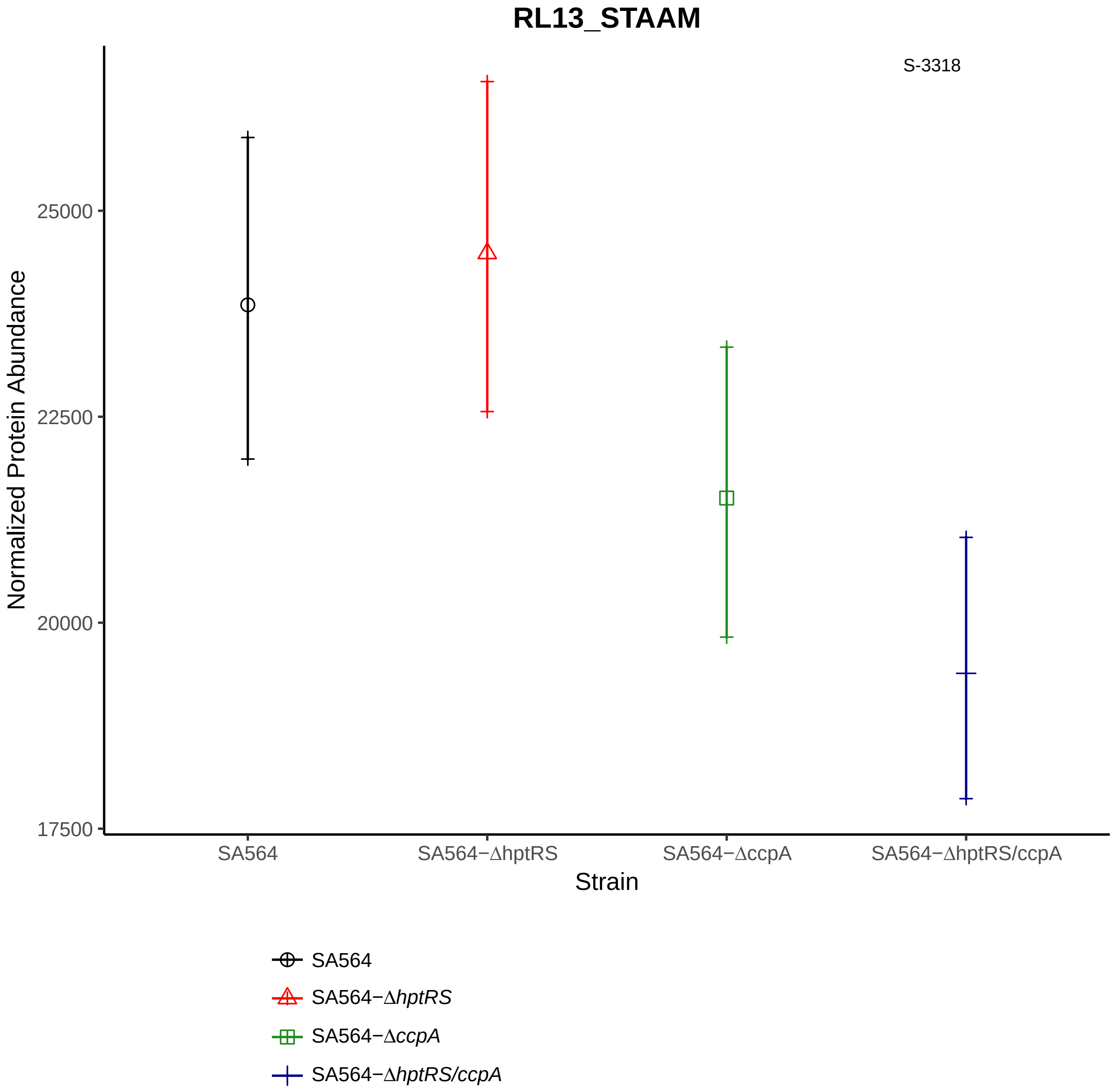

RL13_STAAM
S-3318
Normalized Protein Abundance
25000
22500
20000
17500
SA564
SA564−ΔhptRS
SA564−ΔccpA
SA564−ΔhptRS/ccpA
Strain
SA564
SA564−ΔhptRS
SA564−ΔccpA
SA564−ΔhptRS/ccpA

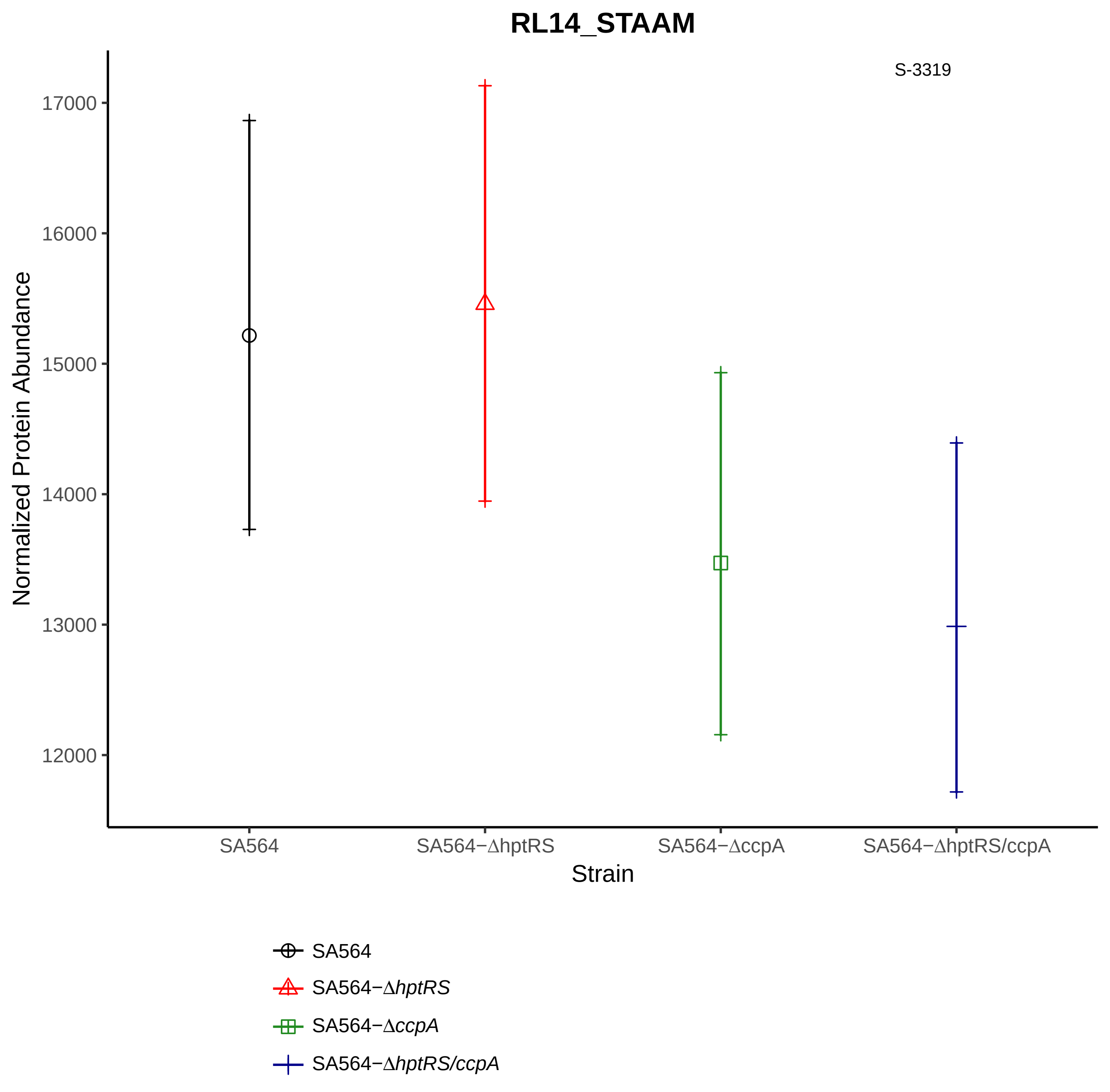

RL14_STAAM
S-3319
Normalized Protein Abundance
17000
16000
15000
14000
13000
12000
SA564
SA564−ΔhptRS
SA564−ΔccpA
SA564−ΔhptRS/ccpA
Strain
SA564
SA564−ΔhptRS
SA564−ΔccpA
SA564−ΔhptRS/ccpA

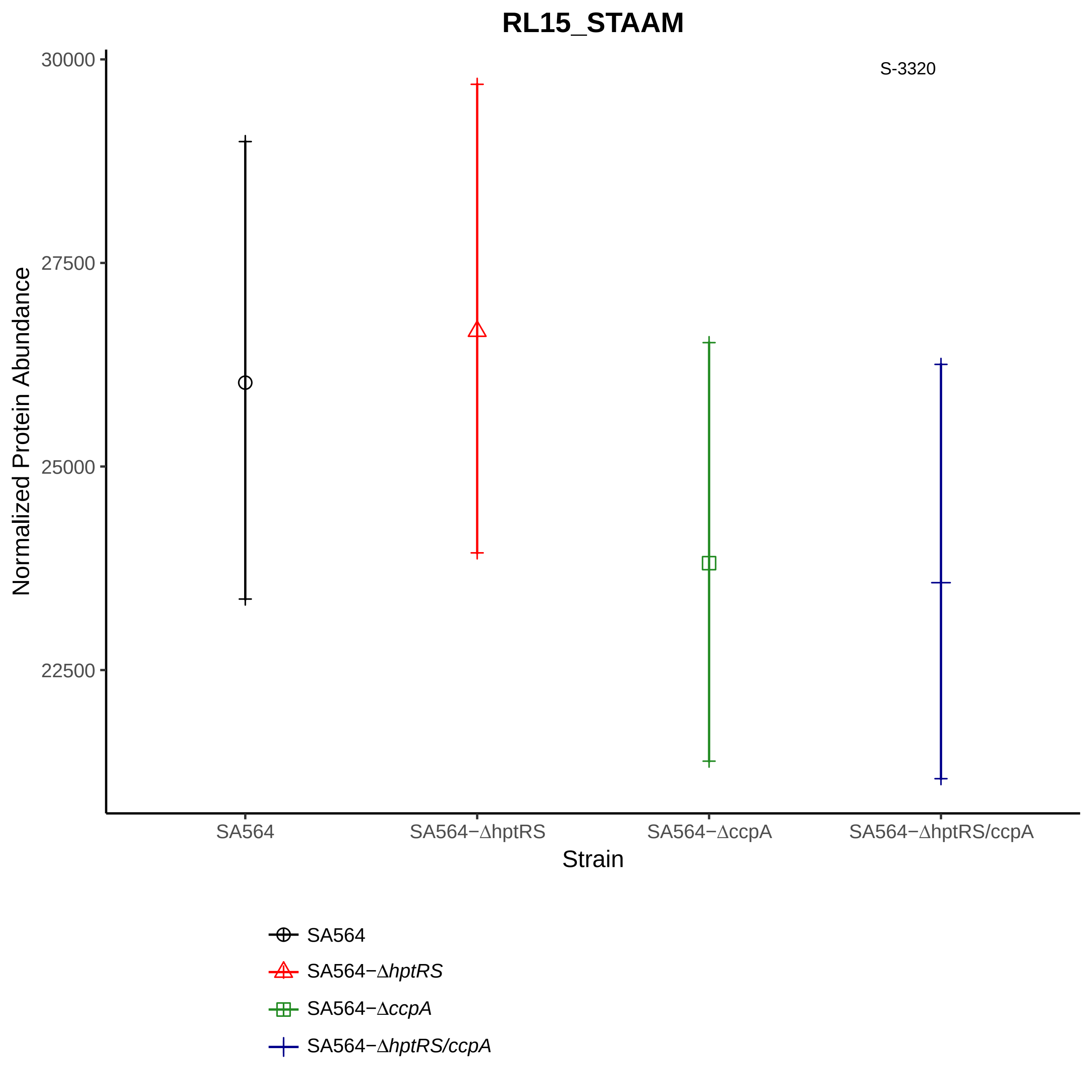

RL15_STAAM
S-3320
Normalized Protein Abundance
30000
27500
25000
22500
SA564
SA564−ΔhptRS
SA564−ΔccpA
SA564−ΔhptRS/ccpA
Strain
SA564
SA564−Δ*hptRS*
SA564−Δ*ccpA*
SA564−Δ*hptRS/ccpA*

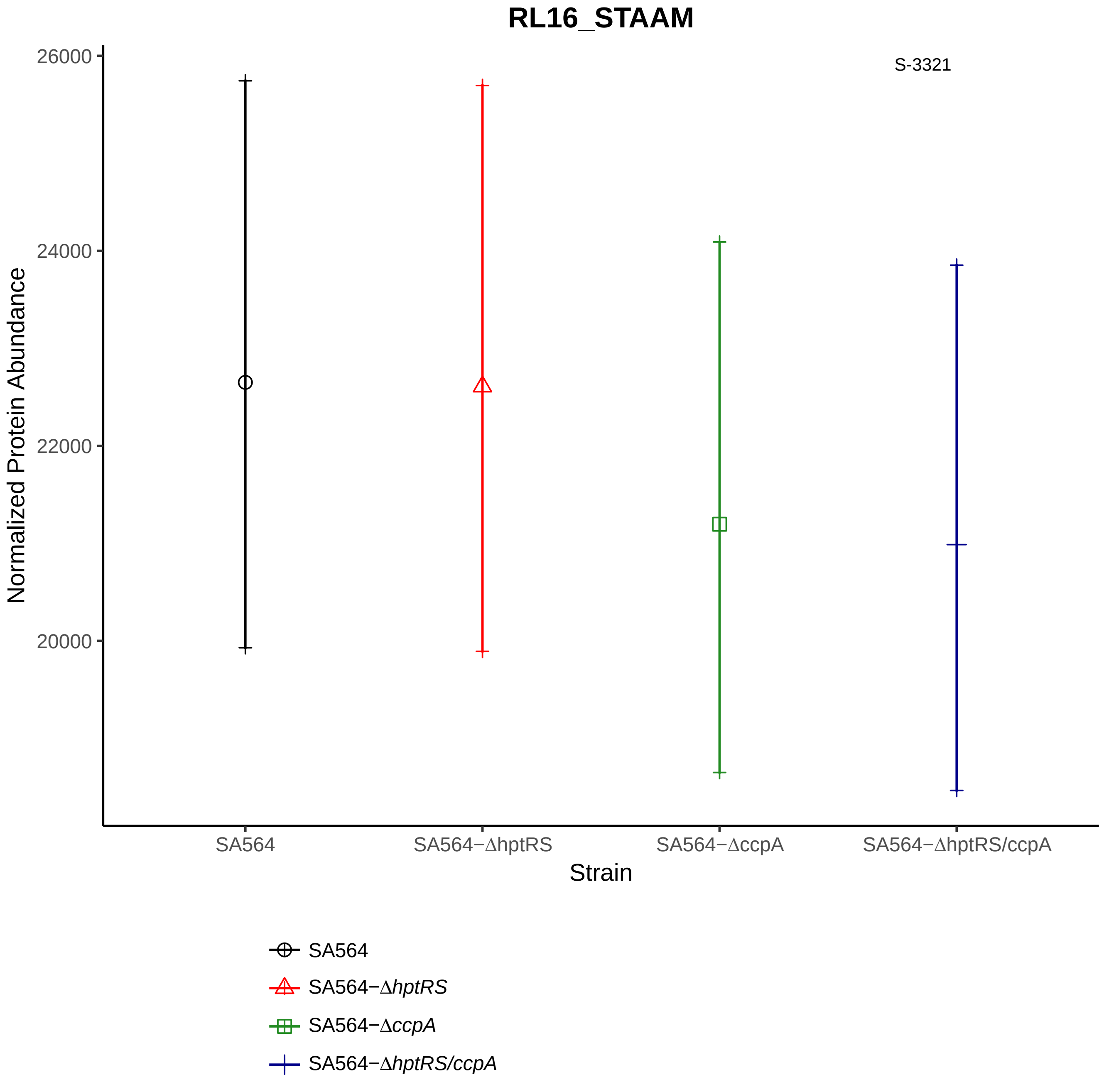
RL16_STAAM
S-3321
Normalized Protein Abundance
26000
24000
22000
20000
SA564
SA564−ΔhptRS
SA564−ΔccpA
SA564−ΔhptRS/ccpA
Strain
SA564
SA564−ΔhptRS
SA564−ΔccpA
SA564−ΔhptRS/ccpA

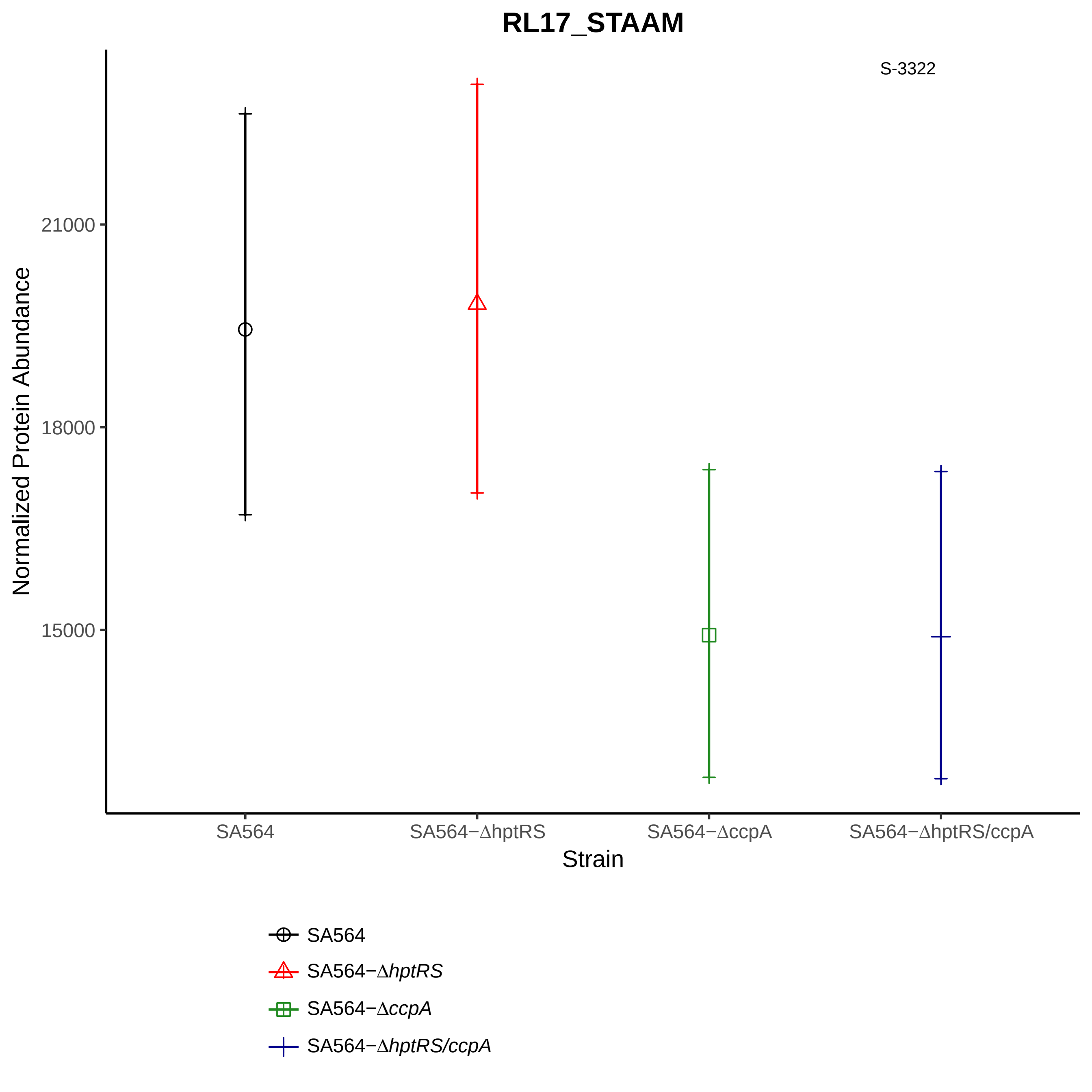

RL17_STAAM
S-3322
Normalized Protein Abundance
21000
18000
15000
SA564
SA564−ΔhptRS
SA564−ΔccpA
SA564−ΔhptRS/ccpA
Strain
SA564
SA564−ΔhptRS
SA564−ΔccpA
SA564−ΔhptRS/ccpA

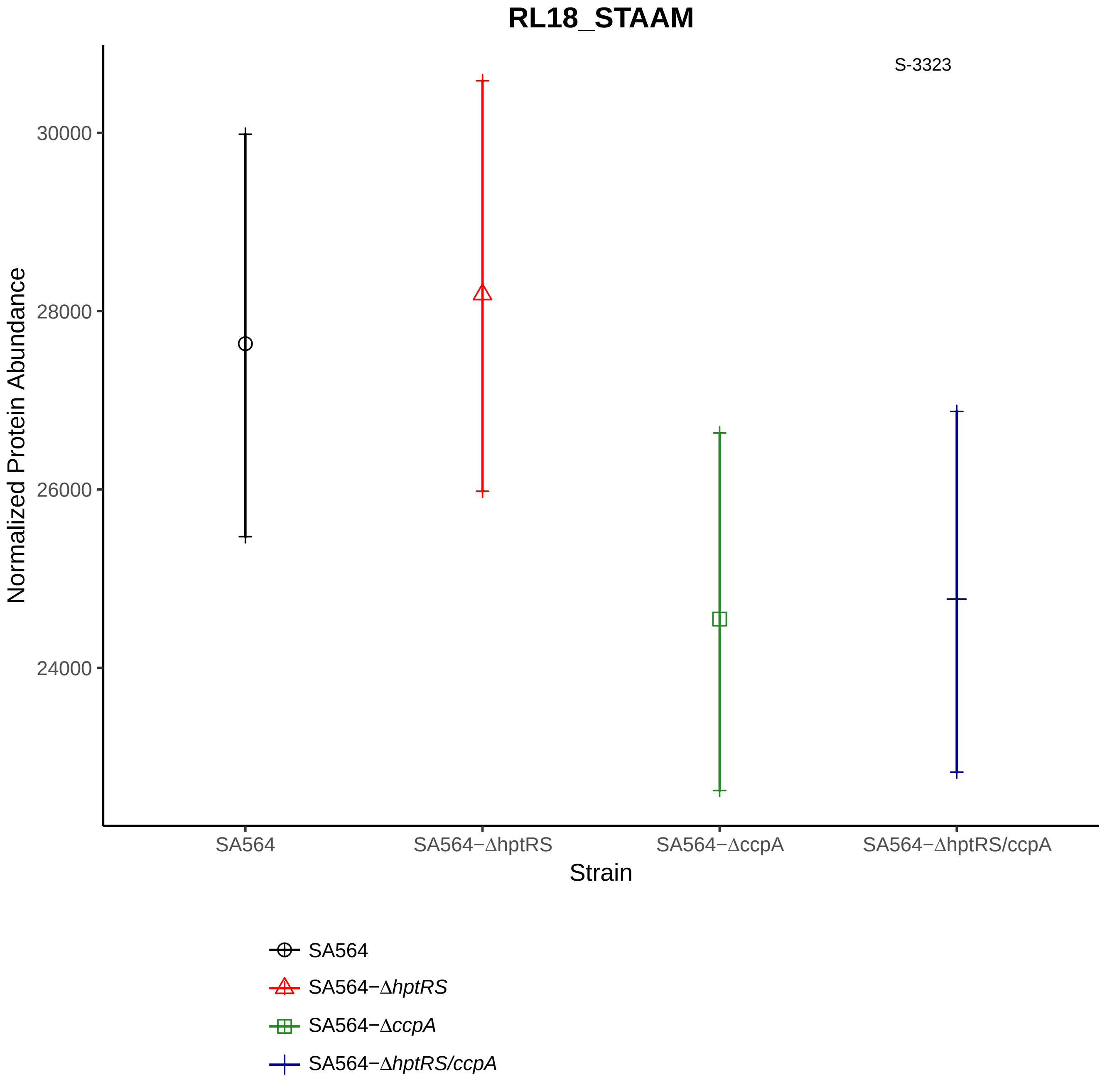

RL18_STAAM
S-3323
Normalized Protein Abundance
30000
28000
26000
24000
SA564
SA564−ΔhptRS
SA564−ΔccpA
SA564−ΔhptRS/ccpA
Strain
SA564
SA564−ΔhptRS
SA564−ΔccpA
SA564−ΔhptRS/ccpA

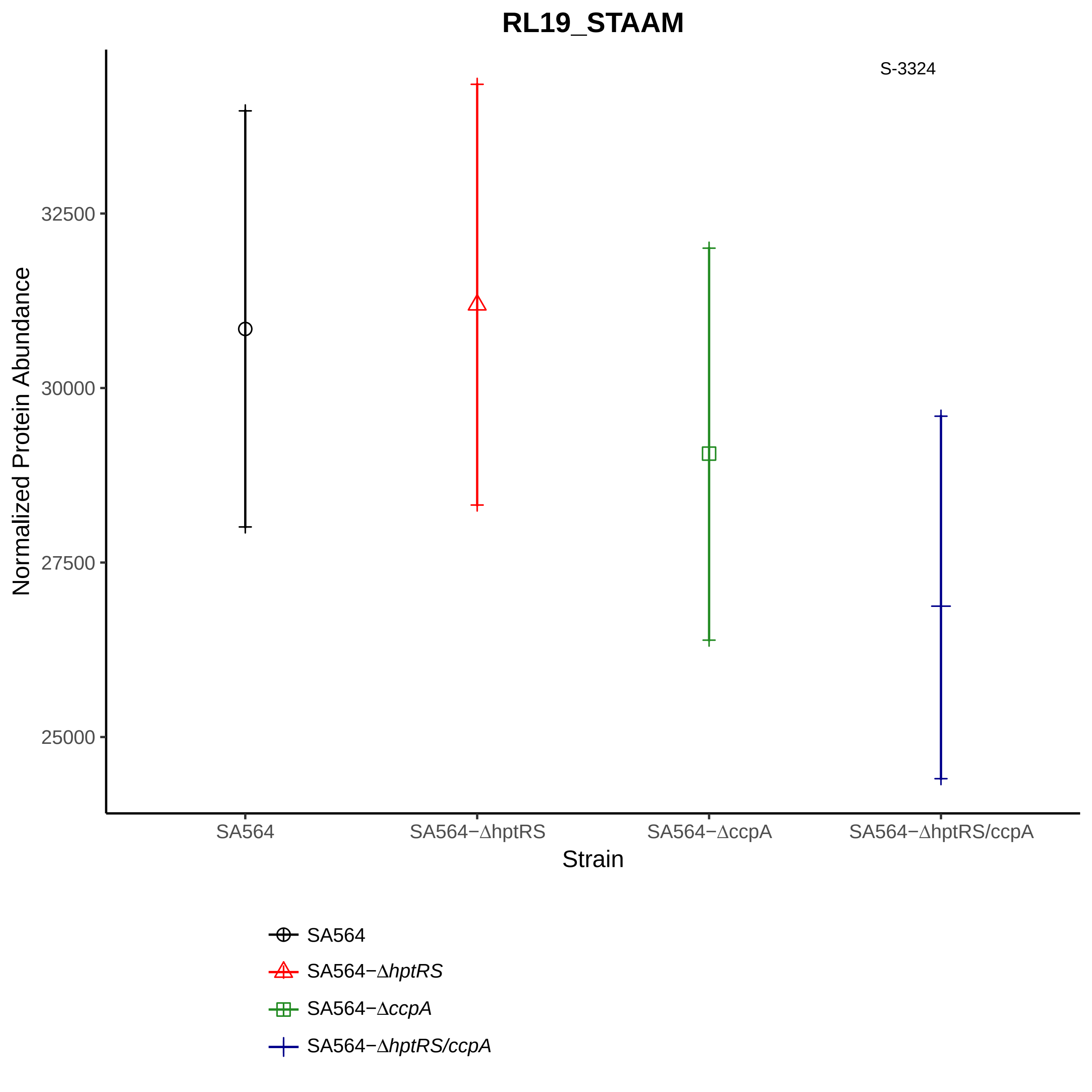

RL19_STAAM
S-3324
Normalized Protein Abundance
32500
30000
27500
25000
SA564
SA564−ΔhptRS
SA564−ΔccpA
SA564−ΔhptRS/ccpA
Strain
SA564
SA564−Δ*hptRS*
SA564−Δ*ccpA*
SA564−Δ*hptRS/ccpA*

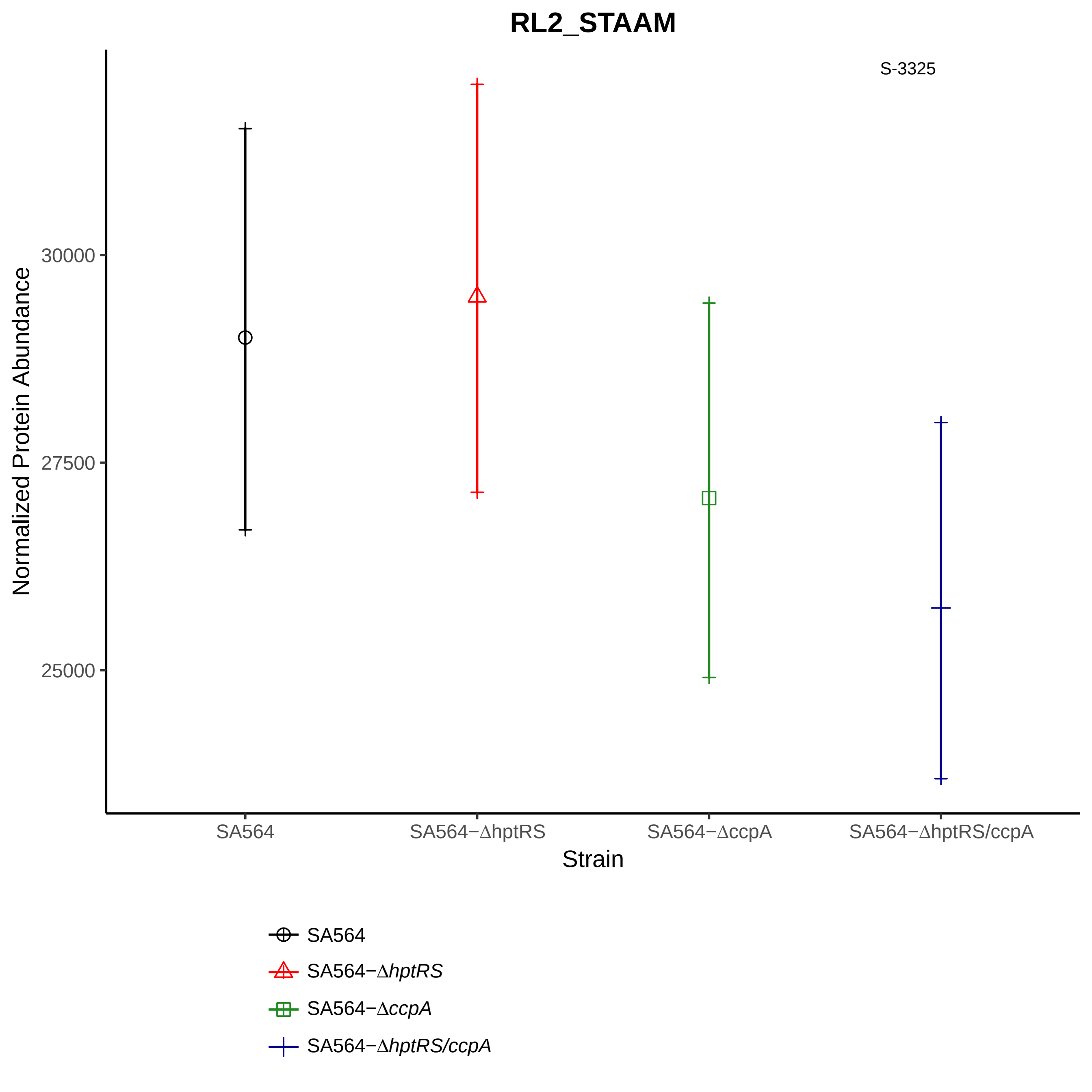

RL2_STAAM
S-3325
Normalized Protein Abundance
30000
27500
25000
SA564
SA564−ΔhptRS
SA564−ΔccpA
SA564−ΔhptRS/ccpA
Strain
SA564
SA564−ΔhptRS
SA564−ΔccpA
SA564−ΔhptRS/ccpA

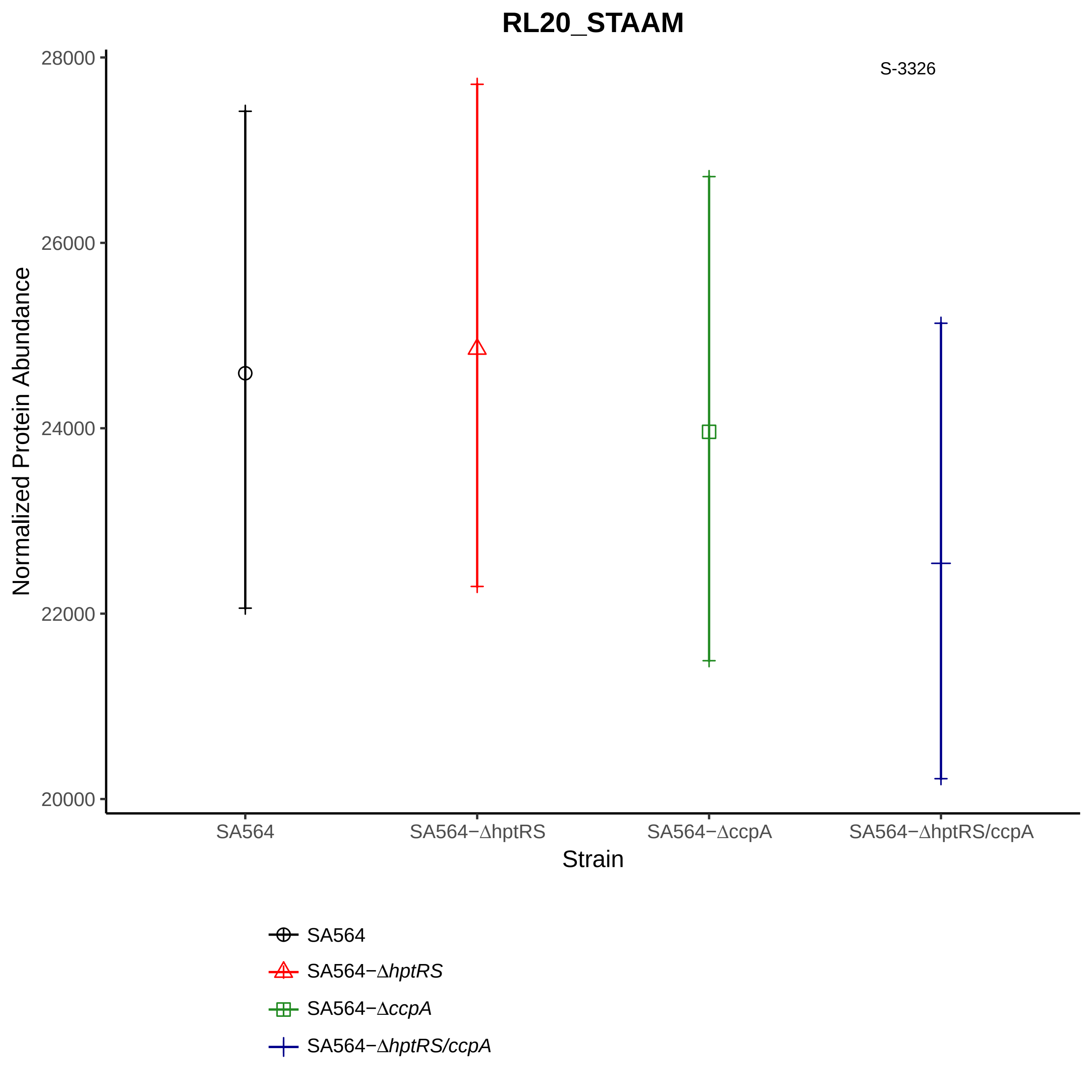

RL20_STAAM
S-3326
Normalized Protein Abundance
28000
26000
24000
22000
20000
SA564
SA564−ΔhptRS
SA564−ΔccpA
SA564−ΔhptRS/ccpA
Strain
SA564
SA564−ΔhptRS
SA564−ΔccpA
SA564−ΔhptRS/ccpA

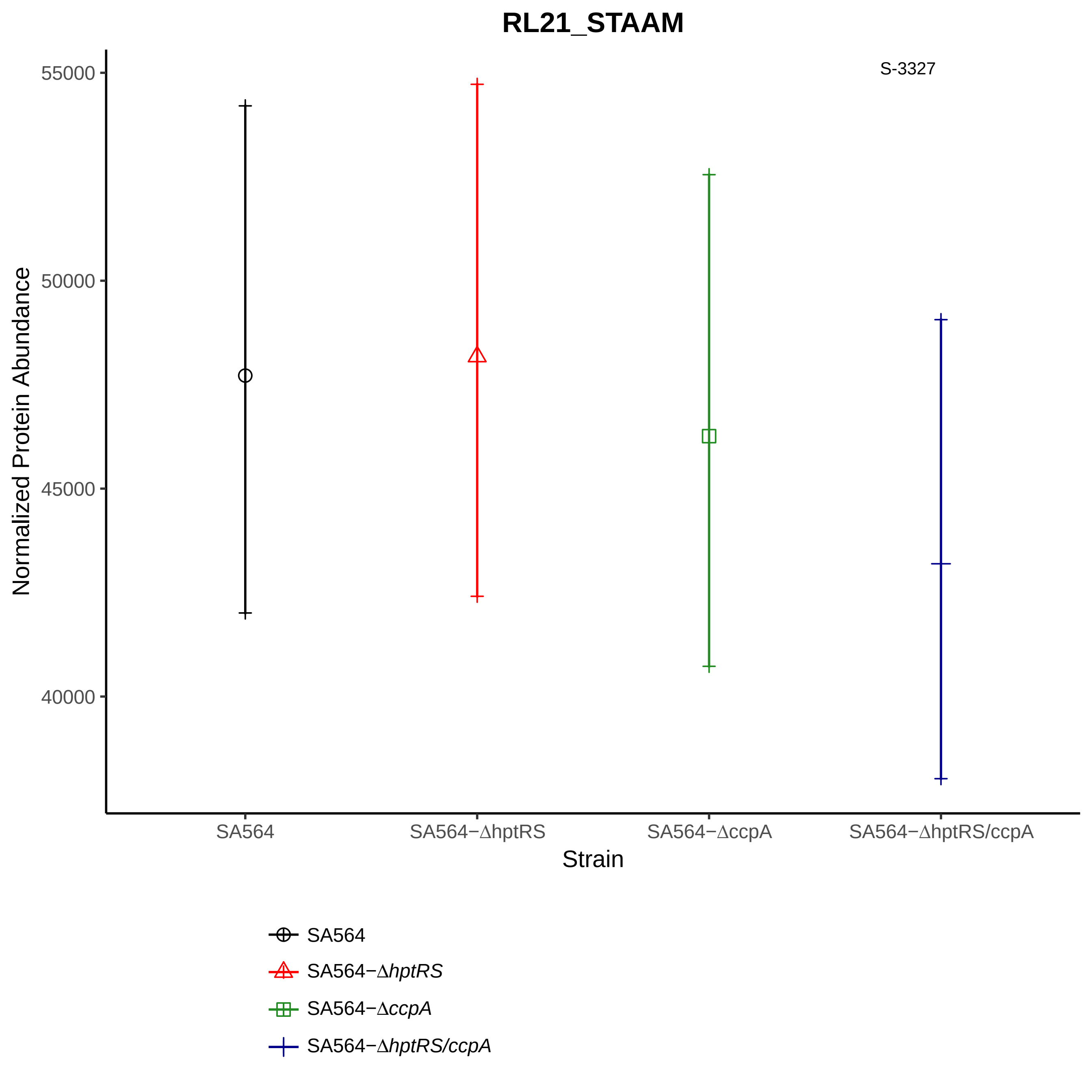

RL21_STAAM
S-3327
55000
50000
45000
40000
Normalized Protein Abundance
SA564
SA564−ΔhptRS
SA564−ΔccpA
SA564−ΔhptRS/ccpA
Strain
SA564
SA564−ΔhptRS
SA564−ΔccpA
SA564−ΔhptRS/ccpA

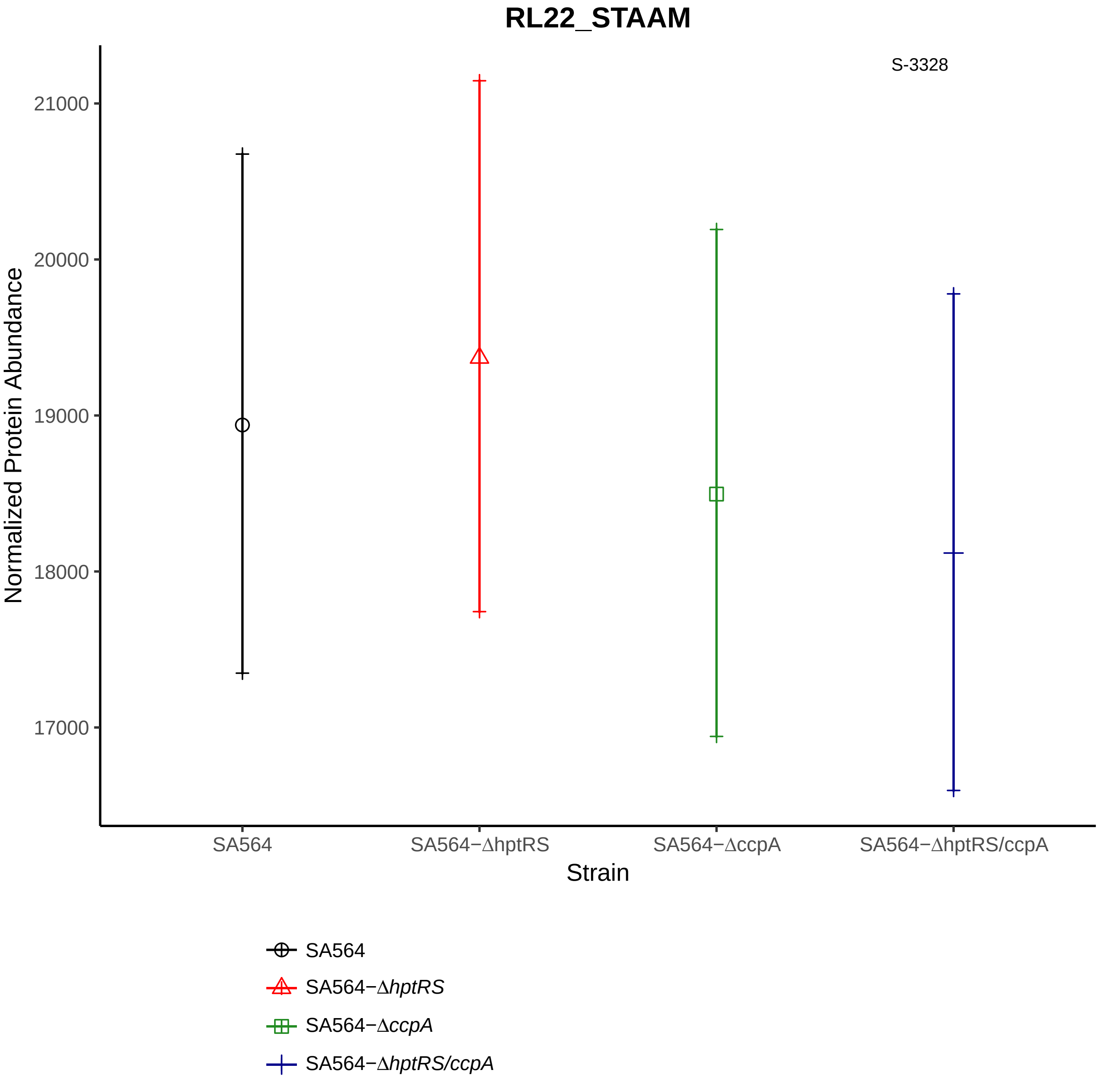

RL22_STAAM
S-3328
Normalized Protein Abundance
21000
20000
19000
18000
17000
SA564
SA564−ΔhptRS
SA564−ΔccpA
SA564−ΔhptRS/ccpA
Strain
SA564
SA564−ΔhptRS
SA564−ΔccpA
SA564−ΔhptRS/ccpA

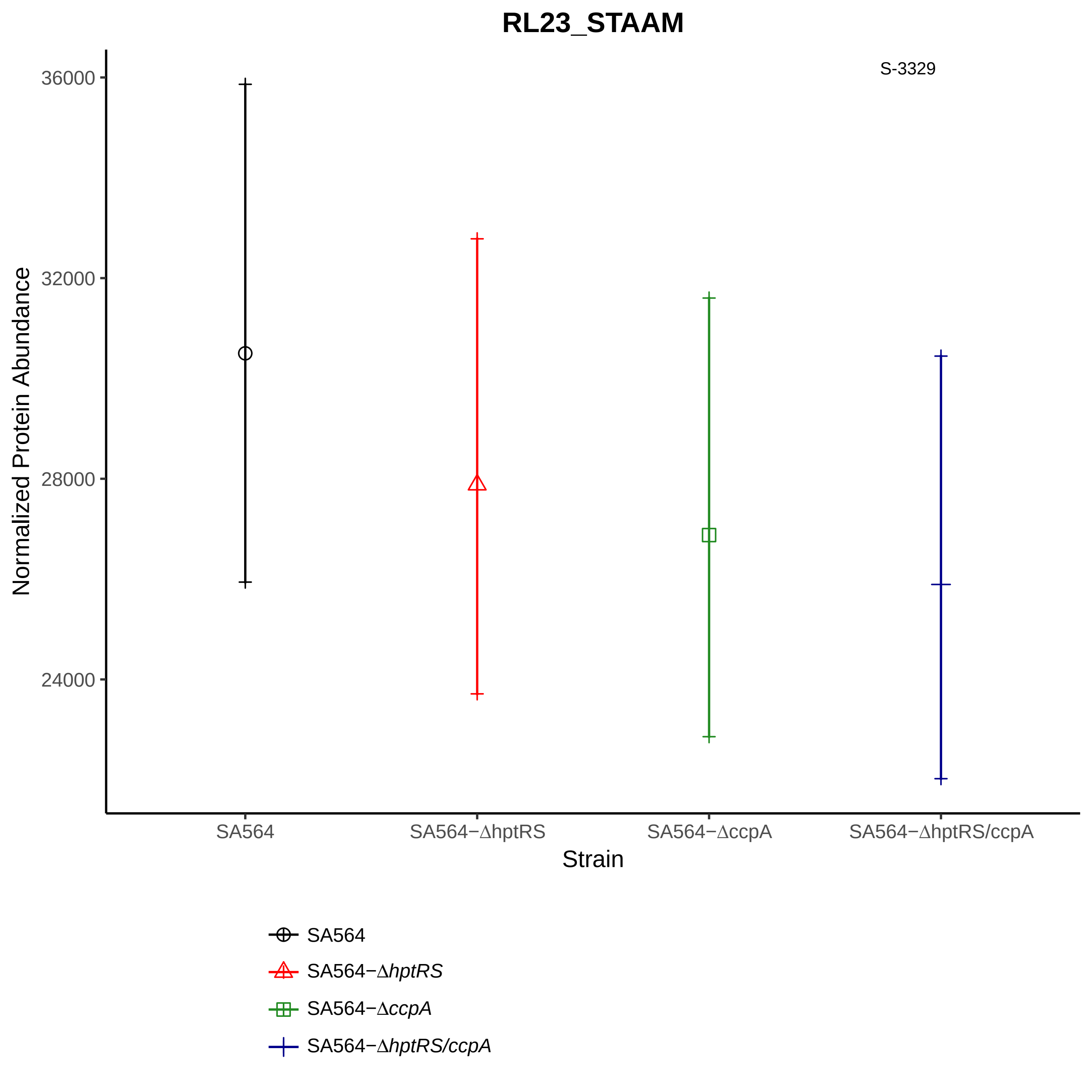
RL23_STAAM
S-3329
Normalized Protein Abundance
36000
32000
28000
24000
SA564
SA564−ΔhptRS
SA564−ΔccpA
SA564−ΔhptRS/ccpA
Strain
SA564
SA564−ΔhptRS
SA564−ΔccpA
SA564−ΔhptRS/ccpA

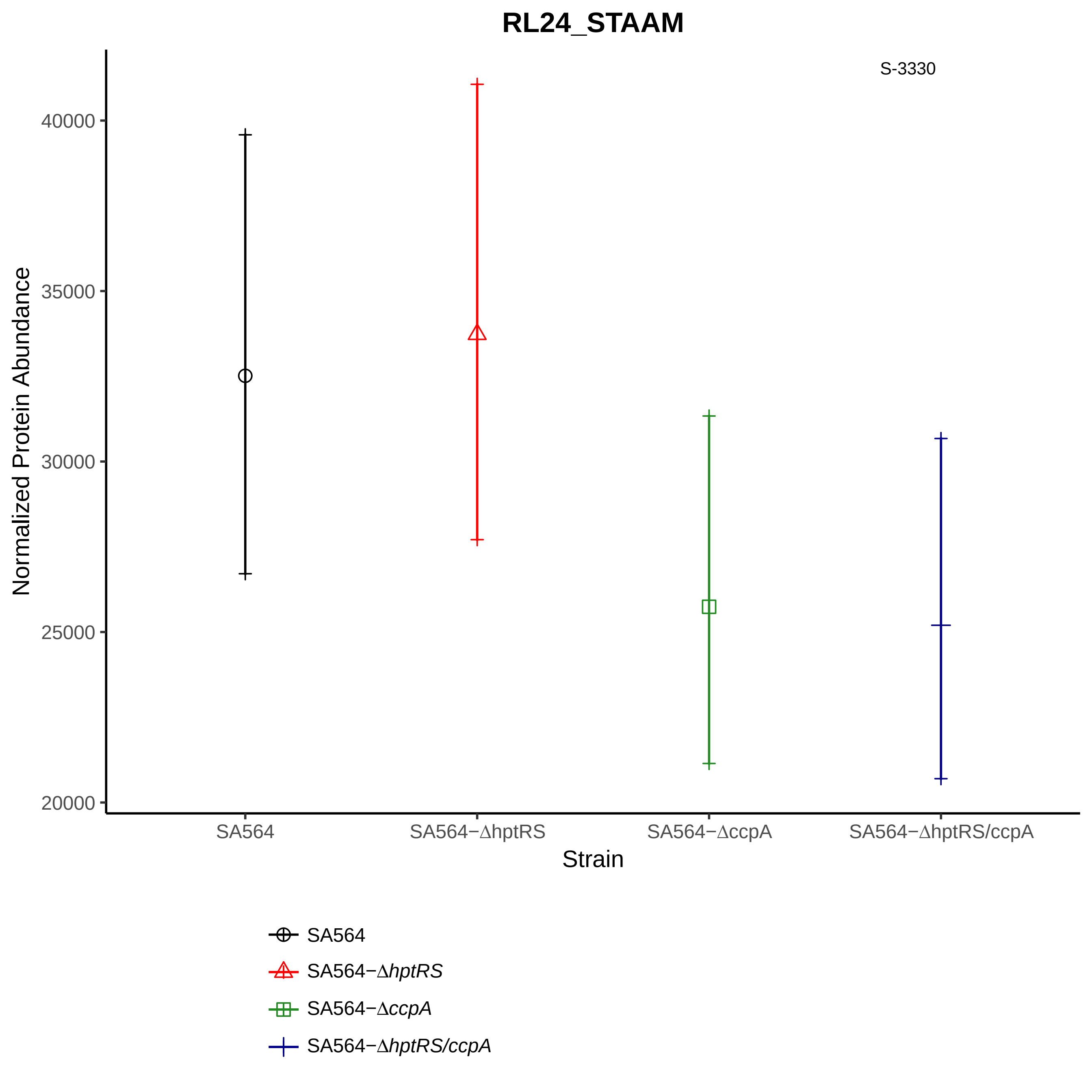
RL24_STAAM
S-3330
Normalized Protein Abundance
40000
35000
30000
25000
20000
SA564
SA564−ΔhptRS
SA564−ΔccpA
SA564−ΔhptRS/ccpA
Strain
SA564
SA564−ΔhptRS
SA564−ΔccpA
SA564−ΔhptRS/ccpA

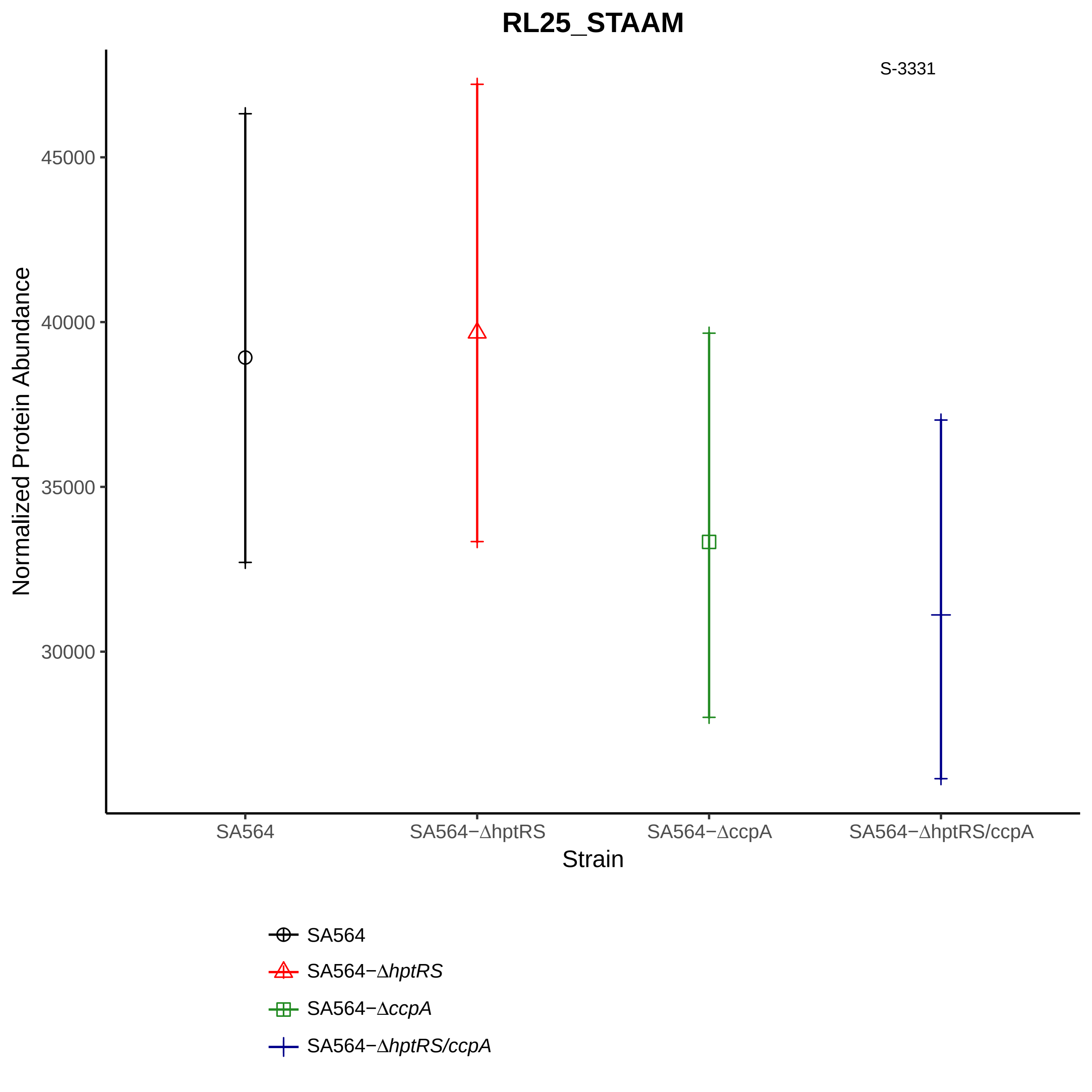
RL25_STAAM
S-3331
Normalized Protein Abundance
45000
40000
35000
30000
SA564
SA564−ΔhptRS
SA564−ΔccpA
SA564−ΔhptRS/ccpA
Strain
SA564
SA564−ΔhptRS
SA564−ΔccpA
SA564−ΔhptRS/ccpA

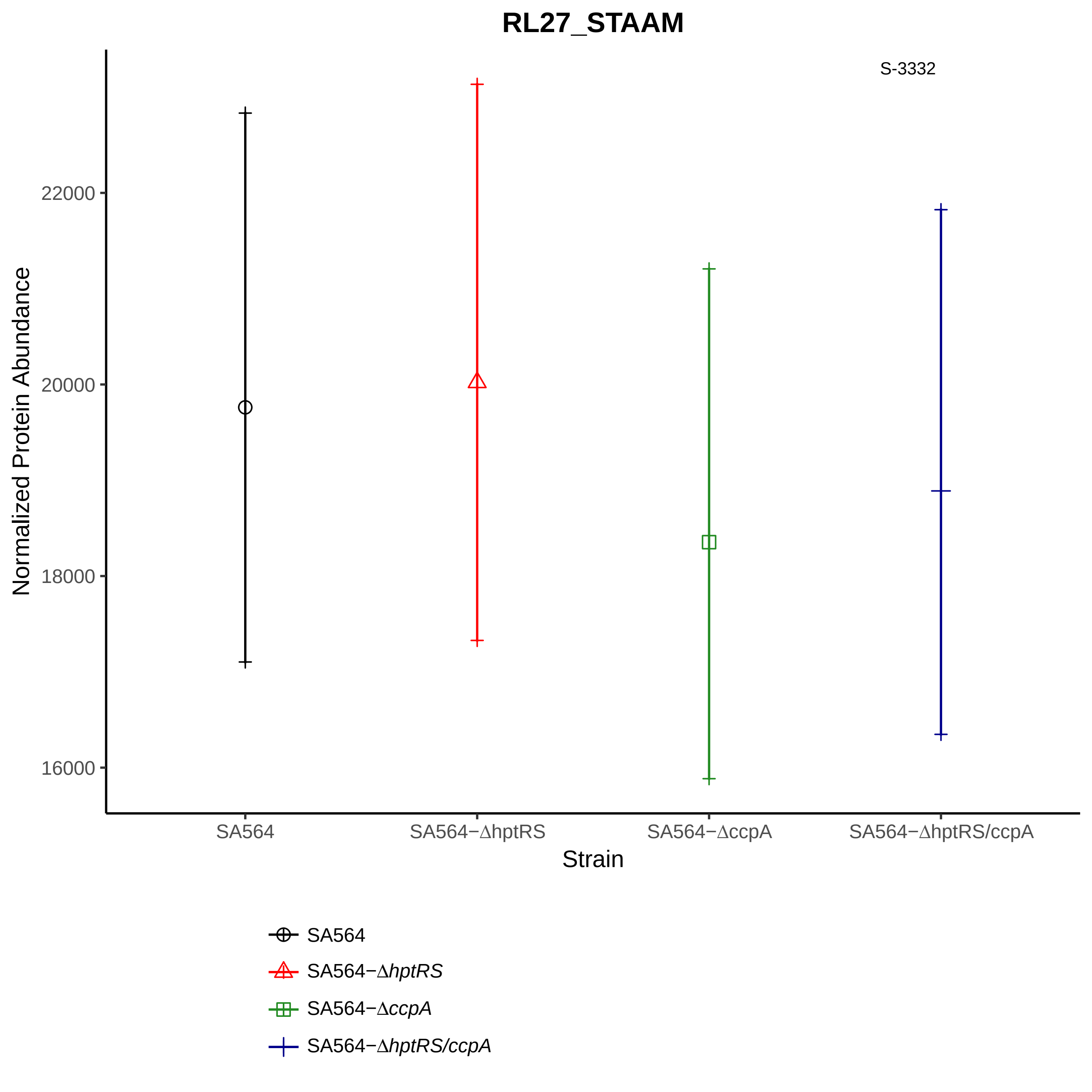

RL27_STAAM
S-3332
Normalized Protein Abundance
22000
20000
18000
16000
SA564
SA564−ΔhptRS
SA564−ΔccpA
SA564−ΔhptRS/ccpA
Strain
SA564
SA564−ΔhptRS
SA564−ΔccpA
SA564−ΔhptRS/ccpA

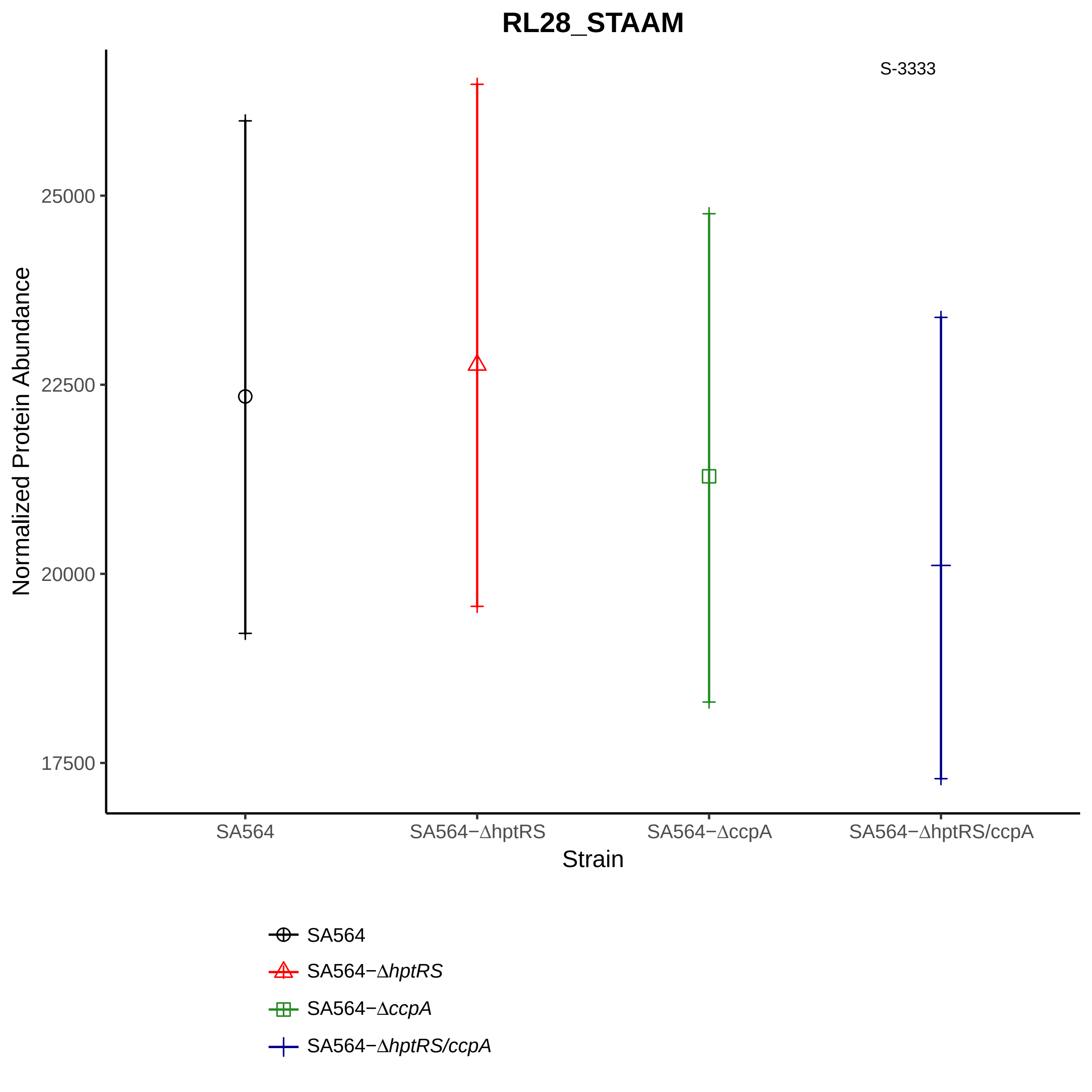

RL28_STAAM
S-3333
Normalized Protein Abundance
25000
22500
20000
17500
SA564
SA564−ΔhptRS
SA564−ΔccpA
SA564−ΔhptRS/ccpA
Strain
SA564
SA564−ΔhptRS
SA564−ΔccpA
SA564−ΔhptRS/ccpA

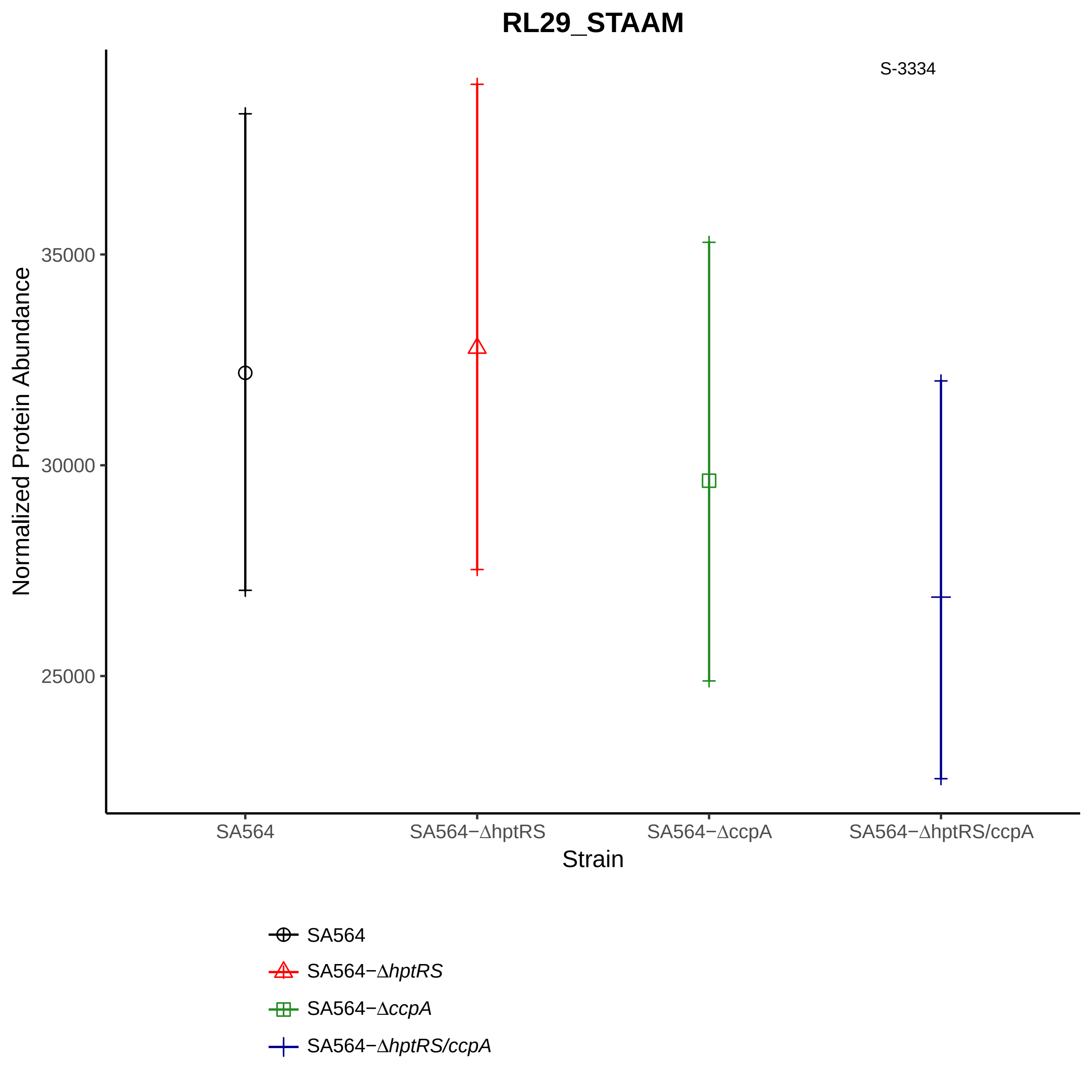
RL29_STAAM
S-3334
Normalized Protein Abundance
35000
30000
25000
SA564
SA564−ΔhptRS
SA564−ΔccpA
SA564−ΔhptRS/ccpA
Strain
SA564
SA564−ΔhptRS
SA564−ΔccpA
SA564−ΔhptRS/ccpA

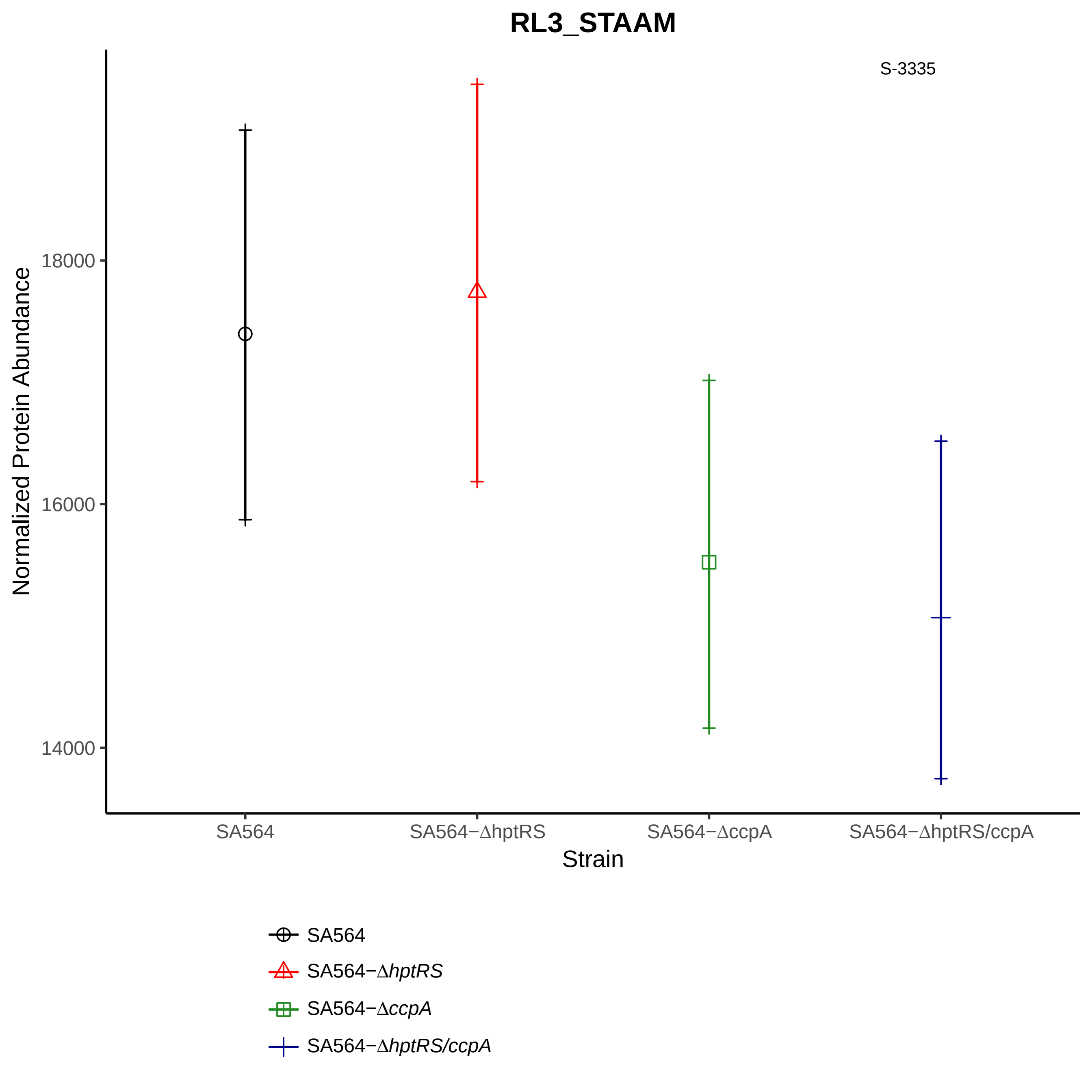
RL3_STAAM
S-3335
Normalized Protein Abundance
18000
16000
14000
SA564
SA564−ΔhptRS
SA564−ΔccpA
SA564−ΔhptRS/ccpA
Strain
SA564
SA564−Δ*hptRS*
SA564−Δ*ccpA*
SA564−Δ*hptRS/ccpA*

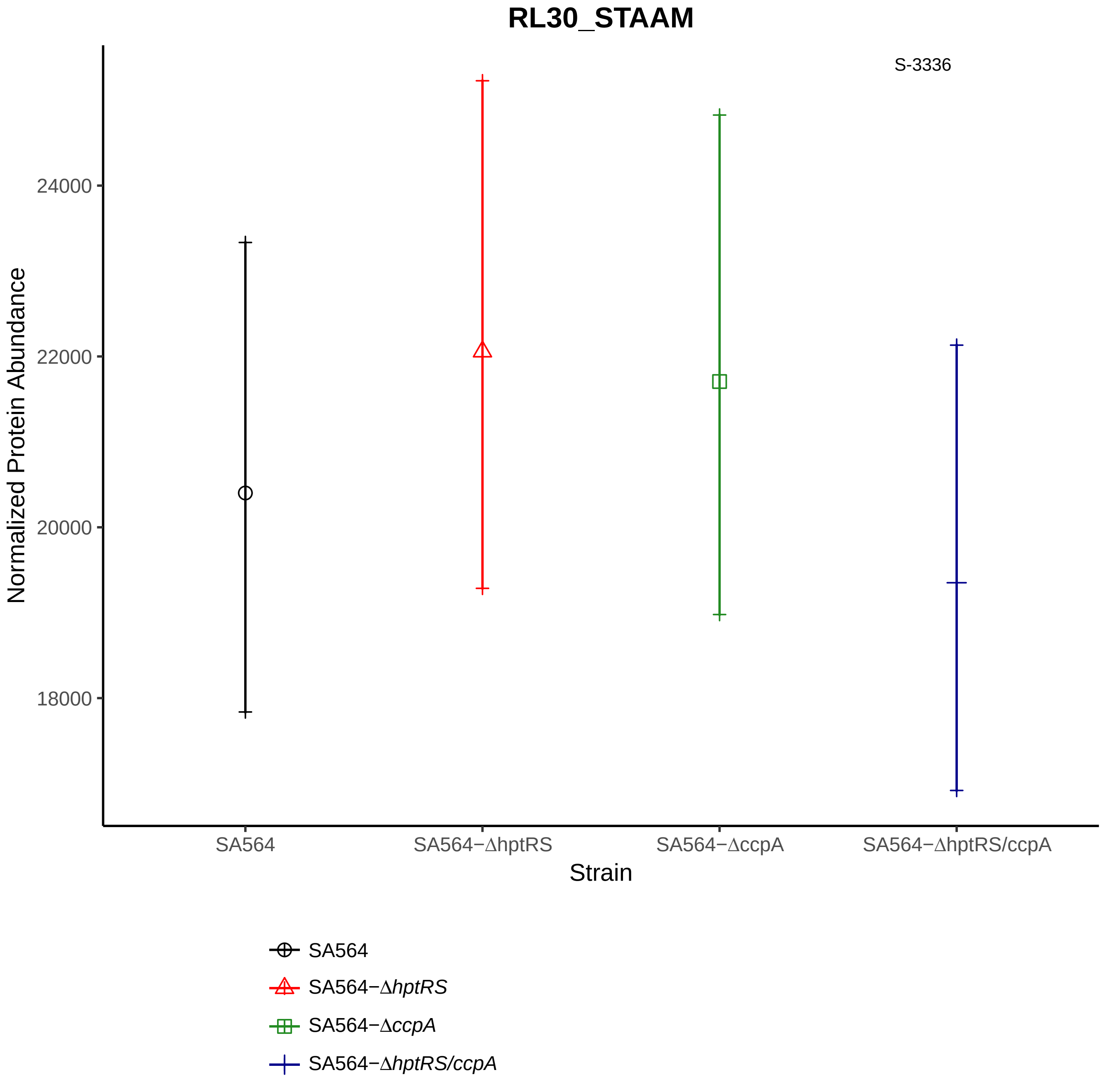

RL30_STAAM
S-3336
Normalized Protein Abundance
24000
22000
20000
18000
SA564
SA564−ΔhptRS
SA564−ΔccpA
SA564−ΔhptRS/ccpA
Strain
SA564
SA564−ΔhptRS
SA564−ΔccpA
SA564−ΔhptRS/ccpA

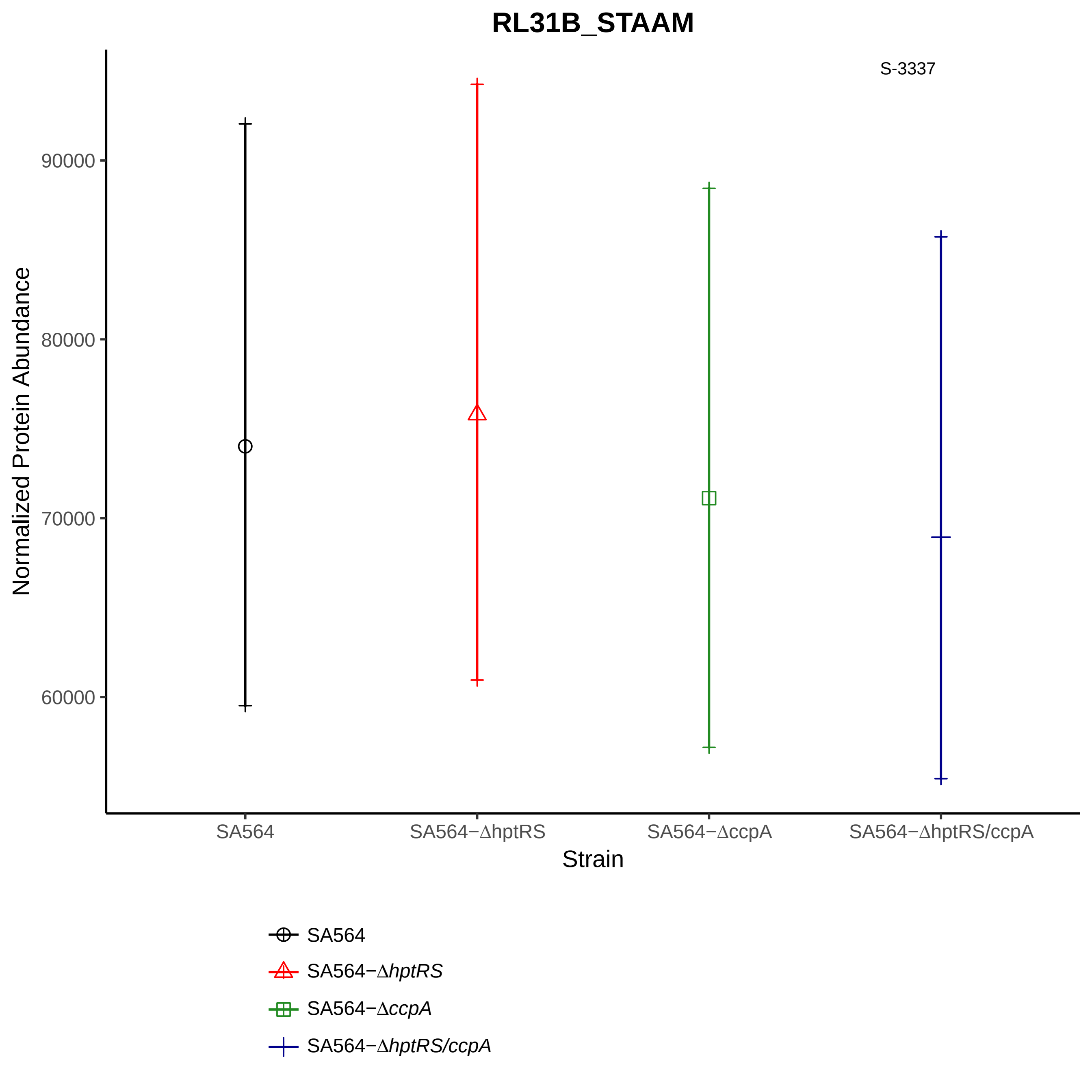
RL31B_STAAM
S-3337
Normalized Protein Abundance
90000
80000
70000
60000
SA564
SA564−ΔhptRS
SA564−ΔccpA
SA564−ΔhptRS/ccpA
Strain
SA564
SA564−ΔhptRS
SA564−ΔccpA
SA564−ΔhptRS/ccpA

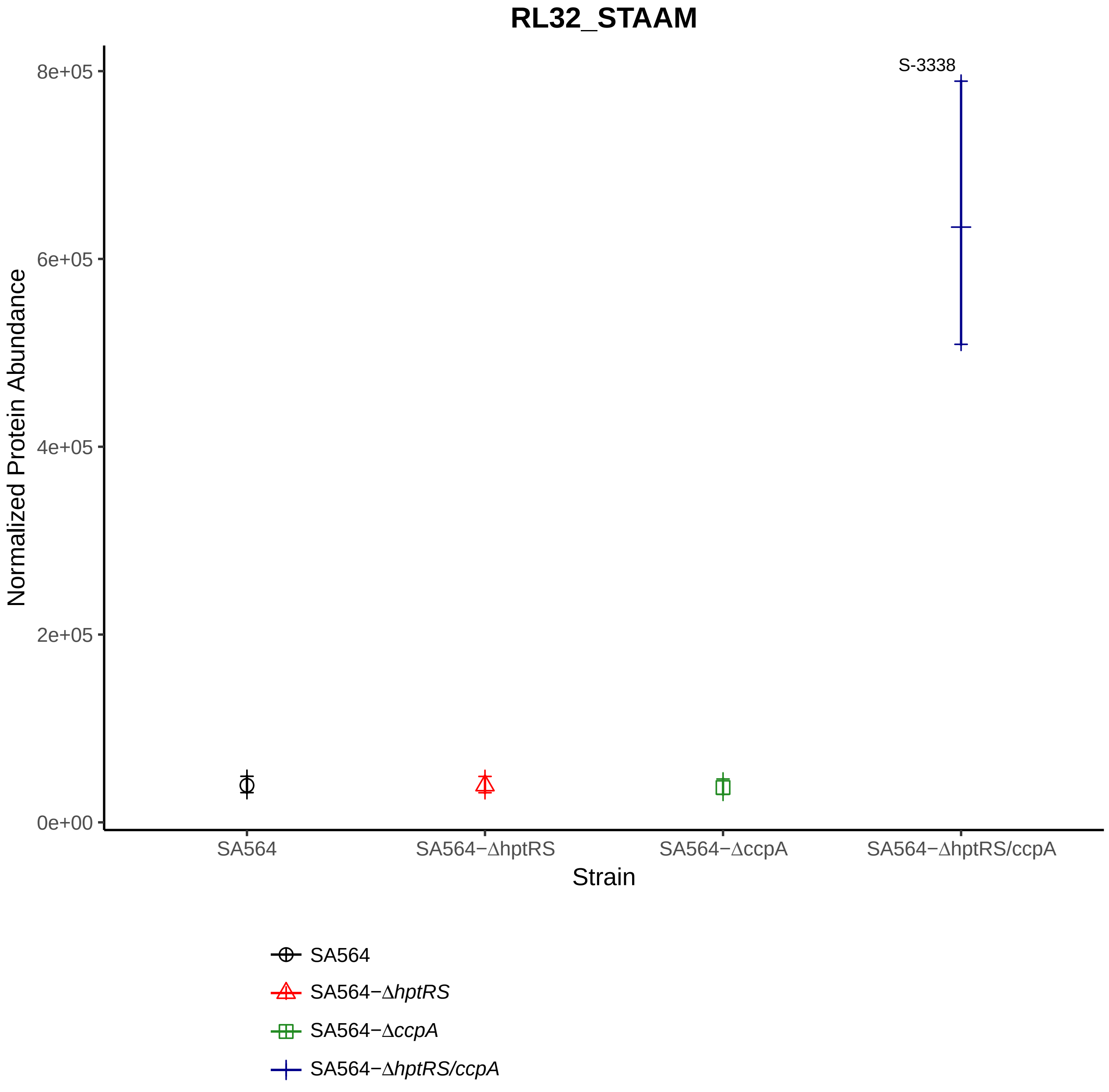

RL32_STAAM
S-3338
8e+05
6e+05
4e+05
2e+05
0e+00
Normalized Protein Abundance
SA564
SA564−ΔhptRS
SA564−ΔccpA
SA564−ΔhptRS/ccpA
Strain
SA564
SA564−ΔhptRS
SA564−ΔccpA
SA564−ΔhptRS/ccpA

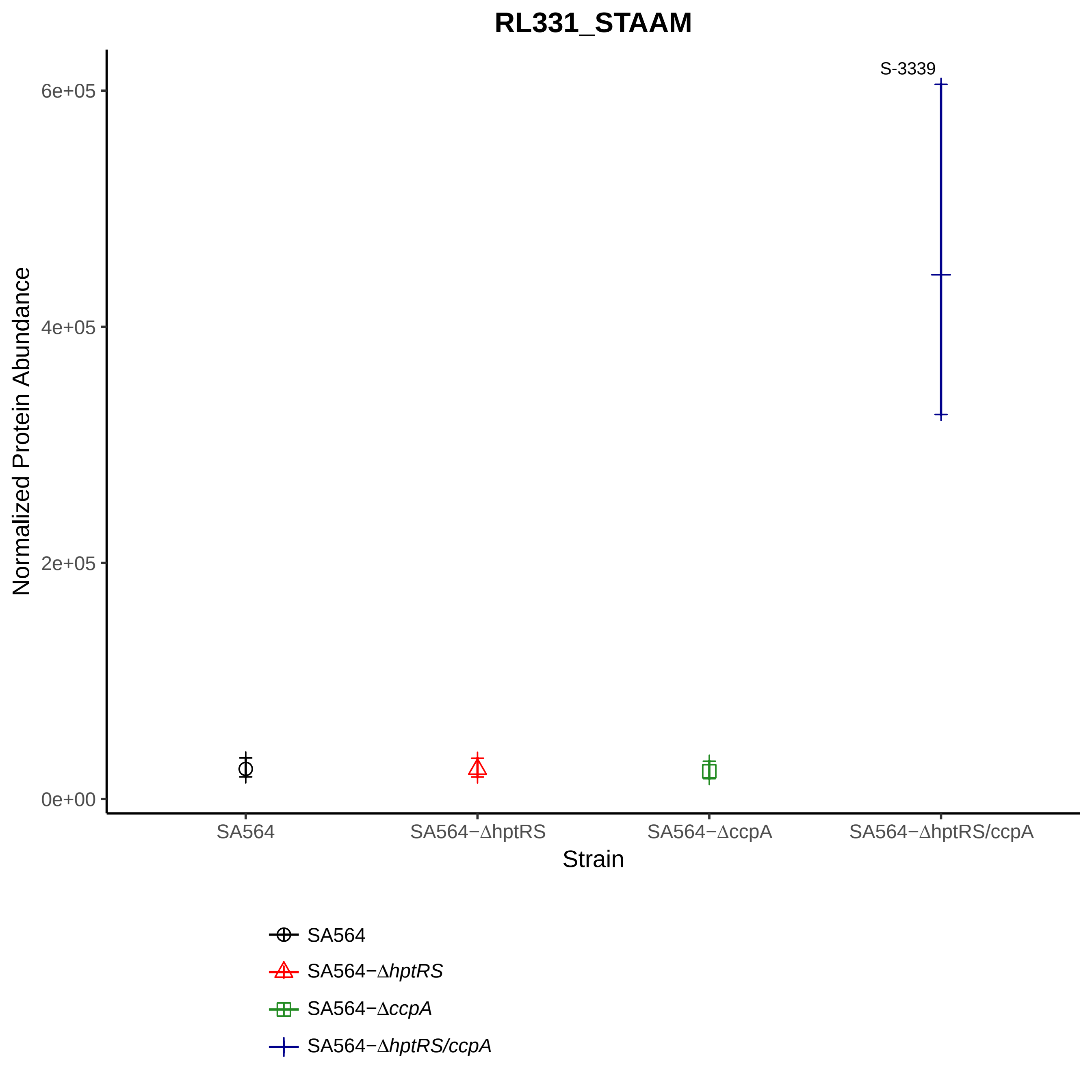
RL331_STAAM
S-3339
Normalized Protein Abundance
6e+05
4e+05
2e+05
0e+00
SA564
SA564−ΔhptRS
SA564−ΔccpA
SA564−ΔhptRS/ccpA
Strain
SA564
SA564−ΔhptRS
SA564−ΔccpA
SA564−ΔhptRS/ccpA

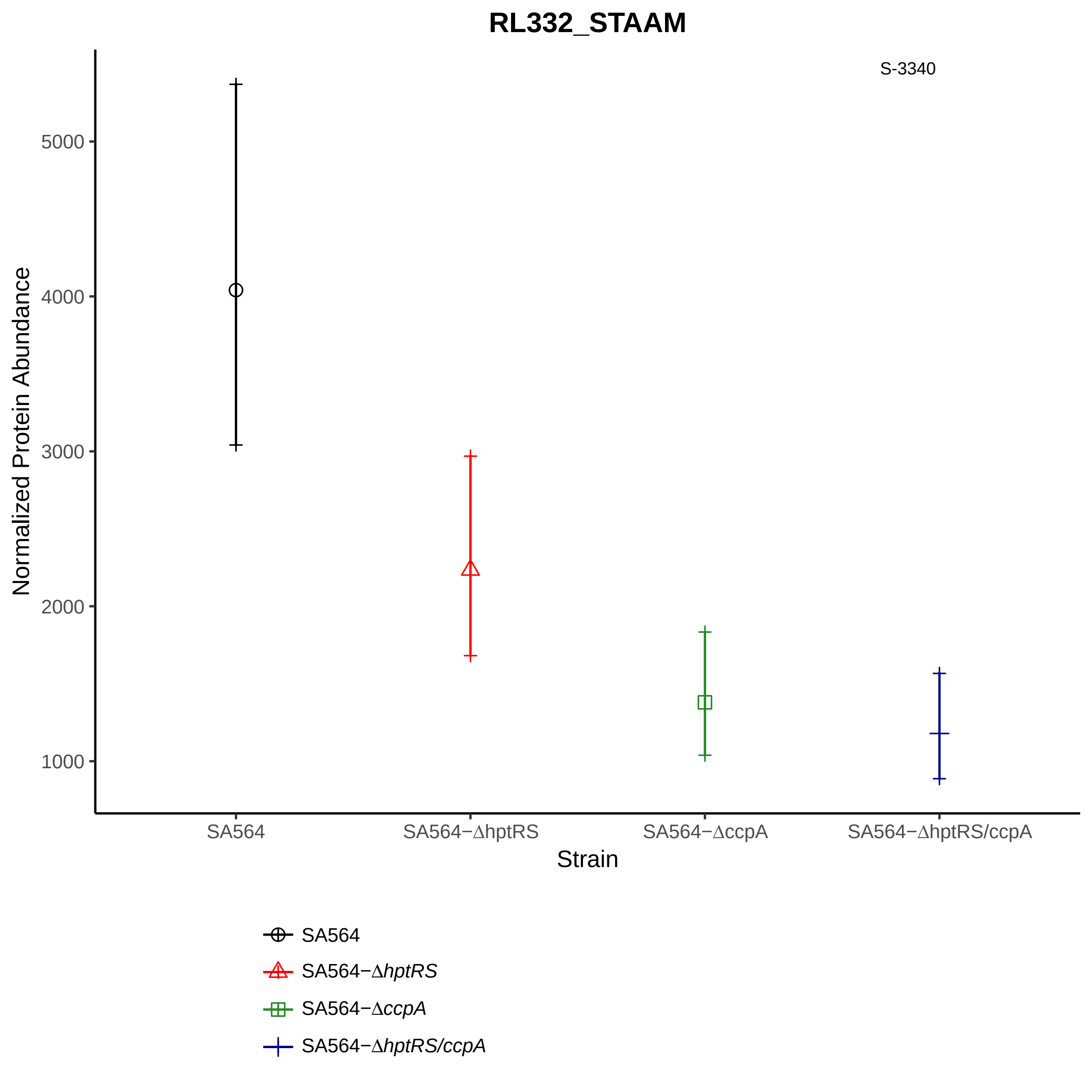
RL332_STAAM
S-3340
Normalized Protein Abundance
5000
4000
3000
2000
1000
SA564
SA564−ΔhptRS
SA564−ΔccpA
SA564−ΔhptRS/ccpA
Strain
SA564
SA564−ΔhptRS
SA564−ΔccpA
SA564−ΔhptRS/ccpA

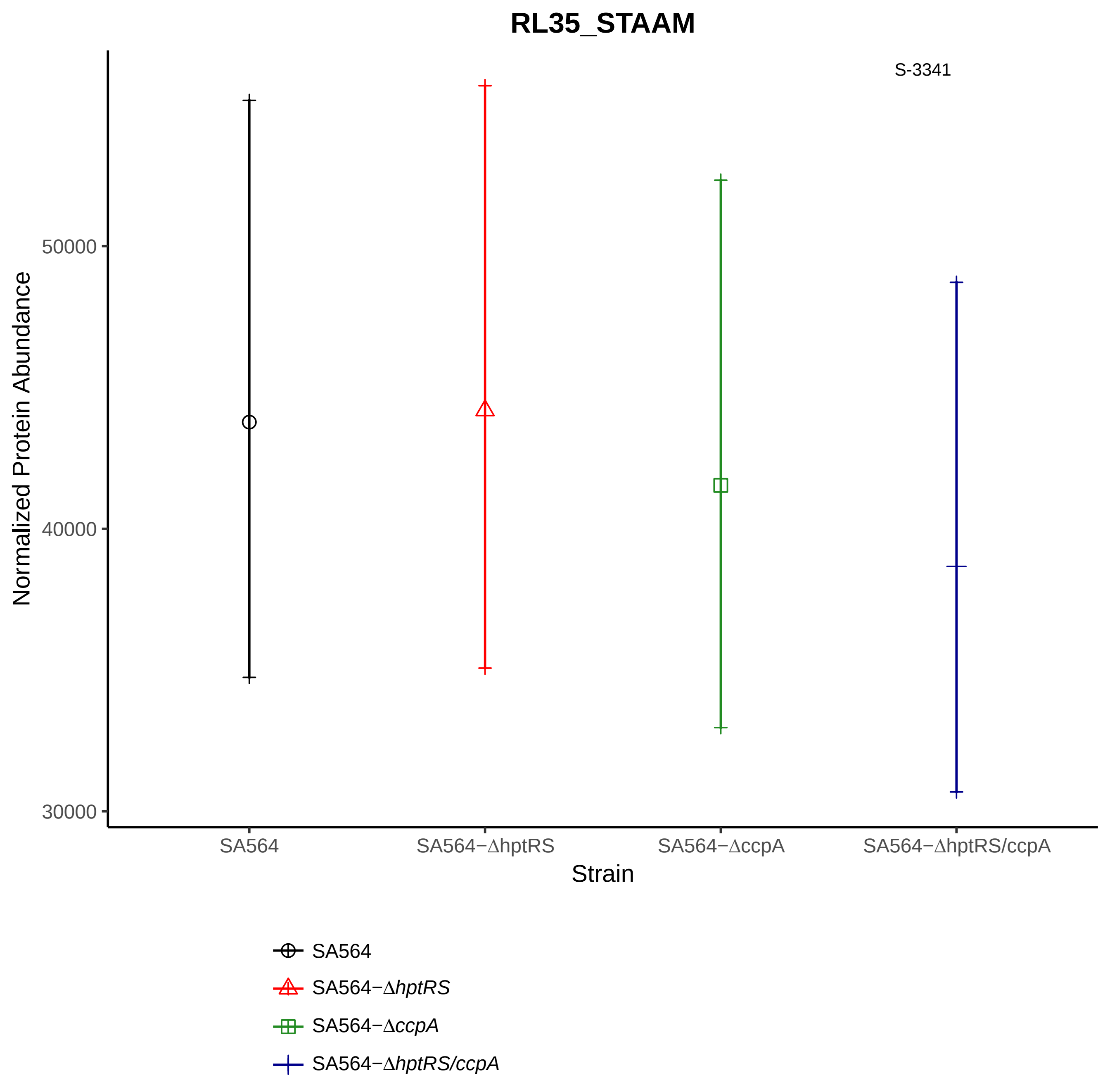
RL35_STAAM
S-3341
Normalized Protein Abundance
50000
40000
30000
SA564
SA564−ΔhptRS
SA564−ΔccpA
SA564−ΔhptRS/ccpA
Strain
SA564
SA564−ΔhptRS
SA564−ΔccpA
SA564−ΔhptRS/ccpA

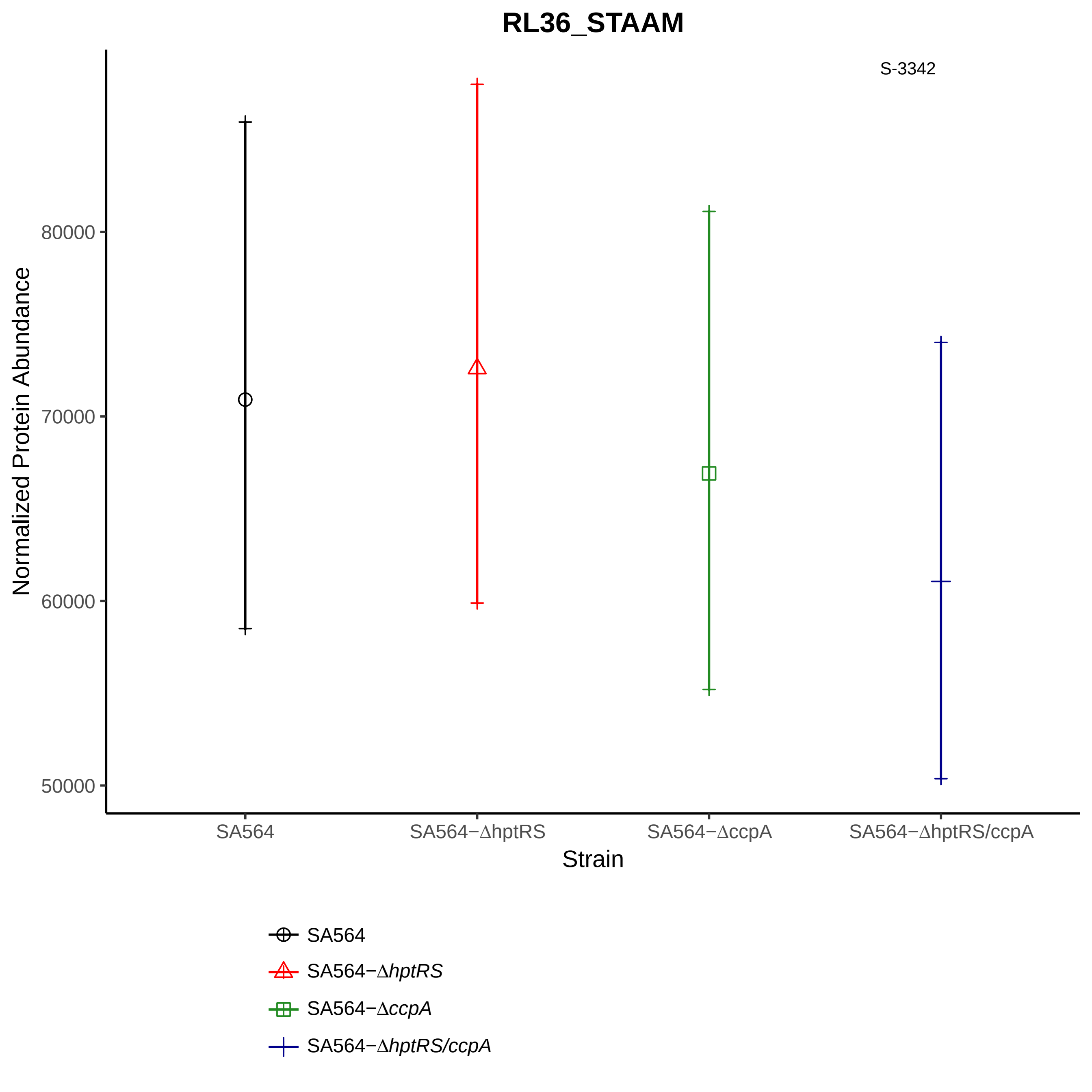

RL36_STAAM
S-3342
Normalized Protein Abundance
80000
70000
60000
50000
SA564
SA564−ΔhptRS
SA564−ΔccpA
SA564−ΔhptRS/ccpA
Strain
SA564
SA564−ΔhptRS
SA564−ΔccpA
SA564−ΔhptRS/ccpA

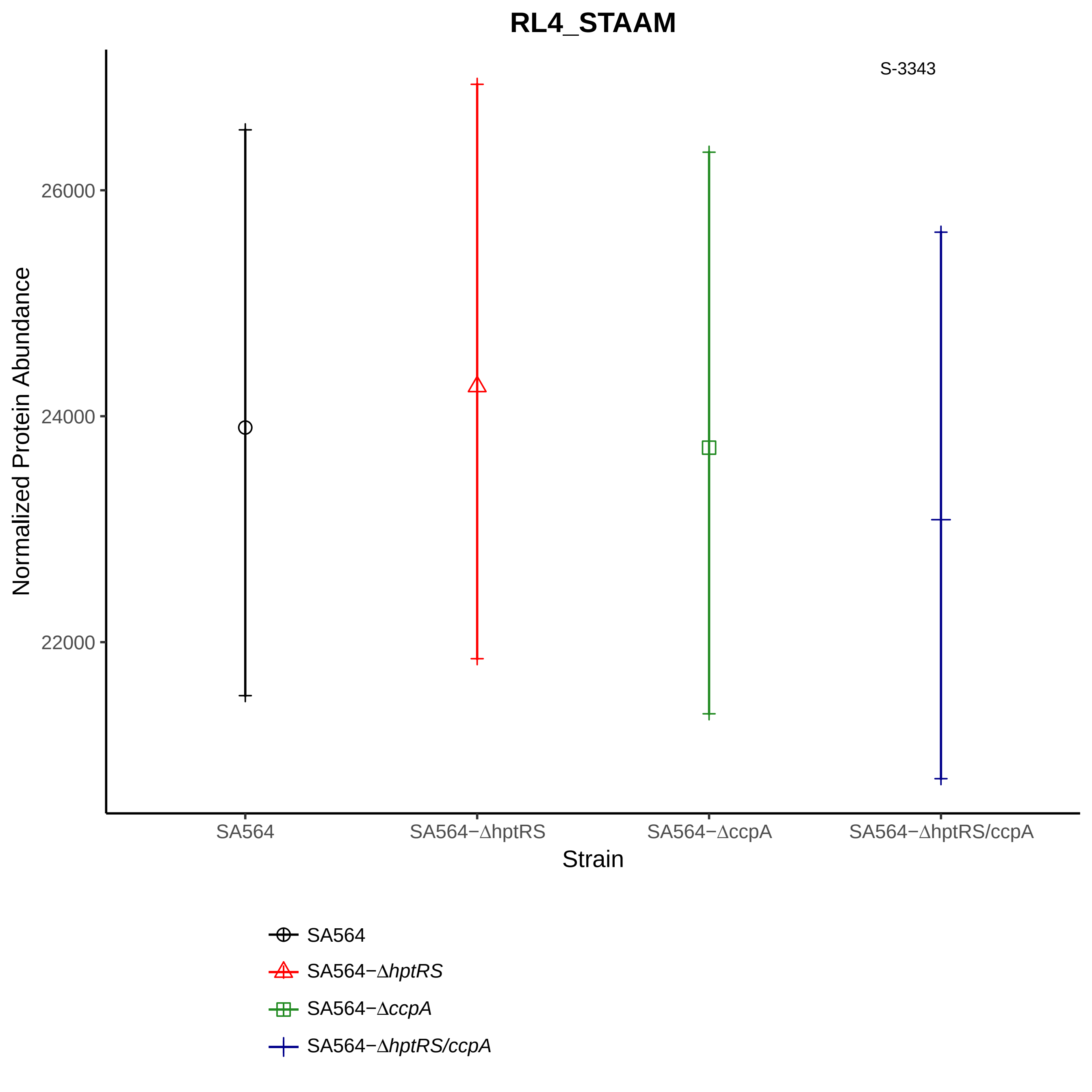
RL4_STAAM
S-3343
Normalized Protein Abundance
26000
24000
22000
SA564
SA564−ΔhptRS
SA564−ΔccpA
SA564−ΔhptRS/ccpA
Strain
SA564
SA564−Δ*hptRS*
SA564−Δ*ccpA*
SA564−Δ*hptRS/ccpA*

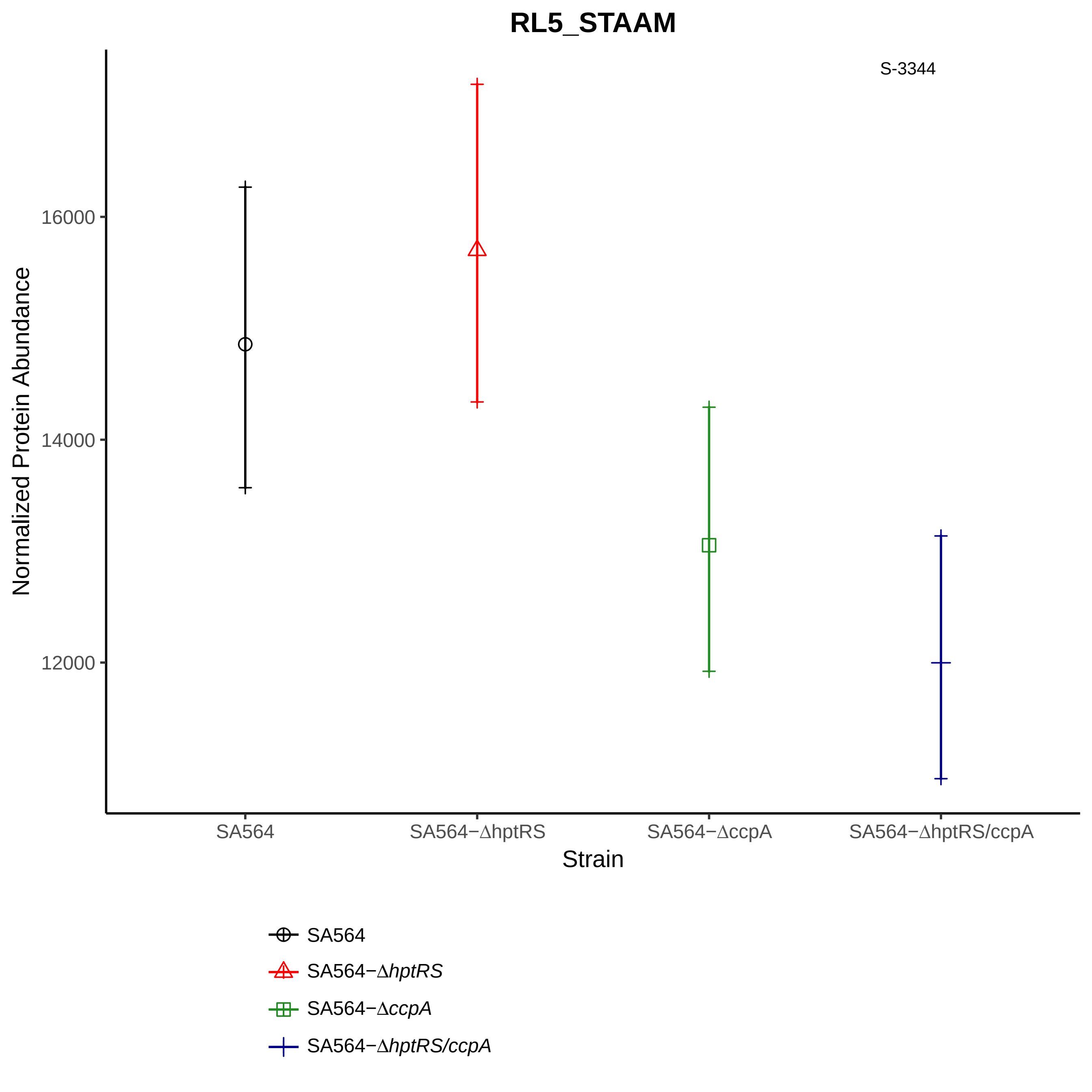
RL5_STAAM
S-3344
Normalized Protein Abundance
16000
14000
12000
SA564
SA564−ΔhptRS
SA564−ΔccpA
SA564−ΔhptRS/ccpA
Strain
SA564
SA564−ΔhptRS
SA564−ΔccpA
SA564−ΔhptRS/ccpA

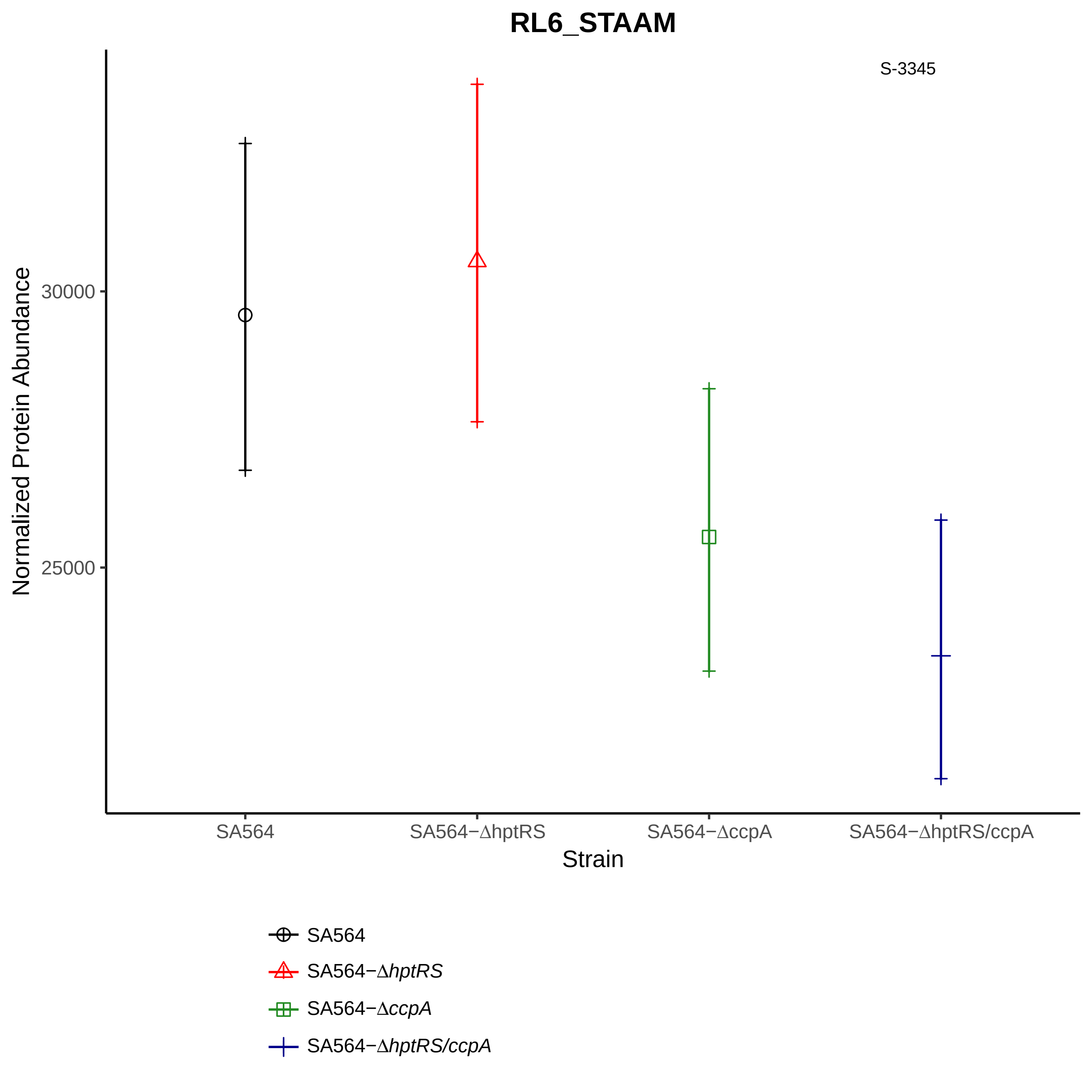

RL6_STAAM
S-3345
Normalized Protein Abundance
30000
25000
SA564
SA564−ΔhptRS
SA564−ΔccpA
SA564−ΔhptRS/ccpA
Strain
SA564
SA564−ΔhptRS
SA564−ΔccpA
SA564−ΔhptRS/ccpA

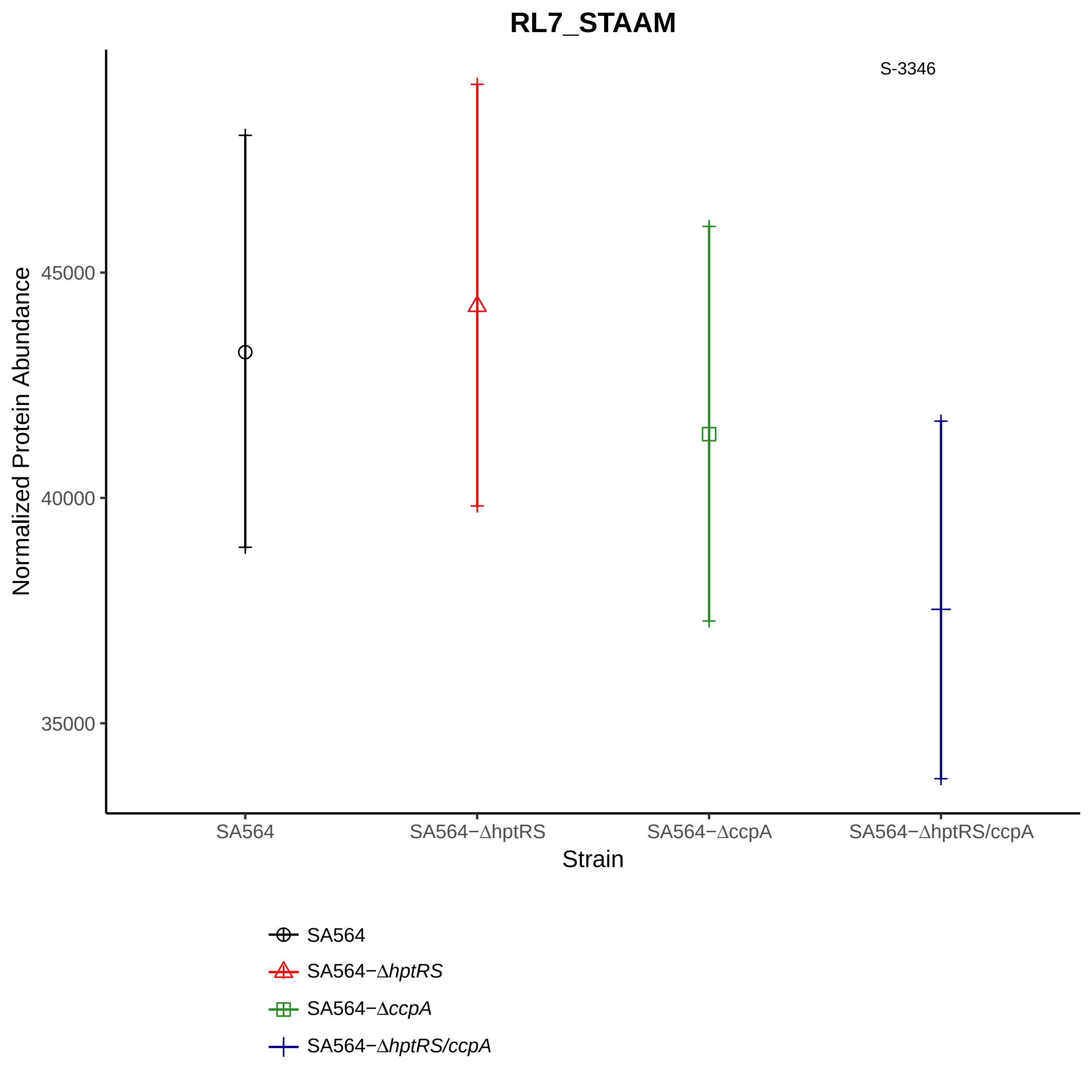

RL7_STAAM
S-3346
Normalized Protein Abundance
45000
40000
35000
SA564
SA564−ΔhptRS
SA564−ΔccpA
SA564−ΔhptRS/ccpA
Strain
SA564
SA564−ΔhptRS
SA564−ΔccpA
SA564−ΔhptRS/ccpA

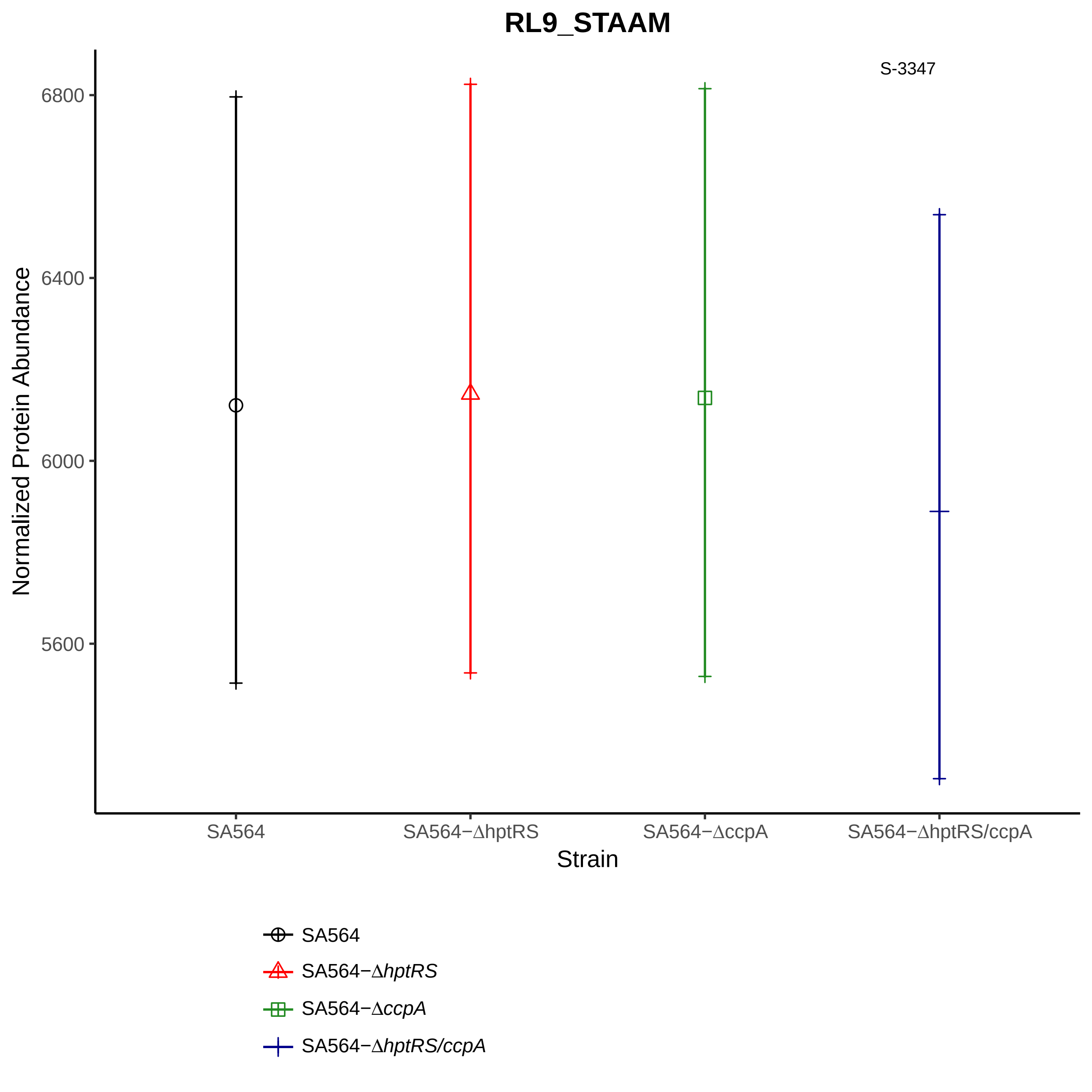

RL9_STAAM
S-3347
Normalized Protein Abundance
6800
6400
6000
5600
SA564
SA564−ΔhptRS
SA564−ΔccpA
SA564−ΔhptRS/ccpA
Strain
SA564
SA564−ΔhptRS
SA564−ΔccpA
SA564−ΔhptRS/ccpA

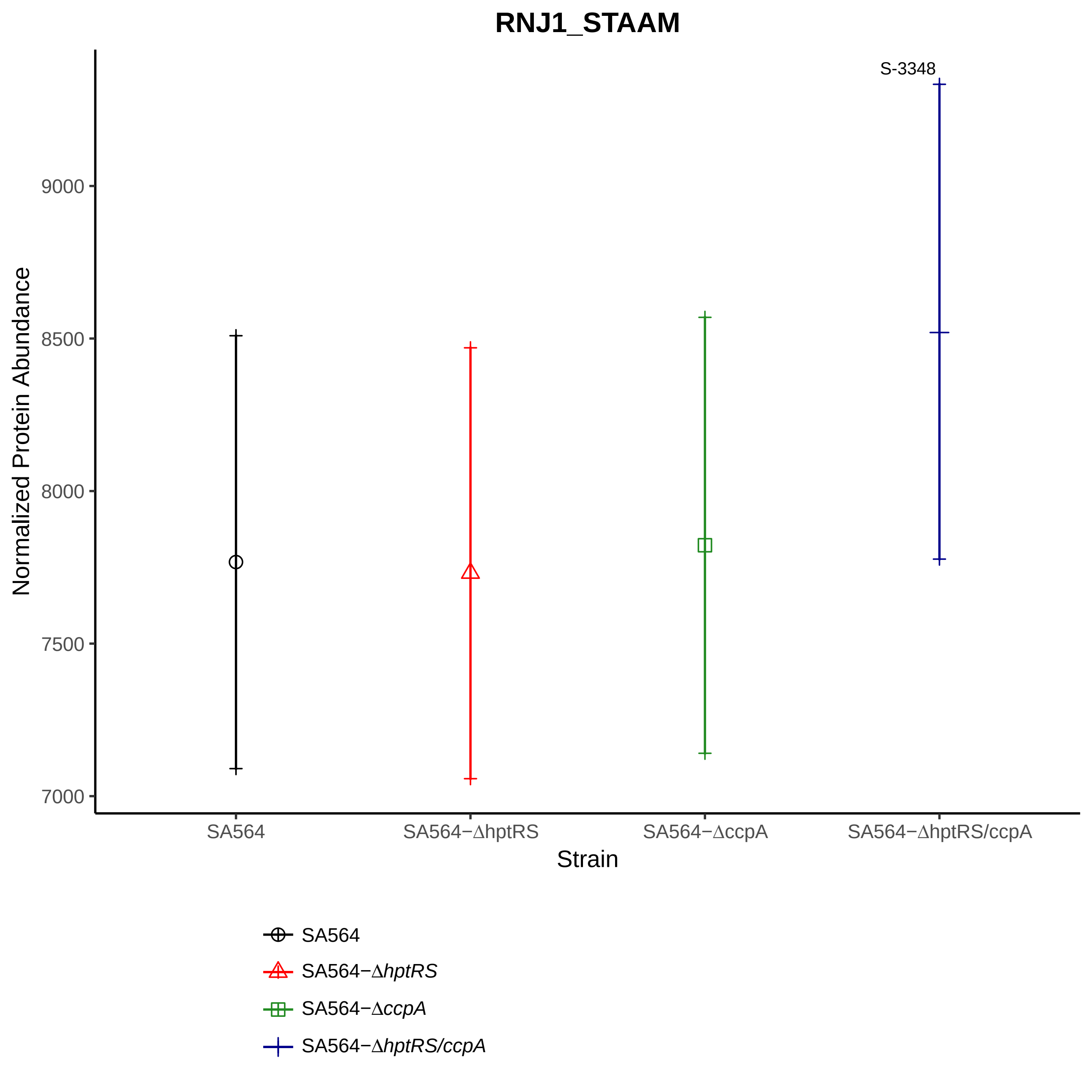

RNJ1_STAAM
S-3348
9000
8500
8000
7500
7000
Normalized Protein Abundance
SA564
SA564−ΔhptRS
SA564−ΔccpA
SA564−ΔhptRS/ccpA
Strain
SA564
SA564−Δ*hptRS*
SA564−Δ*ccpA*
SA564−Δ*hptRS/ccpA*

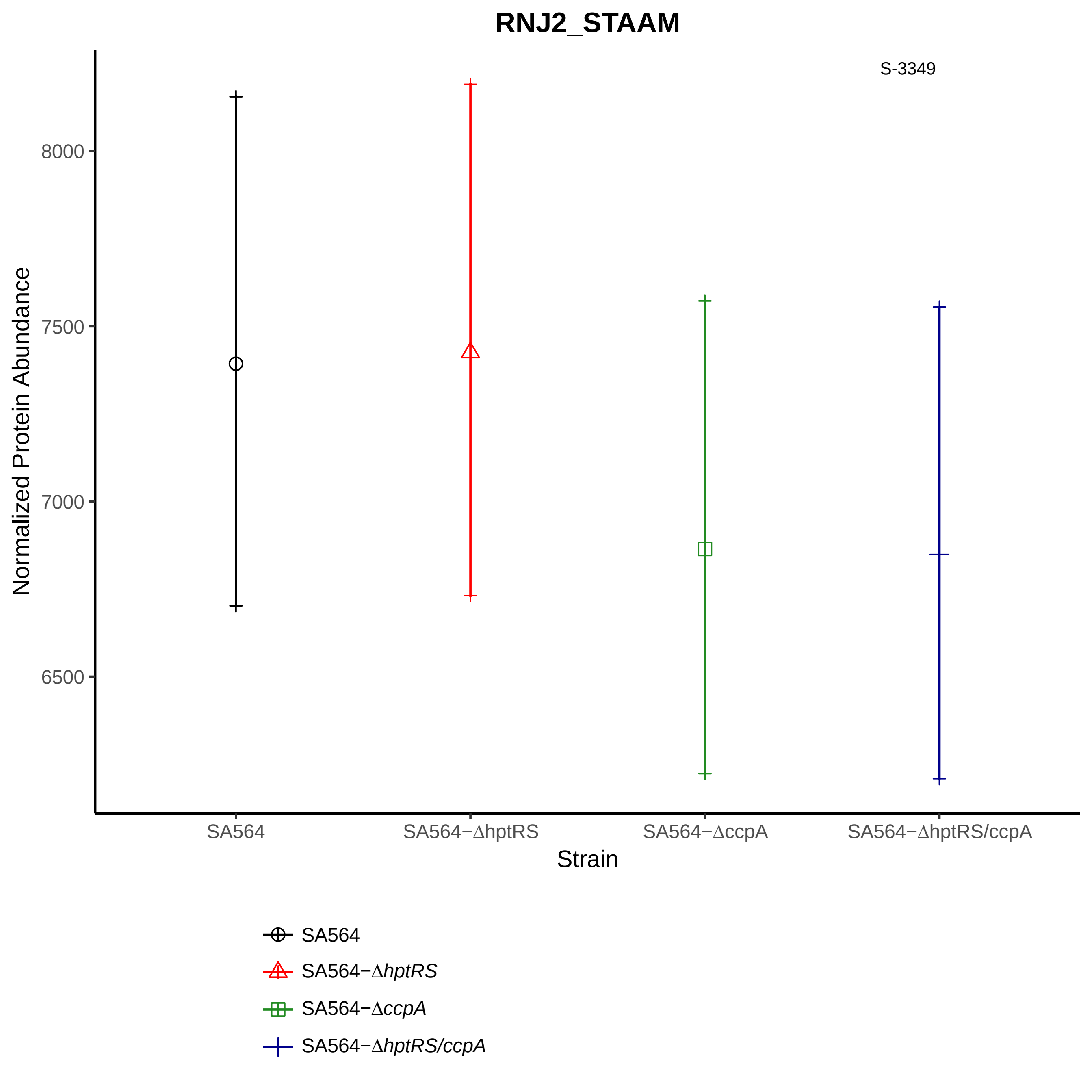
RNJ2_STAAM
S-3349
Normalized Protein Abundance
8000
7500
7000
6500
SA564
SA564−ΔhptRS
SA564−ΔccpA
SA564−ΔhptRS/ccpA
Strain
SA564
SA564−ΔhptRS
SA564−ΔccpA
SA564−ΔhptRS/ccpA

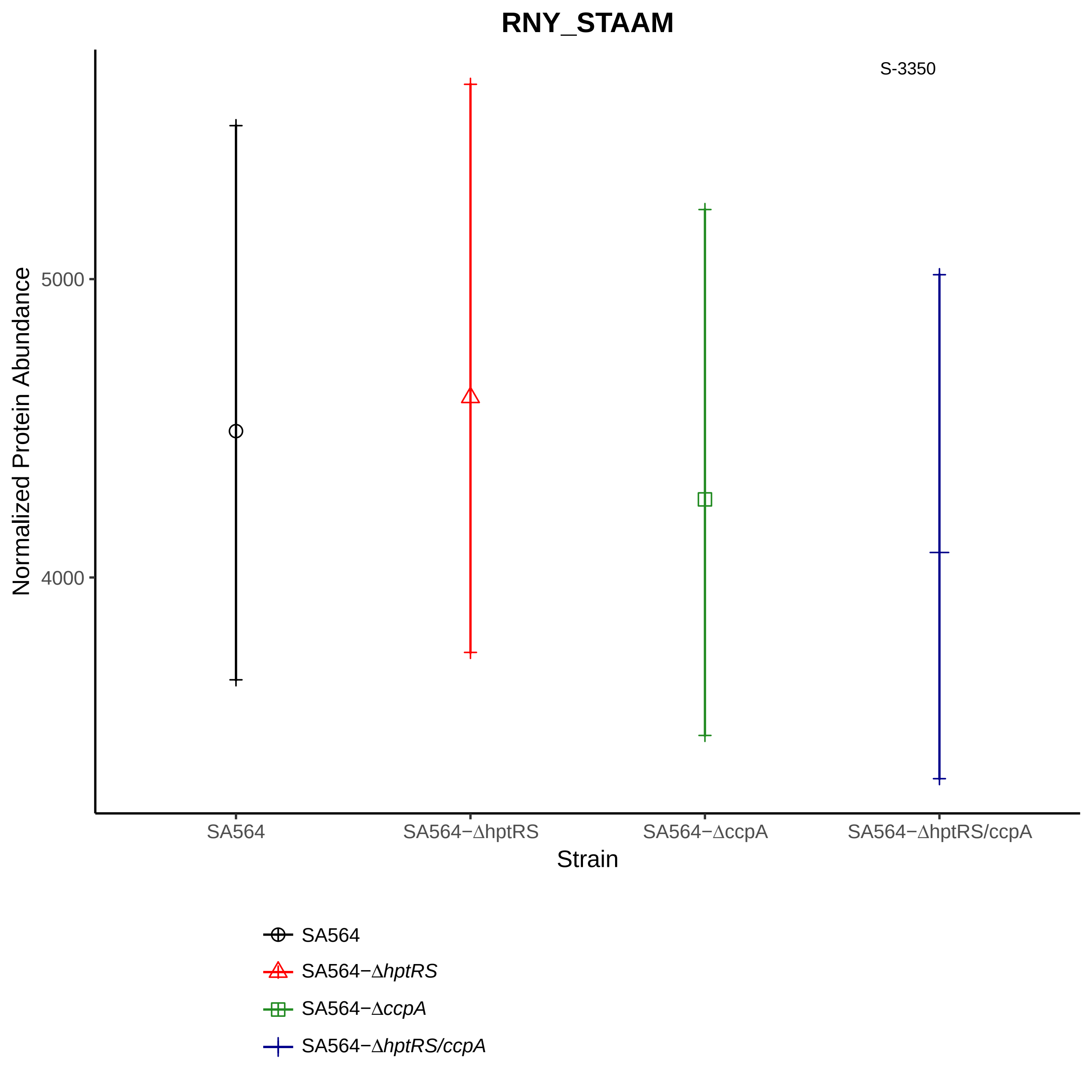

RNY_STAAM
S-3350
Normalized Protein Abundance
5000
4000
SA564
SA564−ΔhptRS
SA564−ΔccpA
SA564−ΔhptRS/ccpA
Strain
SA564
SA564−Δ*hptRS*
SA564−Δ*ccpA*
SA564−Δ*hptRS/ccpA*

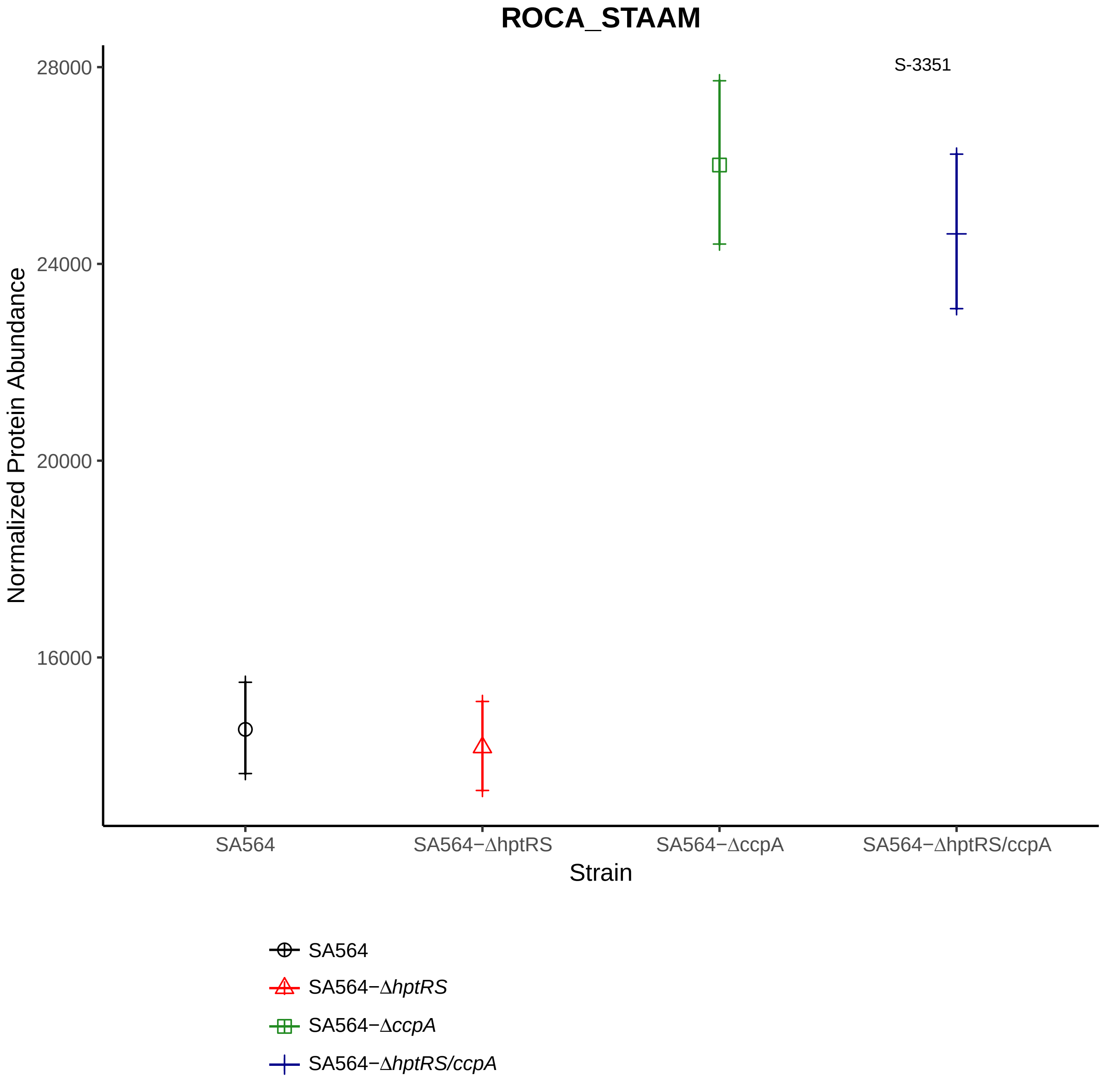

ROCA_STAAM
S-3351
Normalized Protein Abundance
28000
24000
20000
16000
SA564
SA564−ΔhptRS
SA564−ΔccpA
SA564−ΔhptRS/ccpA
Strain
SA564
SA564−ΔhptRS
SA564−ΔccpA
SA564−ΔhptRS/ccpA

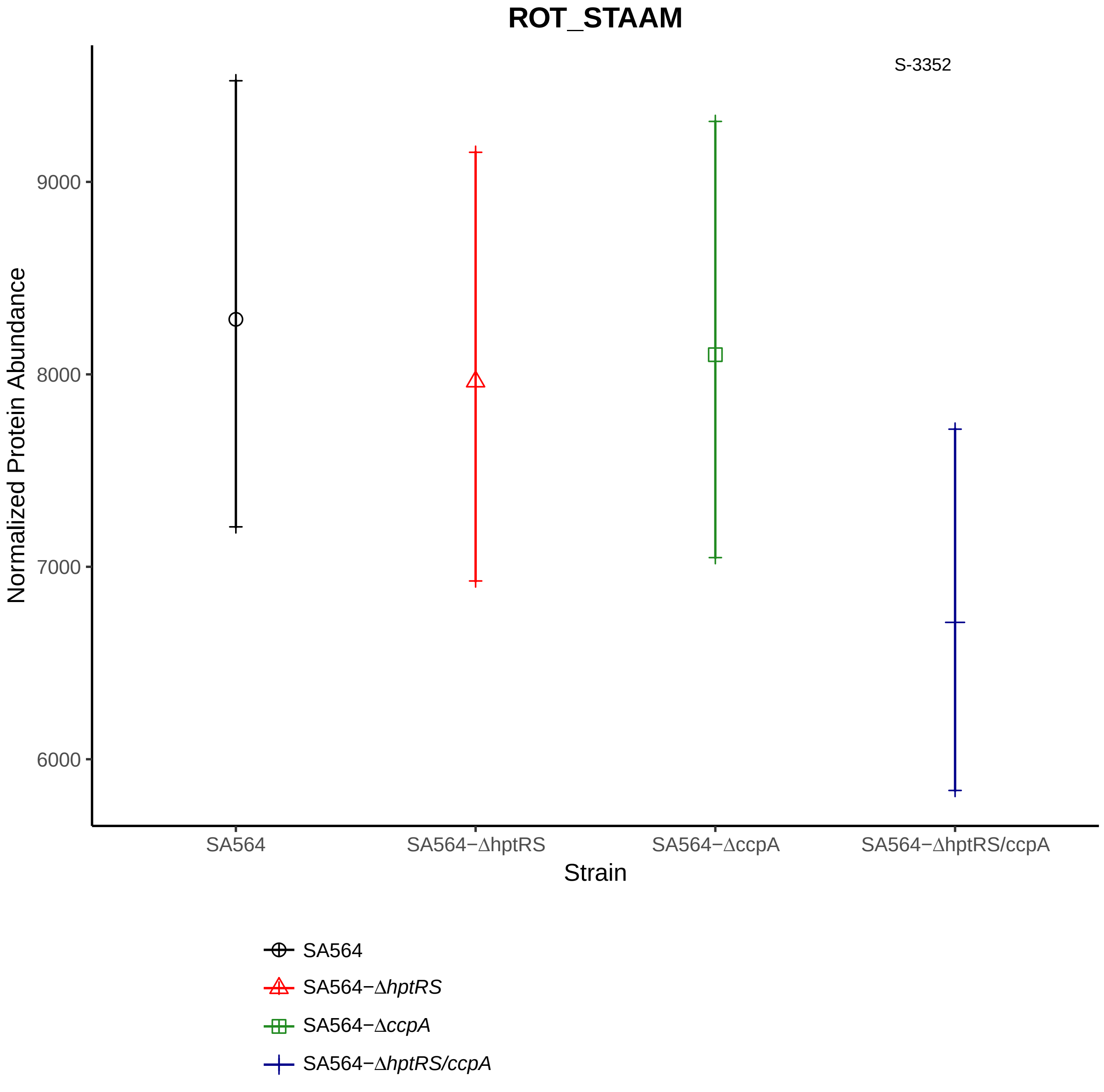

ROT_STAAM
S-3352
Normalized Protein Abundance
9000
8000
7000
6000
SA564
SA564−ΔhptRS
SA564−ΔccpA
SA564−ΔhptRS/ccpA
Strain
SA564
SA564−ΔhptRS
SA564−ΔccpA
SA564−ΔhptRS/ccpA

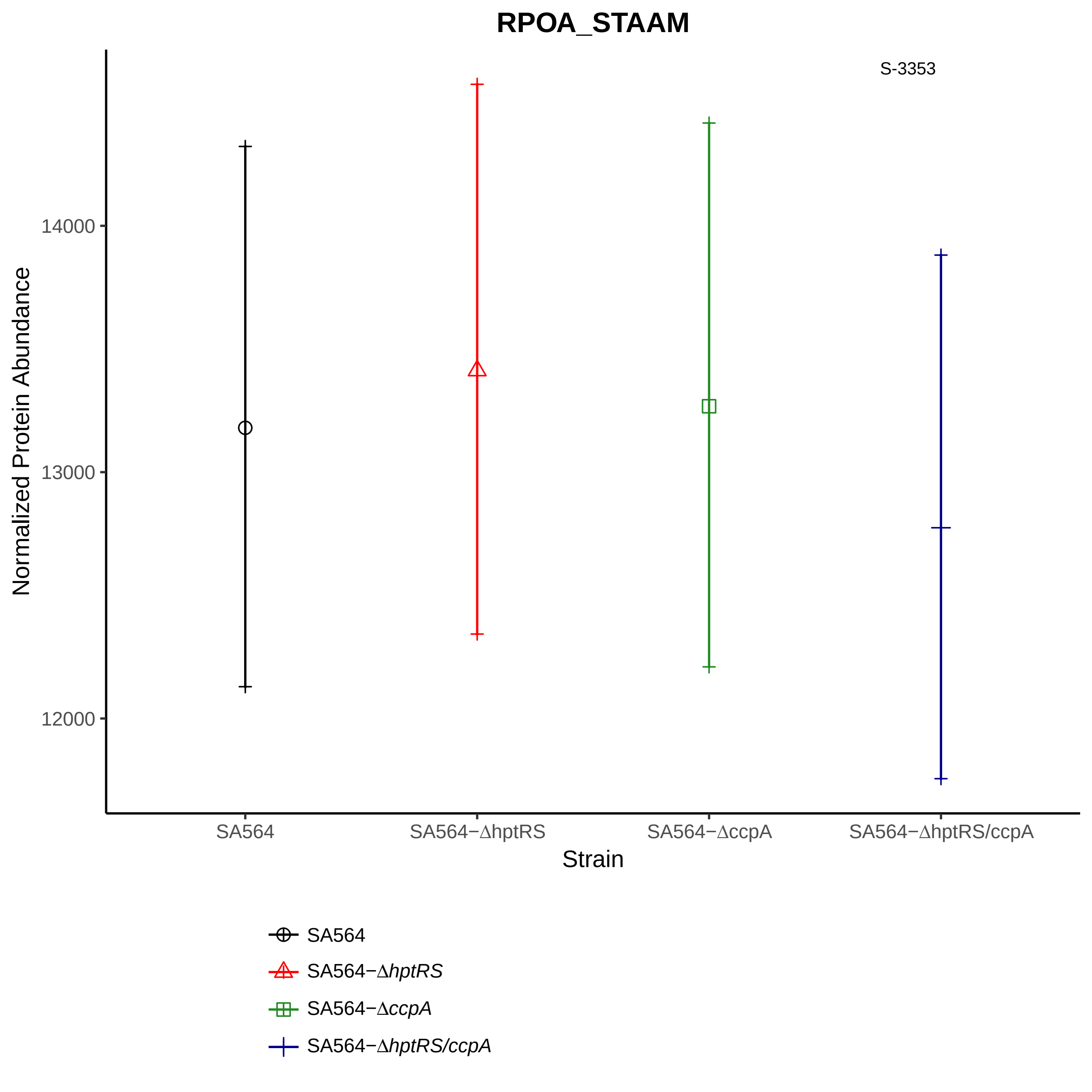

RPOA_STAAM
S-3353
Normalized Protein Abundance
14000
13000
12000
SA564
SA564−ΔhptRS
SA564−ΔccpA
SA564−ΔhptRS/ccpA
Strain
SA564
SA564−ΔhptRS
SA564−ΔccpA
SA564−ΔhptRS/ccpA

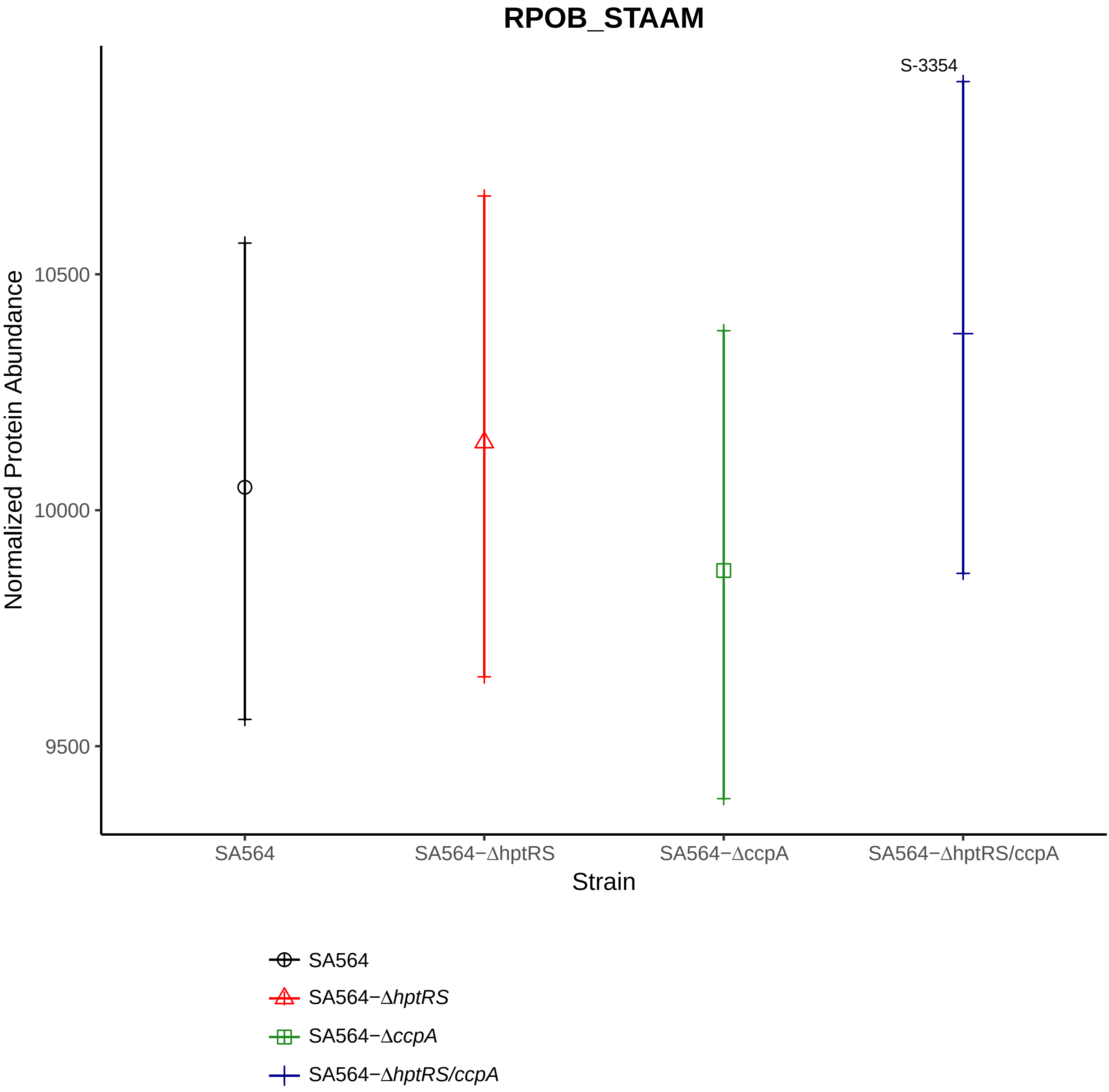
RPOB_STAAM
S-3354
10500
10000
9500
Normalized Protein Abundance
SA564
SA564−ΔhptRS
SA564−ΔccpA
SA564−ΔhptRS/ccpA
Strain
SA564
SA564−ΔhptRS
SA564−ΔccpA
SA564−ΔhptRS/ccpA

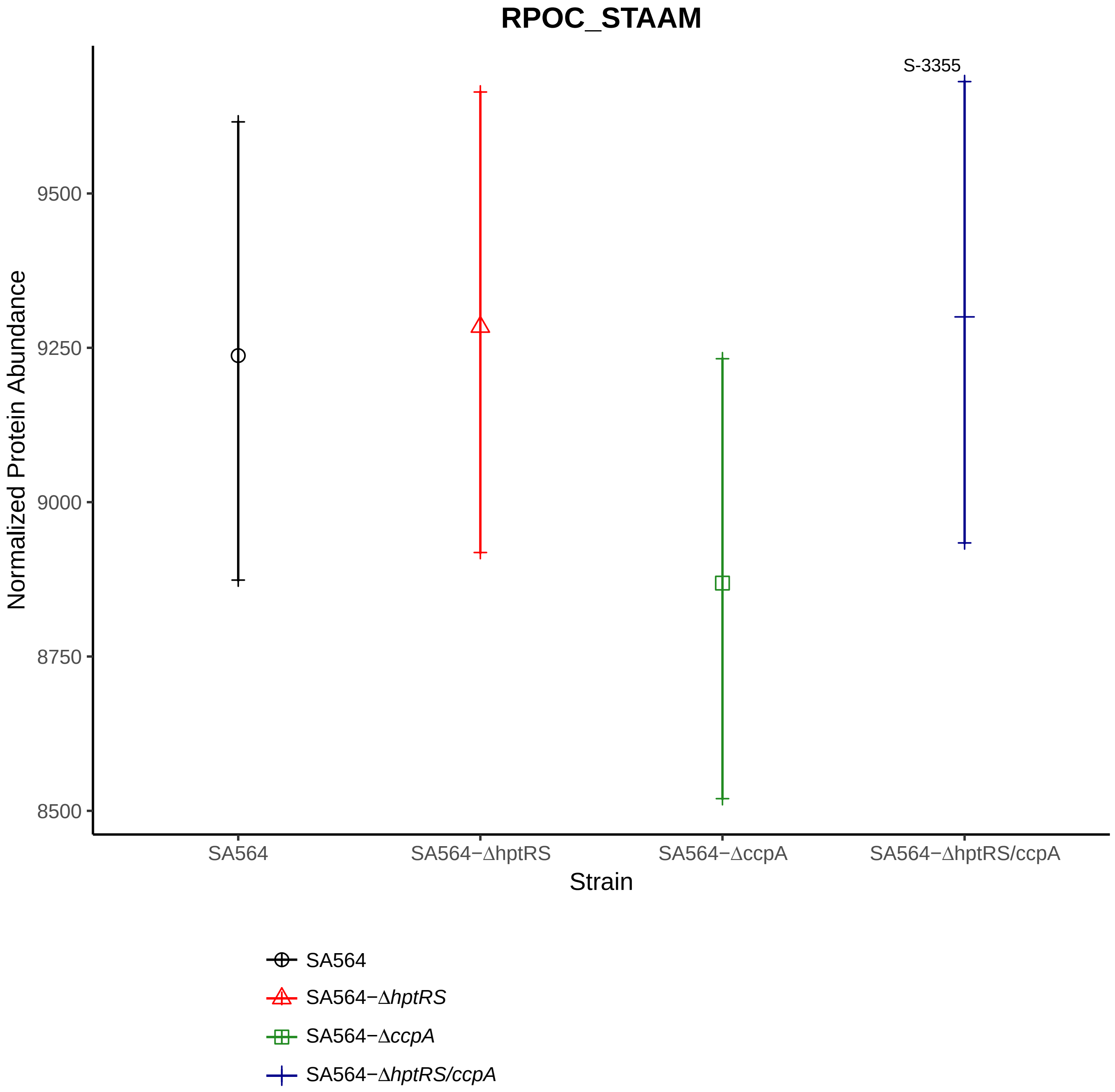
RPOC_STAAM
S-3355
Normalized Protein Abundance
9500
9250
9000
8750
8500
SA564
SA564−ΔhptRS
SA564−ΔccpA
SA564−ΔhptRS/ccpA
Strain
SA564
SA564−ΔhptRS
SA564−ΔccpA
SA564−ΔhptRS/ccpA

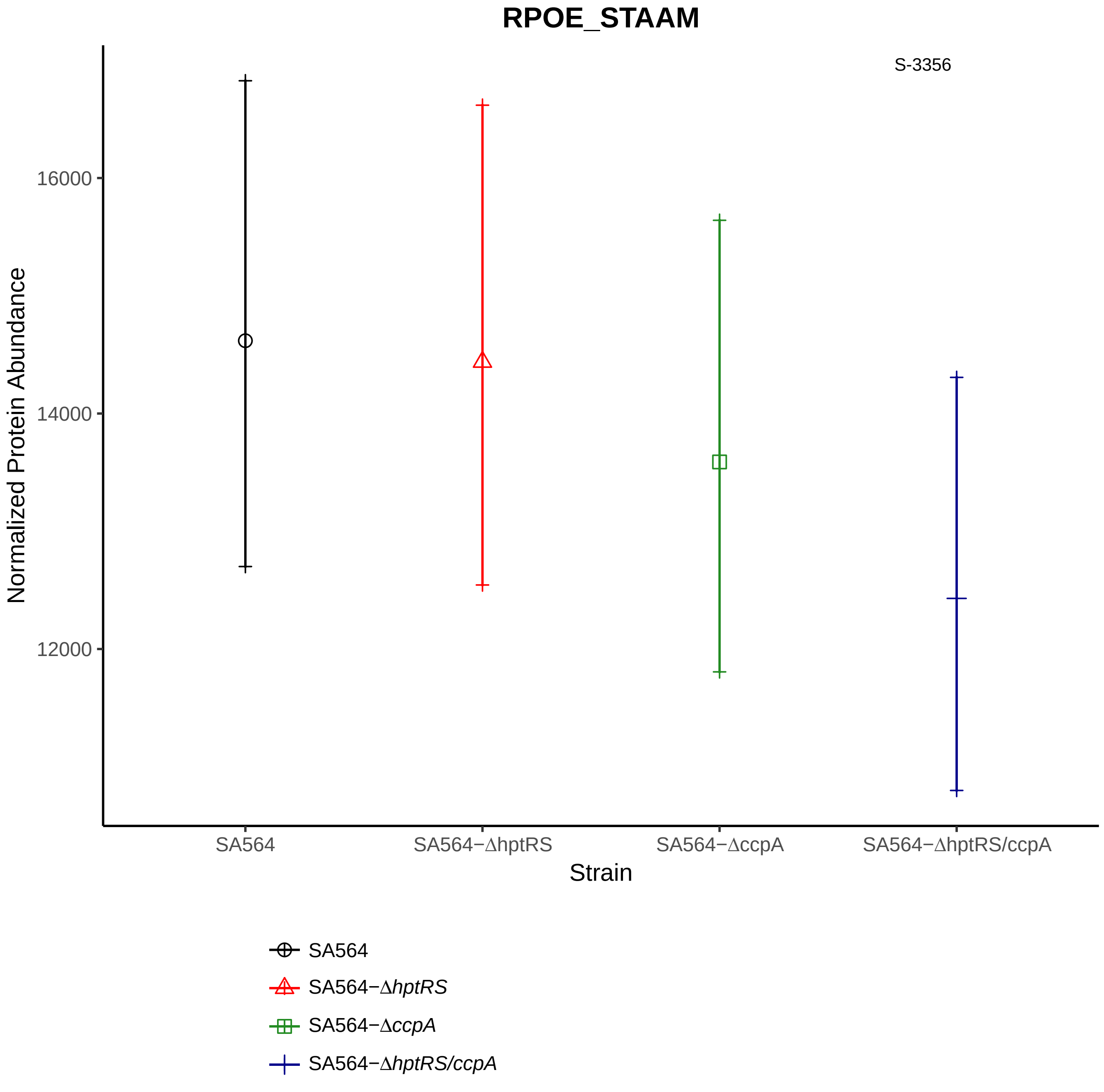
RPOE_STAAM
S-3356
Normalized Protein Abundance
16000
14000
12000
SA564
SA564−ΔhptRS
SA564−ΔccpA
SA564−ΔhptRS/ccpA
Strain
SA564
SA564−ΔhptRS
SA564−ΔccpA
SA564−ΔhptRS/ccpA

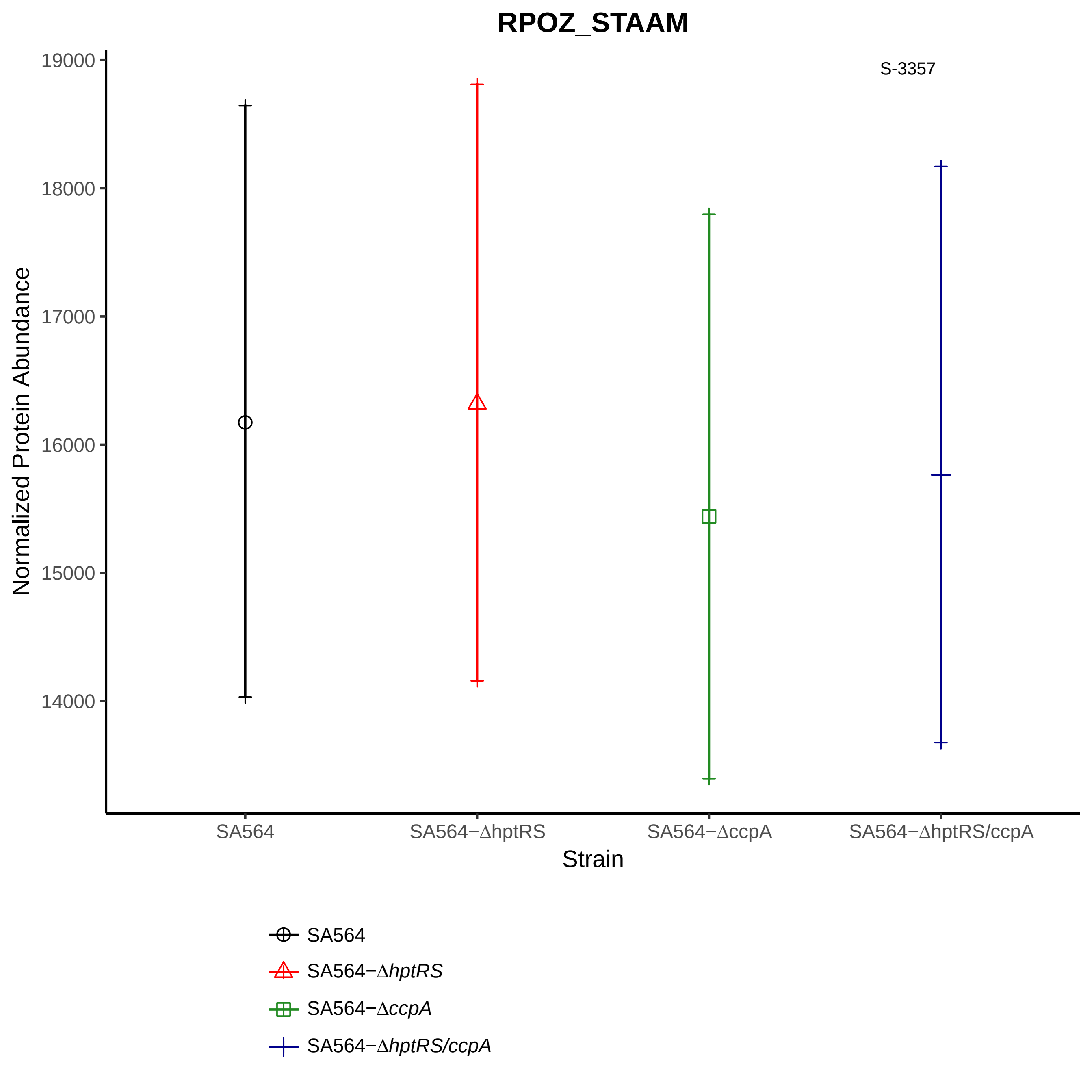

RPOZ_STAAM
S-3357
Normalized Protein Abundance
19000
18000
17000
16000
15000
14000
SA564
SA564−ΔhptRS
SA564−ΔccpA
SA564−ΔhptRS/ccpA
Strain
SA564
SA564−Δ*hptRS*
SA564−Δ*ccpA*
SA564−Δ*hptRS/ccpA*

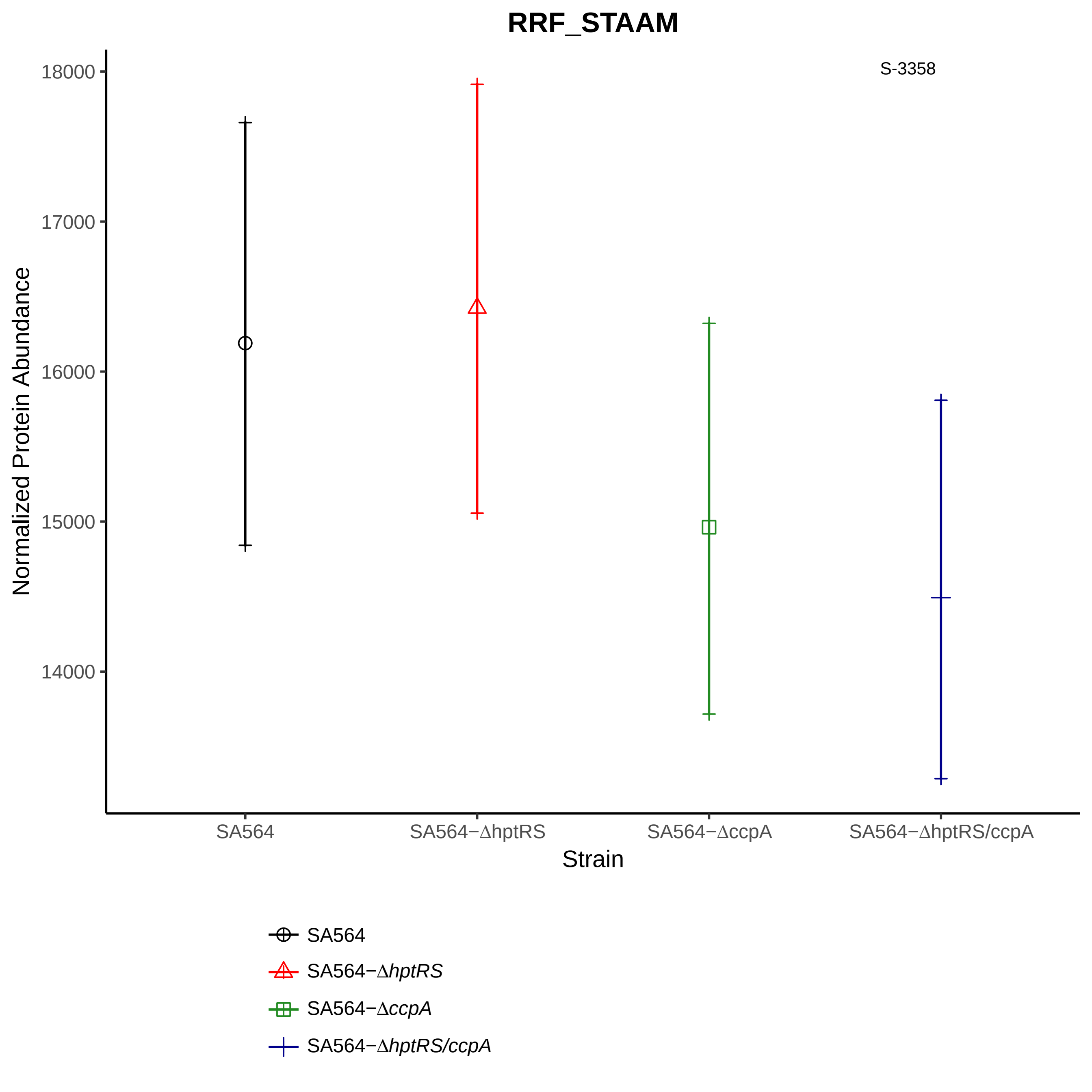

RRF_STAAM
S-3358
Normalized Protein Abundance
18000
17000
16000
15000
14000
SA564
SA564−ΔhptRS
SA564−ΔccpA
SA564−ΔhptRS/ccpA
Strain
SA564
SA564−ΔhptRS
SA564−ΔccpA
SA564−ΔhptRS/ccpA

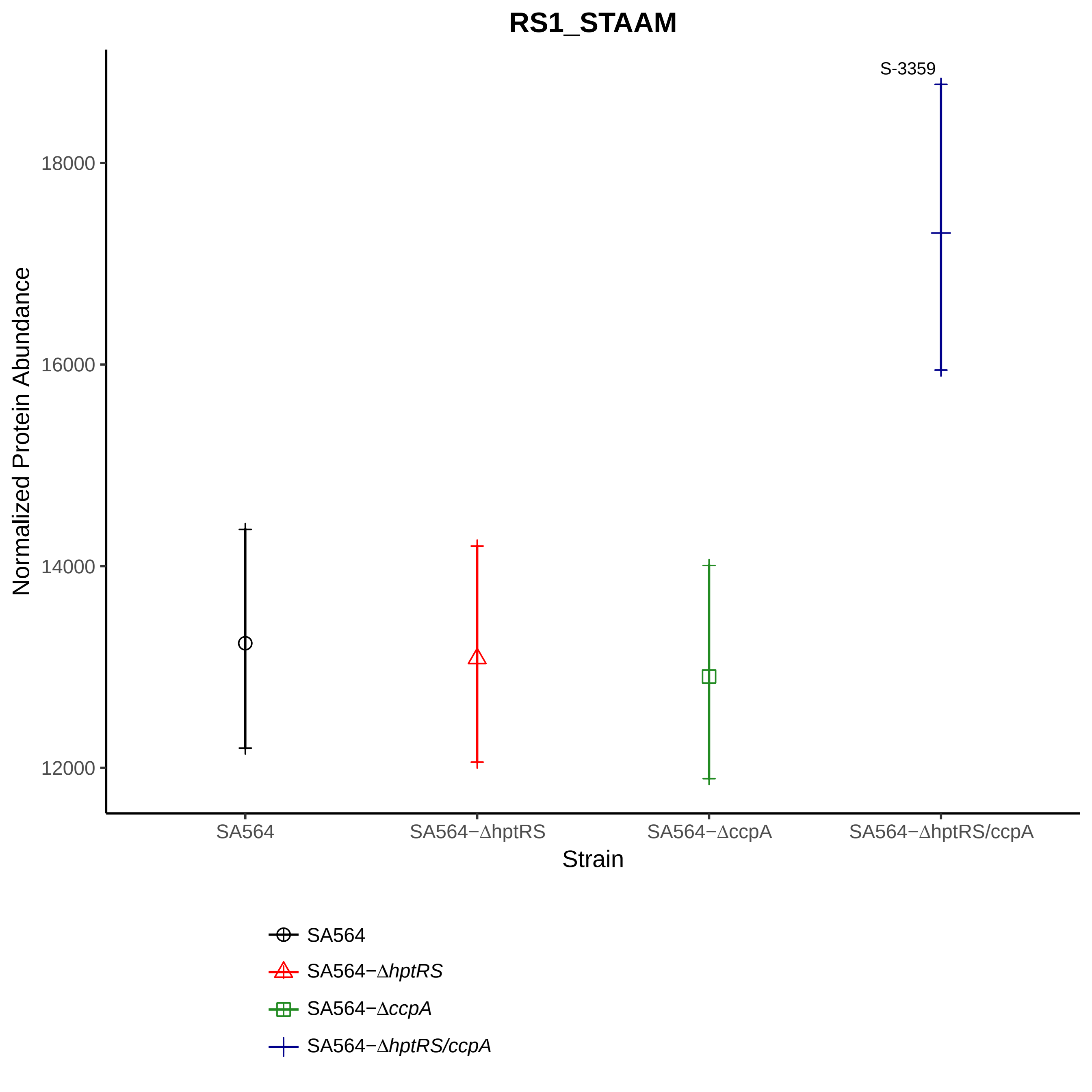

RS1_STAAM
S-3359
Normalized Protein Abundance
18000
16000
14000
12000
SA564
SA564−ΔhptRS
SA564−ΔccpA
SA564−ΔhptRS/ccpA
Strain
SA564
SA564−ΔhptRS
SA564−ΔccpA
SA564−ΔhptRS/ccpA

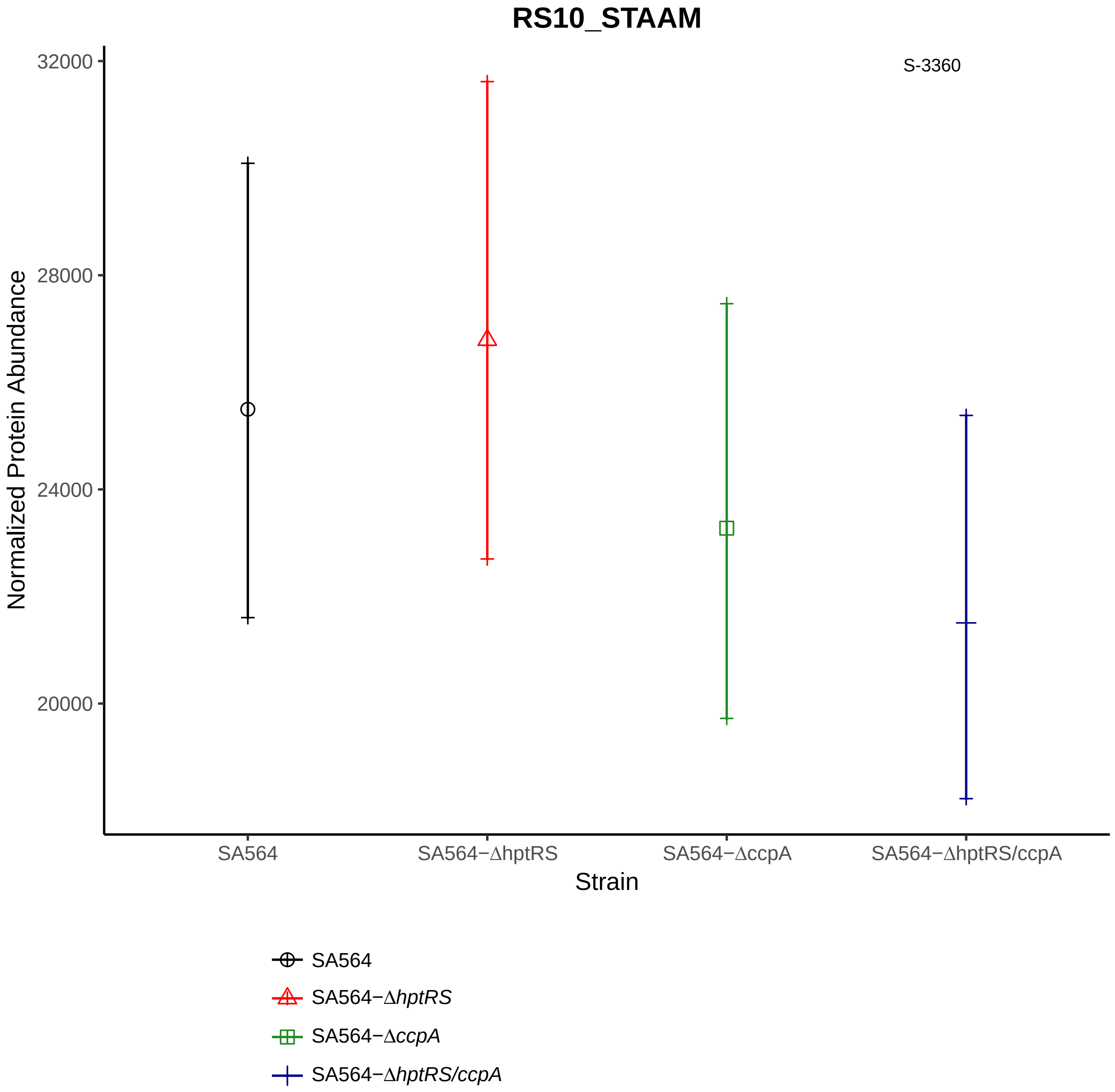
RS10_STAAM
S-3360
Normalized Protein Abundance
32000
28000
24000
20000
SA564
SA564−ΔhptRS
SA564−ΔccpA
SA564−ΔhptRS/ccpA
Strain
SA564
SA564−Δ*hptRS*
SA564−Δ*ccpA*
SA564−Δ*hptRS/ccpA*

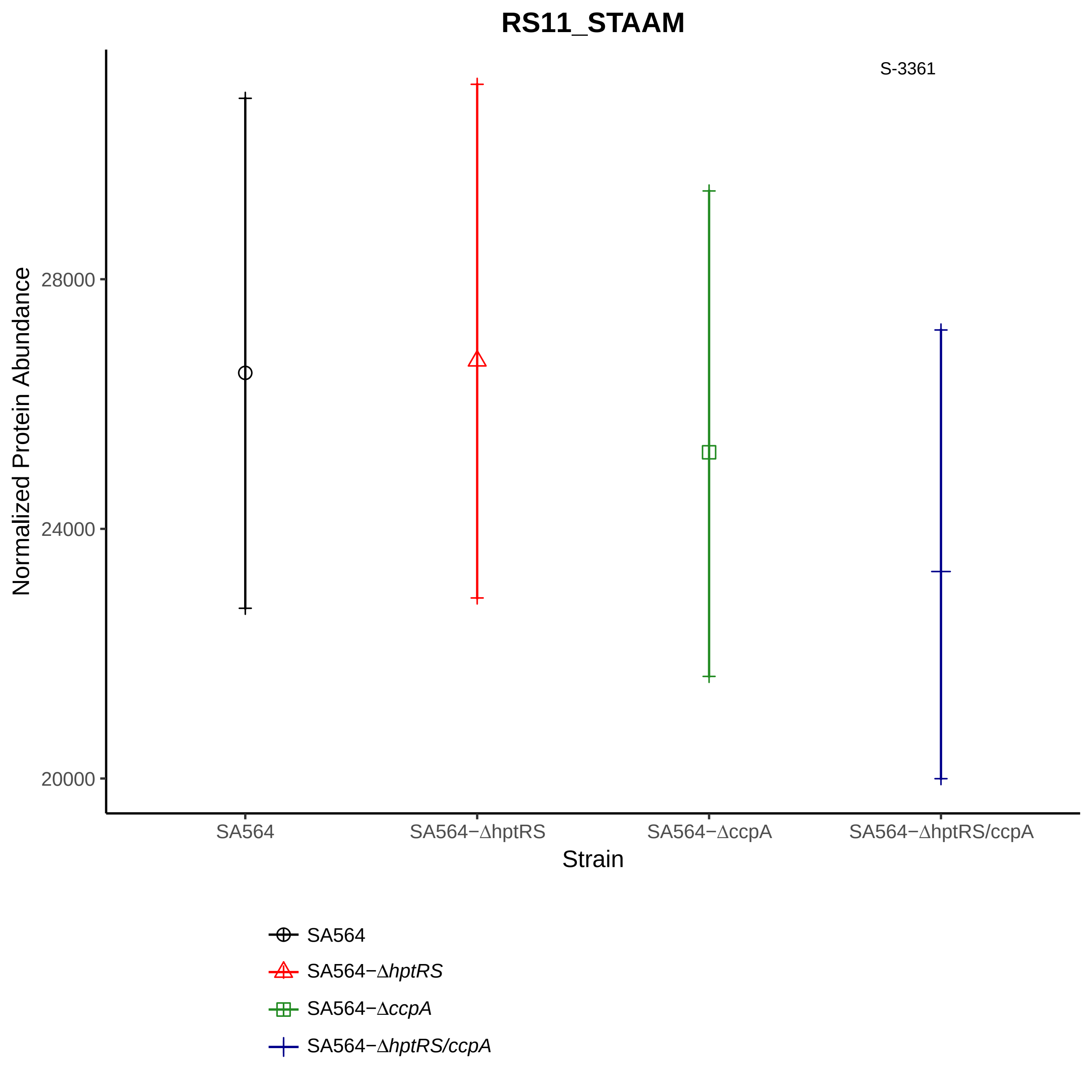
RS11_STAAM
S-3361
Normalized Protein Abundance
28000
24000
20000
SA564
SA564−ΔhptRS
SA564−ΔccpA
SA564−ΔhptRS/ccpA
Strain
SA564
SA564−ΔhptRS
SA564−ΔccpA
SA564−ΔhptRS/ccpA

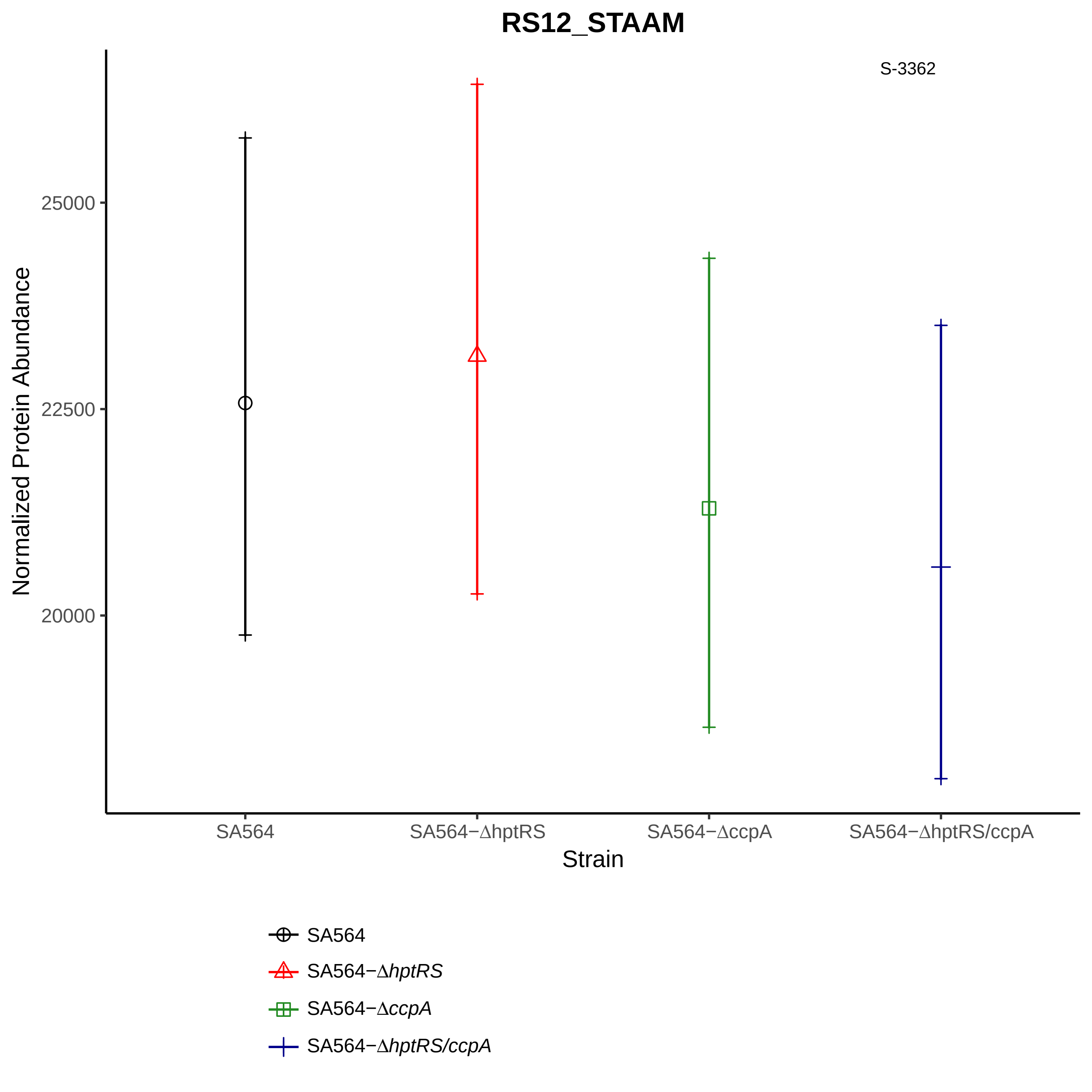

RS12_STAAM
S-3362
Normalized Protein Abundance
25000
22500
20000
SA564
SA564−ΔhptRS
SA564−ΔccpA
SA564−ΔhptRS/ccpA
Strain
SA564
SA564−ΔhptRS
SA564−ΔccpA
SA564−ΔhptRS/ccpA

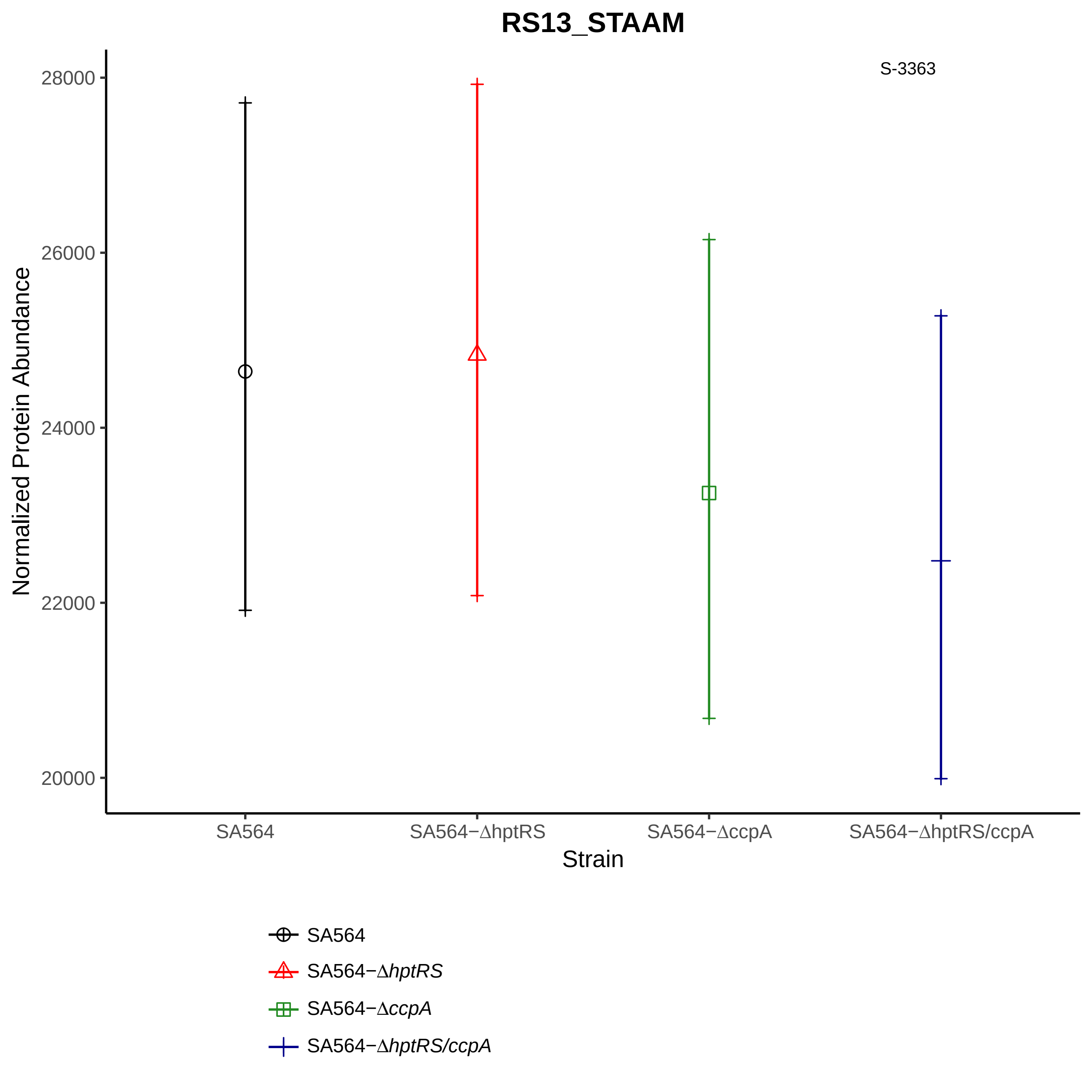
RS13_STAAM
S-3363
Normalized Protein Abundance
28000
26000
24000
22000
20000
SA564
SA564−ΔhptRS
SA564−ΔccpA
SA564−ΔhptRS/ccpA
Strain
SA564
SA564−ΔhptRS
SA564−ΔccpA
SA564−ΔhptRS/ccpA

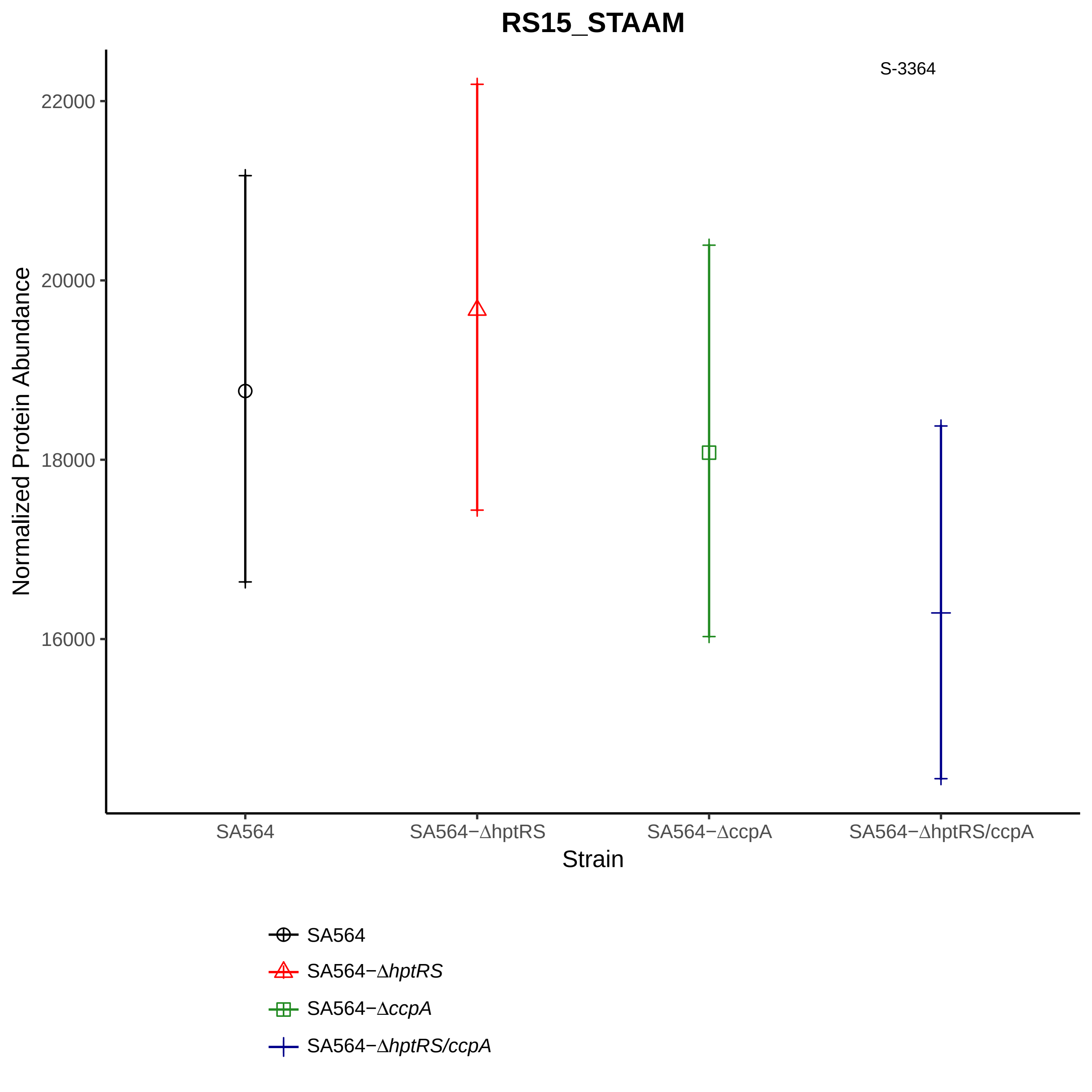

RS15_STAAM
S-3364
Normalized Protein Abundance
22000
20000
18000
16000
SA564
SA564−ΔhptRS
SA564−ΔccpA
SA564−ΔhptRS/ccpA
Strain
SA564
SA564−ΔhptRS
SA564−ΔccpA
SA564−ΔhptRS/ccpA

RS16_STAAM

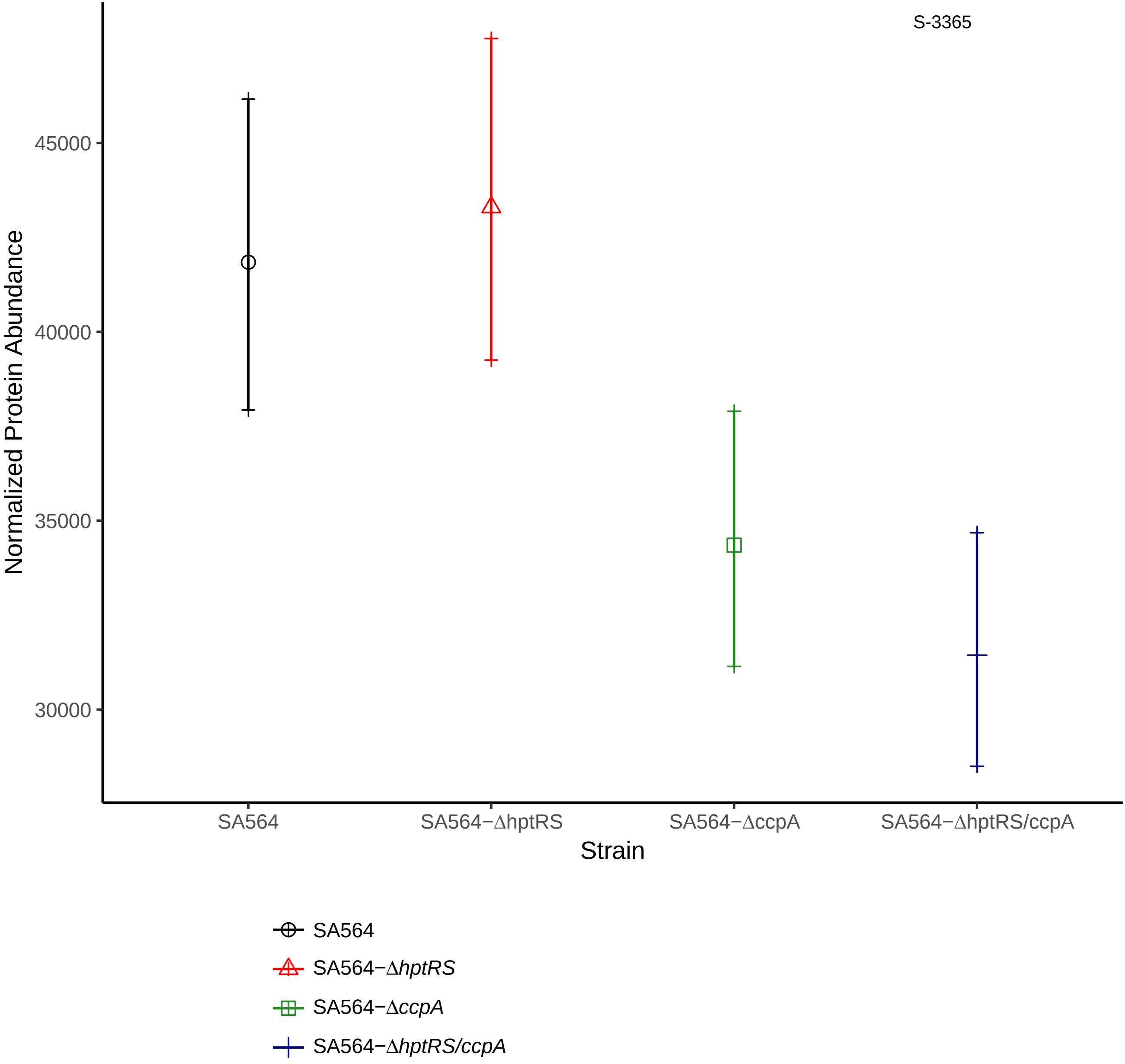

S-3365
Normalized Protein Abundance
45000
40000
35000
30000
SA564
SA564−ΔhptRS
SA564−ΔccpA
SA564−ΔhptRS/ccpA
Strain
SA564
SA564−Δ*hptRS*
SA564−Δ*ccpA*
SA564−Δ*hptRS/ccpA*

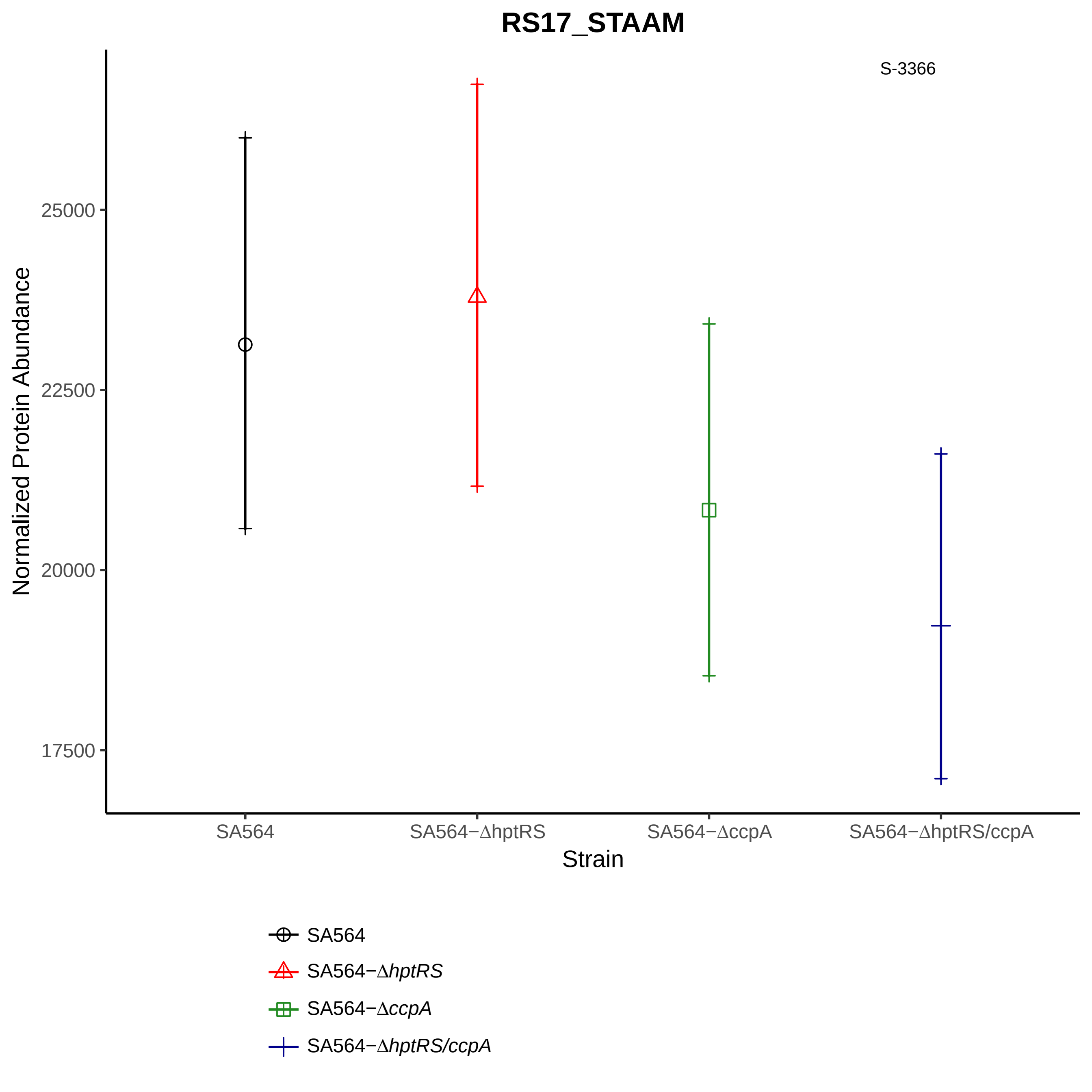
RS17_STAAM
S-3366
Normalized Protein Abundance
25000
22500
20000
17500
SA564
SA564−ΔhptRS
SA564−ΔccpA
SA564−ΔhptRS/ccpA
Strain
SA564
SA564−ΔhptRS
SA564−ΔccpA
SA564−ΔhptRS/ccpA

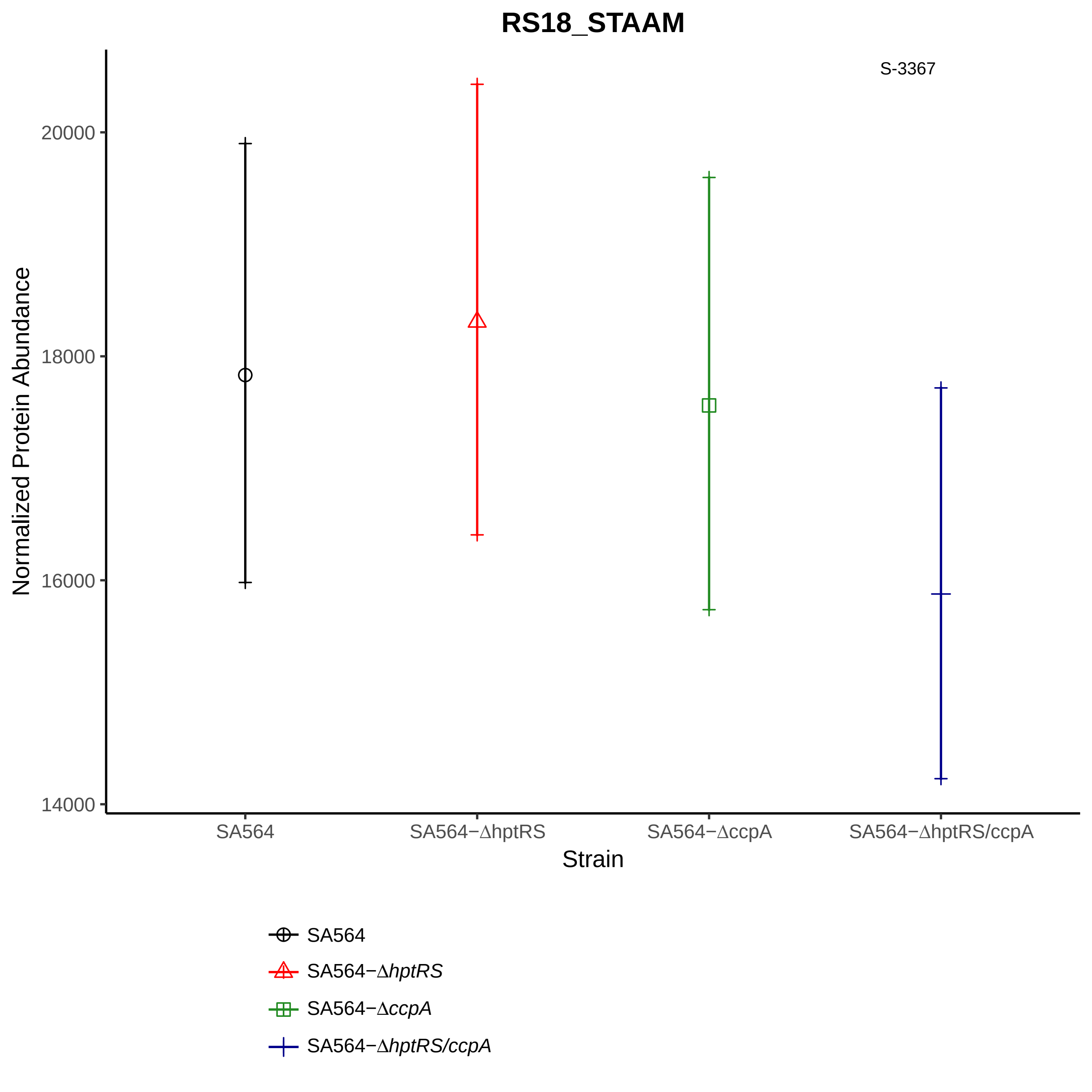

RS18_STAAM
S-3367
Normalized Protein Abundance
20000
18000
16000
14000
SA564
SA564−ΔhptRS
SA564−ΔccpA
SA564−ΔhptRS/ccpA
Strain
SA564
SA564−ΔhptRS
SA564−ΔccpA
SA564−ΔhptRS/ccpA

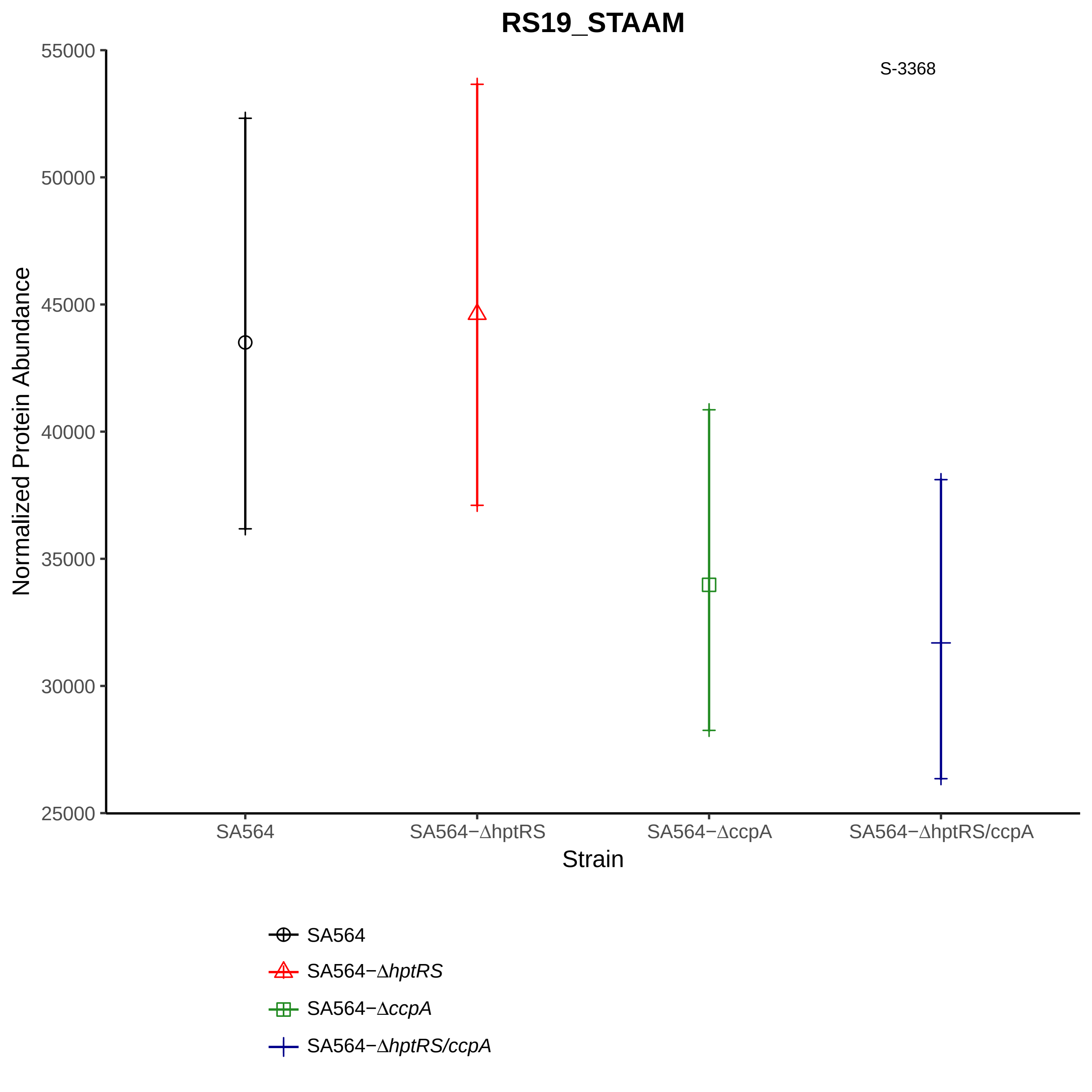
RS19_STAAM
S-3368
Normalized Protein Abundance
55000
50000
45000
40000
35000
30000
25000
SA564
SA564−ΔhptRS
SA564−ΔccpA
SA564−ΔhptRS/ccpA
Strain
SA564
SA564−ΔhptRS
SA564−ΔccpA
SA564−ΔhptRS/ccpA

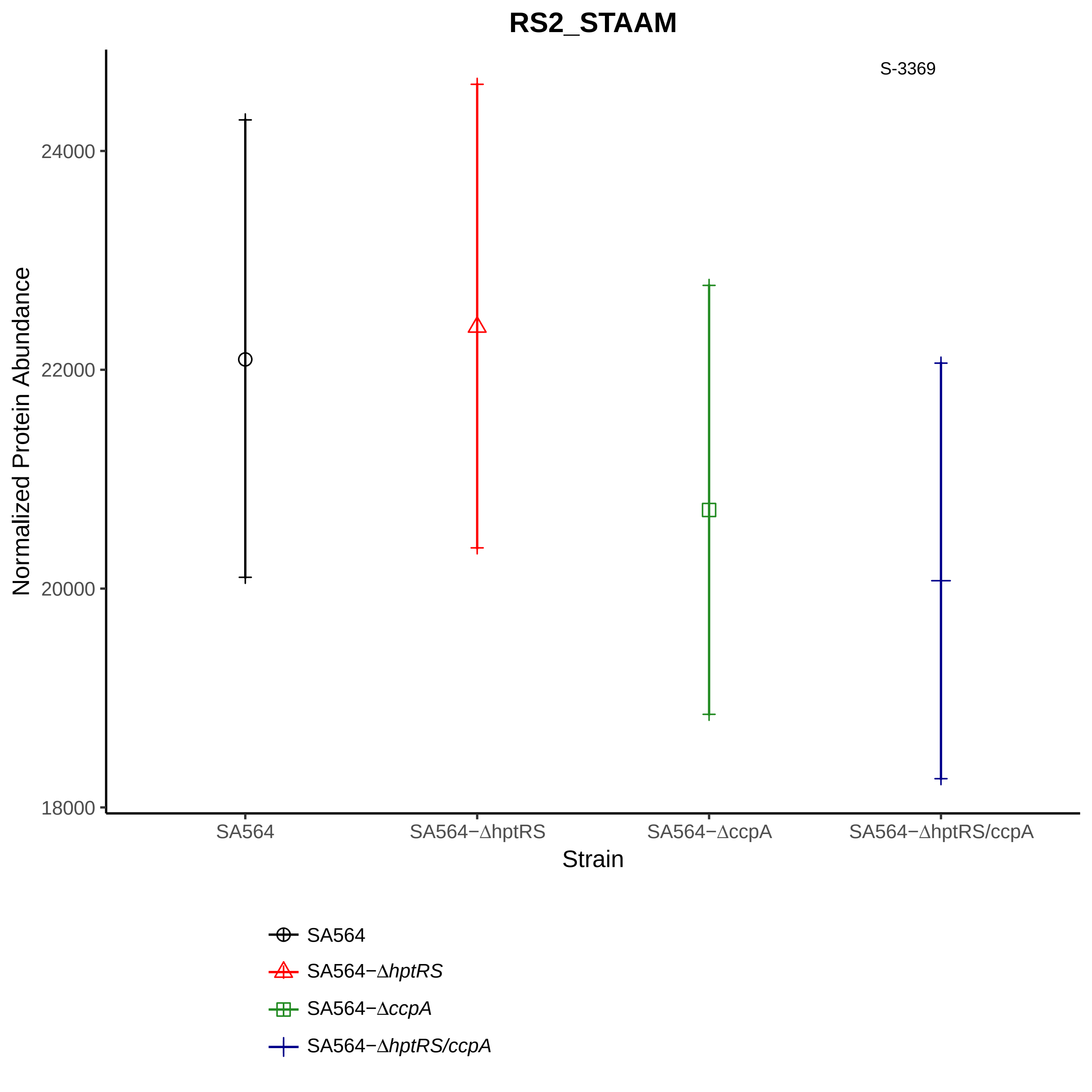
RS2_STAAM
S-3369
Normalized Protein Abundance
24000
22000
20000
18000
SA564
SA564−ΔhptRS
SA564−ΔccpA
SA564−ΔhptRS/ccpA
Strain
SA564
SA564−ΔhptRS
SA564−ΔccpA
SA564−ΔhptRS/ccpA

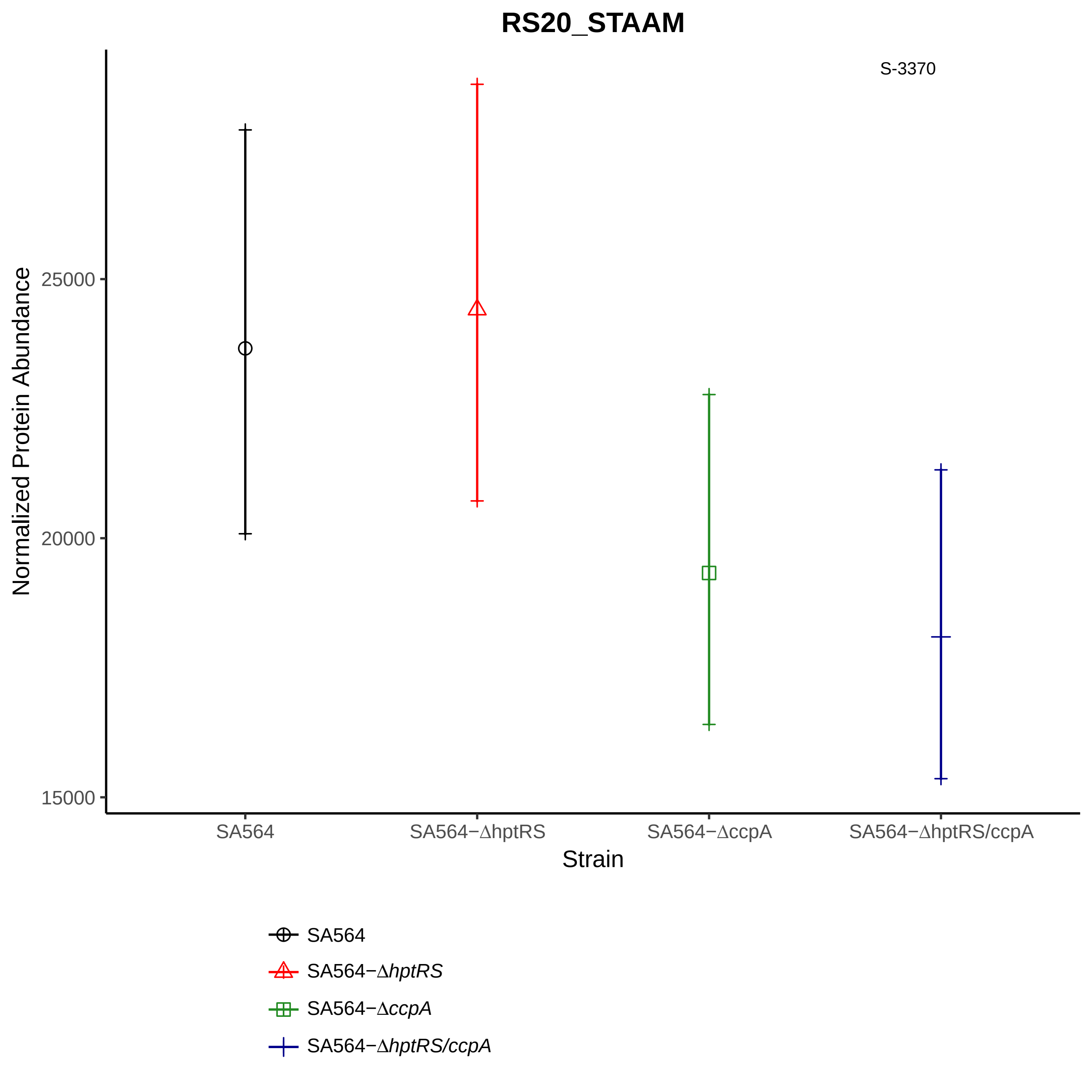

RS20_STAAM
S-3370
Normalized Protein Abundance
25000
20000
15000
SA564
SA564−ΔhptRS
SA564−ΔccpA
SA564−ΔhptRS/ccpA
Strain
SA564
SA564−ΔhptRS
SA564−ΔccpA
SA564−ΔhptRS/ccpA

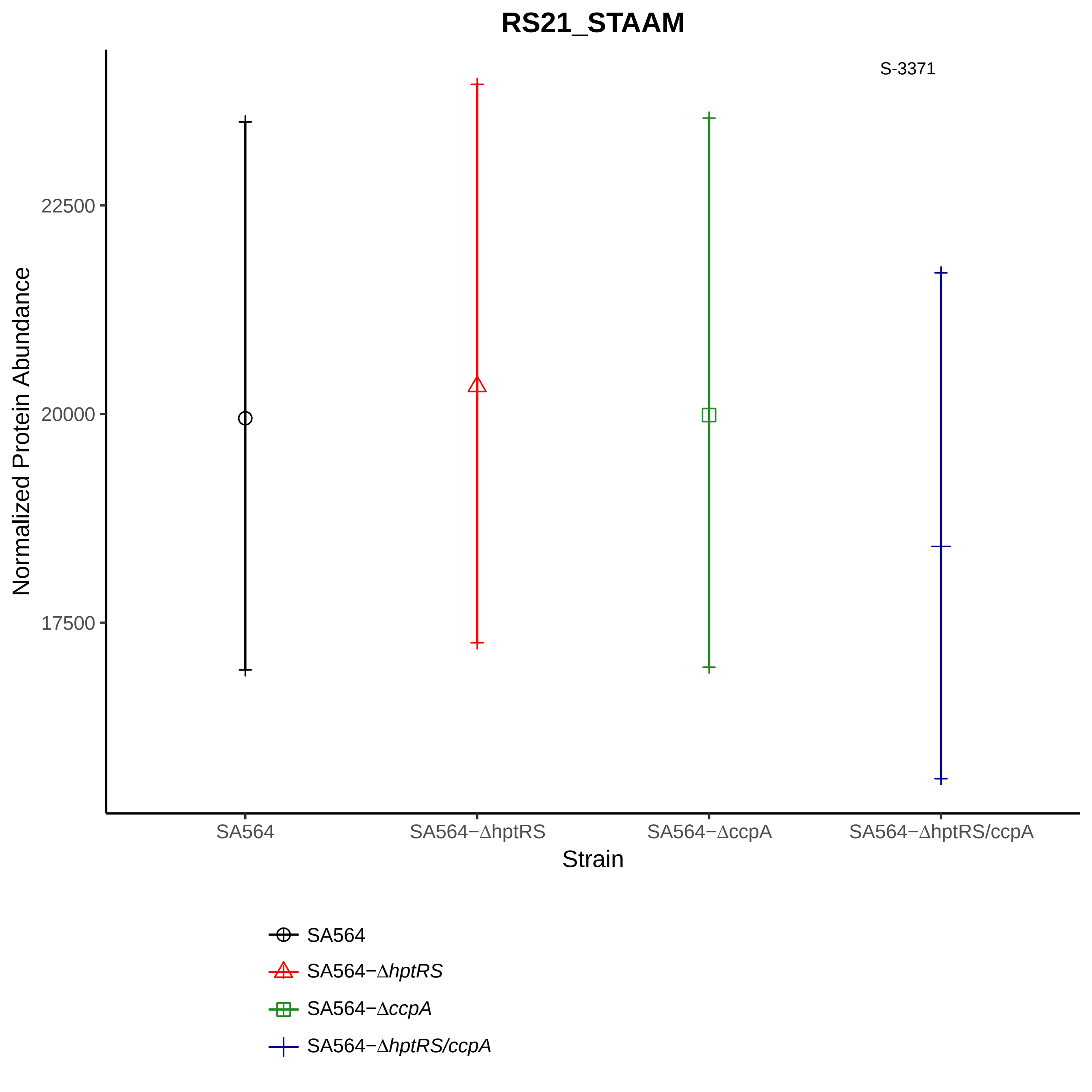
RS21_STAAM
S-3371
Normalized Protein Abundance
22500
20000
17500
SA564
SA564−ΔhptRS
SA564−ΔccpA
SA564−ΔhptRS/ccpA
Strain
SA564
SA564−ΔhptRS
SA564−ΔccpA
SA564−ΔhptRS/ccpA

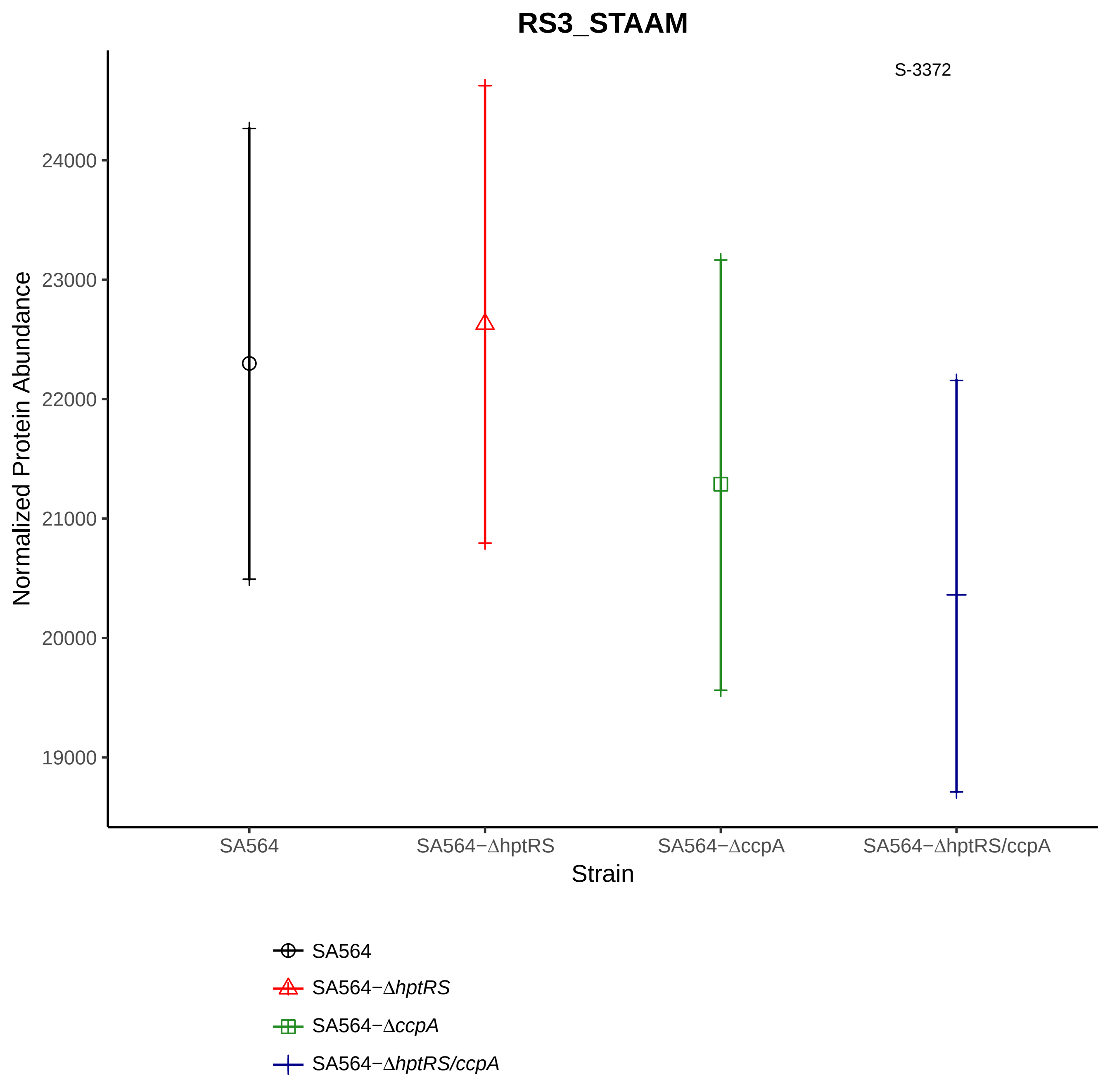

RS3_STAAM
S-3372
Normalized Protein Abundance
24000
23000
22000
21000
20000
19000
SA564
SA564−ΔhptRS
SA564−ΔccpA
SA564−ΔhptRS/ccpA
Strain
SA564
SA564−ΔhptRS
SA564−ΔccpA
SA564−ΔhptRS/ccpA

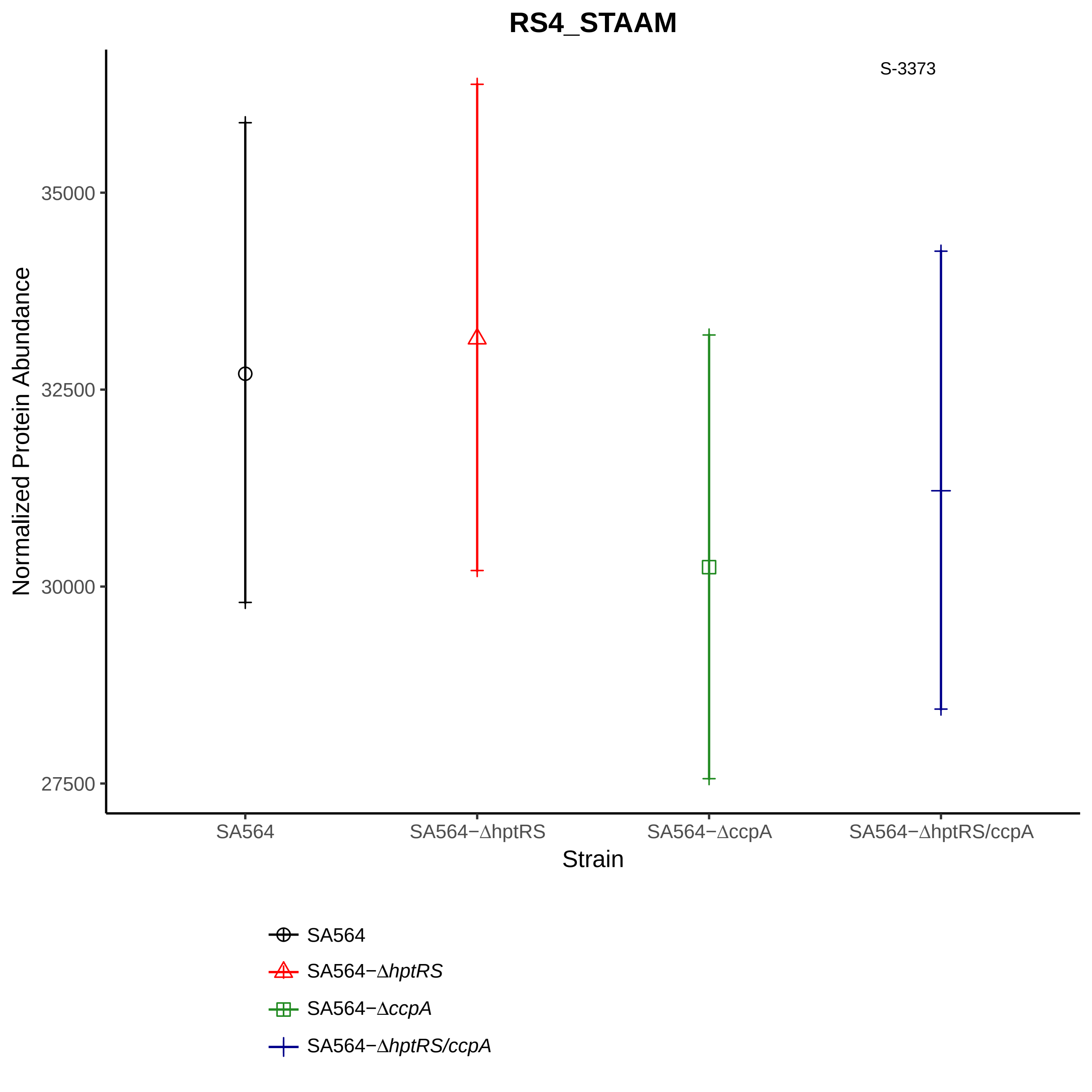

RS4_STAAM
S-3373
Normalized Protein Abundance
35000
32500
30000
27500
SA564
SA564−ΔhptRS
SA564−ΔccpA
SA564−ΔhptRS/ccpA
Strain
SA564
SA564−ΔhptRS
SA564−ΔccpA
SA564−ΔhptRS/ccpA

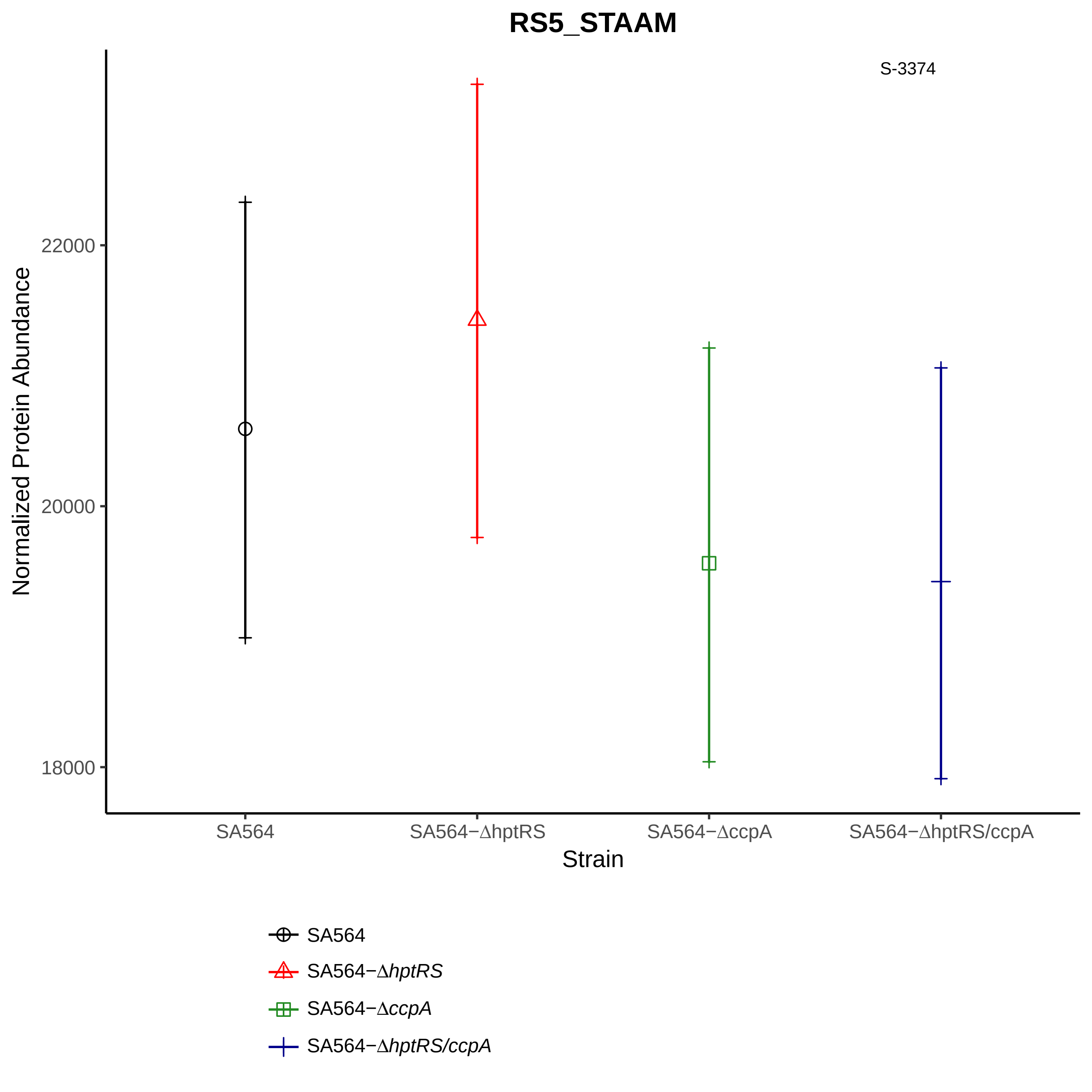

RS5_STAAM
S-3374
Normalized Protein Abundance
22000
20000
18000
SA564
SA564−ΔhptRS
SA564−ΔccpA
SA564−ΔhptRS/ccpA
Strain
SA564
SA564−ΔhptRS
SA564−ΔccpA
SA564−ΔhptRS/ccpA

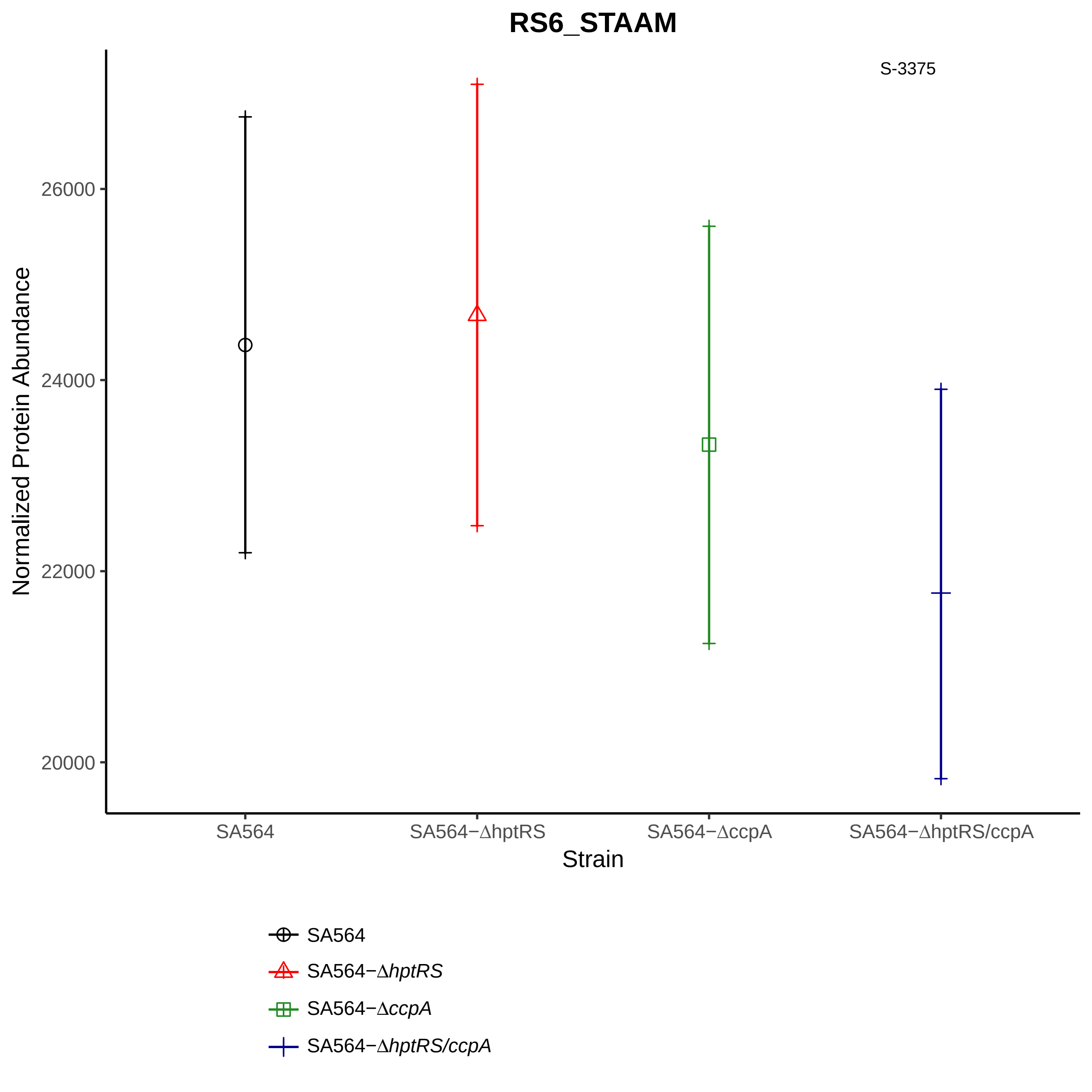

RS6_STAAM
S-3375
Normalized Protein Abundance
26000
24000
22000
20000
SA564
SA564−ΔhptRS
SA564−ΔccpA
SA564−ΔhptRS/ccpA
Strain
SA564
SA564−ΔhptRS
SA564−ΔccpA
SA564−ΔhptRS/ccpA

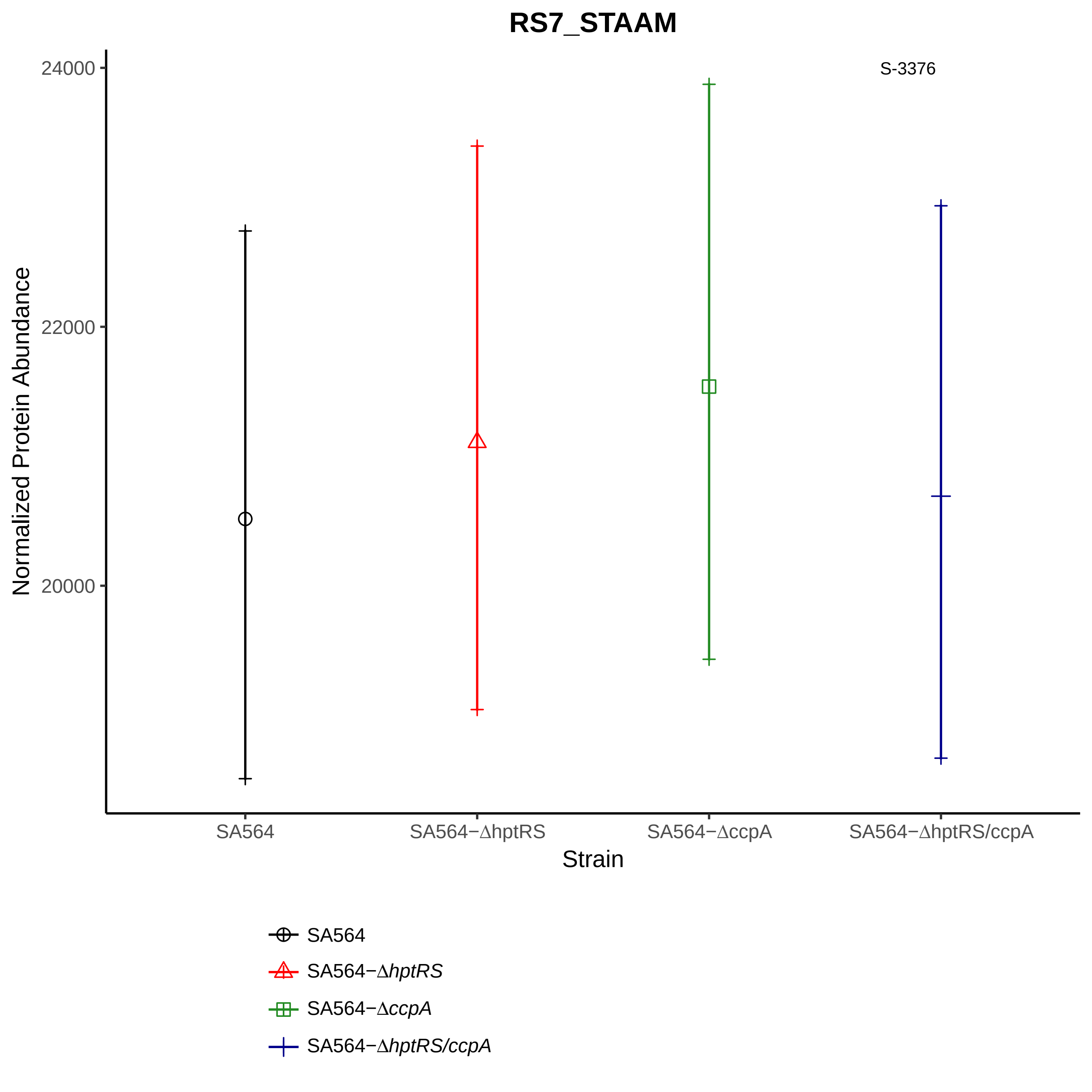
RS7_STAAM
S-3376
Normalized Protein Abundance
24000
22000
20000
SA564
SA564−ΔhptRS
SA564−ΔccpA
SA564−ΔhptRS/ccpA
Strain
SA564
SA564−Δ*hptRS*
SA564−Δ*ccpA*
SA564−Δ*hptRS/ccpA*

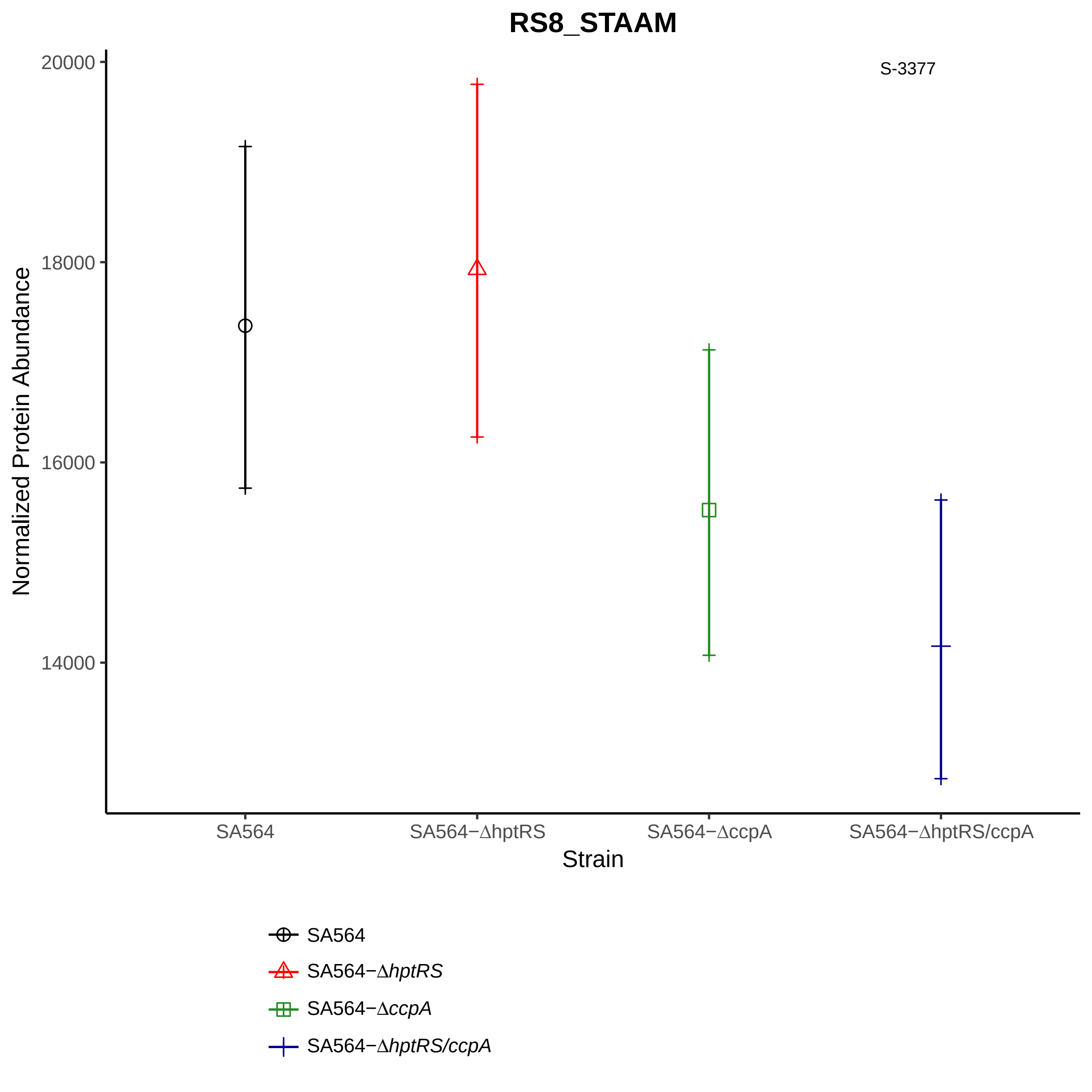
RS8_STAAM
S-3377
Normalized Protein Abundance
20000
18000
16000
14000
SA564
SA564−ΔhptRS
SA564−ΔccpA
SA564−ΔhptRS/ccpA
Strain
SA564
SA564−ΔhptRS
SA564−ΔccpA
SA564−ΔhptRS/ccpA

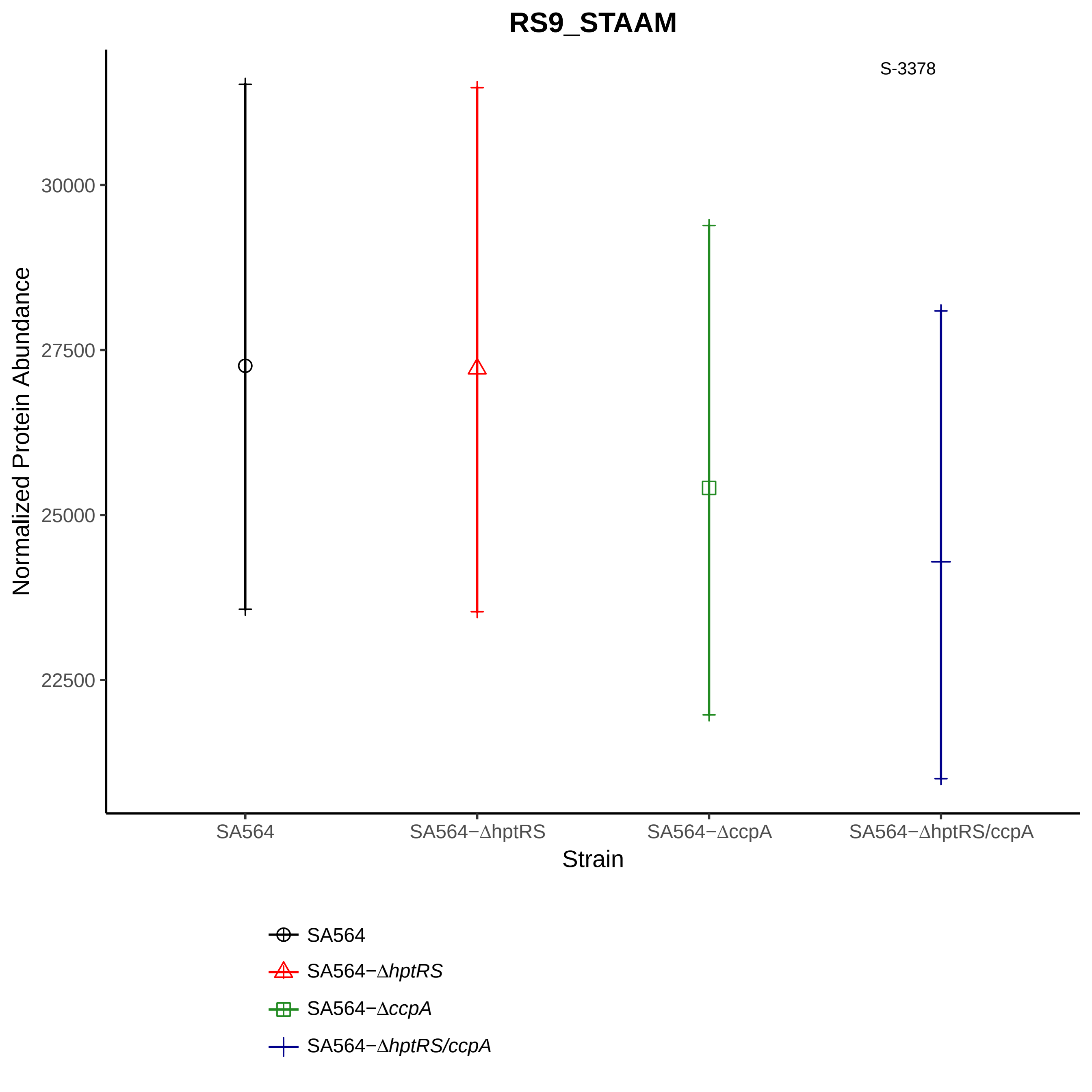
RS9_STAAM
S-3378
Normalized Protein Abundance
30000
27500
25000
22500
SA564
SA564−ΔhptRS
SA564−ΔccpA
SA564−ΔhptRS/ccpA
Strain
SA564
SA564−ΔhptRS
SA564−ΔccpA
SA564−ΔhptRS/ccpA

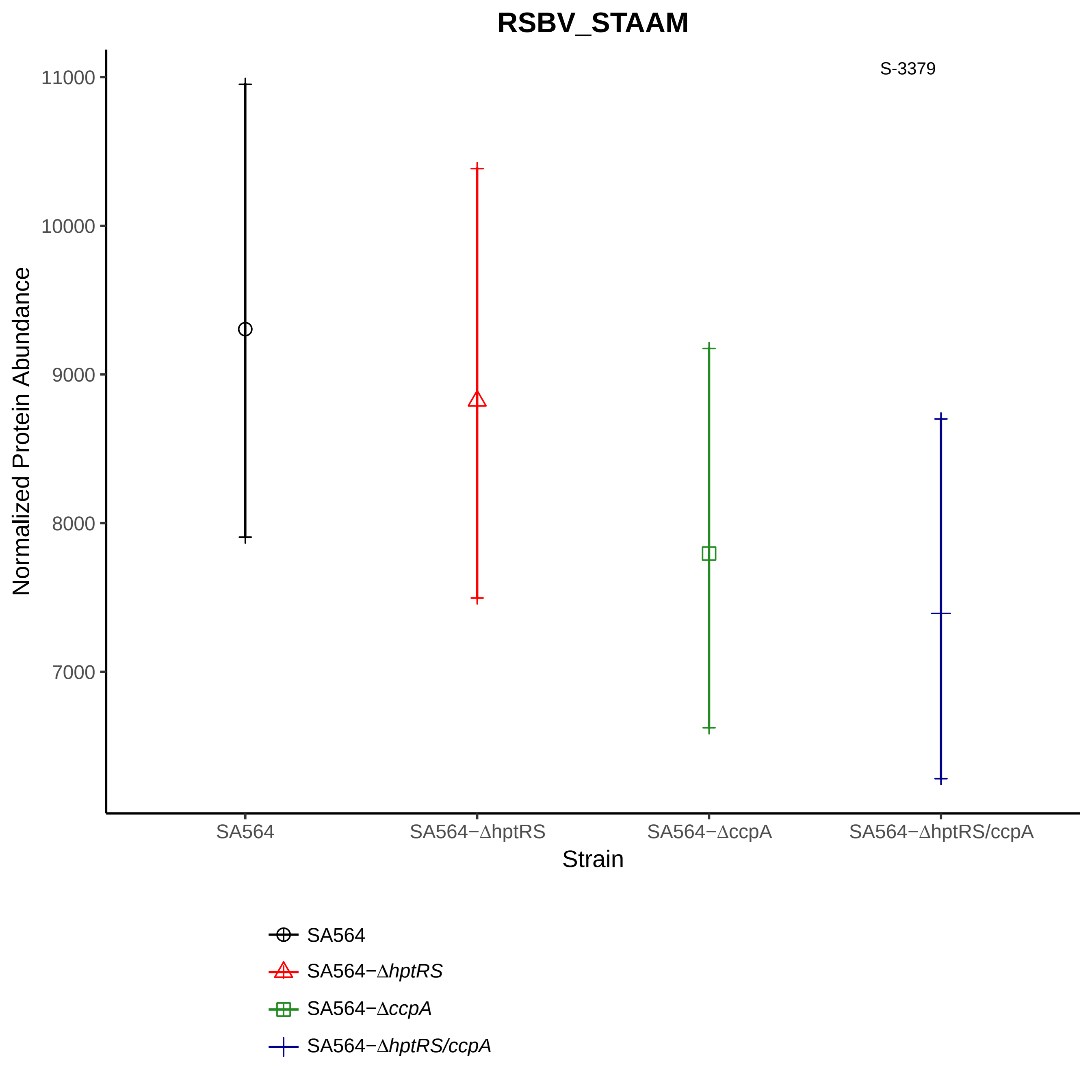

RSBV_STAAM
S-3379
Normalized Protein Abundance
11000
10000
9000
8000
7000
SA564
SA564−ΔhptRS
SA564−ΔccpA
SA564−ΔhptRS/ccpA
Strain
SA564
SA564−Δ*hptRS*
SA564−Δ*ccpA*
SA564−Δ*hptRS/ccpA*

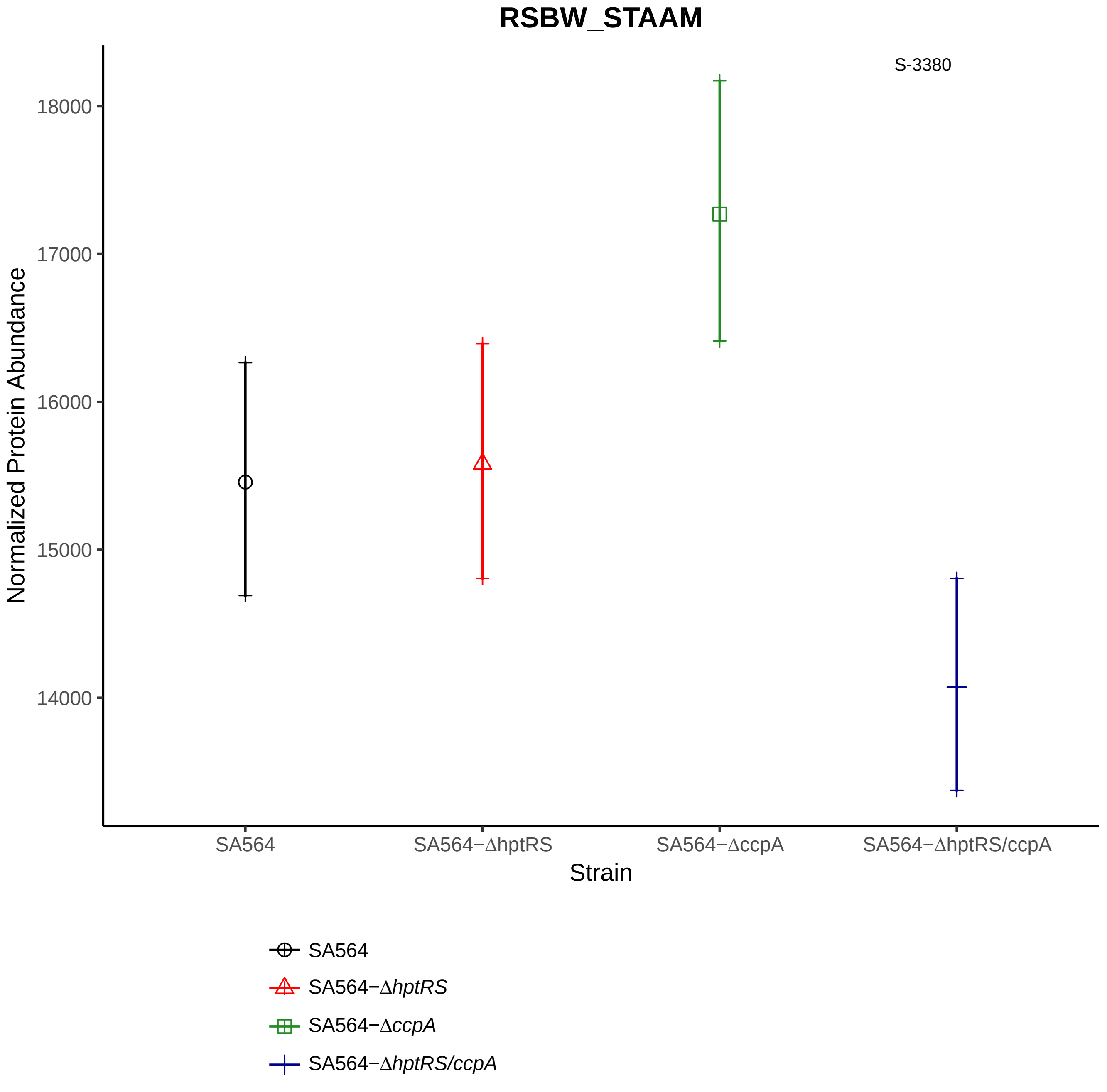
RSBW_STAAM
S-3380
18000
17000
16000
15000
14000
Normalized Protein Abundance
SA564
SA564−ΔhptRS
SA564−ΔccpA
SA564−ΔhptRS/ccpA
Strain
SA564
SA564−Δ*hptRS*
SA564−Δ*ccpA*
SA564−Δ*hptRS/ccpA*

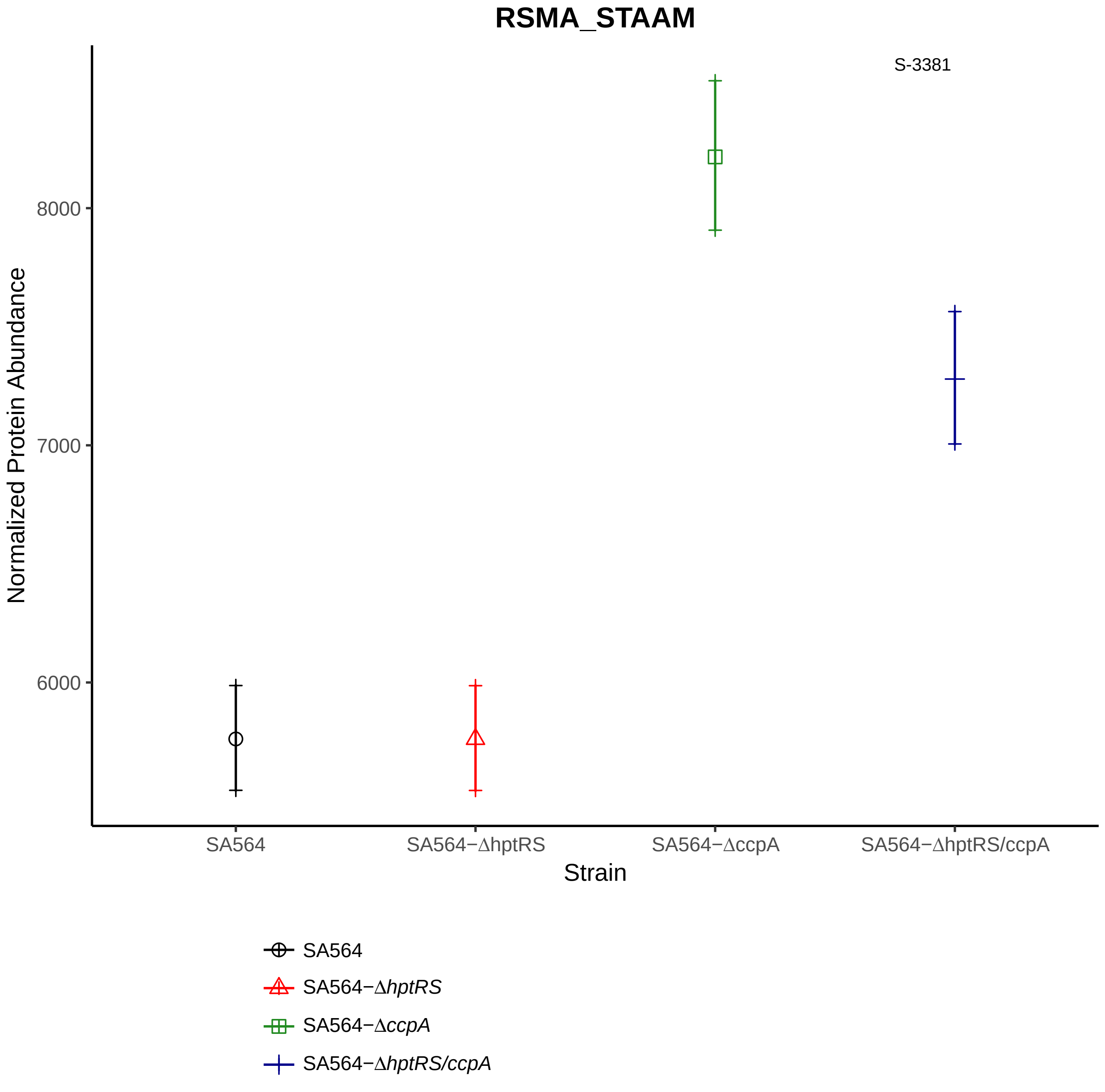

RSMA_STAAM
S-3381
Normalized Protein Abundance
8000
7000
6000
SA564
SA564−ΔhptRS
SA564−ΔccpA
SA564−ΔhptRS/ccpA
Strain
SA564
SA564−ΔhptRS
SA564−ΔccpA
SA564−ΔhptRS/ccpA

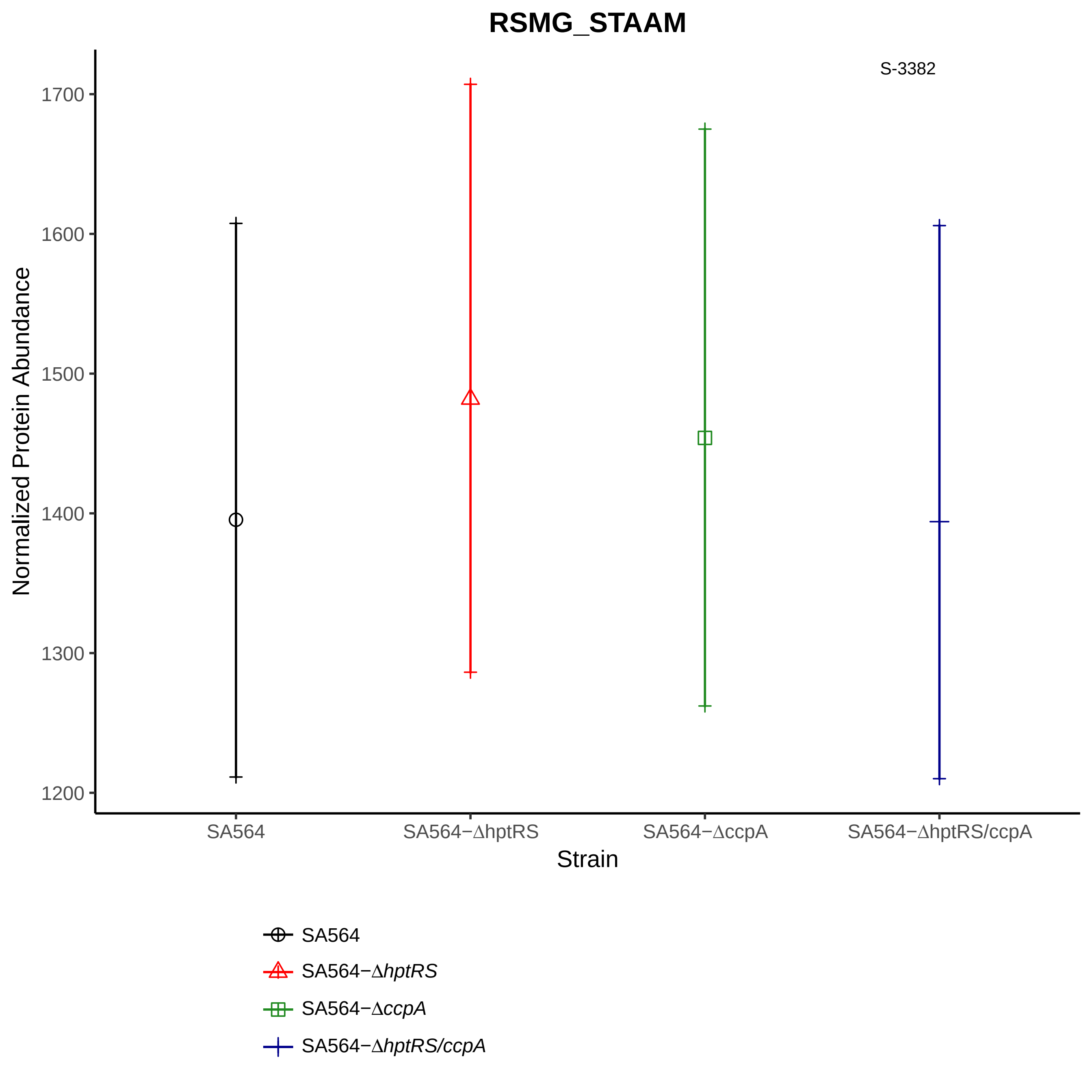

RSMG_STAAM
S-3382
Normalized Protein Abundance
1200
1300
1400
1500
1600
1700
SA564
SA564−ΔhptRS
SA564−ΔccpA
SA564−ΔhptRS/ccpA
Strain
SA564
SA564−ΔhptRS
SA564−ΔccpA
SA564−ΔhptRS/ccpA

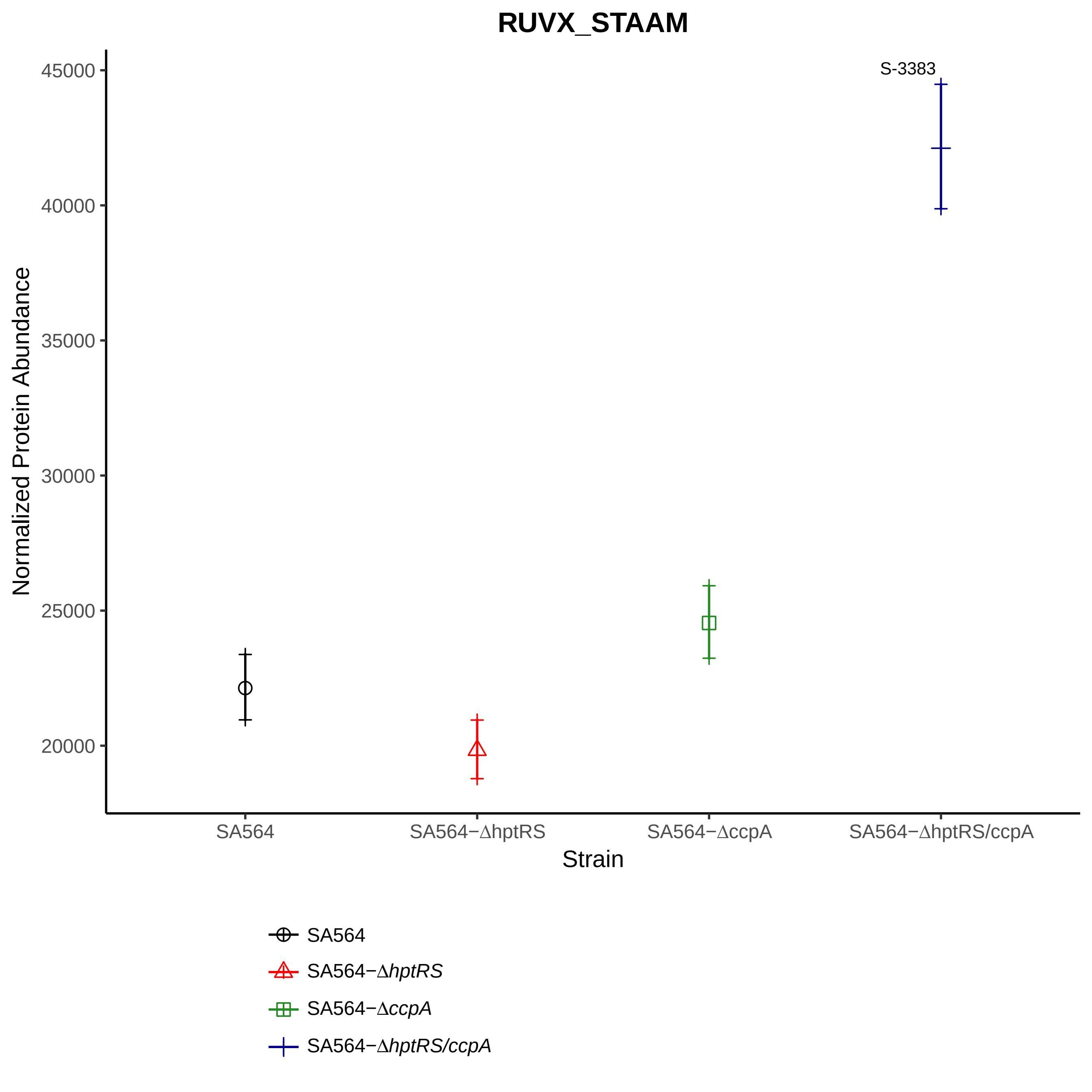

RUVX_STAAM
S-3383
45000
40000
35000
30000
25000
20000
Normalized Protein Abundance
SA564
SA564−ΔhptRS
SA564−ΔccpA
SA564−ΔhptRS/ccpA
Strain
SA564
SA564−ΔhptRS
SA564−ΔccpA
SA564−ΔhptRS/ccpA

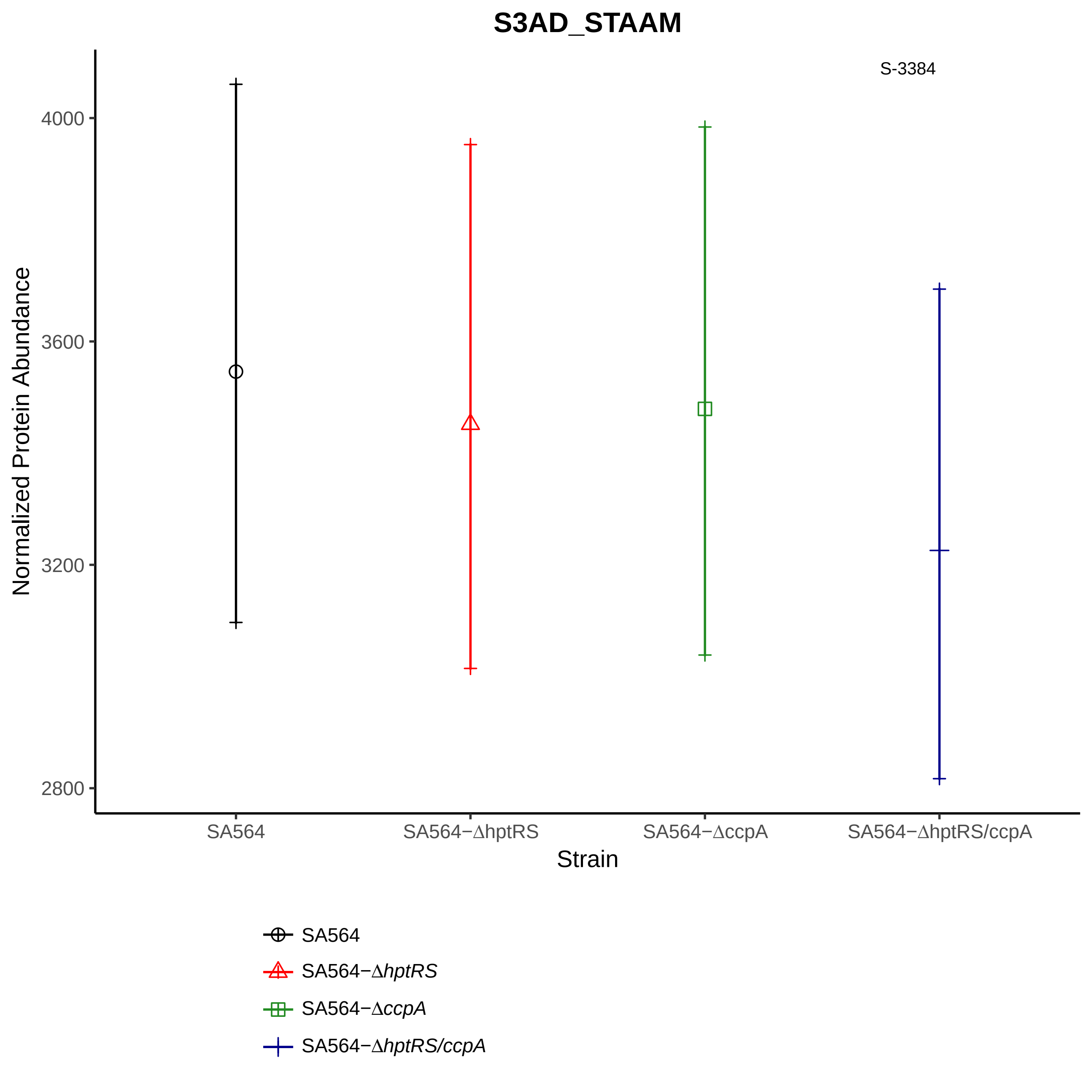

S3AD_STAAM
S-3384
Normalized Protein Abundance
4000
3600
3200
2800
SA564
SA564−ΔhptRS
SA564−ΔccpA
SA564−ΔhptRS/ccpA
Strain
SA564
SA564−ΔhptRS
SA564−ΔccpA
SA564−ΔhptRS/ccpA

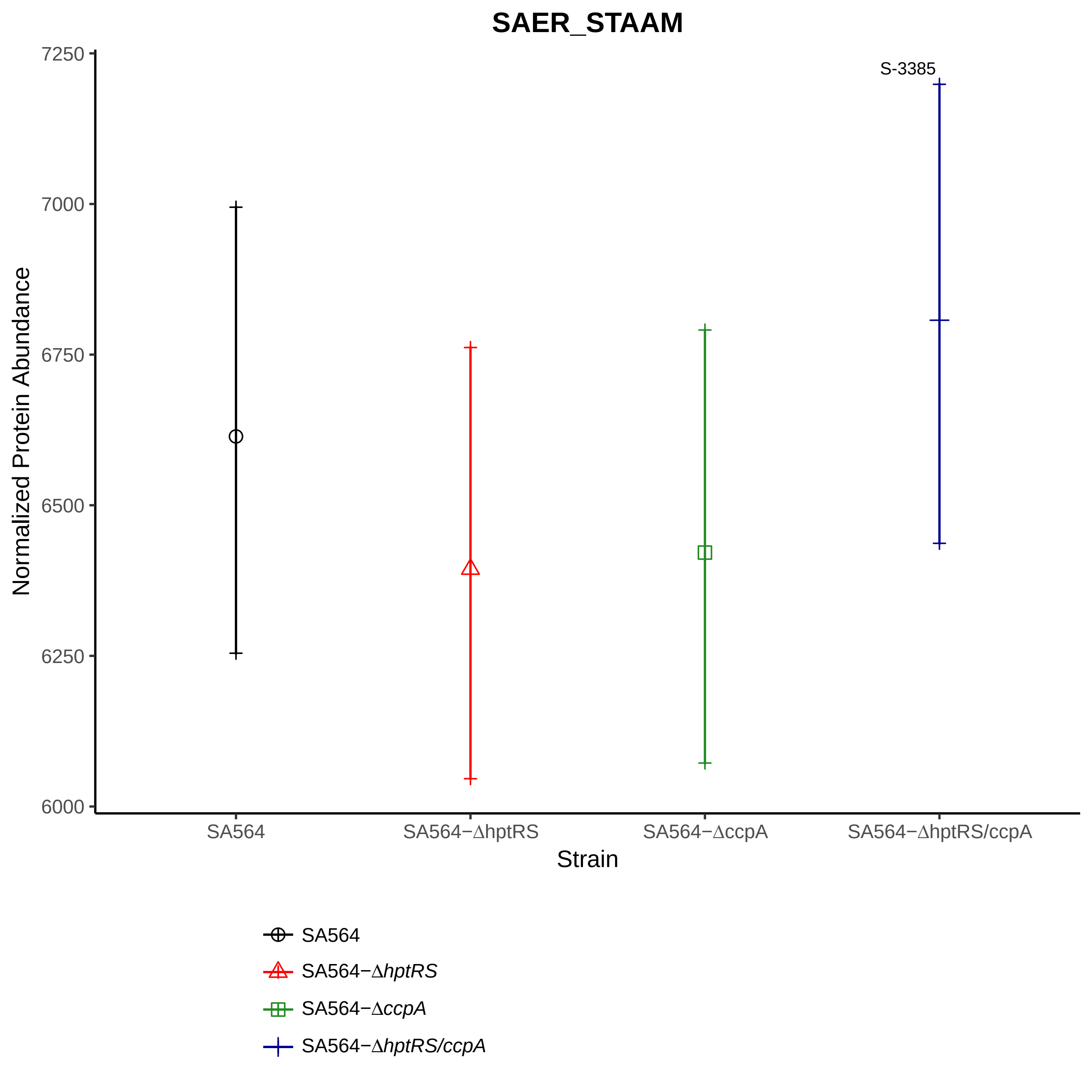

SAER_STAAM
S-3385
Normalized Protein Abundance
7250
7000
6750
6500
6250
6000
SA564
SA564−ΔhptRS
SA564−ΔccpA
SA564−ΔhptRS/ccpA
Strain
SA564
SA564−ΔhptRS
SA564−ΔccpA
SA564−ΔhptRS/ccpA

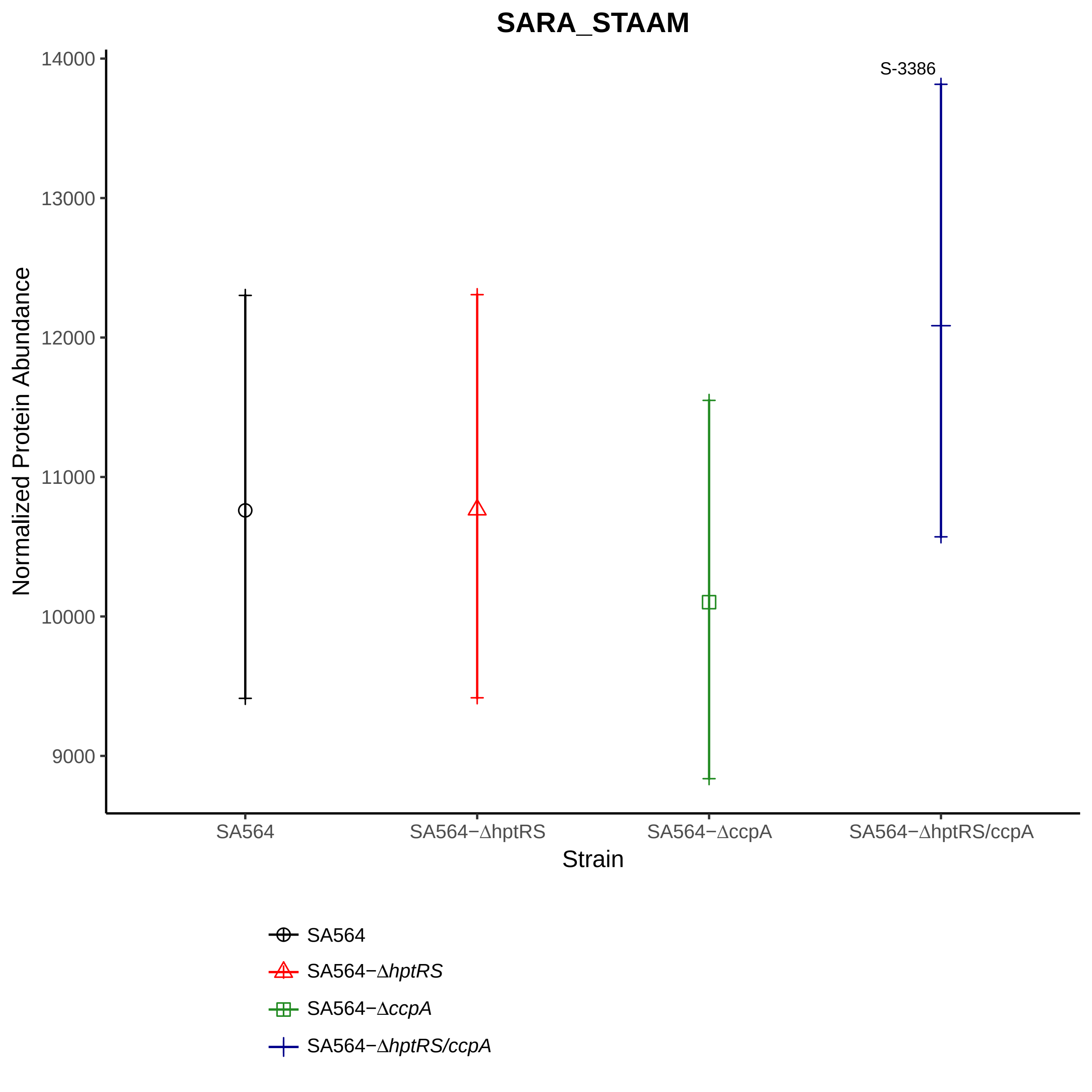
SARA_STAAM
S-3386
Normalized Protein Abundance
14000
13000
12000
11000
10000
9000
SA564
SA564−ΔhptRS
SA564−ΔccpA
SA564−ΔhptRS/ccpA
Strain
SA564
SA564−ΔhptRS
SA564−ΔccpA
SA564−ΔhptRS/ccpA

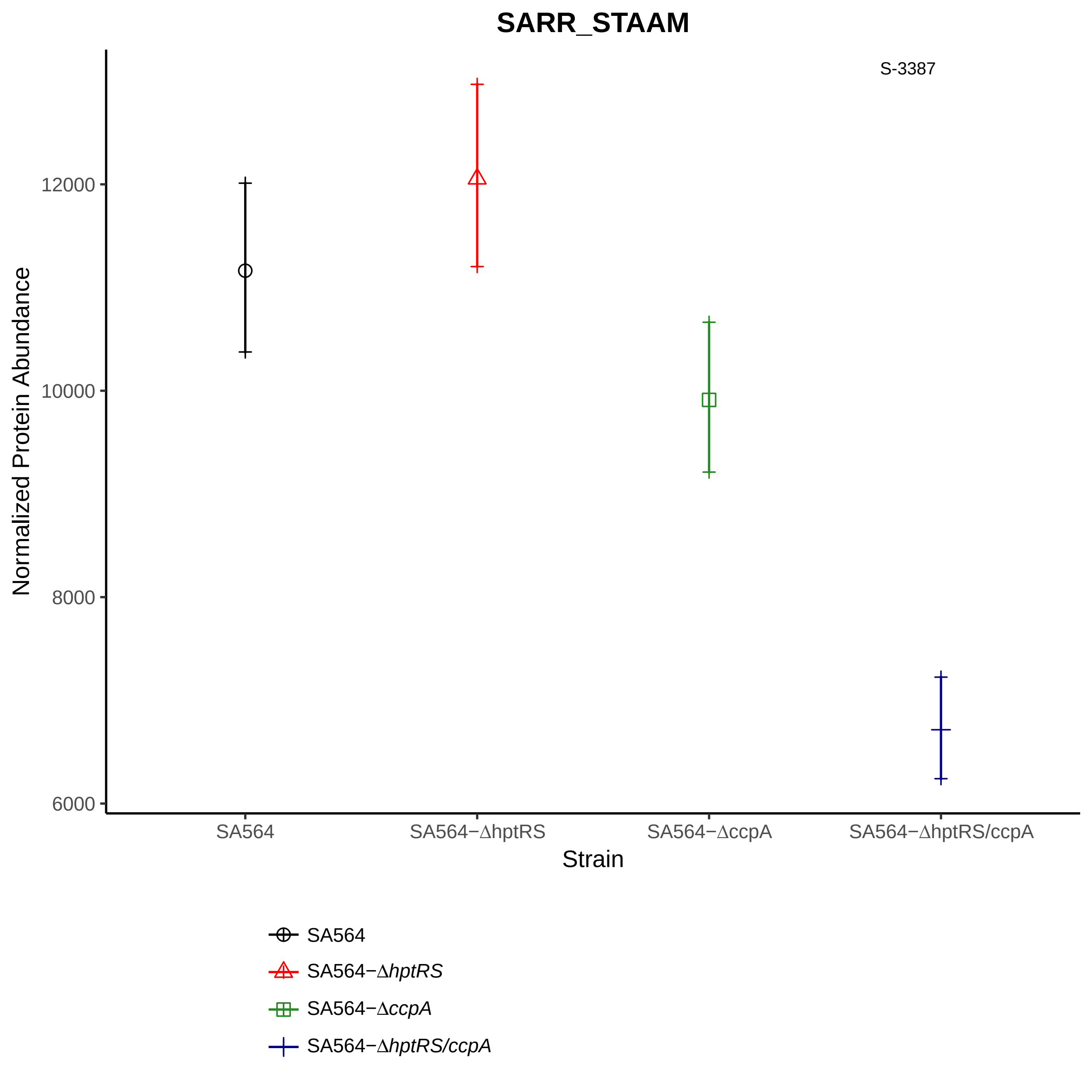

SARR_STAAM
S-3387
Normalized Protein Abundance
12000
10000
8000
6000
SA564
SA564−ΔhptRS
SA564−ΔccpA
SA564−ΔhptRS/ccpA
Strain
SA564
SA564−Δ*hptRS*
SA564−Δ*ccpA*
SA564−Δ*hptRS/ccpA*

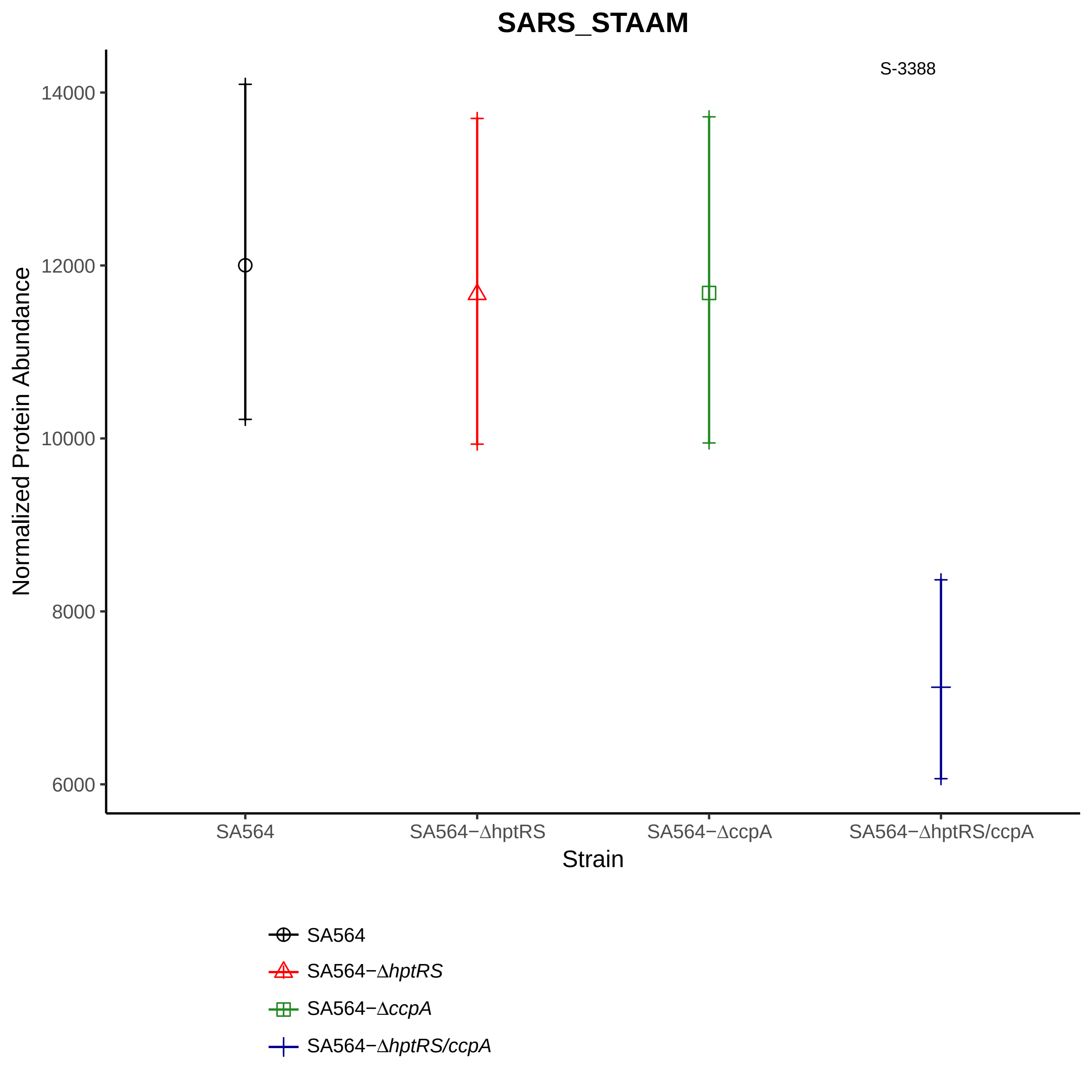
SARS_STAAM
S-3388
Normalized Protein Abundance
14000
12000
10000
8000
6000
SA564
SA564−ΔhptRS
SA564−ΔccpA
SA564−ΔhptRS/ccpA
Strain
SA564
SA564−ΔhptRS
SA564−ΔccpA
SA564−ΔhptRS/ccpA

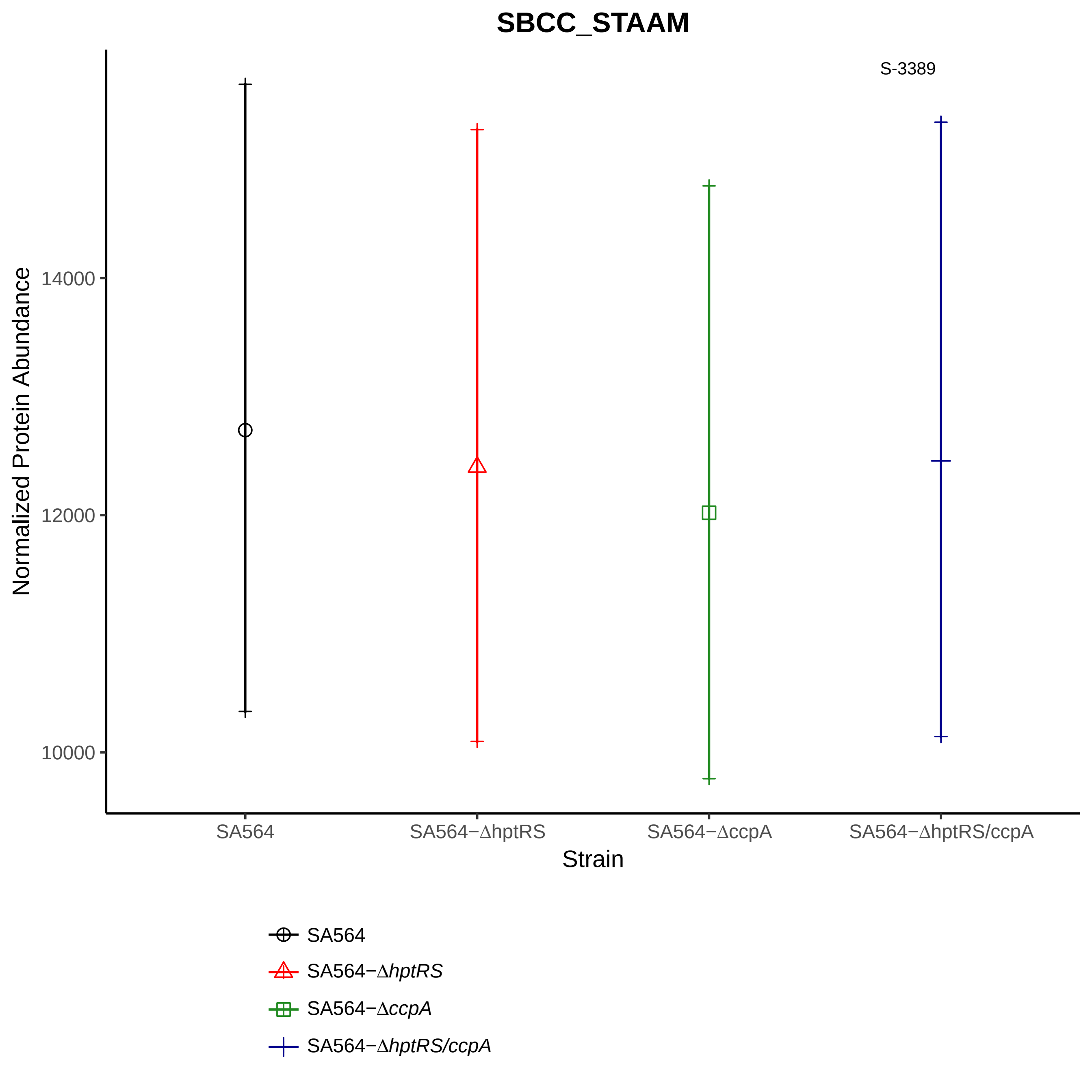

SBCC_STAAM
S-3389
Normalized Protein Abundance
14000
12000
10000
SA564
SA564−ΔhptRS
SA564−ΔccpA
SA564−ΔhptRS/ccpA
Strain
SA564
SA564−ΔhptRS
SA564−ΔccpA
SA564−ΔhptRS/ccpA

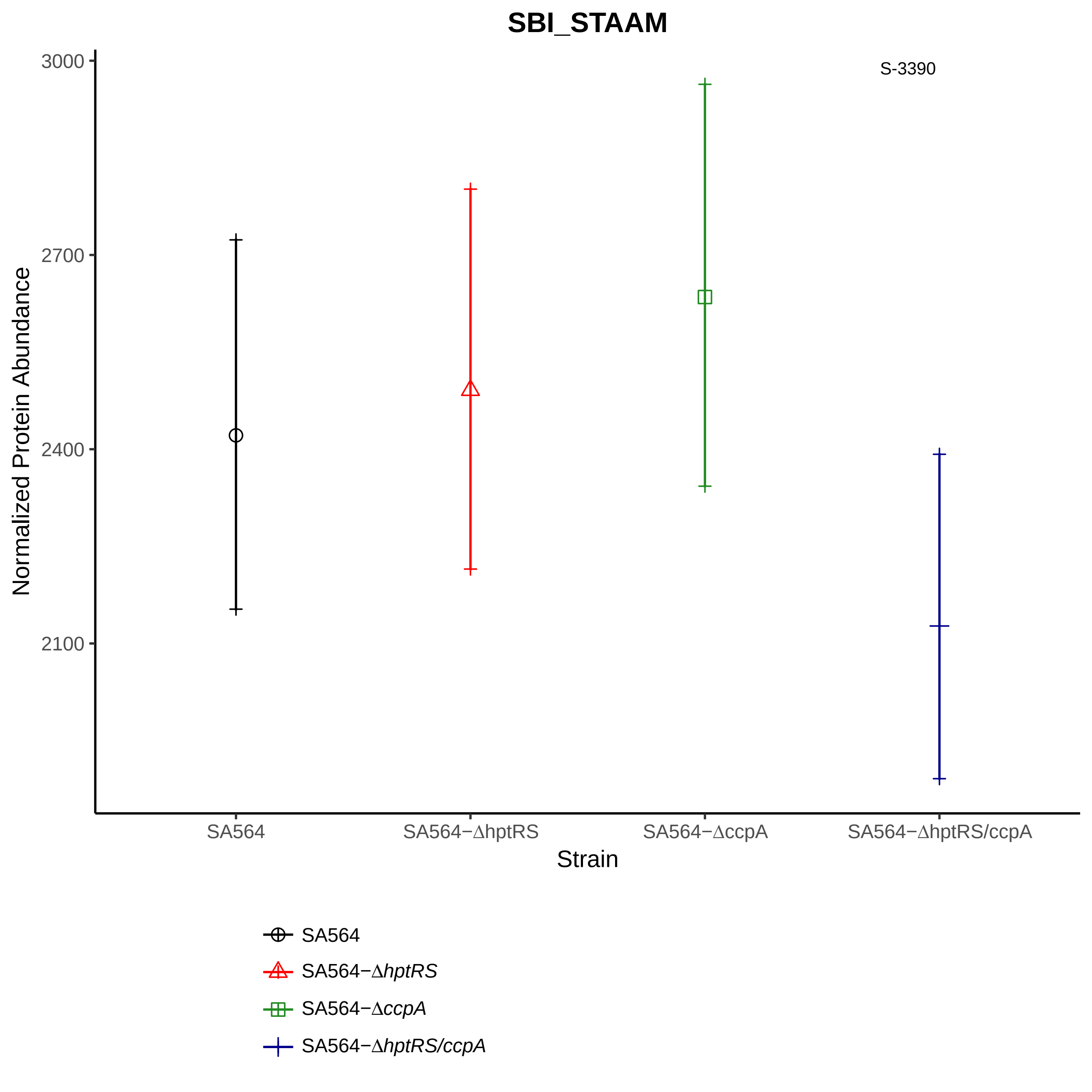
SBI_STAAM
S-3390
Normalized Protein Abundance
3000
2700
2400
2100
SA564
SA564−ΔhptRS
SA564−ΔccpA
SA564−ΔhptRS/ccpA
Strain
SA564
SA564−ΔhptRS
SA564−ΔccpA
SA564−ΔhptRS/ccpA

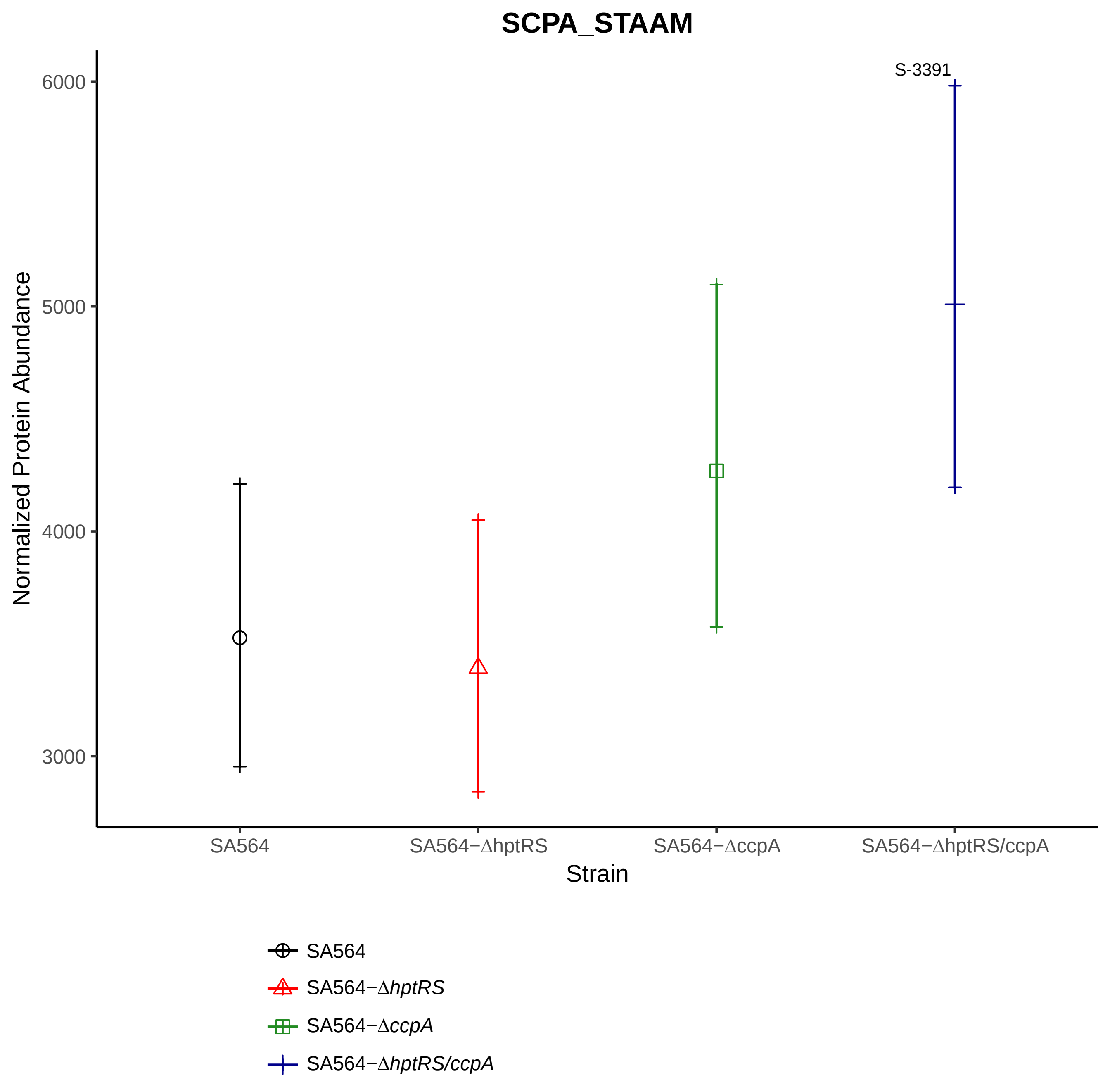
SCPA_STAAM
S-3391
6000
5000
4000
3000
Normalized Protein Abundance
SA564
SA564−ΔhptRS
SA564−ΔccpA
SA564−ΔhptRS/ccpA
Strain
SA564
SA564−ΔhptRS
SA564−ΔccpA
SA564−ΔhptRS/ccpA

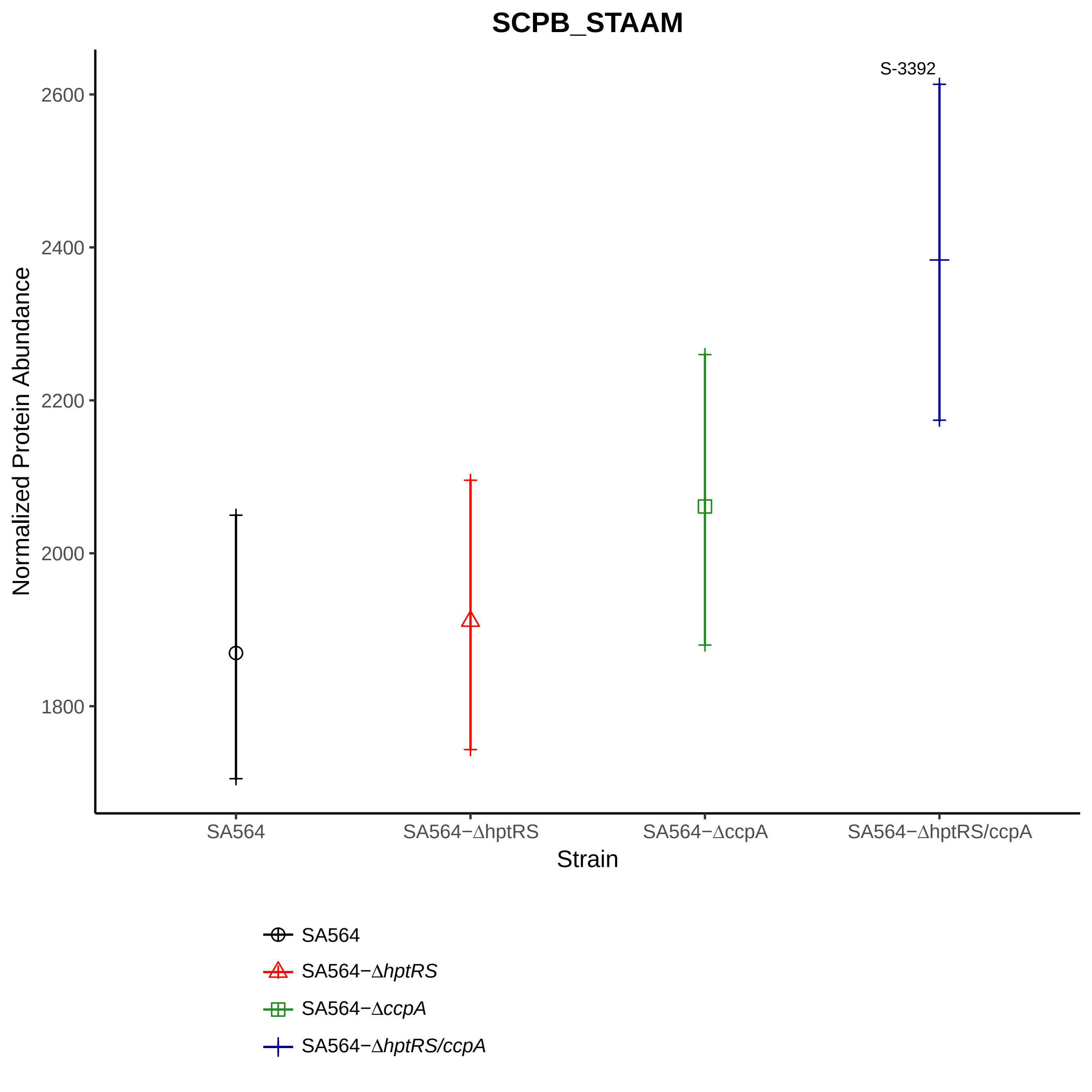

SCPB_STAAM
S-3392
2600
2400
2200
2000
1800
Normalized Protein Abundance
SA564
SA564−ΔhptRS
SA564−ΔccpA
SA564−ΔhptRS/ccpA
Strain
SA564
SA564−ΔhptRS
SA564−ΔccpA
SA564−ΔhptRS/ccpA

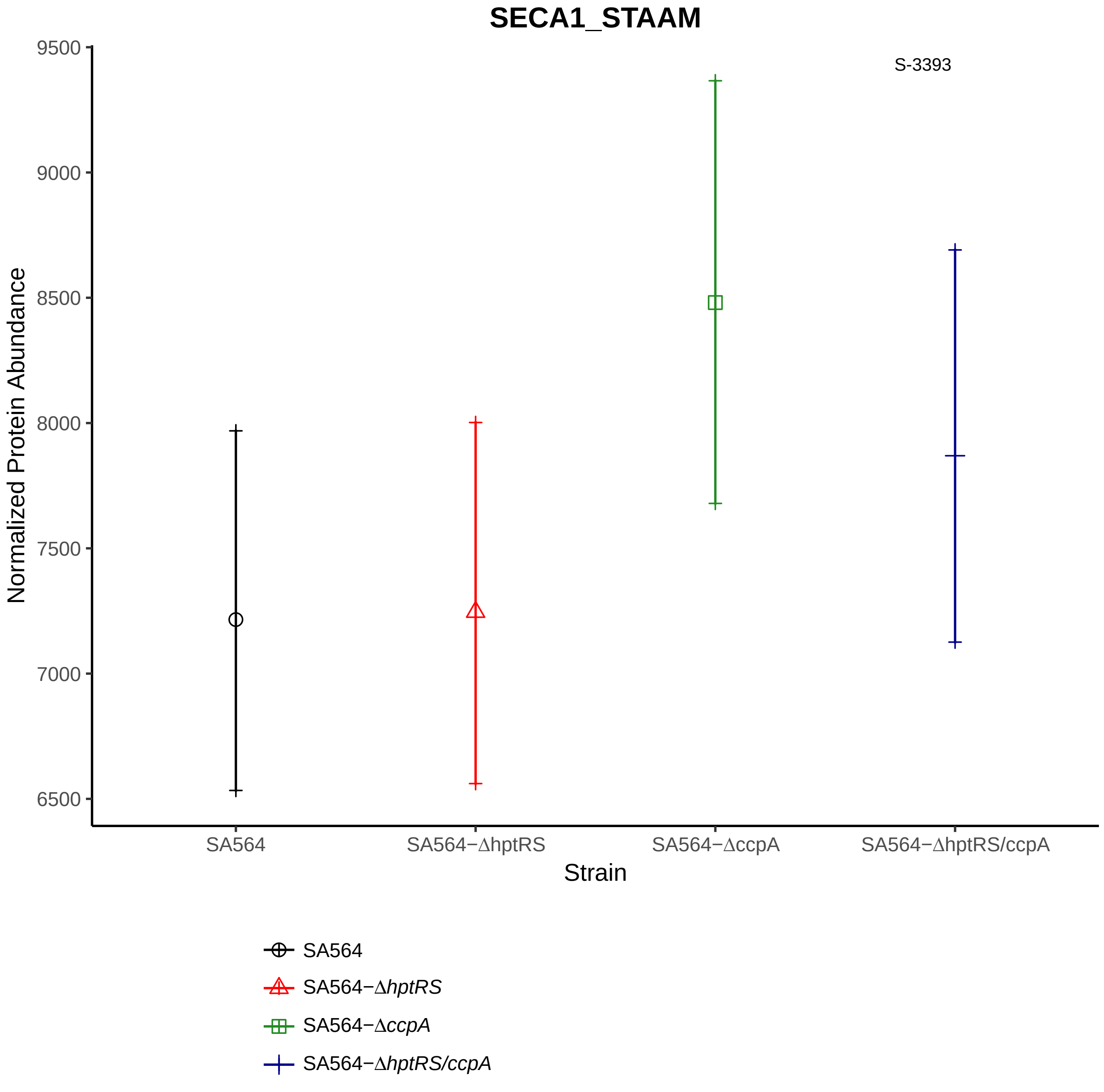

SECA1_STAAM
S-3393
Normalized Protein Abundance
9500
9000
8500
8000
7500
7000
6500
SA564
SA564−ΔhptRS
SA564−ΔccpA
SA564−ΔhptRS/ccpA
Strain
SA564
SA564−ΔhptRS
SA564−ΔccpA
SA564−ΔhptRS/ccpA

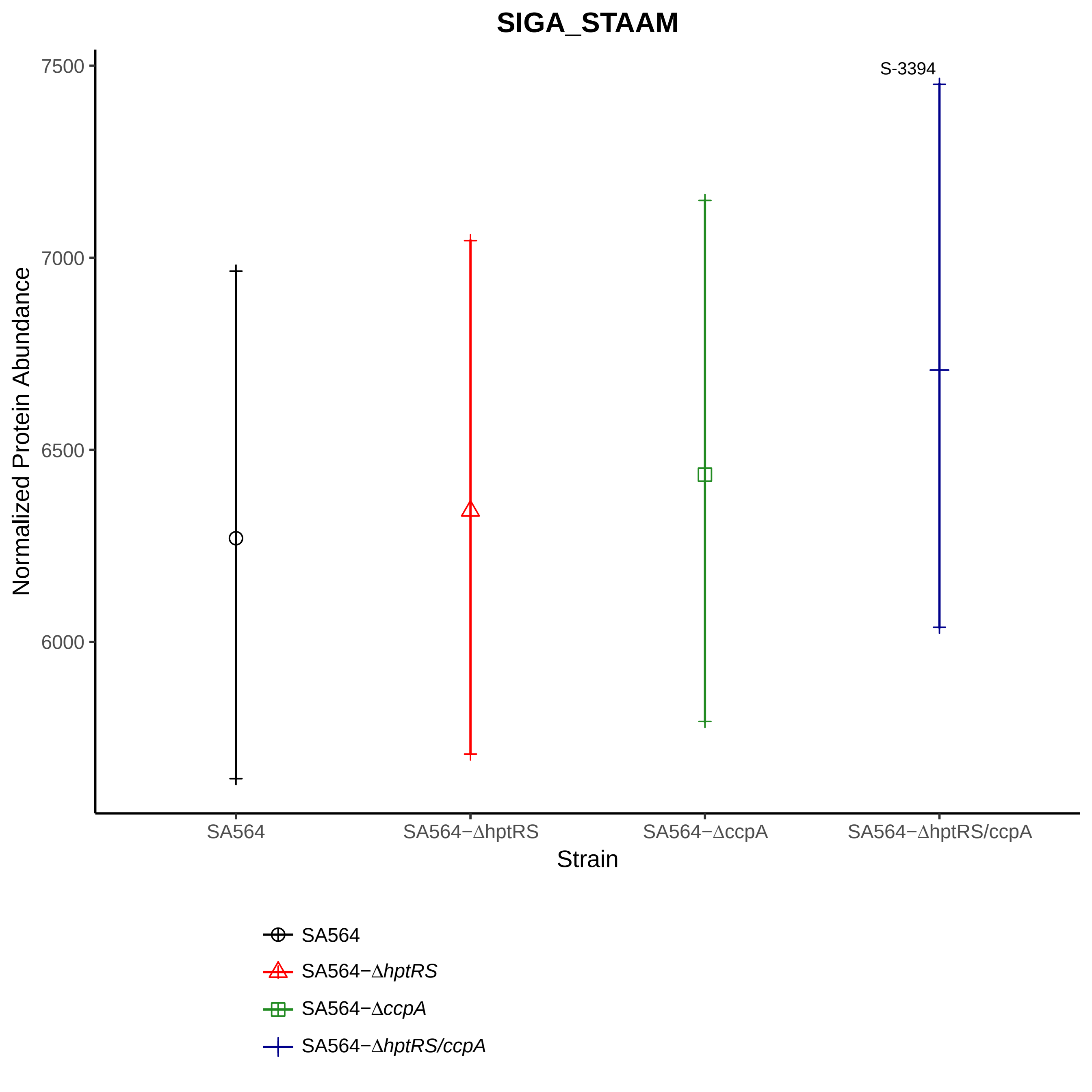
SIGA_STAAM
S-3394
Normalized Protein Abundance
7500
7000
6500
6000
SA564
SA564−ΔhptRS
SA564−ΔccpA
SA564−ΔhptRS/ccpA
Strain
SA564
SA564−ΔhptRS
SA564−ΔccpA
SA564−ΔhptRS/ccpA

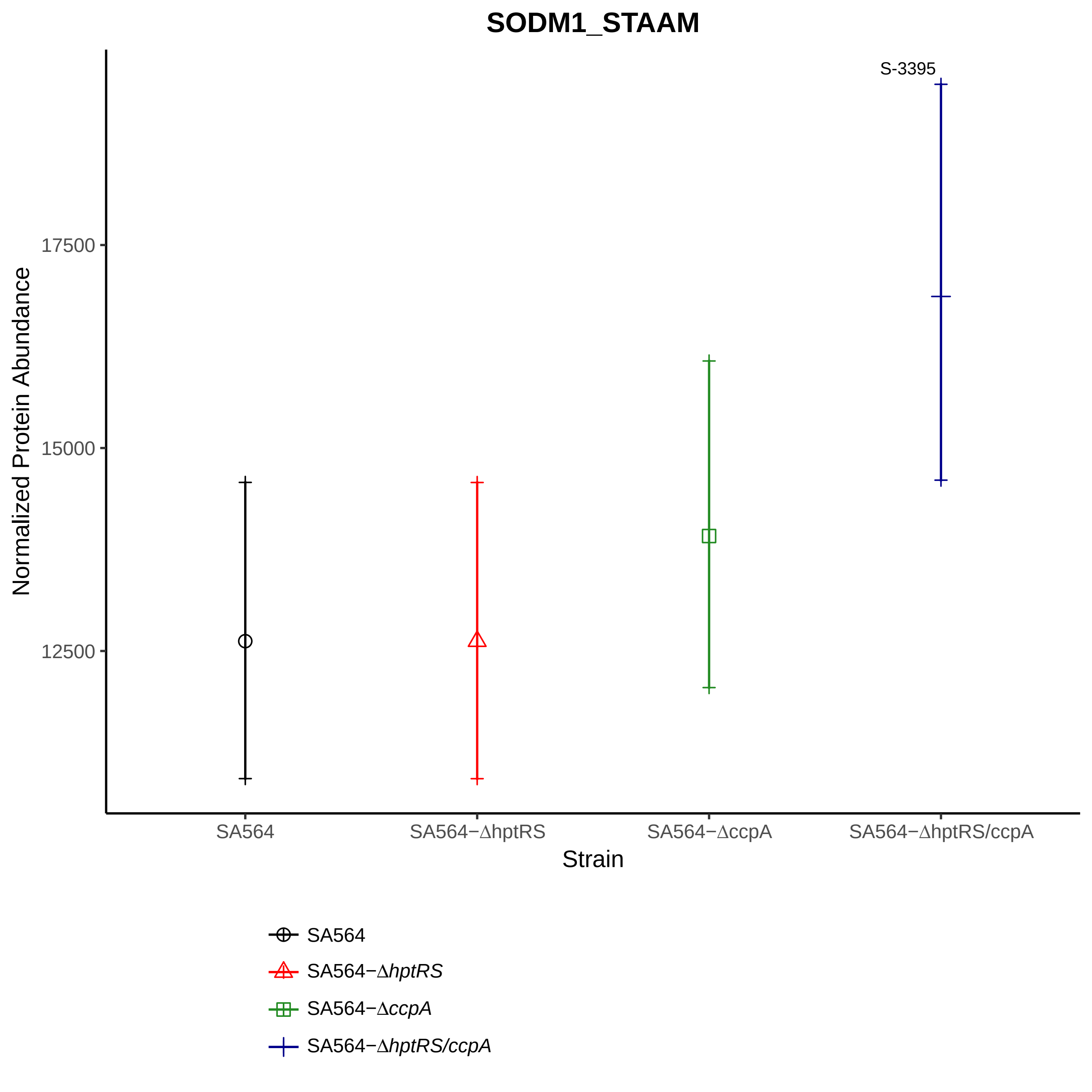

SODM1_STAAM
S-3395
17500
15000
12500
Normalized Protein Abundance
SA564
SA564−ΔhptRS
SA564−ΔccpA
SA564−ΔhptRS/ccpA
Strain
SA564
SA564−ΔhptRS
SA564−ΔccpA
SA564−ΔhptRS/ccpA

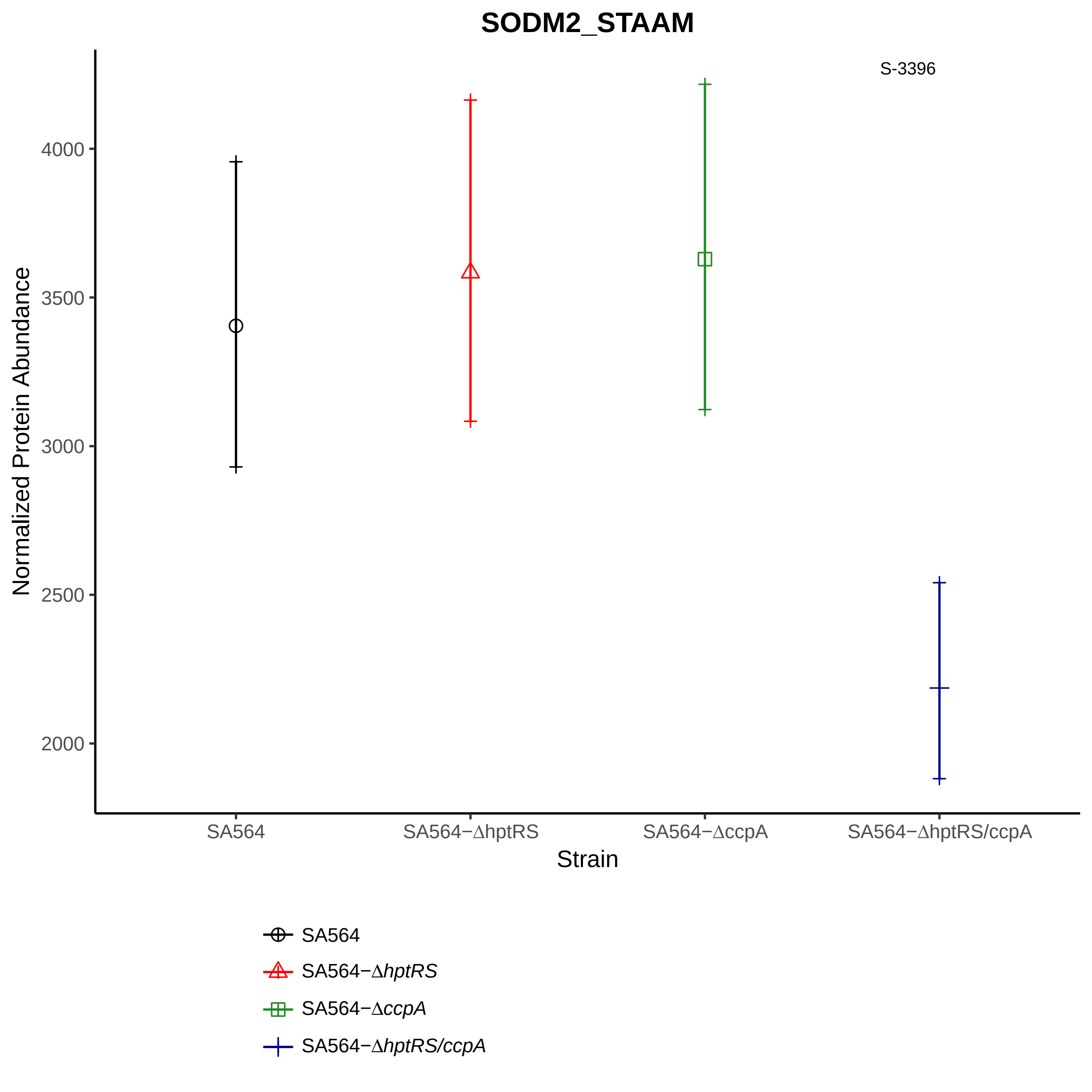

SODM2_STAAM
S-3396
Normalized Protein Abundance
4000
3500
3000
2500
2000
SA564
SA564−ΔhptRS
SA564−ΔccpA
SA564−ΔhptRS/ccpA
Strain
SA564
SA564−Δ*hptRS*
SA564−Δ*ccpA*
SA564−Δ*hptRS/ccpA*

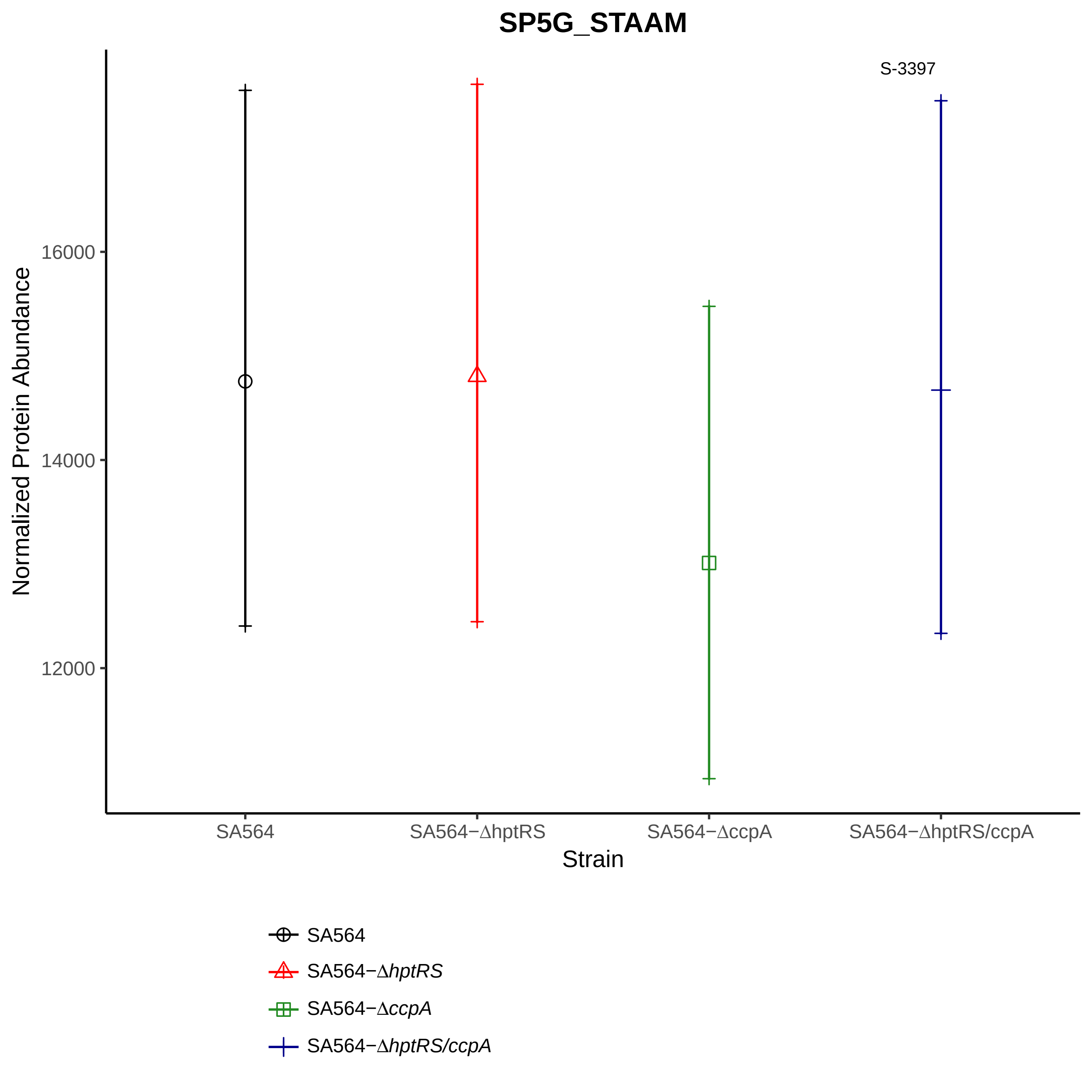

SP5G_STAAM
S-3397
Normalized Protein Abundance
16000
14000
12000
SA564
SA564−ΔhptRS
SA564−ΔccpA
SA564−ΔhptRS/ccpA
Strain
SA564
SA564−ΔhptRS
SA564−ΔccpA
SA564−ΔhptRS/ccpA

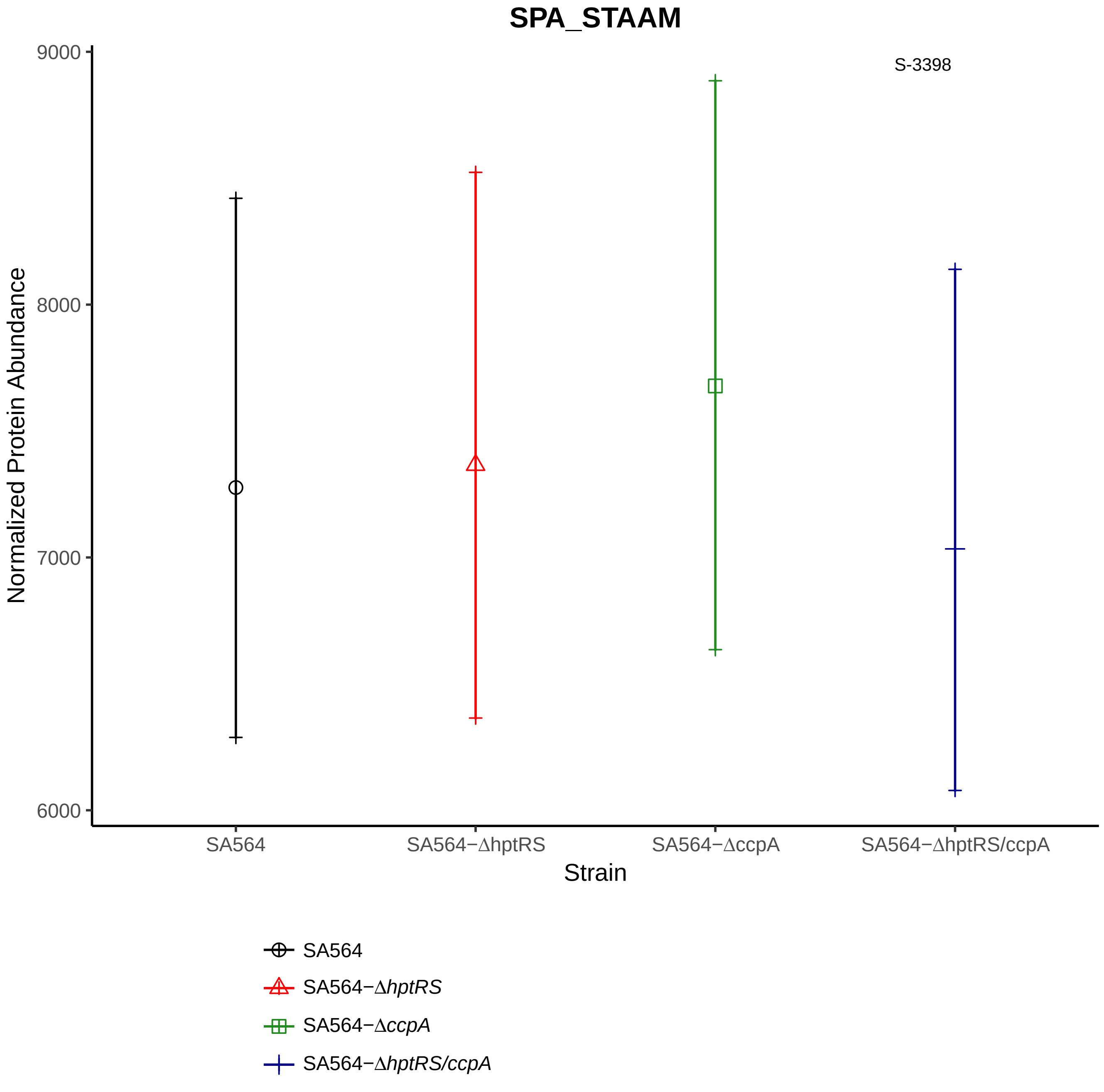
SPA_STAAM
S-3398
Normalized Protein Abundance
9000
8000
7000
6000
SA564
SA564−ΔhptRS
SA564−ΔccpA
SA564−ΔhptRS/ccpA
Strain
SA564
SA564−ΔhptRS
SA564−ΔccpA
SA564−ΔhptRS/ccpA

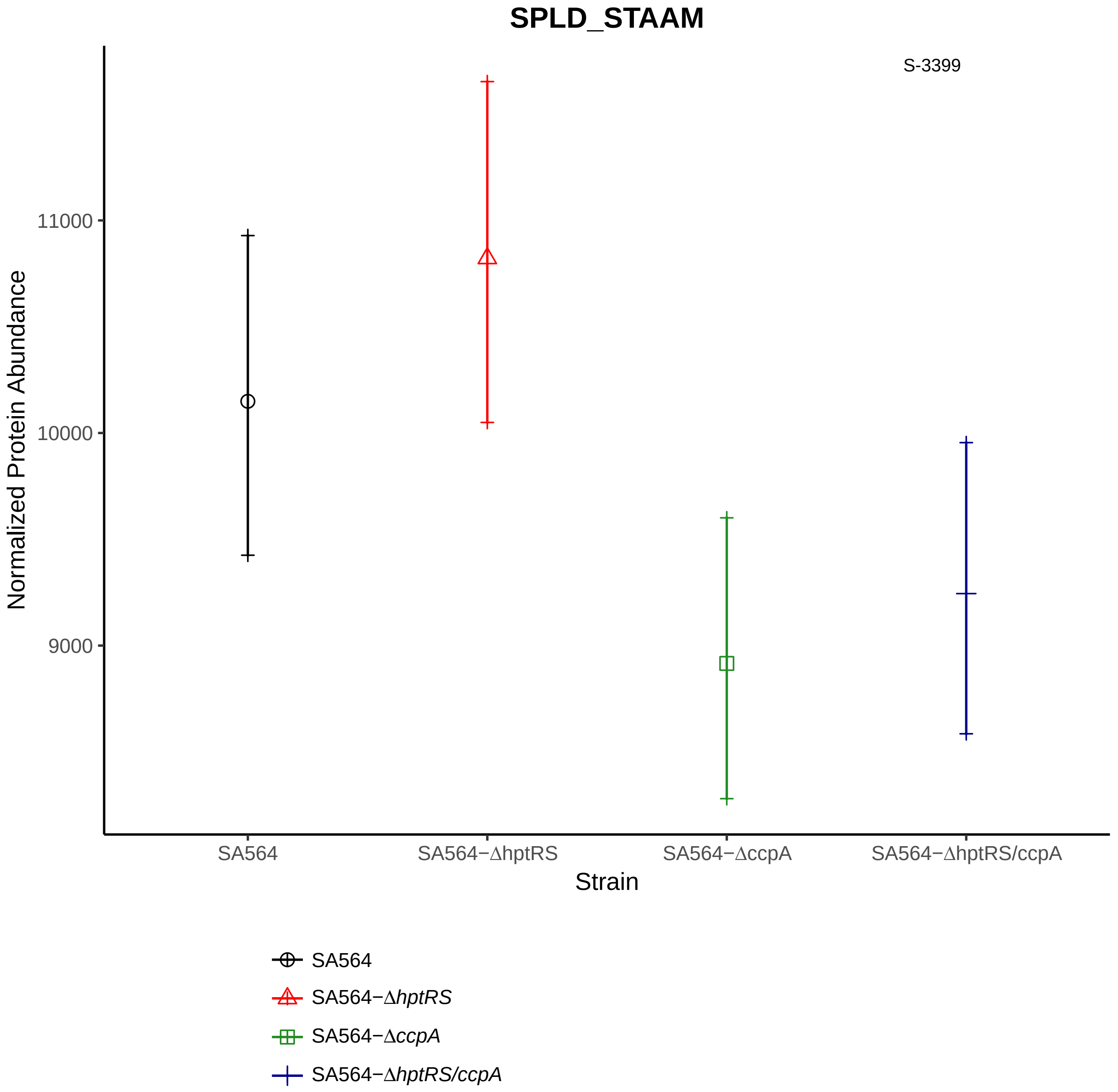

SPLD_STAAM
S-3399
Normalized Protein Abundance
11000
10000
9000
SA564
SA564−ΔhptRS
SA564−ΔccpA
SA564−ΔhptRS/ccpA
Strain
SA564
SA564−ΔhptRS
SA564−ΔccpA
SA564−ΔhptRS/ccpA

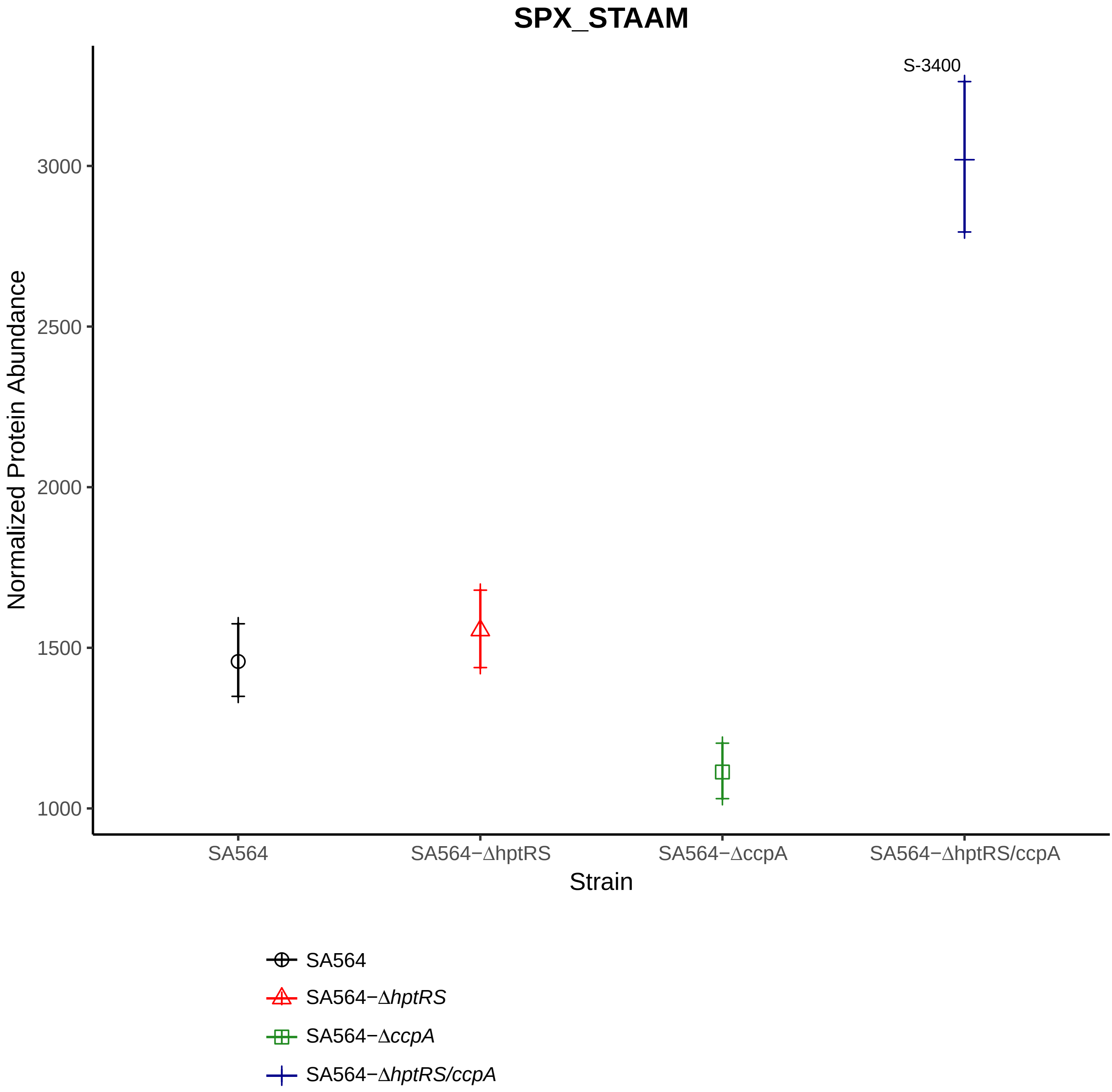
SPX_STAAM
S-3400
Normalized Protein Abundance
3000
2500
2000
1500
1000
SA564
SA564−ΔhptRS
SA564−ΔccpA
SA564−ΔhptRS/ccpA
Strain
SA564
SA564−ΔhptRS
SA564−ΔccpA
SA564−ΔhptRS/ccpA

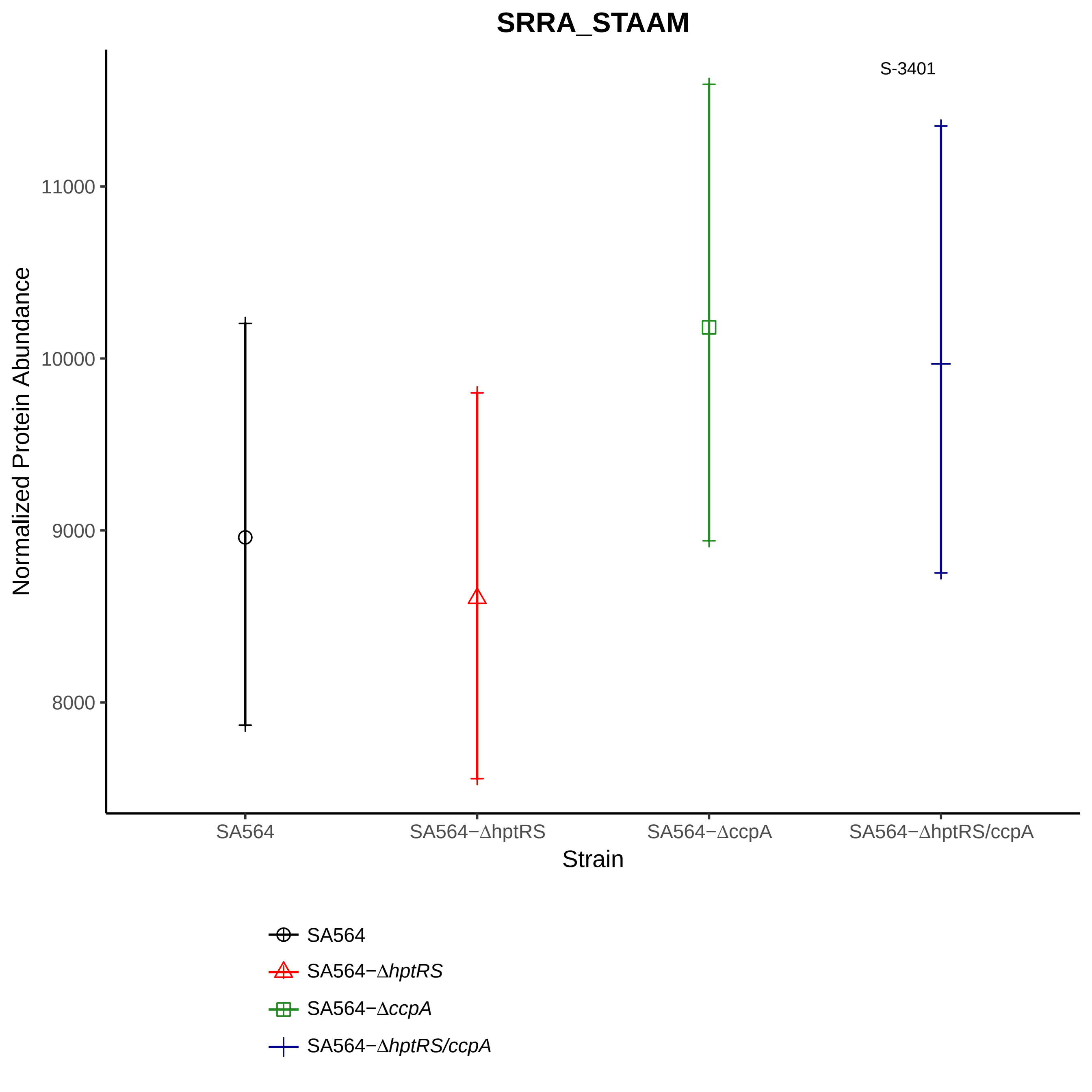

SRRA_STAAM
S-3401
Normalized Protein Abundance
11000
10000
9000
8000
SA564
SA564−ΔhptRS
SA564−ΔccpA
SA564−ΔhptRS/ccpA
Strain
SA564
SA564−ΔhptRS
SA564−ΔccpA
SA564−ΔhptRS/ccpA

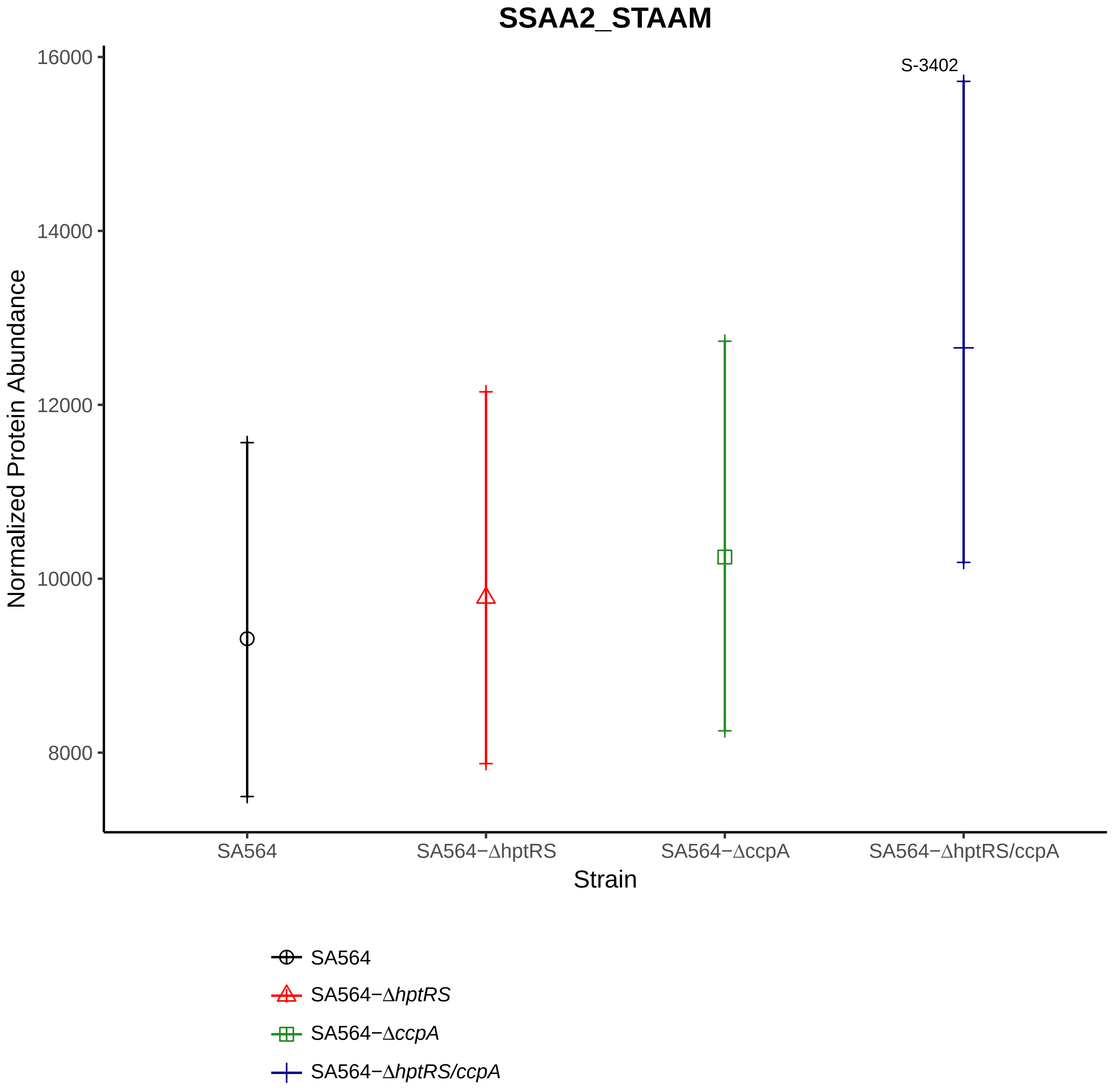
SSAA2_STAAM
S-3402
Normalized Protein Abundance
16000
14000
12000
10000
8000
SA564
SA564−ΔhptRS
SA564−ΔccpA
SA564−ΔhptRS/ccpA
Strain
SA564
SA564−ΔhptRS
SA564−ΔccpA
SA564−ΔhptRS/ccpA

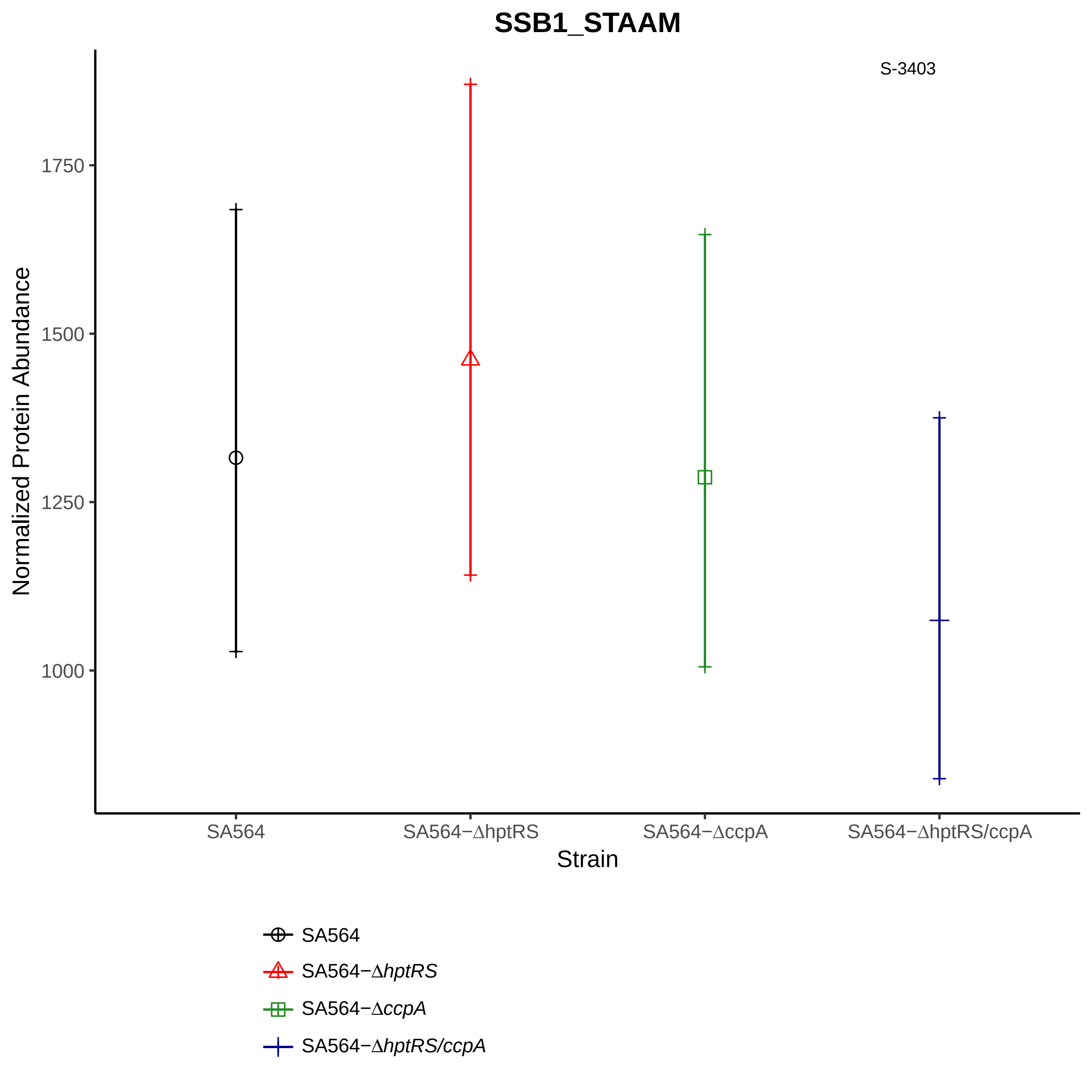

SSB1_STAAM
S-3403
Normalized Protein Abundance
1750
1500
1250
1000
SA564
SA564−ΔhptRS
SA564−ΔccpA
SA564−ΔhptRS/ccpA
Strain
SA564
SA564−ΔhptRS
SA564−ΔccpA
SA564−ΔhptRS/ccpA

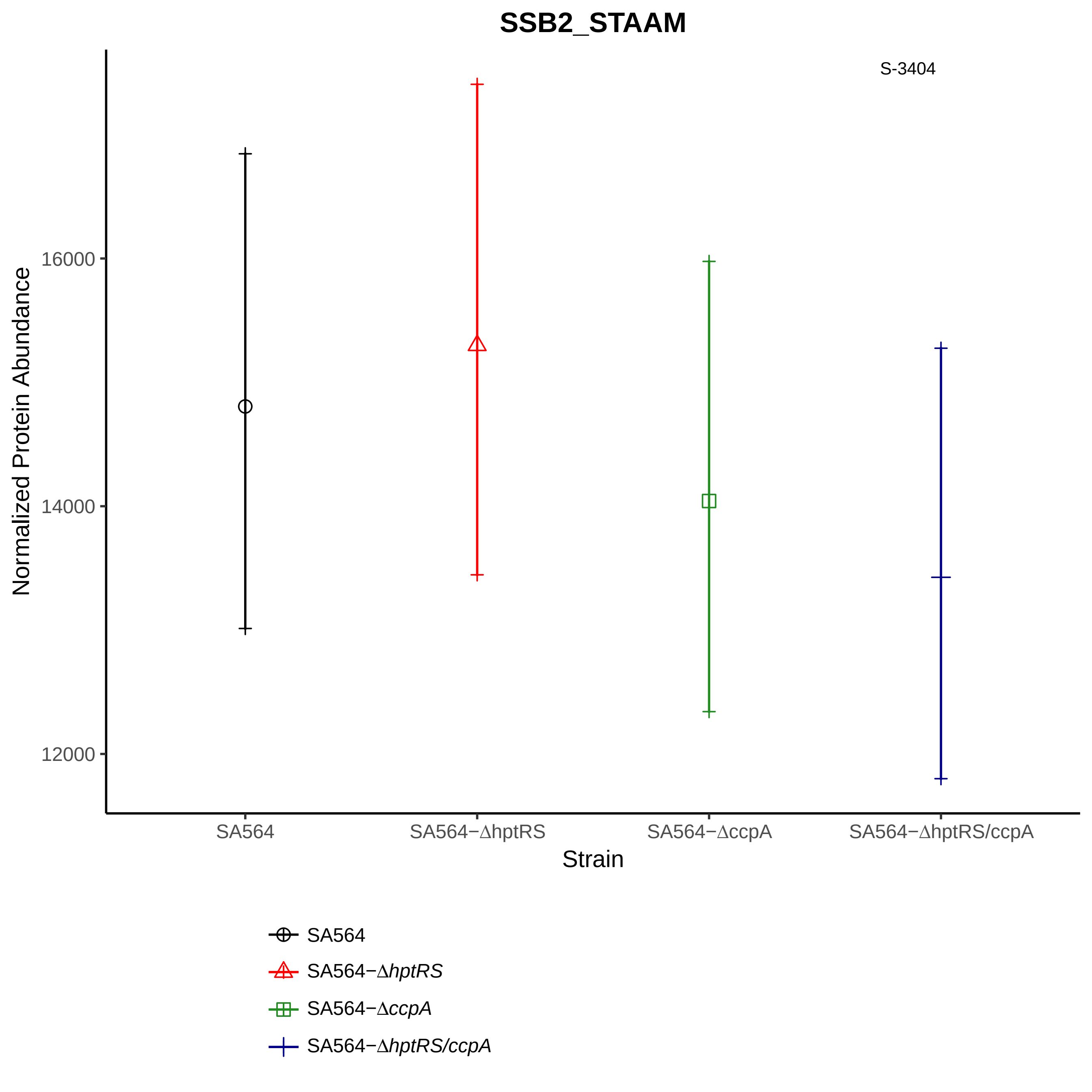
SSB2_STAAM
S-3404
Normalized Protein Abundance
16000
14000
12000
SA564
SA564−ΔhptRS
SA564−ΔccpA
SA564−ΔhptRS/ccpA
Strain
SA564
SA564−ΔhptRS
SA564−ΔccpA
SA564−ΔhptRS/ccpA

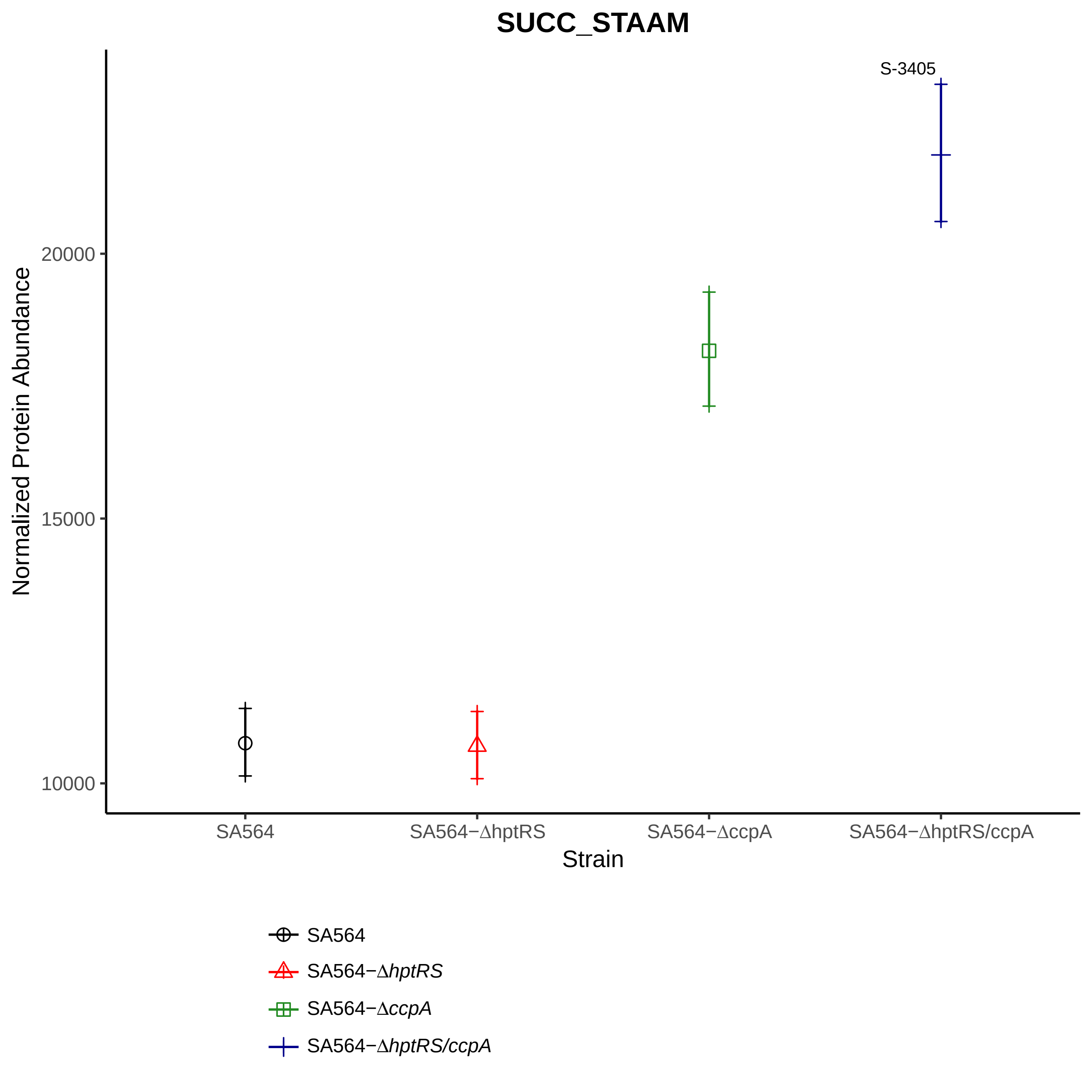

SUCC_STAAM
S-3405
20000
15000
10000
Normalized Protein Abundance
SA564
SA564−ΔhptRS
SA564−ΔccpA
SA564−ΔhptRS/ccpA
Strain
SA564
SA564−ΔhptRS
SA564−ΔccpA
SA564−ΔhptRS/ccpA

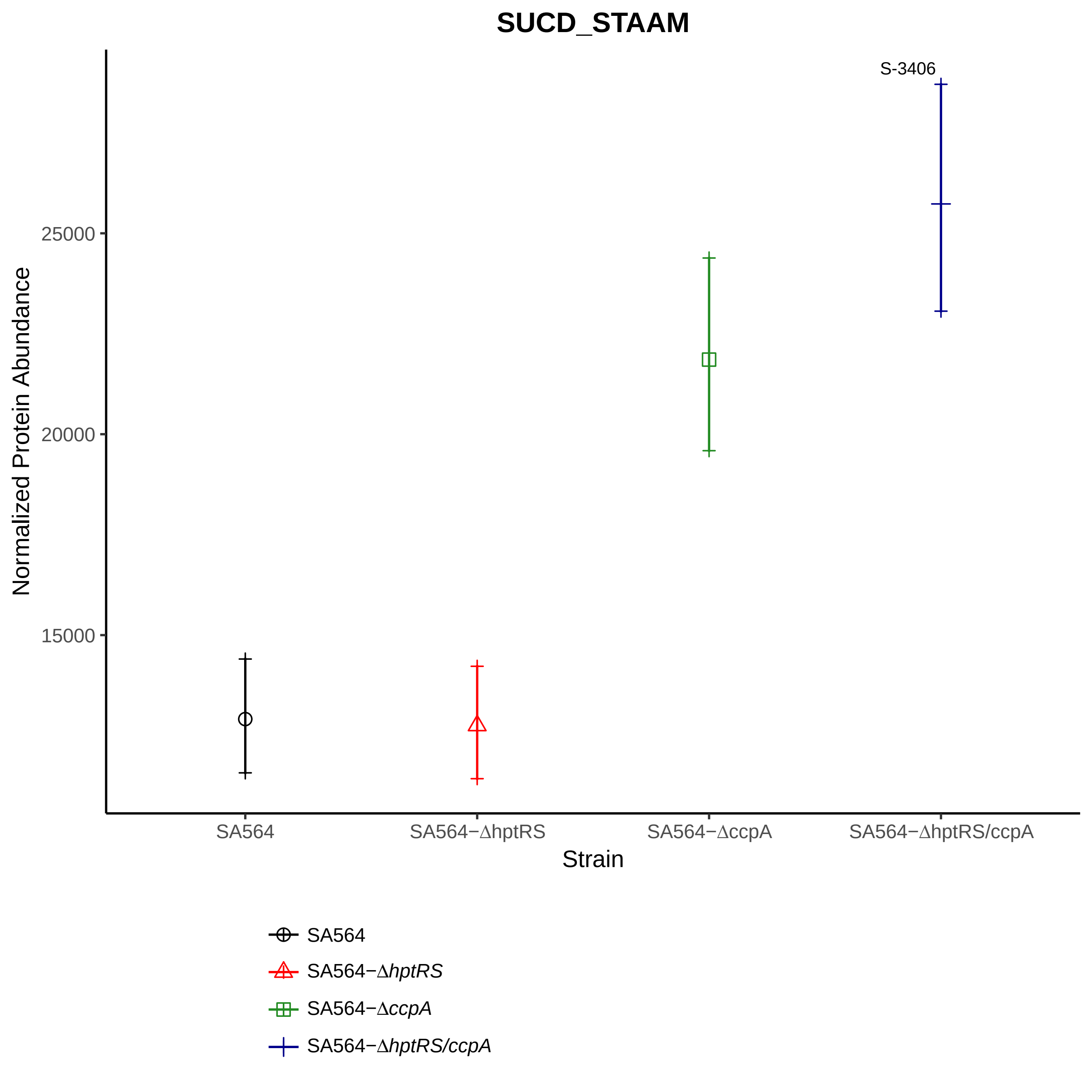

SUCD_STAAM
S-3406
Normalized Protein Abundance
25000
20000
15000
SA564
SA564−ΔhptRS
SA564−ΔccpA
SA564−ΔhptRS/ccpA
Strain
SA564
SA564−ΔhptRS
SA564−ΔccpA
SA564−ΔhptRS/ccpA

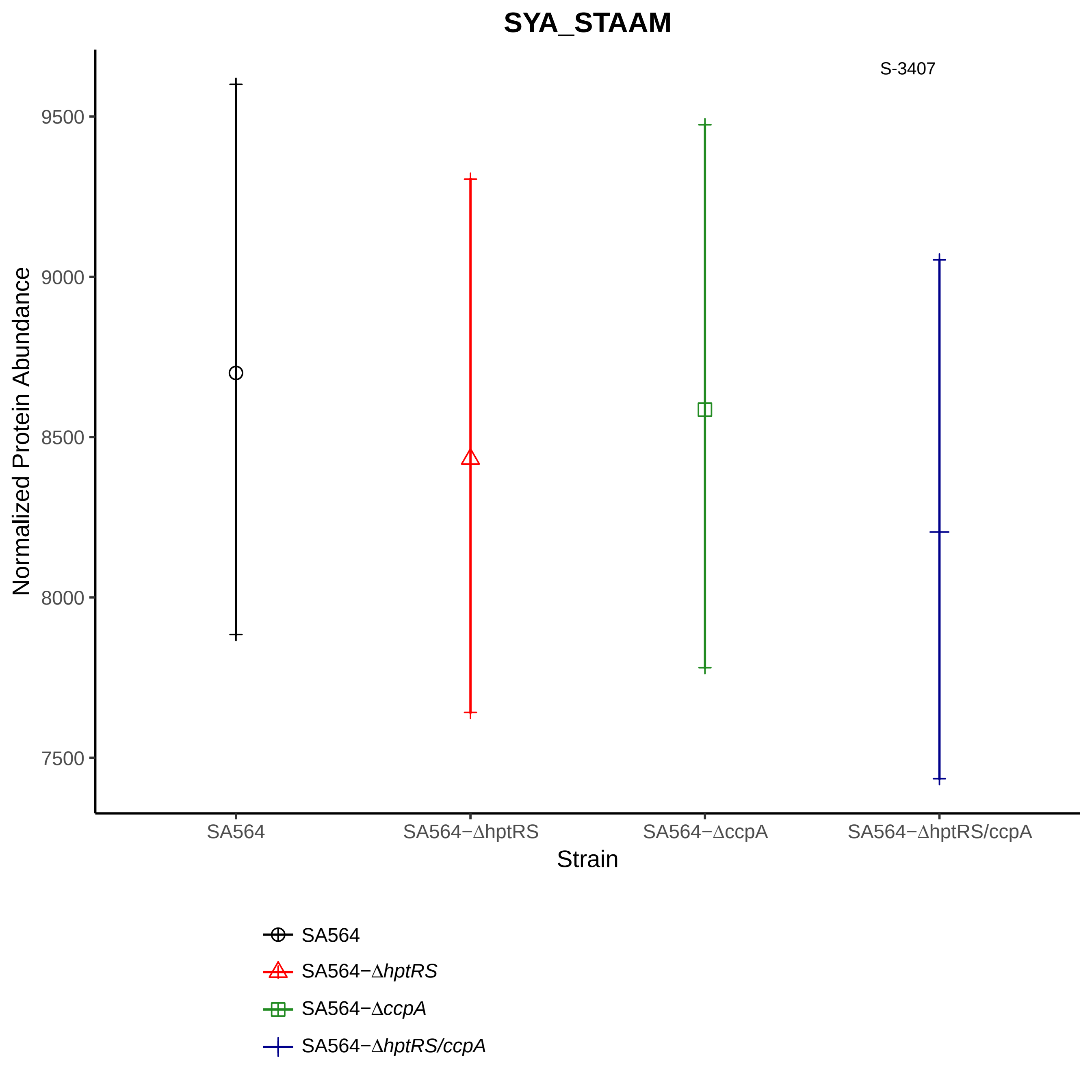

SYA_STAAM
S-3407
9500
9000
8500
8000
7500
Normalized Protein Abundance
SA564
SA564−ΔhptRS
SA564−ΔccpA
SA564−ΔhptRS/ccpA
Strain
SA564
SA564−ΔhptRS
SA564−ΔccpA
SA564−ΔhptRS/ccpA

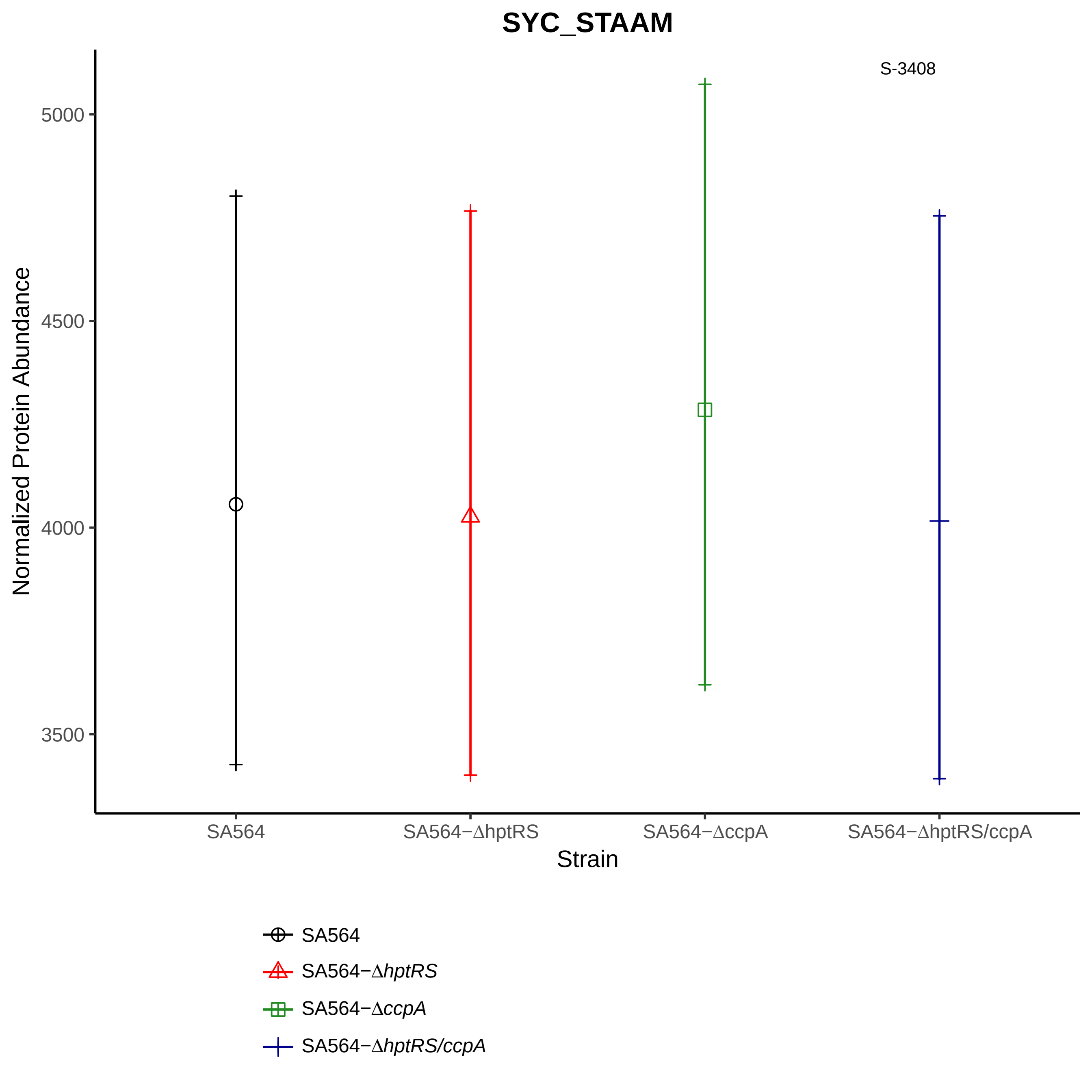

SYC_STAAM
S-3408
Normalized Protein Abundance
5000
4500
4000
3500
SA564
SA564−ΔhptRS
SA564−ΔccpA
SA564−ΔhptRS/ccpA
Strain
SA564
SA564−ΔhptRS
SA564−ΔccpA
SA564−ΔhptRS/ccpA

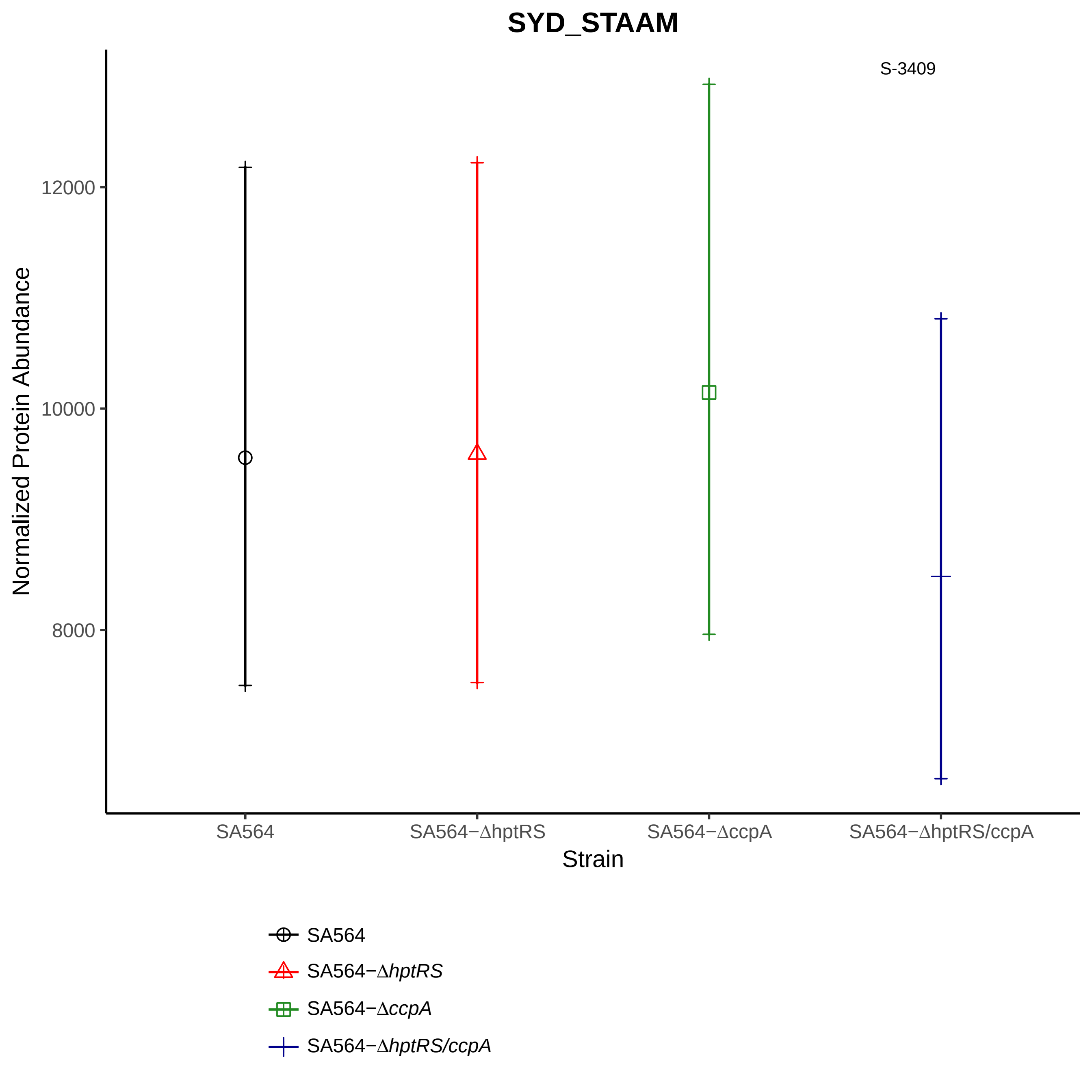

SYD_STAAM
S-3409
Normalized Protein Abundance
12000
10000
8000
SA564
SA564−ΔhptRS
SA564−ΔccpA
SA564−ΔhptRS/ccpA
Strain
SA564
SA564−ΔhptRS
SA564−ΔccpA
SA564−ΔhptRS/ccpA

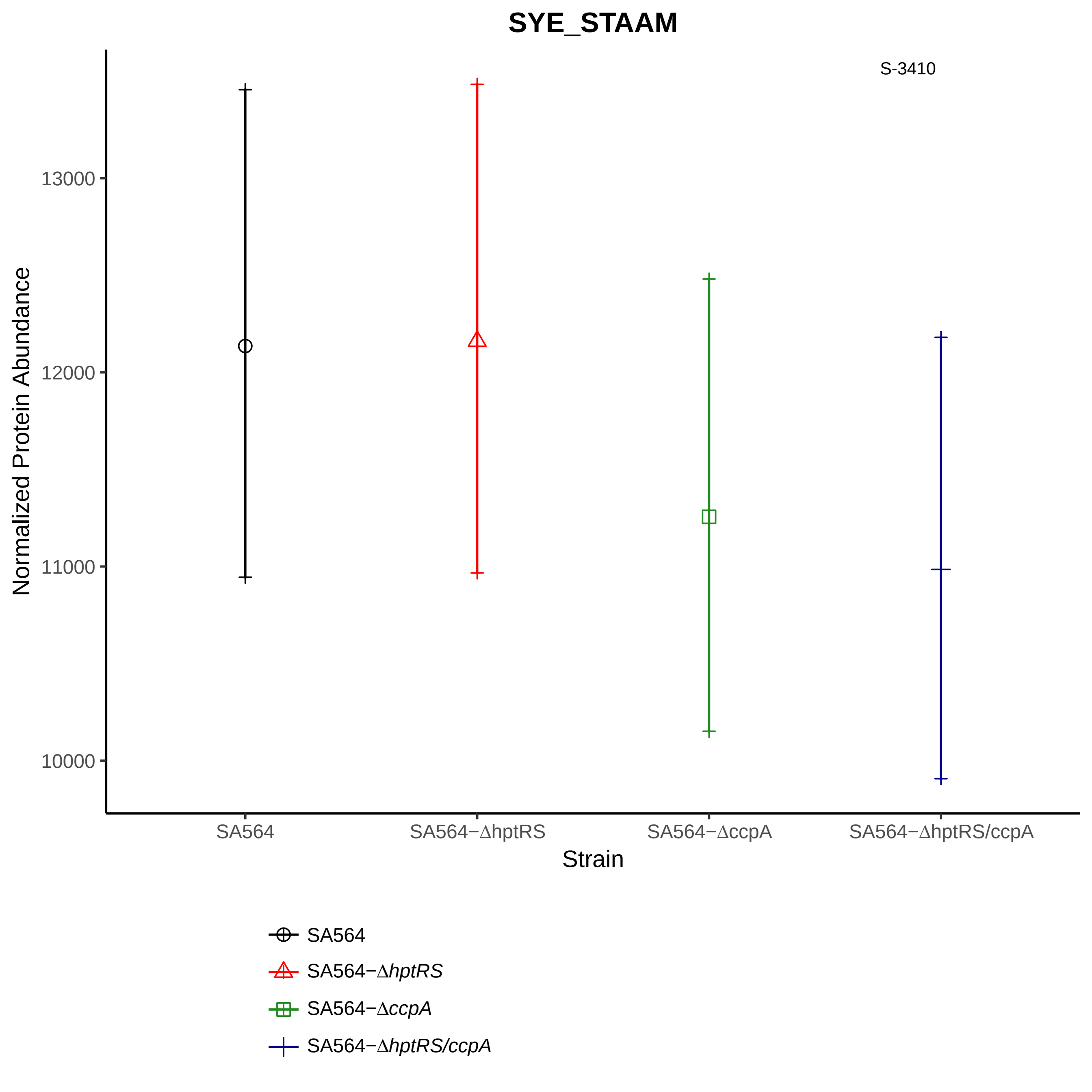
SYE_STAAM
S-3410
Normalized Protein Abundance
13000
12000
11000
10000
SA564
SA564−ΔhptRS
SA564−ΔccpA
SA564−ΔhptRS/ccpA
Strain
SA564
SA564−ΔhptRS
SA564−ΔccpA
SA564−ΔhptRS/ccpA

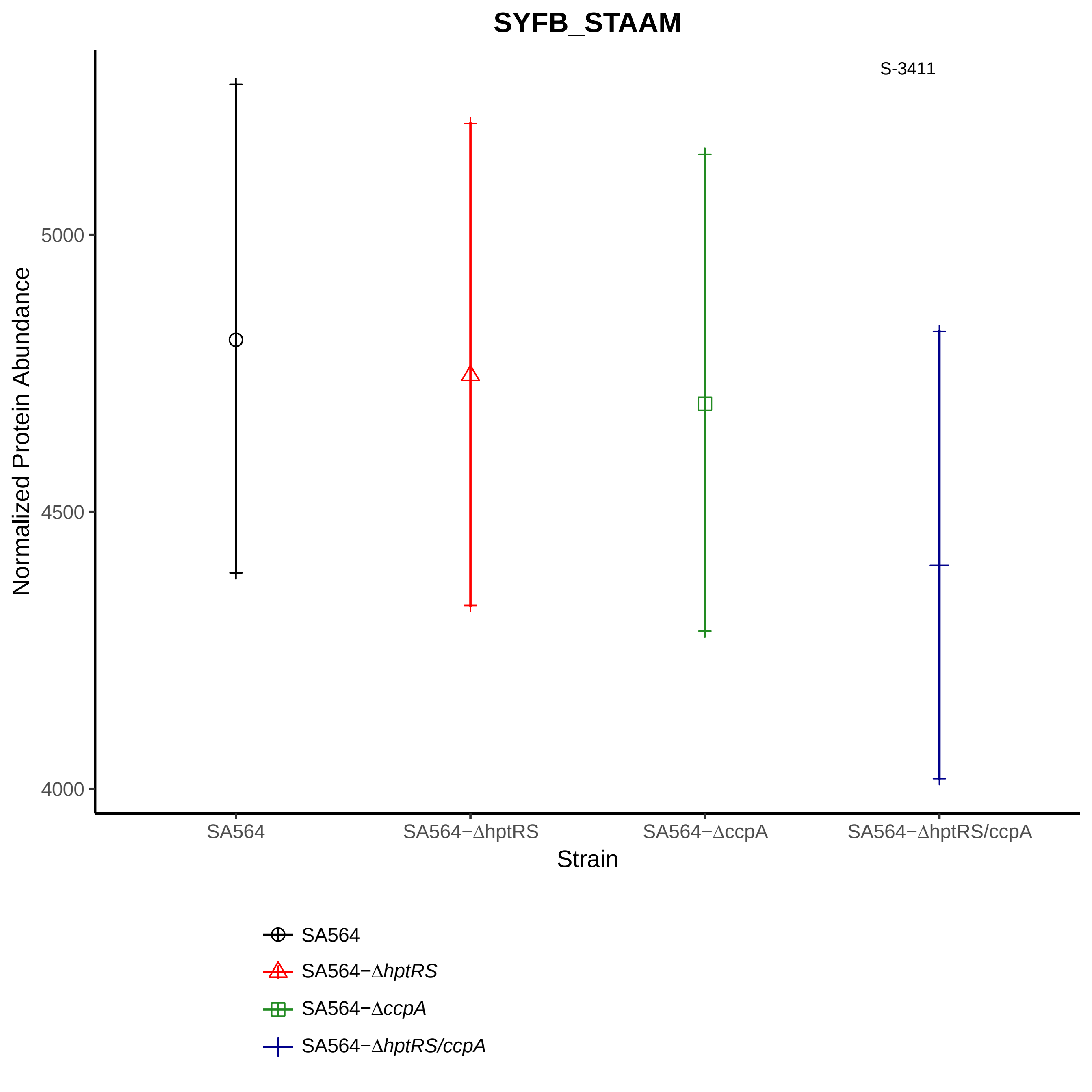

SYFB_STAAM
S-3411
Normalized Protein Abundance
5000
4500
4000
SA564
SA564−ΔhptRS
SA564−ΔccpA
SA564−ΔhptRS/ccpA
Strain
SA564
SA564−ΔhptRS
SA564−ΔccpA
SA564−ΔhptRS/ccpA

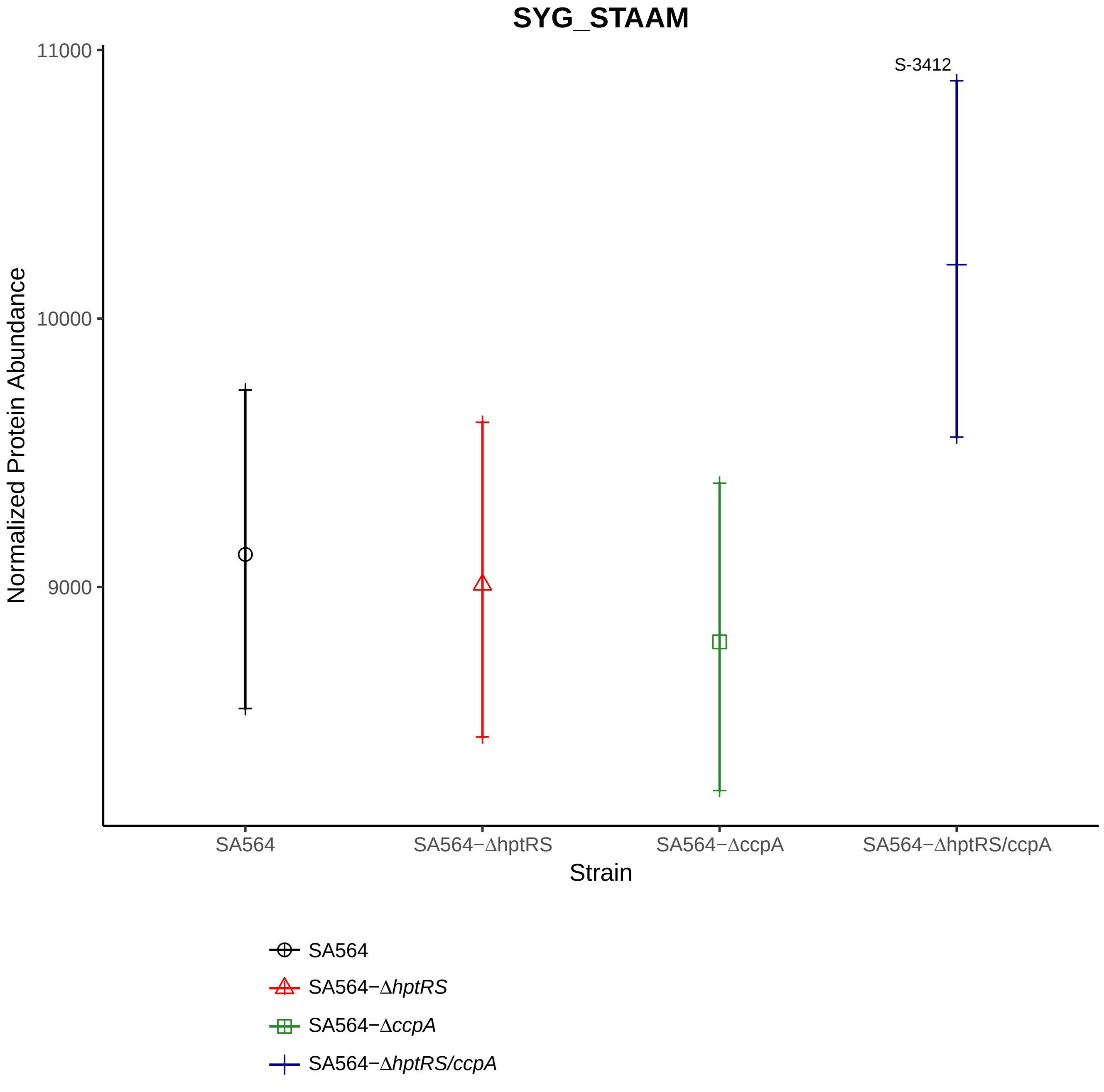

SYG_STAAM
S-3412
11000
10000
9000
Normalized Protein Abundance
SA564
SA564−ΔhptRS
SA564−ΔccpA
SA564−ΔhptRS/ccpA
Strain
SA564
SA564−ΔhptRS
SA564−ΔccpA
SA564−ΔhptRS/ccpA

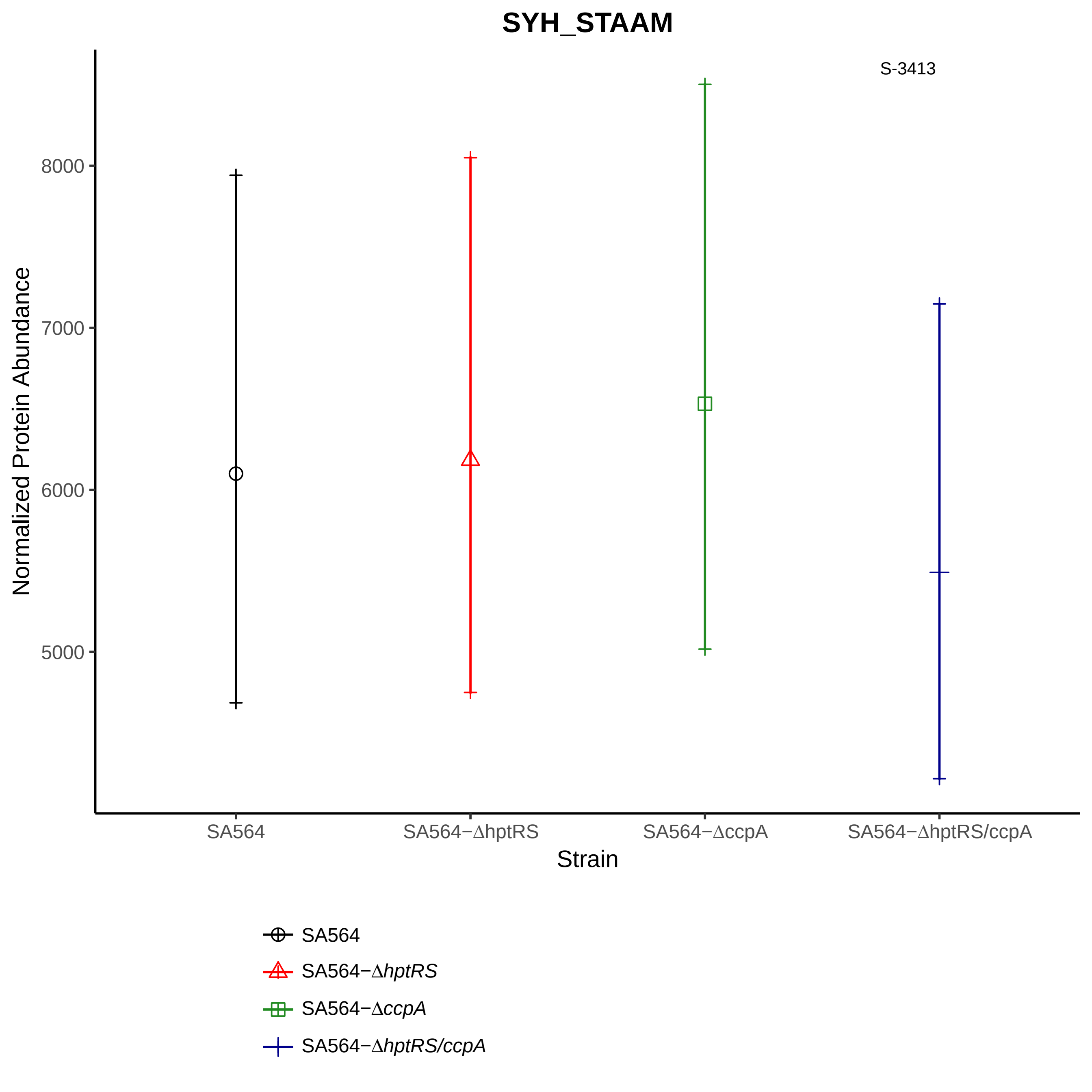
SYH_STAAM
S-3413
Normalized Protein Abundance
8000
7000
6000
5000
SA564
SA564−ΔhptRS
SA564−ΔccpA
SA564−ΔhptRS/ccpA
Strain
SA564
SA564−ΔhptRS
SA564−ΔccpA
SA564−ΔhptRS/ccpA

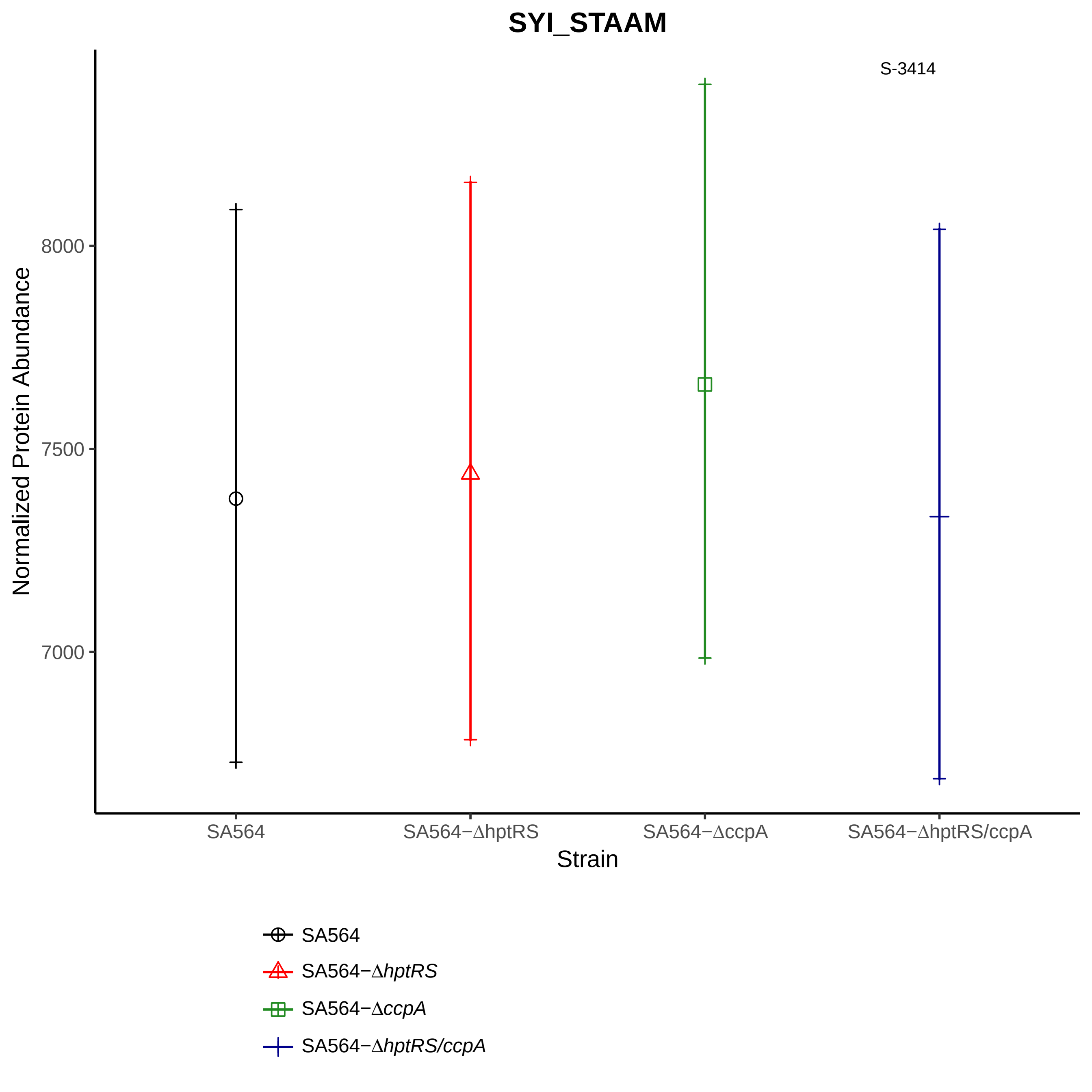

SYI_STAAM
S-3414
8000
7500
7000
Normalized Protein Abundance
SA564
SA564−ΔhptRS
SA564−ΔccpA
SA564−ΔhptRS/ccpA
Strain
SA564
SA564−ΔhptRS
SA564−ΔccpA
SA564−ΔhptRS/ccpA

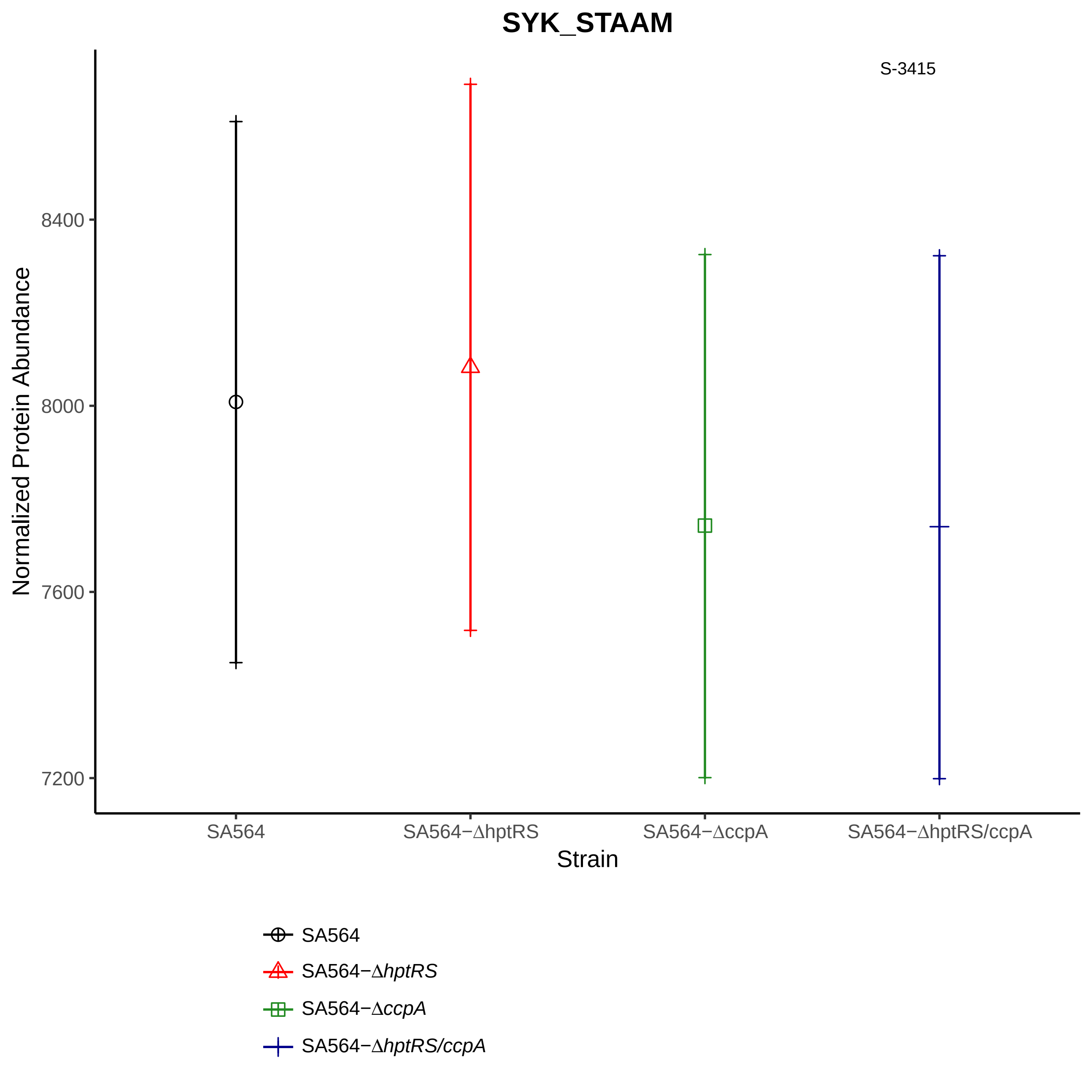

SYK_STAAM
S-3415
Normalized Protein Abundance
8400
8000
7600
7200
SA564
SA564−ΔhptRS
SA564−ΔccpA
SA564−ΔhptRS/ccpA
Strain
SA564
SA564−ΔhptRS
SA564−ΔccpA
SA564−ΔhptRS/ccpA

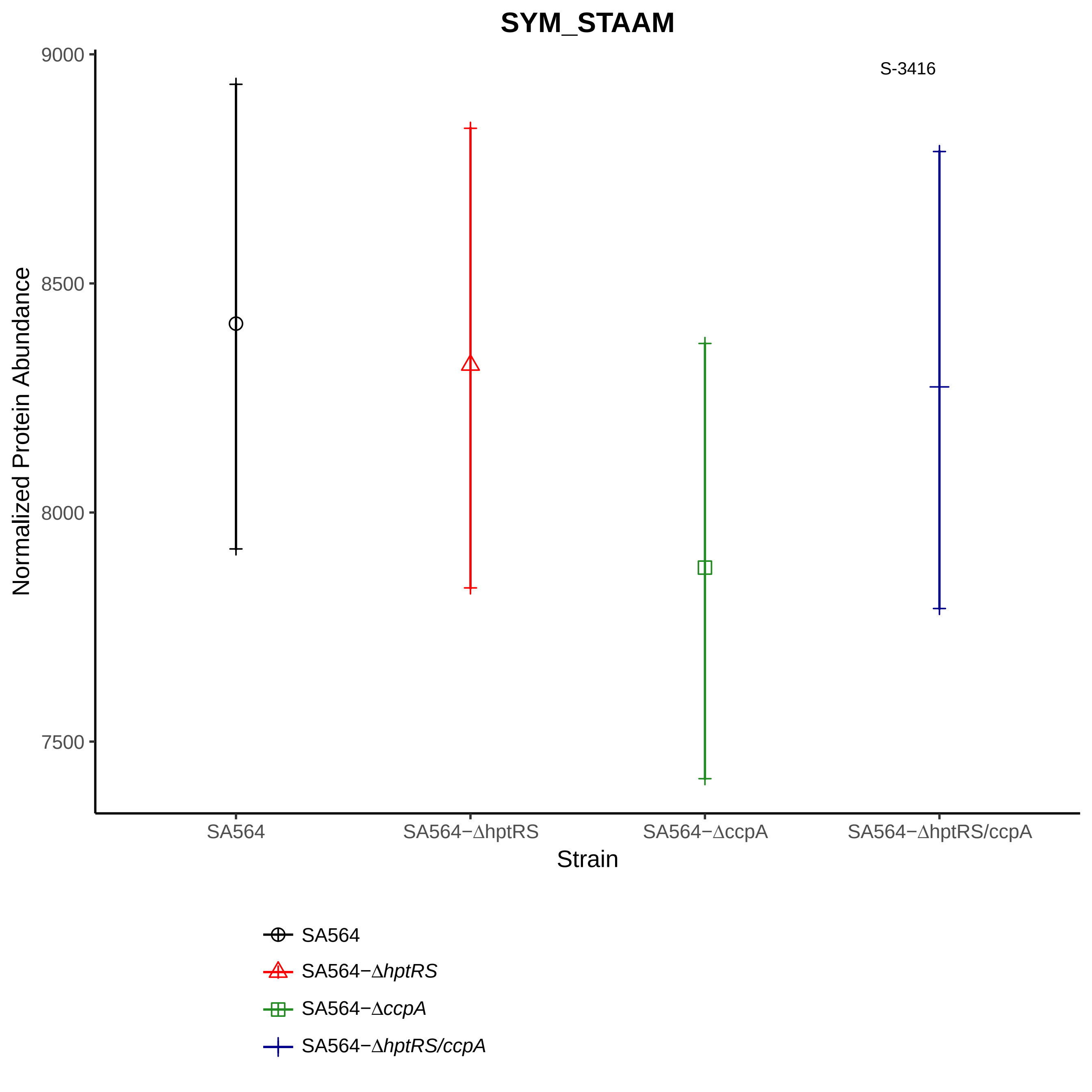

SYM_STAAM
S-3416
Normalized Protein Abundance
9000
8500
8000
7500
SA564
SA564−ΔhptRS
SA564−ΔccpA
SA564−ΔhptRS/ccpA
Strain
SA564
SA564−ΔhptRS
SA564−ΔccpA
SA564−ΔhptRS/ccpA

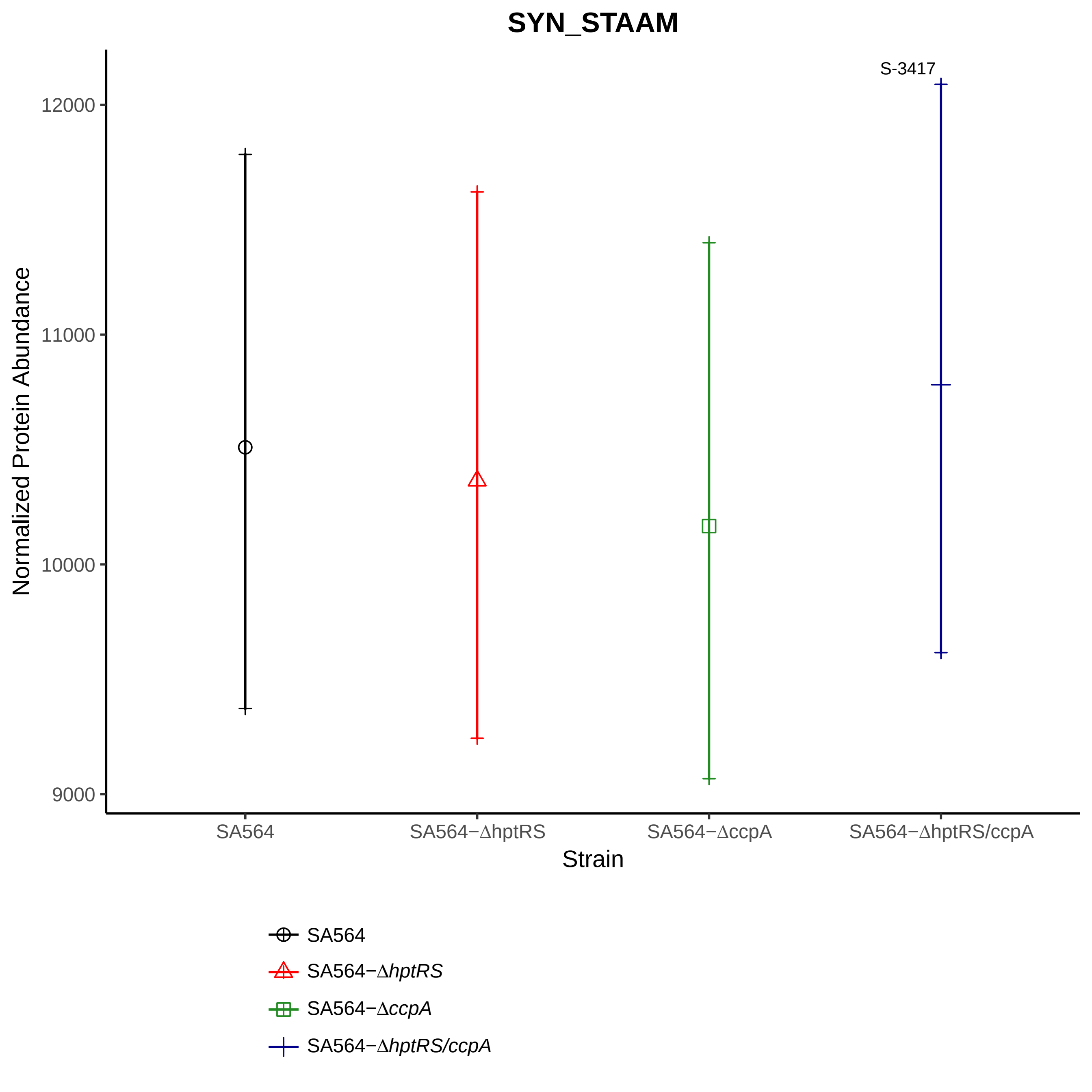
SYN_STAAM
S-3417
Normalized Protein Abundance
12000
11000
10000
9000
SA564
SA564−ΔhptRS
SA564−ΔccpA
SA564−ΔhptRS/ccpA
Strain
SA564
SA564−Δ*hptRS*
SA564−Δ*ccpA*
SA564−Δ*hptRS/ccpA*

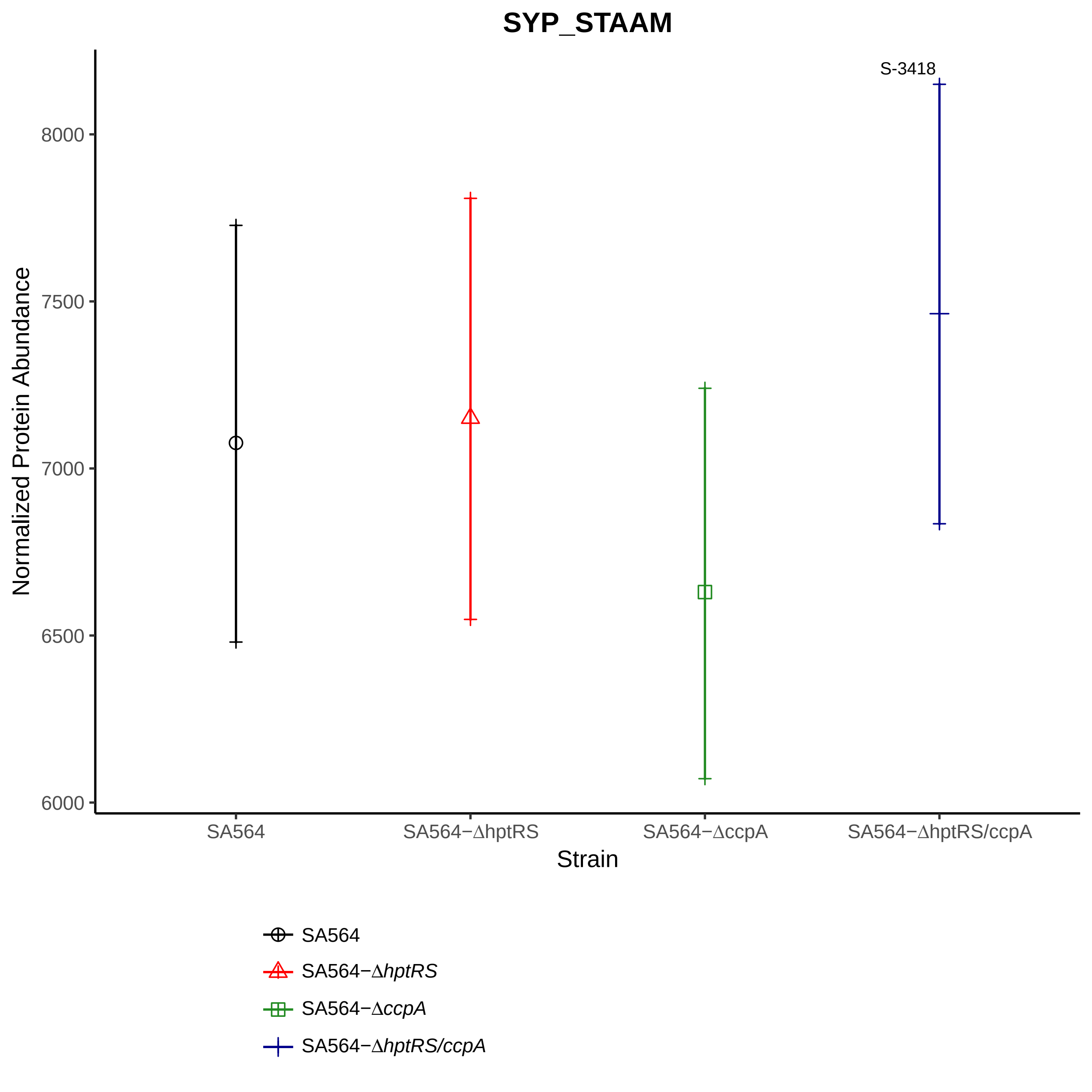

SYP_STAAM
S-3418
8000
7500
7000
6500
6000
Normalized Protein Abundance
SA564
SA564−ΔhptRS
SA564−ΔccpA
SA564−ΔhptRS/ccpA
Strain
SA564
SA564−ΔhptRS
SA564−ΔccpA
SA564−ΔhptRS/ccpA

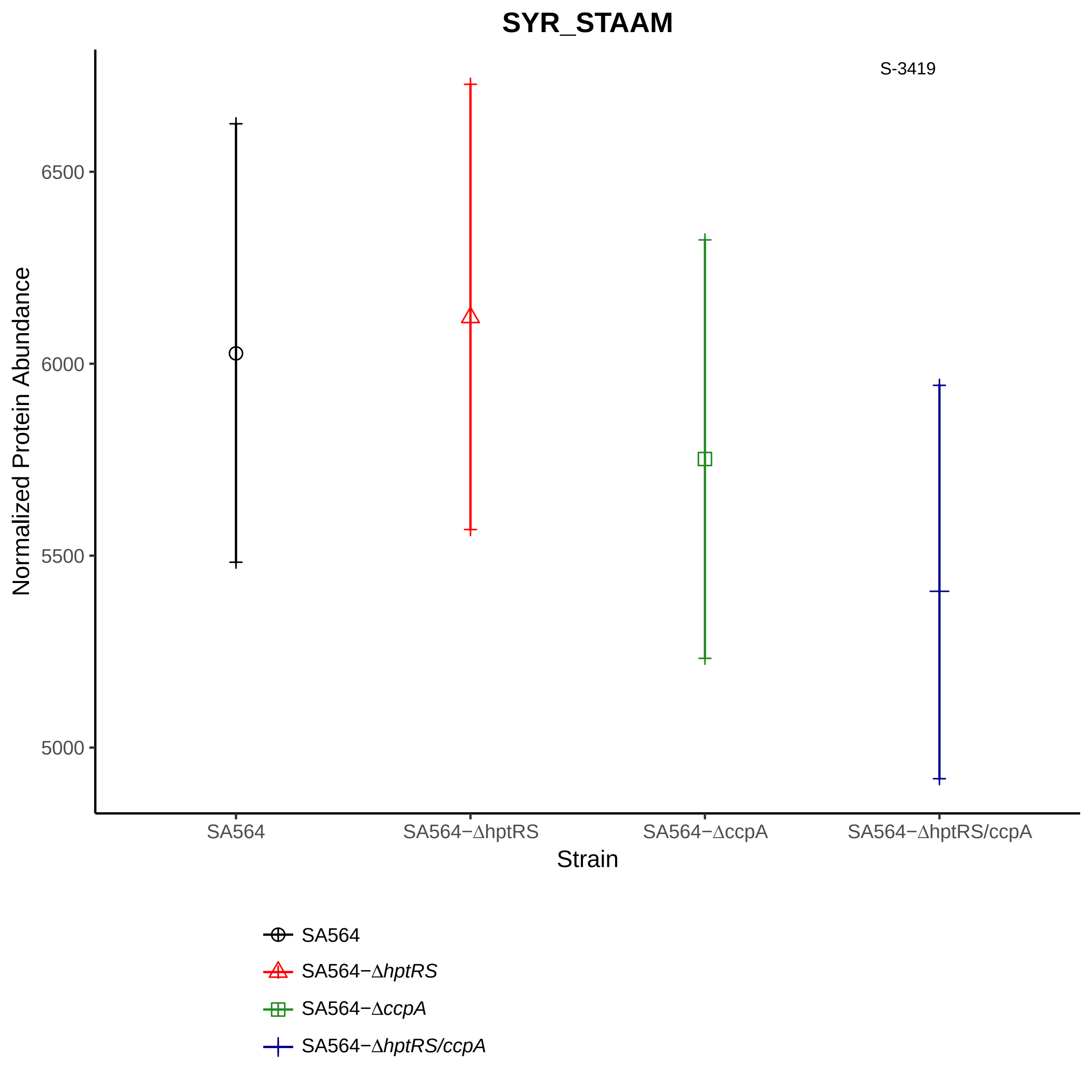
SYR_STAAM
S-3419
Normalized Protein Abundance
6500
6000
5500
5000
SA564
SA564−ΔhptRS
SA564−ΔccpA
SA564−ΔhptRS/ccpA
Strain
SA564
SA564−ΔhptRS
SA564−ΔccpA
SA564−ΔhptRS/ccpA

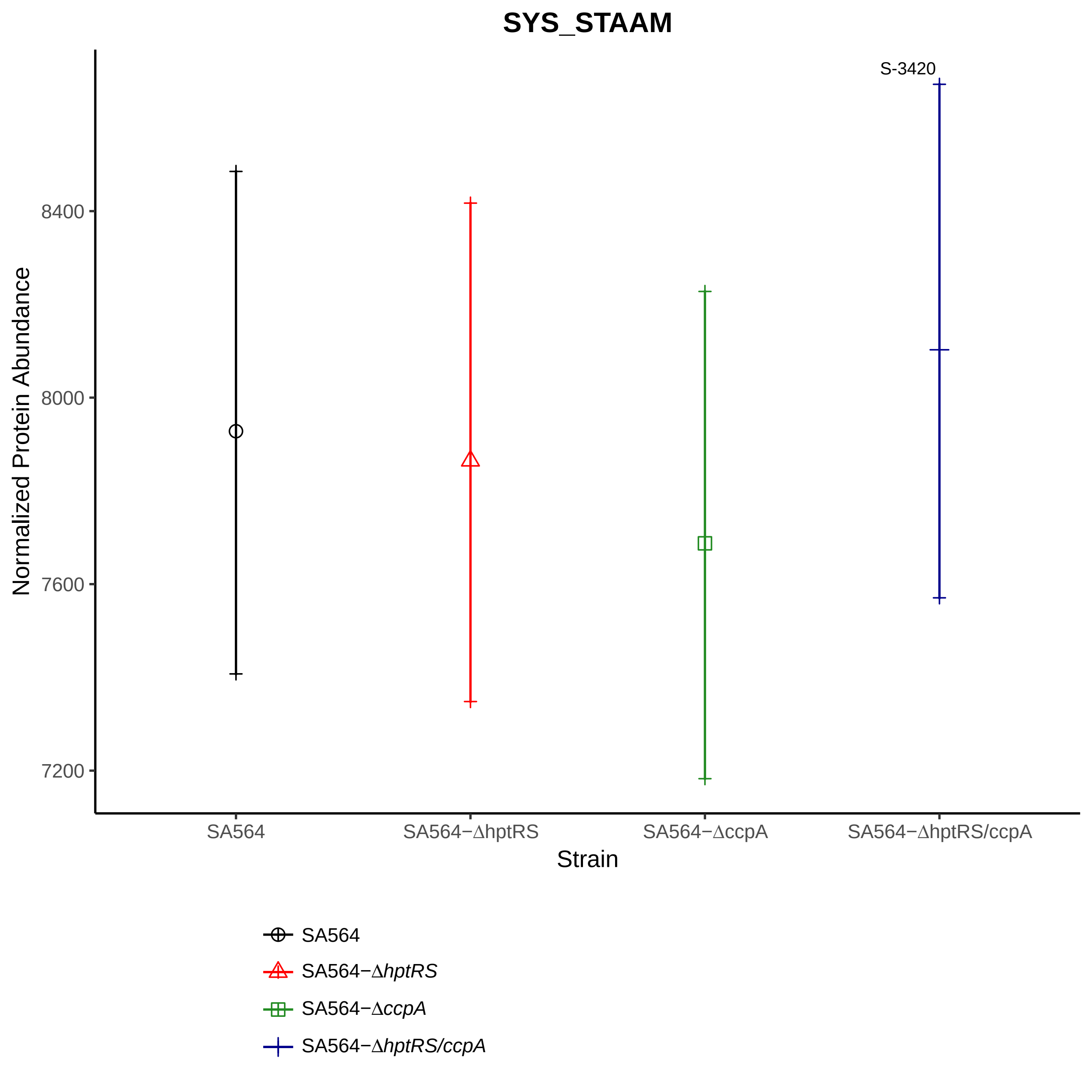
SYS_STAAM
S-3420
8400
8000
7600
7200
Normalized Protein Abundance
SA564
SA564−ΔhptRS
SA564−ΔccpA
SA564−ΔhptRS/ccpA
Strain
SA564
SA564−ΔhptRS
SA564−ΔccpA
SA564−ΔhptRS/ccpA

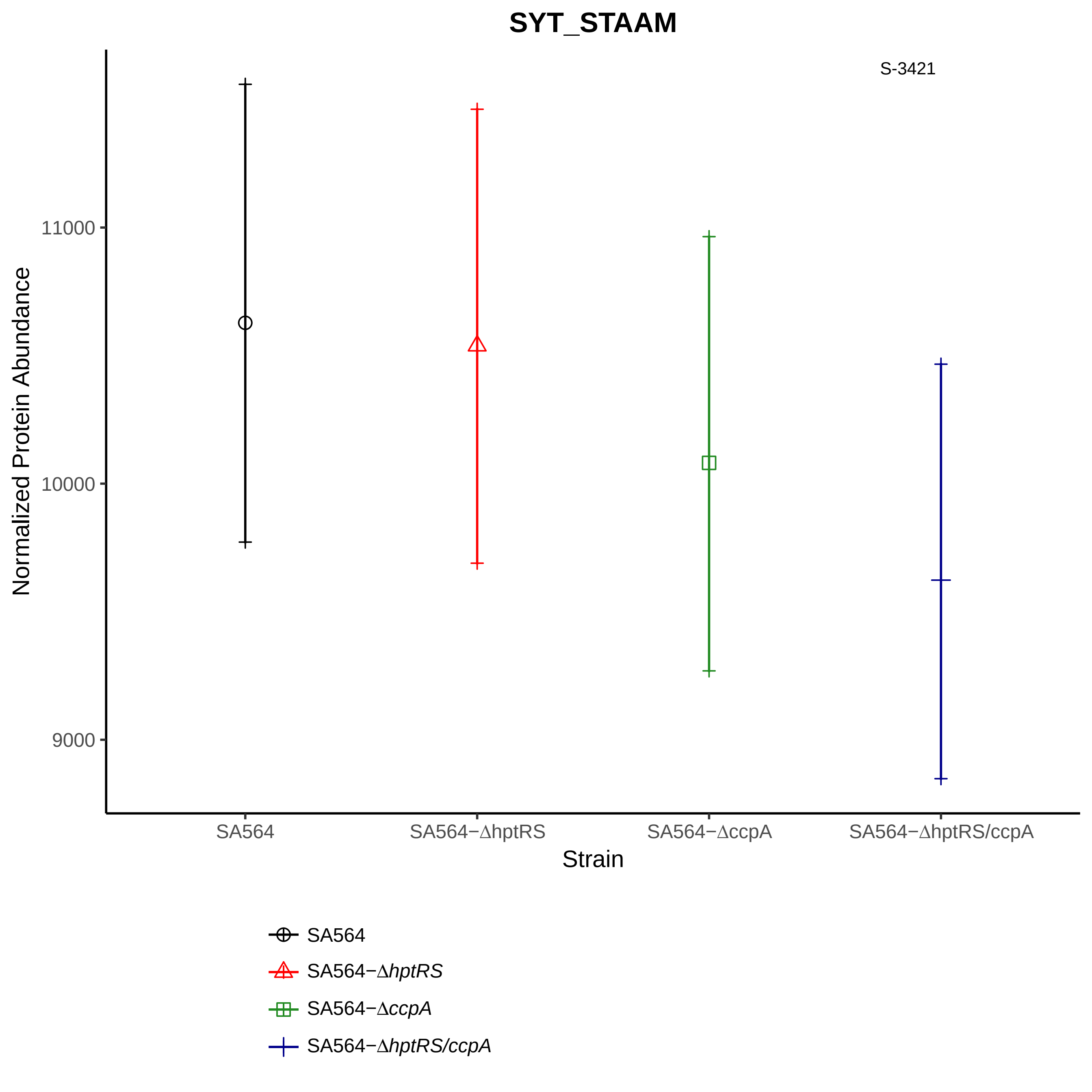
SYT_STAAM
S-3421
Normalized Protein Abundance
11000
10000
9000
SA564
SA564−ΔhptRS
SA564−ΔccpA
SA564−ΔhptRS/ccpA
Strain
SA564
SA564−ΔhptRS
SA564−ΔccpA
SA564−ΔhptRS/ccpA

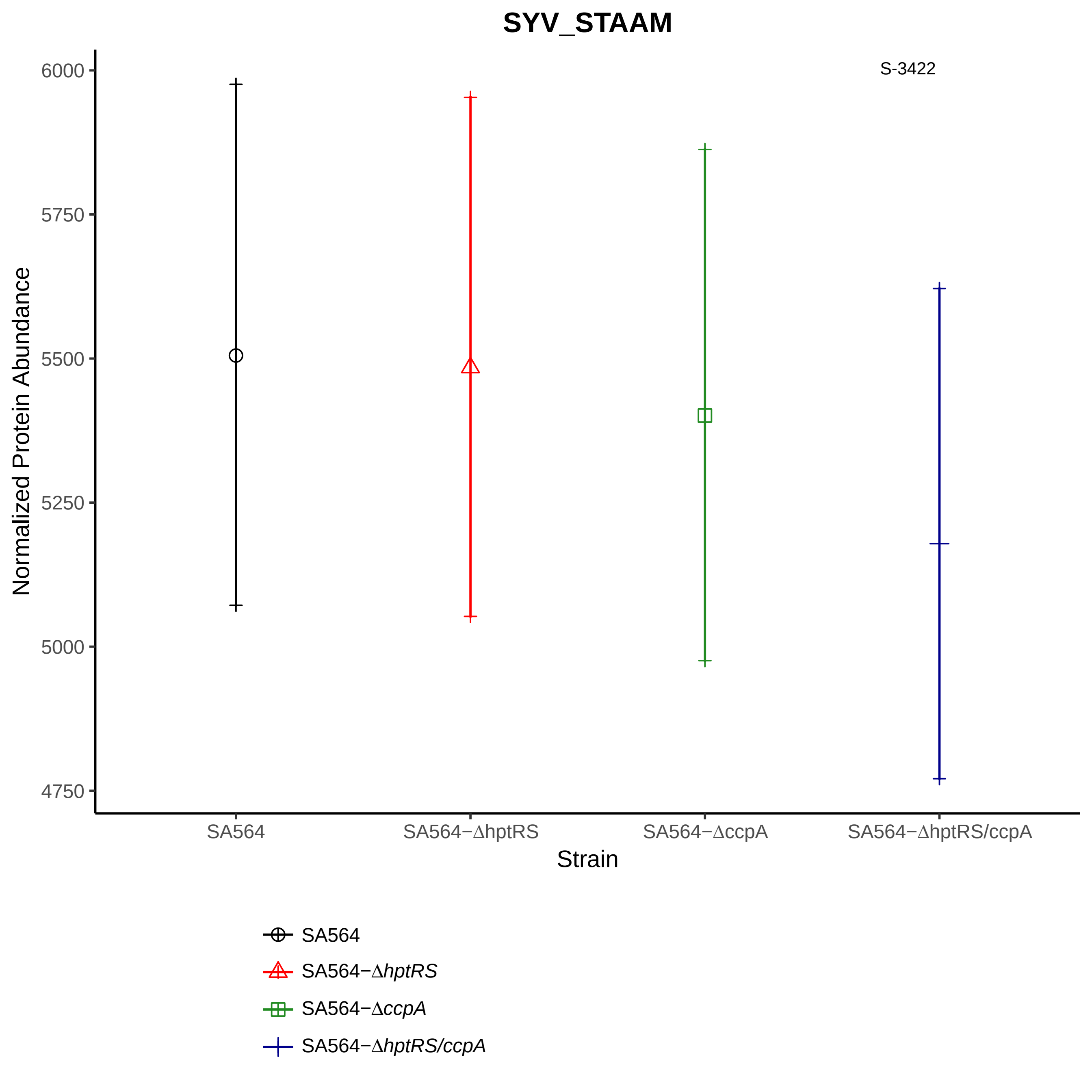

SYV_STAAM
S-3422
Normalized Protein Abundance
6000
5750
5500
5250
5000
4750
SA564
SA564−ΔhptRS
SA564−ΔccpA
SA564−ΔhptRS/ccpA
Strain
SA564
SA564−Δ*hptRS*
SA564−Δ*ccpA*
SA564−Δ*hptRS/ccpA*

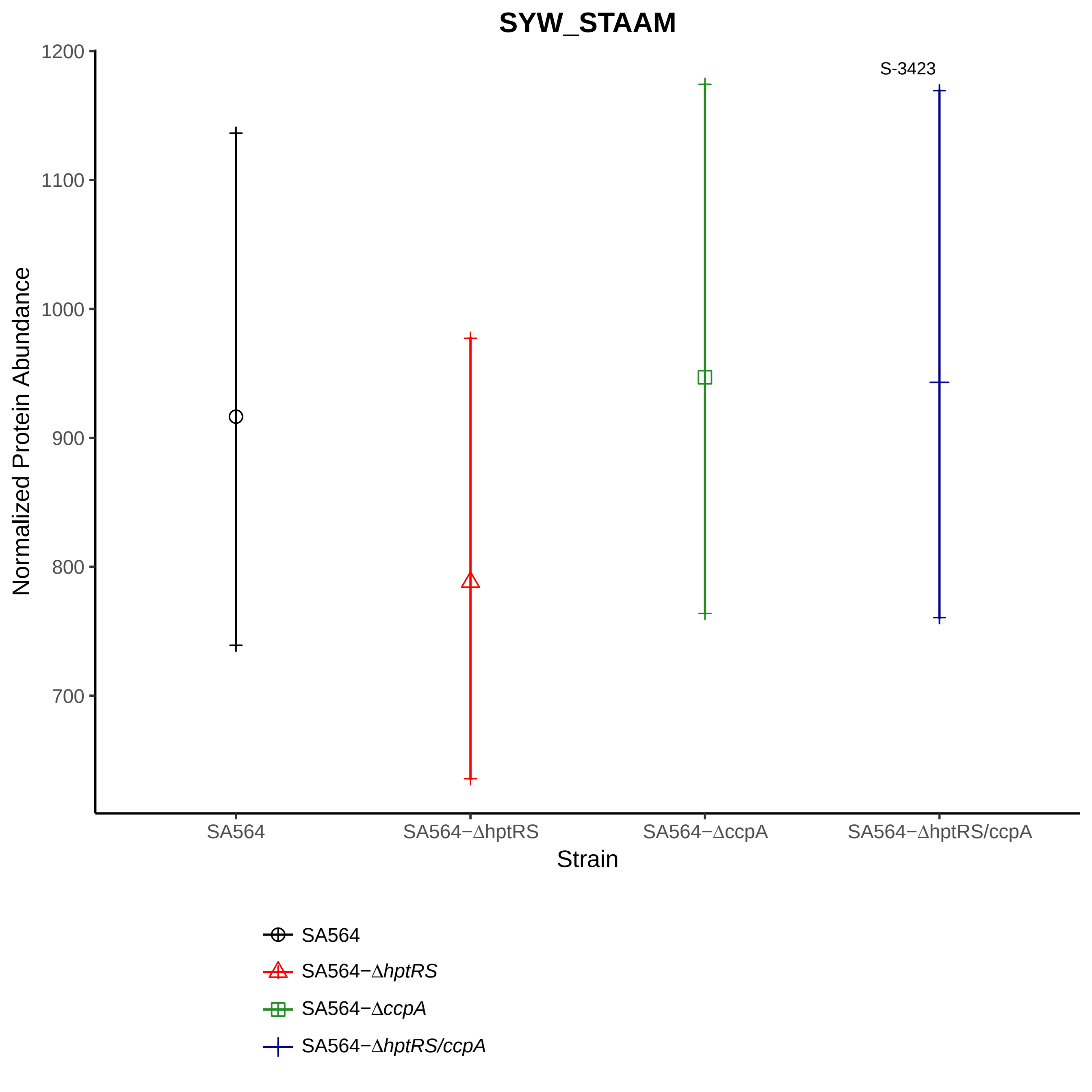

SYW_STAAM
S-3423
Normalized Protein Abundance
1200
1100
1000
900
800
700
SA564
SA564−ΔhptRS
SA564−ΔccpA
SA564−ΔhptRS/ccpA
Strain
SA564
SA564−ΔhptRS
SA564−ΔccpA
SA564−ΔhptRS/ccpA

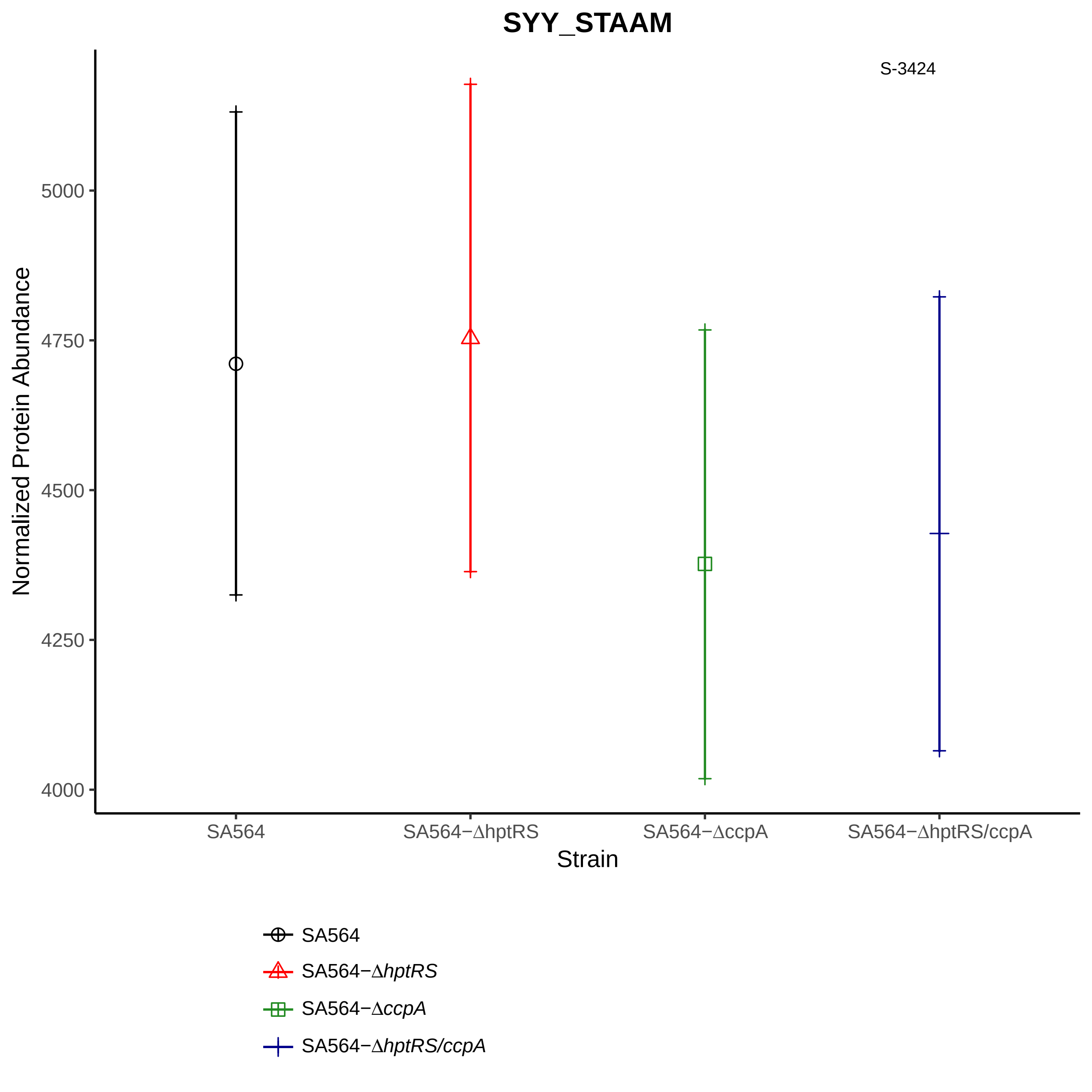

SYY_STAAM
S-3424
Normalized Protein Abundance
5000
4750
4500
4250
4000
SA564
SA564−ΔhptRS
SA564−ΔccpA
SA564−ΔhptRS/ccpA
Strain
SA564
SA564−ΔhptRS
SA564−ΔccpA
SA564−ΔhptRS/ccpA

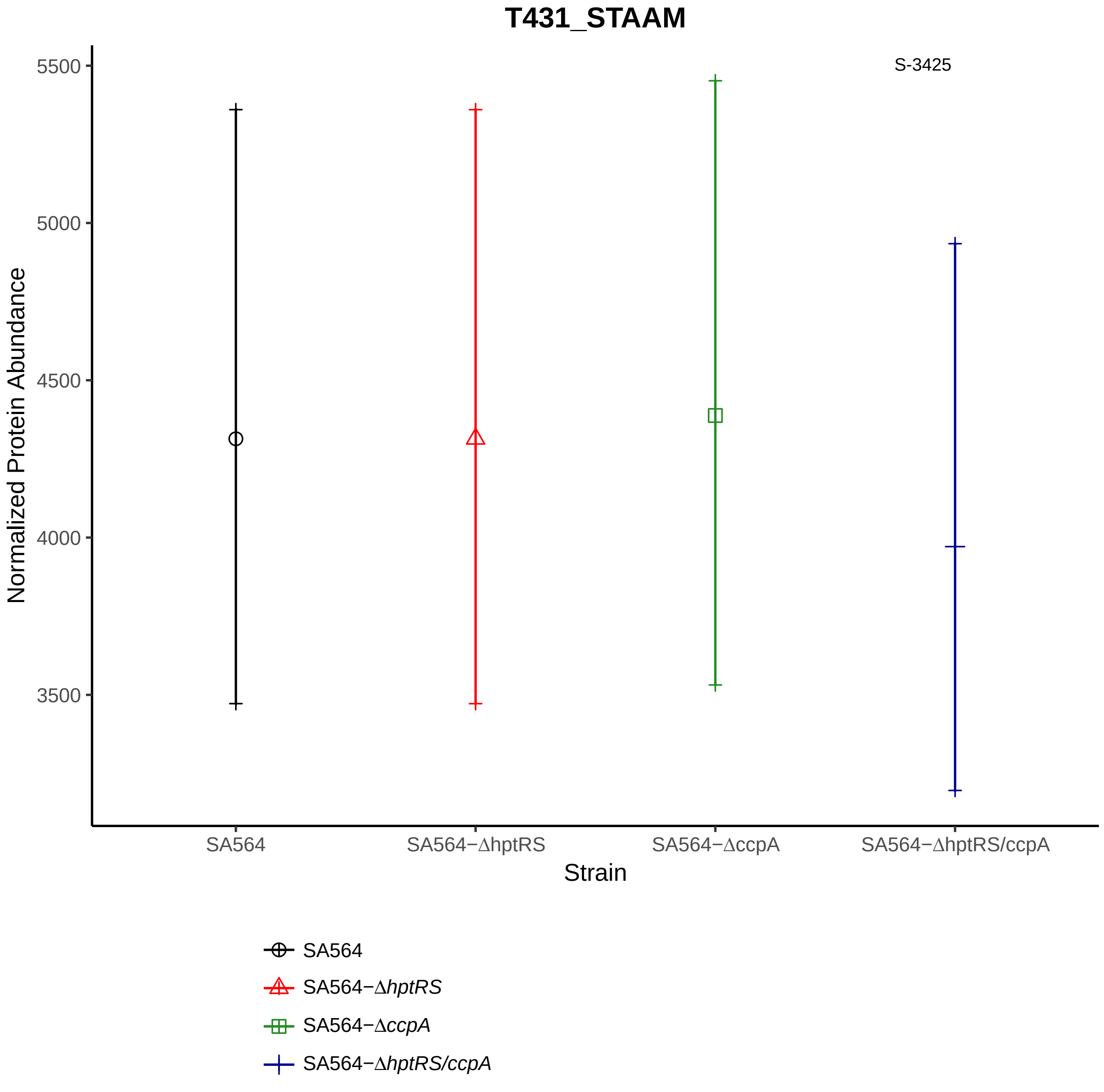

T431_STAAM
S-3425
Normalized Protein Abundance
5500
5000
4500
4000
3500
Strain
SA564
SA564−ΔhptRS
SA564−ΔccpA
SA564−ΔhptRS/ccpA
SA564
SA564−ΔhptRS
SA564−ΔccpA
SA564−ΔhptRS/ccpA

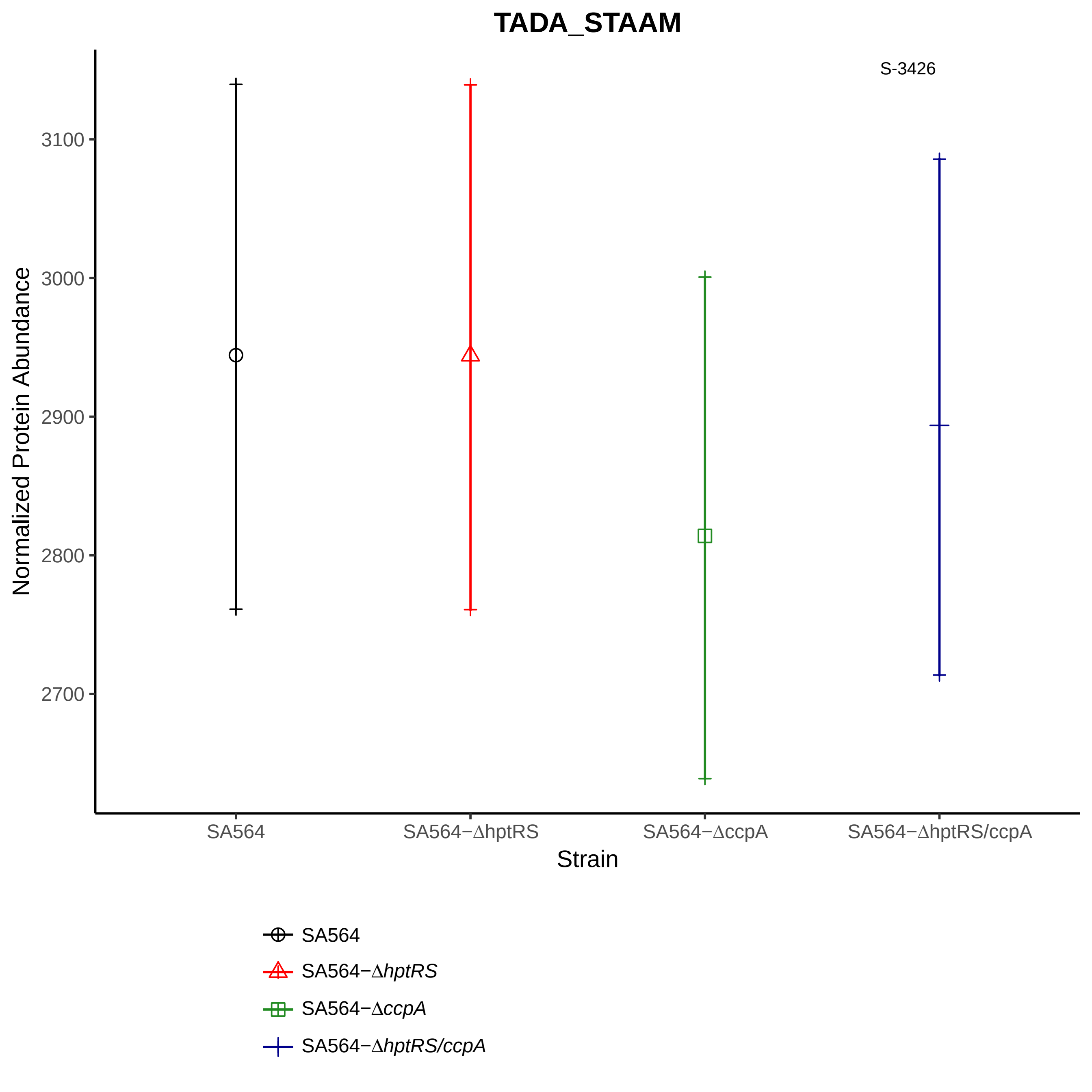
TADA_STAAM
S-3426
Normalized Protein Abundance
3100
3000
2900
2800
2700
SA564
SA564−ΔhptRS
SA564−ΔccpA
SA564−ΔhptRS/ccpA
Strain
SA564
SA564−ΔhptRS
SA564−ΔccpA
SA564−ΔhptRS/ccpA

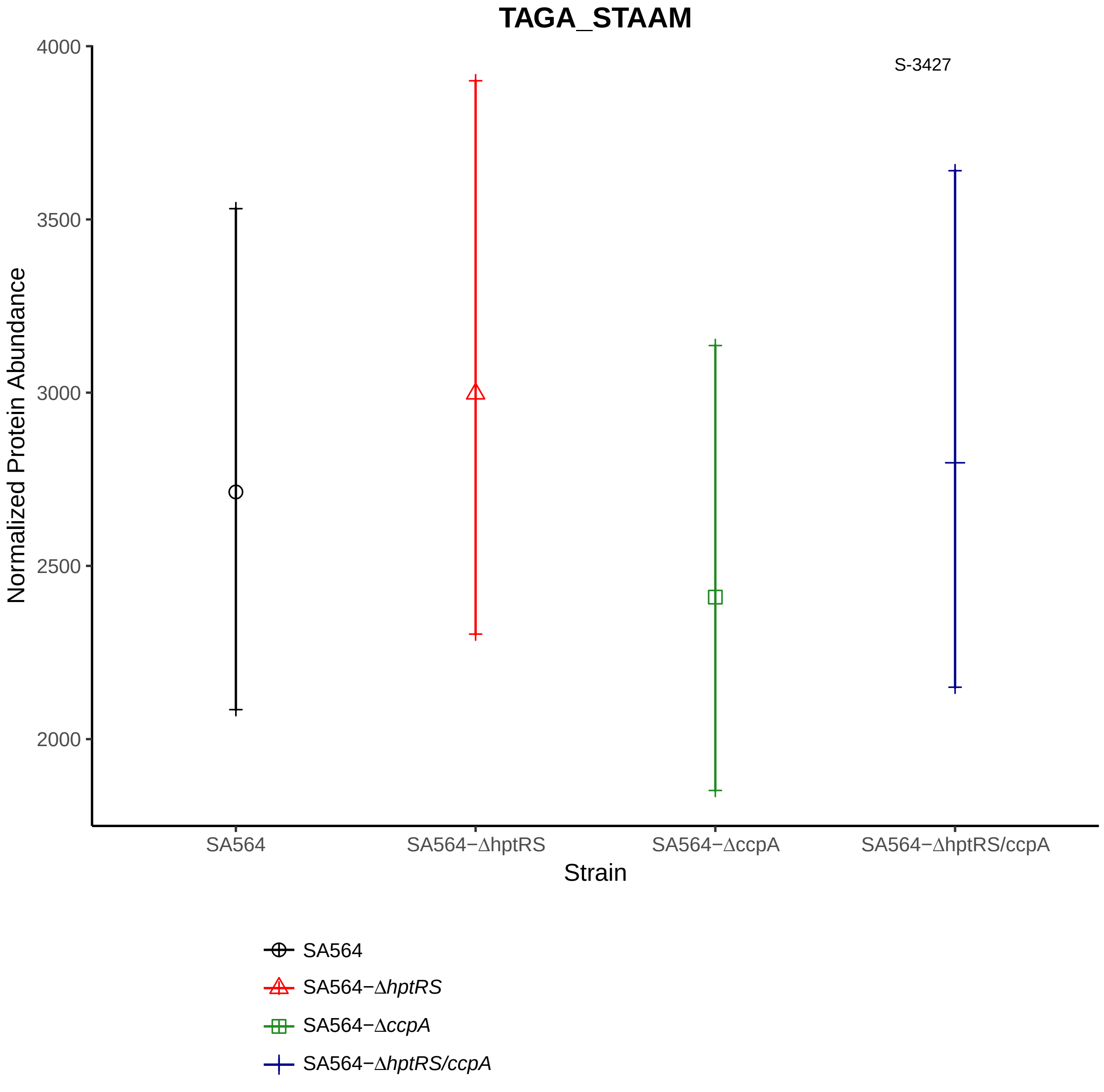
TAGA_STAAM
S-3427
Normalized Protein Abundance
4000
3500
3000
2500
2000
SA564
SA564−ΔhptRS
SA564−ΔccpA
SA564−ΔhptRS/ccpA
Strain
SA564
SA564−ΔhptRS
SA564−ΔccpA
SA564−ΔhptRS/ccpA

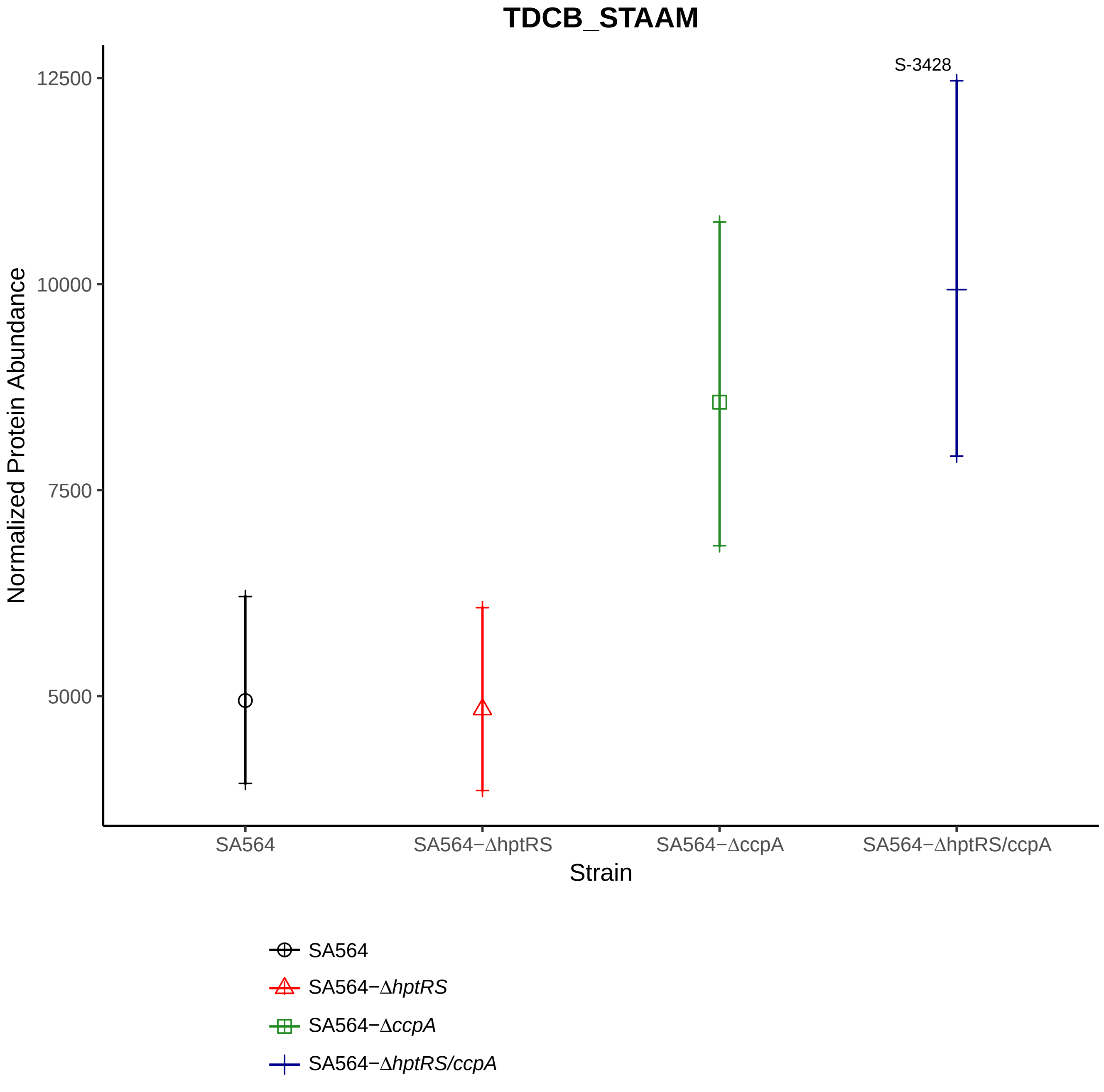

TDCB_STAAM
S-3428
12500
10000
7500
5000
Normalized Protein Abundance
SA564
SA564−ΔhptRS
SA564−ΔccpA
SA564−ΔhptRS/ccpA
Strain
SA564
SA564−Δ*hptRS*
SA564−Δ*ccpA*
SA564−Δ*hptRS/ccpA*

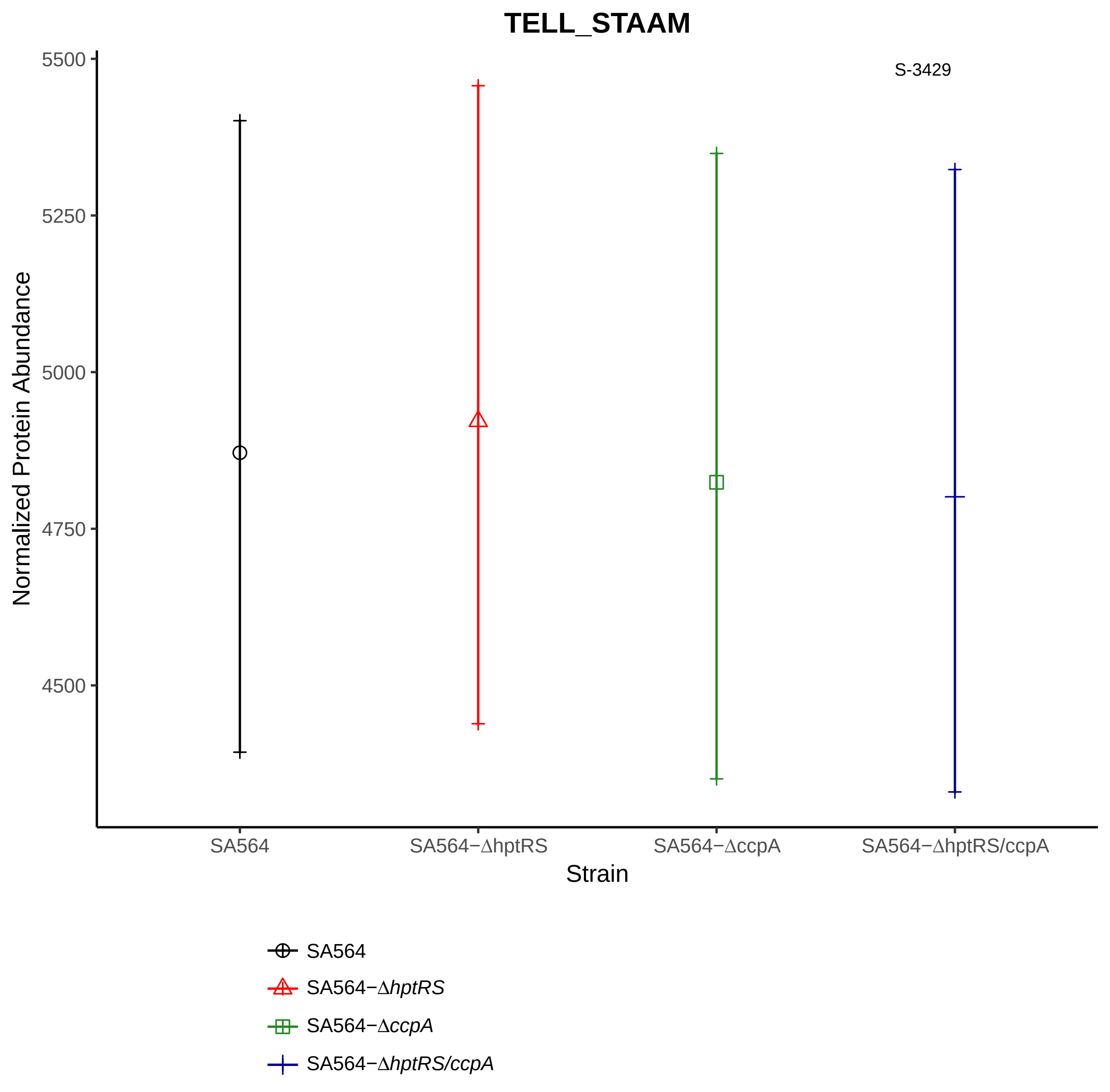

TELL_STAAM
S-3429
Normalized Protein Abundance
5500
5250
5000
4750
4500
SA564
SA564−ΔhptRS
SA564−ΔccpA
SA564−ΔhptRS/ccpA
Strain
SA564
SA564−Δ*hptRS*
SA564−Δ*ccpA*
SA564−Δ*hptRS/ccpA*

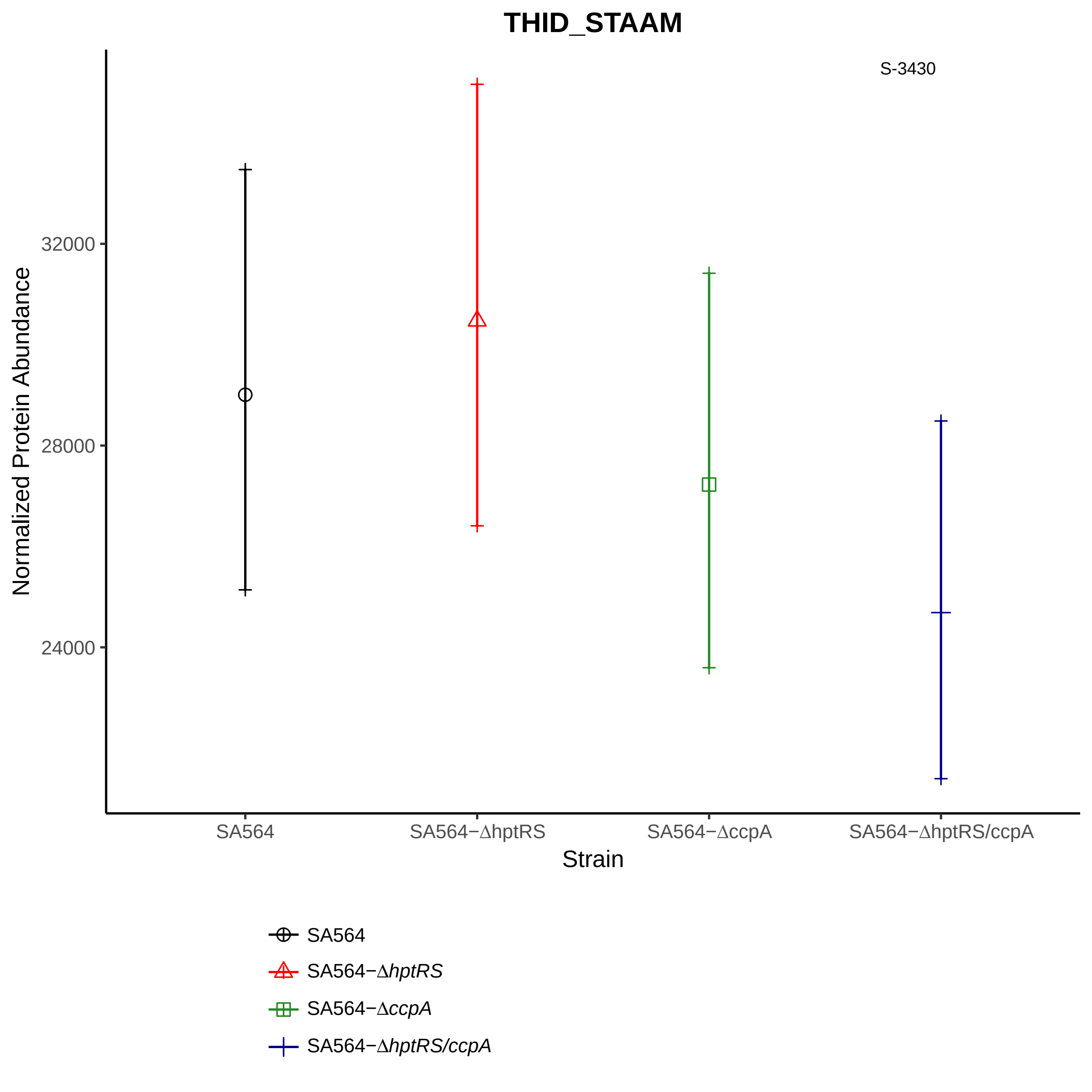
THID_STAAM
S-3430
Normalized Protein Abundance
32000
28000
24000
SA564
SA564−ΔhptRS
SA564−ΔccpA
SA564−ΔhptRS/ccpA
Strain
SA564
SA564−ΔhptRS
SA564−ΔccpA
SA564−ΔhptRS/ccpA

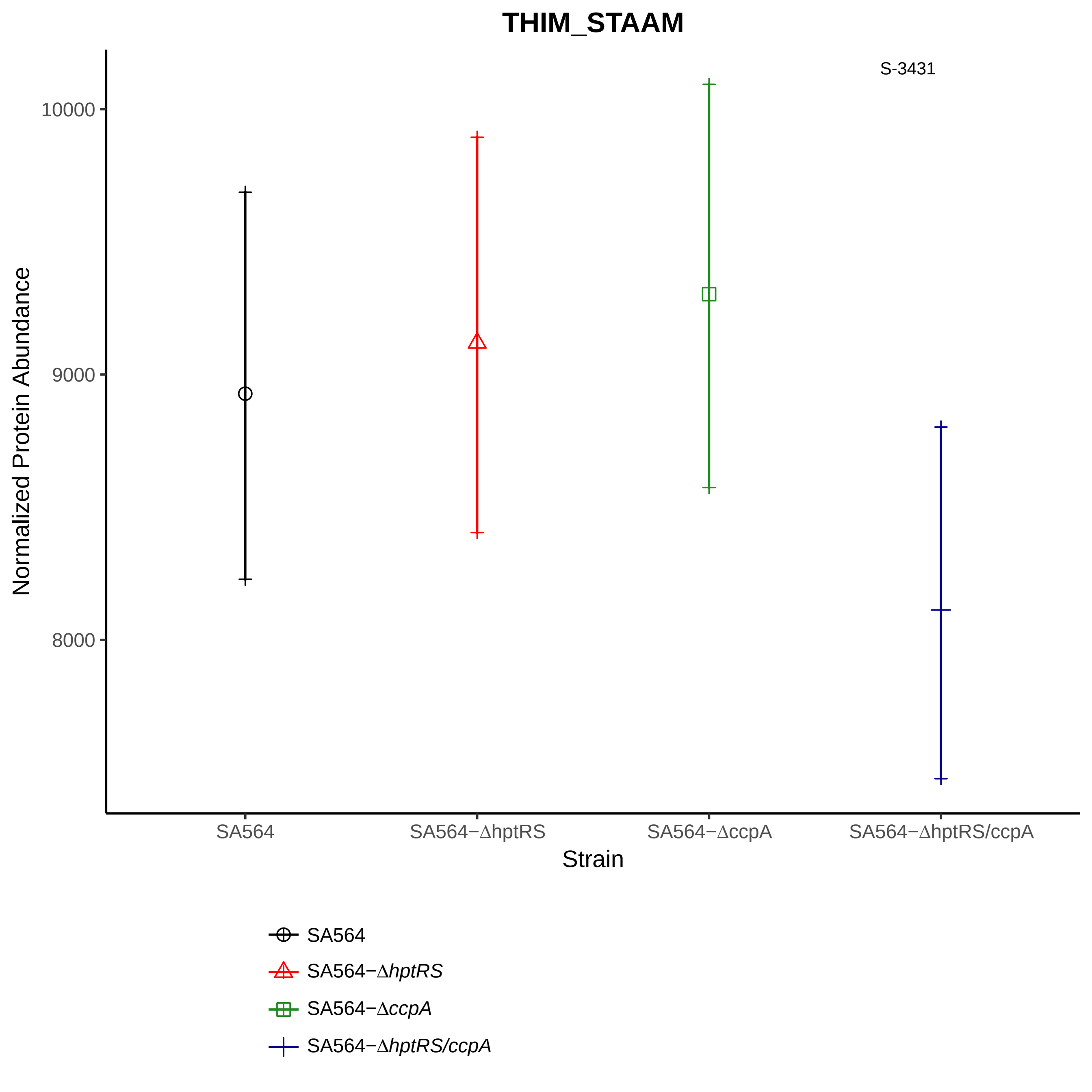
THIM_STAAM
S-3431
10000
9000
8000
Normalized Protein Abundance
SA564
SA564−ΔhptRS
SA564−ΔccpA
SA564−ΔhptRS/ccpA
Strain
SA564
SA564−ΔhptRS
SA564−ΔccpA
SA564−ΔhptRS/ccpA

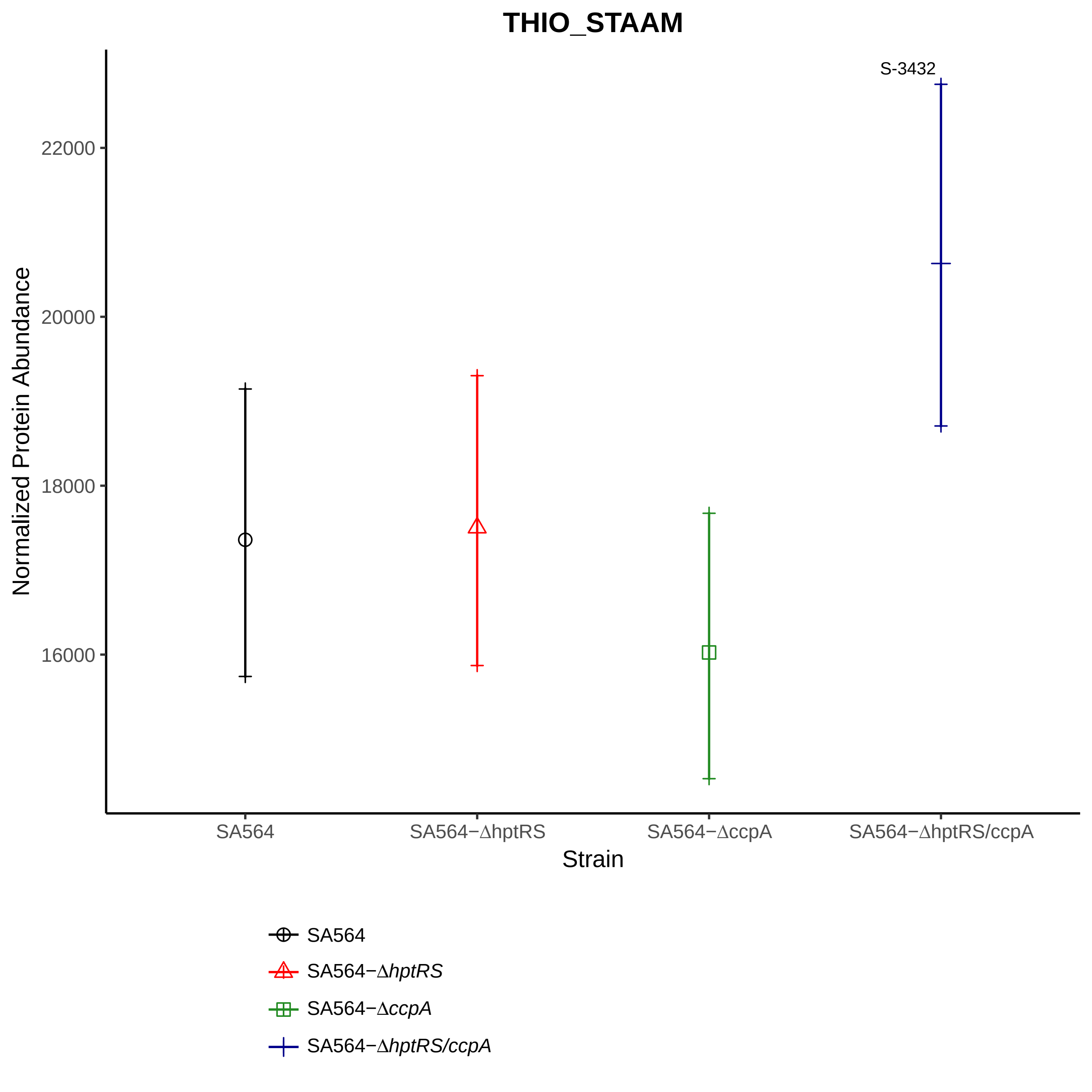
THIO_STAAM
S-3432
Normalized Protein Abundance
22000
20000
18000
16000
SA564
SA564−ΔhptRS
SA564−ΔccpA
SA564−ΔhptRS/ccpA
Strain
SA564
SA564−ΔhptRS
SA564−ΔccpA
SA564−ΔhptRS/ccpA

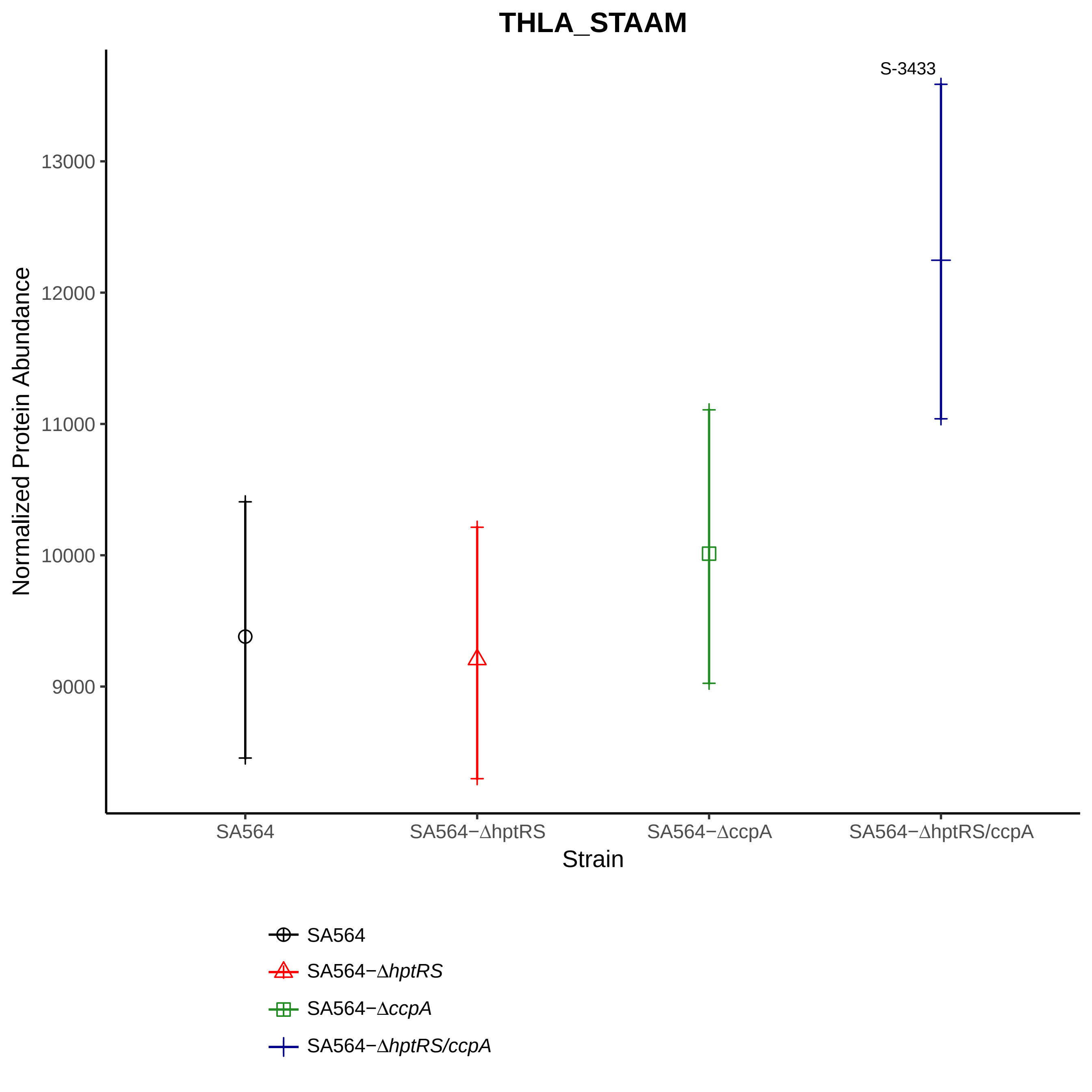
THLA_STAAM
S-3433
Normalized Protein Abundance
13000
12000
11000
10000
9000
SA564
SA564−ΔhptRS
SA564−ΔccpA
SA564−ΔhptRS/ccpA
Strain
SA564
SA564−Δ*hptRS*
SA564−Δ*ccpA*
SA564−Δ*hptRS/ccpA*

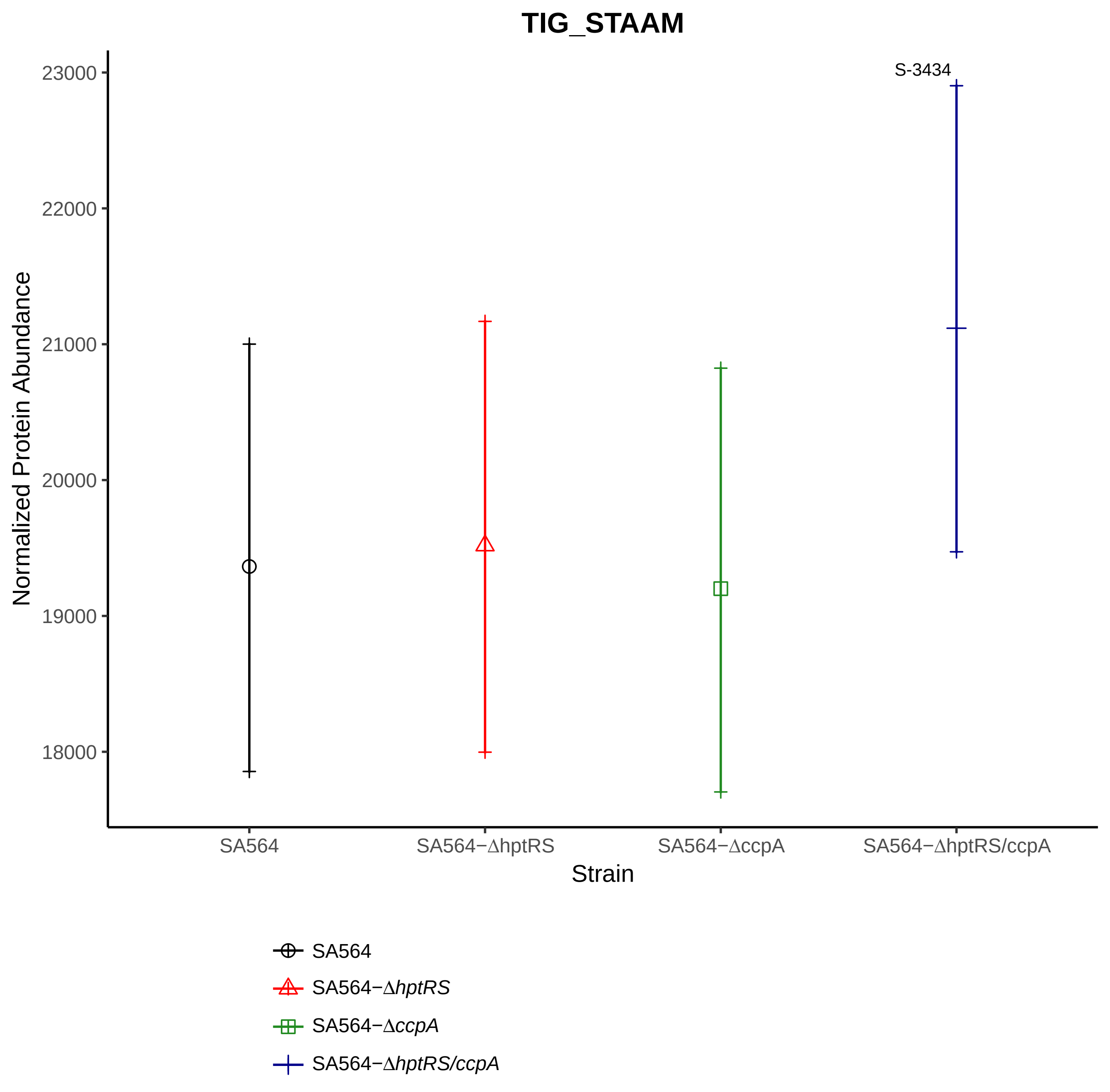
TIG_STAAM
S-3434
23000
22000
21000
20000
19000
18000
Normalized Protein Abundance
SA564
SA564−ΔhptRS
SA564−ΔccpA
SA564−ΔhptRS/ccpA
Strain
SA564
SA564−Δ*hptRS*
SA564−Δ*ccpA*
SA564−Δ*hptRS/ccpA*

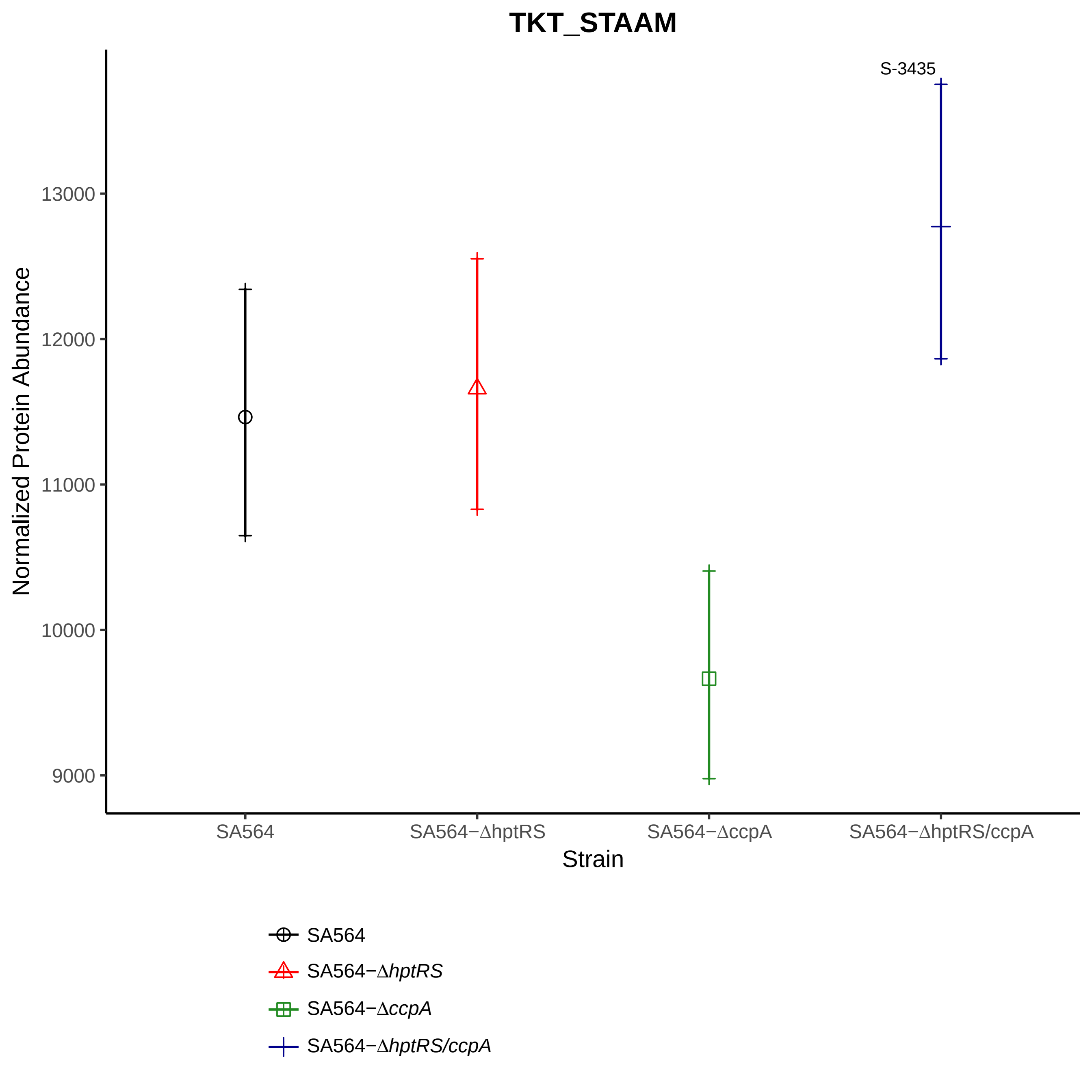

TKT_STAAM
S-3435
Normalized Protein Abundance
13000
12000
11000
10000
9000
SA564
SA564−ΔhptRS
SA564−ΔccpA
SA564−ΔhptRS/ccpA
Strain
SA564
SA564−Δ*hptRS*
SA564−Δ*ccpA*
SA564−Δ*hptRS/ccpA*

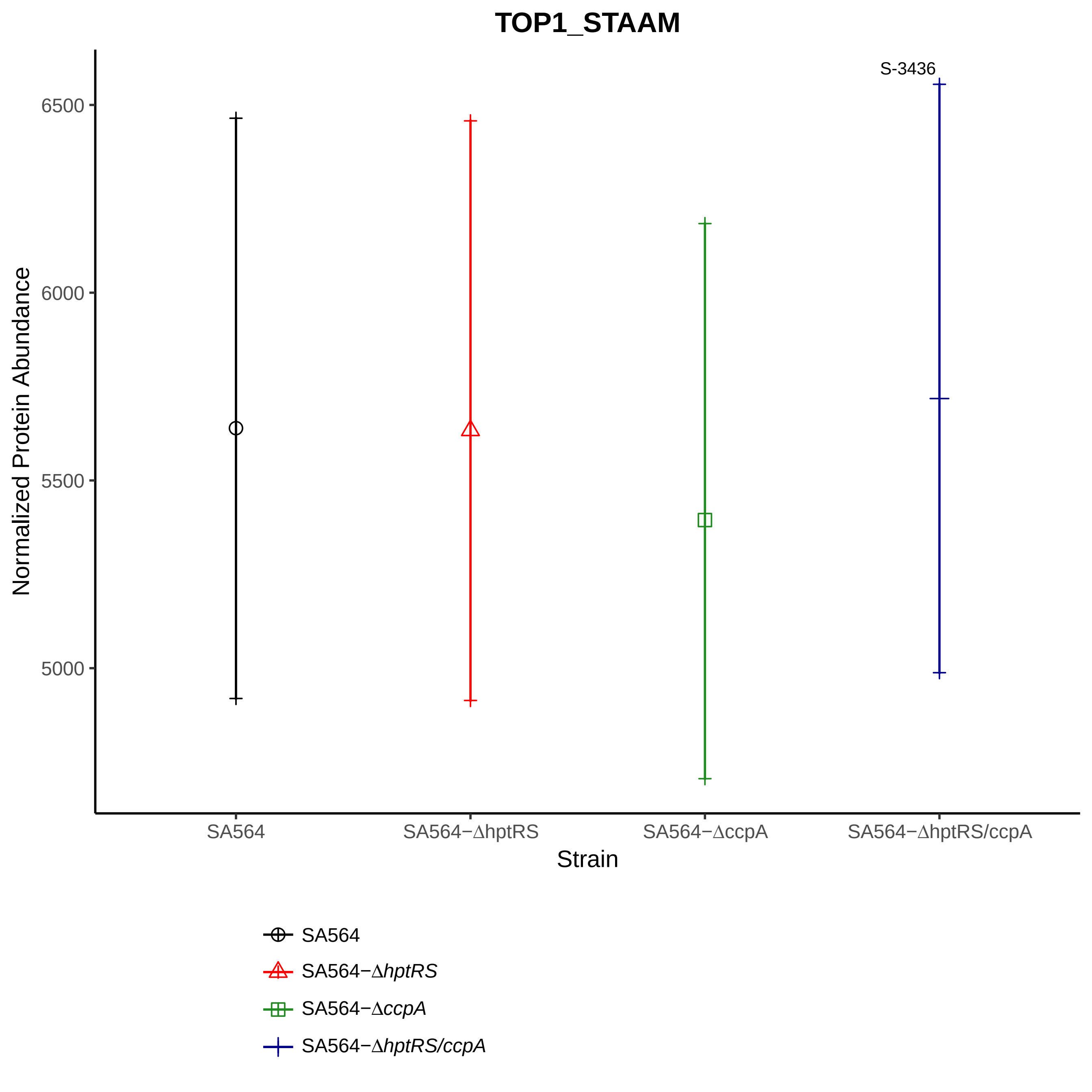

TOP1_STAAM
S-3436
6500
6000
5500
5000
Normalized Protein Abundance
SA564
SA564−ΔhptRS
SA564−ΔccpA
SA564−ΔhptRS/ccpA
Strain
SA564
SA564−ΔhptRS
SA564−ΔccpA
SA564−ΔhptRS/ccpA

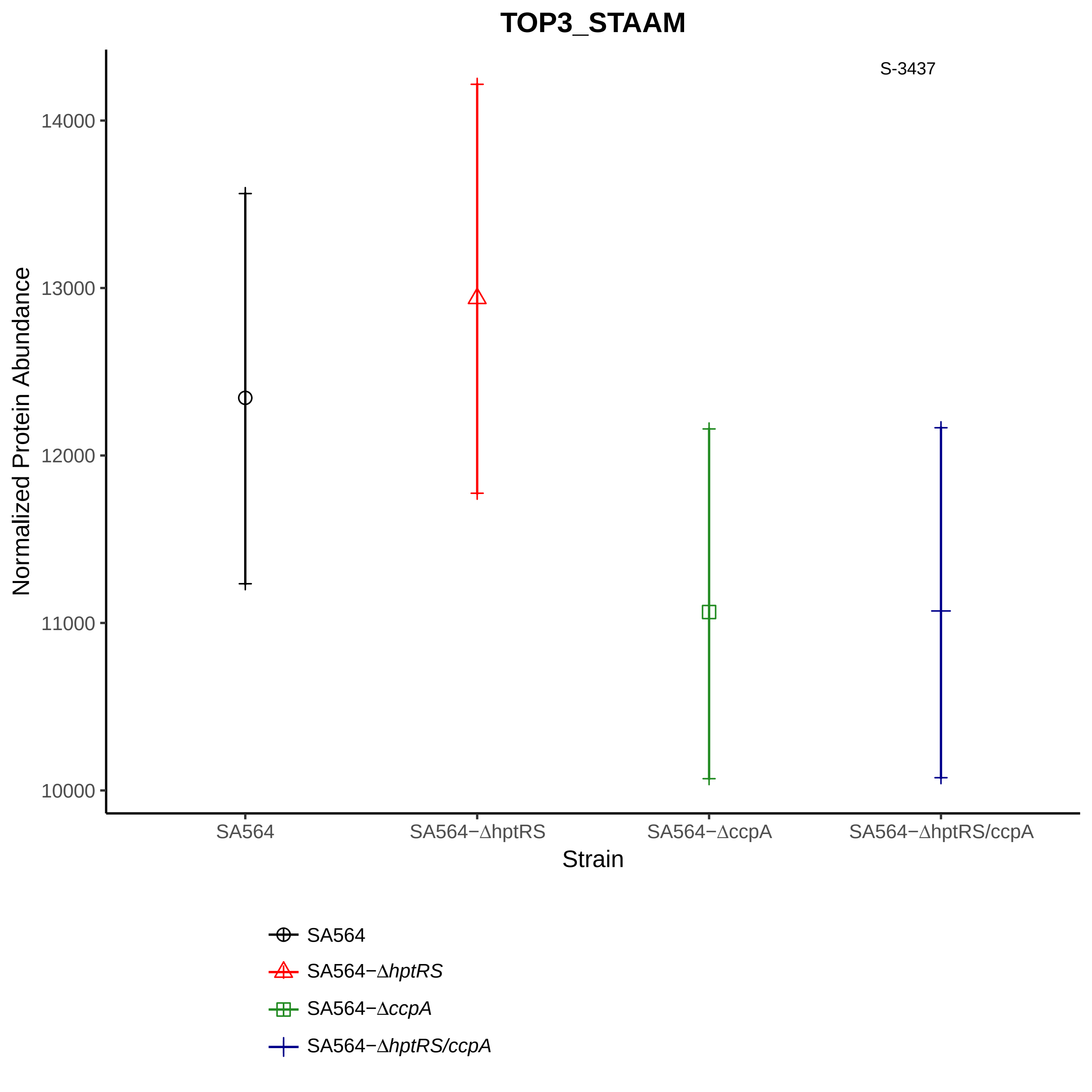

TOP3_STAAM
S-3437
Normalized Protein Abundance
14000
13000
12000
11000
10000
SA564
SA564−ΔhptRS
SA564−ΔccpA
SA564−ΔhptRS/ccpA
Strain
SA564
SA564−Δ*hptRS*
SA564−Δ*ccpA*
SA564−Δ*hptRS/ccpA*

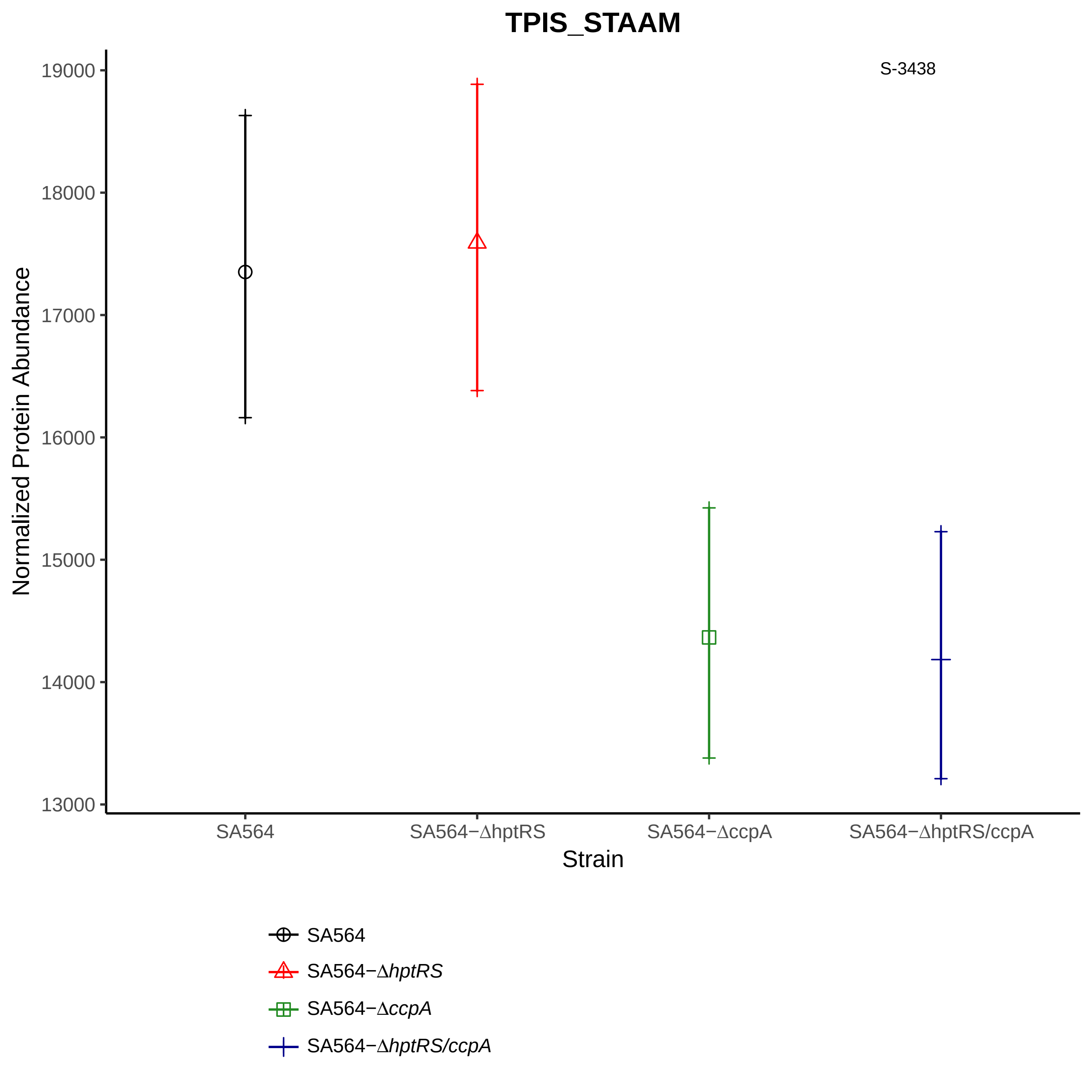

TPIS_STAAM
S-3438
Normalized Protein Abundance
19000
18000
17000
16000
15000
14000
13000
SA564
SA564−ΔhptRS
SA564−ΔccpA
SA564−ΔhptRS/ccpA
Strain
SA564
SA564−ΔhptRS
SA564−ΔccpA
SA564−ΔhptRS/ccpA

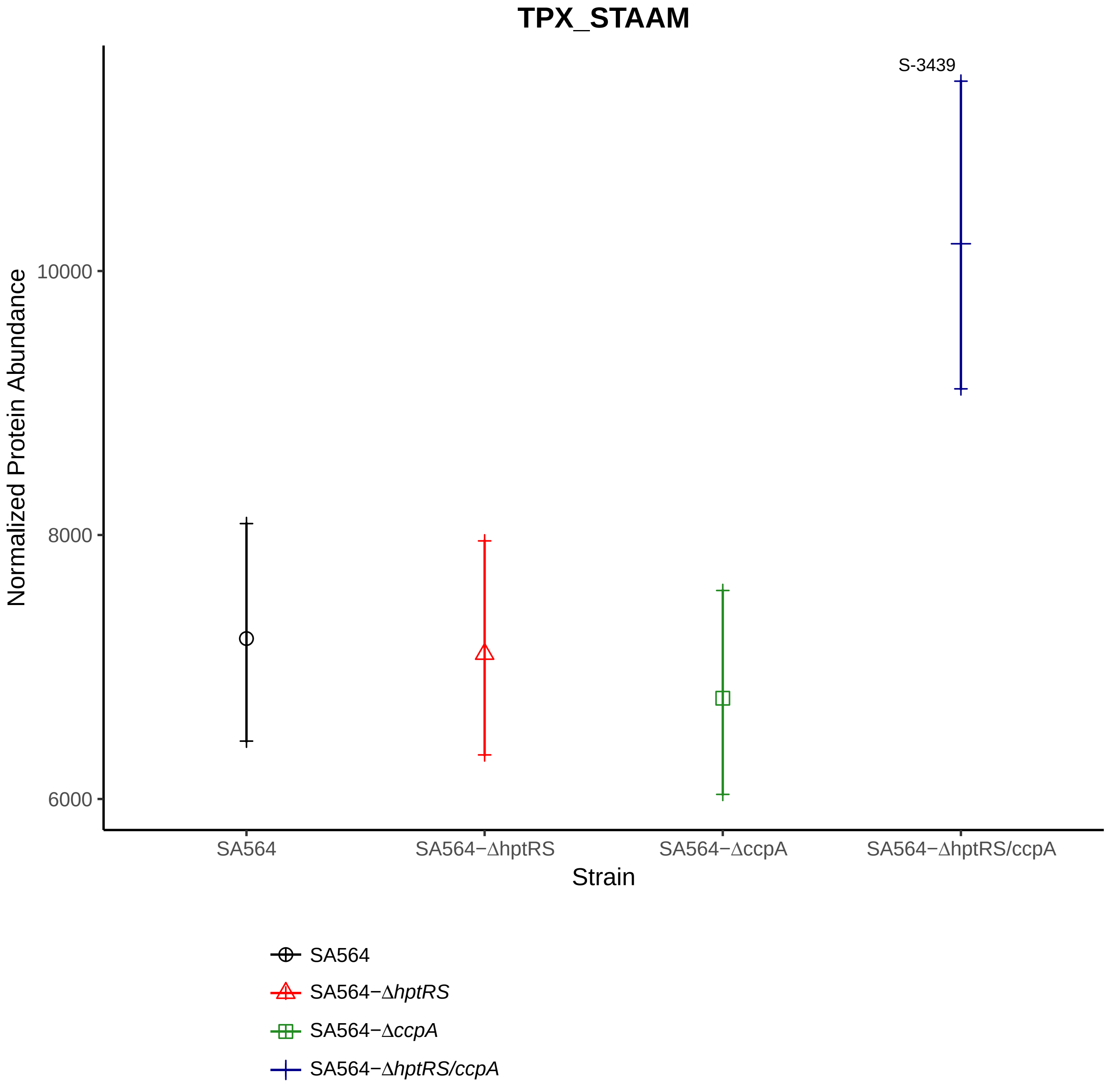
TPX_STAAM
S-3439
Normalized Protein Abundance
10000
8000
6000
SA564
SA564−ΔhptRS
SA564−ΔccpA
SA564−ΔhptRS/ccpA
Strain
SA564
SA564−Δ*hptRS*
SA564−Δ*ccpA*
SA564−Δ*hptRS/ccpA*

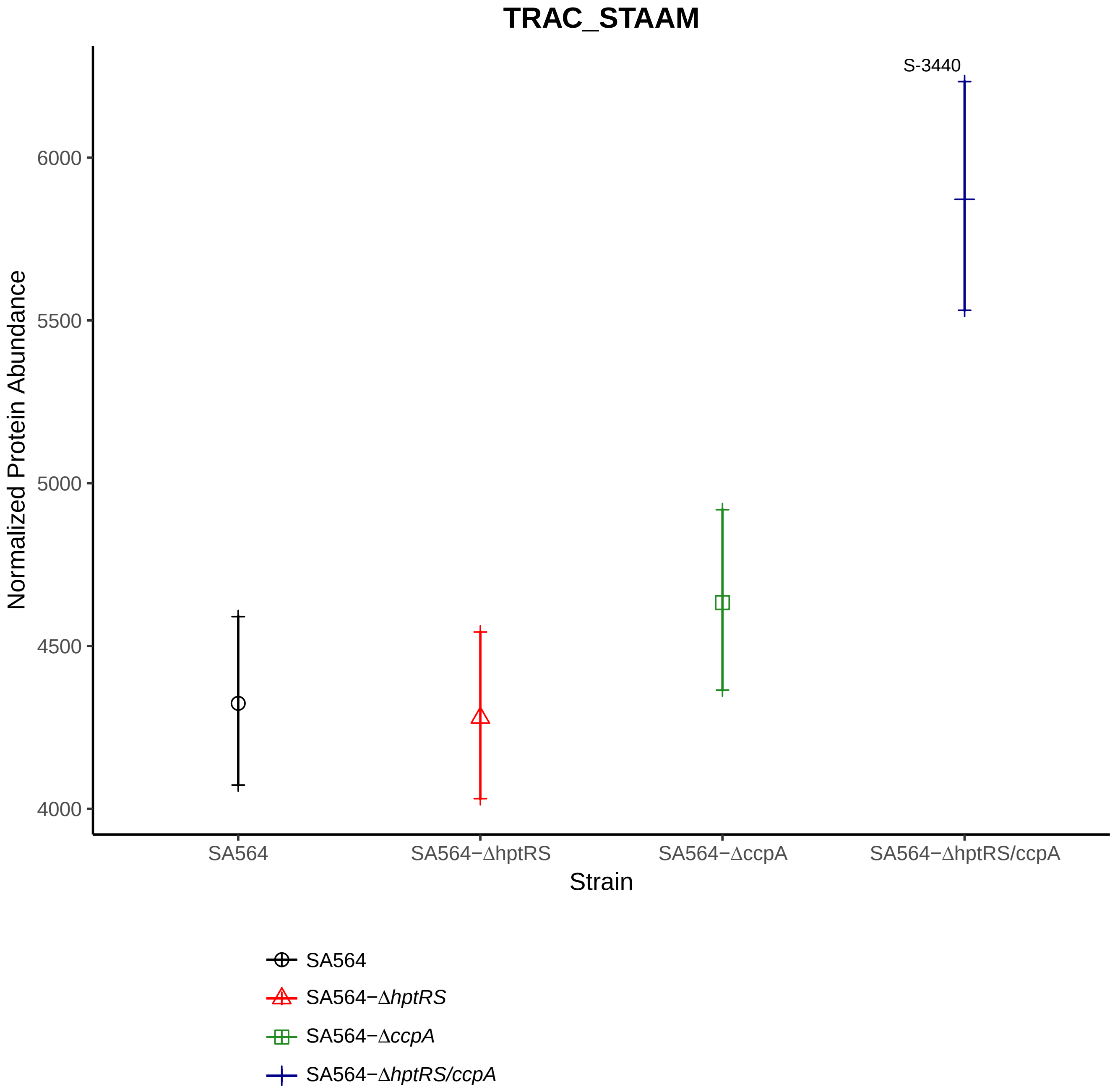

TRAC_STAAM
S-3440
Normalized Protein Abundance
6000
5500
5000
4500
4000
SA564
SA564−ΔhptRS
SA564−ΔccpA
SA564−ΔhptRS/ccpA
Strain
SA564
SA564−ΔhptRS
SA564−ΔccpA
SA564−ΔhptRS/ccpA

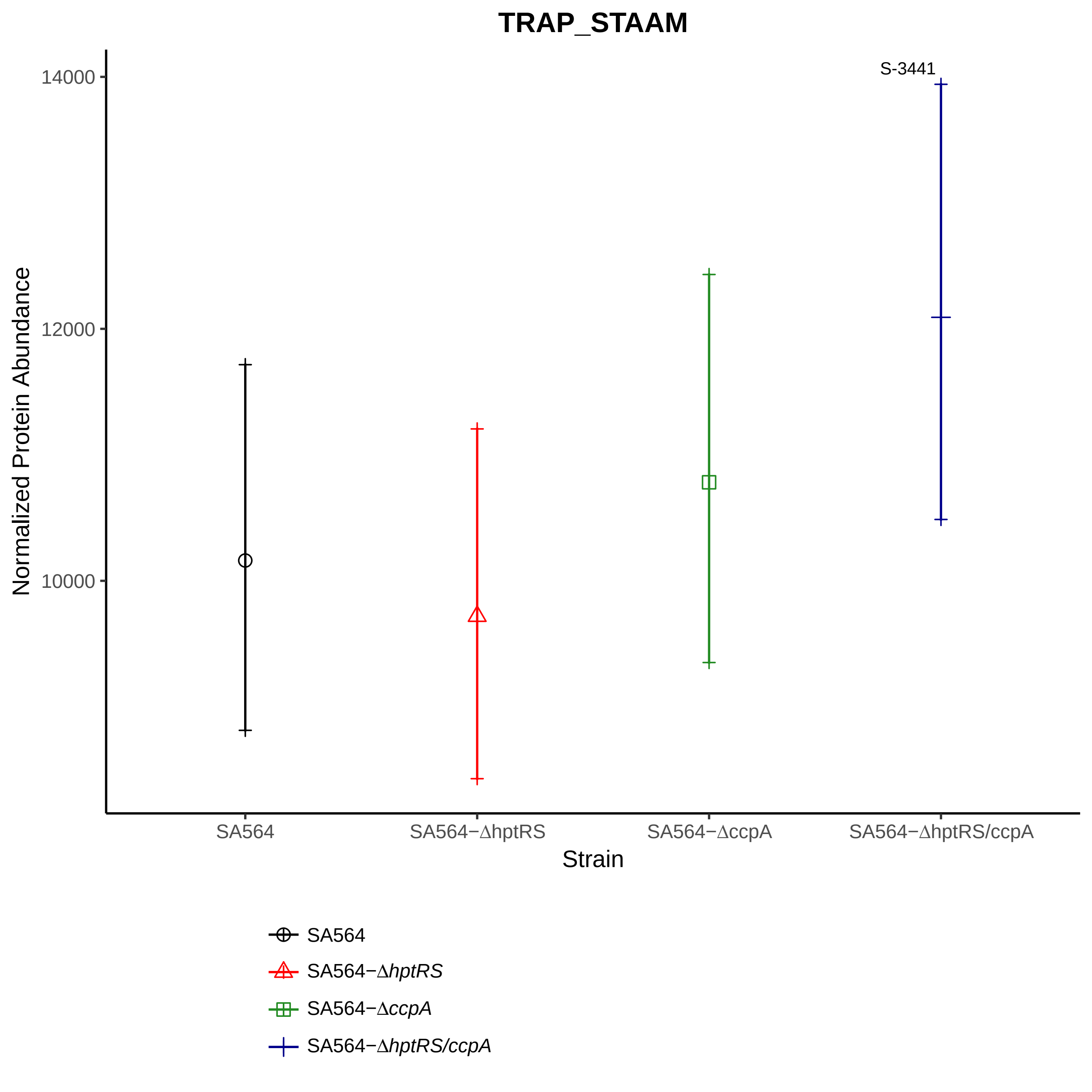

TRAP_STAAM
S-3441
14000
12000
10000
Normalized Protein Abundance
SA564
SA564−ΔhptRS
SA564−ΔccpA
SA564−ΔhptRS/ccpA
Strain
SA564
SA564−Δ*hptRS*
SA564−Δ*ccpA*
SA564−Δ*hptRS/ccpA*

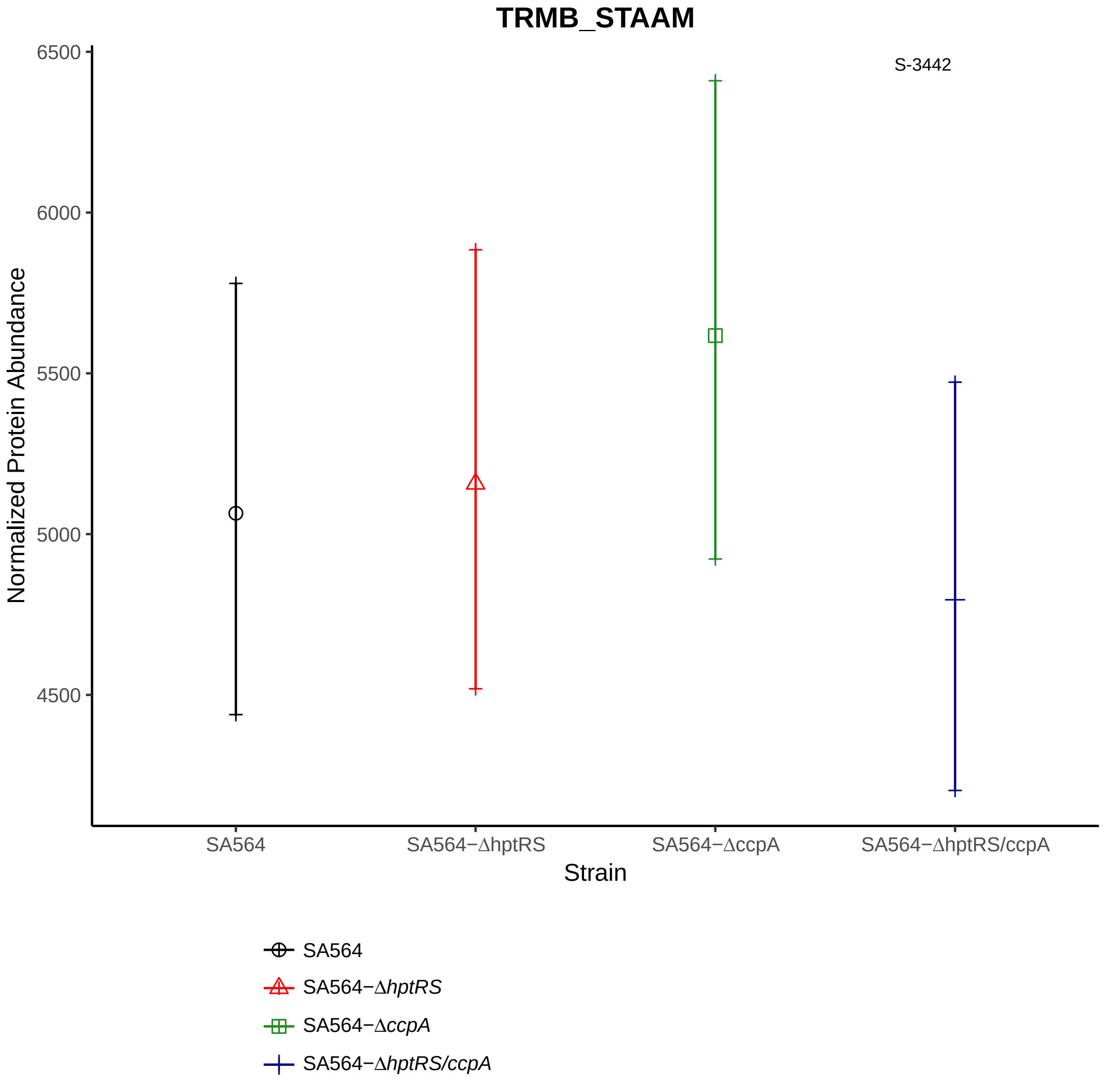

TRMB_STAAM
S-3442
Normalized Protein Abundance
6500
6000
5500
5000
4500
SA564
SA564−ΔhptRS
SA564−ΔccpA
SA564−ΔhptRS/ccpA
Strain
SA564
SA564−ΔhptRS
SA564−ΔccpA
SA564−ΔhptRS/ccpA

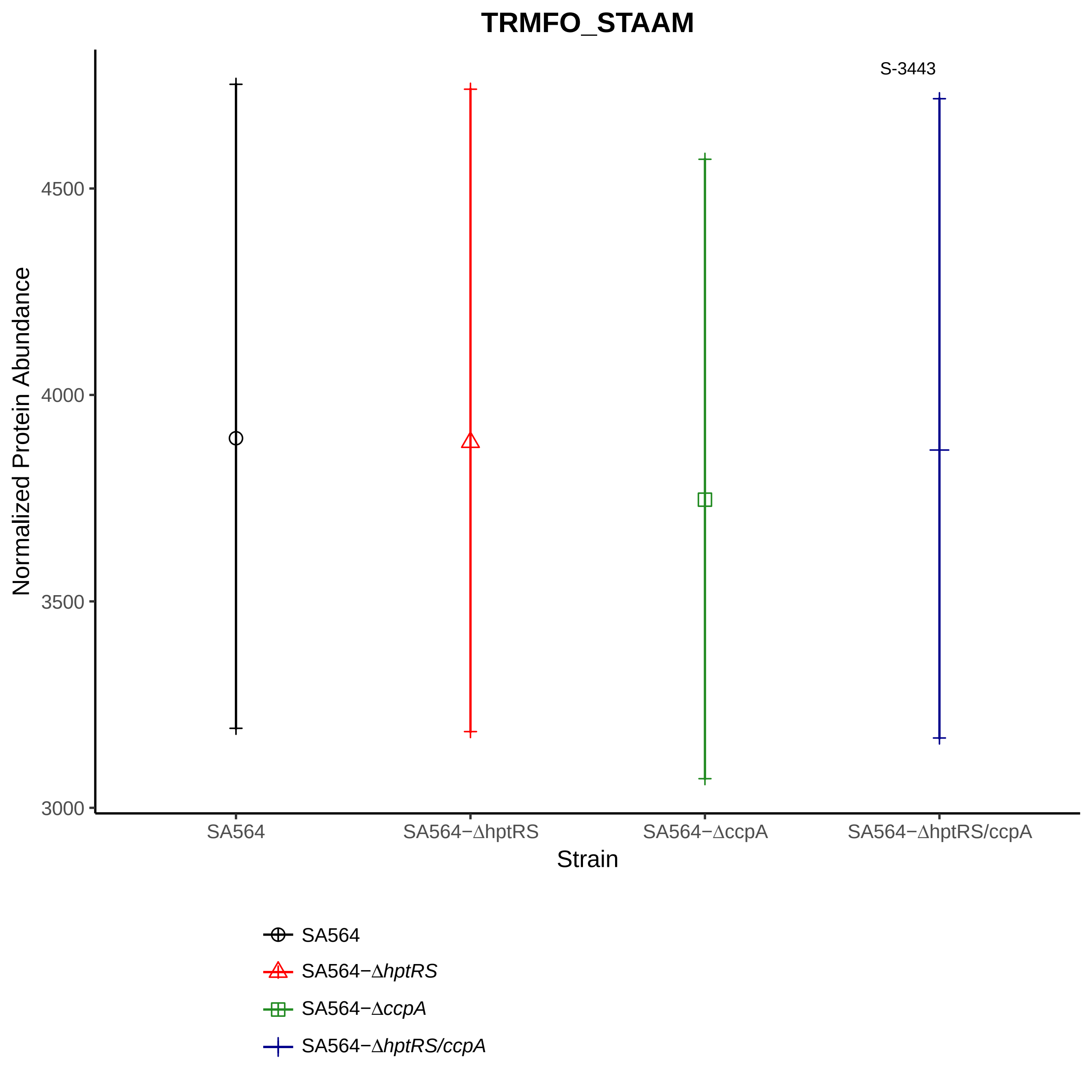
TRMFO_STAAM
S-3443
Normalized Protein Abundance
4500
4000
3500
3000
SA564
SA564−ΔhptRS
SA564−ΔccpA
SA564−ΔhptRS/ccpA
Strain
SA564
SA564−ΔhptRS
SA564−ΔccpA
SA564−ΔhptRS/ccpA

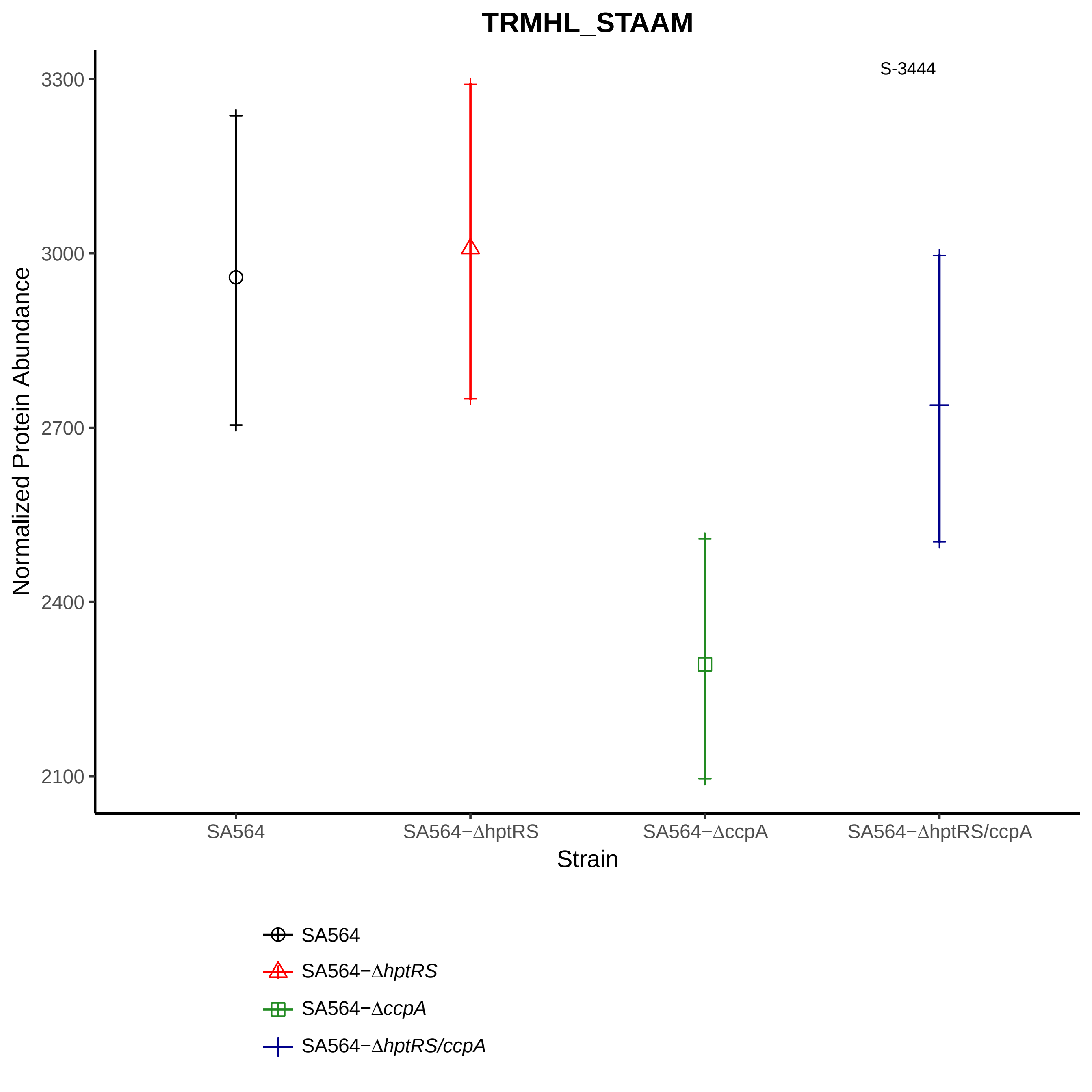

TRMHL_STAAM
S-3444
Normalized Protein Abundance
3300
3000
2700
2400
2100
SA564
SA564−ΔhptRS
SA564−ΔccpA
SA564−ΔhptRS/ccpA
Strain
SA564
SA564−ΔhptRS
SA564−ΔccpA
SA564−ΔhptRS/ccpA

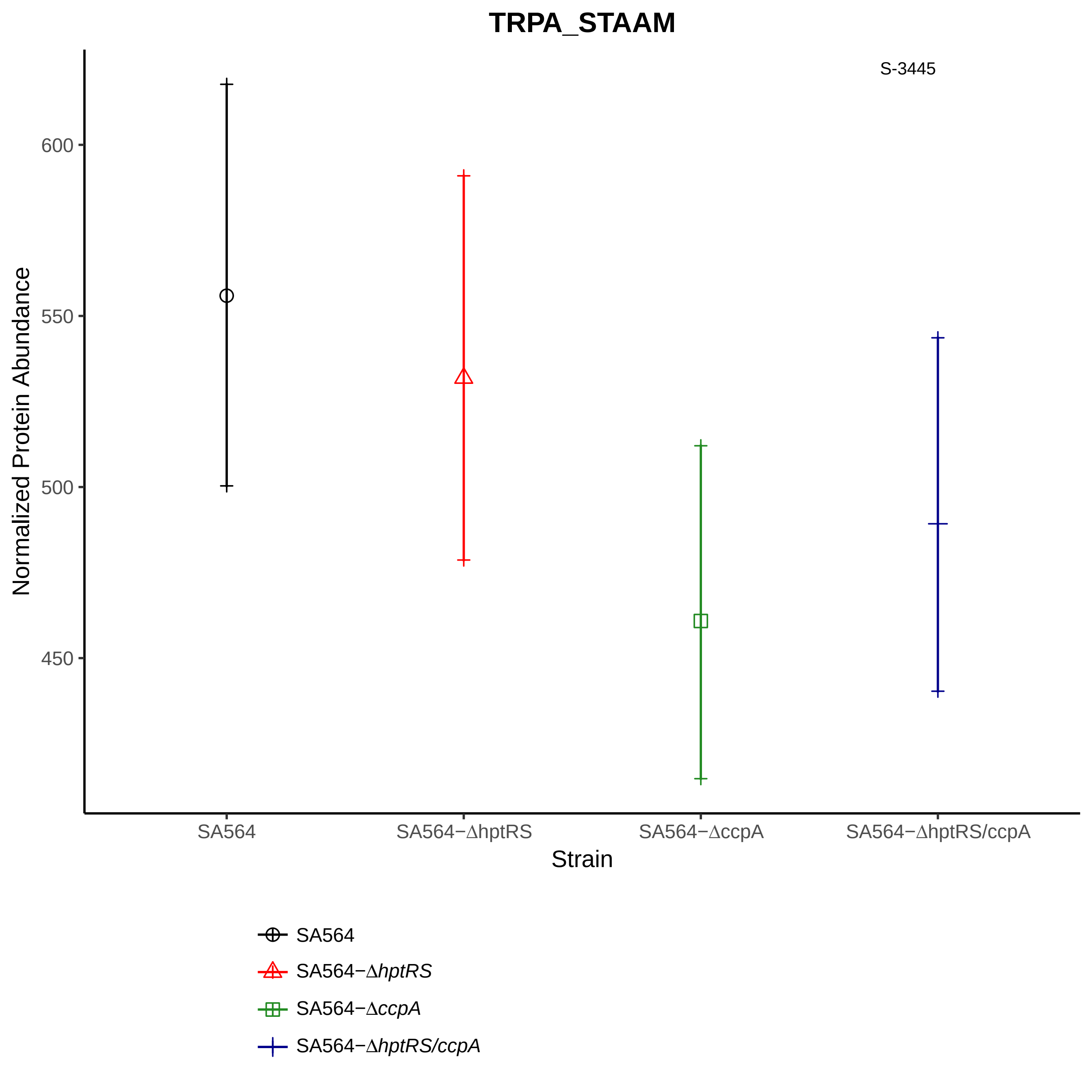

TRPA_STAAM
S-3445
Normalized Protein Abundance
600
550
500
450
SA564
SA564−ΔhptRS
SA564−ΔccpA
SA564−ΔhptRS/ccpA
Strain
SA564
SA564−ΔhptRS
SA564−ΔccpA
SA564−ΔhptRS/ccpA

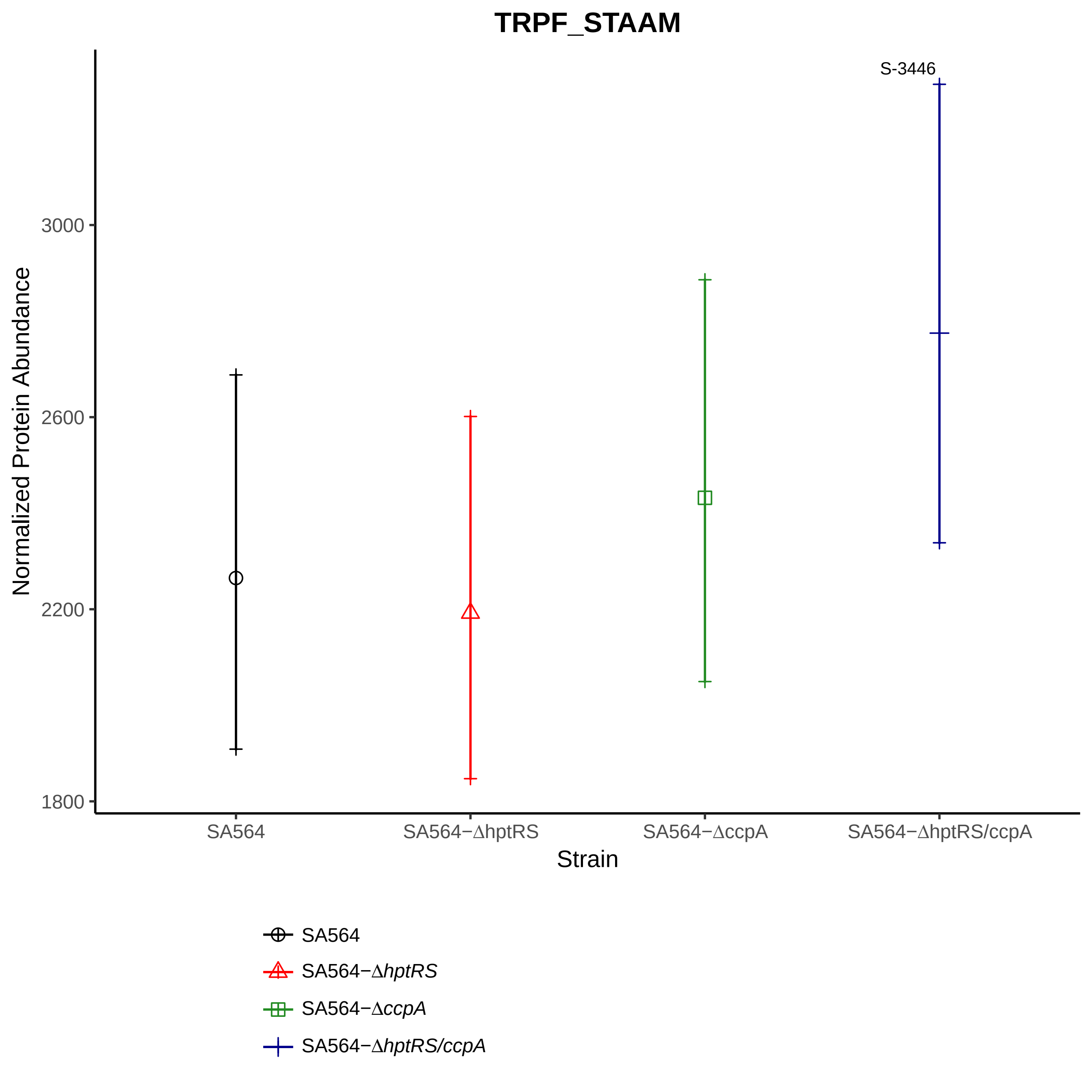

TRPF_STAAM
S-3446
Normalized Protein Abundance
3000
2600
2200
1800
SA564
SA564−ΔhptRS
SA564−ΔccpA
SA564−ΔhptRS/ccpA
Strain
SA564
SA564−ΔhptRS
SA564−ΔccpA
SA564−ΔhptRS/ccpA

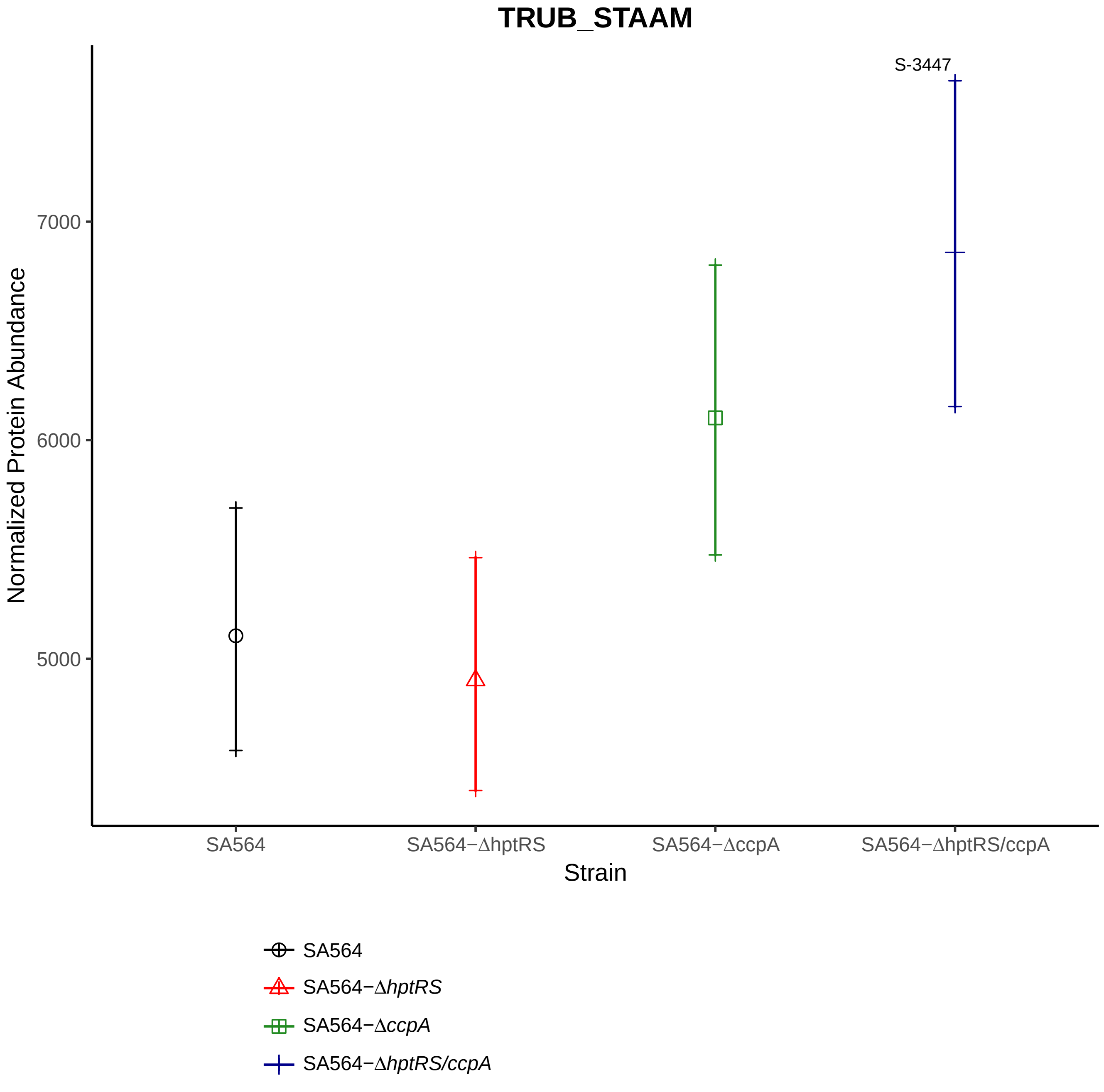

TRUB_STAAM
S-3447
Normalized Protein Abundance
7000
6000
5000
SA564
SA564−ΔhptRS
SA564−ΔccpA
SA564−ΔhptRS/ccpA
Strain
SA564
SA564−ΔhptRS
SA564−ΔccpA
SA564−ΔhptRS/ccpA

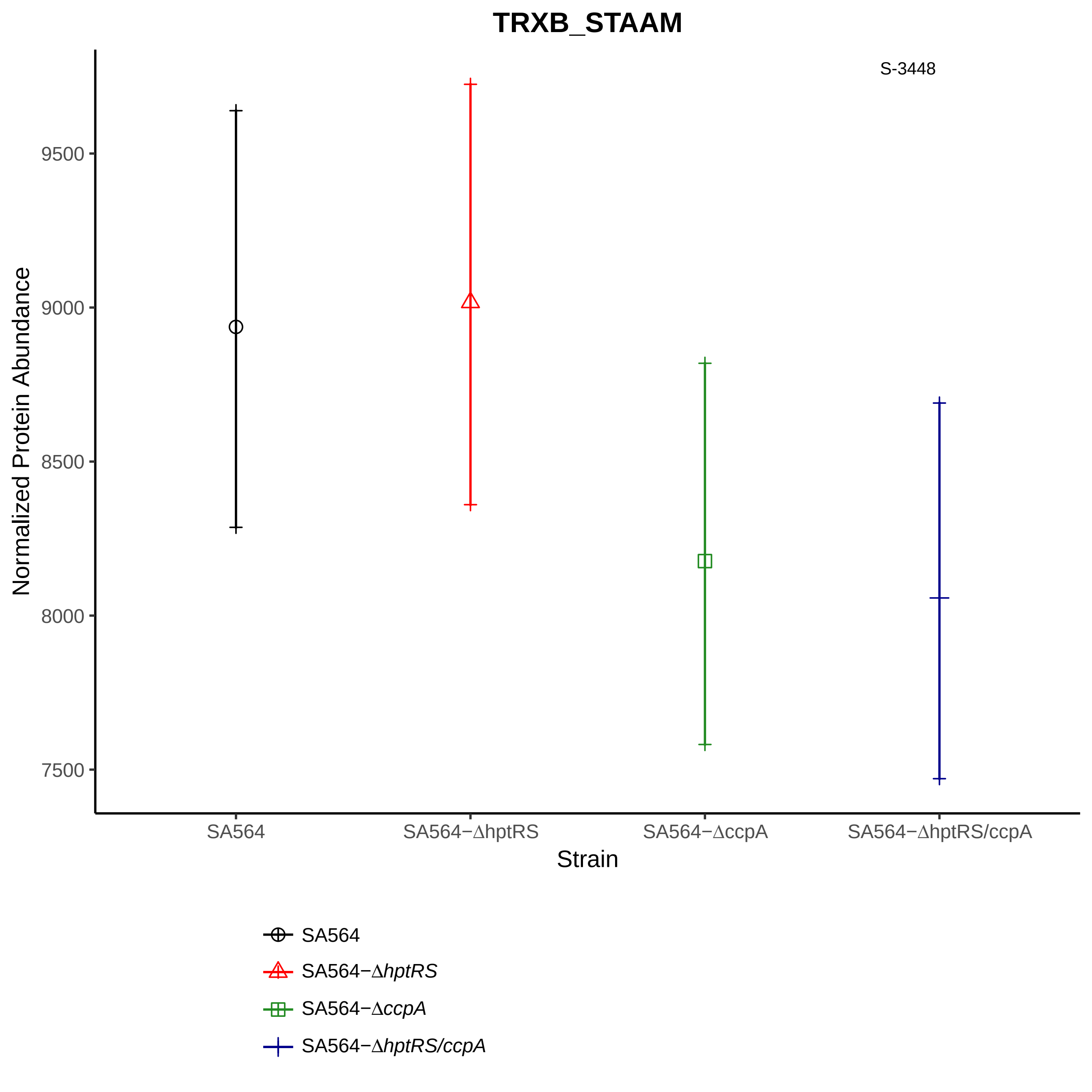

TRXB_STAAM
S-3448
Normalized Protein Abundance
9500
9000
8500
8000
7500
SA564
SA564−ΔhptRS
SA564−ΔccpA
SA564−ΔhptRS/ccpA
Strain
SA564
SA564−ΔhptRS
SA564−ΔccpA
SA564−ΔhptRS/ccpA

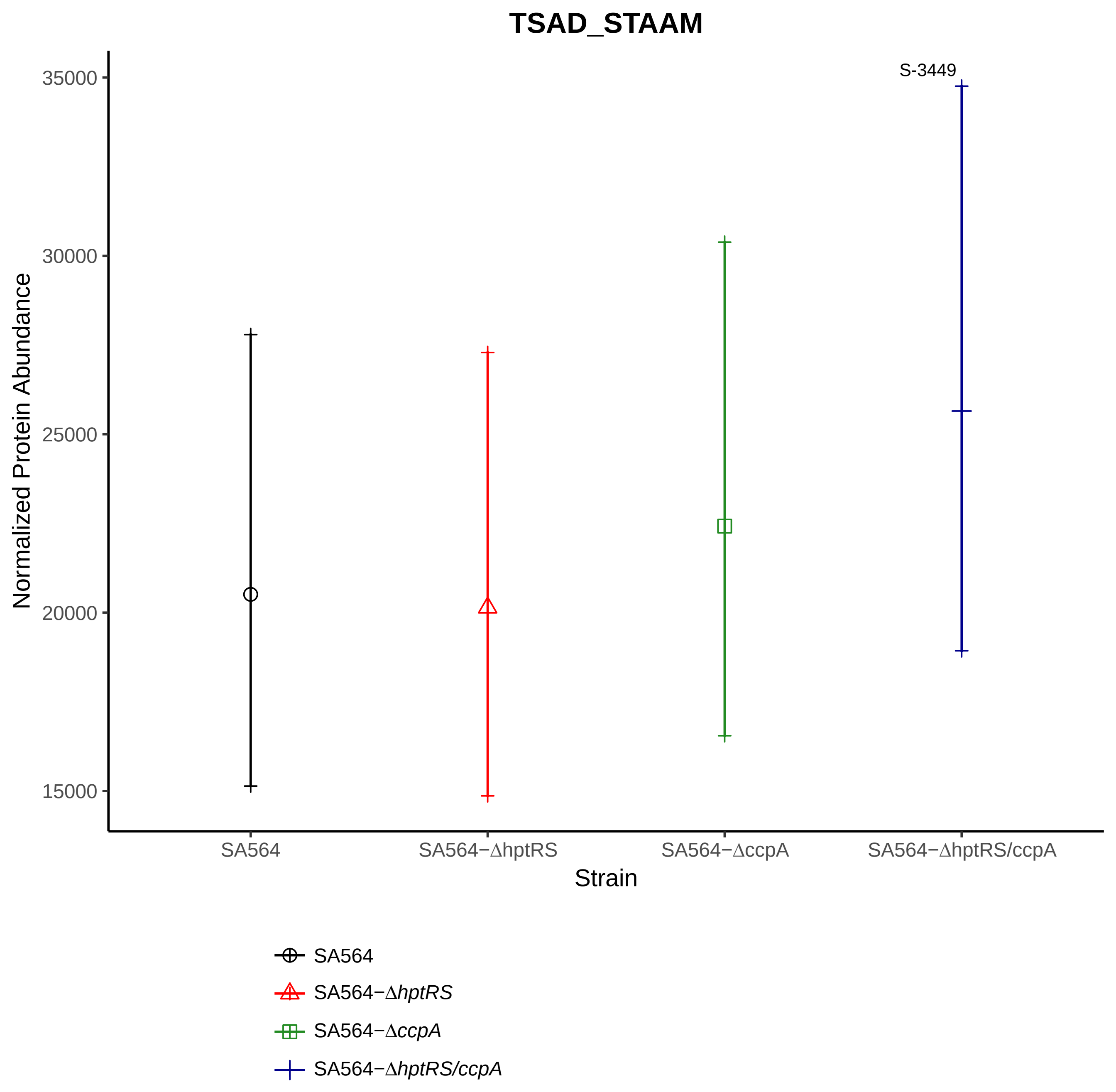
TSAD_STAAM
S-3449
Normalized Protein Abundance
35000
30000
25000
20000
15000
SA564
SA564−ΔhptRS
SA564−ΔccpA
SA564−ΔhptRS/ccpA
Strain
SA564
SA564−ΔhptRS
SA564−ΔccpA
SA564−ΔhptRS/ccpA

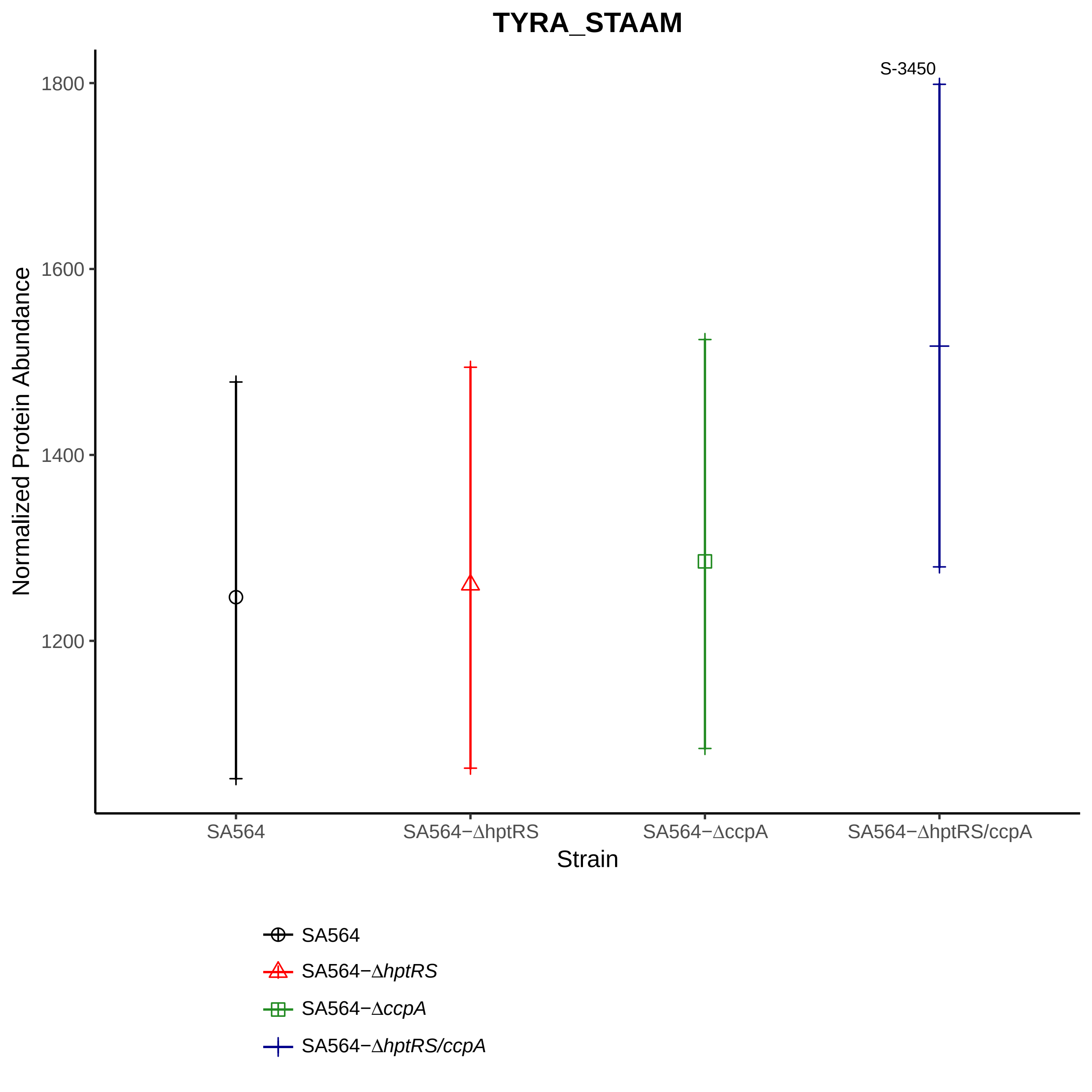

TYRA_STAAM
S-3450
1800
1600
1400
1200
Normalized Protein Abundance
SA564
SA564−ΔhptRS
SA564−ΔccpA
SA564−ΔhptRS/ccpA
Strain
SA564
SA564−ΔhptRS
SA564−ΔccpA
SA564−ΔhptRS/ccpA

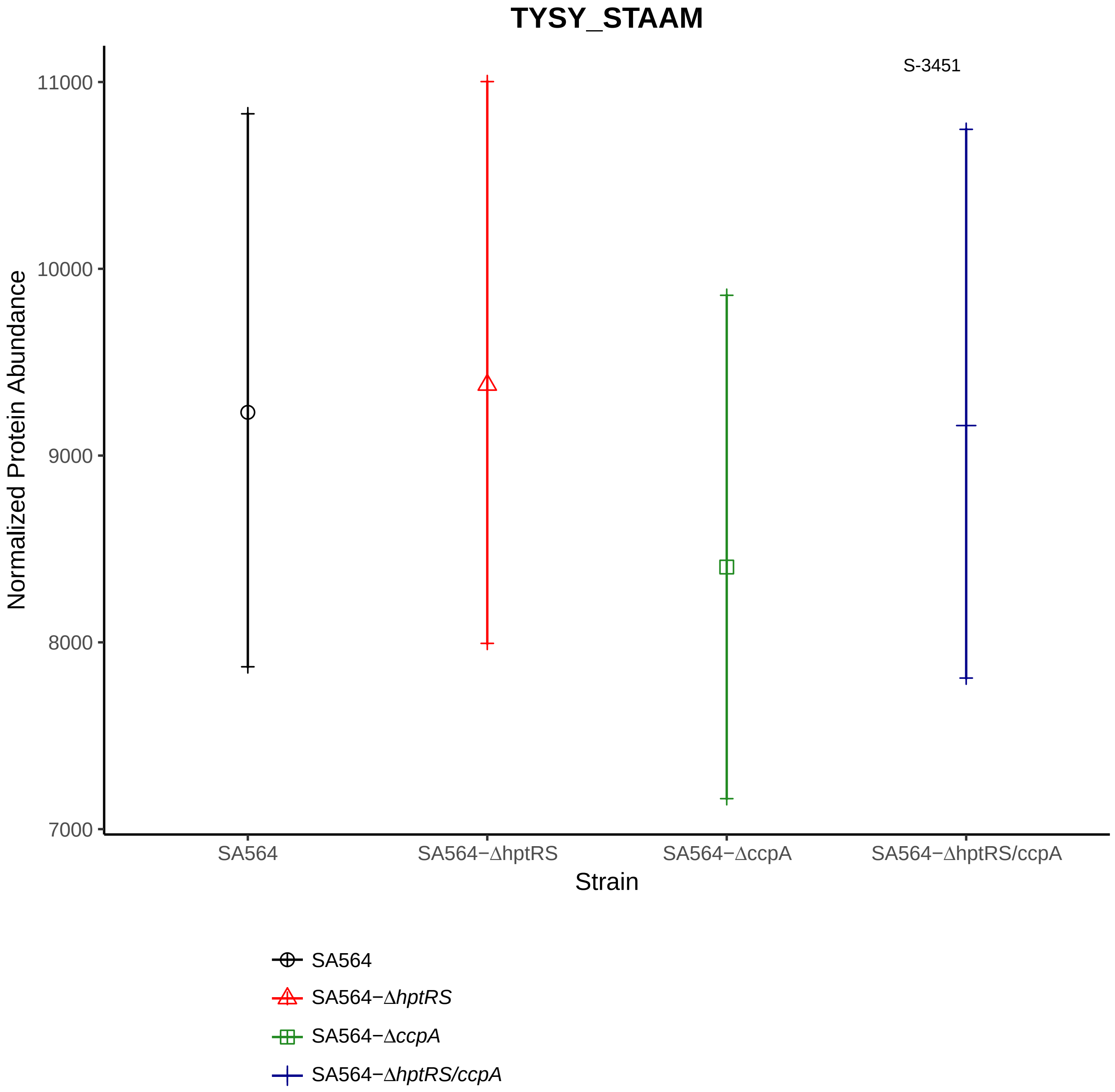
TYSY_STAAM
S-3451
Normalized Protein Abundance
11000
10000
9000
8000
7000
SA564
SA564−ΔhptRS
SA564−ΔccpA
SA564−ΔhptRS/ccpA
Strain
SA564
SA564−ΔhptRS
SA564−ΔccpA
SA564−ΔhptRS/ccpA

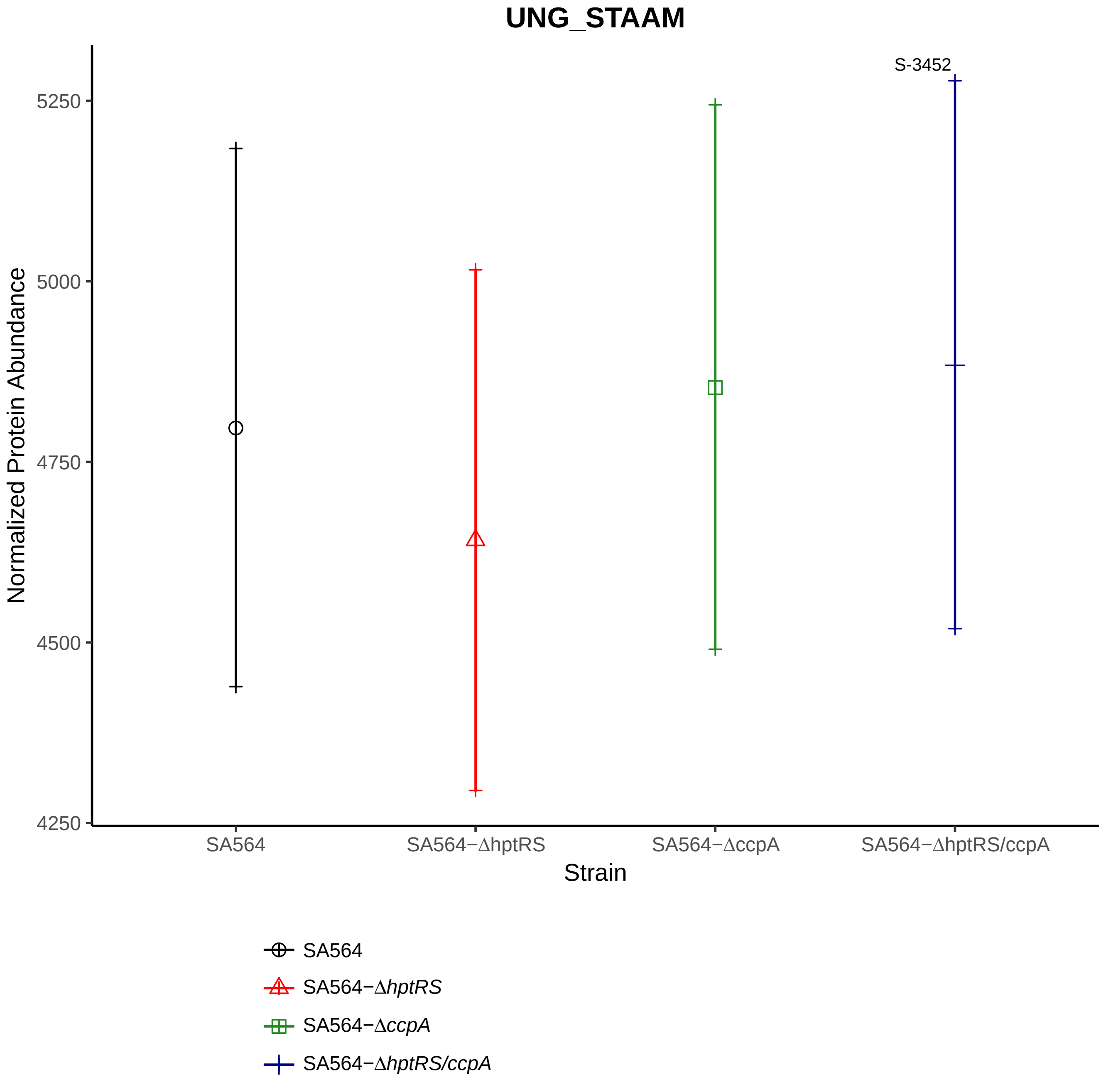

UNG_STAAM
S-3452
Normalized Protein Abundance
5250
5000
4750
4500
4250
SA564
SA564−ΔhptRS
SA564−ΔccpA
SA564−ΔhptRS/ccpA
Strain
SA564
SA564−ΔhptRS
SA564−ΔccpA
SA564−ΔhptRS/ccpA

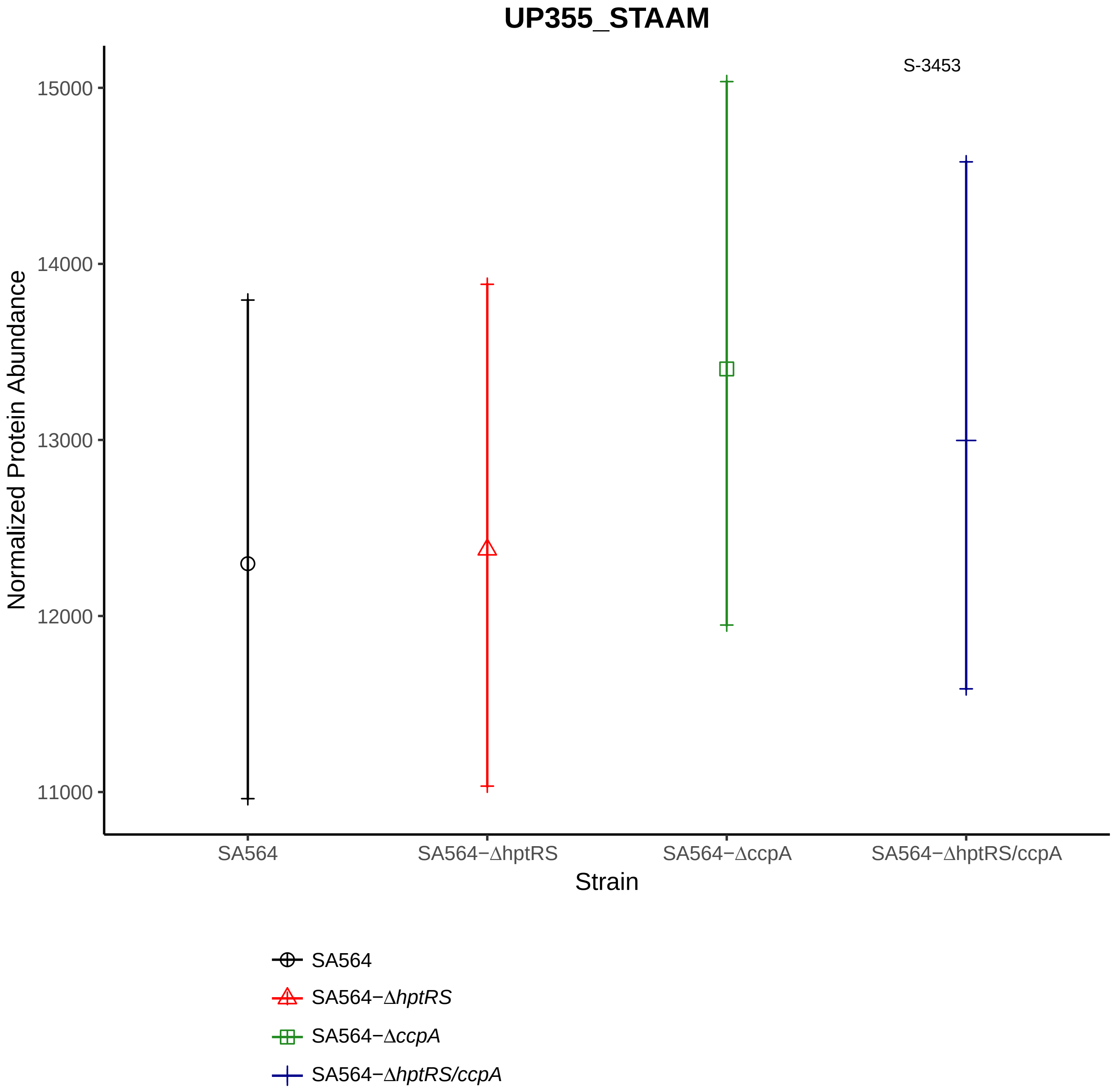

UP355_STAAM
S-3453
Normalized Protein Abundance
15000
14000
13000
12000
11000
SA564
SA564−ΔhptRS
SA564−ΔccpA
SA564−ΔhptRS/ccpA
Strain
SA564
SA564−ΔhptRS
SA564−ΔccpA
SA564−ΔhptRS/ccpA

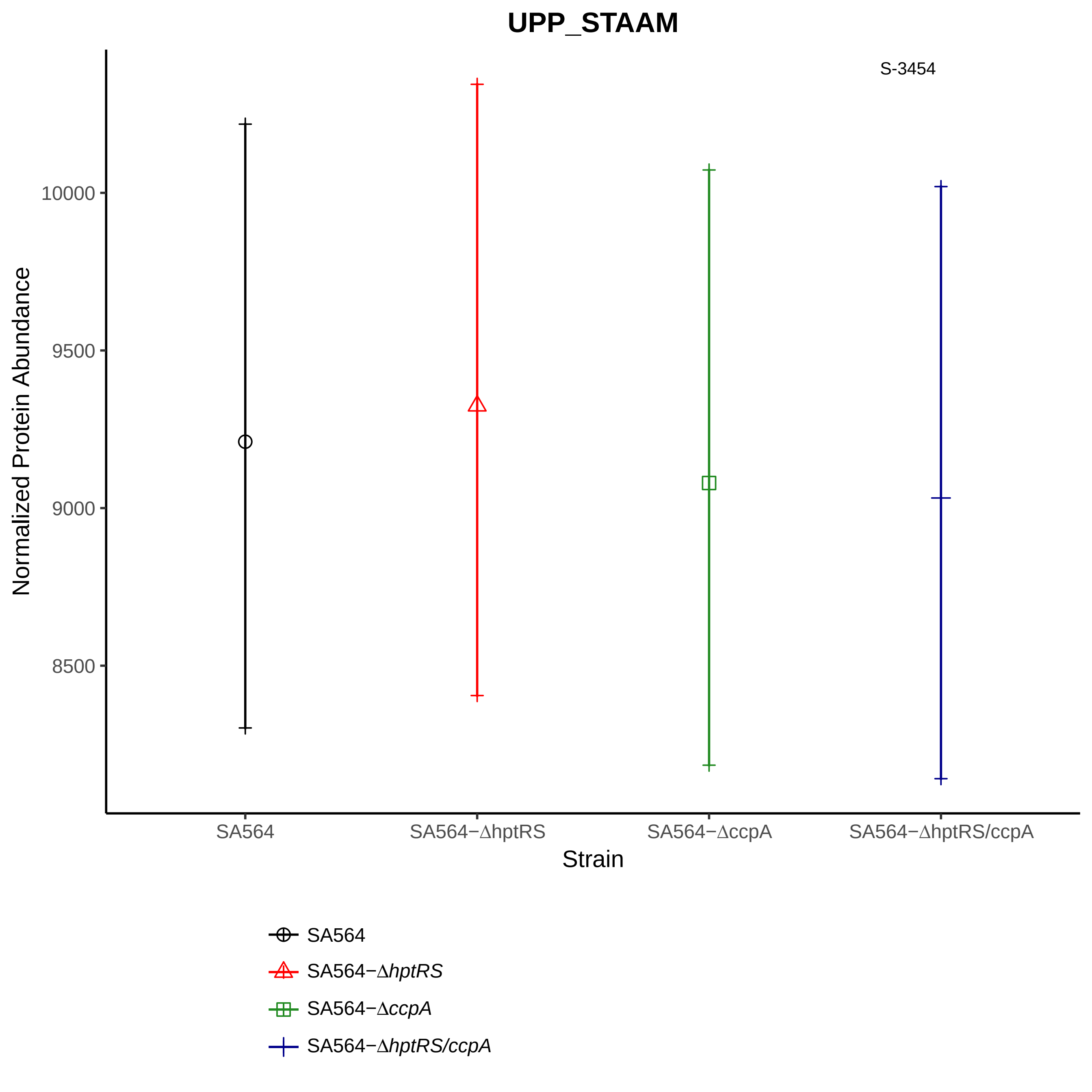
UPP_STAAM
Normalized Protein Abundance
10000
9500
9000
8500
SA564
SA564−ΔhptRS
SA564−ΔccpA
SA564−ΔhptRS/ccpA
Strain
SA564
SA564−Δ*hptRS*
SA564−Δ*ccpA*
SA564−Δ*hptRS/ccpA*

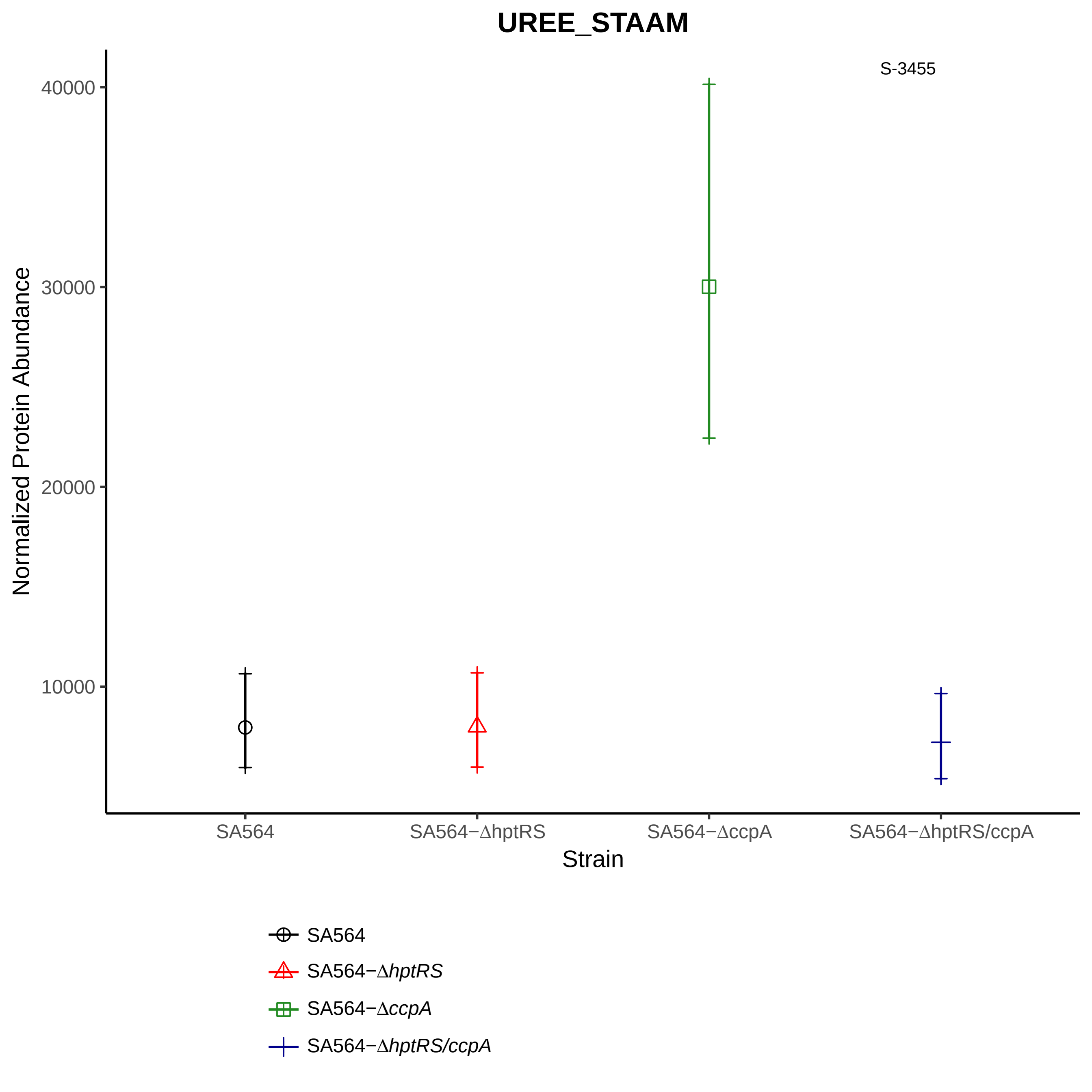
UREE_STAAM
S-3455
Normalized Protein Abundance
40000
30000
20000
10000
SA564
SA564−ΔhptRS
SA564−ΔccpA
SA564−ΔhptRS/ccpA
Strain
SA564
SA564−ΔhptRS
SA564−ΔccpA
SA564−ΔhptRS/ccpA

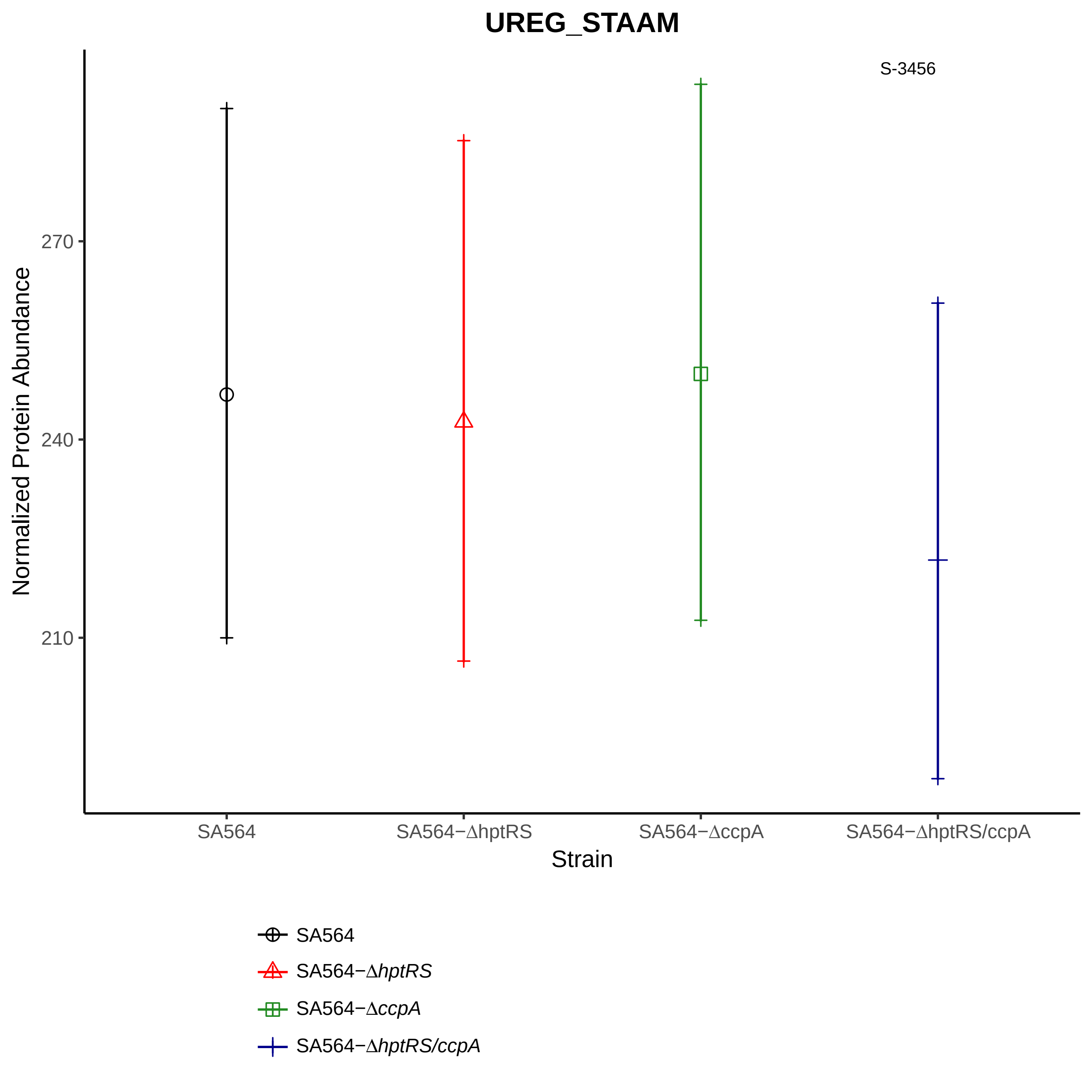
UREG_STAAM
S-3456
Normalized Protein Abundance
270
240
210
SA564
SA564−ΔhptRS
SA564−ΔccpA
SA564−ΔhptRS/ccpA
Strain
SA564
SA564−ΔhptRS
SA564−ΔccpA
SA564−ΔhptRS/ccpA

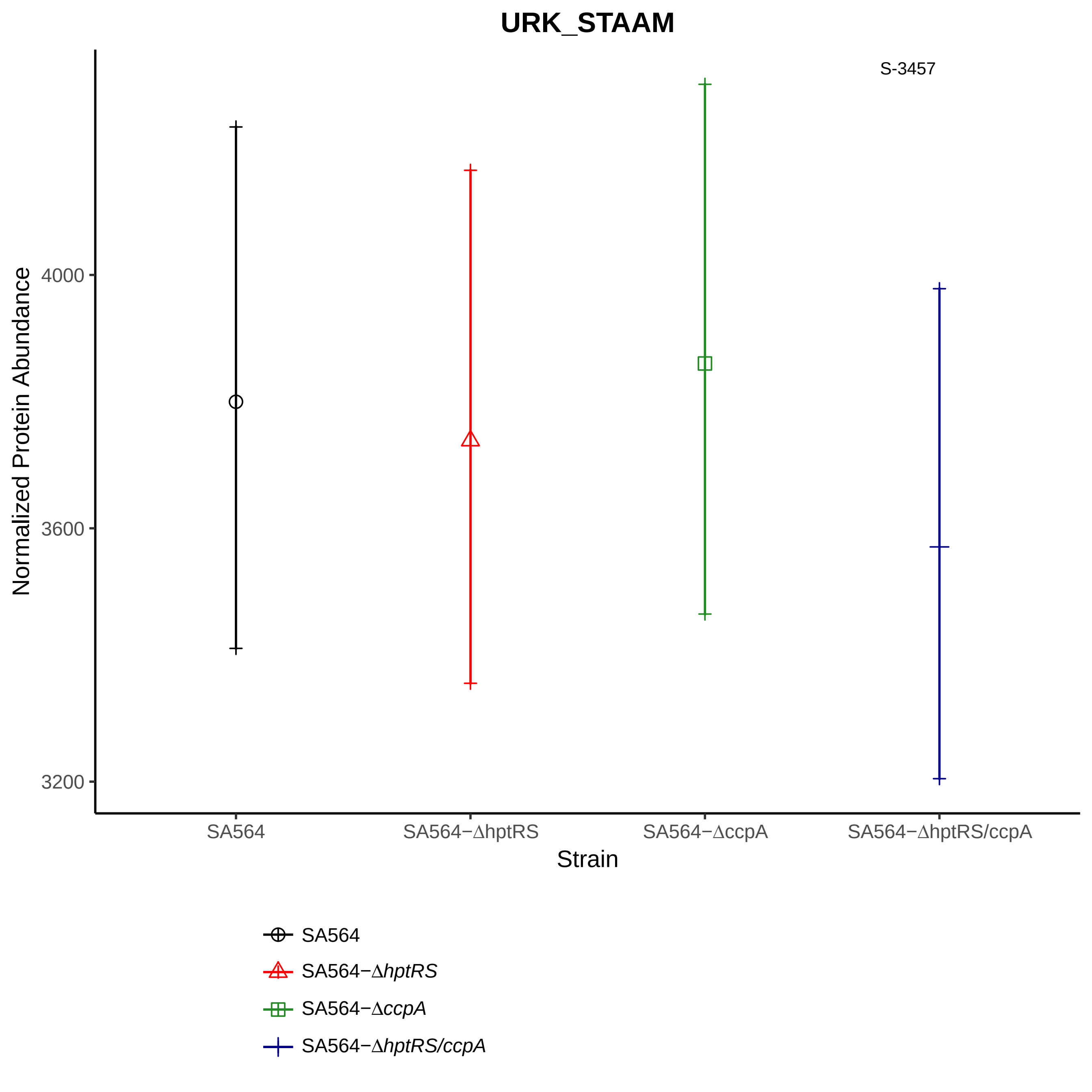

URK_STAAM
S-3457
Normalized Protein Abundance
4000
3600
3200
SA564
SA564−ΔhptRS
SA564−ΔccpA
SA564−ΔhptRS/ccpA
Strain
SA564
SA564−ΔhptRS
SA564−ΔccpA
SA564−ΔhptRS/ccpA

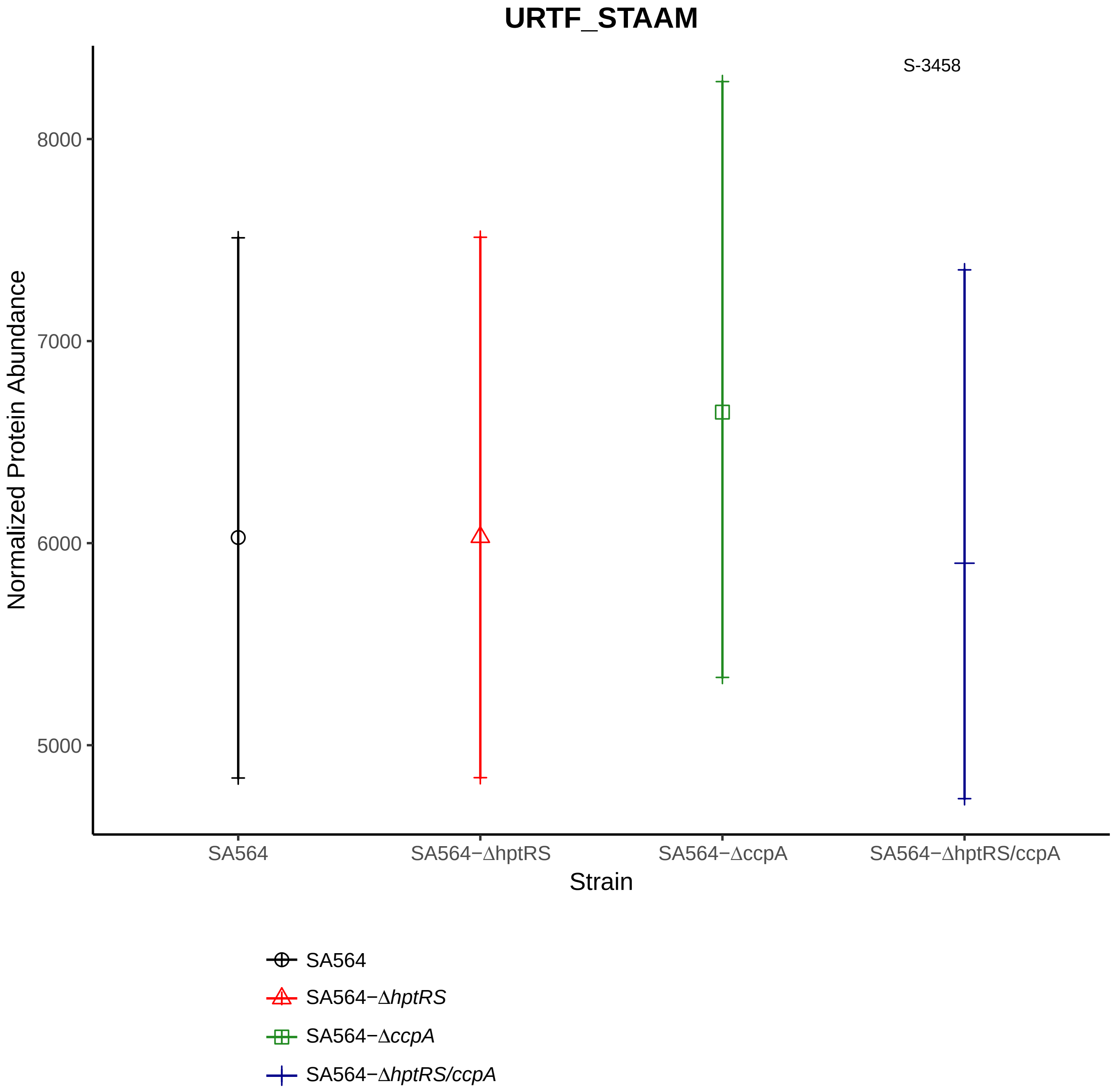
URTF_STAAM
S-3458
Normalized Protein Abundance
8000
7000
6000
5000
SA564
SA564−ΔhptRS
SA564−ΔccpA
SA564−ΔhptRS/ccpA
Strain
SA564
SA564−ΔhptRS
SA564−ΔccpA
SA564−ΔhptRS/ccpA

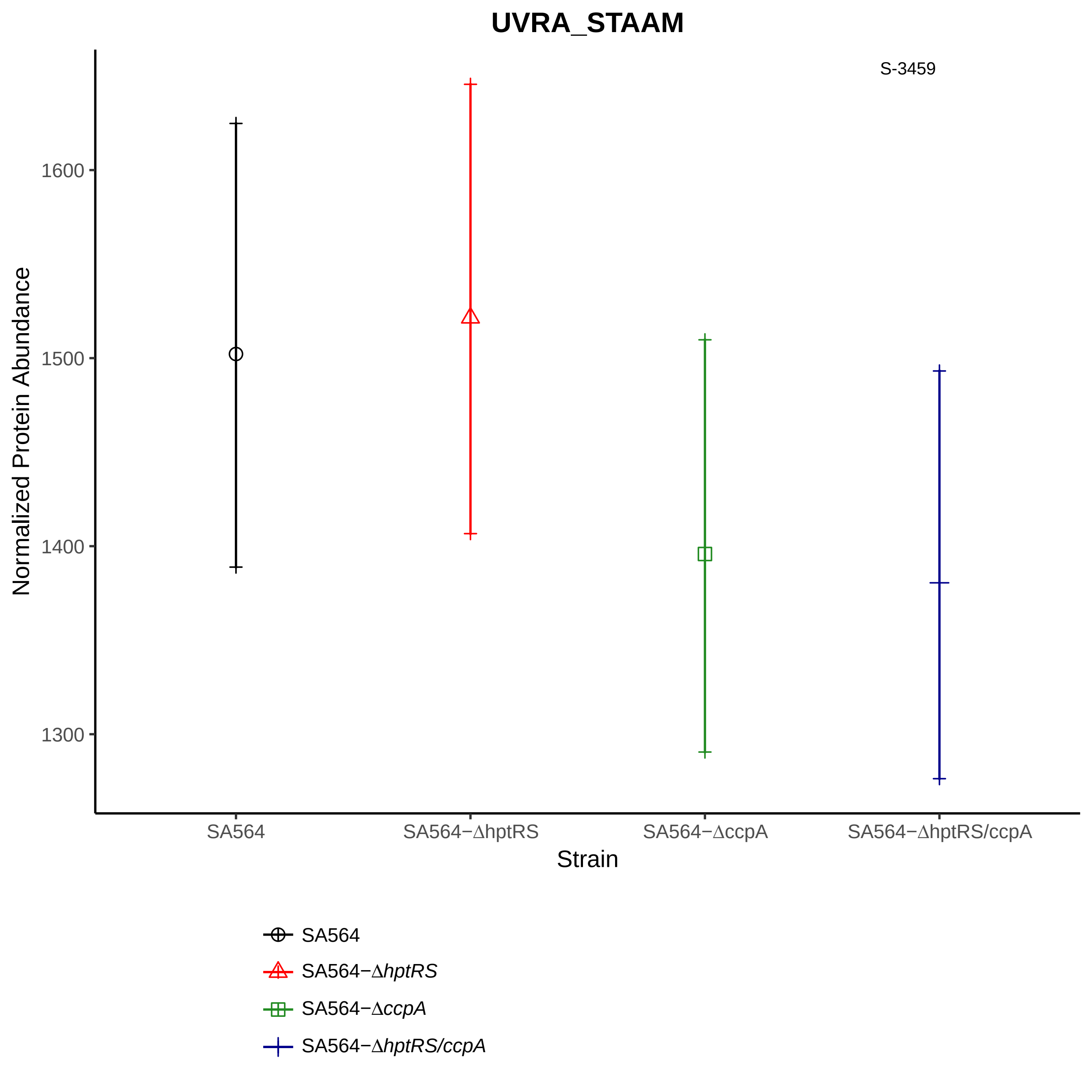

UVRA_STAAM
S-3459
Normalized Protein Abundance
1600
1500
1400
1300
SA564
SA564−ΔhptRS
SA564−ΔccpA
SA564−ΔhptRS/ccpA
Strain
SA564
SA564−ΔhptRS
SA564−ΔccpA
SA564−ΔhptRS/ccpA

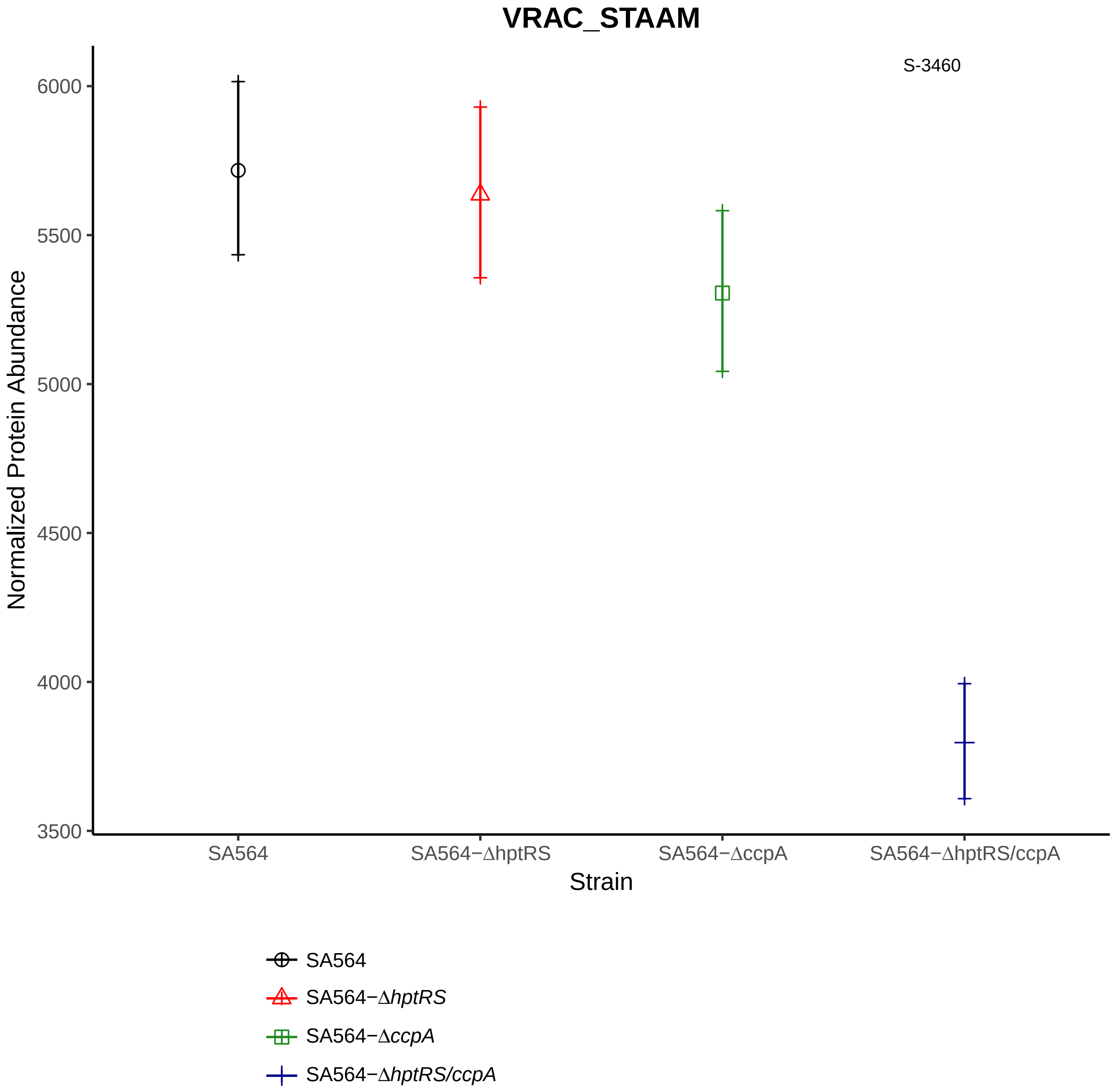
VRAC_STAAM
S-3460
Normalized Protein Abundance
6000
5500
5000
4500
4000
3500
SA564
SA564−ΔhptRS
SA564−ΔccpA
SA564−ΔhptRS/ccpA
Strain
SA564
SA564−ΔhptRS
SA564−ΔccpA
SA564−ΔhptRS/ccpA

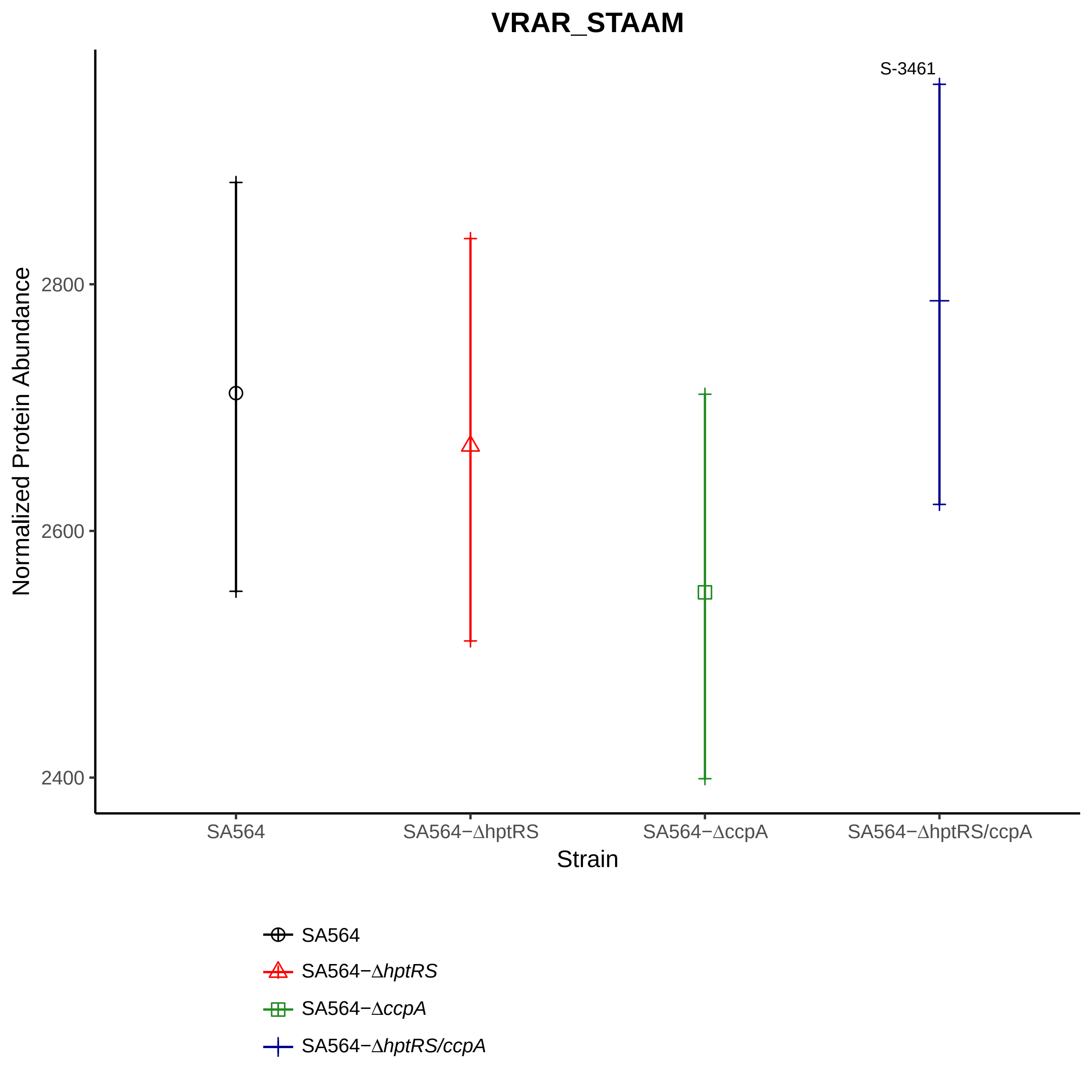

VRAR_STAAM
S-3461
Normalized Protein Abundance
2800
2600
2400
SA564
SA564−ΔhptRS
SA564−ΔccpA
SA564−ΔhptRS/ccpA
Strain
SA564
SA564−ΔhptRS
SA564−ΔccpA
SA564−ΔhptRS/ccpA

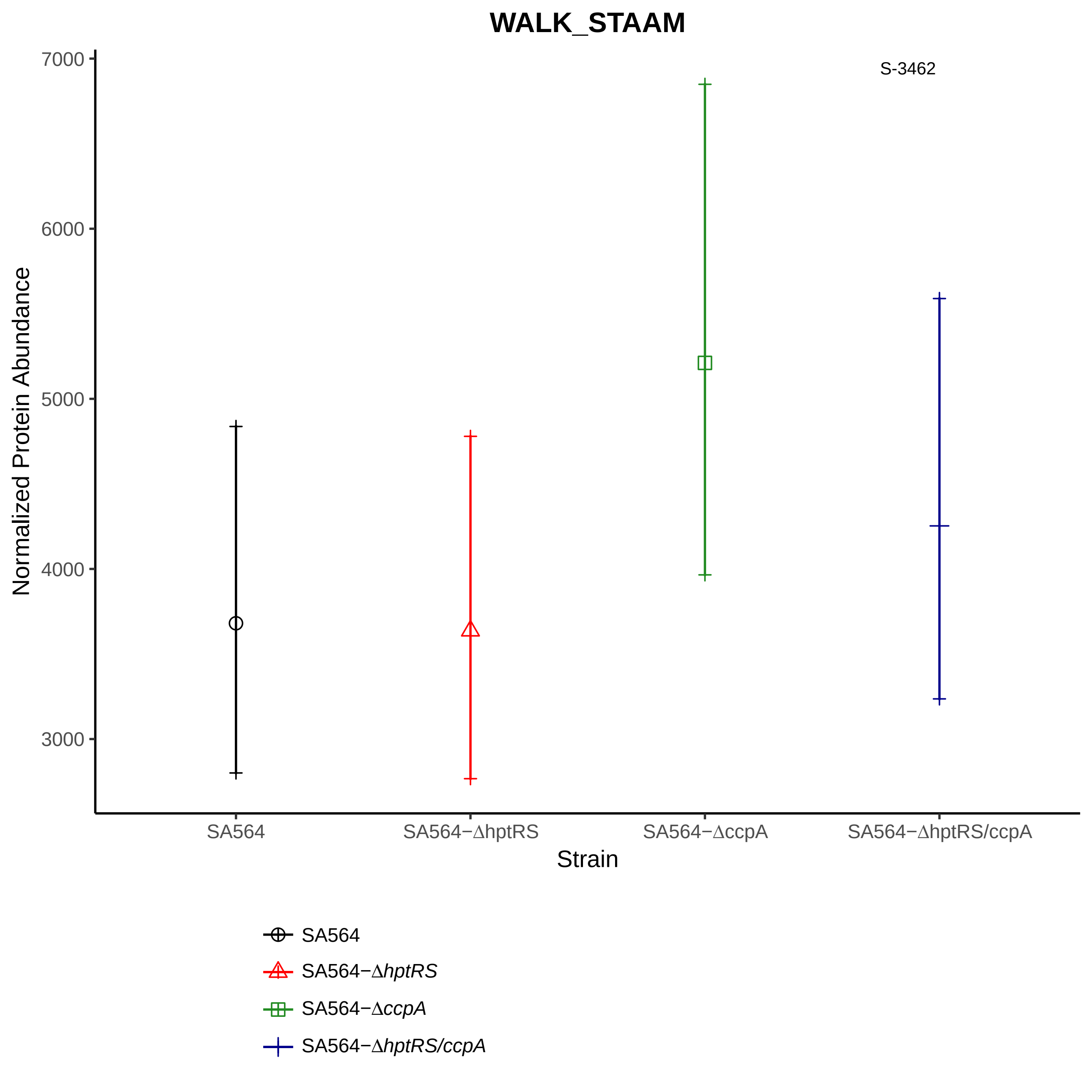

WALK_STAAM
S-3462
Normalized Protein Abundance
7000
6000
5000
4000
3000
SA564
SA564−ΔhptRS
SA564−ΔccpA
SA564−ΔhptRS/ccpA
Strain
SA564
SA564−Δ*hptRS*
SA564−Δ*ccpA*
SA564−Δ*hptRS/ccpA*

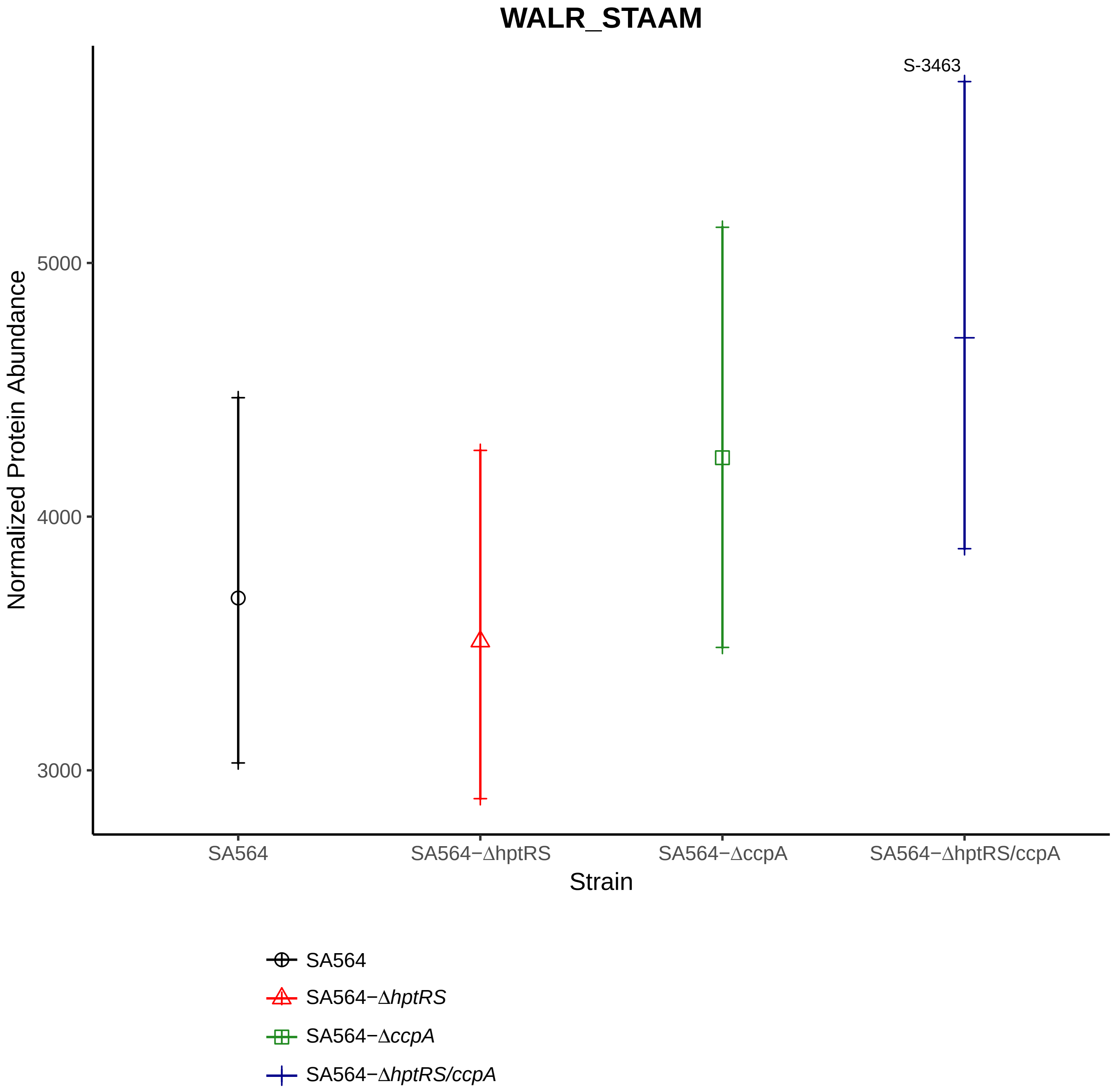

WALR_STAAM
S-3463
Normalized Protein Abundance
5000
4000
3000
SA564
SA564−ΔhptRS
SA564−ΔccpA
SA564−ΔhptRS/ccpA
Strain
SA564
SA564−ΔhptRS
SA564−ΔccpA
SA564−ΔhptRS/ccpA

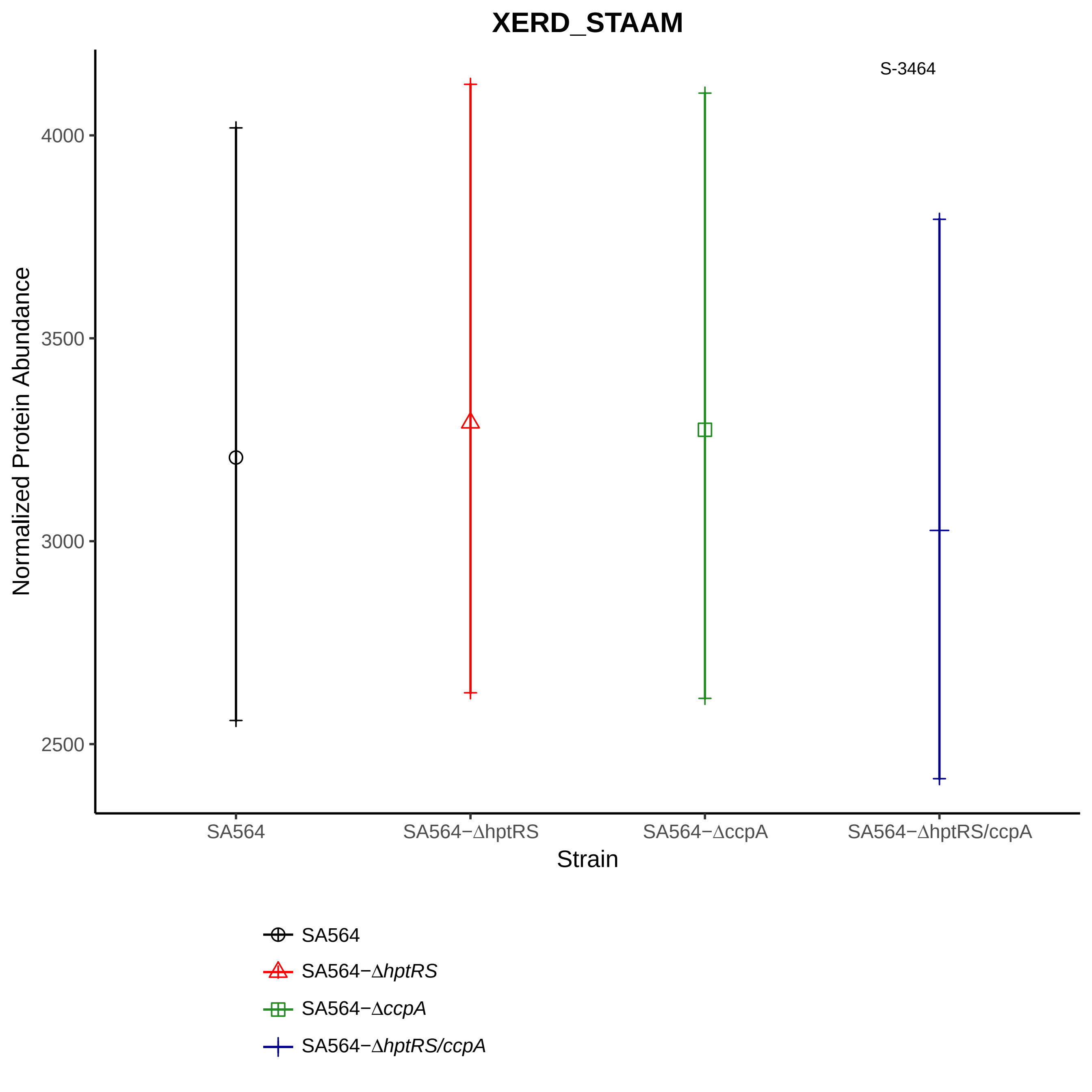
XERD_STAAM
S-3464
Normalized Protein Abundance
4000
3500
3000
2500
SA564
SA564−ΔhptRS
SA564−ΔccpA
SA564−ΔhptRS/ccpA
Strain
SA564
SA564−ΔhptRS
SA564−ΔccpA
SA564−ΔhptRS/ccpA

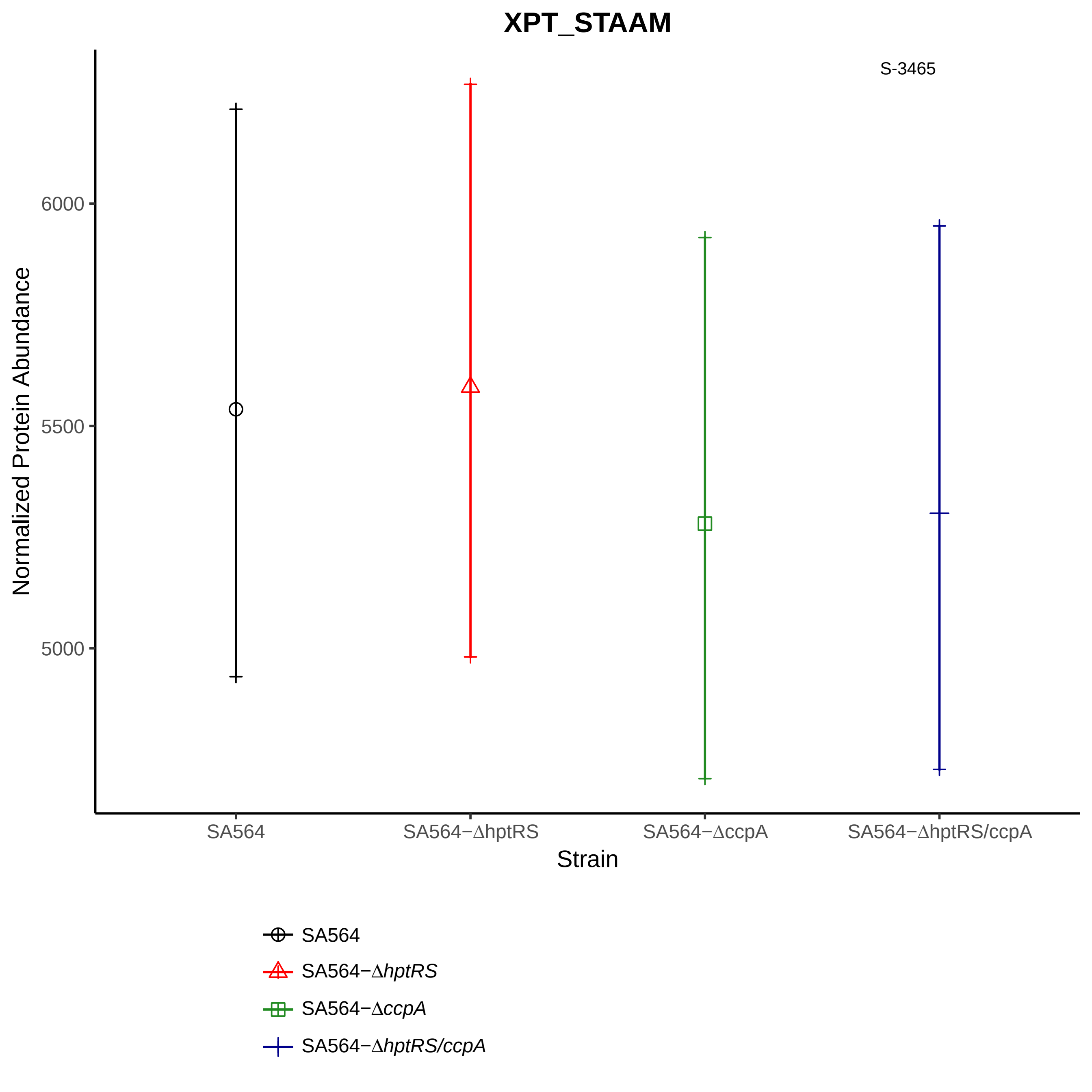

XPT_STAAM
S-3465
Normalized Protein Abundance
6000
5500
5000
SA564
SA564−ΔhptRS
SA564−ΔccpA
SA564−ΔhptRS/ccpA
Strain
SA564
SA564−ΔhptRS
SA564−ΔccpA
SA564−ΔhptRS/ccpA

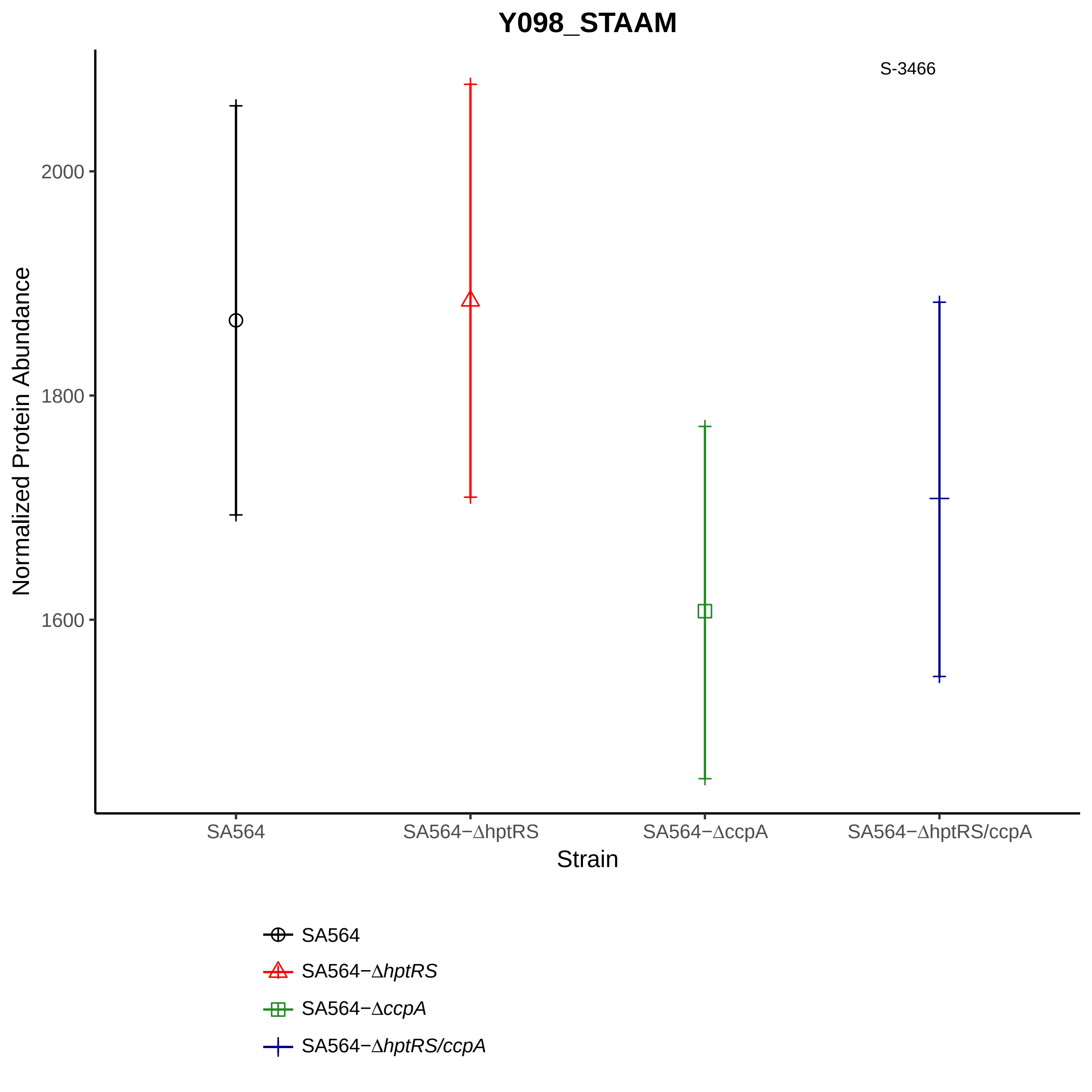

Y098_STAAM
S-3466
Normalized Protein Abundance
2000
1800
1600
SA564
SA564−ΔhptRS
SA564−ΔccpA
SA564−ΔhptRS/ccpA
Strain
SA564
SA564−ΔhptRS
SA564−ΔccpA
SA564−ΔhptRS/ccpA

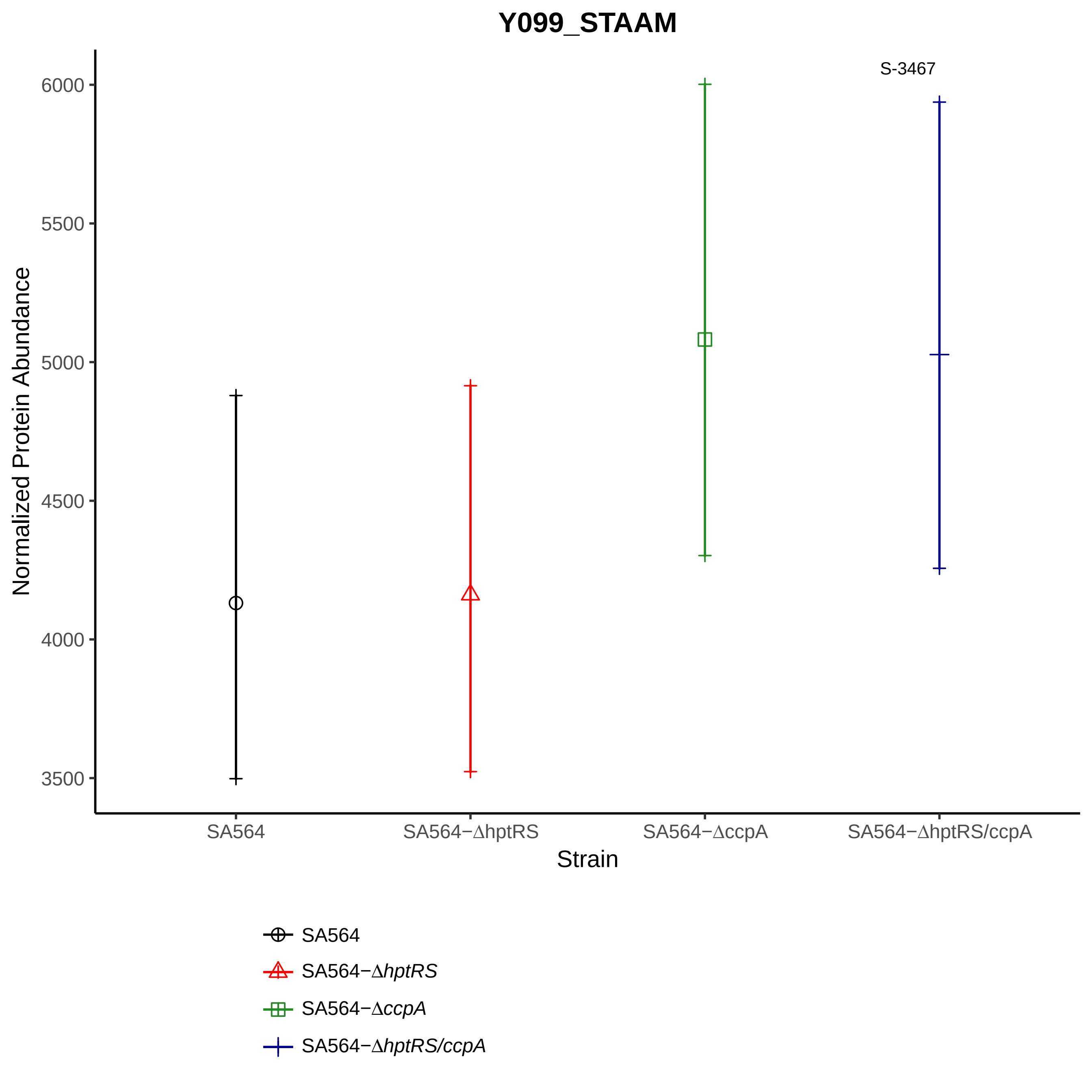

Y099_STAAM
S-3467
Normalized Protein Abundance
6000
5500
5000
4500
4000
3500
SA564
SA564−ΔhptRS
SA564−ΔccpA
SA564−ΔhptRS/ccpA
Strain
SA564
SA564−ΔhptRS
SA564−ΔccpA
SA564−ΔhptRS/ccpA

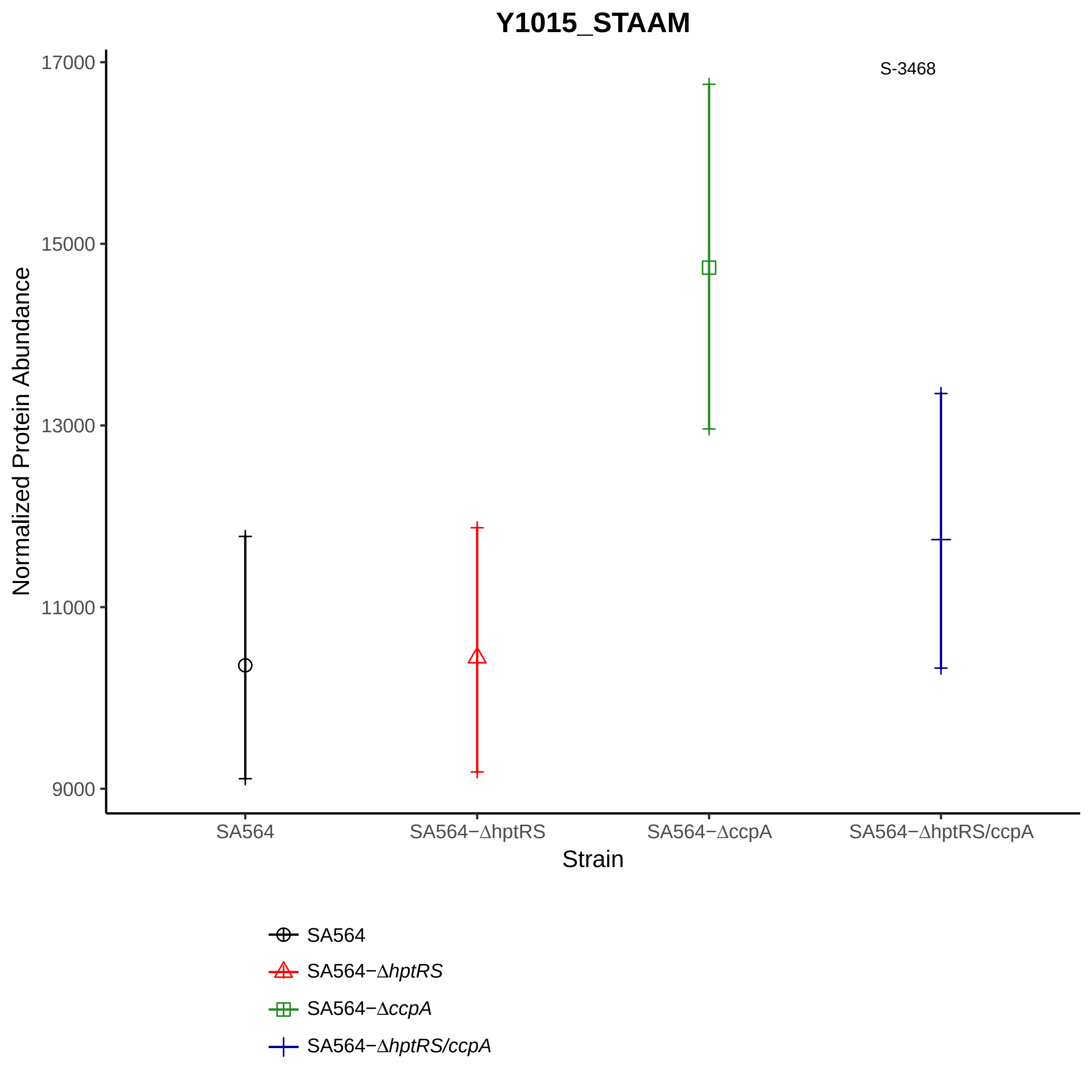

Y1015_STAAM
S-3468
Normalized Protein Abundance
17000
15000
13000
11000
9000
SA564
SA564−ΔhptRS
SA564−ΔccpA
SA564−ΔhptRS/ccpA
Strain
SA564
SA564−Δ*hptRS*
SA564−Δ*ccpA*
SA564−Δ*hptRS/ccpA*

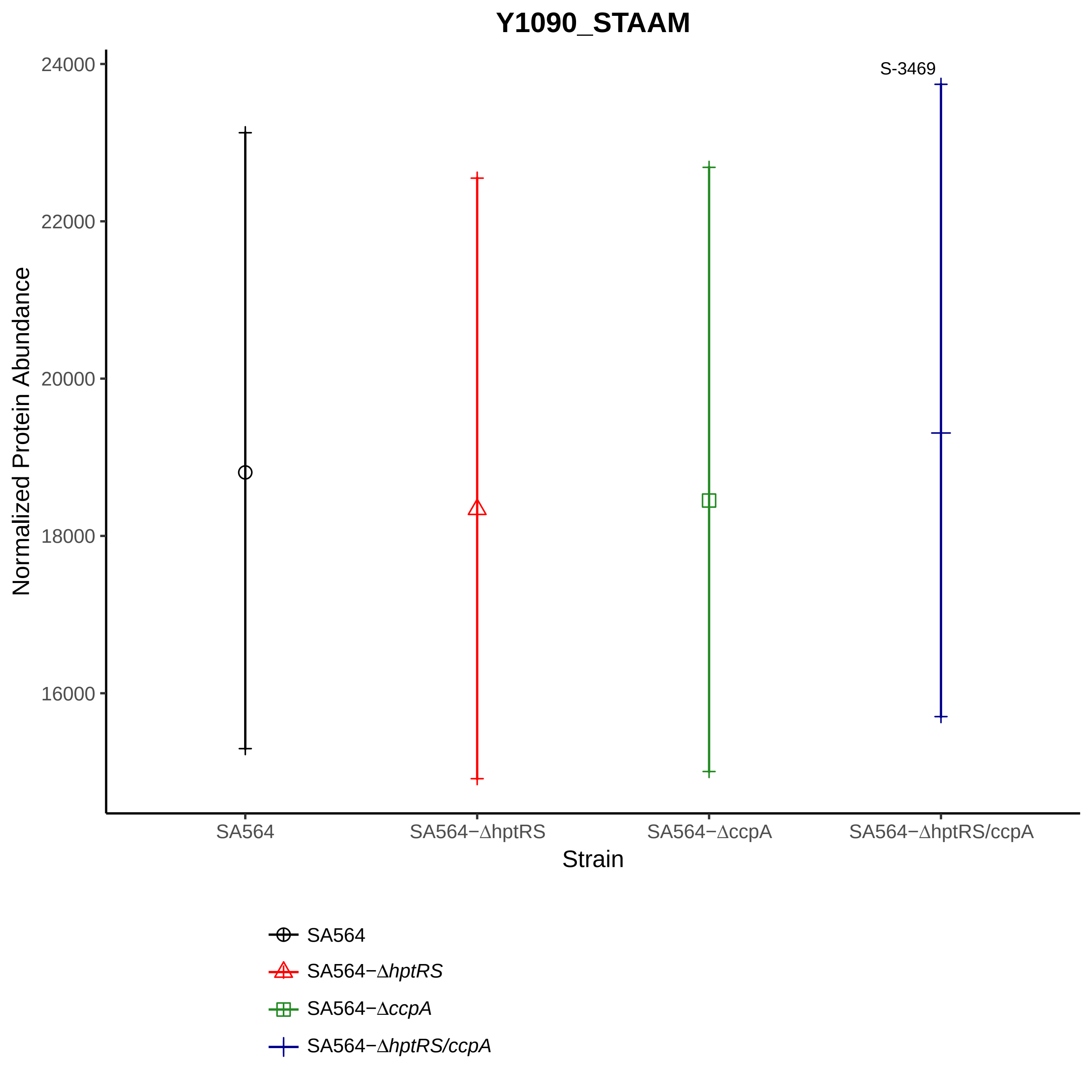

Y1090_STAAM
S-3469
24000
22000
20000
18000
16000
Normalized Protein Abundance
SA564
SA564−ΔhptRS
SA564−ΔccpA
SA564−ΔhptRS/ccpA
Strain
SA564
SA564−Δ*hptRS*
SA564−Δ*ccpA*
SA564−Δ*hptRS/ccpA*

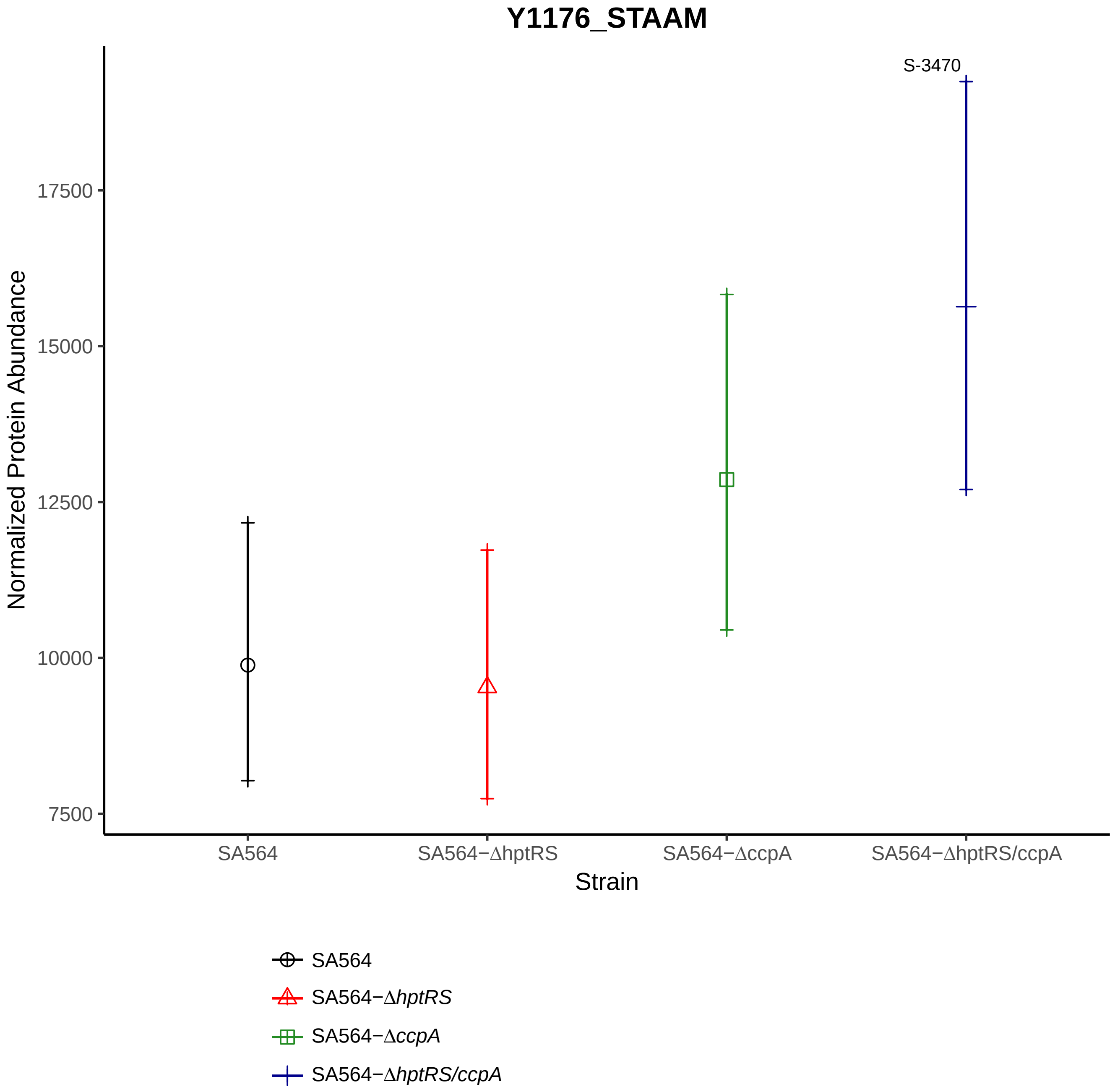

Y1176_STAAM
S-3470
Normalized Protein Abundance
17500
15000
12500
10000
7500
SA564
SA564−ΔhptRS
SA564−ΔccpA
SA564−ΔhptRS/ccpA
Strain
SA564
SA564−Δ*hptRS*
SA564−Δ*ccpA*
SA564−Δ*hptRS/ccpA*

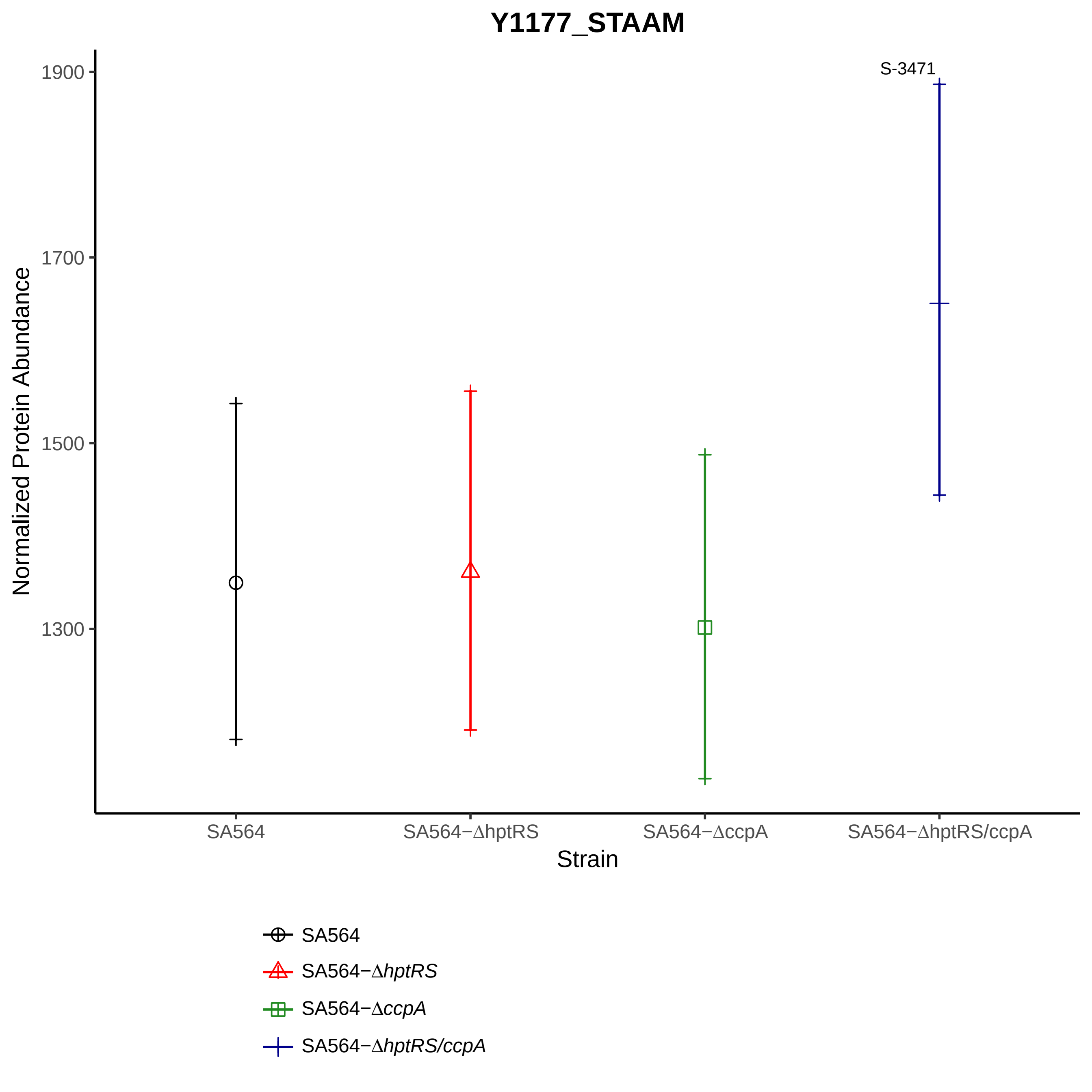

Y1177_STAAM
S-3471
1900
1700
1500
1300
Normalized Protein Abundance
SA564
SA564−ΔhptRS
SA564−ΔccpA
SA564−ΔhptRS/ccpA
Strain
SA564
SA564−Δ*hptRS*
SA564−Δ*ccpA*
SA564−Δ*hptRS/ccpA*

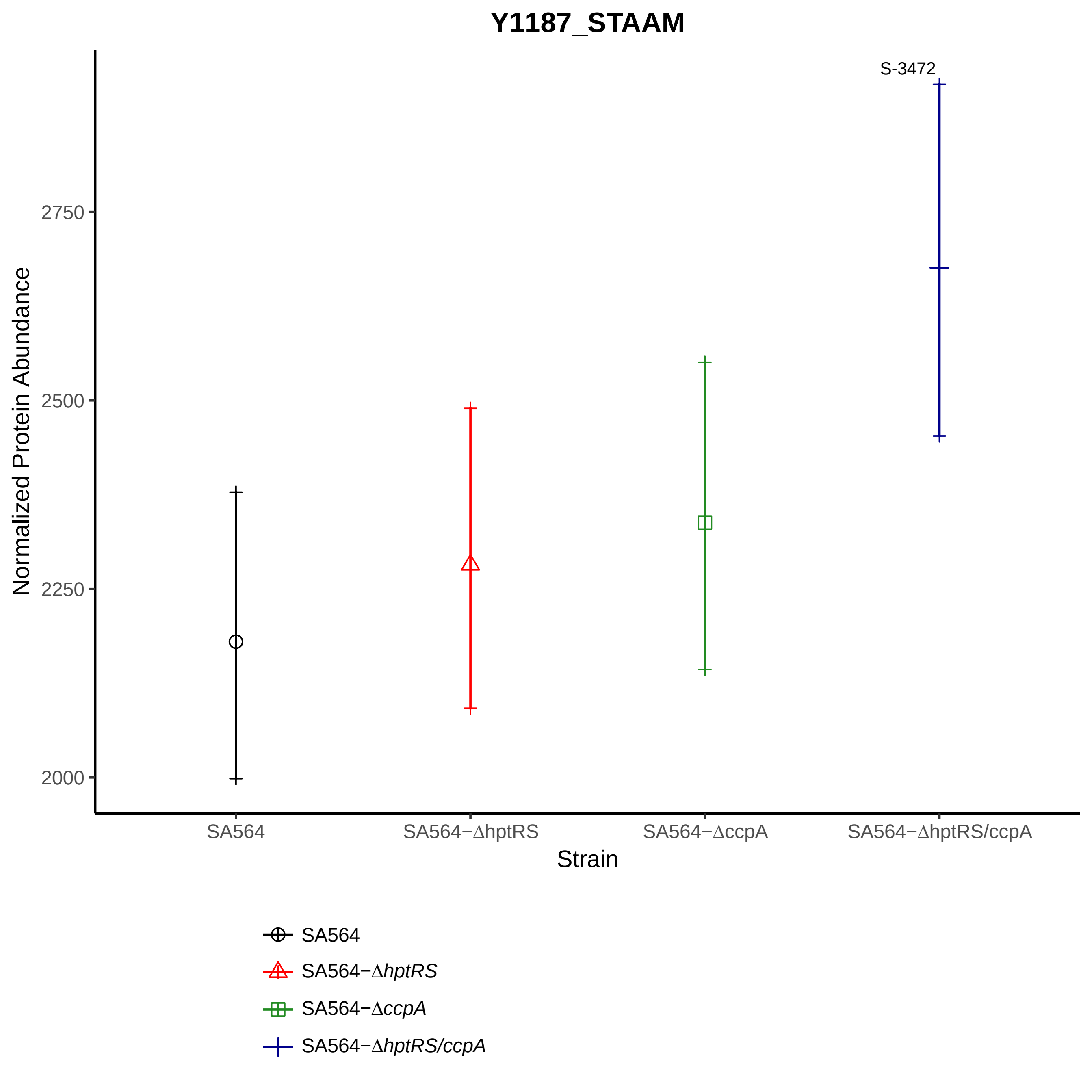

Y1187_STAAM
S-3472
Normalized Protein Abundance
2750
2500
2250
2000
SA564
SA564−ΔhptRS
SA564−ΔccpA
SA564−ΔhptRS/ccpA
Strain
SA564
SA564−ΔhptRS
SA564−ΔccpA
SA564−ΔhptRS/ccpA

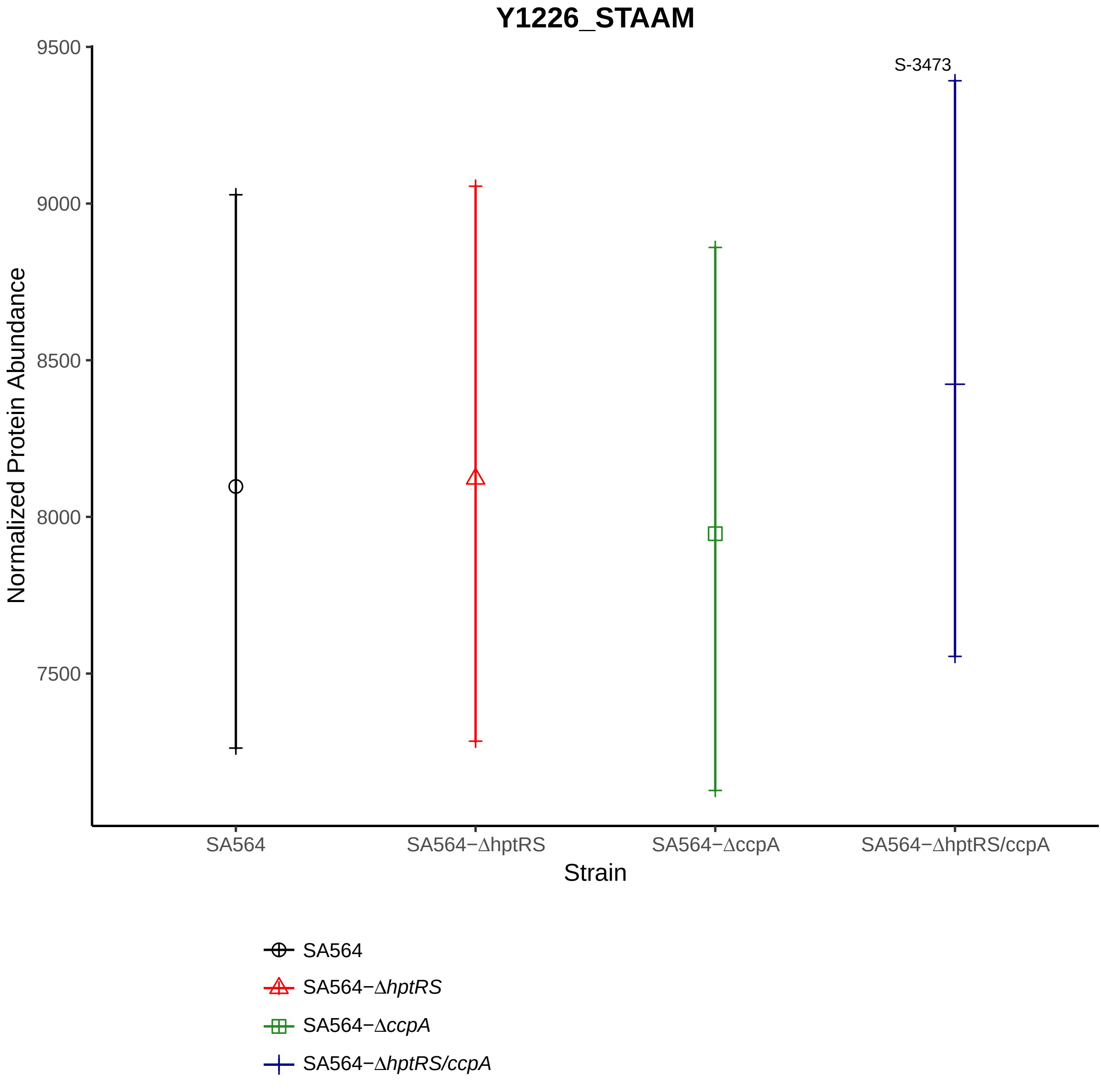

Y1226_STAAM
S-3473
Normalized Protein Abundance
9500
9000
8500
8000
7500
SA564
SA564−ΔhptRS
SA564−ΔccpA
SA564−ΔhptRS/ccpA
Strain
SA564
SA564−Δ*hptRS*
SA564−Δ*ccpA*
SA564−Δ*hptRS/ccpA*

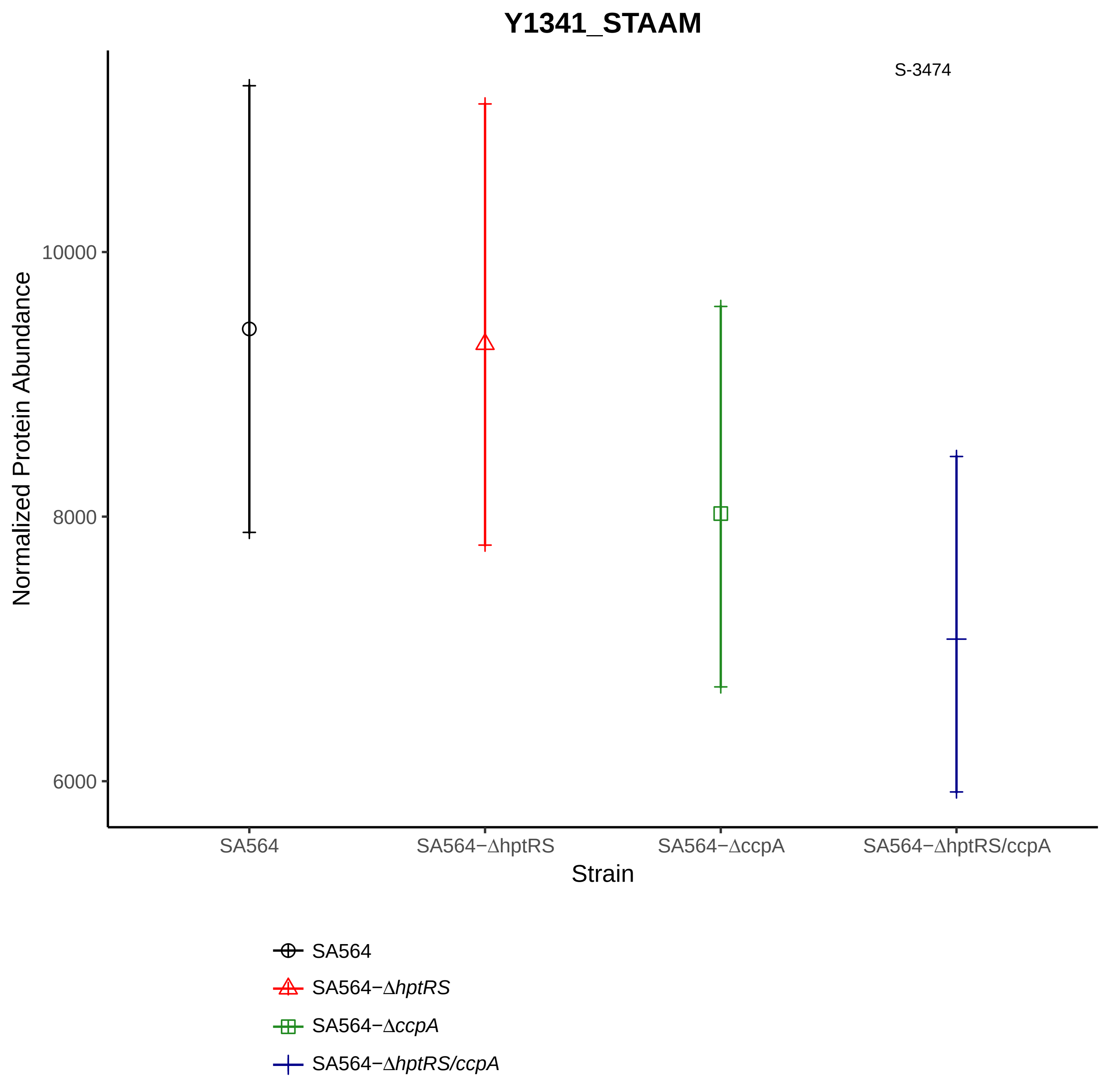

Y1341_STAAM
S-3474
Normalized Protein Abundance
10000
8000
6000
SA564
SA564−ΔhptRS
SA564−ΔccpA
SA564−ΔhptRS/ccpA
Strain
SA564
SA564−Δ*hptRS*
SA564−Δ*ccpA*
SA564−Δ*hptRS/ccpA*

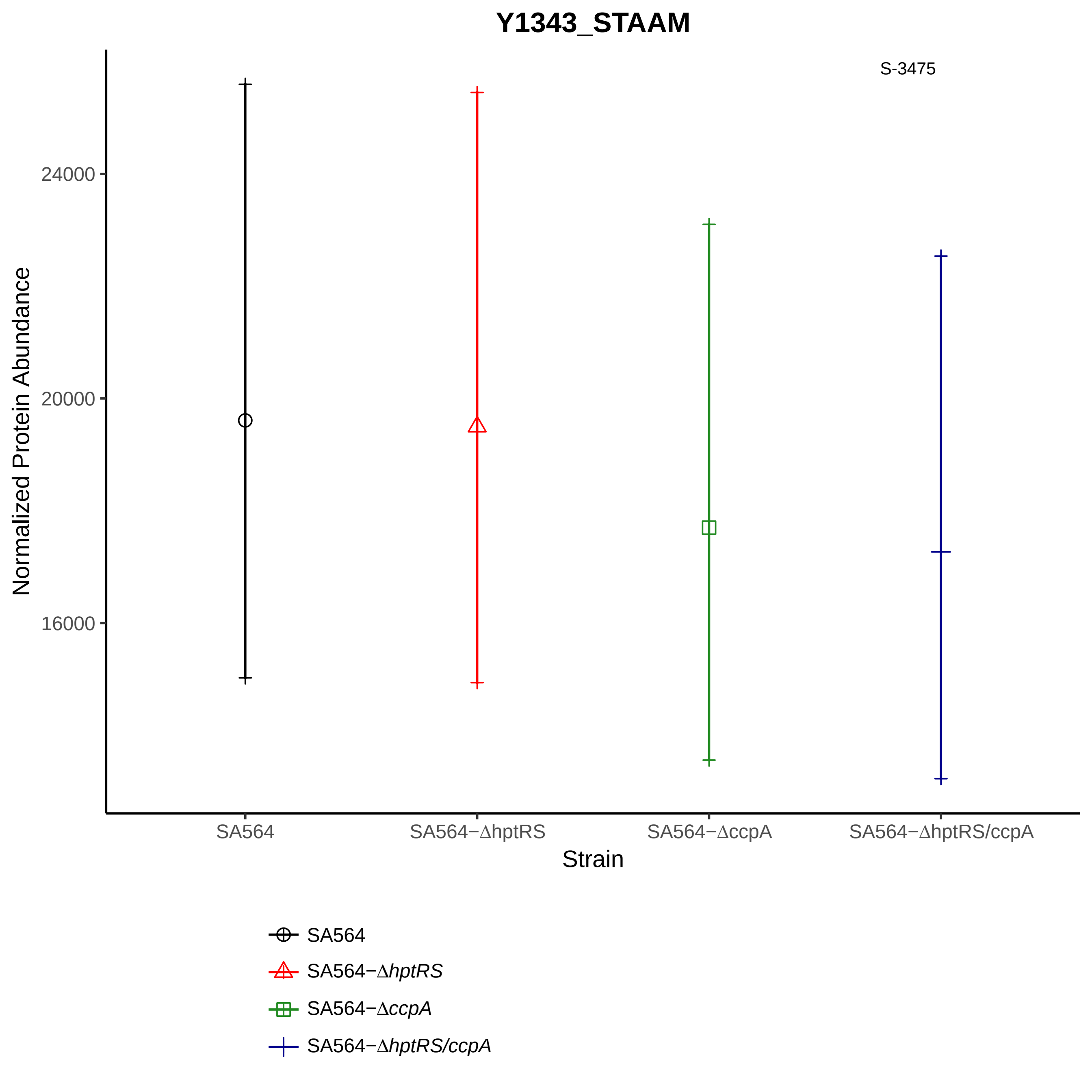
Y1343_STAAM
S-3475
Normalized Protein Abundance
24000
20000
16000
SA564
SA564−ΔhptRS
SA564−ΔccpA
SA564−ΔhptRS/ccpA
Strain
SA564
SA564−ΔhptRS
SA564−ΔccpA
SA564−ΔhptRS/ccpA

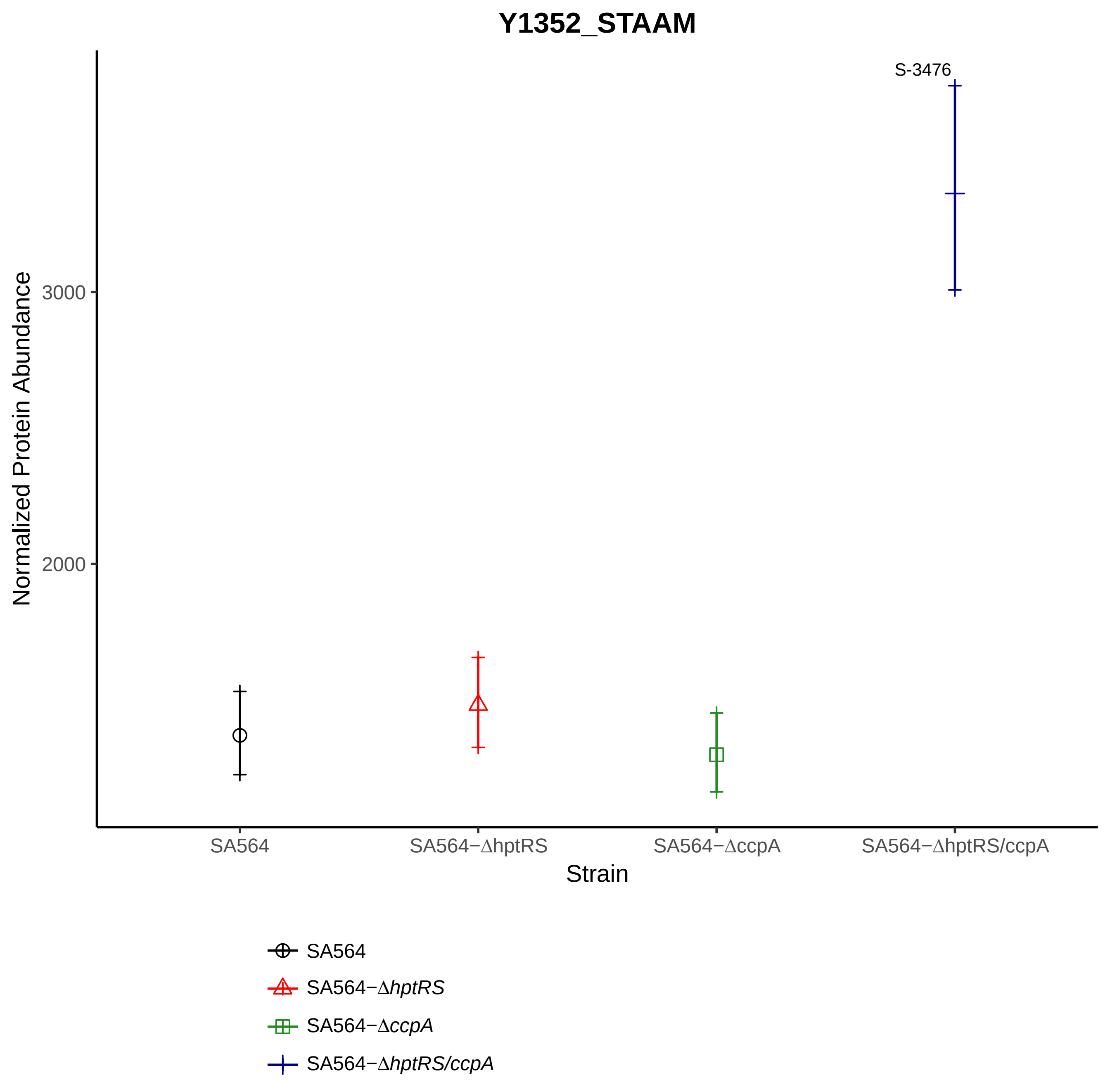
Y1352_STAAM
S-3476
Normalized Protein Abundance
3000
2000
SA564
SA564−ΔhptRS
SA564−ΔccpA
SA564−ΔhptRS/ccpA
Strain
SA564
SA564−ΔhptRS
SA564−ΔccpA
SA564−ΔhptRS/ccpA

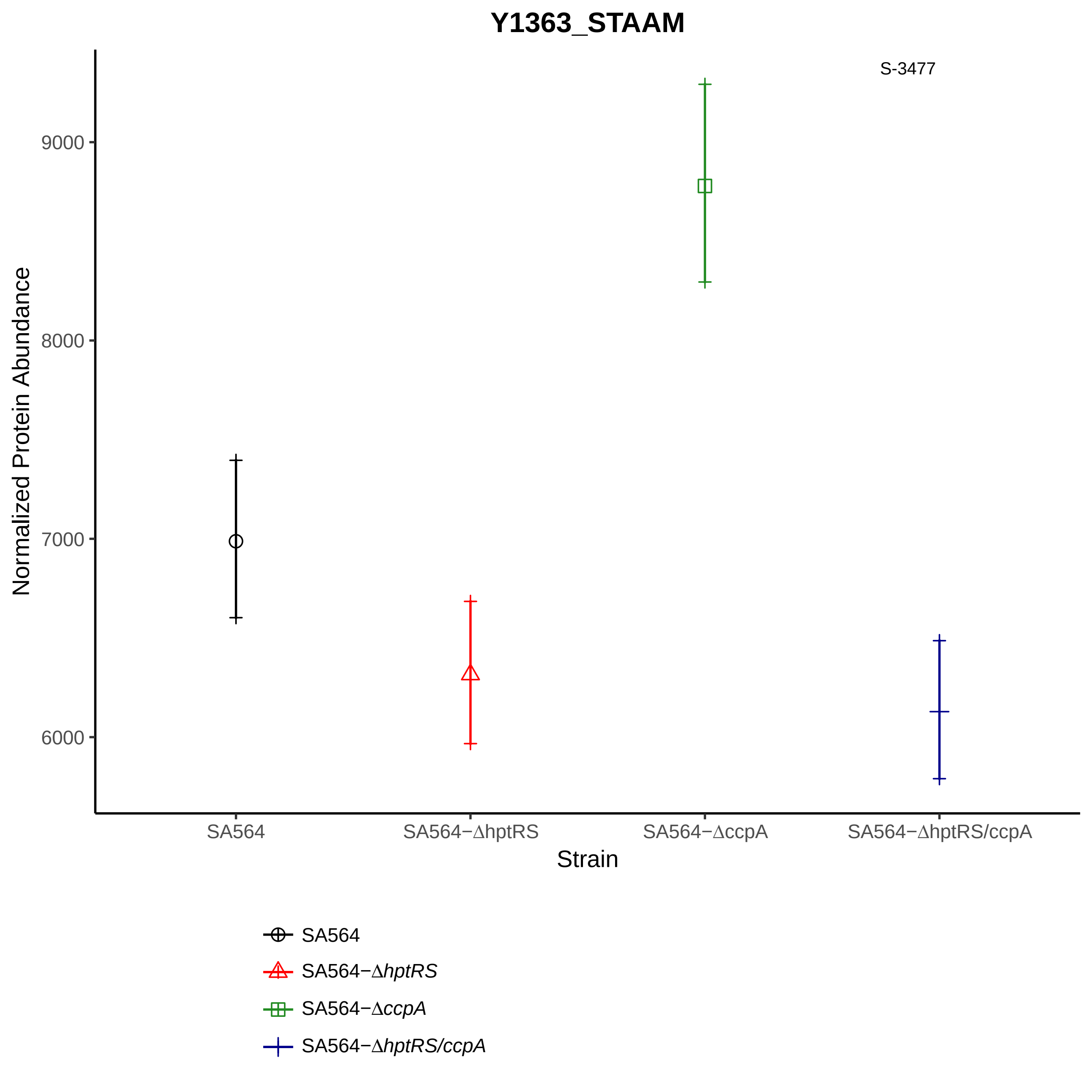
Y1363_STAAM
S-3477
Normalized Protein Abundance
9000
8000
7000
6000
SA564
SA564−ΔhptRS
SA564−ΔccpA
SA564−ΔhptRS/ccpA
Strain
SA564
SA564−Δ*hptRS*
SA564−Δ*ccpA*
SA564−Δ*hptRS/ccpA*

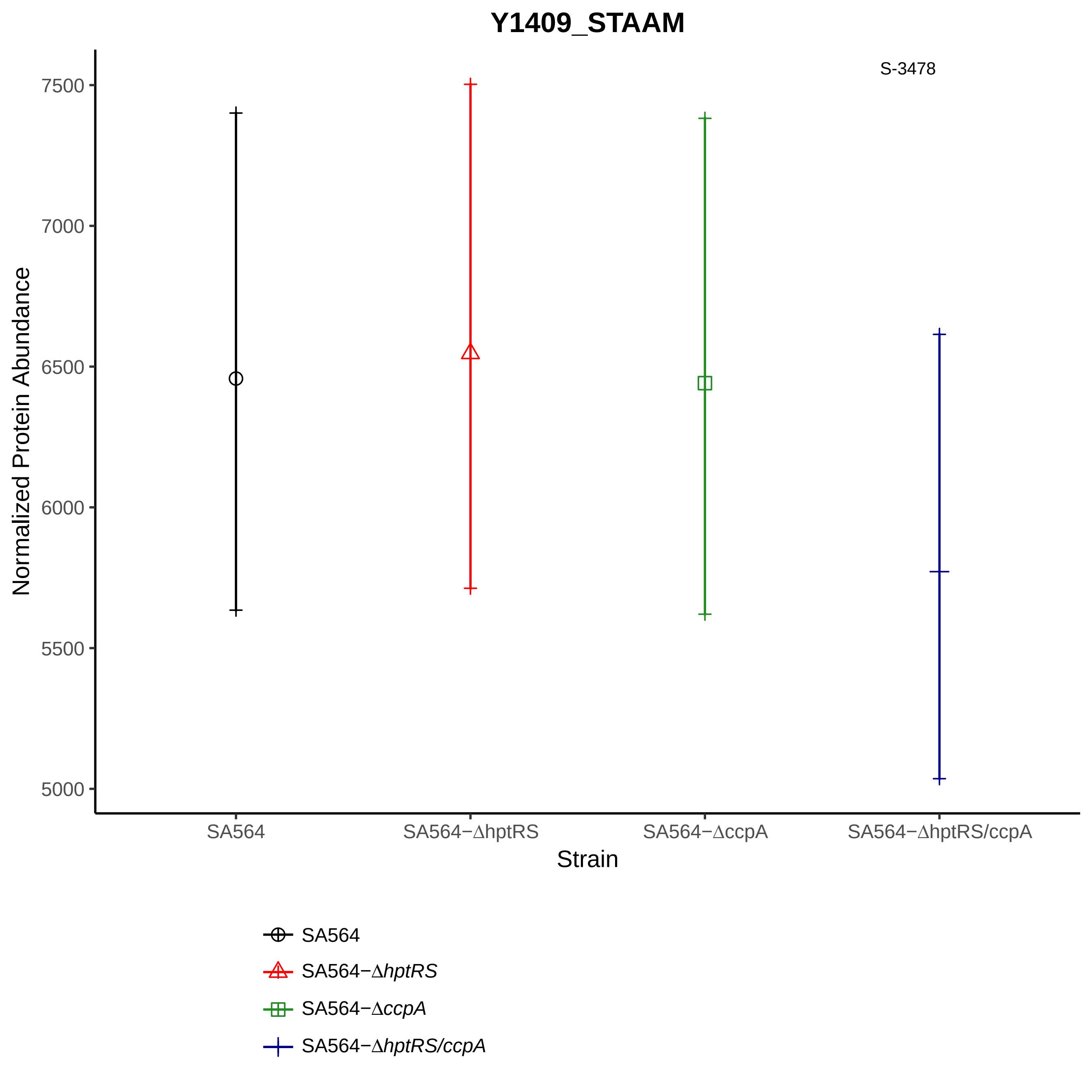

Y1409_STAAM
S-3478
Normalized Protein Abundance
7500
7000
6500
6000
5500
5000
SA564
SA564−ΔhptRS
SA564−ΔccpA
SA564−ΔhptRS/ccpA
Strain
SA564
SA564−ΔhptRS
SA564−ΔccpA
SA564−ΔhptRS/ccpA

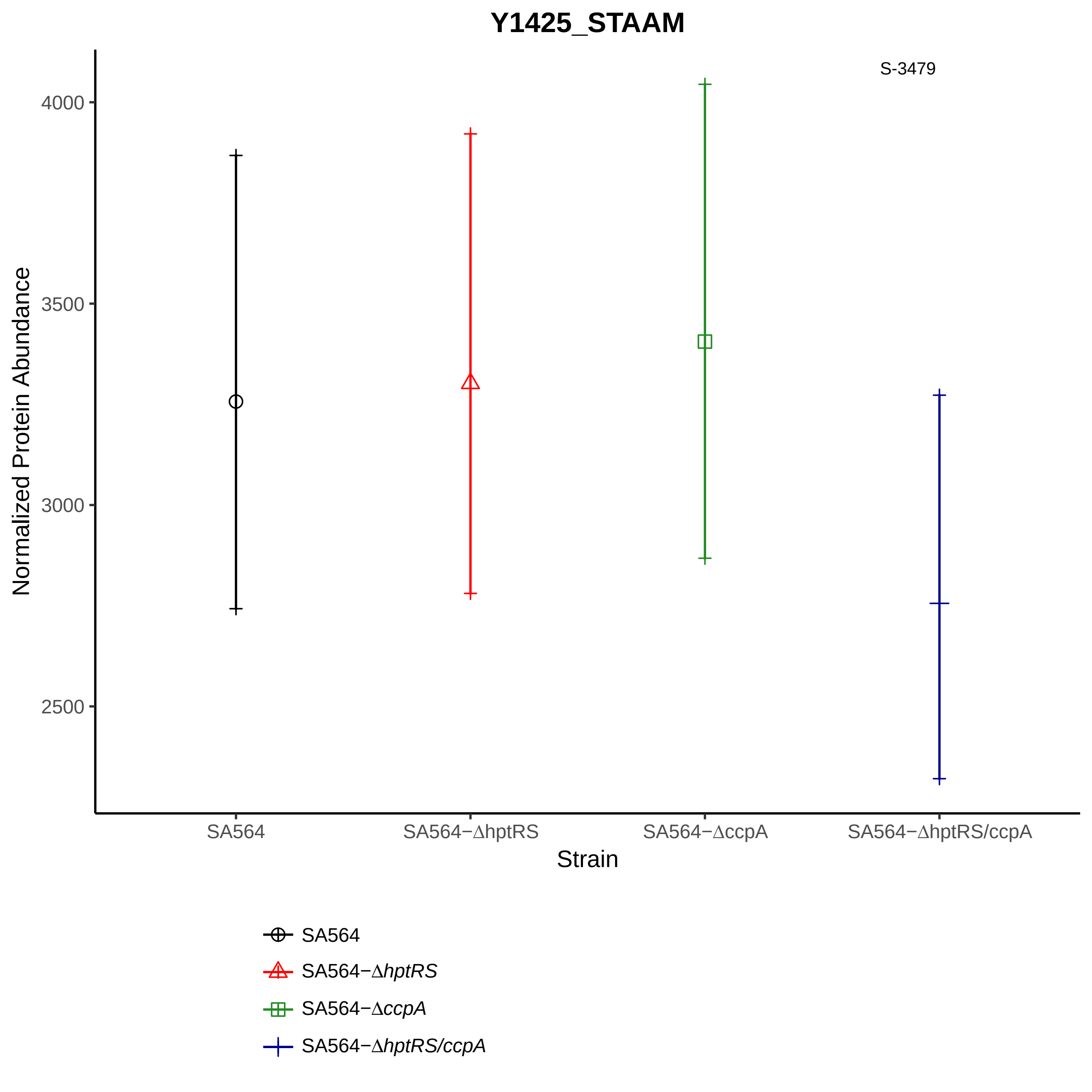

Y1425_STAAM
S-3479
Normalized Protein Abundance
4000
3500
3000
2500
SA564
SA564−ΔhptRS
SA564−ΔccpA
SA564−ΔhptRS/ccpA
Strain
SA564
SA564−ΔhptRS
SA564−ΔccpA
SA564−ΔhptRS/ccpA

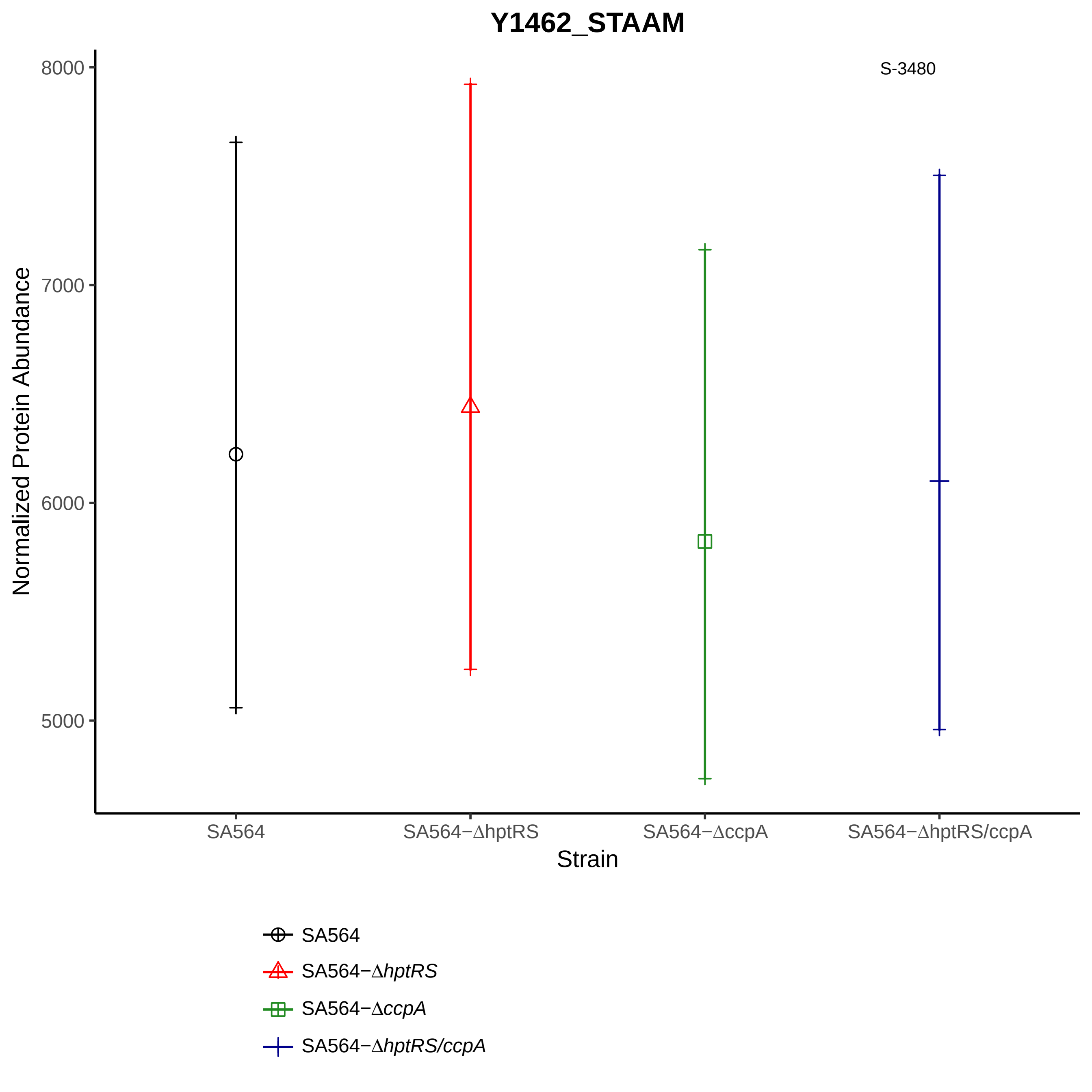
Y1462_STAAM
S-3480
Normalized Protein Abundance
8000
7000
6000
5000
SA564
SA564−ΔhptRS
SA564−ΔccpA
SA564−ΔhptRS/ccpA
Strain
SA564
SA564−ΔhptRS
SA564−ΔccpA
SA564−ΔhptRS/ccpA

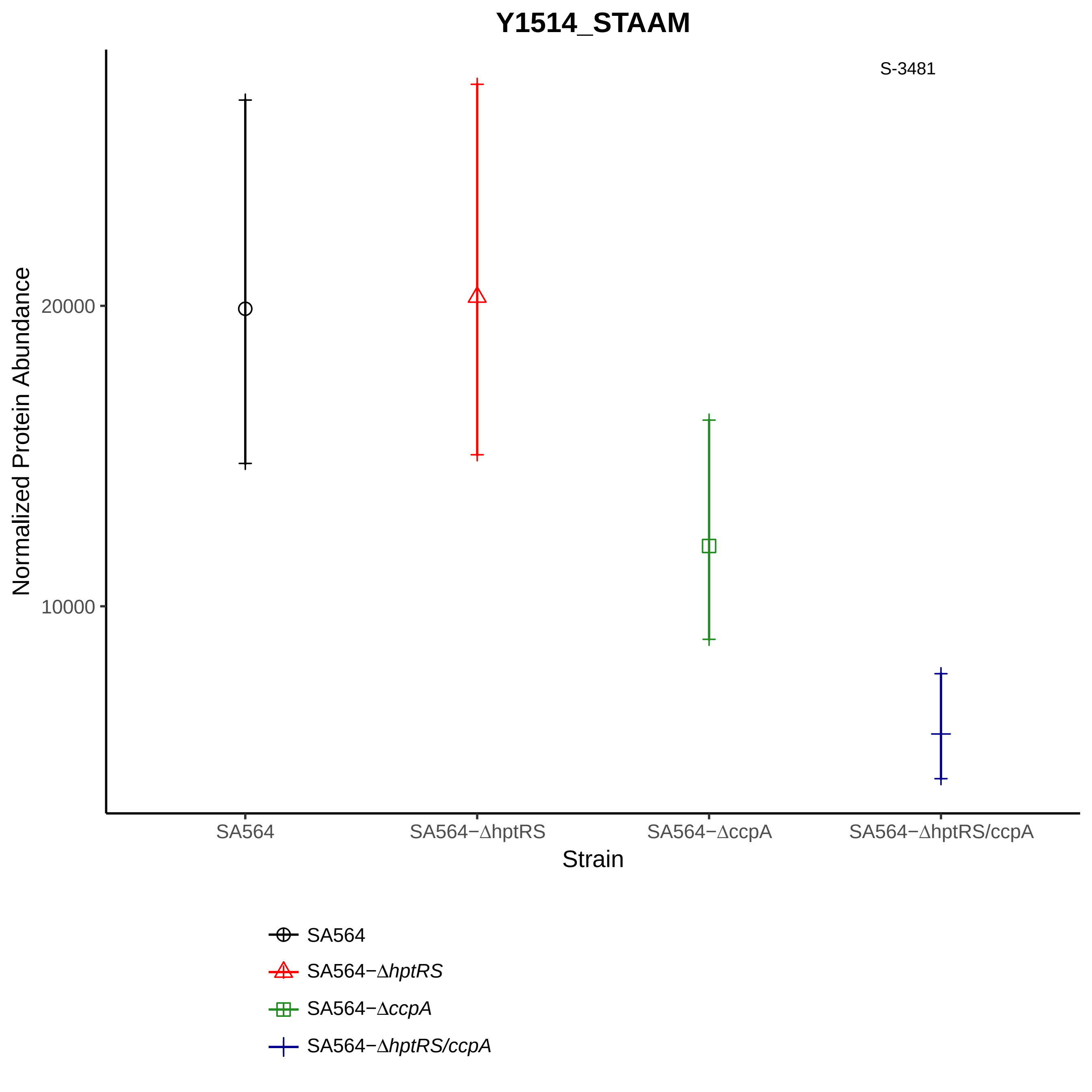

Y1514_STAAM
S-3481
Normalized Protein Abundance
20000
10000
SA564
SA564−ΔhptRS
SA564−ΔccpA
SA564−ΔhptRS/ccpA
Strain
SA564
SA564−ΔhptRS
SA564−ΔccpA
SA564−ΔhptRS/ccpA

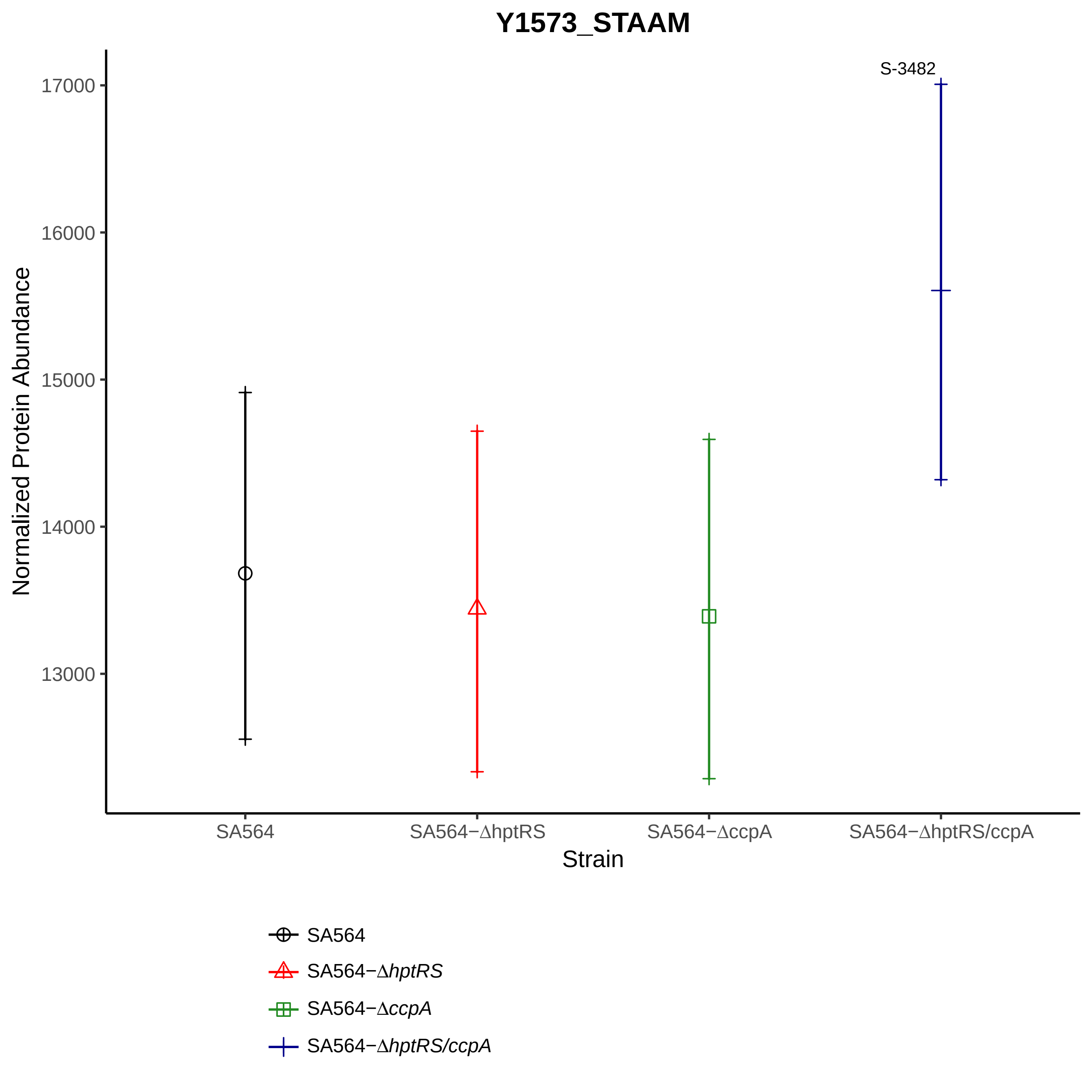

Y1573_STAAM
S-3482
Normalized Protein Abundance
17000
16000
15000
14000
13000
Strain
SA564
SA564−ΔhptRS
SA564−ΔccpA
SA564−ΔhptRS/ccpA
SA564
SA564−Δ*hptRS*
SA564−Δ*ccpA*
SA564−Δ*hptRS/ccpA*

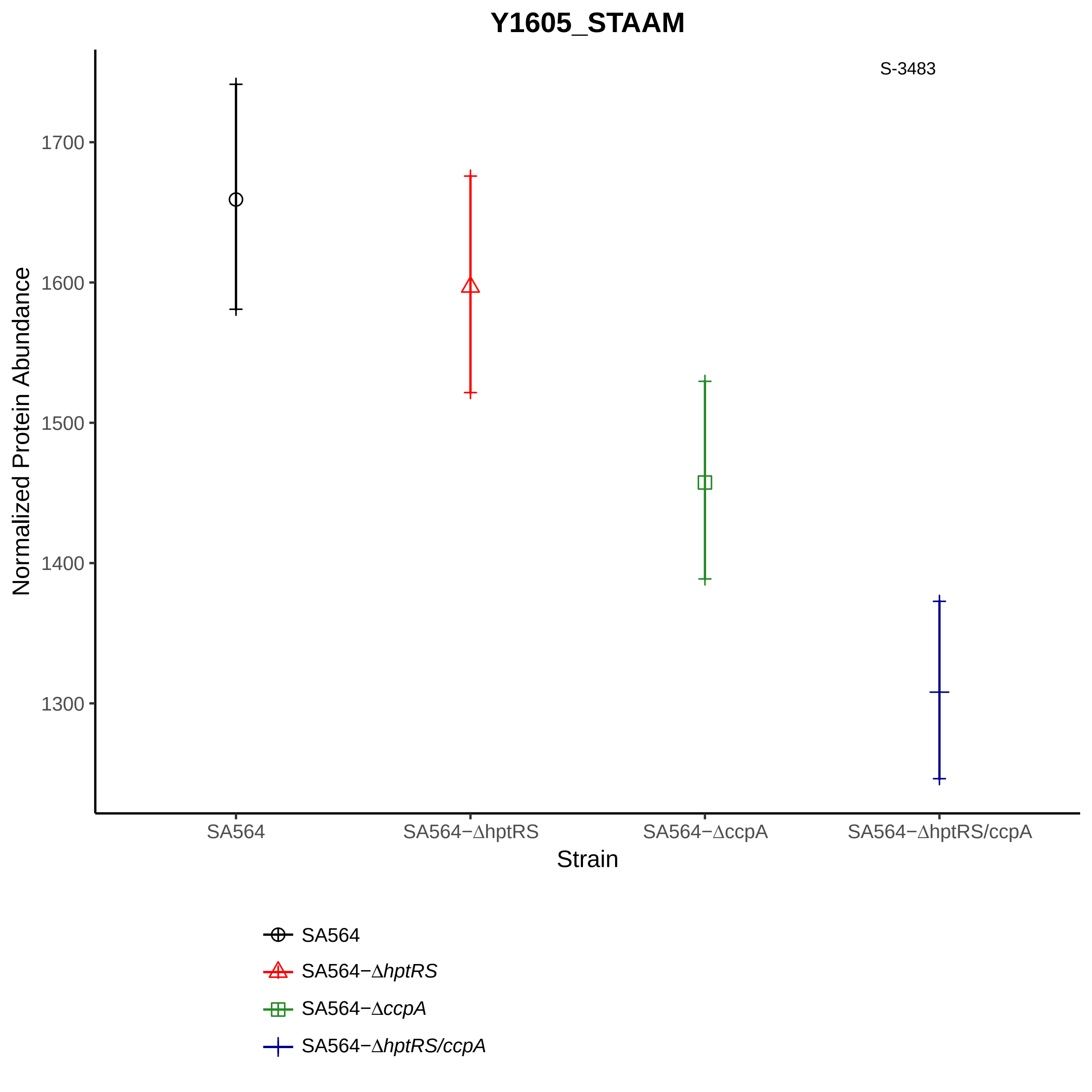

Y1605_STAAM
S-3483
Normalized Protein Abundance
1700
1600
1500
1400
1300
SA564
SA564−ΔhptRS
SA564−ΔccpA
SA564−ΔhptRS/ccpA
Strain
SA564
SA564−ΔhptRS
SA564−ΔccpA
SA564−ΔhptRS/ccpA

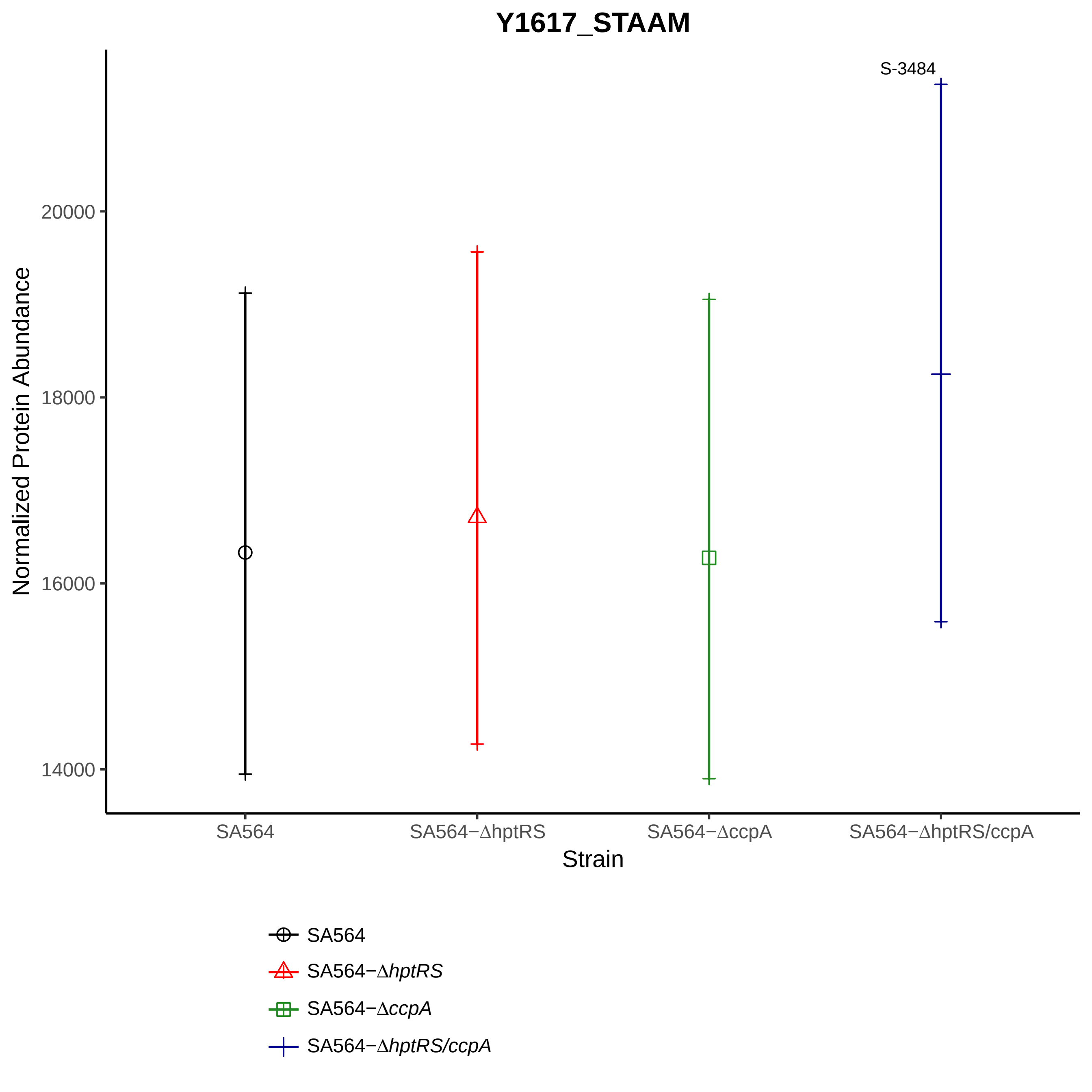

Y1617_STAAM
S-3484
Normalized Protein Abundance
20000
18000
16000
14000
SA564
SA564−ΔhptRS
SA564−ΔccpA
SA564−ΔhptRS/ccpA
Strain
SA564
SA564−ΔhptRS
SA564−ΔccpA
SA564−ΔhptRS/ccpA

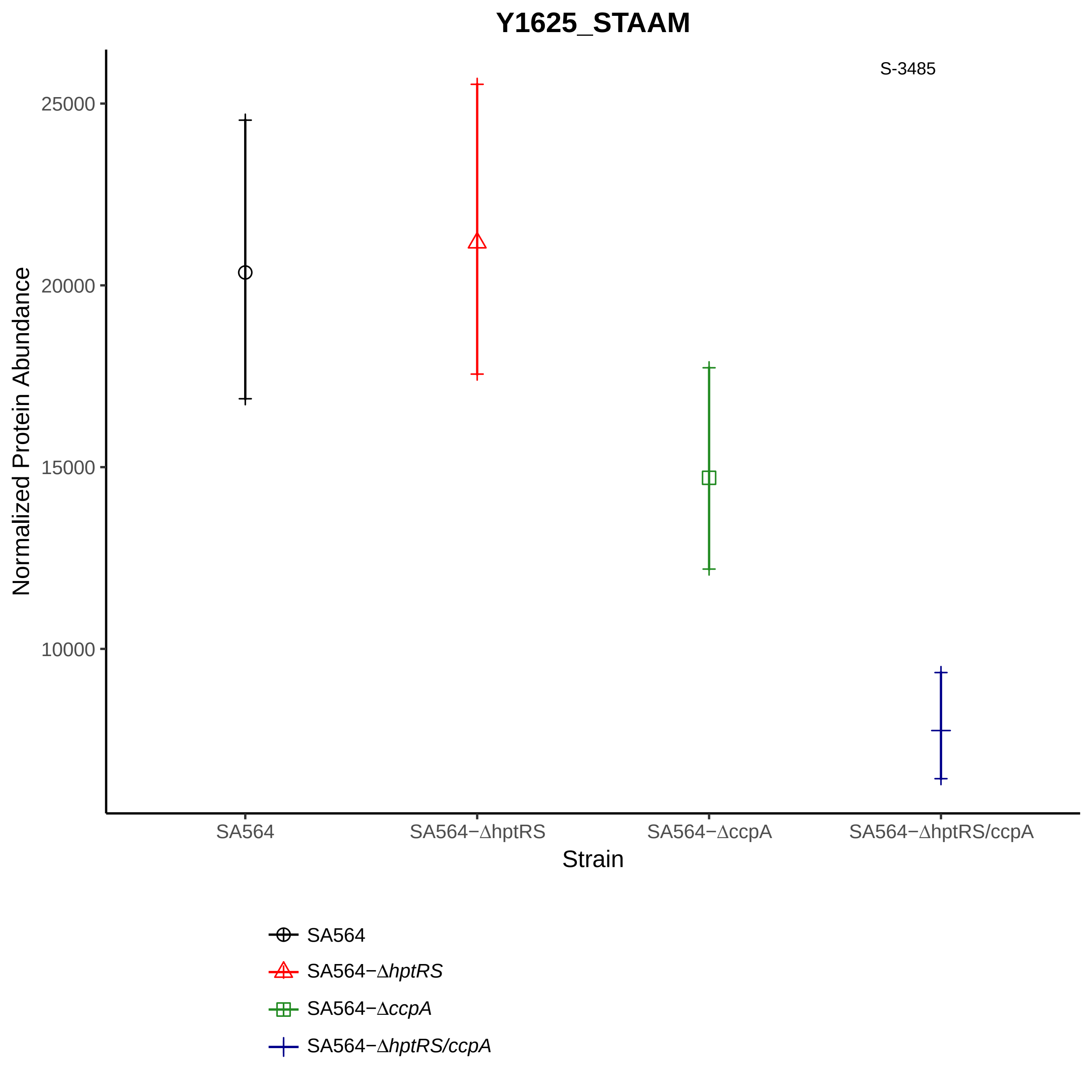

Y1625_STAAM
S-3485
Normalized Protein Abundance
25000
20000
15000
10000
SA564
SA564−ΔhptRS
SA564−ΔccpA
SA564−ΔhptRS/ccpA
Strain
SA564
SA564−Δ*hptRS*
SA564−Δ*ccpA*
SA564−Δ*hptRS/ccpA*

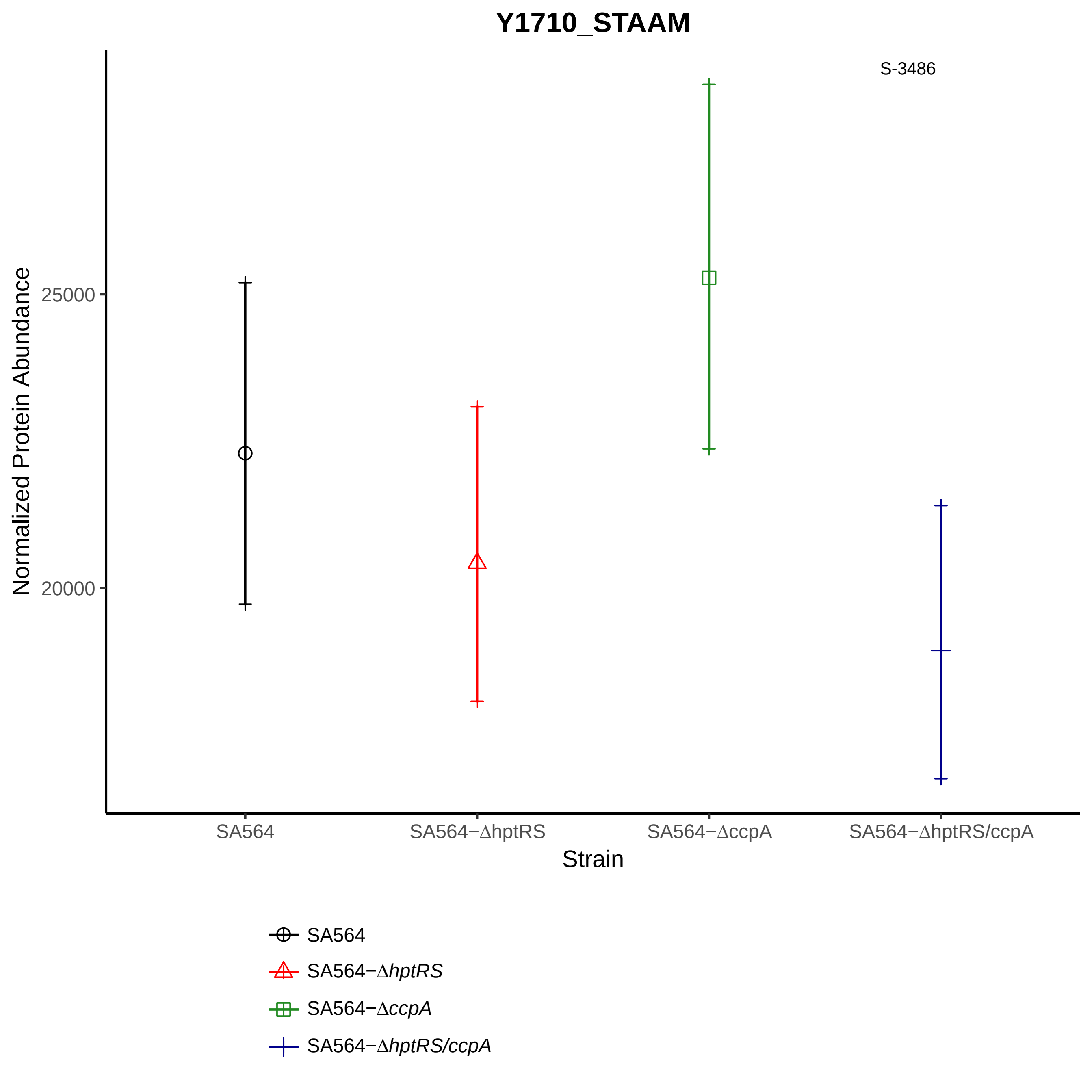

Y1710_STAAM
S-3486
Normalized Protein Abundance
25000
20000
SA564
SA564−ΔhptRS
SA564−ΔccpA
SA564−ΔhptRS/ccpA
Strain
SA564
SA564−ΔhptRS
SA564−ΔccpA
SA564−ΔhptRS/ccpA

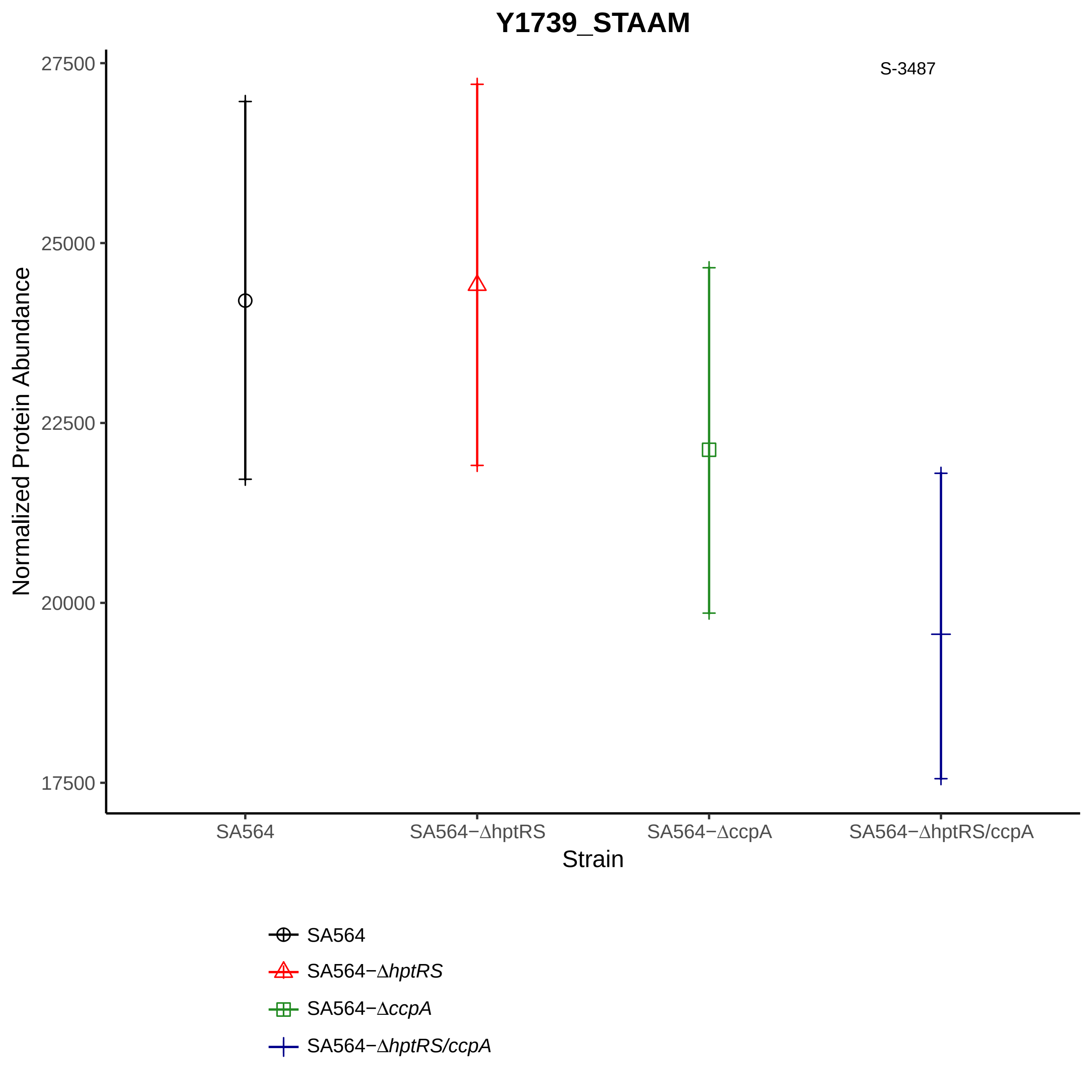

Y1739_STAAM
S-3487
27500
25000
22500
20000
17500
Normalized Protein Abundance
SA564
SA564−ΔhptRS
SA564−ΔccpA
SA564−ΔhptRS/ccpA
Strain
SA564
SA564−ΔhptRS
SA564−ΔccpA
SA564−ΔhptRS/ccpA

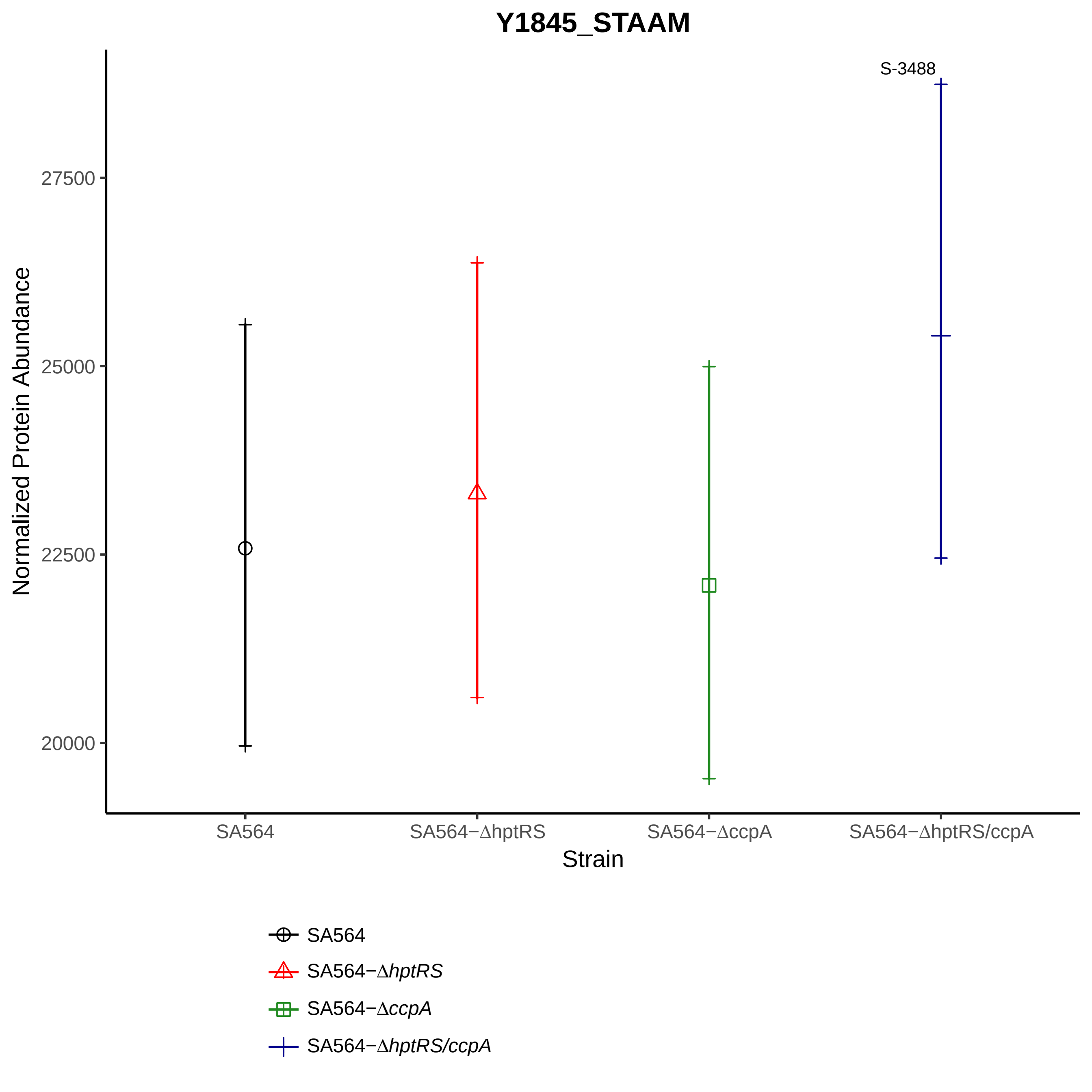

Y1845_STAAM
S-3488
27500
25000
22500
20000
Normalized Protein Abundance
SA564
SA564−ΔhptRS
SA564−ΔccpA
SA564−ΔhptRS/ccpA
Strain
SA564
SA564−Δ*hptRS*
SA564−Δ*ccpA*
SA564−Δ*hptRS/ccpA*

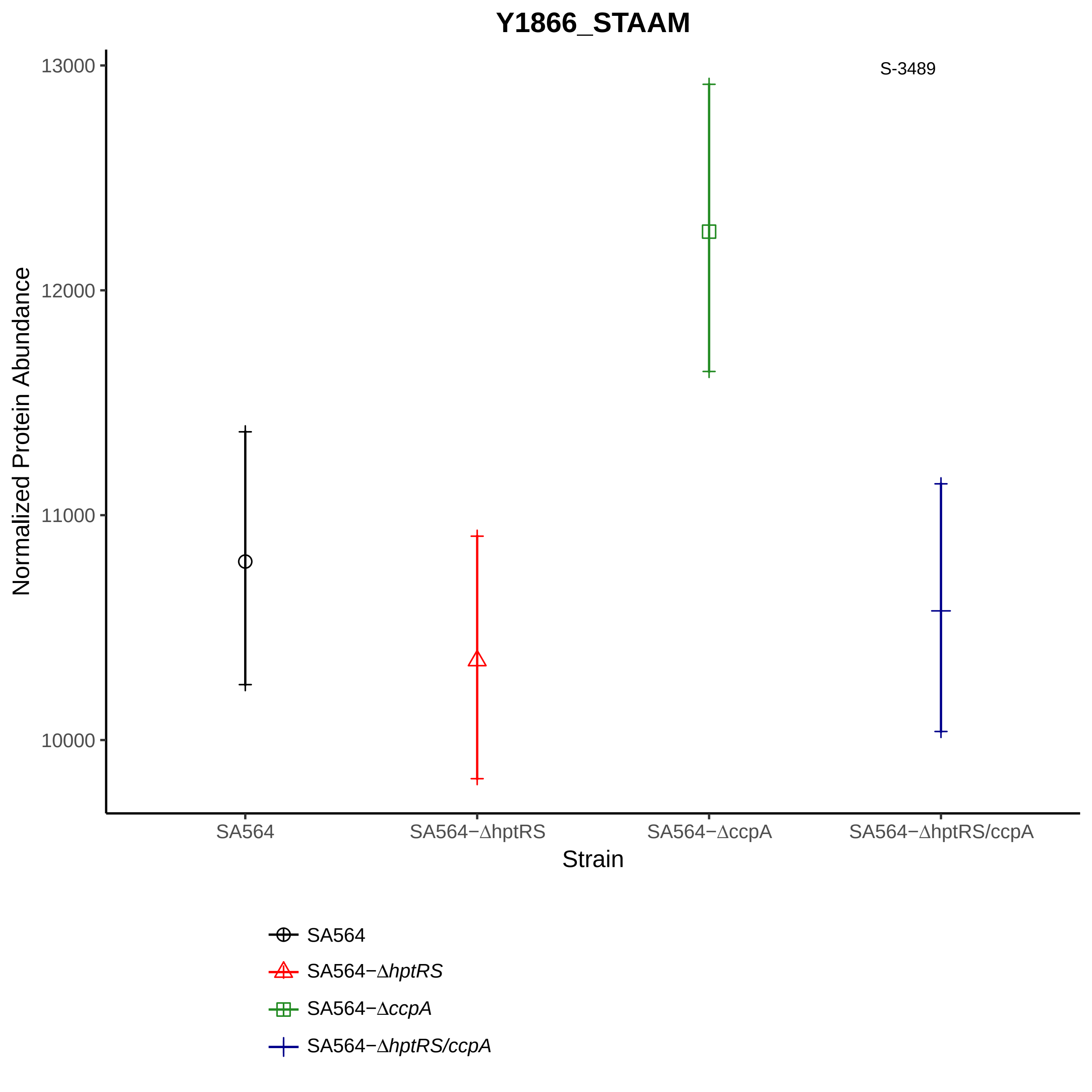
Y1866_STAAM
S-3489
Normalized Protein Abundance
13000
12000
11000
10000
SA564
SA564−ΔhptRS
SA564−ΔccpA
SA564−ΔhptRS/ccpA
Strain
SA564
SA564−ΔhptRS
SA564−ΔccpA
SA564−ΔhptRS/ccpA

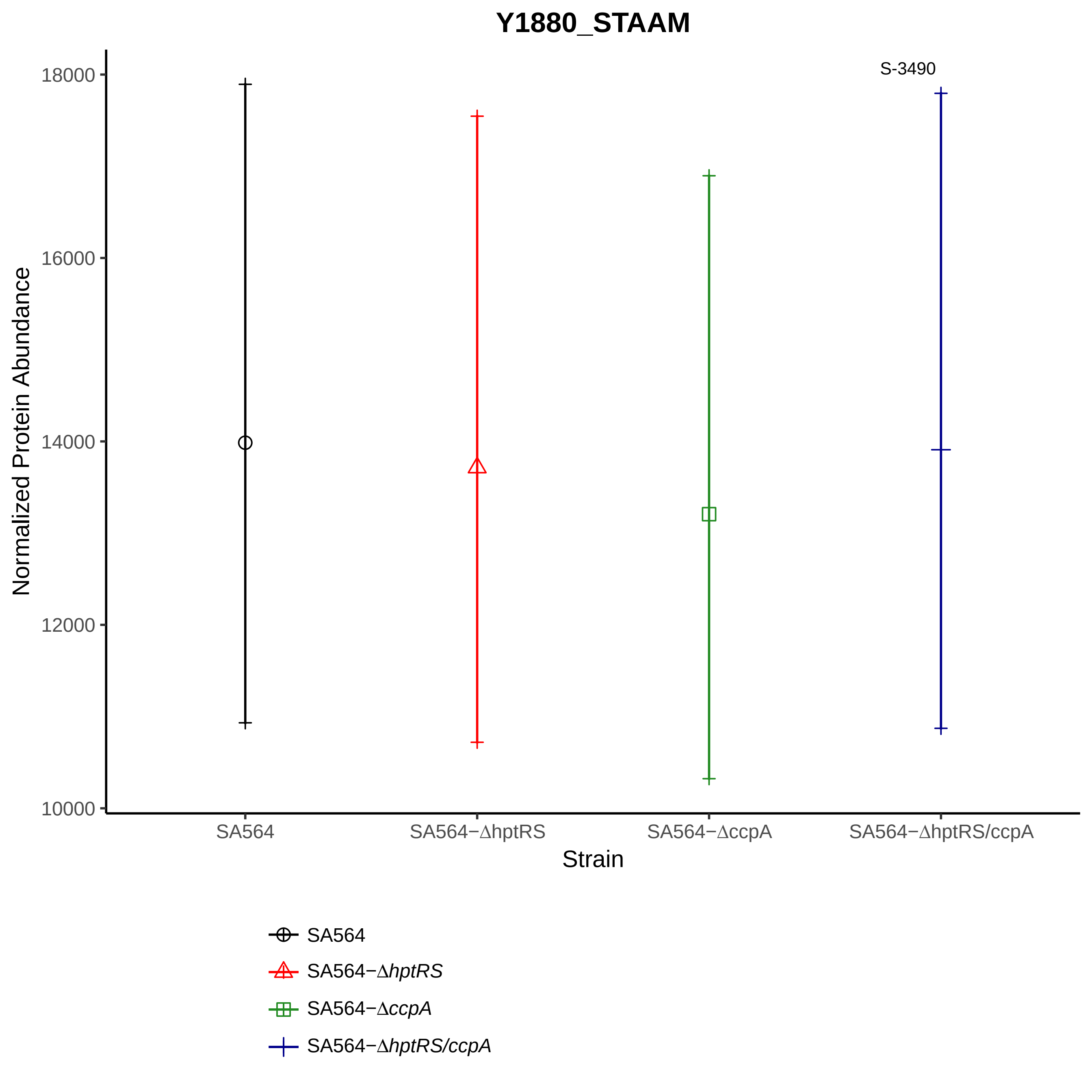

Y1880_STAAM
S-3490
Normalized Protein Abundance
18000
16000
14000
12000
10000
SA564
SA564−ΔhptRS
SA564−ΔccpA
SA564−ΔhptRS/ccpA
Strain
SA564
SA564−Δ*hptRS*
SA564−Δ*ccpA*
SA564−Δ*hptRS/ccpA*

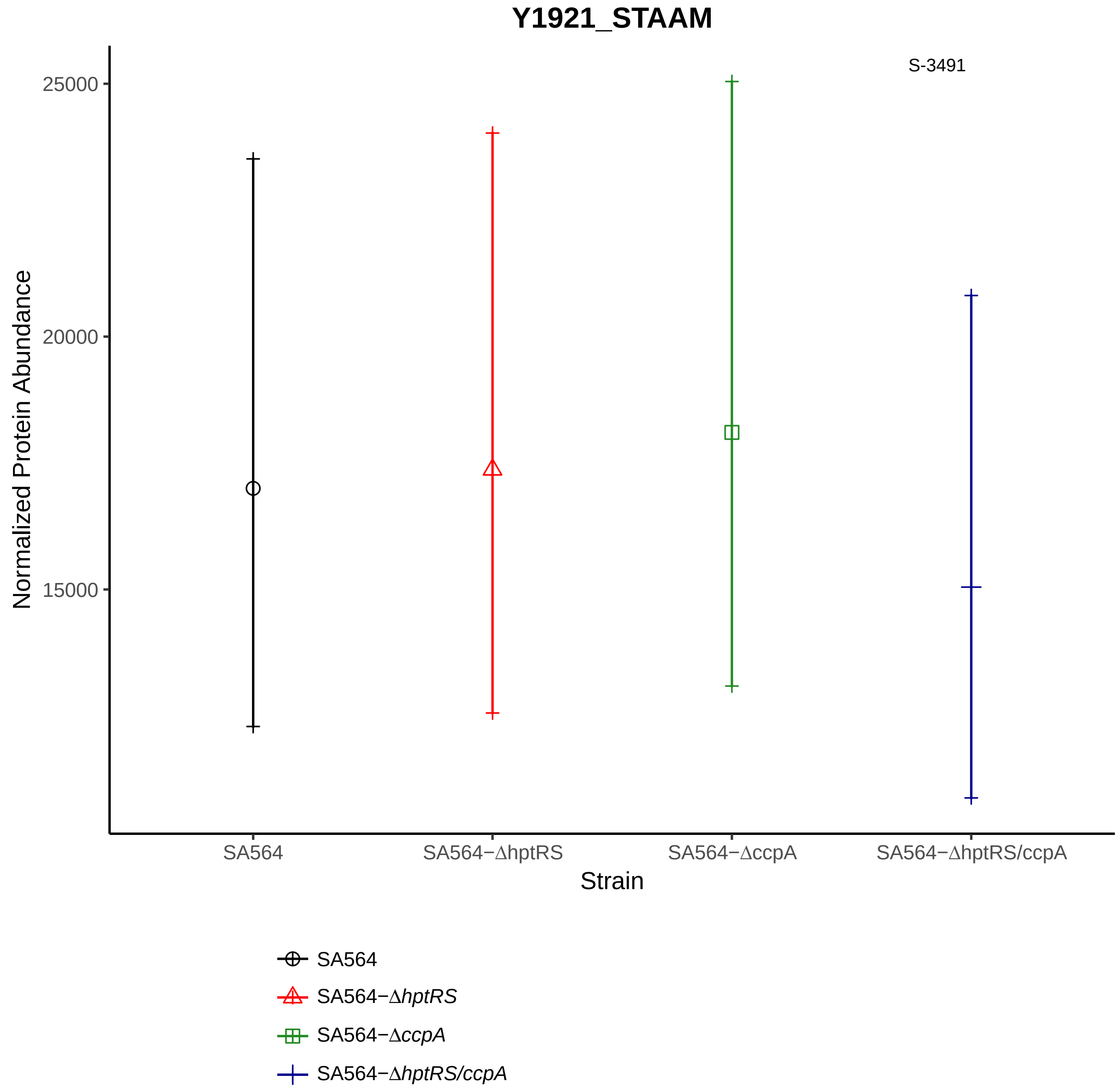
Y1921_STAAM
S-3491
Normalized Protein Abundance
25000
20000
15000
SA564
SA564−ΔhptRS
SA564−ΔccpA
SA564−ΔhptRS/ccpA
Strain
SA564
SA564−ΔhptRS
SA564−ΔccpA
SA564−ΔhptRS/ccpA

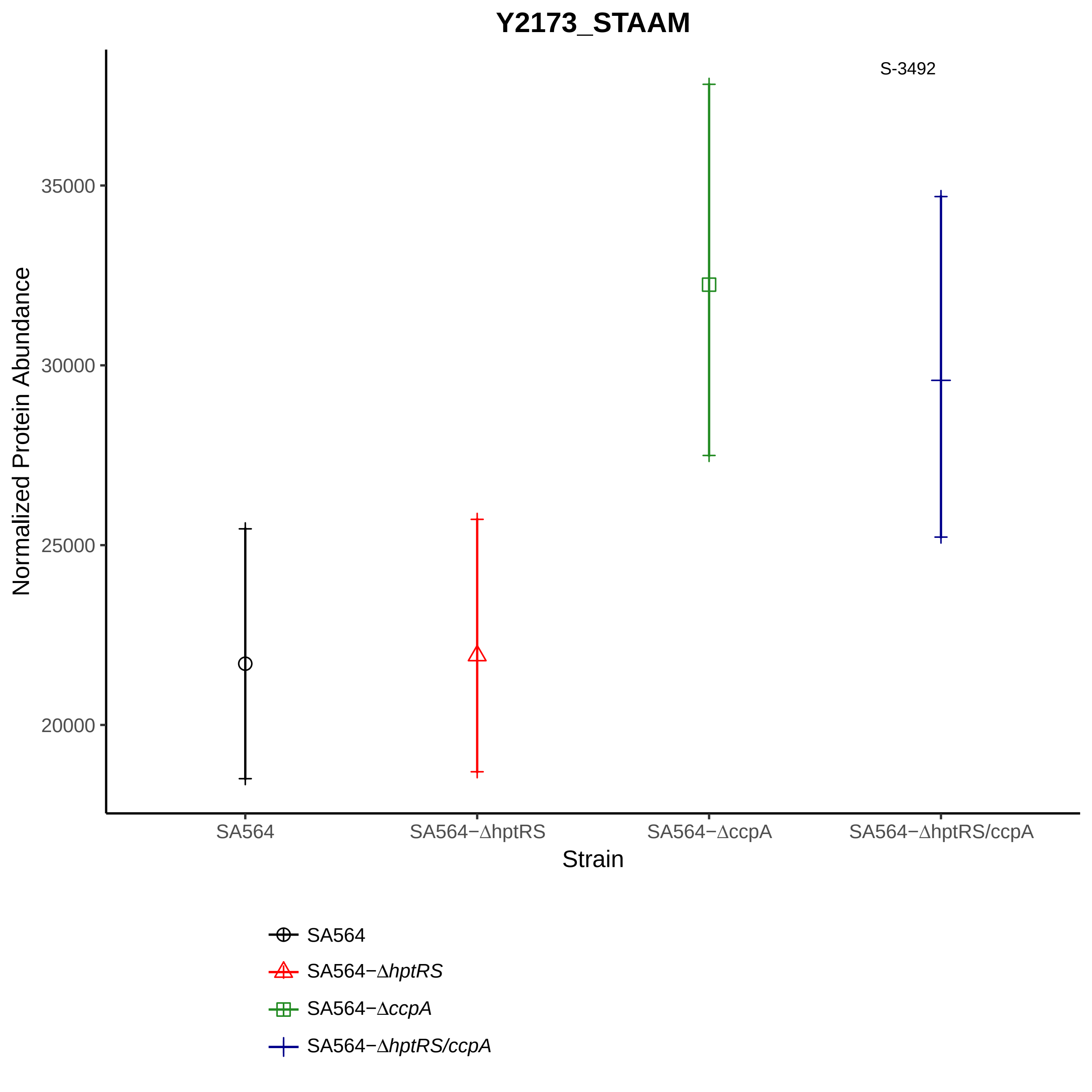

Y2173_STAAM
S-3492
35000
30000
25000
20000
Normalized Protein Abundance
SA564
SA564−ΔhptRS
SA564−ΔccpA
SA564−ΔhptRS/ccpA
Strain
SA564
SA564−Δ*hptRS*
SA564−Δ*ccpA*
SA564−Δ*hptRS/ccpA*

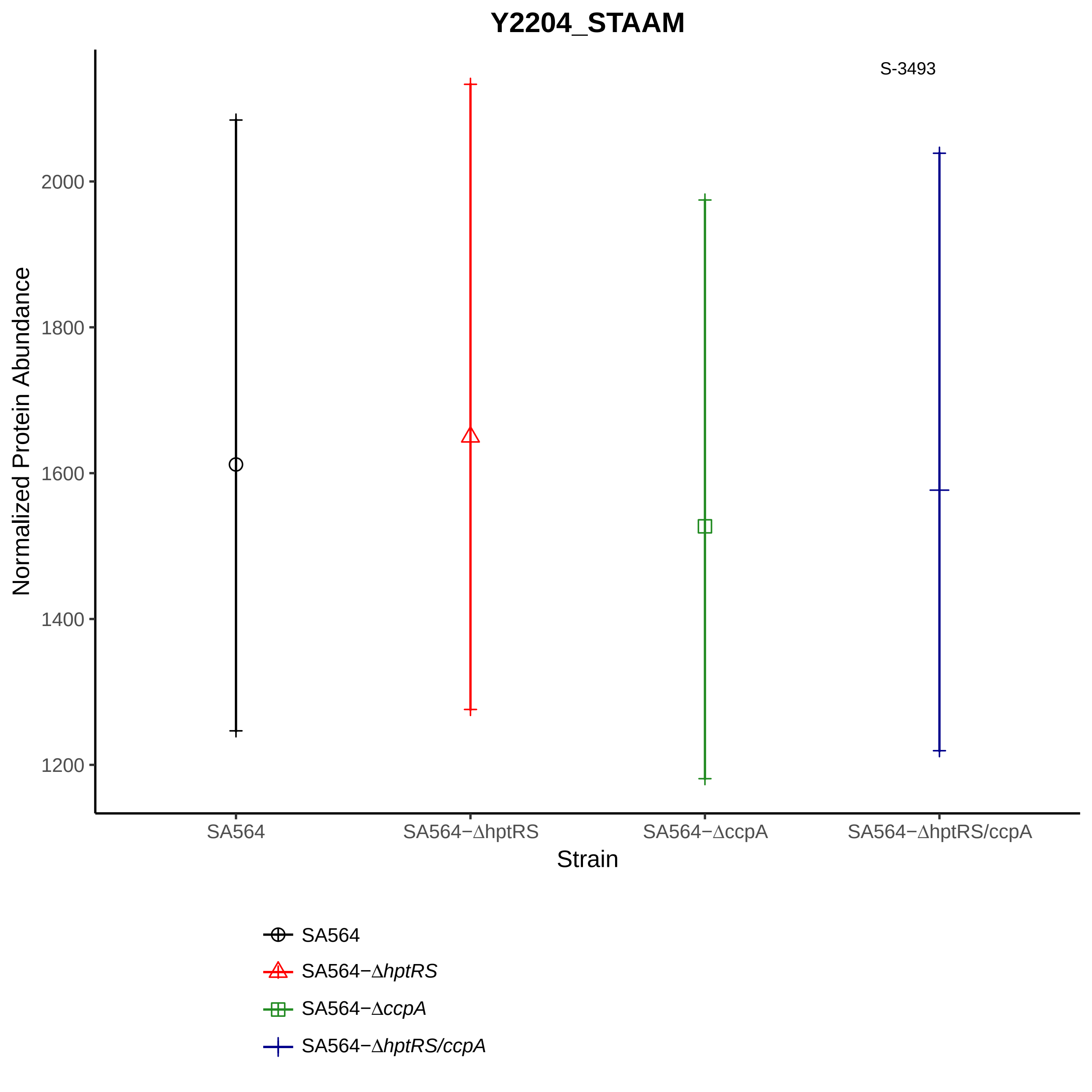
Y2204_STAAM
S-3493
Normalized Protein Abundance
2000
1800
1600
1400
1200
SA564
SA564−ΔhptRS
SA564−ΔccpA
SA564−ΔhptRS/ccpA
Strain
SA564
SA564−Δ*hptRS*
SA564−Δ*ccpA*
SA564−Δ*hptRS/ccpA*

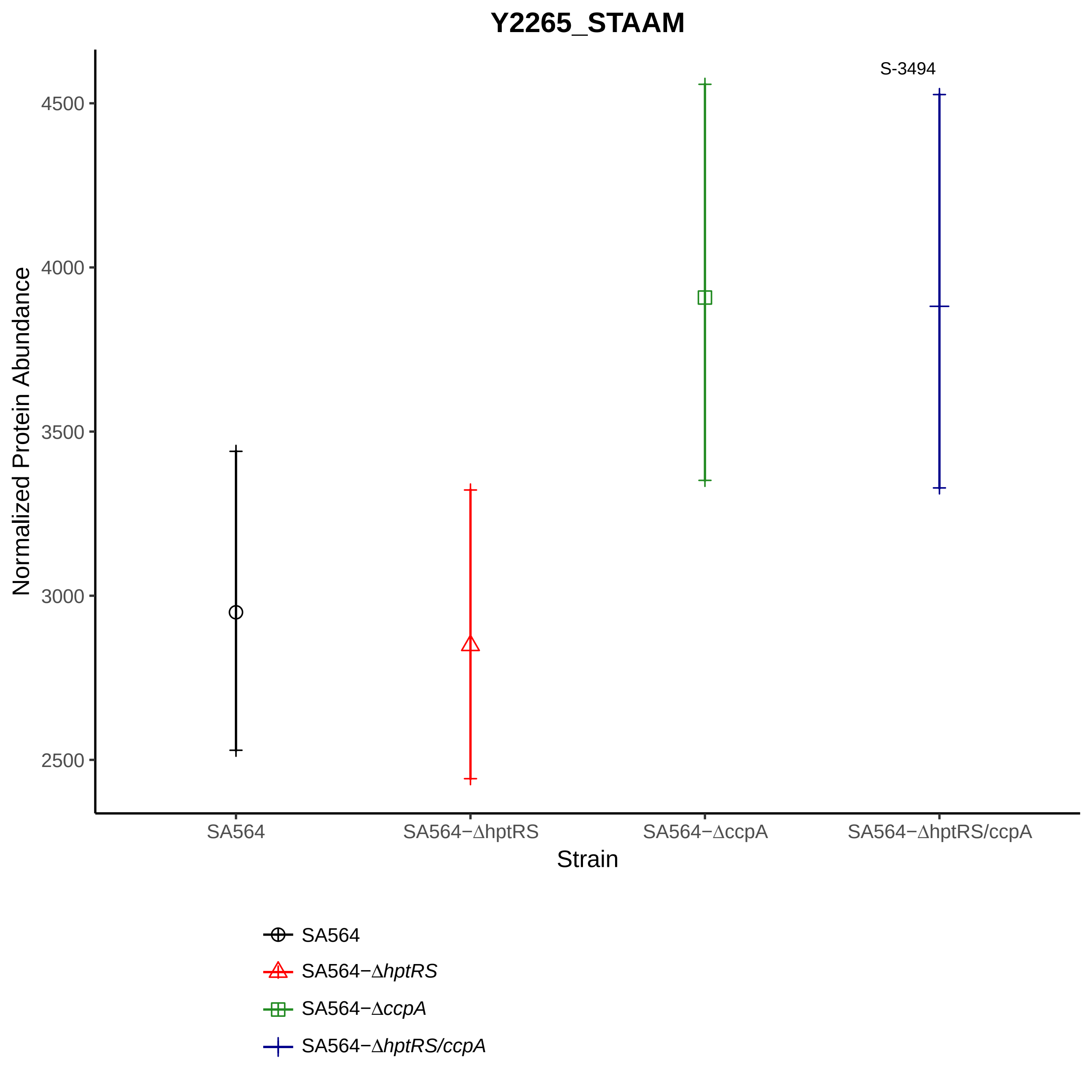

Y2265_STAAM
S-3494
4500
4000
3500
3000
2500
Normalized Protein Abundance
SA564
SA564−ΔhptRS
SA564−ΔccpA
SA564−ΔhptRS/ccpA
Strain
SA564
SA564−ΔhptRS
SA564−ΔccpA
SA564−ΔhptRS/ccpA

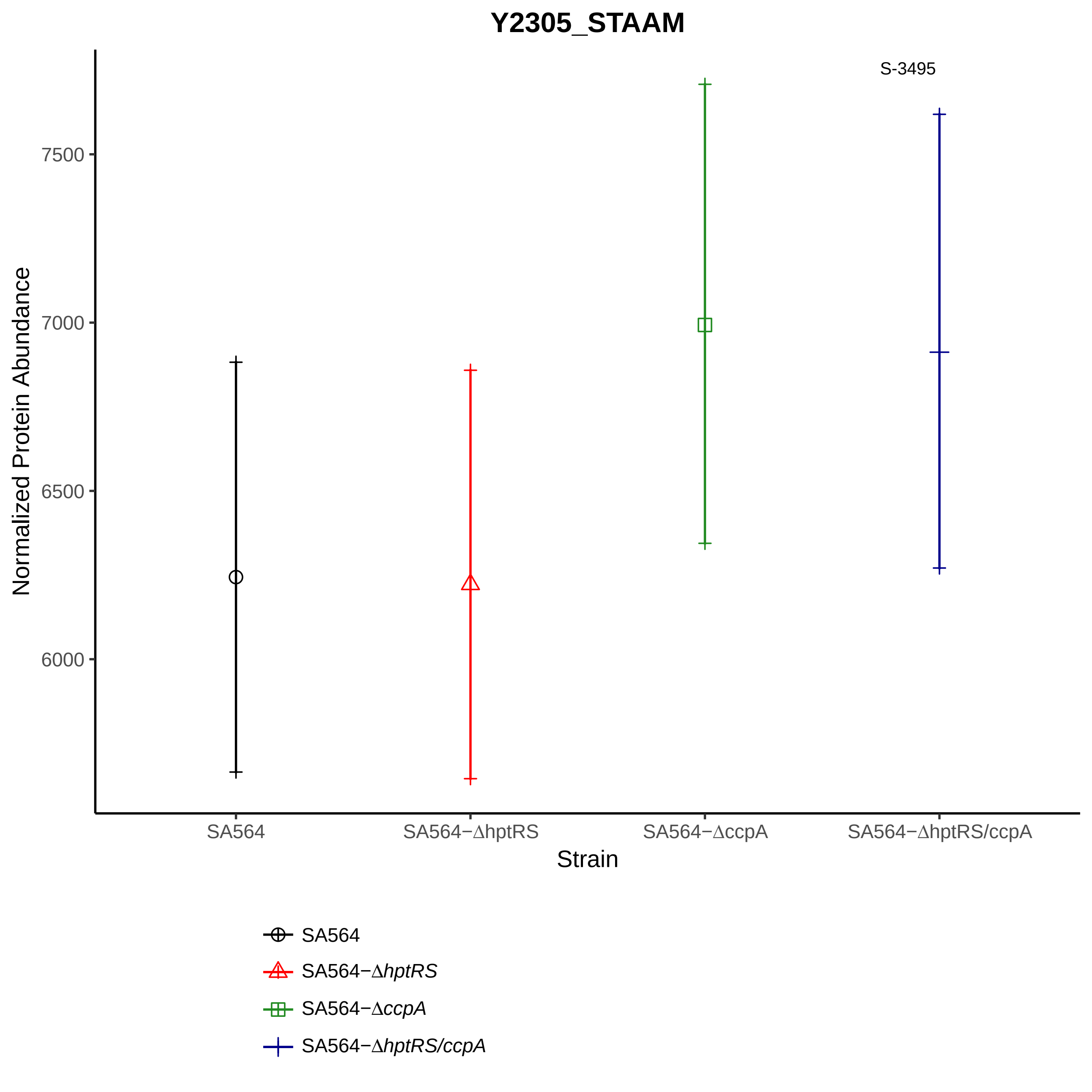

Y2305_STAAM
S-3495
Normalized Protein Abundance
7500
7000
6500
6000
SA564
SA564−ΔhptRS
SA564−ΔccpA
SA564−ΔhptRS/ccpA
Strain
SA564
SA564−Δ*hptRS*
SA564−Δ*ccpA*
SA564−Δ*hptRS/ccpA*

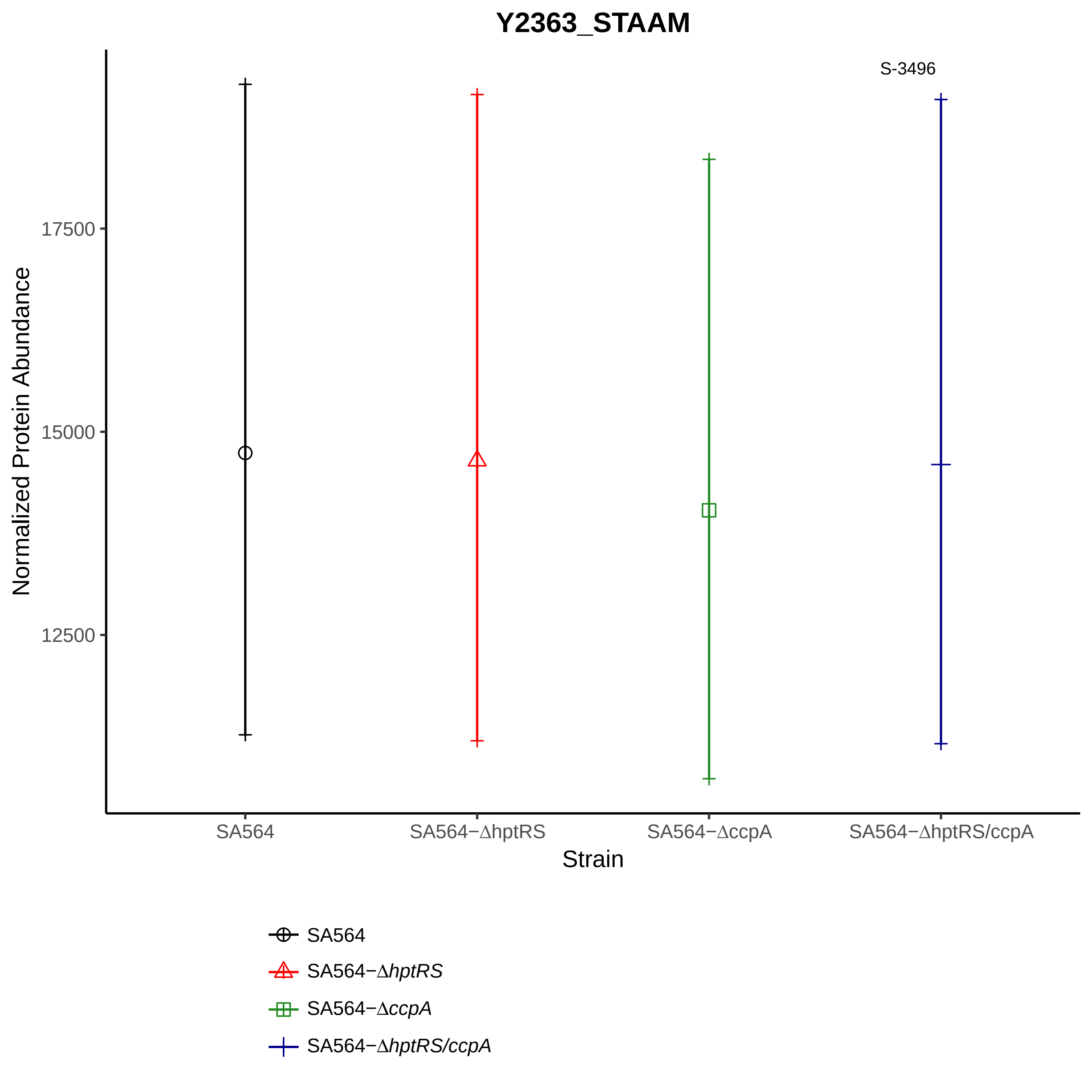

Y2363_STAAM
S-3496
Normalized Protein Abundance
17500
15000
12500
SA564
SA564−ΔhptRS
SA564−ΔccpA
SA564−ΔhptRS/ccpA
Strain
SA564
SA564−ΔhptRS
SA564−ΔccpA
SA564−ΔhptRS/ccpA

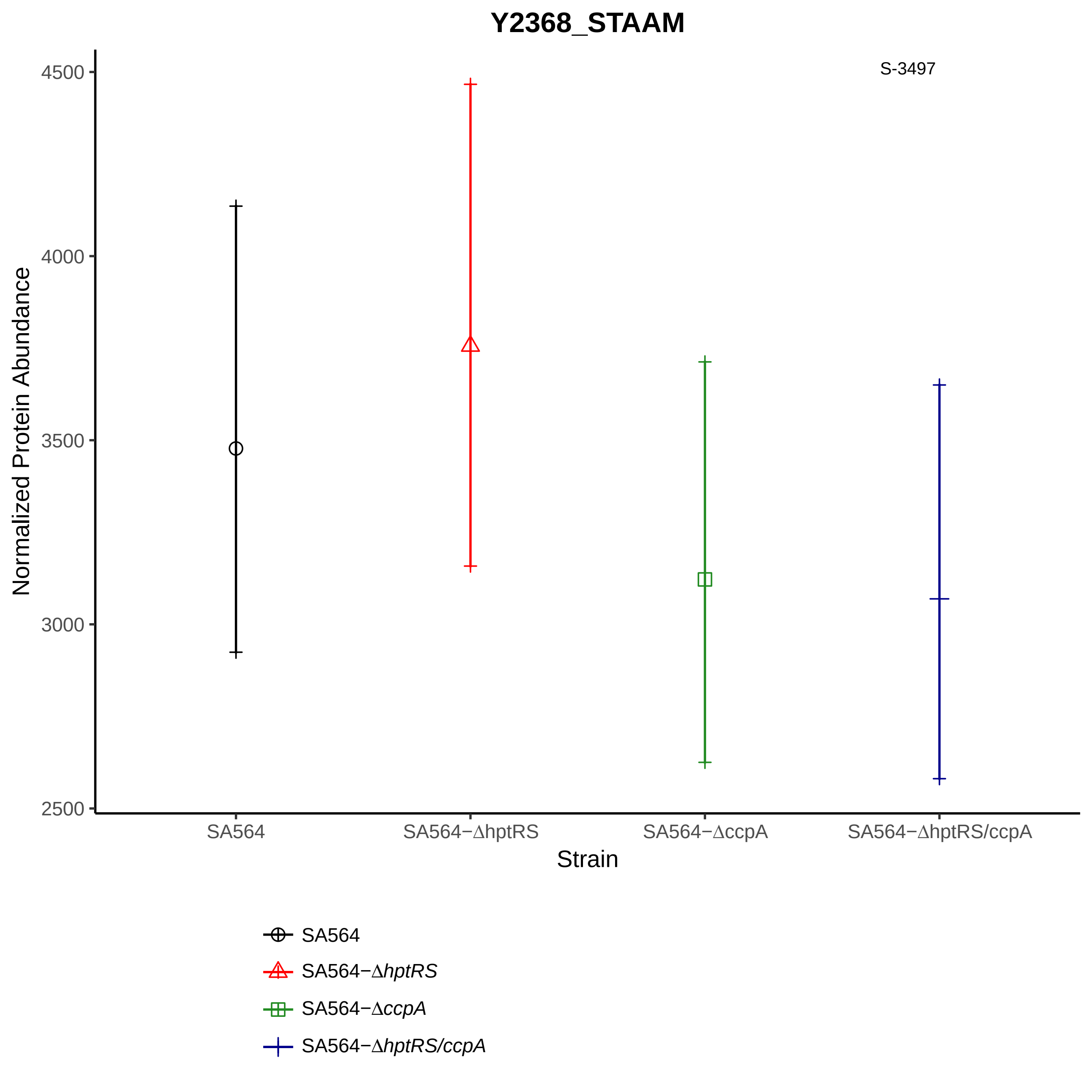

Y2368_STAAM
S-3497
Normalized Protein Abundance
4500
4000
3500
3000
2500
SA564
SA564−ΔhptRS
SA564−ΔccpA
SA564−ΔhptRS/ccpA
Strain
SA564
SA564−ΔhptRS
SA564−ΔccpA
SA564−ΔhptRS/ccpA

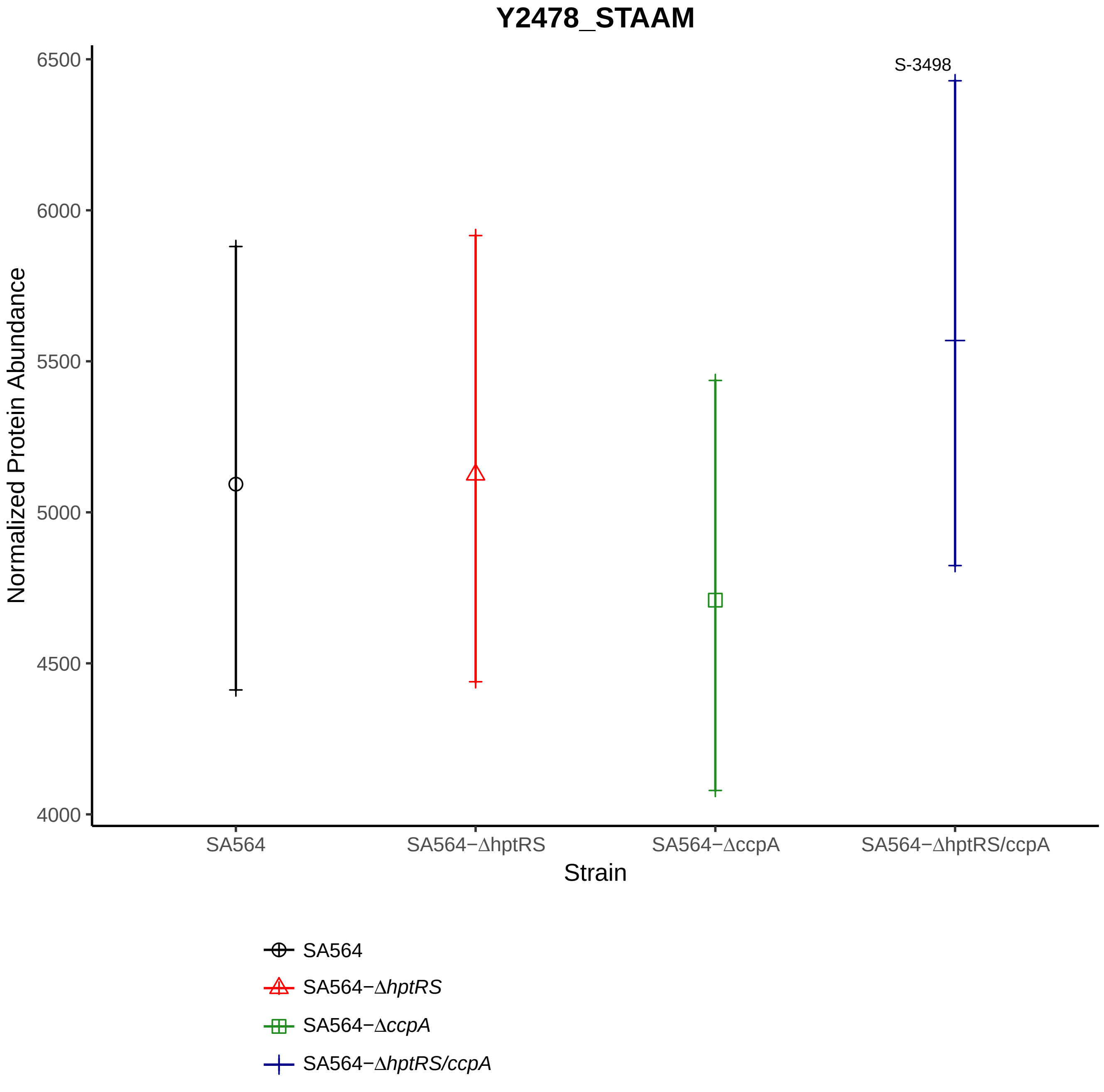

Y2478_STAAM
S-3498
6500
6000
5500
5000
4500
4000
Normalized Protein Abundance
SA564
SA564−ΔhptRS
SA564−ΔccpA
SA564−ΔhptRS/ccpA
Strain
SA564
SA564−Δ*hptRS*
SA564−Δ*ccpA*
SA564−Δ*hptRS/ccpA*

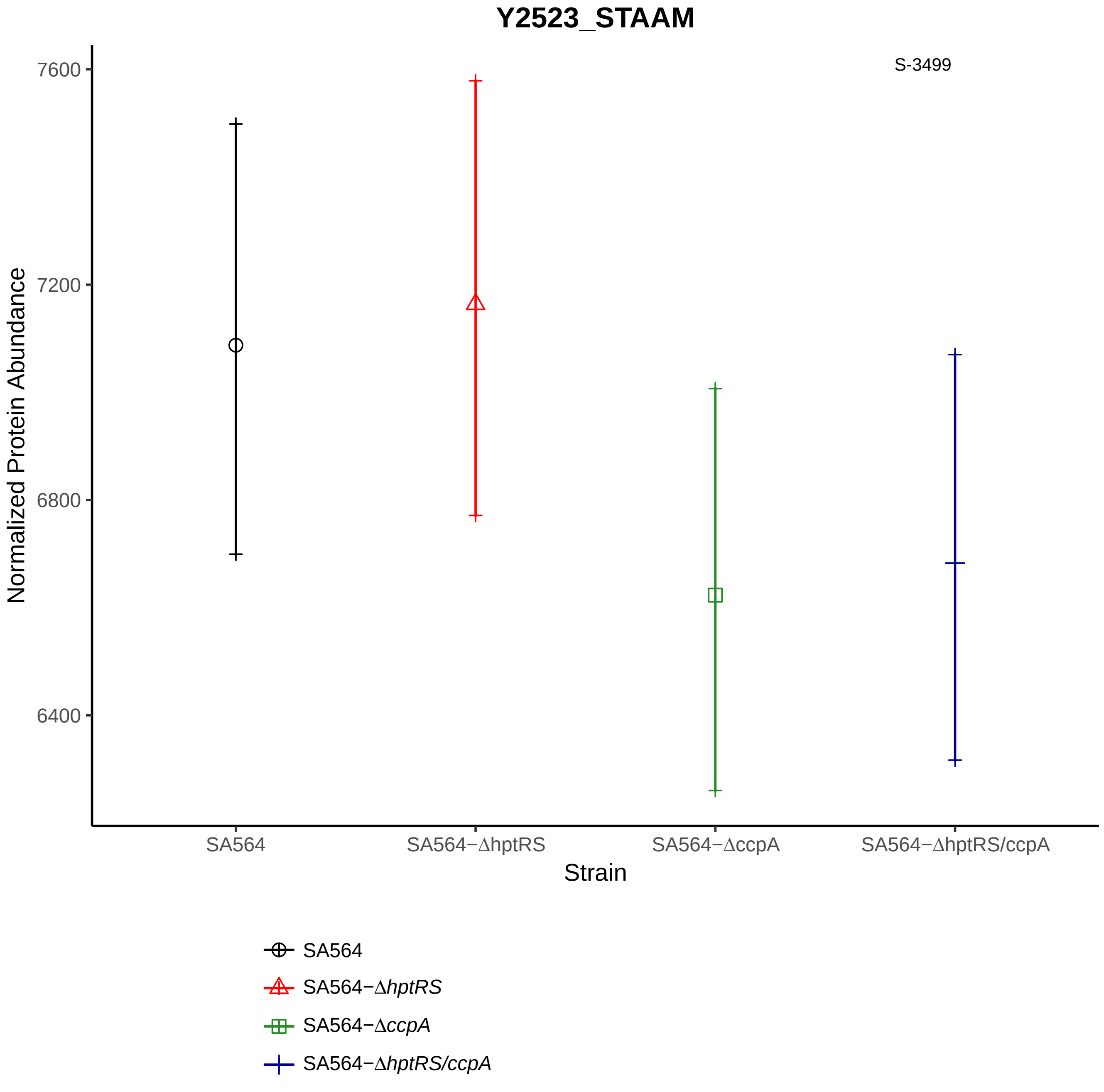

Y2523_STAAM
S-3499
Normalized Protein Abundance
7600
7200
6800
6400
SA564
SA564−ΔhptRS
SA564−ΔccpA
SA564−ΔhptRS/ccpA
Strain
SA564
SA564−ΔhptRS
SA564−ΔccpA
SA564−ΔhptRS/ccpA

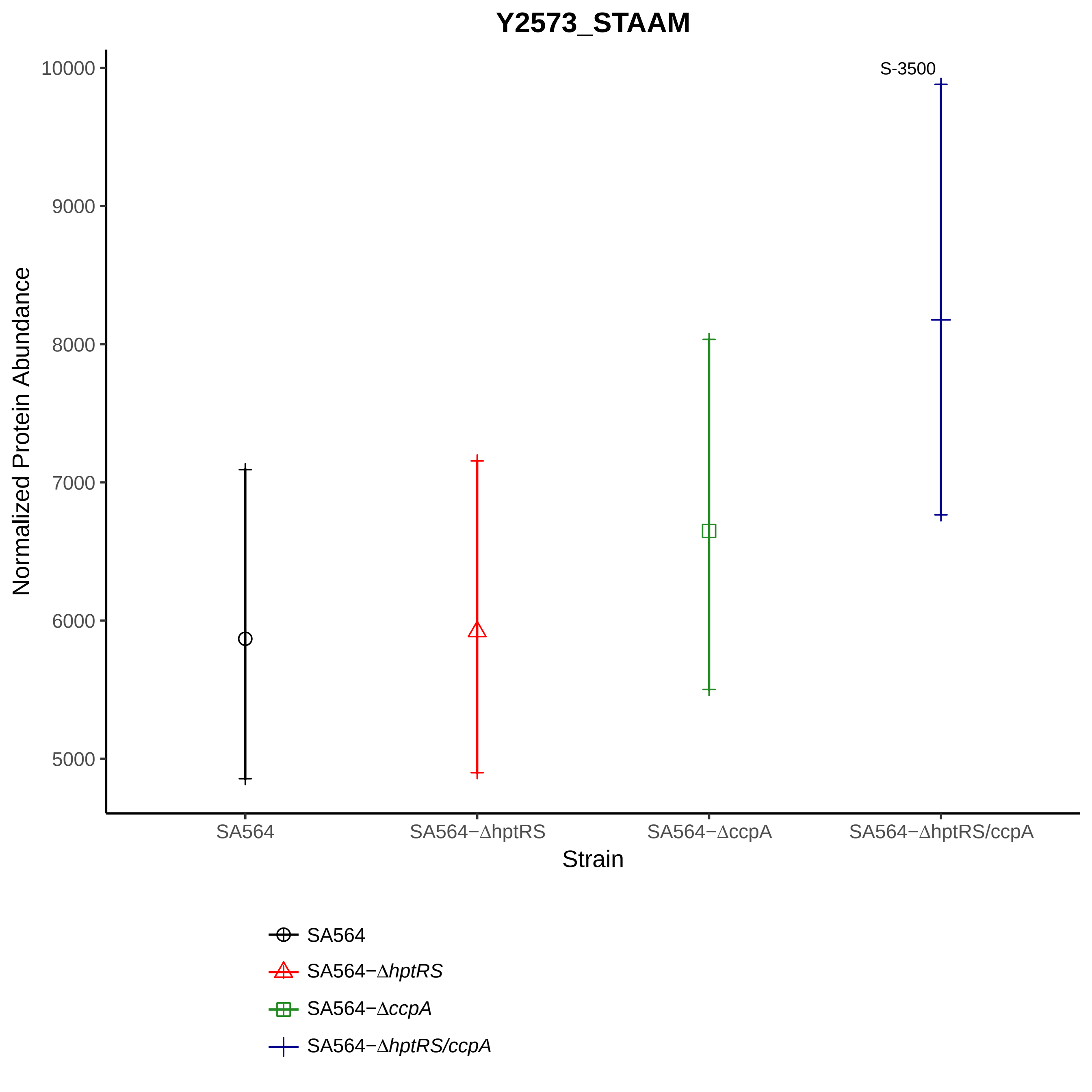
Y2573_STAAM
S-3500
10000
9000
8000
7000
6000
5000
Normalized Protein Abundance
SA564
SA564−ΔhptRS
SA564−ΔccpA
SA564−ΔhptRS/ccpA
Strain
SA564
SA564−Δ*hptRS*
SA564−Δ*ccpA*
SA564−Δ*hptRS/ccpA*

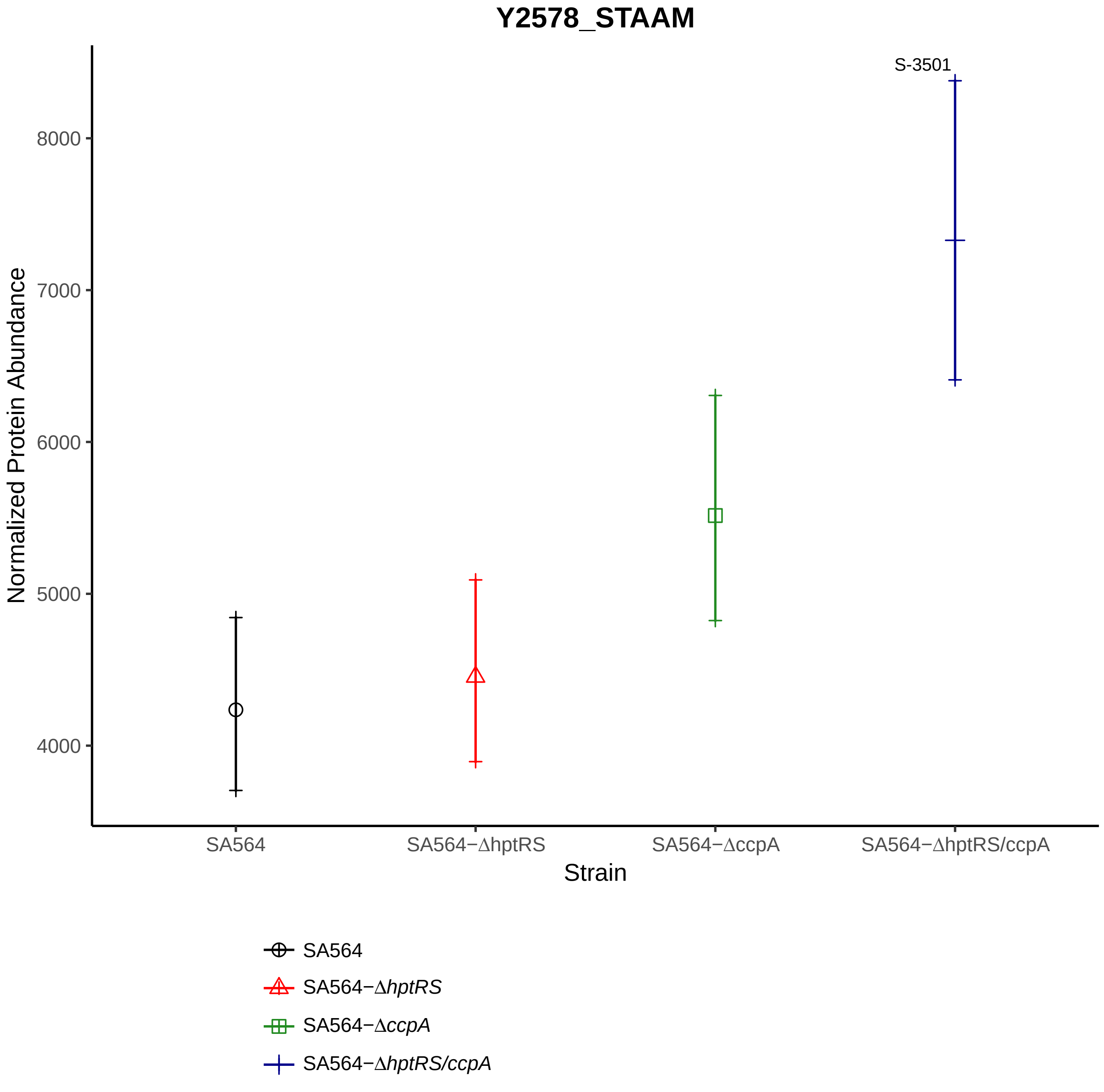
Y2578_STAAM
S-3501
Normalized Protein Abundance
8000
7000
6000
5000
4000
SA564
SA564−ΔhptRS
SA564−ΔccpA
SA564−ΔhptRS/ccpA
Strain
SA564
SA564−ΔhptRS
SA564−ΔccpA
SA564−ΔhptRS/ccpA

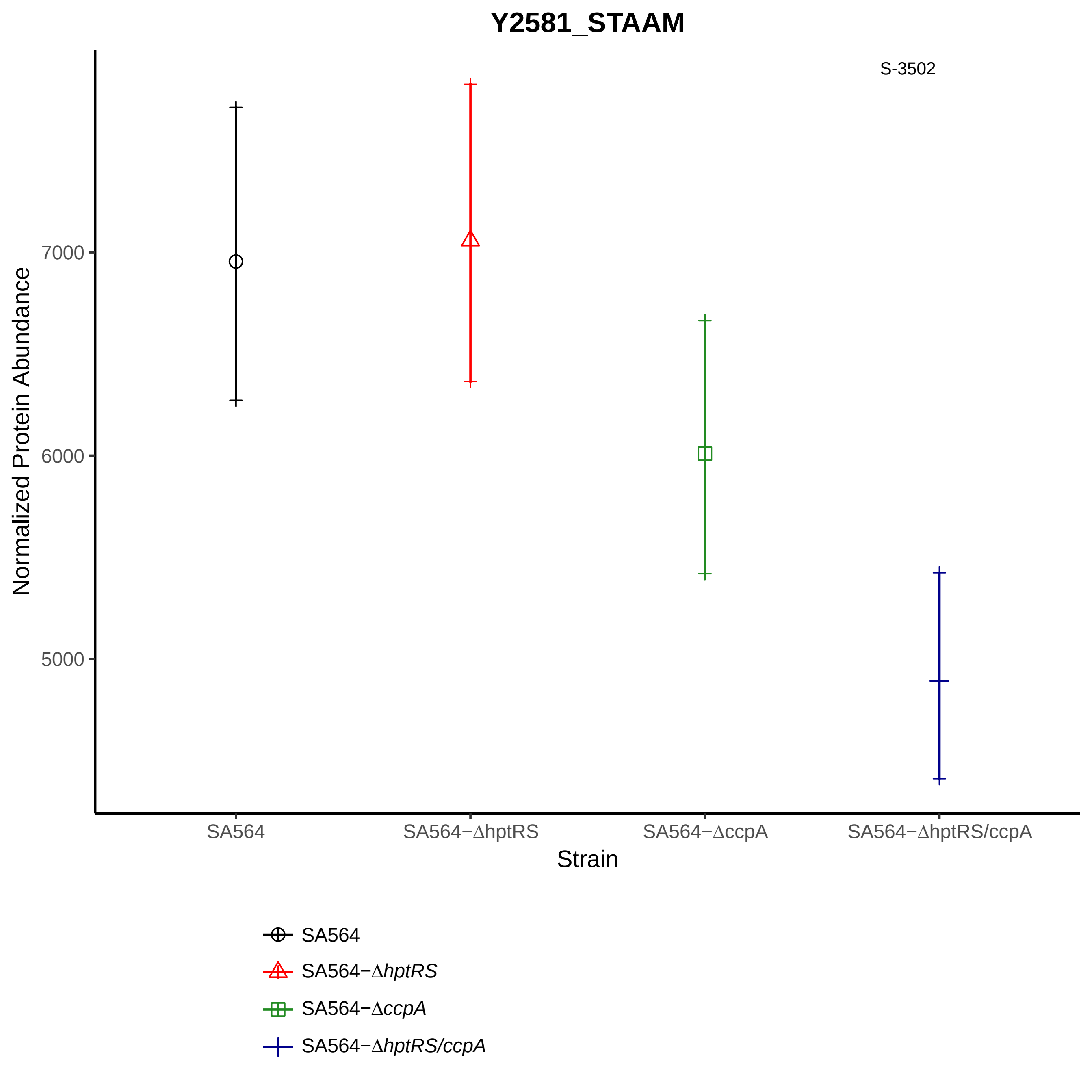

Y2581_STAAM
S-3502
Normalized Protein Abundance
7000
6000
5000
SA564
SA564−ΔhptRS
SA564−ΔccpA
SA564−ΔhptRS/ccpA
Strain
SA564
SA564−ΔhptRS
SA564−ΔccpA
SA564−ΔhptRS/ccpA

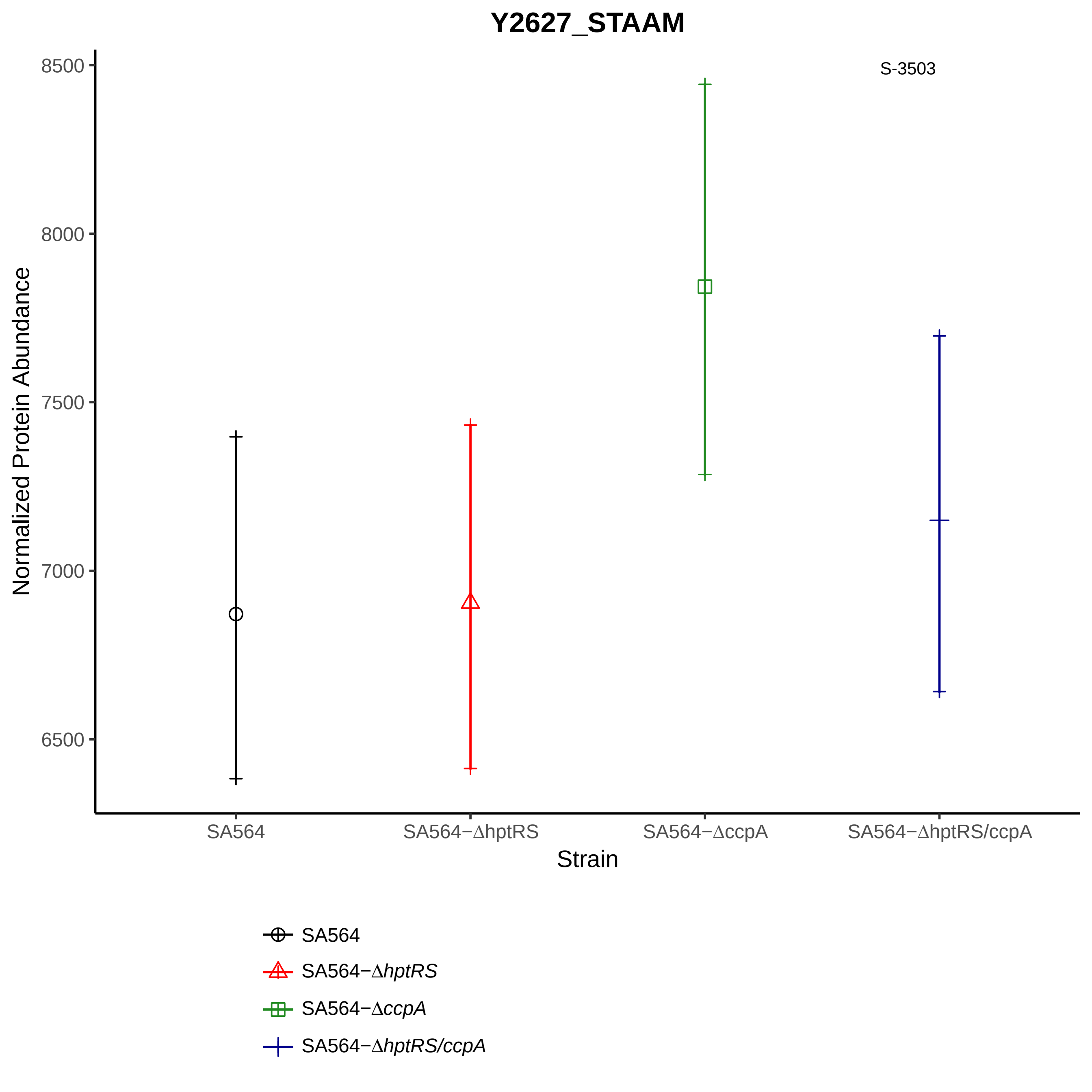

Y2627_STAAM
S-3503
Normalized Protein Abundance
8500
8000
7500
7000
6500
SA564
SA564−ΔhptRS
SA564−ΔccpA
SA564−ΔhptRS/ccpA
Strain
SA564
SA564−ΔhptRS
SA564−ΔccpA
SA564−ΔhptRS/ccpA

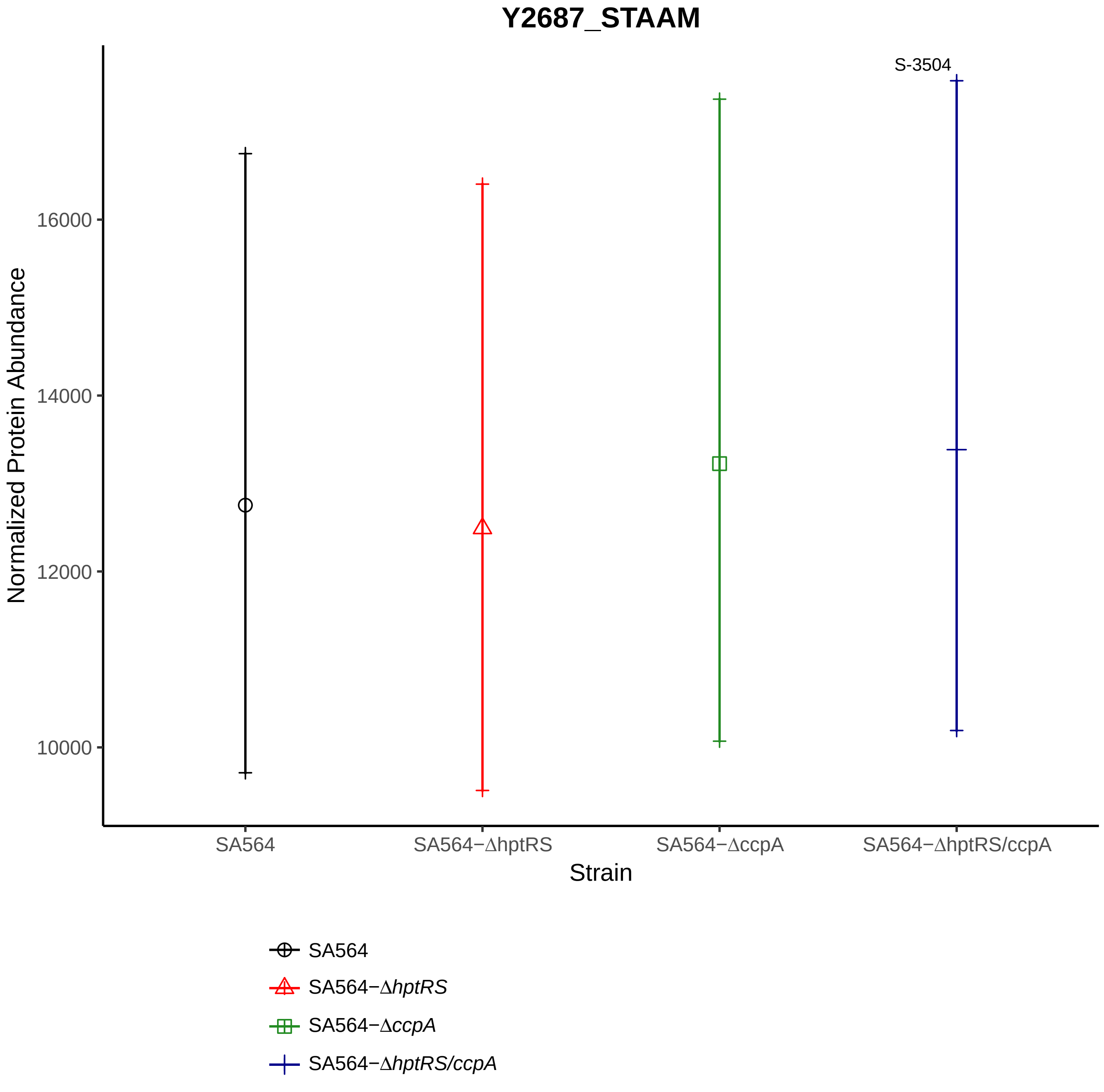
Y2687_STAAM
S-3504
Normalized Protein Abundance
16000
14000
12000
10000
SA564
SA564−ΔhptRS
SA564−ΔccpA
SA564−ΔhptRS/ccpA
Strain
SA564
SA564−ΔhptRS
SA564−ΔccpA
SA564−ΔhptRS/ccpA

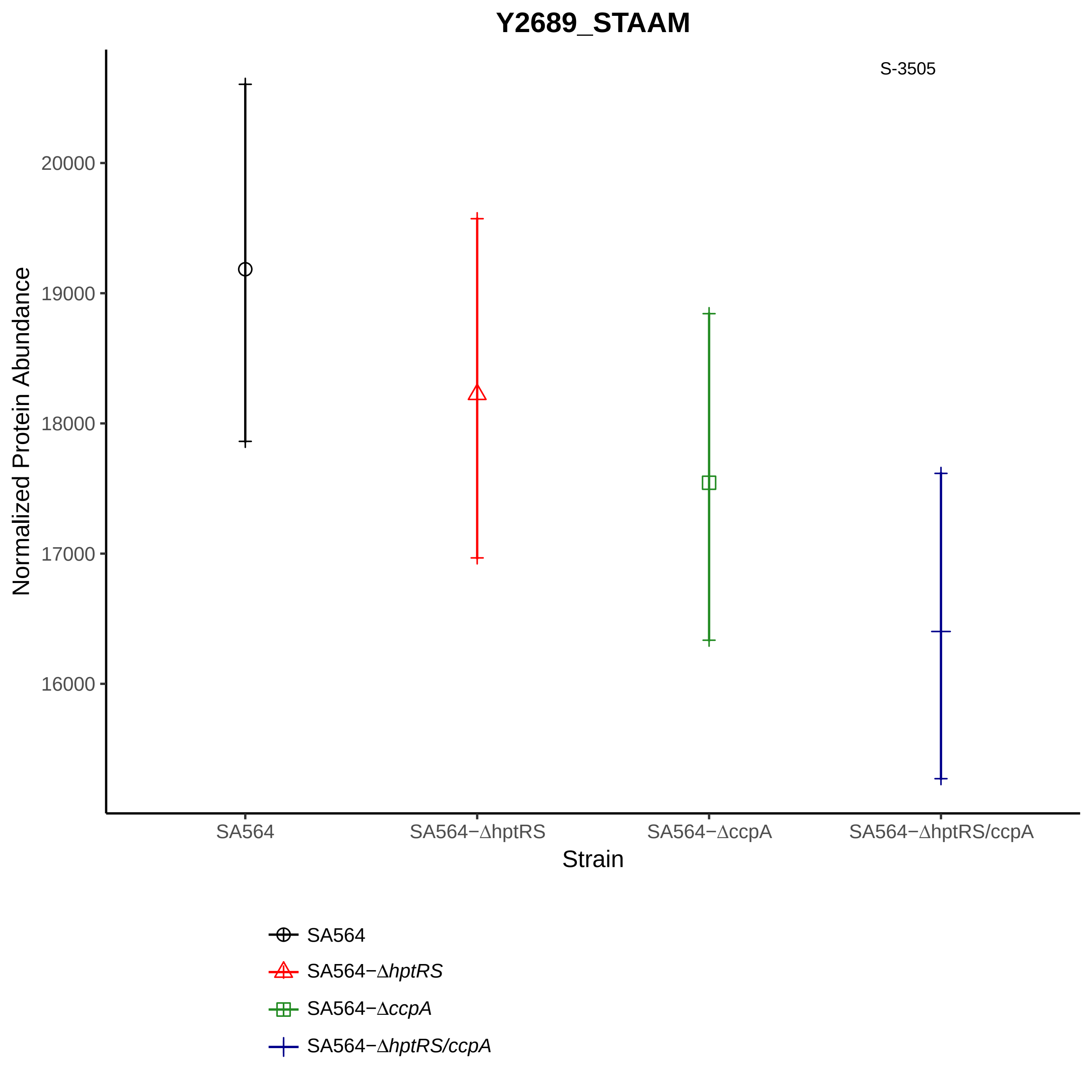

Y2689_STAAM
S-3505
Normalized Protein Abundance
20000
19000
18000
17000
16000
SA564
SA564−ΔhptRS
SA564−ΔccpA
SA564−ΔhptRS/ccpA
Strain
SA564
SA564−ΔhptRS
SA564−ΔccpA
SA564−ΔhptRS/ccpA

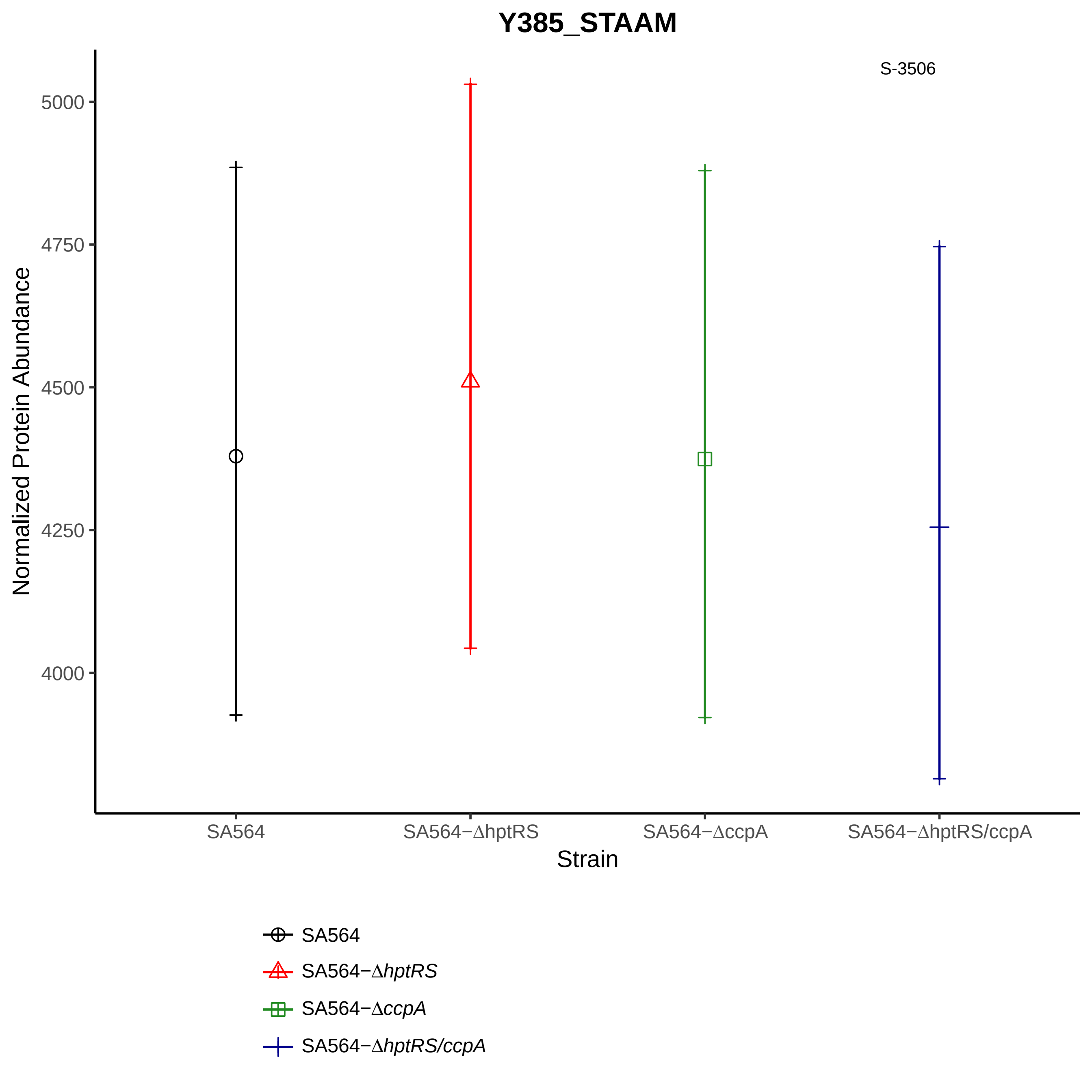
Y385_STAAM
S-3506
Normalized Protein Abundance
5000
4750
4500
4250
4000
SA564
SA564−ΔhptRS
SA564−ΔccpA
SA564−ΔhptRS/ccpA
Strain
SA564
SA564−ΔhptRS
SA564−ΔccpA
SA564−ΔhptRS/ccpA

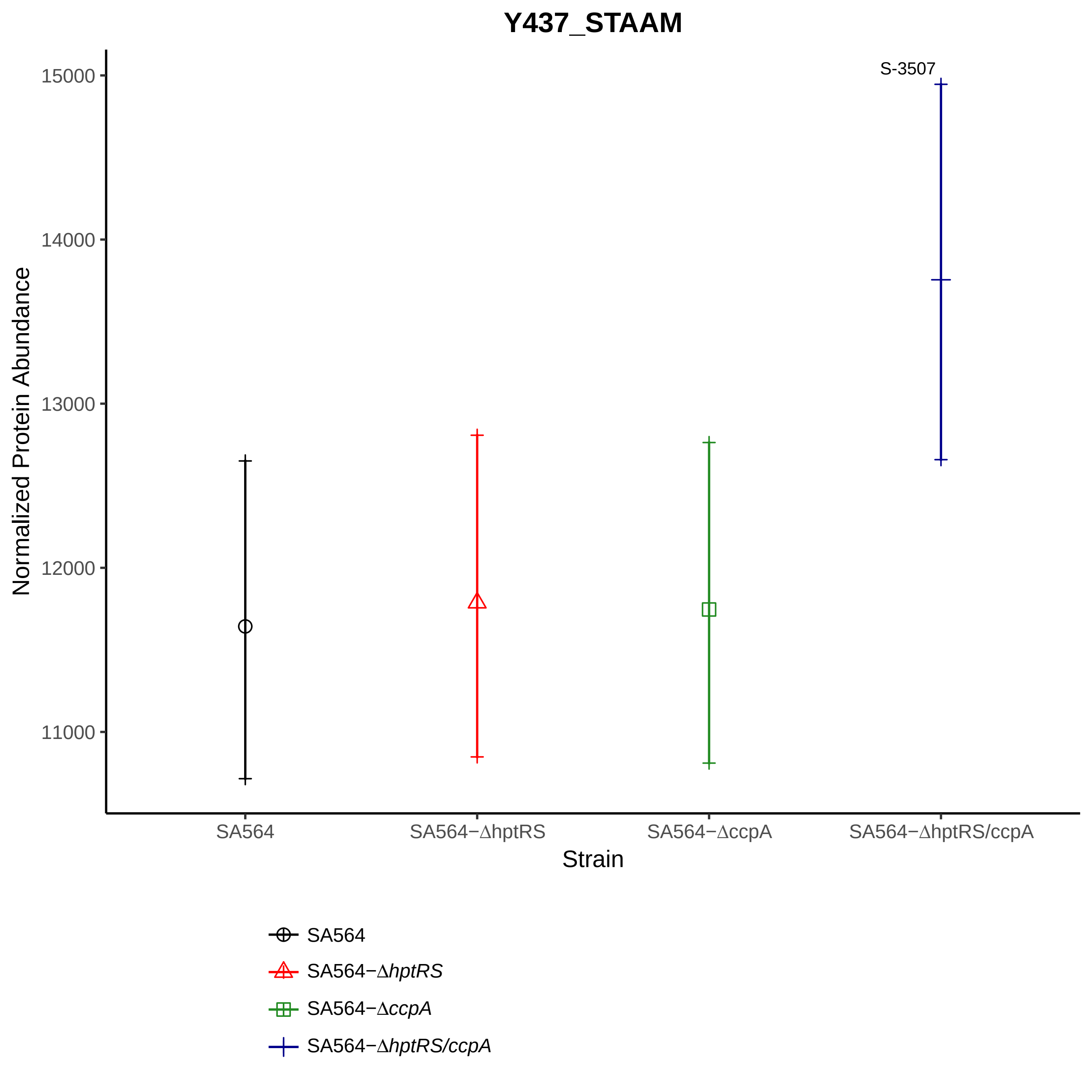

Y437_STAAM
S-3507
15000
14000
13000
12000
11000
Normalized Protein Abundance
SA564
SA564−ΔhptRS
SA564−ΔccpA
SA564−ΔhptRS/ccpA
Strain
SA564
SA564−ΔhptRS
SA564−ΔccpA
SA564−ΔhptRS/ccpA

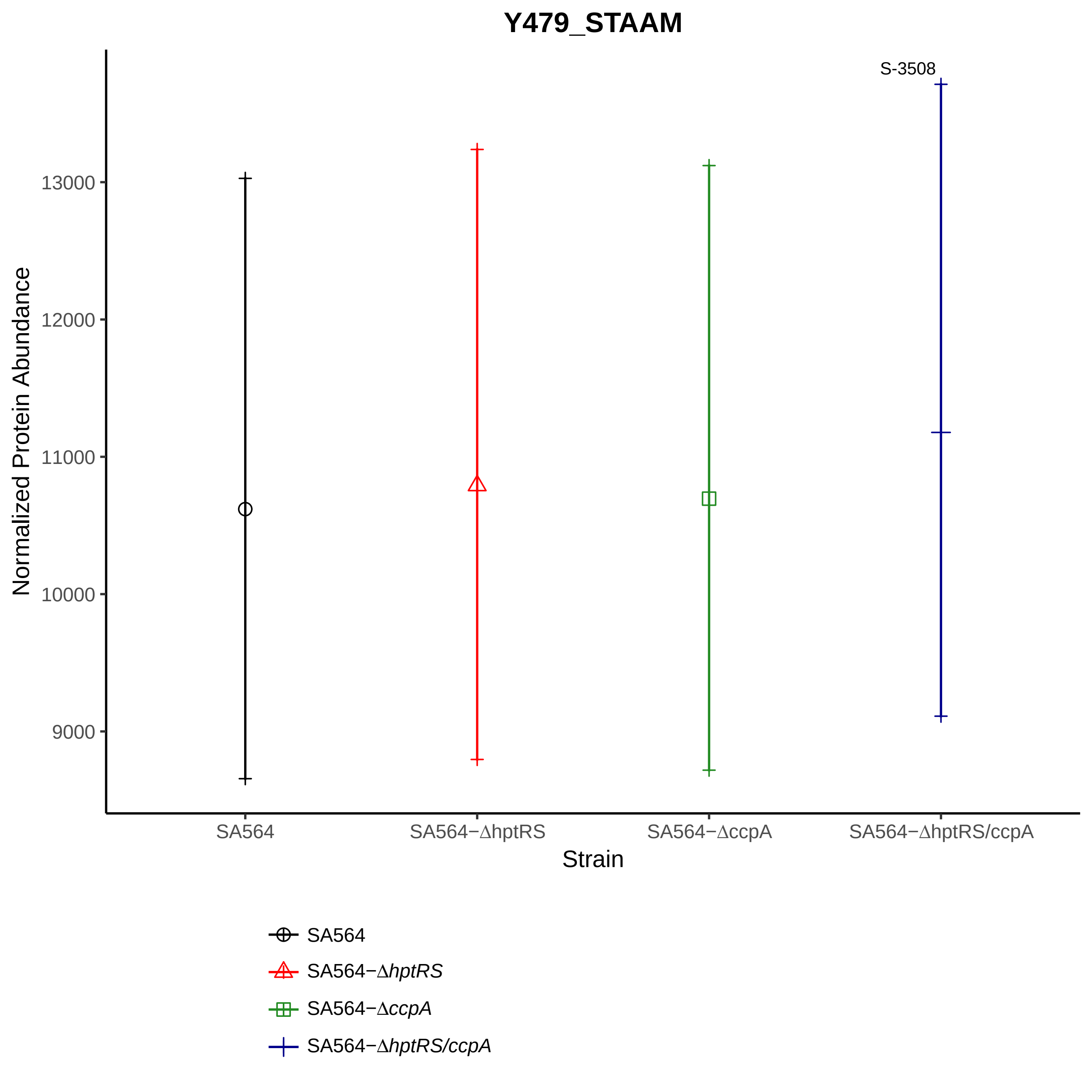

Y479_STAAM
S-3508
Normalized Protein Abundance
13000
12000
11000
10000
9000
SA564
SA564−ΔhptRS
SA564−ΔccpA
SA564−ΔhptRS/ccpA
Strain
SA564
SA564−ΔhptRS
SA564−ΔccpA
SA564−ΔhptRS/ccpA

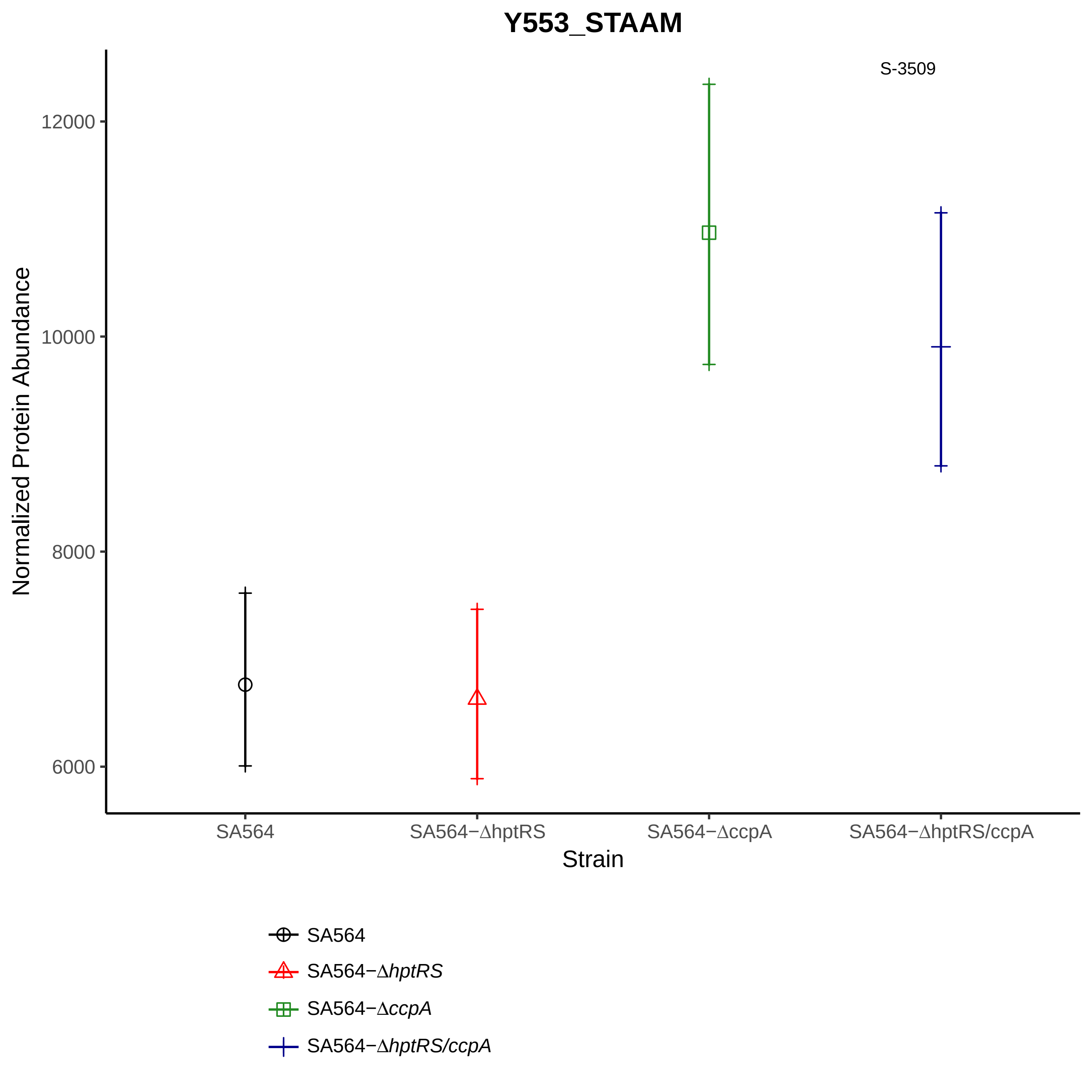

Y553_STAAM
S-3509
Normalized Protein Abundance
12000
10000
8000
6000
SA564
SA564−ΔhptRS
SA564−ΔccpA
SA564−ΔhptRS/ccpA
Strain
SA564
SA564−ΔhptRS
SA564−ΔccpA
SA564−ΔhptRS/ccpA

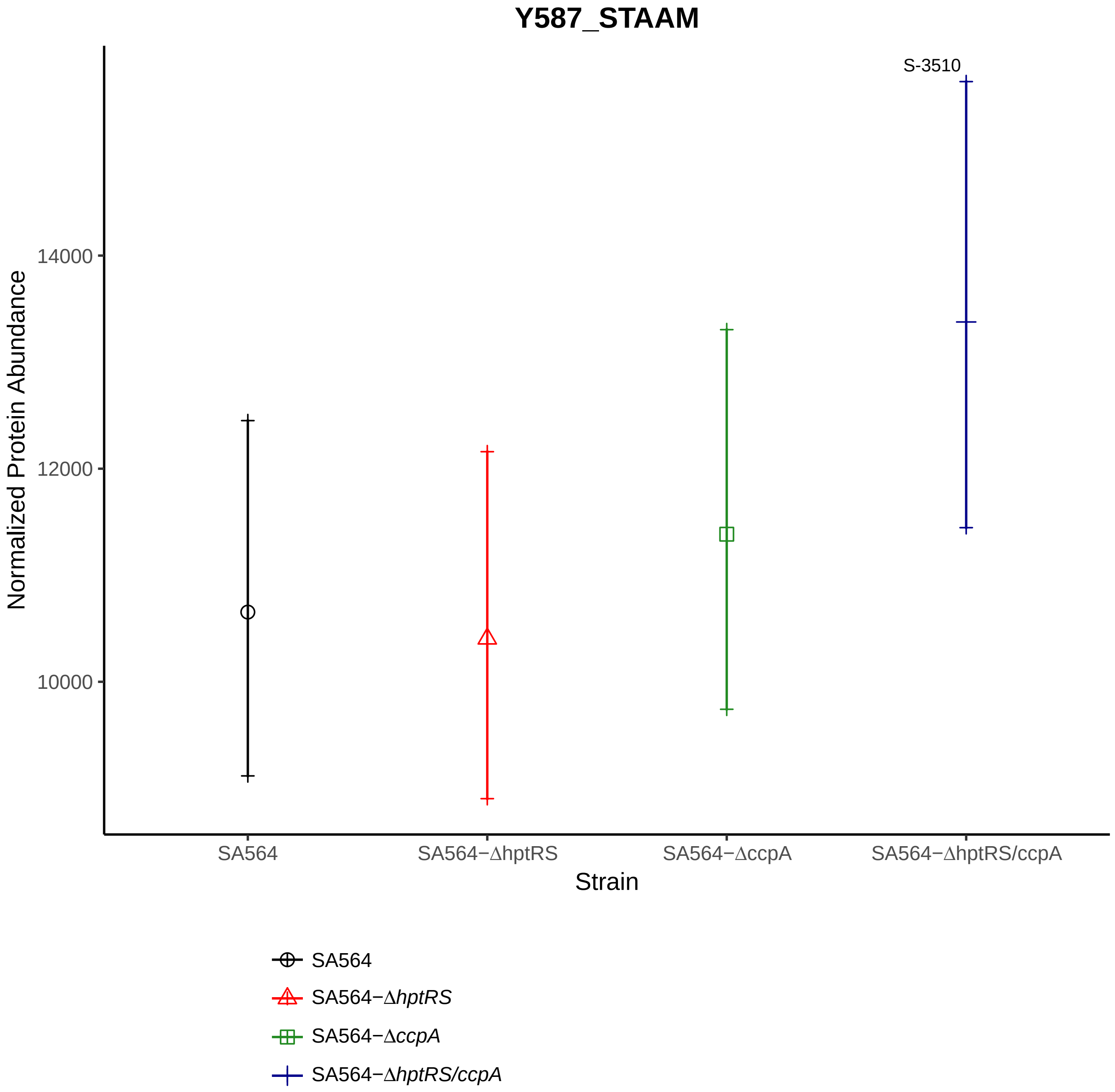

Y587_STAAM
S-3510
14000
12000
10000
Normalized Protein Abundance
SA564
SA564−ΔhptRS
SA564−ΔccpA
SA564−ΔhptRS/ccpA
Strain
SA564
SA564−Δ*hptRS*
SA564−Δ*ccpA*
SA564−Δ*hptRS/ccpA*

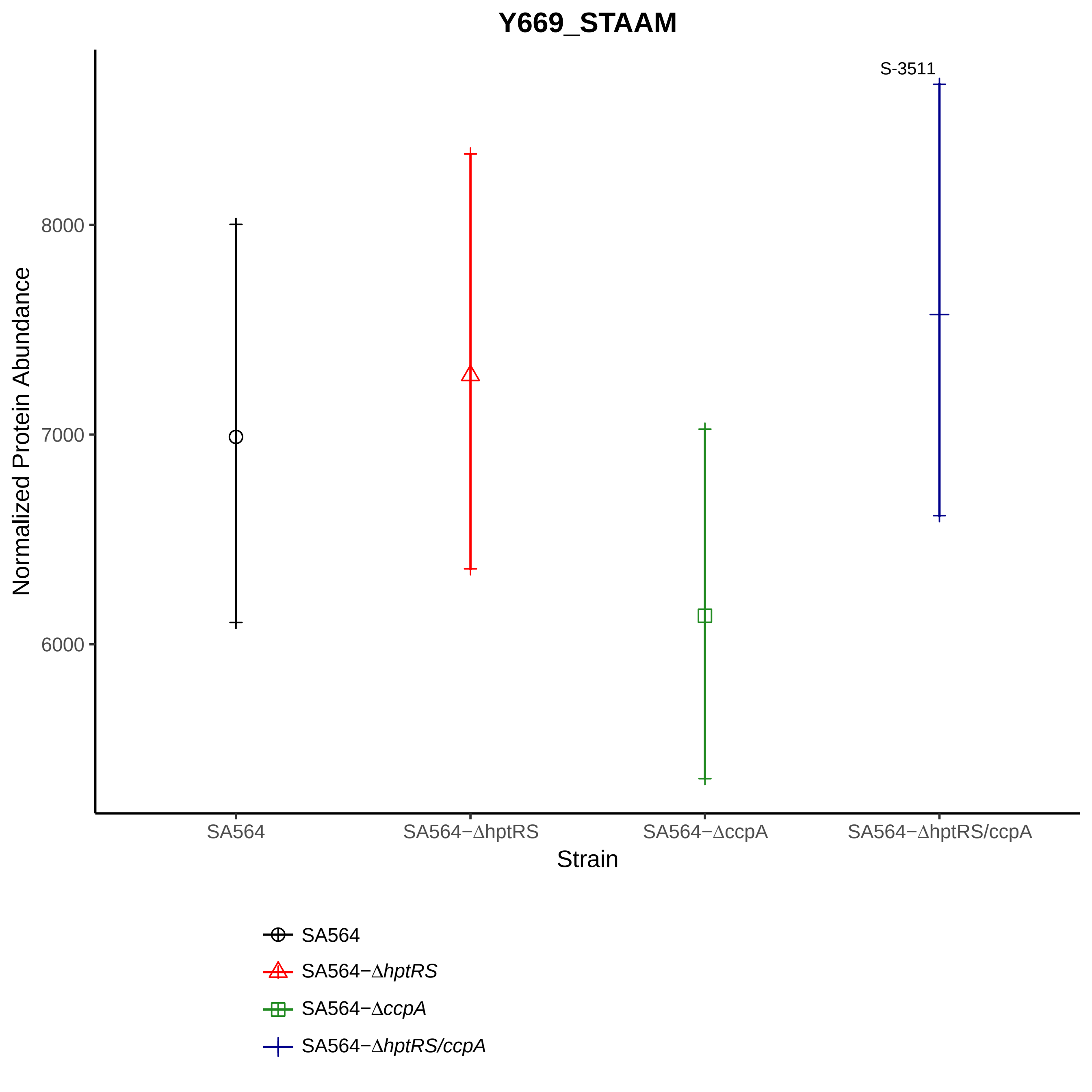

Y669_STAAM
S-3511
8000
7000
6000
Normalized Protein Abundance
SA564
SA564−ΔhptRS
SA564−ΔccpA
SA564−ΔhptRS/ccpA
Strain
SA564
SA564−ΔhptRS
SA564−ΔccpA
SA564−ΔhptRS/ccpA

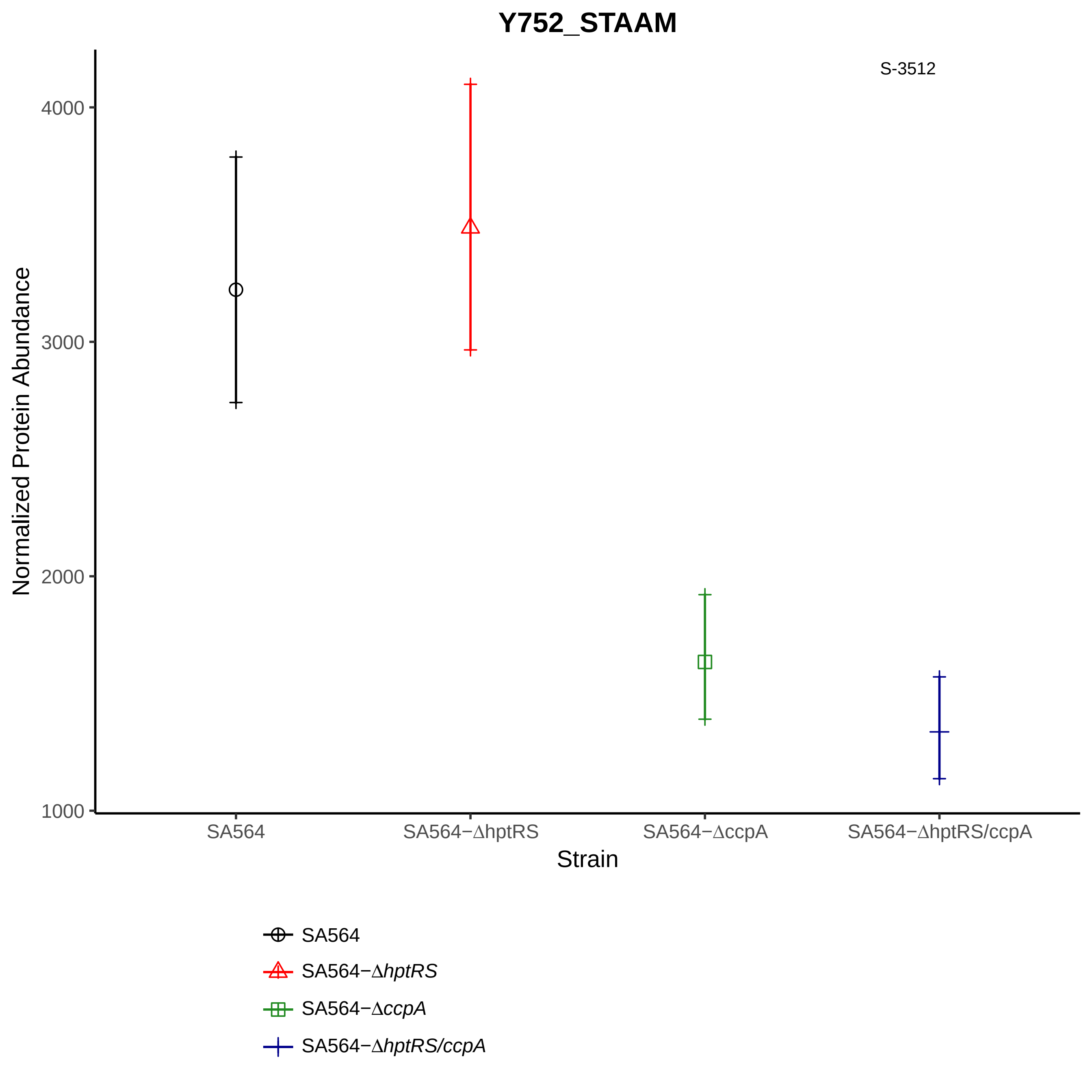
Y752_STAAM
S-3512
Normalized Protein Abundance
4000
3000
2000
1000
SA564
SA564−ΔhptRS
SA564−ΔccpA
SA564−ΔhptRS/ccpA
Strain
SA564
SA564−ΔhptRS
SA564−ΔccpA
SA564−ΔhptRS/ccpA

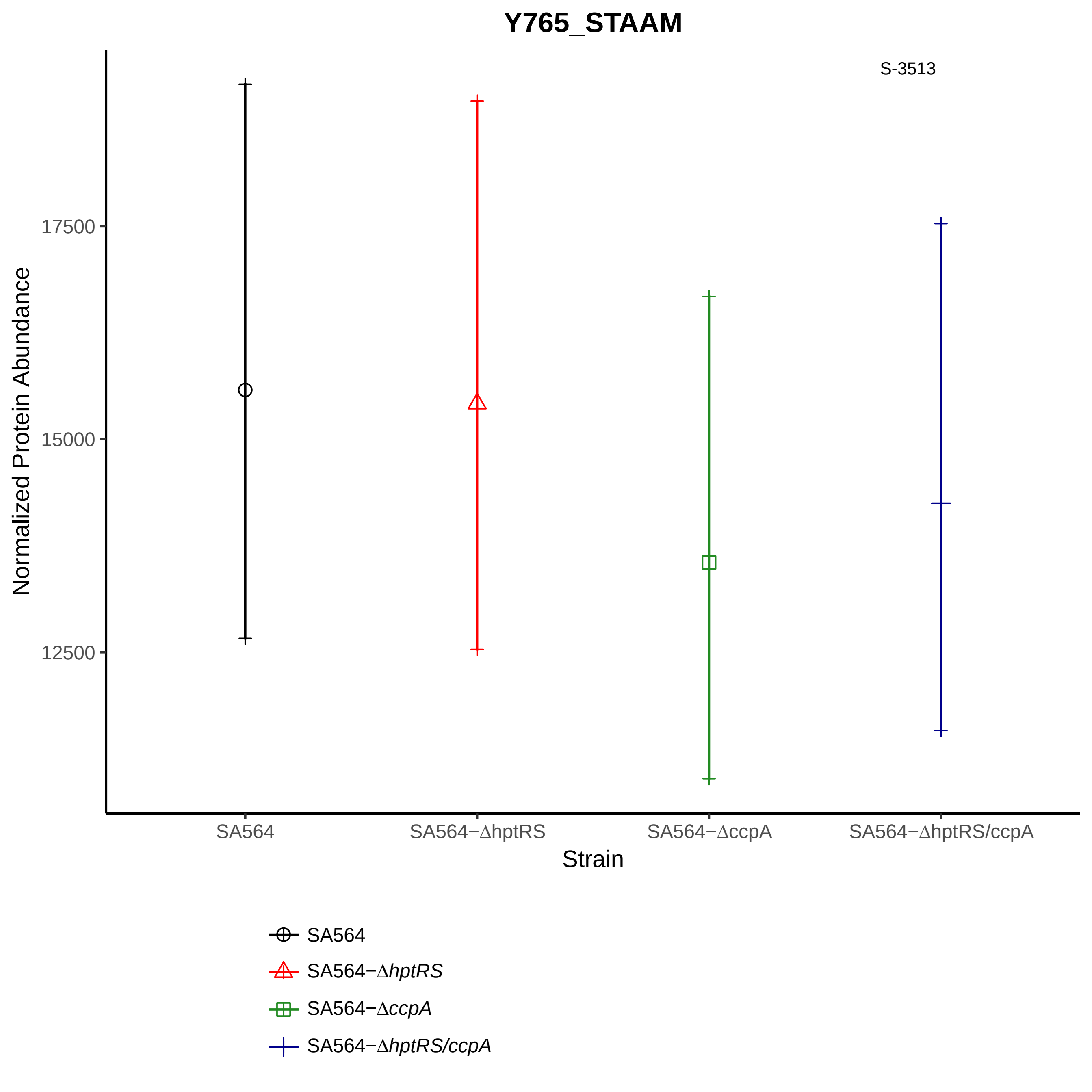

Y765_STAAM
S-3513
Normalized Protein Abundance
17500
15000
12500
SA564
SA564−ΔhptRS
SA564−ΔccpA
SA564−ΔhptRS/ccpA
Strain
SA564
SA564−ΔhptRS
SA564−ΔccpA
SA564−ΔhptRS/ccpA

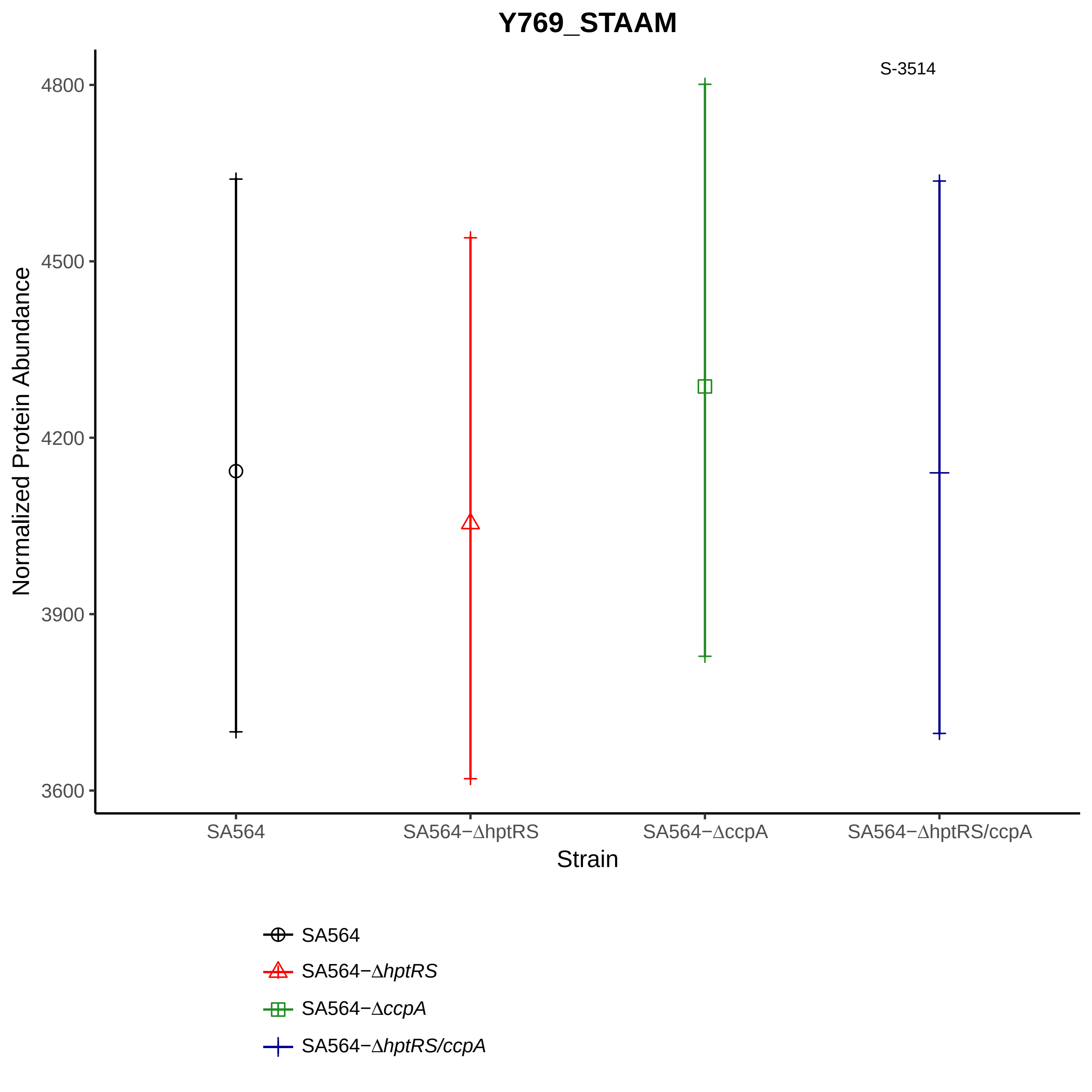
Y769_STAAM
S-3514
Normalized Protein Abundance
4800
4500
4200
3900
3600
SA564
SA564−ΔhptRS
SA564−ΔccpA
SA564−ΔhptRS/ccpA
Strain
SA564
SA564−ΔhptRS
SA564−ΔccpA
SA564−ΔhptRS/ccpA

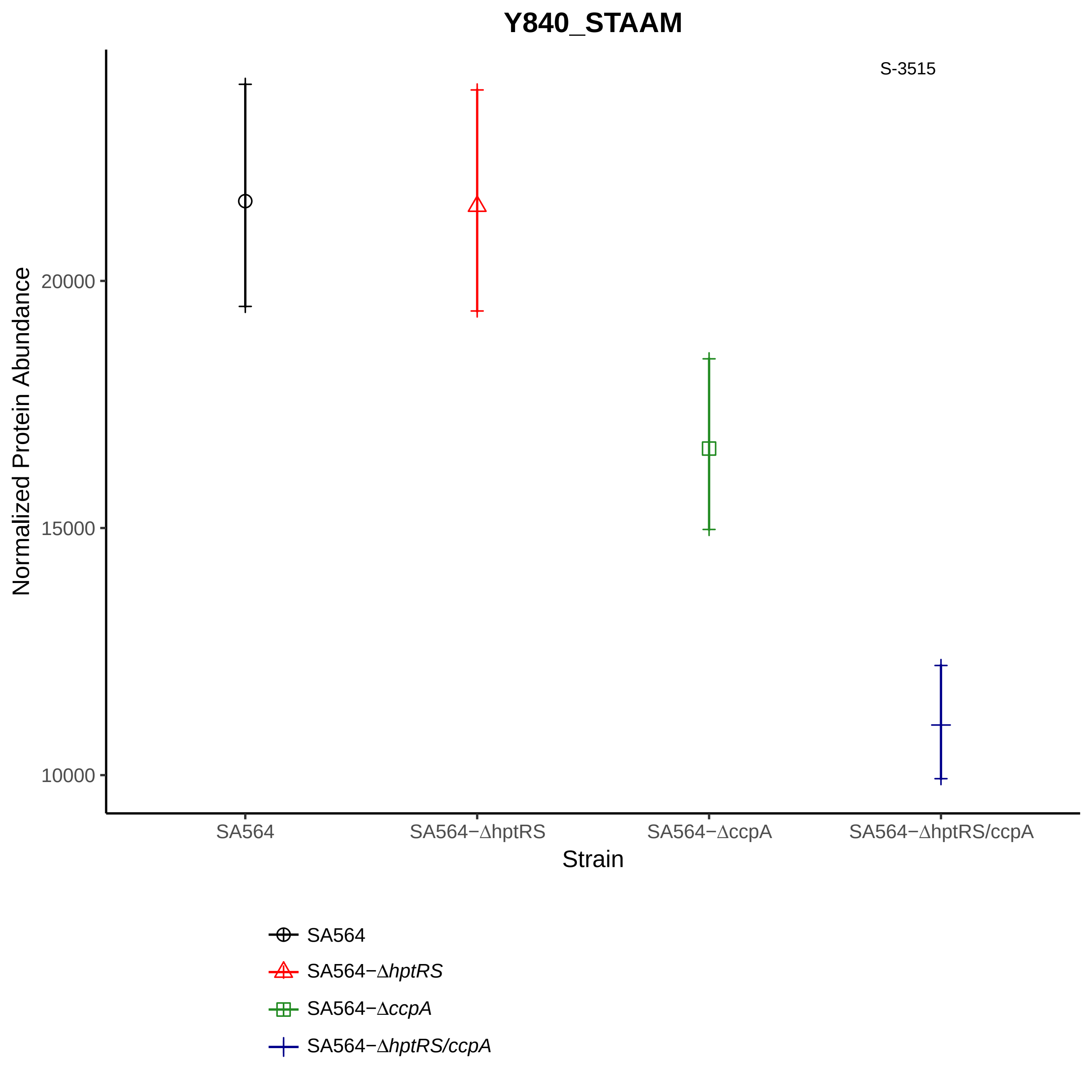
Y840_STAAM
S-3515
Normalized Protein Abundance
20000
15000
10000
SA564
SA564−ΔhptRS
SA564−ΔccpA
SA564−ΔhptRS/ccpA
Strain
SA564
SA564−ΔhptRS
SA564−ΔccpA
SA564−ΔhptRS/ccpA

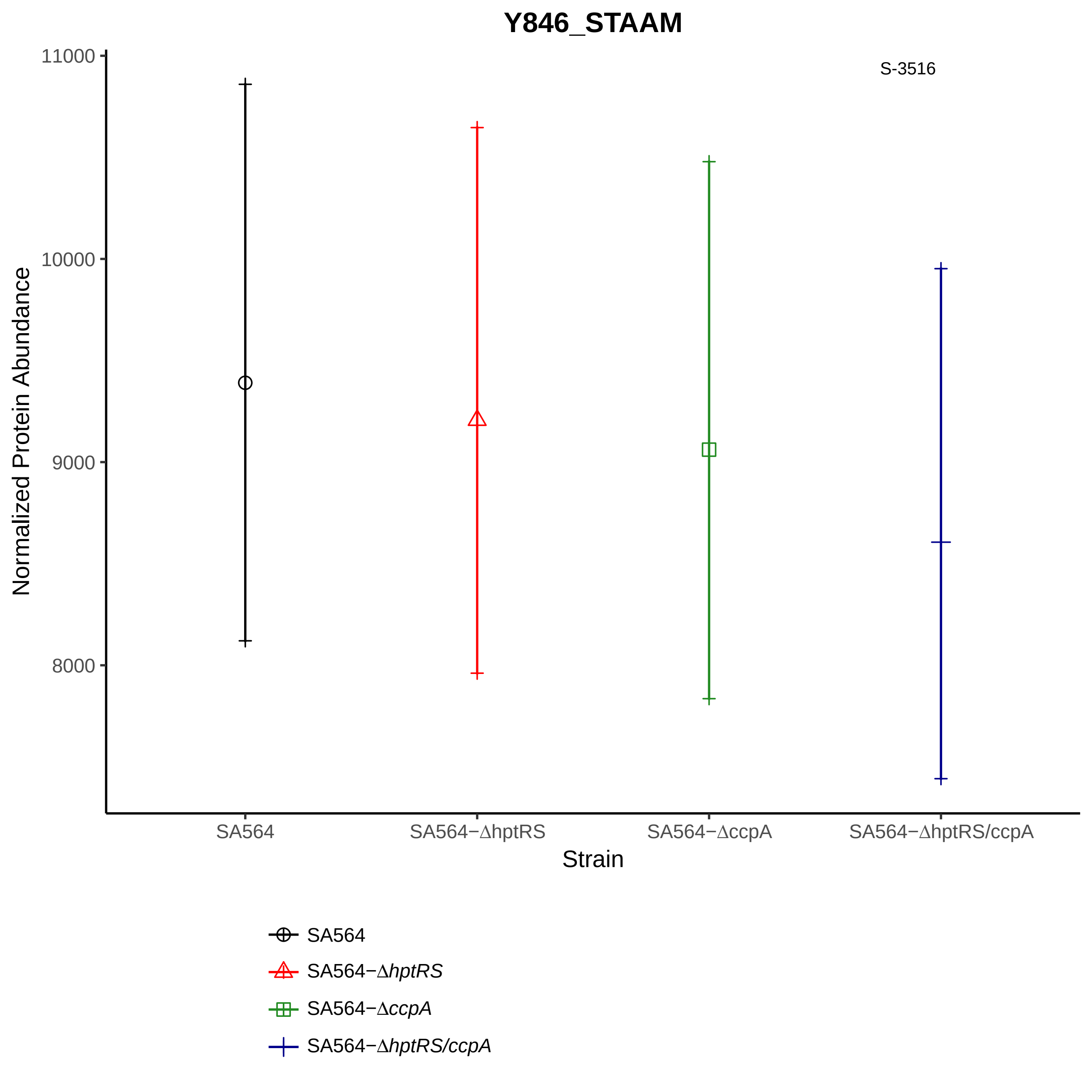

Y846_STAAM
S-3516
Normalized Protein Abundance
11000
10000
9000
8000
SA564
SA564−ΔhptRS
SA564−ΔccpA
SA564−ΔhptRS/ccpA
Strain
SA564
SA564−Δ*hptRS*
SA564−Δ*ccpA*
SA564−Δ*hptRS/ccpA*

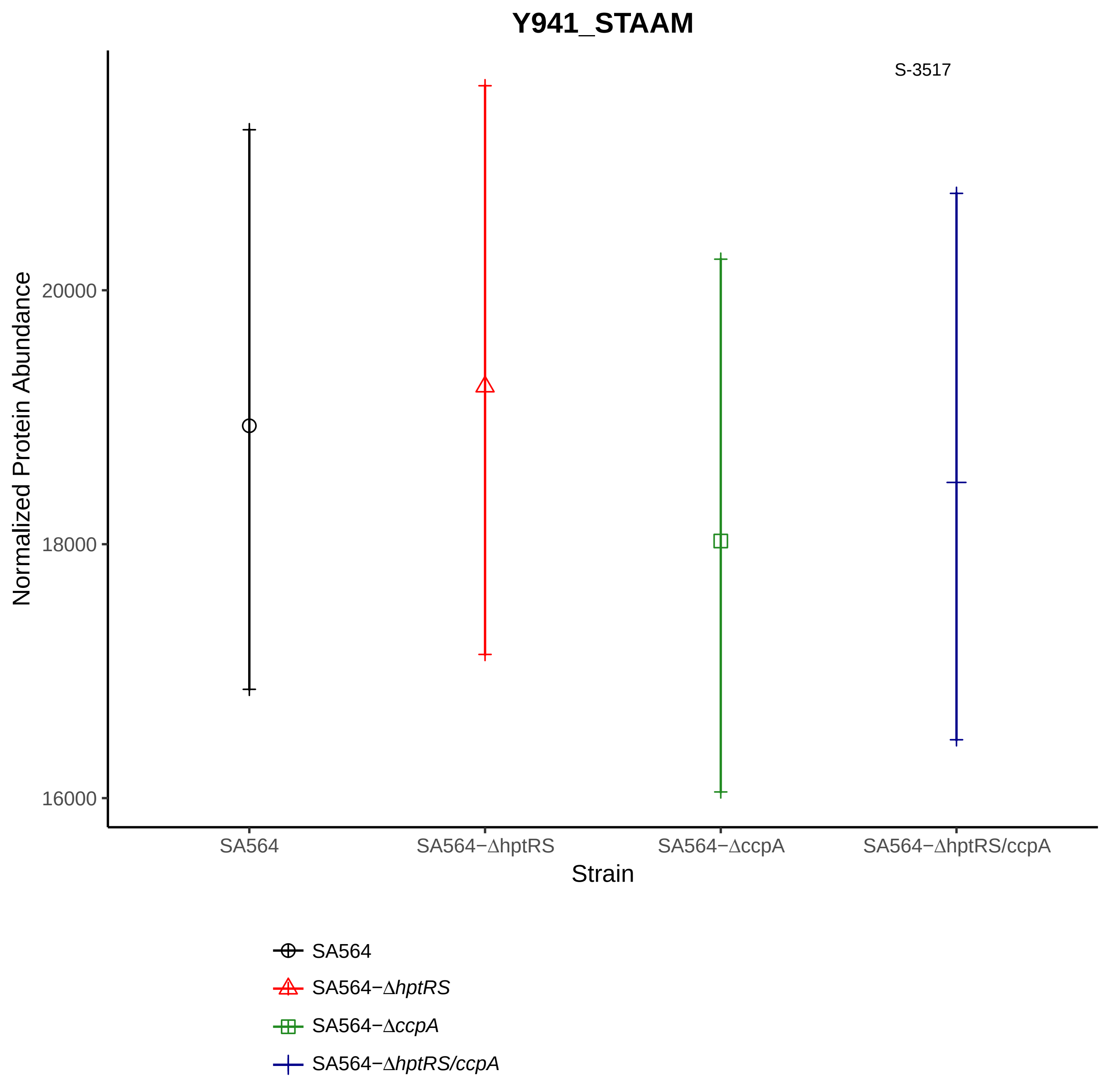

Y941_STAAM
S-3517
Normalized Protein Abundance
20000
18000
16000
SA564
SA564−ΔhptRS
SA564−ΔccpA
SA564−ΔhptRS/ccpA
Strain
SA564
SA564−Δ*hptRS*
SA564−Δ*ccpA*
SA564−Δ*hptRS/ccpA*

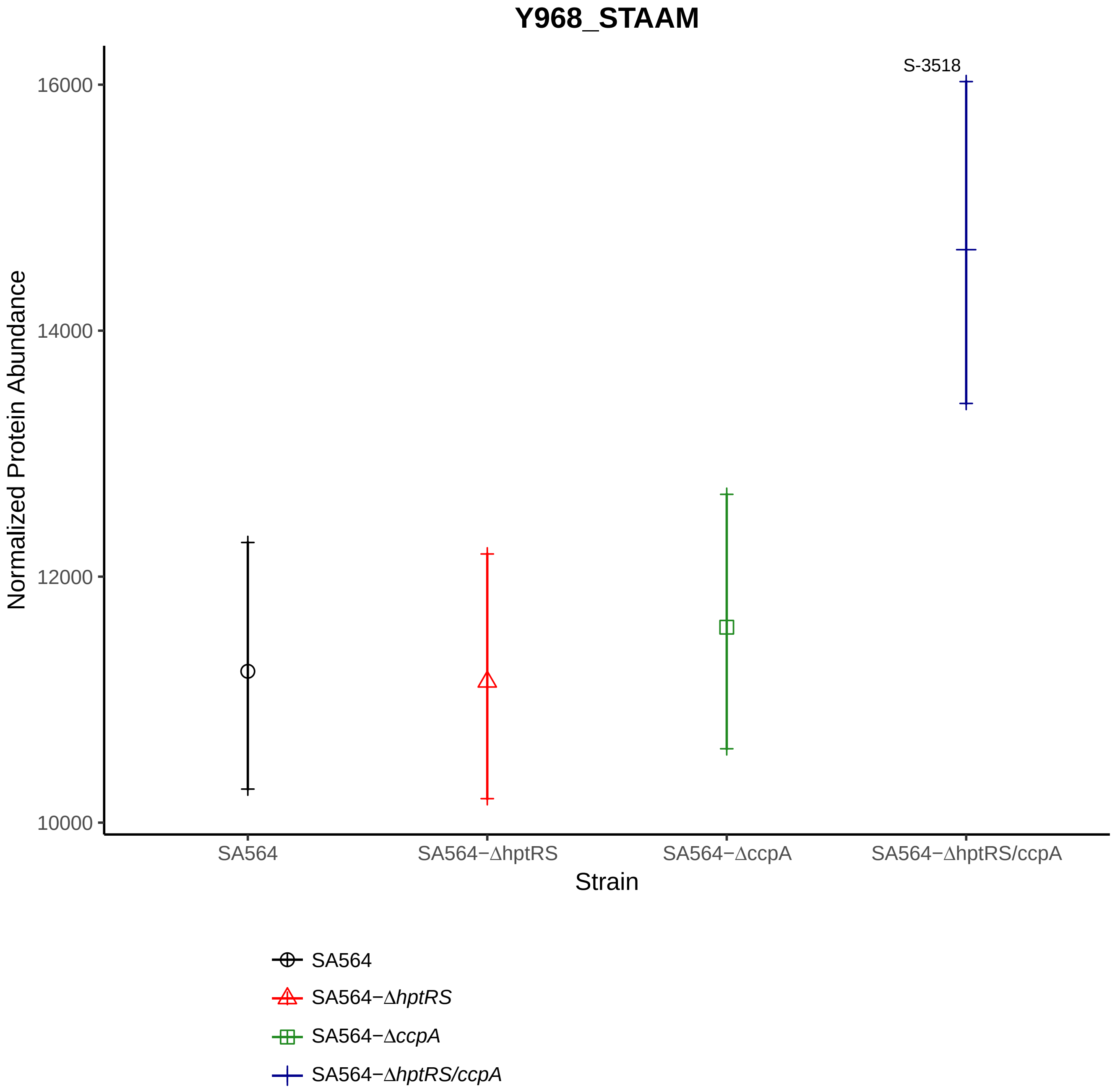
Y968_STAAM
S-3518
16000
14000
12000
10000
Normalized Protein Abundance
SA564
SA564−ΔhptRS
SA564−ΔccpA
SA564−ΔhptRS/ccpA
Strain
SA564
SA564−ΔhptRS
SA564−ΔccpA
SA564−ΔhptRS/ccpA

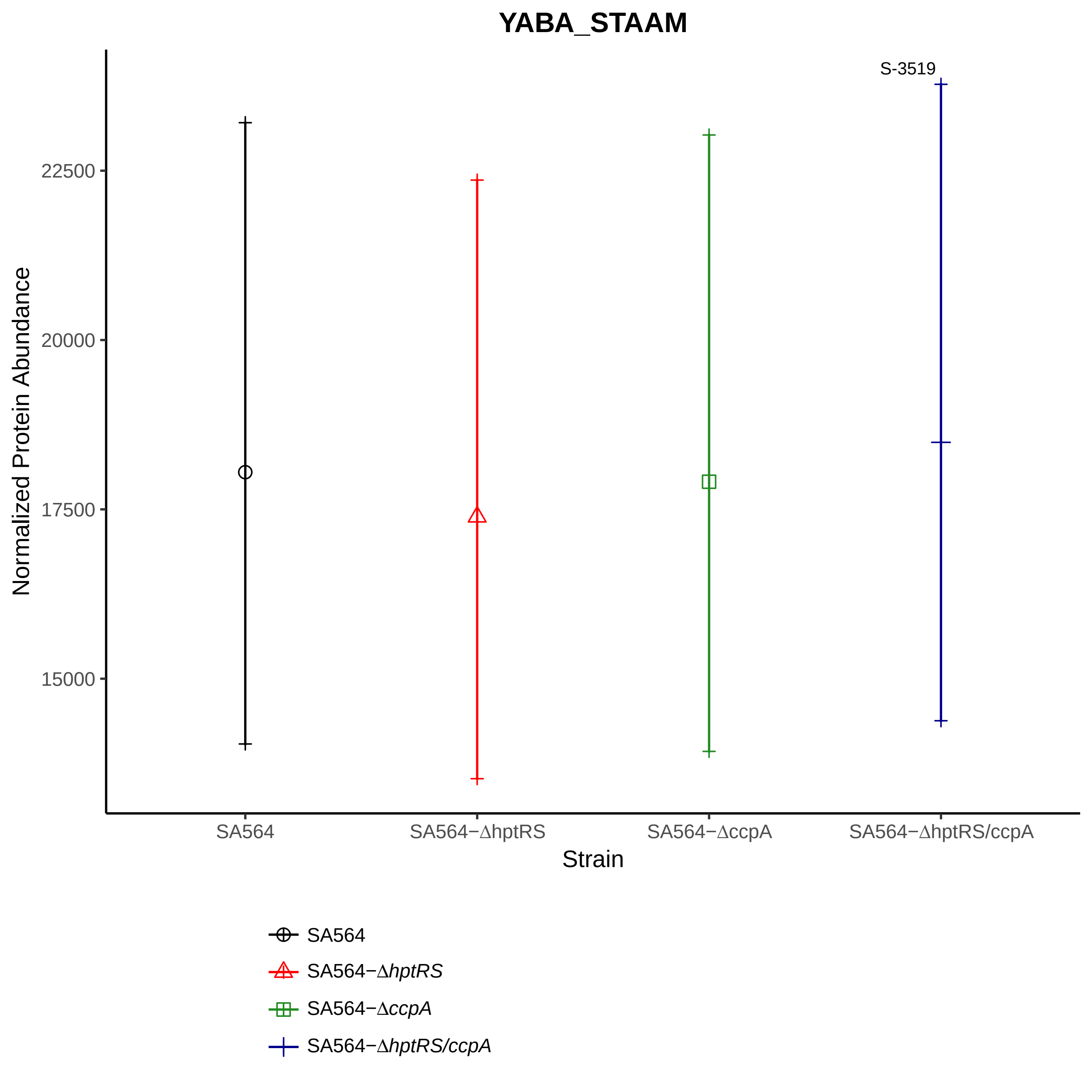

YABA_STAAM
S-3519
22500
20000
17500
15000
Normalized Protein Abundance
SA564
SA564−ΔhptRS
SA564−ΔccpA
SA564−ΔhptRS/ccpA
Strain
SA564
SA564−ΔhptRS
SA564−ΔccpA
SA564−ΔhptRS/ccpA

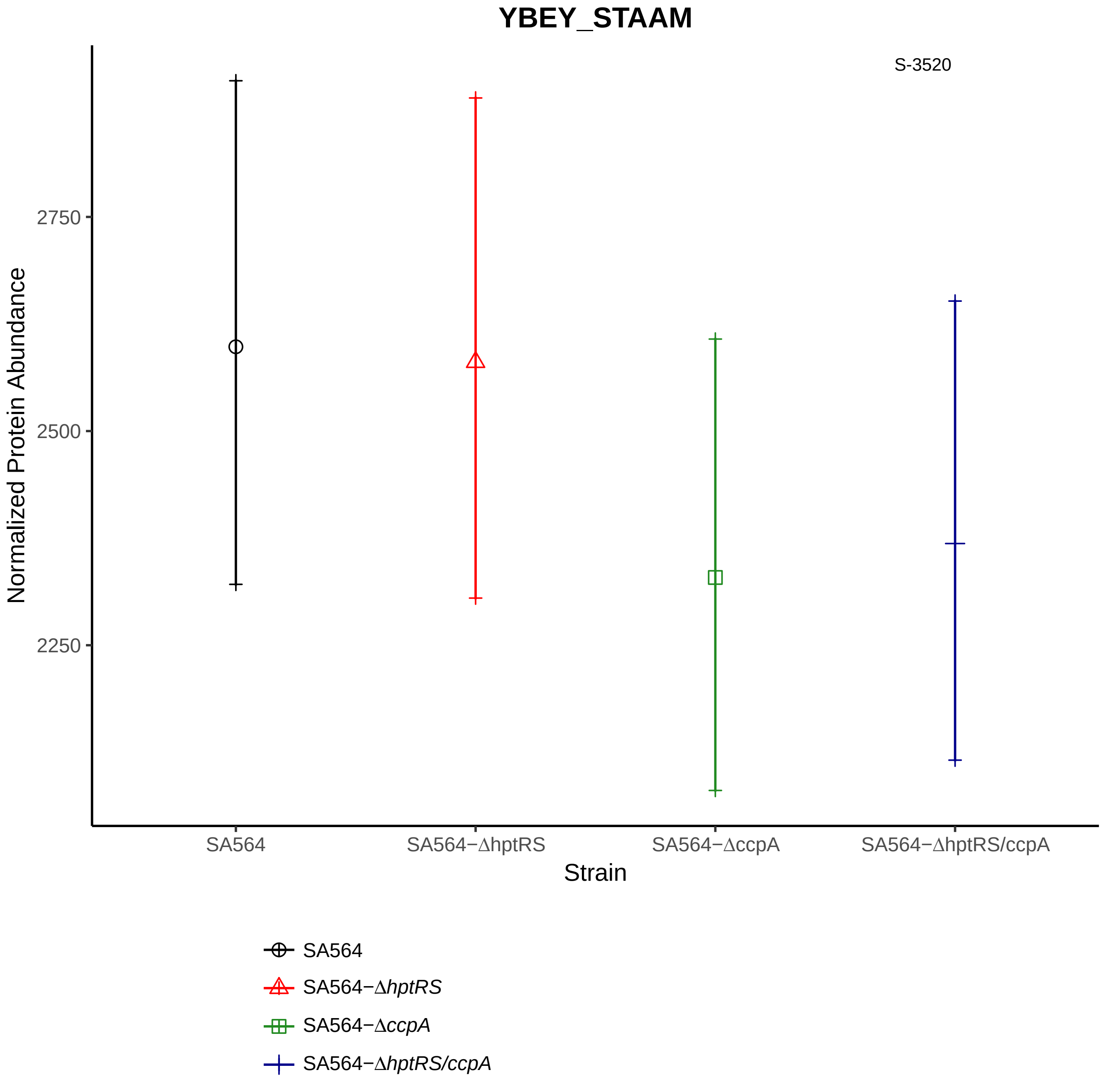

YBEY_STAAM
S-3520
Normalized Protein Abundance
2750
2500
2250
SA564
SA564−ΔhptRS
SA564−ΔccpA
SA564−ΔhptRS/ccpA
Strain
SA564
SA564−ΔhptRS
SA564−ΔccpA
SA564−ΔhptRS/ccpA
