# Supplementary material for: Coordinated regulation of transcription by CcpA and the Staphylococcus aureus two-component system HptRS
Source: PLoS One. 2018 Dec 12;13(12):e0207161. doi: 10.1371/journal.pone.0207161 (PMC6291074; doi:10.1371/journal.pone.0207161)
Supplement: S1 File — (PDF) [file pone.0207161.s001.pdf]

Supporting Information File 1

```
##
########################################
##
## GS_2016Pepion_Analysis.R
## Author  :   Sean Olson
## Date    :   March 2018 (final draft)
## Contact : solson19@huskers.unl.edu
##
########################################

library(data.table)
options(java.parameters = "-Xmx3000m")
library(xlsx)
library(lsmeans)
require(ggplot2)
require(effects)

########################################
##
## Data
##
########################################

data = data.frame(fread(input="GS2016PepIonNB.csv", sep = ",", skip=2, verbose=TRUE))
new.data = stack(data.frame(t(data)))
for(i in 1:nrow(data)){
  new.data$PepNo[((i-1)*80+1):(i*80)] = i
}
new.data = new.data[,c(3,1)]
colnames(new.data) = c("PepNo", "normalized.abundance")
media = c(rep("TSA",40), rep("GSA",40))
strain = rep(c(rep("564",5), rep("0024",5),  rep("ccpA",5), rep("ccpA/0024",5)), 4)
hour = rep(c(rep(2,20), rep(6,20)), 2)
hour = factor(hour)
exp.design = do.call(what="rbind", replicate(length(table(new.data$PepNo)), cbind(media, strain,
hour), simplify=FALSE))
proteins = data.frame(fread(input="protein_information.csv", sep=",",skip=1)); head(proteins)
colnames(proteins) = c("Protein", "PepNo", "Description")
df = merge(cbind(new.data, exp.design), proteins, by="PepNo")
df = df[-which(df$Protein==""),]
df$normalized.abundance[df$normalized.abundance==0] <- 1e-4
df.nona = na.omit(df)
ldf = split(df.nona, df.nona$Protein)
```

```
########################################
##
## GLM by Protein
##
########################################

ldf = split(df.nona, df.nona$Protein)
mods = lapply(X=ldf, FUN = function(x){ tryCatch({
  glm(formula = normalized.abundance ~ media*strain*hour-1, family = Gamma(link="log"),
na.action=na.omit, data=x)},
  error=function(e){cat("ERROR : ",conditionMessage(e),"\n")}) } )

########################################
##
## Multiple Comparisons
##
########################################

## three way
three.way.mcp = lapply(X=mods, FUN=function(x){
  data.frame(summary(pairs(lsmeans(x, ~strain*media*hour))))
})
for(i in 1:length(ldf)){
  three.way.mcp[[i]]$Protein = names(table(ldf[[i]]$Protein))
  three.way.mcp[[i]]$Description = names(table(ldf[[i]]$Description))
  three.way.mcp[[i]]$"Number Peptides/Protein" = length(table(ldf[[i]]$PepNo))
}
three.way.mcp.res = do.call("rbind", three.way.mcp)
three.way.mcp.res = three.way.mcp.res[,-which(colnames(three.way.mcp.res) %in% "df")]

## strain x media
strain.media.mcp = lapply(X=mods, FUN=function(x){
  data.frame(summary(pairs(lsmeans(x, ~strain*media))))
})
for(i in 1:length(ldf)){
  strain.media.mcp[[i]]$Protein = names(table(ldf[[i]]$Protein))
  strain.media.mcp[[i]]$Description = names(table(ldf[[i]]$Description))
  strain.media.mcp[[i]]$"Number Peptides/Protein" = length(table(ldf[[i]]$PepNo))
}
strain.media.mcp.res = do.call("rbind", strain.media.mcp)
strain.media.mcp.res = strain.media.mcp.res[,-which(colnames(strain.media.mcp.res) %in% "df")]

## strain x hour
strain.hour.mcp = lapply(X=mods, FUN=function(x){
  data.frame(summary(pairs(lsmeans(x, ~strain*hour))))
})
for(i in 1:length(ldf)){
  strain.hour.mcp[[i]]$Protein = names(table(ldf[[i]]$Protein))
  strain.hour.mcp[[i]]$Description = names(table(ldf[[i]]$Description))
  strain.hour.mcp[[i]]$"Number Peptides/Protein" = length(table(ldf[[i]]$PepNo))
}
strain.hour.mcp.res = do.call("rbind", strain.hour.mcp)
```

```
strain.hour.mcp.res = strain.hour.mcp.res[,-which(colnames(strain.hour.mcp.res) %in% "df")]

## media x hour
media.hour.mcp = lapply(X=mods, FUN=function(x){
  data.frame(summary(pairs(lsmeans(x, ~media*hour))))
})
for(i in 1:length(ldf)){
  media.hour.mcp[[i]]$Protein = names(table(ldf[[i]]$Protein))
  media.hour.mcp[[i]]$Description = names(table(ldf[[i]]$Description))
  media.hour.mcp[[i]]$"Number Peptides/Protein" = length(table(ldf[[i]]$PepNo))
}
media.hour.mcp.res = do.call("rbind", media.hour.mcp)
media.hour.mcp.res = media.hour.mcp.res[,-which(colnames(media.hour.mcp.res) %in% "df")]

### strain
strain.mcp = lapply(X=mods, FUN=function(x){
  data.frame(summary(pairs(lsmeans(x, ~strain))))
})
for(i in 1:length(ldf)){
  strain.mcp[[i]]$Protein = names(table(ldf[[i]]$Protein))
  strain.mcp[[i]]$Description = names(table(ldf[[i]]$Description))
  strain.mcp[[i]]$"Number Peptides/Protein" = length(table(ldf[[i]]$PepNo))
}
strain.mcp.res = do.call("rbind", strain.mcp)
strain.mcp.res = strain.mcp.res[,-which(colnames(strain.mcp.res) %in% "df")]

### media
media.mcp = lapply(X=mods, FUN=function(x){
  data.frame(summary(pairs(lsmeans(x, ~media))))
})
for(i in 1:length(ldf)){
  media.mcp[[i]]$Protein = names(table(ldf[[i]]$Protein))
  media.mcp[[i]]$Description = names(table(ldf[[i]]$Description))
  media.mcp[[i]]$"Number Peptides/Protein" = length(table(ldf[[i]]$PepNo))
}
media.mcp.res = do.call("rbind", media.mcp)
media.mcp.res = media.mcp.res[,-which(colnames(media.mcp.res) %in% "df")]

### hour
hour.mcp = lapply(X=mods, FUN=function(x){
  data.frame(summary(pairs(lsmeans(x, ~hour))))
})
for(i in 1:length(ldf)){
  hour.mcp[[i]]$Protein = names(table(ldf[[i]]$Protein))
  hour.mcp[[i]]$Description = names(table(ldf[[i]]$Description))
  hour.mcp[[i]]$"Number Peptides/Protein" = length(table(ldf[[i]]$PepNo))
}
hour.mcp.res = do.call("rbind", hour.mcp)
hour.mcp.res = hour.mcp.res[,-which(colnames(hour.mcp.res) %in% "df")]

### write
write.xlsx(x=three.way.mcp.res,
```

```
        file=paste0("GS2016PepIon_MultComp_", Sys.Date(), ".xlsx"),
        sheetName="Three Way", row.names=FALSE, append=FALSE)
write.xlsx(x=strain.media.mcp.res,
        file=paste0("GS2016PepIon_MultComp_", Sys.Date(), ".xlsx"),
        sheetName="Strain X Media", row.names=FALSE, append=TRUE)
write.xlsx(x=strain.hour.mcp.res,
        file=paste0("GS2016PepIon_MultComp_", Sys.Date(), ".xlsx"),
        sheetName="Strain X Hour", row.names=FALSE, append=TRUE)
write.xlsx(x=media.hour.mcp.res,
        file=paste0("GS2016PepIon_MultComp_", Sys.Date(), ".xlsx"),
        sheetName="Media X Hour", row.names=FALSE, append=TRUE)
write.xlsx(x=strain.mcp.res,
        file=paste0("GS2016PepIon_MultComp_", Sys.Date(), ".xlsx"),
        sheetName="Strain", row.names=FALSE, append=TRUE)
write.xlsx(x=media.mcp.res,
        file=paste0("GS2016PepIon_MultComp_", Sys.Date(), ".xlsx"),
        sheetName="Media", row.names=FALSE, append=TRUE)
write.xlsx(x=hour.mcp.res,
        file=paste0("GS2016PepIon_MultComp_", Sys.Date(), ".xlsx"),
        sheetName="Hour", row.names=FALSE, append=TRUE)

## "p-Value table"
n = length(table(df$Protein))
pval.res = matrix(NA, ncol=7, nrow=n)
for(i in 1:length(mods)){
  cat(i,"\n")
  pval.res[i,] = tryCatch({anova(mods[[i]], test="F")$`Pr(>F)`[2:8]},
                   error=function(e){ rep(NA,7) })
}
pval.res.df = data.frame(pval.res)
for(i in 1:length(ldf)){
  pval.res.df$Protein[i] = names(table(ldf[[i]]$Protein))
  pval.res.df$Description[i] = names(table(ldf[[i]]$Description))
  pval.res.df$"Number Peptides/Protein"[i] = length(table(ldf[[i]]$PepNo))
}
colnames(pval.res.df) = c("media pValue", "strain pValue", "hour pValue",
                   "m*s pValue", "m*h pValue", "s*h pValue", "m*s*h pValue",
                   "Protein", "Description",
                   "Number Peptides/Protein")
write.table(pval.res.df, file=paste0("GS2016PepIon_Results_byProtein_", Sys.Date(), ".csv"),
        row.names = FALSE, sep = ",")




##########################################
##
## Effect Plots
##
##########################################
```

```
## three way
plot.3way = function(ests, protein, ...){
  if(class(ests$hour)=="factor"){ests$hour = as.numeric(levels(ests$hour))[ests$hour]}
  levels(ests$media)[levels(ests$media)=="GSA"] = "TSB-dex"
  levels(ests$media)[levels(ests$media)=="TSA"] = "TSB"
  levels(ests$strain)[levels(ests$strain)=="0024"] = "SA564-ΔhptRS"
  levels(ests$strain)[levels(ests$strain)=="564"] = "SA564"
  levels(ests$strain)[levels(ests$strain)=="ccpA"] = "SA564-ΔccpA"
  levels(ests$strain)[levels(ests$strain)=="ccpA/0024"] = "SA564-ΔhptRS/ccpA"
  ests$media = factor(ests$media, levels(ests$media)[c(2,1)])
  ests$strain = factor(ests$strain, levels(ests$strain)[c(2,1,3,4)])
  cols=c("black", "red", "forestgreen", "darkblue")
  names(cols) = levels(ests$strain)
  shps = c(1,2,0,3)
  names(shps) = levels(ests$strain)
  mylabels = c("SA564",
               expression(paste("SA564-", Delta, italic("hptRS"))),
               expression(paste("SA564-", Delta, italic("ccpA"))),
               expression(paste("SA564-",Delta, italic("hptRS/ccpA"))))
  ggplot(ests, aes(x=hour, y=fit, colour=strain, group=strain)) +
    geom_segment(aes(x=hour, xend=hour, y=lower, yend=upper), lineend="square") +
    geom_segment(aes(x=hour-0.05, xend=hour+0.05, y=lower, yend=lower)) +
    geom_segment(aes(x=hour-0.05, xend=hour+0.05, y=upper, yend=upper)) +
    geom_point(aes(shape=strain),cex=2.5) + geom_line() +
    xlab("Time (h)") + ylab("Normalized Protein Abundance") +
    facet_grid(~media) +
    scale_x_discrete(limits=c("1","2"), labels=c("2h","6h")) +
    ggtitle(paste(protein)) +
    theme(text=element_text(family="Helvetica"),
        plot.title=element_text(face="bold", hjust=0.5),
        panel.grid.major = element_blank(),
        panel.grid.minor = element_blank(),
        panel.background = element_blank(),
        axis.line = element_line(colour = "black"),
        legend.position="bottom",
        legend.direction="vertical",
        legend.box.margin = margin(1,200,1,1),
        legend.title=element_blank(),
        legend.key=element_rect(fill="white"),
        legend.text.align=0,
        strip.background=element_rect(colour="white", fill="white")) +
    scale_colour_manual(name="strain", values=cols, labels=mylabels) +
    scale_shape_manual(name="strain", values=shps, labels=mylabels)
}

plot.3way(ests=data.frame(effect(term="media*strain*hour", mod = mods[[i]], se = TRUE,
kr=TRUE)), protein=names(table(ldf[[i]]$Protein)))

## printing plots
plots=list()
is = 1:length(mods)
```

```
for(i in is){
   plots[[i]]=plot.3way(ests=data.frame(effect(term="media*strain*hour", mod = mods[[i]], se =
TRUE, kr=TRUE)), protein=names(table(ldf[[i]]$Protein)))
}

pdf(file=paste0("protein_plots_", Sys.Date(),".pdf"))
for(i in 1:length(plots)){ cat("Now printing plot : ", i, "\n\n"); print(plots[[i]])  }
dev.off()


## two way
plot.2way = function(ests, protein, type, ...){

   if(type=="strain*media"){
      levels(ests$media)[levels(ests$media)=="GSA"] = "TSB-dex"
      levels(ests$media)[levels(ests$media)=="TSA"] = "TSB"
      levels(ests$strain)[levels(ests$strain)=="0024"] = "SA564-ΔhptRS"
      levels(ests$strain)[levels(ests$strain)=="564"] = "SA564"
      levels(ests$strain)[levels(ests$strain)=="ccpA"] = "SA564-ΔccpA"
      levels(ests$strain)[levels(ests$strain)=="ccpA/0024"] = "SA564-ΔhptRS/ccpA"
      ests$media = factor(ests$media, levels(ests$media)[c(2,1)])
      ests$strain = factor(ests$strain, levels(ests$strain)[c(2,1,3,4)])
      cols=c("black", "red", "forestgreen", "darkblue")
      names(cols) = levels(ests$strain)
      shps = c(1,2,0,3)
      names(shps) = levels(ests$strain)
      mylabels = c("SA564",
                expression(paste("SA564-", Delta, italic("hptRS"))),
                expression(paste("SA564-", Delta, italic("ccpA"))),
                expression(paste("SA564-",Delta, italic("hptRS/ccpA"))))
      p = ggplot(ests, aes(x=media, y=fit, colour=strain, group=strain)) +
         geom_segment(aes(x=media, xend=media, y=lower, yend=upper), lineend="square") +
         geom_point(aes(x=media, y=lower), shape=3)+
         geom_point(aes(x=media, y=upper), shape=3)+
         geom_point(aes(shape=strain),cex=2.5) + geom_line() +
         xlab("Media") + ylab("Normalized Protein Abundance") +
         ggtitle(paste(protein)) +
         theme(text=element_text(family="Helvetica"),
              plot.title=element_text(face="bold", hjust=0.5),
              panel.grid.major = element_blank(),
              panel.grid.minor = element_blank(),
              panel.background = element_blank(),
              axis.line = element_line(colour = "black"),
              legend.position="bottom",
              legend.direction="vertical",
              legend.box.margin = margin(1,200,1,1),
              legend.title=element_blank(),
              legend.key=element_rect(fill="white"),
              legend.text.align=0,
              strip.background=element_rect(colour="white", fill="white")) +
         scale_colour_manual(name="strain", values=cols, labels=mylabels) +
         scale_shape_manual(name="strain", values=shps, labels=mylabels)
```

```
  return(p)
}

if(type=="strain*hour"){
  if(class(ests$hour)=="factor"){ests$hour = as.numeric(levels(ests$hour))[ests$hour]}

  levels(ests$strain)[levels(ests$strain)=="0024"] = "SA564-ΔhptRS"
  levels(ests$strain)[levels(ests$strain)=="564"] = "SA564"
  levels(ests$strain)[levels(ests$strain)=="ccpA"] = "SA564-ΔccpA"
  levels(ests$strain)[levels(ests$strain)=="ccpA/0024"] = "SA564-ΔhptRS/ccpA"
  ests$strain = factor(ests$strain, levels(ests$strain)[c(2,1,3,4)])
  cols=c("black", "red", "forestgreen", "darkblue")
  names(cols) = levels(ests$strain)
  shps = c(1,2,0,3)
  names(shps) = levels(ests$strain)
  mylabels = c("SA564",
           expression(paste("SA564-", Delta, italic("hptRS"))),
           expression(paste("SA564-", Delta, italic("ccpA"))),
           expression(paste("SA564-",Delta, italic("hptRS/ccpA"))))
  p = ggplot(ests, aes(x=hour, y=fit, colour=strain, group=strain)) +
    geom_segment(aes(x=hour, xend=hour, y=lower, yend=upper), lineend="square") +
    geom_segment(aes(x=hour-0.05, xend=hour+0.05, y=lower, yend=lower)) +
    geom_segment(aes(x=hour-0.05, xend=hour+0.05, y=upper, yend=upper)) +
    geom_point(aes(shape=strain),cex=2.5) + geom_line() +
    xlab("Time (h)") + ylab("Normalized Protein Abundance") +
    scale_x_discrete(limits=c("1","2"), labels=c("2h","6h")) +
    ggtitle(paste(protein)) +
    theme(text=element_text(family="Helvetica"),
        plot.title=element_text(face="bold", hjust=0.5),
        panel.grid.major = element_blank(),
        panel.grid.minor = element_blank(),
        panel.background = element_blank(),
        axis.line = element_line(colour = "black"),
        legend.position="bottom",
        legend.direction="vertical",
        legend.box.margin = margin(1,200,1,1),
        legend.title=element_blank(),
        legend.key=element_rect(fill="white"),
        legend.text.align=0,
        strip.background=element_rect(colour="white", fill="white")) +
    scale_colour_manual(name="strain", values=cols, labels=mylabels) +
    scale_shape_manual(name="strain", values=shps, labels=mylabels)
  return(p)
}

if(type=="media*hour"){
  if(class(ests$hour)=="factor"){ests$hour = as.numeric(levels(ests$hour))[ests$hour]}
  levels(ests$media)[levels(ests$media)=="GSA"] = "TSB-dex"
  levels(ests$media)[levels(ests$media)=="TSA"] = "TSB"
  ests$media = factor(ests$media, levels(ests$media)[c(2,1)])
  cols=c("black", "red")
  names(cols) = levels(ests$media)
```

```
    shps = c(1,2)
    names(shps) = levels(ests$media)
    mylabels=c("TSB-dex", "TSB")
    p = ggplot(ests, aes(x=hour, y=fit, colour=media, group=media)) +
      geom_segment(aes(x=hour, xend=hour, y=lower, yend=upper), lineend="square") +
      geom_segment(aes(x=hour-0.05, xend=hour+0.05, y=lower, yend=lower)) +
      geom_segment(aes(x=hour-0.05, xend=hour+0.05, y=upper, yend=upper)) +
      geom_point(aes(shape=media),cex=2.5) + geom_line() +
      xlab("Time (h)") + ylab("Normalized Protein Abundance") +
      scale_x_discrete(limits=c("1","2"), labels=c("2h","6h")) +
      ggtitle(paste(protein)) +
      theme(text=element_text(family="Helvetica"),
          plot.title=element_text(face="bold", hjust=0.5),
          panel.grid.major = element_blank(),
          panel.grid.minor = element_blank(),
          panel.background = element_blank(),
          axis.line = element_line(colour = "black"),
          legend.position="bottom",
          legend.direction="vertical",
          legend.box.margin = margin(1,200,1,1),
          legend.title=element_blank(),
          legend.key=element_rect(fill="white"),
          legend.text.align=0,
          strip.background=element_rect(colour="white", fill="white")) +
      scale_colour_manual(name="media", values=cols, labels=mylabels) +
      scale_shape_manual(name="media", values=shps, labels=mylabels)
    return(p)
  }
}

plot.2way(ests=data.frame(effect(term="media*strain", mod = mods[[i]], se = TRUE, kr=TRUE)),
protein=names(table(ldf[[i]]$Protein)), type="strain*media")
plot.2way(ests=data.frame(effect(term="strain*hour", mod = mods[[i]], se = TRUE, kr=TRUE)),
protein=names(table(ldf[[i]]$Protein)), type="strain*hour")
plot.2way(ests=data.frame(effect(term="media*hour", mod = mods[[i]], se = TRUE, kr=TRUE)),
protein=names(table(ldf[[i]]$Protein)), type="media*hour")

plots.sm=list()
is = 1:length(mods)
for(i in is){
  plots.sm[[i]]=plot.2way(ests=data.frame(effect(term="media*strain", mod = mods[[i]], se =
TRUE, kr=TRUE)), protein=names(table(ldf[[i]]$Protein)), type="strain*media")
}
pdf(file=paste0("protein_plots_strain_X_media_", Sys.Date(),".pdf"))
for(i in 1:length(plots.sm)){ cat("Now printing plot : ", i, "\n\n"); print(plots.sm[[i]])  }
dev.off()

plots.sh=list()
is = 1:length(mods)
for(i in is){
  cat("Now saving plot : ", i, "\n\n")
  plots.sh[[i]]=plot.2way(ests=data.frame(effect(term="strain*hour", mod = mods[[i]], se =
```

```
TRUE, kr=TRUE)), protein=names(table(ldf[[i]]$Protein)), type="strain*hour")
}
pdf(file=paste0("protein_plots_strain_X_hour_", Sys.Date(),".pdf"))
for(i in 1:length(plots.sh)){ cat("Now printing plot : ", i, "\n\n"); print(plots.sh[[i]])  }
dev.off()

plots.mh=list()
is = 1:length(mods)
for(i in is){
  cat("Now saving plot : ", i, "\n\n")
  plots.mh[[i]]=plot.2way(ests=data.frame(effect(term="media*hour", mod = mods[[i]], se =
TRUE, kr=TRUE)), protein=names(table(ldf[[i]]$Protein)), type="media*hour")
}
pdf(file=paste0("protein_plots_media_X_hour_", Sys.Date(),".pdf"))
for(i in 1:length(plots.mh)){ cat("Now printing plot : ", i, "\n\n"); print(plots.mh[[i]])  }
dev.off()

## one way

plot.1way = function(ests, protein, type, ...){
  if(type=="strain"){
    levels(ests$strain)[levels(ests$strain)=="0024"] = "SA564-ΔhptRS"
    levels(ests$strain)[levels(ests$strain)=="564"] = "SA564"
    levels(ests$strain)[levels(ests$strain)=="ccpA"] = "SA564-ΔccpA"
    levels(ests$strain)[levels(ests$strain)=="ccpA/0024"] = "SA564-ΔhptRS/ccpA"
    ests$strain = factor(ests$strain, levels(ests$strain)[c(2,1,3,4)])
    cols=c("black", "red", "forestgreen", "darkblue")
    names(cols) = levels(ests$strain)
    shps = c(1,2,0,3)
    names(shps) = levels(ests$strain)
    mylabels = c("SA564",
              expression(paste("SA564-", Delta, italic("hptRS"))),
              expression(paste("SA564-", Delta, italic("ccpA"))),
              expression(paste("SA564-",Delta, italic("hptRS/ccpA"))))
    p = ggplot(ests, aes(x=strain, y=fit, colour=strain, group=strain)) +
      geom_segment(aes(x=strain, xend=strain, y=lower, yend=upper), lineend="square") +
      geom_point(aes(x=strain, y=lower), shape=3) +
      geom_point(aes(x=strain, y=upper), shape=3) +
      geom_point(aes(shape=strain),cex=2.5) +
      xlab("Strain") + ylab("Normalized Protein Abundance") +
      ggtitle(paste(protein)) +
      theme(text=element_text(family="Helvetica"),
          plot.title=element_text(face="bold", hjust=0.5),
          panel.grid.major = element_blank(),
          panel.grid.minor = element_blank(),
          panel.background = element_blank(),
          axis.line = element_line(colour = "black"),
          legend.position="bottom",
          legend.direction="vertical",
          legend.box.margin = margin(1,200,1,1),
          legend.title=element_blank(),
          legend.key=element_rect(fill="white"),
```

```
          legend.text.align=0,
          strip.background=element_rect(colour="white", fill="white")) +
      scale_colour_manual(name="strain", values=cols, labels=mylabels) +
      scale_shape_manual(name="strain", values=shps, labels=mylabels)
   return(p)
}

if(type=="media"){
   levels(ests$media)[levels(ests$media)=="GSA"] = "TSB-dex"
   levels(ests$media)[levels(ests$media)=="TSA"] = "TSB"
   ests$media = factor(ests$media, levels(ests$media)[c(2,1)])
   cols=c("black", "red")
   names(cols) = levels(ests$media)
   shps = c(1,2)
   names(shps) = levels(ests$media)
   mylabels = c("TSB-dex", "TSB")
   p = ggplot(ests, aes(x=media, y=fit, colour=media, group=1)) +
      geom_segment(aes(x=media, xend=media, y=lower, yend=upper), lineend="square") +
      geom_point(aes(x=media, y=lower), shape=3) +
      geom_point(aes(x=media, y=upper), shape=3) +
      geom_point(aes(shape=media),cex=2.5) +
      xlab("Media") + ylab("Normalized Protein Abundance") +
      ggtitle(paste(protein)) +
      theme(text=element_text(family="Helvetica"),
          plot.title=element_text(face="bold", hjust=0.5),
          panel.grid.major = element_blank(),
          panel.grid.minor = element_blank(),
          panel.background = element_blank(),
          axis.line = element_line(colour = "black"),
          legend.position="bottom",
          legend.direction="vertical",
          legend.box.margin = margin(1,200,1,1),
          legend.title=element_blank(),
          legend.key=element_rect(fill="white"),
          legend.text.align=0,
          strip.background=element_rect(colour="white", fill="white")) +
      scale_colour_manual(name="media", values=cols, labels=mylabels) +
      scale_shape_manual(name="media", values=shps, labels=mylabels)
   return(p)
}

if(type=="hour"){
   cols=c("black", "red")
   names(cols) = levels(ests$hour)[!is.na(levels(ests$hour))]
   shps = c(1,2)
   names(shps) = levels(ests$hour)[!is.na(levels(ests$hour))]
   mylabels = c("2h", "6h")
   p = ggplot(ests, aes(x=hour, y=fit, colour=hour, group=1)) +
      geom_segment(aes(x=hour, xend=hour, y=lower, yend=upper), lineend="square") +
      geom_point(aes(x=hour, y=lower), shape=3) +
      geom_point(aes(x=hour, y=upper), shape=3) +
      geom_point(aes(shape=hour),cex=2.5) +
```

```
        xlab("Hour") + ylab("Normalized Protein Abundance") +
        ggtitle(paste(protein)) +
        theme(text=element_text(family="Helvetica"),
              plot.title=element_text(face="bold", hjust=0.5),
              panel.grid.major = element_blank(),
              panel.grid.minor = element_blank(),
              panel.background = element_blank(),
              axis.line = element_line(colour = "black"),
              legend.position="bottom",
              legend.direction="vertical",
              legend.box.margin = margin(1,200,1,1),
              legend.title=element_blank(),
              legend.key=element_rect(fill="white"),
              legend.text.align=0,
              strip.background=element_rect(colour="white", fill="white")) +
        scale_colour_manual(name="hour", values=cols, labels=mylabels) +
        scale_shape_manual(name="hour", values=shps, labels=mylabels)
      return(p)
  }
}

plot.1way(ests=data.frame(effect(term="strain", mod = mods[[i]], se = TRUE, kr=TRUE)),
protein=names(table(ldf[[i]]$Protein)), type="strain")
plot.1way(ests=data.frame(effect(term="media", mod = mods[[i]], se = TRUE, kr=TRUE)),
protein=names(table(ldf[[i]]$Protein)), type="media")
plot.1way(ests=data.frame(effect(term="hour", mod = mods[[i]], se = TRUE, kr=TRUE)),
protein=names(table(ldf[[i]]$Protein)), type="hour")


plots.s=list()
is = 1:length(mods)
for(i in is){
   plots.s[[i]]=plot.1way(ests=data.frame(effect(term="strain", mod = mods[[i]], se = TRUE,
kr=TRUE)), protein=names(table(ldf[[i]]$Protein)), type="strain")
}
pdf(file=paste0("protein_plots_strain_", Sys.Date(),".pdf"))
for(i in 1:length(plots.s)){ cat("Now printing plot : ", i, "\n\n"); print(plots.s[[i]])  }
dev.off()

plots.m=list()
is = 1:length(mods)
for(i in is){
   plots.m[[i]]=plot.1way(ests=data.frame(effect(term="media", mod = mods[[i]], se = TRUE,
kr=TRUE)), protein=names(table(ldf[[i]]$Protein)), type="media")
}
pdf(file=paste0("protein_plots_media_", Sys.Date(),".pdf"))
for(i in 1:length(plots.m)){ cat("Now printing plot : ", i, "\n\n"); print(plots.m[[i]])  }
dev.off()

plots.h=list()
is = 1:length(mods)
for(i in is){
```

```
   plots.h[[i]]=plot.1way(ests=data.frame(effect(term="hour", mod = mods[[i]], se = TRUE,
kr=TRUE)), protein=names(table(ldf[[i]]$Protein)), type="hour")
}
pdf(file=paste0("protein_plots_hour_", Sys.Date(),".pdf"))
for(i in 1:length(plots.h)){ cat("Now printing plot : ", i, "\n\n"); print(plots.h[[i]])  }
dev.off()

########################################
##
## Further Estimates and Fold Changes
##
########################################

three.way.ests = list()
two.way.ests = list()
strain.ests = list()
hour.ests = list()
for(i in 1:length(mods)){
   three.way.ests[[i]] = data.frame(effect(term="media*strain*hour", mod=mods[[i]]),
names(table(ldf[[i]]$Protein)))
   two.way.ests[[i]] = data.frame(effect(term="strain*hour", mod=mods[[i]]),
names(table(ldf[[i]]$Protein)))
   strain.ests[[i]] = data.frame(effect(term="strain", mod=mods[[i]]),
names(table(ldf[[i]]$Protein)))
   hour.ests[[i]] = data.frame(effect(term="hour", mod=mods[[i]]), names(table(ldf[[i]]$Protein)))
}
three.way.ests.df = do.call(what = "rbind.data.frame", args=three.way.ests)
colnames(three.way.ests.df)[ncol(three.way.ests.df)] = "Protein"
two.way.ests.df = do.call(what = "rbind.data.frame", args=two.way.ests)
colnames(two.way.ests.df)[ncol(two.way.ests.df)] = "Protein"
strain.ests.df = do.call(what = "rbind.data.frame", args=strain.ests)
colnames(strain.ests.df)[ncol(strain.ests.df)] = "Protein"
hour.ests.df = do.call(what = "rbind.data.frame", args=hour.ests)
colnames(hour.ests.df)[ncol(hour.ests.df)] = "Protein"

write.xlsx(x=three.way.ests.df, file=paste0("GS2016PepIon_Estimates_byProtein", Sys.Date(),
".xlsx"), sheetName="Strain x Media x Hour", row.names=FALSE)
write.xlsx(x=two.way.ests.df, file=paste0("GS2016PepIon_Estimates_byProtein", Sys.Date(),
".xlsx"), sheetName="Strain x Hour", row.names=FALSE, append = TRUE)
write.xlsx(x=strain.ests.df, file=paste0("GS2016PepIon_Estimates_byProtein", Sys.Date(), ".xlsx"),
sheetName="Strain", row.names=FALSE, append = TRUE)
write.xlsx(x=hour.ests.df, file=paste0("GS2016PepIon_Estimates_byProtein", Sys.Date(), ".xlsx"),
sheetName="Hour", row.names=FALSE, append = TRUE)


fold.change.3way = function(estimates){
   wt.gsa.1 = estimates[which(estimates$media %in% "GSA" & estimates$strain %in% 564 &
estimates$hour %in% 1),]$fit
   wt.tsa.1 = estimates[which(estimates$media %in% "TSA" & estimates$strain %in% 564 &
estimates$hour %in% 1),]$fit
   wt.gsa.2 = estimates[which(estimates$media %in% "GSA" & estimates$strain %in% 564 &
estimates$hour %in% 2),]$fit
```

```
  wt.tsa.2 = estimates[which(estimates$media %in% "TSA" & estimates$strain %in% 564 &
estimates$hour %in% 2),]$fit

  fcs = data.frame(wt.gsa.1 / estimates[1,]$fit, # fc 0024 gsa phase 1
                wt.gsa.1 / estimates[5,]$fit, # fc ccpa gsa phase 1
                wt.gsa.1 / estimates[7,]$fit, # fc ccpA/0024 phase 1
                wt.gsa.2 / estimates[9,]$fit,
                wt.gsa.2 / estimates[13,]$fit,
                wt.gsa.2 / estimates[15,]$fit,
                wt.tsa.1 / estimates[2,]$fit,
                wt.tsa.1 / estimates[6,]$fit,
                wt.tsa.1 / estimates[8,]$fit,
                wt.tsa.2 / estimates[10,]$fit,
                wt.tsa.2 / estimates[14,]$fit,
                wt.tsa.2 / estimates[16,]$fit, estimates[1,ncol(estimates)])
  colnames(fcs) = c("fc 0024 GSA P1",
                "fc ccpA GSA P1",
                "fc ccpA/0024 P1",
                "fc 0024 GSA P2",
                "fc ccpA GSA P2",
                "fc ccpA/0024 P2",
                "fc 0024 TSA P1",
                "fc ccpA TSA P1",
                "fc ccpA/0024 TSA P1",
                "fc 0024 TSA P2",
                "fc ccpA TSA P2",
                "fc ccpA/0024 TSA P2", "Protein")
  fcs

}
ThreewayFCs = do.call(what="rbind.data.frame", args=lapply(X=three.way.ests,
FUN=fold.change.3way))
write.xlsx(x=ThreewayFCs, file=paste0("GS2016PepIon_FCs_byProtein_", Sys.Date(), ".xlsx"),
sheetName="FCs Strain x Media x Hour", row.names=FALSE, append=TRUE)

fold.change.2way = function(estimates){
  wt1 = estimates[which(estimates$strain %in% 564 & estimates$hour %in% 1),]$fit
  wt2 = estimates[which(estimates$strain %in% 564 & estimates$hour %in% 2),]$fit
  fcs = data.frame(wt1 / estimates[1,]$fit, wt1 / estimates[3,]$fit, wt1 / estimates[4,]$fit,
                wt2 / estimates[5,]$fit, wt2 / estimates[7,]$fit, wt2 / estimates[8,]$fit,
estimates[1,ncol(estimates)])
  colnames(fcs) = c("fc 0024 P1", "fc ccpA P1", "fc ccpA/0024 P1",
                "fc 0024 P2", "fc ccpA P2", "fc ccpA/0024 P2", "Protein")
  fcs

}
TwowayFCs = do.call(what="rbind.data.frame", args=lapply(X=two.way.ests,
FUN=fold.change.2way))
write.xlsx(x=TwowayFCs, file=paste0("GS2016PepIon_FCs_byProtein_", Sys.Date(), ".xlsx"),
sheetName="FCs Strain x Hour", row.names=FALSE, append=TRUE)

fold.change.1way = function(estimates){
```

```
   wt = estimates[which(estimates$strain %in% 564),]$fit
   fcs = data.frame(wt / estimates[1,]$fit, wt / estimates[3,]$fit, wt / estimates[4,]$fit,
estimates[1,ncol(estimates)])
   colnames(fcs) = c("fc 0024", "fc ccpA", "fc ccpA/0024", "Protein")
   fcs
}
OnewayFCs = do.call(what="rbind.data.frame", args=lapply(X=strain.ests,
FUN=fold.change.1way))
write.xlsx(x=OnewayFCs, file=paste0("GS2016PepIon_FCs_byProtein_", Sys.Date(), ".xlsx"),
sheetName="FCs Strain", row.names=FALSE, append=TRUE)
```

```
####################

/* GS2016PepIon_Power.sas 8/
/* Author : Sean Olson */
/* Date     : March 2018 (final copy) */

/* sim data */
/* simulate: 2 fold difference between wt and mutant(single); 2 fold differnce between single and
double mutant*/
data power_od_tsb;
  input strain @@;
    input hour @@;
              input mu @@;
              do rep=1 to 2;
      output;
      end;
CARDS;
1 2 1
1 6 2
2 2 1
2 6 2
3 2 3.6931472
3 6 4.6931472
4 2 3.6931472
4 6 4.6931472
;
/*ods results off;
ods html exclude all;*/
proc glimmix data=power_od_tsb method=laplace;
  nloptions maxit=1000;
  class strain hour;
  model mu=strain|hour / dist=gamma noint solution;
  parms (0.15) / hold=1; /* Dispersion parameter must be specified in this line*/
  lsmeans strain*hour / ilink plot=meanplot(sliceby=strain join cl ilink) ilink;
  ods output tests3=fstuff;
run;
data power_ods_tsb_out;
 set fstuff;
```

```
 alpha=0.05;
 ncparm=numdf*fvalue;
 fcrit=finv(1-alpha,numdf,dendf,0);
 power=1-probf(fcrit,numdf,dendf,ncparm);
 ods results on;
 ods html select all;
 proc print data=power_ods_tsb_out; run;

####################
##
##References
##
##R
##
```


```
## R Core Team (2017). R: A language and environment for statistical
## computing. R Foundation for Statistical Computing, Vienna, Austria. URL
## https://www.R-project.org/.
##
## 'data.table' package
## M Dowle, A Srinivasan, T Short, S Lianoglou with contributions from R
## Saporta and E Antonyan (2015). data.table: Extension of Data.frame. R
## package version 1.9.6. https://CRAN.R-project.org/package=data.table
##
## 'xlsx' package
## Adrian A. Dragulescu (2014). xlsx: Read, write, format Excel 2007 and Excel
## 97/2000/XP/2003 files. R package version 0.5.7.
## https://CRAN.R-project.org/package=xlsx
##
## 'lsmeans' package
## Russell V. Lenth (2016). Least-Squares Means: The R Package lsmeans.
## Journal of Statistical Software, 69(1), 1-33. doi:10.18637/jss.v069.i01
##
## 'ggplot2' package
## H. Wickham. ggplot2: Elegant Graphics for Data Analysis. Springer-Verlag
## New York, 2009.
##
## 'effects' package
## John Fox (2003). Effect Displays in R for Generalised Linear Models.
## Journal of Statistical Software, 8(15), 1-27. URL
## http://www.jstatsoft.org/v08/i15/.
##
## SAS 9.4
##
## SAS Institute Inc. SAS/STAT Software, Version 9.4. Cary, NC, 2002-2012. URL
http://www.sas.com/.
##
####################
```
